# Supplementary material for: Cross-cohort transcriptomic meta-analysis identifies conserved melanogenesis signatures and developmental background in black-boned chicken breast muscle
Source: Anim Biosci. 2026 Jun 24;39(10):260430. doi: 10.5713/ab.260430 (PMC13662945; doi:10.5713/ab.260430)
Supplement: Supplementary file 2 [file ab-260430-Supplementary-10.pdf]

Supplement 10. Long-format PRJNA729271 background contrast results across developmental and breed-background comparisons.

cohort_id	background_contrast	background_type	gene_id	symbol	log2FoldChange	lfcSE	stat	pvalue	padj	direction
PRJNA729271	stage_E14_vs_E10	ordinary_muscle_development	ENSGALG00010000007	ND1	-0.238757738	0.243331252	-0.981204577	0.326491868	0.441299853	negative
PRJNA729271	stage_E14_vs_E10	ordinary_muscle_development	ENSGALG00010000011	ND2	-0.334918952	0.253014748	-1.323713165	0.185598325	0.281612867	negative
PRJNA729271	stage_E14_vs_E10	ordinary_muscle_development	ENSGALG00010000017	COX1	-0.124716688	0.272487924	-0.457696205	0.647170715	0.739106486	negative
PRJNA729271	stage_E14_vs_E10	ordinary_muscle_development	ENSGALG00010000020	COX2	-0.016724821	0.239243205	-0.069907193	0.944267528	0.960639742	negative
PRJNA729271	stage_E14_vs_E10	ordinary_muscle_development	ENSGALG00010000022	ATP8	0.132161178	0.272629658	0.484764494	0.627843412	0.721956943	positive
PRJNA729271	stage_E14_vs_E10	ordinary_muscle_development	ENSGALG00010000023	ATP6	-0.129257488	0.198869629	-0.649960927	0.515717461	0.624499172	negative
PRJNA729271	stage_E14_vs_E10	ordinary_muscle_development	ENSGALG00010000024	COX3	0.130352353	0.221543053	0.588383841	0.556274681	0.660485051	positive
PRJNA729271	stage_E14_vs_E10	ordinary_muscle_development	ENSGALG00010000026	ND3	-0.160199409	0.318328762	-0.503251443	0.614787501	0.711143949	negative
PRJNA729271	stage_E14_vs_E10	ordinary_muscle_development	ENSGALG00010000028	ND4L	-0.336217886	0.28418142	-1.183110019	0.236765548	0.341688233	negative
PRJNA729271	stage_E14_vs_E10	ordinary_muscle_development	ENSGALG00010000029	ND4	-0.283215454	0.256644724	-1.103531176	0.269796561	0.379569169	negative
PRJNA729271	stage_E14_vs_E10	ordinary_muscle_development	ENSGALG00010000033	ND5	-0.170205572	0.232494698	-0.732083671	0.4641175	0.57671523	negative
PRJNA729271	stage_E14_vs_E10	ordinary_muscle_development	ENSGALG00010000034	CYTB	-0.250730428	0.219592593	-1.141798205	0.253537909	0.361040572	negative
PRJNA729271	stage_E14_vs_E10	ordinary_muscle_development	ENSGALG00010000037	ND6	0.335968392	0.190774874	1.761072536	0.078226127	0.138684896	positive
PRJNA729271	stage_E14_vs_E10	ordinary_muscle_development	ENSGALG00010000039	PDIA3	-0.089486104	0.065446598	-1.367314826	0.171526647	0.264268992	negative
PRJNA729271	stage_E14_vs_E10	ordinary_muscle_development	ENSGALG00010000042	CKMT1B	3.885006096	0.443986148	8.750286731	2.12811E-18	4.01654E-17	positive
PRJNA729271	stage_E14_vs_E10	ordinary_muscle_development	ENSGALG00010000043	PPIP5K1	0.628941995	0.120675723	5.2118353	1.86982E-07	1.14746E-06	positive
PRJNA729271	stage_E14_vs_E10	ordinary_muscle_development	ENSGALG00010000044	MAP1A	1.363576271	0.132239369	10.31142451	6.2567E-25	1.87378E-23	positive
PRJNA729271	stage_E14_vs_E10	ordinary_muscle_development	ENSGALG00010000045	TP53BP1	-0.258752869	0.070777027	-3.655887773	0.000256293	0.000928279	negative
PRJNA729271	stage_E14_vs_E10	ordinary_muscle_development	ENSGALG00010000046	TUBGCP4	-0.407962193	0.060401789	-6.754140912	1.43684E-11	1.45237E-10	negative
PRJNA729271	stage_E14_vs_E10	ordinary_muscle_development	ENSGALG00010000047	IQGAP1	-0.560683436	0.097039692	-5.777877337	7.56489E-09	5.586E-08	negative
PRJNA729271	stage_E14_vs_E10	ordinary_muscle_development	ENSGALG00010000048	MAN2A2	-0.058859574	0.148998442	-0.395034827	0.692817186	0.775713356	negative
PRJNA729271	stage_E14_vs_E10	ordinary_muscle_development	ENSGALG00010000049	FES	0.242962778	0.194456361	1.249446284	0.211501889	0.312541531	positive
PRJNA729271	stage_E14_vs_E10	ordinary_muscle_development	ENSGALG00010000050	CIB1	0.59574275	0.129339187	4.606049918	4.1039E-06	2.05124E-05	positive
PRJNA729271	stage_E14_vs_E10	ordinary_muscle_development	ENSGALG00010000051	GDPGP1	0.236321819	0.17871092	1.322369215	0.186045235	0.282143302	positive
PRJNA729271	stage_E14_vs_E10	ordinary_muscle_development	ENSGALG00010000052	TTLL13	0.731354524	0.130883526	5.587827166	2.29928E-08	1.60154E-07	positive
PRJNA729271	stage_E14_vs_E10	ordinary_muscle_development	ENSGALG00010000053	HDDC3	0.406085875	0.114318312	3.552238215	0.000381969	0.001331883	positive
PRJNA729271	stage_E14_vs_E10	ordinary_muscle_development	ENSGALG00010000054	VPS33B	0.220023793	0.099055807	2.221210437	0.026336712	0.055102574	positive
PRJNA729271	stage_E14_vs_E10	ordinary_muscle_development	ENSGALG00010000055	PRC1	0.284729219	0.209808904	1.357088348	0.17475314	0.268189176	positive
PRJNA729271	stage_E14_vs_E10	ordinary_muscle_development	ENSGALG00010000056	UNC45A	0.112752601	0.080307599	1.404009114	0.160316136	0.250461563	positive
PRJNA729271	stage_E14_vs_E10	ordinary_muscle_development	ENSGALG00010000058	FURIN	-0.198271388	0.173419156	-1.143307309	0.252911021	0.360360293	negative
PRJNA729271	stage_E14_vs_E10	ordinary_muscle_development	ENSGALG00010000059	MFAP1	0.157001385	0.109441889	1.434563918	0.151411403	0.239257417	positive
PRJNA729271	stage_E14_vs_E10	ordinary_muscle_development	ENSGALG00010000060	HYPK	-0.074006075	0.245233892	-0.301777517	0.762821671	0.830927445	negative
PRJNA729271	stage_E14_vs_E10	ordinary_muscle_development	ENSGALG00010000061	SERINC4	-0.08036219	0.394264191	-0.203828276	0.838487695	0.887273992	negative
PRJNA729271	stage_E14_vs_E10	ordinary_muscle_development	ENSGALG00010000063	SERF2	0.463222347	0.127252385	3.640185977	0.000272441	0.000982347	positive
PRJNA729271	stage_E14_vs_E10	ordinary_muscle_development	ENSGALG00010000069	ENSGALG00010000069	1.498847233	0.233380165	6.422341975	1.34194E-10	1.21593E-09	positive
PRJNA729271	stage_E14_vs_E10	ordinary_muscle_development	ENSGALG00010000076	ENSGALG00010000076	0.161758985	0.196859089	0.821699351	0.411248029	0.525989603	positive
PRJNA729271	stage_E14_vs_E10	ordinary_muscle_development	ENSGALG00010000078	ENSGALG00010000078	0.803563555	0.467173106	1.720055253	0.085422398	0.149360394	positive
PRJNA729271	stage_E14_vs_E10	ordinary_muscle_development	ENSGALG00010000081	ENSGALG00010000081	0.685730983	0.317383872	2.160572866	0.030728349	0.06262759	positive
PRJNA729271	stage_E14_vs_E10	ordinary_muscle_development	ENSGALG00010000084	ENSGALG00010000084	0.781642879	0.263308624	2.968542644	0.002992156	0.008297551	positive
PRJNA729271	stage_E14_vs_E10	ordinary_muscle_development	ENSGALG00010000085	ENSGALG00010000085	-0.058363307	0.144554563	-0.403745866	0.686399608	0.770683783	negative
PRJNA729271	stage_E14_vs_E10	ordinary_muscle_development	ENSGALG00010000089	ENSGALG00010000089	0.193794196	0.313123167	0.618907244	0.535977466	0.642756132	positive
PRJNA729271	stage_E14_vs_E10	ordinary_muscle_development	ENSGALG00010000091	ENSGALG00010000091	3.140544286	0.881319629	3.563456643	0.000366003	0.001284245	positive
PRJNA729271	stage_E14_vs_E10	ordinary_muscle_development	ENSGALG00010000092	INPPL1	0.400311943	0.171226468	2.337909249	0.019391956	0.042296265	positive
PRJNA729271	stage_E14_vs_E10	ordinary_muscle_development	ENSGALG00010000093	ENSGALG00010000093	1.840309429	0.327685675	5.616081414	1.95337E-08	1.3758E-07	positive
PRJNA729271	stage_E14_vs_E10	ordinary_muscle_development	ENSGALG00010000098	ENSGALG00010000098	0.25797156	0.194289785	1.327766978	0.184255099	0.280014433	positive
PRJNA729271	stage_E14_vs_E10	ordinary_muscle_development	ENSGALG00010000105	CACNA1F	0.298092001	0.281490166	1.058978384	0.289609624	0.402058376	positive
PRJNA729271	stage_E14_vs_E10	ordinary_muscle_development	ENSGALG00010000107	ENSGALG00010000107	-0.513046625	0.530014243	-0.967986487	0.333051118	0.448034892	negative
PRJNA729271	stage_E14_vs_E10	ordinary_muscle_development	ENSGALG00010000121	ENSGALG00010000121	-0.164070797	0.134388339	-1.220870781	0.222134949	0.324843229	negative
PRJNA729271	stage_E14_vs_E10	ordinary_muscle_development	ENSGALG00010000123	ENSGALG00010000123	-0.55352235	0.254498028	-2.174957324	0.029633315	0.060788975	negative
PRJNA729271	stage_E14_vs_E10	ordinary_muscle_development	ENSGALG00010000124	ENSGALG00010000124	0.660495905	0.182278862	3.623546353	0.000290591	0.00104312	positive
PRJNA729271	stage_E14_vs_E10	ordinary_muscle_development	ENSGALG00010000126	ENSGALG00010000126	0.574493758	0.198305479	2.897014045	0.003767329	0.010144424	positive
PRJNA729271	stage_E14_vs_E10	ordinary_muscle_development	ENSGALG00010000127	ENSGALG00010000127	0.51577772	0.206752029	2.494668235	0.012607495	0.029187931	positive
PRJNA729271	stage_E14_vs_E10	ordinary_muscle_development	ENSGALG00010000133	USP39	-0.029966373	0.139372216	-0.215009659	0.829759788	0.881061324	negative
PRJNA729271	stage_E14_vs_E10	ordinary_muscle_development	ENSGALG00010000134	ENSGALG00010000134	0.383947808	0.126429352	3.036856559	0.002390592	0.006790443	positive
PRJNA729271	stage_E14_vs_E10	ordinary_muscle_development	ENSGALG00010000137	ENSGALG00010000137	-2.190295928	0.556203239	-3.937941702	8.21836E-05	0.000328712	negative
PRJNA729271	stage_E14_vs_E10	ordinary_muscle_development	ENSGALG00010000140	SEPHS3	0.038391901	0.126279215	0.304023913	0.761109673	0.829807048	positive
PRJNA729271	stage_E14_vs_E10	ordinary_muscle_development	ENSGALG00010000141	ENSGALG00010000141	-0.339303149	0.139593674	-2.430648461	0.01507183	0.034082087	negative
PRJNA729271	stage_E14_vs_E10	ordinary_muscle_development	ENSGALG00010000143	SCARF2	-1.106268866	0.158596607	-6.975362761	3.05083E-12	3.32007E-11	negative
PRJNA729271	stage_E14_vs_E10	ordinary_muscle_development	ENSGALG00010000145	ENSGALG00010000145	1.065477967	0.278353591	3.827785964	0.000129301	0.000499259	positive
PRJNA729271	stage_E14_vs_E10	ordinary_muscle_development	ENSGALG00010000146	ACADM	0.682537278	0.16476814	4.142410535	3.43674E-05	0.000146949	positive
PRJNA729271	stage_E14_vs_E10	ordinary_muscle_development	ENSGALG00010000147	ENSGALG00010000147	0.493662416	0.105871498	4.662845277	3.11867E-06	1.59229E-05	positive
PRJNA729271	stage_E14_vs_E10	ordinary_muscle_development	ENSGALG00010000148	ENSGALG00010000148	0.264271245	0.291954478	0.905179627	0.365370233	0.481414237	positive
PRJNA729271	stage_E14_vs_E10	ordinary_muscle_development	ENSGALG00010000149	ENSGALG00010000149	-0.08162029	0.135517464	-0.602286135	0.546983688	0.652392707	negative
PRJNA729271	stage_E14_vs_E10	ordinary_muscle_development	ENSGALG00010000150	B3GAT3	0.149104923	0.125240848	1.19054546	0.233832074	0.338229309	positive
PRJNA729271	stage_E14_vs_E10	ordinary_muscle_development	ENSGALG00010000151	TBX5	-3.120405323	0.234391754	-13.31277771	1.95091E-40	1.50418E-38	negative
PRJNA729271	stage_E14_vs_E10	ordinary_muscle_development	ENSGALG00010000153	NF2	-0.089276439	0.093360625	-0.956253647	0.338944102	0.454049642	negative
PRJNA729271	stage_E14_vs_E10	ordinary_muscle_development	ENSGALG00010000154	ENSGALG00010000154	5.834060532	3.935234422	1.482519186	0.138202202	0.221941162	positive
PRJNA729271	stage_E14_vs_E10	ordinary_muscle_development	ENSGALG00010000155	MAZ	0.361534115	0.163731035	2.208097655	0.027237466	0.056676296	positive
PRJNA729271	stage_E14_vs_E10	ordinary_muscle_development	ENSGALG00010000156	ENSGALG00010000156	0.182749319	0.513543349	0.355859576	0.72194573	0.798609176	positive
PRJNA729271	stage_E14_vs_E10	ordinary_muscle_development	ENSGALG00010000157	ENSGALG00010000157	7.855953344	5.308015883	1.480016925			positive
PRJNA729271	stage_E14_vs_E10	ordinary_muscle_development	ENSGALG00010000161	CDC42EP2	0.253309124	0.326336162	0.776221434	0.437618253	0.551541307	positive
PRJNA729271	stage_E14_vs_E10	ordinary_muscle_development	ENSGALG00010000166	PFDN6	0.281181182	0.129042269	2.178985107	0.029332775	0.060271425	positive
PRJNA729271	stage_E14_vs_E10	ordinary_muscle_development	ENSGALG00010000171	DPF2	-0.239741898	0.200610168	-1.195063542	0.232062205	0.336374166	negative
PRJNA729271	stage_E14_vs_E10	ordinary_muscle_development	ENSGALG00010000172	ENSGALG00010000172	-1.037560678	1.126607481	-0.920960224	0.357071194	0.47349254	negative
PRJNA729271	stage_E14_vs_E10	ordinary_muscle_development	ENSGALG00010000173	NIPSNAP1	-0.147336823	0.120899741	-1.218669465	0.222969672	0.325768107	negative
PRJNA729271	stage_E14_vs_E10	ordinary_muscle_development	ENSGALG00010000174	ENSGALG00010000174	0.473607235	0.095935528	4.936724147	7.94457E-07	4.44414E-06	positive
PRJNA729271	stage_E14_vs_E10	ordinary_muscle_development	ENSGALG00010000175	ENSGALG00010000175	-0.94358297	0.862878733	-1.09352906	0.274161577	0.384478721	negative
PRJNA729271	stage_E14_vs_E10	ordinary_muscle_development	ENSGALG00010000176	ENSGALG00010000176	-0.772329995	0.508557595	-1.518667705	0.12884616	0.209351539	negative
PRJNA729271	stage_E14_vs_E10	ordinary_muscle_development	ENSGALG00010000177	ENSGALG00010000177	0.219931223	0.208857246	1.053021753	0.292331022	0.404944397	positive
PRJNA729271	stage_E14_vs_E10	ordinary_muscle_development	ENSGALG00010000184	ENSGALG00010000184	5.503978132	3.401661229	1.618026535	0.105656879	0.178288296	positive
PRJNA729271	stage_E14_vs_E10	ordinary_muscle_development	ENSGALG00010000186	THOC5	0.149418167	0.078689112	1.898841704	0.057585292	0.106828925	positive
PRJNA729271	stage_E14_vs_E10	ordinary_muscle_development	ENSGALG00010000188	ENSGALG00010000188	0.660447829	1.328715102	0.497057517	0.619148492	0.714876326	positive
PRJNA729271	stage_E14_vs_E10	ordinary_muscle_development	ENSGALG00010000189	ENSGALG00010000189	0.426484273	0.593805193	0.718222538	0.472620083	0.584524583	positive
PRJNA729271	stage_E14_vs_E10	ordinary_muscle_development	ENSGALG00010000191	NEFH	1.455538406	0.417502185	3.486301294	0.000489749	0.001664848	positive
PRJNA729271	stage_E14_vs_E10	ordinary_muscle_development	ENSGALG00010000192	ENSGALG00010000192	2.620812965	0.660503243	3.967903248	7.25078E-05	0.000293166	positive
PRJNA729271	stage_E14_vs_E10	ordinary_muscle_development	ENSGALG00010000196	KSR2	-2.204409145	0.739871591	-2.979448289	0.00288768	0.008032415	negative
PRJNA729271	stage_E14_vs_E10	ordinary_muscle_development	ENSGALG00010000197	ENSGALG00010000197	2.017552703	0.477112367	4.228674091	2.35073E-05	0.000103411	positive
PRJNA729271	stage_E14_vs_E10	ordinary_muscle_development	ENSGALG00010000200	GAL3ST1	-2.724773857	0.252342806	-10.79790582	3.52144E-27	1.25722E-25	negative
PRJNA729271	stage_E14_vs_E10	ordinary_muscle_development	ENSGALG00010000202	ENSGALG00010000202	-0.10320022	0.288538337	-0.35766554	0.720593624	0.797754875	negative
PRJNA729271	stage_E14_vs_E10	ordinary_muscle_development	ENSGALG00010000205	RNF215	0.137805499	0.111087498	1.240513119	0.214785665	0.316522795	positive
PRJNA729271	stage_E14_vs_E10	ordinary_muscle_development	ENSGALG00010000210	CCDC157	-0.713819494	0.524556284	-1.360806296	0.173574912	0.266748128	negative
PRJNA729271	stage_E14_vs_E10	ordinary_muscle_development	ENSGALG00010000215	SF3A1	-0.194602902	0.089473433	-2.174979716	0.029631636	0.060788975	negative
PRJNA729271	stage_E14_vs_E10	ordinary_muscle_development	ENSGALG00010000221	TBC1D10A	-0.138038414	0.087226353	-1.582531075	0.113528384	0.189279265	negative
PRJNA729271	stage_E14_vs_E10	ordinary_muscle_development	ENSGALG00010000223	CASTOR1	-0.070341311	0.157940648	-0.445365473	0.65605566	0.746518295	negative
PRJNA729271	stage_E14_vs_E10	ordinary_muscle_development	ENSGALG00010000224	MYBPC2	3.722968995	0.255937894	14.54637663	6.15795E-48	7.82977E-46	positive
PRJNA729271	stage_E14_vs_E10	ordinary_muscle_development	ENSGALG00010000225	LIF	0.204201824	0.566131279	0.360696948	0.718326006	0.796339232	positive
PRJNA729271	stage_E14_vs_E10	ordinary_muscle_development	ENSGALG00010000226	ENSGALG00010000226	-0.082635432	0.449011023	-0.184038759	0.853983049	0.898945845	negative
PRJNA729271	stage_E14_vs_E10	ordinary_muscle_development	ENSGALG00010000227	ENSGALG00010000227	0.663594533	0.275463311	2.409012404	0.015995754	0.035858098	positive
PRJNA729271	stage_E14_vs_E10	ordinary_muscle_development	ENSGALG00010000231	ENSGALG00010000231	0.753973214	0.473127277	1.593594899	0.111026798	0.185663756	positive
PRJNA729271	stage_E14_vs_E10	ordinary_muscle_development	ENSGALG00010000232	ENSGALG00010000232	0.200200351	0.161574722	1.239057373	0.215324252	0.317090829	positive
PRJNA729271	stage_E14_vs_E10	ordinary_muscle_development	ENSGALG00010000233	HORMAD2	-0.369820522	0.434038279	-0.852045869	0.394188634	0.509542395	negative
PRJNA729271	stage_E14_vs_E10	ordinary_muscle_development	ENSGALG00010000234	ENSGALG00010000234	2.837681688	4.538017826	0.625313032			positive
PRJNA729271	stage_E14_vs_E10	ordinary_muscle_development	ENSGALG00010000235	ENSGALG00010000235	0.053272428	0.214409232	0.248461448	0.803777395	0.861899232	positive
PRJNA729271	stage_E14_vs_E10	ordinary_muscle_development	ENSGALG00010000239	ENSGALG00010000239	3.821552499	4.617516367	0.82762078	0.407885296	0.522736465	positive
PRJNA729271	stage_E14_vs_E10	ordinary_muscle_development	ENSGALG00010000242	ENSGALG00010000242	0.701565723	0.200858865	3.492829273	0.000477932	0.001629646	positive
PRJNA729271	stage_E14_vs_E10	ordinary_muscle_development	ENSGALG00010000246	ENSGALG00010000246	4.917760442	4.236029519	1.160936301	0.245667805	0.352395332	positive
PRJNA729271	stage_E14_vs_E10	ordinary_muscle_development	ENSGALG00010000247	ENSGALG00010000247	2.009121119	0.225710974	8.901300121	5.51963E-19	1.09001E-17	positive
PRJNA729271	stage_E14_vs_E10	ordinary_muscle_development	ENSGALG00010000249	ENSGALG00010000249	3.503570139	4.380203592	0.799864679			positive
PRJNA729271	stage_E14_vs_E10	ordinary_muscle_development	ENSGALG00010000254	ENSGALG00010000254	-0.884853512	0.583454696	-1.516576211	0.129373715	0.210059204	negative
PRJNA729271	stage_E14_vs_E10	ordinary_muscle_development	ENSGALG00010000255	ENSGALG00010000255	0.190226002	0.144614626	1.315399464	0.188375686	0.285021457	positive
PRJNA729271	stage_E14_vs_E10	ordinary_muscle_development	ENSGALG00010000256	ENSGALG00010000256	-0.114162366	0.37491616	-0.304501056	0.760746189	0.829540605	negative
PRJNA729271	stage_E14_vs_E10	ordinary_muscle_development	ENSGALG00010000262	HNRNPKL	2.152548143	3.46995864	0.620338271	0.535035103	0.642104142	positive
PRJNA729271	stage_E14_vs_E10	ordinary_muscle_development	ENSGALG00010000263	MTMR3	0.377374704	0.091466296	4.125833449	3.69395E-05	0.000157112	positive
PRJNA729271	stage_E14_vs_E10	ordinary_muscle_development	ENSGALG00010000267	ENSGALG00010000267	0.179934834	0.120282643	1.495933492	0.134670987	0.217396466	positive
PRJNA729271	stage_E14_vs_E10	ordinary_muscle_development	ENSGALG00010000269	ENSGALG00010000269	-1.016790091	0.469508012	-2.165650136	0.030337932	0.061962565	negative
PRJNA729271	stage_E14_vs_E10	ordinary_muscle_development	ENSGALG00010000271	RTN4R	-2.714268608	0.374221962	-7.253098127	4.07345E-13	4.86364E-12	negative
PRJNA729271	stage_E14_vs_E10	ordinary_muscle_development	ENSGALG00010000272	ENSGALG00010000272	1.259824153	0.328229602	3.8382405	0.000123919	0.000480655	positive
PRJNA729271	stage_E14_vs_E10	ordinary_muscle_development	ENSGALG00010000277	ENSGALG00010000277	1.176801596	0.60423305	1.947595544	0.051463377	0.09716838	positive
PRJNA729271	stage_E14_vs_E10	ordinary_muscle_development	ENSGALG00010000279	ENSGALG00010000279	-0.03156055	0.124982244	-0.252520273	0.800638946	0.859577582	negative
PRJNA729271	stage_E14_vs_E10	ordinary_muscle_development	ENSGALG00010000281	OSBP2	0.898632843	0.204404058	4.396355196	1.10084E-05	5.12252E-05	positive
PRJNA729271	stage_E14_vs_E10	ordinary_muscle_development	ENSGALG00010000282	ENSGALG00010000282	4.052800447	4.921872102	0.823426607	0.410265437	0.525103533	positive
PRJNA729271	stage_E14_vs_E10	ordinary_muscle_development	ENSGALG00010000284	ENSGALG00010000284	0.097377215	0.148518245	0.65565827	0.512044024	0.620990473	positive
PRJNA729271	stage_E14_vs_E10	ordinary_muscle_development	ENSGALG00010000285	MYLPF	2.727688854	0.145718142	18.71893794	3.47001E-78	1.52418E-75	positive
PRJNA729271	stage_E14_vs_E10	ordinary_muscle_development	ENSGALG00010000286	TCN2	0.147002124	0.144929774	1.014298989	0.310440106	0.424272048	positive
PRJNA729271	stage_E14_vs_E10	ordinary_muscle_development	ENSGALG00010000287	IQCD	-0.361475639	0.178711465	-2.022677385	0.04310642	0.08352193	negative
PRJNA729271	stage_E14_vs_E10	ordinary_muscle_development	ENSGALG00010000288	VARS2	0.067434791	0.153112485	0.440426469	0.659628257	0.749084268	positive
PRJNA729271	stage_E14_vs_E10	ordinary_muscle_development	ENSGALG00010000289	QPRT	-0.292700307	0.45395135	-0.644783428	0.519067543	0.627147123	negative
PRJNA729271	stage_E14_vs_E10	ordinary_muscle_development	ENSGALG00010000292	ABHD16A	-0.010611139	0.134650957	-0.078804781	0.937187901	0.95618629	negative
PRJNA729271	stage_E14_vs_E10	ordinary_muscle_development	ENSGALG00010000293	ENSGALG00010000293	0.601761766	0.366200726	1.643256614	0.100329851	0.170510164	positive

PRJNA729271	stage_E14_vs_E10	ordinary_muscle_development	ENSGALG00010000294	DUSP21	-0.022824171	0.269258931	-0.084766625	0.932446928	0.953697283	negative
PRJNA729271	stage_E14_vs_E10	ordinary_muscle_development	ENSGALG00010000298	SLC35E4	-0.426211646	0.238649054	-1.785934781	0.074109819	0.132407472	negative
PRJNA729271	stage_E14_vs_E10	ordinary_muscle_development	ENSGALG00010000301	UQCR10	0.40111449	0.154922012	2.589138147	0.009621649	0.023048388	positive
PRJNA729271	stage_E14_vs_E10	ordinary_muscle_development	ENSGALG00010000302	ENSGALG00010000302	0.150034808	0.231338409	0.648551221	0.516628493	0.625033805	positive
PRJNA729271	stage_E14_vs_E10	ordinary_muscle_development	ENSGALG00010000303	ENSGALG00010000303	0.077370086	0.112463338	0.687958289	0.491479048	0.601739066	positive
PRJNA729271	stage_E14_vs_E10	ordinary_muscle_development	ENSGALG00010000306	ENSGALG00010000306	0.295606376	0.214845606	1.375901427	0.168852154	0.260956601	positive
PRJNA729271	stage_E14_vs_E10	ordinary_muscle_development	ENSGALG00010000307	ZMAT5	0.425521314	0.111922432	3.801930552	0.000143573	0.000549535	positive
PRJNA729271	stage_E14_vs_E10	ordinary_muscle_development	ENSGALG00010000308	ENSGALG00010000308	0.156665584	0.33095332	0.47337668	0.635944465	0.729504195	positive
PRJNA729271	stage_E14_vs_E10	ordinary_muscle_development	ENSGALG00010000309	ENSGALG00010000309	3.818307027	4.277284886	0.892694111	0.372021005	0.48813655	positive
PRJNA729271	stage_E14_vs_E10	ordinary_muscle_development	ENSGALG00010000312	CABP7	-2.186979002	0.506537324	-4.317508108	1.57801E-05	7.1613E-05	negative
PRJNA729271	stage_E14_vs_E10	ordinary_muscle_development	ENSGALG00010000313	ENSGALG00010000313	5.144327931	3.782147368	1.360160626	0.1737791	0.266956337	positive
PRJNA729271	stage_E14_vs_E10	ordinary_muscle_development	ENSGALG00010000318	ENSGALG00010000318	3.804677267	4.723408221	0.805494061	0.420534632	0.534940719	positive
PRJNA729271	stage_E14_vs_E10	ordinary_muscle_development	ENSGALG00010000323	AP1B1	0.276661704	0.144556637	1.913863731	0.05563757	0.103778995	positive
PRJNA729271	stage_E14_vs_E10	ordinary_muscle_development	ENSGALG00010000324	ENSGALG00010000324	-0.419414588	0.170471917	-2.460314848	0.013881518	0.031731998	negative
PRJNA729271	stage_E14_vs_E10	ordinary_muscle_development	ENSGALG00010000326	ENSGALG00010000326	2.034720592	1.781418581	1.14219118	0.253374561	0.360858128	positive
PRJNA729271	stage_E14_vs_E10	ordinary_muscle_development	ENSGALG00010000331	ENSGALG00010000331	-0.271871737	0.162670579	-1.671302455	0.094661952	0.162689707	negative
PRJNA729271	stage_E14_vs_E10	ordinary_muscle_development	ENSGALG00010000338	ENSGALG00010000338	-0.022919435	0.188870279	-0.121350142	0.903413706	0.933897608	negative
PRJNA729271	stage_E14_vs_E10	ordinary_muscle_development	ENSGALG00010000340	NAA10	-0.028487567	0.132033044	-0.215760891	0.829174135	0.880807662	negative
PRJNA729271	stage_E14_vs_E10	ordinary_muscle_development	ENSGALG00010000341	EWSR1	-0.091790386	0.146603728	-0.626112225	0.531241334	0.638714593	negative
PRJNA729271	stage_E14_vs_E10	ordinary_muscle_development	ENSGALG00010000346	ENSGALG00010000346	-0.100105893	0.112024083	-0.893610471	0.371530342	0.487757658	negative
PRJNA729271	stage_E14_vs_E10	ordinary_muscle_development	ENSGALG00010000349	CHIR-IG1-5	0.454221052	0.461544993	0.984131687	0.325050769	0.439721035	positive
PRJNA729271	stage_E14_vs_E10	ordinary_muscle_development	ENSGALG00010000350	ENSGALG00010000350	-0.027482513	0.165482355	-0.166075185	0.868097782	0.909561877	negative
PRJNA729271	stage_E14_vs_E10	ordinary_muscle_development	ENSGALG00010000353	ENSGALG00010000353	0.617332815	0.110344557	5.594592358	2.21141E-08	1.54479E-07	positive
PRJNA729271	stage_E14_vs_E10	ordinary_muscle_development	ENSGALG00010000356	ENSGALG00010000356	1.231893439	0.117920229	10.44683723	1.51493E-25	4.76333E-24	positive
PRJNA729271	stage_E14_vs_E10	ordinary_muscle_development	ENSGALG00010000366	EMID1	-2.027005118	0.227096406	-8.925747236	4.427E-19	8.82661E-18	negative
PRJNA729271	stage_E14_vs_E10	ordinary_muscle_development	ENSGALG00010000367	HSD17B10	0.158284295	0.138811762	1.140280136	0.254169611	0.361593041	positive
PRJNA729271	stage_E14_vs_E10	ordinary_muscle_development	ENSGALG00010000373	ENSGALG00010000373	1.011342833	0.494586346	2.044825623	0.040872053	0.079736259	positive
PRJNA729271	stage_E14_vs_E10	ordinary_muscle_development	ENSGALG00010000376	ASCC2	-0.111330411	0.081516509	-1.365740663	0.172020376	0.264864601	negative
PRJNA729271	stage_E14_vs_E10	ordinary_muscle_development	ENSGALG00010000377	ENSGALG00010000377	0.075728877	0.240428655	0.314974423	0.752781074	0.823017172	positive
PRJNA729271	stage_E14_vs_E10	ordinary_muscle_development	ENSGALG00010000381	ENSGALG00010000381	0.308339223	0.295917254	1.041977848	0.29742195	0.410857825	positive
PRJNA729271	stage_E14_vs_E10	ordinary_muscle_development	ENSGALG00010000382	ENSGALG00010000382	-1.019235257	0.526227944	-1.936870266	0.052761204	0.099087589	negative
PRJNA729271	stage_E14_vs_E10	ordinary_muscle_development	ENSGALG00010000384	ENSGALG00010000384	-0.44324948	0.14539744	-3.048537022	0.002299586	0.006574457	negative
PRJNA729271	stage_E14_vs_E10	ordinary_muscle_development	ENSGALG00010000386	ENSGALG00010000386	-0.573858292	0.371658484	-1.544047337	0.122576865	0.200990007	negative
PRJNA729271	stage_E14_vs_E10	ordinary_muscle_development	ENSGALG00010000387	DTX1	-2.262653425	0.481346625	-4.70067371	2.59305E-06	1.34108E-05	negative
PRJNA729271	stage_E14_vs_E10	ordinary_muscle_development	ENSGALG00010000388	ENSGALG00010000388	0.087458465	0.182095347	0.480289399	0.631021626	0.724889718	positive
PRJNA729271	stage_E14_vs_E10	ordinary_muscle_development	ENSGALG00010000391	ENSGALG00010000391	0.795783816	0.692731603	1.148762108	0.250654088	0.357812784	positive
PRJNA729271	stage_E14_vs_E10	ordinary_muscle_development	ENSGALG00010000402	PFKFB1	2.615375546	0.331162383	7.897562296	2.84411E-15	4.10203E-14	positive
PRJNA729271	stage_E14_vs_E10	ordinary_muscle_development	ENSGALG00010000405	ENSGALG00010000405	1.337947198	0.406070369	3.294865371	0.000984688	0.003105539	positive
PRJNA729271	stage_E14_vs_E10	ordinary_muscle_development	ENSGALG00010000406	NSMCE3	-0.046491503	0.096469397	-0.481930065	0.629855627	0.723664975	negative
PRJNA729271	stage_E14_vs_E10	ordinary_muscle_development	ENSGALG00010000407	WSB2	0.43159046	0.084914407	5.082652951	3.72199E-07	2.19399E-06	positive
PRJNA729271	stage_E14_vs_E10	ordinary_muscle_development	ENSGALG00010000408	ENSGALG00010000408	3.651225814	0.355196406	10.27945598	8.72185E-25	2.55917E-23	positive
PRJNA729271	stage_E14_vs_E10	ordinary_muscle_development	ENSGALG00010000411	ENSGALG00010000411	1.998768279	0.951773308	2.100046579	0.035724744	0.071169621	positive
PRJNA729271	stage_E14_vs_E10	ordinary_muscle_development	ENSGALG00010000412	RFC5	0.128132362	0.103447426	1.238623003	0.215485145	0.317242353	positive
PRJNA729271	stage_E14_vs_E10	ordinary_muscle_development	ENSGALG00010000422	ITIH6	-2.158905415	0.533458792	-4.046995662	5.18792E-05	0.000215223	negative
PRJNA729271	stage_E14_vs_E10	ordinary_muscle_development	ENSGALG00010000423	ENSGALG00010000423	0.774711165	0.208795344	3.710385249	0.000206944	0.000765609	positive
PRJNA729271	stage_E14_vs_E10	ordinary_muscle_development	ENSGALG00010000428	GNL3L	-0.056995956	0.143210285	-0.397987869	0.690639128	0.774231567	negative
PRJNA729271	stage_E14_vs_E10	ordinary_muscle_development	ENSGALG00010000431	AIFM3	-3.349984286	1.101261573	-3.041951493	0.002350498	0.006701507	negative
PRJNA729271	stage_E14_vs_E10	ordinary_muscle_development	ENSGALG00010000432	ENSGALG00010000432	-0.248535677	0.183745227	-1.352610249	0.176180177	0.26994242	negative
PRJNA729271	stage_E14_vs_E10	ordinary_muscle_development	ENSGALG00010000434	TP53	-0.337063743	0.218513746	-1.542528785	0.122945149	0.201457148	negative
PRJNA729271	stage_E14_vs_E10	ordinary_muscle_development	ENSGALG00010000435	P2RX6	-1.915181205	0.344391184	-5.561063388	2.68136E-08	1.85519E-07	negative
PRJNA729271	stage_E14_vs_E10	ordinary_muscle_development	ENSGALG00010000436	ENSGALG00010000436	0.235871396	0.400770242	0.588545184	0.556166415	0.660410599	positive
PRJNA729271	stage_E14_vs_E10	ordinary_muscle_development	ENSGALG00010000440	ENSGALG00010000440	-2.271886053	0.455127574	-4.991756556	5.98326E-07	3.40508E-06	negative
PRJNA729271	stage_E14_vs_E10	ordinary_muscle_development	ENSGALG00010000441	SLC7A4	-1.445898147	0.204472294	-7.071364618	1.53417E-12	1.71192E-11	negative
PRJNA729271	stage_E14_vs_E10	ordinary_muscle_development	ENSGALG00010000442	DGCR6	-0.548382607	0.12893544	-4.253156525	2.10778E-05	9.34321E-05	negative
PRJNA729271	stage_E14_vs_E10	ordinary_muscle_development	ENSGALG00010000443	TBX3	-1.260655905	0.157209384	-8.018960889	1.06643E-15	1.59033E-14	negative
PRJNA729271	stage_E14_vs_E10	ordinary_muscle_development	ENSGALG00010000444	RBM19	-0.196238527	0.118002966	-1.662996567	0.096313123	0.164843395	negative
PRJNA729271	stage_E14_vs_E10	ordinary_muscle_development	ENSGALG00010000449	PLBD2	0.450806014	0.107812333	4.181395601	2.89725E-05	0.000125698	positive
PRJNA729271	stage_E14_vs_E10	ordinary_muscle_development	ENSGALG00010000450	ENSGALG00010000450	0.507917162	0.186961285	2.716697001	0.006593694	0.016530524	positive
PRJNA729271	stage_E14_vs_E10	ordinary_muscle_development	ENSGALG00010000452	SLC8B1	0.729613124	0.227954851	3.200691368	0.001370983	0.004171368	positive
PRJNA729271	stage_E14_vs_E10	ordinary_muscle_development	ENSGALG00010000453	ACHE	1.211473871	0.241525733	5.015920489	5.27802E-07	3.0323E-06	positive
PRJNA729271	stage_E14_vs_E10	ordinary_muscle_development	ENSGALG00010000457	TPCN1	-0.222275755	0.079816718	-2.784827054	0.005355628	0.0138279	negative
PRJNA729271	stage_E14_vs_E10	ordinary_muscle_development	ENSGALG00010000458	ENSGALG00010000458	0.4504701	0.199091215	2.262631728	0.023658401	0.05032705	positive
PRJNA729271	stage_E14_vs_E10	ordinary_muscle_development	ENSGALG00010000462	ENSGALG00010000462	0.18447024	0.201111584	0.917253183	0.359009984	0.475423967	positive
PRJNA729271	stage_E14_vs_E10	ordinary_muscle_development	ENSGALG00010000465	ENSGALG00010000465	0.233132237	0.192402081	1.211692906	0.225629956	0.328727129	positive
PRJNA729271	stage_E14_vs_E10	ordinary_muscle_development	ENSGALG00010000466	FBXO21	0.097536895	0.077031295	1.266198309	0.205442093	0.305329964	positive
PRJNA729271	stage_E14_vs_E10	ordinary_muscle_development	ENSGALG00010000467	RNASEK	-0.179504193	0.199255377	-0.900875027	0.367654771	0.48376506	negative
PRJNA729271	stage_E14_vs_E10	ordinary_muscle_development	ENSGALG00010000468	TESC	-2.403302241	0.289567567	-8.299625084	1.0444E-16	1.7164E-15	negative
PRJNA729271	stage_E14_vs_E10	ordinary_muscle_development	ENSGALG00010000469	ENSGALG00010000469	0.399200271	0.487932324	0.818146804	0.413273366	0.52811403	positive
PRJNA729271	stage_E14_vs_E10	ordinary_muscle_development	ENSGALG00010000470	FBXW8	-0.061407079	0.107179954	-0.57293437	0.566689132	0.669286969	negative
PRJNA729271	stage_E14_vs_E10	ordinary_muscle_development	ENSGALG00010000471	ENSGALG00010000471	-0.198875695	0.196427835	-1.012461878	0.311317265	0.425103707	negative
PRJNA729271	stage_E14_vs_E10	ordinary_muscle_development	ENSGALG00010000475	RNFT2	0.01166001	0.103230853	0.112950824	0.910069543	0.93805628	positive
PRJNA729271	stage_E14_vs_E10	ordinary_muscle_development	ENSGALG00010000476	ENSGALG00010000476	0.173637498	0.219407063	0.791394293	0.428713939	0.5431051	positive
PRJNA729271	stage_E14_vs_E10	ordinary_muscle_development	ENSGALG00010000479	C12orf49	-0.195229551	0.114220047	-1.709240684	0.087406376	0.152150284	negative
PRJNA729271	stage_E14_vs_E10	ordinary_muscle_development	ENSGALG00010000483	ENSGALG00010000483	-0.379445449	0.261944229	-1.448573424	0.147456747	0.234105756	negative
PRJNA729271	stage_E14_vs_E10	ordinary_muscle_development	ENSGALG00010000485	MED13L	0.11397072	0.126047577	0.904188108	0.365895665	0.481975432	positive
PRJNA729271	stage_E14_vs_E10	ordinary_muscle_development	ENSGALG00010000491	ENSGALG00010000491	0.602537342	0.15520998	3.882078598	0.000103567	0.000406391	positive
PRJNA729271	stage_E14_vs_E10	ordinary_muscle_development	ENSGALG00010000495	ENSGALG00010000495	0.374876773	0.549959825	0.681643924	0.495464134	0.605123095	positive
PRJNA729271	stage_E14_vs_E10	ordinary_muscle_development	ENSGALG00010000501	ENSGALG00010000501	0.20543131	0.161853419	1.269242947	0.204354428	0.304243779	positive
PRJNA729271	stage_E14_vs_E10	ordinary_muscle_development	ENSGALG00010000507	TRPT1	0.663331258	0.294979845	2.248734176	0.024529413	0.051882949	positive
PRJNA729271	stage_E14_vs_E10	ordinary_muscle_development	ENSGALG00010000509	ENSGALG00010000509	1.131945791	0.818669761	1.382664714	0.166767696	0.258230719	positive
PRJNA729271	stage_E14_vs_E10	ordinary_muscle_development	ENSGALG00010000511	FERMT3	0.213004908	0.157133737	1.355564453	0.175237788	0.268809021	positive
PRJNA729271	stage_E14_vs_E10	ordinary_muscle_development	ENSGALG00010000525	ENSGALG00010000525	-0.577859662	0.518694348	-1.114065856	0.265250935	0.374045365	negative
PRJNA729271	stage_E14_vs_E10	ordinary_muscle_development	ENSGALG00010000526	POLD1	-0.022156213	0.151039282	-0.146691729	0.883375347	0.919997532	negative
PRJNA729271	stage_E14_vs_E10	ordinary_muscle_development	ENSGALG00010000529	STIP1	0.090460304	0.192428808	0.470097515	0.638285349	0.731282002	positive
PRJNA729271	stage_E14_vs_E10	ordinary_muscle_development	ENSGALG00010000532	ENSGALG00010000532	1.857697836	0.470108123	3.951639516	7.76176E-05	0.000311826	positive
PRJNA729271	stage_E14_vs_E10	ordinary_muscle_development	ENSGALG00010000546	FLRT1	-2.461959156	0.47971106	-5.132170924	2.86419E-07	1.71129E-06	negative
PRJNA729271	stage_E14_vs_E10	ordinary_muscle_development	ENSGALG00010000555	OTUB1	0.157021704	0.100730863	1.558824162	0.119038004	0.196386964	positive
PRJNA729271	stage_E14_vs_E10	ordinary_muscle_development	ENSGALG00010000561	ENSGALG00010000561	-0.002991573	0.275065628	-0.010875853	0.991322496	0.994478481	negative
PRJNA729271	stage_E14_vs_E10	ordinary_muscle_development	ENSGALG00010000564	NAA40	-0.069343809	0.086914657	-0.797837926	0.42496455	0.539250735	negative
PRJNA729271	stage_E14_vs_E10	ordinary_muscle_development	ENSGALG00010000569	SIPA1	0.117251055	0.229843904	0.510133415	0.609957997	0.707250713	positive
PRJNA729271	stage_E14_vs_E10	ordinary_muscle_development	ENSGALG00010000574	ENSGALG00010000574	1.828118848	0.173952858	10.50927742	7.82911E-26	2.52184E-24	positive
PRJNA729271	stage_E14_vs_E10	ordinary_muscle_development	ENSGALG00010000575	ENSGALG00010000575	0.694137027	0.158866392	4.369313215	1.24638E-05	5.75542E-05	positive
PRJNA729271	stage_E14_vs_E10	ordinary_muscle_development	ENSGALG00010000576	NOS1	0.610097839	0.467271206	1.305661106	0.19166783	0.289007094	positive
PRJNA729271	stage_E14_vs_E10	ordinary_muscle_development	ENSGALG00010000577	ENSGALG00010000577	-0.090199098	0.265149341	-0.340182244	0.733719289	0.808396252	negative
PRJNA729271	stage_E14_vs_E10	ordinary_muscle_development	ENSGALG00010000578	ENSGALG00010000578	-0.673827488	0.423532604	-1.590969576	0.111616423	0.186563659	negative
PRJNA729271	stage_E14_vs_E10	ordinary_muscle_development	ENSGALG00010000579	ENSGALG00010000579	-1.34740706	0.899782097	-1.497481517	0.134268011	0.216848448	negative
PRJNA729271	stage_E14_vs_E10	ordinary_muscle_development	ENSGALG00010000580	ENSGALG00010000580	-1.925731849	0.63632529	-3.026332411	0.0024754	0.00699706	negative
PRJNA729271	stage_E14_vs_E10	ordinary_muscle_development	ENSGALG00010000581	ENSGALG00010000581	0.031124138	0.129558767	0.240231813	0.810150552	0.866587872	positive
PRJNA729271	stage_E14_vs_E10	ordinary_muscle_development	ENSGALG00010000583	CCS	0.084283685	0.139125339	0.605811172	0.54464015	0.650186047	positive
PRJNA729271	stage_E14_vs_E10	ordinary_muscle_development	ENSGALG00010000584	RBM14	0.070658467	0.210767526	0.335243614	0.737441325	0.811386975	positive
PRJNA729271	stage_E14_vs_E10	ordinary_muscle_development	ENSGALG00010000585	SPTBN2	-0.737047816	0.480772253	-1.533049822	0.12526358	0.204562369	negative
PRJNA729271	stage_E14_vs_E10	ordinary_muscle_development	ENSGALG00010000586	ENSGALG00010000586	1.703395242	0.269193675	6.327768437	2.48732E-10	2.19706E-09	positive
PRJNA729271	stage_E14_vs_E10	ordinary_muscle_development	ENSGALG00010000589	ENSGALG00010000589	0.113358057	0.338895728	0.334492434	0.738007999	0.811651532	positive
PRJNA729271	stage_E14_vs_E10	ordinary_muscle_development	ENSGALG00010000590	DNAH5	0.084865509	0.434554563	0.195293103	0.845163496	0.892188834	positive
PRJNA729271	stage_E14_vs_E10	ordinary_muscle_development	ENSGALG00010000591	SBK2	2.63720575	0.306251354	8.611246005	7.22707E-18	1.30782E-16	positive
PRJNA729271	stage_E14_vs_E10	ordinary_muscle_development	ENSGALG00010000592	CCT5	-0.092654691	0.118712128	-0.78049895	0.435097246	0.54919477	negative
PRJNA729271	stage_E14_vs_E10	ordinary_muscle_development	ENSGALG00010000593	ATPSCKMT	-0.115085091	0.134511611	-0.855577376	0.392231588	0.50780608	negative
PRJNA729271	stage_E14_vs_E10	ordinary_muscle_development	ENSGALG00010000595	MYO10	0.405322495	0.137429563	2.949310812	0.003184835	0.008766461	positive
PRJNA729271	stage_E14_vs_E10	ordinary_muscle_development	ENSGALG00010000596	ENSGALG00010000596	-0.705114214	0.665113748	-1.06014079	0.289080553	0.401516158	negative
PRJNA729271	stage_E14_vs_E10	ordinary_muscle_development	ENSGALG00010000597	CDH9	-3.292032096	0.734586029	-4.481479319	7.41274E-06	3.55316E-05	negative
PRJNA729271	stage_E14_vs_E10	ordinary_muscle_development	ENSGALG00010000598	ENSGALG00010000598	-0.243496671	0.501015715	-0.486006054	0.626962874	0.721427868	negative
PRJNA729271	stage_E14_vs_E10	ordinary_muscle_development	ENSGALG00010000599	RETREG1	2.262606741	0.37494761	6.034461037	1.59494E-09	1.27798E-08	positive
PRJNA729271	stage_E14_vs_E10	ordinary_muscle_development	ENSGALG00010000601	ENSGALG00010000601	-1.099849353	0.330815793	-3.324657939	0.000885271	0.00282146	negative
PRJNA729271	stage_E14_vs_E10	ordinary_muscle_development	ENSGALG00010000603	ZNF622	0.167492849	0.107879224	1.552595974	0.120519688	0.198334796	positive
PRJNA729271	stage_E14_vs_E10	ordinary_muscle_development	ENSGALG00010000604	ENSGALG00010000604	0.249254324	0.127512747	1.954740454	0.050613713	0.095751209	positive
PRJNA729271	stage_E14_vs_E10	ordinary_muscle_development	ENSGALG00010000605	MARCHF11	0.070109898	0.575464919	0.121831749	0.903032269	0.933739911	positive
PRJNA729271	stage_E14_vs_E10	ordinary_muscle_development	ENSGALG00010000606	UBE2QL1	-0.652147487	0.156207671	-4.174874916	2.9815E-05	0.000129121	negative
PRJNA729271	stage_E14_vs_E10	ordinary_muscle_development	ENSGALG00010000607	ENSGALG00010000607	-0.216980945	0.247698282	-0.875988897	0.38103607	0.496952682	negative
PRJNA729271	stage_E14_vs_E10	ordinary_muscle_development	ENSGALG00010000608	ENSGALG00010000608	-1.052888903	0.935809388	-1.125110431	0.260542242	0.368768655	negative
PRJNA729271	stage_E14_vs_E10	ordinary_muscle_development	ENSGALG00010000610	ENSGALG00010000610	0.152500312	0.077791031	1.960384239	0.049950897	0.094695036	positive
PRJNA729271	stage_E14_vs_E10	ordinary_muscle_development	ENSGALG00010000611	C7orf57	0.085897251	0.33908541	0.253320398	0.800020635	0.859239708	positive
PRJNA729271	stage_E14_vs_E10	ordinary_muscle_development	ENSGALG00010000612	ENSGALG00010000612	-0.144379413	0.372561892	-0.387531351	0.69836288	0.780656277	negative
PRJNA729271	stage_E14_vs_E10	ordinary_muscle_development	ENSGALG00010000613	ENSGALG00010000613	0.406796496	0.174121468	2.336279961	0.019476655	0.042466022	positive
PRJNA729271	stage_E14_vs_E10	ordinary_muscle_development	ENSGALG00010000614	KAT8	0.321187242	0.184318668	1.742564903	0.081409645	0.143573769	positive
PRJNA729271	stage_E14_vs_E10	ordinary_muscle_development	ENSGALG00010000615	ENSGALG00010000615	-0.658281351	0.584443787	-1.126338181	0.260022403	0.36824863	negative
PRJNA729271	stage_E14_vs_E10	ordinary_muscle_development	ENSGALG00010000616	PSENEN	0.050183791	0.505789041	0.099218819	0.920964533	0.945391163	positive
PRJNA729271	stage_E14_vs_E10	ordinary_muscle_development	ENSGALG00010000619	ENSGALG00010000619	0.265298837	0.289772889	0.915540574	0.359907912	0.476339042	positive
PRJNA729271	stage_E14_vs_E10	ordinary_muscle_development	ENSGALG00010000621	UPP1	-0.571205891	0.23541941	-2.426333033	0.015252267	0.034398881	negative
PRJNA729271	stage_E14_vs_E10	ordinary_muscle_development	ENSGALG00010000622	ENSGALG00010000622	-0.120008247	0.420770955	-0.285210388	0.775482962	0.840986498	negative
PRJNA729271	stage_E14_vs_E10	ordinary_muscle_development	ENSGALG00010000623	ENSGALG00010000623	-0.194044635	0.24123192	-0.804390378	0.421171556	0.535609906	negative
PRJNA729271	stage_E14_vs_E10	ordinary_muscle_development	ENSGALG00010000625	ENSGALG00010000625	1.180260639	0.369913201	3.190642119	0.00141957	0.004303842	positive

PRJNA729271	stage_E14_vs_E10	ordinary_muscle_development	ENSGALG00010000626	ABCA13	-0.84466394	0.642549217	-1.314551349	0.18866073	0.285333606	negative
PRJNA729271	stage_E14_vs_E10	ordinary_muscle_development	ENSGALG00010000627	ENSGALG00010000627	-0.381303417	0.238721058	-1.597276002	0.110204198	0.184543651	negative
PRJNA729271	stage_E14_vs_E10	ordinary_muscle_development	ENSGALG00010000630	ENSGALG00010000630	-0.650820502	0.392026623	-1.66014363	0.096885561	0.165609179	negative
PRJNA729271	stage_E14_vs_E10	ordinary_muscle_development	ENSGALG00010000632	ENSGALG00010000632	0.413612444	0.350491409	1.18009296	0.237963244	0.343211664	positive
PRJNA729271	stage_E14_vs_E10	ordinary_muscle_development	ENSGALG00010000633	VWC2	-2.426552761	0.430952075	-5.630678907	1.79502E-08	1.26762E-07	negative
PRJNA729271	stage_E14_vs_E10	ordinary_muscle_development	ENSGALG00010000634	ENSGALG00010000634	0.073179744	0.193724036	0.377752528	0.705614442	0.786248816	positive
PRJNA729271	stage_E14_vs_E10	ordinary_muscle_development	ENSGALG00010000635	ENSGALG00010000635	-0.103298227	0.123976202	-0.833210128	0.404726232	0.519666648	negative
PRJNA729271	stage_E14_vs_E10	ordinary_muscle_development	ENSGALG00010000636	ENSGALG00010000636	-0.176543243	0.143859262	-1.227194135	0.219749633	0.322102431	negative
PRJNA729271	stage_E14_vs_E10	ordinary_muscle_development	ENSGALG00010000639	SCN1B	-0.083706496	0.290751816	-0.287896728	0.773425791	0.839446413	negative
PRJNA729271	stage_E14_vs_E10	ordinary_muscle_development	ENSGALG00010000641	ENSGALG00010000641	-0.902042911	0.43093853	-2.093205522	0.036330812	0.072168716	negative
PRJNA729271	stage_E14_vs_E10	ordinary_muscle_development	ENSGALG00010000643	VKORC1	0.165028756	0.205159492	0.804392499	0.421170332	0.535609906	positive
PRJNA729271	stage_E14_vs_E10	ordinary_muscle_development	ENSGALG00010000645	ENSGALG00010000645	-0.210888355	0.520284675	-0.405332628	0.685233026	0.769758939	negative
PRJNA729271	stage_E14_vs_E10	ordinary_muscle_development	ENSGALG00010000646	ENSGALG00010000646	0.104991781	0.503120316	0.208681259	0.834697071	0.884813079	positive
PRJNA729271	stage_E14_vs_E10	ordinary_muscle_development	ENSGALG00010000649	PAPD7	0.354149897	0.082076369	4.314882616	1.59688E-05	7.23336E-05	positive
PRJNA729271	stage_E14_vs_E10	ordinary_muscle_development	ENSGALG00010000650	BCKDK	0.547964498	0.169334658	3.235985504	0.001212235	0.003750555	positive
PRJNA729271	stage_E14_vs_E10	ordinary_muscle_development	ENSGALG00010000651	ENSGALG00010000651	1.417821839	0.648534926	2.186191958	0.028801564	0.059368412	positive
PRJNA729271	stage_E14_vs_E10	ordinary_muscle_development	ENSGALG00010000652	SRD5A1	0.378263975	0.22331284	1.693874719	0.090289092	0.15617427	positive
PRJNA729271	stage_E14_vs_E10	ordinary_muscle_development	ENSGALG00010000653	NSUN2	-0.261536526	0.090458304	-2.891238455	0.003837268	0.010298316	negative
PRJNA729271	stage_E14_vs_E10	ordinary_muscle_development	ENSGALG00010000654	ENSGALG00010000654	-0.156515603	0.159733661	-0.979853603	0.327158388	0.4420751	negative
PRJNA729271	stage_E14_vs_E10	ordinary_muscle_development	ENSGALG00010000655	ENSGALG00010000655	-0.807866529	0.789280995	-1.023547425	0.306049089	0.419698071	negative
PRJNA729271	stage_E14_vs_E10	ordinary_muscle_development	ENSGALG00010000656	ENSGALG00010000656	0.555890884	0.410935694	1.352744217	0.176137359	0.269918711	positive
PRJNA729271	stage_E14_vs_E10	ordinary_muscle_development	ENSGALG00010000659	ENSGALG00010000659	1.179973155	0.549934893	2.145659732	0.031900136	0.064634117	positive
PRJNA729271	stage_E14_vs_E10	ordinary_muscle_development	ENSGALG00010000660	ENSGALG00010000660	0.438968984	0.183822702	2.388002014	0.016940249	0.037707142	positive
PRJNA729271	stage_E14_vs_E10	ordinary_muscle_development	ENSGALG00010000661	ENSGALG00010000661	-1.093517773	0.595231872	-1.837129066	0.066190844	0.120260251	negative
PRJNA729271	stage_E14_vs_E10	ordinary_muscle_development	ENSGALG00010000670	ENSGALG00010000670	-0.829598785	0.517731643	-1.602372187	0.109073331	0.183030556	negative
PRJNA729271	stage_E14_vs_E10	ordinary_muscle_development	ENSGALG00010000674	GRB10	0.086323942	0.169542297	0.509158738	0.610640962	0.70778028	positive
PRJNA729271	stage_E14_vs_E10	ordinary_muscle_development	ENSGALG00010000684	COBL	1.943780509	0.13469609	14.43086073	3.30925E-47	4.03089E-45	positive
PRJNA729271	stage_E14_vs_E10	ordinary_muscle_development	ENSGALG00010000686	ENSGALG00010000686	0.183536043	0.130458372	1.406855229	0.159470322	0.249355158	positive
PRJNA729271	stage_E14_vs_E10	ordinary_muscle_development	ENSGALG00010000687	IKZF1	-0.669308939	0.294438735	-2.273168779	0.023016011	0.049148067	negative
PRJNA729271	stage_E14_vs_E10	ordinary_muscle_development	ENSGALG00010000688	ENSGALG00010000688	0.091897195	0.296870282	0.309553367	0.756900623	0.826710461	positive
PRJNA729271	stage_E14_vs_E10	ordinary_muscle_development	ENSGALG00010000695	FIGNL1	-0.040085798	0.135407647	-0.296037918	0.767201124	0.834439881	negative
PRJNA729271	stage_E14_vs_E10	ordinary_muscle_development	ENSGALG00010000697	ENSGALG00010000697	-0.994044573	0.259263594	-3.834107824	0.000126021	0.000488024	negative
PRJNA729271	stage_E14_vs_E10	ordinary_muscle_development	ENSGALG00010000702	DDC	-2.403761276	0.441517704	-5.444314584	5.20052E-08	3.44208E-07	negative
PRJNA729271	stage_E14_vs_E10	ordinary_muscle_development	ENSGALG00010000704	ENSGALG00010000704	0.204959955	0.088507216	2.315742871	0.020572312	0.044553364	positive
PRJNA729271	stage_E14_vs_E10	ordinary_muscle_development	ENSGALG00010000705	ENSGALG00010000705	3.64522565	1.019226578	3.576462515	0.000348275	0.001228883	positive
PRJNA729271	stage_E14_vs_E10	ordinary_muscle_development	ENSGALG00010000710	ENSGALG00010000710	-0.706937235	0.549734942	-1.285960163	0.198456965	0.297142207	negative
PRJNA729271	stage_E14_vs_E10	ordinary_muscle_development	ENSGALG00010000711	SPRY2	0.219679823	0.110451781	1.988920611	0.046709966	0.089582027	positive
PRJNA729271	stage_E14_vs_E10	ordinary_muscle_development	ENSGALG00010000714	FBXL7	-0.674851335	0.092815557	-7.270885987	3.57137E-13	4.27827E-12	negative
PRJNA729271	stage_E14_vs_E10	ordinary_muscle_development	ENSGALG00010000715	PCDH20	-1.094378011	0.219684465	-4.981590358	6.30638E-07	3.57913E-06	negative
PRJNA729271	stage_E14_vs_E10	ordinary_muscle_development	ENSGALG00010000717	NDFIP2	-0.115930194	0.067160391	-1.726169126	0.084316991	0.147802005	negative
PRJNA729271	stage_E14_vs_E10	ordinary_muscle_development	ENSGALG00010000718	EEF1G	0.220394807	0.150844315	1.461074664	0.143994952	0.229590456	positive
PRJNA729271	stage_E14_vs_E10	ordinary_muscle_development	ENSGALG00010000720	RBM26	-0.013161918	0.050468669	-0.260793846	0.794251491	0.855452174	negative
PRJNA729271	stage_E14_vs_E10	ordinary_muscle_development	ENSGALG00010000721	ENSGALG00010000721	-0.10930824	0.14515792	-0.753029801	0.451432006	0.564533425	negative
PRJNA729271	stage_E14_vs_E10	ordinary_muscle_development	ENSGALG00010000722	ANKH	-0.244287224	0.170453129	-1.433163622	0.151811083	0.23975359	negative
PRJNA729271	stage_E14_vs_E10	ordinary_muscle_development	ENSGALG00010000724	OBI1	-0.499956003	0.144451052	-3.461075552	0.000538022	0.001811317	negative
PRJNA729271	stage_E14_vs_E10	ordinary_muscle_development	ENSGALG00010000725	ENSGALG00010000725	-0.852273874	0.547064268	-1.55790448	0.119255893	0.196679277	negative
PRJNA729271	stage_E14_vs_E10	ordinary_muscle_development	ENSGALG00010000727	ENSGALG00010000727	0.113707776	0.145388293	0.782097199	0.434157453	0.548180512	positive
PRJNA729271	stage_E14_vs_E10	ordinary_muscle_development	ENSGALG00010000728	OTULIN	-0.18222956	0.259786778	-0.701458177	0.483017127	0.594542565	negative
PRJNA729271	stage_E14_vs_E10	ordinary_muscle_development	ENSGALG00010000729	EDNRB	-0.814709462	0.156579632	-5.203163742	1.95924E-07	1.19929E-06	negative
PRJNA729271	stage_E14_vs_E10	ordinary_muscle_development	ENSGALG00010000730	INTS5	0.172285874	0.223782021	0.769882554	0.441369563	0.55535172	positive
PRJNA729271	stage_E14_vs_E10	ordinary_muscle_development	ENSGALG00010000731	ENSGALG00010000731	-0.576211884	0.444347344	-1.29676005	0.19471376	0.292815521	negative
PRJNA729271	stage_E14_vs_E10	ordinary_muscle_development	ENSGALG00010000732	ENSGALG00010000732	0.296843971	0.140806039	2.108176417	0.035015732	0.069959068	positive
PRJNA729271	stage_E14_vs_E10	ordinary_muscle_development	ENSGALG00010000733	ENSGALG00010000733	-0.680467299	0.415702577	-1.636909023	0.10164948	0.172328564	negative
PRJNA729271	stage_E14_vs_E10	ordinary_muscle_development	ENSGALG00010000734	SLAIN1	0.57878802	0.280265762	2.065139941	0.038909761	0.076546236	positive
PRJNA729271	stage_E14_vs_E10	ordinary_muscle_development	ENSGALG00010000735	ENSGALG00010000735	0.249407883	0.545309673	0.457369263	0.647405654	0.739316548	positive
PRJNA729271	stage_E14_vs_E10	ordinary_muscle_development	ENSGALG00010000737	ENSGALG00010000737	-1.827777531	0.810405197	-2.255387228	0.024109034	0.051120603	negative
PRJNA729271	stage_E14_vs_E10	ordinary_muscle_development	ENSGALG00010000741	FAM105A	-0.214044387	0.192586499	-1.111419481	0.266387831	0.375283469	negative
PRJNA729271	stage_E14_vs_E10	ordinary_muscle_development	ENSGALG00010000753	TRIO	0.309523395	0.068025271	4.550123642	5.36144E-06	2.63348E-05	positive
PRJNA729271	stage_E14_vs_E10	ordinary_muscle_development	ENSGALG00010000757	SCEL	-0.27225356	0.465660745	-0.584660749	0.558775851	0.662528394	negative
PRJNA729271	stage_E14_vs_E10	ordinary_muscle_development	ENSGALG00010000760	CDH10	-0.441466494	0.275136851	-1.604534223	0.108596347	0.182340127	negative
PRJNA729271	stage_E14_vs_E10	ordinary_muscle_development	ENSGALG00010000762	ENSGALG00010000762	0.219641857	0.296596119	0.740541912	0.45897124	0.571802501	positive
PRJNA729271	stage_E14_vs_E10	ordinary_muscle_development	ENSGALG00010000768	ENSGALG00010000768	0.795063125	0.323630595	2.456699514	0.014021992	0.03200768	positive
PRJNA729271	stage_E14_vs_E10	ordinary_muscle_development	ENSGALG00010000770	ENSGALG00010000770	-2.032043516	0.582089843	-3.490944806	0.000481316	0.00163887	negative
PRJNA729271	stage_E14_vs_E10	ordinary_muscle_development	ENSGALG00010000777	MYCBP2	-0.27246509	0.082482524	-3.303306893	0.000955518	0.003024727	negative
PRJNA729271	stage_E14_vs_E10	ordinary_muscle_development	ENSGALG00010000783	DAP	0.086827028	0.162537664	0.534196356	0.59320569	0.693093381	positive
PRJNA729271	stage_E14_vs_E10	ordinary_muscle_development	ENSGALG00010000784	FBXL3	-0.010564148	0.095446658	-0.110681172	0.911869177	0.939346437	negative
PRJNA729271	stage_E14_vs_E10	ordinary_muscle_development	ENSGALG00010000785	CLN5	0.007503961	0.091922741	0.081633346	0.934938284	0.954677816	positive
PRJNA729271	stage_E14_vs_E10	ordinary_muscle_development	ENSGALG00010000786	ANKRD33B	1.589692671	0.30911251	5.142763947	2.70726E-07	1.6229E-06	positive
PRJNA729271	stage_E14_vs_E10	ordinary_muscle_development	ENSGALG00010000787	SNRNP48	0.190036131	0.102113075	1.861036218	0.062739062	0.114934521	positive
PRJNA729271	stage_E14_vs_E10	ordinary_muscle_development	ENSGALG00010000788	GATD3AL1	0.214048161	0.104488535	2.04853251	0.04050785	0.079142915	positive
PRJNA729271	stage_E14_vs_E10	ordinary_muscle_development	ENSGALG00010000789	ROPN1L	0.263361092	0.22724164	1.158947328	0.246477656	0.353102749	positive
PRJNA729271	stage_E14_vs_E10	ordinary_muscle_development	ENSGALG00010000790	MARCHF6	0.160067959	0.099288767	1.612145706	0.106930237	0.180080607	positive
PRJNA729271	stage_E14_vs_E10	ordinary_muscle_development	ENSGALG00010000791	ACOD1	-0.936160493	0.526435915	-1.77829906	0.075354751	0.13439732	negative
PRJNA729271	stage_E14_vs_E10	ordinary_muscle_development	ENSGALG00010000792	ENSGALG00010000792	6.811397771	1.633236273	4.170491365	3.03944E-05	0.000131512	positive
PRJNA729271	stage_E14_vs_E10	ordinary_muscle_development	ENSGALG00010000793	ENSGALG00010000793	10.85318641	1.144192761	9.48545278	2.41333E-21	5.74404E-20	positive
PRJNA729271	stage_E14_vs_E10	ordinary_muscle_development	ENSGALG00010000795	ENSGALG00010000795	0.027323251	0.217964882	0.125356208	0.900241555	0.931406234	positive
PRJNA729271	stage_E14_vs_E10	ordinary_muscle_development	ENSGALG00010000796	ENSGALG00010000796	-0.14066237	0.195709921	-0.718728868	0.472307993	0.584437797	negative
PRJNA729271	stage_E14_vs_E10	ordinary_muscle_development	ENSGALG00010000797	ENSGALG00010000797	1.829134881	0.415165071	4.405801471	1.05393E-05	4.92164E-05	positive
PRJNA729271	stage_E14_vs_E10	ordinary_muscle_development	ENSGALG00010000798	ENSGALG00010000798	-2.426500907	0.446401564	-5.435690871	5.45845E-08	3.60129E-07	negative
PRJNA729271	stage_E14_vs_E10	ordinary_muscle_development	ENSGALG00010000799	ENSGALG00010000799	-0.944375967	0.292215622	-3.231777827	0.001230227	0.00380054	negative
PRJNA729271	stage_E14_vs_E10	ordinary_muscle_development	ENSGALG00010000800	LMO7	0.58574931	0.151493531	3.866497175	0.00011041	0.000431837	positive
PRJNA729271	stage_E14_vs_E10	ordinary_muscle_development	ENSGALG00010000802	SEMA5A	-0.89699897	0.125985183	-7.119876695	1.08024E-12	1.2252E-11	negative
PRJNA729271	stage_E14_vs_E10	ordinary_muscle_development	ENSGALG00010000803	MTRR	-0.023156998	0.096361087	-0.240314828	0.8100862	0.866582987	negative
PRJNA729271	stage_E14_vs_E10	ordinary_muscle_development	ENSGALG00010000804	ENSGALG00010000804	-1.008467834	0.473551288	-2.12958524	0.033205871	0.066849874	negative
PRJNA729271	stage_E14_vs_E10	ordinary_muscle_development	ENSGALG00010000805	FASTKD3	0.198511919	0.128770604	1.541593453	0.123172418	0.201692746	positive
PRJNA729271	stage_E14_vs_E10	ordinary_muscle_development	ENSGALG00010000806	CFAP90	-0.018950339	0.45430416	-0.041712888	0.96672758	0.977790543	negative
PRJNA729271	stage_E14_vs_E10	ordinary_muscle_development	ENSGALG00010000807	ENSGALG00010000807	2.683500951	0.724191607	3.705512359	0.000210964	0.000779684	positive
PRJNA729271	stage_E14_vs_E10	ordinary_muscle_development	ENSGALG00010000808	ADCY2	0.24502663	0.171367667	1.429829988	0.15276582	0.240951095	positive
PRJNA729271	stage_E14_vs_E10	ordinary_muscle_development	ENSGALG00010000810	UCHL3	-0.115611719	0.137109298	-0.843208454	0.399111871	0.514507877	negative
PRJNA729271	stage_E14_vs_E10	ordinary_muscle_development	ENSGALG00010000811	ENSGALG00010000811	4.617764093	1.047941158	4.406510861	1.05049E-05	4.91031E-05	positive
PRJNA729271	stage_E14_vs_E10	ordinary_muscle_development	ENSGALG00010000812	ENSGALG00010000812	-0.270385053	0.095205709	-2.840008812	0.004511229	0.011897791	negative
PRJNA729271	stage_E14_vs_E10	ordinary_muscle_development	ENSGALG00010000813	ENSGALG00010000813	-1.37771668	0.445764188	-3.09068498	0.001996954	0.005807858	negative
PRJNA729271	stage_E14_vs_E10	ordinary_muscle_development	ENSGALG00010000814	CDH18	-0.90489989	0.280922099	-3.221177307	0.001276651	0.003923889	negative
PRJNA729271	stage_E14_vs_E10	ordinary_muscle_development	ENSGALG00010000815	ENSGALG00010000815	-0.122725535	0.156535187	-0.784012443	0.433032809	0.547141786	negative
PRJNA729271	stage_E14_vs_E10	ordinary_muscle_development	ENSGALG00010000816	TBC1D4	0.167025379	0.104664846	1.595811636	0.110530855	0.184946049	positive
PRJNA729271	stage_E14_vs_E10	ordinary_muscle_development	ENSGALG00010000817	ENSGALG00010000817	0.257668839	0.115448112	2.231901712	0.02562146	0.053886108	positive
PRJNA729271	stage_E14_vs_E10	ordinary_muscle_development	ENSGALG00010000818	KLF12	-0.60630183	0.073553843	-8.242966005	1.67993E-16	2.69963E-15	negative
PRJNA729271	stage_E14_vs_E10	ordinary_muscle_development	ENSGALG00010000820	KLF5	1.679232293	0.191693675	8.759977562	1.95289E-18	3.70028E-17	positive
PRJNA729271	stage_E14_vs_E10	ordinary_muscle_development	ENSGALG00010000821	ENSGALG00010000821	1.631366767	0.148906752	10.95562657	6.24447E-28	2.351E-26	positive
PRJNA729271	stage_E14_vs_E10	ordinary_muscle_development	ENSGALG00010000822	BASP1	-0.726611831	0.164461625	-4.418123885	9.95614E-06	4.66885E-05	negative
PRJNA729271	stage_E14_vs_E10	ordinary_muscle_development	ENSGALG00010000823	PIBF1	1.043213845	0.158481776	6.582547681	4.62454E-11	4.45697E-10	positive
PRJNA729271	stage_E14_vs_E10	ordinary_muscle_development	ENSGALG00010000843	DIS3	-0.351446875	0.081784789	-4.297215665	1.72957E-05	7.78816E-05	negative
PRJNA729271	stage_E14_vs_E10	ordinary_muscle_development	ENSGALG00010000853	ENSGALG00010000853	0.095194298	0.304660568	0.312460187	0.754690816	0.824680161	positive
PRJNA729271	stage_E14_vs_E10	ordinary_muscle_development	ENSGALG00010000857	ENSGALG00010000857	-1.080584555	0.782327604	-1.381243037			negative
PRJNA729271	stage_E14_vs_E10	ordinary_muscle_development	ENSGALG00010000859	BORA	-0.611355626	0.186360971	-3.280491737	0.001036263	0.003258272	negative
PRJNA729271	stage_E14_vs_E10	ordinary_muscle_development	ENSGALG00010000864	MZT1	-0.041005749	0.1276261	-0.321295947	0.747986127	0.81924741	negative
PRJNA729271	stage_E14_vs_E10	ordinary_muscle_development	ENSGALG00010000870	DACH1	0.199211707	0.139112264	1.432021168	0.152137763	0.240143404	positive
PRJNA729271	stage_E14_vs_E10	ordinary_muscle_development	ENSGALG00010000901	PCDH9	-1.51490354	0.240784184	-6.291540885	3.1433E-10	2.72991E-09	negative
PRJNA729271	stage_E14_vs_E10	ordinary_muscle_development	ENSGALG00010001066	SLC33A1	-0.275636875	0.083145888	-3.315099315	0.000916106	0.002909499	negative
PRJNA729271	stage_E14_vs_E10	ordinary_muscle_development	ENSGALG00010001070	C3orf33	-0.026366918	0.176398	-0.14947402	0.881179608	0.918436644	negative
PRJNA729271	stage_E14_vs_E10	ordinary_muscle_development	ENSGALG00010001082	P2RY1	2.440524044	0.180095242	13.55129661	7.78429E-42	6.67652E-40	positive
PRJNA729271	stage_E14_vs_E10	ordinary_muscle_development	ENSGALG00010001104	MBNL1	0.031825305	0.123228021	0.258263538	0.796203524	0.856682774	positive
PRJNA729271	stage_E14_vs_E10	ordinary_muscle_development	ENSGALG00010001111	SUCNR1	0.028312727	0.527848179	0.053638012	0.957223571	0.970548102	positive
PRJNA729271	stage_E14_vs_E10	ordinary_muscle_development	ENSGALG00010001112	TSC22D2	0.37772847	0.088784305	4.25445096	2.09563E-05	9.29217E-05	positive
PRJNA729271	stage_E14_vs_E10	ordinary_muscle_development	ENSGALG00010001113	IGSF10	-2.116979236	0.314922416	-6.722224673	1.78971E-11	1.78909E-10	negative
PRJNA729271	stage_E14_vs_E10	ordinary_muscle_development	ENSGALG00010001114	MED12L	0.621284958	0.101066697	6.147276736	7.88245E-10	6.52892E-09	positive
PRJNA729271	stage_E14_vs_E10	ordinary_muscle_development	ENSGALG00010001115	PFN2	-0.1220158	0.095677226	-1.27528571	0.202208134	0.301605978	negative
PRJNA729271	stage_E14_vs_E10	ordinary_muscle_development	ENSGALG00010001116	RNF13	-0.105199969	0.070092183	-1.500880193	0.133386552	0.215703874	negative
PRJNA729271	stage_E14_vs_E10	ordinary_muscle_development	ENSGALG00010001117	P2RY13	0.343038634	0.31475376	1.0898636	0.275773277	0.386215812	positive
PRJNA729271	stage_E14_vs_E10	ordinary_muscle_development	ENSGALG00010001118	P2RY12	0.033675407	0.311854826	0.107984244	0.914008191	0.940755838	positive
PRJNA729271	stage_E14_vs_E10	ordinary_muscle_development	ENSGALG00010001119	COMMD2	-0.158194112	0.098255622	-1.61002606	0.107392167	0.180774528	negative
PRJNA729271	stage_E14_vs_E10	ordinary_muscle_development	ENSGALG00010001120	GPR87	0.491680273	0.815821809	0.602680963	0.546720949	0.652132995	positive
PRJNA729271	stage_E14_vs_E10	ordinary_muscle_development	ENSGALG00010001121	WWTR1	1.323854009	0.091110879	14.53014197	7.80585E-48	9.83876E-46	positive
PRJNA729271	stage_E14_vs_E10	ordinary_muscle_development	ENSGALG00010001122	P2RY14	2.425255674	0.424131405	5.718170473	1.07677E-08	7.81562E-08	positive
PRJNA729271	stage_E14_vs_E10	ordinary_muscle_development	ENSGALG00010001123	CP	0.234395335	0.149440336	1.568487742	0.116767349	0.193411349	positive
PRJNA729271	stage_E14_vs_E10	ordinary_muscle_development	ENSGALG00010001124	GPR171	0.228274251	0.690462639	0.330610576	0.740938656	0.813690872	positive
PRJNA729271	stage_E14_vs_E10	ordinary_muscle_development	ENSGALG00010001125	CLRN1	1.358266982	0.264224658	5.140576181	2.73897E-07	1.64123E-06	positive
PRJNA729271	stage_E14_vs_E10	ordinary_muscle_development	ENSGALG00010001126	MINDY4B	0.454622395	1.27041963	0.357852149	0.720453962	0.797717976	positive
PRJNA729271	stage_E14_vs_E10	ordinary_muscle_development	ENSGALG00010001127	SIAH2	0.257160291	0.099519794	2.584011482	0.009765854	0.023352956	positive
PRJNA729271	stage_E14_vs_E10	ordinary_muscle_development	ENSGALG00010001129	TM4SF4	-2.334385297	1.061145964	-2.199872003	0.027815978	0.057714371	negative
PRJNA729271	stage_E14_vs_E10	ordinary_muscle_development	ENSGALG00010001131	HPS3	0.506935886	0.09865215	5.138619764	2.76764E-07	1.65567E-06	positive

PRJNA729271	stage_E14_vs_E10	ordinary_muscle_development	ENSGALG00010001132	SELENOT	0.078592101	0.094225525	0.834085041	0.404233064	0.519216505	positive
PRJNA729271	stage_E14_vs_E10	ordinary_muscle_development	ENSGALG00010001133	EIF2A	-0.088611767	0.072559338	-1.221231733	0.221998292	0.32477445	negative
PRJNA729271	stage_E14_vs_E10	ordinary_muscle_development	ENSGALG00010001134	ENSGALG00010001134	0.321299603	0.616832002	0.520886728	0.602445681	0.700782454	positive
PRJNA729271	stage_E14_vs_E10	ordinary_muscle_development	ENSGALG00010001135	GYG1	0.830259539	0.159421074	5.207966054	1.90922E-07	1.17065E-06	positive
PRJNA729271	stage_E14_vs_E10	ordinary_muscle_development	ENSGALG00010001136	SERP1	-0.108113403	0.125196725	-0.863548173	0.387836145	0.503735209	negative
PRJNA729271	stage_E14_vs_E10	ordinary_muscle_development	ENSGALG00010001138	SPTSSB	-2.938378396	0.608872043	-4.825937456	1.39346E-06	7.55357E-06	negative
PRJNA729271	stage_E14_vs_E10	ordinary_muscle_development	ENSGALG00010001140	NMD3	-0.173196641	0.121012315	-1.431231536	0.152363868	0.240421758	negative
PRJNA729271	stage_E14_vs_E10	ordinary_muscle_development	ENSGALG00010001141	MTAP	-0.630062467	0.110825431	-5.685179489	1.30675E-08	9.38157E-08	negative
PRJNA729271	stage_E14_vs_E10	ordinary_muscle_development	ENSGALG00010001142	PPM1L	0.077408993	0.148188988	0.522366703	0.601415035	0.700097213	positive
PRJNA729271	stage_E14_vs_E10	ordinary_muscle_development	ENSGALG00010001143	CDKN2A	-0.325730647	0.516980405	-0.630063816	0.528652832	0.636182881	negative
PRJNA729271	stage_E14_vs_E10	ordinary_muscle_development	ENSGALG00010001144	CDKN2B	0.470388932	1.588289438	0.296160713	0.76710735	0.834400498	positive
PRJNA729271	stage_E14_vs_E10	ordinary_muscle_development	ENSGALG00010001145	TRIM36	1.842240354	0.206904917	8.903801703	5.39658E-19	1.06717E-17	positive
PRJNA729271	stage_E14_vs_E10	ordinary_muscle_development	ENSGALG00010001146	DIPK1C	3.269989036	0.239119764	13.67510985	1.42991E-42	1.30356E-40	positive
PRJNA729271	stage_E14_vs_E10	ordinary_muscle_development	ENSGALG00010001147	PGGT1B	0.11143481	0.110402328	1.009351991	0.312805854	0.426614021	positive
PRJNA729271	stage_E14_vs_E10	ordinary_muscle_development	ENSGALG00010001149	CIDEA	0.638816951	0.62112807	1.028478637	0.303724735	0.41731018	positive
PRJNA729271	stage_E14_vs_E10	ordinary_muscle_development	ENSGALG00010001150	ENSGALG00010001150	1.491294776	0.600810989	2.482136319	0.01305973	0.030090731	positive
PRJNA729271	stage_E14_vs_E10	ordinary_muscle_development	ENSGALG00010001151	CCDC112	-0.055804362	0.21894827	-0.254874642	0.798819922	0.858522635	negative
PRJNA729271	stage_E14_vs_E10	ordinary_muscle_development	ENSGALG00010001152	FEM1C	0.777715659	0.197825985	3.931311946	8.44836E-05	0.000336888	positive
PRJNA729271	stage_E14_vs_E10	ordinary_muscle_development	ENSGALG00010001153	TSHZ1	-1.149212709	0.110609382	-10.38983032	2.75843E-25	8.52527E-24	negative
PRJNA729271	stage_E14_vs_E10	ordinary_muscle_development	ENSGALG00010001154	PTGR3	-0.084204377	0.083673292	-1.00634713	0.314248617	0.428225411	negative
PRJNA729271	stage_E14_vs_E10	ordinary_muscle_development	ENSGALG00010001155	CYB5A	0.416681904	0.092304048	4.514232186	6.35466E-06	3.08579E-05	positive
PRJNA729271	stage_E14_vs_E10	ordinary_muscle_development	ENSGALG00010001156	ALDH7A1	-0.000623928	0.181070352	-0.003445777	0.997250673	0.998283737	negative
PRJNA729271	stage_E14_vs_E10	ordinary_muscle_development	ENSGALG00010001157	TIMM21	0.753401494	0.091758047	8.210740256	2.19829E-16	3.48243E-15	positive
PRJNA729271	stage_E14_vs_E10	ordinary_muscle_development	ENSGALG00010001158	FRMPD1	-0.097124758	0.345020654	-0.281504186	0.778323718	0.842995016	negative
PRJNA729271	stage_E14_vs_E10	ordinary_muscle_development	ENSGALG00010001159	RTTN	0.135148214	0.108612326	1.24431746	0.213382747	0.314903576	positive
PRJNA729271	stage_E14_vs_E10	ordinary_muscle_development	ENSGALG00010001160	TRMT10B	0.285424336	0.125770812	2.269400436	0.023243986	0.04954729	positive
PRJNA729271	stage_E14_vs_E10	ordinary_muscle_development	ENSGALG00010001161	B3GALNT1	-0.333736207	0.098536017	-3.386946407	0.000706752	0.002310413	negative
PRJNA729271	stage_E14_vs_E10	ordinary_muscle_development	ENSGALG00010001162	ENSGALG00010001162	-0.368830072	0.417815534	-0.882758161	0.37736695	0.493634204	negative
PRJNA729271	stage_E14_vs_E10	ordinary_muscle_development	ENSGALG00010001163	ARL14	0.196284203	0.434436587	0.451813243	0.651403529	0.742361361	positive
PRJNA729271	stage_E14_vs_E10	ordinary_muscle_development	ENSGALG00010001164	KPNA4	0.0102603	0.074653239	0.137439447	0.890683453	0.924884064	positive
PRJNA729271	stage_E14_vs_E10	ordinary_muscle_development	ENSGALG00010001165	ENSGALG00010001165	0.62841625	0.159732106	3.934188729	8.34782E-05	0.000333338	positive
PRJNA729271	stage_E14_vs_E10	ordinary_muscle_development	ENSGALG00010001166	TRIM59	0.145153835	0.095669166	1.517247841	0.129204122	0.209862589	positive
PRJNA729271	stage_E14_vs_E10	ordinary_muscle_development	ENSGALG00010001167	SMC4	-0.245812216	0.152693364	-1.609842169	0.107432317	0.180821113	negative
PRJNA729271	stage_E14_vs_E10	ordinary_muscle_development	ENSGALG00010001168	DOK6	-0.450419867	0.135764795	-3.317648491	0.000907786	0.002887506	negative
PRJNA729271	stage_E14_vs_E10	ordinary_muscle_development	ENSGALG00010001169	ENSGALG00010001169	0.118327632	0.102887564	1.150067388	0.250116117	0.357291132	positive
PRJNA729271	stage_E14_vs_E10	ordinary_muscle_development	ENSGALG00010001170	DCAF10	-0.269044966	0.068754347	-3.913133923	9.1106E-05	0.000361506	negative
PRJNA729271	stage_E14_vs_E10	ordinary_muscle_development	ENSGALG00010001171	POLR1E	-0.022095039	0.126691395	-0.174400468	0.861550746	0.904743756	negative
PRJNA729271	stage_E14_vs_E10	ordinary_muscle_development	ENSGALG00010001172	TMEM200C	-1.86643979	0.279302165	-6.682511004	2.34882E-11	2.31607E-10	negative
PRJNA729271	stage_E14_vs_E10	ordinary_muscle_development	ENSGALG00010001173	IFT80	-0.020897835	0.110040835	-0.189909815	0.849379801	0.895596146	negative
PRJNA729271	stage_E14_vs_E10	ordinary_muscle_development	ENSGALG00010001174	ENSGALG00010001174	-0.402751867	0.138566175	-2.906566971	0.003654187	0.009870935	negative
PRJNA729271	stage_E14_vs_E10	ordinary_muscle_development	ENSGALG00010001177	SEH1L	-0.124007026	0.069561258	-1.782702463	0.07463475	0.133263206	negative
PRJNA729271	stage_E14_vs_E10	ordinary_muscle_development	ENSGALG00010001178	ZBTB5	-0.10575705	0.063976395	-1.653063612	0.098317918	0.167739904	negative
PRJNA729271	stage_E14_vs_E10	ordinary_muscle_development	ENSGALG00010001179	GRHPR	0.821446948	0.184549538	4.451091865	8.54348E-06	4.04963E-05	positive
PRJNA729271	stage_E14_vs_E10	ordinary_muscle_development	ENSGALG00010001180	SPIRE1	-0.553212877	0.159968064	-3.458270751	0.000543655	0.00182649	negative
PRJNA729271	stage_E14_vs_E10	ordinary_muscle_development	ENSGALG00010001181	MFSD1	0.266177707	0.104590785	2.544944164	0.010929521	0.025747345	positive
PRJNA729271	stage_E14_vs_E10	ordinary_muscle_development	ENSGALG00010001182	ZCCHC7	-0.083560201	0.082222303	-1.016271713	0.309500015	0.423226671	negative
PRJNA729271	stage_E14_vs_E10	ordinary_muscle_development	ENSGALG00010001183	RARRES1	-0.520422474	0.189879918	-2.74079787	0.00612902	0.015539644	negative
PRJNA729271	stage_E14_vs_E10	ordinary_muscle_development	ENSGALG00010001185	PTPN2	-0.458168314	0.06601685	-6.940172279	3.91622E-12	4.19554E-11	negative
PRJNA729271	stage_E14_vs_E10	ordinary_muscle_development	ENSGALG00010001186	ENSGALG00010001186	-0.244613987	0.352165991	-0.694598552	0.48730692	0.598501424	negative
PRJNA729271	stage_E14_vs_E10	ordinary_muscle_development	ENSGALG00010001187	GFM1	-0.047520781	0.181852954	-0.261314318	0.793850126	0.855173107	negative
PRJNA729271	stage_E14_vs_E10	ordinary_muscle_development	ENSGALG00010001188	PSMG2	-0.084201667	0.110264494	-0.763633549	0.445085651	0.558621224	negative
PRJNA729271	stage_E14_vs_E10	ordinary_muscle_development	ENSGALG00010001190	LXN	-2.021191484	0.463171786	-4.363805278	1.27819E-05	5.8967E-05	negative
PRJNA729271	stage_E14_vs_E10	ordinary_muscle_development	ENSGALG00010001191	TOMM5	0.393561864	0.150956926	2.60711367	0.009130904	0.022007392	positive
PRJNA729271	stage_E14_vs_E10	ordinary_muscle_development	ENSGALG00010001192	ENSGALG00010001192	-0.033582362	0.221152272	-0.151851763	0.879303855	0.917075074	negative
PRJNA729271	stage_E14_vs_E10	ordinary_muscle_development	ENSGALG00010001193	PRELID3A	-0.230244296	0.229184707	-1.004623301	0.315078273	0.429073616	negative
PRJNA729271	stage_E14_vs_E10	ordinary_muscle_development	ENSGALG00010001194	POT1	-0.363925858	0.107118067	-3.397427418	0.000680226	0.002230493	negative
PRJNA729271	stage_E14_vs_E10	ordinary_muscle_development	ENSGALG00010001195	ZNF608	-0.443810412	0.140980643	-3.148023759	0.001643783	0.004900584	negative
PRJNA729271	stage_E14_vs_E10	ordinary_muscle_development	ENSGALG00010001196	LSMEM1	1.903571722	0.209516948	9.085526197	1.03198E-19	2.15232E-18	positive
PRJNA729271	stage_E14_vs_E10	ordinary_muscle_development	ENSGALG00010001197	CEP76	-0.308092199	0.139692577	-2.205501577	0.027418917	0.056947586	negative
PRJNA729271	stage_E14_vs_E10	ordinary_muscle_development	ENSGALG00010001198	IFRD1	-0.084541989	0.176311437	-0.479503716	0.631580325	0.725301602	negative
PRJNA729271	stage_E14_vs_E10	ordinary_muscle_development	ENSGALG00010001199	AFG3L2	-0.035255812	0.090052967	-0.391500833	0.695427073	0.778094591	negative
PRJNA729271	stage_E14_vs_E10	ordinary_muscle_development	ENSGALG00010001200	CTTNBP2	-0.930642928	0.130377583	-7.138059373	9.46577E-13	1.08207E-11	negative
PRJNA729271	stage_E14_vs_E10	ordinary_muscle_development	ENSGALG00010001201	TUBB6	1.141402131	0.164522058	6.937684507	3.98579E-12	4.26062E-11	positive
PRJNA729271	stage_E14_vs_E10	ordinary_muscle_development	ENSGALG00010001202	CFTR	-0.175817922	0.263467039	-0.667324164	0.504565095	0.613610182	negative
PRJNA729271	stage_E14_vs_E10	ordinary_muscle_development	ENSGALG00010001203	TMX3	-0.409518531	0.100015192	-4.094563258	4.22965E-05	0.000178171	negative
PRJNA729271	stage_E14_vs_E10	ordinary_muscle_development	ENSGALG00010001204	SNX2	-0.236789271	0.081413498	-2.908476805	0.003631941	0.009816551	negative
PRJNA729271	stage_E14_vs_E10	ordinary_muscle_development	ENSGALG00010001205	CEP192	-0.179566475	0.103787697	-1.730132567	0.083606593	0.146787496	negative
PRJNA729271	stage_E14_vs_E10	ordinary_muscle_development	ENSGALG00010001206	RSRC1	0.016001821	0.075530547	0.211858935	0.832217088	0.883088338	positive
PRJNA729271	stage_E14_vs_E10	ordinary_muscle_development	ENSGALG00010001208	MOV10L1	-2.970428611	0.776098318	-3.827386998	0.000129511	0.000499936	negative
PRJNA729271	stage_E14_vs_E10	ordinary_muscle_development	ENSGALG00010001209	CDH7	-0.463472591	0.241272585	-1.92095008	0.054738001	0.102285333	negative
PRJNA729271	stage_E14_vs_E10	ordinary_muscle_development	ENSGALG00010001210	PANX2	-1.591282664	0.183051618	-8.693081663	3.52739E-18	6.52994E-17	negative
PRJNA729271	stage_E14_vs_E10	ordinary_muscle_development	ENSGALG00010001213	PPIC	-0.301595431	0.218113	-1.382748536	0.166741984	0.258218489	negative
PRJNA729271	stage_E14_vs_E10	ordinary_muscle_development	ENSGALG00010001214	TES	-1.093221153	0.172311756	-6.344437422	2.2324E-10	1.98033E-09	negative
PRJNA729271	stage_E14_vs_E10	ordinary_muscle_development	ENSGALG00010001215	PRDM6	-3.416569757	0.700249473	-4.879075086	1.06584E-06	5.86734E-06	negative
PRJNA729271	stage_E14_vs_E10	ordinary_muscle_development	ENSGALG00010001216	SHOX2	-2.80295648	0.374676286	-7.481008507	7.37544E-14	9.48598E-13	negative
PRJNA729271	stage_E14_vs_E10	ordinary_muscle_development	ENSGALG00010001218	ENSGALG00010001218	-0.292027814	0.10866237	-2.687478787	0.007199368	0.017868978	negative
PRJNA729271	stage_E14_vs_E10	ordinary_muscle_development	ENSGALG00010001219	TFEC	-1.032978329	0.427840377	-2.414401225	0.015761096	0.035408723	negative
PRJNA729271	stage_E14_vs_E10	ordinary_muscle_development	ENSGALG00010001220	PLXNB2	0.0109188	0.082275703	0.132709896	0.894422816	0.927507808	positive
PRJNA729271	stage_E14_vs_E10	ordinary_muscle_development	ENSGALG00010001221	GRAMD2B	-0.427254405	0.154070769	-2.773104911	0.005552422	0.014267391	negative
PRJNA729271	stage_E14_vs_E10	ordinary_muscle_development	ENSGALG00010001222	ZNF800	0.265794608	0.094811129	2.803411476	0.005056511	0.013156791	positive
PRJNA729271	stage_E14_vs_E10	ordinary_muscle_development	ENSGALG00010001223	GRM8	-2.37454609	0.519530842	-4.570558473	4.86426E-06	2.40312E-05	negative
PRJNA729271	stage_E14_vs_E10	ordinary_muscle_development	ENSGALG00010001224	DNAJB9	-0.281511312	0.189699274	-1.483987291	0.137812292	0.221388582	negative
PRJNA729271	stage_E14_vs_E10	ordinary_muscle_development	ENSGALG00010001225	SOCS6	-0.187301118	0.097419439	-1.922625704	0.054527074	0.101956905	negative
PRJNA729271	stage_E14_vs_E10	ordinary_muscle_development	ENSGALG00010001226	VEPH1	0.582949939	0.487930476	1.194739759	0.232188723	0.336422985	positive
PRJNA729271	stage_E14_vs_E10	ordinary_muscle_development	ENSGALG00010001227	ENSGALG00010001227	3.25945195	0.550889118	5.916711451	3.28442E-09	2.53637E-08	positive
PRJNA729271	stage_E14_vs_E10	ordinary_muscle_development	ENSGALG00010001228	THAP5	-0.060987431	0.108889599	-0.560085003	0.575421459	0.677337507	negative
PRJNA729271	stage_E14_vs_E10	ordinary_muscle_development	ENSGALG00010001229	ZNF407	-0.234958057	0.103171142	-2.277362181	0.022764607	0.048711688	negative
PRJNA729271	stage_E14_vs_E10	ordinary_muscle_development	ENSGALG00010001230	PTX3	-1.105139328	0.581604996	-1.90015446	0.057412853	0.106541966	negative
PRJNA729271	stage_E14_vs_E10	ordinary_muscle_development	ENSGALG00010001231	CNDP1	0.645836779	0.546453445	1.181869718	0.237257399	0.342329882	positive
PRJNA729271	stage_E14_vs_E10	ordinary_muscle_development	ENSGALG00010001232	TSPAN12	1.558194799	0.100330443	15.53062807	2.15261E-54	3.94963E-52	positive
PRJNA729271	stage_E14_vs_E10	ordinary_muscle_development	ENSGALG00010001233	CNDP2	0.257363259	0.095929295	2.682843233	0.00729992	0.018081398	positive
PRJNA729271	stage_E14_vs_E10	ordinary_muscle_development	ENSGALG00010001234	AASS	0.684269348	0.098634753	6.937406238	3.99364E-12	4.26587E-11	positive
PRJNA729271	stage_E14_vs_E10	ordinary_muscle_development	ENSGALG00010001235	CCDC102B	-0.512502894	0.291616426	-1.757455509	0.078840199	0.139671069	negative
PRJNA729271	stage_E14_vs_E10	ordinary_muscle_development	ENSGALG00010001237	MELK	-0.452244402	0.266076254	-1.699679678	0.089191194	0.154681306	negative
PRJNA729271	stage_E14_vs_E10	ordinary_muscle_development	ENSGALG00010001238	GOLIM4	-0.161562455	0.121835512	-1.326070307	0.18481641	0.280664385	negative
PRJNA729271	stage_E14_vs_E10	ordinary_muscle_development	ENSGALG00010001239	ENSGALG00010001239	-0.054463692	0.245663742	-0.221700164	0.824547303	0.877261682	negative
PRJNA729271	stage_E14_vs_E10	ordinary_muscle_development	ENSGALG00010001242	ENSGALG00010001242	-0.00353461	0.620031092	-0.005700698	0.995451526	0.997665992	negative
PRJNA729271	stage_E14_vs_E10	ordinary_muscle_development	ENSGALG00010001244	ENSGALG00010001244	0.45403213	0.149168332	3.043756826	0.002336439	0.006669296	positive
PRJNA729271	stage_E14_vs_E10	ordinary_muscle_development	ENSGALG00010001245	SERPINI1	-0.62910701	0.22262316	-2.825883026	0.004715048	0.012372306	negative
PRJNA729271	stage_E14_vs_E10	ordinary_muscle_development	ENSGALG00010001248	PTPRZ1	-0.58623816	0.239942382	-2.44324556	0.014555829	0.033085581	negative
PRJNA729271	stage_E14_vs_E10	ordinary_muscle_development	ENSGALG00010001249	PDCD10	-0.021584803	0.138844438	-0.155460334	0.876458422	0.915249282	negative
PRJNA729271	stage_E14_vs_E10	ordinary_muscle_development	ENSGALG00010001250	ENSGALG00010001250	-0.547752127	0.652952462	-0.838885154	0.401533759	0.5167568	negative
PRJNA729271	stage_E14_vs_E10	ordinary_muscle_development	ENSGALG00010001251	ZNF277	-0.159548395	0.12074308	-1.321387484	0.186372197	0.282534253	negative
PRJNA729271	stage_E14_vs_E10	ordinary_muscle_development	ENSGALG00010001252	SNCAIP	-0.172391331	0.121730334	-1.416173967	0.156724576	0.245830833	negative
PRJNA729271	stage_E14_vs_E10	ordinary_muscle_development	ENSGALG00010001253	ENSGALG00010001253	-0.999465236	0.80021788	-1.248991382	0.211668226	0.312691698	negative
PRJNA729271	stage_E14_vs_E10	ordinary_muscle_development	ENSGALG00010001254	NETO1	0.016982997	0.388259614	0.043741342	0.965110585	0.976836668	positive
PRJNA729271	stage_E14_vs_E10	ordinary_muscle_development	ENSGALG00010001258	ENSGALG00010001258	-0.592687136	0.124969175	-4.742666643	2.10923E-06	1.10573E-05	negative
PRJNA729271	stage_E14_vs_E10	ordinary_muscle_development	ENSGALG00010001259	ENSGALG00010001259	0.262576611	0.166335325	1.578597998	0.114428294	0.190538555	positive
PRJNA729271	stage_E14_vs_E10	ordinary_muscle_development	ENSGALG00010001260	ENSGALG00010001260	-3.038535913	0.830296273	-3.659580335	0.000252629	0.000916379	negative
PRJNA729271	stage_E14_vs_E10	ordinary_muscle_development	ENSGALG00010001262	ENSGALG00010001262	0.505299504	0.143821649	3.513375813	0.000442451	0.001516871	positive
PRJNA729271	stage_E14_vs_E10	ordinary_muscle_development	ENSGALG00010001264	FAM3C	-0.128805481	0.113164084	-1.138218739	0.255029156	0.362700904	negative
PRJNA729271	stage_E14_vs_E10	ordinary_muscle_development	ENSGALG00010001265	OPN1LW	-0.585101885	0.698481703	-0.837676753	0.402212273	0.517400328	negative
PRJNA729271	stage_E14_vs_E10	ordinary_muscle_development	ENSGALG00010001267	BCHE	1.536046349	0.248745961	6.175160957	6.60961E-10	5.53474E-09	positive
PRJNA729271	stage_E14_vs_E10	ordinary_muscle_development	ENSGALG00010001268	CBLN2	-0.697627416	0.418924263	-1.665282912	0.095856326	0.16427494	negative
PRJNA729271	stage_E14_vs_E10	ordinary_muscle_development	ENSGALG00010001270	DRAP1	0.04659782	0.109905271	0.423981668	0.671579183	0.758322563	positive
PRJNA729271	stage_E14_vs_E10	ordinary_muscle_development	ENSGALG00010001271	ENSGALG00010001271	0.45004094	0.18393203	2.446778525	0.014413938	0.032809364	positive
PRJNA729271	stage_E14_vs_E10	ordinary_muscle_development	ENSGALG00010001273	ENSGALG00010001273	-0.317856164	0.55969969	-0.567904841	0.570099585	0.672219432	negative
PRJNA729271	stage_E14_vs_E10	ordinary_muscle_development	ENSGALG00010001274	DLGAP1	-1.873653151	0.416872986	-4.49454201	6.97198E-06	3.36191E-05	negative
PRJNA729271	stage_E14_vs_E10	ordinary_muscle_development	ENSGALG00010001276	ENSGALG00010001276	-1.775932718	1.192852526	-1.488811633			negative
PRJNA729271	stage_E14_vs_E10	ordinary_muscle_development	ENSGALG00010001277	ENSGALG00010001277	0.200537975	0.161929943	1.238424294	0.215558777	0.31725875	positive
PRJNA729271	stage_E14_vs_E10	ordinary_muscle_development	ENSGALG00010001279	LPIN2	-0.416150925	0.135627531	-3.068336665	0.00215254	0.006203662	negative
PRJNA729271	stage_E14_vs_E10	ordinary_muscle_development	ENSGALG00010001281	LRRN3	-0.533055744	0.145865766	-3.654426656	0.000257757	0.000932416	negative
PRJNA729271	stage_E14_vs_E10	ordinary_muscle_development	ENSGALG00010001283	ENSGALG00010001283	0.53844518	0.177949037	3.025839249	0.002479441	0.007007115	positive
PRJNA729271	stage_E14_vs_E10	ordinary_muscle_development	ENSGALG00010001285	ENSGALG00010001285	0.72317207	0.217453589	3.325638693	0.000882162	0.002812789	positive
PRJNA729271	stage_E14_vs_E10	ordinary_muscle_development	ENSGALG00010001286	SRFBP1	-0.633468875	0.110605611	-5.727276106	1.02056E-08	7.43368E-08	negative
PRJNA729271	stage_E14_vs_E10	ordinary_muscle_development	ENSGALG00010001287	PLPP4	-3.910042603	0.418196151	-9.349781412	8.7829E-21	1.9923E-19	negative
PRJNA729271	stage_E14_vs_E10	ordinary_muscle_development	ENSGALG00010001288	TGIF1	-0.358158908	0.153534347	-2.332760809	0.019660703	0.042813466	negative
PRJNA729271	stage_E14_vs_E10	ordinary_muscle_development	ENSGALG00010001289	ENSGALG00010001289	-0.197687703	0.390144076	-0.506704356	0.612362274	0.709066319	negative
PRJNA729271	stage_E14_vs_E10	ordinary_muscle_development	ENSGALG00010001291	ENSGALG00010001291	0.470301597	0.143266281	3.282709599	0.001028145	0.003233449	positive
PRJNA729271	stage_E14_vs_E10	ordinary_muscle_development	ENSGALG00010001292	LOX	0.220929088	0.142144269	1.554259548	0.120122519	0.197749364	positive
PRJNA729271	stage_E14_vs_E10	ordinary_muscle_development	ENSGALG00010001295	ENSGALG00010001295	0.331510606	0.127320402	2.603750866	0.009220976	0.022194959	positive

PRJNA729271	stage_E14_vs_E10	ordinary_muscle_development	ENSGALG00010001297	MYL12A	0.508048029	0.222723055	2.281075165	0.022543999	0.048310951	positive
PRJNA729271	stage_E14_vs_E10	ordinary_muscle_development	ENSGALG00010001298	DOCK4	-0.036552169	0.13188495	-0.277151936	0.781663444	0.845790655	negative
PRJNA729271	stage_E14_vs_E10	ordinary_muscle_development	ENSGALG00010001300	MRPL49	0.171585378	0.180075531	0.952852266	0.340664928	0.455655452	positive
PRJNA729271	stage_E14_vs_E10	ordinary_muscle_development	ENSGALG00010001301	GMPS	-0.248073587	0.074555816	-3.327353914	0.000876749	0.002799225	negative
PRJNA729271	stage_E14_vs_E10	ordinary_muscle_development	ENSGALG00010001302	ENSGALG00010001302	-0.887117788	0.367278745	-2.415380146	0.015718795	0.035330118	negative
PRJNA729271	stage_E14_vs_E10	ordinary_muscle_development	ENSGALG00010001304	SYVN1	-0.266732369	0.177032671	-1.506684428	0.131891565	0.213605389	negative
PRJNA729271	stage_E14_vs_E10	ordinary_muscle_development	ENSGALG00010001305	WNT16	-1.839280715	0.393860762	-4.66987548	3.01382E-06	1.54311E-05	negative
PRJNA729271	stage_E14_vs_E10	ordinary_muscle_development	ENSGALG00010001306	CEP120	-0.159532215	0.084140966	-1.896011213	0.057958562	0.107403395	negative
PRJNA729271	stage_E14_vs_E10	ordinary_muscle_development	ENSGALG00010001307	CALU	-0.118991424	0.154727567	-0.769038291	0.44187058	0.55578914	negative
PRJNA729271	stage_E14_vs_E10	ordinary_muscle_development	ENSGALG00010001308	MGMT	-0.019453401	0.137430689	-0.141550633	0.887434963	0.922688479	negative
PRJNA729271	stage_E14_vs_E10	ordinary_muscle_development	ENSGALG00010001313	EMILIN2	0.297720495	0.176498434	1.686816632	0.091638616	0.158206497	positive
PRJNA729271	stage_E14_vs_E10	ordinary_muscle_development	ENSGALG00010001320	CPED1	-1.025517799	0.19802071	-5.17884113	2.23269E-07	1.35466E-06	negative
PRJNA729271	stage_E14_vs_E10	ordinary_muscle_development	ENSGALG00010001330	ENSGALG00010001330	0.241758101	0.143406544	1.68582336	0.091829827	0.158443936	positive
PRJNA729271	stage_E14_vs_E10	ordinary_muscle_development	ENSGALG00010001333	PLCH1	0.221266748	0.270934885	0.816678694	0.41411207	0.528938126	positive
PRJNA729271	stage_E14_vs_E10	ordinary_muscle_development	ENSGALG00010001334	EBF3	-1.345614463	0.159068252	-8.459352825	2.68865E-17	4.63401E-16	negative
PRJNA729271	stage_E14_vs_E10	ordinary_muscle_development	ENSGALG00010001343	ENSGALG00010001343	0.273805723	0.267830597	1.02230935	0.306634509	0.4202995	positive
PRJNA729271	stage_E14_vs_E10	ordinary_muscle_development	ENSGALG00010001347	MYOM1	2.065080961	0.16479589	12.53114356	5.04302E-36	2.97149E-34	positive
PRJNA729271	stage_E14_vs_E10	ordinary_muscle_development	ENSGALG00010001349	ING3	0.665696604	0.103865775	6.409200751	1.46284E-10	1.32194E-09	positive
PRJNA729271	stage_E14_vs_E10	ordinary_muscle_development	ENSGALG00010001351	CAPN1	0.356015678	0.156020466	2.281852421	0.022498055	0.048226753	positive
PRJNA729271	stage_E14_vs_E10	ordinary_muscle_development	ENSGALG00010001353	GLRX3	-0.009956298	0.078469649	-0.126880884	0.899034677	0.930423222	negative
PRJNA729271	stage_E14_vs_E10	ordinary_muscle_development	ENSGALG00010001358	TCERG1L	-0.451142803	0.458947907	-0.982993487	0.325610644	0.440231908	negative
PRJNA729271	stage_E14_vs_E10	ordinary_muscle_development	ENSGALG00010001363	HIBADH	0.609295233	0.210619867	2.892866856	0.003817431	0.010256471	positive
PRJNA729271	stage_E14_vs_E10	ordinary_muscle_development	ENSGALG00010001364	SMCHD1	-0.287851557	0.184074713	-1.563775663	0.117870261	0.194904111	negative
PRJNA729271	stage_E14_vs_E10	ordinary_muscle_development	ENSGALG00010001365	ENSGALG00010001365	0.289834817	0.283776511	1.021348865	0.307089183	0.420763561	positive
PRJNA729271	stage_E14_vs_E10	ordinary_muscle_development	ENSGALG00010001367	ENSGALG00010001367	-0.440983746	0.444348508	-0.992427651	0.320988956	0.435486234	negative
PRJNA729271	stage_E14_vs_E10	ordinary_muscle_development	ENSGALG00010001368	WIPF3	1.889698784	0.139406917	13.55527277	7.37375E-42	6.36206E-40	positive
PRJNA729271	stage_E14_vs_E10	ordinary_muscle_development	ENSGALG00010001369	PPP2R2D	-0.021231055	0.12378374	-0.171517316	0.863817016	0.906401306	negative
PRJNA729271	stage_E14_vs_E10	ordinary_muscle_development	ENSGALG00010001372	ENSGALG00010001372	0.187502805	0.29687034	0.631598311	0.527649391	0.635133526	positive
PRJNA729271	stage_E14_vs_E10	ordinary_muscle_development	ENSGALG00010001373	IMPA2	0.648085291	0.139718855	4.63849556	3.50954E-06	1.77374E-05	positive
PRJNA729271	stage_E14_vs_E10	ordinary_muscle_development	ENSGALG00010001378	POLA2	-0.285285597	0.124822371	-2.285532583	0.022281618	0.047819374	negative
PRJNA729271	stage_E14_vs_E10	ordinary_muscle_development	ENSGALG00010001380	SCRN1	-0.638627486	0.153376489	-4.163789951	3.13008E-05	0.000135152	negative
PRJNA729271	stage_E14_vs_E10	ordinary_muscle_development	ENSGALG00010001381	MDFIC	-0.628857706	0.130498866	-4.818874869	1.4437E-06	7.80248E-06	negative
PRJNA729271	stage_E14_vs_E10	ordinary_muscle_development	ENSGALG00010001382	BNIP3	0.444297672	0.193372519	2.297625718	0.021583101	0.046485445	positive
PRJNA729271	stage_E14_vs_E10	ordinary_muscle_development	ENSGALG00010001385	MME	0.135636775	0.256854414	0.528068693	0.597451659	0.697120422	positive
PRJNA729271	stage_E14_vs_E10	ordinary_muscle_development	ENSGALG00010001386	FKBP14	-0.345918517	0.183934413	-1.88066231	0.06001787	0.110597384	negative
PRJNA729271	stage_E14_vs_E10	ordinary_muscle_development	ENSGALG00010001388	MPPE1	0.450280838	0.164050414	2.744771123	0.006055311	0.015387752	positive
PRJNA729271	stage_E14_vs_E10	ordinary_muscle_development	ENSGALG00010001395	GNAL	-0.015332784	0.104848403	-0.146237647	0.883733787	0.920188664	negative
PRJNA729271	stage_E14_vs_E10	ordinary_muscle_development	ENSGALG00010001397	KLHL7	-0.060655272	0.062173315	-0.975583692	0.329270809	0.444062186	negative
PRJNA729271	stage_E14_vs_E10	ordinary_muscle_development	ENSGALG00010001398	WDR11	-0.143605389	0.095834681	-1.498469943	0.134011196	0.216506051	negative
PRJNA729271	stage_E14_vs_E10	ordinary_muscle_development	ENSGALG00010001400	SNX24	0.058015282	0.159860707	0.362911457	0.716671028	0.795051779	positive
PRJNA729271	stage_E14_vs_E10	ordinary_muscle_development	ENSGALG00010001402	NUP42	-0.06862424	0.129296294	-0.530751791	0.595590791	0.695263632	negative
PRJNA729271	stage_E14_vs_E10	ordinary_muscle_development	ENSGALG00010001406	EML3	0.133970968	0.160069798	0.83695344	0.40261874	0.51783129	positive
PRJNA729271	stage_E14_vs_E10	ordinary_muscle_development	ENSGALG00010001407	FOXP2	-0.592829197	0.148478265	-3.992700178	6.53251E-05	0.000266504	negative
PRJNA729271	stage_E14_vs_E10	ordinary_muscle_development	ENSGALG00010001409	GPNMB	2.232474835	0.919631971	2.427574188	0.015200178	0.034302752	positive
PRJNA729271	stage_E14_vs_E10	ordinary_muscle_development	ENSGALG00010001411	FGFR2	-3.710418667	0.364700289	-10.17388464	2.59354E-24	7.37125E-23	negative
PRJNA729271	stage_E14_vs_E10	ordinary_muscle_development	ENSGALG00010001412	ENSGALG00010001412	-0.346502693	0.199441983	-1.737360848	0.082323497	0.144868167	negative
PRJNA729271	stage_E14_vs_E10	ordinary_muscle_development	ENSGALG00010001414	CCDC126	-0.075725138	0.173337099	-0.436866307	0.662208308	0.751425507	negative
PRJNA729271	stage_E14_vs_E10	ordinary_muscle_development	ENSGALG00010001415	MC5R	-0.57459301	0.26631154	-2.157597118	0.030959169	0.063009429	negative
PRJNA729271	stage_E14_vs_E10	ordinary_muscle_development	ENSGALG00010001416	AP4M1	0.301094351	0.090544604	3.325370462	0.000883011	0.002814877	positive
PRJNA729271	stage_E14_vs_E10	ordinary_muscle_development	ENSGALG00010001418	RNMT	-0.270823012	0.063947356	-4.235093165	2.28457E-05	0.000100684	negative
PRJNA729271	stage_E14_vs_E10	ordinary_muscle_development	ENSGALG00010001419	KCND2	-1.592503993	0.222552892	-7.155620305	8.32954E-13	9.58989E-12	negative
PRJNA729271	stage_E14_vs_E10	ordinary_muscle_development	ENSGALG00010001420	FAM221A	-1.520527596	0.393046458	-3.868569645	0.000109476	0.0004283	negative
PRJNA729271	stage_E14_vs_E10	ordinary_muscle_development	ENSGALG00010001422	AP1S1	-0.051257587	0.137942719	-0.37158603	0.710201092	0.789929775	negative
PRJNA729271	stage_E14_vs_E10	ordinary_muscle_development	ENSGALG00010001423	FAM210A	0.120905696	0.137233461	0.881021983	0.378305922	0.494520208	positive
PRJNA729271	stage_E14_vs_E10	ordinary_muscle_development	ENSGALG00010001426	ATE1	0.067205015	0.090752997	0.740526673	0.458980483	0.571802501	positive
PRJNA729271	stage_E14_vs_E10	ordinary_muscle_development	ENSGALG00010001427	ASB11	0.792915893	0.566432378	1.399842106	0.161560606	0.25197127	positive
PRJNA729271	stage_E14_vs_E10	ordinary_muscle_development	ENSGALG00010001432	NSMCE4A	-0.038206538	0.112741308	-0.338886772	0.73469503	0.809286759	negative
PRJNA729271	stage_E14_vs_E10	ordinary_muscle_development	ENSGALG00010001436	ENSGALG00010001436	-3.488586652	1.432728942	-2.434924395	0.014894902	0.03374748	negative
PRJNA729271	stage_E14_vs_E10	ordinary_muscle_development	ENSGALG00010001440	CDCA7L	-0.285302618	0.147619805	-1.932685235	0.053274985	0.099937998	negative
PRJNA729271	stage_E14_vs_E10	ordinary_muscle_development	ENSGALG00010001441	HMGCLL1	-0.602566006	0.297545987	-2.025118915	0.042855155	0.083157359	negative
PRJNA729271	stage_E14_vs_E10	ordinary_muscle_development	ENSGALG00010001442	LDLRAD4	-0.060400019	0.113914934	-0.530220378	0.595959144	0.695637606	negative
PRJNA729271	stage_E14_vs_E10	ordinary_muscle_development	ENSGALG00010001443	ANKRD26	-0.518384139	0.145182678	-3.570564665	0.000356212	0.001254141	negative
PRJNA729271	stage_E14_vs_E10	ordinary_muscle_development	ENSGALG00010001448	GPR37	0.243614649	0.31959969	0.762249328	0.445911211	0.559318362	positive
PRJNA729271	stage_E14_vs_E10	ordinary_muscle_development	ENSGALG00010001450	GPR149	-0.873850957	0.454042609	-1.924601214	0.054279267	0.101559053	negative
PRJNA729271	stage_E14_vs_E10	ordinary_muscle_development	ENSGALG00010001453	TAX1BP1	-0.057238301	0.073651262	-0.777153019	0.437068497	0.55099216	negative
PRJNA729271	stage_E14_vs_E10	ordinary_muscle_development	ENSGALG00010001454	VEGFD	-2.284909856	0.189087453	-12.08387873	1.28511E-33	6.62908E-32	negative
PRJNA729271	stage_E14_vs_E10	ordinary_muscle_development	ENSGALG00010001456	LSM8	-0.28427173	0.109541406	-2.595107555	0.009456132	0.022693152	negative
PRJNA729271	stage_E14_vs_E10	ordinary_muscle_development	ENSGALG00010001457	NDC80	-0.234503	0.224196548	-1.045970609	0.295574602	0.408785849	negative
PRJNA729271	stage_E14_vs_E10	ordinary_muscle_development	ENSGALG00010001458	JAZF1	0.236325364	0.153789772	1.536678035	0.124372172	0.203404563	positive
PRJNA729271	stage_E14_vs_E10	ordinary_muscle_development	ENSGALG00010001460	MALSU1	-0.483114384	0.106368788	-4.541881042	5.57545E-06	2.73212E-05	negative
PRJNA729271	stage_E14_vs_E10	ordinary_muscle_development	ENSGALG00010001461	ASB9	-1.479040192	0.22169165	-6.671609836	2.53012E-11	2.48638E-10	negative
PRJNA729271	stage_E14_vs_E10	ordinary_muscle_development	ENSGALG00010001462	DHX36	-0.192114212	0.108437026	-1.771666186	0.076449986	0.136018479	negative
PRJNA729271	stage_E14_vs_E10	ordinary_muscle_development	ENSGALG00010001463	DSEL	-0.219323221	0.158112386	-1.387134978	0.165400608	0.25660728	negative
PRJNA729271	stage_E14_vs_E10	ordinary_muscle_development	ENSGALG00010001465	TACC2	0.803090079	0.157702475	5.092438012	3.53488E-07	2.09135E-06	positive
PRJNA729271	stage_E14_vs_E10	ordinary_muscle_development	ENSGALG00010001466	PRKX	-0.164493692	0.129682862	-1.268430459	0.20464427	0.304511704	negative
PRJNA729271	stage_E14_vs_E10	ordinary_muscle_development	ENSGALG00010001468	ANOS1	-0.898692573	0.152447612	-5.895091188	3.74475E-09	2.87805E-08	negative
PRJNA729271	stage_E14_vs_E10	ordinary_muscle_development	ENSGALG00010001469	ARHGEF26	0.500812302	0.118897739	4.212126372	2.52978E-05	0.000110783	positive
PRJNA729271	stage_E14_vs_E10	ordinary_muscle_development	ENSGALG00010001470	GPR85	-1.385062199	0.31999088	-4.32844273	1.50167E-05	6.8492E-05	negative
PRJNA729271	stage_E14_vs_E10	ordinary_muscle_development	ENSGALG00010001471	BMP5	-2.362960658	0.369767474	-6.390396188	1.65457E-10	1.48593E-09	negative
PRJNA729271	stage_E14_vs_E10	ordinary_muscle_development	ENSGALG00010001472	ENSGALG00010001472	0.261110392	0.18486712	1.412422025	0.157825735	0.247210291	positive
PRJNA729271	stage_E14_vs_E10	ordinary_muscle_development	ENSGALG00010001475	BMT2	0.060184907	0.135703541	0.443502848	0.657402059	0.747383308	positive
PRJNA729271	stage_E14_vs_E10	ordinary_muscle_development	ENSGALG00010001476	IGF2BP3	-0.427084554	0.193009145	-2.212768486	0.026913611	0.056131337	negative
PRJNA729271	stage_E14_vs_E10	ordinary_muscle_development	ENSGALG00010001477	NLGN4	-1.528182619	0.219743685	-6.954386952	3.54098E-12	3.82463E-11	negative
PRJNA729271	stage_E14_vs_E10	ordinary_muscle_development	ENSGALG00010001479	TMEM168	0.244705888	0.154134485	1.587612846	0.112373912	0.187612511	positive
PRJNA729271	stage_E14_vs_E10	ordinary_muscle_development	ENSGALG00010001480	JAKMIP3	-1.112714176	0.287489362	-3.870453385	0.000108633	0.000425233	negative
PRJNA729271	stage_E14_vs_E10	ordinary_muscle_development	ENSGALG00010001482	TRA2A	0.153637656	0.064766259	2.372186656	0.017683155	0.039102568	positive
PRJNA729271	stage_E14_vs_E10	ordinary_muscle_development	ENSGALG00010001483	ENSGALG00010001483	-1.335668448	0.265810767	-5.02488467	5.03735E-07	2.90324E-06	negative
PRJNA729271	stage_E14_vs_E10	ordinary_muscle_development	ENSGALG00010001486	WNT2	0.293042867	0.266671369	1.098891374	0.27181545	0.381816546	positive
PRJNA729271	stage_E14_vs_E10	ordinary_muscle_development	ENSGALG00010001487	CDH19	-0.494259797	0.233257204	-2.118947615	0.034094894	0.068402144	negative
PRJNA729271	stage_E14_vs_E10	ordinary_muscle_development	ENSGALG00010001488	GRPR	-3.087166015	0.391814134	-7.879159389	3.29591E-15	4.73012E-14	negative
PRJNA729271	stage_E14_vs_E10	ordinary_muscle_development	ENSGALG00010001491	PHF3	-0.135843808	0.088201999	-1.540144331	0.123525176	0.202201855	negative
PRJNA729271	stage_E14_vs_E10	ordinary_muscle_development	ENSGALG00010001492	ENSGALG00010001492	-0.056645385	0.308504811	-0.183612646	0.854317342	0.899101856	negative
PRJNA729271	stage_E14_vs_E10	ordinary_muscle_development	ENSGALG00010001495	CCNL1	-0.030088552	0.109349083	-0.27516053	0.783192909	0.847003001	negative
PRJNA729271	stage_E14_vs_E10	ordinary_muscle_development	ENSGALG00010001496	PTP4A1	-0.196274469	0.190430097	-1.030690377	0.302686039	0.416344101	negative
PRJNA729271	stage_E14_vs_E10	ordinary_muscle_development	ENSGALG00010001497	LEKR1	-1.124789231	0.275252781	-4.086386433	4.38144E-05	0.000184084	negative
PRJNA729271	stage_E14_vs_E10	ordinary_muscle_development	ENSGALG00010001499	TIPARP	-0.20524294	0.187496955	-1.094646793	0.273671407	0.383865594	negative
PRJNA729271	stage_E14_vs_E10	ordinary_muscle_development	ENSGALG00010001501	CAPZA2	-0.06348934	0.106446999	-0.596440865	0.550880748	0.655799642	negative
PRJNA729271	stage_E14_vs_E10	ordinary_muscle_development	ENSGALG00010001502	LGSN	2.465366519	0.902980534	2.730254337	0.006328548	0.015981238	positive
PRJNA729271	stage_E14_vs_E10	ordinary_muscle_development	ENSGALG00010001504	RBMS3	-0.154025882	0.098675091	-1.560939853	0.118537943	0.195740201	negative
PRJNA729271	stage_E14_vs_E10	ordinary_muscle_development	ENSGALG00010001505	SSR3	0.117352513	0.096542595	1.215551669	0.224155755	0.327237151	positive
PRJNA729271	stage_E14_vs_E10	ordinary_muscle_development	ENSGALG00010001506	DPYSL4	2.36834243	0.243118194	9.741526923	2.00518E-22	5.15339E-21	positive
PRJNA729271	stage_E14_vs_E10	ordinary_muscle_development	ENSGALG00010001507	METTL4	-0.022124557	0.092230855	-0.239882375	0.810421444	0.866813669	negative
PRJNA729271	stage_E14_vs_E10	ordinary_muscle_development	ENSGALG00010001508	CD109	-2.766687234	0.29241744	-9.461430332	3.03759E-21	7.14771E-20	negative
PRJNA729271	stage_E14_vs_E10	ordinary_muscle_development	ENSGALG00010001510	AP1S2	-0.244391891	0.097858666	-2.497396506	0.012510898	0.028996716	negative
PRJNA729271	stage_E14_vs_E10	ordinary_muscle_development	ENSGALG00010001512	PLCL2	0.943916171	0.173413555	5.443151034	5.23462E-08	3.46149E-07	positive
PRJNA729271	stage_E14_vs_E10	ordinary_muscle_development	ENSGALG00010001513	ZRSR2	-0.078770509	0.092195568	-0.854384991	0.39289171	0.508342884	negative
PRJNA729271	stage_E14_vs_E10	ordinary_muscle_development	ENSGALG00010001514	MET	0.239127436	0.151962987	1.573589996	0.115582268	0.192000851	positive
PRJNA729271	stage_E14_vs_E10	ordinary_muscle_development	ENSGALG00010001516	CLTRN	-0.405262591	0.491722559	-0.824169208	0.409843421	0.524748731	negative
PRJNA729271	stage_E14_vs_E10	ordinary_muscle_development	ENSGALG00010001517	CAV2	0.982962989	0.164892236	5.961244841	2.50324E-09	1.96344E-08	positive
PRJNA729271	stage_E14_vs_E10	ordinary_muscle_development	ENSGALG00010001518	SLC17A5	-0.559752591	0.165124055	-3.389891264	0.000699204	0.002287284	negative
PRJNA729271	stage_E14_vs_E10	ordinary_muscle_development	ENSGALG00010001520	KCNAB1	0.545472335	0.185616777	2.938701682	0.003295901	0.009024196	positive
PRJNA729271	stage_E14_vs_E10	ordinary_muscle_development	ENSGALG00010001521	TBC1D5	-0.333573493	0.073945012	-4.511102046	6.44917E-06	3.12749E-05	negative
PRJNA729271	stage_E14_vs_E10	ordinary_muscle_development	ENSGALG00010001524	ACE2	-0.905609502	1.65901423	-0.545872052	0.585153891	0.685811274	negative
PRJNA729271	stage_E14_vs_E10	ordinary_muscle_development	ENSGALG00010001525	EEF1A1	0.278459509	0.176693231	1.575948933	0.115037573	0.191311337	positive
PRJNA729271	stage_E14_vs_E10	ordinary_muscle_development	ENSGALG00010001526	PPP1R3A	3.164028608	0.149705357	21.13503939	3.78872E-99	3.92268E-96	positive
PRJNA729271	stage_E14_vs_E10	ordinary_muscle_development	ENSGALG00010001527	STK32C	1.905369164	0.216308666	8.808566017	1.26757E-18	2.42393E-17	positive
PRJNA729271	stage_E14_vs_E10	ordinary_muscle_development	ENSGALG00010001528	NR1D2	-0.244950044	0.183628811	-1.333941246	0.182223113	0.277537461	negative
PRJNA729271	stage_E14_vs_E10	ordinary_muscle_development	ENSGALG00010001531	ENSGALG00010001531	-10.74595401	5.524679758	-1.945081794			negative
PRJNA729271	stage_E14_vs_E10	ordinary_muscle_development	ENSGALG00010001532	ENSGALG00010001532	0.348750494	0.27642924	1.261626643	0.207083165	0.307172839	positive
PRJNA729271	stage_E14_vs_E10	ordinary_muscle_development	ENSGALG00010001533	ENSGALG00010001533	0.04852928	0.14951333	0.324581627	0.745497734	0.817419824	positive
PRJNA729271	stage_E14_vs_E10	ordinary_muscle_development	ENSGALG00010001534	SLITRK3	-2.803926343	0.306221399	-9.156532979	5.35914E-20	1.14573E-18	negative
PRJNA729271	stage_E14_vs_E10	ordinary_muscle_development	ENSGALG00010001535	RAP2B	0.315121256	0.103660494	3.039935892	0.002366285	0.006733388	positive
PRJNA729271	stage_E14_vs_E10	ordinary_muscle_development	ENSGALG00010001540	TRABD	-0.249136431	0.128825807	-1.933901578	0.05312523	0.099695781	negative
PRJNA729271	stage_E14_vs_E10	ordinary_muscle_development	ENSGALG00010001542	MTO1	0.235989974	0.127691512	1.848125767	0.064584151	0.117798826	positive
PRJNA729271	stage_E14_vs_E10	ordinary_muscle_development	ENSGALG00010001545	SELENOO	-0.056599327	0.138205163	-0.409531204	0.682149872	0.767445079	negative
PRJNA729271	stage_E14_vs_E10	ordinary_muscle_development	ENSGALG00010001546	CGAS	-0.595084779	0.161043011	-3.695191575	0.000219721	0.000807315	negative
PRJNA729271	stage_E14_vs_E10	ordinary_muscle_development	ENSGALG00010001547	PWWP2B	0.396042624	0.109702802	3.61014138	0.00030603	0.001093435	positive
PRJNA729271	stage_E14_vs_E10	ordinary_muscle_development	ENSGALG00010001548	AKAP17A	-0.039168313	0.094837551	-0.413004268	0.679603487	0.765649972	negative
PRJNA729271	stage_E14_vs_E10	ordinary_muscle_development	ENSGALG00010001549	ENSGALG00010001549	-0.80803006	0.46692305	-1.730542239	0.083533441	0.14667683	negative
PRJNA729271	stage_E14_vs_E10	ordinary_muscle_development	ENSGALG00010001551	FHIP1B	0.580983671	0.160647476	3.616512909	0.000298598	0.001070008	positive
PRJNA729271	stage_E14_vs_E10	ordinary_muscle_development	ENSGALG00010001552	TUBGCP6	0.043410735	0.107262217	0.404715994	0.685686285	0.770108685	positive

PRJNA729271	stage_E14_vs_E10	ordinary_muscle_development	ENSGALG00010001553	CNGA4	0.08090478	0.397333404	0.203619377	0.83865095	0.887381963	positive
PRJNA729271	stage_E14_vs_E10	ordinary_muscle_development	ENSGALG00010001554	ENSGALG00010001554	-0.087671247	0.182948713	-0.479212158	0.631787706	0.72542481	negative
PRJNA729271	stage_E14_vs_E10	ordinary_muscle_development	ENSGALG00010001556	EPB41L3	0.432397259	0.116846571	3.700555825	0.000215128	0.000792648	positive
PRJNA729271	stage_E14_vs_E10	ordinary_muscle_development	ENSGALG00010001557	THRB	-2.423517069	0.328516641	-7.377151609	1.61712E-13	2.00001E-12	negative
PRJNA729271	stage_E14_vs_E10	ordinary_muscle_development	ENSGALG00010001558	CCKBR	-0.951725402	0.553191321	-1.720427209	0.085354815	0.149279317	negative
PRJNA729271	stage_E14_vs_E10	ordinary_muscle_development	ENSGALG00010001559	HDAC10	-0.915232443	0.122408787	-7.476852465	7.61238E-14	9.7561E-13	negative
PRJNA729271	stage_E14_vs_E10	ordinary_muscle_development	ENSGALG00010001567	INPP5A	0.046080481	0.081586895	0.56480248	0.57220812	0.674157254	positive
PRJNA729271	stage_E14_vs_E10	ordinary_muscle_development	ENSGALG00010001570	SMPD1	0.21206601	0.163523255	1.296855357	0.194680959	0.292815521	positive
PRJNA729271	stage_E14_vs_E10	ordinary_muscle_development	ENSGALG00010001585	ENSGALG00010001585	0.587102976	0.136457379	4.302464119	1.68909E-05	7.61405E-05	positive
PRJNA729271	stage_E14_vs_E10	ordinary_muscle_development	ENSGALG00010001586	ZBTB14	-0.160471753	0.094129961	-1.704789327	0.088233723	0.153355115	negative
PRJNA729271	stage_E14_vs_E10	ordinary_muscle_development	ENSGALG00010001588	ENSGALG00010001588	0.747272577	0.579972806	1.288461406	0.197585395	0.296112521	positive
PRJNA729271	stage_E14_vs_E10	ordinary_muscle_development	ENSGALG00010001595	AKAIN1	-0.013583637	0.273794463	-0.049612532	0.96043116	0.972983622	negative
PRJNA729271	stage_E14_vs_E10	ordinary_muscle_development	ENSGALG00010001599	TBLX1	0.220682494	0.074585362	2.958790946	0.003088485	0.008532037	positive
PRJNA729271	stage_E14_vs_E10	ordinary_muscle_development	ENSGALG00010001604	ENSGALG00010001604	2.436763265	0.444066835	5.487379536	4.0794E-08	2.74389E-07	positive
PRJNA729271	stage_E14_vs_E10	ordinary_muscle_development	ENSGALG00010001605	APBB1	0.589965659	0.111521488	5.290152351	1.22215E-07	7.67547E-07	positive
PRJNA729271	stage_E14_vs_E10	ordinary_muscle_development	ENSGALG00010001606	PIGA	-0.414464289	0.107608313	-3.851601043	0.000117348	0.000456756	negative
PRJNA729271	stage_E14_vs_E10	ordinary_muscle_development	ENSGALG00010001608	ENSGALG00010001608	-0.302515639	0.476984003	-0.634225963	0.525933366	0.633541439	negative
PRJNA729271	stage_E14_vs_E10	ordinary_muscle_development	ENSGALG00010001609	ENSGALG00010001609	0.980922136	0.457725324	2.143036631	0.032110157	0.064977904	positive
PRJNA729271	stage_E14_vs_E10	ordinary_muscle_development	ENSGALG00010001611	MAPK12	-0.492392097	0.158774611	-3.101201719	0.001927369	0.005639326	negative
PRJNA729271	stage_E14_vs_E10	ordinary_muscle_development	ENSGALG00010001612	HPX	-0.693080126	0.991135546	-0.699278852	0.484377781	0.595964344	negative
PRJNA729271	stage_E14_vs_E10	ordinary_muscle_development	ENSGALG00010001616	OPNVA	1.022928947	0.449199701	2.277225351	0.022772772	0.04871602	positive
PRJNA729271	stage_E14_vs_E10	ordinary_muscle_development	ENSGALG00010001617	TRIM3	-0.026095224	0.09180543	-0.284244882	0.776222723	0.841569368	negative
PRJNA729271	stage_E14_vs_E10	ordinary_muscle_development	ENSGALG00010001620	MOSPD2	0.541138821	0.142987957	3.784506275	0.000154014	0.000585479	positive
PRJNA729271	stage_E14_vs_E10	ordinary_muscle_development	ENSGALG00010001621	DLGAP2	1.43821881	0.231315251	6.217570188	5.04912E-10	4.29077E-09	positive
PRJNA729271	stage_E14_vs_E10	ordinary_muscle_development	ENSGALG00010001627	ARFIP2	-0.09216404	0.117729198	-0.782847765	0.433716515	0.547671476	negative
PRJNA729271	stage_E14_vs_E10	ordinary_muscle_development	ENSGALG00010001628	MAPK11	-0.791934871	0.204971748	-3.863629402	0.000111715	0.00043647	negative
PRJNA729271	stage_E14_vs_E10	ordinary_muscle_development	ENSGALG00010001632	FANCB	1.416827256	0.269446797	5.258282046	1.45407E-07	9.03808E-07	positive
PRJNA729271	stage_E14_vs_E10	ordinary_muscle_development	ENSGALG00010001633	B3GAT2	-1.06627894	0.335448928	-3.178662534	0.001479562	0.00446704	negative
PRJNA729271	stage_E14_vs_E10	ordinary_muscle_development	ENSGALG00010001635	TIMM10B	0.483878699	0.14612365	3.311433149	0.000928194	0.00294531	positive
PRJNA729271	stage_E14_vs_E10	ordinary_muscle_development	ENSGALG00010001639	ENSGALG00010001639	-0.99767581	0.159808376	-6.242950678	4.29392E-10	3.68505E-09	negative
PRJNA729271	stage_E14_vs_E10	ordinary_muscle_development	ENSGALG00010001642	ENSGALG00010001642	0.602586916	0.120529556	4.999495041	5.74807E-07	3.27905E-06	positive
PRJNA729271	stage_E14_vs_E10	ordinary_muscle_development	ENSGALG00010001647	ARL8BL	0.985042151	0.149815557	6.575032473	4.86426E-11	4.66936E-10	positive
PRJNA729271	stage_E14_vs_E10	ordinary_muscle_development	ENSGALG00010001649	UBE2E2	-0.165002241	0.211900973	-0.778676181	0.436170491	0.550103688	negative
PRJNA729271	stage_E14_vs_E10	ordinary_muscle_development	ENSGALG00010001654	RPS11	0.46276345	0.169368787	2.732282951	0.00628971	0.015895808	positive
PRJNA729271	stage_E14_vs_E10	ordinary_muscle_development	ENSGALG00010001657	SMAP1	-0.115082162	0.096310777	-1.194904302	0.232124422	0.33639707	negative
PRJNA729271	stage_E14_vs_E10	ordinary_muscle_development	ENSGALG00010001661	TCTN3	-0.140197745	0.10323103	-1.358096934	0.174432928	0.267811174	negative
PRJNA729271	stage_E14_vs_E10	ordinary_muscle_development	ENSGALG00010001664	SPAM1	5.817647998	1.900462771	3.061174409	0.002204706	0.00633946	positive
PRJNA729271	stage_E14_vs_E10	ordinary_muscle_development	ENSGALG00010001666	PIEZO2	-0.855535805	0.182254661	-4.694177921	2.67681E-06	1.38276E-05	negative
PRJNA729271	stage_E14_vs_E10	ordinary_muscle_development	ENSGALG00010001668	UBE2E1	0.185893791	0.084081541	2.210875165	0.027044483	0.056331406	positive
PRJNA729271	stage_E14_vs_E10	ordinary_muscle_development	ENSGALG00010001670	SHOX	-0.092489728	0.157859118	-0.58590045	0.557942415	0.661760519	negative
PRJNA729271	stage_E14_vs_E10	ordinary_muscle_development	ENSGALG00010001672	ENSGALG00010001672	0.955757701	0.576480314	1.65791906	0.097333804	0.166242402	positive
PRJNA729271	stage_E14_vs_E10	ordinary_muscle_development	ENSGALG00010001674	ENSGALG00010001674	0.105161269	0.103779505	1.01331442	0.310910002	0.424714021	positive
PRJNA729271	stage_E14_vs_E10	ordinary_muscle_development	ENSGALG00010001677	SDHAF4	0.069294517	0.106329676	0.651694988	0.514597956	0.623305538	positive
PRJNA729271	stage_E14_vs_E10	ordinary_muscle_development	ENSGALG00010001678	SYAP1	0.213908188	0.058270606	3.670944941	0.000241655	0.000879878	positive
PRJNA729271	stage_E14_vs_E10	ordinary_muscle_development	ENSGALG00010001681	NAPG	-0.234199869	0.091532571	-2.558650619	0.010507929	0.024842999	negative
PRJNA729271	stage_E14_vs_E10	ordinary_muscle_development	ENSGALG00010001686	WASL	-0.110531048	0.085638806	-1.290665448	0.196819711	0.29530087	negative
PRJNA729271	stage_E14_vs_E10	ordinary_muscle_development	ENSGALG00010001688	ENSGALG00010001688	0.182540706	0.164054394	1.112684039	0.265844153	0.374736069	positive
PRJNA729271	stage_E14_vs_E10	ordinary_muscle_development	ENSGALG00010001689	ILK	0.072596971	0.066753401	1.087539675	0.276798375	0.387389441	positive
PRJNA729271	stage_E14_vs_E10	ordinary_muscle_development	ENSGALG00010001691	CTPS2	0.837312127	0.132741938	6.307819054	2.82995E-10	2.47407E-09	positive
PRJNA729271	stage_E14_vs_E10	ordinary_muscle_development	ENSGALG00010001692	ALDH18A1	-0.247082722	0.254041755	-0.972606738	0.330748792	0.445516564	negative
PRJNA729271	stage_E14_vs_E10	ordinary_muscle_development	ENSGALG00010001693	NKIRAS1	0.480434657	0.196312021	2.447301265	0.014393048	0.032775203	positive
PRJNA729271	stage_E14_vs_E10	ordinary_muscle_development	ENSGALG00010001694	ENSGALG00010001694	-0.05671402	0.212917497	-0.266366177	0.789957211	0.852410465	negative
PRJNA729271	stage_E14_vs_E10	ordinary_muscle_development	ENSGALG00010001695	LMOD2	3.241577899	0.468806694	6.914529895	4.69417E-12	4.97747E-11	positive
PRJNA729271	stage_E14_vs_E10	ordinary_muscle_development	ENSGALG00010001696	FAM135A	0.024853656	0.090373301	0.275011043	0.783307754	0.847064003	positive
PRJNA729271	stage_E14_vs_E10	ordinary_muscle_development	ENSGALG00010001697	TAF10	0.277288448	0.115100708	2.409094201	0.01599217	0.035855607	positive
PRJNA729271	stage_E14_vs_E10	ordinary_muscle_development	ENSGALG00010001698	RPL15	0.5574197	0.176413012	3.159742542	0.001579086	0.004725197	positive
PRJNA729271	stage_E14_vs_E10	ordinary_muscle_development	ENSGALG00010001699	ASB15	3.061199976	0.257496634	11.88831064	1.36132E-32	6.66634E-31	positive
PRJNA729271	stage_E14_vs_E10	ordinary_muscle_development	ENSGALG00010001700	ENSGALG00010001700	0.815449909	0.201115807	4.054628638	5.0214E-05	0.000208733	positive
PRJNA729271	stage_E14_vs_E10	ordinary_muscle_development	ENSGALG00010001701	NDUFA5	0.795093825	0.16938824	4.693913979	2.68027E-06	1.38406E-05	positive
PRJNA729271	stage_E14_vs_E10	ordinary_muscle_development	ENSGALG00010001702	PLEKHA1	0.174440006	0.082642407	2.110780813	0.034791156	0.069539135	positive
PRJNA729271	stage_E14_vs_E10	ordinary_muscle_development	ENSGALG00010001704	ENSGALG00010001704	-0.09072232	0.14530323	-0.624365475	0.532387594	0.639738913	negative
PRJNA729271	stage_E14_vs_E10	ordinary_muscle_development	ENSGALG00010001705	HTRA1	0.853951482	0.154863064	5.514236009	3.50298E-08	2.3872E-07	positive
PRJNA729271	stage_E14_vs_E10	ordinary_muscle_development	ENSGALG00010001706	IQUB	0.453702227	0.357895504	1.26769468	0.204907005	0.30477678	positive
PRJNA729271	stage_E14_vs_E10	ordinary_muscle_development	ENSGALG00010001707	ENSGALG00010001707	0.137702113	1.219534926	0.112913628	0.910099032	0.93805628	positive
PRJNA729271	stage_E14_vs_E10	ordinary_muscle_development	ENSGALG00010001708	AADAC	-0.588393851	0.269968486	-2.179490872	0.029295222	0.060220524	negative
PRJNA729271	stage_E14_vs_E10	ordinary_muscle_development	ENSGALG00010001710	LCMT2	-0.226578387	0.077276464	-2.932049084	0.003367335	0.00919158	negative
PRJNA729271	stage_E14_vs_E10	ordinary_muscle_development	ENSGALG00010001711	CREB5	-0.963876775	0.175836696	-5.481658812	4.21356E-08	2.82627E-07	negative
PRJNA729271	stage_E14_vs_E10	ordinary_muscle_development	ENSGALG00010001712	ATP6AP1	0.137062014	0.15931192	0.860337466	0.389603046	0.505228488	positive
PRJNA729271	stage_E14_vs_E10	ordinary_muscle_development	ENSGALG00010001713	PNPLA4	-0.546902863	0.228448148	-2.393991236	0.016666147	0.037182669	negative
PRJNA729271	stage_E14_vs_E10	ordinary_muscle_development	ENSGALG00010001716	TRIL	-1.32879006	0.151285938	-8.783301849	1.58744E-18	3.02365E-17	negative
PRJNA729271	stage_E14_vs_E10	ordinary_muscle_development	ENSGALG00010001717	STARD10	-2.298634173	0.690968293	-3.326685458	0.000878855	0.002804713	negative
PRJNA729271	stage_E14_vs_E10	ordinary_muscle_development	ENSGALG00010001721	TM4SF1	0.486961217	0.160323604	3.037364468	0.002386567	0.006780339	positive
PRJNA729271	stage_E14_vs_E10	ordinary_muscle_development	ENSGALG00010001725	MTA2	-0.439011164	0.185667683	-2.36449961	0.018054446	0.039798773	negative
PRJNA729271	stage_E14_vs_E10	ordinary_muscle_development	ENSGALG00010001727	COL9A1	-4.770217855	0.773428196	-6.167628594	6.93217E-10	5.78479E-09	negative
PRJNA729271	stage_E14_vs_E10	ordinary_muscle_development	ENSGALG00010001728	STS	-0.210496213	0.324980342	-0.647719835	0.517166173	0.625632088	negative
PRJNA729271	stage_E14_vs_E10	ordinary_muscle_development	ENSGALG00010001729	ENSGALG00010001729	0.613731328	0.247441771	2.480306078	0.013126965	0.030226426	positive
PRJNA729271	stage_E14_vs_E10	ordinary_muscle_development	ENSGALG00010001731	ENSGALG00010001731	0.121632337	0.197265422	0.616592284	0.537503685	0.644000324	positive
PRJNA729271	stage_E14_vs_E10	ordinary_muscle_development	ENSGALG00010001738	PUDP	-0.548747143	0.124537948	-4.40626452	1.05169E-05	4.91431E-05	negative
PRJNA729271	stage_E14_vs_E10	ordinary_muscle_development	ENSGALG00010001741	SATB1	-1.257908025	0.199499877	-6.305307284	2.87623E-10	2.50999E-09	negative
PRJNA729271	stage_E14_vs_E10	ordinary_muscle_development	ENSGALG00010001745	CADPS2	-0.832428497	0.10317652	-8.068003237	7.14572E-16	1.08344E-14	negative
PRJNA729271	stage_E14_vs_E10	ordinary_muscle_development	ENSGALG00010001749	VAPA	0.628886173	0.105986395	5.93365002	2.96273E-09	2.30514E-08	positive
PRJNA729271	stage_E14_vs_E10	ordinary_muscle_development	ENSGALG00010001751	AVPR2C	-1.265193672	0.436530905	-2.898291177	0.003752021	0.01010508	negative
PRJNA729271	stage_E14_vs_E10	ordinary_muscle_development	ENSGALG00010001752	ARRB1	0.466543379	0.108603107	4.295856659	1.7402E-05	7.8336E-05	positive
PRJNA729271	stage_E14_vs_E10	ordinary_muscle_development	ENSGALG00010001753	PNPLA8	0.291869926	0.204404758	1.427901824	0.153320119	0.241667586	positive
PRJNA729271	stage_E14_vs_E10	ordinary_muscle_development	ENSGALG00010001754	APCDD1	-2.775614639	0.189472345	-14.64918082	1.3635E-48	1.82999E-46	negative
PRJNA729271	stage_E14_vs_E10	ordinary_muscle_development	ENSGALG00010001755	CUZD1	-1.447740419	1.548644432	-0.934843654	0.349868879	0.465859764	negative
PRJNA729271	stage_E14_vs_E10	ordinary_muscle_development	ENSGALG00010001756	ENSGALG00010001756	0.077110341	0.218587377	0.352766671	0.724263372	0.800298433	positive
PRJNA729271	stage_E14_vs_E10	ordinary_muscle_development	ENSGALG00010001758	COL19A1	2.791655324	0.276281604	10.10438367	5.28267E-24	1.4613E-22	positive
PRJNA729271	stage_E14_vs_E10	ordinary_muscle_development	ENSGALG00010001759	C10orf88	-0.218364449	0.088074655	-2.479310857	0.013163653	0.030301277	negative
PRJNA729271	stage_E14_vs_E10	ordinary_muscle_development	ENSGALG00010001760	RAB5A	-0.064028033	0.095708798	-0.668987956	0.503503161	0.612785753	negative
PRJNA729271	stage_E14_vs_E10	ordinary_muscle_development	ENSGALG00010001761	ELOB	0.229358973	0.148752053	1.541887779	0.123100866	0.201643921	positive
PRJNA729271	stage_E14_vs_E10	ordinary_muscle_development	ENSGALG00010001763	ENSGALG00010001763	-0.545009161	0.269179507	-2.024705254	0.042897639	0.083184117	negative
PRJNA729271	stage_E14_vs_E10	ordinary_muscle_development	ENSGALG00010001764	ENSGALG00010001764	-0.557426283	0.434999854	-1.281440164	0.200039105	0.299109431	negative
PRJNA729271	stage_E14_vs_E10	ordinary_muscle_development	ENSGALG00010001767	ENSGALG00010001767	-0.590793991	0.179247366	-3.295970278	0.000980824	0.003094025	negative
PRJNA729271	stage_E14_vs_E10	ordinary_muscle_development	ENSGALG00010001771	ENSGALG00010001771	0.174271908	0.134689446	1.293879461	0.19570706	0.293996667	positive
PRJNA729271	stage_E14_vs_E10	ordinary_muscle_development	ENSGALG00010001773	NRCAM	-0.650552564	0.23228945	-2.800611758	0.005100584	0.013240145	negative
PRJNA729271	stage_E14_vs_E10	ordinary_muscle_development	ENSGALG00010001776	LMBRD1	0.710202251	0.131253406	5.410924366	6.27003E-08	4.11054E-07	positive
PRJNA729271	stage_E14_vs_E10	ordinary_muscle_development	ENSGALG00010001778	KAT2B	0.696822559	0.148628685	4.688345036	2.75423E-06	1.41771E-05	positive
PRJNA729271	stage_E14_vs_E10	ordinary_muscle_development	ENSGALG00010001779	ENSGALG00010001779	0.251718802	0.605135224	0.415971161	0.677431106	0.76367739	positive
PRJNA729271	stage_E14_vs_E10	ordinary_muscle_development	ENSGALG00010001780	ENSGALG00010001780	0.186069611	0.237214124	0.784395161	0.432808277	0.54690576	positive
PRJNA729271	stage_E14_vs_E10	ordinary_muscle_development	ENSGALG00010001783	SGO1	-0.475616001	0.243046479	-1.956893198	0.050360026	0.095321048	negative
PRJNA729271	stage_E14_vs_E10	ordinary_muscle_development	ENSGALG00010001785	CNTN1	0.094615966	0.212742134	0.444744838	0.656504162	0.746823719	positive
PRJNA729271	stage_E14_vs_E10	ordinary_muscle_development	ENSGALG00010001786	P2RY8	-0.55739858	0.20807721	-2.678806487	0.007388508	0.018269605	negative
PRJNA729271	stage_E14_vs_E10	ordinary_muscle_development	ENSGALG00010001788	HOXA1	-0.813354935	0.351740952	-2.312369171	0.020757348	0.044900426	negative
PRJNA729271	stage_E14_vs_E10	ordinary_muscle_development	ENSGALG00010001790	HOXA2	-1.458003272	0.234271847	-6.223553074	4.8602E-10	4.14892E-09	negative
PRJNA729271	stage_E14_vs_E10	ordinary_muscle_development	ENSGALG00010001793	IKZF5	0.075910004	0.103441355	0.733845801	0.463042722	0.575675809	positive
PRJNA729271	stage_E14_vs_E10	ordinary_muscle_development	ENSGALG00010001794	ENSGALG00010001794	-1.484212619	0.544015833	-2.728252615	0.006367083	0.016058964	negative
PRJNA729271	stage_E14_vs_E10	ordinary_muscle_development	ENSGALG00010001795	ENSGALG00010001795	0.812955084	0.479658492	1.694862278	0.09010155	0.155924304	positive
PRJNA729271	stage_E14_vs_E10	ordinary_muscle_development	ENSGALG00010001796	SLCO2B1	0.555298974	0.158040136	3.513657918	0.000441982	0.001516337	positive
PRJNA729271	stage_E14_vs_E10	ordinary_muscle_development	ENSGALG00010001798	PSTK	0.337358604	0.127189232	2.652414825	0.007991829	0.019610895	positive
PRJNA729271	stage_E14_vs_E10	ordinary_muscle_development	ENSGALG00010001800	ENSGALG00010001800	-0.329410675	0.19023638	-1.73158612	0.083347279	0.146438644	negative
PRJNA729271	stage_E14_vs_E10	ordinary_muscle_development	ENSGALG00010001801	HOXA3	-1.347242407	0.142975091	-9.422916968	4.38727E-21	1.01913E-19	negative
PRJNA729271	stage_E14_vs_E10	ordinary_muscle_development	ENSGALG00010001803	PPP4R1	0.036467234	0.095808914	0.380624647	0.70348179	0.784441623	positive
PRJNA729271	stage_E14_vs_E10	ordinary_muscle_development	ENSGALG00010001805	ENSGALG00010001805	0.115276006	0.263456	0.437553164	0.661710229	0.751036706	positive
PRJNA729271	stage_E14_vs_E10	ordinary_muscle_development	ENSGALG00010001806	ENSGALG00010001806	2.113681602	0.91969587	2.298239744	0.021548148	0.04642396	positive
PRJNA729271	stage_E14_vs_E10	ordinary_muscle_development	ENSGALG00010001807	ASMTL	0.132811029	0.102345496	1.297673414	0.194399583	0.292508711	positive
PRJNA729271	stage_E14_vs_E10	ordinary_muscle_development	ENSGALG00010001808	HOXA4	-1.307007156	0.14761107	-8.854397968	8.41367E-19	1.62825E-17	negative
PRJNA729271	stage_E14_vs_E10	ordinary_muscle_development	ENSGALG00010001811	ACADSB	0.249856789	0.148731761	1.679915488	0.09297376	0.160073008	positive
PRJNA729271	stage_E14_vs_E10	ordinary_muscle_development	ENSGALG00010001813	HOXA5	-0.524482584	0.101839088	-5.150110785	2.60333E-07	1.56643E-06	negative
PRJNA729271	stage_E14_vs_E10	ordinary_muscle_development	ENSGALG00010001817	SLC25A6	-0.13737063	0.112839496	-1.21739847	0.223452645	0.326375059	negative
PRJNA729271	stage_E14_vs_E10	ordinary_muscle_development	ENSGALG00010001818	ADGRB3	-1.536156692	0.236121541	-6.505788014	7.72872E-11	7.21363E-10	negative
PRJNA729271	stage_E14_vs_E10	ordinary_muscle_development	ENSGALG00010001819	HOXA6	0.525550858	0.141609419	3.711270482	0.000206222	0.000763325	positive
PRJNA729271	stage_E14_vs_E10	ordinary_muscle_development	ENSGALG00010001820	PGM2L1	0.009016981	0.15877471	0.056791039	0.954711653	0.96886131	positive
PRJNA729271	stage_E14_vs_E10	ordinary_muscle_development	ENSGALG00010001822	KCNE3	-0.865166618	0.348348556	-2.483623377	0.013005327	0.029998762	negative
PRJNA729271	stage_E14_vs_E10	ordinary_muscle_development	ENSGALG00010001823	RALBP1	-0.011581431	0.075605983	-0.15318141	0.878255217	0.916428798	negative
PRJNA729271	stage_E14_vs_E10	ordinary_muscle_development	ENSGALG00010001825	LIPT2	0.613311051	0.145718094	4.208887402	2.56631E-05	0.000112247	positive
PRJNA729271	stage_E14_vs_E10	ordinary_muscle_development	ENSGALG00010001826	HOXA7	0.649134245	0.133119376	4.876331792	1.08077E-06	5.94525E-06	positive
PRJNA729271	stage_E14_vs_E10	ordinary_muscle_development	ENSGALG00010001827	PDZRN4	-1.613425306	0.176079963	-9.163026157	5.0462E-20	1.08523E-18	negative
PRJNA729271	stage_E14_vs_E10	ordinary_muscle_development	ENSGALG00010001828	ENSGALG00010001828	-2.758939244	1.081834386	-2.550241775	0.010764824	0.025396504	negative
PRJNA729271	stage_E14_vs_E10	ordinary_muscle_development	ENSGALG00010001829	POLD3	0.002977999	0.120897975	0.024632333	0.980348229	0.986987053	positive
PRJNA729271	stage_E14_vs_E10	ordinary_muscle_development	ENSGALG00010001831	CSF2RA	0.162369658	0.254169245	0.638824963	0.522936807	0.630875491	positive

PRJNA729271	stage_E14_vs_E10	ordinary_muscle_development	ENSGALG00010001832	BUB3	-0.207145326	0.085218572	-2.430753306	0.01506747	0.034082087	negative
PRJNA729271	stage_E14_vs_E10	ordinary_muscle_development	ENSGALG00010001834	ZCRB1	0.138028132	0.08298042	1.663381932	0.096236008	0.164750316	positive
PRJNA729271	stage_E14_vs_E10	ordinary_muscle_development	ENSGALG00010001835	MECP2	0.568902369	0.189362716	3.004299802	0.002661929	0.007477647	positive
PRJNA729271	stage_E14_vs_E10	ordinary_muscle_development	ENSGALG00010001836	TWSG1	-0.577430088	0.139767608	-4.131358453	3.60626E-05	0.000153653	negative
PRJNA729271	stage_E14_vs_E10	ordinary_muscle_development	ENSGALG00010001837	TAB3	0.094595893	0.13879962	0.681528468	0.49553716	0.605123095	positive
PRJNA729271	stage_E14_vs_E10	ordinary_muscle_development	ENSGALG00010001838	PPHLN1	-0.199041192	0.060975461	-3.264283498	0.001097413	0.003422332	negative
PRJNA729271	stage_E14_vs_E10	ordinary_muscle_development	ENSGALG00010001839	ENSGALG00010001839	-0.120039688	0.752258355	-0.159572422	0.873217904	0.91296116	negative
PRJNA729271	stage_E14_vs_E10	ordinary_muscle_development	ENSGALG00010001840	HOXA9	0.148274557	0.144012884	1.029592303	0.30320143	0.416882518	positive
PRJNA729271	stage_E14_vs_E10	ordinary_muscle_development	ENSGALG00010001842	GPR26	-0.819870889	0.510283167	-1.606697891	0.108120657	0.181705446	negative
PRJNA729271	stage_E14_vs_E10	ordinary_muscle_development	ENSGALG00010001843	PRICKLE1	-0.946410584	0.120528017	-7.852204073	4.08789E-15	5.79784E-14	negative
PRJNA729271	stage_E14_vs_E10	ordinary_muscle_development	ENSGALG00010001844	HOXA10	-0.284170115	0.160131266	-1.774607306	0.07596275	0.13535096	negative
PRJNA729271	stage_E14_vs_E10	ordinary_muscle_development	ENSGALG00010001845	ANKRD12	0.602196957	0.133738455	4.502795825	6.70653E-06	3.24362E-05	positive
PRJNA729271	stage_E14_vs_E10	ordinary_muscle_development	ENSGALG00010001848	HOXA11	-0.208002876	0.354192337	-0.587259671	0.557029322	0.66116443	negative
PRJNA729271	stage_E14_vs_E10	ordinary_muscle_development	ENSGALG00010001849	NDUFV2	0.66185525	0.0943296	7.016411055	2.2764E-12	2.51114E-11	positive
PRJNA729271	stage_E14_vs_E10	ordinary_muscle_development	ENSGALG00010001850	MYOM2	2.684129327	0.18781032	14.29170308	2.46506E-46	2.85849E-44	positive
PRJNA729271	stage_E14_vs_E10	ordinary_muscle_development	ENSGALG00010001852	CPXM2	-1.198698467	0.380809201	-3.147766557	0.00164523	0.004903889	negative
PRJNA729271	stage_E14_vs_E10	ordinary_muscle_development	ENSGALG00010001853	ENSGALG00010001853	0.140811204	0.188929039	0.745312656	0.456082732	0.569219838	positive
PRJNA729271	stage_E14_vs_E10	ordinary_muscle_development	ENSGALG00010001854	CHRDL2	0.559757055	0.293489051	1.907250211	0.056488194	0.105068186	positive
PRJNA729271	stage_E14_vs_E10	ordinary_muscle_development	ENSGALG00010001856	CHST15	-0.678360758	0.116216352	-5.837050839	5.31329E-09	4.005E-08	negative
PRJNA729271	stage_E14_vs_E10	ordinary_muscle_development	ENSGALG00010001857	HNRNPA2B1	0.004345356	0.187518267	0.023172974	0.981512297	0.987609557	positive
PRJNA729271	stage_E14_vs_E10	ordinary_muscle_development	ENSGALG00010001858	ADAMTS20	1.290057547	0.160322307	8.046650363	8.50908E-16	1.28612E-14	positive
PRJNA729271	stage_E14_vs_E10	ordinary_muscle_development	ENSGALG00010001860	RNF169	0.17731104	0.127242772	1.393486152	0.16347283	0.25421507	positive
PRJNA729271	stage_E14_vs_E10	ordinary_muscle_development	ENSGALG00010001861	CBX3	-0.151245991	0.073783117	-2.049872612	0.040376863	0.078940199	negative
PRJNA729271	stage_E14_vs_E10	ordinary_muscle_development	ENSGALG00010001864	ENSGALG00010001864	0.355594296	0.193941001	1.833517897	0.06672559	0.121019447	positive
PRJNA729271	stage_E14_vs_E10	ordinary_muscle_development	ENSGALG00010001865	CYBB	0.757422199	0.316918296	2.389960468	0.016850186	0.037535493	positive
PRJNA729271	stage_E14_vs_E10	ordinary_muscle_development	ENSGALG00010001866	PUS7L	-0.278040116	0.11746983	-2.366906599	0.017937459	0.039574348	negative
PRJNA729271	stage_E14_vs_E10	ordinary_muscle_development	ENSGALG00010001868	ENSGALG00010001868	0.205230557	0.120938523	1.696982509	0.089699967	0.155359186	positive
PRJNA729271	stage_E14_vs_E10	ordinary_muscle_development	ENSGALG00010001869	CRLF2	1.356167031	1.205753172	1.124746807	0.260696341	0.368950738	positive
PRJNA729271	stage_E14_vs_E10	ordinary_muscle_development	ENSGALG00010001870	PLP2	1.174690799	0.164069609	7.159709891	8.0848E-13	9.33035E-12	positive
PRJNA729271	stage_E14_vs_E10	ordinary_muscle_development	ENSGALG00010001871	ENSGALG00010001871	0.287069811	0.121548776	2.361766371	0.0181881	0.040054165	positive
PRJNA729271	stage_E14_vs_E10	ordinary_muscle_development	ENSGALG00010001873	ENSGALG00010001873	-0.308839514	0.20072564	-1.538615165	0.123898274	0.202766793	negative
PRJNA729271	stage_E14_vs_E10	ordinary_muscle_development	ENSGALG00010001877	OAT	0.329752494	0.136643594	2.413230545	0.015811814	0.03551166	positive
PRJNA729271	stage_E14_vs_E10	ordinary_muscle_development	ENSGALG00010001878	XK	-0.172420205	0.182328481	-0.945657006	0.344323558	0.459573662	negative
PRJNA729271	stage_E14_vs_E10	ordinary_muscle_development	ENSGALG00010001879	IRAK4	-0.319443502	0.166668146	-1.916644	0.055283174	0.103171058	negative
PRJNA729271	stage_E14_vs_E10	ordinary_muscle_development	ENSGALG00010001880	MTCL1	1.028397099	0.144347286	7.124464402	1.04486E-12	1.18786E-11	positive
PRJNA729271	stage_E14_vs_E10	ordinary_muscle_development	ENSGALG00010001883	KBTBD11	-0.938659265	0.087870515	-10.68230071	1.2318E-26	4.26133E-25	negative
PRJNA729271	stage_E14_vs_E10	ordinary_muscle_development	ENSGALG00010001885	SMPX	1.048705293	0.372203459	2.817559232	0.004839019	0.0126518	positive
PRJNA729271	stage_E14_vs_E10	ordinary_muscle_development	ENSGALG00010001886	NEU3	-0.168279656	0.082985107	-2.027829589	0.042577643	0.082663132	negative
PRJNA729271	stage_E14_vs_E10	ordinary_muscle_development	ENSGALG00010001888	SNX10	2.239658569	0.198532884	11.28104585	1.62799E-29	6.82015E-28	positive
PRJNA729271	stage_E14_vs_E10	ordinary_muscle_development	ENSGALG00010001890	KLHL34	1.591900472	0.258129862	6.167052732	6.95746E-10	5.80255E-09	positive
PRJNA729271	stage_E14_vs_E10	ordinary_muscle_development	ENSGALG00010001892	RAB12	0.567586581	0.070662122	8.032402145	9.55825E-16	1.43721E-14	positive
PRJNA729271	stage_E14_vs_E10	ordinary_muscle_development	ENSGALG00010001895	P4HA3	-0.013542056	0.239285031	-0.056593829	0.95486875	0.96886131	negative
PRJNA729271	stage_E14_vs_E10	ordinary_muscle_development	ENSGALG00010001896	ARHGEF10	0.313064259	0.133425561	2.346358942	0.018957841	0.041465806	positive
PRJNA729271	stage_E14_vs_E10	ordinary_muscle_development	ENSGALG00010001897	TLR7	0.557039632	0.181002048	3.077532209	0.002087223	0.006039987	positive
PRJNA729271	stage_E14_vs_E10	ordinary_muscle_development	ENSGALG00010001898	LHPP	0.112086318	0.131056016	0.855255035	0.392409974	0.507930403	positive
PRJNA729271	stage_E14_vs_E10	ordinary_muscle_development	ENSGALG00010001899	TWF1	-0.162083351	0.111944631	-1.447888563	0.147648219	0.234358402	negative
PRJNA729271	stage_E14_vs_E10	ordinary_muscle_development	ENSGALG00010001901	PRPS2	2.196833937	0.18129226	12.11763778	8.51789E-34	4.40953E-32	positive
PRJNA729271	stage_E14_vs_E10	ordinary_muscle_development	ENSGALG00010001902	WDR45	0.273091655	0.140943172	1.937601176	0.052671899	0.098947534	positive
PRJNA729271	stage_E14_vs_E10	ordinary_muscle_development	ENSGALG00010001903	NARS2	-0.061122522	0.114383849	-0.534363227	0.593090256	0.69301437	negative
PRJNA729271	stage_E14_vs_E10	ordinary_muscle_development	ENSGALG00010001906	SKAP2	0.008043625	0.126980691	0.063345261	0.949491575	0.965113501	positive
PRJNA729271	stage_E14_vs_E10	ordinary_muscle_development	ENSGALG00010001908	FAM83B	-1.586383483	0.364540169	-4.351738483	1.35062E-05	6.20157E-05	negative
PRJNA729271	stage_E14_vs_E10	ordinary_muscle_development	ENSGALG00010001910	FAM53B	0.534589587	0.093751398	5.702203906	1.18268E-08	8.53734E-08	positive
PRJNA729271	stage_E14_vs_E10	ordinary_muscle_development	ENSGALG00010001911	USP35	-1.72574423	0.373092996	-4.625506907	3.73683E-06	1.87748E-05	negative
PRJNA729271	stage_E14_vs_E10	ordinary_muscle_development	ENSGALG00010001912	TMEM117	1.794571298	0.209542616	8.564230676	1.088E-17	1.93741E-16	positive
PRJNA729271	stage_E14_vs_E10	ordinary_muscle_development	ENSGALG00010001914	ENSGALG00010001914	0.432526294	0.148281465	2.916927577	0.003534978	0.009588232	positive
PRJNA729271	stage_E14_vs_E10	ordinary_muscle_development	ENSGALG00010001915	EEF1AKMT2	-0.255431518	0.163743455	-1.559949479	0.118771821	0.196014749	negative
PRJNA729271	stage_E14_vs_E10	ordinary_muscle_development	ENSGALG00010001918	CNKSR2	1.882090402	0.141301778	13.31965125	1.77937E-40	1.39416E-38	positive
PRJNA729271	stage_E14_vs_E10	ordinary_muscle_development	ENSGALG00010001921	ENSGALG00010001921	-0.011829556	0.273862996	-0.04319516	0.965545963	0.977134453	negative
PRJNA729271	stage_E14_vs_E10	ordinary_muscle_development	ENSGALG00010001923	RAPGEF5	0.337233616	0.102196726	3.299847557	0.000967374	0.003056917	positive
PRJNA729271	stage_E14_vs_E10	ordinary_muscle_development	ENSGALG00010001925	FRMPD4	0.46994873	0.169772449	2.768109512	0.005638251	0.014457181	positive
PRJNA729271	stage_E14_vs_E10	ordinary_muscle_development	ENSGALG00010001928	KCTD21	-0.962160501	0.172234743	-5.586332244	2.31915E-08	1.61306E-07	negative
PRJNA729271	stage_E14_vs_E10	ordinary_muscle_development	ENSGALG00010001929	POLR2I	0.391684599	0.119648549	3.273625978	0.00106177	0.003325489	positive
PRJNA729271	stage_E14_vs_E10	ordinary_muscle_development	ENSGALG00010001935	ABRAXAS2	-0.035639281	0.129088606	-0.276083864	0.782483654	0.846488586	negative
PRJNA729271	stage_E14_vs_E10	ordinary_muscle_development	ENSGALG00010001938	ALG8	-0.206785913	0.097161535	-2.128269302	0.033314762	0.06702255	negative
PRJNA729271	stage_E14_vs_E10	ordinary_muscle_development	ENSGALG00010001940	PTPRM	-0.198388746	0.110995334	-1.787361134	0.073879139	0.132104027	negative
PRJNA729271	stage_E14_vs_E10	ordinary_muscle_development	ENSGALG00010001947	TINAG	4.116514028	0.305340718	13.48170677	2.00416E-41	1.67921E-39	positive
PRJNA729271	stage_E14_vs_E10	ordinary_muscle_development	ENSGALG00010001948	EMX2	-1.77018327	0.249883445	-7.084035794	1.40016E-12	1.57206E-11	negative
PRJNA729271	stage_E14_vs_E10	ordinary_muscle_development	ENSGALG00010001952	ENSGALG00010001952	0.275036819	0.071406594	3.851700582	0.0001173	0.000456693	positive
PRJNA729271	stage_E14_vs_E10	ordinary_muscle_development	ENSGALG00010001957	NDUFC2	0.382223493	0.11174359	3.42054067	0.000624968	0.002064822	positive
PRJNA729271	stage_E14_vs_E10	ordinary_muscle_development	ENSGALG00010001958	RAB11FIP2	0.169583684	0.111423758	1.521970602	0.128016447	0.208328274	positive
PRJNA729271	stage_E14_vs_E10	ordinary_muscle_development	ENSGALG00010001966	ARHGAP6	0.600353205	0.140962708	4.258950573	2.05389E-05	9.11826E-05	positive
PRJNA729271	stage_E14_vs_E10	ordinary_muscle_development	ENSGALG00010001967	LAMA1	-2.141366091	0.229406555	-9.334371863	1.01592E-20	2.29016E-19	negative
PRJNA729271	stage_E14_vs_E10	ordinary_muscle_development	ENSGALG00010001969	PLEKHA8	-0.209891605	0.108754584	-1.929956397	0.053612241	0.100527065	negative
PRJNA729271	stage_E14_vs_E10	ordinary_muscle_development	ENSGALG00010001971	MLIP	2.361452609	0.206905445	11.41319702	3.59277E-30	1.5592E-28	positive
PRJNA729271	stage_E14_vs_E10	ordinary_muscle_development	ENSGALG00010001972	FAM204A	-0.053816468	0.157805745	-0.341029841	0.733081117	0.807815934	negative
PRJNA729271	stage_E14_vs_E10	ordinary_muscle_development	ENSGALG00010001974	ENSGALG00010001974	0.819492556	0.391331294	2.09411455	0.036249777	0.072027488	positive
PRJNA729271	stage_E14_vs_E10	ordinary_muscle_development	ENSGALG00010001976	THRSP	6.508647206	1.427128049	4.560660981	5.09929E-06	2.50982E-05	positive
PRJNA729271	stage_E14_vs_E10	ordinary_muscle_development	ENSGALG00010001982	DMD	1.227174504	0.121895815	10.06740475	7.69826E-24	2.10144E-22	positive
PRJNA729271	stage_E14_vs_E10	ordinary_muscle_development	ENSGALG00010001987	KCTD14	-3.006987715	0.427220799	-7.038486239	1.94339E-12	2.15363E-11	negative
PRJNA729271	stage_E14_vs_E10	ordinary_muscle_development	ENSGALG00010001991	TOMM7	0.390246764	0.130123852	2.999040977	0.002708309	0.007596978	positive
PRJNA729271	stage_E14_vs_E10	ordinary_muscle_development	ENSGALG00010001995	LRRC1	-0.372807103	0.129716831	-2.874007187	0.004052997	0.010815206	negative
PRJNA729271	stage_E14_vs_E10	ordinary_muscle_development	ENSGALG00010001997	INTS4	-0.094593845	0.076014384	-1.24442034	0.213344901	0.314879782	negative
PRJNA729271	stage_E14_vs_E10	ordinary_muscle_development	ENSGALG00010002005	COLQ	1.690707495	0.209192554	8.082063469	6.36799E-16	9.69581E-15	positive
PRJNA729271	stage_E14_vs_E10	ordinary_muscle_development	ENSGALG00010002006	ENSGALG00010002006	0.559431342	0.433276502	1.29116474	0.196646559	0.295116318	positive
PRJNA729271	stage_E14_vs_E10	ordinary_muscle_development	ENSGALG00010002012	MID1	-1.123094258	0.13659467	-8.222094308	1.9998E-16	3.18539E-15	negative
PRJNA729271	stage_E14_vs_E10	ordinary_muscle_development	ENSGALG00010002017	ARHGAP28	-0.276722621	0.117551871	-2.354046937	0.018570266	0.04075337	negative
PRJNA729271	stage_E14_vs_E10	ordinary_muscle_development	ENSGALG00010002022	ERNI	-1.186094773	0.664790234	-1.784163956	0.074397027	0.132891094	negative
PRJNA729271	stage_E14_vs_E10	ordinary_muscle_development	ENSGALG00010002023	CLCN4	-0.489699211	0.243008324	-2.015154064	0.043888509	0.084821858	negative
PRJNA729271	stage_E14_vs_E10	ordinary_muscle_development	ENSGALG00010002024	KLHL31	2.019123633	0.165829486	12.17590234	4.17748E-34	2.18602E-32	positive
PRJNA729271	stage_E14_vs_E10	ordinary_muscle_development	ENSGALG00010002027	ENSGALG00010002027	-0.08292046	0.738225744	-0.112323989	0.910566521	0.93836021	negative
PRJNA729271	stage_E14_vs_E10	ordinary_muscle_development	ENSGALG00010002034	WWC3	-0.267892597	0.098717156	-2.71373902	0.006652856	0.016666633	negative
PRJNA729271	stage_E14_vs_E10	ordinary_muscle_development	ENSGALG00010002039	AAMDC	0.460075995	0.126841277	3.627178839	0.000286535	0.001029579	positive
PRJNA729271	stage_E14_vs_E10	ordinary_muscle_development	ENSGALG00010002041	GCLC	0.06565967	0.138525037	0.473991357	0.63550607	0.729232148	positive
PRJNA729271	stage_E14_vs_E10	ordinary_muscle_development	ENSGALG00010002046	FAM126A	-0.113956011	0.087406984	-1.303740334	0.192322132	0.289722439	negative
PRJNA729271	stage_E14_vs_E10	ordinary_muscle_development	ENSGALG00010002050	ENSGALG00010002050	-0.710228504	1.101170269	-0.644976098	0.518942675	0.62708318	negative
PRJNA729271	stage_E14_vs_E10	ordinary_muscle_development	ENSGALG00010002054	RSF1	-0.318078963	0.099360984	-3.201246119	0.001368346	0.004166844	negative
PRJNA729271	stage_E14_vs_E10	ordinary_muscle_development	ENSGALG00010002056	PRLHR	-1.609944488	0.4252922	-3.785502037	0.000153399	0.000583599	negative
PRJNA729271	stage_E14_vs_E10	ordinary_muscle_development	ENSGALG00010002062	GSTA4	-0.281426694	0.361240589	-0.779056127	0.435946653	0.550103688	negative
PRJNA729271	stage_E14_vs_E10	ordinary_muscle_development	ENSGALG00010002065	CLNS1A	-0.074557753	0.061700584	-1.208380014	0.226901124	0.330313527	negative
PRJNA729271	stage_E14_vs_E10	ordinary_muscle_development	ENSGALG00010002072	NEURL4	0.132357041	0.159719124	0.828686249	0.407281969	0.522141573	positive
PRJNA729271	stage_E14_vs_E10	ordinary_muscle_development	ENSGALG00010002079	ARX	1.983312487	0.189362216	10.47364426	1.14162E-25	3.62889E-24	positive
PRJNA729271	stage_E14_vs_E10	ordinary_muscle_development	ENSGALG00010002084	HACL1	-0.172961478	0.088405468	-1.956456785	0.050411368	0.095405769	negative
PRJNA729271	stage_E14_vs_E10	ordinary_muscle_development	ENSGALG00010002087	POLA1	-0.26560499	0.071648609	-3.707050197	0.000209687	0.000775198	negative
PRJNA729271	stage_E14_vs_E10	ordinary_muscle_development	ENSGALG00010002088	OZFL1	-0.066317479	0.132514237	-0.500455504	0.61675438	0.712736565	negative
PRJNA729271	stage_E14_vs_E10	ordinary_muscle_development	ENSGALG00010002090	AQP11	-0.549186453	0.29446637	-1.865022662	0.062178228	0.114041247	negative
PRJNA729271	stage_E14_vs_E10	ordinary_muscle_development	ENSGALG00010002097	PCYT1B	-2.970785289	0.47691447	-6.229178344	4.68888E-10	4.00739E-09	negative
PRJNA729271	stage_E14_vs_E10	ordinary_muscle_development	ENSGALG00010002098	ENSGALG00010002098	0.522979759	0.414520154	1.261650983	0.207074402	0.307172839	positive
PRJNA729271	stage_E14_vs_E10	ordinary_muscle_development	ENSGALG00010002100	BTD	0.347910728	0.114366675	3.042063862	0.00234962	0.006700324	positive
PRJNA729271	stage_E14_vs_E10	ordinary_muscle_development	ENSGALG00010002102	PAK1	-3.272229273	0.283366614	-11.54768807	7.5833E-31	3.38215E-29	negative
PRJNA729271	stage_E14_vs_E10	ordinary_muscle_development	ENSGALG00010002106	ENSGALG00010002106	2.90964574	0.834717067	3.485786808	0.000490692	0.001667641	positive
PRJNA729271	stage_E14_vs_E10	ordinary_muscle_development	ENSGALG00010002113	GDPD4	-0.908501048	0.195016195	-4.658592839	3.18378E-06	1.62211E-05	negative
PRJNA729271	stage_E14_vs_E10	ordinary_muscle_development	ENSGALG00010002118	ENSGALG00010002118	0.779165722	0.267196239	2.916080427	0.003544591	0.009608911	positive
PRJNA729271	stage_E14_vs_E10	ordinary_muscle_development	ENSGALG00010002122	ENSGALG00010002122	0.439161134	0.521025414	0.842878528	0.399296384	0.514623799	positive
PRJNA729271	stage_E14_vs_E10	ordinary_muscle_development	ENSGALG00010002126	ENSGALG00010002126	0.762628739	0.301468577	2.529712207	0.011415612	0.026774967	positive
PRJNA729271	stage_E14_vs_E10	ordinary_muscle_development	ENSGALG00010002134	ANKRD28	0.014349034	0.120770725	0.118812183	0.905424156	0.935273797	positive
PRJNA729271	stage_E14_vs_E10	ordinary_muscle_development	ENSGALG00010002135	ELOVL5	-0.132793045	0.167926155	-0.790782383	0.429070992	0.543399098	negative
PRJNA729271	stage_E14_vs_E10	ordinary_muscle_development	ENSGALG00010002136	ACAP1	-0.206955603	0.237754648	-0.870458703	0.384049778	0.500161863	negative
PRJNA729271	stage_E14_vs_E10	ordinary_muscle_development	ENSGALG00010002137	PDK3	0.185885421	0.131499856	1.413578897	0.157485581	0.246783609	positive
PRJNA729271	stage_E14_vs_E10	ordinary_muscle_development	ENSGALG00010002138	MYO7A	-0.394189492	0.168082619	-2.345212689	0.019016228	0.041580967	negative
PRJNA729271	stage_E14_vs_E10	ordinary_muscle_development	ENSGALG00010002140	ENSGALG00010002140	2.001150356	0.395695439	5.057299528	4.25235E-07	2.4784E-06	positive
PRJNA729271	stage_E14_vs_E10	ordinary_muscle_development	ENSGALG00010002142	CLDN34	-0.076727276	0.250104224	-0.306781208	0.759009912	0.828252634	negative
PRJNA729271	stage_E14_vs_E10	ordinary_muscle_development	ENSGALG00010002145	ENSGALG00010002145	2.035728926	0.445308889	4.571498515	4.84249E-06	2.39318E-05	positive
PRJNA729271	stage_E14_vs_E10	ordinary_muscle_development	ENSGALG00010002146	CACUL1	0.033889485	0.087937024	0.385383575	0.69995325	0.782072177	positive
PRJNA729271	stage_E14_vs_E10	ordinary_muscle_development	ENSGALG00010002148	POLR2A	-0.546827706	0.179453382	-3.047185294	0.002309953	0.00660019	negative
PRJNA729271	stage_E14_vs_E10	ordinary_muscle_development	ENSGALG00010002149	ENSGALG00010002149	-1.815357377	0.329186205	-5.514682411	3.4941E-08	2.38339E-07	negative
PRJNA729271	stage_E14_vs_E10	ordinary_muscle_development	ENSGALG00010002150	EIF3A	-0.000169396	0.132391141	-0.001279514	0.998979096	0.999350415	negative
PRJNA729271	stage_E14_vs_E10	ordinary_muscle_development	ENSGALG00010002159	GALNT15	-0.504802304	0.191315181	-2.638589898	0.008325162	0.020349617	negative
PRJNA729271	stage_E14_vs_E10	ordinary_muscle_development	ENSGALG00010002160	CAPN5	0.882238187	0.164814836	5.352905157	8.65532E-08	5.56853E-07	positive
PRJNA729271	stage_E14_vs_E10	ordinary_muscle_development	ENSGALG00010002161	CDKL5	1.07699165	0.172252393	6.252404564	4.04181E-10	3.47485E-09	positive
PRJNA729271	stage_E14_vs_E10	ordinary_muscle_development	ENSGALG00010002173	GCM1	-1.036315668	0.872515166	-1.187733701	0.234938342	0.339456864	negative
PRJNA729271	stage_E14_vs_E10	ordinary_muscle_development	ENSGALG00010002174	ENSGALG00010002174	2.16836384	0.446389228	4.857563099	1.18839E-06	6.50765E-06	positive

PRJNA729271	stage_E14_vs_E10	ordinary_muscle_development	ENSGALG00010002177	FBXO9	0.115458326	0.155206454	0.74390158	0.456936019	0.569892239	positive
PRJNA729271	stage_E14_vs_E10	ordinary_muscle_development	ENSGALG00010002181	FIS1	0.233405033	0.255994147	0.911759256	0.361895463	0.478052924	positive
PRJNA729271	stage_E14_vs_E10	ordinary_muscle_development	ENSGALG00010002185	ENSGALG00010002185	0.393665613	0.31906397	1.233814061	0.217272199	0.319245871	positive
PRJNA729271	stage_E14_vs_E10	ordinary_muscle_development	ENSGALG00010002186	CILK1	-0.07941322	0.072794163	-1.090928415	0.275304383	0.385745483	negative
PRJNA729271	stage_E14_vs_E10	ordinary_muscle_development	ENSGALG00010002192	ENSGALG00010002192	-0.008251675	0.195239328	-0.042264408	0.966287918	0.977618718	negative
PRJNA729271	stage_E14_vs_E10	ordinary_muscle_development	ENSGALG00010002194	SHROOM2	-1.803280041	0.130169663	-13.85330495	1.21491E-43	1.18189E-41	negative
PRJNA729271	stage_E14_vs_E10	ordinary_muscle_development	ENSGALG00010002196	ENSGALG00010002196	-0.307387927	0.099006051	-3.104738797	0.001904471	0.005586989	negative
PRJNA729271	stage_E14_vs_E10	ordinary_muscle_development	ENSGALG00010002197	GSTA2	-0.381487265	0.187722448	-2.032187781	0.042134649	0.081879842	negative
PRJNA729271	stage_E14_vs_E10	ordinary_muscle_development	ENSGALG00010002202	SFXN4	0.289351648	0.099701196	2.902188335	0.003705657	0.009995068	positive
PRJNA729271	stage_E14_vs_E10	ordinary_muscle_development	ENSGALG00010002206	DPH3P1	-0.015452503	0.110287687	-0.140110861	0.888572399	0.923552053	negative
PRJNA729271	stage_E14_vs_E10	ordinary_muscle_development	ENSGALG00010002210	GPR143	-1.864107462	0.95493535	-1.952077135	0.050929047	0.096297488	negative
PRJNA729271	stage_E14_vs_E10	ordinary_muscle_development	ENSGALG00010002211	ACER3	-0.375284612	0.173787691	-2.159442994	0.030815815	0.062770552	negative
PRJNA729271	stage_E14_vs_E10	ordinary_muscle_development	ENSGALG00010002215	YLEC18	2.294389873	0.346892268	6.614128039	3.73748E-11	3.61406E-10	positive
PRJNA729271	stage_E14_vs_E10	ordinary_muscle_development	ENSGALG00010002225	PRDX3	0.242038609	0.10543187	2.295687328	0.021693765	0.046687535	positive
PRJNA729271	stage_E14_vs_E10	ordinary_muscle_development	ENSGALG00010002226	OXNAD1	-0.043978451	0.131720261	-0.333877649	0.738471887	0.812089371	negative
PRJNA729271	stage_E14_vs_E10	ordinary_muscle_development	ENSGALG00010002227	ENSGALG00010002227	2.923631596	1.109253842	2.635674078	0.008397034	0.020490743	positive
PRJNA729271	stage_E14_vs_E10	ordinary_muscle_development	ENSGALG00010002231	TMEM14A	-0.329406514	0.132356058	-2.488790608	0.012817843	0.02963697	negative
PRJNA729271	stage_E14_vs_E10	ordinary_muscle_development	ENSGALG00010002235	MXRA5	0.154434707	0.201206078	0.767544941	0.442757584	0.556518486	positive
PRJNA729271	stage_E14_vs_E10	ordinary_muscle_development	ENSGALG00010002238	ENSGALG00010002238	0.065895702	0.380580599	0.173145196	0.8625373	0.90532065	positive
PRJNA729271	stage_E14_vs_E10	ordinary_muscle_development	ENSGALG00010002239	AGPAT5	0.040467686	0.127823984	0.316589148	0.751555371	0.821906491	positive
PRJNA729271	stage_E14_vs_E10	ordinary_muscle_development	ENSGALG00010002243	ARSH	-1.233912805	0.179194477	-6.885886343	5.74288E-12	6.00599E-11	negative
PRJNA729271	stage_E14_vs_E10	ordinary_muscle_development	ENSGALG00010002247	RFTN1	-0.18247343	0.184401165	-0.989545971	0.322396089	0.436945424	negative
PRJNA729271	stage_E14_vs_E10	ordinary_muscle_development	ENSGALG00010002251	GRK5	-0.632721701	0.149383009	-4.23556672	2.27976E-05	0.000100502	negative
PRJNA729271	stage_E14_vs_E10	ordinary_muscle_development	ENSGALG00010002253	ENSGALG00010002253	-0.165822572	0.320599612	-0.517226365	0.60499815	0.702990063	negative
PRJNA729271	stage_E14_vs_E10	ordinary_muscle_development	ENSGALG00010002254	ENSGALG00010002254	0.861864598	0.532211734	1.619401719	0.105360857	0.177830184	positive
PRJNA729271	stage_E14_vs_E10	ordinary_muscle_development	ENSGALG00010002259	TASL	0.516837975	0.516551496	1.000554599	0.317042189	0.43089794	positive
PRJNA729271	stage_E14_vs_E10	ordinary_muscle_development	ENSGALG00010002261	NROB1	-0.305034889	0.346931054	-0.87923778	0.379272364	0.495418561	negative
PRJNA729271	stage_E14_vs_E10	ordinary_muscle_development	ENSGALG00010002269	MCPH1	-0.226230518	0.129934704	-1.741109271	0.081664427	0.143957057	negative
PRJNA729271	stage_E14_vs_E10	ordinary_muscle_development	ENSGALG00010002270	IMMP2L	-0.171431701	0.242185691	-0.70785231	0.479036978	0.591149412	negative
PRJNA729271	stage_E14_vs_E10	ordinary_muscle_development	ENSGALG00010002271	ENSGALG00010002271	-1.147873759	1.042293482	-1.101296111	0.270767803	0.38052931	negative
PRJNA729271	stage_E14_vs_E10	ordinary_muscle_development	ENSGALG00010002274	ENSGALG00010002274	3.32902383	0.778719267	4.274998672	1.91138E-05	8.53002E-05	positive
PRJNA729271	stage_E14_vs_E10	ordinary_muscle_development	ENSGALG00010002275	IL1RAPL1	-1.342301969	0.228450264	-5.875685787	4.21096E-09	3.221E-08	negative
PRJNA729271	stage_E14_vs_E10	ordinary_muscle_development	ENSGALG00010002277	RGS10	-1.086448836	0.177660834	-6.11529739	9.63771E-10	7.89704E-09	negative
PRJNA729271	stage_E14_vs_E10	ordinary_muscle_development	ENSGALG00010002279	ENSGALG00010002279	5.304955984	0.694984465	7.633200811	2.28996E-14	3.06774E-13	positive
PRJNA729271	stage_E14_vs_E10	ordinary_muscle_development	ENSGALG00010002280	ST7	-0.167268744	0.14669269	-1.140266389	0.254175336	0.361593041	negative
PRJNA729271	stage_E14_vs_E10	ordinary_muscle_development	ENSGALG00010002281	TGFBR2	0.534007919	0.155180433	3.441206524	0.000579126	0.001931532	positive
PRJNA729271	stage_E14_vs_E10	ordinary_muscle_development	ENSGALG00010002282	TSKU	0.086138295	0.164594214	0.52333732	0.600739535	0.699808692	positive
PRJNA729271	stage_E14_vs_E10	ordinary_muscle_development	ENSGALG00010002283	ENSGALG00010002283	4.090634113	0.652863605	6.265679515	3.71203E-10	3.20083E-09	positive
PRJNA729271	stage_E14_vs_E10	ordinary_muscle_development	ENSGALG00010002285	CAV1	1.433749476	0.157537668	9.100994668	8.95083E-20	1.88032E-18	positive
PRJNA729271	stage_E14_vs_E10	ordinary_muscle_development	ENSGALG00010002288	ANGPT2	0.088074149	0.17736114	0.496580868	0.619484648	0.715150523	positive
PRJNA729271	stage_E14_vs_E10	ordinary_muscle_development	ENSGALG00010002289	ENSGALG00010002289	5.29885669	0.93738882	5.652784178	1.57869E-08	1.12283E-07	positive
PRJNA729271	stage_E14_vs_E10	ordinary_muscle_development	ENSGALG00010002291	RNASEH1	0.16807065	0.115340079	1.457174746	0.14506815	0.230996686	positive
PRJNA729271	stage_E14_vs_E10	ordinary_muscle_development	ENSGALG00010002293	GYG2	-0.775391123	0.362359448	-2.139839675	0.032367726	0.06538046	negative
PRJNA729271	stage_E14_vs_E10	ordinary_muscle_development	ENSGALG00010002294	DAZL	-1.370648293	0.925853879	-1.480415348	0.138762437	0.222717475	negative
PRJNA729271	stage_E14_vs_E10	ordinary_muscle_development	ENSGALG00010002295	YAF2	-0.164089891	0.113665926	-1.44361549	0.148847165	0.236003026	negative
PRJNA729271	stage_E14_vs_E10	ordinary_muscle_development	ENSGALG00010002298	ENSGALG00010002298	0.5390096	0.369528128	1.458642951	0.144663406	0.23040282	positive
PRJNA729271	stage_E14_vs_E10	ordinary_muscle_development	ENSGALG00010002303	ENSGALG00010002303	1.249195296	0.810685828	1.540911723	0.123338272	0.201941518	positive
PRJNA729271	stage_E14_vs_E10	ordinary_muscle_development	ENSGALG00010002308	GXYLT1	-0.779270469	0.102221909	-7.623321401	2.4723E-14	3.30591E-13	negative
PRJNA729271	stage_E14_vs_E10	ordinary_muscle_development	ENSGALG00010002309	GADL1	1.117003966	0.33008355	3.384003732	0.00071437	0.002332687	positive
PRJNA729271	stage_E14_vs_E10	ordinary_muscle_development	ENSGALG00010002310	PRRG1	-0.226543969	0.103969264	-2.178951366	0.029335281	0.060271425	negative
PRJNA729271	stage_E14_vs_E10	ordinary_muscle_development	ENSGALG00010002312	ENSGALG00010002312	-0.786765367	0.191492763	-4.108590596	3.98081E-05	0.000168571	negative
PRJNA729271	stage_E14_vs_E10	ordinary_muscle_development	ENSGALG00010002315	ENSGALG00010002315	-0.796350749	0.166071259	-4.795235209	1.62484E-06	8.70043E-06	negative
PRJNA729271	stage_E14_vs_E10	ordinary_muscle_development	ENSGALG00010002324	SNTG2	-1.751699197	0.325884976	-5.375206981	7.64948E-08	4.96326E-07	negative
PRJNA729271	stage_E14_vs_E10	ordinary_muscle_development	ENSGALG00010002328	ENSGALG00010002328	-0.521918936	0.26062672	-2.002553443	0.045225241	0.087196046	negative
PRJNA729271	stage_E14_vs_E10	ordinary_muscle_development	ENSGALG00010002329	STT3B	-0.171409583	0.072338691	-2.369542236	0.017810121	0.039341314	negative
PRJNA729271	stage_E14_vs_E10	ordinary_muscle_development	ENSGALG00010002330	KHDRBS2	-0.232032086	0.278484317	-0.83319624	0.404734063	0.519666648	negative
PRJNA729271	stage_E14_vs_E10	ordinary_muscle_development	ENSGALG00010002331	ENSGALG00010002331	-0.175272162	0.265722891	-0.659605055	0.509507309	0.618327901	negative
PRJNA729271	stage_E14_vs_E10	ordinary_muscle_development	ENSGALG00010002332	ENSGALG00010002332	1.737847632	0.589339017	2.948808042	0.00319002	0.008777401	positive
PRJNA729271	stage_E14_vs_E10	ordinary_muscle_development	ENSGALG00010002333	ENSGALG00010002333	-0.314540343	0.702133666	-0.447977868	0.654169169	0.744927497	negative
PRJNA729271	stage_E14_vs_E10	ordinary_muscle_development	ENSGALG00010002334	OSBPL10	0.055801171	0.166600398	0.3349402	0.737670197	0.811515597	positive
PRJNA729271	stage_E14_vs_E10	ordinary_muscle_development	ENSGALG00010002335	TMEM47	0.827265846	0.133770898	6.184198931	6.24186E-10	5.24802E-09	positive
PRJNA729271	stage_E14_vs_E10	ordinary_muscle_development	ENSGALG00010002336	PRIM2	-0.158675353	0.11440932	-1.38690933	0.165469412	0.256674669	negative
PRJNA729271	stage_E14_vs_E10	ordinary_muscle_development	ENSGALG00010002338	CD99	0.412756132	0.148215451	2.784838761	0.005355435	0.0138279	positive
PRJNA729271	stage_E14_vs_E10	ordinary_muscle_development	ENSGALG00010002339	ZNFY4	0.282012625	0.307869166	0.916014518	0.35965928	0.476096919	positive
PRJNA729271	stage_E14_vs_E10	ordinary_muscle_development	ENSGALG00010002340	RAB23	-0.675554505	0.134130083	-5.036562196	4.73967E-07	2.74367E-06	negative
PRJNA729271	stage_E14_vs_E10	ordinary_muscle_development	ENSGALG00010002341	FAM49A	0.404040113	0.109347563	3.69500795	0.00021988	0.000807694	positive
PRJNA729271	stage_E14_vs_E10	ordinary_muscle_development	ENSGALG00010002342	BAG2	1.194846805	0.124093116	9.628630883	6.05306E-22	1.49725E-20	positive
PRJNA729271	stage_E14_vs_E10	ordinary_muscle_development	ENSGALG00010002343	GPD1L	0.402410545	0.081205142	4.955481072	7.21515E-07	4.06466E-06	positive
PRJNA729271	stage_E14_vs_E10	ordinary_muscle_development	ENSGALG00010002345	SCML2	-0.738830749	0.096271531	-7.674446874	1.66134E-14	2.24637E-13	negative
PRJNA729271	stage_E14_vs_E10	ordinary_muscle_development	ENSGALG00010002346	FBXO15	-0.680204628	0.352717155	-1.928470501	0.05379663	0.100838245	negative
PRJNA729271	stage_E14_vs_E10	ordinary_muscle_development	ENSGALG00010002347	ZNF451	-0.136183253	0.060460229	-2.252443548	0.024294256	0.051459693	negative
PRJNA729271	stage_E14_vs_E10	ordinary_muscle_development	ENSGALG00010002348	THAP12	-0.769748554	0.153446279	-5.016404177	5.26475E-07	3.02588E-06	negative
PRJNA729271	stage_E14_vs_E10	ordinary_muscle_development	ENSGALG00010002349	ZBED1	-0.067434875	0.130022918	-0.518638377	0.604012942	0.702170859	negative
PRJNA729271	stage_E14_vs_E10	ordinary_muscle_development	ENSGALG00010002350	BEND6	-0.089917746	0.247681432	-0.363037897	0.716576575	0.795051779	negative
PRJNA729271	stage_E14_vs_E10	ordinary_muscle_development	ENSGALG00010002352	ENSGALG00010002352	0.121043506	0.106546676	1.136060843	0.255931101	0.363733828	positive
PRJNA729271	stage_E14_vs_E10	ordinary_muscle_development	ENSGALG00010002353	CMTM8	0.20588619	0.136673185	1.506412463	0.131961323	0.21369449	positive
PRJNA729271	stage_E14_vs_E10	ordinary_muscle_development	ENSGALG00010002355	UFM1	-0.198085819	0.09997718	-1.981310332	0.047556482	0.090952792	negative
PRJNA729271	stage_E14_vs_E10	ordinary_muscle_development	ENSGALG00010002356	WNT11	-2.551016729	0.325846364	-7.82889426	4.92179E-15	6.9363E-14	negative
PRJNA729271	stage_E14_vs_E10	ordinary_muscle_development	ENSGALG00010002357	RAB31	0.757346201	0.139371599	5.434006677	5.51025E-08	3.6305E-07	positive
PRJNA729271	stage_E14_vs_E10	ordinary_muscle_development	ENSGALG00010002358	FREM2	2.192570856	0.233691424	9.382333414	6.45286E-21	1.4844E-19	positive
PRJNA729271	stage_E14_vs_E10	ordinary_muscle_development	ENSGALG00010002359	CMTM6	-0.339790956	0.115607055	-2.93918874	0.003290726	0.009015134	negative
PRJNA729271	stage_E14_vs_E10	ordinary_muscle_development	ENSGALG00010002360	DHRSX	0.498363251	0.163302102	3.051787126	0.002274834	0.006512683	positive
PRJNA729271	stage_E14_vs_E10	ordinary_muscle_development	ENSGALG00010002361	ENSGALG00010002361	-0.099265423	0.098940199	-1.003287073	0.315722371	0.429676359	negative
PRJNA729271	stage_E14_vs_E10	ordinary_muscle_development	ENSGALG00010002362	TRPC4	-1.446201209	0.353560337	-4.090394358	4.3064E-05	0.000181036	negative
PRJNA729271	stage_E14_vs_E10	ordinary_muscle_development	ENSGALG00010002364	VSNL1	0.826499193	0.388278598	2.128624128	0.033285371	0.066982014	positive
PRJNA729271	stage_E14_vs_E10	ordinary_muscle_development	ENSGALG00010002365	RAI2	0.856985098	0.137833442	6.217541148	5.05006E-10	4.29077E-09	positive
PRJNA729271	stage_E14_vs_E10	ordinary_muscle_development	ENSGALG00010002366	ENSGALG00010002366	0.56824142	0.756704508	0.750942295	0.452687385	0.565664107	positive
PRJNA729271	stage_E14_vs_E10	ordinary_muscle_development	ENSGALG00010002367	STK31	-3.657790711	0.66033796	-5.539270695	3.03734E-08	2.09151E-07	negative
PRJNA729271	stage_E14_vs_E10	ordinary_muscle_development	ENSGALG00010002368	UVRAG	0.285636664	0.095063381	3.004697083	0.002658455	0.007469335	positive
PRJNA729271	stage_E14_vs_E10	ordinary_muscle_development	ENSGALG00010002369	GEMIN8	-0.456635368	0.118732952	-3.845902604	0.000120109	0.000466502	negative
PRJNA729271	stage_E14_vs_E10	ordinary_muscle_development	ENSGALG00010002370	ENSGALG00010002370	-0.32481528	0.153726105	-2.112948094	0.034605211	0.069329997	negative
PRJNA729271	stage_E14_vs_E10	ordinary_muscle_development	ENSGALG00010002371	BAG3	0.603996251	0.155492226	3.884414453	0.000102577	0.000402941	positive
PRJNA729271	stage_E14_vs_E10	ordinary_muscle_development	ENSGALG00010002372	SMC6	-0.339446118	0.126010004	-2.693802934	0.007064192	0.017563544	negative
PRJNA729271	stage_E14_vs_E10	ordinary_muscle_development	ENSGALG00010002373	POSTN	1.054757121	0.255593081	4.126704519	3.67999E-05	0.000156564	positive
PRJNA729271	stage_E14_vs_E10	ordinary_muscle_development	ENSGALG00010002374	DST	-0.306170342	0.114315956	-2.678281785	0.007400093	0.018295131	negative
PRJNA729271	stage_E14_vs_E10	ordinary_muscle_development	ENSGALG00010002375	NPY	-3.366387504	0.521925616	-6.449937316	1.11896E-10	1.01945E-09	negative
PRJNA729271	stage_E14_vs_E10	ordinary_muscle_development	ENSGALG00010002376	GEN1	-0.318401352	0.191256771	-1.664784733	0.095955711	0.164386955	negative
PRJNA729271	stage_E14_vs_E10	ordinary_muscle_development	ENSGALG00010002377	ART1A	1.147546192	0.269326939	4.260792458	2.03703E-05	9.05452E-05	positive
PRJNA729271	stage_E14_vs_E10	ordinary_muscle_development	ENSGALG00010002378	ENSGALG00010002378	0.348569647	0.447526275	0.778880853	0.436049904	0.550103688	positive
PRJNA729271	stage_E14_vs_E10	ordinary_muscle_development	ENSGALG00010002379	NHS	-2.126158585	0.134484975	-15.80963659	2.67003E-56	5.23E-54	negative
PRJNA729271	stage_E14_vs_E10	ordinary_muscle_development	ENSGALG00010002380	CMPK2	-0.369944699	0.749860137	-0.493351602	0.621764173	0.71701835	negative
PRJNA729271	stage_E14_vs_E10	ordinary_muscle_development	ENSGALG00010002381	TDRD3	0.134195203	0.096218669	1.394689873	0.16310938	0.253813254	positive
PRJNA729271	stage_E14_vs_E10	ordinary_muscle_development	ENSGALG00010002382	ART1D	-1.862273198	0.774227015	-2.405332237	0.016157769	0.036165358	negative
PRJNA729271	stage_E14_vs_E10	ordinary_muscle_development	ENSGALG00010002383	INPP5F	-0.062357526	0.123699123	-0.504106451	0.614186574	0.710740834	negative
PRJNA729271	stage_E14_vs_E10	ordinary_muscle_development	ENSGALG00010002384	ENSGALG00010002384	-2.098753323	0.311708097	-6.733072846	1.66117E-11	1.6675E-10	negative
PRJNA729271	stage_E14_vs_E10	ordinary_muscle_development	ENSGALG00010002385	PALS2	1.071358767	0.122858492	8.720266334	2.77548E-18	5.19775E-17	positive
PRJNA729271	stage_E14_vs_E10	ordinary_muscle_development	ENSGALG00010002386	MHCY2B2	-0.207440395	0.348753797	-0.594804692	0.551974031	0.656723597	negative
PRJNA729271	stage_E14_vs_E10	ordinary_muscle_development	ENSGALG00010002387	MCMBP	0.040073958	0.108319382	0.369961103	0.711411473	0.790972562	positive
PRJNA729271	stage_E14_vs_E10	ordinary_muscle_development	ENSGALG00010002388	ENSGALG00010002388	-0.679343835	1.076344566	-0.631158327	0.527937007	0.635374205	negative
PRJNA729271	stage_E14_vs_E10	ordinary_muscle_development	ENSGALG00010002389	COL21A1	-0.772050193	0.322048812	-2.397308001	0.016516034	0.036864785	negative
PRJNA729271	stage_E14_vs_E10	ordinary_muscle_development	ENSGALG00010002391	REPS2	0.038714897	0.159250631	0.243106711	0.807922733	0.864951556	positive
PRJNA729271	stage_E14_vs_E10	ordinary_muscle_development	ENSGALG00010002392	DFNA5	-0.830266669	0.205568567	-4.038879483	5.37071E-05	0.000222297	negative
PRJNA729271	stage_E14_vs_E10	ordinary_muscle_development	ENSGALG00010002393	GPM6B	-0.079463929	0.106761327	-0.7443138	0.456686655	0.569685485	negative
PRJNA729271	stage_E14_vs_E10	ordinary_muscle_development	ENSGALG00010002395	CLN8	0.218046494	0.089895791	2.425547317	0.015285323	0.034462709	positive
PRJNA729271	stage_E14_vs_E10	ordinary_muscle_development	ENSGALG00010002396	SEC23IP	-0.098330749	0.08729179	-1.126460457	0.259970669	0.368216845	negative
PRJNA729271	stage_E14_vs_E10	ordinary_muscle_development	ENSGALG00010002398	ENSGALG00010002398	-2.529108879	0.413408906	-6.117693261	9.49396E-10	7.78806E-09	negative
PRJNA729271	stage_E14_vs_E10	ordinary_muscle_development	ENSGALG00010002399	CSMD1	-2.113149786	0.394796134	-5.352508814	8.67431E-08	5.57656E-07	negative
PRJNA729271	stage_E14_vs_E10	ordinary_muscle_development	ENSGALG00010002400	RBBP7	-0.266636811	0.152326519	-1.750429365	0.080044253	0.141475606	negative
PRJNA729271	stage_E14_vs_E10	ordinary_muscle_development	ENSGALG00010002401	ZRANB1	0.094214274	0.161323474	0.584008465	0.559214617	0.662835544	positive
PRJNA729271	stage_E14_vs_E10	ordinary_muscle_development	ENSGALG00010002402	TXLNG	0.297225787	0.12541521	2.369934133	0.017791254	0.039317614	positive
PRJNA729271	stage_E14_vs_E10	ordinary_muscle_development	ENSGALG00010002403	ZNFY3	0.097704191	0.260798445	0.374634868	0.707932038	0.787950156	positive
PRJNA729271	stage_E14_vs_E10	ordinary_muscle_development	ENSGALG00010002404	OSBPL3	-1.68536603	0.255241238	-6.60303186	4.02833E-11	3.89271E-10	negative
PRJNA729271	stage_E14_vs_E10	ordinary_muscle_development	ENSGALG00010002405	KCNQ5	-1.288460054	0.203125838	-6.343161783	2.25097E-10	1.99558E-09	negative
PRJNA729271	stage_E14_vs_E10	ordinary_muscle_development	ENSGALG00010002406	DIAPH3	-0.166191535	0.244631813	-0.67935373	0.49691376	0.606446489	negative
PRJNA729271	stage_E14_vs_E10	ordinary_muscle_development	ENSGALG00010002408	OFD1	-0.122282128	0.113787308	-1.074655259	0.282529102	0.393926446	negative
PRJNA729271	stage_E14_vs_E10	ordinary_muscle_development	ENSGALG00010002409	CYCS	0.016035833	0.166852889	0.096107613	0.923435105	0.947355924	positive
PRJNA729271	stage_E14_vs_E10	ordinary_muscle_development	ENSGALG00010002410	RNF121	0.185354699	0.10363168	1.788591075	0.073680696	0.131869542	positive
PRJNA729271	stage_E14_vs_E10	ordinary_muscle_development	ENSGALG00010002411	ENSGALG00010002411	0.106692627	0.164772584	0.647514435	0.517299055	0.625704822	positive
PRJNA729271	stage_E14_vs_E10	ordinary_muscle_development	ENSGALG00010002413	TSSC1	-0.303105955	0.081306092	-3.727961188	0.000193035	0.000720032	negative
PRJNA729271	stage_E14_vs_E10	ordinary_muscle_development	ENSGALG00010002414	ENSGALG00010002414	0.507203036	0.361716026	1.402213338	0.16085155	0.251126636	positive
PRJNA729271	stage_E14_vs_E10	ordinary_muscle_development	ENSGALG00010002415	PCDH17	-2.798345175	0.270603709	-10.34111906	4.59144E-25	1.38652E-23	negative

PRJNA729271	stage_E14_vs_E10	ordinary_muscle_development	ENSGALG00010002416	SH3BGRL2	-0.270038735	0.258447327	-1.044850177	0.29609222	0.409411116	negative
PRJNA729271	stage_E14_vs_E10	ordinary_muscle_development	ENSGALG00010002418	TRAPPC2	-0.415284579	0.082626033	-5.026074293	5.00622E-07	2.88874E-06	negative
PRJNA729271	stage_E14_vs_E10	ordinary_muscle_development	ENSGALG00010002419	CTBP2	0.014552202	0.11269027	0.129134498	0.897251225	0.929240962	positive
PRJNA729271	stage_E14_vs_E10	ordinary_muscle_development	ENSGALG00010002420	TMEM272	0.648012914	0.670963626	0.965794402	0.33414707	0.449342405	positive
PRJNA729271	stage_E14_vs_E10	ordinary_muscle_development	ENSGALG00010002421	ENSGALG00010002421	-0.135962098	0.179700231	-0.756605027	0.449286529	0.56233557	negative
PRJNA729271	stage_E14_vs_E10	ordinary_muscle_development	ENSGALG00010002422	TRAPPC12	0.036578867	0.102158348	0.35806048	0.720298053	0.797610411	positive
PRJNA729271	stage_E14_vs_E10	ordinary_muscle_development	ENSGALG00010002423	ENSGALG00010002423	1.33786007	0.569420397	2.349512028	0.018798037	0.041178411	positive
PRJNA729271	stage_E14_vs_E10	ordinary_muscle_development	ENSGALG00010002424	CASK	-0.071472404	0.056186407	-1.272058627	0.20335229	0.30296962	negative
PRJNA729271	stage_E14_vs_E10	ordinary_muscle_development	ENSGALG00010002425	FDX2	0.264801834	0.132922186	1.992156783	0.046353858	0.089075855	positive
PRJNA729271	stage_E14_vs_E10	ordinary_muscle_development	ENSGALG00010002427	CYB5R4	-0.058004506	0.111225455	-0.52150388	0.602015803	0.700563509	negative
PRJNA729271	stage_E14_vs_E10	ordinary_muscle_development	ENSGALG00010002428	IBSP	-4.128114553	0.726160536	-5.684851143	1.30927E-08	9.39031E-08	negative
PRJNA729271	stage_E14_vs_E10	ordinary_muscle_development	ENSGALG00010002429	ENSGALG00010002429	2.133953532	0.53673607	3.975796769	7.0144E-05	0.000284243	positive
PRJNA729271	stage_E14_vs_E10	ordinary_muscle_development	ENSGALG00010002430	NUMA1	0.343631114	0.174348353	1.97094557	0.048730102	0.092878741	positive
PRJNA729271	stage_E14_vs_E10	ordinary_muscle_development	ENSGALG00010002431	BCKDHB	0.605418051	0.14743286	4.106398332	4.01876E-05	0.000170029	positive
PRJNA729271	stage_E14_vs_E10	ordinary_muscle_development	ENSGALG00010002432	RAVER1	-0.032712672	0.163171947	-0.200479755	0.841105389	0.889264961	negative
PRJNA729271	stage_E14_vs_E10	ordinary_muscle_development	ENSGALG00010002433	GRHL1	0.075423826	0.317890197	0.237263767	0.812452169	0.868526027	positive
PRJNA729271	stage_E14_vs_E10	ordinary_muscle_development	ENSGALG00010002434	GPR34	1.500307682	0.247122847	6.071100661	1.27037E-09	1.02413E-08	positive
PRJNA729271	stage_E14_vs_E10	ordinary_muscle_development	ENSGALG00010002435	ATP7B	-1.574569204	0.654507656	-2.405730763	0.016140155	0.036137094	negative
PRJNA729271	stage_E14_vs_E10	ordinary_muscle_development	ENSGALG00010002436	ENSGALG00010002436	2.693570976	0.874388095	3.0805211	0.002066387	0.005984472	positive
PRJNA729271	stage_E14_vs_E10	ordinary_muscle_development	ENSGALG00010002437	TTK	-0.419791071	0.194144988	-2.162255518	0.030598486	0.06240679	negative
PRJNA729271	stage_E14_vs_E10	ordinary_muscle_development	ENSGALG00010002438	RARB	-0.811191783	0.089581494	-9.055349989	1.36131E-19	2.82291E-18	negative
PRJNA729271	stage_E14_vs_E10	ordinary_muscle_development	ENSGALG00010002439	GPR82	-1.692001585	1.174706321	-1.440361352	0.149765192	0.237017846	negative
PRJNA729271	stage_E14_vs_E10	ordinary_muscle_development	ENSGALG00010002440	LRRC51	0.334987025	0.261971051	1.278717716	0.200996479	0.300169376	positive
PRJNA729271	stage_E14_vs_E10	ordinary_muscle_development	ENSGALG00010002441	USO1	0.949680276	0.06690488	14.1944844	9.91127E-46	1.10511E-43	positive
PRJNA729271	stage_E14_vs_E10	ordinary_muscle_development	ENSGALG00010002442	ENSGALG00010002442	1.100900308	0.43059789	2.556678362	0.010567688	0.024976135	positive
PRJNA729271	stage_E14_vs_E10	ordinary_muscle_development	ENSGALG00010002443	TYK2	0.319775924	0.135625729	2.357782149	0.018384478	0.040412953	positive
PRJNA729271	stage_E14_vs_E10	ordinary_muscle_development	ENSGALG00010002444	KLF11	0.13038498	0.182281612	0.715294202	0.474427271	0.58660951	positive
PRJNA729271	stage_E14_vs_E10	ordinary_muscle_development	ENSGALG00010002445	EDRF1	0.184167067	0.111142721	1.657032196	0.097512966	0.166494371	positive
PRJNA729271	stage_E14_vs_E10	ordinary_muscle_development	ENSGALG00010002446	RIMS1	-2.047377784	0.250418405	-8.17582789	2.93841E-16	4.60457E-15	negative
PRJNA729271	stage_E14_vs_E10	ordinary_muscle_development	ENSGALG00010002447	YLEC12	2.208895525	0.543713369	4.062610288	4.8527E-05	0.000202417	positive
PRJNA729271	stage_E14_vs_E10	ordinary_muscle_development	ENSGALG00010002448	RAB9A	-0.42951432	0.135860216	-3.161442939	0.001569896	0.004701578	negative
PRJNA729271	stage_E14_vs_E10	ordinary_muscle_development	ENSGALG00010002449	ALG11	0.189152281	0.132624937	1.426219577	0.15380497	0.242326418	positive
PRJNA729271	stage_E14_vs_E10	ordinary_muscle_development	ENSGALG00010002453	TOP2B	-0.255856857	0.062548189	-4.090555801	4.30341E-05	0.000180963	negative
PRJNA729271	stage_E14_vs_E10	ordinary_muscle_development	ENSGALG00010002454	NEK5	-2.329868788	0.569531922	-4.090848466	4.29798E-05	0.000180787	negative
PRJNA729271	stage_E14_vs_E10	ordinary_muscle_development	ENSGALG00010002455	NYX	0.433829743	0.358413027	1.210418456	0.226118363	0.329273225	positive
PRJNA729271	stage_E14_vs_E10	ordinary_muscle_development	ENSGALG00010002456	ELOVL4	-1.34107846	0.47624648	-2.815933588	0.004863572	0.012706827	negative
PRJNA729271	stage_E14_vs_E10	ordinary_muscle_development	ENSGALG00010002457	ENSGALG00010002457	1.00503713	0.442323199	2.2721782	0.023075749	0.049246612	positive
PRJNA729271	stage_E14_vs_E10	ordinary_muscle_development	ENSGALG00010002458	ENSGALG00010002458	2.034035331	0.19726498	10.31118313	6.27243E-25	1.87462E-23	positive
PRJNA729271	stage_E14_vs_E10	ordinary_muscle_development	ENSGALG00010002459	G3BP2	-0.151201143	0.072810914	-2.076627473	0.037835955	0.07472846	negative
PRJNA729271	stage_E14_vs_E10	ordinary_muscle_development	ENSGALG00010002460	LAMTOR1	0.225896846	0.181315858	1.245874735	0.212810387	0.314186857	positive
PRJNA729271	stage_E14_vs_E10	ordinary_muscle_development	ENSGALG00010002461	TOMT	0.321433268	0.313069948	1.026713904	0.3045552	0.418200798	positive
PRJNA729271	stage_E14_vs_E10	ordinary_muscle_development	ENSGALG00010002462	RRM2	-0.556573108	0.274356617	-2.028648382	0.042494117	0.082534132	negative
PRJNA729271	stage_E14_vs_E10	ordinary_muscle_development	ENSGALG00010002463	ASRGL1	0.44606101	0.097473934	4.576208132	4.7348E-06	2.34555E-05	positive
PRJNA729271	stage_E14_vs_E10	ordinary_muscle_development	ENSGALG00010002464	NGLY1	0.096832354	0.082987837	1.166825852	0.243280705	0.349641544	positive
PRJNA729271	stage_E14_vs_E10	ordinary_muscle_development	ENSGALG00010002466	UROS	0.16013113	0.176583984	0.906827036	0.364498271	0.480555171	positive
PRJNA729271	stage_E14_vs_E10	ordinary_muscle_development	ENSGALG00010002467	NEK3	-0.560400883	0.209431606	-2.675818108	0.007454709	0.018408179	negative
PRJNA729271	stage_E14_vs_E10	ordinary_muscle_development	ENSGALG00010002468	ENSGALG00010002468	2.06815583	0.533891994	3.873734484	0.00010718	0.000420008	positive
PRJNA729271	stage_E14_vs_E10	ordinary_muscle_development	ENSGALG00010002469	MEPE	-5.232595875	1.384328532	-3.779880103	0.000156904	0.000595216	negative
PRJNA729271	stage_E14_vs_E10	ordinary_muscle_development	ENSGALG00010002470	OXSM	-0.161544048	0.16101678	-1.003274611	0.315728383	0.429676359	negative
PRJNA729271	stage_E14_vs_E10	ordinary_muscle_development	ENSGALG00010002471	OGFRL1	0.831526158	0.149806677	5.550661516	2.84591E-08	1.96529E-07	positive
PRJNA729271	stage_E14_vs_E10	ordinary_muscle_development	ENSGALG00010002472	CDC37	0.032781113	0.214202028	0.153038292	0.878368078	0.916428798	positive
PRJNA729271	stage_E14_vs_E10	ordinary_muscle_development	ENSGALG00010002473	ENSGALG00010002473	1.07545874	0.632408274	1.700576645	0.089022513	0.154461146	positive
PRJNA729271	stage_E14_vs_E10	ordinary_muscle_development	ENSGALG00010002474	HPCAL1	0.165671578	0.139703886	1.185876662	0.235671011	0.340311945	positive
PRJNA729271	stage_E14_vs_E10	ordinary_muscle_development	ENSGALG00010002475	ENSGALG00010002475	-0.000534883	0.180096921	-0.002969972	0.997630308	0.998456903	negative
PRJNA729271	stage_E14_vs_E10	ordinary_muscle_development	ENSGALG00010002476	DDX3X	0.013695595	0.144961801	0.094477267	0.92473004	0.948147551	positive
PRJNA729271	stage_E14_vs_E10	ordinary_muscle_development	ENSGALG00010002478	PPME1	-0.03416846	0.054504062	-0.626897498	0.530726427	0.638307299	negative
PRJNA729271	stage_E14_vs_E10	ordinary_muscle_development	ENSGALG00010002481	CKAP2	-0.108945879	0.244783736	-0.445069925	0.656269222	0.746615052	negative
PRJNA729271	stage_E14_vs_E10	ordinary_muscle_development	ENSGALG00010002483	COQ2	0.875165376	0.136925938	6.391523709	1.64241E-10	1.47593E-09	positive
PRJNA729271	stage_E14_vs_E10	ordinary_muscle_development	ENSGALG00010002487	BCCIP	-0.434067349	0.117984874	-3.679008442	0.000234143	0.000855316	negative
PRJNA729271	stage_E14_vs_E10	ordinary_muscle_development	ENSGALG00010002492	RPL8	0.561662755	0.12954754	4.335572527	1.45381E-05	6.64345E-05	positive
PRJNA729271	stage_E14_vs_E10	ordinary_muscle_development	ENSGALG00010002500	OSR1	-0.638596291	0.196294421	-3.253257468	0.001140901	0.003542707	negative
PRJNA729271	stage_E14_vs_E10	ordinary_muscle_development	ENSGALG00010002501	TCEANC	-0.242343767	0.093877073	-2.581501092	0.009837168	0.02349864	negative
PRJNA729271	stage_E14_vs_E10	ordinary_muscle_development	ENSGALG00010002504	ENSGALG00010002504	0.046259709	0.065628676	0.704870367	0.480890913	0.592831586	positive
PRJNA729271	stage_E14_vs_E10	ordinary_muscle_development	ENSGALG00010002507	C2CD3	0.094259275	0.107081574	0.880256715	0.378720256	0.494866141	positive
PRJNA729271	stage_E14_vs_E10	ordinary_muscle_development	ENSGALG00010002511	VPS36	-0.043404585	0.076370906	-0.56833927	0.569804619	0.671980958	negative
PRJNA729271	stage_E14_vs_E10	ordinary_muscle_development	ENSGALG00010002512	DAXX	-0.294433041	0.232768835	-1.264916074	0.205901415	0.305803781	negative
PRJNA729271	stage_E14_vs_E10	ordinary_muscle_development	ENSGALG00010002518	MAST1	-0.270055388	0.363503663	-0.74292343	0.457528039	0.570434279	negative
PRJNA729271	stage_E14_vs_E10	ordinary_muscle_development	ENSGALG00010002520	TTC32	0.583720086	0.163036451	3.580304165	0.000343194	0.001212726	positive
PRJNA729271	stage_E14_vs_E10	ordinary_muscle_development	ENSGALG00010002522	HPSE	-0.623017595	0.314153818	-1.983160987	0.04734945	0.090592698	negative
PRJNA729271	stage_E14_vs_E10	ordinary_muscle_development	ENSGALG00010002523	DNAJB13	-0.251356558	0.462416265	-0.543572052	0.586735996	0.687085011	negative
PRJNA729271	stage_E14_vs_E10	ordinary_muscle_development	ENSGALG00010002526	ZBTB22	0.293440649	0.10329447	2.840816637	0.004499818	0.011870756	positive
PRJNA729271	stage_E14_vs_E10	ordinary_muscle_development	ENSGALG00010002527	LRRC3B	-0.881220074	0.164645463	-5.352228089	8.68778E-08	5.58198E-07	negative
PRJNA729271	stage_E14_vs_E10	ordinary_muscle_development	ENSGALG00010002528	MED14	-0.194418269	0.115401018	-1.684718839	0.09204283	0.158771965	negative
PRJNA729271	stage_E14_vs_E10	ordinary_muscle_development	ENSGALG00010002530	STAG3	0.475641391	0.120816454	3.936892491	8.25436E-05	0.00033006	positive
PRJNA729271	stage_E14_vs_E10	ordinary_muscle_development	ENSGALG00010002533	LPAR2	-0.673367411	0.141689275	-4.752423312	2.00993E-06	1.05865E-05	negative
PRJNA729271	stage_E14_vs_E10	ordinary_muscle_development	ENSGALG00010002536	RGL2	0.592581234	0.144283808	4.107052908	4.00739E-05	0.000169598	positive
PRJNA729271	stage_E14_vs_E10	ordinary_muscle_development	ENSGALG00010002539	SNAP91	0.068579578	0.188904801	0.363037774	0.716576667	0.795051779	positive
PRJNA729271	stage_E14_vs_E10	ordinary_muscle_development	ENSGALG00010002543	UCP3	0.553528772	0.56634706	0.977366726	0.32838763	0.443242266	positive
PRJNA729271	stage_E14_vs_E10	ordinary_muscle_development	ENSGALG00010002544	THSD1	0.463332139	0.124565509	3.719586111	0.00019955	0.000741763	positive
PRJNA729271	stage_E14_vs_E10	ordinary_muscle_development	ENSGALG00010002546	ATP6AP2	-0.101562346	0.063227185	-1.606308194	0.108206211	0.181807004	negative
PRJNA729271	stage_E14_vs_E10	ordinary_muscle_development	ENSGALG00010002547	PBX4	-0.276592818	0.096627091	-2.862476933	0.004203439	0.01116731	negative
PRJNA729271	stage_E14_vs_E10	ordinary_muscle_development	ENSGALG00010002551	WDR35	-0.004314966	0.10788997	-0.039994136	0.968097802	0.978469436	negative
PRJNA729271	stage_E14_vs_E10	ordinary_muscle_development	ENSGALG00010002553	ENSGALG00010002553	-0.419771655	0.227292955	-1.846830908	0.064771651	0.118125953	negative
PRJNA729271	stage_E14_vs_E10	ordinary_muscle_development	ENSGALG00010002554	EGFL6	0.255813633	0.292716688	0.873929105	0.382156863	0.498195205	positive
PRJNA729271	stage_E14_vs_E10	ordinary_muscle_development	ENSGALG00010002556	ENSGALG00010002556	2.770608835	0.231877934	11.94856617	6.6055E-33	3.31304E-31	positive
PRJNA729271	stage_E14_vs_E10	ordinary_muscle_development	ENSGALG00010002557	ENSGALG00010002557	0.288728893	0.407607932	0.708349545	0.478728216	0.590868997	positive
PRJNA729271	stage_E14_vs_E10	ordinary_muscle_development	ENSGALG00010002559	NEK10	-0.636748945	0.535541496	-1.188981526	0.234446937	0.338949566	negative
PRJNA729271	stage_E14_vs_E10	ordinary_muscle_development	ENSGALG00010002561	PAAF1	0.242544974	0.079702256	3.043138114	0.002341249	0.006680393	positive
PRJNA729271	stage_E14_vs_E10	ordinary_muscle_development	ENSGALG00010002565	MRPS18C	0.119844168	0.114710062	1.044757246	0.296135179	0.40943146	positive
PRJNA729271	stage_E14_vs_E10	ordinary_muscle_development	ENSGALG00010002566	TMSB4X	0.20267444	0.115570316	1.753689414	0.07948374	0.14064275	positive
PRJNA729271	stage_E14_vs_E10	ordinary_muscle_development	ENSGALG00010002567	COA4	0.354631173	0.21124815	1.678742149	0.093202308	0.160390294	positive
PRJNA729271	stage_E14_vs_E10	ordinary_muscle_development	ENSGALG00010002569	GTPBP6	-0.092192404	0.114928963	-0.802168588	0.422455444	0.536865831	negative
PRJNA729271	stage_E14_vs_E10	ordinary_muscle_development	ENSGALG00010002570	ADAM12	0.075973036	0.266491268	0.285086401	0.775577949	0.841026585	positive
PRJNA729271	stage_E14_vs_E10	ordinary_muscle_development	ENSGALG00010002571	BCOR	0.17111287	0.140813784	1.215171304	0.224300763	0.32731502	positive
PRJNA729271	stage_E14_vs_E10	ordinary_muscle_development	ENSGALG00010002573	MVB12A	0.648971675	0.094268689	6.884276036	5.80822E-12	6.06994E-11	positive
PRJNA729271	stage_E14_vs_E10	ordinary_muscle_development	ENSGALG00010002575	BGV	0.237776924	0.398525989	0.596640949	0.550747125	0.655717085	positive
PRJNA729271	stage_E14_vs_E10	ordinary_muscle_development	ENSGALG00010002576	HELQ	-0.113798852	0.102483466	-1.110411817	0.266821608	0.375784999	negative
PRJNA729271	stage_E14_vs_E10	ordinary_muscle_development	ENSGALG00010002577	MRPL48	0.045016881	0.102318539	0.439967983	0.659960296	0.749343921	positive
PRJNA729271	stage_E14_vs_E10	ordinary_muscle_development	ENSGALG00010002579	MATN3	-0.332281301	0.564253982	-0.588886054	0.555937714	0.660193115	negative
PRJNA729271	stage_E14_vs_E10	ordinary_muscle_development	ENSGALG00010002581	ATP13A1	0.015587419	0.076306525	0.20427373	0.838139597	0.887099924	positive
PRJNA729271	stage_E14_vs_E10	ordinary_muscle_development	ENSGALG00010002582	ENSGALG00010002582	-0.515336909	0.374072743	-1.377638224	0.168315013	0.260320755	negative
PRJNA729271	stage_E14_vs_E10	ordinary_muscle_development	ENSGALG00010002584	ENSGALG00010002584	0.192523555	0.442023971	0.435550032	0.663163231	0.752155793	positive
PRJNA729271	stage_E14_vs_E10	ordinary_muscle_development	ENSGALG00010002586	GPR63	-0.167645727	0.146110963	-1.147386367	0.251221973	0.358305864	negative
PRJNA729271	stage_E14_vs_E10	ordinary_muscle_development	ENSGALG00010002587	DOCK1	-0.382343935	0.066284454	-5.768229419	8.01087E-09	5.89729E-08	negative
PRJNA729271	stage_E14_vs_E10	ordinary_muscle_development	ENSGALG00010002588	FAM175A	-0.36372301	0.082138727	-4.428154946	9.50426E-06	4.47432E-05	negative
PRJNA729271	stage_E14_vs_E10	ordinary_muscle_development	ENSGALG00010002589	SLC4A7	0.506051293	0.143949438	3.515479466	0.000438961	0.001507043	positive
PRJNA729271	stage_E14_vs_E10	ordinary_muscle_development	ENSGALG00010002590	ATP6V1C2	-0.269772563	0.250120659	-1.078569695	0.280779597	0.391864073	negative
PRJNA729271	stage_E14_vs_E10	ordinary_muscle_development	ENSGALG00010002591	ENSGALG00010002591	0.333915688	0.211555125	1.578386194	0.114476915	0.19059762	positive
PRJNA729271	stage_E14_vs_E10	ordinary_muscle_development	ENSGALG00010002592	RPS6KA3	-0.076026403	0.101470505	-0.749246333	0.453708747	0.566696105	negative
PRJNA729271	stage_E14_vs_E10	ordinary_muscle_development	ENSGALG00010002597	ENSGALG00010002597	-0.150316352	0.113459067	-1.324850946	0.185220593	0.281128011	negative
PRJNA729271	stage_E14_vs_E10	ordinary_muscle_development	ENSGALG00010002598	NMT2	-0.047478437	0.101887218	-0.465990116	0.641222575	0.73370076	negative
PRJNA729271	stage_E14_vs_E10	ordinary_muscle_development	ENSGALG00010002599	KIFC1	0.076680272	0.218440497	0.351035057	0.725562048	0.801357961	positive
PRJNA729271	stage_E14_vs_E10	ordinary_muscle_development	ENSGALG00010002600	TRPA1	-1.674892397	0.553636574	-3.025256054	0.002484227	0.007019273	negative
PRJNA729271	stage_E14_vs_E10	ordinary_muscle_development	ENSGALG00010002601	PLCXD1	2.167002746	0.186132661	11.64224878	2.51301E-31	1.14188E-29	positive
PRJNA729271	stage_E14_vs_E10	ordinary_muscle_development	ENSGALG00010002603	INSYN2A	-0.442691846	0.257016521	-1.722425637	0.084992443	0.148824047	negative
PRJNA729271	stage_E14_vs_E10	ordinary_muscle_development	ENSGALG00010002604	BLEC3	-0.293919321	0.385275312	-0.762881273	0.445534206	0.558961786	negative
PRJNA729271	stage_E14_vs_E10	ordinary_muscle_development	ENSGALG00010002605	EOMES	-2.749060953	1.060929142	-2.591182432	0.009564677	0.022925674	negative
PRJNA729271	stage_E14_vs_E10	ordinary_muscle_development	ENSGALG00010002608	RPP38	-0.331232996	0.125361543	-2.642221742	0.008236411	0.020156472	negative
PRJNA729271	stage_E14_vs_E10	ordinary_muscle_development	ENSGALG00010002609	PDIA6	-0.267839172	0.12503003	-2.14219874	0.032177493	0.065068744	negative
PRJNA729271	stage_E14_vs_E10	ordinary_muscle_development	ENSGALG00010002611	NPS	0.1120219	0.373559045	0.299877358	0.764270706	0.832251813	positive
PRJNA729271	stage_E14_vs_E10	ordinary_muscle_development	ENSGALG00010002612	JPH1	0.971721848	0.214781842	4.524227176	6.06166E-06	2.95142E-05	positive
PRJNA729271	stage_E14_vs_E10	ordinary_muscle_development	ENSGALG00010002613	ENSGALG00010002613	-0.223985204	0.103361816	-2.16700144	0.030234744	0.061821501	negative
PRJNA729271	stage_E14_vs_E10	ordinary_muscle_development	ENSGALG00010002614	GPAT3	0.071262396	0.144455477	0.493317372	0.621788355	0.71701835	positive
PRJNA729271	stage_E14_vs_E10	ordinary_muscle_development	ENSGALG00010002616	ZNFB3	0.263469392	0.162089896	1.625452285	0.104066223	0.175931876	positive
PRJNA729271	stage_E14_vs_E10	ordinary_muscle_development	ENSGALG00010002619	RAB6A	-0.050892672	0.067014522	-0.759427526	0.447596852	0.560713659	negative
PRJNA729271	stage_E14_vs_E10	ordinary_muscle_development	ENSGALG00010002621	SLC25A15	-0.500627258	0.161306375	-3.103580115	0.001911945	0.005604263	negative
PRJNA729271	stage_E14_vs_E10	ordinary_muscle_development	ENSGALG00010002622	AZI2	0.187822106	0.079128919	2.373621535	0.017614595	0.038992602	positive
PRJNA729271	stage_E14_vs_E10	ordinary_muscle_development	ENSGALG00010002623	MID1IP1	0.131418277	0.136698548	0.961372881	0.336364715	0.451473851	positive
PRJNA729271	stage_E14_vs_E10	ordinary_muscle_development	ENSGALG00010002625	FHL5	0.524620247	0.218487598	2.40114428	0.016343891	0.036514288	positive
PRJNA729271	stage_E14_vs_E10	ordinary_muscle_development	ENSGALG00010002626	CHD7	0.559637072	0.135423576	4.132493677	3.58849E-05	0.000152957	positive
PRJNA729271	stage_E14_vs_E10	ordinary_muscle_development	ENSGALG00010002627	MRPS31	0.000633554	0.146326474	0.004329732	0.996545385	0.998160417	positive

PRJNA729271	stage_E14_vs_E10	ordinary_muscle_development	ENSGALG00010002628	TRIM7.2	0.581376752	0.191192771	3.040788353	0.002359596	0.006723481	positive
PRJNA729271	stage_E14_vs_E10	ordinary_muscle_development	ENSGALG00010002632	HPGD	0.253291156	0.274824447	0.921647104	0.35671268	0.473103696	positive
PRJNA729271	stage_E14_vs_E10	ordinary_muscle_development	ENSGALG00010002633	ENSGALG00010002633	-0.259283998	0.120360056	-2.154236266	0.031221648	0.063454541	negative
PRJNA729271	stage_E14_vs_E10	ordinary_muscle_development	ENSGALG00010002634	RGN	0.382968815	0.183380264	2.088386212	0.03676301	0.072889353	positive
PRJNA729271	stage_E14_vs_E10	ordinary_muscle_development	ENSGALG00010002638	FABP9	0.255820617	0.589638196	0.43386032	0.664389876	0.753183776	positive
PRJNA729271	stage_E14_vs_E10	ordinary_muscle_development	ENSGALG00010002644	KCNF1	1.798306853	0.476217504	3.776230058	0.00015922	0.000603213	positive
PRJNA729271	stage_E14_vs_E10	ordinary_muscle_development	ENSGALG00010002646	FOXO1	0.017826341	0.169791548	0.104989567	0.916384087	0.941788666	positive
PRJNA729271	stage_E14_vs_E10	ordinary_muscle_development	ENSGALG00010002647	FAM168A	-0.346584718	0.097689421	-3.547822431	0.00038843	0.001350838	negative
PRJNA729271	stage_E14_vs_E10	ordinary_muscle_development	ENSGALG00010002649	UFL1	0.055502077	0.078864804	0.703762305	0.481580812	0.593379282	positive
PRJNA729271	stage_E14_vs_E10	ordinary_muscle_development	ENSGALG00010002653	EIF1AX	-0.106243124	0.063279015	-1.678962992	0.093159257	0.160354284	negative
PRJNA729271	stage_E14_vs_E10	ordinary_muscle_development	ENSGALG00010002655	FABP4	5.479443026	0.94643242	5.789576635	7.05641E-09	5.23452E-08	positive
PRJNA729271	stage_E14_vs_E10	ordinary_muscle_development	ENSGALG00010002656	GLRA3	1.100648969	0.571818026	1.924823841	0.0542514	0.101520017	positive
PRJNA729271	stage_E14_vs_E10	ordinary_muscle_development	ENSGALG00010002658	FBXL12	-0.080329027	0.136679677	-0.587717423	0.556721979	0.660907869	negative
PRJNA729271	stage_E14_vs_E10	ordinary_muscle_development	ENSGALG00010002661	ACBD7	-2.193524923	0.202033659	-10.85722514	1.84263E-27	6.76177E-26	negative
PRJNA729271	stage_E14_vs_E10	ordinary_muscle_development	ENSGALG00010002664	ZNFB1	0.433643848	0.134766912	3.217732316	0.001292083	0.003963756	positive
PRJNA729271	stage_E14_vs_E10	ordinary_muscle_development	ENSGALG00010002667	ENSGALG00010002667	1.48241021	1.587097995	0.934038235			positive
PRJNA729271	stage_E14_vs_E10	ordinary_muscle_development	ENSGALG00010002669	IL4I1	1.203191063	1.220116288	0.986128186	0.324070214	0.438713327	positive
PRJNA729271	stage_E14_vs_E10	ordinary_muscle_development	ENSGALG00010002673	SLC66A3	-0.334372941	0.1472576	-2.270666777	0.023167158	0.049412588	negative
PRJNA729271	stage_E14_vs_E10	ordinary_muscle_development	ENSGALG00010002678	CCNI	0.92161658	0.172742823	5.335194622	9.54421E-08	6.10564E-07	positive
PRJNA729271	stage_E14_vs_E10	ordinary_muscle_development	ENSGALG00010002681	PLEKHB1	-0.604473216	0.158471303	-3.814401752	0.000136513	0.000525009	negative
PRJNA729271	stage_E14_vs_E10	ordinary_muscle_development	ENSGALG00010002682	UBL5	0.055824311	0.165478663	0.337350511	0.735852687	0.810254079	positive
PRJNA729271	stage_E14_vs_E10	ordinary_muscle_development	ENSGALG00010002685	CA8	-1.62438471	0.185054278	-8.777882491	1.66581E-18	3.16461E-17	negative
PRJNA729271	stage_E14_vs_E10	ordinary_muscle_development	ENSGALG00010002689	COG6	-0.131203678	0.059133437	-2.218773075	0.026502167	0.055401497	negative
PRJNA729271	stage_E14_vs_E10	ordinary_muscle_development	ENSGALG00010002693	MSC	0.890313669	0.168492188	5.284005628	1.26389E-07	7.91709E-07	positive
PRJNA729271	stage_E14_vs_E10	ordinary_muscle_development	ENSGALG00010002700	LHFPL6	-0.044102946	0.170134741	-0.259223635	0.795462696	0.85631131	negative
PRJNA729271	stage_E14_vs_E10	ordinary_muscle_development	ENSGALG00010002701	TMEM196	1.846275044	0.409105184	4.512959297	6.39293E-06	3.10333E-05	positive
PRJNA729271	stage_E14_vs_E10	ordinary_muscle_development	ENSGALG00010002703	PIN1	0.116000408	0.095600031	1.213392995	0.224979603	0.32809458	positive
PRJNA729271	stage_E14_vs_E10	ordinary_muscle_development	ENSGALG00010002704	KCNB2	-0.614552195	0.259002462	-2.372765848	0.017655453	0.039065148	negative
PRJNA729271	stage_E14_vs_E10	ordinary_muscle_development	ENSGALG00010002705	JADE3	0.320371293	0.051762838	6.189214268	6.04648E-10	5.09557E-09	positive
PRJNA729271	stage_E14_vs_E10	ordinary_muscle_development	ENSGALG00010002706	ROCK2	-0.097846997	0.144355247	-0.677820858	0.497885284	0.607274251	negative
PRJNA729271	stage_E14_vs_E10	ordinary_muscle_development	ENSGALG00010002707	RELT	-0.642009911	0.215243179	-2.982718961	0.002857002	0.007956243	negative
PRJNA729271	stage_E14_vs_E10	ordinary_muscle_development	ENSGALG00010002712	PTPRE	1.275250075	0.121740943	10.47511254	1.12404E-25	3.58875E-24	positive
PRJNA729271	stage_E14_vs_E10	ordinary_muscle_development	ENSGALG00010002713	OLFM2	0.006303724	0.174825917	0.03605715	0.971236789	0.98009449	positive
PRJNA729271	stage_E14_vs_E10	ordinary_muscle_development	ENSGALG00010002716	NHLRC3	-0.297133793	0.107566757	-2.76231989	0.005739222	0.014677138	negative
PRJNA729271	stage_E14_vs_E10	ordinary_muscle_development	ENSGALG00010002722	TRIM7.1	1.644847822	0.149586054	10.99599716	3.99473E-28	1.51978E-26	positive
PRJNA729271	stage_E14_vs_E10	ordinary_muscle_development	ENSGALG00010002725	TSPAN7	-0.44745906	0.178843173	-2.501963336	0.012350671	0.028675794	negative
PRJNA729271	stage_E14_vs_E10	ordinary_muscle_development	ENSGALG00010002728	RP2	-0.085175995	0.111047371	-0.767023962	0.443067269	0.556859453	negative
PRJNA729271	stage_E14_vs_E10	ordinary_muscle_development	ENSGALG00010002730	E2F6	-0.175788225	0.101062037	-1.739409091	0.08196283	0.144373706	negative
PRJNA729271	stage_E14_vs_E10	ordinary_muscle_development	ENSGALG00010002733	RDH8	0.3266316	0.36395084	0.897460766	0.3694731	0.485804843	positive
PRJNA729271	stage_E14_vs_E10	ordinary_muscle_development	ENSGALG00010002739	HEP21	2.379547559	0.605399609	3.930540295	8.47552E-05	0.000337879	positive
PRJNA729271	stage_E14_vs_E10	ordinary_muscle_development	ENSGALG00010002741	PDK4	2.656626534	0.574602042	4.623419931	3.77464E-06	1.89386E-05	positive
PRJNA729271	stage_E14_vs_E10	ordinary_muscle_development	ENSGALG00010002742	PROSER1	-0.034495542	0.118495959	-0.291111545	0.77096601	0.837419852	negative
PRJNA729271	stage_E14_vs_E10	ordinary_muscle_development	ENSGALG00010002747	FUT9	-1.568753709	0.532993665	-2.94328772	0.003247465	0.00891684	negative
PRJNA729271	stage_E14_vs_E10	ordinary_muscle_development	ENSGALG00010002750	ENSGALG00010002750	-0.035235533	0.234380348	-0.150334844	0.880500444	0.917860764	negative
PRJNA729271	stage_E14_vs_E10	ordinary_muscle_development	ENSGALG00010002752	NDUFA4	0.628533792	0.15421274	4.0757579	4.58647E-05	0.000191919	positive
PRJNA729271	stage_E14_vs_E10	ordinary_muscle_development	ENSGALG00010002753	SLC9A7	0.247348263	0.151548692	1.632137229	0.102650581	0.173821754	positive
PRJNA729271	stage_E14_vs_E10	ordinary_muscle_development	ENSGALG00010002758	GREB1	-2.147473977	0.244592249	-8.779812048	1.63748E-18	3.11487E-17	negative
PRJNA729271	stage_E14_vs_E10	ordinary_muscle_development	ENSGALG00010002760	RAB2A	0.241310826	0.092466534	2.609709881	0.009061904	0.021884755	positive
PRJNA729271	stage_E14_vs_E10	ordinary_muscle_development	ENSGALG00010002762	PPAN	-0.076132067	0.142985148	-0.532447377	0.594416179	0.694339795	negative
PRJNA729271	stage_E14_vs_E10	ordinary_muscle_development	ENSGALG00010002763	SEPTIN11	-0.350174594	0.115394655	-3.034582441	0.002408689	0.006831139	negative
PRJNA729271	stage_E14_vs_E10	ordinary_muscle_development	ENSGALG00010002765	MAP7D2	-2.622696193	0.414356422	-6.329565692	2.45852E-10	2.17294E-09	negative
PRJNA729271	stage_E14_vs_E10	ordinary_muscle_development	ENSGALG00010002766	NANOS1	-1.632046804	0.330989091	-4.930817509	8.18862E-07	4.57395E-06	negative
PRJNA729271	stage_E14_vs_E10	ordinary_muscle_development	ENSGALG00010002768	TRIM39.2	0.518441477	1.189325507	0.435912182	0.662900446	0.751916579	positive
PRJNA729271	stage_E14_vs_E10	ordinary_muscle_development	ENSGALG00010002774	TBX18	-1.208399012	0.117707466	-10.26612032	1.00152E-24	2.92092E-23	negative
PRJNA729271	stage_E14_vs_E10	ordinary_muscle_development	ENSGALG00010002779	FBXO8	-0.269080196	0.087298461	-3.082301724	0.002054065	0.005951164	negative
PRJNA729271	stage_E14_vs_E10	ordinary_muscle_development	ENSGALG00010002781	EIF3G	0.576405873	0.137555222	4.1903598	2.78512E-05	0.000121269	positive
PRJNA729271	stage_E14_vs_E10	ordinary_muscle_development	ENSGALG00010002782	GSTA3	-0.096606798	0.146535485	-0.659272381	0.509720875	0.618486912	negative
PRJNA729271	stage_E14_vs_E10	ordinary_muscle_development	ENSGALG00010002784	TRIM27.2	0.115852073	0.528179974	0.219342041	0.826383615	0.878506087	positive
PRJNA729271	stage_E14_vs_E10	ordinary_muscle_development	ENSGALG00010002787	CEP44	-0.280733019	0.127135012	-2.208148762	0.027233905	0.056676296	negative
PRJNA729271	stage_E14_vs_E10	ordinary_muscle_development	ENSGALG00010002788	ENSGALG00010002788	4.954550308	0.629995695	7.864419303	3.70814E-15	5.2799E-14	positive
PRJNA729271	stage_E14_vs_E10	ordinary_muscle_development	ENSGALG00010002790	BCLAF3	0.09680878	0.101588467	0.952950498	0.340615152	0.455630918	positive
PRJNA729271	stage_E14_vs_E10	ordinary_muscle_development	ENSGALG00010002792	ENSGALG00010002792	0.497116918	0.228747747	2.173210117	0.029764507	0.061023554	positive
PRJNA729271	stage_E14_vs_E10	ordinary_muscle_development	ENSGALG00010002796	PRDM8	-0.192143306	0.803833632	-0.239033673	0.811079472	0.867276561	negative
PRJNA729271	stage_E14_vs_E10	ordinary_muscle_development	ENSGALG00010002800	TUBGCP5	-0.166582292	0.074567151	-2.233990313	0.02548371	0.053619739	negative
PRJNA729271	stage_E14_vs_E10	ordinary_muscle_development	ENSGALG00010002801	FGF5	-0.372899098	0.348037534	-1.071433571	0.283974525	0.395561286	negative
PRJNA729271	stage_E14_vs_E10	ordinary_muscle_development	ENSGALG00010002805	FAM171A1	-0.196193429	0.098014875	-2.001669942	0.045320241	0.087321135	negative
PRJNA729271	stage_E14_vs_E10	ordinary_muscle_development	ENSGALG00010002807	P2RY6	1.093281403	0.220597161	4.956008483	7.19561E-07	4.05522E-06	positive
PRJNA729271	stage_E14_vs_E10	ordinary_muscle_development	ENSGALG00010002809	SH3KBP1	0.031399099	0.109487142	0.286783441	0.774278143	0.839994138	positive
PRJNA729271	stage_E14_vs_E10	ordinary_muscle_development	ENSGALG00010002810	TRIM27.1	0.882503653	0.304122168	2.901806398	0.003710178	0.0100054	positive
PRJNA729271	stage_E14_vs_E10	ordinary_muscle_development	ENSGALG00010002812	TRIM13	-0.063981154	0.099244597	-0.644681481	0.519133619	0.62717468	negative
PRJNA729271	stage_E14_vs_E10	ordinary_muscle_development	ENSGALG00010002814	TERF1	-0.460287946	0.148368309	-3.102333306	0.001920016	0.005623487	negative
PRJNA729271	stage_E14_vs_E10	ordinary_muscle_development	ENSGALG00010002817	SHFM1	0.09843843	0.091385859	1.077173551	0.281402739	0.39254477	positive
PRJNA729271	stage_E14_vs_E10	ordinary_muscle_development	ENSGALG00010002819	BMP3	0.881734882	0.342617284	2.573527146	0.010066775	0.023940591	positive
PRJNA729271	stage_E14_vs_E10	ordinary_muscle_development	ENSGALG00010002824	NXPH1	-0.883559807	0.451798298	-1.955651028	0.050506277	0.09557291	negative
PRJNA729271	stage_E14_vs_E10	ordinary_muscle_development	ENSGALG00010002825	TRIM41	0.335747809	0.119853849	2.801310191	0.005089557	0.013225732	positive
PRJNA729271	stage_E14_vs_E10	ordinary_muscle_development	ENSGALG00010002827	SBSPON	-0.216423879	0.336791475	-0.64260498	0.520480451	0.628330485	negative
PRJNA729271	stage_E14_vs_E10	ordinary_muscle_development	ENSGALG00010002832	LPIN1	-0.942541637	0.207712199	-4.537728844	5.68633E-06	2.78175E-05	negative
PRJNA729271	stage_E14_vs_E10	ordinary_muscle_development	ENSGALG00010002834	MRPL53	-0.235285799	0.112279656	-2.095533665	0.036123579	0.071825964	negative
PRJNA729271	stage_E14_vs_E10	ordinary_muscle_development	ENSGALG00010002835	CYFIP1	0.005251355	0.083991425	0.062522518	0.950146731	0.965601687	positive
PRJNA729271	stage_E14_vs_E10	ordinary_muscle_development	ENSGALG00010002837	DNMT1	-0.127778765	0.12931533	-0.98811769	0.323095013	0.437769884	negative
PRJNA729271	stage_E14_vs_E10	ordinary_muscle_development	ENSGALG00010002838	ENSGALG00010002838	0.090751654	0.264993361	0.34246765	0.731998985	0.806930207	positive
PRJNA729271	stage_E14_vs_E10	ordinary_muscle_development	ENSGALG00010002840	SRPX	0.731992047	0.169197666	4.326253806	1.51667E-05	6.90889E-05	positive
PRJNA729271	stage_E14_vs_E10	ordinary_muscle_development	ENSGALG00010002848	TRIB2	-0.185554161	0.106554902	-1.741394874	0.081614386	0.143899833	negative
PRJNA729271	stage_E14_vs_E10	ordinary_muscle_development	ENSGALG00010002853	CEP162	-0.586639319	0.168098183	-3.489861161	0.000483272	0.001645144	negative
PRJNA729271	stage_E14_vs_E10	ordinary_muscle_development	ENSGALG00010002855	RACK1	0.615129561	0.125262202	4.910735664	9.07353E-07	5.02948E-06	positive
PRJNA729271	stage_E14_vs_E10	ordinary_muscle_development	ENSGALG00010002857	DCAF15	-0.119363304	0.139843903	-0.853546712	0.393356195	0.508716813	negative
PRJNA729271	stage_E14_vs_E10	ordinary_muscle_development	ENSGALG00010002860	NIPA2	0.263924029	0.107508051	2.454923388	0.014091462	0.03214095	positive
PRJNA729271	stage_E14_vs_E10	ordinary_muscle_development	ENSGALG00010002864	RNASEH2B	0.159946946	0.091737094	1.743536222	0.081239993	0.143292005	positive
PRJNA729271	stage_E14_vs_E10	ordinary_muscle_development	ENSGALG00010002866	ENSGALG00010002866	1.948490058	0.252470382	7.717697587	1.1845E-14	1.62127E-13	positive
PRJNA729271	stage_E14_vs_E10	ordinary_muscle_development	ENSGALG00010002868	PPP1R9A	0.779486639	0.066445931	11.73114186	8.82599E-32	4.12686E-30	positive
PRJNA729271	stage_E14_vs_E10	ordinary_muscle_development	ENSGALG00010002869	ENSGALG00010002869	-0.525001162	0.311881929	-1.683333058	0.092310634	0.159158159	negative
PRJNA729271	stage_E14_vs_E10	ordinary_muscle_development	ENSGALG00010002870	FABP5	1.571363947	0.216473957	7.258905276	3.90236E-13	4.6632E-12	positive
PRJNA729271	stage_E14_vs_E10	ordinary_muscle_development	ENSGALG00010002878	P2RY2	1.329011512	0.258688913	5.137489264	2.78433E-07	1.66497E-06	positive
PRJNA729271	stage_E14_vs_E10	ordinary_muscle_development	ENSGALG00010002880	RASGEF1B	-0.715961111	0.207601436	-3.448729094	0.000563231	0.001882416	negative
PRJNA729271	stage_E14_vs_E10	ordinary_muscle_development	ENSGALG00010002881	ENSGALG00010002881	1.211666214	0.33553619	3.611134204	0.000304861	0.001090293	positive
PRJNA729271	stage_E14_vs_E10	ordinary_muscle_development	ENSGALG00010002883	MAP3K15	0.124674813	0.430627636	0.289518839	0.772184366	0.838412912	positive
PRJNA729271	stage_E14_vs_E10	ordinary_muscle_development	ENSGALG00010002884	ADHFE1	1.471830018	0.261199472	5.634888952	1.75171E-08	1.23919E-07	positive
PRJNA729271	stage_E14_vs_E10	ordinary_muscle_development	ENSGALG00010002886	HERC2	-0.123466541	0.105796009	-1.167024562	0.243200451	0.34961723	negative
PRJNA729271	stage_E14_vs_E10	ordinary_muscle_development	ENSGALG00010002888	ENSGALG00010002888	-4.004141222	0.366701397	-10.91935088	9.31592E-28	3.44475E-26	negative
PRJNA729271	stage_E14_vs_E10	ordinary_muscle_development	ENSGALG00010002890	VXN	-0.040596149	0.1644089	-0.246921846	0.804968709	0.86289169	negative
PRJNA729271	stage_E14_vs_E10	ordinary_muscle_development	ENSGALG00010002894	FCHSD2	-0.287233865	0.06966567	-4.123033129	3.73916E-05	0.000158942	negative
PRJNA729271	stage_E14_vs_E10	ordinary_muscle_development	ENSGALG00010002896	OCA2	-1.808412023	0.245805528	-7.357084421	1.8797E-13	2.3149E-12	negative
PRJNA729271	stage_E14_vs_E10	ordinary_muscle_development	ENSGALG00010002904	PON1	1.102603401	0.179449384	6.144369925	8.02815E-10	6.64508E-09	positive
PRJNA729271	stage_E14_vs_E10	ordinary_muscle_development	ENSGALG00010002907	GABRG3	-1.24248684	0.345390556	-3.597338782	0.00032149	0.001142715	negative
PRJNA729271	stage_E14_vs_E10	ordinary_muscle_development	ENSGALG00010002908	S1PR2	-0.228499465	0.179083556	-1.275937724	0.201977534	0.301324039	negative
PRJNA729271	stage_E14_vs_E10	ordinary_muscle_development	ENSGALG00010002911	MYBL1	0.331086989	0.217388491	1.52301986	0.127753737	0.207949513	positive
PRJNA729271	stage_E14_vs_E10	ordinary_muscle_development	ENSGALG00010002912	RPS3	0.617728225	0.19226267	3.212938965	0.001313842	0.004020296	positive
PRJNA729271	stage_E14_vs_E10	ordinary_muscle_development	ENSGALG00010002913	NXNL1	-1.172161815	0.602434046	-1.945709779	0.051689611	0.097506626	negative
PRJNA729271	stage_E14_vs_E10	ordinary_muscle_development	ENSGALG00010002914	SLC27A1	-0.053759567	0.307166512	-0.175017668	0.861065751	0.904365485	negative
PRJNA729271	stage_E14_vs_E10	ordinary_muscle_development	ENSGALG00010002916	SDCBP	-0.487134175	0.122465172	-3.977736429	6.95744E-05	0.000282093	negative
PRJNA729271	stage_E14_vs_E10	ordinary_muscle_development	ENSGALG00010002917	PGLS	0.628598789	0.095031835	6.614612757	3.72526E-11	3.60465E-10	positive
PRJNA729271	stage_E14_vs_E10	ordinary_muscle_development	ENSGALG00010002918	LRATD1	-2.788767904	0.396629276	-7.031170102	2.04809E-12	2.26446E-11	negative
PRJNA729271	stage_E14_vs_E10	ordinary_muscle_development	ENSGALG00010002919	ENSGALG00010002919	-0.25399564	0.204586662	-1.241506348	0.214418755	0.316110644	negative
PRJNA729271	stage_E14_vs_E10	ordinary_muscle_development	ENSGALG00010002920	VCPIP1	0.006129886	0.102770863	0.059646142	0.952437467	0.967048269	positive
PRJNA729271	stage_E14_vs_E10	ordinary_muscle_development	ENSGALG00010002922	TMEM106B	-0.023240666	0.090624399	-0.256450425	0.797603056	0.857676941	negative
PRJNA729271	stage_E14_vs_E10	ordinary_muscle_development	ENSGALG00010002923	SERPINH1	0.286911655	0.158422695	1.811051477	0.070132884	0.126314134	positive
PRJNA729271	stage_E14_vs_E10	ordinary_muscle_development	ENSGALG00010002925	INTS6	-0.41997577	0.121849822	-3.446667081	0.000567547	0.001895967	negative
PRJNA729271	stage_E14_vs_E10	ordinary_muscle_development	ENSGALG00010002926	GALNTL6	-2.008089578	0.3339485	-6.013171417	1.81928E-09	1.44813E-08	negative
PRJNA729271	stage_E14_vs_E10	ordinary_muscle_development	ENSGALG00010002927	GABRA5	-5.614444561	0.682534751	-8.225873562	1.93773E-16	3.10016E-15	negative
PRJNA729271	stage_E14_vs_E10	ordinary_muscle_development	ENSGALG00010002928	ASB4	2.021152923	0.613718482	3.293289971	0.000990223	0.003121636	positive
PRJNA729271	stage_E14_vs_E10	ordinary_muscle_development	ENSGALG00010002929	PDHA2	0.215566449	0.112788776	1.911240256	0.055973713	0.10431203	positive
PRJNA729271	stage_E14_vs_E10	ordinary_muscle_development	ENSGALG00010002930	SERAF	-0.490190268	0.277398795	-1.767095881	0.077212172	0.137172501	negative
PRJNA729271	stage_E14_vs_E10	ordinary_muscle_development	ENSGALG00010002934	VWDE	-5.213294259	0.639126028	-8.156911201	3.43701E-16	5.35693E-15	negative
PRJNA729271	stage_E14_vs_E10	ordinary_muscle_development	ENSGALG00010002935	SYTL5	-3.089011861	0.612645797	-5.042084472	4.60488E-07	2.67312E-06	negative
PRJNA729271	stage_E14_vs_E10	ordinary_muscle_development	ENSGALG00010002937	GALNT7	0.307375475	0.107280142	2.865166561	0.0041679	0.011078985	positive
PRJNA729271	stage_E14_vs_E10	ordinary_muscle_development	ENSGALG00010002939	HMGB2	0.137651855	0.17047027	0.807483058	0.419388232	0.533951025	positive
PRJNA729271	stage_E14_vs_E10	ordinary_muscle_development	ENSGALG00010002940	BG1	0.407542252	0.426786812	0.95490826	0.339624092	0.45468285	positive
PRJNA729271	stage_E14_vs_E10	ordinary_muscle_development	ENSGALG00010002942	GABRB3	-3.640945566	0.502016822	-7.252636573	4.08736E-13	4.87623E-12	negative
PRJNA729271	stage_E14_vs_E10	ordinary_muscle_development	ENSGALG00010002943	SAP30	0.037295697	0.146082985	0.255304865	0.798487643	0.858229155	positive
PRJNA729271	stage_E14_vs_E10	ordinary_muscle_development	ENSGALG00010002946	GDPD5	1.434319498	0.114270151	12.55200496	3.87571E-36	2.30239E-34	positive
PRJNA729271	stage_E14_vs_E10	ordinary_muscle_development	ENSGALG00010002947	ENSGALG00010002947	-1.304619795	0.677325351	-1.926134603	0.054087569	0.101291901	negative

PRJNA729271	stage_E14_vs_E10	ordinary_muscle_development	ENSGALG00010002948	DLX6	-4.891229821	1.601259444	-3.054614192	0.002253502	0.006461822	negative
PRJNA729271	stage_E14_vs_E10	ordinary_muscle_development	ENSGALG00010002949	ENSGALG00010002949	0.240013969	1.183033576	0.202880099	0.83922875	0.887734126	positive
PRJNA729271	stage_E14_vs_E10	ordinary_muscle_development	ENSGALG00010002951	ENSGALG00010002951	-1.564881896	4.276978549	-0.365884906	0.71445096	0.793568283	negative
PRJNA729271	stage_E14_vs_E10	ordinary_muscle_development	ENSGALG00010002952	NBAS	-0.089626194	0.064103627	-1.398145436	0.162069398	0.252601712	negative
PRJNA729271	stage_E14_vs_E10	ordinary_muscle_development	ENSGALG00010002953	WDFY2	0.749249659	0.129046986	5.806022143	6.39745E-09	4.77257E-08	positive
PRJNA729271	stage_E14_vs_E10	ordinary_muscle_development	ENSGALG00010002954	CHST7	0.375070083	0.122004349	3.074235367	0.002110429	0.006098617	positive
PRJNA729271	stage_E14_vs_E10	ordinary_muscle_development	ENSGALG00010002955	BLEC1	-0.797188078	0.497425761	-1.602627246	0.109016975	0.182957167	negative
PRJNA729271	stage_E14_vs_E10	ordinary_muscle_development	ENSGALG00010002956	SCRG1	-0.70076315	0.400559011	-1.74946295	0.080211028	0.14175309	negative
PRJNA729271	stage_E14_vs_E10	ordinary_muscle_development	ENSGALG00010002957	ADGRG2	-0.299975018	0.292490038	-1.025590547	0.305084625	0.418808755	negative
PRJNA729271	stage_E14_vs_E10	ordinary_muscle_development	ENSGALG00010002958	ENSGALG00010002958	0.382238345	0.17364307	2.201287651	0.027715667	0.057539185	positive
PRJNA729271	stage_E14_vs_E10	ordinary_muscle_development	ENSGALG00010002959	DLX5	-3.770649993	0.578829397	-6.514268297	7.30448E-11	6.84412E-10	negative
PRJNA729271	stage_E14_vs_E10	ordinary_muscle_development	ENSGALG00010002960	BLEC2	0.149649009	0.768615817	0.194699362	0.845628311	0.892549506	positive
PRJNA729271	stage_E14_vs_E10	ordinary_muscle_development	ENSGALG00010002965	PRSS35	0.634582908	0.211468042	3.000845431	0.002692312	0.007557138	positive
PRJNA729271	stage_E14_vs_E10	ordinary_muscle_development	ENSGALG00010002967	OLFM4	-3.433999739	0.907485664	-3.784081527	0.000154277	0.000586326	negative
PRJNA729271	stage_E14_vs_E10	ordinary_muscle_development	ENSGALG00010002972	ME1	-0.536351757	0.153519851	-3.493696429	0.000476382	0.001624744	negative
PRJNA729271	stage_E14_vs_E10	ordinary_muscle_development	ENSGALG00010002978	DYNLT3	0.177933592	0.102042949	1.743712769	0.081209188	0.143263187	positive
PRJNA729271	stage_E14_vs_E10	ordinary_muscle_development	ENSGALG00010002981	KLHL35	0.718086329	0.22195486	3.235280947	0.001215231	0.003758219	positive
PRJNA729271	stage_E14_vs_E10	ordinary_muscle_development	ENSGALG00010002982	HAND2	-2.952601532	0.295629978	-9.987490266	1.72904E-23	4.64119E-22	negative
PRJNA729271	stage_E14_vs_E10	ordinary_muscle_development	ENSGALG00010002983	NSMAF	0.365721723	0.111345809	3.28455759	0.001021426	0.003215108	positive
PRJNA729271	stage_E14_vs_E10	ordinary_muscle_development	ENSGALG00010002985	ENSGALG00010002985	0.265752785	0.208835481	1.272546142	0.203179141	0.302805022	positive
PRJNA729271	stage_E14_vs_E10	ordinary_muscle_development	ENSGALG00010002987	PCDH8	-2.276968249	0.365471187	-6.230226428	4.65762E-10	3.98537E-09	negative
PRJNA729271	stage_E14_vs_E10	ordinary_muscle_development	ENSGALG00010002991	BLB1	0.276767947	0.313372762	0.883190822	0.37713318	0.493415059	positive
PRJNA729271	stage_E14_vs_E10	ordinary_muscle_development	ENSGALG00010002992	ENSGALG00010002992	-1.382253371	0.252790155	-5.467987345	4.55174E-08	3.04044E-07	negative
PRJNA729271	stage_E14_vs_E10	ordinary_muscle_development	ENSGALG00010002994	DUSP4	-1.147124641	0.12414491	-9.240206807	2.46021E-20	5.39497E-19	negative
PRJNA729271	stage_E14_vs_E10	ordinary_muscle_development	ENSGALG00010002995	RWDD2A	-0.720804911	0.125569323	-5.740294631	9.4512E-09	6.89804E-08	negative
PRJNA729271	stage_E14_vs_E10	ordinary_muscle_development	ENSGALG00010002998	LECT1	-1.572662019	0.27986008	-5.619458199	1.91557E-08	1.3505E-07	negative
PRJNA729271	stage_E14_vs_E10	ordinary_muscle_development	ENSGALG00010002999	CMTM7	-0.205877961	0.199125991	-1.033908028	0.301179161	0.41513807	negative
PRJNA729271	stage_E14_vs_E10	ordinary_muscle_development	ENSGALG00010003001	MAP6	-1.072379308	0.290907268	-3.686326975	0.000227514	0.000834045	negative
PRJNA729271	stage_E14_vs_E10	ordinary_muscle_development	ENSGALG00010003002	PHKA2	-0.160144048	0.184876974	-0.866219539	0.38636978	0.502485892	negative
PRJNA729271	stage_E14_vs_E10	ordinary_muscle_development	ENSGALG00010003006	CYP7B1	0.904196767	0.200038904	4.520104593	6.18091E-06	3.00444E-05	positive
PRJNA729271	stage_E14_vs_E10	ordinary_muscle_development	ENSGALG00010003009	TNKS	0.270540639	0.057444998	4.709559542	2.48253E-06	1.28883E-05	positive
PRJNA729271	stage_E14_vs_E10	ordinary_muscle_development	ENSGALG00010003017	PGM3	0.137728457	0.152166403	0.905117384	0.365403203	0.481414237	positive
PRJNA729271	stage_E14_vs_E10	ordinary_muscle_development	ENSGALG00010003018	MOGAT2	0.642863438	0.794587247	0.809053305	0.418484486	0.532987665	positive
PRJNA729271	stage_E14_vs_E10	ordinary_muscle_development	ENSGALG00010003019	TAPBP	0.019623807	0.256719313	0.076440712	0.939068481	0.957229088	positive
PRJNA729271	stage_E14_vs_E10	ordinary_muscle_development	ENSGALG00010003022	BLB2	0.576973663	0.272312221	2.118794598	0.03410783	0.068418626	positive
PRJNA729271	stage_E14_vs_E10	ordinary_muscle_development	ENSGALG00010003024	SUGT1	0.058727949	0.071725223	0.818790745	0.412905812	0.527737391	positive
PRJNA729271	stage_E14_vs_E10	ordinary_muscle_development	ENSGALG00010003025	MFHAS1	0.265582561	0.123066918	2.158033738	0.030925209	0.062957992	positive
PRJNA729271	stage_E14_vs_E10	ordinary_muscle_development	ENSGALG00010003027	SCIN	-1.007443128	0.254538387	-3.957922181	7.56046E-05	0.00030416	negative
PRJNA729271	stage_E14_vs_E10	ordinary_muscle_development	ENSGALG00010003030	PPEF1	1.033527722	0.598437063	1.727044975	0.084159587	0.147615345	positive
PRJNA729271	stage_E14_vs_E10	ordinary_muscle_development	ENSGALG00010003034	ERI1	-0.101671819	0.059060258	-1.721492983	0.085161404	0.149047882	negative
PRJNA729271	stage_E14_vs_E10	ordinary_muscle_development	ENSGALG00010003035	DGAT2	1.758636245	0.457142491	3.84701987	0.000119563	0.00046463	positive
PRJNA729271	stage_E14_vs_E10	ordinary_muscle_development	ENSGALG00010003042	TOX	-0.621083822	0.28016614	-2.216841127	0.02663395	0.055596069	negative
PRJNA729271	stage_E14_vs_E10	ordinary_muscle_development	ENSGALG00010003052	ENSGALG00010003052	-0.154233716	0.093445923	-1.650513047	0.098838047	0.168528113	negative
PRJNA729271	stage_E14_vs_E10	ordinary_muscle_development	ENSGALG00010003057	DDX1	-0.033006083	0.089387873	-0.369245653	0.71194463	0.791504634	negative
PRJNA729271	stage_E14_vs_E10	ordinary_muscle_development	ENSGALG00010003061	ENSGALG00010003061	-1.760733169	0.342123679	-5.146481453	2.65418E-07	1.59306E-06	negative
PRJNA729271	stage_E14_vs_E10	ordinary_muscle_development	ENSGALG00010003063	GGH	0.269702076	0.087714235	3.074781137	0.002106571	0.006088683	positive
PRJNA729271	stage_E14_vs_E10	ordinary_muscle_development	ENSGALG00010003064	VAV1	2.162021761	0.247266816	8.74367937	2.25639E-18	4.25311E-17	positive
PRJNA729271	stage_E14_vs_E10	ordinary_muscle_development	ENSGALG00010003068	BRD2	-0.064468911	0.126126157	-0.511146244	0.609248657	0.706541266	negative
PRJNA729271	stage_E14_vs_E10	ordinary_muscle_development	ENSGALG00010003072	ELF1	0.328664268	0.074084245	4.436358475	9.14934E-06	4.31986E-05	positive
PRJNA729271	stage_E14_vs_E10	ordinary_muscle_development	ENSGALG00010003074	TTPA	0.550273292	0.355633326	1.547305193	0.12178967	0.199993346	positive
PRJNA729271	stage_E14_vs_E10	ordinary_muscle_development	ENSGALG00010003077	EIF2S3	0.339046049	0.081664825	4.151677913	3.30047E-05	0.000141707	positive
PRJNA729271	stage_E14_vs_E10	ordinary_muscle_development	ENSGALG00010003081	DOP1A	-0.245751881	0.103863564	-2.36610292	0.017976446	0.039642262	negative
PRJNA729271	stage_E14_vs_E10	ordinary_muscle_development	ENSGALG00010003087	TWISTNB	-0.323415906	0.178626579	-1.810569894	0.070207456	0.126432734	negative
PRJNA729271	stage_E14_vs_E10	ordinary_muscle_development	ENSGALG00010003090	DMA	-0.340204363	0.197608711	-1.7216061	0.085140897	0.149047882	negative
PRJNA729271	stage_E14_vs_E10	ordinary_muscle_development	ENSGALG00010003092	ART1F	0.39379818	1.563669603	0.25184232	0.801162945	0.859956819	positive
PRJNA729271	stage_E14_vs_E10	ordinary_muscle_development	ENSGALG00010003097	TFPI2	1.916085593	0.135205039	14.17170252	1.37135E-45	1.50589E-43	positive
PRJNA729271	stage_E14_vs_E10	ordinary_muscle_development	ENSGALG00010003101	KLHL15	-0.058956475	0.070494529	-0.83632696	0.402970991	0.518100453	negative
PRJNA729271	stage_E14_vs_E10	ordinary_muscle_development	ENSGALG00010003103	YTHDF3	0.367129234	0.086103398	4.263818163	2.00963E-05	8.94094E-05	positive
PRJNA729271	stage_E14_vs_E10	ordinary_muscle_development	ENSGALG00010003107	GNG11	2.584243103	0.180213262	14.33991633	1.23204E-46	1.4519E-44	positive
PRJNA729271	stage_E14_vs_E10	ordinary_muscle_development	ENSGALG00010003108	PPP1R3B	1.304984625	0.180031105	7.24866199	4.20909E-13	5.01321E-12	positive
PRJNA729271	stage_E14_vs_E10	ordinary_muscle_development	ENSGALG00010003109	ART5	0.97537486	0.65574792	1.487423491	0.136902996	0.220268474	positive
PRJNA729271	stage_E14_vs_E10	ordinary_muscle_development	ENSGALG00010003110	BF1	0.889588353	0.260431429	3.415825642	0.000635889	0.002096728	positive
PRJNA729271	stage_E14_vs_E10	ordinary_muscle_development	ENSGALG00010003113	WBP4	0.226532596	0.115300789	1.964709855	0.049447829	0.09403651	positive
PRJNA729271	stage_E14_vs_E10	ordinary_muscle_development	ENSGALG00010003115	UBE3D	-0.216369934	0.151039614	-1.432537649	0.151990011	0.239962446	negative
PRJNA729271	stage_E14_vs_E10	ordinary_muscle_development	ENSGALG00010003117	ID2	-1.60233655	0.125733757	-12.74388508	3.37177E-37	2.22153E-35	negative
PRJNA729271	stage_E14_vs_E10	ordinary_muscle_development	ENSGALG00010003121	ARMC1	0.069602474	0.081573158	0.853252173	0.393519476	0.508837182	positive
PRJNA729271	stage_E14_vs_E10	ordinary_muscle_development	ENSGALG00010003125	APOO	0.094840245	0.106383195	0.891496495	0.372662871	0.488713319	positive
PRJNA729271	stage_E14_vs_E10	ordinary_muscle_development	ENSGALG00010003126	ENSGALG00010003126	0.408375312	0.461905705	0.884109695	0.376637	0.492834002	positive
PRJNA729271	stage_E14_vs_E10	ordinary_muscle_development	ENSGALG00010003131	TAP1	-0.021030461	0.371407007	-0.056623759	0.954844908	0.96886131	negative
PRJNA729271	stage_E14_vs_E10	ordinary_muscle_development	ENSGALG00010003132	TPBG	-0.125369189	0.130992867	-0.957068821	0.33853252	0.453598528	negative
PRJNA729271	stage_E14_vs_E10	ordinary_muscle_development	ENSGALG00010003133	SAT1	0.05329294	0.168171674	0.316896054	0.751322477	0.821831085	positive
PRJNA729271	stage_E14_vs_E10	ordinary_muscle_development	ENSGALG00010003136	NUP98	-0.118099549	0.104436404	-1.130827409	0.258127741	0.366352845	negative
PRJNA729271	stage_E14_vs_E10	ordinary_muscle_development	ENSGALG00010003152	ENSGALG00010003152	1.90986932	0.126129828	15.14209085	8.54531E-52	1.30383E-49	positive
PRJNA729271	stage_E14_vs_E10	ordinary_muscle_development	ENSGALG00010003154	MTFR1	-0.200171828	0.080014327	-2.501699832	0.012359867	0.028692548	negative
PRJNA729271	stage_E14_vs_E10	ordinary_muscle_development	ENSGALG00010003159	TAP2	0.340463378	0.277753391	1.225775777	0.220283065	0.322747661	positive
PRJNA729271	stage_E14_vs_E10	ordinary_muscle_development	ENSGALG00010003160	KIDINS220	-0.13458388	0.151376067	-0.88906974	0.373965601	0.49019998	negative
PRJNA729271	stage_E14_vs_E10	ordinary_muscle_development	ENSGALG00010003164	C3	1.276642988	0.461004531	2.769263432	0.005618319	0.014416274	positive
PRJNA729271	stage_E14_vs_E10	ordinary_muscle_development	ENSGALG00010003165	BMP2K	0.961324229	0.109039677	8.816279161	1.18326E-18	2.26571E-17	positive
PRJNA729271	stage_E14_vs_E10	ordinary_muscle_development	ENSGALG00010003166	PGAP2	0.214425695	0.109434256	1.959401945	0.050065733	0.094887919	positive
PRJNA729271	stage_E14_vs_E10	ordinary_muscle_development	ENSGALG00010003169	MTRF1	0.308493519	0.107652119	2.865652077	0.004161513	0.011070477	positive
PRJNA729271	stage_E14_vs_E10	ordinary_muscle_development	ENSGALG00010003170	C4	0.640032053	0.406254672	1.575445395	0.115153673	0.191482447	positive
PRJNA729271	stage_E14_vs_E10	ordinary_muscle_development	ENSGALG00010003171	PAQR3	-0.513791199	0.100726593	-5.100849563	3.38132E-07	2.00295E-06	negative
PRJNA729271	stage_E14_vs_E10	ordinary_muscle_development	ENSGALG00010003176	PRAG1	0.602317827	0.250511469	2.404352302	0.016201152	0.036251264	positive
PRJNA729271	stage_E14_vs_E10	ordinary_muscle_development	ENSGALG00010003178	CENPA	0.24576714	0.188195237	1.305915834	0.191581181	0.28896662	positive
PRJNA729271	stage_E14_vs_E10	ordinary_muscle_development	ENSGALG00010003180	IBTK	0.227244245	0.126538621	1.79584891	0.07251855	0.129997079	positive
PRJNA729271	stage_E14_vs_E10	ordinary_muscle_development	ENSGALG00010003183	NAA16	0.234264603	0.087815661	2.667685908	0.007637562	0.018824428	positive
PRJNA729271	stage_E14_vs_E10	ordinary_muscle_development	ENSGALG00010003184	BET1	0.322850191	0.0787138	4.101570392	4.10356E-05	0.000173364	positive
PRJNA729271	stage_E14_vs_E10	ordinary_muscle_development	ENSGALG00010003186	CYP21A1	0.35808209	0.209505323	1.709178955	0.087417806	0.152151909	positive
PRJNA729271	stage_E14_vs_E10	ordinary_muscle_development	ENSGALG00010003187	RHOG	-0.018136406	0.249970514	-0.072554182	0.942160888	0.959436706	negative
PRJNA729271	stage_E14_vs_E10	ordinary_muscle_development	ENSGALG00010003189	ANTXR2	0.82423472	0.126517851	6.514770139	7.28011E-11	6.83011E-10	positive
PRJNA729271	stage_E14_vs_E10	ordinary_muscle_development	ENSGALG00010003191	CC2D1A	0.312102141	0.16462073	1.895886021	0.057975118	0.107420342	positive
PRJNA729271	stage_E14_vs_E10	ordinary_muscle_development	ENSGALG00010003192	RGCC	-0.41128571	0.309611936	-1.328391005	0.18404897	0.279789178	negative
PRJNA729271	stage_E14_vs_E10	ordinary_muscle_development	ENSGALG00010003193	ACOT9	0.490455568	0.136267917	3.599200603	0.000319197	0.001135122	positive
PRJNA729271	stage_E14_vs_E10	ordinary_muscle_development	ENSGALG00010003198	PRKG2	-2.100311661	0.340578926	-6.166886737	6.96476E-10	5.8053E-09	negative
PRJNA729271	stage_E14_vs_E10	ordinary_muscle_development	ENSGALG00010003201	MBOAT2	-0.471453941	0.126702756	-3.742254362	0.000182377	0.000682912	negative
PRJNA729271	stage_E14_vs_E10	ordinary_muscle_development	ENSGALG00010003202	FAM46A	-0.067636354	0.103229381	-0.655204487	0.512336106	0.621240744	negative
PRJNA729271	stage_E14_vs_E10	ordinary_muscle_development	ENSGALG00010003204	PKD2	-0.504460688	0.143288071	-3.520604932	0.000430564	0.001479265	negative
PRJNA729271	stage_E14_vs_E10	ordinary_muscle_development	ENSGALG00010003205	ENSGALG00010003205	1.408433137	0.266130364	5.292267726	1.20809E-07	7.59048E-07	positive
PRJNA729271	stage_E14_vs_E10	ordinary_muscle_development	ENSGALG00010003206	ENSGALG00010003206	-0.083083817	0.520436639	-0.159642521	0.873162681	0.91296116	negative
PRJNA729271	stage_E14_vs_E10	ordinary_muscle_development	ENSGALG00010003210	EYA1	1.395581546	0.199802476	6.984806053	2.8525E-12	3.12524E-11	positive
PRJNA729271	stage_E14_vs_E10	ordinary_muscle_development	ENSGALG00010003212	LTB4R	1.859287641	0.732417293	2.538563274	0.011130867	0.026183369	positive
PRJNA729271	stage_E14_vs_E10	ordinary_muscle_development	ENSGALG00010003214	PRDX4	-0.09108855	0.094250203	-0.966454681	0.333816713	0.448965517	negative
PRJNA729271	stage_E14_vs_E10	ordinary_muscle_development	ENSGALG00010003218	MAP3K7	0.266551427	0.06034579	4.417067469	1.00049E-05	4.6902E-05	positive
PRJNA729271	stage_E14_vs_E10	ordinary_muscle_development	ENSGALG00010003220	SPP1	-4.476320276	0.954742803	-4.688509053	2.75203E-06	1.41707E-05	negative
PRJNA729271	stage_E14_vs_E10	ordinary_muscle_development	ENSGALG00010003222	ITGA8	-2.191490817	0.309701061	-7.076148887	1.48216E-12	1.65643E-11	negative
PRJNA729271	stage_E14_vs_E10	ordinary_muscle_development	ENSGALG00010003223	AREG	2.423000689	0.620063115	3.907667834	9.31913E-05	0.000368771	positive
PRJNA729271	stage_E14_vs_E10	ordinary_muscle_development	ENSGALG00010003229	STIM1	0.372723642	0.105638652	3.528288502	0.000418256	0.001441422	positive
PRJNA729271	stage_E14_vs_E10	ordinary_muscle_development	ENSGALG00010003239	RRM1	-0.127341909	0.117739736	-1.081554225	0.279450654	0.390347617	negative
PRJNA729271	stage_E14_vs_E10	ordinary_muscle_development	ENSGALG00010003240	PLACL2	0.873374735	0.949196239	0.920120308	0.357509893	0.47390086	positive
PRJNA729271	stage_E14_vs_E10	ordinary_muscle_development	ENSGALG00010003242	ASAP2	-1.015860261	0.121656833	-8.350211306	6.81409E-17	1.13922E-15	negative
PRJNA729271	stage_E14_vs_E10	ordinary_muscle_development	ENSGALG00010003243	VPS50	-0.233544322	0.091310091	-2.557705493	0.010536528	0.024906552	negative
PRJNA729271	stage_E14_vs_E10	ordinary_muscle_development	ENSGALG00010003245	ENSGALG00010003245	-0.926905381	0.432153483	-2.144852276	0.03196466	0.06472866	negative
PRJNA729271	stage_E14_vs_E10	ordinary_muscle_development	ENSGALG00010003246	VWA8	0.22924767	0.109267929	2.098032542	0.035902271	0.071474168	positive
PRJNA729271	stage_E14_vs_E10	ordinary_muscle_development	ENSGALG00010003247	ENSGALG00010003247	-0.257222269	0.217479868	-1.182740594	0.236911971	0.341865507	negative
PRJNA729271	stage_E14_vs_E10	ordinary_muscle_development	ENSGALG00010003248	MRI1	0.05976874	0.095484029	0.625955361	0.531344221	0.63878531	positive
PRJNA729271	stage_E14_vs_E10	ordinary_muscle_development	ENSGALG00010003249	YJU2B	0.431645774	0.33783676	1.277675568	0.201363843	0.300687021	positive
PRJNA729271	stage_E14_vs_E10	ordinary_muscle_development	ENSGALG00010003250	CD1G	-1.318333665	0.880299526	-1.4975967	0.134238065	0.216824242	negative
PRJNA729271	stage_E14_vs_E10	ordinary_muscle_development	ENSGALG00010003251	ELOF1	-0.023644954	0.153517188	-0.154021543	0.87759275	0.916015475	negative
PRJNA729271	stage_E14_vs_E10	ordinary_muscle_development	ENSGALG00010003252	PHEX	-0.840128451	0.195012591	-4.308072851	1.64683E-05	7.43992E-05	negative
PRJNA729271	stage_E14_vs_E10	ordinary_muscle_development	ENSGALG00010003253	ITGB1BP1	-0.159089123	0.112016796	-1.42022562	0.155542007	0.244425563	negative
PRJNA729271	stage_E14_vs_E10	ordinary_muscle_development	ENSGALG00010003254	DGKH	0.14764307	0.168219812	0.877679435	0.380117708	0.4960661	positive
PRJNA729271	stage_E14_vs_E10	ordinary_muscle_development	ENSGALG00010003255	CALCR	-3.141113991	0.352685448	-8.906276138	5.27754E-19	1.04505E-17	negative
PRJNA729271	stage_E14_vs_E10	ordinary_muscle_development	ENSGALG00010003256	ENSGALG00010003256	4.249995849	1.308836368	3.247155987	0.001165645	0.003612577	positive
PRJNA729271	stage_E14_vs_E10	ordinary_muscle_development	ENSGALG00010003257	ENSGALG00010003257	2.857146884	0.792036224	3.607343701	0.000309348	0.001104161	positive
PRJNA729271	stage_E14_vs_E10	ordinary_muscle_development	ENSGALG00010003259	VEGFC	0.245551939	0.141755603	1.732220337	0.083234339	0.146268251	positive
PRJNA729271	stage_E14_vs_E10	ordinary_muscle_development	ENSGALG00010003260	ACP5	-0.096968499	0.269544566	-0.359749411	0.719034538	0.796759087	negative
PRJNA729271	stage_E14_vs_E10	ordinary_muscle_development	ENSGALG00010003261	PEX2	-0.236342719	0.173243912	-1.3642148	0.172498499	0.265403433	negative
PRJNA729271	stage_E14_vs_E10	ordinary_muscle_development	ENSGALG00010003262	ENSGALG00010003262	-0.492443259	0.574427532	-0.857276561	0.391292059	0.506951948	negative
PRJNA729271	stage_E14_vs_E10	ordinary_muscle_development	ENSGALG00010003263	ENSGALG00010003263	0.500471829	0.774890931	0.645860996	0.518369381	0.62672151	positive
PRJNA729271	stage_E14_vs_E10	ordinary_muscle_development	ENSGALG00010003264	ENSGALG00010003264	-0.66639532	0.388140482	-1.71689208	0.085998892	0.150205319	negative
PRJNA729271	stage_E14_vs_E10	ordinary_muscle_development	ENSGALG00010003268	SMS	-0.255378648	0.062793854	-4.06693701	4.76351E-05	0.000198983	negative

PRJNA729271	stage_E14_vs_E10	ordinary_muscle_development	ENSGALG00010003269	ENSGALG00010003269	1.844163369	0.405575142	4.547032548	5.44076E-06	2.67063E-05	positive
PRJNA729271	stage_E14_vs_E10	ordinary_muscle_development	ENSGALG00010003271	AKAP11	-0.336606725	0.083907916	-4.011620609	6.03034E-05	0.000247269	negative
PRJNA729271	stage_E14_vs_E10	ordinary_muscle_development	ENSGALG00010003272	CPSF3	-0.177603528	0.065615578	-2.7067281	0.006794988	0.016991853	negative
PRJNA729271	stage_E14_vs_E10	ordinary_muscle_development	ENSGALG00010003273	NFIX	0.116633288	0.198984639	0.586142169	0.55777998	0.661722045	positive
PRJNA729271	stage_E14_vs_E10	ordinary_muscle_development	ENSGALG00010003274	BACH2	0.011340762	0.147285418	0.076998537	0.938624709	0.957092613	positive
PRJNA729271	stage_E14_vs_E10	ordinary_muscle_development	ENSGALG00010003275	MBTPS2	0.100092491	0.12670368	0.789973037	0.429543515	0.543680864	positive
PRJNA729271	stage_E14_vs_E10	ordinary_muscle_development	ENSGALG00010003276	TNFSF11	-1.02375843	0.379206461	-2.699738888	0.006939392	0.017297761	negative
PRJNA729271	stage_E14_vs_E10	ordinary_muscle_development	ENSGALG00010003278	ENSGALG00010003278	0.311649017	0.499751605	0.623607835	0.532885164	0.64010694	positive
PRJNA729271	stage_E14_vs_E10	ordinary_muscle_development	ENSGALG00010003279	ENSGALG00010003279	0.462738246	0.38110599	1.214198303	0.224672009	0.327726755	positive
PRJNA729271	stage_E14_vs_E10	ordinary_muscle_development	ENSGALG00010003280	ENSGALG00010003280	-0.064698085	0.165684704	-0.390489186	0.69617485	0.778750922	negative
PRJNA729271	stage_E14_vs_E10	ordinary_muscle_development	ENSGALG00010003287	ENSGALG00010003287	0.335627596	1.926856178	0.174184041	0.861720827	0.904791263	positive
PRJNA729271	stage_E14_vs_E10	ordinary_muscle_development	ENSGALG00010003292	GADD45GIP1	0.404952134	0.123697965	3.273717011	0.001061429	0.003325136	positive
PRJNA729271	stage_E14_vs_E10	ordinary_muscle_development	ENSGALG00010003294	ENSGALG00010003294	2.889890949	4.421848195	0.653548205	0.513402923	0.62217	positive
PRJNA729271	stage_E14_vs_E10	ordinary_muscle_development	ENSGALG00010003295	IAH1	0.014133419	0.132871105	0.106369398	0.915289272	0.941362463	positive
PRJNA729271	stage_E14_vs_E10	ordinary_muscle_development	ENSGALG00010003296	ENSGALG00010003296	0.897924658	0.572993681	1.567076022	0.117096923	0.193868635	positive
PRJNA729271	stage_E14_vs_E10	ordinary_muscle_development	ENSGALG00010003298	IL7	4.284629386	1.011583447	4.23556692	2.27976E-05	0.000100502	positive
PRJNA729271	stage_E14_vs_E10	ordinary_muscle_development	ENSGALG00010003299	RAD23A	0.647590919	0.120480091	5.375086562	7.65459E-08	4.96435E-07	positive
PRJNA729271	stage_E14_vs_E10	ordinary_muscle_development	ENSGALG00010003300	CASP8AP2	-0.438899707	0.110877929	-3.958404618	7.54521E-05	0.000303631	negative
PRJNA729271	stage_E14_vs_E10	ordinary_muscle_development	ENSGALG00010003303	MTHFD2L	0.122608377	0.187734721	0.653093774	0.513695826	0.62242088	positive
PRJNA729271	stage_E14_vs_E10	ordinary_muscle_development	ENSGALG00010003304	ENSGALG00010003304	-0.379158819	0.623791892	-0.607829028	0.543300875	0.64891466	negative
PRJNA729271	stage_E14_vs_E10	ordinary_muscle_development	ENSGALG00010003305	CALR	-0.036944413	0.077955426	-0.473917145	0.635558993	0.729235146	negative
PRJNA729271	stage_E14_vs_E10	ordinary_muscle_development	ENSGALG00010003307	COL1A2	0.672942013	0.220380766	3.053542399	0.002261568	0.006481104	positive
PRJNA729271	stage_E14_vs_E10	ordinary_muscle_development	ENSGALG00010003308	MDN1	-0.424372253	0.098979709	-4.287467147	1.80722E-05	8.10258E-05	negative
PRJNA729271	stage_E14_vs_E10	ordinary_muscle_development	ENSGALG00010003311	ENSGALG00010003311	0.129282628	1.168946944	0.110597515	0.911935518	0.93934802	positive
PRJNA729271	stage_E14_vs_E10	ordinary_muscle_development	ENSGALG00010003315	EPGN	-0.008991578	0.325243177	-0.027645708	0.977944726	0.985285939	negative
PRJNA729271	stage_E14_vs_E10	ordinary_muscle_development	ENSGALG00010003317	LYRM2	0.603315413	0.17629403	3.422211247	0.00062114	0.00205417	positive
PRJNA729271	stage_E14_vs_E10	ordinary_muscle_development	ENSGALG00010003320	OR51E2	0.693148904	0.46251461	1.498652991	0.133963678	0.216453407	positive
PRJNA729271	stage_E14_vs_E10	ordinary_muscle_development	ENSGALG00010003323	STMN2	-1.39430657	0.465212542	-2.997138821	0.002725266	0.007633377	negative
PRJNA729271	stage_E14_vs_E10	ordinary_muscle_development	ENSGALG00010003324	ENSGALG00010003324	0.427551116	0.587058659	0.728293689	0.466433825	0.578750068	positive
PRJNA729271	stage_E14_vs_E10	ordinary_muscle_development	ENSGALG00010003330	HEY1	1.464517208	0.118873481	12.31996571	7.07244E-35	3.82519E-33	positive
PRJNA729271	stage_E14_vs_E10	ordinary_muscle_development	ENSGALG00010003331	EPSTI1	-0.344477936	0.634568389	-0.542853918	0.587230386	0.68749733	negative
PRJNA729271	stage_E14_vs_E10	ordinary_muscle_development	ENSGALG00010003333	ENSGALG00010003333	20.13307007	2.686051598	7.495414489	6.60892E-14	8.55238E-13	positive
PRJNA729271	stage_E14_vs_E10	ordinary_muscle_development	ENSGALG00010003334	TAC1	-4.195272092	0.908046004	-4.620109632	3.83537E-06	1.92166E-05	negative
PRJNA729271	stage_E14_vs_E10	ordinary_muscle_development	ENSGALG00010003339	ENSGALG00010003339	-0.187261864	0.456377335	-0.410322444	0.68156943	0.767038744	negative
PRJNA729271	stage_E14_vs_E10	ordinary_muscle_development	ENSGALG00010003344	ZFAND1	-0.003453881	0.189613028	-0.018215422	0.985466999	0.990180518	negative
PRJNA729271	stage_E14_vs_E10	ordinary_muscle_development	ENSGALG00010003346	ENSGALG00010003346	-3.251828013	5.47463579	-0.593980703	0.55252502	0.657271249	negative
PRJNA729271	stage_E14_vs_E10	ordinary_muscle_development	ENSGALG00010003347	GPM6A	1.822621877	0.348848672	5.2246777	1.74459E-07	1.07334E-06	positive
PRJNA729271	stage_E14_vs_E10	ordinary_muscle_development	ENSGALG00010003348	ADAM17	-0.072572617	0.082789218	-0.876595031	0.380706639	0.496655813	negative
PRJNA729271	stage_E14_vs_E10	ordinary_muscle_development	ENSGALG00010003351	ENSGALG00010003351	-1.404083506	1.599635471	-0.87775217	0.380078226	0.496059237	negative
PRJNA729271	stage_E14_vs_E10	ordinary_muscle_development	ENSGALG00010003352	FARSA	0.130247687	0.100831419	1.291737125	0.196448196	0.294889956	positive
PRJNA729271	stage_E14_vs_E10	ordinary_muscle_development	ENSGALG00010003356	ENSGALG00010003356	6.059253238	1.107949731	5.468888223	4.52867E-08	3.02642E-07	positive
PRJNA729271	stage_E14_vs_E10	ordinary_muscle_development	ENSGALG00010003358	DNAJC15	-0.166070109	0.133892936	-1.240320168	0.214856996	0.316563545	negative
PRJNA729271	stage_E14_vs_E10	ordinary_muscle_development	ENSGALG00010003363	HBE1	0.348836766	0.502443162	0.69428105	0.487505972	0.598653443	positive
PRJNA729271	stage_E14_vs_E10	ordinary_muscle_development	ENSGALG00010003364	ANKRD6	-0.467666314	0.144317564	-3.240536363	0.001193051	0.003691987	negative
PRJNA729271	stage_E14_vs_E10	ordinary_muscle_development	ENSGALG00010003366	CASD1	0.131636304	0.107698191	1.222270336	0.221605413	0.32433062	positive
PRJNA729271	stage_E14_vs_E10	ordinary_muscle_development	ENSGALG00010003371	ENSGALG00010003371	0.126941396	0.366623349	0.346244713	0.729158814	0.804839858	positive
PRJNA729271	stage_E14_vs_E10	ordinary_muscle_development	ENSGALG00010003372	ENOX1	-0.386743495	0.139525602	-2.771846091	0.005573938	0.014317604	negative
PRJNA729271	stage_E14_vs_E10	ordinary_muscle_development	ENSGALG00010003374	SYCE2	0.176713111	0.4060205	0.435231992	0.663394043	0.752358709	positive
PRJNA729271	stage_E14_vs_E10	ordinary_muscle_development	ENSGALG00010003375	YWHAQ	-0.179426026	0.085867915	-2.089558444	0.036657483	0.072741992	negative
PRJNA729271	stage_E14_vs_E10	ordinary_muscle_development	ENSGALG00010003379	ENSGALG00010003379	0.053427455	0.431602343	0.123788611	0.90148265	0.932357546	positive
PRJNA729271	stage_E14_vs_E10	ordinary_muscle_development	ENSGALG00010003382	ASNS	-0.630234198	0.599553611	-1.051172382	0.293179425	0.40584813	negative
PRJNA729271	stage_E14_vs_E10	ordinary_muscle_development	ENSGALG00010003383	UBE2J1	0.253115496	0.085490895	2.960730447	0.003069104	0.008483346	positive
PRJNA729271	stage_E14_vs_E10	ordinary_muscle_development	ENSGALG00010003388	PXDN	-0.236416654	0.185862715	-1.271996131	0.203374494	0.302971561	negative
PRJNA729271	stage_E14_vs_E10	ordinary_muscle_development	ENSGALG00010003389	ENSGALG00010003389	0.248810717	0.364733785	0.682170743	0.495130992	0.604975936	positive
PRJNA729271	stage_E14_vs_E10	ordinary_muscle_development	ENSGALG00010003395	ENSGALG00010003395	0.432874661	1.002420617	0.431829367	0.665865434	0.754040583	positive
PRJNA729271	stage_E14_vs_E10	ordinary_muscle_development	ENSGALG00010003397	SPATA4	1.576975319	0.394980267	3.992542032	6.53687E-05	0.000266607	positive
PRJNA729271	stage_E14_vs_E10	ordinary_muscle_development	ENSGALG00010003400	PKIA	1.733572905	0.114299658	15.16691239	5.85678E-52	9.22762E-50	positive
PRJNA729271	stage_E14_vs_E10	ordinary_muscle_development	ENSGALG00010003401	ENSGALG00010003401	0.466161648	1.723631909	0.270453132	0.786811669	0.850069407	positive
PRJNA729271	stage_E14_vs_E10	ordinary_muscle_development	ENSGALG00010003403	GCDH	0.38783991	0.147299753	2.632997702	0.008463492	0.020628605	positive
PRJNA729271	stage_E14_vs_E10	ordinary_muscle_development	ENSGALG00010003405	GABRR2	0.47367861	0.19787085	2.393877679	0.016671308	0.037188459	positive
PRJNA729271	stage_E14_vs_E10	ordinary_muscle_development	ENSGALG00010003406	C1GALT1	-0.533710919	0.105152372	-5.075595617	3.86284E-07	2.26871E-06	negative
PRJNA729271	stage_E14_vs_E10	ordinary_muscle_development	ENSGALG00010003407	CHMP4C	-0.066790498	0.210944603	-0.316625773	0.751527577	0.821906491	negative
PRJNA729271	stage_E14_vs_E10	ordinary_muscle_development	ENSGALG00010003408	ENSGALG00010003408	12.71626137	3.663431577	3.471133855	0.000518266	0.001749886	positive
PRJNA729271	stage_E14_vs_E10	ordinary_muscle_development	ENSGALG00010003412	ASB5	1.403149194	0.146468027	9.579901	9.7138E-22	2.3704E-20	positive
PRJNA729271	stage_E14_vs_E10	ordinary_muscle_development	ENSGALG00010003419	ENSGALG00010003419	-0.149898814	0.894135334	-0.167646673	0.866861251	0.908608998	negative
PRJNA729271	stage_E14_vs_E10	ordinary_muscle_development	ENSGALG00010003420	ENSGALG00010003420	0.363768813	0.631579267	0.575966995	0.56463749	0.66724445	positive
PRJNA729271	stage_E14_vs_E10	ordinary_muscle_development	ENSGALG00010003428	SNX16	-0.426783597	0.156669088	-2.724108514	0.006447532	0.016219537	negative
PRJNA729271	stage_E14_vs_E10	ordinary_muscle_development	ENSGALG00010003431	PM20D2	0.018135732	0.286915198	0.06320938	0.949599776	0.965113501	positive
PRJNA729271	stage_E14_vs_E10	ordinary_muscle_development	ENSGALG00010003432	LACC1	0.328084059	0.320407444	1.023958914	0.305854682	0.419667135	positive
PRJNA729271	stage_E14_vs_E10	ordinary_muscle_development	ENSGALG00010003437	PNRC1	0.781043825	0.178995067	4.363493585	1.28002E-05	5.90135E-05	positive
PRJNA729271	stage_E14_vs_E10	ordinary_muscle_development	ENSGALG00010003438	KCNS3	-0.60155439	0.164430309	-3.658415494	0.000253779	0.000920093	negative
PRJNA729271	stage_E14_vs_E10	ordinary_muscle_development	ENSGALG00010003439	GMIP	-0.572430948	0.232053168	-2.466809452	0.013632288	0.031250991	negative
PRJNA729271	stage_E14_vs_E10	ordinary_muscle_development	ENSGALG00010003442	SPCS3	0.0396477	0.089182338	0.444568973	0.656631274	0.746851092	positive
PRJNA729271	stage_E14_vs_E10	ordinary_muscle_development	ENSGALG00010003447	ZC2HC1A	0.394542417	0.124576623	3.167066244	0.001539852	0.004624624	positive
PRJNA729271	stage_E14_vs_E10	ordinary_muscle_development	ENSGALG00010003449	BORCS6	0.477662915	0.109896652	4.34647376	1.38344E-05	6.34786E-05	positive
PRJNA729271	stage_E14_vs_E10	ordinary_muscle_development	ENSGALG00010003454	BPNT2	-0.643362971	0.119301039	-5.392769201	6.938E-08	4.54024E-07	negative
PRJNA729271	stage_E14_vs_E10	ordinary_muscle_development	ENSGALG00010003456	CDS1	-1.037423067	0.341333441	-3.039324433	0.002371094	0.006745633	negative
PRJNA729271	stage_E14_vs_E10	ordinary_muscle_development	ENSGALG00010003466	TSC22D1	-0.45718159	0.100298828	-4.558194744	5.15952E-06	2.5386E-05	negative
PRJNA729271	stage_E14_vs_E10	ordinary_muscle_development	ENSGALG00010003468	ILVBL	0.690649998	0.13419323	5.146682873	2.65133E-07	1.59201E-06	positive
PRJNA729271	stage_E14_vs_E10	ordinary_muscle_development	ENSGALG00010003470	METTL3	0.085184562	0.186786747	0.456052495	0.64835223	0.740145109	positive
PRJNA729271	stage_E14_vs_E10	ordinary_muscle_development	ENSGALG00010003471	ADI1	0.10775325	0.133492877	0.807183521	0.419560758	0.534062099	positive
PRJNA729271	stage_E14_vs_E10	ordinary_muscle_development	ENSGALG00010003472	FAM110B	-0.04896679	0.128045155	-0.382418142	0.702151236	0.783742659	negative
PRJNA729271	stage_E14_vs_E10	ordinary_muscle_development	ENSGALG00010003473	ENSGALG00010003473	-0.959253765	0.251239442	-3.818085869	0.000134491	0.000517919	negative
PRJNA729271	stage_E14_vs_E10	ordinary_muscle_development	ENSGALG00010003474	RPS7	0.20959823	0.166084568	1.261997021	0.206949858	0.307129947	positive
PRJNA729271	stage_E14_vs_E10	ordinary_muscle_development	ENSGALG00010003475	ENSGALG00010003475	0.472497792	2.232576104	0.211637933	0.832389513	0.883141999	positive
PRJNA729271	stage_E14_vs_E10	ordinary_muscle_development	ENSGALG00010003478	COLEC11	0.278767084	0.179182428	1.555772446	0.119762212	0.197357125	positive
PRJNA729271	stage_E14_vs_E10	ordinary_muscle_development	ENSGALG00010003479	SYDE1	-0.034938219	0.20082471	-0.173973704	0.861886128	0.904839113	negative
PRJNA729271	stage_E14_vs_E10	ordinary_muscle_development	ENSGALG00010003485	RNGTT	-0.04922334	0.101225925	-0.486272067	0.626774281	0.721268117	negative
PRJNA729271	stage_E14_vs_E10	ordinary_muscle_development	ENSGALG00010003489	SGCE	-0.320717993	0.118548145	-2.705381798	0.006822592	0.017050599	negative
PRJNA729271	stage_E14_vs_E10	ordinary_muscle_development	ENSGALG00010003503	NUFIP1	-0.159950935	0.084438823	-1.894281921	0.058187599	0.107731416	negative
PRJNA729271	stage_E14_vs_E10	ordinary_muscle_development	ENSGALG00010003506	DYNC1I1	-1.530505876	0.161403141	-9.48250369	2.48254E-21	5.89908E-20	negative
PRJNA729271	stage_E14_vs_E10	ordinary_muscle_development	ENSGALG00010003507	WDFY3	0.015194446	0.097457826	0.155907909	0.876105611	0.915122205	positive
PRJNA729271	stage_E14_vs_E10	ordinary_muscle_development	ENSGALG00010003509	GPALPP1	-0.350745611	0.069796677	-5.025247986	5.02782E-07	2.90005E-06	negative
PRJNA729271	stage_E14_vs_E10	ordinary_muscle_development	ENSGALG00010003511	UBXN2B	-0.160577072	0.139103711	-1.154369439	0.248348739	0.355221529	negative
PRJNA729271	stage_E14_vs_E10	ordinary_muscle_development	ENSGALG00010003512	ENSGALG00010003512	0.206765009	0.42489223	0.4866293	0.626521056	0.721148471	positive
PRJNA729271	stage_E14_vs_E10	ordinary_muscle_development	ENSGALG00010003517	GTF2F2	-0.292676059	0.085688622	-3.415576672	0.000636471	0.002098169	negative
PRJNA729271	stage_E14_vs_E10	ordinary_muscle_development	ENSGALG00010003519	KCTD4	-0.103221813	0.621770071	-0.166012837	0.868146848	0.909561877	negative
PRJNA729271	stage_E14_vs_E10	ordinary_muscle_development	ENSGALG00010003520	ENSGALG00010003520	0.472522512	1.808471231	0.261282847	0.793874393	0.855173107	positive
PRJNA729271	stage_E14_vs_E10	ordinary_muscle_development	ENSGALG00010003522	ENSGALG00010003522	2.614123147	0.639695351	4.086512658	4.37906E-05	0.000184037	positive
PRJNA729271	stage_E14_vs_E10	ordinary_muscle_development	ENSGALG00010003525	NOL10	-0.236211089	0.064410325	-3.667286071	0.000245138	0.000891888	negative
PRJNA729271	stage_E14_vs_E10	ordinary_muscle_development	ENSGALG00010003528	GDAP1	-0.519989607	0.192527575	-2.700857827	0.00691609	0.017263427	negative
PRJNA729271	stage_E14_vs_E10	ordinary_muscle_development	ENSGALG00010003531	PPM1K	0.516087888	0.149257832	3.457693851	0.00054482	0.001828895	positive
PRJNA729271	stage_E14_vs_E10	ordinary_muscle_development	ENSGALG00010003539	ENSGALG00010003539	0.562405112	0.205073358	2.742458202	0.006098121	0.015474836	positive
PRJNA729271	stage_E14_vs_E10	ordinary_muscle_development	ENSGALG00010003541	CNR1	2.458664884	0.217979651	11.27933215	1.66001E-29	6.93427E-28	positive
PRJNA729271	stage_E14_vs_E10	ordinary_muscle_development	ENSGALG00010003543	SOX11	-0.131362492	0.079060627	-1.661541245	0.096604792	0.165225542	negative
PRJNA729271	stage_E14_vs_E10	ordinary_muscle_development	ENSGALG00010003552	PI15	0.005647417	0.388777793	0.01452608	0.988410273	0.992243708	positive
PRJNA729271	stage_E14_vs_E10	ordinary_muscle_development	ENSGALG00010003556	ENSGALG00010003556	0.472506567	2.007218613	0.235403639	0.813895462	0.869566943	positive
PRJNA729271	stage_E14_vs_E10	ordinary_muscle_development	ENSGALG00010003558	TPT1	0.898387285	0.184666809	4.864909347	1.14509E-06	6.28479E-06	positive
PRJNA729271	stage_E14_vs_E10	ordinary_muscle_development	ENSGALG00010003560	ADGRL3	0.250056217	0.136900248	1.826557814	0.067766281	0.122646054	positive
PRJNA729271	stage_E14_vs_E10	ordinary_muscle_development	ENSGALG00010003571	RPL7	0.514283523	0.157425492	3.266837639	0.00108756	0.003395259	positive
PRJNA729271	stage_E14_vs_E10	ordinary_muscle_development	ENSGALG00010003577	ENSGALG00010003577	0.472520473	1.755449981	0.269173419	0.787796234	0.850712098	positive
PRJNA729271	stage_E14_vs_E10	ordinary_muscle_development	ENSGALG00010003582	RDH10	0.997420745	0.137895145	7.233182483	4.71805E-13	5.57815E-12	positive
PRJNA729271	stage_E14_vs_E10	ordinary_muscle_development	ENSGALG00010003583	RSAD2	-0.096237582	0.817328754	-0.117746477	0.906268539	0.935716909	negative
PRJNA729271	stage_E14_vs_E10	ordinary_muscle_development	ENSGALG00010003585	PNPLA6	0.529011281	0.142207268	3.720001718	0.000199221	0.00074082	positive
PRJNA729271	stage_E14_vs_E10	ordinary_muscle_development	ENSGALG00010003589	SLC25A30	-0.497097422	0.389121044	-1.277487892	0.201430052	0.300754904	negative
PRJNA729271	stage_E14_vs_E10	ordinary_muscle_development	ENSGALG00010003595	SNX13	0.435351114	0.093922868	4.635198286	3.56595E-06	1.79849E-05	positive
PRJNA729271	stage_E14_vs_E10	ordinary_muscle_development	ENSGALG00010003596	CRISPLD1	-0.326639566	0.150363364	-2.172334785	0.02983042	0.06113275	negative
PRJNA729271	stage_E14_vs_E10	ordinary_muscle_development	ENSGALG00010003597	RNF144A	0.724828427	0.14030374	5.166137619	2.38981E-07	1.44575E-06	positive
PRJNA729271	stage_E14_vs_E10	ordinary_muscle_development	ENSGALG00010003604	ODC	-0.273662337	0.21575493	-1.268394361	0.204657154	0.304511704	negative
PRJNA729271	stage_E14_vs_E10	ordinary_muscle_development	ENSGALG00010003617	COG3	-0.219410001	0.081746782	-2.684020041	0.007274275	0.018024036	negative
PRJNA729271	stage_E14_vs_E10	ordinary_muscle_development	ENSGALG00010003619	HNF4G	-0.914095196	0.514061787	-1.778181571	0.075374039	0.134401118	negative
PRJNA729271	stage_E14_vs_E10	ordinary_muscle_development	ENSGALG00010003627	MCOLN1	0.030162265	0.119513137	0.25237615	0.800750334	0.859577582	positive
PRJNA729271	stage_E14_vs_E10	ordinary_muscle_development	ENSGALG00010003632	TECRL	-1.25757407	0.372727318	-3.373978805	0.000740901	0.002411713	negative
PRJNA729271	stage_E14_vs_E10	ordinary_muscle_development	ENSGALG00010003634	CYP4F11	0.296420069	0.313783705	0.94466368	0.344830605	0.460208049	positive
PRJNA729271	stage_E14_vs_E10	ordinary_muscle_development	ENSGALG00010003639	ENSGALG00010003639	-1.197883373	0.800970195	-1.495540509	0.134773436	0.217495096	negative
PRJNA729271	stage_E14_vs_E10	ordinary_muscle_development	ENSGALG00010003641	ENSGALG00010003641	-0.055604186	2.578076982	-0.021568086	0.982792491	0.988726899	negative
PRJNA729271	stage_E14_vs_E10	ordinary_muscle_development	ENSGALG00010003650	SPACA1	3.379062081	0.478099896	7.067690474	1.57533E-12	1.7538E-11	positive
PRJNA729271	stage_E14_vs_E10	ordinary_muscle_development	ENSGALG00010003652	MYCN	0.554814897	0.138090449	4.017764453	5.87529E-05	0.000241253	positive
PRJNA729271	stage_E14_vs_E10	ordinary_muscle_development	ENSGALG00010003660	IGFBP7	1.352645064	0.165100121	8.192877462	2.55054E-16	4.01412E-15	positive
PRJNA729271	stage_E14_vs_E10	ordinary_muscle_development	ENSGALG00010003665	RDH14	0.020355427	0.09458992	0.215196579	0.829614058	0.881008583	positive
PRJNA729271	stage_E14_vs_E10	ordinary_muscle_development	ENSGALG00010003666	SLC25A13	-0.242600142	0.075124876	-3.229291733	0.001240972	0.003830471	negative

PRJNA729271	stage_E14_vs_E10	ordinary_muscle_development	ENSGALG00010003669	ENSGALG00010003669	0.953470736	1.676252672	0.568810868	0.569484499	0.6717674	positive
PRJNA729271	stage_E14_vs_E10	ordinary_muscle_development	ENSGALG00010003671	ENSGALG00010003671	-0.012248772	0.380926981	-0.032155171	0.974348306	0.982618708	negative
PRJNA729271	stage_E14_vs_E10	ordinary_muscle_development	ENSGALG00010003674	AKIRIN2	-0.236499795	0.132225354	-1.78861155	0.073677396	0.131869542	negative
PRJNA729271	stage_E14_vs_E10	ordinary_muscle_development	ENSGALG00010003676	POLR2B	-0.045866405	0.077748646	-0.589931879	0.55523632	0.659782334	negative
PRJNA729271	stage_E14_vs_E10	ordinary_muscle_development	ENSGALG00010003678	ZNFY1	-0.401603653	0.442886091	-0.906787686	0.364519083	0.480555171	negative
PRJNA729271	stage_E14_vs_E10	ordinary_muscle_development	ENSGALG00010003680	TRAPPC5	0.230704496	0.105869991	2.179130209	0.029321997	0.060261214	positive
PRJNA729271	stage_E14_vs_E10	ordinary_muscle_development	ENSGALG00010003681	NT5C1B	-1.327349955	0.131767637	-10.07341395	7.24197E-24	1.98436E-22	negative
PRJNA729271	stage_E14_vs_E10	ordinary_muscle_development	ENSGALG00010003683	ENSGALG00010003683	-0.006090359	1.755426205	-0.003469447	0.997231787	0.998283737	negative
PRJNA729271	stage_E14_vs_E10	ordinary_muscle_development	ENSGALG00010003691	ERICH1	-0.136620878	0.144168996	-0.947643959	0.343310739	0.458517384	negative
PRJNA729271	stage_E14_vs_E10	ordinary_muscle_development	ENSGALG00010003692	ZNFY2	-0.489673113	0.256858609	-1.906391672	0.056599408	0.105248034	negative
PRJNA729271	stage_E14_vs_E10	ordinary_muscle_development	ENSGALG00010003695	NOA1	-0.041320278	0.100704511	-0.410312084	0.681577028	0.767038744	negative
PRJNA729271	stage_E14_vs_E10	ordinary_muscle_development	ENSGALG00010003698	MACC1	-1.388914926	1.077371266	-1.289170196	0.197338924	0.295804313	negative
PRJNA729271	stage_E14_vs_E10	ordinary_muscle_development	ENSGALG00010003700	REST	0.147158616	0.065970716	2.230665745	0.025703278	0.054018997	positive
PRJNA729271	stage_E14_vs_E10	ordinary_muscle_development	ENSGALG00010003701	SARAF	0.668363575	0.081723144	8.178387901	2.87666E-16	4.51269E-15	positive
PRJNA729271	stage_E14_vs_E10	ordinary_muscle_development	ENSGALG00010003703	TDRP	-0.337488078	0.222602575	-1.516101413	0.129493711	0.21019164	negative
PRJNA729271	stage_E14_vs_E10	ordinary_muscle_development	ENSGALG00010003713	SPERT	-0.092085805	0.259555277	-0.35478302	0.72275215	0.799194573	negative
PRJNA729271	stage_E14_vs_E10	ordinary_muscle_development	ENSGALG00010003714	ORC3	-0.473458977	0.105781028	-4.475840186	7.61114E-06	3.64104E-05	negative
PRJNA729271	stage_E14_vs_E10	ordinary_muscle_development	ENSGALG00010003716	YLEC8	1.948449655	0.678585688	2.871339152	0.004087367	0.010902906	positive
PRJNA729271	stage_E14_vs_E10	ordinary_muscle_development	ENSGALG00010003717	LEPROTL1	0.203241074	0.09628418	2.110845976	0.034785553	0.069539135	positive
PRJNA729271	stage_E14_vs_E10	ordinary_muscle_development	ENSGALG00010003719	RPGR	-0.295280889	0.111227214	-2.65475397	0.007936627	0.01949524	negative
PRJNA729271	stage_E14_vs_E10	ordinary_muscle_development	ENSGALG00010003720	ENSGALG00010003720	0.472477963	2.731725995	0.1729595	0.862683262	0.905408289	positive
PRJNA729271	stage_E14_vs_E10	ordinary_muscle_development	ENSGALG00010003722	MHCY11	1.171474164	0.655361394	1.787523913	0.073852851	0.1320949	positive
PRJNA729271	stage_E14_vs_E10	ordinary_muscle_development	ENSGALG00010003724	ENSGALG00010003724	0.725800656	0.209044318	3.47199419	0.000516608	0.0017451	positive
PRJNA729271	stage_E14_vs_E10	ordinary_muscle_development	ENSGALG00010003726	ENPEP	-3.937858464	0.499574672	-7.882422165	3.21095E-15	4.61276E-14	negative
PRJNA729271	stage_E14_vs_E10	ordinary_muscle_development	ENSGALG00010003727	FBXO25	0.13053597	0.152722505	0.854726481	0.392702587	0.508188912	positive
PRJNA729271	stage_E14_vs_E10	ordinary_muscle_development	ENSGALG00010003729	RARS2	-0.070338872	0.100382383	-0.700709334	0.48348443	0.594966195	negative
PRJNA729271	stage_E14_vs_E10	ordinary_muscle_development	ENSGALG00010003731	ENSGALG00010003731	1.825700271	0.574716974	3.176694534	0.001489638	0.004492782	positive
PRJNA729271	stage_E14_vs_E10	ordinary_muscle_development	ENSGALG00010003733	STAU2	0.303220868	0.070168736	4.32131013	1.55105E-05	7.05445E-05	positive
PRJNA729271	stage_E14_vs_E10	ordinary_muscle_development	ENSGALG00010003734	ZC3H13	-0.352024616	0.141716605	-2.484004017	0.012991434	0.029971485	negative
PRJNA729271	stage_E14_vs_E10	ordinary_muscle_development	ENSGALG00010003736	ENSGALG00010003736	0.472457009	3.115575725	0.151643565	0.879468071	0.91718035	positive
PRJNA729271	stage_E14_vs_E10	ordinary_muscle_development	ENSGALG00010003737	SRP72	0.063673587	0.055254543	1.152368355	0.249169739	0.356161508	positive
PRJNA729271	stage_E14_vs_E10	ordinary_muscle_development	ENSGALG00010003740	PITX2	-1.309406052	0.353642719	-3.702624093	0.000213381	0.000786988	negative
PRJNA729271	stage_E14_vs_E10	ordinary_muscle_development	ENSGALG00010003742	ZFHX4	-1.495755582	0.166168776	-9.001423822	2.22809E-19	4.56806E-18	negative
PRJNA729271	stage_E14_vs_E10	ordinary_muscle_development	ENSGALG00010003743	ARL9	0.006933503	0.174445303	0.039746003	0.968295626	0.978557143	positive
PRJNA729271	stage_E14_vs_E10	ordinary_muscle_development	ENSGALG00010003746	HOPX	1.186804253	0.407951469	2.909179995	0.003623782	0.00979976	positive
PRJNA729271	stage_E14_vs_E10	ordinary_muscle_development	ENSGALG00010003749	MHCY15	1.743684071	0.947953893	1.839418651	0.065853633	0.119782709	positive
PRJNA729271	stage_E14_vs_E10	ordinary_muscle_development	ENSGALG00010003753	SLCO5A1	-0.163940479	0.160283898	-1.022813154	0.306396197	0.420012567	negative
PRJNA729271	stage_E14_vs_E10	ordinary_muscle_development	ENSGALG00010003754	FAM110C	-0.360604015	0.248648251	-1.450257593	0.1469867	0.233487366	negative
PRJNA729271	stage_E14_vs_E10	ordinary_muscle_development	ENSGALG00010003755	SLC35A1	-0.37493793	0.086665262	-4.32627702	1.51651E-05	6.90889E-05	negative
PRJNA729271	stage_E14_vs_E10	ordinary_muscle_development	ENSGALG00010003756	MHCY7	0.883154565	0.372221685	2.372657481	0.017660633	0.039070646	positive
PRJNA729271	stage_E14_vs_E10	ordinary_muscle_development	ENSGALG00010003761	UGT8	-0.119464598	0.340705926	-0.350638449	0.725859608	0.801625525	negative
PRJNA729271	stage_E14_vs_E10	ordinary_muscle_development	ENSGALG00010003762	EVI5L	0.46343352	0.098271284	4.715859023	2.40693E-06	1.25183E-05	positive
PRJNA729271	stage_E14_vs_E10	ordinary_muscle_development	ENSGALG00010003764	HDAC9	0.473978364	0.192957925	2.456381945	0.014034391	0.032025339	positive
PRJNA729271	stage_E14_vs_E10	ordinary_muscle_development	ENSGALG00010003765	ENSGALG00010003765	-0.309500298	0.875288214	-0.353598155	0.723640057	0.799783654	negative
PRJNA729271	stage_E14_vs_E10	ordinary_muscle_development	ENSGALG00010003767	ENSGALG00010003767	0.240490837	0.41033469	0.586084587	0.557818673	0.661722045	positive
PRJNA729271	stage_E14_vs_E10	ordinary_muscle_development	ENSGALG00010003768	ARSJ	-0.372085959	0.126571428	-2.939731069	0.003284972	0.009004477	negative
PRJNA729271	stage_E14_vs_E10	ordinary_muscle_development	ENSGALG00010003769	C6orf163	-0.015805101	0.484879783	-0.032595917	0.973996826	0.982400946	negative
PRJNA729271	stage_E14_vs_E10	ordinary_muscle_development	ENSGALG00010003772	ENSGALG00010003772	0.610450379	0.443158698	1.37749836	0.168358221	0.260359802	positive
PRJNA729271	stage_E14_vs_E10	ordinary_muscle_development	ENSGALG00010003775	NCOA2	0.039989881	0.114181466	0.350230932	0.726165395	0.801902133	positive
PRJNA729271	stage_E14_vs_E10	ordinary_muscle_development	ENSGALG00010003777	ENSGALG00010003777	0.460317397	0.873997252	0.526680599	0.59841541	0.697929522	positive
PRJNA729271	stage_E14_vs_E10	ordinary_muscle_development	ENSGALG00010003778	LCA5	-0.32189303	0.123387467	-2.608798433	0.009086075	0.021921213	negative
PRJNA729271	stage_E14_vs_E10	ordinary_muscle_development	ENSGALG00010003785	ENSGALG00010003785	1.085117715	1.223599832	0.886824014	0.375173656	0.491427991	positive
PRJNA729271	stage_E14_vs_E10	ordinary_muscle_development	ENSGALG00010003786	ENSGALG00010003786	1.956558455	0.585975409	3.33897707	0.000840875	0.002700151	positive
PRJNA729271	stage_E14_vs_E10	ordinary_muscle_development	ENSGALG00010003787	SMIM8	0.079219653	0.18962702	0.417765639	0.676118476	0.762333498	positive
PRJNA729271	stage_E14_vs_E10	ordinary_muscle_development	ENSGALG00010003788	NUDT9	-0.053383291	0.125456964	-0.425510782	0.670464363	0.757650343	negative
PRJNA729271	stage_E14_vs_E10	ordinary_muscle_development	ENSGALG00010003790	ELOVL6	-0.332547677	0.180407775	-1.843311229	0.065283583	0.118865159	negative
PRJNA729271	stage_E14_vs_E10	ordinary_muscle_development	ENSGALG00010003791	ITGB8	-1.180306832	0.169544386	-6.961639138	3.36336E-12	3.64637E-11	negative
PRJNA729271	stage_E14_vs_E10	ordinary_muscle_development	ENSGALG00010003793	TRAM1L1	-0.237091579	0.084546012	-2.804290519	0.005042744	0.013127619	negative
PRJNA729271	stage_E14_vs_E10	ordinary_muscle_development	ENSGALG00010003795	LCP1	0.460362907	0.200992684	2.290446082	0.021995471	0.04728227	positive
PRJNA729271	stage_E14_vs_E10	ordinary_muscle_development	ENSGALG00010003796	ENSGALG00010003796	0.990964958	0.307062843	3.227238269	0.001249913	0.003853146	positive
PRJNA729271	stage_E14_vs_E10	ordinary_muscle_development	ENSGALG00010003797	ENSGALG00010003797	-0.058759122	0.173070068	-0.339510598	0.734225115	0.808892076	negative
PRJNA729271	stage_E14_vs_E10	ordinary_muscle_development	ENSGALG00010003803	LACTB2	0.441211739	0.132443219	3.331327523	0.000864328	0.002766882	positive
PRJNA729271	stage_E14_vs_E10	ordinary_muscle_development	ENSGALG00010003804	PSME1	0.764573672	0.265996545	2.874374452	0.004048287	0.010806615	positive
PRJNA729271	stage_E14_vs_E10	ordinary_muscle_development	ENSGALG00010003806	EGF	1.962900313	0.317254899	6.187139482	6.12658E-10	5.15707E-09	positive
PRJNA729271	stage_E14_vs_E10	ordinary_muscle_development	ENSGALG00010003808	TWIST1	-1.692050253	0.161097556	-10.50326458	8.34434E-26	2.67591E-24	negative
PRJNA729271	stage_E14_vs_E10	ordinary_muscle_development	ENSGALG00010003810	HMGN2P46	0.29002867	0.09082394	3.193306397	0.001406536	0.004269684	positive
PRJNA729271	stage_E14_vs_E10	ordinary_muscle_development	ENSGALG00010003812	BG8	0.202373668	0.400699367	0.50505113	0.613522925	0.710134536	positive
PRJNA729271	stage_E14_vs_E10	ordinary_muscle_development	ENSGALG00010003813	LRIT3	0.793033642	0.374982443	2.114855393	0.034442274	0.069022641	positive
PRJNA729271	stage_E14_vs_E10	ordinary_muscle_development	ENSGALG00010003814	TPP1	0.416062277	0.132451355	3.141245901	0.001682307	0.00500514	positive
PRJNA729271	stage_E14_vs_E10	ordinary_muscle_development	ENSGALG00010003815	RASAL3	1.297017443	0.624414664	2.07717326	0.037785571	0.074639118	positive
PRJNA729271	stage_E14_vs_E10	ordinary_muscle_development	ENSGALG00010003816	KLHL8	-0.067492135	0.175971583	-0.383539969	0.701319441	0.783115731	negative
PRJNA729271	stage_E14_vs_E10	ordinary_muscle_development	ENSGALG00010003817	BF2	1.083883932	0.285200435	3.800428751	0.000144446	0.000552585	positive
PRJNA729271	stage_E14_vs_E10	ordinary_muscle_development	ENSGALG00010003818	GAB2	0.940530966	0.154076097	6.104327562	1.03234E-09	8.43507E-09	positive
PRJNA729271	stage_E14_vs_E10	ordinary_muscle_development	ENSGALG00010003819	PHIP	-0.272601626	0.09392492	-2.902335459	0.003703917	0.009992234	negative
PRJNA729271	stage_E14_vs_E10	ordinary_muscle_development	ENSGALG00010003820	SH3YL1	-0.747291356	0.298539058	-2.503161102	0.01230895	0.028592665	negative
PRJNA729271	stage_E14_vs_E10	ordinary_muscle_development	ENSGALG00010003821	RUBCNL	0.635660265	0.155590698	4.08546444	4.39887E-05	0.000184656	positive
PRJNA729271	stage_E14_vs_E10	ordinary_muscle_development	ENSGALG00010003822	ENSGALG00010003822	-0.659548752	0.156235774	-4.221496364	2.42686E-05	0.000106501	negative
PRJNA729271	stage_E14_vs_E10	ordinary_muscle_development	ENSGALG00010003823	PTPN13	-0.608398922	0.149016906	-4.082751005	4.45057E-05	0.000186556	negative
PRJNA729271	stage_E14_vs_E10	ordinary_muscle_development	ENSGALG00010003824	SP4	0.284090013	0.157757778	1.800798777	0.071734602	0.128703188	positive
PRJNA729271	stage_E14_vs_E10	ordinary_muscle_development	ENSGALG00010003826	ENSGALG00010003826	-0.098697074	0.106205381	-0.929303895	0.352731617	0.468982278	negative
PRJNA729271	stage_E14_vs_E10	ordinary_muscle_development	ENSGALG00010003827	RRH	0.414330805	0.66839516	0.619888996	0.535330871	0.64235273	positive
PRJNA729271	stage_E14_vs_E10	ordinary_muscle_development	ENSGALG00010003829	ENSGALG00010003829	-0.223812766	0.125296253	-1.786268632	0.074055773	0.132327223	negative
PRJNA729271	stage_E14_vs_E10	ordinary_muscle_development	ENSGALG00010003830	DMB2	-0.555099484	0.236728398	-2.344879151	0.019033248	0.041596536	negative
PRJNA729271	stage_E14_vs_E10	ordinary_muscle_development	ENSGALG00010003831	IRAK1BP1	0.170411113	0.162480232	1.048811358	0.294264949	0.407195268	positive
PRJNA729271	stage_E14_vs_E10	ordinary_muscle_development	ENSGALG00010003832	ART1B	2.351693988	1.018135103	2.309805429	0.020898929	0.045172975	positive
PRJNA729271	stage_E14_vs_E10	ordinary_muscle_development	ENSGALG00010003833	ACP1	0.070822718	0.157360483	0.450066732	0.652662324	0.743562084	positive
PRJNA729271	stage_E14_vs_E10	ordinary_muscle_development	ENSGALG00010003834	MAPK10	0.239609808	0.408497159	0.586564196	0.557496435	0.661502196	positive
PRJNA729271	stage_E14_vs_E10	ordinary_muscle_development	ENSGALG00010003835	ENSGALG00010003835	0.968387034	0.165755544	5.842260307	5.14973E-09	3.89404E-08	positive
PRJNA729271	stage_E14_vs_E10	ordinary_muscle_development	ENSGALG00010003836	CD1F	0.559178049	0.396153153	1.411519877	0.158091379	0.247561423	positive
PRJNA729271	stage_E14_vs_E10	ordinary_muscle_development	ENSGALG00010003837	ALKAL2	-1.371009399	0.275933114	-4.968629462	6.74278E-07	3.81188E-06	negative
PRJNA729271	stage_E14_vs_E10	ordinary_muscle_development	ENSGALG00010003838	GAR1	-0.482820428	0.178482472	-2.705141977	0.00682752	0.017059973	negative
PRJNA729271	stage_E14_vs_E10	ordinary_muscle_development	ENSGALG00010003839	ENSGALG00010003839	-0.137487872	0.857769185	-0.160285394	0.872656264	0.912768982	negative
PRJNA729271	stage_E14_vs_E10	ordinary_muscle_development	ENSGALG00010003841	UBE2W	0.280449128	0.142448388	1.968777126	0.04897869	0.093266699	positive
PRJNA729271	stage_E14_vs_E10	ordinary_muscle_development	ENSGALG00010003842	ENSGALG00010003842	0.092273771	0.144140334	0.640166205	0.522064551	0.629980492	positive
PRJNA729271	stage_E14_vs_E10	ordinary_muscle_development	ENSGALG00010003843	LRCH1	0.588936696	0.107079853	5.499976679	3.79841E-08	2.568E-07	positive
PRJNA729271	stage_E14_vs_E10	ordinary_muscle_development	ENSGALG00010003844	TMEM18	-0.706918929	0.107551567	-6.572837069	4.93655E-11	4.73249E-10	negative
PRJNA729271	stage_E14_vs_E10	ordinary_muscle_development	ENSGALG00010003845	CFI	0.402109564	0.865604887	0.464541699			positive
PRJNA729271	stage_E14_vs_E10	ordinary_muscle_development	ENSGALG00010003848	ESD	0.957474466	0.123264878	7.767617849	7.99759E-15	1.10721E-13	positive
PRJNA729271	stage_E14_vs_E10	ordinary_muscle_development	ENSGALG00010003850	PLA2G12A	0.238271213	0.098046358	2.430189321	0.015090938	0.034113946	positive
PRJNA729271	stage_E14_vs_E10	ordinary_muscle_development	ENSGALG00010003851	PHF14	-0.252893363	0.061072608	-4.140863967	3.46E-05	0.000147856	negative
PRJNA729271	stage_E14_vs_E10	ordinary_muscle_development	ENSGALG00010003852	TCEB1	-0.046312229	0.1069161	-0.433164215	0.664895474	0.753530876	negative
PRJNA729271	stage_E14_vs_E10	ordinary_muscle_development	ENSGALG00010003853	ENSGALG00010003853	0.24986974	0.226073614	1.105258309	0.269047677	0.378552328	positive
PRJNA729271	stage_E14_vs_E10	ordinary_muscle_development	ENSGALG00010003858	SUCLA2	0.525644987	0.124544539	4.220538218	2.4372E-05	0.00010689	positive
PRJNA729271	stage_E14_vs_E10	ordinary_muscle_development	ENSGALG00010003860	TMEM70	-0.038195564	0.113765566	-0.335739232	0.737067518	0.811098822	negative
PRJNA729271	stage_E14_vs_E10	ordinary_muscle_development	ENSGALG00010003861	NUDT15	-0.145094207	0.098419157	-1.474247606	0.140414936	0.224946342	negative
PRJNA729271	stage_E14_vs_E10	ordinary_muscle_development	ENSGALG00010003862	PCK2	0.088880301	0.14435416	0.615710007	0.538085931	0.644458052	positive
PRJNA729271	stage_E14_vs_E10	ordinary_muscle_development	ENSGALG00010003863	HTR1B	-2.748189139	0.30019577	-9.154656443	5.4531E-20	1.16239E-18	negative
PRJNA729271	stage_E14_vs_E10	ordinary_muscle_development	ENSGALG00010003865	AKAP8L	-0.208076454	0.18892814	-1.101352363	0.27074333	0.38052931	negative
PRJNA729271	stage_E14_vs_E10	ordinary_muscle_development	ENSGALG00010003868	HBBA	0.258983897	0.398825926	0.649365751	0.516101996	0.624691298	positive
PRJNA729271	stage_E14_vs_E10	ordinary_muscle_development	ENSGALG00010003869	LY96	-0.036847854	0.285331668	-0.129140427	0.897246533	0.929240962	negative
PRJNA729271	stage_E14_vs_E10	ordinary_muscle_development	ENSGALG00010003870	MED4	-0.236067598	0.088010107	-2.682278284	0.00731226	0.01810887	negative
PRJNA729271	stage_E14_vs_E10	ordinary_muscle_development	ENSGALG00010003871	ENSGALG00010003871	0.138521347	0.187922306	0.737120303	0.461049193	0.573640177	positive
PRJNA729271	stage_E14_vs_E10	ordinary_muscle_development	ENSGALG00010003874	YES1	-0.078402941	0.09695979	-0.808612943	0.418737818	0.533263457	negative
PRJNA729271	stage_E14_vs_E10	ordinary_muscle_development	ENSGALG00010003875	AKAP8	0.044047635	0.108901971	0.404470507	0.685866761	0.770251701	positive
PRJNA729271	stage_E14_vs_E10	ordinary_muscle_development	ENSGALG00010003878	ARHGAP24	0.777976158	0.0879466	8.846006077	9.07073E-19	1.74841E-17	positive
PRJNA729271	stage_E14_vs_E10	ordinary_muscle_development	ENSGALG00010003879	CASP6	-0.503340087	0.116539908	-4.319036255	1.56712E-05	7.12082E-05	negative
PRJNA729271	stage_E14_vs_E10	ordinary_muscle_development	ENSGALG00010003882	ENSGALG00010003882	0.015450626	0.121287662	0.127388278	0.898633093	0.930211158	positive
PRJNA729271	stage_E14_vs_E10	ordinary_muscle_development	ENSGALG00010003884	TYMS	-0.434520647	0.189723749	-2.29028073	0.022005048	0.047290001	negative
PRJNA729271	stage_E14_vs_E10	ordinary_muscle_development	ENSGALG00010003887	GIPC1	0.065477392	0.140153661	0.46718289	0.640369034	0.733071327	positive
PRJNA729271	stage_E14_vs_E10	ordinary_muscle_development	ENSGALG00010003889	ITM2B	0.22443781	0.087456127	2.566290292	0.010279276	0.024365384	positive
PRJNA729271	stage_E14_vs_E10	ordinary_muscle_development	ENSGALG00010003892	OPRK1	-4.115335527	2.555895768	-1.610134332	0.107368533	0.180755737	negative
PRJNA729271	stage_E14_vs_E10	ordinary_muscle_development	ENSGALG00010003894	COPS4	0.112426688	0.12427451	0.904664101	0.365643365	0.481686865	positive
PRJNA729271	stage_E14_vs_E10	ordinary_muscle_development	ENSGALG00010003895	THSD7A	-0.724654584	0.166063201	-4.36372766	1.27865E-05	5.89691E-05	negative
PRJNA729271	stage_E14_vs_E10	ordinary_muscle_development	ENSGALG00010003896	MCUB	-0.177784378	0.131606641	-1.350876952	0.176734852	0.270637856	negative
PRJNA729271	stage_E14_vs_E10	ordinary_muscle_development	ENSGALG00010003898	NDST4	-3.999684245	0.538251892	-7.430878187	1.07879E-13	1.35974E-12	negative
PRJNA729271	stage_E14_vs_E10	ordinary_muscle_development	ENSGALG00010003899	LIN54	0.026530765	0.16447479	0.161305966	0.871852425	0.911994003	positive
PRJNA729271	stage_E14_vs_E10	ordinary_muscle_development	ENSGALG00010003900	RB1	0.006124656	0.08563394	0.071521363	0.942982822	0.959869102	positive
PRJNA729271	stage_E14_vs_E10	ordinary_muscle_development	ENSGALG00010003902	ENSGALG00010003902	0.594240647	0.124259777	4.782244589	1.73349E-06	9.22764E-06	positive
PRJNA729271	stage_E14_vs_E10	ordinary_muscle_development	ENSGALG00010003903	COL28A1	2.853475822	0.281200726	10.1474696	3.40066E-24	9.57138E-23	positive
PRJNA729271	stage_E14_vs_E10	ordinary_muscle_development	ENSGALG00010003909	DNAJB1	-0.33914022	0.156425974	-2.168055666	0.030154451	0.061700843	negative
PRJNA729271	stage_E14_vs_E10	ordinary_muscle_development	ENSGALG00010003910	THAP9	0.167345974	0.14833521	1.128160834	0.259252001	0.367552598	positive

PRJNA729271	stage_E14_vs_E10	ordinary_muscle_development	ENSGALG00010003913	PSMB5	0.31749159	0.116731532	2.719844281	0.006531267	0.016401717	positive
PRJNA729271	stage_E14_vs_E10	ordinary_muscle_development	ENSGALG00010003914	CAMK2D	0.178437847	0.105837211	1.68596513	0.091802516	0.158443936	positive
PRJNA729271	stage_E14_vs_E10	ordinary_muscle_development	ENSGALG00010003915	MIOS	-0.01292422	0.091695766	-0.140946745	0.887912014	0.923064236	negative
PRJNA729271	stage_E14_vs_E10	ordinary_muscle_development	ENSGALG00010003918	HTR1E	-0.908802305	0.65296353	-1.391811737	0.163979415	0.25486614	negative
PRJNA729271	stage_E14_vs_E10	ordinary_muscle_development	ENSGALG00010003920	LPAR6	0.557847083	0.152628093	3.654943688	0.000257238	0.000931255	positive
PRJNA729271	stage_E14_vs_E10	ordinary_muscle_development	ENSGALG00010003921	COLEC12	-0.392307123	0.168250976	-2.331678135	0.01971763	0.042901088	negative
PRJNA729271	stage_E14_vs_E10	ordinary_muscle_development	ENSGALG00010003923	ENSGALG00010003923	-0.217137251	0.117750484	-1.844045504	0.065176509	0.118752453	negative
PRJNA729271	stage_E14_vs_E10	ordinary_muscle_development	ENSGALG00010003925	SEC31A	-0.376088661	0.085559058	-4.395661551	1.10436E-05	5.13561E-05	negative
PRJNA729271	stage_E14_vs_E10	ordinary_muscle_development	ENSGALG00010003928	ENSGALG00010003928	0.379442929	0.155267681	2.443798518	0.01453354	0.033050466	positive
PRJNA729271	stage_E14_vs_E10	ordinary_muscle_development	ENSGALG00010003929	RCBTB2	0.128340272	0.096820369	1.325550333	0.184988687	0.280864252	positive
PRJNA729271	stage_E14_vs_E10	ordinary_muscle_development	ENSGALG00010003933	TECR	0.340680316	0.207990052	1.637964471	0.101429105	0.172035442	positive
PRJNA729271	stage_E14_vs_E10	ordinary_muscle_development	ENSGALG00010003937	ENSGALG00010003937	-0.048056171	0.207699393	-0.231373671	0.817024517	0.871753432	negative
PRJNA729271	stage_E14_vs_E10	ordinary_muscle_development	ENSGALG00010003948	SCD5	-1.397722469	0.134008994	-10.43006462	1.80765E-25	5.67141E-24	negative
PRJNA729271	stage_E14_vs_E10	ordinary_muscle_development	ENSGALG00010003958	CYSLTR2	0.213755815	0.405318908	0.527376866	0.597931905	0.697490984	positive
PRJNA729271	stage_E14_vs_E10	ordinary_muscle_development	ENSGALG00010003960	ASPH	-0.516378105	0.196111902	-2.633078873	0.00846147	0.020627144	negative
PRJNA729271	stage_E14_vs_E10	ordinary_muscle_development	ENSGALG00010003962	UMAD1	-0.147248565	0.20472813	-0.719239534	0.471993344	0.584148184	negative
PRJNA729271	stage_E14_vs_E10	ordinary_muscle_development	ENSGALG00010003965	SNTG1	-2.748871975	0.519976121	-5.286535025	1.24655E-07	7.8152E-07	negative
PRJNA729271	stage_E14_vs_E10	ordinary_muscle_development	ENSGALG00010003968	DSN1	0.272075778	0.171817715	1.583514121	0.113304331	0.18894918	positive
PRJNA729271	stage_E14_vs_E10	ordinary_muscle_development	ENSGALG00010003972	PENK	0.777691399	0.240364811	3.235462774	0.001214457	0.003756628	positive
PRJNA729271	stage_E14_vs_E10	ordinary_muscle_development	ENSGALG00010003977	MYO6	-0.869722925	0.116634086	-7.456850333	8.86153E-14	1.12772E-12	negative
PRJNA729271	stage_E14_vs_E10	ordinary_muscle_development	ENSGALG00010003978	ANK2	1.126214969	0.119543487	9.420964697	4.46962E-21	1.0366E-19	positive
PRJNA729271	stage_E14_vs_E10	ordinary_muscle_development	ENSGALG00010003980	CHCHD7	-0.240703651	0.119255405	-2.018387765	0.043550893	0.084281734	negative
PRJNA729271	stage_E14_vs_E10	ordinary_muscle_development	ENSGALG00010003981	TMEM150C	0.351573692	0.162706999	2.160777929	0.030712498	0.062604086	positive
PRJNA729271	stage_E14_vs_E10	ordinary_muscle_development	ENSGALG00010003982	GLCC11	-0.725121649	0.120399438	-6.022633192	1.71602E-09	1.3697E-08	negative
PRJNA729271	stage_E14_vs_E10	ordinary_muscle_development	ENSGALG00010003985	PRMT5	0.07825039	0.133392433	0.586617909	0.557460352	0.661502196	positive
PRJNA729271	stage_E14_vs_E10	ordinary_muscle_development	ENSGALG00010003986	FNDC3A	-0.446722149	0.064006355	-6.979340597	2.96569E-12	3.23702E-11	negative
PRJNA729271	stage_E14_vs_E10	ordinary_muscle_development	ENSGALG00010003988	ENOPH1	-0.237409249	0.087910975	-2.70056439	0.006922194	0.01726562	negative
PRJNA729271	stage_E14_vs_E10	ordinary_muscle_development	ENSGALG00010003990	ICA1	-1.050866786	0.169932005	-6.184042778	6.24804E-10	5.25017E-09	negative
PRJNA729271	stage_E14_vs_E10	ordinary_muscle_development	ENSGALG00010003993	ENSGALG00010003993	0.508693776	0.133738411	3.803647525	0.000142581	0.000546605	positive
PRJNA729271	stage_E14_vs_E10	ordinary_muscle_development	ENSGALG00010003994	SDR16C5	0.681326382	0.08489424	8.025590213	1.01039E-15	1.50986E-14	positive
PRJNA729271	stage_E14_vs_E10	ordinary_muscle_development	ENSGALG00010003995	MLNR	-1.052509194	0.201254566	-5.22974069	1.69748E-07	1.04569E-06	negative
PRJNA729271	stage_E14_vs_E10	ordinary_muscle_development	ENSGALG00010004000	ENSGALG00010004000	0.322493561	0.177886175	1.812920882	0.069844025	0.125856432	positive
PRJNA729271	stage_E14_vs_E10	ordinary_muscle_development	ENSGALG00010004001	LARP7	-0.276709253	0.111747942	-2.476191033	0.01327925	0.030543119	negative
PRJNA729271	stage_E14_vs_E10	ordinary_muscle_development	ENSGALG00010004002	CDADC1	-0.073815686	0.12697209	-0.581353632	0.561002141	0.664156425	negative
PRJNA729271	stage_E14_vs_E10	ordinary_muscle_development	ENSGALG00010004003	HNRNPDL	-0.077902204	0.067850744	-1.148140748	0.250910465	0.358052548	negative
PRJNA729271	stage_E14_vs_E10	ordinary_muscle_development	ENSGALG00010004006	NKAIN3	-1.655025391	0.318967554	-5.188695127	2.11773E-07	1.29085E-06	negative
PRJNA729271	stage_E14_vs_E10	ordinary_muscle_development	ENSGALG00010004007	ENSGALG00010004007	0.010149057	0.153217967	0.066239342	0.947187275	0.963201877	positive
PRJNA729271	stage_E14_vs_E10	ordinary_muscle_development	ENSGALG00010004016	ENSGALG00010004016	1.415445804	0.372855233	3.796234246	0.000146911	0.000560977	positive
PRJNA729271	stage_E14_vs_E10	ordinary_muscle_development	ENSGALG00010004018	HNRNPD	-0.252399036	0.160248662	-1.575046134	0.115245796	0.191588748	negative
PRJNA729271	stage_E14_vs_E10	ordinary_muscle_development	ENSGALG00010004029	ARL4A	0.921214421	0.194219112	4.743170814	2.10399E-06	1.10377E-05	positive
PRJNA729271	stage_E14_vs_E10	ordinary_muscle_development	ENSGALG00010004034	NEDD8	0.076436206	0.111323769	0.686611732	0.492327433	0.602523315	positive
PRJNA729271	stage_E14_vs_E10	ordinary_muscle_development	ENSGALG00010004036	ADCYAP1	1.659420266	0.442217274	3.752499878	0.00017508	0.00065848	positive
PRJNA729271	stage_E14_vs_E10	ordinary_muscle_development	ENSGALG00010004038	SENP6	0.018381609	0.087299094	0.210558986	0.833231419	0.883790828	positive
PRJNA729271	stage_E14_vs_E10	ordinary_muscle_development	ENSGALG00010004039	ALPK1	-0.169186007	0.273564888	-0.618449277	0.536279224	0.64301161	negative
PRJNA729271	stage_E14_vs_E10	ordinary_muscle_development	ENSGALG00010004040	LGALS1B	1.184255478	0.224363763	5.278283194	1.304E-07	8.14367E-07	positive
PRJNA729271	stage_E14_vs_E10	ordinary_muscle_development	ENSGALG00010004042	PLAG1	-0.397184124	0.119981178	-3.31038694	0.000931671	0.002955048	negative
PRJNA729271	stage_E14_vs_E10	ordinary_muscle_development	ENSGALG00010004043	CAB39L	0.610963574	0.200570015	3.046136159	0.002318028	0.006620654	positive
PRJNA729271	stage_E14_vs_E10	ordinary_muscle_development	ENSGALG00010004044	RNF138	-0.425453885	0.132308735	-3.215614482	0.001301656	0.003988057	negative
PRJNA729271	stage_E14_vs_E10	ordinary_muscle_development	ENSGALG00010004046	ENSGALG00010004046	0.842432979	0.12014634	7.011724041	2.35399E-12	2.59082E-11	positive
PRJNA729271	stage_E14_vs_E10	ordinary_muscle_development	ENSGALG00010004047	PDE7A	-0.552221107	0.114420042	-4.82626205	1.39119E-06	7.54409E-06	negative
PRJNA729271	stage_E14_vs_E10	ordinary_muscle_development	ENSGALG00010004049	SETDB2	-0.25209966	0.137753294	-1.830080816	0.067237855	0.121857053	negative
PRJNA729271	stage_E14_vs_E10	ordinary_muscle_development	ENSGALG00010004055	FILIP1	1.139851952	0.175359612	6.500082535	8.0276E-11	7.45897E-10	positive
PRJNA729271	stage_E14_vs_E10	ordinary_muscle_development	ENSGALG00010004056	SHROOM3	1.222069282	0.180578613	6.767519473	1.31009E-11	1.32888E-10	positive
PRJNA729271	stage_E14_vs_E10	ordinary_muscle_development	ENSGALG00010004057	ENSGALG00010004057	0.386460213	0.377338089	1.024174937	0.305752655	0.419566859	positive
PRJNA729271	stage_E14_vs_E10	ordinary_muscle_development	ENSGALG00010004058	TMEM30A	-0.23791604	0.061602138	-3.86213935	0.000112398	0.000439023	negative
PRJNA729271	stage_E14_vs_E10	ordinary_muscle_development	ENSGALG00010004060	ETV1	-0.309642397	0.090672689	-3.414946662	0.000637945	0.00210255	negative
PRJNA729271	stage_E14_vs_E10	ordinary_muscle_development	ENSGALG00010004063	ENSGALG00010004063	-0.143350339	0.448539376	-0.319593656	0.749276384	0.820237232	negative
PRJNA729271	stage_E14_vs_E10	ordinary_muscle_development	ENSGALG00010004065	TIFA	-1.173505962	0.311874298	-3.762753036	0.000168053	0.000634357	negative
PRJNA729271	stage_E14_vs_E10	ordinary_muscle_development	ENSGALG00010004070	ARL11	-0.687177045	0.404340878	-1.699499317	0.089225143	0.154696984	negative
PRJNA729271	stage_E14_vs_E10	ordinary_muscle_development	ENSGALG00010004072	SGK3	-0.824559774	0.135473826	-6.086487707	1.15415E-09	9.35645E-09	negative
PRJNA729271	stage_E14_vs_E10	ordinary_muscle_development	ENSGALG00010004076	COX7A2	-0.01442741	0.136240284	-0.105896799	0.915664234	0.941364918	negative
PRJNA729271	stage_E14_vs_E10	ordinary_muscle_development	ENSGALG00010004079	AP1AR	-0.820002333	0.149623031	-5.480455303	4.24233E-08	2.84293E-07	negative
PRJNA729271	stage_E14_vs_E10	ordinary_muscle_development	ENSGALG00010004081	RPS20	0.466277842	0.16791789	2.776820513	0.005489348	0.014122844	positive
PRJNA729271	stage_E14_vs_E10	ordinary_muscle_development	ENSGALG00010004082	FAM241A	0.1409019	0.122651981	1.148794334	0.250640796	0.357812784	positive
PRJNA729271	stage_E14_vs_E10	ordinary_muscle_development	ENSGALG00010004083	PHF11	-0.157299998	0.182112024	-0.863754047	0.387723016	0.503633401	negative
PRJNA729271	stage_E14_vs_E10	ordinary_muscle_development	ENSGALG00010004085	RB1CC1	0.145742731	0.140873305	1.034565991	0.300871643	0.414753635	positive
PRJNA729271	stage_E14_vs_E10	ordinary_muscle_development	ENSGALG00010004086	UTP3	0.020307731	0.256881347	0.079054908	0.936988949	0.95618629	positive
PRJNA729271	stage_E14_vs_E10	ordinary_muscle_development	ENSGALG00010004087	NCOA7	-0.253012149	0.108209509	-2.338169272	0.019378469	0.042277379	negative
PRJNA729271	stage_E14_vs_E10	ordinary_muscle_development	ENSGALG00010004094	DGKB	-0.708443133	0.170828752	-4.147095407	3.3672E-05	0.00014423	negative
PRJNA729271	stage_E14_vs_E10	ordinary_muscle_development	ENSGALG00010004095	ANKRD50	0.50991033	0.113028255	4.511352755	6.44155E-06	3.12484E-05	positive
PRJNA729271	stage_E14_vs_E10	ordinary_muscle_development	ENSGALG00010004096	COL12A1	-1.243814913	0.414045139	-3.004056312	0.00266406	0.007481504	negative
PRJNA729271	stage_E14_vs_E10	ordinary_muscle_development	ENSGALG00010004097	ENSGALG00010004097	-0.230877008	0.114099618	-2.023468716	0.043024846	0.083397317	negative
PRJNA729271	stage_E14_vs_E10	ordinary_muscle_development	ENSGALG00010004101	ARID1B	0.087001243	0.166219308	0.523412376	0.600687314	0.6998041	positive
PRJNA729271	stage_E14_vs_E10	ordinary_muscle_development	ENSGALG00010004102	PCMTD1	0.412187123	0.188459004	2.187144763	0.028731958	0.059258641	positive
PRJNA729271	stage_E14_vs_E10	ordinary_muscle_development	ENSGALG00010004104	SCARB2	0.103186312	0.094769213	1.088816805	0.276234679	0.386712544	positive
PRJNA729271	stage_E14_vs_E10	ordinary_muscle_development	ENSGALG00010004108	PERPB	-0.148123001	0.242167561	-0.611655004	0.540766038	0.64673298	negative
PRJNA729271	stage_E14_vs_E10	ordinary_muscle_development	ENSGALG00010004109	AGMO	-1.197381714	0.500354869	-2.39306498	0.016708282	0.037248007	negative
PRJNA729271	stage_E14_vs_E10	ordinary_muscle_development	ENSGALG00010004113	ECHDC1	0.53473986	0.180646463	2.960145747	0.003074935	0.008497843	positive
PRJNA729271	stage_E14_vs_E10	ordinary_muscle_development	ENSGALG00010004116	SPRY1	0.090640106	0.095066417	0.953439804	0.340367282	0.4553834	positive
PRJNA729271	stage_E14_vs_E10	ordinary_muscle_development	ENSGALG00010004117	STBD1	0.930902493	0.192535052	4.834976705	1.33161E-06	7.24267E-06	positive
PRJNA729271	stage_E14_vs_E10	ordinary_muscle_development	ENSGALG00010004122	TRIM55	2.650834113	0.194666299	13.61732427	3.15932E-42	2.7587E-40	positive
PRJNA729271	stage_E14_vs_E10	ordinary_muscle_development	ENSGALG00010004123	NDUFAF4	-0.069602033	0.109177507	-0.637512565	0.523791028	0.631643174	negative
PRJNA729271	stage_E14_vs_E10	ordinary_muscle_development	ENSGALG00010004125	MANEA	0.117162116	0.092002705	1.273463809	0.202853507	0.302444105	positive
PRJNA729271	stage_E14_vs_E10	ordinary_muscle_development	ENSGALG00010004127	EPHA7	-2.553705997	0.174423685	-14.64082125	1.54194E-48	2.0505E-46	negative
PRJNA729271	stage_E14_vs_E10	ordinary_muscle_development	ENSGALG00010004131	NUDT6	0.46119147	0.157073829	2.936144578	0.003323194	0.009090337	positive
PRJNA729271	stage_E14_vs_E10	ordinary_muscle_development	ENSGALG00010004133	CRH	0.456329473	1.057098601	0.431681087	0.665973215	0.754103723	positive
PRJNA729271	stage_E14_vs_E10	ordinary_muscle_development	ENSGALG00010004135	NUP54	-0.206325096	0.062778542	-3.286554419	0.001014212	0.003193787	negative
PRJNA729271	stage_E14_vs_E10	ordinary_muscle_development	ENSGALG00010004137	CROT	-0.37305738	0.083792059	-4.452180621	8.50026E-06	4.03123E-05	negative
PRJNA729271	stage_E14_vs_E10	ordinary_muscle_development	ENSGALG00010004139	PPEF2	0.77020155	0.613425981	1.255573734	0.209270559	0.309907719	positive
PRJNA729271	stage_E14_vs_E10	ordinary_muscle_development	ENSGALG00010004141	KPNA3	-0.283798483	0.141214176	-2.009702509	0.044462684	0.085828553	negative
PRJNA729271	stage_E14_vs_E10	ordinary_muscle_development	ENSGALG00010004142	RBBP8	-0.655130784	0.109866281	-5.962983169	2.47674E-09	1.94371E-08	negative
PRJNA729271	stage_E14_vs_E10	ordinary_muscle_development	ENSGALG00010004143	TCF24	-3.242699065	0.484119522	-6.698137375	2.11093E-11	2.09002E-10	negative
PRJNA729271	stage_E14_vs_E10	ordinary_muscle_development	ENSGALG00010004146	NAAA	0.659765267	0.177649227	3.71386512	0.000204118	0.000756863	positive
PRJNA729271	stage_E14_vs_E10	ordinary_muscle_development	ENSGALG00010004147	SPRYD7	-0.052670998	0.091505684	-0.575603563	0.564883172	0.66748036	negative
PRJNA729271	stage_E14_vs_E10	ordinary_muscle_development	ENSGALG00010004148	ENSGALG00010004148	-0.079302558	0.153682958	-0.516014003	0.605844629	0.703663293	negative
PRJNA729271	stage_E14_vs_E10	ordinary_muscle_development	ENSGALG00010004149	SDAD1	-0.167029996	0.119515172	-1.397563117	0.162244301	0.25279277	negative
PRJNA729271	stage_E14_vs_E10	ordinary_muscle_development	ENSGALG00010004154	COPS5	-0.10244958	0.129669239	-0.790083912	0.429478764	0.543680864	negative
PRJNA729271	stage_E14_vs_E10	ordinary_muscle_development	ENSGALG00010004158	JCHAIN	1.328626697	0.480779936	2.763481994	0.005718825	0.014627557	positive
PRJNA729271	stage_E14_vs_E10	ordinary_muscle_development	ENSGALG00010004172	ST8SIA6	0.017666921	0.124263417	0.142173148	0.886943241	0.922480644	positive
PRJNA729271	stage_E14_vs_E10	ordinary_muscle_development	ENSGALG00010004177	CSPP1	-0.019297634	0.126386511	-0.152687451	0.878644758	0.916564808	negative
PRJNA729271	stage_E14_vs_E10	ordinary_muscle_development	ENSGALG00010004181	BBS12	-0.530393713	0.130655823	-4.059472428	4.91837E-05	0.000204744	negative
PRJNA729271	stage_E14_vs_E10	ordinary_muscle_development	ENSGALG00010004187	ARFGEF1	-0.127386409	0.094352996	-1.350104544	0.176982451	0.270907119	negative
PRJNA729271	stage_E14_vs_E10	ordinary_muscle_development	ENSGALG00010004188	BMI1	0.123985684	0.114378228	1.083997244	0.278366021	0.389057513	positive
PRJNA729271	stage_E14_vs_E10	ordinary_muscle_development	ENSGALG00010004191	CABLES1	0.167727162	0.125121486	1.340514463	0.180078145	0.274963944	positive
PRJNA729271	stage_E14_vs_E10	ordinary_muscle_development	ENSGALG00010004200	ABCB1	-0.625929934	0.194927186	-3.211096129	0.001322297	0.004041901	negative
PRJNA729271	stage_E14_vs_E10	ordinary_muscle_development	ENSGALG00010004201	CPA6	-2.235713803	0.45458747	-4.918115766	8.73812E-07	4.86348E-06	negative
PRJNA729271	stage_E14_vs_E10	ordinary_muscle_development	ENSGALG00010004210	SYNCRIP	-0.268634844	0.070205927	-3.826384139	0.000130039	0.000501709	negative
PRJNA729271	stage_E14_vs_E10	ordinary_muscle_development	ENSGALG00010004211	RNF146	0.180641389	0.074332601	2.430177166	0.015091444	0.034113946	positive
PRJNA729271	stage_E14_vs_E10	ordinary_muscle_development	ENSGALG00010004212	RSPO3	-1.925544564	0.381985994	-5.040877399	4.63402E-07	2.68896E-06	negative
PRJNA729271	stage_E14_vs_E10	ordinary_muscle_development	ENSGALG00010004214	REPS1	-0.245833164	0.125192648	-1.963638992	0.049571972	0.094186097	negative
PRJNA729271	stage_E14_vs_E10	ordinary_muscle_development	ENSGALG00010004216	SNX14	-0.169016772	0.073467925	-2.300551872	0.021416974	0.046189412	negative
PRJNA729271	stage_E14_vs_E10	ordinary_muscle_development	ENSGALG00010004217	CCDC28A	0.860508095	0.110265376	7.803973689	5.99877E-15	8.38497E-14	positive
PRJNA729271	stage_E14_vs_E10	ordinary_muscle_development	ENSGALG00010004219	FGF2	0.664067793	0.181246576	3.663891523	0.000248412	0.000902038	positive
PRJNA729271	stage_E14_vs_E10	ordinary_muscle_development	ENSGALG00010004222	SPAG6	-1.597089015	0.244386103	-6.535105702	6.35646E-11	5.9985E-10	negative
PRJNA729271	stage_E14_vs_E10	ordinary_muscle_development	ENSGALG00010004224	NT5E	2.246538928	0.257114221	8.737513322	2.38298E-18	4.47425E-17	positive
PRJNA729271	stage_E14_vs_E10	ordinary_muscle_development	ENSGALG00010004225	CETN4	-0.594473032	0.122409747	-4.856419094	1.19528E-06	6.53793E-06	negative
PRJNA729271	stage_E14_vs_E10	ordinary_muscle_development	ENSGALG00010004226	RUNDC3B	-1.547681426	0.202234616	-7.652900653	1.96496E-14	2.64457E-13	negative
PRJNA729271	stage_E14_vs_E10	ordinary_muscle_development	ENSGALG00010004230	TAF1B	-0.118391651	0.114685899	-1.032312188	0.301925894	0.415614039	negative
PRJNA729271	stage_E14_vs_E10	ordinary_muscle_development	ENSGALG00010004231	ARMC3	-1.097515334	0.39841213	-2.754723691	0.005874172	0.014985238	negative
PRJNA729271	stage_E14_vs_E10	ordinary_muscle_development	ENSGALG00010004233	NHSL1	1.086571659	0.1176676	9.234246789	2.6011E-20	5.6953E-19	positive
PRJNA729271	stage_E14_vs_E10	ordinary_muscle_development	ENSGALG00010004235	ADAD1	-2.460243138	0.457520028	-5.377345221	7.55921E-08	4.90909E-07	negative
PRJNA729271	stage_E14_vs_E10	ordinary_muscle_development	ENSGALG00010004236	DBF4	-0.563454281	0.121408072	-4.64099523	3.46735E-06	1.75425E-05	negative
PRJNA729271	stage_E14_vs_E10	ordinary_muscle_development	ENSGALG00010004237	RUFY3	0.048420727	0.091010117	0.532036752	0.594700541	0.694523235	positive
PRJNA729271	stage_E14_vs_E10	ordinary_muscle_development	ENSGALG00010004238	CCNC	-0.11527985	0.123014634	-0.93712306	0.348695261	0.464467727	negative
PRJNA729271	stage_E14_vs_E10	ordinary_muscle_development	ENSGALG00010004240	ENSGALG00010004240	0.516875751	0.174216118	2.966865275	0.003008528	0.00833976	positive
PRJNA729271	stage_E14_vs_E10	ordinary_muscle_development	ENSGALG00010004241	PREX2	-0.746554362	0.11551126	-6.463044069	1.02617E-10	9.38447E-10	negative
PRJNA729271	stage_E14_vs_E10	ordinary_muscle_development	ENSGALG00010004242	ADAM22	-0.734871947	0.171300064	-4.289968893	1.78698E-05	8.02426E-05	negative
PRJNA729271	stage_E14_vs_E10	ordinary_muscle_development	ENSGALG00010004243	BLTP1	-0.174727565	0.112736071	-1.549881619	0.121169932	0.199201335	negative
PRJNA729271	stage_E14_vs_E10	ordinary_muscle_development	ENSGALG00010004244	TMEM241	0.113344612	0.079916093	1.418295213	0.156104594	0.245070517	positive
PRJNA729271	stage_E14_vs_E10	ordinary_muscle_development	ENSGALG00010004245	ARFGEF3	-0.769323363	0.220269268	-3.492649573	0.000478254	0.001630359	negative
PRJNA729271	stage_E14_vs_E10	ordinary_muscle_development	ENSGALG00010004246	C8orf34	-2.348221722	0.312927027	-7.504055318	6.18732E-14	8.02911E-13	negative

PRJNA729271	stage_E14_vs_E10	ordinary_muscle_development	ENSGALG00010004247	MEOX2	-0.349356855	0.103626741	-3.371300219	0.000748143	0.002432009	negative
PRJNA729271	stage_E14_vs_E10	ordinary_muscle_development	ENSGALG00010004248	GRSF1	-0.355424544	0.159147153	-2.233307584	0.025528668	0.053698743	negative
PRJNA729271	stage_E14_vs_E10	ordinary_muscle_development	ENSGALG00010004249	USP45	0.074877807	0.101826931	0.73534385	0.462130109	0.574689081	positive
PRJNA729271	stage_E14_vs_E10	ordinary_muscle_development	ENSGALG00010004250	MOB1B	0.054421565	0.086655344	0.628023186	0.529988751	0.637578799	positive
PRJNA729271	stage_E14_vs_E10	ordinary_muscle_development	ENSGALG00010004251	CCN2	-1.894824134	0.267226529	-7.09070369	1.33432E-12	1.50279E-11	negative
PRJNA729271	stage_E14_vs_E10	ordinary_muscle_development	ENSGALG00010004255	RPS12	0.388542119	0.151055331	2.57218408	0.010105914	0.024025788	positive
PRJNA729271	stage_E14_vs_E10	ordinary_muscle_development	ENSGALG00010004256	ENSGALG00010004256	-1.135760358	0.517200918	-2.195975142	0.028093723	0.058165764	negative
PRJNA729271	stage_E14_vs_E10	ordinary_muscle_development	ENSGALG00010004257	ENSGALG00010004257	1.322619446	0.628878322	2.103140464	0.035453493	0.070702229	positive
PRJNA729271	stage_E14_vs_E10	ordinary_muscle_development	ENSGALG00010004265	PIP4K2A	0.012828742	0.092349364	0.138915323	0.889517062	0.924136311	positive
PRJNA729271	stage_E14_vs_E10	ordinary_muscle_development	ENSGALG00010004275	ADARB2	-2.033489074	0.608876945	-3.339737349	0.000838577	0.002693368	negative
PRJNA729271	stage_E14_vs_E10	ordinary_muscle_development	ENSGALG00010004281	VIM	0.552170518	0.171595323	3.217864612	0.001291488	0.003962767	positive
PRJNA729271	stage_E14_vs_E10	ordinary_muscle_development	ENSGALG00010004283	DCK	-0.091457567	0.124721184	-0.733296173	0.463377809	0.576018449	negative
PRJNA729271	stage_E14_vs_E10	ordinary_muscle_development	ENSGALG00010004285	SLC18B1	-0.469033319	0.117867134	-3.979339305	6.91071E-05	0.000280276	negative
PRJNA729271	stage_E14_vs_E10	ordinary_muscle_development	ENSGALG00010004296	ENSGALG00010004296	0.422041604	0.567008146	0.74433076	0.456676397	0.569685485	positive
PRJNA729271	stage_E14_vs_E10	ordinary_muscle_development	ENSGALG00010004297	SULF1	-1.160892256	0.182962603	-6.344970151	2.22469E-10	1.9747E-09	negative
PRJNA729271	stage_E14_vs_E10	ordinary_muscle_development	ENSGALG00010004300	VNN2	2.109121061	0.245797728	8.580718286	9.42825E-18	1.68719E-16	positive
PRJNA729271	stage_E14_vs_E10	ordinary_muscle_development	ENSGALG00010004302	CRPPA	-0.072491638	0.127286542	-0.56951534	0.569006464	0.67131277	negative
PRJNA729271	stage_E14_vs_E10	ordinary_muscle_development	ENSGALG00010004306	PNISR	-0.191772078	0.143826919	-1.333353164	0.182415935	0.277801951	negative
PRJNA729271	stage_E14_vs_E10	ordinary_muscle_development	ENSGALG00010004307	NPC2	0.63889007	0.140643022	4.542636101	5.55551E-06	2.72419E-05	positive
PRJNA729271	stage_E14_vs_E10	ordinary_muscle_development	ENSGALG00010004308	DSG2	-2.435395598	0.469330714	-5.189082076	2.11333E-07	1.28926E-06	negative
PRJNA729271	stage_E14_vs_E10	ordinary_muscle_development	ENSGALG00010004309	MRPS28	-0.019983827	0.135781844	-0.147175987	0.882993114	0.919863819	negative
PRJNA729271	stage_E14_vs_E10	ordinary_muscle_development	ENSGALG00010004312	PDLIM5	1.200660064	0.181990363	6.597382645	4.1848E-11	4.04121E-10	positive
PRJNA729271	stage_E14_vs_E10	ordinary_muscle_development	ENSGALG00010004319	TAAR1	2.614674083	0.878645879	2.97579963	0.002922257	0.008121342	positive
PRJNA729271	stage_E14_vs_E10	ordinary_muscle_development	ENSGALG00010004323	TRDMT1	-0.071316335	0.157812657	-0.451905038	0.651337395	0.742344357	negative
PRJNA729271	stage_E14_vs_E10	ordinary_muscle_development	ENSGALG00010004325	LTBP2	1.441623118	0.112751888	12.78580029	1.96826E-37	1.32083E-35	positive
PRJNA729271	stage_E14_vs_E10	ordinary_muscle_development	ENSGALG00010004326	ENSGALG00010004326	-0.075758686	0.942192006	-0.080406844	0.935913683	0.955424244	negative
PRJNA729271	stage_E14_vs_E10	ordinary_muscle_development	ENSGALG00010004328	ENSGALG00010004328	-0.204306704	0.685255362	-0.298146816	0.765591111	0.833439216	negative
PRJNA729271	stage_E14_vs_E10	ordinary_muscle_development	ENSGALG00010004329	COQ3	0.605301853	0.120001623	5.044113895	4.55628E-07	2.64809E-06	positive
PRJNA729271	stage_E14_vs_E10	ordinary_muscle_development	ENSGALG00010004330	STX7	-0.058984024	0.102967817	-0.572839415	0.566753429	0.669308372	negative
PRJNA729271	stage_E14_vs_E10	ordinary_muscle_development	ENSGALG00010004332	ENSGALG00010004332	-0.090682792	0.886306565	-0.102315379	0.918506348	0.943434632	negative
PRJNA729271	stage_E14_vs_E10	ordinary_muscle_development	ENSGALG00010004333	DIO2	-1.555389112	0.321525834	-4.837524535	1.31466E-06	7.16123E-06	negative
PRJNA729271	stage_E14_vs_E10	ordinary_muscle_development	ENSGALG00010004334	ENSGALG00010004334	0.55086222	0.228893965	2.406626236	0.016100639	0.036059758	positive
PRJNA729271	stage_E14_vs_E10	ordinary_muscle_development	ENSGALG00010004336	ENSGALG00010004336	-1.543537121	0.443578552	-3.479737945	0.000501904	0.001701381	negative
PRJNA729271	stage_E14_vs_E10	ordinary_muscle_development	ENSGALG00010004337	BMPR1B	0.585180604	0.227854313	2.568222633	0.010222148	0.024253236	positive
PRJNA729271	stage_E14_vs_E10	ordinary_muscle_development	ENSGALG00010004338	ENSGALG00010004338	0.358444837	0.167045538	2.145791151	0.031889645	0.064621893	positive
PRJNA729271	stage_E14_vs_E10	ordinary_muscle_development	ENSGALG00010004339	B4GALT6	-0.893835212	0.110093493	-8.118874102	4.70528E-16	7.24794E-15	negative
PRJNA729271	stage_E14_vs_E10	ordinary_muscle_development	ENSGALG00010004340	SLC4A4	1.224137415	0.168945886	7.245736736	4.30095E-13	5.11421E-12	positive
PRJNA729271	stage_E14_vs_E10	ordinary_muscle_development	ENSGALG00010004341	ENSGALG00010004341	-0.250066097	0.165891786	-1.507404937	0.131706894	0.213364865	negative
PRJNA729271	stage_E14_vs_E10	ordinary_muscle_development	ENSGALG00010004342	ZNF438	0.016684889	0.129087292	0.129252758	0.897157651	0.929240962	positive
PRJNA729271	stage_E14_vs_E10	ordinary_muscle_development	ENSGALG00010004344	SOSTDC1	-4.149740433	0.351823623	-11.79494543	4.14469E-32	1.96331E-30	negative
PRJNA729271	stage_E14_vs_E10	ordinary_muscle_development	ENSGALG00010004345	FAXC	-0.74312877	0.1788039	-4.156110515	3.23711E-05	0.000139234	negative
PRJNA729271	stage_E14_vs_E10	ordinary_muscle_development	ENSGALG00010004346	CEP128	0.149004273	0.095740772	1.556330396	0.119629547	0.197160919	positive
PRJNA729271	stage_E14_vs_E10	ordinary_muscle_development	ENSGALG00010004350	MOXD1	-1.862318341	0.242592043	-7.676749485	1.63176E-14	2.2105E-13	negative
PRJNA729271	stage_E14_vs_E10	ordinary_muscle_development	ENSGALG00010004352	CHMP2A	0.358975309	0.055697649	6.445071057	1.15546E-10	1.05204E-09	positive
PRJNA729271	stage_E14_vs_E10	ordinary_muscle_development	ENSGALG00010004355	ANKMY2	0.060784954	0.122793402	0.495018075	0.620587362	0.716248058	positive
PRJNA729271	stage_E14_vs_E10	ordinary_muscle_development	ENSGALG00010004356	TRAPPC8	-0.099017765	0.077175826	-1.283015291	0.199486719	0.298527748	negative
PRJNA729271	stage_E14_vs_E10	ordinary_muscle_development	ENSGALG00010004358	GC	5.908479028	2.108658927	2.802007927	0.005078563	0.013201895	positive
PRJNA729271	stage_E14_vs_E10	ordinary_muscle_development	ENSGALG00010004362	FBXL4	0.154301717	0.122767746	1.256858765	0.208804782	0.309281148	positive
PRJNA729271	stage_E14_vs_E10	ordinary_muscle_development	ENSGALG00010004364	TSHR	1.016986995	0.449194569	2.264023356	0.023572678	0.050180727	positive
PRJNA729271	stage_E14_vs_E10	ordinary_muscle_development	ENSGALG00010004365	TPD52	-0.619508353	0.154386874	-4.012700916	6.00279E-05	0.000246349	negative
PRJNA729271	stage_E14_vs_E10	ordinary_muscle_development	ENSGALG00010004366	UNC5C	-4.68089467	0.355121902	-13.18109258	1.12746E-39	8.21233E-38	negative
PRJNA729271	stage_E14_vs_E10	ordinary_muscle_development	ENSGALG00010004367	TSPAN13	-1.355373051	0.180127089	-7.524537572	5.29072E-14	6.8903E-13	negative
PRJNA729271	stage_E14_vs_E10	ordinary_muscle_development	ENSGALG00010004368	GJD4	-1.604803971	0.357183978	-4.492933807	7.02486E-06	3.38515E-05	negative
PRJNA729271	stage_E14_vs_E10	ordinary_muscle_development	ENSGALG00010004369	UBE2M	-0.108321871	0.234884088	-0.4611716	0.644675495	0.736895458	negative
PRJNA729271	stage_E14_vs_E10	ordinary_muscle_development	ENSGALG00010004370	ATP6V1H	0.114800941	0.073379156	1.564489798	0.117702587	0.194693484	positive
PRJNA729271	stage_E14_vs_E10	ordinary_muscle_development	ENSGALG00010004372	SYNJ2	0.236510421	0.185210494	1.276981751	0.20160869	0.300990623	positive
PRJNA729271	stage_E14_vs_E10	ordinary_muscle_development	ENSGALG00010004373	MMS22L	-0.2809875	0.117020238	-2.401187233	0.016341973	0.036514288	negative
PRJNA729271	stage_E14_vs_E10	ordinary_muscle_development	ENSGALG00010004374	ILDR1	-3.041373217	0.601328029	-5.057760606	4.24208E-07	2.47341E-06	negative
PRJNA729271	stage_E14_vs_E10	ordinary_muscle_development	ENSGALG00010004375	ENSGALG00010004375	-0.5080689	0.215455434	-2.358115973	0.018367954	0.040382753	negative
PRJNA729271	stage_E14_vs_E10	ordinary_muscle_development	ENSGALG00010004377	AGR2	-4.640837325	0.78236753	-5.931786723	2.99656E-09	2.32896E-08	negative
PRJNA729271	stage_E14_vs_E10	ordinary_muscle_development	ENSGALG00010004378	TRIM28	-0.264387689	0.154099241	-1.715697541	0.086217415	0.150550708	negative
PRJNA729271	stage_E14_vs_E10	ordinary_muscle_development	ENSGALG00010004379	ZBTB10	0.084872743	0.058658755	1.446889619	0.147927841	0.234725129	positive
PRJNA729271	stage_E14_vs_E10	ordinary_muscle_development	ENSGALG00010004380	ISCA2	0.323400083	0.083588797	3.868940512	0.000109309	0.000427764	positive
PRJNA729271	stage_E14_vs_E10	ordinary_muscle_development	ENSGALG00010004381	ENSGALG00010004381	-0.203924992	0.432176778	-0.471855505	0.637029929	0.730405216	negative
PRJNA729271	stage_E14_vs_E10	ordinary_muscle_development	ENSGALG00010004383	ENSGALG00010004383	-1.256550524	0.497404532	-2.526214466	0.011529907	0.027021181	negative
PRJNA729271	stage_E14_vs_E10	ordinary_muscle_development	ENSGALG00010004384	ENSGALG00010004384	1.50513033	0.523189737	2.876834585	0.004016861	0.010728653	positive
PRJNA729271	stage_E14_vs_E10	ordinary_muscle_development	ENSGALG00010004385	ADAMTS3	-2.690333753	0.205537115	-13.0892844	3.79163E-39	2.72078E-37	negative
PRJNA729271	stage_E14_vs_E10	ordinary_muscle_development	ENSGALG00010004386	CFAP44	-0.056137109	0.355780062	-0.157785991	0.874625438	0.914154719	negative
PRJNA729271	stage_E14_vs_E10	ordinary_muscle_development	ENSGALG00010004389	ENSGALG00010004389	0.075939942	0.128870222	0.589274552	0.555677113	0.660020428	positive
PRJNA729271	stage_E14_vs_E10	ordinary_muscle_development	ENSGALG00010004390	PNLDC1	-0.785891927	0.525525056	-1.495441402	0.134799282	0.21751259	negative
PRJNA729271	stage_E14_vs_E10	ordinary_muscle_development	ENSGALG00010004393	ZBTB45	0.346471456	0.210494739	1.645986299	0.09976658	0.169771845	positive
PRJNA729271	stage_E14_vs_E10	ordinary_muscle_development	ENSGALG00010004394	CCNY	-0.524715828	0.084441253	-6.213974951	5.16608E-10	4.38164E-09	negative
PRJNA729271	stage_E14_vs_E10	ordinary_muscle_development	ENSGALG00010004395	BOC	-1.125764198	0.190296809	-5.915833285	3.302E-09	2.54858E-08	negative
PRJNA729271	stage_E14_vs_E10	ordinary_muscle_development	ENSGALG00010004397	ENSGALG00010004397	-0.04068685	0.167734645	-0.242566762	0.808341032	0.865163056	negative
PRJNA729271	stage_E14_vs_E10	ordinary_muscle_development	ENSGALG00010004398	COX18	-0.343961465	0.121265693	-2.836428482	0.004562119	0.012016704	negative
PRJNA729271	stage_E14_vs_E10	ordinary_muscle_development	ENSGALG00010004399	POU3F2	-1.900226056	0.611064819	-3.109696378	0.001872797	0.005503472	negative
PRJNA729271	stage_E14_vs_E10	ordinary_muscle_development	ENSGALG00010004404	RGS20	1.610587334	0.352265277	4.572086547	4.82891E-06	2.38728E-05	positive
PRJNA729271	stage_E14_vs_E10	ordinary_muscle_development	ENSGALG00010004406	ENSGALG00010004406	-0.146567909	0.134780955	-1.087452667	0.276836806	0.387403891	negative
PRJNA729271	stage_E14_vs_E10	ordinary_muscle_development	ENSGALG00010004408	SERAC1	-0.131632096	0.108243916	-1.216069232	0.22395855	0.327015129	negative
PRJNA729271	stage_E14_vs_E10	ordinary_muscle_development	ENSGALG00010004416	STXBP6	-0.653210273	0.212839961	-3.069020832	0.002147616	0.006193732	negative
PRJNA729271	stage_E14_vs_E10	ordinary_muscle_development	ENSGALG00010004420	ENSGALG00010004420	0.753900661	0.287865268	2.618935814	0.008820454	0.021365723	positive
PRJNA729271	stage_E14_vs_E10	ordinary_muscle_development	ENSGALG00010004421	RAP1GDS1	0.246471465	0.073524288	3.352245518	0.000801589	0.002587182	positive
PRJNA729271	stage_E14_vs_E10	ordinary_muscle_development	ENSGALG00010004424	MRPL18	0.183491873	0.145064781	1.264896081	0.205908582	0.305803781	positive
PRJNA729271	stage_E14_vs_E10	ordinary_muscle_development	ENSGALG00010004425	CREM	0.48191292	0.242814649	1.984694588	0.047178461	0.090313232	positive
PRJNA729271	stage_E14_vs_E10	ordinary_muscle_development	ENSGALG00010004429	ENSGALG00010004429	0.126955527	0.348316604	0.364483134	0.715497266	0.794136858	positive
PRJNA729271	stage_E14_vs_E10	ordinary_muscle_development	ENSGALG00010004430	TCEA1	-0.166348154	0.092690129	-1.79466956	0.072706363	0.130301525	negative
PRJNA729271	stage_E14_vs_E10	ordinary_muscle_development	ENSGALG00010004433	AHR1A	-0.41358985	0.158740876	-2.605440141	0.00917563	0.022096821	negative
PRJNA729271	stage_E14_vs_E10	ordinary_muscle_development	ENSGALG00010004434	TCP1	-0.100160497	0.086309581	-1.160479472	0.245853647	0.3525804	negative
PRJNA729271	stage_E14_vs_E10	ordinary_muscle_development	ENSGALG00010004436	NOVA1	-0.062055907	0.150657532	-0.411900463	0.680412382	0.766203968	negative
PRJNA729271	stage_E14_vs_E10	ordinary_muscle_development	ENSGALG00010004440	GTF2H5	0.091365771	0.10422762	0.876598451	0.380704781	0.496655813	positive
PRJNA729271	stage_E14_vs_E10	ordinary_muscle_development	ENSGALG00010004443	ANKRD17	-0.206201405	0.093460262	-2.206300301	0.02736298	0.056872154	negative
PRJNA729271	stage_E14_vs_E10	ordinary_muscle_development	ENSGALG00010004445	EPHA6	-2.326240912	0.502434677	-4.629937017	3.65777E-06	1.83967E-05	negative
PRJNA729271	stage_E14_vs_E10	ordinary_muscle_development	ENSGALG00010004446	ACAT2	-0.202084508	0.123774807	-1.632678836	0.102536561	0.173669952	negative
PRJNA729271	stage_E14_vs_E10	ordinary_muscle_development	ENSGALG00010004451	CLDND1	0.066438018	0.083931663	0.791572756	0.428609838	0.54302068	positive
PRJNA729271	stage_E14_vs_E10	ordinary_muscle_development	ENSGALG00010004454	SRI	-0.037265716	0.163365136	-0.228113031	0.81955837	0.873638473	negative
PRJNA729271	stage_E14_vs_E10	ordinary_muscle_development	ENSGALG00010004457	AGA	0.078622568	0.125970579	0.624134371	0.532539343	0.639827782	positive
PRJNA729271	stage_E14_vs_E10	ordinary_muscle_development	ENSGALG00010004460	ENSGALG00010004460	-2.96095621	0.767908661	-3.855870315	0.000115319	0.00044934	negative
PRJNA729271	stage_E14_vs_E10	ordinary_muscle_development	ENSGALG00010004468	LYPLA1	0.236803077	0.12217994	1.938150225	0.052604897	0.09888011	positive
PRJNA729271	stage_E14_vs_E10	ordinary_muscle_development	ENSGALG00010004471	C1H3ORF52	-0.873965746	0.271492223	-3.219118895	0.001285852	0.003949654	negative
PRJNA729271	stage_E14_vs_E10	ordinary_muscle_development	ENSGALG00010004472	NEIL3	-0.443336282	0.185519213	-2.389705494	0.016861888	0.037555788	negative
PRJNA729271	stage_E14_vs_E10	ordinary_muscle_development	ENSGALG00010004473	JDP2	0.463163156	0.131266352	3.528422546	0.000418044	0.001441378	positive
PRJNA729271	stage_E14_vs_E10	ordinary_muscle_development	ENSGALG00010004477	TULP4	-0.21669084	0.125468845	-1.72704897	0.084158869	0.147615345	negative
PRJNA729271	stage_E14_vs_E10	ordinary_muscle_development	ENSGALG00010004481	ZNF704	0.410700213	0.077379438	5.307614307	1.11069E-07	7.03035E-07	positive
PRJNA729271	stage_E14_vs_E10	ordinary_muscle_development	ENSGALG00010004483	STEAP4	-1.914736437	0.749013728	-2.556343583	0.010577862	0.024996104	negative
PRJNA729271	stage_E14_vs_E10	ordinary_muscle_development	ENSGALG00010004487	ABHD10	-0.00056315	0.098406709	-0.00572268	0.995433987	0.997665992	negative
PRJNA729271	stage_E14_vs_E10	ordinary_muscle_development	ENSGALG00010004489	LIG1	-0.356700694	0.252309122	-1.41374474	0.157436864	0.246734495	negative
PRJNA729271	stage_E14_vs_E10	ordinary_muscle_development	ENSGALG00010004494	WTAP	0.001304549	0.056397727	0.023131239	0.981545587	0.987609557	positive
PRJNA729271	stage_E14_vs_E10	ordinary_muscle_development	ENSGALG00010004495	MRPL15	0.219091251	0.113140201	1.936458034	0.052811628	0.099145777	positive
PRJNA729271	stage_E14_vs_E10	ordinary_muscle_development	ENSGALG00010004501	TAGLN3	-0.989697037	0.312574027	-3.166280472	0.001544018	0.004633654	negative
PRJNA729271	stage_E14_vs_E10	ordinary_muscle_development	ENSGALG00010004503	ENSGALG00010004503	0.84643514	0.178733265	4.735744863	2.18252E-06	1.14167E-05	positive
PRJNA729271	stage_E14_vs_E10	ordinary_muscle_development	ENSGALG00010004504	ZNF804B	-1.82150848	0.621732321	-2.929731043	0.003392555	0.009252133	negative
PRJNA729271	stage_E14_vs_E10	ordinary_muscle_development	ENSGALG00010004505	ENSGALG00010004505	0.455726297	0.132083905	3.450278807	0.000560008	0.00187337	positive
PRJNA729271	stage_E14_vs_E10	ordinary_muscle_development	ENSGALG00010004507	CUL2	0.023602705	0.089970596	0.262337983	0.793060881	0.854699037	positive
PRJNA729271	stage_E14_vs_E10	ordinary_muscle_development	ENSGALG00010004515	ENSGALG00010004515	-1.565812818	0.258843615	-6.049261893	1.45511E-09	1.16964E-08	negative
PRJNA729271	stage_E14_vs_E10	ordinary_muscle_development	ENSGALG00010004516	ENSGALG00010004516	1.092798177	0.279315422	3.91241618	9.13773E-05	0.000362395	positive
PRJNA729271	stage_E14_vs_E10	ordinary_muscle_development	ENSGALG00010004522	TSPAN5	-0.089510256	0.06251735	-1.431766624	0.152210622	0.240232248	negative
PRJNA729271	stage_E14_vs_E10	ordinary_muscle_development	ENSGALG00010004533	PAG1	1.408606155	0.111245321	12.66216093	9.58248E-37	6.03905E-35	positive
PRJNA729271	stage_E14_vs_E10	ordinary_muscle_development	ENSGALG00010004534	CUBN	0.600313315	0.214195561	2.802641249	0.005068602	0.013183094	positive
PRJNA729271	stage_E14_vs_E10	ordinary_muscle_development	ENSGALG00010004542	ERMARD	0.117931735	0.126134574	0.934967554	0.349805021	0.465817526	positive
PRJNA729271	stage_E14_vs_E10	ordinary_muscle_development	ENSGALG00010004545	TMPRSS7	-1.587674707	0.669048896	-2.3730324	0.017642716	0.039042927	negative
PRJNA729271	stage_E14_vs_E10	ordinary_muscle_development	ENSGALG00010004553	BATF	-0.401397443	0.355976157	-1.127596429	0.259490395	0.367710752	negative
PRJNA729271	stage_E14_vs_E10	ordinary_muscle_development	ENSGALG00010004565	ENSGALG00010004565	0.634919323	0.422303902	1.503465442	0.132719062	0.214801563	positive
PRJNA729271	stage_E14_vs_E10	ordinary_muscle_development	ENSGALG00010004568	RSU1	0.162015217	0.066319668	2.442943729	0.014568009	0.033102882	positive
PRJNA729271	stage_E14_vs_E10	ordinary_muscle_development	ENSGALG00010004570	ENSGALG00010004570	-2.011238348	0.663810218	-3.029839395	0.002446838	0.006921864	negative
PRJNA729271	stage_E14_vs_E10	ordinary_muscle_development	ENSGALG00010004576	STEAP2	-0.723845054	0.212955829	-3.399038466	0.000676232	0.002218647	negative
PRJNA729271	stage_E14_vs_E10	ordinary_muscle_development	ENSGALG00010004577	ZPLD1	-4.148618438	0.427956957	-9.69400864	3.19728E-22	8.08805E-21	negative
PRJNA729271	stage_E14_vs_E10	ordinary_muscle_development	ENSGALG00010004583	XKR4	-1.020113308	0.118542617	-8.605456289	7.60138E-18	1.37213E-16	negative
PRJNA729271	stage_E14_vs_E10	ordinary_muscle_development	ENSGALG00010004584	EIF4E	-0.21327163	0.06631766	-3.215910064	0.001300316	0.003985067	negative
PRJNA729271	stage_E14_vs_E10	ordinary_muscle_development	ENSGALG00010004586	ENSGALG00010004586	-2.013864551	0.319940822	-6.294490767	3.08411E-10	2.6801E-09	negative
PRJNA729271	stage_E14_vs_E10	ordinary_muscle_development	ENSGALG00010004588	MINDY3	-0.266618767	0.151101373	-1.764502614	0.077647396	0.13786122	negative
PRJNA729271	stage_E14_vs_E10	ordinary_muscle_development	ENSGALG00010004591	RRS1	0.25211	0.16126067	1.563369421	0.117965728	0.195017476	positive

PRJNA729271	stage_E14_vs_E10	ordinary_muscle_development	ENSGALG00010004602	DYNLT2	-1.21803309	0.342678837	-3.55444503	0.000378778	0.001322346	negative
PRJNA729271	stage_E14_vs_E10	ordinary_muscle_development	ENSGALG00010004609	CFAP69	-3.4148535	0.651595296	-5.240758366	1.59918E-07	9.88491E-07	negative
PRJNA729271	stage_E14_vs_E10	ordinary_muscle_development	ENSGALG00010004610	TGS1	-0.067774687	0.121275571	-0.558848628	0.576265032	0.678196129	negative
PRJNA729271	stage_E14_vs_E10	ordinary_muscle_development	ENSGALG00010004622	ENSGALG00010004622	0.023861292	0.22959205	0.103929085	0.917225626	0.942355851	positive
PRJNA729271	stage_E14_vs_E10	ordinary_muscle_development	ENSGALG00010004623	MBIP	-0.267269395	0.123355096	-2.166666834	0.030260267	0.061847515	negative
PRJNA729271	stage_E14_vs_E10	ordinary_muscle_development	ENSGALG00010004624	PHF10	-0.050444804	0.07568275	-0.666529749	0.505072555	0.614025554	negative
PRJNA729271	stage_E14_vs_E10	ordinary_muscle_development	ENSGALG00010004630	ENSGALG00010004630	0.037995354	0.349269137	0.108785318	0.913372769	0.940627942	positive
PRJNA729271	stage_E14_vs_E10	ordinary_muscle_development	ENSGALG00010004639	HBS1L	0.0842188	0.079378761	1.060973979	0.288701727	0.401066852	positive
PRJNA729271	stage_E14_vs_E10	ordinary_muscle_development	ENSGALG00010004641	METAP1	-0.299008441	0.079742448	-3.749677216	0.000177062	0.000665221	negative
PRJNA729271	stage_E14_vs_E10	ordinary_muscle_development	ENSGALG00010004650	GTPBP10	-0.161488265	0.126997639	-1.271584789	0.203520684	0.303127036	negative
PRJNA729271	stage_E14_vs_E10	ordinary_muscle_development	ENSGALG00010004666	C6orf120	0.106712325	0.116341363	0.917234612	0.359019713	0.475423967	positive
PRJNA729271	stage_E14_vs_E10	ordinary_muscle_development	ENSGALG00010004669	ELMOD1	-2.699081251	0.34480808	-7.827778419	4.96566E-15	6.98131E-14	negative
PRJNA729271	stage_E14_vs_E10	ordinary_muscle_development	ENSGALG00010004673	RAB18	0.222645448	0.084666147	2.629686777	0.008546357	0.020802837	positive
PRJNA729271	stage_E14_vs_E10	ordinary_muscle_development	ENSGALG00010004689	BBX	-0.084629781	0.083614894	-1.012137632	0.311472251	0.42524162	negative
PRJNA729271	stage_E14_vs_E10	ordinary_muscle_development	ENSGALG00010004705	ENSGALG00010004705	0.194732376	0.233286752	0.834733967	0.403867512	0.518884913	positive
PRJNA729271	stage_E14_vs_E10	ordinary_muscle_development	ENSGALG00010004707	OTUD1	0.193898048	0.297930569	0.650816222	0.515165127	0.623888254	positive
PRJNA729271	stage_E14_vs_E10	ordinary_muscle_development	ENSGALG00010004715	WDR27	-1.062674034	0.336368903	-3.159251717	0.001581748	0.004731209	negative
PRJNA729271	stage_E14_vs_E10	ordinary_muscle_development	ENSGALG00010004716	ADH6	2.702472304	0.947427913	2.852430532	0.004338629	0.011486472	positive
PRJNA729271	stage_E14_vs_E10	ordinary_muscle_development	ENSGALG00010004717	ALKBH8	-0.431376553	0.17466379	-2.469753763	0.013520608	0.031034239	negative
PRJNA729271	stage_E14_vs_E10	ordinary_muscle_development	ENSGALG00010004721	LAMA2	1.424207453	0.158905754	8.962592068	3.17134E-19	6.41124E-18	positive
PRJNA729271	stage_E14_vs_E10	ordinary_muscle_development	ENSGALG00010004728	ENSGALG00010004728	0.594257276	0.215505753	2.757500752	0.005824508	0.014869011	positive
PRJNA729271	stage_E14_vs_E10	ordinary_muscle_development	ENSGALG00010004729	ENSGALG00010004729	2.128854555	1.275445082	1.669107188	0.095096139	0.16328104	positive
PRJNA729271	stage_E14_vs_E10	ordinary_muscle_development	ENSGALG00010004730	CLDN12	-0.053752743	0.061100607	-0.879741554	0.378999333	0.495186166	negative
PRJNA729271	stage_E14_vs_E10	ordinary_muscle_development	ENSGALG00010004735	ENSGALG00010004735	0.950757823	0.166918087	5.695954461	1.22684E-08	8.83637E-08	positive
PRJNA729271	stage_E14_vs_E10	ordinary_muscle_development	ENSGALG00010004736	CD47	0.803326108	0.100807182	7.968937254	1.60045E-15	2.35997E-14	positive
PRJNA729271	stage_E14_vs_E10	ordinary_muscle_development	ENSGALG00010004739	ENSGALG00010004739	1.561142916	0.649013585	2.40540869	0.016154388	0.036163376	positive
PRJNA729271	stage_E14_vs_E10	ordinary_muscle_development	ENSGALG00010004746	LYN	0.560406007	0.140969872	3.975360103	7.02728E-05	0.000284686	positive
PRJNA729271	stage_E14_vs_E10	ordinary_muscle_development	ENSGALG00010004751	PTER	0.083955857	0.125288449	0.67010054	0.502793701	0.612127893	positive
PRJNA729271	stage_E14_vs_E10	ordinary_muscle_development	ENSGALG00010004755	PAX9	-5.156389069	0.939884826	-5.486192486	4.10689E-08	2.76111E-07	negative
PRJNA729271	stage_E14_vs_E10	ordinary_muscle_development	ENSGALG00010004757	TRMT10A	0.550474702	0.07224893	7.619139833	2.55372E-14	3.40535E-13	positive
PRJNA729271	stage_E14_vs_E10	ordinary_muscle_development	ENSGALG00010004758	FLT3	-0.120412164	1.044910326	-0.11523684	0.908257399	0.936957583	negative
PRJNA729271	stage_E14_vs_E10	ordinary_muscle_development	ENSGALG00010004760	THBS2	-0.563456499	0.240307632	-2.34472994	0.019040866	0.041603459	negative
PRJNA729271	stage_E14_vs_E10	ordinary_muscle_development	ENSGALG00010004762	IFT57	-0.36721367	0.106408042	-3.450995465	0.000558523	0.001869698	negative
PRJNA729271	stage_E14_vs_E10	ordinary_muscle_development	ENSGALG00010004765	ENSGALG00010004765	1.372663542	0.201210777	6.822018006	8.97704E-12	9.23508E-11	positive
PRJNA729271	stage_E14_vs_E10	ordinary_muscle_development	ENSGALG00010004769	HTR2A	0.190647244	0.36231146	0.526197114	0.598751259	0.698222003	positive
PRJNA729271	stage_E14_vs_E10	ordinary_muscle_development	ENSGALG00010004777	CDK14	-0.854613581	0.123131697	-6.940646479	3.9031E-12	4.18669E-11	negative
PRJNA729271	stage_E14_vs_E10	ordinary_muscle_development	ENSGALG00010004778	ENSGALG00010004778	-0.755751646	0.452573987	-1.669896344	0.094939874	0.163051359	negative
PRJNA729271	stage_E14_vs_E10	ordinary_muscle_development	ENSGALG00010004780	ENSGALG00010004780	1.799068002	0.252957039	7.112148419	1.1425E-12	1.29279E-11	positive
PRJNA729271	stage_E14_vs_E10	ordinary_muscle_development	ENSGALG00010004781	ENSGALG00010004781	-0.319260112	0.0796273	-4.009430322	6.08654E-05	0.000249151	negative
PRJNA729271	stage_E14_vs_E10	ordinary_muscle_development	ENSGALG00010004787	RIOK3	0.147863479	0.114063932	1.296321079	0.194864889	0.29297444	positive
PRJNA729271	stage_E14_vs_E10	ordinary_muscle_development	ENSGALG00010004791	FZD1	-0.412595264	0.11794594	-3.498172663	0.000468458	0.001599658	negative
PRJNA729271	stage_E14_vs_E10	ordinary_muscle_development	ENSGALG00010004798	PTPRK	-1.689313326	0.107674945	-15.68901031	1.79838E-55	3.42993E-53	negative
PRJNA729271	stage_E14_vs_E10	ordinary_muscle_development	ENSGALG00010004800	ZC3H12C	-0.560518668	0.278058879	-2.01582726	0.043818042	0.084696962	negative
PRJNA729271	stage_E14_vs_E10	ordinary_muscle_development	ENSGALG00010004803	RMC1	0.16474554	0.140514155	1.172448002	0.241017237	0.346945267	positive
PRJNA729271	stage_E14_vs_E10	ordinary_muscle_development	ENSGALG00010004808	THEMIS	-2.021006204	1.060281702	-1.906103066	0.056636835	0.10530412	negative
PRJNA729271	stage_E14_vs_E10	ordinary_muscle_development	ENSGALG00010004824	ATM	-0.200284812	0.111046169	-1.803617483	0.071291293	0.12806634	negative
PRJNA729271	stage_E14_vs_E10	ordinary_muscle_development	ENSGALG00010004832	C6orf58	0.040193324	2.003726893	0.020059282	0.983996082	0.989377445	positive
PRJNA729271	stage_E14_vs_E10	ordinary_muscle_development	ENSGALG00010004835	HHLA2	-0.670701726	0.409700047	-1.637055527	0.101618868	0.172296817	negative
PRJNA729271	stage_E14_vs_E10	ordinary_muscle_development	ENSGALG00010004837	CENPC	-0.230058119	0.129836787	-1.771902438	0.076410754	0.135965366	negative
PRJNA729271	stage_E14_vs_E10	ordinary_muscle_development	ENSGALG00010004841	AKAP9	0.414705828	0.194468405	2.132510043	0.03296494	0.066457135	positive
PRJNA729271	stage_E14_vs_E10	ordinary_muscle_development	ENSGALG00010004846	TTLL2	0.819309743	0.344877606	2.375653646	0.017517897	0.038790395	positive
PRJNA729271	stage_E14_vs_E10	ordinary_muscle_development	ENSGALG00010004851	MTCL3	-2.740448651	0.441289544	-6.210091958	5.29536E-10	4.48343E-09	negative
PRJNA729271	stage_E14_vs_E10	ordinary_muscle_development	ENSGALG00010004852	ENSGALG00010004852	2.152983009	0.223038743	9.652985285	4.77591E-22	1.18947E-20	positive
PRJNA729271	stage_E14_vs_E10	ordinary_muscle_development	ENSGALG00010004855	ZEB1	0.724079234	0.091511002	7.912482848	2.52306E-15	3.65717E-14	positive
PRJNA729271	stage_E14_vs_E10	ordinary_muscle_development	ENSGALG00010004859	NPC1	0.661332422	0.19137396	3.455707469	0.000548851	0.001840294	positive
PRJNA729271	stage_E14_vs_E10	ordinary_muscle_development	ENSGALG00010004866	SLC25A21	-0.579429181	0.30089041	-1.925715015	0.054139968	0.101376933	negative
PRJNA729271	stage_E14_vs_E10	ordinary_muscle_development	ENSGALG00010004868	NPAT	-0.284607831	0.112735522	-2.524562151	0.011584252	0.027113654	negative
PRJNA729271	stage_E14_vs_E10	ordinary_muscle_development	ENSGALG00010004871	C11orf87	-2.192454805	0.32381494	-6.77070305	1.28158E-11	1.30361E-10	negative
PRJNA729271	stage_E14_vs_E10	ordinary_muscle_development	ENSGALG00010004872	KIAA0408	0.093433799	0.251703384	0.371205972	0.710484125	0.790123323	positive
PRJNA729271	stage_E14_vs_E10	ordinary_muscle_development	ENSGALG00010004876	ENSGALG00010004876	-0.945436499	0.663124969	-1.425729	0.153946582	0.242523172	negative
PRJNA729271	stage_E14_vs_E10	ordinary_muscle_development	ENSGALG00010004879	ENSGALG00010004879	0.151972893	0.453886232	0.33482596	0.737756377	0.811548811	positive
PRJNA729271	stage_E14_vs_E10	ordinary_muscle_development	ENSGALG00010004880	STAP1	-0.032129916	0.691487032	-0.046464958	0.962939663	0.975047881	negative
PRJNA729271	stage_E14_vs_E10	ordinary_muscle_development	ENSGALG00010004881	MIPOL1	-0.103035685	0.147958484	-0.69638241	0.486189376	0.597483256	negative
PRJNA729271	stage_E14_vs_E10	ordinary_muscle_development	ENSGALG00010004883	KRIT1	-0.242269532	0.073417804	-3.29987439	0.000967281	0.003056917	negative
PRJNA729271	stage_E14_vs_E10	ordinary_muscle_development	ENSGALG00010004887	MYH15	-1.925627071	0.718564054	-2.679826608	0.00736603	0.018220241	negative
PRJNA729271	stage_E14_vs_E10	ordinary_muscle_development	ENSGALG00010004890	ACAT1	0.074584026	0.098099869	0.760286703	0.447083228	0.560304629	positive
PRJNA729271	stage_E14_vs_E10	ordinary_muscle_development	ENSGALG00010004891	ANKIB1	-0.082179383	0.076495419	-1.07430463	0.282686171	0.39407333	negative
PRJNA729271	stage_E14_vs_E10	ordinary_muscle_development	ENSGALG00010004892	HEY2	-1.952578216	0.272132125	-7.175111052	7.22485E-13	8.38465E-12	negative
PRJNA729271	stage_E14_vs_E10	ordinary_muscle_development	ENSGALG00010004896	UBA6	-0.335436971	0.081848459	-4.098268623	4.16252E-05	0.00017565	negative
PRJNA729271	stage_E14_vs_E10	ordinary_muscle_development	ENSGALG00010004899	GATAD1	0.608521302	0.102165585	5.95622588	2.5813E-09	2.02248E-08	positive
PRJNA729271	stage_E14_vs_E10	ordinary_muscle_development	ENSGALG00010004901	CUL5	-0.030267121	0.092616284	-0.326801292	0.743818182	0.816111161	negative
PRJNA729271	stage_E14_vs_E10	ordinary_muscle_development	ENSGALG00010004902	ANKRD29	-0.993401704	0.33284379	-2.984588368	0.002839602	0.007916912	negative
PRJNA729271	stage_E14_vs_E10	ordinary_muscle_development	ENSGALG00010004903	ENSGALG00010004903	0.896783413	0.516620297	1.735865622	0.082587598	0.145229598	positive
PRJNA729271	stage_E14_vs_E10	ordinary_muscle_development	ENSGALG00010004907	ENSGALG00010004907	1.033082821	0.113402077	9.109910901	8.24497E-20	1.73455E-18	positive
PRJNA729271	stage_E14_vs_E10	ordinary_muscle_development	ENSGALG00010004917	ENSGALG00010004917	0.334632439	0.087226921	3.836343568	0.00012488	0.000484251	positive
PRJNA729271	stage_E14_vs_E10	ordinary_muscle_development	ENSGALG00010004921	RRAGD	0.628072532	0.133570302	4.70218695	2.5739E-06	1.33245E-05	positive
PRJNA729271	stage_E14_vs_E10	ordinary_muscle_development	ENSGALG00010004924	ENSGALG00010004924	1.222387523	0.339320512	3.602456909	0.000315224	0.00112237	positive
PRJNA729271	stage_E14_vs_E10	ordinary_muscle_development	ENSGALG00010004927	KIF25	-0.370130239	0.215319358	-1.71898264	0.085617533	0.149629388	negative
PRJNA729271	stage_E14_vs_E10	ordinary_muscle_development	ENSGALG00010004928	HDDC2	-0.103532811	0.120639818	-0.85819767	0.390783323	0.506421101	negative
PRJNA729271	stage_E14_vs_E10	ordinary_muscle_development	ENSGALG00010004929	ENSGALG00010004929	2.985886438	0.157193535	18.99496972	1.87696E-80	9.38155E-78	positive
PRJNA729271	stage_E14_vs_E10	ordinary_muscle_development	ENSGALG00010004933	SPTLC2	-0.09746793	0.089582645	-1.088022466	0.27658519	0.387128459	negative
PRJNA729271	stage_E14_vs_E10	ordinary_muscle_development	ENSGALG00010004934	CIP2A	-0.428305704	0.15907147	-2.692536276	0.007091082	0.017624354	negative
PRJNA729271	stage_E14_vs_E10	ordinary_muscle_development	ENSGALG00010004935	SVIL	0.449998168	0.06902093	6.519734885	7.04318E-11	6.62068E-10	positive
PRJNA729271	stage_E14_vs_E10	ordinary_muscle_development	ENSGALG00010004939	DDX10	-0.347896138	0.108180345	-3.215890454	0.001300405	0.003985067	negative
PRJNA729271	stage_E14_vs_E10	ordinary_muscle_development	ENSGALG00010004942	GABRR1	0.33974649	0.427859356	0.794061146	0.427159836	0.541657057	positive
PRJNA729271	stage_E14_vs_E10	ordinary_muscle_development	ENSGALG00010004944	ALKBH1	-0.124260397	0.095824819	-1.296745448	0.194718786	0.292815521	negative
PRJNA729271	stage_E14_vs_E10	ordinary_muscle_development	ENSGALG00010004946	SH2D1B	0.831046489	0.461403078	1.801129054	0.071682541	0.128625704	positive
PRJNA729271	stage_E14_vs_E10	ordinary_muscle_development	ENSGALG00010004949	RAB39A	-0.912767885	0.326490419	-2.795695771	0.005178813	0.013423978	negative
PRJNA729271	stage_E14_vs_E10	ordinary_muscle_development	ENSGALG00010004950	SLIRP	0.27811335	0.109017712	2.551084082	0.010738841	0.025343456	positive
PRJNA729271	stage_E14_vs_E10	ordinary_muscle_development	ENSGALG00010004951	AFDN	-1.000978042	0.062117774	-16.11419698	2.02781E-58	4.38703E-56	negative
PRJNA729271	stage_E14_vs_E10	ordinary_muscle_development	ENSGALG00010004952	PEX1	0.375782153	0.121247017	3.099310503	0.001939716	0.005669729	positive
PRJNA729271	stage_E14_vs_E10	ordinary_muscle_development	ENSGALG00010004957	ENSGALG00010004957	-0.080181862	0.138374169	-0.579456866	0.562280943	0.665120254	negative
PRJNA729271	stage_E14_vs_E10	ordinary_muscle_development	ENSGALG00010004958	TPD52L1	0.424956042	0.397843278	1.068149357	0.285453145	0.39720105	positive
PRJNA729271	stage_E14_vs_E10	ordinary_muscle_development	ENSGALG00010004962	ENSGALG00010004962	0.01886597	0.093489582	0.201797564	0.840074986	0.888382222	positive
PRJNA729271	stage_E14_vs_E10	ordinary_muscle_development	ENSGALG00010004963	ENSGALG00010004963	-0.094542828	0.24235488	-0.390100782	0.696462025	0.778875521	negative
PRJNA729271	stage_E14_vs_E10	ordinary_muscle_development	ENSGALG00010004965	JCAD	-0.110383751	0.10071229	-1.09603059	0.273065388	0.383200968	negative
PRJNA729271	stage_E14_vs_E10	ordinary_muscle_development	ENSGALG00010004966	LAMA3	-0.708564414	0.212637383	-3.332266442	0.000861417	0.002758782	negative
PRJNA729271	stage_E14_vs_E10	ordinary_muscle_development	ENSGALG00010004967	SLC35F2	-0.943786092	0.266728067	-3.538383122	0.000402585	0.001394379	negative
PRJNA729271	stage_E14_vs_E10	ordinary_muscle_development	ENSGALG00010004968	TRAT1	0.950336337	1.240826633	0.765889699	0.443741936	0.557369095	positive
PRJNA729271	stage_E14_vs_E10	ordinary_muscle_development	ENSGALG00010004969	YTHDC1	-0.042386486	0.07711255	-0.549670404	0.58254546	0.683835151	negative
PRJNA729271	stage_E14_vs_E10	ordinary_muscle_development	ENSGALG00010004970	RBM48	0.159411946	0.165651886	0.962331004	0.33588336	0.451133182	positive
PRJNA729271	stage_E14_vs_E10	ordinary_muscle_development	ENSGALG00010004973	SLN	2.133279489	0.208862101	10.213818	1.71962E-24	4.96358E-23	positive
PRJNA729271	stage_E14_vs_E10	ordinary_muscle_development	ENSGALG00010004974	MTPAP	0.03692466	0.060666838	0.608646532	0.542758754	0.64858105	positive
PRJNA729271	stage_E14_vs_E10	ordinary_muscle_development	ENSGALG00010004975	SNW1	0.117423515	0.06848422	1.714606868	0.086417329	0.150862347	positive
PRJNA729271	stage_E14_vs_E10	ordinary_muscle_development	ENSGALG00010004976	ENSGALG00010004976	0.865471673	0.230601013	3.753113061	0.000174652	0.000657042	positive
PRJNA729271	stage_E14_vs_E10	ordinary_muscle_development	ENSGALG00010004977	RNF217	1.109467792	0.13017165	8.523113863	1.5532E-17	2.73556E-16	positive
PRJNA729271	stage_E14_vs_E10	ordinary_muscle_development	ENSGALG00010004980	FNDC1	0.73228511	0.37741167	1.940282107	0.052345415	0.098491026	positive
PRJNA729271	stage_E14_vs_E10	ordinary_muscle_development	ENSGALG00010004981	ENSGALG00010004981	-0.044545483	0.199409033	-0.22338749	0.82323394	0.876185913	negative
PRJNA729271	stage_E14_vs_E10	ordinary_muscle_development	ENSGALG00010004983	CLXN	1.454302158	0.247815673	5.868483382	4.39799E-09	3.35521E-08	positive
PRJNA729271	stage_E14_vs_E10	ordinary_muscle_development	ENSGALG00010004987	MAP3K8	2.398305928	0.258822641	9.266213802	1.92871E-20	4.2552E-19	positive
PRJNA729271	stage_E14_vs_E10	ordinary_muscle_development	ENSGALG00010004988	HJURP	-0.339550306	0.214912243	-1.579948643	0.114118628	0.190110277	negative
PRJNA729271	stage_E14_vs_E10	ordinary_muscle_development	ENSGALG00010004990	ENSGALG00010004990	-16.87739022	2.51362712	-6.714357147	1.88897E-11	1.88054E-10	negative
PRJNA729271	stage_E14_vs_E10	ordinary_muscle_development	ENSGALG00010004991	FAM133B	-0.154234079	0.062405201	-2.471493985	0.013454981	0.030903178	negative
PRJNA729271	stage_E14_vs_E10	ordinary_muscle_development	ENSGALG00010004993	SULT1B	0.935219671	0.261832006	3.571830984	0.000354494	0.001248395	positive
PRJNA729271	stage_E14_vs_E10	ordinary_muscle_development	ENSGALG00010004995	PIT54	3.429826146	2.285263189	1.500845138	0.133395621	0.215703874	positive
PRJNA729271	stage_E14_vs_E10	ordinary_muscle_development	ENSGALG00010004996	GPR158	1.151925807	0.206587759	5.575963511	2.46164E-08	1.70724E-07	positive
PRJNA729271	stage_E14_vs_E10	ordinary_muscle_development	ENSGALG00010004999	ADCK1	-0.306004333	0.184228761	-1.661001954	0.096713054	0.165386192	negative
PRJNA729271	stage_E14_vs_E10	ordinary_muscle_development	ENSGALG00010005003	EXPH5	0.334454445	0.414657971	0.806579081	0.41990903	0.534379402	positive
PRJNA729271	stage_E14_vs_E10	ordinary_muscle_development	ENSGALG00010005004	CDK6	-0.149949857	0.09547474	-1.570570985	0.116282334	0.192718441	negative
PRJNA729271	stage_E14_vs_E10	ordinary_muscle_development	ENSGALG00010005010	NKAIN2	-2.145714869	0.32752499	-6.551301224	5.70379E-11	5.41785E-10	negative
PRJNA729271	stage_E14_vs_E10	ordinary_muscle_development	ENSGALG00010005012	ENSGALG00010005012	1.619184617	1.123692263	1.440950224	0.149598746	0.236832003	positive
PRJNA729271	stage_E14_vs_E10	ordinary_muscle_development	ENSGALG00010005014	SULT1B1	0.371906923	0.152108768	2.445006481	0.014484954	0.03296066	positive
PRJNA729271	stage_E14_vs_E10	ordinary_muscle_development	ENSGALG00010005015	NRXN3	0.222071506	0.383900862	0.578460556	0.56295322	0.66574259	positive
PRJNA729271	stage_E14_vs_E10	ordinary_muscle_development	ENSGALG00010005016	STX11	-0.165631121	0.169046745	-0.979794796	0.327187421	0.4420751	negative
PRJNA729271	stage_E14_vs_E10	ordinary_muscle_development	ENSGALG00010005017	ENSGALG00010005017	-0.213070012	0.171934041	-1.239254375	0.21525131	0.317047834	negative
PRJNA729271	stage_E14_vs_E10	ordinary_muscle_development	ENSGALG00010005023	POGLUT3	-0.272362146	0.180474515	-1.509144636	0.131261825	0.212728103	negative
PRJNA729271	stage_E14_vs_E10	ordinary_muscle_development	ENSGALG00010005025	THOC1	-0.205269483	0.054961237	-3.73480462	0.000187861	0.000701816	negative
PRJNA729271	stage_E14_vs_E10	ordinary_muscle_development	ENSGALG00010005029	FAT4	-0.896751632	0.197334408	-4.544324745	5.51116E-06	2.70428E-05	negative
PRJNA729271	stage_E14_vs_E10	ordinary_muscle_development	ENSGALG00010005033	USP14	-0.163459665	0.134481023	-1.215484985	0.224181172	0.327241298	negative
PRJNA729271	stage_E14_vs_E10	ordinary_muscle_development	ENSGALG00010005035	NECTIN3	0.108415176	0.097046438	1.117147398	0.263931307	0.3727284	positive
PRJNA729271	stage_E14_vs_E10	ordinary_muscle_development	ENSGALG00010005037	THNSL1	0.388591157	0.141322052	2.749685212	0.005965254	0.015198868	positive

PRJNA729271	stage_E14_vs_E10	ordinary_muscle_development	ENSGALG00010005042	SLC46A3	0.190034271	0.212640802	0.893686765	0.371489508	0.487748227	positive
PRJNA729271	stage_E14_vs_E10	ordinary_muscle_development	ENSGALG00010005045	UTRN	-0.344297658	0.099333774	-3.46606843	0.000528129	0.001781114	negative
PRJNA729271	stage_E14_vs_E10	ordinary_muscle_development	ENSGALG00010005048	ROCK1	-0.190577695	0.114276333	-1.667691725	0.095376939	0.163627498	negative
PRJNA729271	stage_E14_vs_E10	ordinary_muscle_development	ENSGALG00010005057	FLVCR2	0.915700274	0.282182021	3.245069514	0.00117422	0.003636819	positive
PRJNA729271	stage_E14_vs_E10	ordinary_muscle_development	ENSGALG00010005060	POMP	0.1137874	0.13814859	0.823659505	0.410133055	0.524980451	positive
PRJNA729271	stage_E14_vs_E10	ordinary_muscle_development	ENSGALG00010005065	ENKUR	-1.459911696	0.417453181	-3.497186658	0.000470193	0.001604767	negative
PRJNA729271	stage_E14_vs_E10	ordinary_muscle_development	ENSGALG00010005068	PLCXD2	0.649671752	0.263724904	2.463444827	0.013760907	0.031501003	positive
PRJNA729271	stage_E14_vs_E10	ordinary_muscle_development	ENSGALG00010005070	ALB	4.313123388	1.40403116	3.071957028	0.002126603	0.006139238	positive
PRJNA729271	stage_E14_vs_E10	ordinary_muscle_development	ENSGALG00010005075	TRDN	2.016322738	0.168265102	11.98301201	4.36183E-33	2.21065E-31	positive
PRJNA729271	stage_E14_vs_E10	ordinary_muscle_development	ENSGALG00010005077	ERG28	0.386230148	0.09867015	3.914356553	9.06456E-05	0.000359876	positive
PRJNA729271	stage_E14_vs_E10	ordinary_muscle_development	ENSGALG00010005081	GREB1L	0.254523423	0.179161199	1.420639199	0.155421677	0.244262952	positive
PRJNA729271	stage_E14_vs_E10	ordinary_muscle_development	ENSGALG00010005082	ENSGALG00010005082	-0.349722459	0.084278996	-4.149580254	3.33085E-05	0.000142969	negative
PRJNA729271	stage_E14_vs_E10	ordinary_muscle_development	ENSGALG00010005087	ENPP1	-0.737441182	0.217187984	-3.395405065	0.000685271	0.002245255	negative
PRJNA729271	stage_E14_vs_E10	ordinary_muscle_development	ENSGALG00010005092	ESCO1	0.312629912	0.140949278	2.218031319	0.026552697	0.055474394	positive
PRJNA729271	stage_E14_vs_E10	ordinary_muscle_development	ENSGALG00010005104	ENPP3	-0.576633709	0.199820377	-2.885760289	0.003904694	0.010459905	negative
PRJNA729271	stage_E14_vs_E10	ordinary_muscle_development	ENSGALG00010005107	TTLL5	-0.059389045	0.072085801	-0.82386606	0.410015667	0.524883063	negative
PRJNA729271	stage_E14_vs_E10	ordinary_muscle_development	ENSGALG00010005109	ABHD3	-1.275879006	0.383643687	-3.325687483	0.000882008	0.002812789	negative
PRJNA729271	stage_E14_vs_E10	ordinary_muscle_development	ENSGALG00010005111	RASSF6	-0.491414206	0.399074354	-1.231385083	0.218178873	0.320350767	negative
PRJNA729271	stage_E14_vs_E10	ordinary_muscle_development	ENSGALG00010005114	PHLDB2	-0.013481076	0.099420303	-0.135596813	0.892140025	0.925998544	negative
PRJNA729271	stage_E14_vs_E10	ordinary_muscle_development	ENSGALG00010005118	PRTFDC1	-0.534655774	0.136077441	-3.929055164	8.52803E-05	0.00033967	negative
PRJNA729271	stage_E14_vs_E10	ordinary_muscle_development	ENSGALG00010005119	MIB1	-0.536147962	0.055470896	-9.665392206	4.22997E-22	1.05895E-20	negative
PRJNA729271	stage_E14_vs_E10	ordinary_muscle_development	ENSGALG00010005122	IL8L1	-1.215279712	1.13809532	-1.067818917	0.285602203	0.39728269	negative
PRJNA729271	stage_E14_vs_E10	ordinary_muscle_development	ENSGALG00010005123	MTIF3	0.358907195	0.105420674	3.404523815	0.000662795	0.002178013	positive
PRJNA729271	stage_E14_vs_E10	ordinary_muscle_development	ENSGALG00010005126	MED23	-0.247540422	0.057100968	-4.335135324	1.4567E-05	6.65457E-05	negative
PRJNA729271	stage_E14_vs_E10	ordinary_muscle_development	ENSGALG00010005127	GATA6	-1.905932732	0.318309868	-5.987664607	2.12875E-09	1.68246E-08	negative
PRJNA729271	stage_E14_vs_E10	ordinary_muscle_development	ENSGALG00010005131	IL8L2	2.332191908	0.657829392	3.545283834	0.000392191	0.001361955	positive
PRJNA729271	stage_E14_vs_E10	ordinary_muscle_development	ENSGALG00010005132	LNX2	-0.024895664	0.093253302	-0.266968172	0.789493667	0.852037131	negative
PRJNA729271	stage_E14_vs_E10	ordinary_muscle_development	ENSGALG00010005138	ENSGALG00010005138	5.548289585	0.503138487	11.02736071	2.8202E-28	1.0901E-26	positive
PRJNA729271	stage_E14_vs_E10	ordinary_muscle_development	ENSGALG00010005140	DCT	-2.354341387	0.938199045	-2.509426329	0.012092744	0.028162648	negative
PRJNA729271	stage_E14_vs_E10	ordinary_muscle_development	ENSGALG00010005141	CCDC170	-0.629203505	0.307230872	-2.047982678	0.040561697	0.079216086	negative
PRJNA729271	stage_E14_vs_E10	ordinary_muscle_development	ENSGALG00010005143	GTF3A	-0.429285281	0.134211983	-3.198561499	0.001381151	0.004197899	negative
PRJNA729271	stage_E14_vs_E10	ordinary_muscle_development	ENSGALG00010005145	AKAP7	1.645128151	0.17450848	9.42721037	4.2114E-21	9.79844E-20	positive
PRJNA729271	stage_E14_vs_E10	ordinary_muscle_development	ENSGALG00010005146	TGFB3	0.713932675	0.204451432	3.491942657	0.000479521	0.001633912	positive
PRJNA729271	stage_E14_vs_E10	ordinary_muscle_development	ENSGALG00010005148	TTC39C	-0.208380463	0.08113928	-2.568182315	0.010223337	0.024253236	negative
PRJNA729271	stage_E14_vs_E10	ordinary_muscle_development	ENSGALG00010005150	ALCAM	-2.523674977	0.187794895	-13.43846421	3.59827E-41	2.96346E-39	negative
PRJNA729271	stage_E14_vs_E10	ordinary_muscle_development	ENSGALG00010005154	CWF19L2	-0.184788803	0.147183036	-1.255503402	0.209296074	0.309913841	negative
PRJNA729271	stage_E14_vs_E10	ordinary_muscle_development	ENSGALG00010005157	IMPA1	-0.357703091	0.108217941	-3.305395456	0.000948425	0.0030049	negative
PRJNA729271	stage_E14_vs_E10	ordinary_muscle_development	ENSGALG00010005158	IFT43	-0.383167062	0.193475715	-1.980440094	0.047654099	0.091091409	negative
PRJNA729271	stage_E14_vs_E10	ordinary_muscle_development	ENSGALG00010005159	ARHGAP21	0.189061354	0.09805628	1.928090213	0.053843906	0.100913812	positive
PRJNA729271	stage_E14_vs_E10	ordinary_muscle_development	ENSGALG00010005163	GPC6	-3.247926866	0.380909477	-8.526768325	1.50493E-17	2.65376E-16	negative
PRJNA729271	stage_E14_vs_E10	ordinary_muscle_development	ENSGALG00010005165	METTL21C	-0.180700405	0.614128053	-0.294238968	0.768575306	0.835495654	negative
PRJNA729271	stage_E14_vs_E10	ordinary_muscle_development	ENSGALG00010005166	CBLB	-0.437204445	0.123499493	-3.540131494	0.000399928	0.001385836	negative
PRJNA729271	stage_E14_vs_E10	ordinary_muscle_development	ENSGALG00010005167	GUCY1A2	-0.359644256	0.178104837	-2.019284049	0.043457705	0.084135092	negative
PRJNA729271	stage_E14_vs_E10	ordinary_muscle_development	ENSGALG00010005170	GPATCH2L	0.122173329	0.058229961	2.098117988	0.035894724	0.071468959	positive
PRJNA729271	stage_E14_vs_E10	ordinary_muscle_development	ENSGALG00010005172	OSBPL1A	0.129348312	0.081026956	1.59636149	0.110408109	0.184807373	positive
PRJNA729271	stage_E14_vs_E10	ordinary_muscle_development	ENSGALG00010005174	TPP2	0.008081953	0.185129314	0.043655719	0.965178836	0.976837539	positive
PRJNA729271	stage_E14_vs_E10	ordinary_muscle_development	ENSGALG00010005176	BAMBI	-1.307792824	0.302052055	-4.329693515	1.49317E-05	6.81685E-05	negative
PRJNA729271	stage_E14_vs_E10	ordinary_muscle_development	ENSGALG00010005177	AASDHPPT	0.352498375	0.12691136	2.777516331	0.005477609	0.014095143	positive
PRJNA729271	stage_E14_vs_E10	ordinary_muscle_development	ENSGALG00010005178	EPHA5	-0.077493101	0.197418101	-0.392532906	0.694664502	0.777421399	negative
PRJNA729271	stage_E14_vs_E10	ordinary_muscle_development	ENSGALG00010005180	WAC	0.030711959	0.115569783	0.265743846	0.790436493	0.852737196	positive
PRJNA729271	stage_E14_vs_E10	ordinary_muscle_development	ENSGALG00010005181	IMPACT	0.031186316	0.133062205	0.234373959	0.814694674	0.870128674	positive
PRJNA729271	stage_E14_vs_E10	ordinary_muscle_development	ENSGALG00010005182	NEPRO	-0.418883318	0.082614929	-5.070310233	3.97168E-07	2.32886E-06	negative
PRJNA729271	stage_E14_vs_E10	ordinary_muscle_development	ENSGALG00010005184	FGF14	-2.963966049	0.306939182	-9.656525537	4.61242E-22	1.15271E-20	negative
PRJNA729271	stage_E14_vs_E10	ordinary_muscle_development	ENSGALG00010005186	ESR1	-0.606307271	0.194684105	-3.114313172	0.001843737	0.0054308	negative
PRJNA729271	stage_E14_vs_E10	ordinary_muscle_development	ENSGALG00010005187	KBTBD3	0.506295553	0.255812199	1.979168915	0.047796991	0.091352504	positive
PRJNA729271	stage_E14_vs_E10	ordinary_muscle_development	ENSGALG00010005190	ENSGALG00010005190	1.372910119	0.281877673	4.870588375	1.11266E-06	6.11607E-06	positive
PRJNA729271	stage_E14_vs_E10	ordinary_muscle_development	ENSGALG00010005191	MSANTD4	-0.195651034	0.103228123	-1.895326856	0.058049112	0.107488743	negative
PRJNA729271	stage_E14_vs_E10	ordinary_muscle_development	ENSGALG00010005193	EPB41L2	0.460479654	0.098234421	4.687559097	2.76483E-06	1.42266E-05	positive
PRJNA729271	stage_E14_vs_E10	ordinary_muscle_development	ENSGALG00010005194	ENSGALG00010005194	4.179567512	1.099086104	3.802766222	0.000143089	0.000547974	positive
PRJNA729271	stage_E14_vs_E10	ordinary_muscle_development	ENSGALG00010005198	TRPC3	-1.222858385	0.500805892	-2.441781149	0.014615003	0.033194057	negative
PRJNA729271	stage_E14_vs_E10	ordinary_muscle_development	ENSGALG00010005203	ENSGALG00010005203	1.557862592	0.104849164	14.85813076	6.16206E-50	8.42633E-48	positive
PRJNA729271	stage_E14_vs_E10	ordinary_muscle_development	ENSGALG00010005204	ITGBL1	-1.890323937	0.318694946	-5.931452506	3.00266E-09	2.33246E-08	negative
PRJNA729271	stage_E14_vs_E10	ordinary_muscle_development	ENSGALG00010005208	ENSGALG00010005208	-0.284846815	0.108805288	-2.6179501	0.008845974	0.021416802	negative
PRJNA729271	stage_E14_vs_E10	ordinary_muscle_development	ENSGALG00010005211	ESRRB	1.184623124	0.347183818	3.412091985	0.000644664	0.00212228	positive
PRJNA729271	stage_E14_vs_E10	ordinary_muscle_development	ENSGALG00010005212	TAF13	0.344555699	0.099832688	3.451331481	0.000557828	0.001867802	positive
PRJNA729271	stage_E14_vs_E10	ordinary_muscle_development	ENSGALG00010005213	ENSGALG00010005213	-0.619908457	0.16128384	-3.843586905	0.000121249	0.00047055	negative
PRJNA729271	stage_E14_vs_E10	ordinary_muscle_development	ENSGALG00010005214	BBS7	-0.192266807	0.115234251	-1.668486636	0.095219163	0.163434193	negative
PRJNA729271	stage_E14_vs_E10	ordinary_muscle_development	ENSGALG00010005215	VASH1	0.415326576	0.14199062	2.925028256	0.003444249	0.009366698	positive
PRJNA729271	stage_E14_vs_E10	ordinary_muscle_development	ENSGALG00010005216	ENSGALG00010005216	-1.124286896	0.166600221	-6.748411793	1.49472E-11	1.50772E-10	negative
PRJNA729271	stage_E14_vs_E10	ordinary_muscle_development	ENSGALG00010005217	CCNA2	-0.105157621	0.142800988	-0.736392812	0.461491678	0.574092162	negative
PRJNA729271	stage_E14_vs_E10	ordinary_muscle_development	ENSGALG00010005218	GTPBP8	-0.100011272	0.117186737	-0.853435082	0.393418074	0.508751448	negative
PRJNA729271	stage_E14_vs_E10	ordinary_muscle_development	ENSGALG00010005219	NALCN	-1.335403024	0.184471369	-7.239080158	4.51738E-13	5.34963E-12	negative
PRJNA729271	stage_E14_vs_E10	ordinary_muscle_development	ENSGALG00010005220	ANGEL1	0.394938059	0.085269651	4.631636888	3.62786E-06	1.82653E-05	positive
PRJNA729271	stage_E14_vs_E10	ordinary_muscle_development	ENSGALG00010005221	ENSGALG00010005221	-0.377086501	0.313632553	-1.202319395	0.229239805	0.333082496	negative
PRJNA729271	stage_E14_vs_E10	ordinary_muscle_development	ENSGALG00010005222	ABCB1	0.416556733	1.174223315	0.354750861	0.722776244	0.799194573	positive
PRJNA729271	stage_E14_vs_E10	ordinary_muscle_development	ENSGALG00010005223	GRIA4	-2.193863533	0.423903121	-5.175388957	2.27437E-07	1.37707E-06	negative
PRJNA729271	stage_E14_vs_E10	ordinary_muscle_development	ENSGALG00010005224	ENSGALG00010005224	-0.516404151	0.452717819	-1.14067556	0.254004961	0.361528074	negative
PRJNA729271	stage_E14_vs_E10	ordinary_muscle_development	ENSGALG00010005226	MYCT1	0.214599012	0.168776468	1.271498418	0.20355139	0.303141621	positive
PRJNA729271	stage_E14_vs_E10	ordinary_muscle_development	ENSGALG00010005227	EXOSC9	-0.143472265	0.102738578	-1.396478976	0.162570307	0.253086	negative
PRJNA729271	stage_E14_vs_E10	ordinary_muscle_development	ENSGALG00010005228	ZNF521	-1.052632068	0.131595933	-7.998971123	1.25463E-15	1.86905E-14	negative
PRJNA729271	stage_E14_vs_E10	ordinary_muscle_development	ENSGALG00010005229	CIPC	-0.17129698	0.071578939	-2.393119852	0.016705783	0.037248007	negative
PRJNA729271	stage_E14_vs_E10	ordinary_muscle_development	ENSGALG00010005231	ENSGALG00010005231	0.058446704	0.433717044	0.134757683	0.892803464	0.926318424	positive
PRJNA729271	stage_E14_vs_E10	ordinary_muscle_development	ENSGALG00010005232	GATA3	-1.182938171	0.432884303	-2.732688992	0.006281962	0.015880195	negative
PRJNA729271	stage_E14_vs_E10	ordinary_muscle_development	ENSGALG00010005236	TMTC4	-1.50853128	0.090691709	-16.63361839	3.97827E-62	1.01167E-59	negative
PRJNA729271	stage_E14_vs_E10	ordinary_muscle_development	ENSGALG00010005237	RAP2A	-0.595494696	0.107911108	-5.518381821	3.42135E-08	2.33927E-07	negative
PRJNA729271	stage_E14_vs_E10	ordinary_muscle_development	ENSGALG00010005238	TMEM63C	-0.647664809	0.166017928	-3.90117392	9.57273E-05	0.000377878	negative
PRJNA729271	stage_E14_vs_E10	ordinary_muscle_development	ENSGALG00010005239	ENSGALG00010005239	-1.136332035	0.174254522	-6.521104988	6.97913E-11	6.56473E-10	negative
PRJNA729271	stage_E14_vs_E10	ordinary_muscle_development	ENSGALG00010005240	VIP	-0.294534607	0.376117672	-0.783091647	0.433573296	0.547586035	negative
PRJNA729271	stage_E14_vs_E10	ordinary_muscle_development	ENSGALG00010005241	C4orf54	2.316687707	0.18749509	12.35599135	4.52148E-35	2.51107E-33	positive
PRJNA729271	stage_E14_vs_E10	ordinary_muscle_development	ENSGALG00010005242	CCDC80	-0.317049734	0.233918319	-1.355386511	0.175294445	0.268849115	negative
PRJNA729271	stage_E14_vs_E10	ordinary_muscle_development	ENSGALG00010005243	NGB	-2.706312884	0.502728897	-5.38324512	7.31548E-08	4.76575E-07	negative
PRJNA729271	stage_E14_vs_E10	ordinary_muscle_development	ENSGALG00010005244	FBXO5	-0.100303929	0.087591908	-1.145127807	0.252156218	0.359390795	negative
PRJNA729271	stage_E14_vs_E10	ordinary_muscle_development	ENSGALG00010005245	KIAA1217	0.128424199	0.136273181	0.942402591	0.345986561	0.461505734	positive
PRJNA729271	stage_E14_vs_E10	ordinary_muscle_development	ENSGALG00010005246	ENSGALG00010005246	-0.843438212	0.196343738	-4.295722499	1.74125E-05	7.83591E-05	negative
PRJNA729271	stage_E14_vs_E10	ordinary_muscle_development	ENSGALG00010005247	THAP7	0.33202727	0.117131358	2.834657399	0.004587485	0.012072549	positive
PRJNA729271	stage_E14_vs_E10	ordinary_muscle_development	ENSGALG00010005248	TMEM200A	-0.568191036	0.239521261	-2.37219458	0.017682776	0.039102568	negative
PRJNA729271	stage_E14_vs_E10	ordinary_muscle_development	ENSGALG00010005249	PDGFD	0.762607263	0.228694311	3.334614046	0.000854179	0.002737413	positive
PRJNA729271	stage_E14_vs_E10	ordinary_muscle_development	ENSGALG00010005250	SLC35A5	-0.178606966	0.086975948	-2.053521342	0.040022042	0.07832044	negative
PRJNA729271	stage_E14_vs_E10	ordinary_muscle_development	ENSGALG00010005251	MTRF1L	-0.031490937	0.101024473	-0.311715927	0.755256423	0.825225528	negative
PRJNA729271	stage_E14_vs_E10	ordinary_muscle_development	ENSGALG00010005252	KLHL14	-1.434043959	0.207298796	-6.917763098	4.58831E-12	4.87592E-11	negative
PRJNA729271	stage_E14_vs_E10	ordinary_muscle_development	ENSGALG00010005253	POMT2	-0.255267065	0.093894747	-2.718651175	0.006554869	0.016443896	negative
PRJNA729271	stage_E14_vs_E10	ordinary_muscle_development	ENSGALG00010005254	ENSGALG00010005254	-1.3614347	0.749209079	-1.817162575	0.069192224	0.124806033	negative
PRJNA729271	stage_E14_vs_E10	ordinary_muscle_development	ENSGALG00010005255	BZW2	-0.048678077	0.092949915	-0.523702219	0.600485672	0.699625417	negative
PRJNA729271	stage_E14_vs_E10	ordinary_muscle_development	ENSGALG00010005256	OPRM1	-2.86419442	0.389527057	-7.353005061	1.938E-13	2.38263E-12	negative
PRJNA729271	stage_E14_vs_E10	ordinary_muscle_development	ENSGALG00010005258	ITIH5	2.031593792	0.278923902	7.283684822	3.24823E-13	3.91707E-12	positive
PRJNA729271	stage_E14_vs_E10	ordinary_muscle_development	ENSGALG00010005259	SLC27A5	0.441166175	0.684676849	0.644342182	0.519353568	0.627388113	positive
PRJNA729271	stage_E14_vs_E10	ordinary_muscle_development	ENSGALG00010005260	ANXA5	0.404255603	0.149798868	2.698655918	0.006962012	0.017348179	positive
PRJNA729271	stage_E14_vs_E10	ordinary_muscle_development	ENSGALG00010005261	ATG3	-0.026033389	0.108919243	-0.239015513	0.811093554	0.867276561	negative
PRJNA729271	stage_E14_vs_E10	ordinary_muscle_development	ENSGALG00010005262	ENSGALG00010005262	-0.045355628	0.124344159	-0.364758813	0.715291453	0.794068286	negative
PRJNA729271	stage_E14_vs_E10	ordinary_muscle_development	ENSGALG00010005263	ENSGALG00010005263	0.542421581	0.333040487	1.628695619	0.103377471	0.174930707	positive
PRJNA729271	stage_E14_vs_E10	ordinary_muscle_development	ENSGALG00010005264	ITIH2	1.704161276	1.047069534	1.62755311			positive
PRJNA729271	stage_E14_vs_E10	ordinary_muscle_development	ENSGALG00010005265	DYNC2H1	-0.539859689	0.094068056	-5.739033135	9.52186E-09	6.94612E-08	negative
PRJNA729271	stage_E14_vs_E10	ordinary_muscle_development	ENSGALG00010005266	SLC19A2	-0.34162703	0.267686474	-1.276220739	0.201877499	0.301261638	negative
PRJNA729271	stage_E14_vs_E10	ordinary_muscle_development	ENSGALG00010005267	CYP51A1	-0.255938932	0.179998972	-1.421891074	0.155057875	0.243902756	negative
PRJNA729271	stage_E14_vs_E10	ordinary_muscle_development	ENSGALG00010005268	MTTP	1.268557085	0.925734196	1.370325403	0.170585347	0.263186227	positive
PRJNA729271	stage_E14_vs_E10	ordinary_muscle_development	ENSGALG00010005269	KIN	-0.046639512	0.104904416	-0.444590553	0.656615676	0.746851092	negative
PRJNA729271	stage_E14_vs_E10	ordinary_muscle_development	ENSGALG00010005270	GSTZ1	0.018381808	0.21315648	0.086236215	0.931278642	0.953041791	positive
PRJNA729271	stage_E14_vs_E10	ordinary_muscle_development	ENSGALG00010005272	MBNL2	-1.022927926	0.096253401	-10.62744713	2.22109E-26	7.53975E-25	negative
PRJNA729271	stage_E14_vs_E10	ordinary_muscle_development	ENSGALG00010005273	ENSGALG00010005273	1.240812767	0.662756717	1.872199459	0.061179014	0.112465416	positive
PRJNA729271	stage_E14_vs_E10	ordinary_muscle_development	ENSGALG00010005275	L3MBTL3	0.860809877	0.170916739	5.036428164	4.74299E-07	2.7445E-06	positive
PRJNA729271	stage_E14_vs_E10	ordinary_muscle_development	ENSGALG00010005277	ENSGALG00010005277	0.962249182	0.201052637	4.786056023	1.70091E-06	9.0809E-06	positive
PRJNA729271	stage_E14_vs_E10	ordinary_muscle_development	ENSGALG00010005278	DAPP1	0.30066832	0.128121152	2.346750056	0.018937954	0.041447326	positive
PRJNA729271	stage_E14_vs_E10	ordinary_muscle_development	ENSGALG00010005279	CCDC181	-0.046049545	0.313231041	-0.147014627	0.883120475	0.919930383	negative
PRJNA729271	stage_E14_vs_E10	ordinary_muscle_development	ENSGALG00010005280	TMEM244	1.96502484	0.443486208	4.430858965	9.38585E-06	4.42288E-05	positive
PRJNA729271	stage_E14_vs_E10	ordinary_muscle_development	ENSGALG00010005281	TMED8	-0.524160683	0.066650799	-7.864282044	3.7122E-15	5.28051E-14	negative
PRJNA729271	stage_E14_vs_E10	ordinary_muscle_development	ENSGALG00010005282	FLT1	0.633512069	0.126468104	5.009263588	5.46387E-07	3.13039E-06	positive
PRJNA729271	stage_E14_vs_E10	ordinary_muscle_development	ENSGALG00010005283	ATP5C1	0.397321302	0.132092086	3.007911485	0.002630497	0.00739652	positive
PRJNA729271	stage_E14_vs_E10	ordinary_muscle_development	ENSGALG00010005284	ENSGALG00010005284	-0.194266953	0.217456328	-0.893360775	0.371664001	0.487800588	negative
PRJNA729271	stage_E14_vs_E10	ordinary_muscle_development	ENSGALG00010005285	MPP7	0.020200283	0.12797036	0.157851264	0.874574003	0.914154719	positive
PRJNA729271	stage_E14_vs_E10	ordinary_muscle_development	ENSGALG00010005286	ENSGALG00010005286	0.387712319	0.621238182	0.624096087	0.532564484	0.639827782	positive
PRJNA729271	stage_E14_vs_E10	ordinary_muscle_development	ENSGALG00010005287	ENSGALG00010005287	-0.923985596	0.394649898	-2.341279194	0.01921779	0.041971051	negative

PRJNA729271	stage_E14_vs_E10	ordinary_muscle_development	ENSGALG00010005288	ENSGALG00010005288	0.172948702	0.094090378	1.838112533	0.066045825	0.120093392	positive
PRJNA729271	stage_E14_vs_E10	ordinary_muscle_development	ENSGALG00010005291	IFNGR1	0.280885132	0.095741399	2.933789706	0.00334851	0.009147503	positive
PRJNA729271	stage_E14_vs_E10	ordinary_muscle_development	ENSGALG00010005292	TAF3	-0.173407421	0.098371691	-1.762777675	0.077937994	0.138275548	negative
PRJNA729271	stage_E14_vs_E10	ordinary_muscle_development	ENSGALG00010005293	BLZF1	-0.091533417	0.095724634	-0.956215893	0.338963172	0.454049642	negative
PRJNA729271	stage_E14_vs_E10	ordinary_muscle_development	ENSGALG00010005295	ENSGALG00010005295	-0.978654925	0.560396076	-1.746362916	0.08074791	0.142562844	negative
PRJNA729271	stage_E14_vs_E10	ordinary_muscle_development	ENSGALG00010005296	PAN3	0.26266497	0.100907924	2.603016285	0.009240756	0.022235191	positive
PRJNA729271	stage_E14_vs_E10	ordinary_muscle_development	ENSGALG00010005297	IL22RA2	0.066025843	0.550282885	0.119985275	0.904494813	0.934672582	positive
PRJNA729271	stage_E14_vs_E10	ordinary_muscle_development	ENSGALG00010005299	ODAD2	-3.256752804	0.57419482	-5.6718603	1.41255E-08	1.01111E-07	negative
PRJNA729271	stage_E14_vs_E10	ordinary_muscle_development	ENSGALG00010005300	QRFPR	-1.682480572	0.847156185	-1.986033511	0.047029603	0.090075858	negative
PRJNA729271	stage_E14_vs_E10	ordinary_muscle_development	ENSGALG00010005301	ENSGALG00010005301	0.308643427	0.370593457	0.832835606	0.404937451	0.519798826	positive
PRJNA729271	stage_E14_vs_E10	ordinary_muscle_development	ENSGALG00010005302	PSPC1	-0.396784585	0.079060145	-5.018768768	5.20037E-07	2.99243E-06	negative
PRJNA729271	stage_E14_vs_E10	ordinary_muscle_development	ENSGALG00010005303	ENSGALG00010005303	0.984401683	0.290184007	3.392336103	0.000692994	0.002269018	positive
PRJNA729271	stage_E14_vs_E10	ordinary_muscle_development	ENSGALG00010005304	ENSGALG00010005304	0.219828531	0.208899369	1.052317835	0.292653751	0.405236543	positive
PRJNA729271	stage_E14_vs_E10	ordinary_muscle_development	ENSGALG00010005305	SCAF8	-0.081847479	0.085921126	-0.952588533	0.340798589	0.45574384	negative
PRJNA729271	stage_E14_vs_E10	ordinary_muscle_development	ENSGALG00010005307	TIAM2	-1.282541817	0.155276078	-8.259751484	1.45976E-16	2.36416E-15	negative
PRJNA729271	stage_E14_vs_E10	ordinary_muscle_development	ENSGALG00010005308	ENSGALG00010005308	-1.349866779	0.782480002	-1.725113455	0.084507028	0.148065457	negative
PRJNA729271	stage_E14_vs_E10	ordinary_muscle_development	ENSGALG00010005309	ASXL3	-1.144857843	0.137485656	-8.327107564	8.28415E-17	1.37705E-15	negative
PRJNA729271	stage_E14_vs_E10	ordinary_muscle_development	ENSGALG00010005310	LAMTOR3	0.273135263	0.075825239	3.602168165	0.000315574	0.001123342	positive
PRJNA729271	stage_E14_vs_E10	ordinary_muscle_development	ENSGALG00010005311	YME1L1	-0.091529676	0.078137849	-1.171387197	0.241443176	0.347401115	negative
PRJNA729271	stage_E14_vs_E10	ordinary_muscle_development	ENSGALG00010005313	MPHOSPH8	-0.502658422	0.140825993	-3.569358257	0.000357857	0.001258402	negative
PRJNA729271	stage_E14_vs_E10	ordinary_muscle_development	ENSGALG00010005314	IL20RA	-1.377097658	0.633267622	-2.174590348	0.029660828	0.060836806	negative
PRJNA729271	stage_E14_vs_E10	ordinary_muscle_development	ENSGALG00010005315	ENSGALG00010005315	0.46492419	0.543844167	0.854884944	0.392614847	0.508120732	positive
PRJNA729271	stage_E14_vs_E10	ordinary_muscle_development	ENSGALG00010005316	PARP4	0.306644698	0.107466424	2.853400035	0.004325413	0.011459855	positive
PRJNA729271	stage_E14_vs_E10	ordinary_muscle_development	ENSGALG00010005317	CHCHD3	0.106832884	0.109533152	0.975347482	0.329387925	0.44411911	positive
PRJNA729271	stage_E14_vs_E10	ordinary_muscle_development	ENSGALG00010005318	SLC35D3	-1.594390794	0.360236734	-4.425952832	9.60175E-06	4.51728E-05	negative
PRJNA729271	stage_E14_vs_E10	ordinary_muscle_development	ENSGALG00010005319	NOL4	-1.262185582	0.685548843	-1.841131517	0.065602288	0.119400448	negative
PRJNA729271	stage_E14_vs_E10	ordinary_muscle_development	ENSGALG00010005321	HS6ST3	-3.6388532	0.287969481	-12.63624597	1.33266E-36	8.2551E-35	negative
PRJNA729271	stage_E14_vs_E10	ordinary_muscle_development	ENSGALG00010005322	TFB1M	0.167924229	0.121680639	1.380040652	0.167574128	0.259340913	positive
PRJNA729271	stage_E14_vs_E10	ordinary_muscle_development	ENSGALG00010005323	DNAJB14	0.329836711	0.062856069	5.247491856	1.54184E-07	9.55083E-07	positive
PRJNA729271	stage_E14_vs_E10	ordinary_muscle_development	ENSGALG00010005324	CENPJ	-0.265920489	0.176616783	-1.505635449	0.132160783	0.213993583	negative
PRJNA729271	stage_E14_vs_E10	ordinary_muscle_development	ENSGALG00010005326	MASTL	-0.375525629	0.176472146	-2.12795978	0.033340418	0.067064858	negative
PRJNA729271	stage_E14_vs_E10	ordinary_muscle_development	ENSGALG00010005329	VIPAS39	0.072050132	0.116336592	0.619324761	0.535702435	0.64269217	positive
PRJNA729271	stage_E14_vs_E10	ordinary_muscle_development	ENSGALG00010005331	ENSGALG00010005331	0.3217451	0.837722837	0.384071062	0.700925779	0.782796761	positive
PRJNA729271	stage_E14_vs_E10	ordinary_muscle_development	ENSGALG00010005332	ENSGALG00010005332	-0.950059779	0.850292636	-1.117332715	0.263852093	0.372689153	negative
PRJNA729271	stage_E14_vs_E10	ordinary_muscle_development	ENSGALG00010005333	PLXNA4	0.449284535	0.152290455	2.950181846	0.003175869	0.008746766	positive
PRJNA729271	stage_E14_vs_E10	ordinary_muscle_development	ENSGALG00010005334	NME7	0.013446928	0.130668738	0.102908531	0.918035567	0.943017897	positive
PRJNA729271	stage_E14_vs_E10	ordinary_muscle_development	ENSGALG00010005335	AHSA1	0.086993105	0.133586864	0.651210026	0.514910919	0.623632501	positive
PRJNA729271	stage_E14_vs_E10	ordinary_muscle_development	ENSGALG00010005336	PEX7	0.120267747	0.125412487	0.958977448	0.337570106	0.452754265	positive
PRJNA729271	stage_E14_vs_E10	ordinary_muscle_development	ENSGALG00010005337	DCTN1	-0.183049019	0.153711482	-1.190861061	0.233708133	0.338151267	negative
PRJNA729271	stage_E14_vs_E10	ordinary_muscle_development	ENSGALG00010005338	PCGF1	0.778182929	0.111676278	6.968202585	3.21016E-12	3.48549E-11	positive
PRJNA729271	stage_E14_vs_E10	ordinary_muscle_development	ENSGALG00010005341	UGGT2	0.068707682	0.118126278	0.581646037	0.560805126	0.664069137	positive
PRJNA729271	stage_E14_vs_E10	ordinary_muscle_development	ENSGALG00010005342	MAP3K5	-0.254137558	0.145485399	-1.74682518	0.080667667	0.142455877	negative
PRJNA729271	stage_E14_vs_E10	ordinary_muscle_development	ENSGALG00010005343	RNF17	-3.562681944	1.715746093	-2.076462222	0.037851222	0.074738245	negative
PRJNA729271	stage_E14_vs_E10	ordinary_muscle_development	ENSGALG00010005344	ENSGALG00010005344	-0.108157347	0.73969941	-0.146217971	0.883749319	0.920188664	negative
PRJNA729271	stage_E14_vs_E10	ordinary_muscle_development	ENSGALG00010005345	NOX3	-2.087231718	0.658585089	-3.169266588	0.001528242	0.00459773	negative
PRJNA729271	stage_E14_vs_E10	ordinary_muscle_development	ENSGALG00010005346	FBXO30	0.085130101	0.220517985	0.386046067	0.699462552	0.781584157	positive
PRJNA729271	stage_E14_vs_E10	ordinary_muscle_development	ENSGALG00010005347	DOK1	-0.182756575	0.147550647	-1.23860233	0.215492805	0.317242353	negative
PRJNA729271	stage_E14_vs_E10	ordinary_muscle_development	ENSGALG00010005349	ACBD5	0.554499337	0.103499647	5.35749981	8.43815E-08	5.44088E-07	positive
PRJNA729271	stage_E14_vs_E10	ordinary_muscle_development	ENSGALG00010005350	ARHGAP20	-2.343977745	0.284989573	-8.224784216	1.95543E-16	3.12157E-15	negative
PRJNA729271	stage_E14_vs_E10	ordinary_muscle_development	ENSGALG00010005351	DTNA	1.908451724	0.163009863	11.70758438	1.16553E-31	5.39756E-30	positive
PRJNA729271	stage_E14_vs_E10	ordinary_muscle_development	ENSGALG00010005352	ENSGALG00010005352	1.055069127	0.632468935	1.668175413	0.09528091	0.163501455	positive
PRJNA729271	stage_E14_vs_E10	ordinary_muscle_development	ENSGALG00010005353	ENSGALG00010005353	0.554543998	0.100499807	5.517861328	3.4315E-08	2.3451E-07	positive
PRJNA729271	stage_E14_vs_E10	ordinary_muscle_development	ENSGALG00010005354	M1AP	-0.430130978	0.339117004	-1.268385166	0.204660436	0.304511704	negative
PRJNA729271	stage_E14_vs_E10	ordinary_muscle_development	ENSGALG00010005355	FDX1	-0.565461541	0.103872071	-5.443826592	5.2148E-08	3.44995E-07	negative
PRJNA729271	stage_E14_vs_E10	ordinary_muscle_development	ENSGALG00010005356	DNAJC3	0.141282698	0.084042629	1.681083759	0.092746646	0.159738907	positive
PRJNA729271	stage_E14_vs_E10	ordinary_muscle_development	ENSGALG00010005357	TNIP3	-2.043790576	0.511815789	-3.993215177	6.51834E-05	0.000266075	negative
PRJNA729271	stage_E14_vs_E10	ordinary_muscle_development	ENSGALG00010005358	SHPRH	0.030552372	0.088773648	0.344160374	0.730725681	0.80601695	positive
PRJNA729271	stage_E14_vs_E10	ordinary_muscle_development	ENSGALG00010005359	MEIS2	-2.567367181	0.1314954	-19.52438776	6.81286E-85	3.9501E-82	negative
PRJNA729271	stage_E14_vs_E10	ordinary_muscle_development	ENSGALG00010005360	CAMK1D	-0.059328645	0.150583848	-0.393990761	0.69358786	0.77645629	negative
PRJNA729271	stage_E14_vs_E10	ordinary_muscle_development	ENSGALG00010005361	ENSGALG00010005361	0.661756848	0.443559465	1.491923633	0.135719166	0.21870476	positive
PRJNA729271	stage_E14_vs_E10	ordinary_muscle_development	ENSGALG00010005362	ATP1B1	0.93919634	0.174203073	5.391387902	6.99156E-08	4.57322E-07	positive
PRJNA729271	stage_E14_vs_E10	ordinary_muscle_development	ENSGALG00010005363	RDX	0.167746947	0.085396598	1.964328222	0.049492041	0.094071221	positive
PRJNA729271	stage_E14_vs_E10	ordinary_muscle_development	ENSGALG00010005364	MAP7	-2.478842715	0.34354615	-7.215457698	5.37529E-13	6.32425E-12	negative
PRJNA729271	stage_E14_vs_E10	ordinary_muscle_development	ENSGALG00010005366	AUP1	0.260865082	0.113753717	2.29324447	0.021833934	0.046969854	positive
PRJNA729271	stage_E14_vs_E10	ordinary_muscle_development	ENSGALG00010005367	H2AZ1	-0.175880927	0.094651329	-1.858198182	0.063140876	0.115515209	negative
PRJNA729271	stage_E14_vs_E10	ordinary_muscle_development	ENSGALG00010005368	CCDC3	-1.942748184	0.229879411	-8.451162191	2.88417E-17	4.95333E-16	negative
PRJNA729271	stage_E14_vs_E10	ordinary_muscle_development	ENSGALG00010005369	DPT	2.027232541	0.313090416	6.474910886	9.48679E-11	8.70874E-10	positive
PRJNA729271	stage_E14_vs_E10	ordinary_muscle_development	ENSGALG00010005371	GRM1	0.123690851	1.062230339	0.116444472	0.907300291	0.93643675	positive
PRJNA729271	stage_E14_vs_E10	ordinary_muscle_development	ENSGALG00010005372	CDIN1	-0.380865218	0.106433942	-3.578418792	0.000345679	0.001220911	negative
PRJNA729271	stage_E14_vs_E10	ordinary_muscle_development	ENSGALG00010005376	HTRA2	-0.001559056	0.094573979	-0.016485042	0.986847435	0.991301417	negative
PRJNA729271	stage_E14_vs_E10	ordinary_muscle_development	ENSGALG00010005377	EMCN	0.647468667	0.141267384	4.583284904	4.57728E-06	2.27452E-05	positive
PRJNA729271	stage_E14_vs_E10	ordinary_muscle_development	ENSGALG00010005378	ENSGALG00010005378	-0.670110063	0.645190238	-1.038623996	0.298979641	0.412577104	negative
PRJNA729271	stage_E14_vs_E10	ordinary_muscle_development	ENSGALG00010005379	DZIP1	0.001662501	0.165517771	0.010044242	0.991985989	0.99500636	positive
PRJNA729271	stage_E14_vs_E10	ordinary_muscle_development	ENSGALG00010005380	XCL1	-0.247113437	1.30358942	-0.189563856	0.849650912	0.895751689	negative
PRJNA729271	stage_E14_vs_E10	ordinary_muscle_development	ENSGALG00010005381	FGF9	-1.17981115	0.171493048	-6.879644172	6.00022E-12	6.26608E-11	negative
PRJNA729271	stage_E14_vs_E10	ordinary_muscle_development	ENSGALG00010005382	RAB32	0.243020351	0.126459324	1.921727429	0.054640064	0.102128656	positive
PRJNA729271	stage_E14_vs_E10	ordinary_muscle_development	ENSGALG00010005383	OPTN	0.121457697	0.139502952	0.870646077	0.38394743	0.500100414	positive
PRJNA729271	stage_E14_vs_E10	ordinary_muscle_development	ENSGALG00010005384	MAPRE2	-0.336700138	0.172855401	-1.947871673	0.05143032	0.097118615	negative
PRJNA729271	stage_E14_vs_E10	ordinary_muscle_development	ENSGALG00010005385	PDSS1	0.283164287	0.097868728	2.893307124	0.003812083	0.010247802	positive
PRJNA729271	stage_E14_vs_E10	ordinary_muscle_development	ENSGALG00010005386	CPOX	-0.011779797	0.13868792	-0.084937441	0.932311126	0.953694409	negative
PRJNA729271	stage_E14_vs_E10	ordinary_muscle_development	ENSGALG00010005387	CLDN10	-4.219651641	0.617625241	-6.832058274	8.37048E-12	8.6356E-11	negative
PRJNA729271	stage_E14_vs_E10	ordinary_muscle_development	ENSGALG00010005388	ENSGALG00010005388	0.790352881	0.480886919	1.643531669	0.100272978	0.17045348	positive
PRJNA729271	stage_E14_vs_E10	ordinary_muscle_development	ENSGALG00010005389	MCM10	-0.374941069	0.180818269	-2.073579568	0.038118374	0.07522081	negative
PRJNA729271	stage_E14_vs_E10	ordinary_muscle_development	ENSGALG00010005391	ADGB	-0.793270804	0.392708259	-2.020000306	0.043383356	0.084013593	negative
PRJNA729271	stage_E14_vs_E10	ordinary_muscle_development	ENSGALG00010005392	ENSGALG00010005392	0.111084641	0.10553046	1.052631072	0.292510111	0.405115044	positive
PRJNA729271	stage_E14_vs_E10	ordinary_muscle_development	ENSGALG00010005394	PHYH	1.281572805	0.135768722	9.439381828	3.74985E-21	8.7386E-20	positive
PRJNA729271	stage_E14_vs_E10	ordinary_muscle_development	ENSGALG00010005395	ENSGALG00010005395	-0.82273708	0.269813394	-3.049281827	0.002293892	0.006562061	negative
PRJNA729271	stage_E14_vs_E10	ordinary_muscle_development	ENSGALG00010005396	MICU2	0.670441775	0.122211302	5.485922842	4.11316E-08	2.76404E-07	positive
PRJNA729271	stage_E14_vs_E10	ordinary_muscle_development	ENSGALG00010005398	SEPHS1	0.313176673	0.084678974	3.698399469	0.000216963	0.000798599	positive
PRJNA729271	stage_E14_vs_E10	ordinary_muscle_development	ENSGALG00010005399	NDNF	-3.444005278	0.223655728	-15.39869023	1.67018E-53	2.81503E-51	negative
PRJNA729271	stage_E14_vs_E10	ordinary_muscle_development	ENSGALG00010005401	BCLAF1	-0.48337137	0.085672742	-5.642067209	1.6802E-08	1.19241E-07	negative
PRJNA729271	stage_E14_vs_E10	ordinary_muscle_development	ENSGALG00010005402	ABCC4	-1.113756363	0.172644272	-6.451163128	1.10995E-10	1.01187E-09	negative
PRJNA729271	stage_E14_vs_E10	ordinary_muscle_development	ENSGALG00010005403	ENSGALG00010005403	-0.222926055	0.135824641	-1.641278441	0.100739629	0.171106272	negative
PRJNA729271	stage_E14_vs_E10	ordinary_muscle_development	ENSGALG00010005404	BEND7	0.673029547	0.186659129	3.605661028	0.000311359	0.001110301	positive
PRJNA729271	stage_E14_vs_E10	ordinary_muscle_development	ENSGALG00010005405	CEBPD	0.625687608	0.478083454	1.308741398	0.190621964	0.287819309	positive
PRJNA729271	stage_E14_vs_E10	ordinary_muscle_development	ENSGALG00010005407	DPH6	-0.158439782	0.118214182	-1.340277275	0.180155217	0.275023683	negative
PRJNA729271	stage_E14_vs_E10	ordinary_muscle_development	ENSGALG00010005408	APBB1IP	-0.754539568	0.208930111	-3.611444824	0.000304496	0.001089257	negative
PRJNA729271	stage_E14_vs_E10	ordinary_muscle_development	ENSGALG00010005409	PRKCQ	0.995548829	0.218042936	4.565838494	4.97502E-06	2.45476E-05	positive
PRJNA729271	stage_E14_vs_E10	ordinary_muscle_development	ENSGALG00010005411	STXBP5	-0.075086018	0.097009516	-0.77400673	0.438926812	0.55280599	negative
PRJNA729271	stage_E14_vs_E10	ordinary_muscle_development	ENSGALG00010005412	MTFR2	0.062091423	0.154917487	0.400803193	0.688565028	0.772503876	positive
PRJNA729271	stage_E14_vs_E10	ordinary_muscle_development	ENSGALG00010005413	GAD2	-0.756802794	0.246846582	-3.065883221	0.002170281	0.006249149	negative
PRJNA729271	stage_E14_vs_E10	ordinary_muscle_development	ENSGALG00010005414	ENSGALG00010005414	0.281163041	0.203668298	1.380494873	0.167434327	0.25920758	positive
PRJNA729271	stage_E14_vs_E10	ordinary_muscle_development	ENSGALG00010005415	PRKDC	-0.271988605	0.086993099	-3.126553809	0.001768681	0.005230981	negative
PRJNA729271	stage_E14_vs_E10	ordinary_muscle_development	ENSGALG00010005416	PPP3CA	-1.030323443	0.16288861	-6.325325292	2.527E-10	2.22803E-09	negative
PRJNA729271	stage_E14_vs_E10	ordinary_muscle_development	ENSGALG00010005417	LOXL3	-0.66471502	0.165874268	-4.007342606	6.14058E-05	0.000251292	negative
PRJNA729271	stage_E14_vs_E10	ordinary_muscle_development	ENSGALG00010005420	DHRS4	0.98629337	0.116368311	8.475618149	2.33835E-17	4.0592E-16	positive
PRJNA729271	stage_E14_vs_E10	ordinary_muscle_development	ENSGALG00010005421	ENSGALG00010005421	0.504887104	0.331201105	1.524412501	0.127405699	0.207452888	positive
PRJNA729271	stage_E14_vs_E10	ordinary_muscle_development	ENSGALG00010005423	ST3GAL6	-0.287001025	0.150345279	-1.908946045	0.056269052	0.104704312	negative
PRJNA729271	stage_E14_vs_E10	ordinary_muscle_development	ENSGALG00010005424	ENSGALG00010005424	0.252958466	0.134998599	1.873785861	0.060959946	0.112105356	positive
PRJNA729271	stage_E14_vs_E10	ordinary_muscle_development	ENSGALG00010005425	ELAPOR2	-1.839547675	0.255999359	-7.185751096	6.68387E-13	7.78174E-12	negative
PRJNA729271	stage_E14_vs_E10	ordinary_muscle_development	ENSGALG00010005426	ENSGALG00010005426	1.114421788	0.968353243	1.150842212	0.249797155	0.35687235	positive
PRJNA729271	stage_E14_vs_E10	ordinary_muscle_development	ENSGALG00010005427	ENSGALG00010005427	0.307705477	0.138711649	2.218310284	0.026533683	0.055442661	positive
PRJNA729271	stage_E14_vs_E10	ordinary_muscle_development	ENSGALG00010005428	ZNF770	0.013336073	0.101683706	0.131152506	0.895654666	0.928253029	positive
PRJNA729271	stage_E14_vs_E10	ordinary_muscle_development	ENSGALG00010005429	ZDHHC20	-0.904876466	0.116247147	-7.784074607	7.02253E-15	9.76887E-14	negative
PRJNA729271	stage_E14_vs_E10	ordinary_muscle_development	ENSGALG00010005430	MCM4	-0.422669493	0.133127085	-3.174932378	0.001498714	0.004516396	negative
PRJNA729271	stage_E14_vs_E10	ordinary_muscle_development	ENSGALG00010005431	GRM3	-2.179911815	0.646041314	-3.374260699	0.000740142	0.002409785	negative
PRJNA729271	stage_E14_vs_E10	ordinary_muscle_development	ENSGALG00010005433	GPC5	-0.160628394	0.127681649	-1.258038219	0.208377935	0.308838258	negative
PRJNA729271	stage_E14_vs_E10	ordinary_muscle_development	ENSGALG00010005434	PRDM5	-0.463302894	0.218228074	-2.123021508	0.03375205	0.067810353	negative
PRJNA729271	stage_E14_vs_E10	ordinary_muscle_development	ENSGALG00010005435	MAD2L1	-0.192069462	0.165806176	-1.158397511	0.246701854	0.353319176	negative
PRJNA729271	stage_E14_vs_E10	ordinary_muscle_development	ENSGALG00010005436	AQR	-0.388856224	0.05411054	-7.186330509	6.65558E-13	7.75504E-12	negative
PRJNA729271	stage_E14_vs_E10	ordinary_muscle_development	ENSGALG00010005437	PDE7B	0.404541769	0.15934926	2.538711314	0.011126159	0.026176542	positive
PRJNA729271	stage_E14_vs_E10	ordinary_muscle_development	ENSGALG00010005438	GPR180	-0.423544673	0.136561086	-3.101503399	0.001925407	0.00563472	negative
PRJNA729271	stage_E14_vs_E10	ordinary_muscle_development	ENSGALG00010005439	TBX19	0.066480044	0.522223304	0.127301949	0.898701418	0.930211158	positive
PRJNA729271	stage_E14_vs_E10	ordinary_muscle_development	ENSGALG00010005440	ENSGALG00010005440	1.786591444	0.501905078	3.55962018	0.000371392	0.001300319	positive
PRJNA729271	stage_E14_vs_E10	ordinary_muscle_development	ENSGALG00010005441	UBE2V2	-0.263226894	0.095129675	-2.767032424	0.005656913	0.014492215	negative
PRJNA729271	stage_E14_vs_E10	ordinary_muscle_development	ENSGALG00010005442	SASH1	0.514134666	0.097080021	5.295988395	1.18374E-07	7.44721E-07	positive
PRJNA729271	stage_E14_vs_E10	ordinary_muscle_development	ENSGALG00010005443	BANK1	-1.303184009	0.447274004	-2.913614466	0.003572708	0.009670664	negative
PRJNA729271	stage_E14_vs_E10	ordinary_muscle_development	ENSGALG00010005444	ACTC1	1.714661635	0.42654657	4.01986971	5.82303E-05	0.00023948	positive
PRJNA729271	stage_E14_vs_E10	ordinary_muscle_development	ENSGALG00010005445	SFT2D2	-1.145423942	0.102901411	-11.1312754	8.83621E-29	3.49948E-27	negative
PRJNA729271	stage_E14_vs_E10	ordinary_muscle_development	ENSGALG00010005446	ENSGALG00010005446	0.585504516	0.394988087	1.482334621	0.138251281	0.221995382	positive
PRJNA729271	stage_E14_vs_E10	ordinary_muscle_development	ENSGALG00010005447	MYO3A	0.750809186	0.298372213	2.516350897	0.011857706	0.027686445	positive

PRJNA729271	stage_E14_vs_E10	ordinary_muscle_development	ENSGALG00010005448	ENSGALG00010005448	0.113344749	0.14747876	0.768549647	0.442160707	0.556026712	positive
PRJNA729271	stage_E14_vs_E10	ordinary_muscle_development	ENSGALG00010005449	TIPRL	0.060430322	0.085333098	0.708169785	0.478839827	0.590956432	positive
PRJNA729271	stage_E14_vs_E10	ordinary_muscle_development	ENSGALG00010005450	ENSGALG00010005450	1.339240254	0.440476253	3.040436898	0.002362352	0.00673001	positive
PRJNA729271	stage_E14_vs_E10	ordinary_muscle_development	ENSGALG00010005453	UST	-0.429589025	0.114225064	-3.760899851	0.000169303	0.000638909	negative
PRJNA729271	stage_E14_vs_E10	ordinary_muscle_development	ENSGALG00010005454	GPR161	-0.419034916	0.094170272	-4.44975796	8.59671E-06	4.07087E-05	negative
PRJNA729271	stage_E14_vs_E10	ordinary_muscle_development	ENSGALG00010005455	ENSGALG00010005455	0.798383433	0.705667242	1.131387977	0.25789183	0.366089714	positive
PRJNA729271	stage_E14_vs_E10	ordinary_muscle_development	ENSGALG00010005456	RPL7L1	-0.165357009	0.069439832	-2.381299115	0.017251697	0.038288678	negative
PRJNA729271	stage_E14_vs_E10	ordinary_muscle_development	ENSGALG00010005457	SEMA3D	-0.367083064	0.159598002	-2.300047992	0.021445501	0.046244055	negative
PRJNA729271	stage_E14_vs_E10	ordinary_muscle_development	ENSGALG00010005458	PRKD1	-0.155680008	0.174514632	-0.892074241	0.372353141	0.488483915	negative
PRJNA729271	stage_E14_vs_E10	ordinary_muscle_development	ENSGALG00010005459	ZC3H12D	1.403057295	0.551495571	2.544095307	0.010956119	0.025805807	positive
PRJNA729271	stage_E14_vs_E10	ordinary_muscle_development	ENSGALG00010005460	TGDS	-0.589103406	0.097543591	-6.039386082	1.54702E-09	1.24027E-08	negative
PRJNA729271	stage_E14_vs_E10	ordinary_muscle_development	ENSGALG00010005462	AHI1	-0.096987531	0.150726298	-0.643467875	0.519920554	0.627863736	negative
PRJNA729271	stage_E14_vs_E10	ordinary_muscle_development	ENSGALG00010005463	SKA3	-0.301415038	0.243079327	-1.23998631	0.214980458	0.316713257	negative
PRJNA729271	stage_E14_vs_E10	ordinary_muscle_development	ENSGALG00010005464	SLC39A8	-2.788347903	0.261417795	-10.66625133	1.46415E-26	5.04105E-25	negative
PRJNA729271	stage_E14_vs_E10	ordinary_muscle_development	ENSGALG00010005465	ENSGALG00010005465	-0.130871175	0.08600708	-1.521632573	0.128101171	0.208421425	negative
PRJNA729271	stage_E14_vs_E10	ordinary_muscle_development	ENSGALG00010005466	LATS2	0.21126049	0.088809777	2.378797668	0.017369207	0.03853178	positive
PRJNA729271	stage_E14_vs_E10	ordinary_muscle_development	ENSGALG00010005467	ENSGALG00010005467	0.83088247	0.263186419	3.157011193	0.001593952	0.004763781	positive
PRJNA729271	stage_E14_vs_E10	ordinary_muscle_development	ENSGALG00010005468	PPIL4	0.182872939	0.102313461	1.787379069	0.073876242	0.132104027	positive
PRJNA729271	stage_E14_vs_E10	ordinary_muscle_development	ENSGALG00010005470	SEMA3A	-0.582634163	0.140526023	-4.146094443	3.38194E-05	0.000144819	negative
PRJNA729271	stage_E14_vs_E10	ordinary_muscle_development	ENSGALG00010005472	PFKP	-0.380458394	0.113992058	-3.337586854	0.000845093	0.002711293	negative
PRJNA729271	stage_E14_vs_E10	ordinary_muscle_development	ENSGALG00010005473	ENSGALG00010005473	-0.387518987	0.823615969	-0.470509317	0.637991179	0.731157665	negative
PRJNA729271	stage_E14_vs_E10	ordinary_muscle_development	ENSGALG00010005474	COL8A1	-2.12475531	0.34878048	-6.091955922	1.11539E-09	9.06766E-09	negative
PRJNA729271	stage_E14_vs_E10	ordinary_muscle_development	ENSGALG00010005475	TMEM181	-0.115999141	0.090655996	-1.279552875	0.200702432	0.29985382	negative
PRJNA729271	stage_E14_vs_E10	ordinary_muscle_development	ENSGALG00010005476	NFKB1	0.029936618	0.115929053	0.258232235	0.796227682	0.856682774	positive
PRJNA729271	stage_E14_vs_E10	ordinary_muscle_development	ENSGALG00010005477	TMEM161B	-0.289074732	0.161801137	-1.786605077	0.07400134	0.132246262	negative
PRJNA729271	stage_E14_vs_E10	ordinary_muscle_development	ENSGALG00010005479	ENSGALG00010005479	0.05258484	0.611786686	0.085952901	0.931503858	0.953157721	positive
PRJNA729271	stage_E14_vs_E10	ordinary_muscle_development	ENSGALG00010005481	ENSGALG00010005481	-0.302255685	0.547126162	-0.552442391	0.580645294	0.682212331	negative
PRJNA729271	stage_E14_vs_E10	ordinary_muscle_development	ENSGALG00010005482	XPO4	-0.411093269	0.073225792	-5.614050148	1.97645E-08	1.39004E-07	negative
PRJNA729271	stage_E14_vs_E10	ordinary_muscle_development	ENSGALG00010005484	GINM1	1.940941937	0.121899441	15.92248436	4.42454E-57	8.90746E-55	positive
PRJNA729271	stage_E14_vs_E10	ordinary_muscle_development	ENSGALG00010005485	PITRM1	-0.54000092	0.095746216	-5.639919172	1.7013E-08	1.20588E-07	negative
PRJNA729271	stage_E14_vs_E10	ordinary_muscle_development	ENSGALG00010005486	IRF5	0.399816497	0.32670548	1.223782648	0.221034232	0.323658066	positive
PRJNA729271	stage_E14_vs_E10	ordinary_muscle_development	ENSGALG00010005487	ENSGALG00010005487	-0.876266992	0.210999008	-4.152943649	3.28226E-05	0.000141134	negative
PRJNA729271	stage_E14_vs_E10	ordinary_muscle_development	ENSGALG00010005489	G2E3	-0.119714921	0.084314948	-1.419854044	0.155650178	0.244569032	negative
PRJNA729271	stage_E14_vs_E10	ordinary_muscle_development	ENSGALG00010005490	DCBLD2	0.137727207	0.114670824	1.20106582	0.22972567	0.333654669	positive
PRJNA729271	stage_E14_vs_E10	ordinary_muscle_development	ENSGALG00010005491	GGACT	-0.669317152	0.175764195	-3.808040384	0.000140072	0.000537556	negative
PRJNA729271	stage_E14_vs_E10	ordinary_muscle_development	ENSGALG00010005492	TNPO3	0.136383118	0.09638667	1.414958297	0.157080724	0.246336156	positive
PRJNA729271	stage_E14_vs_E10	ordinary_muscle_development	ENSGALG00010005493	KLF6	0.279558222	0.181040605	1.544174144	0.122546151	0.200962378	positive
PRJNA729271	stage_E14_vs_E10	ordinary_muscle_development	ENSGALG00010005494	PPDPFL	-1.119882677	0.451170503	-2.482171747	0.013058432	0.030090731	negative
PRJNA729271	stage_E14_vs_E10	ordinary_muscle_development	ENSGALG00010005495	TXN	0.69818291	0.146291544	4.772544561	1.81913E-06	9.6481E-06	positive
PRJNA729271	stage_E14_vs_E10	ordinary_muscle_development	ENSGALG00010005497	MKX	-2.248394304	0.201880487	-11.13725422	8.26276E-29	3.29942E-27	negative
PRJNA729271	stage_E14_vs_E10	ordinary_muscle_development	ENSGALG00010005498	SMO	-0.108410999	0.136785082	-0.792564494	0.428031602	0.542382907	negative
PRJNA729271	stage_E14_vs_E10	ordinary_muscle_development	ENSGALG00010005499	PDE5A	0.049711146	0.112264462	0.442803935	0.657907557	0.747741103	positive
PRJNA729271	stage_E14_vs_E10	ordinary_muscle_development	ENSGALG00010005500	KATNA1	1.46117726	0.127496254	11.4605505	2.08187E-30	9.08938E-29	positive
PRJNA729271	stage_E14_vs_E10	ordinary_muscle_development	ENSGALG00010005502	SCFD1	-0.232428502	0.070383354	-3.302322047	0.000958879	0.003034706	negative
PRJNA729271	stage_E14_vs_E10	ordinary_muscle_development	ENSGALG00010005503	DYNLT1	-0.383617504	0.137705729	-2.785777365	0.005339953	0.013797259	negative
PRJNA729271	stage_E14_vs_E10	ordinary_muscle_development	ENSGALG00010005504	LATS1	-0.099302026	0.077809097	-1.276226419	0.201875492	0.301261638	negative
PRJNA729271	stage_E14_vs_E10	ordinary_muscle_development	ENSGALG00010005505	FABP2	-2.1514287	1.088003543	-1.977409645	0.047995345	0.091683253	negative
PRJNA729271	stage_E14_vs_E10	ordinary_muscle_development	ENSGALG00010005507	USP53	-0.117803404	0.120792859	-0.975251392	0.329435576	0.44411911	negative
PRJNA729271	stage_E14_vs_E10	ordinary_muscle_development	ENSGALG00010005508	SS18	-0.192479728	0.063206141	-3.045269417	0.00232472	0.006638458	negative
PRJNA729271	stage_E14_vs_E10	ordinary_muscle_development	ENSGALG00010005509	COCH	-0.740933328	0.45657213	-1.622817686	0.104628384	0.176779161	negative
PRJNA729271	stage_E14_vs_E10	ordinary_muscle_development	ENSGALG00010005510	MYOZ2	1.790039879	0.317269191	5.642022384	1.68064E-08	1.19241E-07	positive
PRJNA729271	stage_E14_vs_E10	ordinary_muscle_development	ENSGALG00010005511	AHCYL2	0.037674027	0.098432347	0.382740309	0.701912324	0.783596668	positive
PRJNA729271	stage_E14_vs_E10	ordinary_muscle_development	ENSGALG00010005512	SMARCAD1	-0.915078019	0.107793021	-8.489213997	2.08039E-17	3.62444E-16	negative
PRJNA729271	stage_E14_vs_E10	ordinary_muscle_development	ENSGALG00010005513	TMEM30CP	-0.210376355	0.259144695	-0.81181039	0.41690044	0.531516846	negative
PRJNA729271	stage_E14_vs_E10	ordinary_muscle_development	ENSGALG00010005514	EEF1AKMT1	-0.084984911	0.143576011	-0.591915809	0.553906967	0.658698949	negative
PRJNA729271	stage_E14_vs_E10	ordinary_muscle_development	ENSGALG00010005515	PCCA	0.40659496	0.095271144	4.267766092	1.9744E-05	8.79251E-05	positive
PRJNA729271	stage_E14_vs_E10	ordinary_muscle_development	ENSGALG00010005516	LARP4B	0.963020333	0.131549135	7.32061318	2.4684E-13	3.00921E-12	positive
PRJNA729271	stage_E14_vs_E10	ordinary_muscle_development	ENSGALG00010005518	HPGDS	-3.048087689	0.658179315	-4.631090068	3.63746E-06	1.83073E-05	negative
PRJNA729271	stage_E14_vs_E10	ordinary_muscle_development	ENSGALG00010005519	PTBP3	-2.142311567	0.257747197	-8.311677449	9.43586E-17	1.56133E-15	negative
PRJNA729271	stage_E14_vs_E10	ordinary_muscle_development	ENSGALG00010005521	SYTL3	0.121102203	0.428412223	0.282676815	0.777424591	0.842351773	positive
PRJNA729271	stage_E14_vs_E10	ordinary_muscle_development	ENSGALG00010005522	SYNPO2	1.271656855	0.178134972	7.138726572	9.41995E-13	1.07768E-11	positive
PRJNA729271	stage_E14_vs_E10	ordinary_muscle_development	ENSGALG00010005523	ENSGALG00010005523	1.178055672	0.652248006	1.806146835	0.070895407	0.127466326	positive
PRJNA729271	stage_E14_vs_E10	ordinary_muscle_development	ENSGALG00010005524	STRIP2	-0.447201267	0.278460849	-1.605975375	0.108279321	0.181908757	negative
PRJNA729271	stage_E14_vs_E10	ordinary_muscle_development	ENSGALG00010005526	STRN3	0.040590949	0.069675957	0.582567514	0.56018448	0.663538116	positive
PRJNA729271	stage_E14_vs_E10	ordinary_muscle_development	ENSGALG00010005527	NUP43	-0.019493458	0.093396659	-0.208716864	0.834669275	0.884813079	negative
PRJNA729271	stage_E14_vs_E10	ordinary_muscle_development	ENSGALG00010005529	ENSGALG00010005529	0.359977204	0.379825738	0.947743052	0.343260278	0.458492235	positive
PRJNA729271	stage_E14_vs_E10	ordinary_muscle_development	ENSGALG00010005530	IL17D	1.378937864	0.210743873	6.543193146	6.0219E-11	5.70364E-10	positive
PRJNA729271	stage_E14_vs_E10	ordinary_muscle_development	ENSGALG00010005531	SUSD1	-0.209066284	0.254105012	-0.822755451	0.410647075	0.525415372	negative
PRJNA729271	stage_E14_vs_E10	ordinary_muscle_development	ENSGALG00010005532	GTPBP4	0.024197756	0.097082662	0.249248993	0.803168184	0.861407535	positive
PRJNA729271	stage_E14_vs_E10	ordinary_muscle_development	ENSGALG00010005533	OPN4-1	-1.369634584	0.688998471	-1.987863024	0.046826841	0.08975867	negative
PRJNA729271	stage_E14_vs_E10	ordinary_muscle_development	ENSGALG00010005534	ENSGALG00010005534	-0.337938108	0.161818665	-2.088375325	0.036763992	0.072889353	negative
PRJNA729271	stage_E14_vs_E10	ordinary_muscle_development	ENSGALG00010005535	AP4S1	-0.275318024	0.156209122	-1.762496456	0.077985455	0.138321413	negative
PRJNA729271	stage_E14_vs_E10	ordinary_muscle_development	ENSGALG00010005536	TAF4B	-0.570674021	0.182715702	-3.123289439	0.001788418	0.005283267	negative
PRJNA729271	stage_E14_vs_E10	ordinary_muscle_development	ENSGALG00010005537	GRIN3A	-2.702855955	0.297422144	-9.087608341	1.01241E-19	2.11455E-18	negative
PRJNA729271	stage_E14_vs_E10	ordinary_muscle_development	ENSGALG00010005538	ENSGALG00010005538	0.638966256	0.336508973	1.898808969	0.057589598	0.106828925	positive
PRJNA729271	stage_E14_vs_E10	ordinary_muscle_development	ENSGALG00010005539	IFT88	-0.215977691	0.130271576	-1.65790342	0.097336962	0.166242402	negative
PRJNA729271	stage_E14_vs_E10	ordinary_muscle_development	ENSGALG00010005540	PCMT1	0.694212174	0.137127105	5.062545238	4.13696E-07	2.4199E-06	positive
PRJNA729271	stage_E14_vs_E10	ordinary_muscle_development	ENSGALG00010005542	KCTD1	-0.083441042	0.129779743	-0.642943501	0.520260761	0.628189576	negative
PRJNA729271	stage_E14_vs_E10	ordinary_muscle_development	ENSGALG00010005543	SEC24D	0.195577665	0.201589891	0.970175957	0.331958793	0.446814254	positive
PRJNA729271	stage_E14_vs_E10	ordinary_muscle_development	ENSGALG00010005544	AQP4	6.33031247	0.308830835	20.49766976	2.25853E-93	1.81874E-90	positive
PRJNA729271	stage_E14_vs_E10	ordinary_muscle_development	ENSGALG00010005545	RPL34	0.377010702	0.166898381	2.258923665	0.023888134	0.050771041	positive
PRJNA729271	stage_E14_vs_E10	ordinary_muscle_development	ENSGALG00010005546	NRF1	0.200099695	0.153088751	1.307082943	0.191184543	0.288519921	positive
PRJNA729271	stage_E14_vs_E10	ordinary_muscle_development	ENSGALG00010005547	LRP11	0.654546124	0.214676022	3.048995031	0.002296083	0.006567032	positive
PRJNA729271	stage_E14_vs_E10	ordinary_muscle_development	ENSGALG00010005548	EZR	0.108671239	0.175970655	0.617553186	0.536869912	0.643541037	positive
PRJNA729271	stage_E14_vs_E10	ordinary_muscle_development	ENSGALG00010005549	RNF20	-0.049001501	0.113291831	-0.432524579	0.665360192	0.753762875	negative
PRJNA729271	stage_E14_vs_E10	ordinary_muscle_development	ENSGALG00010005550	ENSGALG00010005550	0.344665937	0.600718116	0.573756523	0.566132573	0.668793125	positive
PRJNA729271	stage_E14_vs_E10	ordinary_muscle_development	ENSGALG00010005551	METTL14	-0.48607868	0.079788066	-6.092122576	1.11423E-09	9.0633E-09	negative
PRJNA729271	stage_E14_vs_E10	ordinary_muscle_development	ENSGALG00010005552	WDR37	-0.463266758	0.086443591	-5.359179953	8.36006E-08	5.39533E-07	negative
PRJNA729271	stage_E14_vs_E10	ordinary_muscle_development	ENSGALG00010005553	PPP1R14C	1.016300561	0.162995921	6.235128799	4.51407E-10	3.8671E-09	positive
PRJNA729271	stage_E14_vs_E10	ordinary_muscle_development	ENSGALG00010005554	CRYL1	0.061800034	0.162856859	0.379474553	0.704335498	0.785092513	positive
PRJNA729271	stage_E14_vs_E10	ordinary_muscle_development	ENSGALG00010005555	PRSS12	-1.589003594	0.148694389	-10.6863723	1.17892E-26	4.09796E-25	negative
PRJNA729271	stage_E14_vs_E10	ordinary_muscle_development	ENSGALG00010005556	IYD	-1.039529032	0.741369175	-1.402174608	0.160863112	0.251126636	negative
PRJNA729271	stage_E14_vs_E10	ordinary_muscle_development	ENSGALG00010005557	CMSS1	-0.415990119	0.165928065	-2.50705098	0.012174315	0.028326678	negative
PRJNA729271	stage_E14_vs_E10	ordinary_muscle_development	ENSGALG00010005558	TAGAP	0.413791882	0.214087557	1.932816128	0.053258853	0.099933602	positive
PRJNA729271	stage_E14_vs_E10	ordinary_muscle_development	ENSGALG00010005559	FILIP1L	-0.407354494	0.130996055	-3.109669929	0.001872965	0.005503472	negative
PRJNA729271	stage_E14_vs_E10	ordinary_muscle_development	ENSGALG00010005560	RSPH3	-1.045947388	0.364366747	-2.870589579	0.004097071	0.010922759	negative
PRJNA729271	stage_E14_vs_E10	ordinary_muscle_development	ENSGALG00010005561	CLYBL	0.219777804	0.198167005	1.109053465	0.267407118	0.376499872	positive
PRJNA729271	stage_E14_vs_E10	ordinary_muscle_development	ENSGALG00010005562	HECTD1	0.05331982	0.085395796	0.624384601	0.532375035	0.639738913	positive
PRJNA729271	stage_E14_vs_E10	ordinary_muscle_development	ENSGALG00010005564	TM9SF2	-0.013946667	0.057711211	-0.241663036	0.809041274	0.865720749	negative
PRJNA729271	stage_E14_vs_E10	ordinary_muscle_development	ENSGALG00010005565	PGAP4	-0.570516798	0.174529054	-3.26889298	0.001079691	0.003372872	negative
PRJNA729271	stage_E14_vs_E10	ordinary_muscle_development	ENSGALG00010005567	NDST3	-0.324775453	0.235946938	-1.376476659	0.168674109	0.260792662	negative
PRJNA729271	stage_E14_vs_E10	ordinary_muscle_development	ENSGALG00010005568	PLEKHG1	-0.133694127	0.131895177	-1.013639239	0.310754927	0.424542193	negative
PRJNA729271	stage_E14_vs_E10	ordinary_muscle_development	ENSGALG00010005572	ENSGALG00010005572	0.45879514	0.154425948	2.970971826	0.00296859	0.008236928	positive
PRJNA729271	stage_E14_vs_E10	ordinary_muscle_development	ENSGALG00010005573	CHST9	-1.198367287	0.285327047	-4.199977879	2.66941E-05	0.000116475	negative
PRJNA729271	stage_E14_vs_E10	ordinary_muscle_development	ENSGALG00010005575	ENSGALG00010005575	1.721470614	0.451749209	3.81067765	0.000138586	0.000532417	positive
PRJNA729271	stage_E14_vs_E10	ordinary_muscle_development	ENSGALG00010005578	HEATR5A	0.09787232	0.120123042	0.814767245	0.415205553	0.529835769	positive
PRJNA729271	stage_E14_vs_E10	ordinary_muscle_development	ENSGALG00010005581	GR42L5	0.459875964	0.850212238	0.540895488	0.588579621	0.688896628	positive
PRJNA729271	stage_E14_vs_E10	ordinary_muscle_development	ENSGALG00010005583	ALDOB	-2.026081188	1.012018288	-2.00202033			negative
PRJNA729271	stage_E14_vs_E10	ordinary_muscle_development	ENSGALG00010005587	LEF1	-2.392976699	0.201577567	-11.87124509	1.66964E-32	8.09413E-31	negative
PRJNA729271	stage_E14_vs_E10	ordinary_muscle_development	ENSGALG00010005588	PLAGL1	0.331301482	0.126202015	2.625167919	0.008660624	0.021027764	positive
PRJNA729271	stage_E14_vs_E10	ordinary_muscle_development	ENSGALG00010005590	TBC1D23	0.183633317	0.055213748	3.325862175	0.000881455	0.002811772	positive
PRJNA729271	stage_E14_vs_E10	ordinary_muscle_development	ENSGALG00010005593	ENSGALG00010005593	0.038141639	1.209814818	0.031526841	0.974849387	0.982882666	positive
PRJNA729271	stage_E14_vs_E10	ordinary_muscle_development	ENSGALG00010005594	MRPL50	0.323356063	0.149951536	2.156403801	0.031052148	0.063163189	positive
PRJNA729271	stage_E14_vs_E10	ordinary_muscle_development	ENSGALG00010005596	PARD3	-0.044341428	0.053143457	-0.834372284	0.40407123	0.519100637	negative
PRJNA729271	stage_E14_vs_E10	ordinary_muscle_development	ENSGALG00010005597	ENSGALG00010005597	0.851536825	0.501024834	1.699590055	0.089208062	0.154692052	positive
PRJNA729271	stage_E14_vs_E10	ordinary_muscle_development	ENSGALG00010005601	TMEM215	0.274243745	0.376516727	0.728370681	0.466386706	0.578741144	positive
PRJNA729271	stage_E14_vs_E10	ordinary_muscle_development	ENSGALG00010005609	GJB6	-1.158997442	0.540777234	-2.143206793	0.032096497	0.06495933	negative
PRJNA729271	stage_E14_vs_E10	ordinary_muscle_development	ENSGALG00010005610	MTHFD1L	-0.721649389	0.190475338	-3.788676257	0.000151452	0.000576345	negative
PRJNA729271	stage_E14_vs_E10	ordinary_muscle_development	ENSGALG00010005617	DTD2	1.170511676	0.207674876	5.636270025	1.73773E-08	1.2305E-07	positive
PRJNA729271	stage_E14_vs_E10	ordinary_muscle_development	ENSGALG00010005618	PHACTR2	0.340538856	0.067365101	5.055122804	4.30114E-07	2.50482E-06	positive
PRJNA729271	stage_E14_vs_E10	ordinary_muscle_development	ENSGALG00010005619	CELF2	0.544746314	0.076121713	7.156253971	8.29115E-13	9.55327E-12	positive
PRJNA729271	stage_E14_vs_E10	ordinary_muscle_development	ENSGALG00010005621	CDH2	-0.135779754	0.139220484	-0.975285752	0.329418536	0.44411911	negative
PRJNA729271	stage_E14_vs_E10	ordinary_muscle_development	ENSGALG00010005629	NRP1	-0.022072027	0.173241181	-0.127406355	0.898618786	0.930211158	negative
PRJNA729271	stage_E14_vs_E10	ordinary_muscle_development	ENSGALG00010005630	NIT2	-0.012241032	0.163496188	-0.074870445	0.940317792	0.958233014	negative
PRJNA729271	stage_E14_vs_E10	ordinary_muscle_development	ENSGALG00010005632	ITGB1	0.349008138	0.093386595	3.737240215	0.000186051	0.000695951	positive
PRJNA729271	stage_E14_vs_E10	ordinary_muscle_development	ENSGALG00010005634	HADH	0.219049741	0.086751376	2.525028992	0.011568874	0.027102852	positive
PRJNA729271	stage_E14_vs_E10	ordinary_muscle_development	ENSGALG00010005636	ENSGALG00010005636	0.923827708	0.619566126	1.491088149	0.135938353	0.219009273	positive
PRJNA729271	stage_E14_vs_E10	ordinary_muscle_development	ENSGALG00010005637	TOPORS	-0.292718399	0.070242159	-4.167275059	3.08262E-05	0.000133222	negative
PRJNA729271	stage_E14_vs_E10	ordinary_muscle_development	ENSGALG00010005643	ENSGALG00010005643	-3.999030862	1.286170668	-3.109253663	0.001875606	0.005510019	negative
PRJNA729271	stage_E14_vs_E10	ordinary_muscle_development	ENSGALG00010005645	FUCA2	-0.240015171	0.156969932	-1.529051884	0.126251582	0.205804845	negative
PRJNA729271	stage_E14_vs_E10	ordinary_muscle_development	ENSGALG00010005649	TOMM70	0.132316995	0.078298917	1.689895594	0.091047931	0.157336642	positive

PRJNA729271	stage_E14_vs_E10	ordinary_muscle_development	ENSGALG00010005651	USP6NL	0.339102778	0.120181885	2.821579797	0.004778775	0.012518305	positive
PRJNA729271	stage_E14_vs_E10	ordinary_muscle_development	ENSGALG00010005656	CYP2U1	-0.479685708	0.135922833	-3.529103223	0.00041697	0.001438017	negative
PRJNA729271	stage_E14_vs_E10	ordinary_muscle_development	ENSGALG00010005660	UBAC2	0.160606107	0.10114928	1.587812647	0.112328711	0.187559574	positive
PRJNA729271	stage_E14_vs_E10	ordinary_muscle_development	ENSGALG00010005668	LNP1	-0.208366032	0.305870749	-0.681222485	0.495730722	0.605308468	negative
PRJNA729271	stage_E14_vs_E10	ordinary_muscle_development	ENSGALG00010005670	SGMS2	-1.395567675	0.207750328	-6.717523335	1.84839E-11	1.8414E-10	negative
PRJNA729271	stage_E14_vs_E10	ordinary_muscle_development	ENSGALG00010005675	GJA3	-1.300040065	0.292916935	-4.438255054	9.06911E-06	4.28337E-05	negative
PRJNA729271	stage_E14_vs_E10	ordinary_muscle_development	ENSGALG00010005679	TMEM45A	-2.338559961	0.206093412	-11.3470874	7.66744E-30	3.26881E-28	negative
PRJNA729271	stage_E14_vs_E10	ordinary_muscle_development	ENSGALG00010005680	ENSGALG00010005680	0.87554608	0.477463514	1.833744476	0.066691933	0.120988682	positive
PRJNA729271	stage_E14_vs_E10	ordinary_muscle_development	ENSGALG00010005681	EPC1	0.068420993	0.065415098	1.045951092	0.295583614	0.408785849	positive
PRJNA729271	stage_E14_vs_E10	ordinary_muscle_development	ENSGALG00010005688	ENSGALG00010005688	1.137250156	0.517756232	2.196497281	0.02805637	0.058113329	positive
PRJNA729271	stage_E14_vs_E10	ordinary_muscle_development	ENSGALG00010005692	SEC24B	-0.084212301	0.144051754	-0.584597541	0.558818361	0.662528394	negative
PRJNA729271	stage_E14_vs_E10	ordinary_muscle_development	ENSGALG00010005694	ENSGALG00010005694	0.13572825	0.070824536	1.916401547	0.055314004	0.103215305	positive
PRJNA729271	stage_E14_vs_E10	ordinary_muscle_development	ENSGALG00010005695	KIF5B	-0.134252754	0.083468507	-1.608424051	0.107742338	0.181195636	negative
PRJNA729271	stage_E14_vs_E10	ordinary_muscle_development	ENSGALG00010005696	NUBPL	-0.457497518	0.137824508	-3.319420639	0.000902044	0.002869871	negative
PRJNA729271	stage_E14_vs_E10	ordinary_muscle_development	ENSGALG00010005699	PRPF18	0.286887553	0.092159114	3.112959106	0.001852217	0.005453561	positive
PRJNA729271	stage_E14_vs_E10	ordinary_muscle_development	ENSGALG00010005704	AKAP12	0.400535287	0.093523279	4.282733578	1.84611E-05	8.26417E-05	positive
PRJNA729271	stage_E14_vs_E10	ordinary_muscle_development	ENSGALG00010005705	ECHDC3	0.099217696	0.124589098	0.796359368	0.425823188	0.540245698	positive
PRJNA729271	stage_E14_vs_E10	ordinary_muscle_development	ENSGALG00010005712	ENSGALG00010005712	0.048268591	0.108046422	0.44673937	0.655063252	0.745652728	positive
PRJNA729271	stage_E14_vs_E10	ordinary_muscle_development	ENSGALG00010005714	ARHGAP5	-0.140213644	0.067715503	-2.070628414	0.038393535	0.075675046	negative
PRJNA729271	stage_E14_vs_E10	ordinary_muscle_development	ENSGALG00010005716	ZBTB2	-0.141826755	0.111201482	-1.275403452	0.202166478	0.301574878	negative
PRJNA729271	stage_E14_vs_E10	ordinary_muscle_development	ENSGALG00010005720	COL25A1	-1.046736781	0.178300012	-5.870648953	4.34093E-09	3.31516E-08	negative
PRJNA729271	stage_E14_vs_E10	ordinary_muscle_development	ENSGALG00010005724	PROSER2	-0.926800559	0.122805416	-7.546902996	4.4573E-14	5.82584E-13	negative
PRJNA729271	stage_E14_vs_E10	ordinary_muscle_development	ENSGALG00010005738	ELAVL2	-2.126345277	0.335702104	-6.334024269	2.38848E-10	2.11232E-09	negative
PRJNA729271	stage_E14_vs_E10	ordinary_muscle_development	ENSGALG00010005740	ENSGALG00010005740	0.484575717	0.338784731	1.430335175	0.152620844	0.240764614	positive
PRJNA729271	stage_E14_vs_E10	ordinary_muscle_development	ENSGALG00010005742	GPR183	0.15602324	0.207899541	0.750474192	0.452969162	0.565821096	positive
PRJNA729271	stage_E14_vs_E10	ordinary_muscle_development	ENSGALG00010005743	ETNPPL	3.5818728	0.223643021	16.01602759	9.87615E-58	2.01627E-55	positive
PRJNA729271	stage_E14_vs_E10	ordinary_muscle_development	ENSGALG00010005748	AIG1	1.051732333	0.177636961	5.920684124	3.20605E-09	2.47981E-08	positive
PRJNA729271	stage_E14_vs_E10	ordinary_muscle_development	ENSGALG00010005752	RMND1	-0.049506284	0.097948615	-0.50543118	0.613256024	0.709995692	negative
PRJNA729271	stage_E14_vs_E10	ordinary_muscle_development	ENSGALG00010005755	ZMYM2	0.054704138	0.097076471	0.563515932	0.573083614	0.674920558	positive
PRJNA729271	stage_E14_vs_E10	ordinary_muscle_development	ENSGALG00010005756	TET2	-0.260828592	0.164119291	-1.589262238	0.1120012	0.187120493	negative
PRJNA729271	stage_E14_vs_E10	ordinary_muscle_development	ENSGALG00010005757	ARHGAP12	0.706244847	0.132878888	5.314951525	1.06686E-07	6.77655E-07	positive
PRJNA729271	stage_E14_vs_E10	ordinary_muscle_development	ENSGALG00010005758	HIVEP2	0.197687628	0.105638628	1.871357401	0.061295559	0.112647394	positive
PRJNA729271	stage_E14_vs_E10	ordinary_muscle_development	ENSGALG00010005760	GPR18	0.255368972	0.308819969	0.826918585	0.408283209	0.523120756	positive
PRJNA729271	stage_E14_vs_E10	ordinary_muscle_development	ENSGALG00010005763	AKAP6	2.16793255	0.142400861	15.22415338	2.44503E-52	3.93786E-50	positive
PRJNA729271	stage_E14_vs_E10	ordinary_muscle_development	ENSGALG00010005768	CXXC4	-2.56206139	0.538185236	-4.760556813	1.9306E-06	1.01982E-05	negative
PRJNA729271	stage_E14_vs_E10	ordinary_muscle_development	ENSGALG00010005769	TFG	0.072473613	0.104602821	0.692845688	0.488406396	0.599426598	positive
PRJNA729271	stage_E14_vs_E10	ordinary_muscle_development	ENSGALG00010005772	SVEP1	0.501655029	0.140890044	3.560613755	0.000369989	0.001296975	positive
PRJNA729271	stage_E14_vs_E10	ordinary_muscle_development	ENSGALG00010005777	ENSGALG00010005777	0.303654252	0.957968552	0.316977266	0.751260853	0.821831085	positive
PRJNA729271	stage_E14_vs_E10	ordinary_muscle_development	ENSGALG00010005779	ARMT1	0.284712754	0.110805476	2.569482699	0.010185048	0.02418219	positive
PRJNA729271	stage_E14_vs_E10	ordinary_muscle_development	ENSGALG00010005784	RASL11A	-2.52228463	0.497711386	-5.067765577	4.02513E-07	2.35734E-06	negative
PRJNA729271	stage_E14_vs_E10	ordinary_muscle_development	ENSGALG00010005785	RPL21	0.275264895	0.139790263	1.969127814	0.048938416	0.093202251	positive
PRJNA729271	stage_E14_vs_E10	ordinary_muscle_development	ENSGALG00010005789	RNASET2	0.416948142	0.154495373	2.698774296	0.006959536	0.017344992	positive
PRJNA729271	stage_E14_vs_E10	ordinary_muscle_development	ENSGALG00010005790	MUSK	1.360889034	0.145730326	9.338406544	9.77948E-21	2.208E-19	positive
PRJNA729271	stage_E14_vs_E10	ordinary_muscle_development	ENSGALG00010005792	ADGRG6	-1.501040083	0.16019228	-9.370239823	7.23679E-21	1.65193E-19	negative
PRJNA729271	stage_E14_vs_E10	ordinary_muscle_development	ENSGALG00010005799	UFSP2	-0.041729	0.096609509	-0.431934709	0.665788868	0.754012785	negative
PRJNA729271	stage_E14_vs_E10	ordinary_muscle_development	ENSGALG00010005800	FRMD4A	0.079443189	0.107651589	0.737965781	0.460535244	0.573148309	positive
PRJNA729271	stage_E14_vs_E10	ordinary_muscle_development	ENSGALG00010005814	UPF2	-0.276166836	0.123822048	-2.230352671	0.025724039	0.054047683	negative
PRJNA729271	stage_E14_vs_E10	ordinary_muscle_development	ENSGALG00010005817	VTA1	0.196868533	0.068930343	2.856050387	0.004289471	0.011370862	positive
PRJNA729271	stage_E14_vs_E10	ordinary_muscle_development	ENSGALG00010005821	DOCK9	0.433195614	0.15396287	2.813636908	0.004898452	0.012786433	positive
PRJNA729271	stage_E14_vs_E10	ordinary_muscle_development	ENSGALG00010005824	NMBR	-5.490720679	1.332630883	-4.120211191	3.78525E-05	0.000160712	negative
PRJNA729271	stage_E14_vs_E10	ordinary_muscle_development	ENSGALG00010005826	LRP2BP	0.391668629	0.201143584	1.947209155	0.051509665	0.097243109	positive
PRJNA729271	stage_E14_vs_E10	ordinary_muscle_development	ENSGALG00010005828	LPAR1	-0.124614546	0.151830073	-0.82075009	0.411788633	0.526588111	negative
PRJNA729271	stage_E14_vs_E10	ordinary_muscle_development	ENSGALG00010005829	NPAS3	-0.206279869	0.131658549	-1.566779149	0.117166323	0.19396138	negative
PRJNA729271	stage_E14_vs_E10	ordinary_muscle_development	ENSGALG00010005830	USP12	-0.324761514	0.183163139	-1.77307244	0.076216704	0.135686702	negative
PRJNA729271	stage_E14_vs_E10	ordinary_muscle_development	ENSGALG00010005831	SEC61A2	0.018035583	0.104937014	0.171870558	0.863539293	0.906175491	positive
PRJNA729271	stage_E14_vs_E10	ordinary_muscle_development	ENSGALG00010005836	NEBL	-1.416068245	0.248424666	-5.700191795	1.19673E-08	8.63442E-08	negative
PRJNA729271	stage_E14_vs_E10	ordinary_muscle_development	ENSGALG00010005839	RPS6KA2	-0.252756123	0.181546512	-1.392238935	0.163850056	0.254719709	negative
PRJNA729271	stage_E14_vs_E10	ordinary_muscle_development	ENSGALG00010005841	ENSGALG00010005841	0.012553712	0.32069003	0.039145939	0.968774035	0.978659112	positive
PRJNA729271	stage_E14_vs_E10	ordinary_muscle_development	ENSGALG00010005843	GPR12	-2.138631304	0.447718684	-4.77673008	1.78169E-06	9.46337E-06	negative
PRJNA729271	stage_E14_vs_E10	ordinary_muscle_development	ENSGALG00010005848	WASF3	-1.105518781	0.201889433	-5.475862514	4.35386E-08	2.91228E-07	negative
PRJNA729271	stage_E14_vs_E10	ordinary_muscle_development	ENSGALG00010005849	SNX25	-0.21130582	0.097174363	-2.17450172	0.029667476	0.060841832	negative
PRJNA729271	stage_E14_vs_E10	ordinary_muscle_development	ENSGALG00010005851	SLC15A1	-2.56424225	1.059815132	-2.419518437	0.015541073	0.034984913	negative
PRJNA729271	stage_E14_vs_E10	ordinary_muscle_development	ENSGALG00010005853	COMMD3	0.036371884	0.137103942	0.265286928	0.790788434	0.852926434	positive
PRJNA729271	stage_E14_vs_E10	ordinary_muscle_development	ENSGALG00010005858	CFAP97	-0.282010828	0.109459056	-2.576404711	0.009983372	0.023794568	negative
PRJNA729271	stage_E14_vs_E10	ordinary_muscle_development	ENSGALG00010005859	ENSGALG00010005859	0.096385686	0.556638093	0.173156827	0.862528158	0.90532065	positive
PRJNA729271	stage_E14_vs_E10	ordinary_muscle_development	ENSGALG00010005860	ABI3BP	-0.411985043	0.212524344	-1.938531062	0.052558465	0.098836916	negative
PRJNA729271	stage_E14_vs_E10	ordinary_muscle_development	ENSGALG00010005861	MPC1L	0.36714275	0.131898761	2.783519324	0.005377266	0.013871413	positive
PRJNA729271	stage_E14_vs_E10	ordinary_muscle_development	ENSGALG00010005866	DNAJC1	-0.120980817	0.148527336	-0.814535692	0.415338134	0.529958296	negative
PRJNA729271	stage_E14_vs_E10	ordinary_muscle_development	ENSGALG00010005871	EGLN3	2.026573902	0.195098486	10.38744045	2.82842E-25	8.70443E-24	positive
PRJNA729271	stage_E14_vs_E10	ordinary_muscle_development	ENSGALG00010005872	STK24	-0.426577581	0.135560375	-3.146771919	0.001650837	0.004918578	negative
PRJNA729271	stage_E14_vs_E10	ordinary_muscle_development	ENSGALG00010005875	CDK8	-0.787094774	0.148595461	-5.296896481	1.17787E-07	7.4179E-07	negative
PRJNA729271	stage_E14_vs_E10	ordinary_muscle_development	ENSGALG00010005879	FAM107B	0.183464077	0.120935239	1.517043986	0.129255579	0.209922646	positive
PRJNA729271	stage_E14_vs_E10	ordinary_muscle_development	ENSGALG00010005880	SLC25A4	0.440923484	0.180169735	2.447267211	0.014394408	0.032775203	positive
PRJNA729271	stage_E14_vs_E10	ordinary_muscle_development	ENSGALG00010005881	CORO2A	-0.822852318	0.278725553	-2.952195481	0.003155231	0.008696535	negative
PRJNA729271	stage_E14_vs_E10	ordinary_muscle_development	ENSGALG00010005884	MYB	-0.783884022	0.263515578	-2.974716063	0.002932599	0.008144858	negative
PRJNA729271	stage_E14_vs_E10	ordinary_muscle_development	ENSGALG00010005885	SLITRK5	0.376570652	0.366688062	1.026950946	0.304443563	0.418126724	positive
PRJNA729271	stage_E14_vs_E10	ordinary_muscle_development	ENSGALG00010005887	CCNH	-0.20214154	0.08365471	-2.41637966	0.015675707	0.03524967	negative
PRJNA729271	stage_E14_vs_E10	ordinary_muscle_development	ENSGALG00010005888	IMPG2	-0.153576258	0.22700003	-0.676547301	0.498693225	0.607748525	negative
PRJNA729271	stage_E14_vs_E10	ordinary_muscle_development	ENSGALG00010005890	SNX32	0.018650733	0.088752333	0.210143585	0.833555609	0.883923047	positive
PRJNA729271	stage_E14_vs_E10	ordinary_muscle_development	ENSGALG00010005891	RNF6	-0.300016312	0.117047453	-2.563202408	0.010371157	0.024551677	negative
PRJNA729271	stage_E14_vs_E10	ordinary_muscle_development	ENSGALG00010005892	ENSGALG00010005892	0.52253701	0.848823342	0.615601603	0.538157493	0.64446405	positive
PRJNA729271	stage_E14_vs_E10	ordinary_muscle_development	ENSGALG00010005893	SFT2D1	0.039004191	0.066122593	0.589876909	0.555273176	0.659782334	positive
PRJNA729271	stage_E14_vs_E10	ordinary_muscle_development	ENSGALG00010005895	MLLT10	0.029888146	0.099091242	0.301622483	0.762939867	0.830927445	positive
PRJNA729271	stage_E14_vs_E10	ordinary_muscle_development	ENSGALG00010005899	ENSGALG00010005899	-0.221709389	0.087659981	-2.529197322	0.011432373	0.026809942	negative
PRJNA729271	stage_E14_vs_E10	ordinary_muscle_development	ENSGALG00010005900	SPTSSA	-0.222249398	0.086447014	-2.57093204	0.010142523	0.024100962	negative
PRJNA729271	stage_E14_vs_E10	ordinary_muscle_development	ENSGALG00010005901	ENSGALG00010005901	0.105517932	0.243593316	0.433172525	0.664889437	0.753530876	positive
PRJNA729271	stage_E14_vs_E10	ordinary_muscle_development	ENSGALG00010005902	EAPP	-0.310361857	0.099076739	-3.132540086	0.001733008	0.005132805	negative
PRJNA729271	stage_E14_vs_E10	ordinary_muscle_development	ENSGALG00010005903	PRR18	-0.255998891	0.232379693	-1.101640546	0.270617974	0.380392507	negative
PRJNA729271	stage_E14_vs_E10	ordinary_muscle_development	ENSGALG00010005905	CITED2	-0.811991273	0.202907664	-4.001777253	6.28685E-05	0.000256988	negative
PRJNA729271	stage_E14_vs_E10	ordinary_muscle_development	ENSGALG00010005909	BRMS1L	-0.249188987	0.100362529	-2.482888683	0.013032181	0.03004158	negative
PRJNA729271	stage_E14_vs_E10	ordinary_muscle_development	ENSGALG00010005910	ENSGALG00010005910	0.17514068	0.4825061	0.362981277	0.716618871	0.795051779	positive
PRJNA729271	stage_E14_vs_E10	ordinary_muscle_development	ENSGALG00010005911	TBXT	-1.148481758	0.397740995	-2.887511657	0.003883022	0.01041147	negative
PRJNA729271	stage_E14_vs_E10	ordinary_muscle_development	ENSGALG00010005913	NFKBIA	0.631407104	0.143729913	4.393011101	1.11791E-05	5.19364E-05	positive
PRJNA729271	stage_E14_vs_E10	ordinary_muscle_development	ENSGALG00010005915	PAPSS1	-1.357324675	0.098682867	-13.75441066	4.79169E-43	4.5101E-41	negative
PRJNA729271	stage_E14_vs_E10	ordinary_muscle_development	ENSGALG00010005926	NUDT5	-0.396936372	0.231546956	-1.714280245	0.08647727	0.150895392	negative
PRJNA729271	stage_E14_vs_E10	ordinary_muscle_development	ENSGALG00010005934	SLITRK6	-3.99547167	0.274353843	-14.56320651	4.81455E-48	6.23097E-46	negative
PRJNA729271	stage_E14_vs_E10	ordinary_muscle_development	ENSGALG00010005935	ATP8A2	2.528983046	0.15458403	16.35992445	3.69586E-60	9.07992E-58	positive
PRJNA729271	stage_E14_vs_E10	ordinary_muscle_development	ENSGALG00010005941	TXLNB	3.29021122	0.181635302	18.11438185	2.45414E-73	9.1212E-71	positive
PRJNA729271	stage_E14_vs_E10	ordinary_muscle_development	ENSGALG00010005943	PROX2	1.571066679	0.647085844	2.427910753	0.01518608	0.034286951	positive
PRJNA729271	stage_E14_vs_E10	ordinary_muscle_development	ENSGALG00010005949	SLITRK1	0.305959164	0.348557728	0.877786203	0.380059753	0.496059237	positive
PRJNA729271	stage_E14_vs_E10	ordinary_muscle_development	ENSGALG00010005956	TXNL4B	0.000477665	0.111327345	0.004290631	0.996576582	0.998160417	positive
PRJNA729271	stage_E14_vs_E10	ordinary_muscle_development	ENSGALG00010005963	UGCG	0.585091583	0.103329986	5.662360038	1.49305E-08	1.06452E-07	positive
PRJNA729271	stage_E14_vs_E10	ordinary_muscle_development	ENSGALG00010005964	HSPA14	-0.124619573	0.112604587	-1.10670068	0.268423361	0.377747245	negative
PRJNA729271	stage_E14_vs_E10	ordinary_muscle_development	ENSGALG00010005966	PLXDC2	-1.649220849	0.156616667	-10.53030233	6.2633E-26	2.04474E-24	negative
PRJNA729271	stage_E14_vs_E10	ordinary_muscle_development	ENSGALG00010005967	GJD2	0.327364568	0.2701097	1.211968944	0.225524269	0.32862752	positive
PRJNA729271	stage_E14_vs_E10	ordinary_muscle_development	ENSGALG00010005969	HECA	-0.021460537	0.065985805	-0.325229598	0.745007309	0.817106609	negative
PRJNA729271	stage_E14_vs_E10	ordinary_muscle_development	ENSGALG00010005970	SHISA2	1.650142149	0.118392531	13.93789058	3.72796E-44	3.8324E-42	positive
PRJNA729271	stage_E14_vs_E10	ordinary_muscle_development	ENSGALG00010005975	ABRACL	-0.527672061	0.103438191	-5.101327246	3.3728E-07	1.99954E-06	negative
PRJNA729271	stage_E14_vs_E10	ordinary_muscle_development	ENSGALG00010005978	VPS16	0.410297057	0.113862345	3.603448145	0.000314023	0.001118371	positive
PRJNA729271	stage_E14_vs_E10	ordinary_muscle_development	ENSGALG00010005979	SUV39H2	-0.037667611	0.091622983	-0.411115308	0.680987985	0.766733016	negative
PRJNA729271	stage_E14_vs_E10	ordinary_muscle_development	ENSGALG00010005981	AREL1	0.229233848	0.097591699	2.348907222	0.018828598	0.041239124	positive
PRJNA729271	stage_E14_vs_E10	ordinary_muscle_development	ENSGALG00010005984	IDH3B	0.736660388	0.117723238	6.25756137	3.91044E-10	3.3679E-09	positive
PRJNA729271	stage_E14_vs_E10	ordinary_muscle_development	ENSGALG00010005985	ENSGALG00010005985	-3.304049913	0.321136425	-10.28861771	7.93067E-25	2.34124E-23	negative
PRJNA729271	stage_E14_vs_E10	ordinary_muscle_development	ENSGALG00010005988	NUP58	-0.071498941	0.088205197	-0.810597832	0.417596662	0.532184246	negative
PRJNA729271	stage_E14_vs_E10	ordinary_muscle_development	ENSGALG00010005989	FCF1	-0.034107037	0.098570665	-0.346016095	0.729330619	0.804968196	negative
PRJNA729271	stage_E14_vs_E10	ordinary_muscle_development	ENSGALG00010005990	SLC2A12	0.455808366	0.152766871	2.983685939	0.002847989	0.007935718	positive
PRJNA729271	stage_E14_vs_E10	ordinary_muscle_development	ENSGALG00010005991	FARP1	-0.893366919	0.088199194	-10.12896917	4.10959E-24	1.14555E-22	negative
PRJNA729271	stage_E14_vs_E10	ordinary_muscle_development	ENSGALG00010005997	ENSGALG00010005997	0.600846863	0.232615471	2.583004738	0.009794398	0.023411906	positive
PRJNA729271	stage_E14_vs_E10	ordinary_muscle_development	ENSGALG00010005998	YLPM1	-0.260737678	0.132993844	-1.960524404	0.049934528	0.094679721	negative
PRJNA729271	stage_E14_vs_E10	ordinary_muscle_development	ENSGALG00010005999	PDE10A	-0.144508114	0.293294198	-0.492707034	0.622219607	0.71733661	negative
PRJNA729271	stage_E14_vs_E10	ordinary_muscle_development	ENSGALG00010006001	NOP56	-0.014102519	0.139407952	-0.101160079	0.919423386	0.94417584	negative
PRJNA729271	stage_E14_vs_E10	ordinary_muscle_development	ENSGALG00010006004	DCLRE1C	-0.536849764	0.135419338	-3.964350832	7.35959E-05	0.000296821	negative
PRJNA729271	stage_E14_vs_E10	ordinary_muscle_development	ENSGALG00010006009	MALRD1	-1.01244545	0.703181857	-1.439805991	0.149922296	0.237240576	negative
PRJNA729271	stage_E14_vs_E10	ordinary_muscle_development	ENSGALG00010006010	IPO5	-0.063702136	0.080637094	-0.789985508	0.429536232	0.543680864	negative
PRJNA729271	stage_E14_vs_E10	ordinary_muscle_development	ENSGALG00010006013	TRMT10C	-0.159491516	0.105029704	-1.518537234	0.12887902	0.209381462	negative
PRJNA729271	stage_E14_vs_E10	ordinary_muscle_development	ENSGALG00010006015	CDC123	-0.275939955	0.111199095	-2.481494622	0.013083268	0.030135385	negative
PRJNA729271	stage_E14_vs_E10	ordinary_muscle_development	ENSGALG00010006016	MTMR6	-0.029296445	0.076165538	-0.384641739	0.700502866	0.782445021	negative
PRJNA729271	stage_E14_vs_E10	ordinary_muscle_development	ENSGALG00010006019	SLC10A2	-2.848588736	1.021071434	-2.789803574	0.005274003	0.013648754	negative
PRJNA729271	stage_E14_vs_E10	ordinary_muscle_development	ENSGALG00010006021	ENSGALG00010006021	0.160542382	0.125249594	1.281779657	0.199919952	0.298992954	positive
PRJNA729271	stage_E14_vs_E10	ordinary_muscle_development	ENSGALG00010006022	AFG2A	-0.403461521	0.088972824	-4.534660175	5.76963E-06	2.8187E-05	negative
PRJNA729271	stage_E14_vs_E10	ordinary_muscle_development	ENSGALG00010006024	AMER2	-3.734501544	0.44754766	-8.344366149	7.15968E-17	1.19562E-15	negative

PRJNA729271	stage_E14_vs_E10	ordinary_muscle_development	ENSGALG00010006025	TNFAIP3	0.573645511	0.148667132	3.858590017	0.000114043	0.000444728	positive
PRJNA729271	stage_E14_vs_E10	ordinary_muscle_development	ENSGALG00010006028	ARL5B	-0.503284194	0.127941127	-3.933717069	8.36422E-05	0.000333809	negative
PRJNA729271	stage_E14_vs_E10	ordinary_muscle_development	ENSGALG00010006029	HSDL2	0.0485621	0.134453083	0.361182492	0.717963029	0.796134022	positive
PRJNA729271	stage_E14_vs_E10	ordinary_muscle_development	ENSGALG00010006031	ERCC5	-0.05725914	0.096786835	-0.591600502	0.554118138	0.658862101	negative
PRJNA729271	stage_E14_vs_E10	ordinary_muscle_development	ENSGALG00010006033	NSUN6	-0.043087826	0.162491025	-0.265170501	0.790878119	0.852959697	negative
PRJNA729271	stage_E14_vs_E10	ordinary_muscle_development	ENSGALG00010006035	ADH1C	2.066625983	0.689127113	2.998903896	0.002709528	0.007598105	positive
PRJNA729271	stage_E14_vs_E10	ordinary_muscle_development	ENSGALG00010006037	METTL21EP	0.88470258	0.765596468	1.155572964	0.247855872	0.354586544	positive
PRJNA729271	stage_E14_vs_E10	ordinary_muscle_development	ENSGALG00010006038	ENSGALG00010006038	0.415508863	0.191886389	2.165389975	0.030357833	0.061968285	positive
PRJNA729271	stage_E14_vs_E10	ordinary_muscle_development	ENSGALG00010006039	PCNP	-0.26850643	0.124969508	-2.148575551	0.031668062	0.064253717	negative
PRJNA729271	stage_E14_vs_E10	ordinary_muscle_development	ENSGALG00010006041	QKI	0.362210578	0.085701802	4.226405644	2.37454E-05	0.000104395	positive
PRJNA729271	stage_E14_vs_E10	ordinary_muscle_development	ENSGALG00010006047	ZBTB11	-0.365932595	0.134924146	-2.712135719	0.006685122	0.016738787	negative
PRJNA729271	stage_E14_vs_E10	ordinary_muscle_development	ENSGALG00010006049	AIMP1	-0.06047065	0.123851204	-0.488252421	0.625371058	0.72016791	negative
PRJNA729271	stage_E14_vs_E10	ordinary_muscle_development	ENSGALG00010006052	RALGAPA1	-0.378167814	0.08130334	-4.651319548	3.29818E-06	1.67392E-05	negative
PRJNA729271	stage_E14_vs_E10	ordinary_muscle_development	ENSGALG00010006053	TMEM243	-0.639374098	0.23008258	-2.77888964	0.005454505	0.014045666	negative
PRJNA729271	stage_E14_vs_E10	ordinary_muscle_development	ENSGALG00010006054	ENSGALG00010006054	2.370599317	2.217495731	1.069043464	0.285050085	0.396754463	positive
PRJNA729271	stage_E14_vs_E10	ordinary_muscle_development	ENSGALG00010006055	ENSGALG00010006055	1.239409747	0.583708889	2.1233354	0.033725756	0.067781958	positive
PRJNA729271	stage_E14_vs_E10	ordinary_muscle_development	ENSGALG00010006059	BIVM	-0.325519791	0.054716655	-5.949190304	2.69472E-09	2.10793E-08	negative
PRJNA729271	stage_E14_vs_E10	ordinary_muscle_development	ENSGALG00010006060	ENSGALG00010006060	-0.102915838	0.371807143	-0.276798979	0.781934465	0.846020756	negative
PRJNA729271	stage_E14_vs_E10	ordinary_muscle_development	ENSGALG00010006061	RPL24	0.395653865	0.158992246	2.488510454	0.012827947	0.029646455	positive
PRJNA729271	stage_E14_vs_E10	ordinary_muscle_development	ENSGALG00010006062	ENSGALG00010006062	0.241241408	0.661922043	0.364455922	0.715517583	0.794136858	positive
PRJNA729271	stage_E14_vs_E10	ordinary_muscle_development	ENSGALG00010006063	POGLUT2	-0.439836538	0.100639348	-4.370423179	1.24006E-05	5.72806E-05	negative
PRJNA729271	stage_E14_vs_E10	ordinary_muscle_development	ENSGALG00010006064	DMTF1	-0.069567502	0.079965642	-0.869967408	0.384318216	0.500331645	negative
PRJNA729271	stage_E14_vs_E10	ordinary_muscle_development	ENSGALG00010006065	WDR54	0.585744056	0.154471235	3.79192964	0.000149481	0.000570042	positive
PRJNA729271	stage_E14_vs_E10	ordinary_muscle_development	ENSGALG00010006066	ARHGAP18	0.468960478	0.120110788	3.9043993	9.44597E-05	0.000373179	positive
PRJNA729271	stage_E14_vs_E10	ordinary_muscle_development	ENSGALG00010006068	PACRG	-0.188162133	0.271726712	-0.692468294	0.488643291	0.599575185	negative
PRJNA729271	stage_E14_vs_E10	ordinary_muscle_development	ENSGALG00010006069	TTR	1.72172402	1.409454678	1.221553305	0.221876596	0.324661948	positive
PRJNA729271	stage_E14_vs_E10	ordinary_muscle_development	ENSGALG00010006070	TMEM242	0.237630812	0.113746349	2.089129129	0.036696101	0.072784617	positive
PRJNA729271	stage_E14_vs_E10	ordinary_muscle_development	ENSGALG00010006071	TEX30	-0.20786937	0.238200516	-0.872665492	0.382845432	0.498906063	negative
PRJNA729271	stage_E14_vs_E10	ordinary_muscle_development	ENSGALG00010006072	ENSGALG00010006072	0.235854671	0.232963208	1.012411674	0.311341259	0.425103707	positive
PRJNA729271	stage_E14_vs_E10	ordinary_muscle_development	ENSGALG00010006073	CEP97	0.075875444	0.102425363	0.740787646	0.458822207	0.571752741	positive
PRJNA729271	stage_E14_vs_E10	ordinary_muscle_development	ENSGALG00010006075	SOX21	-1.951027638	0.362438221	-5.383062616	7.3229E-08	4.76844E-07	negative
PRJNA729271	stage_E14_vs_E10	ordinary_muscle_development	ENSGALG00010006076	PODXL	0.335343623	0.16633965	2.016017367	0.04379816	0.084669822	positive
PRJNA729271	stage_E14_vs_E10	ordinary_muscle_development	ENSGALG00010006077	TBCK	-0.141503928	0.099272047	-1.425415639	0.15403709	0.24258666	negative
PRJNA729271	stage_E14_vs_E10	ordinary_muscle_development	ENSGALG00010006078	SPATA13	-1.458482449	0.162678481	-8.965429502	3.09074E-19	6.25702E-18	negative
PRJNA729271	stage_E14_vs_E10	ordinary_muscle_development	ENSGALG00010006079	ENSGALG00010006079	-2.83438122	0.932258105	-3.040339584	0.002363115	0.006730862	negative
PRJNA729271	stage_E14_vs_E10	ordinary_muscle_development	ENSGALG00010006080	ENSGALG00010006080	-0.333559617	0.204238677	-1.633185357	0.102430019	0.173530051	negative
PRJNA729271	stage_E14_vs_E10	ordinary_muscle_development	ENSGALG00010006081	ZDHHC14	-0.124823235	0.078423114	-1.591663836	0.111460258	0.186345611	negative
PRJNA729271	stage_E14_vs_E10	ordinary_muscle_development	ENSGALG00010006083	CACNB2	-0.22029371	0.165343202	-1.332342107	0.182747797	0.278161221	negative
PRJNA729271	stage_E14_vs_E10	ordinary_muscle_development	ENSGALG00010006084	TMEM68	-0.380503105	0.147768013	-2.575003196	0.010023916	0.023869996	negative
PRJNA729271	stage_E14_vs_E10	ordinary_muscle_development	ENSGALG00010006085	DLST	0.635325241	0.089720148	7.081188091	1.42924E-12	1.59975E-11	positive
PRJNA729271	stage_E14_vs_E10	ordinary_muscle_development	ENSGALG00010006086	ENSGALG00010006086	-0.619682016	0.304400535	-2.035745489	0.041775919	0.081324462	negative
PRJNA729271	stage_E14_vs_E10	ordinary_muscle_development	ENSGALG00010006087	ENSGALG00010006087	-0.876239217	0.587619201	-1.491168458	0.135917273	0.218999652	negative
PRJNA729271	stage_E14_vs_E10	ordinary_muscle_development	ENSGALG00010006091	RTKN	0.663163683	0.100123743	6.623440759	3.50933E-11	3.40708E-10	positive
PRJNA729271	stage_E14_vs_E10	ordinary_muscle_development	ENSGALG00010006096	EDIL3	-0.924695473	0.236518541	-3.909610927	9.24449E-05	0.000366117	negative
PRJNA729271	stage_E14_vs_E10	ordinary_muscle_development	ENSGALG00010006100	RPS6KL1	0.408394619	0.197037756	2.072671889	0.038202826	0.07534013	positive
PRJNA729271	stage_E14_vs_E10	ordinary_muscle_development	ENSGALG00010006101	ENSGALG00010006101	0.297217783	0.114191431	2.602802865	0.00924651	0.022245339	positive
PRJNA729271	stage_E14_vs_E10	ordinary_muscle_development	ENSGALG00010006103	NXPE3	-0.274203669	0.115869901	-2.366478844	0.017958201	0.03961408	negative
PRJNA729271	stage_E14_vs_E10	ordinary_muscle_development	ENSGALG00010006106	SHOC1	1.945078095	0.368280862	5.281507394	1.28125E-07	8.01544E-07	positive
PRJNA729271	stage_E14_vs_E10	ordinary_muscle_development	ENSGALG00010006107	PGF	1.586291225	0.193287838	8.206885879	2.26999E-16	3.58817E-15	positive
PRJNA729271	stage_E14_vs_E10	ordinary_muscle_development	ENSGALG00010006108	MKLN1	0.153247069	0.060633638	2.527426581	0.011490185	0.026932443	positive
PRJNA729271	stage_E14_vs_E10	ordinary_muscle_development	ENSGALG00010006111	WBP1	0.441474469	0.083478953	5.288452421	1.23356E-07	7.73708E-07	positive
PRJNA729271	stage_E14_vs_E10	ordinary_muscle_development	ENSGALG00010006113	EIF2B2	-0.246781598	0.16813624	-1.467747809	0.142172739	0.227229069	negative
PRJNA729271	stage_E14_vs_E10	ordinary_muscle_development	ENSGALG00010006118	NPNT	-0.373032826	0.207019666	-1.801919759	0.071558031	0.128449989	negative
PRJNA729271	stage_E14_vs_E10	ordinary_muscle_development	ENSGALG00010006120	MOGS	0.254555193	0.133844497	1.901872691	0.0571878	0.106161788	positive
PRJNA729271	stage_E14_vs_E10	ordinary_muscle_development	ENSGALG00010006121	ENSGALG00010006121	0.152397042	0.321803157	0.47357224	0.635804975	0.729401909	positive
PRJNA729271	stage_E14_vs_E10	ordinary_muscle_development	ENSGALG00010006123	MLH3	-0.171166796	0.102895971	-1.663493666	0.096213658	0.164731511	negative
PRJNA729271	stage_E14_vs_E10	ordinary_muscle_development	ENSGALG00010006126	ENSGALG00010006126	2.006986704	0.285396395	7.03227771	2.03189E-12	2.24826E-11	positive
PRJNA729271	stage_E14_vs_E10	ordinary_muscle_development	ENSGALG00010006128	ACYP1	0.212705034	0.123583157	1.721149056	0.085223779	0.149085045	positive
PRJNA729271	stage_E14_vs_E10	ordinary_muscle_development	ENSGALG00010006131	ENSGALG00010006131	1.129461316	0.123974909	9.11040243	8.2077E-20	1.72922E-18	positive
PRJNA729271	stage_E14_vs_E10	ordinary_muscle_development	ENSGALG00010006132	MIPEP	0.035765127	0.132365669	0.270199419	0.78700684	0.850176192	positive
PRJNA729271	stage_E14_vs_E10	ordinary_muscle_development	ENSGALG00010006134	K123	0.033558351	0.797890758	0.042058829	0.966451801	0.977655424	positive
PRJNA729271	stage_E14_vs_E10	ordinary_muscle_development	ENSGALG00010006135	ZC2HC1C	-0.601278406	0.144798966	-4.152504824	3.28856E-05	0.000141321	negative
PRJNA729271	stage_E14_vs_E10	ordinary_muscle_development	ENSGALG00010006139	GAREM1	-0.838080027	0.132282598	-6.335527428	2.36531E-10	2.09439E-09	negative
PRJNA729271	stage_E14_vs_E10	ordinary_muscle_development	ENSGALG00010006146	CCDC142	0.368551234	0.180386762	2.043116857	0.041040872	0.079990242	positive
PRJNA729271	stage_E14_vs_E10	ordinary_muscle_development	ENSGALG00010006147	ENSGALG00010006147	1.177562751	0.217116125	5.423654044	5.83928E-08	3.83674E-07	positive
PRJNA729271	stage_E14_vs_E10	ordinary_muscle_development	ENSGALG00010006151	ENSGALG00010006151	-0.19501417	0.155640963	-1.252974572	0.210214961	0.311147336	negative
PRJNA729271	stage_E14_vs_E10	ordinary_muscle_development	ENSGALG00010006155	TNFRSF19	0.229036478	0.192607149	1.189137988	0.234385372	0.33889436	positive
PRJNA729271	stage_E14_vs_E10	ordinary_muscle_development	ENSGALG00010006156	NFKBIZ	0.749294225	0.197449229	3.794870345	0.000147721	0.000563625	positive
PRJNA729271	stage_E14_vs_E10	ordinary_muscle_development	ENSGALG00010006157	SNX9	0.313374011	0.073247267	4.278303131	1.88323E-05	8.41995E-05	positive
PRJNA729271	stage_E14_vs_E10	ordinary_muscle_development	ENSGALG00010006163	ENSGALG00010006163	0.271746118	0.521274968	0.521310507	0.602150483	0.700607742	positive
PRJNA729271	stage_E14_vs_E10	ordinary_muscle_development	ENSGALG00010006172	ENSGALG00010006172	0.174222209	0.192008244	0.907368381	0.364212024	0.480325111	positive
PRJNA729271	stage_E14_vs_E10	ordinary_muscle_development	ENSGALG00010006173	TBPL1	-0.297671483	0.081186731	-3.666504127	0.000245889	0.00089417	negative
PRJNA729271	stage_E14_vs_E10	ordinary_muscle_development	ENSGALG00010006175	IL2RA	1.23053635	0.567070196	2.169989464	0.030007644	0.06144382	positive
PRJNA729271	stage_E14_vs_E10	ordinary_muscle_development	ENSGALG00010006176	MMR1L3	0.57968763	0.219895347	2.636197795	0.008384084	0.020462587	positive
PRJNA729271	stage_E14_vs_E10	ordinary_muscle_development	ENSGALG00010006177	NEK9	-0.011261622	0.113551382	-0.099176442	0.920998179	0.945391163	negative
PRJNA729271	stage_E14_vs_E10	ordinary_muscle_development	ENSGALG00010006178	RPS23	0.277859435	0.140850212	1.972729964	0.048526336	0.09253904	positive
PRJNA729271	stage_E14_vs_E10	ordinary_muscle_development	ENSGALG00010006180	CD200L	2.447946377	1.102645681	2.220066173			positive
PRJNA729271	stage_E14_vs_E10	ordinary_muscle_development	ENSGALG00010006183	PRKN	0.245757719	0.14372233	1.709948053	0.087275479	0.152031976	positive
PRJNA729271	stage_E14_vs_E10	ordinary_muscle_development	ENSGALG00010006184	LBX2	0.540907289	0.27854726	1.941886952	0.052150787	0.098247027	positive
PRJNA729271	stage_E14_vs_E10	ordinary_muscle_development	ENSGALG00010006187	TCF21	0.858043469	0.586390593	1.463262676	0.143395517	0.228760512	positive
PRJNA729271	stage_E14_vs_E10	ordinary_muscle_development	ENSGALG00010006188	TMED10	0.317503696	0.074562848	4.258202386	2.06077E-05	9.14602E-05	positive
PRJNA729271	stage_E14_vs_E10	ordinary_muscle_development	ENSGALG00010006193	RBM17	-0.177953416	0.098107576	-1.813860089	0.069699269	0.125611202	negative
PRJNA729271	stage_E14_vs_E10	ordinary_muscle_development	ENSGALG00010006197	ENSGALG00010006197	-1.163403455	0.434441756	-2.677927336	0.007407929	0.018311379	negative
PRJNA729271	stage_E14_vs_E10	ordinary_muscle_development	ENSGALG00010006198	ENSGALG00010006198	0.490150721	0.697998544	0.702223129	0.482540024	0.594157123	positive
PRJNA729271	stage_E14_vs_E10	ordinary_muscle_development	ENSGALG00010006200	GSTCD	-0.551929894	0.095464294	-5.78153224	7.40233E-09	5.47712E-08	negative
PRJNA729271	stage_E14_vs_E10	ordinary_muscle_development	ENSGALG00010006201	SSTR1	-3.479972052	0.379170679	-9.177851147	4.39779E-20	9.55712E-19	negative
PRJNA729271	stage_E14_vs_E10	ordinary_muscle_development	ENSGALG00010006202	SACS	0.718379973	0.200173848	3.588780351	0.000332229	0.001177421	positive
PRJNA729271	stage_E14_vs_E10	ordinary_muscle_development	ENSGALG00010006203	MMR1L4	1.107790979	0.175174031	6.323945217	2.54968E-10	2.2453E-09	positive
PRJNA729271	stage_E14_vs_E10	ordinary_muscle_development	ENSGALG00010006205	CGGBP1	-0.045117399	0.072891675	-0.618965045	0.535939387	0.642756132	negative
PRJNA729271	stage_E14_vs_E10	ordinary_muscle_development	ENSGALG00010006207	ENSGALG00010006207	0.639699257	0.199400405	3.208114137	0.001336085	0.004082324	positive
PRJNA729271	stage_E14_vs_E10	ordinary_muscle_development	ENSGALG00010006212	FOS	0.444650501	0.371917148	1.195563323	0.231867011	0.336225723	positive
PRJNA729271	stage_E14_vs_E10	ordinary_muscle_development	ENSGALG00010006219	CLEC14A	-0.378400149	0.194919612	-1.941313888	0.052220216	0.098328401	negative
PRJNA729271	stage_E14_vs_E10	ordinary_muscle_development	ENSGALG00010006220	SGCG	1.395964671	0.170864279	8.170020576	3.08337E-16	4.8265E-15	positive
PRJNA729271	stage_E14_vs_E10	ordinary_muscle_development	ENSGALG00010006223	ATG10	-0.18098298	0.111631392	-1.621255244	0.104962909	0.177282378	negative
PRJNA729271	stage_E14_vs_E10	ordinary_muscle_development	ENSGALG00010006227	AGPAT4	-1.32082726	0.14491897	-9.114246806	7.92188E-20	1.67387E-18	negative
PRJNA729271	stage_E14_vs_E10	ordinary_muscle_development	ENSGALG00010006229	INTS12	-0.403902539	0.132527927	-3.047678686	0.002306164	0.006591963	negative
PRJNA729271	stage_E14_vs_E10	ordinary_muscle_development	ENSGALG00010006232	ZNF654	-0.157112939	0.103297376	-1.520977058	0.128265595	0.208618693	negative
PRJNA729271	stage_E14_vs_E10	ordinary_muscle_development	ENSGALG00010006236	C3orf38	-0.066964345	0.083107264	-0.80575802	0.420382388	0.53484093	negative
PRJNA729271	stage_E14_vs_E10	ordinary_muscle_development	ENSGALG00010006237	ENSGALG00010006237	0.641752858	0.310395152	2.067535056	0.038683764	0.076205649	positive
PRJNA729271	stage_E14_vs_E10	ordinary_muscle_development	ENSGALG00010006239	EYA4	0.869309078	0.162308218	5.355915357	8.51244E-08	5.48634E-07	positive
PRJNA729271	stage_E14_vs_E10	ordinary_muscle_development	ENSGALG00010006241	MRC1	-1.355284181	0.254774095	-5.319552534	1.04023E-07	6.6132E-07	negative
PRJNA729271	stage_E14_vs_E10	ordinary_muscle_development	ENSGALG00010006242	ENSGALG00010006242	0.020872755	0.559237458	0.037323599	0.970226989	0.979621079	positive
PRJNA729271	stage_E14_vs_E10	ordinary_muscle_development	ENSGALG00010006244	EPHA3	-0.601190486	0.161894788	-3.713464113	0.000204441	0.00075751	negative
PRJNA729271	stage_E14_vs_E10	ordinary_muscle_development	ENSGALG00010006245	PFKFB3	-0.641675657	0.510054613	-1.258052844	0.208372646	0.308838258	negative
PRJNA729271	stage_E14_vs_E10	ordinary_muscle_development	ENSGALG00010006246	SUPT20H	-0.033739393	0.098470181	-0.342635637	0.731872589	0.806852234	negative
PRJNA729271	stage_E14_vs_E10	ordinary_muscle_development	ENSGALG00010006249	C9orf72	0.030748602	0.104448988	0.294388702	0.768460899	0.835433941	positive
PRJNA729271	stage_E14_vs_E10	ordinary_muscle_development	ENSGALG00010006251	SEMA3E	-3.13639304	0.370948396	-8.455065639	2.7893E-17	4.80178E-16	negative
PRJNA729271	stage_E14_vs_E10	ordinary_muscle_development	ENSGALG00010006252	COX7C	0.203083595	0.136658921	1.486061745	0.13726279	0.220628093	positive
PRJNA729271	stage_E14_vs_E10	ordinary_muscle_development	ENSGALG00010006254	PROS1	0.614493351	0.137293843	4.475753154	7.61424E-06	3.64132E-05	positive
PRJNA729271	stage_E14_vs_E10	ordinary_muscle_development	ENSGALG00010006259	PPA2	-0.003478739	0.08702245	-0.039975185	0.96811291	0.978469436	negative
PRJNA729271	stage_E14_vs_E10	ordinary_muscle_development	ENSGALG00010006260	MAP3K4	-0.543992441	0.160406334	-3.391340157	0.000695517	0.002276766	negative
PRJNA729271	stage_E14_vs_E10	ordinary_muscle_development	ENSGALG00010006261	PCLO	-1.572721493	0.480693681	-3.271774842	0.001068747	0.00334517	negative
PRJNA729271	stage_E14_vs_E10	ordinary_muscle_development	ENSGALG00010006263	EXOSC8	-0.136924143	0.148253178	-0.923583187	0.355703371	0.472110645	negative
PRJNA729271	stage_E14_vs_E10	ordinary_muscle_development	ENSGALG00010006266	CENPW	-0.21317394	0.213661932	-0.997716057	0.318417065	0.432482698	negative
PRJNA729271	stage_E14_vs_E10	ordinary_muscle_development	ENSGALG00010006269	ENSGALG00010006269	-1.185404625	1.39708246	-0.848485798	0.396167479	0.511622203	negative
PRJNA729271	stage_E14_vs_E10	ordinary_muscle_development	ENSGALG00010006270	STAM	-0.048410864	0.092140777	-0.525401077	0.599304405	0.698698411	negative
PRJNA729271	stage_E14_vs_E10	ordinary_muscle_development	ENSGALG00010006273	ARL13B	-0.270256173	0.097532623	-2.770931032	0.005589626	0.014355357	negative
PRJNA729271	stage_E14_vs_E10	ordinary_muscle_development	ENSGALG00010006277	GNG10	-0.700370549	0.156006629	-4.489364035	7.14361E-06	3.43324E-05	negative
PRJNA729271	stage_E14_vs_E10	ordinary_muscle_development	ENSGALG00010006279	HACD1	1.751884203	0.141586702	12.3732256	3.64869E-35	2.042E-33	positive
PRJNA729271	stage_E14_vs_E10	ordinary_muscle_development	ENSGALG00010006281	TRMT11	0.281940726	0.079749756	3.535317703	0.000407285	0.00140931	positive
PRJNA729271	stage_E14_vs_E10	ordinary_muscle_development	ENSGALG00010006283	DNAJC25	-0.309711806	0.107177185	-2.88971767	0.00385588	0.010342519	negative
PRJNA729271	stage_E14_vs_E10	ordinary_muscle_development	ENSGALG00010006285	FZD8	-0.608325108	0.153159088	-3.971851205	7.13163E-05	0.00028859	negative
PRJNA729271	stage_E14_vs_E10	ordinary_muscle_development	ENSGALG00010006287	ECPAS	0.104717426	0.122624655	0.853967137	0.393123198	0.508460851	positive
PRJNA729271	stage_E14_vs_E10	ordinary_muscle_development	ENSGALG00010006288	ARF5	-0.596085475	0.160895064	-3.704808967	0.00021155	0.000781054	negative
PRJNA729271	stage_E14_vs_E10	ordinary_muscle_development	ENSGALG00010006289	SKIDA1	-0.905025756	0.130985041	-6.909382547	4.86768E-12	5.13889E-11	negative
PRJNA729271	stage_E14_vs_E10	ordinary_muscle_development	ENSGALG00010006291	ENSGALG00010006291	3.520169119	0.190196056	18.50810791	1.77631E-76	6.7757E-74	positive
PRJNA729271	stage_E14_vs_E10	ordinary_muscle_development	ENSGALG00010006293	SLC22A3	-2.474567236	0.500377554	-4.945400163	7.59876E-07	4.26419E-06	negative
PRJNA729271	stage_E14_vs_E10	ordinary_muscle_development	ENSGALG00010006300	GCC1	0.350112872	0.117606216	2.976992909	0.002910907	0.008092367	positive
PRJNA729271	stage_E14_vs_E10	ordinary_muscle_development	ENSGALG00010006301	ENSGALG00010006301	0.223507982	0.14521229	1.539180898	0.12376014	0.202563598	positive
PRJNA729271	stage_E14_vs_E10	ordinary_muscle_development	ENSGALG00010006310	ALG5	-0.128213896	0.108973861	-1.176556423	0.239372599	0.345107005	negative
PRJNA729271	stage_E14_vs_E10	ordinary_muscle_development	ENSGALG00010006311	ABCD4	-0.034155658	0.110965475	-0.307804367	0.758231198	0.82777638	negative

PRJNA729271	stage_E14_vs_E10	ordinary_muscle_development	ENSGALG00010006323	ANAPC10	-0.108577045	0.104041529	-1.043593325	0.296673582	0.40998032	negative
PRJNA729271	stage_E14_vs_E10	ordinary_muscle_development	ENSGALG00010006327	DENND6B	0.544167593	0.108611927	5.010201055	5.43732E-07	3.11764E-06	positive
PRJNA729271	stage_E14_vs_E10	ordinary_muscle_development	ENSGALG00010006328	SYNDIG1L	-0.906755344	0.427052267	-2.123288912	0.033729649	0.067781958	negative
PRJNA729271	stage_E14_vs_E10	ordinary_muscle_development	ENSGALG00010006330	STX19	-0.003213635	0.414189024	-0.007758861	0.993809387	0.996501336	negative
PRJNA729271	stage_E14_vs_E10	ordinary_muscle_development	ENSGALG00010006331	SMAD9	-1.592986796	0.156085876	-10.20583567	1.86707E-24	5.35904E-23	negative
PRJNA729271	stage_E14_vs_E10	ordinary_muscle_development	ENSGALG00010006336	ALDH8A1	0.755188013	0.885292881	0.853037485	0.393638517	0.508945706	positive
PRJNA729271	stage_E14_vs_E10	ordinary_muscle_development	ENSGALG00010006339	ENSGALG00010006339	-1.430717131	0.973134675	-1.470214932	0.141503552	0.2263401	negative
PRJNA729271	stage_E14_vs_E10	ordinary_muscle_development	ENSGALG00010006341	HHIP	-2.020457141	0.222237972	-9.091412807	9.77603E-20	2.0507E-18	negative
PRJNA729271	stage_E14_vs_E10	ordinary_muscle_development	ENSGALG00010006342	RFXAP	-0.379313437	0.084541035	-4.486737549	7.23221E-06	3.47007E-05	negative
PRJNA729271	stage_E14_vs_E10	ordinary_muscle_development	ENSGALG00010006343	SEC23A	0.031617513	0.094527502	0.334479519	0.738017743	0.811651532	positive
PRJNA729271	stage_E14_vs_E10	ordinary_muscle_development	ENSGALG00010006344	HINT3	0.103581222	0.104939218	0.987059217	0.323613609	0.438226762	positive
PRJNA729271	stage_E14_vs_E10	ordinary_muscle_development	ENSGALG00010006345	SMC2	-0.352873317	0.249493808	-1.414357012	0.157257105	0.246547061	negative
PRJNA729271	stage_E14_vs_E10	ordinary_muscle_development	ENSGALG00010006346	NSUN3	0.032772971	0.129128297	0.253801617	0.799648824	0.859150842	positive
PRJNA729271	stage_E14_vs_E10	ordinary_muscle_development	ENSGALG00010006347	PPP6R2	0.435155903	0.135825397	3.20378894	0.001356319	0.004137172	positive
PRJNA729271	stage_E14_vs_E10	ordinary_muscle_development	ENSGALG00010006351	IGF2R	0.02920993	0.174170403	0.167708919	0.866812279	0.908608998	positive
PRJNA729271	stage_E14_vs_E10	ordinary_muscle_development	ENSGALG00010006352	GEMIN2	-0.093659084	0.097296876	-0.962611422	0.335742563	0.450985864	negative
PRJNA729271	stage_E14_vs_E10	ordinary_muscle_development	ENSGALG00010006353	ABCE1	-0.369269473	0.080511538	-4.586541041	4.5065E-06	2.24319E-05	negative
PRJNA729271	stage_E14_vs_E10	ordinary_muscle_development	ENSGALG00010006355	PTGR1	0.046837297	0.151461204	0.309236265	0.757141809	0.826786991	positive
PRJNA729271	stage_E14_vs_E10	ordinary_muscle_development	ENSGALG00010006359	SGK1	-0.126017058	0.164490573	-0.766105047	0.4436138	0.557256437	negative
PRJNA729271	stage_E14_vs_E10	ordinary_muscle_development	ENSGALG00010006361	SERTM1	-0.914047057	0.25630463	-3.566252616	0.000362122	0.001270935	negative
PRJNA729271	stage_E14_vs_E10	ordinary_muscle_development	ENSGALG00010006363	TRAPPC6A	-0.062323942	0.097598424	-0.638575286	0.523099263	0.630966449	negative
PRJNA729271	stage_E14_vs_E10	ordinary_muscle_development	ENSGALG00010006372	SBF1	-0.053102415	0.113212926	-0.469049045	0.639034579	0.731890505	negative
PRJNA729271	stage_E14_vs_E10	ordinary_muscle_development	ENSGALG00010006378	CAAP1	-0.509020879	0.100558918	-5.061916832	4.15062E-07	2.42692E-06	negative
PRJNA729271	stage_E14_vs_E10	ordinary_muscle_development	ENSGALG00010006381	SPART	0.710973373	0.156654336	4.538485117	5.66598E-06	2.77367E-05	positive
PRJNA729271	stage_E14_vs_E10	ordinary_muscle_development	ENSGALG00010006382	PNN	-0.411143443	0.19626989	-2.094786128	0.036190009	0.071938313	negative
PRJNA729271	stage_E14_vs_E10	ordinary_muscle_development	ENSGALG00010006387	OTUD4	-0.247346609	0.135297088	-1.828173932	0.067523452	0.122297886	negative
PRJNA729271	stage_E14_vs_E10	ordinary_muscle_development	ENSGALG00010006404	SMAD1	0.285101575	0.108755736	2.621485416	0.008754751	0.021226045	positive
PRJNA729271	stage_E14_vs_E10	ordinary_muscle_development	ENSGALG00010006408	ENSGALG00010006408	-0.068411954	0.64466207	-0.106120644	0.915486632	0.941362463	negative
PRJNA729271	stage_E14_vs_E10	ordinary_muscle_development	ENSGALG00010006411	RGS17	-1.615477564	0.375014441	-4.307774281	1.64906E-05	7.44644E-05	negative
PRJNA729271	stage_E14_vs_E10	ordinary_muscle_development	ENSGALG00010006421	LMF2	0.118448148	0.111955761	1.057990648	0.290059706	0.402472518	positive
PRJNA729271	stage_E14_vs_E10	ordinary_muscle_development	ENSGALG00010006434	MMAA	0.122809151	0.115495728	1.063322022	0.287635942	0.39985451	positive
PRJNA729271	stage_E14_vs_E10	ordinary_muscle_development	ENSGALG00010006446	LSAMP	-0.99128249	0.184175669	-5.382266251	7.35539E-08	4.78744E-07	negative
PRJNA729271	stage_E14_vs_E10	ordinary_muscle_development	ENSGALG00010006454	SOD2	0.181813444	0.105624174	1.72132418	0.085192014	0.149065457	positive
PRJNA729271	stage_E14_vs_E10	ordinary_muscle_development	ENSGALG00010006457	NCAPH2	0.055231316	0.196123015	0.281615677	0.778238218	0.842995016	positive
PRJNA729271	stage_E14_vs_E10	ordinary_muscle_development	ENSGALG00010006463	SCO2	0.322979389	0.132793292	2.432196561	0.01500756	0.033968549	positive
PRJNA729271	stage_E14_vs_E10	ordinary_muscle_development	ENSGALG00010006465	PLAA	-0.248428513	0.115855611	-2.144294176	0.032009323	0.064810049	negative
PRJNA729271	stage_E14_vs_E10	ordinary_muscle_development	ENSGALG00010006472	DCLK1	-0.659567508	0.322508573	-2.0451162	0.040843404	0.079691094	negative
PRJNA729271	stage_E14_vs_E10	ordinary_muscle_development	ENSGALG00010006478	ENSGALG00010006478	-0.251220787	0.132439831	-1.896867325	0.057845451	0.107221203	negative
PRJNA729271	stage_E14_vs_E10	ordinary_muscle_development	ENSGALG00010006480	IFT74	-0.669721937	0.117567714	-5.696478327	1.22307E-08	8.81574E-08	negative
PRJNA729271	stage_E14_vs_E10	ordinary_muscle_development	ENSGALG00010006486	CEP43	0.308450822	0.110419463	2.793446127	0.005214971	0.013512873	positive
PRJNA729271	stage_E14_vs_E10	ordinary_muscle_development	ENSGALG00010006492	GAP43	-2.020023454	0.458475161	-4.405960509	1.05316E-05	4.91962E-05	negative
PRJNA729271	stage_E14_vs_E10	ordinary_muscle_development	ENSGALG00010006496	UBE2H	0.103561699	0.113787567	0.910131935	0.362752934	0.478880126	positive
PRJNA729271	stage_E14_vs_E10	ordinary_muscle_development	ENSGALG00010006499	CCR6	-2.514108258	0.978667952	-2.568908333	0.010201944	0.024214373	negative
PRJNA729271	stage_E14_vs_E10	ordinary_muscle_development	ENSGALG00010006503	LRRC19	0.951238695	1.27540687	0.745831559	0.455769173	0.568877479	positive
PRJNA729271	stage_E14_vs_E10	ordinary_muscle_development	ENSGALG00010006506	ZNF827	-0.070731059	0.103641756	-0.682457163	0.494949921	0.604813615	negative
PRJNA729271	stage_E14_vs_E10	ordinary_muscle_development	ENSGALG00010006515	FBXO33	-0.308912771	0.102787968	-3.005339792	0.002652843	0.007455014	negative
PRJNA729271	stage_E14_vs_E10	ordinary_muscle_development	ENSGALG00010006516	NBEA	-0.998394131	0.126255555	-7.907724396	2.62136E-15	3.7883E-14	negative
PRJNA729271	stage_E14_vs_E10	ordinary_muscle_development	ENSGALG00010006517	ZBTB20	0.53195294	0.157175149	3.384459582	0.000713185	0.002329342	positive
PRJNA729271	stage_E14_vs_E10	ordinary_muscle_development	ENSGALG00010006521	ZNF410	-0.344456628	0.101998895	-3.377062357	0.000732644	0.002388615	negative
PRJNA729271	stage_E14_vs_E10	ordinary_muscle_development	ENSGALG00010006524	TAB2	-0.126693669	0.091187923	-1.389368954	0.164720586	0.255798682	negative
PRJNA729271	stage_E14_vs_E10	ordinary_muscle_development	ENSGALG00010006526	LSM6	-0.311188115	0.158653377	-1.961433923	0.049828426	0.094537046	negative
PRJNA729271	stage_E14_vs_E10	ordinary_muscle_development	ENSGALG00010006528	ZC3HC1	-0.016077771	0.077884532	-0.20643086	0.836454367	0.885833715	negative
PRJNA729271	stage_E14_vs_E10	ordinary_muscle_development	ENSGALG00010006531	FAM161B	-0.343574099	0.130666779	-2.629391351	0.008553786	0.020817181	negative
PRJNA729271	stage_E14_vs_E10	ordinary_muscle_development	ENSGALG00010006532	KLHDC10	-0.03110779	0.10264618	-0.303058431	0.761845333	0.830457115	negative
PRJNA729271	stage_E14_vs_E10	ordinary_muscle_development	ENSGALG00010006537	DRD3	1.193638156	0.224250518	5.322788856	1.02188E-07	6.50514E-07	positive
PRJNA729271	stage_E14_vs_E10	ordinary_muscle_development	ENSGALG00010006538	TEK	-0.142075231	0.106826385	-1.329963857	0.18353018	0.2791469	negative
PRJNA729271	stage_E14_vs_E10	ordinary_muscle_development	ENSGALG00010006542	RFC3	-0.282412314	0.137956281	-2.047114582	0.040646836	0.079328919	negative
PRJNA729271	stage_E14_vs_E10	ordinary_muscle_development	ENSGALG00010006544	MOB3B	-1.079475278	0.132809151	-8.128018826	4.36363E-16	6.73598E-15	negative
PRJNA729271	stage_E14_vs_E10	ordinary_muscle_development	ENSGALG00010006546	SMOC2	-2.872389127	0.209554687	-13.7071099	9.2052E-43	8.49869E-41	negative
PRJNA729271	stage_E14_vs_E10	ordinary_muscle_development	ENSGALG00010006548	TMEM209	-0.246856689	0.07660925	-3.222283056	0.001271734	0.003911264	negative
PRJNA729271	stage_E14_vs_E10	ordinary_muscle_development	ENSGALG00010006549	ZDHHC23	-1.135750953	0.185059449	-6.137222153	8.39769E-10	6.93587E-09	negative
PRJNA729271	stage_E14_vs_E10	ordinary_muscle_development	ENSGALG00010006552	CPA2	0.472507593	1.830263566	0.258163688	0.796280581	0.856682774	positive
PRJNA729271	stage_E14_vs_E10	ordinary_muscle_development	ENSGALG00010006557	COQ6	0.387621611	0.107564948	3.603605254	0.000313834	0.001117969	positive
PRJNA729271	stage_E14_vs_E10	ordinary_muscle_development	ENSGALG00010006558	USP10	-0.075422117	0.088099394	-0.856102571	0.391941047	0.507585182	negative
PRJNA729271	stage_E14_vs_E10	ordinary_muscle_development	ENSGALG00010006564	SLC10A7	-0.761333284	0.143421918	-5.308346847	1.10624E-07	7.00828E-07	negative
PRJNA729271	stage_E14_vs_E10	ordinary_muscle_development	ENSGALG00010006566	CPA5	0.472415352	2.919585631	0.161809041	0.871456233	0.911645359	positive
PRJNA729271	stage_E14_vs_E10	ordinary_muscle_development	ENSGALG00010006568	ENTPD5	-0.821315447	0.199216849	-4.122720798	3.74423E-05	0.000159064	negative
PRJNA729271	stage_E14_vs_E10	ordinary_muscle_development	ENSGALG00010006570	ENSGALG00010006570	0.304476679	0.49107913	0.620015513	0.535247574	0.642305951	positive
PRJNA729271	stage_E14_vs_E10	ordinary_muscle_development	ENSGALG00010006574	CPA1	1.535676946	2.663198497	0.576628797	0.564190241	0.666824653	positive
PRJNA729271	stage_E14_vs_E10	ordinary_muscle_development	ENSGALG00010006575	SSBP2	-0.661816645	0.116344508	-5.688421891	1.28219E-08	9.21433E-08	negative
PRJNA729271	stage_E14_vs_E10	ordinary_muscle_development	ENSGALG00010006576	CCDC191	0.255267115	0.106447107	2.398065312	0.016481926	0.03679432	positive
PRJNA729271	stage_E14_vs_E10	ordinary_muscle_development	ENSGALG00010006580	ALDH6A1	0.297038919	0.138691375	2.141725958	0.032215541	0.065136597	positive
PRJNA729271	stage_E14_vs_E10	ordinary_muscle_development	ENSGALG00010006581	CEP41	-0.35504595	0.085650391	-4.145292819	3.3938E-05	0.000145241	negative
PRJNA729271	stage_E14_vs_E10	ordinary_muscle_development	ENSGALG00010006585	TTC29	-0.926268702	0.185074192	-5.004850712	5.59053E-07	3.1979E-06	negative
PRJNA729271	stage_E14_vs_E10	ordinary_muscle_development	ENSGALG00010006588	ACOT12	-1.692428285	0.683838738	-2.474893845	0.013327578	0.030634832	negative
PRJNA729271	stage_E14_vs_E10	ordinary_muscle_development	ENSGALG00010006592	STARD13	0.696634992	0.114119653	6.104426131	1.03171E-09	8.43463E-09	positive
PRJNA729271	stage_E14_vs_E10	ordinary_muscle_development	ENSGALG00010006598	ENSGALG00010006598	0.012844635	0.151592683	0.084731235	0.932475063	0.953697283	positive
PRJNA729271	stage_E14_vs_E10	ordinary_muscle_development	ENSGALG00010006602	EDNRA	-0.659826208	0.118113037	-5.586396079	2.3183E-08	1.61306E-07	negative
PRJNA729271	stage_E14_vs_E10	ordinary_muscle_development	ENSGALG00010006603	MEST	-0.240019926	0.215378025	-1.114412323	0.265102339	0.373872193	negative
PRJNA729271	stage_E14_vs_E10	ordinary_muscle_development	ENSGALG00010006606	SULT	0.250686763	1.187837698	0.211044626			positive
PRJNA729271	stage_E14_vs_E10	ordinary_muscle_development	ENSGALG00010006608	KL	2.35109241	0.410085935	5.733170069	9.85707E-09	7.18703E-08	positive
PRJNA729271	stage_E14_vs_E10	ordinary_muscle_development	ENSGALG00010006614	TMEM184C	-0.294693088	0.054027871	-5.454464236	4.91207E-08	3.2601E-07	negative
PRJNA729271	stage_E14_vs_E10	ordinary_muscle_development	ENSGALG00010006615	GRAMD1C	-0.26351693	0.099988796	-2.635464563	0.00840222	0.020499946	negative
PRJNA729271	stage_E14_vs_E10	ordinary_muscle_development	ENSGALG00010006616	COPG2	-0.004467145	0.067027069	-0.066646882	0.946862822	0.963012888	negative
PRJNA729271	stage_E14_vs_E10	ordinary_muscle_development	ENSGALG00010006619	LIN52	-0.240039813	0.101319155	-2.369145438	0.017829241	0.039377548	negative
PRJNA729271	stage_E14_vs_E10	ordinary_muscle_development	ENSGALG00010006621	DACT2	-1.545241011	0.209293618	-7.383125315	1.54616E-13	1.91881E-12	negative
PRJNA729271	stage_E14_vs_E10	ordinary_muscle_development	ENSGALG00010006623	ENSGALG00010006623	0.033878082	0.185489994	0.182641024	0.855079693	0.899708199	positive
PRJNA729271	stage_E14_vs_E10	ordinary_muscle_development	ENSGALG00010006627	SF3B5	0.061488912	0.10880345	0.565137517	0.571980232	0.674037911	positive
PRJNA729271	stage_E14_vs_E10	ordinary_muscle_development	ENSGALG00010006633	FBXO18	0.191849309	0.092018037	2.084909827	0.037077487	0.073460657	positive
PRJNA729271	stage_E14_vs_E10	ordinary_muscle_development	ENSGALG00010006634	PRMT9	0.028784237	0.095346832	0.30188981	0.762736064	0.830927445	positive
PRJNA729271	stage_E14_vs_E10	ordinary_muscle_development	ENSGALG00010006635	ENSGALG00010006635	-0.758310138	0.469016354	-1.616809586	0.10591939	0.178669071	negative
PRJNA729271	stage_E14_vs_E10	ordinary_muscle_development	ENSGALG00010006641	ENSGALG00010006641	-3.353176218	0.482404744	-6.950960282	3.62808E-12	3.91287E-11	negative
PRJNA729271	stage_E14_vs_E10	ordinary_muscle_development	ENSGALG00010006651	ENSGALG00010006651	0.441825012	0.109961634	4.017992426	5.86961E-05	0.000241088	positive
PRJNA729271	stage_E14_vs_E10	ordinary_muscle_development	ENSGALG00010006653	CFL2	1.377080125	0.104425779	13.18716637	1.04022E-39	7.65382E-38	positive
PRJNA729271	stage_E14_vs_E10	ordinary_muscle_development	ENSGALG00010006657	PDS5B	-0.18780912	0.087830598	-2.138310837	0.032491524	0.065603097	negative
PRJNA729271	stage_E14_vs_E10	ordinary_muscle_development	ENSGALG00010006664	ENSGALG00010006664	2.450032977	1.347012924	1.818863749	0.06893222	0.124476457	positive
PRJNA729271	stage_E14_vs_E10	ordinary_muscle_development	ENSGALG00010006670	ANKRD16	0.24779847	0.177459742	1.396364418	0.162604784	0.253109573	positive
PRJNA729271	stage_E14_vs_E10	ordinary_muscle_development	ENSGALG00010006673	BAZ1A	0.176345047	0.080596913	2.187987604	0.028670505	0.059173996	positive
PRJNA729271	stage_E14_vs_E10	ordinary_muscle_development	ENSGALG00010006678	ENSGALG00010006678	-3.711970511	1.263380199	-2.938126237	0.003302025	0.009037549	negative
PRJNA729271	stage_E14_vs_E10	ordinary_muscle_development	ENSGALG00010006681	CRISPLD2	-2.96981878	0.222979821	-13.31877819	1.8003E-40	1.40297E-38	negative
PRJNA729271	stage_E14_vs_E10	ordinary_muscle_development	ENSGALG00010006682	ARHGAP10	0.488035633	0.11928098	4.091479083	4.28631E-05	0.000180348	positive
PRJNA729271	stage_E14_vs_E10	ordinary_muscle_development	ENSGALG00010006685	ENSGALG00010006685	-0.416129007	0.11449243	-3.634554756	0.000278461	0.001002806	negative
PRJNA729271	stage_E14_vs_E10	ordinary_muscle_development	ENSGALG00010006694	ENSGALG00010006694	-2.19120321	0.58824243	-3.72500027	0.000195315	0.000727974	negative
PRJNA729271	stage_E14_vs_E10	ordinary_muscle_development	ENSGALG00010006701	LINGO2	-1.972917927	0.276773998	-7.128263277	1.01643E-12	1.15736E-11	negative
PRJNA729271	stage_E14_vs_E10	ordinary_muscle_development	ENSGALG00010006715	IRF8	0.052144267	0.162093116	0.321693287	0.747685064	0.81900609	positive
PRJNA729271	stage_E14_vs_E10	ordinary_muscle_development	ENSGALG00010006716	ENSGALG00010006716	0.187745268	0.097888207	1.917955938	0.055116598	0.102899935	positive
PRJNA729271	stage_E14_vs_E10	ordinary_muscle_development	ENSGALG00010006720	SRP54	-0.219235678	0.090851826	-2.413112501	0.015816937	0.03551766	negative
PRJNA729271	stage_E14_vs_E10	ordinary_muscle_development	ENSGALG00010006728	ENSGALG00010006728	-0.034950588	0.135938426	-0.257106021	0.79709693	0.857370139	negative
PRJNA729271	stage_E14_vs_E10	ordinary_muscle_development	ENSGALG00010006730	HAPLN1	-5.422456763	0.485310085	-11.17317965	5.51699E-29	2.23376E-27	negative
PRJNA729271	stage_E14_vs_E10	ordinary_muscle_development	ENSGALG00010006735	NR3C2	-0.958052808	0.11316824	-8.465739255	2.54533E-17	4.39744E-16	negative
PRJNA729271	stage_E14_vs_E10	ordinary_muscle_development	ENSGALG00010006739	VCAN	-0.025478558	0.253556745	-0.100484639	0.919959578	0.944525717	negative
PRJNA729271	stage_E14_vs_E10	ordinary_muscle_development	ENSGALG00010006740	BRCA2	-0.339698868	0.151598109	-2.240785653	0.025039963	0.052816394	negative
PRJNA729271	stage_E14_vs_E10	ordinary_muscle_development	ENSGALG00010006748	FAM177A1	0.192718127	0.077204205	2.496212817	0.012552727	0.029075067	positive
PRJNA729271	stage_E14_vs_E10	ordinary_muscle_development	ENSGALG00010006752	ZAR1L	-0.960190829	0.434372874	-2.210522082	0.02706895	0.056366101	negative
PRJNA729271	stage_E14_vs_E10	ordinary_muscle_development	ENSGALG00010006760	TASOR2	0.152140699	0.101959918	1.492161833	0.135656725	0.218628445	positive
PRJNA729271	stage_E14_vs_E10	ordinary_muscle_development	ENSGALG00010006761	PPP2R3C	-0.091974684	0.092412241	-0.995265161	0.319607317	0.433977336	negative
PRJNA729271	stage_E14_vs_E10	ordinary_muscle_development	ENSGALG00010006767	ENSGALG00010006767	3.655789314	0.338330341	10.80538417	3.24595E-27	1.16461E-25	positive
PRJNA729271	stage_E14_vs_E10	ordinary_muscle_development	ENSGALG00010006794	PERPA	-1.544387462	0.554706962	-2.784150135	0.005366819	0.013853297	negative
PRJNA729271	stage_E14_vs_E10	ordinary_muscle_development	ENSGALG00010006803	COX4I1	0.48816057	0.103930838	4.696975226	2.64042E-06	1.36494E-05	positive
PRJNA729271	stage_E14_vs_E10	ordinary_muscle_development	ENSGALG00010006804	PSMA6	0.108314576	0.101926091	1.06267762	0.287928173	0.40022237	positive
PRJNA729271	stage_E14_vs_E10	ordinary_muscle_development	ENSGALG00010006814	ENSGALG00010006814	-1.240592193	0.395377385	-3.137741913	0.001702547	0.005057053	negative
PRJNA729271	stage_E14_vs_E10	ordinary_muscle_development	ENSGALG00010006816	ENSGALG00010006816	2.245876664	0.53186964	4.222607378	2.41492E-05	0.000106074	positive
PRJNA729271	stage_E14_vs_E10	ordinary_muscle_development	ENSGALG00010006819	EMC8	0.144849355	0.131464199	1.101815982	0.270541681	0.380322148	positive
PRJNA729271	stage_E14_vs_E10	ordinary_muscle_development	ENSGALG00010006821	XRCC4	-0.087311459	0.122334123	-0.713713045	0.475404639	0.587617484	negative
PRJNA729271	stage_E14_vs_E10	ordinary_muscle_development	ENSGALG00010006828	GTF2A1	-0.158012685	0.10582321	-1.493176072	0.135391104	0.218321732	negative
PRJNA729271	stage_E14_vs_E10	ordinary_muscle_development	ENSGALG00010006833	LTV1	0.120337411	0.084932292	1.416862869	0.156523024	0.245612692	positive
PRJNA729271	stage_E14_vs_E10	ordinary_muscle_development	ENSGALG00010006835	FRY	-1.117750561	0.153920277	-7.261879846	3.81747E-13	4.5693E-12	negative
PRJNA729271	stage_E14_vs_E10	ordinary_muscle_development	ENSGALG00010006838	ASB13	0.010494658	0.126644647	0.08286697	0.933957318	0.954502666	positive
PRJNA729271	stage_E14_vs_E10	ordinary_muscle_development	ENSGALG00010006840	ENSGALG00010006840	0.020400808	0.270720748	0.075357387	0.939930363	0.957972902	positive
PRJNA729271	stage_E14_vs_E10	ordinary_muscle_development	ENSGALG00010006843	STON2	-1.141166615	0.199330086	-5.725009391	1.03428E-08	7.52983E-08	negative
PRJNA729271	stage_E14_vs_E10	ordinary_muscle_development	ENSGALG00010006845	CALML3	-1.737824862	0.508970973	-3.414388944	0.000639252	0.00210638	negative

PRJNA729271	stage_E14_vs_E10	ordinary_muscle_development	ENSGALG00010006847	SEL1L	0.067720235	0.086320759	0.784518531	0.432735913	0.546861993	positive
PRJNA729271	stage_E14_vs_E10	ordinary_muscle_development	ENSGALG00010006850	IRF2BPL	-0.039693858	0.159553177	-0.248781371	0.803529902	0.86173172	negative
PRJNA729271	stage_E14_vs_E10	ordinary_muscle_development	ENSGALG00010006851	TMEM167A	-0.034502198	0.103942812	-0.331934433	0.739938769	0.812900747	negative
PRJNA729271	stage_E14_vs_E10	ordinary_muscle_development	ENSGALG00010006853	NET1	0.141790782	0.083364481	1.700853656	0.088970471	0.15440936	positive
PRJNA729271	stage_E14_vs_E10	ordinary_muscle_development	ENSGALG00010006855	DCLK2	-0.056151107	0.122572473	-0.458105359	0.646876749	0.738945424	negative
PRJNA729271	stage_E14_vs_E10	ordinary_muscle_development	ENSGALG00010006865	EFCAB2	-0.203156924	0.314130703	-0.646727373	0.517808404	0.626170942	negative
PRJNA729271	stage_E14_vs_E10	ordinary_muscle_development	ENSGALG00010006877	GSE1	0.08046925	0.24146869	0.333249209	0.738946176	0.812268447	positive
PRJNA729271	stage_E14_vs_E10	ordinary_muscle_development	ENSGALG00010006883	LRBA	-0.117634402	0.069775425	-1.685900192	0.091815025	0.158443936	negative
PRJNA729271	stage_E14_vs_E10	ordinary_muscle_development	ENSGALG00010006885	RHOU	0.097002441	0.152914243	0.634358443	0.525846924	0.633489957	positive
PRJNA729271	stage_E14_vs_E10	ordinary_muscle_development	ENSGALG00010006901	TUBAL3	1.205854816	0.260862204	4.622573903	3.79008E-06	1.90094E-05	positive
PRJNA729271	stage_E14_vs_E10	ordinary_muscle_development	ENSGALG00010006903	B3GLCT	-0.883908509	0.144610448	-6.112341967	9.81795E-10	8.04018E-09	negative
PRJNA729271	stage_E14_vs_E10	ordinary_muscle_development	ENSGALG00010006912	ENSGALG00010006912	-0.249997372	0.178373123	-1.40154171	0.161052144	0.251340528	negative
PRJNA729271	stage_E14_vs_E10	ordinary_muscle_development	ENSGALG00010006913	HSPH1	-0.792743947	0.184733753	-4.291278303	1.77648E-05	7.98202E-05	negative
PRJNA729271	stage_E14_vs_E10	ordinary_muscle_development	ENSGALG00010006922	SMYD3	-0.202232986	0.155273618	-1.302429788	0.192769505	0.29027571	negative
PRJNA729271	stage_E14_vs_E10	ordinary_muscle_development	ENSGALG00010006923	ENSGALG00010006923	0.056977888	0.219936232	0.25906549	0.795584711	0.856363005	positive
PRJNA729271	stage_E14_vs_E10	ordinary_muscle_development	ENSGALG00010006926	ID4	-2.519417069	0.226006507	-11.14754219	7.36116E-29	2.96389E-27	negative
PRJNA729271	stage_E14_vs_E10	ordinary_muscle_development	ENSGALG00010006927	UCN3	-2.204170774	0.754491115	-2.921400569	0.003484615	0.009469346	negative
PRJNA729271	stage_E14_vs_E10	ordinary_muscle_development	ENSGALG00010006939	FBXO31	0.602781608	0.126977703	4.747145313	2.06308E-06	1.08506E-05	positive
PRJNA729271	stage_E14_vs_E10	ordinary_muscle_development	ENSGALG00010006942	ALOX5AP	-0.408571099	0.184753092	-2.211443898	0.027005113	0.056273593	negative
PRJNA729271	stage_E14_vs_E10	ordinary_muscle_development	ENSGALG00010006944	MAB21L2	-4.544142142	0.468084992	-9.707942393	2.7891E-22	7.13017E-21	negative
PRJNA729271	stage_E14_vs_E10	ordinary_muscle_development	ENSGALG00010006946	PLPPR1	-2.544308314	0.257251631	-9.890348626	4.58429E-23	1.20817E-21	negative
PRJNA729271	stage_E14_vs_E10	ordinary_muscle_development	ENSGALG00010006948	ENSGALG00010006948	-2.936707833	0.233466697	-12.57870123	2.76539E-36	1.65638E-34	negative
PRJNA729271	stage_E14_vs_E10	ordinary_muscle_development	ENSGALG00010006953	MAP1LC3B	-0.010485379	0.128193775	-0.081793198	0.934811165	0.954677816	negative
PRJNA729271	stage_E14_vs_E10	ordinary_muscle_development	ENSGALG00010006957	PALM2AKAP2	0.319077085	0.091198305	3.498717285	0.000467502	0.001597087	positive
PRJNA729271	stage_E14_vs_E10	ordinary_muscle_development	ENSGALG00010006958	KIF26B	-4.064262213	0.233438413	-17.41042601	6.87703E-68	2.16701E-65	negative
PRJNA729271	stage_E14_vs_E10	ordinary_muscle_development	ENSGALG00010006961	ZCCHC14	0.699821437	0.116046416	6.030530377	1.63422E-09	1.30801E-08	positive
PRJNA729271	stage_E14_vs_E10	ordinary_muscle_development	ENSGALG00010006963	ENSGALG00010006963	-0.26126948	0.434107482	-0.601854359	0.547271087	0.652628088	negative
PRJNA729271	stage_E14_vs_E10	ordinary_muscle_development	ENSGALG00010006966	PGBD5	-0.139882786	0.20213705	-0.692019529	0.488925065	0.599688383	negative
PRJNA729271	stage_E14_vs_E10	ordinary_muscle_development	ENSGALG00010006983	GTPBP2	0.262130631	0.124082143	2.112557252	0.034638681	0.069377875	positive
PRJNA729271	stage_E14_vs_E10	ordinary_muscle_development	ENSGALG00010006988	USPL1	-0.263329567	0.103953405	-2.533149994	0.011304257	0.026539554	negative
PRJNA729271	stage_E14_vs_E10	ordinary_muscle_development	ENSGALG00010006992	SLC35B4	-0.474213871	0.101702234	-4.66276748	3.11985E-06	1.59233E-05	negative
PRJNA729271	stage_E14_vs_E10	ordinary_muscle_development	ENSGALG00010006998	POLH	0.148278504	0.120939521	1.226055001	0.220177978	0.322665028	positive
PRJNA729271	stage_E14_vs_E10	ordinary_muscle_development	ENSGALG00010007000	LRGUK	-3.054302233	0.597365803	-5.112951256	3.17164E-07	1.88723E-06	negative
PRJNA729271	stage_E14_vs_E10	ordinary_muscle_development	ENSGALG00010007001	SNX30	-0.677697596	0.092293507	-7.342852326	2.09089E-13	2.56192E-12	negative
PRJNA729271	stage_E14_vs_E10	ordinary_muscle_development	ENSGALG00010007002	EXOC4	0.024261353	0.118963351	0.203939728	0.838400599	0.8872466	positive
PRJNA729271	stage_E14_vs_E10	ordinary_muscle_development	ENSGALG00010007004	ENSGALG00010007004	-0.838499222	0.606207249	-1.383189039	0.16660691	0.258036879	negative
PRJNA729271	stage_E14_vs_E10	ordinary_muscle_development	ENSGALG00010007007	ACSL1	0.150772295	0.217166854	0.694269368	0.487513297	0.598653443	positive
PRJNA729271	stage_E14_vs_E10	ordinary_muscle_development	ENSGALG00010007010	ENSGALG00010007010	-0.895596292	1.047030944	-0.855367549	0.392347703	0.507911043	negative
PRJNA729271	stage_E14_vs_E10	ordinary_muscle_development	ENSGALG00010007011	HMGB1	-0.348016061	0.101559266	-3.426728804	0.000610899	0.002023533	negative
PRJNA729271	stage_E14_vs_E10	ordinary_muscle_development	ENSGALG00010007014	ENSGALG00010007014	-0.756395622	0.58052411	-1.302952985	0.192590813	0.290036762	negative
PRJNA729271	stage_E14_vs_E10	ordinary_muscle_development	ENSGALG00010007017	SRD5A2	-2.656117052	0.407983501	-6.510354086	7.49739E-11	7.00675E-10	negative
PRJNA729271	stage_E14_vs_E10	ordinary_muscle_development	ENSGALG00010007021	SULT6B1	-1.358907643	0.268911101	-5.053371307	4.34079E-07	2.5269E-06	negative
PRJNA729271	stage_E14_vs_E10	ordinary_muscle_development	ENSGALG00010007025	ENSGALG00010007025	0.477902331	0.764439335	0.625167111	0.531861385	0.63935402	positive
PRJNA729271	stage_E14_vs_E10	ordinary_muscle_development	ENSGALG00010007028	KATNAL1	-0.321754873	0.13637934	-2.359264035	0.018311222	0.040270242	negative
PRJNA729271	stage_E14_vs_E10	ordinary_muscle_development	ENSGALG00010007033	CEBPZOS	0.833306876	0.18529558	4.49717623	6.88619E-06	3.32607E-05	positive
PRJNA729271	stage_E14_vs_E10	ordinary_muscle_development	ENSGALG00010007038	CENPU	-0.150172041	0.197340049	-0.760981066	0.446668377	0.56008953	negative
PRJNA729271	stage_E14_vs_E10	ordinary_muscle_development	ENSGALG00010007041	NAE1	-0.071500666	0.078226792	-0.914017614	0.36070759	0.476918409	negative
PRJNA729271	stage_E14_vs_E10	ordinary_muscle_development	ENSGALG00010007049	ENSGALG00010007049	-0.765752979	0.512182064	-1.495079645	0.134893658	0.217640646	negative
PRJNA729271	stage_E14_vs_E10	ordinary_muscle_development	ENSGALG00010007051	E2F3	-0.521659364	0.144272259	-3.615798133	0.000299424	0.0010727	negative
PRJNA729271	stage_E14_vs_E10	ordinary_muscle_development	ENSGALG00010007066	INIP	-0.723008976	0.152021911	-4.755952409	1.97513E-06	1.04145E-05	negative
PRJNA729271	stage_E14_vs_E10	ordinary_muscle_development	ENSGALG00010007067	CASP3	0.156366158	0.105791165	1.478064433	0.139390533	0.22355231	positive
PRJNA729271	stage_E14_vs_E10	ordinary_muscle_development	ENSGALG00010007068	NCAM2	-3.202513494	0.232627037	-13.76672949	4.04097E-43	3.82835E-41	negative
PRJNA729271	stage_E14_vs_E10	ordinary_muscle_development	ENSGALG00010007070	UBL3	0.131953221	0.052633201	2.507033945	0.012174902	0.028326678	positive
PRJNA729271	stage_E14_vs_E10	ordinary_muscle_development	ENSGALG00010007085	PRIMPOL	-0.071610773	0.160207494	-0.446987659	0.654883971	0.745507198	negative
PRJNA729271	stage_E14_vs_E10	ordinary_muscle_development	ENSGALG00010007090	CEBPZ	-0.20027591	0.092347259	-2.168726097	0.030103485	0.061605254	negative
PRJNA729271	stage_E14_vs_E10	ordinary_muscle_development	ENSGALG00010007092	DYNC1LI2	0.078488707	0.060882762	1.289177843	0.197336266	0.295804313	positive
PRJNA729271	stage_E14_vs_E10	ordinary_muscle_development	ENSGALG00010007097	ZCCHC9	0.062627189	0.166676451	0.375741079	0.707109396	0.787397303	positive
PRJNA729271	stage_E14_vs_E10	ordinary_muscle_development	ENSGALG00010007098	NDUFAF7	0.004266957	0.127634117	0.033431165	0.973330757	0.981797448	positive
PRJNA729271	stage_E14_vs_E10	ordinary_muscle_development	ENSGALG00010007102	CMTM3	-0.182061044	0.165087456	-1.102815733	0.270107194	0.379953523	negative
PRJNA729271	stage_E14_vs_E10	ordinary_muscle_development	ENSGALG00010007106	SLC7A1	-0.341858248	0.239136802	-1.429550972	0.152845936	0.241051228	negative
PRJNA729271	stage_E14_vs_E10	ordinary_muscle_development	ENSGALG00010007108	MBOAT1	-0.974294924	0.191586888	-5.085394589	3.66863E-07	2.16341E-06	negative
PRJNA729271	stage_E14_vs_E10	ordinary_muscle_development	ENSGALG00010007110	ARL6	0.054517502	0.102481909	0.531971957	0.594745418	0.694523235	positive
PRJNA729271	stage_E14_vs_E10	ordinary_muscle_development	ENSGALG00010007114	CKLF	0.055421339	0.143268175	0.38683636	0.698877357	0.781110902	positive
PRJNA729271	stage_E14_vs_E10	ordinary_muscle_development	ENSGALG00010007115	ENSGALG00010007115	-0.033552109	0.278311339	-0.120556027	0.9040427	0.934353371	negative
PRJNA729271	stage_E14_vs_E10	ordinary_muscle_development	ENSGALG00010007118	ENSGALG00010007118	-0.382044045	0.119345316	-3.201164985	0.001368731	0.004167142	negative
PRJNA729271	stage_E14_vs_E10	ordinary_muscle_development	ENSGALG00010007123	ENSGALG00010007123	0.615287916	0.249302136	2.468041095	0.013585472	0.031153523	positive
PRJNA729271	stage_E14_vs_E10	ordinary_muscle_development	ENSGALG00010007126	ENSGALG00010007126	0.617982182	1.086765157	0.56864372	0.569597948	0.671817576	positive
PRJNA729271	stage_E14_vs_E10	ordinary_muscle_development	ENSGALG00010007130	IRF2	0.572631102	0.122514347	4.673992185	2.95401E-06	1.51408E-05	positive
PRJNA729271	stage_E14_vs_E10	ordinary_muscle_development	ENSGALG00010007133	BLVRA	1.184087171	0.186345489	6.354257235	2.09436E-10	1.86473E-09	positive
PRJNA729271	stage_E14_vs_E10	ordinary_muscle_development	ENSGALG00010007135	TK2	-0.48783825	0.116206183	-4.19804041	2.69235E-05	0.000117335	negative
PRJNA729271	stage_E14_vs_E10	ordinary_muscle_development	ENSGALG00010007137	ENSGALG00010007137	-0.056507889	0.360907666	-0.156571595	0.87558249	0.914707618	negative
PRJNA729271	stage_E14_vs_E10	ordinary_muscle_development	ENSGALG00010007141	PRKD3	-0.372396941	0.082062992	-4.537940117	5.68064E-06	2.77991E-05	negative
PRJNA729271	stage_E14_vs_E10	ordinary_muscle_development	ENSGALG00010007143	CKMT2	2.383304546	0.326028534	7.310110304	2.66923E-13	3.24857E-12	positive
PRJNA729271	stage_E14_vs_E10	ordinary_muscle_development	ENSGALG00010007146	ENSGALG00010007146	0.173266487	0.142802139	1.213332577	0.225002692	0.32809458	positive
PRJNA729271	stage_E14_vs_E10	ordinary_muscle_development	ENSGALG00010007155	FOXL1	-2.570440321	0.712542905	-3.60741831	0.000309259	0.001104115	negative
PRJNA729271	stage_E14_vs_E10	ordinary_muscle_development	ENSGALG00010007160	ENSGALG00010007160	-0.377843594	0.295132159	-1.280252196	0.200456454	0.299629473	negative
PRJNA729271	stage_E14_vs_E10	ordinary_muscle_development	ENSGALG00010007162	ENSGALG00010007162	0.233832138	0.415194308	0.563187243	0.573307389	0.675123129	positive
PRJNA729271	stage_E14_vs_E10	ordinary_muscle_development	ENSGALG00010007165	ENSGALG00010007165	-0.097061225	0.326587272	-0.29719843	0.76631502	0.833809209	negative
PRJNA729271	stage_E14_vs_E10	ordinary_muscle_development	ENSGALG00010007166	RASGRF2	1.578254346	0.182357736	8.654715637	4.94142E-18	9.05511E-17	positive
PRJNA729271	stage_E14_vs_E10	ordinary_muscle_development	ENSGALG00010007168	MINA	-0.126016268	0.106081585	-1.187918416	0.234865553	0.339385524	negative
PRJNA729271	stage_E14_vs_E10	ordinary_muscle_development	ENSGALG00010007169	QPCT	-1.019282313	0.218932782	-4.655686116	3.22904E-06	1.6417E-05	negative
PRJNA729271	stage_E14_vs_E10	ordinary_muscle_development	ENSGALG00010007175	FOXC2	-4.195134345	0.460236524	-9.115170412	7.85469E-20	1.6621E-18	negative
PRJNA729271	stage_E14_vs_E10	ordinary_muscle_development	ENSGALG00010007181	GJB2	-1.252326098	0.642055783	-1.950494227	0.051117241	0.096615518	negative
PRJNA729271	stage_E14_vs_E10	ordinary_muscle_development	ENSGALG00010007182	ENSGALG00010007182	0.235399319	0.294777941	0.798564907	0.424542743	0.538857011	positive
PRJNA729271	stage_E14_vs_E10	ordinary_muscle_development	ENSGALG00010007183	VOPP1	-0.778027775	0.327447459	-2.376038515	0.017499636	0.038755879	negative
PRJNA729271	stage_E14_vs_E10	ordinary_muscle_development	ENSGALG00010007184	VGLL3	1.129660319	0.091545491	12.33987935	5.52386E-35	3.04442E-33	positive
PRJNA729271	stage_E14_vs_E10	ordinary_muscle_development	ENSGALG00010007187	ENPP6	0.255244685	0.277128536	0.92103357	0.3570329	0.473485077	positive
PRJNA729271	stage_E14_vs_E10	ordinary_muscle_development	ENSGALG00010007188	MAB21L1	-0.947532202	0.206885612	-4.579981149	4.65018E-06	2.30758E-05	negative
PRJNA729271	stage_E14_vs_E10	ordinary_muscle_development	ENSGALG00010007190	ENSGALG00010007190	-1.076529759	0.149844155	-7.184329331	6.75379E-13	7.85684E-12	negative
PRJNA729271	stage_E14_vs_E10	ordinary_muscle_development	ENSGALG00010007201	ENSGALG00010007201	-0.396349163	0.273812324	-1.447521269	0.147750984	0.234470168	negative
PRJNA729271	stage_E14_vs_E10	ordinary_muscle_development	ENSGALG00010007203	SLC30A6	0.039902303	0.103260198	0.386424816	0.699182074	0.781391223	positive
PRJNA729271	stage_E14_vs_E10	ordinary_muscle_development	ENSGALG00010007204	LANCL2	-0.224310158	0.086068232	-2.606189906	0.009155568	0.022059502	negative
PRJNA729271	stage_E14_vs_E10	ordinary_muscle_development	ENSGALG00010007207	EPM2A	0.969155959	0.103111066	9.399145935	5.50077E-21	1.26965E-19	positive
PRJNA729271	stage_E14_vs_E10	ordinary_muscle_development	ENSGALG00010007214	MTHFSD	-0.135225433	0.140536836	-0.96220633	0.33594597	0.451175469	negative
PRJNA729271	stage_E14_vs_E10	ordinary_muscle_development	ENSGALG00010007217	MSH3	-0.303032497	0.142923121	-2.120248245	0.033985116	0.068210226	negative
PRJNA729271	stage_E14_vs_E10	ordinary_muscle_development	ENSGALG00010007223	FOXF1	-0.542092221	0.440298274	-1.231193154	0.218250631	0.320411134	negative
PRJNA729271	stage_E14_vs_E10	ordinary_muscle_development	ENSGALG00010007224	DHFR	-0.571213027	0.213057131	-2.681032197	0.007339545	0.018164028	negative
PRJNA729271	stage_E14_vs_E10	ordinary_muscle_development	ENSGALG00010007226	ENSGALG00010007226	2.149466672	1.48738302	1.445133259	0.148420458	0.235377959	positive
PRJNA729271	stage_E14_vs_E10	ordinary_muscle_development	ENSGALG00010007230	ENSGALG00010007230	-0.653726743	0.346093234	-1.888874669	0.058908624	0.108774588	negative
PRJNA729271	stage_E14_vs_E10	ordinary_muscle_development	ENSGALG00010007243	BEAN1	0.374972253	0.187118907	2.003924989	0.045078096	0.086935472	positive
PRJNA729271	stage_E14_vs_E10	ordinary_muscle_development	ENSGALG00010007251	SPAST	0.264468685	0.061901383	4.272419683	1.93363E-05	8.624E-05	positive
PRJNA729271	stage_E14_vs_E10	ordinary_muscle_development	ENSGALG00010007252	NFATC1	-0.52229503	0.153863455	-3.394535943	0.00068745	0.002251885	negative
PRJNA729271	stage_E14_vs_E10	ordinary_muscle_development	ENSGALG00010007259	ANKRD34B	-0.221926021	0.095131886	-2.332824768	0.019657344	0.042813466	negative
PRJNA729271	stage_E14_vs_E10	ordinary_muscle_development	ENSGALG00010007265	CD200R1A	0.21556809	0.281261338	0.766433423	0.443418452	0.557059323	positive
PRJNA729271	stage_E14_vs_E10	ordinary_muscle_development	ENSGALG00010007270	CDH5	0.253235301	0.108317891	2.337889876	0.019392962	0.042296265	positive
PRJNA729271	stage_E14_vs_E10	ordinary_muscle_development	ENSGALG00010007271	CADM2	-3.04262892	0.298297843	-10.19996958	1.98335E-24	5.67034E-23	negative
PRJNA729271	stage_E14_vs_E10	ordinary_muscle_development	ENSGALG00010007275	DPY30	-0.105062598	0.097849802	-1.073712932	0.282951363	0.394401386	negative
PRJNA729271	stage_E14_vs_E10	ordinary_muscle_development	ENSGALG00010007277	FAM151B	-0.084422851	0.183420035	-0.460270608	0.645321995	0.73749808	negative
PRJNA729271	stage_E14_vs_E10	ordinary_muscle_development	ENSGALG00010007283	STOX2	0.144019433	0.118931629	1.210943076	0.225917222	0.329013376	positive
PRJNA729271	stage_E14_vs_E10	ordinary_muscle_development	ENSGALG00010007284	ENSGALG00010007284	-0.345715783	0.081808373	-4.225921767	2.37965E-05	0.000104588	negative
PRJNA729271	stage_E14_vs_E10	ordinary_muscle_development	ENSGALG00010007287	ENSGALG00010007287	0.124748824	0.62052959	0.20103606	0.840670378	0.888869876	positive
PRJNA729271	stage_E14_vs_E10	ordinary_muscle_development	ENSGALG00010007294	TRAPPC11	-0.029148615	0.064278625	-0.453472905	0.650208249	0.741558478	negative
PRJNA729271	stage_E14_vs_E10	ordinary_muscle_development	ENSGALG00010007300	CCDC125	-1.382380518	0.286765896	-4.820588982	1.43135E-06	7.74736E-06	negative
PRJNA729271	stage_E14_vs_E10	ordinary_muscle_development	ENSGALG00010007302	CDH11	-1.593836297	0.17478667	-9.118751987	7.59944E-20	1.61044E-18	negative
PRJNA729271	stage_E14_vs_E10	ordinary_muscle_development	ENSGALG00010007304	F5	-0.400266018	0.357826239	-1.118604437	0.26330893	0.371958185	negative
PRJNA729271	stage_E14_vs_E10	ordinary_muscle_development	ENSGALG00010007318	RMDN2	0.236509728	0.12492379	1.893232095	0.058327011	0.107948164	positive
PRJNA729271	stage_E14_vs_E10	ordinary_muscle_development	ENSGALG00010007319	ATP9B	-0.084492042	0.100828727	-0.837975888	0.402044245	0.517321912	negative
PRJNA729271	stage_E14_vs_E10	ordinary_muscle_development	ENSGALG00010007320	ENSGALG00010007320	0.090342892	0.303404459	0.29776389	0.765883377	0.83360742	positive
PRJNA729271	stage_E14_vs_E10	ordinary_muscle_development	ENSGALG00010007321	CD200	1.215712526	0.149922478	8.108940997	5.10628E-16	7.84895E-15	positive
PRJNA729271	stage_E14_vs_E10	ordinary_muscle_development	ENSGALG00010007322	AK6	-0.207920475	0.107033278	-1.942577855	0.052067185	0.098129481	negative
PRJNA729271	stage_E14_vs_E10	ordinary_muscle_development	ENSGALG00010007324	ENSGALG00010007324	0.164597876	0.137600659	1.196199767	0.231618612	0.335899127	positive
PRJNA729271	stage_E14_vs_E10	ordinary_muscle_development	ENSGALG00010007326	RWDD4	-0.043961718	0.115936009	-0.379189503	0.704547146	0.785207664	negative
PRJNA729271	stage_E14_vs_E10	ordinary_muscle_development	ENSGALG00010007328	NOVA1	0.076399811	0.133259848	0.573314555	0.566431732	0.669037481	positive
PRJNA729271	stage_E14_vs_E10	ordinary_muscle_development	ENSGALG00010007330	MMP13	-1.164845986	0.708474448	-1.644160899	0.100142971	0.170288067	negative
PRJNA729271	stage_E14_vs_E10	ordinary_muscle_development	ENSGALG00010007333	QTRT2	-0.462360458	0.066159957	-6.988524116	2.77793E-12	3.04815E-11	negative
PRJNA729271	stage_E14_vs_E10	ordinary_muscle_development	ENSGALG00010007337	PRORP	-0.004122848	0.095616576	-0.043118553	0.96560703	0.977134453	negative
PRJNA729271	stage_E14_vs_E10	ordinary_muscle_development	ENSGALG00010007339	ENSGALG00010007339	0.111827239	0.223983292	0.499265987	0.617592014	0.713502352	positive
PRJNA729271	stage_E14_vs_E10	ordinary_muscle_development	ENSGALG00010007344	RAD17	-0.144580004	0.107091363	-1.350062222	0.176996025	0.270907119	negative
PRJNA729271	stage_E14_vs_E10	ordinary_muscle_development	ENSGALG00010007353	ING2	-0.445814566	0.117156846	-3.805279696	0.000141644	0.0005433	negative
PRJNA729271	stage_E14_vs_E10	ordinary_muscle_development	ENSGALG00010007357	NOX4	1.163666546	0.116333801	10.0028241	1.48112E-23	3.99792E-22	positive
PRJNA729271	stage_E14_vs_E10	ordinary_muscle_development	ENSGALG00010007360	ROBO1	-1.636840222	0.185212665	-8.837625757	9.77742E-19	1.87962E-17	negative

PRJNA729271	stage_E14_vs_E10	ordinary_muscle_development	ENSGALG00010007365	MEMO1	0.124407405	0.087323231	1.424677073	0.154250569	0.24289647	positive
PRJNA729271	stage_E14_vs_E10	ordinary_muscle_development	ENSGALG00010007369	ENSGALG00010007369	-0.013037908	0.210721974	-0.061872561	0.950664319	0.965721445	negative
PRJNA729271	stage_E14_vs_E10	ordinary_muscle_development	ENSGALG00010007374	CMIP	-0.308385631	0.133348928	-2.312621752	0.020743445	0.044877049	negative
PRJNA729271	stage_E14_vs_E10	ordinary_muscle_development	ENSGALG00010007375	CHORDC1	-0.115940442	0.073769283	-1.571662852	0.116028762	0.192584095	negative
PRJNA729271	stage_E14_vs_E10	ordinary_muscle_development	ENSGALG00010007376	CDKN2AIP	-0.121852084	0.058315396	-2.089535394	0.036659555	0.072741992	negative
PRJNA729271	stage_E14_vs_E10	ordinary_muscle_development	ENSGALG00010007381	EGFR	-0.087283228	0.097853733	-0.891976473	0.372405543	0.488508447	negative
PRJNA729271	stage_E14_vs_E10	ordinary_muscle_development	ENSGALG00010007382	EIF2AK2	0.052296932	0.257283957	0.203265422	0.838927581	0.88754509	positive
PRJNA729271	stage_E14_vs_E10	ordinary_muscle_development	ENSGALG00010007387	ENSGALG00010007387	-0.98320863	0.481213706	-2.043185008	0.041034127	0.079987853	negative
PRJNA729271	stage_E14_vs_E10	ordinary_muscle_development	ENSGALG00010007388	CYP1B1	-3.005662115	0.199354731	-15.07695407	2.2962E-51	3.32835E-49	negative
PRJNA729271	stage_E14_vs_E10	ordinary_muscle_development	ENSGALG00010007391	NANP	0.238006721	0.138075858	1.723738851	0.084754998	0.148444139	positive
PRJNA729271	stage_E14_vs_E10	ordinary_muscle_development	ENSGALG00010007392	NAALAD2	0.26322797	0.203119538	1.295926389	0.195000847	0.293116956	positive
PRJNA729271	stage_E14_vs_E10	ordinary_muscle_development	ENSGALG00010007393	PLCG2	-0.006563656	0.128776484	-0.050969371	0.959349927	0.97209208	negative
PRJNA729271	stage_E14_vs_E10	ordinary_muscle_development	ENSGALG00010007399	WWC2	0.124560355	0.106219861	1.172665393	0.240930014	0.346869344	positive
PRJNA729271	stage_E14_vs_E10	ordinary_muscle_development	ENSGALG00010007401	SEC61G	0.29269721	0.219814904	1.331562165	0.183004104	0.278434396	positive
PRJNA729271	stage_E14_vs_E10	ordinary_muscle_development	ENSGALG00010007403	SDR42E1	-0.140818197	0.21949761	-0.641547745	0.521166871	0.629001981	negative
PRJNA729271	stage_E14_vs_E10	ordinary_muscle_development	ENSGALG00010007404	FOLH1	0.249176656	0.172967691	1.440596534	0.1496987	0.236964362	positive
PRJNA729271	stage_E14_vs_E10	ordinary_muscle_development	ENSGALG00010007408	HCLS1	0.815268585	0.247092741	3.299443691	0.000968767	0.003060652	positive
PRJNA729271	stage_E14_vs_E10	ordinary_muscle_development	ENSGALG00010007411	ENSGALG00010007411	-0.179623056	0.596918259	-0.300917342	0.763477526	0.831450544	negative
PRJNA729271	stage_E14_vs_E10	ordinary_muscle_development	ENSGALG00010007413	GPATCH11	-0.503943837	0.097366943	-5.175717988	2.27036E-07	1.37579E-06	negative
PRJNA729271	stage_E14_vs_E10	ordinary_muscle_development	ENSGALG00010007414	ROBO2	-2.801038353	0.225093689	-12.44387775	1.50992E-35	8.71962E-34	negative
PRJNA729271	stage_E14_vs_E10	ordinary_muscle_development	ENSGALG00010007416	DCTD	-0.323824149	0.249598562	-1.297379863	0.194500518	0.292578352	negative
PRJNA729271	stage_E14_vs_E10	ordinary_muscle_development	ENSGALG00010007418	NME6	0.344556593	0.145005474	2.376162671	0.017493749	0.03874876	positive
PRJNA729271	stage_E14_vs_E10	ordinary_muscle_development	ENSGALG00010007419	RABL2A	0.022829352	0.143018063	0.159625655	0.873175968	0.91296116	positive
PRJNA729271	stage_E14_vs_E10	ordinary_muscle_development	ENSGALG00010007423	HEATR5B	-0.292069033	0.064348492	-4.538863659	5.65582E-06	2.76963E-05	negative
PRJNA729271	stage_E14_vs_E10	ordinary_muscle_development	ENSGALG00010007425	ARSA	-0.225591972	0.127518542	-1.769091522	0.076878602	0.136646884	negative
PRJNA729271	stage_E14_vs_E10	ordinary_muscle_development	ENSGALG00010007429	C21orf91	-0.516035508	0.20942628	-2.464043704	0.013737936	0.031468297	negative
PRJNA729271	stage_E14_vs_E10	ordinary_muscle_development	ENSGALG00010007430	MEF2C	1.008126302	0.146834739	6.865720665	6.61564E-12	6.8741E-11	positive
PRJNA729271	stage_E14_vs_E10	ordinary_muscle_development	ENSGALG00010007432	NAT	-0.110617252	0.198123622	-0.558324399	0.576622887	0.678366102	negative
PRJNA729271	stage_E14_vs_E10	ordinary_muscle_development	ENSGALG00010007433	RNF144B	0.897583014	0.274400245	3.271072201	0.001071406	0.003350598	positive
PRJNA729271	stage_E14_vs_E10	ordinary_muscle_development	ENSGALG00010007434	ENSGALG00010007434	-0.650053195	0.252132034	-2.578225323	0.009930922	0.023703065	negative
PRJNA729271	stage_E14_vs_E10	ordinary_muscle_development	ENSGALG00010007436	PNAT3	-0.132891308	0.271241245	-0.489937685	0.624177995	0.719241526	negative
PRJNA729271	stage_E14_vs_E10	ordinary_muscle_development	ENSGALG00010007437	TUSC1	0.065990221	0.137643706	0.479427815	0.63163431	0.725306133	positive
PRJNA729271	stage_E14_vs_E10	ordinary_muscle_development	ENSGALG00010007439	HSD17B2	-1.397054351	0.330772451	-4.223611564	2.40418E-05	0.000105634	negative
PRJNA729271	stage_E14_vs_E10	ordinary_muscle_development	ENSGALG00010007441	STRN	0.112072463	0.136307871	0.822200967	0.410962529	0.525719842	positive
PRJNA729271	stage_E14_vs_E10	ordinary_muscle_development	ENSGALG00010007455	TRPC6	0.271519414	0.209863974	1.293787631	0.195738786	0.294013855	positive
PRJNA729271	stage_E14_vs_E10	ordinary_muscle_development	ENSGALG00010007457	SHANK3	-0.174135205	0.178295509	-0.976666245	0.328734411	0.443545127	negative
PRJNA729271	stage_E14_vs_E10	ordinary_muscle_development	ENSGALG00010007465	PNAT10	-0.44075534	0.210964071	-2.089243623	0.036685798	0.072775082	negative
PRJNA729271	stage_E14_vs_E10	ordinary_muscle_development	ENSGALG00010007467	CHODL	-1.848432666	0.331084284	-5.582967098	2.36449E-08	1.64302E-07	negative
PRJNA729271	stage_E14_vs_E10	ordinary_muscle_development	ENSGALG00010007479	NFX1	-0.199973229	0.095941402	-2.084326729	0.037130458	0.073545502	negative
PRJNA729271	stage_E14_vs_E10	ordinary_muscle_development	ENSGALG00010007480	TENM3	-0.881602794	0.105883361	-8.326169309	8.35004E-17	1.38641E-15	negative
PRJNA729271	stage_E14_vs_E10	ordinary_muscle_development	ENSGALG00010007485	CTSC	0.024384509	0.155640529	0.156671977	0.875503374	0.914690889	positive
PRJNA729271	stage_E14_vs_E10	ordinary_muscle_development	ENSGALG00010007500	MPHOSPH6	-0.026789976	0.116359614	-0.230234317	0.817909697	0.872474191	negative
PRJNA729271	stage_E14_vs_E10	ordinary_muscle_development	ENSGALG00010007506	RAB38	-0.55238211	0.553764752	-0.997503197	0.318520323	0.432582412	negative
PRJNA729271	stage_E14_vs_E10	ordinary_muscle_development	ENSGALG00010007513	GALNT12	-0.138056743	0.127266686	-1.084783047	0.278017756	0.388683195	negative
PRJNA729271	stage_E14_vs_E10	ordinary_muscle_development	ENSGALG00010007514	VIT	-4.731132462	0.373197171	-12.67729991	7.90068E-37	5.02283E-35	negative
PRJNA729271	stage_E14_vs_E10	ordinary_muscle_development	ENSGALG00010007521	SLC12A7	-0.877168383	0.136602086	-6.421339578	1.3508E-10	1.22298E-09	negative
PRJNA729271	stage_E14_vs_E10	ordinary_muscle_development	ENSGALG00010007529	CDH13	0.673432146	0.134587504	5.003675117	5.62475E-07	3.21494E-06	positive
PRJNA729271	stage_E14_vs_E10	ordinary_muscle_development	ENSGALG00010007532	DCUN1D5	-0.037964156	0.266123995	-0.142655891	0.886561954	0.922309328	negative
PRJNA729271	stage_E14_vs_E10	ordinary_muscle_development	ENSGALG00010007536	GABBR2	1.768546892	0.189831521	9.316402689	1.20352E-20	2.70046E-19	positive
PRJNA729271	stage_E14_vs_E10	ordinary_muscle_development	ENSGALG00010007539	RPS3A	0.450100377	0.175330735	2.567150459	0.010253811	0.024312984	positive
PRJNA729271	stage_E14_vs_E10	ordinary_muscle_development	ENSGALG00010007542	MRPL36	0.187804936	0.109972859	1.707738963	0.087684789	0.152554689	positive
PRJNA729271	stage_E14_vs_E10	ordinary_muscle_development	ENSGALG00010007544	MLYCD	-0.112466477	0.103657185	-1.084984862	0.27792836	0.388595696	negative
PRJNA729271	stage_E14_vs_E10	ordinary_muscle_development	ENSGALG00010007547	ENSGALG00010007547	-0.840876985	0.628001477	-1.338972943	0.180579481	0.275439291	negative
PRJNA729271	stage_E14_vs_E10	ordinary_muscle_development	ENSGALG00010007548	HSBP1	0.143337732	0.085890714	1.668838516	0.095149387	0.163353117	positive
PRJNA729271	stage_E14_vs_E10	ordinary_muscle_development	ENSGALG00010007549	SH3D19	0.073006348	0.157794791	0.462666398	0.643603508	0.735789851	positive
PRJNA729271	stage_E14_vs_E10	ordinary_muscle_development	ENSGALG00010007551	FEZ2	0.333542969	0.110791729	3.010540337	0.002607833	0.007337061	positive
PRJNA729271	stage_E14_vs_E10	ordinary_muscle_development	ENSGALG00010007555	NDUFS6	0.446149974	0.13017604	3.427281799	0.000609656	0.002020339	positive
PRJNA729271	stage_E14_vs_E10	ordinary_muscle_development	ENSGALG00010007564	IRX4	-5.463621175	0.51068636	-10.6985845	1.03345E-26	3.60092E-25	negative
PRJNA729271	stage_E14_vs_E10	ordinary_muscle_development	ENSGALG00010007566	FHIP1A	-1.362504392	0.1801397	-7.563598658	3.92068E-14	5.13836E-13	negative
PRJNA729271	stage_E14_vs_E10	ordinary_muscle_development	ENSGALG00010007567	TRIP13	-0.380266664	0.1200319	-3.168046701	0.001534669	0.004612279	negative
PRJNA729271	stage_E14_vs_E10	ordinary_muscle_development	ENSGALG00010007569	GPR89A	0.170906115	0.079583099	2.147517709	0.031752089	0.064388155	positive
PRJNA729271	stage_E14_vs_E10	ordinary_muscle_development	ENSGALG00010007574	ENSGALG00010007574	0.156910559	0.184910067	0.8485777	0.39611632	0.511601716	positive
PRJNA729271	stage_E14_vs_E10	ordinary_muscle_development	ENSGALG00010007575	MMP27	-0.766390323	0.330360415	-2.319861246	0.020348385	0.044147558	negative
PRJNA729271	stage_E14_vs_E10	ordinary_muscle_development	ENSGALG00010007577	WFDC1	0.543786018	0.118805148	4.577125057	4.7141E-06	2.3361E-05	positive
PRJNA729271	stage_E14_vs_E10	ordinary_muscle_development	ENSGALG00010007579	GATB	0.559891686	0.178212219	3.141713233	0.001679625	0.00499929	positive
PRJNA729271	stage_E14_vs_E10	ordinary_muscle_development	ENSGALG00010007580	BRD9	-0.296356178	0.099596606	-2.975565021	0.002924493	0.008123904	negative
PRJNA729271	stage_E14_vs_E10	ordinary_muscle_development	ENSGALG00010007581	LPCAT1	-0.421485424	0.112587805	-3.743615239	0.000181391	0.000679749	negative
PRJNA729271	stage_E14_vs_E10	ordinary_muscle_development	ENSGALG00010007584	ELP2	-0.192146527	0.112926405	-1.701519925	0.0888454	0.154229229	negative
PRJNA729271	stage_E14_vs_E10	ordinary_muscle_development	ENSGALG00010007585	KCNG4	-1.279031179	0.614105116	-2.082756105	0.037273461	0.073773643	negative
PRJNA729271	stage_E14_vs_E10	ordinary_muscle_development	ENSGALG00010007586	MMP7	-2.654328934	1.697970734	-1.563235974	0.117997101	0.195047096	negative
PRJNA729271	stage_E14_vs_E10	ordinary_muscle_development	ENSGALG00010007588	TTC27	-0.462969711	0.102215215	-4.529361983	5.91621E-06	2.88447E-05	negative
PRJNA729271	stage_E14_vs_E10	ordinary_muscle_development	ENSGALG00010007590	FBXW7	0.134077149	0.137823754	0.972815977	0.330644771	0.445417839	positive
PRJNA729271	stage_E14_vs_E10	ordinary_muscle_development	ENSGALG00010007593	SEC61B	-0.171813437	0.118178663	-1.453844816	0.145989335	0.232208429	negative
PRJNA729271	stage_E14_vs_E10	ordinary_muscle_development	ENSGALG00010007595	ATP2C2	0.924725398	0.153886934	6.009122258	1.8653E-09	1.48232E-08	positive
PRJNA729271	stage_E14_vs_E10	ordinary_muscle_development	ENSGALG00010007596	ENSGALG00010007596	-1.59020631	0.49485691	-3.213466921	0.001311429	0.004015302	negative
PRJNA729271	stage_E14_vs_E10	ordinary_muscle_development	ENSGALG00010007597	SMKR1	-0.682299217	0.63180309	-1.079923838	0.280176097	0.391097123	negative
PRJNA729271	stage_E14_vs_E10	ordinary_muscle_development	ENSGALG00010007601	TMEM154	0.307592189	0.300525014	1.023516094	0.306063894	0.419698071	positive
PRJNA729271	stage_E14_vs_E10	ordinary_muscle_development	ENSGALG00010007603	ALG2	-0.23488856	0.103819125	-2.262478719	0.023667842	0.050339747	negative
PRJNA729271	stage_E14_vs_E10	ordinary_muscle_development	ENSGALG00010007604	ENSGALG00010007604	1.345882973	0.498940156	2.697483768	0.006986569	0.017400398	positive
PRJNA729271	stage_E14_vs_E10	ordinary_muscle_development	ENSGALG00010007610	TLDC1	0.083421219	0.156823352	0.53194386	0.594764878	0.694523235	positive
PRJNA729271	stage_E14_vs_E10	ordinary_muscle_development	ENSGALG00010007614	BIRC6	-0.180952235	0.087947332	-2.057506809	0.039637501	0.077693789	negative
PRJNA729271	stage_E14_vs_E10	ordinary_muscle_development	ENSGALG00010007624	TPPP	0.950590663	0.24731722	3.843608889	0.000121238	0.00047055	positive
PRJNA729271	stage_E14_vs_E10	ordinary_muscle_development	ENSGALG00010007626	DLK2	1.206347548	0.351380307	3.433167776	0.000596573	0.001981513	positive
PRJNA729271	stage_E14_vs_E10	ordinary_muscle_development	ENSGALG00010007633	ENSGALG00010007633	-0.281609028	0.103441144	-2.722408292	0.006480802	0.016289097	negative
PRJNA729271	stage_E14_vs_E10	ordinary_muscle_development	ENSGALG00010007640	TMEM123	-1.217296682	0.218147172	-5.58016255	2.40294E-08	1.66813E-07	negative
PRJNA729271	stage_E14_vs_E10	ordinary_muscle_development	ENSGALG00010007642	ZNF318	-0.090071192	0.10690456	-0.842538356	0.39948668	0.514807914	negative
PRJNA729271	stage_E14_vs_E10	ordinary_muscle_development	ENSGALG00010007643	COTL1	0.062327891	0.163893686	0.380294644	0.703726711	0.784594929	positive
PRJNA729271	stage_E14_vs_E10	ordinary_muscle_development	ENSGALG00010007650	KLHL36	-0.084183818	0.086840575	-0.969406508	0.332342407	0.447247534	negative
PRJNA729271	stage_E14_vs_E10	ordinary_muscle_development	ENSGALG00010007651	CTNNAL1	-1.14397358	0.136687413	-8.369267892	5.79773E-17	9.77187E-16	negative
PRJNA729271	stage_E14_vs_E10	ordinary_muscle_development	ENSGALG00010007658	BIRC2	-0.208405874	0.119483266	-1.744226454	0.08111961	0.143131923	negative
PRJNA729271	stage_E14_vs_E10	ordinary_muscle_development	ENSGALG00010007660	FAM98A	0.16983045	0.09676855	1.755017	0.0792564	0.14033979	positive
PRJNA729271	stage_E14_vs_E10	ordinary_muscle_development	ENSGALG00010007663	ARFIP1	0.039325858	0.109694615	0.358503085	0.71996686	0.797347896	positive
PRJNA729271	stage_E14_vs_E10	ordinary_muscle_development	ENSGALG00010007671	FAM206A	0.082264058	0.100297438	0.820200989	0.412101538	0.526794142	positive
PRJNA729271	stage_E14_vs_E10	ordinary_muscle_development	ENSGALG00010007673	FHDC1	0.286861885	0.32064771	0.894632572	0.370983534	0.487253489	positive
PRJNA729271	stage_E14_vs_E10	ordinary_muscle_development	ENSGALG00010007678	CDH8	0.069540631	0.283852141	0.244988926	0.806465006	0.86404836	positive
PRJNA729271	stage_E14_vs_E10	ordinary_muscle_development	ENSGALG00010007679	TMEM135	0.170921593	0.142126409	1.202602622	0.229130133	0.332987973	positive
PRJNA729271	stage_E14_vs_E10	ordinary_muscle_development	ENSGALG00010007680	CHMP2B	-0.116220087	0.125516851	-0.92593214	0.354481247	0.47087662	negative
PRJNA729271	stage_E14_vs_E10	ordinary_muscle_development	ENSGALG00010007695	RASGRP3	1.645481631	0.140899129	11.67843719	1.64288E-31	7.55987E-30	positive
PRJNA729271	stage_E14_vs_E10	ordinary_muscle_development	ENSGALG00010007698	ADAMTS18	-2.206624593	0.400650219	-5.507608597	3.63741E-08	2.46753E-07	negative
PRJNA729271	stage_E14_vs_E10	ordinary_muscle_development	ENSGALG00010007699	SLC39A6	-0.3154749	0.103826583	-3.03847907	0.002377756	0.006757956	negative
PRJNA729271	stage_E14_vs_E10	ordinary_muscle_development	ENSGALG00010007707	ENSGALG00010007707	-0.068749061	0.95108375	-0.07228497	0.942375125	0.959452654	negative
PRJNA729271	stage_E14_vs_E10	ordinary_muscle_development	ENSGALG00010007713	SCLT1	-0.230737851	0.211669378	-1.090086121	0.275675209	0.386115776	negative
PRJNA729271	stage_E14_vs_E10	ordinary_muscle_development	ENSGALG00010007719	LTBP1	-2.133897523	0.190906703	-11.17769824	5.24323E-29	2.12887E-27	negative
PRJNA729271	stage_E14_vs_E10	ordinary_muscle_development	ENSGALG00010007724	RBM11	-0.738943181	0.389369018	-1.897796552	0.057722888	0.107035084	negative
PRJNA729271	stage_E14_vs_E10	ordinary_muscle_development	ENSGALG00010007730	COG2	-0.157016578	0.061729632	-2.543617593	0.010971112	0.025836924	negative
PRJNA729271	stage_E14_vs_E10	ordinary_muscle_development	ENSGALG00010007731	ENSGALG00010007731	-0.322762134	0.268214855	-1.203371581	0.22883256	0.332624144	negative
PRJNA729271	stage_E14_vs_E10	ordinary_muscle_development	ENSGALG00010007741	ENSGALG00010007741	-1.081991674	0.207557069	-5.212983964	1.85827E-07	1.14086E-06	negative
PRJNA729271	stage_E14_vs_E10	ordinary_muscle_development	ENSGALG00010007748	CSGALNACT1	-2.946873466	0.268817963	-10.96233836	5.79815E-28	2.18865E-26	negative
PRJNA729271	stage_E14_vs_E10	ordinary_muscle_development	ENSGALG00010007751	HSPA13	-0.360153362	0.09454871	-3.809183237	0.000139427	0.000535361	negative
PRJNA729271	stage_E14_vs_E10	ordinary_muscle_development	ENSGALG00010007753	PRSS23	-0.688890184	0.227885871	-3.022961364	0.002503142	0.007067207	negative
PRJNA729271	stage_E14_vs_E10	ordinary_muscle_development	ENSGALG00010007758	DEK	-0.242268886	0.076280904	-3.17600964	0.00149316	0.004501529	negative
PRJNA729271	stage_E14_vs_E10	ordinary_muscle_development	ENSGALG00010007759	AGT	-2.580104151	1.298903494	-1.986370937			negative
PRJNA729271	stage_E14_vs_E10	ordinary_muscle_development	ENSGALG00010007761	ENSGALG00010007761	0.306729372	0.117945415	2.600604451	0.009305968	0.022377242	positive
PRJNA729271	stage_E14_vs_E10	ordinary_muscle_development	ENSGALG00010007762	SH2D4A	-1.664194459	0.342560074	-4.858109821	1.18512E-06	6.49216E-06	negative
PRJNA729271	stage_E14_vs_E10	ordinary_muscle_development	ENSGALG00010007763	FZD4	0.341437887	0.099557937	3.429539589	0.000604606	0.002004063	positive
PRJNA729271	stage_E14_vs_E10	ordinary_muscle_development	ENSGALG00010007765	NUDT7	0.032934973	0.095498642	0.344873734	0.730189297	0.80563957	positive
PRJNA729271	stage_E14_vs_E10	ordinary_muscle_development	ENSGALG00010007766	ENSGALG00010007766	0.490450197	0.131099071	3.7410654	0.000183242	0.000685974	positive
PRJNA729271	stage_E14_vs_E10	ordinary_muscle_development	ENSGALG00010007767	VAT1L	-0.89151126	0.319509649	-2.790248314	0.005266763	0.013632452	negative
PRJNA729271	stage_E14_vs_E10	ordinary_muscle_development	ENSGALG00010007770	ENSGALG00010007770	-1.637564995	0.434169979	-3.771714014	0.00016213	0.000613596	negative
PRJNA729271	stage_E14_vs_E10	ordinary_muscle_development	ENSGALG00010007771	CAPN9	-0.487807702	0.403873655	-1.207822537	0.227115531	0.330459709	negative
PRJNA729271	stage_E14_vs_E10	ordinary_muscle_development	ENSGALG00010007772	CLEC3A	-4.382096436	0.668303994	-6.557040622	5.48861E-11	5.22373E-10	negative
PRJNA729271	stage_E14_vs_E10	ordinary_muscle_development	ENSGALG00010007774	ENSGALG00010007774	-0.126864201	0.28244273	-0.449167875	0.65331056	0.744066675	negative
PRJNA729271	stage_E14_vs_E10	ordinary_muscle_development	ENSGALG00010007777	KIF13A	0.196299343	0.124478229	1.576977313	0.114800745	0.191049002	positive
PRJNA729271	stage_E14_vs_E10	ordinary_muscle_development	ENSGALG00010007780	SIRT5	0.228462401	0.142272664	1.605806728	0.108316382	0.181949931	positive
PRJNA729271	stage_E14_vs_E10	ordinary_muscle_development	ENSGALG00010007781	MBP	-0.360485061	0.237888929	-1.515350303	0.129683716	0.210452918	negative
PRJNA729271	stage_E14_vs_E10	ordinary_muscle_development	ENSGALG00010007783	NOL7	-0.025230366	0.107437425	-0.234837774	0.814334648	0.869971507	negative
PRJNA729271	stage_E14_vs_E10	ordinary_muscle_development	ENSGALG00010007784	INTS10	-0.323221308	0.093463172	-3.458274535	0.000543647	0.00182649	negative
PRJNA729271	stage_E14_vs_E10	ordinary_muscle_development	ENSGALG00010007786	SAMSN1	0.492913363	0.228536398	2.156826511	0.031019185	0.063113852	positive
PRJNA729271	stage_E14_vs_E10	ordinary_muscle_development	ENSGALG00010007787	C1orf198	-0.409360166	0.119025715	-3.439258212	0.000583311	0.001943698	negative
PRJNA729271	stage_E14_vs_E10	ordinary_muscle_development	ENSGALG00010007788	PALM2AKAP2	1.625807947	0.164550657	9.880288366	5.06868E-23	1.32618E-21	positive
PRJNA729271	stage_E14_vs_E10	ordinary_muscle_development	ENSGALG00010007789	WWOX	-0.047422909	0.109969895	-0.431235375	0.666297235	0.754411687	negative

PRJNA729271	stage_E14_vs_E10	ordinary_muscle_development	ENSGALG00010007792	ENSGALG00010007792	0.369228133	0.22675143	1.628338718	0.103453084	0.175038223	positive
PRJNA729271	stage_E14_vs_E10	ordinary_muscle_development	ENSGALG00010007795	ZNF236	-0.188187555	0.115646899	-1.627259847	0.103681922	0.175384462	negative
PRJNA729271	stage_E14_vs_E10	ordinary_muscle_development	ENSGALG00010007796	MCUR1	-0.384968496	0.100114711	-3.845274022	0.000120418	0.000467575	negative
PRJNA729271	stage_E14_vs_E10	ordinary_muscle_development	ENSGALG00010007798	HGSNAT	-0.059875734	0.097536673	-0.613879192	0.539295161	0.645560785	negative
PRJNA729271	stage_E14_vs_E10	ordinary_muscle_development	ENSGALG00010007800	MAF	0.37216976	0.113876248	3.268194799	0.001082358	0.003380475	positive
PRJNA729271	stage_E14_vs_E10	ordinary_muscle_development	ENSGALG00010007801	TTC13	-0.227540068	0.160061001	-1.421583436	0.155147216	0.243929733	negative
PRJNA729271	stage_E14_vs_E10	ordinary_muscle_development	ENSGALG00010007802	ENSGALG00010007802	5.282814485	0.716021692	7.378008993	1.60674E-13	1.98887E-12	positive
PRJNA729271	stage_E14_vs_E10	ordinary_muscle_development	ENSGALG00010007803	ME3	-0.151710178	0.177653043	-0.853968925	0.393122208	0.508460851	negative
PRJNA729271	stage_E14_vs_E10	ordinary_muscle_development	ENSGALG00010007805	ENSGALG00010007805	6.64479166	1.52414806	4.359675962	1.30255E-05	5.99952E-05	positive
PRJNA729271	stage_E14_vs_E10	ordinary_muscle_development	ENSGALG00010007806	DYNLRB2	0.462541593	0.347619724	1.330596517	0.183321809	0.2788885	positive
PRJNA729271	stage_E14_vs_E10	ordinary_muscle_development	ENSGALG00010007809	ARV1	-0.018531662	0.115945627	-0.159830622	0.873014501	0.91296116	negative
PRJNA729271	stage_E14_vs_E10	ordinary_muscle_development	ENSGALG00010007811	PRCP	0.121388877	0.182510252	0.665107171	0.505981948	0.614904048	positive
PRJNA729271	stage_E14_vs_E10	ordinary_muscle_development	ENSGALG00010007812	POMK	0.115496927	0.120137325	0.961374216	0.336364044	0.451473851	positive
PRJNA729271	stage_E14_vs_E10	ordinary_muscle_development	ENSGALG00010007817	CDYL2	-0.954953846	0.134084861	-7.122010911	1.06364E-12	1.20731E-11	negative
PRJNA729271	stage_E14_vs_E10	ordinary_muscle_development	ENSGALG00010007818	ENSGALG00010007818	0.036788058	0.222058366	0.165668418	0.868417901	0.909714351	positive
PRJNA729271	stage_E14_vs_E10	ordinary_muscle_development	ENSGALG00010007819	NRIP1	0.483666632	0.16654955	2.904040467	0.003683805	0.009945383	positive
PRJNA729271	stage_E14_vs_E10	ordinary_muscle_development	ENSGALG00010007821	FAM89A	-0.298489073	0.123967403	-2.407802897	0.016048843	0.035965983	negative
PRJNA729271	stage_E14_vs_E10	ordinary_muscle_development	ENSGALG00010007826	ENSGALG00010007826	7.519689069	1.37695184	5.461112618	4.7316E-08	3.15186E-07	positive
PRJNA729271	stage_E14_vs_E10	ordinary_muscle_development	ENSGALG00010007830	SMIM19	0.709107759	0.130017331	5.453947965	4.92636E-08	3.26808E-07	positive
PRJNA729271	stage_E14_vs_E10	ordinary_muscle_development	ENSGALG00010007835	GUCY1A1	1.374955903	0.18581894	7.39943896	1.36761E-13	1.70452E-12	positive
PRJNA729271	stage_E14_vs_E10	ordinary_muscle_development	ENSGALG00010007845	TFAP2B	-3.237817581	0.738712506	-4.383055051	1.17027E-05	5.42816E-05	negative
PRJNA729271	stage_E14_vs_E10	ordinary_muscle_development	ENSGALG00010007846	ENSGALG00010007846	0.34037551	0.146087437	2.329943753	0.019809124	0.04307313	positive
PRJNA729271	stage_E14_vs_E10	ordinary_muscle_development	ENSGALG00010007847	CMC2	-0.109356914	0.114724206	-0.953215698	0.340480794	0.455493227	negative
PRJNA729271	stage_E14_vs_E10	ordinary_muscle_development	ENSGALG00010007849	CXADR	-2.816510435	0.313487226	-8.984450414	2.60031E-19	5.29374E-18	negative
PRJNA729271	stage_E14_vs_E10	ordinary_muscle_development	ENSGALG00010007850	ENSGALG00010007850	-1.371260366	0.272410623	-5.033799163	4.80853E-07	2.7791E-06	negative
PRJNA729271	stage_E14_vs_E10	ordinary_muscle_development	ENSGALG00010007851	AvBD7	3.318327349	0.897593773	3.696914406	0.000218236	0.000802782	positive
PRJNA729271	stage_E14_vs_E10	ordinary_muscle_development	ENSGALG00010007852	SLC20A2	0.469560309	0.207637366	2.261444167	0.023731766	0.0504609	positive
PRJNA729271	stage_E14_vs_E10	ordinary_muscle_development	ENSGALG00010007853	C1orf131	-0.166889554	0.110623654	-1.508624496	0.13139477	0.212895953	negative
PRJNA729271	stage_E14_vs_E10	ordinary_muscle_development	ENSGALG00010007854	FAM181B	0.135406406	0.117562568	1.151781628	0.249410819	0.356459261	positive
PRJNA729271	stage_E14_vs_E10	ordinary_muscle_development	ENSGALG00010007855	RNF182	-1.969355618	0.220982026	-8.911836194	5.01943E-19	9.95302E-18	negative
PRJNA729271	stage_E14_vs_E10	ordinary_muscle_development	ENSGALG00010007856	PDCD2	0.076538063	0.129886322	0.589269615	0.555680424	0.660020428	positive
PRJNA729271	stage_E14_vs_E10	ordinary_muscle_development	ENSGALG00010007857	ENSGALG00010007857	-0.80473066	0.337424602	-2.384919933	0.017082839	0.037948774	negative
PRJNA729271	stage_E14_vs_E10	ordinary_muscle_development	ENSGALG00010007859	CENPN	-0.175932145	0.154129356	-1.141457727	0.253679494	0.361206706	negative
PRJNA729271	stage_E14_vs_E10	ordinary_muscle_development	ENSGALG00010007860	DEFB4A	2.555185589	0.891499189	2.866167037	0.00415475	0.011058225	positive
PRJNA729271	stage_E14_vs_E10	ordinary_muscle_development	ENSGALG00010007861	INTU	-0.631598135	0.170838165	-3.697055254	0.000218115	0.000802634	negative
PRJNA729271	stage_E14_vs_E10	ordinary_muscle_development	ENSGALG00010007862	ATMIN	-0.070544354	0.085825171	-0.821954137	0.411103	0.52585051	negative
PRJNA729271	stage_E14_vs_E10	ordinary_muscle_development	ENSGALG00010007863	AvBD1	2.708578012	0.824763903	3.28406469	0.001023214	0.003219338	positive
PRJNA729271	stage_E14_vs_E10	ordinary_muscle_development	ENSGALG00010007864	TYR	-1.396944012	0.938874449	-1.487892246	0.136779313	0.22014392	negative
PRJNA729271	stage_E14_vs_E10	ordinary_muscle_development	ENSGALG00010007865	GABRR3	0.836856598	0.418259188	2.000808643	0.045413016	0.087488259	positive
PRJNA729271	stage_E14_vs_E10	ordinary_muscle_development	ENSGALG00010007866	TBP	-0.039954614	0.083419064	-0.478962626	0.631965217	0.725571154	negative
PRJNA729271	stage_E14_vs_E10	ordinary_muscle_development	ENSGALG00010007867	ZNF516	0.05056444	0.104341933	0.484603253	0.627957806	0.721999556	positive
PRJNA729271	stage_E14_vs_E10	ordinary_muscle_development	ENSGALG00010007868	INHBA	-1.72999423	0.294989744	-5.864591113	4.50241E-09	3.42945E-08	negative
PRJNA729271	stage_E14_vs_E10	ordinary_muscle_development	ENSGALG00010007869	GCSH	-0.619246069	0.122924696	-5.037605054	4.71393E-07	2.73095E-06	negative
PRJNA729271	stage_E14_vs_E10	ordinary_muscle_development	ENSGALG00010007870	AvBD3	-1.246799977	0.562176702	-2.217807982	0.026567928	0.055490218	negative
PRJNA729271	stage_E14_vs_E10	ordinary_muscle_development	ENSGALG00010007871	HSPA4L	-0.376654688	0.087921422	-4.283992211	1.83569E-05	8.22007E-05	negative
PRJNA729271	stage_E14_vs_E10	ordinary_muscle_development	ENSGALG00010007872	KLHL2	-0.102765295	0.075410648	-1.362742504	0.172963677	0.266006207	negative
PRJNA729271	stage_E14_vs_E10	ordinary_muscle_development	ENSGALG00010007873	ENSGALG00010007873	0.233544515	0.767179031	0.304419836	0.760808058	0.829540605	positive
PRJNA729271	stage_E14_vs_E10	ordinary_muscle_development	ENSGALG00010007874	ENSGALG00010007874	-0.773376325	0.556043471	-1.39085587	0.164269137	0.255179632	negative
PRJNA729271	stage_E14_vs_E10	ordinary_muscle_development	ENSGALG00010007875	AvBD4	3.085490029	1.42402336	2.166741161	0.030254596	0.061847224	positive
PRJNA729271	stage_E14_vs_E10	ordinary_muscle_development	ENSGALG00010007877	MSMO1	0.068312788	0.186498766	0.366290833	0.714148069	0.79334582	positive
PRJNA729271	stage_E14_vs_E10	ordinary_muscle_development	ENSGALG00010007878	PSMB1	-0.018463291	0.122025586	-0.151306719	0.879733769	0.917325443	negative
PRJNA729271	stage_E14_vs_E10	ordinary_muscle_development	ENSGALG00010007879	PLK4	-0.247505888	0.186341583	-1.328237551	0.184099643	0.279836863	negative
PRJNA729271	stage_E14_vs_E10	ordinary_muscle_development	ENSGALG00010007880	BCO1	-0.104981072	0.287461296	-0.365200718	0.714961582	0.79388449	negative
PRJNA729271	stage_E14_vs_E10	ordinary_muscle_development	ENSGALG00010007881	AvBD14	-1.484796558	0.617322154	-2.405221567	0.016162663	0.036170727	negative
PRJNA729271	stage_E14_vs_E10	ordinary_muscle_development	ENSGALG00010007882	CPE	0.036795427	0.226807504	0.162231963	0.871123191	0.911494466	positive
PRJNA729271	stage_E14_vs_E10	ordinary_muscle_development	ENSGALG00010007883	DHTKD1	0.569304749	0.119951883	4.74610932	2.07367E-06	1.08905E-05	positive
PRJNA729271	stage_E14_vs_E10	ordinary_muscle_development	ENSGALG00010007884	GLI3	-1.107519383	0.109754946	-10.09083806	6.06495E-24	1.6745E-22	negative
PRJNA729271	stage_E14_vs_E10	ordinary_muscle_development	ENSGALG00010007885	USP38	0.30820012	0.103681071	2.972578471	0.002953097	0.008200219	positive
PRJNA729271	stage_E14_vs_E10	ordinary_muscle_development	ENSGALG00010007886	GRM5	-1.593790107	0.729061792	-2.186083709	0.028809482	0.059376288	negative
PRJNA729271	stage_E14_vs_E10	ordinary_muscle_development	ENSGALG00010007887	CDT1	0.123491067	0.12145657	1.016750823	0.309271982	0.422994657	positive
PRJNA729271	stage_E14_vs_E10	ordinary_muscle_development	ENSGALG00010007889	DCAF6	0.902907126	0.097174072	9.291646469	1.5192E-20	3.38261E-19	positive
PRJNA729271	stage_E14_vs_E10	ordinary_muscle_development	ENSGALG00010007890	ENSGALG00010007890	-0.353516772	0.125349723	-2.820243733	0.004798719	0.012562295	negative
PRJNA729271	stage_E14_vs_E10	ordinary_muscle_development	ENSGALG00010007891	GDI1	-0.212175182	0.055357516	-3.832816133	0.000126685	0.000490463	negative
PRJNA729271	stage_E14_vs_E10	ordinary_muscle_development	ENSGALG00010007892	ENSGALG00010007892	0.053508947	0.443517763	0.120646684	0.903970891	0.934353371	positive
PRJNA729271	stage_E14_vs_E10	ordinary_muscle_development	ENSGALG00010007893	MFSD8	-0.255088689	0.149413107	-1.707271158	0.087771664	0.15265782	negative
PRJNA729271	stage_E14_vs_E10	ordinary_muscle_development	ENSGALG00010007894	CD88	0.609372732	0.262066077	2.325263686	0.020057865	0.043582484	positive
PRJNA729271	stage_E14_vs_E10	ordinary_muscle_development	ENSGALG00010007895	GOLGB1	-0.39406207	0.111624225	-3.530255834	0.000415158	0.001432448	negative
PRJNA729271	stage_E14_vs_E10	ordinary_muscle_development	ENSGALG00010007896	TIMMDC1	0.051375548	0.105009091	0.489248577	0.624665722	0.719650163	positive
PRJNA729271	stage_E14_vs_E10	ordinary_muscle_development	ENSGALG00010007897	EFHC1	-0.324988248	0.34044127	-0.954608845	0.339775542	0.454801596	negative
PRJNA729271	stage_E14_vs_E10	ordinary_muscle_development	ENSGALG00010007898	GAB1	0.169750803	0.088705769	1.913638822	0.055666321	0.103805908	positive
PRJNA729271	stage_E14_vs_E10	ordinary_muscle_development	ENSGALG00010007899	APRT	-0.223545959	0.149948045	-1.490822767	0.136008033	0.219072834	negative
PRJNA729271	stage_E14_vs_E10	ordinary_muscle_development	ENSGALG00010007900	FHOD3	-0.011228929	0.200006558	-0.056142805	0.955228044	0.968996212	negative
PRJNA729271	stage_E14_vs_E10	ordinary_muscle_development	ENSGALG00010007901	FSTL5	-2.511887401	0.325822076	-7.709383695	1.26427E-14	1.72233E-13	negative
PRJNA729271	stage_E14_vs_E10	ordinary_muscle_development	ENSGALG00010007902	C7orf25	-0.149791666	0.14509232	-1.032388658	0.301890083	0.415611764	negative
PRJNA729271	stage_E14_vs_E10	ordinary_muscle_development	ENSGALG00010007903	PSMA2	0.032782438	0.103135736	0.317857214	0.750593249	0.821486254	positive
PRJNA729271	stage_E14_vs_E10	ordinary_muscle_development	ENSGALG00010007904	ENSGALG00010007904	0.048059706	0.121345746	0.396055963	0.692063744	0.775056425	positive
PRJNA729271	stage_E14_vs_E10	ordinary_muscle_development	ENSGALG00010007905	MRPL32	0.214026745	0.153501992	1.394292943	0.163229161	0.253945121	positive
PRJNA729271	stage_E14_vs_E10	ordinary_muscle_development	ENSGALG00010007906	NAF1	-0.368562856	0.107462245	-3.429696217	0.000604257	0.002003587	negative
PRJNA729271	stage_E14_vs_E10	ordinary_muscle_development	ENSGALG00010007907	IRX2	-4.162986273	0.639369828	-6.511077145	7.46138E-11	6.97759E-10	negative
PRJNA729271	stage_E14_vs_E10	ordinary_muscle_development	ENSGALG00010007908	IRX1	-3.969672883	0.595720584	-6.663649015	2.67111E-11	2.6143E-10	negative
PRJNA729271	stage_E14_vs_E10	ordinary_muscle_development	ENSGALG00010007909	GALNS	0.029554772	0.105495139	0.280152924	0.779360183	0.843636615	positive
PRJNA729271	stage_E14_vs_E10	ordinary_muscle_development	ENSGALG00010007910	ANKS6	-0.256450268	0.146919699	-1.745513164	0.080895583	0.142788782	negative
PRJNA729271	stage_E14_vs_E10	ordinary_muscle_development	ENSGALG00010007911	IFI6	-0.408487113	0.828964665	-0.492767823	0.622176649	0.71733661	negative
PRJNA729271	stage_E14_vs_E10	ordinary_muscle_development	ENSGALG00010007912	TRAPPC2L	0.030840677	0.109932709	0.280541412	0.779062159	0.84354269	positive
PRJNA729271	stage_E14_vs_E10	ordinary_muscle_development	ENSGALG00010007914	PAQR8	0.37569358	0.12140473	3.094554729	0.001971086	0.005743075	positive
PRJNA729271	stage_E14_vs_E10	ordinary_muscle_development	ENSGALG00010007915	HECW1	1.789606887	0.128591868	13.91695221	4.99765E-44	5.04623E-42	positive
PRJNA729271	stage_E14_vs_E10	ordinary_muscle_development	ENSGALG00010007916	MCM3	-0.197901773	0.201128048	-0.983959101	0.325135623	0.439794779	negative
PRJNA729271	stage_E14_vs_E10	ordinary_muscle_development	ENSGALG00010007917	POGLUT1	-0.958428607	0.077761853	-12.32517707	6.62977E-35	3.61273E-33	negative
PRJNA729271	stage_E14_vs_E10	ordinary_muscle_development	ENSGALG00010007918	NPY1R	-1.366903999	0.66614116	-2.051973487	0.040172239	0.078603754	negative
PRJNA729271	stage_E14_vs_E10	ordinary_muscle_development	ENSGALG00010007920	FAT3	-0.562619647	0.177650897	-3.166995821	0.001540225	0.004624624	negative
PRJNA729271	stage_E14_vs_E10	ordinary_muscle_development	ENSGALG00010007921	FAM120B	0.040742267	0.099322168	0.410203158	0.681656925	0.767069104	positive
PRJNA729271	stage_E14_vs_E10	ordinary_muscle_development	ENSGALG00010007922	IL17A	2.470664276	0.369683499	6.683187862	2.338E-11	2.30696E-10	positive
PRJNA729271	stage_E14_vs_E10	ordinary_muscle_development	ENSGALG00010007923	YAP1	-1.039374901	0.091178843	-11.39929913	4.21499E-30	1.81834E-28	negative
PRJNA729271	stage_E14_vs_E10	ordinary_muscle_development	ENSGALG00010007924	ABHD18	0.082246028	0.115159565	0.714191897	0.475108527	0.58735165	positive
PRJNA729271	stage_E14_vs_E10	ordinary_muscle_development	ENSGALG00010007925	STK17A	2.218595542	0.234742822	9.451175231	3.35047E-21	7.83309E-20	positive
PRJNA729271	stage_E14_vs_E10	ordinary_muscle_development	ENSGALG00010007926	EIF3E	0.143811241	0.131349807	1.094872109	0.27357267	0.383764236	positive
PRJNA729271	stage_E14_vs_E10	ordinary_muscle_development	ENSGALG00010007927	DLL1	0.057195993	0.160687971	0.355944458	0.721882161	0.798609176	positive
PRJNA729271	stage_E14_vs_E10	ordinary_muscle_development	ENSGALG00010007928	C11orf70	-2.01602747	0.588579325	-3.425243438	0.000614249	0.002033699	negative
PRJNA729271	stage_E14_vs_E10	ordinary_muscle_development	ENSGALG00010007929	LRRC14B	4.671062044	0.334966664	13.94485643	3.38128E-44	3.50083E-42	positive
PRJNA729271	stage_E14_vs_E10	ordinary_muscle_development	ENSGALG00010007930	CBFA2T3	1.12554951	0.195548563	5.755856705	8.62035E-09	6.32028E-08	positive
PRJNA729271	stage_E14_vs_E10	ordinary_muscle_development	ENSGALG00010007931	GBE1	-0.328154767	0.189873008	-1.728285507	0.08393705	0.14729147	negative
PRJNA729271	stage_E14_vs_E10	ordinary_muscle_development	ENSGALG00010007932	NPY5R	0.038422507	0.180067314	0.213378576	0.831031689	0.882153375	positive
PRJNA729271	stage_E14_vs_E10	ordinary_muscle_development	ENSGALG00010007933	CEP126	-0.792602653	0.467006574	-1.697198063	0.089659221	0.155307172	negative
PRJNA729271	stage_E14_vs_E10	ordinary_muscle_development	ENSGALG00010007934	PKHD1	1.22304847	0.330312028	3.702706433	0.000213312	0.000786957	positive
PRJNA729271	stage_E14_vs_E10	ordinary_muscle_development	ENSGALG00010007935	LARP1B	0.1545055	0.12355679	1.250481665	0.211123649	0.312205396	positive
PRJNA729271	stage_E14_vs_E10	ordinary_muscle_development	ENSGALG00010007936	ENSGALG00010007936	2.170378126	0.139010839	15.61301367	5.93653E-55	1.10321E-52	positive
PRJNA729271	stage_E14_vs_E10	ordinary_muscle_development	ENSGALG00010007937	ANGPTL5	-0.076574598	0.416921096	-0.183666883	0.854274791	0.899101856	negative
PRJNA729271	stage_E14_vs_E10	ordinary_muscle_development	ENSGALG00010007938	BCL9	-0.255167716	0.100779948	-2.531929437	0.011343682	0.026619179	negative
PRJNA729271	stage_E14_vs_E10	ordinary_muscle_development	ENSGALG00010007940	GLIPR2	-0.568754629	0.271380586	-2.095782301	0.036101507	0.071791926	negative
PRJNA729271	stage_E14_vs_E10	ordinary_muscle_development	ENSGALG00010007941	MRPL19	-0.104245647	0.071324619	-1.461566137	0.14386014	0.22940074	negative
PRJNA729271	stage_E14_vs_E10	ordinary_muscle_development	ENSGALG00010007942	PGRMC2	0.141085319	0.08366922	1.686227261	0.091752035	0.15838344	positive
PRJNA729271	stage_E14_vs_E10	ordinary_muscle_development	ENSGALG00010007943	BLOC1S4	0.080951564	0.100006357	0.809464183	0.418248197	0.532733534	positive
PRJNA729271	stage_E14_vs_E10	ordinary_muscle_development	ENSGALG00010007944	ACSF3	1.275498439	0.129955971	9.814850569	9.71819E-23	2.51995E-21	positive
PRJNA729271	stage_E14_vs_E10	ordinary_muscle_development	ENSGALG00010007945	ENSGALG00010007945	-0.507314278	0.114159408	-4.443911264	8.83381E-06	4.17768E-05	negative
PRJNA729271	stage_E14_vs_E10	ordinary_muscle_development	ENSGALG00010007946	ENSGALG00010007946	0.120277784	0.144798356	0.830657114	0.40616736	0.521146843	positive
PRJNA729271	stage_E14_vs_E10	ordinary_muscle_development	ENSGALG00010007948	ENSGALG00010007948	-0.399589089	0.447074801	-0.893785757	0.371436531	0.487748227	negative
PRJNA729271	stage_E14_vs_E10	ordinary_muscle_development	ENSGALG00010007950	ARC	-2.146442297	0.585631787	-3.665173828	0.000247171	0.000898155	negative
PRJNA729271	stage_E14_vs_E10	ordinary_muscle_development	ENSGALG00010007951	TGFBR1	-0.221470795	0.113608799	-1.94941586	0.051245782	0.096807977	negative
PRJNA729271	stage_E14_vs_E10	ordinary_muscle_development	ENSGALG00010007952	EMC2	0.053705362	0.108534051	0.494825003	0.620723654	0.716248058	positive
PRJNA729271	stage_E14_vs_E10	ordinary_muscle_development	ENSGALG00010007953	SCGN	-0.909829712	0.424077544	-2.145432422	0.031918289	0.064652823	negative
PRJNA729271	stage_E14_vs_E10	ordinary_muscle_development	ENSGALG00010007954	PSCA	-0.442267147	0.598951016	-0.738402867	0.460269673	0.572916179	negative
PRJNA729271	stage_E14_vs_E10	ordinary_muscle_development	ENSGALG00010007955	TMA16	-0.27015256	0.120897471	-2.234559227	0.0254463	0.053563191	negative
PRJNA729271	stage_E14_vs_E10	ordinary_muscle_development	ENSGALG00010007956	HS1BP3	0.956857009	0.129161628	7.408214236	1.28011E-13	1.60235E-12	positive
PRJNA729271	stage_E14_vs_E10	ordinary_muscle_development	ENSGALG00010007957	CDH15	1.945286614	0.162657668	11.9593908	5.79858E-33	2.91841E-31	positive
PRJNA729271	stage_E14_vs_E10	ordinary_muscle_development	ENSGALG00010007958	ENSGALG00010007958	-0.182885494	0.534727644	-0.342016157	0.732338733	0.807181958	negative
PRJNA729271	stage_E14_vs_E10	ordinary_muscle_development	ENSGALG00010007960	TSNAX	-0.198082385	0.060728622	-3.261763207	0.001107216	0.003449193	negative
PRJNA729271	stage_E14_vs_E10	ordinary_muscle_development	ENSGALG00010007962	ADCY8	-0.539925037	0.438888588	-1.23020979	0.218618555	0.32089883	negative
PRJNA729271	stage_E14_vs_E10	ordinary_muscle_development	ENSGALG00010007963	LDAH	0.184846957	0.082276508	2.246655366	0.024662061	0.052125484	positive
PRJNA729271	stage_E14_vs_E10	ordinary_muscle_development	ENSGALG00010007964	RECQL4	-0.005645454	0.15014329	-0.037600443	0.970006255	0.979466434	negative
PRJNA729271	stage_E14_vs_E10	ordinary_muscle_development	ENSGALG00010007965	EGLN1	-0.383057049	0.157758828	-2.428117996	0.015177404	0.03427762	negative
PRJNA729271	stage_E14_vs_E10	ordinary_muscle_development	ENSGALG00010007966	TMEM74	-1.636366589	0.215908866	-7.578968923	3.48312E-14	4.57732E-13	negative

PRJNA729271	stage_E14_vs_E10	ordinary_muscle_development	ENSGALG00010007968	SLC22A31	1.071609173	0.193870208	5.527456663	3.24906E-08	2.22883E-07	positive
PRJNA729271	stage_E14_vs_E10	ordinary_muscle_development	ENSGALG00010007970	SPRTN	-0.218823427	0.107089562	-2.043368397	0.041015983	0.079973995	negative
PRJNA729271	stage_E14_vs_E10	ordinary_muscle_development	ENSGALG00010007972	EXOC8	-0.437839718	0.111739311	-3.918403601	8.91374E-05	0.000354276	negative
PRJNA729271	stage_E14_vs_E10	ordinary_muscle_development	ENSGALG00010007973	CARMIL1	-0.401410378	0.133907396	-2.997671441	0.002720508	0.007622997	negative
PRJNA729271	stage_E14_vs_E10	ordinary_muscle_development	ENSGALG00010007975	ANKRD11	-0.034835395	0.176032708	-0.197891603	0.843129874	0.890950465	negative
PRJNA729271	stage_E14_vs_E10	ordinary_muscle_development	ENSGALG00010007976	ENSGALG00010007976	0.590391575	0.336631769	1.753820135	0.079461332	0.14064275	positive
PRJNA729271	stage_E14_vs_E10	ordinary_muscle_development	ENSGALG00010007977	APOB	1.023921744	1.378810408	0.742612427	0.457716363	0.570619994	positive
PRJNA729271	stage_E14_vs_E10	ordinary_muscle_development	ENSGALG00010007978	JADE1	0.323625418	0.116617888	2.775092418	0.005518602	0.014190552	positive
PRJNA729271	stage_E14_vs_E10	ordinary_muscle_development	ENSGALG00010007979	RAB30	-0.108866541	0.088380378	-1.231795383	0.218025529	0.32019048	negative
PRJNA729271	stage_E14_vs_E10	ordinary_muscle_development	ENSGALG00010007980	MARCHF1	-0.617317037	0.22435303	-2.751543122	0.005931521	0.015123553	negative
PRJNA729271	stage_E14_vs_E10	ordinary_muscle_development	ENSGALG00010007981	TDRD15	-1.121713695	0.544261337	-2.060983611	0.039304604	0.077208325	negative
PRJNA729271	stage_E14_vs_E10	ordinary_muscle_development	ENSGALG00010007982	RECK	-0.197749548	0.145922697	-1.35516648	0.175364522	0.268928136	negative
PRJNA729271	stage_E14_vs_E10	ordinary_muscle_development	ENSGALG00010007983	RCHY1	0.223233377	0.086587738	2.578117665	0.009934016	0.023706547	positive
PRJNA729271	stage_E14_vs_E10	ordinary_muscle_development	ENSGALG00010007984	EIF3H	0.364663218	0.121966727	2.989858206	0.00279107	0.0077966	positive
PRJNA729271	stage_E14_vs_E10	ordinary_muscle_development	ENSGALG00010007985	APELA	0.573637027	0.29113867	1.970322343	0.048801439	0.092994199	positive
PRJNA729271	stage_E14_vs_E10	ordinary_muscle_development	ENSGALG00010007986	MYC	-0.851215867	0.189662951	-4.488045043	7.18797E-06	3.45228E-05	negative
PRJNA729271	stage_E14_vs_E10	ordinary_muscle_development	ENSGALG00010007987	ENSGALG00010007987	0.778274756	0.284489302	2.735690767	0.006224949	0.015752556	positive
PRJNA729271	stage_E14_vs_E10	ordinary_muscle_development	ENSGALG00010007988	TARBP1	-0.090269645	0.122614843	-0.736204874	0.461606026	0.574135863	negative
PRJNA729271	stage_E14_vs_E10	ordinary_muscle_development	ENSGALG00010007989	SPG7	0.206809355	0.097142016	2.128938273	0.033259368	0.06693898	positive
PRJNA729271	stage_E14_vs_E10	ordinary_muscle_development	ENSGALG00010007990	TRHR	0.384390183	0.823210974	0.466940062	0.640542762	0.733154401	positive
PRJNA729271	stage_E14_vs_E10	ordinary_muscle_development	ENSGALG00010007991	PCF11	-0.111065768	0.109933541	-1.010299194	0.312351967	0.426215986	negative
PRJNA729271	stage_E14_vs_E10	ordinary_muscle_development	ENSGALG00010007992	LAPTM4A	-0.211391907	0.098995406	-2.135370877	0.032730728	0.066058055	negative
PRJNA729271	stage_E14_vs_E10	ordinary_muscle_development	ENSGALG00010007993	LRRC14	0.333814989	0.170025166	1.963326938	0.049608197	0.094220631	positive
PRJNA729271	stage_E14_vs_E10	ordinary_muscle_development	ENSGALG00010007994	FAM65B	1.956697837	0.165672302	11.81065161	3.43886E-32	1.65054E-30	positive
PRJNA729271	stage_E14_vs_E10	ordinary_muscle_development	ENSGALG00010007995	KIAA0319	-1.428307549	0.718092697	-1.989029486	0.046697948	0.089577723	negative
PRJNA729271	stage_E14_vs_E10	ordinary_muscle_development	ENSGALG00010007996	NUDCD1	-0.450823585	0.07391261	-6.09941371	1.06458E-09	8.68869E-09	negative
PRJNA729271	stage_E14_vs_E10	ordinary_muscle_development	ENSGALG00010007997	TMEM192	-0.19387393	0.134902575	-1.437140327	0.150678124	0.238228557	negative
PRJNA729271	stage_E14_vs_E10	ordinary_muscle_development	ENSGALG00010007998	RPL13	0.554891403	0.144782851	3.832576858	0.000126808	0.000490809	positive
PRJNA729271	stage_E14_vs_E10	ordinary_muscle_development	ENSGALG00010007999	SDC1	-1.512312085	0.207700253	-7.281224086	3.30805E-13	3.98257E-12	negative
PRJNA729271	stage_E14_vs_E10	ordinary_muscle_development	ENSGALG00010008000	C8orf82	0.432485626	0.167028692	2.589289431	0.009617422	0.023042072	positive
PRJNA729271	stage_E14_vs_E10	ordinary_muscle_development	ENSGALG00010008001	ALDH5A1	-0.36528138	0.136462893	-2.676781747	0.007433304	0.018364708	negative
PRJNA729271	stage_E14_vs_E10	ordinary_muscle_development	ENSGALG00010008002	GJA8	1.253101857	0.150815719	8.308827941	9.66519E-17	1.59564E-15	positive
PRJNA729271	stage_E14_vs_E10	ordinary_muscle_development	ENSGALG00010008004	ENY2	-0.318346567	0.169965022	-1.873012245	0.061066694	0.112273178	negative
PRJNA729271	stage_E14_vs_E10	ordinary_muscle_development	ENSGALG00010008005	ANKRD42	-0.363963773	0.326336663	-1.11530151	0.264721238	0.373589169	negative
PRJNA729271	stage_E14_vs_E10	ordinary_muscle_development	ENSGALG00010008006	CPNE7	-0.22399538	0.515881154	-0.434199578	0.664143519	0.753086592	negative
PRJNA729271	stage_E14_vs_E10	ordinary_muscle_development	ENSGALG00010008007	CBR4	0.399745272	0.120842489	3.307986076	0.000939695	0.002979194	positive
PRJNA729271	stage_E14_vs_E10	ordinary_muscle_development	ENSGALG00010008008	BTC	2.359565525	0.20256634	11.64835935	2.33919E-31	1.06961E-29	positive
PRJNA729271	stage_E14_vs_E10	ordinary_muscle_development	ENSGALG00010008009	GPLD1	-2.557975954	1.371616193	-1.864935663	0.062190423	0.114041247	negative
PRJNA729271	stage_E14_vs_E10	ordinary_muscle_development	ENSGALG00010008010	CERK	-0.346861947	0.091420592	-3.794133689	0.00014816	0.000565152	negative
PRJNA729271	stage_E14_vs_E10	ordinary_muscle_development	ENSGALG00010008011	ADNP2	-0.061182649	0.099079602	-0.617510038	0.536898362	0.643541037	negative
PRJNA729271	stage_E14_vs_E10	ordinary_muscle_development	ENSGALG00010008012	CCDC90B	0.060205698	0.077729929	0.774549754	0.438605757	0.55244964	positive
PRJNA729271	stage_E14_vs_E10	ordinary_muscle_development	ENSGALG00010008013	ENSGALG00010008013	0.963072797	0.226066215	4.260135902	2.04303E-05	9.07838E-05	positive
PRJNA729271	stage_E14_vs_E10	ordinary_muscle_development	ENSGALG00010008015	ARHGAP39	1.526422083	0.144905562	10.53390955	6.02778E-26	1.97676E-24	positive
PRJNA729271	stage_E14_vs_E10	ordinary_muscle_development	ENSGALG00010008016	GSR	0.004131601	0.141893907	0.029117535	0.976770851	0.9845823	positive
PRJNA729271	stage_E14_vs_E10	ordinary_muscle_development	ENSGALG00010008017	GMNN	-0.090199735	0.13019512	-0.692804267	0.488432394	0.599426598	negative
PRJNA729271	stage_E14_vs_E10	ordinary_muscle_development	ENSGALG00010008018	ENSGALG00010008018	0.088964944	0.206485625	0.430852968	0.666575283	0.754608617	positive
PRJNA729271	stage_E14_vs_E10	ordinary_muscle_development	ENSGALG00010008019	PPP2CB	-0.08637965	0.098834139	-0.873985961	0.382125899	0.498195205	negative
PRJNA729271	stage_E14_vs_E10	ordinary_muscle_development	ENSGALG00010008020	COA6	0.055914662	0.131531787	0.425103795	0.670761011	0.757848548	positive
PRJNA729271	stage_E14_vs_E10	ordinary_muscle_development	ENSGALG00010008022	SLC35F3	-0.491455501	0.380840616	-1.290449288	0.196894709	0.295382819	negative
PRJNA729271	stage_E14_vs_E10	ordinary_muscle_development	ENSGALG00010008023	ATAD2	0.127171507	0.075087121	1.693652738	0.09033129	0.156228619	positive
PRJNA729271	stage_E14_vs_E10	ordinary_muscle_development	ENSGALG00010008024	MRS2	0.187778136	0.094461453	1.987881095	0.046824842	0.08975867	positive
PRJNA729271	stage_E14_vs_E10	ordinary_muscle_development	ENSGALG00010008025	TBC1D22A	-0.075578734	0.096524727	-0.782998684	0.433627885	0.547607265	negative
PRJNA729271	stage_E14_vs_E10	ordinary_muscle_development	ENSGALG00010008026	PURG	-0.660677338	0.123067848	-5.368399204	7.94386E-08	5.13816E-07	negative
PRJNA729271	stage_E14_vs_E10	ordinary_muscle_development	ENSGALG00010008027	C6orf62	0.538564116	0.10697719	5.034382702	4.79391E-07	2.77175E-06	positive
PRJNA729271	stage_E14_vs_E10	ordinary_muscle_development	ENSGALG00010008028	KCNK1	-1.975997968	0.165755625	-11.92115179	9.18301E-33	4.54293E-31	negative
PRJNA729271	stage_E14_vs_E10	ordinary_muscle_development	ENSGALG00010008029	LY6E	-0.364580217	0.314406761	-1.159581353	0.246219297	0.352907021	negative
PRJNA729271	stage_E14_vs_E10	ordinary_muscle_development	ENSGALG00010008030	GJA5	0.529799414	0.142659955	3.713722009	0.000204233	0.000756932	positive
PRJNA729271	stage_E14_vs_E10	ordinary_muscle_development	ENSGALG00010008031	MAP3K21	-1.260579866	0.175855649	-7.168264846	7.59543E-13	8.80766E-12	negative
PRJNA729271	stage_E14_vs_E10	ordinary_muscle_development	ENSGALG00010008032	PUM2	-0.110205644	0.053507508	-2.059629532	0.039433971	0.077389034	negative
PRJNA729271	stage_E14_vs_E10	ordinary_muscle_development	ENSGALG00010008033	ACOT13	-0.053042704	0.119718545	-0.443061719	0.657721093	0.747621334	negative
PRJNA729271	stage_E14_vs_E10	ordinary_muscle_development	ENSGALG00010008034	DPEP1	0.482186667	0.317023023	1.520983121	0.128264073	0.208618693	positive
PRJNA729271	stage_E14_vs_E10	ordinary_muscle_development	ENSGALG00010008035	TDP2	-0.076673344	0.100898935	-0.759902416	0.447312918	0.560542988	negative
PRJNA729271	stage_E14_vs_E10	ordinary_muscle_development	ENSGALG00010008036	SPOCK3	-1.488700828	0.162184845	-9.179037838	4.3496E-20	9.46659E-19	negative
PRJNA729271	stage_E14_vs_E10	ordinary_muscle_development	ENSGALG00010008037	ENSGALG00010008037	5.836365513	0.875680336	6.664949836	2.64756E-11	2.59651E-10	positive
PRJNA729271	stage_E14_vs_E10	ordinary_muscle_development	ENSGALG00010008038	WRN	-0.136702805	0.073758122	-1.853393235	0.063826016	0.116592074	negative
PRJNA729271	stage_E14_vs_E10	ordinary_muscle_development	ENSGALG00010008039	CHMP1A	0.208270058	0.08644341	2.409322561	0.015982166	0.035838721	positive
PRJNA729271	stage_E14_vs_E10	ordinary_muscle_development	ENSGALG00010008040	ENSGALG00010008040	0.324099738	0.263188003	1.231438114	0.218159049	0.320350767	positive
PRJNA729271	stage_E14_vs_E10	ordinary_muscle_development	ENSGALG00010008041	ENSGALG00010008041	-0.460280608	0.188300638	-2.444392184	0.014509644	0.033011661	negative
PRJNA729271	stage_E14_vs_E10	ordinary_muscle_development	ENSGALG00010008042	ENSGALG00010008042	-1.419719112	0.927305703	-1.531015185	0.12576564	0.205220415	negative
PRJNA729271	stage_E14_vs_E10	ordinary_muscle_development	ENSGALG00010008043	IL17REL	-2.466533867	0.357832132	-6.89299156	5.46311E-12	5.73409E-11	negative
PRJNA729271	stage_E14_vs_E10	ordinary_muscle_development	ENSGALG00010008045	DCDC2	-1.85206514	0.838555843	-2.208636616	0.027199926	0.0566307	negative
PRJNA729271	stage_E14_vs_E10	ordinary_muscle_development	ENSGALG00010008046	RHOB	0.876159801	0.167488485	5.231164392	1.68446E-07	1.0381E-06	positive
PRJNA729271	stage_E14_vs_E10	ordinary_muscle_development	ENSGALG00010008047	ASAP1	-0.945504309	0.127012161	-7.444203011	9.75314E-14	1.23577E-12	negative
PRJNA729271	stage_E14_vs_E10	ordinary_muscle_development	ENSGALG00010008048	ENSGALG00010008048	3.355964401	0.708667326	4.73559917	2.18409E-06	1.14208E-05	positive
PRJNA729271	stage_E14_vs_E10	ordinary_muscle_development	ENSGALG00010008050	CDK10	0.097937307	0.149740419	0.654047234	0.513081375	0.621832319	positive
PRJNA729271	stage_E14_vs_E10	ordinary_muscle_development	ENSGALG00010008051	ENSGALG00010008051	0.162483301	0.075085173	2.163986506	0.030465385	0.062161565	positive
PRJNA729271	stage_E14_vs_E10	ordinary_muscle_development	ENSGALG00010008052	RBFA	-0.270846389	0.122519236	-2.2106438	0.027060513	0.056356629	negative
PRJNA729271	stage_E14_vs_E10	ordinary_muscle_development	ENSGALG00010008054	CRISP3	-2.938354422	0.61143795	-4.805646135	1.54253E-06	8.28721E-06	negative
PRJNA729271	stage_E14_vs_E10	ordinary_muscle_development	ENSGALG00010008056	GTF2E2	-0.069441039	0.068041145	-1.020574226	0.307456206	0.421027653	negative
PRJNA729271	stage_E14_vs_E10	ordinary_muscle_development	ENSGALG00010008057	BAG1	-0.157644465	0.13127666	-1.200856761	0.229806769	0.333739017	negative
PRJNA729271	stage_E14_vs_E10	ordinary_muscle_development	ENSGALG00010008058	USP25	0.467243958	0.13881259	3.366005618	0.000762652	0.002471967	positive
PRJNA729271	stage_E14_vs_E10	ordinary_muscle_development	ENSGALG00010008059	ENSGALG00010008059	0.111474776	0.092484242	1.205338052	0.228072826	0.331619581	positive
PRJNA729271	stage_E14_vs_E10	ordinary_muscle_development	ENSGALG00010008062	VPS9D1	-0.136319206	0.135908957	-1.003018557	0.315851908	0.429751539	negative
PRJNA729271	stage_E14_vs_E10	ordinary_muscle_development	ENSGALG00010008063	KCNV1	1.512756348	0.253327436	5.971545645	2.35016E-09	1.84838E-08	positive
PRJNA729271	stage_E14_vs_E10	ordinary_muscle_development	ENSGALG00010008064	DDX60	-0.590638651	0.640561253	-0.922064281	0.356495047	0.472944875	negative
PRJNA729271	stage_E14_vs_E10	ordinary_muscle_development	ENSGALG00010008065	CHMP5	-0.182694264	0.085776958	-2.129875755	0.033181872	0.06681084	negative
PRJNA729271	stage_E14_vs_E10	ordinary_muscle_development	ENSGALG00010008066	PCNX2	-2.523014372	0.164232519	-15.36245313	2.92302E-53	4.87001E-51	negative
PRJNA729271	stage_E14_vs_E10	ordinary_muscle_development	ENSGALG00010008067	MPC2	0.248565164	0.145934285	1.703267764	0.08851797	0.15369765	positive
PRJNA729271	stage_E14_vs_E10	ordinary_muscle_development	ENSGALG00010008069	DLG2	-0.119181158	0.217061459	-0.549066417	0.582959871	0.68410001	negative
PRJNA729271	stage_E14_vs_E10	ordinary_muscle_development	ENSGALG00010008071	SMIM18	-1.151015258	0.331194054	-3.475350008	0.000510187	0.001725423	negative
PRJNA729271	stage_E14_vs_E10	ordinary_muscle_development	ENSGALG00010008072	NRSN1	-0.184249985	0.200211963	-0.920274603	0.357429277	0.473837331	negative
PRJNA729271	stage_E14_vs_E10	ordinary_muscle_development	ENSGALG00010008073	ZNF276	0.011711633	0.080780623	0.144980727	0.884726088	0.920941088	positive
PRJNA729271	stage_E14_vs_E10	ordinary_muscle_development	ENSGALG00010008074	RHAG	-0.079638839	0.430469728	-0.185004504	0.853225503	0.89840939	negative
PRJNA729271	stage_E14_vs_E10	ordinary_muscle_development	ENSGALG00010008075	SDHA	0.307885071	0.136544048	2.254840652	0.024143333	0.051178357	positive
PRJNA729271	stage_E14_vs_E10	ordinary_muscle_development	ENSGALG00010008076	ENSGALG00010008076	-3.536462916	1.941102219	-1.821883918	0.068472601	0.123776207	negative
PRJNA729271	stage_E14_vs_E10	ordinary_muscle_development	ENSGALG00010008077	ENSGALG00010008077	1.017315831	1.485949348	0.684623492	0.493581539	0.603752271	positive
PRJNA729271	stage_E14_vs_E10	ordinary_muscle_development	ENSGALG00010008078	GUCY1B1	-0.147601254	0.141384597	-1.043969837	0.296499345	0.409778605	negative
PRJNA729271	stage_E14_vs_E10	ordinary_muscle_development	ENSGALG00010008079	CCDC127	-0.192909382	0.108403484	-1.779549648	0.075149693	0.134100061	negative
PRJNA729271	stage_E14_vs_E10	ordinary_muscle_development	ENSGALG00010008081	TOP1MT	0.092968176	0.139508333	0.666398731	0.505156274	0.614043871	positive
PRJNA729271	stage_E14_vs_E10	ordinary_muscle_development	ENSGALG00010008083	FANCA	-0.543007729	0.23437554	-2.31682764	0.020513123	0.044451744	negative
PRJNA729271	stage_E14_vs_E10	ordinary_muscle_development	ENSGALG00010008084	CYP2AC1	1.748290365	1.357125099	1.28823081			positive
PRJNA729271	stage_E14_vs_E10	ordinary_muscle_development	ENSGALG00010008086	RHPN1	-0.510280707	0.210110245	-2.428633154	0.015155859	0.034234716	negative
PRJNA729271	stage_E14_vs_E10	ordinary_muscle_development	ENSGALG00010008087	CPNE8	-1.066177383	0.183750261	-5.802317645	6.54045E-09	4.87173E-08	negative
PRJNA729271	stage_E14_vs_E10	ordinary_muscle_development	ENSGALG00010008088	SLC35A4	0.348660081	0.069173797	5.040349004	4.64684E-07	2.69424E-06	positive
PRJNA729271	stage_E14_vs_E10	ordinary_muscle_development	ENSGALG00010008091	ENSGALG00010008091	0.682289111	0.272096094	2.507529968	0.012157827	0.028300579	positive
PRJNA729271	stage_E14_vs_E10	ordinary_muscle_development	ENSGALG00010008092	SPIRE2	0.732197074	0.632261136	1.158061175	0.246839072	0.35348077	positive
PRJNA729271	stage_E14_vs_E10	ordinary_muscle_development	ENSGALG00010008093	EFR3A	-0.313854226	0.092316353	-3.399768466	0.000674429	0.002213735	negative
PRJNA729271	stage_E14_vs_E10	ordinary_muscle_development	ENSGALG00010008094	PIM3	-0.402039098	0.142651856	-2.818323643	0.004827512	0.012623992	negative
PRJNA729271	stage_E14_vs_E10	ordinary_muscle_development	ENSGALG00010008096	CPSF1	-0.084097963	0.144627067	-0.581481493	0.560915988	0.664146156	negative
PRJNA729271	stage_E14_vs_E10	ordinary_muscle_development	ENSGALG00010008098	ENSGALG00010008098	-0.057214887	0.165622037	-0.345454556	0.729752669	0.805250071	negative
PRJNA729271	stage_E14_vs_E10	ordinary_muscle_development	ENSGALG00010008099	PANK3	0.061746878	0.076259703	0.809692093	0.418117163	0.532613436	positive
PRJNA729271	stage_E14_vs_E10	ordinary_muscle_development	ENSGALG00010008100	ENSGALG00010008100	0.252776001	0.238580995	1.059497636	0.289373205	0.401845623	positive
PRJNA729271	stage_E14_vs_E10	ordinary_muscle_development	ENSGALG00010008101	CENPQ	-0.264538607	0.20052622	-1.319222027	0.187094897	0.283420651	negative
PRJNA729271	stage_E14_vs_E10	ordinary_muscle_development	ENSGALG00010008103	RBPMS	0.091984662	0.10675015	0.861681803	0.388862642	0.504706662	positive
PRJNA729271	stage_E14_vs_E10	ordinary_muscle_development	ENSGALG00010008104	TCF25	0.099880014	0.094020906	1.062317077	0.288091764	0.400411365	positive
PRJNA729271	stage_E14_vs_E10	ordinary_muscle_development	ENSGALG00010008105	MMUT	0.337379139	0.09503654	3.549993938	0.00038524	0.001341998	positive
PRJNA729271	stage_E14_vs_E10	ordinary_muscle_development	ENSGALG00010008106	ENSGALG00010008106	-0.071453381	0.372159269	-0.19199678	0.847744727	0.894132273	negative
PRJNA729271	stage_E14_vs_E10	ordinary_muscle_development	ENSGALG00010008107	TUBB3	-0.535756579	0.207496461	-2.582003455	0.00982286	0.023472199	negative
PRJNA729271	stage_E14_vs_E10	ordinary_muscle_development	ENSGALG00010008108	ENSGALG00010008108	-0.448337948	0.367176175	-1.221043137	0.222069688	0.324813332	negative
PRJNA729271	stage_E14_vs_E10	ordinary_muscle_development	ENSGALG00010008110	DEF8	0.110149916	0.15169407	0.726131983	0.467757864	0.580144625	positive
PRJNA729271	stage_E14_vs_E10	ordinary_muscle_development	ENSGALG00010008111	SYTL2	-2.510182875	0.265379733	-9.458834117	3.11396E-21	7.31553E-20	negative
PRJNA729271	stage_E14_vs_E10	ordinary_muscle_development	ENSGALG00010008113	DBNDD1	0.420291718	0.161103331	2.608833194	0.009085152	0.021921213	positive
PRJNA729271	stage_E14_vs_E10	ordinary_muscle_development	ENSGALG00010008115	ENSGALG00010008115	0.002042599	0.198631841	0.010283343	0.991795224	0.99488386	positive
PRJNA729271	stage_E14_vs_E10	ordinary_muscle_development	ENSGALG00010008116	URAH	0.990354313	0.379266074	2.611238862	0.009021486	0.021798039	positive
PRJNA729271	stage_E14_vs_E10	ordinary_muscle_development	ENSGALG00010008117	ENSGALG00010008117	0.135458567	0.194595765	0.696102339	0.486364742	0.597597434	positive
PRJNA729271	stage_E14_vs_E10	ordinary_muscle_development	ENSGALG00010008118	ENSGALG00010008118	0.017213067	0.59899378	0.028736638	0.977074636	0.984683087	positive
PRJNA729271	stage_E14_vs_E10	ordinary_muscle_development	ENSGALG00010008119	CCDC83	-1.605198385	0.537891709	-2.984240804	0.002842829	0.007924387	negative
PRJNA729271	stage_E14_vs_E10	ordinary_muscle_development	ENSGALG00010008120	CDC5L	0.003645493	0.064201987	0.05678162	0.954719156	0.96886131	positive
PRJNA729271	stage_E14_vs_E10	ordinary_muscle_development	ENSGALG00010008121	MPZL1	-0.156401267	0.135149376	-1.157247428	0.247171285	0.353851633	negative
PRJNA729271	stage_E14_vs_E10	ordinary_muscle_development	ENSGALG00010008123	ENSGALG00010008123	-0.057097086	0.159288485	-0.358450807	0.720005975	0.797347896	negative
PRJNA729271	stage_E14_vs_E10	ordinary_muscle_development	ENSGALG00010008124	ENSGALG00010008124	-0.315053111	0.208283738	-1.512615021	0.130377476	0.211507725	negative

PRJNA729271	stage_E14_vs_E10	ordinary_muscle_development	ENSGALG00010008126	CDH3	-2.687743344	0.297731491	-9.027406991	1.75788E-19	3.61938E-18	negative
PRJNA729271	stage_E14_vs_E10	ordinary_muscle_development	ENSGALG00010008127	TBC1D2	-0.184835451	0.129840193	-1.423561127	0.154573555	0.24327293	negative
PRJNA729271	stage_E14_vs_E10	ordinary_muscle_development	ENSGALG00010008128	RARS	0.108775881	0.069161733	1.572775536	0.115770802	0.192243989	positive
PRJNA729271	stage_E14_vs_E10	ordinary_muscle_development	ENSGALG00010008129	TANGO6	0.20263273	0.116637238	1.737290195	0.082335961	0.144872513	positive
PRJNA729271	stage_E14_vs_E10	ordinary_muscle_development	ENSGALG00010008130	RCSD1	1.417800031	0.119366855	11.8776693	1.54619E-32	7.52079E-31	positive
PRJNA729271	stage_E14_vs_E10	ordinary_muscle_development	ENSGALG00010008131	OC90	0.145719309	0.519934294	0.280264854	0.779274313	0.843636615	positive
PRJNA729271	stage_E14_vs_E10	ordinary_muscle_development	ENSGALG00010008132	TDO2	-0.003608848	0.655918679	-0.005501974	0.995610082	0.997743925	negative
PRJNA729271	stage_E14_vs_E10	ordinary_muscle_development	ENSGALG00010008133	CREG1	0.224400966	0.16846045	1.332069141	0.18283747	0.278268492	positive
PRJNA729271	stage_E14_vs_E10	ordinary_muscle_development	ENSGALG00010008134	SLC2A13	-0.342105117	0.073543686	-4.65172658	3.29167E-06	1.67179E-05	negative
PRJNA729271	stage_E14_vs_E10	ordinary_muscle_development	ENSGALG00010008135	WWC1	-0.410313709	0.130197856	-3.151462872	0.001624548	0.004848462	negative
PRJNA729271	stage_E14_vs_E10	ordinary_muscle_development	ENSGALG00010008136	ELP1	-0.075417412	0.113047406	-0.667130853	0.504688554	0.613610182	negative
PRJNA729271	stage_E14_vs_E10	ordinary_muscle_development	ENSGALG00010008137	HHLA1	1.83282738	0.460567823	3.979495071	6.90618E-05	0.000280171	positive
PRJNA729271	stage_E14_vs_E10	ordinary_muscle_development	ENSGALG00010008139	ENSGALG00010008139	0.89802183	0.392930348	2.285447876	0.022286579	0.047822941	positive
PRJNA729271	stage_E14_vs_E10	ordinary_muscle_development	ENSGALG00010008141	APTX	-0.143930112	0.153497264	-0.937672166	0.348412912	0.464219612	negative
PRJNA729271	stage_E14_vs_E10	ordinary_muscle_development	ENSGALG00010008142	CD247	-0.08215354	0.355576828	-0.231043008	0.81728139	0.871954396	negative
PRJNA729271	stage_E14_vs_E10	ordinary_muscle_development	ENSGALG00010008143	DCTN6	0.454721608	0.114603846	3.96776919	7.25485E-05	0.000293182	positive
PRJNA729271	stage_E14_vs_E10	ordinary_muscle_development	ENSGALG00010008144	NTPCR	-0.169778685	0.132333846	-1.28295738	0.199507008	0.298527748	negative
PRJNA729271	stage_E14_vs_E10	ordinary_muscle_development	ENSGALG00010008145	ENSGALG00010008145	0.163287912	0.212289871	0.769174297	0.441789846	0.555735817	positive
PRJNA729271	stage_E14_vs_E10	ordinary_muscle_development	ENSGALG00010008146	SOX4	0.008604808	0.117689085	0.073114748	0.941714806	0.959049822	positive
PRJNA729271	stage_E14_vs_E10	ordinary_muscle_development	ENSGALG00010008147	CDKAL1	0.240283878	0.111322786	2.158442901	0.030893413	0.062902097	positive
PRJNA729271	stage_E14_vs_E10	ordinary_muscle_development	ENSGALG00010008148	ABCD2	0.0277564	0.139026839	0.199647785	0.841756053	0.889861232	positive
PRJNA729271	stage_E14_vs_E10	ordinary_muscle_development	ENSGALG00010008149	DNAJA1	-0.245058369	0.281642343	-0.870104852	0.384243106	0.500285986	negative
PRJNA729271	stage_E14_vs_E10	ordinary_muscle_development	ENSGALG00010008150	TERT	-2.388913124	0.560172055	-4.264606033	2.00255E-05	8.91219E-05	negative
PRJNA729271	stage_E14_vs_E10	ordinary_muscle_development	ENSGALG00010008151	SIPA1L2	-0.065193104	0.120589737	-0.540619013	0.58877021	0.68896619	negative
PRJNA729271	stage_E14_vs_E10	ordinary_muscle_development	ENSGALG00010008152	ENSGALG00010008152	0.994967437	0.165589458	6.008639995	1.87086E-09	1.48592E-08	positive
PRJNA729271	stage_E14_vs_E10	ordinary_muscle_development	ENSGALG00010008153	SMU1	-0.016162733	0.061089451	-0.264574868	0.791336983	0.853391085	negative
PRJNA729271	stage_E14_vs_E10	ordinary_muscle_development	ENSGALG00010008154	KCNQ3	-0.695160697	0.225925033	-3.076952946	0.002091283	0.006049321	negative
PRJNA729271	stage_E14_vs_E10	ordinary_muscle_development	ENSGALG00010008155	MAP10	0.302518579	0.220782409	1.37021142	0.170620914	0.263213086	positive
PRJNA729271	stage_E14_vs_E10	ordinary_muscle_development	ENSGALG00010008156	HAS3	-2.950139522	0.96573706	-3.054806164	0.00225206	0.006458966	negative
PRJNA729271	stage_E14_vs_E10	ordinary_muscle_development	ENSGALG00010008157	CTSO	-0.383737716	0.154668774	-2.481029022	0.013100371	0.030169983	negative
PRJNA729271	stage_E14_vs_E10	ordinary_muscle_development	ENSGALG00010008158	CLPTM1L	-0.397424437	0.074385287	-5.342782896	9.15304E-08	5.86531E-07	negative
PRJNA729271	stage_E14_vs_E10	ordinary_muscle_development	ENSGALG00010008159	SUPT3H	-1.667671481	0.12771439	-13.0578197	5.73468E-39	4.07471E-37	negative
PRJNA729271	stage_E14_vs_E10	ordinary_muscle_development	ENSGALG00010008160	TXNL4A	0.039838149	0.08947381	0.445249278	0.656139619	0.746526199	positive
PRJNA729271	stage_E14_vs_E10	ordinary_muscle_development	ENSGALG00010008161	TMEFF1	-2.555894833	0.35678594	-7.163664673	7.85484E-13	9.07942E-12	negative
PRJNA729271	stage_E14_vs_E10	ordinary_muscle_development	ENSGALG00010008162	HSBP1L1	0.047574231	0.205970141	0.230976349	0.817333176	0.871954396	positive
PRJNA729271	stage_E14_vs_E10	ordinary_muscle_development	ENSGALG00010008163	CHTF8	-0.091666131	0.209954491	-0.436600002	0.662401461	0.751556796	negative
PRJNA729271	stage_E14_vs_E10	ordinary_muscle_development	ENSGALG00010008164	TG	0.031755635	0.292814667	0.108449606	0.913639053	0.940755838	positive
PRJNA729271	stage_E14_vs_E10	ordinary_muscle_development	ENSGALG00010008165	MRPL1	0.291886229	0.083932875	3.477615051	0.000505896	0.001712908	positive
PRJNA729271	stage_E14_vs_E10	ordinary_muscle_development	ENSGALG00010008166	RUNX2	-4.689656936	0.416281827	-11.26558171	1.94066E-29	8.06014E-28	negative
PRJNA729271	stage_E14_vs_E10	ordinary_muscle_development	ENSGALG00010008167	UTP4	-0.103469962	0.173684487	-0.5957352	0.551352139	0.656145259	negative
PRJNA729271	stage_E14_vs_E10	ordinary_muscle_development	ENSGALG00010008168	CAVIN4	3.015907697	0.125057451	24.11617766	1.6913E-128	6.1288E-125	positive
PRJNA729271	stage_E14_vs_E10	ordinary_muscle_development	ENSGALG00010008169	HTR4	2.558909131	0.296421976	8.632656609	5.99432E-18	1.09155E-16	positive
PRJNA729271	stage_E14_vs_E10	ordinary_muscle_development	ENSGALG00010008170	PICALM	-0.29846856	0.070133443	-4.25572375	2.08374E-05	9.24229E-05	negative
PRJNA729271	stage_E14_vs_E10	ordinary_muscle_development	ENSGALG00010008171	KIF21A	-2.301843253	0.1822414	-12.63073733	1.42933E-36	8.8162E-35	negative
PRJNA729271	stage_E14_vs_E10	ordinary_muscle_development	ENSGALG00010008172	SNTB2	-0.135447406	0.127913426	-1.058899055	0.289645755	0.40206444	negative
PRJNA729271	stage_E14_vs_E10	ordinary_muscle_development	ENSGALG00010008174	SLC26A2	-0.590102259	0.121977862	-4.837781608	1.31296E-06	7.15466E-06	negative
PRJNA729271	stage_E14_vs_E10	ordinary_muscle_development	ENSGALG00010008175	ENSGALG00010008175	-1.298743361	0.445228939	-2.917023686	0.003533889	0.009587072	negative
PRJNA729271	stage_E14_vs_E10	ordinary_muscle_development	ENSGALG00010008176	SLA	-0.230736709	0.312261694	-0.738920953	0.459954998	0.572662015	negative
PRJNA729271	stage_E14_vs_E10	ordinary_muscle_development	ENSGALG00010008177	NREP	-0.903284805	0.184596512	-4.893292913	9.91626E-07	5.47775E-06	negative
PRJNA729271	stage_E14_vs_E10	ordinary_muscle_development	ENSGALG00010008178	PGR	-2.488178995	0.345633935	-7.198885135	6.07069E-13	7.11355E-12	negative
PRJNA729271	stage_E14_vs_E10	ordinary_muscle_development	ENSGALG00010008179	SEMA6A	0.158130647	0.094930162	1.665757686	0.095761687	0.164159193	positive
PRJNA729271	stage_E14_vs_E10	ordinary_muscle_development	ENSGALG00010008180	POU2F1	0.30446754	0.156866741	1.940931119	0.052266633	0.098377463	positive
PRJNA729271	stage_E14_vs_E10	ordinary_muscle_development	ENSGALG00010008181	DISC1	-0.610169977	0.130267141	-4.683989929	2.81344E-06	1.44561E-05	negative
PRJNA729271	stage_E14_vs_E10	ordinary_muscle_development	ENSGALG00010008182	CLIC5	0.673086435	0.306634264	2.195079008	0.028157931	0.058257095	positive
PRJNA729271	stage_E14_vs_E10	ordinary_muscle_development	ENSGALG00010008183	ALG10	-0.470595587	0.081315879	-5.787253276	7.15467E-09	5.3047E-08	negative
PRJNA729271	stage_E14_vs_E10	ordinary_muscle_development	ENSGALG00010008184	SLC6A2	0.258049221	0.08386165	3.077082544	0.002090374	0.006047899	positive
PRJNA729271	stage_E14_vs_E10	ordinary_muscle_development	ENSGALG00010008185	PDGFC	-0.256239943	0.086579831	-2.959580089	0.003080586	0.008511837	negative
PRJNA729271	stage_E14_vs_E10	ordinary_muscle_development	ENSGALG00010008186	NOP53	0.393527485	0.12104574	3.251064309	0.001149738	0.003568621	positive
PRJNA729271	stage_E14_vs_E10	ordinary_muscle_development	ENSGALG00010008187	ENSGALG00010008187	0.631883453	0.382678028	1.651214352	0.098694814	0.168323488	positive
PRJNA729271	stage_E14_vs_E10	ordinary_muscle_development	ENSGALG00010008188	VPS4A	0.243784733	0.112804592	2.16112419	0.030685747	0.062567156	positive
PRJNA729271	stage_E14_vs_E10	ordinary_muscle_development	ENSGALG00010008189	EXOC3	0.021974253	0.113765539	0.193153857	0.846838475	0.893480272	positive
PRJNA729271	stage_E14_vs_E10	ordinary_muscle_development	ENSGALG00010008190	TSNARE1	-0.009236526	0.167278529	-0.055216447	0.955966026	0.969476496	negative
PRJNA729271	stage_E14_vs_E10	ordinary_muscle_development	ENSGALG00010008192	HNRNPU	-0.027444263	0.082643554	-0.332079901	0.739828926	0.81284168	negative
PRJNA729271	stage_E14_vs_E10	ordinary_muscle_development	ENSGALG00010008193	CNOT6L	-0.090635516	0.131064926	-0.691531437	0.489231632	0.599862349	negative
PRJNA729271	stage_E14_vs_E10	ordinary_muscle_development	ENSGALG00010008194	PDF	0.239571274	0.163872561	1.461936468	0.14375862	0.229264078	positive
PRJNA729271	stage_E14_vs_E10	ordinary_muscle_development	ENSGALG00010008195	ENSGALG00010008195	1.289571246	0.440234989	2.929279314	0.00339749	0.009258623	positive
PRJNA729271	stage_E14_vs_E10	ordinary_muscle_development	ENSGALG00010008196	PDCD6	-0.115967043	0.146637166	-0.79084345	0.429035351	0.543399098	negative
PRJNA729271	stage_E14_vs_E10	ordinary_muscle_development	ENSGALG00010008197	STARD4	-0.317268294	0.157183967	-2.018452011	0.043544207	0.084280049	negative
PRJNA729271	stage_E14_vs_E10	ordinary_muscle_development	ENSGALG00010008198	GLRB	-0.017089355	0.325125063	-0.052562404	0.958080573	0.971208113	negative
PRJNA729271	stage_E14_vs_E10	ordinary_muscle_development	ENSGALG00010008199	TPRKB	-0.368107041	0.112988476	-3.257916686	0.001122334	0.003491035	negative
PRJNA729271	stage_E14_vs_E10	ordinary_muscle_development	ENSGALG00010008200	COG8	0.006064504	0.125724678	0.048236383	0.961527855	0.973890452	positive
PRJNA729271	stage_E14_vs_E10	ordinary_muscle_development	ENSGALG00010008201	ENPP4	0.279184794	0.242464846	1.151444418	0.249549449	0.356593952	positive
PRJNA729271	stage_E14_vs_E10	ordinary_muscle_development	ENSGALG00010008202	KCNG2	1.245301479	0.192332655	6.474727233	9.49834E-11	8.71382E-10	positive
PRJNA729271	stage_E14_vs_E10	ordinary_muscle_development	ENSGALG00010008203	CXCL13	-0.761611652	1.499324004	-0.507970025	0.611474364	0.708498874	negative
PRJNA729271	stage_E14_vs_E10	ordinary_muscle_development	ENSGALG00010008204	ENSGALG00010008204	-0.057773102	0.153316713	-0.376821947	0.70630593	0.786734736	negative
PRJNA729271	stage_E14_vs_E10	ordinary_muscle_development	ENSGALG00010008205	ENSGALG00010008205	-0.083052137	0.165387081	-0.502168225	0.615549192	0.711968205	negative
PRJNA729271	stage_E14_vs_E10	ordinary_muscle_development	ENSGALG00010008206	CXCL13L2	0.043566802	0.679723933	0.064094847	0.948894705	0.964599814	positive
PRJNA729271	stage_E14_vs_E10	ordinary_muscle_development	ENSGALG00010008207	CAMK4	-0.380300003	0.15426169	-2.465291307	0.01369019	0.031368843	negative
PRJNA729271	stage_E14_vs_E10	ordinary_muscle_development	ENSGALG00010008208	ILDR2	0.953033263	0.152522527	6.248475446	4.14478E-10	3.55916E-09	positive
PRJNA729271	stage_E14_vs_E10	ordinary_muscle_development	ENSGALG00010008209	PPARGC1B	0.90509522	0.240331739	3.766024504	0.000165868	0.000627087	positive
PRJNA729271	stage_E14_vs_E10	ordinary_muscle_development	ENSGALG00010008210	CTDP1	0.071673197	0.084530212	0.847900354	0.396493467	0.511997578	positive
PRJNA729271	stage_E14_vs_E10	ordinary_muscle_development	ENSGALG00010008211	ADGRB1	0.7664142	0.231803394	3.306311381	0.00094533	0.002995749	positive
PRJNA729271	stage_E14_vs_E10	ordinary_muscle_development	ENSGALG00010008212	GTSE1	-0.138252992	0.222130757	-0.622394637	0.533682406	0.641011474	negative
PRJNA729271	stage_E14_vs_E10	ordinary_muscle_development	ENSGALG00010008213	ISCA1	0.179954949	0.117038382	1.537572087	0.124153277	0.203069482	positive
PRJNA729271	stage_E14_vs_E10	ordinary_muscle_development	ENSGALG00010008214	CCNG2	0.282012182	0.135405299	2.082726336	0.037276175	0.073773643	positive
PRJNA729271	stage_E14_vs_E10	ordinary_muscle_development	ENSGALG00010008215	SELENOW	0.250064499	0.12823043	1.950118223	0.05116203	0.096687565	positive
PRJNA729271	stage_E14_vs_E10	ordinary_muscle_development	ENSGALG00010008217	ARHGEF37	2.263785245	0.16483904	13.73330761	6.41373E-43	5.95942E-41	positive
PRJNA729271	stage_E14_vs_E10	ordinary_muscle_development	ENSGALG00010008218	ARHGAP42	-0.366745842	0.101251183	-3.622138853	0.000292177	0.001048035	negative
PRJNA729271	stage_E14_vs_E10	ordinary_muscle_development	ENSGALG00010008219	NIP7	0.35770242	0.117420708	3.04633166	0.002316522	0.006617655	positive
PRJNA729271	stage_E14_vs_E10	ordinary_muscle_development	ENSGALG00010008221	ENSGALG00010008221	0.377703793	0.520212202	0.726057159	0.46780373	0.580151871	positive
PRJNA729271	stage_E14_vs_E10	ordinary_muscle_development	ENSGALG00010008222	STYXL2	1.440937407	0.275013336	5.239518299	1.60996E-07	9.93885E-07	positive
PRJNA729271	stage_E14_vs_E10	ordinary_muscle_development	ENSGALG00010008223	ENSGALG00010008223	-0.151238399	0.149528872	-1.011432756	0.31180935	0.425621672	negative
PRJNA729271	stage_E14_vs_E10	ordinary_muscle_development	ENSGALG00010008224	ENSGALG00010008224	-0.145516341	0.211693066	-0.687393044	0.49183508	0.602073261	negative
PRJNA729271	stage_E14_vs_E10	ordinary_muscle_development	ENSGALG00010008226	COX20	-0.731079754	0.164529909	-4.443445932	8.85294E-06	4.18537E-05	negative
PRJNA729271	stage_E14_vs_E10	ordinary_muscle_development	ENSGALG00010008227	DUSP1	1.228149272	0.178188001	6.892435327	5.48452E-12	5.7524E-11	positive
PRJNA729271	stage_E14_vs_E10	ordinary_muscle_development	ENSGALG00010008228	TMED6	3.206224475	0.649481791	4.936588708	7.95009E-07	4.44414E-06	positive
PRJNA729271	stage_E14_vs_E10	ordinary_muscle_development	ENSGALG00010008229	PIGY	0.278548883	0.1223336	2.276961389	0.022788531	0.048738359	positive
PRJNA729271	stage_E14_vs_E10	ordinary_muscle_development	ENSGALG00010008230	TRMU	-0.068252601	0.113222189	-0.602820006	0.546628436	0.652076305	negative
PRJNA729271	stage_E14_vs_E10	ordinary_muscle_development	ENSGALG00010008231	ZCCHC6	-0.161625302	0.109814691	-1.471800367	0.141074795	0.225803793	negative
PRJNA729271	stage_E14_vs_E10	ordinary_muscle_development	ENSGALG00010008232	PYURF	0.187053103	0.127519544	1.466858318	0.142414602	0.227546258	positive
PRJNA729271	stage_E14_vs_E10	ordinary_muscle_development	ENSGALG00010008233	CYRIB	0.03838645	0.069253278	0.554290722	0.579379899	0.680988998	positive
PRJNA729271	stage_E14_vs_E10	ordinary_muscle_development	ENSGALG00010008234	COMMD10	-0.664167466	0.117746413	-5.640659845	1.694E-08	1.2013E-07	negative
PRJNA729271	stage_E14_vs_E10	ordinary_muscle_development	ENSGALG00010008236	ENSGALG00010008236	0.206237004	0.706989698	0.29171147	0.770507239	0.837030835	positive
PRJNA729271	stage_E14_vs_E10	ordinary_muscle_development	ENSGALG00010008237	GRIA2	-1.050342986	0.340365717	-3.085924738	0.002029202	0.005883833	negative
PRJNA729271	stage_E14_vs_E10	ordinary_muscle_development	ENSGALG00010008238	NTAQ1	-0.019880308	0.15688663	-0.126717666	0.899163863	0.930490483	negative
PRJNA729271	stage_E14_vs_E10	ordinary_muscle_development	ENSGALG00010008240	RCAN2	-0.75742224	0.137987341	-5.489070478	4.04054E-08	2.71902E-07	negative
PRJNA729271	stage_E14_vs_E10	ordinary_muscle_development	ENSGALG00010008241	HERC3	0.063828339	0.082569431	0.773026271	0.439506833	0.553392247	positive
PRJNA729271	stage_E14_vs_E10	ordinary_muscle_development	ENSGALG00010008242	CNTN5	-5.433468862	1.248556717	-4.351799793			
PRJNA729271	stage_E14_vs_E10	ordinary_muscle_development	ENSGALG00010008243	YTHDC2	-0.283485523	0.103192671	-2.74714785	0.006011603	0.015290083	negative
PRJNA729271	stage_E14_vs_E10	ordinary_muscle_development	ENSGALG00010008245	FBXO32	0.947258968	0.492252893	1.924333978	0.054312734	0.101608555	positive
PRJNA729271	stage_E14_vs_E10	ordinary_muscle_development	ENSGALG00010008246	TERF2	0.031358168	0.055212159	0.56795765	0.570063724	0.672219432	positive
PRJNA729271	stage_E14_vs_E10	ordinary_muscle_development	ENSGALG00010008247	GASK1B	0.909243574	0.160390361	5.668941488	1.43682E-08	1.02747E-07	positive
PRJNA729271	stage_E14_vs_E10	ordinary_muscle_development	ENSGALG00010008248	TPK1	0.080491114	0.159800486	0.503697551	0.614473929	0.710951437	positive
PRJNA729271	stage_E14_vs_E10	ordinary_muscle_development	ENSGALG00010008250	KLHL38	0.220621184	0.613008299	0.359899179	0.718922531	0.796756792	positive
PRJNA729271	stage_E14_vs_E10	ordinary_muscle_development	ENSGALG00010008251	AHRR	-0.143565634	0.23774896	-0.603853889	0.545940788	0.651416836	negative
PRJNA729271	stage_E14_vs_E10	ordinary_muscle_development	ENSGALG00010008252	ZFP92	-0.290284414	0.117154472	-2.477792007	0.013219818	0.030416074	negative
PRJNA729271	stage_E14_vs_E10	ordinary_muscle_development	ENSGALG00010008253	ENSGALG00010008253	0.637133131	0.663375536	0.960441101	0.336833261	0.451990198	positive
PRJNA729271	stage_E14_vs_E10	ordinary_muscle_development	ENSGALG00010008255	MSANTD3	-0.956661838	0.123585677	-7.740879515	9.87314E-15	1.36037E-13	negative
PRJNA729271	stage_E14_vs_E10	ordinary_muscle_development	ENSGALG00010008257	DESI2	-0.279133281	0.058538267	-4.768389942	1.85704E-06	9.84198E-06	negative
PRJNA729271	stage_E14_vs_E10	ordinary_muscle_development	ENSGALG00010008258	CCDC82	0.168912235	0.131664206	1.282901708	0.199526514	0.298527748	positive
PRJNA729271	stage_E14_vs_E10	ordinary_muscle_development	ENSGALG00010008259	MCC	0.067233908	0.108082648	0.622060147	0.533902319	0.641116239	positive
PRJNA729271	stage_E14_vs_E10	ordinary_muscle_development	ENSGALG00010008261	CRX	-1.390680187	0.398839138	-3.486819758	0.000488801	0.001662404	negative
PRJNA729271	stage_E14_vs_E10	ordinary_muscle_development	ENSGALG00010008262	CYB5B	-0.035791705	0.070328163	-0.508924211	0.610805349	0.70778028	negative
PRJNA729271	stage_E14_vs_E10	ordinary_muscle_development	ENSGALG00010008263	C1orf101	-0.544268019	0.489634099	-1.111581118	0.266318295	0.375221976	negative
PRJNA729271	stage_E14_vs_E10	ordinary_muscle_development	ENSGALG00010008264	CNTNAP2	-1.360558466	0.326315663	-4.169454985	3.05329E-05	0.000132072	negative
PRJNA729271	stage_E14_vs_E10	ordinary_muscle_development	ENSGALG00010008265	ENSGALG00010008265	-1.745152344	0.673098046	-2.592716402	0.009522125	0.022836401	negative
PRJNA729271	stage_E14_vs_E10	ordinary_muscle_development	ENSGALG00010008266	ECSCR	0.148660329	0.18751701	0.79278317	0.427904164	0.542268828	positive
PRJNA729271	stage_E14_vs_E10	ordinary_muscle_development	ENSGALG00010008267	MAML2	-0.599476431	0.117474654	-5.103027866	3.34262E-07	1.98327E-06	negative
PRJNA729271	stage_E14_vs_E10	ordinary_muscle_development	ENSGALG00010008268	ENSGALG00010008268	1.057984778	0.236805036	4.467746116	7.9048E-06	3.76784E-05	positive
PRJNA729271	stage_E14_vs_E10	ordinary_muscle_development	ENSGALG00010008269	ENSGALG00010008269	0.391056652	0.303999942	1.286370811	0.19831368	0.296958347	positive
PRJNA729271	stage_E14_vs_E10	ordinary_muscle_development	ENSGALG00010008270	ENSGALG00010008270	1.014502	0.158568363	6.39788404	1.57545E-10	1.41663E-09	positive

PRJNA729271	stage_E14_vs_E10	ordinary_muscle_development	ENSGALG00010008272	DCP2	-0.710245144	0.082447426	-8.614521725	7.02342E-18	1.27256E-16	negative
PRJNA729271	stage_E14_vs_E10	ordinary_muscle_development	ENSGALG00010008273	TMEM144	-0.30249005	0.120243954	-2.515636248	0.011881774	0.027726532	negative
PRJNA729271	stage_E14_vs_E10	ordinary_muscle_development	ENSGALG00010008274	NFAT5	-0.376886216	0.109022429	-3.456960364	0.000546305	0.001833031	negative
PRJNA729271	stage_E14_vs_E10	ordinary_muscle_development	ENSGALG00010008275	CELSR1	0.536166104	0.169209455	3.168653339	0.001531469	0.004605529	positive
PRJNA729271	stage_E14_vs_E10	ordinary_muscle_development	ENSGALG00010008276	DNAJC18	0.45353783	0.09278122	4.888250318	1.01736E-06	5.61136E-06	positive
PRJNA729271	stage_E14_vs_E10	ordinary_muscle_development	ENSGALG00010008277	MZB1	2.978051553	1.097843631	2.712637273	0.006675013	0.016716363	positive
PRJNA729271	stage_E14_vs_E10	ordinary_muscle_development	ENSGALG00010008278	CYP39A1	-0.132363462	0.163137948	-0.811359118	0.417159469	0.531767348	negative
PRJNA729271	stage_E14_vs_E10	ordinary_muscle_development	ENSGALG00010008279	MAPK15	-0.118004608	0.158835478	-0.742936085	0.457520378	0.570434279	negative
PRJNA729271	stage_E14_vs_E10	ordinary_muscle_development	ENSGALG00010008282	FAM83H	-0.655251706	0.260824998	-2.512227395	0.011997176	0.027971539	negative
PRJNA729271	stage_E14_vs_E10	ordinary_muscle_development	ENSGALG00010008283	NQO1	-0.421449456	0.191995544	-2.19510019	0.028156412	0.058257095	negative
PRJNA729271	stage_E14_vs_E10	ordinary_muscle_development	ENSGALG00010008284	BCKDHA	0.656569468	0.129268358	5.079119726	3.79188E-07	2.23156E-06	positive
PRJNA729271	stage_E14_vs_E10	ordinary_muscle_development	ENSGALG00010008285	ADSS2	-1.116395533	0.088479156	-12.6176106	1.68868E-36	1.0328E-34	negative
PRJNA729271	stage_E14_vs_E10	ordinary_muscle_development	ENSGALG00010008289	MTMR2	-0.581948881	0.068706469	-8.470074116	2.45237E-17	4.25204E-16	negative
PRJNA729271	stage_E14_vs_E10	ordinary_muscle_development	ENSGALG00010008290	FAM13A	-0.448299446	0.163920943	-2.734851562	0.006240841	0.015784504	negative
PRJNA729271	stage_E14_vs_E10	ordinary_muscle_development	ENSGALG00010008291	PTGFRN	1.224684949	0.160655901	7.623031208	2.47787E-14	3.3103E-13	positive
PRJNA729271	stage_E14_vs_E10	ordinary_muscle_development	ENSGALG00010008292	REEP5	0.186805883	0.113398227	1.647343953	0.099487368	0.169416049	positive
PRJNA729271	stage_E14_vs_E10	ordinary_muscle_development	ENSGALG00010008293	PLA2G7	-0.086367859	0.114772941	-0.752510639	0.451744034	0.564818867	negative
PRJNA729271	stage_E14_vs_E10	ordinary_muscle_development	ENSGALG00010008294	NOB1	0.029467799	0.153962655	0.19139576	0.848215542	0.894498674	positive
PRJNA729271	stage_E14_vs_E10	ordinary_muscle_development	ENSGALG00010008295	DMAC2	0.393244191	0.129823392	3.02907037	0.002453075	0.006938015	positive
PRJNA729271	stage_E14_vs_E10	ordinary_muscle_development	ENSGALG00010008296	PALLD	0.085203267	0.15296069	0.557027211	0.577508837	0.679187878	positive
PRJNA729271	stage_E14_vs_E10	ordinary_muscle_development	ENSGALG00010008297	ENSGALG00010008297	-0.380864865	0.281972	-1.350718744	0.176785546	0.270677774	negative
PRJNA729271	stage_E14_vs_E10	ordinary_muscle_development	ENSGALG00010008298	ENSGALG00010008298	-0.609594872	0.130334359	-4.677161695	2.90873E-06	1.49193E-05	negative
PRJNA729271	stage_E14_vs_E10	ordinary_muscle_development	ENSGALG00010008299	ANKRD66	-0.860755202	0.314896089	-2.733457898	0.006267314	0.015845929	negative
PRJNA729271	stage_E14_vs_E10	ordinary_muscle_development	ENSGALG00010008300	CUL1	-0.023883633	0.084698297	-0.281984812	0.777955157	0.842784753	negative
PRJNA729271	stage_E14_vs_E10	ordinary_muscle_development	ENSGALG00010008301	FAM91A1	-0.235735778	0.104814735	-2.249070975	0.024507981	0.051845181	negative
PRJNA729271	stage_E14_vs_E10	ordinary_muscle_development	ENSGALG00010008302	ENSGALG00010008302	-0.096186914	0.14462728	-0.665067568	0.506007277	0.614904048	negative
PRJNA729271	stage_E14_vs_E10	ordinary_muscle_development	ENSGALG00010008303	TEX10	-0.331168625	0.069649425	-4.75479337	1.98649E-06	1.04706E-05	negative
PRJNA729271	stage_E14_vs_E10	ordinary_muscle_development	ENSGALG00010008306	ENSGALG00010008306	0.12382914	0.286250911	0.432589505	0.665313015	0.753762875	positive
PRJNA729271	stage_E14_vs_E10	ordinary_muscle_development	ENSGALG00010008307	ENSGALG00010008307	-0.743613783	0.976123983	-0.761802595	0.446177831	0.559604366	negative
PRJNA729271	stage_E14_vs_E10	ordinary_muscle_development	ENSGALG00010008308	WWP2	-2.119508473	0.12462796	-17.00668509	7.32678E-65	2.08239E-62	negative
PRJNA729271	stage_E14_vs_E10	ordinary_muscle_development	ENSGALG00010008309	ENSGALG00010008309	1.983511308	0.265822402	7.461791376	8.5354E-14	1.09005E-12	positive
PRJNA729271	stage_E14_vs_E10	ordinary_muscle_development	ENSGALG00010008310	EXOSC5	0.204952579	0.177997878	1.15143271	0.249554263	0.356593952	positive
PRJNA729271	stage_E14_vs_E10	ordinary_muscle_development	ENSGALG00010008311	SRP19	-0.564504651	0.114493299	-4.930460154	8.20361E-07	4.58056E-06	negative
PRJNA729271	stage_E14_vs_E10	ordinary_muscle_development	ENSGALG00010008312	ZBTB18	1.129950013	0.216850756	5.210726652	1.88102E-07	1.15385E-06	positive
PRJNA729271	stage_E14_vs_E10	ordinary_muscle_development	ENSGALG00010008313	PSMD7	-0.171661631	0.178286713	-0.962840288	0.335627679	0.450915118	negative
PRJNA729271	stage_E14_vs_E10	ordinary_muscle_development	ENSGALG00010008314	INVS	-0.149044686	0.104477774	-1.426568356	0.153704351	0.242194213	negative
PRJNA729271	stage_E14_vs_E10	ordinary_muscle_development	ENSGALG00010008316	ADGRF5	0.03418452	0.140267897	0.243708796	0.80745636	0.864663116	positive
PRJNA729271	stage_E14_vs_E10	ordinary_muscle_development	ENSGALG00010008317	CEP57	-0.239450826	0.108896677	-2.198880934	0.02788639	0.057827356	negative
PRJNA729271	stage_E14_vs_E10	ordinary_muscle_development	ENSGALG00010008318	ADPRH	0.01033692	0.1346662	0.076759571	0.938814814	0.957092613	positive
PRJNA729271	stage_E14_vs_E10	ordinary_muscle_development	ENSGALG00010008319	ERP44	0.155728272	0.064007991	2.43295048	0.014976349	0.033908494	positive
PRJNA729271	stage_E14_vs_E10	ordinary_muscle_development	ENSGALG00010008320	GPRIN3	0.450334553	0.114348987	3.938246971	8.20791E-05	0.000328384	positive
PRJNA729271	stage_E14_vs_E10	ordinary_muscle_development	ENSGALG00010008321	ZFHX3	-0.966178826	0.103006491	-9.379785828	6.61069E-21	1.51377E-19	negative
PRJNA729271	stage_E14_vs_E10	ordinary_muscle_development	ENSGALG00010008323	AKT3	0.492590891	0.102957626	4.784404117	1.71495E-06	9.13906E-06	positive
PRJNA729271	stage_E14_vs_E10	ordinary_muscle_development	ENSGALG00010008324	SCRIB	1.090140419	0.103317865	10.55132546	5.00855E-26	1.64998E-24	positive
PRJNA729271	stage_E14_vs_E10	ordinary_muscle_development	ENSGALG00010008325	AADAT	0.193793848	0.129720646	1.49393219	0.135193344	0.21807562	positive
PRJNA729271	stage_E14_vs_E10	ordinary_muscle_development	ENSGALG00010008327	TGFB1	0.112870867	0.127559107	0.884851502	0.376236728	0.492464455	positive
PRJNA729271	stage_E14_vs_E10	ordinary_muscle_development	ENSGALG00010008328	ENSGALG00010008328	-0.411927581	0.44553444	-0.924569559	0.355189853	0.471558612	negative
PRJNA729271	stage_E14_vs_E10	ordinary_muscle_development	ENSGALG00010008330	EZH2	-0.523604001	0.075006578	-6.980774405	2.93557E-12	3.21141E-11	negative
PRJNA729271	stage_E14_vs_E10	ordinary_muscle_development	ENSGALG00010008331	CCDC97	-0.115010787	0.264349328	-0.43507123	0.663510726	0.752432168	negative
PRJNA729271	stage_E14_vs_E10	ordinary_muscle_development	ENSGALG00010008332	DHX38	-0.048830057	0.092823542	-0.526052506	0.598851726	0.698282984	negative
PRJNA729271	stage_E14_vs_E10	ordinary_muscle_development	ENSGALG00010008333	APC	0.42732056	0.09160521	4.664806302	3.08908E-06	1.57829E-05	positive
PRJNA729271	stage_E14_vs_E10	ordinary_muscle_development	ENSGALG00010008334	STX17	0.216919184	0.133098816	1.629760437	0.103152138	0.174590174	positive
PRJNA729271	stage_E14_vs_E10	ordinary_muscle_development	ENSGALG00010008335	HNRNPUL1	-0.405158086	0.187046999	-2.166076373	0.030305351	0.061922197	negative
PRJNA729271	stage_E14_vs_E10	ordinary_muscle_development	ENSGALG00010008336	PAIP2B	0.439667741	0.10894825	4.035564979	5.44711E-05	0.000225138	positive
PRJNA729271	stage_E14_vs_E10	ordinary_muscle_development	ENSGALG00010008339	FAM76B	-0.257228484	0.11082489	-2.321035309	0.020284938	0.044036271	negative
PRJNA729271	stage_E14_vs_E10	ordinary_muscle_development	ENSGALG00010008340	PDIA4	-0.283400706	0.105415461	-2.688416898	0.00717917	0.0178219	negative
PRJNA729271	stage_E14_vs_E10	ordinary_muscle_development	ENSGALG00010008341	DHODH	-0.223356005	0.101501496	-2.20051934	0.027770069	0.057635618	negative
PRJNA729271	stage_E14_vs_E10	ordinary_muscle_development	ENSGALG00010008342	PLD3	0.276728007	0.148386544	1.864913087	0.062193588	0.114041247	positive
PRJNA729271	stage_E14_vs_E10	ordinary_muscle_development	ENSGALG00010008343	TNFRSF21	0.373935038	0.22419644	1.66789017	0.095337531	0.163579252	positive
PRJNA729271	stage_E14_vs_E10	ordinary_muscle_development	ENSGALG00010008344	MFAP3L	-0.059947976	0.300477387	-0.19950911	0.841864518	0.889872835	negative
PRJNA729271	stage_E14_vs_E10	ordinary_muscle_development	ENSGALG00010008346	NR4A3	-0.914402642	0.545601852	-1.675952234	0.093747549	0.161252014	negative
PRJNA729271	stage_E14_vs_E10	ordinary_muscle_development	ENSGALG00010008347	PUF60	0.028728694	0.053393274	0.538058286	0.59053681	0.690531709	positive
PRJNA729271	stage_E14_vs_E10	ordinary_muscle_development	ENSGALG00010008348	ENSGALG00010008348	-0.275015345	0.096120057	-2.861165012	0.004220873	0.011207465	negative
PRJNA729271	stage_E14_vs_E10	ordinary_muscle_development	ENSGALG00010008350	LRRC6	-0.026773225	0.17194896	-0.155704487	0.87626596	0.915157799	negative
PRJNA729271	stage_E14_vs_E10	ordinary_muscle_development	ENSGALG00010008351	IST1	0.040752278	0.067177628	0.606634675	0.544093383	0.649693846	positive
PRJNA729271	stage_E14_vs_E10	ordinary_muscle_development	ENSGALG00010008352	ENSGALG00010008352	-0.012233324	0.152443849	-0.080248067	0.936039961	0.955485862	negative
PRJNA729271	stage_E14_vs_E10	ordinary_muscle_development	ENSGALG00010008354	TMEM71	0.298257582	0.239346361	1.246133766	0.21271529	0.314078449	positive
PRJNA729271	stage_E14_vs_E10	ordinary_muscle_development	ENSGALG00010008355	MAFA	2.910498887	0.177926424	16.35787888	3.8221E-60	9.23357E-58	positive
PRJNA729271	stage_E14_vs_E10	ordinary_muscle_development	ENSGALG00010008356	ENSGALG00010008356	-0.149482427	0.114712248	-1.30310781	0.192537958	0.289987292	negative
PRJNA729271	stage_E14_vs_E10	ordinary_muscle_development	ENSGALG00010008358	HPF1	-0.114678944	0.111082434	-1.032376944	0.301895569	0.415611764	negative
PRJNA729271	stage_E14_vs_E10	ordinary_muscle_development	ENSGALG00010008360	ZC3H3	0.347339104	0.059461498	5.841411985	5.17602E-09	3.90966E-08	positive
PRJNA729271	stage_E14_vs_E10	ordinary_muscle_development	ENSGALG00010008361	MATR3	-0.183804298	0.056420492	-3.257757815	0.001122962	0.003492241	negative
PRJNA729271	stage_E14_vs_E10	ordinary_muscle_development	ENSGALG00010008362	RAMP3	0.820978756	0.259409299	3.164800792	0.001551892	0.004652465	positive
PRJNA729271	stage_E14_vs_E10	ordinary_muscle_development	ENSGALG00010008364	ENSGALG00010008364	-0.621076475	0.589092428	-1.054293768	0.291748441	0.404253289	negative
PRJNA729271	stage_E14_vs_E10	ordinary_muscle_development	ENSGALG00010008366	RPRD1A	-0.082934211	0.106195484	-0.780957979	0.43482721	0.548978347	negative
PRJNA729271	stage_E14_vs_E10	ordinary_muscle_development	ENSGALG00010008367	ENSGALG00010008367	-0.352010028	0.615011778	-0.572363068	0.567076031	0.669580244	negative
PRJNA729271	stage_E14_vs_E10	ordinary_muscle_development	ENSGALG00010008368	SNCA	-1.441723891	0.34389804	-4.192300398	2.7614E-05	0.000120272	negative
PRJNA729271	stage_E14_vs_E10	ordinary_muscle_development	ENSGALG00010008372	CLCN3	0.039498567	0.068340849	0.577964238	0.563288264	0.666084466	positive
PRJNA729271	stage_E14_vs_E10	ordinary_muscle_development	ENSGALG00010008373	MACIR	-0.511957908	0.164039787	-3.120937412	0.001802763	0.005322007	negative
PRJNA729271	stage_E14_vs_E10	ordinary_muscle_development	ENSGALG00010008374	ZNF821	0.068460474	0.085128217	0.804204251	0.421279024	0.535699575	positive
PRJNA729271	stage_E14_vs_E10	ordinary_muscle_development	ENSGALG00010008375	ENSGALG00010008375	-0.492327204	0.097758305	-5.036167539	4.74945E-07	2.74714E-06	negative
PRJNA729271	stage_E14_vs_E10	ordinary_muscle_development	ENSGALG00010008376	MMRN1	-3.687843346	0.277752837	-13.27742819	3.12959E-40	2.36268E-38	negative
PRJNA729271	stage_E14_vs_E10	ordinary_muscle_development	ENSGALG00010008377	AKT2	0.288275672	0.116342074	2.477828201	0.013218478	0.030416074	positive
PRJNA729271	stage_E14_vs_E10	ordinary_muscle_development	ENSGALG00010008380	SIL1	0.676010456	0.169522632	3.987729833	6.67086E-05	0.000271613	positive
PRJNA729271	stage_E14_vs_E10	ordinary_muscle_development	ENSGALG00010008381	C18orf21	0.093952236	0.112179421	0.837517569	0.402301706	0.517469449	positive
PRJNA729271	stage_E14_vs_E10	ordinary_muscle_development	ENSGALG00010008382	ENSGALG00010008382	0.648915908	0.128299619	5.057816311	4.24085E-07	2.47341E-06	positive
PRJNA729271	stage_E14_vs_E10	ordinary_muscle_development	ENSGALG00010008383	PLD5	-3.969926858	0.433929157	-9.148790282	5.75746E-20	1.22547E-18	negative
PRJNA729271	stage_E14_vs_E10	ordinary_muscle_development	ENSGALG00010008385	CTNNA1	0.365136362	0.09092794	4.015667183	5.92778E-05	0.00024334	positive
PRJNA729271	stage_E14_vs_E10	ordinary_muscle_development	ENSGALG00010008386	SESN3	0.003352928	0.227852692	0.01471533	0.988259289	0.992229577	positive
PRJNA729271	stage_E14_vs_E10	ordinary_muscle_development	ENSGALG00010008387	PHF20L1	-0.021650002	0.112074062	-0.193175845	0.846821255	0.893480272	negative
PRJNA729271	stage_E14_vs_E10	ordinary_muscle_development	ENSGALG00010008388	ADCY1	-0.219709586	0.367498408	-0.597851804	0.549938821	0.655054911	negative
PRJNA729271	stage_E14_vs_E10	ordinary_muscle_development	ENSGALG00010008389	GALNT1	-0.363127507	0.088289662	-4.112910827	3.90701E-05	0.000165736	negative
PRJNA729271	stage_E14_vs_E10	ordinary_muscle_development	ENSGALG00010008391	ENSGALG00010008391	0.188397148	0.065397109	2.880817682	0.00396645	0.01060378	positive
PRJNA729271	stage_E14_vs_E10	ordinary_muscle_development	ENSGALG00010008392	CD36	0.538216761	0.370371754	1.453179826	0.146173832	0.232425372	positive
PRJNA729271	stage_E14_vs_E10	ordinary_muscle_development	ENSGALG00010008394	CD2AP	0.100666485	0.110986286	0.907017332	0.364397632	0.480482459	positive
PRJNA729271	stage_E14_vs_E10	ordinary_muscle_development	ENSGALG00010008395	ENDOD1	0.753843369	0.144390291	5.220872985	1.78082E-07	1.0947E-06	positive
PRJNA729271	stage_E14_vs_E10	ordinary_muscle_development	ENSGALG00010008396	LRRTM2	0.301149291	0.292628154	1.029119336	0.303423601	0.417002475	positive
PRJNA729271	stage_E14_vs_E10	ordinary_muscle_development	ENSGALG00010008397	ATXN1L	-0.104538661	0.071046626	-1.47140922	0.141180482	0.225898121	negative
PRJNA729271	stage_E14_vs_E10	ordinary_muscle_development	ENSGALG00010008399	INO80C	-0.112838832	0.109182208	-1.033491022	0.30137417	0.415218764	negative
PRJNA729271	stage_E14_vs_E10	ordinary_muscle_development	ENSGALG00010008400	MAP1LC3C	-0.279426327	0.254983506	-1.095860399	0.273139872	0.383268387	negative
PRJNA729271	stage_E14_vs_E10	ordinary_muscle_development	ENSGALG00010008401	PSMD8	0.091097987	0.155954857	0.584130491	0.559132522	0.662835544	positive
PRJNA729271	stage_E14_vs_E10	ordinary_muscle_development	ENSGALG00010008403	GNAT3	-0.501111106	0.400247793	-1.252002172	0.210569073	0.311607831	negative
PRJNA729271	stage_E14_vs_E10	ordinary_muscle_development	ENSGALG00010008405	LRATD2	0.131303629	0.143931473	0.912264887	0.361629294	0.477879911	positive
PRJNA729271	stage_E14_vs_E10	ordinary_muscle_development	ENSGALG00010008407	GNAI1	-0.264076392	0.194153127	-1.360144938	0.173784063	0.266956337	negative
PRJNA729271	stage_E14_vs_E10	ordinary_muscle_development	ENSGALG00010008408	SMARCA4	-0.155599633	0.15310643	-1.016284116	0.309494111	0.423226671	negative
PRJNA729271	stage_E14_vs_E10	ordinary_muscle_development	ENSGALG00010008409	CWC15	0.097336648	0.084468069	1.152348454	0.249177913	0.356161508	positive
PRJNA729271	stage_E14_vs_E10	ordinary_muscle_development	ENSGALG00010008410	NAPRT	0.056204565	0.130817364	0.42964147	0.667456466	0.755426185	positive
PRJNA729271	stage_E14_vs_E10	ordinary_muscle_development	ENSGALG00010008411	ENSGALG00010008411	-0.237240699	0.396138682	-0.59888294	0.549250949	0.654450678	negative
PRJNA729271	stage_E14_vs_E10	ordinary_muscle_development	ENSGALG00010008412	NEK1	-0.35073244	0.103102022	-3.401799811	0.000669437	0.00219884	negative
PRJNA729271	stage_E14_vs_E10	ordinary_muscle_development	ENSGALG00010008415	ENSGALG00010008415	1.078221178	0.343829815	3.135915305	0.001713187	0.005083448	positive
PRJNA729271	stage_E14_vs_E10	ordinary_muscle_development	ENSGALG00010008416	MSX2	-1.068153913	0.352545434	-3.029833344	0.002446887	0.006921864	negative
PRJNA729271	stage_E14_vs_E10	ordinary_muscle_development	ENSGALG00010008417	IGFBP1	-2.116349747	0.963800537	-2.1958379	0.028103548	0.058177796	negative
PRJNA729271	stage_E14_vs_E10	ordinary_muscle_development	ENSGALG00010008418	TMEM245	0.262893661	0.089848751	2.925957876	0.003433974	0.009348844	positive
PRJNA729271	stage_E14_vs_E10	ordinary_muscle_development	ENSGALG00010008422	EXO1	-0.39255097	0.224081873	-1.751819386	0.07980487	0.141120027	negative
PRJNA729271	stage_E14_vs_E10	ordinary_muscle_development	ENSGALG00010008424	PPIP5K2	-1.023615029	0.120870041	-8.468724122	2.48095E-17	4.29134E-16	negative
PRJNA729271	stage_E14_vs_E10	ordinary_muscle_development	ENSGALG00010008425	MAGI2	-0.643886546	0.105567216	-6.099304045	1.06531E-09	8.68976E-09	negative
PRJNA729271	stage_E14_vs_E10	ordinary_muscle_development	ENSGALG00010008426	AP1G1	-0.168170135	0.054967824	-3.059428625	0.002217596	0.006371468	negative
PRJNA729271	stage_E14_vs_E10	ordinary_muscle_development	ENSGALG00010008429	IGFBP3	0.553315331	0.118746089	4.65965097	3.16746E-06	1.61549E-05	positive
PRJNA729271	stage_E14_vs_E10	ordinary_muscle_development	ENSGALG00010008430	ENSGALG00010008430	-0.054711885	0.383756651	-0.142569218	0.88663041	0.922309328	negative
PRJNA729271	stage_E14_vs_E10	ordinary_muscle_development	ENSGALG00010008431	SH3RF1	-0.158659595	0.124701048	-1.27231966	0.203259566	0.302871216	negative
PRJNA729271	stage_E14_vs_E10	ordinary_muscle_development	ENSGALG00010008433	EBAG9	-0.036107199	0.095594362	-0.377712644	0.705644074	0.786248816	negative
PRJNA729271	stage_E14_vs_E10	ordinary_muscle_development	ENSGALG00010008434	PTCHD4	-1.715559221	0.461928505	-3.713906377	0.000204084	0.000756863	negative
PRJNA729271	stage_E14_vs_E10	ordinary_muscle_development	ENSGALG00010008437	SHKBP1	0.52079591	0.154152505	3.37844598	0.000728968	0.002377674	positive
PRJNA729271	stage_E14_vs_E10	ordinary_muscle_development	ENSGALG00010008438	SYBU	-0.284509869	0.263299618	-1.08055557	0.279894855	0.390742168	negative
PRJNA729271	stage_E14_vs_E10	ordinary_muscle_development	ENSGALG00010008440	ENSGALG00010008440	-0.083554413	0.312488649	-0.267383833	0.789173646	0.851818601	negative
PRJNA729271	stage_E14_vs_E10	ordinary_muscle_development	ENSGALG00010008441	PHLPP2	-0.008876052	0.08529838	-0.104058857	0.917122641	0.942347252	negative
PRJNA729271	stage_E14_vs_E10	ordinary_muscle_development	ENSGALG00010008443	RSFR	2.070656961	0.441201523	4.693222607	2.68935E-06	1.38825E-05	positive
PRJNA729271	stage_E14_vs_E10	ordinary_muscle_development	ENSGALG00010008444	SLC7A11	-2.164638517	0.427912792	-5.058597359	4.22352E-07	2.46655E-06	negative
PRJNA729271	stage_E14_vs_E10	ordinary_muscle_development	ENSGALG00010008447	FRRS1L	-0.272346409	0.154843131	-1.75885367	0.078602366	0.13931784	negative
PRJNA729271	stage_E14_vs_E10	ordinary_muscle_development	ENSGALG00010008448	TNS3	-0.88316717	0.080660209	-10.94922981	6.7015E-28	2.51653E-26	negative
PRJNA729271	stage_E14_vs_E10	ordinary_muscle_development	ENSGALG00010008449	SRA1	0.44949771	0.106967143	4.202203578	2.64329E-05	0.00011544	positive

PRJNA729271	stage_E14_vs_E10	ordinary_muscle_development	ENSGALG00010008450	GIN1	-0.409580529	0.1727661	-2.370722778	0.017753341	0.039245796	negative
PRJNA729271	stage_E14_vs_E10	ordinary_muscle_development	ENSGALG00010008451	OPN3	-0.453595427	0.315762055	-1.436510242	0.150857204	0.23845967	negative
PRJNA729271	stage_E14_vs_E10	ordinary_muscle_development	ENSGALG00010008452	ENSGALG00010008452	-0.249159167	0.424743216	-0.586611293	0.557464796	0.661502196	negative
PRJNA729271	stage_E14_vs_E10	ordinary_muscle_development	ENSGALG00010008453	AMOTL1	0.655801406	0.120012618	5.464437142	4.64378E-08	3.09906E-07	positive
PRJNA729271	stage_E14_vs_E10	ordinary_muscle_development	ENSGALG00010008455	FKBP1B	0.842558456	0.150776001	5.588146988	2.29505E-08	1.59937E-07	positive
PRJNA729271	stage_E14_vs_E10	ordinary_muscle_development	ENSGALG00010008456	NHLRC1	0.00818352	0.127428196	0.064220642	0.948794542	0.964599814	positive
PRJNA729271	stage_E14_vs_E10	ordinary_muscle_development	ENSGALG00010008457	MARVELD3	0.570666921	0.116958621	4.879220669	1.06506E-06	5.86551E-06	positive
PRJNA729271	stage_E14_vs_E10	ordinary_muscle_development	ENSGALG00010008459	EPB41L4B	-1.832869256	0.206046222	-8.895427646	5.81948E-19	1.14145E-17	negative
PRJNA729271	stage_E14_vs_E10	ordinary_muscle_development	ENSGALG00010008464	SF3B6	-0.036627141	0.108474814	-0.337655718	0.735622649	0.810062318	negative
PRJNA729271	stage_E14_vs_E10	ordinary_muscle_development	ENSGALG00010008466	C11orf97	-0.430790098	0.292956707	-1.470490649	0.141428916	0.226245684	negative
PRJNA729271	stage_E14_vs_E10	ordinary_muscle_development	ENSGALG00010008468	EPPK1	-0.374728468	0.284035251	-1.319302682	0.187067942	0.283420651	negative
PRJNA729271	stage_E14_vs_E10	ordinary_muscle_development	ENSGALG00010008469	NOCT	-0.04681485	0.279061058	-0.167758449	0.866773312	0.908608998	negative
PRJNA729271	stage_E14_vs_E10	ordinary_muscle_development	ENSGALG00010008473	CSMD3	-2.930677229	0.348995649	-8.397460659	4.56237E-17	7.74375E-16	negative
PRJNA729271	stage_E14_vs_E10	ordinary_muscle_development	ENSGALG00010008474	ENSGALG00010008474	0.34123671	0.140362684	2.431107046	0.015052767	0.034054917	positive
PRJNA729271	stage_E14_vs_E10	ordinary_muscle_development	ENSGALG00010008476	RNF166	-0.164118992	0.134193796	-1.222999849	0.221329754	0.323968054	negative
PRJNA729271	stage_E14_vs_E10	ordinary_muscle_development	ENSGALG00010008486	CTU2	0.033875053	0.1350662	0.25080333	0.801966167	0.860564079	positive
PRJNA729271	stage_E14_vs_E10	ordinary_muscle_development	ENSGALG00010008488	CCSER1	-0.417666973	0.150185321	-2.781010622	0.005418996	0.013964152	negative
PRJNA729271	stage_E14_vs_E10	ordinary_muscle_development	ENSGALG00010008492	ANKRD49	0.034901342	0.122914677	0.283947718	0.776450447	0.841657884	positive
PRJNA729271	stage_E14_vs_E10	ordinary_muscle_development	ENSGALG00010008497	PAM	-0.285907101	0.128584858	-2.223489653	0.0261828	0.05480845	negative
PRJNA729271	stage_E14_vs_E10	ordinary_muscle_development	ENSGALG00010008498	ENSGALG00010008498	0.484423895	0.148112365	3.27065127	0.001073001	0.003354142	positive
PRJNA729271	stage_E14_vs_E10	ordinary_muscle_development	ENSGALG00010008500	ENSGALG00010008500	0.838488776	0.150118165	5.585525093	2.32995E-08	1.61979E-07	positive
PRJNA729271	stage_E14_vs_E10	ordinary_muscle_development	ENSGALG00010008501	ENSGALG00010008501	-0.798353612	0.396266295	-2.014689672	0.043937175	0.084893275	negative
PRJNA729271	stage_E14_vs_E10	ordinary_muscle_development	ENSGALG00010008504	RNF139	-0.018896087	0.087764345	-0.215304821	0.829529672	0.881008583	negative
PRJNA729271	stage_E14_vs_E10	ordinary_muscle_development	ENSGALG00010008505	PTPN3	-0.752066299	0.270354564	-2.781777704	0.005406206	0.013936146	negative
PRJNA729271	stage_E14_vs_E10	ordinary_muscle_development	ENSGALG00010008509	MGARP	-1.062210156	0.378292257	-2.807908798	0.004986435	0.01299532	negative
PRJNA729271	stage_E14_vs_E10	ordinary_muscle_development	ENSGALG00010008514	PIEZO1	0.715321296	0.166637111	4.292689012	1.76522E-05	7.93884E-05	positive
PRJNA729271	stage_E14_vs_E10	ordinary_muscle_development	ENSGALG00010008515	TAFA5	-0.756689023	0.208349284	-3.631829239	0.000281419	0.001012453	negative
PRJNA729271	stage_E14_vs_E10	ordinary_muscle_development	ENSGALG00010008516	MRE11	-0.308791549	0.075047676	-4.114605087	3.87843E-05	0.00016462	negative
PRJNA729271	stage_E14_vs_E10	ordinary_muscle_development	ENSGALG00010008522	NUDT12	0.200414754	0.149447786	1.341035287	0.179908996	0.274763555	positive
PRJNA729271	stage_E14_vs_E10	ordinary_muscle_development	ENSGALG00010008527	CHUK	0.158856747	0.054573765	2.910862903	0.003604321	0.009750772	positive
PRJNA729271	stage_E14_vs_E10	ordinary_muscle_development	ENSGALG00010008529	GPR83	-0.410761024	0.489214922	-0.839633065	0.401114154	0.516369299	negative
PRJNA729271	stage_E14_vs_E10	ordinary_muscle_development	ENSGALG00010008533	ENSGALG00010008533	-0.48688598	0.554434322	-0.8781671	0.379853044	0.495865577	negative
PRJNA729271	stage_E14_vs_E10	ordinary_muscle_development	ENSGALG00010008534	RAB33B	0.102196251	0.072100989	1.417404289	0.156364759	0.245399219	positive
PRJNA729271	stage_E14_vs_E10	ordinary_muscle_development	ENSGALG00010008542	MOCOS	-0.232677163	0.168087528	-1.38426191	0.166278273	0.25761047	negative
PRJNA729271	stage_E14_vs_E10	ordinary_muscle_development	ENSGALG00010008561	EEF1D	0.686700931	0.11470319	5.986764018	2.14057E-09	1.69088E-08	positive
PRJNA729271	stage_E14_vs_E10	ordinary_muscle_development	ENSGALG00010008580	PANX1	-0.386167698	0.241722717	-1.597564775	0.110139871	0.184457242	negative
PRJNA729271	stage_E14_vs_E10	ordinary_muscle_development	ENSGALG00010008588	PYCR3	-0.098850886	0.177314459	-0.557489146	0.577193273	0.678926924	negative
PRJNA729271	stage_E14_vs_E10	ordinary_muscle_development	ENSGALG00010008592	KIAA0513	0.234265295	0.140552786	1.666742457	0.095565627	0.163912408	positive
PRJNA729271	stage_E14_vs_E10	ordinary_muscle_development	ENSGALG00010008596	ENSGALG00010008596	-0.554872689	0.155513117	-3.568012126	0.0003597	0.001263657	negative
PRJNA729271	stage_E14_vs_E10	ordinary_muscle_development	ENSGALG00010008618	EIF4EBP3	-0.110929975	0.116562841	-0.951675283	0.341261688	0.456158998	negative
PRJNA729271	stage_E14_vs_E10	ordinary_muscle_development	ENSGALG00010008623	TRPS1	-1.567017404	0.131564833	-11.91060992	1.04212E-32	5.13794E-31	negative
PRJNA729271	stage_E14_vs_E10	ordinary_muscle_development	ENSGALG00010008636	AGTPBP1	-0.317223297	0.246891388	-1.284869834	0.198837774	0.297681629	negative
PRJNA729271	stage_E14_vs_E10	ordinary_muscle_development	ENSGALG00010008637	RGS7	-4.326086022	0.764176579	-5.661107842	1.50399E-08	1.0718E-07	negative
PRJNA729271	stage_E14_vs_E10	ordinary_muscle_development	ENSGALG00010008653	ENSGALG00010008653	-1.185436787	0.155802024	-7.608609682	2.7706E-14	3.67764E-13	negative
PRJNA729271	stage_E14_vs_E10	ordinary_muscle_development	ENSGALG00010008656	CCDC6	-0.240660647	0.07134293	-3.373293564	0.000742747	0.00241718	negative
PRJNA729271	stage_E14_vs_E10	ordinary_muscle_development	ENSGALG00010008658	TSTA3	0.016998583	0.11056767	0.153739181	0.87781539	0.916181889	positive
PRJNA729271	stage_E14_vs_E10	ordinary_muscle_development	ENSGALG00010008666	SLC16A9	0.369830303	0.188808503	1.958758724	0.05014105	0.094993401	positive
PRJNA729271	stage_E14_vs_E10	ordinary_muscle_development	ENSGALG00010008668	ENSGALG00010008668	-0.059264624	0.30047151	-0.197238746	0.843640711	0.891230385	negative
PRJNA729271	stage_E14_vs_E10	ordinary_muscle_development	ENSGALG00010008674	RXFP1	-0.074863865	0.278125224	-0.269173232	0.787796378	0.850712098	negative
PRJNA729271	stage_E14_vs_E10	ordinary_muscle_development	ENSGALG00010008686	ENSGALG00010008686	-1.296071326	0.401830321	-3.225419434	0.001257882	0.003876064	negative
PRJNA729271	stage_E14_vs_E10	ordinary_muscle_development	ENSGALG00010008688	HBEGF	-0.161320548	0.243672866	-0.66203739	0.50794726	0.616847816	negative
PRJNA729271	stage_E14_vs_E10	ordinary_muscle_development	ENSGALG00010008690	YIF1B	-0.179490091	0.123052792	-1.458642974	0.144663399	0.23040282	negative
PRJNA729271	stage_E14_vs_E10	ordinary_muscle_development	ENSGALG00010008693	C4orf46	-0.011358217	0.101996656	-0.111358721	0.911331892	0.938926418	negative
PRJNA729271	stage_E14_vs_E10	ordinary_muscle_development	ENSGALG00010008696	PFN4	-0.003714646	0.202900288	-0.01830774	0.985393353	0.990180518	negative
PRJNA729271	stage_E14_vs_E10	ordinary_muscle_development	ENSGALG00010008699	NAA35	-0.035248348	0.09992278	-0.352755881	0.724271462	0.800298433	negative
PRJNA729271	stage_E14_vs_E10	ordinary_muscle_development	ENSGALG00010008700	ENSGALG00010008700	-1.099234605	0.264919555	-4.149314709	3.33472E-05	0.000143093	negative
PRJNA729271	stage_E14_vs_E10	ordinary_muscle_development	ENSGALG00010008708	ZC3H4	0.055227055	0.246658541	0.223900844	0.822834459	0.875825047	positive
PRJNA729271	stage_E14_vs_E10	ordinary_muscle_development	ENSGALG00010008716	TATDN1	-0.447487706	0.110796848	-4.038812599	5.37225E-05	0.000222297	negative
PRJNA729271	stage_E14_vs_E10	ordinary_muscle_development	ENSGALG00010008729	TMEM254	0.010195118	0.117775157	0.086564246	0.931017887	0.952925132	positive
PRJNA729271	stage_E14_vs_E10	ordinary_muscle_development	ENSGALG00010008731	NDUFB9	0.443522357	0.087420218	5.073452891	3.90661E-07	2.29256E-06	positive
PRJNA729271	stage_E14_vs_E10	ordinary_muscle_development	ENSGALG00010008739	VSTM5	-1.109354671	0.249734604	-4.442134386	8.90709E-06	4.20959E-05	negative
PRJNA729271	stage_E14_vs_E10	ordinary_muscle_development	ENSGALG00010008745	GRAMD4	0.202934828	0.152659285	1.329331709	0.183738557	0.279434518	positive
PRJNA729271	stage_E14_vs_E10	ordinary_muscle_development	ENSGALG00010008750	PFDN1	0.329636578	0.165788051	1.988301187	0.04677839	0.089689518	positive
PRJNA729271	stage_E14_vs_E10	ordinary_muscle_development	ENSGALG00010008752	GOLM1	0.265244714	0.172491553	1.537725818	0.124115668	0.203047274	positive
PRJNA729271	stage_E14_vs_E10	ordinary_muscle_development	ENSGALG00010008756	ITSN2	1.433837623	0.07209475	19.88823901	5.14503E-88	3.55129E-85	positive
PRJNA729271	stage_E14_vs_E10	ordinary_muscle_development	ENSGALG00010008762	SAE1	-0.116999771	0.100566934	-1.163401987	0.244666444	0.351167453	negative
PRJNA729271	stage_E14_vs_E10	ordinary_muscle_development	ENSGALG00010008764	ETFDH	0.685223989	0.12356052	5.545654784	2.92856E-08	2.01852E-07	positive
PRJNA729271	stage_E14_vs_E10	ordinary_muscle_development	ENSGALG00010008768	PRXL2C	0.452336774	0.153758494	2.941865276	0.003262418	0.008952812	positive
PRJNA729271	stage_E14_vs_E10	ordinary_muscle_development	ENSGALG00010008775	MED17	0.13699095	0.089077938	1.537877418	0.12407859	0.203017266	positive
PRJNA729271	stage_E14_vs_E10	ordinary_muscle_development	ENSGALG00010008780	KARS1	0.084247031	0.05708574	1.475798181	0.139998079	0.224427357	positive
PRJNA729271	stage_E14_vs_E10	ordinary_muscle_development	ENSGALG00010008782	CCDC9	0.48240481	0.266304322	1.811479464	0.070066665	0.126226238	positive
PRJNA729271	stage_E14_vs_E10	ordinary_muscle_development	ENSGALG00010008790	GPN1	0.040654966	0.094343022	0.430927105	0.666521374	0.754606523	positive
PRJNA729271	stage_E14_vs_E10	ordinary_muscle_development	ENSGALG00010008793	C11orf54	0.256378723	0.11367321	2.255401458	0.024108141	0.051120603	positive
PRJNA729271	stage_E14_vs_E10	ordinary_muscle_development	ENSGALG00010008799	ENSGALG00010008799	0.037134447	0.172153199	0.215705819	0.829217065	0.880807662	positive
PRJNA729271	stage_E14_vs_E10	ordinary_muscle_development	ENSGALG00010008807	BRD1	0.070381411	0.061322199	1.147731361	0.25107948	0.35817312	positive
PRJNA729271	stage_E14_vs_E10	ordinary_muscle_development	ENSGALG00010008811	TERF2IP	0.064841411	0.094416864	0.686756671	0.492236079	0.602462379	positive
PRJNA729271	stage_E14_vs_E10	ordinary_muscle_development	ENSGALG00010008814	ENSGALG00010008814	0.701294478	0.226622789	3.094545259	0.001971149	0.005743075	positive
PRJNA729271	stage_E14_vs_E10	ordinary_muscle_development	ENSGALG00010008821	ENSGALG00010008821	-0.194009364	0.13702221	-1.415897199	0.156805605	0.245931319	negative
PRJNA729271	stage_E14_vs_E10	ordinary_muscle_development	ENSGALG00010008824	GREM2	-2.940257052	0.316393748	-9.293031462	1.49955E-20	3.344E-19	negative
PRJNA729271	stage_E14_vs_E10	ordinary_muscle_development	ENSGALG00010008825	PPID	-0.000443622	0.108539362	-0.004087202	0.996738894	0.998254008	negative
PRJNA729271	stage_E14_vs_E10	ordinary_muscle_development	ENSGALG00010008830	DHX34	-0.068723569	0.194713405	-0.352947291	0.724127956	0.800261873	negative
PRJNA729271	stage_E14_vs_E10	ordinary_muscle_development	ENSGALG00010008832	MTSS1	1.73143028	0.123057867	14.07004954	5.80287E-45	6.13961E-43	positive
PRJNA729271	stage_E14_vs_E10	ordinary_muscle_development	ENSGALG00010008837	ENSGALG00010008837	-0.25061542	0.097130081	-2.580203974	0.009874197	0.023572707	negative
PRJNA729271	stage_E14_vs_E10	ordinary_muscle_development	ENSGALG00010008839	ZNF512	0.09034374	0.073512785	1.228952755	0.21908952	0.321386447	positive
PRJNA729271	stage_E14_vs_E10	ordinary_muscle_development	ENSGALG00010008846	SLC35D2	-1.017094658	0.295088198	-3.446748006	0.000567378	0.001895836	negative
PRJNA729271	stage_E14_vs_E10	ordinary_muscle_development	ENSGALG00010008850	CYSTM1	1.283016342	0.124513945	10.30419803	6.74519E-25	2.00763E-23	positive
PRJNA729271	stage_E14_vs_E10	ordinary_muscle_development	ENSGALG00010008852	KHDRBS3	0.505081619	0.082898146	6.09279751	1.10954E-09	9.03023E-09	positive
PRJNA729271	stage_E14_vs_E10	ordinary_muscle_development	ENSGALG00010008859	ENSGALG00010008859	-0.153603139	0.19573339	-0.784756955	0.432596082	0.546732951	negative
PRJNA729271	stage_E14_vs_E10	ordinary_muscle_development	ENSGALG00010008860	SQLE	-0.273862576	0.242872577	-1.127597769	0.259489829	0.367710752	negative
PRJNA729271	stage_E14_vs_E10	ordinary_muscle_development	ENSGALG00010008862	ZNF367	0.25069681	0.137402999	1.824536665	0.068070978	0.123120643	positive
PRJNA729271	stage_E14_vs_E10	ordinary_muscle_development	ENSGALG00010008864	ADAT1	0.011343555	0.137735089	0.082357768	0.934362218	0.954557224	positive
PRJNA729271	stage_E14_vs_E10	ordinary_muscle_development	ENSGALG00010008866	WASHC5	-0.257008092	0.072311788	-3.554165909	0.00037918	0.001323432	negative
PRJNA729271	stage_E14_vs_E10	ordinary_muscle_development	ENSGALG00010008871	PRR7	-0.425716253	0.183587591	-2.318872698	0.02040194	0.044250504	negative
PRJNA729271	stage_E14_vs_E10	ordinary_muscle_development	ENSGALG00010008877	FNDC4	0.565083211	0.246097701	2.296174275	0.021665919	0.046636099	positive
PRJNA729271	stage_E14_vs_E10	ordinary_muscle_development	ENSGALG00010008879	HABP4	-0.081072158	0.165937673	-0.488569934	0.625146203	0.71996617	negative
PRJNA729271	stage_E14_vs_E10	ordinary_muscle_development	ENSGALG00010008880	FMN2	0.288514102	0.277182567	1.040881123	0.297930724	0.411270066	positive
PRJNA729271	stage_E14_vs_E10	ordinary_muscle_development	ENSGALG00010008884	FAM135B	-0.83858333	0.521057568	-1.609387104	0.107531723	0.180904402	negative
PRJNA729271	stage_E14_vs_E10	ordinary_muscle_development	ENSGALG00010008888	FBXO46	0.697450185	0.141531648	4.927874399	8.3129E-07	4.6398E-06	positive
PRJNA729271	stage_E14_vs_E10	ordinary_muscle_development	ENSGALG00010008892	ENSGALG00010008892	0.505807253	0.113792475	4.444997396	8.78929E-06	4.15799E-05	positive
PRJNA729271	stage_E14_vs_E10	ordinary_muscle_development	ENSGALG00010008893	ENSGALG00010008893	2.367473474	0.139417261	16.98120776	1.13134E-64	3.1536E-62	positive
PRJNA729271	stage_E14_vs_E10	ordinary_muscle_development	ENSGALG00010008897	NSMCE2	0.012785712	0.16274023	0.078565157	0.937378502	0.956313443	positive
PRJNA729271	stage_E14_vs_E10	ordinary_muscle_development	ENSGALG00010008903	ENSGALG00010008903	1.618180508	0.42710389	3.788728096	0.000151421	0.000576345	positive
PRJNA729271	stage_E14_vs_E10	ordinary_muscle_development	ENSGALG00010008905	GABARAPL2	0.189552051	0.093453808	2.028296712	0.042529974	0.082581645	positive
PRJNA729271	stage_E14_vs_E10	ordinary_muscle_development	ENSGALG00010008906	DBN1	-0.512548283	0.209034736	-2.451976606	0.01420739	0.032395173	negative
PRJNA729271	stage_E14_vs_E10	ordinary_muscle_development	ENSGALG00010008910	TRIB1	0.380468333	0.261780877	1.453384747	0.146116959	0.23236043	positive
PRJNA729271	stage_E14_vs_E10	ordinary_muscle_development	ENSGALG00010008918	PLAC9	0.873816042	0.245436017	3.560260035	0.000370488	0.001298409	positive
PRJNA729271	stage_E14_vs_E10	ordinary_muscle_development	ENSGALG00010008921	OPA3	0.369483855	0.125859252	2.935690854	0.003328058	0.009100208	positive
PRJNA729271	stage_E14_vs_E10	ordinary_muscle_development	ENSGALG00010008922	ENSGALG00010008922	0.472530822	1.396105965	0.338463436	0.735013977	0.809576565	positive
PRJNA729271	stage_E14_vs_E10	ordinary_muscle_development	ENSGALG00010008928	CFC1	-2.980959154	0.488973427	-6.096362277	1.08509E-09	8.84116E-09	negative
PRJNA729271	stage_E14_vs_E10	ordinary_muscle_development	ENSGALG00010008931	FNIP2	-0.085500671	0.131559399	-0.649901655	0.515755749	0.624499172	negative
PRJNA729271	stage_E14_vs_E10	ordinary_muscle_development	ENSGALG00010008932	VASP	1.078016586	0.119397632	9.028793709	1.73575E-19	3.57889E-18	positive
PRJNA729271	stage_E14_vs_E10	ordinary_muscle_development	ENSGALG00010008936	IFT172	0.085885746	0.092343095	0.930072203	0.352333702	0.468668167	positive
PRJNA729271	stage_E14_vs_E10	ordinary_muscle_development	ENSGALG00010008938	ZBED4	-0.276251022	0.090227916	-3.061702341	0.002200822	0.006329546	negative
PRJNA729271	stage_E14_vs_E10	ordinary_muscle_development	ENSGALG00010008939	RANBP9	-0.036978116	0.103658194	-0.356731243	0.721293014	0.798207329	negative
PRJNA729271	stage_E14_vs_E10	ordinary_muscle_development	ENSGALG00010008943	ENSGALG00010008943	1.184696165	0.504840594	2.346673741	0.018941833	0.041449557	positive
PRJNA729271	stage_E14_vs_E10	ordinary_muscle_development	ENSGALG00010008945	FER1L6	0.439097963	0.559411831	0.784927917	0.432495833	0.546653915	positive
PRJNA729271	stage_E14_vs_E10	ordinary_muscle_development	ENSGALG00010008946	COL22A1	-0.19444742	0.619292983	-0.313982922	0.75353401	0.823591877	negative
PRJNA729271	stage_E14_vs_E10	ordinary_muscle_development	ENSGALG00010008955	KRTCAP3	-1.444363322	0.349832083	-4.128733162	3.64767E-05	0.000155235	negative
PRJNA729271	stage_E14_vs_E10	ordinary_muscle_development	ENSGALG00010008956	LVRN	-1.46331626	0.485604427	-3.013391515	0.002583454	0.007274119	negative
PRJNA729271	stage_E14_vs_E10	ordinary_muscle_development	ENSGALG00010008960	LRRC2	4.125448941	0.405566414	10.1720675	2.64239E-24	7.4954E-23	positive
PRJNA729271	stage_E14_vs_E10	ordinary_muscle_development	ENSGALG00010008966	NRBP1	0.515461778	0.11463452	4.496566116	6.90595E-06	3.33339E-05	positive
PRJNA729271	stage_E14_vs_E10	ordinary_muscle_development	ENSGALG00010008969	PPP1R37	0.125133346	0.186630988	0.670485363	0.502548434	0.611932086	positive
PRJNA729271	stage_E14_vs_E10	ordinary_muscle_development	ENSGALG00010008971	ANXA11	0.012348247	0.103759456	0.119008397	0.905268703	0.935272263	positive
PRJNA729271	stage_E14_vs_E10	ordinary_muscle_development	ENSGALG00010008973	KCNK9	-0.299192072	0.483226313	-0.619155174	0.535814139	0.64271979	negative
PRJNA729271	stage_E14_vs_E10	ordinary_muscle_development	ENSGALG00010008974	SMCO4	-0.396133884	0.099375107	-3.986248629	6.71262E-05	0.000273083	negative
PRJNA729271	stage_E14_vs_E10	ordinary_muscle_development	ENSGALG00010008980	ENSGALG00010008980	-0.488690462	0.247496861	-1.97453196	0.048321287	0.092196532	negative
PRJNA729271	stage_E14_vs_E10	ordinary_muscle_development	ENSGALG00010008983	ENSGALG00010008983	0.760083176	0.303277162	2.506232819	0.012202524	0.02837728	positive
PRJNA729271	stage_E14_vs_E10	ordinary_muscle_development	ENSGALG00010008988	PPM1G	0.039761485	0.077959864	0.510025065	0.610033902	0.707282147	positive
PRJNA729271	stage_E14_vs_E10	ordinary_muscle_development	ENSGALG00010008991	ENSGALG00010008991	-0.027470134	0.127500565	-0.215451076	0.829415652	0.880954047	negative
PRJNA729271	stage_E14_vs_E10	ordinary_muscle_development	ENSGALG00010008995	ECD	-0.478261344	0.098089621	-4.875758933	1.08391E-06	5.96027E-06	negative
PRJNA729271	stage_E14_vs_E10	ordinary_muscle_development	ENSGALG00010008996	CHRM3	-0.902428636	0.237475647	-3.800089177	0.000144644	0.000553197	negative

PRJNA729271	stage_E14_vs_E10	ordinary_muscle_development	ENSGALG00010008997	CHST6	-0.369199228	0.128029553	-2.883703169	0.00393029	0.010518751	negative
PRJNA729271	stage_E14_vs_E10	ordinary_muscle_development	ENSGALG00010008999	TRAPPC9	0.133701135	0.062760805	2.130328544	0.033144499	0.066781972	positive
PRJNA729271	stage_E14_vs_E10	ordinary_muscle_development	ENSGALG00010009000	FAM149B1	-0.219341846	0.101231991	-2.166724619	0.030255858	0.061847224	negative
PRJNA729271	stage_E14_vs_E10	ordinary_muscle_development	ENSGALG00010009002	ENSGALG00010009002	0.790571551	0.096286914	8.210581427	2.2012E-16	3.48323E-15	positive
PRJNA729271	stage_E14_vs_E10	ordinary_muscle_development	ENSGALG00010009003	ALG12	-0.296915908	0.135098372	-2.197775624	0.027965099	0.057965696	negative
PRJNA729271	stage_E14_vs_E10	ordinary_muscle_development	ENSGALG00010009007	TMEM65	0.397782661	0.098924215	4.021084842	5.79307E-05	0.00023835	positive
PRJNA729271	stage_E14_vs_E10	ordinary_muscle_development	ENSGALG00010009011	ACP6	-0.14394419	0.112197796	-1.282950252	0.199509506	0.298527748	negative
PRJNA729271	stage_E14_vs_E10	ordinary_muscle_development	ENSGALG00010009012	DEUP1	-3.767670018	1.005091474	-3.748584199	0.000177836	0.000667632	negative
PRJNA729271	stage_E14_vs_E10	ordinary_muscle_development	ENSGALG00010009013	LIPG	0.354562556	0.221286727	1.60227665	0.109094447	0.183044798	positive
PRJNA729271	stage_E14_vs_E10	ordinary_muscle_development	ENSGALG00010009015	TMEM231	-0.332540793	0.090518717	-3.673724095	0.000239041	0.000870796	negative
PRJNA729271	stage_E14_vs_E10	ordinary_muscle_development	ENSGALG00010009016	DNAJC9	-0.398013122	0.10775794	-3.693585111	0.000221114	0.000811817	negative
PRJNA729271	stage_E14_vs_E10	ordinary_muscle_development	ENSGALG00010009019	ZNF513	0.152757436	0.133878838	1.141012558	0.253864697	0.361399409	positive
PRJNA729271	stage_E14_vs_E10	ordinary_muscle_development	ENSGALG00010009020	ARHGAP35	0.010864591	0.182659109	0.059480147	0.952569678	0.967114764	positive
PRJNA729271	stage_E14_vs_E10	ordinary_muscle_development	ENSGALG00010009021	HNF4beta	-2.22331611	0.514114289	-4.324556149	1.52839E-05	6.95793E-05	negative
PRJNA729271	stage_E14_vs_E10	ordinary_muscle_development	ENSGALG00010009024	PMAIP1	-0.371411411	0.257845418	-1.440442159	0.149742343	0.237007563	negative
PRJNA729271	stage_E14_vs_E10	ordinary_muscle_development	ENSGALG00010009025	UTP23	-0.024111087	0.094904709	-0.254055751	0.799452488	0.859075084	negative
PRJNA729271	stage_E14_vs_E10	ordinary_muscle_development	ENSGALG00010009026	RAPGEF2	0.2074263	0.136507389	1.519524332	0.128630568	0.209094997	positive
PRJNA729271	stage_E14_vs_E10	ordinary_muscle_development	ENSGALG00010009028	PDLIM7	-0.095416223	0.109486113	-0.871491559	0.383485813	0.499566743	negative
PRJNA729271	stage_E14_vs_E10	ordinary_muscle_development	ENSGALG00010009029	IGSF3	0.059733729	0.162447394	0.367711215	0.713088577	0.792472508	positive
PRJNA729271	stage_E14_vs_E10	ordinary_muscle_development	ENSGALG00010009030	ZDHHC7	0.061955291	0.086934899	0.712663059	0.476054281	0.588269975	positive
PRJNA729271	stage_E14_vs_E10	ordinary_muscle_development	ENSGALG00010009031	SPIN1	-0.207837075	0.079185964	-2.624670621	0.008673283	0.021054971	negative
PRJNA729271	stage_E14_vs_E10	ordinary_muscle_development	ENSGALG00010009034	SNX17	0.150635815	0.119380632	1.26181117	0.207016742	0.307172839	positive
PRJNA729271	stage_E14_vs_E10	ordinary_muscle_development	ENSGALG00010009035	ENSGALG00010009035	0.028430616	0.077152358	0.368499643	0.712500711	0.791964007	positive
PRJNA729271	stage_E14_vs_E10	ordinary_muscle_development	ENSGALG00010009037	ENSGALG00010009037	0.866560733	0.185036724	4.683182417	2.82455E-06	1.45081E-05	positive
PRJNA729271	stage_E14_vs_E10	ordinary_muscle_development	ENSGALG00010009038	RAD21	0.031230983	0.12369507	0.252483649	0.800667251	0.859577582	positive
PRJNA729271	stage_E14_vs_E10	ordinary_muscle_development	ENSGALG00010009039	SLC36A4	-0.574068077	0.167276025	-3.431861066	0.000599455	0.001989717	negative
PRJNA729271	stage_E14_vs_E10	ordinary_muscle_development	ENSGALG00010009040	CRELD2	-0.333474348	0.140251632	-2.377686039	0.017421652	0.03862448	negative
PRJNA729271	stage_E14_vs_E10	ordinary_muscle_development	ENSGALG00010009043	TFAM	0.128836776	0.081656105	1.577797229	0.114612201	0.19075713	positive
PRJNA729271	stage_E14_vs_E10	ordinary_muscle_development	ENSGALG00010009047	SUPT5H	0.008167441	0.109738675	0.074426275	0.940671203	0.958391023	positive
PRJNA729271	stage_E14_vs_E10	ordinary_muscle_development	ENSGALG00010009050	PIK3CG	0.264583814	0.09330578	2.835663714	0.004573057	0.012041137	positive
PRJNA729271	stage_E14_vs_E10	ordinary_muscle_development	ENSGALG00010009051	TROJANZ	0.724517707	0.252614531	2.868076133	0.004129761	0.010997776	positive
PRJNA729271	stage_E14_vs_E10	ordinary_muscle_development	ENSGALG00010009054	RYR2	-1.073909725	0.491273823	-2.185969767	0.028817817	0.059376583	negative
PRJNA729271	stage_E14_vs_E10	ordinary_muscle_development	ENSGALG00010009057	NXNL2	-0.730264814	0.375531963	-1.944614272	0.05182142	0.097742549	negative
PRJNA729271	stage_E14_vs_E10	ordinary_muscle_development	ENSGALG00010009059	MED29	0.822235728	0.125524562	6.550397134	5.73843E-11	5.44362E-10	positive
PRJNA729271	stage_E14_vs_E10	ordinary_muscle_development	ENSGALG00010009065	EIF2B4	0.276155498	0.093852457	2.942442935	0.003256338	0.008937818	positive
PRJNA729271	stage_E14_vs_E10	ordinary_muscle_development	ENSGALG00010009067	ENSGALG00010009067	-0.114424873	1.620086055	-0.070628886	0.94369312	0.960383564	negative
PRJNA729271	stage_E14_vs_E10	ordinary_muscle_development	ENSGALG00010009068	AGO2	-0.31176821	0.080308809	-3.882117205	0.000103551	0.000406391	negative
PRJNA729271	stage_E14_vs_E10	ordinary_muscle_development	ENSGALG00010009069	GRID2	-1.7242267	0.251950021	-6.843526717	7.72669E-12	7.98847E-11	negative
PRJNA729271	stage_E14_vs_E10	ordinary_muscle_development	ENSGALG00010009070	ENSGALG00010009070	-3.967578109	1.255730897	-3.159576721	0.001579985	0.004726911	negative
PRJNA729271	stage_E14_vs_E10	ordinary_muscle_development	ENSGALG00010009073	PRKAR2B	0.196159467	0.404632466	0.484784299	0.627829361	0.721956943	positive
PRJNA729271	stage_E14_vs_E10	ordinary_muscle_development	ENSGALG00010009076	TMEM214	0.027169208	0.114529123	0.237225323	0.812481992	0.868526027	positive
PRJNA729271	stage_E14_vs_E10	ordinary_muscle_development	ENSGALG00010009078	PTTG2	-0.012054846	0.163371789	-0.073787805	0.941179231	0.958639095	negative
PRJNA729271	stage_E14_vs_E10	ordinary_muscle_development	ENSGALG00010009079	KCTD15	-0.003788947	0.09477994	-0.039976251	0.96811206	0.978469436	negative
PRJNA729271	stage_E14_vs_E10	ordinary_muscle_development	ENSGALG00010009080	TLL1	-0.619925912	0.168267065	-3.684178547	0.000229441	0.000839625	negative
PRJNA729271	stage_E14_vs_E10	ordinary_muscle_development	ENSGALG00010009081	HBP1	0.410149569	0.122081709	3.3596316	0.000780465	0.002524623	positive
PRJNA729271	stage_E14_vs_E10	ordinary_muscle_development	ENSGALG00010009083	MED30	0.163033933	0.126553535	1.288260605	0.197655261	0.296186603	positive
PRJNA729271	stage_E14_vs_E10	ordinary_muscle_development	ENSGALG00010009085	PAF1	0.325747664	0.087626814	3.717442743	0.00020125	0.000747786	positive
PRJNA729271	stage_E14_vs_E10	ordinary_muscle_development	ENSGALG00010009086	UBE2D1	0.146075508	0.171447004	0.852015519	0.394205479	0.509542395	positive
PRJNA729271	stage_E14_vs_E10	ordinary_muscle_development	ENSGALG00010009088	ENSGALG00010009088	-0.080351161	0.131835935	-0.609478448	0.542207354	0.648072287	negative
PRJNA729271	stage_E14_vs_E10	ordinary_muscle_development	ENSGALG00010009089	AGBL5	0.851794636	0.146246724	5.824367301	5.73294E-09	4.31011E-08	positive
PRJNA729271	stage_E14_vs_E10	ordinary_muscle_development	ENSGALG00010009091	EXT1	0.035876959	0.110695658	0.324104487	0.74585893	0.817643195	positive
PRJNA729271	stage_E14_vs_E10	ordinary_muscle_development	ENSGALG00010009092	EED	-0.797503891	0.109467457	-7.285305748	3.20942E-13	3.87348E-12	negative
PRJNA729271	stage_E14_vs_E10	ordinary_muscle_development	ENSGALG00010009093	SLU7	0.033201847	0.131493685	0.252497657	0.800656425	0.859577582	positive
PRJNA729271	stage_E14_vs_E10	ordinary_muscle_development	ENSGALG00010009094	DARS	0.130621172	0.094906611	1.376312682	0.168724848	0.260815471	positive
PRJNA729271	stage_E14_vs_E10	ordinary_muscle_development	ENSGALG00010009096	ENSGALG00010009096	0.655209946	1.351712838	0.484725696	0.627870936	0.721956943	positive
PRJNA729271	stage_E14_vs_E10	ordinary_muscle_development	ENSGALG00010009097	C1QTNF2	0.905971573	0.181024451	5.004691723	5.59515E-07	3.19928E-06	positive
PRJNA729271	stage_E14_vs_E10	ordinary_muscle_development	ENSGALG00010009098	CAD	-0.215532057	0.197514737	-1.091220131	0.275176032	0.385602918	negative
PRJNA729271	stage_E14_vs_E10	ordinary_muscle_development	ENSGALG00010009099	CXCR4	0.103162057	0.187831905	0.54922542	0.582850762	0.68410001	positive
PRJNA729271	stage_E14_vs_E10	ordinary_muscle_development	ENSGALG00010009100	ENSGALG00010009100	-0.06599977	0.152162595	-0.433745035	0.664473599	0.753183776	negative
PRJNA729271	stage_E14_vs_E10	ordinary_muscle_development	ENSGALG00010009101	HIKESHI	0.297968678	0.097315681	3.061877322	0.002199536	0.0063283	positive
PRJNA729271	stage_E14_vs_E10	ordinary_muscle_development	ENSGALG00010009102	HTRA3	-0.911481488	0.218943961	-4.1630812	3.13982E-05	0.000135492	negative
PRJNA729271	stage_E14_vs_E10	ordinary_muscle_development	ENSGALG00010009103	AP3S1	-0.602889602	0.122891942	-4.905851356	9.3023E-07	5.15037E-06	negative
PRJNA729271	stage_E14_vs_E10	ordinary_muscle_development	ENSGALG00010009104	MTR	-0.108729944	0.12274332	-0.88583186	0.375708139	0.491950268	negative
PRJNA729271	stage_E14_vs_E10	ordinary_muscle_development	ENSGALG00010009106	THSD7B	-0.814547416	0.237340083	-3.431984202	0.000599182	0.001989269	negative
PRJNA729271	stage_E14_vs_E10	ordinary_muscle_development	ENSGALG00010009109	ENSGALG00010009109	-0.408517817	0.21346759	-1.913722905	0.055655571	0.103799215	negative
PRJNA729271	stage_E14_vs_E10	ordinary_muscle_development	ENSGALG00010009111	LRFN1	-0.531021879	0.247506944	-2.14548275	0.031914269	0.064652823	negative
PRJNA729271	stage_E14_vs_E10	ordinary_muscle_development	ENSGALG00010009112	ENSGALG00010009112	0.69316065	0.279664913	2.478539912	0.013192135	0.030362019	positive
PRJNA729271	stage_E14_vs_E10	ordinary_muscle_development	ENSGALG00010009113	RPLP1	0.666622085	0.17633701	3.780386685	0.000156585	0.000594162	positive
PRJNA729271	stage_E14_vs_E10	ordinary_muscle_development	ENSGALG00010009114	SETD7	0.616109293	0.149940464	4.109026194	3.97331E-05	0.000168303	positive
PRJNA729271	stage_E14_vs_E10	ordinary_muscle_development	ENSGALG00010009115	COG5	-0.151166905	0.114066797	-1.325248968	0.185088589	0.280957074	negative
PRJNA729271	stage_E14_vs_E10	ordinary_muscle_development	ENSGALG00010009116	S1PR3	-0.302418645	0.183747048	-1.645842196	0.099796252	0.169802404	negative
PRJNA729271	stage_E14_vs_E10	ordinary_muscle_development	ENSGALG00010009117	ERGIC1	0.106580913	0.066555257	1.601389844	0.109290601	0.183331178	positive
PRJNA729271	stage_E14_vs_E10	ordinary_muscle_development	ENSGALG00010009118	ACTN2	2.168912674	0.143924822	15.06976102	2.56038E-51	3.67452E-49	positive
PRJNA729271	stage_E14_vs_E10	ordinary_muscle_development	ENSGALG00010009119	CISD1	0.339642968	0.153077284	2.218767929	0.026502517	0.055401497	positive
PRJNA729271	stage_E14_vs_E10	ordinary_muscle_development	ENSGALG00010009120	ATG12	0.228144619	0.087477687	2.608032151	0.00910644	0.021959383	positive
PRJNA729271	stage_E14_vs_E10	ordinary_muscle_development	ENSGALG00010009121	SYCN	-0.277875513	0.772343638	-0.359782225	0.719009997	0.796759087	negative
PRJNA729271	stage_E14_vs_E10	ordinary_muscle_development	ENSGALG00010009123	ENSGALG00010009123	0.462790181	0.131450059	3.520654036	0.000430484	0.001479265	positive
PRJNA729271	stage_E14_vs_E10	ordinary_muscle_development	ENSGALG00010009125	CD72L1	2.914017089	1.015856075	2.86853341	0.004123796	0.010985927	positive
PRJNA729271	stage_E14_vs_E10	ordinary_muscle_development	ENSGALG00010009126	JPH3	-0.246752445	0.137339263	-1.796663526	0.072389053	0.129780992	negative
PRJNA729271	stage_E14_vs_E10	ordinary_muscle_development	ENSGALG00010009127	SCOC	-0.39075787	0.074302863	-5.258988038	1.4485E-07	9.00732E-07	negative
PRJNA729271	stage_E14_vs_E10	ordinary_muscle_development	ENSGALG00010009129	ENSGALG00010009129	0.848004312	0.370893204	2.286384065	0.0222318	0.04773366	positive
PRJNA729271	stage_E14_vs_E10	ordinary_muscle_development	ENSGALG00010009130	HEATR1	-0.366415333	0.072141506	-5.079119514	3.79188E-07	2.23156E-06	negative
PRJNA729271	stage_E14_vs_E10	ordinary_muscle_development	ENSGALG00010009131	CDKL2	-2.645984129	0.308311984	-8.582164382	9.31045E-18	1.67023E-16	negative
PRJNA729271	stage_E14_vs_E10	ordinary_muscle_development	ENSGALG00010009133	BOD1L1	-0.162214614	0.129106232	-1.256442944	0.20895542	0.309472649	negative
PRJNA729271	stage_E14_vs_E10	ordinary_muscle_development	ENSGALG00010009135	TENM4	-1.464831358	0.185136057	-7.912188374	2.52903E-15	3.66217E-14	negative
PRJNA729271	stage_E14_vs_E10	ordinary_muscle_development	ENSGALG00010009136	KHK	1.218227536	0.344633604	3.53484838	0.000408009	0.001410805	positive
PRJNA729271	stage_E14_vs_E10	ordinary_muscle_development	ENSGALG00010009137	IPMK	0.286065753	0.091247546	3.135051481	0.00171824	0.005094269	positive
PRJNA729271	stage_E14_vs_E10	ordinary_muscle_development	ENSGALG00010009138	ENSGALG00010009138	0.138081233	0.10318555	1.338183818	0.180836523	0.275802336	positive
PRJNA729271	stage_E14_vs_E10	ordinary_muscle_development	ENSGALG00010009139	CKS2	-0.537449781	0.280196362	-1.918118338	0.055096008	0.102887997	negative
PRJNA729271	stage_E14_vs_E10	ordinary_muscle_development	ENSGALG00010009141	MAML3	0.257404883	0.114748638	2.243206427	0.024883506	0.052516951	positive
PRJNA729271	stage_E14_vs_E10	ordinary_muscle_development	ENSGALG00010009142	LGALS8	-0.18367306	0.132523592	-1.385965001	0.165757591	0.256996072	negative
PRJNA729271	stage_E14_vs_E10	ordinary_muscle_development	ENSGALG00010009143	MCCC2	0.183044244	0.135035498	1.355526855	0.175249759	0.268809021	positive
PRJNA729271	stage_E14_vs_E10	ordinary_muscle_development	ENSGALG00010009144	CREBZF	0.463106754	0.149995819	3.08746442	0.002018719	0.005860472	positive
PRJNA729271	stage_E14_vs_E10	ordinary_muscle_development	ENSGALG00010009147	HNMT	3.021096372	0.226651888	13.32923543	1.56493E-40	1.24636E-38	positive
PRJNA729271	stage_E14_vs_E10	ordinary_muscle_development	ENSGALG00010009149	FUT4	-0.385035505	0.178429336	-2.157915917	0.03093437	0.062967798	negative
PRJNA729271	stage_E14_vs_E10	ordinary_muscle_development	ENSGALG00010009150	ENSGALG00010009150	1.832986613	0.381550036	4.80405305	1.55486E-06	8.35037E-06	positive
PRJNA729271	stage_E14_vs_E10	ordinary_muscle_development	ENSGALG00010009151	CCNJL	-0.225155639	0.3677035	-0.612329333	0.540319886	0.646359392	negative
PRJNA729271	stage_E14_vs_E10	ordinary_muscle_development	ENSGALG00010009152	RPP25L	0.151550286	0.122557523	1.23656453	0.216248795	0.318064565	positive
PRJNA729271	stage_E14_vs_E10	ordinary_muscle_development	ENSGALG00010009153	SECISBP2	-0.155070337	0.124084681	-1.249713795	0.211404116	0.312460759	negative
PRJNA729271	stage_E14_vs_E10	ordinary_muscle_development	ENSGALG00010009155	PAK4	0.089619984	0.217234376	0.412549735	0.679936535	0.765854863	positive
PRJNA729271	stage_E14_vs_E10	ordinary_muscle_development	ENSGALG00010009157	ABHD1	0.361717072	0.137901043	2.623019121	0.008715438	0.021139604	positive
PRJNA729271	stage_E14_vs_E10	ordinary_muscle_development	ENSGALG00010009159	PTK2	-0.231879657	0.098823446	-2.346403269	0.018955586	0.041465806	negative
PRJNA729271	stage_E14_vs_E10	ordinary_muscle_development	ENSGALG00010009160	ENSGALG00010009160	0.214489183	0.390779287	0.548875518	0.583090881	0.684142975	positive
PRJNA729271	stage_E14_vs_E10	ordinary_muscle_development	ENSGALG00010009163	SAMD12	-0.109504824	0.175260359	-0.624812275	0.532094273	0.639527901	negative
PRJNA729271	stage_E14_vs_E10	ordinary_muscle_development	ENSGALG00010009164	TMEM128	0.111295585	0.100166787	1.111102681	0.266524154	0.37543903	positive
PRJNA729271	stage_E14_vs_E10	ordinary_muscle_development	ENSGALG00010009166	CLGN	-0.3573246	0.226098063	-1.580396553	0.11401608	0.189961274	negative
PRJNA729271	stage_E14_vs_E10	ordinary_muscle_development	ENSGALG00010009167	DCTN3	0.171372597	0.110065862	1.557000448	0.119470379	0.197010597	positive
PRJNA729271	stage_E14_vs_E10	ordinary_muscle_development	ENSGALG00010009168	MRPS12	0.203569589	0.174762456	1.164835937	0.244085409	0.350541762	positive
PRJNA729271	stage_E14_vs_E10	ordinary_muscle_development	ENSGALG00010009170	RPL26L1	0.589549264	0.191263401	3.082394554	0.002053425	0.005950498	positive
PRJNA729271	stage_E14_vs_E10	ordinary_muscle_development	ENSGALG00010009172	SPOPL	-0.023373801	0.132011716	-0.177058536	0.859462411	0.903270566	negative
PRJNA729271	stage_E14_vs_E10	ordinary_muscle_development	ENSGALG00010009173	LYAR	-0.714277542	0.142054523	-5.028192885	4.95124E-07	2.86043E-06	negative
PRJNA729271	stage_E14_vs_E10	ordinary_muscle_development	ENSGALG00010009178	ENSGALG00010009178	-0.048515855	0.255691031	-0.189744064	0.84950969	0.895667949	negative
PRJNA729271	stage_E14_vs_E10	ordinary_muscle_development	ENSGALG00010009180	NFKBIB	0.21133289	0.146487276	1.442670624	0.149113278	0.236230167	positive
PRJNA729271	stage_E14_vs_E10	ordinary_muscle_development	ENSGALG00010009181	ARID3C	-1.30709856	0.453023678	-2.885276471	0.003910701	0.010474059	negative
PRJNA729271	stage_E14_vs_E10	ordinary_muscle_development	ENSGALG00010009186	PREB	0.490639164	0.094669291	5.182664389	2.18738E-07	1.3294E-06	positive
PRJNA729271	stage_E14_vs_E10	ordinary_muscle_development	ENSGALG00010009188	ENSGALG00010009188	-2.23603347	0.513207163	-4.356980243	1.31869E-05	6.06422E-05	negative
PRJNA729271	stage_E14_vs_E10	ordinary_muscle_development	ENSGALG00010009189	ENSGALG00010009189	-0.2223514	0.202832668	-1.096230712	0.272977822	0.383115176	negative
PRJNA729271	stage_E14_vs_E10	ordinary_muscle_development	ENSGALG00010009190	ZBTB49	-0.066649919	0.137424456	-0.48499314	0.627681212	0.721956943	negative
PRJNA729271	stage_E14_vs_E10	ordinary_muscle_development	ENSGALG00010009192	LSM14A	0.070550456	0.058778195	1.200282786	0.230029533	0.333928701	positive
PRJNA729271	stage_E14_vs_E10	ordinary_muscle_development	ENSGALG00010009193	EDARADD	-0.675934853	0.211693042	-3.19299514	0.001408053	0.0042725	negative
PRJNA729271	stage_E14_vs_E10	ordinary_muscle_development	ENSGALG00010009195	ENSGALG00010009195	0.30406792	0.757568496	0.401373502	0.688145156	0.772271498	positive
PRJNA729271	stage_E14_vs_E10	ordinary_muscle_development	ENSGALG00010009197	FH	0.340363637	0.102439445	3.322583759	0.000891879	0.002841271	positive
PRJNA729271	stage_E14_vs_E10	ordinary_muscle_development	ENSGALG00010009198	KCNN2	0.629982397	0.166500068	3.783676515	0.000154529	0.000587128	positive
PRJNA729271	stage_E14_vs_E10	ordinary_muscle_development	ENSGALG00010009199	SEMA4D	-0.58467765	0.142760271	-4.095520743	4.21221E-05	0.00017754	negative
PRJNA729271	stage_E14_vs_E10	ordinary_muscle_development	ENSGALG00010009200	ADGRA3	-0.295522996	0.088467525	-3.340468668	0.000836371	0.002688071	negative
PRJNA729271	stage_E14_vs_E10	ordinary_muscle_development	ENSGALG00010009202	FABP6	-0.277143397	0.299961494	-0.923929912	0.355522808	0.471944181	negative
PRJNA729271	stage_E14_vs_E10	ordinary_muscle_development	ENSGALG00010009205	SIRT2	0.24645148	0.097232713	2.534656017	0.011255779	0.026442872	positive
PRJNA729271	stage_E14_vs_E10	ordinary_muscle_development	ENSGALG00010009208	SLC5A6	-0.533419525	0.191543222	-2.784852008	0.005355216	0.0138279	negative
PRJNA729271	stage_E14_vs_E10	ordinary_muscle_development	ENSGALG00010009209	SLC6A19	2.127478959	1.833103323	1.160588676	0.245809213	0.352563283	positive
PRJNA729271	stage_E14_vs_E10	ordinary_muscle_development	ENSGALG00010009210	PCDH15	-0.388998032	0.28332323	-1.37298319	0.169757573	0.262132312	negative
PRJNA729271	stage_E14_vs_E10	ordinary_muscle_development	ENSGALG00010009212	NXPH2	-0.926497578	0.261805511	-3.538877296	0.000401833	0.001392104	negative

PRJNA729271	stage_E14_vs_E10	ordinary_muscle_development	ENSGALG00010009213	DUS4L	-0.480472628	0.087003208	-5.52247028	3.34266E-08	2.29094E-07	negative
PRJNA729271	stage_E14_vs_E10	ordinary_muscle_development	ENSGALG00010009214	PCDH10	-2.52456406	0.239290773	-10.55019393	5.06924E-26	1.66618E-24	negative
PRJNA729271	stage_E14_vs_E10	ordinary_muscle_development	ENSGALG00010009216	NSG1	0.014383943	0.20174368	0.071298112	0.943160498	0.959982545	positive
PRJNA729271	stage_E14_vs_E10	ordinary_muscle_development	ENSGALG00010009219	TNFRSF11B	-1.128047053	0.184440309	-6.1160549	9.59203E-10	7.86405E-09	negative
PRJNA729271	stage_E14_vs_E10	ordinary_muscle_development	ENSGALG00010009220	GADD45G	1.128398175	0.189100322	5.967193294	2.41369E-09	1.89629E-08	positive
PRJNA729271	stage_E14_vs_E10	ordinary_muscle_development	ENSGALG00010009223	ENSGALG00010009223	-0.180400013	0.108485694	-1.662892191	0.096334018	0.164859692	negative
PRJNA729271	stage_E14_vs_E10	ordinary_muscle_development	ENSGALG00010009224	ENSGALG00010009224	1.592427147	0.4972108	3.202720346	0.001361361	0.004149933	positive
PRJNA729271	stage_E14_vs_E10	ordinary_muscle_development	ENSGALG00010009225	HNRNPL	0.081904736	0.123539232	0.662985631	0.507339757	0.616213321	positive
PRJNA729271	stage_E14_vs_E10	ordinary_muscle_development	ENSGALG00010009227	ENSGALG00010009227	0.31706773	0.172842367	1.834432927	0.066589756	0.120848693	positive
PRJNA729271	stage_E14_vs_E10	ordinary_muscle_development	ENSGALG00010009228	ATP6V0E1	-0.120181958	0.100544144	-1.19531534	0.231963849	0.336332172	negative
PRJNA729271	stage_E14_vs_E10	ordinary_muscle_development	ENSGALG00010009232	ECH1	0.201540867	0.164074343	1.228350901	0.219315271	0.32159196	positive
PRJNA729271	stage_E14_vs_E10	ordinary_muscle_development	ENSGALG00010009234	PCDH18	-0.998926936	0.12765474	-7.825223996	5.06754E-15	7.11643E-14	negative
PRJNA729271	stage_E14_vs_E10	ordinary_muscle_development	ENSGALG00010009235	KLHDC4	-0.099757573	0.130404867	-0.764983511	0.444281364	0.557949954	negative
PRJNA729271	stage_E14_vs_E10	ordinary_muscle_development	ENSGALG00010009236	DTWD2	-0.384271019	0.11481171	-3.346967118	0.000817009	0.002631677	negative
PRJNA729271	stage_E14_vs_E10	ordinary_muscle_development	ENSGALG00010009237	DENND3	-0.222536892	0.175647163	-1.26695409	0.205171706	0.305084518	negative
PRJNA729271	stage_E14_vs_E10	ordinary_muscle_development	ENSGALG00010009239	STX18	0.045300248	0.081436312	0.556265961	0.578029053	0.679634257	positive
PRJNA729271	stage_E14_vs_E10	ordinary_muscle_development	ENSGALG00010009243	BCAP29	-0.008035294	0.099195854	-0.081004335	0.935438505	0.955073687	negative
PRJNA729271	stage_E14_vs_E10	ordinary_muscle_development	ENSGALG00010009246	RUSC2	1.929013696	0.141541256	13.62863205	2.70605E-42	2.39172E-40	positive
PRJNA729271	stage_E14_vs_E10	ordinary_muscle_development	ENSGALG00010009250	ERO1B	-0.00825358	0.107597606	-0.07670784	0.938855967	0.957092613	negative
PRJNA729271	stage_E14_vs_E10	ordinary_muscle_development	ENSGALG00010009252	SIRT1	-0.121188968	0.09605804	-1.261622329	0.207084718	0.307172839	negative
PRJNA729271	stage_E14_vs_E10	ordinary_muscle_development	ENSGALG00010009253	SLC45A4	0.068225059	0.136659244	0.499234863	0.617613937	0.713502352	positive
PRJNA729271	stage_E14_vs_E10	ordinary_muscle_development	ENSGALG00010009254	SLC26A4	-2.569402558	0.960188851	-2.67593459	0.007452119	0.018404918	negative
PRJNA729271	stage_E14_vs_E10	ordinary_muscle_development	ENSGALG00010009256	CREBRF	0.305816833	0.174410011	1.753436239	0.079527155	0.140698903	positive
PRJNA729271	stage_E14_vs_E10	ordinary_muscle_development	ENSGALG00010009257	ENSGALG00010009257	2.825775081	0.186026868	15.19014488	4.1101E-52	6.54681E-50	positive
PRJNA729271	stage_E14_vs_E10	ordinary_muscle_development	ENSGALG00010009258	DNAJC12	-1.142859876	0.168822333	-6.76960123	1.29138E-11	1.31174E-10	negative
PRJNA729271	stage_E14_vs_E10	ordinary_muscle_development	ENSGALG00010009259	MGAT4D	1.186657766	0.798959276	1.485254381	0.137476451	0.220898034	positive
PRJNA729271	stage_E14_vs_E10	ordinary_muscle_development	ENSGALG00010009263	COLEC10	0.27126506	0.218475766	1.241625397	0.214374807	0.316078001	positive
PRJNA729271	stage_E14_vs_E10	ordinary_muscle_development	ENSGALG00010009266	PCDH7	-1.524780483	0.235078065	-6.486272901	8.79857E-11	8.12327E-10	negative
PRJNA729271	stage_E14_vs_E10	ordinary_muscle_development	ENSGALG00010009268	PWWP2A	-0.105770892	0.13140234	-0.804939184	0.420854775	0.53530098	negative
PRJNA729271	stage_E14_vs_E10	ordinary_muscle_development	ENSGALG00010009269	ENSGALG00010009269	-2.824917319	0.44924359	-6.288163883	3.21243E-10	2.78327E-09	negative
PRJNA729271	stage_E14_vs_E10	ordinary_muscle_development	ENSGALG00010009273	MAL2	-0.501579774	0.481139136	-1.042483841	0.297187413	0.410572966	negative
PRJNA729271	stage_E14_vs_E10	ordinary_muscle_development	ENSGALG00010009275	CBLL1	-0.316381112	0.14121483	-2.24042413	0.025063401	0.052842764	negative
PRJNA729271	stage_E14_vs_E10	ordinary_muscle_development	ENSGALG00010009276	ENSGALG00010009276	-0.303400648	0.308472097	-0.983559455	0.325332169	0.439978521	negative
PRJNA729271	stage_E14_vs_E10	ordinary_muscle_development	ENSGALG00010009277	ACTN4	-0.126302604	0.127953385	-0.987098576	0.323594316	0.438226762	negative
PRJNA729271	stage_E14_vs_E10	ordinary_muscle_development	ENSGALG00010009279	ELMOD2	-0.620980863	0.112254998	-5.531877217	3.16822E-08	2.17646E-07	negative
PRJNA729271	stage_E14_vs_E10	ordinary_muscle_development	ENSGALG00010009280	HSD17B4	-0.247703902	0.083038239	-2.98301006	0.002854286	0.007950207	negative
PRJNA729271	stage_E14_vs_E10	ordinary_muscle_development	ENSGALG00010009284	CCN3	-1.992526857	0.675344978	-2.950383764	0.003173794	0.008742712	negative
PRJNA729271	stage_E14_vs_E10	ordinary_muscle_development	ENSGALG00010009286	ENSGALG00010009286	0.271785169	0.181330819	1.498836052	0.133916169	0.216400766	positive
PRJNA729271	stage_E14_vs_E10	ordinary_muscle_development	ENSGALG00010009289	GPR137B	0.396856598	0.142304629	2.788782077	0.005290665	0.013684545	positive
PRJNA729271	stage_E14_vs_E10	ordinary_muscle_development	ENSGALG00010009294	ACOX3	0.132106522	0.13967563	0.94580939	0.344245815	0.45951221	positive
PRJNA729271	stage_E14_vs_E10	ordinary_muscle_development	ENSGALG00010009297	CTNNA3	1.45364922	0.209442189	6.940575008	3.90507E-12	4.18669E-11	positive
PRJNA729271	stage_E14_vs_E10	ordinary_muscle_development	ENSGALG00010009299	MAP4K1	0.573393043	0.319754303	1.793230105	0.072936138	0.130632562	positive
PRJNA729271	stage_E14_vs_E10	ordinary_muscle_development	ENSGALG00010009303	TTC1	-0.021382244	0.095945335	-0.222858606	0.823645554	0.876559641	negative
PRJNA729271	stage_E14_vs_E10	ordinary_muscle_development	ENSGALG00010009306	TBC1D9	0.29248826	0.105463749	2.773353523	0.005548181	0.014259022	positive
PRJNA729271	stage_E14_vs_E10	ordinary_muscle_development	ENSGALG00010009307	DIRAS2	0.27093911	0.320746464	0.844714253	0.398270399	0.51360558	positive
PRJNA729271	stage_E14_vs_E10	ordinary_muscle_development	ENSGALG00010009309	TRIM54	2.078251459	0.148918288	13.95564967	2.90641E-44	3.03082E-42	positive
PRJNA729271	stage_E14_vs_E10	ordinary_muscle_development	ENSGALG00010009311	BNIP1	0.049238415	0.108885607	0.452203159	0.651122633	0.742216307	positive
PRJNA729271	stage_E14_vs_E10	ordinary_muscle_development	ENSGALG00010009313	UNC13B	1.415697406	0.167571227	8.448332288	2.95493E-17	5.06286E-16	positive
PRJNA729271	stage_E14_vs_E10	ordinary_muscle_development	ENSGALG00010009317	LRRTM3	-3.266007873	0.499701724	-6.535914758	6.32219E-11	5.97004E-10	negative
PRJNA729271	stage_E14_vs_E10	ordinary_muscle_development	ENSGALG00010009320	GPR20	2.341677238	0.203282708	11.51931349	1.05448E-30	4.65996E-29	positive
PRJNA729271	stage_E14_vs_E10	ordinary_muscle_development	ENSGALG00010009322	ENSGALG00010009322	1.21515713	0.168004589	7.232880589	4.72855E-13	5.58601E-12	positive
PRJNA729271	stage_E14_vs_E10	ordinary_muscle_development	ENSGALG00010009323	RNF150	0.125493588	0.106968552	1.173182078	0.240722798	0.346703825	positive
PRJNA729271	stage_E14_vs_E10	ordinary_muscle_development	ENSGALG00010009326	DLD	0.195915162	0.131749369	1.487029224	0.137007093	0.220363716	positive
PRJNA729271	stage_E14_vs_E10	ordinary_muscle_development	ENSGALG00010009327	NID1	1.314838764	0.163070952	8.062985743	7.44532E-16	1.12769E-14	positive
PRJNA729271	stage_E14_vs_E10	ordinary_muscle_development	ENSGALG00010009328	ENSGALG00010009328	-0.066207319	0.124451607	-0.531992478	0.594731205	0.694523235	negative
PRJNA729271	stage_E14_vs_E10	ordinary_muscle_development	ENSGALG00010009330	EIF3K	0.385506499	0.141384883	2.726645803	0.006398168	0.016115189	positive
PRJNA729271	stage_E14_vs_E10	ordinary_muscle_development	ENSGALG00010009332	ENSGALG00010009332	0.358409054	0.162398528	2.206972313	0.027315994	0.056782637	positive
PRJNA729271	stage_E14_vs_E10	ordinary_muscle_development	ENSGALG00010009333	ADRA1B	0.995320289	0.320022091	3.110161195	0.001869853	0.005498786	positive
PRJNA729271	stage_E14_vs_E10	ordinary_muscle_development	ENSGALG00010009334	SS18L2	0.465767526	0.162740186	2.86203142	0.004209352	0.011178922	positive
PRJNA729271	stage_E14_vs_E10	ordinary_muscle_development	ENSGALG00010009335	PTP4A3	0.333307602	0.195407362	1.705706474	0.088062744	0.153090606	positive
PRJNA729271	stage_E14_vs_E10	ordinary_muscle_development	ENSGALG00010009336	LAMB1	0.908186303	0.109879657	8.265281582	1.39365E-16	2.26215E-15	positive
PRJNA729271	stage_E14_vs_E10	ordinary_muscle_development	ENSGALG00010009337	ZNF330	-0.239153463	0.099489516	-2.403805682	0.016225395	0.036299908	negative
PRJNA729271	stage_E14_vs_E10	ordinary_muscle_development	ENSGALG00010009338	KYNU	0.177866081	0.560105122	0.31755839	0.750819942	0.821554696	positive
PRJNA729271	stage_E14_vs_E10	ordinary_muscle_development	ENSGALG00010009340	SYK	0.1657778	0.141576815	1.170938896	0.241623339	0.347625836	positive
PRJNA729271	stage_E14_vs_E10	ordinary_muscle_development	ENSGALG00010009344	ENPP2	-2.067768981	0.259351808	-7.972834247	1.55076E-15	2.29136E-14	negative
PRJNA729271	stage_E14_vs_E10	ordinary_muscle_development	ENSGALG00010009345	ZFAT	-0.168056566	0.124765121	-1.346983557	0.177985529	0.272227523	negative
PRJNA729271	stage_E14_vs_E10	ordinary_muscle_development	ENSGALG00010009346	STC2	-0.632870122	0.306997127	-2.061485489	0.039256746	0.077156141	negative
PRJNA729271	stage_E14_vs_E10	ordinary_muscle_development	ENSGALG00010009347	TESK1	2.124113929	0.14127241	15.03558928	4.29212E-51	6.09944E-49	positive
PRJNA729271	stage_E14_vs_E10	ordinary_muscle_development	ENSGALG00010009348	ERGIC2	-0.233184705	0.081913015	-2.846735718	0.004417001	0.011683291	negative
PRJNA729271	stage_E14_vs_E10	ordinary_muscle_development	ENSGALG00010009351	GTF3C2	0.004513703	0.121558174	0.037132038	0.970379727	0.979707052	positive
PRJNA729271	stage_E14_vs_E10	ordinary_muscle_development	ENSGALG00010009352	HSF1	0.497703303	0.180067229	2.763986022	0.005709999	0.014612717	positive
PRJNA729271	stage_E14_vs_E10	ordinary_muscle_development	ENSGALG00010009355	GARRE1	0.106315959	0.071728097	1.482208005	0.138284957	0.222000272	positive
PRJNA729271	stage_E14_vs_E10	ordinary_muscle_development	ENSGALG00010009357	LYST	-0.462855224	0.116661009	-3.967522904	7.26235E-05	0.000293389	negative
PRJNA729271	stage_E14_vs_E10	ordinary_muscle_development	ENSGALG00010009359	REEP3	-0.240612976	0.109900605	-2.18936899	0.028570032	0.058991825	negative
PRJNA729271	stage_E14_vs_E10	ordinary_muscle_development	ENSGALG00010009360	TNFAIP8	-0.89631014	0.129440062	-6.924518791	4.3746E-12	4.65906E-11	negative
PRJNA729271	stage_E14_vs_E10	ordinary_muscle_development	ENSGALG00010009361	LRRK2	0.273147787	0.088004738	3.103785026	0.001910621	0.005601629	positive
PRJNA729271	stage_E14_vs_E10	ordinary_muscle_development	ENSGALG00010009362	IL15	0.479424982	0.32641287	1.468768626	0.141895557	0.226841964	positive
PRJNA729271	stage_E14_vs_E10	ordinary_muscle_development	ENSGALG00010009363	TAF2	-0.100237834	0.076602156	-1.308551067	0.190686466	0.287886712	negative
PRJNA729271	stage_E14_vs_E10	ordinary_muscle_development	ENSGALG00010009365	CA5B	-0.825040177	0.162475925	-5.077922638	3.81584E-07	2.24384E-06	negative
PRJNA729271	stage_E14_vs_E10	ordinary_muscle_development	ENSGALG00010009367	DMXL1	-0.268222408	0.119328347	-2.247767738	0.024591004	0.052004751	negative
PRJNA729271	stage_E14_vs_E10	ordinary_muscle_development	ENSGALG00010009368	DSCC1	-0.355937349	0.193776528	-1.836844493	0.066232855	0.120321499	negative
PRJNA729271	stage_E14_vs_E10	ordinary_muscle_development	ENSGALG00010009370	ENSGALG00010009370	0.684732779	0.452976806	1.511628786	0.130628324	0.211819841	positive
PRJNA729271	stage_E14_vs_E10	ordinary_muscle_development	ENSGALG00010009371	PHTF2	0.476932291	0.15541473	3.068771476	0.002149409	0.00619767	positive
PRJNA729271	stage_E14_vs_E10	ordinary_muscle_development	ENSGALG00010009372	RAB28	-0.325425261	0.096744608	-3.363756056	0.000768895	0.002490532	negative
PRJNA729271	stage_E14_vs_E10	ordinary_muscle_development	ENSGALG00010009373	ARHGAP15	-0.547977097	0.218593274	-2.506834207	0.012181784	0.028333593	negative
PRJNA729271	stage_E14_vs_E10	ordinary_muscle_development	ENSGALG00010009376	CDO1	-0.97810441	0.250423554	-3.905800367	9.3914E-05	0.000371225	negative
PRJNA729271	stage_E14_vs_E10	ordinary_muscle_development	ENSGALG00010009378	ENSGALG00010009378	-0.340588761	0.081727942	-4.167347832	3.08164E-05	0.000133219	negative
PRJNA729271	stage_E14_vs_E10	ordinary_muscle_development	ENSGALG00010009379	RYR1	1.592307223	0.219552636	7.252507866	4.09124E-13	4.87686E-12	positive
PRJNA729271	stage_E14_vs_E10	ordinary_muscle_development	ENSGALG00010009381	INPP4B	-1.298186876	0.114928904	-11.29556474	1.38014E-29	5.8324E-28	negative
PRJNA729271	stage_E14_vs_E10	ordinary_muscle_development	ENSGALG00010009383	ASXL2	0.147921751	0.111395223	1.327900305	0.184211044	0.279976836	positive
PRJNA729271	stage_E14_vs_E10	ordinary_muscle_development	ENSGALG00010009385	GPI	0.550000219	0.178007984	3.089750285	0.002003248	0.005824892	positive
PRJNA729271	stage_E14_vs_E10	ordinary_muscle_development	ENSGALG00010009386	TMEM60	-0.038412207	0.110566059	-0.347414096	0.728280245	0.803931319	negative
PRJNA729271	stage_E14_vs_E10	ordinary_muscle_development	ENSGALG00010009387	DEPTOR	1.49611527	0.13869496	10.78709181	3.96127E-27	1.40732E-25	positive
PRJNA729271	stage_E14_vs_E10	ordinary_muscle_development	ENSGALG00010009388	TMED7	-0.144100242	0.07324956	-1.967250627	0.049154324	0.09357656	negative
PRJNA729271	stage_E14_vs_E10	ordinary_muscle_development	ENSGALG00010009389	CPEB4	1.334758695	0.1254199	10.64231988	1.89355E-26	6.47335E-25	positive
PRJNA729271	stage_E14_vs_E10	ordinary_muscle_development	ENSGALG00010009391	RSBN1L	0.084851814	0.106041502	0.800175518	0.423609112	0.53800176	positive
PRJNA729271	stage_E14_vs_E10	ordinary_muscle_development	ENSGALG00010009392	MAPRE3	0.147949107	0.106542966	1.38863326	0.164944298	0.256103567	positive
PRJNA729271	stage_E14_vs_E10	ordinary_muscle_development	ENSGALG00010009393	MARVELD2	-0.331549699	0.128280089	-2.584576463	0.009749868	0.023324697	negative
PRJNA729271	stage_E14_vs_E10	ordinary_muscle_development	ENSGALG00010009394	BANP	0.392937238	0.089566596	4.387095795	1.14874E-05	5.33003E-05	positive
PRJNA729271	stage_E14_vs_E10	ordinary_muscle_development	ENSGALG00010009396	AUH	0.292177686	0.111424573	2.62220152	0.008736376	0.021186844	positive
PRJNA729271	stage_E14_vs_E10	ordinary_muscle_development	ENSGALG00010009398	WTIP	-0.393619089	0.138285299	-2.846427576	0.004421279	0.011689473	negative
PRJNA729271	stage_E14_vs_E10	ordinary_muscle_development	ENSGALG00010009400	CNTFR	-0.11211578	0.281617046	-0.398114326	0.690545915	0.774186947	negative
PRJNA729271	stage_E14_vs_E10	ordinary_muscle_development	ENSGALG00010009404	GNG4	-0.131255259	0.395637386	-0.331756462	0.740073163	0.812986775	negative
PRJNA729271	stage_E14_vs_E10	ordinary_muscle_development	ENSGALG00010009407	PTPN12	-0.080854221	0.086380733	-0.936021478	0.34926213	0.465180058	negative
PRJNA729271	stage_E14_vs_E10	ordinary_muscle_development	ENSGALG00010009408	ENSGALG00010009408	0.018206712	0.088189557	0.206449754	0.836439609	0.885833715	positive
PRJNA729271	stage_E14_vs_E10	ordinary_muscle_development	ENSGALG00010009410	JMJD1C	0.145131196	0.107833498	1.345882295	0.178340479	0.272657278	positive
PRJNA729271	stage_E14_vs_E10	ordinary_muscle_development	ENSGALG00010009411	SMARCA5	-0.576056723	0.095685965	-6.020284439	1.74111E-09	1.38896E-08	negative
PRJNA729271	stage_E14_vs_E10	ordinary_muscle_development	ENSGALG00010009415	HMP19	-4.086288799	0.429877358	-9.505708369	1.98691E-21	4.7841E-20	negative
PRJNA729271	stage_E14_vs_E10	ordinary_muscle_development	ENSGALG00010009416	COL14A1	0.584920517	0.473460557	1.235415514	0.216675902	0.31859578	positive
PRJNA729271	stage_E14_vs_E10	ordinary_muscle_development	ENSGALG00010009424	ENSGALG00010009424	6.569046691	0.977845611	6.717877157	1.84391E-11	1.8382E-10	positive
PRJNA729271	stage_E14_vs_E10	ordinary_muscle_development	ENSGALG00010009431	SMN	-0.326173703	0.149315459	-2.184460366	0.028928437	0.059562172	negative
PRJNA729271	stage_E14_vs_E10	ordinary_muscle_development	ENSGALG00010009432	BOP1	-0.556635937	0.179606708	-3.099193476	0.001940483	0.005670826	negative
PRJNA729271	stage_E14_vs_E10	ordinary_muscle_development	ENSGALG00010009435	GTDC1	-0.419905913	0.088174782	-4.762199636	1.91494E-06	1.01192E-05	negative
PRJNA729271	stage_E14_vs_E10	ordinary_muscle_development	ENSGALG00010009437	DPYSL5	1.941804325	0.138069013	14.06401255	6.31988E-45	6.63817E-43	positive
PRJNA729271	stage_E14_vs_E10	ordinary_muscle_development	ENSGALG00010009439	B3GALNT2	-0.025867166	0.098856254	-0.26166444	0.793580158	0.855110347	negative
PRJNA729271	stage_E14_vs_E10	ordinary_muscle_development	ENSGALG00010009440	DOK3	0.121631016	0.201189195	0.60456038	0.545471139	0.651017222	positive
PRJNA729271	stage_E14_vs_E10	ordinary_muscle_development	ENSGALG00010009441	ZNF518B	-0.505132449	0.098155635	-5.146239952	2.65759E-07	1.59445E-06	negative
PRJNA729271	stage_E14_vs_E10	ordinary_muscle_development	ENSGALG00010009444	NFIL3	0.496953368	0.221559334	2.242980964	0.024898042	0.05253998	positive
PRJNA729271	stage_E14_vs_E10	ordinary_muscle_development	ENSGALG00010009446	ENSGALG00010009446	0.230504291	0.249690382	0.923160472	0.355923585	0.47231643	positive
PRJNA729271	stage_E14_vs_E10	ordinary_muscle_development	ENSGALG00010009447	DDX41	0.021291653	0.104212051	0.204310852	0.83811059	0.887099924	positive
PRJNA729271	stage_E14_vs_E10	ordinary_muscle_development	ENSGALG00010009451	ROR2	-2.270822515	0.140746717	-16.13410645	1.46918E-58	3.22664E-56	negative
PRJNA729271	stage_E14_vs_E10	ordinary_muscle_development	ENSGALG00010009452	TBCE	-0.151931359	0.084119147	-1.806144805	0.070895724	0.127466326	negative
PRJNA729271	stage_E14_vs_E10	ordinary_muscle_development	ENSGALG00010009453	GSAP	0.156760036	0.113187398	1.384960146	0.166064654	0.257326206	positive
PRJNA729271	stage_E14_vs_E10	ordinary_muscle_development	ENSGALG00010009457	BDP1	-0.401129381	0.129569844	-3.095854467	0.001962467	0.00572238	negative
PRJNA729271	stage_E14_vs_E10	ordinary_muscle_development	ENSGALG00010009458	SCX	-2.606688014	0.442796722	-5.886872879	3.93571E-09	3.02002E-08	negative
PRJNA729271	stage_E14_vs_E10	ordinary_muscle_development	ENSGALG00010009461	ZEB2	0.564066973	0.125299432	4.501752036	6.73956E-06	3.2585E-05	positive
PRJNA729271	stage_E14_vs_E10	ordinary_muscle_development	ENSGALG00010009462	NRBF2	0.175529484	0.120571577	1.45581146	0.145444747	0.231469214	positive
PRJNA729271	stage_E14_vs_E10	ordinary_muscle_development	ENSGALG00010009463	MRPL13	0.07576144	0.108663267	0.69721298	0.485669517	0.597046871	positive
PRJNA729271	stage_E14_vs_E10	ordinary_muscle_development	ENSGALG00010009464	SPTLC1	-0.229937886	0.147701403	-1.556775236	0.119523859	0.197071791	negative
PRJNA729271	stage_E14_vs_E10	ordinary_muscle_development	ENSGALG00010009465	GGPS1	-0.298899319	0.099737729	-2.996853061	0.002727822	0.007637584	negative
PRJNA729271	stage_E14_vs_E10	ordinary_muscle_development	ENSGALG00010009466	FAM193B	-0.104917782	0.10730352	-0.977766451	0.328189848	0.443099091	negative

PRJNA729271	stage_E14_vs_E10	ordinary_muscle_development	ENSGALG00010009467	EGR2	0.166009761	0.314749938	0.527433815	0.597892366	0.697490984	positive
PRJNA729271	stage_E14_vs_E10	ordinary_muscle_development	ENSGALG00010009469	DNMT3A	-0.238654267	0.210743267	-1.13244077	0.257449173	0.365598443	negative
PRJNA729271	stage_E14_vs_E10	ordinary_muscle_development	ENSGALG00010009470	ZNF469	-0.396966044	0.210523936	-1.885610023	0.059347526	0.109487385	negative
PRJNA729271	stage_E14_vs_E10	ordinary_muscle_development	ENSGALG00010009472	ADO	0.184078643	0.083390693	2.207424311	0.027284429	0.056733296	positive
PRJNA729271	stage_E14_vs_E10	ordinary_muscle_development	ENSGALG00010009474	ACVR2A	0.295342994	0.095163685	3.103526233	0.001912293	0.005604263	positive
PRJNA729271	stage_E14_vs_E10	ordinary_muscle_development	ENSGALG00010009477	ZNF365	0.042761953	0.243870024	0.175347313	0.860806737	0.904224484	positive
PRJNA729271	stage_E14_vs_E10	ordinary_muscle_development	ENSGALG00010009479	MTBP	-0.487334821	0.117367786	-4.152202575	3.29291E-05	0.000141466	negative
PRJNA729271	stage_E14_vs_E10	ordinary_muscle_development	ENSGALG00010009482	ARID4B	-0.12567905	0.10767954	-1.167158125	0.243146518	0.349574368	negative
PRJNA729271	stage_E14_vs_E10	ordinary_muscle_development	ENSGALG00010009484	RTKN2	0.283415127	0.127228217	2.227612191	0.025906387	0.054335563	positive
PRJNA729271	stage_E14_vs_E10	ordinary_muscle_development	ENSGALG00010009486	B4GALT1	0.368761365	0.127365559	2.895298909	0.003787976	0.010192448	positive
PRJNA729271	stage_E14_vs_E10	ordinary_muscle_development	ENSGALG00010009489	MROH1	0.021854536	0.13740603	0.159050776	0.87362887	0.913324952	positive
PRJNA729271	stage_E14_vs_E10	ordinary_muscle_development	ENSGALG00010009490	SNTB1	-0.041235088	0.159700293	-0.258202959	0.796250275	0.856682774	negative
PRJNA729271	stage_E14_vs_E10	ordinary_muscle_development	ENSGALG00010009491	RBM34	0.090750406	0.101026989	0.898278836	0.369036912	0.485363401	positive
PRJNA729271	stage_E14_vs_E10	ordinary_muscle_development	ENSGALG00010009492	CDC42SE2	-0.789009051	0.123713443	-6.377714766	1.7975E-10	1.6113E-09	negative
PRJNA729271	stage_E14_vs_E10	ordinary_muscle_development	ENSGALG00010009493	ENSGALG00010009493	0.102632556	0.395574127	0.259452146	0.7952864	0.856248709	positive
PRJNA729271	stage_E14_vs_E10	ordinary_muscle_development	ENSGALG00010009494	UBA2	-0.294680578	0.074047164	-3.979633568	6.90216E-05	0.000280086	negative
PRJNA729271	stage_E14_vs_E10	ordinary_muscle_development	ENSGALG00010009497	HS3ST1	-0.853761884	0.198084408	-4.31009131	1.63187E-05	7.38034E-05	negative
PRJNA729271	stage_E14_vs_E10	ordinary_muscle_development	ENSGALG00010009498	ORC4	-0.442195132	0.113069912	-3.910811675	9.19865E-05	0.000364501	negative
PRJNA729271	stage_E14_vs_E10	ordinary_muscle_development	ENSGALG00010009499	MXD3	0.844334845	0.23786508	3.549637656	0.000385762	0.001342847	positive
PRJNA729271	stage_E14_vs_E10	ordinary_muscle_development	ENSGALG00010009500	CCDC146	-1.006287154	0.654904017	-1.536541428	0.124405645	0.203436353	negative
PRJNA729271	stage_E14_vs_E10	ordinary_muscle_development	ENSGALG00010009501	HGH1	0.448176627	0.108261012	4.139778648	3.47641E-05	0.000148513	positive
PRJNA729271	stage_E14_vs_E10	ordinary_muscle_development	ENSGALG00010009502	ARID5B	-0.384923563	0.09364329	-4.11053011	3.94752E-05	0.000167308	negative
PRJNA729271	stage_E14_vs_E10	ordinary_muscle_development	ENSGALG00010009503	IL11RA	-1.412687403	0.13317786	-10.6075244	2.74937E-26	9.24644E-25	negative
PRJNA729271	stage_E14_vs_E10	ordinary_muscle_development	ENSGALG00010009506	HAS2	-0.529118995	0.257380896	-2.055781928	0.039803541	0.077955996	negative
PRJNA729271	stage_E14_vs_E10	ordinary_muscle_development	ENSGALG00010009508	TOMM20	0.428216709	0.08996107	4.76002243	1.93571E-06	1.02216E-05	positive
PRJNA729271	stage_E14_vs_E10	ordinary_muscle_development	ENSGALG00010009511	ZFPM1	-0.980113686	0.251069793	-3.903749923	9.47136E-05	0.00037408	negative
PRJNA729271	stage_E14_vs_E10	ordinary_muscle_development	ENSGALG00010009514	PRELID1	-0.343176724	0.090048513	-3.811020456	0.000138394	0.000531961	negative
PRJNA729271	stage_E14_vs_E10	ordinary_muscle_development	ENSGALG00010009515	MBD5	-0.049441495	0.095990322	-0.515067497	0.606505854	0.704205572	negative
PRJNA729271	stage_E14_vs_E10	ordinary_muscle_development	ENSGALG00010009518	FGL2	0.116030413	0.307968583	0.376760552	0.706351559	0.786734736	positive
PRJNA729271	stage_E14_vs_E10	ordinary_muscle_development	ENSGALG00010009520	SPINK4	0.079292244	0.583438215	0.135905126	0.891896283	0.925811846	positive
PRJNA729271	stage_E14_vs_E10	ordinary_muscle_development	ENSGALG00010009522	TAF5L	-0.105087578	0.080927924	-1.29853298	0.194104251	0.292137121	negative
PRJNA729271	stage_E14_vs_E10	ordinary_muscle_development	ENSGALG00010009524	FAM185A	0.161631918	0.16147743	1.000956714	0.316847736	0.430714427	positive
PRJNA729271	stage_E14_vs_E10	ordinary_muscle_development	ENSGALG00010009525	ENSGALG00010009525	0.504460871	0.155418485	3.245822853	0.001171117	0.003627984	positive
PRJNA729271	stage_E14_vs_E10	ordinary_muscle_development	ENSGALG00010009526	ENSGALG00010009526	-0.462424156	0.224671202	-2.058226206	0.039568424	0.077594443	negative
PRJNA729271	stage_E14_vs_E10	ordinary_muscle_development	ENSGALG00010009527	ENSGALG00010009527	-1.419748974	0.474980046	-2.989070778	0.002798273	0.007815215	negative
PRJNA729271	stage_E14_vs_E10	ordinary_muscle_development	ENSGALG00010009530	KIF3C	-0.419434847	0.147692721	-2.839915492	0.004512549	0.011899107	negative
PRJNA729271	stage_E14_vs_E10	ordinary_muscle_development	ENSGALG00010009531	FBXL13	-0.313390814	0.42264736	-0.741494786	0.458393492	0.571316738	negative
PRJNA729271	stage_E14_vs_E10	ordinary_muscle_development	ENSGALG00010009534	XPA	-0.12888283	0.131030089	-0.983612471	0.325306091	0.439978521	negative
PRJNA729271	stage_E14_vs_E10	ordinary_muscle_development	ENSGALG00010009538	CRIM1	-0.069537431	0.075976029	-0.915254879	0.360057841	0.476406974	negative
PRJNA729271	stage_E14_vs_E10	ordinary_muscle_development	ENSGALG00010009540	RAB10	0.268496444	0.089876648	2.987388261	0.002813721	0.007853821	positive
PRJNA729271	stage_E14_vs_E10	ordinary_muscle_development	ENSGALG00010009541	PMPCB	0.092067042	0.091498254	1.00621638	0.314311494	0.428270832	positive
PRJNA729271	stage_E14_vs_E10	ordinary_muscle_development	ENSGALG00010009542	WDR1	-0.064252116	0.143034721	-0.449206426	0.653282753	0.744066675	negative
PRJNA729271	stage_E14_vs_E10	ordinary_muscle_development	ENSGALG00010009543	MAF1	0.493554569	0.129933687	3.798511218	0.000145568	0.000556289	positive
PRJNA729271	stage_E14_vs_E10	ordinary_muscle_development	ENSGALG00010009546	GAREM2	-2.195914843	0.554705015	-3.958707394	7.53565E-05	0.00030333	negative
PRJNA729271	stage_E14_vs_E10	ordinary_muscle_development	ENSGALG00010009548	PIK3C3	-0.055529153	0.081150343	-0.684275028	0.493801512	0.603970376	negative
PRJNA729271	stage_E14_vs_E10	ordinary_muscle_development	ENSGALG00010009551	HADHA	0.220002686	0.130138397	1.690528633	0.090926865	0.157200778	positive
PRJNA729271	stage_E14_vs_E10	ordinary_muscle_development	ENSGALG00010009554	ABCB10	0.088151108	0.101562903	0.867945934	0.385423931	0.50159094	positive
PRJNA729271	stage_E14_vs_E10	ordinary_muscle_development	ENSGALG00010009555	EPC2	0.028767898	0.075332573	0.381878608	0.702551408	0.783961113	positive
PRJNA729271	stage_E14_vs_E10	ordinary_muscle_development	ENSGALG00010009557	CIDEC	2.718829647	1.056390024	2.573698715	0.010061785	0.023936578	positive
PRJNA729271	stage_E14_vs_E10	ordinary_muscle_development	ENSGALG00010009558	TMEM26	-1.037954925	0.197485515	-5.255853455	1.4734E-07	9.14641E-07	negative
PRJNA729271	stage_E14_vs_E10	ordinary_muscle_development	ENSGALG00010009560	RAB24	0.659472595	0.141866757	4.64853508	3.34301E-06	1.69451E-05	positive
PRJNA729271	stage_E14_vs_E10	ordinary_muscle_development	ENSGALG00010009562	HADHB	0.169587456	0.189572232	0.894579621	0.37101185	0.487253489	positive
PRJNA729271	stage_E14_vs_E10	ordinary_muscle_development	ENSGALG00010009565	TMEM8B	0.478653153	0.212124833	2.256469201	0.024041262	0.051029154	positive
PRJNA729271	stage_E14_vs_E10	ordinary_muscle_development	ENSGALG00010009567	NSD1	0.069355213	0.106169906	0.653247382	0.513596808	0.622352929	positive
PRJNA729271	stage_E14_vs_E10	ordinary_muscle_development	ENSGALG00010009569	ZC3H18	0.015644784	0.142279819	0.109957858	0.912442797	0.939779207	positive
PRJNA729271	stage_E14_vs_E10	ordinary_muscle_development	ENSGALG00010009570	SHARPIN	0.613631162	0.136648226	4.490590037	7.10261E-06	3.41807E-05	positive
PRJNA729271	stage_E14_vs_E10	ordinary_muscle_development	ENSGALG00010009571	DNAJC2	-0.457689418	0.185291678	-2.470102389	0.013507438	0.03100892	negative
PRJNA729271	stage_E14_vs_E10	ordinary_muscle_development	ENSGALG00010009574	TAF1C	-0.044938968	0.134715974	-0.333583065	0.738694199	0.812149	negative
PRJNA729271	stage_E14_vs_E10	ordinary_muscle_development	ENSGALG00010009575	RHOBTB1	-0.953788705	0.240384827	-3.967757519	7.25521E-05	0.000293182	negative
PRJNA729271	stage_E14_vs_E10	ordinary_muscle_development	ENSGALG00010009578	KIF5C	-0.348533572	0.155064634	-2.247666425	0.024597468	0.052004751	negative
PRJNA729271	stage_E14_vs_E10	ordinary_muscle_development	ENSGALG00010009582	TIGD5	0.182623215	0.124330531	1.46885253	0.141872792	0.22683059	positive
PRJNA729271	stage_E14_vs_E10	ordinary_muscle_development	ENSGALG00010009583	LYPD6B	-2.346447254	0.290005305	-8.091049419	5.91528E-16	9.04452E-15	negative
PRJNA729271	stage_E14_vs_E10	ordinary_muscle_development	ENSGALG00010009591	LYPD6	-1.052563639	0.431319331	-2.440334952	0.014673649	0.033311597	negative
PRJNA729271	stage_E14_vs_E10	ordinary_muscle_development	ENSGALG00010009593	NCBP1	-0.1073634	0.061898077	-1.734519152	0.082826011	0.145628703	negative
PRJNA729271	stage_E14_vs_E10	ordinary_muscle_development	ENSGALG00010009594	HINT2	0.769371501	0.160555225	4.791943089	1.65174E-06	8.83466E-06	positive
PRJNA729271	stage_E14_vs_E10	ordinary_muscle_development	ENSGALG00010009595	ENSGALG00010009595	0.321271097	0.186065302	1.726657756	0.084229147	0.14768374	positive
PRJNA729271	stage_E14_vs_E10	ordinary_muscle_development	ENSGALG00010009596	URB2	-0.514756982	0.095666483	-5.380745332	7.41781E-08	4.82258E-07	negative
PRJNA729271	stage_E14_vs_E10	ordinary_muscle_development	ENSGALG00010009597	CDK1	-0.041273126	0.258536687	-0.159641273	0.873163665	0.91296116	negative
PRJNA729271	stage_E14_vs_E10	ordinary_muscle_development	ENSGALG00010009600	LRRC17	0.028646572	0.217038007	0.131988733	0.894993203	0.927896894	positive
PRJNA729271	stage_E14_vs_E10	ordinary_muscle_development	ENSGALG00010009601	ZHX2	0.262818218	0.13560871	1.938062955	0.052615542	0.09888011	positive
PRJNA729271	stage_E14_vs_E10	ordinary_muscle_development	ENSGALG00010009602	TSTD2	-0.097103672	0.079917008	-1.215056396	0.224344583	0.32731502	negative
PRJNA729271	stage_E14_vs_E10	ordinary_muscle_development	ENSGALG00010009603	HINT1	0.162998642	0.177205553	0.919828074	0.35766261	0.473973262	positive
PRJNA729271	stage_E14_vs_E10	ordinary_muscle_development	ENSGALG00010009605	ENSGALG00010009605	0.957953661	0.154531637	6.199077937	5.67949E-10	4.79745E-09	positive
PRJNA729271	stage_E14_vs_E10	ordinary_muscle_development	ENSGALG00010009606	CHSY3	-0.824650933	0.213429262	-3.863813818	0.00011163	0.000436258	negative
PRJNA729271	stage_E14_vs_E10	ordinary_muscle_development	ENSGALG00010009608	AFAP1	0.462810617	0.106317121	4.353114648	1.34217E-05	6.16758E-05	positive
PRJNA729271	stage_E14_vs_E10	ordinary_muscle_development	ENSGALG00010009609	MMADHC	0.022933331	0.071254851	0.321849401	0.747566787	0.81900609	positive
PRJNA729271	stage_E14_vs_E10	ordinary_muscle_development	ENSGALG00010009612	DERL1	-0.131631134	0.063004364	-2.08923835	0.036686273	0.072775082	negative
PRJNA729271	stage_E14_vs_E10	ordinary_muscle_development	ENSGALG00010009615	ENSGALG00010009615	0.642280391	0.396036157	1.621772101	0.104852154	0.177115951	positive
PRJNA729271	stage_E14_vs_E10	ordinary_muscle_development	ENSGALG00010009616	ARMC10	-0.535140535	0.175125888	-3.055747723	0.002245001	0.006441268	negative
PRJNA729271	stage_E14_vs_E10	ordinary_muscle_development	ENSGALG00010009617	SLC25A46	0.156146941	0.09765601	1.598948611	0.109832022	0.184069274	positive
PRJNA729271	stage_E14_vs_E10	ordinary_muscle_development	ENSGALG00010009619	ENSGALG00010009619	0.323860249	0.187333114	1.728793391	0.083846079	0.147172308	positive
PRJNA729271	stage_E14_vs_E10	ordinary_muscle_development	ENSGALG00010009620	TMOD1	1.830038196	0.231800557	7.894882666	2.90588E-15	4.18696E-14	positive
PRJNA729271	stage_E14_vs_E10	ordinary_muscle_development	ENSGALG00010009623	XPO5	-0.327658931	0.110528682	-2.96446973	0.003032051	0.008396938	negative
PRJNA729271	stage_E14_vs_E10	ordinary_muscle_development	ENSGALG00010009629	ENSGALG00010009629	0.305012449	0.545473589	0.559169968	0.576045727	0.678017281	positive
PRJNA729271	stage_E14_vs_E10	ordinary_muscle_development	ENSGALG00010009634	TBC1D31	-0.243987391	0.100806117	-2.420362946	0.015505023	0.034914604	negative
PRJNA729271	stage_E14_vs_E10	ordinary_muscle_development	ENSGALG00010009635	TDRD7	-0.00615916	0.139648629	-0.044104694	0.964820951	0.9766785	negative
PRJNA729271	stage_E14_vs_E10	ordinary_muscle_development	ENSGALG00010009637	CYBA	0.439523611	0.13506102	3.254259531	0.001136884	0.003531746	positive
PRJNA729271	stage_E14_vs_E10	ordinary_muscle_development	ENSGALG00010009638	RND3	-0.892867557	0.149634017	-5.967009196	2.41642E-09	1.8974E-08	negative
PRJNA729271	stage_E14_vs_E10	ordinary_muscle_development	ENSGALG00010009640	NPR2	0.489083604	0.187122864	2.613703074	0.008956684	0.021655902	positive
PRJNA729271	stage_E14_vs_E10	ordinary_muscle_development	ENSGALG00010009643	PSMC2	0.260578585	0.108497661	2.401697724	0.016319187	0.036475963	positive
PRJNA729271	stage_E14_vs_E10	ordinary_muscle_development	ENSGALG00010009644	SLC27A6	-1.188321553	0.225581538	-5.26781387	1.38058E-07	8.6071E-07	negative
PRJNA729271	stage_E14_vs_E10	ordinary_muscle_development	ENSGALG00010009646	MVD	0.483250631	0.166485001	2.902667673	0.00369999	0.0099835	positive
PRJNA729271	stage_E14_vs_E10	ordinary_muscle_development	ENSGALG00010009647	GALNT2	-0.515227442	0.076687657	-6.718518496	1.83581E-11	1.83139E-10	negative
PRJNA729271	stage_E14_vs_E10	ordinary_muscle_development	ENSGALG00010009649	TENM2	-1.266791077	0.312254875	-4.056913684	4.97254E-05	0.000206813	negative
PRJNA729271	stage_E14_vs_E10	ordinary_muscle_development	ENSGALG00010009650	KLHL29	0.504776706	0.189569402	2.66275412	0.007750402	0.019089564	positive
PRJNA729271	stage_E14_vs_E10	ordinary_muscle_development	ENSGALG00010009652	CIAO2B	0.789009357	0.077810291	10.14016716	3.66472E-24	1.02946E-22	positive
PRJNA729271	stage_E14_vs_E10	ordinary_muscle_development	ENSGALG00010009653	ABLIM2	0.887624761	0.18507511	4.796024502	1.61845E-06	8.67264E-06	positive
PRJNA729271	stage_E14_vs_E10	ordinary_muscle_development	ENSGALG00010009654	NDUFA12	0.475005785	0.13983618	3.396873304	0.000681605	0.002234056	positive
PRJNA729271	stage_E14_vs_E10	ordinary_muscle_development	ENSGALG00010009657	MSMP	1.191516442	0.696548114	1.710601778	0.087154651	0.151857995	positive
PRJNA729271	stage_E14_vs_E10	ordinary_muscle_development	ENSGALG00010009658	NAPEPLD	0.213226748	0.123614255	1.724936567	0.084538905	0.148083556	positive
PRJNA729271	stage_E14_vs_E10	ordinary_muscle_development	ENSGALG00010009659	APOLD1	0.216875407	0.253455108	0.855675818	0.39217712	0.507780916	positive
PRJNA729271	stage_E14_vs_E10	ordinary_muscle_development	ENSGALG00010009663	FER	-0.121128306	0.121932257	-0.993406582	0.320511855	0.435001811	negative
PRJNA729271	stage_E14_vs_E10	ordinary_muscle_development	ENSGALG00010009665	DGKQ	-0.180525293	0.170061142	-1.061531696	0.288448336	0.400753248	negative
PRJNA729271	stage_E14_vs_E10	ordinary_muscle_development	ENSGALG00010009666	RRAD	0.890360428	0.364289221	2.444103136	0.014521275	0.033032937	positive
PRJNA729271	stage_E14_vs_E10	ordinary_muscle_development	ENSGALG00010009668	TFAP2A	-2.991219314	0.526020759	-5.686504311	1.29666E-08	9.31373E-08	negative
PRJNA729271	stage_E14_vs_E10	ordinary_muscle_development	ENSGALG00010009671	MINAR2	1.568423119	0.84746062	1.850732744	0.064208011	0.117201249	positive
PRJNA729271	stage_E14_vs_E10	ordinary_muscle_development	ENSGALG00010009672	DDX47	-0.311170791	0.118613427	-2.623402754	0.008705629	0.021122882	negative
PRJNA729271	stage_E14_vs_E10	ordinary_muscle_development	ENSGALG00010009676	ACO1	-0.018654544	0.094447385	-0.197512547	0.843426464	0.891136178	negative
PRJNA729271	stage_E14_vs_E10	ordinary_muscle_development	ENSGALG00010009677	SLC26A5	-0.437216244	0.163794444	-2.669298377	0.007600989	0.018743848	negative
PRJNA729271	stage_E14_vs_E10	ordinary_muscle_development	ENSGALG00010009678	JARID2	-0.51822187	0.099693724	-5.198139358	2.01293E-07	1.2306E-06	negative
PRJNA729271	stage_E14_vs_E10	ordinary_muscle_development	ENSGALG00010009679	ANK3	1.16153042	0.102866462	11.29163382	1.44329E-29	6.08152E-28	positive
PRJNA729271	stage_E14_vs_E10	ordinary_muscle_development	ENSGALG00010009683	ADAMTS19	1.549683348	0.238459326	6.498732397	8.09996E-11	7.52139E-10	positive
PRJNA729271	stage_E14_vs_E10	ordinary_muscle_development	ENSGALG00010009686	MAT2B	0.370301156	0.086899725	4.261246587	2.0329E-05	9.03891E-05	positive
PRJNA729271	stage_E14_vs_E10	ordinary_muscle_development	ENSGALG00010009689	ENSGALG00010009689	-0.423853638	0.4311726	-0.983025448	0.325594914	0.440231908	negative
PRJNA729271	stage_E14_vs_E10	ordinary_muscle_development	ENSGALG00010009690	RBM43	-0.536378638	0.18626784	-2.879609487	0.00398168	0.010640571	negative
PRJNA729271	stage_E14_vs_E10	ordinary_muscle_development	ENSGALG00010009695	ATAD2B	0.171679873	0.075328764	2.279074593	0.022662632	0.048514967	positive
PRJNA729271	stage_E14_vs_E10	ordinary_muscle_development	ENSGALG00010009697	ENSGALG00010009697	0.20221343	0.253735186	0.796946743	0.425481962	0.539860035	positive
PRJNA729271	stage_E14_vs_E10	ordinary_muscle_development	ENSGALG00010009699	POLR1C	-0.230033637	0.106640076	-2.157103084	0.030997633	0.063078857	negative
PRJNA729271	stage_E14_vs_E10	ordinary_muscle_development	ENSGALG00010009700	SH3TC1	0.735317337	0.205278481	3.582047826	0.000340911	0.001205835	positive
PRJNA729271	stage_E14_vs_E10	ordinary_muscle_development	ENSGALG00010009702	ENSGALG00010009702	0.039833648	0.119040617	0.33462232	0.737910006	0.811651532	positive
PRJNA729271	stage_E14_vs_E10	ordinary_muscle_development	ENSGALG00010009704	RGP1	0.025593077	0.129653261	0.197396319	0.84351741	0.891165077	positive
PRJNA729271	stage_E14_vs_E10	ordinary_muscle_development	ENSGALG00010009705	DTNBP1	-0.245062341	0.068646845	-3.569899575	0.000357118	0.001257025	negative
PRJNA729271	stage_E14_vs_E10	ordinary_muscle_development	ENSGALG00010009710	ENSGALG00010009710	0.012902238	0.10854896	0.118860996	0.905385483	0.935273797	positive
PRJNA729271	stage_E14_vs_E10	ordinary_muscle_development	ENSGALG00010009712	UBXN2A	0.838966187	0.178887792	4.689901862	2.73336E-06	1.40846E-05	positive
PRJNA729271	stage_E14_vs_E10	ordinary_muscle_development	ENSGALG00010009715	NUDCD2	0.003572119	0.091332074	0.03911133	0.968801628	0.978659112	positive
PRJNA729271	stage_E14_vs_E10	ordinary_muscle_development	ENSGALG00010009717	EDN1	-0.068839354	0.282329418	-0.243826359	0.807365305	0.864629486	negative
PRJNA729271	stage_E14_vs_E10	ordinary_muscle_development	ENSGALG00010009718	ENSGALG00010009718	-0.166866729	0.176108082	-0.947524536	0.34337156	0.458556362	negative
PRJNA729271	stage_E14_vs_E10	ordinary_muscle_development	ENSGALG00010009719	YIPF3	-0.061021599	0.081143428	-0.752021466	0.45203815	0.565047687	negative
PRJNA729271	stage_E14_vs_E10	ordinary_muscle_development	ENSGALG00010009720	RELN	-1.390836939	0.16890305	-8.234528285	1.80267E-16	2.89045E-15	negative
PRJNA729271	stage_E14_vs_E10	ordinary_muscle_development	ENSGALG00010009721	ZHX1	-0.33174945	0.156313671	-2.122331645	0.033809898	0.067896158	negative
PRJNA729271	stage_E14_vs_E10	ordinary_muscle_development	ENSGALG00010009722	GBA2	0.117656804	0.095291822	1.234699912	0.216942209	0.318855924	positive

PRJNA729271	stage_E14_vs_E10	ordinary_muscle_development	ENSGALG00010009724	ISOC1	-0.087522833	0.143010675	-0.612002096	0.540536371	0.646565002	negative
PRJNA729271	stage_E14_vs_E10	ordinary_muscle_development	ENSGALG00010009726	PRR16	-0.488014732	0.144689352	-3.372844838	0.000743959	0.002420579	negative
PRJNA729271	stage_E14_vs_E10	ordinary_muscle_development	ENSGALG00010009727	CDH20	2.639148954	0.212991192	12.39088307	2.92801E-35	1.65142E-33	positive
PRJNA729271	stage_E14_vs_E10	ordinary_muscle_development	ENSGALG00010009728	CCDC149	-0.299703718	0.098581745	-3.040154338	0.002364569	0.006732358	negative
PRJNA729271	stage_E14_vs_E10	ordinary_muscle_development	ENSGALG00010009730	C8orf76	0.206349118	0.110547853	1.866604478	0.061956842	0.113736629	positive
PRJNA729271	stage_E14_vs_E10	ordinary_muscle_development	ENSGALG00010009731	NMI	-0.557821781	0.242609051	-2.299262036	0.021490064	0.04631259	negative
PRJNA729271	stage_E14_vs_E10	ordinary_muscle_development	ENSGALG00010009732	HMMR	-0.3807196	0.24010153	-1.585660865	0.112816261	0.188221882	negative
PRJNA729271	stage_E14_vs_E10	ordinary_muscle_development	ENSGALG00010009736	WISP1	0.038766783	0.326423594	0.118762196	0.90546376	0.935273797	positive
PRJNA729271	stage_E14_vs_E10	ordinary_muscle_development	ENSGALG00010009738	ENSGALG00010009738	0.057393676	0.255654679	0.224496873	0.8223707	0.875588607	positive
PRJNA729271	stage_E14_vs_E10	ordinary_muscle_development	ENSGALG00010009740	LRRC73	0.766582072	0.285944657	2.680875659	0.007342979	0.018169424	positive
PRJNA729271	stage_E14_vs_E10	ordinary_muscle_development	ENSGALG00010009741	C2H6orf52	-0.037235017	0.06885754	-0.54075439	0.588676884	0.688912597	negative
PRJNA729271	stage_E14_vs_E10	ordinary_muscle_development	ENSGALG00010009743	CCNG1	0.112282777	0.172922957	0.649322562	0.516129905	0.624691298	positive
PRJNA729271	stage_E14_vs_E10	ordinary_muscle_development	ENSGALG00010009744	DAPK1	-0.382289429	0.101954137	-3.749621533	0.000177102	0.000665221	negative
PRJNA729271	stage_E14_vs_E10	ordinary_muscle_development	ENSGALG00010009749	NDRG1	0.01332983	0.167394274	0.079631336	0.936530472	0.955851936	positive
PRJNA729271	stage_E14_vs_E10	ordinary_muscle_development	ENSGALG00010009751	ENSGALG00010009751	0.095085611	0.19961493	0.476345185	0.63382848	0.727479912	positive
PRJNA729271	stage_E14_vs_E10	ordinary_muscle_development	ENSGALG00010009761	PDP2	0.254203496	0.115881519	2.193650015	0.02826058	0.058427771	positive
PRJNA729271	stage_E14_vs_E10	ordinary_muscle_development	ENSGALG00010009762	MAN2A1	-0.00251808	0.102403519	-0.024589779	0.980382172	0.986987053	negative
PRJNA729271	stage_E14_vs_E10	ordinary_muscle_development	ENSGALG00010009765	STX10	-0.212516803	0.096228324	-2.208464151	0.027211934	0.056647564	negative
PRJNA729271	stage_E14_vs_E10	ordinary_muscle_development	ENSGALG00010009769	TMCC3	-0.292633233	0.237328666	-1.23302944	0.217564781	0.319610975	negative
PRJNA729271	stage_E14_vs_E10	ordinary_muscle_development	ENSGALG00010009772	ENSGALG00010009772	0.017932342	0.557617871	0.032158837	0.974345382	0.982618708	positive
PRJNA729271	stage_E14_vs_E10	ordinary_muscle_development	ENSGALG00010009774	PIAS2	-0.375120617	0.059046229	-6.352998666	2.11158E-10	1.8789E-09	negative
PRJNA729271	stage_E14_vs_E10	ordinary_muscle_development	ENSGALG00010009775	KIAA1614	-0.18726588	0.132487156	-1.413464405	0.15751922	0.246783609	negative
PRJNA729271	stage_E14_vs_E10	ordinary_muscle_development	ENSGALG00010009777	ST3GAL1	-0.88650708	0.191021762	-4.640869562	3.46946E-06	1.7547E-05	negative
PRJNA729271	stage_E14_vs_E10	ordinary_muscle_development	ENSGALG00010009778	ENSGALG00010009778	0.167220751	0.109961632	1.520719071	0.128330351	0.208677186	positive
PRJNA729271	stage_E14_vs_E10	ordinary_muscle_development	ENSGALG00010009779	PPP1R3E	0.448271105	0.375809983	1.192813189	0.232942543	0.337245522	positive
PRJNA729271	stage_E14_vs_E10	ordinary_muscle_development	ENSGALG00010009780	MFSD2B	-0.258984905	0.248723424	-1.041256596	0.297756475	0.411202373	negative
PRJNA729271	stage_E14_vs_E10	ordinary_muscle_development	ENSGALG00010009784	ENSGALG00010009784	-0.062484641	0.176247817	-0.35452718	0.722943839	0.799318912	negative
PRJNA729271	stage_E14_vs_E10	ordinary_muscle_development	ENSGALG00010009786	DIPK2B	-0.006449413	0.160009451	-0.04030645	0.967848812	0.978469436	negative
PRJNA729271	stage_E14_vs_E10	ordinary_muscle_development	ENSGALG00010009788	PAK1IP1	0.033384575	0.091589774	0.364501115	0.715483841	0.794136858	positive
PRJNA729271	stage_E14_vs_E10	ordinary_muscle_development	ENSGALG00010009791	GABRG2	-1.156047997	0.302457215	-3.822186876	0.000132273	0.000509785	negative
PRJNA729271	stage_E14_vs_E10	ordinary_muscle_development	ENSGALG00010009793	TNFAIP6	0.359281783	0.287083209	1.251490062	0.210755737	0.31178857	positive
PRJNA729271	stage_E14_vs_E10	ordinary_muscle_development	ENSGALG00010009795	NDUFA6	0.448474708	0.114628787	3.912409087	9.13799E-05	0.000362395	positive
PRJNA729271	stage_E14_vs_E10	ordinary_muscle_development	ENSGALG00010009796	ENSGALG00010009796	-0.093628173	0.19294963	-0.485246711	0.627501351	0.721818275	negative
PRJNA729271	stage_E14_vs_E10	ordinary_muscle_development	ENSGALG00010009798	YIPF4	-0.095441782	0.077637099	-1.229332159	0.218947295	0.321251243	negative
PRJNA729271	stage_E14_vs_E10	ordinary_muscle_development	ENSGALG00010009801	ENSGALG00010009801	-0.780713038	0.59867655	-1.304064837	0.192211477	0.289615941	negative
PRJNA729271	stage_E14_vs_E10	ordinary_muscle_development	ENSGALG00010009804	XPR1	-0.264989972	0.117410539	-2.256952177	0.024011063	0.050987454	negative
PRJNA729271	stage_E14_vs_E10	ordinary_muscle_development	ENSGALG00010009806	WDCP	0.205513602	0.171579023	1.197778136	0.231003401	0.335174605	positive
PRJNA729271	stage_E14_vs_E10	ordinary_muscle_development	ENSGALG00010009809	SMDT1	0.149849893	0.090314481	1.659201176	0.097075261	0.165912735	positive
PRJNA729271	stage_E14_vs_E10	ordinary_muscle_development	ENSGALG00010009811	ENSGALG00010009811	-0.062783815	0.086747243	-0.723755744	0.469215703	0.581554649	negative
PRJNA729271	stage_E14_vs_E10	ordinary_muscle_development	ENSGALG00010009822	ENSGALG00010009822	-0.23372401	0.199015013	-1.174403912	0.240233279	0.346072489	negative
PRJNA729271	stage_E14_vs_E10	ordinary_muscle_development	ENSGALG00010009830	RNASEL	-0.54281292	0.190694392	-2.846506988	0.004420176	0.011689473	negative
PRJNA729271	stage_E14_vs_E10	ordinary_muscle_development	ENSGALG00010009831	N6AMT1	-0.483525715	0.119358216	-4.051046761	5.0989E-05	0.000211894	negative
PRJNA729271	stage_E14_vs_E10	ordinary_muscle_development	ENSGALG00010009833	SOD3	1.807624122	0.312672708	5.781202126	7.41687E-09	5.48508E-08	positive
PRJNA729271	stage_E14_vs_E10	ordinary_muscle_development	ENSGALG00010009840	ENSGALG00010009840	0.069506799	0.175020531	0.397135116	0.691267826	0.77463681	positive
PRJNA729271	stage_E14_vs_E10	ordinary_muscle_development	ENSGALG00010009841	GABRA1	-1.317587288	0.241497217	-5.455910846	4.87224E-08	3.23811E-07	negative
PRJNA729271	stage_E14_vs_E10	ordinary_muscle_development	ENSGALG00010009842	PHETA2	0.218750519	0.089873124	2.43399261	0.0149333	0.033821591	positive
PRJNA729271	stage_E14_vs_E10	ordinary_muscle_development	ENSGALG00010009847	ENSGALG00010009847	-0.240796326	0.116924082	-2.059424561	0.039453585	0.077417045	negative
PRJNA729271	stage_E14_vs_E10	ordinary_muscle_development	ENSGALG00010009850	RIF1	-0.358429398	0.10401842	-3.44582621	0.000569316	0.001901438	negative
PRJNA729271	stage_E14_vs_E10	ordinary_muscle_development	ENSGALG00010009852	ENSGALG00010009852	0.146362114	0.241830999	0.605224783	0.545029651	0.650543873	positive
PRJNA729271	stage_E14_vs_E10	ordinary_muscle_development	ENSGALG00010009857	ORC5	-0.259106948	0.134583711	-1.925247466	0.054198406	0.101433945	negative
PRJNA729271	stage_E14_vs_E10	ordinary_muscle_development	ENSGALG00010009858	ST8SIA5	-1.468469071	0.249901832	-5.876183699	4.19832E-09	3.21303E-08	negative
PRJNA729271	stage_E14_vs_E10	ordinary_muscle_development	ENSGALG00010009866	NAGA	0.112150592	0.114774182	0.977141291	0.328499208	0.443351585	positive
PRJNA729271	stage_E14_vs_E10	ordinary_muscle_development	ENSGALG00010009868	LTN1	0.090195907	0.094871537	0.950716199	0.341748463	0.456725426	positive
PRJNA729271	stage_E14_vs_E10	ordinary_muscle_development	ENSGALG00010009876	SELENOI	0.155450523	0.106495046	1.459697221	0.144373306	0.230117778	positive
PRJNA729271	stage_E14_vs_E10	ordinary_muscle_development	ENSGALG00010009883	ENSGALG00010009883	-0.058534725	0.199522742	-0.293373698	0.769236527	0.836066408	negative
PRJNA729271	stage_E14_vs_E10	ordinary_muscle_development	ENSGALG00010009884	GFOD1	0.246311307	0.115669921	2.129432654	0.033218481	0.066865975	positive
PRJNA729271	stage_E14_vs_E10	ordinary_muscle_development	ENSGALG00010009910	ENSGALG00010009910	-0.520912118	0.177220918	-2.93933765	0.003289145	0.009012507	negative
PRJNA729271	stage_E14_vs_E10	ordinary_muscle_development	ENSGALG00010009914	RWDD2B	-0.081688922	0.119256979	-0.684982324	0.493355075	0.603577128	negative
PRJNA729271	stage_E14_vs_E10	ordinary_muscle_development	ENSGALG00010009941	CACNA1E	-0.797025722	0.354269063	-2.249775118	0.024463224	0.051765611	negative
PRJNA729271	stage_E14_vs_E10	ordinary_muscle_development	ENSGALG00010009943	DHX15	-0.194463913	0.068256531	-2.849015481	0.004385475	0.01160661	negative
PRJNA729271	stage_E14_vs_E10	ordinary_muscle_development	ENSGALG00010009948	WBP2NL	0.178377645	0.064885569	2.749111203	0.005975711	0.015219045	positive
PRJNA729271	stage_E14_vs_E10	ordinary_muscle_development	ENSGALG00010009972	PTK2B	0.278663948	0.270985109	1.028336757	0.303791447	0.41731018	positive
PRJNA729271	stage_E14_vs_E10	ordinary_muscle_development	ENSGALG00010009985	USP16	-0.227155288	0.113995561	-1.992667839	0.046297831	0.088991787	negative
PRJNA729271	stage_E14_vs_E10	ordinary_muscle_development	ENSGALG00010009989	TBC1D7	-0.017864792	0.132444647	-0.13488497	0.892702823	0.926317375	negative
PRJNA729271	stage_E14_vs_E10	ordinary_muscle_development	ENSGALG00010009991	LHFPL3	-3.115529329	0.518346172	-6.01051864	1.84931E-09	1.47042E-08	negative
PRJNA729271	stage_E14_vs_E10	ordinary_muscle_development	ENSGALG00010009993	ENSGALG00010009993	-0.690561664	0.360090741	-1.917743463	0.055143548	0.10293699	negative
PRJNA729271	stage_E14_vs_E10	ordinary_muscle_development	ENSGALG00010009995	SEPTIN3	-0.712686442	0.388803054	-1.83302686	0.066798577	0.121119119	negative
PRJNA729271	stage_E14_vs_E10	ordinary_muscle_development	ENSGALG00010009999	GABRB2	0.615664342	0.225057657	2.735584957	0.006226951	0.015754871	positive
PRJNA729271	stage_E14_vs_E10	ordinary_muscle_development	ENSGALG00010010006	PPARGC1A	1.550182557	0.265349778	5.842034502	5.15671E-09	3.89711E-08	positive
PRJNA729271	stage_E14_vs_E10	ordinary_muscle_development	ENSGALG00010010007	CTIF	0.239936774	0.094212692	2.546756393	0.010872931	0.025626525	positive
PRJNA729271	stage_E14_vs_E10	ordinary_muscle_development	ENSGALG00010010009	ELF2	0.100614121	0.087555135	1.149151569	0.250493487	0.357689203	positive
PRJNA729271	stage_E14_vs_E10	ordinary_muscle_development	ENSGALG00010010011	EPB41L4A	0.102786908	0.122895216	0.836378436	0.40294204	0.518100453	positive
PRJNA729271	stage_E14_vs_E10	ordinary_muscle_development	ENSGALG00010010014	GLUL	0.771204365	0.392564058	1.964531264	0.049468514	0.094063507	positive
PRJNA729271	stage_E14_vs_E10	ordinary_muscle_development	ENSGALG00010010017	CCDC59	-0.397388448	0.10427652	-3.810910156	0.000138456	0.000532058	negative
PRJNA729271	stage_E14_vs_E10	ordinary_muscle_development	ENSGALG00010010022	KMT2E	-0.273211321	0.101403536	-2.69429777	0.007053712	0.017549528	negative
PRJNA729271	stage_E14_vs_E10	ordinary_muscle_development	ENSGALG00010010035	ATP10B	-0.042975226	0.282459692	-0.152146402	0.879071467	0.916898677	negative
PRJNA729271	stage_E14_vs_E10	ordinary_muscle_development	ENSGALG00010010037	NAA15	-0.034443548	0.13816575	-0.249291503	0.803135304	0.861407535	negative
PRJNA729271	stage_E14_vs_E10	ordinary_muscle_development	ENSGALG00010010042	METTL25	-0.489252082	0.111278232	-4.396655777	1.09931E-05	5.11707E-05	negative
PRJNA729271	stage_E14_vs_E10	ordinary_muscle_development	ENSGALG00010010049	ZBTB7C	-0.708647295	0.282202869	-2.511127179	0.012034633	0.028045339	negative
PRJNA729271	stage_E14_vs_E10	ordinary_muscle_development	ENSGALG00010010052	ZNF346	0.154192581	0.111647728	1.381063309	0.167259496	0.258964579	positive
PRJNA729271	stage_E14_vs_E10	ordinary_muscle_development	ENSGALG00010010053	CHRNA2	-5.33981752	0.745376969	-7.163915369	7.84048E-13	9.07005E-12	negative
PRJNA729271	stage_E14_vs_E10	ordinary_muscle_development	ENSGALG00010010059	ENSGALG00010010059	-0.107522845	0.118448531	-0.907760052	0.364005008	0.480139466	negative
PRJNA729271	stage_E14_vs_E10	ordinary_muscle_development	ENSGALG00010010061	CYP2D6	1.542527393	0.202538698	7.615963793	2.61731E-14	3.48694E-13	positive
PRJNA729271	stage_E14_vs_E10	ordinary_muscle_development	ENSGALG00010010062	RGS7BP	-0.13310372	0.168985828	-0.787662025	0.430894424	0.545010007	negative
PRJNA729271	stage_E14_vs_E10	ordinary_muscle_development	ENSGALG00010010064	ENSGALG00010010064	-0.604120353	0.088443959	-6.83054398	8.45932E-12	8.72105E-11	negative
PRJNA729271	stage_E14_vs_E10	ordinary_muscle_development	ENSGALG00010010071	GLT8D2	-0.839315709	0.267819966	-3.133880272	0.001725112	0.005112554	negative
PRJNA729271	stage_E14_vs_E10	ordinary_muscle_development	ENSGALG00010010077	CCT8	0.05332806	0.115052311	0.463511421	0.642997831	0.735267715	positive
PRJNA729271	stage_E14_vs_E10	ordinary_muscle_development	ENSGALG00010010080	CDC42EP3	-0.332919462	0.118991845	-2.797834278	0.00514465	0.013342584	negative
PRJNA729271	stage_E14_vs_E10	ordinary_muscle_development	ENSGALG00010010084	HK3	0.747473143	0.211338813	3.536847448	0.000404933	0.001401507	positive
PRJNA729271	stage_E14_vs_E10	ordinary_muscle_development	ENSGALG00010010085	TDG	-0.20013935	0.102183166	-1.958633282	0.050155749	0.095008832	negative
PRJNA729271	stage_E14_vs_E10	ordinary_muscle_development	ENSGALG00010010094	SLC2A9	0.70174877	0.24828003	2.826440649	0.004706847	0.012355259	positive
PRJNA729271	stage_E14_vs_E10	ordinary_muscle_development	ENSGALG00010010095	C12orf73	0.322138646	0.097823629	3.293055576	0.000991049	0.00312356	positive
PRJNA729271	stage_E14_vs_E10	ordinary_muscle_development	ENSGALG00010010100	IRF2BP2	0.361754477	0.16231249	2.228753172	0.025830332	0.054230977	positive
PRJNA729271	stage_E14_vs_E10	ordinary_muscle_development	ENSGALG00010010106	C7orf73	0.07802418	0.2059877	0.378780769	0.704850669	0.785485543	positive
PRJNA729271	stage_E14_vs_E10	ordinary_muscle_development	ENSGALG00010010107	MGST2	0.526358854	0.300144434	1.753685209	0.079484461	0.14064275	positive
PRJNA729271	stage_E14_vs_E10	ordinary_muscle_development	ENSGALG00010010111	CNOT4	-0.123814261	0.135745582	-0.912105269	0.361713305	0.477879911	negative
PRJNA729271	stage_E14_vs_E10	ordinary_muscle_development	ENSGALG00010010115	NEB	1.85282157	0.171705969	10.79066488	3.81023E-27	1.35698E-25	positive
PRJNA729271	stage_E14_vs_E10	ordinary_muscle_development	ENSGALG00010010116	DRD5	-1.486756729	0.431069871	-3.44899245	0.000562682	0.001881015	negative
PRJNA729271	stage_E14_vs_E10	ordinary_muscle_development	ENSGALG00010010119	SRPK2	-0.008289925	0.123343467	-0.067210085	0.946414457	0.962686144	negative
PRJNA729271	stage_E14_vs_E10	ordinary_muscle_development	ENSGALG00010010122	THBS4	-0.850103634	0.245820045	-3.45823561	0.000543726	0.00182649	negative
PRJNA729271	stage_E14_vs_E10	ordinary_muscle_development	ENSGALG00010010124	ENSGALG00010010124	-0.123232099	0.147267622	-0.836790175	0.402710521	0.51790338	negative
PRJNA729271	stage_E14_vs_E10	ordinary_muscle_development	ENSGALG00010010125	AHCTF1	-0.108335613	0.06628744	-1.634330919	0.102189385	0.173162863	negative
PRJNA729271	stage_E14_vs_E10	ordinary_muscle_development	ENSGALG00010010127	FAM13C	-0.02873939	0.364517324	-0.078842316	0.937158046	0.95618629	negative
PRJNA729271	stage_E14_vs_E10	ordinary_muscle_development	ENSGALG00010010136	EPHX2	-0.096769592	0.115507051	-0.837780822	0.402153811	0.517371039	negative
PRJNA729271	stage_E14_vs_E10	ordinary_muscle_development	ENSGALG00010010142	ENSGALG00010010142	-0.674741456	0.324745028	-2.07775762	0.037731689	0.074553003	negative
PRJNA729271	stage_E14_vs_E10	ordinary_muscle_development	ENSGALG00010010146	PHACTR1	-1.084398683	0.302080041	-3.589772689	0.000330966	0.001173522	negative
PRJNA729271	stage_E14_vs_E10	ordinary_muscle_development	ENSGALG00010010150	NTRK2	-0.539501177	0.298082833	-1.809903546	0.070310746	0.126587289	negative
PRJNA729271	stage_E14_vs_E10	ordinary_muscle_development	ENSGALG00010010153	ENSGALG00010010153	-0.218477463	0.454185579	-0.481031264	0.630494278	0.72434133	negative
PRJNA729271	stage_E14_vs_E10	ordinary_muscle_development	ENSGALG00010010154	IQSEC3	-1.240075517	0.257469072	-4.81640574	1.46167E-06	7.88498E-06	negative
PRJNA729271	stage_E14_vs_E10	ordinary_muscle_development	ENSGALG00010010155	BRINP3	-1.119562764	0.335571249	-3.33628929	0.000849048	0.002723378	negative
PRJNA729271	stage_E14_vs_E10	ordinary_muscle_development	ENSGALG00010010156	ENSGALG00010010156	-0.11880787	0.0972263	-1.22197256	0.221718004	0.324462642	negative
PRJNA729271	stage_E14_vs_E10	ordinary_muscle_development	ENSGALG00010010161	SEPSECS	0.280993818	0.062164902	4.520136088	6.17999E-06	3.00444E-05	positive
PRJNA729271	stage_E14_vs_E10	ordinary_muscle_development	ENSGALG00010010164	ENSGALG00010010164	0.218190729	0.416058364	0.524423367	0.599984107	0.69915344	positive
PRJNA729271	stage_E14_vs_E10	ordinary_muscle_development	ENSGALG00010010167	PUS7	-0.557929266	0.066875769	-8.342771617	7.25692E-17	1.20907E-15	negative
PRJNA729271	stage_E14_vs_E10	ordinary_muscle_development	ENSGALG00010010168	SNCB	0.904048291	0.223571138	4.043671734	5.26206E-05	0.000218111	positive
PRJNA729271	stage_E14_vs_E10	ordinary_muscle_development	ENSGALG00010010171	ENSGALG00010010171	0.081573269	0.396565326	0.205699448	0.83702569	0.886309254	positive
PRJNA729271	stage_E14_vs_E10	ordinary_muscle_development	ENSGALG00010010178	LGI2	-1.423788803	0.174445103	-8.161815828	3.30025E-16	5.15486E-15	negative
PRJNA729271	stage_E14_vs_E10	ordinary_muscle_development	ENSGALG00010010179	CDHR2	-1.603573245	0.942855461	-1.700762536	0.088987587	0.154420576	negative
PRJNA729271	stage_E14_vs_E10	ordinary_muscle_development	ENSGALG00010010183	NKX3-2	-5.274526548	0.448522688	-11.75977647	6.29006E-32	2.96985E-30	negative
PRJNA729271	stage_E14_vs_E10	ordinary_muscle_development	ENSGALG00010010185	PHYHIPL	-1.049085784	0.324815676	-3.229788034	0.00123882	0.003825458	negative
PRJNA729271	stage_E14_vs_E10	ordinary_muscle_development	ENSGALG00010010186	TNPO1	0.210938676	0.072016264	2.929042208	0.003400082	0.009263947	positive
PRJNA729271	stage_E14_vs_E10	ordinary_muscle_development	ENSGALG00010010188	DSP	-1.713500455	0.345633483	-4.957564994	7.13822E-07	4.02444E-06	negative
PRJNA729271	stage_E14_vs_E10	ordinary_muscle_development	ENSGALG00010010190	RNF44	-0.019183163	0.177549007	-0.10804433	0.913960528	0.940755838	negative
PRJNA729271	stage_E14_vs_E10	ordinary_muscle_development	ENSGALG00010010191	RINT1	-0.067915256	0.090798439	-0.747978235	0.454473286	0.567553225	negative
PRJNA729271	stage_E14_vs_E10	ordinary_muscle_development	ENSGALG00010010195	ENSGALG00010010195	1.976661342	0.284860081	6.939060515	3.94716E-12	4.22556E-11	positive
PRJNA729271	stage_E14_vs_E10	ordinary_muscle_development	ENSGALG00010010196	EFCAB10	0.75902771	0.323765818	2.344372597	0.019059121	0.041637069	positive
PRJNA729271	stage_E14_vs_E10	ordinary_muscle_development	ENSGALG00010010197	SCCPDH	-0.285213495	0.120978272	-2.357559669	0.018395499	0.040431047	negative
PRJNA729271	stage_E14_vs_E10	ordinary_muscle_development	ENSGALG00010010201	RIOK1	-0.413334453	0.097239253	-4.250695459	2.13108E-05	9.4407E-05	negative
PRJNA729271	stage_E14_vs_E10	ordinary_muscle_development	ENSGALG00010010205	ARL5A	0.157298302	0.082362515	1.909828787	0.056155262	0.104583132	positive
PRJNA729271	stage_E14_vs_E10	ordinary_muscle_development	ENSGALG00010010206	SCARA5	0.446122001	0.158631212	2.812321712	0.004918528	0.012831647	positive
PRJNA729271	stage_E14_vs_E10	ordinary_muscle_development	ENSGALG00010010214	FAF2	-0.273829537	0.073867453	-3.707039083	0.000209697	0.000775198	negative

PRJNA729271	stage_E14_vs_E10	ordinary_muscle_development	ENSGALG00010010215	ENSGALG00010010215	0.179329211	0.12337251	1.453558908	0.146068637	0.232309073	positive
PRJNA729271	stage_E14_vs_E10	ordinary_muscle_development	ENSGALG00010010216	ATXN7L1	0.110787854	0.128124492	0.864689117	0.387209444	0.503236878	positive
PRJNA729271	stage_E14_vs_E10	ordinary_muscle_development	ENSGALG00010010218	PBK	-0.154963907	0.215415176	-0.719373212	0.471910998	0.584146022	negative
PRJNA729271	stage_E14_vs_E10	ordinary_muscle_development	ENSGALG00010010220	LHX9	-5.92161059	0.501689451	-11.80333885	3.75126E-32	1.78864E-30	negative
PRJNA729271	stage_E14_vs_E10	ordinary_muscle_development	ENSGALG00010010222	PAH	1.208740589	1.057682662	1.142819706	0.253113455	0.360577841	positive
PRJNA729271	stage_E14_vs_E10	ordinary_muscle_development	ENSGALG00010010223	ENSGALG00010010223	0.359900161	0.207325088	1.735921906	0.082577644	0.145229598	positive
PRJNA729271	stage_E14_vs_E10	ordinary_muscle_development	ENSGALG00010010226	BICC1	-0.606903937	0.164037669	-3.699783962	0.000215783	0.000794658	negative
PRJNA729271	stage_E14_vs_E10	ordinary_muscle_development	ENSGALG00010010231	C1orf53	0.398299827	0.226574018	1.757923659	0.0787605	0.139546931	positive
PRJNA729271	stage_E14_vs_E10	ordinary_muscle_development	ENSGALG00010010234	ENSGALG00010010234	1.498316333	0.268113629	5.58836318	2.2922E-08	1.59814E-07	positive
PRJNA729271	stage_E14_vs_E10	ordinary_muscle_development	ENSGALG00010010243	ESCO2	0.275132905	0.103103565	2.668510105	0.007618849	0.018781499	positive
PRJNA729271	stage_E14_vs_E10	ordinary_muscle_development	ENSGALG00010010246	CLTB	0.563923014	0.06627018	8.509453471	1.74755E-17	3.05927E-16	positive
PRJNA729271	stage_E14_vs_E10	ordinary_muscle_development	ENSGALG00010010252	WDR7	0.135127385	0.096010615	1.407421302	0.159302498	0.249119615	positive
PRJNA729271	stage_E14_vs_E10	ordinary_muscle_development	ENSGALG00010010253	IL10RB	0.257654432	0.122924067	2.096045462	0.036078158	0.071765185	positive
PRJNA729271	stage_E14_vs_E10	ordinary_muscle_development	ENSGALG00010010254	WDR41	-0.182429836	0.114359416	-1.595232314	0.110660295	0.185136309	negative
PRJNA729271	stage_E14_vs_E10	ordinary_muscle_development	ENSGALG00010010255	CACNB4	0.225486366	0.173981852	1.296033826	0.194963831	0.293092796	positive
PRJNA729271	stage_E14_vs_E10	ordinary_muscle_development	ENSGALG00010010257	ENSGALG00010010257	0.038941075	0.139008856	0.280133774	0.779374874	0.843636615	positive
PRJNA729271	stage_E14_vs_E10	ordinary_muscle_development	ENSGALG00010010259	IGF1	0.547990889	0.395482428	1.385626391	0.165861015	0.257101424	positive
PRJNA729271	stage_E14_vs_E10	ordinary_muscle_development	ENSGALG00010010262	HSPA9	-0.052313635	0.145624798	-0.35923576	0.719418727	0.797062941	negative
PRJNA729271	stage_E14_vs_E10	ordinary_muscle_development	ENSGALG00010010263	RAB4A	-0.644975579	0.136349377	-4.730315565	2.24171E-06	1.1701E-05	negative
PRJNA729271	stage_E14_vs_E10	ordinary_muscle_development	ENSGALG00010010266	EFNA5	-2.154848809	0.217049284	-9.927924068	3.1474E-23	8.34031E-22	negative
PRJNA729271	stage_E14_vs_E10	ordinary_muscle_development	ENSGALG00010010267	CCSER2	0.625512042	0.106499229	5.873395011	4.26959E-09	3.26412E-08	positive
PRJNA729271	stage_E14_vs_E10	ordinary_muscle_development	ENSGALG00010010270	SSR1	-0.174294675	0.077374644	-2.252607139	0.024283931	0.051446298	negative
PRJNA729271	stage_E14_vs_E10	ordinary_muscle_development	ENSGALG00010010271	HUNK	-0.871465096	0.105412004	-8.267228239	1.37109E-16	2.23303E-15	negative
PRJNA729271	stage_E14_vs_E10	ordinary_muscle_development	ENSGALG00010010274	MIS18A	-0.055471868	0.087766494	-0.632039237	0.527361238	0.634892122	negative
PRJNA729271	stage_E14_vs_E10	ordinary_muscle_development	ENSGALG00010010275	PDE8B	-1.891397554	0.864578087	-2.187653819	0.028694828	0.059207336	negative
PRJNA729271	stage_E14_vs_E10	ordinary_muscle_development	ENSGALG00010010279	CCSAP	-0.096013313	0.240987307	-0.398416473	0.690323217	0.774057015	negative
PRJNA729271	stage_E14_vs_E10	ordinary_muscle_development	ENSGALG00010010280	HSP90B1	-0.323297226	0.135372402	-2.388206318	0.016930834	0.037697763	negative
PRJNA729271	stage_E14_vs_E10	ordinary_muscle_development	ENSGALG00010010284	CCDC25	0.19350168	0.094055897	2.057305143	0.039656883	0.077700259	positive
PRJNA729271	stage_E14_vs_E10	ordinary_muscle_development	ENSGALG00010010285	ENSGALG00010010285	-1.722707584	0.714472402	-2.411160431	0.015901852	0.035691753	negative
PRJNA729271	stage_E14_vs_E10	ordinary_muscle_development	ENSGALG00010010287	STAM2	-0.119984521	0.096828633	-1.239142979	0.215292553	0.317076362	negative
PRJNA729271	stage_E14_vs_E10	ordinary_muscle_development	ENSGALG00010010288	ENSGALG00010010288	0.008422367	0.163369829	0.051553991	0.95888408	0.971755907	positive
PRJNA729271	stage_E14_vs_E10	ordinary_muscle_development	ENSGALG00010010290	ENSGALG00010010290	0.573731072	0.163611193	3.506673728	0.000453745	0.001552286	positive
PRJNA729271	stage_E14_vs_E10	ordinary_muscle_development	ENSGALG00010010291	TCF20	0.096808238	0.117031012	0.827201581	0.408122816	0.522961476	positive
PRJNA729271	stage_E14_vs_E10	ordinary_muscle_development	ENSGALG00010010293	ENSGALG00010010293	-2.945390081	0.795644081	-3.70189404	0.000213996	0.000788678	negative
PRJNA729271	stage_E14_vs_E10	ordinary_muscle_development	ENSGALG00010010295	ENSGALG00010010295	2.021305826	0.181516151	11.1356803	8.41004E-29	3.33982E-27	positive
PRJNA729271	stage_E14_vs_E10	ordinary_muscle_development	ENSGALG00010010298	KCNIP4	-1.324795428	0.405142081	-3.269952667	0.001075655	0.003360987	negative
PRJNA729271	stage_E14_vs_E10	ordinary_muscle_development	ENSGALG00010010300	DENND1B	0.360481705	0.09336918	3.860821155	0.000113007	0.000441161	positive
PRJNA729271	stage_E14_vs_E10	ordinary_muscle_development	ENSGALG00010010301	ENSGALG00010010301	0.228720504	0.503179512	0.454550509	0.649432646	0.740930831	positive
PRJNA729271	stage_E14_vs_E10	ordinary_muscle_development	ENSGALG00010010302	WDR36	-0.284363608	0.103662895	-2.743157118	0.006085156	0.015447345	negative
PRJNA729271	stage_E14_vs_E10	ordinary_muscle_development	ENSGALG00010010303	ETF1	0.280819658	0.184516904	1.521918329	0.128029546	0.208328274	positive
PRJNA729271	stage_E14_vs_E10	ordinary_muscle_development	ENSGALG00010010305	URB1	-0.610483772	0.10141532	-6.01964057	1.74805E-09	1.39373E-08	negative
PRJNA729271	stage_E14_vs_E10	ordinary_muscle_development	ENSGALG00010010308	PACRGL	-1.002166709	0.215262352	-4.6555596	3.23102E-06	1.64213E-05	negative
PRJNA729271	stage_E14_vs_E10	ordinary_muscle_development	ENSGALG00010010310	NT5DC3	-0.57859769	0.121893249	-4.746757455	2.06704E-06	1.08636E-05	negative
PRJNA729271	stage_E14_vs_E10	ordinary_muscle_development	ENSGALG00010010311	CTSV	-0.072489044	0.189531564	-0.382464234	0.702117053	0.783742659	negative
PRJNA729271	stage_E14_vs_E10	ordinary_muscle_development	ENSGALG00010010313	GRID1	-1.14750842	0.181562789	-6.320174019	2.61269E-10	2.29382E-09	negative
PRJNA729271	stage_E14_vs_E10	ordinary_muscle_development	ENSGALG00010010314	CRB1	1.904624662	0.275752297	6.907012866	4.94965E-12	5.21783E-11	positive
PRJNA729271	stage_E14_vs_E10	ordinary_muscle_development	ENSGALG00010010317	ENSGALG00010010317	0.033776783	0.105994279	0.318666096	0.749979727	0.820945185	positive
PRJNA729271	stage_E14_vs_E10	ordinary_muscle_development	ENSGALG00010010320	SYPL1	-2.110752388	0.670676825	-3.147197444	0.001648436	0.004912434	negative
PRJNA729271	stage_E14_vs_E10	ordinary_muscle_development	ENSGALG00010010321	NUP133	-0.1426234	0.070168975	-2.032570668	0.042095917	0.081815543	negative
PRJNA729271	stage_E14_vs_E10	ordinary_muscle_development	ENSGALG00010010322	ENSGALG00010010322	-0.027177717	0.066678301	-0.407594617	0.683571317	0.768388231	negative
PRJNA729271	stage_E14_vs_E10	ordinary_muscle_development	ENSGALG00010010323	FBP2	0.884833143	0.253639636	3.488544436	0.000485658	0.00165228	positive
PRJNA729271	stage_E14_vs_E10	ordinary_muscle_development	ENSGALG00010010327	FBP1	-0.854760174	0.578199487	-1.478313615	0.139323855	0.223470098	negative
PRJNA729271	stage_E14_vs_E10	ordinary_muscle_development	ENSGALG00010010330	ALPK2	1.231036715	0.153396202	8.025209891	1.01353E-15	1.51298E-14	positive
PRJNA729271	stage_E14_vs_E10	ordinary_muscle_development	ENSGALG00010010333	WAPL	0.082615867	0.062843585	1.314626903	0.188635324	0.285324953	positive
PRJNA729271	stage_E14_vs_E10	ordinary_muscle_development	ENSGALG00010010334	ZBTB41	0.87111626	0.085957811	10.13423042	3.89428E-24	1.08972E-22	positive
PRJNA729271	stage_E14_vs_E10	ordinary_muscle_development	ENSGALG00010010336	FMNL2	-0.478799979	0.086870488	-5.511652925	3.55479E-08	2.41796E-07	negative
PRJNA729271	stage_E14_vs_E10	ordinary_muscle_development	ENSGALG00010010337	SCARA3	-0.000677706	0.15351264	-0.004414661	0.996477621	0.998160417	negative
PRJNA729271	stage_E14_vs_E10	ordinary_muscle_development	ENSGALG00010010338	CNST	-0.227758994	0.15776418	-1.443667335	0.148832574	0.236003026	negative
PRJNA729271	stage_E14_vs_E10	ordinary_muscle_development	ENSGALG00010010345	ENSGALG00010010345	-0.214353599	0.135300552	-1.584277339	0.11313062	0.188702916	negative
PRJNA729271	stage_E14_vs_E10	ordinary_muscle_development	ENSGALG00010010346	TFB2M	-0.082386487	0.139507484	-0.590552453	0.554820331	0.659460492	negative
PRJNA729271	stage_E14_vs_E10	ordinary_muscle_development	ENSGALG00010010347	FBXW11	0.006904438	0.083520538	0.082667549	0.934115889	0.954530124	positive
PRJNA729271	stage_E14_vs_E10	ordinary_muscle_development	ENSGALG00010010349	RREB1	0.727173453	0.081731955	8.897052006	5.73496E-19	1.1264E-17	positive
PRJNA729271	stage_E14_vs_E10	ordinary_muscle_development	ENSGALG00010010351	NAMPTP1	0.179615285	0.213064477	0.843009063	0.399223375	0.514605853	positive
PRJNA729271	stage_E14_vs_E10	ordinary_muscle_development	ENSGALG00010010352	MALT1	-0.112355697	0.114741675	-0.97920566	0.327478373	0.442385742	negative
PRJNA729271	stage_E14_vs_E10	ordinary_muscle_development	ENSGALG00010010356	STAB2	-2.246645207	0.305207703	-7.361037052	1.82487E-13	2.24928E-12	negative
PRJNA729271	stage_E14_vs_E10	ordinary_muscle_development	ENSGALG00010010358	ASPM	0.155736678	0.180926175	0.86077472	0.389362131	0.505138063	positive
PRJNA729271	stage_E14_vs_E10	ordinary_muscle_development	ENSGALG00010010361	PIGM	-0.403026948	0.155651326	-2.589293378	0.009617312	0.023042072	negative
PRJNA729271	stage_E14_vs_E10	ordinary_muscle_development	ENSGALG00010010366	CLU	-0.382766147	0.236823383	-1.616251498	0.106039949	0.178809802	negative
PRJNA729271	stage_E14_vs_E10	ordinary_muscle_development	ENSGALG00010010368	ENSGALG00010010368	-0.031752461	0.079300418	-0.400407228	0.688856601	0.772651585	negative
PRJNA729271	stage_E14_vs_E10	ordinary_muscle_development	ENSGALG00010010369	ENSGALG00010010369	-0.395479009	0.239635846	-1.650333271	0.098874791	0.168570936	negative
PRJNA729271	stage_E14_vs_E10	ordinary_muscle_development	ENSGALG00010010371	SOCS1L	-0.742048607	0.202745257	-3.660004772	0.000252211	0.000915092	negative
PRJNA729271	stage_E14_vs_E10	ordinary_muscle_development	ENSGALG00010010374	CMTM4	-0.669396277	0.111951037	-5.979366473	2.24007E-09	1.76371E-08	negative
PRJNA729271	stage_E14_vs_E10	ordinary_muscle_development	ENSGALG00010010379	SLC35F6	0.129340472	0.187681692	0.68914805	0.490730105	0.601093108	positive
PRJNA729271	stage_E14_vs_E10	ordinary_muscle_development	ENSGALG00010010381	MAST4	-0.266752071	0.132208673	-2.017659386	0.043626747	0.084383467	negative
PRJNA729271	stage_E14_vs_E10	ordinary_muscle_development	ENSGALG00010010382	SEC11C	-0.103201847	0.152417241	-0.677100875	0.498341954	0.607626735	negative
PRJNA729271	stage_E14_vs_E10	ordinary_muscle_development	ENSGALG00010010383	SLIT2	-1.101190081	0.159911098	-6.88626426	5.72766E-12	5.99439E-11	negative
PRJNA729271	stage_E14_vs_E10	ordinary_muscle_development	ENSGALG00010010386	PRPF40A	-0.080686193	0.146303274	-0.551499573	0.581291259	0.682754998	negative
PRJNA729271	stage_E14_vs_E10	ordinary_muscle_development	ENSGALG00010010390	KCNK3	2.013632353	0.411447572	4.894019284	9.87972E-07	5.45964E-06	positive
PRJNA729271	stage_E14_vs_E10	ordinary_muscle_development	ENSGALG00010010393	FANCC	-0.564906604	0.138398942	-4.081726335	4.47024E-05	0.000187326	negative
PRJNA729271	stage_E14_vs_E10	ordinary_muscle_development	ENSGALG00010010394	MRPL42	0.270587357	0.122555376	2.207878315	0.027252757	0.056691836	positive
PRJNA729271	stage_E14_vs_E10	ordinary_muscle_development	ENSGALG00010010395	GRP	-0.713539205	0.851687658	-0.837794464	0.402146148	0.517371039	negative
PRJNA729271	stage_E14_vs_E10	ordinary_muscle_development	ENSGALG00010010397	PICK1	-0.072430234	0.089842251	-0.806193454	0.420131313	0.534595788	negative
PRJNA729271	stage_E14_vs_E10	ordinary_muscle_development	ENSGALG00010010399	FGF18	0.189174022	0.179242064	1.055410868	0.291237455	0.403622421	positive
PRJNA729271	stage_E14_vs_E10	ordinary_muscle_development	ENSGALG00010010403	ENSGALG00010010403	-1.221744155	0.439701891	-2.778573802	0.005459811	0.014054335	negative
PRJNA729271	stage_E14_vs_E10	ordinary_muscle_development	ENSGALG00010010404	COP1	-0.013101978	0.11583271	-0.113111207	0.909942391	0.938028231	negative
PRJNA729271	stage_E14_vs_E10	ordinary_muscle_development	ENSGALG00010010405	EVA1C	-0.942985756	0.278828393	-3.381957427	0.000719713	0.002349074	negative
PRJNA729271	stage_E14_vs_E10	ordinary_muscle_development	ENSGALG00010010406	PTCH1	-1.765702636	0.094904181	-18.605109	2.92097E-77	1.2097E-74	negative
PRJNA729271	stage_E14_vs_E10	ordinary_muscle_development	ENSGALG00010010407	SOX10	-0.47747477	0.289389882	-1.649935951	0.098956037	0.168669773	negative
PRJNA729271	stage_E14_vs_E10	ordinary_muscle_development	ENSGALG00010010408	GAS8	0.128125846	0.112155786	1.142391758	0.253291216	0.360795614	positive
PRJNA729271	stage_E14_vs_E10	ordinary_muscle_development	ENSGALG00010010410	CACNA2D1	0.772606147	0.158250085	4.882184706	1.04917E-06	5.7824E-06	positive
PRJNA729271	stage_E14_vs_E10	ordinary_muscle_development	ENSGALG00010010411	CD180	-0.103142828	0.451992657	-0.228195805	0.819494022	0.873638473	negative
PRJNA729271	stage_E14_vs_E10	ordinary_muscle_development	ENSGALG00010010416	SLC35B3	-0.594975553	0.126210669	-4.714146257	2.42726E-06	1.26105E-05	negative
PRJNA729271	stage_E14_vs_E10	ordinary_muscle_development	ENSGALG00010010417	OTOF	-0.29122602	0.660212395	-0.441109592	0.65913366	0.748734537	negative
PRJNA729271	stage_E14_vs_E10	ordinary_muscle_development	ENSGALG00010010418	BRINP2	1.730629771	0.405304039	4.269954411	1.95513E-05	8.71719E-05	positive
PRJNA729271	stage_E14_vs_E10	ordinary_muscle_development	ENSGALG00010010420	NPM1	0.00531171	0.112211416	0.047336631	0.962244933	0.974412485	positive
PRJNA729271	stage_E14_vs_E10	ordinary_muscle_development	ENSGALG00010010421	POLR2F	0.299635513	0.090195999	3.322048827	0.000893591	0.002844224	positive
PRJNA729271	stage_E14_vs_E10	ordinary_muscle_development	ENSGALG00010010422	CDH1	-1.655085944	0.892717472	-1.85398628	0.063741123	0.11646507	negative
PRJNA729271	stage_E14_vs_E10	ordinary_muscle_development	ENSGALG00010010423	C21orf59	-0.137987774	0.09658327	-1.428692301	0.153092692	0.241387856	negative
PRJNA729271	stage_E14_vs_E10	ordinary_muscle_development	ENSGALG00010010425	BLOC1S5	0.367990271	0.078209014	4.7052156	2.53598E-06	1.31564E-05	positive
PRJNA729271	stage_E14_vs_E10	ordinary_muscle_development	ENSGALG00010010430	ASTN1	-2.988906215	0.336318299	-8.887135258	6.27047E-19	1.22165E-17	negative
PRJNA729271	stage_E14_vs_E10	ordinary_muscle_development	ENSGALG00010010431	SOCS2	-1.637670945	0.249021334	-6.576428275	4.81883E-11	4.62883E-10	negative
PRJNA729271	stage_E14_vs_E10	ordinary_muscle_development	ENSGALG00010010433	ENSGALG00010010433	-1.707135461	1.617615837	-1.055340472	0.291269637	0.403628431	negative
PRJNA729271	stage_E14_vs_E10	ordinary_muscle_development	ENSGALG00010010434	PROM1	-0.943719824	0.14481726	-6.516625317	7.19067E-11	6.75057E-10	negative
PRJNA729271	stage_E14_vs_E10	ordinary_muscle_development	ENSGALG00010010435	GAS1	-0.336889392	0.165160943	-2.039764277	0.041373809	0.080606635	negative
PRJNA729271	stage_E14_vs_E10	ordinary_muscle_development	ENSGALG00010010438	LDB3	1.844801527	0.185377147	9.951612479	2.4813E-23	6.61147E-22	positive
PRJNA729271	stage_E14_vs_E10	ordinary_muscle_development	ENSGALG00010010439	HGF	-0.438300509	0.24261763	-1.80654847	0.07083271	0.127416249	negative
PRJNA729271	stage_E14_vs_E10	ordinary_muscle_development	ENSGALG00010010444	TXNDC5	-0.386930461	0.139273057	-2.778214743	0.005465848	0.014067377	negative
PRJNA729271	stage_E14_vs_E10	ordinary_muscle_development	ENSGALG00010010445	LMAN1L	-0.108900136	0.082545456	-1.319274746	0.187077278	0.283420651	negative
PRJNA729271	stage_E14_vs_E10	ordinary_muscle_development	ENSGALG00010010446	C22orf23	0.092984959	0.596846883	0.155793657	0.87619567	0.915150327	positive
PRJNA729271	stage_E14_vs_E10	ordinary_muscle_development	ENSGALG00010010447	BTF3	0.183118055	0.095894302	1.909582223	0.056187026	0.10460197	positive
PRJNA729271	stage_E14_vs_E10	ordinary_muscle_development	ENSGALG00010010448	GALNT13	-0.137314107	0.356726121	-0.38492866	0.700290272	0.78226784	negative
PRJNA729271	stage_E14_vs_E10	ordinary_muscle_development	ENSGALG00010010449	PAPPA2	-3.072583294	0.750197468	-4.095699366	4.20896E-05	0.000177454	negative
PRJNA729271	stage_E14_vs_E10	ordinary_muscle_development	ENSGALG00010010451	RANBP17	-0.252468631	0.464267206	-0.543800269	0.586578923	0.686956572	negative
PRJNA729271	stage_E14_vs_E10	ordinary_muscle_development	ENSGALG00010010453	DRC1	-0.642708406	0.38551903	-1.667124984	0.095489555	0.163801314	negative
PRJNA729271	stage_E14_vs_E10	ordinary_muscle_development	ENSGALG00010010456	BMP6	-1.561453876	0.179080356	-8.719291789	2.79947E-18	5.23592E-17	negative
PRJNA729271	stage_E14_vs_E10	ordinary_muscle_development	ENSGALG00010010457	AGGF1	-0.336509969	0.089374973	-3.765147654	0.000166451	0.000629127	negative
PRJNA729271	stage_E14_vs_E10	ordinary_muscle_development	ENSGALG00010010460	SEMA3C	0.719516281	0.141968534	5.068139128	4.01724E-07	2.35367E-06	positive
PRJNA729271	stage_E14_vs_E10	ordinary_muscle_development	ENSGALG00010010461	SLC7A5	-0.353597339	0.26175752	-1.350858377	0.176740804	0.270637856	negative
PRJNA729271	stage_E14_vs_E10	ordinary_muscle_development	ENSGALG00010010463	ACAA2	0.910751504	0.127997446	7.115388111	1.11598E-12	1.26376E-11	positive
PRJNA729271	stage_E14_vs_E10	ordinary_muscle_development	ENSGALG00010010465	BMPR1A	-0.276979834	0.097354238	-2.845072192	0.004440136	0.011731639	negative
PRJNA729271	stage_E14_vs_E10	ordinary_muscle_development	ENSGALG00010010466	CRADD	0.235225185	0.123765852	1.900566114	0.057358867	0.106455414	positive
PRJNA729271	stage_E14_vs_E10	ordinary_muscle_development	ENSGALG00010010467	FGFBP2	-7.001577225	0.495997735	-14.11614757	3.02058E-45	3.24321E-43	negative
PRJNA729271	stage_E14_vs_E10	ordinary_muscle_development	ENSGALG00010010468	ENSGALG00010010468	2.96145436	1.334719325	2.218784357	0.026501399	0.055401497	positive
PRJNA729271	stage_E14_vs_E10	ordinary_muscle_development	ENSGALG00010010471	KCNJ3	2.041074453	0.142746309	14.29861457	2.23209E-46	2.6092E-44	positive
PRJNA729271	stage_E14_vs_E10	ordinary_muscle_development	ENSGALG00010010472	MMRN2	0.371172334	0.137024726	2.708798218	0.006752739	0.016899337	positive
PRJNA729271	stage_E14_vs_E10	ordinary_muscle_development	ENSGALG00010010473	TRIM35	-0.076172138	0.179073504	-0.425367997	0.670568432	0.757708873	negative
PRJNA729271	stage_E14_vs_E10	ordinary_muscle_development	ENSGALG00010010475	CCDC71L	-0.038908549	0.108245442	-0.359447462	0.719260374	0.796948412	negative
PRJNA729271	stage_E14_vs_E10	ordinary_muscle_development	ENSGALG00010010478	ENSGALG00010010478	0.296038253	0.208636235	1.418920603	0.155922165	0.244890213	positive
PRJNA729271	stage_E14_vs_E10	ordinary_muscle_development	ENSGALG00010010483	FGFBP1	0.642674854	0.714706694	0.899214824	0.368538246	0.484795524	positive
PRJNA729271	stage_E14_vs_E10	ordinary_muscle_development	ENSGALG00010010486	ENSGALG00010010486	-0.345492041	0.469255224	-0.736256142	0.461574832	0.574135863	negative
PRJNA729271	stage_E14_vs_E10	ordinary_muscle_development	ENSGALG00010010494	ENSGALG00010010494	0.287339131	0.263306525	1.091272352	0.27515306	0.385602918	positive

PRJNA729271	stage_E14_vs_E10	ordinary_muscle_development	ENSGALG00010010496	MRPL17	0.324909587	0.142019358	2.287783801	0.022150115	0.047572368	positive
PRJNA729271	stage_E14_vs_E10	ordinary_muscle_development	ENSGALG00010010499	NR4A2	0.492968307	0.483847414	1.018850763	0.308273822	0.421901276	positive
PRJNA729271	stage_E14_vs_E10	ordinary_muscle_development	ENSGALG00010010505	CD38	-1.529833379	0.232555573	-6.578356118	4.75678E-11	4.57832E-10	negative
PRJNA729271	stage_E14_vs_E10	ordinary_muscle_development	ENSGALG00010010511	GABRP	-0.557958849	0.358734644	-1.555352568	0.119862124	0.197476868	negative
PRJNA729271	stage_E14_vs_E10	ordinary_muscle_development	ENSGALG00010010512	PLXNC1	-0.698549483	0.199193998	-3.506880178	0.000453393	0.001551448	negative
PRJNA729271	stage_E14_vs_E10	ordinary_muscle_development	ENSGALG00010010515	RXRG	-0.683775836	0.371608738	-1.840042407	0.065762012	0.119661105	negative
PRJNA729271	stage_E14_vs_E10	ordinary_muscle_development	ENSGALG00010010517	STMN4	-2.334419413	0.206842537	-11.28597359	1.53928E-29	6.4672E-28	negative
PRJNA729271	stage_E14_vs_E10	ordinary_muscle_development	ENSGALG00010010521	SNCG	2.396299296	0.378743581	6.326970061	2.50022E-10	2.20711E-09	positive
PRJNA729271	stage_E14_vs_E10	ordinary_muscle_development	ENSGALG00010010526	ENSGALG00010010526	0.102025576	0.24973987	0.408527385	0.68288653	0.767797103	positive
PRJNA729271	stage_E14_vs_E10	ordinary_muscle_development	ENSGALG00010010528	GPD2	0.857901827	0.124770961	6.875813269	6.16372E-12	6.42756E-11	positive
PRJNA729271	stage_E14_vs_E10	ordinary_muscle_development	ENSGALG00010010534	SYNJ1	-0.133066591	0.121275086	-1.097229409	0.27254112	0.382613418	negative
PRJNA729271	stage_E14_vs_E10	ordinary_muscle_development	ENSGALG00010010535	LMX1A	-1.128847125	0.414805651	-2.721388008	0.006500841	0.016333799	negative
PRJNA729271	stage_E14_vs_E10	ordinary_muscle_development	ENSGALG00010010537	ENSGALG00010010537	0.15369789	0.205190006	0.74905154	0.453826141	0.566793893	positive
PRJNA729271	stage_E14_vs_E10	ordinary_muscle_development	ENSGALG00010010544	ENSGALG00010010544	0.440600567	0.154962435	2.843273381	0.004465275	0.011791613	positive
PRJNA729271	stage_E14_vs_E10	ordinary_muscle_development	ENSGALG00010010546	MICALL1	1.107194469	0.166459463	6.651436029	2.90247E-11	2.83308E-10	positive
PRJNA729271	stage_E14_vs_E10	ordinary_muscle_development	ENSGALG00010010547	BST1	0.429409717	0.164795802	2.605707862	0.009168462	0.022083227	positive
PRJNA729271	stage_E14_vs_E10	ordinary_muscle_development	ENSGALG00010010567	ERMN	-3.370431777	0.776514139	-4.340464145	1.42182E-05	6.50751E-05	negative
PRJNA729271	stage_E14_vs_E10	ordinary_muscle_development	ENSGALG00010010572	AQP3	-0.135725226	0.148367066	-0.914793485	0.360300056	0.476553455	negative
PRJNA729271	stage_E14_vs_E10	ordinary_muscle_development	ENSGALG00010010573	CEP83	-0.199671947	0.149009766	-1.339992357	0.18024783	0.275107117	negative
PRJNA729271	stage_E14_vs_E10	ordinary_muscle_development	ENSGALG00010010575	PAXBP1	-0.153895578	0.12283634	-1.252850568	0.210260095	0.311182364	negative
PRJNA729271	stage_E14_vs_E10	ordinary_muscle_development	ENSGALG00010010577	PBX1	-0.860236291	0.145739785	-5.902549453	3.57927E-09	2.75526E-08	negative
PRJNA729271	stage_E14_vs_E10	ordinary_muscle_development	ENSGALG00010010578	GLUD2	0.133093529	0.111351436	1.195256507	0.231986827	0.336332172	positive
PRJNA729271	stage_E14_vs_E10	ordinary_muscle_development	ENSGALG00010010585	FBXL5	-0.050803715	0.163559196	-0.310613628	0.756094366	0.826016569	negative
PRJNA729271	stage_E14_vs_E10	ordinary_muscle_development	ENSGALG00010010586	CYTIP	-2.703417084	0.332331789	-8.134693019	4.12983E-16	6.39551E-15	negative
PRJNA729271	stage_E14_vs_E10	ordinary_muscle_development	ENSGALG00010010588	SLC25A3	0.402214551	0.100983343	3.982979171	6.80567E-05	0.00027648	positive
PRJNA729271	stage_E14_vs_E10	ordinary_muscle_development	ENSGALG00010010593	EIF3L	0.480150627	0.103644105	4.632686302	3.60951E-06	1.81856E-05	positive
PRJNA729271	stage_E14_vs_E10	ordinary_muscle_development	ENSGALG00010010596	SHLD2	-0.244459484	0.13374977	-1.827737594	0.067588943	0.122386225	negative
PRJNA729271	stage_E14_vs_E10	ordinary_muscle_development	ENSGALG00010010599	F2RL1	-1.924521588	0.201510704	-9.550468287	1.29111E-21	3.13479E-20	negative
PRJNA729271	stage_E14_vs_E10	ordinary_muscle_development	ENSGALG00010010604	HIVEP1	-0.131408293	0.099793104	-1.316807353	0.187903208	0.284395635	negative
PRJNA729271	stage_E14_vs_E10	ordinary_muscle_development	ENSGALG00010010605	NUF2	-0.139445702	0.265514562	-0.52519041	0.599450831	0.698756718	negative
PRJNA729271	stage_E14_vs_E10	ordinary_muscle_development	ENSGALG00010010614	IKBIP	0.017554244	0.139041517	0.126251819	0.899532594	0.930739164	positive
PRJNA729271	stage_E14_vs_E10	ordinary_muscle_development	ENSGALG00010010618	APAF1	0.255431651	0.134698882	1.896316042	0.057918266	0.107342446	positive
PRJNA729271	stage_E14_vs_E10	ordinary_muscle_development	ENSGALG00010010619	LRRC52	0.656805449	0.251569124	2.610834903	0.009032149	0.021820166	positive
PRJNA729271	stage_E14_vs_E10	ordinary_muscle_development	ENSGALG00010010620	ANKRD54	0.228496573	0.110217602	2.073140482	0.038159207	0.075274593	positive
PRJNA729271	stage_E14_vs_E10	ordinary_muscle_development	ENSGALG00010010623	SMAD2	-0.038737574	0.076108154	-0.508980603	0.610765821	0.70778028	negative
PRJNA729271	stage_E14_vs_E10	ordinary_muscle_development	ENSGALG00010010625	FAM169A	-2.567922752	0.24797705	-10.35548551	3.95177E-25	1.20086E-23	negative
PRJNA729271	stage_E14_vs_E10	ordinary_muscle_development	ENSGALG00010010626	ADTRP	0.45025832	0.614390324	0.732853859	0.463647568	0.576180684	positive
PRJNA729271	stage_E14_vs_E10	ordinary_muscle_development	ENSGALG00010010629	ENSGALG00010010629	-0.788826353	0.320117409	-2.464178239	0.01373278	0.031461459	negative
PRJNA729271	stage_E14_vs_E10	ordinary_muscle_development	ENSGALG00010010633	EPCIP	-0.087854174	0.343316662	-0.255898368	0.798029315	0.857927384	negative
PRJNA729271	stage_E14_vs_E10	ordinary_muscle_development	ENSGALG00010010635	ALDH9A1	-0.001496868	0.075368208	-0.019860741	0.984154463	0.989410386	negative
PRJNA729271	stage_E14_vs_E10	ordinary_muscle_development	ENSGALG00010010638	ACVR1C	0.596556722	0.434110942	1.374203376	0.169378553	0.261630661	positive
PRJNA729271	stage_E14_vs_E10	ordinary_muscle_development	ENSGALG00010010642	CC2D2A	-0.340126279	0.118691122	-2.865642132	0.004161644	0.011070477	negative
PRJNA729271	stage_E14_vs_E10	ordinary_muscle_development	ENSGALG00010010645	FAM219A	0.052384155	0.084595419	0.61923158	0.535763811	0.642712607	positive
PRJNA729271	stage_E14_vs_E10	ordinary_muscle_development	ENSGALG00010010646	MCM3AP	-0.263527558	0.057234182	-4.604373618	4.13709E-06	2.06641E-05	negative
PRJNA729271	stage_E14_vs_E10	ordinary_muscle_development	ENSGALG00010010648	CENPM	0.194480351	0.232613108	0.836067894	0.403116709	0.518241836	positive
PRJNA729271	stage_E14_vs_E10	ordinary_muscle_development	ENSGALG00010010651	NUAK1	-0.753635801	0.134263419	-5.613113418	1.98718E-08	1.39691E-07	negative
PRJNA729271	stage_E14_vs_E10	ordinary_muscle_development	ENSGALG00010010655	C1QTNF7	-1.966678693	0.263617204	-7.460357919	8.62878E-14	1.10004E-12	negative
PRJNA729271	stage_E14_vs_E10	ordinary_muscle_development	ENSGALG00010010657	TMEM170B	-0.611983322	0.175816219	-3.480812673	0.000499895	0.001695362	negative
PRJNA729271	stage_E14_vs_E10	ordinary_muscle_development	ENSGALG00010010659	ADAMTS1	-0.875187294	0.152375123	-5.74363635	9.26647E-09	6.76662E-08	negative
PRJNA729271	stage_E14_vs_E10	ordinary_muscle_development	ENSGALG00010010661	CPEB2	-0.246280834	0.180478669	-1.364598018	0.172379428	0.265276548	negative
PRJNA729271	stage_E14_vs_E10	ordinary_muscle_development	ENSGALG00010010662	MGST3	0.370456159	0.150816834	2.456331633	0.014036356	0.032025339	positive
PRJNA729271	stage_E14_vs_E10	ordinary_muscle_development	ENSGALG00010010663	EFR3B	0.279099826	0.163583677	1.706159388	0.087978408	0.152980689	positive
PRJNA729271	stage_E14_vs_E10	ordinary_muscle_development	ENSGALG00010010666	C12orf75	0.230787531	0.377519331	0.611326394	0.540983521	0.646939703	positive
PRJNA729271	stage_E14_vs_E10	ordinary_muscle_development	ENSGALG00010010671	SIGMAR1	0.002929568	0.137183571	0.021355095	0.982962394	0.988760576	positive
PRJNA729271	stage_E14_vs_E10	ordinary_muscle_development	ENSGALG00010010678	KCNIP1	-0.239414935	0.347599237	-0.688767148	0.490969813	0.601318752	negative
PRJNA729271	stage_E14_vs_E10	ordinary_muscle_development	ENSGALG00010010680	ENSGALG00010010680	-1.33739837	0.612561153	-2.183289558	0.029014493	0.059705434	negative
PRJNA729271	stage_E14_vs_E10	ordinary_muscle_development	ENSGALG00010010682	SMIM13	-0.270156819	0.104848061	-2.576650602	0.009976274	0.023791722	negative
PRJNA729271	stage_E14_vs_E10	ordinary_muscle_development	ENSGALG00010010685	STIM2	-0.457858334	0.108202429	-4.23149776	2.3214E-05	0.000102183	negative
PRJNA729271	stage_E14_vs_E10	ordinary_muscle_development	ENSGALG00010010688	IQGAP2	-1.044885352	0.169357719	-6.169694292	6.84222E-10	5.71301E-09	negative
PRJNA729271	stage_E14_vs_E10	ordinary_muscle_development	ENSGALG00010010689	POFUT2	0.027320858	0.149406587	0.182862469	0.854905931	0.89959067	positive
PRJNA729271	stage_E14_vs_E10	ordinary_muscle_development	ENSGALG00010010690	MTPN	-0.090213959	0.134992576	-0.668288297	0.503949582	0.613123149	negative
PRJNA729271	stage_E14_vs_E10	ordinary_muscle_development	ENSGALG00010010693	KCNMB1	-0.790018355	0.346557022	-2.279620105	0.02263023	0.048459917	negative
PRJNA729271	stage_E14_vs_E10	ordinary_muscle_development	ENSGALG00010010697	NEDD9	-1.206616918	0.139284194	-8.662985271	4.59568E-18	8.46434E-17	negative
PRJNA729271	stage_E14_vs_E10	ordinary_muscle_development	ENSGALG00010010698	GPRIN2	-1.40158746	0.171003979	-8.196227163	2.48049E-16	3.91238E-15	negative
PRJNA729271	stage_E14_vs_E10	ordinary_muscle_development	ENSGALG00010010699	DNAJC27	0.21160671	0.149177141	1.41849286	0.156046922	0.245006513	positive
PRJNA729271	stage_E14_vs_E10	ordinary_muscle_development	ENSGALG00010010706	SREBF2	0.069804581	0.138474746	0.504096112	0.614193839	0.710740834	positive
PRJNA729271	stage_E14_vs_E10	ordinary_muscle_development	ENSGALG00010010710	UCK2	-0.089068556	0.097932968	-0.909484901	0.363094224	0.479199744	negative
PRJNA729271	stage_E14_vs_E10	ordinary_muscle_development	ENSGALG00010010711	ENSGALG00010010711	-1.843555196	0.429601912	-4.2913105	1.77622E-05	7.98202E-05	negative
PRJNA729271	stage_E14_vs_E10	ordinary_muscle_development	ENSGALG00010010712	ADAMTS5	0.132062737	0.145176866	0.909667912	0.362997672	0.47911594	positive
PRJNA729271	stage_E14_vs_E10	ordinary_muscle_development	ENSGALG00010010713	NSA2	0.102938599	0.090538408	1.136960564	0.255554772	0.363234597	positive
PRJNA729271	stage_E14_vs_E10	ordinary_muscle_development	ENSGALG00010010719	SH3BP4	0.445628408	0.164624463	2.706939175	0.00679067	0.016985463	positive
PRJNA729271	stage_E14_vs_E10	ordinary_muscle_development	ENSGALG00010010722	TBC1D19	-0.06423104	0.1631865	-0.393605106	0.693872611	0.776715074	negative
PRJNA729271	stage_E14_vs_E10	ordinary_muscle_development	ENSGALG00010010724	ADCY3	0.245456054	0.141702947	1.732187368	0.083240207	0.146268251	positive
PRJNA729271	stage_E14_vs_E10	ordinary_muscle_development	ENSGALG00010010728	ELOVL2	-2.550170873	1.119344926	-2.278270812	0.022710448	0.048602973	negative
PRJNA729271	stage_E14_vs_E10	ordinary_muscle_development	ENSGALG00010010732	CCKAR	-3.889252499	0.913614369	-4.256995766	2.07192E-05	9.19269E-05	negative
PRJNA729271	stage_E14_vs_E10	ordinary_muscle_development	ENSGALG00010010733	GFM2	-0.398552431	0.125836434	-3.167226043	0.001539006	0.004623399	negative
PRJNA729271	stage_E14_vs_E10	ordinary_muscle_development	ENSGALG00010010734	AMN1	0.146406348	0.143303796	1.021650174	0.306946501	0.420607821	positive
PRJNA729271	stage_E14_vs_E10	ordinary_muscle_development	ENSGALG00010010740	CCDC134	0.406218184	0.095534938	4.252037979	2.11834E-05	9.38714E-05	positive
PRJNA729271	stage_E14_vs_E10	ordinary_muscle_development	ENSGALG00010010742	LCP2	0.546409193	0.264250557	2.067769314	0.038661721	0.076172576	positive
PRJNA729271	stage_E14_vs_E10	ordinary_muscle_development	ENSGALG00010010743	KLHL20	0.102332313	0.082539985	1.239790792	0.215052785	0.316787613	positive
PRJNA729271	stage_E14_vs_E10	ordinary_muscle_development	ENSGALG00010010745	ATVR1	-0.044136784	0.062774154	-0.703104403	0.481990688	0.593682446	negative
PRJNA729271	stage_E14_vs_E10	ordinary_muscle_development	ENSGALG00010010748	SF3B1	0.003203951	0.071578718	0.044761227	0.964297631	0.976286524	positive
PRJNA729271	stage_E14_vs_E10	ordinary_muscle_development	ENSGALG00010010749	NCOA4	-0.010446768	0.098049244	-0.106546134	0.915149054	0.941362463	negative
PRJNA729271	stage_E14_vs_E10	ordinary_muscle_development	ENSGALG00010010751	SLC30A5	-0.323523301	0.078004937	-4.147472121	3.36166E-05	0.000144036	negative
PRJNA729271	stage_E14_vs_E10	ordinary_muscle_development	ENSGALG00010010752	ENSGALG00010010752	-0.223697786	0.250950779	-0.891401044	0.372714058	0.488736229	negative
PRJNA729271	stage_E14_vs_E10	ordinary_muscle_development	ENSGALG00010010753	ENSGALG00010010753	0.028340812	1.161307577	0.024404225			positive
PRJNA729271	stage_E14_vs_E10	ordinary_muscle_development	ENSGALG00010010754	DNAI1	-1.008457554	0.252662904	-3.991316253	6.57076E-05	0.000267838	negative
PRJNA729271	stage_E14_vs_E10	ordinary_muscle_development	ENSGALG00010010756	CENPL	-0.181560919	0.188768033	-0.96182026	0.336139899	0.451321974	negative
PRJNA729271	stage_E14_vs_E10	ordinary_muscle_development	ENSGALG00010010759	ENSGALG00010010759	0.475017891	0.248489478	1.911621755	0.055924727	0.104234141	positive
PRJNA729271	stage_E14_vs_E10	ordinary_muscle_development	ENSGALG00010010760	FOXI1	-0.891467193	0.888048363	-1.003849824	0.315451005	0.429419827	negative
PRJNA729271	stage_E14_vs_E10	ordinary_muscle_development	ENSGALG00010010761	MEI1	-0.010531857	0.26555256	-0.039660159	0.968364065	0.978558082	negative
PRJNA729271	stage_E14_vs_E10	ordinary_muscle_development	ENSGALG00010010762	MRPL39	0.02933201	0.109804373	0.267129702	0.789369299	0.851966343	positive
PRJNA729271	stage_E14_vs_E10	ordinary_muscle_development	ENSGALG00010010765	ENSGALG00010010765	-1.00890693	1.118384475	-0.902110994	0.366997909	0.483163914	negative
PRJNA729271	stage_E14_vs_E10	ordinary_muscle_development	ENSGALG00010010766	CENPO	-0.351564956	0.226194802	-1.554257452	0.120123018	0.197749364	negative
PRJNA729271	stage_E14_vs_E10	ordinary_muscle_development	ENSGALG00010010768	ENSGALG00010010768	0.135786145	0.09145475	1.484735835	0.137613815	0.221094242	positive
PRJNA729271	stage_E14_vs_E10	ordinary_muscle_development	ENSGALG00010010769	HEXB	-0.536425922	0.120414227	-4.454838393	8.39564E-06	3.98607E-05	negative
PRJNA729271	stage_E14_vs_E10	ordinary_muscle_development	ENSGALG00010010771	ENSGALG00010010771	-0.081140765	0.071129605	-1.140745325	0.253975919	0.361522238	negative
PRJNA729271	stage_E14_vs_E10	ordinary_muscle_development	ENSGALG00010010774	KPTN	0.018376447	0.133267752	0.137891173	0.890326426	0.924683995	positive
PRJNA729271	stage_E14_vs_E10	ordinary_muscle_development	ENSGALG00010010776	GCAT	-0.028161386	0.194152021	-0.145048121	0.884672878	0.920941088	negative
PRJNA729271	stage_E14_vs_E10	ordinary_muscle_development	ENSGALG00010010778	UPP2	0.590698531	0.389504366	1.516538919	0.129383137	0.210059204	positive
PRJNA729271	stage_E14_vs_E10	ordinary_muscle_development	ENSGALG00010010779	DOCK2	-0.34602102	0.188094904	-1.839608693	0.065825707	0.119746941	negative
PRJNA729271	stage_E14_vs_E10	ordinary_muscle_development	ENSGALG00010010780	PARG	-0.037720025	0.048153968	-0.783321211	0.43343851	0.547463507	negative
PRJNA729271	stage_E14_vs_E10	ordinary_muscle_development	ENSGALG00010010781	NAPA	0.223364337	0.097212884	2.29768243	0.02157987	0.046485394	positive
PRJNA729271	stage_E14_vs_E10	ordinary_muscle_development	ENSGALG00010010783	F2RL2	-0.507938843	0.14181806	-3.581623111	0.000341466	0.001207502	negative
PRJNA729271	stage_E14_vs_E10	ordinary_muscle_development	ENSGALG00010010784	CCDC148	0.541054046	0.295177351	1.832979546	0.066805613	0.121119119	positive
PRJNA729271	stage_E14_vs_E10	ordinary_muscle_development	ENSGALG00010010786	NABP1	-0.02190065	0.154164553	-0.14206022	0.88703244	0.922480644	negative
PRJNA729271	stage_E14_vs_E10	ordinary_muscle_development	ENSGALG00010010789	ENSGALG00010010789	-0.492504208	0.264318192	-1.863300462	0.062420005	0.114427466	negative
PRJNA729271	stage_E14_vs_E10	ordinary_muscle_development	ENSGALG00010010791	H-10	0.203231246	0.292978275	0.693673432	0.487887031	0.599061628	positive
PRJNA729271	stage_E14_vs_E10	ordinary_muscle_development	ENSGALG00010010792	XRCC6	-0.061433115	0.143776225	-0.427282847	0.669173325	0.75684046	negative
PRJNA729271	stage_E14_vs_E10	ordinary_muscle_development	ENSGALG00010010796	CAVIN2	1.47229687	0.146023422	10.08260761	6.59522E-24	1.81745E-22	positive
PRJNA729271	stage_E14_vs_E10	ordinary_muscle_development	ENSGALG00010010799	DARS2	0.170273604	0.104135859	1.635110187	0.102025951	0.172926352	positive
PRJNA729271	stage_E14_vs_E10	ordinary_muscle_development	ENSGALG00010010800	BICRA	-0.121779744	0.191386991	-0.636301055	0.524580217	0.632279249	negative
PRJNA729271	stage_E14_vs_E10	ordinary_muscle_development	ENSGALG00010010805	PTRHD1	0.069112823	0.153481508	0.45030065	0.652493671	0.743428373	positive
PRJNA729271	stage_E14_vs_E10	ordinary_muscle_development	ENSGALG00010010806	INSYN2B	-2.571194079	0.985524274	-2.608960678	0.009081768	0.021918119	negative
PRJNA729271	stage_E14_vs_E10	ordinary_muscle_development	ENSGALG00010010809	CENPH	-0.470640277	0.148084291	-3.178191786	0.001481967	0.004473367	negative
PRJNA729271	stage_E14_vs_E10	ordinary_muscle_development	ENSGALG00010010810	GALT	0.305799731	0.109410964	2.794964239	0.005190546	0.013451986	positive
PRJNA729271	stage_E14_vs_E10	ordinary_muscle_development	ENSGALG00010010813	EHD2	0.904328127	0.149714108	6.040366781	1.53764E-09	1.23343E-08	positive
PRJNA729271	stage_E14_vs_E10	ordinary_muscle_development	ENSGALG00010010816	AP2S1	0.002845554	0.10951414	0.025983437	0.979270549	0.986210423	positive
PRJNA729271	stage_E14_vs_E10	ordinary_muscle_development	ENSGALG00010010821	RGS8	-2.904221336	0.447611623	-6.48826167	8.68324E-11	8.02189E-10	negative
PRJNA729271	stage_E14_vs_E10	ordinary_muscle_development	ENSGALG00010010822	OGDHL	-1.545106328	0.359110366	-4.302594622	1.6881E-05	7.61324E-05	negative
PRJNA729271	stage_E14_vs_E10	ordinary_muscle_development	ENSGALG00010010823	TMEFF2	-1.589954099	0.166303879	-9.560535262	1.17149E-21	2.85391E-20	negative
PRJNA729271	stage_E14_vs_E10	ordinary_muscle_development	ENSGALG00010010824	PKP4	-0.220594652	0.109229415	-2.01955354	0.043429718	0.084092141	negative
PRJNA729271	stage_E14_vs_E10	ordinary_muscle_development	ENSGALG00010010825	MAK	-0.613301743	0.445005522	-1.378189062	0.168144924	0.260085441	negative
PRJNA729271	stage_E14_vs_E10	ordinary_muscle_development	ENSGALG00010010826	NCOA1	0.615617801	0.161058375	3.822327164	0.000132198	0.000509631	positive
PRJNA729271	stage_E14_vs_E10	ordinary_muscle_development	ENSGALG00010010828	SPDL1	-0.552956055	0.122140865	-4.527199448	5.97705E-06	2.91218E-05	negative
PRJNA729271	stage_E14_vs_E10	ordinary_muscle_development	ENSGALG00010010829	LAMB4	-0.584464946	0.24128458	-2.422305424	0.015422382	0.034744705	negative
PRJNA729271	stage_E14_vs_E10	ordinary_muscle_development	ENSGALG00010010831	DESI1	0.197096773	0.091891525	2.144885204	0.031962026	0.06472866	positive
PRJNA729271	stage_E14_vs_E10	ordinary_muscle_development	ENSGALG00010010834	KGD4	0.333181529	0.125219345	2.66078319	0.007795914	0.01918536	positive
PRJNA729271	stage_E14_vs_E10	ordinary_muscle_development	ENSGALG00010010836	DIP2A	-0.242577458	0.105316944	-2.303308929	0.021261468	0.045895007	negative
PRJNA729271	stage_E14_vs_E10	ordinary_muscle_development	ENSGALG00010010837	RBPJ	0.145255895	0.122363994	1.187080372	0.23519592	0.33972744	positive
PRJNA729271	stage_E14_vs_E10	ordinary_muscle_development	ENSGALG00010010838	JAM2	0.156074184	0.175137167	0.89115398	0.372846569	0.488821546	positive

PRJNA729271	stage_E14_vs_E10	ordinary_muscle_development	ENSGALG00010010840	RGS16	-2.388074923	0.791632134	-3.016647279	0.00255587	0.007203449	negative
PRJNA729271	stage_E14_vs_E10	ordinary_muscle_development	ENSGALG00010010841	PMM1	0.184814907	0.132231414	1.397662638	0.162214399	0.252773351	positive
PRJNA729271	stage_E14_vs_E10	ordinary_muscle_development	ENSGALG00010010843	ZNF395	0.613971862	0.178733422	3.435126206	0.000592278	0.001969504	positive
PRJNA729271	stage_E14_vs_E10	ordinary_muscle_development	ENSGALG00010010844	CDC14B	-0.001659541	0.17461852	-0.009503808	0.992417172	0.995369977	negative
PRJNA729271	stage_E14_vs_E10	ordinary_muscle_development	ENSGALG00010010845	FKRP	0.207263004	0.251090932	0.825449978	0.40911617	0.52386386	positive
PRJNA729271	stage_E14_vs_E10	ordinary_muscle_development	ENSGALG00010010848	RGR	1.644666238	0.555390922	2.961276772	0.003063665	0.008471542	positive
PRJNA729271	stage_E14_vs_E10	ordinary_muscle_development	ENSGALG00010010849	ACBD6	-0.17823575	0.107350772	-1.660311768	0.09685175	0.165589303	negative
PRJNA729271	stage_E14_vs_E10	ordinary_muscle_development	ENSGALG00010010850	ENSGALG00010010850	0.22021414	0.106856395	2.060841936	0.039318122	0.077224415	positive
PRJNA729271	stage_E14_vs_E10	ordinary_muscle_development	ENSGALG00010010851	PNOC	5.201765708	0.748628672	6.94839231	3.69472E-12	3.97587E-11	positive
PRJNA729271	stage_E14_vs_E10	ordinary_muscle_development	ENSGALG00010010853	SMIM20	0.246879423	0.145641623	1.695115852	0.090053446	0.155859665	positive
PRJNA729271	stage_E14_vs_E10	ordinary_muscle_development	ENSGALG00010010854	LRIT1	1.112092238	0.558602233	1.990848178	0.046497581	0.089247064	positive
PRJNA729271	stage_E14_vs_E10	ordinary_muscle_development	ENSGALG00010010855	ATP5PF	0.440933395	0.148480737	2.969633666	0.002981551	0.008269724	positive
PRJNA729271	stage_E14_vs_E10	ordinary_muscle_development	ENSGALG00010010856	CSDC2	-2.351454051	0.446099877	-5.271138084	1.3558E-07	8.45627E-07	negative
PRJNA729271	stage_E14_vs_E10	ordinary_muscle_development	ENSGALG00010010857	SEL1L3	-0.013586515	0.060403577	-0.224928979	0.822034525	0.875423587	negative
PRJNA729271	stage_E14_vs_E10	ordinary_muscle_development	ENSGALG00010010859	ENSGALG00010010859	0.024847353	0.262795649	0.094550091	0.924672194	0.948147551	positive
PRJNA729271	stage_E14_vs_E10	ordinary_muscle_development	ENSGALG00010010861	ENSGALG00010010861	-0.775543191	0.243539145	-3.184470373	0.001450192	0.004386587	negative
PRJNA729271	stage_E14_vs_E10	ordinary_muscle_development	ENSGALG00010010862	STRN4	-0.019763752	0.149987801	-0.131769064	0.895166956	0.928010659	negative
PRJNA729271	stage_E14_vs_E10	ordinary_muscle_development	ENSGALG00010010865	ENSGALG00010010865	0.455649519	0.156662969	2.908469838	0.003632022	0.009816551	positive
PRJNA729271	stage_E14_vs_E10	ordinary_muscle_development	ENSGALG00010010867	QSOX1	0.113385044	0.160797876	0.705140185	0.480723001	0.59268609	positive
PRJNA729271	stage_E14_vs_E10	ordinary_muscle_development	ENSGALG00010010868	SLC39A10	-0.721347713	0.106648197	-6.763805967	1.34413E-11	1.36246E-10	negative
PRJNA729271	stage_E14_vs_E10	ordinary_muscle_development	ENSGALG00010010869	ENSGALG00010010869	2.1320665	0.174294482	12.23255303	2.08288E-34	1.10591E-32	positive
PRJNA729271	stage_E14_vs_E10	ordinary_muscle_development	ENSGALG00010010870	NOL6	-0.447514641	0.120938419	-3.700351324	0.000215301	0.000793085	negative
PRJNA729271	stage_E14_vs_E10	ordinary_muscle_development	ENSGALG00010010871	GCNT2	-2.651051389	0.961319539	-2.757721322	0.00582058	0.014864218	negative
PRJNA729271	stage_E14_vs_E10	ordinary_muscle_development	ENSGALG00010010874	ENSGALG00010010874	-0.427414238	0.277424886	-1.540648514	0.123402354	0.202023618	negative
PRJNA729271	stage_E14_vs_E10	ordinary_muscle_development	ENSGALG00010010876	ENSGALG00010010876	0.064450802	0.157801494	0.408429603	0.682958304	0.767818243	positive
PRJNA729271	stage_E14_vs_E10	ordinary_muscle_development	ENSGALG00010010880	ELP3	0.072442607	0.10508404	0.689377831	0.490585531	0.601060681	positive
PRJNA729271	stage_E14_vs_E10	ordinary_muscle_development	ENSGALG00010010882	CEP350	1.675590304	0.098059192	17.08753937	1.83766E-65	5.32738E-63	positive
PRJNA729271	stage_E14_vs_E10	ordinary_muscle_development	ENSGALG00010010884	DNAH7	-2.137045512	0.301708801	-7.08313945	1.40925E-12	1.58104E-11	negative
PRJNA729271	stage_E14_vs_E10	ordinary_muscle_development	ENSGALG00010010885	SNRPD2	-0.478682319	0.272213845	-1.758478958	0.078666049	0.139396623	negative
PRJNA729271	stage_E14_vs_E10	ordinary_muscle_development	ENSGALG00010010887	GABPA	0.039556791	0.065845483	0.600751774	0.548005329	0.653288695	positive
PRJNA729271	stage_E14_vs_E10	ordinary_muscle_development	ENSGALG00010010888	GNPAT	0.186637992	0.118782099	1.571263624	0.116121427	0.192630469	positive
PRJNA729271	stage_E14_vs_E10	ordinary_muscle_development	ENSGALG00010010889	CCL19	0.286717983	0.648847102	0.441888363	0.658569992	0.748234209	positive
PRJNA729271	stage_E14_vs_E10	ordinary_muscle_development	ENSGALG00010010890	NOL12	-0.186130412	0.132459507	-1.405187262	0.159965601	0.250075646	negative
PRJNA729271	stage_E14_vs_E10	ordinary_muscle_development	ENSGALG00010010891	POLR3H	0.220382365	0.101683176	2.16734344	0.030208677	0.06179435	positive
PRJNA729271	stage_E14_vs_E10	ordinary_muscle_development	ENSGALG00010010893	CCL21	-3.439717852	0.523156527	-6.574930592	4.86759E-11	4.66947E-10	negative
PRJNA729271	stage_E14_vs_E10	ordinary_muscle_development	ENSGALG00010010894	SPAG1	-0.445458999	0.142013618	-3.136734381	0.001708408	0.005070307	negative
PRJNA729271	stage_E14_vs_E10	ordinary_muscle_development	ENSGALG00010010896	LY86	1.961308606	0.292834314	6.697673427	2.11764E-11	2.09523E-10	positive
PRJNA729271	stage_E14_vs_E10	ordinary_muscle_development	ENSGALG00010010897	CEP170	-0.294879886	0.09039779	-3.262025386	0.001106192	0.003446745	negative
PRJNA729271	stage_E14_vs_E10	ordinary_muscle_development	ENSGALG00010010898	PHF24	-5.303862226	3.569493153	-1.485886651	0.137309105	0.220678066	negative
PRJNA729271	stage_E14_vs_E10	ordinary_muscle_development	ENSGALG00010010900	SLC34A2	-1.282609721	1.024632077	-1.25177588	0.210651541	0.311698049	negative
PRJNA729271	stage_E14_vs_E10	ordinary_muscle_development	ENSGALG00010010904	TOR1AIP1	0.219674705	0.079734923	2.755062597	0.005868091	0.01497236	positive
PRJNA729271	stage_E14_vs_E10	ordinary_muscle_development	ENSGALG00010010905	ENC1	0.069283313	0.147562175	0.469519459	0.638698379	0.731621069	positive
PRJNA729271	stage_E14_vs_E10	ordinary_muscle_development	ENSGALG00010010906	ATP6V1C1	-0.070695469	0.139299464	-0.507507112	0.611799047	0.708705122	negative
PRJNA729271	stage_E14_vs_E10	ordinary_muscle_development	ENSGALG00010010908	STK17B	0.613875237	0.12223181	5.022221596	5.10772E-07	2.94029E-06	positive
PRJNA729271	stage_E14_vs_E10	ordinary_muscle_development	ENSGALG00010010909	SLIT3	-2.149348455	0.328978005	-6.533410819	6.42885E-11	6.06286E-10	negative
PRJNA729271	stage_E14_vs_E10	ordinary_muscle_development	ENSGALG00010010911	ENSGALG00010010911	-1.173636679	0.339270594	-3.459293846	0.000541594	0.001821439	negative
PRJNA729271	stage_E14_vs_E10	ordinary_muscle_development	ENSGALG00010010912	BAALC	-0.646183184	0.335305336	-1.927148524	0.053961123	0.101094284	negative
PRJNA729271	stage_E14_vs_E10	ordinary_muscle_development	ENSGALG00010010913	IFT56	-0.893584486	0.142058727	-6.290247025	3.16961E-10	2.75111E-09	negative
PRJNA729271	stage_E14_vs_E10	ordinary_muscle_development	ENSGALG00010010915	APP	-0.45173064	0.110045176	-4.104956302	4.04391E-05	0.000171043	negative
PRJNA729271	stage_E14_vs_E10	ordinary_muscle_development	ENSGALG00010010916	ENSGALG00010010916	-1.722018798	0.521077418	-3.304727358	0.000950688	0.003010755	negative
PRJNA729271	stage_E14_vs_E10	ordinary_muscle_development	ENSGALG00010010917	TANC1	-0.158245789	0.068485217	-2.310656173	0.020851854	0.045084669	negative
PRJNA729271	stage_E14_vs_E10	ordinary_muscle_development	ENSGALG00010010918	TOR1AIP2	0.009614677	0.120282276	0.07993428	0.936289525	0.95567331	positive
PRJNA729271	stage_E14_vs_E10	ordinary_muscle_development	ENSGALG00010010919	CDHR1	-0.363488039	0.466889399	-0.778531361	0.436255826	0.550159057	negative
PRJNA729271	stage_E14_vs_E10	ordinary_muscle_development	ENSGALG00010010920	UBN2	0.047033289	0.113800213	0.413297024	0.67938901	0.76547433	positive
PRJNA729271	stage_E14_vs_E10	ordinary_muscle_development	ENSGALG00010010921	ENSGALG00010010921	0.032374219	0.125898469	0.257145451	0.797066492	0.857370139	positive
PRJNA729271	stage_E14_vs_E10	ordinary_muscle_development	ENSGALG00010010922	DYM	0.180841663	0.101696364	1.778251021	0.075362637	0.13439732	positive
PRJNA729271	stage_E14_vs_E10	ordinary_muscle_development	ENSGALG00010010923	F13A1	1.318348436	0.230996764	5.70721604	1.14839E-08	8.29806E-08	positive
PRJNA729271	stage_E14_vs_E10	ordinary_muscle_development	ENSGALG00010010924	ENSGALG00010010924	0.648453797	0.182796639	3.547405472	0.000389045	0.001352005	positive
PRJNA729271	stage_E14_vs_E10	ordinary_muscle_development	ENSGALG00010010928	HECW2	0.316072777	0.115546386	2.735462254	0.006229273	0.015757995	positive
PRJNA729271	stage_E14_vs_E10	ordinary_muscle_development	ENSGALG00010010929	FMC1	0.753826264	0.146692211	5.138829532	2.76455E-07	1.65451E-06	positive
PRJNA729271	stage_E14_vs_E10	ordinary_muscle_development	ENSGALG00010010930	FAM163A	-1.609495205	0.410084426	-3.924789876	8.68055E-05	0.000345482	negative
PRJNA729271	stage_E14_vs_E10	ordinary_muscle_development	ENSGALG00010010931	CYYR1	-0.858388936	0.189471817	-4.53043069	5.88636E-06	2.87318E-05	negative
PRJNA729271	stage_E14_vs_E10	ordinary_muscle_development	ENSGALG00010010932	GHITM	0.417175376	0.08815828	4.732117912	2.22189E-06	1.16059E-05	positive
PRJNA729271	stage_E14_vs_E10	ordinary_muscle_development	ENSGALG00010010933	ANAPC4	-0.077052747	0.109884378	-0.701216569	0.483167871	0.594677617	negative
PRJNA729271	stage_E14_vs_E10	ordinary_muscle_development	ENSGALG00010010934	ENSGALG00010010934	-0.133652401	0.113162233	-1.18106896	0.237575329	0.342686277	negative
PRJNA729271	stage_E14_vs_E10	ordinary_muscle_development	ENSGALG00010010935	LGALS1A	0.837605166	0.227330813	3.684521054	0.000229133	0.000839344	positive
PRJNA729271	stage_E14_vs_E10	ordinary_muscle_development	ENSGALG00010010937	CDK7	0.153785951	0.114130068	1.347462195	0.177831421	0.27204923	positive
PRJNA729271	stage_E14_vs_E10	ordinary_muscle_development	ENSGALG00010010938	FZD6	-0.081186828	0.120119089	-0.675886143	0.499112936	0.608157533	negative
PRJNA729271	stage_E14_vs_E10	ordinary_muscle_development	ENSGALG00010010939	ZCCHC4	-0.101623265	0.13517577	-0.751786102	0.452179701	0.565175887	negative
PRJNA729271	stage_E14_vs_E10	ordinary_muscle_development	ENSGALG00010010940	NRG3	-0.592170332	0.322227886	-1.837737694	0.066101067	0.120157382	negative
PRJNA729271	stage_E14_vs_E10	ordinary_muscle_development	ENSGALG00010010941	RIPK4	-2.036271857	0.633678017	-3.213417228	0.001311656	0.004015302	negative
PRJNA729271	stage_E14_vs_E10	ordinary_muscle_development	ENSGALG00010010942	NRN1	1.173343142	0.339143832	3.459721307	0.000540735	0.001818972	positive
PRJNA729271	stage_E14_vs_E10	ordinary_muscle_development	ENSGALG00010010943	WDSUB1	-0.02269815	0.102642277	-0.221138407	0.824984666	0.877412076	negative
PRJNA729271	stage_E14_vs_E10	ordinary_muscle_development	ENSGALG00010010945	PI4K2B	-0.681949886	0.097346916	-7.005356897	2.46357E-12	2.70937E-11	negative
PRJNA729271	stage_E14_vs_E10	ordinary_muscle_development	ENSGALG00010010946	SH2D4B	0.894495976	0.301865212	2.963229752	0.003044293	0.008424402	positive
PRJNA729271	stage_E14_vs_E10	ordinary_muscle_development	ENSGALG00010010947	LUC7L2	-0.060036121	0.06955915	-0.863094507	0.388085508	0.503923626	negative
PRJNA729271	stage_E14_vs_E10	ordinary_muscle_development	ENSGALG00010010948	PRDM15	-0.141231444	0.08186821	-1.725107271	0.084508142	0.148065457	negative
PRJNA729271	stage_E14_vs_E10	ordinary_muscle_development	ENSGALG00010010949	FZD3	-0.619389929	0.221252943	-2.799465269	0.005118732	0.013280119	negative
PRJNA729271	stage_E14_vs_E10	ordinary_muscle_development	ENSGALG00010010951	ENSGALG00010010951	-0.330833677	0.127238317	-2.600110436	0.009319376	0.022405766	negative
PRJNA729271	stage_E14_vs_E10	ordinary_muscle_development	ENSGALG00010010952	ENSGALG00010010952	-0.963984442	0.519666212	-1.855006962	0.063595232	0.116242812	negative
PRJNA729271	stage_E14_vs_E10	ordinary_muscle_development	ENSGALG00010010953	EXTL3	0.464130718	0.109421002	4.241696827	2.21836E-05	9.80639E-05	positive
PRJNA729271	stage_E14_vs_E10	ordinary_muscle_development	ENSGALG00010010954	MTERF2	0.331595524	0.225744122	1.468899927	0.141859934	0.22683059	positive
PRJNA729271	stage_E14_vs_E10	ordinary_muscle_development	ENSGALG00010010955	GTF3C3	0.01384812	0.080836664	0.171309893	0.863980102	0.906502728	positive
PRJNA729271	stage_E14_vs_E10	ordinary_muscle_development	ENSGALG00010010956	AXDND1	1.761785054	0.593215724	2.969889336	0.00297907	0.008264426	positive
PRJNA729271	stage_E14_vs_E10	ordinary_muscle_development	ENSGALG00010010957	ZMAT2	0.194305888	0.074873624	2.595117993	0.009455845	0.022693152	positive
PRJNA729271	stage_E14_vs_E10	ordinary_muscle_development	ENSGALG00010010958	LCORL	-0.46785381	0.1605488	-2.914090982	0.003567259	0.009661327	negative
PRJNA729271	stage_E14_vs_E10	ordinary_muscle_development	ENSGALG00010010959	TMEM263	0.033059729	0.08915149	0.370826434	0.710766811	0.790377056	positive
PRJNA729271	stage_E14_vs_E10	ordinary_muscle_development	ENSGALG00010010960	POLDIP3	0.067951566	0.068910822	0.986079741	0.324093984	0.438713327	positive
PRJNA729271	stage_E14_vs_E10	ordinary_muscle_development	ENSGALG00010010961	C2orf66	-0.465600182	0.389122508	-1.196538807	0.231486364	0.335808131	negative
PRJNA729271	stage_E14_vs_E10	ordinary_muscle_development	ENSGALG00010010962	ANKDD1B	-0.159201806	0.275659575	-0.577530476	0.563581157	0.666286175	negative
PRJNA729271	stage_E14_vs_E10	ordinary_muscle_development	ENSGALG00010010963	TSPAN14	0.402229547	0.072399419	5.555701315	2.76499E-08	1.91215E-07	positive
PRJNA729271	stage_E14_vs_E10	ordinary_muscle_development	ENSGALG00010010964	INTS9	-0.168795578	0.088688426	-1.903242469	0.057008914	0.105914407	negative
PRJNA729271	stage_E14_vs_E10	ordinary_muscle_development	ENSGALG00010010965	BAZ2B	-0.126382795	0.131860019	-0.95846183	0.33782993	0.452950221	negative
PRJNA729271	stage_E14_vs_E10	ordinary_muscle_development	ENSGALG00010010966	ENSGALG00010010966	1.800615216	0.529585529	3.400046109	0.000673745	0.002211989	positive
PRJNA729271	stage_E14_vs_E10	ordinary_muscle_development	ENSGALG00010010967	PRXL2A	-0.192499706	0.389960995	-0.493638359	0.621561605	0.71701835	negative
PRJNA729271	stage_E14_vs_E10	ordinary_muscle_development	ENSGALG00010010968	RRP7A	0.123774886	0.245908127	0.503337922	0.614726709	0.711143949	positive
PRJNA729271	stage_E14_vs_E10	ordinary_muscle_development	ENSGALG00010010970	TPM2	1.359325451	0.352421633	3.857099914	0.00011474	0.000447206	positive
PRJNA729271	stage_E14_vs_E10	ordinary_muscle_development	ENSGALG00010010971	LCORL	-0.245151973	0.124942991	-1.962110653	0.049749603	0.09442079	negative
PRJNA729271	stage_E14_vs_E10	ordinary_muscle_development	ENSGALG00010010972	EXOSC3	0.158795098	0.158627024	1.001059559	0.316798015	0.430714427	positive
PRJNA729271	stage_E14_vs_E10	ordinary_muscle_development	ENSGALG00010010973	PGAP1	0.008833844	0.111798675	0.07901564	0.937020183	0.95618629	positive
PRJNA729271	stage_E14_vs_E10	ordinary_muscle_development	ENSGALG00010010974	NCAPG	-0.158273427	0.189608085	-0.834739864	0.403864191	0.518884913	negative
PRJNA729271	stage_E14_vs_E10	ordinary_muscle_development	ENSGALG00010010975	ATP8B1	-2.42747731	0.882498905	-2.750685916	0.005947063	0.015160514	negative
PRJNA729271	stage_E14_vs_E10	ordinary_muscle_development	ENSGALG00010010976	DYDC2	4.284439559	0.588079633	7.285475165	3.20539E-13	3.87184E-12	positive
PRJNA729271	stage_E14_vs_E10	ordinary_muscle_development	ENSGALG00010010977	FARS2	0.711539254	0.07959533	8.939459771	3.91077E-19	7.85134E-18	positive
PRJNA729271	stage_E14_vs_E10	ordinary_muscle_development	ENSGALG00010010978	SERHL	-0.197194496	0.133444355	-1.477728272	0.139480524	0.223671888	negative
PRJNA729271	stage_E14_vs_E10	ordinary_muscle_development	ENSGALG00010010979	ENSGALG00010010979	-0.886350752	0.327357917	-2.707589173	0.006777386	0.016955162	negative
PRJNA729271	stage_E14_vs_E10	ordinary_muscle_development	ENSGALG00010010980	POLK	-0.216444357	0.125808743	-1.720423813	0.085355431	0.149279317	negative
PRJNA729271	stage_E14_vs_E10	ordinary_muscle_development	ENSGALG00010010981	RIC8B	-0.066623426	0.06043844	-1.102335296	0.27031593	0.380152266	negative
PRJNA729271	stage_E14_vs_E10	ordinary_muscle_development	ENSGALG00010010982	ZBTB21	0.127592054	0.119436188	1.068286385	0.285391348	0.397153186	positive
PRJNA729271	stage_E14_vs_E10	ordinary_muscle_development	ENSGALG00010010983	MED28	0.118509006	0.087965722	1.347218022	0.177910025	0.272140758	positive
PRJNA729271	stage_E14_vs_E10	ordinary_muscle_development	ENSGALG00010010984	MAT1A	3.205709616	0.389899083	8.221895766	2.00311E-16	3.18717E-15	positive
PRJNA729271	stage_E14_vs_E10	ordinary_muscle_development	ENSGALG00010010985	CERT1	0.054218297	0.108353273	0.500384488	0.616804374	0.712737516	positive
PRJNA729271	stage_E14_vs_E10	ordinary_muscle_development	ENSGALG00010010986	NARS	-0.026556544	0.129582869	-0.204938691	0.837620026	0.886679491	negative
PRJNA729271	stage_E14_vs_E10	ordinary_muscle_development	ENSGALG00010010987	MARCHF7	0.129222025	0.080303279	1.609174945	0.107578093	0.180944493	positive
PRJNA729271	stage_E14_vs_E10	ordinary_muscle_development	ENSGALG00010010988	ANKRD44	-0.03104161	0.084917298	-0.365551079	0.714700085	0.793684877	negative
PRJNA729271	stage_E14_vs_E10	ordinary_muscle_development	ENSGALG00010010989	TRIQK	-0.285985887	0.176551523	-1.619843784	0.105265838	0.177731896	negative
PRJNA729271	stage_E14_vs_E10	ordinary_muscle_development	ENSGALG00010010990	MED18	0.184432547	0.119107919	1.548449077	0.121514213	0.199631477	positive
PRJNA729271	stage_E14_vs_E10	ordinary_muscle_development	ENSGALG00010010991	C2CD2	0.74994846	0.160992398	4.658284933	3.18855E-06	1.62347E-05	positive
PRJNA729271	stage_E14_vs_E10	ordinary_muscle_development	ENSGALG00010010992	PCDHA6	-0.13791919	0.295467328	-0.466783218	0.640654984	0.73322495	negative
PRJNA729271	stage_E14_vs_E10	ordinary_muscle_development	ENSGALG00010010993	ENSGALG00010010993	4.019765109	0.329289304	12.20739652	2.83827E-34	1.50148E-32	positive
PRJNA729271	stage_E14_vs_E10	ordinary_muscle_development	ENSGALG00010010994	NAA20	0.173729322	0.149175031	1.164600545	0.244180723	0.350609171	positive
PRJNA729271	stage_E14_vs_E10	ordinary_muscle_development	ENSGALG00010010995	HEBP1	0.312229286	0.182840205	1.707662086	0.087699061	0.152554689	positive
PRJNA729271	stage_E14_vs_E10	ordinary_muscle_development	ENSGALG00010010996	RFX4	1.257211312	0.539766934	2.329174375	0.01984983	0.043149863	positive
PRJNA729271	stage_E14_vs_E10	ordinary_muscle_development	ENSGALG00010010997	LAP3	-0.074500583	0.148659756	-0.501148294	0.61626676	0.712343436	negative
PRJNA729271	stage_E14_vs_E10	ordinary_muscle_development	ENSGALG00010010998	SOAT1	0.257986694	0.165953925	1.554568197	0.120048943	0.197739708	positive
PRJNA729271	stage_E14_vs_E10	ordinary_muscle_development	ENSGALG00010011001	ANGPT1	-1.577814691	0.249608333	-6.321161915	2.59604E-10	2.28196E-09	negative
PRJNA729271	stage_E14_vs_E10	ordinary_muscle_development	ENSGALG00010011002	RPRM	-0.951394543	0.580118132	-1.64000139	0.101004878	0.171442149	negative
PRJNA729271	stage_E14_vs_E10	ordinary_muscle_development	ENSGALG00010011003	FECH	-0.00338253	0.111551249	-0.030322655	0.975809729	0.983681899	negative
PRJNA729271	stage_E14_vs_E10	ordinary_muscle_development	ENSGALG00010011004	UMODL1	0.457835368	0.378943054	1.208190421	0.226974025	0.330353298	positive
PRJNA729271	stage_E14_vs_E10	ordinary_muscle_development	ENSGALG00010011006	TMEM64	0.026533991	0.100009119	0.265315718	0.790766257	0.852926434	positive
PRJNA729271	stage_E14_vs_E10	ordinary_muscle_development	ENSGALG00010011007	ENSGALG00010011007	0.5704765	0.161414823	3.534226224	0.000408971	0.001413456	positive

PRJNA729271	stage_E14_vs_E10	ordinary_muscle_development	ENSGALG00010011009	SFTPA1	1.624157785	1.164790722	1.394377337	0.163203688	0.253932746	positive
PRJNA729271	stage_E14_vs_E10	ordinary_muscle_development	ENSGALG00010011013	ENSGALG00010011013	0.494006733	0.400213034	1.234359433	0.217068999	0.318979636	positive
PRJNA729271	stage_E14_vs_E10	ordinary_muscle_development	ENSGALG00010011015	LYRM4	0.654437025	0.181042046	3.614834446	0.00030054	0.001076432	positive
PRJNA729271	stage_E14_vs_E10	ordinary_muscle_development	ENSGALG00010011018	QDPR	0.234005428	0.102633321	2.280014189	0.022606847	0.048416999	positive
PRJNA729271	stage_E14_vs_E10	ordinary_muscle_development	ENSGALG00010011019	NECAB1	-1.11567191	0.205267638	-5.435206052	5.47331E-08	3.60945E-07	negative
PRJNA729271	stage_E14_vs_E10	ordinary_muscle_development	ENSGALG00010011020	SFTPA2	0.450605686	0.870781532	0.517472717	0.604826209	0.702874682	positive
PRJNA729271	stage_E14_vs_E10	ordinary_muscle_development	ENSGALG00010011021	PPP1R3G	0.990593449	0.189057201	5.239649398	1.60882E-07	9.93602E-07	positive
PRJNA729271	stage_E14_vs_E10	ordinary_muscle_development	ENSGALG00010011022	C5orf22	-0.508226944	0.19426779	-2.61611533	0.008893651	0.021517856	negative
PRJNA729271	stage_E14_vs_E10	ordinary_muscle_development	ENSGALG00010011025	POLR3B	-0.244401281	0.090021004	-2.714936193	0.006628854	0.016612248	negative
PRJNA729271	stage_E14_vs_E10	ordinary_muscle_development	ENSGALG00010011027	FAM234B	-0.160584386	0.093289703	-1.721351669	0.085187029	0.149065457	negative
PRJNA729271	stage_E14_vs_E10	ordinary_muscle_development	ENSGALG00010011028	HMBOX1	-0.210138375	0.082638442	-2.542864662	0.010994781	0.02588005	negative
PRJNA729271	stage_E14_vs_E10	ordinary_muscle_development	ENSGALG00010011029	HMGCR	-0.284217361	0.125015917	-2.273449402	0.022999112	0.049119217	negative
PRJNA729271	stage_E14_vs_E10	ordinary_muscle_development	ENSGALG00010011033	DROSHA	-0.296878687	0.097196	-3.054433178	0.002254862	0.006464444	negative
PRJNA729271	stage_E14_vs_E10	ordinary_muscle_development	ENSGALG00010011034	FAM180A	-1.7697069	0.476921987	-3.710684241	0.0002067	0.0007649	negative
PRJNA729271	stage_E14_vs_E10	ordinary_muscle_development	ENSGALG00010011038	GSG1	-0.571022585	0.361088036	-1.581394364	0.113787895	0.189668301	negative
PRJNA729271	stage_E14_vs_E10	ordinary_muscle_development	ENSGALG00010011040	CDH6	-2.743922418	0.219528291	-12.49917448	7.54305E-36	4.40873E-34	negative
PRJNA729271	stage_E14_vs_E10	ordinary_muscle_development	ENSGALG00010011042	SLC13A4	0.95025892	0.462684493	2.053794614	0.039995574	0.078289784	positive
PRJNA729271	stage_E14_vs_E10	ordinary_muscle_development	ENSGALG00010011044	MBL2	1.518879246	0.606574498	2.504027536	0.012278848	0.028527312	positive
PRJNA729271	stage_E14_vs_E10	ordinary_muscle_development	ENSGALG00010011045	ENSGALG00010011045	-0.571652657	0.397540285	-1.437974161	0.150441386	0.237906163	negative
PRJNA729271	stage_E14_vs_E10	ordinary_muscle_development	ENSGALG00010011049	ABL2	0.982921195	0.127159366	7.729837154	1.07684E-14	1.47811E-13	positive
PRJNA729271	stage_E14_vs_E10	ordinary_muscle_development	ENSGALG00010011050	SYT10	1.831575048	0.411392082	4.452139764	8.50188E-06	4.03123E-05	positive
PRJNA729271	stage_E14_vs_E10	ordinary_muscle_development	ENSGALG00010011055	TOR3A	1.402278876	0.128144194	10.94297628	7.18033E-28	2.67555E-26	positive
PRJNA729271	stage_E14_vs_E10	ordinary_muscle_development	ENSGALG00010011056	ENSGALG00010011056	-0.752396139	0.521459525	-1.442865849	0.149058265	0.236208544	negative
PRJNA729271	stage_E14_vs_E10	ordinary_muscle_development	ENSGALG00010011059	ENSGALG00010011059	-0.120991084	0.206309941	-0.586453001	0.557571136	0.66153668	negative
PRJNA729271	stage_E14_vs_E10	ordinary_muscle_development	ENSGALG00010011061	KIF13B	-0.042941	0.154728531	-0.277524772	0.781377187	0.845607162	negative
PRJNA729271	stage_E14_vs_E10	ordinary_muscle_development	ENSGALG00010011062	TMEM55A	-0.230946086	0.119540382	-1.931950369	0.053365632	0.100095087	negative
PRJNA729271	stage_E14_vs_E10	ordinary_muscle_development	ENSGALG00010011064	MYLIP	-0.567886379	0.097925348	-5.799176496	6.66414E-09	4.95876E-08	negative
PRJNA729271	stage_E14_vs_E10	ordinary_muscle_development	ENSGALG00010011067	TMTC2	-0.410907141	0.101863916	-4.033883211	5.48626E-05	0.000226566	negative
PRJNA729271	stage_E14_vs_E10	ordinary_muscle_development	ENSGALG00010011069	ENSGALG00010011069	-0.470033931	0.512061845	-0.917924144	0.358658581	0.47507595	negative
PRJNA729271	stage_E14_vs_E10	ordinary_muscle_development	ENSGALG00010011076	PKP2	0.281969158	0.286351692	0.984695274	0.324773775	0.439387331	positive
PRJNA729271	stage_E14_vs_E10	ordinary_muscle_development	ENSGALG00010011077	RASGEF1A	0.584331729	0.143913779	4.060290359	4.90117E-05	0.000204145	positive
PRJNA729271	stage_E14_vs_E10	ordinary_muscle_development	ENSGALG00010011080	EMP1	0.067164322	0.254852622	0.263541812	0.792133001	0.854122431	positive
PRJNA729271	stage_E14_vs_E10	ordinary_muscle_development	ENSGALG00010011085	UGT1A1	-0.460246333	1.439598114	-0.319704734	0.74919217	0.820206987	negative
PRJNA729271	stage_E14_vs_E10	ordinary_muscle_development	ENSGALG00010011086	OTUD6A	-0.153110644	0.113199921	-1.352568469	0.176193531	0.26994242	negative
PRJNA729271	stage_E14_vs_E10	ordinary_muscle_development	ENSGALG00010011090	GRIN2B	0.538966175	0.293262022	1.837831481	0.066087241	0.120147317	positive
PRJNA729271	stage_E14_vs_E10	ordinary_muscle_development	ENSGALG00010011091	YARS2	-0.177979037	0.099553288	-1.78777659	0.073812059	0.13207083	negative
PRJNA729271	stage_E14_vs_E10	ordinary_muscle_development	ENSGALG00010011094	CSGALNACT2	-0.187851844	0.089274119	-2.104213913	0.035359791	0.070539523	negative
PRJNA729271	stage_E14_vs_E10	ordinary_muscle_development	ENSGALG00010011095	TNN	-3.388637702	0.479218497	-7.071174684	1.53628E-12	1.71295E-11	negative
PRJNA729271	stage_E14_vs_E10	ordinary_muscle_development	ENSGALG00010011096	LDB2	-1.466513991	0.115359026	-12.71260728	5.0328E-37	3.27132E-35	negative
PRJNA729271	stage_E14_vs_E10	ordinary_muscle_development	ENSGALG00010011099	CCBE1	-0.455318785	0.197348122	-2.307185799	0.021044464	0.045460434	negative
PRJNA729271	stage_E14_vs_E10	ordinary_muscle_development	ENSGALG00010011101	ENSGALG00010011101	-1.373969577	0.829775712	-1.655832482	0.097755748	0.166839699	negative
PRJNA729271	stage_E14_vs_E10	ordinary_muscle_development	ENSGALG00010011104	ABCG1	0.617300298	0.246814513	2.501069696	0.012381881	0.028734448	positive
PRJNA729271	stage_E14_vs_E10	ordinary_muscle_development	ENSGALG00010011106	USP40	0.142614675	0.115160757	1.238396472	0.215569088	0.31725875	positive
PRJNA729271	stage_E14_vs_E10	ordinary_muscle_development	ENSGALG00010011107	ENSGALG00010011107	0.532438712	0.1875062	2.839579241	0.004517307	0.011907323	positive
PRJNA729271	stage_E14_vs_E10	ordinary_muscle_development	ENSGALG00010011109	ENSGALG00010011109	-0.967775417	1.249153207	-0.774745173	0.438490252	0.552442087	negative
PRJNA729271	stage_E14_vs_E10	ordinary_muscle_development	ENSGALG00010011111	MRPS14	0.205017884	0.116737436	1.756230825	0.079049003	0.139989652	positive
PRJNA729271	stage_E14_vs_E10	ordinary_muscle_development	ENSGALG00010011116	TAPT1	0.440778492	0.127313111	3.462161032	0.000535856	0.001805075	positive
PRJNA729271	stage_E14_vs_E10	ordinary_muscle_development	ENSGALG00010011117	SLC6A15	-3.431776984	0.444759648	-7.716025938	1.20013E-14	1.63957E-13	negative
PRJNA729271	stage_E14_vs_E10	ordinary_muscle_development	ENSGALG00010011118	GMPR2	1.568917642	0.13591009	11.54379079	7.93495E-31	3.52813E-29	positive
PRJNA729271	stage_E14_vs_E10	ordinary_muscle_development	ENSGALG00010011119	ENSGALG00010011119	0.209367585	0.2879473	0.727103832	0.467162352	0.5795548	positive
PRJNA729271	stage_E14_vs_E10	ordinary_muscle_development	ENSGALG00010011120	ENSGALG00010011120	-0.562377134	3.022257254	-0.186078512	0.852383192	0.897747345	negative
PRJNA729271	stage_E14_vs_E10	ordinary_muscle_development	ENSGALG00010011121	NUP35	-0.304208228	0.100323224	-3.03228121	0.00242713	0.00687671	negative
PRJNA729271	stage_E14_vs_E10	ordinary_muscle_development	ENSGALG00010011124	CACYBP	0.216234925	0.067181935	3.218646889	0.00128797	0.003954015	positive
PRJNA729271	stage_E14_vs_E10	ordinary_muscle_development	ENSGALG00010011126	ATXN1	0.44773024	0.138903658	3.223314973	0.001267161	0.003901058	positive
PRJNA729271	stage_E14_vs_E10	ordinary_muscle_development	ENSGALG00010011128	GCNT4	-1.266078012	0.254308041	-4.978521355	6.40719E-07	3.63208E-06	negative
PRJNA729271	stage_E14_vs_E10	ordinary_muscle_development	ENSGALG00010011129	ENSGALG00010011129	-0.540945443	0.11878963	-4.553810337	5.26829E-06	2.58948E-05	negative
PRJNA729271	stage_E14_vs_E10	ordinary_muscle_development	ENSGALG00010011130	DNM1L	0.208940653	0.114915817	1.818206216	0.06903262	0.124626707	positive
PRJNA729271	stage_E14_vs_E10	ordinary_muscle_development	ENSGALG00010011131	RET	-1.320677665	0.496013317	-2.662585095	0.007754296	0.019095909	negative
PRJNA729271	stage_E14_vs_E10	ordinary_muscle_development	ENSGALG00010011133	CDC37L1	-0.21396448	0.119719504	-1.78721489	0.073902764	0.132104027	negative
PRJNA729271	stage_E14_vs_E10	ordinary_muscle_development	ENSGALG00010011136	ENSGALG00010011136	-0.555127317	0.386959925	-1.434586067	0.151405087	0.239257417	negative
PRJNA729271	stage_E14_vs_E10	ordinary_muscle_development	ENSGALG00010011139	UBASH3A	2.181669473	0.618655213	3.526470684	0.000421138	0.001451008	positive
PRJNA729271	stage_E14_vs_E10	ordinary_muscle_development	ENSGALG00010011141	MSX1	-4.719347571	0.813093997	-5.804184499	6.468E-09	4.82272E-08	negative
PRJNA729271	stage_E14_vs_E10	ordinary_muscle_development	ENSGALG00010011142	DUSP19	-0.274086756	0.149972738	-1.827577197	0.067613031	0.122414549	negative
PRJNA729271	stage_E14_vs_E10	ordinary_muscle_development	ENSGALG00010011143	AK3	0.264893372	0.167252802	1.583790339	0.113241438	0.188866028	positive
PRJNA729271	stage_E14_vs_E10	ordinary_muscle_development	ENSGALG00010011144	ATF7IP	0.001303763	0.060825774	0.021434378	0.98289915	0.988760576	positive
PRJNA729271	stage_E14_vs_E10	ordinary_muscle_development	ENSGALG00010011146	BMS1	-0.418700014	0.12849813	-3.258413291	0.001120371	0.003485679	negative
PRJNA729271	stage_E14_vs_E10	ordinary_muscle_development	ENSGALG00010011148	ENSGALG00010011148	-0.118846071	0.183073644	-0.649170837	0.516227959	0.62470565	negative
PRJNA729271	stage_E14_vs_E10	ordinary_muscle_development	ENSGALG00010011150	FGD4	-0.161341596	0.17049458	-0.946315103	0.343987892	0.45925251	negative
PRJNA729271	stage_E14_vs_E10	ordinary_muscle_development	ENSGALG00010011152	SLC18A3	0.977450234	0.473237907	2.065452111	0.038880242	0.076512853	positive
PRJNA729271	stage_E14_vs_E10	ordinary_muscle_development	ENSGALG00010011154	SLC26A7	-3.46426787	0.495205919	-6.995610793	2.64106E-12	2.90016E-11	negative
PRJNA729271	stage_E14_vs_E10	ordinary_muscle_development	ENSGALG00010011157	ENSGALG00010011157	-5.619228264	1.574356484	-3.569222295	0.000358043	0.00125875	negative
PRJNA729271	stage_E14_vs_E10	ordinary_muscle_development	ENSGALG00010011161	ENSGALG00010011161	-0.128676211	0.094249756	-1.365268382	0.172168713	0.265064842	negative
PRJNA729271	stage_E14_vs_E10	ordinary_muscle_development	ENSGALG00010011166	SOX7	0.249979618	0.141703117	1.76410811	0.077713779	0.137962184	positive
PRJNA729271	stage_E14_vs_E10	ordinary_muscle_development	ENSGALG00010011170	RBM24	1.74800927	0.142674741	12.25170807	1.64494E-34	8.83091E-33	positive
PRJNA729271	stage_E14_vs_E10	ordinary_muscle_development	ENSGALG00010011175	GPR52	-0.73249126	0.191331156	-3.828395103	0.000128982	0.000498158	negative
PRJNA729271	stage_E14_vs_E10	ordinary_muscle_development	ENSGALG00010011178	RSPO2	-3.669542586	0.322364879	-11.38319594	5.07068E-30	2.18099E-28	negative
PRJNA729271	stage_E14_vs_E10	ordinary_muscle_development	ENSGALG00010011181	ENSGALG00010011181	-0.203903375	0.268063454	-0.760653391	0.446864121	0.560197934	negative
PRJNA729271	stage_E14_vs_E10	ordinary_muscle_development	ENSGALG00010011184	FAM8A1	0.466816133	0.135318904	3.44974811	0.00056111	0.001876191	positive
PRJNA729271	stage_E14_vs_E10	ordinary_muscle_development	ENSGALG00010011187	STK32B	-3.425476418	0.348502813	-9.829121287	8.43515E-23	2.19117E-21	negative
PRJNA729271	stage_E14_vs_E10	ordinary_muscle_development	ENSGALG00010011190	ENSGALG00010011190	-0.09591123	0.210755595	-0.455082721	0.649049728	0.740785497	negative
PRJNA729271	stage_E14_vs_E10	ordinary_muscle_development	ENSGALG00010011192	RPL17	0.424413639	0.12346707	3.437464244	0.000587188	0.001953924	positive
PRJNA729271	stage_E14_vs_E10	ordinary_muscle_development	ENSGALG00010011193	FAM20B	-0.427156822	0.072137432	-5.921430944	3.19152E-09	2.47121E-08	negative
PRJNA729271	stage_E14_vs_E10	ordinary_muscle_development	ENSGALG00010011198	PINX1	-0.03430103	0.0626587	-0.54742645	0.584085788	0.684930944	negative
PRJNA729271	stage_E14_vs_E10	ordinary_muscle_development	ENSGALG00010011199	ENSGALG00010011199	-4.973107133	1.718897299	-2.893196199	0.00381343	0.010249521	negative
PRJNA729271	stage_E14_vs_E10	ordinary_muscle_development	ENSGALG00010011200	PCDHB4	-0.086102143	0.57236978	-0.150430973	0.880424606	0.917847718	negative
PRJNA729271	stage_E14_vs_E10	ordinary_muscle_development	ENSGALG00010011203	ENSGALG00010011203	-0.242860265	0.103478395	-2.3469659	0.018926987	0.041430329	negative
PRJNA729271	stage_E14_vs_E10	ordinary_muscle_development	ENSGALG00010011205	CYTL1	-4.748653969	0.828604532	-5.730905139	9.98961E-09	7.28001E-08	negative
PRJNA729271	stage_E14_vs_E10	ordinary_muscle_development	ENSGALG00010011210	NUP153	-0.156099775	0.097169577	-1.606467577	0.108171213	0.181769272	negative
PRJNA729271	stage_E14_vs_E10	ordinary_muscle_development	ENSGALG00010011211	SLC37A1	0.46645515	0.149144759	3.127532961	0.001762801	0.005215155	positive
PRJNA729271	stage_E14_vs_E10	ordinary_muscle_development	ENSGALG00010011212	NCKAP1	-0.093153439	0.062573921	-1.488694293	0.136567889	0.21985246	negative
PRJNA729271	stage_E14_vs_E10	ordinary_muscle_development	ENSGALG00010011213	XKR6	0.063266361	0.176402741	0.358647266	0.719858982	0.797333104	positive
PRJNA729271	stage_E14_vs_E10	ordinary_muscle_development	ENSGALG00010011218	ENSGALG00010011218	-0.325828875	0.570935004	-0.570693463	0.568207451	0.670697638	negative
PRJNA729271	stage_E14_vs_E10	ordinary_muscle_development	ENSGALG00010011222	RCL1	-0.020926903	0.088224772	-0.237199848	0.812501754	0.868526027	negative
PRJNA729271	stage_E14_vs_E10	ordinary_muscle_development	ENSGALG00010011231	PLBD1	-0.102619462	0.205923416	-0.498337994	0.618245833	0.714061621	negative
PRJNA729271	stage_E14_vs_E10	ordinary_muscle_development	ENSGALG00010011235	ENSGALG00010011235	-0.217085967	0.090213777	-2.406350485	0.016112798	0.036081416	negative
PRJNA729271	stage_E14_vs_E10	ordinary_muscle_development	ENSGALG00010011237	ENSGALG00010011237	0.018658792	0.252313137	0.073950933	0.941049428	0.958622161	positive
PRJNA729271	stage_E14_vs_E10	ordinary_muscle_development	ENSGALG00010011239	RALGPS2	0.541272149	0.072999611	7.414726435	1.21876E-13	1.52687E-12	positive
PRJNA729271	stage_E14_vs_E10	ordinary_muscle_development	ENSGALG00010011243	ALX1	-3.832969034	0.50033976	-7.660732454	1.84876E-14	2.49513E-13	negative
PRJNA729271	stage_E14_vs_E10	ordinary_muscle_development	ENSGALG00010011244	PDE9A	-0.364168711	0.166159797	-2.191677637	0.028402792	0.058688306	negative
PRJNA729271	stage_E14_vs_E10	ordinary_muscle_development	ENSGALG00010011246	DNAJB5	0.936427636	0.233457437	4.01112788	6.04294E-05	0.000247631	positive
PRJNA729271	stage_E14_vs_E10	ordinary_muscle_development	ENSGALG00010011247	ENSGALG00010011247	-0.190914908	0.093865859	-2.033912122	0.041960457	0.081585086	negative
PRJNA729271	stage_E14_vs_E10	ordinary_muscle_development	ENSGALG00010011250	ENSGALG00010011250	-0.397670823	0.099143204	-4.01107496	6.04429E-05	0.000247631	negative
PRJNA729271	stage_E14_vs_E10	ordinary_muscle_development	ENSGALG00010011251	FRZB	0.065122341	0.175199442	0.371704043	0.710113215	0.789892644	positive
PRJNA729271	stage_E14_vs_E10	ordinary_muscle_development	ENSGALG00010011253	KDM4C	0.322788303	0.108296836	2.980588496	0.002876951	0.008005645	positive
PRJNA729271	stage_E14_vs_E10	ordinary_muscle_development	ENSGALG00010011262	ENSGALG00010011262	-0.14245347	0.197601453	-0.720913073	0.470962999	0.583224783	negative
PRJNA729271	stage_E14_vs_E10	ordinary_muscle_development	ENSGALG00010011267	EVC2	-0.784032916	0.163935691	-4.782563896	1.73073E-06	9.2186E-06	negative
PRJNA729271	stage_E14_vs_E10	ordinary_muscle_development	ENSGALG00010011271	ANGPTL1	-1.302960719	0.160310955	-8.127708549	4.37482E-16	6.74606E-15	negative
PRJNA729271	stage_E14_vs_E10	ordinary_muscle_development	ENSGALG00010011273	RUNX1T1	-0.38112483	0.097792852	-3.897266758	9.72844E-05	0.00038319	negative
PRJNA729271	stage_E14_vs_E10	ordinary_muscle_development	ENSGALG00010011274	ENSGALG00010011274	0.026159174	0.514179675	0.05087555	0.959424688	0.972099878	positive
PRJNA729271	stage_E14_vs_E10	ordinary_muscle_development	ENSGALG00010011275	WDR4	-0.370213682	0.102540879	-3.610400902	0.000305724	0.001093111	negative
PRJNA729271	stage_E14_vs_E10	ordinary_muscle_development	ENSGALG00010011278	ENSGALG00010011278	1.484915757	0.523997741	2.83382091	0.00459951	0.01209541	positive
PRJNA729271	stage_E14_vs_E10	ordinary_muscle_development	ENSGALG00010011280	FAM199X	-0.278446758	0.080604733	-3.454471549	0.000551372	0.001847492	negative
PRJNA729271	stage_E14_vs_E10	ordinary_muscle_development	ENSGALG00010011281	ENSGALG00010011281	0.403958223	0.582762729	0.693177863	0.488197939	0.599240336	positive
PRJNA729271	stage_E14_vs_E10	ordinary_muscle_development	ENSGALG00010011282	CRY1	0.014927435	0.127149299	0.117400848	0.906542412	0.935854445	positive
PRJNA729271	stage_E14_vs_E10	ordinary_muscle_development	ENSGALG00010011283	LONRF3	0.293230692	0.142760275	2.054007614	0.039974955	0.07825999	positive
PRJNA729271	stage_E14_vs_E10	ordinary_muscle_development	ENSGALG00010011285	ENSGALG00010011285	0.50235884	0.271413619	1.850897693	0.064184273	0.117172674	positive
PRJNA729271	stage_E14_vs_E10	ordinary_muscle_development	ENSGALG00010011287	ENSGALG00010011287	-0.497588626	0.117476505	-4.235643778	2.27898E-05	0.000100502	negative
PRJNA729271	stage_E14_vs_E10	ordinary_muscle_development	ENSGALG00010011288	ENSGALG00010011288	0.099008319	0.286382058	0.345721098	0.729552327	0.805090292	positive
PRJNA729271	stage_E14_vs_E10	ordinary_muscle_development	ENSGALG00010011293	NDUFV3	0.394876959	0.167611017	2.355912904	0.018477249	0.040573812	positive
PRJNA729271	stage_E14_vs_E10	ordinary_muscle_development	ENSGALG00010011295	FAM167A	-1.714254513	0.38426816	-4.461089134	8.15442E-06	3.88299E-05	negative
PRJNA729271	stage_E14_vs_E10	ordinary_muscle_development	ENSGALG00010011297	OSR2	-0.159768619	0.183622094	-0.870094745	0.384248629	0.500285986	negative
PRJNA729271	stage_E14_vs_E10	ordinary_muscle_development	ENSGALG00010011300	EVC	-0.544271912	0.160931094	-3.382018341	0.000719553	0.002349074	negative
PRJNA729271	stage_E14_vs_E10	ordinary_muscle_development	ENSGALG00010011304	COMMD5	-0.086630747	0.090652309	-0.955637512	0.339255403	0.454315139	negative
PRJNA729271	stage_E14_vs_E10	ordinary_muscle_development	ENSGALG00010011306	ECI2	-0.09523059	0.13815557	-0.689299679	0.4906347	0.601060681	negative
PRJNA729271	stage_E14_vs_E10	ordinary_muscle_development	ENSGALG00010011307	ENSGALG00010011307	-0.743162031	0.456072737	-1.629481376	0.103211154	0.174669665	negative
PRJNA729271	stage_E14_vs_E10	ordinary_muscle_development	ENSGALG00010011308	SPIC	0.496537971	1.578162927	0.314630361	0.753042325	0.823240704	positive
PRJNA729271	stage_E14_vs_E10	ordinary_muscle_development	ENSGALG00010011311	DOCK8	-0.345488317	0.19113658	-1.807546818	0.070677062	0.127215201	negative
PRJNA729271	stage_E14_vs_E10	ordinary_muscle_development	ENSGALG00010011312	VPS13B	0.03082024	0.052180631	0.590645213	0.554758164	0.659460492	positive
PRJNA729271	stage_E14_vs_E10	ordinary_muscle_development	ENSGALG00010011313	RASSF9	-1.684497637	0.253739532	-6.638688205	3.16487E-11	3.07885E-10	negative
PRJNA729271	stage_E14_vs_E10	ordinary_muscle_development	ENSGALG00010011316	PARPBP	-0.256624353	0.184274159	-1.392622567	0.163733955	0.25459383	negative
PRJNA729271	stage_E14_vs_E10	ordinary_muscle_development	ENSGALG00010011317	BLK	0.110485675	0.371108072	0.297718329	0.765918153	0.83360742	positive

PRJNA729271	stage_E14_vs_E10	ordinary_muscle_development	ENSGALG00010011318	PRP4K	-0.277914707	0.097414999	-2.85289441	0.004332301	0.01147391	negative
PRJNA729271	stage_E14_vs_E10	ordinary_muscle_development	ENSGALG00010011321	MAP2	-0.866072165	0.147875224	-5.856776694	4.71937E-09	3.58341E-08	negative
PRJNA729271	stage_E14_vs_E10	ordinary_muscle_development	ENSGALG00010011322	GATA4	-2.925253002	1.243577197	-2.352289032	0.01865827	0.040909338	negative
PRJNA729271	stage_E14_vs_E10	ordinary_muscle_development	ENSGALG00010011323	GUCY2C	-2.506295778	0.944682312	-2.653056742	0.007976646	0.019583585	negative
PRJNA729271	stage_E14_vs_E10	ordinary_muscle_development	ENSGALG00010011326	COX6C	0.30360546	0.135545639	2.239876274	0.025098956	0.052910031	positive
PRJNA729271	stage_E14_vs_E10	ordinary_muscle_development	ENSGALG00010011329	PCDHGC5	-0.0364808	0.116721634	-0.312545317	0.754626129	0.824680161	negative
PRJNA729271	stage_E14_vs_E10	ordinary_muscle_development	ENSGALG00010011333	ENSGALG00010011333	1.189187388	0.129744872	9.165582951	4.92799E-20	1.06455E-18	positive
PRJNA729271	stage_E14_vs_E10	ordinary_muscle_development	ENSGALG00010011335	VCP	-0.036575056	0.17366461	-0.210607426	0.833193617	0.883790828	negative
PRJNA729271	stage_E14_vs_E10	ordinary_muscle_development	ENSGALG00010011339	ENSGALG00010011339	-0.007600163	0.291771747	-0.026048318	0.979218799	0.986210423	negative
PRJNA729271	stage_E14_vs_E10	ordinary_muscle_development	ENSGALG00010011340	PKNOX1	0.134223156	0.107235162	1.251671132	0.210689723	0.311722725	positive
PRJNA729271	stage_E14_vs_E10	ordinary_muscle_development	ENSGALG00010011342	RASAL2	0.895924982	0.064714131	13.84434844	1.37623E-43	1.3299E-41	positive
PRJNA729271	stage_E14_vs_E10	ordinary_muscle_development	ENSGALG00010011343	PXDC1	-0.632188424	0.140531205	-4.498562625	6.84144E-06	3.30556E-05	negative
PRJNA729271	stage_E14_vs_E10	ordinary_muscle_development	ENSGALG00010011345	CRMP1	-1.163242781	0.223543787	-5.203646204	1.95416E-07	1.19668E-06	negative
PRJNA729271	stage_E14_vs_E10	ordinary_muscle_development	ENSGALG00010011347	RGS22	-2.757904068	0.436170058	-6.323001818	2.5653E-10	2.25631E-09	negative
PRJNA729271	stage_E14_vs_E10	ordinary_muscle_development	ENSGALG00010011348	SLC22A23	-0.525931835	0.163166925	-3.223274793	0.001267339	0.003901058	negative
PRJNA729271	stage_E14_vs_E10	ordinary_muscle_development	ENSGALG00010011349	ENSGALG00010011349	-0.370767331	0.088251646	-4.201251153	2.65444E-05	0.000115892	negative
PRJNA729271	stage_E14_vs_E10	ordinary_muscle_development	ENSGALG00010011351	KANK1	-0.094344988	0.108118912	-0.872603934	0.382878996	0.498906063	negative
PRJNA729271	stage_E14_vs_E10	ordinary_muscle_development	ENSGALG00010011352	NTS	-2.804649946	0.605761152	-4.629960073	3.65736E-06	1.83967E-05	negative
PRJNA729271	stage_E14_vs_E10	ordinary_muscle_development	ENSGALG00010011353	ENSGALG00010011353	-3.212952377	0.212654477	-15.10879253	1.41713E-51	2.11766E-49	negative
PRJNA729271	stage_E14_vs_E10	ordinary_muscle_development	ENSGALG00010011357	UNC80	-0.531770338	0.410148759	-1.296530407	0.194792811	0.292896452	negative
PRJNA729271	stage_E14_vs_E10	ordinary_muscle_development	ENSGALG00010011359	RNF19A	0.215175704	0.086015094	2.501604013	0.012363212	0.028695718	positive
PRJNA729271	stage_E14_vs_E10	ordinary_muscle_development	ENSGALG00010011360	TCF4	0.038584366	0.17133038	0.225204463	0.821820218	0.875323981	positive
PRJNA729271	stage_E14_vs_E10	ordinary_muscle_development	ENSGALG00010011361	NUP37	-0.298003405	0.088263717	-3.376284316	0.00073472	0.002393742	negative
PRJNA729271	stage_E14_vs_E10	ordinary_muscle_development	ENSGALG00010011363	FDFT1	0.097737381	0.146464904	0.667309218	0.50457464	0.613610182	positive
PRJNA729271	stage_E14_vs_E10	ordinary_muscle_development	ENSGALG00010011365	PSMG4	0.395031306	0.177584682	2.22446723	0.026117025	0.054714016	positive
PRJNA729271	stage_E14_vs_E10	ordinary_muscle_development	ENSGALG00010011367	IER3IP1	0.425470768	0.111716992	3.808469617	0.00013983	0.000536766	positive
PRJNA729271	stage_E14_vs_E10	ordinary_muscle_development	ENSGALG00010011368	ENSGALG00010011368	2.337901821	0.254800139	9.175433848	4.4976E-20	9.75938E-19	positive
PRJNA729271	stage_E14_vs_E10	ordinary_muscle_development	ENSGALG00010011370	MGAT4C	-4.212448941	1.521227821	-2.769111163	0.005620945	0.014420461	negative
PRJNA729271	stage_E14_vs_E10	ordinary_muscle_development	ENSGALG00010011371	ENSGALG00010011371	0.829336472	0.191090506	4.340019232	1.4247E-05	6.51864E-05	positive
PRJNA729271	stage_E14_vs_E10	ordinary_muscle_development	ENSGALG00010011372	VMA21	-0.182191848	0.091958237	-1.981245547	0.047563744	0.090954679	negative
PRJNA729271	stage_E14_vs_E10	ordinary_muscle_development	ENSGALG00010011374	ENSGALG00010011374	0.302502807	0.09092421	3.326977578	0.000877934	0.002802391	positive
PRJNA729271	stage_E14_vs_E10	ordinary_muscle_development	ENSGALG00010011375	C12orf50	-0.15841663	0.137448672	-1.152551188	0.249094648	0.356112726	negative
PRJNA729271	stage_E14_vs_E10	ordinary_muscle_development	ENSGALG00010011377	SEC16B	1.567761421	0.287391894	5.455134443	4.89358E-08	3.25055E-07	positive
PRJNA729271	stage_E14_vs_E10	ordinary_muscle_development	ENSGALG00010011378	TUBB2B	0.348680109	0.190591335	1.829464642	0.067330031	0.121993601	positive
PRJNA729271	stage_E14_vs_E10	ordinary_muscle_development	ENSGALG00010011379	CELF4	0.286914233	0.414368217	0.692413707	0.48867756	0.599575185	positive
PRJNA729271	stage_E14_vs_E10	ordinary_muscle_development	ENSGALG00010011381	RLIG1	-0.179102727	0.115645504	-1.548721921	0.121448582	0.199568892	negative
PRJNA729271	stage_E14_vs_E10	ordinary_muscle_development	ENSGALG00010011382	ENSGALG00010011382	0.018203221	0.192407542	0.094607628	0.924626491	0.948147551	positive
PRJNA729271	stage_E14_vs_E10	ordinary_muscle_development	ENSGALG00010011384	PUM3	-0.467631783	0.079727661	-5.865364341	4.48148E-09	3.4153E-08	negative
PRJNA729271	stage_E14_vs_E10	ordinary_muscle_development	ENSGALG00010011386	CTSB	0.219174572	0.12626072	1.735888815	0.082583496	0.145229598	positive
PRJNA729271	stage_E14_vs_E10	ordinary_muscle_development	ENSGALG00010011388	GPR50	-0.317850368	0.163325535	-1.946115573	0.051640858	0.097440021	negative
PRJNA729271	stage_E14_vs_E10	ordinary_muscle_development	ENSGALG00010011389	RC3H1	0.155691671	0.085619755	1.818408259	0.069001757	0.124586506	positive
PRJNA729271	stage_E14_vs_E10	ordinary_muscle_development	ENSGALG00010011394	SERPINC1	0.91722643	0.924946669	0.991653315	0.321366672	0.43587629	positive
PRJNA729271	stage_E14_vs_E10	ordinary_muscle_development	ENSGALG00010011398	ENSGALG00010011398	0.162242592	0.183177328	0.885713281	0.37577205	0.49198951	positive
PRJNA729271	stage_E14_vs_E10	ordinary_muscle_development	ENSGALG00010011399	JAKMIP1	2.320677708	0.240340149	9.655805415	4.64495E-22	1.15884E-20	positive
PRJNA729271	stage_E14_vs_E10	ordinary_muscle_development	ENSGALG00010011400	BPHL	-0.16372195	0.200671415	-0.81587081	0.414574029	0.529356109	negative
PRJNA729271	stage_E14_vs_E10	ordinary_muscle_development	ENSGALG00010011401	ANKRD46	-0.112352899	0.066791654	-1.682139791	0.092541736	0.159480735	negative
PRJNA729271	stage_E14_vs_E10	ordinary_muscle_development	ENSGALG00010011403	SV2C	-1.093815559	0.484579931	-2.25724486	0.023992779	0.050963559	negative
PRJNA729271	stage_E14_vs_E10	ordinary_muscle_development	ENSGALG00010011406	UBE2R2	0.009456825	0.11501839	0.082220112	0.934471681	0.954557224	positive
PRJNA729271	stage_E14_vs_E10	ordinary_muscle_development	ENSGALG00010011407	CEP290	-0.691440332	0.179424285	-3.853660785	0.000116365	0.000453294	negative
PRJNA729271	stage_E14_vs_E10	ordinary_muscle_development	ENSGALG00010011411	ENSGALG00010011411	-0.289163341	0.207665198	-1.392449695	0.163786265	0.25464785	negative
PRJNA729271	stage_E14_vs_E10	ordinary_muscle_development	ENSGALG00010011412	KANSL1L	0.328624646	0.119988073	2.738810925	0.006166182	0.015628399	positive
PRJNA729271	stage_E14_vs_E10	ordinary_muscle_development	ENSGALG00010011413	ZBTB37	0.604196691	0.103341364	5.846610358	5.01691E-09	3.80065E-08	positive
PRJNA729271	stage_E14_vs_E10	ordinary_muscle_development	ENSGALG00010011414	PABPC1	-0.01573744	0.114080504	-0.137950301	0.890279696	0.924683995	negative
PRJNA729271	stage_E14_vs_E10	ordinary_muscle_development	ENSGALG00010011416	ENSGALG00010011416	0.882302469	0.425504323	2.073545253	0.038121564	0.07522081	positive
PRJNA729271	stage_E14_vs_E10	ordinary_muscle_development	ENSGALG00010011417	WFS1	0.600253784	0.144173276	4.163419203	3.13517E-05	0.000135331	positive
PRJNA729271	stage_E14_vs_E10	ordinary_muscle_development	ENSGALG00010011418	RIPK1	-0.426158673	0.084378519	-5.050558805	4.40519E-07	2.56336E-06	negative
PRJNA729271	stage_E14_vs_E10	ordinary_muscle_development	ENSGALG00010011419	RNF180	0.235539929	0.146876939	1.603654936	0.108790134	0.182639912	positive
PRJNA729271	stage_E14_vs_E10	ordinary_muscle_development	ENSGALG00010011420	HMGB3	-0.314758075	0.099054059	-3.177639339	0.001484793	0.004480529	negative
PRJNA729271	stage_E14_vs_E10	ordinary_muscle_development	ENSGALG00010011421	ENSGALG00010011421	0.266028179	0.116887337	2.275936696	0.022849799	0.048850713	positive
PRJNA729271	stage_E14_vs_E10	ordinary_muscle_development	ENSGALG00010011422	KATNAL2	-0.203794847	0.205318404	-0.99257954	0.3209149	0.435426516	negative
PRJNA729271	stage_E14_vs_E10	ordinary_muscle_development	ENSGALG00010011423	GDF6	-2.69062332	0.41509393	-6.48196258	9.05371E-11	8.33758E-10	negative
PRJNA729271	stage_E14_vs_E10	ordinary_muscle_development	ENSGALG00010011424	ACADL	0.714362694	0.097118339	7.355590143	1.90085E-13	2.33895E-12	positive
PRJNA729271	stage_E14_vs_E10	ordinary_muscle_development	ENSGALG00010011425	WASHC3	-0.342640741	0.079249071	-4.323593165	1.53508E-05	6.984E-05	negative
PRJNA729271	stage_E14_vs_E10	ordinary_muscle_development	ENSGALG00010011426	NQO2	0.579563395	0.126078353	4.596850925	4.28924E-06	2.14093E-05	positive
PRJNA729271	stage_E14_vs_E10	ordinary_muscle_development	ENSGALG00010011427	TMTC3	-0.207249371	0.086832646	-2.38676788	0.016997219	0.037804845	negative
PRJNA729271	stage_E14_vs_E10	ordinary_muscle_development	ENSGALG00010011428	UQCRB	0.113599876	0.163022283	0.696836491	0.485905126	0.597235208	positive
PRJNA729271	stage_E14_vs_E10	ordinary_muscle_development	ENSGALG00010011429	UBAP2	0.05452195	0.106944066	0.509817435	0.61017937	0.707394223	positive
PRJNA729271	stage_E14_vs_E10	ordinary_muscle_development	ENSGALG00010011430	ENSGALG00010011430	-0.398972344	0.329998057	-1.209014221	0.226657382	0.329991839	negative
PRJNA729271	stage_E14_vs_E10	ordinary_muscle_development	ENSGALG00010011431	DRAM1	-0.392426711	0.436071233	-0.899914236	0.368165893	0.48439363	negative
PRJNA729271	stage_E14_vs_E10	ordinary_muscle_development	ENSGALG00010011433	RNASE6	1.876088313	0.339147312	5.531779989	3.16997E-08	2.17664E-07	positive
PRJNA729271	stage_E14_vs_E10	ordinary_muscle_development	ENSGALG00010011434	NPL	0.450292041	0.179780636	2.504674868	0.0122564	0.028484291	positive
PRJNA729271	stage_E14_vs_E10	ordinary_muscle_development	ENSGALG00010011436	UBAP2L2	2.690499213	2.23679981	1.202834157			positive
PRJNA729271	stage_E14_vs_E10	ordinary_muscle_development	ENSGALG00010011437	DMRT1	-0.257656651	0.486519531	-0.529591588	0.596395128	0.696000712	negative
PRJNA729271	stage_E14_vs_E10	ordinary_muscle_development	ENSGALG00010011438	ENSGALG00010011438	-0.249799683	0.463927688	-0.5384453	0.590269659	0.690275007	negative
PRJNA729271	stage_E14_vs_E10	ordinary_muscle_development	ENSGALG00010011439	ENSGALG00010011439	-0.197568837	0.313638105	-0.629926128	0.528742917	0.636238468	negative
PRJNA729271	stage_E14_vs_E10	ordinary_muscle_development	ENSGALG00010011440	FUNDC1	-0.332064945	0.11149106	-2.978399739	0.002897578	0.008056857	negative
PRJNA729271	stage_E14_vs_E10	ordinary_muscle_development	ENSGALG00010011441	CD99L2	-0.143545601	0.080030186	-1.793643226	0.072870132	0.130530471	negative
PRJNA729271	stage_E14_vs_E10	ordinary_muscle_development	ENSGALG00010011442	ENSGALG00010011442	2.502212415	2.049968971	1.220609897	0.222233757	0.324954939	positive
PRJNA729271	stage_E14_vs_E10	ordinary_muscle_development	ENSGALG00010011443	ENSGALG00010011443	1.19418706	0.16124533	7.40602569	1.30141E-13	1.6262E-12	positive
PRJNA729271	stage_E14_vs_E10	ordinary_muscle_development	ENSGALG00010011445	MTERF3	-0.131193812	0.079531224	-1.649588753	0.099027077	0.168731337	negative
PRJNA729271	stage_E14_vs_E10	ordinary_muscle_development	ENSGALG00010011446	ENSGALG00010011446	-0.155959018	0.221996744	-0.702528405	0.482349694	0.593973223	negative
PRJNA729271	stage_E14_vs_E10	ordinary_muscle_development	ENSGALG00010011447	LAMC1	0.855483164	0.130345048	6.563219536	5.26582E-11	5.02159E-10	positive
PRJNA729271	stage_E14_vs_E10	ordinary_muscle_development	ENSGALG00010011448	AvBD10	-0.234319996	2.732083914	-0.085766032	0.93165241	0.953157721	negative
PRJNA729271	stage_E14_vs_E10	ordinary_muscle_development	ENSGALG00010011449	DUSP22	0.66153576	0.14449543	4.578246955	4.68889E-06	2.3252E-05	positive
PRJNA729271	stage_E14_vs_E10	ordinary_muscle_development	ENSGALG00010011450	PTDSS1	-0.34862494	0.067031007	-5.200950351	1.98272E-07	1.21264E-06	negative
PRJNA729271	stage_E14_vs_E10	ordinary_muscle_development	ENSGALG00010011451	CRHBP	-4.575100715	0.489691629	-9.342819931	9.38019E-21	2.12115E-19	negative
PRJNA729271	stage_E14_vs_E10	ordinary_muscle_development	ENSGALG00010011452	KITLG	-0.132924738	0.18977023	-0.700450947	0.483645729	0.595114163	negative
PRJNA729271	stage_E14_vs_E10	ordinary_muscle_development	ENSGALG00010011453	GNPTAB	-0.614398965	0.124458017	-4.936596131	7.94979E-07	4.44414E-06	negative
PRJNA729271	stage_E14_vs_E10	ordinary_muscle_development	ENSGALG00010011456	KIAA1328	-0.23861014	0.09166679	-2.603016212	0.009240758	0.022235191	negative
PRJNA729271	stage_E14_vs_E10	ordinary_muscle_development	ENSGALG00010011457	ENSGALG00010011457	-0.35247806	0.279241701	-1.262268705	0.206852114	0.307016321	negative
PRJNA729271	stage_E14_vs_E10	ordinary_muscle_development	ENSGALG00010011458	TPGS2	-0.678121723	0.124678705	-5.438953853	5.35943E-08	3.53918E-07	negative
PRJNA729271	stage_E14_vs_E10	ordinary_muscle_development	ENSGALG00010011459	ENSGALG00010011459	-0.047633194	0.273805617	-0.17396719	0.861891248	0.904839113	negative
PRJNA729271	stage_E14_vs_E10	ordinary_muscle_development	ENSGALG00010011460	LAMC2	-0.788525476	0.585665552	-1.346375031	0.178181599	0.272469911	negative
PRJNA729271	stage_E14_vs_E10	ordinary_muscle_development	ENSGALG00010011461	IRF4	0.388210307	0.353931483	1.096851582	0.272706277	0.382808202	positive
PRJNA729271	stage_E14_vs_E10	ordinary_muscle_development	ENSGALG00010011463	IMP3	0.135301929	0.124501044	1.086753368	0.277145822	0.387688543	positive
PRJNA729271	stage_E14_vs_E10	ordinary_muscle_development	ENSGALG00010011464	FANCG	0.036557304	0.149609682	0.24435119	0.806958841	0.864513555	positive
PRJNA729271	stage_E14_vs_E10	ordinary_muscle_development	ENSGALG00010011465	PPP2R2C	1.499937501	0.332888536	4.50582504	6.61155E-06	3.19982E-05	positive
PRJNA729271	stage_E14_vs_E10	ordinary_muscle_development	ENSGALG00010011466	MTMR1	-0.175344289	0.065630223	-2.671700331	0.007546801	0.018626065	negative
PRJNA729271	stage_E14_vs_E10	ordinary_muscle_development	ENSGALG00010011467	ENSGALG00010011467	3.246478695	3.967765024	0.818213446			positive
PRJNA729271	stage_E14_vs_E10	ordinary_muscle_development	ENSGALG00010011468	EFHC2	0.635902313	0.246137269	2.583527137	0.009779577	0.023380335	positive
PRJNA729271	stage_E14_vs_E10	ordinary_muscle_development	ENSGALG00010011469	CPS1	0.84045076	0.331331897	2.536582704	0.01119403	0.026319134	positive
PRJNA729271	stage_E14_vs_E10	ordinary_muscle_development	ENSGALG00010011471	NMNAT2	-2.306609626	0.385418125	-5.984694222	2.16797E-09	1.71065E-08	negative
PRJNA729271	stage_E14_vs_E10	ordinary_muscle_development	ENSGALG00010011475	NDP	-2.418817215	0.304376653	-7.946789594	1.91407E-15	2.79964E-14	negative
PRJNA729271	stage_E14_vs_E10	ordinary_muscle_development	ENSGALG00010011477	SDC2	-0.157714959	0.16724238	-0.943032256	0.345664404	0.461150993	negative
PRJNA729271	stage_E14_vs_E10	ordinary_muscle_development	ENSGALG00010011478	HDAC3	-0.117740832	0.075384486	-1.561870852	0.118318416	0.195431931	negative
PRJNA729271	stage_E14_vs_E10	ordinary_muscle_development	ENSGALG00010011480	BLTP3B	-0.471176107	0.064000362	-7.362084988	1.8106E-13	2.23358E-12	negative
PRJNA729271	stage_E14_vs_E10	ordinary_muscle_development	ENSGALG00010011482	DMRT2	1.019580636	0.146113005	6.978027987	2.99352E-12	3.26494E-11	positive
PRJNA729271	stage_E14_vs_E10	ordinary_muscle_development	ENSGALG00010011484	CHPT1	0.083058748	0.078417745	1.059183078	0.28951641	0.401967468	positive
PRJNA729271	stage_E14_vs_E10	ordinary_muscle_development	ENSGALG00010011485	MTM1	0.033874545	0.076940471	0.440269527	0.659741909	0.749154639	positive
PRJNA729271	stage_E14_vs_E10	ordinary_muscle_development	ENSGALG00010011487	MAN2B2	-0.266720141	0.157975811	-1.688360636	0.091342022	0.157769615	negative
PRJNA729271	stage_E14_vs_E10	ordinary_muscle_development	ENSGALG00010011493	ENSGALG00010011493	0.138677204	0.26559294	0.522141907	0.60157153	0.700215155	positive
PRJNA729271	stage_E14_vs_E10	ordinary_muscle_development	ENSGALG00010011496	ACTR6	-0.314798213	0.085445941	-3.684179846	0.00022944	0.000839625	negative
PRJNA729271	stage_E14_vs_E10	ordinary_muscle_development	ENSGALG00010011497	CPQ	0.679547526	0.248183333	2.738086869	0.006179775	0.015646435	positive
PRJNA729271	stage_E14_vs_E10	ordinary_muscle_development	ENSGALG00010011499	SMG7	-0.099930493	0.146333243	-0.68289673	0.4946721	0.604640808	negative
PRJNA729271	stage_E14_vs_E10	ordinary_muscle_development	ENSGALG00010011503	NCF2	0.515510011	0.376327885	1.369842711	0.170736006	0.263266399	positive
PRJNA729271	stage_E14_vs_E10	ordinary_muscle_development	ENSGALG00010011505	MYL1	1.94100007	0.152278473	12.74638521	3.26539E-37	2.16127E-35	positive
PRJNA729271	stage_E14_vs_E10	ordinary_muscle_development	ENSGALG00010011509	EXOC2	0.102814888	0.066495507	1.546193005	0.122057962	0.200365817	positive
PRJNA729271	stage_E14_vs_E10	ordinary_muscle_development	ENSGALG00010011510	ENSGALG00010011510	0.3368625	0.261521361	1.288087898	0.197715368	0.296246047	positive
PRJNA729271	stage_E14_vs_E10	ordinary_muscle_development	ENSGALG00010011515	SERINC5	0.362750077	0.084997752	4.267760816	1.97445E-05	8.79251E-05	positive
PRJNA729271	stage_E14_vs_E10	ordinary_muscle_development	ENSGALG00010011523	PIGO	-0.085557761	0.118946349	-0.719297081	0.471957894	0.584148184	negative
PRJNA729271	stage_E14_vs_E10	ordinary_muscle_development	ENSGALG00010011525	ENSGALG00010011525	-3.054044292	0.25588436	-11.9352519	7.75239E-33	3.86154E-31	negative
PRJNA729271	stage_E14_vs_E10	ordinary_muscle_development	ENSGALG00010011529	SCYL2	-0.012013893	0.081788038	-0.146890589	0.88321838	0.919966256	negative
PRJNA729271	stage_E14_vs_E10	ordinary_muscle_development	ENSGALG00010011530	ARPC5	-0.088163972	0.119485153	-0.737865501	0.460596185	0.57317494	negative
PRJNA729271	stage_E14_vs_E10	ordinary_muscle_development	ENSGALG00010011531	MTDH	0.246923592	0.071689166	3.444364123	0.000572404	0.001911311	positive
PRJNA729271	stage_E14_vs_E10	ordinary_muscle_development	ENSGALG00010011533	ENSGALG00010011533	-1.066093626	0.273538942	-3.897410801	9.72266E-05	0.000383066	negative
PRJNA729271	stage_E14_vs_E10	ordinary_muscle_development	ENSGALG00010011536	FCHSD1	-1.080818878	0.173746051	-6.220681677	4.95E-10	4.21564E-09	negative
PRJNA729271	stage_E14_vs_E10	ordinary_muscle_development	ENSGALG00010011538	ENSGALG00010011538	0.58776211	0.258280689	2.275671918	0.022865653	0.048870192	positive
PRJNA729271	stage_E14_vs_E10	ordinary_muscle_development	ENSGALG00010011539	LANCL1	0.194916188	0.105682398	1.844358116	0.065130967	0.118706572	positive
PRJNA729271	stage_E14_vs_E10	ordinary_muscle_development	ENSGALG00010011544	STOML2	0.001471298	0.090997221	0.0161686	0.987099886	0.991389635	positive
PRJNA729271	stage_E14_vs_E10	ordinary_muscle_development	ENSGALG00010011545	USP12-like	-0.185031939	0.075153556	-2.462051683	0.013814476	0.031598679	negative
PRJNA729271	stage_E14_vs_E10	ordinary_muscle_development	ENSGALG00010011546	MAOA	1.460707168	0.126005578	11.59240087	4.5033E-31	2.03985E-29	positive

PRJNA729271	stage_E14_vs_E10	ordinary_muscle_development	ENSGALG00010011549	KIAA0232	0.432527085	0.143730004	3.009302663	0.002618481	0.00736559	positive
PRJNA729271	stage_E14_vs_E10	ordinary_muscle_development	ENSGALG00010011551	GMDS	-0.826406368	0.152270924	-5.427210553	5.72416E-08	3.7646E-07	negative
PRJNA729271	stage_E14_vs_E10	ordinary_muscle_development	ENSGALG00010011555	ENSGALG00010011555	2.387538618	4.208931852	0.567255233	0.570540787	0.672678324	positive
PRJNA729271	stage_E14_vs_E10	ordinary_muscle_development	ENSGALG00010011556	LAPTM4B	1.78896248	0.142474089	12.55640584	3.66611E-36	2.18684E-34	positive
PRJNA729271	stage_E14_vs_E10	ordinary_muscle_development	ENSGALG00010011558	MYBPC1	2.990922637	0.55984243	5.342436508			positive
PRJNA729271	stage_E14_vs_E10	ordinary_muscle_development	ENSGALG00010011559	MTX3	-0.297002572	0.126311374	-2.351352564	0.018705301	0.040993852	negative
PRJNA729271	stage_E14_vs_E10	ordinary_muscle_development	ENSGALG00010011562	ENSGALG00010011562	-3.605512133	0.364681474	-9.88674333	4.75237E-23	1.24567E-21	negative
PRJNA729271	stage_E14_vs_E10	ordinary_muscle_development	ENSGALG00010011564	ARAP3	0.503699613	0.121776729	4.136255082	3.5302E-05	0.000150684	positive
PRJNA729271	stage_E14_vs_E10	ordinary_muscle_development	ENSGALG00010011570	RGL1	-0.480830286	0.136428542	-3.52441123	0.000424425	0.001460598	negative
PRJNA729271	stage_E14_vs_E10	ordinary_muscle_development	ENSGALG00010011571	ATOSB	0.587394301	0.113723171	5.165124182	2.40279E-07	1.4524E-06	positive
PRJNA729271	stage_E14_vs_E10	ordinary_muscle_development	ENSGALG00010011572	PTCD2	0.031417986	0.102431655	0.306721455	0.759055398	0.828252634	positive
PRJNA729271	stage_E14_vs_E10	ordinary_muscle_development	ENSGALG00010011573	CMAS	-0.54215777	0.178133226	-3.043552194	0.002338029	0.00667252	negative
PRJNA729271	stage_E14_vs_E10	ordinary_muscle_development	ENSGALG00010011574	GAS2L3	0.073780498	0.138247786	0.533683036	0.593560848	0.693396558	positive
PRJNA729271	stage_E14_vs_E10	ordinary_muscle_development	ENSGALG00010011575	ENSGALG00010011575	-1.00654212	0.431390221	-2.333252053	0.01963492	0.042778922	negative
PRJNA729271	stage_E14_vs_E10	ordinary_muscle_development	ENSGALG00010011577	ENSGALG00010011577	-0.051356703	0.159873194	-0.321233986	0.748033079	0.81924741	negative
PRJNA729271	stage_E14_vs_E10	ordinary_muscle_development	ENSGALG00010011578	MYLK4	1.50707758	0.328671702	4.585358493	4.53208E-06	2.25515E-05	positive
PRJNA729271	stage_E14_vs_E10	ordinary_muscle_development	ENSGALG00010011584	ENSGALG00010011584	-0.246629474	0.942823764	-0.261585976	0.793640657	0.85511197	negative
PRJNA729271	stage_E14_vs_E10	ordinary_muscle_development	ENSGALG00010011586	ENSGALG00010011586	1.707273209	0.252005328	6.774750457	1.24621E-11	1.27062E-10	positive
PRJNA729271	stage_E14_vs_E10	ordinary_muscle_development	ENSGALG00010011588	COLGALT2	0.265628425	0.195720342	1.357183535	0.174722901	0.268189176	positive
PRJNA729271	stage_E14_vs_E10	ordinary_muscle_development	ENSGALG00010011589	RERG	-0.645676597	0.22406738	-2.881618	0.003956391	0.01058079	negative
PRJNA729271	stage_E14_vs_E10	ordinary_muscle_development	ENSGALG00010011593	WRNIP1	-0.485716781	0.097153277	-4.999489429	5.74823E-07	3.27905E-06	negative
PRJNA729271	stage_E14_vs_E10	ordinary_muscle_development	ENSGALG00010011595	ENSGALG00010011595	-0.013773253	0.162798885	-0.084602873	0.932577115	0.953697283	negative
PRJNA729271	stage_E14_vs_E10	ordinary_muscle_development	ENSGALG00010011597	TBC1D14	-0.146281512	0.13000588	-1.125191514	0.260507889	0.368756039	negative
PRJNA729271	stage_E14_vs_E10	ordinary_muscle_development	ENSGALG00010011601	ANO4	-5.265189528	0.879717679	-5.985090052	2.1627E-09	1.70743E-08	negative
PRJNA729271	stage_E14_vs_E10	ordinary_muscle_development	ENSGALG00010011603	ENSGALG00010011603	-0.095867076	0.27932965	-0.343204083	0.731444933	0.80659694	negative
PRJNA729271	stage_E14_vs_E10	ordinary_muscle_development	ENSGALG00010011605	APPL2	-0.159250529	0.140735646	-1.131557877	0.257820358	0.366024103	negative
PRJNA729271	stage_E14_vs_E10	ordinary_muscle_development	ENSGALG00010011607	TSEN15	-0.196187863	0.156817595	-1.251057723	0.210913418	0.311945342	negative
PRJNA729271	stage_E14_vs_E10	ordinary_muscle_development	ENSGALG00010011609	ENSGALG00010011609	-0.791665271	0.852302297	-0.928855024	0.352964224	0.469205468	negative
PRJNA729271	stage_E14_vs_E10	ordinary_muscle_development	ENSGALG00010011612	ENSGALG00010011612	0.00631349	0.440857969	0.014320915	0.988573954	0.992300616	positive
PRJNA729271	stage_E14_vs_E10	ordinary_muscle_development	ENSGALG00010011617	MAMLD1	0.809880876	0.095847162	8.449711617	2.92023E-17	5.00932E-16	positive
PRJNA729271	stage_E14_vs_E10	ordinary_muscle_development	ENSGALG00010011619	PCDH1	0.474770672	0.158557069	2.994320431	0.002750569	0.007695329	positive
PRJNA729271	stage_E14_vs_E10	ordinary_muscle_development	ENSGALG00010011621	WASHC4	0.209835751	0.087582158	2.395873266	0.016580822	0.037003698	positive
PRJNA729271	stage_E14_vs_E10	ordinary_muscle_development	ENSGALG00010011622	BACH1	0.283210293	0.159883991	1.771348657	0.076502741	0.136061677	positive
PRJNA729271	stage_E14_vs_E10	ordinary_muscle_development	ENSGALG00010011625	ERBB4	0.517594152	0.217623809	2.378389364	0.017388454	0.038562675	positive
PRJNA729271	stage_E14_vs_E10	ordinary_muscle_development	ENSGALG00010011626	SERPINB1	-1.001036083	0.645069665	-1.551826317	0.120703787	0.198535604	negative
PRJNA729271	stage_E14_vs_E10	ordinary_muscle_development	ENSGALG00010011628	MATN2	1.72509264	0.19169041	8.999368531	2.2702E-19	4.64126E-18	positive
PRJNA729271	stage_E14_vs_E10	ordinary_muscle_development	ENSGALG00010011630	DELE1	0.362390121	0.100288259	3.613485024	0.000302109	0.001081518	positive
PRJNA729271	stage_E14_vs_E10	ordinary_muscle_development	ENSGALG00010011633	TADA2B	0.118541472	0.082427098	1.438137146	0.150395145	0.237858988	positive
PRJNA729271	stage_E14_vs_E10	ordinary_muscle_development	ENSGALG00010011634	AVD	1.521209611	0.899098988	1.69192673	0.090659945	0.156778323	positive
PRJNA729271	stage_E14_vs_E10	ordinary_muscle_development	ENSGALG00010011635	PCDH12	0.579027408	0.193897169	2.986260249	0.002824122	0.007876784	positive
PRJNA729271	stage_E14_vs_E10	ordinary_muscle_development	ENSGALG00010011636	GRIK1	-0.873976578	0.398850777	-2.191236998	0.028434646	0.058737381	negative
PRJNA729271	stage_E14_vs_E10	ordinary_muscle_development	ENSGALG00010011637	CECR2	0.434491436	0.152276716	2.853301847	0.00432675	0.011461302	positive
PRJNA729271	stage_E14_vs_E10	ordinary_muscle_development	ENSGALG00010011638	GRPEL1	0.141038405	0.081012371	1.740948988	0.081692521	0.143985054	positive
PRJNA729271	stage_E14_vs_E10	ordinary_muscle_development	ENSGALG00010011639	ENSGALG00010011639	0.548648172	0.14199609	3.8638259	0.000111625	0.000436258	positive
PRJNA729271	stage_E14_vs_E10	ordinary_muscle_development	ENSGALG00010011641	ENSGALG00010011641	-1.68435054	0.522374719	-3.22441052	0.001262323	0.003888094	negative
PRJNA729271	stage_E14_vs_E10	ordinary_muscle_development	ENSGALG00010011643	C1orf21	0.104447331	0.080438897	1.298467963	0.194126578	0.292137121	positive
PRJNA729271	stage_E14_vs_E10	ordinary_muscle_development	ENSGALG00010011647	FBXO38	0.061984821	0.082244024	0.753669607	0.451047636	0.564101423	positive
PRJNA729271	stage_E14_vs_E10	ordinary_muscle_development	ENSGALG00010011648	ENSGALG00010011648	0.182870736	0.219437013	0.833363222	0.40463991	0.519646983	positive
PRJNA729271	stage_E14_vs_E10	ordinary_muscle_development	ENSGALG00010011649	MRPS27	-0.157921847	0.136855715	-1.1539295	0.248529074	0.355444393	negative
PRJNA729271	stage_E14_vs_E10	ordinary_muscle_development	ENSGALG00010011650	RPL30	0.300805557	0.155301034	1.936919216	0.052755219	0.099087589	positive
PRJNA729271	stage_E14_vs_E10	ordinary_muscle_development	ENSGALG00010011651	SERPINB6	-0.014511202	0.127234324	-0.114051002	0.909197364	0.93739354	negative
PRJNA729271	stage_E14_vs_E10	ordinary_muscle_development	ENSGALG00010011653	NR2C1	-0.146197853	0.087451293	-1.671763195	0.094571027	0.162552714	negative
PRJNA729271	stage_E14_vs_E10	ordinary_muscle_development	ENSGALG00010011654	CLDN8	1.925719969	1.091841057	1.76373654	0.077776346	0.138056348	positive
PRJNA729271	stage_E14_vs_E10	ordinary_muscle_development	ENSGALG00010011655	SORCS2	0.303084997	0.18404678	1.646782395	0.099602781	0.169552826	positive
PRJNA729271	stage_E14_vs_E10	ordinary_muscle_development	ENSGALG00010011657	ALDH1L2	2.49295271	0.142053795	17.54935662	6.01576E-69	2.02787E-66	positive
PRJNA729271	stage_E14_vs_E10	ordinary_muscle_development	ENSGALG00010011658	TENM1	-1.614374881	0.293370726	-5.50284926	3.73702E-08	2.5324E-07	negative
PRJNA729271	stage_E14_vs_E10	ordinary_muscle_development	ENSGALG00010011659	CD14	0.217144957	0.155449683	1.3968826	0.162448878	0.253040194	positive
PRJNA729271	stage_E14_vs_E10	ordinary_muscle_development	ENSGALG00010011660	ENSGALG00010011660	-0.3870472	0.084250385	-4.594011041	4.34806E-06	2.1673E-05	negative
PRJNA729271	stage_E14_vs_E10	ordinary_muscle_development	ENSGALG00010011662	RIDA	0.008365597	0.165435682	0.050567069	0.959670505	0.972280979	positive
PRJNA729271	stage_E14_vs_E10	ordinary_muscle_development	ENSGALG00010011663	UTP20	-0.268271948	0.136654169	-1.963145003	0.049629328	0.094245658	negative
PRJNA729271	stage_E14_vs_E10	ordinary_muscle_development	ENSGALG00010011664	SERPINB10B	3.388157421	0.539186913	6.283827258	3.30338E-10	2.86036E-09	positive
PRJNA729271	stage_E14_vs_E10	ordinary_muscle_development	ENSGALG00010011667	ENSGALG00010011667	-1.459236473	0.413923775	-3.525374867	0.000422884	0.00145631	negative
PRJNA729271	stage_E14_vs_E10	ordinary_muscle_development	ENSGALG00010011669	ENSGALG00010011669	-0.106154513	0.748704767	-0.141784208	0.887250458	0.922641179	negative
PRJNA729271	stage_E14_vs_E10	ordinary_muscle_development	ENSGALG00010011670	TMCO6	0.191912396	0.169455688	1.132522595	0.257414791	0.365591553	positive
PRJNA729271	stage_E14_vs_E10	ordinary_muscle_development	ENSGALG00010011671	SLC41A2	-1.198622289	0.193117601	-6.206696235	5.411E-10	4.57599E-09	negative
PRJNA729271	stage_E14_vs_E10	ordinary_muscle_development	ENSGALG00010011673	POP1	-0.627207531	0.118797532	-5.279634349	1.29442E-07	8.08733E-07	negative
PRJNA729271	stage_E14_vs_E10	ordinary_muscle_development	ENSGALG00010011674	ENSGALG00010011674	-2.514383086	0.51519183	-4.880479346	1.05828E-06	5.83041E-06	negative
PRJNA729271	stage_E14_vs_E10	ordinary_muscle_development	ENSGALG00010011677	EDEM3	-0.203872822	0.078885704	-2.584407706	0.009754641	0.023332264	negative
PRJNA729271	stage_E14_vs_E10	ordinary_muscle_development	ENSGALG00010011679	OVAL	-3.910832328	0.92630166	-4.22198566	2.4216E-05	0.000106335	negative
PRJNA729271	stage_E14_vs_E10	ordinary_muscle_development	ENSGALG00010011681	MAP1B	-1.044573879	0.35863572	-2.912632012	0.003583967	0.009699327	negative
PRJNA729271	stage_E14_vs_E10	ordinary_muscle_development	ENSGALG00010011682	USP15	0.062968495	0.122792697	0.512803261	0.608088949	0.705478575	positive
PRJNA729271	stage_E14_vs_E10	ordinary_muscle_development	ENSGALG00010011683	NIPAL2	0.001534519	0.267774739	0.005730635	0.99542764	0.997665992	positive
PRJNA729271	stage_E14_vs_E10	ordinary_muscle_development	ENSGALG00010011685	NDUFA2	0.516149352	0.111592291	4.625313678	3.74032E-06	1.87858E-05	positive
PRJNA729271	stage_E14_vs_E10	ordinary_muscle_development	ENSGALG00010011688	PDE3A	-0.433268179	0.147589406	-2.935631984	0.00332869	0.009100218	negative
PRJNA729271	stage_E14_vs_E10	ordinary_muscle_development	ENSGALG00010011692	ARL1	-0.025386235	0.088404952	-0.287158518	0.773990947	0.839808277	negative
PRJNA729271	stage_E14_vs_E10	ordinary_muscle_development	ENSGALG00010011696	ZFC3H1	-0.241954724	0.109128376	-2.217156823	0.026612377	0.055559038	negative
PRJNA729271	stage_E14_vs_E10	ordinary_muscle_development	ENSGALG00010011699	TIAM1	-1.356554329	0.144005217	-9.42017488	4.50337E-21	1.04275E-19	negative
PRJNA729271	stage_E14_vs_E10	ordinary_muscle_development	ENSGALG00010011702	NUP205	-0.020956778	0.083366071	-0.251382582	0.801518333	0.860210887	negative
PRJNA729271	stage_E14_vs_E10	ordinary_muscle_development	ENSGALG00010011703	YWHAZ	-0.36743022	0.093971885	-3.910001583	9.22955E-05	0.000365625	negative
PRJNA729271	stage_E14_vs_E10	ordinary_muscle_development	ENSGALG00010011708	IK	0.121847895	0.091187521	1.336234319	0.181472697	0.276597975	positive
PRJNA729271	stage_E14_vs_E10	ordinary_muscle_development	ENSGALG00010011712	DCAF12L2	0.207322834	0.075174186	2.75789931	0.005817411	0.014858745	positive
PRJNA729271	stage_E14_vs_E10	ordinary_muscle_development	ENSGALG00010011713	AVPR1A	-1.838496435	0.610685203	-3.010546882	0.002607777	0.007337061	negative
PRJNA729271	stage_E14_vs_E10	ordinary_muscle_development	ENSGALG00010011715	SLCO1B3	0.629664035	1.761297307	0.35750014	0.720717421	0.797830993	positive
PRJNA729271	stage_E14_vs_E10	ordinary_muscle_development	ENSGALG00010011720	IKZF2	-0.88104621	0.1814558	-4.855431521	1.20125E-06	6.56565E-06	negative
PRJNA729271	stage_E14_vs_E10	ordinary_muscle_development	ENSGALG00010011723	SPRY3	0.807126016	0.156586278	5.154513068	2.54291E-07	1.53262E-06	positive
PRJNA729271	stage_E14_vs_E10	ordinary_muscle_development	ENSGALG00010011724	ARHGEF28	-1.777153557	0.156189989	-11.3781528	5.37256E-30	2.304E-28	negative
PRJNA729271	stage_E14_vs_E10	ordinary_muscle_development	ENSGALG00010011726	WDR55	-0.218700277	0.082900093	-2.638118585	0.008336742	0.020369596	negative
PRJNA729271	stage_E14_vs_E10	ordinary_muscle_development	ENSGALG00010011729	FAM129A	0.047403674	0.232385243	0.203987453	0.838363304	0.8872466	positive
PRJNA729271	stage_E14_vs_E10	ordinary_muscle_development	ENSGALG00010011734	ENSGALG00010011734	-0.200878814	0.286207997	-0.701863038	0.482764581	0.594294155	negative
PRJNA729271	stage_E14_vs_E10	ordinary_muscle_development	ENSGALG00010011738	FGD6	-0.189923557	0.117618727	-1.614739102	0.106367206	0.179299064	negative
PRJNA729271	stage_E14_vs_E10	ordinary_muscle_development	ENSGALG00010011745	AFAP1L1	1.596517656	0.190243304	8.39197817	4.7803E-17	8.09467E-16	positive
PRJNA729271	stage_E14_vs_E10	ordinary_muscle_development	ENSGALG00010011746	SOD1	-0.111918901	0.142377761	-0.7860701	0.43182642	0.546093523	negative
PRJNA729271	stage_E14_vs_E10	ordinary_muscle_development	ENSGALG00010011747	UTP15	-0.149176421	0.11537051	-1.293020379	0.196004011	0.294312355	negative
PRJNA729271	stage_E14_vs_E10	ordinary_muscle_development	ENSGALG00010011751	ZNF706	0.385258076	0.109465471	3.519448408	0.000432445	0.001485377	positive
PRJNA729271	stage_E14_vs_E10	ordinary_muscle_development	ENSGALG00010011752	RNF2	-1.227396569	0.119429125	-10.27719635	8.92872E-25	2.61458E-23	negative
PRJNA729271	stage_E14_vs_E10	ordinary_muscle_development	ENSGALG00010011758	SERPINB11	1.504502541	0.431617946	3.48572749	0.000490801	0.001667641	positive
PRJNA729271	stage_E14_vs_E10	ordinary_muscle_development	ENSGALG00010011759	CREB3	-0.065294826	0.130193893	-0.501519881	0.61600529	0.712316214	negative
PRJNA729271	stage_E14_vs_E10	ordinary_muscle_development	ENSGALG00010011764	PCYOX1L	0.091491866	0.125175324	0.730909761	0.464834274	0.577308954	positive
PRJNA729271	stage_E14_vs_E10	ordinary_muscle_development	ENSGALG00010011770	CHST11	-2.384603058	0.290373706	-8.212186596	2.17196E-16	3.44826E-15	negative
PRJNA729271	stage_E14_vs_E10	ordinary_muscle_development	ENSGALG00010011772	GRHL2	-2.263804141	0.450642768	-5.023500441	5.07381E-07	2.92309E-06	negative
PRJNA729271	stage_E14_vs_E10	ordinary_muscle_development	ENSGALG00010011774	ANKRA2	-0.036269214	0.091181077	-0.397771284	0.690798786	0.774300896	negative
PRJNA729271	stage_E14_vs_E10	ordinary_muscle_development	ENSGALG00010011775	VEZT	0.0322524	0.07944516	0.405970614	0.684764192	0.769490423	positive
PRJNA729271	stage_E14_vs_E10	ordinary_muscle_development	ENSGALG00010011776	IL17RA	-0.402652183	0.136438486	-2.951162792	0.0031658	0.008722348	negative
PRJNA729271	stage_E14_vs_E10	ordinary_muscle_development	ENSGALG00010011780	SERPINB5	1.169213602	0.622243431	1.879029243	0.0602405	0.110923663	positive
PRJNA729271	stage_E14_vs_E10	ordinary_muscle_development	ENSGALG00010011784	GRPEL2	0.154259621	0.133706838	1.153715278	0.248616918	0.35550701	positive
PRJNA729271	stage_E14_vs_E10	ordinary_muscle_development	ENSGALG00010011789	TXNRD1	-0.934572602	0.134331563	-6.957207813	3.47083E-12	3.75726E-11	negative
PRJNA729271	stage_E14_vs_E10	ordinary_muscle_development	ENSGALG00010011790	AMY2A	-1.130010981	1.123225244	-1.006041297	0.314395705	0.428305051	negative
PRJNA729271	stage_E14_vs_E10	ordinary_muscle_development	ENSGALG00010011792	HDHD5	0.560287511	0.141231428	3.96715886	7.27345E-05	0.000293755	positive
PRJNA729271	stage_E14_vs_E10	ordinary_muscle_development	ENSGALG00010011793	TLN1	0.022459968	0.113975945	0.197058842	0.843781491	0.891314146	positive
PRJNA729271	stage_E14_vs_E10	ordinary_muscle_development	ENSGALG00010011796	METAP2	0.043909363	0.089151404	0.492525759	0.622347717	0.717370191	positive
PRJNA729271	stage_E14_vs_E10	ordinary_muscle_development	ENSGALG00010011797	SCAF4	-0.07990216	0.155533627	-0.513729163	0.607441359	0.704952963	negative
PRJNA729271	stage_E14_vs_E10	ordinary_muscle_development	ENSGALG00010011802	VPS4B	0.010398791	0.092257359	0.112715026	0.910256488	0.938151863	positive
PRJNA729271	stage_E14_vs_E10	ordinary_muscle_development	ENSGALG00010011808	SMARCA2	0.07102611	0.122611485	0.579277786	0.562401752	0.665144696	positive
PRJNA729271	stage_E14_vs_E10	ordinary_muscle_development	ENSGALG00010011810	FGFR4	1.030078049	0.125170427	8.229404269	1.88147E-16	3.01347E-15	positive
PRJNA729271	stage_E14_vs_E10	ordinary_muscle_development	ENSGALG00010011814	ENSGALG00010011814	1.052499327	0.530698346	1.983234609	0.04734123	0.090588927	positive
PRJNA729271	stage_E14_vs_E10	ordinary_muscle_development	ENSGALG00010011817	KDSR	-0.335039375	0.09042573	-3.705133229	0.00021128	0.000780453	negative
PRJNA729271	stage_E14_vs_E10	ordinary_muscle_development	ENSGALG00010011819	USP44	-0.425472877	0.141100213	-3.015395001	0.002566448	0.00722764	negative
PRJNA729271	stage_E14_vs_E10	ordinary_muscle_development	ENSGALG00010011823	ENSGALG00010011823	-1.706928858	0.595730847	-2.865268547	0.004166557	0.011078985	negative
PRJNA729271	stage_E14_vs_E10	ordinary_muscle_development	ENSGALG00010011825	TAFA2	-5.149149974	0.388262862	-13.26202033	3.84398E-40	2.87209E-38	negative
PRJNA729271	stage_E14_vs_E10	ordinary_muscle_development	ENSGALG00010011837	VLDLR	1.046216633	0.134498141	7.778669844	7.32911E-15	1.01856E-13	positive
PRJNA729271	stage_E14_vs_E10	ordinary_muscle_development	ENSGALG00010011839	CBS	-0.594058109	0.45990758	-1.291690187	0.196464457	0.294889956	negative
PRJNA729271	stage_E14_vs_E10	ordinary_muscle_development	ENSGALG00010011841	KCNMB4	-0.717028621	0.236822395	-3.027706152	0.002464176	0.006968051	negative
PRJNA729271	stage_E14_vs_E10	ordinary_muscle_development	ENSGALG00010011843	BCL2	0.183001296	0.111733688	1.637834559	0.10145621	0.172061281	positive
PRJNA729271	stage_E14_vs_E10	ordinary_muscle_development	ENSGALG00010011846	UBAP1	0.111165595	0.104543376	1.063344219	0.287625879	0.39985451	positive
PRJNA729271	stage_E14_vs_E10	ordinary_muscle_development	ENSGALG00010011850	SPAG16	-0.793017863	0.342099121	-2.31809383	0.020444222	0.044328946	negative
PRJNA729271	stage_E14_vs_E10	ordinary_muscle_development	ENSGALG00010011856	NFYB	-0.022862779	0.128269355	-0.178240383	0.858534197	0.902491347	negative
PRJNA729271	stage_E14_vs_E10	ordinary_muscle_development	ENSGALG00010011857	ENSGALG00010011857	0.232880346	0.244135658	0.953897305	0.340135628	0.455135542	positive
PRJNA729271	stage_E14_vs_E10	ordinary_muscle_development	ENSGALG00010011859	NTN4	0.324518185	0.206229262	1.57357972	0.115584645	0.192000851	positive
PRJNA729271	stage_E14_vs_E10	ordinary_muscle_development	ENSGALG00010011860	CECR1	0.112148387	0.155710857	0.720234854	0.471380408	0.583588914	positive
PRJNA729271	stage_E14_vs_E10	ordinary_muscle_development	ENSGALG00010011861	ENSGALG00010011861	-0.882772332	0.394759819	-2.236226408	0.025336944	0.053341904	negative
PRJNA729271	stage_E14_vs_E10	ordinary_muscle_development	ENSGALG00010011866	KLF8	0.690420606	0.185358971	3.724775786	0.000195489	0.000728248	positive

PRJNA729271	stage_E14_vs_E10	ordinary_muscle_development	ENSGALG00010011867	ENSGALG00010011867	-0.135179921	0.273358464	-0.494515221	0.62094236	0.716433933	negative
PRJNA729271	stage_E14_vs_E10	ordinary_muscle_development	ENSGALG00010011868	SNRPF	-0.10606003	0.123160914	-0.861150079	0.38915539	0.504996185	negative
PRJNA729271	stage_E14_vs_E10	ordinary_muscle_development	ENSGALG00010011870	AvBD6	2.523181087	1.395824262	1.80766387	0.070658831	0.127198181	positive
PRJNA729271	stage_E14_vs_E10	ordinary_muscle_development	ENSGALG00010011871	CCT2	-0.18387553	0.097280846	-1.890151428	0.058737711	0.108625047	negative
PRJNA729271	stage_E14_vs_E10	ordinary_muscle_development	ENSGALG00010011873	RFX3	-0.357283893	0.191152604	-1.869102935	0.061608495	0.113154477	negative
PRJNA729271	stage_E14_vs_E10	ordinary_muscle_development	ENSGALG00010011874	KCNS2	-1.44813703	0.754872961	-1.918385085	0.055062201	0.102838114	negative
PRJNA729271	stage_E14_vs_E10	ordinary_muscle_development	ENSGALG00010011875	PSD2	0.749900212	0.284359142	2.637158792	0.008360369	0.020415017	positive
PRJNA729271	stage_E14_vs_E10	ordinary_muscle_development	ENSGALG00010011876	AMDHD1	-0.607254482	0.451217559	-1.345813056	0.178362814	0.272660724	negative
PRJNA729271	stage_E14_vs_E10	ordinary_muscle_development	ENSGALG00010011880	U2AF1	-0.176981541	0.155893871	-1.135269399	0.25626246	0.364097663	negative
PRJNA729271	stage_E14_vs_E10	ordinary_muscle_development	ENSGALG00010011881	RIOK2	-0.337273029	0.094807737	-3.557442021	0.000374484	0.001309248	negative
PRJNA729271	stage_E14_vs_E10	ordinary_muscle_development	ENSGALG00010011882	ENSGALG00010011882	-0.073900891	0.279975593	-0.263954762	0.791814777	0.853842821	negative
PRJNA729271	stage_E14_vs_E10	ordinary_muscle_development	ENSGALG00010011883	HCFC2	0.143347639	0.106994942	1.339760896	0.180323093	0.275193012	positive
PRJNA729271	stage_E14_vs_E10	ordinary_muscle_development	ENSGALG00010011885	NCALD	-0.236014417	0.210802963	-1.119597246	0.26288543	0.371468542	negative
PRJNA729271	stage_E14_vs_E10	ordinary_muscle_development	ENSGALG00010011886	NTNG1	-1.341934094	0.159224735	-8.427924789	3.51849E-17	5.99301E-16	negative
PRJNA729271	stage_E14_vs_E10	ordinary_muscle_development	ENSGALG00010011887	PGM5	0.566791216	0.132643897	4.273028982	1.92835E-05	8.60311E-05	positive
PRJNA729271	stage_E14_vs_E10	ordinary_muscle_development	ENSGALG00010011888	CAND1	-0.431964219	0.068606647	-6.296244427	3.04943E-10	2.65315E-09	negative
PRJNA729271	stage_E14_vs_E10	ordinary_muscle_development	ENSGALG00010011891	HAL	-1.00225826	0.579928611	-1.728244203	0.083944452	0.14729147	negative
PRJNA729271	stage_E14_vs_E10	ordinary_muscle_development	ENSGALG00010011893	LIX1	-3.557270813	0.479136633	-7.424334867	1.13348E-13	1.42496E-12	negative
PRJNA729271	stage_E14_vs_E10	ordinary_muscle_development	ENSGALG00010011894	VWC2L	-1.328443719	0.321592871	-4.130824522	3.61464E-05	0.000153965	negative
PRJNA729271	stage_E14_vs_E10	ordinary_muscle_development	ENSGALG00010011895	PHF21B	-1.115147406	0.610621614	-1.82624948	0.067812691	0.122688711	negative
PRJNA729271	stage_E14_vs_E10	ordinary_muscle_development	ENSGALG00010011896	SIK1	1.708187707	0.298412397	5.724251829	1.03891E-08	7.55591E-08	positive
PRJNA729271	stage_E14_vs_E10	ordinary_muscle_development	ENSGALG00010011898	ENSGALG00010011898	0.070089624	0.110205213	0.635991911	0.524781695	0.632416916	positive
PRJNA729271	stage_E14_vs_E10	ordinary_muscle_development	ENSGALG00010011899	PHLPP1	0.271492513	0.08319141	3.263468093	0.001100576	0.003431457	positive
PRJNA729271	stage_E14_vs_E10	ordinary_muscle_development	ENSGALG00010011902	TP53I3	-0.467849872	0.245067466	-1.909065615	0.056253628	0.104699067	negative
PRJNA729271	stage_E14_vs_E10	ordinary_muscle_development	ENSGALG00010011903	LTA4H	-0.013913048	0.115424593	-0.12053799	0.904056987	0.934353371	negative
PRJNA729271	stage_E14_vs_E10	ordinary_muscle_development	ENSGALG00010011904	KIF24	1.151835327	0.333103827	3.457886797	0.00054443	0.001828432	positive
PRJNA729271	stage_E14_vs_E10	ordinary_muscle_development	ENSGALG00010011906	ENSGALG00010011906	0.616821201	0.933006886	0.661111092	0.508541072	0.617362047	positive
PRJNA729271	stage_E14_vs_E10	ordinary_muscle_development	ENSGALG00010011907	BIK	-0.390418882	0.212404778	-1.838088984	0.066049294	0.120093392	negative
PRJNA729271	stage_E14_vs_E10	ordinary_muscle_development	ENSGALG00010011908	ENSGALG00010011908	-0.245679845	0.175006602	-1.403831871	0.160368921	0.250499379	negative
PRJNA729271	stage_E14_vs_E10	ordinary_muscle_development	ENSGALG00010011909	C8orf48	-0.581129764	0.200041244	-2.905049744	0.003671947	0.009917063	negative
PRJNA729271	stage_E14_vs_E10	ordinary_muscle_development	ENSGALG00010011911	NUDT2	0.40429725	0.136804651	2.955288783	0.003123765	0.008619624	positive
PRJNA729271	stage_E14_vs_E10	ordinary_muscle_development	ENSGALG00010011912	TCP11X2	0.287898063	0.125818153	2.288207669	0.02212543	0.0475357	positive
PRJNA729271	stage_E14_vs_E10	ordinary_muscle_development	ENSGALG00010011913	CXXC5	0.91561967	0.110201735	8.308577635	9.6856E-17	1.59719E-15	positive
PRJNA729271	stage_E14_vs_E10	ordinary_muscle_development	ENSGALG00010011915	RRM2B	0.125025626	0.121166474	1.031849999	0.302142392	0.415833078	positive
PRJNA729271	stage_E14_vs_E10	ordinary_muscle_development	ENSGALG00010011916	MCAT	0.315070913	0.095939276	3.28406598	0.00102321	0.003219338	positive
PRJNA729271	stage_E14_vs_E10	ordinary_muscle_development	ENSGALG00010011917	HSF2BP	-0.359048088	0.17510547	-2.050467574	0.040318825	0.078837362	negative
PRJNA729271	stage_E14_vs_E10	ordinary_muscle_development	ENSGALG00010011921	MEGF10	0.916092177	0.167086546	5.482740517	4.18787E-08	2.81294E-07	positive
PRJNA729271	stage_E14_vs_E10	ordinary_muscle_development	ENSGALG00010011922	PIK3R1	-0.165994351	0.173064059	-0.959149765	0.337483303	0.452752623	negative
PRJNA729271	stage_E14_vs_E10	ordinary_muscle_development	ENSGALG00010011923	BARD1	-0.470081449	0.145406683	-3.232873751	0.001225517	0.003787606	negative
PRJNA729271	stage_E14_vs_E10	ordinary_muscle_development	ENSGALG00010011925	RRP1B	-0.209212046	0.446683125	-0.468367921	0.639521504	0.732332454	negative
PRJNA729271	stage_E14_vs_E10	ordinary_muscle_development	ENSGALG00010011926	DYRK2	0.96153377	0.194774031	4.936663095	7.94706E-07	4.44414E-06	positive
PRJNA729271	stage_E14_vs_E10	ordinary_muscle_development	ENSGALG00010011927	ENSGALG00010011927	-1.298337183	0.49318467	-2.632557868	0.008474459	0.020651862	negative
PRJNA729271	stage_E14_vs_E10	ordinary_muscle_development	ENSGALG00010011929	ELK3	-0.633602426	0.133638267	-4.741175098	2.12482E-06	1.1135E-05	negative
PRJNA729271	stage_E14_vs_E10	ordinary_muscle_development	ENSGALG00010011930	TSPO	0.129849954	0.149797547	0.866836322	0.386031697	0.502156462	positive
PRJNA729271	stage_E14_vs_E10	ordinary_muscle_development	ENSGALG00010011931	ENSGALG00010011931	2.661904155	1.297338215	2.051819738			positive
PRJNA729271	stage_E14_vs_E10	ordinary_muscle_development	ENSGALG00010011932	VAV3	-1.097109095	0.167854497	-6.536072097	6.31555E-11	5.96766E-10	negative
PRJNA729271	stage_E14_vs_E10	ordinary_muscle_development	ENSGALG00010011935	ST8SIA4	-0.353791571	0.124883096	-2.832982063	0.004611597	0.012120599	negative
PRJNA729271	stage_E14_vs_E10	ordinary_muscle_development	ENSGALG00010011936	CKAP4	-0.436896973	0.175308358	-2.492162828	0.012696782	0.029380563	negative
PRJNA729271	stage_E14_vs_E10	ordinary_muscle_development	ENSGALG00010011937	SLC1A1	-0.123343616	0.288046264	-0.428207658	0.668499941	0.756196867	negative
PRJNA729271	stage_E14_vs_E10	ordinary_muscle_development	ENSGALG00010011941	ENSGALG00010011941	-2.970733857	0.240979322	-12.32775425	6.42114E-35	3.51224E-33	negative
PRJNA729271	stage_E14_vs_E10	ordinary_muscle_development	ENSGALG00010011943	TTLL12	-0.278146365	0.053533321	-5.195761393	2.03884E-07	1.24538E-06	negative
PRJNA729271	stage_E14_vs_E10	ordinary_muscle_development	ENSGALG00010011945	ENSGALG00010011945	-1.054522807	0.517600849	-2.037328203	0.041617163	0.081037182	negative
PRJNA729271	stage_E14_vs_E10	ordinary_muscle_development	ENSGALG00010011947	THAP1	-0.107067242	0.11519998	-0.929403303	0.352680117	0.46895682	negative
PRJNA729271	stage_E14_vs_E10	ordinary_muscle_development	ENSGALG00010011949	ABCA12	1.966909925	0.413822939	4.753022948	2.00398E-06	1.05589E-05	positive
PRJNA729271	stage_E14_vs_E10	ordinary_muscle_development	ENSGALG00010011950	PDXK	0.762324514	0.164938971	4.621858062	3.80318E-06	1.90685E-05	positive
PRJNA729271	stage_E14_vs_E10	ordinary_muscle_development	ENSGALG00010011952	UBE2D2	0.159878955	0.173686407	0.920503554	0.357309677	0.473765438	positive
PRJNA729271	stage_E14_vs_E10	ordinary_muscle_development	ENSGALG00010011953	CDK17	-0.010785365	0.057822166	-0.186526478	0.852031917	0.897582902	negative
PRJNA729271	stage_E14_vs_E10	ordinary_muscle_development	ENSGALG00010011954	ENSGALG00010011954	-0.594384925	0.534503205	-1.112032481	0.266124185	0.375021395	negative
PRJNA729271	stage_E14_vs_E10	ordinary_muscle_development	ENSGALG00010011957	ENSGALG00010011957	1.15092008	0.21111523	5.451620341	4.99129E-08	3.30511E-07	positive
PRJNA729271	stage_E14_vs_E10	ordinary_muscle_development	ENSGALG00010011960	CFAP54	-1.735150559	0.30019818	-5.780016913	7.46931E-09	5.52104E-08	negative
PRJNA729271	stage_E14_vs_E10	ordinary_muscle_development	ENSGALG00010011961	AGPAT3	1.131439993	0.102700644	11.01687339	3.16875E-28	1.22157E-26	positive
PRJNA729271	stage_E14_vs_E10	ordinary_muscle_development	ENSGALG00010011962	CPSF6	-0.147728863	0.057340791	-2.576331106	0.009985498	0.023794568	negative
PRJNA729271	stage_E14_vs_E10	ordinary_muscle_development	ENSGALG00010011963	ZCCHC2	-0.023543102	0.099867948	-0.235742322	0.813632627	0.869414297	negative
PRJNA729271	stage_E14_vs_E10	ordinary_muscle_development	ENSGALG00010011964	ENSGALG00010011964	2.836829925	4.307618204	0.658561133	0.510177628	0.618985915	positive
PRJNA729271	stage_E14_vs_E10	ordinary_muscle_development	ENSGALG00010011965	ATIC	0.235210304	0.146723191	1.603088799	0.108915051	0.182807279	positive
PRJNA729271	stage_E14_vs_E10	ordinary_muscle_development	ENSGALG00010011966	SLC25A24	-0.131234683	0.137774021	-0.952535769	0.340825335	0.45574384	negative
PRJNA729271	stage_E14_vs_E10	ordinary_muscle_development	ENSGALG00010011967	MTMR9	-0.145225763	0.137154626	-1.058846991	0.289669469	0.40206444	negative
PRJNA729271	stage_E14_vs_E10	ordinary_muscle_development	ENSGALG00010011968	ENSGALG00010011968	1.851601141	0.244731143	7.565858253	3.85312E-14	5.05439E-13	positive
PRJNA729271	stage_E14_vs_E10	ordinary_muscle_development	ENSGALG00010011969	TMEM173	-0.011301032	0.307128142	-0.036795821	0.970647806	0.979841211	negative
PRJNA729271	stage_E14_vs_E10	ordinary_muscle_development	ENSGALG00010011971	RNF170	-0.357762755	0.088483941	-4.043250673	5.27152E-05	0.000218379	negative
PRJNA729271	stage_E14_vs_E10	ordinary_muscle_development	ENSGALG00010011972	IPO11	-0.196693641	0.080543542	-2.442078365	0.014602976	0.033171939	negative
PRJNA729271	stage_E14_vs_E10	ordinary_muscle_development	ENSGALG00010011973	TRAPPC10	0.042313565	0.049152783	0.860857979	0.389316268	0.505138063	positive
PRJNA729271	stage_E14_vs_E10	ordinary_muscle_development	ENSGALG00010011976	TNFRSF11A	-0.006392334	0.089099292	-0.071743942	0.942805686	0.959756192	negative
PRJNA729271	stage_E14_vs_E10	ordinary_muscle_development	ENSGALG00010011977	GATD3A	0.247302206	0.118444569	2.087915115	0.036805492	0.072951677	positive
PRJNA729271	stage_E14_vs_E10	ordinary_muscle_development	ENSGALG00010011978	ENSGALG00010011978	1.86801723	5.422081401	0.344520322	0.730455016	0.805779715	positive
PRJNA729271	stage_E14_vs_E10	ordinary_muscle_development	ENSGALG00010011979	NEDD1	0.011351087	0.076445461	0.148486079	0.881959172	0.919050913	positive
PRJNA729271	stage_E14_vs_E10	ordinary_muscle_development	ENSGALG00010011980	AvBD9	16.71861929	2.346796356	7.124017917	1.04825E-12	1.19079E-11	positive
PRJNA729271	stage_E14_vs_E10	ordinary_muscle_development	ENSGALG00010011981	SCUBE1	-3.728239467	0.343479866	-10.85431734	1.90223E-27	6.96283E-26	negative
PRJNA729271	stage_E14_vs_E10	ordinary_muscle_development	ENSGALG00010011982	STK3	0.303184382	0.104565996	2.899454833	0.003738122	0.010073262	positive
PRJNA729271	stage_E14_vs_E10	ordinary_muscle_development	ENSGALG00010011983	LYZ	0.094776394	0.553961667	0.171088362	0.864154286	0.906502728	positive
PRJNA729271	stage_E14_vs_E10	ordinary_muscle_development	ENSGALG00010011984	FN1	-0.329835158	0.240230286	-1.372995733	0.169753673	0.262132312	negative
PRJNA729271	stage_E14_vs_E10	ordinary_muscle_development	ENSGALG00010011987	H2BC32	-0.033886123	0.374810651	-0.090408645	0.927962487	0.9504189	negative
PRJNA729271	stage_E14_vs_E10	ordinary_muscle_development	ENSGALG00010011988	HINTW	2.696519153	3.134324947	0.860318952	0.389613249	0.505228488	positive
PRJNA729271	stage_E14_vs_E10	ordinary_muscle_development	ENSGALG00010011989	H1-010	0.570255938	0.136294771	4.183989834	2.86437E-05	0.000124383	positive
PRJNA729271	stage_E14_vs_E10	ordinary_muscle_development	ENSGALG00010011991	CSNK1A1	0.468531772	0.056037528	8.361035736	6.217E-17	1.043E-15	positive
PRJNA729271	stage_E14_vs_E10	ordinary_muscle_development	ENSGALG00010011992	YEATS4	-0.430772575	0.078946107	-5.456539789	4.85503E-08	3.22963E-07	negative
PRJNA729271	stage_E14_vs_E10	ordinary_muscle_development	ENSGALG00010011993	RELCH	-0.097477191	0.049795711	-1.957541893	0.050283791	0.095189181	negative
PRJNA729271	stage_E14_vs_E10	ordinary_muscle_development	ENSGALG00010011996	LRRC70	-0.260449224	0.221666181	-1.174961479	0.240010127	0.345785389	negative
PRJNA729271	stage_E14_vs_E10	ordinary_muscle_development	ENSGALG00010011997	DENND5B	1.237248259	0.128204871	9.650555812	4.889E-22	1.21554E-20	positive
PRJNA729271	stage_E14_vs_E10	ordinary_muscle_development	ENSGALG00010011998	MPPED1	-0.4655632	0.07413461	-6.279970971	3.38636E-10	2.93047E-09	negative
PRJNA729271	stage_E14_vs_E10	ordinary_muscle_development	ENSGALG00010012001	EEIG2	-0.460251472	0.253271707	-1.817224186	0.069182793	0.124804553	negative
PRJNA729271	stage_E14_vs_E10	ordinary_muscle_development	ENSGALG00010012004	LGR5	-2.461774828	0.192344574	-12.79877448	1.66559E-37	1.13346E-35	negative
PRJNA729271	stage_E14_vs_E10	ordinary_muscle_development	ENSGALG00010012007	MREG	-0.000964897	0.182524079	-0.005286412	0.995782073	0.997778318	negative
PRJNA729271	stage_E14_vs_E10	ordinary_muscle_development	ENSGALG00010012008	ZNF366	0.391804294	0.196442711	1.994496471	0.046097825	0.088666133	positive
PRJNA729271	stage_E14_vs_E10	ordinary_muscle_development	ENSGALG00010012010	HENMT1	-0.124755738	0.150915289	-0.826660699	0.408429403	0.523199648	negative
PRJNA729271	stage_E14_vs_E10	ordinary_muscle_development	ENSGALG00010012011	HOOK3	-0.079302052	0.068386035	-1.159623472	0.24620214	0.352907021	negative
PRJNA729271	stage_E14_vs_E10	ordinary_muscle_development	ENSGALG00010012013	FOXD1	-4.998925172	0.82302126	-6.073871253	1.24863E-09	1.00773E-08	negative
PRJNA729271	stage_E14_vs_E10	ordinary_muscle_development	ENSGALG00010012015	ENSGALG00010012015	0.152158967	0.215481071	0.706136118	0.480103492	0.592163046	positive
PRJNA729271	stage_E14_vs_E10	ordinary_muscle_development	ENSGALG00010012016	WDR91	-0.054098799	0.06175753	-0.875987085	0.381037055	0.496952682	negative
PRJNA729271	stage_E14_vs_E10	ordinary_muscle_development	ENSGALG00010012017	N4BP3	0.191467511	0.126065775	1.518790577	0.128815219	0.20932473	positive
PRJNA729271	stage_E14_vs_E10	ordinary_muscle_development	ENSGALG00010012019	ENSGALG00010012019	0.172693799	0.32254449	0.535410787	0.592365833	0.692446996	positive
PRJNA729271	stage_E14_vs_E10	ordinary_muscle_development	ENSGALG00010012021	ENSGALG00010012021	-0.147309312	0.129904152	-1.133984628	0.256800996	0.364755555	negative
PRJNA729271	stage_E14_vs_E10	ordinary_muscle_development	ENSGALG00010012024	UBR5	0.019611544	0.075635742	0.259289368	0.795411982	0.85631131	positive
PRJNA729271	stage_E14_vs_E10	ordinary_muscle_development	ENSGALG00010012026	SYT1	-1.442195123	0.235472649	-6.124682111	9.08648E-10	7.49196E-09	negative
PRJNA729271	stage_E14_vs_E10	ordinary_muscle_development	ENSGALG00010012028	TMEM140	1.28313776	0.179544211	7.146639565	8.8928E-13	1.02059E-11	positive
PRJNA729271	stage_E14_vs_E10	ordinary_muscle_development	ENSGALG00010012031	GRK6	-0.100943728	0.105779706	-0.954282553	0.339940637	0.454980566	negative
PRJNA729271	stage_E14_vs_E10	ordinary_muscle_development	ENSGALG00010012034	ARHGEF9	0.563212277	0.150652451	3.738487325	0.000185131	0.000692687	positive
PRJNA729271	stage_E14_vs_E10	ordinary_muscle_development	ENSGALG00010012035	ENSGALG00010012035	0.234557236	0.453521066	0.517191489	0.605022493	0.702990063	positive
PRJNA729271	stage_E14_vs_E10	ordinary_muscle_development	ENSGALG00010012039	ODF1	0.242736986	0.561818054	0.432056222	0.665700552	0.753971675	positive
PRJNA729271	stage_E14_vs_E10	ordinary_muscle_development	ENSGALG00010012040	B4GALT7	-0.256692373	0.150143826	-1.709643208	0.087331871	0.152078676	negative
PRJNA729271	stage_E14_vs_E10	ordinary_muscle_development	ENSGALG00010012041	ICOSLG	-0.216041386	0.150824849	-1.432399157	0.15202962	0.239998839	negative
PRJNA729271	stage_E14_vs_E10	ordinary_muscle_development	ENSGALG00010012042	FNDC7	-1.644159346	0.277111408	-5.933206998	2.97074E-09	2.31013E-08	negative
PRJNA729271	stage_E14_vs_E10	ordinary_muscle_development	ENSGALG00010012043	NUP50	-0.392742962	0.095255671	-4.12304019	3.73904E-05	0.000158942	negative
PRJNA729271	stage_E14_vs_E10	ordinary_muscle_development	ENSGALG00010012044	ENSGALG00010012044	1.153634473	0.654954695	1.761395837	0.078171429	0.138604876	positive
PRJNA729271	stage_E14_vs_E10	ordinary_muscle_development	ENSGALG00010012045	ENSGALG00010012045	0.076319211	0.300702794	0.253802799	0.799647911	0.859150842	positive
PRJNA729271	stage_E14_vs_E10	ordinary_muscle_development	ENSGALG00010012046	LOXHD1	2.559347551	0.26523053	9.649520938	4.93859E-22	1.22577E-20	positive
PRJNA729271	stage_E14_vs_E10	ordinary_muscle_development	ENSGALG00010012047	LMAN2	0.082219967	0.089927819	0.91428845	0.360565298	0.476773763	positive
PRJNA729271	stage_E14_vs_E10	ordinary_muscle_development	ENSGALG00010012048	PDE6H	3.301354757	0.444215465	7.431877133	1.07067E-13	1.35069E-12	positive
PRJNA729271	stage_E14_vs_E10	ordinary_muscle_development	ENSGALG00010012049	IFNAR1	-0.265501067	0.145381852	-1.826232529	0.067815243	0.122688711	negative
PRJNA729271	stage_E14_vs_E10	ordinary_muscle_development	ENSGALG00010012051	ARK2C	0.742460943	0.182991458	4.057353022	4.9632E-05	0.000206491	positive
PRJNA729271	stage_E14_vs_E10	ordinary_muscle_development	ENSGALG00010012052	KLF10	0.467766657	0.124073479	3.770077694	0.000163197	0.000617311	positive
PRJNA729271	stage_E14_vs_E10	ordinary_muscle_development	ENSGALG00010012054	FAM60A	-0.207318964	0.085411882	-2.427284818	0.015212308	0.034324783	negative
PRJNA729271	stage_E14_vs_E10	ordinary_muscle_development	ENSGALG00010012055	ENSGALG00010012055	0.352912233	0.156419575	2.256189691	0.024058754	0.05105133	positive
PRJNA729271	stage_E14_vs_E10	ordinary_muscle_development	ENSGALG00010012058	ENSGALG00010012058	-0.745984721	0.279242077	-2.671462441	0.007552152	0.018634148	negative
PRJNA729271	stage_E14_vs_E10	ordinary_muscle_development	ENSGALG00010012059	ARHGDIB	0.643619075	0.143873172	4.473516941	7.69435E-06	3.67478E-05	positive
PRJNA729271	stage_E14_vs_E10	ordinary_muscle_development	ENSGALG00010012060	ENSGALG00010012060	0.431411147	0.124363004	3.468966908	0.000522464	0.001763239	positive
PRJNA729271	stage_E14_vs_E10	ordinary_muscle_development	ENSGALG00010012061	AZIN1	0.092126826	0.120700684	0.763266809	0.445304293	0.558847249	positive
PRJNA729271	stage_E14_vs_E10	ordinary_muscle_development	ENSGALG00010012062	HMGA2	-1.857200808	0.149838958	-12.39464575	2.79378E-35	1.58187E-33	negative
PRJNA729271	stage_E14_vs_E10	ordinary_muscle_development	ENSGALG00010012063	PTPRD	-0.200533243	0.195937598	-1.023454639	0.306092937	0.419698071	negative
PRJNA729271	stage_E14_vs_E10	ordinary_muscle_development	ENSGALG00010012064	RGS14	-1.234307355	0.173756359	-7.103667232	1.21489E-12	1.37255E-11	negative
PRJNA729271	stage_E14_vs_E10	ordinary_muscle_development	ENSGALG00010012066	STXBP3	-0.689636915	0.120609885	-5.717913726	1.0784E-08	7.82352E-08	negative

PRJNA729271	stage_E14_vs_E10	ordinary_muscle_development	ENSGALG00010012067	ENSGALG00010012067	0.039981794	0.082872378	0.482450181	0.629486181	0.723328487	positive
PRJNA729271	stage_E14_vs_E10	ordinary_muscle_development	ENSGALG00010012069	ERP27	0.987806022	0.416533497	2.3714924	0.017716411	0.039170131	positive
PRJNA729271	stage_E14_vs_E10	ordinary_muscle_development	ENSGALG00010012070	DNAJC28	0.045461455	0.161196167	0.282025654	0.777923839	0.842784753	positive
PRJNA729271	stage_E14_vs_E10	ordinary_muscle_development	ENSGALG00010012071	ENSGALG00010012071	-0.43226964	0.150153762	-2.878846549	0.003991325	0.010662413	negative
PRJNA729271	stage_E14_vs_E10	ordinary_muscle_development	ENSGALG00010012072	CAPRIN2	-0.1624803	0.149109766	-1.089669067	0.275858947	0.386261151	negative
PRJNA729271	stage_E14_vs_E10	ordinary_muscle_development	ENSGALG00010012074	KIAA0930	0.21033337	0.086424421	2.433726109	0.014944298	0.033841213	positive
PRJNA729271	stage_E14_vs_E10	ordinary_muscle_development	ENSGALG00010012075	PIGN	-0.095879358	0.092380547	-1.037873888	0.299328772	0.412980254	negative
PRJNA729271	stage_E14_vs_E10	ordinary_muscle_development	ENSGALG00010012077	IFNGR2	-0.120641409	0.068868407	-1.751767089	0.079813865	0.141120027	negative
PRJNA729271	stage_E14_vs_E10	ordinary_muscle_development	ENSGALG00010012078	TMBIM4	-0.043971509	0.081523254	-0.539373817	0.589628944	0.689692668	negative
PRJNA729271	stage_E14_vs_E10	ordinary_muscle_development	ENSGALG00010012079	ENSGALG00010012079	0.208598988	0.362628338	0.575241827	0.565127759	0.667668858	positive
PRJNA729271	stage_E14_vs_E10	ordinary_muscle_development	ENSGALG00010012081	CHIC1	-0.341122952	0.12434276	-2.743408235	0.006080504	0.015440944	negative
PRJNA729271	stage_E14_vs_E10	ordinary_muscle_development	ENSGALG00010012082	HAUS1	-0.425003396	0.19689094	-2.158572639	0.030883337	0.062890415	negative
PRJNA729271	stage_E14_vs_E10	ordinary_muscle_development	ENSGALG00010012083	MGP	0.950519923	0.277845235	3.421040934	0.000623819	0.00206209	positive
PRJNA729271	stage_E14_vs_E10	ordinary_muscle_development	ENSGALG00010012084	TMEM50B	0.186991004	0.105393783	1.774212854	0.076027949	0.135450482	positive
PRJNA729271	stage_E14_vs_E10	ordinary_muscle_development	ENSGALG00010012085	IPO8	0.184331078	0.096210637	1.91591163	0.055376345	0.103318332	positive
PRJNA729271	stage_E14_vs_E10	ordinary_muscle_development	ENSGALG00010012086	CIBAR1	-0.924740955	0.129617596	-7.134378236	9.72256E-13	1.10967E-11	negative
PRJNA729271	stage_E14_vs_E10	ordinary_muscle_development	ENSGALG00010012087	TMEM171	-0.012070091	0.169697317	-0.071127179	0.943296537	0.960053595	negative
PRJNA729271	stage_E14_vs_E10	ordinary_muscle_development	ENSGALG00010012088	ATP5F1AZ	0.115997337	0.169881584	0.682812901	0.494725077	0.604640808	positive
PRJNA729271	stage_E14_vs_E10	ordinary_muscle_development	ENSGALG00010012089	ENSGALG00010012089	-0.470317627	0.227327525	-2.068898729	0.038555591	0.075984132	negative
PRJNA729271	stage_E14_vs_E10	ordinary_muscle_development	ENSGALG00010012090	HELB	-0.312010442	0.223581773	-1.395509291	0.162862315	0.253483223	negative
PRJNA729271	stage_E14_vs_E10	ordinary_muscle_development	ENSGALG00010012091	ENSGALG00010012091	0.287162897	0.442070083	0.649586814	0.515959153	0.624641103	positive
PRJNA729271	stage_E14_vs_E10	ordinary_muscle_development	ENSGALG00010012092	OC3	-0.232581267	0.908313329	-0.25605841	0.797905735	0.857858154	negative
PRJNA729271	stage_E14_vs_E10	ordinary_muscle_development	ENSGALG00010012093	ENSGALG00010012093	-0.216835902	0.067157777	-3.2287534	0.001243311	0.003835239	negative
PRJNA729271	stage_E14_vs_E10	ordinary_muscle_development	ENSGALG00010012094	ENSGALG00010012094	-0.278253449	0.336616341	-0.82661896	0.408453068	0.523199648	negative
PRJNA729271	stage_E14_vs_E10	ordinary_muscle_development	ENSGALG00010012095	RNF38	0.257241602	0.084593056	3.040930476	0.002358483	0.00672163	positive
PRJNA729271	stage_E14_vs_E10	ordinary_muscle_development	ENSGALG00010012096	ENSGALG00010012096	-0.142019137	0.16157847	-0.878948391	0.379429259	0.495524561	negative
PRJNA729271	stage_E14_vs_E10	ordinary_muscle_development	ENSGALG00010012097	GART	-0.354023128	0.192016154	-1.843715338	0.065224637	0.118772752	negative
PRJNA729271	stage_E14_vs_E10	ordinary_muscle_development	ENSGALG00010012098	ART4	0.201557521	0.572764301	0.351903079	0.72491095	0.800821905	positive
PRJNA729271	stage_E14_vs_E10	ordinary_muscle_development	ENSGALG00010012099	UPK3A	0.598811357	0.465008213	1.287743614	0.197835226	0.296394998	positive
PRJNA729271	stage_E14_vs_E10	ordinary_muscle_development	ENSGALG00010012100	UBE2F	0.237203625	0.11127487	2.131690885	0.033032267	0.066574347	positive
PRJNA729271	stage_E14_vs_E10	ordinary_muscle_development	ENSGALG00010012101	ENSGALG00010012101	-0.199074016	0.133380231	-1.492530148	0.13556022	0.218545811	negative
PRJNA729271	stage_E14_vs_E10	ordinary_muscle_development	ENSGALG00010012102	SON	-0.102560619	0.104837579	-0.978281066	0.327935332	0.442920484	negative
PRJNA729271	stage_E14_vs_E10	ordinary_muscle_development	ENSGALG00010012103	ENSGALG00010012103	0.523628948	0.207739433	2.520604488	0.011715345	0.02738051	positive
PRJNA729271	stage_E14_vs_E10	ordinary_muscle_development	ENSGALG00010012105	DONSON	-0.517087774	0.198064508	-2.610703856	0.00903561	0.021824891	negative
PRJNA729271	stage_E14_vs_E10	ordinary_muscle_development	ENSGALG00010012106	TMTC1	0.13094624	0.138001303	0.948876839	0.342683255	0.457848077	positive
PRJNA729271	stage_E14_vs_E10	ordinary_muscle_development	ENSGALG00010012107	CLCC1	-0.227197648	0.084663572	-2.683534892	0.007284837	0.018047123	negative
PRJNA729271	stage_E14_vs_E10	ordinary_muscle_development	ENSGALG00010012108	PSTPIP2	0.35300143	0.333443155	1.058655501	0.289756701	0.402147011	positive
PRJNA729271	stage_E14_vs_E10	ordinary_muscle_development	ENSGALG00010012110	ENSGALG00010012110	0.349161734	0.11264195	3.099748673	0.001936849	0.005663633	positive
PRJNA729271	stage_E14_vs_E10	ordinary_muscle_development	ENSGALG00010012111	DRD1	-1.292026463	0.339699365	-3.803440916	0.0001427	0.000546832	negative
PRJNA729271	stage_E14_vs_E10	ordinary_muscle_development	ENSGALG00010012112	MBD2	0.950637485	0.190072767	5.001439709	5.69038E-07	3.24988E-06	positive
PRJNA729271	stage_E14_vs_E10	ordinary_muscle_development	ENSGALG00010012113	WDR47	-0.133097605	0.093873999	-1.417832482	0.156239678	0.245256025	negative
PRJNA729271	stage_E14_vs_E10	ordinary_muscle_development	ENSGALG00010012114	TAL2	0.84572069	0.30382101	2.783614894	0.005375682	0.013869795	positive
PRJNA729271	stage_E14_vs_E10	ordinary_muscle_development	ENSGALG00010012116	RBM12B	-0.058083984	0.077292684	-0.751481004	0.452363229	0.565356527	negative
PRJNA729271	stage_E14_vs_E10	ordinary_muscle_development	ENSGALG00010012118	ENSGALG00010012118	0.401600271	0.719202953	0.558396304	0.576573796	0.678363407	positive
PRJNA729271	stage_E14_vs_E10	ordinary_muscle_development	ENSGALG00010012119	EPG5	-0.00592145	0.122475079	-0.048348207	0.961438736	0.973890452	negative
PRJNA729271	stage_E14_vs_E10	ordinary_muscle_development	ENSGALG00010012120	ENSGALG00010012120	0.235042953	0.186008124	1.263616597	0.206367674	0.30636004	positive
PRJNA729271	stage_E14_vs_E10	ordinary_muscle_development	ENSGALG00010012121	SREK1	-0.327137597	0.100003293	-3.27126824	0.001070663	0.003348999	negative
PRJNA729271	stage_E14_vs_E10	ordinary_muscle_development	ENSGALG00010012122	GRIP1	-1.570527312	0.234007424	-6.711442258	1.9271E-11	1.91587E-10	negative
PRJNA729271	stage_E14_vs_E10	ordinary_muscle_development	ENSGALG00010012123	ENSGALG00010012123	-1.855272048	0.20869833	-8.889731174	6.12569E-19	1.19666E-17	negative
PRJNA729271	stage_E14_vs_E10	ordinary_muscle_development	ENSGALG00010012124	ERAP1	0.937971568	0.147499459	6.359152578	2.0287E-10	1.81183E-09	positive
PRJNA729271	stage_E14_vs_E10	ordinary_muscle_development	ENSGALG00010012125	RNF152	0.395104736	0.147052438	2.686828864	0.00721339	0.017900717	positive
PRJNA729271	stage_E14_vs_E10	ordinary_muscle_development	ENSGALG00010012127	ENSGALG00010012127	0.196764955	0.115289595	1.706701765	0.087877499	0.152823557	positive
PRJNA729271	stage_E14_vs_E10	ordinary_muscle_development	ENSGALG00010012128	ENSGALG00010012128	-2.04322771	1.103714672	-1.851228186	0.064136732	0.117100634	negative
PRJNA729271	stage_E14_vs_E10	ordinary_muscle_development	ENSGALG00010012129	CRYZL1	-0.156176331	0.094875036	-1.646126719	0.099737673	0.169742581	negative
PRJNA729271	stage_E14_vs_E10	ordinary_muscle_development	ENSGALG00010012130	TMEM67	-0.767442709	0.16435914	-4.669303503	3.02223E-06	1.54686E-05	negative
PRJNA729271	stage_E14_vs_E10	ordinary_muscle_development	ENSGALG00010012131	SLC14A1	-2.713987863	0.278427694	-9.747549986	1.88974E-22	4.86532E-21	negative
PRJNA729271	stage_E14_vs_E10	ordinary_muscle_development	ENSGALG00010012132	MC4R	-2.201664886	0.873338558	-2.520975247	0.011703008	0.027356089	negative
PRJNA729271	stage_E14_vs_E10	ordinary_muscle_development	ENSGALG00010012133	RAB3IP	-1.585368621	0.229472511	-6.9087518	4.88937E-12	5.15803E-11	negative
PRJNA729271	stage_E14_vs_E10	ordinary_muscle_development	ENSGALG00010012135	CAMSAP2	0.098120811	0.091057877	1.077565325	0.281227783	0.392338472	positive
PRJNA729271	stage_E14_vs_E10	ordinary_muscle_development	ENSGALG00010012136	ENSGALG00010012136	0.336445326	0.173256673	1.941889567	0.052150471	0.098247027	positive
PRJNA729271	stage_E14_vs_E10	ordinary_muscle_development	ENSGALG00010012137	ENSGALG00010012137	-1.695661347	1.029402496	-1.647228712	0.099511044	0.16943646	negative
PRJNA729271	stage_E14_vs_E10	ordinary_muscle_development	ENSGALG00010012138	DDX11	-0.316458032	0.155490768	-2.035220715	0.04182867	0.081394357	negative
PRJNA729271	stage_E14_vs_E10	ordinary_muscle_development	ENSGALG00010012140	GLOD5	2.519733858	0.548519112	4.593702942	4.35449E-06	2.16976E-05	positive
PRJNA729271	stage_E14_vs_E10	ordinary_muscle_development	ENSGALG00010012141	ENSGALG00010012141	-0.240517744	0.09388214	-2.561911593	0.010409781	0.024627025	negative
PRJNA729271	stage_E14_vs_E10	ordinary_muscle_development	ENSGALG00010012144	H1-01	0.542069382	0.157399009	3.443918646	0.000573348	0.001913581	positive
PRJNA729271	stage_E14_vs_E10	ordinary_muscle_development	ENSGALG00010012145	ENSGALG00010012145	1.663250059	1.076747135	1.544698847	0.122419124	0.200776782	positive
PRJNA729271	stage_E14_vs_E10	ordinary_muscle_development	ENSGALG00010012147	ARFGAP3	-0.012321392	0.122186818	-0.100840603	0.919676994	0.94436937	negative
PRJNA729271	stage_E14_vs_E10	ordinary_muscle_development	ENSGALG00010012148	SETBP1	-0.509355076	0.109146057	-4.66672904	3.06032E-06	1.56526E-05	negative
PRJNA729271	stage_E14_vs_E10	ordinary_muscle_development	ENSGALG00010012149	SFXN1	0.107379715	0.172186815	0.623623331	0.532874985	0.64010694	positive
PRJNA729271	stage_E14_vs_E10	ordinary_muscle_development	ENSGALG00010012151	MYRFL	-0.237389427	0.477575269	-0.497072278	0.619138083	0.714876326	negative
PRJNA729271	stage_E14_vs_E10	ordinary_muscle_development	ENSGALG00010012154	CWC27	0.053960096	0.112391383	0.480108837	0.631150005	0.724979738	positive
PRJNA729271	stage_E14_vs_E10	ordinary_muscle_development	ENSGALG00010012156	ENSGALG00010012156	0.703836931	0.503881605	1.396829977	0.162464705	0.253040194	positive
PRJNA729271	stage_E14_vs_E10	ordinary_muscle_development	ENSGALG00010012157	H2BC32	0.198715168	0.198125599	1.002975733	0.31587257	0.429751539	positive
PRJNA729271	stage_E14_vs_E10	ordinary_muscle_development	ENSGALG00010012159	WASHC1	0.204182225	0.099523801	2.051591912	0.040209339	0.078644497	positive
PRJNA729271	stage_E14_vs_E10	ordinary_muscle_development	ENSGALG00010012160	SYT4	-7.14286062	0.708992191	-10.07466755	7.15022E-24	1.96292E-22	negative
PRJNA729271	stage_E14_vs_E10	ordinary_muscle_development	ENSGALG00010012163	PDP1	0.090001904	0.221071091	0.407117474	0.68392171	0.768722487	positive
PRJNA729271	stage_E14_vs_E10	ordinary_muscle_development	ENSGALG00010012168	ENSGALG00010012168	-0.838995396	0.564080347	-1.487368599	0.136917486	0.220268474	negative
PRJNA729271	stage_E14_vs_E10	ordinary_muscle_development	ENSGALG00010012169	H3C33	0.311753823	0.227718656	1.369030667	0.170989689	0.263585615	positive
PRJNA729271	stage_E14_vs_E10	ordinary_muscle_development	ENSGALG00010012171	CDH17	-0.095616761	0.388675794	-0.246006473	0.805677222	0.863395781	negative
PRJNA729271	stage_E14_vs_E10	ordinary_muscle_development	ENSGALG00010012172	CNOT2	-0.030350949	0.088088572	-0.344550355	0.730432434	0.805779715	negative
PRJNA729271	stage_E14_vs_E10	ordinary_muscle_development	ENSGALG00010012175	PACSIN2	-0.235057172	0.154584761	-1.520571437	0.128367419	0.208714048	negative
PRJNA729271	stage_E14_vs_E10	ordinary_muscle_development	ENSGALG00010012179	ENSGALG00010012179	0.579073759	0.180156278	3.214285761	0.001307695	0.004005713	positive
PRJNA729271	stage_E14_vs_E10	ordinary_muscle_development	ENSGALG00010012180	DDX59	-0.396611375	0.104995011	-3.777430681	0.000158455	0.000600785	negative
PRJNA729271	stage_E14_vs_E10	ordinary_muscle_development	ENSGALG00010012186	KIF20A	0.039959709	0.246932192	0.161824622	0.871443963	0.911645359	positive
PRJNA729271	stage_E14_vs_E10	ordinary_muscle_development	ENSGALG00010012187	ENSGALG00010012187	-0.517253826	0.834829189	-0.619592406	0.535526167	0.642533879	negative
PRJNA729271	stage_E14_vs_E10	ordinary_muscle_development	ENSGALG00010012188	ADAMTS6	-0.17326398	0.18093583	-0.957599056	0.338264976	0.453407696	negative
PRJNA729271	stage_E14_vs_E10	ordinary_muscle_development	ENSGALG00010012190	CAST	0.295147764	0.132387891	2.229416616	0.025786198	0.054154004	positive
PRJNA729271	stage_E14_vs_E10	ordinary_muscle_development	ENSGALG00010012191	BEST3	-0.049709859	0.421366902	-0.11797286	0.906089162	0.935716909	negative
PRJNA729271	stage_E14_vs_E10	ordinary_muscle_development	ENSGALG00010012193	TTLL1	-1.138647207	0.21369669	-5.328333385	9.91181E-08	6.32079E-07	negative
PRJNA729271	stage_E14_vs_E10	ordinary_muscle_development	ENSGALG00010012194	LRRFIP1	1.026104526	0.159958892	6.414801422	1.41007E-10	1.27504E-09	positive
PRJNA729271	stage_E14_vs_E10	ordinary_muscle_development	ENSGALG00010012195	ITSN1	-0.411212596	0.072033335	-5.70864307	1.1388E-08	8.2329E-08	negative
PRJNA729271	stage_E14_vs_E10	ordinary_muscle_development	ENSGALG00010012196	CENPK	-0.240826726	0.258972036	-0.929933324	0.352405608	0.468720801	negative
PRJNA729271	stage_E14_vs_E10	ordinary_muscle_development	ENSGALG00010012198	TMEM5	-0.055173364	0.100961252	-0.546480582	0.584735632	0.685517873	negative
PRJNA729271	stage_E14_vs_E10	ordinary_muscle_development	ENSGALG00010012199	ENSGALG00010012199	-0.051611303	0.296126055	-0.174288288	0.861638904	0.904770784	negative
PRJNA729271	stage_E14_vs_E10	ordinary_muscle_development	ENSGALG00010012200	GEM	0.596623568	0.238919065	2.497178565	0.01251859	0.029005269	positive
PRJNA729271	stage_E14_vs_E10	ordinary_muscle_development	ENSGALG00010012205	SLC6A13	-1.210732564	0.194610364	-6.221315974	4.93002E-10	4.2011E-09	negative
PRJNA729271	stage_E14_vs_E10	ordinary_muscle_development	ENSGALG00010012206	RIBC2	-2.361582395	0.668152269	-3.534497306	0.000408552	0.001412343	negative
PRJNA729271	stage_E14_vs_E10	ordinary_muscle_development	ENSGALG00010012207	GRIA3	0.692560488	0.252544367	2.742331958	0.006100466	0.015478076	positive
PRJNA729271	stage_E14_vs_E10	ordinary_muscle_development	ENSGALG00010012209	KIF14	-0.030650068	0.225078235	-0.136175177	0.891682798	0.925656532	negative
PRJNA729271	stage_E14_vs_E10	ordinary_muscle_development	ENSGALG00010012210	PPWD1	-0.392342779	0.073551193	-5.334281658	9.59235E-08	6.12786E-07	negative
PRJNA729271	stage_E14_vs_E10	ordinary_muscle_development	ENSGALG00010012211	ENSGALG00010012211	2.188873626	3.990860623	0.548471579	0.583368139	0.684295527	positive
PRJNA729271	stage_E14_vs_E10	ordinary_muscle_development	ENSGALG00010012212	RAD54B	0.188863072	0.178829092	1.056109322	0.290918272	0.403232484	positive
PRJNA729271	stage_E14_vs_E10	ordinary_muscle_development	ENSGALG00010012215	RAB17	0.73091866	0.930022133	0.785915339	0.431917088	0.546097907	positive
PRJNA729271	stage_E14_vs_E10	ordinary_muscle_development	ENSGALG00010012216	BRD8	0.160528667	0.056027646	2.865168878	0.004167869	0.011078985	positive
PRJNA729271	stage_E14_vs_E10	ordinary_muscle_development	ENSGALG00010012219	KDM5A	-0.370142403	0.076674279	-4.827465079	1.38282E-06	7.50429E-06	negative
PRJNA729271	stage_E14_vs_E10	ordinary_muscle_development	ENSGALG00010012226	SLCO4C1	-0.528947575	0.523580083	-1.01025152	0.312374802	0.426215986	negative
PRJNA729271	stage_E14_vs_E10	ordinary_muscle_development	ENSGALG00010012228	NHP2	-0.219314024	0.109564358	-2.001691324	0.04531794	0.087321135	negative
PRJNA729271	stage_E14_vs_E10	ordinary_muscle_development	ENSGALG00010012229	FSBP	0.998123329	0.200772867	4.97140547	6.64693E-07	3.76062E-06	positive
PRJNA729271	stage_E14_vs_E10	ordinary_muscle_development	ENSGALG00010012230	PTPRQ	0.743829424	0.387804312	1.918053513	0.055104226	0.102890088	positive
PRJNA729271	stage_E14_vs_E10	ordinary_muscle_development	ENSGALG00010012232	PCSK1	-0.191441583	0.431565638	-0.443597835	0.657333371	0.747383308	negative
PRJNA729271	stage_E14_vs_E10	ordinary_muscle_development	ENSGALG00010012234	C1GALT1C1	-0.339589117	0.060557139	-5.60774706	2.04977E-08	1.43951E-07	negative
PRJNA729271	stage_E14_vs_E10	ordinary_muscle_development	ENSGALG00010012236	PRLH	-0.703988128	0.48984265	-1.437171974	0.150669134	0.238228557	negative
PRJNA729271	stage_E14_vs_E10	ordinary_muscle_development	ENSGALG00010012240	TRIM14	-0.309127313	0.22217208	-1.391386862	0.164108146	0.255011533	negative
PRJNA729271	stage_E14_vs_E10	ordinary_muscle_development	ENSGALG00010012242	CCDC77	-0.387624335	0.122278485	-3.17001258	0.001524323	0.004587846	negative
PRJNA729271	stage_E14_vs_E10	ordinary_muscle_development	ENSGALG00010012243	ENSGALG00010012243	1.470064423	1.468455818	1.00109544	0.316780669	0.430714427	positive
PRJNA729271	stage_E14_vs_E10	ordinary_muscle_development	ENSGALG00010012244	ENSGALG00010012244	-0.017310699	0.079319598	-0.21823987	0.827242222	0.879160936	negative
PRJNA729271	stage_E14_vs_E10	ordinary_muscle_development	ENSGALG00010012245	ENSGALG00010012245	0.294115346	0.077031681	3.818108872	0.000134479	0.000517919	positive
PRJNA729271	stage_E14_vs_E10	ordinary_muscle_development	ENSGALG00010012247	TRIM23	0.431027492	0.125608673	3.431510602	0.00060023	0.001991378	positive
PRJNA729271	stage_E14_vs_E10	ordinary_muscle_development	ENSGALG00010012248	H2AC39	0.574936791	0.209750576	2.741049882	0.006124321	0.015530446	positive
PRJNA729271	stage_E14_vs_E10	ordinary_muscle_development	ENSGALG00010012250	NANS	-0.436560856	0.110393259	-3.954597047	7.66637E-05	0.000308079	negative
PRJNA729271	stage_E14_vs_E10	ordinary_muscle_development	ENSGALG00010012253	MYF6	1.342249273	0.289584259	4.635090592	3.56781E-06	1.7988E-05	positive
PRJNA729271	stage_E14_vs_E10	ordinary_muscle_development	ENSGALG00010012259	IL1RAPL2	-0.121556795	0.21695397	-0.56028841	0.575282731	0.677229204	negative
PRJNA729271	stage_E14_vs_E10	ordinary_muscle_development	ENSGALG00010012260	ENSGALG00010012260	1.020947417	0.303075876	3.368619866	0.000755455	0.002450286	positive
PRJNA729271	stage_E14_vs_E10	ordinary_muscle_development	ENSGALG00010012263	SHLD3	-0.071564068	0.127312223	-0.562114666	0.574037897	0.675873553	negative
PRJNA729271	stage_E14_vs_E10	ordinary_muscle_development	ENSGALG00010012264	MYF5	1.559085096	0.15426833	10.10631994	5.17934E-24	1.43546E-22	positive
PRJNA729271	stage_E14_vs_E10	ordinary_muscle_development	ENSGALG00010012266	H3C33	0.377200788	0.202687152	1.860999988	0.062744178	0.114934521	positive
PRJNA729271	stage_E14_vs_E10	ordinary_muscle_development	ENSGALG00010012268	ENSGALG00010012268	0.91104184	0.162033969	5.62253609	1.88174E-08	1.32729E-07	positive
PRJNA729271	stage_E14_vs_E10	ordinary_muscle_development	ENSGALG00010012271	ENSGALG00010012271	0.749314231	0.258270925	2.901272111	0.00371651	0.010018749	positive
PRJNA729271	stage_E14_vs_E10	ordinary_muscle_development	ENSGALG00010012274	B4GALNT3	0.3111165	0.276759241	1.124141325	0.260953077	0.369133878	positive
PRJNA729271	stage_E14_vs_E10	ordinary_muscle_development	ENSGALG00010012275	VIRMA	0.020177315	0.072981783	0.27647057	0.782186661	0.846230456	positive
PRJNA729271	stage_E14_vs_E10	ordinary_muscle_development	ENSGALG00010012277	H3C33	0.459927922	0.209752846	2.192713621	0.028328019	0.058542149	positive

PRJNA729271	stage_E14_vs_E10	ordinary_muscle_development	ENSGALG00010012282	ENSGALG00010012282	1.370125884	1.47332367	0.929955795			positive
PRJNA729271	stage_E14_vs_E10	ordinary_muscle_development	ENSGALG00010012283	CUPIN1A	0.00332607	0.754974394	0.00440554	0.996484899	0.998160417	positive
PRJNA729271	stage_E14_vs_E10	ordinary_muscle_development	ENSGALG00010012285	FBLN1	-0.648445254	0.198629719	-3.264593314	0.001096213	0.003419327	negative
PRJNA729271	stage_E14_vs_E10	ordinary_muscle_development	ENSGALG00010012286	ENSGALG00010012286	1.992750868	2.668998224	0.746628773	0.455287675	0.568374374	positive
PRJNA729271	stage_E14_vs_E10	ordinary_muscle_development	ENSGALG00010012288	MCTS1	-0.226961615	0.113717217	-1.995842146	0.045951108	0.088430869	negative
PRJNA729271	stage_E14_vs_E10	ordinary_muscle_development	ENSGALG00010012290	ENSGALG00010012290	0.245463899	0.093070295	2.637403245	0.008354345	0.020403747	positive
PRJNA729271	stage_E14_vs_E10	ordinary_muscle_development	ENSGALG00010012291	NR5A2	1.69450481	0.185699641	9.124976227	7.1752E-20	1.52276E-18	positive
PRJNA729271	stage_E14_vs_E10	ordinary_muscle_development	ENSGALG00010012293	SRGAP1	0.051560176	0.104519288	0.493307758	0.621795147	0.71701835	positive
PRJNA729271	stage_E14_vs_E10	ordinary_muscle_development	ENSGALG00010012294	ENSGALG00010012294	0.094825615	0.487583484	0.194480777	0.845799446	0.892600143	positive
PRJNA729271	stage_E14_vs_E10	ordinary_muscle_development	ENSGALG00010012295	CUL4B	-0.155176508	0.065021941	-2.386525323	0.017008436	0.037812466	negative
PRJNA729271	stage_E14_vs_E10	ordinary_muscle_development	ENSGALG00010012297	ABLIM3	2.403953359	0.184051919	13.06127844	5.47997E-39	3.91291E-37	positive
PRJNA729271	stage_E14_vs_E10	ordinary_muscle_development	ENSGALG00010012300	NINJ2	-3.118158117	0.224137948	-13.91178132	5.37246E-44	5.33382E-42	negative
PRJNA729271	stage_E14_vs_E10	ordinary_muscle_development	ENSGALG00010012303	CA9	-0.282284836	0.213383024	-1.322902031	0.18586796	0.281933453	negative
PRJNA729271	stage_E14_vs_E10	ordinary_muscle_development	ENSGALG00010012304	ESRP1	-1.067955259	0.565290344	-1.889215463	0.058862964	0.108726767	negative
PRJNA729271	stage_E14_vs_E10	ordinary_muscle_development	ENSGALG00010012307	ENSGALG00010012307	0.362858478	0.172637	2.101858113	0.035565707	0.070872274	positive
PRJNA729271	stage_E14_vs_E10	ordinary_muscle_development	ENSGALG00010012308	TRABD2A	1.167830552	0.343694893	3.397869958	0.000679127	0.002227641	positive
PRJNA729271	stage_E14_vs_E10	ordinary_muscle_development	ENSGALG00010012309	TRAPPC13	-0.112616201	0.123769573	-0.909885994	0.362882637	0.479007725	negative
PRJNA729271	stage_E14_vs_E10	ordinary_muscle_development	ENSGALG00010012311	ENSGALG00010012311	-2.795281951	0.514691953	-5.430980479	5.60453E-08	3.69094E-07	negative
PRJNA729271	stage_E14_vs_E10	ordinary_muscle_development	ENSGALG00010012312	SH3TC2	0.403189031	0.179504933	2.246116711	0.024696533	0.05217906	positive
PRJNA729271	stage_E14_vs_E10	ordinary_muscle_development	ENSGALG00010012314	ADRB2	1.67049561	0.268612645	6.218976075	5.0041E-10	4.25921E-09	positive
PRJNA729271	stage_E14_vs_E10	ordinary_muscle_development	ENSGALG00010012315	ATP6V1E1	-0.130407725	0.100722295	-1.294725507	0.195414938	0.293618692	negative
PRJNA729271	stage_E14_vs_E10	ordinary_muscle_development	ENSGALG00010012316	ENSGALG00010012316	0.535269635	0.766712476	0.698136071	0.485092102	0.596647539	positive
PRJNA729271	stage_E14_vs_E10	ordinary_muscle_development	ENSGALG00010012317	ENSGALG00010012317	-0.226130691	1.148044865	-0.196970256	0.843850814	0.891322418	negative
PRJNA729271	stage_E14_vs_E10	ordinary_muscle_development	ENSGALG00010012318	POLR1D	0.132663769	0.132246525	1.003155049	0.315786058	0.429714505	positive
PRJNA729271	stage_E14_vs_E10	ordinary_muscle_development	ENSGALG00010012320	DPY19L4	-0.127211003	0.060398275	-2.106202573	0.03518676	0.070233005	negative
PRJNA729271	stage_E14_vs_E10	ordinary_muscle_development	ENSGALG00010012322	ENSGALG00010012322	-1.214485423	0.266178533	-4.562672314	5.05066E-06	2.48842E-05	negative
PRJNA729271	stage_E14_vs_E10	ordinary_muscle_development	ENSGALG00010012323	SMAD7B	3.092418259	3.510131915	0.880997733	0.378319047	0.494520208	positive
PRJNA729271	stage_E14_vs_E10	ordinary_muscle_development	ENSGALG00010012324	ENSGALG00010012324	0.377936383	0.162802269	2.321444206	0.020262882	0.044001812	positive
PRJNA729271	stage_E14_vs_E10	ordinary_muscle_development	ENSGALG00010012325	MLPH	-0.112279162	0.496145975	-0.226302677	0.820966017	0.87471082	negative
PRJNA729271	stage_E14_vs_E10	ordinary_muscle_development	ENSGALG00010012326	SGTB	-0.326232533	0.115796861	-2.817283044	0.004843182	0.012660402	negative
PRJNA729271	stage_E14_vs_E10	ordinary_muscle_development	ENSGALG00010012327	UBTD2	-0.180254001	0.08462004	-2.130157363	0.033158624	0.066801147	negative
PRJNA729271	stage_E14_vs_E10	ordinary_muscle_development	ENSGALG00010012329	ATXN10	-0.063898432	0.10532325	-0.606688763	0.544057481	0.649693846	negative
PRJNA729271	stage_E14_vs_E10	ordinary_muscle_development	ENSGALG00010012331	ENSGALG00010012331	0.521584046	0.251705205	2.072202064	0.038246601	0.075416199	positive
PRJNA729271	stage_E14_vs_E10	ordinary_muscle_development	ENSGALG00010012334	ARHGEF39	-0.089629211	0.215507935	-0.415897497	0.677485012	0.763678765	negative
PRJNA729271	stage_E14_vs_E10	ordinary_muscle_development	ENSGALG00010012336	ENSGALG00010012336	3.726193554	4.094052487	0.910147968	0.36274448	0.478880126	positive
PRJNA729271	stage_E14_vs_E10	ordinary_muscle_development	ENSGALG00010012337	ACSS3	0.615061949	0.151255915	4.066366248	4.77519E-05	0.000199413	positive
PRJNA729271	stage_E14_vs_E10	ordinary_muscle_development	ENSGALG00010012338	H3C33	0.701562554	0.224805905	3.120747895	0.001803924	0.005323265	positive
PRJNA729271	stage_E14_vs_E10	ordinary_muscle_development	ENSGALG00010012339	WNK1	-0.260472525	0.155010755	-1.680351307	0.092888984	0.15996505	negative
PRJNA729271	stage_E14_vs_E10	ordinary_muscle_development	ENSGALG00010012340	ENHO	0.54397638	0.15765542	3.450413427	0.000559729	0.001872869	positive
PRJNA729271	stage_E14_vs_E10	ordinary_muscle_development	ENSGALG00010012341	GOPC	-0.023008059	0.079893745	-0.287983229	0.773359577	0.839437402	negative
PRJNA729271	stage_E14_vs_E10	ordinary_muscle_development	ENSGALG00010012342	ENSGALG00010012342	2.024216284	1.486639791	1.361605075	0.173322553	0.266416798	positive
PRJNA729271	stage_E14_vs_E10	ordinary_muscle_development	ENSGALG00010012343	BCL2L13	0.625887329	0.170335525	3.674438022	0.000238373	0.00086902	positive
PRJNA729271	stage_E14_vs_E10	ordinary_muscle_development	ENSGALG00010012345	H2BC32	0.435934343	0.164955431	2.642740159	0.008223812	0.020129037	positive
PRJNA729271	stage_E14_vs_E10	ordinary_muscle_development	ENSGALG00010012346	NLN	-0.301152756	0.137125529	-2.196182998	0.028078848	0.058151579	negative
PRJNA729271	stage_E14_vs_E10	ordinary_muscle_development	ENSGALG00010012347	ENSGALG00010012347	-1.536911611	0.472134521	-3.255240919	0.001132962	0.003520319	negative
PRJNA729271	stage_E14_vs_E10	ordinary_muscle_development	ENSGALG00010012350	H2AC32	0.49275599	0.192032072	2.566008816	0.010287621	0.024377811	positive
PRJNA729271	stage_E14_vs_E10	ordinary_muscle_development	ENSGALG00010012352	PTPRC	0.389872399	0.195087158	1.998452403	0.045667635	0.087932037	positive
PRJNA729271	stage_E14_vs_E10	ordinary_muscle_development	ENSGALG00010012353	PIGG	0.334790475	0.15160256	2.208343149	0.027220362	0.05665697	positive
PRJNA729271	stage_E14_vs_E10	ordinary_muscle_development	ENSGALG00010012354	DCBLD1	-0.059445038	0.149767246	-0.396916147	0.691429297	0.774686268	negative
PRJNA729271	stage_E14_vs_E10	ordinary_muscle_development	ENSGALG00010012358	STK10	-0.438032282	0.149414916	-2.931650283	0.003371662	0.009200346	negative
PRJNA729271	stage_E14_vs_E10	ordinary_muscle_development	ENSGALG00010012360	ROS1	2.085148072	0.372361084	5.599801272	2.14598E-08	1.50274E-07	positive
PRJNA729271	stage_E14_vs_E10	ordinary_muscle_development	ENSGALG00010012361	WIF1	-4.864256092	0.349525774	-13.91673077	5.01315E-44	5.04623E-42	negative
PRJNA729271	stage_E14_vs_E10	ordinary_muscle_development	ENSGALG00010012362	COL6A3	0.90334876	0.292500842	3.088362936	0.002012625	0.005847433	positive
PRJNA729271	stage_E14_vs_E10	ordinary_muscle_development	ENSGALG00010012366	INTS8	-0.062557829	0.066615561	-0.939087317	0.347685916	0.463463983	negative
PRJNA729271	stage_E14_vs_E10	ordinary_muscle_development	ENSGALG00010012367	RAD52	-0.225575243	0.123477179	-1.826857756	0.067721159	0.122579696	negative
PRJNA729271	stage_E14_vs_E10	ordinary_muscle_development	ENSGALG00010012371	ATP6V1G3	-4.310553962	0.719817038	-5.988402239	2.11912E-09	1.67576E-08	negative
PRJNA729271	stage_E14_vs_E10	ordinary_muscle_development	ENSGALG00010012372	LEMD3	-0.060498148	0.07232172	-0.836514237	0.402865671	0.518056946	negative
PRJNA729271	stage_E14_vs_E10	ordinary_muscle_development	ENSGALG00010012373	WNT7B	-3.96079612	0.520796298	-7.605269344	2.84311E-14	3.76699E-13	negative
PRJNA729271	stage_E14_vs_E10	ordinary_muscle_development	ENSGALG00010012376	VGLL2	2.055859139	0.185600421	11.07680215	1.62576E-28	6.35185E-27	positive
PRJNA729271	stage_E14_vs_E10	ordinary_muscle_development	ENSGALG00010012378	RPS19BP1	-0.000107768	0.089859	-0.001199297	0.9990431	0.999350415	negative
PRJNA729271	stage_E14_vs_E10	ordinary_muscle_development	ENSGALG00010012382	SH3PXD2B	-0.300477635	0.1321539	-2.273694795	0.022984343	0.049102145	negative
PRJNA729271	stage_E14_vs_E10	ordinary_muscle_development	ENSGALG00010012384	NRK	0.051382838	0.121760965	0.421997623	0.673026753	0.75948017	positive
PRJNA729271	stage_E14_vs_E10	ordinary_muscle_development	ENSGALG00010012387	ERBIN	0.040320578	0.088645216	0.454853399	0.649214711	0.740872377	positive
PRJNA729271	stage_E14_vs_E10	ordinary_muscle_development	ENSGALG00010012390	MMP16	-0.943864899	0.174269398	-5.416125317	6.09044E-08	3.99642E-07	negative
PRJNA729271	stage_E14_vs_E10	ordinary_muscle_development	ENSGALG00010012391	NEURL1B	0.742637162	0.155818166	4.766049946	1.87873E-06	9.94237E-06	positive
PRJNA729271	stage_E14_vs_E10	ordinary_muscle_development	ENSGALG00010012393	ENSGALG00010012393	1.823707291	1.603321111	1.137456046	0.255347689	0.363011451	positive
PRJNA729271	stage_E14_vs_E10	ordinary_muscle_development	ENSGALG00010012398	ENSGALG00010012398	-1.897443068	0.491712934	-3.858843115	0.000113925	0.000444387	negative
PRJNA729271	stage_E14_vs_E10	ordinary_muscle_development	ENSGALG00010012399	ERC1	-0.711633285	0.084258091	-8.445874795	3.01777E-17	5.16441E-16	negative
PRJNA729271	stage_E14_vs_E10	ordinary_muscle_development	ENSGALG00010012401	RIPK2	-0.407897598	0.132781007	-3.071957414	0.002126601	0.006139238	negative
PRJNA729271	stage_E14_vs_E10	ordinary_muscle_development	ENSGALG00010012404	VAMP7	0.173231176	0.051094791	3.390388177	0.000697937	0.002283657	positive
PRJNA729271	stage_E14_vs_E10	ordinary_muscle_development	ENSGALG00010012405	MSRB3	1.485651894	0.125858188	11.80417355	3.71422E-32	1.77682E-30	positive
PRJNA729271	stage_E14_vs_E10	ordinary_muscle_development	ENSGALG00010012406	ENSGALG00010012406	1.929352637	0.245193698	7.8686877	3.58381E-15	5.11796E-14	positive
PRJNA729271	stage_E14_vs_E10	ordinary_muscle_development	ENSGALG00010012407	ATF4	0.303960362	0.128831601	2.359361836	0.018306396	0.040265738	positive
PRJNA729271	stage_E14_vs_E10	ordinary_muscle_development	ENSGALG00010012410	CACNA1I	-1.454510647	0.387014905	-3.758280695	0.000171085	0.000644793	negative
PRJNA729271	stage_E14_vs_E10	ordinary_muscle_development	ENSGALG00010012412	PURA	0.453024588	0.132502433	3.4189907	0.000628539	0.002074294	positive
PRJNA729271	stage_E14_vs_E10	ordinary_muscle_development	ENSGALG00010012413	NEK7	0.060985843	0.072183464	0.844872769	0.39818188	0.513537357	positive
PRJNA729271	stage_E14_vs_E10	ordinary_muscle_development	ENSGALG00010012414	ENSGALG00010012414	3.861478732	0.203395811	18.98504549	2.2674E-80	1.09553E-77	positive
PRJNA729271	stage_E14_vs_E10	ordinary_muscle_development	ENSGALG00010012415	BID	-0.124575918	0.112178516	-1.110514939	0.266777194	0.375758958	negative
PRJNA729271	stage_E14_vs_E10	ordinary_muscle_development	ENSGALG00010012418	ENSGALG00010012418	-0.06407304	0.784125297	-0.081712757	0.934875134	0.954677816	negative
PRJNA729271	stage_E14_vs_E10	ordinary_muscle_development	ENSGALG00010012419	HACE1	-0.201193594	0.096654474	-2.081575604	0.037381252	0.073951309	negative
PRJNA729271	stage_E14_vs_E10	ordinary_muscle_development	ENSGALG00010012426	MIEF1	0.081667398	0.079323388	1.029550047	0.303221275	0.416882518	positive
PRJNA729271	stage_E14_vs_E10	ordinary_muscle_development	ENSGALG00010012427	C7	-1.886198526	0.461706637	-4.085274877	4.40247E-05	0.000184753	negative
PRJNA729271	stage_E14_vs_E10	ordinary_muscle_development	ENSGALG00010012428	CCNE2	-0.46772838	0.206170659	-2.268646677	0.023289821	0.049637693	negative
PRJNA729271	stage_E14_vs_E10	ordinary_muscle_development	ENSGALG00010012429	CXCR7	-0.173982167	0.101785925	-1.709294945	0.087396329	0.152150284	negative
PRJNA729271	stage_E14_vs_E10	ordinary_muscle_development	ENSGALG00010012431	LAMP2	-0.116917683	0.079507294	-1.470527768	0.141418871	0.226245684	negative
PRJNA729271	stage_E14_vs_E10	ordinary_muscle_development	ENSGALG00010012433	ENSGALG00010012433	-0.526809123	0.743112303	-0.708922623	0.478372495	0.590480228	negative
PRJNA729271	stage_E14_vs_E10	ordinary_muscle_development	ENSGALG00010012436	ENSGALG00010012436	1.397502506	0.200421179	6.972828498	3.10631E-12	3.37779E-11	positive
PRJNA729271	stage_E14_vs_E10	ordinary_muscle_development	ENSGALG00010012437	ENSGALG00010012437	3.772443491	3.784099674	0.996919695	0.318803491	0.43292642	positive
PRJNA729271	stage_E14_vs_E10	ordinary_muscle_development	ENSGALG00010012439	TENT2	-0.12696229	0.101561144	-1.250106921	0.211260492	0.31231217	negative
PRJNA729271	stage_E14_vs_E10	ordinary_muscle_development	ENSGALG00010012441	FST	-0.37647216	0.239159884	-1.574144264	0.115454101	0.19184996	negative
PRJNA729271	stage_E14_vs_E10	ordinary_muscle_development	ENSGALG00010012442	CPLX1	-4.09991289	0.325917388	-12.57960773	2.73384E-36	1.64427E-34	negative
PRJNA729271	stage_E14_vs_E10	ordinary_muscle_development	ENSGALG00010012445	SOBP	-0.743184214	0.16334005	-4.549920323	5.36662E-06	2.63514E-05	negative
PRJNA729271	stage_E14_vs_E10	ordinary_muscle_development	ENSGALG00010012447	COPS8	0.155620126	0.116375772	1.337220994	0.181150511	0.276194031	positive
PRJNA729271	stage_E14_vs_E10	ordinary_muscle_development	ENSGALG00010012449	SUB1	-0.830264709	0.109272799	-7.598091378	3.0053E-14	3.971E-13	negative
PRJNA729271	stage_E14_vs_E10	ordinary_muscle_development	ENSGALG00010012450	PPFIA2	-1.306133984	0.248538903	-5.255249653	1.47824E-07	9.16862E-07	negative
PRJNA729271	stage_E14_vs_E10	ordinary_muscle_development	ENSGALG00010012454	IQCA1	0.451275213	0.446711561	1.010216105	0.312391765	0.426215986	positive
PRJNA729271	stage_E14_vs_E10	ordinary_muscle_development	ENSGALG00010012456	ENSGALG00010012456	0.086933047	0.084261131	1.031709941	0.302208019	0.415883911	positive
PRJNA729271	stage_E14_vs_E10	ordinary_muscle_development	ENSGALG00010012458	MRPS30	0.195091064	0.107391069	1.816641417	0.069272037	0.124934451	positive
PRJNA729271	stage_E14_vs_E10	ordinary_muscle_development	ENSGALG00010012464	ENSGALG00010012464	0.248852407	0.237731096	1.046781053	0.295200571	0.408372998	positive
PRJNA729271	stage_E14_vs_E10	ordinary_muscle_development	ENSGALG00010012466	PDSS2	0.022896347	0.120949745	0.189304631	0.849854066	0.895848763	positive
PRJNA729271	stage_E14_vs_E10	ordinary_muscle_development	ENSGALG00010012469	CETN2	-0.464079617	0.128891001	-3.600558728	0.000317534	0.001129764	negative
PRJNA729271	stage_E14_vs_E10	ordinary_muscle_development	ENSGALG00010012470	CHD1	-0.412439081	0.10554604	-3.90766986	9.31905E-05	0.000368771	negative
PRJNA729271	stage_E14_vs_E10	ordinary_muscle_development	ENSGALG00010012474	ASB18	2.775120539	0.92909013	2.986922851	0.002818008	0.007861245	positive
PRJNA729271	stage_E14_vs_E10	ordinary_muscle_development	ENSGALG00010012477	ENSGALG00010012477	-0.052153611	0.254478323	-0.204943235	0.837616476	0.886679491	negative
PRJNA729271	stage_E14_vs_E10	ordinary_muscle_development	ENSGALG00010012483	ENSGALG00010012483	-0.047672377	0.253550537	-0.188019232	0.850861575	0.896636753	negative
PRJNA729271	stage_E14_vs_E10	ordinary_muscle_development	ENSGALG00010012485	C6	-0.081547901	1.424183809	-0.057259394	0.954338566	0.968639277	negative
PRJNA729271	stage_E14_vs_E10	ordinary_muscle_development	ENSGALG00010012486	RADX	-0.03100755	0.1078581	-0.287484662	0.773741244	0.839663048	negative
PRJNA729271	stage_E14_vs_E10	ordinary_muscle_development	ENSGALG00010012489	GBX2	-2.821350345	0.866417308	-3.256341164	0.001128581	0.003508208	negative
PRJNA729271	stage_E14_vs_E10	ordinary_muscle_development	ENSGALG00010012497	PLCXD3	-0.599436879	0.407745665	-1.470124469	0.141528046	0.226354301	negative
PRJNA729271	stage_E14_vs_E10	ordinary_muscle_development	ENSGALG00010012498	KCNT2	0.195248066	0.21442606	0.910561271	0.362526585	0.47867087	positive
PRJNA729271	stage_E14_vs_E10	ordinary_muscle_development	ENSGALG00010012506	TP53INP1	-0.364803543	0.125188896	-2.914024776	0.003568016	0.009661571	negative
PRJNA729271	stage_E14_vs_E10	ordinary_muscle_development	ENSGALG00010012508	MDM1	-0.219720472	0.065756265	-3.341437824	0.000833457	0.002679298	negative
PRJNA729271	stage_E14_vs_E10	ordinary_muscle_development	ENSGALG00010012509	MICAL3	0.165402818	0.09337856	1.771314727	0.07650838	0.136061677	positive
PRJNA729271	stage_E14_vs_E10	ordinary_muscle_development	ENSGALG00010012511	OXCT1	-0.333806937	0.122065414	-2.734656162	0.006244547	0.015791121	negative
PRJNA729271	stage_E14_vs_E10	ordinary_muscle_development	ENSGALG00010012517	MGAT3	-0.556741381	0.175881103	-3.165441709	0.001548477	0.004644149	negative
PRJNA729271	stage_E14_vs_E10	ordinary_muscle_development	ENSGALG00010012519	ENSGALG00010012519	1.547747176	1.670106304	0.926735724	0.354063764	0.47036516	positive
PRJNA729271	stage_E14_vs_E10	ordinary_muscle_development	ENSGALG00010012521	HOMER1	1.02531269	0.129619733	7.910158965	2.5706E-15	3.71865E-14	positive
PRJNA729271	stage_E14_vs_E10	ordinary_muscle_development	ENSGALG00010012529	BEND3	-0.196802323	0.123965431	-1.587558091	0.112386301	0.187612511	negative
PRJNA729271	stage_E14_vs_E10	ordinary_muscle_development	ENSGALG00010012532	FAM174A	-0.055209069	0.15202695	-0.363153169	0.716490468	0.795051779	negative
PRJNA729271	stage_E14_vs_E10	ordinary_muscle_development	ENSGALG00010012535	JMY	-0.336561067	0.102608782	-3.280041531	0.001037918	0.003262768	negative
PRJNA729271	stage_E14_vs_E10	ordinary_muscle_development	ENSGALG00010012536	FBXO4	-0.180254716	0.133077604	-1.354508279	0.175574274	0.269192839	negative
PRJNA729271	stage_E14_vs_E10	ordinary_muscle_development	ENSGALG00010012538	RAP1B	-0.25673363	0.087638935	-2.929447166	0.003395655	0.009256467	negative
PRJNA729271	stage_E14_vs_E10	ordinary_muscle_development	ENSGALG00010012547	ENSGALG00010012547	0.696688884	0.334230392	2.084457013	0.037118616	0.073532096	positive
PRJNA729271	stage_E14_vs_E10	ordinary_muscle_development	ENSGALG00010012550	TAB1	0.289662016	0.093069433	3.112321683	0.001856221	0.005462154	positive
PRJNA729271	stage_E14_vs_E10	ordinary_muscle_development	ENSGALG00010012551	PCGF3	-0.74405727	0.156216266	-4.762994845	1.90741E-06	1.00831E-05	negative
PRJNA729271	stage_E14_vs_E10	ordinary_muscle_development	ENSGALG00010012553	ENSGALG00010012553	-0.355201355	0.224748767	-1.580437391	0.114006734	0.189961274	negative
PRJNA729271	stage_E14_vs_E10	ordinary_muscle_development	ENSGALG00010012555	ENSGALG00010012555	3.10939902	0.21285953	14.60775104	2.50656E-48	3.30297E-46	positive
PRJNA729271	stage_E14_vs_E10	ordinary_muscle_development	ENSGALG00010012561	OSGIN2	-0.617102382	0.122739206	-5.027752776	4.96261E-07	2.86586E-06	negative
PRJNA729271	stage_E14_vs_E10	ordinary_muscle_development	ENSGALG00010012565	SYNGR1	-0.006855514	0.122283057	-0.056062666	0.955291885	0.968996212	negative
PRJNA729271	stage_E14_vs_E10	ordinary_muscle_development	ENSGALG00010012569	C6orf203	0.131540485	0.121551225	1.082181482	0.279171895	0.390033409	positive
PRJNA729271	stage_E14_vs_E10	ordinary_muscle_development	ENSGALG00010012571	AGAP1	0.853991707	0.080873336	10.55962017	4.58513E-26	1.51393E-24	positive

PRJNA729271	stage_E14_vs_E10	ordinary_muscle_development	ENSGALG00010012574	PHAX	-0.264599204	0.104664486	-2.528070559	0.01146913	0.02688744	negative
PRJNA729271	stage_E14_vs_E10	ordinary_muscle_development	ENSGALG00010012578	NDUFS4	0.236195616	0.15282562	1.54552369	0.122219643	0.200563085	positive
PRJNA729271	stage_E14_vs_E10	ordinary_muscle_development	ENSGALG00010012582	CD24	-2.581684421	0.223553828	-11.54837937	7.52255E-31	3.36541E-29	negative
PRJNA729271	stage_E14_vs_E10	ordinary_muscle_development	ENSGALG00010012583	NDUFAF6	0.042512546	0.100490929	0.423048592	0.672259809	0.758910119	positive
PRJNA729271	stage_E14_vs_E10	ordinary_muscle_development	ENSGALG00010012586	CDC73	-0.227847235	0.133815716	-1.702694131	0.088625324	0.153865621	negative
PRJNA729271	stage_E14_vs_E10	ordinary_muscle_development	ENSGALG00010012587	MBLAC2	-0.153319402	0.148187696	-1.034629771	0.300841845	0.414752002	negative
PRJNA729271	stage_E14_vs_E10	ordinary_muscle_development	ENSGALG00010012588	PEX26	0.278777873	0.103938572	2.682140683	0.007315269	0.018113226	positive
PRJNA729271	stage_E14_vs_E10	ordinary_muscle_development	ENSGALG00010012593	TUBA8B	0.059354378	0.991677505	0.0598525	0.952273111	0.966949124	positive
PRJNA729271	stage_E14_vs_E10	ordinary_muscle_development	ENSGALG00010012599	DMGDH	2.53037513	0.196596584	12.87090078	6.56322E-38	4.48745E-36	positive
PRJNA729271	stage_E14_vs_E10	ordinary_muscle_development	ENSGALG00010012600	NUP107	-0.298766377	0.098690609	-3.027303006	0.002467465	0.00697599	negative
PRJNA729271	stage_E14_vs_E10	ordinary_muscle_development	ENSGALG00010012601	TUBA8A	0.00078891	0.249279676	0.00316476	0.997474891	0.998370291	positive
PRJNA729271	stage_E14_vs_E10	ordinary_muscle_development	ENSGALG00010012604	ENSGALG00010012604	1.520885127	2.125833995	0.715429864			positive
PRJNA729271	stage_E14_vs_E10	ordinary_muscle_development	ENSGALG00010012606	ATP5PO	0.215706591	0.114117906	1.890208101	0.058730134	0.108624893	positive
PRJNA729271	stage_E14_vs_E10	ordinary_muscle_development	ENSGALG00010012607	LMNB1	-0.185607448	0.163044894	-1.138382461	0.254960814	0.36263929	negative
PRJNA729271	stage_E14_vs_E10	ordinary_muscle_development	ENSGALG00010012610	RPL3	0.422699592	0.154942854	2.728099948	0.00637003	0.016060809	positive
PRJNA729271	stage_E14_vs_E10	ordinary_muscle_development	ENSGALG00010012614	LHFPL2	-0.240361459	0.144279514	-1.665943087	0.095724751	0.164139069	negative
PRJNA729271	stage_E14_vs_E10	ordinary_muscle_development	ENSGALG00010012618	B3GALT2	0.120545376	0.328009988	0.3675052	0.713242214	0.792582482	positive
PRJNA729271	stage_E14_vs_E10	ordinary_muscle_development	ENSGALG00010012624	PDGFB	0.251307727	0.182986076	1.373370767	0.169637113	0.262002126	positive
PRJNA729271	stage_E14_vs_E10	ordinary_muscle_development	ENSGALG00010012629	NUS1	0.232262802	0.10947732	2.1215609	0.033874629	0.068007305	positive
PRJNA729271	stage_E14_vs_E10	ordinary_muscle_development	ENSGALG00010012631	GLRX2	0.131104791	0.154715413	0.847393208	0.396775989	0.512316761	positive
PRJNA729271	stage_E14_vs_E10	ordinary_muscle_development	ENSGALG00010012633	USP18	-0.414956441	0.54287417	-0.76436947	0.444647097	0.558264155	negative
PRJNA729271	stage_E14_vs_E10	ordinary_muscle_development	ENSGALG00010012640	PRRC1	-0.183092034	0.072597624	-2.522011371	0.011668594	0.027284443	negative
PRJNA729271	stage_E14_vs_E10	ordinary_muscle_development	ENSGALG00010012648	MRPS6	-0.168465831	0.130008016	-1.295811112	0.195040569	0.293116956	negative
PRJNA729271	stage_E14_vs_E10	ordinary_muscle_development	ENSGALG00010012650	DSE	-0.760444882	0.104441629	-7.281051519	3.31228E-13	3.98436E-12	negative
PRJNA729271	stage_E14_vs_E10	ordinary_muscle_development	ENSGALG00010012653	CBX7	0.48888908	0.251780822	1.941724857	0.052170418	0.098260162	positive
PRJNA729271	stage_E14_vs_E10	ordinary_muscle_development	ENSGALG00010012654	TENT5B	0.166760106	0.31055769	0.536969818	0.591288463	0.691342498	positive
PRJNA729271	stage_E14_vs_E10	ordinary_muscle_development	ENSGALG00010012662	SLC35E3	-0.489226143	0.107253469	-4.561401589	5.08133E-06	2.50183E-05	negative
PRJNA729271	stage_E14_vs_E10	ordinary_muscle_development	ENSGALG00010012667	SCAMP1	-0.086186763	0.088177415	-0.977424468	0.328359054	0.443242266	negative
PRJNA729271	stage_E14_vs_E10	ordinary_muscle_development	ENSGALG00010012669	ERCC8	-0.062175595	0.14709196	-0.422698799	0.672515032	0.759139116	negative
PRJNA729271	stage_E14_vs_E10	ordinary_muscle_development	ENSGALG00010012670	ENSGALG00010012670	-0.566705964	0.209702043	-2.702434164	0.006883381	0.017190664	negative
PRJNA729271	stage_E14_vs_E10	ordinary_muscle_development	ENSGALG00010012671	NBN	-0.993929283	0.18450491	-5.387007218	7.16406E-08	4.67761E-07	negative
PRJNA729271	stage_E14_vs_E10	ordinary_muscle_development	ENSGALG00010012672	RO60	-0.329076684	0.087608336	-3.756225702	0.000172495	0.000649602	negative
PRJNA729271	stage_E14_vs_E10	ordinary_muscle_development	ENSGALG00010012673	ENSGALG00010012673	-0.532641084	0.316399125	-1.683446765	0.092288637	0.159139161	negative
PRJNA729271	stage_E14_vs_E10	ordinary_muscle_development	ENSGALG00010012674	ENSGALG00010012674	0.25432661	0.128625968	1.977257118	0.048012574	0.09170408	positive
PRJNA729271	stage_E14_vs_E10	ordinary_muscle_development	ENSGALG00010012676	ENSGALG00010012676	0.50556907	0.207429379	2.437306965	0.014797112	0.033555091	positive
PRJNA729271	stage_E14_vs_E10	ordinary_muscle_development	ENSGALG00010012680	TRPM8	2.660510461	0.254153897	10.46810807	1.21038E-25	3.83905E-24	positive
PRJNA729271	stage_E14_vs_E10	ordinary_muscle_development	ENSGALG00010012682	ENSGALG00010012682	-0.463399718	0.110726745	-4.185074877	2.85072E-05	0.000123902	negative
PRJNA729271	stage_E14_vs_E10	ordinary_muscle_development	ENSGALG00010012683	MDM2	0.387240953	0.085892864	4.50841824	6.53127E-06	3.16413E-05	positive
PRJNA729271	stage_E14_vs_E10	ordinary_muscle_development	ENSGALG00010012685	NPTXR	-0.49965474	0.261722892	-1.909098343	0.056249407	0.104699067	negative
PRJNA729271	stage_E14_vs_E10	ordinary_muscle_development	ENSGALG00010012688	NDUFAF2	-0.004662769	0.112621258	-0.041402213	0.966975249	0.977972804	negative
PRJNA729271	stage_E14_vs_E10	ordinary_muscle_development	ENSGALG00010012690	NT5DC1	-0.513547703	0.336244273	-1.527305427	0.126685081	0.206395442	negative
PRJNA729271	stage_E14_vs_E10	ordinary_muscle_development	ENSGALG00010012693	UCHL5	-0.046647757	0.076745944	-0.607820486	0.543306541	0.64891466	negative
PRJNA729271	stage_E14_vs_E10	ordinary_muscle_development	ENSGALG00010012696	DNAL4	-0.206177034	0.087264197	-2.362676116	0.018143518	0.039980283	negative
PRJNA729271	stage_E14_vs_E10	ordinary_muscle_development	ENSGALG00010012699	RNF128	0.716858345	0.141372011	5.070723262	3.96307E-07	2.32475E-06	positive
PRJNA729271	stage_E14_vs_E10	ordinary_muscle_development	ENSGALG00010012702	PTPRO	-2.994658005	0.343682442	-8.713444849	2.94777E-18	5.49909E-17	negative
PRJNA729271	stage_E14_vs_E10	ordinary_muscle_development	ENSGALG00010012704	SMIM15	0.121635275	0.066402182	1.831796366	0.066981765	0.121423297	positive
PRJNA729271	stage_E14_vs_E10	ordinary_muscle_development	ENSGALG00010012705	CPM	0.466297469	0.185626579	2.512018867	0.012004267	0.027983572	positive
PRJNA729271	stage_E14_vs_E10	ordinary_muscle_development	ENSGALG00010012706	TBC1D8B	0.318349481	0.10404798	3.059641166	0.002216023	0.006368211	positive
PRJNA729271	stage_E14_vs_E10	ordinary_muscle_development	ENSGALG00010012708	SPP2	1.990751759	1.096294455	1.815891479	0.069387021	0.125110693	positive
PRJNA729271	stage_E14_vs_E10	ordinary_muscle_development	ENSGALG00010012709	PPP1R8	-0.174092474	0.055520249	-3.135657299	0.001714694	0.005085839	negative
PRJNA729271	stage_E14_vs_E10	ordinary_muscle_development	ENSGALG00010012711	ELL2	-0.17224392	0.122955566	-1.400863143	0.161255002	0.251575854	negative
PRJNA729271	stage_E14_vs_E10	ordinary_muscle_development	ENSGALG00010012712	EEF1E1	-0.096038998	0.133020819	-0.721984718	0.470303873	0.582654243	negative
PRJNA729271	stage_E14_vs_E10	ordinary_muscle_development	ENSGALG00010012714	RGS2	1.090099753	0.171792443	6.345446496	2.21781E-10	1.97101E-09	positive
PRJNA729271	stage_E14_vs_E10	ordinary_muscle_development	ENSGALG00010012719	COL10A1	-4.406469367	0.780794444	-5.64357162	1.66558E-08	1.18288E-07	negative
PRJNA729271	stage_E14_vs_E10	ordinary_muscle_development	ENSGALG00010012720	ACO2	0.385631758	0.119909922	3.216012095	0.001299854	0.003985063	positive
PRJNA729271	stage_E14_vs_E10	ordinary_muscle_development	ENSGALG00010012722	ENSGALG00010012722	-0.303558076	0.098013261	-3.097112292	0.001954158	0.005703603	negative
PRJNA729271	stage_E14_vs_E10	ordinary_muscle_development	ENSGALG00010012725	ENSGALG00010012725	-1.897516316	0.31666042	-5.992274994	2.06926E-09	1.63722E-08	negative
PRJNA729271	stage_E14_vs_E10	ordinary_muscle_development	ENSGALG00010012726	IL7R	1.632350281	0.519670697	3.141124353	0.001683006	0.00500619	positive
PRJNA729271	stage_E14_vs_E10	ordinary_muscle_development	ENSGALG00010012730	CAP2	1.337970221	0.167795021	7.973837427	1.53822E-15	2.27747E-14	positive
PRJNA729271	stage_E14_vs_E10	ordinary_muscle_development	ENSGALG00010012732	MGST1	-0.448068068	0.173860268	-2.577173457	0.009961195	0.023759671	negative
PRJNA729271	stage_E14_vs_E10	ordinary_muscle_development	ENSGALG00010012733	ENSGALG00010012733	-0.160267944	0.978822901	-0.163735384	0.869939461	0.910847478	negative
PRJNA729271	stage_E14_vs_E10	ordinary_muscle_development	ENSGALG00010012734	TMEM19	-0.4719733	0.144567372	-3.264729057	0.001095688	0.003418425	negative
PRJNA729271	stage_E14_vs_E10	ordinary_muscle_development	ENSGALG00010012738	RAB21	0.222240634	0.068421573	3.248107628	0.001161753	0.003602827	positive
PRJNA729271	stage_E14_vs_E10	ordinary_muscle_development	ENSGALG00010012741	ENSGALG00010012741	0.174622622	0.149703534	1.166456243	0.24343003	0.349808495	positive
PRJNA729271	stage_E14_vs_E10	ordinary_muscle_development	ENSGALG00010012742	ENSGALG00010012742	1.552785436	0.173719141	8.938482146	3.94552E-19	7.91014E-18	positive
PRJNA729271	stage_E14_vs_E10	ordinary_muscle_development	ENSGALG00010012743	ENSGALG00010012743	-0.185272698	0.511290156	-0.362363124	0.717080693	0.795386238	negative
PRJNA729271	stage_E14_vs_E10	ordinary_muscle_development	ENSGALG00010012744	ARL4C	0.022174165	0.230018575	0.096401628	0.923201599	0.947183408	positive
PRJNA729271	stage_E14_vs_E10	ordinary_muscle_development	ENSGALG00010012745	MARCHF3	0.87216666	0.280230878	3.112314628	0.001856266	0.005462154	positive
PRJNA729271	stage_E14_vs_E10	ordinary_muscle_development	ENSGALG00010012747	TOB2	0.316748072	0.145468648	2.177431884	0.029448359	0.060473106	positive
PRJNA729271	stage_E14_vs_E10	ordinary_muscle_development	ENSGALG00010012748	ENSGALG00010012748	-0.04152538	0.211678672	-0.196171772	0.844475721	0.89172256	negative
PRJNA729271	stage_E14_vs_E10	ordinary_muscle_development	ENSGALG00010012749	RGS4	0.477012587	0.179738175	2.653930293	0.007956026	0.019536269	positive
PRJNA729271	stage_E14_vs_E10	ordinary_muscle_development	ENSGALG00010012751	AP3B1	-0.261174188	0.074341354	-3.513175008	0.000442786	0.001517659	negative
PRJNA729271	stage_E14_vs_E10	ordinary_muscle_development	ENSGALG00010012753	GTPBP1	0.095045446	0.051542619	1.844016615	0.065180719	0.118752453	positive
PRJNA729271	stage_E14_vs_E10	ordinary_muscle_development	ENSGALG00010012755	CLVS2	-1.568176561	0.375221187	-4.17933906	2.92358E-05	0.000126726	negative
PRJNA729271	stage_E14_vs_E10	ordinary_muscle_development	ENSGALG00010012756	RPA2	0.031877524	0.100707154	0.316536836	0.75159507	0.821906491	positive
PRJNA729271	stage_E14_vs_E10	ordinary_muscle_development	ENSGALG00010012758	NAB1	-0.160193193	0.128220243	-1.249359613	0.211533573	0.312556487	negative
PRJNA729271	stage_E14_vs_E10	ordinary_muscle_development	ENSGALG00010012759	ATP1B4	2.761674151	0.198710307	13.89799143	6.51439E-44	6.42354E-42	positive
PRJNA729271	stage_E14_vs_E10	ordinary_muscle_development	ENSGALG00010012760	PLEKHF2	0.32703188	0.105619829	3.096311384	0.001959445	0.00571674	positive
PRJNA729271	stage_E14_vs_E10	ordinary_muscle_development	ENSGALG00010012761	SMPDL3A	-0.467570836	0.229605418	-2.036410291	0.041709173	0.081205435	negative
PRJNA729271	stage_E14_vs_E10	ordinary_muscle_development	ENSGALG00010012763	SMPDL3B	-0.038862524	0.127984133	-0.303651107	0.761393711	0.83005429	negative
PRJNA729271	stage_E14_vs_E10	ordinary_muscle_development	ENSGALG00010012765	PHF5A	-0.030633855	0.106460104	-0.287749626	0.7735384	0.839505773	negative
PRJNA729271	stage_E14_vs_E10	ordinary_muscle_development	ENSGALG00010012767	JOSD1	0.187670986	0.068710396	2.731333213	0.006307866	0.015931786	positive
PRJNA729271	stage_E14_vs_E10	ordinary_muscle_development	ENSGALG00010012768	EPS8	-0.451158463	0.115731703	-3.898313519	9.6865E-05	0.000381745	negative
PRJNA729271	stage_E14_vs_E10	ordinary_muscle_development	ENSGALG00010012769	NEMP2	-0.225728665	0.077634984	-2.907563745	0.003642561	0.009841365	negative
PRJNA729271	stage_E14_vs_E10	ordinary_muscle_development	ENSGALG00010012771	ITGA2	-1.504879395	0.185619678	-8.107326804	5.17455E-16	7.94546E-15	negative
PRJNA729271	stage_E14_vs_E10	ordinary_muscle_development	ENSGALG00010012772	TOMM22	0.026563075	0.073331393	0.362233338	0.717177669	0.795386238	positive
PRJNA729271	stage_E14_vs_E10	ordinary_muscle_development	ENSGALG00010012773	XKR8	-0.184363741	0.117315469	-1.571521151	0.116061645	0.192594568	negative
PRJNA729271	stage_E14_vs_E10	ordinary_muscle_development	ENSGALG00010012775	KCNE2	0.479597473	0.146409753	3.275720805	0.001053927	0.003305923	positive
PRJNA729271	stage_E14_vs_E10	ordinary_muscle_development	ENSGALG00010012778	TBC1D15	-0.087741847	0.151403908	-0.579521679	0.562237223	0.665120254	negative
PRJNA729271	stage_E14_vs_E10	ordinary_muscle_development	ENSGALG00010012780	FABP7	-1.346652253	0.241539405	-5.575290095	2.47118E-08	1.71304E-07	negative
PRJNA729271	stage_E14_vs_E10	ordinary_muscle_development	ENSGALG00010012781	SPINT1L	1.583175136	3.183547785	0.497299002	0.618978215	0.714793597	positive
PRJNA729271	stage_E14_vs_E10	ordinary_muscle_development	ENSGALG00010012786	TEF	-0.077849172	0.088202066	-0.882623	0.377439998	0.493638254	negative
PRJNA729271	stage_E14_vs_E10	ordinary_muscle_development	ENSGALG00010012788	RGS1	-1.324627164	0.771918245	-1.716020022	0.086158378	0.150465745	negative
PRJNA729271	stage_E14_vs_E10	ordinary_muscle_development	ENSGALG00010012790	PITX1	-2.885102809	0.413331351	-6.980120927	2.94926E-12	3.22152E-11	negative
PRJNA729271	stage_E14_vs_E10	ordinary_muscle_development	ENSGALG00010012791	CFAP418	-0.25516472	0.191151836	-1.334879778	0.181915697	0.27721489	negative
PRJNA729271	stage_E14_vs_E10	ordinary_muscle_development	ENSGALG00010012792	CBY1	-0.800675605	0.143887898	-5.564579184	2.62786E-08	1.81991E-07	negative
PRJNA729271	stage_E14_vs_E10	ordinary_muscle_development	ENSGALG00010012795	TBCA	-0.013129546	0.135393318	-0.096973365	0.922747546	0.946784574	negative
PRJNA729271	stage_E14_vs_E10	ordinary_muscle_development	ENSGALG00010012796	MOCS2	-0.361144981	0.122205344	-2.955230675	0.003124354	0.008619624	negative
PRJNA729271	stage_E14_vs_E10	ordinary_muscle_development	ENSGALG00010012797	ZC3H7B	0.0828356	0.069947201	1.184258975	0.236310563	0.341065579	positive
PRJNA729271	stage_E14_vs_E10	ordinary_muscle_development	ENSGALG00010012798	MFSD6	-0.255316845	0.096434381	-2.647570742	0.00810724	0.019873911	negative
PRJNA729271	stage_E14_vs_E10	ordinary_muscle_development	ENSGALG00010012801	DMC1	0.56966907	0.456244835	1.248603877	0.211809994	0.31286924	positive
PRJNA729271	stage_E14_vs_E10	ordinary_muscle_development	ENSGALG00010012803	NUP155	-0.320339699	0.089451556	-3.581152912	0.000342081	0.001209088	negative
PRJNA729271	stage_E14_vs_E10	ordinary_muscle_development	ENSGALG00010012804	ENSGALG00010012804	-0.157093407	0.160594387	-0.978199865	0.327975483	0.442933442	negative
PRJNA729271	stage_E14_vs_E10	ordinary_muscle_development	ENSGALG00010012808	EYA3	-0.218078291	0.091727387	-2.37746107	0.017432283	0.038642138	negative
PRJNA729271	stage_E14_vs_E10	ordinary_muscle_development	ENSGALG00010012809	RHOBTB3	-0.035236215	0.26002576	-0.135510479	0.892208279	0.92599946	negative
PRJNA729271	stage_E14_vs_E10	ordinary_muscle_development	ENSGALG00010012812	DECR1	0.231214931	0.239723931	0.964505006	0.3347928	0.450002006	positive
PRJNA729271	stage_E14_vs_E10	ordinary_muscle_development	ENSGALG00010012818	RGS18	0.310878212	0.194913602	1.594953917	0.110722541	0.185219069	positive
PRJNA729271	stage_E14_vs_E10	ordinary_muscle_development	ENSGALG00010012819	RANGAP1	-0.230737923	0.066752947	-3.456595293	0.000547046	0.001834666	negative
PRJNA729271	stage_E14_vs_E10	ordinary_muscle_development	ENSGALG00010012821	ZBTB33	0.146292126	0.05113888	2.860683023	0.004227295	0.011222461	positive
PRJNA729271	stage_E14_vs_E10	ordinary_muscle_development	ENSGALG00010012824	HIBCH	0.010376072	0.117494996	0.088310753	0.929629693	0.95169026	positive
PRJNA729271	stage_E14_vs_E10	ordinary_muscle_development	ENSGALG00010012827	ENSGALG00010012827	0.312497694	0.184920454	1.689903346	0.091046447	0.157336642	positive
PRJNA729271	stage_E14_vs_E10	ordinary_muscle_development	ENSGALG00010012829	RGS5	1.056460388	0.182854534	5.777600163	7.57736E-09	5.59236E-08	positive
PRJNA729271	stage_E14_vs_E10	ordinary_muscle_development	ENSGALG00010012830	ARRDC3	0.08560521	0.149288723	0.573420472	0.566360033	0.669007308	positive
PRJNA729271	stage_E14_vs_E10	ordinary_muscle_development	ENSGALG00010012831	ENSGALG00010012831	0.412493013	0.379432522	1.087131411	0.276978738	0.387529615	positive
PRJNA729271	stage_E14_vs_E10	ordinary_muscle_development	ENSGALG00010012832	CHADL	-4.168938988	0.372582451	-11.18930583	4.60021E-29	1.87303E-27	negative
PRJNA729271	stage_E14_vs_E10	ordinary_muscle_development	ENSGALG00010012833	JAK2	0.027308423	0.105434571	0.259008246	0.795628878	0.856363005	positive
PRJNA729271	stage_E14_vs_E10	ordinary_muscle_development	ENSGALG00010012834	TPH2	-3.925633362	0.639248891	-6.141009261	8.19988E-10	6.77635E-09	negative
PRJNA729271	stage_E14_vs_E10	ordinary_muscle_development	ENSGALG00010012836	ENSGALG00010012836	0.36529082	1.152114496	0.317061214			positive
PRJNA729271	stage_E14_vs_E10	ordinary_muscle_development	ENSGALG00010012837	PKIB	-0.232583717	0.276089829	-0.842420445	0.399552653	0.514847161	negative
PRJNA729271	stage_E14_vs_E10	ordinary_muscle_development	ENSGALG00010012838	C2orf88	0.500590659	0.222909027	2.245717303	0.024722121	0.052221968	positive
PRJNA729271	stage_E14_vs_E10	ordinary_muscle_development	ENSGALG00010012839	TGFBI	-2.086298608	0.180164702	-11.57995206	5.20752E-31	2.34419E-29	negative
PRJNA729271	stage_E14_vs_E10	ordinary_muscle_development	ENSGALG00010012841	L3MBTL2	-0.213818085	0.09042942	-2.364474799	0.018055656	0.039798773	negative
PRJNA729271	stage_E14_vs_E10	ordinary_muscle_development	ENSGALG00010012842	DDX17	0.31444038	0.048692096	6.457729453	1.06286E-10	9.70157E-10	positive
PRJNA729271	stage_E14_vs_E10	ordinary_muscle_development	ENSGALG00010012843	ENSGALG00010012843	-0.425075626	0.158217468	-2.686654207	0.007217163	0.017907013	negative
PRJNA729271	stage_E14_vs_E10	ordinary_muscle_development	ENSGALG00010012845	HDHD2	0.128777339	0.101164573	1.27294897	0.203036151	0.302654155	positive
PRJNA729271	stage_E14_vs_E10	ordinary_muscle_development	ENSGALG00010012847	RLN3	-1.039512526	0.749014897	-1.387839588	0.165185897	0.256342728	negative
PRJNA729271	stage_E14_vs_E10	ordinary_muscle_development	ENSGALG00010012848	SERINC1	0.365701613	0.092447525	3.95577507	7.62869E-05	0.00030682	positive
PRJNA729271	stage_E14_vs_E10	ordinary_muscle_development	ENSGALG00010012849	TRHDE	-2.291178813	0.321929594	-7.117018298	1.10287E-12	1.24989E-11	negative
PRJNA729271	stage_E14_vs_E10	ordinary_muscle_development	ENSGALG00010012850	CALB1	-0.831886045	1.202671373	-0.69169855	0.489126658	0.599821565	negative
PRJNA729271	stage_E14_vs_E10	ordinary_muscle_development	ENSGALG00010012851	ENSGALG00010012851	0.458778079	0.23230253	1.974916414	0.048277634	0.0921375	positive
PRJNA729271	stage_E14_vs_E10	ordinary_muscle_development	ENSGALG00010012852	MSTN	0.972222854	0.418833727	2.321262094	0.020272703	0.044016301	positive

PRJNA729271	stage_E14_vs_E10	ordinary_muscle_development	ENSGALG00010012854	LECT2	2.940842783	0.746207677	3.941051365	8.11253E-05	0.000324747	positive
PRJNA729271	stage_E14_vs_E10	ordinary_muscle_development	ENSGALG00010012856	AVR2	-0.366534987	1.121558495	-0.326808623	0.743812637	0.816111161	negative
PRJNA729271	stage_E14_vs_E10	ordinary_muscle_development	ENSGALG00010012857	NSDHL	-0.187238132	0.212367457	-0.881670545	0.377954994	0.49422261	negative
PRJNA729271	stage_E14_vs_E10	ordinary_muscle_development	ENSGALG00010012859	FBXL21	0.173131258	0.111410518	1.553993834	0.120185887	0.197830393	positive
PRJNA729271	stage_E14_vs_E10	ordinary_muscle_development	ENSGALG00010012860	ENSGALG00010012860	-0.230602388	0.269128692	-0.856848025	0.39152888	0.507213434	negative
PRJNA729271	stage_E14_vs_E10	ordinary_muscle_development	ENSGALG00010012861	WDR70	0.056957985	0.081062303	0.702644547	0.482277294	0.593967874	positive
PRJNA729271	stage_E14_vs_E10	ordinary_muscle_development	ENSGALG00010012863	KCNE1	0.980439869	0.398301309	2.46155322	0.013833687	0.03163763	positive
PRJNA729271	stage_E14_vs_E10	ordinary_muscle_development	ENSGALG00010012865	EP300	-0.313819486	0.123256627	-2.546065828	0.010894464	0.02566893	negative
PRJNA729271	stage_E14_vs_E10	ordinary_muscle_development	ENSGALG00010012867	A4GALT	0.357332264	0.102033906	3.502093363	0.000461618	0.001578102	positive
PRJNA729271	stage_E14_vs_E10	ordinary_muscle_development	ENSGALG00010012868	CTHRC1	-1.328241439	0.594652354	-2.233643625	0.025506531	0.053659966	negative
PRJNA729271	stage_E14_vs_E10	ordinary_muscle_development	ENSGALG00010012869	ENSGALG00010012869	-0.627167854	0.220135694	-2.849005734	0.004385609	0.01160661	negative
PRJNA729271	stage_E14_vs_E10	ordinary_muscle_development	ENSGALG00010012870	KCNC2	-2.843321482	0.207956465	-13.67267653	1.47855E-42	1.33947E-40	negative
PRJNA729271	stage_E14_vs_E10	ordinary_muscle_development	ENSGALG00010012871	ENSGALG00010012871	-1.065114492	0.364301911	-2.923173708	0.003458827	0.009404558	negative
PRJNA729271	stage_E14_vs_E10	ordinary_muscle_development	ENSGALG00010012873	PTAFR	0.618691682	0.224109267	2.760669783	0.005768297	0.014743689	positive
PRJNA729271	stage_E14_vs_E10	ordinary_muscle_development	ENSGALG00010012875	HSF2	-0.174547407	0.132333643	-1.319198973	0.187102601	0.283420651	negative
PRJNA729271	stage_E14_vs_E10	ordinary_muscle_development	ENSGALG00010012876	KDELR3	-0.109063701	0.159503023	-0.683772001	0.49411915	0.604256903	negative
PRJNA729271	stage_E14_vs_E10	ordinary_muscle_development	ENSGALG00010012877	SLC25A32	-0.080643425	0.093732498	-0.860357154	0.389592196	0.505228488	negative
PRJNA729271	stage_E14_vs_E10	ordinary_muscle_development	ENSGALG00010012878	GDNF	-0.42470017	0.317694746	-1.336818363	0.181281934	0.276336274	negative
PRJNA729271	stage_E14_vs_E10	ordinary_muscle_development	ENSGALG00010012879	RCAN1	1.782140547	0.173058813	10.29788957	7.2024E-25	2.13495E-23	positive
PRJNA729271	stage_E14_vs_E10	ordinary_muscle_development	ENSGALG00010012880	CLK4	-0.031999472	0.14052357	-0.227716049	0.819866995	0.873885734	negative
PRJNA729271	stage_E14_vs_E10	ordinary_muscle_development	ENSGALG00010012882	ENSGALG00010012882	-1.527774303	0.776871768	-1.966572047	0.049232569	0.093700904	negative
PRJNA729271	stage_E14_vs_E10	ordinary_muscle_development	ENSGALG00010012883	DCAF13	0.070406332	0.065581639	1.073567741	0.283016462	0.394454194	positive
PRJNA729271	stage_E14_vs_E10	ordinary_muscle_development	ENSGALG00010012884	ENSGALG00010012884	0.131113899	0.205873599	0.636866018	0.52421212	0.632019284	positive
PRJNA729271	stage_E14_vs_E10	ordinary_muscle_development	ENSGALG00010012885	PMS1	0.20214879	0.128694957	1.570759211	0.11623859	0.192709467	positive
PRJNA729271	stage_E14_vs_E10	ordinary_muscle_development	ENSGALG00010012887	KCNJ4	-0.626964794	0.434433814	-1.443176781	0.148970679	0.236121376	negative
PRJNA729271	stage_E14_vs_E10	ordinary_muscle_development	ENSGALG00010012888	ENSGALG00010012888	2.850191841	3.604463319	0.790739588	0.429095969	0.543399098	positive
PRJNA729271	stage_E14_vs_E10	ordinary_muscle_development	ENSGALG00010012889	RIC1	-0.033914496	0.104904688	-0.323288658	0.746476643	0.818222848	negative
PRJNA729271	stage_E14_vs_E10	ordinary_muscle_development	ENSGALG00010012890	MFAP3	0.130979812	0.069242068	1.891621893	0.058541377	0.108289594	positive
PRJNA729271	stage_E14_vs_E10	ordinary_muscle_development	ENSGALG00010012891	CAPS2	-2.405174663	0.297576828	-8.082533441	6.34349E-16	9.67884E-15	negative
PRJNA729271	stage_E14_vs_E10	ordinary_muscle_development	ENSGALG00010012892	ORMDL1	0.082452765	0.159045417	0.518422767	0.604163334	0.702273258	positive
PRJNA729271	stage_E14_vs_E10	ordinary_muscle_development	ENSGALG00010012893	ENSGALG00010012893	-0.071172523	0.189239674	-0.376097259	0.706844592	0.787162904	negative
PRJNA729271	stage_E14_vs_E10	ordinary_muscle_development	ENSGALG00010012894	CLIC6	0.704100279	0.648455611	1.085811067	0.277562589	0.388121718	positive
PRJNA729271	stage_E14_vs_E10	ordinary_muscle_development	ENSGALG00010012896	STK38L	0.101122877	0.098785844	1.023657566	0.305997045	0.419698071	positive
PRJNA729271	stage_E14_vs_E10	ordinary_muscle_development	ENSGALG00010012897	ENSGALG00010012897	-0.221457138	0.310669568	-0.712838209	0.475945879	0.588186163	negative
PRJNA729271	stage_E14_vs_E10	ordinary_muscle_development	ENSGALG00010012898	FAM114A2	-0.028745014	0.11501729	-0.249919068	0.802649937	0.861042839	negative
PRJNA729271	stage_E14_vs_E10	ordinary_muscle_development	ENSGALG00010012899	ENSGALG00010012899	-0.281095554	0.133421503	-2.106823469	0.035132884	0.070144788	negative
PRJNA729271	stage_E14_vs_E10	ordinary_muscle_development	ENSGALG00010012900	RBX1	0.152256458	0.171800608	0.886239343	0.375488567	0.491796041	positive
PRJNA729271	stage_E14_vs_E10	ordinary_muscle_development	ENSGALG00010012903	RGMB	0.560025118	0.091458129	6.123295149	9.16597E-10	7.54033E-09	positive
PRJNA729271	stage_E14_vs_E10	ordinary_muscle_development	ENSGALG00010012904	ERMP1	0.181056554	0.133106537	1.360237882	0.173754658	0.266956337	positive
PRJNA729271	stage_E14_vs_E10	ordinary_muscle_development	ENSGALG00010012905	NKAIN1	-0.13281075	0.181787257	-0.730583389	0.465033661	0.577507104	negative
PRJNA729271	stage_E14_vs_E10	ordinary_muscle_development	ENSGALG00010012908	STRAP	-0.09781045	0.082300272	-1.188458405	0.234652858	0.339179615	negative
PRJNA729271	stage_E14_vs_E10	ordinary_muscle_development	ENSGALG00010012910	XPNPEP3	0.067526439	0.098227691	0.687448093	0.4918004	0.602073261	positive
PRJNA729271	stage_E14_vs_E10	ordinary_muscle_development	ENSGALG00010012911	CSNK1E	0.216505311	0.082444936	2.626059556	0.00863797	0.020986822	positive
PRJNA729271	stage_E14_vs_E10	ordinary_muscle_development	ENSGALG00010012912	NKAPL	0.005552022	0.098152548	0.056565238	0.954891526	0.96886131	positive
PRJNA729271	stage_E14_vs_E10	ordinary_muscle_development	ENSGALG00010012913	EGFLAM	-0.341823795	0.15278291	-2.237316954	0.025265632	0.053214957	negative
PRJNA729271	stage_E14_vs_E10	ordinary_muscle_development	ENSGALG00010012915	ENSGALG00010012915	0.989783657	0.298426219	3.316677942	0.000910946	0.002895648	positive
PRJNA729271	stage_E14_vs_E10	ordinary_muscle_development	ENSGALG00010012917	RUNX1	-1.746786495	0.260423823	-6.707475817	1.9802E-11	1.9673E-10	negative
PRJNA729271	stage_E14_vs_E10	ordinary_muscle_development	ENSGALG00010012918	GLRX	-0.042336232	0.28066781	-0.150841067	0.88010109	0.917576444	negative
PRJNA729271	stage_E14_vs_E10	ordinary_muscle_development	ENSGALG00010012921	EBF1	0.000541652	0.139603858	0.003879925	0.996904276	0.998281691	positive
PRJNA729271	stage_E14_vs_E10	ordinary_muscle_development	ENSGALG00010012922	SNRNP40	-0.195762292	0.127969009	-1.529763282	0.126075333	0.205649395	negative
PRJNA729271	stage_E14_vs_E10	ordinary_muscle_development	ENSGALG00010012923	MLANA	-2.530261721	0.831062549	-3.044610449	0.002329819	0.006651708	negative
PRJNA729271	stage_E14_vs_E10	ordinary_muscle_development	ENSGALG00010012924	GLIPR1	-0.529676772	0.202333011	-2.617846536	0.008848659	0.021419725	negative
PRJNA729271	stage_E14_vs_E10	ordinary_muscle_development	ENSGALG00010012925	SETD4	-0.669212054	0.177783012	-3.764206983	0.000167078	0.000631171	negative
PRJNA729271	stage_E14_vs_E10	ordinary_muscle_development	ENSGALG00010012926	OSGEPL1	0.332756051	0.163652586	2.033307621	0.042021454	0.081681772	positive
PRJNA729271	stage_E14_vs_E10	ordinary_muscle_development	ENSGALG00010012927	ENSGALG00010012927	3.700291921	3.683121043	1.004662046			positive
PRJNA729271	stage_E14_vs_E10	ordinary_muscle_development	ENSGALG00010012930	IL6ST	0.271860695	0.090812697	2.993641908	0.002756693	0.007709485	positive
PRJNA729271	stage_E14_vs_E10	ordinary_muscle_development	ENSGALG00010012932	ATF6	0.297929133	0.117474147	2.536125096	0.011208669	0.026349279	positive
PRJNA729271	stage_E14_vs_E10	ordinary_muscle_development	ENSGALG00010012933	ZCCHC17	-0.118209472	0.142658341	-0.828619425	0.407319793	0.522141573	negative
PRJNA729271	stage_E14_vs_E10	ordinary_muscle_development	ENSGALG00010012934	NDUFA1	0.430211064	0.151721267	2.835535667	0.004574891	0.012043778	positive
PRJNA729271	stage_E14_vs_E10	ordinary_muscle_development	ENSGALG00010012938	UPF3B	-0.155234904	0.150200303	-1.033519244	0.301360969	0.415218764	negative
PRJNA729271	stage_E14_vs_E10	ordinary_muscle_development	ENSGALG00010012939	GJA1	-1.395421268	0.20686559	-6.745545583	1.52453E-11	1.53459E-10	negative
PRJNA729271	stage_E14_vs_E10	ordinary_muscle_development	ENSGALG00010012940	FBN2	-0.895658062	0.223355029	-4.010019676	6.07137E-05	0.000248671	negative
PRJNA729271	stage_E14_vs_E10	ordinary_muscle_development	ENSGALG00010012943	ASNSD1	-0.270989211	0.121437775	-2.231506713	0.025647583	0.053917581	negative
PRJNA729271	stage_E14_vs_E10	ordinary_muscle_development	ENSGALG00010012944	NCK1	0.400081406	0.050363066	7.94394459	1.95851E-15	2.86176E-14	positive
PRJNA729271	stage_E14_vs_E10	ordinary_muscle_development	ENSGALG00010012945	CLINT1	0.13898401	0.0623661	2.228518533	0.025845957	0.054255922	positive
PRJNA729271	stage_E14_vs_E10	ordinary_muscle_development	ENSGALG00010012948	ST13P5	0.00766937	0.094960626	0.080763688	0.935629887	0.955201804	positive
PRJNA729271	stage_E14_vs_E10	ordinary_muscle_development	ENSGALG00010012949	ENSGALG00010012949	-0.535892307	0.168988909	-3.171168511	0.00151827	0.004570577	negative
PRJNA729271	stage_E14_vs_E10	ordinary_muscle_development	ENSGALG00010012952	BRD10	-0.312363284	0.186962604	-1.670726005	0.09477581	0.162856306	negative
PRJNA729271	stage_E14_vs_E10	ordinary_muscle_development	ENSGALG00010012954	RIMS2	-0.552199675	0.319958211	-1.725849366	0.084374516	0.147884959	negative
PRJNA729271	stage_E14_vs_E10	ordinary_muscle_development	ENSGALG00010012955	RPL39L	0.415378752	0.155140512	2.677435739	0.007418808	0.018332019	positive
PRJNA729271	stage_E14_vs_E10	ordinary_muscle_development	ENSGALG00010012956	FABP3	2.825095355	0.168224616	16.79359074	2.71886E-63	7.16543E-61	positive
PRJNA729271	stage_E14_vs_E10	ordinary_muscle_development	ENSGALG00010012957	DERA	-0.496842683	0.227732573	-2.181693536	0.029132158	0.059922043	negative
PRJNA729271	stage_E14_vs_E10	ordinary_muscle_development	ENSGALG00010012958	OLFML2B	0.585721993	0.20481564	2.859752274	0.004239721	0.011249268	positive
PRJNA729271	stage_E14_vs_E10	ordinary_muscle_development	ENSGALG00010012959	TBC1D32	-0.28383539	0.145824528	-1.94641734	0.051604628	0.097384336	negative
PRJNA729271	stage_E14_vs_E10	ordinary_muscle_development	ENSGALG00010012962	SLC25A17	-0.294542812	0.208256678	-1.414325891	0.157266238	0.246547061	negative
PRJNA729271	stage_E14_vs_E10	ordinary_muscle_development	ENSGALG00010012963	UIMC1	-0.012682374	0.087220935	-0.145405158	0.884390993	0.920724516	negative
PRJNA729271	stage_E14_vs_E10	ordinary_muscle_development	ENSGALG00010012964	CBR3	-0.110513982	0.071429929	-1.547166328	0.121823143	0.200025652	negative
PRJNA729271	stage_E14_vs_E10	ordinary_muscle_development	ENSGALG00010012965	ENSGALG00010012965	-0.208410928	0.083420656	-2.49831322	0.012478588	0.02893883	negative
PRJNA729271	stage_E14_vs_E10	ordinary_muscle_development	ENSGALG00010012966	GLIPR1L	-0.36719273	0.270312802	-1.358399333	0.174337006	0.2677007	negative
PRJNA729271	stage_E14_vs_E10	ordinary_muscle_development	ENSGALG00010012967	ECRG4	0.233587652	0.20876907	1.11888055	0.263191102	0.371827974	positive
PRJNA729271	stage_E14_vs_E10	ordinary_muscle_development	ENSGALG00010012968	LSM11	-0.263233733	0.075150554	-3.502751709	0.000460478	0.001574578	negative
PRJNA729271	stage_E14_vs_E10	ordinary_muscle_development	ENSGALG00010012969	SLC12A2	-0.966556808	0.142700894	-6.773305897	1.25872E-11	1.28216E-10	negative
PRJNA729271	stage_E14_vs_E10	ordinary_muscle_development	ENSGALG00010012970	UNC5A	0.954984837	0.163341472	5.846554629	5.01859E-09	3.80065E-08	positive
PRJNA729271	stage_E14_vs_E10	ordinary_muscle_development	ENSGALG00010012971	ENSGALG00010012971	1.237195036	0.411589617	3.005894667	0.002648007	0.00744431	positive
PRJNA729271	stage_E14_vs_E10	ordinary_muscle_development	ENSGALG00010012973	GLDC	-2.033162252	0.310461037	-6.548848347	5.79825E-11	5.49677E-10	negative
PRJNA729271	stage_E14_vs_E10	ordinary_muscle_development	ENSGALG00010012974	SLC49A3	-0.53041543	0.212529175	-2.495729967	0.012569825	0.029107781	negative
PRJNA729271	stage_E14_vs_E10	ordinary_muscle_development	ENSGALG00010012975	KRR1	-0.28335027	0.091678039	-3.090710399	0.001996783	0.005807858	negative
PRJNA729271	stage_E14_vs_E10	ordinary_muscle_development	ENSGALG00010012976	SLC9A2	1.254198833	0.236943987	5.293229199	1.20175E-07	7.55394E-07	positive
PRJNA729271	stage_E14_vs_E10	ordinary_muscle_development	ENSGALG00010012977	NKRF	-0.221044828	0.098011974	-2.255283903	0.024115514	0.051126866	negative
PRJNA729271	stage_E14_vs_E10	ordinary_muscle_development	ENSGALG00010012978	HSD17B7	-0.141628547	0.207681669	-0.681950161	0.495270466	0.605052289	negative
PRJNA729271	stage_E14_vs_E10	ordinary_muscle_development	ENSGALG00010012979	HTR1A	-0.019893863	0.548990422	-0.036237178	0.971093242	0.980017861	negative
PRJNA729271	stage_E14_vs_E10	ordinary_muscle_development	ENSGALG00010012980	KICS2	0.056797286	0.073598982	0.77171293	0.440284471	0.554226957	positive
PRJNA729271	stage_E14_vs_E10	ordinary_muscle_development	ENSGALG00010012982	DDR2	-0.478538074	0.242990105	-1.969372677	0.048910311	0.093160967	negative
PRJNA729271	stage_E14_vs_E10	ordinary_muscle_development	ENSGALG00010012985	ARNTL2	-0.938710616	0.170946114	-5.491266191	3.99062E-08	2.68917E-07	negative
PRJNA729271	stage_E14_vs_E10	ordinary_muscle_development	ENSGALG00010012986	TMEM184B	0.152130645	0.140045116	1.086297395	0.277347439	0.38789571	positive
PRJNA729271	stage_E14_vs_E10	ordinary_muscle_development	ENSGALG00010012988	RPL18A	0.372995357	0.134843806	2.766128961	0.00567261	0.01452986	positive
PRJNA729271	stage_E14_vs_E10	ordinary_muscle_development	ENSGALG00010012991	MFSD9	-0.055114567	0.100402186	-0.548937925	0.58304805	0.684142975	negative
PRJNA729271	stage_E14_vs_E10	ordinary_muscle_development	ENSGALG00010012992	HARS1	-0.1158344	0.126387118	-0.916504804	0.359402189	0.475800048	negative
PRJNA729271	stage_E14_vs_E10	ordinary_muscle_development	ENSGALG00010012993	DOP1B	-0.334133083	0.098469813	-3.393253964	0.000690676	0.00226194	negative
PRJNA729271	stage_E14_vs_E10	ordinary_muscle_development	ENSGALG00010012995	SERINC2	0.735464159	0.147726991	4.978536128	6.4067E-07	3.63208E-06	positive
PRJNA729271	stage_E14_vs_E10	ordinary_muscle_development	ENSGALG00010012997	UBE2A	0.018482734	0.057560923	0.321098635	0.748135645	0.819297837	positive
PRJNA729271	stage_E14_vs_E10	ordinary_muscle_development	ENSGALG00010012998	MAFF	0.56523543	0.198971041	2.840792447	0.004500159	0.011870756	positive
PRJNA729271	stage_E14_vs_E10	ordinary_muscle_development	ENSGALG00010013001	ADGRV1	-2.237450361	0.335950884	-6.660052005	2.73731E-11	2.67547E-10	negative
PRJNA729271	stage_E14_vs_E10	ordinary_muscle_development	ENSGALG00010013004	UAP1	-0.075336375	0.152245971	-0.494833288	0.620717805	0.716248058	negative
PRJNA729271	stage_E14_vs_E10	ordinary_muscle_development	ENSGALG00010013005	SLC40A1	-0.228378675	0.091006198	-2.509484859	0.01209074	0.028162648	negative
PRJNA729271	stage_E14_vs_E10	ordinary_muscle_development	ENSGALG00010013006	GRIK2	-5.078655702	1.061319178	-4.78522937	1.70792E-06	9.10829E-06	negative
PRJNA729271	stage_E14_vs_E10	ordinary_muscle_development	ENSGALG00010013008	STEEP1	-0.328617769	0.108263446	-3.035352947	0.002402544	0.006818735	negative
PRJNA729271	stage_E14_vs_E10	ordinary_muscle_development	ENSGALG00010013012	TMEM182	2.266473111	0.151408277	14.96928149	1.16574E-50	1.60927E-48	positive
PRJNA729271	stage_E14_vs_E10	ordinary_muscle_development	ENSGALG00010013013	DCSTAMP	-0.992221573	1.33892397	-0.741058936	0.458657706	0.571596892	negative
PRJNA729271	stage_E14_vs_E10	ordinary_muscle_development	ENSGALG00010013015	PLA2G6	0.220631311	0.149056062	1.480190123	0.138822516	0.22276457	positive
PRJNA729271	stage_E14_vs_E10	ordinary_muscle_development	ENSGALG00010013016	QRSL1	0.059871678	0.122464825	0.488888778	0.624920441	0.71981925	positive
PRJNA729271	stage_E14_vs_E10	ordinary_muscle_development	ENSGALG00010013017	SCMH1	0.593579977	0.157382799	3.771568323	0.000162225	0.000613795	positive
PRJNA729271	stage_E14_vs_E10	ordinary_muscle_development	ENSGALG00010013018	ID1	-0.986676111	0.193193167	-5.10719983	3.26968E-07	1.94158E-06	negative
PRJNA729271	stage_E14_vs_E10	ordinary_muscle_development	ENSGALG00010013019	DPYS	-2.059796718	0.981919193	-2.097725284	0.03592942	0.071508574	negative
PRJNA729271	stage_E14_vs_E10	ordinary_muscle_development	ENSGALG00010013021	SLC25A5	-0.430957287	0.160847986	-2.679283071	0.007377999	0.018246732	negative
PRJNA729271	stage_E14_vs_E10	ordinary_muscle_development	ENSGALG00010013027	UHMK1	0.10309243	0.125652767	0.820454911	0.411956823	0.526732242	positive
PRJNA729271	stage_E14_vs_E10	ordinary_muscle_development	ENSGALG00010013029	MYLK2	1.429461004	0.265488193	5.384273363	7.27379E-08	4.74285E-07	positive
PRJNA729271	stage_E14_vs_E10	ordinary_muscle_development	ENSGALG00010013032	BAIAP2L2	1.155733773	0.387752237	2.980598593	0.002876856	0.008005645	positive
PRJNA729271	stage_E14_vs_E10	ordinary_muscle_development	ENSGALG00010013033	MAP3K1	0.109876949	0.124970345	0.879224182	0.379279735	0.495418561	positive
PRJNA729271	stage_E14_vs_E10	ordinary_muscle_development	ENSGALG00010013036	ENSGALG00010013036	0.329349264	0.424950462	0.775029782	0.438322061	0.552332285	positive
PRJNA729271	stage_E14_vs_E10	ordinary_muscle_development	ENSGALG00010013037	MORC4	0.443714454	0.089398342	4.963340954	6.92908E-07	3.91415E-06	positive
PRJNA729271	stage_E14_vs_E10	ordinary_muscle_development	ENSGALG00010013040	SLC25A43	0.47162219	0.205149777	2.298916418	0.021509686	0.046347985	positive
PRJNA729271	stage_E14_vs_E10	ordinary_muscle_development	ENSGALG00010013041	LRP12	-0.053714915	0.07476422	-0.718457501	0.472475243	0.584483906	negative
PRJNA729271	stage_E14_vs_E10	ordinary_muscle_development	ENSGALG00010013042	SLC25A48	0.818738576	0.295996864	2.766038008	0.005674192	0.014531345	positive
PRJNA729271	stage_E14_vs_E10	ordinary_muscle_development	ENSGALG00010013044	ENSGALG00010013044	0.635287382	0.124865328	5.087780526	3.62278E-07	2.13725E-06	positive
PRJNA729271	stage_E14_vs_E10	ordinary_muscle_development	ENSGALG00010013045	FSD1L	0.601628564	0.144627656	4.159844545	3.18464E-05	0.000137263	positive
PRJNA729271	stage_E14_vs_E10	ordinary_muscle_development	ENSGALG00010013046	SLC12A5	-0.214221856	0.359680487	-0.595589319	0.551449614	0.656207384	negative
PRJNA729271	stage_E14_vs_E10	ordinary_muscle_development	ENSGALG00010013047	CTPS1	-0.178085091	0.099868423	-1.783197179	0.074554212	0.133135801	negative
PRJNA729271	stage_E14_vs_E10	ordinary_muscle_development	ENSGALG00010013048	WDR75	-0.369473336	0.086136544	-4.289391231	1.79164E-05	8.04017E-05	negative
PRJNA729271	stage_E14_vs_E10	ordinary_muscle_development	ENSGALG00010013051	XPOT	-0.086371735	0.078528602	-1.09987613	0.271386096	0.381287338	negative
PRJNA729271	stage_E14_vs_E10	ordinary_muscle_development	ENSGALG00010013053	MRTFA	0.335057048	0.174197133	1.923436068	0.054425308	0.101792883	positive

PRJNA729271	stage_E14_vs_E10	ordinary_muscle_development	ENSGALG00010013056	ENSGALG00010013056	1.804434946	0.563984945	3.199438143	0.001376957	0.004186909	positive
PRJNA729271	stage_E14_vs_E10	ordinary_muscle_development	ENSGALG00010013058	ENSGALG00010013058	0.172015793	0.273979771	0.627841219	0.530107961	0.637669285	positive
PRJNA729271	stage_E14_vs_E10	ordinary_muscle_development	ENSGALG00010013062	ENSGALG00010013062	-0.151966604	0.10221753	-1.486698068	0.137094573	0.220431041	negative
PRJNA729271	stage_E14_vs_E10	ordinary_muscle_development	ENSGALG00010013063	PARP12	-0.227687538	0.218557906	-1.041772142	0.297517333	0.410950423	negative
PRJNA729271	stage_E14_vs_E10	ordinary_muscle_development	ENSGALG00010013065	FKTN	-0.019452113	0.101428643	-0.191781261	0.84791355	0.894245264	negative
PRJNA729271	stage_E14_vs_E10	ordinary_muscle_development	ENSGALG00010013071	ENSGALG00010013071	-0.130881336	0.225748295	-0.579766665	0.56207198	0.665080274	negative
PRJNA729271	stage_E14_vs_E10	ordinary_muscle_development	ENSGALG00010013076	RTN4IP1	-0.003136061	0.078455846	-0.039972302	0.968115208	0.978469436	negative
PRJNA729271	stage_E14_vs_E10	ordinary_muscle_development	ENSGALG00010013078	COL5A2	-0.151074794	0.24197397	-0.624343164	0.532402243	0.639738913	negative
PRJNA729271	stage_E14_vs_E10	ordinary_muscle_development	ENSGALG00010013079	ENSGALG00010013079	0.018280813	0.20440422	0.089434616	0.928736514	0.950977378	positive
PRJNA729271	stage_E14_vs_E10	ordinary_muscle_development	ENSGALG00010013080	ARHGEF7	0.012051623	0.09872267	0.122075541	0.902839192	0.933627316	positive
PRJNA729271	stage_E14_vs_E10	ordinary_muscle_development	ENSGALG00010013081	NCOA5	-0.161446554	0.098314437	-1.642144932	0.100559972	0.17082114	negative
PRJNA729271	stage_E14_vs_E10	ordinary_muscle_development	ENSGALG00010013082	CHAF1B	-0.455824626	0.110884101	-4.110820424	3.94256E-05	0.000167146	negative
PRJNA729271	stage_E14_vs_E10	ordinary_muscle_development	ENSGALG00010013084	SGSM3	0.062013434	0.115981976	0.53468165	0.592870012	0.69283827	positive
PRJNA729271	stage_E14_vs_E10	ordinary_muscle_development	ENSGALG00010013085	LYSMD3	-0.20105186	0.087982488	-2.285134975	0.022304915	0.047855201	negative
PRJNA729271	stage_E14_vs_E10	ordinary_muscle_development	ENSGALG00010013086	CXCL14	-3.067728229	0.517210035	-5.931300673	3.00544E-09	2.33336E-08	negative
PRJNA729271	stage_E14_vs_E10	ordinary_muscle_development	ENSGALG00010013087	GPN2	-0.023636257	0.090085757	-0.262375073	0.793032289	0.854699037	negative
PRJNA729271	stage_E14_vs_E10	ordinary_muscle_development	ENSGALG00010013089	TBK1	0.101259178	0.075705162	1.337546546	0.181044299	0.276061132	positive
PRJNA729271	stage_E14_vs_E10	ordinary_muscle_development	ENSGALG00010013093	GPATCH3	0.268870183	0.154782081	1.737088569	0.082371539	0.144917521	positive
PRJNA729271	stage_E14_vs_E10	ordinary_muscle_development	ENSGALG00010013095	ADSL	1.980024673	0.127616604	15.51541573	2.72862E-54	4.94391E-52	positive
PRJNA729271	stage_E14_vs_E10	ordinary_muscle_development	ENSGALG00010013096	CLDN14	-1.166852437	0.405959217	-2.874309508	0.004049119	0.010806847	negative
PRJNA729271	stage_E14_vs_E10	ordinary_muscle_development	ENSGALG00010013097	ENSGALG00010013097	-1.111116617	0.430587083	-2.580468996	0.009866622	0.023561232	negative
PRJNA729271	stage_E14_vs_E10	ordinary_muscle_development	ENSGALG00010013099	SIM2	0.311122173	0.201791771	1.541798121	0.123122659	0.201650184	positive
PRJNA729271	stage_E14_vs_E10	ordinary_muscle_development	ENSGALG00010013100	ZFPM2	0.253381098	0.143395365	1.767010382	0.07722649	0.137181123	positive
PRJNA729271	stage_E14_vs_E10	ordinary_muscle_development	ENSGALG00010013103	AIRIM	0.201083267	0.138415754	1.45274841	0.146293621	0.232539317	positive
PRJNA729271	stage_E14_vs_E10	ordinary_muscle_development	ENSGALG00010013104	HLCS	0.118115721	0.108556184	1.088060733	0.276568297	0.387128459	positive
PRJNA729271	stage_E14_vs_E10	ordinary_muscle_development	ENSGALG00010013106	SETD9	0.151223807	0.178530781	0.84704613	0.39696941	0.512475204	positive
PRJNA729271	stage_E14_vs_E10	ordinary_muscle_development	ENSGALG00010013109	ENSGALG00010013109	-1.365018533	1.562623062	-0.873543061	0.382367146	0.498418325	negative
PRJNA729271	stage_E14_vs_E10	ordinary_muscle_development	ENSGALG00010013110	ANKRD10	-0.322903455	0.156217964	-2.067005911	0.038733597	0.076272719	negative
PRJNA729271	stage_E14_vs_E10	ordinary_muscle_development	ENSGALG00010013111	RFESD	0.710013778	0.284325804	2.497183749	0.012518407	0.029005269	positive
PRJNA729271	stage_E14_vs_E10	ordinary_muscle_development	ENSGALG00010013112	CD40	0.634333318	0.158092214	4.012426047	6.00979E-05	0.000246566	positive
PRJNA729271	stage_E14_vs_E10	ordinary_muscle_development	ENSGALG00010013114	PIGP	-0.248995175	0.120795763	-2.061290631	0.039275322	0.077171721	negative
PRJNA729271	stage_E14_vs_E10	ordinary_muscle_development	ENSGALG00010013115	ENSGALG00010013115	0.059563156	0.362447381	0.164336009	0.869466636	0.910549736	positive
PRJNA729271	stage_E14_vs_E10	ordinary_muscle_development	ENSGALG00010013116	TARS	-0.316618188	0.167862731	-1.886173216	0.059271617	0.109375186	negative
PRJNA729271	stage_E14_vs_E10	ordinary_muscle_development	ENSGALG00010013117	ARSK	-0.510294337	0.131705578	-3.874508172	0.00010684	0.000419007	negative
PRJNA729271	stage_E14_vs_E10	ordinary_muscle_development	ENSGALG00010013119	RNF114	0.028444231	0.078756892	0.361164973	0.717976125	0.796134022	positive
PRJNA729271	stage_E14_vs_E10	ordinary_muscle_development	ENSGALG00010013121	PCBD2	-0.249840042	0.163382718	-1.529170562	0.126222166	0.205804845	negative
PRJNA729271	stage_E14_vs_E10	ordinary_muscle_development	ENSGALG00010013123	PPFIBP1	0.106996918	0.108088952	0.9898969	0.322224515	0.436753726	positive
PRJNA729271	stage_E14_vs_E10	ordinary_muscle_development	ENSGALG00010013125	POU3F3	-4.515961784	0.396027272	-11.40315859	4.03219E-30	1.74468E-28	negative
PRJNA729271	stage_E14_vs_E10	ordinary_muscle_development	ENSGALG00010013129	RASSF3	1.499513917	0.110461987	13.57493163	5.63963E-42	4.895E-40	positive
PRJNA729271	stage_E14_vs_E10	ordinary_muscle_development	ENSGALG00010013131	SPATA2	0.151083396	0.115674402	1.306109157	0.191515439	0.288927591	positive
PRJNA729271	stage_E14_vs_E10	ordinary_muscle_development	ENSGALG00010013132	SKIC3	-0.296774464	0.089172491	-3.328094375	0.000874422	0.002793642	negative
PRJNA729271	stage_E14_vs_E10	ordinary_muscle_development	ENSGALG00010013133	TXNDC15	0.122425599	0.115881002	1.056476882	0.290750397	0.403135403	positive
PRJNA729271	stage_E14_vs_E10	ordinary_muscle_development	ENSGALG00010013134	ENSGALG00010013134	1.966005703	5.373424983	0.365875714	0.714457819	0.793568283	positive
PRJNA729271	stage_E14_vs_E10	ordinary_muscle_development	ENSGALG00010013135	TTC3	-0.414052392	0.084371316	-4.907501862	9.22438E-07	5.11114E-06	negative
PRJNA729271	stage_E14_vs_E10	ordinary_muscle_development	ENSGALG00010013137	TMEM121	-0.924794788	0.142312914	-6.49831954	8.12221E-11	7.53723E-10	negative
PRJNA729271	stage_E14_vs_E10	ordinary_muscle_development	ENSGALG00010013139	TMEM185A	0.424823995	0.081788793	5.194158998	2.05647E-07	1.25563E-06	positive
PRJNA729271	stage_E14_vs_E10	ordinary_muscle_development	ENSGALG00010013143	ENSGALG00010013143	-0.83025493	0.444736038	-1.866848778	0.061922709	0.113688367	negative
PRJNA729271	stage_E14_vs_E10	ordinary_muscle_development	ENSGALG00010013144	TEDC1	-0.36641933	0.163377781	-2.242773329	0.024911435	0.05256059	negative
PRJNA729271	stage_E14_vs_E10	ordinary_muscle_development	ENSGALG00010013145	ENSGALG00010013145	-0.369827199	0.278769863	-1.32663981	0.18462786	0.2805221	negative
PRJNA729271	stage_E14_vs_E10	ordinary_muscle_development	ENSGALG00010013146	TNRC6B	0.069739041	0.119411358	0.584023514	0.559204492	0.662835544	positive
PRJNA729271	stage_E14_vs_E10	ordinary_muscle_development	ENSGALG00010013149	MCTP1	0.133856994	0.197412012	0.678059011	0.497734278	0.607141156	positive
PRJNA729271	stage_E14_vs_E10	ordinary_muscle_development	ENSGALG00010013150	GNS	0.313255008	0.103074115	3.039123908	0.002372672	0.0067488	positive
PRJNA729271	stage_E14_vs_E10	ordinary_muscle_development	ENSGALG00010013152	TBXAS1	-0.025589838	0.221016619	-0.115782414	0.907824988	0.936801895	negative
PRJNA729271	stage_E14_vs_E10	ordinary_muscle_development	ENSGALG00010013154	ENSGALG00010013154	-0.275588658	0.349179492	-0.789246404	0.429968005	0.544123121	negative
PRJNA729271	stage_E14_vs_E10	ordinary_muscle_development	ENSGALG00010013156	ABCC9	0.240360754	0.130215934	1.845862851	0.064912122	0.118367242	positive
PRJNA729271	stage_E14_vs_E10	ordinary_muscle_development	ENSGALG00010013158	CRIP1	0.423280454	0.18751924	2.257264123	0.023991576	0.050963559	positive
PRJNA729271	stage_E14_vs_E10	ordinary_muscle_development	ENSGALG00010013159	ENSGALG00010013159	-0.612719231	1.403753545	-0.436486329	0.662483916	0.751561741	negative
PRJNA729271	stage_E14_vs_E10	ordinary_muscle_development	ENSGALG00010013160	CRYBG1	-0.469330514	0.543268737	-0.8639012	0.387642168	0.503618645	negative
PRJNA729271	stage_E14_vs_E10	ordinary_muscle_development	ENSGALG00010013162	IDS	0.110876021	0.10933459	1.01409829	0.310535854	0.424282892	positive
PRJNA729271	stage_E14_vs_E10	ordinary_muscle_development	ENSGALG00010013163	ENSGALG00010013163	-0.342993624	0.449863245	-0.762439758	0.445797585	0.559224232	negative
PRJNA729271	stage_E14_vs_E10	ordinary_muscle_development	ENSGALG00010013164	FAM83F	-0.832738635	0.404910699	-2.056598248	0.039724887	0.077822981	negative
PRJNA729271	stage_E14_vs_E10	ordinary_muscle_development	ENSGALG00010013165	NUDC	-0.034246818	0.080592614	-0.42493742	0.670882295	0.757848548	negative
PRJNA729271	stage_E14_vs_E10	ordinary_muscle_development	ENSGALG00010013168	VPS26C	0.177269269	0.137461213	1.289594833	0.19719137	0.295736049	positive
PRJNA729271	stage_E14_vs_E10	ordinary_muscle_development	ENSGALG00010013170	COL3A1	1.168295332	0.234908634	4.973403122	6.57877E-07	3.72642E-06	positive
PRJNA729271	stage_E14_vs_E10	ordinary_muscle_development	ENSGALG00010013171	SLC9A8	-0.379143696	0.13658552	-2.775870346	0.005505416	0.014161669	negative
PRJNA729271	stage_E14_vs_E10	ordinary_muscle_development	ENSGALG00010013172	TNFSF13B	-0.438104014	0.481138022	-0.910557873	0.362528376	0.47867087	negative
PRJNA729271	stage_E14_vs_E10	ordinary_muscle_development	ENSGALG00010013173	KCNJ8	-0.433977983	0.155876377	-2.784116438	0.005367376	0.013853297	negative
PRJNA729271	stage_E14_vs_E10	ordinary_muscle_development	ENSGALG00010013174	ADAMTS12	0.126838864	0.165213786	0.767725665	0.442650185	0.556431743	positive
PRJNA729271	stage_E14_vs_E10	ordinary_muscle_development	ENSGALG00010013175	HIPK2	0.08383696	0.163975771	0.511276512	0.60915745	0.706498865	positive
PRJNA729271	stage_E14_vs_E10	ordinary_muscle_development	ENSGALG00010013177	OXR1	-0.023405142	0.069621505	-0.336176907	0.736737466	0.810889379	negative
PRJNA729271	stage_E14_vs_E10	ordinary_muscle_development	ENSGALG00010013179	TBC1D30	-1.166122372	0.191468424	-6.090416089	1.12618E-09	9.15018E-09	negative
PRJNA729271	stage_E14_vs_E10	ordinary_muscle_development	ENSGALG00010013180	C5orf24	-0.075060722	0.058667695	-1.279421688	0.200748601	0.299891886	negative
PRJNA729271	stage_E14_vs_E10	ordinary_muscle_development	ENSGALG00010013181	DYRK1A	0.761199304	0.078726051	9.668963379	4.08495E-22	1.02619E-20	positive
PRJNA729271	stage_E14_vs_E10	ordinary_muscle_development	ENSGALG00010013183	CLTA	-0.320838644	0.136812723	-2.345093621	0.019022302	0.041587975	negative
PRJNA729271	stage_E14_vs_E10	ordinary_muscle_development	ENSGALG00010013184	LDHB	-0.218767906	0.113980353	-1.919347499	0.054940371	0.102637024	negative
PRJNA729271	stage_E14_vs_E10	ordinary_muscle_development	ENSGALG00010013185	ENSGALG00010013185	-0.475129625	0.312960112	-1.518179498	0.128969154	0.209504415	negative
PRJNA729271	stage_E14_vs_E10	ordinary_muscle_development	ENSGALG00010013186	DDX46	-0.267208515	0.079740043	-3.350995355	0.000805217	0.002596918	negative
PRJNA729271	stage_E14_vs_E10	ordinary_muscle_development	ENSGALG00010013187	KDF1	-1.931881432	0.218001245	-8.861790828	7.87392E-19	1.52993E-17	negative
PRJNA729271	stage_E14_vs_E10	ordinary_muscle_development	ENSGALG00010013188	ENSGALG00010013188	-0.740932247	0.365937262	-2.024752123	0.042892823	0.083184117	negative
PRJNA729271	stage_E14_vs_E10	ordinary_muscle_development	ENSGALG00010013190	SLC45A2	-1.302905351	0.366447978	-3.555498815	0.000377263	0.001317961	negative
PRJNA729271	stage_E14_vs_E10	ordinary_muscle_development	ENSGALG00010013191	ABRA	4.024130423	0.419498041	9.592727562	8.57866E-22	2.10402E-20	positive
PRJNA729271	stage_E14_vs_E10	ordinary_muscle_development	ENSGALG00010013192	B4GALT5	0.769794952	0.080671034	9.542396017	1.3957E-21	3.38305E-20	positive
PRJNA729271	stage_E14_vs_E10	ordinary_muscle_development	ENSGALG00010013194	GRAP2	-7.385064325	0.893411909	-8.266135975	1.38371E-16	2.24852E-15	negative
PRJNA729271	stage_E14_vs_E10	ordinary_muscle_development	ENSGALG00010013196	MRPS9	-0.143771431	0.113499562	-1.266713527	0.205257741	0.305149842	negative
PRJNA729271	stage_E14_vs_E10	ordinary_muscle_development	ENSGALG00010013198	CAMLG	-0.281704125	0.085356764	-3.30031402	0.000965767	0.003053172	negative
PRJNA729271	stage_E14_vs_E10	ordinary_muscle_development	ENSGALG00010013199	RALYL	-5.348360924	0.833106856	-6.419777828	1.36473E-10	1.23482E-09	negative
PRJNA729271	stage_E14_vs_E10	ordinary_muscle_development	ENSGALG00010013202	ENSGALG00010013202	-0.521056596	0.650174509	-0.801410373	0.422894112	0.537234895	negative
PRJNA729271	stage_E14_vs_E10	ordinary_muscle_development	ENSGALG00010013203	AMACR	0.244455609	0.119313603	2.048849435	0.04047684	0.07909299	positive
PRJNA729271	stage_E14_vs_E10	ordinary_muscle_development	ENSGALG00010013204	ENSGALG00010013204	-0.107313071	0.354683982	-0.302559677	0.762225448	0.830648663	negative
PRJNA729271	stage_E14_vs_E10	ordinary_muscle_development	ENSGALG00010013205	KCNJ6	3.085247411	0.325068084	9.491080669	2.28651E-21	5.47818E-20	positive
PRJNA729271	stage_E14_vs_E10	ordinary_muscle_development	ENSGALG00010013206	AFF2	-0.667213074	0.111271275	-5.996274182	2.01896E-09	1.5983E-08	negative
PRJNA729271	stage_E14_vs_E10	ordinary_muscle_development	ENSGALG00010013207	C1QTNF3	-0.04847875	0.198616669	-0.244081982	0.807167326	0.864603531	negative
PRJNA729271	stage_E14_vs_E10	ordinary_muscle_development	ENSGALG00010013208	ENSGALG00010013208	-0.177989316	0.306354587	-0.58099119	0.561246391	0.664320304	negative
PRJNA729271	stage_E14_vs_E10	ordinary_muscle_development	ENSGALG00010013209	KIAA1549	-0.523405104	0.101548668	-5.154229143	2.54676E-07	1.5343E-06	negative
PRJNA729271	stage_E14_vs_E10	ordinary_muscle_development	ENSGALG00010013212	ENSGALG00010013212	0.390407398	0.152080959	2.567102416	0.010255232	0.024312984	positive
PRJNA729271	stage_E14_vs_E10	ordinary_muscle_development	ENSGALG00010013213	SH2D1A	0.162707488	0.3866718	0.42078964	0.673908697	0.760357014	positive
PRJNA729271	stage_E14_vs_E10	ordinary_muscle_development	ENSGALG00010013214	RAI14	-0.490843719	0.16923525	-2.900363368	0.003727303	0.010045976	negative
PRJNA729271	stage_E14_vs_E10	ordinary_muscle_development	ENSGALG00010013215	SF3A3	-0.073897345	0.080711521	-0.915573682	0.35989054	0.476339042	negative
PRJNA729271	stage_E14_vs_E10	ordinary_muscle_development	ENSGALG00010013217	TGFBRAP1	0.229344707	0.081814125	2.80324097	0.005059185	0.013160964	positive
PRJNA729271	stage_E14_vs_E10	ordinary_muscle_development	ENSGALG00010013220	CSTF1	-0.036937509	0.08598974	-0.42955757	0.667517799	0.755426185	negative
PRJNA729271	stage_E14_vs_E10	ordinary_muscle_development	ENSGALG00010013221	C2orf49	-0.004317865	0.11696758	-0.036915057	0.970552734	0.979813475	negative
PRJNA729271	stage_E14_vs_E10	ordinary_muscle_development	ENSGALG00010013224	ATP6V0A4	0.363554568	0.71096383	0.51135452	0.609102835	0.706498865	positive
PRJNA729271	stage_E14_vs_E10	ordinary_muscle_development	ENSGALG00010013225	GNE	-0.441698914	0.095085981	-4.645258025	3.39652E-06	1.71961E-05	negative
PRJNA729271	stage_E14_vs_E10	ordinary_muscle_development	ENSGALG00010013226	DUSP6	-0.720031143	0.112847635	-6.38056033	1.76441E-10	1.58262E-09	negative
PRJNA729271	stage_E14_vs_E10	ordinary_muscle_development	ENSGALG00010013227	CITED4	0.913093593	0.16209177	5.633189111	1.76907E-08	1.25086E-07	positive
PRJNA729271	stage_E14_vs_E10	ordinary_muscle_development	ENSGALG00010013228	RTFDC1	0.229519622	0.084487007	2.716626276	0.006595103	0.016530524	positive
PRJNA729271	stage_E14_vs_E10	ordinary_muscle_development	ENSGALG00010013230	ENSGALG00010013230	0.780866965	0.345969931	2.257037085	0.024005758	0.050983657	positive
PRJNA729271	stage_E14_vs_E10	ordinary_muscle_development	ENSGALG00010013232	FHL2	1.571101445	0.420923545	3.732510246	0.000189581	0.000708059	positive
PRJNA729271	stage_E14_vs_E10	ordinary_muscle_development	ENSGALG00010013233	LRRCC1	-0.228552161	0.12393326	-1.844155154	0.065160532	0.118745525	negative
PRJNA729271	stage_E14_vs_E10	ordinary_muscle_development	ENSGALG00010013235	GCNT7	0.276747478	0.247719812	1.117179431	0.263917613	0.3727284	positive
PRJNA729271	stage_E14_vs_E10	ordinary_muscle_development	ENSGALG00010013236	MON2	-0.115555523	0.082468298	-1.401211445	0.161150853	0.251440432	negative
PRJNA729271	stage_E14_vs_E10	ordinary_muscle_development	ENSGALG00010013238	RAD1	-0.253999994	0.130314728	-1.949127303	0.051280224	0.096860418	negative
PRJNA729271	stage_E14_vs_E10	ordinary_muscle_development	ENSGALG00010013241	SEC24A	-0.231691213	0.135604458	-1.708581098	0.087528573	0.152326409	negative
PRJNA729271	stage_E14_vs_E10	ordinary_muscle_development	ENSGALG00010013242	SULT4A1	-0.055231277	0.155536813	-0.355100994	0.722513932	0.799087399	negative
PRJNA729271	stage_E14_vs_E10	ordinary_muscle_development	ENSGALG00010013244	TRIM24	-0.109044071	0.147291134	-0.740330175	0.459099676	0.571901839	negative
PRJNA729271	stage_E14_vs_E10	ordinary_muscle_development	ENSGALG00010013246	LNPEP	0.24176831	0.17516725	1.380214115	0.167520729	0.259285955	positive
PRJNA729271	stage_E14_vs_E10	ordinary_muscle_development	ENSGALG00010013247	ATG5	-0.199235974	0.091710723	-2.172439235	0.029822548	0.06112526	negative
PRJNA729271	stage_E14_vs_E10	ordinary_muscle_development	ENSGALG00010013248	ENSGALG00010013248	0.532500387	0.679585536	0.783566393	0.433294582	0.54737711	positive
PRJNA729271	stage_E14_vs_E10	ordinary_muscle_development	ENSGALG00010013249	AURKA	-0.590524587	0.223676598	-2.640082121	0.008288594	0.020270486	negative
PRJNA729271	stage_E14_vs_E10	ordinary_muscle_development	ENSGALG00010013250	E2F5	-0.715002085	0.09562807	-7.476905935	7.60928E-14	9.7561E-13	negative
PRJNA729271	stage_E14_vs_E10	ordinary_muscle_development	ENSGALG00010013251	MTA1	-0.159117694	0.081041137	-1.963418825	0.049597528	0.094220631	negative
PRJNA729271	stage_E14_vs_E10	ordinary_muscle_development	ENSGALG00010013252	PPM1H	-0.01232791	0.175134254	-0.0703912	0.943882295	0.960424036	negative
PRJNA729271	stage_E14_vs_E10	ordinary_muscle_development	ENSGALG00010013253	SHB	1.241138493	0.096990042	12.79655591	1.71385E-37	1.15545E-35	positive
PRJNA729271	stage_E14_vs_E10	ordinary_muscle_development	ENSGALG00010013254	SAR1B	0.101029805	0.141852	0.712219815	0.476328668	0.588508698	positive
PRJNA729271	stage_E14_vs_E10	ordinary_muscle_development	ENSGALG00010013256	BRIX1	-0.26887178	0.117041715	-2.297230361	0.021605632	0.046520148	negative
PRJNA729271	stage_E14_vs_E10	ordinary_muscle_development	ENSGALG00010013257	STX12	-0.046265198	0.08889023	-0.520475626	0.602732112	0.701059377	negative
PRJNA729271	stage_E14_vs_E10	ordinary_muscle_development	ENSGALG00010013258	RBIS	0.110134511	0.1450348	0.7593661	0.447633587	0.560713659	positive
PRJNA729271	stage_E14_vs_E10	ordinary_muscle_development	ENSGALG00010013259	PTPRB	0.18331462	0.140468086	1.305026821	0.191883715	0.289212275	positive
PRJNA729271	stage_E14_vs_E10	ordinary_muscle_development	ENSGALG00010013260	FAM210B	-0.534689995	0.081453425	-6.564364776	5.22551E-11	4.98643E-10	negative
PRJNA729271	stage_E14_vs_E10	ordinary_muscle_development	ENSGALG00010013261	JADE2	0.021436797	0.198988679	0.107728726	0.914210882	0.940755838	positive
PRJNA729271	stage_E14_vs_E10	ordinary_muscle_development	ENSGALG00010013262	CA13	0.939701458	0.262115781	3.585062506	0.000336997	0.001192573	positive

PRJNA729271	stage_E14_vs_E10	ordinary_muscle_development	ENSGALG00010013263	ATP11A	-0.18104688	0.112368643	-1.611186851	0.107139003	0.180406787	negative
PRJNA729271	stage_E14_vs_E10	ordinary_muscle_development	ENSGALG00010013264	DNAJC21	-0.43030573	0.122030491	-3.526214853	0.000421545	0.001452066	negative
PRJNA729271	stage_E14_vs_E10	ordinary_muscle_development	ENSGALG00010013265	PACS2	1.729424274	0.12149719	14.23427387	5.61431E-46	6.40782E-44	positive
PRJNA729271	stage_E14_vs_E10	ordinary_muscle_development	ENSGALG00010013266	KIAA1755	1.508297072	0.187798988	8.031444089	9.6332E-16	1.44451E-14	positive
PRJNA729271	stage_E14_vs_E10	ordinary_muscle_development	ENSGALG00010013267	PNPLA3	-0.458233773	0.179358483	-2.554848624	0.010623399	0.025095529	negative
PRJNA729271	stage_E14_vs_E10	ordinary_muscle_development	ENSGALG00010013268	PTPRR	-0.770526513	0.289933342	-2.657598842	0.007869951	0.019344572	negative
PRJNA729271	stage_E14_vs_E10	ordinary_muscle_development	ENSGALG00010013269	INPP5B	0.070099393	0.10139606	0.691342382	0.489350404	0.599923775	positive
PRJNA729271	stage_E14_vs_E10	ordinary_muscle_development	ENSGALG00010013271	GULP1	0.29823066	0.138897715	2.147124306	0.031783387	0.064433593	positive
PRJNA729271	stage_E14_vs_E10	ordinary_muscle_development	ENSGALG00010013273	STAG2	0.22008661	0.116264279	1.892985641	0.058359779	0.107981235	positive
PRJNA729271	stage_E14_vs_E10	ordinary_muscle_development	ENSGALG00010013274	MTF1	0.20816464	0.120031107	1.734255771	0.082872711	0.145693141	positive
PRJNA729271	stage_E14_vs_E10	ordinary_muscle_development	ENSGALG00010013275	MAPRE1	1.046273773	0.075084783	13.93456477	3.90574E-44	3.98688E-42	positive
PRJNA729271	stage_E14_vs_E10	ordinary_muscle_development	ENSGALG00010013276	POC1B	-0.372333092	0.105270021	-3.536933775	0.000404801	0.001401384	negative
PRJNA729271	stage_E14_vs_E10	ordinary_muscle_development	ENSGALG00010013277	YRDC	-0.166374025	0.086485802	-1.923714898	0.05439033	0.101740589	negative
PRJNA729271	stage_E14_vs_E10	ordinary_muscle_development	ENSGALG00010013278	SAMM50	0.276514805	0.10277427	2.690506147	0.007134372	0.017722832	positive
PRJNA729271	stage_E14_vs_E10	ordinary_muscle_development	ENSGALG00010013280	FRS2	-0.325574724	0.11042919	-2.948266885	0.003195611	0.008789445	negative
PRJNA729271	stage_E14_vs_E10	ordinary_muscle_development	ENSGALG00010013281	BIRC8	0.47851888	0.139326236	3.434520971	0.000593602	0.001973002	positive
PRJNA729271	stage_E14_vs_E10	ordinary_muscle_development	ENSGALG00010013282	SEC22B	-0.02895205	0.088470395	-0.327251277	0.743477841	0.815878668	negative
PRJNA729271	stage_E14_vs_E10	ordinary_muscle_development	ENSGALG00010013283	PAWR	-1.546973416	0.280880501	-5.507585641	3.63788E-08	2.46753E-07	negative
PRJNA729271	stage_E14_vs_E10	ordinary_muscle_development	ENSGALG00010013284	MANEAL	-1.384204003	0.309599109	-4.470956036	7.78707E-06	3.71417E-05	negative
PRJNA729271	stage_E14_vs_E10	ordinary_muscle_development	ENSGALG00010013285	ATP2B1	-0.206773858	0.107322159	-1.9266651	0.05402138	0.101194094	negative
PRJNA729271	stage_E14_vs_E10	ordinary_muscle_development	ENSGALG00010013286	RNF14	0.046550376	0.119752006	0.38872314	0.697480963	0.779799615	positive
PRJNA729271	stage_E14_vs_E10	ordinary_muscle_development	ENSGALG00010013288	CA3A	2.605443597	0.778175484	3.348144025	0.000813547	0.002621965	positive
PRJNA729271	stage_E14_vs_E10	ordinary_muscle_development	ENSGALG00010013289	TM9SF4	-0.382191231	0.072446411	-5.275502584	1.32393E-07	8.26101E-07	negative
PRJNA729271	stage_E14_vs_E10	ordinary_muscle_development	ENSGALG00010013290	AKR1D1	-2.996659589	0.338271996	-8.858727959	8.09326E-19	1.56834E-17	negative
PRJNA729271	stage_E14_vs_E10	ordinary_muscle_development	ENSGALG00010013292	PRDM1	-1.361719756	0.283566159	-4.802123651	1.56992E-06	8.42501E-06	negative
PRJNA729271	stage_E14_vs_E10	ordinary_muscle_development	ENSGALG00010013293	TUBGCP3	-0.331848194	0.054429512	-6.096843104	1.08184E-09	8.81958E-09	negative
PRJNA729271	stage_E14_vs_E10	ordinary_muscle_development	ENSGALG00010013295	CA3B	-0.763388035	0.400878257	-1.90428895	0.056872563	0.105674631	negative
PRJNA729271	stage_E14_vs_E10	ordinary_muscle_development	ENSGALG00010013296	AGXT2	-1.761202785	0.43797708	-4.021221354	5.78972E-05	0.000238279	negative
PRJNA729271	stage_E14_vs_E10	ordinary_muscle_development	ENSGALG00010013297	FCHO2	0.163911781	0.124496955	1.316592689	0.187975191	0.284474879	positive
PRJNA729271	stage_E14_vs_E10	ordinary_muscle_development	ENSGALG00010013298	EPYC	-3.535829936	0.357614664	-9.887262158	4.72781E-23	1.24161E-21	negative
PRJNA729271	stage_E14_vs_E10	ordinary_muscle_development	ENSGALG00010013299	PLAGL2	-0.560724099	0.107520442	-5.215046426	1.83771E-07	1.12919E-06	negative
PRJNA729271	stage_E14_vs_E10	ordinary_muscle_development	ENSGALG00010013300	GNPDA1	0.008999761	0.096795154	0.092977393	0.925921521	0.949137308	positive
PRJNA729271	stage_E14_vs_E10	ordinary_muscle_development	ENSGALG00010013301	PARVB	0.321796706	0.140772402	2.285936026	0.022258001	0.047775764	positive
PRJNA729271	stage_E14_vs_E10	ordinary_muscle_development	ENSGALG00010013302	ENSGALG00010013302	-0.281211017	0.681190802	-0.412822687	0.679736528	0.765740341	negative
PRJNA729271	stage_E14_vs_E10	ordinary_muscle_development	ENSGALG00010013303	TFPI	-1.264914742	0.235578345	-5.369401591	7.89983E-08	5.11425E-07	negative
PRJNA729271	stage_E14_vs_E10	ordinary_muscle_development	ENSGALG00010013304	KCNJ15	0.878134669	0.231333602	3.795966789	0.000147069	0.000561435	positive
PRJNA729271	stage_E14_vs_E10	ordinary_muscle_development	ENSGALG00010013305	THOC2	-0.330635613	0.090749892	-3.643371985	0.00026909	0.000970503	negative
PRJNA729271	stage_E14_vs_E10	ordinary_muscle_development	ENSGALG00010013306	ARSB	-0.210814056	0.149382493	-1.411236695	0.158174833	0.247609611	negative
PRJNA729271	stage_E14_vs_E10	ordinary_muscle_development	ENSGALG00010013307	POFUT1	-0.210774625	0.084902231	-2.482556966	0.013044321	0.030064785	negative
PRJNA729271	stage_E14_vs_E10	ordinary_muscle_development	ENSGALG00010013309	ENSGALG00010013309	0.550356643	0.286453911	1.921274672	0.054697089	0.10222206	positive
PRJNA729271	stage_E14_vs_E10	ordinary_muscle_development	ENSGALG00010013310	MYO16	1.568611875	0.29350371	5.344436272	9.06989E-08	5.81717E-07	positive
PRJNA729271	stage_E14_vs_E10	ordinary_muscle_development	ENSGALG00010013311	RSPO1	-4.029803724	0.26038189	-15.47651308	4.99841E-54	8.72915E-52	negative
PRJNA729271	stage_E14_vs_E10	ordinary_muscle_development	ENSGALG00010013312	ENSGALG00010013312	-1.859618748	0.63674085	-2.920526848	0.003494401	0.009490602	negative
PRJNA729271	stage_E14_vs_E10	ordinary_muscle_development	ENSGALG00010013313	GAK	-0.217058302	0.093312741	-2.326137879	0.020011196	0.043494121	negative
PRJNA729271	stage_E14_vs_E10	ordinary_muscle_development	ENSGALG00010013314	KIT	-0.645104563	0.206547928	-3.123268147	0.001788547	0.005283267	negative
PRJNA729271	stage_E14_vs_E10	ordinary_muscle_development	ENSGALG00010013316	KDM7A	0.273295053	0.130395157	2.0958988	0.036091169	0.071781215	positive
PRJNA729271	stage_E14_vs_E10	ordinary_muscle_development	ENSGALG00010013317	PREP	-0.118206568	0.109013296	-1.084331665	0.27821777	0.388887809	negative
PRJNA729271	stage_E14_vs_E10	ordinary_muscle_development	ENSGALG00010013318	KERA	0.579947845	0.485486534	1.194570403	0.232254919	0.336485262	positive
PRJNA729271	stage_E14_vs_E10	ordinary_muscle_development	ENSGALG00010013319	ENSGALG00010013319	2.162197106	1.558925605	1.386979019			positive
PRJNA729271	stage_E14_vs_E10	ordinary_muscle_development	ENSGALG00010013320	CALCRL	0.303905924	0.123345878	2.463851478	0.013745306	0.031480203	positive
PRJNA729271	stage_E14_vs_E10	ordinary_muscle_development	ENSGALG00010013322	IRS2	-0.139017748	0.513762412	-0.270587618	0.78670822	0.850043656	negative
PRJNA729271	stage_E14_vs_E10	ordinary_muscle_development	ENSGALG00010013323	NOTCH2	0.002899892	0.108550057	0.026714794	0.978687213	0.985828433	positive
PRJNA729271	stage_E14_vs_E10	ordinary_muscle_development	ENSGALG00010013324	PARVG	0.899411931	0.146875336	6.123641695	9.14604E-10	7.52822E-09	positive
PRJNA729271	stage_E14_vs_E10	ordinary_muscle_development	ENSGALG00010013325	CA2	0.164423839	0.317736862	0.517484304	0.604818123	0.702874682	positive
PRJNA729271	stage_E14_vs_E10	ordinary_muscle_development	ENSGALG00010013326	FGF20	-5.968428889	0.888078856	-6.720606899	1.80969E-11	1.80782E-10	negative
PRJNA729271	stage_E14_vs_E10	ordinary_muscle_development	ENSGALG00010013327	SLF1	-0.433242147	0.09450415	-4.584371685	4.55354E-06	2.26363E-05	negative
PRJNA729271	stage_E14_vs_E10	ordinary_muscle_development	ENSGALG00010013328	SLC37A3	0.191799663	0.075965152	2.524837473	0.011575181	0.027104315	positive
PRJNA729271	stage_E14_vs_E10	ordinary_muscle_development	ENSGALG00010013329	LIFR	-0.317540892	0.122484105	-2.592506947	0.009527926	0.022846532	negative
PRJNA729271	stage_E14_vs_E10	ordinary_muscle_development	ENSGALG00010013330	ERG	-0.994336702	0.129315175	-7.689249967	1.48E-14	2.01433E-13	negative
PRJNA729271	stage_E14_vs_E10	ordinary_muscle_development	ENSGALG00010013331	KIF3B	-0.389334827	0.149312993	-2.60750802	0.009120393	0.021989372	negative
PRJNA729271	stage_E14_vs_E10	ordinary_muscle_development	ENSGALG00010013332	LUM	0.524231353	0.320661011	1.634845947	0.102081346	0.173000013	positive
PRJNA729271	stage_E14_vs_E10	ordinary_muscle_development	ENSGALG00010013333	PPP1R12A	0.268003877	0.097638662	2.744854043	0.006053782	0.015386562	positive
PRJNA729271	stage_E14_vs_E10	ordinary_muscle_development	ENSGALG00010013334	SH3BGRL3	0.542056882	0.120658479	4.492488947	7.03956E-06	3.38998E-05	positive
PRJNA729271	stage_E14_vs_E10	ordinary_muscle_development	ENSGALG00010013335	POPDC3	0.969102348	0.176759169	5.482614314	4.19086E-08	2.81364E-07	positive
PRJNA729271	stage_E14_vs_E10	ordinary_muscle_development	ENSGALG00010013336	MTNR1A	-0.003744811	0.701343856	-0.00533948	0.995739732	0.997778318	negative
PRJNA729271	stage_E14_vs_E10	ordinary_muscle_development	ENSGALG00010013337	RAB19	0.233849483	0.253517876	0.922418122	0.356310521	0.472743341	positive
PRJNA729271	stage_E14_vs_E10	ordinary_muscle_development	ENSGALG00010013339	ATP6V0D2	-3.402985859	0.789052371	-4.312750314	1.61236E-05	7.29893E-05	negative
PRJNA729271	stage_E14_vs_E10	ordinary_muscle_development	ENSGALG00010013340	LIN28B	-2.397840341	0.33092397	-7.245895003	4.29593E-13	5.11244E-12	negative
PRJNA729271	stage_E14_vs_E10	ordinary_muscle_development	ENSGALG00010013343	SHISAL1	-3.642293079	0.351180233	-10.37157772	3.33965E-25	1.02127E-23	negative
PRJNA729271	stage_E14_vs_E10	ordinary_muscle_development	ENSGALG00010013344	OSMR	0.646626526	0.12321268	5.248051778	1.53716E-07	9.52593E-07	positive
PRJNA729271	stage_E14_vs_E10	ordinary_muscle_development	ENSGALG00010013345	DCN	0.278742522	0.213769308	1.303940799	0.192253768	0.289649555	positive
PRJNA729271	stage_E14_vs_E10	ordinary_muscle_development	ENSGALG00010013346	HMGCS2	0.486365943	0.690150852	0.704724108	0.480981946	0.592893384	positive
PRJNA729271	stage_E14_vs_E10	ordinary_muscle_development	ENSGALG00010013347	ENSGALG00010013347	0.52862498	0.311581872	1.696584519	0.089775239	0.155452405	positive
PRJNA729271	stage_E14_vs_E10	ordinary_muscle_development	ENSGALG00010013348	MKRN4P	0.107940817	0.069855423	1.545203122	0.122297139	0.200667539	positive
PRJNA729271	stage_E14_vs_E10	ordinary_muscle_development	ENSGALG00010013349	ETS2	-0.009103086	0.100397926	-0.090670056	0.927754765	0.950335704	negative
PRJNA729271	stage_E14_vs_E10	ordinary_muscle_development	ENSGALG00010013351	EDAR	-3.057571326	0.391637106	-7.807154324	5.84936E-15	8.19193E-14	negative
PRJNA729271	stage_E14_vs_E10	ordinary_muscle_development	ENSGALG00010013352	ASXL1	-0.241593277	0.085812559	-2.815360354	0.004872257	0.012727223	negative
PRJNA729271	stage_E14_vs_E10	ordinary_muscle_development	ENSGALG00010013353	NDFIP1	0.153554129	0.084333763	1.820790686	0.068638681	0.124039107	positive
PRJNA729271	stage_E14_vs_E10	ordinary_muscle_development	ENSGALG00010013354	WWP1	0.582154365	0.146007109	3.987164531	6.68677E-05	0.000272107	positive
PRJNA729271	stage_E14_vs_E10	ordinary_muscle_development	ENSGALG00010013355	FAM171B	-1.578345572	0.147270347	-10.71733454	8.44006E-27	2.95504E-25	negative
PRJNA729271	stage_E14_vs_E10	ordinary_muscle_development	ENSGALG00010013356	AMER1	-0.218080582	0.06408199	-3.40314933	0.000666139	0.002188504	negative
PRJNA729271	stage_E14_vs_E10	ordinary_muscle_development	ENSGALG00010013357	RICTOR	0.295395967	0.109386116	2.700488668	0.00692377	0.01726562	positive
PRJNA729271	stage_E14_vs_E10	ordinary_muscle_development	ENSGALG00010013358	RPS16	0.48610519	0.174125852	2.791688794	0.005243376	0.013581437	positive
PRJNA729271	stage_E14_vs_E10	ordinary_muscle_development	ENSGALG00010013359	BTG1	0.222295683	0.132476294	1.678003488	0.093346418	0.160600158	positive
PRJNA729271	stage_E14_vs_E10	ordinary_muscle_development	ENSGALG00010013360	PRR5	1.218678952	0.110640468	11.01476679	3.24376E-28	1.24059E-26	positive
PRJNA729271	stage_E14_vs_E10	ordinary_muscle_development	ENSGALG00010013361	ENSGALG00010013361	-0.874352381	0.233192057	-3.7494947	0.000177191	0.000665385	negative
PRJNA729271	stage_E14_vs_E10	ordinary_muscle_development	ENSGALG00010013362	ENSGALG00010013362	2.904536592	0.359822583	8.072135342	6.90792E-16	1.04958E-14	positive
PRJNA729271	stage_E14_vs_E10	ordinary_muscle_development	ENSGALG00010013363	HS3ST5	0.327927817	0.241713903	1.356677517	0.174883699	0.268361128	positive
PRJNA729271	stage_E14_vs_E10	ordinary_muscle_development	ENSGALG00010013364	OTOGL	0.096620004	0.416871932	0.23177383	0.816713685	0.871550089	positive
PRJNA729271	stage_E14_vs_E10	ordinary_muscle_development	ENSGALG00010013365	CEP85	-0.031381146	0.228227836	-0.137499203	0.890636223	0.924884064	negative
PRJNA729271	stage_E14_vs_E10	ordinary_muscle_development	ENSGALG00010013367	NOL4L	-0.176538916	0.169037571	-1.044376793	0.296311096	0.409635607	negative
PRJNA729271	stage_E14_vs_E10	ordinary_muscle_development	ENSGALG00010013369	MTMR8	-0.218633087	0.061300395	-3.56658526	0.000361663	0.001269631	negative
PRJNA729271	stage_E14_vs_E10	ordinary_muscle_development	ENSGALG00010013370	CNKSR1	0.632329863	0.245438705	2.57632496	0.009985675	0.023794568	positive
PRJNA729271	stage_E14_vs_E10	ordinary_muscle_development	ENSGALG00010013371	FAT1	-0.673763622	0.156456941	-4.306383725	1.65945E-05	7.49104E-05	negative
PRJNA729271	stage_E14_vs_E10	ordinary_muscle_development	ENSGALG00010013372	SPRY4	-0.431652403	0.294337489	-1.466522002	0.142506132	0.227667406	negative
PRJNA729271	stage_E14_vs_E10	ordinary_muscle_development	ENSGALG00010013373	ENSGALG00010013373	-0.810588571	0.57539386	-1.408754295	0.158907834	0.24860972	negative
PRJNA729271	stage_E14_vs_E10	ordinary_muscle_development	ENSGALG00010013374	RMDN1	1.370326022	0.16312466	8.400483536	4.44644E-17	7.55582E-16	positive
PRJNA729271	stage_E14_vs_E10	ordinary_muscle_development	ENSGALG00010013375	PSMG1	-0.066933047	0.086854086	-0.770637867	0.44092161	0.554950608	negative
PRJNA729271	stage_E14_vs_E10	ordinary_muscle_development	ENSGALG00010013376	ARHGAP8	-4.556739377	0.9068246	-5.02494019	5.03589E-07	2.90324E-06	negative
PRJNA729271	stage_E14_vs_E10	ordinary_muscle_development	ENSGALG00010013378	SH3RF3	0.613777907	0.141042392	4.351726455	1.3507E-05	6.20157E-05	positive
PRJNA729271	stage_E14_vs_E10	ordinary_muscle_development	ENSGALG00010013379	ZNF593	0.509747364	0.131590856	3.873729373	0.000107182	0.000420008	positive
PRJNA729271	stage_E14_vs_E10	ordinary_muscle_development	ENSGALG00010013380	PHGDH	0.795762922	0.609436871	1.305734785	0.191642764	0.288999362	positive
PRJNA729271	stage_E14_vs_E10	ordinary_muscle_development	ENSGALG00010013381	DENND2A	0.128874682	0.088069105	1.463335892	0.143375491	0.228760512	positive
PRJNA729271	stage_E14_vs_E10	ordinary_muscle_development	ENSGALG00010013382	ASB12	2.044223852	0.220260162	9.280951369	1.67972E-20	3.71718E-19	positive
PRJNA729271	stage_E14_vs_E10	ordinary_muscle_development	ENSGALG00010013383	SEPTIN10	0.084312157	0.139042989	0.606374743	0.544265936	0.649846354	positive
PRJNA729271	stage_E14_vs_E10	ordinary_muscle_development	ENSGALG00010013385	PGRMC1	-0.355486536	0.155441325	-2.286949981	0.022198743	0.047669745	negative
PRJNA729271	stage_E14_vs_E10	ordinary_muscle_development	ENSGALG00010013386	ITGAV	0.066953355	0.12176353	0.549863777	0.582412811	0.683764331	positive
PRJNA729271	stage_E14_vs_E10	ordinary_muscle_development	ENSGALG00010013387	NDST1	0.081183064	0.091364084	0.888566497	0.374236103	0.490510201	positive
PRJNA729271	stage_E14_vs_E10	ordinary_muscle_development	ENSGALG00010013388	SOWAHC	0.094524941	0.207289351	0.456004811	0.648386519	0.740145109	positive
PRJNA729271	stage_E14_vs_E10	ordinary_muscle_development	ENSGALG00010013389	NAP1L1	-0.515785335	0.0616734	-8.363173328	6.10534E-17	1.02546E-15	negative
PRJNA729271	stage_E14_vs_E10	ordinary_muscle_development	ENSGALG00010013390	SLC16A2	-1.833288637	0.134311048	-13.64957437	2.03061E-42	1.8169E-40	negative
PRJNA729271	stage_E14_vs_E10	ordinary_muscle_development	ENSGALG00010013391	HMGN1	-0.175919674	0.087382631	-2.013210992	0.044092437	0.08517056	negative
PRJNA729271	stage_E14_vs_E10	ordinary_muscle_development	ENSGALG00010013392	HDAC2	-0.339064775	0.068538341	-4.947081706	7.53344E-07	4.23738E-06	negative
PRJNA729271	stage_E14_vs_E10	ordinary_muscle_development	ENSGALG00010013393	ADCK2	-0.035006086	0.159455282	-0.219535445	0.826232972	0.878410366	negative
PRJNA729271	stage_E14_vs_E10	ordinary_muscle_development	ENSGALG00010013394	DNMT3B	-0.906287201	0.114103528	-7.94267468	1.97867E-15	2.88831E-14	negative
PRJNA729271	stage_E14_vs_E10	ordinary_muscle_development	ENSGALG00010013396	FYB1	0.475497682	0.18671371	2.546667207	0.01087571	0.025628907	positive
PRJNA729271	stage_E14_vs_E10	ordinary_muscle_development	ENSGALG00010013397	MATN1	-6.025840786	0.441613992	-13.64504045	2.16094E-42	1.92164E-40	negative
PRJNA729271	stage_E14_vs_E10	ordinary_muscle_development	ENSGALG00010013398	CHAMP1	-0.062490451	0.155877589	-0.400894391	0.688497879	0.772488332	negative
PRJNA729271	stage_E14_vs_E10	ordinary_muscle_development	ENSGALG00010013399	FGF1	2.463110412	0.324684468	7.586166443	3.29509E-14	4.34203E-13	positive
PRJNA729271	stage_E14_vs_E10	ordinary_muscle_development	ENSGALG00010013400	BBS10	-0.090874518	0.163300591	-0.556486155	0.577878556	0.679512426	negative
PRJNA729271	stage_E14_vs_E10	ordinary_muscle_development	ENSGALG00010013402	MARCKS	0.360286196	0.15493968	2.325332005	0.020054214	0.043581085	positive
PRJNA729271	stage_E14_vs_E10	ordinary_muscle_development	ENSGALG00010013403	CPNE3	0.985807404	0.102590236	9.609173742	7.31335E-22	1.79673E-20	positive
PRJNA729271	stage_E14_vs_E10	ordinary_muscle_development	ENSGALG00010013404	ENSGALG00010013404	-0.022785109	0.218275497	-0.104386929	0.916862295	0.942146531	negative
PRJNA729271	stage_E14_vs_E10	ordinary_muscle_development	ENSGALG00010013405	RPS14	0.421001974	0.17532064	2.401325794	0.016335785	0.036507432	positive
PRJNA729271	stage_E14_vs_E10	ordinary_muscle_development	ENSGALG00010013406	ENSGALG00010013406	0.278377578	0.269451345	1.033127438	0.301544265	0.415389805	positive
PRJNA729271	stage_E14_vs_E10	ordinary_muscle_development	ENSGALG00010013407	ZC3H15	-0.006571271	0.112762109	-0.058275525	0.953529163	0.967885519	negative
PRJNA729271	stage_E14_vs_E10	ordinary_muscle_development	ENSGALG00010013408	RLIM	0.031675905	0.064766553	0.48907814	0.624786377	0.71972332	positive
PRJNA729271	stage_E14_vs_E10	ordinary_muscle_development	ENSGALG00010013409	DAB2	-0.362333351	0.14726909	-2.460348945	0.013880199	0.031731998	negative
PRJNA729271	stage_E14_vs_E10	ordinary_muscle_development	ENSGALG00010013410	KIAA0825	-0.448122085	0.100197952	-4.472367708	7.73583E-06	3.69337E-05	negative
PRJNA729271	stage_E14_vs_E10	ordinary_muscle_development	ENSGALG00010013411	ENSGALG00010013411	-0.312957871	0.456936004	-0.684905255	0.49340371	0.603585684	negative
PRJNA729271	stage_E14_vs_E10	ordinary_muscle_development	ENSGALG00010013412	COMMD7	0.004302696	0.09740769	0.044172033	0.964767275	0.9766785	positive

PRJNA729271	stage_E14_vs_E10	ordinary_muscle_development	ENSGALG00010013413	WRB	-0.098561501	0.082305491	-1.1975082	0.231108533	0.335293583	negative
PRJNA729271	stage_E14_vs_E10	ordinary_muscle_development	ENSGALG00010013414	LAPTM5	0.485530526	0.173923337	2.791635285	0.005244243	0.013581437	positive
PRJNA729271	stage_E14_vs_E10	ordinary_muscle_development	ENSGALG00010013416	NDUFB2	0.422982567	0.110315898	3.834284762	0.00012593	0.000487803	positive
PRJNA729271	stage_E14_vs_E10	ordinary_muscle_development	ENSGALG00010013417	AKR1B1	0.376158607	0.087942222	4.277338006	1.89142E-05	8.45012E-05	positive
PRJNA729271	stage_E14_vs_E10	ordinary_muscle_development	ENSGALG00010013418	HCN1	-2.528426908	0.82579196	-3.061820689	0.002199952	0.0063283	negative
PRJNA729271	stage_E14_vs_E10	ordinary_muscle_development	ENSGALG00010013419	ENSGALG00010013419	0.773513235	0.340414942	2.272265816	0.02307046	0.049242574	positive
PRJNA729271	stage_E14_vs_E10	ordinary_muscle_development	ENSGALG00010013420	ENSGALG00010013420	2.885153031	4.195323005	0.687707008			positive
PRJNA729271	stage_E14_vs_E10	ordinary_muscle_development	ENSGALG00010013423	ZNF804A	-0.333345584	0.381437011	-0.873920396	0.382161606	0.498195205	negative
PRJNA729271	stage_E14_vs_E10	ordinary_muscle_development	ENSGALG00010013425	ENSGALG00010013425	-0.424975082	0.224952148	-1.88917992	0.058867724	0.108726767	negative
PRJNA729271	stage_E14_vs_E10	ordinary_muscle_development	ENSGALG00010013427	NEXMIF	-0.64217522	0.165419009	-3.882112598	0.000103553	0.000406391	negative
PRJNA729271	stage_E14_vs_E10	ordinary_muscle_development	ENSGALG00010013428	BPGM	0.243188688	0.153152878	1.587881933	0.11231304	0.187555013	positive
PRJNA729271	stage_E14_vs_E10	ordinary_muscle_development	ENSGALG00010013429	PDE4DIP	1.499957436	0.187534202	7.998314013	1.26134E-15	1.87712E-14	positive
PRJNA729271	stage_E14_vs_E10	ordinary_muscle_development	ENSGALG00010013431	TMEM175	-0.389847685	0.086476562	-4.508131163	6.54011E-06	3.16736E-05	negative
PRJNA729271	stage_E14_vs_E10	ordinary_muscle_development	ENSGALG00010013434	CORIN	-2.979520078	0.408723634	-7.289815983	3.10379E-13	3.75537E-12	negative
PRJNA729271	stage_E14_vs_E10	ordinary_muscle_development	ENSGALG00010013435	SDC3	-3.074109648	0.181184431	-16.96674282	1.44741E-64	3.95852E-62	negative
PRJNA729271	stage_E14_vs_E10	ordinary_muscle_development	ENSGALG00010013436	NPAS2	-1.610082273	0.228775768	-7.037818231	1.95273E-12	2.16233E-11	negative
PRJNA729271	stage_E14_vs_E10	ordinary_muscle_development	ENSGALG00010013437	ENSGALG00010013437	-0.058891858	0.124285519	-0.473843279	0.63561167	0.729237862	negative
PRJNA729271	stage_E14_vs_E10	ordinary_muscle_development	ENSGALG00010013438	RPL37	0.321414137	0.18748472	1.714348441	0.086464752	0.150891714	positive
PRJNA729271	stage_E14_vs_E10	ordinary_muscle_development	ENSGALG00010013440	EEA1	-0.3073663	0.098181601	-3.130589625	0.001744558	0.005165957	negative
PRJNA729271	stage_E14_vs_E10	ordinary_muscle_development	ENSGALG00010013442	ENSGALG00010013442	0.33499933	0.104499234	3.20575871	0.001347069	0.004114152	positive
PRJNA729271	stage_E14_vs_E10	ordinary_muscle_development	ENSGALG00010013444	OSBPL8	-0.449004657	0.102613702	-4.375679347	1.21055E-05	5.60425E-05	negative
PRJNA729271	stage_E14_vs_E10	ordinary_muscle_development	ENSGALG00010013446	ATP5I	0.490132094	0.145358527	3.371884016	0.000746559	0.002427405	positive
PRJNA729271	stage_E14_vs_E10	ordinary_muscle_development	ENSGALG00010013449	LCA5L	-0.804131449	0.219503314	-3.663413701	0.000248876	0.000903446	negative
PRJNA729271	stage_E14_vs_E10	ordinary_muscle_development	ENSGALG00010013450	CD74	0.948960393	0.190966694	4.969245558	6.72139E-07	3.80127E-06	positive
PRJNA729271	stage_E14_vs_E10	ordinary_muscle_development	ENSGALG00010013451	PRKAB2	1.096822091	0.169306052	6.47833954	9.27375E-11	8.53479E-10	positive
PRJNA729271	stage_E14_vs_E10	ordinary_muscle_development	ENSGALG00010013452	LSS	-0.313706365	0.223398694	-1.404244397	0.160246085	0.25037911	negative
PRJNA729271	stage_E14_vs_E10	ordinary_muscle_development	ENSGALG00010013453	PUM1	-0.015579429	0.088995874	-0.175057878	0.861034156	0.904365485	negative
PRJNA729271	stage_E14_vs_E10	ordinary_muscle_development	ENSGALG00010013457	SH3BGR	1.995098495	0.147669604	13.51055623	1.35494E-41	1.14853E-39	positive
PRJNA729271	stage_E14_vs_E10	ordinary_muscle_development	ENSGALG00010013458	LAMA4	1.159099099	0.143634357	8.069789996	7.04192E-16	1.06882E-14	positive
PRJNA729271	stage_E14_vs_E10	ordinary_muscle_development	ENSGALG00010013459	ATP10D	-1.076741491	0.14422449	-7.465732677	8.28374E-14	1.05978E-12	negative
PRJNA729271	stage_E14_vs_E10	ordinary_muscle_development	ENSGALG00010013461	S100B	0.755356794	0.608979858	1.240364166	0.214840729	0.316563545	positive
PRJNA729271	stage_E14_vs_E10	ordinary_muscle_development	ENSGALG00010013463	ENSGALG00010013463	-0.534277292	0.215489243	-2.479368733	0.013161517	0.030301173	negative
PRJNA729271	stage_E14_vs_E10	ordinary_muscle_development	ENSGALG00010013464	ABCB7	-0.233288949	0.07381028	-3.160656589	0.00157414	0.004713314	negative
PRJNA729271	stage_E14_vs_E10	ordinary_muscle_development	ENSGALG00010013465	CALD1	-1.274195712	0.157224602	-8.104302354	5.3049E-16	8.13699E-15	negative
PRJNA729271	stage_E14_vs_E10	ordinary_muscle_development	ENSGALG00010013466	BRAF	0.148551189	0.060307399	2.463233244	0.013769031	0.031509647	positive
PRJNA729271	stage_E14_vs_E10	ordinary_muscle_development	ENSGALG00010013468	DEPDC1B	-0.324098061	0.26667811	-1.215315578	0.224245753	0.327291488	negative
PRJNA729271	stage_E14_vs_E10	ordinary_muscle_development	ENSGALG00010013470	RPL31	0.291436736	0.146297066	1.992088737	0.046361323	0.08907839	positive
PRJNA729271	stage_E14_vs_E10	ordinary_muscle_development	ENSGALG00010013472	ENSGALG00010013472	0.973038543	0.224328269	4.337565421	1.4407E-05	6.58559E-05	positive
PRJNA729271	stage_E14_vs_E10	ordinary_muscle_development	ENSGALG00010013476	ZDHHC17	-0.429981701	0.128805771	-3.33821768	0.000843177	0.002706343	negative
PRJNA729271	stage_E14_vs_E10	ordinary_muscle_development	ENSGALG00010013477	RFC1	-0.210779482	0.088912084	-2.370650566	0.01775681	0.039247478	negative
PRJNA729271	stage_E14_vs_E10	ordinary_muscle_development	ENSGALG00010013480	TBC1D8	0.425787231	0.152691222	2.78855082	0.005294444	0.01369017	positive
PRJNA729271	stage_E14_vs_E10	ordinary_muscle_development	ENSGALG00010013482	ENSGALG00010013482	0.738573817	0.232041888	3.182933149	0.001457913	0.004406265	positive
PRJNA729271	stage_E14_vs_E10	ordinary_muscle_development	ENSGALG00010013483	ENSGALG00010013483	0.673569354	0.20131132	3.345908993	0.000820133	0.002641153	positive
PRJNA729271	stage_E14_vs_E10	ordinary_muscle_development	ENSGALG00010013484	TCOF1	0.023113691	0.100832004	0.229229715	0.818690378	0.872969689	positive
PRJNA729271	stage_E14_vs_E10	ordinary_muscle_development	ENSGALG00010013486	PLPP6	0.519362549	0.16208517	3.204257059	0.001354115	0.004132189	positive
PRJNA729271	stage_E14_vs_E10	ordinary_muscle_development	ENSGALG00010013487	ENSGALG00010013487	-0.362533293	0.092340633	-3.926042962	8.63547E-05	0.000343782	negative
PRJNA729271	stage_E14_vs_E10	ordinary_muscle_development	ENSGALG00010013488	UPRT	-0.373007442	0.117328356	-3.179175559	0.001476946	0.004460069	negative
PRJNA729271	stage_E14_vs_E10	ordinary_muscle_development	ENSGALG00010013491	CNOT11	-0.016480776	0.110858051	-0.148665576	0.881817526	0.918969375	negative
PRJNA729271	stage_E14_vs_E10	ordinary_muscle_development	ENSGALG00010013492	B3GALT5	-3.268422076	0.655670225	-4.98485664	6.20078E-07	3.52333E-06	negative
PRJNA729271	stage_E14_vs_E10	ordinary_muscle_development	ENSGALG00010013493	ENSGALG00010013493	0.337185207	0.127118252	2.652531797	0.00798906	0.019607421	positive
PRJNA729271	stage_E14_vs_E10	ordinary_muscle_development	ENSGALG00010013494	ASB1	-1.10345952	0.14511419	-7.604077319	2.86944E-14	3.7984E-13	negative
PRJNA729271	stage_E14_vs_E10	ordinary_muscle_development	ENSGALG00010013497	FAM172A	0.148719176	0.106714814	1.39361322	0.163434434	0.254182631	positive
PRJNA729271	stage_E14_vs_E10	ordinary_muscle_development	ENSGALG00010013498	KDR	0.251383663	0.125113354	2.009247251	0.044510919	0.085910222	positive
PRJNA729271	stage_E14_vs_E10	ordinary_muscle_development	ENSGALG00010013501	AKR1B10	-2.967565783	0.142299172	-20.85441355	1.38992E-96	1.34312E-93	negative
PRJNA729271	stage_E14_vs_E10	ordinary_muscle_development	ENSGALG00010013502	PAFAH2	1.246974362	0.200627352	6.215375661	5.1202E-10	4.34782E-09	positive
PRJNA729271	stage_E14_vs_E10	ordinary_muscle_development	ENSGALG00010013504	IGSF5	-1.418015664	0.779532506	-1.819059055	0.068902421	0.124453656	negative
PRJNA729271	stage_E14_vs_E10	ordinary_muscle_development	ENSGALG00010013506	RNF149	-0.614312744	0.103969217	-5.908602173	3.45023E-09	2.66016E-08	negative
PRJNA729271	stage_E14_vs_E10	ordinary_muscle_development	ENSGALG00010013508	TUBE1	-0.301855848	0.191608298	-1.575379833	0.115168797	0.191485628	negative
PRJNA729271	stage_E14_vs_E10	ordinary_muscle_development	ENSGALG00010013509	ENSGALG00010013509	-0.452758726	0.193428247	-2.34070635	0.019247299	0.042022834	negative
PRJNA729271	stage_E14_vs_E10	ordinary_muscle_development	ENSGALG00010013510	MRPS33	0.064350174	0.087078369	0.738991497	0.459912161	0.572662015	positive
PRJNA729271	stage_E14_vs_E10	ordinary_muscle_development	ENSGALG00010013511	FAM78B	0.48759141	0.226664738	2.151156877	0.031463819	0.063875079	positive
PRJNA729271	stage_E14_vs_E10	ordinary_muscle_development	ENSGALG00010013512	ENSGALG00010013512	4.210284369	3.426711418	1.228666163			positive
PRJNA729271	stage_E14_vs_E10	ordinary_muscle_development	ENSGALG00010013514	LMBRD2	-0.31675404	0.137096362	-2.310448189	0.020863354	0.045102806	negative
PRJNA729271	stage_E14_vs_E10	ordinary_muscle_development	ENSGALG00010013516	CSRP2	1.442458415	0.132524532	10.88446342	1.36701E-27	5.02915E-26	positive
PRJNA729271	stage_E14_vs_E10	ordinary_muscle_development	ENSGALG00010013517	STMN1	0.589592006	0.217516865	2.710557662	0.006717017	0.016815744	positive
PRJNA729271	stage_E14_vs_E10	ordinary_muscle_development	ENSGALG00010013520	CREG2	-0.400620437	0.182319053	-2.197359138	0.027994807	0.058010683	negative
PRJNA729271	stage_E14_vs_E10	ordinary_muscle_development	ENSGALG00010013523	TMEM178B	-1.157126838	0.174016455	-6.649525415	2.9404E-11	2.86432E-10	negative
PRJNA729271	stage_E14_vs_E10	ordinary_muscle_development	ENSGALG00010013524	ZDHHC15	-0.126289114	0.121950015	-1.035580969	0.300397674	0.414257853	negative
PRJNA729271	stage_E14_vs_E10	ordinary_muscle_development	ENSGALG00010013526	SIRPA	1.02558432	0.219467011	4.673068256	2.96733E-06	1.52038E-05	positive
PRJNA729271	stage_E14_vs_E10	ordinary_muscle_development	ENSGALG00010013527	ADARB1	-0.099665954	0.098690178	-1.00988726	0.312549307	0.426310549	negative
PRJNA729271	stage_E14_vs_E10	ordinary_muscle_development	ENSGALG00010013528	PRDX6	0.398250274	0.131088341	3.038029697	0.002381305	0.006766715	positive
PRJNA729271	stage_E14_vs_E10	ordinary_muscle_development	ENSGALG00010013531	ARSI	-2.077548637	0.231881495	-8.959527508	3.26073E-19	6.58276E-18	negative
PRJNA729271	stage_E14_vs_E10	ordinary_muscle_development	ENSGALG00010013532	WDR19	-0.167193785	0.07270873	-2.299500834	0.021476516	0.046297159	negative
PRJNA729271	stage_E14_vs_E10	ordinary_muscle_development	ENSGALG00010013533	SKP2	-0.424122574	0.080025632	-5.299834089	1.15908E-07	7.31108E-07	negative
PRJNA729271	stage_E14_vs_E10	ordinary_muscle_development	ENSGALG00010013537	LMO3	-2.156578469	0.380383461	-5.669485369	1.43227E-08	1.02472E-07	negative
PRJNA729271	stage_E14_vs_E10	ordinary_muscle_development	ENSGALG00010013538	E2F7	-0.562543825	0.259228234	-2.170071587	0.030001423	0.061439761	negative
PRJNA729271	stage_E14_vs_E10	ordinary_muscle_development	ENSGALG00010013539	NSFL1C	0.289228267	0.091377093	3.165216335	0.001549677	0.004646786	positive
PRJNA729271	stage_E14_vs_E10	ordinary_muscle_development	ENSGALG00010013544	ENSGALG00010013544	-0.476060247	0.286429055	-1.662052917	0.096502165	0.165127952	negative
PRJNA729271	stage_E14_vs_E10	ordinary_muscle_development	ENSGALG00010013546	IDUA	-0.597704294	0.131770189	-4.535959919	5.73421E-06	2.80328E-05	negative
PRJNA729271	stage_E14_vs_E10	ordinary_muscle_development	ENSGALG00010013549	PCP4	-2.254331173	0.268501424	-8.395974723	4.62045E-17	7.83315E-16	negative
PRJNA729271	stage_E14_vs_E10	ordinary_muscle_development	ENSGALG00010013550	KLHL5	-0.268174874	0.124348014	-2.156647828	0.031033115	0.063133333	negative
PRJNA729271	stage_E14_vs_E10	ordinary_muscle_development	ENSGALG00010013551	UBE2N	0.071958617	0.11147442	0.645516853	0.5185923	0.626834172	positive
PRJNA729271	stage_E14_vs_E10	ordinary_muscle_development	ENSGALG00010013552	FGF16	-0.738555598	0.405250078	-1.822468739	0.068383893	0.123655755	negative
PRJNA729271	stage_E14_vs_E10	ordinary_muscle_development	ENSGALG00010013553	TADA1	0.174439771	0.109835301	1.588194042	0.112242468	0.18746166	positive
PRJNA729271	stage_E14_vs_E10	ordinary_muscle_development	ENSGALG00010013554	ENSGALG00010013554	2.057681942	5.404005616	0.380769764	0.703374097	0.784441623	positive
PRJNA729271	stage_E14_vs_E10	ordinary_muscle_development	ENSGALG00010013556	RASL11B	-1.955917103	0.163026971	-11.99750626	3.66163E-33	1.86885E-31	negative
PRJNA729271	stage_E14_vs_E10	ordinary_muscle_development	ENSGALG00010013558	SNAPC3	-0.029526393	0.080440237	-0.367059992	0.713574269	0.792829912	negative
PRJNA729271	stage_E14_vs_E10	ordinary_muscle_development	ENSGALG00010013561	AGK	0.157422295	0.107157982	1.469067376	0.141814515	0.226787445	positive
PRJNA729271	stage_E14_vs_E10	ordinary_muscle_development	ENSGALG00010013562	RERGL	0.129457277	0.158191108	0.818360012	0.413151648	0.528005038	positive
PRJNA729271	stage_E14_vs_E10	ordinary_muscle_development	ENSGALG00010013563	FKBP1A	0.69761818	0.098450513	7.085978149	1.38066E-12	1.55377E-11	positive
PRJNA729271	stage_E14_vs_E10	ordinary_muscle_development	ENSGALG00010013565	ENSGALG00010013565	4.077731775	5.264507666	0.774570394	0.438593557	0.55244964	positive
PRJNA729271	stage_E14_vs_E10	ordinary_muscle_development	ENSGALG00010013569	ENSGALG00010013569	1.215571866	0.533000249	2.280621571	0.02257085	0.04836134	positive
PRJNA729271	stage_E14_vs_E10	ordinary_muscle_development	ENSGALG00010013572	DENND11	0.365291272	0.101588011	3.595810857	0.000323383	0.001149116	positive
PRJNA729271	stage_E14_vs_E10	ordinary_muscle_development	ENSGALG00010013573	ENSGALG00010013573	3.933105312	0.456943512	8.607421286	7.47224E-18	1.3505E-16	positive
PRJNA729271	stage_E14_vs_E10	ordinary_muscle_development	ENSGALG00010013574	NR2F1	-0.136393259	0.144175996	-0.94601919	0.344138798	0.459411667	negative
PRJNA729271	stage_E14_vs_E10	ordinary_muscle_development	ENSGALG00010013576	CAMK2A	1.512412564	0.185360141	8.159319247	3.36918E-16	5.25686E-15	positive
PRJNA729271	stage_E14_vs_E10	ordinary_muscle_development	ENSGALG00010013579	H2AC32	0.425750661	0.176479882	2.412460034	0.015845275	0.035575783	positive
PRJNA729271	stage_E14_vs_E10	ordinary_muscle_development	ENSGALG00010013580	RANBP3L	-0.603458749	0.307508731	-1.962411756	0.049714565	0.094370432	negative
PRJNA729271	stage_E14_vs_E10	ordinary_muscle_development	ENSGALG00010013582	MAP4K4	-0.29173041	0.106311358	-2.744113286	0.00606746	0.015410518	negative
PRJNA729271	stage_E14_vs_E10	ordinary_muscle_development	ENSGALG00010013583	FASLG	0.377131494	0.332451533	1.134395413	0.256628722	0.364582312	positive
PRJNA729271	stage_E14_vs_E10	ordinary_muscle_development	ENSGALG00010013587	SLC26A1	0.008319624	0.456490717	0.018225177	0.985459217	0.990180518	positive
PRJNA729271	stage_E14_vs_E10	ordinary_muscle_development	ENSGALG00010013589	DSCAM	-1.542825933	0.449405585	-3.433036844	0.000596861	0.001982016	negative
PRJNA729271	stage_E14_vs_E10	ordinary_muscle_development	ENSGALG00010013592	H3C33	0.417211715	0.183569049	2.272778108	0.023039555	0.049191096	positive
PRJNA729271	stage_E14_vs_E10	ordinary_muscle_development	ENSGALG00010013596	SDCBP2	0.142925489	0.11967478	1.194282441	0.232367504	0.336573402	positive
PRJNA729271	stage_E14_vs_E10	ordinary_muscle_development	ENSGALG00010013598	PAQR7	0.048256506	0.232381158	0.207661012	0.835493662	0.885151494	positive
PRJNA729271	stage_E14_vs_E10	ordinary_muscle_development	ENSGALG00010013604	FYN	0.387114794	0.110667059	3.498012841	0.000468739	0.00160018	positive
PRJNA729271	stage_E14_vs_E10	ordinary_muscle_development	ENSGALG00010013609	PIGC	0.725032653	0.16218428	4.470424969	7.80643E-06	3.72218E-05	positive
PRJNA729271	stage_E14_vs_E10	ordinary_muscle_development	ENSGALG00010013612	TWIST2	-2.280375299	0.286012137	-7.973001851	1.54866E-15	2.29059E-14	negative
PRJNA729271	stage_E14_vs_E10	ordinary_muscle_development	ENSGALG00010013614	ATRX	-0.42457695	0.086429921	-4.912383901	8.99757E-07	4.9931E-06	negative
PRJNA729271	stage_E14_vs_E10	ordinary_muscle_development	ENSGALG00010013617	PIK3C2G	-0.929167504	0.166300833	-5.587269089	2.30668E-08	1.60592E-07	negative
PRJNA729271	stage_E14_vs_E10	ordinary_muscle_development	ENSGALG00010013619	IL1R2	0.103279303	0.197717911	0.522356835	0.601421904	0.700097213	positive
PRJNA729271	stage_E14_vs_E10	ordinary_muscle_development	ENSGALG00010013621	PDGFRA	-0.474724119	0.164653884	-2.883163812	0.003937026	0.01053289	negative
PRJNA729271	stage_E14_vs_E10	ordinary_muscle_development	ENSGALG00010013624	SST1	-8.103925146	1.740043611	-4.657311515	3.20366E-06	1.62994E-05	negative
PRJNA729271	stage_E14_vs_E10	ordinary_muscle_development	ENSGALG00010013625	SSBP1	-0.061063338	0.11119304	-0.549165109	0.582892147	0.68410001	negative
PRJNA729271	stage_E14_vs_E10	ordinary_muscle_development	ENSGALG00010013627	MYCBP	0.165652485	0.146609893	1.129886132	0.258524208	0.366807792	positive
PRJNA729271	stage_E14_vs_E10	ordinary_muscle_development	ENSGALG00010013632	SLC1A3	-4.236754197	0.514049159	-8.241924186	1.69463E-16	2.72023E-15	negative
PRJNA729271	stage_E14_vs_E10	ordinary_muscle_development	ENSGALG00010013635	BCL6	1.050784241	0.157274006	6.68123278	2.36941E-11	2.33477E-10	positive
PRJNA729271	stage_E14_vs_E10	ordinary_muscle_development	ENSGALG00010013638	COMMD8	-0.245712671	0.11482396	-2.13990766	0.03236223	0.06537847	negative
PRJNA729271	stage_E14_vs_E10	ordinary_muscle_development	ENSGALG00010013639	ENSGALG00010013639	0.722274941	0.447657677	1.613453711	0.106645972	0.179706274	positive
PRJNA729271	stage_E14_vs_E10	ordinary_muscle_development	ENSGALG00010013640	SNPH	-1.610437176	0.545447242	-2.952507687	0.003152042	0.00869105	negative
PRJNA729271	stage_E14_vs_E10	ordinary_muscle_development	ENSGALG00010013645	RRAGC	-0.115015937	0.119735402	-0.960584207	0.336761273	0.451935437	negative
PRJNA729271	stage_E14_vs_E10	ordinary_muscle_development	ENSGALG00010013646	CLDN1	-0.298338456	0.336038268	-0.887810957	0.374642448	0.490998398	negative
PRJNA729271	stage_E14_vs_E10	ordinary_muscle_development	ENSGALG00010013648	NAV3	0.874064759	0.117409954	7.444554163	9.72724E-14	1.23356E-12	positive
PRJNA729271	stage_E14_vs_E10	ordinary_muscle_development	ENSGALG00010013650	BACE2	-0.913332173	0.159787399	-5.715921151	1.09111E-08	7.90389E-08	negative
PRJNA729271	stage_E14_vs_E10	ordinary_muscle_development	ENSGALG00010013651	ENSGALG00010013651	-0.854813191	0.162454929	-5.261848297	1.42614E-07	8.8759E-07	negative
PRJNA729271	stage_E14_vs_E10	ordinary_muscle_development	ENSGALG00010013654	SLC6A7	-0.882272695	0.400016246	-2.20559216	0.027412568	0.05694256	negative
PRJNA729271	stage_E14_vs_E10	ordinary_muscle_development	ENSGALG00010013657	MAGT1	0.02433576	0.103848281	0.234339559	0.814721377	0.870128674	positive
PRJNA729271	stage_E14_vs_E10	ordinary_muscle_development	ENSGALG00010013661	GABRA4	-1.232175929	0.355821144	-3.46290812	0.000534371	0.001800908	negative
PRJNA729271	stage_E14_vs_E10	ordinary_muscle_development	ENSGALG00010013663	OPA1	0.061165586	0.106110259	0.576434235	0.56432171	0.666925657	positive
PRJNA729271	stage_E14_vs_E10	ordinary_muscle_development	ENSGALG00010013664	REV3L	-0.252990631	0.096179567	-2.630398941	0.008528472	0.020773014	negative
PRJNA729271	stage_E14_vs_E10	ordinary_muscle_development	ENSGALG00010013666	COX7B	0.246571029	0.143459098	1.71875491	0.085659009	0.149665783	positive

PRJNA729271	stage_E14_vs_E10	ordinary_muscle_development	ENSGALG00010013667	SOWAHA	-2.372925375	0.511656312	-4.637733025	3.52251E-06	1.77781E-05	negative
PRJNA729271	stage_E14_vs_E10	ordinary_muscle_development	ENSGALG00010013669	NPR3	-1.621943325	0.258998627	-6.262362635	3.79188E-10	3.26773E-09	negative
PRJNA729271	stage_E14_vs_E10	ordinary_muscle_development	ENSGALG00010013672	ENSGALG00010013672	-1.854491863	0.460820921	-4.024322201	5.71396E-05	0.000235563	negative
PRJNA729271	stage_E14_vs_E10	ordinary_muscle_development	ENSGALG00010013675	CHRNA6	-0.645881144	0.430511276	-1.500265337	0.133545685	0.215874283	negative
PRJNA729271	stage_E14_vs_E10	ordinary_muscle_development	ENSGALG00010013677	GABRG1	-2.890232106	0.418185564	-6.911362686	4.8002E-12	5.07875E-11	negative
PRJNA729271	stage_E14_vs_E10	ordinary_muscle_development	ENSGALG00010013678	ATP7A	-0.307027604	0.101781603	-3.016533389	0.002556831	0.007204755	negative
PRJNA729271	stage_E14_vs_E10	ordinary_muscle_development	ENSGALG00010013679	MFSD4B	0.246438827	0.214561151	1.148571519	0.250732707	0.357889767	positive
PRJNA729271	stage_E14_vs_E10	ordinary_muscle_development	ENSGALG00010013686	UQCRQ	0.619619398	0.138329299	4.479307003	7.48858E-06	3.58833E-05	positive
PRJNA729271	stage_E14_vs_E10	ordinary_muscle_development	ENSGALG00010013690	CREB3L2	-0.435576473	0.101239571	-4.302433034	1.68933E-05	7.61405E-05	negative
PRJNA729271	stage_E14_vs_E10	ordinary_muscle_development	ENSGALG00010013696	CETN3	-0.234182745	0.10697881	-2.189057313	0.028592675	0.059030169	negative
PRJNA729271	stage_E14_vs_E10	ordinary_muscle_development	ENSGALG00010013697	HDAC4	0.061251932	0.066450754	0.921764295	0.356651536	0.473065886	positive
PRJNA729271	stage_E14_vs_E10	ordinary_muscle_development	ENSGALG00010013698	TFAP2E	-1.240151415	0.491719224	-2.522072261	0.011666574	0.027284443	negative
PRJNA729271	stage_E14_vs_E10	ordinary_muscle_development	ENSGALG00010013709	PSMF1	0.437462653	0.099934553	4.377491484	1.20053E-05	5.56142E-05	positive
PRJNA729271	stage_E14_vs_E10	ordinary_muscle_development	ENSGALG00010013715	IL1R1	-0.003624074	0.233602459	-0.015513853	0.987622233	0.991796056	negative
PRJNA729271	stage_E14_vs_E10	ordinary_muscle_development	ENSGALG00010013719	NCDN	0.199552323	0.138051897	1.445487728	0.148320938	0.235245868	positive
PRJNA729271	stage_E14_vs_E10	ordinary_muscle_development	ENSGALG00010013720	SLC16A10	-0.050424907	0.189920339	-0.265505567	0.790620021	0.852871713	negative
PRJNA729271	stage_E14_vs_E10	ordinary_muscle_development	ENSGALG00010013732	FNTA	0.122782305	0.074675185	1.644218296	0.100131119	0.170288067	positive
PRJNA729271	stage_E14_vs_E10	ordinary_muscle_development	ENSGALG00010013737	AFF4	0.204081889	0.084407855	2.417806847	0.015614363	0.035133528	positive
PRJNA729271	stage_E14_vs_E10	ordinary_muscle_development	ENSGALG00010013740	GNPDA2	-0.856360124	0.116868144	-7.32757527	2.34354E-13	2.8594E-12	negative
PRJNA729271	stage_E14_vs_E10	ordinary_muscle_development	ENSGALG00010013742	RSPO4	-1.433896829	1.143388363	-1.254076809	0.209814089	0.310617426	negative
PRJNA729271	stage_E14_vs_E10	ordinary_muscle_development	ENSGALG00010013744	ENSGALG00010013744	-0.797575265	0.311610415	-2.559526981	0.010481472	0.024791641	negative
PRJNA729271	stage_E14_vs_E10	ordinary_muscle_development	ENSGALG00010013746	ENSGALG00010013746	-0.236797285	0.08666749	-2.732250402	0.006290331	0.015895808	negative
PRJNA729271	stage_E14_vs_E10	ordinary_muscle_development	ENSGALG00010013749	NELL2	-0.568403892	0.462891139	-1.227942908	0.2194684	0.321722741	negative
PRJNA729271	stage_E14_vs_E10	ordinary_muscle_development	ENSGALG00010013751	FUT10	-0.274596894	0.16955148	-1.619548794	0.105329237	0.177797518	negative
PRJNA729271	stage_E14_vs_E10	ordinary_muscle_development	ENSGALG00010013753	PLEKHA5	-0.600337915	0.124428265	-4.824771261	1.40164E-06	7.59506E-06	negative
PRJNA729271	stage_E14_vs_E10	ordinary_muscle_development	ENSGALG00010013755	ANGPT4	1.269943298	0.153425918	8.277240993	1.26062E-16	2.05773E-15	positive
PRJNA729271	stage_E14_vs_E10	ordinary_muscle_development	ENSGALG00010013757	DGKI	0.308370688	0.211876461	1.455426838	0.145551131	0.231587667	positive
PRJNA729271	stage_E14_vs_E10	ordinary_muscle_development	ENSGALG00010013758	FAM3B	-2.103258438	0.491401359	-4.28012336	1.8679E-05	8.35912E-05	negative
PRJNA729271	stage_E14_vs_E10	ordinary_muscle_development	ENSGALG00010013760	RPF2	-0.331727233	0.113834321	-2.914123184	0.003566891	0.009661327	negative
PRJNA729271	stage_E14_vs_E10	ordinary_muscle_development	ENSGALG00010013766	DNM3	-0.771727465	0.109379767	-7.055486473	1.71998E-12	1.91043E-11	negative
PRJNA729271	stage_E14_vs_E10	ordinary_muscle_development	ENSGALG00010013767	PGK2	-0.109616156	0.172127044	-0.636832851	0.524233725	0.632019284	negative
PRJNA729271	stage_E14_vs_E10	ordinary_muscle_development	ENSGALG00010013768	FAM110A	1.016756652	0.215086033	4.727209087	2.27627E-06	1.18728E-05	positive
PRJNA729271	stage_E14_vs_E10	ordinary_muscle_development	ENSGALG00010013771	PELO	-0.104015531	0.102515824	-1.014629026	0.310282698	0.424176903	negative
PRJNA729271	stage_E14_vs_E10	ordinary_muscle_development	ENSGALG00010013772	KIAA0319L	0.508376954	0.143200762	3.550099494	0.000385086	0.001341783	positive
PRJNA729271	stage_E14_vs_E10	ordinary_muscle_development	ENSGALG00010013775	DBX2	-1.044189001	0.353067608	-2.957476072	0.003101688	0.008566753	negative
PRJNA729271	stage_E14_vs_E10	ordinary_muscle_development	ENSGALG00010013777	CPLANE1	-0.6394445	0.093585888	-6.832702189	8.33298E-12	8.60303E-11	negative
PRJNA729271	stage_E14_vs_E10	ordinary_muscle_development	ENSGALG00010013778	ENSGALG00010013778	0.652653162	0.660648762	0.98789735	0.323202923	0.437847211	positive
PRJNA729271	stage_E14_vs_E10	ordinary_muscle_development	ENSGALG00010013779	SLC52A3	-0.168964805	0.228668173	-0.738908273	0.459962698	0.572662015	negative
PRJNA729271	stage_E14_vs_E10	ordinary_muscle_development	ENSGALG00010013783	NDUFA10	0.230260292	0.095650962	2.407297198	0.016071085	0.036004696	positive
PRJNA729271	stage_E14_vs_E10	ordinary_muscle_development	ENSGALG00010013784	ANO6	1.092838014	0.094364987	11.58096925	5.14609E-31	2.32376E-29	positive
PRJNA729271	stage_E14_vs_E10	ordinary_muscle_development	ENSGALG00010013787	ZCCHC10	-0.290600298	0.102016588	-2.848559275	0.004391767	0.011620786	negative
PRJNA729271	stage_E14_vs_E10	ordinary_muscle_development	ENSGALG00010013791	AEBP2	0.045755282	0.062642824	0.730415372	0.465136325	0.577553152	positive
PRJNA729271	stage_E14_vs_E10	ordinary_muscle_development	ENSGALG00010013792	FGF10	-1.148460176	0.236314136	-4.859887746	1.17452E-06	6.439E-06	negative
PRJNA729271	stage_E14_vs_E10	ordinary_muscle_development	ENSGALG00010013793	METTL13	-0.065804665	0.125270068	-0.525302382	0.599373002	0.698722186	negative
PRJNA729271	stage_E14_vs_E10	ordinary_muscle_development	ENSGALG00010013794	GTF3C6	-0.205942343	0.176501306	-1.166803513	0.243289728	0.349641544	negative
PRJNA729271	stage_E14_vs_E10	ordinary_muscle_development	ENSGALG00010013797	IL1RL1	-0.281514413	0.320576079	-0.878151651	0.379861426	0.495865577	negative
PRJNA729271	stage_E14_vs_E10	ordinary_muscle_development	ENSGALG00010013802	PYROXD1	-0.004038969	0.139933496	-0.028863488	0.976973466	0.984683087	negative
PRJNA729271	stage_E14_vs_E10	ordinary_muscle_development	ENSGALG00010013805	HSPA4	-0.100706552	0.145225204	-0.693450925	0.488026613	0.599131511	negative
PRJNA729271	stage_E14_vs_E10	ordinary_muscle_development	ENSGALG00010013808	PLAAT1	0.375311654	0.137586584	2.72782158	0.006375408	0.016071572	positive
PRJNA729271	stage_E14_vs_E10	ordinary_muscle_development	ENSGALG00010013809	MX1	-0.601549284	1.244870147	-0.483222516	0.62893775	0.722839572	negative
PRJNA729271	stage_E14_vs_E10	ordinary_muscle_development	ENSGALG00010013814	AMD1	0.390497518	0.109474049	3.567032745	0.000361046	0.00126808	positive
PRJNA729271	stage_E14_vs_E10	ordinary_muscle_development	ENSGALG00010013815	SRXN1	0.394449595	0.249292472	1.582276403	0.113586485	0.189354353	positive
PRJNA729271	stage_E14_vs_E10	ordinary_muscle_development	ENSGALG00010013820	MASP1	-2.07679188	0.466369582	-4.453103208	8.4638E-06	4.01581E-05	negative
PRJNA729271	stage_E14_vs_E10	ordinary_muscle_development	ENSGALG00010013823	GUF1	0.243254293	0.109638973	2.218684521	0.026508195	0.055405376	positive
PRJNA729271	stage_E14_vs_E10	ordinary_muscle_development	ENSGALG00010013828	PTN	-4.398036933	0.27367761	-16.07013788	4.13166E-58	8.67949E-56	negative
PRJNA729271	stage_E14_vs_E10	ordinary_muscle_development	ENSGALG00010013831	TCERG1	-0.130555317	0.112681728	-1.158620113	0.246611067	0.35325896	negative
PRJNA729271	stage_E14_vs_E10	ordinary_muscle_development	ENSGALG00010013833	TMPRSS2	-4.109341879	1.329591661	-3.090679642	0.00199699	0.005807858	negative
PRJNA729271	stage_E14_vs_E10	ordinary_muscle_development	ENSGALG00010013836	IL18R1	-0.057998209	0.256128161	-0.226442141	0.820857555	0.87468428	negative
PRJNA729271	stage_E14_vs_E10	ordinary_muscle_development	ENSGALG00010013837	IFI27L2	-0.63866873	0.341574892	-1.86977657	0.061514851	0.113011124	negative
PRJNA729271	stage_E14_vs_E10	ordinary_muscle_development	ENSGALG00010013842	CDK19	0.647706902	0.110140432	5.880736915	4.08444E-09	3.13083E-08	positive
PRJNA729271	stage_E14_vs_E10	ordinary_muscle_development	ENSGALG00010013846	YIPF7	3.671391784	0.327091508	11.22435679	3.09631E-29	1.27866E-27	positive
PRJNA729271	stage_E14_vs_E10	ordinary_muscle_development	ENSGALG00010013848	ITPA	-0.066460467	0.094152986	-0.705877414	0.480264374	0.592260686	negative
PRJNA729271	stage_E14_vs_E10	ordinary_muscle_development	ENSGALG00010013857	IL18RAP	-2.296394803	0.901022725	-2.548653589	0.010813966	0.02550202	negative
PRJNA729271	stage_E14_vs_E10	ordinary_muscle_development	ENSGALG00010013859	MYOC	-0.42451532	0.254273431	-1.669522915	0.095013793	0.163158977	negative
PRJNA729271	stage_E14_vs_E10	ordinary_muscle_development	ENSGALG00010013861	TCF15	1.083709667	0.156733327	6.91435373	4.70001E-12	4.98002E-11	positive
PRJNA729271	stage_E14_vs_E10	ordinary_muscle_development	ENSGALG00010013866	KCTD8	-2.389469753	0.387101528	-6.172721049	6.71246E-10	5.6079E-09	negative
PRJNA729271	stage_E14_vs_E10	ordinary_muscle_development	ENSGALG00010013867	RECQL	-0.264864502	0.12076247	-2.193268333	0.028288052	0.058476228	negative
PRJNA729271	stage_E14_vs_E10	ordinary_muscle_development	ENSGALG00010013871	ENSGALG00010013871	0.641890981	0.220282319	2.913946899	0.003568906	0.009662177	positive
PRJNA729271	stage_E14_vs_E10	ordinary_muscle_development	ENSGALG00010013872	GLS2	-0.250206785	0.197311738	-1.26807856	0.204769897	0.304643299	negative
PRJNA729271	stage_E14_vs_E10	ordinary_muscle_development	ENSGALG00010013878	ARID2	-0.017410315	0.081219578	-0.214361067	0.8302655	0.881533726	negative
PRJNA729271	stage_E14_vs_E10	ordinary_muscle_development	ENSGALG00010013880	ZMYM4	-0.146999993	0.088290285	-1.664962262	0.095920285	0.164365118	negative
PRJNA729271	stage_E14_vs_E10	ordinary_muscle_development	ENSGALG00010013889	ENSGALG00010013889	0.434071178	0.419214399	1.035439571	0.300463674	0.414309451	positive
PRJNA729271	stage_E14_vs_E10	ordinary_muscle_development	ENSGALG00010013890	SFPQ	-0.306109371	0.071568155	-4.27717285	1.89282E-05	8.45237E-05	negative
PRJNA729271	stage_E14_vs_E10	ordinary_muscle_development	ENSGALG00010013893	CSNK2A1	0.001880663	0.078274604	0.024026478	0.980831489	0.987370819	positive
PRJNA729271	stage_E14_vs_E10	ordinary_muscle_development	ENSGALG00010013897	AMOT	-0.498775363	0.073695255	-6.768079762	1.30503E-11	1.32468E-10	negative
PRJNA729271	stage_E14_vs_E10	ordinary_muscle_development	ENSGALG00010013898	EMB	-2.151891256	0.295695383	-7.277392131	3.40335E-13	4.08712E-12	negative
PRJNA729271	stage_E14_vs_E10	ordinary_muscle_development	ENSGALG00010013899	WNT5B	-0.831208007	0.142142228	-5.847720384	4.98355E-09	3.77807E-08	negative
PRJNA729271	stage_E14_vs_E10	ordinary_muscle_development	ENSGALG00010013901	NFXL1	-0.462452906	0.120703512	-3.831312754	0.000127461	0.000493075	negative
PRJNA729271	stage_E14_vs_E10	ordinary_muscle_development	ENSGALG00010013908	TBC1D20	0.484341069	0.115398449	4.197119396	2.70331E-05	0.000117777	positive
PRJNA729271	stage_E14_vs_E10	ordinary_muscle_development	ENSGALG00010013910	ADIPOR2	0.353128155	0.208148234	1.696522466	0.089786979	0.155454165	positive
PRJNA729271	stage_E14_vs_E10	ordinary_muscle_development	ENSGALG00010013911	TNIK	0.427675719	0.147704512	2.895481758	0.00378577	0.010188403	positive
PRJNA729271	stage_E14_vs_E10	ordinary_muscle_development	ENSGALG00010013914	NRG1	1.322961369	0.226705068	5.835605627	5.35955E-09	4.03358E-08	positive
PRJNA729271	stage_E14_vs_E10	ordinary_muscle_development	ENSGALG00010013915	ENSGALG00010013915	0.66188654	0.190563914	3.473304714	0.000514091	0.001737411	positive
PRJNA729271	stage_E14_vs_E10	ordinary_muscle_development	ENSGALG00010013917	TRPC5	-2.256549123	0.549378809	-4.107455708	4.00041E-05	0.000169352	negative
PRJNA729271	stage_E14_vs_E10	ordinary_muscle_development	ENSGALG00010013921	PPP2R2B	1.160567281	0.104200579	11.13781984	8.21047E-29	3.28759E-27	positive
PRJNA729271	stage_E14_vs_E10	ordinary_muscle_development	ENSGALG00010013922	ENSGALG00010013922	-2.117239676	0.797481655	-2.654907061	0.007933026	0.019493001	negative
PRJNA729271	stage_E14_vs_E10	ordinary_muscle_development	ENSGALG00010013923	FMR1NB	0.009597112	0.550949299	0.017419229	0.986102169	0.990750048	positive
PRJNA729271	stage_E14_vs_E10	ordinary_muscle_development	ENSGALG00010013928	CCNO	-1.267817588	0.583224893	-2.173805686	0.029719731	0.060940374	negative
PRJNA729271	stage_E14_vs_E10	ordinary_muscle_development	ENSGALG00010013930	SLC22A16	3.213893903	0.303008644	10.60660797	2.77646E-26	9.31594E-25	positive
PRJNA729271	stage_E14_vs_E10	ordinary_muscle_development	ENSGALG00010013934	STAT1	0.319497144	0.282758753	1.129928396	0.258506398	0.366807792	positive
PRJNA729271	stage_E14_vs_E10	ordinary_muscle_development	ENSGALG00010013936	DLGAP3	-1.106007063	0.347838327	-3.17965841	0.001474488	0.004453573	negative
PRJNA729271	stage_E14_vs_E10	ordinary_muscle_development	ENSGALG00010013938	ENSGALG00010013938	0.092113751	0.414948091	0.221988614	0.824322747	0.877087148	positive
PRJNA729271	stage_E14_vs_E10	ordinary_muscle_development	ENSGALG00010013940	UPF3A	0.512038952	0.217504375	2.354154727	0.018564882	0.040747723	positive
PRJNA729271	stage_E14_vs_E10	ordinary_muscle_development	ENSGALG00010013942	CACNA2D4	0.966416952	0.485379103	1.991055951	0.046474736	0.089240004	positive
PRJNA729271	stage_E14_vs_E10	ordinary_muscle_development	ENSGALG00010013944	METTL24	-1.12064881	0.211026274	-5.310470531	1.09343E-07	6.93316E-07	negative
PRJNA729271	stage_E14_vs_E10	ordinary_muscle_development	ENSGALG00010013945	SCAF11	-0.060630717	0.076454674	-0.793028266	0.427761355	0.542155716	negative
PRJNA729271	stage_E14_vs_E10	ordinary_muscle_development	ENSGALG00010013947	ENSGALG00010013947	0.311848168	0.167726719	1.859263502	0.062989798	0.115282464	positive
PRJNA729271	stage_E14_vs_E10	ordinary_muscle_development	ENSGALG00010013949	PRRC2C	-0.55754606	0.126118675	-4.420804925	9.83339E-06	4.62026E-05	negative
PRJNA729271	stage_E14_vs_E10	ordinary_muscle_development	ENSGALG00010013953	DHX29	-0.246928854	0.08052032	-3.066665081	0.002164612	0.006234067	negative
PRJNA729271	stage_E14_vs_E10	ordinary_muscle_development	ENSGALG00010013954	LPL	0.523926596	0.334093915	1.568201549	0.116834103	0.193499808	positive
PRJNA729271	stage_E14_vs_E10	ordinary_muscle_development	ENSGALG00010013958	TUSC3	0.878016739	0.171408299	5.12237006	3.01719E-07	1.79976E-06	positive
PRJNA729271	stage_E14_vs_E10	ordinary_muscle_development	ENSGALG00010013959	STK32A	-0.713823859	0.418771033	-1.704568377	0.088274953	0.153385932	negative
PRJNA729271	stage_E14_vs_E10	ordinary_muscle_development	ENSGALG00010013964	SLC38A1	-0.71136216	0.1596467	-4.455852585	8.35604E-06	3.96857E-05	negative
PRJNA729271	stage_E14_vs_E10	ordinary_muscle_development	ENSGALG00010013965	SLC2A2	1.383997992	1.01045913	1.36967241			positive
PRJNA729271	stage_E14_vs_E10	ordinary_muscle_development	ENSGALG00010013967	MLLT3	-1.069131643	0.154736094	-6.909387579	4.8675E-12	5.13889E-11	negative
PRJNA729271	stage_E14_vs_E10	ordinary_muscle_development	ENSGALG00010013971	ENSGALG00010013971	-0.745612657	0.356044512	-2.094155736	0.036246109	0.072027488	negative
PRJNA729271	stage_E14_vs_E10	ordinary_muscle_development	ENSGALG00010013973	ENSGALG00010013973	0.237684142	0.240104644	0.989918969	0.322213727	0.436753726	positive
PRJNA729271	stage_E14_vs_E10	ordinary_muscle_development	ENSGALG00010013976	CDC40	-0.156827526	0.074114122	-2.116027571	0.034342463	0.068851175	negative
PRJNA729271	stage_E14_vs_E10	ordinary_muscle_development	ENSGALG00010013977	SLC38A2	-0.552327295	0.192745429	-2.86557922	0.004162471	0.011070646	negative
PRJNA729271	stage_E14_vs_E10	ordinary_muscle_development	ENSGALG00010013980	FMO4	-0.007414707	0.390915664	-0.018967537	0.984867002	0.989880573	negative
PRJNA729271	stage_E14_vs_E10	ordinary_muscle_development	ENSGALG00010013981	RLF	0.064031682	0.106835162	0.599350262	0.548939337	0.654294523	positive
PRJNA729271	stage_E14_vs_E10	ordinary_muscle_development	ENSGALG00010013989	ENSGALG00010013989	0.336114491	0.210489821	1.59682064	0.110305694	0.184670944	positive
PRJNA729271	stage_E14_vs_E10	ordinary_muscle_development	ENSGALG00010013994	CHRM2	-2.478433565	0.403445958	-6.143161219	8.08951E-10	6.68896E-09	negative
PRJNA729271	stage_E14_vs_E10	ordinary_muscle_development	ENSGALG00010013995	REM1	1.510764222	0.206795811	7.305584266	2.76065E-13	3.3542E-12	positive
PRJNA729271	stage_E14_vs_E10	ordinary_muscle_development	ENSGALG00010014000	STAT4	1.476772778	1.222212945	1.208277808	0.226940422	0.330337559	positive
PRJNA729271	stage_E14_vs_E10	ordinary_muscle_development	ENSGALG00010014001	CDC16	-0.28265676	0.082198563	-3.438706833	0.0005845	0.001946319	negative
PRJNA729271	stage_E14_vs_E10	ordinary_muscle_development	ENSGALG00010014002	ZDHHC18	-0.195408021	0.267174247	-0.731387936	0.464542232	0.577144051	negative
PRJNA729271	stage_E14_vs_E10	ordinary_muscle_development	ENSGALG00010014010	EIF5A2	-0.075976468	0.102099262	-0.744143169	0.456789865	0.569758979	negative
PRJNA729271	stage_E14_vs_E10	ordinary_muscle_development	ENSGALG00010014011	SFN	0.451598	0.437538395	1.03213342	0.30200962	0.415689815	positive
PRJNA729271	stage_E14_vs_E10	ordinary_muscle_development	ENSGALG00010014014	PARP8	-1.100979604	0.208427409	-5.28231681	1.2756E-07	7.98354E-07	negative
PRJNA729271	stage_E14_vs_E10	ordinary_muscle_development	ENSGALG00010014018	DPYSL3	0.140273986	0.238143839	0.589030507	0.555840809	0.66013212	positive
PRJNA729271	stage_E14_vs_E10	ordinary_muscle_development	ENSGALG00010014019	IRAG2	-0.473278729	0.239950537	-1.972401204	0.048563825	0.092598347	negative
PRJNA729271	stage_E14_vs_E10	ordinary_muscle_development	ENSGALG00010014020	PDXP	0.023919777	0.097487775	0.24536181	0.806176297	0.863802884	positive
PRJNA729271	stage_E14_vs_E10	ordinary_muscle_development	ENSGALG00010014026	SH3BP1	0.12155451	0.374340342	0.324716566	0.745395596	0.817408773	positive
PRJNA729271	stage_E14_vs_E10	ordinary_muscle_development	ENSGALG00010014030	WASF1	-0.964695921	0.316407331	-3.048905088	0.00229677	0.006567703	negative
PRJNA729271	stage_E14_vs_E10	ordinary_muscle_development	ENSGALG00010014031	RPL22L1	0.300788731	0.188240018	1.597900033	0.110065227	0.184374837	positive
PRJNA729271	stage_E14_vs_E10	ordinary_muscle_development	ENSGALG00010014032	PSD3	-0.447418123	0.076915816	-5.8169847	5.99186E-09	4.48848E-08	negative
PRJNA729271	stage_E14_vs_E10	ordinary_muscle_development	ENSGALG00010014033	SLC38A4	0.240512737	0.477290786	0.503912381	0.61432295	0.710833492	positive
PRJNA729271	stage_E14_vs_E10	ordinary_muscle_development	ENSGALG00010014035	PSIP1	-0.675405427	0.116717286	-5.786678674	7.17917E-09	5.32015E-08	negative
PRJNA729271	stage_E14_vs_E10	ordinary_muscle_development	ENSGALG00010014041	SKIV2L2	0.054082255	0.098255581	0.550424258	0.582028414	0.6834496	positive
PRJNA729271	stage_E14_vs_E10	ordinary_muscle_development	ENSGALG00010014042	SGCZ	0.402129205	0.310836888	1.293698466	0.195769596	0.294029664	positive

PRJNA729271	stage_E14_vs_E10	ordinary_muscle_development	ENSGALG00010014045	GOLT1B	-0.34213614	0.109016563	-3.138386787	0.001698805	0.005048008	negative
PRJNA729271	stage_E14_vs_E10	ordinary_muscle_development	ENSGALG00010014047	HM13	-0.229246488	0.167001031	-1.372724989	0.169837858	0.26220042	negative
PRJNA729271	stage_E14_vs_E10	ordinary_muscle_development	ENSGALG00010014056	FMO3	-0.271805446	0.267209543	-1.01719962	0.309058477	0.422742533	negative
PRJNA729271	stage_E14_vs_E10	ordinary_muscle_development	ENSGALG00010014062	GGA1	0.133795221	0.103736304	1.289762753	0.197133044	0.295679167	positive
PRJNA729271	stage_E14_vs_E10	ordinary_muscle_development	ENSGALG00010014065	CHUNK-1	-0.158909299	0.582998515	-0.272572392	0.785181937	0.848710826	negative
PRJNA729271	stage_E14_vs_E10	ordinary_muscle_development	ENSGALG00010014066	BCL2L1	-0.451477069	0.116627009	-3.871119336	0.000108337	0.000424187	negative
PRJNA729271	stage_E14_vs_E10	ordinary_muscle_development	ENSGALG00010014076	PRRX1	-1.861265268	0.245008737	-7.596730169	3.03707E-14	4.00932E-13	negative
PRJNA729271	stage_E14_vs_E10	ordinary_muscle_development	ENSGALG00010014077	SLITRK2	0.100926638	0.099616889	1.013147861	0.31098954	0.424782641	positive
PRJNA729271	stage_E14_vs_E10	ordinary_muscle_development	ENSGALG00010014080	MYO1B	-0.159489885	0.160578052	-0.993223441	0.320601077	0.435082166	negative
PRJNA729271	stage_E14_vs_E10	ordinary_muscle_development	ENSGALG00010014082	HNRNPR	0.015045135	0.042144553	0.356988845	0.721100157	0.798132771	positive
PRJNA729271	stage_E14_vs_E10	ordinary_muscle_development	ENSGALG00010014083	ENSGALG00010014083	0.002717479	0.14353861	0.018932043	0.984895317	0.989880573	positive
PRJNA729271	stage_E14_vs_E10	ordinary_muscle_development	ENSGALG00010014089	FIG4	-0.175219943	0.111879433	-1.566149733	0.117313567	0.194160786	negative
PRJNA729271	stage_E14_vs_E10	ordinary_muscle_development	ENSGALG00010014091	CHIC2	0.024206019	0.09825266	0.246365023	0.805399679	0.863289828	positive
PRJNA729271	stage_E14_vs_E10	ordinary_muscle_development	ENSGALG00010014092	TYRP1	1.484804149	0.894384723	1.66014033	0.096886225	0.165609179	positive
PRJNA729271	stage_E14_vs_E10	ordinary_muscle_development	ENSGALG00010014093	PLD1	0.977513877	0.091927719	10.63350524	2.08139E-26	7.08211E-25	positive
PRJNA729271	stage_E14_vs_E10	ordinary_muscle_development	ENSGALG00010014096	JAKMIP2	-0.794047294	0.150359607	-5.280988096	1.28489E-07	8.03472E-07	negative
PRJNA729271	stage_E14_vs_E10	ordinary_muscle_development	ENSGALG00010014099	TPX2	-0.198305898	0.142556988	-1.391064025	0.164206013	0.255126923	negative
PRJNA729271	stage_E14_vs_E10	ordinary_muscle_development	ENSGALG00010014102	TRIM2	-0.348332507	0.137675293	-2.530101795	0.011402944	0.026749583	negative
PRJNA729271	stage_E14_vs_E10	ordinary_muscle_development	ENSGALG00010014104	ALDH1A1	-1.793953355	0.578778319	-3.099551755	0.001938137	0.005666256	negative
PRJNA729271	stage_E14_vs_E10	ordinary_muscle_development	ENSGALG00010014105	AHR1B	-0.350853257	0.415382446	-0.844651143	0.398305645	0.51360558	negative
PRJNA729271	stage_E14_vs_E10	ordinary_muscle_development	ENSGALG00010014109	AMIGO2	-1.472588445	0.164193644	-8.968608123	3.00285E-19	6.08759E-18	negative
PRJNA729271	stage_E14_vs_E10	ordinary_muscle_development	ENSGALG00010014111	GORAB	-0.009671121	0.079703469	-0.121338767	0.903422715	0.933897608	negative
PRJNA729271	stage_E14_vs_E10	ordinary_muscle_development	ENSGALG00010014114	ANXA1	0.880470705	0.332376094	2.649019355	0.008072571	0.01979897	positive
PRJNA729271	stage_E14_vs_E10	ordinary_muscle_development	ENSGALG00010014120	SOX5	-1.649407267	0.130379499	-12.65125429	1.10103E-36	6.87906E-35	negative
PRJNA729271	stage_E14_vs_E10	ordinary_muscle_development	ENSGALG00010014122	ABCA1	-0.269216952	0.140786125	-1.912240656	0.055845334	0.104126333	negative
PRJNA729271	stage_E14_vs_E10	ordinary_muscle_development	ENSGALG00010014125	PLPP1	2.081692341	0.138871054	14.99010979	8.52122E-51	1.18765E-48	positive
PRJNA729271	stage_E14_vs_E10	ordinary_muscle_development	ENSGALG00010014128	COL4A1	1.922617597	0.144556486	13.30011295	2.31119E-40	1.77252E-38	positive
PRJNA729271	stage_E14_vs_E10	ordinary_muscle_development	ENSGALG00010014131	CDC23	-0.241516812	0.114313665	-2.11275539	0.03462171	0.069353466	negative
PRJNA729271	stage_E14_vs_E10	ordinary_muscle_development	ENSGALG00010014134	RPAP3	-0.327157199	0.113142374	-2.891553247	0.003833426	0.010293259	negative
PRJNA729271	stage_E14_vs_E10	ordinary_muscle_development	ENSGALG00010014135	ZBTB24	-0.091442656	0.153389659	-0.596146162	0.551077588	0.655926231	negative
PRJNA729271	stage_E14_vs_E10	ordinary_muscle_development	ENSGALG00010014142	PPIL6	2.489969893	0.465198954	5.352483857	8.6755E-08	5.57656E-07	positive
PRJNA729271	stage_E14_vs_E10	ordinary_muscle_development	ENSGALG00010014143	NFATC2	-0.595144458	0.152751876	-3.896151542	9.77333E-05	0.000384748	negative
PRJNA729271	stage_E14_vs_E10	ordinary_muscle_development	ENSGALG00010014145	NTMT2	3.90715239	0.252077554	15.49980282	3.47958E-54	6.22674E-52	positive
PRJNA729271	stage_E14_vs_E10	ordinary_muscle_development	ENSGALG00010014147	ENSGALG00010014147	0.755210863	0.332172088	2.273553048	0.022992873	0.04911313	positive
PRJNA729271	stage_E14_vs_E10	ordinary_muscle_development	ENSGALG00010014148	KCNG1	-0.723965461	0.357485712	-2.025159151	0.042851025	0.083157359	negative
PRJNA729271	stage_E14_vs_E10	ordinary_muscle_development	ENSGALG00010014149	LGALS2	0.615029747	0.603995447	1.018268846	0.30855021	0.422126974	positive
PRJNA729271	stage_E14_vs_E10	ordinary_muscle_development	ENSGALG00010014151	ATP23	-0.161898431	0.089573045	-1.807445874	0.070692787	0.127227706	negative
PRJNA729271	stage_E14_vs_E10	ordinary_muscle_development	ENSGALG00010014153	FOCAD	-0.139956273	0.132478066	-1.056448639	0.290763295	0.403135403	negative
PRJNA729271	stage_E14_vs_E10	ordinary_muscle_development	ENSGALG00010014154	DNAI7	-0.926561338	0.381604681	-2.428065963	0.015179582	0.03427762	negative
PRJNA729271	stage_E14_vs_E10	ordinary_muscle_development	ENSGALG00010014155	ENSGALG00010014155	-6.243034003	1.43208803	-4.359392631	1.30424E-05	6.00402E-05	negative
PRJNA729271	stage_E14_vs_E10	ordinary_muscle_development	ENSGALG00010014156	CD164	0.118570831	0.092155523	1.28663836	0.198220367	0.296863369	positive
PRJNA729271	stage_E14_vs_E10	ordinary_muscle_development	ENSGALG00010014159	CDC42EP1	-0.144589886	0.149034599	-0.970176636	0.331958454	0.446814254	negative
PRJNA729271	stage_E14_vs_E10	ordinary_muscle_development	ENSGALG00010014161	GFRA3	0.027100623	0.25014381	0.108340169	0.91372586	0.940755838	positive
PRJNA729271	stage_E14_vs_E10	ordinary_muscle_development	ENSGALG00010014162	COL18A1	0.597522013	0.188333501	3.172680428	0.001510387	0.004548232	positive
PRJNA729271	stage_E14_vs_E10	ordinary_muscle_development	ENSGALG00010014165	LUZP1	-0.287266925	0.116261132	-2.470876716	0.013478228	0.030951665	negative
PRJNA729271	stage_E14_vs_E10	ordinary_muscle_development	ENSGALG00010014166	FGF12	-0.331018464	0.192283939	-1.72150865	0.085158564	0.149047882	negative
PRJNA729271	stage_E14_vs_E10	ordinary_muscle_development	ENSGALG00010014167	SLC38A9	-0.121979416	0.10743904	-1.135336054	0.256234541	0.364093684	negative
PRJNA729271	stage_E14_vs_E10	ordinary_muscle_development	ENSGALG00010014170	CEMIP2	-1.263361989	0.106566007	-11.85520625	2.02226E-32	9.77087E-31	negative
PRJNA729271	stage_E14_vs_E10	ordinary_muscle_development	ENSGALG00010014171	DLC1	-0.117720735	0.132708182	-0.887064633	0.375044104	0.491347097	negative
PRJNA729271	stage_E14_vs_E10	ordinary_muscle_development	ENSGALG00010014173	MND1	-0.539111011	0.200448181	-2.689528075	0.007155313	0.017768761	negative
PRJNA729271	stage_E14_vs_E10	ordinary_muscle_development	ENSGALG00010014176	ABHD17B	-0.540581839	0.088827741	-6.085732138	1.1596E-09	9.39543E-09	negative
PRJNA729271	stage_E14_vs_E10	ordinary_muscle_development	ENSGALG00010014178	LRTM2	0.103393844	0.349939471	0.295462079	0.767640916	0.834792939	positive
PRJNA729271	stage_E14_vs_E10	ordinary_muscle_development	ENSGALG00010014179	CTBPL	-0.150409845	0.041708166	-3.606244496	0.00031066	0.0011083	negative
PRJNA729271	stage_E14_vs_E10	ordinary_muscle_development	ENSGALG00010014180	SLC19A1	-0.371154862	0.143004004	-2.595415878	0.009447653	0.022680312	negative
PRJNA729271	stage_E14_vs_E10	ordinary_muscle_development	ENSGALG00010014184	SLC44A1	-0.334102609	0.132512202	-2.521296927	0.011692314	0.027335499	negative
PRJNA729271	stage_E14_vs_E10	ordinary_muscle_development	ENSGALG00010014186	MOCS3	-0.178976982	0.105360981	-1.698702684	0.089375217	0.154889261	negative
PRJNA729271	stage_E14_vs_E10	ordinary_muscle_development	ENSGALG00010014189	DDX4	0.905022953	0.470771322	1.922425836	0.054552197	0.101977573	positive
PRJNA729271	stage_E14_vs_E10	ordinary_muscle_development	ENSGALG00010014190	HACD4	0.117968053	0.227465951	0.51861851	0.604026798	0.702170859	positive
PRJNA729271	stage_E14_vs_E10	ordinary_muscle_development	ENSGALG00010014192	KDM1A	-0.211637681	0.06010552	-3.52110223	0.000429757	0.001477194	negative
PRJNA729271	stage_E14_vs_E10	ordinary_muscle_development	ENSGALG00010014193	CARD10	-2.091781756	0.356541449	-5.866868386	4.44103E-09	3.38626E-08	negative
PRJNA729271	stage_E14_vs_E10	ordinary_muscle_development	ENSGALG00010014194	LRIG3	-0.832369548	0.09059466	-9.187843424	4.00796E-20	8.73614E-19	negative
PRJNA729271	stage_E14_vs_E10	ordinary_muscle_development	ENSGALG00010014197	SH3GL2	-1.445437459	0.327842221	-4.408942366	1.03877E-05	4.86178E-05	negative
PRJNA729271	stage_E14_vs_E10	ordinary_muscle_development	ENSGALG00010014198	KDM3B	-0.123369737	0.044039498	-2.801342928	0.005089041	0.013225732	negative
PRJNA729271	stage_E14_vs_E10	ordinary_muscle_development	ENSGALG00010014199	LNX1	0.090357397	0.222463269	0.406167712	0.684619376	0.769387336	positive
PRJNA729271	stage_E14_vs_E10	ordinary_muscle_development	ENSGALG00010014200	DCP1B	0.377951809	0.08753417	4.317763103	1.57618E-05	7.15528E-05	positive
PRJNA729271	stage_E14_vs_E10	ordinary_muscle_development	ENSGALG00010014203	TMEM38B	1.137410091	0.168945942	6.73239071	1.66898E-11	1.67417E-10	positive
PRJNA729271	stage_E14_vs_E10	ordinary_muscle_development	ENSGALG00010014204	KIFAP3	-0.122954979	0.082738329	-1.486070371	0.137260508	0.220628093	negative
PRJNA729271	stage_E14_vs_E10	ordinary_muscle_development	ENSGALG00010014205	DPM1	-0.146996128	0.084104743	-1.747774541	0.080503075	0.142182537	negative
PRJNA729271	stage_E14_vs_E10	ordinary_muscle_development	ENSGALG00010014209	CCDC50	0.519615209	0.107296081	4.842816308	1.28012E-06	6.98618E-06	positive
PRJNA729271	stage_E14_vs_E10	ordinary_muscle_development	ENSGALG00010014210	ENSGALG00010014210	-0.251028479	0.250046341	-1.003927823	0.315413405	0.42940897	negative
PRJNA729271	stage_E14_vs_E10	ordinary_muscle_development	ENSGALG00010014213	ETFRF1	0.298134726	0.151379794	1.969448622	0.048901597	0.09315661	positive
PRJNA729271	stage_E14_vs_E10	ordinary_muscle_development	ENSGALG00010014214	REEP2	-0.665134749	0.134674619	-4.938827789	7.85936E-07	4.40191E-06	negative
PRJNA729271	stage_E14_vs_E10	ordinary_muscle_development	ENSGALG00010014217	RASA3	0.372074019	0.109402868	3.400953077	0.000671514	0.002205163	positive
PRJNA729271	stage_E14_vs_E10	ordinary_muscle_development	ENSGALG00010014218	IL31RA	0.452269039	0.167601751	2.698474433	0.006965809	0.017354658	positive
PRJNA729271	stage_E14_vs_E10	ordinary_muscle_development	ENSGALG00010014220	C9orf85	-0.264541059	0.130021567	-2.034593692	0.041891773	0.081495268	negative
PRJNA729271	stage_E14_vs_E10	ordinary_muscle_development	ENSGALG00010014221	SCYL3	0.163471081	0.131485783	1.243260505	0.213771853	0.31541358	positive
PRJNA729271	stage_E14_vs_E10	ordinary_muscle_development	ENSGALG00010014222	TBX22	-1.861358315	0.404717502	-4.599154488	4.24209E-06	2.11812E-05	negative
PRJNA729271	stage_E14_vs_E10	ordinary_muscle_development	ENSGALG00010014224	NMUR2	-1.526520715	0.53218601	-2.868396924	0.004125576	0.010988648	negative
PRJNA729271	stage_E14_vs_E10	ordinary_muscle_development	ENSGALG00010014225	ZMPSTE24	-0.045905469	0.074824733	-0.613506625	0.539541403	0.645684607	negative
PRJNA729271	stage_E14_vs_E10	ordinary_muscle_development	ENSGALG00010014226	SLC16A7	-0.399338844	0.260795052	-1.531236275	0.125711009	0.205154365	negative
PRJNA729271	stage_E14_vs_E10	ordinary_muscle_development	ENSGALG00010014234	GLRA1	1.802848296	0.602362391	2.992962911	0.002762833	0.007723677	positive
PRJNA729271	stage_E14_vs_E10	ordinary_muscle_development	ENSGALG00010014236	WDR44	0.09609126	0.080946976	1.187088941	0.23519254	0.33972744	positive
PRJNA729271	stage_E14_vs_E10	ordinary_muscle_development	ENSGALG00010014238	PHLDA1	-0.733323207	0.11766887	-6.232091836	4.60248E-10	3.94051E-09	negative
PRJNA729271	stage_E14_vs_E10	ordinary_muscle_development	ENSGALG00010014240	PIGV	0.284130422	0.126618653	2.243985506	0.024833334	0.052441175	positive
PRJNA729271	stage_E14_vs_E10	ordinary_muscle_development	ENSGALG00010014241	ENSGALG00010014241	2.655010564	0.860318486	3.086078709	0.002028151	0.00588314	positive
PRJNA729271	stage_E14_vs_E10	ordinary_muscle_development	ENSGALG00010014243	ENSGALG00010014243	0.351682641	0.14368165	2.447651742	0.014379056	0.032750538	positive
PRJNA729271	stage_E14_vs_E10	ordinary_muscle_development	ENSGALG00010014244	SESN1	0.468462021	0.370175921	1.265511868	0.205687897	0.305601278	positive
PRJNA729271	stage_E14_vs_E10	ordinary_muscle_development	ENSGALG00010014246	KRAS	-0.446566612	0.114097318	-3.913909821	9.08136E-05	0.000360444	negative
PRJNA729271	stage_E14_vs_E10	ordinary_muscle_development	ENSGALG00010014249	ENSGALG00010014249	0.310440959	0.100407093	3.091823008	0.001989314	0.005791346	positive
PRJNA729271	stage_E14_vs_E10	ordinary_muscle_development	ENSGALG00010014250	ENSGALG00010014250	2.262631077	0.254279376	8.898209189	5.6755E-19	1.11623E-17	positive
PRJNA729271	stage_E14_vs_E10	ordinary_muscle_development	ENSGALG00010014257	NOP16	-0.211090437	0.123116851	-1.714553569	0.086427108	0.150862347	negative
PRJNA729271	stage_E14_vs_E10	ordinary_muscle_development	ENSGALG00010014258	ENSGALG00010014258	-2.805025254	0.616385328	-4.550765776	5.3451E-06	2.62635E-05	negative
PRJNA729271	stage_E14_vs_E10	ordinary_muscle_development	ENSGALG00010014260	ADNP	-0.230787187	0.07519053	-3.069365075	0.002145143	0.00618783	negative
PRJNA729271	stage_E14_vs_E10	ordinary_muscle_development	ENSGALG00010014263	ZNF131	-0.237988079	0.118123307	-2.014742779	0.043931607	0.084893275	negative
PRJNA729271	stage_E14_vs_E10	ordinary_muscle_development	ENSGALG00010014265	ZNF462	-0.518649519	0.139527144	-3.717194397	0.000201447	0.000748329	negative
PRJNA729271	stage_E14_vs_E10	ordinary_muscle_development	ENSGALG00010014267	SCFD2	-0.551842827	0.073346254	-7.523803857	5.32051E-14	6.92288E-13	negative
PRJNA729271	stage_E14_vs_E10	ordinary_muscle_development	ENSGALG00010014272	PARD6B	-0.656082875	0.2497582	-2.626872208	0.008617369	0.02094034	negative
PRJNA729271	stage_E14_vs_E10	ordinary_muscle_development	ENSGALG00010014274	FIRRM	-0.28319495	0.145956754	-1.940266157	0.052347352	0.098491026	negative
PRJNA729271	stage_E14_vs_E10	ordinary_muscle_development	ENSGALG00010014278	G3BP1	-0.195624211	0.139352165	-1.403811782	0.160374904	0.250499379	negative
PRJNA729271	stage_E14_vs_E10	ordinary_muscle_development	ENSGALG00010014284	TMEM131L	-0.12985918	0.083116063	-1.562383668	0.118197632	0.19526723	negative
PRJNA729271	stage_E14_vs_E10	ordinary_muscle_development	ENSGALG00010014288	ARMC2	-0.15420307	0.238443153	-0.646707897	0.517821011	0.626170942	negative
PRJNA729271	stage_E14_vs_E10	ordinary_muscle_development	ENSGALG00010014291	OSTN	-3.894851718	0.497930148	-7.822084549	5.19557E-15	7.28334E-14	negative
PRJNA729271	stage_E14_vs_E10	ordinary_muscle_development	ENSGALG00010014292	NIM1K	-0.013212049	0.466847827	-0.028300546	0.977422445	0.984896652	negative
PRJNA729271	stage_E14_vs_E10	ordinary_muscle_development	ENSGALG00010014294	TMPO	0.274895144	0.101726564	2.702294584	0.006886272	0.017194921	positive
PRJNA729271	stage_E14_vs_E10	ordinary_muscle_development	ENSGALG00010014295	CACNA1C	-0.332097091	0.17458221	-1.902239014	0.057139914	0.106116984	negative
PRJNA729271	stage_E14_vs_E10	ordinary_muscle_development	ENSGALG00010014298	METTL18	-0.356225513	0.223166436	-1.596232474	0.1104369	0.184826563	negative
PRJNA729271	stage_E14_vs_E10	ordinary_muscle_development	ENSGALG00010014300	ELFN2	-0.997754608	0.447752375	-2.228362516	0.025856351	0.054262024	negative
PRJNA729271	stage_E14_vs_E10	ordinary_muscle_development	ENSGALG00010014301	ARID1A	-0.216456771	0.119004182	-1.818900542	0.068926605	0.124476457	negative
PRJNA729271	stage_E14_vs_E10	ordinary_muscle_development	ENSGALG00010014302	FOXO3	0.93008426	0.233426087	3.984491499	6.76248E-05	0.00027488	positive
PRJNA729271	stage_E14_vs_E10	ordinary_muscle_development	ENSGALG00010014303	GABRA2	-3.922811669	0.53120198	-7.384783597	1.52702E-13	1.89667E-12	negative
PRJNA729271	stage_E14_vs_E10	ordinary_muscle_development	ENSGALG00010014305	ARL10	-1.361650969	0.282550068	-4.81914932	1.44172E-06	7.79764E-06	negative
PRJNA729271	stage_E14_vs_E10	ordinary_muscle_development	ENSGALG00010014306	ENSGALG00010014306	-0.663060928	0.314516165	-2.108193477	0.035014257	0.069959068	negative
PRJNA729271	stage_E14_vs_E10	ordinary_muscle_development	ENSGALG00010014308	ENSGALG00010014308	0.081787901	0.202655709	0.403580543	0.686521196	0.770747792	positive
PRJNA729271	stage_E14_vs_E10	ordinary_muscle_development	ENSGALG00010014310	COL6A1	0.711695114	0.237745993	2.993510448	0.002757881	0.007711319	positive
PRJNA729271	stage_E14_vs_E10	ordinary_muscle_development	ENSGALG00010014312	KIAA1191	0.209597398	0.080391875	2.607196291	0.009128701	0.022005742	positive
PRJNA729271	stage_E14_vs_E10	ordinary_muscle_development	ENSGALG00010014314	ENSGALG00010014314	-0.19417523	0.163403751	-1.188315623	0.234709085	0.33922706	negative
PRJNA729271	stage_E14_vs_E10	ordinary_muscle_development	ENSGALG00010014317	RAD23B	-0.115344355	0.146354309	-0.788117251	0.430628125	0.544768256	negative
PRJNA729271	stage_E14_vs_E10	ordinary_muscle_development	ENSGALG00010014318	ENSGALG00010014318	-1.607648987	0.484920587	-3.31528302	0.000915504	0.002908224	negative
PRJNA729271	stage_E14_vs_E10	ordinary_muscle_development	ENSGALG00010014319	ENSGALG00010014319	0.220808203	0.11480339	1.92335961	0.054434903	0.101797693	positive
PRJNA729271	stage_E14_vs_E10	ordinary_muscle_development	ENSGALG00010014320	UTS2B	-2.133236145	0.880386614	-2.423067446	0.015390069	0.034688081	negative
PRJNA729271	stage_E14_vs_E10	ordinary_muscle_development	ENSGALG00010014323	KLF4	-0.484781801	0.177603989	-2.729565951	0.006341776	0.016009064	negative
PRJNA729271	stage_E14_vs_E10	ordinary_muscle_development	ENSGALG00010014324	COL4A2	1.744109395	0.142248879	12.26097107	1.46731E-34	7.90656E-33	positive
PRJNA729271	stage_E14_vs_E10	ordinary_muscle_development	ENSGALG00010014328	RASSF8	-0.771204202	0.090824028	-8.49119132	2.0453E-17	3.56758E-16	negative
PRJNA729271	stage_E14_vs_E10	ordinary_muscle_development	ENSGALG00010014329	IER5	0.384932829	0.154550974	2.49065288	0.012750863	0.029500998	positive
PRJNA729271	stage_E14_vs_E10	ordinary_muscle_development	ENSGALG00010014334	HMGCS1	-0.15574435	0.157959616	-0.985975747	0.324145014	0.438741431	negative
PRJNA729271	stage_E14_vs_E10	ordinary_muscle_development	ENSGALG00010014338	BHLHE41	0.000288628	0.229476513	0.001257769	0.998996446	0.999350415	positive
PRJNA729271	stage_E14_vs_E10	ordinary_muscle_development	ENSGALG00010014340	EFNB1	-0.624079502	0.136809796	-4.561658024	5.07513E-06	2.49962E-05	negative
PRJNA729271	stage_E14_vs_E10	ordinary_muscle_development	ENSGALG00010014344	LSG1	0.047214236	0.084799675	0.556773791	0.577681993	0.679336401	positive
PRJNA729271	stage_E14_vs_E10	ordinary_muscle_development	ENSGALG00010014346	AFG1L	0.184096252	0.159101895	1.157096541	0.24723292	0.353904915	positive
PRJNA729271	stage_E14_vs_E10	ordinary_muscle_development	ENSGALG00010014347	KIAA0040	-0.464007595	0.40501132	-1.145665742	0.251933484	0.359179291	negative
PRJNA729271	stage_E14_vs_E10	ordinary_muscle_development	ENSGALG00010014350	PTGER4	-0.927144178	0.172349229	-5.379450669	7.47135E-08	4.8542E-07	negative

PRJNA729271	stage_E14_vs_E10	ordinary_muscle_development	ENSGALG00010014355	RAB20	0.783563764	0.164710221	4.757226121	1.96271E-06	1.03566E-05	positive
PRJNA729271	stage_E14_vs_E10	ordinary_muscle_development	ENSGALG00010014359	TMEM271	0.369853042	0.362378826	1.02062542	0.307431941	0.421027653	positive
PRJNA729271	stage_E14_vs_E10	ordinary_muscle_development	ENSGALG00010014361	SSPN	1.954089641	0.149031693	13.11190664	2.81433E-39	2.02954E-37	positive
PRJNA729271	stage_E14_vs_E10	ordinary_muscle_development	ENSGALG00010014362	NAXD	0.586763788	0.107050712	5.48117597	4.22508E-08	2.83268E-07	positive
PRJNA729271	stage_E14_vs_E10	ordinary_muscle_development	ENSGALG00010014367	OPHN1	-0.041997204	0.10873056	-0.386250232	0.699311355	0.781475452	negative
PRJNA729271	stage_E14_vs_E10	ordinary_muscle_development	ENSGALG00010014371	TTC33	0.147768184	0.097483617	1.515825816	0.129563402	0.210281213	positive
PRJNA729271	stage_E14_vs_E10	ordinary_muscle_development	ENSGALG00010014372	CCDC171	-0.068743544	0.123787636	-0.555334493	0.57866589	0.680217507	negative
PRJNA729271	stage_E14_vs_E10	ordinary_muscle_development	ENSGALG00010014375	H2BC35	0.335359904	0.246743448	1.359144108	0.174100928	0.267414746	positive
PRJNA729271	stage_E14_vs_E10	ordinary_muscle_development	ENSGALG00010014379	CARS2	0.027508834	0.08541519	0.32206021	0.747407081	0.818933075	positive
PRJNA729271	stage_E14_vs_E10	ordinary_muscle_development	ENSGALG00010014380	SNX3	0.167098642	0.117410424	1.423201072	0.154677875	0.243410682	positive
PRJNA729271	stage_E14_vs_E10	ordinary_muscle_development	ENSGALG00010014388	MFNG	0.817061156	0.235192058	3.474016785	0.000512729	0.00173321	positive
PRJNA729271	stage_E14_vs_E10	ordinary_muscle_development	ENSGALG00010014389	COL6A2	1.000181938	0.264478256	3.781717078	0.00015575	0.000591305	positive
PRJNA729271	stage_E14_vs_E10	ordinary_muscle_development	ENSGALG00010014390	SPARC	1.006043655	0.159035278	6.325915015	2.51736E-10	2.22089E-09	positive
PRJNA729271	stage_E14_vs_E10	ordinary_muscle_development	ENSGALG00010014393	ATP8A1	0.237083611	0.144605361	1.639521591	0.101104678	0.171545394	positive
PRJNA729271	stage_E14_vs_E10	ordinary_muscle_development	ENSGALG00010014396	ING1	0.12333203	0.116774161	1.056158563	0.290895778	0.403232484	positive
PRJNA729271	stage_E14_vs_E10	ordinary_muscle_development	ENSGALG00010014397	PRKAA1	-0.24785412	0.075693177	-3.274457878	0.001058649	0.003317864	negative
PRJNA729271	stage_E14_vs_E10	ordinary_muscle_development	ENSGALG00010014399	GAS6	1.200832738	0.190662915	6.298197724	3.01126E-10	2.62309E-09	positive
PRJNA729271	stage_E14_vs_E10	ordinary_muscle_development	ENSGALG00010014400	RIPOR3	-0.856466818	0.100548147	-8.517977105	1.62364E-17	2.85269E-16	negative
PRJNA729271	stage_E14_vs_E10	ordinary_muscle_development	ENSGALG00010014403	H2BC37	0.839713613	0.187586897	4.476398011	7.59129E-06	3.63274E-05	positive
PRJNA729271	stage_E14_vs_E10	ordinary_muscle_development	ENSGALG00010014407	YIPF6	0.028917464	0.088658092	0.326168359	0.744296979	0.81650893	positive
PRJNA729271	stage_E14_vs_E10	ordinary_muscle_development	ENSGALG00010014408	ENSGALG00010014408	0.011767501	0.293701855	0.040066144	0.968040394	0.978469436	positive
PRJNA729271	stage_E14_vs_E10	ordinary_muscle_development	ENSGALG00010014411	ATP13A3	-0.006864216	0.079962958	-0.085842441	0.931591668	0.953157721	negative
PRJNA729271	stage_E14_vs_E10	ordinary_muscle_development	ENSGALG00010014414	TMEM255B	0.120252733	0.515355929	0.233339185	0.815498033	0.870765671	positive
PRJNA729271	stage_E14_vs_E10	ordinary_muscle_development	ENSGALG00010014416	H2BC32	0.378518341	0.26972395	1.403354581	0.16051113	0.250631135	positive
PRJNA729271	stage_E14_vs_E10	ordinary_muscle_development	ENSGALG00010014418	RAC2	0.22176595	0.307861921	0.720342254	0.471314295	0.583556907	positive
PRJNA729271	stage_E14_vs_E10	ordinary_muscle_development	ENSGALG00010014427	TMEM267	-0.277404345	0.132687159	-2.090664592	0.036558141	0.072560627	negative
PRJNA729271	stage_E14_vs_E10	ordinary_muscle_development	ENSGALG00010014431	ENSGALG00010014431	0.597005125	0.156634493	3.811453748	0.000138152	0.00053117	positive
PRJNA729271	stage_E14_vs_E10	ordinary_muscle_development	ENSGALG00010014439	ENSGALG00010014439	0.970824348	0.839461907	1.1564841			positive
PRJNA729271	stage_E14_vs_E10	ordinary_muscle_development	ENSGALG00010014442	SHISA3	-2.325271409	0.650450067	-3.574865353	0.000350408	0.001235506	negative
PRJNA729271	stage_E14_vs_E10	ordinary_muscle_development	ENSGALG00010014449	ENSGALG00010014449	0.339151585	0.154225426	2.199064014	0.027873371	0.057808629	positive
PRJNA729271	stage_E14_vs_E10	ordinary_muscle_development	ENSGALG00010014453	NR2E1	0.199538103	0.345890086	0.576882979	0.564018509	0.666676041	positive
PRJNA729271	stage_E14_vs_E10	ordinary_muscle_development	ENSGALG00010014455	C4orf19	-1.654889512	0.228604417	-7.239096829	4.51682E-13	5.34963E-12	negative
PRJNA729271	stage_E14_vs_E10	ordinary_muscle_development	ENSGALG00010014465	SIMC1	0.195525585	0.153635368	1.272659981	0.203138724	0.302775919	positive
PRJNA729271	stage_E14_vs_E10	ordinary_muscle_development	ENSGALG00010014469	TMCO3	-0.46753711	0.125058466	-3.738548247	0.000185086	0.000692687	negative
PRJNA729271	stage_E14_vs_E10	ordinary_muscle_development	ENSGALG00010014470	RPS6KA1	-0.312238132	0.127901024	-2.441248108	0.014636595	0.033237889	negative
PRJNA729271	stage_E14_vs_E10	ordinary_muscle_development	ENSGALG00010014474	PTPN1	-0.099676754	0.07670285	-1.299518253	0.193766134	0.29171584	negative
PRJNA729271	stage_E14_vs_E10	ordinary_muscle_development	ENSGALG00010014476	C5orf34	-0.089753141	0.173926042	-0.516041989	0.605825083	0.703663293	negative
PRJNA729271	stage_E14_vs_E10	ordinary_muscle_development	ENSGALG00010014477	ITPR2	-0.272097595	0.098157762	-2.772043587	0.005570558	0.014311456	negative
PRJNA729271	stage_E14_vs_E10	ordinary_muscle_development	ENSGALG00010014482	THOC3	-0.054305276	0.077668613	-0.69919204	0.484432025	0.595980495	negative
PRJNA729271	stage_E14_vs_E10	ordinary_muscle_development	ENSGALG00010014487	OSTM1	0.01170419	0.082723965	0.141484871	0.887486911	0.922688479	positive
PRJNA729271	stage_E14_vs_E10	ordinary_muscle_development	ENSGALG00010014488	ENSGALG00010014488	1.442587833	0.409358952	3.52401682	0.000425057	0.001462427	positive
PRJNA729271	stage_E14_vs_E10	ordinary_muscle_development	ENSGALG00010014492	CPLX2	-1.687308757	0.312171623	-5.405067697	6.47839E-08	4.2433E-07	negative
PRJNA729271	stage_E14_vs_E10	ordinary_muscle_development	ENSGALG00010014494	KLHL9	-0.065874343	0.065105543	-1.011808519	0.311629616	0.425416395	negative
PRJNA729271	stage_E14_vs_E10	ordinary_muscle_development	ENSGALG00010014504	SEC63	-0.215332515	0.045067193	-4.778032516	1.77019E-06	9.41264E-06	negative
PRJNA729271	stage_E14_vs_E10	ordinary_muscle_development	ENSGALG00010014505	CYTH4	0.803686331	0.216294571	3.715702753	0.00020264	0.00075218	positive
PRJNA729271	stage_E14_vs_E10	ordinary_muscle_development	ENSGALG00010014512	HMGN4	-0.177996872	0.110319756	-1.613463246	0.106643902	0.179706274	negative
PRJNA729271	stage_E14_vs_E10	ordinary_muscle_development	ENSGALG00010014515	ETNK1	-0.79074798	0.11251575	-7.027887029	2.09685E-12	2.3166E-11	negative
PRJNA729271	stage_E14_vs_E10	ordinary_muscle_development	ENSGALG00010014517	DCUN1D2	0.806099628	0.127681972	6.313339432	2.73078E-10	2.39459E-09	positive
PRJNA729271	stage_E14_vs_E10	ordinary_muscle_development	ENSGALG00010014522	FAT2	-1.7634246	0.40074261	-4.400392068	1.08055E-05	5.03601E-05	negative
PRJNA729271	stage_E14_vs_E10	ordinary_muscle_development	ENSGALG00010014537	GKAP1	0.693580931	0.122060194	5.682286012	1.32906E-08	9.52757E-08	positive
PRJNA729271	stage_E14_vs_E10	ordinary_muscle_development	ENSGALG00010014539	PAIP1	-0.117055217	0.084427052	-1.386465767	0.165604726	0.256814005	negative
PRJNA729271	stage_E14_vs_E10	ordinary_muscle_development	ENSGALG00010014547	DHDDS	0.113283718	0.105762476	1.07111446	0.284117967	0.395723065	positive
PRJNA729271	stage_E14_vs_E10	ordinary_muscle_development	ENSGALG00010014550	SCML4	-2.648464939	0.588942291	-4.496985491	6.89237E-06	3.32794E-05	negative
PRJNA729271	stage_E14_vs_E10	ordinary_muscle_development	ENSGALG00010014555	VGLL1	-3.234746343	0.719521675	-4.495689922	6.93447E-06	3.34604E-05	negative
PRJNA729271	stage_E14_vs_E10	ordinary_muscle_development	ENSGALG00010014567	UTP11	-0.283599016	0.115331191	-2.45899667	0.013932591	0.031828669	negative
PRJNA729271	stage_E14_vs_E10	ordinary_muscle_development	ENSGALG00010014570	KIF27	-0.905279671	0.161469815	-5.606494767	2.06465E-08	1.44926E-07	negative
PRJNA729271	stage_E14_vs_E10	ordinary_muscle_development	ENSGALG00010014572	IL1RAP	-0.177578144	0.123953329	-1.432621016	0.151966172	0.239950944	negative
PRJNA729271	stage_E14_vs_E10	ordinary_muscle_development	ENSGALG00010014573	CEBPB	0.831902046	0.351722383	2.365223504	0.018019193	0.039730483	positive
PRJNA729271	stage_E14_vs_E10	ordinary_muscle_development	ENSGALG00010014576	HRH2	0.7953263	0.260996695	3.047265794	0.002309334	0.006599724	positive
PRJNA729271	stage_E14_vs_E10	ordinary_muscle_development	ENSGALG00010014578	ENSGALG00010014578	2.490612499	0.313405161	7.946941572	1.91173E-15	2.79904E-14	positive
PRJNA729271	stage_E14_vs_E10	ordinary_muscle_development	ENSGALG00010014579	NNT	0.208183423	0.144598115	1.43973816	0.149941493	0.237245054	positive
PRJNA729271	stage_E14_vs_E10	ordinary_muscle_development	ENSGALG00010014592	QNG1	-0.03417399	0.091614335	-0.373020118	0.709133471	0.789045034	negative
PRJNA729271	stage_E14_vs_E10	ordinary_muscle_development	ENSGALG00010014599	SSTR3	-1.801248595	0.402641111	-4.473583417	7.69195E-06	3.67478E-05	negative
PRJNA729271	stage_E14_vs_E10	ordinary_muscle_development	ENSGALG00010014602	FRK	-5.352296095	1.364705842	-3.921941219	8.78384E-05	0.000349305	negative
PRJNA729271	stage_E14_vs_E10	ordinary_muscle_development	ENSGALG00010014603	ENSGALG00010014603	1.707811571	0.222980055	7.659032864	1.87338E-14	2.52367E-13	positive
PRJNA729271	stage_E14_vs_E10	ordinary_muscle_development	ENSGALG00010014611	TMEM189	-0.013583375	0.103571551	-0.131149673	0.895656907	0.928253029	negative
PRJNA729271	stage_E14_vs_E10	ordinary_muscle_development	ENSGALG00010014614	GHR	0.949186948	0.090111015	10.53352856	6.05224E-26	1.9803E-24	positive
PRJNA729271	stage_E14_vs_E10	ordinary_muscle_development	ENSGALG00010014617	GRTP1	1.770340317	0.201042474	8.805802493	1.2992E-18	2.48114E-17	positive
PRJNA729271	stage_E14_vs_E10	ordinary_muscle_development	ENSGALG00010014624	GPRC6A	-0.309890608	0.42966506	-0.721237626	0.470763326	0.583073947	negative
PRJNA729271	stage_E14_vs_E10	ordinary_muscle_development	ENSGALG00010014626	C2CD5	-0.326519327	0.090571152	-3.605114009	0.000312016	0.001112041	negative
PRJNA729271	stage_E14_vs_E10	ordinary_muscle_development	ENSGALG00010014632	NWD2	-1.268434435	0.165807103	-7.650060904	2.00884E-14	2.69862E-13	negative
PRJNA729271	stage_E14_vs_E10	ordinary_muscle_development	ENSGALG00010014634	CCDC152	1.11071979	0.462856603	2.399706048	0.016408242	0.03664676	positive
PRJNA729271	stage_E14_vs_E10	ordinary_muscle_development	ENSGALG00010014641	DOCK11	0.106984889	0.095957343	1.114921339	0.264884132	0.373673527	positive
PRJNA729271	stage_E14_vs_E10	ordinary_muscle_development	ENSGALG00010014645	SLC36A1	-0.228614637	0.116403098	-1.963991001	0.049531136	0.094133186	negative
PRJNA729271	stage_E14_vs_E10	ordinary_muscle_development	ENSGALG00010014648	C1QTNF6	-1.139179674	0.275332418	-4.137470195	3.51156E-05	0.000149971	negative
PRJNA729271	stage_E14_vs_E10	ordinary_muscle_development	ENSGALG00010014653	FAM162B	-0.069088545	0.51331036	-0.134594098	0.892932809	0.926357076	negative
PRJNA729271	stage_E14_vs_E10	ordinary_muscle_development	ENSGALG00010014654	LAMP1	0.181917639	0.104550407	1.739999337	0.081859134	0.144261174	positive
PRJNA729271	stage_E14_vs_E10	ordinary_muscle_development	ENSGALG00010014655	FHL3	0.224725322	0.133913707	1.678135327	0.093320684	0.160574942	positive
PRJNA729271	stage_E14_vs_E10	ordinary_muscle_development	ENSGALG00010014658	SELENOP1	0.870174696	0.173594032	5.012699392	5.36717E-07	3.07986E-06	positive
PRJNA729271	stage_E14_vs_E10	ordinary_muscle_development	ENSGALG00010014668	GP5	0.095377907	0.142717354	0.668299295	0.503942563	0.613123149	positive
PRJNA729271	stage_E14_vs_E10	ordinary_muscle_development	ENSGALG00010014672	ISL1	-2.641938551	1.02744975	-2.571355486	0.010130128	0.024075457	negative
PRJNA729271	stage_E14_vs_E10	ordinary_muscle_development	ENSGALG00010014676	UBE2V1	0.044796641	0.056275681	0.796021302	0.426019654	0.540447653	positive
PRJNA729271	stage_E14_vs_E10	ordinary_muscle_development	ENSGALG00010014677	ENSGALG00010014677	-0.199509726	0.089654664	-2.225313414	0.026060206	0.054610768	negative
PRJNA729271	stage_E14_vs_E10	ordinary_muscle_development	ENSGALG00010014682	IL13RA1	0.292535547	0.125433879	2.332189275	0.019690736	0.042856588	positive
PRJNA729271	stage_E14_vs_E10	ordinary_muscle_development	ENSGALG00010014687	CUL4A	0.219434632	0.115996584	1.891733577	0.058526487	0.108275869	positive
PRJNA729271	stage_E14_vs_E10	ordinary_muscle_development	ENSGALG00010014690	CSF3R	1.872834179	0.370283028	5.057845047	4.24021E-07	2.47341E-06	positive
PRJNA729271	stage_E14_vs_E10	ordinary_muscle_development	ENSGALG00010014691	PABIR2	0.121548475	0.053386707	2.276755407	0.022800836	0.048753225	positive
PRJNA729271	stage_E14_vs_E10	ordinary_muscle_development	ENSGALG00010014696	SPINK5	-0.353503222	0.63265757	-0.558759175	0.576326088	0.678196129	negative
PRJNA729271	stage_E14_vs_E10	ordinary_muscle_development	ENSGALG00010014697	ENSGALG00010014697	0.107011777	0.688474542	0.155433165	0.87647984	0.915249282	positive
PRJNA729271	stage_E14_vs_E10	ordinary_muscle_development	ENSGALG00010014702	ENSGALG00010014702	0.283016619	0.259815571	1.089298145	0.276022431	0.386452732	positive
PRJNA729271	stage_E14_vs_E10	ordinary_muscle_development	ENSGALG00010014703	PCID2	0.012096826	0.07880452	0.153504215	0.878000667	0.916243316	positive
PRJNA729271	stage_E14_vs_E10	ordinary_muscle_development	ENSGALG00010014705	ARAP2	-1.4440394	0.220447656	-6.55048653	5.73499E-11	5.44362E-10	negative
PRJNA729271	stage_E14_vs_E10	ordinary_muscle_development	ENSGALG00010014707	KPNA5	0.099719177	0.12975375	0.768526359	0.442174537	0.556026712	positive
PRJNA729271	stage_E14_vs_E10	ordinary_muscle_development	ENSGALG00010014710	NPEPL1	0.155625843	0.099782333	1.559625278	0.118841839	0.196107975	positive
PRJNA729271	stage_E14_vs_E10	ordinary_muscle_development	ENSGALG00010014713	ST8SIA1	-0.470238535	0.129251803	-3.638158407	0.000274595	0.000989373	negative
PRJNA729271	stage_E14_vs_E10	ordinary_muscle_development	ENSGALG00010014716	TMEM35A	-0.951449427	0.111330499	-8.546170491	1.2724E-17	2.25746E-16	negative
PRJNA729271	stage_E14_vs_E10	ordinary_muscle_development	ENSGALG00010014717	ELOVL7	0.613137569	0.184541532	3.322490936	0.000892176	0.002841592	positive
PRJNA729271	stage_E14_vs_E10	ordinary_muscle_development	ENSGALG00010014719	ENSGALG00010014719	0.324553566	1.057724224	0.306841385	0.758964106	0.828252634	positive
PRJNA729271	stage_E14_vs_E10	ordinary_muscle_development	ENSGALG00010014722	KCTD17	-0.461751927	0.3601195	-1.282218615	0.199765968	0.298824325	negative
PRJNA729271	stage_E14_vs_E10	ordinary_muscle_development	ENSGALG00010014726	PWP2	-0.66527936	0.057035321	-11.66433973	1.93902E-31	8.89431E-30	negative
PRJNA729271	stage_E14_vs_E10	ordinary_muscle_development	ENSGALG00010014728	INTS13	-0.130925732	0.111932478	-1.169684922	0.242127788	0.348247895	negative
PRJNA729271	stage_E14_vs_E10	ordinary_muscle_development	ENSGALG00010014729	MRPS15	0.02433911	0.095189179	0.255691988	0.798188682	0.858035075	positive
PRJNA729271	stage_E14_vs_E10	ordinary_muscle_development	ENSGALG00010014734	ZUP1	-0.107177723	0.091061911	-1.176976428	0.239204915	0.344899557	negative
PRJNA729271	stage_E14_vs_E10	ordinary_muscle_development	ENSGALG00010014735	STX16	0.050932967	0.104649616	0.486699985	0.626470956	0.72114807	positive
PRJNA729271	stage_E14_vs_E10	ordinary_muscle_development	ENSGALG00010014736	KIF2A	-0.187415633	0.087513574	-2.141560723	0.032228847	0.065148141	negative
PRJNA729271	stage_E14_vs_E10	ordinary_muscle_development	ENSGALG00010014737	RMI1	-0.353731192	0.1133465	-3.120795023	0.001803635	0.005323265	negative
PRJNA729271	stage_E14_vs_E10	ordinary_muscle_development	ENSGALG00010014738	BRWD1	-0.02327173	0.086454922	-0.269177614	0.787793006	0.850712098	negative
PRJNA729271	stage_E14_vs_E10	ordinary_muscle_development	ENSGALG00010014739	TRMT2B	0.085637469	0.176320072	0.485693252	0.627184668	0.721568519	positive
PRJNA729271	stage_E14_vs_E10	ordinary_muscle_development	ENSGALG00010014740	TRMT9B	-1.496228054	0.414975166	-3.605584562	0.000311451	0.001110301	negative
PRJNA729271	stage_E14_vs_E10	ordinary_muscle_development	ENSGALG00010014742	PROZ	16.2102488	2.894792406	5.599796644	2.14603E-08	1.50274E-07	positive
PRJNA729271	stage_E14_vs_E10	ordinary_muscle_development	ENSGALG00010014744	IL2RB	-0.759572204	0.576727269	-1.317038823	0.187825611	0.284313611	negative
PRJNA729271	stage_E14_vs_E10	ordinary_muscle_development	ENSGALG00010014745	LSM10	0.84577589	0.181945612	4.648509401	3.34342E-06	1.69451E-05	positive
PRJNA729271	stage_E14_vs_E10	ordinary_muscle_development	ENSGALG00010014748	FGFR1OP2	-0.044048628	0.107166744	-0.41102889	0.681051351	0.766741334	negative
PRJNA729271	stage_E14_vs_E10	ordinary_muscle_development	ENSGALG00010014750	LONRF1	0.042475322	0.18147126	0.234060875	0.81493772	0.870295605	positive
PRJNA729271	stage_E14_vs_E10	ordinary_muscle_development	ENSGALG00010014753	ENSGALG00010014753	-1.397923962	0.390833273	-3.576778278	0.000347855	0.001227699	negative
PRJNA729271	stage_E14_vs_E10	ordinary_muscle_development	ENSGALG00010014754	DIMT1	-0.468514677	0.141941437	-3.300760433	0.000964232	0.003049649	negative
PRJNA729271	stage_E14_vs_E10	ordinary_muscle_development	ENSGALG00010014758	FXN	-0.217701413	0.098422283	-2.211911844	0.026972757	0.056228371	negative
PRJNA729271	stage_E14_vs_E10	ordinary_muscle_development	ENSGALG00010014762	STK40	-0.089736883	0.103748411	-0.864947053	0.38706785	0.503097963	negative
PRJNA729271	stage_E14_vs_E10	ordinary_muscle_development	ENSGALG00010014764	ENSGALG00010014764	4.969304643	2.324329344	2.137952032	0.032520637	0.065652734	positive
PRJNA729271	stage_E14_vs_E10	ordinary_muscle_development	ENSGALG00010014766	F10	0.259795435	0.211440886	1.228690629	0.219187821	0.321441468	positive
PRJNA729271	stage_E14_vs_E10	ordinary_muscle_development	ENSGALG00010014767	LRRC15	2.578922972	0.242402898	10.63899396	1.96237E-26	6.69282E-25	positive
PRJNA729271	stage_E14_vs_E10	ordinary_muscle_development	ENSGALG00010014769	CCDC69	3.106064743	0.320692007	9.68550719	3.4748E-22	8.75951E-21	positive
PRJNA729271	stage_E14_vs_E10	ordinary_muscle_development	ENSGALG00010014777	APCDD1L	-4.359910448	0.24663948	-17.67726093	6.27726E-70	2.1664E-67	negative
PRJNA729271	stage_E14_vs_E10	ordinary_muscle_development	ENSGALG00010014780	EVA1B	0.200301024	0.12465771	1.606808156	0.108096459	0.181705446	positive
PRJNA729271	stage_E14_vs_E10	ordinary_muscle_development	ENSGALG00010014783	ENSGALG00010014783	0.067502337	0.118501579	0.56963239	0.568927055	0.671273721	positive
PRJNA729271	stage_E14_vs_E10	ordinary_muscle_development	ENSGALG00010014789	F7	-1.011315259	0.934506878	-1.082191349	0.279167512	0.390033409	negative
PRJNA729271	stage_E14_vs_E10	ordinary_muscle_development	ENSGALG00010014792	ENSGALG00010014792	0.62522619	0.270276392	2.313284504	0.020707002	0.044824969	positive
PRJNA729271	stage_E14_vs_E10	ordinary_muscle_development	ENSGALG00010014793	TLR2A	0.350507132	0.175497653	1.997218348	0.045801469	0.088166307	positive
PRJNA729271	stage_E14_vs_E10	ordinary_muscle_development	ENSGALG00010014795	THRAP3	0.132070955	0.095036119	1.389692212	0.164622361	0.255673537	positive
PRJNA729271	stage_E14_vs_E10	ordinary_muscle_development	ENSGALG00010014796	PRLR	-1.408403754	0.249180313	-5.652146982	1.58456E-08	1.12644E-07	negative
PRJNA729271	stage_E14_vs_E10	ordinary_muscle_development	ENSGALG00010014797	ZDHHC9	0.204351998	0.09165538	2.229569055	0.025776066	0.054140571	positive

PRJNA729271	stage_E14_vs_E10	ordinary_muscle_development	ENSGALG00010014801	MPST	0.436800734	0.085286732	5.1215555	3.03026E-07	1.80681E-06	positive
PRJNA729271	stage_E14_vs_E10	ordinary_muscle_development	ENSGALG00010014802	ENSGALG00010014802	-0.024437691	0.392175753	-0.06231311	0.950313489	0.965647222	negative
PRJNA729271	stage_E14_vs_E10	ordinary_muscle_development	ENSGALG00010014805	SPEF2	-1.597032541	0.362583209	-4.404595974	1.05981E-05	4.9459E-05	negative
PRJNA729271	stage_E14_vs_E10	ordinary_muscle_development	ENSGALG00010014806	MAP7D1	1.153987329	0.226210084	5.101396496	3.37156E-07	1.99954E-06	positive
PRJNA729271	stage_E14_vs_E10	ordinary_muscle_development	ENSGALG00010014807	HES1	0.565611399	0.223129719	2.534899432	0.011247961	0.02643736	positive
PRJNA729271	stage_E14_vs_E10	ordinary_muscle_development	ENSGALG00010014810	ENTREP1	0.889305437	0.224289323	3.964992291	7.33983E-05	0.000296106	positive
PRJNA729271	stage_E14_vs_E10	ordinary_muscle_development	ENSGALG00010014812	RWDD1	-0.657496502	0.082798207	-7.940950919	2.00637E-15	2.92579E-14	negative
PRJNA729271	stage_E14_vs_E10	ordinary_muscle_development	ENSGALG00010014813	SASH3	0.245686408	0.2696992	0.910964541	0.362314057	0.47847506	positive
PRJNA729271	stage_E14_vs_E10	ordinary_muscle_development	ENSGALG00010014816	TST	0.538735973	0.107765353	4.999157497	5.75814E-07	3.28341E-06	positive
PRJNA729271	stage_E14_vs_E10	ordinary_muscle_development	ENSGALG00010014818	ENSGALG00010014818	0.38870802	0.417626959	0.930754138	0.351980758	0.46832762	positive
PRJNA729271	stage_E14_vs_E10	ordinary_muscle_development	ENSGALG00010014821	C3AR1L	0.887697186	0.230618514	3.849201738	0.000118503	0.000461006	positive
PRJNA729271	stage_E14_vs_E10	ordinary_muscle_development	ENSGALG00010014824	ANXA6	0.996254324	0.090443073	11.01526399	3.2259E-28	1.23702E-26	positive
PRJNA729271	stage_E14_vs_E10	ordinary_muscle_development	ENSGALG00010014829	ENSGALG00010014829	1.185533537	0.405624145	2.922739074	0.003469671	0.009432274	positive
PRJNA729271	stage_E14_vs_E10	ordinary_muscle_development	ENSGALG00010014830	TRAPPC3L	-1.121538137	0.382637923	-2.931068958	0.003377978	0.009215846	negative
PRJNA729271	stage_E14_vs_E10	ordinary_muscle_development	ENSGALG00010014831	VAPB	0.002530744	0.060184619	0.04204968	0.966459094	0.977655424	positive
PRJNA729271	stage_E14_vs_E10	ordinary_muscle_development	ENSGALG00010014837	APBA1	0.050887602	0.11045368	0.460714412	0.645003514	0.737212264	positive
PRJNA729271	stage_E14_vs_E10	ordinary_muscle_development	ENSGALG00010014839	TM7SF3	-0.151297244	0.108212192	-1.398153406	0.162067005	0.252601712	negative
PRJNA729271	stage_E14_vs_E10	ordinary_muscle_development	ENSGALG00010014840	NCF4	0.476544352	0.214053481	2.226286392	0.025995004	0.054497771	positive
PRJNA729271	stage_E14_vs_E10	ordinary_muscle_development	ENSGALG00010014847	PAICS	-0.238432587	0.090669237	-2.629696627	0.008546109	0.020802837	negative
PRJNA729271	stage_E14_vs_E10	ordinary_muscle_development	ENSGALG00010014851	CALHM4	0.48139347	0.43427069	1.108510155	0.267641556	0.376720174	positive
PRJNA729271	stage_E14_vs_E10	ordinary_muscle_development	ENSGALG00010014856	MMGT1	0.119503753	0.071173145	1.679056801	0.093140974	0.160341855	positive
PRJNA729271	stage_E14_vs_E10	ordinary_muscle_development	ENSGALG00010014859	TRAPPC3	0.076919901	0.128140505	0.600277803	0.548321109	0.653611388	positive
PRJNA729271	stage_E14_vs_E10	ordinary_muscle_development	ENSGALG00010014861	MED21	-0.053561183	0.092321258	-0.580160889	0.561806125	0.664928537	negative
PRJNA729271	stage_E14_vs_E10	ordinary_muscle_development	ENSGALG00010014862	ENSGALG00010014862	-0.574178324	0.318390465	-1.803377884	0.071328888	0.128102123	negative
PRJNA729271	stage_E14_vs_E10	ordinary_muscle_development	ENSGALG00010014865	TLR2	0.778071661	0.37804491	2.058146109	0.03957611	0.077594443	positive
PRJNA729271	stage_E14_vs_E10	ordinary_muscle_development	ENSGALG00010014866	FAM26E	0.558510659	0.148757908	3.754493906	0.000173692	0.00065377	positive
PRJNA729271	stage_E14_vs_E10	ordinary_muscle_development	ENSGALG00010014867	TP63	-0.811210265	0.271554431	-2.987284214	0.002814679	0.007854498	negative
PRJNA729271	stage_E14_vs_E10	ordinary_muscle_development	ENSGALG00010014870	ENSGALG00010014870	1.633547087	1.969460421	0.829438901	0.406856097	0.521782128	positive
PRJNA729271	stage_E14_vs_E10	ordinary_muscle_development	ENSGALG00010014873	CALHM6	-1.098203124	0.74586926	-1.472380192	0.140918239	0.225614571	negative
PRJNA729271	stage_E14_vs_E10	ordinary_muscle_development	ENSGALG00010014876	PCNT	-0.188389159	0.10301799	-1.828701553	0.06744433	0.122185422	negative
PRJNA729271	stage_E14_vs_E10	ordinary_muscle_development	ENSGALG00010014877	ENSGALG00010014877	-0.46860012	0.09081162	-5.160133888	2.46773E-07	1.48979E-06	negative
PRJNA729271	stage_E14_vs_E10	ordinary_muscle_development	ENSGALG00010014878	TMCO1	0.231273744	0.129404893	1.787210196	0.073903522	0.132104027	positive
PRJNA729271	stage_E14_vs_E10	ordinary_muscle_development	ENSGALG00010014880	SFRP2	-0.008426878	0.292408718	-0.028818833	0.97700908	0.984683087	negative
PRJNA729271	stage_E14_vs_E10	ordinary_muscle_development	ENSGALG00010014881	ENSGALG00010014881	1.037866171	0.392518216	2.644122303	0.008190306	0.020057185	positive
PRJNA729271	stage_E14_vs_E10	ordinary_muscle_development	ENSGALG00010014882	XPNPEP2	0.471222503	0.592233279	0.795670422	0.426223623	0.540659089	positive
PRJNA729271	stage_E14_vs_E10	ordinary_muscle_development	ENSGALG00010014883	PPAT	-0.255380005	0.119992759	-2.128295139	0.033312621	0.06702255	negative
PRJNA729271	stage_E14_vs_E10	ordinary_muscle_development	ENSGALG00010014886	RABGAP1L	0.10058026	0.069353281	1.450259584	0.146986145	0.233487366	positive
PRJNA729271	stage_E14_vs_E10	ordinary_muscle_development	ENSGALG00010014887	MTMR12	0.377244036	0.130677766	2.886826488	0.003891488	0.010428381	positive
PRJNA729271	stage_E14_vs_E10	ordinary_muscle_development	ENSGALG00010014889	PVALB	-1.091698016	0.438345528	-2.490496528	0.012756474	0.029509271	negative
PRJNA729271	stage_E14_vs_E10	ordinary_muscle_development	ENSGALG00010014893	SLC9A6	0.284759127	0.085511402	3.330072032	0.000868235	0.002777548	positive
PRJNA729271	stage_E14_vs_E10	ordinary_muscle_development	ENSGALG00010014894	RAB22A	-0.07824632	0.07517316	-1.040881078	0.297930745	0.411270066	negative
PRJNA729271	stage_E14_vs_E10	ordinary_muscle_development	ENSGALG00010014897	COL8A2	-2.991422318	0.24976939	-11.97673707	4.70479E-33	2.37616E-31	negative
PRJNA729271	stage_E14_vs_E10	ordinary_muscle_development	ENSGALG00010014898	ENSGALG00010014898	1.762986651	0.663927538	2.65539016	0.007921673	0.019468404	positive
PRJNA729271	stage_E14_vs_E10	ordinary_muscle_development	ENSGALG00010014900	UBLCP1	-0.195333313	0.081894605	-2.385179244	0.017070802	0.037927846	negative
PRJNA729271	stage_E14_vs_E10	ordinary_muscle_development	ENSGALG00010014902	AASDH	-0.00734183	0.125666365	-0.05842319	0.953411544	0.967833905	negative
PRJNA729271	stage_E14_vs_E10	ordinary_muscle_development	ENSGALG00010014903	MCF2L	-0.541671057	0.155834688	-3.475933793	0.000509078	0.001722073	negative
PRJNA729271	stage_E14_vs_E10	ordinary_muscle_development	ENSGALG00010014906	IFT27	-0.038171467	0.107019342	-0.356678208	0.721332722	0.798207329	negative
PRJNA729271	stage_E14_vs_E10	ordinary_muscle_development	ENSGALG00010014907	ENSGALG00010014907	-0.105751105	0.069374444	-1.524352463	0.127420688	0.20745399	negative
PRJNA729271	stage_E14_vs_E10	ordinary_muscle_development	ENSGALG00010014908	ENSGALG00010014908	0.003945552	0.101196091	0.038989179	0.968899016	0.978689285	positive
PRJNA729271	stage_E14_vs_E10	ordinary_muscle_development	ENSGALG00010014909	FBXL14	-0.265994819	0.096007158	-2.770572772	0.005595779	0.014368613	negative
PRJNA729271	stage_E14_vs_E10	ordinary_muscle_development	ENSGALG00010014912	ENSGALG00010014912	1.268482442	0.324837818	3.90497156	9.42365E-05	0.000372398	positive
PRJNA729271	stage_E14_vs_E10	ordinary_muscle_development	ENSGALG00010014914	PTAR1	-0.002616648	0.179261943	-0.014596787	0.988353862	0.992243708	negative
PRJNA729271	stage_E14_vs_E10	ordinary_muscle_development	ENSGALG00010014916	DYNLT2B	-0.239207906	0.149721348	-1.597687365	0.110112572	0.184432832	negative
PRJNA729271	stage_E14_vs_E10	ordinary_muscle_development	ENSGALG00010014917	EIF3D	0.364254833	0.104922464	3.47165724	0.000517256	0.001746885	positive
PRJNA729271	stage_E14_vs_E10	ordinary_muscle_development	ENSGALG00010014918	PLRG1	0.037828819	0.083626056	0.452356848	0.651011928	0.742216307	positive
PRJNA729271	stage_E14_vs_E10	ordinary_muscle_development	ENSGALG00010014922	MAN1A1	-0.952712194	0.165336993	-5.7622446	8.30026E-09	6.10103E-08	negative
PRJNA729271	stage_E14_vs_E10	ordinary_muscle_development	ENSGALG00010014923	CIMIP1	-0.198267738	0.335593773	-0.590796834	0.554656557	0.65942805	negative
PRJNA729271	stage_E14_vs_E10	ordinary_muscle_development	ENSGALG00010014928	SUCO	0.402275699	0.079807659	5.040565066	4.64159E-07	2.69227E-06	positive
PRJNA729271	stage_E14_vs_E10	ordinary_muscle_development	ENSGALG00010014929	FHL1	2.031554498	0.28480733	7.133083622	9.81449E-13	1.11928E-11	positive
PRJNA729271	stage_E14_vs_E10	ordinary_muscle_development	ENSGALG00010014931	RNF145	-0.107120711	0.107791796	-0.993774242	0.320332789	0.434880938	negative
PRJNA729271	stage_E14_vs_E10	ordinary_muscle_development	ENSGALG00010014934	MAMDC2	1.890305492	0.203594315	9.284667367	1.62213E-20	3.60073E-19	positive
PRJNA729271	stage_E14_vs_E10	ordinary_muscle_development	ENSGALG00010014935	PCYT1A	0.46416651	0.130975164	3.543927698	0.000394213	0.001367625	positive
PRJNA729271	stage_E14_vs_E10	ordinary_muscle_development	ENSGALG00010014936	FOXRED2	-0.114132698	0.167790444	-0.680209761	0.496371653	0.605937941	negative
PRJNA729271	stage_E14_vs_E10	ordinary_muscle_development	ENSGALG00010014940	VAMP4	-0.287260671	0.083534101	-3.438843165	0.000584206	0.001946233	negative
PRJNA729271	stage_E14_vs_E10	ordinary_muscle_development	ENSGALG00010014942	PMEPA1	-0.648283519	0.144505387	-4.486223892	7.24966E-06	3.47729E-05	negative
PRJNA729271	stage_E14_vs_E10	ordinary_muscle_development	ENSGALG00010014943	APLN	0.788773363	0.300088611	2.628468173	0.008577038	0.02086326	positive
PRJNA729271	stage_E14_vs_E10	ordinary_muscle_development	ENSGALG00010014944	FGB	0.721530333	0.916449718	0.787310333			positive
PRJNA729271	stage_E14_vs_E10	ordinary_muscle_development	ENSGALG00010014945	TXN2	0.448951431	0.104165049	4.310000655	1.63254E-05	7.38106E-05	positive
PRJNA729271	stage_E14_vs_E10	ordinary_muscle_development	ENSGALG00010014946	PCK1	2.533429888	1.048906787	2.415305076	0.015722035	0.035331922	positive
PRJNA729271	stage_E14_vs_E10	ordinary_muscle_development	ENSGALG00010014948	RBM38	1.415651048	0.259312463	5.459248016	4.78155E-08	3.18368E-07	positive
PRJNA729271	stage_E14_vs_E10	ordinary_muscle_development	ENSGALG00010014949	AGO3	-0.385290392	0.108578017	-3.548511969	0.000387414	0.001347953	negative
PRJNA729271	stage_E14_vs_E10	ordinary_muscle_development	ENSGALG00010014952	C21orf58	0.562797358	0.493557881	1.140286436	0.254166987	0.361593041	positive
PRJNA729271	stage_E14_vs_E10	ordinary_muscle_development	ENSGALG00010014953	ENSGALG00010014953	0.409041989	0.351055349	1.165178054	0.243946927	0.350377597	positive
PRJNA729271	stage_E14_vs_E10	ordinary_muscle_development	ENSGALG00010014956	FGG	1.215525369	1.537792377	0.790435294	0.429273597	0.54352907	positive
PRJNA729271	stage_E14_vs_E10	ordinary_muscle_development	ENSGALG00010014965	ABHD13	-0.084901791	0.098770849	-0.859583489	0.39001868	0.505589367	negative
PRJNA729271	stage_E14_vs_E10	ordinary_muscle_development	ENSGALG00010014967	HNRNPH1	-0.075639504	0.107566857	-0.703185966	0.481939864	0.593670292	negative
PRJNA729271	stage_E14_vs_E10	ordinary_muscle_development	ENSGALG00010014970	SLC51A	0.010030697	0.516530807	0.019419358	0.984506568	0.989695728	positive
PRJNA729271	stage_E14_vs_E10	ordinary_muscle_development	ENSGALG00010014971	SMC5	-0.127806853	0.123791852	-1.032433484	0.301869093	0.415611764	negative
PRJNA729271	stage_E14_vs_E10	ordinary_muscle_development	ENSGALG00010014972	FGA	1.231344835	1.664019786	0.739982087	0.459310863	0.572115748	positive
PRJNA729271	stage_E14_vs_E10	ordinary_muscle_development	ENSGALG00010014974	LIG4	-0.180691768	0.111758263	-1.61680902	0.105919512	0.178669071	negative
PRJNA729271	stage_E14_vs_E10	ordinary_muscle_development	ENSGALG00010014976	ITGB2	0.678191275	0.212850769	3.186228912	0.001441405	0.00436183	positive
PRJNA729271	stage_E14_vs_E10	ordinary_muscle_development	ENSGALG00010014980	ENSGALG00010014980	0.181918779	0.727412781	0.25009016	0.802517625	0.86096462	positive
PRJNA729271	stage_E14_vs_E10	ordinary_muscle_development	ENSGALG00010014982	ENSGALG00010014982	-0.681268561	0.367705647	-1.852755229	0.06391745	0.116744385	negative
PRJNA729271	stage_E14_vs_E10	ordinary_muscle_development	ENSGALG00010014984	LPP	-0.015677798	0.092508856	-0.169473488	0.865424224	0.9074965	negative
PRJNA729271	stage_E14_vs_E10	ordinary_muscle_development	ENSGALG00010014985	ENSGALG00010014985	-0.104226302	0.107234605	-0.971946529	0.331077151	0.445793154	negative
PRJNA729271	stage_E14_vs_E10	ordinary_muscle_development	ENSGALG00010014987	KLF9	0.093562461	0.339478494	0.275606445	0.782850359	0.846695713	positive
PRJNA729271	stage_E14_vs_E10	ordinary_muscle_development	ENSGALG00010014990	AGO1	-0.448145789	0.04617216	-9.70597411	2.84347E-22	7.25636E-21	negative
PRJNA729271	stage_E14_vs_E10	ordinary_muscle_development	ENSGALG00010014992	ARL15	-0.863128442	0.096714505	-8.92449837	4.47724E-19	8.9145E-18	negative
PRJNA729271	stage_E14_vs_E10	ordinary_muscle_development	ENSGALG00010014994	ENSGALG00010014994	0.424688799	0.118237753	3.591820609	0.000328376	0.001165192	positive
PRJNA729271	stage_E14_vs_E10	ordinary_muscle_development	ENSGALG00010014995	LRAT	-1.219456848	0.239556205	-5.090483254	3.57152E-07	2.11044E-06	negative
PRJNA729271	stage_E14_vs_E10	ordinary_muscle_development	ENSGALG00010014997	RAE1	-0.26842147	0.088553838	-3.031166971	0.002436105	0.006896573	negative
PRJNA729271	stage_E14_vs_E10	ordinary_muscle_development	ENSGALG00010014998	NALF1	-0.701421837	0.489438229	-1.433116163	0.151824643	0.23975359	negative
PRJNA729271	stage_E14_vs_E10	ordinary_muscle_development	ENSGALG00010015002	ADAM19	0.544301044	0.199609605	2.726827923	0.006394638	0.016111642	positive
PRJNA729271	stage_E14_vs_E10	ordinary_muscle_development	ENSGALG00010015006	HSPB3	2.090142241	0.331696038	6.301378373	2.9501E-10	2.57291E-09	positive
PRJNA729271	stage_E14_vs_E10	ordinary_muscle_development	ENSGALG00010015009	AGO4	0.157287167	0.104011367	1.512211324	0.130480111	0.211641793	positive
PRJNA729271	stage_E14_vs_E10	ordinary_muscle_development	ENSGALG00010015013	SNX18	-0.722216003	0.107309822	-6.730194795	1.69436E-11	1.69846E-10	negative
PRJNA729271	stage_E14_vs_E10	ordinary_muscle_development	ENSGALG00010015016	ARGLU1	-0.178847244	0.079818252	-2.240681044	0.025046743	0.052817304	negative
PRJNA729271	stage_E14_vs_E10	ordinary_muscle_development	ENSGALG00010015017	FAM184A	-0.70496397	0.191342882	-3.68429682	0.000229335	0.000839625	negative
PRJNA729271	stage_E14_vs_E10	ordinary_muscle_development	ENSGALG00010015018	MAP7D3	0.08732982	0.067686249	1.290215089	0.196975989	0.295474175	positive
PRJNA729271	stage_E14_vs_E10	ordinary_muscle_development	ENSGALG00010015020	NIPAL4	1.708411822	0.724775104	2.357161295	0.018415246	0.040456046	positive
PRJNA729271	stage_E14_vs_E10	ordinary_muscle_development	ENSGALG00010015021	RBM46	-0.351162039	0.727298304	-0.482830824	0.629215863	0.723101873	negative
PRJNA729271	stage_E14_vs_E10	ordinary_muscle_development	ENSGALG00010015024	CRACD	-1.232431779	0.374352275	-3.292171202	0.000994171	0.003132718	negative
PRJNA729271	stage_E14_vs_E10	ordinary_muscle_development	ENSGALG00010015025	TRAF3IP1	-0.792565579	0.184507656	-4.295570157	1.74245E-05	7.83886E-05	negative
PRJNA729271	stage_E14_vs_E10	ordinary_muscle_development	ENSGALG00010015027	CLSPN	-0.244744373	0.173380772	-1.411600436	0.158067644	0.247561423	negative
PRJNA729271	stage_E14_vs_E10	ordinary_muscle_development	ENSGALG00010015028	MCM9	-0.286767201	0.264450038	-1.084390844	0.278191542	0.388887809	negative
PRJNA729271	stage_E14_vs_E10	ordinary_muscle_development	ENSGALG00010015029	TFRC	-1.02734863	0.182318173	-5.634921692	1.75138E-08	1.23919E-07	negative
PRJNA729271	stage_E14_vs_E10	ordinary_muscle_development	ENSGALG00010015031	MYH9	0.012561443	0.109516431	0.114699165	0.908683576	0.937197406	positive
PRJNA729271	stage_E14_vs_E10	ordinary_muscle_development	ENSGALG00010015032	C1orf216	-1.615332615	0.248676209	-6.495726406	8.26337E-11	7.65352E-10	negative
PRJNA729271	stage_E14_vs_E10	ordinary_muscle_development	ENSGALG00010015033	BMP7	-1.809153108	0.324413987	-5.576680359	2.45152E-08	1.70104E-07	negative
PRJNA729271	stage_E14_vs_E10	ordinary_muscle_development	ENSGALG00010015035	SLC35F1	-0.981374254	0.112281318	-8.740316455	2.32458E-18	4.37028E-17	negative
PRJNA729271	stage_E14_vs_E10	ordinary_muscle_development	ENSGALG00010015037	CYFIP2	0.817729783	0.188901039	4.328879232	1.4987E-05	6.83779E-05	positive
PRJNA729271	stage_E14_vs_E10	ordinary_muscle_development	ENSGALG00010015038	PCSK5	-0.309103373	0.205821884	-1.501800329	0.133148683	0.21537665	negative
PRJNA729271	stage_E14_vs_E10	ordinary_muscle_development	ENSGALG00010015039	FAM114A1	0.339919716	0.126099985	2.69563646	0.007025429	0.017485165	positive
PRJNA729271	stage_E14_vs_E10	ordinary_muscle_development	ENSGALG00010015040	ZC4H2	-0.87922098	0.085667864	-10.26313649	1.03296E-24	3.00658E-23	negative
PRJNA729271	stage_E14_vs_E10	ordinary_muscle_development	ENSGALG00010015041	TFAP2C	-2.178460095	0.468612283	-4.648747319	3.33957E-06	1.69433E-05	negative
PRJNA729271	stage_E14_vs_E10	ordinary_muscle_development	ENSGALG00010015042	EFNB2	-0.220722956	0.132270851	-1.668719554	0.095172972	0.163374257	negative
PRJNA729271	stage_E14_vs_E10	ordinary_muscle_development	ENSGALG00010015043	COQ10B	0.538275776	0.222448166	2.419780691	0.01552987	0.034965124	positive
PRJNA729271	stage_E14_vs_E10	ordinary_muscle_development	ENSGALG00010015044	ZC3H12B	-0.696313941	0.128137781	-5.434103301	5.50727E-08	3.63019E-07	negative
PRJNA729271	stage_E14_vs_E10	ordinary_muscle_development	ENSGALG00010015046	PSMB2	0.238854767	0.09333831	2.559021759	0.010496717	0.024820541	positive
PRJNA729271	stage_E14_vs_E10	ordinary_muscle_development	ENSGALG00010015047	NPY2R	-1.893675439	0.200257998	-9.456178818	3.19402E-21	7.47938E-20	negative
PRJNA729271	stage_E14_vs_E10	ordinary_muscle_development	ENSGALG00010015049	DCK2	0.054581079	0.248054014	0.220037071	0.825842284	0.878123819	positive
PRJNA729271	stage_E14_vs_E10	ordinary_muscle_development	ENSGALG00010015050	HIVEP3	-0.542838303	0.175785684	-3.088068906	0.002014618	0.005850908	negative
PRJNA729271	stage_E14_vs_E10	ordinary_muscle_development	ENSGALG00010015052	ENSGALG00010015052	-0.129825552	0.106741596	-1.216260168	0.22388583	0.32694188	negative
PRJNA729271	stage_E14_vs_E10	ordinary_muscle_development	ENSGALG00010015054	ENSGALG00010015054	-0.019830269	0.173618727	-0.114217341	0.909065506	0.937340396	negative
PRJNA729271	stage_E14_vs_E10	ordinary_muscle_development	ENSGALG00010015055	ZNF335	0.014973749	0.182102649	0.082226968	0.934466229	0.954557224	positive
PRJNA729271	stage_E14_vs_E10	ordinary_muscle_development	ENSGALG00010015056	ENSGALG00010015056	-1.050166839	0.440798451	-2.382419535	0.01719929	0.038195758	negative
PRJNA729271	stage_E14_vs_E10	ordinary_muscle_development	ENSGALG00010015057	LAS1L	-0.163869935	0.112269349	-1.459614189	0.144396137	0.230128863	negative
PRJNA729271	stage_E14_vs_E10	ordinary_muscle_development	ENSGALG00010015059	ENSGALG00010015059	0.144708632	0.162252313	0.891874077	0.372460431	0.488516931	positive
PRJNA729271	stage_E14_vs_E10	ordinary_muscle_development	ENSGALG00010015061	MMP9	0.167951242	0.460012449	0.365101516	0.715035629	0.793905894	positive
PRJNA729271	stage_E14_vs_E10	ordinary_muscle_development	ENSGALG00010015062	ENSGALG00010015062	-0.024285558	0.228413306	-0.106322871	0.915326186	0.941362463	negative
PRJNA729271	stage_E14_vs_E10	ordinary_muscle_development	ENSGALG00010015063	EEF1AKMT4	-0.11879207	0.204868675	-0.579844969	0.562019169	0.665072076	negative

PRJNA729271	stage_E14_vs_E10	ordinary_muscle_development	ENSGALG00010015064	RFK	-0.490802714	0.146397233	-3.352540913	0.000800734	0.002584998	negative
PRJNA729271	stage_E14_vs_E10	ordinary_muscle_development	ENSGALG00010015065	F11	0.427604493	1.451205115	0.294654759	0.768257627	0.8352756	positive
PRJNA729271	stage_E14_vs_E10	ordinary_muscle_development	ENSGALG00010015066	RUFY1	-0.203124408	0.069141617	-2.937802386	0.003305476	0.009045286	negative
PRJNA729271	stage_E14_vs_E10	ordinary_muscle_development	ENSGALG00010015068	ESM1	-0.271153032	0.325683605	-0.832565802	0.405089652	0.519898371	negative
PRJNA729271	stage_E14_vs_E10	ordinary_muscle_development	ENSGALG00010015069	HSPD1	0.044348557	0.102249473	0.433728953	0.66448528	0.753183776	positive
PRJNA729271	stage_E14_vs_E10	ordinary_muscle_development	ENSGALG00010015070	EDN2	-0.659498207	0.450579767	-1.463665828	0.143285277	0.228660144	negative
PRJNA729271	stage_E14_vs_E10	ordinary_muscle_development	ENSGALG00010015071	SNAI1	-0.97872984	0.121716323	-8.041072992	8.90552E-16	1.34324E-14	negative
PRJNA729271	stage_E14_vs_E10	ordinary_muscle_development	ENSGALG00010015072	MAP9	-0.499044592	0.136585493	-3.653715935	0.000258472	0.000934769	negative
PRJNA729271	stage_E14_vs_E10	ordinary_muscle_development	ENSGALG00010015075	ENSGALG00010015075	0.722642054	0.168644454	4.285003367	1.82736E-05	8.18783E-05	positive
PRJNA729271	stage_E14_vs_E10	ordinary_muscle_development	ENSGALG00010015076	ITK	-3.271678824	0.999806482	-3.272312075	0.001066718	0.00333954	negative
PRJNA729271	stage_E14_vs_E10	ordinary_muscle_development	ENSGALG00010015080	ASF1A	-0.18082678	0.076076792	-2.376898081	0.017458911	0.038683418	negative
PRJNA729271	stage_E14_vs_E10	ordinary_muscle_development	ENSGALG00010015083	CEP135	-0.439323951	0.183643563	-2.392264358	0.016744777	0.037323626	negative
PRJNA729271	stage_E14_vs_E10	ordinary_muscle_development	ENSGALG00010015084	FOXO6	-0.437497458	0.20387884	-2.145869863	0.031883363	0.064618197	negative
PRJNA729271	stage_E14_vs_E10	ordinary_muscle_development	ENSGALG00010015085	PSMD2	0.034919335	0.16337613	0.213735845	0.830753057	0.881986783	positive
PRJNA729271	stage_E14_vs_E10	ordinary_muscle_development	ENSGALG00010015086	FOXN3	0.078291567	0.095800703	0.817233747	0.41379486	0.528594034	positive
PRJNA729271	stage_E14_vs_E10	ordinary_muscle_development	ENSGALG00010015087	GABRA3	-2.222973958	0.418982509	-5.30564859	1.12273E-07	7.09724E-07	negative
PRJNA729271	stage_E14_vs_E10	ordinary_muscle_development	ENSGALG00010015088	ENSGALG00010015088	-0.120810511	1.08077766	-0.111781096			negative
PRJNA729271	stage_E14_vs_E10	ordinary_muscle_development	ENSGALG00010015090	GZMA	-0.886542646	0.56515962	-1.56865862	0.116727449	0.193389459	negative
PRJNA729271	stage_E14_vs_E10	ordinary_muscle_development	ENSGALG00010015091	MSN	-0.008060499	0.121179062	-0.066517257	0.946966019	0.963044443	negative
PRJNA729271	stage_E14_vs_E10	ordinary_muscle_development	ENSGALG00010015092	HSPE1	-0.047962451	0.122310426	-0.392137059	0.694956947	0.777688639	negative
PRJNA729271	stage_E14_vs_E10	ordinary_muscle_development	ENSGALG00010015094	CDK5RAP1	0.5584082	0.213275669	2.61824616	0.008838302	0.021405378	positive
PRJNA729271	stage_E14_vs_E10	ordinary_muscle_development	ENSGALG00010015095	RBFOX2	0.674270717	0.132988048	5.070160276	3.97481E-07	2.32976E-06	positive
PRJNA729271	stage_E14_vs_E10	ordinary_muscle_development	ENSGALG00010015096	GABRE	-5.344025292	1.209021987	-4.420122503	9.8645E-06	4.63337E-05	negative
PRJNA729271	stage_E14_vs_E10	ordinary_muscle_development	ENSGALG00010015097	MFSD2A	0.410467946	0.157035348	2.613857018	0.008952649	0.021649759	positive
PRJNA729271	stage_E14_vs_E10	ordinary_muscle_development	ENSGALG00010015098	HAVCR1	0.764428205	0.215446882	3.548105209	0.000388013	0.001349712	positive
PRJNA729271	stage_E14_vs_E10	ordinary_muscle_development	ENSGALG00010015100	MOB4	0.275864683	0.088678321	3.110846917	0.001865516	0.00548826	positive
PRJNA729271	stage_E14_vs_E10	ordinary_muscle_development	ENSGALG00010015101	CAP1	-0.308564177	0.098953097	-3.118287218	0.001819054	0.005365729	negative
PRJNA729271	stage_E14_vs_E10	ordinary_muscle_development	ENSGALG00010015103	ITPK1	0.253968522	0.090010511	2.821542934	0.004779324	0.012518305	positive
PRJNA729271	stage_E14_vs_E10	ordinary_muscle_development	ENSGALG00010015104	CNGA2	-0.655430977	0.331272291	-1.978526414	0.047869352	0.091454627	negative
PRJNA729271	stage_E14_vs_E10	ordinary_muscle_development	ENSGALG00010015105	ENSGALG00010015105	1.291895866	1.179446126	1.095341142	0.27336721	0.383513134	positive
PRJNA729271	stage_E14_vs_E10	ordinary_muscle_development	ENSGALG00010015107	MB	-3.50768905	0.59838447	-5.861931963	4.57512E-09	3.47753E-08	negative
PRJNA729271	stage_E14_vs_E10	ordinary_muscle_development	ENSGALG00010015108	ENSGALG00010015108	-0.094630252	0.08838569	-1.070651273	0.28432626	0.395861026	negative
PRJNA729271	stage_E14_vs_E10	ordinary_muscle_development	ENSGALG00010015109	PPT1	0.397788191	0.107521707	3.699608208	0.000215933	0.000795007	positive
PRJNA729271	stage_E14_vs_E10	ordinary_muscle_development	ENSGALG00010015110	ENSGALG00010015110	0.449305345	0.853174874	0.526627494	0.598452296	0.697929522	positive
PRJNA729271	stage_E14_vs_E10	ordinary_muscle_development	ENSGALG00010015111	ADAMTS2	0.627417245	0.142286318	4.409540223	1.0359E-05	4.84994E-05	positive
PRJNA729271	stage_E14_vs_E10	ordinary_muscle_development	ENSGALG00010015112	CBLN4	-3.582162889	0.419034719	-8.548606421	1.24584E-17	2.21304E-16	negative
PRJNA729271	stage_E14_vs_E10	ordinary_muscle_development	ENSGALG00010015115	CLDN2	-0.035993682	0.393332606	-0.09150953	0.927087734	0.949893031	negative
PRJNA729271	stage_E14_vs_E10	ordinary_muscle_development	ENSGALG00010015117	RASD2	-0.534787078	0.204929331	-2.609617059	0.009064363	0.021887046	negative
PRJNA729271	stage_E14_vs_E10	ordinary_muscle_development	ENSGALG00010015118	GPX8	-0.423906949	0.18465711	-2.295643795	0.021696257	0.046687535	negative
PRJNA729271	stage_E14_vs_E10	ordinary_muscle_development	ENSGALG00010015119	TLR1A	0.241160403	0.216111143	1.115909154	0.264461023	0.373258279	positive
PRJNA729271	stage_E14_vs_E10	ordinary_muscle_development	ENSGALG00010015121	UNC79	-3.512382614	0.263741935	-13.3174977	1.83144E-40	1.41961E-38	negative
PRJNA729271	stage_E14_vs_E10	ordinary_muscle_development	ENSGALG00010015122	ENSGALG00010015122	-1.973185562	0.203348403	-9.703472146	2.91409E-22	7.41049E-21	negative
PRJNA729271	stage_E14_vs_E10	ordinary_muscle_development	ENSGALG00010015123	CEP85L	0.076874407	0.251208539	0.306018289	0.75959072	0.828587259	positive
PRJNA729271	stage_E14_vs_E10	ordinary_muscle_development	ENSGALG00010015124	UXS1	0.01694943	0.09515253	0.178129047	0.85862163	0.902517805	positive
PRJNA729271	stage_E14_vs_E10	ordinary_muscle_development	ENSGALG00010015126	MCM5	-0.205649819	0.177963392	-1.155573722	0.247855562	0.354586544	negative
PRJNA729271	stage_E14_vs_E10	ordinary_muscle_development	ENSGALG00010015127	RPL11	0.286385483	0.151542949	1.889797472	0.058785052	0.108672011	positive
PRJNA729271	stage_E14_vs_E10	ordinary_muscle_development	ENSGALG00010015128	OCRL	0.228484739	0.106136875	2.152736635	0.031339383	0.063649201	positive
PRJNA729271	stage_E14_vs_E10	ordinary_muscle_development	ENSGALG00010015129	PRRG3	-0.268019288	0.125570866	-2.134406624	0.03280951	0.066171399	negative
PRJNA729271	stage_E14_vs_E10	ordinary_muscle_development	ENSGALG00010015131	DOK5	-0.596468026	0.247642952	-2.408580662	0.016014687	0.035894988	negative
PRJNA729271	stage_E14_vs_E10	ordinary_muscle_development	ENSGALG00010015132	GCNT1	-2.077653352	0.293383861	-7.081689301	1.42408E-12	1.59538E-11	negative
PRJNA729271	stage_E14_vs_E10	ordinary_muscle_development	ENSGALG00010015133	RFTN2	1.455152613	0.127285237	11.43221825	2.8864E-30	1.25641E-28	positive
PRJNA729271	stage_E14_vs_E10	ordinary_muscle_development	ENSGALG00010015134	PLN	0.727728997	0.530123929	1.372752591	0.169829274	0.26220042	positive
PRJNA729271	stage_E14_vs_E10	ordinary_muscle_development	ENSGALG00010015136	TCEB3	0.239933794	0.090215652	2.659558395	0.007824317	0.01924219	positive
PRJNA729271	stage_E14_vs_E10	ordinary_muscle_development	ENSGALG00010015137	AMN	0.739455542	0.43122045	1.71479702	0.086382448	0.150820716	positive
PRJNA729271	stage_E14_vs_E10	ordinary_muscle_development	ENSGALG00010015138	ENSGALG00010015138	-2.291671259	0.485024821	-4.724853575	2.30281E-06	1.20069E-05	negative
PRJNA729271	stage_E14_vs_E10	ordinary_muscle_development	ENSGALG00010015140	ASCC3	-0.135262272	0.072727887	-1.859840533	0.062908091	0.115162008	negative
PRJNA729271	stage_E14_vs_E10	ordinary_muscle_development	ENSGALG00010015141	PFDN4	0.223065885	0.135614402	1.644853948	0.099999934	0.170109029	positive
PRJNA729271	stage_E14_vs_E10	ordinary_muscle_development	ENSGALG00010015142	HNRNPAB	0.000447327	0.128452242	0.003482435	0.997221425	0.998283737	positive
PRJNA729271	stage_E14_vs_E10	ordinary_muscle_development	ENSGALG00010015143	EXOC1	0.084267027	0.078581545	1.07235136	0.283562246	0.395119411	positive
PRJNA729271	stage_E14_vs_E10	ordinary_muscle_development	ENSGALG00010015145	CLIC2	0.513343907	0.125710184	4.08355068	4.43528E-05	0.000186022	positive
PRJNA729271	stage_E14_vs_E10	ordinary_muscle_development	ENSGALG00010015146	ENSGALG00010015146	-0.109103909	0.129799876	-0.840554795	0.400597392	0.515918187	negative
PRJNA729271	stage_E14_vs_E10	ordinary_muscle_development	ENSGALG00010015148	ENSGALG00010015148	-0.067980975	0.074616797	-0.911067987	0.362259552	0.478446671	negative
PRJNA729271	stage_E14_vs_E10	ordinary_muscle_development	ENSGALG00010015149	ENSGALG00010015149	0.777463277	0.138450782	5.615448805	1.96053E-08	1.38018E-07	positive
PRJNA729271	stage_E14_vs_E10	ordinary_muscle_development	ENSGALG00010015151	ENSGALG00010015151	-0.2604933	0.340560119	-0.764896667	0.444333079	0.557966558	negative
PRJNA729271	stage_E14_vs_E10	ordinary_muscle_development	ENSGALG00010015152	BOLL	2.149252151	0.278347121	7.721481511	1.14985E-14	1.57534E-13	positive
PRJNA729271	stage_E14_vs_E10	ordinary_muscle_development	ENSGALG00010015153	ERCC6L	-0.224139176	0.23812211	-0.941278305	0.346562258	0.462093637	negative
PRJNA729271	stage_E14_vs_E10	ordinary_muscle_development	ENSGALG00010015154	CYP24A1	-2.494954829	0.700559566	-3.561374292	0.000368919	0.001293849	negative
PRJNA729271	stage_E14_vs_E10	ordinary_muscle_development	ENSGALG00010015155	HMOX1	-0.058753207	0.177490875	-0.331020997	0.740628625	0.813535308	negative
PRJNA729271	stage_E14_vs_E10	ordinary_muscle_development	ENSGALG00010015156	CNOT6	0.348722488	0.073317872	4.75630945	1.97164E-06	1.03999E-05	positive
PRJNA729271	stage_E14_vs_E10	ordinary_muscle_development	ENSGALG00010015157	CDC42BPB	-0.121836737	0.069569478	-1.751295829	0.079894966	0.141246192	negative
PRJNA729271	stage_E14_vs_E10	ordinary_muscle_development	ENSGALG00010015158	PIN4	0.142904874	0.163677898	0.873085958	0.382616227	0.498653319	positive
PRJNA729271	stage_E14_vs_E10	ordinary_muscle_development	ENSGALG00010015160	SIM1	-4.910523928	0.523517525	-9.379865411	6.6057E-21	1.51377E-19	negative
PRJNA729271	stage_E14_vs_E10	ordinary_muscle_development	ENSGALG00010015161	GFPT2	-0.502916498	0.200408505	-2.509456865	0.012091698	0.028162648	negative
PRJNA729271	stage_E14_vs_E10	ordinary_muscle_development	ENSGALG00010015163	PLK2	-0.681304247	0.20920358	-3.256656731	0.001127327	0.003505062	negative
PRJNA729271	stage_E14_vs_E10	ordinary_muscle_development	ENSGALG00010015165	KLF3	-0.177250942	0.118110205	-1.500725048	0.133426692	0.21573005	negative
PRJNA729271	stage_E14_vs_E10	ordinary_muscle_development	ENSGALG00010015166	YTHDF2	-0.356379249	0.180911507	-1.969909237	0.048848774	0.093068214	negative
PRJNA729271	stage_E14_vs_E10	ordinary_muscle_development	ENSGALG00010015167	RAB3C	-2.474911458	0.395722113	-6.254165174	3.99648E-10	3.43996E-09	negative
PRJNA729271	stage_E14_vs_E10	ordinary_muscle_development	ENSGALG00010015169	ENSGALG00010015169	-0.874648427	0.598662616	-1.461003918	0.144014366	0.229596155	negative
PRJNA729271	stage_E14_vs_E10	ordinary_muscle_development	ENSGALG00010015170	EXOC1L	1.679066954	0.559984971	2.998414318	0.002713885	0.00760885	positive
PRJNA729271	stage_E14_vs_E10	ordinary_muscle_development	ENSGALG00010015171	NHSL2	1.544758474	0.198778234	7.771265715	7.77057E-15	1.07681E-13	positive
PRJNA729271	stage_E14_vs_E10	ordinary_muscle_development	ENSGALG00010015172	PPP4R3A	-0.093417042	0.076213536	-1.225727699	0.220301163	0.322747661	negative
PRJNA729271	stage_E14_vs_E10	ordinary_muscle_development	ENSGALG00010015173	ENS-1	-1.463009518	0.697826366	-2.096523704	0.036035759	0.071700526	negative
PRJNA729271	stage_E14_vs_E10	ordinary_muscle_development	ENSGALG00010015174	ST6GAL2	-1.411770001	0.212305434	-6.64971204	2.93667E-11	2.86261E-10	negative
PRJNA729271	stage_E14_vs_E10	ordinary_muscle_development	ENSGALG00010015175	MAPK9	0.149783717	0.068161086	2.197496048	0.027985038	0.057998732	positive
PRJNA729271	stage_E14_vs_E10	ordinary_muscle_development	ENSGALG00010015177	TC2N	-0.143373108	0.157856109	-0.908251885	0.363745157	0.479884049	negative
PRJNA729271	stage_E14_vs_E10	ordinary_muscle_development	ENSGALG00010015180	PITHD1	0.553789843	0.124426328	4.450744888	8.55729E-06	4.05353E-05	positive
PRJNA729271	stage_E14_vs_E10	ordinary_muscle_development	ENSGALG00010015181	VPS13A	0.095116629	0.120223909	0.791162339	0.428849265	0.543229057	positive
PRJNA729271	stage_E14_vs_E10	ordinary_muscle_development	ENSGALG00010015182	PHYKPL	-0.918917626	0.322901958	-2.84581001	0.004429862	0.011706626	negative
PRJNA729271	stage_E14_vs_E10	ordinary_muscle_development	ENSGALG00010015183	SLC5A7	-5.465929425	0.754526117	-7.244188506	4.35036E-13	5.16873E-12	negative
PRJNA729271	stage_E14_vs_E10	ordinary_muscle_development	ENSGALG00010015186	ENSGALG00010015186	4.519457405	0.277486308	16.28713658	1.218E-59	2.84756E-57	positive
PRJNA729271	stage_E14_vs_E10	ordinary_muscle_development	ENSGALG00010015188	SULT1C3	-2.157784175	1.202846405	-1.793898345	0.072829396	0.130489752	negative
PRJNA729271	stage_E14_vs_E10	ordinary_muscle_development	ENSGALG00010015190	FBLN5	-1.396943855	0.192867448	-7.243025538	4.38784E-13	5.20473E-12	negative
PRJNA729271	stage_E14_vs_E10	ordinary_muscle_development	ENSGALG00010015191	TOM1	0.734805699	0.188904177	3.889832994	0.000100313	0.000394476	positive
PRJNA729271	stage_E14_vs_E10	ordinary_muscle_development	ENSGALG00010015192	ENSGALG00010015192	-0.241940037	0.250332805	-0.966473559	0.333807271	0.448965517	negative
PRJNA729271	stage_E14_vs_E10	ordinary_muscle_development	ENSGALG00010015193	LYPLA2	-0.098342783	0.10548818	-0.932263529	0.351200351	0.467364842	negative
PRJNA729271	stage_E14_vs_E10	ordinary_muscle_development	ENSGALG00010015194	TMLHE	0.123912526	0.112875926	1.09777639	0.272302143	0.382351988	positive
PRJNA729271	stage_E14_vs_E10	ordinary_muscle_development	ENSGALG00010015196	PDE4D	-1.17241709	0.221269814	-5.298585779	1.16703E-07	7.35803E-07	negative
PRJNA729271	stage_E14_vs_E10	ordinary_muscle_development	ENSGALG00010015198	NME5	0.445658173	0.217475511	2.04923382	0.040439257	0.07904822	positive
PRJNA729271	stage_E14_vs_E10	ordinary_muscle_development	ENSGALG00010015199	ENSGALG00010015199	-0.324539286	0.165117652	-1.965503278	0.049356017	0.093874224	negative
PRJNA729271	stage_E14_vs_E10	ordinary_muscle_development	ENSGALG00010015200	TBC1D1	-0.443147482	0.085405673	-5.188735881	2.11726E-07	1.29085E-06	negative
PRJNA729271	stage_E14_vs_E10	ordinary_muscle_development	ENSGALG00010015201	GCC2	-0.199043516	0.10849751	-1.834544543	0.066573202	0.120835898	negative
PRJNA729271	stage_E14_vs_E10	ordinary_muscle_development	ENSGALG00010015203	HMGXB4	-0.01889549	0.063311823	-0.298451205	0.765358812	0.833311499	negative
PRJNA729271	stage_E14_vs_E10	ordinary_muscle_development	ENSGALG00010015204	ENSGALG00010015204	0.271989537	0.136856548	1.987406093	0.046877413	0.089829631	positive
PRJNA729271	stage_E14_vs_E10	ordinary_muscle_development	ENSGALG00010015207	AKR1E2	-0.377665156	0.17809458	-2.120587587	0.033956524	0.068162278	negative
PRJNA729271	stage_E14_vs_E10	ordinary_muscle_development	ENSGALG00010015208	GNA14	-2.07420312	0.860593271	-2.410201414	0.015943716	0.035763566	negative
PRJNA729271	stage_E14_vs_E10	ordinary_muscle_development	ENSGALG00010015212	GNAQ	-0.124420745	0.097214048	-1.279863837	0.200593028	0.29975985	negative
PRJNA729271	stage_E14_vs_E10	ordinary_muscle_development	ENSGALG00010015213	RUBCN	0.40361919	0.116877364	3.453356373	0.000553657	0.001854265	positive
PRJNA729271	stage_E14_vs_E10	ordinary_muscle_development	ENSGALG00010015218	PLCL1	-1.385544288	0.155668122	-8.900629548	5.55308E-19	1.09513E-17	negative
PRJNA729271	stage_E14_vs_E10	ordinary_muscle_development	ENSGALG00010015227	FYTTD1	-0.157227542	0.074915412	-2.09873426	0.035840333	0.071380273	negative
PRJNA729271	stage_E14_vs_E10	ordinary_muscle_development	ENSGALG00010015233	ENSGALG00010015233	2.634011275	0.590888072	4.457716107	8.28375E-06	3.93682E-05	positive
PRJNA729271	stage_E14_vs_E10	ordinary_muscle_development	ENSGALG00010015234	TRIP11	-0.470968937	0.143742445	-3.276477854	0.001051106	0.003297787	negative
PRJNA729271	stage_E14_vs_E10	ordinary_muscle_development	ENSGALG00010015241	ENSGALG00010015241	1.048477953	0.147160554	7.12472142	1.04291E-12	1.18658E-11	positive
PRJNA729271	stage_E14_vs_E10	ordinary_muscle_development	ENSGALG00010015248	ENSGALG00010015248	-0.254492041	0.13645253	-1.865059156	0.062173113	0.114041247	negative
PRJNA729271	stage_E14_vs_E10	ordinary_muscle_development	ENSGALG00010015250	RORB	-1.406581957	0.435563882	-3.229335613	0.001240782	0.003830471	negative
PRJNA729271	stage_E14_vs_E10	ordinary_muscle_development	ENSGALG00010015253	ENSGALG00010015253	0.735893809	0.070696185	10.40924362	2.25002E-25	6.98374E-24	positive
PRJNA729271	stage_E14_vs_E10	ordinary_muscle_development	ENSGALG00010015260	RASGEF1C	1.498624538	0.138381562	10.82965474	2.49093E-27	8.98159E-26	positive
PRJNA729271	stage_E14_vs_E10	ordinary_muscle_development	ENSGALG00010015261	IQCG	0.203609125	0.373948167	0.544484884	0.586107847	0.686571298	positive
PRJNA729271	stage_E14_vs_E10	ordinary_muscle_development	ENSGALG00010015283	ENSGALG00010015283	1.432743564	0.47361992	3.025091433	0.00248558	0.007020619	positive
PRJNA729271	stage_E14_vs_E10	ordinary_muscle_development	ENSGALG00010015285	ALG13	-0.299544535	0.405200365	-0.739250408	0.459754957	0.572570504	negative
PRJNA729271	stage_E14_vs_E10	ordinary_muscle_development	ENSGALG00010015291	ZFP64	-0.073545962	0.091617554	-0.802749672	0.422119436	0.53648586	negative
PRJNA729271	stage_E14_vs_E10	ordinary_muscle_development	ENSGALG00010015296	RPL35A	0.447549181	0.195350758	2.291003047	0.021963237	0.047226988	positive
PRJNA729271	stage_E14_vs_E10	ordinary_muscle_development	ENSGALG00010015298	ENSGALG00010015298	0.964680212	0.248697297	3.878933232	0.000104916	0.00041157	positive
PRJNA729271	stage_E14_vs_E10	ordinary_muscle_development	ENSGALG00010015299	GALE	0.026213592	0.093264973	0.281065777	0.778659949	0.843296172	positive
PRJNA729271	stage_E14_vs_E10	ordinary_muscle_development	ENSGALG00010015300	ATXN3	0.136554812	0.126873347	1.07630811	0.281789483	0.392970805	positive
PRJNA729271	stage_E14_vs_E10	ordinary_muscle_development	ENSGALG00010015306	BCAT1	0.592967115	0.090664787	6.540214061	6.14308E-11	5.81227E-10	positive
PRJNA729271	stage_E14_vs_E10	ordinary_muscle_development	ENSGALG00010015310	PGM2	-0.220046893	0.090196059	-2.439650861	0.014701463	0.033361764	negative
PRJNA729271	stage_E14_vs_E10	ordinary_muscle_development	ENSGALG00010015314	GMEB1	-0.109869597	0.064971719	-1.691037242	0.09082969	0.157053126	negative
PRJNA729271	stage_E14_vs_E10	ordinary_muscle_development	ENSGALG00010015318	SALL4	0.856035021	0.160450978	5.335181066	9.54493E-08	6.10564E-07	positive
PRJNA729271	stage_E14_vs_E10	ordinary_muscle_development	ENSGALG00010015320	TRPM6	-1.822727132	0.396686042	-4.594885979	4.32986E-06	2.15897E-05	negative
PRJNA729271	stage_E14_vs_E10	ordinary_muscle_development	ENSGALG00010015327	LARGE1	0.295122841	0.124977661	2.361404733	0.018205849	0.040081072	positive
PRJNA729271	stage_E14_vs_E10	ordinary_muscle_development	ENSGALG00010015328	ENSGALG00010015328	0.719107939	0.385928126	1.863320888	0.062417133	0.114427466	positive

PRJNA729271	stage_E14_vs_E10	ordinary_muscle_development	ENSGALG00010015333	MED7	0.105036514	0.065193834	1.611141835	0.107148812	0.180406787	positive
PRJNA729271	stage_E14_vs_E10	ordinary_muscle_development	ENSGALG00010015339	ENSGALG00010015339	-0.238626674	0.790950492	-0.301696094	0.762883746	0.830927445	negative
PRJNA729271	stage_E14_vs_E10	ordinary_muscle_development	ENSGALG00010015348	CPSF2	-0.15273898	0.059333369	-2.574250907	0.010045739	0.02390233	negative
PRJNA729271	stage_E14_vs_E10	ordinary_muscle_development	ENSGALG00010015353	RELL1	-0.00788937	0.091246336	-0.086462323	0.931098906	0.952925132	negative
PRJNA729271	stage_E14_vs_E10	ordinary_muscle_development	ENSGALG00010015356	ENSGALG00010015356	0.097641182	0.082333562	1.185922003	0.235653103	0.340311945	positive
PRJNA729271	stage_E14_vs_E10	ordinary_muscle_development	ENSGALG00010015362	ATP9A	1.309211984	0.12486938	10.4846519	1.01619E-25	3.25159E-24	positive
PRJNA729271	stage_E14_vs_E10	ordinary_muscle_development	ENSGALG00010015364	CLOCK	0.124154434	0.203311168	0.610662149	0.541423269	0.647305369	positive
PRJNA729271	stage_E14_vs_E10	ordinary_muscle_development	ENSGALG00010015370	ENSGALG00010015370	0.928178306	0.142743878	6.502403584	7.90467E-11	7.36837E-10	positive
PRJNA729271	stage_E14_vs_E10	ordinary_muscle_development	ENSGALG00010015374	LIMS1	-0.624773164	0.150260184	-4.157942217	3.21127E-05	0.000138246	negative
PRJNA729271	stage_E14_vs_E10	ordinary_muscle_development	ENSGALG00010015384	TAF12	0.386691881	0.115115787	3.359155948	0.000781809	0.002528408	positive
PRJNA729271	stage_E14_vs_E10	ordinary_muscle_development	ENSGALG00010015386	SLC24A4	-0.522136769	0.25709723	-2.030892239	0.042265927	0.082112936	negative
PRJNA729271	stage_E14_vs_E10	ordinary_muscle_development	ENSGALG00010015388	PSMD1	-0.019882204	0.122225741	-0.162667894	0.870779927	0.911424589	negative
PRJNA729271	stage_E14_vs_E10	ordinary_muscle_development	ENSGALG00010015390	CCNB3	-0.050660353	0.202089439	-0.250682831	0.802059336	0.860600346	negative
PRJNA729271	stage_E14_vs_E10	ordinary_muscle_development	ENSGALG00010015391	SYN3	-0.665612687	0.355235753	-1.873720992	0.060968891	0.112107583	negative
PRJNA729271	stage_E14_vs_E10	ordinary_muscle_development	ENSGALG00010015392	TMEM165	-0.2123817	0.080702469	-2.631662987	0.00849681	0.020699372	negative
PRJNA729271	stage_E14_vs_E10	ordinary_muscle_development	ENSGALG00010015393	DCX	0.180628336	0.377339718	0.478688904	0.632159961	0.725737259	positive
PRJNA729271	stage_E14_vs_E10	ordinary_muscle_development	ENSGALG00010015397	SRD5A3	-0.280378903	0.080448603	-3.485192923	0.000491782	0.001670585	negative
PRJNA729271	stage_E14_vs_E10	ordinary_muscle_development	ENSGALG00010015404	BEND4	-1.341085479	0.231415798	-5.795133665	6.82668E-09	5.0693E-08	negative
PRJNA729271	stage_E14_vs_E10	ordinary_muscle_development	ENSGALG00010015407	TIMP3	-0.111003176	0.175517071	-0.632435208	0.527102534	0.634686096	negative
PRJNA729271	stage_E14_vs_E10	ordinary_muscle_development	ENSGALG00010015409	FAM13B	0.566465635	0.131665179	4.302319258	1.6902E-05	7.6156E-05	positive
PRJNA729271	stage_E14_vs_E10	ordinary_muscle_development	ENSGALG00010015410	HTR2B	-0.550685716	0.195255958	-2.82032733	0.004797468	0.012562295	negative
PRJNA729271	stage_E14_vs_E10	ordinary_muscle_development	ENSGALG00010015413	CARNMT1	0.011431108	0.106258305	0.107578494	0.914330058	0.940811683	positive
PRJNA729271	stage_E14_vs_E10	ordinary_muscle_development	ENSGALG00010015416	CAPN6	0.430320163	0.162806795	2.64313392	0.008214254	0.020109037	positive
PRJNA729271	stage_E14_vs_E10	ordinary_muscle_development	ENSGALG00010015422	ENSGALG00010015422	-0.777410529	0.485644707	-1.600780402	0.109425566	0.183472941	negative
PRJNA729271	stage_E14_vs_E10	ordinary_muscle_development	ENSGALG00010015430	HMGCL	0.281542713	0.130286912	2.160943945	0.03069967	0.062586739	positive
PRJNA729271	stage_E14_vs_E10	ordinary_muscle_development	ENSGALG00010015431	RIN3	-0.305921081	0.156605482	-1.953450659	0.050766218	0.096014658	negative
PRJNA729271	stage_E14_vs_E10	ordinary_muscle_development	ENSGALG00010015436	SLC30A9	-0.202558471	0.098021793	-2.066463639	0.038784722	0.07636302	negative
PRJNA729271	stage_E14_vs_E10	ordinary_muscle_development	ENSGALG00010015440	ENSGALG00010015440	0.755333848	0.356968917	2.115965317	0.034347757	0.068852267	positive
PRJNA729271	stage_E14_vs_E10	ordinary_muscle_development	ENSGALG00010015443	PAK3	0.371134789	0.100817617	3.681249386	0.000232094	0.000848474	positive
PRJNA729271	stage_E14_vs_E10	ordinary_muscle_development	ENSGALG00010015449	HDX	-0.042626928	0.090502109	-0.471004808	0.637637302	0.730809891	negative
PRJNA729271	stage_E14_vs_E10	ordinary_muscle_development	ENSGALG00010015466	NMRK1	-0.109770742	0.124902129	-0.878854049	0.379480416	0.495546724	negative
PRJNA729271	stage_E14_vs_E10	ordinary_muscle_development	ENSGALG00010015469	TMEM33	-0.232048462	0.072321231	-3.208580085	0.001333922	0.004076574	negative
PRJNA729271	stage_E14_vs_E10	ordinary_muscle_development	ENSGALG00010015472	LGMN	-0.196373498	0.134200421	-1.463285258	0.14338934	0.228760512	negative
PRJNA729271	stage_E14_vs_E10	ordinary_muscle_development	ENSGALG00010015477	ARMC9	-0.524923652	0.099231146	-5.289908173	1.22378E-07	7.6824E-07	negative
PRJNA729271	stage_E14_vs_E10	ordinary_muscle_development	ENSGALG00010015478	FBXO7	0.113983834	0.061284309	1.859918735	0.062897024	0.115162008	positive
PRJNA729271	stage_E14_vs_E10	ordinary_muscle_development	ENSGALG00010015481	GOLGA5	0.032071625	0.069834437	0.459252282	0.64605301	0.738120784	positive
PRJNA729271	stage_E14_vs_E10	ordinary_muscle_development	ENSGALG00010015483	SGCD	0.225046779	0.162311021	1.386515694	0.165589491	0.256814005	positive
PRJNA729271	stage_E14_vs_E10	ordinary_muscle_development	ENSGALG00010015485	BCAS1	-0.313127025	0.193696893	-1.616582592	0.105968412	0.178719197	negative
PRJNA729271	stage_E14_vs_E10	ordinary_muscle_development	ENSGALG00010015486	MTFR1L	0.616277307	0.119386494	5.162035393	2.44279E-07	1.47596E-06	positive
PRJNA729271	stage_E14_vs_E10	ordinary_muscle_development	ENSGALG00010015487	RPS6KA6	-0.018333807	0.116464237	-0.157420062	0.874913804	0.914272625	negative
PRJNA729271	stage_E14_vs_E10	ordinary_muscle_development	ENSGALG00010015490	RANBP2	-0.187774321	0.082846783	-2.266525199	0.023419246	0.049884199	negative
PRJNA729271	stage_E14_vs_E10	ordinary_muscle_development	ENSGALG00010015493	OSTF1	-0.134649421	0.141352314	-0.952580238	0.340802794	0.45574384	negative
PRJNA729271	stage_E14_vs_E10	ordinary_muscle_development	ENSGALG00010015494	CHRDL1	-2.225284249	0.242693022	-9.169131563	4.76845E-20	1.03162E-18	negative
PRJNA729271	stage_E14_vs_E10	ordinary_muscle_development	ENSGALG00010015498	MID2	1.263937289	0.131924341	9.580773951	9.63204E-22	2.35593E-20	positive
PRJNA729271	stage_E14_vs_E10	ordinary_muscle_development	ENSGALG00010015502	BPIFB6	4.794363656	1.214940079	3.946172934	7.94103E-05	0.00031841	positive
PRJNA729271	stage_E14_vs_E10	ordinary_muscle_development	ENSGALG00010015505	CHGA	-0.667505426	0.452876895	-1.473922456	0.140502471	0.22503683	negative
PRJNA729271	stage_E14_vs_E10	ordinary_muscle_development	ENSGALG00010015507	AMMECR1	-0.259364902	0.067385099	-3.848994916	0.000118603	0.000461147	negative
PRJNA729271	stage_E14_vs_E10	ordinary_muscle_development	ENSGALG00010015508	FUCA1	0.072046745	0.104470379	0.68963802	0.490421851	0.601053922	positive
PRJNA729271	stage_E14_vs_E10	ordinary_muscle_development	ENSGALG00010015511	BPIFB4	1.691755649	0.272753582	6.202505698	5.55712E-10	4.69682E-09	positive
PRJNA729271	stage_E14_vs_E10	ordinary_muscle_development	ENSGALG00010015516	SELENON	0.001814579	0.112322635	0.016155064	0.987110684	0.991389635	positive
PRJNA729271	stage_E14_vs_E10	ordinary_muscle_development	ENSGALG00010015520	UBE3A	-0.037388517	0.078444825	-0.476621846	0.633631424	0.727311332	negative
PRJNA729271	stage_E14_vs_E10	ordinary_muscle_development	ENSGALG00010015521	DKC1	-0.109680041	0.08124337	-1.350018357	0.177010095	0.270907119	negative
PRJNA729271	stage_E14_vs_E10	ordinary_muscle_development	ENSGALG00010015523	MRPL22	-0.144094851	0.144130484	-0.999752772	0.317430167	0.431310261	negative
PRJNA729271	stage_E14_vs_E10	ordinary_muscle_development	ENSGALG00010015532	NXT2	-0.324388456	0.110290224	-2.941225828	0.003269161	0.008968336	negative
PRJNA729271	stage_E14_vs_E10	ordinary_muscle_development	ENSGALG00010015533	PSAT1	0.0702463	0.27640556	0.254142139	0.79938575	0.859067055	positive
PRJNA729271	stage_E14_vs_E10	ordinary_muscle_development	ENSGALG00010015540	ACSL4	-0.8358138	0.153632513	-5.440344514	5.31776E-08	3.51326E-07	negative
PRJNA729271	stage_E14_vs_E10	ordinary_muscle_development	ENSGALG00010015541	RASEF	-1.906302906	0.335040305	-5.689771878	1.27209E-08	9.15086E-08	negative
PRJNA729271	stage_E14_vs_E10	ordinary_muscle_development	ENSGALG00010015542	CNGA3	-0.16621125	0.269464328	-0.616820976	0.537352815	0.643926012	negative
PRJNA729271	stage_E14_vs_E10	ordinary_muscle_development	ENSGALG00010015544	RTCB	-0.102622213	0.072849494	-1.408688067	0.158927425	0.248613536	negative
PRJNA729271	stage_E14_vs_E10	ordinary_muscle_development	ENSGALG00010015551	VWA3B	-0.124882155	0.344091293	-0.362933204	0.716654782	0.795051779	negative
PRJNA729271	stage_E14_vs_E10	ordinary_muscle_development	ENSGALG00010015553	ASCL4	-5.432491867	0.873658419	-6.218095936	5.03224E-10	4.28065E-09	negative
PRJNA729271	stage_E14_vs_E10	ordinary_muscle_development	ENSGALG00010015555	PNRC2	-0.083359817	0.101987103	-0.817356456	0.413724752	0.528551056	negative
PRJNA729271	stage_E14_vs_E10	ordinary_muscle_development	ENSGALG00010015557	IRS4	-0.252000597	0.192244011	-1.310837178	0.189912779	0.28689794	negative
PRJNA729271	stage_E14_vs_E10	ordinary_muscle_development	ENSGALG00010015558	MPP1	-0.055327763	0.259637436	-0.213096246	0.831251893	0.882193307	negative
PRJNA729271	stage_E14_vs_E10	ordinary_muscle_development	ENSGALG00010015562	IRF9	0.151355614	0.280146659	0.540272777	0.589008929	0.689078646	positive
PRJNA729271	stage_E14_vs_E10	ordinary_muscle_development	ENSGALG00010015563	B3GNT7	-2.212243553	0.317987458	-6.957015115	3.47558E-12	3.75959E-11	negative
PRJNA729271	stage_E14_vs_E10	ordinary_muscle_development	ENSGALG00010015564	PKD2L2	-0.103031179	0.285454444	-0.36093738	0.718146259	0.796261763	negative
PRJNA729271	stage_E14_vs_E10	ordinary_muscle_development	ENSGALG00010015565	ENSGALG00010015565	-0.456118158	0.451942721	-1.009238863	0.312860093	0.426614021	negative
PRJNA729271	stage_E14_vs_E10	ordinary_muscle_development	ENSGALG00010015567	ENSGALG00010015567	0.283587472	0.551749406	0.513978753	0.607266845	0.704952963	positive
PRJNA729271	stage_E14_vs_E10	ordinary_muscle_development	ENSGALG00010015570	LIMCH1	1.624076351	0.127776859	12.71025412	5.18656E-37	3.35622E-35	positive
PRJNA729271	stage_E14_vs_E10	ordinary_muscle_development	ENSGALG00010015571	DUSP15	-0.472139631	0.307370274	-1.536061459	0.124523308	0.203582827	negative
PRJNA729271	stage_E14_vs_E10	ordinary_muscle_development	ENSGALG00010015573	MAN1C1	-0.059619288	0.124274193	-0.479739893	0.631412358	0.725223623	negative
PRJNA729271	stage_E14_vs_E10	ordinary_muscle_development	ENSGALG00010015574	C14orf132	-0.775619281	0.26018259	-2.981057573	0.002872548	0.007996463	negative
PRJNA729271	stage_E14_vs_E10	ordinary_muscle_development	ENSGALG00010015580	GEMIN5	-0.299789828	0.070823784	-4.232897652	2.307E-05	0.000101579	negative
PRJNA729271	stage_E14_vs_E10	ordinary_muscle_development	ENSGALG00010015583	PRDM4	-0.306439428	0.135826259	-2.256113293	0.024063537	0.051054006	negative
PRJNA729271	stage_E14_vs_E10	ordinary_muscle_development	ENSGALG00010015584	TTLL9	-0.206826107	0.258268548	-0.80081802	0.423237006	0.537623381	negative
PRJNA729271	stage_E14_vs_E10	ordinary_muscle_development	ENSGALG00010015585	BDKRB1	-1.138285301	0.328391859	-3.466240924	0.00052779	0.0017808	negative
PRJNA729271	stage_E14_vs_E10	ordinary_muscle_development	ENSGALG00010015588	BDKRB2	-2.739355869	0.517213871	-5.296369688	1.18128E-07	7.43491E-07	negative
PRJNA729271	stage_E14_vs_E10	ordinary_muscle_development	ENSGALG00010015589	FRMD3	1.952648366	0.178724104	10.92548976	8.70694E-28	3.23608E-26	positive
PRJNA729271	stage_E14_vs_E10	ordinary_muscle_development	ENSGALG00010015596	UCHL1	-0.12356256	0.503182373	-0.24556218	0.806021169	0.863700513	negative
PRJNA729271	stage_E14_vs_E10	ordinary_muscle_development	ENSGALG00010015605	LDLRAP1	-0.384870856	0.056990366	-6.753261705	1.44558E-11	1.46019E-10	negative
PRJNA729271	stage_E14_vs_E10	ordinary_muscle_development	ENSGALG00010015606	TMEM131	0.239983078	0.108287921	2.21615741	0.026680724	0.055677669	positive
PRJNA729271	stage_E14_vs_E10	ordinary_muscle_development	ENSGALG00010015610	ENSGALG00010015610	1.909094141	0.286417877	6.665415435	2.63918E-11	2.59004E-10	positive
PRJNA729271	stage_E14_vs_E10	ordinary_muscle_development	ENSGALG00010015613	PWP1	-0.153035913	0.079296505	-1.929920038	0.053616746	0.100527065	negative
PRJNA729271	stage_E14_vs_E10	ordinary_muscle_development	ENSGALG00010015614	ATG2B	-0.128175389	0.107250483	-1.195103134	0.232046738	0.336374166	negative
PRJNA729271	stage_E14_vs_E10	ordinary_muscle_development	ENSGALG00010015616	NADK2	-0.649949243	0.117860321	-5.514572163	3.49629E-08	2.38376E-07	negative
PRJNA729271	stage_E14_vs_E10	ordinary_muscle_development	ENSGALG00010015617	SRSF10	-0.022625933	0.055133569	-0.410383964	0.681524308	0.767038744	negative
PRJNA729271	stage_E14_vs_E10	ordinary_muscle_development	ENSGALG00010015618	NCL	-0.431105987	0.100789262	-4.277300753	1.89173E-05	8.45012E-05	negative
PRJNA729271	stage_E14_vs_E10	ordinary_muscle_development	ENSGALG00010015620	PDRG1	0.222583188	0.143985676	1.545870352	0.122135882	0.200448326	positive
PRJNA729271	stage_E14_vs_E10	ordinary_muscle_development	ENSGALG00010015621	IDNK	-0.526676783	0.158946591	-3.313545632	0.000921211	0.00292379	negative
PRJNA729271	stage_E14_vs_E10	ordinary_muscle_development	ENSGALG00010015624	ITGA1	-0.010613474	0.129880933	-0.081716955	0.934871795	0.954677816	negative
PRJNA729271	stage_E14_vs_E10	ordinary_muscle_development	ENSGALG00010015626	XKR7	0.052788388	0.496969073	0.106220671	0.915407271	0.941362463	positive
PRJNA729271	stage_E14_vs_E10	ordinary_muscle_development	ENSGALG00010015628	UBQLN1	0.174721974	0.118284291	1.477135905	0.139639211	0.223901588	positive
PRJNA729271	stage_E14_vs_E10	ordinary_muscle_development	ENSGALG00010015631	GSKIP	-0.364741704	0.103974909	-3.507978107	0.000451526	0.001546155	negative
PRJNA729271	stage_E14_vs_E10	ordinary_muscle_development	ENSGALG00010015632	COL4A5	-0.331265738	0.161780843	-2.04762029	0.04059722	0.079253427	negative
PRJNA729271	stage_E14_vs_E10	ordinary_muscle_development	ENSGALG00010015633	CCM2L	0.221124392	0.186700467	1.184380495	0.236262478	0.341030135	positive
PRJNA729271	stage_E14_vs_E10	ordinary_muscle_development	ENSGALG00010015634	ENSGALG00010015634	2.127850964	1.062292031	2.003075333	0.045169203	0.087099588	positive
PRJNA729271	stage_E14_vs_E10	ordinary_muscle_development	ENSGALG00010015635	ABTB3	1.334340413	0.122946213	10.85304198	1.92897E-27	7.04291E-26	positive
PRJNA729271	stage_E14_vs_E10	ordinary_muscle_development	ENSGALG00010015636	APBB2	-0.153206742	0.12421188	-1.233430667	0.217415129	0.319423505	negative
PRJNA729271	stage_E14_vs_E10	ordinary_muscle_development	ENSGALG00010015642	ENSGALG00010015642	1.33287796	0.638368859	2.087943265	0.036802953	0.072951677	positive
PRJNA729271	stage_E14_vs_E10	ordinary_muscle_development	ENSGALG00010015643	ENSGALG00010015643	-2.079483603	0.120780294	-17.21707678	1.97726E-66	5.84905E-64	negative
PRJNA729271	stage_E14_vs_E10	ordinary_muscle_development	ENSGALG00010015645	MACO1	0.600008284	0.093425546	6.422314978	1.34217E-10	1.21593E-09	positive
PRJNA729271	stage_E14_vs_E10	ordinary_muscle_development	ENSGALG00010015647	PDZD2	-1.667010064	0.140117655	-11.89721642	1.22361E-32	6.01227E-31	negative
PRJNA729271	stage_E14_vs_E10	ordinary_muscle_development	ENSGALG00010015650	ENSGALG00010015650	1.768227865	1.218917028	1.450654823	0.146876001	0.23336267	positive
PRJNA729271	stage_E14_vs_E10	ordinary_muscle_development	ENSGALG00010015653	HCK	0.703016368	0.532251007	1.320836145	0.186556005	0.282769977	positive
PRJNA729271	stage_E14_vs_E10	ordinary_muscle_development	ENSGALG00010015655	ENSGALG00010015655	-0.092322872	0.478336191	-0.193008335	0.84695244	0.893491676	negative
PRJNA729271	stage_E14_vs_E10	ordinary_muscle_development	ENSGALG00010015657	NMUR1	-2.182374672	0.974545435	-2.239377041	0.025131394	0.052963006	negative
PRJNA729271	stage_E14_vs_E10	ordinary_muscle_development	ENSGALG00010015660	KLHL3	0.191261634	0.121582522	1.573101386	0.115695344	0.192140698	positive
PRJNA729271	stage_E14_vs_E10	ordinary_muscle_development	ENSGALG00010015668	ZNF217	0.100364666	0.134774904	0.744683636	0.456462994	0.569569387	positive
PRJNA729271	stage_E14_vs_E10	ordinary_muscle_development	ENSGALG00010015669	INPP4A	-0.009353216	0.130197259	-0.071838812	0.942730185	0.959746736	negative
PRJNA729271	stage_E14_vs_E10	ordinary_muscle_development	ENSGALG00010015670	ENSGALG00010015670	-0.844549301	0.130105164	-6.491281921	8.5109E-11	7.87272E-10	negative
PRJNA729271	stage_E14_vs_E10	ordinary_muscle_development	ENSGALG00010015674	RFKL	-0.245819888	0.194211696	-1.265731637	0.205609178	0.305542275	negative
PRJNA729271	stage_E14_vs_E10	ordinary_muscle_development	ENSGALG00010015676	TGM2	1.121165443	0.192588709	5.821553357	5.83032E-09	4.37425E-08	positive
PRJNA729271	stage_E14_vs_E10	ordinary_muscle_development	ENSGALG00010015677	GRHL3	-0.239098226	0.233283568	-1.024925278	0.305398446	0.419160163	negative
PRJNA729271	stage_E14_vs_E10	ordinary_muscle_development	ENSGALG00010015680	ENSGALG00010015680	-0.086960188	0.102746846	-0.84635384	0.397355381	0.512790799	negative
PRJNA729271	stage_E14_vs_E10	ordinary_muscle_development	ENSGALG00010015684	PDE6D	0.07502047	0.095762699	0.7833997	0.433392432	0.547453011	positive
PRJNA729271	stage_E14_vs_E10	ordinary_muscle_development	ENSGALG00010015686	CNOT8	-0.118785874	0.050369165	-2.358305405	0.018358582	0.040368272	negative
PRJNA729271	stage_E14_vs_E10	ordinary_muscle_development	ENSGALG00010015691	EXOC3L4	-2.626307155	0.86525237	-3.03530767	0.002402904	0.006818735	negative
PRJNA729271	stage_E14_vs_E10	ordinary_muscle_development	ENSGALG00010015693	COA5	0.132141312	0.106758697	1.237756885	0.215806219	0.317510267	positive
PRJNA729271	stage_E14_vs_E10	ordinary_muscle_development	ENSGALG00010015695	ASAH1	-0.110145885	0.094229463	-1.16891132	0.242439362	0.34866143	negative
PRJNA729271	stage_E14_vs_E10	ordinary_muscle_development	ENSGALG00010015697	FAXDC2	-0.130969885	0.350631986	-0.373525207	0.708757588	0.788747888	negative
PRJNA729271	stage_E14_vs_E10	ordinary_muscle_development	ENSGALG00010015698	TNFAIP2	-0.612279571	0.200709159	-3.050581123	0.00228399	0.006537605	negative
PRJNA729271	stage_E14_vs_E10	ordinary_muscle_development	ENSGALG00010015701	UNC50	0.145951297	0.082276084	1.773921301	0.076076169	0.135515798	positive
PRJNA729271	stage_E14_vs_E10	ordinary_muscle_development	ENSGALG00010015703	GK5	0.276623312	0.185380955	1.492188407	0.135649761	0.218628445	positive
PRJNA729271	stage_E14_vs_E10	ordinary_muscle_development	ENSGALG00010015705	NPY6R	-3.357965532	0.930150312	-3.610132136	0.000306041	0.001093435	negative
PRJNA729271	stage_E14_vs_E10	ordinary_muscle_development	ENSGALG00010015706	ENSGALG00010015706	0.060842008	0.165675392	0.367236242	0.713442808	0.792744615	positive
PRJNA729271	stage_E14_vs_E10	ordinary_muscle_development	ENSGALG00010015709	HNRNPA0	0.105122728	0.111456601	0.943171843	0.345593013	0.461098189	positive
PRJNA729271	stage_E14_vs_E10	ordinary_muscle_development	ENSGALG00010015711	LBFABP	3.329701969	0.663526477	5.018190057	5.21606E-07	3.00027E-06	positive
PRJNA729271	stage_E14_vs_E10	ordinary_muscle_development	ENSGALG00010015716	MGAT4A	-1.156482515	0.13288013	-8.703201273	3.22651E-18	5.98825E-17	negative
PRJNA729271	stage_E14_vs_E10	ordinary_muscle_development	ENSGALG00010015719	XRN1	0.107219252	0.099132866	1.081571198	0.279443108	0.390347617	positive
PRJNA729271	stage_E14_vs_E10	ordinary_muscle_development	ENSGALG00010015729	RHCE	-0.083121535	0.196686082	-0.422610151	0.672579719	0.759153016	negative

PRJNA729271	stage_E14_vs_E10	ordinary_muscle_development	ENSGALG00010015731	PHF6	-0.399407204	0.067262482	-5.938038424	2.88452E-09	2.25276E-08	negative
PRJNA729271	stage_E14_vs_E10	ordinary_muscle_development	ENSGALG00010015733	COL4A6	-0.110070265	0.152696483	-0.720843486	0.471005817	0.583224783	negative
PRJNA729271	stage_E14_vs_E10	ordinary_muscle_development	ENSGALG00010015734	TSHZ2	-1.190787358	0.165426647	-7.198280205	6.09767E-13	7.1394E-12	negative
PRJNA729271	stage_E14_vs_E10	ordinary_muscle_development	ENSGALG00010015738	CRACDL	-3.742995757	0.438673061	-8.53254072	1.43168E-17	2.52768E-16	negative
PRJNA729271	stage_E14_vs_E10	ordinary_muscle_development	ENSGALG00010015742	HPRT1	0.764619131	0.085475397	8.945487872	3.7031E-19	7.44473E-18	positive
PRJNA729271	stage_E14_vs_E10	ordinary_muscle_development	ENSGALG00010015746	PAPOLB	-0.125472337	0.071881013	-1.745556042	0.080888126	0.142788782	negative
PRJNA729271	stage_E14_vs_E10	ordinary_muscle_development	ENSGALG00010015749	ATR	-0.273127003	0.13288139	-2.055419516	0.039838502	0.078013927	negative
PRJNA729271	stage_E14_vs_E10	ordinary_muscle_development	ENSGALG00010015751	MYOM3	2.934795097	0.537994378	5.455066482	4.89545E-08	3.25055E-07	positive
PRJNA729271	stage_E14_vs_E10	ordinary_muscle_development	ENSGALG00010015753	RPRD1B	-0.025030922	0.077802882	-0.321722302	0.747663081	0.81900609	negative
PRJNA729271	stage_E14_vs_E10	ordinary_muscle_development	ENSGALG00010015756	PCM1	-0.367625286	0.103032386	-3.568055639	0.00035964	0.001263657	negative
PRJNA729271	stage_E14_vs_E10	ordinary_muscle_development	ENSGALG00010015758	PCDH19	0.107870636	0.169444024	0.636615169	0.524375542	0.632137681	positive
PRJNA729271	stage_E14_vs_E10	ordinary_muscle_development	ENSGALG00010015759	EIF5	-0.030183598	0.091587665	-0.329559641	0.741732723	0.814439498	negative
PRJNA729271	stage_E14_vs_E10	ordinary_muscle_development	ENSGALG00010015764	TTI1	0.043618164	0.094937619	0.459440261	0.645918042	0.738082934	positive
PRJNA729271	stage_E14_vs_E10	ordinary_muscle_development	ENSGALG00010015765	ENSGALG00010015765	0.108950773	0.371433039	0.293325475	0.769273383	0.836066408	positive
PRJNA729271	stage_E14_vs_E10	ordinary_muscle_development	ENSGALG00010015766	TLE1	-0.568218372	0.09781938	-5.808852702	6.29024E-09	4.69743E-08	negative
PRJNA729271	stage_E14_vs_E10	ordinary_muscle_development	ENSGALG00010015769	TMEM50A	0.068398552	0.09932627	0.688624996	0.491059287	0.601377523	positive
PRJNA729271	stage_E14_vs_E10	ordinary_muscle_development	ENSGALG00010015774	PLS1	-0.454382424	0.336519999	-1.350238992	0.176939335	0.270884628	negative
PRJNA729271	stage_E14_vs_E10	ordinary_muscle_development	ENSGALG00010015776	SPOCK1	-3.536767131	0.189877521	-18.62657098	1.95666E-77	8.34168E-75	negative
PRJNA729271	stage_E14_vs_E10	ordinary_muscle_development	ENSGALG00010015778	PABIR3	-0.44100658	0.086273117	-5.111749683	3.19189E-07	1.89772E-06	negative
PRJNA729271	stage_E14_vs_E10	ordinary_muscle_development	ENSGALG00010015782	ATG4A	-0.059742507	0.081482067	-0.73319822	0.463437541	0.576018449	negative
PRJNA729271	stage_E14_vs_E10	ordinary_muscle_development	ENSGALG00010015785	FGL1	-0.564534497	0.194561472	-2.901573947	0.003712931	0.010010964	negative
PRJNA729271	stage_E14_vs_E10	ordinary_muscle_development	ENSGALG00010015788	TRPC1	-0.430298205	0.127987082	-3.362044039	0.000773678	0.002504346	negative
PRJNA729271	stage_E14_vs_E10	ordinary_muscle_development	ENSGALG00010015792	MARK3	-0.01205184	0.081213765	-0.148396522	0.882029845	0.919058486	negative
PRJNA729271	stage_E14_vs_E10	ordinary_muscle_development	ENSGALG00010015793	RSRP1	0.402531824	0.233161762	1.726405821	0.084274429	0.147745265	positive
PRJNA729271	stage_E14_vs_E10	ordinary_muscle_development	ENSGALG00010015795	PSMD10	0.330057283	0.132906431	2.483380831	0.013014187	0.030004874	positive
PRJNA729271	stage_E14_vs_E10	ordinary_muscle_development	ENSGALG00010015796	LARP1	-0.18369975	0.140937261	-1.303415071	0.192433094	0.289859472	negative
PRJNA729271	stage_E14_vs_E10	ordinary_muscle_development	ENSGALG00010015797	PIP5K1B	-0.266357476	0.162144704	-1.642714627	0.100441991	0.170640724	negative
PRJNA729271	stage_E14_vs_E10	ordinary_muscle_development	ENSGALG00010015798	IL22RA1	1.035099035	0.256601395	4.033879219	5.48635E-05	0.000226566	positive
PRJNA729271	stage_E14_vs_E10	ordinary_muscle_development	ENSGALG00010015800	BVES	1.057539736	0.181932483	5.81281428	6.14312E-09	4.59703E-08	positive
PRJNA729271	stage_E14_vs_E10	ordinary_muscle_development	ENSGALG00010015801	VSTM2L	0.484160868	0.21005586	2.304914839	0.021171343	0.045720891	positive
PRJNA729271	stage_E14_vs_E10	ordinary_muscle_development	ENSGALG00010015802	TRPC7	-5.039848032	1.079569623	-4.668386296	3.03575E-06	1.55324E-05	negative
PRJNA729271	stage_E14_vs_E10	ordinary_muscle_development	ENSGALG00010015803	SYF2	0.190816666	0.159088176	1.199439651	0.230357038	0.334303691	positive
PRJNA729271	stage_E14_vs_E10	ordinary_muscle_development	ENSGALG00010015804	CKB	-1.249683726	0.160118329	-7.804751219	5.9619E-15	8.34149E-14	negative
PRJNA729271	stage_E14_vs_E10	ordinary_muscle_development	ENSGALG00010015807	ENSGALG00010015807	0.327761108	0.939575021	0.348839742	0.727209626	0.802810626	positive
PRJNA729271	stage_E14_vs_E10	ordinary_muscle_development	ENSGALG00010015809	PCOLCE2	-2.629968085	0.256756813	-10.24303134	1.27188E-24	3.68717E-23	negative
PRJNA729271	stage_E14_vs_E10	ordinary_muscle_development	ENSGALG00010015810	LIPT1	0.003213907	0.102071398	0.031486855	0.974881275	0.982882666	positive
PRJNA729271	stage_E14_vs_E10	ordinary_muscle_development	ENSGALG00010015811	CTNNBL1	0.240525455	0.052184398	4.609144969	4.04328E-06	2.02164E-05	positive
PRJNA729271	stage_E14_vs_E10	ordinary_muscle_development	ENSGALG00010015813	ENSGALG00010015813	0.249131423	0.138562954	1.797965591	0.072182461	0.129458645	positive
PRJNA729271	stage_E14_vs_E10	ordinary_muscle_development	ENSGALG00010015814	PAQR9	-2.069599966	0.53083335	-3.898775327	9.66804E-05	0.000381231	negative
PRJNA729271	stage_E14_vs_E10	ordinary_muscle_development	ENSGALG00010015816	MITD1	-0.409845949	0.21284821	-1.925531578	0.054162889	0.101393656	negative
PRJNA729271	stage_E14_vs_E10	ordinary_muscle_development	ENSGALG00010015820	ENSGALG00010015820	-1.069781623	0.536402173	-1.994364819	0.0461122	0.08867025	negative
PRJNA729271	stage_E14_vs_E10	ordinary_muscle_development	ENSGALG00010015822	WFDC2	-0.843262163	0.500209405	-1.685818289	0.091830804	0.158443936	negative
PRJNA729271	stage_E14_vs_E10	ordinary_muscle_development	ENSGALG00010015825	RUNX3	-2.872714184	0.497238033	-5.777342028	7.58899E-09	5.59809E-08	negative
PRJNA729271	stage_E14_vs_E10	ordinary_muscle_development	ENSGALG00010015827	ENSGALG00010015827	0.823328998	0.150398896	5.47430213	4.39239E-08	2.9367E-07	positive
PRJNA729271	stage_E14_vs_E10	ordinary_muscle_development	ENSGALG00010015829	SMAD5	0.401912163	0.047356126	8.487015284	2.12012E-17	3.68921E-16	positive
PRJNA729271	stage_E14_vs_E10	ordinary_muscle_development	ENSGALG00010015831	LYG2	2.483149547	0.627878443	3.954825292	7.65906E-05	0.00030787	positive
PRJNA729271	stage_E14_vs_E10	ordinary_muscle_development	ENSGALG00010015832	RAB39B	-0.193466051	0.084006179	-2.30299786	0.021278964	0.045919097	negative
PRJNA729271	stage_E14_vs_E10	ordinary_muscle_development	ENSGALG00010015834	STARD8	1.042096314	0.132806521	7.846725475	4.27041E-15	6.05079E-14	positive
PRJNA729271	stage_E14_vs_E10	ordinary_muscle_development	ENSGALG00010015837	GDA	0.295582394	0.170482844	1.733795534	0.082954368	0.145819012	positive
PRJNA729271	stage_E14_vs_E10	ordinary_muscle_development	ENSGALG00010015838	IFNLR1	0.189209325	1.265804853	0.149477484	0.881176875	0.918436644	positive
PRJNA729271	stage_E14_vs_E10	ordinary_muscle_development	ENSGALG00010015839	U2SURP	-0.199276367	0.070196892	-2.838820387	0.004528064	0.011933506	negative
PRJNA729271	stage_E14_vs_E10	ordinary_muscle_development	ENSGALG00010015840	MTUS1	-0.703415164	0.148125179	-4.748788624	2.04639E-06	1.07667E-05	negative
PRJNA729271	stage_E14_vs_E10	ordinary_muscle_development	ENSGALG00010015843	ENSGALG00010015843	0.12414992	0.361778729	0.343165338	0.731474079	0.80659694	positive
PRJNA729271	stage_E14_vs_E10	ordinary_muscle_development	ENSGALG00010015845	SAP30L	0.053212199	0.125480561	0.424067266	0.671516757	0.758322563	positive
PRJNA729271	stage_E14_vs_E10	ordinary_muscle_development	ENSGALG00010015848	UBE2B	0.42343318	0.162068366	2.612682478	0.008983472	0.021717049	positive
PRJNA729271	stage_E14_vs_E10	ordinary_muscle_development	ENSGALG00010015852	ZFAND5	0.078376951	0.285950761	0.274092473	0.784013558	0.84770077	positive
PRJNA729271	stage_E14_vs_E10	ordinary_muscle_development	ENSGALG00010015855	NIPAL3	-0.349781757	0.079489869	-4.400331291	1.08086E-05	5.03601E-05	negative
PRJNA729271	stage_E14_vs_E10	ordinary_muscle_development	ENSGALG00010015856	TXNDC9	0.046025726	0.133106487	0.345781237	0.729507127	0.805090292	positive
PRJNA729271	stage_E14_vs_E10	ordinary_muscle_development	ENSGALG00010015858	CHST2	-0.200698492	0.089714095	-2.23708986	0.025280468	0.053232746	negative
PRJNA729271	stage_E14_vs_E10	ordinary_muscle_development	ENSGALG00010015861	PPP2CA	-0.054299053	0.066689378	-0.814208414	0.415525566	0.530150786	negative
PRJNA729271	stage_E14_vs_E10	ordinary_muscle_development	ENSGALG00010015862	PDGFRL	-0.70450766	0.209935167	-3.355834431	0.000791259	0.002556118	negative
PRJNA729271	stage_E14_vs_E10	ordinary_muscle_development	ENSGALG00010015863	ENSGALG00010015863	-0.827184068	0.529730909	-1.561517469	0.118401705	0.195537509	negative
PRJNA729271	stage_E14_vs_E10	ordinary_muscle_development	ENSGALG00010015865	VRK1	-0.208321439	0.135460635	-1.537874372	0.124079335	0.203017266	negative
PRJNA729271	stage_E14_vs_E10	ordinary_muscle_development	ENSGALG00010015866	ARHGEF6	-0.140826319	0.070732875	-1.990959926	0.046485293	0.089245606	negative
PRJNA729271	stage_E14_vs_E10	ordinary_muscle_development	ENSGALG00010015867	PICALML	0.00722116	0.056572096	0.127645266	0.898429707	0.930195615	positive
PRJNA729271	stage_E14_vs_E10	ordinary_muscle_development	ENSGALG00010015869	SLC7A2	0.924586668	0.195056783	4.740089792	2.13624E-06	1.11907E-05	positive
PRJNA729271	stage_E14_vs_E10	ordinary_muscle_development	ENSGALG00010015870	EIF5B	-0.33249533	0.156100031	-2.130014505	0.033170416	0.06681084	negative
PRJNA729271	stage_E14_vs_E10	ordinary_muscle_development	ENSGALG00010015872	TMC1	-0.312388067	0.337428724	-0.925789787	0.354555236	0.470931746	negative
PRJNA729271	stage_E14_vs_E10	ordinary_muscle_development	ENSGALG00010015874	CLIC4	0.242578709	0.114271603	2.12282582	0.03376845	0.067831719	positive
PRJNA729271	stage_E14_vs_E10	ordinary_muscle_development	ENSGALG00010015875	DNTTIP1	0.302241017	0.139347362	2.168975523	0.030084542	0.061583878	positive
PRJNA729271	stage_E14_vs_E10	ordinary_muscle_development	ENSGALG00010015876	SKP1	-0.086824175	0.077139408	-1.125548899	0.260356509	0.36864976	negative
PRJNA729271	stage_E14_vs_E10	ordinary_muscle_development	ENSGALG00010015877	REV1	-0.074016769	0.103349416	-0.716179855	0.4738803	0.58598319	negative
PRJNA729271	stage_E14_vs_E10	ordinary_muscle_development	ENSGALG00010015879	TRMT61A	0.165885268	0.117265546	1.414612165	0.15718224	0.24646869	positive
PRJNA729271	stage_E14_vs_E10	ordinary_muscle_development	ENSGALG00010015881	GAB3	0.373167996	0.18095798	2.062180381	0.039190566	0.077036514	positive
PRJNA729271	stage_E14_vs_E10	ordinary_muscle_development	ENSGALG00010015886	AFF3	-2.051570287	0.211468045	-9.701561687	2.96919E-22	7.53737E-21	negative
PRJNA729271	stage_E14_vs_E10	ordinary_muscle_development	ENSGALG00010015887	SLC9A9	0.407040659	0.180070978	2.260445645	0.023793606	0.05058497	positive
PRJNA729271	stage_E14_vs_E10	ordinary_muscle_development	ENSGALG00010015889	SH3BP5	0.574509221	0.165055458	3.480704179	0.000500097	0.001695652	positive
PRJNA729271	stage_E14_vs_E10	ordinary_muscle_development	ENSGALG00010015892	UBE2C	0.317861575	0.21878176	1.452870544	0.146259701	0.232510897	positive
PRJNA729271	stage_E14_vs_E10	ordinary_muscle_development	ENSGALG00010015893	TCF7	-0.603045246	0.414642545	-1.45437378	0.145842705	0.23200066	negative
PRJNA729271	stage_E14_vs_E10	ordinary_muscle_development	ENSGALG00010015894	CEP78	-0.555083241	0.107568648	-5.160269765	2.46594E-07	1.48933E-06	negative
PRJNA729271	stage_E14_vs_E10	ordinary_muscle_development	ENSGALG00010015895	RBMXL1	-0.094670956	0.084303894	-1.122972523	0.261449164	0.369763454	negative
PRJNA729271	stage_E14_vs_E10	ordinary_muscle_development	ENSGALG00010015896	LONRF2	0.119708975	0.077850137	1.537684844	0.124125691	0.203047274	positive
PRJNA729271	stage_E14_vs_E10	ordinary_muscle_development	ENSGALG00010015898	ENSGALG00010015898	0.973185044	0.21818314	4.460404433	8.18051E-06	3.89414E-05	positive
PRJNA729271	stage_E14_vs_E10	ordinary_muscle_development	ENSGALG00010015899	SMARCA1	-0.287331874	0.115045907	-2.497541036	0.012505799	0.028989534	negative
PRJNA729271	stage_E14_vs_E10	ordinary_muscle_development	ENSGALG00010015900	ENSGALG00010015900	-0.116278296	0.164380432	-0.707373098	0.47933465	0.59141605	negative
PRJNA729271	stage_E14_vs_E10	ordinary_muscle_development	ENSGALG00010015902	ENSGALG00010015902	-0.321322169	0.538347852	-0.596867189	0.550596055	0.655622264	negative
PRJNA729271	stage_E14_vs_E10	ordinary_muscle_development	ENSGALG00010015904	MTMR7	-0.481864716	0.262884708	-1.83298876	0.066804243	0.121119119	negative
PRJNA729271	stage_E14_vs_E10	ordinary_muscle_development	ENSGALG00010015905	ANPEP	-0.070272488	0.217725422	-0.322757388	0.746878987	0.818540066	negative
PRJNA729271	stage_E14_vs_E10	ordinary_muscle_development	ENSGALG00010015906	BAG5	0.472762221	0.10052325	4.703013683	2.56349E-06	1.32849E-05	positive
PRJNA729271	stage_E14_vs_E10	ordinary_muscle_development	ENSGALG00010015908	CHRD	0.352062297	0.137189807	2.56624239	0.010280696	0.024365384	positive
PRJNA729271	stage_E14_vs_E10	ordinary_muscle_development	ENSGALG00010015909	TNNC2	2.369510212	0.106601618	22.22771332	1.8534E-109	2.6865E-106	positive
PRJNA729271	stage_E14_vs_E10	ordinary_muscle_development	ENSGALG00010015910	C5orf15	-0.104475904	0.092900833	-1.124595984	0.260760276	0.368962283	negative
PRJNA729271	stage_E14_vs_E10	ordinary_muscle_development	ENSGALG00010015911	TM9SF2L	0.046605298	0.077995014	0.597542021	0.550145561	0.655229167	positive
PRJNA729271	stage_E14_vs_E10	ordinary_muscle_development	ENSGALG00010015912	ENSGALG00010015912	-0.081921851	0.262193913	-0.31244757	0.754700403	0.824680161	negative
PRJNA729271	stage_E14_vs_E10	ordinary_muscle_development	ENSGALG00010015913	CHST10	0.022360634	0.103004536	0.217083973	0.828142904	0.880053621	positive
PRJNA729271	stage_E14_vs_E10	ordinary_muscle_development	ENSGALG00010015914	SRRM1	-0.176711031	0.117237696	-1.507288497	0.131736725	0.213378458	negative
PRJNA729271	stage_E14_vs_E10	ordinary_muscle_development	ENSGALG00010015916	COA8	0.544353976	0.144859984	3.757793985	0.000171418	0.000645881	positive
PRJNA729271	stage_E14_vs_E10	ordinary_muscle_development	ENSGALG00010015917	TMEM63A	-0.131232501	0.109073429	-1.203157377	0.228915425	0.332711229	negative
PRJNA729271	stage_E14_vs_E10	ordinary_muscle_development	ENSGALG00010015918	BICD1	-1.27774123	0.187741541	-6.805852472	1.00453E-11	1.03047E-10	negative
PRJNA729271	stage_E14_vs_E10	ordinary_muscle_development	ENSGALG00010015919	ENSGALG00010015919	-1.051458574	0.246861192	-4.259310937	2.05058E-05	9.10636E-05	negative
PRJNA729271	stage_E14_vs_E10	ordinary_muscle_development	ENSGALG00010015921	SNX21	1.321331573	0.109523153	12.06440411	1.62837E-33	8.36992E-32	positive
PRJNA729271	stage_E14_vs_E10	ordinary_muscle_development	ENSGALG00010015923	RCAN3	-0.56547422	0.119921114	-4.715384967	2.41254E-06	1.2543E-05	negative
PRJNA729271	stage_E14_vs_E10	ordinary_muscle_development	ENSGALG00010015927	GLA	0.234278744	0.172728868	1.356338094	0.17499162	0.268469891	positive
PRJNA729271	stage_E14_vs_E10	ordinary_muscle_development	ENSGALG00010015928	CAPN7	0.140527712	0.120841204	1.16291221	0.244865123	0.351417818	positive
PRJNA729271	stage_E14_vs_E10	ordinary_muscle_development	ENSGALG00010015929	SLC6A12	-1.740003813	0.803108938	-2.166585043	0.030266509	0.06185155	negative
PRJNA729271	stage_E14_vs_E10	ordinary_muscle_development	ENSGALG00010015931	RBM47	-0.251402318	0.282533939	-0.88981281	0.373566407	0.489720997	negative
PRJNA729271	stage_E14_vs_E10	ordinary_muscle_development	ENSGALG00010015932	ACOT8	0.014478434	0.146286038	0.098973449	0.921159351	0.945489647	positive
PRJNA729271	stage_E14_vs_E10	ordinary_muscle_development	ENSGALG00010015933	ENSGALG00010015933	6.615764488	0.649890264	10.17981782	2.44018E-24	6.96267E-23	positive
PRJNA729271	stage_E14_vs_E10	ordinary_muscle_development	ENSGALG00010015934	TMC2	-2.053338282	1.288779615	-1.593242365	0.111105831	0.185774485	negative
PRJNA729271	stage_E14_vs_E10	ordinary_muscle_development	ENSGALG00010015935	EPHX1	0.844320997	0.107898745	7.825123458	5.07159E-15	7.11643E-14	positive
PRJNA729271	stage_E14_vs_E10	ordinary_muscle_development	ENSGALG00010015936	RPL36AL	0.310817234	0.139479914	2.228401394	0.02585376	0.054262024	positive
PRJNA729271	stage_E14_vs_E10	ordinary_muscle_development	ENSGALG00010015937	ENSGALG00010015937	0.61835371	0.191204461	3.233992061	0.001220728	0.003774415	positive
PRJNA729271	stage_E14_vs_E10	ordinary_muscle_development	ENSGALG00010015939	SUN2	0.112572575	0.079754611	1.411486732	0.158101145	0.247561423	positive
PRJNA729271	stage_E14_vs_E10	ordinary_muscle_development	ENSGALG00010015942	AP3S2	0.443628585	0.129754965	3.418971952	0.000628582	0.002074294	positive
PRJNA729271	stage_E14_vs_E10	ordinary_muscle_development	ENSGALG00010015946	TLE4	0.579653951	0.097286405	5.958221491	2.54998E-09	1.99902E-08	positive
PRJNA729271	stage_E14_vs_E10	ordinary_muscle_development	ENSGALG00010015954	CTSA	0.214700731	0.096611052	2.222320594	0.026261647	0.054961389	positive
PRJNA729271	stage_E14_vs_E10	ordinary_muscle_development	ENSGALG00010015957	ENSGALG00010015957	0.559145796	0.219381501	2.548737211	0.010811373	0.02550202	positive
PRJNA729271	stage_E14_vs_E10	ordinary_muscle_development	ENSGALG00010015959	TRNAU1AP	0.465006491	0.129699594	3.585257874	0.000336745	0.001191971	positive
PRJNA729271	stage_E14_vs_E10	ordinary_muscle_development	ENSGALG00010015960	CHRNA9	-1.894135296	0.901045669	-2.102152378	0.03553993	0.070830645	negative
PRJNA729271	stage_E14_vs_E10	ordinary_muscle_development	ENSGALG00010015963	DIPK2A	-0.454206292	0.108536376	-4.18483009	2.85379E-05	0.000123998	negative
PRJNA729271	stage_E14_vs_E10	ordinary_muscle_development	ENSGALG00010015964	KLC1	0.347470374	0.086273962	4.027523071	5.63675E-05	0.000232512	positive
PRJNA729271	stage_E14_vs_E10	ordinary_muscle_development	ENSGALG00010015967	ZSWIM1	-0.153603156	0.199587835	-0.769601793	0.441536141	0.555513095	negative
PRJNA729271	stage_E14_vs_E10	ordinary_muscle_development	ENSGALG00010015970	THPO	0.333885947	0.376777211	0.886162798	0.375529807	0.491805616	positive
PRJNA729271	stage_E14_vs_E10	ordinary_muscle_development	ENSGALG00010015971	ENSGALG00010015971	-0.633215732	0.155373862	-4.0754328	4.59288E-05	0.000192132	negative
PRJNA729271	stage_E14_vs_E10	ordinary_muscle_development	ENSGALG00010015972	FSTL4	0.210147696	0.297378066	0.706668447	0.479772542	0.591855575	positive
PRJNA729271	stage_E14_vs_E10	ordinary_muscle_development	ENSGALG00010015974	VPS37A	0.064664025	0.165719921	0.390200677	0.696388161	0.77886932	positive
PRJNA729271	stage_E14_vs_E10	ordinary_muscle_development	ENSGALG00010015975	BTK	0.591974801	0.176617649	3.351730733	0.000803081	0.002590843	positive
PRJNA729271	stage_E14_vs_E10	ordinary_muscle_development	ENSGALG00010015979	ENSGALG00010015979	2.539256001	0.203152042	12.49928861	7.53223E-36	4.40873E-34	positive
PRJNA729271	stage_E14_vs_E10	ordinary_muscle_development	ENSGALG00010015980	EXOSC7	-0.095898505	0.084895968	-1.129600229	0.258644715	0.366942854	negative
PRJNA729271	stage_E14_vs_E10	ordinary_muscle_development	ENSGALG00010015983	CNOT7	0.04008302	0.064427402	0.622142424	0.533848221	0.641104389	positive
PRJNA729271	stage_E14_vs_E10	ordinary_muscle_development	ENSGALG00010015984	PDCL3	0.922039458	0.107252397	8.596912374	8.18895E-18	1.47452E-16	positive

PRJNA729271	stage_E14_vs_E10	ordinary_muscle_development	ENSGALG00010015985	TIMM8A	0.021289801	0.110252243	0.193100846	0.84687999	0.893480272	positive
PRJNA729271	stage_E14_vs_E10	ordinary_muscle_development	ENSGALG00010015986	NEURL2	1.199176419	0.241708412	4.961252322	7.00401E-07	3.95339E-06	positive
PRJNA729271	stage_E14_vs_E10	ordinary_muscle_development	ENSGALG00010015987	POLR2H	0.002229105	0.094259602	0.023648571	0.981132929	0.987446602	positive
PRJNA729271	stage_E14_vs_E10	ordinary_muscle_development	ENSGALG00010015989	ENAH	-0.103108576	0.162902048	-0.632948314	0.526767396	0.63433525	negative
PRJNA729271	stage_E14_vs_E10	ordinary_muscle_development	ENSGALG00010015992	ATP10A	-1.778765877	0.158480924	-11.22384844	3.11417E-29	1.28238E-27	negative
PRJNA729271	stage_E14_vs_E10	ordinary_muscle_development	ENSGALG00010015994	ENSGALG00010015994	0.151505282	0.102668598	1.475673046	0.140031685	0.224456406	positive
PRJNA729271	stage_E14_vs_E10	ordinary_muscle_development	ENSGALG00010016004	XRCC3	-0.431502052	0.167890904	-2.570133586	0.010165931	0.024148667	negative
PRJNA729271	stage_E14_vs_E10	ordinary_muscle_development	ENSGALG00010016006	PLTP	-1.072494891	0.31844812	-3.367879491	0.000757487	0.002456325	negative
PRJNA729271	stage_E14_vs_E10	ordinary_muscle_development	ENSGALG00010016008	TAF7	-0.072463423	0.091233469	-0.794263592	0.427041995	0.541555005	negative
PRJNA729271	stage_E14_vs_E10	ordinary_muscle_development	ENSGALG00010016011	ADD1	0.049631233	0.080481551	0.616678387	0.53744688	0.643985494	positive
PRJNA729271	stage_E14_vs_E10	ordinary_muscle_development	ENSGALG00010016015	ZDHHC3	-0.13084216	0.082478482	-1.586379347	0.112653283	0.188036542	negative
PRJNA729271	stage_E14_vs_E10	ordinary_muscle_development	ENSGALG00010016016	ZDHHC2	0.504636596	0.274912436	1.835626654	0.066412892	0.120574661	positive
PRJNA729271	stage_E14_vs_E10	ordinary_muscle_development	ENSGALG00010016017	RAPGEF6	0.182639043	0.071098379	2.568821484	0.010204501	0.024216477	positive
PRJNA729271	stage_E14_vs_E10	ordinary_muscle_development	ENSGALG00010016022	EPHB3	-0.620988655	0.164469571	-3.77570544	0.000159555	0.000604326	negative
PRJNA729271	stage_E14_vs_E10	ordinary_muscle_development	ENSGALG00010016025	PCIF1	0.312581489	0.107440068	2.909356774	0.003621733	0.009796048	positive
PRJNA729271	stage_E14_vs_E10	ordinary_muscle_development	ENSGALG00010016027	COPS2	-0.430688025	0.15010307	-2.869281923	0.004114049	0.010964418	negative
PRJNA729271	stage_E14_vs_E10	ordinary_muscle_development	ENSGALG00010016030	TGM4	-0.532857323	0.728755422	-0.731188143	0.464664242	0.577246159	negative
PRJNA729271	stage_E14_vs_E10	ordinary_muscle_development	ENSGALG00010016040	RPSA2	0.175637588	0.105887247	1.658722772	0.097171669	0.166018781	positive
PRJNA729271	stage_E14_vs_E10	ordinary_muscle_development	ENSGALG00010016041	ENSGALG00010016041	0.068753972	0.090419576	0.760388125	0.44702262	0.560304629	positive
PRJNA729271	stage_E14_vs_E10	ordinary_muscle_development	ENSGALG00010016043	ADAMTSL1	-0.408852839	0.192248502	-2.126689338	0.033445904	0.067267709	negative
PRJNA729271	stage_E14_vs_E10	ordinary_muscle_development	ENSGALG00010016047	SRP9	0.230521056	0.088381891	2.608238567	0.00910095	0.021949796	positive
PRJNA729271	stage_E14_vs_E10	ordinary_muscle_development	ENSGALG00010016049	ZFYVE21	0.150405178	0.121809306	1.23475934	0.216920084	0.318855924	positive
PRJNA729271	stage_E14_vs_E10	ordinary_muscle_development	ENSGALG00010016053	DRP2	-1.605536172	0.528703235	-3.036743611	0.002391488	0.006791657	negative
PRJNA729271	stage_E14_vs_E10	ordinary_muscle_development	ENSGALG00010016057	SOHO1	-1.704315138	0.580055364	-2.938193908	0.003301305	0.009037283	negative
PRJNA729271	stage_E14_vs_E10	ordinary_muscle_development	ENSGALG00010016059	EPDR1	-0.51972827	0.201228457	-2.582777198	0.009800859	0.023423488	negative
PRJNA729271	stage_E14_vs_E10	ordinary_muscle_development	ENSGALG00010016060	ENSGALG00010016060	2.264249336	1.360444939	1.664344709	0.096043563	0.164518015	positive
PRJNA729271	stage_E14_vs_E10	ordinary_muscle_development	ENSGALG00010016066	C14orf2	0.362231394	0.149533074	2.422416554	0.015417666	0.03473948	positive
PRJNA729271	stage_E14_vs_E10	ordinary_muscle_development	ENSGALG00010016067	AHSG	5.546796925	0.846086662	6.555825982	5.53347E-11	5.26297E-10	positive
PRJNA729271	stage_E14_vs_E10	ordinary_muscle_development	ENSGALG00010016069	GALK2	0.494900571	0.144547713	3.423786929	0.00061755	0.002043696	positive
PRJNA729271	stage_E14_vs_E10	ordinary_muscle_development	ENSGALG00010016073	SAXO1	0.939858727	1.14959408	0.817581693	0.413596084	0.528433252	positive
PRJNA729271	stage_E14_vs_E10	ordinary_muscle_development	ENSGALG00010016076	RAB3GAP2	0.052774994	0.090147257	0.585430945	0.558257987	0.662080635	positive
PRJNA729271	stage_E14_vs_E10	ordinary_muscle_development	ENSGALG00010016083	CDH22	0.599301715	0.308338076	1.943651344	0.05193751	0.097948765	positive
PRJNA729271	stage_E14_vs_E10	ordinary_muscle_development	ENSGALG00010016086	TDRD9	-0.128671704	0.324833506	-0.396115861	0.692019558	0.775056425	negative
PRJNA729271	stage_E14_vs_E10	ordinary_muscle_development	ENSGALG00010016087	IARS2	0.087280742	0.089785047	0.972107768	0.330996938	0.445767966	positive
PRJNA729271	stage_E14_vs_E10	ordinary_muscle_development	ENSGALG00010016091	HMX1	-1.125176978	0.622775366	-1.806714007	0.070806883	0.127385598	negative
PRJNA729271	stage_E14_vs_E10	ordinary_muscle_development	ENSGALG00010016094	FGF7	0.159901121	0.184834025	0.865106523	0.386980325	0.503071924	positive
PRJNA729271	stage_E14_vs_E10	ordinary_muscle_development	ENSGALG00010016097	FNIP1	0.087968594	0.110818253	0.793809607	0.427306279	0.541700587	positive
PRJNA729271	stage_E14_vs_E10	ordinary_muscle_development	ENSGALG00010016100	SFRP4	-0.537197378	0.406267934	-1.322273634	0.186077049	0.282162028	negative
PRJNA729271	stage_E14_vs_E10	ordinary_muscle_development	ENSGALG00010016103	BPNT1	-0.180566023	0.084092742	-2.147224823	0.031775387	0.064426387	negative
PRJNA729271	stage_E14_vs_E10	ordinary_muscle_development	ENSGALG00010016107	UVSSA	-0.013737905	0.088917718	-0.154501326	0.877214466	0.915752516	negative
PRJNA729271	stage_E14_vs_E10	ordinary_muscle_development	ENSGALG00010016109	MLH1	-0.043240871	0.097738611	-0.442413404	0.658190081	0.747931605	negative
PRJNA729271	stage_E14_vs_E10	ordinary_muscle_development	ENSGALG00010016111	EPB41	0.035718306	0.101725572	0.351124165	0.7254952	0.801357961	positive
PRJNA729271	stage_E14_vs_E10	ordinary_muscle_development	ENSGALG00010016113	DTWD1	0.003100213	0.147808113	0.020974582	0.983265932	0.988928649	positive
PRJNA729271	stage_E14_vs_E10	ordinary_muscle_development	ENSGALG00010016114	HAUS6	-0.300917038	0.150860159	-1.994675325	0.046078302	0.088640344	negative
PRJNA729271	stage_E14_vs_E10	ordinary_muscle_development	ENSGALG00010016115	RD3L	0.305222823	0.456610602	0.668453212	0.503844339	0.613098026	positive
PRJNA729271	stage_E14_vs_E10	ordinary_muscle_development	ENSGALG00010016117	ENSGALG00010016117	0.115358517	0.233927334	0.493138253	0.621914904	0.717099398	positive
PRJNA729271	stage_E14_vs_E10	ordinary_muscle_development	ENSGALG00010016118	EPRS	0.006404848	0.087467185	0.073225719	0.941626501	0.959027272	positive
PRJNA729271	stage_E14_vs_E10	ordinary_muscle_development	ENSGALG00010016119	FETUB	1.536324674	1.416154874	1.084856397			positive
PRJNA729271	stage_E14_vs_E10	ordinary_muscle_development	ENSGALG00010016121	SLC35C2	-0.329590727	0.09635248	-3.42067717	0.000624654	0.002064379	negative
PRJNA729271	stage_E14_vs_E10	ordinary_muscle_development	ENSGALG00010016122	AFG2B	-0.056959955	0.130374711	-0.436894195	0.662188082	0.751425507	negative
PRJNA729271	stage_E14_vs_E10	ordinary_muscle_development	ENSGALG00010016127	ENSGALG00010016127	0.816571403	0.160480598	5.088287379	3.61312E-07	2.13241E-06	positive
PRJNA729271	stage_E14_vs_E10	ordinary_muscle_development	ENSGALG00010016129	CENPI	-0.101448077	0.230191659	-0.440711349	0.659421979	0.748908688	negative
PRJNA729271	stage_E14_vs_E10	ordinary_muscle_development	ENSGALG00010016131	SMYD1	1.887391338	0.21735171	8.683581742	3.83497E-18	7.09029E-17	positive
PRJNA729271	stage_E14_vs_E10	ordinary_muscle_development	ENSGALG00010016137	STARD3NL	-0.329523577	0.140056524	-2.352789917	0.018633158	0.04086664	negative
PRJNA729271	stage_E14_vs_E10	ordinary_muscle_development	ENSGALG00010016139	LURAP1L	-0.729191259	0.192361095	-3.790741874	0.000150198	0.000572173	negative
PRJNA729271	stage_E14_vs_E10	ordinary_muscle_development	ENSGALG00010016141	RRP15	-0.405637701	0.1756244	-2.309688757	0.020905392	0.045180208	negative
PRJNA729271	stage_E14_vs_E10	ordinary_muscle_development	ENSGALG00010016147	FABP1	1.751169718	0.805890705	2.172961802	0.029783192	0.061053228	positive
PRJNA729271	stage_E14_vs_E10	ordinary_muscle_development	ENSGALG00010016150	POLG	-0.016738365	0.093790301	-0.178465841	0.858357145	0.902436123	negative
PRJNA729271	stage_E14_vs_E10	ordinary_muscle_development	ENSGALG00010016159	ASPG	-1.829620701	0.190046866	-9.627207939	6.13744E-22	1.51554E-20	negative
PRJNA729271	stage_E14_vs_E10	ordinary_muscle_development	ENSGALG00010016165	KK34	-0.741763035	1.205649192	-0.615239524	0.538396549	0.644697066	negative
PRJNA729271	stage_E14_vs_E10	ordinary_muscle_development	ENSGALG00010016168	PLIN2	0.146117895	0.229389592	0.636985721	0.524134145	0.632004361	positive
PRJNA729271	stage_E14_vs_E10	ordinary_muscle_development	ENSGALG00010016169	RHOH	-0.400974155	0.121485433	-3.30059452	0.000964802	0.003050787	negative
PRJNA729271	stage_E14_vs_E10	ordinary_muscle_development	ENSGALG00010016173	ENSGALG00010016173	-0.717915922	0.297701685	-2.411527912	0.015885836	0.035661327	negative
PRJNA729271	stage_E14_vs_E10	ordinary_muscle_development	ENSGALG00010016177	ENSGALG00010016177	0.129387202	1.673406657	0.077319641	0.938369267	0.957054779	positive
PRJNA729271	stage_E14_vs_E10	ordinary_muscle_development	ENSGALG00010016181	FANCI	-0.419667643	0.170390289	-2.462978648	0.013778812	0.031527053	negative
PRJNA729271	stage_E14_vs_E10	ordinary_muscle_development	ENSGALG00010016182	ELMO2	0.053235509	0.061459774	0.866184586	0.386388945	0.502485892	positive
PRJNA729271	stage_E14_vs_E10	ordinary_muscle_development	ENSGALG00010016190	THNSL2	0.33112924	0.152095727	2.177110732	0.029472307	0.060510068	positive
PRJNA729271	stage_E14_vs_E10	ordinary_muscle_development	ENSGALG00010016193	TCAIM	-0.075778545	0.134259743	-0.564417475	0.572470049	0.674411034	negative
PRJNA729271	stage_E14_vs_E10	ordinary_muscle_development	ENSGALG00010016197	SNRPN	-0.139939587	0.114424607	-1.222985079	0.221335332	0.323968054	negative
PRJNA729271	stage_E14_vs_E10	ordinary_muscle_development	ENSGALG00010016199	RLBP1	-3.058907805	0.361845706	-8.453624728	2.82396E-17	4.85567E-16	negative
PRJNA729271	stage_E14_vs_E10	ordinary_muscle_development	ENSGALG00010016200	MICU3	0.089285242	0.117436493	0.760285324	0.447084052	0.560304629	positive
PRJNA729271	stage_E14_vs_E10	ordinary_muscle_development	ENSGALG00010016205	CD40LG	1.743772299	1.260155951	1.383774998	0.166427361	0.257786343	positive
PRJNA729271	stage_E14_vs_E10	ordinary_muscle_development	ENSGALG00010016206	KNG1	0.073596266	1.184589601	0.06212807	0.950460843	0.965647222	positive
PRJNA729271	stage_E14_vs_E10	ordinary_muscle_development	ENSGALG00010016209	ADAMTS7	0.038966041	0.164741954	0.236527735	0.813023189	0.868891183	positive
PRJNA729271	stage_E14_vs_E10	ordinary_muscle_development	ENSGALG00010016211	TACC3	-0.25803916	0.160819934	-1.604522236	0.108598987	0.182340127	negative
PRJNA729271	stage_E14_vs_E10	ordinary_muscle_development	ENSGALG00010016215	SPATA17	-0.654307423	0.309820581	-2.111891408	0.034695765	0.069434503	negative
PRJNA729271	stage_E14_vs_E10	ordinary_muscle_development	ENSGALG00010016218	N4BP2	0.048881595	0.075261998	0.649485752	0.516024453	0.624667985	positive
PRJNA729271	stage_E14_vs_E10	ordinary_muscle_development	ENSGALG00010016221	TMEM200B	0.716298326	0.200202108	3.577876049	0.000346398	0.001222853	positive
PRJNA729271	stage_E14_vs_E10	ordinary_muscle_development	ENSGALG00010016224	MOSPD1	0.552229338	0.115568438	4.778375042	1.76718E-06	9.40008E-06	positive
PRJNA729271	stage_E14_vs_E10	ordinary_muscle_development	ENSGALG00010016225	P4HA2	0.555654631	0.156462679	3.551355733	0.000383252	0.001336036	positive
PRJNA729271	stage_E14_vs_E10	ordinary_muscle_development	ENSGALG00010016229	ENSGALG00010016229	0.282656184	0.22075896	1.280383748	0.200410207	0.299602511	positive
PRJNA729271	stage_E14_vs_E10	ordinary_muscle_development	ENSGALG00010016235	DDX27	-0.251288573	0.122940454	-2.043986052	0.040954925	0.079876432	negative
PRJNA729271	stage_E14_vs_E10	ordinary_muscle_development	ENSGALG00010016236	GPATCH2	-0.075939139	0.063591178	-1.19417726	0.232408636	0.336573402	negative
PRJNA729271	stage_E14_vs_E10	ordinary_muscle_development	ENSGALG00010016240	EIF4A2	0.368641302	0.100319549	3.674670658	0.000238156	0.000868666	positive
PRJNA729271	stage_E14_vs_E10	ordinary_muscle_development	ENSGALG00010016241	TOPAZ1	-0.757002037	0.820211597	-0.922935056	0.35604105	0.472429058	negative
PRJNA729271	stage_E14_vs_E10	ordinary_muscle_development	ENSGALG00010016242	PDS5A	-0.13838241	0.092166234	-1.501443682	0.133240843	0.215501676	negative
PRJNA729271	stage_E14_vs_E10	ordinary_muscle_development	ENSGALG00010016245	FAM96A	0.071224059	0.102059477	0.697868156	0.485259651	0.596745452	positive
PRJNA729271	stage_E14_vs_E10	ordinary_muscle_development	ENSGALG00010016247	KIF26A	-1.804459612	0.166486136	-10.83849776	2.26154E-27	8.17482E-26	negative
PRJNA729271	stage_E14_vs_E10	ordinary_muscle_development	ENSGALG00010016251	MPDZ	-0.336440683	0.072543952	-4.637749567	3.52223E-06	1.77781E-05	negative
PRJNA729271	stage_E14_vs_E10	ordinary_muscle_development	ENSGALG00010016253	EDN3	-0.558601739	0.203441478	-2.745761314	0.006037067	0.015352155	negative
PRJNA729271	stage_E14_vs_E10	ordinary_muscle_development	ENSGALG00010016257	ENSGALG00010016257	-0.078250017	0.106409847	-0.73536444	0.462117573	0.574689081	negative
PRJNA729271	stage_E14_vs_E10	ordinary_muscle_development	ENSGALG00010016258	CTNNB1	-0.500942901	0.076918381	-6.512655301	7.38338E-11	6.90911E-10	negative
PRJNA729271	stage_E14_vs_E10	ordinary_muscle_development	ENSGALG00010016260	INTS6L	-0.1889214	0.144741015	-1.305237496	0.19181199	0.289150555	negative
PRJNA729271	stage_E14_vs_E10	ordinary_muscle_development	ENSGALG00010016262	SPG11	0.460855916	0.059995405	7.681520251	1.57212E-14	2.13369E-13	positive
PRJNA729271	stage_E14_vs_E10	ordinary_muscle_development	ENSGALG00010016263	HGFAC	0.610785544	1.348423588	0.452962667			positive
PRJNA729271	stage_E14_vs_E10	ordinary_muscle_development	ENSGALG00010016266	PDLIM4	0.029033457	0.120333844	0.241274241	0.809342574	0.865979229	positive
PRJNA729271	stage_E14_vs_E10	ordinary_muscle_development	ENSGALG00010016270	RFC4	-0.101447094	0.10810132	-0.938444545	0.348016003	0.46381868	negative
PRJNA729271	stage_E14_vs_E10	ordinary_muscle_development	ENSGALG00010016274	ZNF831	-0.240858452	0.540528537	-0.445598032	0.655887632	0.746428391	negative
PRJNA729271	stage_E14_vs_E10	ordinary_muscle_development	ENSGALG00010016276	ACTR2	-0.377863827	0.107187719	-3.525252987	0.000423079	0.00145631	negative
PRJNA729271	stage_E14_vs_E10	ordinary_muscle_development	ENSGALG00010016279	PRELID3B	-0.138734392	0.105564478	-1.314214727	0.188773954	0.285415506	negative
PRJNA729271	stage_E14_vs_E10	ordinary_muscle_development	ENSGALG00010016281	SLC22A4	-0.140573922	0.201362315	-0.698114351	0.485105684	0.596647539	negative
PRJNA729271	stage_E14_vs_E10	ordinary_muscle_development	ENSGALG00010016286	ULK4	-0.442027879	0.131283054	-3.366983524	0.000759952	0.002463768	negative
PRJNA729271	stage_E14_vs_E10	ordinary_muscle_development	ENSGALG00010016287	BCL11B	-4.292546495	0.343699387	-12.48924687	8.54588E-36	4.9748E-34	negative
PRJNA729271	stage_E14_vs_E10	ordinary_muscle_development	ENSGALG00010016289	IRF1	0.073073193	0.272954997	0.267711505	0.788921393	0.85160974	positive
PRJNA729271	stage_E14_vs_E10	ordinary_muscle_development	ENSGALG00010016292	PHKA1	0.059677382	0.15035235	0.396916855	0.691428774	0.774686268	positive
PRJNA729271	stage_E14_vs_E10	ordinary_muscle_development	ENSGALG00010016295	UBE2K	-0.230658265	0.092874821	-2.483539264	0.013008399	0.030001074	negative
PRJNA729271	stage_E14_vs_E10	ordinary_muscle_development	ENSGALG00010016299	SRSF4	0.168514679	0.105299055	1.600343708	0.109522356	0.183613988	positive
PRJNA729271	stage_E14_vs_E10	ordinary_muscle_development	ENSGALG00010016303	ENSGALG00010016303	0.376120313	0.203360556	1.849524416	0.064382126	0.117459901	positive
PRJNA729271	stage_E14_vs_E10	ordinary_muscle_development	ENSGALG00010016304	EIF3J	0.173627214	0.099797891	1.739788397	0.081896181	0.144287216	positive
PRJNA729271	stage_E14_vs_E10	ordinary_muscle_development	ENSGALG00010016305	DENND4C	0.233540768	0.096194799	2.427789956	0.015191138	0.034293031	positive
PRJNA729271	stage_E14_vs_E10	ordinary_muscle_development	ENSGALG00010016307	RAD50	-0.471600961	0.127275635	-3.705351477	0.000211098	0.00077998	negative
PRJNA729271	stage_E14_vs_E10	ordinary_muscle_development	ENSGALG00010016308	RPS6	0.230068503	0.140697356	1.635201321	0.102006851	0.172914198	positive
PRJNA729271	stage_E14_vs_E10	ordinary_muscle_development	ENSGALG00010016310	HDAC8	0.026713523	0.0912611	0.292715333	0.769739749	0.836385132	positive
PRJNA729271	stage_E14_vs_E10	ordinary_muscle_development	ENSGALG00010016312	MECR	0.140296062	0.143208062	0.979665952	0.327251038	0.442119843	positive
PRJNA729271	stage_E14_vs_E10	ordinary_muscle_development	ENSGALG00010016314	ACER2	-0.200539031	0.156208396	-1.283791623	0.199214876	0.298184595	negative
PRJNA729271	stage_E14_vs_E10	ordinary_muscle_development	ENSGALG00010016315	RPS4Y1	0.546480878	0.13639971	4.006466575	6.16338E-05	0.000252154	positive
PRJNA729271	stage_E14_vs_E10	ordinary_muscle_development	ENSGALG00010016317	CTDSPL2	-0.144172133	0.05599508	-2.574728565	0.010031878	0.023877187	negative
PRJNA729271	stage_E14_vs_E10	ordinary_muscle_development	ENSGALG00010016319	RBM41	0.225762898	0.184919094	1.22087391	0.222133764	0.324843229	positive
PRJNA729271	stage_E14_vs_E10	ordinary_muscle_development	ENSGALG00010016322	EBF4	0.065042479	0.13050934	0.498374129	0.618220368	0.714061621	positive
PRJNA729271	stage_E14_vs_E10	ordinary_muscle_development	ENSGALG00010016324	TUBB1	-0.256029188	0.210264593	-1.217652411	0.223356089	0.326266906	negative
PRJNA729271	stage_E14_vs_E10	ordinary_muscle_development	ENSGALG00010016328	USH2A	-0.345783675	0.633487768	-0.545841123	0.585175153	0.685811274	negative
PRJNA729271	stage_E14_vs_E10	ordinary_muscle_development	ENSGALG00010016332	ENSGALG00010016332	-0.660469782	0.232004848	-2.846793026	0.004416206	0.011683291	negative
PRJNA729271	stage_E14_vs_E10	ordinary_muscle_development	ENSGALG00010016335	ABHD4	0.622181626	0.184654681	3.369433264	0.000753229	0.002445802	positive
PRJNA729271	stage_E14_vs_E10	ordinary_muscle_development	ENSGALG00010016336	SLC24A2	-0.770429068	0.359391833	-2.143702213	0.032056755	0.06489702	negative
PRJNA729271	stage_E14_vs_E10	ordinary_muscle_development	ENSGALG00010016338	PTPRU	-0.264473989	0.148829371	-1.777028205	0.075563599	0.134705986	negative
PRJNA729271	stage_E14_vs_E10	ordinary_muscle_development	ENSGALG00010016339	SETD3	1.257834435	0.121544278	10.34877543	4.23872E-25	1.28536E-23	positive
PRJNA729271	stage_E14_vs_E10	ordinary_muscle_development	ENSGALG00010016340	RGS12	-0.97209319	0.095871839	-10.13950703	3.68957E-24	1.03443E-22	negative
PRJNA729271	stage_E14_vs_E10	ordinary_muscle_development	ENSGALG00010016341	SLX4	-0.133827592	0.061970731	-2.159529029	0.030809148	0.06276579	negative
PRJNA729271	stage_E14_vs_E10	ordinary_muscle_development	ENSGALG00010016344	SMIM14	0.052546741	0.111701051	0.47042298	0.638052849	0.731170531	positive
PRJNA729271	stage_E14_vs_E10	ordinary_muscle_development	ENSGALG00010016345	CTSZ	-0.696338026	0.111386251	-6.251561745	4.06368E-10	3.49159E-09	negative
PRJNA729271	stage_E14_vs_E10	ordinary_muscle_development	ENSGALG00010016346	TRAP1	0.008103612	0.103573595	0.078240134	0.937637038	0.956509878	positive

PRJNA729271	stage_E14_vs_E10	ordinary_muscle_development	ENSGALG00010016347	ANO10	0.019693212	0.089034261	0.221186895	0.824946913	0.877412076	positive
PRJNA729271	stage_E14_vs_E10	ordinary_muscle_development	ENSGALG00010016350	CASC4	0.133044479	0.062575703	2.126136387	0.033491905	0.06734154	positive
PRJNA729271	stage_E14_vs_E10	ordinary_muscle_development	ENSGALG00010016353	C1orf232	0.511306578	0.136895855	3.735004093	0.000187712	0.000701526	positive
PRJNA729271	stage_E14_vs_E10	ordinary_muscle_development	ENSGALG00010016355	MCF2L2	0.402730946	0.228432024	1.763023147	0.077896586	0.138219	positive
PRJNA729271	stage_E14_vs_E10	ordinary_muscle_development	ENSGALG00010016361	NELFCD	-0.239220304	0.081516553	-2.934622428	0.003339538	0.009124713	negative
PRJNA729271	stage_E14_vs_E10	ordinary_muscle_development	ENSGALG00010016362	NUP62	0.151389702	0.167443533	0.904123911	0.365929701	0.481976466	positive
PRJNA729271	stage_E14_vs_E10	ordinary_muscle_development	ENSGALG00010016366	KIF3A	0.026538926	0.097297047	0.272761887	0.785036259	0.848679935	positive
PRJNA729271	stage_E14_vs_E10	ordinary_muscle_development	ENSGALG00010016368	CCNK	-0.207486173	0.14622789	-1.418923389	0.155921353	0.244890213	negative
PRJNA729271	stage_E14_vs_E10	ordinary_muscle_development	ENSGALG00010016369	MSANTD1	-0.434428879	0.245258175	-1.771312533	0.076508745	0.136061677	negative
PRJNA729271	stage_E14_vs_E10	ordinary_muscle_development	ENSGALG00010016370	TMEM255A	1.000008216	0.1867952	5.353500608	8.62687E-08	5.55516E-07	positive
PRJNA729271	stage_E14_vs_E10	ordinary_muscle_development	ENSGALG00010016372	DNAAF6	0.016881243	0.195113177	0.086520259	0.931052852	0.952925132	positive
PRJNA729271	stage_E14_vs_E10	ordinary_muscle_development	ENSGALG00010016373	SRL	2.52454408	0.131638705	19.17782522	5.67029E-82	3.16119E-79	positive
PRJNA729271	stage_E14_vs_E10	ordinary_muscle_development	ENSGALG00010016374	FRMD5	0.170171919	0.375874443	0.452736072	0.650738802	0.742070564	positive
PRJNA729271	stage_E14_vs_E10	ordinary_muscle_development	ENSGALG00010016375	CCNI2	0.030399552	0.130490808	0.232963165	0.815790009	0.870952765	positive
PRJNA729271	stage_E14_vs_E10	ordinary_muscle_development	ENSGALG00010016377	FRMPD3	3.438171955	0.277172679	12.40444032	2.47234E-35	1.40536E-33	positive
PRJNA729271	stage_E14_vs_E10	ordinary_muscle_development	ENSGALG00010016378	WDR76	0.164068565	0.170668276	0.961330188	0.336386174	0.451473851	positive
PRJNA729271	stage_E14_vs_E10	ordinary_muscle_development	ENSGALG00010016379	TFAP4	0.319684494	0.149277613	2.141543441	0.032230239	0.065148141	positive
PRJNA729271	stage_E14_vs_E10	ordinary_muscle_development	ENSGALG00010016382	UGDH	0.007945058	0.185915243	0.04273484	0.965912907	0.977375748	positive
PRJNA729271	stage_E14_vs_E10	ordinary_muscle_development	ENSGALG00010016383	FAM110D	0.143409543	0.17592409	0.815178538	0.414970121	0.529675229	positive
PRJNA729271	stage_E14_vs_E10	ordinary_muscle_development	ENSGALG00010016384	GNAS	-0.171359161	0.073948796	-2.31726778	0.020489149	0.04441482	negative
PRJNA729271	stage_E14_vs_E10	ordinary_muscle_development	ENSGALG00010016385	HTT	-0.150211688	0.106478526	-1.410722833	0.158326356	0.247806989	negative
PRJNA729271	stage_E14_vs_E10	ordinary_muscle_development	ENSGALG00010016387	CCDC85C	-0.573455046	0.155743948	-3.682037419	0.000231377	0.000846282	negative
PRJNA729271	stage_E14_vs_E10	ordinary_muscle_development	ENSGALG00010016390	ENSGALG00010016390	0.076538356	0.363961334	0.210292547	0.833439352	0.883923047	positive
PRJNA729271	stage_E14_vs_E10	ordinary_muscle_development	ENSGALG00010016392	STMN3	0.959196347	0.304889163	3.146049327	0.001654921	0.00492872	positive
PRJNA729271	stage_E14_vs_E10	ordinary_muscle_development	ENSGALG00010016393	BLM	-0.081573529	0.118372479	-0.689125797	0.490744108	0.601093108	negative
PRJNA729271	stage_E14_vs_E10	ordinary_muscle_development	ENSGALG00010016394	PDIK1L	0.081833983	0.095361496	0.858144916	0.390812448	0.506421101	positive
PRJNA729271	stage_E14_vs_E10	ordinary_muscle_development	ENSGALG00010016395	ENSGALG00010016395	-0.695118792	0.159955838	-4.345691903	1.38837E-05	6.36649E-05	negative
PRJNA729271	stage_E14_vs_E10	ordinary_muscle_development	ENSGALG00010016397	LIAS	0.113657241	0.103118491	1.102200397	0.27037456	0.380159817	positive
PRJNA729271	stage_E14_vs_E10	ordinary_muscle_development	ENSGALG00010016398	SEPTIN6	-0.175218113	0.067699928	-2.588158046	0.00964907	0.023102621	negative
PRJNA729271	stage_E14_vs_E10	ordinary_muscle_development	ENSGALG00010016399	ACSM5	3.440762313	0.336900665	10.21298758	1.73441E-24	4.98814E-23	positive
PRJNA729271	stage_E14_vs_E10	ordinary_muscle_development	ENSGALG00010016400	ENSGALG00010016400	0.857415206	0.086517372	9.910324257	3.75426E-23	9.93028E-22	positive
PRJNA729271	stage_E14_vs_E10	ordinary_muscle_development	ENSGALG00010016402	SEPTIN8	0.096962703	0.07576259	1.279822975	0.200607402	0.29975985	positive
PRJNA729271	stage_E14_vs_E10	ordinary_muscle_development	ENSGALG00010016403	GRK4	-0.944473303	0.133586133	-7.070144773	1.54772E-12	1.72438E-11	negative
PRJNA729271	stage_E14_vs_E10	ordinary_muscle_development	ENSGALG00010016404	ESRRG	1.35483168	0.14869769	9.111316251	8.13885E-20	1.71721E-18	positive
PRJNA729271	stage_E14_vs_E10	ordinary_muscle_development	ENSGALG00010016407	RPL9	0.407053292	0.18070219	2.252619586	0.024283145	0.051446298	positive
PRJNA729271	stage_E14_vs_E10	ordinary_muscle_development	ENSGALG00010016409	GMEB2	0.631008382	0.206633737	3.053752938	0.002259981	0.006477838	positive
PRJNA729271	stage_E14_vs_E10	ordinary_muscle_development	ENSGALG00010016410	TRAK1	0.34749445	0.096840208	3.588328228	0.000332805	0.001179061	positive
PRJNA729271	stage_E14_vs_E10	ordinary_muscle_development	ENSGALG00010016411	CTSH	0.219387699	0.123328018	1.778895844	0.07525684	0.13424168	positive
PRJNA729271	stage_E14_vs_E10	ordinary_muscle_development	ENSGALG00010016412	TMSB15B	0.697525846	0.214650276	3.249592121	0.001155706	0.003586376	positive
PRJNA729271	stage_E14_vs_E10	ordinary_muscle_development	ENSGALG00010016415	RASGRF1	-0.187500193	0.635247322	-0.295160934	0.767870943	0.834980444	negative
PRJNA729271	stage_E14_vs_E10	ordinary_muscle_development	ENSGALG00010016416	CYP46A1	-0.091065129	0.113257325	-0.804055091	0.421365159	0.535762104	negative
PRJNA729271	stage_E14_vs_E10	ordinary_muscle_development	ENSGALG00010016417	NOP14	-0.646812004	0.105668992	-6.121114568	9.29231E-10	7.62871E-09	negative
PRJNA729271	stage_E14_vs_E10	ordinary_muscle_development	ENSGALG00010016418	NFIB	0.087703496	0.097762248	0.897110056	0.369660193	0.485830492	positive
PRJNA729271	stage_E14_vs_E10	ordinary_muscle_development	ENSGALG00010016421	ENSGALG00010016421	0.598029078	0.336080079	1.779424356	0.075170216	0.134120173	positive
PRJNA729271	stage_E14_vs_E10	ordinary_muscle_development	ENSGALG00010016422	KCTD3	1.088486116	0.08978252	12.12358613	7.92155E-34	4.11552E-32	positive
PRJNA729271	stage_E14_vs_E10	ordinary_muscle_development	ENSGALG00010016423	TRMT44	-0.393123395	0.104719631	-3.75405635	0.000173996	0.000654742	negative
PRJNA729271	stage_E14_vs_E10	ordinary_muscle_development	ENSGALG00010016425	TRIM63	1.915451553	0.414137323	4.625160411	3.74309E-06	1.87932E-05	positive
PRJNA729271	stage_E14_vs_E10	ordinary_muscle_development	ENSGALG00010016426	HELZ2	-0.742198465	0.372843544	-1.9906432	0.046520127	0.089277009	negative
PRJNA729271	stage_E14_vs_E10	ordinary_muscle_development	ENSGALG00010016428	ADRA1D	0.653898738	0.198275432	3.297931218	0.000974	0.003075845	positive
PRJNA729271	stage_E14_vs_E10	ordinary_muscle_development	ENSGALG00010016429	ENSGALG00010016429	0.642497152	0.341600257	1.880845047	0.059993	0.110565612	positive
PRJNA729271	stage_E14_vs_E10	ordinary_muscle_development	ENSGALG00010016431	B3GNT5	-1.123263931	0.157108483	-7.149607138	8.70266E-13	1.00036E-11	negative
PRJNA729271	stage_E14_vs_E10	ordinary_muscle_development	ENSGALG00010016433	TMED3	0.298388988	0.141300848	2.11172822	0.034709767	0.069434503	positive
PRJNA729271	stage_E14_vs_E10	ordinary_muscle_development	ENSGALG00010016434	GPR139	-1.191039234	0.457272617	-2.604658992	0.009196574	0.022143578	negative
PRJNA729271	stage_E14_vs_E10	ordinary_muscle_development	ENSGALG00010016436	SLC30A2	0.029485065	0.612667303	0.048125736	0.961616036	0.973911713	positive
PRJNA729271	stage_E14_vs_E10	ordinary_muscle_development	ENSGALG00010016441	ENSGALG00010016441	1.274036904	0.55628208	2.290271338	0.022005592	0.047290001	positive
PRJNA729271	stage_E14_vs_E10	ordinary_muscle_development	ENSGALG00010016445	ZDHHC21	-0.16819391	0.135507244	-1.241217106	0.214525557	0.316173043	negative
PRJNA729271	stage_E14_vs_E10	ordinary_muscle_development	ENSGALG00010016447	ID3	-1.176359628	0.112429795	-10.46305947	1.27666E-25	4.04042E-24	negative
PRJNA729271	stage_E14_vs_E10	ordinary_muscle_development	ENSGALG00010016449	FMR1	0.02657969	0.106606105	0.249326151	0.803108504	0.861407535	positive
PRJNA729271	stage_E14_vs_E10	ordinary_muscle_development	ENSGALG00010016450	ENSGALG00010016450	-0.651119075	0.261580495	-2.489172886	0.012804069	0.029609919	negative
PRJNA729271	stage_E14_vs_E10	ordinary_muscle_development	ENSGALG00010016451	USP46	-0.51280686	0.147753391	-3.47069435	0.000519115	0.001752344	negative
PRJNA729271	stage_E14_vs_E10	ordinary_muscle_development	ENSGALG00010016452	GLCE	-0.298173723	0.113743491	-2.621457455	0.008755469	0.021226045	negative
PRJNA729271	stage_E14_vs_E10	ordinary_muscle_development	ENSGALG00010016453	MYCL	0.524151103	0.17266895	3.035584001	0.002400703	0.006815158	positive
PRJNA729271	stage_E14_vs_E10	ordinary_muscle_development	ENSGALG00010016454	ANKS3	0.045742954	0.092686319	0.493524331	0.621642153	0.71701835	positive
PRJNA729271	stage_E14_vs_E10	ordinary_muscle_development	ENSGALG00010016455	RBM27	-0.170328723	0.090364951	-1.884898091	0.059443598	0.109650668	negative
PRJNA729271	stage_E14_vs_E10	ordinary_muscle_development	ENSGALG00010016456	LAMP3	-1.749609056	0.371135636	-4.714203885	2.42658E-06	1.26105E-05	negative
PRJNA729271	stage_E14_vs_E10	ordinary_muscle_development	ENSGALG00010016457	SMOX	-0.567933964	0.180026884	-3.154717515	0.001606536	0.004798421	negative
PRJNA729271	stage_E14_vs_E10	ordinary_muscle_development	ENSGALG00010016458	ZC3H12A	0.267936263	0.089531754	2.9926395	0.002765762	0.007730375	positive
PRJNA729271	stage_E14_vs_E10	ordinary_muscle_development	ENSGALG00010016459	PAQR5	0.058455543	0.311375382	0.187733348	0.851085687	0.896807722	positive
PRJNA729271	stage_E14_vs_E10	ordinary_muscle_development	ENSGALG00010016460	LBR	-0.23013098	0.068531824	-3.358016276	0.00078504	0.002537158	negative
PRJNA729271	stage_E14_vs_E10	ordinary_muscle_development	ENSGALG00010016461	HPCAL4	-0.615786773	0.226630695	-2.717137562	0.006584923	0.016513575	negative
PRJNA729271	stage_E14_vs_E10	ordinary_muscle_development	ENSGALG00010016462	RNF24	-0.265077538	0.152214964	-1.741468321	0.081601521	0.143894654	negative
PRJNA729271	stage_E14_vs_E10	ordinary_muscle_development	ENSGALG00010016463	FREM1	0.63381829	0.142129018	4.459457327	8.21674E-06	3.9101E-05	positive
PRJNA729271	stage_E14_vs_E10	ordinary_muscle_development	ENSGALG00010016464	PANK2	0.017905	0.08273268	0.216419922	0.828660439	0.880474495	positive
PRJNA729271	stage_E14_vs_E10	ordinary_muscle_development	ENSGALG00010016465	STK35	-0.913241009	0.174895564	-5.221636197	1.77349E-07	1.09066E-06	negative
PRJNA729271	stage_E14_vs_E10	ordinary_muscle_development	ENSGALG00010016466	TMEM164	-0.441593018	0.233671535	-1.889802362	0.058784398	0.108672011	negative
PRJNA729271	stage_E14_vs_E10	ordinary_muscle_development	ENSGALG00010016468	TTC39B	0.328793432	0.156602154	2.099546033	0.035768794	0.071247585	positive
PRJNA729271	stage_E14_vs_E10	ordinary_muscle_development	ENSGALG00010016469	MAVS	0.699299456	0.101107459	6.916398249	4.63271E-12	4.9195E-11	positive
PRJNA729271	stage_E14_vs_E10	ordinary_muscle_development	ENSGALG00010016470	TNIP1	0.571647283	0.158287549	3.611448186	0.000304492	0.001089257	positive
PRJNA729271	stage_E14_vs_E10	ordinary_muscle_development	ENSGALG00010016471	POMGNT2	0.344090622	0.135010256	2.548625808	0.010814827	0.02550202	positive
PRJNA729271	stage_E14_vs_E10	ordinary_muscle_development	ENSGALG00010016472	TFDP1	-0.115705211	0.120659765	-0.958937817	0.337590072	0.452754265	negative
PRJNA729271	stage_E14_vs_E10	ordinary_muscle_development	ENSGALG00010016473	RAB9B	0.183311242	0.158007701	1.160141189	0.245991327	0.352719782	positive
PRJNA729271	stage_E14_vs_E10	ordinary_muscle_development	ENSGALG00010016474	MAPK8IP3	0.420122592	0.109150456	3.849022766	0.00011859	0.000461147	positive
PRJNA729271	stage_E14_vs_E10	ordinary_muscle_development	ENSGALG00010016475	MCCC1	0.551089427	0.083242342	6.620301788	3.58466E-11	3.47556E-10	positive
PRJNA729271	stage_E14_vs_E10	ordinary_muscle_development	ENSGALG00010016477	CNIH3	0.250017499	0.177922106	1.405207619	0.159959549	0.250075646	positive
PRJNA729271	stage_E14_vs_E10	ordinary_muscle_development	ENSGALG00010016479	ENSGALG00010016479	-1.692303207	0.387059132	-4.372208449	1.22996E-05	5.68504E-05	negative
PRJNA729271	stage_E14_vs_E10	ordinary_muscle_development	ENSGALG00010016480	GPX3	2.82508398	0.133327859	21.18899976	1.20633E-99	1.34505E-96	positive
PRJNA729271	stage_E14_vs_E10	ordinary_muscle_development	ENSGALG00010016482	EML1	-0.022357991	0.140829826	-0.158758917	0.873858817	0.913499463	negative
PRJNA729271	stage_E14_vs_E10	ordinary_muscle_development	ENSGALG00010016483	WDR26	0.10005498	0.102411661	0.976988161	0.328575014	0.443371329	positive
PRJNA729271	stage_E14_vs_E10	ordinary_muscle_development	ENSGALG00010016489	CCK	-3.517106492	0.490786382	-7.166267485	7.70701E-13	8.92277E-12	negative
PRJNA729271	stage_E14_vs_E10	ordinary_muscle_development	ENSGALG00010016491	DNAJC8	0.038109387	0.052582836	0.724749544	0.468605695	0.580897934	positive
PRJNA729271	stage_E14_vs_E10	ordinary_muscle_development	ENSGALG00010016496	PLP1	0.826392596	0.343262774	2.407463491	0.016063768	0.035993866	positive
PRJNA729271	stage_E14_vs_E10	ordinary_muscle_development	ENSGALG00010016498	KIF23	-0.334066245	0.205447037	-1.626045571	0.103939961	0.175759419	negative
PRJNA729271	stage_E14_vs_E10	ordinary_muscle_development	ENSGALG00010016500	ENSGALG00010016500	0.426927223	0.106667909	4.00239612	6.27042E-05	0.000256433	positive
PRJNA729271	stage_E14_vs_E10	ordinary_muscle_development	ENSGALG00010016502	YIPF5	-0.103122682	0.131220834	-0.785871264	0.431942911	0.546097907	negative
PRJNA729271	stage_E14_vs_E10	ordinary_muscle_development	ENSGALG00010016504	SNRK	0.259772874	0.08466333	3.068304486	0.002152771	0.006203662	positive
PRJNA729271	stage_E14_vs_E10	ordinary_muscle_development	ENSGALG00010016505	KCTD16	1.585117706	0.257127721	6.164709513	7.06126E-10	5.8756E-09	positive
PRJNA729271	stage_E14_vs_E10	ordinary_muscle_development	ENSGALG00010016507	SPATA18	-0.308219193	0.140865038	-2.188046078	0.028666246	0.059173631	negative
PRJNA729271	stage_E14_vs_E10	ordinary_muscle_development	ENSGALG00010016508	CREBBP	0.096011138	0.119541201	0.803163573	0.421880197	0.536278755	positive
PRJNA729271	stage_E14_vs_E10	ordinary_muscle_development	ENSGALG00010016510	THG1L	-0.066567083	0.10844891	-0.613810529	0.539340539	0.645560785	negative
PRJNA729271	stage_E14_vs_E10	ordinary_muscle_development	ENSGALG00010016514	GASK1A	-0.795767192	0.237474461	-3.350959043	0.000805322	0.002596918	negative
PRJNA729271	stage_E14_vs_E10	ordinary_muscle_development	ENSGALG00010016518	TMEM42	-0.322911111	0.141592879	-2.280560391	0.022574473	0.048361956	negative
PRJNA729271	stage_E14_vs_E10	ordinary_muscle_development	ENSGALG00010016519	MEX3B	-0.655595	0.17580156	-3.729176236	0.000192107	0.000716754	negative
PRJNA729271	stage_E14_vs_E10	ordinary_muscle_development	ENSGALG00010016520	CHMP1B	0.160002526	0.082426568	1.941152352	0.052239801	0.098352502	positive
PRJNA729271	stage_E14_vs_E10	ordinary_muscle_development	ENSGALG00010016526	EIF1B	0.125279822	0.116258448	1.07759758	0.281213383	0.392338472	positive
PRJNA729271	stage_E14_vs_E10	ordinary_muscle_development	ENSGALG00010016527	MRPS34	0.110690701	0.095617341	1.157642534	0.247009944	0.353655585	positive
PRJNA729271	stage_E14_vs_E10	ordinary_muscle_development	ENSGALG00010016529	DCUN1D1	-0.09024397	0.097117679	-0.929222891	0.352773587	0.46899506	negative
PRJNA729271	stage_E14_vs_E10	ordinary_muscle_development	ENSGALG00010016533	CNIH4	-0.057236767	0.109479013	-0.522810401	0.601106201	0.69993248	negative
PRJNA729271	stage_E14_vs_E10	ordinary_muscle_development	ENSGALG00010016534	TRIT1	-0.327586549	0.142950332	-2.291610965	0.021928102	0.047165431	negative
PRJNA729271	stage_E14_vs_E10	ordinary_muscle_development	ENSGALG00010016535	BNC2	-0.699816397	0.12980626	-5.391237638	6.99741E-08	4.57498E-07	negative
PRJNA729271	stage_E14_vs_E10	ordinary_muscle_development	ENSGALG00010016541	UBE2J2	-0.131103638	0.078801497	-1.663720149	0.096168368	0.164692875	negative
PRJNA729271	stage_E14_vs_E10	ordinary_muscle_development	ENSGALG00010016542	ENOX2	0.211657174	0.075530908	2.802259102	0.00507461	0.013195337	positive
PRJNA729271	stage_E14_vs_E10	ordinary_muscle_development	ENSGALG00010016543	TCEA3	3.430180005	0.174599026	19.64604321	6.24993E-86	3.9027E-83	positive
PRJNA729271	stage_E14_vs_E10	ordinary_muscle_development	ENSGALG00010016544	ADCY9	0.631329199	0.135570952	4.656817618	3.21135E-06	1.63328E-05	positive
PRJNA729271	stage_E14_vs_E10	ordinary_muscle_development	ENSGALG00010016546	C1QTNF12	-1.961549831	0.421605845	-4.65256792	3.27827E-06	1.66556E-05	negative
PRJNA729271	stage_E14_vs_E10	ordinary_muscle_development	ENSGALG00010016547	SGCB	1.158257753	0.143309984	8.082184646	6.36167E-16	9.69581E-15	positive
PRJNA729271	stage_E14_vs_E10	ordinary_muscle_development	ENSGALG00010016548	EVL	0.5659136	0.107952136	5.242264037	1.58618E-07	9.81293E-07	positive
PRJNA729271	stage_E14_vs_E10	ordinary_muscle_development	ENSGALG00010016549	PTPRA	-0.153237547	0.102015578	-1.502099482	0.133071419	0.215323757	negative
PRJNA729271	stage_E14_vs_E10	ordinary_muscle_development	ENSGALG00010016550	FBLIM1	-1.086704191	0.125704319	-8.644923237	5.38415E-18	9.84152E-17	negative
PRJNA729271	stage_E14_vs_E10	ordinary_muscle_development	ENSGALG00010016552	ENSGALG00010016552	1.036038742	0.46288455	2.238222778	0.025206531	0.053105911	positive
PRJNA729271	stage_E14_vs_E10	ordinary_muscle_development	ENSGALG00010016553	SLC25A14	0.024014403	0.093517441	0.25679063	0.797340403	0.857568387	positive
PRJNA729271	stage_E14_vs_E10	ordinary_muscle_development	ENSGALG00010016556	ASAP3	-0.250597078	0.195719205	-1.28039084	0.200407714	0.299602511	negative
PRJNA729271	stage_E14_vs_E10	ordinary_muscle_development	ENSGALG00010016558	NR2F2	-0.336988723	0.109005662	-3.091479081	0.00199162	0.005796894	negative
PRJNA729271	stage_E14_vs_E10	ordinary_muscle_development	ENSGALG00010016559	ROGDI	-0.065167001	0.11046556	-0.589930487	0.555237254	0.659782334	negative
PRJNA729271	stage_E14_vs_E10	ordinary_muscle_development	ENSGALG00010016560	DCUN1D4	0.42114801	0.169377219	2.486450146	0.012902465	0.029804179	positive
PRJNA729271	stage_E14_vs_E10	ordinary_muscle_development	ENSGALG00010016561	DEGS2	0.883467904	0.584312276	1.511979022	0.130539199	0.211699004	positive
PRJNA729271	stage_E14_vs_E10	ordinary_muscle_development	ENSGALG00010016562	GLIS2	-0.247914843	0.222042428	-1.116520142	0.264199553	0.372962661	negative
PRJNA729271	stage_E14_vs_E10	ordinary_muscle_development	ENSGALG00010016564	SPEN	0.012732349	0.166019252	0.076692003	0.938868566	0.957092613	positive
PRJNA729271	stage_E14_vs_E10	ordinary_muscle_development	ENSGALG00010016565	NVL	-0.373585198	0.096028765	-3.890346798	0.000100101	0.000393749	negative
PRJNA729271	stage_E14_vs_E10	ordinary_muscle_development	ENSGALG00010016566	CNTLN	-0.126556559	0.202507288	-0.624948171	0.532005075	0.639473718	negative
PRJNA729271	stage_E14_vs_E10	ordinary_muscle_development	ENSGALG00010016567	E2F2	1.11393324	0.260948371	4.268787868	1.96538E-05	8.75751E-05	positive
PRJNA729271	stage_E14_vs_E10	ordinary_muscle_development	ENSGALG00010016568	PAM16	0.620483654	0.135190721	4.589691125	4.43902E-06	2.21112E-05	positive

PRJNA729271	stage_E14_vs_E10	ordinary_muscle_development	ENSGALG00010016569	YY1	0.085211203	0.086525149	0.984814285	0.324715302	0.439349231	positive
PRJNA729271	stage_E14_vs_E10	ordinary_muscle_development	ENSGALG00010016572	RAB33A	0.063447102	0.113553554	0.558741662	0.576338042	0.678196129	positive
PRJNA729271	stage_E14_vs_E10	ordinary_muscle_development	ENSGALG00010016573	DEGS1	-0.225921471	0.128103255	-1.76358884	0.077801228	0.138083605	negative
PRJNA729271	stage_E14_vs_E10	ordinary_muscle_development	ENSGALG00010016575	CWH43	-0.250131164	0.383541098	-0.652162611	0.514296277	0.622992189	negative
PRJNA729271	stage_E14_vs_E10	ordinary_muscle_development	ENSGALG00010016576	MRPS26	0.143547802	0.106116846	1.352733409	0.176140813	0.269918711	positive
PRJNA729271	stage_E14_vs_E10	ordinary_muscle_development	ENSGALG00010016577	ZBTB17	0.372152966	0.185678777	2.004283806	0.045039668	0.086872918	positive
PRJNA729271	stage_E14_vs_E10	ordinary_muscle_development	ENSGALG00010016580	MARS2	-0.050748144	0.103817601	-0.488820233	0.624968972	0.71981925	negative
PRJNA729271	stage_E14_vs_E10	ordinary_muscle_development	ENSGALG00010016581	H2AFYMACROH2A1	0.045599061	0.075590495	0.603238026	0.546350354	0.651798221	positive
PRJNA729271	stage_E14_vs_E10	ordinary_muscle_development	ENSGALG00010016583	VPS41	-0.237493971	0.082656562	-2.873262142	0.004062569	0.010838751	negative
PRJNA729271	stage_E14_vs_E10	ordinary_muscle_development	ENSGALG00010016584	EHBP1	-0.361202852	0.160518855	-2.250220714	0.024434938	0.05172221	negative
PRJNA729271	stage_E14_vs_E10	ordinary_muscle_development	ENSGALG00010016588	ATP11B	-0.08575375	0.100148988	-0.856261779	0.391852998	0.507542597	negative
PRJNA729271	stage_E14_vs_E10	ordinary_muscle_development	ENSGALG00010016589	FBXO28	0.060749532	0.075995676	0.799381434	0.424069272	0.538444648	positive
PRJNA729271	stage_E14_vs_E10	ordinary_muscle_development	ENSGALG00010016591	HOMER2	-2.834490196	0.212308193	-13.35082811	1.17139E-40	9.43298E-39	negative
PRJNA729271	stage_E14_vs_E10	ordinary_muscle_development	ENSGALG00010016592	TMEM129	-0.177362887	0.103189033	-1.718815276	0.085648013	0.149664611	negative
PRJNA729271	stage_E14_vs_E10	ordinary_muscle_development	ENSGALG00010016597	FRYL	0.085114446	0.125341674	0.679059433	0.497100205	0.606571889	positive
PRJNA729271	stage_E14_vs_E10	ordinary_muscle_development	ENSGALG00010016598	AIFM1	0.088071898	0.088094526	0.999743138	0.31743483	0.431310261	positive
PRJNA729271	stage_E14_vs_E10	ordinary_muscle_development	ENSGALG00010016599	ENSGALG00010016599	-1.078496539	0.28721539	-3.755009579	0.000173335	0.000652594	negative
PRJNA729271	stage_E14_vs_E10	ordinary_muscle_development	ENSGALG00010016600	GNL2	-0.177511427	0.108794672	-1.631618747	0.102759826	0.173966793	negative
PRJNA729271	stage_E14_vs_E10	ordinary_muscle_development	ENSGALG00010016602	HSPB7	0.44674931	0.521857253	0.856075694	0.391955912	0.507585182	positive
PRJNA729271	stage_E14_vs_E10	ordinary_muscle_development	ENSGALG00010016603	ENSGALG00010016603	-0.189569476	0.125466163	-1.510921121	0.130808549	0.212088358	negative
PRJNA729271	stage_E14_vs_E10	ordinary_muscle_development	ENSGALG00010016604	TP53BP2	-0.336544288	0.061727522	-5.452094588	4.978E-08	3.29781E-07	negative
PRJNA729271	stage_E14_vs_E10	ordinary_muscle_development	ENSGALG00010016605	WDPCP	0.250044692	0.157207869	1.590535468	0.111714157	0.18670549	positive
PRJNA729271	stage_E14_vs_E10	ordinary_muscle_development	ENSGALG00010016606	VASN	-0.747233127	0.178635463	-4.183005522	2.8768E-05	0.000124848	negative
PRJNA729271	stage_E14_vs_E10	ordinary_muscle_development	ENSGALG00010016607	FAM103A1	-0.066769865	0.113593098	-0.58779861	0.556667477	0.660897296	negative
PRJNA729271	stage_E14_vs_E10	ordinary_muscle_development	ENSGALG00010016609	SOX2	-1.365633649	0.489948131	-2.787302496	0.005314884	0.013739833	negative
PRJNA729271	stage_E14_vs_E10	ordinary_muscle_development	ENSGALG00010016611	DNALI1	-0.181058126	0.198513266	-0.912070663	0.36173152	0.47787991	negative
PRJNA729271	stage_E14_vs_E10	ordinary_muscle_development	ENSGALG00010016612	GRIA1	-1.268207548	0.331897627	-3.82108049	0.000132868	0.000511942	negative
PRJNA729271	stage_E14_vs_E10	ordinary_muscle_development	ENSGALG00010016613	SNIP1	0.103336907	0.073632442	1.403415465	0.160492984	0.2506298	positive
PRJNA729271	stage_E14_vs_E10	ordinary_muscle_development	ENSGALG00010016614	DNAJC19	-0.096420096	0.139520013	-0.691084341	0.48951254	0.600040953	negative
PRJNA729271	stage_E14_vs_E10	ordinary_muscle_development	ENSGALG00010016615	BCORL1	0.398806391	0.125273423	3.183487618	0.001455123	0.004399669	positive
PRJNA729271	stage_E14_vs_E10	ordinary_muscle_development	ENSGALG00010016616	SLBP	-0.36973331	0.231490061	-1.597188706	0.11022365	0.184554904	negative
PRJNA729271	stage_E14_vs_E10	ordinary_muscle_development	ENSGALG00010016618	TRPM3	-2.4697079	0.4953899	-4.985382019	6.18395E-07	3.51515E-06	negative
PRJNA729271	stage_E14_vs_E10	ordinary_muscle_development	ENSGALG00010016619	SLC25A47	1.660954632	0.322024736	5.157847977	2.49804E-07	1.50746E-06	positive
PRJNA729271	stage_E14_vs_E10	ordinary_muscle_development	ENSGALG00010016620	ENSGALG00010016620	0.099642073	0.120296939	0.828306939	0.407496694	0.52229769	positive
PRJNA729271	stage_E14_vs_E10	ordinary_muscle_development	ENSGALG00010016622	KIAA1143	-0.356999479	0.133319461	-2.677774682	0.007411306	0.018316603	negative
PRJNA729271	stage_E14_vs_E10	ordinary_muscle_development	ENSGALG00010016623	DNAJA3	0.317399133	0.141305931	2.246184092	0.024692219	0.05217906	positive
PRJNA729271	stage_E14_vs_E10	ordinary_muscle_development	ENSGALG00010016624	MDH1	0.226685357	0.154709648	1.465230902	0.142857931	0.228078612	positive
PRJNA729271	stage_E14_vs_E10	ordinary_muscle_development	ENSGALG00010016626	CAPN2	-1.894633622	0.23307668	-8.128799587	4.33562E-16	6.69988E-15	negative
PRJNA729271	stage_E14_vs_E10	ordinary_muscle_development	ENSGALG00010016629	LARS1	-0.07570283	0.070696096	-1.070820522	0.284250138	0.395810994	negative
PRJNA729271	stage_E14_vs_E10	ordinary_muscle_development	ENSGALG00010016630	ENSGALG00010016630	0.308294561	0.362941572	0.849433036	0.395640377	0.511169201	positive
PRJNA729271	stage_E14_vs_E10	ordinary_muscle_development	ENSGALG00010016631	FAM53A	1.687892553	0.09095865	18.55670188	7.19909E-77	2.82029E-74	positive
PRJNA729271	stage_E14_vs_E10	ordinary_muscle_development	ENSGALG00010016637	POU6F2	-1.933573768	0.605197682	-3.194945758	0.001398571	0.004248175	negative
PRJNA729271	stage_E14_vs_E10	ordinary_muscle_development	ENSGALG00010016640	OCIAD1	0.176460605	0.103841743	1.699322448	0.089258445	0.15472389	positive
PRJNA729271	stage_E14_vs_E10	ordinary_muscle_development	ENSGALG00010016643	BTBD1	0.165998403	0.173375679	0.957449185	0.338340583	0.453467106	positive
PRJNA729271	stage_E14_vs_E10	ordinary_muscle_development	ENSGALG00010016644	NMRAL1	-0.305903181	0.195432316	-1.565264063	0.117521006	0.194437505	negative
PRJNA729271	stage_E14_vs_E10	ordinary_muscle_development	ENSGALG00010016647	EGR1	0.640133954	0.205160999	3.120154205	0.001807564	0.00533292	positive
PRJNA729271	stage_E14_vs_E10	ordinary_muscle_development	ENSGALG00010016648	ADRA2C	-2.192074752	0.283373684	-7.735632769	1.0289E-14	1.41364E-13	negative
PRJNA729271	stage_E14_vs_E10	ordinary_muscle_development	ENSGALG00010016651	ENSGALG00010016651	2.607992451	0.178909345	14.57717289	3.92435E-48	5.12463E-46	positive
PRJNA729271	stage_E14_vs_E10	ordinary_muscle_development	ENSGALG00010016652	FXR1	0.841659375	0.098966269	8.504507462	1.82368E-17	3.18485E-16	positive
PRJNA729271	stage_E14_vs_E10	ordinary_muscle_development	ENSGALG00010016653	EPHA2	-0.533721787	0.207257012	-2.575168786	0.010019118	0.02386249	negative
PRJNA729271	stage_E14_vs_E10	ordinary_muscle_development	ENSGALG00010016654	SMIM3	1.145215627	0.181420977	6.312476364	2.74605E-10	2.40653E-09	positive
PRJNA729271	stage_E14_vs_E10	ordinary_muscle_development	ENSGALG00010016655	TRANK1	-0.56834012	0.323878231	-1.754795679	0.079294263	0.140389684	negative
PRJNA729271	stage_E14_vs_E10	ordinary_muscle_development	ENSGALG00010016656	CAPN8	-4.352574752	1.093028274	-3.982124575	6.8302E-05	0.000277399	negative
PRJNA729271	stage_E14_vs_E10	ordinary_muscle_development	ENSGALG00010016657	SLC25A29	1.186345	0.279272741	4.247979933	2.15707E-05	9.55E-05	positive
PRJNA729271	stage_E14_vs_E10	ordinary_muscle_development	ENSGALG00010016658	ZAR1	-0.982961325	0.501681561	-1.959333174	0.050073781	0.094890765	negative
PRJNA729271	stage_E14_vs_E10	ordinary_muscle_development	ENSGALG00010016661	UBE4B	-0.473137193	0.128160931	-3.691742785	0.000222723	0.000817101	negative
PRJNA729271	stage_E14_vs_E10	ordinary_muscle_development	ENSGALG00010016664	TM6SF1	1.064419669	0.249465038	4.266809004	1.98289E-05	8.82738E-05	positive
PRJNA729271	stage_E14_vs_E10	ordinary_muscle_development	ENSGALG00010016665	ENSGALG00010016665	0.998729717	1.263440185	0.790484369	0.429244947	0.54352907	positive
PRJNA729271	stage_E14_vs_E10	ordinary_muscle_development	ENSGALG00010016667	WARS1	-0.311211066	0.142793208	-2.179452855	0.029298043	0.060220524	negative
PRJNA729271	stage_E14_vs_E10	ordinary_muscle_development	ENSGALG00010016671	CASS4	1.500748548	0.24299247	6.176111343	6.56997E-10	5.5079E-09	positive
PRJNA729271	stage_E14_vs_E10	ordinary_muscle_development	ENSGALG00010016672	DCTN4	0.108369714	0.080084565	1.353191017	0.175994614	0.269751711	positive
PRJNA729271	stage_E14_vs_E10	ordinary_muscle_development	ENSGALG00010016673	LRPAP1	-0.126434885	0.079021243	-1.600011338	0.109596068	0.183716319	negative
PRJNA729271	stage_E14_vs_E10	ordinary_muscle_development	ENSGALG00010016674	SLC10A4	0.027371256	0.325975805	0.083967138	0.933082559	0.953944964	positive
PRJNA729271	stage_E14_vs_E10	ordinary_muscle_development	ENSGALG00010016675	SKIL	-0.48438072	0.139208325	-3.479538459	0.000502278	0.001701852	negative
PRJNA729271	stage_E14_vs_E10	ordinary_muscle_development	ENSGALG00010016676	NLRC3	-0.477051358	0.499048731	-0.955921392	0.33911195	0.45420696	negative
PRJNA729271	stage_E14_vs_E10	ordinary_muscle_development	ENSGALG00010016677	MEAF6	0.178798307	0.08775835	2.037393669	0.041610608	0.081035303	positive
PRJNA729271	stage_E14_vs_E10	ordinary_muscle_development	ENSGALG00010016678	NOX1	2.090136532	0.317051693	6.592415625	4.32727E-11	4.17602E-10	positive
PRJNA729271	stage_E14_vs_E10	ordinary_muscle_development	ENSGALG00010016680	BPIFB3	3.744742444	0.727461345	5.14768581	2.6372E-07	1.58483E-06	positive
PRJNA729271	stage_E14_vs_E10	ordinary_muscle_development	ENSGALG00010016681	SUSD4	0.102257876	0.302302409	0.338263517	0.735164614	0.809680958	positive
PRJNA729271	stage_E14_vs_E10	ordinary_muscle_development	ENSGALG00010016682	GPR141	0.545959147	0.443829175	1.230110994	0.218655544	0.320920626	positive
PRJNA729271	stage_E14_vs_E10	ordinary_muscle_development	ENSGALG00010016683	RBM22	-0.027407095	0.072859655	-0.376162836	0.706795842	0.787162904	negative
PRJNA729271	stage_E14_vs_E10	ordinary_muscle_development	ENSGALG00010016691	ENSGALG00010016691	0.662115292	0.292262553	2.265481108	0.023483172	0.050005668	positive
PRJNA729271	stage_E14_vs_E10	ordinary_muscle_development	ENSGALG00010016692	PRKCI	-0.387863347	0.07293474	-5.31795061	1.04943E-07	6.66875E-07	negative
PRJNA729271	stage_E14_vs_E10	ordinary_muscle_development	ENSGALG00010016696	DOK7	1.729798784	0.128673316	13.44333723	3.36894E-41	2.79044E-39	positive
PRJNA729271	stage_E14_vs_E10	ordinary_muscle_development	ENSGALG00010016699	SYNPO	-0.773535201	0.146904801	-5.265554276	1.39767E-07	8.70616E-07	negative
PRJNA729271	stage_E14_vs_E10	ordinary_muscle_development	ENSGALG00010016700	CLUAP1	-0.277319756	0.120470587	-2.301970647	0.021336828	0.046037112	negative
PRJNA729271	stage_E14_vs_E10	ordinary_muscle_development	ENSGALG00010016702	WDR25	0.117911199	0.093789886	1.257184584	0.208686804	0.309201188	positive
PRJNA729271	stage_E14_vs_E10	ordinary_muscle_development	ENSGALG00010016704	CSTF2	0.172998509	0.068635576	2.520537002	0.011717592	0.027381347	positive
PRJNA729271	stage_E14_vs_E10	ordinary_muscle_development	ENSGALG00010016706	RTN4	0.672844496	0.12550225	5.36121462	8.26642E-08	5.33727E-07	positive
PRJNA729271	stage_E14_vs_E10	ordinary_muscle_development	ENSGALG00010016707	KIF1B	1.087488176	0.130418995	8.338418591	7.52906E-17	1.25297E-15	positive
PRJNA729271	stage_E14_vs_E10	ordinary_muscle_development	ENSGALG00010016712	ENSGALG00010016712	-0.462404018	0.159733138	-2.894853409	0.003793356	0.01020503	negative
PRJNA729271	stage_E14_vs_E10	ordinary_muscle_development	ENSGALG00010016720	INTS15	-0.1648298	0.095719891	-1.722001538	0.08506924	0.148940528	negative
PRJNA729271	stage_E14_vs_E10	ordinary_muscle_development	ENSGALG00010016724	PGD	-0.449872293	0.108763428	-4.136245993	3.53034E-05	0.000150684	negative
PRJNA729271	stage_E14_vs_E10	ordinary_muscle_development	ENSGALG00010016729	ELMO1	0.039765587	0.094726112	0.419795408	0.67463492	0.760989066	positive
PRJNA729271	stage_E14_vs_E10	ordinary_muscle_development	ENSGALG00010016731	C4orf48	-0.326804423	0.268954921	-1.215089957	0.224331784	0.32731502	negative
PRJNA729271	stage_E14_vs_E10	ordinary_muscle_development	ENSGALG00010016732	ARHGAP26	0.409684137	0.074186125	5.5223822	3.34434E-08	2.29094E-07	positive
PRJNA729271	stage_E14_vs_E10	ordinary_muscle_development	ENSGALG00010016733	PHC3	-0.050665672	0.156108485	-0.324554247	0.745518459	0.817419824	negative
PRJNA729271	stage_E14_vs_E10	ordinary_muscle_development	ENSGALG00010016735	ENSGALG00010016735	2.522937687	0.316986483	7.959133348	1.73248E-15	2.54689E-14	positive
PRJNA729271	stage_E14_vs_E10	ordinary_muscle_development	ENSGALG00010016736	CENPS	-0.360543566	0.264891834	-1.361097323	0.173482935	0.266635049	negative
PRJNA729271	stage_E14_vs_E10	ordinary_muscle_development	ENSGALG00010016738	NAA60	1.070902238	0.148797179	7.197060092	6.15247E-13	7.19774E-12	positive
PRJNA729271	stage_E14_vs_E10	ordinary_muscle_development	ENSGALG00010016741	NELFA	-0.107934109	0.089048603	-1.212080889	0.225481418	0.328609809	negative
PRJNA729271	stage_E14_vs_E10	ordinary_muscle_development	ENSGALG00010016742	TRMT12	0.139044564	0.129008107	1.077797108	0.28112431	0.392307392	positive
PRJNA729271	stage_E14_vs_E10	ordinary_muscle_development	ENSGALG00010016744	CRYBG2	-1.697083911	0.630206908	-2.692899571	0.00708336	0.017608181	negative
PRJNA729271	stage_E14_vs_E10	ordinary_muscle_development	ENSGALG00010016745	BEGAIN	-0.393689209	0.370696531	-1.06202561	0.288224059	0.40051843	negative
PRJNA729271	stage_E14_vs_E10	ordinary_muscle_development	ENSGALG00010016746	HDGFL3	-0.717945231	0.122102371	-5.87986312	4.10606E-09	3.14574E-08	negative
PRJNA729271	stage_E14_vs_E10	ordinary_muscle_development	ENSGALG00010016750	SYTL4	0.201478768	0.124912242	1.612962548	0.106752645	0.179827905	positive
PRJNA729271	stage_E14_vs_E10	ordinary_muscle_development	ENSGALG00010016751	DFFA	-0.217577997	0.083390511	-2.609145755	0.009076857	0.021909916	negative
PRJNA729271	stage_E14_vs_E10	ordinary_muscle_development	ENSGALG00010016752	ENSGALG00010016752	-0.247836484	0.177037607	-1.399908687	0.161540664	0.25196728	negative
PRJNA729271	stage_E14_vs_E10	ordinary_muscle_development	ENSGALG00010016754	ENSGALG00010016754	-0.045220311	0.18363244	-0.246254482	0.805485244	0.863317702	negative
PRJNA729271	stage_E14_vs_E10	ordinary_muscle_development	ENSGALG00010016756	NUDT16L1	0.143720315	0.106653125	1.347549027	0.177803475	0.272035187	positive
PRJNA729271	stage_E14_vs_E10	ordinary_muscle_development	ENSGALG00010016757	SRPX2	-0.365478582	0.276592512	-1.321361085	0.186380995	0.282534253	negative
PRJNA729271	stage_E14_vs_E10	ordinary_muscle_development	ENSGALG00010016758	NR3C1	0.434325694	0.067866711	6.39968678	1.55696E-10	1.40088E-09	positive
PRJNA729271	stage_E14_vs_E10	ordinary_muscle_development	ENSGALG00010016760	DLK1	0.218813249	0.260925322	0.838604881	0.401691071	0.516913359	positive
PRJNA729271	stage_E14_vs_E10	ordinary_muscle_development	ENSGALG00010016761	GPR160	0.038048841	0.352670129	0.107887903	0.914084613	0.940755838	positive
PRJNA729271	stage_E14_vs_E10	ordinary_muscle_development	ENSGALG00010016762	EML6	0.085010456	0.11976023	0.709838778	0.477804119	0.589879116	positive
PRJNA729271	stage_E14_vs_E10	ordinary_muscle_development	ENSGALG00010016763	UBALD1	0.836150744	0.172196949	4.855781241	1.19913E-06	6.55654E-06	positive
PRJNA729271	stage_E14_vs_E10	ordinary_muscle_development	ENSGALG00010016766	AOAH	-0.298061872	0.343653371	-0.867332891	0.38575964	0.501892647	negative
PRJNA729271	stage_E14_vs_E10	ordinary_muscle_development	ENSGALG00010016767	SLAIN2	0.075108579	0.092685829	0.810356655	0.417735222	0.532300363	positive
PRJNA729271	stage_E14_vs_E10	ordinary_muscle_development	ENSGALG00010016768	GALNT10	0.399289494	0.097916382	4.077861996	4.54517E-05	0.000190356	positive
PRJNA729271	stage_E14_vs_E10	ordinary_muscle_development	ENSGALG00010016769	BNC1	-4.211701997	0.240403093	-17.51933362	1.02013E-68	3.36064E-66	negative
PRJNA729271	stage_E14_vs_E10	ordinary_muscle_development	ENSGALG00010016772	TSPAN6	-0.53761828	0.125249949	-4.29236326	1.76781E-05	7.94804E-05	negative
PRJNA729271	stage_E14_vs_E10	ordinary_muscle_development	ENSGALG00010016773	RPS27A	0.483273534	0.161240698	2.997218079	0.002724557	0.007632868	positive
PRJNA729271	stage_E14_vs_E10	ordinary_muscle_development	ENSGALG00010016774	PEX14	-0.417806621	0.117699301	-3.549779962	0.000385553	0.001342444	negative
PRJNA729271	stage_E14_vs_E10	ordinary_muscle_development	ENSGALG00010016777	TNMD	-0.921066253	0.48745449	-1.88954307	0.058819097	0.108706211	negative
PRJNA729271	stage_E14_vs_E10	ordinary_muscle_development	ENSGALG00010016778	MTIF2	-0.323820393	0.107873466	-3.00185398	0.002683409	0.007533606	negative
PRJNA729271	stage_E14_vs_E10	ordinary_muscle_development	ENSGALG00010016779	CTNNA2	-0.868799046	0.148877472	-5.835664953	5.35765E-09	4.03358E-08	negative
PRJNA729271	stage_E14_vs_E10	ordinary_muscle_development	ENSGALG00010016780	PRORSD1P	-0.233563331	0.137433724	-1.699461561	0.089232251	0.154696984	negative
PRJNA729271	stage_E14_vs_E10	ordinary_muscle_development	ENSGALG00010016781	CDIP1	0.687131254	0.143944279	4.773591955	1.80969E-06	9.60155E-06	positive
PRJNA729271	stage_E14_vs_E10	ordinary_muscle_development	ENSGALG00010016783	CLBA1	-0.362728648	0.297456768	-1.219433168	0.222679828	0.325443043	negative
PRJNA729271	stage_E14_vs_E10	ordinary_muscle_development	ENSGALG00010016785	PDGFRB	-0.078464722	0.194349323	-0.403730357	0.686411014	0.770683783	negative
PRJNA729271	stage_E14_vs_E10	ordinary_muscle_development	ENSGALG00010016786	DISP1	-0.327020547	0.110632467	-2.955918423	0.003117395	0.008603702	negative
PRJNA729271	stage_E14_vs_E10	ordinary_muscle_development	ENSGALG00010016787	PPP2R5C	-0.264547729	0.074870328	-3.53341217	0.000410232	0.001416802	negative
PRJNA729271	stage_E14_vs_E10	ordinary_muscle_development	ENSGALG00010016788	ANLN	-0.309748427	0.287739902	-1.076487569	0.281709257	0.39292382	negative
PRJNA729271	stage_E14_vs_E10	ordinary_muscle_development	ENSGALG00010016789	DISP3	0.241834857	0.113028212	2.139597294	0.032387326	0.065410935	positive
PRJNA729271	stage_E14_vs_E10	ordinary_muscle_development	ENSGALG00010016791	LRRTM1	-1.912187142	0.432744931	-4.418739551	9.92782E-06	4.6601E-05	negative
PRJNA729271	stage_E14_vs_E10	ordinary_muscle_development	ENSGALG00010016792	HMOX2	-0.453604141	0.120746617	-3.756661281	0.000172195	0.000648641	negative
PRJNA729271	stage_E14_vs_E10	ordinary_muscle_development	ENSGALG00010016793	SLC9A1	0.523623207	0.154150295	3.39683557	0.000681699	0.002234056	positive
PRJNA729271	stage_E14_vs_E10	ordinary_muscle_development	ENSGALG00010016795	GUCA2B	-0.482620201	1.279137985	-0.377301125	0.705949836	0.786468594	negative
PRJNA729271	stage_E14_vs_E10	ordinary_muscle_development	ENSGALG00010016796	BROX	0.008867715	0.08394436	0.105638009	0.915869566	0.941393374	positive
PRJNA729271	stage_E14_vs_E10	ordinary_muscle_development	ENSGALG00010016797	SEC62	-0.172911308	0.108724803	-1.590357518	0.11175424	0.18672942	negative
PRJNA729271	stage_E14_vs_E10	ordinary_muscle_development	ENSGALG00010016799	WDTC1	0.238586159	0.132342981	1.802786649	0.071421728	0.128252966	positive

PRJNA729271	stage_E14_vs_E10	ordinary_muscle_development	ENSGALG00010016800	RPL14	0.374821933	0.169004249	2.217825504	0.026566732	0.055490218	positive
PRJNA729271	stage_E14_vs_E10	ordinary_muscle_development	ENSGALG00010016802	TMEM222	-0.057200462	0.081938236	-0.698092435	0.485119389	0.596647539	negative
PRJNA729271	stage_E14_vs_E10	ordinary_muscle_development	ENSGALG00010016804	CSF1R	0.818511582	0.136237159	6.007990669	1.87837E-09	1.49107E-08	positive
PRJNA729271	stage_E14_vs_E10	ordinary_muscle_development	ENSGALG00010016805	ENSGALG00010016805	-2.141684395	0.693482157	-3.088304974	0.002013018	0.005847433	negative
PRJNA729271	stage_E14_vs_E10	ordinary_muscle_development	ENSGALG00010016807	VSIG4	1.670523413	0.242831031	6.879365495	6.01197E-12	6.27383E-11	positive
PRJNA729271	stage_E14_vs_E10	ordinary_muscle_development	ENSGALG00010016808	ENTPD3	-1.045461863	0.533256195	-1.960524553	0.049934511	0.094679721	negative
PRJNA729271	stage_E14_vs_E10	ordinary_muscle_development	ENSGALG00010016809	SYTL1	-0.082367079	0.492133612	-0.16736731	0.867081045	0.908707956	negative
PRJNA729271	stage_E14_vs_E10	ordinary_muscle_development	ENSGALG00010016810	SH3GL3	0.07148216	0.193676568	0.369080063	0.712068049	0.791581132	positive
PRJNA729271	stage_E14_vs_E10	ordinary_muscle_development	ENSGALG00010016812	LRRC31	-0.003000941	0.120652629	-0.02487257	0.980156606	0.986974181	negative
PRJNA729271	stage_E14_vs_E10	ordinary_muscle_development	ENSGALG00010016813	ENSGALG00010016813	1.528467244	0.385732572	3.962504993	7.41675E-05	0.000298876	positive
PRJNA729271	stage_E14_vs_E10	ordinary_muscle_development	ENSGALG00010016814	HMGXB3	0.081609022	0.099380251	0.821179471	0.411544048	0.526321773	positive
PRJNA729271	stage_E14_vs_E10	ordinary_muscle_development	ENSGALG00010016816	MAP3K6	-0.320767679	0.316185421	-1.014492313	0.310347896	0.42418602	negative
PRJNA729271	stage_E14_vs_E10	ordinary_muscle_development	ENSGALG00010016817	GGCT	-0.812116442	0.097839356	-8.3005089	1.03666E-16	1.70755E-15	negative
PRJNA729271	stage_E14_vs_E10	ordinary_muscle_development	ENSGALG00010016820	EIF2AK3	-0.22871647	0.107705603	-2.123533629	0.033709161	0.067759573	negative
PRJNA729271	stage_E14_vs_E10	ordinary_muscle_development	ENSGALG00010016823	HSF3	0.533119292	0.07887026	6.759446363	1.3852E-11	1.40115E-10	positive
PRJNA729271	stage_E14_vs_E10	ordinary_muscle_development	ENSGALG00010016825	ENSGALG00010016825	-0.497148029	0.300934948	-1.65201161	0.098532184	0.168085678	negative
PRJNA729271	stage_E14_vs_E10	ordinary_muscle_development	ENSGALG00010016828	LACTBL1	2.929856601	0.384235239	7.625163711	2.43725E-14	3.26204E-13	positive
PRJNA729271	stage_E14_vs_E10	ordinary_muscle_development	ENSGALG00010016831	AIDA	-0.139298155	0.066031735	-2.109563764	0.034895948	0.069738973	negative
PRJNA729271	stage_E14_vs_E10	ordinary_muscle_development	ENSGALG00010016832	DYNC1H1	-0.134967787	0.094148398	-1.433564358	0.151696621	0.239635975	negative
PRJNA729271	stage_E14_vs_E10	ordinary_muscle_development	ENSGALG00010016834	ENSGALG00010016834	0.743100962	0.074315685	9.999247962	1.53559E-23	4.12956E-22	positive
PRJNA729271	stage_E14_vs_E10	ordinary_muscle_development	ENSGALG00010016835	CCDC88A	-0.620596116	0.151219351	-4.103946431	4.06162E-05	0.000171742	negative
PRJNA729271	stage_E14_vs_E10	ordinary_muscle_development	ENSGALG00010016836	HEPH	0.562699668	0.150229303	3.745605267	0.000179959	0.00067508	positive
PRJNA729271	stage_E14_vs_E10	ordinary_muscle_development	ENSGALG00010016840	LRRC34	-0.450502169	0.202191641	-2.228094923	0.025874186	0.054289725	negative
PRJNA729271	stage_E14_vs_E10	ordinary_muscle_development	ENSGALG00010016842	NPY7R	0.454711455	0.424649703	1.070791882	0.284263018	0.395810994	positive
PRJNA729271	stage_E14_vs_E10	ordinary_muscle_development	ENSGALG00010016845	MIA3	-0.142127787	0.137927358	-1.030453923	0.302796972	0.416457169	negative
PRJNA729271	stage_E14_vs_E10	ordinary_muscle_development	ENSGALG00010016847	EPHB2	-0.193760402	0.105670443	-1.833629125	0.066709066	0.121004619	negative
PRJNA729271	stage_E14_vs_E10	ordinary_muscle_development	ENSGALG00010016848	ENSGALG00010016848	0.246797977	0.167298647	1.475194101	0.140160367	0.224588162	positive
PRJNA729271	stage_E14_vs_E10	ordinary_muscle_development	ENSGALG00010016849	WASF2	0.43250162	0.152646912	2.833346677	0.00460634	0.012108977	positive
PRJNA729271	stage_E14_vs_E10	ordinary_muscle_development	ENSGALG00010016851	TEC	0.315529813	0.141471147	2.230347453	0.025724385	0.054047683	positive
PRJNA729271	stage_E14_vs_E10	ordinary_muscle_development	ENSGALG00010016852	AHDC1	0.104397383	0.168731187	0.618720135	0.536100743	0.642850784	positive
PRJNA729271	stage_E14_vs_E10	ordinary_muscle_development	ENSGALG00010016854	TAF1A	-0.019136725	0.102621266	-0.186479135	0.85206904	0.897582902	negative
PRJNA729271	stage_E14_vs_E10	ordinary_muscle_development	ENSGALG00010016856	NOD1	-0.71645264	0.138274454	-5.181381077	2.20249E-07	1.33746E-06	negative
PRJNA729271	stage_E14_vs_E10	ordinary_muscle_development	ENSGALG00010016858	ENSGALG00010016858	-0.277504896	0.163800422	-1.694164718	0.090233987	0.156116214	negative
PRJNA729271	stage_E14_vs_E10	ordinary_muscle_development	ENSGALG00010016859	RPIA	1.133792768	0.130037178	8.718989369	2.80696E-18	5.24315E-17	positive
PRJNA729271	stage_E14_vs_E10	ordinary_muscle_development	ENSGALG00010016860	VSX1	-0.951571533	0.442196794	-2.151918661	0.031403761	0.063762084	negative
PRJNA729271	stage_E14_vs_E10	ordinary_muscle_development	ENSGALG00010016861	FGR	0.251875766	0.12190507	2.066163169	0.038813075	0.076408465	positive
PRJNA729271	stage_E14_vs_E10	ordinary_muscle_development	ENSGALG00010016862	TXK	1.224347954	0.592027022	2.06806093	0.038634294	0.076128887	positive
PRJNA729271	stage_E14_vs_E10	ordinary_muscle_development	ENSGALG00010016863	MYNN	-0.192225925	0.080586005	-2.385351214	0.017062823	0.037921744	negative
PRJNA729271	stage_E14_vs_E10	ordinary_muscle_development	ENSGALG00010016864	ENSGALG00010016864	0.102015156	0.265363693	0.384435243	0.700655884	0.782555635	positive
PRJNA729271	stage_E14_vs_E10	ordinary_muscle_development	ENSGALG00010016865	CFAP36	-0.555312358	0.135912746	-4.085800451	4.39251E-05	0.00018449	negative
PRJNA729271	stage_E14_vs_E10	ordinary_muscle_development	ENSGALG00010016866	ZNRF2	1.027297767	0.097700109	10.51480672	7.3832E-26	2.38883E-24	positive
PRJNA729271	stage_E14_vs_E10	ordinary_muscle_development	ENSGALG00010016867	FLT4	-0.476869121	0.119554758	-3.988708845	6.64339E-05	0.000270722	negative
PRJNA729271	stage_E14_vs_E10	ordinary_muscle_development	ENSGALG00010016868	NIPAL1	1.610581594	0.480507868	3.351831885	0.000802788	0.002590473	positive
PRJNA729271	stage_E14_vs_E10	ordinary_muscle_development	ENSGALG00010016869	HSP90AA1	-0.236107765	0.156348008	-1.510142453	0.131007079	0.212362739	negative
PRJNA729271	stage_E14_vs_E10	ordinary_muscle_development	ENSGALG00010016872	EDA2R	0.938030772	0.280443127	3.344816404	0.000823371	0.002650401	positive
PRJNA729271	stage_E14_vs_E10	ordinary_muscle_development	ENSGALG00010016873	ENTPD6	0.103128575	0.139069319	0.741562382	0.458352523	0.571316738	positive
PRJNA729271	stage_E14_vs_E10	ordinary_muscle_development	ENSGALG00010016874	ADAMTSL3	0.224745172	0.14490737	1.5509575	0.120911869	0.198799631	positive
PRJNA729271	stage_E14_vs_E10	ordinary_muscle_development	ENSGALG00010016875	SESN2	1.35494668	0.218788336	6.192956637	5.9046E-10	4.98469E-09	positive
PRJNA729271	stage_E14_vs_E10	ordinary_muscle_development	ENSGALG00010016876	MTURN	0.393218989	0.18517174	2.12353672	0.033708902	0.067759573	positive
PRJNA729271	stage_E14_vs_E10	ordinary_muscle_development	ENSGALG00010016877	UBE2D3	-0.056727938	0.135576252	-0.418420901	0.675639407	0.761894602	negative
PRJNA729271	stage_E14_vs_E10	ordinary_muscle_development	ENSGALG00010016878	SUCLG1	0.250409206	0.125538656	1.994678082	0.046078001	0.088640344	positive
PRJNA729271	stage_E14_vs_E10	ordinary_muscle_development	ENSGALG00010016879	RNF130	-0.205830728	0.099927118	-2.059808512	0.039416851	0.07736591	negative
PRJNA729271	stage_E14_vs_E10	ordinary_muscle_development	ENSGALG00010016880	AR	0.420014773	0.20185215	2.080804062	0.037451844	0.074060639	positive
PRJNA729271	stage_E14_vs_E10	ordinary_muscle_development	ENSGALG00010016881	C1QB	0.681163284	0.292357596	2.32989768	0.01981156	0.04307313	positive
PRJNA729271	stage_E14_vs_E10	ordinary_muscle_development	ENSGALG00010016882	ENSGALG00010016882	-0.222880412	0.047971157	-4.646133729	3.38214E-06	1.71353E-05	negative
PRJNA729271	stage_E14_vs_E10	ordinary_muscle_development	ENSGALG00010016883	TMEM251	-0.116757037	0.076699075	-1.522274383	0.127940344	0.208229877	negative
PRJNA729271	stage_E14_vs_E10	ordinary_muscle_development	ENSGALG00010016884	SBK1	0.370080048	0.245245868	1.509016444	0.131294581	0.2127574	positive
PRJNA729271	stage_E14_vs_E10	ordinary_muscle_development	ENSGALG00010016885	CDCA4	-0.118366368	0.153164278	-0.772806616	0.439636838	0.553507857	negative
PRJNA729271	stage_E14_vs_E10	ordinary_muscle_development	ENSGALG00010016887	TJP2	0.31209461	0.138448631	2.254226761	0.024181906	0.051245136	positive
PRJNA729271	stage_E14_vs_E10	ordinary_muscle_development	ENSGALG00010016888	ENSGALG00010016888	0.323057619	0.077931635	4.145397663	3.39224E-05	0.000145217	positive
PRJNA729271	stage_E14_vs_E10	ordinary_muscle_development	ENSGALG00010016889	FUNDC2	0.536272597	0.087046094	6.160788719	7.23835E-10	6.0126E-09	positive
PRJNA729271	stage_E14_vs_E10	ordinary_muscle_development	ENSGALG00010016890	UBR7	-0.201178573	0.074939328	-2.684552651	0.007262694	0.018001497	negative
PRJNA729271	stage_E14_vs_E10	ordinary_muscle_development	ENSGALG00010016891	ENSGALG00010016891	-0.16365794	1.164965991	-0.14048302			negative
PRJNA729271	stage_E14_vs_E10	ordinary_muscle_development	ENSGALG00010016892	MAL	4.146052165	0.396440048	10.45820721	1.34374E-25	4.24347E-24	positive
PRJNA729271	stage_E14_vs_E10	ordinary_muscle_development	ENSGALG00010016893	TBC1D9B	0.013984607	0.050337343	0.277817748	0.781152266	0.845490001	positive
PRJNA729271	stage_E14_vs_E10	ordinary_muscle_development	ENSGALG00010016894	BTBD7	0.010470649	0.091460387	0.114482886	0.908855012	0.937240567	positive
PRJNA729271	stage_E14_vs_E10	ordinary_muscle_development	ENSGALG00010016895	ENSGALG00010016895	0.478360985	0.15515148	3.083186731	0.002047966	0.00593587	positive
PRJNA729271	stage_E14_vs_E10	ordinary_muscle_development	ENSGALG00010016896	SPTBN1	0.303596966	0.09868799	3.07633145	0.002095647	0.006060736	positive
PRJNA729271	stage_E14_vs_E10	ordinary_muscle_development	ENSGALG00010016898	ENSGALG00010016898	-2.900845872	0.801731831	-3.61822465	0.000296631	0.001063484	negative
PRJNA729271	stage_E14_vs_E10	ordinary_muscle_development	ENSGALG00010016899	SAXO2	-0.617061791	0.511444407	-1.206508042	0.22762166	0.331029995	negative
PRJNA729271	stage_E14_vs_E10	ordinary_muscle_development	ENSGALG00010016900	CISD2	-0.269226325	0.088559947	-3.040046144	0.002365419	0.006733388	negative
PRJNA729271	stage_E14_vs_E10	ordinary_muscle_development	ENSGALG00010016901	MECOM	-0.926340708	0.192028025	-4.823987057	1.40717E-06	7.62215E-06	negative
PRJNA729271	stage_E14_vs_E10	ordinary_muscle_development	ENSGALG00010016902	MRNIP	0.035227044	0.137317084	0.256537958	0.797535475	0.857676941	positive
PRJNA729271	stage_E14_vs_E10	ordinary_muscle_development	ENSGALG00010016903	PPM1A	0.166324191	0.147405701	1.128342995	0.259175092	0.367515453	positive
PRJNA729271	stage_E14_vs_E10	ordinary_muscle_development	ENSGALG00010016904	EAF1	0.40197536	0.069722207	5.765384866	8.14717E-09	5.99154E-08	positive
PRJNA729271	stage_E14_vs_E10	ordinary_muscle_development	ENSGALG00010016905	C1QC	0.511988009	0.376715809	1.359082888	0.174120325	0.267416201	positive
PRJNA729271	stage_E14_vs_E10	ordinary_muscle_development	ENSGALG00010016907	SQSTM1	0.005054033	0.162313742	0.031137431	0.975159938	0.983095236	positive
PRJNA729271	stage_E14_vs_E10	ordinary_muscle_development	ENSGALG00010016908	CMC4	0.353938089	0.160827993	2.200724402	0.02775554	0.057613713	positive
PRJNA729271	stage_E14_vs_E10	ordinary_muscle_development	ENSGALG00010016909	MRPS5	0.068626799	0.133053084	0.515785108	0.606004505	0.703792588	positive
PRJNA729271	stage_E14_vs_E10	ordinary_muscle_development	ENSGALG00010016910	NSD2	-0.225779333	0.109659208	-2.058918151	0.03950208	0.077491223	negative
PRJNA729271	stage_E14_vs_E10	ordinary_muscle_development	ENSGALG00010016911	AXIN1	-0.385815407	0.098018592	-3.936145153	8.28009E-05	0.000330907	negative
PRJNA729271	stage_E14_vs_E10	ordinary_muscle_development	ENSGALG00010016912	CCDC88C	0.20368997	0.159795335	1.274692849	0.202417979	0.3018258	positive
PRJNA729271	stage_E14_vs_E10	ordinary_muscle_development	ENSGALG00010016913	BRCC3	-0.079369502	0.108577129	-0.73099651	0.464781285	0.577292607	negative
PRJNA729271	stage_E14_vs_E10	ordinary_muscle_development	ENSGALG00010016915	PLOD2	-1.235710561	0.167799429	-7.36421197	1.78196E-13	2.20013E-12	negative
PRJNA729271	stage_E14_vs_E10	ordinary_muscle_development	ENSGALG00010016916	ACYP2	0.862310316	0.158992913	5.423577049	5.8418E-08	3.83674E-07	positive
PRJNA729271	stage_E14_vs_E10	ordinary_muscle_development	ENSGALG00010016917	METTL6	-0.042971928	0.170728249	-0.251697818	0.801274643	0.860013029	negative
PRJNA729271	stage_E14_vs_E10	ordinary_muscle_development	ENSGALG00010016918	RCOR1	-0.300298212	0.073576981	-4.081415257	4.47623E-05	0.000187523	negative
PRJNA729271	stage_E14_vs_E10	ordinary_muscle_development	ENSGALG00010016919	ARHGDIG	0.542105317	0.194745101	2.783666001	0.005374835	0.013869795	positive
PRJNA729271	stage_E14_vs_E10	ordinary_muscle_development	ENSGALG00010016920	C1QA	0.491668279	0.3222671	1.525654585	0.12709591	0.206994969	positive
PRJNA729271	stage_E14_vs_E10	ordinary_muscle_development	ENSGALG00010016921	EFL1	-0.229950084	0.07398934	-3.107881261	0.001884338	0.005531283	negative
PRJNA729271	stage_E14_vs_E10	ordinary_muscle_development	ENSGALG00010016922	ENSGALG00010016922	0.657653167	0.190015822	3.46104425	0.000538084	0.001811317	positive
PRJNA729271	stage_E14_vs_E10	ordinary_muscle_development	ENSGALG00010016923	ENSGALG00010016923	-0.082068967	0.15894766	-0.516326991	0.605626048	0.703522164	negative
PRJNA729271	stage_E14_vs_E10	ordinary_muscle_development	ENSGALG00010016924	LETM1	-0.267876526	0.117070513	-2.28816394	0.022127976	0.0475357	negative
PRJNA729271	stage_E14_vs_E10	ordinary_muscle_development	ENSGALG00010016925	PHACTR4	-0.124662022	0.132967045	-0.93754074	0.348480478	0.464224292	negative
PRJNA729271	stage_E14_vs_E10	ordinary_muscle_development	ENSGALG00010016926	ENSGALG00010016926	0.657081178	0.179306058	3.664578805	0.000247746	0.000900019	positive
PRJNA729271	stage_E14_vs_E10	ordinary_muscle_development	ENSGALG00010016927	MGAT4B	0.095847051	0.115980022	0.826410003	0.408571551	0.523305172	positive
PRJNA729271	stage_E14_vs_E10	ordinary_muscle_development	ENSGALG00010016928	RGS11	0.225640396	0.463352002	0.48697404	0.626276728	0.721096288	positive
PRJNA729271	stage_E14_vs_E10	ordinary_muscle_development	ENSGALG00010016930	C10H15ORF48	0.472910083	0.454834965	1.039739948	0.298460735	0.411939474	positive
PRJNA729271	stage_E14_vs_E10	ordinary_muscle_development	ENSGALG00010016931	SLC30A4	0.303520784	0.081681925	3.71588677	0.000202492	0.000751825	positive
PRJNA729271	stage_E14_vs_E10	ordinary_muscle_development	ENSGALG00010016932	OGT	-0.020850143	0.092153321	-0.226254925	0.821003154	0.87471082	negative
PRJNA729271	stage_E14_vs_E10	ordinary_muscle_development	ENSGALG00010016934	SLC25A38	-0.253438462	0.138633473	-1.828118827	0.06753172	0.122297886	negative
PRJNA729271	stage_E14_vs_E10	ordinary_muscle_development	ENSGALG00010016935	PRIMA1	0.051544117	0.248229898	0.20764669	0.835504846	0.885151494	positive
PRJNA729271	stage_E14_vs_E10	ordinary_muscle_development	ENSGALG00010016936	RCC1	0.623403488	0.160905885	3.874336165	0.000106916	0.00041919	positive
PRJNA729271	stage_E14_vs_E10	ordinary_muscle_development	ENSGALG00010016937	DHRS7	-0.300726749	0.171153099	-1.757062836	0.078907099	0.139755426	negative
PRJNA729271	stage_E14_vs_E10	ordinary_muscle_development	ENSGALG00010016938	PSME4	-0.135391762	0.166612742	-0.81261349	0.416439695	0.531130082	negative
PRJNA729271	stage_E14_vs_E10	ordinary_muscle_development	ENSGALG00010016939	FAM234A	0.477728767	0.078501671	6.085587246	1.16065E-09	9.39868E-09	positive
PRJNA729271	stage_E14_vs_E10	ordinary_muscle_development	ENSGALG00010016940	FGFR3	-2.911701536	0.213010066	-13.66931427	1.54848E-42	1.39411E-40	negative
PRJNA729271	stage_E14_vs_E10	ordinary_muscle_development	ENSGALG00010016941	SLC8A1	-0.281018507	0.176315285	-1.593840867	0.110971682	0.185614416	negative
PRJNA729271	stage_E14_vs_E10	ordinary_muscle_development	ENSGALG00010016942	ZBTB40	0.262036799	0.151499959	1.729616306	0.083698851	0.146931675	positive
PRJNA729271	stage_E14_vs_E10	ordinary_muscle_development	ENSGALG00010016943	PCNX4	-0.365923424	0.108250232	-3.3803477	0.000723942	0.002361813	negative
PRJNA729271	stage_E14_vs_E10	ordinary_muscle_development	ENSGALG00010016944	OPRD1	-1.329967715	0.384747187	-3.456731484	0.000546769	0.001834164	negative
PRJNA729271	stage_E14_vs_E10	ordinary_muscle_development	ENSGALG00010016945	BDH2	0.167849912	0.145233503	1.155724458	0.247793881	0.354567848	positive
PRJNA729271	stage_E14_vs_E10	ordinary_muscle_development	ENSGALG00010016946	TAF1	-0.21081501	0.106429765	-1.980789958	0.047614833	0.091028358	negative
PRJNA729271	stage_E14_vs_E10	ordinary_muscle_development	ENSGALG00010016947	CANX	-0.072241513	0.070772943	-1.020750445	0.307372688	0.420992829	negative
PRJNA729271	stage_E14_vs_E10	ordinary_muscle_development	ENSGALG00010016948	CCR8	-1.931297554	0.791931113	-2.43871913	0.014739419	0.033429492	negative
PRJNA729271	stage_E14_vs_E10	ordinary_muscle_development	ENSGALG00010016949	LUC7L	-0.11158256	0.093708372	-1.1907427	0.233754609	0.338184755	negative
PRJNA729271	stage_E14_vs_E10	ordinary_muscle_development	ENSGALG00010016950	ENSGALG00010016950	3.398578398	0.978717439	3.472481702	0.00051567	0.00174234	positive
PRJNA729271	stage_E14_vs_E10	ordinary_muscle_development	ENSGALG00010016952	GINS1	0.033751133	0.197813977	0.170620569	0.864522123	0.906747336	positive
PRJNA729271	stage_E14_vs_E10	ordinary_muscle_development	ENSGALG00010016953	GJA4	0.951704526	0.160728937	5.921177245	3.19645E-09	2.47371E-08	positive
PRJNA729271	stage_E14_vs_E10	ordinary_muscle_development	ENSGALG00010016955	ENSGALG00010016955	-3.196192208	0.215824732	-14.80920275	1.27746E-49	1.73055E-47	negative
PRJNA729271	stage_E14_vs_E10	ordinary_muscle_development	ENSGALG00010016956	CENPE	-0.373607895	0.255417707	-1.462732946	0.143540467	0.228966554	negative
PRJNA729271	stage_E14_vs_E10	ordinary_muscle_development	ENSGALG00010016957	WNT4	0.046638916	0.202898386	0.229863414	0.818197906	0.872684227	positive
PRJNA729271	stage_E14_vs_E10	ordinary_muscle_development	ENSGALG00010016958	TLR15	-0.796448127	0.569581031	-1.398305218	0.162021431	0.25258127	negative
PRJNA729271	stage_E14_vs_E10	ordinary_muscle_development	ENSGALG00010016959	PERCC1	-0.330664284	1.240011522	-0.266662267	0.789729208	0.852227879	negative
PRJNA729271	stage_E14_vs_E10	ordinary_muscle_development	ENSGALG00010016960	GJB3	-1.92172055	0.705655779	-2.723311572	0.006463107	0.016253078	negative
PRJNA729271	stage_E14_vs_E10	ordinary_muscle_development	ENSGALG00010016961	EIF4A3	0.119646814	0.073996942	1.61691567	0.105896485	0.178669071	positive
PRJNA729271	stage_E14_vs_E10	ordinary_muscle_development	ENSGALG00010016962	SQOR	0.81614497	0.137766129	5.924133713	3.13948E-09	2.43352E-08	positive
PRJNA729271	stage_E14_vs_E10	ordinary_muscle_development	ENSGALG00010016963	CD8A	-2.170712825	1.052574292	-2.062289419	0.03918019	0.077026563	negative
PRJNA729271	stage_E14_vs_E10	ordinary_muscle_development	ENSGALG00010016964	MAML1	0.010976518	0.084407277	0.130042317	0.896532946	0.928696138	positive
PRJNA729271	stage_E14_vs_E10	ordinary_muscle_development	ENSGALG00010016965	SLC25A33	0.318038921	0.337776328	0.941566637	0.346414558	0.461981692	positive
PRJNA729271	stage_E14_vs_E10	ordinary_muscle_development	ENSGALG00010016966	KDM3A	0.067442964	0.158102135	0.426578449	0.669686397	0.757066318	positive

PRJNA729271	stage_E14_vs_E10	ordinary_muscle_development	ENSGALG00010016967	ERLEC1	0.104706585	0.07419255	1.411281658	0.15816158	0.247609611	positive
PRJNA729271	stage_E14_vs_E10	ordinary_muscle_development	ENSGALG00010016968	C15orf61	0.180324526	0.147136067	1.225563045	0.220363152	0.32280585	positive
PRJNA729271	stage_E14_vs_E10	ordinary_muscle_development	ENSGALG00010016969	CHMP3	0.245912303	0.083651013	2.939740874	0.003284868	0.009004477	positive
PRJNA729271	stage_E14_vs_E10	ordinary_muscle_development	ENSGALG00010016970	SYNE3	0.714240695	0.09889591	7.222145958	5.11735E-13	6.03548E-12	positive
PRJNA729271	stage_E14_vs_E10	ordinary_muscle_development	ENSGALG00010016972	ASB3	-0.321517627	0.100125817	-3.211136127	0.001322113	0.004041901	negative
PRJNA729271	stage_E14_vs_E10	ordinary_muscle_development	ENSGALG00010016973	PLSCR1	-0.311194902	0.1290771	-2.410922641	0.015912223	0.035703974	negative
PRJNA729271	stage_E14_vs_E10	ordinary_muscle_development	ENSGALG00010016974	CFAP96	-0.968494278	0.591544691	-1.637229263	0.101582575	0.172255431	negative
PRJNA729271	stage_E14_vs_E10	ordinary_muscle_development	ENSGALG00010016975	MALL	-0.223114787	0.235693215	-0.946632202	0.343826229	0.45907896	negative
PRJNA729271	stage_E14_vs_E10	ordinary_muscle_development	ENSGALG00010016976	ENSGALG00010016976	2.44799729	1.001859759	2.443453056	0.014547462	0.033076935	positive
PRJNA729271	stage_E14_vs_E10	ordinary_muscle_development	ENSGALG00010016977	CLCN7	-0.208106993	0.119061838	-1.747889981	0.08048308	0.142164544	negative
PRJNA729271	stage_E14_vs_E10	ordinary_muscle_development	ENSGALG00010016978	FAM181A	0.804502564	0.364439569	2.207506078	0.027278723	0.056729568	positive
PRJNA729271	stage_E14_vs_E10	ordinary_muscle_development	ENSGALG00010016979	PLSCR5	0.291810496	0.449775177	0.64879191	0.516472887	0.624949871	positive
PRJNA729271	stage_E14_vs_E10	ordinary_muscle_development	ENSGALG00010016980	NPHP1	0.001207223	0.099904654	0.012083752	0.990358795	0.993758406	positive
PRJNA729271	stage_E14_vs_E10	ordinary_muscle_development	ENSGALG00010016981	ENSGALG00010016981	-0.078496977	0.480314016	-0.163428453	0.870181102	0.911034675	negative
PRJNA729271	stage_E14_vs_E10	ordinary_muscle_development	ENSGALG00010016982	GLRX5	-0.143991203	0.140681057	-1.023529439	0.306057588	0.419698071	negative
PRJNA729271	stage_E14_vs_E10	ordinary_muscle_development	ENSGALG00010016983	ZFAND2A	-0.1606374	0.284411833	-0.564805615	0.572205987	0.674157254	negative
PRJNA729271	stage_E14_vs_E10	ordinary_muscle_development	ENSGALG00010016984	RNF103	0.149979608	0.11594441	1.293547556	0.195821747	0.294077521	positive
PRJNA729271	stage_E14_vs_E10	ordinary_muscle_development	ENSGALG00010016985	ENSGALG00010016985	0.181972325	0.197791253	0.920022109	0.357561206	0.473925537	positive
PRJNA729271	stage_E14_vs_E10	ordinary_muscle_development	ENSGALG00010016986	GFER	-0.163320773	0.085760844	-1.904374606	0.056861414	0.105667461	negative
PRJNA729271	stage_E14_vs_E10	ordinary_muscle_development	ENSGALG00010016987	ASB2	1.538635294	0.318666765	4.828351947	1.37668E-06	7.47655E-06	positive
PRJNA729271	stage_E14_vs_E10	ordinary_muscle_development	ENSGALG00010016988	ECI1	1.022296378	0.109120704	9.36849138	7.35766E-21	1.67688E-19	positive
PRJNA729271	stage_E14_vs_E10	ordinary_muscle_development	ENSGALG00010016989	RHOG2	-0.071069717	0.092506687	-0.768265729	0.442329331	0.556124872	negative
PRJNA729271	stage_E14_vs_E10	ordinary_muscle_development	ENSGALG00010016991	PDLIM3	0.128864197	0.19967697	0.645363346	0.518691751	0.626849836	positive
PRJNA729271	stage_E14_vs_E10	ordinary_muscle_development	ENSGALG00010016993	ENSGALG00010016993	1.087477766	0.179741862	6.050219774	1.44648E-09	1.16353E-08	positive
PRJNA729271	stage_E14_vs_E10	ordinary_muscle_development	ENSGALG00010016994	ATL2	0.299081319	0.115589624	2.587440879	0.009669179	0.023139301	positive
PRJNA729271	stage_E14_vs_E10	ordinary_muscle_development	ENSGALG00010016998	DNASE1L2	0.646615675	0.338162111	1.912147028	0.055857338	0.104135321	positive
PRJNA729271	stage_E14_vs_E10	ordinary_muscle_development	ENSGALG00010016999	ITGB1BP2	2.081582978	0.172674761	12.05493476	1.82677E-33	9.35655E-32	positive
PRJNA729271	stage_E14_vs_E10	ordinary_muscle_development	ENSGALG00010017001	TPCN3	-0.708123553	0.114693373	-6.174058127	6.65591E-10	5.56707E-09	negative
PRJNA729271	stage_E14_vs_E10	ordinary_muscle_development	ENSGALG00010017003	CPZ	1.505270651	0.39700308	3.791584313	0.000149689	0.000570536	positive
PRJNA729271	stage_E14_vs_E10	ordinary_muscle_development	ENSGALG00010017004	CX3CR1	-1.801761654	0.425121659	-4.238225969	2.25293E-05	9.9501E-05	negative
PRJNA729271	stage_E14_vs_E10	ordinary_muscle_development	ENSGALG00010017006	DNAH3	-0.178185351	0.245093072	-0.727010965	0.467219239	0.57957577	negative
PRJNA729271	stage_E14_vs_E10	ordinary_muscle_development	ENSGALG00010017008	RTN1	-3.666991159	0.186496836	-19.66248454	4.52038E-86	2.97831E-83	negative
PRJNA729271	stage_E14_vs_E10	ordinary_muscle_development	ENSGALG00010017010	NONO	0.078399747	0.0544048	1.441044675	0.149572062	0.236827553	positive
PRJNA729271	stage_E14_vs_E10	ordinary_muscle_development	ENSGALG00010017012	SYNGR3	-0.197310011	0.183916147	-1.072825927	0.283349226	0.394880014	negative
PRJNA729271	stage_E14_vs_E10	ordinary_muscle_development	ENSGALG00010017016	ZIC1	-6.241405108	0.82340194	-7.580022347	3.45495E-14	4.54443E-13	negative
PRJNA729271	stage_E14_vs_E10	ordinary_muscle_development	ENSGALG00010017017	ENSGALG00010017017	0.217591817	0.268528637	0.810311403	0.417761223	0.532300363	positive
PRJNA729271	stage_E14_vs_E10	ordinary_muscle_development	ENSGALG00010017018	HP1BP3	-0.047417053	0.083162159	-0.570175823	0.568558453	0.671057306	negative
PRJNA729271	stage_E14_vs_E10	ordinary_muscle_development	ENSGALG00010017020	GJB5	0.427681668	0.300317513	1.424098327	0.154418011	0.243107317	positive
PRJNA729271	stage_E14_vs_E10	ordinary_muscle_development	ENSGALG00010017022	CCR4	-2.555387758	0.885013707	-2.887399074	0.003884412	0.01041327	negative
PRJNA729271	stage_E14_vs_E10	ordinary_muscle_development	ENSGALG00010017024	BUB1	-0.300624031	0.215173264	-1.397125393	0.162375867	0.252970571	negative
PRJNA729271	stage_E14_vs_E10	ordinary_muscle_development	ENSGALG00010017033	LDAF1	0.157205115	0.110814996	1.418626725	0.15600787	0.244971734	positive
PRJNA729271	stage_E14_vs_E10	ordinary_muscle_development	ENSGALG00010017034	ZMYM3	0.071359465	0.088330031	0.807873201	0.41916358	0.533711884	positive
PRJNA729271	stage_E14_vs_E10	ordinary_muscle_development	ENSGALG00010017035	PIK3CD	0.219443988	0.20307814	1.080588917	0.279880015	0.390742168	positive
PRJNA729271	stage_E14_vs_E10	ordinary_muscle_development	ENSGALG00010017038	ZNF598	-0.221559775	0.131127704	-1.689648861	0.091095152	0.157377993	negative
PRJNA729271	stage_E14_vs_E10	ordinary_muscle_development	ENSGALG00010017039	MAP2K5	0.010512955	0.076064505	0.138211052	0.890073621	0.924581993	positive
PRJNA729271	stage_E14_vs_E10	ordinary_muscle_development	ENSGALG00010017041	JKAMP	-0.012270374	0.087972823	-0.139479139	0.889071539	0.923872103	negative
PRJNA729271	stage_E14_vs_E10	ordinary_muscle_development	ENSGALG00010017042	HNRNPLL	-0.012084876	0.068387893	-0.176710757	0.859735591	0.903492162	negative
PRJNA729271	stage_E14_vs_E10	ordinary_muscle_development	ENSGALG00010017045	GLB1	0.070221992	0.09833335	0.71412183	0.475151848	0.587355112	positive
PRJNA729271	stage_E14_vs_E10	ordinary_muscle_development	ENSGALG00010017049	TRAF3	-0.193333937	0.087947702	-2.198282988	0.027928946	0.057899038	negative
PRJNA729271	stage_E14_vs_E10	ordinary_muscle_development	ENSGALG00010017050	L3HYPDH	0.093176792	0.152144877	0.612421489	0.540258928	0.646339812	positive
PRJNA729271	stage_E14_vs_E10	ordinary_muscle_development	ENSGALG00010017054	GALM	0.274342034	0.14759192	1.858787624	0.063057247	0.115391339	positive
PRJNA729271	stage_E14_vs_E10	ordinary_muscle_development	ENSGALG00010017055	GPR135	0.041940035	0.268849157	0.155998387	0.876034293	0.915113655	positive
PRJNA729271	stage_E14_vs_E10	ordinary_muscle_development	ENSGALG00010017056	AGTR1	0.759270096	0.203427522	3.732386298	0.000189674	0.000708225	positive
PRJNA729271	stage_E14_vs_E10	ordinary_muscle_development	ENSGALG00010017057	NDUFAB1	0.289934958	0.121478808	2.386712238	0.016999792	0.037804845	positive
PRJNA729271	stage_E14_vs_E10	ordinary_muscle_development	ENSGALG00010017060	CLSTN1	-1.133496569	0.102090331	-11.10287882	1.21467E-28	4.78442E-27	negative
PRJNA729271	stage_E14_vs_E10	ordinary_muscle_development	ENSGALG00010017063	TMPPE	0.119875022	0.105388225	1.137461247	0.255345516	0.363011451	positive
PRJNA729271	stage_E14_vs_E10	ordinary_muscle_development	ENSGALG00010017065	VRK2	-0.380509778	0.131964205	-2.883431737	0.003933679	0.010525877	negative
PRJNA729271	stage_E14_vs_E10	ordinary_muscle_development	ENSGALG00010017068	ZP2	-3.227246738	1.013648158	-3.183793816	0.001453585	0.004395935	negative
PRJNA729271	stage_E14_vs_E10	ordinary_muscle_development	ENSGALG00010017070	BLOC1S6	-0.045807338	0.104189247	-0.439655143	0.660186895	0.749542496	negative
PRJNA729271	stage_E14_vs_E10	ordinary_muscle_development	ENSGALG00010017077	CTNNBIP1	0.538378136	0.10348451	5.202499754	1.96626E-07	1.20308E-06	positive
PRJNA729271	stage_E14_vs_E10	ordinary_muscle_development	ENSGALG00010017080	PALB2	-0.250928447	0.093049482	-2.696720522	0.007002601	0.017431343	negative
PRJNA729271	stage_E14_vs_E10	ordinary_muscle_development	ENSGALG00010017083	ENSGALG00010017083	0.911974665	0.381069753	2.39319615	0.016702309	0.037246149	positive
PRJNA729271	stage_E14_vs_E10	ordinary_muscle_development	ENSGALG00010017085	ENSGALG00010017085	-0.009722999	0.11752052	-0.082734479	0.934062668	0.954530124	negative
PRJNA729271	stage_E14_vs_E10	ordinary_muscle_development	ENSGALG00010017089	CRTAP	1.168939582	0.156431698	7.472523762	7.86712E-14	1.00737E-12	positive
PRJNA729271	stage_E14_vs_E10	ordinary_muscle_development	ENSGALG00010017094	SORBS2	-0.127766515	0.147648324	-0.865343481	0.386850292	0.502995603	negative
PRJNA729271	stage_E14_vs_E10	ordinary_muscle_development	ENSGALG00010017096	DCTN5	0.054771988	0.08966137	0.610876095	0.541281612	0.647189389	positive
PRJNA729271	stage_E14_vs_E10	ordinary_muscle_development	ENSGALG00010017098	DAAM1	0.328164675	0.121756066	2.695263455	0.0070333	0.017501747	positive
PRJNA729271	stage_E14_vs_E10	ordinary_muscle_development	ENSGALG00010017100	SUSD5	-5.460752429	0.57385159	-9.515966368	1.80032E-21	4.34203E-20	negative
PRJNA729271	stage_E14_vs_E10	ordinary_muscle_development	ENSGALG00010017101	CSMD2	-0.591724883	0.233075453	-2.538769638	0.011124304	0.026176427	negative
PRJNA729271	stage_E14_vs_E10	ordinary_muscle_development	ENSGALG00010017104	FANCL	-0.51603207	0.173637305	-2.971896338	0.002959666	0.008216885	negative
PRJNA729271	stage_E14_vs_E10	ordinary_muscle_development	ENSGALG00010017105	PLK1	-0.222702406	0.232561295	-0.957607353	0.33826079	0.453407696	negative
PRJNA729271	stage_E14_vs_E10	ordinary_muscle_development	ENSGALG00010017106	CRYM	0.268109961	0.111576865	2.402917143	0.016264872	0.036371386	positive
PRJNA729271	stage_E14_vs_E10	ordinary_muscle_development	ENSGALG00010017109	ITGB1BP3	0.460070754	0.127438122	3.610150136	0.00030602	0.001093435	positive
PRJNA729271	stage_E14_vs_E10	ordinary_muscle_development	ENSGALG00010017110	BRF1	0.028089952	0.070132436	0.40052725	0.688768217	0.772633148	positive
PRJNA729271	stage_E14_vs_E10	ordinary_muscle_development	ENSGALG00010017111	GJB1	-0.873349624	0.452629581	-1.929501872	0.053668588	0.10061125	negative
PRJNA729271	stage_E14_vs_E10	ordinary_muscle_development	ENSGALG00010017112	LZIC	-0.086733113	0.062099383	-1.396682372	0.162509107	0.253042165	negative
PRJNA729271	stage_E14_vs_E10	ordinary_muscle_development	ENSGALG00010017115	LGALSL	0.006478208	0.193680239	0.033447955	0.973317368	0.981797448	positive
PRJNA729271	stage_E14_vs_E10	ordinary_muscle_development	ENSGALG00010017116	ENSGALG00010017116	0.439527758	0.251427049	1.748132346	0.080441113	0.142107731	positive
PRJNA729271	stage_E14_vs_E10	ordinary_muscle_development	ENSGALG00010017119	BTBD6	1.026551016	0.149014504	6.888933554	5.62122E-12	5.88725E-11	positive
PRJNA729271	stage_E14_vs_E10	ordinary_muscle_development	ENSGALG00010017120	ERN2	0.76664989	0.141173725	5.430542336	5.6183E-08	3.69833E-07	positive
PRJNA729271	stage_E14_vs_E10	ordinary_muscle_development	ENSGALG00010017122	NCBP2	-0.326796764	0.192110847	-1.701084394	0.088927141	0.154352641	negative
PRJNA729271	stage_E14_vs_E10	ordinary_muscle_development	ENSGALG00010017127	SOX8	0.832418932	0.131886985	6.311607845	2.76151E-10	2.41716E-09	positive
PRJNA729271	stage_E14_vs_E10	ordinary_muscle_development	ENSGALG00010017131	TLR3	1.155874909	0.383210489	3.016292457	0.002558863	0.007209081	positive
PRJNA729271	stage_E14_vs_E10	ordinary_muscle_development	ENSGALG00010017132	TMEM108	0.614741435	0.258899086	2.374444207	0.017575392	0.038911762	positive
PRJNA729271	stage_E14_vs_E10	ordinary_muscle_development	ENSGALG00010017133	WDR20	-0.023291343	0.112123579	-0.207729215	0.835440405	0.885151494	negative
PRJNA729271	stage_E14_vs_E10	ordinary_muscle_development	ENSGALG00010017135	ENSGALG00010017135	0.548360231	0.13157635	4.167620017	3.07796E-05	0.0001331	positive
PRJNA729271	stage_E14_vs_E10	ordinary_muscle_development	ENSGALG00010017136	ENSGALG00010017136	0.376805202	0.380129905	0.991253772	0.32156168	0.436099976	positive
PRJNA729271	stage_E14_vs_E10	ordinary_muscle_development	ENSGALG00010017139	COL9A2	-4.418241814	0.559003519	-7.903781758	2.70567E-15	3.90624E-14	negative
PRJNA729271	stage_E14_vs_E10	ordinary_muscle_development	ENSGALG00010017140	UQCRC2	0.644356383	0.114976164	5.604260584	2.09146E-08	1.46665E-07	positive
PRJNA729271	stage_E14_vs_E10	ordinary_muscle_development	ENSGALG00010017141	AFTPH	-0.003951216	0.075663124	-0.052221155	0.958352476	0.971352992	negative
PRJNA729271	stage_E14_vs_E10	ordinary_muscle_development	ENSGALG00010017142	RBP7	1.482400142	0.694503999	2.134473156	0.032804069	0.066171399	positive
PRJNA729271	stage_E14_vs_E10	ordinary_muscle_development	ENSGALG00010017143	FAM149A	-1.028495932	0.276270123	-3.722791019	0.000197033	0.000733245	negative
PRJNA729271	stage_E14_vs_E10	ordinary_muscle_development	ENSGALG00010017144	CPB1	0.161095852	0.746142601	0.21590491	0.829061869	0.880771899	positive
PRJNA729271	stage_E14_vs_E10	ordinary_muscle_development	ENSGALG00010017147	SRSF7L	0.02265722	0.069577923	0.325638061	0.744698212	0.816829404	positive
PRJNA729271	stage_E14_vs_E10	ordinary_muscle_development	ENSGALG00010017148	MOK	-0.826554397	0.475049983	-1.739931431	0.081871059	0.144264649	negative
PRJNA729271	stage_E14_vs_E10	ordinary_muscle_development	ENSGALG00010017152	CYP4V2	0.301314865	0.208783944	1.44318983	0.148967003	0.236121376	positive
PRJNA729271	stage_E14_vs_E10	ordinary_muscle_development	ENSGALG00010017153	SLC35F4	0.857186837	0.553381959	1.548996715	0.12138251	0.199505554	positive
PRJNA729271	stage_E14_vs_E10	ordinary_muscle_development	ENSGALG00010017154	PIGZ	-0.122260773	0.188330561	-0.649181804	0.516220872	0.62470565	negative
PRJNA729271	stage_E14_vs_E10	ordinary_muscle_development	ENSGALG00010017162	BCL11A	-1.192470646	0.191960479	-6.212063311	5.22933E-10	4.43011E-09	negative
PRJNA729271	stage_E14_vs_E10	ordinary_muscle_development	ENSGALG00010017163	CDV3	-0.212576768	0.124485187	-1.707647097	0.087701844	0.152554689	negative
PRJNA729271	stage_E14_vs_E10	ordinary_muscle_development	ENSGALG00010017167	SMAP2	0.109591348	0.09546255	1.14800357	0.25096709	0.358083273	positive
PRJNA729271	stage_E14_vs_E10	ordinary_muscle_development	ENSGALG00010017169	CCDC198	0.032560307	0.237899703	0.13686569	0.891136959	0.925222421	positive
PRJNA729271	stage_E14_vs_E10	ordinary_muscle_development	ENSGALG00010017174	DHX57	-0.382970801	0.095307222	-4.018276839	5.86253E-05	0.000240866	negative
PRJNA729271	stage_E14_vs_E10	ordinary_muscle_development	ENSGALG00010017180	ENSGALG00010017180	-1.599908129	0.44238639	-3.616540125	0.000298567	0.001070008	negative
PRJNA729271	stage_E14_vs_E10	ordinary_muscle_development	ENSGALG00010017183	NFYC	0.271497707	0.093096442	2.916305937	0.00354203	0.009605561	positive
PRJNA729271	stage_E14_vs_E10	ordinary_muscle_development	ENSGALG00010017184	CARD11	0.542312221	0.212408788	2.553153411	0.010675246	0.025201579	positive
PRJNA729271	stage_E14_vs_E10	ordinary_muscle_development	ENSGALG00010017185	NAA30	0.003958786	0.063802031	0.062047967	0.950524633	0.965647222	positive
PRJNA729271	stage_E14_vs_E10	ordinary_muscle_development	ENSGALG00010017187	ZNF839	0.100049698	0.138690123	0.721390216	0.470669465	0.583007511	positive
PRJNA729271	stage_E14_vs_E10	ordinary_muscle_development	ENSGALG00010017188	ENSGALG00010017188	-0.035474923	0.159804925	-0.221988922	0.824322507	0.877087148	negative
PRJNA729271	stage_E14_vs_E10	ordinary_muscle_development	ENSGALG00010017190	STK26	-0.675411471	0.067728686	-9.97231032	2.01487E-23	5.39842E-22	negative
PRJNA729271	stage_E14_vs_E10	ordinary_muscle_development	ENSGALG00010017198	TOPBP1	-0.080697427	0.065143444	-1.238765139	0.215432488	0.317217992	negative
PRJNA729271	stage_E14_vs_E10	ordinary_muscle_development	ENSGALG00010017200	KCNQ4	0.937389048	0.304421995	3.079242184	0.002075279	0.006006623	positive
PRJNA729271	stage_E14_vs_E10	ordinary_muscle_development	ENSGALG00010017201	AP5M1	-0.672100061	0.148644277	-4.521533377	6.13933E-06	2.98723E-05	negative
PRJNA729271	stage_E14_vs_E10	ordinary_muscle_development	ENSGALG00010017207	ENSGALG00010017207	1.220441483	0.301505261	4.047828155	5.16951E-05	0.000214521	positive
PRJNA729271	stage_E14_vs_E10	ordinary_muscle_development	ENSGALG00010017208	CINP	-0.145942654	0.168855734	-0.864303812	0.387421017	0.503421572	negative
PRJNA729271	stage_E14_vs_E10	ordinary_muscle_development	ENSGALG00010017211	UBFD1	0.45958326	0.128078631	3.588289924	0.000332854	0.001179061	positive
PRJNA729271	stage_E14_vs_E10	ordinary_muscle_development	ENSGALG00010017212	TINAGL1	0.978698461	0.138131802	7.085250811	1.38793E-12	1.56074E-11	positive
PRJNA729271	stage_E14_vs_E10	ordinary_muscle_development	ENSGALG00010017215	FRMD7	0.666107832	0.65247861	1.02088838	0.307307325	0.42094308	positive
PRJNA729271	stage_E14_vs_E10	ordinary_muscle_development	ENSGALG00010017219	EARS2	-0.201841845	0.129175461	-1.562540153	0.118160794	0.195228624	negative
PRJNA729271	stage_E14_vs_E10	ordinary_muscle_development	ENSGALG00010017221	SPPL2A	0.507163074	0.075485684	6.718665703	1.83396E-11	1.8308E-10	positive
PRJNA729271	stage_E14_vs_E10	ordinary_muscle_development	ENSGALG00010017223	NOC2L	-0.221540017	0.073896908	-2.997960565	0.002717928	0.007617241	negative
PRJNA729271	stage_E14_vs_E10	ordinary_muscle_development	ENSGALG00010017226	PEF1	0.387703681	0.072212986	5.368891445	7.92221E-08	5.12645E-07	positive
PRJNA729271	stage_E14_vs_E10	ordinary_muscle_development	ENSGALG00010017228	RAP2C	-0.176503032	0.071310829	-2.475122418	0.013319051	0.030620087	negative
PRJNA729271	stage_E14_vs_E10	ordinary_muscle_development	ENSGALG00010017240	MELTF	-0.316867826	0.236636814	-1.339047045	0.180555358	0.275431479	negative
PRJNA729271	stage_E14_vs_E10	ordinary_muscle_development	ENSGALG00010017246	GGA2	0.159056861	0.129435561	1.228849788	0.219128131	0.321386447	positive
PRJNA729271	stage_E14_vs_E10	ordinary_muscle_development	ENSGALG00010017248	TECPR2	-0.073794507	0.084117482	-0.877279075	0.380335075	0.496305088	negative
PRJNA729271	stage_E14_vs_E10	ordinary_muscle_development	ENSGALG00010017250	COL16A1	-0.324460836	0.169728999	-1.911640542	0.055922315	0.104234141	negative
PRJNA729271	stage_E14_vs_E10	ordinary_muscle_development	ENSGALG00010017252	EXOC5	-0.146446737	0.087507883	-1.673526217	0.09422375	0.162032656	negative
PRJNA729271	stage_E14_vs_E10	ordinary_muscle_development	ENSGALG00010017256	CARHSP1	-0.132303251	0.128558395	-1.029129616	0.303418771	0.417002475	negative
PRJNA729271	stage_E14_vs_E10	ordinary_muscle_development	ENSGALG00010017263	COG7	0.092164134	0.068930245	1.337063777	0.18120182	0.276243203	positive
PRJNA729271	stage_E14_vs_E10	ordinary_muscle_development	ENSGALG00010017264	PIAS1	0.047372332	0.085898296	0.551493269	0.58129558	0.682754998	positive

PRJNA729271	stage_E14_vs_E10	ordinary_muscle_development	ENSGALG00010017266	PMM2	-0.11990922	0.106437081	-1.126573732	0.25992275	0.368216845	negative
PRJNA729271	stage_E14_vs_E10	ordinary_muscle_development	ENSGALG00010017272	NRXN1	1.077341884	0.134123885	8.032438711	9.5554E-16	1.43721E-14	positive
PRJNA729271	stage_E14_vs_E10	ordinary_muscle_development	ENSGALG00010017278	CCR5	0.558703673	0.229056952	2.439147417	0.014721961	0.033400349	positive
PRJNA729271	stage_E14_vs_E10	ordinary_muscle_development	ENSGALG00010017280	SOS1	-0.187550539	0.155961126	-1.202546714	0.229151779	0.332987973	negative
PRJNA729271	stage_E14_vs_E10	ordinary_muscle_development	ENSGALG00010017283	INF2	-0.863056952	0.139749571	-6.175739537	6.58545E-10	5.51769E-09	negative
PRJNA729271	stage_E14_vs_E10	ordinary_muscle_development	ENSGALG00010017284	ENSGALG00010017284	-0.359098667	0.199118083	-1.803445788	0.071318232	0.128098857	negative
PRJNA729271	stage_E14_vs_E10	ordinary_muscle_development	ENSGALG00010017285	IL21R	0.098201564	1.058444697	0.092779117	0.926079042	0.949195002	positive
PRJNA729271	stage_E14_vs_E10	ordinary_muscle_development	ENSGALG00010017289	ADGRB2	-1.470604879	0.287935686	-5.107407488	3.26609E-07	1.94024E-06	negative
PRJNA729271	stage_E14_vs_E10	ordinary_muscle_development	ENSGALG00010017290	DACT1	-0.434540494	0.156163304	-2.782603106	0.005392473	0.01390493	negative
PRJNA729271	stage_E14_vs_E10	ordinary_muscle_development	ENSGALG00010017296	SCNN1G	1.006346697	0.226094461	4.451001103	8.54709E-06	4.05002E-05	positive
PRJNA729271	stage_E14_vs_E10	ordinary_muscle_development	ENSGALG00010017299	USP31	0.806192893	0.109992668	7.329514816	2.30987E-13	2.82307E-12	positive
PRJNA729271	stage_E14_vs_E10	ordinary_muscle_development	ENSGALG00010017300	TRPM7	-0.570456906	0.103890137	-5.490963088	3.99748E-08	2.69254E-07	negative
PRJNA729271	stage_E14_vs_E10	ordinary_muscle_development	ENSGALG00010017301	LHCGR	-1.91045296	0.485834418	-3.93231292	8.41324E-05	0.000335581	negative
PRJNA729271	stage_E14_vs_E10	ordinary_muscle_development	ENSGALG00010017304	ANKRD9	0.243314481	0.304533211	0.798975193	0.424304796	0.538649327	positive
PRJNA729271	stage_E14_vs_E10	ordinary_muscle_development	ENSGALG00010017305	HS3ST2	1.232374618	0.525206272	2.346458305	0.018952787	0.041465806	positive
PRJNA729271	stage_E14_vs_E10	ordinary_muscle_development	ENSGALG00010017307	TMEM186	-0.091617726	0.157269474	-0.582552502	0.560194588	0.663538116	negative
PRJNA729271	stage_E14_vs_E10	ordinary_muscle_development	ENSGALG00010017310	SAMD11	-0.950243768	0.17527054	-5.421582937	5.90736E-08	3.87804E-07	negative
PRJNA729271	stage_E14_vs_E10	ordinary_muscle_development	ENSGALG00010017312	PTP4A2	0.353623974	0.154754476	2.285064588	0.022309041	0.04785697	positive
PRJNA729271	stage_E14_vs_E10	ordinary_muscle_development	ENSGALG00010017313	MAP4K3	-0.146714425	0.08261273	-1.775929986	0.075744458	0.134995195	negative
PRJNA729271	stage_E14_vs_E10	ordinary_muscle_development	ENSGALG00010017314	CALML4	0.352171494	0.219833845	1.601989422	0.109157949	0.183130147	positive
PRJNA729271	stage_E14_vs_E10	ordinary_muscle_development	ENSGALG00010017315	HS3ST4	-3.363842112	0.985714247	-3.412593581	0.000643478	0.002118859	negative
PRJNA729271	stage_E14_vs_E10	ordinary_muscle_development	ENSGALG00010017318	ADSS1	0.657687985	0.124457098	5.284455417	1.26079E-07	7.90108E-07	positive
PRJNA729271	stage_E14_vs_E10	ordinary_muscle_development	ENSGALG00010017322	KHDRBS1	0.037710532	0.068480545	0.550675117	0.581856405	0.683302973	positive
PRJNA729271	stage_E14_vs_E10	ordinary_muscle_development	ENSGALG00010017323	ENSGALG00010017323	1.269931923	0.103763515	12.23871336	1.93069E-34	1.02887E-32	positive
PRJNA729271	stage_E14_vs_E10	ordinary_muscle_development	ENSGALG00010017326	STON1	-0.325089824	0.121211046	-2.682014846	0.007318021	0.018116946	negative
PRJNA729271	stage_E14_vs_E10	ordinary_muscle_development	ENSGALG00010017327	CLN6	-0.330647715	0.132983648	-2.48637873	0.012905054	0.029805412	negative
PRJNA729271	stage_E14_vs_E10	ordinary_muscle_development	ENSGALG00010017332	FGFR1	0.136972531	0.158548097	0.863917849	0.387633021	0.503618645	positive
PRJNA729271	stage_E14_vs_E10	ordinary_muscle_development	ENSGALG00010017334	KDM8	-0.177999181	0.148956297	-1.194975867	0.232096459	0.336390178	negative
PRJNA729271	stage_E14_vs_E10	ordinary_muscle_development	ENSGALG00010017335	THUMPD2	-0.061529997	0.136034304	-0.452312361	0.651043972	0.742216307	negative
PRJNA729271	stage_E14_vs_E10	ordinary_muscle_development	ENSGALG00010017337	ABAT	-0.725215881	0.203016688	-3.572198371	0.000353997	0.001246947	negative
PRJNA729271	stage_E14_vs_E10	ordinary_muscle_development	ENSGALG00010017338	MBNL3	-0.24228251	0.074669238	-3.244743298	0.001175565	0.00364021	negative
PRJNA729271	stage_E14_vs_E10	ordinary_muscle_development	ENSGALG00010017340	SIVA1	-0.325746109	0.101730064	-3.20206336	0.00136447	0.004157024	negative
PRJNA729271	stage_E14_vs_E10	ordinary_muscle_development	ENSGALG00010017341	TA3	-0.07337879	0.077864725	-0.942388085	0.345993985	0.461505734	negative
PRJNA729271	stage_E14_vs_E10	ordinary_muscle_development	ENSGALG00010017343	DLG1	-0.018204914	0.099056923	-0.183782345	0.854184207	0.899092301	negative
PRJNA729271	stage_E14_vs_E10	ordinary_muscle_development	ENSGALG00010017344	RERE	0.260110453	0.153915478	1.689956439	0.091036289	0.157336642	positive
PRJNA729271	stage_E14_vs_E10	ordinary_muscle_development	ENSGALG00010017346	GM2A	0.595580895	0.146580867	4.063155777	4.84137E-05	0.000202076	positive
PRJNA729271	stage_E14_vs_E10	ordinary_muscle_development	ENSGALG00010017347	TMEM39B	-0.11330584	0.111728484	-1.014117756	0.310526566	0.424282892	negative
PRJNA729271	stage_E14_vs_E10	ordinary_muscle_development	ENSGALG00010017349	ENSGALG00010017349	1.192743252	0.111130635	10.73280337	7.13967E-27	2.51188E-25	positive
PRJNA729271	stage_E14_vs_E10	ordinary_muscle_development	ENSGALG00010017351	CCR2	1.20054688	0.207196809	5.794234417	6.86335E-09	5.09392E-08	positive
PRJNA729271	stage_E14_vs_E10	ordinary_muscle_development	ENSGALG00010017352	GPCPD1	0.090604291	0.238488228	0.379910956	0.704011515	0.784852092	positive
PRJNA729271	stage_E14_vs_E10	ordinary_muscle_development	ENSGALG00010017354	FEM1B	0.105369734	0.104593968	1.00741693	0.313734459	0.427603833	positive
PRJNA729271	stage_E14_vs_E10	ordinary_muscle_development	ENSGALG00010017356	NSMCE1	-0.046067532	0.115116701	-0.400181132	0.68902311	0.772718761	negative
PRJNA729271	stage_E14_vs_E10	ordinary_muscle_development	ENSGALG00010017359	EHD3	0.23153527	0.095310827	2.42926516	0.015129463	0.034180418	positive
PRJNA729271	stage_E14_vs_E10	ordinary_muscle_development	ENSGALG00010017361	METTL22	0.190776819	0.150496356	1.267650757	0.204922697	0.30477678	positive
PRJNA729271	stage_E14_vs_E10	ordinary_muscle_development	ENSGALG00010017362	ENO1	0.70385453	0.130118297	5.409343246	6.32563E-08	4.14512E-07	positive
PRJNA729271	stage_E14_vs_E10	ordinary_muscle_development	ENSGALG00010017363	TIMM9	0.123983526	0.140240762	0.884076239	0.376655059	0.492834002	positive
PRJNA729271	stage_E14_vs_E10	ordinary_muscle_development	ENSGALG00010017364	AKT1	-0.523438182	0.072946356	-7.175659059	7.19597E-13	8.35782E-12	negative
PRJNA729271	stage_E14_vs_E10	ordinary_muscle_development	ENSGALG00010017367	TIMD4	0.087671028	0.307240607	0.285349741	0.775376208	0.840986498	positive
PRJNA729271	stage_E14_vs_E10	ordinary_muscle_development	ENSGALG00010017368	ERLIN2	-0.004461668	0.0502436	-0.088800715	0.92924029	0.951415503	negative
PRJNA729271	stage_E14_vs_E10	ordinary_muscle_development	ENSGALG00010017369	CAPN14	0.667847815	0.46988273	1.42130743	0.155227405	0.244010544	positive
PRJNA729271	stage_E14_vs_E10	ordinary_muscle_development	ENSGALG00010017371	TMEM114	0.922420038	1.086563686	0.848933248	0.395918437	0.511437283	positive
PRJNA729271	stage_E14_vs_E10	ordinary_muscle_development	ENSGALG00010017372	ENSGALG00010017372	0.07544483	0.110525031	0.682604017	0.494857095	0.604751167	positive
PRJNA729271	stage_E14_vs_E10	ordinary_muscle_development	ENSGALG00010017373	RP1-2705.3	0.106735886	0.104258423	1.023762711	0.305947367	0.419698071	positive
PRJNA729271	stage_E14_vs_E10	ordinary_muscle_development	ENSGALG00010017375	BDH1A	-0.379849404	0.161136737	-2.357311007	0.018407823	0.040452	negative
PRJNA729271	stage_E14_vs_E10	ordinary_muscle_development	ENSGALG00010017376	GALNT14	0.382863029	0.26280924	1.456809621	0.14516894	0.231106401	positive
PRJNA729271	stage_E14_vs_E10	ordinary_muscle_development	ENSGALG00010017377	USP8	0.061318369	0.075126979	0.816196396	0.414387818	0.529211579	positive
PRJNA729271	stage_E14_vs_E10	ordinary_muscle_development	ENSGALG00010017378	ENSGALG00010017378	2.83870824	0.530268091	5.353345393	8.63428E-08	5.55746E-07	positive
PRJNA729271	stage_E14_vs_E10	ordinary_muscle_development	ENSGALG00010017380	MARCKSL1	0.040751896	0.133829178	0.304506809	0.760741806	0.829540605	positive
PRJNA729271	stage_E14_vs_E10	ordinary_muscle_development	ENSGALG00010017381	CA6	0.291154188	0.638978748	0.455655511	0.648637719	0.740373552	positive
PRJNA729271	stage_E14_vs_E10	ordinary_muscle_development	ENSGALG00010017383	ENSGALG00010017383	-0.193943807	0.150476122	-1.28886766	0.197444099	0.295931363	negative
PRJNA729271	stage_E14_vs_E10	ordinary_muscle_development	ENSGALG00010017384	ARID4A	-0.19655874	0.092147277	-2.133093316	0.032917072	0.066369865	negative
PRJNA729271	stage_E14_vs_E10	ordinary_muscle_development	ENSGALG00010017386	ENSGALG00010017386	2.258756982	0.489707611	4.612460435	3.9793E-06	1.99171E-05	positive
PRJNA729271	stage_E14_vs_E10	ordinary_muscle_development	ENSGALG00010017387	HS6ST2	-0.454401252	0.11305667	-4.019234338	5.83876E-05	0.000239957	negative
PRJNA729271	stage_E14_vs_E10	ordinary_muscle_development	ENSGALG00010017388	PLPBP	0.505094028	0.08180881	6.174078679	6.65504E-10	5.56707E-09	positive
PRJNA729271	stage_E14_vs_E10	ordinary_muscle_development	ENSGALG00010017390	DDX24	-0.11276786	0.101167321	-1.11466686	0.264993207	0.37375465	negative
PRJNA729271	stage_E14_vs_E10	ordinary_muscle_development	ENSGALG00010017391	CRAMP1	-0.124428011	0.11638226	-1.069132106	0.285010146	0.396752104	negative
PRJNA729271	stage_E14_vs_E10	ordinary_muscle_development	ENSGALG00010017393	VDAC1	1.816507777	0.16375883	11.09257911	1.363E-28	5.33966E-27	positive
PRJNA729271	stage_E14_vs_E10	ordinary_muscle_development	ENSGALG00010017395	BRF2	-0.185051251	0.120559727	-1.534934222	0.124799986	0.203897182	negative
PRJNA729271	stage_E14_vs_E10	ordinary_muscle_development	ENSGALG00010017396	CCR8L	4.537704395	0.269634701	16.82908163	1.49401E-63	4.01032E-61	positive
PRJNA729271	stage_E14_vs_E10	ordinary_muscle_development	ENSGALG00010017398	SLC22A5	-0.20330801	0.182739351	-1.112557363	0.265898581	0.374776345	negative
PRJNA729271	stage_E14_vs_E10	ordinary_muscle_development	ENSGALG00010017399	CPXM1	0.168681278	0.290163838	0.581331151	0.561017289	0.664156425	positive
PRJNA729271	stage_E14_vs_E10	ordinary_muscle_development	ENSGALG00010017400	GPC4	-0.962749351	0.118857983	-8.099997363	5.49604E-16	8.42125E-15	negative
PRJNA729271	stage_E14_vs_E10	ordinary_muscle_development	ENSGALG00010017402	SLC2A5	1.21259073	0.228310738	5.311141925	1.0894E-07	6.91371E-07	positive
PRJNA729271	stage_E14_vs_E10	ordinary_muscle_development	ENSGALG00010017403	ITGA11	-0.038183467	0.291794568	-0.130857361	0.895888146	0.928359928	negative
PRJNA729271	stage_E14_vs_E10	ordinary_muscle_development	ENSGALG00010017405	XCR1	0.86892662	1.151705452	0.754469486	0.450567362	0.563792637	positive
PRJNA729271	stage_E14_vs_E10	ordinary_muscle_development	ENSGALG00010017406	IFT140	-0.348881101	0.121516395	-2.871061977	0.004090953	0.010910463	negative
PRJNA729271	stage_E14_vs_E10	ordinary_muscle_development	ENSGALG00010017407	EPN2	-0.076260212	0.084908255	-0.89814838	0.369106449	0.485410813	negative
PRJNA729271	stage_E14_vs_E10	ordinary_muscle_development	ENSGALG00010017409	GPC3	-0.862578591	0.309330557	-2.788533405	0.005294729	0.01369017	negative
PRJNA729271	stage_E14_vs_E10	ordinary_muscle_development	ENSGALG00010017411	RAB11FIP1	0.374819659	0.136645513	2.743007453	0.00608793	0.015451681	positive
PRJNA729271	stage_E14_vs_E10	ordinary_muscle_development	ENSGALG00010017413	ZIC3	-4.004780226	0.71321824	-5.615083857	1.96467E-08	1.38242E-07	negative
PRJNA729271	stage_E14_vs_E10	ordinary_muscle_development	ENSGALG00010017414	ENSGALG00010017414	0.29423745	0.196854141	1.494697794	0.13499333	0.21777722	positive
PRJNA729271	stage_E14_vs_E10	ordinary_muscle_development	ENSGALG00010017415	HDAC1	-0.246891372	0.06142079	-4.019671059	5.82795E-05	0.00023958	negative
PRJNA729271	stage_E14_vs_E10	ordinary_muscle_development	ENSGALG00010017418	CHGB	-0.147025958	0.285214822	-0.515491994	0.606209262	0.703923041	negative
PRJNA729271	stage_E14_vs_E10	ordinary_muscle_development	ENSGALG00010017419	B9D1	-0.404045813	0.097718649	-4.134787161	3.55284E-05	0.0001516	negative
PRJNA729271	stage_E14_vs_E10	ordinary_muscle_development	ENSGALG00010017421	GPR157	1.300113531	0.262024424	4.961802848	6.98419E-07	3.94374E-06	positive
PRJNA729271	stage_E14_vs_E10	ordinary_muscle_development	ENSGALG00010017422	ENSGALG00010017422	0.413041958	0.219349255	1.88303333	0.059695851	0.110073955	positive
PRJNA729271	stage_E14_vs_E10	ordinary_muscle_development	ENSGALG00010017423	TMEM204	-0.013978415	0.132484724	-0.105509635	0.915971425	0.941431313	negative
PRJNA729271	stage_E14_vs_E10	ordinary_muscle_development	ENSGALG00010017424	APOD	2.198415705	0.487245079	4.511930034	6.42404E-06	3.11739E-05	positive
PRJNA729271	stage_E14_vs_E10	ordinary_muscle_development	ENSGALG00010017425	H6PD	0.684521732	0.124176081	5.512508747	3.53755E-08	2.40736E-07	positive
PRJNA729271	stage_E14_vs_E10	ordinary_muscle_development	ENSGALG00010017428	PSMA3	0.085271357	0.107663041	0.792020697	0.428348608	0.542737157	positive
PRJNA729271	stage_E14_vs_E10	ordinary_muscle_development	ENSGALG00010017429	TELO2	-0.010730544	0.100813756	-0.106439282	0.915233828	0.941362463	negative
PRJNA729271	stage_E14_vs_E10	ordinary_muscle_development	ENSGALG00010017430	CACNA1H	0.80206084	0.197637835	4.058235315	4.94449E-05	0.000205772	positive
PRJNA729271	stage_E14_vs_E10	ordinary_muscle_development	ENSGALG00010017431	FYCO1	1.119429809	0.118094622	9.479092157	2.56505E-21	6.08517E-20	positive
PRJNA729271	stage_E14_vs_E10	ordinary_muscle_development	ENSGALG00010017433	RNPS1	-0.065146493	0.077987168	-0.835348879	0.403521304	0.518669975	negative
PRJNA729271	stage_E14_vs_E10	ordinary_muscle_development	ENSGALG00010017434	FGF13	1.877767851	0.148410836	12.65249833	1.08373E-36	6.80029E-35	positive
PRJNA729271	stage_E14_vs_E10	ordinary_muscle_development	ENSGALG00010017435	ACTR10	0.061520565	0.089231577	0.689448374	0.490541151	0.601060681	positive
PRJNA729271	stage_E14_vs_E10	ordinary_muscle_development	ENSGALG00010017437	DRG2	0.238229981	0.139065088	1.713082589	0.086697347	0.15117022	positive
PRJNA729271	stage_E14_vs_E10	ordinary_muscle_development	ENSGALG00010017438	ADRB3	0.968024293	0.858353876	1.127768301	0.259417784	0.367710752	positive
PRJNA729271	stage_E14_vs_E10	ordinary_muscle_development	ENSGALG00010017439	PPP4R4	-2.348613761	0.275033043	-8.539387615	1.34935E-17	2.38522E-16	negative
PRJNA729271	stage_E14_vs_E10	ordinary_muscle_development	ENSGALG00010017441	SPSB1	-0.121452202	0.256761757	-0.473015156	0.636202367	0.729742289	negative
PRJNA729271	stage_E14_vs_E10	ordinary_muscle_development	ENSGALG00010017442	LCK	0.566643609	0.255430066	2.218390414	0.026528224	0.055439246	positive
PRJNA729271	stage_E14_vs_E10	ordinary_muscle_development	ENSGALG00010017445	F9	4.433729758	1.161545167	3.8170963	0.000135031	0.000519862	positive
PRJNA729271	stage_E14_vs_E10	ordinary_muscle_development	ENSGALG00010017447	CAPN13	0.318002275	0.493698989	0.644121786	0.519496464	0.627508437	positive
PRJNA729271	stage_E14_vs_E10	ordinary_muscle_development	ENSGALG00010017449	TRMT6	-0.169624592	0.082301651	-2.061010819	0.039302008	0.077208325	negative
PRJNA729271	stage_E14_vs_E10	ordinary_muscle_development	ENSGALG00010017450	ENSGALG00010017450	1.259716804	0.210028513	5.997837087	1.99963E-09	1.58386E-08	positive
PRJNA729271	stage_E14_vs_E10	ordinary_muscle_development	ENSGALG00010017451	GABPB1	-0.091594054	0.075842227	-1.207692035	0.227165743	0.330499593	negative
PRJNA729271	stage_E14_vs_E10	ordinary_muscle_development	ENSGALG00010017452	ARMH4	-1.642202192	0.432173708	-3.799866026	0.000144774	0.000553549	negative
PRJNA729271	stage_E14_vs_E10	ordinary_muscle_development	ENSGALG00010017453	PPP1R2	-0.047721526	0.138388592	-0.34483714	0.73021681	0.80563957	negative
PRJNA729271	stage_E14_vs_E10	ordinary_muscle_development	ENSGALG00010017454	ENSGALG00010017454	0.744573652	0.157775965	4.719183012	2.36794E-06	1.23261E-05	positive
PRJNA729271	stage_E14_vs_E10	ordinary_muscle_development	ENSGALG00010017455	CCR9	0.703668178	0.765874653	0.918777211	0.358212115	0.47461468	positive
PRJNA729271	stage_E14_vs_E10	ordinary_muscle_development	ENSGALG00010017457	ZMAT4	-0.731249959	0.246658565	-2.964624234	0.003030529	0.008394327	negative
PRJNA729271	stage_E14_vs_E10	ordinary_muscle_development	ENSGALG00010017458	ENSGALG00010017458	0.093467898	0.172131687	0.543002278	0.587128234	0.687433259	positive
PRJNA729271	stage_E14_vs_E10	ordinary_muscle_development	ENSGALG00010017459	FAM167B	0.659637263	0.298360312	2.210874695	0.027044515	0.056331406	positive
PRJNA729271	stage_E14_vs_E10	ordinary_muscle_development	ENSGALG00010017462	MCM8	0.030354932	0.115773364	0.262192707	0.793172876	0.854735026	positive
PRJNA729271	stage_E14_vs_E10	ordinary_muscle_development	ENSGALG00010017463	PPP1R36	0.680961594	0.323989922	2.10179869	0.035570914	0.070872907	positive
PRJNA729271	stage_E14_vs_E10	ordinary_muscle_development	ENSGALG00010017464	SH3BP2	-0.335461323	0.130195743	-2.576592111	0.009977962	0.023791833	negative
PRJNA729271	stage_E14_vs_E10	ordinary_muscle_development	ENSGALG00010017465	TMEM234	0.139022894	0.223414437	0.622264594	0.533767898	0.64106104	positive
PRJNA729271	stage_E14_vs_E10	ordinary_muscle_development	ENSGALG00010017466	SPIA4	5.452750816	2.732737245	1.995343982			positive
PRJNA729271	stage_E14_vs_E10	ordinary_muscle_development	ENSGALG00010017467	SFRP1	-0.96436259	0.105276285	-9.160302214	5.17522E-20	1.11133E-18	negative
PRJNA729271	stage_E14_vs_E10	ordinary_muscle_development	ENSGALG00010017468	AMDHD2	0.127642056	0.126300108	1.010625073	0.312195911	0.426069083	positive
PRJNA729271	stage_E14_vs_E10	ordinary_muscle_development	ENSGALG00010017470	HSPA2	1.423554465	0.17000674	8.373517831	5.59232E-17	9.43665E-16	positive
PRJNA729271	stage_E14_vs_E10	ordinary_muscle_development	ENSGALG00010017471	CRLS1	0.228092349	0.088985587	2.563250495	0.01036972	0.024551677	positive
PRJNA729271	stage_E14_vs_E10	ordinary_muscle_development	ENSGALG00010017472	LZTFL1	-0.793669919	0.167639201	-4.73439334	2.19711E-06	1.14847E-05	negative
PRJNA729271	stage_E14_vs_E10	ordinary_muscle_development	ENSGALG00010017473	EIF3I	0.293213103	0.104328796	2.810471461	0.004946898	0.012901274	positive
PRJNA729271	stage_E14_vs_E10	ordinary_muscle_development	ENSGALG00010017475	STUM	2.719148327	0.246529966	11.02968685	2.7482E-28	1.06511E-26	positive
PRJNA729271	stage_E14_vs_E10	ordinary_muscle_development	ENSGALG00010017476	ATP6V0C	0.091009404	0.112632343	0.808021937	0.419077954	0.533649735	positive
PRJNA729271	stage_E14_vs_E10	ordinary_muscle_development	ENSGALG00010017477	MCF2	0.966657104	0.182087434	5.308752396	1.10378E-07	6.99576E-07	positive
PRJNA729271	stage_E14_vs_E10	ordinary_muscle_development	ENSGALG00010017479	SSTR5	2.087870859	0.466647414	4.474193571	7.67002E-06	3.66558E-05	positive
PRJNA729271	stage_E14_vs_E10	ordinary_muscle_development	ENSGALG00010017480	DCDC2B	0.116859483	0.196804457	0.593784735	0.5526561	0.657373229	positive
PRJNA729271	stage_E14_vs_E10	ordinary_muscle_development	ENSGALG00010017481	SPIA1	5.457730585	2.497801039	2.185014138			positive
PRJNA729271	stage_E14_vs_E10	ordinary_muscle_development	ENSGALG00010017482	CORO2B	0.766670948	0.139153928	5.509517102	3.59819E-08	2.44519E-07	positive

PRJNA729271	stage_E14_vs_E10	ordinary_muscle_development	ENSGALG00010017484	IQCC	0.255572253	0.127255047	2.008346692	0.044606463	0.08608317	positive
PRJNA729271	stage_E14_vs_E10	ordinary_muscle_development	ENSGALG00010017485	LBH	0.291954449	0.107446094	2.717217885	0.006583325	0.016512424	positive
PRJNA729271	stage_E14_vs_E10	ordinary_muscle_development	ENSGALG00010017487	C1QTNF8	-1.655883073	0.512915948	-3.228371195	0.001244973	0.003838733	negative
PRJNA729271	stage_E14_vs_E10	ordinary_muscle_development	ENSGALG00010017489	TNFRSF1B	0.362255919	0.177991314	2.035244924	0.041826235	0.081394357	positive
PRJNA729271	stage_E14_vs_E10	ordinary_muscle_development	ENSGALG00010017491	CCDC28B	0.243825113	0.20196808	1.207245781	0.227337504	0.330716291	positive
PRJNA729271	stage_E14_vs_E10	ordinary_muscle_development	ENSGALG00010017493	YPEL5	0.325749698	0.134048752	2.430083787	0.015095333	0.034113946	positive
PRJNA729271	stage_E14_vs_E10	ordinary_muscle_development	ENSGALG00010017494	TBC1D24	-0.049873443	0.090829875	-0.549086334	0.582946203	0.68410001	negative
PRJNA729271	stage_E14_vs_E10	ordinary_muscle_development	ENSGALG00010017495	NT5M	-0.243449383	0.133943922	-1.817547072	0.069133388	0.124777544	negative
PRJNA729271	stage_E14_vs_E10	ordinary_muscle_development	ENSGALG00010017496	PARP1	-0.374811452	0.103014589	-3.638430793	0.000274304	0.000988819	negative
PRJNA729271	stage_E14_vs_E10	ordinary_muscle_development	ENSGALG00010017499	SNRPD1	-0.01392154	0.097213607	-0.14320567	0.886127752	0.922168075	negative
PRJNA729271	stage_E14_vs_E10	ordinary_muscle_development	ENSGALG00010017500	ACAP2	0.038045333	0.100291252	0.379348474	0.704429108	0.78513648	positive
PRJNA729271	stage_E14_vs_E10	ordinary_muscle_development	ENSGALG00010017502	NAT8B	0.419496135	0.117081403	3.582944213	0.000339743	0.001201996	positive
PRJNA729271	stage_E14_vs_E10	ordinary_muscle_development	ENSGALG00010017503	MED9	0.109652867	0.125570874	0.873234872	0.382535071	0.49859238	positive
PRJNA729271	stage_E14_vs_E10	ordinary_muscle_development	ENSGALG00010017505	TNFRSF8	-0.463602047	0.326077845	-1.42175267	0.155098064	0.243913034	negative
PRJNA729271	stage_E14_vs_E10	ordinary_muscle_development	ENSGALG00010017509	RASD1	-0.129070747	0.302625752	-0.426502856	0.669741468	0.757069529	negative
PRJNA729271	stage_E14_vs_E10	ordinary_muscle_development	ENSGALG00010017510	HDC	0.841231173	0.425878017	1.975286679	0.048235624	0.092069446	positive
PRJNA729271	stage_E14_vs_E10	ordinary_muscle_development	ENSGALG00010017513	TNIP2	-0.291066579	0.145942351	-1.99439421	0.046108991	0.08867025	negative
PRJNA729271	stage_E14_vs_E10	ordinary_muscle_development	ENSGALG00010017514	TXLNA	-0.07819062	0.095904319	-0.815298221	0.414901627	0.529634442	negative
PRJNA729271	stage_E14_vs_E10	ordinary_muscle_development	ENSGALG00010017516	AAK1	0.155407902	0.079512356	1.954512611	0.050640625	0.09578962	positive
PRJNA729271	stage_E14_vs_E10	ordinary_muscle_development	ENSGALG00010017517	LIN9	-0.093706586	0.095992684	-0.976184665	0.328972961	0.443784371	negative
PRJNA729271	stage_E14_vs_E10	ordinary_muscle_development	ENSGALG00010017518	PEMT	0.425242133	0.147221224	2.888456713	0.003871373	0.010382156	positive
PRJNA729271	stage_E14_vs_E10	ordinary_muscle_development	ENSGALG00010017519	NTN3	0.334419006	0.185529731	1.802508977	0.071465364	0.128315428	positive
PRJNA729271	stage_E14_vs_E10	ordinary_muscle_development	ENSGALG00010017520	ZBTB1	0.022271281	0.080286081	0.277399026	0.781473729	0.845648503	positive
PRJNA729271	stage_E14_vs_E10	ordinary_muscle_development	ENSGALG00010017521	MIIP	-0.034216976	0.1154532	-0.296370965	0.766946796	0.834351082	negative
PRJNA729271	stage_E14_vs_E10	ordinary_muscle_development	ENSGALG00010017522	XXYLT1	-0.019247162	0.106062061	-0.18147075	0.85599809	0.900478432	negative
PRJNA729271	stage_E14_vs_E10	ordinary_muscle_development	ENSGALG00010017524	ACBD3	0.065826901	0.058500539	1.125235798	0.260489127	0.368756039	positive
PRJNA729271	stage_E14_vs_E10	ordinary_muscle_development	ENSGALG00010017525	ENSGALG00010017525	-0.077264133	0.522128401	-0.14797918	0.882359199	0.91933558	negative
PRJNA729271	stage_E14_vs_E10	ordinary_muscle_development	ENSGALG00010017526	PLEKHA2	0.988680835	0.170953112	5.783345059	7.32296E-09	5.42393E-08	positive
PRJNA729271	stage_E14_vs_E10	ordinary_muscle_development	ENSGALG00010017528	RAI1	0.096966922	0.176358929	0.549827118	0.582437957	0.683764331	positive
PRJNA729271	stage_E14_vs_E10	ordinary_muscle_development	ENSGALG00010017529	MFN2	0.156848685	0.11844318	1.324252575	0.185419175	0.281399952	positive
PRJNA729271	stage_E14_vs_E10	ordinary_muscle_development	ENSGALG00010017530	TLR1B	0.515664479	0.211684163	2.436008781	0.014850324	0.033654698	positive
PRJNA729271	stage_E14_vs_E10	ordinary_muscle_development	ENSGALG00010017531	SACM1L	-0.321183849	0.111325465	-2.885088779	0.003913033	0.010478369	negative
PRJNA729271	stage_E14_vs_E10	ordinary_muscle_development	ENSGALG00010017532	MIXL1	0.265822886	0.314206018	0.846014623	0.397544587	0.512943634	positive
PRJNA729271	stage_E14_vs_E10	ordinary_muscle_development	ENSGALG00010017533	FAM193A	-0.199339629	0.077003055	-2.588723654	0.009633237	0.023070429	negative
PRJNA729271	stage_E14_vs_E10	ordinary_muscle_development	ENSGALG00010017534	ATP11C	-0.338019803	0.083574545	-4.044530605	5.24281E-05	0.000217433	negative
PRJNA729271	stage_E14_vs_E10	ordinary_muscle_development	ENSGALG00010017535	LIMD1	0.37998289	0.088638975	4.286860159	1.81216E-05	8.12223E-05	positive
PRJNA729271	stage_E14_vs_E10	ordinary_muscle_development	ENSGALG00010017536	GSC	-4.944512523	0.511563884	-9.665483979	4.22618E-22	1.05895E-20	negative
PRJNA729271	stage_E14_vs_E10	ordinary_muscle_development	ENSGALG00010017537	MANBA	0.101367959	0.187765613	0.539864344	0.589290589	0.689352521	positive
PRJNA729271	stage_E14_vs_E10	ordinary_muscle_development	ENSGALG00010017538	H3-3A	-0.017217123	0.075704844	-0.227424322	0.82009381	0.874063219	negative
PRJNA729271	stage_E14_vs_E10	ordinary_muscle_development	ENSGALG00010017539	KPNA6	0.286258211	0.138473744	2.067238185	0.038711716	0.07624208	positive
PRJNA729271	stage_E14_vs_E10	ordinary_muscle_development	ENSGALG00010017540	EIF4G1	-0.156185066	0.164675534	-0.94844123	0.342904878	0.458101955	negative
PRJNA729271	stage_E14_vs_E10	ordinary_muscle_development	ENSGALG00010017541	SREBF1	0.228977756	0.228992545	0.999935417	0.317341763	0.431264659	positive
PRJNA729271	stage_E14_vs_E10	ordinary_muscle_development	ENSGALG00010017542	PLOD1	0.380194982	0.125066985	3.039930825	0.002366325	0.006733388	positive
PRJNA729271	stage_E14_vs_E10	ordinary_muscle_development	ENSGALG00010017543	ZBTB25	0.521785072	0.173901371	3.000465552	0.002695672	0.007565106	positive
PRJNA729271	stage_E14_vs_E10	ordinary_muscle_development	ENSGALG00010017544	SDE2	0.045057978	0.068120918	0.661441142	0.508329449	0.617156828	positive
PRJNA729271	stage_E14_vs_E10	ordinary_muscle_development	ENSGALG00010017545	DICER1	-0.674857616	0.120651786	-5.593432475	2.22624E-08	1.55365E-07	negative
PRJNA729271	stage_E14_vs_E10	ordinary_muscle_development	ENSGALG00010017546	NFU1	0.807443477	0.107726486	7.495310669	6.61415E-14	8.55238E-13	positive
PRJNA729271	stage_E14_vs_E10	ordinary_muscle_development	ENSGALG00010017547	RNF4	-0.159537965	0.065058047	-2.452240284	0.014196982	0.032376536	negative
PRJNA729271	stage_E14_vs_E10	ordinary_muscle_development	ENSGALG00010017550	TM2D2	0.158783272	0.075396366	2.10598044	0.035206051	0.070261836	positive
PRJNA729271	stage_E14_vs_E10	ordinary_muscle_development	ENSGALG00010017551	NALF2	-0.033514375	0.273837804	-0.122387685	0.902591991	0.933438278	negative
PRJNA729271	stage_E14_vs_E10	ordinary_muscle_development	ENSGALG00010017552	LARS2	0.04956011	0.101889844	0.486408735	0.626677399	0.721245204	positive
PRJNA729271	stage_E14_vs_E10	ordinary_muscle_development	ENSGALG00010017553	EDA	0.606631642	0.108249812	5.60399719	2.09464E-08	1.46817E-07	positive
PRJNA729271	stage_E14_vs_E10	ordinary_muscle_development	ENSGALG00010017554	FAM131A	-0.882360649	0.290762686	-3.034641968	0.002408214	0.006831127	negative
PRJNA729271	stage_E14_vs_E10	ordinary_muscle_development	ENSGALG00010017555	CELA2A	-0.118492753	1.244704976	-0.09519746	0.924157987	0.947855721	negative
PRJNA729271	stage_E14_vs_E10	ordinary_muscle_development	ENSGALG00010017556	TOM1L2	0.367304404	0.114000437	3.221956095	0.001273186	0.0039149	positive
PRJNA729271	stage_E14_vs_E10	ordinary_muscle_development	ENSGALG00010017558	ENSGALG00010017558	-0.007511886	0.073728716	-0.101885478	0.918847577	0.943718247	negative
PRJNA729271	stage_E14_vs_E10	ordinary_muscle_development	ENSGALG00010017559	DUSP10	0.379796254	0.150425012	2.524821175	0.011575718	0.027104315	positive
PRJNA729271	stage_E14_vs_E10	ordinary_muscle_development	ENSGALG00010017561	CFAP99	-0.264652511	0.563000678	-0.470074942	0.638301476	0.731282002	negative
PRJNA729271	stage_E14_vs_E10	ordinary_muscle_development	ENSGALG00010017562	SLC30A10	-1.41205765	0.568413791	-2.484207232	0.012984022	0.029959154	negative
PRJNA729271	stage_E14_vs_E10	ordinary_muscle_development	ENSGALG00010017563	ALK	3.052207513	0.150651976	20.25998985	2.90022E-91	2.10194E-88	positive
PRJNA729271	stage_E14_vs_E10	ordinary_muscle_development	ENSGALG00010017565	ENSGALG00010017565	0.664380437	0.184116555	3.608477449	0.000307999	0.00110016	positive
PRJNA729271	stage_E14_vs_E10	ordinary_muscle_development	ENSGALG00010017568	CDCP1	-2.563912166	0.242362355	-10.57883831	3.73561E-26	1.24477E-24	negative
PRJNA729271	stage_E14_vs_E10	ordinary_muscle_development	ENSGALG00010017569	CLMN	-1.269716791	0.245331212	-5.175520796	2.27276E-07	1.37667E-06	negative
PRJNA729271	stage_E14_vs_E10	ordinary_muscle_development	ENSGALG00010017570	DRC3	-0.280433011	0.345460681	-0.811765351	0.416926287	0.531516846	negative
PRJNA729271	stage_E14_vs_E10	ordinary_muscle_development	ENSGALG00010017571	ADAM9	0.092531093	0.150205407	0.616030372	0.537874475	0.644338059	positive
PRJNA729271	stage_E14_vs_E10	ordinary_muscle_development	ENSGALG00010017573	ENSGALG00010017573	-0.283768912	0.229434364	-1.236819573	0.216154074	0.317957509	negative
PRJNA729271	stage_E14_vs_E10	ordinary_muscle_development	ENSGALG00010017574	DYNC1LI1	-0.061834554	0.054953767	-1.125210469	0.260499858	0.368756039	negative
PRJNA729271	stage_E14_vs_E10	ordinary_muscle_development	ENSGALG00010017575	CLCN2	-0.541774249	0.925727504	-0.585241604	0.558385274	0.662177415	negative
PRJNA729271	stage_E14_vs_E10	ordinary_muscle_development	ENSGALG00010017576	ENSGALG00010017576	-0.708964909	0.279705198	-2.53468621	0.011254809	0.026442872	negative
PRJNA729271	stage_E14_vs_E10	ordinary_muscle_development	ENSGALG00010017577	CLPX	0.288455213	0.137711454	2.094634867	0.036203463	0.07195519	positive
PRJNA729271	stage_E14_vs_E10	ordinary_muscle_development	ENSGALG00010017578	BSDC1	0.248962882	0.099563507	2.500543808	0.01240029	0.028772564	positive
PRJNA729271	stage_E14_vs_E10	ordinary_muscle_development	ENSGALG00010017579	P3H2	0.681432688	0.140831538	4.838636981	1.30733E-06	7.12662E-06	positive
PRJNA729271	stage_E14_vs_E10	ordinary_muscle_development	ENSGALG00010017580	LYPLAL1	0.164149671	0.152797047	1.07429871	0.282688823	0.39407333	positive
PRJNA729271	stage_E14_vs_E10	ordinary_muscle_development	ENSGALG00010017581	EFCAB11	-0.83541942	0.37251114	-2.242669627	0.024918127	0.052567057	negative
PRJNA729271	stage_E14_vs_E10	ordinary_muscle_development	ENSGALG00010017582	CLCN6	-0.212283401	0.101734208	-2.086647211	0.036920036	0.073158705	negative
PRJNA729271	stage_E14_vs_E10	ordinary_muscle_development	ENSGALG00010017583	MTHFD1	-0.378613865	0.104753699	-3.614324533	0.000301132	0.001078286	negative
PRJNA729271	stage_E14_vs_E10	ordinary_muscle_development	ENSGALG00010017584	WNT11B	-1.552417266	0.670779983	-2.314346441	0.020648726	0.044705495	negative
PRJNA729271	stage_E14_vs_E10	ordinary_muscle_development	ENSGALG00010017585	ENSGALG00010017585	0.256220857	0.110957062	2.309189264	0.020933081	0.045233305	positive
PRJNA729271	stage_E14_vs_E10	ordinary_muscle_development	ENSGALG00010017586	ZFYVE28	0.686867454	0.156138052	4.399103524	1.08699E-05	5.06133E-05	positive
PRJNA729271	stage_E14_vs_E10	ordinary_muscle_development	ENSGALG00010017587	MB21D2	-0.188763252	0.144348702	-1.307689293	0.190978716	0.288297906	negative
PRJNA729271	stage_E14_vs_E10	ordinary_muscle_development	ENSGALG00010017588	NOX5	-1.389407737	0.474296078	-2.929410135	0.00339606	0.009256467	negative
PRJNA729271	stage_E14_vs_E10	ordinary_muscle_development	ENSGALG00010017589	ATPAF2	-0.05561923	0.14565247	-0.381862593	0.702563288	0.783961113	negative
PRJNA729271	stage_E14_vs_E10	ordinary_muscle_development	ENSGALG00010017590	ENSGALG00010017590	0.495213613	0.187826468	2.636548606	0.00837542	0.020444883	positive
PRJNA729271	stage_E14_vs_E10	ordinary_muscle_development	ENSGALG00010017591	ESR2	-2.026795168	0.255757348	-7.924680114	2.28734E-15	3.32882E-14	negative
PRJNA729271	stage_E14_vs_E10	ordinary_muscle_development	ENSGALG00010017592	GID4	0.14971974	0.111574932	1.341876152	0.179636155	0.274433608	positive
PRJNA729271	stage_E14_vs_E10	ordinary_muscle_development	ENSGALG00010017593	MTHFR	-0.31868573	0.133248508	-2.39166454	0.016772164	0.037373178	negative
PRJNA729271	stage_E14_vs_E10	ordinary_muscle_development	ENSGALG00010017594	CEP68	0.506522032	0.0992969	5.101086042	3.3771E-07	2.00127E-06	positive
PRJNA729271	stage_E14_vs_E10	ordinary_muscle_development	ENSGALG00010017595	CCNF	-0.351721459	0.122670542	-2.86720393	0.004141161	0.011024082	negative
PRJNA729271	stage_E14_vs_E10	ordinary_muscle_development	ENSGALG00010017596	TDP1	-0.116569498	0.091152631	-1.278838549	0.200953916	0.300136735	negative
PRJNA729271	stage_E14_vs_E10	ordinary_muscle_development	ENSGALG00010017597	CNOT10	-0.01269542	0.133744967	-0.094922602	0.924376304	0.947986028	negative
PRJNA729271	stage_E14_vs_E10	ordinary_muscle_development	ENSGALG00010017598	ENSGALG00010017598	0.343650992	0.117147803	2.933482181	0.003351829	0.009154845	positive
PRJNA729271	stage_E14_vs_E10	ordinary_muscle_development	ENSGALG00010017599	KCNK13	1.137334733	0.214805239	5.294725297	1.19195E-07	7.49561E-07	positive
PRJNA729271	stage_E14_vs_E10	ordinary_muscle_development	ENSGALG00010017600	MXD4	0.578814568	0.102893349	5.625383707	1.85096E-08	1.30622E-07	positive
PRJNA729271	stage_E14_vs_E10	ordinary_muscle_development	ENSGALG00010017601	ENSGALG00010017601	0.949450601	0.11365075	8.354107635	6.59292E-17	1.10479E-15	positive
PRJNA729271	stage_E14_vs_E10	ordinary_muscle_development	ENSGALG00010017603	CYP3A5	-1.237528443	1.398799981	-0.88470722	0.376314561	0.492521856	negative
PRJNA729271	stage_E14_vs_E10	ordinary_muscle_development	ENSGALG00010017604	GATM	2.664703742	0.241199699	11.04770759	2.24884E-28	8.73913E-27	positive
PRJNA729271	stage_E14_vs_E10	ordinary_muscle_development	ENSGALG00010017605	GFPT1	-0.167139151	0.070744593	-2.362571371	0.018148647	0.039985504	negative
PRJNA729271	stage_E14_vs_E10	ordinary_muscle_development	ENSGALG00010017606	ENSGALG00010017606	-0.010465341	0.255693407	-0.040929255	0.967352295	0.97821763	negative
PRJNA729271	stage_E14_vs_E10	ordinary_muscle_development	ENSGALG00010017608	RBBP4	-0.10885361	0.080398033	-1.353933745	0.175757516	0.269441327	negative
PRJNA729271	stage_E14_vs_E10	ordinary_muscle_development	ENSGALG00010017609	TRIM71	-0.138957361	0.195404766	-0.711125749	0.477006315	0.589145069	negative
PRJNA729271	stage_E14_vs_E10	ordinary_muscle_development	ENSGALG00010017610	PSMC1	-0.00668779	0.118951532	-0.056222811	0.955164309	0.968996212	negative
PRJNA729271	stage_E14_vs_E10	ordinary_muscle_development	ENSGALG00010017612	CLDN11	-0.989756239	0.417936699	-2.368196524	0.017875038	0.039460658	negative
PRJNA729271	stage_E14_vs_E10	ordinary_muscle_development	ENSGALG00010017613	HAUS3	-0.093011864	0.117149564	-0.793958261	0.427219731	0.541685619	negative
PRJNA729271	stage_E14_vs_E10	ordinary_muscle_development	ENSGALG00010017615	AGTRAP	0.032379316	0.1343274	0.24104774	0.809518116	0.866103121	positive
PRJNA729271	stage_E14_vs_E10	ordinary_muscle_development	ENSGALG00010017616	PDCD7	0.337430667	0.15558911	2.16872933	0.030103239	0.061605254	positive
PRJNA729271	stage_E14_vs_E10	ordinary_muscle_development	ENSGALG00010017619	POLN	0.434489277	0.318942486	1.362280963	0.173109233	0.266117121	positive
PRJNA729271	stage_E14_vs_E10	ordinary_muscle_development	ENSGALG00010017620	UBAP1L	-0.337179507	0.332842906	-1.013028974	0.311046322	0.424820167	negative
PRJNA729271	stage_E14_vs_E10	ordinary_muscle_development	ENSGALG00010017621	SYNE2	-0.522097655	0.156048242	-3.345745182	0.000820618	0.002642127	negative
PRJNA729271	stage_E14_vs_E10	ordinary_muscle_development	ENSGALG00010017622	RAB1B	-0.067644443	0.112486175	-0.601357838	0.547601674	0.652861183	negative
PRJNA729271	stage_E14_vs_E10	ordinary_muscle_development	ENSGALG00010017625	YBX1	0.275380018	0.091231681	3.018469189	0.002540552	0.007163063	positive
PRJNA729271	stage_E14_vs_E10	ordinary_muscle_development	ENSGALG00010017626	COPS7B	-0.11548352	0.139178955	-0.829748434	0.406681031	0.521667394	negative
PRJNA729271	stage_E14_vs_E10	ordinary_muscle_development	ENSGALG00010017628	NRDE2	-0.153336395	0.102760594	-1.492171165	0.13565428	0.218628445	negative
PRJNA729271	stage_E14_vs_E10	ordinary_muscle_development	ENSGALG00010017629	ENSGALG00010017629	-0.115358636	0.139669585	-0.825939563	0.408838377	0.52360066	negative
PRJNA729271	stage_E14_vs_E10	ordinary_muscle_development	ENSGALG00010017631	NAT8L	-0.148969571	0.249692363	-0.596612443	0.550766162	0.655717085	negative
PRJNA729271	stage_E14_vs_E10	ordinary_muscle_development	ENSGALG00010017632	AWAT1	0.699847934	0.589030686	1.188134933	0.234780254	0.339296089	positive
PRJNA729271	stage_E14_vs_E10	ordinary_muscle_development	ENSGALG00010017633	HLX	-0.266521652	0.15395456	-1.731170887	0.08342129	0.146515401	negative
PRJNA729271	stage_E14_vs_E10	ordinary_muscle_development	ENSGALG00010017634	PPIH	0.033165246	0.095064873	0.34886962	0.727187194	0.802810626	positive
PRJNA729271	stage_E14_vs_E10	ordinary_muscle_development	ENSGALG00010017636	ZBTB8OS	-0.116664265	0.156925265	-0.743438384	0.457216313	0.570143708	negative
PRJNA729271	stage_E14_vs_E10	ordinary_muscle_development	ENSGALG00010017638	GFRA2	-0.363808408	0.258293993	-1.408505104	0.158981557	0.248644547	negative
PRJNA729271	stage_E14_vs_E10	ordinary_muscle_development	ENSGALG00010017639	ENSGALG00010017639	-0.535991824	0.121069714	-4.427133822	9.54935E-06	4.49408E-05	negative
PRJNA729271	stage_E14_vs_E10	ordinary_muscle_development	ENSGALG00010017641	GPRC5B	1.403998323	0.159086714	8.825365024	1.09103E-18	2.09187E-17	positive
PRJNA729271	stage_E14_vs_E10	ordinary_muscle_development	ENSGALG00010017642	MTARC1	0.585169231	0.122250916	4.786624511	1.6961E-06	9.05856E-06	positive
PRJNA729271	stage_E14_vs_E10	ordinary_muscle_development	ENSGALG00010017643	CALM1	-0.323400925	0.060972324	-5.304060971	1.13254E-07	7.15304E-07	negative
PRJNA729271	stage_E14_vs_E10	ordinary_muscle_development	ENSGALG00010017644	RBBP6	-0.108324267	0.0970084	-1.11664832	0.264144723	0.372957116	negative
PRJNA729271	stage_E14_vs_E10	ordinary_muscle_development	ENSGALG00010017645	SYNC	2.008873153	0.268358665	7.485777126	7.1125E-14	9.17222E-13	positive
PRJNA729271	stage_E14_vs_E10	ordinary_muscle_development	ENSGALG00010017646	MAEA	0.135119496	0.086076426	1.569761924	0.116470509	0.192985828	positive
PRJNA729271	stage_E14_vs_E10	ordinary_muscle_development	ENSGALG00010017647	ITGA9	-0.111644022	0.166744052	-0.669553248	0.503142626	0.612487306	negative
PRJNA729271	stage_E14_vs_E10	ordinary_muscle_development	ENSGALG00010017648	ENSGALG00010017648	-0.449264199	0.141880335	-3.16650084	0.001542849	0.004631103	negative
PRJNA729271	stage_E14_vs_E10	ordinary_muscle_development	ENSGALG00010017649	ENSGALG00010017649	0.340299368	0.103801757	3.278358465	0.001044127	0.003278731	positive
PRJNA729271	stage_E14_vs_E10	ordinary_muscle_development	ENSGALG00010017651	PPCS	-0.302730224	0.122307464	-2.4751574	0.013317747	0.030620087	negative
PRJNA729271	stage_E14_vs_E10	ordinary_muscle_development	ENSGALG00010017652	C1orf115	0.395443453	0.174235785	2.269588031	0.023232591	0.049530284	positive

PRJNA729271	stage_E14_vs_E10	ordinary_muscle_development	ENSGALG00010017653	ENSGALG00010017653	1.376138079	0.171364849	8.03045714	9.71101E-16	1.45414E-14	positive
PRJNA729271	stage_E14_vs_E10	ordinary_muscle_development	ENSGALG00010017654	KBTBD13	3.269021996	0.328239772	9.959250154	2.29789E-23	6.13406E-22	positive
PRJNA729271	stage_E14_vs_E10	ordinary_muscle_development	ENSGALG00010017655	ENSGALG00010017655	-0.452513828	0.104036743	-4.349557819	1.36412E-05	6.26123E-05	negative
PRJNA729271	stage_E14_vs_E10	ordinary_muscle_development	ENSGALG00010017656	FSD2	4.637001148	0.209621461	22.12083212	1.9922E-108	2.6252E-105	positive
PRJNA729271	stage_E14_vs_E10	ordinary_muscle_development	ENSGALG00010017657	DIS3L2	0.386813717	0.09287683	4.164803183	3.11621E-05	0.000134593	positive
PRJNA729271	stage_E14_vs_E10	ordinary_muscle_development	ENSGALG00010017658	WHAMM	0.368097905	0.163185507	2.255702195	0.024089288	0.051093683	positive
PRJNA729271	stage_E14_vs_E10	ordinary_muscle_development	ENSGALG00010017659	TNRC6A	0.088136864	0.078159816	1.127649328	0.259468045	0.367710752	positive
PRJNA729271	stage_E14_vs_E10	ordinary_muscle_development	ENSGALG00010017660	CLIP4	1.438902108	0.247737582	5.808170478	6.31592E-09	4.71417E-08	positive
PRJNA729271	stage_E14_vs_E10	ordinary_muscle_development	ENSGALG00010017661	NHSL3	0.358484725	0.166913979	2.147721395	0.031735895	0.06436432	positive
PRJNA729271	stage_E14_vs_E10	ordinary_muscle_development	ENSGALG00010017662	GOLGA4	-0.041938157	0.16552861	-0.253358966	0.799990834	0.859239708	negative
PRJNA729271	stage_E14_vs_E10	ordinary_muscle_development	ENSGALG00010017663	ZMYND12	-0.093284697	0.208135809	-0.448191484	0.654015008	0.74481046	negative
PRJNA729271	stage_E14_vs_E10	ordinary_muscle_development	ENSGALG00010017664	ENSGALG00010017664	0.197742203	0.074085175	2.669119741	0.007605033	0.018750631	positive
PRJNA729271	stage_E14_vs_E10	ordinary_muscle_development	ENSGALG00010017665	CTBP1	0.214022351	0.062448645	3.427173632	0.000609899	0.002020682	positive
PRJNA729271	stage_E14_vs_E10	ordinary_muscle_development	ENSGALG00010017667	SLC2A1	-0.43380602	0.10937952	-3.966062562	7.30697E-05	0.000294888	negative
PRJNA729271	stage_E14_vs_E10	ordinary_muscle_development	ENSGALG00010017668	ENSGALG00010017668	-0.899747731	0.920283077	-0.977685837	0.328229729	0.443111662	negative
PRJNA729271	stage_E14_vs_E10	ordinary_muscle_development	ENSGALG00010017669	ENSGALG00010017669	0.387652691	0.174560892	2.220730462	0.026369223	0.055154673	positive
PRJNA729271	stage_E14_vs_E10	ordinary_muscle_development	ENSGALG00010017670	FAM179A	1.771437043	0.332186567	5.332657067	9.6786E-08	6.17751E-07	positive
PRJNA729271	stage_E14_vs_E10	ordinary_muscle_development	ENSGALG00010017671	ANTXR1	0.286220622	0.183982997	1.555690614	0.119781679	0.197366766	positive
PRJNA729271	stage_E14_vs_E10	ordinary_muscle_development	ENSGALG00010017672	SPRED2	-0.271367757	0.076806434	-3.533138324	0.000410658	0.001417933	negative
PRJNA729271	stage_E14_vs_E10	ordinary_muscle_development	ENSGALG00010017673	WDR89	-0.053539688	0.090778186	-0.589785828	0.555334245	0.65980081	negative
PRJNA729271	stage_E14_vs_E10	ordinary_muscle_development	ENSGALG00010017675	ENSGALG00010017675	0.802509101	0.454228198	1.766753152	0.077269578	0.137240844	positive
PRJNA729271	stage_E14_vs_E10	ordinary_muscle_development	ENSGALG00010017676	SPON2	1.706535038	0.262741445	6.495111712	8.29718E-11	7.67993E-10	positive
PRJNA729271	stage_E14_vs_E10	ordinary_muscle_development	ENSGALG00010017677	WDR43	-0.237296217	0.109783658	-2.161489429	0.030657553	0.062518462	negative
PRJNA729271	stage_E14_vs_E10	ordinary_muscle_development	ENSGALG00010017678	YARS	-0.045241041	0.136787461	-0.330739677	0.740841129	0.813645414	negative
PRJNA729271	stage_E14_vs_E10	ordinary_muscle_development	ENSGALG00010017679	RASL12	2.295873903	0.180875442	12.69312121	6.45607E-37	4.1225E-35	positive
PRJNA729271	stage_E14_vs_E10	ordinary_muscle_development	ENSGALG00010017681	ENSGALG00010017681	1.310605987	1.328238037	0.986725233	0.323777356	0.438407546	positive
PRJNA729271	stage_E14_vs_E10	ordinary_muscle_development	ENSGALG00010017684	TRMT61B	0.229075631	0.093165196	2.458811231	0.013939789	0.031840095	positive
PRJNA729271	stage_E14_vs_E10	ordinary_muscle_development	ENSGALG00010017685	PHC2	0.097628308	0.183624067	0.531674906	0.594951175	0.694684814	positive
PRJNA729271	stage_E14_vs_E10	ordinary_muscle_development	ENSGALG00010017687	TTC7B	0.225438044	0.096641664	2.332721049	0.019662791	0.042813466	positive
PRJNA729271	stage_E14_vs_E10	ordinary_muscle_development	ENSGALG00010017688	SPDYA	-0.624405554	0.510454464	-1.223234583	0.221241107	0.323928267	negative
PRJNA729271	stage_E14_vs_E10	ordinary_muscle_development	ENSGALG00010017691	LZTS1	1.562651198	0.220876057	7.074787625	1.49678E-12	1.67148E-11	positive
PRJNA729271	stage_E14_vs_E10	ordinary_muscle_development	ENSGALG00010017692	EPCAM	-1.517380383	0.984188695	-1.541757583	0.123132513	0.201650184	negative
PRJNA729271	stage_E14_vs_E10	ordinary_muscle_development	ENSGALG00010017693	MTFMT	0.161924107	0.115691894	1.399614977	0.161628647	0.252050267	positive
PRJNA729271	stage_E14_vs_E10	ordinary_muscle_development	ENSGALG00010017694	ATP6V1B2	-0.233853359	0.07030366	-3.326332657	0.000879968	0.002807647	negative
PRJNA729271	stage_E14_vs_E10	ordinary_muscle_development	ENSGALG00010017695	FNDC5	1.938921731	0.20407211	9.501159817	2.07565E-21	4.98949E-20	positive
PRJNA729271	stage_E14_vs_E10	ordinary_muscle_development	ENSGALG00010017698	ENSGALG00010017698	-0.978686102	0.896795412	-1.091314796	0.27513439	0.385602918	negative
PRJNA729271	stage_E14_vs_E10	ordinary_muscle_development	ENSGALG00010017699	RNF19B	0.43539781	0.238725507	1.823842852	0.068175833	0.12329491	positive
PRJNA729271	stage_E14_vs_E10	ordinary_muscle_development	ENSGALG00010017700	VPS35L	0.031956556	0.078150506	0.408910418	0.682605403	0.767540557	positive
PRJNA729271	stage_E14_vs_E10	ordinary_muscle_development	ENSGALG00010017701	PPP2R5E	-0.114823586	0.098586062	-1.164704052	0.244138808	0.350583716	negative
PRJNA729271	stage_E14_vs_E10	ordinary_muscle_development	ENSGALG00010017703	RPS6KA5	0.381151036	0.139858963	2.725252843	0.006425226	0.016174653	positive
PRJNA729271	stage_E14_vs_E10	ordinary_muscle_development	ENSGALG00010017704	ZNF362	-0.516461153	0.196683268	-2.625852	0.008643239	0.020992585	negative
PRJNA729271	stage_E14_vs_E10	ordinary_muscle_development	ENSGALG00010017705	MAK16	0.110456654	0.103316649	1.069107976	0.285021018	0.396752104	positive
PRJNA729271	stage_E14_vs_E10	ordinary_muscle_development	ENSGALG00010017706	FGFRL1	-1.069191723	0.143458807	-7.452952836	9.12739E-14	1.16054E-12	negative
PRJNA729271	stage_E14_vs_E10	ordinary_muscle_development	ENSGALG00010017707	AK2	0.058916187	0.094905068	0.620790734	0.534737321	0.641799906	positive
PRJNA729271	stage_E14_vs_E10	ordinary_muscle_development	ENSGALG00010017708	MEIS1	-0.922759537	0.103130906	-8.947458832	3.6376E-19	7.32319E-18	negative
PRJNA729271	stage_E14_vs_E10	ordinary_muscle_development	ENSGALG00010017710	RAB41	-0.177404065	0.114321509	-1.551799543	0.120710195	0.198535604	negative
PRJNA729271	stage_E14_vs_E10	ordinary_muscle_development	ENSGALG00010017711	LRRFIP2	0.230805628	0.134346731	1.717984695	0.085799407	0.149893022	positive
PRJNA729271	stage_E14_vs_E10	ordinary_muscle_development	ENSGALG00010017712	MSH2	-0.112067475	0.070981407	-1.578828588	0.114375379	0.190494212	negative
PRJNA729271	stage_E14_vs_E10	ordinary_muscle_development	ENSGALG00010017713	ALKBH5	0.114798919	0.149753047	0.766588207	0.443326389	0.557040223	positive
PRJNA729271	stage_E14_vs_E10	ordinary_muscle_development	ENSGALG00010017714	ATAD3A	-0.259250292	0.151640905	-1.709632978	0.087333763	0.152078676	negative
PRJNA729271	stage_E14_vs_E10	ordinary_muscle_development	ENSGALG00010017715	CILP	0.700814418	0.168970157	4.147563283	3.36033E-05	0.000144021	positive
PRJNA729271	stage_E14_vs_E10	ordinary_muscle_development	ENSGALG00010017717	LLGL1	0.009110231	0.149275975	0.061029454	0.951335752	0.966200373	positive
PRJNA729271	stage_E14_vs_E10	ordinary_muscle_development	ENSGALG00010017718	ATOH8	0.086934552	0.298807416	0.2909384	0.771098432	0.837484772	positive
PRJNA729271	stage_E14_vs_E10	ordinary_muscle_development	ENSGALG00010017719	ENSGALG00010017719	-1.69644752	1.940268166	-0.874336625	0.381934959	0.498034116	negative
PRJNA729271	stage_E14_vs_E10	ordinary_muscle_development	ENSGALG00010017720	ENSGALG00010017720	-0.124676926	0.280801316	-0.444004065	0.657039645	0.747198309	negative
PRJNA729271	stage_E14_vs_E10	ordinary_muscle_development	ENSGALG00010017721	VWA1	1.42264223	0.119761668	11.87894468	1.52278E-32	7.43187E-31	positive
PRJNA729271	stage_E14_vs_E10	ordinary_muscle_development	ENSGALG00010017723	KCNK12	-1.440343239	0.333751594	-4.315614568	1.59159E-05	7.21168E-05	negative
PRJNA729271	stage_E14_vs_E10	ordinary_muscle_development	ENSGALG00010017724	FLII	0.125260125	0.128207864	0.977008128	0.328565128	0.443371329	positive
PRJNA729271	stage_E14_vs_E10	ordinary_muscle_development	ENSGALG00010017725	ENSGALG00010017725	0.563828839	0.397322948	1.419069406	0.155878782	0.244875144	positive
PRJNA729271	stage_E14_vs_E10	ordinary_muscle_development	ENSGALG00010017726	TTI2	0.40652524	0.116869417	3.478456981	0.000504309	0.001707936	positive
PRJNA729271	stage_E14_vs_E10	ordinary_muscle_development	ENSGALG00010017727	AZIN2	0.750119595	0.146520125	5.119566992	3.06238E-07	1.82371E-06	positive
PRJNA729271	stage_E14_vs_E10	ordinary_muscle_development	ENSGALG00010017729	MIEF2	0.545594876	0.141580743	3.853595238	0.000116396	0.000453294	positive
PRJNA729271	stage_E14_vs_E10	ordinary_muscle_development	ENSGALG00010017730	TARP	0.897039381	0.603584632	1.486186582	0.137229776	0.220623957	positive
PRJNA729271	stage_E14_vs_E10	ordinary_muscle_development	ENSGALG00010017732	RHOJ	-0.030386453	0.076300359	-0.398247842	0.690447503	0.774136491	negative
PRJNA729271	stage_E14_vs_E10	ordinary_muscle_development	ENSGALG00010017733	TRIM62	0.151007158	0.128399764	1.176070362	0.23956676	0.345318237	positive
PRJNA729271	stage_E14_vs_E10	ordinary_muscle_development	ENSGALG00010017734	TOP3A	-0.080056764	0.114684435	-0.698061286	0.485138868	0.596647539	negative
PRJNA729271	stage_E14_vs_E10	ordinary_muscle_development	ENSGALG00010017736	ENSGALG00010017736	0.47224261	4.395975581	0.107426122	0.914450932	0.940869269	positive
PRJNA729271	stage_E14_vs_E10	ordinary_muscle_development	ENSGALG00010017737	ENSGALG00010017737	-0.588948813	0.188632381	-3.122204196	0.001795024	0.005301319	negative
PRJNA729271	stage_E14_vs_E10	ordinary_muscle_development	ENSGALG00010017738	NDUFS5	0.375086853	0.111680282	3.358577244	0.000783448	0.002533143	positive
PRJNA729271	stage_E14_vs_E10	ordinary_muscle_development	ENSGALG00010017739	MRPL20	0.011444471	0.097983977	0.116799411	0.907019011	0.9362131	positive
PRJNA729271	stage_E14_vs_E10	ordinary_muscle_development	ENSGALG00010017741	SMCR8	0.09125662	0.139226975	0.65545215	0.512176685	0.621099393	positive
PRJNA729271	stage_E14_vs_E10	ordinary_muscle_development	ENSGALG00010017742	DGLUCY	1.277491797	0.237241448	5.384774901	7.25354E-08	4.73177E-07	positive
PRJNA729271	stage_E14_vs_E10	ordinary_muscle_development	ENSGALG00010017743	KCNH5	-0.266630071	0.281489333	-0.947211991	0.343530766	0.458726712	negative
PRJNA729271	stage_E14_vs_E10	ordinary_muscle_development	ENSGALG00010017744	ETAA1	-0.164949942	0.097626601	-1.689600387	0.091104432	0.157377993	negative
PRJNA729271	stage_E14_vs_E10	ordinary_muscle_development	ENSGALG00010017745	SHMT1	0.26272865	0.182538186	1.439307883	0.15006331	0.237385975	positive
PRJNA729271	stage_E14_vs_E10	ordinary_muscle_development	ENSGALG00010017746	MSH6	-0.190753352	0.088361627	-2.158780446	0.030867204	0.062866394	negative
PRJNA729271	stage_E14_vs_E10	ordinary_muscle_development	ENSGALG00010017747	DUSP26	2.093723015	0.118358421	17.68968358	5.03574E-70	1.78032E-67	positive
PRJNA729271	stage_E14_vs_E10	ordinary_muscle_development	ENSGALG00010017748	ENSGALG00010017748	0.869219537	0.417142527	2.083747114	0.037183176	0.073639861	positive
PRJNA729271	stage_E14_vs_E10	ordinary_muscle_development	ENSGALG00010017751	ST3GAL5	-0.211058334	0.125347035	-1.683791991	0.092221876	0.159042961	negative
PRJNA729271	stage_E14_vs_E10	ordinary_muscle_development	ENSGALG00010017753	GNRHR	0.729457232	0.329154036	2.216157639	0.026680708	0.055677669	positive
PRJNA729271	stage_E14_vs_E10	ordinary_muscle_development	ENSGALG00010017754	PDZD11	-0.49198607	0.158200878	-3.109882038	0.001871621	0.005502869	negative
PRJNA729271	stage_E14_vs_E10	ordinary_muscle_development	ENSGALG00010017755	C1D	-0.50992476	0.138666699	-3.677341167	0.000235678	0.000860708	negative
PRJNA729271	stage_E14_vs_E10	ordinary_muscle_development	ENSGALG00010017756	EIF2B5	0.145618799	0.117882786	1.235284673	0.216724576	0.318602711	positive
PRJNA729271	stage_E14_vs_E10	ordinary_muscle_development	ENSGALG00010017757	ENSGALG00010017757	0.764887682	0.138505424	5.52243848	3.34327E-08	2.29094E-07	positive
PRJNA729271	stage_E14_vs_E10	ordinary_muscle_development	ENSGALG00010017759	ENSGALG00010017759	-0.349791738	1.057138581	-0.330885415	0.740731039	0.813586149	negative
PRJNA729271	stage_E14_vs_E10	ordinary_muscle_development	ENSGALG00010017761	PARP16	0.709354435	0.105878327	6.699713317	2.08829E-11	2.07043E-10	positive
PRJNA729271	stage_E14_vs_E10	ordinary_muscle_development	ENSGALG00010017762	FBXO11	0.03185698	0.054692019	0.582479509	0.56024374	0.663538116	positive
PRJNA729271	stage_E14_vs_E10	ordinary_muscle_development	ENSGALG00010017763	POLR1A	-0.360849102	0.085862279	-4.202649923	2.63808E-05	0.000115247	negative
PRJNA729271	stage_E14_vs_E10	ordinary_muscle_development	ENSGALG00010017764	IGDCC3	-0.024889724	0.215758501	-0.115359181	0.908160432	0.936924232	negative
PRJNA729271	stage_E14_vs_E10	ordinary_muscle_development	ENSGALG00010017765	SYT16	0.1158047	0.289746487	0.39967594	0.689395216	0.773076253	positive
PRJNA729271	stage_E14_vs_E10	ordinary_muscle_development	ENSGALG00010017766	DNAAF10	0.045081566	0.153850206	0.293022461	0.769504984	0.836255416	positive
PRJNA729271	stage_E14_vs_E10	ordinary_muscle_development	ENSGALG00010017767	KIF4B	-0.339997197	0.219616528	-1.548140301	0.121588521	0.199730917	negative
PRJNA729271	stage_E14_vs_E10	ordinary_muscle_development	ENSGALG00010017768	FOXN2	0.104483535	0.079703478	1.310903087	0.189890508	0.286894196	positive
PRJNA729271	stage_E14_vs_E10	ordinary_muscle_development	ENSGALG00010017770	PTCD3	0.127581918	0.0688849	1.852102839	0.064011057	0.116900626	positive
PRJNA729271	stage_E14_vs_E10	ordinary_muscle_development	ENSGALG00010017771	PNO1	-0.006927731	0.157370043	-0.044021915	0.964886935	0.9766785	negative
PRJNA729271	stage_E14_vs_E10	ordinary_muscle_development	ENSGALG00010017772	ENSGALG00010017772	0.309877051	0.390350792	0.793842507	0.427287123	0.541700587	positive
PRJNA729271	stage_E14_vs_E10	ordinary_muscle_development	ENSGALG00010017773	DVL3	-0.008541884	0.077719788	-0.109906167	0.912483791	0.939779207	negative
PRJNA729271	stage_E14_vs_E10	ordinary_muscle_development	ENSGALG00010017774	ASH2L	0.074408675	0.163048368	0.456359518	0.648131471	0.740028804	positive
PRJNA729271	stage_E14_vs_E10	ordinary_muscle_development	ENSGALG00010017775	PRPSAP2	-0.161811171	0.084260359	-1.920371264	0.054811021	0.102408577	negative
PRJNA729271	stage_E14_vs_E10	ordinary_muscle_development	ENSGALG00010017776	GPR68	-0.570255769	0.366316501	-1.556729676	0.11953468	0.197071791	negative
PRJNA729271	stage_E14_vs_E10	ordinary_muscle_development	ENSGALG00010017778	PPP3R1	0.741550706	0.078521994	9.443859893	3.59294E-21	8.38643E-20	positive
PRJNA729271	stage_E14_vs_E10	ordinary_muscle_development	ENSGALG00010017779	SNAPC1	-0.084999521	0.123458028	-0.688489215	0.491144759	0.601431384	negative
PRJNA729271	stage_E14_vs_E10	ordinary_muscle_development	ENSGALG00010017780	TAF9	-0.016932263	0.136405326	-0.124131975	0.901210781	0.932209397	negative
PRJNA729271	stage_E14_vs_E10	ordinary_muscle_development	ENSGALG00010017781	CYP26C1	0.529382285	0.308179573	1.717772142	0.085838185	0.149942696	positive
PRJNA729271	stage_E14_vs_E10	ordinary_muscle_development	ENSGALG00010017782	IGDCC4	0.424079008	0.113514952	3.735886771	0.000187055	0.000699345	positive
PRJNA729271	stage_E14_vs_E10	ordinary_muscle_development	ENSGALG00010017783	STAR	0.054903462	0.384865141	0.142656365	0.88656158	0.922309328	positive
PRJNA729271	stage_E14_vs_E10	ordinary_muscle_development	ENSGALG00010017786	KAZALD1	0.555434947	0.164864324	3.369042694	0.000754297	0.002448173	positive
PRJNA729271	stage_E14_vs_E10	ordinary_muscle_development	ENSGALG00010017787	CNRIP1	1.393107863	0.23532454	5.919942999	3.22053E-09	2.48969E-08	positive
PRJNA729271	stage_E14_vs_E10	ordinary_muscle_development	ENSGALG00010017789	IMMT	0.234034323	0.130226498	1.797132891	0.072314525	0.129679456	positive
PRJNA729271	stage_E14_vs_E10	ordinary_muscle_development	ENSGALG00010017790	R3HCC1L	0.700014912	0.1710881	4.09154647	4.28506E-05	0.000180348	positive
PRJNA729271	stage_E14_vs_E10	ordinary_muscle_development	ENSGALG00010017791	CCNL2	0.023377181	0.11125862	0.210115688	0.833577381	0.883923047	positive
PRJNA729271	stage_E14_vs_E10	ordinary_muscle_development	ENSGALG00010017792	LSM1	-0.060372919	0.120445075	-0.501248551	0.616196209	0.712343436	negative
PRJNA729271	stage_E14_vs_E10	ordinary_muscle_development	ENSGALG00010017793	FAM83G	-1.61300552	0.304281275	-5.301034454	1.15148E-07	7.26633E-07	negative
PRJNA729271	stage_E14_vs_E10	ordinary_muscle_development	ENSGALG00010017795	MACF1	0.392365638	0.114060858	3.439967435	0.000581784	0.001939504	positive
PRJNA729271	stage_E14_vs_E10	ordinary_muscle_development	ENSGALG00010017796	MXI1	0.410947829	0.133097698	3.087565275	0.002018034	0.005859657	positive
PRJNA729271	stage_E14_vs_E10	ordinary_muscle_development	ENSGALG00010017797	PPP1R21	-0.057521449	0.090642438	-0.634597325	0.525691074	0.633354843	negative
PRJNA729271	stage_E14_vs_E10	ordinary_muscle_development	ENSGALG00010017798	BAG4	-0.100622743	0.157544945	-0.638692296	0.523023125	0.630927114	negative
PRJNA729271	stage_E14_vs_E10	ordinary_muscle_development	ENSGALG00010017799	GRAPL	-0.780951151	0.371351968	-2.102994513	0.035466249	0.070712968	negative
PRJNA729271	stage_E14_vs_E10	ordinary_muscle_development	ENSGALG00010017801	SMNDC1	-0.043903836	0.07066522	-0.621293419	0.534406583	0.641467785	negative
PRJNA729271	stage_E14_vs_E10	ordinary_muscle_development	ENSGALG00010017803	ENSGALG00010017803	-0.080263141	0.118467411	-0.677512409	0.498080899	0.607410621	negative
PRJNA729271	stage_E14_vs_E10	ordinary_muscle_development	ENSGALG00010017804	DUSP5	-0.012500643	0.240186894	-0.052045481	0.958492454	0.971426941	negative
PRJNA729271	stage_E14_vs_E10	ordinary_muscle_development	ENSGALG00010017805	DPP8	-0.099269943	0.106707952	-0.930295646	0.352218032	0.468557302	negative
PRJNA729271	stage_E14_vs_E10	ordinary_muscle_development	ENSGALG00010017806	ENSGALG00010017806	0.945853089	0.764584109	1.237081804	0.216056714	0.317846552	positive
PRJNA729271	stage_E14_vs_E10	ordinary_muscle_development	ENSGALG00010017807	MRPL35	0.124159856	0.14662777	0.846769042	0.397123867	0.512628947	positive
PRJNA729271	stage_E14_vs_E10	ordinary_muscle_development	ENSGALG00010017808	SFRP5	1.04841822	0.341769928	3.067614008	0.002157751	0.006216777	positive
PRJNA729271	stage_E14_vs_E10	ordinary_muscle_development	ENSGALG00010017809	MOSMO	0.240441866	0.096243153	2.498275027	0.012479933	0.02893883	positive
PRJNA729271	stage_E14_vs_E10	ordinary_muscle_development	ENSGALG00010017810	HIF1A	-0.987765833	0.078448786	-12.59121883	2.36001E-36	1.43131E-34	negative
PRJNA729271	stage_E14_vs_E10	ordinary_muscle_development	ENSGALG00010017811	HACD3	-0.422091828	0.064809109	-6.512847227	7.37395E-11	6.90474E-10	negative
PRJNA729271	stage_E14_vs_E10	ordinary_muscle_development	ENSGALG00010017812	ENSGALG00010017812	0.530870375	1.860911575	0.285274369	0.775433948	0.840986498	positive
PRJNA729271	stage_E14_vs_E10	ordinary_muscle_development	ENSGALG00010017813	DDHD2	-0.01728851	0.159646379	-0.108292531	0.913763647	0.940755838	negative
PRJNA729271	stage_E14_vs_E10	ordinary_muscle_development	ENSGALG00010017814	ENSGALG00010017814	0.684706311	0.232598014	2.943732404	0.003242802	0.008907414	positive

PRJNA729271	stage_E14_vs_E10	ordinary_muscle_development	ENSGALG00010017815	VWA9	-0.18177364	0.082396978	-2.206071701	0.02737898	0.056889093	negative
PRJNA729271	stage_E14_vs_E10	ordinary_muscle_development	ENSGALG00010017816	PRKCH	-1.246874823	0.294167486	-4.238656143	2.24862E-05	9.93408E-05	negative
PRJNA729271	stage_E14_vs_E10	ordinary_muscle_development	ENSGALG00010017817	REEP1	2.214035275	0.116440747	19.01426547	1.29944E-80	6.72693E-78	positive
PRJNA729271	stage_E14_vs_E10	ordinary_muscle_development	ENSGALG00010017818	SLC38A6	0.151126787	0.135326477	1.116756978	0.264098248	0.372927824	positive
PRJNA729271	stage_E14_vs_E10	ordinary_muscle_development	ENSGALG00010017820	FLRT2	-1.356216055	0.135213404	-10.0301894	1.12301E-23	3.03695E-22	negative
PRJNA729271	stage_E14_vs_E10	ordinary_muscle_development	ENSGALG00010017821	ENSGALG00010017821	-1.754301765	0.334989564	-5.23688483	1.6331E-07	1.0069E-06	negative
PRJNA729271	stage_E14_vs_E10	ordinary_muscle_development	ENSGALG00010017822	VWA3A	2.371674269	0.258772397	9.165097564	4.95022E-20	1.06776E-18	positive
PRJNA729271	stage_E14_vs_E10	ordinary_muscle_development	ENSGALG00010017823	PPIE	0.100344418	0.088023578	1.139972039	0.25429795	0.361731971	positive
PRJNA729271	stage_E14_vs_E10	ordinary_muscle_development	ENSGALG00010017824	YAE1	-0.145126144	0.122469841	-1.184994953	0.236019443	0.340713258	negative
PRJNA729271	stage_E14_vs_E10	ordinary_muscle_development	ENSGALG00010017825	PLPP5	-0.581905919	0.138989974	-4.186675502	2.8307E-05	0.000123179	negative
PRJNA729271	stage_E14_vs_E10	ordinary_muscle_development	ENSGALG00010017826	PABPC4	0.328529865	0.141604052	2.320059768	0.020337644	0.044137469	positive
PRJNA729271	stage_E14_vs_E10	ordinary_muscle_development	ENSGALG00010017828	ENSGALG00010017828	0.474889996	0.23603313	2.011963306	0.044223805	0.085390177	positive
PRJNA729271	stage_E14_vs_E10	ordinary_muscle_development	ENSGALG00010017830	DENND4A	-0.098148411	0.128053405	-0.766464673	0.443399864	0.557059323	negative
PRJNA729271	stage_E14_vs_E10	ordinary_muscle_development	ENSGALG00010017831	PLEK	0.443005444	0.183856362	2.409519254	0.015973553	0.03582495	positive
PRJNA729271	stage_E14_vs_E10	ordinary_muscle_development	ENSGALG00010017832	POU3F1	-0.967128237	0.408110231	-2.369772092	0.017799053	0.03932483	negative
PRJNA729271	stage_E14_vs_E10	ordinary_muscle_development	ENSGALG00010017834	ENSGALG00010017834	2.065127005	0.300977249	6.861405683	6.81862E-12	7.06981E-11	positive
PRJNA729271	stage_E14_vs_E10	ordinary_muscle_development	ENSGALG00010017838	B3GNT2	-0.343899266	0.123435344	-2.786068041	0.005335167	0.01378735	negative
PRJNA729271	stage_E14_vs_E10	ordinary_muscle_development	ENSGALG00010017841	GALC	-1.07361835	0.265136642	-4.049302049	5.13706E-05	0.000213296	negative
PRJNA729271	stage_E14_vs_E10	ordinary_muscle_development	ENSGALG00010017843	NSD3	-0.093611214	0.070634472	-1.325290776	0.185074727	0.280957074	negative
PRJNA729271	stage_E14_vs_E10	ordinary_muscle_development	ENSGALG00010017848	CYP3A4	1.023306233	0.195077843	5.245630255	1.55749E-07	9.63954E-07	positive
PRJNA729271	stage_E14_vs_E10	ordinary_muscle_development	ENSGALG00010017849	FBXO48	0.566869498	0.184558951	3.071482016	0.00212999	0.006146567	positive
PRJNA729271	stage_E14_vs_E10	ordinary_muscle_development	ENSGALG00010017850	TRMT5	-0.151332652	0.086788911	-1.743686499	0.081213771	0.143263187	negative
PRJNA729271	stage_E14_vs_E10	ordinary_muscle_development	ENSGALG00010017852	LETM2	-0.188790642	0.114664851	-1.646456096	0.099669892	0.169647145	negative
PRJNA729271	stage_E14_vs_E10	ordinary_muscle_development	ENSGALG00010017855	SDR42E2	2.463190924	0.233992449	10.52679662	6.50092E-26	2.11755E-24	positive
PRJNA729271	stage_E14_vs_E10	ordinary_muscle_development	ENSGALG00010017856	FASTKD5	0.013224503	0.114624294	0.115372601	0.908149795	0.936924232	positive
PRJNA729271	stage_E14_vs_E10	ordinary_muscle_development	ENSGALG00010017858	GPR65	0.299672532	0.292814542	1.023420934	0.306108866	0.419698071	positive
PRJNA729271	stage_E14_vs_E10	ordinary_muscle_development	ENSGALG00010017859	EXOC6	0.398974385	0.134640974	2.96324644	0.003044127	0.008424402	positive
PRJNA729271	stage_E14_vs_E10	ordinary_muscle_development	ENSGALG00010017860	CYSLTR1	-0.766898724	0.20711114	-3.702836663	0.000213202	0.000786753	negative
PRJNA729271	stage_E14_vs_E10	ordinary_muscle_development	ENSGALG00010017861	PRSS56	1.767205847	0.460871519	3.834487	0.000125827	0.000487662	positive
PRJNA729271	stage_E14_vs_E10	ordinary_muscle_development	ENSGALG00010017865	UBOX5	0.112602155	0.132403324	0.850448097	0.395076005	0.510531042	positive
PRJNA729271	stage_E14_vs_E10	ordinary_muscle_development	ENSGALG00010017866	RALA	0.404492001	0.094116342	4.297787098	1.72512E-05	7.77053E-05	positive
PRJNA729271	stage_E14_vs_E10	ordinary_muscle_development	ENSGALG00010017867	APLF	0.202541251	0.109444407	1.850631357	0.064222605	0.117213128	positive
PRJNA729271	stage_E14_vs_E10	ordinary_muscle_development	ENSGALG00010017868	CHRND	2.235413119	0.23999356	9.314471269	1.22561E-20	2.74579E-19	positive
PRJNA729271	stage_E14_vs_E10	ordinary_muscle_development	ENSGALG00010017870	RAB11A	0.234295382	0.073340089	3.194642732	0.00140004	0.004251746	positive
PRJNA729271	stage_E14_vs_E10	ordinary_muscle_development	ENSGALG00010017873	HSPG2	0.426640954	0.109685928	3.889659889	0.000100385	0.000394651	positive
PRJNA729271	stage_E14_vs_E10	ordinary_muscle_development	ENSGALG00010017876	COMMD1	0.054746432	0.136358685	0.401488411	0.688060569	0.772236775	positive
PRJNA729271	stage_E14_vs_E10	ordinary_muscle_development	ENSGALG00010017878	CHRNG	3.124197541	0.244477574	12.77907616	2.14603E-37	1.43349E-35	positive
PRJNA729271	stage_E14_vs_E10	ordinary_muscle_development	ENSGALG00010017880	CDK13	0.047311921	0.083936639	0.56366232	0.572983965	0.674906759	positive
PRJNA729271	stage_E14_vs_E10	ordinary_muscle_development	ENSGALG00010017881	KCNK10	-2.007668265	0.486240049	-4.12896525	3.64399E-05	0.00015517	negative
PRJNA729271	stage_E14_vs_E10	ordinary_muscle_development	ENSGALG00010017882	LZTS3	1.698405282	0.199284724	8.522506135	1.56137E-17	2.74662E-16	positive
PRJNA729271	stage_E14_vs_E10	ordinary_muscle_development	ENSGALG00010017886	DDRGK1	-0.157034908	0.101642751	-1.544969088	0.122353741	0.200714969	negative
PRJNA729271	stage_E14_vs_E10	ordinary_muscle_development	ENSGALG00010017887	PROKR1	-0.096442093	0.4448421	-0.216800731	0.828363642	0.880223663	negative
PRJNA729271	stage_E14_vs_E10	ordinary_muscle_development	ENSGALG00010017888	MPLKIP	-0.355808863	0.107274277	-3.316814355	0.000910501	0.00289487	negative
PRJNA729271	stage_E14_vs_E10	ordinary_muscle_development	ENSGALG00010017890	ANAPC1	-0.307427436	0.064427858	-4.771653819	1.82719E-06	9.68733E-06	negative
PRJNA729271	stage_E14_vs_E10	ordinary_muscle_development	ENSGALG00010017891	GOLGA7B	2.266830155	0.335991948	6.746680002	1.51266E-11	1.5237E-10	positive
PRJNA729271	stage_E14_vs_E10	ordinary_muscle_development	ENSGALG00010017894	KAT6A	0.068243004	0.080722685	0.84540057	0.397887227	0.513294353	positive
PRJNA729271	stage_E14_vs_E10	ordinary_muscle_development	ENSGALG00010017895	MNAT1	0.234727273	0.079705616	2.944927648	0.003230302	0.008878124	positive
PRJNA729271	stage_E14_vs_E10	ordinary_muscle_development	ENSGALG00010017898	NHERF2	0.047336049	0.114750235	0.41251374	0.679962912	0.765854863	positive
PRJNA729271	stage_E14_vs_E10	ordinary_muscle_development	ENSGALG00010017901	HTR7L	-3.231374467	0.744364454	-4.34111872	1.41759E-05	6.4902E-05	negative
PRJNA729271	stage_E14_vs_E10	ordinary_muscle_development	ENSGALG00010017903	ENSGALG00010017903	-1.290781719	0.320871939	-4.0227317	5.7527E-05	0.000236905	negative
PRJNA729271	stage_E14_vs_E10	ordinary_muscle_development	ENSGALG00010017904	MEGF11	-1.583518542	0.260554229	-6.077500821	1.2207E-09	9.86841E-09	negative
PRJNA729271	stage_E14_vs_E10	ordinary_muscle_development	ENSGALG00010017905	EIF4E2	0.158714741	0.086118984	1.842970426	0.065333329	0.118940794	positive
PRJNA729271	stage_E14_vs_E10	ordinary_muscle_development	ENSGALG00010017909	NTHL1	-0.171341511	0.133664592	-1.281876581	0.199885944	0.29897294	negative
PRJNA729271	stage_E14_vs_E10	ordinary_muscle_development	ENSGALG00010017915	BFSP1	0.04556365	0.540426032	0.084310614	0.932809474	0.953751562	positive
PRJNA729271	stage_E14_vs_E10	ordinary_muscle_development	ENSGALG00010017918	SIX4	0.192597721	0.200858907	0.958870702	0.337623886	0.452757723	positive
PRJNA729271	stage_E14_vs_E10	ordinary_muscle_development	ENSGALG00010017920	AP3M2	-0.45861832	0.079726936	-5.752363529	8.80043E-09	6.44254E-08	negative
PRJNA729271	stage_E14_vs_E10	ordinary_muscle_development	ENSGALG00010017921	SPATA7	0.27181172	0.202164467	1.344507886	0.178784208	0.273276083	positive
PRJNA729271	stage_E14_vs_E10	ordinary_muscle_development	ENSGALG00010017922	MERTK	0.073787565	0.130821712	0.564031487	0.572732703	0.674665627	positive
PRJNA729271	stage_E14_vs_E10	ordinary_muscle_development	ENSGALG00010017927	PCSK2	-0.982348405	0.129898261	-7.562444607	3.95564E-14	5.17949E-13	negative
PRJNA729271	stage_E14_vs_E10	ordinary_muscle_development	ENSGALG00010017930	TSC2	-0.071753321	0.076854537	-0.933625058	0.350497335	0.466525149	negative
PRJNA729271	stage_E14_vs_E10	ordinary_muscle_development	ENSGALG00010017933	SIX1	0.44744132	0.195716855	2.28616651	0.022244519	0.047753896	positive
PRJNA729271	stage_E14_vs_E10	ordinary_muscle_development	ENSGALG00010017937	PTPN21	1.636636583	0.119005018	13.75266863	4.9085E-43	4.59024E-41	positive
PRJNA729271	stage_E14_vs_E10	ordinary_muscle_development	ENSGALG00010017941	DIS3L	-0.124836534	0.136428708	-0.915031273	0.360175213	0.476475286	negative
PRJNA729271	stage_E14_vs_E10	ordinary_muscle_development	ENSGALG00010017946	PTGDR	0.34312473	0.251429969	1.364693044	0.172349547	0.265258726	positive
PRJNA729271	stage_E14_vs_E10	ordinary_muscle_development	ENSGALG00010017948	PLAT	-0.784008565	0.162820407	-4.815173831	1.47072E-06	7.92788E-06	negative
PRJNA729271	stage_E14_vs_E10	ordinary_muscle_development	ENSGALG00010017949	SFXN3	0.57839641	0.138163447	4.186320063	2.83513E-05	0.000123317	positive
PRJNA729271	stage_E14_vs_E10	ordinary_muscle_development	ENSGALG00010017950	FBLN7	-2.427747872	0.318871868	-7.613553025	2.66662E-14	3.54611E-13	negative
PRJNA729271	stage_E14_vs_E10	ordinary_muscle_development	ENSGALG00010017954	PTGER2	0.011188075	0.267167171	0.041876681	0.966597006	0.977726699	positive
PRJNA729271	stage_E14_vs_E10	ordinary_muscle_development	ENSGALG00010017955	IKBKB	0.179062144	0.062726824	2.854634296	0.004308641	0.011417505	positive
PRJNA729271	stage_E14_vs_E10	ordinary_muscle_development	ENSGALG00010017956	TIPIN	-0.259412035	0.078661296	-3.29783577	0.000974331	0.003076014	negative
PRJNA729271	stage_E14_vs_E10	ordinary_muscle_development	ENSGALG00010017957	THUMPD1	0.059212768	0.097351936	0.608234111	0.543032214	0.648849211	positive
PRJNA729271	stage_E14_vs_E10	ordinary_muscle_development	ENSGALG00010017958	CAPN10	-0.190140426	0.08758644	-2.170888858	0.029939575	0.061339101	negative
PRJNA729271	stage_E14_vs_E10	ordinary_muscle_development	ENSGALG00010017959	PDZD7	0.718292271	0.281893468	2.548098321	0.010831194	0.025532307	positive
PRJNA729271	stage_E14_vs_E10	ordinary_muscle_development	ENSGALG00010017962	TXNDC16	-0.190311471	0.149638029	-1.271812202	0.203439853	0.303037783	negative
PRJNA729271	stage_E14_vs_E10	ordinary_muscle_development	ENSGALG00010017963	LPAR4	-0.865656095	0.168893582	-5.125452882	2.96823E-07	1.77202E-06	negative
PRJNA729271	stage_E14_vs_E10	ordinary_muscle_development	ENSGALG00010017970	AMPH	-0.354579243	0.731832868	-0.484508497	0.628025036	0.722019582	negative
PRJNA729271	stage_E14_vs_E10	ordinary_muscle_development	ENSGALG00010017972	RNPEPL1	0.116074976	0.173159674	0.670334919	0.502644312	0.611997421	positive
PRJNA729271	stage_E14_vs_E10	ordinary_muscle_development	ENSGALG00010017973	PKD1	0.458244846	0.129166958	3.547694028	0.000388619	0.001351172	positive
PRJNA729271	stage_E14_vs_E10	ordinary_muscle_development	ENSGALG00010017974	MXRA8	-0.522982545	0.184048816	-2.84154256	0.004489586	0.011849335	negative
PRJNA729271	stage_E14_vs_E10	ordinary_muscle_development	ENSGALG00010017976	GPR137C	-0.46309393	0.103118627	-4.490885331	7.09277E-06	3.41447E-05	negative
PRJNA729271	stage_E14_vs_E10	ordinary_muscle_development	ENSGALG00010017979	ENSGALG00010017979	-0.86461645	0.164971054	-5.241019134	1.59692E-07	9.87516E-07	negative
PRJNA729271	stage_E14_vs_E10	ordinary_muscle_development	ENSGALG00010017983	SLC4A11	-0.083120563	0.205699461	-0.40408741	0.686148443	0.770508342	negative
PRJNA729271	stage_E14_vs_E10	ordinary_muscle_development	ENSGALG00010017985	ABCC5	0.620357136	0.26755399	2.31862412	0.020415426	0.04427313	positive
PRJNA729271	stage_E14_vs_E10	ordinary_muscle_development	ENSGALG00010017987	ERI2	-0.355610683	0.208609049	-1.704675253	0.088255008	0.15336966	negative
PRJNA729271	stage_E14_vs_E10	ordinary_muscle_development	ENSGALG00010017991	RAB26	1.456285909	0.239683717	6.075865005	1.23321E-09	9.95844E-09	positive
PRJNA729271	stage_E14_vs_E10	ordinary_muscle_development	ENSGALG00010017992	ENSGALG00010017992	0.165078811	0.720210734	0.22920904	0.818706447	0.872969689	positive
PRJNA729271	stage_E14_vs_E10	ordinary_muscle_development	ENSGALG00010017993	REXO5	-0.383599255	0.089537536	-4.284228382	1.83374E-05	8.21388E-05	negative
PRJNA729271	stage_E14_vs_E10	ordinary_muscle_development	ENSGALG00010017996	ZC3H14	-0.017875689	0.090986189	-0.196465961	0.844245473	0.891544378	negative
PRJNA729271	stage_E14_vs_E10	ordinary_muscle_development	ENSGALG00010017997	ENSGALG00010017997	-0.513378879	0.332690675	-1.54311172	0.122803672	0.201248075	negative
PRJNA729271	stage_E14_vs_E10	ordinary_muscle_development	ENSGALG00010017999	GPR174	-1.999833648	0.961445259	-2.080028612	0.037522909	0.074170811	negative
PRJNA729271	stage_E14_vs_E10	ordinary_muscle_development	ENSGALG00010018000	MAP2K1	0.156936765	0.072859538	2.153963215	0.031243057	0.063480251	positive
PRJNA729271	stage_E14_vs_E10	ordinary_muscle_development	ENSGALG00010018001	TRAF7	0.413451017	0.083648184	4.942737521	7.70332E-07	4.32119E-06	positive
PRJNA729271	stage_E14_vs_E10	ordinary_muscle_development	ENSGALG00010018002	ZC3H8	-0.014247183	0.079099227	-0.180117855	0.857060046	0.901333916	negative
PRJNA729271	stage_E14_vs_E10	ordinary_muscle_development	ENSGALG00010018004	CDC42BPA	0.287338282	0.061671437	4.659179286	3.17473E-06	1.61806E-05	positive
PRJNA729271	stage_E14_vs_E10	ordinary_muscle_development	ENSGALG00010018006	DCUN1D3	-0.014385551	0.101256797	-0.14206998	0.88702473	0.922480644	negative
PRJNA729271	stage_E14_vs_E10	ordinary_muscle_development	ENSGALG00010018007	DNAAF9	-0.067200776	0.179449798	-0.374482318	0.70804551	0.788015945	negative
PRJNA729271	stage_E14_vs_E10	ordinary_muscle_development	ENSGALG00010018008	ABCF3	0.106667949	0.132402627	0.805633178	0.42045439	0.534885587	positive
PRJNA729271	stage_E14_vs_E10	ordinary_muscle_development	ENSGALG00010018010	ENSGALG00010018010	0.025004605	0.119030222	0.210069377	0.833613526	0.883923047	positive
PRJNA729271	stage_E14_vs_E10	ordinary_muscle_development	ENSGALG00010018011	LIPA	-0.325295829	0.185676872	-1.751945867	0.079783117	0.141100083	negative
PRJNA729271	stage_E14_vs_E10	ordinary_muscle_development	ENSGALG00010018012	DVL1	0.565929123	0.138149933	4.096484964	4.19471E-05	0.000176905	positive
PRJNA729271	stage_E14_vs_E10	ordinary_muscle_development	ENSGALG00010018014	LYRM1	0.092245444	0.094246093	0.978772074	0.327692611	0.442633902	positive
PRJNA729271	stage_E14_vs_E10	ordinary_muscle_development	ENSGALG00010018016	IFIT5	-0.34749241	0.878040631	-0.395758919	0.692282886	0.775175004	negative
PRJNA729271	stage_E14_vs_E10	ordinary_muscle_development	ENSGALG00010018019	NAPB	-0.280173655	0.35245339	-0.794923989	0.426657719	0.541115026	negative
PRJNA729271	stage_E14_vs_E10	ordinary_muscle_development	ENSGALG00010018020	SNAPC5	0.103203529	0.102048641	1.011317038	0.311864714	0.425657159	positive
PRJNA729271	stage_E14_vs_E10	ordinary_muscle_development	ENSGALG00010018021	ATRN	-0.16797837	0.071284874	-2.356437783	0.018451158	0.040528798	negative
PRJNA729271	stage_E14_vs_E10	ordinary_muscle_development	ENSGALG00010018022	XPO6	-0.359731132	0.115155036	-3.123885358	0.0017848	0.005276499	negative
PRJNA729271	stage_E14_vs_E10	ordinary_muscle_development	ENSGALG00010018025	CPTP	-0.016576572	0.131033616	-0.126506257	0.899331197	0.930597208	negative
PRJNA729271	stage_E14_vs_E10	ordinary_muscle_development	ENSGALG00010018026	CCDC39	-1.041987177	0.644571778	-1.616557245	0.105973887	0.178719197	negative
PRJNA729271	stage_E14_vs_E10	ordinary_muscle_development	ENSGALG00010018027	EML5	-0.804868968	0.283968523	-2.834359807	0.00459176	0.012081605	negative
PRJNA729271	stage_E14_vs_E10	ordinary_muscle_development	ENSGALG00010018030	POLB	-0.072578723	0.100953138	-0.718934787	0.472181102	0.584330664	negative
PRJNA729271	stage_E14_vs_E10	ordinary_muscle_development	ENSGALG00010018032	ERO1A	0.240101776	0.121542496	1.975455358	0.048216496	0.092065839	positive
PRJNA729271	stage_E14_vs_E10	ordinary_muscle_development	ENSGALG00010018033	COQ8A	0.688888641	0.213352791	3.228871	0.001242799	0.003834578	positive
PRJNA729271	stage_E14_vs_E10	ordinary_muscle_development	ENSGALG00010018035	ITM2A	0.489070287	0.086366248	5.662747867	1.48968E-08	1.06264E-07	positive
PRJNA729271	stage_E14_vs_E10	ordinary_muscle_development	ENSGALG00010018036	RPL4	0.480485013	0.107016636	4.489816102	7.12847E-06	3.42824E-05	positive
PRJNA729271	stage_E14_vs_E10	ordinary_muscle_development	ENSGALG00010018037	GZF1	-0.177598673	0.08152877	-2.178355854	0.029379554	0.060353831	negative
PRJNA729271	stage_E14_vs_E10	ordinary_muscle_development	ENSGALG00010018039	TTC14	0.030423374	0.131036313	0.232175137	0.816401988	0.871281609	positive
PRJNA729271	stage_E14_vs_E10	ordinary_muscle_development	ENSGALG00010018042	ZWILCH	-0.186135824	0.135704914	-1.371621842	0.170181191	0.262646546	negative
PRJNA729271	stage_E14_vs_E10	ordinary_muscle_development	ENSGALG00010018043	PSMC6	0.415645367	0.090784668	4.578365229	4.68624E-06	2.32468E-05	positive
PRJNA729271	stage_E14_vs_E10	ordinary_muscle_development	ENSGALG00010018044	INTS11	-0.149485709	0.057115286	-2.617262733	0.008863809	0.021452815	negative
PRJNA729271	stage_E14_vs_E10	ordinary_muscle_development	ENSGALG00010018045	PSEN2	0.37695618	0.073204471	5.149360047	2.61377E-07	1.5714E-06	positive
PRJNA729271	stage_E14_vs_E10	ordinary_muscle_development	ENSGALG00010018046	CAPN15	0.718038033	0.101128922	7.10022437	1.24555E-12	1.40609E-11	positive
PRJNA729271	stage_E14_vs_E10	ordinary_muscle_development	ENSGALG00010018048	GFRA4	-3.160007778	0.160860898	-19.64434994	6.46188E-86	3.9027E-83	negative
PRJNA729271	stage_E14_vs_E10	ordinary_muscle_development	ENSGALG00010018049	SUGCT	-0.396096665	0.156293862	-2.534307238	0.01126699	0.026460631	negative
PRJNA729271	stage_E14_vs_E10	ordinary_muscle_development	ENSGALG00010018051	GNPNAT1	-0.311246888	0.132377246	-2.351211379	0.0187124	0.041003211	negative
PRJNA729271	stage_E14_vs_E10	ordinary_muscle_development	ENSGALG00010018052	PPP1RL	0.111116991	0.130866543	0.849086314	0.395833265	0.511372833	positive
PRJNA729271	stage_E14_vs_E10	ordinary_muscle_development	ENSGALG00010018053	DIAPH2	0.136392283	0.092015844	1.482269533	0.138268592	0.221998586	positive
PRJNA729271	stage_E14_vs_E10	ordinary_muscle_development	ENSGALG00010018054	SMAD6	-1.555648022	0.160415998	-9.697586508	3.08715E-22	7.82311E-21	negative
PRJNA729271	stage_E14_vs_E10	ordinary_muscle_development	ENSGALG00010018055	PEX5L	0.20338103	0.464749972	0.437613862	0.661666221	0.751036706	positive
PRJNA729271	stage_E14_vs_E10	ordinary_muscle_development	ENSGALG00010018057	SSTR4	0.892245885	0.17237175	5.176288374	2.26344E-07	1.37274E-06	positive
PRJNA729271	stage_E14_vs_E10	ordinary_muscle_development	ENSGALG00010018058	ITPKB	-0.081336039	0.090006245	-0.903671062	0.366169848	0.48224895	negative
PRJNA729271	stage_E14_vs_E10	ordinary_muscle_development	ENSGALG00010018060	CASKIN1	-2.345467143	0.331087103	-7.08413926	1.39911E-12	1.57206E-11	negative

PRJNA729271	stage_E14_vs_E10	ordinary_muscle_development	ENSGALG00010018061	NDUFAF5	0.206717493	0.135133929	1.529723102	0.126085283	0.205649395	positive
PRJNA729271	stage_E14_vs_E10	ordinary_muscle_development	ENSGALG00010018062	GOLGA7	-0.309062169	0.092661908	-3.335374552	0.000851846	0.002731749	negative
PRJNA729271	stage_E14_vs_E10	ordinary_muscle_development	ENSGALG00010018063	DECR2	0.4280738	0.097401078	4.394959574	1.10793E-05	5.15058E-05	positive
PRJNA729271	stage_E14_vs_E10	ordinary_muscle_development	ENSGALG00010018064	TTC8	-0.848637237	0.159361315	-5.325239923	1.0082E-07	6.42367E-07	negative
PRJNA729271	stage_E14_vs_E10	ordinary_muscle_development	ENSGALG00010018067	ENSGALG00010018067	1.004117891	0.177607815	5.653568177	1.57151E-08	1.11826E-07	positive
PRJNA729271	stage_E14_vs_E10	ordinary_muscle_development	ENSGALG00010018068	ADAM33	0.50413131	0.1375143	3.666028254	0.000246347	0.000895385	positive
PRJNA729271	stage_E14_vs_E10	ordinary_muscle_development	ENSGALG00010018069	ENSGALG00010018069	-0.497134621	0.357250269	-1.391558422	0.164056157	0.254958078	negative
PRJNA729271	stage_E14_vs_E10	ordinary_muscle_development	ENSGALG00010018071	GPAT4	0.043200916	0.078034385	0.553613843	0.57984315	0.681380338	positive
PRJNA729271	stage_E14_vs_E10	ordinary_muscle_development	ENSGALG00010018075	MLST8	0.765053292	0.102486077	7.46494857	8.33322E-14	1.06517E-12	positive
PRJNA729271	stage_E14_vs_E10	ordinary_muscle_development	ENSGALG00010018076	FOXA2	-4.385731366	1.409052025	-3.112540408	0.001854846	0.005460194	negative
PRJNA729271	stage_E14_vs_E10	ordinary_muscle_development	ENSGALG00010018078	NKX6-3	2.129700344	0.77185086	2.759212246	0.005794089	0.014804393	positive
PRJNA729271	stage_E14_vs_E10	ordinary_muscle_development	ENSGALG00010018080	BRICD5	0.728618416	0.327358725	2.22574919	0.026030986	0.054565313	positive
PRJNA729271	stage_E14_vs_E10	ordinary_muscle_development	ENSGALG00010018081	DIO3	-0.748493277	0.237609134	-3.15010313	0.001632128	0.00486784	negative
PRJNA729271	stage_E14_vs_E10	ordinary_muscle_development	ENSGALG00010018082	RAB11FIP3	-0.838523318	0.176984155	-4.737844	2.16004E-06	1.13073E-05	negative
PRJNA729271	stage_E14_vs_E10	ordinary_muscle_development	ENSGALG00010018083	PAX1	-7.545223273	1.06928221	-7.056344152	1.7094E-12	1.90014E-11	negative
PRJNA729271	stage_E14_vs_E10	ordinary_muscle_development	ENSGALG00010018085	PGP	0.584346686	0.160114042	3.649565504	0.000262684	0.000948346	positive
PRJNA729271	stage_E14_vs_E10	ordinary_muscle_development	ENSGALG00010018087	ENSGALG00010018087	-0.153340792	0.053417194	-2.870626122	0.004096597	0.010922759	negative
PRJNA729271	stage_E14_vs_E10	ordinary_muscle_development	ENSGALG00010018089	TASP1	-0.334547078	0.093566487	-3.575501132	0.000349558	0.001233107	negative
PRJNA729271	stage_E14_vs_E10	ordinary_muscle_development	ENSGALG00010018092	ENSGALG00010018092	-2.049457998	0.210791419	-9.722682282	2.41339E-22	6.18057E-21	negative
PRJNA729271	stage_E14_vs_E10	ordinary_muscle_development	ENSGALG00010018095	E4F1	0.046015644	0.067335774	0.683375881	0.494369357	0.604409904	positive
PRJNA729271	stage_E14_vs_E10	ordinary_muscle_development	ENSGALG00010018099	SMAD3	-0.270978543	0.130391725	-2.078188189	0.03769203	0.074484795	negative
PRJNA729271	stage_E14_vs_E10	ordinary_muscle_development	ENSGALG00010018102	ZDHHC4	0.040797735	0.167855361	0.243052915	0.807964406	0.864951556	positive
PRJNA729271	stage_E14_vs_E10	ordinary_muscle_development	ENSGALG00010018103	BTRC	0.06285201	0.106611817	0.589540748	0.555498586	0.659941972	positive
PRJNA729271	stage_E14_vs_E10	ordinary_muscle_development	ENSGALG00010018111	NKX2-2	1.280552876	0.586023099	2.185157683	0.028877287	0.059482205	positive
PRJNA729271	stage_E14_vs_E10	ordinary_muscle_development	ENSGALG00010018115	KLHL4	-0.667887676	0.185455427	-3.601337999	0.000316584	0.001126659	negative
PRJNA729271	stage_E14_vs_E10	ordinary_muscle_development	ENSGALG00010018119	GRID2IP	-0.08668758	0.482374302	-0.179710196	0.857380089	0.901568156	negative
PRJNA729271	stage_E14_vs_E10	ordinary_muscle_development	ENSGALG00010018121	FERMT2	0.687922859	0.159629397	4.309499834	1.63624E-05	7.39549E-05	positive
PRJNA729271	stage_E14_vs_E10	ordinary_muscle_development	ENSGALG00010018123	USP13	1.500439949	0.141310881	10.61800716	2.45751E-26	8.30342E-25	positive
PRJNA729271	stage_E14_vs_E10	ordinary_muscle_development	ENSGALG00010018128	SEPTIN12	0.142212402	0.186739304	0.76155581	0.446325157	0.559740712	positive
PRJNA729271	stage_E14_vs_E10	ordinary_muscle_development	ENSGALG00010018137	NDUFB5	0.35236911	0.127612355	2.761246037	0.005758128	0.01472289	positive
PRJNA729271	stage_E14_vs_E10	ordinary_muscle_development	ENSGALG00010018140	CHPF	-0.036323517	0.100281882	-0.36221415	0.717192006	0.795386238	negative
PRJNA729271	stage_E14_vs_E10	ordinary_muscle_development	ENSGALG00010018142	DDHD1	-0.54767294	0.140337256	-3.902548459	9.51852E-05	0.00037584	negative
PRJNA729271	stage_E14_vs_E10	ordinary_muscle_development	ENSGALG00010018143	MRPL47	0.170455868	0.122906316	1.386876384	0.16547946	0.256674669	positive
PRJNA729271	stage_E14_vs_E10	ordinary_muscle_development	ENSGALG00010018145	ENSGALG00010018145	0.301994859	0.349040858	0.865213491	0.386921622	0.503043225	positive
PRJNA729271	stage_E14_vs_E10	ordinary_muscle_development	ENSGALG00010018147	ISM1	-1.448600597	0.288710778	-5.017480154	5.23536E-07	3.01018E-06	negative
PRJNA729271	stage_E14_vs_E10	ordinary_muscle_development	ENSGALG00010018148	XRN2	-0.018444404	0.054065134	-0.341151551	0.732989495	0.80777638	negative
PRJNA729271	stage_E14_vs_E10	ordinary_muscle_development	ENSGALG00010018149	ENSGALG00010018149	-0.572951689	0.29502697	-1.942031569	0.052133279	0.098241273	negative
PRJNA729271	stage_E14_vs_E10	ordinary_muscle_development	ENSGALG00010018150	AAGAB	-0.217553731	0.078059429	-2.787026932	0.005319406	0.01374907	negative
PRJNA729271	stage_E14_vs_E10	ordinary_muscle_development	ENSGALG00010018154	SLC16A12	-1.058987535	0.226478966	-4.675875876	2.92702E-06	1.50078E-05	negative
PRJNA729271	stage_E14_vs_E10	ordinary_muscle_development	ENSGALG00010018157	FBXL2	0.6339316	0.150473941	4.212899558	2.52113E-05	0.000110438	positive
PRJNA729271	stage_E14_vs_E10	ordinary_muscle_development	ENSGALG00010018160	ENSGALG00010018160	0.039496767	0.1647775	0.239697571	0.810564718	0.866902943	positive
PRJNA729271	stage_E14_vs_E10	ordinary_muscle_development	ENSGALG00010018162	ENSGALG00010018162	-0.151692463	0.334543822	-0.453430772	0.650238582	0.741558478	negative
PRJNA729271	stage_E14_vs_E10	ordinary_muscle_development	ENSGALG00010018163	CCT7	-0.049290722	0.080576268	-0.61172753	0.540718044	0.646728942	negative
PRJNA729271	stage_E14_vs_E10	ordinary_muscle_development	ENSGALG00010018165	DACH2	0.013832582	0.185152764	0.074709022	0.940446229	0.958296526	positive
PRJNA729271	stage_E14_vs_E10	ordinary_muscle_development	ENSGALG00010018166	MSS51	2.522738287	0.24385006	10.34544869	4.38856E-25	1.32802E-23	positive
PRJNA729271	stage_E14_vs_E10	ordinary_muscle_development	ENSGALG00010018168	ENSGALG00010018168	0.093208151	0.200151416	0.465688191	0.641438706	0.733832699	positive
PRJNA729271	stage_E14_vs_E10	ordinary_muscle_development	ENSGALG00010018172	ACTL6A	-0.473283965	0.091319584	-5.182721412	2.18672E-07	1.3294E-06	negative
PRJNA729271	stage_E14_vs_E10	ordinary_muscle_development	ENSGALG00010018174	BMP4	-0.796962991	0.206421063	-3.86086081	0.000112988	0.000441161	negative
PRJNA729271	stage_E14_vs_E10	ordinary_muscle_development	ENSGALG00010018177	KIF20B	-0.440651758	0.184722887	-2.385474615	0.0170571	0.037914838	negative
PRJNA729271	stage_E14_vs_E10	ordinary_muscle_development	ENSGALG00010018179	PRADC1	-0.022059845	0.117217563	-0.188195735	0.850723216	0.89655613	negative
PRJNA729271	stage_E14_vs_E10	ordinary_muscle_development	ENSGALG00010018183	CYP17A1	2.977605988	0.278676866	10.68479789	1.1991E-26	4.15812E-25	positive
PRJNA729271	stage_E14_vs_E10	ordinary_muscle_development	ENSGALG00010018185	GNB4	-0.259572204	0.069185493	-3.751829949	0.000175549	0.0006599	negative
PRJNA729271	stage_E14_vs_E10	ordinary_muscle_development	ENSGALG00010018186	SPTLC3	-0.141797429	0.54648587	-0.259471355	0.79527158	0.856248709	negative
PRJNA729271	stage_E14_vs_E10	ordinary_muscle_development	ENSGALG00010018188	UBP1	0.202785559	0.051207241	3.960095402	7.49198E-05	0.00030174	positive
PRJNA729271	stage_E14_vs_E10	ordinary_muscle_development	ENSGALG00010018189	MIS18BP1	0.016001801	0.116085757	0.13784465	0.890363195	0.924683995	positive
PRJNA729271	stage_E14_vs_E10	ordinary_muscle_development	ENSGALG00010018192	IQCH	-0.630914995	0.20015071	-3.152199641	0.001620454	0.004837999	negative
PRJNA729271	stage_E14_vs_E10	ordinary_muscle_development	ENSGALG00010018193	ANK1	2.5639765	0.246690542	10.39349332	2.65449E-25	8.22154E-24	positive
PRJNA729271	stage_E14_vs_E10	ordinary_muscle_development	ENSGALG00010018198	BTBD3	-0.024142241	0.100949923	-0.239150661	0.810988759	0.867276561	negative
PRJNA729271	stage_E14_vs_E10	ordinary_muscle_development	ENSGALG00010018199	BORCS7	0.36621096	0.1179443	3.104948345	0.001903123	0.005584162	positive
PRJNA729271	stage_E14_vs_E10	ordinary_muscle_development	ENSGALG00010018206	KIZ	-0.215586783	0.123310607	-1.748323097	0.080408096	0.142066717	negative
PRJNA729271	stage_E14_vs_E10	ordinary_muscle_development	ENSGALG00010018209	GMPPA	0.124401013	0.130617044	0.95241026	0.340888958	0.455786869	positive
PRJNA729271	stage_E14_vs_E10	ordinary_muscle_development	ENSGALG00010018215	CFAP70	1.019066649	0.281250726	3.623338733	0.000290825	0.001043699	positive
PRJNA729271	stage_E14_vs_E10	ordinary_muscle_development	ENSGALG00010018216	FANCM	0.665661918	0.082851011	8.034445278	9.40032E-16	1.4164E-14	positive
PRJNA729271	stage_E14_vs_E10	ordinary_muscle_development	ENSGALG00010018217	ENSGALG00010018217	0.800492391	0.368031522	2.175064752	0.029625264	0.060788975	positive
PRJNA729271	stage_E14_vs_E10	ordinary_muscle_development	ENSGALG00010018219	SLC24A5	-2.165708637	0.509511976	-4.250554924	2.13242E-05	9.44374E-05	negative
PRJNA729271	stage_E14_vs_E10	ordinary_muscle_development	ENSGALG00010018226	JAG1	-0.515394911	0.101895468	-5.058074896	4.2351E-07	2.47232E-06	negative
PRJNA729271	stage_E14_vs_E10	ordinary_muscle_development	ENSGALG00010018229	AS3MT	1.440941315	0.1867341	7.716540878	1.19529E-14	1.63451E-13	positive
PRJNA729271	stage_E14_vs_E10	ordinary_muscle_development	ENSGALG00010018231	SMYD5	0.169622517	0.074827116	2.266858937	0.023398844	0.049848067	positive
PRJNA729271	stage_E14_vs_E10	ordinary_muscle_development	ENSGALG00010018233	RASSF2	0.055668846	0.18371597	0.303015826	0.761877801	0.830457115	positive
PRJNA729271	stage_E14_vs_E10	ordinary_muscle_development	ENSGALG00010018240	SOUL	-1.095674479	0.680797049	-1.609399572	0.107528999	0.180904402	negative
PRJNA729271	stage_E14_vs_E10	ordinary_muscle_development	ENSGALG00010018241	RALGAPA2	-0.155571155	0.091272215	-1.704474414	0.088292492	0.153398019	negative
PRJNA729271	stage_E14_vs_E10	ordinary_muscle_development	ENSGALG00010018244	CHM	0.097120793	0.08142623	1.192745783	0.232968949	0.337250067	positive
PRJNA729271	stage_E14_vs_E10	ordinary_muscle_development	ENSGALG00010018251	USP48	0.090453572	0.16593778	0.545105351	0.585681062	0.686182266	positive
PRJNA729271	stage_E14_vs_E10	ordinary_muscle_development	ENSGALG00010018255	MFN1	0.326535183	0.112767515	2.89564938	0.003783749	0.010184854	positive
PRJNA729271	stage_E14_vs_E10	ordinary_muscle_development	ENSGALG00010018258	OSCP1	-0.49118435	0.274902759	-1.786756713	0.073976818	0.13221874	negative
PRJNA729271	stage_E14_vs_E10	ordinary_muscle_development	ENSGALG00010018259	WDR90	0.184762882	0.126842598	1.456631168	0.145218219	0.231159118	positive
PRJNA729271	stage_E14_vs_E10	ordinary_muscle_development	ENSGALG00010018261	PRNP	0.319595455	0.194552828	1.642718118	0.100441268	0.170640724	positive
PRJNA729271	stage_E14_vs_E10	ordinary_muscle_development	ENSGALG00010018263	SLX4IP	-0.277203241	0.100596076	-2.755606889	0.005858336	0.014952128	negative
PRJNA729271	stage_E14_vs_E10	ordinary_muscle_development	ENSGALG00010018264	CFAP61	-1.027654793	0.319401706	-3.217436768	0.001293415	0.003967003	negative
PRJNA729271	stage_E14_vs_E10	ordinary_muscle_development	ENSGALG00010018265	PDCD4	-0.260208861	0.065097014	-3.997247265	6.40834E-05	0.000261806	negative
PRJNA729271	stage_E14_vs_E10	ordinary_muscle_development	ENSGALG00010018267	ADPRHL2	0.066173049	0.075466803	0.876849773	0.380568241	0.496564646	positive
PRJNA729271	stage_E14_vs_E10	ordinary_muscle_development	ENSGALG00010018273	PROM2	1.506810043	0.688999745	2.186952976	0.028745957	0.059279079	positive
PRJNA729271	stage_E14_vs_E10	ordinary_muscle_development	ENSGALG00010018274	ZNF711	-0.08702982	0.097008857	-0.897132722	0.3696481	0.485830492	negative
PRJNA729271	stage_E14_vs_E10	ordinary_muscle_development	ENSGALG00010018275	ZNF639	-0.081733332	0.082631788	-0.989126987	0.322601015	0.437182284	negative
PRJNA729271	stage_E14_vs_E10	ordinary_muscle_development	ENSGALG00010018276	ARHGAP17	0.020184167	0.071206864	0.283458168	0.776825644	0.841812778	positive
PRJNA729271	stage_E14_vs_E10	ordinary_muscle_development	ENSGALG00010018278	PUSL1	0.096783284	0.149877555	0.645749016	0.518441911	0.626756923	positive
PRJNA729271	stage_E14_vs_E10	ordinary_muscle_development	ENSGALG00010018283	POF1B	1.41119806	0.171589137	8.224285568	1.96358E-16	3.13114E-15	positive
PRJNA729271	stage_E14_vs_E10	ordinary_muscle_development	ENSGALG00010018284	ENSGALG00010018284	1.556245274	0.167184979	9.308523299	1.29622E-20	2.89949E-19	positive
PRJNA729271	stage_E14_vs_E10	ordinary_muscle_development	ENSGALG00010018285	BBIP1	0.14475064	0.189509685	0.763816585	0.444976552	0.558532657	positive
PRJNA729271	stage_E14_vs_E10	ordinary_muscle_development	ENSGALG00010018287	MKKS	-0.176796058	0.102390948	-1.726676646	0.084225752	0.14768374	negative
PRJNA729271	stage_E14_vs_E10	ordinary_muscle_development	ENSGALG00010018288	CRNKL1	-0.140012729	0.060787572	-2.303311758	0.021261309	0.045895007	negative
PRJNA729271	stage_E14_vs_E10	ordinary_muscle_development	ENSGALG00010018289	HEYL	0.032437968	0.203039001	0.159762252	0.87306836	0.91296116	positive
PRJNA729271	stage_E14_vs_E10	ordinary_muscle_development	ENSGALG00010018290	RHOT2	-0.089895425	0.131975581	-0.681151953	0.495775346	0.605311964	negative
PRJNA729271	stage_E14_vs_E10	ordinary_muscle_development	ENSGALG00010018294	FKBP3	1.065718203	0.177561804	6.001956379	1.94954E-09	1.54587E-08	positive
PRJNA729271	stage_E14_vs_E10	ordinary_muscle_development	ENSGALG00010018296	PIK3CA	-0.085091014	0.096743484	-0.879552924	0.379101551	0.495275077	negative
PRJNA729271	stage_E14_vs_E10	ordinary_muscle_development	ENSGALG00010018298	ENSGALG00010018298	-0.12072898	0.196678217	-0.613840118	0.539320984	0.645560785	negative
PRJNA729271	stage_E14_vs_E10	ordinary_muscle_development	ENSGALG00010018299	SHOC2	-0.209166881	0.082667203	-2.530228124	0.011398839	0.026744281	negative
PRJNA729271	stage_E14_vs_E10	ordinary_muscle_development	ENSGALG00010018301	ENSGALG00010018301	-0.185672648	0.301507727	-0.615813896	0.538017355	0.644455959	negative
PRJNA729271	stage_E14_vs_E10	ordinary_muscle_development	ENSGALG00010018302	RAB11FIP5	0.174091281	0.100924465	1.724966113	0.08453358	0.148083556	positive
PRJNA729271	stage_E14_vs_E10	ordinary_muscle_development	ENSGALG00010018306	DES	1.921395963	0.150980363	12.72613157	4.23298E-37	2.77633E-35	positive
PRJNA729271	stage_E14_vs_E10	ordinary_muscle_development	ENSGALG00010018308	ACAP3	0.508936064	0.173991263	2.925066782	0.003443823	0.009366698	positive
PRJNA729271	stage_E14_vs_E10	ordinary_muscle_development	ENSGALG00010018309	DPYSL2	-0.085780237	0.123742187	-0.693217398	0.488173132	0.599240336	negative
PRJNA729271	stage_E14_vs_E10	ordinary_muscle_development	ENSGALG00010018310	APOOL	0.099922849	0.111803391	0.893737196	0.371462519	0.487748227	positive
PRJNA729271	stage_E14_vs_E10	ordinary_muscle_development	ENSGALG00010018311	NKX2-3	1.278688429	0.757797472	1.687374894	0.091531288	0.158058854	positive
PRJNA729271	stage_E14_vs_E10	ordinary_muscle_development	ENSGALG00010018312	PRPF39	-0.010867788	0.078334292	-0.138736016	0.889658757	0.924217278	negative
PRJNA729271	stage_E14_vs_E10	ordinary_muscle_development	ENSGALG00010018313	MYEF2	-0.334177685	0.080829198	-4.134368451	3.55932E-05	0.000151832	negative
PRJNA729271	stage_E14_vs_E10	ordinary_muscle_development	ENSGALG00010018314	SLC24A3	0.422946624	0.122152857	3.462437415	0.000535306	0.001803642	positive
PRJNA729271	stage_E14_vs_E10	ordinary_muscle_development	ENSGALG00010018315	CLASP2	0.638954621	0.117165607	5.453431563	4.9407E-08	3.2746E-07	positive
PRJNA729271	stage_E14_vs_E10	ordinary_muscle_development	ENSGALG00010018317	ZMAT3	-0.23972743	0.128668432	-1.863140989	0.062442433	0.114454105	negative
PRJNA729271	stage_E14_vs_E10	ordinary_muscle_development	ENSGALG00010018319	STUB1	0.261928963	0.20158004	1.299379453	0.19381374	0.291757208	positive
PRJNA729271	stage_E14_vs_E10	ordinary_muscle_development	ENSGALG00010018322	NT5C1A	0.985365176	0.250199843	3.938312528	8.20567E-05	0.000328384	positive
PRJNA729271	stage_E14_vs_E10	ordinary_muscle_development	ENSGALG00010018323	GOT1	0.340955027	0.142831671	2.387110821	0.016981372	0.037781271	positive
PRJNA729271	stage_E14_vs_E10	ordinary_muscle_development	ENSGALG00010018325	ENSGALG00010018325	0.363484791	0.193397457	1.879470375	0.060180295	0.110854413	positive
PRJNA729271	stage_E14_vs_E10	ordinary_muscle_development	ENSGALG00010018326	LCMT1	-0.053556772	0.106753101	-0.501688212	0.615886858	0.712300807	negative
PRJNA729271	stage_E14_vs_E10	ordinary_muscle_development	ENSGALG00010018327	SNAP25	-1.244880153	0.510442869	-2.438823672	0.014735156	0.033425053	negative
PRJNA729271	stage_E14_vs_E10	ordinary_muscle_development	ENSGALG00010018328	ENSGALG00010018328	0.858666388	0.583458067	1.471684834	0.141106005	0.225816414	positive
PRJNA729271	stage_E14_vs_E10	ordinary_muscle_development	ENSGALG00010018329	ENSGALG00010018329	0.58345751	0.230179205	2.534796791	0.011251257	0.026440819	positive
PRJNA729271	stage_E14_vs_E10	ordinary_muscle_development	ENSGALG00010018333	ENSGALG00010018333	0.579277415	0.212585153	2.724919435	0.006431718	0.016188184	positive
PRJNA729271	stage_E14_vs_E10	ordinary_muscle_development	ENSGALG00010018336	JMJD8	-0.539273173	0.145575769	-3.704415773	0.000211878	0.000782067	negative
PRJNA729271	stage_E14_vs_E10	ordinary_muscle_development	ENSGALG00010018337	TMEM151B	-0.110808034	0.313197115	-0.353796472	0.723491418	0.799741333	negative
PRJNA729271	stage_E14_vs_E10	ordinary_muscle_development	ENSGALG00010018338	PDCD6IP	0.238712052	0.084553957	2.82319958	0.004754809	0.012465358	positive
PRJNA729271	stage_E14_vs_E10	ordinary_muscle_development	ENSGALG00010018339	FAM76A	0.052869254	0.115140366	0.45917219	0.64611052	0.738128309	positive
PRJNA729271	stage_E14_vs_E10	ordinary_muscle_development	ENSGALG00010018340	DNPEP	0.339075826	0.09729927	3.484875323	0.000492366	0.001672177	positive
PRJNA729271	stage_E14_vs_E10	ordinary_muscle_development	ENSGALG00010018342	BNIP3L	-0.032783555	0.105975308	-0.309350877	0.757054634	0.826775238	negative
PRJNA729271	stage_E14_vs_E10	ordinary_muscle_development	ENSGALG00010018344	SFXN5	0.032748387	0.181635876	0.18029691	0.856919482	0.901251479	positive
PRJNA729271	stage_E14_vs_E10	ordinary_muscle_development	ENSGALG00010018345	WDR24	0.028521618	0.170907618	0.166883248	0.867461914	0.909041386	positive
PRJNA729271	stage_E14_vs_E10	ordinary_muscle_development	ENSGALG00010018347	CTXN2	-2.249963381	0.573943644	-3.920181718	8.84822E-05	0.000351769	negative
PRJNA729271	stage_E14_vs_E10	ordinary_muscle_development	ENSGALG00010018348	TOGARAM1	-0.11831438	0.107495733	-1.100642573	0.271052249	0.380892132	negative
PRJNA729271	stage_E14_vs_E10	ordinary_muscle_development	ENSGALG00010018349	KCNMB2	-1.013761445	0.204945345	-4.946496575	7.55611E-07	4.24354E-06	negative
PRJNA729271	stage_E14_vs_E10	ordinary_muscle_development	ENSGALG00010018350	CNNM1	-2.092908415	0.271042914	-7.721686528	1.148E-14	1.5743E-13	negative
PRJNA729271	stage_E14_vs_E10	ordinary_muscle_development	ENSGALG00010018351	RIN2	0.169790375	0.086675397	1.958922385	0.050121877	0.094969491	positive

PRJNA729271	stage_E14_vs_E10	ordinary_muscle_development	ENSGALG00010018354	AARS2	0.53243384	0.114467822	4.6513844	3.29714E-06	1.67392E-05	positive
PRJNA729271	stage_E14_vs_E10	ordinary_muscle_development	ENSGALG00010018355	PRKCB	0.588417638	0.150843488	3.90084879	9.5856E-05	0.00037818	positive
PRJNA729271	stage_E14_vs_E10	ordinary_muscle_development	ENSGALG00010018356	ADRA1A	-1.662036439	0.372744737	-4.458913224	8.23763E-06	3.91747E-05	negative
PRJNA729271	stage_E14_vs_E10	ordinary_muscle_development	ENSGALG00010018359	CACNG3	-3.779939118	0.639796904	-5.908029714	3.46224E-09	2.668E-08	negative
PRJNA729271	stage_E14_vs_E10	ordinary_muscle_development	ENSGALG00010018361	ASIC4	1.772209482	0.195834058	9.049546826	1.43563E-19	2.96854E-18	positive
PRJNA729271	stage_E14_vs_E10	ordinary_muscle_development	ENSGALG00010018363	TCTE1	-0.269145707	0.383481786	-0.701847434	0.482774314	0.594294155	negative
PRJNA729271	stage_E14_vs_E10	ordinary_muscle_development	ENSGALG00010018365	HPSE2	-1.926130964	0.257457624	-7.481351425	7.35622E-14	9.46966E-13	negative
PRJNA729271	stage_E14_vs_E10	ordinary_muscle_development	ENSGALG00010018367	PAK5	0.449188405	0.200185939	2.243855917	0.024841673	0.052441175	positive
PRJNA729271	stage_E14_vs_E10	ordinary_muscle_development	ENSGALG00010018370	ZBTB8A	-0.234780013	0.109672089	-2.140745345	0.03229458	0.065269093	negative
PRJNA729271	stage_E14_vs_E10	ordinary_muscle_development	ENSGALG00010018371	ENSGALG00010018371	0.189101857	0.136247891	1.387925019	0.165159878	0.256342728	positive
PRJNA729271	stage_E14_vs_E10	ordinary_muscle_development	ENSGALG00010018372	NLGN3	-0.843438247	0.464585046	-1.815465765	0.069452362	0.125212935	negative
PRJNA729271	stage_E14_vs_E10	ordinary_muscle_development	ENSGALG00010018374	HPS1	0.160896695	0.128949258	1.247752007	0.212121889	0.313266101	positive
PRJNA729271	stage_E14_vs_E10	ordinary_muscle_development	ENSGALG00010018376	PYGB	-1.127803179	0.143177324	-7.876967857	3.35421E-15	4.80903E-14	negative
PRJNA729271	stage_E14_vs_E10	ordinary_muscle_development	ENSGALG00010018377	RAP1GAP	0.280486255	0.299806312	0.935558205	0.349500702	0.465455042	positive
PRJNA729271	stage_E14_vs_E10	ordinary_muscle_development	ENSGALG00010018378	ARPP21	0.66437431	0.144544222	4.596339445	4.29978E-06	2.14545E-05	positive
PRJNA729271	stage_E14_vs_E10	ordinary_muscle_development	ENSGALG00010018379	SRBD1	0.357270958	0.125931682	2.83702204	0.004553647	0.011996567	positive
PRJNA729271	stage_E14_vs_E10	ordinary_muscle_development	ENSGALG00010018380	SLC20A1	-0.776447546	0.131736763	-5.893932153	3.77112E-09	2.89679E-08	negative
PRJNA729271	stage_E14_vs_E10	ordinary_muscle_development	ENSGALG00010018381	HAPSTR1	0.065223329	0.067309565	0.969005346	0.332542522	0.447475293	positive
PRJNA729271	stage_E14_vs_E10	ordinary_muscle_development	ENSGALG00010018383	METRN	0.727439546	0.157126394	4.629645765	3.66292E-06	1.84162E-05	positive
PRJNA729271	stage_E14_vs_E10	ordinary_muscle_development	ENSGALG00010018384	LAMP5	1.001171536	0.23392091	4.279957426	1.86929E-05	8.36277E-05	positive
PRJNA729271	stage_E14_vs_E10	ordinary_muscle_development	ENSGALG00010018385	SPR	-0.113342852	0.134655797	-0.841722782	0.399943143	0.515304521	negative
PRJNA729271	stage_E14_vs_E10	ordinary_muscle_development	ENSGALG00010018387	NT5DC4	-0.467897057	0.311198774	-1.503531168	0.132702126	0.214798137	negative
PRJNA729271	stage_E14_vs_E10	ordinary_muscle_development	ENSGALG00010018388	SMC3	-0.204719731	0.064862468	-3.156212468	0.001598324	0.004774877	negative
PRJNA729271	stage_E14_vs_E10	ordinary_muscle_development	ENSGALG00010018391	STAC	-1.836997869	0.217676693	-8.439111417	3.19759E-17	5.45283E-16	negative
PRJNA729271	stage_E14_vs_E10	ordinary_muscle_development	ENSGALG00010018393	PRKCE	-0.387434426	0.075847711	-5.108056923	3.25489E-07	1.93438E-06	negative
PRJNA729271	stage_E14_vs_E10	ordinary_muscle_development	ENSGALG00010018394	KLHL28	-0.127497354	0.096309148	-1.32383431	0.185558079	0.281581276	negative
PRJNA729271	stage_E14_vs_E10	ordinary_muscle_development	ENSGALG00010018395	MED12	-0.116592284	0.07824385	-1.490114355	0.13619417	0.219323908	negative
PRJNA729271	stage_E14_vs_E10	ordinary_muscle_development	ENSGALG00010018396	TBL1XR1	-0.177249216	0.074220373	-2.388147725	0.016933534	0.037697984	negative
PRJNA729271	stage_E14_vs_E10	ordinary_muscle_development	ENSGALG00010018397	FAM173A	0.508796526	0.161093626	3.158390169	0.001586431	0.004743258	positive
PRJNA729271	stage_E14_vs_E10	ordinary_muscle_development	ENSGALG00010018398	LRFN5	-0.08461243	0.182881774	-0.462661906	0.643606728	0.735789851	negative
PRJNA729271	stage_E14_vs_E10	ordinary_muscle_development	ENSGALG00010018399	ABHD12	-0.150241287	0.162712344	-0.923355189	0.355822136	0.472225037	negative
PRJNA729271	stage_E14_vs_E10	ordinary_muscle_development	ENSGALG00010018401	ENSGALG00010018401	0.05911538	0.100098317	0.590573162	0.554806451	0.659460492	positive
PRJNA729271	stage_E14_vs_E10	ordinary_muscle_development	ENSGALG00010018404	IL2RG	0.742130766	0.258944445	2.865984501	0.004157146	0.011062572	positive
PRJNA729271	stage_E14_vs_E10	ordinary_muscle_development	ENSGALG00010018405	DCLK3	-0.17018252	0.188392371	-0.903340822	0.366345037	0.482392016	negative
PRJNA729271	stage_E14_vs_E10	ordinary_muscle_development	ENSGALG00010018406	TMEM260	-0.073967652	0.120140443	-0.615676541	0.538108023	0.644458052	negative
PRJNA729271	stage_E14_vs_E10	ordinary_muscle_development	ENSGALG00010018410	EXOC6B	0.06591376	0.097476523	0.676201388	0.498912791	0.607964768	positive
PRJNA729271	stage_E14_vs_E10	ordinary_muscle_development	ENSGALG00010018413	SLC15A2	-0.338155317	0.354472628	-0.95396736	0.340100165	0.455135542	negative
PRJNA729271	stage_E14_vs_E10	ordinary_muscle_development	ENSGALG00010018414	EPAS1	-0.178062094	0.138095897	-1.289409009	0.197255931	0.295802268	negative
PRJNA729271	stage_E14_vs_E10	ordinary_muscle_development	ENSGALG00010018415	PELI2	-0.822847027	0.126570519	-6.501095455	7.97372E-11	7.41367E-10	negative
PRJNA729271	stage_E14_vs_E10	ordinary_muscle_development	ENSGALG00010018416	DUT	-0.01944695	0.132512106	-0.146756028	0.883324594	0.919997532	negative
PRJNA729271	stage_E14_vs_E10	ordinary_muscle_development	ENSGALG00010018418	PLCB4	-0.499995624	0.117935184	-4.23957981	2.23939E-05	9.89631E-05	negative
PRJNA729271	stage_E14_vs_E10	ordinary_muscle_development	ENSGALG00010018419	USP7	-0.253714745	0.133731253	-1.897198602	0.057801731	0.107153868	negative
PRJNA729271	stage_E14_vs_E10	ordinary_muscle_development	ENSGALG00010018420	RHOQ	0.237467881	0.132144789	1.797027969	0.07233118	0.129693277	positive
PRJNA729271	stage_E14_vs_E10	ordinary_muscle_development	ENSGALG00010018422	ENSGALG00010018422	0.932279954	0.211752399	4.402688974	1.06917E-05	4.98799E-05	positive
PRJNA729271	stage_E14_vs_E10	ordinary_muscle_development	ENSGALG00010018425	CYP26B1	0.071188406	0.226398544	0.314438445	0.753188061	0.823275842	positive
PRJNA729271	stage_E14_vs_E10	ordinary_muscle_development	ENSGALG00010018426	ENSGALG00010018426	0.533449549	0.361729729	1.47471857	0.140288221	0.224768185	positive
PRJNA729271	stage_E14_vs_E10	ordinary_muscle_development	ENSGALG00010018427	PIGF	-0.273901753	0.141067881	-1.941630871	0.052181803	0.098268836	negative
PRJNA729271	stage_E14_vs_E10	ordinary_muscle_development	ENSGALG00010018428	PPP2R5D	0.033400158	0.061368195	0.544258436	0.586263645	0.686698305	positive
PRJNA729271	stage_E14_vs_E10	ordinary_muscle_development	ENSGALG00010018430	LOXL4	-1.205093504	0.246332861	-4.89213456	9.97482E-07	5.5059E-06	negative
PRJNA729271	stage_E14_vs_E10	ordinary_muscle_development	ENSGALG00010018434	HSPBAP1	0.543034541	0.160859731	3.375826496	0.000735943	0.00239719	positive
PRJNA729271	stage_E14_vs_E10	ordinary_muscle_development	ENSGALG00010018436	FOXO4	1.045164305	0.143982004	7.25899261	3.89984E-13	4.6632E-12	positive
PRJNA729271	stage_E14_vs_E10	ordinary_muscle_development	ENSGALG00010018438	PPP2R2A	-0.037446668	0.098333306	-0.380813681	0.703341507	0.784441623	negative
PRJNA729271	stage_E14_vs_E10	ordinary_muscle_development	ENSGALG00010018440	MEA1	0.204421797	0.100948411	2.025012539	0.042866077	0.083167418	positive
PRJNA729271	stage_E14_vs_E10	ordinary_muscle_development	ENSGALG00010018441	CRIPT	0.162903044	0.093361719	1.744859086	0.081009401	0.142960698	positive
PRJNA729271	stage_E14_vs_E10	ordinary_muscle_development	ENSGALG00010018443	PLCB1	0.812536826	0.146739893	5.53725923	3.07242E-08	2.11365E-07	positive
PRJNA729271	stage_E14_vs_E10	ordinary_muscle_development	ENSGALG00010018444	NUBP2	-0.188341374	0.094876166	-1.985128415	0.047130186	0.090232736	negative
PRJNA729271	stage_E14_vs_E10	ordinary_muscle_development	ENSGALG00010018446	FBN1	0.712569006	0.232085323	3.070288964	0.002138518	0.006169947	positive
PRJNA729271	stage_E14_vs_E10	ordinary_muscle_development	ENSGALG00010018448	KLHDC3	0.818815265	0.165527378	4.946705955	7.54799E-07	4.24227E-06	positive
PRJNA729271	stage_E14_vs_E10	ordinary_muscle_development	ENSGALG00010018451	PIK3R4	-0.387698876	0.111200225	-3.486493637	0.000489397	0.001664042	negative
PRJNA729271	stage_E14_vs_E10	ordinary_muscle_development	ENSGALG00010018452	SPSB3	0.257567232	0.118361905	2.176099085	0.029547852	0.060647992	positive
PRJNA729271	stage_E14_vs_E10	ordinary_muscle_development	ENSGALG00010018453	TMX4	-0.022339391	0.091873309	-0.243154311	0.80788586	0.864951556	negative
PRJNA729271	stage_E14_vs_E10	ordinary_muscle_development	ENSGALG00010018455	RBM20	2.059399655	0.246824976	8.343562676	7.20852E-17	1.20239E-15	positive
PRJNA729271	stage_E14_vs_E10	ordinary_muscle_development	ENSGALG00010018458	SEMA5B	-0.556929061	0.10575382	-5.266278437	1.39217E-07	8.67563E-07	negative
PRJNA729271	stage_E14_vs_E10	ordinary_muscle_development	ENSGALG00010018459	SNX12	0.419309788	0.101999433	4.11090313	3.94115E-05	0.000167135	positive
PRJNA729271	stage_E14_vs_E10	ordinary_muscle_development	ENSGALG00010018462	KTN1	-0.160076699	0.116438944	-1.374769419	0.169202942	0.261415119	negative
PRJNA729271	stage_E14_vs_E10	ordinary_muscle_development	ENSGALG00010018464	ENSGALG00010018464	-1.778371531	0.950302241	-1.871374658	0.061293169	0.112647394	negative
PRJNA729271	stage_E14_vs_E10	ordinary_muscle_development	ENSGALG00010018465	NAALADL2	-0.949696886	0.197205073	-4.815783248	1.46624E-06	7.90666E-06	negative
PRJNA729271	stage_E14_vs_E10	ordinary_muscle_development	ENSGALG00010018466	DYSF	0.596395171	0.182790501	3.262725186	0.001103465	0.003438985	positive
PRJNA729271	stage_E14_vs_E10	ordinary_muscle_development	ENSGALG00010018467	TLX1	-0.970293481	0.83528994	-1.161624766	0.245387918	0.352098383	negative
PRJNA729271	stage_E14_vs_E10	ordinary_muscle_development	ENSGALG00010018469	KLHDC7A	0.319684681	0.19613116	1.629953549	0.103111315	0.174541458	positive
PRJNA729271	stage_E14_vs_E10	ordinary_muscle_development	ENSGALG00010018470	IQCB1	0.59255626	0.157615944	3.759494419	0.000170257	0.000642174	positive
PRJNA729271	stage_E14_vs_E10	ordinary_muscle_development	ENSGALG00010018471	HHEX	0.362231451	0.181907177	1.991298294	0.046448103	0.08921391	positive
PRJNA729271	stage_E14_vs_E10	ordinary_muscle_development	ENSGALG00010018472	LMF1	0.589570554	0.138858315	4.245842624	2.17773E-05	9.63855E-05	positive
PRJNA729271	stage_E14_vs_E10	ordinary_muscle_development	ENSGALG00010018476	ENSGALG00010018476	-0.02045567	0.375565982	-0.054466247	0.9565637	0.969946892	negative
PRJNA729271	stage_E14_vs_E10	ordinary_muscle_development	ENSGALG00010018480	EME2	0.164529726	0.241327988	0.681768111	0.495385591	0.605123095	positive
PRJNA729271	stage_E14_vs_E10	ordinary_muscle_development	ENSGALG00010018482	TWNK	-0.006724993	0.127157752	-0.05288701	0.957821935	0.97101895	negative
PRJNA729271	stage_E14_vs_E10	ordinary_muscle_development	ENSGALG00010018484	NLGN1	0.790976937	0.232818581	3.397396099	0.000680304	0.002230493	positive
PRJNA729271	stage_E14_vs_E10	ordinary_muscle_development	ENSGALG00010018485	ATP2C1	0.076769816	0.06597292	1.163656485	0.244563251	0.351054102	positive
PRJNA729271	stage_E14_vs_E10	ordinary_muscle_development	ENSGALG00010018486	ENSGALG00010018486	0.07414878	0.100358503	0.738839046	0.460004739	0.572662015	positive
PRJNA729271	stage_E14_vs_E10	ordinary_muscle_development	ENSGALG00010018487	ENSGALG00010018487	-0.168633598	0.117396738	-1.436441932	0.150876629	0.238464369	negative
PRJNA729271	stage_E14_vs_E10	ordinary_muscle_development	ENSGALG00010018488	ENSGALG00010018488	0.479727289	0.153587192	3.123484991	0.00178723	0.005281528	positive
PRJNA729271	stage_E14_vs_E10	ordinary_muscle_development	ENSGALG00010018489	SLC7A3	-0.453900741	0.205241195	-2.211547937	0.026997916	0.056266685	negative
PRJNA729271	stage_E14_vs_E10	ordinary_muscle_development	ENSGALG00010018490	NME3	0.065873636	0.119256811	0.552367912	0.580696311	0.682216974	positive
PRJNA729271	stage_E14_vs_E10	ordinary_muscle_development	ENSGALG00010018491	TIA1	-0.068234852	0.119524669	-0.570885095	0.568077535	0.670598898	negative
PRJNA729271	stage_E14_vs_E10	ordinary_muscle_development	ENSGALG00010018492	ENSGALG00010018492	0.948676299	0.169553129	5.595156556	2.20423E-08	1.54122E-07	positive
PRJNA729271	stage_E14_vs_E10	ordinary_muscle_development	ENSGALG00010018494	HAO1	-0.156544764	0.289748694	-0.540277719	0.589005522	0.689078646	negative
PRJNA729271	stage_E14_vs_E10	ordinary_muscle_development	ENSGALG00010018495	ENSGALG00010018495	0.888503205	0.632616175	1.404490178	0.160172934	0.25031878	positive
PRJNA729271	stage_E14_vs_E10	ordinary_muscle_development	ENSGALG00010018496	GNG13	0.200843301	0.408058343	0.492192609	0.622583189	0.717584552	positive
PRJNA729271	stage_E14_vs_E10	ordinary_muscle_development	ENSGALG00010018497	ASTE1	-0.473345846	0.098456093	-4.807684645	1.52688E-06	8.21231E-06	negative
PRJNA729271	stage_E14_vs_E10	ordinary_muscle_development	ENSGALG00010018498	ENSGALG00010018498	2.817000183	0.380749981	7.39855633	1.37673E-13	1.71441E-12	positive
PRJNA729271	stage_E14_vs_E10	ordinary_muscle_development	ENSGALG00010018501	ATG14	0.092712195	0.134495658	0.689332251	0.490614207	0.601060681	positive
PRJNA729271	stage_E14_vs_E10	ordinary_muscle_development	ENSGALG00010018502	ARHGEF10L	0.538532073	0.1218652	4.419080038	9.91219E-06	4.65427E-05	positive
PRJNA729271	stage_E14_vs_E10	ordinary_muscle_development	ENSGALG00010018503	BMP2	-2.804677926	0.181424625	-15.45919096	6.5415E-54	1.1288E-51	negative
PRJNA729271	stage_E14_vs_E10	ordinary_muscle_development	ENSGALG00010018504	SLCO3A1	0.118382352	0.179833801	0.658287549	0.510353377	0.619147322	positive
PRJNA729271	stage_E14_vs_E10	ordinary_muscle_development	ENSGALG00010018505	CHTF18	-0.28558669	0.141175533	-2.022919156	0.043081483	0.083484773	negative
PRJNA729271	stage_E14_vs_E10	ordinary_muscle_development	ENSGALG00010018506	ENSGALG00010018506	0.938749715	0.138566668	6.774715213	1.24652E-11	1.27062E-10	positive
PRJNA729271	stage_E14_vs_E10	ordinary_muscle_development	ENSGALG00010018508	ENSGALG00010018508	-0.005445909	0.099864394	-0.05453304	0.956510485	0.969946892	negative
PRJNA729271	stage_E14_vs_E10	ordinary_muscle_development	ENSGALG00010018509	ENSGALG00010018509	-0.032753341	0.108150444	-0.302849803	0.762004327	0.830532576	negative
PRJNA729271	stage_E14_vs_E10	ordinary_muscle_development	ENSGALG00010018510	ENSGALG00010018510	0.55339687	0.125253913	4.418200269	9.95262E-06	4.66871E-05	positive
PRJNA729271	stage_E14_vs_E10	ordinary_muscle_development	ENSGALG00010018511	SOCS5	-0.149234335	0.100316598	-1.487633539	0.136847563	0.220229314	negative
PRJNA729271	stage_E14_vs_E10	ordinary_muscle_development	ENSGALG00010018512	FERMT1	-0.609410194	0.171611619	-3.551101004	0.000383623	0.001337008	negative
PRJNA729271	stage_E14_vs_E10	ordinary_muscle_development	ENSGALG00010018513	RPUSD1	0.153298262	0.150530557	1.018386327	0.308494397	0.422090456	positive
PRJNA729271	stage_E14_vs_E10	ordinary_muscle_development	ENSGALG00010018514	EAF2	-0.164287812	0.240850604	-0.68211501	0.49516623	0.604975936	negative
PRJNA729271	stage_E14_vs_E10	ordinary_muscle_development	ENSGALG00010018515	SEMA4G	0.774490663	0.16801676	4.609603618	4.03437E-06	2.01788E-05	positive
PRJNA729271	stage_E14_vs_E10	ordinary_muscle_development	ENSGALG00010018516	LRRN4	0.545923736	0.15322888	3.562799232	0.000366921	0.001287155	positive
PRJNA729271	stage_E14_vs_E10	ordinary_muscle_development	ENSGALG00010018517	ECT2	-0.282785061	0.195124569	-1.449253993	0.147266664	0.233880826	negative
PRJNA729271	stage_E14_vs_E10	ordinary_muscle_development	ENSGALG00010018518	ENSGALG00010018518	-1.188498051	0.858171122	-1.384919651	0.166077038	0.257326206	negative
PRJNA729271	stage_E14_vs_E10	ordinary_muscle_development	ENSGALG00010018519	OGDH	0.021472898	0.136706231	0.157073292	0.875187086	0.914426354	positive
PRJNA729271	stage_E14_vs_E10	ordinary_muscle_development	ENSGALG00010018520	ENSGALG00010018520	0.340514299	0.122374329	2.782563159	0.005393137	0.01390493	positive
PRJNA729271	stage_E14_vs_E10	ordinary_muscle_development	ENSGALG00010018521	ENSGALG00010018521	0.29923009	0.158610709	1.886569272	0.059218283	0.109304599	positive
PRJNA729271	stage_E14_vs_E10	ordinary_muscle_development	ENSGALG00010018522	NEK11	-0.093989669	0.24698192	-0.380552831	0.703535088	0.784441623	negative
PRJNA729271	stage_E14_vs_E10	ordinary_muscle_development	ENSGALG00010018523	TEX11	-0.943460316	0.175839203	-5.365471984	8.07378E-08	5.21987E-07	negative
PRJNA729271	stage_E14_vs_E10	ordinary_muscle_development	ENSGALG00010018524	SEC22A	0.110499435	0.11567485	0.95525894	0.339446766	0.454487426	positive
PRJNA729271	stage_E14_vs_E10	ordinary_muscle_development	ENSGALG00010018525	ST8SIA2	0.047486433	0.289980814	0.163757154	0.869922322	0.910847478	positive
PRJNA729271	stage_E14_vs_E10	ordinary_muscle_development	ENSGALG00010018526	ENSGALG00010018526	0.070995049	0.100552163	0.70605193	0.480155844	0.592177228	positive
PRJNA729271	stage_E14_vs_E10	ordinary_muscle_development	ENSGALG00010018527	CCT4	-0.060243324	0.080901055	-0.744654375	0.456480688	0.569569387	negative
PRJNA729271	stage_E14_vs_E10	ordinary_muscle_development	ENSGALG00010018528	ENSGALG00010018528	2.24366763	0.583272476	3.846688677	0.000119725	0.000465133	positive
PRJNA729271	stage_E14_vs_E10	ordinary_muscle_development	ENSGALG00010018529	UBR2	-0.038435194	0.092771859	-0.414297978	0.678655895	0.764820183	negative
PRJNA729271	stage_E14_vs_E10	ordinary_muscle_development	ENSGALG00010018530	FAM161A	0.20885437	0.20290686	1.029311527	0.303333308	0.41695745	positive
PRJNA729271	stage_E14_vs_E10	ordinary_muscle_development	ENSGALG00010018531	ADCY5	0.098548653	0.135055748	0.729688698	0.465580491	0.577889126	positive
PRJNA729271	stage_E14_vs_E10	ordinary_muscle_development	ENSGALG00010018532	MFSD13A	0.190683194	0.122872475	1.551878837	0.120691218	0.198535604	positive
PRJNA729271	stage_E14_vs_E10	ordinary_muscle_development	ENSGALG00010018533	PCYOX1	-0.106440384	0.173750998	-0.612603008	0.540138869	0.646249517	negative
PRJNA729271	stage_E14_vs_E10	ordinary_muscle_development	ENSGALG00010018534	NCEH1	-0.260356386	0.196212444	-1.326910672	0.184538234	0.280415316	negative
PRJNA729271	stage_E14_vs_E10	ordinary_muscle_development	ENSGALG00010018535	FAM174B	-0.174758862	0.102313325	-1.708075281	0.087622375	0.152471348	negative
PRJNA729271	stage_E14_vs_E10	ordinary_muscle_development	ENSGALG00010018536	ENSGALG00010018536	0.041987785	0.245251412	0.171203032	0.864064124	0.906502728	positive
PRJNA729271	stage_E14_vs_E10	ordinary_muscle_development	ENSGALG00010018537	ENSGALG00010018537	-1.5200263	0.695936821	-2.184144096	0.028951661	0.059601524	negative
PRJNA729271	stage_E14_vs_E10	ordinary_muscle_development	ENSGALG00010018539	CHD2	0.092529693	0.087894336	1.052737837	0.292461162	0.405085958	positive
PRJNA729271	stage_E14_vs_E10	ordinary_muscle_development	ENSGALG00010018540	TNFSF10	0.32065576	0.10892498	2.943822072	0.003241863	0.008906521	positive
PRJNA729271	stage_E14_vs_E10	ordinary_muscle_development	ENSGALG00010018542	ZMIZ2	0.719035051	0.2631961	2.731936569	0.006296326	0.015908183	positive
PRJNA729271	stage_E14_vs_E10	ordinary_muscle_development	ENSGALG00010018543	MRPL3	-0.13911547	0.076671322	-1.814439425	0.069610101	0.125466104	negative
PRJNA729271	stage_E14_vs_E10	ordinary_muscle_development	ENSGALG00010018544	HACD2	-0.778069985	0.100291207	-7.758107692	8.62059E-15	1.19119E-13	negative

PRJNA729271	stage_E14_vs_E10	ordinary_muscle_development	ENSGALG00010018546	SNRPGP15	-0.168674415	0.13518247	-1.247753605	0.212121304	0.313266101	negative
PRJNA729271	stage_E14_vs_E10	ordinary_muscle_development	ENSGALG00010018548	RGMA	0.440363521	0.123071169	3.578120895	0.000346073	0.001222006	positive
PRJNA729271	stage_E14_vs_E10	ordinary_muscle_development	ENSGALG00010018549	GHSR	0.115380343	0.37419962	0.308339018	0.757824379	0.827407681	positive
PRJNA729271	stage_E14_vs_E10	ordinary_muscle_development	ENSGALG00010018551	FBXO34	0.260846303	0.111769579	2.333786211	0.019606919	0.042724338	positive
PRJNA729271	stage_E14_vs_E10	ordinary_muscle_development	ENSGALG00010018552	DTD1	0.172806165	0.102742192	1.681939633	0.092580546	0.159491827	positive
PRJNA729271	stage_E14_vs_E10	ordinary_muscle_development	ENSGALG00010018553	ENSGALG00010018553	0.452144364	0.387720506	1.166160566	0.243549533	0.349945527	positive
PRJNA729271	stage_E14_vs_E10	ordinary_muscle_development	ENSGALG00010018554	PPIA	0.202354882	0.123416166	1.639614073	0.101085435	0.171532824	positive
PRJNA729271	stage_E14_vs_E10	ordinary_muscle_development	ENSGALG00010018555	UBN1	-0.241425165	0.071262421	-3.387832781	0.000704472	0.002303999	negative
PRJNA729271	stage_E14_vs_E10	ordinary_muscle_development	ENSGALG00010018556	MRPL2	0.693517936	0.126100269	5.499733991	3.80365E-08	2.57034E-07	positive
PRJNA729271	stage_E14_vs_E10	ordinary_muscle_development	ENSGALG00010018557	TBCC	0.28488651	0.101458863	2.807901661	0.004986545	0.01299532	positive
PRJNA729271	stage_E14_vs_E10	ordinary_muscle_development	ENSGALG00010018558	ENSGALG00010018558	1.522588673	0.218956752	6.953832921	3.55493E-12	3.83683E-11	positive
PRJNA729271	stage_E14_vs_E10	ordinary_muscle_development	ENSGALG00010018559	DLG3	-0.668201954	0.124184765	-5.380707983	7.41935E-08	4.82258E-07	negative
PRJNA729271	stage_E14_vs_E10	ordinary_muscle_development	ENSGALG00010018560	ENSGALG00010018560	0.685522532	0.079978514	8.571333689	1.02293E-17	1.82379E-16	positive
PRJNA729271	stage_E14_vs_E10	ordinary_muscle_development	ENSGALG00010018561	ACTR1A	0.452213552	0.058840602	7.68539976	1.5252E-14	2.0739E-13	positive
PRJNA729271	stage_E14_vs_E10	ordinary_muscle_development	ENSGALG00010018562	ENSGALG00010018562	0.952056452	0.755558817	1.260069277	0.207644372	0.307908458	positive
PRJNA729271	stage_E14_vs_E10	ordinary_muscle_development	ENSGALG00010018564	DLGAP5	-0.274434273	0.151970851	-1.80583494	0.070944126	0.12753753	negative
PRJNA729271	stage_E14_vs_E10	ordinary_muscle_development	ENSGALG00010018566	ENSGALG00010018566	0.309255152	0.14681924	2.106366657	0.035172515	0.070214242	positive
PRJNA729271	stage_E14_vs_E10	ordinary_muscle_development	ENSGALG00010018568	FAM136A	-0.251361671	0.192494423	-1.305812743	0.191616246	0.288989437	negative
PRJNA729271	stage_E14_vs_E10	ordinary_muscle_development	ENSGALG00010018573	SEC23B	-0.182961605	0.078176755	-2.340358131	0.019265256	0.042055706	negative
PRJNA729271	stage_E14_vs_E10	ordinary_muscle_development	ENSGALG00010018574	ENSGALG00010018574	0.027064967	0.200268306	0.135143536	0.892498389	0.926171545	positive
PRJNA729271	stage_E14_vs_E10	ordinary_muscle_development	ENSGALG00010018576	PTK7	-0.55320849	0.11207359	-4.936118208	7.96928E-07	4.45315E-06	negative
PRJNA729271	stage_E14_vs_E10	ordinary_muscle_development	ENSGALG00010018577	NARFL	0.535329849	0.080043169	6.688014136	2.26219E-11	2.23673E-10	positive
PRJNA729271	stage_E14_vs_E10	ordinary_muscle_development	ENSGALG00010018579	FNDC3B	0.068590448	0.101300021	0.677102007	0.498341236	0.607626735	positive
PRJNA729271	stage_E14_vs_E10	ordinary_muscle_development	ENSGALG00010018581	SUFU	-0.261618061	0.091239587	-2.867374458	0.00413893	0.011020167	negative
PRJNA729271	stage_E14_vs_E10	ordinary_muscle_development	ENSGALG00010018582	RCC2	-0.134892644	0.110190568	-1.224175949	0.22088586	0.323473484	negative
PRJNA729271	stage_E14_vs_E10	ordinary_muscle_development	ENSGALG00010018583	PPL	1.176297016	0.213241176	5.516275226	3.4626E-08	2.36524E-07	positive
PRJNA729271	stage_E14_vs_E10	ordinary_muscle_development	ENSGALG00010018585	GDPD2	0.898154214	0.400279081	2.243820015	0.024843984	0.052441175	positive
PRJNA729271	stage_E14_vs_E10	ordinary_muscle_development	ENSGALG00010018587	SRF	0.87868271	0.173189348	5.073537832	3.90487E-07	2.29247E-06	positive
PRJNA729271	stage_E14_vs_E10	ordinary_muscle_development	ENSGALG00010018588	POLR3F	-0.079767225	0.10238016	-0.77912776	0.435904459	0.550098827	negative
PRJNA729271	stage_E14_vs_E10	ordinary_muscle_development	ENSGALG00010018590	TGFA	-2.284455215	0.621508412	-3.675662582	0.000237233	0.000865733	negative
PRJNA729271	stage_E14_vs_E10	ordinary_muscle_development	ENSGALG00010018593	LGALS3	-2.234328088	0.352044397	-6.346722475	2.1995E-10	1.95594E-09	negative
PRJNA729271	stage_E14_vs_E10	ordinary_muscle_development	ENSGALG00010018594	BICRAL	-0.303334475	0.104323958	-2.90762046	0.0036419	0.009841365	negative
PRJNA729271	stage_E14_vs_E10	ordinary_muscle_development	ENSGALG00010018598	ENSGALG00010018598	0.245859561	0.077131302	3.187545833	0.001434857	0.004343829	positive
PRJNA729271	stage_E14_vs_E10	ordinary_muscle_development	ENSGALG00010018601	CPNE4	-0.891439341	0.261630826	-3.407241243	0.000656231	0.002157421	negative
PRJNA729271	stage_E14_vs_E10	ordinary_muscle_development	ENSGALG00010018602	MCTP2	-0.254838179	0.219446245	-1.161278379	0.245528709	0.352230665	negative
PRJNA729271	stage_E14_vs_E10	ordinary_muscle_development	ENSGALG00010018604	FAM43A	0.133929853	0.12406605	1.07950445	0.280362911	0.391320211	positive
PRJNA729271	stage_E14_vs_E10	ordinary_muscle_development	ENSGALG00010018606	DZANK1	-0.547465436	0.230319776	-2.376979715	0.017455048	0.038680771	negative
PRJNA729271	stage_E14_vs_E10	ordinary_muscle_development	ENSGALG00010018607	HAGHL	0.649532271	0.114740445	5.660883292	1.50596E-08	1.07214E-07	positive
PRJNA729271	stage_E14_vs_E10	ordinary_muscle_development	ENSGALG00010018608	TRIM8	0.81409969	0.124924809	6.516717532	7.18626E-11	6.75057E-10	positive
PRJNA729271	stage_E14_vs_E10	ordinary_muscle_development	ENSGALG00010018609	ENSGALG00010018609	0.142748925	0.124038968	1.150839346	0.249798334	0.35687235	positive
PRJNA729271	stage_E14_vs_E10	ordinary_muscle_development	ENSGALG00010018611	NUDCD3	0.276557781	0.112258288	2.463584517	0.013755546	0.031498679	positive
PRJNA729271	stage_E14_vs_E10	ordinary_muscle_development	ENSGALG00010018613	PADI3	1.281907418	0.373249824	3.434448824	0.00059376	0.001973075	positive
PRJNA729271	stage_E14_vs_E10	ordinary_muscle_development	ENSGALG00010018615	HTR2C	0.797063758	0.233608686	3.411961133	0.000644973	0.002122817	positive
PRJNA729271	stage_E14_vs_E10	ordinary_muscle_development	ENSGALG00010018616	ENSGALG00010018616	-0.558906445	0.849616742	-0.657833606	0.510645057	0.619397498	negative
PRJNA729271	stage_E14_vs_E10	ordinary_muscle_development	ENSGALG00010018618	FAHD1	0.506710869	0.169415332	2.990938669	0.002781213	0.007770565	positive
PRJNA729271	stage_E14_vs_E10	ordinary_muscle_development	ENSGALG00010018619	MAPK1IP1L	-0.088241021	0.075005516	-1.176460419	0.23941094	0.345127954	negative
PRJNA729271	stage_E14_vs_E10	ordinary_muscle_development	ENSGALG00010018623	ARRDC4	0.372078316	0.131959393	2.819642525	0.004807718	0.012579511	positive
PRJNA729271	stage_E14_vs_E10	ordinary_muscle_development	ENSGALG00010018624	ENSGALG00010018624	0.842631211	0.386988185	2.177408108	0.029450131	0.060473106	positive
PRJNA729271	stage_E14_vs_E10	ordinary_muscle_development	ENSGALG00010018627	MYLK	-1.09354233	0.179970892	-6.076217769	1.2305E-09	9.94211E-09	negative
PRJNA729271	stage_E14_vs_E10	ordinary_muscle_development	ENSGALG00010018628	XDH	-3.051743672	0.756142661	-4.035936379	5.4385E-05	0.000224846	negative
PRJNA729271	stage_E14_vs_E10	ordinary_muscle_development	ENSGALG00010018631	KAT14	0.047765367	0.050179489	0.951890267	0.341152636	0.456055286	positive
PRJNA729271	stage_E14_vs_E10	ordinary_muscle_development	ENSGALG00010018633	ENSGALG00010018633	0.415853236	0.182303495	2.281104027	0.022542292	0.048310951	positive
PRJNA729271	stage_E14_vs_E10	ordinary_muscle_development	ENSGALG00010018638	SEC14L5	0.666059532	0.160766397	4.143027043	3.42752E-05	0.000146597	positive
PRJNA729271	stage_E14_vs_E10	ordinary_muscle_development	ENSGALG00010018641	ENSGALG00010018641	-1.938210997	0.664838439	-2.915311273	0.003553339	0.009628436	negative
PRJNA729271	stage_E14_vs_E10	ordinary_muscle_development	ENSGALG00010018644	ESF1	-0.196406495	0.09531635	-2.060575074	0.039343597	0.07726398	negative
PRJNA729271	stage_E14_vs_E10	ordinary_muscle_development	ENSGALG00010018649	HAGH	0.960460861	0.106635601	9.006943754	2.11879E-19	4.35012E-18	positive
PRJNA729271	stage_E14_vs_E10	ordinary_muscle_development	ENSGALG00010018654	ENSGALG00010018654	-0.252886697	0.226812533	-1.114959099	0.264867949	0.373673527	negative
PRJNA729271	stage_E14_vs_E10	ordinary_muscle_development	ENSGALG00010018655	BIRC5	0.055916793	0.206248498	0.271113699	0.786303586	0.84973313	positive
PRJNA729271	stage_E14_vs_E10	ordinary_muscle_development	ENSGALG00010018662	PADI1	3.878995977	0.749446841	5.175812034	2.26922E-07	1.37567E-06	positive
PRJNA729271	stage_E14_vs_E10	ordinary_muscle_development	ENSGALG00010018665	IL13RA2	-1.055607109	0.329817374	-3.200580661	0.00137151	0.004172095	negative
PRJNA729271	stage_E14_vs_E10	ordinary_muscle_development	ENSGALG00010018668	OVOL2	-2.025921653	0.723734049	-2.799262595	0.005121947	0.01328608	negative
PRJNA729271	stage_E14_vs_E10	ordinary_muscle_development	ENSGALG00010018670	MRAS	1.432762369	0.212920483	6.729096008	1.7072E-11	1.71015E-10	positive
PRJNA729271	stage_E14_vs_E10	ordinary_muscle_development	ENSGALG00010018672	PKDCC	-1.024863646	0.097560284	-10.50492683	8.19863E-26	2.63502E-24	negative
PRJNA729271	stage_E14_vs_E10	ordinary_muscle_development	ENSGALG00010018673	NAGPA	-0.176042556	0.08915505	-1.974566278	0.048317389	0.092196532	negative
PRJNA729271	stage_E14_vs_E10	ordinary_muscle_development	ENSGALG00010018684	PADI2	1.001296722	1.176518822	0.851067321	0.394731957	0.510131929	positive
PRJNA729271	stage_E14_vs_E10	ordinary_muscle_development	ENSGALG00010018685	ARL3	0.064498104	0.102005205	0.632302086	0.527189501	0.634738086	positive
PRJNA729271	stage_E14_vs_E10	ordinary_muscle_development	ENSGALG00010018693	ENSGALG00010018693	0.199625083	0.164781318	1.211454583	0.225721231	0.32882706	positive
PRJNA729271	stage_E14_vs_E10	ordinary_muscle_development	ENSGALG00010018694	MGME1	0.039255627	0.097489624	0.402664666	0.687194931	0.771384692	positive
PRJNA729271	stage_E14_vs_E10	ordinary_muscle_development	ENSGALG00010018698	MRPS16	0.325290958	0.114281813	2.84639306	0.004421758	0.011689473	positive
PRJNA729271	stage_E14_vs_E10	ordinary_muscle_development	ENSGALG00010018701	SFXN2	0.153257993	0.114260478	1.341303626	0.179821893	0.274688412	positive
PRJNA729271	stage_E14_vs_E10	ordinary_muscle_development	ENSGALG00010018704	PGPEP1L	0.100533513	0.446949373	0.224932664	0.822031659	0.875423587	positive
PRJNA729271	stage_E14_vs_E10	ordinary_muscle_development	ENSGALG00010018707	SLC45A1	0.036809617	0.099019981	0.371739283	0.710086974	0.789892644	positive
PRJNA729271	stage_E14_vs_E10	ordinary_muscle_development	ENSGALG00010018709	SNX5	0.184763403	0.111422711	1.658220316	0.097273006	0.16617233	positive
PRJNA729271	stage_E14_vs_E10	ordinary_muscle_development	ENSGALG00010018713	ENSGALG00010018713	-0.185224552	0.132617484	-1.396682741	0.162508997	0.253042165	negative
PRJNA729271	stage_E14_vs_E10	ordinary_muscle_development	ENSGALG00010018715	DNAJC13	-0.20668421	0.102970915	-2.007209619	0.044727348	0.086293479	negative
PRJNA729271	stage_E14_vs_E10	ordinary_muscle_development	ENSGALG00010018717	MEIOB	-2.073213993	0.62663968	-3.308462676	0.000938097	0.002974779	negative
PRJNA729271	stage_E14_vs_E10	ordinary_muscle_development	ENSGALG00010018719	ERRFI1	-0.461750466	0.083822773	-5.508651752	3.61592E-08	2.45493E-07	negative
PRJNA729271	stage_E14_vs_E10	ordinary_muscle_development	ENSGALG00010018721	LRCH2	0.002617863	0.188488008	0.013888753	0.988918735	0.992531561	positive
PRJNA729271	stage_E14_vs_E10	ordinary_muscle_development	ENSGALG00010018724	RRBP1	0.054245911	0.14085428	0.385120785	0.700147929	0.782169113	positive
PRJNA729271	stage_E14_vs_E10	ordinary_muscle_development	ENSGALG00010018726	EBF2	-0.813316616	0.155555024	-5.228481819	1.70908E-07	1.05238E-06	negative
PRJNA729271	stage_E14_vs_E10	ordinary_muscle_development	ENSGALG00010018727	PARK7	0.026694947	0.132295507	0.201782722	0.840086589	0.888382222	positive
PRJNA729271	stage_E14_vs_E10	ordinary_muscle_development	ENSGALG00010018729	HS3ST6	-5.069547899	0.417033143	-12.15622302	5.31602E-34	2.77179E-32	negative
PRJNA729271	stage_E14_vs_E10	ordinary_muscle_development	ENSGALG00010018730	IGF1R	-0.384692636	0.086813616	-4.431247701	9.36894E-06	4.41635E-05	negative
PRJNA729271	stage_E14_vs_E10	ordinary_muscle_development	ENSGALG00010018732	ENSGALG00010018732	1.13113859	0.220388091	5.132485084	2.85941E-07	1.70916E-06	positive
PRJNA729271	stage_E14_vs_E10	ordinary_muscle_development	ENSGALG00010018736	EEF2KMT	0.060617875	0.12933658	0.468683146	0.639296136	0.732132219	positive
PRJNA729271	stage_E14_vs_E10	ordinary_muscle_development	ENSGALG00010018747	FLRT3	-1.28361688	0.120991567	-10.60914335	2.70215E-26	9.10876E-25	negative
PRJNA729271	stage_E14_vs_E10	ordinary_muscle_development	ENSGALG00010018750	SOCS4	0.024751073	0.12577785	0.196784039	0.843996542	0.891411387	positive
PRJNA729271	stage_E14_vs_E10	ordinary_muscle_development	ENSGALG00010018752	PLS3	1.01426603	0.140612862	7.21318104	5.46597E-13	6.42572E-12	positive
PRJNA729271	stage_E14_vs_E10	ordinary_muscle_development	ENSGALG00010018753	SYNM	-0.119433379	0.154410072	-0.773481787	0.439237304	0.553148976	negative
PRJNA729271	stage_E14_vs_E10	ordinary_muscle_development	ENSGALG00010018756	EML4	-0.645458791	0.061621148	-10.47463107	1.12977E-25	3.59913E-24	negative
PRJNA729271	stage_E14_vs_E10	ordinary_muscle_development	ENSGALG00010018758	ENSGALG00010018758	-1.036270899	0.129839251	-7.981183607	1.44937E-15	2.15252E-14	negative
PRJNA729271	stage_E14_vs_E10	ordinary_muscle_development	ENSGALG00010018764	TMEM177	0.493156599	0.088632382	5.564067956	2.63557E-08	1.82438E-07	positive
PRJNA729271	stage_E14_vs_E10	ordinary_muscle_development	ENSGALG00010018767	NDUFB6	0.707624388	0.169133043	4.183832886	2.86635E-05	0.000124432	positive
PRJNA729271	stage_E14_vs_E10	ordinary_muscle_development	ENSGALG00010018774	MSRB1	0.262488034	0.123166856	2.131158024	0.033076126	0.066653475	positive
PRJNA729271	stage_E14_vs_E10	ordinary_muscle_development	ENSGALG00010018780	WDHD1	-0.250503461	0.20150244	-1.243178304	0.213802136	0.315426154	negative
PRJNA729271	stage_E14_vs_E10	ordinary_muscle_development	ENSGALG00010018783	ENSGALG00010018783	-0.27395157	0.178449405	-1.535177832	0.124740152	0.203822399	negative
PRJNA729271	stage_E14_vs_E10	ordinary_muscle_development	ENSGALG00010018786	DSTN	-0.321513225	0.114648471	-2.804339417	0.00504198	0.013127619	negative
PRJNA729271	stage_E14_vs_E10	ordinary_muscle_development	ENSGALG00010018796	PER3	-0.448678009	0.311361834	-1.441018004	0.149579596	0.236827553	negative
PRJNA729271	stage_E14_vs_E10	ordinary_muscle_development	ENSGALG00010018799	ENSGALG00010018799	-3.05551092	0.383860244	-7.959956704	1.72099E-15	2.53257E-14	negative
PRJNA729271	stage_E14_vs_E10	ordinary_muscle_development	ENSGALG00010018806	GCH1	-1.153604412	0.318409973	-3.62301595	0.000291188	0.001044745	negative
PRJNA729271	stage_E14_vs_E10	ordinary_muscle_development	ENSGALG00010018807	RD3	-1.692540142	0.226910812	-7.459054633	8.71455E-14	1.10999E-12	negative
PRJNA729271	stage_E14_vs_E10	ordinary_muscle_development	ENSGALG00010018808	MATCAP2	-0.397781223	0.08781741	-4.529639642	5.90844E-06	2.88166E-05	negative
PRJNA729271	stage_E14_vs_E10	ordinary_muscle_development	ENSGALG00010018811	VAMP3	0.455185622	0.123251506	3.693144493	0.000221498	0.00081302	positive
PRJNA729271	stage_E14_vs_E10	ordinary_muscle_development	ENSGALG00010018815	ENSGALG00010018815	0.500668288	0.170325046	2.939487172	0.003287559	0.009009863	positive
PRJNA729271	stage_E14_vs_E10	ordinary_muscle_development	ENSGALG00010018819	LRRC28	0.256837378	0.07689348	3.340171057	0.000837268	0.002689761	positive
PRJNA729271	stage_E14_vs_E10	ordinary_muscle_development	ENSGALG00010018822	TRAF5	0.112326885	0.198649559	0.565452476	0.57176604	0.673910291	positive
PRJNA729271	stage_E14_vs_E10	ordinary_muscle_development	ENSGALG00010018823	RAB40C	0.186657146	0.072469333	2.575670809	0.010004585	0.023835709	positive
PRJNA729271	stage_E14_vs_E10	ordinary_muscle_development	ENSGALG00010018829	PTPN4	0.577683244	0.13879121	4.162246608	3.15132E-05	0.000135907	positive
PRJNA729271	stage_E14_vs_E10	ordinary_muscle_development	ENSGALG00010018831	WBP1L	0.728666298	0.240200504	3.033575225	0.002416745	0.006849963	positive
PRJNA729271	stage_E14_vs_E10	ordinary_muscle_development	ENSGALG00010018832	EEPD1	1.588111687	0.183837	8.638694552	5.6859E-18	1.038E-16	positive
PRJNA729271	stage_E14_vs_E10	ordinary_muscle_development	ENSGALG00010018834	SAMD4A	0.572670447	0.171576472	3.337697991	0.000844755	0.002710809	positive
PRJNA729271	stage_E14_vs_E10	ordinary_muscle_development	ENSGALG00010018835	CAMK2B	0.494798406	0.175779234	2.814885448	0.004879462	0.012743749	positive
PRJNA729271	stage_E14_vs_E10	ordinary_muscle_development	ENSGALG00010018837	RCOR3	-0.437005	0.093781938	-4.659799205	3.16518E-06	1.6149E-05	negative
PRJNA729271	stage_E14_vs_E10	ordinary_muscle_development	ENSGALG00010018838	WFIKKN1	-0.156306977	0.208252644	-0.750564191	0.452914979	0.565802173	negative
PRJNA729271	stage_E14_vs_E10	ordinary_muscle_development	ENSGALG00010018839	RPL3L	0.752443923	0.273199823	2.75418891	0.00588378	0.015007106	positive
PRJNA729271	stage_E14_vs_E10	ordinary_muscle_development	ENSGALG00010018843	MCRIP2	1.356417741	0.236000579	5.747518692	9.05626E-09	6.61979E-08	positive
PRJNA729271	stage_E14_vs_E10	ordinary_muscle_development	ENSGALG00010018847	CGRRF1	-0.071971864	0.153961897	-0.467465427	0.640166922	0.732955729	negative
PRJNA729271	stage_E14_vs_E10	ordinary_muscle_development	ENSGALG00010018848	MEF2A	0.98287975	0.114536347	8.58137851	9.37429E-18	1.67961E-16	positive
PRJNA729271	stage_E14_vs_E10	ordinary_muscle_development	ENSGALG00010018849	KCNH1	-0.584450954	0.2829676	-2.065434182	0.038881937	0.076512853	negative
PRJNA729271	stage_E14_vs_E10	ordinary_muscle_development	ENSGALG00010018852	NDUFB10	0.360238983	0.116124256	3.102185497	0.001920975	0.005625159	positive
PRJNA729271	stage_E14_vs_E10	ordinary_muscle_development	ENSGALG00010018854	LYSMD4	0.349747188	0.170758274	2.048200533	0.040540354	0.079185074	positive
PRJNA729271	stage_E14_vs_E10	ordinary_muscle_development	ENSGALG00010018855	SYNDIG1	-2.933477422	0.679135525	-4.319428621	1.56434E-05	7.1104E-05	negative
PRJNA729271	stage_E14_vs_E10	ordinary_muscle_development	ENSGALG00010018857	ENSGALG00010018857	0.086627589	0.081902318	1.05769398	0.290194981	0.402562565	positive
PRJNA729271	stage_E14_vs_E10	ordinary_muscle_development	ENSGALG00010018858	EPB41L5	0.015697821	0.112364452	0.139704511	0.888893462	0.923753279	positive
PRJNA729271	stage_E14_vs_E10	ordinary_muscle_development	ENSGALG00010018860	AGTR2	0.085421538	0.215607277	0.396190421	0.691964557	0.775056425	positive
PRJNA729271	stage_E14_vs_E10	ordinary_muscle_development	ENSGALG00010018864	ABCA3	0.1311691	0.119224185	1.100188695	0.271249915	0.381132951	positive
PRJNA729271	stage_E14_vs_E10	ordinary_muscle_development	ENSGALG00010018865	ENSGALG00010018865	-0.3466434	0.22948815	-1.51050675	0.130914168	0.212235865	negative
PRJNA729271	stage_E14_vs_E10	ordinary_muscle_development	ENSGALG00010018867	RPS2	0.363759618	0.155912318	2.333103782	0.019642699	0.042789438	positive
PRJNA729271	stage_E14_vs_E10	ordinary_muscle_development	ENSGALG00010018872	KCTD9	0.056980169	0.100459543	0.567195185	0.570581579	0.672678324	positive
PRJNA729271	stage_E14_vs_E10	ordinary_muscle_development	ENSGALG00010018874	ENSGALG00010018874	-0.255716744	0.11257466	-2.271530228	0.023114899	0.049315641	negative
PRJNA729271	stage_E14_vs_E10	ordinary_muscle_development	ENSGALG00010018876	GMFB	-0.367791999	0.097373396	-3.777130257	0.000158646	0.000601352	negative

PRJNA729271	stage_E14_vs_E10	ordinary_muscle_development	ENSGALG00010018877	CST7	2.512731519	0.840671982	2.988955946	0.002799325	0.007816647	positive
PRJNA729271	stage_E14_vs_E10	ordinary_muscle_development	ENSGALG00010018880	TMEM17	-0.149419526	0.249493641	-0.598891118	0.549245495	0.654450678	negative
PRJNA729271	stage_E14_vs_E10	ordinary_muscle_development	ENSGALG00010018881	ENSGALG00010018881	0.798876833	0.63059114	1.266869739	0.205201871	0.305098073	positive
PRJNA729271	stage_E14_vs_E10	ordinary_muscle_development	ENSGALG00010018882	HHAT	-0.235870276	0.152820799	-1.543443545	0.122723195	0.201184428	negative
PRJNA729271	stage_E14_vs_E10	ordinary_muscle_development	ENSGALG00010018883	TBL3	-0.113656037	0.073026486	-1.556367325	0.11962077	0.197160919	negative
PRJNA729271	stage_E14_vs_E10	ordinary_muscle_development	ENSGALG00010018885	EFEMP1	0.737608152	0.20727683	3.558565386	0.000372886	0.001303976	positive
PRJNA729271	stage_E14_vs_E10	ordinary_muscle_development	ENSGALG00010018887	ADAMTS17	-0.101608624	0.198823289	-0.511049908	0.609316111	0.706562962	negative
PRJNA729271	stage_E14_vs_E10	ordinary_muscle_development	ENSGALG00010018891	GNRH1	0.143121101	0.303317634	0.471852227	0.63703227	0.730405216	positive
PRJNA729271	stage_E14_vs_E10	ordinary_muscle_development	ENSGALG00010018892	APMAP	-0.368240713	0.12295639	-2.994888777	0.002745449	0.007682488	negative
PRJNA729271	stage_E14_vs_E10	ordinary_muscle_development	ENSGALG00010018894	SERTAD4	-0.205053187	0.090663815	-2.261687177	0.023716738	0.050436343	negative
PRJNA729271	stage_E14_vs_E10	ordinary_muscle_development	ENSGALG00010018895	SEPTIN7	-0.1337151	0.089518119	-1.493721058	0.135248542	0.218140382	negative
PRJNA729271	stage_E14_vs_E10	ordinary_muscle_development	ENSGALG00010018897	CCDC85A	-1.023838362	0.167194053	-6.123652992	9.1454E-10	7.52822E-09	negative
PRJNA729271	stage_E14_vs_E10	ordinary_muscle_development	ENSGALG00010018899	ACSS1	-0.362549454	0.098774824	-3.670464191	0.00024211	0.000881313	negative
PRJNA729271	stage_E14_vs_E10	ordinary_muscle_development	ENSGALG00010018901	TCF7L2	-0.135853569	0.146896815	-0.924823105	0.35505793	0.471461377	negative
PRJNA729271	stage_E14_vs_E10	ordinary_muscle_development	ENSGALG00010018902	NOXO1	-1.048319015	0.544464137	-1.925414264	0.054177552	0.101408009	negative
PRJNA729271	stage_E14_vs_E10	ordinary_muscle_development	ENSGALG00010018903	CAMTA1	-0.012563994	0.094585549	-0.132832066	0.894326194	0.927507808	negative
PRJNA729271	stage_E14_vs_E10	ordinary_muscle_development	ENSGALG00010018904	UGP2	0.414093441	0.271327608	1.526175108	0.126966261	0.206830293	positive
PRJNA729271	stage_E14_vs_E10	ordinary_muscle_development	ENSGALG00010018908	ENSGALG00010018908	0.003830901	0.231271907	0.016564488	0.986784055	0.991301417	positive
PRJNA729271	stage_E14_vs_E10	ordinary_muscle_development	ENSGALG00010018909	TTBK1	-0.054970351	0.129152961	-0.425622073	0.670383254	0.75761775	negative
PRJNA729271	stage_E14_vs_E10	ordinary_muscle_development	ENSGALG00010018911	CNIH1	0.044440252	0.093412171	0.475743697	0.634256986	0.727914094	positive
PRJNA729271	stage_E14_vs_E10	ordinary_muscle_development	ENSGALG00010018913	ENSGALG00010018913	0.152609781	0.222526394	0.685805302	0.492835892	0.60304375	positive
PRJNA729271	stage_E14_vs_E10	ordinary_muscle_development	ENSGALG00010018915	DOCK5	-1.230720497	0.189431011	-6.496932545	8.19742E-11	7.59729E-10	negative
PRJNA729271	stage_E14_vs_E10	ordinary_muscle_development	ENSGALG00010018916	CERS3	0.203391359	0.308090701	0.660167147	0.509146572	0.617941854	positive
PRJNA729271	stage_E14_vs_E10	ordinary_muscle_development	ENSGALG00010018917	DNAJC11	-0.052776566	0.104865983	-0.503276316	0.614770015	0.711143949	negative
PRJNA729271	stage_E14_vs_E10	ordinary_muscle_development	ENSGALG00010018919	RALB	-0.095797225	0.107415421	-0.891838663	0.372479415	0.488516931	negative
PRJNA729271	stage_E14_vs_E10	ordinary_muscle_development	ENSGALG00010018920	ACAD11	-0.391240471	0.114405267	-3.419776733	0.000626726	0.002069336	negative
PRJNA729271	stage_E14_vs_E10	ordinary_muscle_development	ENSGALG00010018922	EEF2K	1.205894911	0.114041519	10.57417441	3.9262E-26	1.30528E-24	positive
PRJNA729271	stage_E14_vs_E10	ordinary_muscle_development	ENSGALG00010018923	VPS54	-0.324410182	0.091160926	-3.558653857	0.00037276	0.001303852	negative
PRJNA729271	stage_E14_vs_E10	ordinary_muscle_development	ENSGALG00010018925	LINS1	-0.487220968	0.125835421	-3.871890481	0.000107994	0.000422961	negative
PRJNA729271	stage_E14_vs_E10	ordinary_muscle_development	ENSGALG00010018926	SLC22A7	1.437832773	0.894437502	1.607527379	0.107938728	0.181483803	positive
PRJNA729271	stage_E14_vs_E10	ordinary_muscle_development	ENSGALG00010018932	CDKN3	0.067054097	0.22544724	0.297427002	0.766140531	0.833786846	positive
PRJNA729271	stage_E14_vs_E10	ordinary_muscle_development	ENSGALG00010018933	PHF13	0.136516423	0.08608612	1.585812249	0.112781906	0.188186224	positive
PRJNA729271	stage_E14_vs_E10	ordinary_muscle_development	ENSGALG00010018936	SYT14	1.131519332	0.217334715	5.206344197	1.92597E-07	1.18042E-06	positive
PRJNA729271	stage_E14_vs_E10	ordinary_muscle_development	ENSGALG00010018937	INHBB	-1.958002885	0.30285462	-6.465157717	1.01193E-10	9.26592E-10	negative
PRJNA729271	stage_E14_vs_E10	ordinary_muscle_development	ENSGALG00010018941	KLHL21	1.063947309	0.143751434	7.401298764	1.34859E-13	1.6837E-12	positive
PRJNA729271	stage_E14_vs_E10	ordinary_muscle_development	ENSGALG00010018943	ACKR4	0.452645281	0.358038689	1.264235667	0.206145453	0.306061491	positive
PRJNA729271	stage_E14_vs_E10	ordinary_muscle_development	ENSGALG00010018944	NEFL	-0.25191044	0.133787554	-1.882913859	0.059712043	0.110089806	negative
PRJNA729271	stage_E14_vs_E10	ordinary_muscle_development	ENSGALG00010018945	HABP2	-0.43371802	0.677467754	-0.64020467	0.522039547	0.629980492	negative
PRJNA729271	stage_E14_vs_E10	ordinary_muscle_development	ENSGALG00010018947	ENSGALG00010018947	0.240943309	0.213535754	1.12835113	0.259171658	0.367515453	positive
PRJNA729271	stage_E14_vs_E10	ordinary_muscle_development	ENSGALG00010018948	POLR3E	-0.110344681	0.108345728	-1.018449757	0.308464267	0.422089072	negative
PRJNA729271	stage_E14_vs_E10	ordinary_muscle_development	ENSGALG00010018950	TTL	0.670145325	0.096827523	6.921021051	4.484E-12	4.76857E-11	positive
PRJNA729271	stage_E14_vs_E10	ordinary_muscle_development	ENSGALG00010018951	YKT6	0.229803031	0.084508856	2.719277501	0.006542469	0.016424159	positive
PRJNA729271	stage_E14_vs_E10	ordinary_muscle_development	ENSGALG00010018955	ZBTB48	0.436728721	0.141226103	3.092407941	0.001985398	0.005781106	positive
PRJNA729271	stage_E14_vs_E10	ordinary_muscle_development	ENSGALG00010018958	PELI3	-0.048000275	0.117241094	-0.409415101	0.682235059	0.767481349	negative
PRJNA729271	stage_E14_vs_E10	ordinary_muscle_development	ENSGALG00010018959	UBA5	-0.306735878	0.114824928	-2.671335251	0.007555015	0.018636818	negative
PRJNA729271	stage_E14_vs_E10	ordinary_muscle_development	ENSGALG00010018960	RBFOX1	-1.120599375	0.220118048	-5.090901845	3.56364E-07	2.10751E-06	negative
PRJNA729271	stage_E14_vs_E10	ordinary_muscle_development	ENSGALG00010018961	ASB7	0.027720924	0.096866234	0.286177365	0.774742279	0.840371873	positive
PRJNA729271	stage_E14_vs_E10	ordinary_muscle_development	ENSGALG00010018968	GLI2	-0.595578981	0.194921161	-3.055486527	0.002246957	0.006445605	negative
PRJNA729271	stage_E14_vs_E10	ordinary_muscle_development	ENSGALG00010018969	DIEXF	-0.263331806	0.087863482	-2.997056358	0.002726003	0.007633967	negative
PRJNA729271	stage_E14_vs_E10	ordinary_muscle_development	ENSGALG00010018971	POLR1B	0.351043595	0.098250166	3.572956776	0.000352973	0.001243945	positive
PRJNA729271	stage_E14_vs_E10	ordinary_muscle_development	ENSGALG00010018973	NEFM	-3.104220721	0.359984565	-8.623205044	6.51057E-18	1.18407E-16	negative
PRJNA729271	stage_E14_vs_E10	ordinary_muscle_development	ENSGALG00010018974	TAS1R1	-0.096701994	0.300621171	-0.32167393	0.74769973	0.81900609	negative
PRJNA729271	stage_E14_vs_E10	ordinary_muscle_development	ENSGALG00010018977	NPHP3	-0.173504296	0.109747635	-1.580938823	0.113892026	0.189820044	negative
PRJNA729271	stage_E14_vs_E10	ordinary_muscle_development	ENSGALG00010018983	MDGA2	-1.393143604	0.167519056	-8.316329134	9.07296E-17	1.50472E-15	negative
PRJNA729271	stage_E14_vs_E10	ordinary_muscle_development	ENSGALG00010018985	NOL9	0.015058676	0.088140801	0.170847956	0.86434332	0.906625401	positive
PRJNA729271	stage_E14_vs_E10	ordinary_muscle_development	ENSGALG00010018986	CTDSP1	0.199464929	0.192441568	1.036496069	0.299970772	0.413747868	positive
PRJNA729271	stage_E14_vs_E10	ordinary_muscle_development	ENSGALG00010018987	MRPS10	0.096005526	0.106033303	0.905428044	0.365238664	0.481284949	positive
PRJNA729271	stage_E14_vs_E10	ordinary_muscle_development	ENSGALG00010018990	GCK	-0.322264501	0.388015813	-0.830544761	0.406230851	0.521182173	negative
PRJNA729271	stage_E14_vs_E10	ordinary_muscle_development	ENSGALG00010018991	RYBP	-1.24435209	0.130230224	-9.555017662	1.23563E-21	3.00511E-20	negative
PRJNA729271	stage_E14_vs_E10	ordinary_muscle_development	ENSGALG00010018993	ENSGALG00010018993	1.032164842	0.438044067	2.356303669	0.018457822	0.040537292	positive
PRJNA729271	stage_E14_vs_E10	ordinary_muscle_development	ENSGALG00010018998	RPS29	1.389746061	0.439936024	3.158973089	0.001583261	0.004734757	positive
PRJNA729271	stage_E14_vs_E10	ordinary_muscle_development	ENSGALG00010018999	THUMPD3	-0.233938023	0.070541969	-3.316295635	0.000912193	0.002898342	negative
PRJNA729271	stage_E14_vs_E10	ordinary_muscle_development	ENSGALG00010019000	TRERF1	0.041664864	0.117796316	0.353702606	0.72356177	0.79975811	positive
PRJNA729271	stage_E14_vs_E10	ordinary_muscle_development	ENSGALG00010019001	ALDH1A3	0.673157691	0.15972878	4.214379458	2.50466E-05	0.000109749	positive
PRJNA729271	stage_E14_vs_E10	ordinary_muscle_development	ENSGALG00010019004	ECE2	-0.188940807	0.19417155	-0.973061229	0.330522872	0.445295011	negative
PRJNA729271	stage_E14_vs_E10	ordinary_muscle_development	ENSGALG00010019005	ENSGALG00010019005	-0.280006707	0.217184265	-1.289258715	0.197308159	0.295804313	negative
PRJNA729271	stage_E14_vs_E10	ordinary_muscle_development	ENSGALG00010019006	ENSGALG00010019006	-0.257065689	0.076541838	-3.358499032	0.00078367	0.002533295	negative
PRJNA729271	stage_E14_vs_E10	ordinary_muscle_development	ENSGALG00010019008	HERPUD2	0.439515342	0.043698529	10.05789798	8.47893E-24	2.30586E-22	positive
PRJNA729271	stage_E14_vs_E10	ordinary_muscle_development	ENSGALG00010019009	NRAP	1.110921214	0.218649726	5.080826012	3.75797E-07	2.2134E-06	positive
PRJNA729271	stage_E14_vs_E10	ordinary_muscle_development	ENSGALG00010019011	VIL1	0.418168261	0.415973179	1.005276982	0.314763496	0.428725509	positive
PRJNA729271	stage_E14_vs_E10	ordinary_muscle_development	ENSGALG00010019012	ADAM28	0.518886553	0.353939318	1.466032527	0.142639426	0.227805032	positive
PRJNA729271	stage_E14_vs_E10	ordinary_muscle_development	ENSGALG00010019013	LRRK1	-0.298570597	0.100125076	-2.981976227	0.002863942	0.007974039	negative
PRJNA729271	stage_E14_vs_E10	ordinary_muscle_development	ENSGALG00010019015	GSG1L	-2.406198711	0.792535273	-3.036077753	0.002396775	0.00680534	negative
PRJNA729271	stage_E14_vs_E10	ordinary_muscle_development	ENSGALG00010019016	PLEKHG5	0.026747317	0.144228648	0.185450791	0.852875474	0.898161038	positive
PRJNA729271	stage_E14_vs_E10	ordinary_muscle_development	ENSGALG00010019017	IRAK2	0.042015952	0.082179885	0.511268078	0.609163354	0.706498865	positive
PRJNA729271	stage_E14_vs_E10	ordinary_muscle_development	ENSGALG00010019018	SH3BGRL	-0.028202524	0.10941659	-0.257753635	0.796597049	0.856959643	negative
PRJNA729271	stage_E14_vs_E10	ordinary_muscle_development	ENSGALG00010019019	TBX20	1.131052369	0.312054981	3.624529131	0.000289488	0.001039419	positive
PRJNA729271	stage_E14_vs_E10	ordinary_muscle_development	ENSGALG00010019020	KCNK2	1.315035272	0.440087669	2.988121148	0.002806983	0.00783652	positive
PRJNA729271	stage_E14_vs_E10	ordinary_muscle_development	ENSGALG00010019021	LRR1	-0.270909258	0.139872994	-1.936823183	0.052766961	0.099087589	negative
PRJNA729271	stage_E14_vs_E10	ordinary_muscle_development	ENSGALG00010019023	AP2M1	0.18663788	0.071325935	2.616690281	0.008878686	0.021485235	positive
PRJNA729271	stage_E14_vs_E10	ordinary_muscle_development	ENSGALG00010019024	CNPY3	0.137402112	0.100494165	1.367264579	0.17154239	0.264268992	positive
PRJNA729271	stage_E14_vs_E10	ordinary_muscle_development	ENSGALG00010019025	DNAAF2	-0.294406876	0.232696457	-1.265197075	0.205800691	0.305737523	negative
PRJNA729271	stage_E14_vs_E10	ordinary_muscle_development	ENSGALG00010019026	CHSY1	-0.202597493	0.123225765	-1.644116335	0.100152174	0.170288067	negative
PRJNA729271	stage_E14_vs_E10	ordinary_muscle_development	ENSGALG00010019027	GNLY	-0.455034765	0.325768832	-1.396802641	0.162472928	0.253040194	negative
PRJNA729271	stage_E14_vs_E10	ordinary_muscle_development	ENSGALG00010019028	TNFRSF25	1.62599944	0.820758408	1.981093858	0.047580749	0.090975195	positive
PRJNA729271	stage_E14_vs_E10	ordinary_muscle_development	ENSGALG00010019029	CENPF	-0.302563529	0.25914861	-1.167529045	0.242996786	0.349393752	negative
PRJNA729271	stage_E14_vs_E10	ordinary_muscle_development	ENSGALG00010019030	CDR2	-0.680770352	0.149192764	-4.56302528	5.04217E-06	2.48593E-05	negative
PRJNA729271	stage_E14_vs_E10	ordinary_muscle_development	ENSGALG00010019032	GNMT	2.146532789	0.186922171	11.48356443	1.59564E-30	6.98755E-29	positive
PRJNA729271	stage_E14_vs_E10	ordinary_muscle_development	ENSGALG00010019033	CYP2AB4	0.75761011	0.844282017	0.897342469	0.369536201	0.485827832	positive
PRJNA729271	stage_E14_vs_E10	ordinary_muscle_development	ENSGALG00010019034	CASP7	-0.301711572	0.170497801	-1.769592159	0.076795106	0.136515215	negative
PRJNA729271	stage_E14_vs_E10	ordinary_muscle_development	ENSGALG00010019035	ENSGALG00010019035	-0.976522288	0.439190375	-2.223460131	0.026184789	0.05480845	negative
PRJNA729271	stage_E14_vs_E10	ordinary_muscle_development	ENSGALG00010019036	REL	-0.049374789	0.109731073	-0.449961779	0.652738	0.743589855	negative
PRJNA729271	stage_E14_vs_E10	ordinary_muscle_development	ENSGALG00010019038	PLEKHS1	-4.402848575	0.537676289	-8.188660477	2.6415E-16	4.15276E-15	negative
PRJNA729271	stage_E14_vs_E10	ordinary_muscle_development	ENSGALG00010019040	HMGN5	-0.364636879	0.081254445	-4.487592997	7.20324E-06	3.45846E-05	negative
PRJNA729271	stage_E14_vs_E10	ordinary_muscle_development	ENSGALG00010019041	KATNIP	-0.191693388	0.086304935	-2.221117351	0.026343014	0.055107806	negative
PRJNA729271	stage_E14_vs_E10	ordinary_muscle_development	ENSGALG00010019042	PEX6	0.494349903	0.105083777	4.704340844	2.54687E-06	1.32082E-05	positive
PRJNA729271	stage_E14_vs_E10	ordinary_muscle_development	ENSGALG00010019043	METTL9	-0.474603703	0.10089744	-4.703823064	2.55335E-06	1.3237E-05	negative
PRJNA729271	stage_E14_vs_E10	ordinary_muscle_development	ENSGALG00010019045	LBX1	1.590944464	0.263744007	6.032153981	1.61789E-09	1.29565E-08	positive
PRJNA729271	stage_E14_vs_E10	ordinary_muscle_development	ENSGALG00010019046	SELENOS	0.106355683	0.080834828	1.315716078	0.188269356	0.284890313	positive
PRJNA729271	stage_E14_vs_E10	ordinary_muscle_development	ENSGALG00010019047	GHRL	-0.552303967	0.409728496	-1.347975484	0.177666268	0.271853959	negative
PRJNA729271	stage_E14_vs_E10	ordinary_muscle_development	ENSGALG00010019049	POLD2	-0.150817754	0.160273254	-0.941003878	0.346702872	0.462238607	negative
PRJNA729271	stage_E14_vs_E10	ordinary_muscle_development	ENSGALG00010019050	TNS1	-0.283389252	0.13421368	-2.11147815	0.034731234	0.069467261	negative
PRJNA729271	stage_E14_vs_E10	ordinary_muscle_development	ENSGALG00010019051	IGSF6	0.576309441	0.261732193	2.201905064	0.027672015	0.057456791	positive
PRJNA729271	stage_E14_vs_E10	ordinary_muscle_development	ENSGALG00010019059	BRWD3	0.363945515	0.076936316	4.73047757	2.23992E-06	1.16959E-05	positive
PRJNA729271	stage_E14_vs_E10	ordinary_muscle_development	ENSGALG00010019062	PUS10	-0.595387936	0.094679065	-6.28848561	3.20578E-10	2.77917E-09	negative
PRJNA729271	stage_E14_vs_E10	ordinary_muscle_development	ENSGALG00010019064	ENSGALG00010019064	-0.018209042	0.171778034	-0.106003321	0.915579717	0.941362463	negative
PRJNA729271	stage_E14_vs_E10	ordinary_muscle_development	ENSGALG00010019065	CAND2	0.849227597	0.121718149	6.977000613	3.01548E-12	3.28642E-11	positive
PRJNA729271	stage_E14_vs_E10	ordinary_muscle_development	ENSGALG00010019067	GTF3C1	-0.157290553	0.056181105	-2.799705595	0.005114923	0.013272612	negative
PRJNA729271	stage_E14_vs_E10	ordinary_muscle_development	ENSGALG00010019068	SNRPA1	0.046347793	0.163061875	0.284234394	0.776230759	0.841569368	positive
PRJNA729271	stage_E14_vs_E10	ordinary_muscle_development	ENSGALG00010019072	AEBP1	0.203569828	0.211612264	0.961994472	0.336052381	0.451276567	positive
PRJNA729271	stage_E14_vs_E10	ordinary_muscle_development	ENSGALG00010019076	ESPN	-1.691931819	0.430066886	-3.934113212	8.35044E-05	0.000333351	negative
PRJNA729271	stage_E14_vs_E10	ordinary_muscle_development	ENSGALG00010019079	CAPG	0.37533111	0.24544826	1.529165899	0.126223322	0.205804845	positive
PRJNA729271	stage_E14_vs_E10	ordinary_muscle_development	ENSGALG00010019081	PCSK6	0.393984438	0.136486869	2.886610564	0.003894159	0.010433611	positive
PRJNA729271	stage_E14_vs_E10	ordinary_muscle_development	ENSGALG00010019082	POLR3K	0.052262787	0.178995685	0.291977918	0.770303508	0.836917023	positive
PRJNA729271	stage_E14_vs_E10	ordinary_muscle_development	ENSGALG00010019083	OTOA	-1.466913853	0.235762601	-6.22199554	4.90871E-10	4.1854E-09	negative
PRJNA729271	stage_E14_vs_E10	ordinary_muscle_development	ENSGALG00010019087	PEX13	0.068424128	0.086626397	0.789876179	0.429600084	0.543704987	positive
PRJNA729271	stage_E14_vs_E10	ordinary_muscle_development	ENSGALG00010019093	RPL32	0.354394216	0.151731623	2.335664832	0.019508717	0.042529344	positive
PRJNA729271	stage_E14_vs_E10	ordinary_muscle_development	ENSGALG00010019094	SNRNP25	0.350081987	0.166850329	2.098179789	0.035889266	0.07146791	positive
PRJNA729271	stage_E14_vs_E10	ordinary_muscle_development	ENSGALG00010019097	STC1	-0.815824405	0.241001489	-3.385142593	0.000711413	0.002324077	negative
PRJNA729271	stage_E14_vs_E10	ordinary_muscle_development	ENSGALG00010019101	POLE2	-0.226758635	0.11897849	-1.905879242	0.056665875	0.105344601	negative
PRJNA729271	stage_E14_vs_E10	ordinary_muscle_development	ENSGALG00010019104	POLL	0.915337778	0.117745386	7.773873871	7.61215E-15	1.05587E-13	positive
PRJNA729271	stage_E14_vs_E10	ordinary_muscle_development	ENSGALG00010019108	KDELR2	-0.016153365	0.099322201	-0.162635994	0.870805045	0.911424589	negative
PRJNA729271	stage_E14_vs_E10	ordinary_muscle_development	ENSGALG00010019110	MBD4	0.175974114	0.108269479	1.625334452	0.104091314	0.175933256	positive
PRJNA729271	stage_E14_vs_E10	ordinary_muscle_development	ENSGALG00010019113	KLHDC1	0.445620559	0.133826993	3.329825686	0.000869004	0.002779044	positive
PRJNA729271	stage_E14_vs_E10	ordinary_muscle_development	ENSGALG00010019114	TENT5D	-0.847961377	0.331301764	-2.559483436	0.010482785	0.024791641	negative
PRJNA729271	stage_E14_vs_E10	ordinary_muscle_development	ENSGALG00010019115	DBNL	-0.007457721	0.100022275	-0.074560603	0.940564321	0.958349489	negative
PRJNA729271	stage_E14_vs_E10	ordinary_muscle_development	ENSGALG00010019120	DAGLB	-0.270491001	0.082816629	-3.266143581	0.0010923	0.003402859	negative
PRJNA729271	stage_E14_vs_E10	ordinary_muscle_development	ENSGALG00010019123	PLEKHO2	0.431639501	0.118159356	3.65302855	0.000259165	0.000936809	positive
PRJNA729271	stage_E14_vs_E10	ordinary_muscle_development	ENSGALG00010019124	BMPER	-0.598939432	0.248868425	-2.406650955	0.016099549	0.036059758	negative
PRJNA729271	stage_E14_vs_E10	ordinary_muscle_development	ENSGALG00010019125	ENSGALG00010019125	0.038834611	0.20516081	0.189288641	0.849866598	0.895848763	positive
PRJNA729271	stage_E14_vs_E10	ordinary_muscle_development	ENSGALG00010019127	TMBIM1	0.067225247	0.122521986	0.548679053	0.583225724	0.684245801	positive

PRJNA729271	stage_E14_vs_E10	ordinary_muscle_development	ENSGALG00010019130	RPS6KC1	0.05151658	0.081337003	0.633372003	0.526490744	0.634061009	positive
PRJNA729271	stage_E14_vs_E10	ordinary_muscle_development	ENSGALG00010019132	ENSGALG00010019132	0.404435342	0.152698294	2.648591096	0.008082806	0.019817367	positive
PRJNA729271	stage_E14_vs_E10	ordinary_muscle_development	ENSGALG00010019135	KIAA1841	-1.26783396	0.191646323	-6.615488032	3.70328E-11	3.58577E-10	negative
PRJNA729271	stage_E14_vs_E10	ordinary_muscle_development	ENSGALG00010019139	DPCD	-0.092910907	0.126775982	-0.732874682	0.463634866	0.576180684	negative
PRJNA729271	stage_E14_vs_E10	ordinary_muscle_development	ENSGALG00010019141	ACOT7	-0.974460852	0.152927509	-6.372044224	1.86525E-10	1.671E-09	negative
PRJNA729271	stage_E14_vs_E10	ordinary_muscle_development	ENSGALG00010019144	IFT122	-0.450540593	0.071400479	-6.310049999	2.78945E-10	2.44014E-09	negative
PRJNA729271	stage_E14_vs_E10	ordinary_muscle_development	ENSGALG00010019145	RAC1	-0.137325523	0.07623542	-1.801334903	0.07165011	0.12858887	negative
PRJNA729271	stage_E14_vs_E10	ordinary_muscle_development	ENSGALG00010019146	KLHDC2	0.240426472	0.08549195	2.812270312	0.004919314	0.012831647	positive
PRJNA729271	stage_E14_vs_E10	ordinary_muscle_development	ENSGALG00010019148	ENSGALG00010019148	-0.231618807	0.218070954	-1.062125892	0.288178537	0.400493566	negative
PRJNA729271	stage_E14_vs_E10	ordinary_muscle_development	ENSGALG00010019149	COX5B	0.340220078	0.180507161	1.884801005	0.059456709	0.109660898	positive
PRJNA729271	stage_E14_vs_E10	ordinary_muscle_development	ENSGALG00010019158	ENSGALG00010019158	0.936541787	0.260654734	3.593035789	0.000326848	0.001160337	positive
PRJNA729271	stage_E14_vs_E10	ordinary_muscle_development	ENSGALG00010019166	PNPT1	-0.177534938	0.108231443	-1.640326815	0.100937233	0.171361583	negative
PRJNA729271	stage_E14_vs_E10	ordinary_muscle_development	ENSGALG00010019169	GPR153	-1.392337181	0.178581	-7.796670329	6.3562E-15	8.85895E-14	negative
PRJNA729271	stage_E14_vs_E10	ordinary_muscle_development	ENSGALG00010019172	NEMF	0.071883139	0.08616932	0.834208039	0.404163762	0.519173496	positive
PRJNA729271	stage_E14_vs_E10	ordinary_muscle_development	ENSGALG00010019173	RHO	-0.896683497	0.689384178	-1.300702171	0.193360419	0.291135273	negative
PRJNA729271	stage_E14_vs_E10	ordinary_muscle_development	ENSGALG00010019174	ENSGALG00010019174	-0.45590596	0.441303795	-1.033088691	0.301562395	0.415389805	negative
PRJNA729271	stage_E14_vs_E10	ordinary_muscle_development	ENSGALG00010019183	ANGEL2	-0.038905841	0.078169542	-0.497710993	0.618687759	0.714515103	negative
PRJNA729271	stage_E14_vs_E10	ordinary_muscle_development	ENSGALG00010019184	PNKD	-0.177215144	0.17704413	-1.000965942	0.316843274	0.430714427	negative
PRJNA729271	stage_E14_vs_E10	ordinary_muscle_development	ENSGALG00010019185	ANKDD1A	-0.412763578	0.211669552	-1.950037562	0.051171642	0.096693124	negative
PRJNA729271	stage_E14_vs_E10	ordinary_muscle_development	ENSGALG00010019188	ENSGALG00010019188	1.360719049	0.372536133	3.652582738	0.000259616	0.000937969	positive
PRJNA729271	stage_E14_vs_E10	ordinary_muscle_development	ENSGALG00010019189	ICMT	0.009929007	0.091996014	0.107928669	0.914052275	0.940755838	positive
PRJNA729271	stage_E14_vs_E10	ordinary_muscle_development	ENSGALG00010019193	ENSGALG00010019193	4.551247225	1.004483999	4.530930536	5.87244E-06	2.86796E-05	positive
PRJNA729271	stage_E14_vs_E10	ordinary_muscle_development	ENSGALG00010019194	ENSGALG00010019194	0.568614962	0.185799954	3.060361153	0.002210702	0.00635544	positive
PRJNA729271	stage_E14_vs_E10	ordinary_muscle_development	ENSGALG00010019204	ARF6	-0.266297961	0.229681902	-1.15942074	0.246284727	0.352931005	negative
PRJNA729271	stage_E14_vs_E10	ordinary_muscle_development	ENSGALG00010019205	ENSGALG00010019205	-0.529074684	0.260679254	-2.029600269	0.042397188	0.082356906	negative
PRJNA729271	stage_E14_vs_E10	ordinary_muscle_development	ENSGALG00010019206	RNF207	0.1806669	0.283082678	0.638212485	0.523335371	0.631198719	positive
PRJNA729271	stage_E14_vs_E10	ordinary_muscle_development	ENSGALG00010019208	PLXND1	0.446205095	0.157929502	2.825343524	0.004722995	0.012390916	positive
PRJNA729271	stage_E14_vs_E10	ordinary_muscle_development	ENSGALG00010019209	VCPKMT	0.176780081	0.083308546	2.121992147	0.033838398	0.067943977	positive
PRJNA729271	stage_E14_vs_E10	ordinary_muscle_development	ENSGALG00010019212	RPL22	0.300205189	0.113753863	2.639076869	0.008313213	0.020327264	positive
PRJNA729271	stage_E14_vs_E10	ordinary_muscle_development	ENSGALG00010019213	RHBDF1	0.567121955	0.108971117	5.204332777	1.94695E-07	1.19277E-06	positive
PRJNA729271	stage_E14_vs_E10	ordinary_muscle_development	ENSGALG00010019215	ENSGALG00010019215	0.117483506	0.099425724	1.181620827	0.237356186	0.342404251	positive
PRJNA729271	stage_E14_vs_E10	ordinary_muscle_development	ENSGALG00010019218	RMND5A	0.030727194	0.137949704	0.222742008	0.823736305	0.876591861	positive
PRJNA729271	stage_E14_vs_E10	ordinary_muscle_development	ENSGALG00010019221	VASH2	1.149348898	0.231497777	4.964837732	6.87585E-07	3.88559E-06	positive
PRJNA729271	stage_E14_vs_E10	ordinary_muscle_development	ENSGALG00010019223	AAMP	0.363137834	0.124110936	2.925913263	0.003434467	0.009348844	positive
PRJNA729271	stage_E14_vs_E10	ordinary_muscle_development	ENSGALG00010019225	PROK2	-4.04749718	0.529541439	-7.643400274	2.11559E-14	2.8394E-13	negative
PRJNA729271	stage_E14_vs_E10	ordinary_muscle_development	ENSGALG00010019227	CLCN5	-0.243977168	0.072378591	-3.370847161	0.000749374	0.002434375	negative
PRJNA729271	stage_E14_vs_E10	ordinary_muscle_development	ENSGALG00010019230	SPG21	0.050094657	0.096677818	0.518160815	0.604346072	0.702429342	positive
PRJNA729271	stage_E14_vs_E10	ordinary_muscle_development	ENSGALG00010019231	ENSGALG00010019231	0.952169172	0.311935514	3.052455169	0.002269776	0.006502057	positive
PRJNA729271	stage_E14_vs_E10	ordinary_muscle_development	ENSGALG00010019232	SOS2	0.01651064	0.093774342	0.176067777	0.860240696	0.903826391	positive
PRJNA729271	stage_E14_vs_E10	ordinary_muscle_development	ENSGALG00010019233	ARPC2	-0.192871693	0.052814462	-3.651872695	0.000260335	0.000940333	negative
PRJNA729271	stage_E14_vs_E10	ordinary_muscle_development	ENSGALG00010019236	MPG	0.129713344	0.205463697	0.631320016	0.527831302	0.635299736	positive
PRJNA729271	stage_E14_vs_E10	ordinary_muscle_development	ENSGALG00010019237	SORCS3	-2.42365896	0.33694215	-7.19310114	6.3336E-13	7.40367E-12	negative
PRJNA729271	stage_E14_vs_E10	ordinary_muscle_development	ENSGALG00010019239	CYTH3	0.199561676	0.080841914	2.468542203	0.013566465	0.031129637	positive
PRJNA729271	stage_E14_vs_E10	ordinary_muscle_development	ENSGALG00010019240	ENSGALG00010019240	0.358499407	0.192707964	1.860324809	0.062839586	0.115080201	positive
PRJNA729271	stage_E14_vs_E10	ordinary_muscle_development	ENSGALG00010019243	SERTAD2	-0.030282033	0.154723064	-0.19571764	0.844831177	0.892032919	negative
PRJNA729271	stage_E14_vs_E10	ordinary_muscle_development	ENSGALG00010019246	IL17RC	0.604780338	0.134727634	4.488910846	7.15882E-06	3.43942E-05	positive
PRJNA729271	stage_E14_vs_E10	ordinary_muscle_development	ENSGALG00010019247	HBZ	-3.652800427	0.44410848	-8.225018408	1.95161E-16	3.11892E-15	negative
PRJNA729271	stage_E14_vs_E10	ordinary_muscle_development	ENSGALG00010019248	RMI2	0.443575011	0.160746308	2.759472458	0.005789477	0.014795216	positive
PRJNA729271	stage_E14_vs_E10	ordinary_muscle_development	ENSGALG00010019250	CXCR1	0.352003129	0.236439387	1.488766882	0.136548766	0.219846092	positive
PRJNA729271	stage_E14_vs_E10	ordinary_muscle_development	ENSGALG00010019252	TATDN3	0.113633043	0.115027428	0.987877803	0.323212498	0.437847211	positive
PRJNA729271	stage_E14_vs_E10	ordinary_muscle_development	ENSGALG00010019253	HBAD	-0.059819559	0.426281494	-0.140328772	0.888400232	0.923505549	negative
PRJNA729271	stage_E14_vs_E10	ordinary_muscle_development	ENSGALG00010019255	ENSGALG00010019255	0.490268434	0.200155757	2.449434585	0.01430807	0.032593976	positive
PRJNA729271	stage_E14_vs_E10	ordinary_muscle_development	ENSGALG00010019256	BBS9	-0.541152379	0.100864891	-5.365121354	8.08948E-08	5.22769E-07	negative
PRJNA729271	stage_E14_vs_E10	ordinary_muscle_development	ENSGALG00010019257	SLC1A4	-0.372238926	0.157259495	-2.367036253	0.017931176	0.039566509	negative
PRJNA729271	stage_E14_vs_E10	ordinary_muscle_development	ENSGALG00010019259	L2HGDH	-0.076177589	0.108184479	-0.704145266	0.481342313	0.593135835	negative
PRJNA729271	stage_E14_vs_E10	ordinary_muscle_development	ENSGALG00010019260	TRAIL-LIKE	-0.234986729	0.17564116	-1.337879629	0.180935679	0.27592453	negative
PRJNA729271	stage_E14_vs_E10	ordinary_muscle_development	ENSGALG00010019262	TMC3	-1.937716874	0.44856893	-4.319775054	1.56188E-05	7.10147E-05	negative
PRJNA729271	stage_E14_vs_E10	ordinary_muscle_development	ENSGALG00010019263	SNRPB2	-0.202222795	0.101670743	-1.988996928	0.046701542	0.089577723	negative
PRJNA729271	stage_E14_vs_E10	ordinary_muscle_development	ENSGALG00010019264	HK2	0.113273014	0.214648013	0.527715177	0.597697038	0.697329247	positive
PRJNA729271	stage_E14_vs_E10	ordinary_muscle_development	ENSGALG00010019265	RUFY4	-0.499969828	0.959286934	-0.521189032	0.602235094	0.700649947	negative
PRJNA729271	stage_E14_vs_E10	ordinary_muscle_development	ENSGALG00010019266	ATP5S	0.006538791	0.154459382	0.0423334	0.96623292	0.977618718	positive
PRJNA729271	stage_E14_vs_E10	ordinary_muscle_development	ENSGALG00010019267	STARD5	-1.603455091	0.19343289	-8.2894646	1.13759E-16	1.86321E-15	negative
PRJNA729271	stage_E14_vs_E10	ordinary_muscle_development	ENSGALG00010019268	NSL1	-0.073267437	0.091720282	-0.798813909	0.424398324	0.538720879	negative
PRJNA729271	stage_E14_vs_E10	ordinary_muscle_development	ENSGALG00010019269	SOCS1	-0.251590671	0.254156195	-0.98990572	0.322220203	0.436753726	negative
PRJNA729271	stage_E14_vs_E10	ordinary_muscle_development	ENSGALG00010019270	ENSGALG00010019270	-3.488345615	0.242591727	-14.37949124	6.96043E-47	8.40762E-45	negative
PRJNA729271	stage_E14_vs_E10	ordinary_muscle_development	ENSGALG00010019271	CDKL1	-0.070379321	0.181698858	-0.387340468	0.69850417	0.780754006	negative
PRJNA729271	stage_E14_vs_E10	ordinary_muscle_development	ENSGALG00010019274	ENSGALG00010019274	0.25745355	0.566342899	0.454589526	0.649404572	0.740930831	positive
PRJNA729271	stage_E14_vs_E10	ordinary_muscle_development	ENSGALG00010019275	CTAGE1	-0.529353556	0.611003203	-0.866367891	0.386288447	0.502445355	negative
PRJNA729271	stage_E14_vs_E10	ordinary_muscle_development	ENSGALG00010019277	IGFBP5	-0.466039387	0.132843609	-3.508180699	0.000451182	0.001545343	negative
PRJNA729271	stage_E14_vs_E10	ordinary_muscle_development	ENSGALG00010019278	JPT2	-0.417860864	0.072443539	-5.768090136	8.01749E-09	5.89917E-08	negative
PRJNA729271	stage_E14_vs_E10	ordinary_muscle_development	ENSGALG00010019280	TLNRD1	-0.135832405	0.11590485	-1.171930296	0.241225042	0.34712171	negative
PRJNA729271	stage_E14_vs_E10	ordinary_muscle_development	ENSGALG00010019281	ENSGALG00010019281	-4.863275125	1.378356235	-3.528315107	0.000418214	0.001441422	negative
PRJNA729271	stage_E14_vs_E10	ordinary_muscle_development	ENSGALG00010019282	IGFBP2	-0.647968925	0.152925388	-4.237157296	2.26368E-05	9.99147E-05	negative
PRJNA729271	stage_E14_vs_E10	ordinary_muscle_development	ENSGALG00010019283	NT5C3A	0.260688545	0.2558178	1.019039902	0.308184024	0.421825063	positive
PRJNA729271	stage_E14_vs_E10	ordinary_muscle_development	ENSGALG00010019284	CHD5	0.328855496	0.132358869	2.484574692	0.012970629	0.029933016	positive
PRJNA729271	stage_E14_vs_E10	ordinary_muscle_development	ENSGALG00010019285	BATF3	1.123819899	0.260345756	4.316643819	1.58419E-05	7.18489E-05	positive
PRJNA729271	stage_E14_vs_E10	ordinary_muscle_development	ENSGALG00010019287	AHSA2	-0.318689784	0.093439151	-3.410666508	0.000648043	0.002132437	negative
PRJNA729271	stage_E14_vs_E10	ordinary_muscle_development	ENSGALG00010019288	RPL37A	0.265825935	0.156694146	1.696463722	0.089798095	0.155454841	positive
PRJNA729271	stage_E14_vs_E10	ordinary_muscle_development	ENSGALG00010019289	FKBP9	-0.358355629	0.166280562	-2.155126396	0.031151945	0.06333952	negative
PRJNA729271	stage_E14_vs_E10	ordinary_muscle_development	ENSGALG00010019291	ADD2	-0.497923896	0.266870485	-1.865788553	0.062070955	0.113917256	negative
PRJNA729271	stage_E14_vs_E10	ordinary_muscle_development	ENSGALG00010019292	ENSGALG00010019292	-0.146980389	0.091323549	-1.609446742	0.107518692	0.180904402	negative
PRJNA729271	stage_E14_vs_E10	ordinary_muscle_development	ENSGALG00010019294	SMARCAL1	-0.150378212	0.09556832	-1.573515279	0.115599553	0.192003613	negative
PRJNA729271	stage_E14_vs_E10	ordinary_muscle_development	ENSGALG00010019295	MAP4K5	-0.182815727	0.129344213	-1.413404762	0.157536746	0.246784301	negative
PRJNA729271	stage_E14_vs_E10	ordinary_muscle_development	ENSGALG00010019296	ENSGALG00010019296	-0.190916383	0.101310677	-1.884464587	0.059502161	0.109730766	negative
PRJNA729271	stage_E14_vs_E10	ordinary_muscle_development	ENSGALG00010019297	SLC25A26	0.407184484	0.146439639	2.780561922	0.005426491	0.013978494	positive
PRJNA729271	stage_E14_vs_E10	ordinary_muscle_development	ENSGALG00010019298	PGAP6	0.699165998	0.091270297	7.660389184	1.8537E-14	2.49948E-13	positive
PRJNA729271	stage_E14_vs_E10	ordinary_muscle_development	ENSGALG00010019300	MESDC2	-0.124695862	0.065395329	-1.906800756	0.056546393	0.105162942	negative
PRJNA729271	stage_E14_vs_E10	ordinary_muscle_development	ENSGALG00010019302	USP34	-0.334779101	0.094409438	-3.546034253	0.000391075	0.001358408	negative
PRJNA729271	stage_E14_vs_E10	ordinary_muscle_development	ENSGALG00010019305	SNRNP27	0.141155315	0.117840834	1.197847213	0.230976503	0.335169127	positive
PRJNA729271	stage_E14_vs_E10	ordinary_muscle_development	ENSGALG00010019307	ATF3	0.047851062	0.487827568	0.098090115	0.921860737	0.94614256	positive
PRJNA729271	stage_E14_vs_E10	ordinary_muscle_development	ENSGALG00010019310	CRELD1	0.562701173	0.096687387	5.819799179	5.89184E-09	4.41812E-08	positive
PRJNA729271	stage_E14_vs_E10	ordinary_muscle_development	ENSGALG00010019312	KBTBD2	-0.113012849	0.071815843	-1.573647885	0.115568876	0.192000851	negative
PRJNA729271	stage_E14_vs_E10	ordinary_muscle_development	ENSGALG00010019313	MRPL28	0.175413244	0.098536274	1.780189548	0.075044945	0.133946125	positive
PRJNA729271	stage_E14_vs_E10	ordinary_muscle_development	ENSGALG00010019314	GKN2	1.92850243	1.199057377	1.608348747	0.10775882	0.181202331	positive
PRJNA729271	stage_E14_vs_E10	ordinary_muscle_development	ENSGALG00010019316	CLEC16A	-0.069958007	0.087510392	-0.799425127	0.424043946	0.538444648	negative
PRJNA729271	stage_E14_vs_E10	ordinary_muscle_development	ENSGALG00010019318	NOG2	-0.223964827	0.277664963	-0.806600964	0.419896418	0.534379402	negative
PRJNA729271	stage_E14_vs_E10	ordinary_muscle_development	ENSGALG00010019319	ACTR3	-0.184704458	0.065670449	-2.812596237	0.004914332	0.012825574	negative
PRJNA729271	stage_E14_vs_E10	ordinary_muscle_development	ENSGALG00010019322	EGR3	0.536512477	0.318981675	1.681953914	0.092577777	0.159491827	positive
PRJNA729271	stage_E14_vs_E10	ordinary_muscle_development	ENSGALG00010019323	LRIG1	0.715163337	0.118844629	6.017632786	1.76986E-09	1.41034E-08	positive
PRJNA729271	stage_E14_vs_E10	ordinary_muscle_development	ENSGALG00010019325	SLITRK4	-2.023793302	0.203732561	-9.933578095	2.97388E-23	7.89495E-22	negative
PRJNA729271	stage_E14_vs_E10	ordinary_muscle_development	ENSGALG00010019331	CEMIP	-0.853639448	0.311026267	-2.744589571	0.006058662	0.015393568	negative
PRJNA729271	stage_E14_vs_E10	ordinary_muscle_development	ENSGALG00010019334	ATL1	-0.111097239	0.12829777	-0.865932736	0.38652705	0.502620399	negative
PRJNA729271	stage_E14_vs_E10	ordinary_muscle_development	ENSGALG00010019335	HIPK3	0.88788589	0.115140502	7.711325537	1.24518E-14	1.69791E-13	positive
PRJNA729271	stage_E14_vs_E10	ordinary_muscle_development	ENSGALG00010019336	DEXI	0.449729373	0.239943423	1.874314235	0.060887127	0.112014076	positive
PRJNA729271	stage_E14_vs_E10	ordinary_muscle_development	ENSGALG00010019337	XPO1	-0.053077037	0.08380178	-0.63336408	0.526495916	0.634061009	negative
PRJNA729271	stage_E14_vs_E10	ordinary_muscle_development	ENSGALG00010019344	DNAJC24	-0.275081484	0.082802566	-3.322137202	0.000893308	0.002843948	negative
PRJNA729271	stage_E14_vs_E10	ordinary_muscle_development	ENSGALG00010019345	ENSGALG00010019345	0.233411987	0.184282944	1.266595716	0.205299885	0.305181195	positive
PRJNA729271	stage_E14_vs_E10	ordinary_muscle_development	ENSGALG00010019346	POU3F4	-0.607099405	0.493981737	-1.228991599	0.219074956	0.321386447	negative
PRJNA729271	stage_E14_vs_E10	ordinary_muscle_development	ENSGALG00010019350	SAV1	-0.244333894	0.088099778	-2.773376959	0.005547782	0.014259022	negative
PRJNA729271	stage_E14_vs_E10	ordinary_muscle_development	ENSGALG00010019355	ENTPD4	0.039426479	0.097298429	0.40521188	0.685321773	0.769758939	positive
PRJNA729271	stage_E14_vs_E10	ordinary_muscle_development	ENSGALG00010019356	AVL9	-0.147451515	0.093845155	-1.571221396	0.116131232	0.192630469	negative
PRJNA729271	stage_E14_vs_E10	ordinary_muscle_development	ENSGALG00010019365	ENSGALG00010019365	-0.148709746	0.175738309	-0.846199935	0.397441218	0.512855912	negative
PRJNA729271	stage_E14_vs_E10	ordinary_muscle_development	ENSGALG00010019371	TMEM87A	0.289384951	0.07665268	3.775275088	0.000159831	0.000605212	positive
PRJNA729271	stage_E14_vs_E10	ordinary_muscle_development	ENSGALG00010019373	DENND5A	0.034273791	0.060822157	0.563508307	0.573088805	0.674920558	positive
PRJNA729271	stage_E14_vs_E10	ordinary_muscle_development	ENSGALG00010019376	ENSGALG00010019376	-0.955751809	0.392522057	-2.434899627	0.014895921	0.03374748	negative
PRJNA729271	stage_E14_vs_E10	ordinary_muscle_development	ENSGALG00010019388	GPR75	-0.106754454	0.130564796	-0.817635819	0.413565168	0.528433252	negative
PRJNA729271	stage_E14_vs_E10	ordinary_muscle_development	ENSGALG00010019390	TVP23A	-0.220973348	0.202777148	-1.089734966	0.275829909	0.386257804	negative
PRJNA729271	stage_E14_vs_E10	ordinary_muscle_development	ENSGALG00010019391	GANC	-0.043193154	0.134718419	-0.320618028	0.748499874	0.819510966	negative
PRJNA729271	stage_E14_vs_E10	ordinary_muscle_development	ENSGALG00010019392	NIN	0.236861725	0.098726993	2.399158708	0.016432791	0.03669028	positive
PRJNA729271	stage_E14_vs_E10	ordinary_muscle_development	ENSGALG00010019398	SLC35F5	0.135560186	0.120372851	1.126169112	0.260093945	0.368313964	positive
PRJNA729271	stage_E14_vs_E10	ordinary_muscle_development	ENSGALG00010019399	CD93	0.545286132	0.151131114	3.608033574	0.000308527	0.001101772	positive
PRJNA729271	stage_E14_vs_E10	ordinary_muscle_development	ENSGALG00010019403	PACC1	-0.277345991	0.10270153	-2.700504968	0.00692343	0.01726562	negative
PRJNA729271	stage_E14_vs_E10	ordinary_muscle_development	ENSGALG00010019411	LSM5	-0.366343998	0.156223945	-2.344992618	0.019027456	0.041592969	negative
PRJNA729271	stage_E14_vs_E10	ordinary_muscle_development	ENSGALG00010019416	GPR39	0.344374772	0.460780906	0.747372054	0.45483901	0.567912089	positive
PRJNA729271	stage_E14_vs_E10	ordinary_muscle_development	ENSGALG00010019418	ZNF703	0.341526963	0.227380877	1.502003893	0.133096103	0.21533431	positive
PRJNA729271	stage_E14_vs_E10	ordinary_muscle_development	ENSGALG00010019422	IMMP1L	0.527484834	0.137728377	3.829892192	0.000128199	0.000495666	positive
PRJNA729271	stage_E14_vs_E10	ordinary_muscle_development	ENSGALG00010019423	LYPD1	0.369218416	0.177426538	2.080964999	0.03743711	0.074046973	positive
PRJNA729271	stage_E14_vs_E10	ordinary_muscle_development	ENSGALG00010019426	GRM7	-0.523610735	0.24786608	-2.112474339	0.034645785	0.069382516	negative
PRJNA729271	stage_E14_vs_E10	ordinary_muscle_development	ENSGALG00010019430	ENSGALG00010019430	-0.405672985	0.404960833	-1.001758571	0.316460211	0.430429835	negative
PRJNA729271	stage_E14_vs_E10	ordinary_muscle_development	ENSGALG00010019441	LMCD1	-0.29109794	0.143767724	-2.024779491	0.042890012	0.083184117	negative
PRJNA729271	stage_E14_vs_E10	ordinary_muscle_development	ENSGALG00010019443	NCKAP5	-0.201624787	0.13176768	-1.53015358	0.125978718	0.205544971	negative

PRJNA729271	stage_E14_vs_E10	ordinary_muscle_development	ENSGALG00010019451	CAV3	3.402584199	0.142860995	23.81744713	2.2029E-125	6.3862E-122	positive
PRJNA729271	stage_E14_vs_E10	ordinary_muscle_development	ENSGALG00010019458	ELP4	-0.084901509	0.098847926	-0.85891038	0.390389962	0.505964101	negative
PRJNA729271	stage_E14_vs_E10	ordinary_muscle_development	ENSGALG00010019461	MGAT5	-0.147524413	0.070007903	-2.107253711	0.035095593	0.070081824	negative
PRJNA729271	stage_E14_vs_E10	ordinary_muscle_development	ENSGALG00010019470	TGFB2	-0.061378729	0.199917648	-0.307020066	0.758828099	0.828191649	negative
PRJNA729271	stage_E14_vs_E10	ordinary_muscle_development	ENSGALG00010019471	NUBP1	0.338888344	0.091169905	3.71710757	0.000201517	0.000748395	positive
PRJNA729271	stage_E14_vs_E10	ordinary_muscle_development	ENSGALG00010019472	CAPN3	4.142891977	0.256559247	16.14789577	1.17502E-58	2.6203E-56	positive
PRJNA729271	stage_E14_vs_E10	ordinary_muscle_development	ENSGALG00010019473	OXTR	-1.224053014	0.477200566	-2.565070329	0.010315489	0.024439853	negative
PRJNA729271	stage_E14_vs_E10	ordinary_muscle_development	ENSGALG00010019474	TMEM163	-0.963306735	0.53311327	-1.806945708	0.070770745	0.127336389	negative
PRJNA729271	stage_E14_vs_E10	ordinary_muscle_development	ENSGALG00010019477	NCAPH	-0.108700394	0.172875357	-0.628779001	0.529493749	0.637074086	negative
PRJNA729271	stage_E14_vs_E10	ordinary_muscle_development	ENSGALG00010019481	PYGL	0.137614597	0.097330039	1.413896463	0.157392305	0.246691334	positive
PRJNA729271	stage_E14_vs_E10	ordinary_muscle_development	ENSGALG00010019483	SCUBE2	-1.70943955	0.259829042	-6.579093464	4.73325E-11	4.5587E-10	negative
PRJNA729271	stage_E14_vs_E10	ordinary_muscle_development	ENSGALG00010019488	TEKT5	-0.979395083	1.072074623	-0.913551223	0.360952706	0.477155447	negative
PRJNA729271	stage_E14_vs_E10	ordinary_muscle_development	ENSGALG00010019495	EMP2	-0.561961191	0.197009359	-2.85245936	0.004338236	0.011486472	negative
PRJNA729271	stage_E14_vs_E10	ordinary_muscle_development	ENSGALG00010019498	ENSGALG00010019498	-0.352755518	0.269810652	-1.307418794	0.191070518	0.288406452	negative
PRJNA729271	stage_E14_vs_E10	ordinary_muscle_development	ENSGALG00010019509	ZNF106	1.892005481	0.22215689	8.516528472	1.64407E-17	2.88509E-16	positive
PRJNA729271	stage_E14_vs_E10	ordinary_muscle_development	ENSGALG00010019515	ENSGALG00010019515	-0.174612607	0.108494735	-1.60941087	0.10752653	0.180904402	negative
PRJNA729271	stage_E14_vs_E10	ordinary_muscle_development	ENSGALG00010019520	CD6	1.233170141	1.144364866	1.077602238	0.281211303	0.392338472	positive
PRJNA729271	stage_E14_vs_E10	ordinary_muscle_development	ENSGALG00010019524	HHIPL2	-0.509209308	0.39021811	-1.304935099	0.191914948	0.289229276	negative
PRJNA729271	stage_E14_vs_E10	ordinary_muscle_development	ENSGALG00010019529	SNAP23	-0.122705	0.066886098	-1.834536671	0.066574369	0.120835898	negative
PRJNA729271	stage_E14_vs_E10	ordinary_muscle_development	ENSGALG00010019534	PPP2R5A	0.415187208	0.104413665	3.976368483	6.99757E-05	0.00028364	positive
PRJNA729271	stage_E14_vs_E10	ordinary_muscle_development	ENSGALG00010019540	UNC5D	-1.803219977	0.378545089	-4.763554007	1.90213E-06	1.00589E-05	negative
PRJNA729271	stage_E14_vs_E10	ordinary_muscle_development	ENSGALG00010019546	HSD17B12	-0.004873305	0.076030915	-0.064096364	0.948893497	0.964599814	negative
PRJNA729271	stage_E14_vs_E10	ordinary_muscle_development	ENSGALG00010019557	TRIM9	-0.830585412	0.329064387	-2.524081747	0.011600095	0.02714617	negative
PRJNA729271	stage_E14_vs_E10	ordinary_muscle_development	ENSGALG00010019572	NFKBIE	-0.187937239	0.183574698	-1.02376439	0.305946574	0.419698071	negative
PRJNA729271	stage_E14_vs_E10	ordinary_muscle_development	ENSGALG00010019577	MXD1	-0.385559567	0.187681262	-2.054331711	0.039943598	0.078209165	negative
PRJNA729271	stage_E14_vs_E10	ordinary_muscle_development	ENSGALG00010019580	CD5	0.8867143	0.48459112	1.829819542	0.067276928	0.121912622	positive
PRJNA729271	stage_E14_vs_E10	ordinary_muscle_development	ENSGALG00010019581	SLC35B2	-0.24586425	0.132436096	-1.856474614	0.063385938	0.11593428	negative
PRJNA729271	stage_E14_vs_E10	ordinary_muscle_development	ENSGALG00010019584	RAD18	-0.206339777	0.113409171	-1.819427606	0.068846218	0.12438314	negative
PRJNA729271	stage_E14_vs_E10	ordinary_muscle_development	ENSGALG00010019585	PDE1C	-1.491944958	0.212720279	-7.013647043	2.32185E-12	2.55738E-11	negative
PRJNA729271	stage_E14_vs_E10	ordinary_muscle_development	ENSGALG00010019586	GLYR1	0.016730772	0.089929538	0.186043117	0.852410949	0.897747345	positive
PRJNA729271	stage_E14_vs_E10	ordinary_muscle_development	ENSGALG00010019587	CCNT2	-0.193774424	0.127000231	-1.525780089	0.127064641	0.206967296	negative
PRJNA729271	stage_E14_vs_E10	ordinary_muscle_development	ENSGALG00010019588	CHMP7	0.33301088	0.092612163	3.595757491	0.000323449	0.001149116	positive
PRJNA729271	stage_E14_vs_E10	ordinary_muscle_development	ENSGALG00010019591	ENSGALG00010019591	-0.354189465	0.227008311	-1.560248886	0.118701077	0.195920305	negative
PRJNA729271	stage_E14_vs_E10	ordinary_muscle_development	ENSGALG00010019592	ALKBH3	0.005217056	0.130623855	0.039939531	0.968141335	0.978469436	positive
PRJNA729271	stage_E14_vs_E10	ordinary_muscle_development	ENSGALG00010019594	TMX1	0.233536722	0.310963478	0.75101013	0.45264656	0.565661857	positive
PRJNA729271	stage_E14_vs_E10	ordinary_muscle_development	ENSGALG00010019595	GRIN2A	-0.64557059	0.218931518	-2.948732996	0.003190795	0.008777866	negative
PRJNA729271	stage_E14_vs_E10	ordinary_muscle_development	ENSGALG00010019598	KCNAB2	-0.404232359	0.131524687	-3.073433336	0.00211611	0.006112596	negative
PRJNA729271	stage_E14_vs_E10	ordinary_muscle_development	ENSGALG00010019600	MAP3K19	-0.192906756	0.201938482	-0.955274864	0.339438715	0.454487426	negative
PRJNA729271	stage_E14_vs_E10	ordinary_muscle_development	ENSGALG00010019602	SLC29A1	0.478746237	0.194348052	2.463344666	0.013764752	0.03150483	positive
PRJNA729271	stage_E14_vs_E10	ordinary_muscle_development	ENSGALG00010019610	CORO7	0.564335218	0.099077267	5.695910212	1.22715E-08	8.83637E-08	positive
PRJNA729271	stage_E14_vs_E10	ordinary_muscle_development	ENSGALG00010019613	FRMD6	-0.35826651	0.122892995	-2.915272025	0.003553786	0.009628436	negative
PRJNA729271	stage_E14_vs_E10	ordinary_muscle_development	ENSGALG00010019614	MICALL2	-0.280869312	0.157429453	-1.784096347	0.074408011	0.132891094	negative
PRJNA729271	stage_E14_vs_E10	ordinary_muscle_development	ENSGALG00010019617	ANO3	-0.899278438	0.141268479	-6.365740227	1.9435E-10	1.73895E-09	negative
PRJNA729271	stage_E14_vs_E10	ordinary_muscle_development	ENSGALG00010019618	NPHP4	0.016904729	0.099166762	0.170467694	0.864642339	0.906807807	positive
PRJNA729271	stage_E14_vs_E10	ordinary_muscle_development	ENSGALG00010019621	ENSGALG00010019621	1.865798863	0.151029265	12.35388959	4.6412E-35	2.56772E-33	positive
PRJNA729271	stage_E14_vs_E10	ordinary_muscle_development	ENSGALG00010019622	C2orf42	0.355711639	0.141502491	2.513818928	0.011943174	0.027859077	positive
PRJNA729271	stage_E14_vs_E10	ordinary_muscle_development	ENSGALG00010019624	SRGAP3	0.156387232	0.129115684	1.211217936	0.225811891	0.328926073	positive
PRJNA729271	stage_E14_vs_E10	ordinary_muscle_development	ENSGALG00010019625	INTS1	0.056958116	0.075730382	0.752117118	0.451980631	0.565024514	positive
PRJNA729271	stage_E14_vs_E10	ordinary_muscle_development	ENSGALG00010019630	AJAP1	-1.284768966	0.373539744	-3.439443821	0.000582911	0.001942812	negative
PRJNA729271	stage_E14_vs_E10	ordinary_muscle_development	ENSGALG00010019634	GNG2	-0.517243351	0.261369924	-1.97897043	0.047819336	0.09138316	negative
PRJNA729271	stage_E14_vs_E10	ordinary_muscle_development	ENSGALG00010019635	LRRC57	0.058323725	0.078097972	0.746802047	0.45518306	0.568292718	positive
PRJNA729271	stage_E14_vs_E10	ordinary_muscle_development	ENSGALG00010019640	MGRN1	0.077468777	0.115754514	0.669250591	0.503335639	0.61263331	positive
PRJNA729271	stage_E14_vs_E10	ordinary_muscle_development	ENSGALG00010019641	CAPN11	0.815687467	0.081559087	10.00118434	1.50585E-23	4.05712E-22	positive
PRJNA729271	stage_E14_vs_E10	ordinary_muscle_development	ENSGALG00010019643	RAB3GAP1	0.019729938	0.069801528	0.282657685	0.777439256	0.842351773	positive
PRJNA729271	stage_E14_vs_E10	ordinary_muscle_development	ENSGALG00010019645	ENSGALG00010019645	0.076227706	0.167681533	0.454598099	0.649398403	0.740930831	positive
PRJNA729271	stage_E14_vs_E10	ordinary_muscle_development	ENSGALG00010019646	VPS37C	0.105586768	0.126428833	0.835147851	0.403634468	0.518677448	positive
PRJNA729271	stage_E14_vs_E10	ordinary_muscle_development	ENSGALG00010019648	PRDM16	-1.45600145	0.187744979	-7.755208454	8.81985E-15	1.21756E-13	negative
PRJNA729271	stage_E14_vs_E10	ordinary_muscle_development	ENSGALG00010019649	TMEM63B	0.175113315	0.07485294	2.339431349	0.019313121	0.042141154	positive
PRJNA729271	stage_E14_vs_E10	ordinary_muscle_development	ENSGALG00010019650	KBTBD8	0.104851231	0.086872763	1.206951721	0.227450737	0.330847811	positive
PRJNA729271	stage_E14_vs_E10	ordinary_muscle_development	ENSGALG00010019652	TNFRSF10B	-0.219939516	0.324003966	-0.678817358	0.497253594	0.606707984	negative
PRJNA729271	stage_E14_vs_E10	ordinary_muscle_development	ENSGALG00010019655	RTRAF	0.214578662	0.098247758	2.184056569	0.028958092	0.059606296	positive
PRJNA729271	stage_E14_vs_E10	ordinary_muscle_development	ENSGALG00010019657	PPP1R17	-2.012202136	0.270417162	-7.441103677	9.98475E-14	1.26401E-12	negative
PRJNA729271	stage_E14_vs_E10	ordinary_muscle_development	ENSGALG00010019659	MRPL14	0.107613885	0.125268053	0.859068873	0.390302519	0.505896004	positive
PRJNA729271	stage_E14_vs_E10	ordinary_muscle_development	ENSGALG00010019660	ENSGALG00010019660	2.945875509	0.436517889	6.748579112	1.493E-11	1.50704E-10	positive
PRJNA729271	stage_E14_vs_E10	ordinary_muscle_development	ENSGALG00010019667	ITPRID1	0.57934395	0.601514147	0.963142685	0.335475923	0.450753013	positive
PRJNA729271	stage_E14_vs_E10	ordinary_muscle_development	ENSGALG00010019670	ENSGALG00010019670	-0.420563448	0.114406005	-3.676060968	0.000236863	0.000864818	negative
PRJNA729271	stage_E14_vs_E10	ordinary_muscle_development	ENSGALG00010019673	NID2	1.024622339	0.13241315	7.738070863	1.00937E-14	1.38944E-13	positive
PRJNA729271	stage_E14_vs_E10	ordinary_muscle_development	ENSGALG00010019675	MAFK	0.925504597	0.137148472	6.748194728	1.49696E-11	1.50893E-10	positive
PRJNA729271	stage_E14_vs_E10	ordinary_muscle_development	ENSGALG00010019679	RHOBTB2	-0.802797669	0.164399169	-4.883222185	1.04366E-06	5.75423E-06	negative
PRJNA729271	stage_E14_vs_E10	ordinary_muscle_development	ENSGALG00010019680	CCDC34	0.142585839	0.197614975	0.721533574	0.470581291	0.582948109	positive
PRJNA729271	stage_E14_vs_E10	ordinary_muscle_development	ENSGALG00010019682	TMEM158	-0.291298435	0.346263806	-0.841261576	0.400201411	0.515499818	negative
PRJNA729271	stage_E14_vs_E10	ordinary_muscle_development	ENSGALG00010019683	TMEM184A	1.147079626	0.266265035	4.308037012	1.6471E-05	7.43992E-05	positive
PRJNA729271	stage_E14_vs_E10	ordinary_muscle_development	ENSGALG00010019684	PIGQ	0.194045592	0.123127628	1.575971173	0.115032447	0.191311337	positive
PRJNA729271	stage_E14_vs_E10	ordinary_muscle_development	ENSGALG00010019690	ENSGALG00010019690	0.399034996	0.088807125	4.493276824	7.01355E-06	3.38083E-05	positive
PRJNA729271	stage_E14_vs_E10	ordinary_muscle_development	ENSGALG00010019692	MMEL1	-1.384755407	0.303913671	-4.556410384	5.20352E-06	2.55938E-05	negative
PRJNA729271	stage_E14_vs_E10	ordinary_muscle_development	ENSGALG00010019693	HAUS2	-0.277945513	0.116462494	-2.386566734	0.017006521	0.037812466	negative
PRJNA729271	stage_E14_vs_E10	ordinary_muscle_development	ENSGALG00010019695	PGA5	-15.21873674	2.525620648	-6.025741335	1.68336E-09	1.34437E-08	negative
PRJNA729271	stage_E14_vs_E10	ordinary_muscle_development	ENSGALG00010019697	EIF4EBP1	0.804093353	0.145308648	5.53369233	3.13559E-08	2.15609E-07	positive
PRJNA729271	stage_E14_vs_E10	ordinary_muscle_development	ENSGALG00010019700	HES5B	-0.26823635	0.243236233	-1.102781224	0.270122184	0.379953523	negative
PRJNA729271	stage_E14_vs_E10	ordinary_muscle_development	ENSGALG00010019706	NPRL3	0.174522788	0.097535395	1.789327748	0.073562048	0.131721048	positive
PRJNA729271	stage_E14_vs_E10	ordinary_muscle_development	ENSGALG00010019713	NUDT14	0.435251735	0.113112181	3.847965194	0.000119103	0.000462965	positive
PRJNA729271	stage_E14_vs_E10	ordinary_muscle_development	ENSGALG00010019716	VEGFA	0.629497849	0.129574808	4.85818083	1.18469E-06	6.49216E-06	positive
PRJNA729271	stage_E14_vs_E10	ordinary_muscle_development	ENSGALG00010019717	ZRANB3	-0.1886279	0.136985586	-1.376990856	0.168515075	0.260574569	negative
PRJNA729271	stage_E14_vs_E10	ordinary_muscle_development	ENSGALG00010019724	HES5A	-0.733526115	0.230202561	-3.186437675	0.001440365	0.004359593	negative
PRJNA729271	stage_E14_vs_E10	ordinary_muscle_development	ENSGALG00010019726	PEBP4	0.944150622	0.246401201	3.831761454	0.000127229	0.000492308	positive
PRJNA729271	stage_E14_vs_E10	ordinary_muscle_development	ENSGALG00010019731	PANK4	0.017205391	0.134465763	0.127953692	0.89818562	0.930130197	positive
PRJNA729271	stage_E14_vs_E10	ordinary_muscle_development	ENSGALG00010019732	HBA1	0.489074729	0.403462435	1.212193967	0.22543814	0.328579773	positive
PRJNA729271	stage_E14_vs_E10	ordinary_muscle_development	ENSGALG00010019735	ENSGALG00010019735	1.153537267	0.171095702	6.742058688	1.56158E-11	1.56861E-10	positive
PRJNA729271	stage_E14_vs_E10	ordinary_muscle_development	ENSGALG00010019738	ADRA2A	-0.235201912	0.351307208	-0.669504944	0.503173428	0.612487306	negative
PRJNA729271	stage_E14_vs_E10	ordinary_muscle_development	ENSGALG00010019743	KCNU1	-1.294627943	0.689431325	-1.877820018	0.060405791	0.111199129	negative
PRJNA729271	stage_E14_vs_E10	ordinary_muscle_development	ENSGALG00010019744	KBP	-1.1221547	0.472044521	-2.377222167	0.017443578	0.038661264	negative
PRJNA729271	stage_E14_vs_E10	ordinary_muscle_development	ENSGALG00010019752	GPAM	-0.58830205	0.167443345	-3.513439433	0.000442345	0.001516866	negative
PRJNA729271	stage_E14_vs_E10	ordinary_muscle_development	ENSGALG00010019761	MRPS18A	0.237139167	0.086562129	2.739525595	0.006152792	0.015597189	positive
PRJNA729271	stage_E14_vs_E10	ordinary_muscle_development	ENSGALG00010019767	SUCLG2	-0.387246584	0.076797626	-5.042429087	4.59659E-07	2.66937E-06	negative
PRJNA729271	stage_E14_vs_E10	ordinary_muscle_development	ENSGALG00010019771	VWCE	-1.822971662	0.356964448	-5.106871772	3.27536E-07	1.94416E-06	negative
PRJNA729271	stage_E14_vs_E10	ordinary_muscle_development	ENSGALG00010019772	ENSGALG00010019772	-0.272922858	0.164782239	-1.656263809	0.097668406	0.166710262	negative
PRJNA729271	stage_E14_vs_E10	ordinary_muscle_development	ENSGALG00010019778	ABHD17C	-0.010703892	0.129741184	-0.082501884	0.93424762	0.954535588	negative
PRJNA729271	stage_E14_vs_E10	ordinary_muscle_development	ENSGALG00010019785	ACSL5	-0.232468977	0.226550857	-1.0261227	0.304833752	0.418543642	negative
PRJNA729271	stage_E14_vs_E10	ordinary_muscle_development	ENSGALG00010019786	R3HDM1	-0.069484074	0.087373026	-0.795257718	0.426463603	0.540916165	negative
PRJNA729271	stage_E14_vs_E10	ordinary_muscle_development	ENSGALG00010019789	ARNT2	-0.966228435	0.17058656	-5.66415335	1.47752E-08	1.05501E-07	negative
PRJNA729271	stage_E14_vs_E10	ordinary_muscle_development	ENSGALG00010019794	RSPH9	-1.137703177	0.476217255	-2.389042323	0.016892356	0.037617868	negative
PRJNA729271	stage_E14_vs_E10	ordinary_muscle_development	ENSGALG00010019799	DOK2	0.300293195	0.268365819	1.118969606	0.263153106	0.371810534	positive
PRJNA729271	stage_E14_vs_E10	ordinary_muscle_development	ENSGALG00010019800	EXT2	-0.160290081	0.05949694	-2.694089518	0.007058121	0.017556678	negative
PRJNA729271	stage_E14_vs_E10	ordinary_muscle_development	ENSGALG00010019801	FIBIN	-2.363769229	0.186097183	-12.70180017	5.7785E-37	3.70617E-35	negative
PRJNA729271	stage_E14_vs_E10	ordinary_muscle_development	ENSGALG00010019803	FAH	-1.50032299	0.489694222	-3.063795575	0.002185482	0.006289172	negative
PRJNA729271	stage_E14_vs_E10	ordinary_muscle_development	ENSGALG00010019804	ELFN1	-0.98291983	0.268959017	-3.654533842	0.00025765	0.000932416	negative
PRJNA729271	stage_E14_vs_E10	ordinary_muscle_development	ENSGALG00010019807	ZDHHC6	0.107301317	0.078336331	1.369751622	0.170764448	0.263266399	positive
PRJNA729271	stage_E14_vs_E10	ordinary_muscle_development	ENSGALG00010019810	ZFAND6	0.098955017	0.124682296	0.793657319	0.427394954	0.541765619	positive
PRJNA729271	stage_E14_vs_E10	ordinary_muscle_development	ENSGALG00010019811	UBXN4	0.868597507	0.110339429	7.872050052	3.48877E-15	4.99207E-14	positive
PRJNA729271	stage_E14_vs_E10	ordinary_muscle_development	ENSGALG00010019813	ENSGALG00010019813	1.719931209	0.222318374	7.73634305	1.02317E-14	1.4071E-13	positive
PRJNA729271	stage_E14_vs_E10	ordinary_muscle_development	ENSGALG00010019816	ENSGALG00010019816	1.488029551	0.743749667	2.000712897	0.045423339	0.087496518	positive
PRJNA729271	stage_E14_vs_E10	ordinary_muscle_development	ENSGALG00010019820	BBOX1	-0.610059155	0.154394814	-3.951293056	7.77301E-05	0.000312191	negative
PRJNA729271	stage_E14_vs_E10	ordinary_muscle_development	ENSGALG00010019823	LCT	0.042751393	0.194121774	0.220229768	0.825692214	0.878043204	positive
PRJNA729271	stage_E14_vs_E10	ordinary_muscle_development	ENSGALG00010019825	BCL2A1	0.248896086	0.292984757	0.849518893	0.395592622	0.511153063	positive
PRJNA729271	stage_E14_vs_E10	ordinary_muscle_development	ENSGALG00010019830	JAG2	1.214355428	0.097540539	12.44975109	1.40282E-35	8.13355E-34	positive
PRJNA729271	stage_E14_vs_E10	ordinary_muscle_development	ENSGALG00010019832	MAD2L1BP	-0.333672041	0.132794817	-2.512688745	0.011981499	0.027942415	negative
PRJNA729271	stage_E14_vs_E10	ordinary_muscle_development	ENSGALG00010019833	MTHFS	-0.039611972	0.12468586	-0.317694177	0.750716928	0.821503992	negative
PRJNA729271	stage_E14_vs_E10	ordinary_muscle_development	ENSGALG00010019834	PLCH2	-1.080284723	0.228914892	-4.719154423	2.36827E-06	1.23261E-05	negative
PRJNA729271	stage_E14_vs_E10	ordinary_muscle_development	ENSGALG00010019836	MINAR1	-1.081345715	0.519189376	-2.082757785	0.037273307	0.073773643	negative
PRJNA729271	stage_E14_vs_E10	ordinary_muscle_development	ENSGALG00010019838	ALX4	-1.420401965	0.169760403	-8.367098228	5.90545E-17	9.94186E-16	negative
PRJNA729271	stage_E14_vs_E10	ordinary_muscle_development	ENSGALG00010019843	MCM6	-0.159737895	0.108491538	-1.472353492	0.140925445	0.225614571	negative
PRJNA729271	stage_E14_vs_E10	ordinary_muscle_development	ENSGALG00010019846	ENSGALG00010019846	-0.003953159	0.071073956	-0.055620354	0.95564425	0.969217982	negative
PRJNA729271	stage_E14_vs_E10	ordinary_muscle_development	ENSGALG00010019848	MAD1L1	0.220258272	0.123151473	1.788515118	0.073692939	0.131873969	positive
PRJNA729271	stage_E14_vs_E10	ordinary_muscle_development	ENSGALG00010019854	CCDC14	-0.48267338	0.145236342	-3.323365038	0.000889384	0.002833948	negative
PRJNA729271	stage_E14_vs_E10	ordinary_muscle_development	ENSGALG00010019855	TTC7A	0.013103139	0.125029342	0.104800509	0.916534106	0.941876063	positive
PRJNA729271	stage_E14_vs_E10	ordinary_muscle_development	ENSGALG00010019859	BIN3	0.336767788	0.134068635	2.511905852	0.012008112	0.027988034	positive
PRJNA729271	stage_E14_vs_E10	ordinary_muscle_development	ENSGALG00010019865	ENSGALG00010019865	0.038223378	0.134527761	0.284130037	0.776310729	0.841569368	positive
PRJNA729271	stage_E14_vs_E10	ordinary_muscle_development	ENSGALG00010019867	PEX11A	0.16035448	0.371036069	0.4321803	0.665610376	0.753928452	positive
PRJNA729271	stage_E14_vs_E10	ordinary_muscle_development	ENSGALG00010019871	TAFA1	-2.582569587	0.550428548	-4.69192522	2.70646E-06	1.39626E-05	negative
PRJNA729271	stage_E14_vs_E10	ordinary_muscle_development	ENSGALG00010019872	VTI1A	0.230444467	0.107920468	2.135317534	0.032735082	0.066058055	positive
PRJNA729271	stage_E14_vs_E10	ordinary_muscle_development	ENSGALG00010019873	LGR4	-0.358287484	0.097029538	-3.692560965	0.000222007	0.000814682	negative

PRJNA729271	stage_E14_vs_E10	ordinary_muscle_development	ENSGALG00010019875	PLEKHH1	-2.224602216	0.265897126	-8.366401871	5.94043E-17	9.98917E-16	negative
PRJNA729271	stage_E14_vs_E10	ordinary_muscle_development	ENSGALG00010019876	MRM2	0.137691259	0.223471249	0.616147535	0.537797151	0.644298678	positive
PRJNA729271	stage_E14_vs_E10	ordinary_muscle_development	ENSGALG00010019878	MCFD2	0.072956457	0.149875106	0.486781691	0.626413047	0.72114807	positive
PRJNA729271	stage_E14_vs_E10	ordinary_muscle_development	ENSGALG00010019891	PIGH	-0.135100383	0.101854436	-1.32640647	0.184705097	0.280580631	negative
PRJNA729271	stage_E14_vs_E10	ordinary_muscle_development	ENSGALG00010019896	DDB1	0.125419063	0.090629535	1.383865226	0.166399726	0.257771084	positive
PRJNA729271	stage_E14_vs_E10	ordinary_muscle_development	ENSGALG00010019898	NUDT1	-0.451528026	0.18417659	-2.451603796	0.014222116	0.032423651	negative
PRJNA729271	stage_E14_vs_E10	ordinary_muscle_development	ENSGALG00010019899	ENSGALG00010019899	0.126018894	0.443448587	0.284179265	0.776273005	0.841569368	positive
PRJNA729271	stage_E14_vs_E10	ordinary_muscle_development	ENSGALG00010019905	ENSGALG00010019905	0.303915909	0.124009593	2.450745163	0.014256084	0.032490871	positive
PRJNA729271	stage_E14_vs_E10	ordinary_muscle_development	ENSGALG00010019906	ENSGALG00010019906	1.095359726	0.232408344	4.713082616	2.43997E-06	1.26719E-05	positive
PRJNA729271	stage_E14_vs_E10	ordinary_muscle_development	ENSGALG00010019910	ENSGALG00010019910	0.276977066	0.236295072	1.172166069	0.241130388	0.347035624	positive
PRJNA729271	stage_E14_vs_E10	ordinary_muscle_development	ENSGALG00010019912	ARG2	-1.672010144	0.184451214	-9.064782524	1.24853E-19	2.59648E-18	negative
PRJNA729271	stage_E14_vs_E10	ordinary_muscle_development	ENSGALG00010019916	NOC3L	-0.097882057	0.112900882	-0.866973358	0.385956607	0.502103843	negative
PRJNA729271	stage_E14_vs_E10	ordinary_muscle_development	ENSGALG00010019919	LIN7C	-0.34239457	0.10696612	-3.200962777	0.001369692	0.004169191	negative
PRJNA729271	stage_E14_vs_E10	ordinary_muscle_development	ENSGALG00010019921	CALM2	0.012862094	0.112630155	0.114197604	0.909081151	0.937340396	positive
PRJNA729271	stage_E14_vs_E10	ordinary_muscle_development	ENSGALG00010019925	RDH11	-0.814829382	0.137120803	-5.942419841	2.80845E-09	2.19453E-08	negative
PRJNA729271	stage_E14_vs_E10	ordinary_muscle_development	ENSGALG00010019926	KIF7	-0.3372948	0.098435299	-3.426563465	0.000611271	0.002024302	negative
PRJNA729271	stage_E14_vs_E10	ordinary_muscle_development	ENSGALG00010019931	ZFYVE26	-0.108897059	0.13491724	-0.807139689	0.419586008	0.534062099	negative
PRJNA729271	stage_E14_vs_E10	ordinary_muscle_development	ENSGALG00010019932	KALRN	-0.525978494	0.116727915	-4.506021504	6.60544E-06	3.19792E-05	negative
PRJNA729271	stage_E14_vs_E10	ordinary_muscle_development	ENSGALG00010019934	TKFC	-0.106935206	0.150367161	-0.711160641	0.476984695	0.589145069	negative
PRJNA729271	stage_E14_vs_E10	ordinary_muscle_development	ENSGALG00010019935	ENSGALG00010019935	3.424441758	1.237255536	2.767772428	0.005644085	0.014467023	positive
PRJNA729271	stage_E14_vs_E10	ordinary_muscle_development	ENSGALG00010019938	TAFA4	-3.000480943	0.33744373	-8.891796387	6.01288E-19	1.17779E-17	negative
PRJNA729271	stage_E14_vs_E10	ordinary_muscle_development	ENSGALG00010019947	ENSGALG00010019947	-0.582869871	0.283059999	-2.059174283	0.039477546	0.077453577	negative
PRJNA729271	stage_E14_vs_E10	ordinary_muscle_development	ENSGALG00010019948	TICRR	-0.106485838	0.125448187	-0.848843182	0.395968558	0.511456448	negative
PRJNA729271	stage_E14_vs_E10	ordinary_muscle_development	ENSGALG00010019954	CYB561A3	0.14772798	0.130456689	1.132391001	0.257470087	0.365598443	positive
PRJNA729271	stage_E14_vs_E10	ordinary_muscle_development	ENSGALG00010019956	SLC29A3	-0.527776084	0.169749473	-3.109147112	0.001876283	0.005510019	negative
PRJNA729271	stage_E14_vs_E10	ordinary_muscle_development	ENSGALG00010019961	HTATSF1	-0.321056137	0.068723855	-4.67168405	2.9874E-06	1.53012E-05	negative
PRJNA729271	stage_E14_vs_E10	ordinary_muscle_development	ENSGALG00010019962	SNX8	0.101059718	0.142083111	0.711271855	0.476915789	0.58913366	positive
PRJNA729271	stage_E14_vs_E10	ordinary_muscle_development	ENSGALG00010019967	CEP170B	-0.372245385	0.106465958	-3.496379429	0.000471618	0.001609251	negative
PRJNA729271	stage_E14_vs_E10	ordinary_muscle_development	ENSGALG00010019968	RHCG	2.675203593	0.809636496	3.304203313	0.000952467	0.00301573	positive
PRJNA729271	stage_E14_vs_E10	ordinary_muscle_development	ENSGALG00010019972	PPP1CB	0.090061847	0.091376159	0.985616473	0.324321351	0.438939121	positive
PRJNA729271	stage_E14_vs_E10	ordinary_muscle_development	ENSGALG00010019975	ENSGALG00010019975	-0.533281326	0.197715019	-2.697222137	0.006992061	0.017411085	negative
PRJNA729271	stage_E14_vs_E10	ordinary_muscle_development	ENSGALG00010019977	ZMIZ1	-0.34142986	0.087416004	-3.905804951	9.39122E-05	0.000371225	negative
PRJNA729271	stage_E14_vs_E10	ordinary_muscle_development	ENSGALG00010019979	ENSGALG00010019979	0.482086769	0.261304519	1.844923199	0.065048711	0.118576092	positive
PRJNA729271	stage_E14_vs_E10	ordinary_muscle_development	ENSGALG00010019982	PLCE1	0.395646743	0.112078977	3.530070953	0.000415448	0.001433109	positive
PRJNA729271	stage_E14_vs_E10	ordinary_muscle_development	ENSGALG00010019985	UMPS	-0.497767043	0.082569504	-6.028461127	1.65528E-09	1.32413E-08	negative
PRJNA729271	stage_E14_vs_E10	ordinary_muscle_development	ENSGALG00010019989	ENSGALG00010019989	0.14253611	0.280795693	0.507615014	0.611723359	0.70867408	positive
PRJNA729271	stage_E14_vs_E10	ordinary_muscle_development	ENSGALG00010019990	MARCHF8	0.76859878	0.105429734	7.290151941	3.09606E-13	3.74915E-12	positive
PRJNA729271	stage_E14_vs_E10	ordinary_muscle_development	ENSGALG00010019991	EIF3B	0.16393768	0.075097459	2.182999036	0.029035881	0.059740965	positive
PRJNA729271	stage_E14_vs_E10	ordinary_muscle_development	ENSGALG00010019992	CHAC2	-0.448959359	0.135490347	-3.313589271	0.000921067	0.00292379	negative
PRJNA729271	stage_E14_vs_E10	ordinary_muscle_development	ENSGALG00010019994	PLD4	-0.691587047	0.757774974	-0.912654905	0.361424068	0.477691425	negative
PRJNA729271	stage_E14_vs_E10	ordinary_muscle_development	ENSGALG00010019996	ITGB5	0.195076883	0.124145252	1.571359999	0.116099052	0.192630469	positive
PRJNA729271	stage_E14_vs_E10	ordinary_muscle_development	ENSGALG00010019997	FSHR	1.406832328	0.404889898	3.474604663	0.000511607	0.00172982	positive
PRJNA729271	stage_E14_vs_E10	ordinary_muscle_development	ENSGALG00010019998	PLEK2	-3.887835626	0.624371276	-6.226800902	4.76055E-10	4.06625E-09	negative
PRJNA729271	stage_E14_vs_E10	ordinary_muscle_development	ENSGALG00010019999	ERLIN1	-0.272582358	0.062221382	-4.380847037	1.18219E-05	5.4782E-05	negative
PRJNA729271	stage_E14_vs_E10	ordinary_muscle_development	ENSGALG00010020003	ENSGALG00010020003	0.738352573	0.186950365	3.949457773	7.83284E-05	0.000314333	positive
PRJNA729271	stage_E14_vs_E10	ordinary_muscle_development	ENSGALG00010020005	EOGT	-0.310406794	0.16376367	-1.895455772	0.058032046	0.107470871	negative
PRJNA729271	stage_E14_vs_E10	ordinary_muscle_development	ENSGALG00010020006	PLB1	-0.667335153	0.368590106	-1.810507504	0.070217122	0.126434432	negative
PRJNA729271	stage_E14_vs_E10	ordinary_muscle_development	ENSGALG00010020008	EIF2S1	-0.049640538	0.078317923	-0.633833688	0.526189365	0.633797146	negative
PRJNA729271	stage_E14_vs_E10	ordinary_muscle_development	ENSGALG00010020009	MACROD2	-0.920852933	0.11671397	-7.889826137	3.02608E-15	4.35581E-14	negative
PRJNA729271	stage_E14_vs_E10	ordinary_muscle_development	ENSGALG00010020010	SORBS3	0.71047574	0.213464851	3.328303163	0.000873767	0.002792164	positive
PRJNA729271	stage_E14_vs_E10	ordinary_muscle_development	ENSGALG00010020011	TLE3	-0.357301853	0.20265668	-1.763089447	0.077885405	0.13821608	negative
PRJNA729271	stage_E14_vs_E10	ordinary_muscle_development	ENSGALG00010020013	ADAM8	-1.093721661	0.551665851	-1.982579959	0.047414368	0.090704931	negative
PRJNA729271	stage_E14_vs_E10	ordinary_muscle_development	ENSGALG00010020015	ATP6V1D	0.391258412	0.078101512	5.009613775	5.45394E-07	3.12593E-06	positive
PRJNA729271	stage_E14_vs_E10	ordinary_muscle_development	ENSGALG00010020017	ENSGALG00010020017	0.409418145	0.32469316	1.260938622	0.207330962	0.307506629	positive
PRJNA729271	stage_E14_vs_E10	ordinary_muscle_development	ENSGALG00010020018	ENSGALG00010020018	0.728846478	0.204884847	3.557346913	0.000374619	0.001309406	positive
PRJNA729271	stage_E14_vs_E10	ordinary_muscle_development	ENSGALG00010020019	PEX10	-0.010201398	0.098614914	-0.103446806	0.917608366	0.942645873	negative
PRJNA729271	stage_E14_vs_E10	ordinary_muscle_development	ENSGALG00010020023	FOSL2	-1.296906892	0.210637391	-6.15705924	7.41081E-10	6.14529E-09	negative
PRJNA729271	stage_E14_vs_E10	ordinary_muscle_development	ENSGALG00010020025	LOXL2	-0.646904318	0.139575835	-4.634787391	3.57305E-06	1.80081E-05	negative
PRJNA729271	stage_E14_vs_E10	ordinary_muscle_development	ENSGALG00010020027	RER1	-0.078270595	0.06984014	-1.120710729	0.262411014	0.370942817	negative
PRJNA729271	stage_E14_vs_E10	ordinary_muscle_development	ENSGALG00010020030	MRPL33	0.051269049	0.1347027	0.380608918	0.703493463	0.784441623	positive
PRJNA729271	stage_E14_vs_E10	ordinary_muscle_development	ENSGALG00010020032	CHST12	0.104317618	0.121193927	0.860749546	0.389375998	0.505138063	positive
PRJNA729271	stage_E14_vs_E10	ordinary_muscle_development	ENSGALG00010020036	TMF1	-0.155905182	0.08356948	-1.865575583	0.062100768	0.113943119	negative
PRJNA729271	stage_E14_vs_E10	ordinary_muscle_development	ENSGALG00010020037	MPP5	-0.14136308	0.08806133	-1.605279865	0.108432228	0.182102323	negative
PRJNA729271	stage_E14_vs_E10	ordinary_muscle_development	ENSGALG00010020038	SKI	-0.187457855	0.090435342	-2.072838459	0.038187316	0.075319791	negative
PRJNA729271	stage_E14_vs_E10	ordinary_muscle_development	ENSGALG00010020041	CDH23	0.894824973	0.151640031	5.900981198	3.61346E-09	2.77863E-08	positive
PRJNA729271	stage_E14_vs_E10	ordinary_muscle_development	ENSGALG00010020046	BDNF	0.144750023	0.224837452	0.643798541	0.51970608	0.627657027	positive
PRJNA729271	stage_E14_vs_E10	ordinary_muscle_development	ENSGALG00010020048	ENSGALG00010020048	0.131374323	0.262019566	0.501391269	0.616095783	0.712316214	positive
PRJNA729271	stage_E14_vs_E10	ordinary_muscle_development	ENSGALG00010020053	LFNG	0.589387214	0.134296974	4.388685732	1.14038E-05	5.29291E-05	positive
PRJNA729271	stage_E14_vs_E10	ordinary_muscle_development	ENSGALG00010020055	R3HCC1	0.130241093	0.083346185	1.56265213	0.11813444	0.19520733	positive
PRJNA729271	stage_E14_vs_E10	ordinary_muscle_development	ENSGALG00010020056	UACA	-0.619847835	0.165191321	-3.752302662	0.000175218	0.000658828	negative
PRJNA729271	stage_E14_vs_E10	ordinary_muscle_development	ENSGALG00010020057	RBKS	-0.133312161	0.090863089	-1.467176197	0.14232813	0.227433166	negative
PRJNA729271	stage_E14_vs_E10	ordinary_muscle_development	ENSGALG00010020059	PRKCZ	-0.669280188	0.140460611	-4.764895894	1.88951E-06	9.99579E-06	negative
PRJNA729271	stage_E14_vs_E10	ordinary_muscle_development	ENSGALG00010020061	UBA3	0.116249337	0.071799528	1.619082197	0.105429579	0.177925456	positive
PRJNA729271	stage_E14_vs_E10	ordinary_muscle_development	ENSGALG00010020063	ENSGALG00010020063	0.655742245	0.12904713	5.081416733	3.7463E-07	2.20742E-06	positive
PRJNA729271	stage_E14_vs_E10	ordinary_muscle_development	ENSGALG00010020064	TMEM138	0.212079099	0.128552051	1.649752746	0.098993517	0.168713819	positive
PRJNA729271	stage_E14_vs_E10	ordinary_muscle_development	ENSGALG00010020065	TTYH3	-0.435509064	0.110680074	-3.934846167	8.325E-05	0.000332518	negative
PRJNA729271	stage_E14_vs_E10	ordinary_muscle_development	ENSGALG00010020066	ENSGALG00010020066	4.098095149	0.365262328	11.21959433	3.26766E-29	1.34178E-27	positive
PRJNA729271	stage_E14_vs_E10	ordinary_muscle_development	ENSGALG00010020069	TMEM216	-0.247684005	0.180032902	-1.375770776	0.168892612	0.260991302	negative
PRJNA729271	stage_E14_vs_E10	ordinary_muscle_development	ENSGALG00010020070	ARL6IP5	0.930842843	0.114245083	8.147771584	3.70692E-16	5.76521E-15	positive
PRJNA729271	stage_E14_vs_E10	ordinary_muscle_development	ENSGALG00010020071	ENSGALG00010020071	0.034500988	0.143347855	0.240680186	0.809802997	0.866343969	positive
PRJNA729271	stage_E14_vs_E10	ordinary_muscle_development	ENSGALG00010020074	ENSGALG00010020074	-0.267795501	0.207311261	-1.291755692	0.196441764	0.294889956	negative
PRJNA729271	stage_E14_vs_E10	ordinary_muscle_development	ENSGALG00010020075	CPSF7	-0.123413146	0.137645167	-0.896603558	0.369930499	0.486141667	negative
PRJNA729271	stage_E14_vs_E10	ordinary_muscle_development	ENSGALG00010020080	SLC4A1AP	0.037976413	0.101220815	0.375183832	0.707523754	0.787616682	positive
PRJNA729271	stage_E14_vs_E10	ordinary_muscle_development	ENSGALG00010020083	CFAP74	-0.345112806	0.417016884	-0.827575139	0.407911152	0.522736465	negative
PRJNA729271	stage_E14_vs_E10	ordinary_muscle_development	ENSGALG00010020084	ENSGALG00010020084	0.118007796	0.085981675	1.372476119	0.169915268	0.262291993	positive
PRJNA729271	stage_E14_vs_E10	ordinary_muscle_development	ENSGALG00010020087	ENSGALG00010020087	-0.680118198	0.547867069	-1.241392733	0.214460702	0.316140332	negative
PRJNA729271	stage_E14_vs_E10	ordinary_muscle_development	ENSGALG00010020088	LMOD3	2.167669995	0.194269013	11.15808418	6.53854E-29	2.64E-27	positive
PRJNA729271	stage_E14_vs_E10	ordinary_muscle_development	ENSGALG00010020090	MORF4L2	-0.10875928	0.052611196	-2.067226911	0.038712778	0.07624208	negative
PRJNA729271	stage_E14_vs_E10	ordinary_muscle_development	ENSGALG00010020091	SUPT7L	-0.070185609	0.081991606	-0.856009687	0.391992421	0.507587113	negative
PRJNA729271	stage_E14_vs_E10	ordinary_muscle_development	ENSGALG00010020092	SDHAF2	0.513288023	0.137059713	3.744995614	0.000180397	0.000676372	positive
PRJNA729271	stage_E14_vs_E10	ordinary_muscle_development	ENSGALG00010020094	CLEC3B	3.102030869	0.227667723	13.62525538	2.83417E-42	2.48978E-40	positive
PRJNA729271	stage_E14_vs_E10	ordinary_muscle_development	ENSGALG00010020095	LRFN2	-1.618828312	0.328780911	-4.92372963	8.49101E-07	4.73192E-06	negative
PRJNA729271	stage_E14_vs_E10	ordinary_muscle_development	ENSGALG00010020096	GPHN	0.00448552	0.11678986	0.038406758	0.969363373	0.979021885	positive
PRJNA729271	stage_E14_vs_E10	ordinary_muscle_development	ENSGALG00010020097	RETSAT	0.090634327	0.228960483	0.3958514	0.692214656	0.775158486	positive
PRJNA729271	stage_E14_vs_E10	ordinary_muscle_development	ENSGALG00010020098	GABRD	1.087525447	0.330724239	3.288314912	0.00100789	0.00317457	positive
PRJNA729271	stage_E14_vs_E10	ordinary_muscle_development	ENSGALG00010020100	MOCS1	0.128270921	0.158341581	0.810089936	0.417888488	0.532370881	positive
PRJNA729271	stage_E14_vs_E10	ordinary_muscle_development	ENSGALG00010020101	KIF15	-0.455916145	0.204299607	-2.231605593	0.025641042	0.053911648	negative
PRJNA729271	stage_E14_vs_E10	ordinary_muscle_development	ENSGALG00010020102	TMEM127	-0.326558345	0.205334698	-1.590370981	0.111751207	0.18672942	negative
PRJNA729271	stage_E14_vs_E10	ordinary_muscle_development	ENSGALG00010020103	PSAP	0.032698229	0.105039404	0.311294882	0.755576457	0.825512983	positive
PRJNA729271	stage_E14_vs_E10	ordinary_muscle_development	ENSGALG00010020104	BRAT1	-0.007159016	0.111334212	-0.064302034	0.948729734	0.964599814	negative
PRJNA729271	stage_E14_vs_E10	ordinary_muscle_development	ENSGALG00010020106	ENSGALG00010020106	0.535925557	0.163532196	3.277186816	0.00104847	0.003291488	positive
PRJNA729271	stage_E14_vs_E10	ordinary_muscle_development	ENSGALG00010020108	RPAP1	-0.057683806	0.114995088	-0.501619736	0.615935034	0.712300807	negative
PRJNA729271	stage_E14_vs_E10	ordinary_muscle_development	ENSGALG00010020109	WDFY4	-0.750571119	0.362472209	-2.070699767	0.038386862	0.075672184	negative
PRJNA729271	stage_E14_vs_E10	ordinary_muscle_development	ENSGALG00010020111	ADRA2B	0.364432382	0.12363489	2.947649981	0.003201994	0.008803661	positive
PRJNA729271	stage_E14_vs_E10	ordinary_muscle_development	ENSGALG00010020113	CIAO1	0.176510703	0.102663934	1.719305847	0.085558696	0.14958029	positive
PRJNA729271	stage_E14_vs_E10	ordinary_muscle_development	ENSGALG00010020116	GNA12	-0.065231493	0.070760892	-0.921857982	0.356602659	0.473044343	negative
PRJNA729271	stage_E14_vs_E10	ordinary_muscle_development	ENSGALG00010020117	AKAP13	0.427662351	0.101843907	4.199194269	2.67866E-05	0.000116844	positive
PRJNA729271	stage_E14_vs_E10	ordinary_muscle_development	ENSGALG00010020118	GNB1	-0.246898392	0.093074452	-2.652697782	0.007985133	0.019601101	negative
PRJNA729271	stage_E14_vs_E10	ordinary_muscle_development	ENSGALG00010020120	DAAM2	-1.045776205	0.101720836	-10.28084552	8.59701E-25	2.52766E-23	negative
PRJNA729271	stage_E14_vs_E10	ordinary_muscle_development	ENSGALG00010020121	ENSGALG00010020121	-0.194465763	0.214619796	-0.906094251	0.364885968	0.48095136	negative
PRJNA729271	stage_E14_vs_E10	ordinary_muscle_development	ENSGALG00010020123	ENSGALG00010020123	0.342798594	0.141872619	2.416242088	0.015681631	0.035257522	positive
PRJNA729271	stage_E14_vs_E10	ordinary_muscle_development	ENSGALG00010020125	NAV2	0.856899517	0.129389771	6.622621806	3.52883E-11	3.42372E-10	positive
PRJNA729271	stage_E14_vs_E10	ordinary_muscle_development	ENSGALG00010020126	NADK	-0.023813883	0.104186468	-0.228569832	0.819203275	0.873435194	negative
PRJNA729271	stage_E14_vs_E10	ordinary_muscle_development	ENSGALG00010020127	FRMD4B	0.247008164	0.138102411	1.788586911	0.073681367	0.131869542	positive
PRJNA729271	stage_E14_vs_E10	ordinary_muscle_development	ENSGALG00010020128	KIF6	-1.090578662	0.535777962	-2.035504892	0.041800097	0.081360602	negative
PRJNA729271	stage_E14_vs_E10	ordinary_muscle_development	ENSGALG00010020129	MGAT2	0.146556213	0.114153711	1.283849751	0.199194532	0.298184595	positive
PRJNA729271	stage_E14_vs_E10	ordinary_muscle_development	ENSGALG00010020130	PDXDC1	-0.027699357	0.072530611	-0.381898849	0.702536394	0.783961113	negative
PRJNA729271	stage_E14_vs_E10	ordinary_muscle_development	ENSGALG00010020131	SNRNP200	-0.192893652	0.124340279	-1.551336814	0.120820989	0.198672743	negative
PRJNA729271	stage_E14_vs_E10	ordinary_muscle_development	ENSGALG00010020132	SHC4	-0.547401057	0.229295515	-2.387316898	0.016971855	0.037765894	negative
PRJNA729271	stage_E14_vs_E10	ordinary_muscle_development	ENSGALG00010020133	ENSGALG00010020133	1.165806898	0.370687332	3.144987158	0.001660941	0.004945633	positive
PRJNA729271	stage_E14_vs_E10	ordinary_muscle_development	ENSGALG00010020136	KCNK17	-0.16494162	0.291886542	-0.56508813	0.572013822	0.674037911	negative
PRJNA729271	stage_E14_vs_E10	ordinary_muscle_development	ENSGALG00010020137	NTAN1	-0.316468675	0.080203422	-3.945825076	7.95257E-05	0.000318756	negative
PRJNA729271	stage_E14_vs_E10	ordinary_muscle_development	ENSGALG00010020138	ENSGALG00010020138	0.092615976	0.084762787	1.092649023	0.27454793	0.384946048	positive
PRJNA729271	stage_E14_vs_E10	ordinary_muscle_development	ENSGALG00010020140	SECISBP2L	0.259361973	0.094363884	2.748530071	0.005986314	0.015239134	positive
PRJNA729271	stage_E14_vs_E10	ordinary_muscle_development	ENSGALG00010020141	THOC7	-0.022868269	0.127290163	-0.179654645	0.857423702	0.901568156	negative
PRJNA729271	stage_E14_vs_E10	ordinary_muscle_development	ENSGALG00010020142	SLC35E2B	0.12413218	0.067893571	1.828334836	0.067499315	0.122269753	positive
PRJNA729271	stage_E14_vs_E10	ordinary_muscle_development	ENSGALG00010020143	KCNK5	-0.712811374	0.177201806	-4.022596545	5.756E-05	0.000236959	negative
PRJNA729271	stage_E14_vs_E10	ordinary_muscle_development	ENSGALG00010020147	ABHD2	0.116012794	0.147067391	0.788841042	0.430204918	0.544375407	positive
PRJNA729271	stage_E14_vs_E10	ordinary_muscle_development	ENSGALG00010020149	FAHD2A	0.340747095	0.17250394	1.975300364	0.048234072	0.092069446	positive
PRJNA729271	stage_E14_vs_E10	ordinary_muscle_development	ENSGALG00010020152	ATXN7	0.146817278	0.107426635	1.366674827	0.171727252	0.264525665	positive
PRJNA729271	stage_E14_vs_E10	ordinary_muscle_development	ENSGALG00010020153	AXL	0.382107341	0.120283136	3.176732446	0.001489444	0.004492782	positive

PRJNA729271	stage_E14_vs_E10	ordinary_muscle_development	ENSGALG00010020155	MFGE8	0.066334383	0.135090432	0.491036871	0.62340037	0.718412177	positive
PRJNA729271	stage_E14_vs_E10	ordinary_muscle_development	ENSGALG00010020160	SYT7	-1.712158506	0.25267665	-6.776085193	1.23476E-11	1.26041E-10	negative
PRJNA729271	stage_E14_vs_E10	ordinary_muscle_development	ENSGALG00010020163	CDC2L1	0.160504207	0.094526071	1.697988778	0.08950988	0.15508555	positive
PRJNA729271	stage_E14_vs_E10	ordinary_muscle_development	ENSGALG00010020164	C10orf71	2.439754837	0.14811845	16.47164707	5.86515E-61	1.46578E-58	positive
PRJNA729271	stage_E14_vs_E10	ordinary_muscle_development	ENSGALG00010020166	HAPLN3	0.037431512	0.170740748	0.219230107	0.826470804	0.878534343	positive
PRJNA729271	stage_E14_vs_E10	ordinary_muscle_development	ENSGALG00010020168	COX7A2L	0.246994465	0.114979176	2.148166947	0.031700495	0.064301523	positive
PRJNA729271	stage_E14_vs_E10	ordinary_muscle_development	ENSGALG00010020170	SORCS1	-1.406656499	0.306070004	-4.595865263	4.30957E-06	2.1496E-05	negative
PRJNA729271	stage_E14_vs_E10	ordinary_muscle_development	ENSGALG00010020171	RRN3	-0.245303851	0.074903256	-3.274942407	0.001056835	0.003312895	negative
PRJNA729271	stage_E14_vs_E10	ordinary_muscle_development	ENSGALG00010020175	ACAN	-5.239125281	0.501456336	-10.4478195	1.49933E-25	4.72451E-24	negative
PRJNA729271	stage_E14_vs_E10	ordinary_muscle_development	ENSGALG00010020176	ENSGALG00010020176	-0.416580958	0.250126642	-1.665480154	0.095817	0.164226961	negative
PRJNA729271	stage_E14_vs_E10	ordinary_muscle_development	ENSGALG00010020177	KCNG3	0.434732949	0.228980107	1.898562081	0.057622078	0.106861825	positive
PRJNA729271	stage_E14_vs_E10	ordinary_muscle_development	ENSGALG00010020179	PSMD6	-0.144146053	0.134871874	-1.068762883	0.285176528	0.396892346	negative
PRJNA729271	stage_E14_vs_E10	ordinary_muscle_development	ENSGALG00010020180	TCF7L1	-0.091095963	0.200283615	-0.454834826	0.649228074	0.740872377	negative
PRJNA729271	stage_E14_vs_E10	ordinary_muscle_development	ENSGALG00010020181	LEUTX	2.910804473	0.210611461	13.82073161	1.91118E-43	1.82253E-41	positive
PRJNA729271	stage_E14_vs_E10	ordinary_muscle_development	ENSGALG00010020183	XPNPEP1	-0.143488697	0.067225889	-2.13442619	0.03280791	0.066171399	negative
PRJNA729271	stage_E14_vs_E10	ordinary_muscle_development	ENSGALG00010020184	RSL1D1	-0.487232856	0.068136799	-7.150803397	8.62715E-13	9.92464E-12	negative
PRJNA729271	stage_E14_vs_E10	ordinary_muscle_development	ENSGALG00010020186	MGA	0.027611924	0.094586004	0.291923998	0.770344734	0.836917023	positive
PRJNA729271	stage_E14_vs_E10	ordinary_muscle_development	ENSGALG00010020187	MMP23B	-0.200154213	0.207289505	-0.965578133	0.334255322	0.449446279	negative
PRJNA729271	stage_E14_vs_E10	ordinary_muscle_development	ENSGALG00010020193	VSTM4	0.544938209	0.176068032	3.095043452	0.001967841	0.005736897	positive
PRJNA729271	stage_E14_vs_E10	ordinary_muscle_development	ENSGALG00010020194	PRICKLE2	-0.919613385	0.153832102	-5.97803303	2.25848E-09	1.77723E-08	negative
PRJNA729271	stage_E14_vs_E10	ordinary_muscle_development	ENSGALG00010020199	DET1	-0.074678658	0.081817417	-0.912747682	0.36137526	0.477670471	negative
PRJNA729271	stage_E14_vs_E10	ordinary_muscle_development	ENSGALG00010020202	MAPKBP1	1.291256757	0.089830872	14.37430948	7.50145E-47	8.98624E-45	positive
PRJNA729271	stage_E14_vs_E10	ordinary_muscle_development	ENSGALG00010020204	GSPT2	-0.013287038	0.086244823	-0.154061863	0.877560958	0.916015475	negative
PRJNA729271	stage_E14_vs_E10	ordinary_muscle_development	ENSGALG00010020206	MRPS11	0.065597756	0.08097616	0.810087265	0.417890023	0.532370881	positive
PRJNA729271	stage_E14_vs_E10	ordinary_muscle_development	ENSGALG00010020211	ADD3	-0.324242966	0.083165413	-3.898771768	9.66818E-05	0.000381231	negative
PRJNA729271	stage_E14_vs_E10	ordinary_muscle_development	ENSGALG00010020212	JMJD7	0.072792064	0.136148945	0.534650222	0.592891749	0.69283827	positive
PRJNA729271	stage_E14_vs_E10	ordinary_muscle_development	ENSGALG00010020215	PRMT3	-0.108488586	0.078648217	-1.379415713	0.167766617	0.259583381	negative
PRJNA729271	stage_E14_vs_E10	ordinary_muscle_development	ENSGALG00010020224	MRPL46	0.180160059	0.08529174	2.11228025	0.034662419	0.069396652	positive
PRJNA729271	stage_E14_vs_E10	ordinary_muscle_development	ENSGALG00010020226	TCIM	0.028853241	0.19975665	0.144441957	0.885151487	0.921317737	positive
PRJNA729271	stage_E14_vs_E10	ordinary_muscle_development	ENSGALG00010020228	TM9SF3	-0.178397953	0.082833846	-2.153684294	0.031264939	0.063515807	negative
PRJNA729271	stage_E14_vs_E10	ordinary_muscle_development	ENSGALG00010020230	ENSGALG00010020230	-0.077989294	0.217202344	-0.359062856	0.719548068	0.797145311	negative
PRJNA729271	stage_E14_vs_E10	ordinary_muscle_development	ENSGALG00010020236	SLC35G1	-1.264939788	0.319084551	-3.964277759	7.36185E-05	0.000296829	negative
PRJNA729271	stage_E14_vs_E10	ordinary_muscle_development	ENSGALG00010020237	MIB2	0.045334766	0.07468186	0.607038521	0.543825348	0.649480796	positive
PRJNA729271	stage_E14_vs_E10	ordinary_muscle_development	ENSGALG00010020240	SV2B	-0.296460085	0.343375025	-0.863371133	0.387933446	0.503815079	negative
PRJNA729271	stage_E14_vs_E10	ordinary_muscle_development	ENSGALG00010020244	SLC25A37	-0.383953523	0.212865468	-1.803737954	0.071272397	0.128065472	negative
PRJNA729271	stage_E14_vs_E10	ordinary_muscle_development	ENSGALG00010020246	ADAMTS9	-0.309352986	0.128796298	-2.401877926	0.016311151	0.036463623	negative
PRJNA729271	stage_E14_vs_E10	ordinary_muscle_development	ENSGALG00010020248	NOLC1	-0.376590578	0.080263978	-4.691900255	2.70679E-06	1.39626E-05	negative
PRJNA729271	stage_E14_vs_E10	ordinary_muscle_development	ENSGALG00010020250	ENSGALG00010020250	-0.246958679	0.244503254	-1.010042501	0.312474928	0.426289325	negative
PRJNA729271	stage_E14_vs_E10	ordinary_muscle_development	ENSGALG00010020252	DMTN	0.580068687	0.187890979	3.087262035	0.002020094	0.005862116	positive
PRJNA729271	stage_E14_vs_E10	ordinary_muscle_development	ENSGALG00010020254	ENSGALG00010020254	-0.708596434	0.132825981	-5.334772842	9.56642E-08	6.11399E-07	negative
PRJNA729271	stage_E14_vs_E10	ordinary_muscle_development	ENSGALG00010020257	ZNF710	0.189040436	0.161195729	1.172738497	0.240900689	0.346869344	positive
PRJNA729271	stage_E14_vs_E10	ordinary_muscle_development	ENSGALG00010020259	ENSGALG00010020259	-0.305494388	0.167291854	-1.826116343	0.067832739	0.122705048	negative
PRJNA729271	stage_E14_vs_E10	ordinary_muscle_development	ENSGALG00010020261	SSU72	-0.097080687	0.091090097	-1.065765536	0.286529644	0.398431234	negative
PRJNA729271	stage_E14_vs_E10	ordinary_muscle_development	ENSGALG00010020265	ENSGALG00010020265	-1.779423415	0.228896237	-7.773930397	7.60876E-15	1.05587E-13	negative
PRJNA729271	stage_E14_vs_E10	ordinary_muscle_development	ENSGALG00010020268	CPLANE2	0.36165674	0.096218995	3.75868339	0.00017081	0.000643924	positive
PRJNA729271	stage_E14_vs_E10	ordinary_muscle_development	ENSGALG00010020269	ENSGALG00010020269	1.577421797	0.53009742	2.97572057	0.002923011	0.008121342	positive
PRJNA729271	stage_E14_vs_E10	ordinary_muscle_development	ENSGALG00010020272	ENSGALG00010020272	0.421235044	0.086495707	4.870011016	1.11592E-06	6.12932E-06	positive
PRJNA729271	stage_E14_vs_E10	ordinary_muscle_development	ENSGALG00010020275	ELOVL3	-1.520858868	0.454864731	-3.343540981	0.000827165	0.002661432	negative
PRJNA729271	stage_E14_vs_E10	ordinary_muscle_development	ENSGALG00010020277	IDH2	0.764480807	0.124659443	6.132554324	8.64792E-10	7.13847E-09	positive
PRJNA729271	stage_E14_vs_E10	ordinary_muscle_development	ENSGALG00010020282	MAGI1	-0.938459166	0.136945871	-6.852774471	7.24312E-12	7.49387E-11	negative
PRJNA729271	stage_E14_vs_E10	ordinary_muscle_development	ENSGALG00010020283	FBXO42	-0.414857997	0.222286954	-1.866317341	0.06199698	0.1137959	negative
PRJNA729271	stage_E14_vs_E10	ordinary_muscle_development	ENSGALG00010020284	HEG1	0.014864228	0.118818017	0.12510079	0.900443758	0.931548945	positive
PRJNA729271	stage_E14_vs_E10	ordinary_muscle_development	ENSGALG00010020285	ADAMTS14	-1.030660057	0.25938318	-3.973503823	7.0823E-05	0.000286674	negative
PRJNA729271	stage_E14_vs_E10	ordinary_muscle_development	ENSGALG00010020287	GALNT16	1.119381841	0.159565481	7.015187965	2.2964E-12	2.53128E-11	positive
PRJNA729271	stage_E14_vs_E10	ordinary_muscle_development	ENSGALG00010020290	NELL1	-2.900422976	0.566819192	-5.117016182	3.10407E-07	1.84778E-06	negative
PRJNA729271	stage_E14_vs_E10	ordinary_muscle_development	ENSGALG00010020291	SNX29	-0.359040822	0.145330316	-2.470515655	0.013491841	0.03097802	negative
PRJNA729271	stage_E14_vs_E10	ordinary_muscle_development	ENSGALG00010020292	NPM2	-0.29444723	0.23007457	-1.279790416	0.200618855	0.29975985	negative
PRJNA729271	stage_E14_vs_E10	ordinary_muscle_development	ENSGALG00010020293	SEMA4B	0.092574054	0.109550073	0.845038722	0.398089219	0.513463538	positive
PRJNA729271	stage_E14_vs_E10	ordinary_muscle_development	ENSGALG00010020295	LGI1	-0.719795772	0.147051091	-4.894868624	9.83714E-07	5.43819E-06	negative
PRJNA729271	stage_E14_vs_E10	ordinary_muscle_development	ENSGALG00010020298	ENSGALG00010020298	0.330986228	0.173248973	1.910465743	0.056073273	0.10445728	positive
PRJNA729271	stage_E14_vs_E10	ordinary_muscle_development	ENSGALG00010020300	EHD4	0.032710049	0.128954283	0.253656169	0.799761199	0.859152048	positive
PRJNA729271	stage_E14_vs_E10	ordinary_muscle_development	ENSGALG00010020302	HAAO	-0.130786228	1.550906213	-0.084328909	0.932794929	0.953751562	negative
PRJNA729271	stage_E14_vs_E10	ordinary_muscle_development	ENSGALG00010020303	EXD2	-0.224366445	0.070007158	-3.20490718	0.00135106	0.004124604	negative
PRJNA729271	stage_E14_vs_E10	ordinary_muscle_development	ENSGALG00010020304	ZFP36L2	0.180059184	0.155348219	1.15906822	0.246428378	0.35306705	positive
PRJNA729271	stage_E14_vs_E10	ordinary_muscle_development	ENSGALG00010020305	XPO7	0.018498159	0.137687043	0.134349308	0.893126364	0.926491566	positive
PRJNA729271	stage_E14_vs_E10	ordinary_muscle_development	ENSGALG00010020307	SHQ1	-0.488018908	0.225551401	-2.163670473	0.030489649	0.062202316	negative
PRJNA729271	stage_E14_vs_E10	ordinary_muscle_development	ENSGALG00010020309	GXYLT2	0.250310042	0.159429162	1.570039256	0.116405979	0.192900956	positive
PRJNA729271	stage_E14_vs_E10	ordinary_muscle_development	ENSGALG00010020311	SGPL1	0.008890581	0.133412707	0.066639684	0.946868553	0.963012888	positive
PRJNA729271	stage_E14_vs_E10	ordinary_muscle_development	ENSGALG00010020313	FRA10AC1	-0.065110376	0.113904165	-0.57162419	0.567576604	0.670116712	negative
PRJNA729271	stage_E14_vs_E10	ordinary_muscle_development	ENSGALG00010020314	THADA	-0.579762171	0.078906713	-7.347437913	2.02042E-13	2.48186E-12	negative
PRJNA729271	stage_E14_vs_E10	ordinary_muscle_development	ENSGALG00010020315	ANO5	-0.785326995	0.108586649	-7.232261067	4.75018E-13	5.60699E-12	negative
PRJNA729271	stage_E14_vs_E10	ordinary_muscle_development	ENSGALG00010020316	PDPK1	0.121300056	0.112069774	1.082361922	0.279091741	0.389996605	positive
PRJNA729271	stage_E14_vs_E10	ordinary_muscle_development	ENSGALG00010020319	NTRK3	0.858301983	0.157385677	5.453494859	4.93894E-08	3.2746E-07	positive
PRJNA729271	stage_E14_vs_E10	ordinary_muscle_development	ENSGALG00010020321	SZRD1	0.203490319	0.169549746	1.200180617	0.230069202	0.333952842	positive
PRJNA729271	stage_E14_vs_E10	ordinary_muscle_development	ENSGALG00010020323	ARHGAP25	2.121555831	0.149247935	14.21497615	7.39766E-46	8.37728E-44	positive
PRJNA729271	stage_E14_vs_E10	ordinary_muscle_development	ENSGALG00010020324	PLEKHH2	-0.915381479	0.186991735	-4.895304494	9.81536E-07	5.42822E-06	negative
PRJNA729271	stage_E14_vs_E10	ordinary_muscle_development	ENSGALG00010020329	PPP4R2	-0.101250742	0.105293286	-0.961606814	0.336247147	0.451412652	negative
PRJNA729271	stage_E14_vs_E10	ordinary_muscle_development	ENSGALG00010020334	CABP4	0.04544346	0.475342017	0.095601605	0.92383699	0.947701145	positive
PRJNA729271	stage_E14_vs_E10	ordinary_muscle_development	ENSGALG00010020336	OSBP	0.089379854	0.134278658	0.665629636	0.505647859	0.614570327	positive
PRJNA729271	stage_E14_vs_E10	ordinary_muscle_development	ENSGALG00010020338	NECAP2	-0.328400368	0.165674341	-1.982204166	0.047456395	0.090773349	negative
PRJNA729271	stage_E14_vs_E10	ordinary_muscle_development	ENSGALG00010020340	DCAF5	0.228061743	0.096313074	2.367920907	0.01788836	0.039484052	positive
PRJNA729271	stage_E14_vs_E10	ordinary_muscle_development	ENSGALG00010020341	DYNC2LI1	-0.395708393	0.126021538	-3.140006066	0.001689443	0.005021218	negative
PRJNA729271	stage_E14_vs_E10	ordinary_muscle_development	ENSGALG00010020342	CPPED1	0.038678853	0.105427372	0.366876763	0.713710945	0.792920989	positive
PRJNA729271	stage_E14_vs_E10	ordinary_muscle_development	ENSGALG00010020343	PATL1	0.107011187	0.175660785	0.609192237	0.542397025	0.648202233	positive
PRJNA729271	stage_E14_vs_E10	ordinary_muscle_development	ENSGALG00010020345	AGBL1	1.282562006	0.452338159	2.835405285	0.004576758	0.012046507	positive
PRJNA729271	stage_E14_vs_E10	ordinary_muscle_development	ENSGALG00010020346	PCBD1	-0.106979384	0.240224875	-0.445330167	0.65608117	0.746518295	negative
PRJNA729271	stage_E14_vs_E10	ordinary_muscle_development	ENSGALG00010020350	PDZRN3	0.183775594	0.107849133	1.704006223	0.088379925	0.15349869	positive
PRJNA729271	stage_E14_vs_E10	ordinary_muscle_development	ENSGALG00010020351	CROCC	-0.523641331	0.147036683	-3.561297229	0.000369027	0.001293916	negative
PRJNA729271	stage_E14_vs_E10	ordinary_muscle_development	ENSGALG00010020352	ABCG5	-0.781134882	0.614809119	-1.270532362	0.203895063	0.303591056	negative
PRJNA729271	stage_E14_vs_E10	ordinary_muscle_development	ENSGALG00010020356	STX3	0.520651504	0.351741642	1.480210023	0.138817207	0.22276457	positive
PRJNA729271	stage_E14_vs_E10	ordinary_muscle_development	ENSGALG00010020357	RBP4	-0.287096472	0.73444336	-0.390903489	0.695868575	0.77852848	negative
PRJNA729271	stage_E14_vs_E10	ordinary_muscle_development	ENSGALG00010020362	CDS2	0.08687244	0.126410254	0.687226212	0.491940189	0.602151076	positive
PRJNA729271	stage_E14_vs_E10	ordinary_muscle_development	ENSGALG00010020364	MRPL16	0.499868322	0.110409725	4.527393954	5.97156E-06	2.91048E-05	positive
PRJNA729271	stage_E14_vs_E10	ordinary_muscle_development	ENSGALG00010020365	UNC5B	-0.251293858	0.13653743	-1.84047596	0.06569839	0.119560348	negative
PRJNA729271	stage_E14_vs_E10	ordinary_muscle_development	ENSGALG00010020369	CNTN3	-2.221237916	0.272728486	-8.144502796	3.80844E-16	5.91041E-15	negative
PRJNA729271	stage_E14_vs_E10	ordinary_muscle_development	ENSGALG00010020370	CEP55	-0.094535845	0.216545305	-0.436563818	0.662427708	0.751556796	negative
PRJNA729271	stage_E14_vs_E10	ordinary_muscle_development	ENSGALG00010020373	MS4A4A	-1.157237782	0.215273087	-5.375673293	7.6297E-08	4.95264E-07	negative
PRJNA729271	stage_E14_vs_E10	ordinary_muscle_development	ENSGALG00010020374	PCNA	-0.186726862	0.095131155	-1.962836067	0.049665225	0.094301473	negative
PRJNA729271	stage_E14_vs_E10	ordinary_muscle_development	ENSGALG00010020375	LRPPRC	-0.181367142	0.101655129	-1.784141583	0.074400662	0.132891094	negative
PRJNA729271	stage_E14_vs_E10	ordinary_muscle_development	ENSGALG00010020377	TMEM230	-0.121951067	0.094109789	-1.295838278	0.195031207	0.293116956	negative
PRJNA729271	stage_E14_vs_E10	ordinary_muscle_development	ENSGALG00010020379	COL13A1	-0.206581185	0.19345077	-1.067874712	0.285577031	0.39728269	negative
PRJNA729271	stage_E14_vs_E10	ordinary_muscle_development	ENSGALG00010020382	MFAP2	-1.581661592	0.145745004	-10.85225256	1.9457E-27	7.06841E-26	negative
PRJNA729271	stage_E14_vs_E10	ordinary_muscle_development	ENSGALG00010020383	BAAT	0.031756228	0.102662851	0.309325405	0.757074008	0.826775238	positive
PRJNA729271	stage_E14_vs_E10	ordinary_muscle_development	ENSGALG00010020385	SLC12A8	-1.068643364	0.353581955	-3.022335696	0.002508322	0.007079075	negative
PRJNA729271	stage_E14_vs_E10	ordinary_muscle_development	ENSGALG00010020386	PPM1B	0.6061985	0.071776852	8.445598867	3.02491E-17	5.17052E-16	positive
PRJNA729271	stage_E14_vs_E10	ordinary_muscle_development	ENSGALG00010020388	MYOF	-0.919055232	0.168232603	-5.463003108	4.68146E-08	3.12277E-07	negative
PRJNA729271	stage_E14_vs_E10	ordinary_muscle_development	ENSGALG00010020389	ACTN1	-0.615907909	0.156711559	-3.930200884	8.48749E-05	0.00033817	negative
PRJNA729271	stage_E14_vs_E10	ordinary_muscle_development	ENSGALG00010020390	KLHL25	0.861419442	0.22169334	3.885635177	0.000102063	0.000401138	positive
PRJNA729271	stage_E14_vs_E10	ordinary_muscle_development	ENSGALG00010020392	SLC23A2	-0.151518078	0.07802816	-1.941838404	0.052156666	0.098247027	negative
PRJNA729271	stage_E14_vs_E10	ordinary_muscle_development	ENSGALG00010020393	SHISA9	-1.594008621	0.304896918	-5.22802471	1.71331E-07	1.05454E-06	negative
PRJNA729271	stage_E14_vs_E10	ordinary_muscle_development	ENSGALG00010020396	CHL1	-2.039178672	0.20068471	-10.1611063	2.95702E-24	8.35516E-23	negative
PRJNA729271	stage_E14_vs_E10	ordinary_muscle_development	ENSGALG00010020399	ATP13A2	-0.900353432	0.132951884	-6.772024601	1.26993E-11	1.29267E-10	negative
PRJNA729271	stage_E14_vs_E10	ordinary_muscle_development	ENSGALG00010020402	SLC3A1	1.403680837	0.327279562	4.288935216	1.79532E-05	8.0542E-05	positive
PRJNA729271	stage_E14_vs_E10	ordinary_muscle_development	ENSGALG00010020405	UBE2I	0.042203022	0.080524802	0.524099668	0.60020922	0.699359537	positive
PRJNA729271	stage_E14_vs_E10	ordinary_muscle_development	ENSGALG00010020413	CYP26A1	1.023471889	0.293881406	3.482601716	0.000496567	0.001686046	positive
PRJNA729271	stage_E14_vs_E10	ordinary_muscle_development	ENSGALG00010020415	TSR3	0.15272658	0.089628589	1.703994014	0.088382205	0.15349869	positive
PRJNA729271	stage_E14_vs_E10	ordinary_muscle_development	ENSGALG00010020416	ENSGALG00010020416	-0.768837362	0.74393304	-1.033476564	0.301380932	0.415218764	negative
PRJNA729271	stage_E14_vs_E10	ordinary_muscle_development	ENSGALG00010020417	ENSGALG00010020417	0.246762907	0.817789017	0.301743973	0.762847244	0.830927445	positive
PRJNA729271	stage_E14_vs_E10	ordinary_muscle_development	ENSGALG00010020422	ERCC4	-0.253735714	0.087536811	-2.898617289	0.003748121	0.010096453	negative
PRJNA729271	stage_E14_vs_E10	ordinary_muscle_development	ENSGALG00010020428	SEMA4C	-0.259105351	0.148997072	-1.738996259	0.082035421	0.144484013	negative
PRJNA729271	stage_E14_vs_E10	ordinary_muscle_development	ENSGALG00010020429	ZNF148	-0.279287351	0.101229162	-2.758961397	0.005798538	0.01481315	negative
PRJNA729271	stage_E14_vs_E10	ordinary_muscle_development	ENSGALG00010020433	GNPTG	-0.126532182	0.06962201	-1.817416383	0.069153381	0.124782554	negative
PRJNA729271	stage_E14_vs_E10	ordinary_muscle_development	ENSGALG00010020436	CNTN4	-2.792617299	0.265686	-10.51096896	7.68994E-26	2.48253E-24	negative
PRJNA729271	stage_E14_vs_E10	ordinary_muscle_development	ENSGALG00010020437	SNX4	-0.657683773	0.083938963	-7.835262098	4.67866E-15	6.60342E-14	negative
PRJNA729271	stage_E14_vs_E10	ordinary_muscle_development	ENSGALG00010020438	HTR7	-0.923125728	0.286691962	-3.219921901	0.001282255	0.003940278	negative
PRJNA729271	stage_E14_vs_E10	ordinary_muscle_development	ENSGALG00010020440	PAX7	1.021177928	0.185230556	5.513010124	3.52748E-08	2.40276E-07	positive
PRJNA729271	stage_E14_vs_E10	ordinary_muscle_development	ENSGALG00010020443	PREPL	-0.252779453	0.135302792	-1.86825009	0.06172722	0.113358172	negative
PRJNA729271	stage_E14_vs_E10	ordinary_muscle_development	ENSGALG00010020444	ENSGALG00010020444	1.839047958	0.881266491	2.08682388	0.036904058	0.073137041	positive
PRJNA729271	stage_E14_vs_E10	ordinary_muscle_development	ENSGALG00010020449	PRPF19	0.1753725	0.096828971	1.811157325	0.070116502	0.126300323	positive
PRJNA729271	stage_E14_vs_E10	ordinary_muscle_development	ENSGALG00010020450	ANKRD39	0.604932961	0.179543189	3.36928939	0.000753623	0.002446531	positive
PRJNA729271	stage_E14_vs_E10	ordinary_muscle_development	ENSGALG00010020453	ENSGALG00010020453	-0.589167935	0.13962229	-4.219726915	2.44598E-05	0.000107243	negative

PRJNA729271	stage_E14_vs_E10	ordinary_muscle_development	ENSGALG00010020455	ENSGALG00010020455	-0.334905537	0.677627145	-0.494232764	0.621141803	0.716607007	negative
PRJNA729271	stage_E14_vs_E10	ordinary_muscle_development	ENSGALG00010020457	IL5RA	0.458560156	0.984694819	0.465687589	0.641439137	0.733832699	positive
PRJNA729271	stage_E14_vs_E10	ordinary_muscle_development	ENSGALG00010020460	MAPK8	-0.001680325	0.140643727	-0.01194739	0.990467589	0.993758406	negative
PRJNA729271	stage_E14_vs_E10	ordinary_muscle_development	ENSGALG00010020462	CNNM4	-0.238887941	0.182073129	-1.312043914	0.189505318	0.286371933	negative
PRJNA729271	stage_E14_vs_E10	ordinary_muscle_development	ENSGALG00010020467	ALDH4A1	0.191052671	0.130927084	1.459229559	0.144501936	0.230221539	positive
PRJNA729271	stage_E14_vs_E10	ordinary_muscle_development	ENSGALG00010020468	ENSGALG00010020468	-0.333340624	0.441939758	-0.754267111	0.450688847	0.56379864	negative
PRJNA729271	stage_E14_vs_E10	ordinary_muscle_development	ENSGALG00010020473	TMEM109	0.723594905	0.163650197	4.421595077	9.79749E-06	4.60489E-05	positive
PRJNA729271	stage_E14_vs_E10	ordinary_muscle_development	ENSGALG00010020476	TMEM132A	0.032797257	0.108493149	0.302297954	0.762424938	0.830741147	positive
PRJNA729271	stage_E14_vs_E10	ordinary_muscle_development	ENSGALG00010020478	ENSGALG00010020478	-1.517350488	0.967377644	-1.568519283	0.116759993	0.193411349	negative
PRJNA729271	stage_E14_vs_E10	ordinary_muscle_development	ENSGALG00010020481	RPP30	-0.748186363	0.122512629	-6.107014159	1.01512E-09	8.30372E-09	negative
PRJNA729271	stage_E14_vs_E10	ordinary_muscle_development	ENSGALG00010020484	TRNT1	-0.209731675	0.12406658	-1.690476802	0.090936773	0.157200778	negative
PRJNA729271	stage_E14_vs_E10	ordinary_muscle_development	ENSGALG00010020485	LMAN2L	-0.070230425	0.129853595	-0.540843131	0.588615711	0.688896628	negative
PRJNA729271	stage_E14_vs_E10	ordinary_muscle_development	ENSGALG00010020486	MRTFB	-0.159036613	0.113215766	-1.404721437	0.160104129	0.25023823	negative
PRJNA729271	stage_E14_vs_E10	ordinary_muscle_development	ENSGALG00010020489	IFFO2	-1.040744382	0.182093279	-5.715446436	1.09416E-08	7.92203E-08	negative
PRJNA729271	stage_E14_vs_E10	ordinary_muscle_development	ENSGALG00010020493	DEPDC7	0.650503246	0.142520785	4.564269322	5.01237E-06	2.47208E-05	positive
PRJNA729271	stage_E14_vs_E10	ordinary_muscle_development	ENSGALG00010020497	OSBPL11	-0.193489104	0.068759384	-2.814002875	0.004892879	0.012776488	negative
PRJNA729271	stage_E14_vs_E10	ordinary_muscle_development	ENSGALG00010020498	FRMPD2	-2.080045867	0.588413911	-3.535004576	0.000407768	0.001410644	negative
PRJNA729271	stage_E14_vs_E10	ordinary_muscle_development	ENSGALG00010020507	ANKRD1	-0.374603983	0.587239888	-0.637906231	0.523534721	0.63138662	negative
PRJNA729271	stage_E14_vs_E10	ordinary_muscle_development	ENSGALG00010020509	CRBN	0.178768632	0.102755642	1.739745167	0.081903775	0.144287216	positive
PRJNA729271	stage_E14_vs_E10	ordinary_muscle_development	ENSGALG00010020514	CAMKMT	-0.140534149	0.133009884	-1.056569219	0.290708235	0.403135403	negative
PRJNA729271	stage_E14_vs_E10	ordinary_muscle_development	ENSGALG00010020517	UBR4	0.02009948	0.094104404	0.213587024	0.830869119	0.882045399	positive
PRJNA729271	stage_E14_vs_E10	ordinary_muscle_development	ENSGALG00010020520	ARHGAP22	0.664407817	0.197674878	3.361114083	0.000776288	0.002511671	positive
PRJNA729271	stage_E14_vs_E10	ordinary_muscle_development	ENSGALG00010020521	ENSGALG00010020521	0.429237502	0.719802654	0.596326646	0.550957034	0.655836594	positive
PRJNA729271	stage_E14_vs_E10	ordinary_muscle_development	ENSGALG00010020524	ENSGALG00010020524	0.954691341	0.399807385	2.387878208	0.016945957	0.037714055	positive
PRJNA729271	stage_E14_vs_E10	ordinary_muscle_development	ENSGALG00010020525	ENSGALG00010020525	0.74285326	0.722599133	1.028029548	0.303935929	0.417469088	positive
PRJNA729271	stage_E14_vs_E10	ordinary_muscle_development	ENSGALG00010020530	SRSF5A	-0.093509375	0.068933966	-1.356506536	0.174938057	0.268416125	negative
PRJNA729271	stage_E14_vs_E10	ordinary_muscle_development	ENSGALG00010020533	ENSGALG00010020533	-0.099503693	0.095869228	-1.037910658	0.299311651	0.412980254	negative
PRJNA729271	stage_E14_vs_E10	ordinary_muscle_development	ENSGALG00010020535	LMLN	-0.446814675	0.145265292	-3.07585295	0.002099013	0.006068049	negative
PRJNA729271	stage_E14_vs_E10	ordinary_muscle_development	ENSGALG00010020537	CCDC167	-0.010632491	0.095443932	-0.111400392	0.91129885	0.938926418	negative
PRJNA729271	stage_E14_vs_E10	ordinary_muscle_development	ENSGALG00010020540	PCGF5	-0.042384467	0.163117086	-0.259840755	0.794986613	0.856116712	negative
PRJNA729271	stage_E14_vs_E10	ordinary_muscle_development	ENSGALG00010020544	BMERB1	-0.790836961	0.163492154	-4.837155449	1.3171E-06	7.17184E-06	negative
PRJNA729271	stage_E14_vs_E10	ordinary_muscle_development	ENSGALG00010020545	STYX	0.872200768	0.141467216	6.165391494	7.0309E-10	5.85547E-09	positive
PRJNA729271	stage_E14_vs_E10	ordinary_muscle_development	ENSGALG00010020546	LRRC20	1.347567397	0.238423919	5.651980742	1.58609E-08	1.12698E-07	positive
PRJNA729271	stage_E14_vs_E10	ordinary_muscle_development	ENSGALG00010020548	CMTR1	-0.214454861	0.184890873	-1.159899664	0.24608966	0.352790982	negative
PRJNA729271	stage_E14_vs_E10	ordinary_muscle_development	ENSGALG00010020550	HECTD2	-0.951156181	0.217024238	-4.382718677	1.17207E-05	5.43481E-05	negative
PRJNA729271	stage_E14_vs_E10	ordinary_muscle_development	ENSGALG00010020554	ZFP36L1	-0.123748195	0.210902093	-0.586756599	0.557367189	0.661502196	negative
PRJNA729271	stage_E14_vs_E10	ordinary_muscle_development	ENSGALG00010020556	EMC1	-0.066043738	0.085396333	-0.773379086	0.439298064	0.553177433	negative
PRJNA729271	stage_E14_vs_E10	ordinary_muscle_development	ENSGALG00010020557	ENSGALG00010020557	-0.409011277	0.267735376	-1.527669904	0.126594516	0.206271078	negative
PRJNA729271	stage_E14_vs_E10	ordinary_muscle_development	ENSGALG00010020558	EIF4EBP2	0.694121058	0.099439768	6.980316519	2.94516E-12	3.21946E-11	positive
PRJNA729271	stage_E14_vs_E10	ordinary_muscle_development	ENSGALG00010020562	LRRN1	-1.233990982	0.226157509	-5.456334337	4.86064E-08	3.23188E-07	negative
PRJNA729271	stage_E14_vs_E10	ordinary_muscle_development	ENSGALG00010020568	PLA2G4F	-1.740764515	0.428462398	-4.062817467	4.84839E-05	0.000202296	negative
PRJNA729271	stage_E14_vs_E10	ordinary_muscle_development	ENSGALG00010020569	DTX3L	0.495696657	0.256476873	1.932714829	0.053271337	0.099937998	positive
PRJNA729271	stage_E14_vs_E10	ordinary_muscle_development	ENSGALG00010020571	MRTO4	0.046039915	0.079422458	0.57968384	0.562127843	0.665092081	positive
PRJNA729271	stage_E14_vs_E10	ordinary_muscle_development	ENSGALG00010020572	PPP1R3C	-0.855272015	0.274427687	-3.116566058	0.001829707	0.005392761	negative
PRJNA729271	stage_E14_vs_E10	ordinary_muscle_development	ENSGALG00010020574	DTL	-0.321469917	0.175957123	-1.826978701	0.067702972	0.12256208	negative
PRJNA729271	stage_E14_vs_E10	ordinary_muscle_development	ENSGALG00010020576	AKR7L	0.348787341	0.103695811	3.363562491	0.000769434	0.002491722	positive
PRJNA729271	stage_E14_vs_E10	ordinary_muscle_development	ENSGALG00010020578	TNKS2	0.016844203	0.062609552	0.269035671	0.787902233	0.850763026	positive
PRJNA729271	stage_E14_vs_E10	ordinary_muscle_development	ENSGALG00010020580	ENSGALG00010020580	0.148849741	0.11336734	1.312986101	0.189187632	0.285981304	positive
PRJNA729271	stage_E14_vs_E10	ordinary_muscle_development	ENSGALG00010020582	KANSL3	0.152730193	0.148108227	1.031206679	0.302443909	0.416129517	positive
PRJNA729271	stage_E14_vs_E10	ordinary_muscle_development	ENSGALG00010020584	PARP9	0.684619743	0.291110369	2.351753202	0.018685168	0.040955921	positive
PRJNA729271	stage_E14_vs_E10	ordinary_muscle_development	ENSGALG00010020586	C11orf16	0.120859752	0.097332175	1.241724559	0.214338205	0.316056184	positive
PRJNA729271	stage_E14_vs_E10	ordinary_muscle_development	ENSGALG00010020588	PALD1	0.011767728	0.109197061	0.107765978	0.914181331	0.940755838	positive
PRJNA729271	stage_E14_vs_E10	ordinary_muscle_development	ENSGALG00010020590	INTS7	-0.186565318	0.073935335	-2.523357988	0.011624	0.027193331	negative
PRJNA729271	stage_E14_vs_E10	ordinary_muscle_development	ENSGALG00010020594	PQLC2	-0.807924434	0.151438873	-5.334987084	9.55514E-08	6.10947E-07	negative
PRJNA729271	stage_E14_vs_E10	ordinary_muscle_development	ENSGALG00010020595	ZFYVE27	0.646599628	0.128637382	5.026529757	4.99435E-07	2.88304E-06	positive
PRJNA729271	stage_E14_vs_E10	ordinary_muscle_development	ENSGALG00010020596	SUMF1	0.130161648	0.119700397	1.087395287	0.276862153	0.387403891	positive
PRJNA729271	stage_E14_vs_E10	ordinary_muscle_development	ENSGALG00010020597	ARID5A	0.248357883	0.241487083	1.028452038	0.303737241	0.41731018	positive
PRJNA729271	stage_E14_vs_E10	ordinary_muscle_development	ENSGALG00010020599	PARN	-0.128448761	0.061956908	-2.073195134	0.038154123	0.075274593	negative
PRJNA729271	stage_E14_vs_E10	ordinary_muscle_development	ENSGALG00010020601	AKIP1	-0.439505178	0.159951311	-2.747743517	0.006000693	0.015267693	negative
PRJNA729271	stage_E14_vs_E10	ordinary_muscle_development	ENSGALG00010020602	DENND2B	-0.414616106	0.095025915	-4.363189835	1.2818E-05	5.90767E-05	negative
PRJNA729271	stage_E14_vs_E10	ordinary_muscle_development	ENSGALG00010020603	VPS39	-0.172959382	0.092912431	-1.861531132	0.062669208	0.11484073	negative
PRJNA729271	stage_E14_vs_E10	ordinary_muscle_development	ENSGALG00010020605	STAMBP	0.081531622	0.085403339	0.954665513	0.339746875	0.454801596	positive
PRJNA729271	stage_E14_vs_E10	ordinary_muscle_development	ENSGALG00010020607	ENSGALG00010020607	1.232412362	0.239182646	5.15259942	2.569E-07	1.54642E-06	positive
PRJNA729271	stage_E14_vs_E10	ordinary_muscle_development	ENSGALG00010020608	CAPZB	0.426385888	0.099380945	4.290418944	1.78336E-05	8.01049E-05	positive
PRJNA729271	stage_E14_vs_E10	ordinary_muscle_development	ENSGALG00010020609	AVPI1	0.488694335	0.225948813	2.162854186	0.030552396	0.062321557	positive
PRJNA729271	stage_E14_vs_E10	ordinary_muscle_development	ENSGALG00010020611	LPGAT1	-0.193834537	0.099123252	-1.955490089	0.050525252	0.095596336	negative
PRJNA729271	stage_E14_vs_E10	ordinary_muscle_development	ENSGALG00010020612	RPL27A	0.451088605	0.192769197	2.340045046	0.019281414	0.042078304	positive
PRJNA729271	stage_E14_vs_E10	ordinary_muscle_development	ENSGALG00010020613	ACTG2	0.59142964	0.240325982	2.460947569	0.013857061	0.031686087	positive
PRJNA729271	stage_E14_vs_E10	ordinary_muscle_development	ENSGALG00010020614	FAIM	-0.313154302	0.107263832	-2.919477114	0.003506191	0.00952084	negative
PRJNA729271	stage_E14_vs_E10	ordinary_muscle_development	ENSGALG00010020616	PI4K2A	0.407239264	0.130448663	3.121835481	0.001797273	0.005306881	positive
PRJNA729271	stage_E14_vs_E10	ordinary_muscle_development	ENSGALG00010020617	DGUOK	0.237321722	0.109589917	2.165543399	0.030346096	0.061962953	positive
PRJNA729271	stage_E14_vs_E10	ordinary_muscle_development	ENSGALG00010020618	ENSGALG00010020618	0.314447132	0.296827594	1.059359501	0.289436086	0.401894441	positive
PRJNA729271	stage_E14_vs_E10	ordinary_muscle_development	ENSGALG00010020619	QARS	-0.029943575	0.09896521	-0.302566675	0.762220114	0.830648663	negative
PRJNA729271	stage_E14_vs_E10	ordinary_muscle_development	ENSGALG00010020620	MARF1	-0.313634559	0.103343093	-3.0348865	0.002406262	0.006826927	negative
PRJNA729271	stage_E14_vs_E10	ordinary_muscle_development	ENSGALG00010020622	ABCG2	0.286673302	0.117635012	2.436972609	0.014810801	0.03358088	positive
PRJNA729271	stage_E14_vs_E10	ordinary_muscle_development	ENSGALG00010020623	NEK2	0.126952131	0.213242624	0.595341256	0.551615384	0.656350763	positive
PRJNA729271	stage_E14_vs_E10	ordinary_muscle_development	ENSGALG00010020624	MINOS1	0.294119858	0.13580157	2.165806023	0.030326013	0.061954552	positive
PRJNA729271	stage_E14_vs_E10	ordinary_muscle_development	ENSGALG00010020625	SLC30A1	0.234615739	0.074881579	3.133156934	0.00172937	0.005124123	positive
PRJNA729271	stage_E14_vs_E10	ordinary_muscle_development	ENSGALG00010020626	MORN4	0.078761671	0.116233274	0.677617241	0.498014411	0.607380639	positive
PRJNA729271	stage_E14_vs_E10	ordinary_muscle_development	ENSGALG00010020629	TRIM66	-1.27156318	0.22983898	-5.53240874	3.15863E-08	2.1709E-07	negative
PRJNA729271	stage_E14_vs_E10	ordinary_muscle_development	ENSGALG00010020630	QRICH1	-0.060725225	0.125635289	-0.483345289	0.628850588	0.722796707	negative
PRJNA729271	stage_E14_vs_E10	ordinary_muscle_development	ENSGALG00010020632	BLOC1S2	-0.017121672	0.110266783	-0.155274974	0.876604544	0.915313562	negative
PRJNA729271	stage_E14_vs_E10	ordinary_muscle_development	ENSGALG00010020633	HOGA1	-0.358003709	0.355388914	-1.00735756	0.313762978	0.427603833	negative
PRJNA729271	stage_E14_vs_E10	ordinary_muscle_development	ENSGALG00010020635	NDE1	-0.066328019	0.13303304	-0.498583049	0.618073149	0.713975956	negative
PRJNA729271	stage_E14_vs_E10	ordinary_muscle_development	ENSGALG00010020636	NBL1	0.633367756	0.178134875	3.555551701	0.000377187	0.001317961	positive
PRJNA729271	stage_E14_vs_E10	ordinary_muscle_development	ENSGALG00010020638	IMPDH2	-0.225271327	0.111688355	-2.016963421	0.043699331	0.084501307	negative
PRJNA729271	stage_E14_vs_E10	ordinary_muscle_development	ENSGALG00010020640	PTPN14	-0.234327119	0.091190584	-2.569641622	0.010180377	0.02417506	negative
PRJNA729271	stage_E14_vs_E10	ordinary_muscle_development	ENSGALG00010020642	ANKRD2	-0.640162236	0.701749773	-0.912237183	0.361643874	0.477879911	negative
PRJNA729271	stage_E14_vs_E10	ordinary_muscle_development	ENSGALG00010020646	CEP70	-0.288527887	0.114830035	-2.512651732	0.011982756	0.027942415	negative
PRJNA729271	stage_E14_vs_E10	ordinary_muscle_development	ENSGALG00010020648	TET3	-0.418452553	0.145864572	-2.868774425	0.004120656	0.010979578	negative
PRJNA729271	stage_E14_vs_E10	ordinary_muscle_development	ENSGALG00010020649	MYH11	1.568121932	0.212883007	7.366120749	1.75665E-13	2.17072E-12	positive
PRJNA729271	stage_E14_vs_E10	ordinary_muscle_development	ENSGALG00010020651	LMO1	0.15432601	0.165547485	0.932215977	0.35122492	0.467364842	positive
PRJNA729271	stage_E14_vs_E10	ordinary_muscle_development	ENSGALG00010020653	NDUFAF3	0.507302846	0.117681553	4.310810247	1.62657E-05	7.35868E-05	positive
PRJNA729271	stage_E14_vs_E10	ordinary_muscle_development	ENSGALG00010020654	SAYSD1	0.034022268	0.146501471	0.23223158	0.816358151	0.871281609	positive
PRJNA729271	stage_E14_vs_E10	ordinary_muscle_development	ENSGALG00010020655	RIC3	-0.520648773	0.130713792	-3.983120414	6.80163E-05	0.000276394	negative
PRJNA729271	stage_E14_vs_E10	ordinary_muscle_development	ENSGALG00010020657	DALRD3	0.450344603	0.090937407	4.95224809	7.3361E-07	4.12798E-06	positive
PRJNA729271	stage_E14_vs_E10	ordinary_muscle_development	ENSGALG00010020659	RAD51B	-0.65890597	0.130648374	-5.043353766	4.57442E-07	2.65757E-06	negative
PRJNA729271	stage_E14_vs_E10	ordinary_muscle_development	ENSGALG00010020662	BFAR	-0.065471022	0.085171247	-0.768698645	0.44207223	0.555994531	negative
PRJNA729271	stage_E14_vs_E10	ordinary_muscle_development	ENSGALG00010020664	ESYT3	0.942357188	0.4351612	2.165535871	0.030346671	0.061962953	positive
PRJNA729271	stage_E14_vs_E10	ordinary_muscle_development	ENSGALG00010020666	TMCO4	-0.063769833	0.208014347	-0.306564592	0.759174808	0.828278014	negative
PRJNA729271	stage_E14_vs_E10	ordinary_muscle_development	ENSGALG00010020667	UBTD1	-0.250099581	0.152411844	-1.640945839	0.100808659	0.1711834	negative
PRJNA729271	stage_E14_vs_E10	ordinary_muscle_development	ENSGALG00010020670	ENSGALG00010020670	0.05086814	0.128177234	0.396857839	0.691472296	0.774686268	positive
PRJNA729271	stage_E14_vs_E10	ordinary_muscle_development	ENSGALG00010020673	MMS19	0.004019941	0.126502809	0.031777482	0.974649504	0.982854081	positive
PRJNA729271	stage_E14_vs_E10	ordinary_muscle_development	ENSGALG00010020674	TRPV4	-5.477833512	0.541941444	-10.10779592	5.10191E-24	1.41671E-22	negative
PRJNA729271	stage_E14_vs_E10	ordinary_muscle_development	ENSGALG00010020675	SMYD2	-0.596910335	0.177774487	-3.357682781	0.000785988	0.002539654	negative
PRJNA729271	stage_E14_vs_E10	ordinary_muscle_development	ENSGALG00010020676	CYP2C23a	4.218537727	1.404295137	3.004025019	0.002664334	0.007481504	positive
PRJNA729271	stage_E14_vs_E10	ordinary_muscle_development	ENSGALG00010020677	CYR61	-1.445483495	0.237410088	-6.088551268	1.13937E-09	9.24184E-09	negative
PRJNA729271	stage_E14_vs_E10	ordinary_muscle_development	ENSGALG00010020679	OTUD3	-0.185737546	0.108047214	-1.719040585	0.085606982	0.149628989	negative
PRJNA729271	stage_E14_vs_E10	ordinary_muscle_development	ENSGALG00010020680	CFAP221	-1.396931394	0.588564781	-2.373453933	0.017622591	0.039004346	negative
PRJNA729271	stage_E14_vs_E10	ordinary_muscle_development	ENSGALG00010020681	BCL10	0.245002131	0.08508843	2.879382445	0.003984548	0.010646272	positive
PRJNA729271	stage_E14_vs_E10	ordinary_muscle_development	ENSGALG00010020682	ZDHHC16	-0.042505384	0.168088214	-0.252875455	0.800364457	0.859481613	negative
PRJNA729271	stage_E14_vs_E10	ordinary_muscle_development	ENSGALG00010020684	P4HTM	0.273457701	0.12386173	2.20776587	0.027260598	0.056700011	positive
PRJNA729271	stage_E14_vs_E10	ordinary_muscle_development	ENSGALG00010020685	RPL6	0.251399911	0.116000947	2.167222928	0.03021786	0.061804414	positive
PRJNA729271	stage_E14_vs_E10	ordinary_muscle_development	ENSGALG00010020686	C1orf52	-0.034108455	0.121148764	-0.281541912	0.778294787	0.842995016	negative
PRJNA729271	stage_E14_vs_E10	ordinary_muscle_development	ENSGALG00010020691	ENSGALG00010020691	0.459972897	0.163104632	2.820109348	0.004800729	0.012565288	positive
PRJNA729271	stage_E14_vs_E10	ordinary_muscle_development	ENSGALG00010020692	TUB	1.124988053	0.152108362	7.39596455	1.40385E-13	1.74668E-12	positive
PRJNA729271	stage_E14_vs_E10	ordinary_muscle_development	ENSGALG00010020693	PTGFR	-0.537637669	0.189073424	-2.843539072	0.004461554	0.011783933	negative
PRJNA729271	stage_E14_vs_E10	ordinary_muscle_development	ENSGALG00010020696	MOB1A	-0.114921589	0.098913038	-1.1618447	0.245298553	0.352005002	negative
PRJNA729271	stage_E14_vs_E10	ordinary_muscle_development	ENSGALG00010020699	PLA2G10	-0.702426021	0.417354802	-1.683042864	0.092366794	0.15921711	negative
PRJNA729271	stage_E14_vs_E10	ordinary_muscle_development	ENSGALG00010020700	MTHFD2	1.143091338	0.131333326	8.703741616	3.21118E-18	5.96744E-17	positive
PRJNA729271	stage_E14_vs_E10	ordinary_muscle_development	ENSGALG00010020701	PTPN11	-0.137039867	0.084347958	-1.624696926	0.104227153	0.176142308	negative
PRJNA729271	stage_E14_vs_E10	ordinary_muscle_development	ENSGALG00010020704	PROX1	-0.57577063	0.240708268	-2.39198526	0.016757515	0.037346277	negative
PRJNA729271	stage_E14_vs_E10	ordinary_muscle_development	ENSGALG00010020705	EXOSC1	0.368874221	0.174049272	2.119366641	0.034059494	0.068340582	positive
PRJNA729271	stage_E14_vs_E10	ordinary_muscle_development	ENSGALG00010020707	SLC4A5	-1.403952109	0.402065057	-3.491853086	0.000479682	0.001634075	negative
PRJNA729271	stage_E14_vs_E10	ordinary_muscle_development	ENSGALG00010020708	FAM214A	-0.387433102	0.133986018	-2.891593524	0.003832935	0.010293259	negative
PRJNA729271	stage_E14_vs_E10	ordinary_muscle_development	ENSGALG00010020712	ZC3H7A	-0.192759837	0.069603551	-2.769396617	0.005616022	0.014412933	negative
PRJNA729271	stage_E14_vs_E10	ordinary_muscle_development	ENSGALG00010020713	SCTR	-2.18359639	1.15286225	-1.894065305	0.058216342	0.107770866	negative
PRJNA729271	stage_E14_vs_E10	ordinary_muscle_development	ENSGALG00010020714	PGAM1	0.660219476	0.144569867	4.566784819	4.95262E-06	2.4451E-05	positive
PRJNA729271	stage_E14_vs_E10	ordinary_muscle_development	ENSGALG00010020715	ENSGALG00010020715	7.675580237	0.68921805	11.13665006	8.31899E-29	3.31274E-27	positive
PRJNA729271	stage_E14_vs_E10	ordinary_muscle_development	ENSGALG00010020716	RRAS	0.578776709	0.179146553	3.230744316	0.001234683	0.003813496	positive
PRJNA729271	stage_E14_vs_E10	ordinary_muscle_development	ENSGALG00010020719	RPH3A	-0.617944146	0.640102612	-0.965382947	0.33435304	0.449535972	negative

PRJNA729271	stage_E14_vs_E10	ordinary_muscle_development	ENSGALG00010020720	ENSGALG00010020720	-1.812166865	0.263695255	-6.872201254	6.32187E-12	6.58774E-11	negative
PRJNA729271	stage_E14_vs_E10	ordinary_muscle_development	ENSGALG00010020721	PTDSS2	-0.140931259	0.16991993	-0.829398052	0.406879203	0.521782128	negative
PRJNA729271	stage_E14_vs_E10	ordinary_muscle_development	ENSGALG00010020723	RPS24	-0.070684422	0.190111651	-0.371804787	0.710038199	0.789892644	negative
PRJNA729271	stage_E14_vs_E10	ordinary_muscle_development	ENSGALG00010020724	TACR1	-0.976710101	0.257170013	-3.797915982	0.000145918	0.000557333	negative
PRJNA729271	stage_E14_vs_E10	ordinary_muscle_development	ENSGALG00010020725	FOPNL	0.074924079	0.105251405	0.71185823	0.47655257	0.588735149	positive
PRJNA729271	stage_E14_vs_E10	ordinary_muscle_development	ENSGALG00010020728	TMEM37	0.340587258	0.211000327	1.614155121	0.106493784	0.179491558	positive
PRJNA729271	stage_E14_vs_E10	ordinary_muscle_development	ENSGALG00010020730	RRP12	-0.045041576	0.461772098	-0.097540704	0.922297011	0.946389294	negative
PRJNA729271	stage_E14_vs_E10	ordinary_muscle_development	ENSGALG00010020732	ARIH2	-0.021723819	0.143471307	-0.151415773	0.879647748	0.917301734	negative
PRJNA729271	stage_E14_vs_E10	ordinary_muscle_development	ENSGALG00010020733	TMEM229B	-1.169367468	0.108454362	-10.78211557	4.18156E-27	1.48195E-25	negative
PRJNA729271	stage_E14_vs_E10	ordinary_muscle_development	ENSGALG00010020736	LRRTM4	0.350055327	0.218323333	1.603380279	0.108850722	0.182720465	positive
PRJNA729271	stage_E14_vs_E10	ordinary_muscle_development	ENSGALG00010020737	POLR3A	-0.61960775	0.115831419	-5.34922006	8.83341E-08	5.66801E-07	negative
PRJNA729271	stage_E14_vs_E10	ordinary_muscle_development	ENSGALG00010020739	PKP3	-0.343864188	0.637813409	-0.539129757	0.589797324	0.689833956	negative
PRJNA729271	stage_E14_vs_E10	ordinary_muscle_development	ENSGALG00010020742	THAP10	-0.988251551	0.197443095	-5.005247469	5.57903E-07	3.19258E-06	negative
PRJNA729271	stage_E14_vs_E10	ordinary_muscle_development	ENSGALG00010020743	PURB	0.454588176	0.165713834	2.743211989	0.006084139	0.015447345	positive
PRJNA729271	stage_E14_vs_E10	ordinary_muscle_development	ENSGALG00010020744	CHST3	-0.240755026	0.111467701	-2.159863559	0.030783234	0.06273063	negative
PRJNA729271	stage_E14_vs_E10	ordinary_muscle_development	ENSGALG00010020745	ABCC1	-0.543842911	0.166809535	-3.260262737	0.001113091	0.003464515	negative
PRJNA729271	stage_E14_vs_E10	ordinary_muscle_development	ENSGALG00010020747	DBI	0.083544334	0.120474657	0.693459817	0.488021035	0.599131511	positive
PRJNA729271	stage_E14_vs_E10	ordinary_muscle_development	ENSGALG00010020750	AIFM2	-0.759957177	0.29054879	-2.615592299	0.008907284	0.021543549	negative
PRJNA729271	stage_E14_vs_E10	ordinary_muscle_development	ENSGALG00010020753	SYDE2	0.628354394	0.096645311	6.501654197	7.94415E-11	7.39567E-10	positive
PRJNA729271	stage_E14_vs_E10	ordinary_muscle_development	ENSGALG00010020754	SLC25A20	0.186309414	0.114466837	1.627627876	0.103603815	0.175272793	positive
PRJNA729271	stage_E14_vs_E10	ordinary_muscle_development	ENSGALG00010020756	MACROH2A2	-0.19025444	0.147809624	-1.287158676	0.198038987	0.29663895	negative
PRJNA729271	stage_E14_vs_E10	ordinary_muscle_development	ENSGALG00010020759	ARHGAP19	0.056567847	0.164885396	0.343073724	0.731542998	0.806611575	positive
PRJNA729271	stage_E14_vs_E10	ordinary_muscle_development	ENSGALG00010020760	TXNDC11	-0.289245623	0.093652643	-3.088493977	0.002011738	0.00584723	negative
PRJNA729271	stage_E14_vs_E10	ordinary_muscle_development	ENSGALG00010020762	TTBK2	-0.494845054	0.141557184	-3.49572548	0.000472775	0.00161282	negative
PRJNA729271	stage_E14_vs_E10	ordinary_muscle_development	ENSGALG00010020764	PRKAR2A	0.050474102	0.098277576	0.51358717	0.607540651	0.705011748	positive
PRJNA729271	stage_E14_vs_E10	ordinary_muscle_development	ENSGALG00010020765	GLT1D1	-1.720604804	0.185926705	-9.25421016	2.1582E-20	4.74706E-19	negative
PRJNA729271	stage_E14_vs_E10	ordinary_muscle_development	ENSGALG00010020766	C2orf76	0.270261218	0.118933892	2.27236503	0.023064472	0.049237043	positive
PRJNA729271	stage_E14_vs_E10	ordinary_muscle_development	ENSGALG00010020767	UBXN10	-0.265700278	0.171571019	-1.548631469	0.121470337	0.199582014	negative
PRJNA729271	stage_E14_vs_E10	ordinary_muscle_development	ENSGALG00010020768	DNAH8	-1.194013165	0.678984882	-1.758526878	0.078657902	0.139396623	negative
PRJNA729271	stage_E14_vs_E10	ordinary_muscle_development	ENSGALG00010020770	B4GALNT4	-0.869151003	0.196181594	-4.430339184	9.4085E-06	4.43212E-05	negative
PRJNA729271	stage_E14_vs_E10	ordinary_muscle_development	ENSGALG00010020771	LINGO1	1.980630029	0.200218534	9.892341088	4.49394E-23	1.18651E-21	positive
PRJNA729271	stage_E14_vs_E10	ordinary_muscle_development	ENSGALG00010020773	ZCCHC24	0.524202597	0.08943667	5.86115962	4.59646E-09	3.49191E-08	positive
PRJNA729271	stage_E14_vs_E10	ordinary_muscle_development	ENSGALG00010020775	UBR1	0.078510065	0.081067758	0.968449932	0.332819712	0.447765151	positive
PRJNA729271	stage_E14_vs_E10	ordinary_muscle_development	ENSGALG00010020776	MCOLN3	-0.976821955	0.44808869	-2.179974582	0.029259346	0.060158045	negative
PRJNA729271	stage_E14_vs_E10	ordinary_muscle_development	ENSGALG00010020778	SLIT1	-2.168372663	0.22928507	-9.457103611	3.16591E-21	7.42554E-20	negative
PRJNA729271	stage_E14_vs_E10	ordinary_muscle_development	ENSGALG00010020779	SLC15A4	-0.062441011	0.064768873	-0.964058934	0.33501638	0.450260772	negative
PRJNA729271	stage_E14_vs_E10	ordinary_muscle_development	ENSGALG00010020781	SNN	0.103498657	0.134362884	0.770292015	0.441126692	0.555094314	positive
PRJNA729271	stage_E14_vs_E10	ordinary_muscle_development	ENSGALG00010020782	PPIF	0.411955125	0.156397304	2.634029585	0.008437814	0.020572936	positive
PRJNA729271	stage_E14_vs_E10	ordinary_muscle_development	ENSGALG00010020786	DDX19	-0.414147512	0.131052458	-3.160165934	0.001576793	0.00471931	negative
PRJNA729271	stage_E14_vs_E10	ordinary_muscle_development	ENSGALG00010020787	TMEM132C	-3.109191425	0.293692936	-10.58653799	3.44083E-26	1.15184E-24	negative
PRJNA729271	stage_E14_vs_E10	ordinary_muscle_development	ENSGALG00010020790	PRPH2L	-0.228602457	0.350109018	-0.652946497	0.513790772	0.622483888	negative
PRJNA729271	stage_E14_vs_E10	ordinary_muscle_development	ENSGALG00010020794	STEAP3	1.736937183	0.129950931	13.36610036	9.54135E-41	7.72636E-39	positive
PRJNA729271	stage_E14_vs_E10	ordinary_muscle_development	ENSGALG00010020797	DGCR8	-0.486541628	0.065307002	-7.450068309	9.32919E-14	1.18516E-12	negative
PRJNA729271	stage_E14_vs_E10	ordinary_muscle_development	ENSGALG00010020798	C1QL2	-0.90513162	0.550387698	-1.644534614	0.100065821	0.170201135	negative
PRJNA729271	stage_E14_vs_E10	ordinary_muscle_development	ENSGALG00010020803	ABCC6	0.469250452	0.162894282	2.880705494	0.003967862	0.010605598	positive
PRJNA729271	stage_E14_vs_E10	ordinary_muscle_development	ENSGALG00010020806	HTD2	0.15571597	0.172508789	0.902655283	0.366708876	0.482827247	positive
PRJNA729271	stage_E14_vs_E10	ordinary_muscle_development	ENSGALG00010020809	ENSGALG00010020809	1.079669363	0.220660782	4.892891955	9.9365E-07	5.48684E-06	positive
PRJNA729271	stage_E14_vs_E10	ordinary_muscle_development	ENSGALG00010020810	IFITM5	-0.540672066	0.243811713	-2.217580356	0.026583459	0.05550666	negative
PRJNA729271	stage_E14_vs_E10	ordinary_muscle_development	ENSGALG00010020811	MCOLN2	-1.974758793	0.735210976	-2.68597567	0.007231835	0.017937278	negative
PRJNA729271	stage_E14_vs_E10	ordinary_muscle_development	ENSGALG00010020813	NPY4R	-0.253130521	0.545761677	-0.463811462	0.642782831	0.735079853	negative
PRJNA729271	stage_E14_vs_E10	ordinary_muscle_development	ENSGALG00010020816	TRMT2A	0.419400007	0.087783782	4.777649592	1.77356E-06	9.42712E-06	positive
PRJNA729271	stage_E14_vs_E10	ordinary_muscle_development	ENSGALG00010020818	TMEM62	-0.520056657	0.196681106	-2.644161746	0.008189351	0.020057185	negative
PRJNA729271	stage_E14_vs_E10	ordinary_muscle_development	ENSGALG00010020820	VWA5B1	-0.880227807	0.454174166	-1.938084269	0.052612942	0.09888011	negative
PRJNA729271	stage_E14_vs_E10	ordinary_muscle_development	ENSGALG00010020827	ENSGALG00010020827	-0.131776527	0.091289453	-1.443502209	0.148879051	0.236027763	negative
PRJNA729271	stage_E14_vs_E10	ordinary_muscle_development	ENSGALG00010020828	CAMK2N1	0.007156128	0.208330414	0.034349896	0.972598137	0.981195017	positive
PRJNA729271	stage_E14_vs_E10	ordinary_muscle_development	ENSGALG00010020831	MUL1	0.34197625	0.143657588	2.380495558	0.017289369	0.038366413	positive
PRJNA729271	stage_E14_vs_E10	ordinary_muscle_development	ENSGALG00010020833	RANBP1	-0.514336921	0.081382341	-6.320006451	2.61552E-10	2.29492E-09	negative
PRJNA729271	stage_E14_vs_E10	ordinary_muscle_development	ENSGALG00010020835	PTGR2	0.544528938	0.11385827	4.782515486	1.73115E-06	9.2186E-06	positive
PRJNA729271	stage_E14_vs_E10	ordinary_muscle_development	ENSGALG00010020839	PGGHG	-0.704233753	0.139506783	-5.048025191	4.464E-07	2.59654E-06	negative
PRJNA729271	stage_E14_vs_E10	ordinary_muscle_development	ENSGALG00010020840	LPAR3	1.330005599	0.237240071	5.606159177	2.06866E-08	1.45136E-07	positive
PRJNA729271	stage_E14_vs_E10	ordinary_muscle_development	ENSGALG00010020843	HMG20A	-0.20191046	0.082182043	-2.456868343	0.014015404	0.031997682	negative
PRJNA729271	stage_E14_vs_E10	ordinary_muscle_development	ENSGALG00010020845	RAB35	-0.158033163	0.091865934	-1.720258609	0.085385444	0.14931379	negative
PRJNA729271	stage_E14_vs_E10	ordinary_muscle_development	ENSGALG00010020847	MIDEAS	0.359614002	0.088915011	4.044468957	5.24419E-05	0.000217433	positive
PRJNA729271	stage_E14_vs_E10	ordinary_muscle_development	ENSGALG00010020854	ENSGALG00010020854	6.408364429	0.382875328	16.73747029	6.98961E-63	1.80919E-60	positive
PRJNA729271	stage_E14_vs_E10	ordinary_muscle_development	ENSGALG00010020855	DNAL1	0.428940838	0.097457623	4.401306195	1.07601E-05	5.01666E-05	positive
PRJNA729271	stage_E14_vs_E10	ordinary_muscle_development	ENSGALG00010020856	VTG2	0.019467623	0.55809068	0.03488254	0.972173399	0.98083479	positive
PRJNA729271	stage_E14_vs_E10	ordinary_muscle_development	ENSGALG00010020861	CDA	-0.076073338	0.184068073	-0.413289155	0.679394775	0.76547433	negative
PRJNA729271	stage_E14_vs_E10	ordinary_muscle_development	ENSGALG00010020865	BICDL1	-1.372016202	0.728988563	-1.882081931	0.059824892	0.110283837	negative
PRJNA729271	stage_E14_vs_E10	ordinary_muscle_development	ENSGALG00010020866	LCOR	0.063793431	0.093821313	0.679946051	0.496538622	0.606039687	positive
PRJNA729271	stage_E14_vs_E10	ordinary_muscle_development	ENSGALG00010020870	RIOX1	-0.034706924	0.146595103	-0.236753641	0.812847918	0.868767923	negative
PRJNA729271	stage_E14_vs_E10	ordinary_muscle_development	ENSGALG00010020873	PINK1	0.116916048	0.138246738	0.845705654	0.397716968	0.513120379	positive
PRJNA729271	stage_E14_vs_E10	ordinary_muscle_development	ENSGALG00010020875	ENSGALG00010020875	0.856225709	0.171845088	4.982543984	6.27537E-07	3.56432E-06	positive
PRJNA729271	stage_E14_vs_E10	ordinary_muscle_development	ENSGALG00010020878	XYLT1	-0.114616954	0.12343043	-0.928595596	0.353098705	0.469341195	negative
PRJNA729271	stage_E14_vs_E10	ordinary_muscle_development	ENSGALG00010020880	PXK	0.070368066	0.168447545	0.41774468	0.676133801	0.762333498	positive
PRJNA729271	stage_E14_vs_E10	ordinary_muscle_development	ENSGALG00010020886	GBF1	-0.03678148	0.085890436	-0.4282372	0.668478435	0.756196867	negative
PRJNA729271	stage_E14_vs_E10	ordinary_muscle_development	ENSGALG00010020887	ARPP19	-0.345385788	0.070914191	-4.870474927	1.1133E-06	6.11726E-06	negative
PRJNA729271	stage_E14_vs_E10	ordinary_muscle_development	ENSGALG00010020889	PPRC1	-0.317701879	0.28146044	-1.128762104	0.258998203	0.367408392	negative
PRJNA729271	stage_E14_vs_E10	ordinary_muscle_development	ENSGALG00010020890	RPS15A	0.396926567	0.188338138	2.107520922	0.03507245	0.070053075	positive
PRJNA729271	stage_E14_vs_E10	ordinary_muscle_development	ENSGALG00010020894	PSMD13	0.271680719	0.120716288	2.250572173	0.024412648	0.051688771	positive
PRJNA729271	stage_E14_vs_E10	ordinary_muscle_development	ENSGALG00010020896	MARCO	1.967803507	0.392386491	5.014962422	5.30438E-07	3.04624E-06	positive
PRJNA729271	stage_E14_vs_E10	ordinary_muscle_development	ENSGALG00010020898	ENSGALG00010020898	0.532917887	0.121406717	4.389525556	1.13598E-05	5.2742E-05	positive
PRJNA729271	stage_E14_vs_E10	ordinary_muscle_development	ENSGALG00010020900	PDHB	0.279843259	0.115940923	2.413671127	0.01579271	0.035474249	positive
PRJNA729271	stage_E14_vs_E10	ordinary_muscle_development	ENSGALG00010020903	AGMAT	-0.302562282	0.398848692	-0.758589131	0.448098377	0.56123615	negative
PRJNA729271	stage_E14_vs_E10	ordinary_muscle_development	ENSGALG00010020905	KCTD6	-0.090717459	0.083206695	-1.090266345	0.275595836	0.386079215	negative
PRJNA729271	stage_E14_vs_E10	ordinary_muscle_development	ENSGALG00010020906	SIRT3	0.201691176	0.11106488	1.815976179	0.069374026	0.125102826	positive
PRJNA729271	stage_E14_vs_E10	ordinary_muscle_development	ENSGALG00010020912	PDE8A	0.248700207	0.144374998	1.722598863	0.084961091	0.148787122	positive
PRJNA729271	stage_E14_vs_E10	ordinary_muscle_development	ENSGALG00010020913	TMEM132B	-1.652559274	0.242546926	-6.813358967	9.53458E-12	9.7878E-11	negative
PRJNA729271	stage_E14_vs_E10	ordinary_muscle_development	ENSGALG00010020915	EN1	-3.513116177	0.349231654	-10.05955828	8.33716E-24	2.27156E-22	negative
PRJNA729271	stage_E14_vs_E10	ordinary_muscle_development	ENSGALG00010020916	ARL6IP1	0.719162375	0.096786215	7.430421547	1.08252E-13	1.36326E-12	positive
PRJNA729271	stage_E14_vs_E10	ordinary_muscle_development	ENSGALG00010020918	ENSGALG00010020918	-1.941858706	0.355540159	-5.461714118	4.71559E-08	3.14264E-07	negative
PRJNA729271	stage_E14_vs_E10	ordinary_muscle_development	ENSGALG00010020920	ACOX2	0.762721887	0.206973055	3.685126479	0.000228589	0.000837775	positive
PRJNA729271	stage_E14_vs_E10	ordinary_muscle_development	ENSGALG00010020921	LDB1	-0.369992648	0.148896642	-2.484895848	0.012958934	0.029910789	negative
PRJNA729271	stage_E14_vs_E10	ordinary_muscle_development	ENSGALG00010020922	INSIG2	-0.146243284	0.080068379	-1.826479896	0.067778007	0.122651961	negative
PRJNA729271	stage_E14_vs_E10	ordinary_muscle_development	ENSGALG00010020924	DNAJC16	-0.021985688	0.119994978	-0.183221733	0.854624042	0.899359336	negative
PRJNA729271	stage_E14_vs_E10	ordinary_muscle_development	ENSGALG00010020925	RIC8A	1.057817481	0.102887605	10.28129168	8.5573E-25	2.5211E-23	positive
PRJNA729271	stage_E14_vs_E10	ordinary_muscle_development	ENSGALG00010020927	ENSGALG00010020927	-0.057157877	0.114194044	-0.500532904	0.616699893	0.712730422	negative
PRJNA729271	stage_E14_vs_E10	ordinary_muscle_development	ENSGALG00010020928	AACS	-0.316763504	0.150032275	-2.111302404	0.034746328	0.069478275	negative
PRJNA729271	stage_E14_vs_E10	ordinary_muscle_development	ENSGALG00010020932	CASP9	0.102087029	0.115014695	0.88759987	0.374756024	0.491058449	positive
PRJNA729271	stage_E14_vs_E10	ordinary_muscle_development	ENSGALG00010020934	ABCD3	-0.318722745	0.153622683	-2.074711483	0.038013283	0.075037795	negative
PRJNA729271	stage_E14_vs_E10	ordinary_muscle_development	ENSGALG00010020935	BRI3BP	-0.133028542	0.064418793	-2.065057959	0.038917517	0.076546236	negative
PRJNA729271	stage_E14_vs_E10	ordinary_muscle_development	ENSGALG00010020936	BET1L	0.261083523	0.12498628	2.088897458	0.036716955	0.072816016	positive
PRJNA729271	stage_E14_vs_E10	ordinary_muscle_development	ENSGALG00010020938	SMG1	-0.027394013	0.07978416	-0.343351521	0.731334026	0.806565221	negative
PRJNA729271	stage_E14_vs_E10	ordinary_muscle_development	ENSGALG00010020939	CTRC	1.912308269	2.243200524	0.852491005	0.393941629	0.509292179	positive
PRJNA729271	stage_E14_vs_E10	ordinary_muscle_development	ENSGALG00010020940	ENSGALG00010020940	-0.362520565	0.129437306	-2.800742518	0.005098518	0.013239249	negative
PRJNA729271	stage_E14_vs_E10	ordinary_muscle_development	ENSGALG00010020941	ENSGALG00010020941	0.722997375	0.195990378	3.688943214	0.000225187	0.000825934	positive
PRJNA729271	stage_E14_vs_E10	ordinary_muscle_development	ENSGALG00010020943	FAM107A	3.416638146	0.328868504	10.38907073	2.78049E-25	8.57514E-24	positive
PRJNA729271	stage_E14_vs_E10	ordinary_muscle_development	ENSGALG00010020944	MYO5A	-0.898683726	0.155623062	-5.774746446	7.70691E-09	5.68218E-08	negative
PRJNA729271	stage_E14_vs_E10	ordinary_muscle_development	ENSGALG00010020945	NUMB	0.51353488	0.084893067	6.049196951	1.4557E-09	1.16964E-08	positive
PRJNA729271	stage_E14_vs_E10	ordinary_muscle_development	ENSGALG00010020946	CTBS	0.181360007	0.2155636	0.841329459	0.400163392	0.515496655	positive
PRJNA729271	stage_E14_vs_E10	ordinary_muscle_development	ENSGALG00010020949	HPS6	0.066327067	0.109761785	0.604281964	0.545656196	0.651184468	positive
PRJNA729271	stage_E14_vs_E10	ordinary_muscle_development	ENSGALG00010020950	EFHD2	0.394407077	0.237150931	1.66310575	0.096291269	0.164825454	positive
PRJNA729271	stage_E14_vs_E10	ordinary_muscle_development	ENSGALG00010020951	ENSGALG00010020951	-4.143545287	0.373156146	-11.10405209	1.19883E-28	4.73487E-27	negative
PRJNA729271	stage_E14_vs_E10	ordinary_muscle_development	ENSGALG00010020952	GNG5	0.178692408	0.09761983	1.830492928	0.067176263	0.121760652	positive
PRJNA729271	stage_E14_vs_E10	ordinary_muscle_development	ENSGALG00010020954	PLIN1	8.886805321	1.406063849	6.320342656	2.60984E-10	2.2927E-09	positive
PRJNA729271	stage_E14_vs_E10	ordinary_muscle_development	ENSGALG00010020955	FHAD1	-0.856651056	0.913691187	-0.937571762	0.348464529	0.464224292	negative
PRJNA729271	stage_E14_vs_E10	ordinary_muscle_development	ENSGALG00010020957	RPF1	-0.219543479	0.100551606	-2.183391077	0.029007022	0.059698536	negative
PRJNA729271	stage_E14_vs_E10	ordinary_muscle_development	ENSGALG00010020959	ENSGALG00010020959	-0.179623163	0.349619426	-0.513767685	0.607414424	0.704952963	negative
PRJNA729271	stage_E14_vs_E10	ordinary_muscle_development	ENSGALG00010020960	CLEC19A	4.523410842	0.365806951	12.36556831	4.01363E-35	2.2376E-33	positive
PRJNA729271	stage_E14_vs_E10	ordinary_muscle_development	ENSGALG00010020962	MYO5C	-1.157365412	0.324325209	-3.568533619	0.000358985	0.001261757	negative
PRJNA729271	stage_E14_vs_E10	ordinary_muscle_development	ENSGALG00010020963	CCDC93	0.03267583	0.10004581	0.326608678	0.743963879	0.816209236	positive
PRJNA729271	stage_E14_vs_E10	ordinary_muscle_development	ENSGALG00010020965	DHX37	-0.292547578	0.091283776	-3.204814578	0.001351495	0.004125063	negative
PRJNA729271	stage_E14_vs_E10	ordinary_muscle_development	ENSGALG00010020966	PAPLN	1.225136493	0.246342296	4.973309547	6.58194E-07	3.72677E-06	positive
PRJNA729271	stage_E14_vs_E10	ordinary_muscle_development	ENSGALG00010020967	VPS51	0.115899636	0.07338359	1.579367204	0.114251855	0.190310347	positive
PRJNA729271	stage_E14_vs_E10	ordinary_muscle_development	ENSGALG00010020969	RBP3	-3.939002783	0.451022802	-8.733489229	2.46934E-18	4.63042E-17	negative
PRJNA729271	stage_E14_vs_E10	ordinary_muscle_development	ENSGALG00010020970	ARMH3	0.173167914	0.078253109	2.212920553	0.026903123	0.056117538	positive
PRJNA729271	stage_E14_vs_E10	ordinary_muscle_development	ENSGALG00010020971	TMEM51	-0.292685274	0.272945377	-1.072321786	0.283575525	0.395119411	negative
PRJNA729271	stage_E14_vs_E10	ordinary_muscle_development	ENSGALG00010020973	GNB5	0.261325575	0.159260428	1.640869473	0.100824513	0.171190268	positive
PRJNA729271	stage_E14_vs_E10	ordinary_muscle_development	ENSGALG00010020975	HCCS	-0.058090974	0.130370987	-0.445582068	0.655899166	0.746428391	negative
PRJNA729271	stage_E14_vs_E10	ordinary_muscle_development	ENSGALG00010020976	GDF10	-3.679930138	0.334163098	-11.01237737	3.33096E-28	1.27059E-26	negative
PRJNA729271	stage_E14_vs_E10	ordinary_muscle_development	ENSGALG00010020978	BCL2L10	1.190338709	0.115351625	10.31921927	5.76903E-25	1.73131E-23	positive
PRJNA729271	stage_E14_vs_E10	ordinary_muscle_development	ENSGALG00010020979	ENSGALG00010020979	0.060611264	0.245354711	0.247035256	0.804880939	0.862861417	positive

PRJNA729271	stage_E14_vs_E10	ordinary_muscle_development	ENSGALG00010020981	CIT	-0.302494287	0.233112558	-1.297631881	0.194413862	0.292508711	negative
PRJNA729271	stage_E14_vs_E10	ordinary_muscle_development	ENSGALG00010020982	TLCD4	-0.050204544	0.151083954	-0.332295671	0.739666008	0.812724287	negative
PRJNA729271	stage_E14_vs_E10	ordinary_muscle_development	ENSGALG00010020984	PSEN1	-0.174477926	0.057150883	-3.052934918	0.002266151	0.006492954	negative
PRJNA729271	stage_E14_vs_E10	ordinary_muscle_development	ENSGALG00010020986	CFAP20DC	-1.310058472	0.304288159	-4.305321894	1.66743E-05	7.52472E-05	negative
PRJNA729271	stage_E14_vs_E10	ordinary_muscle_development	ENSGALG00010020987	NADSYN1	0.166274062	0.090176113	1.843881446	0.06520042	0.118755305	positive
PRJNA729271	stage_E14_vs_E10	ordinary_muscle_development	ENSGALG00010020989	MAPK4	-0.142783148	0.15290241	-0.933818815	0.350397362	0.466434913	negative
PRJNA729271	stage_E14_vs_E10	ordinary_muscle_development	ENSGALG00010020990	UBB	-0.141280031	0.106354221	-1.328391388	0.184048843	0.279789178	negative
PRJNA729271	stage_E14_vs_E10	ordinary_muscle_development	ENSGALG00010020991	ALG14	0.401408621	0.13616418	2.947975163	0.003198628	0.008796074	positive
PRJNA729271	stage_E14_vs_E10	ordinary_muscle_development	ENSGALG00010020992	KCNH7	-1.960357153	0.550719133	-3.559631466	0.000371376	0.001300319	negative
PRJNA729271	stage_E14_vs_E10	ordinary_muscle_development	ENSGALG00010020994	DHCR7	-0.039466272	0.140514688	-0.28086937	0.778810595	0.843341832	negative
PRJNA729271	stage_E14_vs_E10	ordinary_muscle_development	ENSGALG00010020998	SYT17	-0.536108358	0.283738837	-1.889442996	0.058832494	0.108706293	negative
PRJNA729271	stage_E14_vs_E10	ordinary_muscle_development	ENSGALG00010020999	IFIH1	-0.446482457	0.340459933	-1.311409696	0.189719385	0.286665535	negative
PRJNA729271	stage_E14_vs_E10	ordinary_muscle_development	ENSGALG00010021000	NFKB2	0.367024126	0.114593146	3.202845365	0.00136077	0.004149004	positive
PRJNA729271	stage_E14_vs_E10	ordinary_muscle_development	ENSGALG00010021002	SLC44A3	-0.458111682	0.158683832	-2.886946184	0.003890008	0.010426342	negative
PRJNA729271	stage_E14_vs_E10	ordinary_muscle_development	ENSGALG00010021003	KAZN	-0.069773048	0.115946796	-0.601767795	0.547328715	0.652643117	negative
PRJNA729271	stage_E14_vs_E10	ordinary_muscle_development	ENSGALG00010021004	PSD	1.183215974	0.163358682	7.243055319	4.38688E-13	5.20473E-12	positive
PRJNA729271	stage_E14_vs_E10	ordinary_muscle_development	ENSGALG00010021010	LEO1	-0.161419396	0.110787278	-1.457021041	0.145110572	0.231038856	negative
PRJNA729271	stage_E14_vs_E10	ordinary_muscle_development	ENSGALG00010021011	SCARB1	0.184598944	0.105457739	1.75045421	0.080039969	0.141475606	positive
PRJNA729271	stage_E14_vs_E10	ordinary_muscle_development	ENSGALG00010021012	KCNIP2	-2.83746362	0.826083821	-3.434837419	0.000592909	0.001971152	negative
PRJNA729271	stage_E14_vs_E10	ordinary_muscle_development	ENSGALG00010021013	FAP	0.744206942	0.296577458	2.509317284	0.012096478	0.028166819	positive
PRJNA729271	stage_E14_vs_E10	ordinary_muscle_development	ENSGALG00010021014	FBXL15	-0.076158202	0.090696765	-0.839701421	0.401075817	0.516369299	negative
PRJNA729271	stage_E14_vs_E10	ordinary_muscle_development	ENSGALG00010021015	RBM25	-0.424922837	0.135055089	-3.146292665	0.001653545	0.004925633	negative
PRJNA729271	stage_E14_vs_E10	ordinary_muscle_development	ENSGALG00010021016	FHIT	0.120082595	0.20097602	0.59749713	0.550175522	0.655229167	positive
PRJNA729271	stage_E14_vs_E10	ordinary_muscle_development	ENSGALG00010021017	COQ7	0.523342336	0.156339744	3.347468289	0.000815533	0.002627507	positive
PRJNA729271	stage_E14_vs_E10	ordinary_muscle_development	ENSGALG00010021018	F3	0.116302757	0.220561057	0.527304131	0.597982406	0.697493761	positive
PRJNA729271	stage_E14_vs_E10	ordinary_muscle_development	ENSGALG00010021019	VSIG10	-0.063871706	0.090785662	-0.703543986	0.481716805	0.593496395	negative
PRJNA729271	stage_E14_vs_E10	ordinary_muscle_development	ENSGALG00010021020	ZDHHC13	0.363971393	0.116187772	3.132613585	0.001732574	0.005132568	positive
PRJNA729271	stage_E14_vs_E10	ordinary_muscle_development	ENSGALG00010021021	GCG	0.423103645	0.352081569	1.201720518	0.229471829	0.333319387	positive
PRJNA729271	stage_E14_vs_E10	ordinary_muscle_development	ENSGALG00010021022	ZFYVE1	0.452071749	0.078319061	5.772180415	7.82523E-09	5.76355E-08	positive
PRJNA729271	stage_E14_vs_E10	ordinary_muscle_development	ENSGALG00010021023	MGEA5	0.44250873	0.096982068	4.562789194	5.04785E-06	2.48788E-05	positive
PRJNA729271	stage_E14_vs_E10	ordinary_muscle_development	ENSGALG00010021024	ENSGALG00010021024	-0.336779918	0.187292429	-1.798150195	0.07215321	0.129422197	negative
PRJNA729271	stage_E14_vs_E10	ordinary_muscle_development	ENSGALG00010021025	CUEDC2	0.30890523	0.114668482	2.693898318	0.007062171	0.017561531	positive
PRJNA729271	stage_E14_vs_E10	ordinary_muscle_development	ENSGALG00010021026	ADGRA2	-0.302990452	0.13318823	-2.274904109	0.022911683	0.048961351	negative
PRJNA729271	stage_E14_vs_E10	ordinary_muscle_development	ENSGALG00010021027	PLA2G4A	0.562165706	0.166064592	3.385223186	0.000711204	0.002323918	positive
PRJNA729271	stage_E14_vs_E10	ordinary_muscle_development	ENSGALG00010021028	ENSGALG00010021028	0.191130817	0.319061456	0.599040758	0.549145705	0.654434068	positive
PRJNA729271	stage_E14_vs_E10	ordinary_muscle_development	ENSGALG00010021029	PRDM2	0.259672778	0.073741483	3.521393487	0.000429285	0.001475922	positive
PRJNA729271	stage_E14_vs_E10	ordinary_muscle_development	ENSGALG00010021030	CSRP3	2.632244809	0.547221872	4.810196642	1.50782E-06	8.11579E-06	positive
PRJNA729271	stage_E14_vs_E10	ordinary_muscle_development	ENSGALG00010021031	DDX54	-0.026965715	0.109007255	-0.247375415	0.804617699	0.862706824	negative
PRJNA729271	stage_E14_vs_E10	ordinary_muscle_development	ENSGALG00010021032	TMC7	0.29657054	0.114941485	2.580187121	0.009874679	0.023572707	positive
PRJNA729271	stage_E14_vs_E10	ordinary_muscle_development	ENSGALG00010021033	RNF122	1.277176755	0.230062044	5.551444874	2.83318E-08	1.95743E-07	positive
PRJNA729271	stage_E14_vs_E10	ordinary_muscle_development	ENSGALG00010021034	NPM3	-1.257510308	0.27840478	-4.516841653	6.27688E-06	3.05006E-05	negative
PRJNA729271	stage_E14_vs_E10	ordinary_muscle_development	ENSGALG00010021036	E2F8	-0.022638501	0.192353207	-0.117692351	0.906311427	0.935716909	negative
PRJNA729271	stage_E14_vs_E10	ordinary_muscle_development	ENSGALG00010021038	PTGS2	-0.68019884	0.301043879	-2.259467432	0.023854324	0.050706618	negative
PRJNA729271	stage_E14_vs_E10	ordinary_muscle_development	ENSGALG00010021040	FGF8	-2.019935277	0.50647749	-3.988203456	6.65756E-05	0.000271147	negative
PRJNA729271	stage_E14_vs_E10	ordinary_muscle_development	ENSGALG00010021041	GLTP	-0.699756987	0.183919287	-3.804696065	0.000141978	0.000544438	negative
PRJNA729271	stage_E14_vs_E10	ordinary_muscle_development	ENSGALG00010021042	LRRC38	0.805837885	0.094316544	8.543971732	1.29686E-17	2.29523E-16	positive
PRJNA729271	stage_E14_vs_E10	ordinary_muscle_development	ENSGALG00010021043	PTPRG	-0.000510157	0.08972615	-0.005685706	0.995463487	0.997665992	negative
PRJNA729271	stage_E14_vs_E10	ordinary_muscle_development	ENSGALG00010021044	EIF4G2	-0.091523302	0.068002655	-1.34587836	0.178341749	0.272657278	negative
PRJNA729271	stage_E14_vs_E10	ordinary_muscle_development	ENSGALG00010021045	DPP4	0.094617777	0.263841706	0.358615696	0.719882603	0.797333104	positive
PRJNA729271	stage_E14_vs_E10	ordinary_muscle_development	ENSGALG00010021046	ENSGALG00010021046	-1.353234519	0.251045433	-5.390396888	7.03022E-08	4.59437E-07	negative
PRJNA729271	stage_E14_vs_E10	ordinary_muscle_development	ENSGALG00010021047	TMC5	-2.176941484	0.546913634	-3.980411803	6.8796E-05	0.000279249	negative
PRJNA729271	stage_E14_vs_E10	ordinary_muscle_development	ENSGALG00010021048	API5	0.083733007	0.075310962	1.111830272	0.266211134	0.375107454	positive
PRJNA729271	stage_E14_vs_E10	ordinary_muscle_development	ENSGALG00010021050	CTR9	-0.09162435	0.094048503	-0.974224438	0.329945113	0.444723304	negative
PRJNA729271	stage_E14_vs_E10	ordinary_muscle_development	ENSGALG00010021051	GDE1	-0.067769674	0.135226549	-0.50115657	0.616260937	0.712343436	negative
PRJNA729271	stage_E14_vs_E10	ordinary_muscle_development	ENSGALG00010021052	CEP15	-0.537835997	0.144148532	-3.731123656	0.000190628	0.000711418	negative
PRJNA729271	stage_E14_vs_E10	ordinary_muscle_development	ENSGALG00010021053	ENSGALG00010021053	-0.448116862	0.323379632	-1.385730012	0.16582936	0.257079848	negative
PRJNA729271	stage_E14_vs_E10	ordinary_muscle_development	ENSGALG00010021055	IRAG1	-1.499262649	0.224536814	-6.677135122	2.43658E-11	2.39771E-10	negative
PRJNA729271	stage_E14_vs_E10	ordinary_muscle_development	ENSGALG00010021057	ZDHHC8	0.298005845	0.0767291	3.883869926	0.000102807	0.000403735	positive
PRJNA729271	stage_E14_vs_E10	ordinary_muscle_development	ENSGALG00010021058	CCP110	0.065710404	0.092909191	0.707254069	0.479408603	0.59145695	positive
PRJNA729271	stage_E14_vs_E10	ordinary_muscle_development	ENSGALG00010021059	ENSGALG00010021059	2.375786332	1.124862241	2.112068701	0.034680557	0.069413791	positive
PRJNA729271	stage_E14_vs_E10	ordinary_muscle_development	ENSGALG00010021062	MYO9A	0.086736123	0.100267552	0.865046787	0.38701311	0.503071924	positive
PRJNA729271	stage_E14_vs_E10	ordinary_muscle_development	ENSGALG00010021064	LYVE1	-1.518478413	0.321827813	-4.718294542	2.3783E-06	1.23738E-05	negative
PRJNA729271	stage_E14_vs_E10	ordinary_muscle_development	ENSGALG00010021065	CNNM2	-0.019515248	0.108661644	-0.179596471	0.857469376	0.901568156	negative
PRJNA729271	stage_E14_vs_E10	ordinary_muscle_development	ENSGALG00010021067	SLC4A10	-2.170862082	0.241480427	-8.989805544	2.47668E-19	5.04916E-18	negative
PRJNA729271	stage_E14_vs_E10	ordinary_muscle_development	ENSGALG00010021069	PGAM5	0.339608574	0.07046726	4.819381031	1.44004E-06	7.7915E-06	positive
PRJNA729271	stage_E14_vs_E10	ordinary_muscle_development	ENSGALG00010021070	RNF141	0.013881159	0.085532545	0.162290968	0.871076727	0.911494466	positive
PRJNA729271	stage_E14_vs_E10	ordinary_muscle_development	ENSGALG00010021071	HIF1AN	0.258431743	0.095481819	2.706606832	0.006797471	0.016993677	positive
PRJNA729271	stage_E14_vs_E10	ordinary_muscle_development	ENSGALG00010021072	ENSGALG00010021072	-0.054324043	0.08992852	-0.604080258	0.545790284	0.651290868	negative
PRJNA729271	stage_E14_vs_E10	ordinary_muscle_development	ENSGALG00010021073	NDUFB8	0.339204907	0.107335518	3.160229839	0.001576447	0.004719249	positive
PRJNA729271	stage_E14_vs_E10	ordinary_muscle_development	ENSGALG00010021074	TTC17	0.185752719	0.06883857	2.698381422	0.006967756	0.017356525	positive
PRJNA729271	stage_E14_vs_E10	ordinary_muscle_development	ENSGALG00010021075	DHRS3	0.334933968	0.131114959	2.55450613	0.010633856	0.025116138	positive
PRJNA729271	stage_E14_vs_E10	ordinary_muscle_development	ENSGALG00010021077	SEC31B	0.099174265	0.078432181	1.264458845	0.206065383	0.306004306	positive
PRJNA729271	stage_E14_vs_E10	ordinary_muscle_development	ENSGALG00010021078	ENSGALG00010021078	-1.640653816	0.216313771	-7.584601784	3.3351E-14	4.39076E-13	negative
PRJNA729271	stage_E14_vs_E10	ordinary_muscle_development	ENSGALG00010021080	TTLL7	1.012090882	0.174570699	5.797598835	6.72711E-09	5.00049E-08	positive
PRJNA729271	stage_E14_vs_E10	ordinary_muscle_development	ENSGALG00010021081	KCNH6	1.012811533	0.197026232	5.140490812	2.74022E-07	1.6413E-06	positive
PRJNA729271	stage_E14_vs_E10	ordinary_muscle_development	ENSGALG00010021082	SENP8	-0.260345223	0.163365927	-1.593632333	0.111018408	0.185663756	negative
PRJNA729271	stage_E14_vs_E10	ordinary_muscle_development	ENSGALG00010021083	SCD	-1.935750768	0.563330497	-3.436261265			negative
PRJNA729271	stage_E14_vs_E10	ordinary_muscle_development	ENSGALG00010021084	ENSGALG00010021084	-1.667501176	0.59612363	-2.797240525	0.005154115	0.013362349	negative
PRJNA729271	stage_E14_vs_E10	ordinary_muscle_development	ENSGALG00010021085	AMPD3	2.267501932	0.183868779	12.33217485	6.07837E-35	3.33735E-33	positive
PRJNA729271	stage_E14_vs_E10	ordinary_muscle_development	ENSGALG00010021086	PSMD14	-0.020715744	0.10895238	-0.190135763	0.849202746	0.895474597	negative
PRJNA729271	stage_E14_vs_E10	ordinary_muscle_development	ENSGALG00010021087	THBS1	-3.790194703	0.356840349	-10.62154184	2.3662E-26	8.01357E-25	negative
PRJNA729271	stage_E14_vs_E10	ordinary_muscle_development	ENSGALG00010021088	ANKLE2	-0.125662052	0.086586686	-1.451286081	0.146700216	0.233108926	negative
PRJNA729271	stage_E14_vs_E10	ordinary_muscle_development	ENSGALG00010021090	ADM	0.653438113	0.274548132	2.380049387	0.017310318	0.038407019	positive
PRJNA729271	stage_E14_vs_E10	ordinary_muscle_development	ENSGALG00010021091	TANK	-0.060060429	0.081370798	-0.738107907	0.460448879	0.573090031	negative
PRJNA729271	stage_E14_vs_E10	ordinary_muscle_development	ENSGALG00010021092	VPS13D	0.077420704	0.09488937	0.815904917	0.41455452	0.529356109	positive
PRJNA729271	stage_E14_vs_E10	ordinary_muscle_development	ENSGALG00010021093	GRAMD2	-0.119573493	0.22508917	-0.531227216	0.595261335	0.694990983	negative
PRJNA729271	stage_E14_vs_E10	ordinary_muscle_development	ENSGALG00010021094	ENSGALG00010021094	0.364883996	0.323350993	1.128445571	0.259131791	0.367515453	positive
PRJNA729271	stage_E14_vs_E10	ordinary_muscle_development	ENSGALG00010021095	NT5C2	1.357566525	0.104809018	12.95276449	2.26643E-38	1.59475E-36	positive
PRJNA729271	stage_E14_vs_E10	ordinary_muscle_development	ENSGALG00010021096	ZFAND4	-0.076064442	0.098008026	-0.776104215	0.437687455	0.551580565	negative
PRJNA729271	stage_E14_vs_E10	ordinary_muscle_development	ENSGALG00010021097	KATNBL1	0.446014052	0.128275693	3.476995852	0.000507066	0.001715667	positive
PRJNA729271	stage_E14_vs_E10	ordinary_muscle_development	ENSGALG00010021098	SDK1	-0.290734205	0.141703076	-2.051714134	0.040197452	0.078642471	negative
PRJNA729271	stage_E14_vs_E10	ordinary_muscle_development	ENSGALG00010021099	HMCN1	-0.957045137	0.264046115	-3.624537849	0.000289479	0.001039419	negative
PRJNA729271	stage_E14_vs_E10	ordinary_muscle_development	ENSGALG00010021100	EMC7	0.252768887	0.08546934	2.957421789	0.003102234	0.008566753	positive
PRJNA729271	stage_E14_vs_E10	ordinary_muscle_development	ENSGALG00010021101	TMEM240	0.608637473	0.240178035	2.534109633	0.011273346	0.026471269	positive
PRJNA729271	stage_E14_vs_E10	ordinary_muscle_development	ENSGALG00010021102	GOLGA3	-0.055837978	0.112431148	-0.496641535	0.619441858	0.715150523	negative
PRJNA729271	stage_E14_vs_E10	ordinary_muscle_development	ENSGALG00010021103	RBMS1	0.226016712	0.073727788	3.065556646	0.002172652	0.006254736	positive
PRJNA729271	stage_E14_vs_E10	ordinary_muscle_development	ENSGALG00010021104	INA	2.613548034	0.253765535	10.29906617	7.11486E-25	2.11332E-23	positive
PRJNA729271	stage_E14_vs_E10	ordinary_muscle_development	ENSGALG00010021105	ENSGALG00010021105	-0.596254973	0.551417348	-1.081313411	0.279557724	0.39042193	negative
PRJNA729271	stage_E14_vs_E10	ordinary_muscle_development	ENSGALG00010021106	ITGB6	-1.886987275	0.199373313	-9.464593076	2.94706E-21	6.95728E-20	negative
PRJNA729271	stage_E14_vs_E10	ordinary_muscle_development	ENSGALG00010021108	PCGF6	0.092483726	0.104769889	0.882731931	0.377381126	0.493634204	positive
PRJNA729271	stage_E14_vs_E10	ordinary_muscle_development	ENSGALG00010021110	PKLR	-0.170345155	0.16891258	-1.008481164	0.313223526	0.42706942	negative
PRJNA729271	stage_E14_vs_E10	ordinary_muscle_development	ENSGALG00010021111	AVEN	0.275751837	0.077741294	3.54704462	0.000389579	0.001353534	positive
PRJNA729271	stage_E14_vs_E10	ordinary_muscle_development	ENSGALG00010021112	TAF5	-0.253129956	0.069184989	-3.658741009	0.000253457	0.000919155	negative
PRJNA729271	stage_E14_vs_E10	ordinary_muscle_development	ENSGALG00010021113	SYP	-1.461593638	0.30476083	-4.795871041	1.61969E-06	8.67608E-06	negative
PRJNA729271	stage_E14_vs_E10	ordinary_muscle_development	ENSGALG00010021114	SBF2	-0.148346912	0.097930533	-1.514817777	0.129818557	0.210648157	negative
PRJNA729271	stage_E14_vs_E10	ordinary_muscle_development	ENSGALG00010021115	ALPL	-1.350979045	0.245436141	-5.504401434	3.70425E-08	2.51137E-07	negative
PRJNA729271	stage_E14_vs_E10	ordinary_muscle_development	ENSGALG00010021116	FAM21C	0.078954009	0.063755106	1.238395077	0.215569605	0.31725875	positive
PRJNA729271	stage_E14_vs_E10	ordinary_muscle_development	ENSGALG00010021118	USMG5	0.401011791	0.179805733	2.230250307	0.02573083	0.054053389	positive
PRJNA729271	stage_E14_vs_E10	ordinary_muscle_development	ENSGALG00010021120	PDCD11	-0.334961784	0.085918435	-3.898602026	9.67496E-05	0.000381394	negative
PRJNA729271	stage_E14_vs_E10	ordinary_muscle_development	ENSGALG00010021121	PLA2R1	0.427123087	0.09155894	4.665006889	3.08607E-06	1.57731E-05	positive
PRJNA729271	stage_E14_vs_E10	ordinary_muscle_development	ENSGALG00010021122	CYP1A1	0.574881029	0.978372282	0.587589243	0.556808033	0.660955895	positive
PRJNA729271	stage_E14_vs_E10	ordinary_muscle_development	ENSGALG00010021123	RYR3	1.389145967	0.216867829	6.405495805	1.49882E-10	1.35108E-09	positive
PRJNA729271	stage_E14_vs_E10	ordinary_muscle_development	ENSGALG00010021124	CALHM2	0.283811362	0.129352524	2.194092182	0.028228783	0.058370358	positive
PRJNA729271	stage_E14_vs_E10	ordinary_muscle_development	ENSGALG00010021125	LY75	0.08284492	0.124739003	0.664146076	0.506596822	0.615498934	positive
PRJNA729271	stage_E14_vs_E10	ordinary_muscle_development	ENSGALG00010021126	ECE1	0.327127365	0.103850764	3.149975511	0.001632841	0.004868965	positive
PRJNA729271	stage_E14_vs_E10	ordinary_muscle_development	ENSGALG00010021127	SWAP70	-0.324792723	0.077002539	-4.217948225	2.46535E-05	0.00010806	negative
PRJNA729271	stage_E14_vs_E10	ordinary_muscle_development	ENSGALG00010021129	TXNRD3	-0.156054201	0.084920693	-1.837646348	0.066114535	0.120166793	negative
PRJNA729271	stage_E14_vs_E10	ordinary_muscle_development	ENSGALG00010021130	ENSGALG00010021130	0.688412934	1.126899662	0.610891064	0.541271701	0.647189389	positive
PRJNA729271	stage_E14_vs_E10	ordinary_muscle_development	ENSGALG00010021131	EDC3	-0.089615435	0.115232105	-0.77769503	0.436748823	0.550637052	negative
PRJNA729271	stage_E14_vs_E10	ordinary_muscle_development	ENSGALG00010021133	ENSGALG00010021133	0.319167117	0.099957254	3.193036061	0.001407854	0.0042725	positive
PRJNA729271	stage_E14_vs_E10	ordinary_muscle_development	ENSGALG00010021134	CHFR	-0.407020216	0.092589754	-4.395953099	1.10288E-05	5.13036E-05	negative
PRJNA729271	stage_E14_vs_E10	ordinary_muscle_development	ENSGALG00010021135	CLK3	0.449554613	0.105066716	4.278753836	1.87943E-05	8.40552E-05	positive
PRJNA729271	stage_E14_vs_E10	ordinary_muscle_development	ENSGALG00010021136	WEE1	-0.332511644	0.147244614	-2.258226191	0.023931561	0.050855883	negative
PRJNA729271	stage_E14_vs_E10	ordinary_muscle_development	ENSGALG00010021137	PLAU	-1.207313647	0.197851738	-6.102112932	1.04675E-09	8.548E-09	negative
PRJNA729271	stage_E14_vs_E10	ordinary_muscle_development	ENSGALG00010021138	DDX18	-0.480556367	0.098948324	-4.856639803	1.19394E-06	6.53312E-06	negative
PRJNA729271	stage_E14_vs_E10	ordinary_muscle_development	ENSGALG00010021139	ARHGAP29	0.00253754	0.095940495	0.026449099	0.978899133	0.985973381	positive
PRJNA729271	stage_E14_vs_E10	ordinary_muscle_development	ENSGALG00010021140	SLC11A1	0.153397879	0.430648269	0.356202244	0.721689112	0.798540739	positive
PRJNA729271	stage_E14_vs_E10	ordinary_muscle_development	ENSGALG00010021141	SGSM1	0.278964231	0.142054772	1.96377937	0.049555683	0.094167492	positive
PRJNA729271	stage_E14_vs_E10	ordinary_muscle_development	ENSGALG00010021142	WNT6	-3.076242544	0.472210437	-6.514558561	7.29037E-11	6.83531E-10	negative
PRJNA729271	stage_E14_vs_E10	ordinary_muscle_development	ENSGALG00010021143	ABCA4	0.131528053	0.328550657	0.400328078	0.68891489	0.772657175	positive
PRJNA729271	stage_E14_vs_E10	ordinary_muscle_development	ENSGALG00010021144	FOXK1	0.233670967	0.107666123	2.17032954	0.02998189	0.061408436	positive

PRJNA729271	stage_E14_vs_E10	ordinary_muscle_development	ENSGALG00010021145	EIF4G3	-0.506244265	0.157570074	-3.212819878	0.001314387	0.004021114	negative
PRJNA729271	stage_E14_vs_E10	ordinary_muscle_development	ENSGALG00010021146	AP5Z1	-0.072156167	0.084237515	-0.856579962	0.391677064	0.507360057	negative
PRJNA729271	stage_E14_vs_E10	ordinary_muscle_development	ENSGALG00010021147	ARID3B	-0.186343507	0.082023543	-2.271829528	0.023096809	0.049284299	negative
PRJNA729271	stage_E14_vs_E10	ordinary_muscle_development	ENSGALG00010021148	GPATCH8	0.130113565	0.104350719	1.2468871	0.212438898	0.313702304	positive
PRJNA729271	stage_E14_vs_E10	ordinary_muscle_development	ENSGALG00010021149	ENSGALG00010021149	0.659706008	1.085167273	0.607930247	0.543233738	0.64891466	positive
PRJNA729271	stage_E14_vs_E10	ordinary_muscle_development	ENSGALG00010021150	ENSGALG00010021150	0.799720215	0.249040398	3.211206781	0.001321788	0.004041901	positive
PRJNA729271	stage_E14_vs_E10	ordinary_muscle_development	ENSGALG00010021151	WNT10A	-2.114190091	0.520495976	-4.061875955	4.86799E-05	0.000202938	negative
PRJNA729271	stage_E14_vs_E10	ordinary_muscle_development	ENSGALG00010021152	STK11IP	0.1491339	0.12773343	1.167540082	0.242992331	0.349393752	positive
PRJNA729271	stage_E14_vs_E10	ordinary_muscle_development	ENSGALG00010021153	PTPRF	-0.844048592	0.156636117	-5.388594958	7.10106E-08	4.63857E-07	negative
PRJNA729271	stage_E14_vs_E10	ordinary_muscle_development	ENSGALG00010021154	CHST13	-0.908136338	0.207564928	-4.375191631	1.21326E-05	5.61321E-05	negative
PRJNA729271	stage_E14_vs_E10	ordinary_muscle_development	ENSGALG00010021155	RADIL	-1.584493569	0.211916241	-7.47698036	7.60498E-14	9.7561E-13	negative
PRJNA729271	stage_E14_vs_E10	ordinary_muscle_development	ENSGALG00010021156	DDOST	-0.175292367	0.080142872	-2.187248378	0.028724397	0.059258641	negative
PRJNA729271	stage_E14_vs_E10	ordinary_muscle_development	ENSGALG00010021157	ENSGALG00010021157	0.433428968	1.074558291	0.403355473	0.686686738	0.770873937	positive
PRJNA729271	stage_E14_vs_E10	ordinary_muscle_development	ENSGALG00010021158	ACTG1L	-0.267246471	0.127672237	-2.093223064	0.036329247	0.072168716	negative
PRJNA729271	stage_E14_vs_E10	ordinary_muscle_development	ENSGALG00010021159	MMD2	0.630484719	0.239253624	2.635214918	0.008408402	0.020511578	positive
PRJNA729271	stage_E14_vs_E10	ordinary_muscle_development	ENSGALG00010021160	ZNF143	0.043007988	0.163957058	0.262312515	0.793080514	0.854699037	positive
PRJNA729271	stage_E14_vs_E10	ordinary_muscle_development	ENSGALG00010021161	ENSGALG00010021161	-0.196264157	0.10192659	-1.92554423	0.054161308	0.101393656	negative
PRJNA729271	stage_E14_vs_E10	ordinary_muscle_development	ENSGALG00010021162	WIPI2	0.174734344	0.094182709	1.855269894	0.063557695	0.116189783	positive
PRJNA729271	stage_E14_vs_E10	ordinary_muscle_development	ENSGALG00010021163	INHA	2.373678579	0.287569006	8.254292104	1.52805E-16	2.46375E-15	positive
PRJNA729271	stage_E14_vs_E10	ordinary_muscle_development	ENSGALG00010021164	SLC41A3	-0.807705279	0.214804023	-3.760196238	0.00016978	0.000640542	negative
PRJNA729271	stage_E14_vs_E10	ordinary_muscle_development	ENSGALG00010021165	PTRF	2.181348038	0.105648567	20.6472089	1.03425E-94	9.36967E-92	positive
PRJNA729271	stage_E14_vs_E10	ordinary_muscle_development	ENSGALG00010021166	NEURL1	-0.448229005	0.301438683	-1.486965773	0.137023851	0.220366218	negative
PRJNA729271	stage_E14_vs_E10	ordinary_muscle_development	ENSGALG00010021167	KIAA1671	0.144823969	0.125529669	1.15370311	0.248621908	0.35550701	positive
PRJNA729271	stage_E14_vs_E10	ordinary_muscle_development	ENSGALG00010021168	IVNS1ABP	-0.182125195	0.105932913	-1.719250327	0.0855688	0.14958029	negative
PRJNA729271	stage_E14_vs_E10	ordinary_muscle_development	ENSGALG00010021169	CAMK2G	0.612059494	0.140444156	4.35802749	1.3124E-05	6.0372E-05	positive
PRJNA729271	stage_E14_vs_E10	ordinary_muscle_development	ENSGALG00010021170	UBL7	-0.173328553	0.13592927	-1.27513782	0.202260465	0.301652993	negative
PRJNA729271	stage_E14_vs_E10	ordinary_muscle_development	ENSGALG00010021171	ENSGALG00010021171	-0.190035815	0.21160973	-0.898048566	0.369159658	0.485436746	negative
PRJNA729271	stage_E14_vs_E10	ordinary_muscle_development	ENSGALG00010021172	SLC29A4	-0.232604116	0.260976948	-0.891282231	0.372777779	0.488775568	negative
PRJNA729271	stage_E14_vs_E10	ordinary_muscle_development	ENSGALG00010021173	KLF15	1.369741365	0.287337559	4.767011217	1.86979E-06	9.90592E-06	positive
PRJNA729271	stage_E14_vs_E10	ordinary_muscle_development	ENSGALG00010021175	IPO7	0.162915548	0.097558243	1.669931142	0.094932988	0.163051359	positive
PRJNA729271	stage_E14_vs_E10	ordinary_muscle_development	ENSGALG00010021176	ENSGALG00010021176	-0.124639632	0.1177892	-1.058158406	0.289983231	0.402422875	negative
PRJNA729271	stage_E14_vs_E10	ordinary_muscle_development	ENSGALG00010021177	OBSL1	2.317342637	0.186468684	12.42751646	1.85302E-35	1.06586E-33	positive
PRJNA729271	stage_E14_vs_E10	ordinary_muscle_development	ENSGALG00010021178	NDST2	0.073226069	0.095858176	0.763900084	0.444926787	0.558518557	positive
PRJNA729271	stage_E14_vs_E10	ordinary_muscle_development	ENSGALG00010021180	TMEM198	-0.446642116	0.140092402	-3.188196561	0.001431632	0.004335876	negative
PRJNA729271	stage_E14_vs_E10	ordinary_muscle_development	ENSGALG00010021181	DAGLA	-0.089411203	0.127257419	-0.702601108	0.482304372	0.593967874	negative
PRJNA729271	stage_E14_vs_E10	ordinary_muscle_development	ENSGALG00010021182	ZSWIM8	0.418581418	0.067591906	6.192774334	5.91143E-10	4.98756E-09	positive
PRJNA729271	stage_E14_vs_E10	ordinary_muscle_development	ENSGALG00010021183	CFAP100	-0.674093616	0.511107308	-1.318888628	0.187206349	0.283548174	negative
PRJNA729271	stage_E14_vs_E10	ordinary_muscle_development	ENSGALG00010021184	DRAXIN	-1.83083887	0.450726405	-4.061973849	4.86595E-05	0.000202911	negative
PRJNA729271	stage_E14_vs_E10	ordinary_muscle_development	ENSGALG00010021186	SWT1	-0.03778373	0.09001801	-0.419735226	0.674678888	0.760989066	negative
PRJNA729271	stage_E14_vs_E10	ordinary_muscle_development	ENSGALG00010021187	SEMA7A	1.150019114	0.278191535	4.133911239	3.56641E-05	0.000152089	positive
PRJNA729271	stage_E14_vs_E10	ordinary_muscle_development	ENSGALG00010021188	TNRC18	-0.208379861	0.133418962	-1.561845913	0.118324293	0.195431931	negative
PRJNA729271	stage_E14_vs_E10	ordinary_muscle_development	ENSGALG00010021189	ENSGALG00010021189	0.759146036	0.417288419	1.819235814	0.068875461	0.124420465	positive
PRJNA729271	stage_E14_vs_E10	ordinary_muscle_development	ENSGALG00010021190	ENSGALG00010021190	0.024804019	0.102108474	0.242918324	0.80806867	0.864999289	positive
PRJNA729271	stage_E14_vs_E10	ordinary_muscle_development	ENSGALG00010021191	MYRF	-1.117963224	0.202912907	-5.509571762	3.59708E-08	2.44519E-07	negative
PRJNA729271	stage_E14_vs_E10	ordinary_muscle_development	ENSGALG00010021192	ENSGALG00010021192	0.987170108	0.169367853	5.828556542	5.59089E-09	4.20549E-08	positive
PRJNA729271	stage_E14_vs_E10	ordinary_muscle_development	ENSGALG00010021193	USP37	-0.066661465	0.156040892	-0.427205099	0.669229948	0.756845447	negative
PRJNA729271	stage_E14_vs_E10	ordinary_muscle_development	ENSGALG00010021194	CYP11A1	-1.275670762	0.377333526	-3.380751175	0.00072288	0.002358879	negative
PRJNA729271	stage_E14_vs_E10	ordinary_muscle_development	ENSGALG00010021195	GCLM	-0.253619777	0.089928257	-2.820245667	0.00479869	0.012562295	negative
PRJNA729271	stage_E14_vs_E10	ordinary_muscle_development	ENSGALG00010021196	ATP6V0A1	0.434163307	0.120799643	3.594077747	0.000325543	0.001155987	positive
PRJNA729271	stage_E14_vs_E10	ordinary_muscle_development	ENSGALG00010021197	CHCHD1	-0.041569942	0.104575223	-0.397512347	0.690989683	0.774444867	negative
PRJNA729271	stage_E14_vs_E10	ordinary_muscle_development	ENSGALG00010021199	TMEM258	0.362508554	0.133059037	2.724418882	0.006441475	0.016209928	positive
PRJNA729271	stage_E14_vs_E10	ordinary_muscle_development	ENSGALG00010021200	FTO	-0.65436816	0.162014468	-4.038948923	5.36913E-05	0.000222295	negative
PRJNA729271	stage_E14_vs_E10	ordinary_muscle_development	ENSGALG00010021201	TRMT1L	-0.108910909	0.088164048	-1.23532111	0.21671102	0.318602711	negative
PRJNA729271	stage_E14_vs_E10	ordinary_muscle_development	ENSGALG00010021202	UROC1	-0.194222519	0.508989635	-0.381584429	0.702769636	0.784131004	negative
PRJNA729271	stage_E14_vs_E10	ordinary_muscle_development	ENSGALG00010021203	FUT11	0.236950583	0.079320794	2.987244202	0.002815048	0.007854498	positive
PRJNA729271	stage_E14_vs_E10	ordinary_muscle_development	ENSGALG00010021204	NAGLU	0.218046398	0.093300179	2.337041585	0.019437022	0.042385984	positive
PRJNA729271	stage_E14_vs_E10	ordinary_muscle_development	ENSGALG00010021206	HSD17B1	-0.13831606	0.418807481	-0.330261675	0.741202249	0.813918682	negative
PRJNA729271	stage_E14_vs_E10	ordinary_muscle_development	ENSGALG00010021207	MAD2L2	-0.120207103	0.119898411	-1.002574619	0.316066147	0.429934197	negative
PRJNA729271	stage_E14_vs_E10	ordinary_muscle_development	ENSGALG00010021210	ENSGALG00010021210	-0.067136681	0.062883071	-1.067643157	0.285681509	0.397328101	negative
PRJNA729271	stage_E14_vs_E10	ordinary_muscle_development	ENSGALG00010021211	FEN1	-0.073960001	0.152724854	-0.484269579	0.628194562	0.722157204	negative
PRJNA729271	stage_E14_vs_E10	ordinary_muscle_development	ENSGALG00010021212	MYO18B	0.92053312	0.161403049	5.703319275	1.17497E-08	8.48587E-08	positive
PRJNA729271	stage_E14_vs_E10	ordinary_muscle_development	ENSGALG00010021213	FBXO44	-0.461884704	0.205847217	-2.243822927	0.024843797	0.052441175	negative
PRJNA729271	stage_E14_vs_E10	ordinary_muscle_development	ENSGALG00010021214	FBXL18	-0.032557545	0.112779075	-0.288684269	0.772823002	0.838917803	negative
PRJNA729271	stage_E14_vs_E10	ordinary_muscle_development	ENSGALG00010021215	SH3PXD2A	0.409911435	0.111378546	3.680344652	0.000232919	0.000851276	positive
PRJNA729271	stage_E14_vs_E10	ordinary_muscle_development	ENSGALG00010021217	FBXO2	-1.047831524	0.19352428	-5.41447061	6.14703E-08	4.03173E-07	negative
PRJNA729271	stage_E14_vs_E10	ordinary_muscle_development	ENSGALG00010021218	DPF3	-0.655960845	0.17562664	-3.734973487	0.000187735	0.000701526	negative
PRJNA729271	stage_E14_vs_E10	ordinary_muscle_development	ENSGALG00010021219	STRA6	1.087729751	0.209914657	5.181771319	2.19789E-07	1.33522E-06	positive
PRJNA729271	stage_E14_vs_E10	ordinary_muscle_development	ENSGALG00010021220	SYNPO2L	2.234233976	0.205872325	10.85252219	1.93997E-27	7.06529E-26	positive
PRJNA729271	stage_E14_vs_E10	ordinary_muscle_development	ENSGALG00010021222	ENSGALG00010021222	0.077878461	0.34455195	0.22602821	0.821179479	0.874770085	positive
PRJNA729271	stage_E14_vs_E10	ordinary_muscle_development	ENSGALG00010021223	DFFB	0.075034235	0.108430381	0.692003795	0.488934946	0.599688383	positive
PRJNA729271	stage_E14_vs_E10	ordinary_muscle_development	ENSGALG00010021224	MYOZ1	3.311699864	0.141783154	23.35749898	1.1564E-120	2.3945E-117	positive
PRJNA729271	stage_E14_vs_E10	ordinary_muscle_development	ENSGALG00010021227	CHCHD4	-0.14238869	0.091487511	-1.55637297	0.119619429	0.197160919	negative
PRJNA729271	stage_E14_vs_E10	ordinary_muscle_development	ENSGALG00010021228	ESRP2	-1.823614094	0.896365397	-2.034453918	0.04190585	0.081505013	negative
PRJNA729271	stage_E14_vs_E10	ordinary_muscle_development	ENSGALG00010021229	GLO1	-0.15789381	0.178386922	-0.885119877	0.37609198	0.492319448	negative
PRJNA729271	stage_E14_vs_E10	ordinary_muscle_development	ENSGALG00010021230	C1orf174	0.116233096	0.057449753	2.023213156	0.043051176	0.083437197	positive
PRJNA729271	stage_E14_vs_E10	ordinary_muscle_development	ENSGALG00010021231	STN1	0.025725908	0.11056999	0.232666276	0.816020559	0.871131094	positive
PRJNA729271	stage_E14_vs_E10	ordinary_muscle_development	ENSGALG00010021232	ACTB	-0.641399963	0.109279489	-5.869353604	4.37497E-09	3.3394E-08	negative
PRJNA729271	stage_E14_vs_E10	ordinary_muscle_development	ENSGALG00010021233	COASY	0.103813847	0.061543609	1.686833915	0.091635292	0.158206497	positive
PRJNA729271	stage_E14_vs_E10	ordinary_muscle_development	ENSGALG00010021234	ISLR2	1.513365149	0.322936156	4.686267304	2.78233E-06	1.43064E-05	positive
PRJNA729271	stage_E14_vs_E10	ordinary_muscle_development	ENSGALG00010021235	XRCC5	0.022740779	0.13864435	0.164022397	0.869713513	0.910742477	positive
PRJNA729271	stage_E14_vs_E10	ordinary_muscle_development	ENSGALG00010021236	BTBD9	0.015674411	0.073487812	0.213292656	0.831098701	0.882159906	positive
PRJNA729271	stage_E14_vs_E10	ordinary_muscle_development	ENSGALG00010021237	FSCN1	-0.465979769	0.222228805	-2.096846848	0.036007135	0.071653408	negative
PRJNA729271	stage_E14_vs_E10	ordinary_muscle_development	ENSGALG00010021238	NDUFS8	0.409857648	0.14738909	2.780786888	0.005422732	0.013971295	positive
PRJNA729271	stage_E14_vs_E10	ordinary_muscle_development	ENSGALG00010021239	USP54	0.241767098	0.06887454	3.510253553	0.00044768	0.001533707	positive
PRJNA729271	stage_E14_vs_E10	ordinary_muscle_development	ENSGALG00010021240	SLK	-0.123477273	0.078398443	-1.574996499	0.115257253	0.191588748	negative
PRJNA729271	stage_E14_vs_E10	ordinary_muscle_development	ENSGALG00010021241	TMEM43	0.391535646	0.171115753	2.288133254	0.022129762	0.0475357	positive
PRJNA729271	stage_E14_vs_E10	ordinary_muscle_development	ENSGALG00010021242	BCAR3	1.297323288	0.226935386	5.716707788	1.08608E-08	7.87134E-08	positive
PRJNA729271	stage_E14_vs_E10	ordinary_muscle_development	ENSGALG00010021243	DYL1	-0.187261342	0.094231796	-1.987241576	0.046895633	0.089843007	negative
PRJNA729271	stage_E14_vs_E10	ordinary_muscle_development	ENSGALG00010021244	PPP3CB	0.290684318	0.086979558	3.34198431	0.000831818	0.002675216	positive
PRJNA729271	stage_E14_vs_E10	ordinary_muscle_development	ENSGALG00010021245	ZFAND3	0.486042472	0.07022388	6.921327518	4.47431E-12	4.76175E-11	positive
PRJNA729271	stage_E14_vs_E10	ordinary_muscle_development	ENSGALG00010021246	PECR	0.208928424	0.113311749	1.843837248	0.065206863	0.118755305	positive
PRJNA729271	stage_E14_vs_E10	ordinary_muscle_development	ENSGALG00010021247	GNAO1	-0.493874696	0.217574611	-2.269909583	0.02321307	0.049495946	negative
PRJNA729271	stage_E14_vs_E10	ordinary_muscle_development	ENSGALG00010021248	ANXA7	0.154594578	0.167969951	0.920370441	0.35737921	0.473814291	positive
PRJNA729271	stage_E14_vs_E10	ordinary_muscle_development	ENSGALG00010021249	ENSGALG00010021249	-0.028592758	0.335550466	-0.085211499	0.932093249	0.953538828	negative
PRJNA729271	stage_E14_vs_E10	ordinary_muscle_development	ENSGALG00010021250	ISLR	-0.324597505	0.145938929	-2.224200955	0.026134927	0.05472779	negative
PRJNA729271	stage_E14_vs_E10	ordinary_muscle_development	ENSGALG00010021251	RNF216	-0.002924645	0.082817799	-0.035314203	0.971829198	0.980555773	negative
PRJNA729271	stage_E14_vs_E10	ordinary_muscle_development	ENSGALG00010021252	XPC	0.100672956	0.131319632	0.766625326	0.443304313	0.557040223	positive
PRJNA729271	stage_E14_vs_E10	ordinary_muscle_development	ENSGALG00010021253	TMEM169	0.155987459	0.109731142	1.421542291	0.155159168	0.243929733	positive
PRJNA729271	stage_E14_vs_E10	ordinary_muscle_development	ENSGALG00010021254	FNBP1L	-0.454762048	0.108006597	-4.210502496	2.54803E-05	0.000111549	negative
PRJNA729271	stage_E14_vs_E10	ordinary_muscle_development	ENSGALG00010021255	EIF3F	0.484119076	0.091693879	5.279731681	1.29373E-07	8.08652E-07	positive
PRJNA729271	stage_E14_vs_E10	ordinary_muscle_development	ENSGALG00010021256	ENSGALG00010021256	-0.294489651	0.291580418	-1.009977466	0.312506086	0.426291711	negative
PRJNA729271	stage_E14_vs_E10	ordinary_muscle_development	ENSGALG00010021257	RGS6	-1.142658196	0.309290707	-3.694447229	0.000220366	0.000809273	negative
PRJNA729271	stage_E14_vs_E10	ordinary_muscle_development	ENSGALG00010021258	LSM3	-0.15254202	0.108590929	-1.404739984	0.160098612	0.25023823	negative
PRJNA729271	stage_E14_vs_E10	ordinary_muscle_development	ENSGALG00010021259	CEP104	-0.501556278	0.132328942	-3.790223593	0.000150512	0.000573218	negative
PRJNA729271	stage_E14_vs_E10	ordinary_muscle_development	ENSGALG00010021260	KDM4A	0.246585096	0.067004117	3.680148408	0.000233098	0.000851716	positive
PRJNA729271	stage_E14_vs_E10	ordinary_muscle_development	ENSGALG00010021261	JUP	0.635852773	0.127900651	4.971458447	6.64511E-07	3.76062E-06	positive
PRJNA729271	stage_E14_vs_E10	ordinary_muscle_development	ENSGALG00010021262	MDGA1	-0.106097824	0.29954613	-0.354195274	0.723192545	0.79947193	negative
PRJNA729271	stage_E14_vs_E10	ordinary_muscle_development	ENSGALG00010021263	STOML1	-0.825371372	0.245414856	-3.363167932	0.000770535	0.002494729	negative
PRJNA729271	stage_E14_vs_E10	ordinary_muscle_development	ENSGALG00010021264	CBFB	-0.553646799	0.169525582	-3.265859889	0.001091322	0.003405537	negative
PRJNA729271	stage_E14_vs_E10	ordinary_muscle_development	ENSGALG00010021265	SIPA1L1	0.729154184	0.088292775	8.258367464	1.47678E-16	2.38906E-15	positive
PRJNA729271	stage_E14_vs_E10	ordinary_muscle_development	ENSGALG00010021266	COQ5	0.358140529	0.098045984	3.652781241	0.000259415	0.000937478	positive
PRJNA729271	stage_E14_vs_E10	ordinary_muscle_development	ENSGALG00010021267	LRRC47	0.045658286	0.07792374	0.585935513	0.557918851	0.661760519	positive
PRJNA729271	stage_E14_vs_E10	ordinary_muscle_development	ENSGALG00010021269	NUDT13	0.912584193	0.158443335	5.759688111	8.42695E-09	6.19101E-08	positive
PRJNA729271	stage_E14_vs_E10	ordinary_muscle_development	ENSGALG00010021271	ENSGALG00010021271	-0.985675749	0.460717667	-2.139435534	0.032400412	0.065428248	negative
PRJNA729271	stage_E14_vs_E10	ordinary_muscle_development	ENSGALG00010021272	ENSGALG00010021272	-0.351547177	0.360460118	-0.975273435	0.329424644	0.44411911	negative
PRJNA729271	stage_E14_vs_E10	ordinary_muscle_development	ENSGALG00010021275	MAP3K2	-0.086071982	0.090779653	-0.948141786	0.343057278	0.458263316	negative
PRJNA729271	stage_E14_vs_E10	ordinary_muscle_development	ENSGALG00010021277	ENSGALG00010021277	0.32235774	0.224887711	1.43341643	0.151738866	0.239670356	positive
PRJNA729271	stage_E14_vs_E10	ordinary_muscle_development	ENSGALG00010021287	COL17A1	-2.465296651	0.400185247	-6.160388645	7.25666E-10	6.02436E-09	negative
PRJNA729271	stage_E14_vs_E10	ordinary_muscle_development	ENSGALG00010021290	ERCC3	-0.044380068	0.067083831	-0.661561319	0.508252405	0.617114978	negative
PRJNA729271	stage_E14_vs_E10	ordinary_muscle_development	ENSGALG00010021293	PCNX1	-0.039925187	0.068624262	-0.581794042	0.560705418	0.664005313	negative
PRJNA729271	stage_E14_vs_E10	ordinary_muscle_development	ENSGALG00010021296	GATC	0.145312008	0.120884235	1.202075761	0.229334177	0.33315471	positive
PRJNA729271	stage_E14_vs_E10	ordinary_muscle_development	ENSGALG00010021298	RPGRIP1L	-0.922197024	0.141939604	-6.497108624	8.18783E-11	7.59326E-10	negative
PRJNA729271	stage_E14_vs_E10	ordinary_muscle_development	ENSGALG00010021299	CYP27C1	-0.654876543	0.151353435	-4.326803311	1.51289E-05	6.89602E-05	negative
PRJNA729271	stage_E14_vs_E10	ordinary_muscle_development	ENSGALG00010021301	SMURF1	-0.078959414	0.079622884	-0.991667346	0.321359825	0.43587629	negative
PRJNA729271	stage_E14_vs_E10	ordinary_muscle_development	ENSGALG00010021302	P4HA1	0.076880075	0.059459701	1.292977825	0.196018729	0.294312355	positive
PRJNA729271	stage_E14_vs_E10	ordinary_muscle_development	ENSGALG00010021307	MAP3K9	-0.341617956	0.129668306	-2.634552459	0.008424828	0.020548189	negative
PRJNA729271	stage_E14_vs_E10	ordinary_muscle_development	ENSGALG00010021309	ST3GAL3	0.4363968	0.126171343	3.458763231	0.000542662	0.001824607	positive
PRJNA729271	stage_E14_vs_E10	ordinary_muscle_development	ENSGALG00010021311	TRIAP1	0.04752667	0.120002473	0.39604742	0.692070046	0.775056425	positive
PRJNA729271	stage_E14_vs_E10	ordinary_muscle_development	ENSGALG00010021313	KPNA7	4.157094616	1.208911282	3.438709423	0.000584494	0.001946319	positive
PRJNA729271	stage_E14_vs_E10	ordinary_muscle_development	ENSGALG00010021315	COX6A1	0.513335181	0.134862961	3.806346661	0.000141035	0.000541106	positive
PRJNA729271	stage_E14_vs_E10	ordinary_muscle_development	ENSGALG00010021318	TP73	-1.568183251	0.264138102	-5.936982347	2.90316E-09	2.26487E-08	negative

PRJNA729271	stage_E14_vs_E10	ordinary_muscle_development	ENSGALG00010021320	SLC6A6	0.63023381	0.195187502	3.228863553	0.001242832	0.003834578	positive
PRJNA729271	stage_E14_vs_E10	ordinary_muscle_development	ENSGALG00010021325	ARPC1A	-0.218711767	0.063505522	-3.443980299	0.000573217	0.001913581	negative
PRJNA729271	stage_E14_vs_E10	ordinary_muscle_development	ENSGALG00010021327	IGSF1	0.997595586	0.454552684	2.194675382	0.028186892	0.058308691	positive
PRJNA729271	stage_E14_vs_E10	ordinary_muscle_development	ENSGALG00010021329	PSMC3IP	-0.127188914	0.151491619	-0.839577232	0.40114547	0.516369299	negative
PRJNA729271	stage_E14_vs_E10	ordinary_muscle_development	ENSGALG00010021330	COMMD4	0.199441795	0.128313545	1.554331578	0.120105345	0.197749364	positive
PRJNA729271	stage_E14_vs_E10	ordinary_muscle_development	ENSGALG00010021332	OIT3	0.936088875	0.125108967	7.48218853	7.3095E-14	9.41788E-13	positive
PRJNA729271	stage_E14_vs_E10	ordinary_muscle_development	ENSGALG00010021335	NFATC3	-0.055519267	0.101686859	-0.545982714	0.585077819	0.685808102	negative
PRJNA729271	stage_E14_vs_E10	ordinary_muscle_development	ENSGALG00010021338	PDIA5	0.310206259	0.157292301	1.972164285	0.048590855	0.092637702	positive
PRJNA729271	stage_E14_vs_E10	ordinary_muscle_development	ENSGALG00010021341	L3MBTL1	-0.388797406	0.150190492	-2.5886952	0.009634033	0.023070429	negative
PRJNA729271	stage_E14_vs_E10	ordinary_muscle_development	ENSGALG00010021342	SFR1	-0.441580101	0.170173491	-2.594881838	0.009462344	0.022704301	negative
PRJNA729271	stage_E14_vs_E10	ordinary_muscle_development	ENSGALG00010021345	NEIL1	0.06750458	0.152210859	0.443493849	0.657408567	0.747383308	positive
PRJNA729271	stage_E14_vs_E10	ordinary_muscle_development	ENSGALG00010021346	RPS21	0.764257509	0.278421574	2.744965117	0.006051733	0.015384053	positive
PRJNA729271	stage_E14_vs_E10	ordinary_muscle_development	ENSGALG00010021348	SLC4A3	0.477829154	0.114154721	4.185802839	2.8416E-05	0.000123542	positive
PRJNA729271	stage_E14_vs_E10	ordinary_muscle_development	ENSGALG00010021350	CABLES2	-0.324940432	0.133192963	-2.439621623	0.014702653	0.033361764	negative
PRJNA729271	stage_E14_vs_E10	ordinary_muscle_development	ENSGALG00010021352	CFAP43	-0.330167083	0.218769365	-1.509201635	0.131247263	0.212728103	negative
PRJNA729271	stage_E14_vs_E10	ordinary_muscle_development	ENSGALG00010021353	IPO13	0.677359372	0.17832178	3.798522935	0.000145561	0.000556289	positive
PRJNA729271	stage_E14_vs_E10	ordinary_muscle_development	ENSGALG00010021354	ENSGALG00010021354	0.622426738	0.111170344	5.598855896	2.15771E-08	1.51019E-07	positive
PRJNA729271	stage_E14_vs_E10	ordinary_muscle_development	ENSGALG00010021358	MMP15	0.353387905	0.129127662	2.736732786	0.006205267	0.015706172	positive
PRJNA729271	stage_E14_vs_E10	ordinary_muscle_development	ENSGALG00010021360	PTPRN	-0.114171322	0.249395867	-0.457791557	0.647102202	0.739086472	negative
PRJNA729271	stage_E14_vs_E10	ordinary_muscle_development	ENSGALG00010021361	WRAP73	0.398873929	0.084815922	4.702818974	2.56594E-06	1.32928E-05	positive
PRJNA729271	stage_E14_vs_E10	ordinary_muscle_development	ENSGALG00010021362	ARPC1B	-0.037142649	0.120692895	-0.307745117	0.758276286	0.82777638	negative
PRJNA729271	stage_E14_vs_E10	ordinary_muscle_development	ENSGALG00010021363	ENSGALG00010021363	-0.042074191	0.073143072	-0.575231383	0.565134821	0.667668858	negative
PRJNA729271	stage_E14_vs_E10	ordinary_muscle_development	ENSGALG00010021367	GRIP2	2.231327283	0.175567496	12.70922772	5.25508E-37	3.38544E-35	positive
PRJNA729271	stage_E14_vs_E10	ordinary_muscle_development	ENSGALG00010021372	CD86	1.50931322	0.602739661	2.504088111	0.012276746	0.028527	positive
PRJNA729271	stage_E14_vs_E10	ordinary_muscle_development	ENSGALG00010021373	CEP250	-0.301695392	0.152044685	-1.984254778	0.047227445	0.090395063	negative
PRJNA729271	stage_E14_vs_E10	ordinary_muscle_development	ENSGALG00010021375	TUBG1	-0.2842943	0.115467091	-2.462124035	0.013811689	0.031597291	negative
PRJNA729271	stage_E14_vs_E10	ordinary_muscle_development	ENSGALG00010021378	KIF11	-0.139989603	0.184824774	-0.75741796	0.4487995	0.561871545	negative
PRJNA729271	stage_E14_vs_E10	ordinary_muscle_development	ENSGALG00010021381	DNAJB2	0.520773937	0.147380317	3.533537905	0.000410037	0.001416466	positive
PRJNA729271	stage_E14_vs_E10	ordinary_muscle_development	ENSGALG00010021382	TPRG1L	0.535028199	0.086590057	6.178864121	6.45644E-10	5.419E-09	positive
PRJNA729271	stage_E14_vs_E10	ordinary_muscle_development	ENSGALG00010021383	MSI1	-0.516426367	0.118138824	-4.371351851	1.2348E-05	5.70557E-05	negative
PRJNA729271	stage_E14_vs_E10	ordinary_muscle_development	ENSGALG00010021384	ARTN	-1.309309365	0.484807261	-2.700680187	0.006919784	0.01726562	negative
PRJNA729271	stage_E14_vs_E10	ordinary_muscle_development	ENSGALG00010021385	GDF5	-5.212477161	0.392421226	-13.28286243	2.91052E-40	2.22042E-38	negative
PRJNA729271	stage_E14_vs_E10	ordinary_muscle_development	ENSGALG00010021387	SLC2A14	0.096252125	0.094271196	1.021013092	0.307248236	0.420901917	positive
PRJNA729271	stage_E14_vs_E10	ordinary_muscle_development	ENSGALG00010021388	DPH2	-0.075308753	0.141023506	-0.53401561	0.593330734	0.693183605	negative
PRJNA729271	stage_E14_vs_E10	ordinary_muscle_development	ENSGALG00010021390	GSTO2	-0.575178243	0.212939359	-2.701136344	0.0069103	0.017251947	negative
PRJNA729271	stage_E14_vs_E10	ordinary_muscle_development	ENSGALG00010021391	ENSGALG00010021391	0.804194545	0.367246289	2.189796248	0.028539017	0.058936181	positive
PRJNA729271	stage_E14_vs_E10	ordinary_muscle_development	ENSGALG00010021393	CCDC174	-0.102834216	0.11235322	-0.915276089	0.360046709	0.476406974	negative
PRJNA729271	stage_E14_vs_E10	ordinary_muscle_development	ENSGALG00010021394	MAN2C1	-0.03207703	0.095336882	-0.336459821	0.736524145	0.810808649	negative
PRJNA729271	stage_E14_vs_E10	ordinary_muscle_development	ENSGALG00010021396	MED6	-0.102699061	0.078203888	-1.313221929	0.189108177	0.285891012	negative
PRJNA729271	stage_E14_vs_E10	ordinary_muscle_development	ENSGALG00010021397	ENSGALG00010021397	1.544507398	1.827668116	0.845069947	0.398071787	0.513463538	positive
PRJNA729271	stage_E14_vs_E10	ordinary_muscle_development	ENSGALG00010021400	B4GALT2	0.542036032	0.091479771	5.925200995	3.11916E-09	2.41906E-08	positive
PRJNA729271	stage_E14_vs_E10	ordinary_muscle_development	ENSGALG00010021401	PDAP1	0.073169592	0.093934247	0.778944781	0.436012243	0.550103688	positive
PRJNA729271	stage_E14_vs_E10	ordinary_muscle_development	ENSGALG00010021402	ENSGALG00010021402	0.183954456	0.086368452	2.129880211	0.033181504	0.06681084	positive
PRJNA729271	stage_E14_vs_E10	ordinary_muscle_development	ENSGALG00010021404	UQCC1	0.269652302	0.093009962	2.899176575	0.003741441	0.010080333	positive
PRJNA729271	stage_E14_vs_E10	ordinary_muscle_development	ENSGALG00010021405	IDE	-0.006065185	0.114193764	-0.053113105	0.95764179	0.970904228	negative
PRJNA729271	stage_E14_vs_E10	ordinary_muscle_development	ENSGALG00010021407	SIN3A	-0.042910603	0.110518974	-0.388264579	0.697820247	0.780109863	negative
PRJNA729271	stage_E14_vs_E10	ordinary_muscle_development	ENSGALG00010021408	TTC9	1.295384725	0.197724923	6.551448866	5.69815E-11	5.41604E-10	positive
PRJNA729271	stage_E14_vs_E10	ordinary_muscle_development	ENSGALG00010021409	MEGF6	0.7199933	0.232011984	3.103259098	0.00191402	0.005608191	positive
PRJNA729271	stage_E14_vs_E10	ordinary_muscle_development	ENSGALG00010021411	MCU	0.563173649	0.128933369	4.367943338	1.25422E-05	5.78978E-05	positive
PRJNA729271	stage_E14_vs_E10	ordinary_muscle_development	ENSGALG00010021412	ZNF319	0.092641322	0.123118667	0.752455534	0.451777161	0.564818867	positive
PRJNA729271	stage_E14_vs_E10	ordinary_muscle_development	ENSGALG00010021413	SYNJ2BP	0.445674765	0.151828018	2.935392096	0.003331264	0.00910554	positive
PRJNA729271	stage_E14_vs_E10	ordinary_muscle_development	ENSGALG00010021414	BUD31	0.30370655	0.110974712	2.736718534	0.006205536	0.015706172	positive
PRJNA729271	stage_E14_vs_E10	ordinary_muscle_development	ENSGALG00010021415	SIRT4	0.085531832	0.149792085	0.571003681	0.567997148	0.670558613	positive
PRJNA729271	stage_E14_vs_E10	ordinary_muscle_development	ENSGALG00010021416	PLEKHH3	0.311622237	0.123047357	2.532539049	0.011323976	0.026581543	positive
PRJNA729271	stage_E14_vs_E10	ordinary_muscle_development	ENSGALG00010021417	STK16	0.027539773	0.156187671	0.176324881	0.860038716	0.90367968	positive
PRJNA729271	stage_E14_vs_E10	ordinary_muscle_development	ENSGALG00010021419	MANSC1	-0.817149563	0.19885359	-4.109302531	3.96856E-05	0.00016815	negative
PRJNA729271	stage_E14_vs_E10	ordinary_muscle_development	ENSGALG00010021420	PLXNB3	-0.991493411	0.188572525	-5.257889037	1.45718E-07	9.05353E-07	negative
PRJNA729271	stage_E14_vs_E10	ordinary_muscle_development	ENSGALG00010021421	GLB1L	0.502865385	0.161850905	3.106966772	0.001890176	0.005547299	positive
PRJNA729271	stage_E14_vs_E10	ordinary_muscle_development	ENSGALG00010021424	PTCD1	-0.097679225	0.106761628	-0.914928207	0.360229321	0.476503377	negative
PRJNA729271	stage_E14_vs_E10	ordinary_muscle_development	ENSGALG00010021425	PTPN9	0.715323619	0.120491915	5.9366939	2.90827E-09	2.26642E-08	positive
PRJNA729271	stage_E14_vs_E10	ordinary_muscle_development	ENSGALG00010021426	SLC38A7	-0.208842891	0.14378358	-1.452480814	0.14636796	0.232631972	negative
PRJNA729271	stage_E14_vs_E10	ordinary_muscle_development	ENSGALG00010021427	RPLP0	0.451111208	0.13537415	3.332329018	0.000861224	0.002758771	positive
PRJNA729271	stage_E14_vs_E10	ordinary_muscle_development	ENSGALG00010021429	MED8	0.297970703	0.113745284	2.619631271	0.008802489	0.021325769	positive
PRJNA729271	stage_E14_vs_E10	ordinary_muscle_development	ENSGALG00010021431	CPSF4	-0.223687601	0.105944589	-2.111364088	0.034741029	0.069477265	negative
PRJNA729271	stage_E14_vs_E10	ordinary_muscle_development	ENSGALG00010021432	SYT9	-2.372008877	0.315482676	-7.518666032	5.53379E-14	7.19393E-13	negative
PRJNA729271	stage_E14_vs_E10	ordinary_muscle_development	ENSGALG00010021433	ENSGALG00010021433	3.620073442	0.271688133	13.32437086	1.67035E-40	1.31585E-38	positive
PRJNA729271	stage_E14_vs_E10	ordinary_muscle_development	ENSGALG00010021434	ANKZF1	0.183169251	0.176565545	1.037400877	0.29954907	0.413244862	positive
PRJNA729271	stage_E14_vs_E10	ordinary_muscle_development	ENSGALG00010021435	GCN1	-0.037993239	0.088969098	-0.427038596	0.669351216	0.756864489	negative
PRJNA729271	stage_E14_vs_E10	ordinary_muscle_development	ENSGALG00010021438	GOT2	0.113432813	0.110241966	1.028944026	0.303505979	0.417076143	positive
PRJNA729271	stage_E14_vs_E10	ordinary_muscle_development	ENSGALG00010021439	ITPRIP	-0.888387784	0.186667209	-4.759206439	1.94356E-06	1.02592E-05	negative
PRJNA729271	stage_E14_vs_E10	ordinary_muscle_development	ENSGALG00010021441	ENSGALG00010021441	-0.269640423	0.319663851	-0.843512404	0.398941932	0.514380297	negative
PRJNA729271	stage_E14_vs_E10	ordinary_muscle_development	ENSGALG00010021443	SNUPN	0.177583818	0.130853524	1.357119117	0.174743365	0.268189176	positive
PRJNA729271	stage_E14_vs_E10	ordinary_muscle_development	ENSGALG00010021444	ATG9A	0.228063949	0.246852767	0.92388654	0.355545392	0.471944181	positive
PRJNA729271	stage_E14_vs_E10	ordinary_muscle_development	ENSGALG00010021446	HOXB8	-1.672471905	0.608098149	-2.750332174	0.005953488	0.015174223	negative
PRJNA729271	stage_E14_vs_E10	ordinary_muscle_development	ENSGALG00010021447	OCSTAMP	-0.735290102	0.714237197	-1.029476069	0.30325602	0.416890744	negative
PRJNA729271	stage_E14_vs_E10	ordinary_muscle_development	ENSGALG00010021448	SZT2	-0.016234768	0.100071445	-0.162231771	0.871123342	0.911494466	negative
PRJNA729271	stage_E14_vs_E10	ordinary_muscle_development	ENSGALG00010021450	OLFML1	-0.760175	0.24864805	-3.057232906	0.002233906	0.006411975	negative
PRJNA729271	stage_E14_vs_E10	ordinary_muscle_development	ENSGALG00010021452	SLC25A1	-0.214112153	0.113055456	-1.893868375	0.058242483	0.10780549	negative
PRJNA729271	stage_E14_vs_E10	ordinary_muscle_development	ENSGALG00010021454	ATP5J2	0.273778852	0.174261059	1.571084524	0.116163017	0.192630469	positive
PRJNA729271	stage_E14_vs_E10	ordinary_muscle_development	ENSGALG00010021455	CFAP58	-1.047338194	0.52157008	-2.008048839	0.044638102	0.08613276	negative
PRJNA729271	stage_E14_vs_E10	ordinary_muscle_development	ENSGALG00010021457	USB1	0.39095942	0.112440629	3.477029817	0.000507001	0.001715667	positive
PRJNA729271	stage_E14_vs_E10	ordinary_muscle_development	ENSGALG00010021458	USP30	-0.171217291	0.079414431	-2.155997193	0.031083884	0.063215602	negative
PRJNA729271	stage_E14_vs_E10	ordinary_muscle_development	ENSGALG00010021459	ARHGEF16	0.185404618	0.121008855	1.532157441	0.125483588	0.204852417	positive
PRJNA729271	stage_E14_vs_E10	ordinary_muscle_development	ENSGALG00010021461	SNX33	0.239342126	0.109128607	2.193211593	0.028292138	0.058476335	positive
PRJNA729271	stage_E14_vs_E10	ordinary_muscle_development	ENSGALG00010021462	SETD5	-0.130733301	0.093983376	-1.391025793	0.164217606	0.255126923	negative
PRJNA729271	stage_E14_vs_E10	ordinary_muscle_development	ENSGALG00010021464	ENSGALG00010021464	-0.100163428	0.436281749	-0.229584273	0.818414829	0.872851368	negative
PRJNA729271	stage_E14_vs_E10	ordinary_muscle_development	ENSGALG00010021465	ABCB6	-0.131263834	0.213935285	-0.61356795	0.539500868	0.645684607	negative
PRJNA729271	stage_E14_vs_E10	ordinary_muscle_development	ENSGALG00010021466	HES4	-0.207927677	0.178330721	-1.165966669	0.243627922	0.350023464	negative
PRJNA729271	stage_E14_vs_E10	ordinary_muscle_development	ENSGALG00010021467	LHFPL4	-0.061966274	0.251813124	-0.246080399	0.805619996	0.863395781	negative
PRJNA729271	stage_E14_vs_E10	ordinary_muscle_development	ENSGALG00010021469	CSPG4	-0.219831048	0.161592526	-1.360403563	0.173702251	0.266915524	negative
PRJNA729271	stage_E14_vs_E10	ordinary_muscle_development	ENSGALG00010021470	ENSGALG00010021470	-1.55592385	0.593901324	-2.61983563	0.008797216	0.021316557	negative
PRJNA729271	stage_E14_vs_E10	ordinary_muscle_development	ENSGALG00010021473	SLC8A3	0.986311858	0.131392968	7.50658023	6.06919E-14	7.88288E-13	positive
PRJNA729271	stage_E14_vs_E10	ordinary_muscle_development	ENSGALG00010021474	PPFIBP2	1.718359039	0.180292384	9.530957459	1.55839E-21	3.7711E-20	positive
PRJNA729271	stage_E14_vs_E10	ordinary_muscle_development	ENSGALG00010021476	ALKBH2	0.159058171	0.116006753	1.371111318	0.170340257	0.262836069	positive
PRJNA729271	stage_E14_vs_E10	ordinary_muscle_development	ENSGALG00010021477	ZFAND2B	0.316450349	0.116485043	2.716660788	0.006594416	0.016530524	positive
PRJNA729271	stage_E14_vs_E10	ordinary_muscle_development	ENSGALG00010021478	SLC13A3	-0.652984064	0.19503035	-3.348115126	0.000813632	0.002621965	negative
PRJNA729271	stage_E14_vs_E10	ordinary_muscle_development	ENSGALG00010021479	SKAP1	0.484679509	0.422666112	1.146719588	0.251497532	0.358628306	positive
PRJNA729271	stage_E14_vs_E10	ordinary_muscle_development	ENSGALG00010021480	ENSGALG00010021480	-0.129604836	0.074579686	-1.737803453	0.082245452	0.144801145	negative
PRJNA729271	stage_E14_vs_E10	ordinary_muscle_development	ENSGALG00010021481	MICU1	0.473705675	0.174185585	2.7195458	0.006537164	0.016413683	positive
PRJNA729271	stage_E14_vs_E10	ordinary_muscle_development	ENSGALG00010021483	UNG	0.085018938	0.152580514	0.557207046	0.577385976	0.679098485	positive
PRJNA729271	stage_E14_vs_E10	ordinary_muscle_development	ENSGALG00010021484	MARCHF5	0.076879407	0.070986052	1.083021311	0.278798964	0.389625047	positive
PRJNA729271	stage_E14_vs_E10	ordinary_muscle_development	ENSGALG00010021485	SMOC1	-3.053541854	0.215450788	-14.1728043	1.35E-45	1.49376E-43	negative
PRJNA729271	stage_E14_vs_E10	ordinary_muscle_development	ENSGALG00010021487	KLHL17	-0.636048968	0.131690651	-4.829871845	1.36621E-06	7.42527E-06	negative
PRJNA729271	stage_E14_vs_E10	ordinary_muscle_development	ENSGALG00010021488	HYI	-0.613319621	0.130771276	-4.690017875	2.73181E-06	1.40817E-05	negative
PRJNA729271	stage_E14_vs_E10	ordinary_muscle_development	ENSGALG00010021489	CNPPD1	0.351648046	0.085293553	4.122797457	3.74299E-05	0.000159058	positive
PRJNA729271	stage_E14_vs_E10	ordinary_muscle_development	ENSGALG00010021490	ACACB	1.934664372	0.334566117	5.782607004	7.35518E-09	5.44501E-08	positive
PRJNA729271	stage_E14_vs_E10	ordinary_muscle_development	ENSGALG00010021492	PARP6	0.111694754	0.155489813	0.7183413	0.472546871	0.584483906	positive
PRJNA729271	stage_E14_vs_E10	ordinary_muscle_development	ENSGALG00010021493	UNC93B1	0.102429689	0.130386815	0.785583181	0.432111721	0.546263683	positive
PRJNA729271	stage_E14_vs_E10	ordinary_muscle_development	ENSGALG00010021494	SLC2A10	-0.019496486	0.231308439	-0.08428783	0.932827588	0.953751562	negative
PRJNA729271	stage_E14_vs_E10	ordinary_muscle_development	ENSGALG00010021496	MTMR14	0.065778706	0.115619506	0.568923945	0.569407755	0.671731538	positive
PRJNA729271	stage_E14_vs_E10	ordinary_muscle_development	ENSGALG00010021498	B3GALT6	-0.584664024	0.103139049	-5.668697049	1.43888E-08	1.02843E-07	negative
PRJNA729271	stage_E14_vs_E10	ordinary_muscle_development	ENSGALG00010021501	SLC23A3	-0.231867142	0.719044802	-0.322465501	0.747100069	0.818720458	negative
PRJNA729271	stage_E14_vs_E10	ordinary_muscle_development	ENSGALG00010021502	DNAJB12	0.074469989	0.146141578	0.509574274	0.610349751	0.70753516	positive
PRJNA729271	stage_E14_vs_E10	ordinary_muscle_development	ENSGALG00010021503	KCNS1	1.824578056	0.394529053	4.624698842	3.75143E-06	1.88286E-05	positive
PRJNA729271	stage_E14_vs_E10	ordinary_muscle_development	ENSGALG00010021504	ODR4	-0.200048209	0.074911827	-2.670448931	0.007574989	0.018682911	negative
PRJNA729271	stage_E14_vs_E10	ordinary_muscle_development	ENSGALG00010021505	IHH	-5.39370862	0.575968441	-9.364590547	7.63457E-21	1.73725E-19	negative
PRJNA729271	stage_E14_vs_E10	ordinary_muscle_development	ENSGALG00010021506	MAX	-0.477124533	0.256617192	-1.859285147	0.062986731	0.115282464	negative
PRJNA729271	stage_E14_vs_E10	ordinary_muscle_development	ENSGALG00010021509	DDIT4	1.161447763	0.277868391	4.179848457	2.91703E-05	0.000126518	positive
PRJNA729271	stage_E14_vs_E10	ordinary_muscle_development	ENSGALG00010021510	NTSR1	-2.007796489	0.423343596	-4.742711379	2.10877E-06	1.10573E-05	negative
PRJNA729271	stage_E14_vs_E10	ordinary_muscle_development	ENSGALG00010021512	CPNE9	2.220901982	0.397001748	5.594186904	2.21658E-08	1.54766E-07	positive
PRJNA729271	stage_E14_vs_E10	ordinary_muscle_development	ENSGALG00010021513	SDF4	-0.031380165	0.096833955	-0.324061586	0.745891409	0.817643195	negative
PRJNA729271	stage_E14_vs_E10	ordinary_muscle_development	ENSGALG00010021514	TPR	-0.322143958	0.07900006	-4.077768519	4.547E-05	0.000190378	negative
PRJNA729271	stage_E14_vs_E10	ordinary_muscle_development	ENSGALG00010021516	SRSF5	0.330119764	0.15104064	2.185635364	0.028842293	0.059418567	positive
PRJNA729271	stage_E14_vs_E10	ordinary_muscle_development	ENSGALG00010021518	CPEB3	0.946414481	0.146559132	6.457560653	1.06404E-10	9.70628E-10	positive
PRJNA729271	stage_E14_vs_E10	ordinary_muscle_development	ENSGALG00010021519	FNTB	0.185151763	0.099275704	1.865025942	0.062177768	0.114041247	positive
PRJNA729271	stage_E14_vs_E10	ordinary_muscle_development	ENSGALG00010021521	ENSGALG00010021521	-0.290426581	0.276004025	-1.052254875	0.292682629	0.405237817	negative
PRJNA729271	stage_E14_vs_E10	ordinary_muscle_development	ENSGALG00010021523	CELF6	-0.26642412	0.140547677	-1.895613829	0.058011127	0.10745599	negative
PRJNA729271	stage_E14_vs_E10	ordinary_muscle_development	ENSGALG00010021525	ENSGALG00010021525	-1.200095235	0.822548783	-1.458995819	0.144566258	0.230298705	negative
PRJNA729271	stage_E14_vs_E10	ordinary_muscle_development	ENSGALG00010021526	KCNJ12	-0.383395872	0.221459899	-1.731220299	0.08341248	0.146515401	negative
PRJNA729271	stage_E14_vs_E10	ordinary_muscle_development	ENSGALG00010021528	BRPF1	0.162164344	0.166075677	0.976448492	0.328842261	0.443649346	positive
PRJNA729271	stage_E14_vs_E10	ordinary_muscle_development	ENSGALG00010021529	BTAF1	-0.093733901	0.075420458	-1.242817974	0.213934918	0.315589928	negative

PRJNA729271	stage_E14_vs_E10	ordinary_muscle_development	ENSGALG00010021530	MLF2	0.008726787	0.059768934	0.146008753	0.883914479	0.920294524	positive
PRJNA729271	stage_E14_vs_E10	ordinary_muscle_development	ENSGALG00010021531	RAB15	-0.478041577	0.243487017	-1.963314442	0.049609648	0.094220631	negative
PRJNA729271	stage_E14_vs_E10	ordinary_muscle_development	ENSGALG00010021532	DPP10	0.335066332	0.18073251	1.853935027	0.063748456	0.11646507	positive
PRJNA729271	stage_E14_vs_E10	ordinary_muscle_development	ENSGALG00010021533	SSX2IP	-0.042710064	0.298326058	-0.143165717	0.886159304	0.922168075	negative
PRJNA729271	stage_E14_vs_E10	ordinary_muscle_development	ENSGALG00010021534	KIFC3	1.344436401	0.168934273	7.958340121	1.74363E-15	2.56067E-14	positive
PRJNA729271	stage_E14_vs_E10	ordinary_muscle_development	ENSGALG00010021535	SUSD6	-0.043179609	0.085598085	-0.504445971	0.61394802	0.71056983	negative
PRJNA729271	stage_E14_vs_E10	ordinary_muscle_development	ENSGALG00010021538	TNFRSF18	-0.69987089	0.47770317	-1.46507483	0.142900503	0.228121453	negative
PRJNA729271	stage_E14_vs_E10	ordinary_muscle_development	ENSGALG00010021539	HEXA	-0.367524316	0.118700321	-3.09623691	0.001959937	0.00571674	negative
PRJNA729271	stage_E14_vs_E10	ordinary_muscle_development	ENSGALG00010021540	STK4	0.359882989	0.074859511	4.807445102	1.52871E-06	8.2191E-06	positive
PRJNA729271	stage_E14_vs_E10	ordinary_muscle_development	ENSGALG00010021541	CYP27A1	0.908731727	0.278848475	3.258872857	0.001118558	0.003480785	positive
PRJNA729271	stage_E14_vs_E10	ordinary_muscle_development	ENSGALG00010021543	PLEKHD1	0.597575915	0.257987789	2.316295345	0.020542148	0.044501336	positive
PRJNA729271	stage_E14_vs_E10	ordinary_muscle_development	ENSGALG00010021545	CYB5R2	-0.739646344	0.132353149	-5.588430284	2.29131E-08	1.59814E-07	negative
PRJNA729271	stage_E14_vs_E10	ordinary_muscle_development	ENSGALG00010021546	OGG1	0.191411933	0.1619688	1.181782741	0.237291917	0.34234561	positive
PRJNA729271	stage_E14_vs_E10	ordinary_muscle_development	ENSGALG00010021549	ANAPC16	0.101467198	0.090224764	1.124604755	0.260756558	0.368962283	positive
PRJNA729271	stage_E14_vs_E10	ordinary_muscle_development	ENSGALG00010021550	MAP2K3	0.305594982	0.172282204	1.773804693	0.076095462	0.135520791	positive
PRJNA729271	stage_E14_vs_E10	ordinary_muscle_development	ENSGALG00010021552	ENSGALG00010021552	1.538039455	0.170886276	9.000368502	2.24961E-19	4.60567E-18	positive
PRJNA729271	stage_E14_vs_E10	ordinary_muscle_development	ENSGALG00010021553	SLC39A9	-0.179517946	0.08620417	-2.082474038	0.037299192	0.073809118	negative
PRJNA729271	stage_E14_vs_E10	ordinary_muscle_development	ENSGALG00010021554	SNX11	0.443306402	0.146703913	3.021776255	0.002512962	0.00709079	positive
PRJNA729271	stage_E14_vs_E10	ordinary_muscle_development	ENSGALG00010021556	CAMK1	0.184886126	0.140839668	1.312741847	0.189269952	0.286075907	positive
PRJNA729271	stage_E14_vs_E10	ordinary_muscle_development	ENSGALG00010021558	CHURC1	0.575345118	0.179671075	3.202213358	0.001363759	0.00415637	positive
PRJNA729271	stage_E14_vs_E10	ordinary_muscle_development	ENSGALG00010021559	MYO1H	0.902496541	0.532221943	1.695714641	0.089939934	0.155681795	positive
PRJNA729271	stage_E14_vs_E10	ordinary_muscle_development	ENSGALG00010021561	TGIF2	0.292072081	0.095551428	3.05670034	0.002237879	0.006422105	positive
PRJNA729271	stage_E14_vs_E10	ordinary_muscle_development	ENSGALG00010021562	LRP6	-0.322315233	0.082034836	-3.929004422	8.52983E-05	0.00033967	negative
PRJNA729271	stage_E14_vs_E10	ordinary_muscle_development	ENSGALG00010021564	PRKAG3	5.277303395	0.63943827	8.253030269	1.54428E-16	2.48715E-15	positive
PRJNA729271	stage_E14_vs_E10	ordinary_muscle_development	ENSGALG00010021565	CBX1	-0.024286386	0.103608157	-0.234406114	0.814669712	0.870128674	negative
PRJNA729271	stage_E14_vs_E10	ordinary_muscle_development	ENSGALG00010021566	ENSGALG00010021566	-0.145451682	0.094914711	-1.532446139	0.125412379	0.204759229	negative
PRJNA729271	stage_E14_vs_E10	ordinary_muscle_development	ENSGALG00010021568	TADA3	0.319918059	0.141032192	2.268404495	0.023304564	0.049661814	positive
PRJNA729271	stage_E14_vs_E10	ordinary_muscle_development	ENSGALG00010021570	KATNB1	-0.049400808	0.094133601	-0.524794626	0.599725968	0.699021221	negative
PRJNA729271	stage_E14_vs_E10	ordinary_muscle_development	ENSGALG00010021572	MYL9	0.967714402	0.111600336	8.671249924	4.27402E-18	7.88193E-17	positive
PRJNA729271	stage_E14_vs_E10	ordinary_muscle_development	ENSGALG00010021573	NATD1	0.51332997	0.195692551	2.623145176	0.008712214	0.021135321	positive
PRJNA729271	stage_E14_vs_E10	ordinary_muscle_development	ENSGALG00010021574	TTLL4	-0.248667074	0.228971494	-1.086017608	0.277471202	0.388031363	negative
PRJNA729271	stage_E14_vs_E10	ordinary_muscle_development	ENSGALG00010021575	ADGRL2	-0.473068444	0.082656301	-5.723319782	1.04462E-08	7.59369E-08	negative
PRJNA729271	stage_E14_vs_E10	ordinary_muscle_development	ENSGALG00010021576	ERH	-0.024086449	0.121216224	-0.198706481	0.842492354	0.890406611	negative
PRJNA729271	stage_E14_vs_E10	ordinary_muscle_development	ENSGALG00010021577	ARIH1	0.096528277	0.076343036	1.264401861	0.206085825	0.306004306	positive
PRJNA729271	stage_E14_vs_E10	ordinary_muscle_development	ENSGALG00010021578	ENSGALG00010021578	1.332049016	1.091533219	1.220346749	0.222333455	0.325035141	positive
PRJNA729271	stage_E14_vs_E10	ordinary_muscle_development	ENSGALG00010021579	C20H20ORF24	0.019616896	0.111789483	0.175480697	0.860701937	0.904179923	positive
PRJNA729271	stage_E14_vs_E10	ordinary_muscle_development	ENSGALG00010021580	KCTD10	-0.370237281	0.108380319	-3.416093306	0.000635265	0.002095145	negative
PRJNA729271	stage_E14_vs_E10	ordinary_muscle_development	ENSGALG00010021582	STK36	0.045009532	0.138024275	0.326098665	0.744349707	0.81650893	positive
PRJNA729271	stage_E14_vs_E10	ordinary_muscle_development	ENSGALG00010021583	NFE2L1	0.807240306	0.220942888	3.653615245	0.000258574	0.000934903	positive
PRJNA729271	stage_E14_vs_E10	ordinary_muscle_development	ENSGALG00010021584	OVCH2	-1.087130376	0.398706887	-2.726640574	0.006398269	0.016115189	negative
PRJNA729271	stage_E14_vs_E10	ordinary_muscle_development	ENSGALG00010021586	BBS4	0.404023242	0.119725164	3.374589158	0.00073926	0.002407452	positive
PRJNA729271	stage_E14_vs_E10	ordinary_muscle_development	ENSGALG00010021588	RNF25	0.2058293	0.138124349	1.490173896	0.136178518	0.219323069	positive
PRJNA729271	stage_E14_vs_E10	ordinary_muscle_development	ENSGALG00010021593	DCAF7	-0.189413551	0.05363772	-3.531349804	0.000413445	0.001427216	negative
PRJNA729271	stage_E14_vs_E10	ordinary_muscle_development	ENSGALG00010021594	ADPGK	-0.229055115	0.089057938	-2.571978642	0.010111913	0.024036107	negative
PRJNA729271	stage_E14_vs_E10	ordinary_muscle_development	ENSGALG00010021595	UBE3B	0.13183167	0.083608948	1.576765091	0.114849587	0.191091398	positive
PRJNA729271	stage_E14_vs_E10	ordinary_muscle_development	ENSGALG00010021597	GGT7	-0.000114819	0.184566953	-0.000622098	0.999503638	0.999503638	negative
PRJNA729271	stage_E14_vs_E10	ordinary_muscle_development	ENSGALG00010021599	ARPC4	0.236411781	0.102753973	2.300755617	0.021405448	0.046171425	positive
PRJNA729271	stage_E14_vs_E10	ordinary_muscle_development	ENSGALG00010021600	FMN1	-0.105804474	0.128972292	-0.820365928	0.412007533	0.526732242	negative
PRJNA729271	stage_E14_vs_E10	ordinary_muscle_development	ENSGALG00010021601	LPAR5	-0.355080866	0.385975799	-0.919956294	0.357595599	0.473927787	negative
PRJNA729271	stage_E14_vs_E10	ordinary_muscle_development	ENSGALG00010021602	PLEKHG4	-0.61079943	0.098163391	-6.222273126	4.90003E-10	4.18046E-09	negative
PRJNA729271	stage_E14_vs_E10	ordinary_muscle_development	ENSGALG00010021604	SPTB	1.753120179	0.189300498	9.261043659	2.02444E-20	4.45961E-19	positive
PRJNA729271	stage_E14_vs_E10	ordinary_muscle_development	ENSGALG00010021605	MAP3K3	-0.533533284	0.112323127	-4.749986023	2.03431E-06	1.07071E-05	negative
PRJNA729271	stage_E14_vs_E10	ordinary_muscle_development	ENSGALG00010021606	RNF8	-0.150605861	0.093374465	-1.612923423	0.106761146	0.179827905	negative
PRJNA729271	stage_E14_vs_E10	ordinary_muscle_development	ENSGALG00010021607	SLC9A5	-0.798302179	0.201088249	-3.96990965	7.18999E-05	0.00029087	negative
PRJNA729271	stage_E14_vs_E10	ordinary_muscle_development	ENSGALG00010021609	ENSGALG00010021609	1.54725868	0.203253951	7.612440855	2.68967E-14	3.57349E-13	positive
PRJNA729271	stage_E14_vs_E10	ordinary_muscle_development	ENSGALG00010021610	TMEM11	0.511180783	0.087936166	5.813089275	6.13303E-09	4.59186E-08	positive
PRJNA729271	stage_E14_vs_E10	ordinary_muscle_development	ENSGALG00010021611	GREM1	-1.993103358	0.601982415	-3.310899635	0.000929966	0.002950285	negative
PRJNA729271	stage_E14_vs_E10	ordinary_muscle_development	ENSGALG00010021612	BCS1L	0.076128151	0.130539681	0.583180151	0.559772032	0.663225078	positive
PRJNA729271	stage_E14_vs_E10	ordinary_muscle_development	ENSGALG00010021613	ADGRL4	0.610371592	0.150354286	4.059555668	4.91662E-05	0.00020473	positive
PRJNA729271	stage_E14_vs_E10	ordinary_muscle_development	ENSGALG00010021615	FLVCR1	-1.140729614	0.174867393	-6.52339806	6.87321E-11	6.4735E-10	negative
PRJNA729271	stage_E14_vs_E10	ordinary_muscle_development	ENSGALG00010021617	SORBS1	-0.316503603	0.11213306	-2.822571707	0.004764017	0.012487237	negative
PRJNA729271	stage_E14_vs_E10	ordinary_muscle_development	ENSGALG00010021618	NEO1	-0.910285521	0.106105508	-8.579059979	9.56516E-18	1.70958E-16	negative
PRJNA729271	stage_E14_vs_E10	ordinary_muscle_development	ENSGALG00010021619	FHOD1	0.260957404	0.182600801	1.429114233	0.152971403	0.241222856	positive
PRJNA729271	stage_E14_vs_E10	ordinary_muscle_development	ENSGALG00010021621	ZNF518A	-0.081479142	0.136730174	-0.595911932	0.551234062	0.656058603	negative
PRJNA729271	stage_E14_vs_E10	ordinary_muscle_development	ENSGALG00010021622	PTHLH	-2.033299764	0.258226706	-7.874087832	3.43238E-15	4.91624E-14	negative
PRJNA729271	stage_E14_vs_E10	ordinary_muscle_development	ENSGALG00010021623	FAR1	-0.236626764	0.129870191	-1.822025232	0.068451157	0.123761946	negative
PRJNA729271	stage_E14_vs_E10	ordinary_muscle_development	ENSGALG00010021624	HSD11B2	0.029527899	0.378228005	0.078069045	0.937773131	0.956514076	positive
PRJNA729271	stage_E14_vs_E10	ordinary_muscle_development	ENSGALG00010021625	HCN4	-3.161964025	0.958361705	-3.299343043	0.000969114	0.003061083	negative
PRJNA729271	stage_E14_vs_E10	ordinary_muscle_development	ENSGALG00010021626	ZNF142	-0.044406562	0.213459808	-0.208032427	0.835203648	0.885041185	negative
PRJNA729271	stage_E14_vs_E10	ordinary_muscle_development	ENSGALG00010021627	NCOA6	-0.029365503	0.103558769	-0.283563655	0.776744793	0.841788095	negative
PRJNA729271	stage_E14_vs_E10	ordinary_muscle_development	ENSGALG00010021628	REC114	-0.474820959	0.532131002	-0.892300875	0.372231685	0.48836878	negative
PRJNA729271	stage_E14_vs_E10	ordinary_muscle_development	ENSGALG00010021629	DHRS7B	1.003412604	0.100936766	9.941002069	2.76036E-23	7.34155E-22	positive
PRJNA729271	stage_E14_vs_E10	ordinary_muscle_development	ENSGALG00010021630	VTG1	-2.768717362	0.334320278	-8.281631561	1.21499E-16	1.98773E-15	negative
PRJNA729271	stage_E14_vs_E10	ordinary_muscle_development	ENSGALG00010021632	LIMD2	-2.135654999	0.160096053	-13.33983545	1.35759E-40	1.0872E-38	negative
PRJNA729271	stage_E14_vs_E10	ordinary_muscle_development	ENSGALG00010021633	CCNJ	-0.216399094	0.099489933	-2.175085347	0.029623721	0.060788975	negative
PRJNA729271	stage_E14_vs_E10	ordinary_muscle_development	ENSGALG00010021634	PLCD4	1.954040653	0.248678398	7.857701627	3.91246E-15	5.55991E-14	positive
PRJNA729271	stage_E14_vs_E10	ordinary_muscle_development	ENSGALG00010021635	FOXJ2	0.142067197	0.113720882	1.24926218	0.211569196	0.31257726	positive
PRJNA729271	stage_E14_vs_E10	ordinary_muscle_development	ENSGALG00010021636	C1orf159	-0.014820534	0.192950152	-0.07681017	0.93877456	0.957092613	negative
PRJNA729271	stage_E14_vs_E10	ordinary_muscle_development	ENSGALG00010021637	ATP6V0D1	0.08807228	0.102339307	0.86059094	0.389463377	0.505169723	positive
PRJNA729271	stage_E14_vs_E10	ordinary_muscle_development	ENSGALG00010021638	SRC	-0.524726168	0.170834398	-3.071548667	0.002129514	0.006146418	negative
PRJNA729271	stage_E14_vs_E10	ordinary_muscle_development	ENSGALG00010021639	SCG5	-0.694561811	0.346965472	-2.001818243	0.045304283	0.087321135	negative
PRJNA729271	stage_E14_vs_E10	ordinary_muscle_development	ENSGALG00010021640	PLEKHG3	0.001593345	0.104070365	0.015310266	0.987784652	0.991890442	positive
PRJNA729271	stage_E14_vs_E10	ordinary_muscle_development	ENSGALG00010021642	NPTN	-0.120489598	0.089698446	-1.343274081	0.179183242	0.273799398	negative
PRJNA729271	stage_E14_vs_E10	ordinary_muscle_development	ENSGALG00010021643	ENSGALG00010021643	-0.014620386	0.128373001	-0.113889883	0.909325085	0.937458543	negative
PRJNA729271	stage_E14_vs_E10	ordinary_muscle_development	ENSGALG00010021644	TARDBP	-0.069580217	0.153297672	-0.453889585	0.6499083	0.741356796	negative
PRJNA729271	stage_E14_vs_E10	ordinary_muscle_development	ENSGALG00010021645	ASCC1	0.472087645	0.136528635	3.457792171	0.000544621	0.001828651	positive
PRJNA729271	stage_E14_vs_E10	ordinary_muscle_development	ENSGALG00010021646	RPUSD3	0.330393466	0.164321303	2.010655107	0.044361899	0.085645408	positive
PRJNA729271	stage_E14_vs_E10	ordinary_muscle_development	ENSGALG00010021647	ARHGAP11B	-0.462137739	0.132897637	-3.477396208	0.000506309	0.001713907	negative
PRJNA729271	stage_E14_vs_E10	ordinary_muscle_development	ENSGALG00010021648	BRE	-0.106015414	0.066836992	-1.586178709	0.112698776	0.188090818	negative
PRJNA729271	stage_E14_vs_E10	ordinary_muscle_development	ENSGALG00010021649	PXN	0.358088317	0.096063778	3.727610192	0.000193304	0.00072085	positive
PRJNA729271	stage_E14_vs_E10	ordinary_muscle_development	ENSGALG00010021650	CD276	-0.627360301	0.107932142	-5.812543768	6.15306E-09	4.60209E-08	negative
PRJNA729271	stage_E14_vs_E10	ordinary_muscle_development	ENSGALG00010021651	RNF113B	-0.229018829	0.153390835	-1.493041148	0.135426416	0.218354383	negative
PRJNA729271	stage_E14_vs_E10	ordinary_muscle_development	ENSGALG00010021652	BORCS5	-0.016353451	0.095112152	-0.171938612	0.86348579	0.906175491	negative
PRJNA729271	stage_E14_vs_E10	ordinary_muscle_development	ENSGALG00010021655	USP22	-0.301474476	0.091814615	-3.28351295	0.001025219	0.003224947	negative
PRJNA729271	stage_E14_vs_E10	ordinary_muscle_development	ENSGALG00010021656	SETD6	-0.386314647	0.078657213	-4.911369654	9.04424E-07	5.01516E-06	negative
PRJNA729271	stage_E14_vs_E10	ordinary_muscle_development	ENSGALG00010021657	JAGN1	0.14125536	0.141965256	0.994999508	0.319736504	0.434112085	positive
PRJNA729271	stage_E14_vs_E10	ordinary_muscle_development	ENSGALG00010021658	STRADA	-0.166172122	0.089272179	-1.861409948	0.062686307	0.114857542	negative
PRJNA729271	stage_E14_vs_E10	ordinary_muscle_development	ENSGALG00010021659	SPOCK2	0.278418418	0.144822379	1.922482013	0.054545135	0.101977522	positive
PRJNA729271	stage_E14_vs_E10	ordinary_muscle_development	ENSGALG00010021660	RNF223	0.446451119	0.485802319	0.918997506	0.358096877	0.474505369	positive
PRJNA729271	stage_E14_vs_E10	ordinary_muscle_development	ENSGALG00010021661	DUSP16	-0.386797357	0.120978558	-3.197238936	0.001387499	0.004216311	negative
PRJNA729271	stage_E14_vs_E10	ordinary_muscle_development	ENSGALG00010021662	RASGRP1	-0.508234507	0.167016681	-3.043016455	0.002342195	0.006681779	negative
PRJNA729271	stage_E14_vs_E10	ordinary_muscle_development	ENSGALG00010021663	CNOT9	0.106404308	0.139481674	0.762855113	0.445549808	0.558961786	positive
PRJNA729271	stage_E14_vs_E10	ordinary_muscle_development	ENSGALG00010021664	TYSND1	-0.397135856	0.162132133	-2.449458036	0.014307138	0.032593976	negative
PRJNA729271	stage_E14_vs_E10	ordinary_muscle_development	ENSGALG00010021665	CNOT1	0.056795847	0.091313923	0.621984529	0.533952041	0.641122833	positive
PRJNA729271	stage_E14_vs_E10	ordinary_muscle_development	ENSGALG00010021666	CCDC47	0.334724184	0.09206145	3.635877809	0.000277036	0.00099792	positive
PRJNA729271	stage_E14_vs_E10	ordinary_muscle_development	ENSGALG00010021667	TBX15	0.060346725	0.191489324	0.31514407	0.752652269	0.822938421	positive
PRJNA729271	stage_E14_vs_E10	ordinary_muscle_development	ENSGALG00010021668	PYGO1	-0.293154715	0.126532478	-2.316833752	0.02051279	0.044451744	negative
PRJNA729271	stage_E14_vs_E10	ordinary_muscle_development	ENSGALG00010021669	ENSGALG00010021669	0.035293424	0.072913125	0.484047608	0.628352082	0.722281002	positive
PRJNA729271	stage_E14_vs_E10	ordinary_muscle_development	ENSGALG00010021670	BLCAP	0.322238957	0.108834899	2.96080541	0.003068357	0.008482899	positive
PRJNA729271	stage_E14_vs_E10	ordinary_muscle_development	ENSGALG00010021671	TNFRSF13B	1.592741897	0.753189311	2.114663438	0.034458643	0.069045898	positive
PRJNA729271	stage_E14_vs_E10	ordinary_muscle_development	ENSGALG00010021674	ING4	0.052030226	0.117509823	0.442773417	0.657929633	0.747741103	positive
PRJNA729271	stage_E14_vs_E10	ordinary_muscle_development	ENSGALG00010021675	DDX42	0.380429482	0.057832218	6.578158277	4.76311E-11	4.58137E-10	positive
PRJNA729271	stage_E14_vs_E10	ordinary_muscle_development	ENSGALG00010021676	TP53INP2	0.423546329	0.281590483	1.504121604	0.132550058	0.214599922	positive
PRJNA729271	stage_E14_vs_E10	ordinary_muscle_development	ENSGALG00010021677	ENSGALG00010021677	-0.410007525	0.152617723	-2.686500081	0.007220493	0.017912211	negative
PRJNA729271	stage_E14_vs_E10	ordinary_muscle_development	ENSGALG00010021678	DNAAF4	0.738204428	0.151991269	4.856887065	1.19246E-06	6.52743E-06	positive
PRJNA729271	stage_E14_vs_E10	ordinary_muscle_development	ENSGALG00010021679	MASP2	-2.182913888	1.065320012	-2.049068696	0.040455398	0.079061749	negative
PRJNA729271	stage_E14_vs_E10	ordinary_muscle_development	ENSGALG00010021680	PIGT	0.135678655	0.117674155	1.15300301	0.248909149	0.355882631	positive
PRJNA729271	stage_E14_vs_E10	ordinary_muscle_development	ENSGALG00010021681	BTBD10	0.018098642	0.078187796	0.231476562	0.816944592	0.871732322	positive
PRJNA729271	stage_E14_vs_E10	ordinary_muscle_development	ENSGALG00010021682	MYL4	-1.546251126	0.188860507	-8.187265557	2.67228E-16	4.19661E-15	negative
PRJNA729271	stage_E14_vs_E10	ordinary_muscle_development	ENSGALG00010021683	HFM1	-1.124596205	0.428006679	-2.627520224	0.008600973	0.020914461	negative
PRJNA729271	stage_E14_vs_E10	ordinary_muscle_development	ENSGALG00010021684	BIN1	1.615129851	0.121202618	13.32586603	1.63722E-40	1.2968E-38	positive
PRJNA729271	stage_E14_vs_E10	ordinary_muscle_development	ENSGALG00010021685	DBNDD2	0.343365655	0.140589198	2.44233312	0.014592674	0.033153733	positive
PRJNA729271	stage_E14_vs_E10	ordinary_muscle_development	ENSGALG00010021686	CCPG1	0.086409615	0.165663915	0.521595874	0.601951736	0.700545196	positive
PRJNA729271	stage_E14_vs_E10	ordinary_muscle_development	ENSGALG00010021687	ZNF384	-0.154361578	0.105288231	-1.46608578	0.142624919	0.227805032	negative
PRJNA729271	stage_E14_vs_E10	ordinary_muscle_development	ENSGALG00010021688	ITPR1	-0.214455907	0.116629714	-1.83877589	0.065948156	0.119939588	negative
PRJNA729271	stage_E14_vs_E10	ordinary_muscle_development	ENSGALG00010021689	MPRIP	0.476585816	0.108969347	4.373576895	1.22227E-05	5.6531E-05	positive
PRJNA729271	stage_E14_vs_E10	ordinary_muscle_development	ENSGALG00010021691	PIGB	-0.288458308	0.142462751	-2.024798109	0.042888099	0.083184117	negative
PRJNA729271	stage_E14_vs_E10	ordinary_muscle_development	ENSGALG00010021693	PPA1	0.089811395	0.090405588	0.993427471	0.32050168	0.435001811	positive
PRJNA729271	stage_E14_vs_E10	ordinary_muscle_development	ENSGALG00010021694	ENSGALG00010021694	-1.808907895	1.356017962	-1.333985202	0.182208706	0.277537461	negative
PRJNA729271	stage_E14_vs_E10	ordinary_muscle_development	ENSGALG00010021695	FNBP4	-0.015685183	0.069948115	-0.224240248	0.822570368	0.875672553	negative
PRJNA729271	stage_E14_vs_E10	ordinary_muscle_development	ENSGALG00010021697	PIANP	-1.623646264	0.222940701	-7.282861565	3.26813E-13	3.93778E-12	negative

PRJNA729271	stage_E14_vs_E10	ordinary_muscle_development	ENSGALG00010021698	GYPC	0.404568922	0.13446793	3.008664756	0.002623985	0.007379639	positive
PRJNA729271	stage_E14_vs_E10	ordinary_muscle_development	ENSGALG00010021699	SYS1	-0.098125898	0.08143843	-1.204909005	0.228238432	0.331827088	negative
PRJNA729271	stage_E14_vs_E10	ordinary_muscle_development	ENSGALG00010021700	FLCN	-0.034650073	0.078115808	-0.443573128	0.657351237	0.747383308	negative
PRJNA729271	stage_E14_vs_E10	ordinary_muscle_development	ENSGALG00010021701	DR1	-0.160497015	0.075607458	-2.122766968	0.033773384	0.06783223	negative
PRJNA729271	stage_E14_vs_E10	ordinary_muscle_development	ENSGALG00010021702	PLD6	-0.207839325	0.29427281	-0.706281103	0.480013343	0.592102238	negative
PRJNA729271	stage_E14_vs_E10	ordinary_muscle_development	ENSGALG00010021703	CD82	0.486625546	0.173035245	2.812291479	0.004918991	0.012831647	positive
PRJNA729271	stage_E14_vs_E10	ordinary_muscle_development	ENSGALG00010021704	COPS7A	0.472006618	0.102298962	4.613992267	3.95007E-06	1.97776E-05	positive
PRJNA729271	stage_E14_vs_E10	ordinary_muscle_development	ENSGALG00010021705	CCDC18	-0.531946177	0.189052008	-2.813755758	0.004896642	0.01278401	negative
PRJNA729271	stage_E14_vs_E10	ordinary_muscle_development	ENSGALG00010021706	ENSGALG00010021706	0.547630084	0.160124746	3.420021565	0.000626162	0.002067946	positive
PRJNA729271	stage_E14_vs_E10	ordinary_muscle_development	ENSGALG00010021707	DLX2	-1.885950032	0.84786805	-2.224343789	0.026125323	0.05472349	negative
PRJNA729271	stage_E14_vs_E10	ordinary_muscle_development	ENSGALG00010021708	CNTNAP5	-3.342849368	0.326318584	-10.24412808	1.25754E-24	3.6529E-23	negative
PRJNA729271	stage_E14_vs_E10	ordinary_muscle_development	ENSGALG00010021710	ENSGALG00010021710	-0.16273443	0.316738769	-0.513781215	0.607404963	0.704952963	negative
PRJNA729271	stage_E14_vs_E10	ordinary_muscle_development	ENSGALG00010021711	ENSGALG00010021711	-0.634463285	0.229423131	-2.765472175	0.005684045	0.014554008	negative
PRJNA729271	stage_E14_vs_E10	ordinary_muscle_development	ENSGALG00010021713	RAB27A	0.203601165	0.07945772	2.562383682	0.01039564	0.024598319	positive
PRJNA729271	stage_E14_vs_E10	ordinary_muscle_development	ENSGALG00010021714	TSPAN18	0.4400476	0.11500718	3.826261968	0.000130104	0.000501824	positive
PRJNA729271	stage_E14_vs_E10	ordinary_muscle_development	ENSGALG00010021715	MMAB	0.503155177	0.125078435	4.022717244	5.75305E-05	0.000236905	positive
PRJNA729271	stage_E14_vs_E10	ordinary_muscle_development	ENSGALG00010021716	TFCP2L1	0.113818203	0.386036929	0.294837605	0.768117938	0.835186371	positive
PRJNA729271	stage_E14_vs_E10	ordinary_muscle_development	ENSGALG00010021717	SDC4	0.872607236	0.19720426	4.424890401	9.64913E-06	4.53662E-05	positive
PRJNA729271	stage_E14_vs_E10	ordinary_muscle_development	ENSGALG00010021718	SST7	-2.828597566	0.424481423	-6.663654546	2.67101E-11	2.6143E-10	negative
PRJNA729271	stage_E14_vs_E10	ordinary_muscle_development	ENSGALG00010021719	BHLHE40	1.307540806	0.215812547	6.058687613	1.37237E-09	1.10514E-08	positive
PRJNA729271	stage_E14_vs_E10	ordinary_muscle_development	ENSGALG00010021720	CACNB1	1.087415541	0.260647068	4.171984545	3.01958E-05	0.000130692	positive
PRJNA729271	stage_E14_vs_E10	ordinary_muscle_development	ENSGALG00010021721	TP53I11	0.650880619	0.142296365	4.574119774	4.78226E-06	2.36745E-05	positive
PRJNA729271	stage_E14_vs_E10	ordinary_muscle_development	ENSGALG00010021722	AARS1	0.022089623	0.158835772	0.139072093	0.88939318	0.92407384	positive
PRJNA729271	stage_E14_vs_E10	ordinary_muscle_development	ENSGALG00010021723	COPS3	-0.310265509	0.106851859	-2.903697816	0.003687839	0.009952565	negative
PRJNA729271	stage_E14_vs_E10	ordinary_muscle_development	ENSGALG00010021725	SH2D6	0.215376952	0.214178151	1.005597212	0.314609366	0.428555846	positive
PRJNA729271	stage_E14_vs_E10	ordinary_muscle_development	ENSGALG00010021726	ARNTL	0.249972658	0.145889762	1.713435232	0.086632499	0.151075322	positive
PRJNA729271	stage_E14_vs_E10	ordinary_muscle_development	ENSGALG00010021727	MVK	0.313372637	0.099795654	3.140143135	0.001688653	0.005019899	positive
PRJNA729271	stage_E14_vs_E10	ordinary_muscle_development	ENSGALG00010021728	TMED5	-0.393717614	0.064760168	-6.079626206	1.20463E-09	9.74393E-09	negative
PRJNA729271	stage_E14_vs_E10	ordinary_muscle_development	ENSGALG00010021729	RBPJL	-3.655900624	0.629318701	-5.809299192	6.27349E-09	4.68733E-08	negative
PRJNA729271	stage_E14_vs_E10	ordinary_muscle_development	ENSGALG00010021730	ARL8B	0.149308317	0.071998189	2.073778788	0.03809986	0.075198458	positive
PRJNA729271	stage_E14_vs_E10	ordinary_muscle_development	ENSGALG00010021731	SRM	-0.33219337	0.117238505	-2.833483494	0.004604369	0.01210599	negative
PRJNA729271	stage_E14_vs_E10	ordinary_muscle_development	ENSGALG00010021732	CASR	3.334042365	0.248234931	13.43099601	3.98022E-41	3.25951E-39	positive
PRJNA729271	stage_E14_vs_E10	ordinary_muscle_development	ENSGALG00010021733	ENSGALG00010021733	0.619783115	0.616019262	1.006109959	0.314362679	0.428300313	positive
PRJNA729271	stage_E14_vs_E10	ordinary_muscle_development	ENSGALG00010021734	PARD3B	0.333021107	0.062871331	5.296867487	1.17806E-07	7.4179E-07	positive
PRJNA729271	stage_E14_vs_E10	ordinary_muscle_development	ENSGALG00010021735	PIGBOS1	0.678434857	0.144740045	4.687264383	2.76881E-06	1.4242E-05	positive
PRJNA729271	stage_E14_vs_E10	ordinary_muscle_development	ENSGALG00010021736	EDEM1	0.326513411	0.082161567	3.974040676	7.06635E-05	0.000286108	positive
PRJNA729271	stage_E14_vs_E10	ordinary_muscle_development	ENSGALG00010021738	PRDM11	0.17111821	0.071210281	2.402998657	0.016261247	0.03636889	positive
PRJNA729271	stage_E14_vs_E10	ordinary_muscle_development	ENSGALG00010021739	EXOSC10	0.073492365	0.10991975	0.668600185	0.503750555	0.61303537	positive
PRJNA729271	stage_E14_vs_E10	ordinary_muscle_development	ENSGALG00010021740	PDLIM1	1.013717452	0.157753653	6.425952298	1.31046E-10	1.19017E-09	positive
PRJNA729271	stage_E14_vs_E10	ordinary_muscle_development	ENSGALG00010021741	RPL19	0.374324935	0.146000888	2.563853832	0.010351713	0.02451766	positive
PRJNA729271	stage_E14_vs_E10	ordinary_muscle_development	ENSGALG00010021742	MATN4	-5.682076158	0.483587101	-11.74985053	7.07439E-32	3.32933E-30	negative
PRJNA729271	stage_E14_vs_E10	ordinary_muscle_development	ENSGALG00010021743	SYT13	-0.703732701	0.319966997	-2.199391521	0.027850095	0.057768622	negative
PRJNA729271	stage_E14_vs_E10	ordinary_muscle_development	ENSGALG00010021744	LITAF	0.774061625	0.361279252	2.142557649	0.032148635	0.065028532	positive
PRJNA729271	stage_E14_vs_E10	ordinary_muscle_development	ENSGALG00010021745	RSL24D1	-0.049232176	0.125264177	-0.393026782	0.6942997	0.777093168	negative
PRJNA729271	stage_E14_vs_E10	ordinary_muscle_development	ENSGALG00010021746	MTOR	0.444396199	0.110195223	4.032808194	5.51143E-05	0.000227537	positive
PRJNA729271	stage_E14_vs_E10	ordinary_muscle_development	ENSGALG00010021747	AKTIP	-0.038323414	0.116720915	-0.328333738	0.742659337	0.815271667	negative
PRJNA729271	stage_E14_vs_E10	ordinary_muscle_development	ENSGALG00010021748	CDCA7	0.051119506	0.17606154	0.290350214	0.771548327	0.837847843	positive
PRJNA729271	stage_E14_vs_E10	ordinary_muscle_development	ENSGALG00010021749	CLASP1	0.642453627	0.090601621	7.090972767	1.33173E-12	1.50104E-11	positive
PRJNA729271	stage_E14_vs_E10	ordinary_muscle_development	ENSGALG00010021750	HELLS	-0.750717002	0.145967266	-5.143050371	2.70313E-07	1.6211E-06	negative
PRJNA729271	stage_E14_vs_E10	ordinary_muscle_development	ENSGALG00010021751	MTCH2	0.220669649	0.091163081	2.420603244	0.015494779	0.034896957	positive
PRJNA729271	stage_E14_vs_E10	ordinary_muscle_development	ENSGALG00010021752	CSTB	-0.315478264	0.109127708	-2.890908926	0.003841294	0.010307212	negative
PRJNA729271	stage_E14_vs_E10	ordinary_muscle_development	ENSGALG00010021753	MTF2	-0.3569255	0.095870392	-3.723000307	0.000196869	0.000733013	negative
PRJNA729271	stage_E14_vs_E10	ordinary_muscle_development	ENSGALG00010021754	SLC17A9	-0.779261447	0.448469248	-1.737602859	0.082280816	0.144811528	negative
PRJNA729271	stage_E14_vs_E10	ordinary_muscle_development	ENSGALG00010021755	ENSGALG00010021755	0.324404711	0.154246221	2.103161478	0.035451656	0.070702229	positive
PRJNA729271	stage_E14_vs_E10	ordinary_muscle_development	ENSGALG00010021756	USP42	-0.74315031	0.12061236	-6.161477228	7.20694E-10	5.99338E-09	negative
PRJNA729271	stage_E14_vs_E10	ordinary_muscle_development	ENSGALG00010021757	ZP4	2.157769691	0.29233698	7.381104123	1.56982E-13	1.9465E-12	positive
PRJNA729271	stage_E14_vs_E10	ordinary_muscle_development	ENSGALG00010021758	RASSF10	-1.022996285	0.317985556	-3.217115581	0.001294864	0.003970607	negative
PRJNA729271	stage_E14_vs_E10	ordinary_muscle_development	ENSGALG00010021759	NIFK	-0.161820684	0.092500838	-1.749396953	0.080222427	0.141755953	negative
PRJNA729271	stage_E14_vs_E10	ordinary_muscle_development	ENSGALG00010021760	CSTA	0.234454294	0.396322035	0.591575218	0.554135073	0.658862101	positive
PRJNA729271	stage_E14_vs_E10	ordinary_muscle_development	ENSGALG00010021761	MAFB	-0.59312243	0.169872693	-3.491570175	0.00048019	0.001635423	negative
PRJNA729271	stage_E14_vs_E10	ordinary_muscle_development	ENSGALG00010021762	LAMB2	1.108089644	0.199740603	5.547643426	2.89546E-08	1.9976E-07	positive
PRJNA729271	stage_E14_vs_E10	ordinary_muscle_development	ENSGALG00010021764	FAM222A	-1.07001959	0.743786728	-1.43861076	0.150260836	0.237672503	negative
PRJNA729271	stage_E14_vs_E10	ordinary_muscle_development	ENSGALG00010021765	EIF2AK1	0.163124993	0.103623167	1.574213549	0.115438088	0.191845343	positive
PRJNA729271	stage_E14_vs_E10	ordinary_muscle_development	ENSGALG00010021766	AGRN	0.121647451	0.148323691	0.820148487	0.412131464	0.526794142	positive
PRJNA729271	stage_E14_vs_E10	ordinary_muscle_development	ENSGALG00010021767	CCDC58	0.165536405	0.212039584	0.780686335	0.434986999	0.549132255	positive
PRJNA729271	stage_E14_vs_E10	ordinary_muscle_development	ENSGALG00010021768	DIPK1A	0.480998964	0.149435113	3.218781415	0.001287366	0.003953468	positive
PRJNA729271	stage_E14_vs_E10	ordinary_muscle_development	ENSGALG00010021769	ENSGALG00010021769	-0.149535694	0.16707333	-0.895030308	0.370770887	0.487113569	negative
PRJNA729271	stage_E14_vs_E10	ordinary_muscle_development	ENSGALG00010021770	RALGAPB	-0.122787118	0.097557291	-1.258615492	0.208169248	0.308623633	negative
PRJNA729271	stage_E14_vs_E10	ordinary_muscle_development	ENSGALG00010021771	ANKRD61	0.261031685	0.143622975	1.817478605	0.069143861	0.12478091	positive
PRJNA729271	stage_E14_vs_E10	ordinary_muscle_development	ENSGALG00010021772	TSN	-0.406266857	0.082651957	-4.915393088	8.86045E-07	4.92833E-06	negative
PRJNA729271	stage_E14_vs_E10	ordinary_muscle_development	ENSGALG00010021773	ENSGALG00010021773	-1.867290277	0.949771322	-1.966041967	0.049293763	0.09379274	negative
PRJNA729271	stage_E14_vs_E10	ordinary_muscle_development	ENSGALG00010021774	FBXL20	0.62269361	0.071129971	8.754307112	2.05361E-18	3.88605E-17	positive
PRJNA729271	stage_E14_vs_E10	ordinary_muscle_development	ENSGALG00010021775	CCDC36	0.668092248	0.340688029	1.961008872	0.049877988	0.094618693	positive
PRJNA729271	stage_E14_vs_E10	ordinary_muscle_development	ENSGALG00010021776	PMS2	0.074840411	0.091853391	0.814781138	0.415197599	0.529835769	positive
PRJNA729271	stage_E14_vs_E10	ordinary_muscle_development	ENSGALG00010021778	RBL2	0.016891378	0.103798146	0.162732943	0.870728708	0.911424589	positive
PRJNA729271	stage_E14_vs_E10	ordinary_muscle_development	ENSGALG00010021779	UNC13C	-1.720378201	0.248805408	-6.914553069	4.69341E-12	4.97747E-11	negative
PRJNA729271	stage_E14_vs_E10	ordinary_muscle_development	ENSGALG00010021781	RAPGEF4	-1.746205662	0.272606094	-6.405600239	1.49779E-10	1.35099E-09	negative
PRJNA729271	stage_E14_vs_E10	ordinary_muscle_development	ENSGALG00010021782	ADAM11	-1.414226809	0.311683977	-4.537374114	5.6959E-06	2.7855E-05	negative
PRJNA729271	stage_E14_vs_E10	ordinary_muscle_development	ENSGALG00010021783	GIT2	-0.088102144	0.047126441	-1.869484347	0.061555459	0.113071396	negative
PRJNA729271	stage_E14_vs_E10	ordinary_muscle_development	ENSGALG00010021784	ANGPTL7	-0.682537337	0.941365227	-0.725050509	0.468421045	0.580768373	negative
PRJNA729271	stage_E14_vs_E10	ordinary_muscle_development	ENSGALG00010021785	CCDC71	-0.048952899	0.089387997	-0.547645106	0.583935611	0.684856921	negative
PRJNA729271	stage_E14_vs_E10	ordinary_muscle_development	ENSGALG00010021786	FAM162A	0.324688144	0.172147067	1.886109074	0.059280258	0.109377207	positive
PRJNA729271	stage_E14_vs_E10	ordinary_muscle_development	ENSGALG00010021788	RPL5	0.332651146	0.123891534	2.685019199	0.007252564	0.017979462	positive
PRJNA729271	stage_E14_vs_E10	ordinary_muscle_development	ENSGALG00010021789	CHD9	-0.196579599	0.090708651	-2.167153827	0.030223127	0.061806465	negative
PRJNA729271	stage_E14_vs_E10	ordinary_muscle_development	ENSGALG00010021790	PHF21A	-0.00489096	0.127954868	-0.038224102	0.969509004	0.979040653	negative
PRJNA729271	stage_E14_vs_E10	ordinary_muscle_development	ENSGALG00010021791	PLEKHM2	0.413461586	0.184258512	2.243921223	0.02483747	0.052441175	positive
PRJNA729271	stage_E14_vs_E10	ordinary_muscle_development	ENSGALG00010021792	KLHDC8B	0.399970002	0.282815033	1.414245906	0.157289714	0.246557197	positive
PRJNA729271	stage_E14_vs_E10	ordinary_muscle_development	ENSGALG00010021793	ENSGALG00010021793	1.834401633	0.156283114	11.73768291	8.1694E-32	3.83222E-30	positive
PRJNA729271	stage_E14_vs_E10	ordinary_muscle_development	ENSGALG00010021794	ENSGALG00010021794	0.064062351	0.177942735	0.360016672	0.718834666	0.796720331	positive
PRJNA729271	stage_E14_vs_E10	ordinary_muscle_development	ENSGALG00010021795	NDRG3	-0.328569383	0.105937975	-3.101526002	0.00192526	0.00563472	negative
PRJNA729271	stage_E14_vs_E10	ordinary_muscle_development	ENSGALG00010021797	SLC25A34	0.760216801	0.164906215	4.609994849	4.02679E-06	2.01478E-05	positive
PRJNA729271	stage_E14_vs_E10	ordinary_muscle_development	ENSGALG00010021798	RANBP10	0.094168835	0.181168465	0.519786021	0.603212724	0.701449533	positive
PRJNA729271	stage_E14_vs_E10	ordinary_muscle_development	ENSGALG00010021799	ENSGALG00010021799	-0.575645548	0.211626338	-2.72010352	0.006526148	0.016391703	negative
PRJNA729271	stage_E14_vs_E10	ordinary_muscle_development	ENSGALG00010021800	TMEM82	0.259786648	0.338822704	0.766733294	0.443240104	0.557028377	positive
PRJNA729271	stage_E14_vs_E10	ordinary_muscle_development	ENSGALG00010021801	VAMP1	-0.288691612	0.25726014	-1.122177777	0.26178686	0.370168816	negative
PRJNA729271	stage_E14_vs_E10	ordinary_muscle_development	ENSGALG00010021803	TLL2	0.988726449	0.157066519	6.294953583	3.07492E-10	2.67372E-09	positive
PRJNA729271	stage_E14_vs_E10	ordinary_muscle_development	ENSGALG00010021805	FUT8	-0.84277026	0.108263387	-7.784443892	7.00205E-15	9.74973E-14	negative
PRJNA729271	stage_E14_vs_E10	ordinary_muscle_development	ENSGALG00010021807	CDC42	0.158161843	0.082771932	1.910814929	0.056028368	0.104400463	positive
PRJNA729271	stage_E14_vs_E10	ordinary_muscle_development	ENSGALG00010021810	ANKRD13A	-0.218785321	0.096075804	-2.27721562	0.022773353	0.04871602	negative
PRJNA729271	stage_E14_vs_E10	ordinary_muscle_development	ENSGALG00010021812	TEAD1	0.392488854	0.082938636	4.732280046	2.22012E-06	1.16008E-05	positive
PRJNA729271	stage_E14_vs_E10	ordinary_muscle_development	ENSGALG00010021817	EVI5	-0.12579241	0.100375535	-1.253217823	0.210126445	0.311048083	negative
PRJNA729271	stage_E14_vs_E10	ordinary_muscle_development	ENSGALG00010021821	PPP1R19L	-0.104771784	0.118902136	-0.881159812	0.378231328	0.494494732	negative
PRJNA729271	stage_E14_vs_E10	ordinary_muscle_development	ENSGALG00010021823	ENSGALG00010021823	0.461624335	0.242376391	1.904576324	0.056835167	0.105639939	positive
PRJNA729271	stage_E14_vs_E10	ordinary_muscle_development	ENSGALG00010021826	FBXO40	1.470772666	0.214549629	6.85516293	7.12313E-12	7.38025E-11	positive
PRJNA729271	stage_E14_vs_E10	ordinary_muscle_development	ENSGALG00010021827	CCZ1	-0.030061778	0.094596287	-0.317790252	0.750644046	0.821486254	negative
PRJNA729271	stage_E14_vs_E10	ordinary_muscle_development	ENSGALG00010021829	ERCC6	-0.362557369	0.096310915	-3.764447376	0.000166918	0.000630728	negative
PRJNA729271	stage_E14_vs_E10	ordinary_muscle_development	ENSGALG00010021831	ENSGALG00010021831	-0.170633464	0.386524785	-0.441455428	0.658883323	0.748531528	negative
PRJNA729271	stage_E14_vs_E10	ordinary_muscle_development	ENSGALG00010021835	NUDT8	0.470771515	0.169149362	2.783170496	0.005383051	0.013883866	positive
PRJNA729271	stage_E14_vs_E10	ordinary_muscle_development	ENSGALG00010021837	ENSGALG00010021837	0.677204452	0.317578969	2.132397037	0.032974221	0.066466601	positive
PRJNA729271	stage_E14_vs_E10	ordinary_muscle_development	ENSGALG00010021839	C1orf50	0.121730086	0.117783214	1.033509631	0.301365466	0.415218764	positive
PRJNA729271	stage_E14_vs_E10	ordinary_muscle_development	ENSGALG00010021840	ENSGALG00010021840	0.485504061	0.213227666	2.276928081	0.022790521	0.048738359	positive
PRJNA729271	stage_E14_vs_E10	ordinary_muscle_development	ENSGALG00010021841	CENPT	-0.009239123	0.08550973	-0.108047621	0.913957917	0.940755838	negative
PRJNA729271	stage_E14_vs_E10	ordinary_muscle_development	ENSGALG00010021842	ENSGALG00010021842	-0.876925625	0.179122044	-4.895687925	9.79624E-07	5.41972E-06	negative
PRJNA729271	stage_E14_vs_E10	ordinary_muscle_development	ENSGALG00010021843	ENSGALG00010021843	0.333972087	0.387389253	0.86210984	0.388627077	0.504446089	positive
PRJNA729271	stage_E14_vs_E10	ordinary_muscle_development	ENSGALG00010021847	P3H1	0.011108746	0.15033799	0.073891812	0.941096471	0.958622161	positive
PRJNA729271	stage_E14_vs_E10	ordinary_muscle_development	ENSGALG00010021848	LARGE2	-0.050398416	0.139125833	-0.362250598	0.717164772	0.795386238	negative
PRJNA729271	stage_E14_vs_E10	ordinary_muscle_development	ENSGALG00010021851	ENSGALG00010021851	0.40892678	0.220451125	1.854954379	0.063602742	0.116242812	positive
PRJNA729271	stage_E14_vs_E10	ordinary_muscle_development	ENSGALG00010021853	ENSGALG00010021853	0.132551242	0.570706703	0.232258078	0.816337571	0.871281609	positive
PRJNA729271	stage_E14_vs_E10	ordinary_muscle_development	ENSGALG00010021854	TAPBPL	0.092450159	0.156592897	0.590385393	0.554932301	0.659539497	positive
PRJNA729271	stage_E14_vs_E10	ordinary_muscle_development	ENSGALG00010021860	ENSGALG00010021860	0.71872048	0.26005677	2.763706095	0.005714899	0.014620095	positive
PRJNA729271	stage_E14_vs_E10	ordinary_muscle_development	ENSGALG00010021862	PDK1	0.43347765	0.10589273	4.093554405	4.2481E-05	0.000178897	positive
PRJNA729271	stage_E14_vs_E10	ordinary_muscle_development	ENSGALG00010021863	CLDN19	0.659125888	0.294637335	2.237075243	0.025281423	0.053232746	positive
PRJNA729271	stage_E14_vs_E10	ordinary_muscle_development	ENSGALG00010021864	GFI1	-0.595803268	0.612974476	-0.971987075	0.331056979	0.445793154	negative
PRJNA729271	stage_E14_vs_E10	ordinary_muscle_development	ENSGALG00010021866	SCNN1A	-0.156199881	0.400008818	-0.390491094	0.696173439	0.778750922	negative
PRJNA729271	stage_E14_vs_E10	ordinary_muscle_development	ENSGALG00010021867	CXCL12	0.204829132	0.182143614	1.124547429	0.260780861	0.368962283	positive
PRJNA729271	stage_E14_vs_E10	ordinary_muscle_development	ENSGALG00010021869	GJC3	-0.017277856	0.120945295	-0.142856781	0.886403292	0.92228955	negative
PRJNA729271	stage_E14_vs_E10	ordinary_muscle_development	ENSGALG00010021870	ENSGALG00010021870	0.176593285	0.167652294	1.053330562	0.292189517	0.404787063	positive
PRJNA729271	stage_E14_vs_E10	ordinary_muscle_development	ENSGALG00010021871	ENSGALG00010021871	2.958390589	0.254085219	11.64330061	2.4822E-31	1.13143E-29	positive
PRJNA729271	stage_E14_vs_E10	ordinary_muscle_development	ENSGALG00010021874	TMCC1	0.756863667	0.078135613	9.686539016	3.43988E-22	8.6866E-21	positive
PRJNA729271	stage_E14_vs_E10	ordinary_muscle_development	ENSGALG00010021877	PLEKHN1	1.047344027	0.144721856	7.236944397	4.58906E-13	5.43008E-12	positive
PRJNA729271	stage_E14_vs_E10	ordinary_muscle_development	ENSGALG00010021879	CREBL2	-0.324686864	0.157755729	-2.058162113	0.039574574	0.077594443	negative

PRJNA729271	stage_E14_vs_E10	ordinary_muscle_development	ENSGALG00010021881	PHF20	-0.113544263	0.047236682	-2.403730707	0.016228723	0.03630175	negative
PRJNA729271	stage_E14_vs_E10	ordinary_muscle_development	ENSGALG00010021887	NUTF2	-0.119890135	0.109711062	-1.092780738	0.274490081	0.384902169	negative
PRJNA729271	stage_E14_vs_E10	ordinary_muscle_development	ENSGALG00010021892	NDUFV1	0.421446783	0.121860852	3.458426369	0.000543341	0.001826467	positive
PRJNA729271	stage_E14_vs_E10	ordinary_muscle_development	ENSGALG00010021897	ENSGALG00010021897	0.00309719	0.205265517	0.015088703	0.987961414	0.991999217	positive
PRJNA729271	stage_E14_vs_E10	ordinary_muscle_development	ENSGALG00010021902	LMTK2	0.136358577	0.119162683	1.144306033	0.252496742	0.359840751	positive
PRJNA729271	stage_E14_vs_E10	ordinary_muscle_development	ENSGALG00010021903	MORC2	0.554454619	0.075632823	7.330872973	2.28658E-13	2.79696E-12	positive
PRJNA729271	stage_E14_vs_E10	ordinary_muscle_development	ENSGALG00010021905	RBSN	-0.342099028	0.072749293	-4.702437803	2.57074E-06	1.33129E-05	negative
PRJNA729271	stage_E14_vs_E10	ordinary_muscle_development	ENSGALG00010021908	EDC4	-0.04530684	0.108072768	-0.419225314	0.675051473	0.761290835	negative
PRJNA729271	stage_E14_vs_E10	ordinary_muscle_development	ENSGALG00010021913	ENSGALG00010021913	0.966158288	0.175641818	5.500730409	3.78221E-08	2.55944E-07	positive
PRJNA729271	stage_E14_vs_E10	ordinary_muscle_development	ENSGALG00010021916	MRPS25	-0.153501813	0.148515975	-1.033571054	0.301336737	0.415218764	negative
PRJNA729271	stage_E14_vs_E10	ordinary_muscle_development	ENSGALG00010021919	PARVA	0.472516984	0.124240354	3.803248854	0.000142811	0.000547051	positive
PRJNA729271	stage_E14_vs_E10	ordinary_muscle_development	ENSGALG00010021922	MRPL51	0.159100737	0.137334793	1.158488198	0.246664865	0.353301109	positive
PRJNA729271	stage_E14_vs_E10	ordinary_muscle_development	ENSGALG00010021924	ENSGALG00010021924	-1.39855765	0.491411059	-2.846003612	0.004427169	0.011701645	negative
PRJNA729271	stage_E14_vs_E10	ordinary_muscle_development	ENSGALG00010021928	ENSGALG00010021928	-1.366074779	1.032124632	-1.323556029	0.185650537	0.281662605	negative
PRJNA729271	stage_E14_vs_E10	ordinary_muscle_development	ENSGALG00010021931	ITGA6	2.309712414	0.121376589	19.02930728	9.75335E-81	5.2361E-78	positive
PRJNA729271	stage_E14_vs_E10	ordinary_muscle_development	ENSGALG00010021932	RPAP2	-0.155535325	0.162740973	-0.955723208	0.339212094	0.454299113	negative
PRJNA729271	stage_E14_vs_E10	ordinary_muscle_development	ENSGALG00010021933	PRR14L	-0.080166622	0.088974562	-0.901006085	0.367585085	0.483717276	negative
PRJNA729271	stage_E14_vs_E10	ordinary_muscle_development	ENSGALG00010021934	EFTUD2	-0.117216037	0.065839824	-1.780321241	0.075023403	0.133924166	negative
PRJNA729271	stage_E14_vs_E10	ordinary_muscle_development	ENSGALG00010021935	CETP	3.400086436	0.299792369	11.34147092	8.17586E-30	3.46518E-28	positive
PRJNA729271	stage_E14_vs_E10	ordinary_muscle_development	ENSGALG00010021938	CABP2	0.014290085	0.156365583	0.091388942	0.927183548	0.949924054	positive
PRJNA729271	stage_E14_vs_E10	ordinary_muscle_development	ENSGALG00010021943	OCM3	1.949634391	0.568280129	3.430762916			positive
PRJNA729271	stage_E14_vs_E10	ordinary_muscle_development	ENSGALG00010021945	C1QTNF4	0.148618682	0.204596474	0.726399039	0.46759418	0.579991241	positive
PRJNA729271	stage_E14_vs_E10	ordinary_muscle_development	ENSGALG00010021947	CDK2AP2	0.278511973	0.248031578	1.122889169	0.261484568	0.369777445	positive
PRJNA729271	stage_E14_vs_E10	ordinary_muscle_development	ENSGALG00010021949	ENSGALG00010021949	-1.105258693	0.244400627	-4.522323473	6.11645E-06	2.9771E-05	negative
PRJNA729271	stage_E14_vs_E10	ordinary_muscle_development	ENSGALG00010021951	PSKH1	-0.183707048	0.142492358	-1.289241406	0.197314174	0.295804313	negative
PRJNA729271	stage_E14_vs_E10	ordinary_muscle_development	ENSGALG00010021953	PITPNM1	-0.599832809	0.17914504	-3.348308217	0.000813065	0.002621304	negative
PRJNA729271	stage_E14_vs_E10	ordinary_muscle_development	ENSGALG00010021958	BHLHA15	0.267762378	0.377127028	0.710005801	0.477700539	0.589801475	positive
PRJNA729271	stage_E14_vs_E10	ordinary_muscle_development	ENSGALG00010021960	CDKN1B	0.93563055	0.144490987	6.47535578	9.45888E-11	8.68862E-10	positive
PRJNA729271	stage_E14_vs_E10	ordinary_muscle_development	ENSGALG00010021965	PEX16	0.178245017	0.100629828	1.771294071	0.076511813	0.136061677	positive
PRJNA729271	stage_E14_vs_E10	ordinary_muscle_development	ENSGALG00010021966	ADGRA1	0.962627683	0.158094479	6.088939288	1.13661E-09	9.22463E-09	positive
PRJNA729271	stage_E14_vs_E10	ordinary_muscle_development	ENSGALG00010021969	CCDC103	-0.013789245	0.247725838	-0.055663329	0.955610013	0.969217982	negative
PRJNA729271	stage_E14_vs_E10	ordinary_muscle_development	ENSGALG00010021970	ERGIC3	0.046470975	0.089124439	0.521416746	0.602076488	0.700577883	positive
PRJNA729271	stage_E14_vs_E10	ordinary_muscle_development	ENSGALG00010021971	ICOS	-1.914481183	0.3986005	-4.803007484	1.563E-06	8.391E-06	negative
PRJNA729271	stage_E14_vs_E10	ordinary_muscle_development	ENSGALG00010021972	PISD	-0.451924562	0.183695388	-2.460184583	0.013886558	0.031738513	negative
PRJNA729271	stage_E14_vs_E10	ordinary_muscle_development	ENSGALG00010021974	GFAP	2.097796581	0.853219563	2.458683171	0.013944762	0.031846435	positive
PRJNA729271	stage_E14_vs_E10	ordinary_muscle_development	ENSGALG00010021978	NR2C2	0.148127947	0.073397559	2.018159029	0.043574702	0.0843106	positive
PRJNA729271	stage_E14_vs_E10	ordinary_muscle_development	ENSGALG00010021980	GLMN	-0.468766293	0.13234373	-3.542036286	0.000397051	0.001376855	negative
PRJNA729271	stage_E14_vs_E10	ordinary_muscle_development	ENSGALG00010021983	CTLA4	-5.198634304	0.80477774	-6.459714334	1.04901E-10	9.58121E-10	negative
PRJNA729271	stage_E14_vs_E10	ordinary_muscle_development	ENSGALG00010021988	FAM180B	3.390250019	0.262824487	12.89929281	4.54247E-38	3.13538E-36	positive
PRJNA729271	stage_E14_vs_E10	ordinary_muscle_development	ENSGALG00010021989	NCAPD2	-0.088789353	0.149553146	-0.59369766	0.552714349	0.657388568	negative
PRJNA729271	stage_E14_vs_E10	ordinary_muscle_development	ENSGALG00010021994	ENSGALG00010021994	0.678015937	0.322386994	2.103111944	0.035455985	0.070702229	positive
PRJNA729271	stage_E14_vs_E10	ordinary_muscle_development	ENSGALG00010021995	CD28	1.691689546	0.495513602	3.414012327	0.000640137	0.002108814	positive
PRJNA729271	stage_E14_vs_E10	ordinary_muscle_development	ENSGALG00010021999	ANKRD22	-1.237626824	0.44698118	-2.768856673	0.005625338	0.014429175	negative
PRJNA729271	stage_E14_vs_E10	ordinary_muscle_development	ENSGALG00010022002	KIF18B	-0.030211527	0.197936471	-0.152632443	0.878688139	0.916564808	negative
PRJNA729271	stage_E14_vs_E10	ordinary_muscle_development	ENSGALG00010022005	NLRC5	1.559028772	0.185900242	8.386373011	5.01371E-17	8.48001E-16	positive
PRJNA729271	stage_E14_vs_E10	ordinary_muscle_development	ENSGALG00010022006	SFI1	-0.086119067	0.106087205	-0.811776182	0.416920071	0.531516846	negative
PRJNA729271	stage_E14_vs_E10	ordinary_muscle_development	ENSGALG00010022012	ENSGALG00010022012	5.694742442	2.882807615	1.97541536	0.048221031	0.092065839	positive
PRJNA729271	stage_E14_vs_E10	ordinary_muscle_development	ENSGALG00010022017	CNP1	1.918745096	0.58653099	3.271344784	0.001070373	0.003348815	positive
PRJNA729271	stage_E14_vs_E10	ordinary_muscle_development	ENSGALG00010022019	BTBD8	-0.040288387	0.153103564	-0.263144673	0.792439073	0.854325352	negative
PRJNA729271	stage_E14_vs_E10	ordinary_muscle_development	ENSGALG00010022021	TECPR1	0.614464197	0.078207037	7.856891457	3.93784E-15	5.5905E-14	positive
PRJNA729271	stage_E14_vs_E10	ordinary_muscle_development	ENSGALG00010022022	STAMBPL1	-0.812864265	0.242252022	-3.355448829	0.000792363	0.002559114	negative
PRJNA729271	stage_E14_vs_E10	ordinary_muscle_development	ENSGALG00010022024	MICAL2	1.011225765	0.183441771	5.512516362	3.53739E-08	2.40736E-07	positive
PRJNA729271	stage_E14_vs_E10	ordinary_muscle_development	ENSGALG00010022027	EIF4ENIF1	-0.161911949	0.065440421	-2.474188667	0.013353915	0.030689958	negative
PRJNA729271	stage_E14_vs_E10	ordinary_muscle_development	ENSGALG00010022028	FGD5	-0.726070076	0.115739773	-6.273297929	3.5348E-10	3.05345E-09	negative
PRJNA729271	stage_E14_vs_E10	ordinary_muscle_development	ENSGALG00010022029	ENSGALG00010022029	0.299297719	0.634741393	0.471527022	0.637264427	0.730554747	positive
PRJNA729271	stage_E14_vs_E10	ordinary_muscle_development	ENSGALG00010022030	ZNF341	0.046642624	0.152358491	0.306137348	0.759500073	0.82855073	positive
PRJNA729271	stage_E14_vs_E10	ordinary_muscle_development	ENSGALG00010022032	KBTBD4	0.108089322	0.080697106	1.339444845	0.180425899	0.275292424	positive
PRJNA729271	stage_E14_vs_E10	ordinary_muscle_development	ENSGALG00010022033	RAPH1	0.559081196	0.113757699	4.914666886	8.89335E-07	4.94473E-06	positive
PRJNA729271	stage_E14_vs_E10	ordinary_muscle_development	ENSGALG00010022034	ENSGALG00010022034	0.685842999	0.105694007	6.488948789	8.64373E-11	7.99049E-10	positive
PRJNA729271	stage_E14_vs_E10	ordinary_muscle_development	ENSGALG00010022037	DRG1	0.089433215	0.093414898	0.95737636	0.338377326	0.453474421	positive
PRJNA729271	stage_E14_vs_E10	ordinary_muscle_development	ENSGALG00010022038	GAPDH	1.029406559	0.137190369	7.503489968	6.21407E-14	8.05662E-13	positive
PRJNA729271	stage_E14_vs_E10	ordinary_muscle_development	ENSGALG00010022041	MAPK8IP1	-1.143735512	0.153300624	-7.460736162	8.60404E-14	1.09785E-12	negative
PRJNA729271	stage_E14_vs_E10	ordinary_muscle_development	ENSGALG00010022042	AIP	0.058399264	0.083876924	0.69624947	0.486272611	0.597534885	positive
PRJNA729271	stage_E14_vs_E10	ordinary_muscle_development	ENSGALG00010022044	PXMP4	-0.017520147	0.058950531	-0.297200828	0.76631319	0.833809209	negative
PRJNA729271	stage_E14_vs_E10	ordinary_muscle_development	ENSGALG00010022046	CRY2	0.315724362	0.103508595	3.050223626	0.00228671	0.0065441	positive
PRJNA729271	stage_E14_vs_E10	ordinary_muscle_development	ENSGALG00010022049	EPHX4	-2.387062975	0.612688488	-3.896046721	9.77755E-05	0.00038481	negative
PRJNA729271	stage_E14_vs_E10	ordinary_muscle_development	ENSGALG00010022050	ENSGALG00010022050	0.51189115	0.194741138	2.628572244	0.008574414	0.020860378	positive
PRJNA729271	stage_E14_vs_E10	ordinary_muscle_development	ENSGALG00010022051	BAIAP2L1	0.058089628	0.109391914	0.531023056	0.595402802	0.695100163	positive
PRJNA729271	stage_E14_vs_E10	ordinary_muscle_development	ENSGALG00010022052	IFFO1	1.079061351	0.101965273	10.58263583	3.58722E-26	1.19808E-24	positive
PRJNA729271	stage_E14_vs_E10	ordinary_muscle_development	ENSGALG00010022054	ENSGALG00010022054	-0.00953809	0.508983736	-0.018739478	0.985048935	0.989966325	negative
PRJNA729271	stage_E14_vs_E10	ordinary_muscle_development	ENSGALG00010022055	E2F1	0.585297572	0.158839	3.684848001	0.000228839	0.000838479	positive
PRJNA729271	stage_E14_vs_E10	ordinary_muscle_development	ENSGALG00010022058	NLRP3	-0.231993507	0.124247243	-1.867192388	0.061874726	0.113614664	negative
PRJNA729271	stage_E14_vs_E10	ordinary_muscle_development	ENSGALG00010022059	NPTX2	0.366921486	0.246736242	1.487100081	0.13698838	0.22035807	positive
PRJNA729271	stage_E14_vs_E10	ordinary_muscle_development	ENSGALG00010022062	MITF	-0.242948662	0.176010328	-1.380309125	0.167491487	0.259268379	negative
PRJNA729271	stage_E14_vs_E10	ordinary_muscle_development	ENSGALG00010022065	PABPC1L	0.13090657	0.240355866	0.544636466	0.586003569	0.686504626	positive
PRJNA729271	stage_E14_vs_E10	ordinary_muscle_development	ENSGALG00010022066	DAD1	0.089113484	0.137134745	0.649824257	0.515805748	0.624507545	positive
PRJNA729271	stage_E14_vs_E10	ordinary_muscle_development	ENSGALG00010022067	TMEM130	-1.011957953	0.231185176	-4.377261429	1.2018E-05	5.56551E-05	negative
PRJNA729271	stage_E14_vs_E10	ordinary_muscle_development	ENSGALG00010022069	NOP2	-0.139917672	0.134093751	-1.043431714	0.296748392	0.410044608	negative
PRJNA729271	stage_E14_vs_E10	ordinary_muscle_development	ENSGALG00010022071	ACTA2	-0.129140196	0.197934807	-0.65243803	0.514118638	0.622829056	negative
PRJNA729271	stage_E14_vs_E10	ordinary_muscle_development	ENSGALG00010022072	SMTN	1.586022806	0.312387637	5.077098508	3.83242E-07	2.25267E-06	positive
PRJNA729271	stage_E14_vs_E10	ordinary_muscle_development	ENSGALG00010022073	PTPMT1	0.364452354	0.132535333	2.74985051	0.005962246	0.015193874	positive
PRJNA729271	stage_E14_vs_E10	ordinary_muscle_development	ENSGALG00010022076	YWHAB	-0.483012984	0.069170862	-6.982896715	2.89155E-12	3.16564E-11	negative
PRJNA729271	stage_E14_vs_E10	ordinary_muscle_development	ENSGALG00010022079	ABI2	0.006563521	0.074769516	0.087783382	0.930048846	0.95205212	positive
PRJNA729271	stage_E14_vs_E10	ordinary_muscle_development	ENSGALG00010022081	FAS	-0.643413703	0.190807536	-3.37205603	0.000746093	0.002426702	negative
PRJNA729271	stage_E14_vs_E10	ordinary_muscle_development	ENSGALG00010022082	TGFBR3	0.078361794	0.144492317	0.542324987	0.587594645	0.687812677	positive
PRJNA729271	stage_E14_vs_E10	ordinary_muscle_development	ENSGALG00010022084	SELENOM	0.13922481	0.200197848	0.695436096	0.486782046	0.597992927	positive
PRJNA729271	stage_E14_vs_E10	ordinary_muscle_development	ENSGALG00010022085	MLLT6	0.377812057	0.176878731	2.135994841	0.032679834	0.065964935	positive
PRJNA729271	stage_E14_vs_E10	ordinary_muscle_development	ENSGALG00010022087	SLC35C1	0.128524556	0.07252119	1.772234524	0.076355635	0.135900654	positive
PRJNA729271	stage_E14_vs_E10	ordinary_muscle_development	ENSGALG00010022088	DKK3	0.282878598	0.269637108	1.049108556	0.294128159	0.407047328	positive
PRJNA729271	stage_E14_vs_E10	ordinary_muscle_development	ENSGALG00010022090	TRRAP	-0.306582052	0.090193077	-3.399175018	0.000675895	0.002218042	negative
PRJNA729271	stage_E14_vs_E10	ordinary_muscle_development	ENSGALG00010022092	CTRL	0.472509544	1.945796587	0.242836043	0.808132413	0.865003642	positive
PRJNA729271	stage_E14_vs_E10	ordinary_muscle_development	ENSGALG00010022093	CHD4	-0.561401392	0.136229687	-4.120991565	3.77245E-05	0.000160216	negative
PRJNA729271	stage_E14_vs_E10	ordinary_muscle_development	ENSGALG00010022097	INPP5J	1.613859158	0.261204819	6.178519848	6.47054E-10	5.42769E-09	positive
PRJNA729271	stage_E14_vs_E10	ordinary_muscle_development	ENSGALG00010022098	CDC7	-0.35050787	0.151655185	-2.311215863	0.020820935	0.045031251	negative
PRJNA729271	stage_E14_vs_E10	ordinary_muscle_development	ENSGALG00010022100	ENTPD1	-0.855782644	0.166075074	-5.15298669	2.5637E-07	1.54387E-06	negative
PRJNA729271	stage_E14_vs_E10	ordinary_muscle_development	ENSGALG00010022101	ENSGALG00010022101	0.403900891	0.183206437	2.204621733	0.027480649	0.057067624	positive
PRJNA729271	stage_E14_vs_E10	ordinary_muscle_development	ENSGALG00010022104	EXOC3L1	0.050004738	0.140871792	0.354966297	0.722614841	0.799138027	positive
PRJNA729271	stage_E14_vs_E10	ordinary_muscle_development	ENSGALG00010022105	DLG5	-0.545423904	0.083045	-6.567811452	5.10601E-11	4.88526E-10	negative
PRJNA729271	stage_E14_vs_E10	ordinary_muscle_development	ENSGALG00010022108	USP47	0.178903149	0.087983097	2.033380886	0.042014057	0.081678347	positive
PRJNA729271	stage_E14_vs_E10	ordinary_muscle_development	ENSGALG00010022111	PCGF2	-0.274103979	0.154082471	-1.7789433	0.075249058	0.13424168	negative
PRJNA729271	stage_E14_vs_E10	ordinary_muscle_development	ENSGALG00010022115	E2F4	-0.284095263	0.184218371	-1.542165756	0.123033319	0.201578836	negative
PRJNA729271	stage_E14_vs_E10	ordinary_muscle_development	ENSGALG00010022116	CYP20A1	-0.054969178	0.099179785	-0.554237722	0.579416165	0.680988998	negative
PRJNA729271	stage_E14_vs_E10	ordinary_muscle_development	ENSGALG00010022117	FAM20C	0.32792577	0.120049136	2.731596258	0.006302833	0.015921847	positive
PRJNA729271	stage_E14_vs_E10	ordinary_muscle_development	ENSGALG00010022120	FOXL3	-1.591696527	0.464842037	-3.424166491	0.000616688	0.00204131	negative
PRJNA729271	stage_E14_vs_E10	ordinary_muscle_development	ENSGALG00010022122	PSMB3	0.27199388	0.157515125	1.726779441	0.084207282	0.147681132	positive
PRJNA729271	stage_E14_vs_E10	ordinary_muscle_development	ENSGALG00010022124	ENSGALG00010022124	-0.13660155	0.155472932	-0.878619501	0.379607619	0.495640967	negative
PRJNA729271	stage_E14_vs_E10	ordinary_muscle_development	ENSGALG00010022125	CBFA2T2	-0.076653897	0.186523553	-0.410960952	0.681101167	0.766741334	negative
PRJNA729271	stage_E14_vs_E10	ordinary_muscle_development	ENSGALG00010022129	ELMO3	-0.006692784	0.239702097	-0.027921257	0.977724954	0.985132991	negative
PRJNA729271	stage_E14_vs_E10	ordinary_muscle_development	ENSGALG00010022134	CH25H	-1.405080376	0.245680607	-5.71913426	1.07068E-08	7.77921E-08	negative
PRJNA729271	stage_E14_vs_E10	ordinary_muscle_development	ENSGALG00010022135	CHST1	-1.788723331	0.243561927	-7.344018643	2.07274E-13	2.54183E-12	negative
PRJNA729271	stage_E14_vs_E10	ordinary_muscle_development	ENSGALG00010022137	PDDC1	-0.089773792	0.125610251	-0.714701155	0.474793724	0.587012541	negative
PRJNA729271	stage_E14_vs_E10	ordinary_muscle_development	ENSGALG00010022138	SPAG17	0.113560093	0.210163092	0.5403427	0.588960716	0.689078646	positive
PRJNA729271	stage_E14_vs_E10	ordinary_muscle_development	ENSGALG00010022142	TMEM208	0.302227128	0.105141311	2.874485068	0.004046869	0.01080482	positive
PRJNA729271	stage_E14_vs_E10	ordinary_muscle_development	ENSGALG00010022143	MATCAP1	0.209248336	0.191516358	1.092587273	0.274575053	0.384946842	positive
PRJNA729271	stage_E14_vs_E10	ordinary_muscle_development	ENSGALG00010022144	SNTA1	1.364713899	0.187055352	7.29577574	2.96943E-13	3.59882E-12	positive
PRJNA729271	stage_E14_vs_E10	ordinary_muscle_development	ENSGALG00010022148	TALDO1	-0.446013049	0.085543971	-5.213845514	1.84966E-07	1.13605E-06	negative
PRJNA729271	stage_E14_vs_E10	ordinary_muscle_development	ENSGALG00010022149	NBEAL1	-0.155369999	0.10610228	-1.464341763	0.143100592	0.228415711	negative
PRJNA729271	stage_E14_vs_E10	ordinary_muscle_development	ENSGALG00010022150	CPNE2	0.112898562	0.126429247	0.892978199	0.371868848	0.487981075	positive
PRJNA729271	stage_E14_vs_E10	ordinary_muscle_development	ENSGALG00010022151	PIP4K2B	0.623080542	0.153416992	4.061352884	4.87892E-05	0.000203335	positive
PRJNA729271	stage_E14_vs_E10	ordinary_muscle_development	ENSGALG00010022152	ZNF644	-0.23643589	0.100123767	-2.361436221	0.018204303	0.040081072	negative
PRJNA729271	stage_E14_vs_E10	ordinary_muscle_development	ENSGALG00010022154	CPN1	-1.28281745	0.200334364	-6.403381951	1.51972E-10	1.36907E-09	negative
PRJNA729271	stage_E14_vs_E10	ordinary_muscle_development	ENSGALG00010022157	EPS8L2	-1.341016037	0.141350775	-9.487150229	2.37437E-21	5.6606E-20	negative
PRJNA729271	stage_E14_vs_E10	ordinary_muscle_development	ENSGALG00010022158	CWC25	-0.060845779	0.075763513	-0.803101343	0.421916162	0.536278755	negative
PRJNA729271	stage_E14_vs_E10	ordinary_muscle_development	ENSGALG00010022159	KIAA2013	0.154155001	0.089174178	1.728695506	0.083863606	0.147185249	positive
PRJNA729271	stage_E14_vs_E10	ordinary_muscle_development	ENSGALG00010022161	CELF1	0.307063469	0.093972876	3.267575538	0.001084729	0.00338715	positive
PRJNA729271	stage_E14_vs_E10	ordinary_muscle_development	ENSGALG00010022162	POLE	-0.197039211	0.150964107	-1.30520569	0.191822817	0.289150555	negative
PRJNA729271	stage_E14_vs_E10	ordinary_muscle_development	ENSGALG00010022164	PDGFA	0.37977425	0.106982405	3.549875787	0.000385413	0.001342278	positive
PRJNA729271	stage_E14_vs_E10	ordinary_muscle_development	ENSGALG00010022165	FAM192A	-0.018683131	0.088501414	-0.211105446	0.83280499	0.883518139	negative
PRJNA729271	stage_E14_vs_E10	ordinary_muscle_development	ENSGALG00010022166	SDHB	0.518917832	0.107946414	4.807179895	1.53074E-06	8.22696E-06	positive
PRJNA729271	stage_E14_vs_E10	ordinary_muscle_development	ENSGALG00010022167	WDR3	-0.352826778	0.070236408	-5.023417202	5.07601E-07	2.92319E-06	negative
PRJNA729271	stage_E14_vs_E10	ordinary_muscle_development	ENSGALG00010022169	TOX2	-0.607644364	0.228924767	-2.654340871	0.007946351	0.019515818	negative

PRJNA729271	stage_E14_vs_E10	ordinary_muscle_development	ENSGALG00010022170	RSPRY1	-0.456602055	0.138068805	-3.307061697	0.000942801	0.002988389	negative
PRJNA729271	stage_E14_vs_E10	ordinary_muscle_development	ENSGALG00010022171	FOXP1	-0.817800463	0.094410575	-8.66217014	4.62867E-18	8.51428E-17	negative
PRJNA729271	stage_E14_vs_E10	ordinary_muscle_development	ENSGALG00010022172	GIPC2	-0.137408838	0.185712009	-0.739902816	0.459358966	0.5721265	negative
PRJNA729271	stage_E14_vs_E10	ordinary_muscle_development	ENSGALG00010022173	RPL23	0.294731152	0.104003924	2.833846469	0.004599142	0.01209541	positive
PRJNA729271	stage_E14_vs_E10	ordinary_muscle_development	ENSGALG00010022174	KCNMA1	1.088011624	0.124326348	8.751255406	2.10992E-18	3.9874E-17	positive
PRJNA729271	stage_E14_vs_E10	ordinary_muscle_development	ENSGALG00010022176	ARL2BPL	-0.020909649	0.127000367	-0.16464243	0.869225432	0.910362908	negative
PRJNA729271	stage_E14_vs_E10	ordinary_muscle_development	ENSGALG00010022178	ENSGALG00010022178	0.311911259	0.324298419	0.961803207	0.336148467	0.451321974	positive
PRJNA729271	stage_E14_vs_E10	ordinary_muscle_development	ENSGALG00010022180	PLLP	0.162472733	0.397137396	0.40910963	0.682459209	0.767540557	positive
PRJNA729271	stage_E14_vs_E10	ordinary_muscle_development	ENSGALG00010022181	ENSGALG00010022181	-0.582083804	0.229386299	-2.537570064	0.011162502	0.026249265	negative
PRJNA729271	stage_E14_vs_E10	ordinary_muscle_development	ENSGALG00010022182	CNTNAP1	0.096658845	0.301164136	0.320950715	0.74824774	0.819358691	positive
PRJNA729271	stage_E14_vs_E10	ordinary_muscle_development	ENSGALG00010022184	PRKAR1B	0.356305454	0.090363229	3.943035862	8.04566E-05	0.00032216	positive
PRJNA729271	stage_E14_vs_E10	ordinary_muscle_development	ENSGALG00010022186	DNAJB4	0.178193957	0.112730517	1.580707345	0.113944968	0.189886447	positive
PRJNA729271	stage_E14_vs_E10	ordinary_muscle_development	ENSGALG00010022190	ACVR1B	0.199727174	0.16406922	1.21733482	0.223476851	0.326377527	positive
PRJNA729271	stage_E14_vs_E10	ordinary_muscle_development	ENSGALG00010022193	GDAP2	-0.092745026	0.079283593	-1.169788377	0.242086142	0.34822255	negative
PRJNA729271	stage_E14_vs_E10	ordinary_muscle_development	ENSGALG00010022194	ENSGALG00010022194	0.35747215	0.088036604	4.06049454	4.89689E-05	0.000204025	positive
PRJNA729271	stage_E14_vs_E10	ordinary_muscle_development	ENSGALG00010022196	PLXNA1	0.468914622	0.107913137	4.345296908	1.39087E-05	6.37594E-05	positive
PRJNA729271	stage_E14_vs_E10	ordinary_muscle_development	ENSGALG00010022199	RBM39	-0.046654109	0.060963533	-0.765278948	0.444105457	0.557777367	negative
PRJNA729271	stage_E14_vs_E10	ordinary_muscle_development	ENSGALG00010022200	DNMBP	-0.124959087	0.109090882	-1.145458586	0.252019241	0.359266218	negative
PRJNA729271	stage_E14_vs_E10	ordinary_muscle_development	ENSGALG00010022202	CX3CL1	1.117371123	0.161168997	6.932916027	4.12253E-12	4.4003E-11	positive
PRJNA729271	stage_E14_vs_E10	ordinary_muscle_development	ENSGALG00010022203	DNAAF5	-0.09926672	0.130507324	-0.760621835	0.446882974	0.560197934	negative
PRJNA729271	stage_E14_vs_E10	ordinary_muscle_development	ENSGALG00010022204	ENSGALG00010022204	0.19813315	0.330751533	0.599039251	0.54914671	0.654434068	positive
PRJNA729271	stage_E14_vs_E10	ordinary_muscle_development	ENSGALG00010022205	GALNT18	0.043861273	0.10725686	0.408936762	0.68258607	0.767540557	positive
PRJNA729271	stage_E14_vs_E10	ordinary_muscle_development	ENSGALG00010022207	ENSGALG00010022207	0.375919035	0.307775441	1.221406859	0.221932012	0.324710256	positive
PRJNA729271	stage_E14_vs_E10	ordinary_muscle_development	ENSGALG00010022208	CCL17	-3.188949656	1.064769749	-2.994966432	0.00274475	0.007682015	negative
PRJNA729271	stage_E14_vs_E10	ordinary_muscle_development	ENSGALG00010022209	ZFYVE19	-0.373168416	0.119318264	-3.127504576	0.001762971	0.005215155	negative
PRJNA729271	stage_E14_vs_E10	ordinary_muscle_development	ENSGALG00010022210	EZH1	0.201987237	0.083315604	2.424362619	0.015335284	0.034569976	positive
PRJNA729271	stage_E14_vs_E10	ordinary_muscle_development	ENSGALG00010022211	ENSGALG00010022211	0.474632467	0.478436173	0.992049711	0.321173276	0.435695521	positive
PRJNA729271	stage_E14_vs_E10	ordinary_muscle_development	ENSGALG00010022213	IDH1	-0.225654889	0.079757597	-2.82925887	0.004665594	0.012251411	negative
PRJNA729271	stage_E14_vs_E10	ordinary_muscle_development	ENSGALG00010022214	ENSGALG00010022214	-1.816069399	0.261605638	-6.942011701	3.86555E-12	4.15353E-11	negative
PRJNA729271	stage_E14_vs_E10	ordinary_muscle_development	ENSGALG00010022215	ENSGALG00010022215	0.387929029	0.130743079	2.967109472	0.003006139	0.008334733	positive
PRJNA729271	stage_E14_vs_E10	ordinary_muscle_development	ENSGALG00010022216	ROMO1	0.195103213	0.144106344	1.353883585	0.175773521	0.269441327	positive
PRJNA729271	stage_E14_vs_E10	ordinary_muscle_development	ENSGALG00010022218	ENSGALG00010022218	1.72611199	0.248374133	6.949644744	3.66207E-12	3.9466E-11	positive
PRJNA729271	stage_E14_vs_E10	ordinary_muscle_development	ENSGALG00010022219	EIF4E3	-0.25262993	0.137506528	-1.83722136	0.066177224	0.120260251	negative
PRJNA729271	stage_E14_vs_E10	ordinary_muscle_development	ENSGALG00010022221	CUTC	0.385313122	0.080388006	4.793166819	1.64169E-06	8.78416E-06	positive
PRJNA729271	stage_E14_vs_E10	ordinary_muscle_development	ENSGALG00010022222	ENSGALG00010022222	0.039133323	0.106392762	0.367819409	0.713007896	0.792443601	positive
PRJNA729271	stage_E14_vs_E10	ordinary_muscle_development	ENSGALG00010022223	DOK4	-0.635042583	0.169506774	-3.746414178	0.00017938	0.000673083	negative
PRJNA729271	stage_E14_vs_E10	ordinary_muscle_development	ENSGALG00010022224	NHEJ1	-0.120368186	0.150842378	-0.797973267	0.424886004	0.53924548	negative
PRJNA729271	stage_E14_vs_E10	ordinary_muscle_development	ENSGALG00010022225	ENSGALG00010022225	-1.815599581	0.633915258	-2.864104561	0.004181899	0.011112123	negative
PRJNA729271	stage_E14_vs_E10	ordinary_muscle_development	ENSGALG00010022226	FBRSL1	-0.288410558	0.131898436	-2.186610899	0.028770941	0.059313724	negative
PRJNA729271	stage_E14_vs_E10	ordinary_muscle_development	ENSGALG00010022227	TMEM80	-0.119021005	0.157736066	-0.754557966	0.450514253	0.56377485	negative
PRJNA729271	stage_E14_vs_E10	ordinary_muscle_development	ENSGALG00010022228	FUBP1	-0.396457145	0.054508885	-7.273257223	3.50921E-13	4.20728E-12	negative
PRJNA729271	stage_E14_vs_E10	ordinary_muscle_development	ENSGALG00010022229	RAPSN	1.976099398	0.177329494	11.14365891	7.68939E-29	3.08747E-27	positive
PRJNA729271	stage_E14_vs_E10	ordinary_muscle_development	ENSGALG00010022230	NFS1	0.04259632	0.086478753	0.492563998	0.622320692	0.717370191	positive
PRJNA729271	stage_E14_vs_E10	ordinary_muscle_development	ENSGALG00010022231	ZNF503	-1.911220796	0.203707423	-9.382185351	6.46193E-21	1.4844E-19	negative
PRJNA729271	stage_E14_vs_E10	ordinary_muscle_development	ENSGALG00010022232	GPR27	-0.662074994	0.199452195	-3.31946708	0.000901894	0.002869871	negative
PRJNA729271	stage_E14_vs_E10	ordinary_muscle_development	ENSGALG00010022233	FAM46C	2.19916339	0.164157779	13.39664436	6.32593E-41	5.15137E-39	positive
PRJNA729271	stage_E14_vs_E10	ordinary_muscle_development	ENSGALG00010022234	COX15	0.070490662	0.093026837	0.757745445	0.448603387	0.561674536	positive
PRJNA729271	stage_E14_vs_E10	ordinary_muscle_development	ENSGALG00010022235	RAMP2	0.161300245	0.113303654	1.423610263	0.154559323	0.24327293	positive
PRJNA729271	stage_E14_vs_E10	ordinary_muscle_development	ENSGALG00010022236	SHF	1.64779543	0.181305157	9.088519342	1.00397E-19	2.10297E-18	positive
PRJNA729271	stage_E14_vs_E10	ordinary_muscle_development	ENSGALG00010022237	SUN1	0.347874394	0.090862566	3.828577722	0.000128886	0.000497973	positive
PRJNA729271	stage_E14_vs_E10	ordinary_muscle_development	ENSGALG00010022238	COMTD1	-0.94448513	0.167696141	-5.632122021	1.78006E-08	1.25802E-07	negative
PRJNA729271	stage_E14_vs_E10	ordinary_muscle_development	ENSGALG00010022239	DEAF1	0.357820564	0.111172037	3.2186202	0.00128809	0.003954015	positive
PRJNA729271	stage_E14_vs_E10	ordinary_muscle_development	ENSGALG00010022240	ENSGALG00010022240	-0.321788798	0.129455094	-2.485717552	0.012929054	0.029851327	negative
PRJNA729271	stage_E14_vs_E10	ordinary_muscle_development	ENSGALG00010022241	POLR2C	0.132432663	0.093963023	1.409412547	0.158713216	0.248385669	positive
PRJNA729271	stage_E14_vs_E10	ordinary_muscle_development	ENSGALG00010022242	USP19	0.062960745	0.126049818	0.49949096	0.617433554	0.713407681	positive
PRJNA729271	stage_E14_vs_E10	ordinary_muscle_development	ENSGALG00010022243	ENSGALG00010022243	-1.98814089	0.893533507	-2.225032273	0.026079072	0.054642403	negative
PRJNA729271	stage_E14_vs_E10	ordinary_muscle_development	ENSGALG00010022244	ENTPD7	-0.161127871	0.164037607	-0.98226177	0.325970903	0.440677882	negative
PRJNA729271	stage_E14_vs_E10	ordinary_muscle_development	ENSGALG00010022245	PIKFYVE	-0.212414409	0.10058462	-2.111798095	0.034703771	0.069434503	negative
PRJNA729271	stage_E14_vs_E10	ordinary_muscle_development	ENSGALG00010022246	RHOV	-1.439412995	0.426232608	-3.377059772	0.000732651	0.002388615	negative
PRJNA729271	stage_E14_vs_E10	ordinary_muscle_development	ENSGALG00010022247	GALNT9	-0.719825363	0.185278702	-3.885095031	0.00010229	0.000401922	negative
PRJNA729271	stage_E14_vs_E10	ordinary_muscle_development	ENSGALG00010022248	FIGNL2	1.176701734	0.214028596	5.497871576	3.84403E-08	2.59521E-07	positive
PRJNA729271	stage_E14_vs_E10	ordinary_muscle_development	ENSGALG00010022250	SLC25A28	0.322199075	0.128633147	2.504790435	0.012252397	0.028479552	positive
PRJNA729271	stage_E14_vs_E10	ordinary_muscle_development	ENSGALG00010022251	WNK4	-1.099622571	0.152715587	-7.200460629	6.00095E-13	7.03752E-12	negative
PRJNA729271	stage_E14_vs_E10	ordinary_muscle_development	ENSGALG00010022252	COQ9	0.982793111	0.132795435	7.40080492	1.35361E-13	1.68852E-12	positive
PRJNA729271	stage_E14_vs_E10	ordinary_muscle_development	ENSGALG00010022253	ALPK3	1.186724142	0.217429693	5.457967244	4.81616E-08	3.20525E-07	positive
PRJNA729271	stage_E14_vs_E10	ordinary_muscle_development	ENSGALG00010022255	NOC4L	-0.245602026	0.090707246	-2.707634033	0.00677647	0.016955162	negative
PRJNA729271	stage_E14_vs_E10	ordinary_muscle_development	ENSGALG00010022256	PSMC3	0.300668287	0.091837885	3.273902561	0.001060732	0.003323672	positive
PRJNA729271	stage_E14_vs_E10	ordinary_muscle_development	ENSGALG00010022257	KCNK18	4.770213161	0.815276889	5.851034445	4.88525E-09	3.70742E-08	positive
PRJNA729271	stage_E14_vs_E10	ordinary_muscle_development	ENSGALG00010022258	ENSGALG00010022258	2.791805468	0.704446324	3.963120219	7.39765E-05	0.00029819	positive
PRJNA729271	stage_E14_vs_E10	ordinary_muscle_development	ENSGALG00010022259	GET4	0.074628902	0.08489939	0.87902754	0.379386343	0.49551316	positive
PRJNA729271	stage_E14_vs_E10	ordinary_muscle_development	ENSGALG00010022264	SCN8A	-0.850641932	0.2491697	-3.413905998	0.000640387	0.002109158	negative
PRJNA729271	stage_E14_vs_E10	ordinary_muscle_development	ENSGALG00010022266	COA3	0.53296872	0.143762915	3.707275418	0.000209501	0.000774871	positive
PRJNA729271	stage_E14_vs_E10	ordinary_muscle_development	ENSGALG00010022269	CNTD1	0.291936416	0.349551219	0.835174932	0.403619222	0.518677448	positive
PRJNA729271	stage_E14_vs_E10	ordinary_muscle_development	ENSGALG00010022271	SLC18A2	-2.160425818	0.244302061	-8.843256625	9.29684E-19	1.78961E-17	negative
PRJNA729271	stage_E14_vs_E10	ordinary_muscle_development	ENSGALG00010022274	CCDC91	0.833317266	0.173785361	4.795094726	1.62598E-06	8.70332E-06	positive
PRJNA729271	stage_E14_vs_E10	ordinary_muscle_development	ENSGALG00010022275	VDAC2	0.684799082	0.124088273	5.518644644	3.41624E-08	2.33688E-07	positive
PRJNA729271	stage_E14_vs_E10	ordinary_muscle_development	ENSGALG00010022276	BECN1	-0.270926304	0.088774573	-3.051845765	0.002274389	0.006512683	negative
PRJNA729271	stage_E14_vs_E10	ordinary_muscle_development	ENSGALG00010022279	EP400	-0.127209444	0.14894895	-0.854047269	0.393078799	0.508460851	negative
PRJNA729271	stage_E14_vs_E10	ordinary_muscle_development	ENSGALG00010022280	PDZD8	-0.011197626	0.066554282	-0.168248026	0.86638816	0.908310168	negative
PRJNA729271	stage_E14_vs_E10	ordinary_muscle_development	ENSGALG00010022282	TMT1A	1.948689051	0.222928581	8.741315448	2.30412E-18	4.33742E-17	positive
PRJNA729271	stage_E14_vs_E10	ordinary_muscle_development	ENSGALG00010022290	NEXN	0.810518983	0.220121022	3.682151639	0.000231274	0.000846116	positive
PRJNA729271	stage_E14_vs_E10	ordinary_muscle_development	ENSGALG00010022293	DNAJC10	-0.521457107	0.062828141	-8.299737919	1.04341E-16	1.7164E-15	negative
PRJNA729271	stage_E14_vs_E10	ordinary_muscle_development	ENSGALG00010022294	ENSGALG00010022294	-0.689463369	0.718845264	-0.95912626	0.337495143	0.452752623	negative
PRJNA729271	stage_E14_vs_E10	ordinary_muscle_development	ENSGALG00010022296	CIAPIN1	0.00880804	0.096991016	0.090812951	0.927641219	0.950325781	positive
PRJNA729271	stage_E14_vs_E10	ordinary_muscle_development	ENSGALG00010022297	VPS18	0.015100715	0.055460445	0.272279006	0.785407498	0.848891334	positive
PRJNA729271	stage_E14_vs_E10	ordinary_muscle_development	ENSGALG00010022299	MRGBP	0.279223959	0.078710355	3.54748697	0.000388925	0.001351911	positive
PRJNA729271	stage_E14_vs_E10	ordinary_muscle_development	ENSGALG00010022302	ENSGALG00010022302	-0.728544651	0.151429238	-4.811122755	1.50085E-06	8.08127E-06	negative
PRJNA729271	stage_E14_vs_E10	ordinary_muscle_development	ENSGALG00010022303	DUSP13A	2.746147562	0.208577349	13.16608717	1.37543E-39	9.96846E-38	positive
PRJNA729271	stage_E14_vs_E10	ordinary_muscle_development	ENSGALG00010022304	ADAP1	-1.324387243	0.187773065	-7.053126832	1.74941E-12	1.94163E-11	negative
PRJNA729271	stage_E14_vs_E10	ordinary_muscle_development	ENSGALG00010022307	ENSGALG00010022307	4.79622583	0.644805351	7.438253766	1.02025E-13	1.29045E-12	positive
PRJNA729271	stage_E14_vs_E10	ordinary_muscle_development	ENSGALG00010022308	OGFR	-0.13127997	0.180959238	-0.72546708	0.468165533	0.580501232	negative
PRJNA729271	stage_E14_vs_E10	ordinary_muscle_development	ENSGALG00010022309	WSCD2	-0.648841478	0.321539231	-2.017923214	0.043599259	0.084341553	negative
PRJNA729271	stage_E14_vs_E10	ordinary_muscle_development	ENSGALG00010022310	DCLRE1A	-0.327599974	0.094808133	-3.455399477	0.000549478	0.001841971	negative
PRJNA729271	stage_E14_vs_E10	ordinary_muscle_development	ENSGALG00010022311	DLL4	0.242295202	0.14168502	1.71009752	0.087247841	0.152002099	positive
PRJNA729271	stage_E14_vs_E10	ordinary_muscle_development	ENSGALG00010022312	ZNF592	-0.235134005	0.05409923	-4.346346621	1.38424E-05	6.34953E-05	negative
PRJNA729271	stage_E14_vs_E10	ordinary_muscle_development	ENSGALG00010022313	ENSGALG00010022313	-0.836454804	0.390375374	-2.142693568	0.032137712	0.065024587	negative
PRJNA729271	stage_E14_vs_E10	ordinary_muscle_development	ENSGALG00010022314	DUSP13B	1.626220173	0.610999707	2.661572755	0.007777653	0.019146923	positive
PRJNA729271	stage_E14_vs_E10	ordinary_muscle_development	ENSGALG00010022316	INO80	0.008967422	0.068664178	0.130598251	0.896093126	0.92843959	positive
PRJNA729271	stage_E14_vs_E10	ordinary_muscle_development	ENSGALG00010022317	PSME3	-0.274264672	0.072661929	-3.774530584	0.000160309	0.000606864	negative
PRJNA729271	stage_E14_vs_E10	ordinary_muscle_development	ENSGALG00010022320	COL9A3	-4.802569642	0.542855769	-8.846861199	9.00152E-19	1.73738E-17	negative
PRJNA729271	stage_E14_vs_E10	ordinary_muscle_development	ENSGALG00010022322	ENSGALG00010022322	-1.288899032	0.444995559	-2.896431224	0.003774334	0.010161398	negative
PRJNA729271	stage_E14_vs_E10	ordinary_muscle_development	ENSGALG00010022323	DUSP29	1.898406569	0.389096721	4.879009421	1.0662E-06	5.86734E-06	positive
PRJNA729271	stage_E14_vs_E10	ordinary_muscle_development	ENSGALG00010022325	MIGA1	-0.409336112	0.106907983	-3.828863866	0.000128736	0.000497608	negative
PRJNA729271	stage_E14_vs_E10	ordinary_muscle_development	ENSGALG00010022326	COX19	0.37651836	0.094483016	3.985037487	6.74695E-05	0.000274325	positive
PRJNA729271	stage_E14_vs_E10	ordinary_muscle_development	ENSGALG00010022328	ENSGALG00010022328	-0.514243412	0.217791437	-2.361173694	0.018217196	0.040099963	negative
PRJNA729271	stage_E14_vs_E10	ordinary_muscle_development	ENSGALG00010022332	KAT6B	-1.052476815	0.060821569	-17.30433504	4.36289E-67	1.34553E-64	negative
PRJNA729271	stage_E14_vs_E10	ordinary_muscle_development	ENSGALG00010022333	ENSGALG00010022333	0.83312122	0.13129819	6.345260534	2.22049E-10	1.97218E-09	positive
PRJNA729271	stage_E14_vs_E10	ordinary_muscle_development	ENSGALG00010022334	PDE1A	-0.239746959	0.118682425	-2.020071291	0.043375993	0.084013593	negative
PRJNA729271	stage_E14_vs_E10	ordinary_muscle_development	ENSGALG00010022340	GFOD2	0.267308948	0.092767371	2.881497514	0.003957904	0.010582884	positive
PRJNA729271	stage_E14_vs_E10	ordinary_muscle_development	ENSGALG00010022343	GATA5	-3.934404812	0.625535322	-6.289660513	3.18161E-10	2.75987E-09	negative
PRJNA729271	stage_E14_vs_E10	ordinary_muscle_development	ENSGALG00010022345	USP33	-0.244176134	0.122252003	-1.997318054	0.045790643	0.088157176	negative
PRJNA729271	stage_E14_vs_E10	ordinary_muscle_development	ENSGALG00010022347	SEC11A	-0.080335231	0.101879912	-0.788528664	0.430387539	0.544558954	negative
PRJNA729271	stage_E14_vs_E10	ordinary_muscle_development	ENSGALG00010022348	G6PC1	1.572852041	0.62741015	2.506896074	0.012179652	0.028333181	positive
PRJNA729271	stage_E14_vs_E10	ordinary_muscle_development	ENSGALG00010022353	SLC39A13	0.233695446	0.094862394	2.463520427	0.013758006	0.031499335	positive
PRJNA729271	stage_E14_vs_E10	ordinary_muscle_development	ENSGALG00010022354	FAR2	-0.179461065	0.135969453	-1.319863104	0.186880728	0.283232555	negative
PRJNA729271	stage_E14_vs_E10	ordinary_muscle_development	ENSGALG00010022356	TCFL5	-0.612428553	0.199298248	-3.072924926	0.002119718	0.0061218	negative
PRJNA729271	stage_E14_vs_E10	ordinary_muscle_development	ENSGALG00010022357	ENSGALG00010022357	-3.446506616	0.56241674	-6.128029928	8.89739E-10	7.34022E-09	negative
PRJNA729271	stage_E14_vs_E10	ordinary_muscle_development	ENSGALG00010022359	ZZZ3	-0.261357912	0.078374863	-3.334716009	0.000853866	0.002737413	negative
PRJNA729271	stage_E14_vs_E10	ordinary_muscle_development	ENSGALG00010022363	KIF18A	-0.339917086	0.198864906	-1.709286438	0.087397904	0.152150284	negative
PRJNA729271	stage_E14_vs_E10	ordinary_muscle_development	ENSGALG00010022365	PLCG1	-0.990810836	0.108215523	-9.155903001	5.39051E-20	1.15074E-18	negative
PRJNA729271	stage_E14_vs_E10	ordinary_muscle_development	ENSGALG00010022366	AARSD1	0.094278943	0.11287253	0.835269152	0.403566182	0.518677448	positive
PRJNA729271	stage_E14_vs_E10	ordinary_muscle_development	ENSGALG00010022373	AK5	-1.831542571	0.171927497	-10.65299387	1.68846E-26	5.78586E-25	negative
PRJNA729271	stage_E14_vs_E10	ordinary_muscle_development	ENSGALG00010022375	NMB	-0.611367521	0.403030758	-1.516925218	0.129285566	0.209947824	negative
PRJNA729271	stage_E14_vs_E10	ordinary_muscle_development	ENSGALG00010022377	ZHX3	0.031996132	0.090297155	0.354342633	0.723082121	0.799410827	positive
PRJNA729271	stage_E14_vs_E10	ordinary_muscle_development	ENSGALG00010022379	METTL15	-0.25230867	0.109524028	-2.303683257	0.02124043	0.045863256	negative
PRJNA729271	stage_E14_vs_E10	ordinary_muscle_development	ENSGALG00010022382	CALB2	-3.743206426	0.807176528	-4.637407426	3.52807E-06	1.78E-05	negative
PRJNA729271	stage_E14_vs_E10	ordinary_muscle_development	ENSGALG00010022385	ENSGALG00010022385	0.599540879	0.688645992	0.870608245	0.383968093	0.500100414	positive
PRJNA729271	stage_E14_vs_E10	ordinary_muscle_development	ENSGALG00010022386	SPI1	0.536022405	0.245319866	2.184993877	0.028889296	0.059498485	positive
PRJNA729271	stage_E14_vs_E10	ordinary_muscle_development	ENSGALG00010022388	CYP2W1	-0.628262174	0.428676726	-1.465584986	0.142761384	0.227949577	negative
PRJNA729271	stage_E14_vs_E10	ordinary_muscle_development	ENSGALG00010022391	ENSGALG00010022391	0.350086829	0.17839363	1.962440183	0.049711258	0.094370432	positive
PRJNA729271	stage_E14_vs_E10	ordinary_muscle_development	ENSGALG00010022392	EMILIN3	-0.94647307	0.184742096	-5.123212797	3.00373E-07	1.79247E-06	negative

PRJNA729271	stage_E14_vs_E10	ordinary_muscle_development	ENSGALG00010022395	PTGES3L	0.703858603	0.133832355	5.259255922	1.44639E-07	8.99807E-07	positive
PRJNA729271	stage_E14_vs_E10	ordinary_muscle_development	ENSGALG00010022396	PPP1R1C	2.759769865	0.320115927	8.621157618	6.62806E-18	1.20242E-16	positive
PRJNA729271	stage_E14_vs_E10	ordinary_muscle_development	ENSGALG00010022397	ATF1	0.06712377	0.046715215	1.436871693	0.150754454	0.238323243	positive
PRJNA729271	stage_E14_vs_E10	ordinary_muscle_development	ENSGALG00010022400	NHLRC2	0.460665947	0.070628648	6.52236678	6.92065E-11	6.51395E-10	positive
PRJNA729271	stage_E14_vs_E10	ordinary_muscle_development	ENSGALG00010022401	LCAT	1.184293989	0.43770333	2.705700207	0.006816055	0.017037199	positive
PRJNA729271	stage_E14_vs_E10	ordinary_muscle_development	ENSGALG00010022406	ITPRID2	0.364167592	0.10410253	3.498162762	0.000468475	0.001599658	positive
PRJNA729271	stage_E14_vs_E10	ordinary_muscle_development	ENSGALG00010022407	RUNDC1	0.193790961	0.095064006	2.038531398	0.041496818	0.080835422	positive
PRJNA729271	stage_E14_vs_E10	ordinary_muscle_development	ENSGALG00010022408	RAD51AP1	-0.092662806	0.135700638	-0.682847242	0.494703374	0.604640808	negative
PRJNA729271	stage_E14_vs_E10	ordinary_muscle_development	ENSGALG00010022409	CARNS1	-1.060883728	0.488965579	-2.169649097	0.030033439	0.061487952	negative
PRJNA729271	stage_E14_vs_E10	ordinary_muscle_development	ENSGALG00010022411	ADRB1	0.419410842	0.28949801	1.448752071	0.147406833	0.234052146	positive
PRJNA729271	stage_E14_vs_E10	ordinary_muscle_development	ENSGALG00010022412	CHAC1	-0.443176924	0.657854649	-0.673669974	0.500521155	0.609770898	negative
PRJNA729271	stage_E14_vs_E10	ordinary_muscle_development	ENSGALG00010022415	DIP2B	-0.311996341	0.108925143	-2.864318859	0.004179071	0.011106644	negative
PRJNA729271	stage_E14_vs_E10	ordinary_muscle_development	ENSGALG00010022416	CCDC186	0.030619513	0.085581095	0.357783607	0.72050526	0.797717976	positive
PRJNA729271	stage_E14_vs_E10	ordinary_muscle_development	ENSGALG00010022419	DUOX2	0.984782869	0.73881843	1.33291595	0.182559388	0.27799121	positive
PRJNA729271	stage_E14_vs_E10	ordinary_muscle_development	ENSGALG00010022421	CMKLR1	0.354055512	0.178341558	1.985266448	0.047114835	0.090222998	positive
PRJNA729271	stage_E14_vs_E10	ordinary_muscle_development	ENSGALG00010022422	RPS6KB2	0.031026544	0.157088593	0.197509846	0.843428577	0.891136178	positive
PRJNA729271	stage_E14_vs_E10	ordinary_muscle_development	ENSGALG00010022425	VWA2	-0.364275645	0.286311935	-1.272303387	0.203265346	0.302871216	negative
PRJNA729271	stage_E14_vs_E10	ordinary_muscle_development	ENSGALG00010022426	EXD1	-0.645770127	0.229070425	-2.819089922	0.004816002	0.012596167	negative
PRJNA729271	stage_E14_vs_E10	ordinary_muscle_development	ENSGALG00010022427	RPL27	0.462776402	0.138620043	3.338452307	0.000842465	0.002704658	positive
PRJNA729271	stage_E14_vs_E10	ordinary_muscle_development	ENSGALG00010022429	ADK	0.933256585	0.129835497	7.187992535	6.57509E-13	7.67358E-12	positive
PRJNA729271	stage_E14_vs_E10	ordinary_muscle_development	ENSGALG00010022431	LARP4	-0.259993057	0.132615056	-1.960509351	0.049936286	0.094679721	negative
PRJNA729271	stage_E14_vs_E10	ordinary_muscle_development	ENSGALG00010022433	LNPK	0.183471584	0.107893365	1.700489958	0.089038804	0.154461146	positive
PRJNA729271	stage_E14_vs_E10	ordinary_muscle_development	ENSGALG00010022435	PIGK	-0.036999268	0.163682685	-0.226042651	0.821168248	0.874770085	negative
PRJNA729271	stage_E14_vs_E10	ordinary_muscle_development	ENSGALG00010022436	AFAP1L2	0.662125212	0.154382889	4.288851037	1.796E-05	8.05476E-05	positive
PRJNA729271	stage_E14_vs_E10	ordinary_muscle_development	ENSGALG00010022438	RPN2	-0.400265205	0.100855808	-3.968687717	7.22695E-05	0.000292284	negative
PRJNA729271	stage_E14_vs_E10	ordinary_muscle_development	ENSGALG00010022439	IFI35	0.107002657	0.206103744	0.519168915	0.603642954	0.70183722	positive
PRJNA729271	stage_E14_vs_E10	ordinary_muscle_development	ENSGALG00010022440	SORD	0.775618414	0.123004224	6.305624203	2.87035E-10	2.50788E-09	positive
PRJNA729271	stage_E14_vs_E10	ordinary_muscle_development	ENSGALG00010022441	FICD	-0.013048748	0.116388798	-0.112113438	0.910733463	0.938443275	negative
PRJNA729271	stage_E14_vs_E10	ordinary_muscle_development	ENSGALG00010022448	FERRY3	-0.244247865	0.119676646	-2.040898301	0.041260937	0.080397537	negative
PRJNA729271	stage_E14_vs_E10	ordinary_muscle_development	ENSGALG00010022449	ENSGALG00010022449	0.103291723	0.100011214	1.032801412	0.301696843	0.415496032	positive
PRJNA729271	stage_E14_vs_E10	ordinary_muscle_development	ENSGALG00010022451	CHP1	-0.317755872	0.120956784	-2.627019848	0.008613631	0.020938216	negative
PRJNA729271	stage_E14_vs_E10	ordinary_muscle_development	ENSGALG00010022452	AP3M1	0.523530481	0.080859607	6.474561301	9.50878E-11	8.71789E-10	positive
PRJNA729271	stage_E14_vs_E10	ordinary_muscle_development	ENSGALG00010022453	ENSGALG00010022453	0.272942214	0.240327189	1.135710926	0.256077566	0.363906306	positive
PRJNA729271	stage_E14_vs_E10	ordinary_muscle_development	ENSGALG00010022454	ABLIM1	1.101616713	0.1405916	7.835579888	4.66684E-15	6.59316E-14	positive
PRJNA729271	stage_E14_vs_E10	ordinary_muscle_development	ENSGALG00010022455	C7orf50	-0.434291107	0.150099762	-2.893349743	0.003811566	0.010247802	negative
PRJNA729271	stage_E14_vs_E10	ordinary_muscle_development	ENSGALG00010022456	ATP5G3	0.097680821	0.158145679	0.617663548	0.536797146	0.643526145	positive
PRJNA729271	stage_E14_vs_E10	ordinary_muscle_development	ENSGALG00010022459	FGF6	2.863608657	0.728053464	3.93323952	8.38087E-05	0.000334381	positive
PRJNA729271	stage_E14_vs_E10	ordinary_muscle_development	ENSGALG00010022460	VAT1	-0.177990122	0.114917444	-1.548852074	0.121417284	0.199540083	negative
PRJNA729271	stage_E14_vs_E10	ordinary_muscle_development	ENSGALG00010022462	ITGA5	0.110854493	0.128556953	0.862298698	0.388523169	0.50435638	positive
PRJNA729271	stage_E14_vs_E10	ordinary_muscle_development	ENSGALG00010022463	DUSP12	0.610857992	0.111034996	5.501490654	3.76594E-08	2.54961E-07	positive
PRJNA729271	stage_E14_vs_E10	ordinary_muscle_development	ENSGALG00010022466	NEDD4	-0.506568345	0.137604695	-3.681330378	0.00023202	0.000848419	negative
PRJNA729271	stage_E14_vs_E10	ordinary_muscle_development	ENSGALG00010022467	SART3	-0.30653671	0.115466443	-2.654768794	0.007936278	0.01949524	negative
PRJNA729271	stage_E14_vs_E10	ordinary_muscle_development	ENSGALG00010022468	MYBPC3	1.522786835	0.42908185	3.548942554	0.000386781	0.001346074	positive
PRJNA729271	stage_E14_vs_E10	ordinary_muscle_development	ENSGALG00010022470	ENSGALG00010022470	-0.300325962	0.170594242	-1.760469515	0.078328232	0.138848933	negative
PRJNA729271	stage_E14_vs_E10	ordinary_muscle_development	ENSGALG00010022471	ST6GALNAC5	-0.496140551	0.134544879	-3.687546894	0.000226426	0.000830268	negative
PRJNA729271	stage_E14_vs_E10	ordinary_muscle_development	ENSGALG00010022472	QSER1	-0.130874453	0.087379745	-1.497766485	0.134193931	0.216777113	negative
PRJNA729271	stage_E14_vs_E10	ordinary_muscle_development	ENSGALG00010022473	ZNF385A	0.986209477	0.41794185	2.359681081	0.018290652	0.040237213	positive
PRJNA729271	stage_E14_vs_E10	ordinary_muscle_development	ENSGALG00010022474	VCL	0.221265477	0.119575389	1.850426567	0.064252093	0.117252183	positive
PRJNA729271	stage_E14_vs_E10	ordinary_muscle_development	ENSGALG00010022476	OIP5	0.081391156	0.108415154	0.750735976	0.452811567	0.565721743	positive
PRJNA729271	stage_E14_vs_E10	ordinary_muscle_development	ENSGALG00010022478	ATP6V1A	0.050343637	0.095975459	0.524546981	0.599898152	0.69910948	positive
PRJNA729271	stage_E14_vs_E10	ordinary_muscle_development	ENSGALG00010022479	ENSGALG00010022479	1.23547439	0.639535368	1.931831218	0.053380342	0.100109723	positive
PRJNA729271	stage_E14_vs_E10	ordinary_muscle_development	ENSGALG00010022480	PIF1	-0.126616213	0.230878331	-0.548410984	0.583409736	0.684295527	negative
PRJNA729271	stage_E14_vs_E10	ordinary_muscle_development	ENSGALG00010022481	CORO1B	-0.47940286	0.146365167	-3.275389009	0.001055166	0.003308377	negative
PRJNA729271	stage_E14_vs_E10	ordinary_muscle_development	ENSGALG00010022482	PRTG	-1.006523785	0.303731999	-3.31385494	0.000920192	0.002921837	negative
PRJNA729271	stage_E14_vs_E10	ordinary_muscle_development	ENSGALG00010022483	TOP2A	-0.280082622	0.238195352	-1.175852592	0.239653786	0.345384082	negative
PRJNA729271	stage_E14_vs_E10	ordinary_muscle_development	ENSGALG00010022485	ATF2	-0.16724239	0.056455695	-2.962365261	0.003052854	0.00844487	negative
PRJNA729271	stage_E14_vs_E10	ordinary_muscle_development	ENSGALG00010022486	FAM183A	0.046444768	0.556008379	0.083532496	0.933428139	0.954230967	positive
PRJNA729271	stage_E14_vs_E10	ordinary_muscle_development	ENSGALG00010022489	NUSAP1	0.152941058	0.237487488	0.643996278	0.519577848	0.627554446	positive
PRJNA729271	stage_E14_vs_E10	ordinary_muscle_development	ENSGALG00010022493	ENSGALG00010022493	0.186861015	0.15676227	1.192002483	0.233260272	0.337638071	positive
PRJNA729271	stage_E14_vs_E10	ordinary_muscle_development	ENSGALG00010022494	FHIP2A	-0.314847403	0.065363892	-4.816839886	1.4585E-06	7.87078E-06	negative
PRJNA729271	stage_E14_vs_E10	ordinary_muscle_development	ENSGALG00010022496	TUBGCP2	0.264166844	0.071160477	3.712269153	0.000205409	0.000760707	positive
PRJNA729271	stage_E14_vs_E10	ordinary_muscle_development	ENSGALG00010022497	CORO1C	-0.228171404	0.148860232	-1.532789524	0.125327723	0.204644062	negative
PRJNA729271	stage_E14_vs_E10	ordinary_muscle_development	ENSGALG00010022498	NAA50	0.28526826	0.113784477	2.507092952	0.01217287	0.028326678	positive
PRJNA729271	stage_E14_vs_E10	ordinary_muscle_development	ENSGALG00010022500	ENSGALG00010022500	0.372368408	0.409722041	0.90883177	0.363438934	0.479566934	positive
PRJNA729271	stage_E14_vs_E10	ordinary_muscle_development	ENSGALG00010022502	ENSGALG00010022502	-1.041162285	0.4388651	-2.372397087	0.017673086	0.039092229	negative
PRJNA729271	stage_E14_vs_E10	ordinary_muscle_development	ENSGALG00010022504	RAD9A	-0.003461696	0.145972999	-0.023714629	0.981080237	0.987446602	negative
PRJNA729271	stage_E14_vs_E10	ordinary_muscle_development	ENSGALG00010022505	ZNF511	0.207749625	0.12117567	1.71444999	0.086446114	0.150877354	positive
PRJNA729271	stage_E14_vs_E10	ordinary_muscle_development	ENSGALG00010022506	EBNA1BP2	-0.163632506	0.083585122	-1.957675037	0.050268156	0.095179748	negative
PRJNA729271	stage_E14_vs_E10	ordinary_muscle_development	ENSGALG00010022507	ENSGALG00010022507	-0.175753895	0.204199142	-0.8606985	0.38940412	0.505138063	negative
PRJNA729271	stage_E14_vs_E10	ordinary_muscle_development	ENSGALG00010022508	USF3	-0.049957696	0.122162627	-0.408944186	0.682580622	0.767540557	negative
PRJNA729271	stage_E14_vs_E10	ordinary_muscle_development	ENSGALG00010022511	IGFBP4	-0.073975883	0.271309469	-0.27266237	0.785112764	0.848699344	negative
PRJNA729271	stage_E14_vs_E10	ordinary_muscle_development	ENSGALG00010022512	PRAP1	1.365704965	0.845625166	1.615024031	0.106305491	0.179215875	positive
PRJNA729271	stage_E14_vs_E10	ordinary_muscle_development	ENSGALG00010022513	NDUFAF1	0.00939118	0.084599001	0.11100817	0.911609868	0.939146058	positive
PRJNA729271	stage_E14_vs_E10	ordinary_muscle_development	ENSGALG00010022515	CHRNA1	0.629076033	0.168091945	3.742451975	0.000182233	0.000682551	positive
PRJNA729271	stage_E14_vs_E10	ordinary_muscle_development	ENSGALG00010022516	TRUB1	-0.235578264	0.149218403	-1.578748058	0.114393857	0.190503097	negative
PRJNA729271	stage_E14_vs_E10	ordinary_muscle_development	ENSGALG00010022517	HNRNPA1	0.052776026	0.181303534	0.291092096	0.770980885	0.837419852	positive
PRJNA729271	stage_E14_vs_E10	ordinary_muscle_development	ENSGALG00010022518	ECHS1	0.76216165	0.111501779	6.83542142	8.17643E-12	8.44742E-11	positive
PRJNA729271	stage_E14_vs_E10	ordinary_muscle_development	ENSGALG00010022520	TMOD2	-0.658169096	0.1984524	-3.316508624	0.000911498	0.002896768	negative
PRJNA729271	stage_E14_vs_E10	ordinary_muscle_development	ENSGALG00010022521	RTF1	-0.142970914	0.091008648	-1.570959646	0.116192023	0.192656528	negative
PRJNA729271	stage_E14_vs_E10	ordinary_muscle_development	ENSGALG00010022522	TNS4	-0.612384739	0.339965258	-1.801315649	0.071653143	0.12858887	negative
PRJNA729271	stage_E14_vs_E10	ordinary_muscle_development	ENSGALG00010022524	SOX18	0.070435519	0.229775368	0.306540771	0.759192942	0.828278014	positive
PRJNA729271	stage_E14_vs_E10	ordinary_muscle_development	ENSGALG00010022525	ISCU	-0.03014765	0.194311123	-0.155151439	0.876701931	0.915349311	negative
PRJNA729271	stage_E14_vs_E10	ordinary_muscle_development	ENSGALG00010022526	PPP1CA	0.233504685	0.137548974	1.697611249	0.089581158	0.155190497	positive
PRJNA729271	stage_E14_vs_E10	ordinary_muscle_development	ENSGALG00010022528	SIDT1	-0.853828731	0.440605283	-1.937854047	0.052641032	0.098902366	negative
PRJNA729271	stage_E14_vs_E10	ordinary_muscle_development	ENSGALG00010022529	CCR7	-0.702232404	0.343626755	-2.043590593	0.040994009	0.079941903	negative
PRJNA729271	stage_E14_vs_E10	ordinary_muscle_development	ENSGALG00010022531	ENSGALG00010022531	-0.53722337	0.392191604	-1.369798241	0.170749891	0.263266399	negative
PRJNA729271	stage_E14_vs_E10	ordinary_muscle_development	ENSGALG00010022532	CBX5	-0.499351855	0.169809528	-2.940658636	0.003275152	0.008982656	negative
PRJNA729271	stage_E14_vs_E10	ordinary_muscle_development	ENSGALG00010022533	TMEM119	-0.754912495	0.132128542	-5.713470244	1.10695E-08	8.01062E-08	negative
PRJNA729271	stage_E14_vs_E10	ordinary_muscle_development	ENSGALG00010022534	CFAP57	-0.538862845	0.49429084	-1.090173642	0.275636662	0.386099093	negative
PRJNA729271	stage_E14_vs_E10	ordinary_muscle_development	ENSGALG00010022536	SMUG1	0.588772446	0.097391772	6.045402355	1.49038E-09	1.19618E-08	positive
PRJNA729271	stage_E14_vs_E10	ordinary_muscle_development	ENSGALG00010022538	WIPF1	1.324461652	0.156373549	8.469857334	2.45694E-17	4.25488E-16	positive
PRJNA729271	stage_E14_vs_E10	ordinary_muscle_development	ENSGALG00010022539	LYSMD2	0.599024278	0.101073701	5.926608723	3.09255E-09	2.3997E-08	positive
PRJNA729271	stage_E14_vs_E10	ordinary_muscle_development	ENSGALG00010022540	GPR146	-0.788790402	0.242900041	-3.247386863	0.0011647	0.003610419	negative
PRJNA729271	stage_E14_vs_E10	ordinary_muscle_development	ENSGALG00010022541	ITPKA	-1.208127779	0.124231657	-9.724798103	2.36374E-22	6.06414E-21	negative
PRJNA729271	stage_E14_vs_E10	ordinary_muscle_development	ENSGALG00010022542	ENSGALG00010022542	0.797980837	0.712017609	1.120731885	0.262402006	0.370942817	positive
PRJNA729271	stage_E14_vs_E10	ordinary_muscle_development	ENSGALG00010022544	SPICE1	0.205128409	0.067445634	3.041389005	0.002354894	0.006712721	positive
PRJNA729271	stage_E14_vs_E10	ordinary_muscle_development	ENSGALG00010022545	CSTF3	-0.036456839	0.064430224	-0.565834438	0.571506334	0.673658966	negative
PRJNA729271	stage_E14_vs_E10	ordinary_muscle_development	ENSGALG00010022546	MTG1	0.657663736	0.156997625	4.189004347	2.80181E-05	0.000121959	positive
PRJNA729271	stage_E14_vs_E10	ordinary_muscle_development	ENSGALG00010022547	SMARCE1	0.040166135	0.061166472	0.656669138	0.511393683	0.620253655	positive
PRJNA729271	stage_E14_vs_E10	ordinary_muscle_development	ENSGALG00010022548	ENSGALG00010022548	0.522411948	0.112790304	4.631709719	3.62658E-06	1.82652E-05	positive
PRJNA729271	stage_E14_vs_E10	ordinary_muscle_development	ENSGALG00010022550	TRIM45	1.490309285	0.157339543	9.471930909	2.74718E-21	6.50659E-20	positive
PRJNA729271	stage_E14_vs_E10	ordinary_muscle_development	ENSGALG00010022551	GPER1	-2.431660604	0.654738746	-3.713940285	0.000204057	0.000756863	negative
PRJNA729271	stage_E14_vs_E10	ordinary_muscle_development	ENSGALG00010022552	GPR155	0.34225286	0.105248388	3.251858467	0.001146531	0.003559427	positive
PRJNA729271	stage_E14_vs_E10	ordinary_muscle_development	ENSGALG00010022553	PRPF6	-0.157819016	0.169293672	-0.932220409	0.35122263	0.467364842	negative
PRJNA729271	stage_E14_vs_E10	ordinary_muscle_development	ENSGALG00010022554	SCG3	-0.863688159	0.347304465	-2.48683287	0.012888593	0.029776882	negative
PRJNA729271	stage_E14_vs_E10	ordinary_muscle_development	ENSGALG00010022557	WNT1	-0.342465732	0.383330269	-0.893396006	0.371645141	0.487800588	negative
PRJNA729271	stage_E14_vs_E10	ordinary_muscle_development	ENSGALG00010022558	NYAP2	-3.218715873	0.703987646	-4.572119826	4.82815E-06	2.38728E-05	negative
PRJNA729271	stage_E14_vs_E10	ordinary_muscle_development	ENSGALG00010022559	SSH1	-0.148329219	0.085964576	-1.725469089	0.08444297	0.147987045	negative
PRJNA729271	stage_E14_vs_E10	ordinary_muscle_development	ENSGALG00010022560	SAMD10	-1.601161139	1.254715546	-1.276114848	0.201914923	0.301261638	negative
PRJNA729271	stage_E14_vs_E10	ordinary_muscle_development	ENSGALG00010022562	GHRH	-3.156769272	0.989772238	-3.189389588	0.001425736	0.004319825	negative
PRJNA729271	stage_E14_vs_E10	ordinary_muscle_development	ENSGALG00010022565	ARF3	-0.551564433	0.161913764	-3.406532089	0.000657938	0.002162543	negative
PRJNA729271	stage_E14_vs_E10	ordinary_muscle_development	ENSGALG00010022567	PAOX	0.466269734	0.123203705	3.784543117	0.000153991	0.000585479	positive
PRJNA729271	stage_E14_vs_E10	ordinary_muscle_development	ENSGALG00010022568	SCRN3	0.329760936	0.167745871	1.965836379	0.049317514	0.093813303	positive
PRJNA729271	stage_E14_vs_E10	ordinary_muscle_development	ENSGALG00010022569	KIAA1549L	-1.512209791	0.21902314	-6.904338	5.04381E-12	5.30551E-11	negative
PRJNA729271	stage_E14_vs_E10	ordinary_muscle_development	ENSGALG00010022571	RIPOR1	0.336545139	0.10724584	3.138071733	0.001700633	0.005052402	positive
PRJNA729271	stage_E14_vs_E10	ordinary_muscle_development	ENSGALG00010022572	ENSGALG00010022572	-3.261972994	0.347786192	-9.379248133	6.64448E-21	1.51911E-19	negative
PRJNA729271	stage_E14_vs_E10	ordinary_muscle_development	ENSGALG00010022573	AP4E1	-0.115039616	0.080913743	-1.421756197	0.15509704	0.243913034	negative
PRJNA729271	stage_E14_vs_E10	ordinary_muscle_development	ENSGALG00010022574	ITPRIPL2	0.323347636	0.103839704	3.113911371	0.001846249	0.005437096	positive
PRJNA729271	stage_E14_vs_E10	ordinary_muscle_development	ENSGALG00010022575	TMEM41B	-0.497729835	0.109878945	-4.529801725	5.90391E-06	2.88041E-05	negative
PRJNA729271	stage_E14_vs_E10	ordinary_muscle_development	ENSGALG00010022578	ZNF512B	0.097017221	0.134180599	0.723034639	0.469658603	0.582023729	positive
PRJNA729271	stage_E14_vs_E10	ordinary_muscle_development	ENSGALG00010022579	TNFAIP8L3	0.303942881	0.146004701	2.081733522	0.037366817	0.073932843	positive
PRJNA729271	stage_E14_vs_E10	ordinary_muscle_development	ENSGALG00010022581	TMEM125	-1.217472369	0.566132269	-2.1505087	0.031514999	0.063970019	negative
PRJNA729271	stage_E14_vs_E10	ordinary_muscle_development	ENSGALG00010022582	ENSGALG00010022582	-0.386287577	0.217332099	-1.777406919	0.075501313	0.134611505	negative
PRJNA729271	stage_E14_vs_E10	ordinary_muscle_development	ENSGALG00010022583	FKBP11	0.303913342	0.115108377	2.640236522	0.008284818	0.020264672	positive
PRJNA729271	stage_E14_vs_E10	ordinary_muscle_development	ENSGALG00010022585	CIR1	-0.273798948	0.093548259	-2.926820365	0.003424466	0.009325124	negative
PRJNA729271	stage_E14_vs_E10	ordinary_muscle_development	ENSGALG00010022587	ENSGALG00010022587	-1.197407333	0.838161771	-1.428611248	0.153116	0.241398348	negative
PRJNA729271	stage_E14_vs_E10	ordinary_muscle_development	ENSGALG00010022588	TCP11L1	-0.050325266	0.076481446	-0.658006208	0.510534142	0.619314787	negative
PRJNA729271	stage_E14_vs_E10	ordinary_muscle_development	ENSGALG00010022590	ENSGALG00010022590	-0.209060031	0.155963889	-1.340438688	0.180102765	0.27497257	negative
PRJNA729271	stage_E14_vs_E10	ordinary_muscle_development	ENSGALG00010022592	CCDC65	0.365246622	0.363993065	1.003443905	0.315646729	0.429645914	positive
PRJNA729271	stage_E14_vs_E10	ordinary_muscle_development	ENSGALG00010022593	MANBAL	-0.418996119	0.132301965	-3.166968235	0.001540371	0.004624624	negative
PRJNA729271	stage_E14_vs_E10	ordinary_muscle_development	ENSGALG00010022597	SLCO4A1	0.236803409	0.239149208	0.990191067	0.322080738	0.436681349	positive

PRJNA729271	stage_E14_vs_E10	ordinary_muscle_development	ENSGALG00010022599	RNLS	-0.543194813	0.26103397	-2.080935337	0.037439825	0.074046973	negative
PRJNA729271	stage_E14_vs_E10	ordinary_muscle_development	ENSGALG00010022603	ATRNL1	-0.156259922	0.168933631	-0.924978177	0.354977259	0.471405967	negative
PRJNA729271	stage_E14_vs_E10	ordinary_muscle_development	ENSGALG00010022608	P3H4	-0.391523088	0.20495044	-1.91033056	0.056090665	0.104476252	negative
PRJNA729271	stage_E14_vs_E10	ordinary_muscle_development	ENSGALG00010022609	RBBP8NL	-2.45823116	0.677369228	-3.629085966	0.000284427	0.001022764	negative
PRJNA729271	stage_E14_vs_E10	ordinary_muscle_development	ENSGALG00010022614	DAO	0.08485168	0.198661682	0.427116491	0.669294482	0.756859379	positive
PRJNA729271	stage_E14_vs_E10	ordinary_muscle_development	ENSGALG00010022616	GLDN	-0.256524634	0.351316227	-0.730181569	0.465279207	0.577663563	negative
PRJNA729271	stage_E14_vs_E10	ordinary_muscle_development	ENSGALG00010022619	MADD	-0.828225022	0.132450119	-6.253108915	4.02361E-10	3.46126E-09	negative
PRJNA729271	stage_E14_vs_E10	ordinary_muscle_development	ENSGALG00010022622	MPPED2	-0.458907669	0.133549564	-3.436234869	0.000589859	0.001962362	negative
PRJNA729271	stage_E14_vs_E10	ordinary_muscle_development	ENSGALG00010022623	TOP1	-0.151300592	0.125714973	-1.203520859	0.228774824	0.332573571	negative
PRJNA729271	stage_E14_vs_E10	ordinary_muscle_development	ENSGALG00010022625	ENSGALG00010022625	0.645051908	0.12086945	5.336765457	9.46193E-08	6.05789E-07	positive
PRJNA729271	stage_E14_vs_E10	ordinary_muscle_development	ENSGALG00010022627	CTCF	-0.116647093	0.097875786	-1.191787043	0.233344757	0.337726636	negative
PRJNA729271	stage_E14_vs_E10	ordinary_muscle_development	ENSGALG00010022628	SVOP	-1.540736582	0.637469311	-2.416958048	0.015650821	0.035202667	negative
PRJNA729271	stage_E14_vs_E10	ordinary_muscle_development	ENSGALG00010022630	ENSGALG00010022630	-1.665689066	0.269114558	-6.189516763	6.03489E-10	5.08876E-09	negative
PRJNA729271	stage_E14_vs_E10	ordinary_muscle_development	ENSGALG00010022637	DMXL2	-0.707262053	0.109416411	-6.463948557	1.02006E-10	9.33441E-10	negative
PRJNA729271	stage_E14_vs_E10	ordinary_muscle_development	ENSGALG00010022642	SEC13	0.107126368	0.067950784	1.576528798	0.114903987	0.191154975	positive
PRJNA729271	stage_E14_vs_E10	ordinary_muscle_development	ENSGALG00010022643	SP3	0.184232375	0.076299447	2.414596452	0.015752652	0.035395239	positive
PRJNA729271	stage_E14_vs_E10	ordinary_muscle_development	ENSGALG00010022644	ARMC8	0.705563959	0.084495488	8.350315224	6.8081E-17	1.13922E-15	positive
PRJNA729271	stage_E14_vs_E10	ordinary_muscle_development	ENSGALG00010022645	ENSGALG00010022645	-0.543814091	0.635880789	-0.855213902	0.392432741	0.507930403	negative
PRJNA729271	stage_E14_vs_E10	ordinary_muscle_development	ENSGALG00010022647	SVIP	-0.625649296	0.133421083	-4.689283611	2.74163E-06	1.41222E-05	negative
PRJNA729271	stage_E14_vs_E10	ordinary_muscle_development	ENSGALG00010022649	PATZ1	-0.690687162	0.076995854	-8.970446201	2.95316E-19	5.99524E-18	negative
PRJNA729271	stage_E14_vs_E10	ordinary_muscle_development	ENSGALG00010022650	NR1H3	0.220191615	0.098935362	2.225610847	0.026040259	0.054576859	positive
PRJNA729271	stage_E14_vs_E10	ordinary_muscle_development	ENSGALG00010022652	CARMIL2	-0.786055349	0.259328728	-3.031115581	0.00243652	0.006896573	negative
PRJNA729271	stage_E14_vs_E10	ordinary_muscle_development	ENSGALG00010022655	NRIP3	-5.590048938	0.510686666	-10.94614234	6.93381E-28	2.59704E-26	negative
PRJNA729271	stage_E14_vs_E10	ordinary_muscle_development	ENSGALG00010022656	FKBP10	-0.493664418	0.210531503	-2.344848205	0.019034827	0.041596536	negative
PRJNA729271	stage_E14_vs_E10	ordinary_muscle_development	ENSGALG00010022657	MRPS35	0.2148146	0.110983155	1.935560403	0.052921564	0.099326437	positive
PRJNA729271	stage_E14_vs_E10	ordinary_muscle_development	ENSGALG00010022659	TIE1	0.337817928	0.151214636	2.234029311	0.025481145	0.053619739	positive
PRJNA729271	stage_E14_vs_E10	ordinary_muscle_development	ENSGALG00010022660	METAP1D	0.139526509	0.182465108	0.764675016	0.444465087	0.558083977	positive
PRJNA729271	stage_E14_vs_E10	ordinary_muscle_development	ENSGALG00010022661	ENSGALG00010022661	-0.980821212	0.372980807	-2.629682797	0.008546457	0.020802837	negative
PRJNA729271	stage_E14_vs_E10	ordinary_muscle_development	ENSGALG00010022662	GFRA1	-2.387250325	0.148217585	-16.10639062	2.30067E-58	4.90415E-56	negative
PRJNA729271	stage_E14_vs_E10	ordinary_muscle_development	ENSGALG00010022664	TRPC4AP	-0.005894494	0.097469273	-0.060475409	0.951777001	0.966580791	negative
PRJNA729271	stage_E14_vs_E10	ordinary_muscle_development	ENSGALG00010022666	ENSGALG00010022666	-0.163779159	0.098837141	-1.657060872	0.097507169	0.166494371	negative
PRJNA729271	stage_E14_vs_E10	ordinary_muscle_development	ENSGALG00010022667	PTEN	-0.105502843	0.041931611	-2.516069403	0.011867181	0.027704105	negative
PRJNA729271	stage_E14_vs_E10	ordinary_muscle_development	ENSGALG00010022669	MANSC4	-6.225374428	1.104193861	-5.637936097	1.721E-08	1.21925E-07	negative
PRJNA729271	stage_E14_vs_E10	ordinary_muscle_development	ENSGALG00010022671	DBR1	-0.178007054	0.054838027	-3.246051416	0.001170177	0.003625848	negative
PRJNA729271	stage_E14_vs_E10	ordinary_muscle_development	ENSGALG00010022673	KLHL42	0.181570676	0.146921662	1.235833255	0.216520551	0.318431959	positive
PRJNA729271	stage_E14_vs_E10	ordinary_muscle_development	ENSGALG00010022674	ENSGALG00010022674	0.836360544	0.355581726	2.352090906	0.018668212	0.040924944	positive
PRJNA729271	stage_E14_vs_E10	ordinary_muscle_development	ENSGALG00010022681	PPP2R3A	0.307185627	0.093037245	3.301748973	0.00096084	0.003040249	positive
PRJNA729271	stage_E14_vs_E10	ordinary_muscle_development	ENSGALG00010022682	EIF1	-0.149428249	0.155975417	-0.958024359	0.338050476	0.453204	negative
PRJNA729271	stage_E14_vs_E10	ordinary_muscle_development	ENSGALG00010022684	ENSGALG00010022684	0.417439105	0.536033803	0.778755189	0.436123939	0.550103688	positive
PRJNA729271	stage_E14_vs_E10	ordinary_muscle_development	ENSGALG00010022685	ATAD1	-0.072672186	0.107984534	-0.672986983	0.500955572	0.610197565	negative
PRJNA729271	stage_E14_vs_E10	ordinary_muscle_development	ENSGALG00010022689	MPL	-0.236108302	0.423279672	-0.557806853	0.576976282	0.678726766	negative
PRJNA729271	stage_E14_vs_E10	ordinary_muscle_development	ENSGALG00010022691	ASIC2	-2.54354956	0.479370033	-5.306025377	1.12041E-07	7.08878E-07	negative
PRJNA729271	stage_E14_vs_E10	ordinary_muscle_development	ENSGALG00010022695	PIK3IP1	-0.782561123	0.204020903	-3.835690902	0.000125212	0.000485409	negative
PRJNA729271	stage_E14_vs_E10	ordinary_muscle_development	ENSGALG00010022698	SEMA6D	-0.194860533	0.11713081	-1.663614657	0.096189461	0.164709538	negative
PRJNA729271	stage_E14_vs_E10	ordinary_muscle_development	ENSGALG00010022702	CDC20	-0.048967782	0.23023537	-0.212685748	0.831572084	0.882468508	negative
PRJNA729271	stage_E14_vs_E10	ordinary_muscle_development	ENSGALG00010022707	SALL1	-2.8747319	0.442008614	-6.503791574	7.83204E-11	7.30537E-10	negative
PRJNA729271	stage_E14_vs_E10	ordinary_muscle_development	ENSGALG00010022708	SPRED1	0.010803201	0.130067313	0.08305854	0.933804992	0.954414283	positive
PRJNA729271	stage_E14_vs_E10	ordinary_muscle_development	ENSGALG00010022709	RNF5	0.058152896	0.082696233	0.703210939	0.481924303	0.593670292	positive
PRJNA729271	stage_E14_vs_E10	ordinary_muscle_development	ENSGALG00010022711	LRRC49	-0.356621157	0.190049103	-1.876468507	0.060590975	0.111525865	negative
PRJNA729271	stage_E14_vs_E10	ordinary_muscle_development	ENSGALG00010022712	DDX23	0.043519985	0.20128644	0.216209225	0.828824663	0.880584438	positive
PRJNA729271	stage_E14_vs_E10	ordinary_muscle_development	ENSGALG00010022713	PNLIPRP1	0.472417208	2.907171448	0.162500636	0.870911628	0.911470328	positive
PRJNA729271	stage_E14_vs_E10	ordinary_muscle_development	ENSGALG00010022717	ELOVL1	0.045981432	0.171052518	0.268814706	0.788072276	0.850883251	positive
PRJNA729271	stage_E14_vs_E10	ordinary_muscle_development	ENSGALG00010022718	C12orf43	-0.029490363	0.128110747	-0.230194298	0.817940792	0.872474191	negative
PRJNA729271	stage_E14_vs_E10	ordinary_muscle_development	ENSGALG00010022719	MSL2	-0.146653333	0.096525895	-1.519315968	0.128682982	0.209156742	negative
PRJNA729271	stage_E14_vs_E10	ordinary_muscle_development	ENSGALG00010022724	LUZP2	-3.050872141	0.415389035	-7.344614049	2.06354E-13	2.53268E-12	negative
PRJNA729271	stage_E14_vs_E10	ordinary_muscle_development	ENSGALG00010022725	KRT222	-3.970157721	0.533217873	-7.445657629	9.64627E-14	1.22437E-12	negative
PRJNA729271	stage_E14_vs_E10	ordinary_muscle_development	ENSGALG00010022727	ACP2	-0.243813903	0.083156038	-2.932004812	0.003367815	0.00919158	negative
PRJNA729271	stage_E14_vs_E10	ordinary_muscle_development	ENSGALG00010022729	ENSGALG00010022729	-0.185762711	0.130552239	-1.422899464	0.154765302	0.243495392	negative
PRJNA729271	stage_E14_vs_E10	ordinary_muscle_development	ENSGALG00010022731	LRRC8B	-0.850259231	0.176158994	-4.826658069	1.38843E-06	7.53193E-06	negative
PRJNA729271	stage_E14_vs_E10	ordinary_muscle_development	ENSGALG00010022733	PCCB	0.404181735	0.114703531	3.523707877	0.000425553	0.001463786	positive
PRJNA729271	stage_E14_vs_E10	ordinary_muscle_development	ENSGALG00010022734	ENSGALG00010022734	0.325771887	0.118967806	2.738319709	0.006175401	0.015638092	positive
PRJNA729271	stage_E14_vs_E10	ordinary_muscle_development	ENSGALG00010022737	CYLD	0.219262139	0.078618912	2.788923608	0.005288354	0.013681008	positive
PRJNA729271	stage_E14_vs_E10	ordinary_muscle_development	ENSGALG00010022742	IFT46	-0.169289764	0.119327889	-1.418694027	0.155988239	0.244967446	negative
PRJNA729271	stage_E14_vs_E10	ordinary_muscle_development	ENSGALG00010022743	ZNF326	-0.534796722	0.151047193	-3.540593578	0.000399228	0.001383742	negative
PRJNA729271	stage_E14_vs_E10	ordinary_muscle_development	ENSGALG00010022745	PAPSS2	-1.80630738	0.238976835	-7.55850408	4.07731E-14	5.33399E-13	negative
PRJNA729271	stage_E14_vs_E10	ordinary_muscle_development	ENSGALG00010022747	NT5C3B	-0.471461418	0.136478734	-3.454467983	0.00055138	0.001847492	negative
PRJNA729271	stage_E14_vs_E10	ordinary_muscle_development	ENSGALG00010022748	ENSGALG00010022748	-0.806704534	0.319417947	-2.525545425	0.011551885	0.02706831	negative
PRJNA729271	stage_E14_vs_E10	ordinary_muscle_development	ENSGALG00010022749	HSPA12A	-0.468998918	0.128545475	-3.648505845	0.00026377	0.000952028	negative
PRJNA729271	stage_E14_vs_E10	ordinary_muscle_development	ENSGALG00010022751	SLC25A12	-0.584119212	0.192758594	-3.03031476	0.00244299	0.006913538	negative
PRJNA729271	stage_E14_vs_E10	ordinary_muscle_development	ENSGALG00010022754	SNX20	-0.777809065	0.186327604	-4.17441672	2.9875E-05	0.000129343	negative
PRJNA729271	stage_E14_vs_E10	ordinary_muscle_development	ENSGALG00010022755	MINPP1	-0.179087152	0.114220672	-1.567904907	0.116903326	0.193592336	negative
PRJNA729271	stage_E14_vs_E10	ordinary_muscle_development	ENSGALG00010022759	THSD4	-0.182094928	0.120101524	-1.516175002	0.129475107	0.210184979	negative
PRJNA729271	stage_E14_vs_E10	ordinary_muscle_development	ENSGALG00010022760	ELAVL4	-0.953446675	0.1095929	-8.69989455	3.32193E-18	6.15746E-17	negative
PRJNA729271	stage_E14_vs_E10	ordinary_muscle_development	ENSGALG00010022764	MFAP5	1.644951406	0.222922088	7.379041802	1.59433E-13	1.97519E-12	positive
PRJNA729271	stage_E14_vs_E10	ordinary_muscle_development	ENSGALG00010022767	CACNB3	-0.28012355	0.459222551	-0.609995195	0.54186499	0.647780044	negative
PRJNA729271	stage_E14_vs_E10	ordinary_muscle_development	ENSGALG00010022768	ENSGALG00010022768	-1.91851917	0.748648248	-2.562644308	0.010387841	0.024587158	negative
PRJNA729271	stage_E14_vs_E10	ordinary_muscle_development	ENSGALG00010022769	FXYD6	1.698856529	0.097027687	17.50898717	1.22352E-68	3.94108E-66	positive
PRJNA729271	stage_E14_vs_E10	ordinary_muscle_development	ENSGALG00010022770	HAT1	-0.391689734	0.08952361	-4.375267435	1.21284E-05	5.61305E-05	negative
PRJNA729271	stage_E14_vs_E10	ordinary_muscle_development	ENSGALG00010022771	SGMS1	0.47675971	0.219637258	2.170668644	0.029956229	0.061364548	positive
PRJNA729271	stage_E14_vs_E10	ordinary_muscle_development	ENSGALG00010022776	CDH4	-1.030277714	0.178306778	-5.778118607	7.55406E-09	5.58084E-08	negative
PRJNA729271	stage_E14_vs_E10	ordinary_muscle_development	ENSGALG00010022780	DYNC1I2	0.02922156	0.056844348	0.514062721	0.60720814	0.704952963	positive
PRJNA729271	stage_E14_vs_E10	ordinary_muscle_development	ENSGALG00010022782	TMPRSS13	-0.362016619	0.235663614	-1.536158311	0.124499558	0.203566959	negative
PRJNA729271	stage_E14_vs_E10	ordinary_muscle_development	ENSGALG00010022783	STAG1	0.265249519	0.054005023	4.911571264	9.03494E-07	5.01192E-06	positive
PRJNA729271	stage_E14_vs_E10	ordinary_muscle_development	ENSGALG00010022784	DMRTA2	-0.694021673	0.604869857	-1.14739008	0.251220439	0.358305864	negative
PRJNA729271	stage_E14_vs_E10	ordinary_muscle_development	ENSGALG00010022786	KRT40	0.776435387	0.529013721	1.467703685	0.142184729	0.227229069	positive
PRJNA729271	stage_E14_vs_E10	ordinary_muscle_development	ENSGALG00010022787	ENO4	-0.847040031	0.299982235	-2.823633974	0.004748258	0.012454939	negative
PRJNA729271	stage_E14_vs_E10	ordinary_muscle_development	ENSGALG00010022788	CYBRD1	1.249976647	0.173459275	7.206167808	5.75485E-13	6.75437E-12	positive
PRJNA729271	stage_E14_vs_E10	ordinary_muscle_development	ENSGALG00010022789	ENSGALG00010022789	-2.59244875	0.349459077	-7.418461614	1.18489E-13	1.48701E-12	negative
PRJNA729271	stage_E14_vs_E10	ordinary_muscle_development	ENSGALG00010022792	DDB2	-0.349292523	0.123878832	-2.819630425	0.004807899	0.012579511	negative
PRJNA729271	stage_E14_vs_E10	ordinary_muscle_development	ENSGALG00010022795	TAF4	0.055665279	0.109865522	0.506667408	0.612388202	0.709066319	positive
PRJNA729271	stage_E14_vs_E10	ordinary_muscle_development	ENSGALG00010022796	RIMKLB	-0.247507045	0.134957785	-1.833959001	0.06666008	0.120961175	negative
PRJNA729271	stage_E14_vs_E10	ordinary_muscle_development	ENSGALG00010022797	EFHD1	0.723733238	0.131767651	5.492495542	3.96294E-08	2.67176E-07	positive
PRJNA729271	stage_E14_vs_E10	ordinary_muscle_development	ENSGALG00010022799	DCAF17	0.270370247	0.150725093	1.793797188	0.072845546	0.130502557	positive
PRJNA729271	stage_E14_vs_E10	ordinary_muscle_development	ENSGALG00010022800	KLHL10	-0.907401748	0.443231395	-2.047241592	0.04063437	0.079315269	negative
PRJNA729271	stage_E14_vs_E10	ordinary_muscle_development	ENSGALG00010022801	LSM14B	0.199027786	0.102013849	1.950987921	0.051058483	0.096517046	positive
PRJNA729271	stage_E14_vs_E10	ordinary_muscle_development	ENSGALG00010022803	ENSGALG00010022803	-0.415239636	0.160522074	-2.586807068	0.009686981	0.023178078	negative
PRJNA729271	stage_E14_vs_E10	ordinary_muscle_development	ENSGALG00010022804	SNX19	0.015226775	0.110984529	0.137197275	0.890874865	0.925016559	positive
PRJNA729271	stage_E14_vs_E10	ordinary_muscle_development	ENSGALG00010022805	ENSGALG00010022805	-0.07965053	0.268895885	-0.296213271	0.767067215	0.834400498	negative
PRJNA729271	stage_E14_vs_E10	ordinary_muscle_development	ENSGALG00010022806	SPPL3	-0.190377057	0.06123679	-3.108867353	0.00187806	0.005513972	negative
PRJNA729271	stage_E14_vs_E10	ordinary_muscle_development	ENSGALG00010022807	ADCY6	-0.322445774	0.147424907	-2.18718655	0.028728908	0.059258641	negative
PRJNA729271	stage_E14_vs_E10	ordinary_muscle_development	ENSGALG00010022808	PSMA7	-0.028764132	0.13661967	-0.210541656	0.833244943	0.883790828	negative
PRJNA729271	stage_E14_vs_E10	ordinary_muscle_development	ENSGALG00010022809	MYO1E	-0.517536978	0.154732135	-3.344728472	0.000823632	0.002650653	negative
PRJNA729271	stage_E14_vs_E10	ordinary_muscle_development	ENSGALG00010022811	GIGYF2	0.200575747	0.097490527	2.057387057	0.039649009	0.077700259	positive
PRJNA729271	stage_E14_vs_E10	ordinary_muscle_development	ENSGALG00010022813	USP24	0.080628577	0.122017324	0.660796144	0.508743056	0.617555531	positive
PRJNA729271	stage_E14_vs_E10	ordinary_muscle_development	ENSGALG00010022815	LRRC8D	-0.462796047	0.237391852	-1.949502655	0.051235426	0.09680103	negative
PRJNA729271	stage_E14_vs_E10	ordinary_muscle_development	ENSGALG00010022816	METTL8	-0.554687611	0.141989801	-3.906531367	9.36305E-05	0.000370408	negative
PRJNA729271	stage_E14_vs_E10	ordinary_muscle_development	ENSGALG00010022817	KRT23	0.908445886	0.660053783	1.376321006	0.168722272	0.260815471	positive
PRJNA729271	stage_E14_vs_E10	ordinary_muscle_development	ENSGALG00010022819	PACSIN3	2.434658155	0.108075088	22.52746857	2.2336E-112	3.5974E-109	positive
PRJNA729271	stage_E14_vs_E10	ordinary_muscle_development	ENSGALG00010022820	SHTN1	-1.368233673	0.169934071	-8.051555921	8.1748E-16	1.23689E-14	negative
PRJNA729271	stage_E14_vs_E10	ordinary_muscle_development	ENSGALG00010022821	ENSGALG00010022821	1.75586958	1.384973763	1.267799887			positive
PRJNA729271	stage_E14_vs_E10	ordinary_muscle_development	ENSGALG00010022823	IGHMBP2	0.20854733	0.113039535	1.844906121	0.065051196	0.118576092	positive
PRJNA729271	stage_E14_vs_E10	ordinary_muscle_development	ENSGALG00010022824	PUS1	-0.25284963	0.081617122	-3.09799739	0.001948331	0.005689175	negative
PRJNA729271	stage_E14_vs_E10	ordinary_muscle_development	ENSGALG00010022825	SS18L1	0.44387025	0.267875411	1.657002587	0.097518952	0.166494371	positive
PRJNA729271	stage_E14_vs_E10	ordinary_muscle_development	ENSGALG00010022826	SNORC	-5.077286579	0.48927307	-10.37720424	3.14861E-25	9.64885E-24	negative
PRJNA729271	stage_E14_vs_E10	ordinary_muscle_development	ENSGALG00010022827	CMTR2	-0.10910848	0.092835956	-1.175282555	0.239881691	0.345634704	negative
PRJNA729271	stage_E14_vs_E10	ordinary_muscle_development	ENSGALG00010022828	PRKG1	-0.091825005	0.169088126	-0.543060046	0.587088461	0.687433259	negative
PRJNA729271	stage_E14_vs_E10	ordinary_muscle_development	ENSGALG00010022830	MTG2	-0.282282217	0.126910699	-2.224258625	0.026131049	0.054727576	negative
PRJNA729271	stage_E14_vs_E10	ordinary_muscle_development	ENSGALG00010022831	LRRC8C	-0.082467225	0.089930611	-0.917009505	0.35913766	0.475536711	negative
PRJNA729271	stage_E14_vs_E10	ordinary_muscle_development	ENSGALG00010022833	HRH3	-0.565376151	0.50698356	-1.115176498	0.264774795	0.373628374	negative
PRJNA729271	stage_E14_vs_E10	ordinary_muscle_development	ENSGALG00010022834	CHRNA7	-0.935955152	0.226687074	-4.128842174	3.64595E-05	0.000155207	negative
PRJNA729271	stage_E14_vs_E10	ordinary_muscle_development	ENSGALG00010022837	ITGB3	0.093639225	0.146549908	0.638957925	0.522850303	0.630844228	positive
PRJNA729271	stage_E14_vs_E10	ordinary_muscle_development	ENSGALG00010022838	ARFGAP2	0.334169249	0.078937912	4.233317538	2.30269E-05	0.000101451	positive
PRJNA729271	stage_E14_vs_E10	ordinary_muscle_development	ENSGALG00010022839	SYT12	-1.530109443	0.294383903	-5.197666818	2.01805E-07	1.23321E-06	negative
PRJNA729271	stage_E14_vs_E10	ordinary_muscle_development	ENSGALG00010022840	MARVELD1	-0.124082795	0.185960937	-0.667251936	0.504611222	0.613610182	negative
PRJNA729271	stage_E14_vs_E10	ordinary_muscle_development	ENSGALG00010022841	ENSGALG00010022841	-4.754171687	0.335575258	-14.16722946	1.46154E-45	1.59286E-43	negative
PRJNA729271	stage_E14_vs_E10	ordinary_muscle_development	ENSGALG00010022842	PHC1	-0.354161465	0.138564789	-2.555926848	0.010590538	0.02502198	negative
PRJNA729271	stage_E14_vs_E10	ordinary_muscle_development	ENSGALG00010022845	HYDIN	1.043051158	0.329716859	3.163475356	0.001558976	0.004669839	positive
PRJNA729271	stage_E14_vs_E10	ordinary_muscle_development	ENSGALG00010022848	SGIP1	-0.653879598	0.670055064	-0.975859497	0.329134096	0.443919114	negative
PRJNA729271	stage_E14_vs_E10	ordinary_muscle_development	ENSGALG00010022850	ULK1	0.617857726	0.25034255	2.468049184	0.013585165	0.031153523	positive
PRJNA729271	stage_E14_vs_E10	ordinary_muscle_development	ENSGALG00010022858	HERC4	0.283404771	0.126993137	2.231654229	0.025637825	0.053911648	positive
PRJNA729271	stage_E14_vs_E10	ordinary_muscle_development	ENSGALG00010022860	MOB3C	-0.241116571	0.126362523	-1.908133557	0.056373957	0.104882621	negative

PRJNA729271	stage_E14_vs_E10	ordinary_muscle_development	ENSGALG00010022862	ACADS	0.524061117	0.122548868	4.276344015	1.89988E-05	8.48127E-05	positive
PRJNA729271	stage_E14_vs_E10	ordinary_muscle_development	ENSGALG00010022863	FAM81A	0.975920968	0.285607832	3.41699652	0.000633161	0.002088682	positive
PRJNA729271	stage_E14_vs_E10	ordinary_muscle_development	ENSGALG00010022864	CSTPP1	-0.300129287	0.192341145	-1.560400852	0.118665184	0.195883366	negative
PRJNA729271	stage_E14_vs_E10	ordinary_muscle_development	ENSGALG00010022866	KLHL11	-0.208212639	0.097757409	-2.129891137	0.033180602	0.06681084	negative
PRJNA729271	stage_E14_vs_E10	ordinary_muscle_development	ENSGALG00010022867	ATPAF1	-0.929913103	0.133981208	-6.940623345	3.90374E-12	4.18669E-11	negative
PRJNA729271	stage_E14_vs_E10	ordinary_muscle_development	ENSGALG00010022868	VAC14	0.310980308	0.098155843	3.168230204	0.0015337	0.004610325	positive
PRJNA729271	stage_E14_vs_E10	ordinary_muscle_development	ENSGALG00010022872	KRT13	-0.005575933	0.463702367	-0.012024811	0.99040582	0.993758406	negative
PRJNA729271	stage_E14_vs_E10	ordinary_muscle_development	ENSGALG00010022873	NGEF	-1.726867119	0.387274481	-4.459026363	8.23328E-06	3.91668E-05	negative
PRJNA729271	stage_E14_vs_E10	ordinary_muscle_development	ENSGALG00010022874	EFCAB14	0.469272621	0.076023151	6.172759427	6.71083E-10	5.6079E-09	positive
PRJNA729271	stage_E14_vs_E10	ordinary_muscle_development	ENSGALG00010022875	SPON1	-2.08522574	0.245042892	-8.509635707	1.74481E-17	3.05816E-16	negative
PRJNA729271	stage_E14_vs_E10	ordinary_muscle_development	ENSGALG00010022877	KYAT3	0.153325639	0.099916575	1.534536586	0.1248977	0.204023994	positive
PRJNA729271	stage_E14_vs_E10	ordinary_muscle_development	ENSGALG00010022879	ENSGALG00010022879	0.387719447	0.279899403	1.385209981	0.16598827	0.257271168	positive
PRJNA729271	stage_E14_vs_E10	ordinary_muscle_development	ENSGALG00010022881	CCNB1	-0.006646711	0.210300572	-0.031605769	0.974786443	0.982882666	negative
PRJNA729271	stage_E14_vs_E10	ordinary_muscle_development	ENSGALG00010022885	M6PR	0.321646508	0.099029593	3.24798376	0.001162259	0.003603625	positive
PRJNA729271	stage_E14_vs_E10	ordinary_muscle_development	ENSGALG00010022886	PLA2G15	-0.586249322	0.281796891	-2.080396697	0.037489162	0.074114212	negative
PRJNA729271	stage_E14_vs_E10	ordinary_muscle_development	ENSGALG00010022888	OSBPL2	0.271460739	0.090732934	2.991865526	0.002772784	0.007748506	positive
PRJNA729271	stage_E14_vs_E10	ordinary_muscle_development	ENSGALG00010022890	MYPN	1.813418798	0.16767219	10.81526281	2.91456E-27	1.0483E-25	positive
PRJNA729271	stage_E14_vs_E10	ordinary_muscle_development	ENSGALG00010022891	NTM	0.948634235	0.202679657	4.680461021	2.86231E-06	1.46968E-05	positive
PRJNA729271	stage_E14_vs_E10	ordinary_muscle_development	ENSGALG00010022900	MMP17	-0.000230665	0.198888341	-0.00115977	0.999074637	0.999350415	negative
PRJNA729271	stage_E14_vs_E10	ordinary_muscle_development	ENSGALG00010022911	SMPD3	-2.215680988	0.336234455	-6.589690476	4.40744E-11	4.25056E-10	negative
PRJNA729271	stage_E14_vs_E10	ordinary_muscle_development	ENSGALG00010022917	CYP4B7	-1.673869387	0.48349019	-3.462054496	0.000536069	0.00180537	negative
PRJNA729271	stage_E14_vs_E10	ordinary_muscle_development	ENSGALG00010022919	ACLY	-0.455171625	0.123845894	-3.675306544	0.000237564	0.000866723	negative
PRJNA729271	stage_E14_vs_E10	ordinary_muscle_development	ENSGALG00010022921	ENSGALG00010022921	0.083341393	0.198332116	0.420211284	0.674331112	0.76071519	positive
PRJNA729271	stage_E14_vs_E10	ordinary_muscle_development	ENSGALG00010022922	ATOH7	-0.510481355	0.441263118	-1.156863863	0.247327985	0.353984018	negative
PRJNA729271	stage_E14_vs_E10	ordinary_muscle_development	ENSGALG00010022923	CD63	0.388438746	0.126594033	3.068381161	0.002152219	0.006203662	positive
PRJNA729271	stage_E14_vs_E10	ordinary_muscle_development	ENSGALG00010022928	RNF111	0.007451167	0.082453146	0.090368496	0.927994391	0.9504189	positive
PRJNA729271	stage_E14_vs_E10	ordinary_muscle_development	ENSGALG00010022929	LRP4	-0.586707314	0.115285027	-5.089189176	3.59598E-07	2.12316E-06	negative
PRJNA729271	stage_E14_vs_E10	ordinary_muscle_development	ENSGALG00010022930	GTF2B	-0.193396954	0.116565541	-1.659126295	0.097090346	0.165918953	negative
PRJNA729271	stage_E14_vs_E10	ordinary_muscle_development	ENSGALG00010022931	RDH5	0.187188448	0.123787547	1.512175112	0.130489321	0.211641793	positive
PRJNA729271	stage_E14_vs_E10	ordinary_muscle_development	ENSGALG00010022932	OPCML	0.940313067	0.175029039	5.372326073	7.77274E-08	5.03872E-07	positive
PRJNA729271	stage_E14_vs_E10	ordinary_muscle_development	ENSGALG00010022933	UNC119B	-0.306121702	0.074654428	-4.100516328	4.12229E-05	0.000174104	negative
PRJNA729271	stage_E14_vs_E10	ordinary_muscle_development	ENSGALG00010022937	BLOC1S1	0.535257983	0.160747828	3.329799162	0.000869086	0.002779044	positive
PRJNA729271	stage_E14_vs_E10	ordinary_muscle_development	ENSGALG00010022942	NEU2	-0.050373366	0.475954102	-0.105836605	0.915711993	0.941364918	negative
PRJNA729271	stage_E14_vs_E10	ordinary_muscle_development	ENSGALG00010022945	KCNA4	-3.08119052	0.374807074	-8.22073738	2.02256E-16	3.21458E-15	negative
PRJNA729271	stage_E14_vs_E10	ordinary_muscle_development	ENSGALG00010022946	KRT17	-1.469850209	0.471490019	-3.117457739	0.001824181	0.005378662	negative
PRJNA729271	stage_E14_vs_E10	ordinary_muscle_development	ENSGALG00010022949	INSC	0.399600906	0.256484321	1.557993504	0.119234788	0.196666847	positive
PRJNA729271	stage_E14_vs_E10	ordinary_muscle_development	ENSGALG00010022950	ITGA7	1.344841548	0.155522324	8.647257253	5.2752E-18	9.65454E-17	positive
PRJNA729271	stage_E14_vs_E10	ordinary_muscle_development	ENSGALG00010022953	ENSGALG00010022953	6.291963547	0.819700751	7.675927515	1.64226E-14	2.22265E-13	positive
PRJNA729271	stage_E14_vs_E10	ordinary_muscle_development	ENSGALG00010022954	ADRM1	0.058995205	0.143353942	0.411535283	0.680680075	0.766445871	positive
PRJNA729271	stage_E14_vs_E10	ordinary_muscle_development	ENSGALG00010022956	PBLD	0.100851111	0.122561864	0.822858823	0.410588281	0.525415372	positive
PRJNA729271	stage_E14_vs_E10	ordinary_muscle_development	ENSGALG00010022957	SLTM	-0.176604776	0.14262855	-1.238214765	0.215636438	0.317324891	negative
PRJNA729271	stage_E14_vs_E10	ordinary_muscle_development	ENSGALG00010022958	SFSWAP	0.035427265	0.096924261	0.365514939	0.714727057	0.793684877	positive
PRJNA729271	stage_E14_vs_E10	ordinary_muscle_development	ENSGALG00010022959	ADAMTS8	0.82720211	0.263684225	3.137093661	0.001706316	0.005066172	positive
PRJNA729271	stage_E14_vs_E10	ordinary_muscle_development	ENSGALG00010022967	ADAMTS15	0.021204356	0.161629937	0.131190773	0.895624395	0.928253029	positive
PRJNA729271	stage_E14_vs_E10	ordinary_muscle_development	ENSGALG00010022969	TTN	1.994717555	0.575498355	3.466069951	0.000528126	0.001781114	positive
PRJNA729271	stage_E14_vs_E10	ordinary_muscle_development	ENSGALG00010022971	CKAP5	-0.070370511	0.057659259	-1.220454667	0.222292565	0.325008143	negative
PRJNA729271	stage_E14_vs_E10	ordinary_muscle_development	ENSGALG00010022972	ENSGALG00010022972	-1.340432958	1.09335623	-1.225980079			negative
PRJNA729271	stage_E14_vs_E10	ordinary_muscle_development	ENSGALG00010022974	INPP5D	0.614484096	0.155550983	3.950371036	7.80301E-05	0.000313309	positive
PRJNA729271	stage_E14_vs_E10	ordinary_muscle_development	ENSGALG00010022976	MINDY2	0.19326304	0.064775894	2.983564223	0.002849122	0.007937349	positive
PRJNA729271	stage_E14_vs_E10	ordinary_muscle_development	ENSGALG00010022978	ARL14EP	0.080070522	0.084981472	0.942211528	0.346084353	0.461583796	positive
PRJNA729271	stage_E14_vs_E10	ordinary_muscle_development	ENSGALG00010022979	LAMA5	0.582684511	0.148541167	3.922713964	8.75571E-05	0.000348282	positive
PRJNA729271	stage_E14_vs_E10	ordinary_muscle_development	ENSGALG00010022981	HNRNPH3	-0.085255028	0.129317706	-0.659268021	0.509723674	0.618486912	negative
PRJNA729271	stage_E14_vs_E10	ordinary_muscle_development	ENSGALG00010022983	PKN2	0.122410207	0.060347645	2.028417286	0.042517677	0.082568828	positive
PRJNA729271	stage_E14_vs_E10	ordinary_muscle_development	ENSGALG00010022986	ALG9	0.014888804	0.100731062	0.147807475	0.88249471	0.919410682	positive
PRJNA729271	stage_E14_vs_E10	ordinary_muscle_development	ENSGALG00010022987	F2	2.085432208	1.387610444	1.502894575			positive
PRJNA729271	stage_E14_vs_E10	ordinary_muscle_development	ENSGALG00010022990	SLC25A22	0.314159484	0.096333946	3.261150386	0.001109612	0.003455171	positive
PRJNA729271	stage_E14_vs_E10	ordinary_muscle_development	ENSGALG00010022991	TAL1	0.398386583	0.175779154	2.266404033	0.023426657	0.049892652	positive
PRJNA729271	stage_E14_vs_E10	ordinary_muscle_development	ENSGALG00010022992	SLC7A6	-0.463241718	0.107278819	-4.318109806	1.57371E-05	7.14853E-05	negative
PRJNA729271	stage_E14_vs_E10	ordinary_muscle_development	ENSGALG00010022994	LIPC	-2.686820223	0.867141272	-3.098480384	0.001945158	0.005682199	negative
PRJNA729271	stage_E14_vs_E10	ordinary_muscle_development	ENSGALG00010022997	ARHGAP1	-0.194019161	0.093654105	-2.071656783	0.03829746	0.075506214	negative
PRJNA729271	stage_E14_vs_E10	ordinary_muscle_development	ENSGALG00010022999	ENSGALG00010022999	1.114739505	0.489142028	2.278968972	0.02266891	0.048521242	positive
PRJNA729271	stage_E14_vs_E10	ordinary_muscle_development	ENSGALG00010023002	ATG16L1	0.129206621	0.134109385	0.963442049	0.335325733	0.450634758	positive
PRJNA729271	stage_E14_vs_E10	ordinary_muscle_development	ENSGALG00010023003	ENSGALG00010023003	-5.008296132	0.613290227	-8.166274155	3.1806E-16	4.97333E-15	negative
PRJNA729271	stage_E14_vs_E10	ordinary_muscle_development	ENSGALG00010023005	IRF7	0.013809771	0.356931595	0.038690246	0.96913735	0.978861814	positive
PRJNA729271	stage_E14_vs_E10	ordinary_muscle_development	ENSGALG00010023007	LMO4	-0.367019704	0.142149658	-2.581924646	0.009825103	0.02347369	negative
PRJNA729271	stage_E14_vs_E10	ordinary_muscle_development	ENSGALG00010023008	CDHR5	0.283233281	0.840584716	0.336947932	0.736156152	0.810526655	positive
PRJNA729271	stage_E14_vs_E10	ordinary_muscle_development	ENSGALG00010023012	ENSGALG00010023012	-0.649515667	0.557193397	-1.165691608	0.243739154	0.350148567	negative
PRJNA729271	stage_E14_vs_E10	ordinary_muscle_development	ENSGALG00010023013	ENSGALG00010023013	-0.815505435	0.460722451	-1.770057945	0.076717488	0.136393965	negative
PRJNA729271	stage_E14_vs_E10	ordinary_muscle_development	ENSGALG00010023015	METTL2B	0.130327735	0.099723743	1.306887716	0.191250848	0.288588491	positive
PRJNA729271	stage_E14_vs_E10	ordinary_muscle_development	ENSGALG00010023017	BUB1B	-0.158054781	0.19808857	-0.797899553	0.424928783	0.539250735	negative
PRJNA729271	stage_E14_vs_E10	ordinary_muscle_development	ENSGALG00010023019	CABP1	-0.045491721	0.311060895	-0.146246993	0.883726409	0.920188664	negative
PRJNA729271	stage_E14_vs_E10	ordinary_muscle_development	ENSGALG00010023024	PRMT7	-0.071555	0.111297174	-0.642918397	0.520277051	0.628189576	negative
PRJNA729271	stage_E14_vs_E10	ordinary_muscle_development	ENSGALG00010023026	PHRF1	0.039065974	0.091005279	0.42927151	0.667725648	0.755497874	positive
PRJNA729271	stage_E14_vs_E10	ordinary_muscle_development	ENSGALG00010023028	TMEM61	-1.025076721	0.268713099	-3.814762748	0.000136314	0.000524381	negative
PRJNA729271	stage_E14_vs_E10	ordinary_muscle_development	ENSGALG00010023030	HS2ST1	-0.343138992	0.084738115	-4.049405542	5.13479E-05	0.000213263	negative
PRJNA729271	stage_E14_vs_E10	ordinary_muscle_development	ENSGALG00010023041	CD151	0.625115868	0.119367966	5.236881269	1.63313E-07	1.0069E-06	positive
PRJNA729271	stage_E14_vs_E10	ordinary_muscle_development	ENSGALG00010023042	ENSGALG00010023042	-0.105234402	0.635151515	-0.165683935	0.868405689	0.909714351	negative
PRJNA729271	stage_E14_vs_E10	ordinary_muscle_development	ENSGALG00010023043	CNP	0.761684911	0.145089323	5.249765433	1.52293E-07	9.44178E-07	positive
PRJNA729271	stage_E14_vs_E10	ordinary_muscle_development	ENSGALG00010023046	SLC7A6OS	-0.086207369	0.07113226	-1.211930685	0.225538915	0.32862752	negative
PRJNA729271	stage_E14_vs_E10	ordinary_muscle_development	ENSGALG00010023048	ARFGAP1	0.054245824	0.082873199	0.654564136	0.512748421	0.621532728	positive
PRJNA729271	stage_E14_vs_E10	ordinary_muscle_development	ENSGALG00010023050	RUFY2	0.112281434	0.090865397	1.235689695	0.216573929	0.318478148	positive
PRJNA729271	stage_E14_vs_E10	ordinary_muscle_development	ENSGALG00010023052	RAD54L	-0.147110122	0.12488166	-1.177996211	0.238798117	0.344347264	negative
PRJNA729271	stage_E14_vs_E10	ordinary_muscle_development	ENSGALG00010023053	MAN1A2	-0.759998278	0.046707161	-16.27155784	1.57109E-59	3.61476E-57	negative
PRJNA729271	stage_E14_vs_E10	ordinary_muscle_development	ENSGALG00010023054	AQP9	2.651934076	0.229431885	11.5586989	6.67119E-31	2.99377E-29	positive
PRJNA729271	stage_E14_vs_E10	ordinary_muscle_development	ENSGALG00010023056	SAG	0.176350066	0.450036988	0.391856827	0.695164004	0.777860294	positive
PRJNA729271	stage_E14_vs_E10	ordinary_muscle_development	ENSGALG00010023057	PAFAH1B2	-0.291802982	0.07424186	-3.930437374	8.47915E-05	0.00033793	negative
PRJNA729271	stage_E14_vs_E10	ordinary_muscle_development	ENSGALG00010023058	PPARA	0.74367243	0.203397384	3.656253661	0.000255928	0.000927188	positive
PRJNA729271	stage_E14_vs_E10	ordinary_muscle_development	ENSGALG00010023059	SELENOF	-0.055076994	0.092706005	-0.594103848	0.552442657	0.657227209	negative
PRJNA729271	stage_E14_vs_E10	ordinary_muscle_development	ENSGALG00010023060	ALDH1A2	0.071294082	0.167049579	0.426783964	0.669536687	0.75695611	positive
PRJNA729271	stage_E14_vs_E10	ordinary_muscle_development	ENSGALG00010023062	STX2	0.217325365	0.103132555	2.107243093	0.035096513	0.070081824	positive
PRJNA729271	stage_E14_vs_E10	ordinary_muscle_development	ENSGALG00010023063	ENSGALG00010023063	-0.316223581	0.103605426	-3.052191313	0.002271773	0.00650649	negative
PRJNA729271	stage_E14_vs_E10	ordinary_muscle_development	ENSGALG00010023064	ATG13	0.195832232	0.068475932	2.85986954	0.004238153	0.011247168	positive
PRJNA729271	stage_E14_vs_E10	ordinary_muscle_development	ENSGALG00010023065	DNA2	-0.094069993	0.221875085	-0.423977271	0.671582389	0.758322563	negative
PRJNA729271	stage_E14_vs_E10	ordinary_muscle_development	ENSGALG00010023066	TLR21	0.291623368	0.188618661	1.546100297	0.122080347	0.20037987	positive
PRJNA729271	stage_E14_vs_E10	ordinary_muscle_development	ENSGALG00010023068	CDPF1	-0.11653875	0.122661217	-0.950086367	0.34206837	0.457110816	negative
PRJNA729271	stage_E14_vs_E10	ordinary_muscle_development	ENSGALG00010023069	ENSGALG00010023069	1.166717801	0.260879416	4.472249353	7.74011E-06	3.6942E-05	positive
PRJNA729271	stage_E14_vs_E10	ordinary_muscle_development	ENSGALG00010023070	SIDT2	0.027120906	0.202791026	0.133738195	0.893609602	0.926926519	positive
PRJNA729271	stage_E14_vs_E10	ordinary_muscle_development	ENSGALG00010023071	POLR2M	-0.092592623	0.081891749	-1.130670956	0.25819361	0.366410454	negative
PRJNA729271	stage_E14_vs_E10	ordinary_muscle_development	ENSGALG00010023073	POMGNT1	0.224851492	0.08504279	2.643980672	0.008193733	0.02006219	positive
PRJNA729271	stage_E14_vs_E10	ordinary_muscle_development	ENSGALG00010023074	CCNT1	0.169734027	0.164660076	1.030814701	0.302627723	0.416303392	positive
PRJNA729271	stage_E14_vs_E10	ordinary_muscle_development	ENSGALG00010023075	PKDREJ	-0.794631417	0.256336354	-3.099955991	0.001935494	0.005660812	negative
PRJNA729271	stage_E14_vs_E10	ordinary_muscle_development	ENSGALG00010023076	DNAJC7	0.533181095	0.079901962	6.672941221	2.50727E-11	2.46559E-10	positive
PRJNA729271	stage_E14_vs_E10	ordinary_muscle_development	ENSGALG00010023077	KANSL2	-0.057396732	0.151111693	-0.379829854	0.70407172	0.784858847	negative
PRJNA729271	stage_E14_vs_E10	ordinary_muscle_development	ENSGALG00010023078	TAGLN	0.091365483	0.201452528	0.453533564	0.650164581	0.741558478	positive
PRJNA729271	stage_E14_vs_E10	ordinary_muscle_development	ENSGALG00010023079	SLC25A16	-0.105113987	0.095843416	-1.096726215	0.272761093	0.382848073	negative
PRJNA729271	stage_E14_vs_E10	ordinary_muscle_development	ENSGALG00010023080	MCRS1	0.174596268	0.111606788	1.564387537	0.117726586	0.194710961	positive
PRJNA729271	stage_E14_vs_E10	ordinary_muscle_development	ENSGALG00010023081	PRLHR2	-1.000604979	0.635454764	-1.574628181	0.115342294	0.191708124	negative
PRJNA729271	stage_E14_vs_E10	ordinary_muscle_development	ENSGALG00010023083	HARBI1	-0.18706406	0.155948942	-1.199521189	0.230325351	0.334291174	negative
PRJNA729271	stage_E14_vs_E10	ordinary_muscle_development	ENSGALG00010023084	ENSGALG00010023084	0.084759	0.241433077	0.35106623	0.725538662	0.801357961	positive
PRJNA729271	stage_E14_vs_E10	ordinary_muscle_development	ENSGALG00010023086	PCSK7	-0.083659454	0.091149924	-0.917822533	0.358711784	0.475103006	negative
PRJNA729271	stage_E14_vs_E10	ordinary_muscle_development	ENSGALG00010023087	SH3GLB1	0.447866795	0.117806497	3.80171559	0.000143698	0.000549867	positive
PRJNA729271	stage_E14_vs_E10	ordinary_muscle_development	ENSGALG00010023089	ENSGALG00010023089	0.990953534	0.727387832	1.362345493	0.173088877	0.266114051	positive
PRJNA729271	stage_E14_vs_E10	ordinary_muscle_development	ENSGALG00010023090	LRRC41	0.225205648	0.08837537	2.548285225	0.010825392	0.025522781	positive
PRJNA729271	stage_E14_vs_E10	ordinary_muscle_development	ENSGALG00010023091	ENSGALG00010023091	-0.283998061	0.131726844	-2.155961935	0.031086638	0.063215602	negative
PRJNA729271	stage_E14_vs_E10	ordinary_muscle_development	ENSGALG00010023093	POP5	0.149663294	0.127004382	1.178410474	0.238633004	0.344143408	positive
PRJNA729271	stage_E14_vs_E10	ordinary_muscle_development	ENSGALG00010023094	CCDC32	0.009508861	0.101166071	0.093992593	0.925115041	0.94847521	positive
PRJNA729271	stage_E14_vs_E10	ordinary_muscle_development	ENSGALG00010023096	SPATS2	-0.120731178	0.170272497	-0.709046853	0.478295402	0.590435348	negative
PRJNA729271	stage_E14_vs_E10	ordinary_muscle_development	ENSGALG00010023097	LURAP1	0.763862538	0.20832247	3.666731382	0.000245671	0.0008936	positive
PRJNA729271	stage_E14_vs_E10	ordinary_muscle_development	ENSGALG00010023101	CLCA2	1.225317666	1.191501637	1.028381018	0.303770634	0.41731018	positive
PRJNA729271	stage_E14_vs_E10	ordinary_muscle_development	ENSGALG00010023102	RNF10	0.148157181	0.101474828	1.460038752	0.144279424	0.229993429	positive
PRJNA729271	stage_E14_vs_E10	ordinary_muscle_development	ENSGALG00010023103	DGKD	0.094404564	0.125142199	0.75437834	0.450622074	0.56379864	positive
PRJNA729271	stage_E14_vs_E10	ordinary_muscle_development	ENSGALG00010023104	ENSGALG00010023104	0.96797369	0.157002391	6.165343628	7.03302E-10	5.85547E-09	positive
PRJNA729271	stage_E14_vs_E10	ordinary_muscle_development	ENSGALG00010023107	ZC3HAV1	-0.187542262	0.292115217	-0.642014695	0.520863642	0.628688357	negative
PRJNA729271	stage_E14_vs_E10	ordinary_muscle_development	ENSGALG00010023108	TET1	0.156423933	0.061002008	2.564242379	0.010340131	0.024494231	positive
PRJNA729271	stage_E14_vs_E10	ordinary_muscle_development	ENSGALG00010023110	LIMA1	-0.195690305	0.154401693	-1.267410359	0.205008598	0.304873256	negative
PRJNA729271	stage_E14_vs_E10	ordinary_muscle_development	ENSGALG00010023112	PCMT1L	0.204493797	0.133377429	1.533196417	0.125227467	0.204526438	positive
PRJNA729271	stage_E14_vs_E10	ordinary_muscle_development	ENSGALG00010023113	MYZAP	-0.864422051	0.13331195	-6.484205287	8.92007E-11	8.22496E-10	negative
PRJNA729271	stage_E14_vs_E10	ordinary_muscle_development	ENSGALG00010023114	UQCRHL	0.60120704	0.16742617	3.590878535	0.000329565	0.00116884	positive
PRJNA729271	stage_E14_vs_E10	ordinary_muscle_development	ENSGALG00010023115	RPLP2	0.371953747	0.136310819	2.728717713	0.00635811	0.016039125	positive
PRJNA729271	stage_E14_vs_E10	ordinary_muscle_development	ENSGALG00010023116	PROC	1.529680442	1.071158231	1.428062071	0.153273994	0.241621157	positive
PRJNA729271	stage_E14_vs_E10	ordinary_muscle_development	ENSGALG00010023117	RPUSD2	-0.230895889	0.101259493	-2.280239438	0.022593491	0.048395546	negative

PRJNA729271	stage_E14_vs_E10	ordinary_muscle_development	ENSGALG00010023118	PIDD1	-0.200099395	0.13980058	-1.431320205	0.152338466	0.240407846	negative
PRJNA729271	stage_E14_vs_E10	ordinary_muscle_development	ENSGALG00010023119	NDRG4	-0.948257698	0.172405636	-5.500154876	3.79458E-08	2.56661E-07	negative
PRJNA729271	stage_E14_vs_E10	ordinary_muscle_development	ENSGALG00010023120	CHRM4	0.206274756	0.239440595	0.861486151	0.388970345	0.50480125	positive
PRJNA729271	stage_E14_vs_E10	ordinary_muscle_development	ENSGALG00010023122	ENSGALG00010023122	0.155866386	0.70486358	0.221129861	0.82499132	0.877412076	positive
PRJNA729271	stage_E14_vs_E10	ordinary_muscle_development	ENSGALG00010023123	RNF214	-0.011777348	0.14276405	-0.082495192	0.934252942	0.954535588	negative
PRJNA729271	stage_E14_vs_E10	ordinary_muscle_development	ENSGALG00010023124	NSUN4	0.318176426	0.135084438	2.355389202	0.018503314	0.040624892	positive
PRJNA729271	stage_E14_vs_E10	ordinary_muscle_development	ENSGALG00010023125	COL20A1	3.65099081	0.282157777	12.93953633	2.6925E-38	1.8854E-36	positive
PRJNA729271	stage_E14_vs_E10	ordinary_muscle_development	ENSGALG00010023126	CGNL1	0.063474689	0.13711878	0.462917545	0.643423471	0.735696396	positive
PRJNA729271	stage_E14_vs_E10	ordinary_muscle_development	ENSGALG00010023127	CERS5	-0.213903543	0.09596401	-2.22899756	0.025814067	0.05420468	negative
PRJNA729271	stage_E14_vs_E10	ordinary_muscle_development	ENSGALG00010023128	IWS1	-0.005645951	0.136969556	-0.041220483	0.967120124	0.978051085	negative
PRJNA729271	stage_E14_vs_E10	ordinary_muscle_development	ENSGALG00010023130	DYNLT5	0.300649555	0.479850677	0.626548153	0.530955463	0.638476807	positive
PRJNA729271	stage_E14_vs_E10	ordinary_muscle_development	ENSGALG00010023131	SOX6	0.369409263	0.136983082	2.696751002	0.007001961	0.017431343	positive
PRJNA729271	stage_E14_vs_E10	ordinary_muscle_development	ENSGALG00010023134	KNL1	-0.267053053	0.214449827	-1.245293857	0.213023753	0.314469834	negative
PRJNA729271	stage_E14_vs_E10	ordinary_muscle_development	ENSGALG00010023135	GINS3	-0.293951201	0.065272655	-4.503435664	6.68636E-06	3.23494E-05	negative
PRJNA729271	stage_E14_vs_E10	ordinary_muscle_development	ENSGALG00010023136	MDK	-1.296682045	0.404954105	-3.202046924	0.001364548	0.004157024	negative
PRJNA729271	stage_E14_vs_E10	ordinary_muscle_development	ENSGALG00010023137	ETV6	0.094671133	0.111764801	0.84705678	0.396963474	0.512475204	positive
PRJNA729271	stage_E14_vs_E10	ordinary_muscle_development	ENSGALG00010023140	CCAR1	-0.312673598	0.083177745	-3.759101632	0.000170525	0.000643016	negative
PRJNA729271	stage_E14_vs_E10	ordinary_muscle_development	ENSGALG00010023142	TLK2	-0.055226195	0.168421914	-0.327903852	0.742984361	0.815566703	negative
PRJNA729271	stage_E14_vs_E10	ordinary_muscle_development	ENSGALG00010023144	C11orf58	0.034460212	0.106076241	0.32486268	0.745285003	0.817349332	positive
PRJNA729271	stage_E14_vs_E10	ordinary_muscle_development	ENSGALG00010023147	RAD51	-0.430715364	0.186620995	-2.307968429	0.021000893	0.045373072	negative
PRJNA729271	stage_E14_vs_E10	ordinary_muscle_development	ENSGALG00010023149	ADGRD1	-0.644440865	0.184532001	-3.492298691	0.000478882	0.001632119	negative
PRJNA729271	stage_E14_vs_E10	ordinary_muscle_development	ENSGALG00010023151	ENSGALG00010023151	-1.58304124	0.504919245	-3.135236489	0.001717156	0.005092099	negative
PRJNA729271	stage_E14_vs_E10	ordinary_muscle_development	ENSGALG00010023155	CFAP263	0.634270104	0.310425062	2.043230978	0.041029579	0.079987853	positive
PRJNA729271	stage_E14_vs_E10	ordinary_muscle_development	ENSGALG00010023157	STOX1	-2.722386712	0.363979318	-7.479509351	7.46006E-14	9.58631E-13	negative
PRJNA729271	stage_E14_vs_E10	ordinary_muscle_development	ENSGALG00010023158	ENSGALG00010023158	-0.824126087	0.61498931	-1.340065713	0.180223982	0.275099686	negative
PRJNA729271	stage_E14_vs_E10	ordinary_muscle_development	ENSGALG00010023159	DAW1	-2.156964446	0.579899183	-3.719550755	0.000199577	0.000741763	negative
PRJNA729271	stage_E14_vs_E10	ordinary_muscle_development	ENSGALG00010023160	ENSGALG00010023160	-0.170417573	0.108810228	-1.566190754	0.117303967	0.194160786	negative
PRJNA729271	stage_E14_vs_E10	ordinary_muscle_development	ENSGALG00010023161	ODF2L	-1.10678735	0.406737043	-2.721137326	0.006505773	0.016343358	negative
PRJNA729271	stage_E14_vs_E10	ordinary_muscle_development	ENSGALG00010023162	CHRNA4	0.401900975	0.259348497	1.549656077	0.121224086	0.199267762	positive
PRJNA729271	stage_E14_vs_E10	ordinary_muscle_development	ENSGALG00010023163	CRTAC1	-0.985715694	0.280400564	-3.515384132	0.000439118	0.001507227	negative
PRJNA729271	stage_E14_vs_E10	ordinary_muscle_development	ENSGALG00010023164	NKIRAS2	0.723104071	0.132371763	5.462676138	4.6901E-08	3.12709E-07	positive
PRJNA729271	stage_E14_vs_E10	ordinary_muscle_development	ENSGALG00010023165	CSNK2A2	0.476681459	0.128941568	3.696879641	0.000218266	0.000802782	positive
PRJNA729271	stage_E14_vs_E10	ordinary_muscle_development	ENSGALG00010023166	KDM2A	-0.038433757	0.112130159	-0.342760208	0.731778864	0.806810271	negative
PRJNA729271	stage_E14_vs_E10	ordinary_muscle_development	ENSGALG00010023167	GPD1	2.219717715	0.218282459	10.16901553	2.7265E-24	7.71888E-23	positive
PRJNA729271	stage_E14_vs_E10	ordinary_muscle_development	ENSGALG00010023168	SPHKAP	-1.020645919	0.266184386	-3.834356837	0.000125893	0.00048779	negative
PRJNA729271	stage_E14_vs_E10	ordinary_muscle_development	ENSGALG00010023169	RAN	-0.215201531	0.096627341	-2.227128771	0.025938669	0.054387783	negative
PRJNA729271	stage_E14_vs_E10	ordinary_muscle_development	ENSGALG00010023170	TCF12	0.068627262	0.064633127	1.061797022	0.288327842	0.40062424	positive
PRJNA729271	stage_E14_vs_E10	ordinary_muscle_development	ENSGALG00010023171	ENSGALG00010023171	-0.332638619	0.113984703	-2.91827421	0.003519747	0.009555859	negative
PRJNA729271	stage_E14_vs_E10	ordinary_muscle_development	ENSGALG00010023172	DHX58	0.620864714	0.334723686	1.854857424	0.063616589	0.116253462	positive
PRJNA729271	stage_E14_vs_E10	ordinary_muscle_development	ENSGALG00010023174	KCNQ2	-0.798031308	0.31079727	-2.567690851	0.010237842	0.024279698	negative
PRJNA729271	stage_E14_vs_E10	ordinary_muscle_development	ENSGALG00010023175	GRK2	0.035697839	0.136613967	0.261304459	0.793857728	0.855173107	positive
PRJNA729271	stage_E14_vs_E10	ordinary_muscle_development	ENSGALG00010023177	LZTS2	0.334151761	0.17040814	1.960890841	0.049891758	0.09463243	positive
PRJNA729271	stage_E14_vs_E10	ordinary_muscle_development	ENSGALG00010023178	CFAP20	0.088056595	0.097211344	0.905826323	0.365027786	0.481050801	positive
PRJNA729271	stage_E14_vs_E10	ordinary_muscle_development	ENSGALG00010023179	KAT2A	-0.408262459	0.125970389	-3.240939886	0.001191363	0.003687552	negative
PRJNA729271	stage_E14_vs_E10	ordinary_muscle_development	ENSGALG00010023180	EEF1A2	3.696882852	0.121708634	30.37486119	1.1801E-202	1.7106E-198	positive
PRJNA729271	stage_E14_vs_E10	ordinary_muscle_development	ENSGALG00010023181	PLEKHA7	0.389016867	0.163338528	2.381660177	0.017234794	0.038266689	positive
PRJNA729271	stage_E14_vs_E10	ordinary_muscle_development	ENSGALG00010023182	EPHB6	-1.062457761	0.139438707	-7.619532523	2.54596E-14	3.39813E-13	negative
PRJNA729271	stage_E14_vs_E10	ordinary_muscle_development	ENSGALG00010023183	ENSGALG00010023183	5.353905775	0.405897152	13.19030141	9.97855E-40	7.37954E-38	positive
PRJNA729271	stage_E14_vs_E10	ordinary_muscle_development	ENSGALG00010023184	SMARCD1	-0.16059795	0.051143156	-3.140165033	0.001688527	0.005019899	negative
PRJNA729271	stage_E14_vs_E10	ordinary_muscle_development	ENSGALG00010023185	FBXW4	1.006762706	0.117411253	8.574669646	9.93716E-18	1.77388E-16	positive
PRJNA729271	stage_E14_vs_E10	ordinary_muscle_development	ENSGALG00010023186	MIER1	-0.265105952	0.098404331	-2.694047615	0.007059008	0.017556678	negative
PRJNA729271	stage_E14_vs_E10	ordinary_muscle_development	ENSGALG00010023187	ENSGALG00010023187	0.686252878	0.23247689	2.951918697	0.003158061	0.008702679	positive
PRJNA729271	stage_E14_vs_E10	ordinary_muscle_development	ENSGALG00010023188	KIF1BP	0.300039996	0.056992931	5.264512494	1.40562E-07	8.75191E-07	positive
PRJNA729271	stage_E14_vs_E10	ordinary_muscle_development	ENSGALG00010023189	RMDN3	-0.18567087	0.083367887	-2.227126978	0.025938789	0.054387783	negative
PRJNA729271	stage_E14_vs_E10	ordinary_muscle_development	ENSGALG00010023190	SF3B3	-0.136092256	0.055402573	-2.456424832	0.014032716	0.032025339	negative
PRJNA729271	stage_E14_vs_E10	ordinary_muscle_development	ENSGALG00010023193	PPDPF	0.426693874	0.314119207	1.358381993	0.174342505	0.2677007	positive
PRJNA729271	stage_E14_vs_E10	ordinary_muscle_development	ENSGALG00010023194	MRC2	-0.426350821	0.193248702	-2.20622864	0.027367995	0.056874421	negative
PRJNA729271	stage_E14_vs_E10	ordinary_muscle_development	ENSGALG00010023195	ENSGALG00010023195	-2.067087498	0.184667748	-11.19354909	4.38522E-29	1.79053E-27	negative
PRJNA729271	stage_E14_vs_E10	ordinary_muscle_development	ENSGALG00010023196	DGKZ	0.064865995	0.128029129	0.506650284	0.612400219	0.709066319	positive
PRJNA729271	stage_E14_vs_E10	ordinary_muscle_development	ENSGALG00010023198	PTK6	1.416783056	0.423787369	3.343146019	0.000828343	0.002664632	positive
PRJNA729271	stage_E14_vs_E10	ordinary_muscle_development	ENSGALG00010023199	RPS13	0.429158075	0.196657419	2.182262312	0.029090179	0.059844187	positive
PRJNA729271	stage_E14_vs_E10	ordinary_muscle_development	ENSGALG00010023200	VPS26A	0.05252283	0.08220414	0.63893169	0.522867372	0.630844228	positive
PRJNA729271	stage_E14_vs_E10	ordinary_muscle_development	ENSGALG00010023201	ZNF280D	-0.276440074	0.117772463	-2.34723863	0.018913137	0.041416802	negative
PRJNA729271	stage_E14_vs_E10	ordinary_muscle_development	ENSGALG00010023202	PID1	-0.267660946	0.156389543	-1.711501556	0.086988564	0.151605054	negative
PRJNA729271	stage_E14_vs_E10	ordinary_muscle_development	ENSGALG00010023203	COG4	-0.055103113	0.062559158	-0.880816096	0.378417366	0.494604123	negative
PRJNA729271	stage_E14_vs_E10	ordinary_muscle_development	ENSGALG00010023204	SRGN	0.681878204	0.35373618	1.9276462	0.053899148	0.101004286	positive
PRJNA729271	stage_E14_vs_E10	ordinary_muscle_development	ENSGALG00010023205	DNAJC17	0.044745051	0.108481361	0.41246764	0.679996694	0.765854863	positive
PRJNA729271	stage_E14_vs_E10	ordinary_muscle_development	ENSGALG00010023206	HSPB9	-0.719146301	0.318943223	-2.254778433	0.02414724	0.051179155	negative
PRJNA729271	stage_E14_vs_E10	ordinary_muscle_development	ENSGALG00010023207	MNS1	-0.377548004	0.145330447	-2.597858966	0.009380701	0.022538251	negative
PRJNA729271	stage_E14_vs_E10	ordinary_muscle_development	ENSGALG00010023208	BACE1	0.071765297	0.077369397	0.927566978	0.353632234	0.469834943	positive
PRJNA729271	stage_E14_vs_E10	ordinary_muscle_development	ENSGALG00010023209	PIK3C2A	-0.344438277	0.105855457	-3.253854705	0.001138505	0.003536025	negative
PRJNA729271	stage_E14_vs_E10	ordinary_muscle_development	ENSGALG00010023210	ZNHIT6	-0.266685406	0.133853223	-1.99237194	0.046330264	0.089042319	negative
PRJNA729271	stage_E14_vs_E10	ordinary_muscle_development	ENSGALG00010023211	TLK1	-0.095965204	0.075654257	-1.268470634	0.204629931	0.304511704	negative
PRJNA729271	stage_E14_vs_E10	ordinary_muscle_development	ENSGALG00010023212	RIMBP2	1.835826158	0.213833979	8.585287353	9.06097E-18	1.62749E-16	positive
PRJNA729271	stage_E14_vs_E10	ordinary_muscle_development	ENSGALG00010023213	FCSK	0.181231068	0.113567647	1.595798389	0.110533813	0.184946049	positive
PRJNA729271	stage_E14_vs_E10	ordinary_muscle_development	ENSGALG00010023214	SUPV3L1	-0.198298471	0.088166627	-2.249133018	0.024504034	0.051844399	negative
PRJNA729271	stage_E14_vs_E10	ordinary_muscle_development	ENSGALG00010023215	TRIP12	0.010483652	0.056124788	0.186791836	0.851823848	0.897455057	positive
PRJNA729271	stage_E14_vs_E10	ordinary_muscle_development	ENSGALG00010023216	SLC35D1	-1.189740358	0.124180849	-9.580707226	9.63827E-22	2.35593E-20	negative
PRJNA729271	stage_E14_vs_E10	ordinary_muscle_development	ENSGALG00010023217	CREB3L1	0.869788908	0.205325384	4.236148942	2.27386E-05	0.000100334	positive
PRJNA729271	stage_E14_vs_E10	ordinary_muscle_development	ENSGALG00010023218	SSH3	0.359433118	0.128476056	2.797666176	0.005147328	0.013347142	positive
PRJNA729271	stage_E14_vs_E10	ordinary_muscle_development	ENSGALG00010023219	TEX9	-0.382707523	0.149305721	-2.563247546	0.010369808	0.024551677	negative
PRJNA729271	stage_E14_vs_E10	ordinary_muscle_development	ENSGALG00010023220	ST3GAL2	0.03897134	0.084853791	0.459276353	0.646035727	0.738120784	positive
PRJNA729271	stage_E14_vs_E10	ordinary_muscle_development	ENSGALG00010023221	AMY1A	0.643411432	0.137361535	4.684072807	2.8123E-06	1.44554E-05	positive
PRJNA729271	stage_E14_vs_E10	ordinary_muscle_development	ENSGALG00010023222	ENSGALG00010023222	-0.519549474	0.514777129	-1.0092707	0.312844828	0.426614021	negative
PRJNA729271	stage_E14_vs_E10	ordinary_muscle_development	ENSGALG00010023223	CEP164	-0.115428305	0.09169647	-1.258808601	0.208099473	0.308551745	negative
PRJNA729271	stage_E14_vs_E10	ordinary_muscle_development	ENSGALG00010023225	PDPR	0.253242878	0.119284907	2.123008548	0.033753136	0.067810353	positive
PRJNA729271	stage_E14_vs_E10	ordinary_muscle_development	ENSGALG00010023226	ENSGALG00010023226	-0.189939142	0.201373972	-0.943215947	0.345570458	0.461098189	negative
PRJNA729271	stage_E14_vs_E10	ordinary_muscle_development	ENSGALG00010023227	RNPC3	-0.238293246	0.123041093	-1.936696442	0.052782461	0.099103856	negative
PRJNA729271	stage_E14_vs_E10	ordinary_muscle_development	ENSGALG00010023228	GORASP2	0.275076127	0.10162787	2.70669973	0.006795569	0.016991853	positive
PRJNA729271	stage_E14_vs_E10	ordinary_muscle_development	ENSGALG00010023229	HKDC1	-2.002621298	0.462953258	-4.325752683	1.52012E-05	6.92244E-05	negative
PRJNA729271	stage_E14_vs_E10	ordinary_muscle_development	ENSGALG00010023230	ENSGALG00010023230	0.397473077	0.083055486	4.785633021	1.70449E-06	9.0951E-06	positive
PRJNA729271	stage_E14_vs_E10	ordinary_muscle_development	ENSGALG00010023231	ASIC1	-1.033537209	0.200415554	-5.156971036	2.50977E-07	1.51327E-06	negative
PRJNA729271	stage_E14_vs_E10	ordinary_muscle_development	ENSGALG00010023233	SEZ6L	-1.544889677	1.086247388	-1.422226368	0.154960546	0.243776114	negative
PRJNA729271	stage_E14_vs_E10	ordinary_muscle_development	ENSGALG00010023234	ENSGALG00010023234	-0.235715015	0.09287518	-2.537976388	0.011149551	0.026223063	negative
PRJNA729271	stage_E14_vs_E10	ordinary_muscle_development	ENSGALG00010023235	ENSGALG00010023235	-2.479354549	0.381328323	-6.501889309	7.93175E-11	7.38886E-10	negative
PRJNA729271	stage_E14_vs_E10	ordinary_muscle_development	ENSGALG00010023237	GAD1	-4.978975785	0.681469252	-7.306236889	2.74728E-13	3.34075E-12	negative
PRJNA729271	stage_E14_vs_E10	ordinary_muscle_development	ENSGALG00010023238	NUCB2	-0.250061437	0.07797419	-3.206977035	0.001341377	0.004097631	negative
PRJNA729271	stage_E14_vs_E10	ordinary_muscle_development	ENSGALG00010023239	RBPMS2	0.595657251	0.197155121	3.021261876	0.002517235	0.007101465	positive
PRJNA729271	stage_E14_vs_E10	ordinary_muscle_development	ENSGALG00010023240	PIWIL1	-1.542037095	0.408137916	-3.778225534	0.00015795	0.000599027	negative
PRJNA729271	stage_E14_vs_E10	ordinary_muscle_development	ENSGALG00010023243	HK1	0.315663246	0.114340231	2.76073647	0.005767119	0.014743279	positive
PRJNA729271	stage_E14_vs_E10	ordinary_muscle_development	ENSGALG00010023244	PPIB	-0.106728004	0.118400666	-0.901413883	0.367368303	0.483475899	negative
PRJNA729271	stage_E14_vs_E10	ordinary_muscle_development	ENSGALG00010023245	TANC2	-0.067386824	0.142732492	-0.472119721	0.636841337	0.730301834	negative
PRJNA729271	stage_E14_vs_E10	ordinary_muscle_development	ENSGALG00010023246	SP5	-4.092508973	0.661767117	-6.184213251	6.2413E-10	5.24802E-09	negative
PRJNA729271	stage_E14_vs_E10	ordinary_muscle_development	ENSGALG00010023248	PRDM10	-0.023309921	0.119828425	-0.194527478	0.845762882	0.892600143	negative
PRJNA729271	stage_E14_vs_E10	ordinary_muscle_development	ENSGALG00010023249	IL12RB2	-1.742340971	0.621052722	-2.805463869	0.005024421	0.013084619	negative
PRJNA729271	stage_E14_vs_E10	ordinary_muscle_development	ENSGALG00010023250	FZD10	-3.050985403	0.387930425	-7.864774737	3.69762E-15	5.27011E-14	negative
PRJNA729271	stage_E14_vs_E10	ordinary_muscle_development	ENSGALG00010023251	ENSGALG00010023251	1.899940607	1.209607939	1.570707785	0.11625054	0.192709467	positive
PRJNA729271	stage_E14_vs_E10	ordinary_muscle_development	ENSGALG00010023252	GLG1	-0.140647159	0.079550754	-1.768017937	0.077057905	0.136915676	negative
PRJNA729271	stage_E14_vs_E10	ordinary_muscle_development	ENSGALG00010023254	MYO3B	-1.259630086	0.306257286	-4.112979978	3.90584E-05	0.000165735	negative
PRJNA729271	stage_E14_vs_E10	ordinary_muscle_development	ENSGALG00010023255	SNX22	2.035942043	0.305507435	6.664132552	2.66233E-11	2.60923E-10	positive
PRJNA729271	stage_E14_vs_E10	ordinary_muscle_development	ENSGALG00010023257	PAK6	-0.748680836	0.302403168	-2.475770475	0.013294902	0.030574266	negative
PRJNA729271	stage_E14_vs_E10	ordinary_muscle_development	ENSGALG00010023258	ENSGALG00010023258	0.315567981	0.329073861	0.958957908	0.33757995	0.452754265	positive
PRJNA729271	stage_E14_vs_E10	ordinary_muscle_development	ENSGALG00010023259	TSPAN15	1.006002019	0.288910875	3.482049673	0.000497591	0.001688735	positive
PRJNA729271	stage_E14_vs_E10	ordinary_muscle_development	ENSGALG00010023260	ENSGALG00010023260	-1.55176185	0.217310933	-7.140744504	9.28268E-13	1.06281E-11	negative
PRJNA729271	stage_E14_vs_E10	ordinary_muscle_development	ENSGALG00010023261	SNX1	0.655684343	0.095423554	6.871305015	6.36172E-12	6.62451E-11	positive
PRJNA729271	stage_E14_vs_E10	ordinary_muscle_development	ENSGALG00010023262	POLD4	0.182676757	0.216588659	0.843427157	0.398989589	0.514395988	positive
PRJNA729271	stage_E14_vs_E10	ordinary_muscle_development	ENSGALG00010023263	SRP14	-0.298981941	0.071214002	-4.198358946	2.68856E-05	0.000117205	negative
PRJNA729271	stage_E14_vs_E10	ordinary_muscle_development	ENSGALG00010023264	RACGAP1	0.046854315	0.226399106	0.206954503	0.83604539	0.885594704	positive
PRJNA729271	stage_E14_vs_E10	ordinary_muscle_development	ENSGALG00010023265	RAB5C	-0.029587065	0.070209324	-0.421412195	0.673454115	0.75990327	negative
PRJNA729271	stage_E14_vs_E10	ordinary_muscle_development	ENSGALG00010023266	RFWD3	-0.000133297	0.132694926	-0.001004534	0.999198498	0.999405343	negative
PRJNA729271	stage_E14_vs_E10	ordinary_muscle_development	ENSGALG00010023267	DSCAML1	0.113786472	0.675425261	0.168466415	0.866216363	0.908195746	positive
PRJNA729271	stage_E14_vs_E10	ordinary_muscle_development	ENSGALG00010023268	KCNJ11	2.702714285	0.17982995	15.0292779	4.72124E-51	6.64412E-49	positive
PRJNA729271	stage_E14_vs_E10	ordinary_muscle_development	ENSGALG00010023269	OPRL1	-2.624956248	0.529727023	-4.955299867	7.22188E-07	4.06687E-06	negative
PRJNA729271	stage_E14_vs_E10	ordinary_muscle_development	ENSGALG00010023271	PATL2	0.276410922	0.410765018	0.672917385	0.500999851	0.610200222	positive
PRJNA729271	stage_E14_vs_E10	ordinary_muscle_development	ENSGALG00010023273	HDAC7	0.398670916	0.113089971	3.525254372	0.000423076	0.00145631	positive
PRJNA729271	stage_E14_vs_E10	ordinary_muscle_development	ENSGALG00010023274	ZBTB38	0.137305984	0.114924664	1.194747754	0.232185599	0.336422985	positive
PRJNA729271	stage_E14_vs_E10	ordinary_muscle_development	ENSGALG00010023275	TWIST3	-5.422834063	0.436652872	-12.4190963	2.05874E-35	1.17486E-33	negative
PRJNA729271	stage_E14_vs_E10	ordinary_muscle_development	ENSGALG00010023276	ABCC8	2.437697664	0.161431577	15.10050087	1.60708E-51	2.377E-49	positive
PRJNA729271	stage_E14_vs_E10	ordinary_muscle_development	ENSGALG00010023278	ENSGALG00010023278	0.188949485	0.243177036	0.777003813	0.437156521	0.551007284	positive
PRJNA729271	stage_E14_vs_E10	ordinary_muscle_development	ENSGALG00010023279	FA2H	1.408881661	0.406488751	3.465979455	0.000528304	0.001781289	positive
PRJNA729271	stage_E14_vs_E10	ordinary_muscle_development	ENSGALG00010023281	B2M	0.560986163	0.200277554	2.801043607	0.005093764	0.013231918	positive

PRJNA729271	stage_E14_vs_E10	ordinary_muscle_development	ENSGALG00010023282	SERBP1	0.16211876	0.087165893	1.859887562	0.062901435	0.115162008	positive
PRJNA729271	stage_E14_vs_E10	ordinary_muscle_development	ENSGALG00010023285	SLC48A1	0.530987563	0.112401803	4.72401287	2.31236E-06	1.2048E-05	positive
PRJNA729271	stage_E14_vs_E10	ordinary_muscle_development	ENSGALG00010023286	HCRT	0.75866115	0.446342274	1.699729542	0.08918181	0.154681306	positive
PRJNA729271	stage_E14_vs_E10	ordinary_muscle_development	ENSGALG00010023287	RGS19	-0.182636028	0.197449035	-0.924978077	0.354977311	0.471405967	negative
PRJNA729271	stage_E14_vs_E10	ordinary_muscle_development	ENSGALG00010023289	NFRKB	-0.033906773	0.085902468	-0.394712444	0.693055118	0.775919822	negative
PRJNA729271	stage_E14_vs_E10	ordinary_muscle_development	ENSGALG00010023290	SLC16A14	-0.603917502	0.225947005	-2.672828088	0.007521478	0.018566729	negative
PRJNA729271	stage_E14_vs_E10	ordinary_muscle_development	ENSGALG00010023293	COL11A1	-2.60495054	0.48910446	-5.325959488	1.00422E-07	6.4011E-07	negative
PRJNA729271	stage_E14_vs_E10	ordinary_muscle_development	ENSGALG00010023295	ENSGALG00010023295	-0.197427939	0.526076775	-0.375283511	0.707449628	0.787594651	negative
PRJNA729271	stage_E14_vs_E10	ordinary_muscle_development	ENSGALG00010023299	PASK	-0.174100103	0.133006115	-1.308963144	0.190546836	0.287735845	negative
PRJNA729271	stage_E14_vs_E10	ordinary_muscle_development	ENSGALG00010023304	ENSGALG00010023304	-0.326686821	0.174311607	-1.874154153	0.060909181	0.112040429	negative
PRJNA729271	stage_E14_vs_E10	ordinary_muscle_development	ENSGALG00010023305	PLCB2	-0.200737761	0.16281828	-1.232894494	0.21761513	0.319652545	negative
PRJNA729271	stage_E14_vs_E10	ordinary_muscle_development	ENSGALG00010023309	PIK3R3	-0.615332166	0.199801231	-3.079721592	0.002071942	0.005998162	negative
PRJNA729271	stage_E14_vs_E10	ordinary_muscle_development	ENSGALG00010023311	ETS1	0.035885207	0.13325779	0.269291626	0.787705276	0.850712098	positive
PRJNA729271	stage_E14_vs_E10	ordinary_muscle_development	ENSGALG00010023312	UBR3	0.299754815	0.11441016	2.620001719	0.008792932	0.021313019	positive
PRJNA729271	stage_E14_vs_E10	ordinary_muscle_development	ENSGALG00010023317	RAPGEF3	0.883457634	0.148467169	5.950525227	2.67283E-09	2.09194E-08	positive
PRJNA729271	stage_E14_vs_E10	ordinary_muscle_development	ENSGALG00010023319	EIF2AK4	-0.161036039	0.060896132	-2.644437911	0.008182672	0.020045264	negative
PRJNA729271	stage_E14_vs_E10	ordinary_muscle_development	ENSGALG00010023320	ENSGALG00010023320	-0.240063575	0.140063357	-1.71396416	0.086535308	0.150942153	negative
PRJNA729271	stage_E14_vs_E10	ordinary_muscle_development	ENSGALG00010023322	PRKAB1	0.036768755	0.090177036	0.407739674	0.683464807	0.768328089	positive
PRJNA729271	stage_E14_vs_E10	ordinary_muscle_development	ENSGALG00010023324	LAG3	-1.388007328	1.25175392	-1.108849994	0.267494899	0.376550312	negative
PRJNA729271	stage_E14_vs_E10	ordinary_muscle_development	ENSGALG00010023328	TCEA2	-0.263574639	0.106782614	-2.468329139	0.013574544	0.031138315	negative
PRJNA729271	stage_E14_vs_E10	ordinary_muscle_development	ENSGALG00010023330	TSPAN1	3.10293017	1.328530823	2.335610223	0.019511565	0.042529344	positive
PRJNA729271	stage_E14_vs_E10	ordinary_muscle_development	ENSGALG00010023331	CD4	-2.841129639	0.332033881	-8.556746164	1.16098E-17	2.06483E-16	negative
PRJNA729271	stage_E14_vs_E10	ordinary_muscle_development	ENSGALG00010023332	PPP1R7	0.299181472	0.074370355	4.022859263	5.74959E-05	0.000236897	positive
PRJNA729271	stage_E14_vs_E10	ordinary_muscle_development	ENSGALG00010023333	ENDOU	0.47327188	0.459358305	1.030289156	0.302874288	0.416523985	positive
PRJNA729271	stage_E14_vs_E10	ordinary_muscle_development	ENSGALG00010023334	RPS17	0.550672127	0.151603627	3.632315003	0.00028089	0.00101105	positive
PRJNA729271	stage_E14_vs_E10	ordinary_muscle_development	ENSGALG00010023335	WDR59	0.234224351	0.06614677	3.5409794	0.000398645	0.001382051	positive
PRJNA729271	stage_E14_vs_E10	ordinary_muscle_development	ENSGALG00010023336	GPR162	-0.480540801	0.149069464	-3.223603212	0.001265887	0.003898243	negative
PRJNA729271	stage_E14_vs_E10	ordinary_muscle_development	ENSGALG00010023337	FLI1	-0.828397448	0.117328431	-7.060500504	1.65904E-12	1.84557E-11	negative
PRJNA729271	stage_E14_vs_E10	ordinary_muscle_development	ENSGALG00010023338	HDLBP	0.121954698	0.1240362	0.983218591	0.325499867	0.440164247	positive
PRJNA729271	stage_E14_vs_E10	ordinary_muscle_development	ENSGALG00010023340	P3H3	-0.08228492	0.157355606	-0.522923342	0.6010276	0.69993248	negative
PRJNA729271	stage_E14_vs_E10	ordinary_muscle_development	ENSGALG00010023342	FITM2	0.769219234	0.134372669	5.724521519	1.03726E-08	7.54771E-08	positive
PRJNA729271	stage_E14_vs_E10	ordinary_muscle_development	ENSGALG00010023343	TMEM233	3.966885458	0.334853765	11.84662043	2.24045E-32	1.07892E-30	positive
PRJNA729271	stage_E14_vs_E10	ordinary_muscle_development	ENSGALG00010023347	STAT5A	1.210144586	0.169278742	7.148827865	8.7522E-13	1.00525E-11	positive
PRJNA729271	stage_E14_vs_E10	ordinary_muscle_development	ENSGALG00010023349	ENSGALG00010023349	0.704515409	0.614744062	1.146030441	0.251782557	0.358999426	positive
PRJNA729271	stage_E14_vs_E10	ordinary_muscle_development	ENSGALG00010023350	ZNRF1	0.412682942	0.230866387	1.787540179	0.073850224	0.1320949	positive
PRJNA729271	stage_E14_vs_E10	ordinary_muscle_development	ENSGALG00010023351	GNB3	-0.045096005	0.371020844	-0.121545746	0.903258783	0.933861345	negative
PRJNA729271	stage_E14_vs_E10	ordinary_muscle_development	ENSGALG00010023354	SEPTIN2	-0.440920838	0.171361733	-2.573041421	0.010080914	0.023970284	negative
PRJNA729271	stage_E14_vs_E10	ordinary_muscle_development	ENSGALG00010023356	ENSGALG00010023356	-0.282118965	0.188115926	-1.499708028	0.13369005	0.21608355	negative
PRJNA729271	stage_E14_vs_E10	ordinary_muscle_development	ENSGALG00010023359	CDCA3	0.114432558	0.265847522	0.43044433	0.666872455	0.754886078	positive
PRJNA729271	stage_E14_vs_E10	ordinary_muscle_development	ENSGALG00010023362	GADD45A	1.080064564	0.148172787	7.289223518	3.11746E-13	3.76878E-12	positive
PRJNA729271	stage_E14_vs_E10	ordinary_muscle_development	ENSGALG00010023363	CPEB1	-0.340505557	0.197109421	-1.727495094	0.084078786	0.147509319	negative
PRJNA729271	stage_E14_vs_E10	ordinary_muscle_development	ENSGALG00010023364	CTRB1	1.937858567	2.34738193	0.825540378	0.409064868	0.523844445	positive
PRJNA729271	stage_E14_vs_E10	ordinary_muscle_development	ENSGALG00010023366	GDAP1L1	-2.234656762	0.431147884	-5.183040073	2.18298E-07	1.32784E-06	negative
PRJNA729271	stage_E14_vs_E10	ordinary_muscle_development	ENSGALG00010023368	ENSGALG00010023368	-1.465512868	0.213412661	-6.867038077	6.55486E-12	6.81583E-11	negative
PRJNA729271	stage_E14_vs_E10	ordinary_muscle_development	ENSGALG00010023369	ACE	1.032147332	0.476653801	2.1654025	0.030356875	0.061968285	positive
PRJNA729271	stage_E14_vs_E10	ordinary_muscle_development	ENSGALG00010023374	ENSGALG00010023374	-1.062179452	0.202102625	-5.255644032	1.47507E-07	9.15291E-07	negative
PRJNA729271	stage_E14_vs_E10	ordinary_muscle_development	ENSGALG00010023375	ST14	1.026559463	0.143985682	7.129594066	1.00665E-12	1.14713E-11	positive
PRJNA729271	stage_E14_vs_E10	ordinary_muscle_development	ENSGALG00010023377	OSER1	0.289506057	0.150192274	1.927569567	0.053908687	0.101009103	positive
PRJNA729271	stage_E14_vs_E10	ordinary_muscle_development	ENSGALG00010023379	CCDC60	0.984654332	0.480857842	2.047703596	0.040589051	0.079248154	positive
PRJNA729271	stage_E14_vs_E10	ordinary_muscle_development	ENSGALG00010023380	USP5	-0.029336188	0.096354045	-0.304462444	0.760775601	0.829540605	negative
PRJNA729271	stage_E14_vs_E10	ordinary_muscle_development	ENSGALG00010023381	HOXB7	-0.971315045	0.164325123	-5.910934533	3.40172E-09	2.62416E-08	negative
PRJNA729271	stage_E14_vs_E10	ordinary_muscle_development	ENSGALG00010023383	HOXB6	-1.33081363	0.174756229	-7.615257195	2.63167E-14	3.50285E-13	negative
PRJNA729271	stage_E14_vs_E10	ordinary_muscle_development	ENSGALG00010023384	AP3B2	-1.847772616	0.223474681	-8.268375669	1.35796E-16	2.21414E-15	negative
PRJNA729271	stage_E14_vs_E10	ordinary_muscle_development	ENSGALG00010023387	ENSGALG00010023387	1.920617142	0.182524848	10.52249687	6.8046E-26	2.2115E-24	positive
PRJNA729271	stage_E14_vs_E10	ordinary_muscle_development	ENSGALG00010023388	FARP2	-0.506203897	0.082164103	-6.160888706	7.23378E-10	6.01225E-09	negative
PRJNA729271	stage_E14_vs_E10	ordinary_muscle_development	ENSGALG00010023389	GNG12	-0.01800355	0.108825708	-0.165434712	0.868601834	0.909841276	negative
PRJNA729271	stage_E14_vs_E10	ordinary_muscle_development	ENSGALG00010023390	HSPB8	1.810765572	0.250801559	7.219913541	5.20206E-13	6.12542E-12	positive
PRJNA729271	stage_E14_vs_E10	ordinary_muscle_development	ENSGALG00010023392	TPI1	0.396614231	0.149614901	2.650900607	0.008027746	0.019695697	positive
PRJNA729271	stage_E14_vs_E10	ordinary_muscle_development	ENSGALG00010023394	HOXB5	-2.352248978	0.182151286	-12.9137105	3.76702E-38	2.61258E-36	negative
PRJNA729271	stage_E14_vs_E10	ordinary_muscle_development	ENSGALG00010023396	ENSGALG00010023396	-0.312088219	0.141486527	-2.205780474	0.027399374	0.056923309	negative
PRJNA729271	stage_E14_vs_E10	ordinary_muscle_development	ENSGALG00010023397	ENSGALG00010023397	-1.278715154	0.257758077	-4.96091207	7.01629E-07	3.95879E-06	negative
PRJNA729271	stage_E14_vs_E10	ordinary_muscle_development	ENSGALG00010023401	LRRC23	0.58192328	0.376258497	1.54660502	0.121958519	0.200225249	positive
PRJNA729271	stage_E14_vs_E10	ordinary_muscle_development	ENSGALG00010023405	DNAJA2	-0.216998108	0.076310723	-2.843612282	0.004460529	0.011783373	negative
PRJNA729271	stage_E14_vs_E10	ordinary_muscle_development	ENSGALG00010023408	ZBTB44	0.18112143	0.126344642	1.433550542	0.151700566	0.239635975	positive
PRJNA729271	stage_E14_vs_E10	ordinary_muscle_development	ENSGALG00010023411	ENSGALG00010023411	1.651122401	0.182157992	9.064232528	1.25485E-19	2.60587E-18	positive
PRJNA729271	stage_E14_vs_E10	ordinary_muscle_development	ENSGALG00010023412	GSS	0.071098292	0.104036773	0.683395778	0.494356787	0.604409904	positive
PRJNA729271	stage_E14_vs_E10	ordinary_muscle_development	ENSGALG00010023414	SUDS3	0.038012102	0.07484403	0.507884222	0.61153454	0.708511962	positive
PRJNA729271	stage_E14_vs_E10	ordinary_muscle_development	ENSGALG00010023415	HOXB4	-1.026694076	0.167695636	-6.122366094	9.21959E-10	7.57585E-09	negative
PRJNA729271	stage_E14_vs_E10	ordinary_muscle_development	ENSGALG00010023421	OLFM3	-0.886644183	0.252826276	-3.506930521	0.000453307	0.001551448	negative
PRJNA729271	stage_E14_vs_E10	ordinary_muscle_development	ENSGALG00010023422	WLS	-0.696617782	0.154982279	-4.494822163	6.96281E-06	3.3586E-05	negative
PRJNA729271	stage_E14_vs_E10	ordinary_muscle_development	ENSGALG00010023426	KCNJ1	0.569621518	0.426910584	1.334287646	0.182109605	0.277422882	positive
PRJNA729271	stage_E14_vs_E10	ordinary_muscle_development	ENSGALG00010023428	HOXB3	-1.224036731	0.177225269	-6.906671598	4.96157E-12	5.22659E-11	negative
PRJNA729271	stage_E14_vs_E10	ordinary_muscle_development	ENSGALG00010023429	BMF	0.25216509	0.146506704	1.72118465	0.085217322	0.149085045	positive
PRJNA729271	stage_E14_vs_E10	ordinary_muscle_development	ENSGALG00010023431	BCAR1	0.133474888	0.173218586	0.77055754	0.440969237	0.554950608	positive
PRJNA729271	stage_E14_vs_E10	ordinary_muscle_development	ENSGALG00010023432	TMOD3	-0.46276387	0.17487216	-2.646298123	0.008137806	0.019945469	negative
PRJNA729271	stage_E14_vs_E10	ordinary_muscle_development	ENSGALG00010023434	ATN1	-0.626537517	0.185935824	-3.369643916	0.000752654	0.00244448	negative
PRJNA729271	stage_E14_vs_E10	ordinary_muscle_development	ENSGALG00010023437	KCNJ5	1.694614293	0.244508523	6.930696206	4.18774E-12	4.46662E-11	positive
PRJNA729271	stage_E14_vs_E10	ordinary_muscle_development	ENSGALG00010023438	HOXB2	-1.324529069	0.249799682	-5.302364902	1.14312E-07	7.2167E-07	negative
PRJNA729271	stage_E14_vs_E10	ordinary_muscle_development	ENSGALG00010023439	ACSS2	0.651748361	0.132390602	4.92292014	8.52623E-07	4.74972E-06	positive
PRJNA729271	stage_E14_vs_E10	ordinary_muscle_development	ENSGALG00010023441	ENSGALG00010023441	-0.894000522	0.166963369	-5.354471009	8.58071E-08	5.52788E-07	negative
PRJNA729271	stage_E14_vs_E10	ordinary_muscle_development	ENSGALG00010023442	STK25	-0.188008461	0.112361807	-1.673241696	0.094279725	0.162109682	negative
PRJNA729271	stage_E14_vs_E10	ordinary_muscle_development	ENSGALG00010023443	ENSGALG00010023443	0.53039928	0.17907727	2.961845917	0.003058008	0.008457512	positive
PRJNA729271	stage_E14_vs_E10	ordinary_muscle_development	ENSGALG00010023447	C12orf57	0.290914942	0.113227264	2.569301163	0.010190385	0.0241909	positive
PRJNA729271	stage_E14_vs_E10	ordinary_muscle_development	ENSGALG00010023448	FAM219B	0.389456939	0.112653869	3.457111079	0.000546	0.00183243	positive
PRJNA729271	stage_E14_vs_E10	ordinary_muscle_development	ENSGALG00010023453	ARHGAP32	0.304460173	0.17035475	1.787212706	0.073903116	0.132104027	positive
PRJNA729271	stage_E14_vs_E10	ordinary_muscle_development	ENSGALG00010023454	HOXB1	0.178445856	0.409063909	0.436229773	0.662670028	0.751714045	positive
PRJNA729271	stage_E14_vs_E10	ordinary_muscle_development	ENSGALG00010023455	CFDP1	-0.011923074	0.093231122	-0.12788727	0.898238185	0.930130197	negative
PRJNA729271	stage_E14_vs_E10	ordinary_muscle_development	ENSGALG00010023456	MPI	-0.017050018	0.144592953	-0.117917353	0.906133143	0.935716909	negative
PRJNA729271	stage_E14_vs_E10	ordinary_muscle_development	ENSGALG00010023469	ENSGALG00010023469	0.579396363	0.211577692	2.738456765	0.006172828	0.015636443	positive
PRJNA729271	stage_E14_vs_E10	ordinary_muscle_development	ENSGALG00010023472	PTPN6	0.55126274	0.195355741	2.82184049	0.004774892	0.012511218	positive
PRJNA729271	stage_E14_vs_E10	ordinary_muscle_development	ENSGALG00010023473	BOK	-0.093275028	0.197313634	-0.472724697	0.636409605	0.729922236	negative
PRJNA729271	stage_E14_vs_E10	ordinary_muscle_development	ENSGALG00010023475	ENSGALG00010023475	-1.61440643	0.500870812	-3.22319926	0.001267673	0.003901258	negative
PRJNA729271	stage_E14_vs_E10	ordinary_muscle_development	ENSGALG00010023476	SCAMP2	0.145025949	0.150274195	0.965075534	0.33450698	0.449701232	positive
PRJNA729271	stage_E14_vs_E10	ordinary_muscle_development	ENSGALG00010023478	TAOK3	0.253521733	0.123070519	2.059971267	0.039401288	0.077345838	positive
PRJNA729271	stage_E14_vs_E10	ordinary_muscle_development	ENSGALG00010023479	MCCC2L	0.387909127	0.156029476	2.486127213	0.012914179	0.029821735	positive
PRJNA729271	stage_E14_vs_E10	ordinary_muscle_development	ENSGALG00010023482	MTCL2	1.569596429	0.096982015	16.18440719	6.49726E-59	1.47153E-56	positive
PRJNA729271	stage_E14_vs_E10	ordinary_muscle_development	ENSGALG00010023485	BARX2	-0.858876181	0.297763043	-2.884428414	0.003921249	0.010496492	negative
PRJNA729271	stage_E14_vs_E10	ordinary_muscle_development	ENSGALG00010023487	GRN	0.011512044	0.145845176	0.078933319	0.937085661	0.95618629	positive
PRJNA729271	stage_E14_vs_E10	ordinary_muscle_development	ENSGALG00010023489	ETV5	-0.188366782	0.21681227	-0.868801302	0.384955821	0.501026724	negative
PRJNA729271	stage_E14_vs_E10	ordinary_muscle_development	ENSGALG00010023490	PHB2	0.175131463	0.102757858	1.704312118	0.088322792	0.153432272	positive
PRJNA729271	stage_E14_vs_E10	ordinary_muscle_development	ENSGALG00010023496	PLEKHA3	-0.316143014	0.103985229	-3.040268492	0.002363673	0.006731129	negative
PRJNA729271	stage_E14_vs_E10	ordinary_muscle_development	ENSGALG00010023499	PEBP1	0.072229123	0.139700919	0.517026825	0.605137433	0.703067257	positive
PRJNA729271	stage_E14_vs_E10	ordinary_muscle_development	ENSGALG00010023501	ENSGALG00010023501	0.472407215	3.076410128	0.153557944	0.877958299	0.916243316	positive
PRJNA729271	stage_E14_vs_E10	ordinary_muscle_development	ENSGALG00010023506	RPE65	-0.405425479	0.499752155	-0.811253088	0.417220344	0.531798179	negative
PRJNA729271	stage_E14_vs_E10	ordinary_muscle_development	ENSGALG00010023510	ASPHD2	0.748894887	0.242463895	3.088686204	0.002010437	0.005844621	positive
PRJNA729271	stage_E14_vs_E10	ordinary_muscle_development	ENSGALG00010023513	CDCA9	-0.146341083	0.210444357	-0.695390863	0.486810385	0.597992927	negative
PRJNA729271	stage_E14_vs_E10	ordinary_muscle_development	ENSGALG00010023514	ULK3	0.197285071	0.104395047	1.889793391	0.058785598	0.108672011	positive
PRJNA729271	stage_E14_vs_E10	ordinary_muscle_development	ENSGALG00010023518	METTL5	0.348315686	0.118331765	2.94355184	0.003244695	0.008910922	positive
PRJNA729271	stage_E14_vs_E10	ordinary_muscle_development	ENSGALG00010023519	DGKG	0.409975003	0.231141331	1.773698377	0.076113055	0.135535471	positive
PRJNA729271	stage_E14_vs_E10	ordinary_muscle_development	ENSGALG00010023522	EMG1	-0.233029022	0.157858207	-1.476192	0.139892356	0.22428268	negative
PRJNA729271	stage_E14_vs_E10	ordinary_muscle_development	ENSGALG00010023526	KNSTRN	-0.113861385	0.183968432	-0.618918061	0.53597034	0.642756132	negative
PRJNA729271	stage_E14_vs_E10	ordinary_muscle_development	ENSGALG00010023527	TMEM98	-0.971076235	0.226384137	-4.289506531	1.79071E-05	8.03849E-05	negative
PRJNA729271	stage_E14_vs_E10	ordinary_muscle_development	ENSGALG00010023533	FKBP7	-0.003428377	0.162099808	-0.021149793	0.983126165	0.98885669	negative
PRJNA729271	stage_E14_vs_E10	ordinary_muscle_development	ENSGALG00010023536	ENSGALG00010023536	-0.205871546	0.185125875	-1.112062514	0.266111273	0.375021395	negative
PRJNA729271	stage_E14_vs_E10	ordinary_muscle_development	ENSGALG00010023541	PRSS2	0.472437361	2.614160467	0.180722403	0.856585473	0.900965564	positive
PRJNA729271	stage_E14_vs_E10	ordinary_muscle_development	ENSGALG00010023543	ENSGALG00010023543	0.774075269	0.583887472	1.325726797	0.184930208	0.280804878	positive
PRJNA729271	stage_E14_vs_E10	ordinary_muscle_development	ENSGALG00010023544	SSB	-0.502422548	0.101742344	-4.938185303	7.88529E-07	4.41473E-06	negative
PRJNA729271	stage_E14_vs_E10	ordinary_muscle_development	ENSGALG00010023545	PTPRT	1.912538019	0.126480761	15.12117735	1.17424E-51	1.77298E-49	positive
PRJNA729271	stage_E14_vs_E10	ordinary_muscle_development	ENSGALG00010023546	SLC11A2	-0.034825023	0.155115997	-0.224509549	0.822360838	0.875588607	negative
PRJNA729271	stage_E14_vs_E10	ordinary_muscle_development	ENSGALG00010023551	ENSGALG00010023551	-0.095051056	0.174775685	-0.543845993	0.586547456	0.686956572	negative
PRJNA729271	stage_E14_vs_E10	ordinary_muscle_development	ENSGALG00010023552	SGPP2	1.660278229	0.308276299	5.385682371	7.21703E-08	4.71008E-07	positive
PRJNA729271	stage_E14_vs_E10	ordinary_muscle_development	ENSGALG00010023555	HPS4	-0.247476155	0.164925272	-1.500535073	0.133475855	0.21578547	negative
PRJNA729271	stage_E14_vs_E10	ordinary_muscle_development	ENSGALG00010023556	IVD	0.448665712	0.120838636	3.712932594	0.000204871	0.000758909	positive
PRJNA729271	stage_E14_vs_E10	ordinary_muscle_development	ENSGALG00010023559	PJVK	-1.115363806	0.769530397	-1.44940838	0.14722357	0.233838006	negative
PRJNA729271	stage_E14_vs_E10	ordinary_muscle_development	ENSGALG00010023569	LPCAT3	-0.510672136	0.103251528	-4.945903902	7.57913E-07	4.25482E-06	negative
PRJNA729271	stage_E14_vs_E10	ordinary_muscle_development	ENSGALG00010023571	APLP2	1.085409868	0.098536253	11.01533532	3.22334E-28	1.23702E-26	positive
PRJNA729271	stage_E14_vs_E10	ordinary_muscle_development	ENSGALG00010023572	MYBL2	0.329309915	0.1264506	2.604257429	0.009207357	0.02216586	positive
PRJNA729271	stage_E14_vs_E10	ordinary_muscle_development	ENSGALG00010023574	DEPDC1	-0.070196732	0.197462679	-0.355493668	0.722219788	0.798823031	negative
PRJNA729271	stage_E14_vs_E10	ordinary_muscle_development	ENSGALG00010023578	SRRD	-0.020434651	0.098958052	-0.206498112	0.836401839	0.885833715	negative
PRJNA729271	stage_E14_vs_E10	ordinary_muscle_development	ENSGALG00010023580	RDM1	-0.625873949	0.158665181	-3.944620634	7.99264E-05	0.000320125	negative
PRJNA729271	stage_E14_vs_E10	ordinary_muscle_development	ENSGALG00010023585	TFIP11	-0.069641791	0.095401074	-0.729989592	0.465396547	0.577759759	negative

PRJNA729271	stage_E14_vs_E10	ordinary_muscle_development	ENSGALG00010023588	FARSB	-0.148235418	0.134068722	-1.105667423	0.268870495	0.378339756	negative
PRJNA729271	stage_E14_vs_E10	ordinary_muscle_development	ENSGALG00010023590	S1PR1	-0.530429811	0.115868683	-4.577853126	4.69772E-06	2.32878E-05	negative
PRJNA729271	stage_E14_vs_E10	ordinary_muscle_development	ENSGALG00010023592	BAHD1	-0.035182739	0.103680868	-0.33933685	0.734355986	0.808974769	negative
PRJNA729271	stage_E14_vs_E10	ordinary_muscle_development	ENSGALG00010023593	CSK	-0.204450767	0.080673007	-2.534314452	0.011266758	0.026460631	negative
PRJNA729271	stage_E14_vs_E10	ordinary_muscle_development	ENSGALG00010023597	MOGAT1	0.975809418	0.336000339	2.904191766	0.003682026	0.009942429	positive
PRJNA729271	stage_E14_vs_E10	ordinary_muscle_development	ENSGALG00010023598	IL10RA	-0.358722302	0.479636449	-0.74790459	0.454517709	0.567559802	negative
PRJNA729271	stage_E14_vs_E10	ordinary_muscle_development	ENSGALG00010023600	CHST14	-0.609389547	0.082033172	-7.428574733	1.09774E-13	1.38123E-12	negative
PRJNA729271	stage_E14_vs_E10	ordinary_muscle_development	ENSGALG00010023601	TPST2	0.592092847	0.104346683	5.674285283	1.39269E-08	9.97383E-08	positive
PRJNA729271	stage_E14_vs_E10	ordinary_muscle_development	ENSGALG00010023603	C1S	-1.626051005	0.398267331	-4.082812918	4.44938E-05	0.000186556	negative
PRJNA729271	stage_E14_vs_E10	ordinary_muscle_development	ENSGALG00010023604	ENSGALG00010023604	0.210640189	0.397658839	0.529700759	0.596319421	0.696000712	positive
PRJNA729271	stage_E14_vs_E10	ordinary_muscle_development	ENSGALG00010023605	INSYN1	-0.479489262	0.337466361	-1.42085054	0.155360214	0.244192833	negative
PRJNA729271	stage_E14_vs_E10	ordinary_muscle_development	ENSGALG00010023606	LRRC7	-1.742628481	0.26632938	-6.543132717	6.02433E-11	5.70364E-10	negative
PRJNA729271	stage_E14_vs_E10	ordinary_muscle_development	ENSGALG00010023612	GPT2	0.355454664	0.186305671	1.907911135	0.056402703	0.104922636	positive
PRJNA729271	stage_E14_vs_E10	ordinary_muscle_development	ENSGALG00010023613	COPB1	-0.037098996	0.050553129	-0.733861517	0.463033143	0.575675809	negative
PRJNA729271	stage_E14_vs_E10	ordinary_muscle_development	ENSGALG00010023615	PRSS3	0.472283028	5.150527732	0.091696046	0.926939539	0.949808329	positive
PRJNA729271	stage_E14_vs_E10	ordinary_muscle_development	ENSGALG00010023617	ENSGALG00010023617	0.220730817	0.299704324	0.73649527	0.461429345	0.574063888	positive
PRJNA729271	stage_E14_vs_E10	ordinary_muscle_development	ENSGALG00010023620	IRS1	0.415617915	0.14130841	2.941211458	0.003269313	0.008968336	positive
PRJNA729271	stage_E14_vs_E10	ordinary_muscle_development	ENSGALG00010023621	CD79B	1.980265711	0.961993614	2.058501929	0.039541976	0.077558991	positive
PRJNA729271	stage_E14_vs_E10	ordinary_muscle_development	ENSGALG00010023623	OSBPL6	-1.43957336	0.222428492	-6.472072641	9.66676E-11	8.85713E-10	negative
PRJNA729271	stage_E14_vs_E10	ordinary_muscle_development	ENSGALG00010023624	PSMA1	0.17697569	0.070392464	2.514128346	0.0119327	0.027839125	positive
PRJNA729271	stage_E14_vs_E10	ordinary_muscle_development	ENSGALG00010023626	LOXL1	-0.01374046	0.1953522	-0.070336857	0.943925548	0.960424036	negative
PRJNA729271	stage_E14_vs_E10	ordinary_muscle_development	ENSGALG00010023627	IFT52	-0.065977609	0.100713868	-0.655099542	0.512403667	0.621270695	negative
PRJNA729271	stage_E14_vs_E10	ordinary_muscle_development	ENSGALG00010023631	ENSGALG00010023631	1.552991572	0.474921828	3.269994092	0.001075497	0.003360987	positive
PRJNA729271	stage_E14_vs_E10	ordinary_muscle_development	ENSGALG00010023635	SCN4A	3.496862002	0.325842295	10.73176215	7.22059E-27	2.5342E-25	positive
PRJNA729271	stage_E14_vs_E10	ordinary_muscle_development	ENSGALG00010023636	PDE3B	0.152273852	0.0805818	1.889680448	0.058800711	0.108686088	positive
PRJNA729271	stage_E14_vs_E10	ordinary_muscle_development	ENSGALG00010023637	RHBDD1	0.527275868	0.102981387	5.12010845	3.0536E-07	1.81998E-06	positive
PRJNA729271	stage_E14_vs_E10	ordinary_muscle_development	ENSGALG00010023638	DCTN2	0.155399788	0.130732997	1.188680678	0.234565347	0.339086935	positive
PRJNA729271	stage_E14_vs_E10	ordinary_muscle_development	ENSGALG00010023639	KLHL23	0.985753569	0.215546742	4.573270548	4.80169E-06	2.37626E-05	positive
PRJNA729271	stage_E14_vs_E10	ordinary_muscle_development	ENSGALG00010023641	LRRC40	-0.336452226	0.10837468	-3.104527978	0.001905829	0.00558984	negative
PRJNA729271	stage_E14_vs_E10	ordinary_muscle_development	ENSGALG00010023642	PAX6	4.353045883	0.489797272	8.887444124	6.25306E-19	1.21989E-17	positive
PRJNA729271	stage_E14_vs_E10	ordinary_muscle_development	ENSGALG00010023643	SCN4B	1.295410507	0.159251957	8.134345905	4.14168E-16	6.40701E-15	positive
PRJNA729271	stage_E14_vs_E10	ordinary_muscle_development	ENSGALG00010023646	DPH5	-0.094396289	0.142446683	-0.662678041	0.507536776	0.61640097	negative
PRJNA729271	stage_E14_vs_E10	ordinary_muscle_development	ENSGALG00010023648	SCN2B	1.364636396	0.360811317	3.782133023	0.00015549	0.000590472	positive
PRJNA729271	stage_E14_vs_E10	ordinary_muscle_development	ENSGALG00010023652	SLC30A7	-0.300422094	0.094691154	-3.172652155	0.001510534	0.004548232	negative
PRJNA729271	stage_E14_vs_E10	ordinary_muscle_development	ENSGALG00010023655	C1R	0.004582979	0.185788474	0.024667725	0.980319999	0.986987053	positive
PRJNA729271	stage_E14_vs_E10	ordinary_muscle_development	ENSGALG00010023658	ENSGALG00010023658	0.632413428	0.267907076	2.360570085	0.01824687	0.040159183	positive
PRJNA729271	stage_E14_vs_E10	ordinary_muscle_development	ENSGALG00010023660	CRYBB1	1.079772325	0.443253759	2.436013913	0.014850113	0.033654698	positive
PRJNA729271	stage_E14_vs_E10	ordinary_muscle_development	ENSGALG00010023663	PHOSPHO2	0.313861375	0.089091845	3.522896773	0.000426858	0.001467924	positive
PRJNA729271	stage_E14_vs_E10	ordinary_muscle_development	ENSGALG00010023664	SLC6A9	0.361070349	0.323919635	1.114691149	0.264982795	0.37375465	positive
PRJNA729271	stage_E14_vs_E10	ordinary_muscle_development	ENSGALG00010023665	C16orf87	0.010400472	0.135551575	0.076727047	0.938840687	0.957092613	positive
PRJNA729271	stage_E14_vs_E10	ordinary_muscle_development	ENSGALG00010023666	ENSGALG00010023666	0.658643811	0.253645688	2.59670809	0.009412187	0.02260266	positive
PRJNA729271	stage_E14_vs_E10	ordinary_muscle_development	ENSGALG00010023668	RCN1	0.129221214	0.160901163	0.803109264	0.421911584	0.536278755	positive
PRJNA729271	stage_E14_vs_E10	ordinary_muscle_development	ENSGALG00010023670	EXTL2	0.155327693	0.126164014	1.231156875	0.218264197	0.320411134	positive
PRJNA729271	stage_E14_vs_E10	ordinary_muscle_development	ENSGALG00010023671	ENSGALG00010023671	1.424972906	0.714029965	1.995676617	0.045969134	0.088453816	positive
PRJNA729271	stage_E14_vs_E10	ordinary_muscle_development	ENSGALG00010023676	ENSGALG00010023676	0.25869428	0.253823309	1.019190402	0.308112582	0.421767105	positive
PRJNA729271	stage_E14_vs_E10	ordinary_muscle_development	ENSGALG00010023677	MN1	0.179313066	0.161000345	1.113743363	0.265389301	0.374204077	positive
PRJNA729271	stage_E14_vs_E10	ordinary_muscle_development	ENSGALG00010023680	MPZL3	-2.365266134	0.350807586	-6.742346019	1.55849E-11	1.5666E-10	negative
PRJNA729271	stage_E14_vs_E10	ordinary_muscle_development	ENSGALG00010023686	MYLK3	0.648831919	0.218978348	2.962995772	0.003046608	0.0084292	positive
PRJNA729271	stage_E14_vs_E10	ordinary_muscle_development	ENSGALG00010023689	WT1	-2.100354092	0.233961677	-8.977342419	2.77386E-19	5.63914E-18	negative
PRJNA729271	stage_E14_vs_E10	ordinary_muscle_development	ENSGALG00010023693	SRSF11	-0.213922081	0.068091	-3.141708626	0.001679651	0.00499929	negative
PRJNA729271	stage_E14_vs_E10	ordinary_muscle_development	ENSGALG00010023694	PPCDC	0.544541864	0.15329259	3.552303894	0.000381874	0.001331871	positive
PRJNA729271	stage_E14_vs_E10	ordinary_muscle_development	ENSGALG00010023695	CLSTN3	-0.21336623	0.128379936	-1.661990472	0.096514685	0.165129882	negative
PRJNA729271	stage_E14_vs_E10	ordinary_muscle_development	ENSGALG00010023696	LRRC4C	-1.979646053	0.533049953	-3.71380964	0.000204162	0.000756863	negative
PRJNA729271	stage_E14_vs_E10	ordinary_muscle_development	ENSGALG00010023698	ENSGALG00010023698	-0.134457887	0.12915042	-1.04109524	0.297831349	0.41126659	negative
PRJNA729271	stage_E14_vs_E10	ordinary_muscle_development	ENSGALG00010023699	PPIG	-0.575650272	0.202980712	-2.835985088	0.004568458	0.012031213	negative
PRJNA729271	stage_E14_vs_E10	ordinary_muscle_development	ENSGALG00010023702	COL4A4	0.551927931	0.253627841	2.176133067	0.029545312	0.060647992	positive
PRJNA729271	stage_E14_vs_E10	ordinary_muscle_development	ENSGALG00010023703	PITPNB	-0.146516916	0.07874555	-1.860637402	0.062795399	0.115013812	negative
PRJNA729271	stage_E14_vs_E10	ordinary_muscle_development	ENSGALG00010023704	ANKRD13C	0.135024827	0.067808121	1.991278122	0.046450319	0.08921391	positive
PRJNA729271	stage_E14_vs_E10	ordinary_muscle_development	ENSGALG00010023706	CDK5RAP3	0.325888072	0.125467502	2.597390295	0.009393512	0.022561551	positive
PRJNA729271	stage_E14_vs_E10	ordinary_muscle_development	ENSGALG00010023707	MPZL2	-1.935191942	0.277546669	-6.972492056	3.11375E-12	3.38335E-11	negative
PRJNA729271	stage_E14_vs_E10	ordinary_muscle_development	ENSGALG00010023710	CDC14A	-0.206890262	0.120692726	-1.714189983	0.08649384	0.150905465	negative
PRJNA729271	stage_E14_vs_E10	ordinary_muscle_development	ENSGALG00010023711	ENSGALG00010023711	-0.343759481	0.337059691	-1.019877163	0.307786722	0.421400636	negative
PRJNA729271	stage_E14_vs_E10	ordinary_muscle_development	ENSGALG00010023712	ARHGAP40	0.244312664	0.164322555	1.486787152	0.137071036	0.220417646	positive
PRJNA729271	stage_E14_vs_E10	ordinary_muscle_development	ENSGALG00010023714	EIF3M	0.209766632	0.110292848	1.901906034	0.05718344	0.106161788	positive
PRJNA729271	stage_E14_vs_E10	ordinary_muscle_development	ENSGALG00010023715	CD3E	-2.903736215	0.425558624	-6.823351823	8.89405E-12	9.15619E-11	negative
PRJNA729271	stage_E14_vs_E10	ordinary_muscle_development	ENSGALG00010023716	FASTKD1	-0.209147072	0.101462045	-2.061333099	0.039271273	0.077171721	negative
PRJNA729271	stage_E14_vs_E10	ordinary_muscle_development	ENSGALG00010023720	SCAMP5	-0.440312616	0.093222887	-4.723224411	2.32134E-06	1.20905E-05	negative
PRJNA729271	stage_E14_vs_E10	ordinary_muscle_development	ENSGALG00010023724	CYP2R1	-0.878702571	0.12408119	-7.081674253	1.42423E-12	1.59538E-11	negative
PRJNA729271	stage_E14_vs_E10	ordinary_muscle_development	ENSGALG00010023726	PNPO	0.326295521	0.179308185	1.819746937	0.068797552	0.12431071	positive
PRJNA729271	stage_E14_vs_E10	ordinary_muscle_development	ENSGALG00010023730	SP2	0.169285953	0.186736931	0.906547794	0.36464598	0.480678744	positive
PRJNA729271	stage_E14_vs_E10	ordinary_muscle_development	ENSGALG00010023731	ACTR5	0.210556157	0.095023977	2.215821356	0.026703739	0.055717677	positive
PRJNA729271	stage_E14_vs_E10	ordinary_muscle_development	ENSGALG00010023732	TM2D1	-0.079650127	0.077126396	-1.032722023	0.301734004	0.415507732	negative
PRJNA729271	stage_E14_vs_E10	ordinary_muscle_development	ENSGALG00010023733	ORC6	-0.324598073	0.186033134	-1.744840104	0.081012706	0.142960698	negative
PRJNA729271	stage_E14_vs_E10	ordinary_muscle_development	ENSGALG00010023736	KIF5A	-2.970271691	0.617113898	-4.813166098	1.48558E-06	8.00202E-06	negative
PRJNA729271	stage_E14_vs_E10	ordinary_muscle_development	ENSGALG00010023738	ENSGALG00010023738	0.302331893	0.389066714	0.777069542	0.437117743	0.55100632	positive
PRJNA729271	stage_E14_vs_E10	ordinary_muscle_development	ENSGALG00010023740	PPP1R16B	0.03795099	0.101216374	0.374949115	0.707698311	0.7877505	positive
PRJNA729271	stage_E14_vs_E10	ordinary_muscle_development	ENSGALG00010023743	CD3D	-0.366456155	0.239188765	-1.532079297	0.125502868	0.204860819	negative
PRJNA729271	stage_E14_vs_E10	ordinary_muscle_development	ENSGALG00010023744	MT4	2.515127402	0.31530898	7.976707179	1.50289E-15	2.22744E-14	positive
PRJNA729271	stage_E14_vs_E10	ordinary_muscle_development	ENSGALG00010023746	TTC28	-0.440870441	0.088061971	-5.006365803	5.54673E-07	3.17535E-06	negative
PRJNA729271	stage_E14_vs_E10	ordinary_muscle_development	ENSGALG00010023748	ENSGALG00010023748	-0.572177051	0.167844817	-3.408964671	0.000652099	0.002145174	negative
PRJNA729271	stage_E14_vs_E10	ordinary_muscle_development	ENSGALG00010023749	RPP25	0.456956483	0.240271946	1.901830362	0.057193336	0.106161788	positive
PRJNA729271	stage_E14_vs_E10	ordinary_muscle_development	ENSGALG00010023750	ENSGALG00010023750	-0.060156127	0.160195909	-0.375516002	0.707276748	0.787523157	negative
PRJNA729271	stage_E14_vs_E10	ordinary_muscle_development	ENSGALG00010023751	PRRG4	-0.302193127	0.261453372	-1.155820345	0.247754649	0.354546712	negative
PRJNA729271	stage_E14_vs_E10	ordinary_muscle_development	ENSGALG00010023752	C1QL1	-0.344048152	0.251140488	-1.369942995	0.170704697	0.263262364	negative
PRJNA729271	stage_E14_vs_E10	ordinary_muscle_development	ENSGALG00010023759	CALCA	-1.261200516	0.263219434	-4.791441482	1.65587E-06	8.85352E-06	negative
PRJNA729271	stage_E14_vs_E10	ordinary_muscle_development	ENSGALG00010023760	ZW10	-0.107026821	0.089985639	-1.189376676	0.234291475	0.338807619	negative
PRJNA729271	stage_E14_vs_E10	ordinary_muscle_development	ENSGALG00010023762	BBS5	1.713974494	0.152115252	11.26760448	1.8966E-29	7.89977E-28	positive
PRJNA729271	stage_E14_vs_E10	ordinary_muscle_development	ENSGALG00010023764	COX5A	0.092918179	0.178072019	0.521801119	0.601808811	0.700435098	positive
PRJNA729271	stage_E14_vs_E10	ordinary_muscle_development	ENSGALG00010023766	ENSGALG00010023766	1.158675744	0.330940492	3.501160393	0.000463237	0.00158289	positive
PRJNA729271	stage_E14_vs_E10	ordinary_muscle_development	ENSGALG00010023767	ENSGALG00010023767	-0.626129342	0.214355196	-2.920989809	0.003489212	0.009480063	negative
PRJNA729271	stage_E14_vs_E10	ordinary_muscle_development	ENSGALG00010023770	SCRN2	0.287739613	0.134809275	2.134419999	0.032808416	0.066171399	positive
PRJNA729271	stage_E14_vs_E10	ordinary_muscle_development	ENSGALG00010023773	OVOA	1.800859812	0.499357166	3.606356203	0.000310527	0.001108096	positive
PRJNA729271	stage_E14_vs_E10	ordinary_muscle_development	ENSGALG00010023775	ENSGALG00010023775	0.463528348	0.139149647	3.331150024	0.00086488	0.002768035	positive
PRJNA729271	stage_E14_vs_E10	ordinary_muscle_development	ENSGALG00010023777	OAZ2	0.187352636	0.132163635	1.417580836	0.156313178	0.245344832	positive
PRJNA729271	stage_E14_vs_E10	ordinary_muscle_development	ENSGALG00010023778	FANCF	-0.472454042	0.199669695	-2.366178008	0.017972801	0.039640253	negative
PRJNA729271	stage_E14_vs_E10	ordinary_muscle_development	ENSGALG00010023781	COL4A3	1.429083364	0.234995956	6.081310452	1.19204E-09	9.64749E-09	positive
PRJNA729271	stage_E14_vs_E10	ordinary_muscle_development	ENSGALG00010023783	VPS35	0.34917122	0.062224236	5.611498696	2.00582E-08	1.40932E-07	positive
PRJNA729271	stage_E14_vs_E10	ordinary_muscle_development	ENSGALG00010023786	ENSGALG00010023786	-0.277042292	0.276274492	-1.002779121	0.315967445	0.429840274	negative
PRJNA729271	stage_E14_vs_E10	ordinary_muscle_development	ENSGALG00010023788	RTCA	-0.458224831	0.104010044	-4.405582494	1.055E-05	4.92503E-05	negative
PRJNA729271	stage_E14_vs_E10	ordinary_muscle_development	ENSGALG00010023791	VTI1B	-0.067717615	0.080342926	-0.842857216	0.399308304	0.514623799	negative
PRJNA729271	stage_E14_vs_E10	ordinary_muscle_development	ENSGALG00010023792	ZNF609	-0.11387226	0.176426448	-0.645437582	0.518643655	0.626843974	negative
PRJNA729271	stage_E14_vs_E10	ordinary_muscle_development	ENSGALG00010023793	RBM45	-0.013210555	0.063507818	-0.208014624	0.835217548	0.885041185	negative
PRJNA729271	stage_E14_vs_E10	ordinary_muscle_development	ENSGALG00010023794	MT3	0.735611191	0.466129509	1.578126201	0.11453662	0.190675124	positive
PRJNA729271	stage_E14_vs_E10	ordinary_muscle_development	ENSGALG00010023798	ARCN1	-0.004585422	0.056211311	-0.081574713	0.934984911	0.954677816	negative
PRJNA729271	stage_E14_vs_E10	ordinary_muscle_development	ENSGALG00010023799	NMT1	0.33161397	0.126156997	2.628581674	0.008574176	0.020860378	positive
PRJNA729271	stage_E14_vs_E10	ordinary_muscle_development	ENSGALG00010023800	PEX5	0.116918472	0.071589105	1.633188073	0.102429448	0.173530051	positive
PRJNA729271	stage_E14_vs_E10	ordinary_muscle_development	ENSGALG00010023802	ENSGALG00010023802	-0.285098673	0.306732292	-0.929470682	0.352645212	0.46895682	negative
PRJNA729271	stage_E14_vs_E10	ordinary_muscle_development	ENSGALG00010023805	GALNT6	-1.064968057	0.32551996	-3.271590646	0.001069443	0.003346627	negative
PRJNA729271	stage_E14_vs_E10	ordinary_muscle_development	ENSGALG00010023806	PATJ	-1.377495096	0.171504602	-8.031825866	9.60326E-16	1.44248E-14	negative
PRJNA729271	stage_E14_vs_E10	ordinary_muscle_development	ENSGALG00010023807	MRPL10	0.549356554	0.124620687	4.408229218	1.04219E-05	4.87466E-05	positive
PRJNA729271	stage_E14_vs_E10	ordinary_muscle_development	ENSGALG00010023808	DHX35	-0.13728988	0.113214454	-1.212653284	0.225262408	0.328422712	negative
PRJNA729271	stage_E14_vs_E10	ordinary_muscle_development	ENSGALG00010023809	CDON	-0.633686101	0.096352114	-6.576774251	4.80764E-11	4.62114E-10	negative
PRJNA729271	stage_E14_vs_E10	ordinary_muscle_development	ENSGALG00010023811	MRPL44	0.392866245	0.083964677	4.678946638	2.88352E-06	1.48005E-05	positive
PRJNA729271	stage_E14_vs_E10	ordinary_muscle_development	ENSGALG00010023813	CELA1	0.472228656	5.322023468	0.088731036	0.929295667	0.951415503	positive
PRJNA729271	stage_E14_vs_E10	ordinary_muscle_development	ENSGALG00010023815	PLCD3	0.296693156	0.135723474	2.18601209	0.028814721	0.059376583	positive
PRJNA729271	stage_E14_vs_E10	ordinary_muscle_development	ENSGALG00010023817	BBS2	0.043821125	0.115048342	0.380893145	0.703282539	0.784441623	positive
PRJNA729271	stage_E14_vs_E10	ordinary_muscle_development	ENSGALG00010023818	ALOX5	0.834670631	0.187723326	4.446280852	8.73697E-06	4.13459E-05	positive
PRJNA729271	stage_E14_vs_E10	ordinary_muscle_development	ENSGALG00010023819	BIN2	0.545059118	0.233505732	2.334242993	0.019583002	0.042678637	positive
PRJNA729271	stage_E14_vs_E10	ordinary_muscle_development	ENSGALG00010023820	CYP1C1	-1.217919577	0.901089035	-1.351608475	0.176500598	0.270355718	negative
PRJNA729271	stage_E14_vs_E10	ordinary_muscle_development	ENSGALG00010023821	KANK4	-0.771721124	0.123871089	-6.230034204	4.66333E-10	3.98791E-09	negative
PRJNA729271	stage_E14_vs_E10	ordinary_muscle_development	ENSGALG00010023822	CHEK2	-0.618641456	0.119183011	-5.190684899	2.09522E-07	1.27875E-06	negative
PRJNA729271	stage_E14_vs_E10	ordinary_muscle_development	ENSGALG00010023823	MUC2	0.994361532	0.541257801	1.837131086	0.066190546	0.120260251	positive
PRJNA729271	stage_E14_vs_E10	ordinary_muscle_development	ENSGALG00010023826	DBT	0.609606017	0.126975753	4.800963974	1.57904E-06	8.47081E-06	positive
PRJNA729271	stage_E14_vs_E10	ordinary_muscle_development	ENSGALG00010023828	CHEK1	0.121741076	0.157126941	0.774794416	0.438461149	0.552442087	positive
PRJNA729271	stage_E14_vs_E10	ordinary_muscle_development	ENSGALG00010023829	USP1	-0.153163724	0.099225867	-1.543586658	0.122688499	0.201150299	negative
PRJNA729271	stage_E14_vs_E10	ordinary_muscle_development	ENSGALG00010023830	AGFG1	-0.024796625	0.113221754	-0.219009368	0.82664275	0.878652685	negative
PRJNA729271	stage_E14_vs_E10	ordinary_muscle_development	ENSGALG00010023831	SATB2	-3.185355293	0.512508365	-6.21522596	5.12508E-10	4.34942E-09	negative
PRJNA729271	stage_E14_vs_E10	ordinary_muscle_development	ENSGALG00010023832	ENSGALG00010023832	-0.320891156	0.463405271	-0.692463326	0.488646409	0.599575185	negative
PRJNA729271	stage_E14_vs_E10	ordinary_muscle_development	ENSGALG00010023833	PIP4K2C	0.338752087	0.105271029	3.217904201	0.001291309	0.003962767	positive
PRJNA729271	stage_E14_vs_E10	ordinary_muscle_development	ENSGALG00010023835	INAFM2	0.062634711	0.120554071	0.519556999	0.603372375	0.701578901	positive
PRJNA729271	stage_E14_vs_E10	ordinary_muscle_development	ENSGALG00010023836	NDUFS3	0.50764401	0.14776492	3.435483955	0.000591496	0.001967357	positive

PRJNA729271	stage_E14_vs_E10	ordinary_muscle_development	ENSGALG00010023837	STT3A	-0.105359343	0.091668077	-1.149356959	0.25040882	0.357603532	negative
PRJNA729271	stage_E14_vs_E10	ordinary_muscle_development	ENSGALG00010023840	SMAGP	-0.100946128	0.568760344	-0.17748447	0.859127863	0.902984437	negative
PRJNA729271	stage_E14_vs_E10	ordinary_muscle_development	ENSGALG00010023841	OSBPL7	0.041895837	0.145786524	0.28737798	0.77382292	0.839688818	positive
PRJNA729271	stage_E14_vs_E10	ordinary_muscle_development	ENSGALG00010023847	PIK3AP1	0.101950552	0.184309232	0.553149458	0.580161072	0.681698666	positive
PRJNA729271	stage_E14_vs_E10	ordinary_muscle_development	ENSGALG00010023850	DAZAP2	0.218379922	0.210643385	1.036728128	0.299862579	0.413637998	positive
PRJNA729271	stage_E14_vs_E10	ordinary_muscle_development	ENSGALG00010023851	HSCB	0.049531821	0.148624183	0.333268923	0.738931296	0.812268447	positive
PRJNA729271	stage_E14_vs_E10	ordinary_muscle_development	ENSGALG00010023852	LRRC39	1.677251055	0.190825762	8.789437208	1.50311E-18	2.86679E-17	positive
PRJNA729271	stage_E14_vs_E10	ordinary_muscle_development	ENSGALG00010023854	SHCBP1	-0.065239467	0.215806988	-0.302304701	0.762419795	0.830741147	negative
PRJNA729271	stage_E14_vs_E10	ordinary_muscle_development	ENSGALG00010023857	ACBD4	0.280449836	0.151440649	1.851879519	0.064043126	0.116944458	positive
PRJNA729271	stage_E14_vs_E10	ordinary_muscle_development	ENSGALG00010023859	ENSGALG00010023859	1.056544244	0.879919845	1.200727828	0.229856796	0.333744792	positive
PRJNA729271	stage_E14_vs_E10	ordinary_muscle_development	ENSGALG00010023860	TRIP4	0.160440716	0.113272275	1.416416464	0.156653606	0.245746106	positive
PRJNA729271	stage_E14_vs_E10	ordinary_muscle_development	ENSGALG00010023861	ENSGALG00010023861	-0.47056447	0.148731993	-3.163841614	0.001557015	0.00466493	negative
PRJNA729271	stage_E14_vs_E10	ordinary_muscle_development	ENSGALG00010023862	TRMT13	-0.153606116	0.12639604	-1.215276335	0.224260715	0.327291488	negative
PRJNA729271	stage_E14_vs_E10	ordinary_muscle_development	ENSGALG00010023863	EYA2	-0.230510885	0.228824078	-1.007371632	0.313756218	0.427603833	negative
PRJNA729271	stage_E14_vs_E10	ordinary_muscle_development	ENSGALG00010023864	EPHA1	-0.747175629	0.548151009	-1.36308356	0.172856177	0.265869089	negative
PRJNA729271	stage_E14_vs_E10	ordinary_muscle_development	ENSGALG00010023866	AMBRA1	0.0714455	0.07356586	0.971177391	0.331459951	0.446267136	positive
PRJNA729271	stage_E14_vs_E10	ordinary_muscle_development	ENSGALG00010023867	ENSGALG00010023867	0.430140763	0.488522553	0.880493153	0.378592213	0.494788038	positive
PRJNA729271	stage_E14_vs_E10	ordinary_muscle_development	ENSGALG00010023869	DOCK7	-0.306971771	0.045808287	-6.701227857	2.06675E-11	2.05049E-10	negative
PRJNA729271	stage_E14_vs_E10	ordinary_muscle_development	ENSGALG00010023871	UQCRFS1	0.231576532	0.140383323	1.64960144	0.09902448	0.168731337	positive
PRJNA729271	stage_E14_vs_E10	ordinary_muscle_development	ENSGALG00010023874	HEXIM1	0.230404039	0.22476676	1.025080571	0.305325172	0.419099278	positive
PRJNA729271	stage_E14_vs_E10	ordinary_muscle_development	ENSGALG00010023878	ENSGALG00010023878	1.166335132	1.533246884	0.760696235	0.446838524	0.560197934	positive
PRJNA729271	stage_E14_vs_E10	ordinary_muscle_development	ENSGALG00010023879	EI24	0.097414448	0.116830247	0.83381188	0.404387	0.519368205	positive
PRJNA729271	stage_E14_vs_E10	ordinary_muscle_development	ENSGALG00010023880	ENSGALG00010023880	0.003886124	0.190390828	0.020411301	0.983715269	0.989226124	positive
PRJNA729271	stage_E14_vs_E10	ordinary_muscle_development	ENSGALG00010023881	VSTM2B	0.751675492	0.208041344	3.613106313	0.000302551	0.001082832	positive
PRJNA729271	stage_E14_vs_E10	ordinary_muscle_development	ENSGALG00010023883	TBX21	3.539515364	0.349914475	10.1153728	4.72216E-24	1.31378E-22	positive
PRJNA729271	stage_E14_vs_E10	ordinary_muscle_development	ENSGALG00010023884	C2orf69	-0.723434804	0.123859725	-5.840759017	5.19635E-09	3.92298E-08	negative
PRJNA729271	stage_E14_vs_E10	ordinary_muscle_development	ENSGALG00010023887	RASA2	-0.602642316	0.128045546	-4.706468402	2.52045E-06	1.30805E-05	negative
PRJNA729271	stage_E14_vs_E10	ordinary_muscle_development	ENSGALG00010023888	CTH	0.050357066	0.258220056	0.195016092	0.84538035	0.892352765	positive
PRJNA729271	stage_E14_vs_E10	ordinary_muscle_development	ENSGALG00010023889	SAMD8	0.253148851	0.191975264	1.318653488	0.187284983	0.283637637	positive
PRJNA729271	stage_E14_vs_E10	ordinary_muscle_development	ENSGALG00010023890	SASS6	-0.107097303	0.117419224	-0.912093428	0.361719538	0.477879911	negative
PRJNA729271	stage_E14_vs_E10	ordinary_muscle_development	ENSGALG00010023891	ENSGALG00010023891	0.249673758	0.320641182	0.778670276	0.43617397	0.550103688	positive
PRJNA729271	stage_E14_vs_E10	ordinary_muscle_development	ENSGALG00010023896	POP4	-0.304002215	0.122871953	-2.474138382	0.013355795	0.030689958	negative
PRJNA729271	stage_E14_vs_E10	ordinary_muscle_development	ENSGALG00010023902	PTGER3	1.340208515	0.232802542	5.756846578	8.56997E-09	6.28653E-08	positive
PRJNA729271	stage_E14_vs_E10	ordinary_muscle_development	ENSGALG00010023903	TBKBP1	-0.870542364	0.396410971	-2.196060222	0.028087633	0.058161463	negative
PRJNA729271	stage_E14_vs_E10	ordinary_muscle_development	ENSGALG00010023909	FOXRED1	0.108094878	0.152135793	0.710515758	0.477384362	0.589514356	positive
PRJNA729271	stage_E14_vs_E10	ordinary_muscle_development	ENSGALG00010023910	RNF7	0.145003123	0.100483764	1.443050274	0.14900631	0.236152029	positive
PRJNA729271	stage_E14_vs_E10	ordinary_muscle_development	ENSGALG00010023911	TFCP2	-0.117684898	0.120828073	-0.973986379	0.330063302	0.444799885	negative
PRJNA729271	stage_E14_vs_E10	ordinary_muscle_development	ENSGALG00010023913	ZMYND8	-0.359914238	0.082464501	-4.364474813	1.27429E-05	5.88054E-05	negative
PRJNA729271	stage_E14_vs_E10	ordinary_muscle_development	ENSGALG00010023914	CCDC117	-0.087668355	0.063567872	-1.379129932	0.167854697	0.259691944	negative
PRJNA729271	stage_E14_vs_E10	ordinary_muscle_development	ENSGALG00010023917	ZRANB2	0.166272697	0.078720262	2.112196945	0.03466956	0.069401364	positive
PRJNA729271	stage_E14_vs_E10	ordinary_muscle_development	ENSGALG00010023918	MFSD14A	-0.27827119	0.079152003	-3.515655699	0.000438669	0.0015064	negative
PRJNA729271	stage_E14_vs_E10	ordinary_muscle_development	ENSGALG00010023920	ENSGALG00010023920	-2.112540946	0.440063598	-4.800535552	1.58242E-06	8.48582E-06	negative
PRJNA729271	stage_E14_vs_E10	ordinary_muscle_development	ENSGALG00010023921	GRK7	-0.283351525	0.182301392	-1.554302586	0.120112257	0.197749364	negative
PRJNA729271	stage_E14_vs_E10	ordinary_muscle_development	ENSGALG00010023923	CSNK1G1	-0.038408273	0.093868856	-0.409169505	0.682415272	0.767540557	negative
PRJNA729271	stage_E14_vs_E10	ordinary_muscle_development	ENSGALG00010023925	NCOA3	-0.09462075	0.076187238	-1.241950124	0.214254965	0.315965583	negative
PRJNA729271	stage_E14_vs_E10	ordinary_muscle_development	ENSGALG00010023926	ZYX	0.182375329	0.136857241	1.332595398	0.182664616	0.278122228	positive
PRJNA729271	stage_E14_vs_E10	ordinary_muscle_development	ENSGALG00010023927	KPNB1	-0.365225311	0.130007491	-2.809263588	0.004965497	0.012947452	negative
PRJNA729271	stage_E14_vs_E10	ordinary_muscle_development	ENSGALG00010023930	SRPRA	-0.06471137	0.131142201	-0.493444286	0.621698697	0.71701835	negative
PRJNA729271	stage_E14_vs_E10	ordinary_muscle_development	ENSGALG00010023931	CSRNP2	-0.403063047	0.138244879	-2.915573075	0.00355036	0.009622749	negative
PRJNA729271	stage_E14_vs_E10	ordinary_muscle_development	ENSGALG00010023937	XBP1	-0.000148152	0.10973493	-0.001350093	0.998922782	0.999350415	negative
PRJNA729271	stage_E14_vs_E10	ordinary_muscle_development	ENSGALG00010023940	ATP1B3	-0.135786665	0.071392802	-1.901965756	0.057175632	0.106161788	negative
PRJNA729271	stage_E14_vs_E10	ordinary_muscle_development	ENSGALG00010023942	NEGR1	1.553659492	0.220405521	7.049095159	1.80085E-12	1.99719E-11	positive
PRJNA729271	stage_E14_vs_E10	ordinary_muscle_development	ENSGALG00010023945	LETMD1	0.112164507	0.209737836	0.534784323	0.592799005	0.69283827	positive
PRJNA729271	stage_E14_vs_E10	ordinary_muscle_development	ENSGALG00010023946	ZNRF3	-0.426766576	0.124003098	-3.441579936	0.000578328	0.001929311	negative
PRJNA729271	stage_E14_vs_E10	ordinary_muscle_development	ENSGALG00010023947	ENSGALG00010023947	-1.111342647	0.446334588	-2.489931716	0.012776764	0.029551491	negative
PRJNA729271	stage_E14_vs_E10	ordinary_muscle_development	ENSGALG00010023948	SULF2	-1.91185099	0.220851413	-8.6567297	4.85492E-18	8.90786E-17	negative
PRJNA729271	stage_E14_vs_E10	ordinary_muscle_development	ENSGALG00010023953	SLC35A3	-0.587456018	0.154667793	-3.798179347	0.000145763	0.000556888	negative
PRJNA729271	stage_E14_vs_E10	ordinary_muscle_development	ENSGALG00010023954	PLEKHF1	0.837816374	0.094226641	8.89150206	6.02883E-19	1.17932E-17	positive
PRJNA729271	stage_E14_vs_E10	ordinary_muscle_development	ENSGALG00010023956	KREMEN1	-0.244239751	0.129453086	-1.88670474	0.05920005	0.10928486	negative
PRJNA729271	stage_E14_vs_E10	ordinary_muscle_development	ENSGALG00010023957	YBX3	1.356420279	0.13884131	9.769572754	1.52092E-22	3.92972E-21	positive
PRJNA729271	stage_E14_vs_E10	ordinary_muscle_development	ENSGALG00010023962	FPGT	-0.092985923	0.112112517	-0.829398224	0.406879106	0.521782128	negative
PRJNA729271	stage_E14_vs_E10	ordinary_muscle_development	ENSGALG00010023966	PEAK1	0.254648049	0.097994369	2.598598799	0.00936051	0.022493468	positive
PRJNA729271	stage_E14_vs_E10	ordinary_muscle_development	ENSGALG00010023967	PRPH	-1.081246051	0.264695662	-4.084865023	4.41024E-05	0.000185026	negative
PRJNA729271	stage_E14_vs_E10	ordinary_muscle_development	ENSGALG00010023968	TYW5	0.063547884	0.113412095	0.560327225	0.575256261	0.677229204	positive
PRJNA729271	stage_E14_vs_E10	ordinary_muscle_development	ENSGALG00010023972	ENSGALG00010023972	-0.496221333	0.528903177	-0.938208266	0.348137391	0.463895154	negative
PRJNA729271	stage_E14_vs_E10	ordinary_muscle_development	ENSGALG00010023974	PREX1	-0.500619455	0.103009971	-4.8599126	1.17438E-06	6.439E-06	negative
PRJNA729271	stage_E14_vs_E10	ordinary_muscle_development	ENSGALG00010023978	TFDP2	-0.039907811	0.139261784	-0.28656685	0.774444	0.840111194	negative
PRJNA729271	stage_E14_vs_E10	ordinary_muscle_development	ENSGALG00010023979	FAM118B	0.085476875	0.111105655	0.769329649	0.44169764	0.555668052	positive
PRJNA729271	stage_E14_vs_E10	ordinary_muscle_development	ENSGALG00010023981	ENSGALG00010023981	-0.551290371	0.372882009	-1.478457952	0.139285244	0.223457621	negative
PRJNA729271	stage_E14_vs_E10	ordinary_muscle_development	ENSGALG00010023984	FMNL1	0.495102026	0.210954683	2.34695916	0.018927329	0.041430329	positive
PRJNA729271	stage_E14_vs_E10	ordinary_muscle_development	ENSGALG00010023986	SYCE3	-0.080969482	0.571829175	-0.141597326	0.88739808	0.922688479	negative
PRJNA729271	stage_E14_vs_E10	ordinary_muscle_development	ENSGALG00010023987	TNNI3K	-0.140082916	0.426647603	-0.328334005	0.742659136	0.815271667	negative
PRJNA729271	stage_E14_vs_E10	ordinary_muscle_development	ENSGALG00010023989	ENSGALG00010023989	-0.240757646	0.099031832	-2.431113719	0.015052489	0.034054917	negative
PRJNA729271	stage_E14_vs_E10	ordinary_muscle_development	ENSGALG00010023991	TSPAN3	0.130627678	0.105241824	1.241214504	0.214526518	0.316173043	positive
PRJNA729271	stage_E14_vs_E10	ordinary_muscle_development	ENSGALG00010023992	FAM131B	0.47187711	0.128483518	3.672666486	0.000240033	0.000874189	positive
PRJNA729271	stage_E14_vs_E10	ordinary_muscle_development	ENSGALG00010023993	C19orf12	0.190651987	0.174531803	1.092362447	0.274673822	0.385010836	positive
PRJNA729271	stage_E14_vs_E10	ordinary_muscle_development	ENSGALG00010023996	MYO7B	1.685052348	0.506410005	3.327446794	0.000876457	0.002799034	positive
PRJNA729271	stage_E14_vs_E10	ordinary_muscle_development	ENSGALG00010023997	ARFGEF2	-0.211153279	0.061759871	-3.418939793	0.000628656	0.002074294	negative
PRJNA729271	stage_E14_vs_E10	ordinary_muscle_development	ENSGALG00010023999	DGCR14	0.231535321	0.089924348	2.574778995	0.010030416	0.023877187	positive
PRJNA729271	stage_E14_vs_E10	ordinary_muscle_development	ENSGALG00010024001	ENSGALG00010024001	3.129429143	0.452724821	6.912431125	4.76417E-12	5.04432E-11	positive
PRJNA729271	stage_E14_vs_E10	ordinary_muscle_development	ENSGALG00010024002	DGCR2	-0.182959386	0.059260546	-3.08737259	0.002019343	0.005861109	negative
PRJNA729271	stage_E14_vs_E10	ordinary_muscle_development	ENSGALG00010024003	ERICH3	-1.509772003	0.301258626	-5.011547799	5.3994E-07	3.09712E-06	negative
PRJNA729271	stage_E14_vs_E10	ordinary_muscle_development	ENSGALG00010024004	EIF4B	0.454132162	0.124983693	3.633531319	0.000279568	0.001006544	positive
PRJNA729271	stage_E14_vs_E10	ordinary_muscle_development	ENSGALG00010024005	STYK1	-0.000962907	0.258847202	-0.003719982	0.997031891	0.998283737	negative
PRJNA729271	stage_E14_vs_E10	ordinary_muscle_development	ENSGALG00010024008	CCNE1	-0.620302588	0.103696699	-5.981893286	2.20559E-09	1.73939E-08	negative
PRJNA729271	stage_E14_vs_E10	ordinary_muscle_development	ENSGALG00010024009	CSE1L	-0.109222579	0.060553622	-1.803733216	0.07127314	0.128065472	negative
PRJNA729271	stage_E14_vs_E10	ordinary_muscle_development	ENSGALG00010024010	ENSGALG00010024010	0.696572118	0.088509225	7.870051042	3.54497E-15	5.06748E-14	positive
PRJNA729271	stage_E14_vs_E10	ordinary_muscle_development	ENSGALG00010024011	AGPS	0.209828041	0.069686108	3.011045475	0.002603499	0.007327711	positive
PRJNA729271	stage_E14_vs_E10	ordinary_muscle_development	ENSGALG00010024013	PTPRJ	-0.170000209	0.119986903	-1.416823045	0.15653467	0.245612692	negative
PRJNA729271	stage_E14_vs_E10	ordinary_muscle_development	ENSGALG00010024016	CRYZ	0.31988275	0.101532961	3.150531071	0.001629739	0.004861714	positive
PRJNA729271	stage_E14_vs_E10	ordinary_muscle_development	ENSGALG00010024017	TYW3	0.059512889	0.115798471	0.513935014	0.607297427	0.704952963	positive
PRJNA729271	stage_E14_vs_E10	ordinary_muscle_development	ENSGALG00010024018	KRT18	-2.424104127	0.930046626	-2.606432902	0.009149075	0.022047521	negative
PRJNA729271	stage_E14_vs_E10	ordinary_muscle_development	ENSGALG00010024019	MAP3K14	-0.046155338	0.107879834	-0.427840278	0.66876741	0.756440391	negative
PRJNA729271	stage_E14_vs_E10	ordinary_muscle_development	ENSGALG00010024020	MAIP1	-0.088070842	0.121879424	-0.722606324	0.469921782	0.582230638	negative
PRJNA729271	stage_E14_vs_E10	ordinary_muscle_development	ENSGALG00010024021	IGLL1	3.153093156	1.334091636	2.363475696	0.018104414	0.03990018	positive
PRJNA729271	stage_E14_vs_E10	ordinary_muscle_development	ENSGALG00010024023	LHX8	-3.113078498	0.473907867	-6.568952986	5.06703E-11	4.85384E-10	negative
PRJNA729271	stage_E14_vs_E10	ordinary_muscle_development	ENSGALG00010024024	AGL	0.621972272	0.12293355	5.059418475	4.20537E-07	2.45695E-06	positive
PRJNA729271	stage_E14_vs_E10	ordinary_muscle_development	ENSGALG00010024025	CLCN1	0.449569995	0.395003924	1.138140579	0.255061786	0.362711722	positive
PRJNA729271	stage_E14_vs_E10	ordinary_muscle_development	ENSGALG00010024026	TIRAP	0.115906445	0.103155533	1.123608602	0.261179105	0.369417557	positive
PRJNA729271	stage_E14_vs_E10	ordinary_muscle_development	ENSGALG00010024029	SCAPER	0.075764468	0.146611236	0.516771221	0.605315872	0.703218207	positive
PRJNA729271	stage_E14_vs_E10	ordinary_muscle_development	ENSGALG00010024030	STAU1	0.397556516	0.089174573	4.458182456	8.26575E-06	3.92955E-05	positive
PRJNA729271	stage_E14_vs_E10	ordinary_muscle_development	ENSGALG00010024031	LIMS2	0.909952782	0.114987406	7.913499537	2.50253E-15	3.63468E-14	positive
PRJNA729271	stage_E14_vs_E10	ordinary_muscle_development	ENSGALG00010024034	LRP2	-0.780405421	0.244726701	-3.188885464	0.001428225	0.004326461	negative
PRJNA729271	stage_E14_vs_E10	ordinary_muscle_development	ENSGALG00010024035	OSBPL5	-0.548606221	0.136474738	-4.019837132	5.82384E-05	0.00023948	negative
PRJNA729271	stage_E14_vs_E10	ordinary_muscle_development	ENSGALG00010024038	NKD1	0.124388948	0.160981334	0.772691745	0.439704833	0.553545385	positive
PRJNA729271	stage_E14_vs_E10	ordinary_muscle_development	ENSGALG00010024039	MRPL45	-0.163577553	0.105709114	-1.547430929	0.121759368	0.199966241	negative
PRJNA729271	stage_E14_vs_E10	ordinary_muscle_development	ENSGALG00010024040	ENSGALG00010024040	-2.766609541	0.630270484	-4.389559104	1.13581E-05	5.2742E-05	negative
PRJNA729271	stage_E14_vs_E10	ordinary_muscle_development	ENSGALG00010024042	DCPS	0.158076504	0.128516472	1.230009672	0.218693483	0.320943813	positive
PRJNA729271	stage_E14_vs_E10	ordinary_muscle_development	ENSGALG00010024045	PSTPIP1	1.142797694	0.245987978	4.645746116	3.3885E-06	1.71615E-05	positive
PRJNA729271	stage_E14_vs_E10	ordinary_muscle_development	ENSGALG00010024046	ENSGALG00010024046	0.269149871	0.531226633	0.506657337	0.61239527	0.709066319	positive
PRJNA729271	stage_E14_vs_E10	ordinary_muscle_development	ENSGALG00010024049	RCN2	0.117104216	0.143550612	0.815769536	0.41463196	0.529369778	positive
PRJNA729271	stage_E14_vs_E10	ordinary_muscle_development	ENSGALG00010024051	ENSGALG00010024051	-1.718491208	0.630194195	-2.726923258	0.006392791	0.016109788	negative
PRJNA729271	stage_E14_vs_E10	ordinary_muscle_development	ENSGALG00010024052	ENSGALG00010024052	-1.385574944	0.177004605	-7.827903381	4.96073E-15	6.98114E-14	negative
PRJNA729271	stage_E14_vs_E10	ordinary_muscle_development	ENSGALG00010024053	ISL2	-1.701699823	0.566147237	-3.005754884	0.002649225	0.007446289	negative
PRJNA729271	stage_E14_vs_E10	ordinary_muscle_development	ENSGALG00010024054	ENSGALG00010024054	0.501081781	0.617242059	0.811807578	0.416902053	0.531516846	positive
PRJNA729271	stage_E14_vs_E10	ordinary_muscle_development	ENSGALG00010024057	HSD3B1	-2.259135355	0.70755987	-3.192853991	0.001408742	0.004273694	negative
PRJNA729271	stage_E14_vs_E10	ordinary_muscle_development	ENSGALG00010024058	SLC44A5	0.079258462	0.104896498	0.755587302	0.449896672	0.563050618	positive
PRJNA729271	stage_E14_vs_E10	ordinary_muscle_development	ENSGALG00010024060	FRRS1	-0.556985234	0.162314342	-3.431521997	0.000600204	0.001991378	negative
PRJNA729271	stage_E14_vs_E10	ordinary_muscle_development	ENSGALG00010024061	ENSGALG00010024061	9.150561386	1.191411798	7.680435433	1.58549E-14	2.14983E-13	positive
PRJNA729271	stage_E14_vs_E10	ordinary_muscle_development	ENSGALG00010024062	ST3GAL4	0.283153014	0.130783592	2.165049991	0.030383857	0.062012674	positive
PRJNA729271	stage_E14_vs_E10	ordinary_muscle_development	ENSGALG00010024063	BRD7	-0.120691326	0.1037085	-1.163755388	0.244523156	0.351031311	negative
PRJNA729271	stage_E14_vs_E10	ordinary_muscle_development	ENSGALG00010024064	GPR17	-0.457821643	0.211391015	-2.165757343	0.030329735	0.061954552	negative
PRJNA729271	stage_E14_vs_E10	ordinary_muscle_development	ENSGALG00010024066	ENSGALG00010024066	0.47249712	2.266538334	0.208466414	0.834864804	0.884874292	positive
PRJNA729271	stage_E14_vs_E10	ordinary_muscle_development	ENSGALG00010024068	CASP2	-0.655429402	0.082598095	-7.935163613	2.10218E-15	3.06242E-14	negative
PRJNA729271	stage_E14_vs_E10	ordinary_muscle_development	ENSGALG00010024069	ENSGALG00010024069	1.43336855	2.364835876	0.60611756	0.544436691	0.649996692	positive
PRJNA729271	stage_E14_vs_E10	ordinary_muscle_development	ENSGALG00010024073	RABGGTB	-0.258183448	0.1250264	-2.065031448	0.038920025	0.076546236	negative
PRJNA729271	stage_E14_vs_E10	ordinary_muscle_development	ENSGALG00010024074	ADCY7	-1.322878705	0.226556188	-5.839075584	5.24913E-09	3.95869E-08	negative
PRJNA729271	stage_E14_vs_E10	ordinary_muscle_development	ENSGALG00010024075	SPATS2L	0.311791251	0.135886773	2.294493007	0.021762196	0.046822478	positive
PRJNA729271	stage_E14_vs_E10	ordinary_muscle_development	ENSGALG00010024077	SFT2D3	0.286172227	0.114600947	2.49711922	0.012520685	0.029005487	positive
PRJNA729271	stage_E14_vs_E10	ordinary_muscle_development	ENSGALG00010024078	KIRREL3	1.508965633	0.224464612	6.722510161	1.7862E-11	1.78682E-10	positive
PRJNA729271	stage_E14_vs_E10	ordinary_muscle_development	ENSGALG00010024080	TENT4B	-0.199969098	0.086871273	-2.301901315	0.021340738	0.046038696	negative

PRJNA729271	stage_E14_vs_E10	ordinary_muscle_development	ENSGALG00010024081	CARS	-0.238977957	0.098436768	-2.427730631	0.015193623	0.034293299	negative
PRJNA729271	stage_E14_vs_E10	ordinary_muscle_development	ENSGALG00010024083	PALMD	1.274387204	0.189396151	6.728685877	1.71202E-11	1.7138E-10	positive
PRJNA729271	stage_E14_vs_E10	ordinary_muscle_development	ENSGALG00010024086	GABARAPL1	-0.001738149	0.214936261	-0.008086813	0.993547727	0.99636601	negative
PRJNA729271	stage_E14_vs_E10	ordinary_muscle_development	ENSGALG00010024089	ENSGALG00010024089	0.472506212	1.936858367	0.243954963	0.8072657	0.864603531	positive
PRJNA729271	stage_E14_vs_E10	ordinary_muscle_development	ENSGALG00010024091	ETFA	0.772595824	0.115899179	6.666102639	2.62686E-11	2.57969E-10	positive
PRJNA729271	stage_E14_vs_E10	ordinary_muscle_development	ENSGALG00010024095	RBM12	-0.18430225	0.05186077	-3.553789285	0.000379723	0.00132501	negative
PRJNA729271	stage_E14_vs_E10	ordinary_muscle_development	ENSGALG00010024096	MYO1D	-0.98651592	0.221088933	-4.46207735	8.11689E-06	3.86639E-05	negative
PRJNA729271	stage_E14_vs_E10	ordinary_muscle_development	ENSGALG00010024099	ENSGALG00010024099	0.472504096	2.081202727	0.227034152	0.820397186	0.874259106	positive
PRJNA729271	stage_E14_vs_E10	ordinary_muscle_development	ENSGALG00010024101	CNEP1R1	-0.038301752	0.089177156	-0.429501841	0.667558054	0.755426185	negative
PRJNA729271	stage_E14_vs_E10	ordinary_muscle_development	ENSGALG00010024102	SGF29	0.11639578	0.089708097	1.297494697	0.194461029	0.292549311	positive
PRJNA729271	stage_E14_vs_E10	ordinary_muscle_development	ENSGALG00010024104	PLPPR5	1.397365957	0.156437658	8.932414189	4.16812E-19	8.33336E-18	positive
PRJNA729271	stage_E14_vs_E10	ordinary_muscle_development	ENSGALG00010024106	WDR33	-0.000134151	0.062260046	-0.002154692	0.998280806	0.999038959	negative
PRJNA729271	stage_E14_vs_E10	ordinary_muscle_development	ENSGALG00010024107	NFE2L2	0.107698624	0.063334697	1.700467978	0.089042935	0.154461146	positive
PRJNA729271	stage_E14_vs_E10	ordinary_muscle_development	ENSGALG00010024110	CPNE1	-0.42383133	0.125514605	-3.37674911	0.000733479	0.002390776	negative
PRJNA729271	stage_E14_vs_E10	ordinary_muscle_development	ENSGALG00010024112	NAP1L4	0.258518362	0.090325249	2.862083015	0.004208667	0.011178922	positive
PRJNA729271	stage_E14_vs_E10	ordinary_muscle_development	ENSGALG00010024113	ENSGALG00010024113	0.5455962	0.163789469	3.331082291	0.00086509	0.002768098	positive
PRJNA729271	stage_E14_vs_E10	ordinary_muscle_development	ENSGALG00010024115	POLR2D	0.004871846	0.069693417	0.069903966	0.944270096	0.960639742	positive
PRJNA729271	stage_E14_vs_E10	ordinary_muscle_development	ENSGALG00010024116	MCAM	0.491020966	0.182493321	2.690624303	0.007131846	0.017719594	positive
PRJNA729271	stage_E14_vs_E10	ordinary_muscle_development	ENSGALG00010024118	TMEM266	-0.249597611	0.121683774	-2.051198802	0.040247591	0.078708692	negative
PRJNA729271	stage_E14_vs_E10	ordinary_muscle_development	ENSGALG00010024119	AMMECR1L	0.016946776	0.072187324	0.2347611	0.814394162	0.869971507	positive
PRJNA729271	stage_E14_vs_E10	ordinary_muscle_development	ENSGALG00010024121	NRG4	-0.206379115	0.169201989	-1.21972039	0.222570889	0.325316632	negative
PRJNA729271	stage_E14_vs_E10	ordinary_muscle_development	ENSGALG00010024122	SOCS7	0.527474199	0.101683301	5.187422088	2.13225E-07	1.29807E-06	positive
PRJNA729271	stage_E14_vs_E10	ordinary_muscle_development	ENSGALG00010024123	PLPPR4	-2.213154291	0.429768919	-5.149637842	2.6099E-07	1.56973E-06	negative
PRJNA729271	stage_E14_vs_E10	ordinary_muscle_development	ENSGALG00010024124	DIDO1	-0.045340039	0.066520735	-0.68159257	0.495496615	0.605123095	negative
PRJNA729271	stage_E14_vs_E10	ordinary_muscle_development	ENSGALG00010024125	ENSGALG00010024125	3.018850082	1.461798225	2.065161956	0.038907679	0.076546236	positive
PRJNA729271	stage_E14_vs_E10	ordinary_muscle_development	ENSGALG00010024126	CDK5R1	0.868323055	0.260862051	3.328667588	0.000872625	0.002789128	positive
PRJNA729271	stage_E14_vs_E10	ordinary_muscle_development	ENSGALG00010024128	PHLDA2	-1.405116293	0.231981406	-6.057021192	1.38665E-09	1.11602E-08	negative
PRJNA729271	stage_E14_vs_E10	ordinary_muscle_development	ENSGALG00010024129	GID8	0.011578897	0.10069763	0.114986791	0.908455592	0.937095346	positive
PRJNA729271	stage_E14_vs_E10	ordinary_muscle_development	ENSGALG00010024131	ENSGALG00010024131	10.95443516	1.001015921	10.94331762	7.15334E-28	2.67236E-26	positive
PRJNA729271	stage_E14_vs_E10	ordinary_muscle_development	ENSGALG00010024132	ENSGALG00010024132	-0.923486896	0.145244354	-6.358160379	2.04184E-10	1.82244E-09	negative
PRJNA729271	stage_E14_vs_E10	ordinary_muscle_development	ENSGALG00010024133	PSMD11	0.221067514	0.140670606	1.571525993	0.116060522	0.192594568	positive
PRJNA729271	stage_E14_vs_E10	ordinary_muscle_development	ENSGALG00010024135	FBXO22	0.071056362	0.08735041	0.813463409	0.415952417	0.530648679	positive
PRJNA729271	stage_E14_vs_E10	ordinary_muscle_development	ENSGALG00010024136	SAP130	-0.236194776	0.173943822	-1.357879649	0.174501876	0.267888656	negative
PRJNA729271	stage_E14_vs_E10	ordinary_muscle_development	ENSGALG00010024137	ENSGALG00010024137	1.126365356	0.721402422	1.561355109			positive
PRJNA729271	stage_E14_vs_E10	ordinary_muscle_development	ENSGALG00010024138	CBL	0.407329931	0.147915014	2.753810575	0.005890585	0.015021821	positive
PRJNA729271	stage_E14_vs_E10	ordinary_muscle_development	ENSGALG00010024139	SLC22A18	-1.226735179	0.678817372	-1.807165269	0.070736514	0.127290599	negative
PRJNA729271	stage_E14_vs_E10	ordinary_muscle_development	ENSGALG00010024140	KRT6A	3.096232564	0.647785084	4.779721918	1.75538E-06	9.34075E-06	positive
PRJNA729271	stage_E14_vs_E10	ordinary_muscle_development	ENSGALG00010024141	YTHDF1	-0.276213801	0.094671777	-2.917593927	0.003527434	0.009573411	negative
PRJNA729271	stage_E14_vs_E10	ordinary_muscle_development	ENSGALG00010024142	DNAJC6	-2.098369974	0.262736298	-7.986600989	1.3871E-15	2.06216E-14	negative
PRJNA729271	stage_E14_vs_E10	ordinary_muscle_development	ENSGALG00010024143	UBE2Q2	0.578187883	0.116985969	4.942369469	7.71788E-07	4.32768E-06	positive
PRJNA729271	stage_E14_vs_E10	ordinary_muscle_development	ENSGALG00010024144	HNRNPA3	-0.08041802	0.126548855	-0.635470148	0.52512183	0.632721607	negative
PRJNA729271	stage_E14_vs_E10	ordinary_muscle_development	ENSGALG00010024145	RAP1GAP3	0.851594461	0.176222173	4.832504595	1.34826E-06	7.33047E-06	positive
PRJNA729271	stage_E14_vs_E10	ordinary_muscle_development	ENSGALG00010024146	CDKN1C	-2.141315808	0.241667075	-8.86060217	7.95834E-19	1.54426E-17	negative
PRJNA729271	stage_E14_vs_E10	ordinary_muscle_development	ENSGALG00010024147	ENSGALG00010024147	2.179178051	0.238434476	9.139525824	6.27268E-20	1.33317E-18	positive
PRJNA729271	stage_E14_vs_E10	ordinary_muscle_development	ENSGALG00010024148	UGGT1	-0.181127232	0.057901694	-3.128185363	0.001758892	0.005205215	negative
PRJNA729271	stage_E14_vs_E10	ordinary_muscle_development	ENSGALG00010024149	TCIRG1	0.175596804	0.152843093	1.148869737	0.250609698	0.357812784	positive
PRJNA729271	stage_E14_vs_E10	ordinary_muscle_development	ENSGALG00010024150	NHERF4	0.849251634	0.347602747	2.443167213	0.01455899	0.033087576	positive
PRJNA729271	stage_E14_vs_E10	ordinary_muscle_development	ENSGALG00010024151	LEPROT	0.049824492	0.148059135	0.336517512	0.736480648	0.810808649	positive
PRJNA729271	stage_E14_vs_E10	ordinary_muscle_development	ENSGALG00010024152	ZNF423	-0.204446126	0.107056604	-1.909701206	0.056171696	0.104586863	negative
PRJNA729271	stage_E14_vs_E10	ordinary_muscle_development	ENSGALG00010024153	KCNQ1	-1.498961694	0.220493792	-6.79820363	1.05932E-11	1.08438E-10	negative
PRJNA729271	stage_E14_vs_E10	ordinary_muscle_development	ENSGALG00010024154	VPS29L	0.698927016	0.460167193	1.518854508	0.128799122	0.20932204	positive
PRJNA729271	stage_E14_vs_E10	ordinary_muscle_development	ENSGALG00010024155	SNX7	-0.117745869	0.164374233	-0.716328019	0.47378883	0.585920066	negative
PRJNA729271	stage_E14_vs_E10	ordinary_muscle_development	ENSGALG00010024157	CHRNB4	1.309692209	0.359242268	3.645707441	0.000266657	0.00096221	positive
PRJNA729271	stage_E14_vs_E10	ordinary_muscle_development	ENSGALG00010024158	ENSGALG00010024158	2.021132268	0.315372797	6.408708328	1.46758E-10	1.32457E-09	positive
PRJNA729271	stage_E14_vs_E10	ordinary_muscle_development	ENSGALG00010024159	NLRX1	-0.059945545	0.161457333	-0.371277934	0.710430531	0.790123323	negative
PRJNA729271	stage_E14_vs_E10	ordinary_muscle_development	ENSGALG00010024162	SRCIN1	2.248504001	0.264325283	8.506579382	1.7914E-17	3.13225E-16	positive
PRJNA729271	stage_E14_vs_E10	ordinary_muscle_development	ENSGALG00010024163	HS6ST1	0.268735326	0.095089364	2.826134425	0.004711349	0.012364838	positive
PRJNA729271	stage_E14_vs_E10	ordinary_muscle_development	ENSGALG00010024164	SNAP29	0.279801701	0.112446909	2.488300514	0.012835522	0.029658998	positive
PRJNA729271	stage_E14_vs_E10	ordinary_muscle_development	ENSGALG00010024165	LEPR	-1.66367417	0.275987897	-6.028069297	1.6593E-09	1.32662E-08	negative
PRJNA729271	stage_E14_vs_E10	ordinary_muscle_development	ENSGALG00010024166	ENSGALG00010024166	-1.07118569	1.207675722	-0.886981224	0.375089009	0.491361517	negative
PRJNA729271	stage_E14_vs_E10	ordinary_muscle_development	ENSGALG00010024167	CHRNA3	2.268147749	0.387733875	5.849753902	4.92301E-09	3.73412E-08	positive
PRJNA729271	stage_E14_vs_E10	ordinary_muscle_development	ENSGALG00010024168	BIRC7	-0.289858934	0.283602604	-1.022060197	0.30675241	0.420421349	negative
PRJNA729271	stage_E14_vs_E10	ordinary_muscle_development	ENSGALG00010024169	FAM168B	-0.19094071	0.071819723	-2.658611046	0.007846349	0.019289828	negative
PRJNA729271	stage_E14_vs_E10	ordinary_muscle_development	ENSGALG00010024170	NUP160	0.141940962	0.083209038	1.705835859	0.088038645	0.153067069	positive
PRJNA729271	stage_E14_vs_E10	ordinary_muscle_development	ENSGALG00010024171	ENSGALG00010024171	-0.632578501	0.413779052	-1.528783293	0.126318175	0.205890246	negative
PRJNA729271	stage_E14_vs_E10	ordinary_muscle_development	ENSGALG00010024172	ABCG4	-2.461669748	0.378297331	-6.507235305	7.65465E-11	7.1491E-10	negative
PRJNA729271	stage_E14_vs_E10	ordinary_muscle_development	ENSGALG00010024173	CBLN1	0.610820252	0.391369427	1.560725518	0.118588528	0.195801425	positive
PRJNA729271	stage_E14_vs_E10	ordinary_muscle_development	ENSGALG00010024174	PLEKHB2	0.224293937	0.101404668	2.211869937	0.026975653	0.056228371	positive
PRJNA729271	stage_E14_vs_E10	ordinary_muscle_development	ENSGALG00010024175	DHRS9	0.176757608	0.518994461	0.340577061	0.733422001	0.808130133	positive
PRJNA729271	stage_E14_vs_E10	ordinary_muscle_development	ENSGALG00010024177	ENSGALG00010024177	-0.654802001	0.458852236	-1.427043283	0.153567419	0.242031069	negative
PRJNA729271	stage_E14_vs_E10	ordinary_muscle_development	ENSGALG00010024178	N4BP1	0.096973999	0.120918955	0.801975169	0.422567322	0.536913861	positive
PRJNA729271	stage_E14_vs_E10	ordinary_muscle_development	ENSGALG00010024179	ATG4C	-0.123452992	0.113626855	-1.086477238	0.277267906	0.387821895	negative
PRJNA729271	stage_E14_vs_E10	ordinary_muscle_development	ENSGALG00010024180	HINFP	0.137372691	0.138593871	0.991188789	0.321593404	0.436102197	positive
PRJNA729271	stage_E14_vs_E10	ordinary_muscle_development	ENSGALG00010024181	FOXD3	2.861438199	0.31222272	9.164734079	4.96693E-20	1.06977E-18	positive
PRJNA729271	stage_E14_vs_E10	ordinary_muscle_development	ENSGALG00010024183	MTX2	0.298792303	0.10608171	2.816624127	0.004853129	0.012681828	positive
PRJNA729271	stage_E14_vs_E10	ordinary_muscle_development	ENSGALG00010024184	ENSGALG00010024184	-1.841744016	0.217646283	-8.462097267	2.62611E-17	4.53161E-16	negative
PRJNA729271	stage_E14_vs_E10	ordinary_muscle_development	ENSGALG00010024185	CHRNA5	0.147116701	0.153702876	0.957149953	0.338491573	0.453585593	positive
PRJNA729271	stage_E14_vs_E10	ordinary_muscle_development	ENSGALG00010024186	HAO2	-1.006850551	0.697948321	-1.442586107	0.149137099	0.236230167	negative
PRJNA729271	stage_E14_vs_E10	ordinary_muscle_development	ENSGALG00010024188	TSSC4	0.022228823	0.081887983	0.271454029	0.786041852	0.849513618	positive
PRJNA729271	stage_E14_vs_E10	ordinary_muscle_development	ENSGALG00010024189	KAT7	0.057395154	0.139824038	0.41048131	0.681452911	0.767038744	positive
PRJNA729271	stage_E14_vs_E10	ordinary_muscle_development	ENSGALG00010024190	WARS2	-0.142920889	0.100378899	-1.42381407	0.154500302	0.243210455	negative
PRJNA729271	stage_E14_vs_E10	ordinary_muscle_development	ENSGALG00010024191	DPYD	-0.183860733	0.106210128	-1.731103586	0.083433291	0.146518725	negative
PRJNA729271	stage_E14_vs_E10	ordinary_muscle_development	ENSGALG00010024192	ALG6	0.319908428	0.087223114	3.667702449	0.00024474	0.000890661	positive
PRJNA729271	stage_E14_vs_E10	ordinary_muscle_development	ENSGALG00010024193	GSTK1	1.114687494	0.125673875	8.869683501	7.33541E-19	1.4272E-17	positive
PRJNA729271	stage_E14_vs_E10	ordinary_muscle_development	ENSGALG00010024194	NKAIN4	1.561363084	0.08405722	18.57500267	5.12026E-77	2.06161E-74	positive
PRJNA729271	stage_E14_vs_E10	ordinary_muscle_development	ENSGALG00010024195	PSMA4	0.179665334	0.120513733	1.49082871	0.136006472	0.219072834	positive
PRJNA729271	stage_E14_vs_E10	ordinary_muscle_development	ENSGALG00010024196	MRPL21	0.17538614	0.107870789	1.625890952	0.103972855	0.175794534	positive
PRJNA729271	stage_E14_vs_E10	ordinary_muscle_development	ENSGALG00010024197	KRT7	-2.107297001	0.302105946	-6.975357573	3.05094E-12	3.32007E-11	negative
PRJNA729271	stage_E14_vs_E10	ordinary_muscle_development	ENSGALG00010024198	PI4KA	-0.205202984	0.064277575	-3.192450633	0.001410711	0.004278772	negative
PRJNA729271	stage_E14_vs_E10	ordinary_muscle_development	ENSGALG00010024199	SLC35B1	-0.127985089	0.1011195	-1.26568158	0.205627106	0.305542275	negative
PRJNA729271	stage_E14_vs_E10	ordinary_muscle_development	ENSGALG00010024200	GCHFR	-2.338917503	0.612859704	-3.816399555	0.000135413	0.000521193	negative
PRJNA729271	stage_E14_vs_E10	ordinary_muscle_development	ENSGALG00010024201	IREB2	-0.14431111	0.069282431	-2.082939481	0.03725674	0.073773643	negative
PRJNA729271	stage_E14_vs_E10	ordinary_muscle_development	ENSGALG00010024203	SPOP	0.11609746	0.071134565	1.632082244	0.102662162	0.173821754	positive
PRJNA729271	stage_E14_vs_E10	ordinary_muscle_development	ENSGALG00010024204	PTBP2	0.452255955	0.106972001	4.227797452	2.3599E-05	0.000103783	positive
PRJNA729271	stage_E14_vs_E10	ordinary_muscle_development	ENSGALG00010024205	ANKRD13D	-0.213890976	0.168687068	-1.267974951	0.204806896	0.304667073	negative
PRJNA729271	stage_E14_vs_E10	ordinary_muscle_development	ENSGALG00010024206	KRT80	1.237814437	0.528922073	2.340258615	0.019270391	0.042060581	positive
PRJNA729271	stage_E14_vs_E10	ordinary_muscle_development	ENSGALG00010024207	KCTD18	-0.120352602	0.119733361	-1.005171833	0.314814116	0.428754168	negative
PRJNA729271	stage_E14_vs_E10	ordinary_muscle_development	ENSGALG00010024208	SERPIND1	0.413012409	0.592343887	0.69725107	0.485645683	0.597046871	positive
PRJNA729271	stage_E14_vs_E10	ordinary_muscle_development	ENSGALG00010024209	RWDD3	-0.071113829	0.143283782	-0.496314574	0.619672486	0.715253439	negative
PRJNA729271	stage_E14_vs_E10	ordinary_muscle_development	ENSGALG00010024210	NXPH3	-3.046365529	1.097626741	-2.775411182	0.005513196	0.014179164	negative
PRJNA729271	stage_E14_vs_E10	ordinary_muscle_development	ENSGALG00010024211	TRPM5	1.470264718	0.670034334	2.194312506	0.028212951	0.058345944	positive
PRJNA729271	stage_E14_vs_E10	ordinary_muscle_development	ENSGALG00010024212	RIMS4	0.341717674	0.196858116	1.735857685	0.082589001	0.145229598	positive
PRJNA729271	stage_E14_vs_E10	ordinary_muscle_development	ENSGALG00010024213	ENSGALG00010024213	-0.952321028	0.189929671	-5.014071921	5.32901E-07	3.05917E-06	negative
PRJNA729271	stage_E14_vs_E10	ordinary_muscle_development	ENSGALG00010024214	CRABP1	-4.138785671	0.40288803	-10.27279384	9.34582E-25	2.7312E-23	negative
PRJNA729271	stage_E14_vs_E10	ordinary_muscle_development	ENSGALG00010024215	ENSGALG00010024215	0.049279839	0.159136952	0.309669367	0.7568124	0.826676394	positive
PRJNA729271	stage_E14_vs_E10	ordinary_muscle_development	ENSGALG00010024218	NGFR	-0.539057707	0.22761781	-2.36825803	0.017872067	0.039460108	negative
PRJNA729271	stage_E14_vs_E10	ordinary_muscle_development	ENSGALG00010024220	SIAH1	0.625967009	0.200808718	3.117230241	0.001825589	0.00538172	positive
PRJNA729271	stage_E14_vs_E10	ordinary_muscle_development	ENSGALG00010024222	ENSGALG00010024222	1.033520457	0.277466092	3.724853194	0.000195429	0.000728211	positive
PRJNA729271	stage_E14_vs_E10	ordinary_muscle_development	ENSGALG00010024223	ENSGALG00010024223	-1.366292115	0.278050192	-4.913832661	8.9313E-07	4.96393E-06	negative
PRJNA729271	stage_E14_vs_E10	ordinary_muscle_development	ENSGALG00010024225	BCDIN3D	0.182992294	0.172967427	1.05795812	0.290074536	0.402472518	positive
PRJNA729271	stage_E14_vs_E10	ordinary_muscle_development	ENSGALG00010024226	POU2AF1	-1.726984714	1.489806763	-1.159200479	0.246374476	0.353024716	negative
PRJNA729271	stage_E14_vs_E10	ordinary_muscle_development	ENSGALG00010024227	MUC6	1.149942199	0.431563274	2.664596987	0.007708064	0.018994965	positive
PRJNA729271	stage_E14_vs_E10	ordinary_muscle_development	ENSGALG00010024230	LONP2	0.118054255	0.150302338	0.785445233	0.432192569	0.546318243	positive
PRJNA729271	stage_E14_vs_E10	ordinary_muscle_development	ENSGALG00010024233	FAIM2	-1.43577374	0.218940106	-6.55783798	5.45935E-11	5.19929E-10	negative
PRJNA729271	stage_E14_vs_E10	ordinary_muscle_development	ENSGALG00010024234	ENSGALG00010024234	0.35936733	0.270369916	1.329169071	0.183792197	0.27948677	positive
PRJNA729271	stage_E14_vs_E10	ordinary_muscle_development	ENSGALG00010024238	ATF7	0.71038309	0.204154684	3.479631609	0.000502104	0.001701658	positive
PRJNA729271	stage_E14_vs_E10	ordinary_muscle_development	ENSGALG00010024240	WDR61	-0.227746281	0.125265938	-1.818102232	0.069048509	0.124639868	negative
PRJNA729271	stage_E14_vs_E10	ordinary_muscle_development	ENSGALG00010024242	KMT2A	-0.293980245	0.124924779	-2.353258069	0.018609713	0.040821398	negative
PRJNA729271	stage_E14_vs_E10	ordinary_muscle_development	ENSGALG00010024243	MPP3	0.367262132	0.200075198	1.835620491	0.066413805	0.120574661	positive
PRJNA729271	stage_E14_vs_E10	ordinary_muscle_development	ENSGALG00010024245	TARBP2	-0.036331104	0.164226344	-0.221225795	0.824916625	0.877412076	negative
PRJNA729271	stage_E14_vs_E10	ordinary_muscle_development	ENSGALG00010024250	AP2A2	0.092116521	0.053143978	1.733338837	0.083035462	0.145943861	positive
PRJNA729271	stage_E14_vs_E10	ordinary_muscle_development	ENSGALG00010024251	ENSGALG00010024251	0.274438159	0.208376936	1.317027524	0.187829399	0.284313611	positive
PRJNA729271	stage_E14_vs_E10	ordinary_muscle_development	ENSGALG00010024254	ENSGALG00010024254	2.681912262	0.300745738	8.917540385	4.76758E-19	9.46659E-18	positive
PRJNA729271	stage_E14_vs_E10	ordinary_muscle_development	ENSGALG00010024255	CFAP97D1	-0.50350329	0.68993827	-0.729780201	0.465524548	0.577869173	negative
PRJNA729271	stage_E14_vs_E10	ordinary_muscle_development	ENSGALG00010024258	DNAJA4	-0.649730183	0.152517023	-4.260050258	2.04381E-05	9.07907E-05	negative
PRJNA729271	stage_E14_vs_E10	ordinary_muscle_development	ENSGALG00010024259	ENSGALG00010024259	0.521146461	0.134801728	3.866022102	0.000110625	0.000432562	positive
PRJNA729271	stage_E14_vs_E10	ordinary_muscle_development	ENSGALG00010024263	PHKB	1.035712433	0.131672698	7.865810042	3.66717E-15	5.23185E-14	positive
PRJNA729271	stage_E14_vs_E10	ordinary_muscle_development	ENSGALG00010024264	AMER3	-0.174526386	0.304794793	-0.572602913	0.566913587	0.669442969	negative
PRJNA729271	stage_E14_vs_E10	ordinary_muscle_development	ENSGALG00010024266	ENSGALG00010024266	0.163944723	0.239889178	0.683418587	0.494342378	0.604409904	positive
PRJNA729271	stage_E14_vs_E10	ordinary_muscle_development	ENSGALG00010024267	MAP3K12	-0.893514222	0.180873453	-4.939996484	7.8124E-07	4.3773E-06	negative
PRJNA729271	stage_E14_vs_E10	ordinary_muscle_development	ENSGALG00010024270	RAB18L	0.698352345	0.774210463	0.902018739	0.367046913	0.483184543	positive

PRJNA729271	stage_E14_vs_E10	ordinary_muscle_development	ENSGALG00010024274	ACSBG1	-0.000481599	0.311741277	-0.001544867	0.998767375	0.999350415	negative
PRJNA729271	stage_E14_vs_E10	ordinary_muscle_development	ENSGALG00010024277	ITGB3BP	-0.144023898	0.182692983	-0.788338423	0.430498779	0.54465216	negative
PRJNA729271	stage_E14_vs_E10	ordinary_muscle_development	ENSGALG00010024280	ENSGALG00010024280	1.034917437	0.12522407	8.264524828	1.40252E-16	2.274E-15	positive
PRJNA729271	stage_E14_vs_E10	ordinary_muscle_development	ENSGALG00010024282	HIC2	-0.654653495	0.15119485	-4.329866371	1.492E-05	6.81365E-05	negative
PRJNA729271	stage_E14_vs_E10	ordinary_muscle_development	ENSGALG00010024283	UBE4A	0.258184665	0.110079304	2.345442381	0.019004516	0.041561626	positive
PRJNA729271	stage_E14_vs_E10	ordinary_muscle_development	ENSGALG00010024285	CCND2	-0.320835066	0.096725459	-3.316966077	0.000910007	0.002893933	negative
PRJNA729271	stage_E14_vs_E10	ordinary_muscle_development	ENSGALG00010024286	ADA	0.250579018	0.180020555	1.391946704	0.163938538	0.254829931	positive
PRJNA729271	stage_E14_vs_E10	ordinary_muscle_development	ENSGALG00010024288	CHID1	0.0985528	0.132528866	0.743632709	0.457098709	0.570046097	positive
PRJNA729271	stage_E14_vs_E10	ordinary_muscle_development	ENSGALG00010024290	SOST	-0.976995435	0.469961768	-2.078882797	0.037628124	0.074368648	negative
PRJNA729271	stage_E14_vs_E10	ordinary_muscle_development	ENSGALG00010024291	ENSGALG00010024291	2.555934463	0.507220438	5.039099908	4.67726E-07	2.71079E-06	positive
PRJNA729271	stage_E14_vs_E10	ordinary_muscle_development	ENSGALG00010024300	MEOX1	-2.483655671	0.208122241	-11.93363892	7.90412E-33	3.92364E-31	negative
PRJNA729271	stage_E14_vs_E10	ordinary_muscle_development	ENSGALG00010024304	PKIG	1.358027531	0.130413364	10.41325439	2.15719E-25	6.70996E-24	positive
PRJNA729271	stage_E14_vs_E10	ordinary_muscle_development	ENSGALG00010024305	ENSGALG00010024305	1.192987637	0.81747731	1.4593526	0.144468085	0.230218216	positive
PRJNA729271	stage_E14_vs_E10	ordinary_muscle_development	ENSGALG00010024309	ABCB11	-0.629869227	0.405199765	-1.554465923	0.120073319	0.197749364	negative
PRJNA729271	stage_E14_vs_E10	ordinary_muscle_development	ENSGALG00010024311	KLHL6	0.258556872	0.133341201	1.939062119	0.052493775	0.098751409	positive
PRJNA729271	stage_E14_vs_E10	ordinary_muscle_development	ENSGALG00010024313	SERINC3	0.565871549	0.109549699	5.165432264	2.39884E-07	1.45061E-06	positive
PRJNA729271	stage_E14_vs_E10	ordinary_muscle_development	ENSGALG00010024314	ITFG1	-0.285742596	0.146443432	-1.951214834	0.051031495	0.096478613	negative
PRJNA729271	stage_E14_vs_E10	ordinary_muscle_development	ENSGALG00010024321	UBE2L3	-0.10061093	0.082025003	-1.226588553	0.219977274	0.322403497	negative
PRJNA729271	stage_E14_vs_E10	ordinary_muscle_development	ENSGALG00010024322	IDH3A	0.766378614	0.158886499	4.823434469	1.41107E-06	7.64045E-06	positive
PRJNA729271	stage_E14_vs_E10	ordinary_muscle_development	ENSGALG00010024323	TTPAL	-0.276890581	0.097417963	-2.842294909	0.004479004	0.011825712	negative
PRJNA729271	stage_E14_vs_E10	ordinary_muscle_development	ENSGALG00010024325	PCBP2	-0.290735429	0.169836207	-1.711857764	0.086922884	0.151508802	negative
PRJNA729271	stage_E14_vs_E10	ordinary_muscle_development	ENSGALG00010024326	KLHL24	1.060542704	0.178620957	5.93739234	2.89591E-09	2.26043E-08	positive
PRJNA729271	stage_E14_vs_E10	ordinary_muscle_development	ENSGALG00010024327	PPP1R1B	-0.143932425	0.472269359	-0.304767656	0.760543117	0.829501315	negative
PRJNA729271	stage_E14_vs_E10	ordinary_muscle_development	ENSGALG00010024328	YDJC	0.133930922	0.147533108	0.907802479	0.363982588	0.480139466	positive
PRJNA729271	stage_E14_vs_E10	ordinary_muscle_development	ENSGALG00010024330	SDF2L1	-0.481222141	0.099879556	-4.818024418	1.44987E-06	7.83003E-06	negative
PRJNA729271	stage_E14_vs_E10	ordinary_muscle_development	ENSGALG00010024332	EFCAB7	0.023286841	0.162815004	0.143026381	0.886269346	0.92221638	positive
PRJNA729271	stage_E14_vs_E10	ordinary_muscle_development	ENSGALG00010024333	ETV4	-1.571646464	0.226925609	-6.925822388	4.3345E-12	4.61975E-11	negative
PRJNA729271	stage_E14_vs_E10	ordinary_muscle_development	ENSGALG00010024334	USP28	1.140831903	0.122603236	9.305071709	1.33902E-20	2.99061E-19	positive
PRJNA729271	stage_E14_vs_E10	ordinary_muscle_development	ENSGALG00010024336	TSPAN4	0.679463927	0.116347747	5.839940573	5.22194E-09	3.94024E-08	positive
PRJNA729271	stage_E14_vs_E10	ordinary_muscle_development	ENSGALG00010024338	NDUFA9	0.05586699	0.114042345	0.489879351	0.624219275	0.719241526	positive
PRJNA729271	stage_E14_vs_E10	ordinary_muscle_development	ENSGALG00010024339	TOP3B	-0.151729872	0.085254851	-1.779721275	0.075121587	0.134066412	negative
PRJNA729271	stage_E14_vs_E10	ordinary_muscle_development	ENSGALG00010024340	NETO2	-0.24218286	0.369863417	-0.654789983	0.512602981	0.621408397	negative
PRJNA729271	stage_E14_vs_E10	ordinary_muscle_development	ENSGALG00010024342	HNF4A	0.641171025	1.375601771	0.466102209	0.641142342	0.73366687	positive
PRJNA729271	stage_E14_vs_E10	ordinary_muscle_development	ENSGALG00010024343	POLR2L	0.263832379	0.126066397	2.092804943	0.03636657	0.07221006	positive
PRJNA729271	stage_E14_vs_E10	ordinary_muscle_development	ENSGALG00010024344	YEATS2	0.189883382	0.13245386	1.43358134	0.151691772	0.239635975	positive
PRJNA729271	stage_E14_vs_E10	ordinary_muscle_development	ENSGALG00010024345	STARD3	0.548212307	0.089531264	6.123138267	9.175E-10	7.54348E-09	positive
PRJNA729271	stage_E14_vs_E10	ordinary_muscle_development	ENSGALG00010024346	PPM1F	0.295244584	0.08952958	3.297732276	0.00097469	0.003076014	positive
PRJNA729271	stage_E14_vs_E10	ordinary_muscle_development	ENSGALG00010024347	OTOG	0.735495532	0.26671177	2.757641827	0.005821995	0.014865214	positive
PRJNA729271	stage_E14_vs_E10	ordinary_muscle_development	ENSGALG00010024349	R3HDML	-0.431233904	0.38099861	-1.131851646	0.257696811	0.36588454	negative
PRJNA729271	stage_E14_vs_E10	ordinary_muscle_development	ENSGALG00010024350	PGM1	0.580614859	0.136999629	4.238076151	2.25443E-05	9.9537E-05	positive
PRJNA729271	stage_E14_vs_E10	ordinary_muscle_development	ENSGALG00010024351	SP1	-0.023892411	0.154215116	-0.154929112	0.876877203	0.915466368	negative
PRJNA729271	stage_E14_vs_E10	ordinary_muscle_development	ENSGALG00010024354	C2CD2L	-0.224382462	0.076781299	-2.922358234	0.003473917	0.009442046	negative
PRJNA729271	stage_E14_vs_E10	ordinary_muscle_development	ENSGALG00010024356	PARL	0.349543469	0.117098133	2.985047327	0.002835345	0.007906564	positive
PRJNA729271	stage_E14_vs_E10	ordinary_muscle_development	ENSGALG00010024358	MAPK3	-0.045136708	0.061746243	-0.731003306	0.464777134	0.577292607	negative
PRJNA729271	stage_E14_vs_E10	ordinary_muscle_development	ENSGALG00010024360	FTL	-1.102802935	0.240523688	-4.585007583	4.5397E-06	2.25817E-05	negative
PRJNA729271	stage_E14_vs_E10	ordinary_muscle_development	ENSGALG00010024361	H2AX	0.143041448	0.212419114	0.673392544	0.500697591	0.609934581	positive
PRJNA729271	stage_E14_vs_E10	ordinary_muscle_development	ENSGALG00010024362	DHX8	-0.083575905	0.056638785	-1.475594946	0.140052663	0.224465208	negative
PRJNA729271	stage_E14_vs_E10	ordinary_muscle_development	ENSGALG00010024363	EDEM2	-0.357425967	0.110585898	-3.232111626	0.00122879	0.003796913	negative
PRJNA729271	stage_E14_vs_E10	ordinary_muscle_development	ENSGALG00010024365	ENSGALG00010024365	0.030727537	0.103535285	0.296783239	0.766632002	0.834071221	positive
PRJNA729271	stage_E14_vs_E10	ordinary_muscle_development	ENSGALG00010024366	PARD6A	0.410454701	0.408820501	1.003997353	0.315379889	0.429403672	positive
PRJNA729271	stage_E14_vs_E10	ordinary_muscle_development	ENSGALG00010024367	ENSGALG00010024367	-0.829317155	0.230388465	-3.599647031	0.000318649	0.001133454	negative
PRJNA729271	stage_E14_vs_E10	ordinary_muscle_development	ENSGALG00010024368	DPAGT1	0.031747423	0.07834738	0.405213588	0.685320518	0.769758939	positive
PRJNA729271	stage_E14_vs_E10	ordinary_muscle_development	ENSGALG00010024369	MYOD1	1.331636831	0.275378441	4.83566115	1.32704E-06	7.22321E-06	positive
PRJNA729271	stage_E14_vs_E10	ordinary_muscle_development	ENSGALG00010024370	HERPUD1	0.096397451	0.091127213	1.057833855	0.290131195	0.402512603	positive
PRJNA729271	stage_E14_vs_E10	ordinary_muscle_development	ENSGALG00010024371	YPEL1	1.272824199	0.103943685	12.24532492	1.77963E-34	9.51872E-33	positive
PRJNA729271	stage_E14_vs_E10	ordinary_muscle_development	ENSGALG00010024373	TCAP	2.595192264	0.515236456	5.036895653	4.73143E-07	2.73999E-06	positive
PRJNA729271	stage_E14_vs_E10	ordinary_muscle_development	ENSGALG00010024374	ENSGALG00010024374	0.068397529	0.591293709	0.115674373	0.907910617	0.936801895	positive
PRJNA729271	stage_E14_vs_E10	ordinary_muscle_development	ENSGALG00010024377	CIB2	0.935174973	0.167583115	5.580365126	2.40014E-08	1.66699E-07	positive
PRJNA729271	stage_E14_vs_E10	ordinary_muscle_development	ENSGALG00010024378	ZDHHC1	-0.426011835	0.171994621	-2.476890463	0.013253257	0.03048817	negative
PRJNA729271	stage_E14_vs_E10	ordinary_muscle_development	ENSGALG00010024382	ENSGALG00010024382	0.706143585	0.422708278	1.670522255	0.09481608	0.162896655	positive
PRJNA729271	stage_E14_vs_E10	ordinary_muscle_development	ENSGALG00010024383	DUSP28	1.038906135	0.188201182	5.520189208	3.38635E-08	2.31862E-07	positive
PRJNA729271	stage_E14_vs_E10	ordinary_muscle_development	ENSGALG00010024386	TPPP3	-1.255613367	0.273632045	-4.588692706	4.4603E-06	2.22096E-05	negative
PRJNA729271	stage_E14_vs_E10	ordinary_muscle_development	ENSGALG00010024387	PPIL2	0.255637411	0.063476149	4.027298674	5.64213E-05	0.000232668	positive
PRJNA729271	stage_E14_vs_E10	ordinary_muscle_development	ENSGALG00010024389	AAAS	0.175083914	0.106304071	1.647010441	0.0995559	0.169492926	positive
PRJNA729271	stage_E14_vs_E10	ordinary_muscle_development	ENSGALG00010024391	TBC1D2B	-0.438354326	0.110284491	-3.974759481	7.04504E-05	0.000285325	negative
PRJNA729271	stage_E14_vs_E10	ordinary_muscle_development	ENSGALG00010024392	CHMP4B	0.069827669	0.084470369	0.826652826	0.408433866	0.523199648	positive
PRJNA729271	stage_E14_vs_E10	ordinary_muscle_development	ENSGALG00010024393	OGFOD1	-0.144881939	0.100179305	-1.446226229	0.148113759	0.234968692	negative
PRJNA729271	stage_E14_vs_E10	ordinary_muscle_development	ENSGALG00010024396	ENSGALG00010024396	-0.562536585	0.335858843	-1.674919674	0.093949993	0.161581057	negative
PRJNA729271	stage_E14_vs_E10	ordinary_muscle_development	ENSGALG00010024398	PHB	0.140724069	0.087580638	1.606794291	0.108099501	0.181705446	positive
PRJNA729271	stage_E14_vs_E10	ordinary_muscle_development	ENSGALG00010024399	ROR1	-0.966351617	0.168007241	-5.751845038	8.82747E-09	6.45907E-08	negative
PRJNA729271	stage_E14_vs_E10	ordinary_muscle_development	ENSGALG00010024400	KCNA1	-3.690182991	0.397925882	-9.273543538	1.80062E-20	3.97866E-19	negative
PRJNA729271	stage_E14_vs_E10	ordinary_muscle_development	ENSGALG00010024401	KCNC1	2.165491141	0.210413175	10.29161385	7.68769E-25	2.27414E-23	positive
PRJNA729271	stage_E14_vs_E10	ordinary_muscle_development	ENSGALG00010024402	GPC1	0.432566455	0.119142971	3.630650244	0.000282708	0.001016837	positive
PRJNA729271	stage_E14_vs_E10	ordinary_muscle_development	ENSGALG00010024403	HMBS	-0.099945076	0.074629268	-1.339220919	0.180498764	0.275374128	negative
PRJNA729271	stage_E14_vs_E10	ordinary_muscle_development	ENSGALG00010024406	NUDT21	-0.126523427	0.063732373	-1.985230114	0.047118875	0.090222998	negative
PRJNA729271	stage_E14_vs_E10	ordinary_muscle_development	ENSGALG00010024407	HIRA	0.41295243	0.079642601	5.185069597	2.15934E-07	1.31401E-06	positive
PRJNA729271	stage_E14_vs_E10	ordinary_muscle_development	ENSGALG00010024408	ENSGALG00010024408	1.887852747	1.136191682	1.661561845	0.096600659	0.165225542	positive
PRJNA729271	stage_E14_vs_E10	ordinary_muscle_development	ENSGALG00010024410	MYG1	0.22550517	0.1049608	2.148470389	0.031676406	0.064261653	positive
PRJNA729271	stage_E14_vs_E10	ordinary_muscle_development	ENSGALG00010024412	PIGU	-0.202863266	0.09899655	-2.049195299	0.040443022	0.07904822	negative
PRJNA729271	stage_E14_vs_E10	ordinary_muscle_development	ENSGALG00010024413	AMFR	0.100992361	0.092441988	1.092494466	0.274615821	0.384966763	positive
PRJNA729271	stage_E14_vs_E10	ordinary_muscle_development	ENSGALG00010024415	SIK2	0.273504971	0.059685703	4.582420227	4.59625E-06	2.28238E-05	positive
PRJNA729271	stage_E14_vs_E10	ordinary_muscle_development	ENSGALG00010024416	VDR	-1.047683998	0.303597155	-3.450901891	0.000558717	0.001869914	negative
PRJNA729271	stage_E14_vs_E10	ordinary_muscle_development	ENSGALG00010024419	PGAP3	0.285839599	0.123130185	2.321442131	0.020262994	0.044001812	positive
PRJNA729271	stage_E14_vs_E10	ordinary_muscle_development	ENSGALG00010024420	TMEM106C	0.12765424	0.157063435	0.81275594	0.416358002	0.531072618	positive
PRJNA729271	stage_E14_vs_E10	ordinary_muscle_development	ENSGALG00010024421	DAPK2	1.529359418	0.260247571	5.876555969	4.1889E-09	3.20751E-08	positive
PRJNA729271	stage_E14_vs_E10	ordinary_muscle_development	ENSGALG00010024423	SERGEF	0.544783164	0.116017156	4.695712095	2.6568E-06	1.37291E-05	positive
PRJNA729271	stage_E14_vs_E10	ordinary_muscle_development	ENSGALG00010024424	SAMHD1	-0.047847909	0.179899869	-0.265969669	0.790262568	0.852676487	negative
PRJNA729271	stage_E14_vs_E10	ordinary_muscle_development	ENSGALG00010024425	MRPL40	0.50698806	0.088032855	5.759077791	8.45747E-09	6.21029E-08	positive
PRJNA729271	stage_E14_vs_E10	ordinary_muscle_development	ENSGALG00010024426	PHAF1	0.422484146	0.104772971	4.032377275	5.52155E-05	0.00022789	positive
PRJNA729271	stage_E14_vs_E10	ordinary_muscle_development	ENSGALG00010024427	C22orf39	0.657616979	0.133516107	4.925375638	8.41984E-07	4.69406E-06	positive
PRJNA729271	stage_E14_vs_E10	ordinary_muscle_development	ENSGALG00010024428	SAAL1	-0.163811443	0.061840224	-2.648946456	0.008074312	0.019799891	negative
PRJNA729271	stage_E14_vs_E10	ordinary_muscle_development	ENSGALG00010024430	ZNF652	-0.080143073	0.063280874	-1.266465964	0.205346308	0.305218902	negative
PRJNA729271	stage_E14_vs_E10	ordinary_muscle_development	ENSGALG00010024431	FEZ1	0.197656622	0.148564244	1.330445449	0.183371549	0.278934893	positive
PRJNA729271	stage_E14_vs_E10	ordinary_muscle_development	ENSGALG00010024433	ENSGALG00010024433	-0.885062207	0.168970339	-5.237973803	1.62349E-07	1.00181E-06	negative
PRJNA729271	stage_E14_vs_E10	ordinary_muscle_development	ENSGALG00010024434	B3GNT9	-0.219979346	0.157117535	-1.400094172	0.16148512	0.25190775	negative
PRJNA729271	stage_E14_vs_E10	ordinary_muscle_development	ENSGALG00010024437	HERC1	0.004179341	0.081434648	0.051321416	0.959069403	0.971875769	positive
PRJNA729271	stage_E14_vs_E10	ordinary_muscle_development	ENSGALG00010024438	RBL1	-0.300512924	0.119263223	-2.519745117	0.011743984	0.027429753	negative
PRJNA729271	stage_E14_vs_E10	ordinary_muscle_development	ENSGALG00010024439	UFD1	0.223226028	0.112126495	1.990841031	0.046498367	0.089247064	positive
PRJNA729271	stage_E14_vs_E10	ordinary_muscle_development	ENSGALG00010024440	PKNOX2	1.650574908	0.205515241	8.031399045	9.63674E-16	1.44451E-14	positive
PRJNA729271	stage_E14_vs_E10	ordinary_muscle_development	ENSGALG00010024441	SAA	2.143166067	0.796368717	2.691173099	0.007120124	0.017693502	positive
PRJNA729271	stage_E14_vs_E10	ordinary_muscle_development	ENSGALG00010024446	GRM4	-1.639580757	0.504745495	-3.248331632	0.001160839	0.003601532	negative
PRJNA729271	stage_E14_vs_E10	ordinary_muscle_development	ENSGALG00010024449	CACHD1	-0.634950381	0.083600242	-7.595078225	3.07607E-14	4.0571E-13	negative
PRJNA729271	stage_E14_vs_E10	ordinary_muscle_development	ENSGALG00010024451	PHOSPHO1	-1.174573102	0.277730148	-4.229188338	2.34536E-05	0.000103206	negative
PRJNA729271	stage_E14_vs_E10	ordinary_muscle_development	ENSGALG00010024452	SLC16A1	-0.800025773	0.252589075	-3.167301563	0.001538607	0.004623156	negative
PRJNA729271	stage_E14_vs_E10	ordinary_muscle_development	ENSGALG00010024453	HTR3A	5.633540021	0.546617465	10.30618372	6.60732E-25	1.97064E-23	positive
PRJNA729271	stage_E14_vs_E10	ordinary_muscle_development	ENSGALG00010024454	HPS5	0.010889612	0.120146994	0.090635741	0.927782031	0.950335704	positive
PRJNA729271	stage_E14_vs_E10	ordinary_muscle_development	ENSGALG00010024457	CHD6	0.058060835	0.100212627	0.579376437	0.5623352	0.665120254	positive
PRJNA729271	stage_E14_vs_E10	ordinary_muscle_development	ENSGALG00010024459	AHCYL1	-0.263446429	0.090194649	-2.920865406	0.003490606	0.009482071	negative
PRJNA729271	stage_E14_vs_E10	ordinary_muscle_development	ENSGALG00010024460	IMP4	0.518183628	0.15415842	3.361370899	0.000775566	0.002509897	positive
PRJNA729271	stage_E14_vs_E10	ordinary_muscle_development	ENSGALG00010024462	ZBTB32	-0.11604673	0.457355242	-0.253734338	0.799700804	0.859150842	negative
PRJNA729271	stage_E14_vs_E10	ordinary_muscle_development	ENSGALG00010024463	FBXL22	1.465042274	0.228019022	6.425087971	1.31793E-10	1.19621E-09	positive
PRJNA729271	stage_E14_vs_E10	ordinary_muscle_development	ENSGALG00010024465	SENP1	-0.437672084	0.15612182	-2.803401116	0.005056674	0.013156791	negative
PRJNA729271	stage_E14_vs_E10	ordinary_muscle_development	ENSGALG00010024466	ADGRG3	0.472657482	0.857887663	0.550954981	0.581664536	0.683132997	positive
PRJNA729271	stage_E14_vs_E10	ordinary_muscle_development	ENSGALG00010024467	ENSGALG00010024467	0.09312916	0.294587068	0.316134585	0.751900356	0.822178309	positive
PRJNA729271	stage_E14_vs_E10	ordinary_muscle_development	ENSGALG00010024469	GTF2H1	0.090405974	0.127240589	0.710512066	0.477386651	0.589514356	positive
PRJNA729271	stage_E14_vs_E10	ordinary_muscle_development	ENSGALG00010024471	CCDC61	0.262738383	0.112727004	2.330749278	0.019766584	0.042994693	positive
PRJNA729271	stage_E14_vs_E10	ordinary_muscle_development	ENSGALG00010024472	LDHA	0.365053966	0.201502256	1.811661929	0.07003845	0.126191091	positive
PRJNA729271	stage_E14_vs_E10	ordinary_muscle_development	ENSGALG00010024473	RAVER2	-0.274587123	0.056982443	-4.818802189	1.44423E-06	7.80248E-06	negative
PRJNA729271	stage_E14_vs_E10	ordinary_muscle_development	ENSGALG00010024474	STRIP1	0.227462452	0.102873368	2.21109172	0.027029486	0.056316286	positive
PRJNA729271	stage_E14_vs_E10	ordinary_muscle_development	ENSGALG00010024475	ADGRG1	-0.000264585	0.158440522	-0.001669932	0.998667588	0.999350415	negative
PRJNA729271	stage_E14_vs_E10	ordinary_muscle_development	ENSGALG00010024476	KCNB1	-0.478362692	0.165321717	-2.893526034	0.003809427	0.010244461	negative
PRJNA729271	stage_E14_vs_E10	ordinary_muscle_development	ENSGALG00010024479	ERBB2	0.741822005	0.127107809	5.8361639	5.34164E-09	4.02427E-08	positive
PRJNA729271	stage_E14_vs_E10	ordinary_muscle_development	ENSGALG00010024480	CDC45	-0.319603119	0.21351948	-1.496833541	0.134436576	0.217072315	negative
PRJNA729271	stage_E14_vs_E10	ordinary_muscle_development	ENSGALG00010024482	USP3	-0.163053441	0.055276667	-2.949769758	0.003180108	0.008755113	negative
PRJNA729271	stage_E14_vs_E10	ordinary_muscle_development	ENSGALG00010024486	GPR37L1	-1.35402212	0.311748448	-4.343316313	1.40348E-05	6.42965E-05	negative
PRJNA729271	stage_E14_vs_E10	ordinary_muscle_development	ENSGALG00010024488	ADGRG5	0.350939969	0.462157686	0.759351146	0.447642529	0.560713659	positive
PRJNA729271	stage_E14_vs_E10	ordinary_muscle_development	ENSGALG00010024490	TGM3	5.280941378	0.804811228	6.561714346	5.31926E-11	5.06921E-10	positive
PRJNA729271	stage_E14_vs_E10	ordinary_muscle_development	ENSGALG00010024492	NTF3	-1.406845587	0.244367591	-5.75708743	8.55776E-09	6.28075E-08	negative
PRJNA729271	stage_E14_vs_E10	ordinary_muscle_development	ENSGALG00010024493	JAK1	-0.044748941	0.060064323	-0.745016979	0.456261456	0.56939387	negative
PRJNA729271	stage_E14_vs_E10	ordinary_muscle_development	ENSGALG00010024494	DDAH1	-1.453118728	0.182273604	-7.972184087	1.55894E-15	2.30111E-14	negative
PRJNA729271	stage_E14_vs_E10	ordinary_muscle_development	ENSGALG00010024495	CLDN5	0.583391699	0.166313687	3.507779234	0.000451864	0.001546945	positive

PRJNA729271	stage_E14_vs_E10	ordinary_muscle_development	ENSGALG00010024496	UEVLD	-0.004891722	0.20811339	-0.023505081	0.981247386	0.987446602	negative
PRJNA729271	stage_E14_vs_E10	ordinary_muscle_development	ENSGALG00010024497	ABI3	-0.466956177	0.197149199	-2.368542089	0.017858348	0.039435826	negative
PRJNA729271	stage_E14_vs_E10	ordinary_muscle_development	ENSGALG00010024498	CCDC102A	-0.044129119	0.115901524	-0.380746663	0.70339124	0.784441623	negative
PRJNA729271	stage_E14_vs_E10	ordinary_muscle_development	ENSGALG00010024499	CA12	-3.719305443	0.723710543	-5.139216885	2.75886E-07	1.65178E-06	negative
PRJNA729271	stage_E14_vs_E10	ordinary_muscle_development	ENSGALG00010024501	ARL8A	0.261402401	0.08906873	2.93483919	0.003337206	0.00912006	positive
PRJNA729271	stage_E14_vs_E10	ordinary_muscle_development	ENSGALG00010024502	VPS8	-0.009934301	0.07292433	-0.13622752	0.89164142	0.925656532	negative
PRJNA729271	stage_E14_vs_E10	ordinary_muscle_development	ENSGALG00010024504	CASC3	0.13519272	0.186477093	0.724982988	0.468462467	0.580770053	positive
PRJNA729271	stage_E14_vs_E10	ordinary_muscle_development	ENSGALG00010024505	DNASE2B	0.116597328	0.211353134	0.551670685	0.581173998	0.682722838	positive
PRJNA729271	stage_E14_vs_E10	ordinary_muscle_development	ENSGALG00010024506	RAB8B	-0.027159772	0.08103929	-0.335143264	0.737517018	0.811408666	negative
PRJNA729271	stage_E14_vs_E10	ordinary_muscle_development	ENSGALG00010024508	RPS27L	0.427212206	0.190069611	2.247661811	0.024597763	0.052004751	positive
PRJNA729271	stage_E14_vs_E10	ordinary_muscle_development	ENSGALG00010024509	SEPTIN5	0.791465223	0.106710379	7.416946959	1.19851E-13	1.5028E-12	positive
PRJNA729271	stage_E14_vs_E10	ordinary_muscle_development	ENSGALG00010024510	CNN3	-0.043798041	0.100886183	-0.434133196	0.664191721	0.753086592	negative
PRJNA729271	stage_E14_vs_E10	ordinary_muscle_development	ENSGALG00010024511	ENSGALG00010024511	0.003628705	0.167962946	0.0216042	0.982763683	0.988726899	positive
PRJNA729271	stage_E14_vs_E10	ordinary_muscle_development	ENSGALG00010024512	CCNDBP1	0.314623331	0.114519528	2.747333453	0.006008201	0.015284114	positive
PRJNA729271	stage_E14_vs_E10	ordinary_muscle_development	ENSGALG00010024513	SPTY2D1	-0.060748079	0.138740053	-0.437855381	0.661491122	0.750905616	negative
PRJNA729271	stage_E14_vs_E10	ordinary_muscle_development	ENSGALG00010024514	FAF1	0.255354086	0.067838658	3.764138232	0.000167124	0.00063118	positive
PRJNA729271	stage_E14_vs_E10	ordinary_muscle_development	ENSGALG00010024515	C3orf70	0.230123599	0.153042653	1.503656621	0.132669804	0.214769802	positive
PRJNA729271	stage_E14_vs_E10	ordinary_muscle_development	ENSGALG00010024516	EHHADH	0.062204025	0.239559935	0.25965955	0.795126397	0.856203565	positive
PRJNA729271	stage_E14_vs_E10	ordinary_muscle_development	ENSGALG00010024517	LACTB	-0.486203427	0.097439829	-4.989781174	6.04477E-07	3.43874E-06	negative
PRJNA729271	stage_E14_vs_E10	ordinary_muscle_development	ENSGALG00010024518	ENSGALG00010024518	7.119770652	1.05282312	6.762551576	1.35582E-11	1.37335E-10	positive
PRJNA729271	stage_E14_vs_E10	ordinary_muscle_development	ENSGALG00010024519	MIEN1	0.345065338	0.138663564	2.488507625	0.012828049	0.029646455	positive
PRJNA729271	stage_E14_vs_E10	ordinary_muscle_development	ENSGALG00010024520	MAP3K13	-0.437653453	0.130042406	-3.365467198	0.000764142	0.002476242	negative
PRJNA729271	stage_E14_vs_E10	ordinary_muscle_development	ENSGALG00010024521	HEPACAM	-2.757382222	0.557403202	-4.94683599	7.54295E-07	4.24108E-06	negative
PRJNA729271	stage_E14_vs_E10	ordinary_muscle_development	ENSGALG00010024522	DNTTIP2	-0.061338089	0.074602633	-0.822197369	0.410964576	0.525719842	negative
PRJNA729271	stage_E14_vs_E10	ordinary_muscle_development	ENSGALG00010024523	TOX3	-0.883393105	0.233137587	-3.789149216	0.000151164	0.000575551	negative
PRJNA729271	stage_E14_vs_E10	ordinary_muscle_development	ENSGALG00010024524	TMEM41A	0.163979494	0.132815142	1.23464457	0.216962814	0.318855924	positive
PRJNA729271	stage_E14_vs_E10	ordinary_muscle_development	ENSGALG00010024525	GP1BB	0.159128148	0.187965386	0.846582185	0.397228047	0.512717769	positive
PRJNA729271	stage_E14_vs_E10	ordinary_muscle_development	ENSGALG00010024526	TGM6	4.936790436	1.363078996	3.621793344	0.000292568	0.001049176	positive
PRJNA729271	stage_E14_vs_E10	ordinary_muscle_development	ENSGALG00010024527	ATP6V0B	0.17254192	0.099293973	1.73768775	0.082265848	0.144811528	positive
PRJNA729271	stage_E14_vs_E10	ordinary_muscle_development	ENSGALG00010024528	TBX1	-1.372000345	0.212328108	-6.461699113	1.03534E-10	9.46231E-10	negative
PRJNA729271	stage_E14_vs_E10	ordinary_muscle_development	ENSGALG00010024529	GRB7	-0.714288021	0.893842833	-0.799120376	0.424220616	0.538589632	negative
PRJNA729271	stage_E14_vs_E10	ordinary_muscle_development	ENSGALG00010024530	ENSGALG00010024530	-0.463640191	0.551648364	-0.840463275	0.400648684	0.515938404	negative
PRJNA729271	stage_E14_vs_E10	ordinary_muscle_development	ENSGALG00010024531	CEP63	-0.20350097	0.122238368	-1.664788007	0.095955057	0.164386955	negative
PRJNA729271	stage_E14_vs_E10	ordinary_muscle_development	ENSGALG00010024532	TMEM86A	0.022887961	0.214562319	0.106672788	0.915048571	0.941362463	positive
PRJNA729271	stage_E14_vs_E10	ordinary_muscle_development	ENSGALG00010024533	CDKN2C	2.399527161	0.221263634	10.84465223	2.11438E-27	7.662E-26	positive
PRJNA729271	stage_E14_vs_E10	ordinary_muscle_development	ENSGALG00010024534	VWF	0.260775193	0.126570267	2.060319524	0.039368005	0.07730144	positive
PRJNA729271	stage_E14_vs_E10	ordinary_muscle_development	ENSGALG00010024535	ENSGALG00010024535	0.524428761	0.156327838	3.354672907	0.000794589	0.002565731	positive
PRJNA729271	stage_E14_vs_E10	ordinary_muscle_development	ENSGALG00010024536	URI1	0.254961483	0.060644693	4.204184598	2.62025E-05	0.000114503	positive
PRJNA729271	stage_E14_vs_E10	ordinary_muscle_development	ENSGALG00010024537	EPB42	-0.00081399	0.176237989	-0.004618697	0.996314826	0.998160417	negative
PRJNA729271	stage_E14_vs_E10	ordinary_muscle_development	ENSGALG00010024540	PTPN5	-1.922110595	0.278116425	-6.911172529	4.80664E-12	5.08186E-11	negative
PRJNA729271	stage_E14_vs_E10	ordinary_muscle_development	ENSGALG00010024541	WIPF2	0.247462358	0.201973985	1.225218968	0.22049273	0.322930395	positive
PRJNA729271	stage_E14_vs_E10	ordinary_muscle_development	ENSGALG00010024542	ZNFX1	0.311799893	0.425994776	0.731933607	0.464209092	0.576779598	positive
PRJNA729271	stage_E14_vs_E10	ordinary_muscle_development	ENSGALG00010024544	ZNF536	-1.853882093	0.245792668	-7.542462946	4.61177E-14	6.01689E-13	negative
PRJNA729271	stage_E14_vs_E10	ordinary_muscle_development	ENSGALG00010024545	PHACTR3	-1.306923357	0.283033482	-4.617557428	3.88283E-06	1.94477E-05	negative
PRJNA729271	stage_E14_vs_E10	ordinary_muscle_development	ENSGALG00010024546	KY	3.477879396	0.402744505	8.635448409	5.84971E-18	1.06656E-16	positive
PRJNA729271	stage_E14_vs_E10	ordinary_muscle_development	ENSGALG00010024547	PFKM	2.681530018	0.198620061	13.50080151	1.54684E-41	1.30357E-39	positive
PRJNA729271	stage_E14_vs_E10	ordinary_muscle_development	ENSGALG00010024548	CDC6	-0.13822274	0.273524145	-0.505340178	0.613319928	0.710012966	negative
PRJNA729271	stage_E14_vs_E10	ordinary_muscle_development	ENSGALG00010024549	GNB1L	0.058460705	0.130557833	0.447776312	0.654314641	0.74503462	positive
PRJNA729271	stage_E14_vs_E10	ordinary_muscle_development	ENSGALG00010024551	RNF11	0.24869459	0.126979444	1.958542133	0.050166432	0.095012504	positive
PRJNA729271	stage_E14_vs_E10	ordinary_muscle_development	ENSGALG00010024552	CD81	1.010373503	0.066136479	15.27709854	1.08673E-52	1.79001E-50	positive
PRJNA729271	stage_E14_vs_E10	ordinary_muscle_development	ENSGALG00010024553	ASB8	0.338604003	0.110704208	3.058637155	0.002223463	0.006385899	positive
PRJNA729271	stage_E14_vs_E10	ordinary_muscle_development	ENSGALG00010024554	ST6GALNAC3	-1.200265507	0.20987661	-5.718910312	1.07209E-08	7.78557E-08	negative
PRJNA729271	stage_E14_vs_E10	ordinary_muscle_development	ENSGALG00010024555	EPHB1	1.043239021	0.162058926	6.437405511	1.21533E-10	1.10516E-09	positive
PRJNA729271	stage_E14_vs_E10	ordinary_muscle_development	ENSGALG00010024556	ENSGALG00010024556	-0.372412167	0.200647994	-1.856047294	0.063446817	0.116015426	negative
PRJNA729271	stage_E14_vs_E10	ordinary_muscle_development	ENSGALG00010024557	TSPAN32	1.05190186	0.278501801	3.777002002	0.000158727	0.000601504	positive
PRJNA729271	stage_E14_vs_E10	ordinary_muscle_development	ENSGALG00010024558	PFDN5	0.21783579	0.107836978	2.020047244	0.043378487	0.084013593	positive
PRJNA729271	stage_E14_vs_E10	ordinary_muscle_development	ENSGALG00010024559	ENSGALG00010024559	-1.112866869	0.279579076	-3.980508432	6.8768E-05	0.000279214	negative
PRJNA729271	stage_E14_vs_E10	ordinary_muscle_development	ENSGALG00010024560	A4GNT	-0.271256818	0.464955305	-0.58340407	0.559621319	0.663100712	negative
PRJNA729271	stage_E14_vs_E10	ordinary_muscle_development	ENSGALG00010024561	ROBO3	-0.442455151	0.514418462	-0.860107448	0.389729816	0.505329877	negative
PRJNA729271	stage_E14_vs_E10	ordinary_muscle_development	ENSGALG00010024563	ESPL1	0.002136437	0.158386393	0.013488767	0.989237847	0.992730725	positive
PRJNA729271	stage_E14_vs_E10	ordinary_muscle_development	ENSGALG00010024564	PPP1R3D	0.677162948	0.203269066	3.33136251	0.00086422	0.002766882	positive
PRJNA729271	stage_E14_vs_E10	ordinary_muscle_development	ENSGALG00010024565	FAM217B	0.440657689	0.137626637	3.201834315	0.001365555	0.004159219	positive
PRJNA729271	stage_E14_vs_E10	ordinary_muscle_development	ENSGALG00010024566	MTSS2	1.703028352	0.214294422	7.947142703	1.90863E-15	2.79732E-14	positive
PRJNA729271	stage_E14_vs_E10	ordinary_muscle_development	ENSGALG00010024567	IKZF3	-0.306698747	0.461756621	-0.664199998	0.506562315	0.615498934	negative
PRJNA729271	stage_E14_vs_E10	ordinary_muscle_development	ENSGALG00010024568	DZIP1L	0.521488688	0.259115644	2.012571218	0.044159757	0.085289232	positive
PRJNA729271	stage_E14_vs_E10	ordinary_muscle_development	ENSGALG00010024569	ASCL2	-0.560966686	0.26102554	-2.149087349	0.031627476	0.064180354	negative
PRJNA729271	stage_E14_vs_E10	ordinary_muscle_development	ENSGALG00010024570	TPM1	1.485417147	0.164206723	9.046019088	1.48276E-19	3.06162E-18	positive
PRJNA729271	stage_E14_vs_E10	ordinary_muscle_development	ENSGALG00010024571	KCNA3	-4.267079273	0.427988955	-9.970068666	2.06086E-23	5.51148E-22	negative
PRJNA729271	stage_E14_vs_E10	ordinary_muscle_development	ENSGALG00010024573	ENSGALG00010024573	0.037014746	0.283705025	0.130469125	0.89619528	0.928479064	positive
PRJNA729271	stage_E14_vs_E10	ordinary_muscle_development	ENSGALG00010024575	TXNRD2	0.376055879	0.122018516	3.081957489	0.002056442	0.00595686	positive
PRJNA729271	stage_E14_vs_E10	ordinary_muscle_development	ENSGALG00010024576	CSAD	0.394368792	0.149100263	2.644990588	0.008169319	0.020015936	positive
PRJNA729271	stage_E14_vs_E10	ordinary_muscle_development	ENSGALG00010024578	ENSGALG00010024578	3.038056378	0.550793592	5.51580176	3.47236E-08	2.36967E-07	positive
PRJNA729271	stage_E14_vs_E10	ordinary_muscle_development	ENSGALG00010024579	ZNF740	0.107022986	0.104232071	1.026775964	0.30452597	0.418200278	positive
PRJNA729271	stage_E14_vs_E10	ordinary_muscle_development	ENSGALG00010024580	IGF2	-1.735996822	0.196546812	-8.832485249	1.02375E-18	1.96547E-17	negative
PRJNA729271	stage_E14_vs_E10	ordinary_muscle_development	ENSGALG00010024581	RARA	0.220322856	0.171915316	1.281577819	0.199990785	0.299068032	positive
PRJNA729271	stage_E14_vs_E10	ordinary_muscle_development	ENSGALG00010024582	TLN2	0.40650697	0.107413316	3.784511858	0.000154011	0.000585479	positive
PRJNA729271	stage_E14_vs_E10	ordinary_muscle_development	ENSGALG00010024583	ITGB7	0.876707725	0.517533931	1.694010136	0.090263357	0.15614839	positive
PRJNA729271	stage_E14_vs_E10	ordinary_muscle_development	ENSGALG00010024585	TBCCD1	-0.163062237	0.117496028	-1.38781063	0.165194717	0.256342728	negative
PRJNA729271	stage_E14_vs_E10	ordinary_muscle_development	ENSGALG00010024586	DLGAP4	0.746899649	0.094387594	7.913112532	2.51032E-15	3.64235E-14	positive
PRJNA729271	stage_E14_vs_E10	ordinary_muscle_development	ENSGALG00010024587	ENSGALG00010024587	0.871888147	0.184544514	4.724541141	2.30636E-06	1.20211E-05	positive
PRJNA729271	stage_E14_vs_E10	ordinary_muscle_development	ENSGALG00010024588	TTC39A	-2.277702234	0.346931325	-6.565282719	5.19342E-11	4.95907E-10	negative
PRJNA729271	stage_E14_vs_E10	ordinary_muscle_development	ENSGALG00010024589	ADORA1	1.913282098	0.453189145	4.221818017	2.4234E-05	0.000106381	positive
PRJNA729271	stage_E14_vs_E10	ordinary_muscle_development	ENSGALG00010024590	MRPL23	0.037145258	0.103559812	0.358684098	0.719831426	0.797333104	positive
PRJNA729271	stage_E14_vs_E10	ordinary_muscle_development	ENSGALG00010024592	COMT	1.053991184	0.197637536	5.332950438	9.66297E-08	6.17025E-07	positive
PRJNA729271	stage_E14_vs_E10	ordinary_muscle_development	ENSGALG00010024593	SRSF6	-0.115917611	0.074177784	-1.562699832	0.118123214	0.19520733	negative
PRJNA729271	stage_E14_vs_E10	ordinary_muscle_development	ENSGALG00010024594	TNNI2	2.539264203	0.183243542	13.85731893	1.14886E-43	1.12518E-41	positive
PRJNA729271	stage_E14_vs_E10	ordinary_muscle_development	ENSGALG00010024595	MYBPHL	1.603396491	0.317160855	5.055467801	4.29337E-07	2.5013E-06	positive
PRJNA729271	stage_E14_vs_E10	ordinary_muscle_development	ENSGALG00010024596	EPS15	0.322905208	0.066699035	4.841227588	1.29039E-06	7.03962E-06	positive
PRJNA729271	stage_E14_vs_E10	ordinary_muscle_development	ENSGALG00010024597	DNAJB11	-0.485341051	0.099232291	-4.890958817	1.00346E-06	5.53679E-06	negative
PRJNA729271	stage_E14_vs_E10	ordinary_muscle_development	ENSGALG00010024598	LRRC3C	0.120955595	0.235910449	0.512718261	0.608148414	0.705491098	positive
PRJNA729271	stage_E14_vs_E10	ordinary_muscle_development	ENSGALG00010024599	ENSGALG00010024599	0.231469366	0.202480942	1.143166184	0.2529696	0.36040833	positive
PRJNA729271	stage_E14_vs_E10	ordinary_muscle_development	ENSGALG00010024600	CD9	-0.175065991	0.334877731	-0.52277585	0.601130247	0.69993248	negative
PRJNA729271	stage_E14_vs_E10	ordinary_muscle_development	ENSGALG00010024601	ENSGALG00010024601	-0.654358837	0.505485664	-1.294515125	0.195487548	0.293697348	negative
PRJNA729271	stage_E14_vs_E10	ordinary_muscle_development	ENSGALG00010024602	PROCR	-0.422487354	0.251007864	-1.683163815	0.092343384	0.159195688	negative
PRJNA729271	stage_E14_vs_E10	ordinary_muscle_development	ENSGALG00010024603	ENSGALG00010024603	3.98547691	0.939505653	4.242099977	2.21438E-05	9.79177E-05	positive
PRJNA729271	stage_E14_vs_E10	ordinary_muscle_development	ENSGALG00010024604	MFSD5	0.172396871	0.169389735	1.017752762	0.308795466	0.422422639	positive
PRJNA729271	stage_E14_vs_E10	ordinary_muscle_development	ENSGALG00010024605	LSP1P1	0.784861328	0.195621187	4.01214888	6.01685E-05	0.000246786	positive
PRJNA729271	stage_E14_vs_E10	ordinary_muscle_development	ENSGALG00010024607	RARG	-0.469665972	0.140551957	-3.341582588	0.000833022	0.002678496	negative
PRJNA729271	stage_E14_vs_E10	ordinary_muscle_development	ENSGALG00010024608	DENND2D	0.041726446	0.337043094	0.123801517	0.901472431	0.932357546	positive
PRJNA729271	stage_E14_vs_E10	ordinary_muscle_development	ENSGALG00010024609	PRR33	4.171554018	0.867510015	4.808652289	1.51951E-06	8.1757E-06	positive
PRJNA729271	stage_E14_vs_E10	ordinary_muscle_development	ENSGALG00010024610	PFKL	-0.279705618	0.133334194	-2.097778603	0.035924707	0.071508574	negative
PRJNA729271	stage_E14_vs_E10	ordinary_muscle_development	ENSGALG00010024611	ENSGALG00010024611	-0.211875028	0.214284534	-0.988755575	0.322782745	0.437387668	negative
PRJNA729271	stage_E14_vs_E10	ordinary_muscle_development	ENSGALG00010024612	ENSGALG00010024612	-1.353891446	0.530774943	-2.55078252	0.010748137	0.025361265	negative
PRJNA729271	stage_E14_vs_E10	ordinary_muscle_development	ENSGALG00010024613	ENSGALG00010024613	-0.042817972	0.094889973	-0.45123811	0.651817946	0.742775246	negative
PRJNA729271	stage_E14_vs_E10	ordinary_muscle_development	ENSGALG00010024614	ENSGALG00010024614	-2.63117711	0.693258402	-3.795377166	0.000147419	0.000562623	negative
PRJNA729271	stage_E14_vs_E10	ordinary_muscle_development	ENSGALG00010024615	SYT8	-1.519640131	0.58647403	-2.591146502	0.009565676	0.022925674	negative
PRJNA729271	stage_E14_vs_E10	ordinary_muscle_development	ENSGALG00010024616	ARVCF	0.297266982	0.097467784	3.049899874	0.002289177	0.006549865	positive
PRJNA729271	stage_E14_vs_E10	ordinary_muscle_development	ENSGALG00010024618	VPS13C	-0.847317038	0.1229606	-6.890963774	5.54157E-12	5.80803E-11	negative
PRJNA729271	stage_E14_vs_E10	ordinary_muscle_development	ENSGALG00010024619	ENSGALG00010024619	-1.288542017	0.218351216	-5.901235828	3.60789E-09	2.77581E-08	negative
PRJNA729271	stage_E14_vs_E10	ordinary_muscle_development	ENSGALG00010024620	MSANTD2	-0.251318712	0.122728941	-2.047754257	0.040584085	0.079248154	negative
PRJNA729271	stage_E14_vs_E10	ordinary_muscle_development	ENSGALG00010024621	THRA	0.71340437	0.184807289	3.860260996	0.000113266	0.000442054	positive
PRJNA729271	stage_E14_vs_E10	ordinary_muscle_development	ENSGALG00010024622	PRMT8	-0.231742783	0.210156416	-1.102715718	0.270150639	0.379956677	negative
PRJNA729271	stage_E14_vs_E10	ordinary_muscle_development	ENSGALG00010024623	NRDC	0.091119096	0.109451394	0.832507404	0.405122601	0.519898371	positive
PRJNA729271	stage_E14_vs_E10	ordinary_muscle_development	ENSGALG00010024624	CTSD	0.016758507	0.128716799	0.130196733	0.896410779	0.928635952	positive
PRJNA729271	stage_E14_vs_E10	ordinary_muscle_development	ENSGALG00010024625	SPRYD3	0.169410459	0.078664862	2.153572182	0.031273738	0.06352478	positive
PRJNA729271	stage_E14_vs_E10	ordinary_muscle_development	ENSGALG00010024626	TSHZ3	-1.981869426	0.09716508	-20.39693098	1.78045E-92	1.3583E-89	negative
PRJNA729271	stage_E14_vs_E10	ordinary_muscle_development	ENSGALG00010024627	CEPT1	0.004102207	0.078160146	0.052484641	0.958142533	0.971208113	positive
PRJNA729271	stage_E14_vs_E10	ordinary_muscle_development	ENSGALG00010024628	TANGO2	0.498130156	0.109826686	4.535602176	5.74394E-06	2.80709E-05	positive
PRJNA729271	stage_E14_vs_E10	ordinary_muscle_development	ENSGALG00010024629	ENSGALG00010024629	0.695479482	0.171726945	4.049914715	5.12363E-05	0.00021286	positive
PRJNA729271	stage_E14_vs_E10	ordinary_muscle_development	ENSGALG00010024630	CFAP410	-0.238031951	0.095488088	-2.492792099	0.012674303	0.029333231	negative
PRJNA729271	stage_E14_vs_E10	ordinary_muscle_development	ENSGALG00010024631	EPB41L1	0.181564847	0.149102175	1.217720984	0.22333002	0.326261706	positive
PRJNA729271	stage_E14_vs_E10	ordinary_muscle_development	ENSGALG00010024632	IFITM10	0.386778536	0.337741437	1.145191242	0.252129946	0.359388687	positive
PRJNA729271	stage_E14_vs_E10	ordinary_muscle_development	ENSGALG00010024633	NRGN	-0.897262231	0.310037963	-2.894039884	0.003803199	0.010229609	negative
PRJNA729271	stage_E14_vs_E10	ordinary_muscle_development	ENSGALG00010024634	ENSGALG00010024634	0.268925855	0.167938693	1.601333501	0.109303073	0.183331178	positive
PRJNA729271	stage_E14_vs_E10	ordinary_muscle_development	ENSGALG00010024635	ENSGALG00010024635	-0.552962096	0.169858692	-3.255424205	0.001132231	0.003518802	negative
PRJNA729271	stage_E14_vs_E10	ordinary_muscle_development	ENSGALG00010024636	PANX3	-1.5647842	0.292424162	-5.351076968	8.74323E-08	5.61512E-07	negative
PRJNA729271	stage_E14_vs_E10	ordinary_muscle_development	ENSGALG00010024637	DUSP8	0.307804118	0.27789225	1.107638365	0.268018028	0.377213449	positive
PRJNA729271	stage_E14_vs_E10	ordinary_muscle_development	ENSGALG00010024638	ZNF507	-0.259104332	0.129399068	-2.002366302	0.04524535	0.087223214	negative
PRJNA729271	stage_E14_vs_E10	ordinary_muscle_development	ENSGALG00010024639	AAR2	-0.044353593	0.074119267	-0.59840842	0.549567449	0.654666352	negative
PRJNA729271	stage_E14_vs_E10	ordinary_muscle_development	ENSGALG00010024640	GSDMA	-0.338050802	0.289711944	-1.16685145	0.243270365	0.349641544	negative
PRJNA729271	stage_E14_vs_E10	ordinary_muscle_development	ENSGALG00010024641	TRA2B	-0.083296891	0.085904929	-0.969640413	0.332225761	0.447132071	negative

PRJNA729271	stage_E14_vs_E10	ordinary_muscle_development	ENSGALG00010024642	RAB3B	-0.328896118	0.227194094	-1.447643787	0.147716698	0.234441426	negative
PRJNA729271	stage_E14_vs_E10	ordinary_muscle_development	ENSGALG00010024643	ETV7	0.804088656	0.39523982	2.034432302	0.041908028	0.081505013	positive
PRJNA729271	stage_E14_vs_E10	ordinary_muscle_development	ENSGALG00010024644	EIF6	-0.189109556	0.074895357	-2.524983706	0.011570365	0.027102852	negative
PRJNA729271	stage_E14_vs_E10	ordinary_muscle_development	ENSGALG00010024645	PARP11	-0.190808054	0.11812368	-1.615324329	0.106240478	0.179127105	negative
PRJNA729271	stage_E14_vs_E10	ordinary_muscle_development	ENSGALG00010024646	RORA	-0.000772889	0.194018824	-0.003983575	0.996821575	0.998267841	negative
PRJNA729271	stage_E14_vs_E10	ordinary_muscle_development	ENSGALG00010024647	RPUSD4	-0.048429135	0.128419921	-0.377115438	0.706087819	0.786561861	negative
PRJNA729271	stage_E14_vs_E10	ordinary_muscle_development	ENSGALG00010024648	MMP24	-0.552009068	0.118920275	-4.641841498	3.45318E-06	1.74769E-05	negative
PRJNA729271	stage_E14_vs_E10	ordinary_muscle_development	ENSGALG00010024649	NCOR2	0.142549172	0.12933831	1.102141913	0.27039998	0.380159817	positive
PRJNA729271	stage_E14_vs_E10	ordinary_muscle_development	ENSGALG00010024650	PRKAG1	0.317430005	0.120913485	2.625265529	0.008658142	0.021025258	positive
PRJNA729271	stage_E14_vs_E10	ordinary_muscle_development	ENSGALG00010024651	PSMD3	0.119842514	0.127542033	0.939631518	0.347406603	0.463134251	positive
PRJNA729271	stage_E14_vs_E10	ordinary_muscle_development	ENSGALG00010024652	PUS3	-0.108019629	0.120889323	-0.893541515	0.37156725	0.487761936	negative
PRJNA729271	stage_E14_vs_E10	ordinary_muscle_development	ENSGALG00010024653	RFLNA	-1.871558402	0.249740992	-7.493997642	6.68069E-14	8.63072E-13	negative
PRJNA729271	stage_E14_vs_E10	ordinary_muscle_development	ENSGALG00010024654	CCDC92	-0.809259698	0.272365789	-2.971223736	0.002966156	0.00823175	negative
PRJNA729271	stage_E14_vs_E10	ordinary_muscle_development	ENSGALG00010024655	IGF2BP2	-0.393494919	0.165140915	-2.382782717	0.017182333	0.038163946	negative
PRJNA729271	stage_E14_vs_E10	ordinary_muscle_development	ENSGALG00010024656	HYLS1	0.235734123	0.17082705	1.379957822	0.167599632	0.259352692	positive
PRJNA729271	stage_E14_vs_E10	ordinary_muscle_development	ENSGALG00010024657	DPY19L3	0.135607563	0.127204112	1.066062737	0.286395283	0.398282608	positive
PRJNA729271	stage_E14_vs_E10	ordinary_muscle_development	ENSGALG00010024659	BEND5	0.082511516	0.107425925	0.768078244	0.442440702	0.55621665	positive
PRJNA729271	stage_E14_vs_E10	ordinary_muscle_development	ENSGALG00010024660	KMT2D	-0.227875128	0.158024149	-1.442027246	0.149294688	0.236443944	negative
PRJNA729271	stage_E14_vs_E10	ordinary_muscle_development	ENSGALG00010024662	MOB2	0.013117254	0.071006306	0.184733642	0.853437958	0.898502556	positive
PRJNA729271	stage_E14_vs_E10	ordinary_muscle_development	ENSGALG00010024663	ICE2	-0.428906693	0.122491365	-3.501525953	0.000462602	0.001581093	negative
PRJNA729271	stage_E14_vs_E10	ordinary_muscle_development	ENSGALG00010024664	DNAH10	-1.426809561	0.214739615	-6.644370493	3.04516E-11	2.96438E-10	negative
PRJNA729271	stage_E14_vs_E10	ordinary_muscle_development	ENSGALG00010024665	MAP1LC3A	0.939993774	0.148381565	6.334976794	2.37377E-10	2.1006E-09	positive
PRJNA729271	stage_E14_vs_E10	ordinary_muscle_development	ENSGALG00010024666	MED24	-0.292329556	0.11203373	-2.609299504	0.00907278	0.021903721	negative
PRJNA729271	stage_E14_vs_E10	ordinary_muscle_development	ENSGALG00010024667	RBM7	0.432453953	0.135163212	3.199494494	0.001376688	0.004186909	positive
PRJNA729271	stage_E14_vs_E10	ordinary_muscle_development	ENSGALG00010024668	ENSGALG00010024668	0.533386175	0.184578526	2.889752052	0.003855458	0.010342519	positive
PRJNA729271	stage_E14_vs_E10	ordinary_muscle_development	ENSGALG00010024669	STIL	-0.229649566	0.174190594	-1.318380979	0.187376145	0.283716413	negative
PRJNA729271	stage_E14_vs_E10	ordinary_muscle_development	ENSGALG00010024670	SENP2	0.217999716	0.093768675	2.324867187	0.020079063	0.043615468	positive
PRJNA729271	stage_E14_vs_E10	ordinary_muscle_development	ENSGALG00010024671	KCTD20	-0.066378838	0.2803269	-0.236790826	0.812819069	0.868767923	negative
PRJNA729271	stage_E14_vs_E10	ordinary_muscle_development	ENSGALG00010024672	DYNLRB1	-0.205264112	0.104852826	-1.957640241	0.050272242	0.095179748	negative
PRJNA729271	stage_E14_vs_E10	ordinary_muscle_development	ENSGALG00010024673	REXO2	0.106005545	0.098056033	1.081071117	0.27966548	0.390534792	positive
PRJNA729271	stage_E14_vs_E10	ordinary_muscle_development	ENSGALG00010024674	DHH	0.265383151	0.364906976	0.727262476	0.46706518	0.579483848	positive
PRJNA729271	stage_E14_vs_E10	ordinary_muscle_development	ENSGALG00010024675	NR1D1	0.911364766	0.469109826	1.942753521	0.052045946	0.098113946	positive
PRJNA729271	stage_E14_vs_E10	ordinary_muscle_development	ENSGALG00010024676	ENSGALG00010024676	0.36094168	0.164170816	2.198573952	0.027908231	0.057864369	positive
PRJNA729271	stage_E14_vs_E10	ordinary_muscle_development	ENSGALG00010024677	LMBR1L	-0.78631127	0.286132148	-2.74807034	0.005994715	0.015257838	negative
PRJNA729271	stage_E14_vs_E10	ordinary_muscle_development	ENSGALG00010024678	AMOTL2	0.854836062	0.090089449	9.488747793	2.33826E-21	5.58371E-20	positive
PRJNA729271	stage_E14_vs_E10	ordinary_muscle_development	ENSGALG00010024679	MSL1	-0.031702281	0.110502457	-0.286892093	0.774194945	0.839966746	negative
PRJNA729271	stage_E14_vs_E10	ordinary_muscle_development	ENSGALG00010024680	BRSK1	0.762163324	0.246264839	3.094892987	0.001968839	0.005738654	positive
PRJNA729271	stage_E14_vs_E10	ordinary_muscle_development	ENSGALG00010024681	ATP6V0A2	0.024059979	0.085665656	0.280859099	0.778818473	0.843341832	positive
PRJNA729271	stage_E14_vs_E10	ordinary_muscle_development	ENSGALG00010024682	ENSGALG00010024682	0.476850453	0.179036779	2.663421763	0.00773504	0.019054963	positive
PRJNA729271	stage_E14_vs_E10	ordinary_muscle_development	ENSGALG00010024683	SHISA4	0.841219556	0.181366646	4.63822635	3.51412E-06	1.77543E-05	positive
PRJNA729271	stage_E14_vs_E10	ordinary_muscle_development	ENSGALG00010024684	ENSGALG00010024684	-0.870286462	0.317372978	-2.742156777	0.00610372	0.015483623	negative
PRJNA729271	stage_E14_vs_E10	ordinary_muscle_development	ENSGALG00010024685	ENSGALG00010024685	-0.025744066	0.215670484	-0.119367593	0.904984135	0.935070933	negative
PRJNA729271	stage_E14_vs_E10	ordinary_muscle_development	ENSGALG00010024686	CMPK1	-0.337609168	0.132740784	-2.543371808	0.010978833	0.02584671	negative
PRJNA729271	stage_E14_vs_E10	ordinary_muscle_development	ENSGALG00010024687	RYK	-0.199898003	0.106623235	-1.874807154	0.060819259	0.111903421	negative
PRJNA729271	stage_E14_vs_E10	ordinary_muscle_development	ENSGALG00010024688	CADM1	1.383329568	0.128087181	10.79990638	3.44555E-27	1.23317E-25	positive
PRJNA729271	stage_E14_vs_E10	ordinary_muscle_development	ENSGALG00010024689	FOXD2	0.523616917	0.301250926	1.7381421	0.082185778	0.144713661	positive
PRJNA729271	stage_E14_vs_E10	ordinary_muscle_development	ENSGALG00010024690	TUBA1C	-1.004080072	0.648843233	-1.547492555	0.121744518	0.199964509	negative
PRJNA729271	stage_E14_vs_E10	ordinary_muscle_development	ENSGALG00010024691	TCTN2	-0.248802065	0.149541188	-1.663769479	0.096158506	0.164692875	negative
PRJNA729271	stage_E14_vs_E10	ordinary_muscle_development	ENSGALG00010024692	DRAM2	0.757453733	0.106023196	7.144226586	9.0504E-13	1.03786E-11	positive
PRJNA729271	stage_E14_vs_E10	ordinary_muscle_development	ENSGALG00010024693	ANXA2	0.639586119	0.201359449	3.176340228	0.001491459	0.004497337	positive
PRJNA729271	stage_E14_vs_E10	ordinary_muscle_development	ENSGALG00010024694	GTF2H3	-0.024748395	0.094343553	-0.262322058	0.793073158	0.854699037	negative
PRJNA729271	stage_E14_vs_E10	ordinary_muscle_development	ENSGALG00010024695	TRABD2B	-1.034834136	0.195309221	-5.298439731	1.16796E-07	7.36071E-07	negative
PRJNA729271	stage_E14_vs_E10	ordinary_muscle_development	ENSGALG00010024696	TRADD	0.148742904	0.112723784	1.31953434	0.186990538	0.283369351	positive
PRJNA729271	stage_E14_vs_E10	ordinary_muscle_development	ENSGALG00010024697	IGF2BP1	-0.683593443	0.119963932	-5.698324764	1.2099E-08	8.72515E-08	negative
PRJNA729271	stage_E14_vs_E10	ordinary_muscle_development	ENSGALG00010024698	ENSGALG00010024698	-1.345957771	0.328059211	-4.102789149	4.08199E-05	0.000172503	negative
PRJNA729271	stage_E14_vs_E10	ordinary_muscle_development	ENSGALG00010024699	FBXL8	-0.147009689	0.130506923	-1.12645127	0.259974556	0.368216845	negative
PRJNA729271	stage_E14_vs_E10	ordinary_muscle_development	ENSGALG00010024700	TOLLIP	0.220127225	0.084019652	2.619949255	0.008794285	0.021313019	positive
PRJNA729271	stage_E14_vs_E10	ordinary_muscle_development	ENSGALG00010024701	EIF2B1	0.024098834	0.098940419	0.243569152	0.807564522	0.864715059	positive
PRJNA729271	stage_E14_vs_E10	ordinary_muscle_development	ENSGALG00010024702	INAVA	0.439861055	0.636278841	0.691302346	0.489375558	0.599923775	positive
PRJNA729271	stage_E14_vs_E10	ordinary_muscle_development	ENSGALG00010024703	HSF4	-0.258134033	0.548818032	-0.470345394	0.638108271	0.731176236	negative
PRJNA729271	stage_E14_vs_E10	ordinary_muscle_development	ENSGALG00010024705	SLCO2A1	-1.220181171	0.281191208	-4.339329023	1.42918E-05	6.53707E-05	negative
PRJNA729271	stage_E14_vs_E10	ordinary_muscle_development	ENSGALG00010024706	ENSGALG00010024706	2.394037707	0.916526194	2.61207778	0.008999378	0.021748246	positive
PRJNA729271	stage_E14_vs_E10	ordinary_muscle_development	ENSGALG00010024707	ENSGALG00010024707	-0.07624564	0.375429244	-0.203089241	0.839065281	0.887625985	negative
PRJNA729271	stage_E14_vs_E10	ordinary_muscle_development	ENSGALG00010024708	BNIP2	0.429159383	0.062489183	6.867738756	6.52275E-12	6.78732E-11	positive
PRJNA729271	stage_E14_vs_E10	ordinary_muscle_development	ENSGALG00010024709	TEAD4	1.971512561	0.095716874	20.59733542	2.89965E-94	2.47238E-91	positive
PRJNA729271	stage_E14_vs_E10	ordinary_muscle_development	ENSGALG00010024710	HSDL1	0.01856531	0.123034917	0.150894648	0.880058823	0.917576444	positive
PRJNA729271	stage_E14_vs_E10	ordinary_muscle_development	ENSGALG00010024711	DDX55	-0.07931309	0.089356275	-0.887605147	0.374753184	0.491058449	negative
PRJNA729271	stage_E14_vs_E10	ordinary_muscle_development	ENSGALG00010024712	LRIF1	0.105924999	0.097953852	1.081376555	0.279529646	0.390420334	positive
PRJNA729271	stage_E14_vs_E10	ordinary_muscle_development	ENSGALG00010024713	RND2	-0.432861621	0.368201182	-1.175611709	0.239750074	0.345479404	negative
PRJNA729271	stage_E14_vs_E10	ordinary_muscle_development	ENSGALG00010024714	SRPRB	-0.161229026	0.174344468	-0.924772827	0.355084088	0.471461377	negative
PRJNA729271	stage_E14_vs_E10	ordinary_muscle_development	ENSGALG00010024715	DNAAF1	-0.023542575	0.16839764	-0.139803476	0.888815267	0.923738244	negative
PRJNA729271	stage_E14_vs_E10	ordinary_muscle_development	ENSGALG00010024716	GTF2A2	0.077667356	0.075334553	1.030965915	0.302556804	0.416245337	positive
PRJNA729271	stage_E14_vs_E10	ordinary_muscle_development	ENSGALG00010024717	TF	1.09188462	0.652775674	1.672679702	0.094390367	0.162280675	positive
PRJNA729271	stage_E14_vs_E10	ordinary_muscle_development	ENSGALG00010024718	ENSGALG00010024718	0.703321392	0.192280314	3.65779198	0.000254397	0.000922103	positive
PRJNA729271	stage_E14_vs_E10	ordinary_muscle_development	ENSGALG00010024719	PYM1	0.034609903	0.129270435	0.267732546	0.788905196	0.85160974	positive
PRJNA729271	stage_E14_vs_E10	ordinary_muscle_development	ENSGALG00010024720	BUD13	-0.055095097	0.10502964	-0.524567135	0.599884139	0.69910948	negative
PRJNA729271	stage_E14_vs_E10	ordinary_muscle_development	ENSGALG00010024721	ENSGALG00010024721	1.767193313	0.73144883	2.41601769	0.015691299	0.035273787	positive
PRJNA729271	stage_E14_vs_E10	ordinary_muscle_development	ENSGALG00010024722	BRCA1	-0.24888189	0.260174196	-0.956597132	0.338770637	0.453875624	negative
PRJNA729271	stage_E14_vs_E10	ordinary_muscle_development	ENSGALG00010024723	ITCH	0.112144048	0.10012006	1.12009569	0.262672987	0.371240732	positive
PRJNA729271	stage_E14_vs_E10	ordinary_muscle_development	ENSGALG00010024724	HOXD3	-0.531568601	0.229832314	-2.312854065	0.020730664	0.044856094	negative
PRJNA729271	stage_E14_vs_E10	ordinary_muscle_development	ENSGALG00010024726	TMED2	-0.02921713	0.058268705	-0.501420617	0.616075133	0.712316214	negative
PRJNA729271	stage_E14_vs_E10	ordinary_muscle_development	ENSGALG00010024728	SLC5A9	-1.061556353	0.697840057	-1.52120295	0.128208915	0.208573314	negative
PRJNA729271	stage_E14_vs_E10	ordinary_muscle_development	ENSGALG00010024729	FOXM1	-0.208981033	0.164677731	-1.269030319	0.204430251	0.304325407	negative
PRJNA729271	stage_E14_vs_E10	ordinary_muscle_development	ENSGALG00010024731	AOX1	0.90627568	0.199649858	4.539325437	5.64345E-06	2.76451E-05	positive
PRJNA729271	stage_E14_vs_E10	ordinary_muscle_development	ENSGALG00010024732	AHCY	1.683750283	0.224819617	7.489338804	6.92214E-14	8.93468E-13	positive
PRJNA729271	stage_E14_vs_E10	ordinary_muscle_development	ENSGALG00010024733	MBTPS1	-0.141283571	0.087596375	-1.612892896	0.106767779	0.179827905	negative
PRJNA729271	stage_E14_vs_E10	ordinary_muscle_development	ENSGALG00010024734	ZPR1	0.04609067	0.113254247	0.406966373	0.684032686	0.768787608	positive
PRJNA729271	stage_E14_vs_E10	ordinary_muscle_development	ENSGALG00010024735	OTUD7A	1.21501892	0.179166527	6.781506215	1.18929E-11	1.21657E-10	positive
PRJNA729271	stage_E14_vs_E10	ordinary_muscle_development	ENSGALG00010024736	DGKA	0.140002911	0.139803611	1.001425577	0.316621104	0.430608266	positive
PRJNA729271	stage_E14_vs_E10	ordinary_muscle_development	ENSGALG00010024737	STK38	-0.25735623	0.060967943	-4.221172897	2.43034E-05	0.000106622	negative
PRJNA729271	stage_E14_vs_E10	ordinary_muscle_development	ENSGALG00010024738	NBR1	-0.071170645	0.078083259	-0.911471241	0.362047132	0.478209694	negative
PRJNA729271	stage_E14_vs_E10	ordinary_muscle_development	ENSGALG00010024739	ASIP	-3.90588315	0.590330052	-6.616439627	3.67953E-11	3.56516E-10	negative
PRJNA729271	stage_E14_vs_E10	ordinary_muscle_development	ENSGALG00010024741	RILPL2	-0.573598667	0.154816478	-3.705023365	0.000211371	0.000780593	negative
PRJNA729271	stage_E14_vs_E10	ordinary_muscle_development	ENSGALG00010024742	PAK2	-0.04383327	0.069399697	-0.631606072	0.527644318	0.635133526	negative
PRJNA729271	stage_E14_vs_E10	ordinary_muscle_development	ENSGALG00010024744	KLF13	0.288036071	0.142801474	2.017038495	0.043691496	0.08449743	positive
PRJNA729271	stage_E14_vs_E10	ordinary_muscle_development	ENSGALG00010024745	PMEL	-2.101621089	0.554299346	-3.791491192	0.000149745	0.000570599	negative
PRJNA729271	stage_E14_vs_E10	ordinary_muscle_development	ENSGALG00010024746	HOXD4	-1.211860693	0.295669752	-4.098696883	4.15483E-05	0.000175427	negative
PRJNA729271	stage_E14_vs_E10	ordinary_muscle_development	ENSGALG00010024747	TULP3	0.014597363	0.085845513	0.17004223	0.864976925	0.907093079	positive
PRJNA729271	stage_E14_vs_E10	ordinary_muscle_development	ENSGALG00010024748	TRPM1	0.845559582	0.385137566	2.195474178	0.028129601	0.058215101	positive
PRJNA729271	stage_E14_vs_E10	ordinary_muscle_development	ENSGALG00010024749	APOA5	0.584366373	0.699312194	0.835630178	0.403362986	0.518512459	positive
PRJNA729271	stage_E14_vs_E10	ordinary_muscle_development	ENSGALG00010024750	APOA4	-0.109605571	1.657365328	-0.066132415	0.947272404	0.96322087	negative
PRJNA729271	stage_E14_vs_E10	ordinary_muscle_development	ENSGALG00010024751	MTMR10	0.153200449	0.056729775	2.700529802	0.006922913	0.01726562	positive
PRJNA729271	stage_E14_vs_E10	ordinary_muscle_development	ENSGALG00010024752	MPP2	-0.375572895	0.212362799	-1.768543721	0.07697005	0.136792652	negative
PRJNA729271	stage_E14_vs_E10	ordinary_muscle_development	ENSGALG00010024753	HOXD8	0.221101479	0.165928284	1.332512304	0.182691901	0.278134556	positive
PRJNA729271	stage_E14_vs_E10	ordinary_muscle_development	ENSGALG00010024754	EIF2S2	-0.073663542	0.144728618	-0.508977029	0.610768325	0.70778028	negative
PRJNA729271	stage_E14_vs_E10	ordinary_muscle_development	ENSGALG00010024755	PIGX	0.351781391	0.155893358	2.256551509	0.024036113	0.051025697	positive
PRJNA729271	stage_E14_vs_E10	ordinary_muscle_development	ENSGALG00010024756	CDK2	-0.338533248	0.161750895	-2.092929672	0.036355433	0.072206938	negative
PRJNA729271	stage_E14_vs_E10	ordinary_muscle_development	ENSGALG00010024757	PPFIA1	-0.274916697	0.074824323	-3.674162178	0.000238631	0.000869522	negative
PRJNA729271	stage_E14_vs_E10	ordinary_muscle_development	ENSGALG00010024758	SPATA6	0.114162573	0.104187272	1.095743947	0.273190845	0.383302806	positive
PRJNA729271	stage_E14_vs_E10	ordinary_muscle_development	ENSGALG00010024759	APOC3	1.465516069	0.971994135	1.507741678			positive
PRJNA729271	stage_E14_vs_E10	ordinary_muscle_development	ENSGALG00010024760	ENSGALG00010024760	0.585747788	0.194241019	3.015572056	0.00256495	0.007224825	positive
PRJNA729271	stage_E14_vs_E10	ordinary_muscle_development	ENSGALG00010024761	NECAB2	-0.584408219	0.235319146	-2.483470762	0.013010901	0.030002071	negative
PRJNA729271	stage_E14_vs_E10	ordinary_muscle_development	ENSGALG00010024762	FAN1	-0.403121422	0.086515208	-4.65954407	3.16911E-06	1.61576E-05	negative
PRJNA729271	stage_E14_vs_E10	ordinary_muscle_development	ENSGALG00010024763	HOXD9	-0.875371346	0.293704133	-2.980452938	0.002878225	0.008007652	negative
PRJNA729271	stage_E14_vs_E10	ordinary_muscle_development	ENSGALG00010024764	ENSGALG00010024764	-0.534498674	0.26381114	-2.026065593	0.042758063	0.08299118	negative
PRJNA729271	stage_E14_vs_E10	ordinary_muscle_development	ENSGALG00010024765	SNRNP35	0.011357922	0.13412775	0.08467988	0.932515892	0.953697283	positive
PRJNA729271	stage_E14_vs_E10	ordinary_muscle_development	ENSGALG00010024767	CTTN	-0.303638396	0.068515283	-4.431688751	9.3498E-06	4.40876E-05	negative
PRJNA729271	stage_E14_vs_E10	ordinary_muscle_development	ENSGALG00010024769	RALY	1.976016231	0.204189023	9.677387181	3.76207E-22	9.46724E-21	positive
PRJNA729271	stage_E14_vs_E10	ordinary_muscle_development	ENSGALG00010024770	CDC27	-0.021433091	0.071921521	-0.298006646	0.765698091	0.833493078	negative
PRJNA729271	stage_E14_vs_E10	ordinary_muscle_development	ENSGALG00010024771	AOX2	0.332260505	0.285104136	1.16540051	0.243856911	0.350283017	positive
PRJNA729271	stage_E14_vs_E10	ordinary_muscle_development	ENSGALG00010024772	ELAPOR1	-0.444416462	0.223621454	-1.987360575	0.046882453	0.089829631	negative
PRJNA729271	stage_E14_vs_E10	ordinary_muscle_development	ENSGALG00010024773	APOA1	-0.312121553	0.265878742	-1.173924438	0.240425293	0.34631468	negative
PRJNA729271	stage_E14_vs_E10	ordinary_muscle_development	ENSGALG00010024775	ITFG2	0.312896192	0.093832773	3.334615217	0.000854175	0.002737413	positive
PRJNA729271	stage_E14_vs_E10	ordinary_muscle_development	ENSGALG00010024776	MPHOSPH10	-0.317052848	0.105837566	-2.995655144	0.00273856	0.007666168	negative
PRJNA729271	stage_E14_vs_E10	ordinary_muscle_development	ENSGALG00010024778	RAB5B	0.158378392	0.139296087	1.136990961	0.255542065	0.363234597	positive
PRJNA729271	stage_E14_vs_E10	ordinary_muscle_development	ENSGALG00010024779	ENSGALG00010024779	-3.139890297	0.952119808	-3.29778907	0.000974493	0.003076014	negative
PRJNA729271	stage_E14_vs_E10	ordinary_muscle_development	ENSGALG00010024780	HOXD11	-3.125791366	0.78800424	-3.96671897	7.28688E-05	0.000294216	negative
PRJNA729271	stage_E14_vs_E10	ordinary_muscle_development	ENSGALG00010024781	CEP19	0.040250091	0.143686251	0.28012486	0.779381712	0.843636615	positive
PRJNA729271	stage_E14_vs_E10	ordinary_muscle_development	ENSGALG00010024782	HOXD12	-5.540697025	2.178365665	-2.543510998	0.01097446	0.025840611	negative
PRJNA729271	stage_E14_vs_E10	ordinary_muscle_development	ENSGALG00010024783	UBE2T	0.038482592	0.229921514	0.16737273	0.867076781	0.908707956	positive
PRJNA729271	stage_E14_vs_E10	ordinary_muscle_development	ENSGALG00010024784	ENSGALG00010024784	0.18263174	0.137684866	1.326447454	0.184691529	0.280580631	positive

PRJNA729271	stage_E14_vs_E10	ordinary_muscle_development	ENSGALG00010024785	MCEE	0.557457044	0.199095632	2.799946127	0.005111114	0.013265101	positive
PRJNA729271	stage_E14_vs_E10	ordinary_muscle_development	ENSGALG00010024786	PDCD5	-0.135192149	0.097358099	-1.388607116	0.164952252	0.256103567	negative
PRJNA729271	stage_E14_vs_E10	ordinary_muscle_development	ENSGALG00010024787	SARS1	0.020015467	0.099369911	0.201423816	0.840367194	0.888614129	positive
PRJNA729271	stage_E14_vs_E10	ordinary_muscle_development	ENSGALG00010024788	SIK3	0.223231745	0.165760081	1.346715953	0.178071732	0.272330635	positive
PRJNA729271	stage_E14_vs_E10	ordinary_muscle_development	ENSGALG00010024789	SUOX	0.203966282	0.174012035	1.172138943	0.241141277	0.347035624	positive
PRJNA729271	stage_E14_vs_E10	ordinary_muscle_development	ENSGALG00010024791	SHANK2	1.971992025	0.321998206	6.124232961	9.11215E-10	7.50885E-09	positive
PRJNA729271	stage_E14_vs_E10	ordinary_muscle_development	ENSGALG00010024792	BZW1	-0.083175866	0.068611199	-1.212278283	0.225405874	0.328570727	negative
PRJNA729271	stage_E14_vs_E10	ordinary_muscle_development	ENSGALG00010024793	FKBP4	-0.473333961	0.116675076	-4.056855838	4.97378E-05	0.000206813	negative
PRJNA729271	stage_E14_vs_E10	ordinary_muscle_development	ENSGALG00010024794	DDX6	0.004388595	0.063914543	0.068663478	0.94525749	0.961576764	positive
PRJNA729271	stage_E14_vs_E10	ordinary_muscle_development	ENSGALG00010024795	NRROS	0.5226679	0.208581686	2.505818746	0.012216823	0.028401419	positive
PRJNA729271	stage_E14_vs_E10	ordinary_muscle_development	ENSGALG00010024797	IKZF4	0.240232287	0.23528786	1.021014372	0.30724763	0.420901917	positive
PRJNA729271	stage_E14_vs_E10	ordinary_muscle_development	ENSGALG00010024798	FBXO45	-0.07253595	0.084116788	-0.862324292	0.388509089	0.50435638	negative
PRJNA729271	stage_E14_vs_E10	ordinary_muscle_development	ENSGALG00010024799	ENSGALG00010024799	0.018106408	0.185599439	0.097556373	0.922284568	0.946389294	positive
PRJNA729271	stage_E14_vs_E10	ordinary_muscle_development	ENSGALG00010024800	ENSGALG00010024800	-0.112095401	0.130335824	-0.860050582	0.389761161	0.505329877	negative
PRJNA729271	stage_E14_vs_E10	ordinary_muscle_development	ENSGALG00010024801	CXCR5	1.474775048	1.106822094	1.332440919	0.182715344	0.278141032	positive
PRJNA729271	stage_E14_vs_E10	ordinary_muscle_development	ENSGALG00010024802	CD59	-0.223843512	0.164182277	-1.36338414	0.172761477	0.265779835	negative
PRJNA729271	stage_E14_vs_E10	ordinary_muscle_development	ENSGALG00010024803	CLK1	-0.423681371	0.13772925	-3.076190209	0.00209664	0.006062399	negative
PRJNA729271	stage_E14_vs_E10	ordinary_muscle_development	ENSGALG00010024804	WDR53	0.028014411	0.104291016	0.268617687	0.7882239	0.850983572	positive
PRJNA729271	stage_E14_vs_E10	ordinary_muscle_development	ENSGALG00010024805	ENSGALG00010024805	-0.044020744	0.378359826	-0.116346241	0.907378139	0.936450418	negative
PRJNA729271	stage_E14_vs_E10	ordinary_muscle_development	ENSGALG00010024806	ANKRD27	-0.160715201	0.074603742	-2.154251204	0.031220477	0.063454541	negative
PRJNA729271	stage_E14_vs_E10	ordinary_muscle_development	ENSGALG00010024807	PPIL3	-0.317756673	0.189143004	-1.679981107	0.092960992	0.160070038	negative
PRJNA729271	stage_E14_vs_E10	ordinary_muscle_development	ENSGALG00010024808	CELSR2	0.381793254	0.234898763	1.625352341	0.104087505	0.175933256	positive
PRJNA729271	stage_E14_vs_E10	ordinary_muscle_development	ENSGALG00010024809	KANSL1	0.054682171	0.170483474	0.320747633	0.748401647	0.819465317	positive
PRJNA729271	stage_E14_vs_E10	ordinary_muscle_development	ENSGALG00010024810	ADIPOR1	0.050011143	0.113387022	0.441065847	0.659165327	0.748734537	positive
PRJNA729271	stage_E14_vs_E10	ordinary_muscle_development	ENSGALG00010024811	NIF3L1	-0.090210627	0.133336672	-0.676562759	0.498683414	0.607748525	negative
PRJNA729271	stage_E14_vs_E10	ordinary_muscle_development	ENSGALG00010024812	SBNO1	-0.220105198	0.111874791	-1.967424435	0.0491343	0.093550725	negative
PRJNA729271	stage_E14_vs_E10	ordinary_muscle_development	ENSGALG00010024813	RNF168	-0.267869875	0.094871788	-2.823493465	0.004750339	0.012458145	negative
PRJNA729271	stage_E14_vs_E10	ordinary_muscle_development	ENSGALG00010024814	APBA2	-1.16265699	0.140166507	-8.294827425	1.08743E-16	1.78508E-15	negative
PRJNA729271	stage_E14_vs_E10	ordinary_muscle_development	ENSGALG00010024816	NFIA	-0.437007582	0.122430236	-3.569441639	0.000357743	0.001258307	negative
PRJNA729271	stage_E14_vs_E10	ordinary_muscle_development	ENSGALG00010024817	UCKL1	0.282677003	0.224878413	1.257021513	0.208745846	0.309257056	positive
PRJNA729271	stage_E14_vs_E10	ordinary_muscle_development	ENSGALG00010024818	FBXO3	0.206480604	0.075803556	2.723890719	0.006451785	0.016227421	positive
PRJNA729271	stage_E14_vs_E10	ordinary_muscle_development	ENSGALG00010024820	NUDT19	0.129464801	0.139529325	0.927868034	0.353476028	0.469670458	positive
PRJNA729271	stage_E14_vs_E10	ordinary_muscle_development	ENSGALG00010024821	NECAP1	0.087918757	0.105530766	0.833110192	0.404782586	0.519666648	positive
PRJNA729271	stage_E14_vs_E10	ordinary_muscle_development	ENSGALG00010024822	RPS26	0.31039085	0.137100059	2.26397313	0.023575767	0.050180727	positive
PRJNA729271	stage_E14_vs_E10	ordinary_muscle_development	ENSGALG00010024823	ASB10	3.784145532	0.96812195	3.908748821	9.27754E-05	0.000367326	positive
PRJNA729271	stage_E14_vs_E10	ordinary_muscle_development	ENSGALG00010024824	DNAJC5	-0.196511531	0.100947079	-1.946678724	0.051573264	0.09733782	negative
PRJNA729271	stage_E14_vs_E10	ordinary_muscle_development	ENSGALG00010024825	UBXN7	-0.128012416	0.073671971	-1.737599995	0.082281321	0.144811528	negative
PRJNA729271	stage_E14_vs_E10	ordinary_muscle_development	ENSGALG00010024826	RBM33	-0.000715877	0.111501177	-0.006420358	0.994877331	0.997354375	negative
PRJNA729271	stage_E14_vs_E10	ordinary_muscle_development	ENSGALG00010024827	ENSGALG00010024827	-0.437332728	0.362083019	-1.207824462	0.22711479	0.330459709	negative
PRJNA729271	stage_E14_vs_E10	ordinary_muscle_development	ENSGALG00010024828	TIMM17B	0.19545124	0.133124929	1.468179115	0.142055576	0.227072736	positive
PRJNA729271	stage_E14_vs_E10	ordinary_muscle_development	ENSGALG00010024829	ERBB3	1.449847304	0.198519784	7.303288761	2.80818E-13	3.40909E-12	positive
PRJNA729271	stage_E14_vs_E10	ordinary_muscle_development	ENSGALG00010024830	ORC2	-0.411484484	0.103057892	-3.992750846	6.53112E-05	0.000266504	negative
PRJNA729271	stage_E14_vs_E10	ordinary_muscle_development	ENSGALG00010024833	MAPT	0.482479704	0.205337989	2.349685537	0.018789277	0.041165444	positive
PRJNA729271	stage_E14_vs_E10	ordinary_muscle_development	ENSGALG00010024834	INSIG1	0.177878557	0.260073431	0.683955129	0.494003501	0.604166448	positive
PRJNA729271	stage_E14_vs_E10	ordinary_muscle_development	ENSGALG00010024835	ENTREP2	-0.619740906	0.134589934	-4.604660166	4.1314E-06	2.06428E-05	negative
PRJNA729271	stage_E14_vs_E10	ordinary_muscle_development	ENSGALG00010024836	TXNDC12	0.006571554	0.105573434	0.06224628	0.950366708	0.965647222	positive
PRJNA729271	stage_E14_vs_E10	ordinary_muscle_development	ENSGALG00010024837	TNFRSF1A	1.376766393	0.155609374	8.847580045	8.94375E-19	1.72853E-17	positive
PRJNA729271	stage_E14_vs_E10	ordinary_muscle_development	ENSGALG00010024838	EN2	-3.427684789	0.739064019	-4.637872633	3.52014E-06	1.77781E-05	negative
PRJNA729271	stage_E14_vs_E10	ordinary_muscle_development	ENSGALG00010024839	PA2G4	0.305790071	0.098705418	3.098006958	0.001948269	0.005689175	positive
PRJNA729271	stage_E14_vs_E10	ordinary_muscle_development	ENSGALG00010024840	BTF3L4	-0.128953834	0.101482819	-1.270696217	0.203836742	0.303535399	negative
PRJNA729271	stage_E14_vs_E10	ordinary_muscle_development	ENSGALG00010024841	ENSGALG00010024841	-0.218833219	0.190802087	-1.146912077	0.251417961	0.358550112	negative
PRJNA729271	stage_E14_vs_E10	ordinary_muscle_development	ENSGALG00010024842	ZFYVE9	-0.096081621	0.075116329	-1.279104319	0.200860323	0.300027863	negative
PRJNA729271	stage_E14_vs_E10	ordinary_muscle_development	ENSGALG00010024843	BCL9L	-0.379885724	0.134150558	-2.831786379	0.004628876	0.012163807	negative
PRJNA729271	stage_E14_vs_E10	ordinary_muscle_development	ENSGALG00010024844	TM4SF19	-0.178341913	0.41626733	-0.428431202	0.668337212	0.756130806	negative
PRJNA729271	stage_E14_vs_E10	ordinary_muscle_development	ENSGALG00010024845	HYCC2	-0.140741033	0.110550277	-1.273095253	0.202984243	0.3026079	negative
PRJNA729271	stage_E14_vs_E10	ordinary_muscle_development	ENSGALG00010024846	LMO2	-0.200611814	0.143154377	-1.401366962	0.161104367	0.251394961	negative
PRJNA729271	stage_E14_vs_E10	ordinary_muscle_development	ENSGALG00010024847	PPIL1	0.199497673	0.148428008	1.344070272	0.178925664	0.273463465	positive
PRJNA729271	stage_E14_vs_E10	ordinary_muscle_development	ENSGALG00010024848	ESYT1	0.311644671	0.08478155	3.675854826	0.000237054	0.000865299	positive
PRJNA729271	stage_E14_vs_E10	ordinary_muscle_development	ENSGALG00010024849	CENATAC	-0.504730859	0.195590879	-2.580543941	0.00986448	0.02356	negative
PRJNA729271	stage_E14_vs_E10	ordinary_muscle_development	ENSGALG00010024850	CDK2AP1	0.021166001	0.116191925	0.182164127	0.855453925	0.900036629	positive
PRJNA729271	stage_E14_vs_E10	ordinary_muscle_development	ENSGALG00010024851	CPNE5	-1.706002106	0.625328911	-2.728167652	0.006368723	0.016060306	negative
PRJNA729271	stage_E14_vs_E10	ordinary_muscle_development	ENSGALG00010024852	CEP89	-0.251255648	0.089479786	-2.807959863	0.004985644	0.01299532	negative
PRJNA729271	stage_E14_vs_E10	ordinary_muscle_development	ENSGALG00010024853	TJP1	0.418672057	0.067071415	6.242183129	4.31505E-10	3.70099E-09	positive
PRJNA729271	stage_E14_vs_E10	ordinary_muscle_development	ENSGALG00010024854	C12orf65	0.403116917	0.150115361	2.685380855	0.00724472	0.017963089	positive
PRJNA729271	stage_E14_vs_E10	ordinary_muscle_development	ENSGALG00010024855	CAPRIN1	-0.139187306	0.071708544	-1.941014259	0.052256548	0.098371255	negative
PRJNA729271	stage_E14_vs_E10	ordinary_muscle_development	ENSGALG00010024856	SORT1	1.231164375	0.13311928	9.248580456	2.27494E-20	4.99625E-19	positive
PRJNA729271	stage_E14_vs_E10	ordinary_muscle_development	ENSGALG00010024857	SLC37A4	0.386524995	0.141612553	2.729454317	0.006343924	0.016011697	positive
PRJNA729271	stage_E14_vs_E10	ordinary_muscle_development	ENSGALG00010024858	SLC25A39	-0.130381996	0.12173521	-1.071029456	0.284156185	0.395738269	negative
PRJNA729271	stage_E14_vs_E10	ordinary_muscle_development	ENSGALG00010024859	TARSL2	-0.102146668	0.102909281	-0.992589456	0.320910065	0.435426516	negative
PRJNA729271	stage_E14_vs_E10	ordinary_muscle_development	ENSGALG00010024860	NEU4	-1.472608398	0.436820489	-3.371198088	0.00074842	0.002432366	negative
PRJNA729271	stage_E14_vs_E10	ordinary_muscle_development	ENSGALG00010024861	ORC1	-0.077626556	0.062496339	-1.242097653	0.214200534	0.315917455	negative
PRJNA729271	stage_E14_vs_E10	ordinary_muscle_development	ENSGALG00010024862	METTL16	0.130404574	0.119246742	1.093569282	0.274143928	0.384478721	positive
PRJNA729271	stage_E14_vs_E10	ordinary_muscle_development	ENSGALG00010024863	LMOD1	-1.148502376	0.313466396	-3.66387718	0.000248426	0.000902038	negative
PRJNA729271	stage_E14_vs_E10	ordinary_muscle_development	ENSGALG00010024864	MPHOSPH9	-0.26376858	0.125878257	-2.095426065	0.036133134	0.07183511	negative
PRJNA729271	stage_E14_vs_E10	ordinary_muscle_development	ENSGALG00010024865	SNAP47	0.073720472	0.143011791	0.515485273	0.606213958	0.703923041	positive
PRJNA729271	stage_E14_vs_E10	ordinary_muscle_development	ENSGALG00010024866	TRAPPC4	0.159602538	0.084471231	1.889430699	0.058834141	0.108706293	positive
PRJNA729271	stage_E14_vs_E10	ordinary_muscle_development	ENSGALG00010024867	TM2D3	0.045828502	0.10481274	0.437241714	0.661936061	0.7512342	positive
PRJNA729271	stage_E14_vs_E10	ordinary_muscle_development	ENSGALG00010024868	FAAP24	-0.285886465	0.188943885	-1.513076041	0.130260345	0.211341358	negative
PRJNA729271	stage_E14_vs_E10	ordinary_muscle_development	ENSGALG00010024869	NDUFB3	0.583870102	0.122005766	4.785594347	1.70482E-06	9.0951E-06	positive
PRJNA729271	stage_E14_vs_E10	ordinary_muscle_development	ENSGALG00010024870	TPD52L2	0.229764759	0.082331689	2.790720817	0.005259081	0.013617432	positive
PRJNA729271	stage_E14_vs_E10	ordinary_muscle_development	ENSGALG00010024871	POLQ	-0.00178246	0.128945536	-0.013823354	0.98897091	0.992531561	negative
PRJNA729271	stage_E14_vs_E10	ordinary_muscle_development	ENSGALG00010024872	RUNDC3A	-1.636526797	0.203503072	-8.041779332	8.85432E-16	1.33691E-14	negative
PRJNA729271	stage_E14_vs_E10	ordinary_muscle_development	ENSGALG00010024873	HYOU1	-0.42312203	0.10815245	-3.912274091	9.14311E-05	0.000362498	negative
PRJNA729271	stage_E14_vs_E10	ordinary_muscle_development	ENSGALG00010024874	JMJD4	0.344562445	0.080827594	4.262930856	2.01763E-05	8.97378E-05	positive
PRJNA729271	stage_E14_vs_E10	ordinary_muscle_development	ENSGALG00010024876	CFLAR	-0.188196635	0.095103255	-1.978866391	0.047831051	0.091393499	negative
PRJNA729271	stage_E14_vs_E10	ordinary_muscle_development	ENSGALG00010024877	PRPF38A	-0.166271203	0.076356103	-2.177575815	0.029437632	0.06046457	negative
PRJNA729271	stage_E14_vs_E10	ordinary_muscle_development	ENSGALG00010024878	RHPN2	-1.40111397	0.266558506	-5.256309365	1.46975E-07	9.12769E-07	negative
PRJNA729271	stage_E14_vs_E10	ordinary_muscle_development	ENSGALG00010024879	D2HGDH	-0.18894155	0.149886656	-1.260562849	0.207466391	0.307676011	negative
PRJNA729271	stage_E14_vs_E10	ordinary_muscle_development	ENSGALG00010024880	NAT10	-0.387754853	0.136829774	-2.833848533	0.004599113	0.01209541	negative
PRJNA729271	stage_E14_vs_E10	ordinary_muscle_development	ENSGALG00010024881	CASP10	-0.500524912	0.291998471	-1.714135387	0.086503864	0.150905465	negative
PRJNA729271	stage_E14_vs_E10	ordinary_muscle_development	ENSGALG00010024882	MYL6	0.428412176	0.163999054	2.612284426	0.008993939	0.021738728	positive
PRJNA729271	stage_E14_vs_E10	ordinary_muscle_development	ENSGALG00010024883	ING5	0.00475465	0.124421603	0.03821402	0.969517043	0.979040653	positive
PRJNA729271	stage_E14_vs_E10	ordinary_muscle_development	ENSGALG00010024884	ZBTB46	0.352049365	0.133450247	2.638057045	0.008338255	0.020369596	positive
PRJNA729271	stage_E14_vs_E10	ordinary_muscle_development	ENSGALG00010024885	SLC4A1	0.125103117	0.232136221	0.538921143	0.589941268	0.689946641	positive
PRJNA729271	stage_E14_vs_E10	ordinary_muscle_development	ENSGALG00010024886	ABTB2	-1.761482847	0.139052853	-12.66772174	8.92696E-37	5.65049E-35	negative
PRJNA729271	stage_E14_vs_E10	ordinary_muscle_development	ENSGALG00010024887	CASP18	0.734975857	0.256154341	2.869269575	0.00411421	0.010964418	positive
PRJNA729271	stage_E14_vs_E10	ordinary_muscle_development	ENSGALG00010024888	PITPNM2	-0.294257843	0.128077693	-2.297494876	0.021590555	0.046494591	negative
PRJNA729271	stage_E14_vs_E10	ordinary_muscle_development	ENSGALG00010024889	SLC2A4RG	-0.053020701	0.462260196	-0.114698825	0.908683845	0.937197406	negative
PRJNA729271	stage_E14_vs_E10	ordinary_muscle_development	ENSGALG00010024890	SMARCC2	0.199828749	0.144976917	1.37834872	0.168095648	0.26003697	positive
PRJNA729271	stage_E14_vs_E10	ordinary_muscle_development	ENSGALG00010024891	PKP1	0.896525905	0.443680715	2.020655564	0.043315433	0.083915691	positive
PRJNA729271	stage_E14_vs_E10	ordinary_muscle_development	ENSGALG00010024893	ARL6IP4	0.096005528	0.137626059	0.697582484	0.485438339	0.59691455	positive
PRJNA729271	stage_E14_vs_E10	ordinary_muscle_development	ENSGALG00010024894	TUT4	-0.046788344	0.12220543	-0.382866328	0.701818879	0.783574347	negative
PRJNA729271	stage_E14_vs_E10	ordinary_muscle_development	ENSGALG00010024895	ENSGALG00010024895	0.142739916	0.428809145	0.332875167	0.739228515	0.81248994	positive
PRJNA729271	stage_E14_vs_E10	ordinary_muscle_development	ENSGALG00010024896	RNF41	0.245636742	0.100029703	2.455638014	0.014063474	0.032082161	positive
PRJNA729271	stage_E14_vs_E10	ordinary_muscle_development	ENSGALG00010024897	ADAL	0.371846103	0.191644897	1.940287009	0.052344819	0.098491026	positive
PRJNA729271	stage_E14_vs_E10	ordinary_muscle_development	ENSGALG00010024898	NABP2	-0.005077086	0.308184869	-0.016474157	0.986856119	0.991301417	negative
PRJNA729271	stage_E14_vs_E10	ordinary_muscle_development	ENSGALG00010024899	OGFOD2	0.095695369	0.119543471	0.800506869	0.423417186	0.537805127	positive
PRJNA729271	stage_E14_vs_E10	ordinary_muscle_development	ENSGALG00010024900	DTYMK	0.178741033	0.099001685	1.805434264	0.071006753	0.127634287	positive
PRJNA729271	stage_E14_vs_E10	ordinary_muscle_development	ENSGALG00010024901	ZGPAT	-0.334249038	0.129810911	-2.574891699	0.010027148	0.023873769	negative
PRJNA729271	stage_E14_vs_E10	ordinary_muscle_development	ENSGALG00010024902	LARP6	-0.189857634	0.118793773	-1.598212004	0.109995803	0.184279839	negative
PRJNA729271	stage_E14_vs_E10	ordinary_muscle_development	ENSGALG00010024903	CASP8	-0.634165713	0.108018599	-5.870893681	4.33452E-09	3.31201E-08	negative
PRJNA729271	stage_E14_vs_E10	ordinary_muscle_development	ENSGALG00010024904	ALS2CL	0.021315321	0.1622784	0.13135033	0.895498179	0.928253029	positive
PRJNA729271	stage_E14_vs_E10	ordinary_muscle_development	ENSGALG00010024905	GPX7	-0.134399526	0.207176121	-0.648721123	0.516518648	0.624953073	negative
PRJNA729271	stage_E14_vs_E10	ordinary_muscle_development	ENSGALG00010024907	PHLDB1	0.249161566	0.133191704	1.870698842	0.061386835	0.112790237	positive
PRJNA729271	stage_E14_vs_E10	ordinary_muscle_development	ENSGALG00010024908	ENSGALG00010024908	-0.000927441	0.127057855	-0.007299358	0.994176007	0.996789183	negative
PRJNA729271	stage_E14_vs_E10	ordinary_muscle_development	ENSGALG00010024910	UBTF	-0.123303762	0.123150593	-1.001243754	0.316708978	0.430687366	negative
PRJNA729271	stage_E14_vs_E10	ordinary_muscle_development	ENSGALG00010024911	CAT	0.542959335	0.153210711	3.543873219	0.000394295	0.001367625	positive
PRJNA729271	stage_E14_vs_E10	ordinary_muscle_development	ENSGALG00010024915	STXBP5L	-0.418168093	0.172105096	-2.429725228	0.015110273	0.034142387	negative
PRJNA729271	stage_E14_vs_E10	ordinary_muscle_development	ENSGALG00010024917	TRAK2	0.23006252	0.122675387	1.875376358	0.060740965	0.111787747	positive
PRJNA729271	stage_E14_vs_E10	ordinary_muscle_development	ENSGALG00010024918	ATG4B	0.677844991	0.072701221	9.323708451	1.12343E-20	2.5286E-19	positive
PRJNA729271	stage_E14_vs_E10	ordinary_muscle_development	ENSGALG00010024919	ANKRD52	-0.191877649	0.122070377	-1.571860872	0.115982821	0.192529888	negative
PRJNA729271	stage_E14_vs_E10	ordinary_muscle_development	ENSGALG00010024920	VIPR2	-3.19434609	0.317185897	-10.07089572	7.42983E-24	2.03199E-22	negative
PRJNA729271	stage_E14_vs_E10	ordinary_muscle_development	ENSGALG00010024921	STRADB	-0.560431715	0.108021272	-5.188160651	2.12381E-07	1.29402E-06	negative
PRJNA729271	stage_E14_vs_E10	ordinary_muscle_development	ENSGALG00010024924	MNX1	-0.718380739	0.487680828	-1.473055117	0.140736174	0.225386239	negative
PRJNA729271	stage_E14_vs_E10	ordinary_muscle_development	ENSGALG00010024925	GTF2E1	-0.020624239	0.080741049	-0.25543684	0.79838572	0.858183241	negative
PRJNA729271	stage_E14_vs_E10	ordinary_muscle_development	ENSGALG00010024926	ELF5	-2.000372183	0.584268929	-3.42371823	0.000617706	0.002043746	negative
PRJNA729271	stage_E14_vs_E10	ordinary_muscle_development	ENSGALG00010024927	NOM1	-0.123764001	0.111006327	-1.114927451	0.264881512	0.373673527	negative
PRJNA729271	stage_E14_vs_E10	ordinary_muscle_development	ENSGALG00010024928	THAP4	0.23557694	0.085096165	2.768361421	0.005633894	0.014448566	positive
PRJNA729271	stage_E14_vs_E10	ordinary_muscle_development	ENSGALG00010024930	TMEM237	0.111527627	0.099002931	1.126508331	0.259950416	0.368216845	positive
PRJNA729271	stage_E14_vs_E10	ordinary_muscle_development	ENSGALG00010024931	UBE3C	-0.05688416	0.088498868	-0.642767098	0.520375235	0.628255792	negative
PRJNA729271	stage_E14_vs_E10	ordinary_muscle_development	ENSGALG00010024932	ANKK1	4.140157824	1.556293202	2.660268528	0.007807837	0.019208181	positive

PRJNA729271	stage_E14_vs_E10	ordinary_muscle_development	ENSGALG00010024933	CS	0.283553358	0.156963763	1.806489297	0.070841944	0.127417047	positive
PRJNA729271	stage_E14_vs_E10	ordinary_muscle_development	ENSGALG00010024934	G6PC3	-0.471184276	0.224106595	-2.102500717	0.035509437	0.070779605	negative
PRJNA729271	stage_E14_vs_E10	ordinary_muscle_development	ENSGALG00010024935	KLHL30	2.302276196	0.387958676	5.934333568	2.95041E-09	2.29679E-08	positive
PRJNA729271	stage_E14_vs_E10	ordinary_muscle_development	ENSGALG00010024936	EHF	1.474459542	0.506254313	2.912487859	0.003585621	0.009701994	positive
PRJNA729271	stage_E14_vs_E10	ordinary_muscle_development	ENSGALG00010024937	ILKAP	-0.096906323	0.123661534	-0.783641608	0.433250434	0.547369044	negative
PRJNA729271	stage_E14_vs_E10	ordinary_muscle_development	ENSGALG00010024938	ENSGALG00010024938	0.012314639	0.174543712	0.070553326	0.943753259	0.960383564	positive
PRJNA729271	stage_E14_vs_E10	ordinary_muscle_development	ENSGALG00010024939	COQ10A	0.273864491	0.206151986	1.328459149	0.18402647	0.279789178	positive
PRJNA729271	stage_E14_vs_E10	ordinary_muscle_development	ENSGALG00010024940	RABL3	-0.07777129	0.089648121	-0.867517233	0.385658674	0.501806326	negative
PRJNA729271	stage_E14_vs_E10	ordinary_muscle_development	ENSGALG00010024941	PDHX	0.369404071	0.117837169	3.134868849	0.00171931	0.005096399	positive
PRJNA729271	stage_E14_vs_E10	ordinary_muscle_development	ENSGALG00010024942	DNAJB6	0.169097091	0.065704093	2.573615782	0.010064197	0.023938387	positive
PRJNA729271	stage_E14_vs_E10	ordinary_muscle_development	ENSGALG00010024943	ABCB9	-0.974676381	0.191474561	-5.090370112	3.57365E-07	2.11084E-06	negative
PRJNA729271	stage_E14_vs_E10	ordinary_muscle_development	ENSGALG00010024944	ENSGALG00010024944	0.255655978	0.111596861	2.290888612	0.021969857	0.047234214	positive
PRJNA729271	stage_E14_vs_E10	ordinary_muscle_development	ENSGALG00010024945	APIP	0.031100184	0.08071513	0.385307981	0.700009249	0.782074462	positive
PRJNA729271	stage_E14_vs_E10	ordinary_muscle_development	ENSGALG00010024946	KIF1A	-1.238256913	0.172920629	-7.160839754	8.01843E-13	9.26113E-12	negative
PRJNA729271	stage_E14_vs_E10	ordinary_muscle_development	ENSGALG00010024947	MPP4	0.307311643	0.315697076	0.973438358	0.330335483	0.445083921	positive
PRJNA729271	stage_E14_vs_E10	ordinary_muscle_development	ENSGALG00010024949	PAN2	-0.137446569	0.111899944	-1.228298823	0.219334813	0.32159196	negative
PRJNA729271	stage_E14_vs_E10	ordinary_muscle_development	ENSGALG00010024950	NCAM1	1.603059215	0.154433513	10.38025483	3.0496E-25	9.36525E-24	positive
PRJNA729271	stage_E14_vs_E10	ordinary_muscle_development	ENSGALG00010024953	NCAPG2	-0.140282809	0.216921762	-0.646697717	0.517827601	0.626170942	negative
PRJNA729271	stage_E14_vs_E10	ordinary_muscle_development	ENSGALG00010024956	VPS37B	0.337707076	0.138011507	2.446948692	0.014407135	0.032799029	positive
PRJNA729271	stage_E14_vs_E10	ordinary_muscle_development	ENSGALG00010024960	ARFRP1	-0.317177727	0.09852139	-3.219379344	0.001284684	0.003946905	negative
PRJNA729271	stage_E14_vs_E10	ordinary_muscle_development	ENSGALG00010024961	LSM12	-0.196383928	0.203604106	-0.964538148	0.334776192	0.450002006	negative
PRJNA729271	stage_E14_vs_E10	ordinary_muscle_development	ENSGALG00010024963	TNFRSF6B	-0.97664529	0.594477741	-1.64286267	0.10041135	0.170628666	negative
PRJNA729271	stage_E14_vs_E10	ordinary_muscle_development	ENSGALG00010024964	ITM2C	-0.019460716	0.168240676	-0.115671882	0.907912592	0.936801895	negative
PRJNA729271	stage_E14_vs_E10	ordinary_muscle_development	ENSGALG00010024965	STAT2	-0.076630044	0.219566425	-0.349006202	0.727084654	0.802794946	negative
PRJNA729271	stage_E14_vs_E10	ordinary_muscle_development	ENSGALG00010024966	AGAP3	0.046722228	0.112933627	0.413714047	0.679083538	0.765242625	positive
PRJNA729271	stage_E14_vs_E10	ordinary_muscle_development	ENSGALG00010024968	TMEM101	0.322616971	0.131871776	2.446444428	0.014427304	0.032834631	positive
PRJNA729271	stage_E14_vs_E10	ordinary_muscle_development	ENSGALG00010024970	CAB39	-0.137829162	0.077725104	-1.773290153	0.07618064	0.135639157	negative
PRJNA729271	stage_E14_vs_E10	ordinary_muscle_development	ENSGALG00010024973	RTEL1	-0.416036448	0.077930339	-5.338568416	9.36834E-08	6.00062E-07	negative
PRJNA729271	stage_E14_vs_E10	ordinary_muscle_development	ENSGALG00010024974	CD44	0.507584644	0.165507256	3.066842251	0.00216333	0.006231611	positive
PRJNA729271	stage_E14_vs_E10	ordinary_muscle_development	ENSGALG00010024977	LRP3	0.149117902	0.105498181	1.413464205	0.157519279	0.246783609	positive
PRJNA729271	stage_E14_vs_E10	ordinary_muscle_development	ENSGALG00010024983	APOF	-0.40246871	0.606033296	-0.664103297	0.5066242	0.615498934	negative
PRJNA729271	stage_E14_vs_E10	ordinary_muscle_development	ENSGALG00010024984	SHISAL2A	-1.187023464	0.522589565	-2.271425881	0.023121209	0.049321843	negative
PRJNA729271	stage_E14_vs_E10	ordinary_muscle_development	ENSGALG00010024986	PTS	0.360475902	0.156379	2.305142645	0.021158586	0.045700149	positive
PRJNA729271	stage_E14_vs_E10	ordinary_muscle_development	ENSGALG00010024989	TIMELESS	-0.058971299	0.151114851	-0.390241588	0.696357912	0.77886932	negative
PRJNA729271	stage_E14_vs_E10	ordinary_muscle_development	ENSGALG00010024994	NDUFB4	0.294112024	0.104212245	2.822240553	0.004768939	0.01249788	positive
PRJNA729271	stage_E14_vs_E10	ordinary_muscle_development	ENSGALG00010024996	COA7	-0.101294161	0.124038519	-0.816634723	0.414137206	0.528938126	negative
PRJNA729271	stage_E14_vs_E10	ordinary_muscle_development	ENSGALG00010024997	HES6	0.377363655	0.241419451	1.563103774	0.118028188	0.195076236	positive
PRJNA729271	stage_E14_vs_E10	ordinary_muscle_development	ENSGALG00010025003	ENSGALG00010025003	3.089391835	0.246419589	12.53711949	4.67688E-36	2.767E-34	positive
PRJNA729271	stage_E14_vs_E10	ordinary_muscle_development	ENSGALG00010025005	HIP1R	-0.774950124	0.12178795	-6.363110013	1.97709E-10	1.76683E-09	negative
PRJNA729271	stage_E14_vs_E10	ordinary_muscle_development	ENSGALG00010025006	ENSGALG00010025006	0.418044753	0.273528725	1.528339496	0.126428268	0.20604652	positive
PRJNA729271	stage_E14_vs_E10	ordinary_muscle_development	ENSGALG00010025007	ZYG11B	0.042503452	0.101041818	0.420652091	0.67400915	0.760411164	positive
PRJNA729271	stage_E14_vs_E10	ordinary_muscle_development	ENSGALG00010025008	ENSGALG00010025008	-0.526034333	0.593682883	-0.886052719	0.375589119	0.491838855	negative
PRJNA729271	stage_E14_vs_E10	ordinary_muscle_development	ENSGALG00010025012	ENSGALG00010025012	-0.39041861	0.974823287	-0.400501933	0.68878686	0.772633148	negative
PRJNA729271	stage_E14_vs_E10	ordinary_muscle_development	ENSGALG00010025026	DENR	-0.015289189	0.115211046	-0.132705934	0.89442595	0.927507808	negative
PRJNA729271	stage_E14_vs_E10	ordinary_muscle_development	ENSGALG00010025028	HES6	0.39758183	0.131157778	3.031324838	0.002434831	0.006894488	positive
PRJNA729271	stage_E14_vs_E10	ordinary_muscle_development	ENSGALG00010025031	ECHDC2	0.255719825	0.1207632	2.117531051	0.034214803	0.068614218	positive
PRJNA729271	stage_E14_vs_E10	ordinary_muscle_development	ENSGALG00010025032	FSTL1	-0.27825882	0.181233316	-1.535362408	0.124694832	0.203817275	negative
PRJNA729271	stage_E14_vs_E10	ordinary_muscle_development	ENSGALG00010025039	CEBPA	0.048661796	0.308526362	0.1577233	0.874674839	0.914154719	positive
PRJNA729271	stage_E14_vs_E10	ordinary_muscle_development	ENSGALG00010025042	BCO2	-5.783931579	0.941349665	-6.144296636	8.03186E-10	6.64508E-09	negative
PRJNA729271	stage_E14_vs_E10	ordinary_muscle_development	ENSGALG00010025044	TMEM9	1.350538035	0.210126999	6.427246589	1.29936E-10	1.18083E-09	positive
PRJNA729271	stage_E14_vs_E10	ordinary_muscle_development	ENSGALG00010025047	PER2	-0.361463325	0.29332102	-1.232313066	0.217832161	0.319938917	negative
PRJNA729271	stage_E14_vs_E10	ordinary_muscle_development	ENSGALG00010025048	ESYT2	-0.047865444	0.082882824	-0.577507397	0.563596743	0.666286175	negative
PRJNA729271	stage_E14_vs_E10	ordinary_muscle_development	ENSGALG00010025055	ALS2	0.239143506	0.15413989	1.551470583	0.120788952	0.198642597	positive
PRJNA729271	stage_E14_vs_E10	ordinary_muscle_development	ENSGALG00010025056	RBMS2	-0.504991309	0.173088832	-2.917526806	0.003528193	0.009573411	negative
PRJNA729271	stage_E14_vs_E10	ordinary_muscle_development	ENSGALG00010025057	SCP2	-0.070845707	0.066348832	-1.067776259	0.28562145	0.39728269	negative
PRJNA729271	stage_E14_vs_E10	ordinary_muscle_development	ENSGALG00010025060	GPR156	0.735372926	0.151566425	4.851819439	1.22334E-06	6.68387E-06	positive
PRJNA729271	stage_E14_vs_E10	ordinary_muscle_development	ENSGALG00010025064	GPN3	-0.056725291	0.081993385	-0.691827647	0.489045572	0.599772871	negative
PRJNA729271	stage_E14_vs_E10	ordinary_muscle_development	ENSGALG00010025067	SLC1A2	-0.198284309	0.339656677	-0.583778629	0.559369259	0.662964628	negative
PRJNA729271	stage_E14_vs_E10	ordinary_muscle_development	ENSGALG00010025069	PODN	-0.288446484	0.199926824	-1.442760294	0.149088008	0.236229851	negative
PRJNA729271	stage_E14_vs_E10	ordinary_muscle_development	ENSGALG00010025071	CDK15	0.136324107	0.319302298	0.426943707	0.66942033	0.756883595	positive
PRJNA729271	stage_E14_vs_E10	ordinary_muscle_development	ENSGALG00010025072	ENSGALG00010025072	-2.677459381	0.873720753	-3.064433769	0.002180825	0.006277016	negative
PRJNA729271	stage_E14_vs_E10	ordinary_muscle_development	ENSGALG00010025073	IL18	-1.144233389	0.206458567	-5.542193804	2.98705E-08	2.05786E-07	negative
PRJNA729271	stage_E14_vs_E10	ordinary_muscle_development	ENSGALG00010025080	BAZ2A	0.303736973	0.204807234	1.483038308	0.138064233	0.221744162	positive
PRJNA729271	stage_E14_vs_E10	ordinary_muscle_development	ENSGALG00010025092	COL1A1	1.055440839	0.220985911	4.776054887	1.78768E-06	9.48823E-06	positive
PRJNA729271	stage_E14_vs_E10	ordinary_muscle_development	ENSGALG00010025097	LMBR1	0.108237978	0.120823702	0.895833984	0.370341437	0.486593467	positive
PRJNA729271	stage_E14_vs_E10	ordinary_muscle_development	ENSGALG00010025098	ATP5B	0.501497212	0.138214352	3.628401863	0.000285181	0.001024969	positive
PRJNA729271	stage_E14_vs_E10	ordinary_muscle_development	ENSGALG00010025102	ARPC3	-0.147054862	0.084461075	-1.741096273	0.081666705	0.143957057	negative
PRJNA729271	stage_E14_vs_E10	ordinary_muscle_development	ENSGALG00010025103	GSK3B	0.144708819	0.099596421	1.452951995	0.146237083	0.232500441	positive
PRJNA729271	stage_E14_vs_E10	ordinary_muscle_development	ENSGALG00010025113	SDHD	0.327093998	0.12013135	2.722802971	0.006473065	0.016272473	positive
PRJNA729271	stage_E14_vs_E10	ordinary_muscle_development	ENSGALG00010025114	CEBPG	0.094811488	0.096944812	0.977994455	0.328077067	0.442988084	positive
PRJNA729271	stage_E14_vs_E10	ordinary_muscle_development	ENSGALG00010025115	MGAT4F	0.074180746	0.248642086	0.298343481	0.765441021	0.833338411	positive
PRJNA729271	stage_E14_vs_E10	ordinary_muscle_development	ENSGALG00010025116	ANAPC7	-0.15051165	0.068704937	-2.190696262	0.02847378	0.058809837	negative
PRJNA729271	stage_E14_vs_E10	ordinary_muscle_development	ENSGALG00010025121	DYNC2I1	-0.54853188	0.15131399	-3.625123353	0.000288824	0.001037546	negative
PRJNA729271	stage_E14_vs_E10	ordinary_muscle_development	ENSGALG00010025125	PIK3CB	0.535296884	0.130817099	4.091948891	4.27763E-05	0.000180088	positive
PRJNA729271	stage_E14_vs_E10	ordinary_muscle_development	ENSGALG00010025126	ENSGALG00010025126	-0.100579074	0.129831574	-0.774688859	0.438523536	0.552442087	negative
PRJNA729271	stage_E14_vs_E10	ordinary_muscle_development	ENSGALG00010025127	PAMR1	-0.634257466	0.157130604	-4.036498632	5.42548E-05	0.000224372	negative
PRJNA729271	stage_E14_vs_E10	ordinary_muscle_development	ENSGALG00010025130	ATP2A2	0.201250269	0.12506512	1.609163842	0.10758052	0.180944493	positive
PRJNA729271	stage_E14_vs_E10	ordinary_muscle_development	ENSGALG00010025131	SUMO1	0.247254864	0.080123057	3.085938961	0.002029105	0.005883833	positive
PRJNA729271	stage_E14_vs_E10	ordinary_muscle_development	ENSGALG00010025138	CFAP91	-0.625466953	0.262229689	-2.38518741	0.017070423	0.037927846	negative
PRJNA729271	stage_E14_vs_E10	ordinary_muscle_development	ENSGALG00010025139	COPB2	-0.135972307	0.046206696	-2.942697018	0.003253667	0.008932178	negative
PRJNA729271	stage_E14_vs_E10	ordinary_muscle_development	ENSGALG00010025144	PIH1D2	0.385040022	0.14880019	2.587631253	0.009663837	0.023130337	positive
PRJNA729271	stage_E14_vs_E10	ordinary_muscle_development	ENSGALG00010025145	CPT2	0.473570114	0.093517977	5.063947363	4.10663E-07	2.40313E-06	positive
PRJNA729271	stage_E14_vs_E10	ordinary_muscle_development	ENSGALG00010025147	COX17	0.673067261	0.113970666	5.905618377	3.51326E-09	2.70589E-08	positive
PRJNA729271	stage_E14_vs_E10	ordinary_muscle_development	ENSGALG00010025149	IFT81	-0.385833055	0.139268344	-2.770428983	0.005598251	0.014369868	negative
PRJNA729271	stage_E14_vs_E10	ordinary_muscle_development	ENSGALG00010025154	TMEM183A	0.232311552	0.121581221	1.910751918	0.056036469	0.104402137	positive
PRJNA729271	stage_E14_vs_E10	ordinary_muscle_development	ENSGALG00010025155	CZ1B	0.115795122	0.090821084	1.274980629	0.202316098	0.301704922	positive
PRJNA729271	stage_E14_vs_E10	ordinary_muscle_development	ENSGALG00010025156	DLAT	0.356764052	0.11522618	3.096206529	0.001960138	0.00571674	positive
PRJNA729271	stage_E14_vs_E10	ordinary_muscle_development	ENSGALG00010025157	NOP58	-0.405922513	0.139550021	-2.908795779	0.003628238	0.009809981	negative
PRJNA729271	stage_E14_vs_E10	ordinary_muscle_development	ENSGALG00010025160	MRPS22	0.012404285	0.130345171	0.095164899	0.92418385	0.947855721	positive
PRJNA729271	stage_E14_vs_E10	ordinary_muscle_development	ENSGALG00010025161	ENSGALG00010025161	0.002452472	0.102947104	0.023822642	0.980994079	0.987446602	positive
PRJNA729271	stage_E14_vs_E10	ordinary_muscle_development	ENSGALG00010025163	LDLRAD3	-0.955993703	0.133848541	-7.142354327	9.17457E-13	1.05127E-11	negative
PRJNA729271	stage_E14_vs_E10	ordinary_muscle_development	ENSGALG00010025164	C11orf52	1.375126862	0.123880501	11.10043029	1.24841E-28	4.904E-27	positive
PRJNA729271	stage_E14_vs_E10	ordinary_muscle_development	ENSGALG00010025167	PTGES3	-0.385296891	0.188337364	-2.045780418	0.04077798	0.079574154	negative
PRJNA729271	stage_E14_vs_E10	ordinary_muscle_development	ENSGALG00010025168	POPDC2	2.42665804	0.210918769	11.50517829	1.24232E-30	5.45681E-29	positive
PRJNA729271	stage_E14_vs_E10	ordinary_muscle_development	ENSGALG00010025169	RBP2	0.950638935	2.24302561	0.42381992	0.671697149	0.758333996	positive
PRJNA729271	stage_E14_vs_E10	ordinary_muscle_development	ENSGALG00010025170	COMMD9	-0.094807869	0.094415241	-1.004158521	0.315302212	0.429338239	negative
PRJNA729271	stage_E14_vs_E10	ordinary_muscle_development	ENSGALG00010025172	RBP1	1.810212208	1.032237561	1.753677908	0.079485713	0.14064275	positive
PRJNA729271	stage_E14_vs_E10	ordinary_muscle_development	ENSGALG00010025182	PLA1A	1.086439175	0.201906725	5.380896412	7.41158E-08	4.82185E-07	positive
PRJNA729271	stage_E14_vs_E10	ordinary_muscle_development	ENSGALG00010025186	TMEM120B	-0.151557517	0.203622714	-0.744305555	0.456691641	0.569685485	negative
PRJNA729271	stage_E14_vs_E10	ordinary_muscle_development	ENSGALG00010025188	PPFIA4	-1.810067276	0.288636333	-6.271099881	3.58507E-10	3.09319E-09	negative
PRJNA729271	stage_E14_vs_E10	ordinary_muscle_development	ENSGALG00010025190	PRR5L	0.154843469	0.239562349	0.646359786	0.518046378	0.626383237	positive
PRJNA729271	stage_E14_vs_E10	ordinary_muscle_development	ENSGALG00010025192	CRYAB	1.446533387	0.181718653	7.960291142	1.71635E-15	2.5283E-14	positive
PRJNA729271	stage_E14_vs_E10	ordinary_muscle_development	ENSGALG00010025195	P2RX7	0.044792777	0.480543066	0.093212825	0.925734486	0.949043169	positive
PRJNA729271	stage_E14_vs_E10	ordinary_muscle_development	ENSGALG00010025200	PEPD	0.009879517	0.079497762	0.124274146	0.901098217	0.932159481	positive
PRJNA729271	stage_E14_vs_E10	ordinary_muscle_development	ENSGALG00010025204	TRAF6	0.143230852	0.117823818	1.215635808	0.224123687	0.327223293	positive
PRJNA729271	stage_E14_vs_E10	ordinary_muscle_development	ENSGALG00010025207	BMPR2	-0.312302526	0.097472104	-3.204019537	0.001355233	0.004134729	negative
PRJNA729271	stage_E14_vs_E10	ordinary_muscle_development	ENSGALG00010025210	HSPB2	1.477325588	0.295268898	5.003322726	5.63505E-07	3.21955E-06	positive
PRJNA729271	stage_E14_vs_E10	ordinary_muscle_development	ENSGALG00010025212	P2RX4	-0.408790208	0.184164367	-2.219703055	0.026438931	0.055292499	negative
PRJNA729271	stage_E14_vs_E10	ordinary_muscle_development	ENSGALG00010025214	LRP8	-1.160764734	0.194112333	-5.979860828	2.23328E-09	1.75932E-08	negative
PRJNA729271	stage_E14_vs_E10	ordinary_muscle_development	ENSGALG00010025215	MYOG	1.552644515	0.211514502	7.340605509	2.1263E-13	2.6031E-12	positive
PRJNA729271	stage_E14_vs_E10	ordinary_muscle_development	ENSGALG00010025216	DPP6	0.547689055	0.243394572	2.25021064	0.024435577	0.05172221	positive
PRJNA729271	stage_E14_vs_E10	ordinary_muscle_development	ENSGALG00010025219	NACA	-0.388819438	0.125392486	-3.100819269	0.00192986	0.005645475	negative
PRJNA729271	stage_E14_vs_E10	ordinary_muscle_development	ENSGALG00010025222	FAM117B	-0.825337984	0.135062477	-6.110786687	9.91412E-10	8.11435E-09	negative
PRJNA729271	stage_E14_vs_E10	ordinary_muscle_development	ENSGALG00010025225	DMRTB1	-3.315930322	1.048003532	-3.164044988	0.001555927	0.004662636	negative
PRJNA729271	stage_E14_vs_E10	ordinary_muscle_development	ENSGALG00010025228	FDXACB1	0.312635584	0.103559099	3.018909857	0.00253686	0.007154044	positive
PRJNA729271	stage_E14_vs_E10	ordinary_muscle_development	ENSGALG00010025229	ENSGALG00010025229	-0.820066105	0.165357523	-4.959351639	7.07288E-07	3.98916E-06	negative
PRJNA729271	stage_E14_vs_E10	ordinary_muscle_development	ENSGALG00010025230	CHST8	-0.587935692	0.21971119	-2.67594787	0.007451823	0.018404918	negative
PRJNA729271	stage_E14_vs_E10	ordinary_muscle_development	ENSGALG00010025232	PRIM1	0.035088376	0.148765481	0.235863694	0.813538442	0.869377743	positive
PRJNA729271	stage_E14_vs_E10	ordinary_muscle_development	ENSGALG00010025233	WDR12	-0.041319356	0.073113049	-0.565143388	0.571976239	0.674037911	negative
PRJNA729271	stage_E14_vs_E10	ordinary_muscle_development	ENSGALG00010025236	ENSGALG00010025236	-0.041033803	0.260875071	-0.157292925	0.875013996	0.91431141	negative
PRJNA729271	stage_E14_vs_E10	ordinary_muscle_development	ENSGALG00010025238	IGSF9B	0.840681298	0.279050801	3.012646062	0.002589808	0.007290593	positive
PRJNA729271	stage_E14_vs_E10	ordinary_muscle_development	ENSGALG00010025239	RAG2	-1.349544098	0.594467098	-2.270174586	0.023196992	0.049468943	negative
PRJNA729271	stage_E14_vs_E10	ordinary_muscle_development	ENSGALG00010025242	CLSTN2	-1.457470795	0.300911259	-4.843523639	1.27557E-06	6.96396E-06	negative
PRJNA729271	stage_E14_vs_E10	ordinary_muscle_development	ENSGALG00010025243	PAXIP1	-0.174384806	0.086408988	-2.018132715	0.043577441	0.0843106	negative
PRJNA729271	stage_E14_vs_E10	ordinary_muscle_development	ENSGALG00010025245	ENSGALG00010025245	0.574825702	0.068108384	8.439866935	3.17699E-17	5.42409E-16	positive
PRJNA729271	stage_E14_vs_E10	ordinary_muscle_development	ENSGALG00010025247	SGCA	2.032567522	0.140522662	14.46433978	2.03549E-47	2.50037E-45	positive
PRJNA729271	stage_E14_vs_E10	ordinary_muscle_development	ENSGALG00010025248	C11orf74	-0.377841194	0.211361705	-1.787652092	0.073832155	0.132090483	negative
PRJNA729271	stage_E14_vs_E10	ordinary_muscle_development	ENSGALG00010025250	GLIS1	-2.752463495	0.427210422	-6.442875341	1.17231E-10	1.06671E-09	negative
PRJNA729271	stage_E14_vs_E10	ordinary_muscle_development	ENSGALG00010025252	PPP1R9B	-0.126310541	0.144151505	-0.876234634	0.380902492	0.496866597	negative
PRJNA729271	stage_E14_vs_E10	ordinary_muscle_development	ENSGALG00010025256	ANAPC5	-0.350550228	0.118192715	-2.965920776	0.003017782	0.008363815	negative
PRJNA729271	stage_E14_vs_E10	ordinary_muscle_development	ENSGALG00010025257	LPCAT2	-0.982152076	0.167474667	-5.864481441	4.50538E-09	3.42991E-08	negative

PRJNA729271	stage_E14_vs_E10	ordinary_muscle_development	ENSGALG00010025259	SHH	-2.139046438	0.668596029	-3.199310712	0.001377566	0.004187882	negative
PRJNA729271	stage_E14_vs_E10	ordinary_muscle_development	ENSGALG00010025262	CARF	-0.210496147	0.10857242	-1.938762596	0.052530253	0.09879668	negative
PRJNA729271	stage_E14_vs_E10	ordinary_muscle_development	ENSGALG00010025263	SAMD14	-2.286912428	0.816389335	-2.801252208	0.005090472	0.013225737	negative
PRJNA729271	stage_E14_vs_E10	ordinary_muscle_development	ENSGALG00010025268	ZBTB39	0.388229443	0.101439928	3.827185715	0.000129617	0.000500211	positive
PRJNA729271	stage_E14_vs_E10	ordinary_muscle_development	ENSGALG00010025270	NDC1	-0.32260636	0.116728358	-2.763735963	0.005714376	0.014620095	negative
PRJNA729271	stage_E14_vs_E10	ordinary_muscle_development	ENSGALG00010025271	RNF34	0.100207706	0.117718578	0.85124801	0.394631598	0.510047706	positive
PRJNA729271	stage_E14_vs_E10	ordinary_muscle_development	ENSGALG00010025272	NCAPD3	-0.149738251	0.180055528	-0.831622629	0.405621985	0.520493155	negative
PRJNA729271	stage_E14_vs_E10	ordinary_muscle_development	ENSGALG00010025274	PDK2	1.033308656	0.271767063	3.802185019	0.000143426	0.000549116	positive
PRJNA729271	stage_E14_vs_E10	ordinary_muscle_development	ENSGALG00010025280	HSPB11	0.206057967	0.146015262	1.411208426	0.158183166	0.247609611	positive
PRJNA729271	stage_E14_vs_E10	ordinary_muscle_development	ENSGALG00010025286	ITGA3	-0.51388391	0.174067599	-2.952208873	0.003155094	0.008696535	negative
PRJNA729271	stage_E14_vs_E10	ordinary_muscle_development	ENSGALG00010025289	KDM2B	-0.384471606	0.0717424	-5.359056895	8.36575E-08	5.3966E-07	negative
PRJNA729271	stage_E14_vs_E10	ordinary_muscle_development	ENSGALG00010025291	DUS2	-0.324805645	0.091268713	-3.558784118	0.000372576	0.001303835	negative
PRJNA729271	stage_E14_vs_E10	ordinary_muscle_development	ENSGALG00010025292	ATP1A1	0.448110523	0.127475167	3.51527701	0.000439296	0.001507478	positive
PRJNA729271	stage_E14_vs_E10	ordinary_muscle_development	ENSGALG00010025297	SPSB4	-0.657178705	0.14221297	-4.621088389	3.81732E-06	1.91328E-05	negative
PRJNA729271	stage_E14_vs_E10	ordinary_muscle_development	ENSGALG00010025298	ENSGALG00010025298	-2.034381781	0.320044623	-6.356556654	2.06326E-10	1.84043E-09	negative
PRJNA729271	stage_E14_vs_E10	ordinary_muscle_development	ENSGALG00010025301	MORN3	-0.832635584	0.262742223	-3.169020856	0.001529534	0.004600664	negative
PRJNA729271	stage_E14_vs_E10	ordinary_muscle_development	ENSGALG00010025303	TMCC2	0.909944839	0.188902422	4.817009926	1.45725E-06	7.867E-06	positive
PRJNA729271	stage_E14_vs_E10	ordinary_muscle_development	ENSGALG00010025304	DDX28	-0.088853194	0.089698727	-0.99057363	0.32189382	0.436468748	negative
PRJNA729271	stage_E14_vs_E10	ordinary_muscle_development	ENSGALG00010025305	GPR182	0.774516212	0.431469224	1.795067107	0.072643009	0.130204082	positive
PRJNA729271	stage_E14_vs_E10	ordinary_muscle_development	ENSGALG00010025311	ENSGALG00010025311	2.047447316	0.553012032	3.702355819	0.000213607	0.000787444	positive
PRJNA729271	stage_E14_vs_E10	ordinary_muscle_development	ENSGALG00010025312	PLEKHM3	0.572839063	0.111890602	5.119635193	3.06127E-07	1.82371E-06	positive
PRJNA729271	stage_E14_vs_E10	ordinary_muscle_development	ENSGALG00010025313	DIO1	-0.59077458	0.847612865	-0.696986331	0.485811348	0.597170581	negative
PRJNA729271	stage_E14_vs_E10	ordinary_muscle_development	ENSGALG00010025315	LRRC42	0.067481533	0.116868292	0.577415243	0.563658979	0.666305407	positive
PRJNA729271	stage_E14_vs_E10	ordinary_muscle_development	ENSGALG00010025316	SLC25A36	-0.3738594	0.109688399	-3.408376845	0.000653506	0.002148949	negative
PRJNA729271	stage_E14_vs_E10	ordinary_muscle_development	ENSGALG00010025318	NUAK2	-0.830154398	0.411144704	-2.019129491	0.043473762	0.084154939	negative
PRJNA729271	stage_E14_vs_E10	ordinary_muscle_development	ENSGALG00010025319	LDLRAD1	-0.416710396	0.355429196	-1.17241465	0.24103062	0.346945267	negative
PRJNA729271	stage_E14_vs_E10	ordinary_muscle_development	ENSGALG00010025320	JAM3	0.640026678	0.143907051	4.447500481	8.68753E-06	4.11253E-05	positive
PRJNA729271	stage_E14_vs_E10	ordinary_muscle_development	ENSGALG00010025323	KLHDC8A	1.891693826	0.164398051	11.50678987	1.21933E-30	5.3721E-29	positive
PRJNA729271	stage_E14_vs_E10	ordinary_muscle_development	ENSGALG00010025325	DPEP2	-0.287375122	0.246925903	-1.16381116	0.244500549	0.351031311	negative
PRJNA729271	stage_E14_vs_E10	ordinary_muscle_development	ENSGALG00010025327	TMEM59	-0.17185307	0.07152836	-2.402586475	0.016279585	0.036398671	negative
PRJNA729271	stage_E14_vs_E10	ordinary_muscle_development	ENSGALG00010025329	MYO1A	0.631943633	0.201913611	3.129772322	0.001749418	0.005179293	positive
PRJNA729271	stage_E14_vs_E10	ordinary_muscle_development	ENSGALG00010025330	USH1C	-0.680444286	0.359432672	-1.893106383	0.058343724	0.10796531	negative
PRJNA729271	stage_E14_vs_E10	ordinary_muscle_development	ENSGALG00010025331	ENSGALG00010025331	-0.403954137	0.36067654	-1.119990053	0.262718001	0.371268151	negative
PRJNA729271	stage_E14_vs_E10	ordinary_muscle_development	ENSGALG00010025333	FZD5	0.083645295	0.198062002	0.42231874	0.672792382	0.759333923	positive
PRJNA729271	stage_E14_vs_E10	ordinary_muscle_development	ENSGALG00010025334	RHOF	-0.387983253	0.232305925	-1.670139292	0.094891808	0.163007437	negative
PRJNA729271	stage_E14_vs_E10	ordinary_muscle_development	ENSGALG00010025336	ADAM10	0.054753786	0.06291025	0.870347612	0.384110467	0.500195959	positive
PRJNA729271	stage_E14_vs_E10	ordinary_muscle_development	ENSGALG00010025337	NEMP1	-0.130284296	0.13948824	-0.934016339	0.350295465	0.466342098	negative
PRJNA729271	stage_E14_vs_E10	ordinary_muscle_development	ENSGALG00010025338	FADD	-0.04326056	0.092428158	-0.468045242	0.639752236	0.7325388	negative
PRJNA729271	stage_E14_vs_E10	ordinary_muscle_development	ENSGALG00010025340	METTL21A	-0.017939844	0.117457972	-0.152734157	0.878607924	0.916564808	negative
PRJNA729271	stage_E14_vs_E10	ordinary_muscle_development	ENSGALG00010025341	VPS26B	-0.285294035	0.129931625	-2.195724371	0.028111678	0.058186316	negative
PRJNA729271	stage_E14_vs_E10	ordinary_muscle_development	ENSGALG00010025342	TCEANC2	-0.4113439	0.116044668	-3.544703134	0.000393056	0.001364305	negative
PRJNA729271	stage_E14_vs_E10	ordinary_muscle_development	ENSGALG00010025343	NAB2	-0.269965681	0.299400521	-0.901687413	0.367222941	0.483372369	negative
PRJNA729271	stage_E14_vs_E10	ordinary_muscle_development	ENSGALG00010025344	ENSGALG00010025344	-0.070077474	0.536415937	-0.13064018	0.896059956	0.92843959	negative
PRJNA729271	stage_E14_vs_E10	ordinary_muscle_development	ENSGALG00010025345	CCM2	0.09079577	0.088037621	1.031329201	0.302386469	0.416089982	positive
PRJNA729271	stage_E14_vs_E10	ordinary_muscle_development	ENSGALG00010025346	PXYLP1	-1.645094282	0.124705407	-13.19184404	9.77642E-40	7.26714E-38	negative
PRJNA729271	stage_E14_vs_E10	ordinary_muscle_development	ENSGALG00010025348	HIPK1	-0.126641987	0.092892333	-1.363320125	0.172781642	0.265782649	negative
PRJNA729271	stage_E14_vs_E10	ordinary_muscle_development	ENSGALG00010025349	SETD1A	0.085130689	0.12583115	0.676547012	0.498693409	0.607748525	positive
PRJNA729271	stage_E14_vs_E10	ordinary_muscle_development	ENSGALG00010025351	CREB1	-0.042060676	0.066109348	-0.636228879	0.524627253	0.632283365	negative
PRJNA729271	stage_E14_vs_E10	ordinary_muscle_development	ENSGALG00010025353	SLC12A4	0.0245688	0.077177655	0.318340844	0.750226405	0.821153194	positive
PRJNA729271	stage_E14_vs_E10	ordinary_muscle_development	ENSGALG00010025354	ENSGALG00010025354	0.093644494	1.508525029	0.062076858	0.950501626	0.965647222	positive
PRJNA729271	stage_E14_vs_E10	ordinary_muscle_development	ENSGALG00010025355	STAT6	0.005584729	0.14151808	0.039463004	0.968521249	0.978580476	positive
PRJNA729271	stage_E14_vs_E10	ordinary_muscle_development	ENSGALG00010025357	OLFML3	0.437157942	0.119986969	3.643378502	0.000269083	0.000970503	positive
PRJNA729271	stage_E14_vs_E10	ordinary_muscle_development	ENSGALG00010025358	INCENP	-0.154106928	0.202868704	-0.759638743	0.447470554	0.560692054	negative
PRJNA729271	stage_E14_vs_E10	ordinary_muscle_development	ENSGALG00010025359	CDCP2	-0.159733357	0.406443566	-0.393002547	0.6943176	0.777093168	negative
PRJNA729271	stage_E14_vs_E10	ordinary_muscle_development	ENSGALG00010025360	THYN1	1.12749069	0.110390477	10.21365904	1.72244E-24	4.96358E-23	positive
PRJNA729271	stage_E14_vs_E10	ordinary_muscle_development	ENSGALG00010025362	G6PC2	0.17553656	0.231609428	0.75789903	0.448511431	0.561656432	positive
PRJNA729271	stage_E14_vs_E10	ordinary_muscle_development	ENSGALG00010025364	EPHA4	-2.229069451	0.180031202	-12.38157288	3.28834E-35	1.84746E-33	negative
PRJNA729271	stage_E14_vs_E10	ordinary_muscle_development	ENSGALG00010025365	SIKE1	-0.190728538	0.091166922	-2.09208047	0.036431318	0.072328716	negative
PRJNA729271	stage_E14_vs_E10	ordinary_muscle_development	ENSGALG00010025368	LRP1	-0.376708951	0.138850886	-2.713046791	0.006666769	0.016698604	negative
PRJNA729271	stage_E14_vs_E10	ordinary_muscle_development	ENSGALG00010025370	HPD	0.716122677	0.495374658	1.445618313	0.148284287	0.235213476	positive
PRJNA729271	stage_E14_vs_E10	ordinary_muscle_development	ENSGALG00010025373	CYB5RL	-0.105968788	0.086861366	-1.219976076	0.222473944	0.325207727	negative
PRJNA729271	stage_E14_vs_E10	ordinary_muscle_development	ENSGALG00010025377	MYO1G	0.747785141	0.293121604	2.551108931	0.010738076	0.025343456	positive
PRJNA729271	stage_E14_vs_E10	ordinary_muscle_development	ENSGALG00010025378	PAX3	-0.772409057	0.254171774	-3.038925392	0.002374236	0.006750251	negative
PRJNA729271	stage_E14_vs_E10	ordinary_muscle_development	ENSGALG00010025379	ACAD8	1.12461385	0.185162681	6.073652866	1.25033E-09	1.00854E-08	positive
PRJNA729271	stage_E14_vs_E10	ordinary_muscle_development	ENSGALG00010025380	SPC25	-0.220628454	0.222027883	-0.993697058	0.320370375	0.434891234	negative
PRJNA729271	stage_E14_vs_E10	ordinary_muscle_development	ENSGALG00010025381	MRPL37	0.438861386	0.12173665	3.605006271	0.000312145	0.001112229	positive
PRJNA729271	stage_E14_vs_E10	ordinary_muscle_development	ENSGALG00010025382	MAB21L3	-0.119689268	0.290512815	-0.411993074	0.680344499	0.76618705	negative
PRJNA729271	stage_E14_vs_E10	ordinary_muscle_development	ENSGALG00010025386	TPCN2	-0.23479277	0.095783352	-2.451289971	0.014234523	0.032446833	negative
PRJNA729271	stage_E14_vs_E10	ordinary_muscle_development	ENSGALG00010025387	PSMD9	0.13691389	0.0676385	2.024200573	0.042949519	0.083273578	positive
PRJNA729271	stage_E14_vs_E10	ordinary_muscle_development	ENSGALG00010025389	SNED1	-0.230281088	0.147104858	-1.565421372	0.11748414	0.1943987	negative
PRJNA729271	stage_E14_vs_E10	ordinary_muscle_development	ENSGALG00010025390	BCL7A	-0.061018816	0.085645976	-0.712453982	0.476183698	0.588379738	negative
PRJNA729271	stage_E14_vs_E10	ordinary_muscle_development	ENSGALG00010025392	SLC6A2	-0.469207925	0.23861885	-1.966348952	0.049258316	0.093737598	negative
PRJNA729271	stage_E14_vs_E10	ordinary_muscle_development	ENSGALG00010025398	TSPAN2	0.035540488	0.143062827	0.248425736	0.803805023	0.861899232	positive
PRJNA729271	stage_E14_vs_E10	ordinary_muscle_development	ENSGALG00010025400	SLC22A15	-0.347983231	0.1518656	-2.291389437	0.0219409	0.047185956	negative
PRJNA729271	stage_E14_vs_E10	ordinary_muscle_development	ENSGALG00010025403	ENSGALG00010025403	0.299698834	0.127317897	2.353941121	0.018575553	0.040758802	positive
PRJNA729271	stage_E14_vs_E10	ordinary_muscle_development	ENSGALG00010025404	CERS6	0.988635047	0.27477258	3.598012021	0.000320659	0.001140042	positive
PRJNA729271	stage_E14_vs_E10	ordinary_muscle_development	ENSGALG00010025405	TAMALIN	0.595519859	0.165973817	3.588034966	0.00033318	0.001179926	positive
PRJNA729271	stage_E14_vs_E10	ordinary_muscle_development	ENSGALG00010025406	ENSGALG00010025406	-0.027330137	0.147424281	-0.185384228	0.852927678	0.898161038	negative
PRJNA729271	stage_E14_vs_E10	ordinary_muscle_development	ENSGALG00010025407	MLXIP	1.098815729	0.127906252	8.590789848	8.63735E-18	1.55333E-16	positive
PRJNA729271	stage_E14_vs_E10	ordinary_muscle_development	ENSGALG00010025413	NECAB3	1.121207725	0.119838065	9.356023244	8.27944E-21	1.88104E-19	positive
PRJNA729271	stage_E14_vs_E10	ordinary_muscle_development	ENSGALG00010025414	MMP2	-0.280063019	0.221379576	-1.265080653	0.205842418	0.305768174	negative
PRJNA729271	stage_E14_vs_E10	ordinary_muscle_development	ENSGALG00010025415	ENSGALG00010025415	1.307589339	0.29143103	4.486788307	7.23049E-06	3.47007E-05	positive
PRJNA729271	stage_E14_vs_E10	ordinary_muscle_development	ENSGALG00010025416	ENSGALG00010025416	-1.400339319	0.754355971	-1.856337556	0.063405459	0.115955353	negative
PRJNA729271	stage_E14_vs_E10	ordinary_muscle_development	ENSGALG00010025417	B3GAT1	-0.189157965	0.178379177	-1.060426269	0.288950717	0.401374282	negative
PRJNA729271	stage_E14_vs_E10	ordinary_muscle_development	ENSGALG00010025418	CASQ2	2.449465972	0.209440282	11.69529544	1.34718E-31	6.21889E-30	positive
PRJNA729271	stage_E14_vs_E10	ordinary_muscle_development	ENSGALG00010025419	CCND1	-0.039900419	0.134266173	-0.297174025	0.766333652	0.833809209	negative
PRJNA729271	stage_E14_vs_E10	ordinary_muscle_development	ENSGALG00010025420	STK39	0.804570454	0.203174374	3.959999678	7.49499E-05	0.000301777	positive
PRJNA729271	stage_E14_vs_E10	ordinary_muscle_development	ENSGALG00010025421	NR4A1	0.961997249	0.434838734	2.212308087	0.026945385	0.05618952	positive
PRJNA729271	stage_E14_vs_E10	ordinary_muscle_development	ENSGALG00010025422	SSBP3	-0.651617873	0.156656978	-4.159520258	3.18917E-05	0.000137417	negative
PRJNA729271	stage_E14_vs_E10	ordinary_muscle_development	ENSGALG00010025423	NGF	-0.45362864	0.338233653	-1.341169443	0.179865445	0.274725988	negative
PRJNA729271	stage_E14_vs_E10	ordinary_muscle_development	ENSGALG00010025424	IRX6	-4.648388116	0.420613724	-11.05144185	2.15722E-28	8.40562E-27	negative
PRJNA729271	stage_E14_vs_E10	ordinary_muscle_development	ENSGALG00010025425	ENSGALG00010025425	-0.15385685	0.138749997	-1.108878227	0.267482718	0.376550312	negative
PRJNA729271	stage_E14_vs_E10	ordinary_muscle_development	ENSGALG00010025426	ENSGALG00010025426	1.409184991	0.152732669	9.226480482	2.7967E-20	6.11436E-19	positive
PRJNA729271	stage_E14_vs_E10	ordinary_muscle_development	ENSGALG00010025427	SLC35G2	0.911129538	0.128242997	7.104711849	1.20574E-12	1.36327E-11	positive
PRJNA729271	stage_E14_vs_E10	ordinary_muscle_development	ENSGALG00010025428	VANGL1	-0.38718411	0.105742164	-3.661586787	0.000250658	0.000909686	negative
PRJNA729271	stage_E14_vs_E10	ordinary_muscle_development	ENSGALG00010025429	SCN3B	-2.804772364	0.22174288	-12.64875953	1.13655E-36	7.07052E-35	negative
PRJNA729271	stage_E14_vs_E10	ordinary_muscle_development	ENSGALG00010025430	IRX5	-3.931451271	0.766051811	-5.132095786	2.86534E-07	1.71129E-06	negative
PRJNA729271	stage_E14_vs_E10	ordinary_muscle_development	ENSGALG00010025432	IRX3	-4.859628795	0.726889747	-6.685510169	2.30122E-11	2.27222E-10	negative
PRJNA729271	stage_E14_vs_E10	ordinary_muscle_development	ENSGALG00010025433	CHPF2	0.338203103	0.121429739	2.785175257	0.00534988	0.013820443	positive
PRJNA729271	stage_E14_vs_E10	ordinary_muscle_development	ENSGALG00010025434	SYT6	0.672825556	0.233900048	2.876551597	0.004020465	0.0107363	positive
PRJNA729271	stage_E14_vs_E10	ordinary_muscle_development	ENSGALG00010025435	SCLY	0.338321952	0.095047429	3.5595066	0.000371552	0.001300567	positive
PRJNA729271	stage_E14_vs_E10	ordinary_muscle_development	ENSGALG00010025438	EXOSC6	0.234433978	0.117647743	1.992677218	0.046296804	0.088991787	positive
PRJNA729271	stage_E14_vs_E10	ordinary_muscle_development	ENSGALG00010025442	ABCF2	0.217537764	0.147145427	1.478386165	0.139304446	0.223463695	positive
PRJNA729271	stage_E14_vs_E10	ordinary_muscle_development	ENSGALG00010025445	B3GNT4	0.713324478	0.300150087	2.37655929	0.017474953	0.038713043	positive
PRJNA729271	stage_E14_vs_E10	ordinary_muscle_development	ENSGALG00010025448	CHD1L	-0.090487719	0.105461032	-0.858020424	0.390881186	0.506464896	negative
PRJNA729271	stage_E14_vs_E10	ordinary_muscle_development	ENSGALG00010025449	ENSGALG00010025449	-0.080217568	0.11500576	-0.697509135	0.485484225	0.596920336	negative
PRJNA729271	stage_E14_vs_E10	ordinary_muscle_development	ENSGALG00010025451	LRRC43	1.308061826	0.321933846	4.063138575	4.84173E-05	0.000202076	positive
PRJNA729271	stage_E14_vs_E10	ordinary_muscle_development	ENSGALG00010025452	TRIM33	0.09094286	0.125795262	0.722943443	0.469714632	0.582023729	positive
PRJNA729271	stage_E14_vs_E10	ordinary_muscle_development	ENSGALG00010025458	BCAS2	0.484615793	0.114480039	4.233190332	2.30399E-05	0.000101478	positive
PRJNA729271	stage_E14_vs_E10	ordinary_muscle_development	ENSGALG00010025459	DIABLO	-0.02936987	0.076900991	-0.381917966	0.702522214	0.783961113	negative
PRJNA729271	stage_E14_vs_E10	ordinary_muscle_development	ENSGALG00010025464	VPS33A	-0.187195152	0.088685165	-2.110783157	0.034790954	0.069539135	negative
PRJNA729271	stage_E14_vs_E10	ordinary_muscle_development	ENSGALG00010025465	ENSGALG00010025465	0.136399906	0.08111789	1.681502135	0.092665422	0.159617979	positive
PRJNA729271	stage_E14_vs_E10	ordinary_muscle_development	ENSGALG00010025470	ORAOV1	-0.118513146	0.123517123	-0.959487589	0.337313169	0.452592278	negative
PRJNA729271	stage_E14_vs_E10	ordinary_muscle_development	ENSGALG00010025472	ZCCHC8	0.065241615	0.096376627	0.676944364	0.498441255	0.607645584	positive
PRJNA729271	stage_E14_vs_E10	ordinary_muscle_development	ENSGALG00010025473	PARS2	0.257680558	0.078418065	3.285984639	0.001016266	0.003199559	positive
PRJNA729271	stage_E14_vs_E10	ordinary_muscle_development	ENSGALG00010025477	ENSGALG00010025477	0.556227594	0.070207297	7.922646517	2.32508E-15	3.38034E-14	positive
PRJNA729271	stage_E14_vs_E10	ordinary_muscle_development	ENSGALG00010025480	FGF19	1.562530965	0.335431232	4.658275129	3.1887E-06	1.62347E-05	positive
PRJNA729271	stage_E14_vs_E10	ordinary_muscle_development	ENSGALG00010025481	TMEM39A	-0.194901265	0.088711325	-2.197027996	0.028018447	0.058043074	negative
PRJNA729271	stage_E14_vs_E10	ordinary_muscle_development	ENSGALG00010025483	FGF4	1.142947617	0.359338063	3.180702896	0.001469182	0.004438473	positive
PRJNA729271	stage_E14_vs_E10	ordinary_muscle_development	ENSGALG00010025485	DENND2C	1.515885743	0.159161586	9.524193509	1.66328E-21	4.01822E-20	positive
PRJNA729271	stage_E14_vs_E10	ordinary_muscle_development	ENSGALG00010025486	B3GALT1	-0.340430102	0.180957877	-1.881267104	0.059935594	0.110473859	negative
PRJNA729271	stage_E14_vs_E10	ordinary_muscle_development	ENSGALG00010025487	FGF3	0.160365666	0.435218718	0.368471436	0.71252174	0.791964007	positive
PRJNA729271	stage_E14_vs_E10	ordinary_muscle_development	ENSGALG00010025488	SLC4A8	-0.83251568	0.173478181	-4.798964764	1.59488E-06	8.54947E-06	negative
PRJNA729271	stage_E14_vs_E10	ordinary_muscle_development	ENSGALG00010025491	ZMYND11	-0.301538614	0.063648406	-4.737567433	2.16299E-06	1.13186E-05	negative
PRJNA729271	stage_E14_vs_E10	ordinary_muscle_development	ENSGALG00010025496	ATG101	0.170076638	0.110786124	1.535179966	0.124739628	0.203822399	positive
PRJNA729271	stage_E14_vs_E10	ordinary_muscle_development	ENSGALG00010025499	STAC3	3.015774612	0.159188181	18.94471436	4.88233E-80	2.28288E-77	positive
PRJNA729271	stage_E14_vs_E10	ordinary_muscle_development	ENSGALG00010025504	AMPD1	3.180003197	0.197917883	16.06728584	4.32618E-58	8.95828E-56	positive
PRJNA729271	stage_E14_vs_E10	ordinary_muscle_development	ENSGALG00010025505	ENSGALG00010025505	-1.881091111	0.265699421	-7.079771209	1.44393E-12	1.61495E-11	negative
PRJNA729271	stage_E14_vs_E10	ordinary_muscle_development	ENSGALG00010025506	R3HDM2	0.102564557	0.177013917	0.579415218	0.562309038	0.665120254	positive
PRJNA729271	stage_E14_vs_E10	ordinary_muscle_development	ENSGALG00010025508	ENSGALG00010025508	-0.082130784	0.13216828	-0.6214107	0.534329434	0.641467785	negative
PRJNA729271	stage_E14_vs_E10	ordinary_muscle_development	ENSGALG00010025512	NRAS	0.202060641	0.076514218	2.640824746	0.008270449	0.020232938	positive

PRJNA729271	stage_E14_vs_E10	ordinary_muscle_development	ENSGALG00010025513	RSRC2	-0.237659843	0.087128183	-2.72770342	0.006377692	0.016074534	negative
PRJNA729271	stage_E14_vs_E10	ordinary_muscle_development	ENSGALG00010025520	ACOT11	0.759125539	0.295399436	2.569827314	0.010174922	0.024166065	positive
PRJNA729271	stage_E14_vs_E10	ordinary_muscle_development	ENSGALG00010025522	SUMO3	0.06728118	0.10391021	0.647493452	0.517312631	0.625704822	positive
PRJNA729271	stage_E14_vs_E10	ordinary_muscle_development	ENSGALG00010025523	B4GALT4	-0.711244992	0.128610056	-5.530244025	3.19786E-08	2.19474E-07	negative
PRJNA729271	stage_E14_vs_E10	ordinary_muscle_development	ENSGALG00010025527	KNTC1	-0.164638305	0.237864477	-0.692151713	0.488842059	0.599688383	negative
PRJNA729271	stage_E14_vs_E10	ordinary_muscle_development	ENSGALG00010025531	ENSGALG00010025531	0.01911295	0.092773346	0.206017682	0.836777099	0.886110758	positive
PRJNA729271	stage_E14_vs_E10	ordinary_muscle_development	ENSGALG00010025533	INHBC	-1.635120307	0.608858058	-2.685552547	0.007240999	0.017956934	negative
PRJNA729271	stage_E14_vs_E10	ordinary_muscle_development	ENSGALG00010025538	ANO1	-0.464458457	0.21676957	-2.142636796	0.032142274	0.06502474	negative
PRJNA729271	stage_E14_vs_E10	ordinary_muscle_development	ENSGALG00010025541	TTC21B	-0.050291938	0.126814984	-0.396577249	0.691679232	0.774858217	negative
PRJNA729271	stage_E14_vs_E10	ordinary_muscle_development	ENSGALG00010025544	CSDE1	0.004851474	0.085968192	0.056433359	0.954996583	0.968900082	positive
PRJNA729271	stage_E14_vs_E10	ordinary_muscle_development	ENSGALG00010025548	ENSGALG00010025548	-0.837512026	0.195762358	-4.278207688	1.88404E-05	8.42096E-05	negative
PRJNA729271	stage_E14_vs_E10	ordinary_muscle_development	ENSGALG00010025549	DHCR24	-0.407350876	0.244183625	-1.668215371	0.095272981	0.163501455	negative
PRJNA729271	stage_E14_vs_E10	ordinary_muscle_development	ENSGALG00010025553	UBE2G2	0.044363815	0.095500226	0.464541464	0.64225986	0.734655671	positive
PRJNA729271	stage_E14_vs_E10	ordinary_muscle_development	ENSGALG00010025554	DIP2C	0.718346935	0.069557788	10.32734009	5.30102E-25	1.59415E-23	positive
PRJNA729271	stage_E14_vs_E10	ordinary_muscle_development	ENSGALG00010025557	VPS29	-0.114091278	0.076795294	-1.485654541	0.137370521	0.220752295	negative
PRJNA729271	stage_E14_vs_E10	ordinary_muscle_development	ENSGALG00010025558	GALNT3	-1.139860447	0.209689811	-5.435936264	5.45094E-08	3.59797E-07	negative
PRJNA729271	stage_E14_vs_E10	ordinary_muscle_development	ENSGALG00010025559	SLC6A17	-2.295476408	0.930702424	-2.46639135	0.013648213	0.031277604	negative
PRJNA729271	stage_E14_vs_E10	ordinary_muscle_development	ENSGALG00010025562	GRAMD1B	0.65369635	0.181999577	3.591746531	0.000328469	0.001165238	positive
PRJNA729271	stage_E14_vs_E10	ordinary_muscle_development	ENSGALG00010025563	TSPEAR	0.337810846	0.22529777	1.4993972	0.133770619	0.216189668	positive
PRJNA729271	stage_E14_vs_E10	ordinary_muscle_development	ENSGALG00010025565	ENSGALG00010025565	0.15262703	0.247230778	0.617346397	0.53700627	0.643584143	positive
PRJNA729271	stage_E14_vs_E10	ordinary_muscle_development	ENSGALG00010025568	MYL3	1.437954437	0.960432915	1.497194041	0.134342776	0.216945024	positive
PRJNA729271	stage_E14_vs_E10	ordinary_muscle_development	ENSGALG00010025569	PPTC7	0.031302581	0.193216251	0.162008012	0.871299545	0.911613029	positive
PRJNA729271	stage_E14_vs_E10	ordinary_muscle_development	ENSGALG00010025571	SRSF3	-0.133018343	0.228378155	-0.582447751	0.560265126	0.663538116	negative
PRJNA729271	stage_E14_vs_E10	ordinary_muscle_development	ENSGALG00010025572	ENSGALG00010025572	0.152373434	0.110813477	1.375044242	0.169117729	0.261311319	positive
PRJNA729271	stage_E14_vs_E10	ordinary_muscle_development	ENSGALG00010025573	CLMP	-0.641822713	0.167797211	-3.82499035	0.000130777	0.000504287	negative
PRJNA729271	stage_E14_vs_E10	ordinary_muscle_development	ENSGALG00010025574	ARHGAP31	0.442607121	0.098586343	4.489537889	7.13779E-06	3.43158E-05	positive
PRJNA729271	stage_E14_vs_E10	ordinary_muscle_development	ENSGALG00010025575	TRPM2	-0.959998057	0.446684684	-2.149162691	0.031621506	0.064177223	negative
PRJNA729271	stage_E14_vs_E10	ordinary_muscle_development	ENSGALG00010025577	PTH2R	-1.616290148	0.18567812	-8.704795935	3.18147E-18	5.91981E-17	negative
PRJNA729271	stage_E14_vs_E10	ordinary_muscle_development	ENSGALG00010025578	ORMDL2	0.187038781	0.144042569	1.298496566	0.194116756	0.292137121	positive
PRJNA729271	stage_E14_vs_E10	ordinary_muscle_development	ENSGALG00010025579	CSRNP3	-0.842498725	0.210901421	-3.9947513	6.47622E-05	0.000264505	negative
PRJNA729271	stage_E14_vs_E10	ordinary_muscle_development	ENSGALG00010025580	RASSF7	0.516977564	0.142336483	3.632080488	0.000281145	0.001011719	positive
PRJNA729271	stage_E14_vs_E10	ordinary_muscle_development	ENSGALG00010025582	DMAP1	-0.068552578	0.113212355	-0.605522055	0.544832175	0.650361721	negative
PRJNA729271	stage_E14_vs_E10	ordinary_muscle_development	ENSGALG00010025585	SARNP	-0.126713168	0.094943476	-1.334616899	0.182001764	0.27731689	negative
PRJNA729271	stage_E14_vs_E10	ordinary_muscle_development	ENSGALG00010025586	TCTN1	0.114149462	0.124493958	0.91690765	0.359191035	0.475563943	positive
PRJNA729271	stage_E14_vs_E10	ordinary_muscle_development	ENSGALG00010025587	LRRC56	-1.281201455	0.21582245	-5.936367855	2.91406E-09	2.26971E-08	negative
PRJNA729271	stage_E14_vs_E10	ordinary_muscle_development	ENSGALG00010025588	ENSGALG00010025588	-1.209795997	0.309248596	-3.91205009	9.15159E-05	0.000362735	negative
PRJNA729271	stage_E14_vs_E10	ordinary_muscle_development	ENSGALG00010025590	HRAS	0.905267174	0.11344615	7.979708194	1.4668E-15	2.17617E-14	positive
PRJNA729271	stage_E14_vs_E10	ordinary_muscle_development	ENSGALG00010025591	OTOS	0.522948484	0.342808115	1.525484552	0.127138284	0.207040717	positive
PRJNA729271	stage_E14_vs_E10	ordinary_muscle_development	ENSGALG00010025592	HSPA8	-0.516714596	0.208180729	-2.482048161	0.013062962	0.030093394	negative
PRJNA729271	stage_E14_vs_E10	ordinary_muscle_development	ENSGALG00010025594	UPK1B	0.598131752	0.40211989	1.487446322	0.13689697	0.220268474	positive
PRJNA729271	stage_E14_vs_E10	ordinary_muscle_development	ENSGALG00010025595	ERI3	0.029986254	0.09123461	0.328671913	0.742403686	0.815114485	positive
PRJNA729271	stage_E14_vs_E10	ordinary_muscle_development	ENSGALG00010025597	SCN2A	-1.414105914	0.48907636	-2.89138063	0.003835533	0.010295564	negative
PRJNA729271	stage_E14_vs_E10	ordinary_muscle_development	ENSGALG00010025598	ENSGALG00010025598	0.763904045	0.430121456	1.776019388	0.075729722	0.134985528	positive
PRJNA729271	stage_E14_vs_E10	ordinary_muscle_development	ENSGALG00010025599	COPS9	0.398241649	0.141869648	2.807095486	0.004999042	0.013025547	positive
PRJNA729271	stage_E14_vs_E10	ordinary_muscle_development	ENSGALG00010025600	ENSGALG00010025600	-0.121898774	0.108808047	-1.120310282	0.262581562	0.371147708	negative
PRJNA729271	stage_E14_vs_E10	ordinary_muscle_development	ENSGALG00010025604	HVCN1	-0.150754235	0.132098947	-1.141222077	0.253777519	0.361310789	negative
PRJNA729271	stage_E14_vs_E10	ordinary_muscle_development	ENSGALG00010025606	CCDC12	-0.227475406	0.130230691	-1.74671119	0.080687448	0.142473451	negative
PRJNA729271	stage_E14_vs_E10	ordinary_muscle_development	ENSGALG00010025608	ENSGALG00010025608	-2.362958601	0.518720004	-4.555364327	5.22949E-06	2.57128E-05	negative
PRJNA729271	stage_E14_vs_E10	ordinary_muscle_development	ENSGALG00010025610	RNH1	0.477823852	0.133130445	3.589140343	0.00033177	0.001176084	positive
PRJNA729271	stage_E14_vs_E10	ordinary_muscle_development	ENSGALG00010025612	TMEM53	-0.019597088	0.147570683	-0.132797978	0.894353154	0.927507808	negative
PRJNA729271	stage_E14_vs_E10	ordinary_muscle_development	ENSGALG00010025613	CDKN1A	-0.039073367	0.280316297	-0.139390278	0.889141755	0.923878834	negative
PRJNA729271	stage_E14_vs_E10	ordinary_muscle_development	ENSGALG00010025618	ADIPOQ	3.452634015	0.226644252	15.23371531	2.11239E-52	3.44034E-50	positive
PRJNA729271	stage_E14_vs_E10	ordinary_muscle_development	ENSGALG00010025621	PPP1CC	-0.311547434	0.117013313	-2.662495623	0.007756358	0.019097742	negative
PRJNA729271	stage_E14_vs_E10	ordinary_muscle_development	ENSGALG00010025623	ENSGALG00010025623	-0.593922586	0.434252337	-1.367690017	0.171409126	0.264119834	negative
PRJNA729271	stage_E14_vs_E10	ordinary_muscle_development	ENSGALG00010025624	ARMH1	0.127303254	0.357554115	0.356039123	0.721811266	0.798609176	positive
PRJNA729271	stage_E14_vs_E10	ordinary_muscle_development	ENSGALG00010025628	ENSGALG00010025628	-0.782267681	0.264617034	-2.95622572	0.003114291	0.008596771	negative
PRJNA729271	stage_E14_vs_E10	ordinary_muscle_development	ENSGALG00010025629	ST6GAL1	0.693517367	0.145194152	4.776482795	1.78388E-06	9.47154E-06	positive
PRJNA729271	stage_E14_vs_E10	ordinary_muscle_development	ENSGALG00010025635	CHKA	0.037522953	0.161447661	0.232415586	0.816215245	0.871274761	positive
PRJNA729271	stage_E14_vs_E10	ordinary_muscle_development	ENSGALG00010025637	C6orf89	0.266056188	0.12691245	2.096375797	0.036048868	0.071716763	positive
PRJNA729271	stage_E14_vs_E10	ordinary_muscle_development	ENSGALG00010025639	MYL2	-0.662420099	0.433015321	-1.529784439	0.126070095	0.205649395	negative
PRJNA729271	stage_E14_vs_E10	ordinary_muscle_development	ENSGALG00010025640	SLC38A11	-1.746931756	0.564059934	-3.097067616	0.001954453	0.005703603	negative
PRJNA729271	stage_E14_vs_E10	ordinary_muscle_development	ENSGALG00010025642	RPS8	0.289071526	0.117520481	2.459754448	0.01390321	0.031771565	positive
PRJNA729271	stage_E14_vs_E10	ordinary_muscle_development	ENSGALG00010025644	ENSGALG00010025644	-0.010601389	0.288869111	-0.036699627	0.970724506	0.979850398	negative
PRJNA729271	stage_E14_vs_E10	ordinary_muscle_development	ENSGALG00010025645	NBEAL2	-0.067937659	0.188437671	-0.360531198	0.718449931	0.796415704	negative
PRJNA729271	stage_E14_vs_E10	ordinary_muscle_development	ENSGALG00010025647	JHY	-1.522207364	0.304924463	-4.992080158	5.97325E-07	3.40071E-06	negative
PRJNA729271	stage_E14_vs_E10	ordinary_muscle_development	ENSGALG00010025648	ENSGALG00010025648	-0.472863724	0.580324377	-0.814826574	0.415171588	0.529835769	negative
PRJNA729271	stage_E14_vs_E10	ordinary_muscle_development	ENSGALG00010025650	PPP1R1A	0.213366844	0.47439941	0.449762036	0.652882035	0.743695489	positive
PRJNA729271	stage_E14_vs_E10	ordinary_muscle_development	ENSGALG00010025652	KMT5B	-0.025963121	0.07590632	-0.342041624	0.732319568	0.807181958	negative
PRJNA729271	stage_E14_vs_E10	ordinary_muscle_development	ENSGALG00010025653	IGSF11	2.183186919	0.192269705	11.35481497	7.01883E-30	3.00112E-28	positive
PRJNA729271	stage_E14_vs_E10	ordinary_muscle_development	ENSGALG00010025654	CRTAM	-0.900459537	0.985428585	-0.913774524	0.360835336	0.477043797	negative
PRJNA729271	stage_E14_vs_E10	ordinary_muscle_development	ENSGALG00010025658	PDE1B	-3.67093705	0.447052744	-8.211418235	2.18591E-16	3.46661E-15	negative
PRJNA729271	stage_E14_vs_E10	ordinary_muscle_development	ENSGALG00010025659	COBLL1	-0.066867713	0.131359586	-0.509043268	0.610721896	0.70778028	negative
PRJNA729271	stage_E14_vs_E10	ordinary_muscle_development	ENSGALG00010025660	KIF2C	-0.23886713	0.195970172	-1.218895343	0.222883917	0.325675643	negative
PRJNA729271	stage_E14_vs_E10	ordinary_muscle_development	ENSGALG00010025661	SNRPE	0.077421742	0.127040895	0.609423779	0.54224358	0.648072287	positive
PRJNA729271	stage_E14_vs_E10	ordinary_muscle_development	ENSGALG00010025664	GPR55	-0.522135215	0.820446597	-0.636403658	0.524513357	0.632251236	negative
PRJNA729271	stage_E14_vs_E10	ordinary_muscle_development	ENSGALG00010025666	CUX2	-2.19901139	0.307245416	-7.157182095	8.23523E-13	9.49639E-12	negative
PRJNA729271	stage_E14_vs_E10	ordinary_muscle_development	ENSGALG00010025674	GRB14	0.038818283	0.474573978	0.081796062	0.934808888	0.954677816	positive
PRJNA729271	stage_E14_vs_E10	ordinary_muscle_development	ENSGALG00010025679	ENSGALG00010025679	6.959705842	1.087350343	6.400610332	1.54757E-10	1.39329E-09	positive
PRJNA729271	stage_E14_vs_E10	ordinary_muscle_development	ENSGALG00010025685	NCKAP1L	0.835669038	0.193814989	4.311684268	1.62016E-05	7.33193E-05	positive
PRJNA729271	stage_E14_vs_E10	ordinary_muscle_development	ENSGALG00010025688	BEST4	0.59353128	0.531590923	1.11651884	0.264200111	0.372962661	positive
PRJNA729271	stage_E14_vs_E10	ordinary_muscle_development	ENSGALG00010025691	ENSGALG00010025691	0.894107685	0.162302931	5.508881951	3.6112E-08	2.45287E-07	positive
PRJNA729271	stage_E14_vs_E10	ordinary_muscle_development	ENSGALG00010025692	ENSGALG00010025692	-1.426915798	1.253922065	-1.137962109	0.255136305	0.362782101	negative
PRJNA729271	stage_E14_vs_E10	ordinary_muscle_development	ENSGALG00010025696	MTCH1	0.041399711	0.102063999	0.405625018	0.685018144	0.769596806	positive
PRJNA729271	stage_E14_vs_E10	ordinary_muscle_development	ENSGALG00010025701	ENSGALG00010025701	9.208680089	3.147562296	2.925654593	0.003437323	0.009353107	positive
PRJNA729271	stage_E14_vs_E10	ordinary_muscle_development	ENSGALG00010025702	GTSF2	-0.092474398	0.172328259	-0.536617721	0.591531701	0.691527704	negative
PRJNA729271	stage_E14_vs_E10	ordinary_muscle_development	ENSGALG00010025703	UBASH3B	-0.448109776	0.14138515	-3.169426036	0.001527403	0.004596162	negative
PRJNA729271	stage_E14_vs_E10	ordinary_muscle_development	ENSGALG00010025705	ACSL3	-0.368297104	0.081659497	-4.510156441	6.47798E-06	3.13936E-05	negative
PRJNA729271	stage_E14_vs_E10	ordinary_muscle_development	ENSGALG00010025706	ENSGALG00010025706	7.155736898	2.943900952	2.430698932	0.015069731	0.034082087	positive
PRJNA729271	stage_E14_vs_E10	ordinary_muscle_development	ENSGALG00010025708	HOXC8	-2.604324811	0.238929834	-10.89995654	1.15312E-27	4.25306E-26	negative
PRJNA729271	stage_E14_vs_E10	ordinary_muscle_development	ENSGALG00010025710	ENSGALG00010025710	6.538828822	1.889538918	3.460542019	0.000539089	0.001814278	positive
PRJNA729271	stage_E14_vs_E10	ordinary_muscle_development	ENSGALG00010025719	HOXC11	-5.217359888	2.622970316	-1.989103672	0.046689761	0.089577723	negative
PRJNA729271	stage_E14_vs_E10	ordinary_muscle_development	ENSGALG00010025720	PHETA1	-0.282412831	0.246031426	-1.147873002	0.251020995	0.358124933	negative
PRJNA729271	stage_E14_vs_E10	ordinary_muscle_development	ENSGALG00010025723	SETD2	-0.04989559	0.121976078	-0.409060454	0.682495297	0.767540557	negative
PRJNA729271	stage_E14_vs_E10	ordinary_muscle_development	ENSGALG00010025727	GLI1	-1.898122658	0.206348692	-9.198617328	3.62585E-20	7.91517E-19	negative
PRJNA729271	stage_E14_vs_E10	ordinary_muscle_development	ENSGALG00010025734	PLK3	-0.008506832	0.178584616	-0.047634744	0.962007342	0.974239951	negative
PRJNA729271	stage_E14_vs_E10	ordinary_muscle_development	ENSGALG00010025736	KIF9	0.812874615	0.322597366	2.519780695	0.011742797	0.027429753	positive
PRJNA729271	stage_E14_vs_E10	ordinary_muscle_development	ENSGALG00010025737	ENSGALG00010025737	0.929406905	0.14893304	6.240434649	4.36357E-10	3.74038E-09	positive
PRJNA729271	stage_E14_vs_E10	ordinary_muscle_development	ENSGALG00010025738	SOX13	0.621923984	0.149909367	4.148666605	3.34417E-05	0.000143414	positive
PRJNA729271	stage_E14_vs_E10	ordinary_muscle_development	ENSGALG00010025743	SH2B3	-0.075457701	0.108584721	-0.694920056	0.487105403	0.598304619	negative
PRJNA729271	stage_E14_vs_E10	ordinary_muscle_development	ENSGALG00010025745	COX14	0.429980999	0.227595289	1.889235057	0.058860339	0.108726767	positive
PRJNA729271	stage_E14_vs_E10	ordinary_muscle_development	ENSGALG00010025748	ETNK2	2.547683621	0.191834572	13.28062818	2.99868E-40	2.2757E-38	positive
PRJNA729271	stage_E14_vs_E10	ordinary_muscle_development	ENSGALG00010025753	SORL1	-0.680517127	0.170382374	-3.994058258	6.49519E-05	0.000265205	negative
PRJNA729271	stage_E14_vs_E10	ordinary_muscle_development	ENSGALG00010025755	ENSGALG00010025755	-0.018882622	0.252117215	-0.074896201	0.940297299	0.958233014	negative
PRJNA729271	stage_E14_vs_E10	ordinary_muscle_development	ENSGALG00010025782	ENSGALG00010025782	7.399160195	4.489544385	1.648087102	0.099334799	0.169195994	positive
PRJNA729271	stage_E14_vs_E10	ordinary_muscle_development	ENSGALG00010025784	ENSGALG00010025784	6.416790336	2.318823369	2.767261372	0.005652941	0.0144846	positive
PRJNA729271	stage_E14_vs_E10	ordinary_muscle_development	ENSGALG00010025787	KCNE4	1.775926236	0.290132931	6.121077778	9.29445E-10	7.62871E-09	positive
PRJNA729271	stage_E14_vs_E10	ordinary_muscle_development	ENSGALG00010025790	LRP5	0.24921159	0.07733168	3.222632549	0.001270184	0.003908154	positive
PRJNA729271	stage_E14_vs_E10	ordinary_muscle_development	ENSGALG00010025791	FIGN	-0.283226468	0.237158004	-1.194252199	0.23237933	0.336573402	negative
PRJNA729271	stage_E14_vs_E10	ordinary_muscle_development	ENSGALG00010025794	ENSGALG00010025794	6.643372129	4.009495013	1.656909937	0.097537685	0.166506742	positive
PRJNA729271	stage_E14_vs_E10	ordinary_muscle_development	ENSGALG00010025799	SC5D	-0.27658262	0.27256641	-1.014734793	0.310232264	0.42414796	negative
PRJNA729271	stage_E14_vs_E10	ordinary_muscle_development	ENSGALG00010025800	ARL2BP	0.100761874	0.133612989	0.754132321	0.450769771	0.563851211	positive
PRJNA729271	stage_E14_vs_E10	ordinary_muscle_development	ENSGALG00010025801	PTPN7	-0.726782925	0.402828976	-1.804197235	0.071200393	0.127966484	negative
PRJNA729271	stage_E14_vs_E10	ordinary_muscle_development	ENSGALG00010025804	ENSGALG00010025804	8.37372853	2.100097233	3.987305158	6.6828E-05	0.000272023	positive
PRJNA729271	stage_E14_vs_E10	ordinary_muscle_development	ENSGALG00010025805	ATXN2	-0.155383405	0.09366044	-1.659007846	0.097114211	0.165940174	negative
PRJNA729271	stage_E14_vs_E10	ordinary_muscle_development	ENSGALG00010025809	ENSGALG00010025809	0.194824892	0.199188697	0.978092106	0.328028772	0.442964137	positive
PRJNA729271	stage_E14_vs_E10	ordinary_muscle_development	ENSGALG00010025822	KLF7	0.015664707	0.095987037	0.163196068	0.870364062	0.911160413	positive
PRJNA729271	stage_E14_vs_E10	ordinary_muscle_development	ENSGALG00010025829	ENSGALG00010025829	0.797094502	0.257848618	3.09132742	0.001992638	0.005798692	positive
PRJNA729271	stage_E14_vs_E10	ordinary_muscle_development	ENSGALG00010025830	TBCEL	0.590218175	0.189301386	3.117875611	0.001821597	0.005372135	positive
PRJNA729271	stage_E14_vs_E10	ordinary_muscle_development	ENSGALG00010025831	BRAP	0.125626052	0.068506242	1.833789857	0.066685194	0.120988682	positive
PRJNA729271	stage_E14_vs_E10	ordinary_muscle_development	ENSGALG00010025832	ENKD1	0.350383382	0.146514616	2.391456851	0.016781656	0.037388581	positive
PRJNA729271	stage_E14_vs_E10	ordinary_muscle_development	ENSGALG00010025835	PPP6R3	0.032474931	0.119010422	0.27287468	0.78494955	0.848649491	positive
PRJNA729271	stage_E14_vs_E10	ordinary_muscle_development	ENSGALG00010025836	ENSGALG00010025836	0.071147064	0.11354219	0.626613452	0.530912648	0.638476807	positive
PRJNA729271	stage_E14_vs_E10	ordinary_muscle_development	ENSGALG00010025837	ENSGALG00010025837	0.570647257	0.188147004	3.032986143	0.002421467	0.006862007	positive
PRJNA729271	stage_E14_vs_E10	ordinary_muscle_development	ENSGALG00010025846	GRIK4	-0.684203303	0.219550141	-3.116387451	0.001830816	0.005394932	negative
PRJNA729271	stage_E14_vs_E10	ordinary_muscle_development	ENSGALG00010025847	DEF6	-0.205093657	0.125079108	-1.639711536	0.101065159	0.171518497	negative
PRJNA729271	stage_E14_vs_E10	ordinary_muscle_development	ENSGALG00010025849	OBSCN	1.975607241	0.229110886	8.622930487	6.52621E-18	1.18543E-16	positive
PRJNA729271	stage_E14_vs_E10	ordinary_muscle_development	ENSGALG00010025852	BTBD19	-0.675692184	0.242228654	-2.789480815	0.005279262	0.013659927	negative
PRJNA729271	stage_E14_vs_E10	ordinary_muscle_development	ENSGALG00010025858	ENSGALG00010025858	9.367981901	2.858584621	3.277139964	0.001048644	0.003291488	positive
PRJNA729271	stage_E14_vs_E10	ordinary_muscle_development	ENSGALG00010025862	PTCH2	-2.524875537	0.266033172	-9.490829731	2.29203E-21	5.48233E-20	negative
PRJNA729271	stage_E14_vs_E10	ordinary_muscle_development	ENSGALG00010025865	LGR6	-1.623688915	0.481985413	-3.36875115	0.000755096	0.002450215	negative
PRJNA729271	stage_E14_vs_E10	ordinary_muscle_development	ENSGALG00010025869	FASTKD2	-0.016304985	0.091453002	-0.178288134	0.858496697	0.902491347	negative

PRJNA729271	stage_E14_vs_E10	ordinary_muscle_development	ENSGALG00010025870	ENSGALG00010025870	1.020594195	0.164937748	6.187753898	6.10275E-10	5.14E-09	positive
PRJNA729271	stage_E14_vs_E10	ordinary_muscle_development	ENSGALG00010025871	ENSGALG00010025871	7.042876029	1.365608485	5.157317128	2.50513E-07	1.51111E-06	positive
PRJNA729271	stage_E14_vs_E10	ordinary_muscle_development	ENSGALG00010025874	MDH1B	-0.424632604	0.276723068	-1.534503816	0.124905755	0.204023994	negative
PRJNA729271	stage_E14_vs_E10	ordinary_muscle_development	ENSGALG00010025876	ENSGALG00010025876	9.985348849	1.579206017	6.323018494	2.56503E-10	2.25631E-09	positive
PRJNA729271	stage_E14_vs_E10	ordinary_muscle_development	ENSGALG00010025877	ENSGALG00010025877	6.976983342	3.405990672	2.048444642	0.040516451	0.079149051	positive
PRJNA729271	stage_E14_vs_E10	ordinary_muscle_development	ENSGALG00010025881	GAL	-1.233176619	0.786597019	-1.567736196	0.116942711	0.193635434	negative
PRJNA729271	stage_E14_vs_E10	ordinary_muscle_development	ENSGALG00010025882	LDHD	1.703220381	0.173699012	9.805584755	1.06528E-22	2.75737E-21	positive
PRJNA729271	stage_E14_vs_E10	ordinary_muscle_development	ENSGALG00010025886	ALDH2	0.096841855	0.109783893	0.882113508	0.377715428	0.49395391	positive
PRJNA729271	stage_E14_vs_E10	ordinary_muscle_development	ENSGALG00010025887	ZNF76	0.065169594	0.109873345	0.593133798	0.553091613	0.657783306	positive
PRJNA729271	stage_E14_vs_E10	ordinary_muscle_development	ENSGALG00010025889	TESMIN	0.004870103	0.628777619	0.007745351	0.993820166	0.996501336	positive
PRJNA729271	stage_E14_vs_E10	ordinary_muscle_development	ENSGALG00010025890	SCG2	-0.709144093	0.5160365	-1.374213051	0.16937555	0.261630661	negative
PRJNA729271	stage_E14_vs_E10	ordinary_muscle_development	ENSGALG00010025891	NUP93	-0.012634022	0.105869507	-0.119335801	0.905009321	0.935070933	negative
PRJNA729271	stage_E14_vs_E10	ordinary_muscle_development	ENSGALG00010025892	ZC3H11A	0.050031142	0.085718645	0.583666973	0.559444392	0.662999465	positive
PRJNA729271	stage_E14_vs_E10	ordinary_muscle_development	ENSGALG00010025894	DYTN	-0.514744561	0.459145827	-1.121091667	0.262248845	0.370785897	negative
PRJNA729271	stage_E14_vs_E10	ordinary_muscle_development	ENSGALG00010025897	MAPKAPK5	-0.178882074	0.120731127	-1.481656625	0.138431684	0.222211214	negative
PRJNA729271	stage_E14_vs_E10	ordinary_muscle_development	ENSGALG00010025898	ENSGALG00010025898	9.079434752	1.822469557	4.981940421	6.29498E-07	3.57406E-06	positive
PRJNA729271	stage_E14_vs_E10	ordinary_muscle_development	ENSGALG00010025899	ENSGALG00010025899	-0.061052407	0.166964517	-0.365660968	0.714618075	0.793684877	negative
PRJNA729271	stage_E14_vs_E10	ordinary_muscle_development	ENSGALG00010025900	FAM237A	-0.042994549	0.578564062	-0.074312513	0.940761721	0.95841588	negative
PRJNA729271	stage_E14_vs_E10	ordinary_muscle_development	ENSGALG00010025902	ENSGALG00010025902	7.046045334	1.683879199	4.184412598	2.85904E-05	0.000124189	positive
PRJNA729271	stage_E14_vs_E10	ordinary_muscle_development	ENSGALG00010025904	RPL10A	0.364448993	0.138790473	2.625893435	0.008642187	0.020992585	positive
PRJNA729271	stage_E14_vs_E10	ordinary_muscle_development	ENSGALG00010025905	CPT1A	-0.294508225	0.175016663	-1.682743915	0.092424676	0.159297941	negative
PRJNA729271	stage_E14_vs_E10	ordinary_muscle_development	ENSGALG00010025907	ARHGEF12	0.07915595	0.071309742	1.110029947	0.266986122	0.375980165	positive
PRJNA729271	stage_E14_vs_E10	ordinary_muscle_development	ENSGALG00010025910	HEATR3	-0.399244358	0.112615778	-3.545190251	0.00039233	0.001362113	negative
PRJNA729271	stage_E14_vs_E10	ordinary_muscle_development	ENSGALG00010025913	EIF2B3	-0.182282734	0.07653801	-2.381597524	0.017237726	0.038266689	negative
PRJNA729271	stage_E14_vs_E10	ordinary_muscle_development	ENSGALG00010025914	ENSGALG00010025914	-4.445920277	1.708337107	-2.602484169	0.009255109	0.02226233	negative
PRJNA729271	stage_E14_vs_E10	ordinary_muscle_development	ENSGALG00010025917	SCUBE3	-4.172936637	0.338626816	-12.32311334	6.80168E-35	3.69252E-33	negative
PRJNA729271	stage_E14_vs_E10	ordinary_muscle_development	ENSGALG00010025919	RGS9BP	-0.270036812	0.295287097	-0.914489034	0.360459937	0.476721422	negative
PRJNA729271	stage_E14_vs_E10	ordinary_muscle_development	ENSGALG00010025920	GTSF1	0.114758271	0.260274626	0.440912249	0.659276525	0.748802165	positive
PRJNA729271	stage_E14_vs_E10	ordinary_muscle_development	ENSGALG00010025924	TMEM116	2.386385457	0.167077581	14.28309798	2.78921E-46	3.2087E-44	positive
PRJNA729271	stage_E14_vs_E10	ordinary_muscle_development	ENSGALG00010025925	KLHL18	-0.023071502	0.091203817	-0.25296641	0.80029417	0.859469808	negative
PRJNA729271	stage_E14_vs_E10	ordinary_muscle_development	ENSGALG00010025926	ENSGALG00010025926	0.008045026	0.420780516	0.019119292	0.984745941	0.989867713	positive
PRJNA729271	stage_E14_vs_E10	ordinary_muscle_development	ENSGALG00010025927	ENSGALG00010025927	7.928750757	0.844297478	9.390944503	5.94646E-21	1.37033E-19	positive
PRJNA729271	stage_E14_vs_E10	ordinary_muscle_development	ENSGALG00010025929	NELFE	0.431618757	0.087761955	4.918062241	8.74051E-07	4.86348E-06	positive
PRJNA729271	stage_E14_vs_E10	ordinary_muscle_development	ENSGALG00010025931	TMEM136	-0.405411014	0.166393298	-2.436462405	0.014831711	0.03362303	negative
PRJNA729271	stage_E14_vs_E10	ordinary_muscle_development	ENSGALG00010025932	AP1S3	-1.387589074	0.316700088	-4.381397816	1.1792E-05	5.46612E-05	negative
PRJNA729271	stage_E14_vs_E10	ordinary_muscle_development	ENSGALG00010025933	SKIC2	0.202539544	0.101328676	1.998837372	0.045625953	0.087875124	positive
PRJNA729271	stage_E14_vs_E10	ordinary_muscle_development	ENSGALG00010025935	CATH3	-0.098593089	0.37493757	-0.26295868	0.792582427	0.854416353	negative
PRJNA729271	stage_E14_vs_E10	ordinary_muscle_development	ENSGALG00010025936	GOSR2	-0.003213074	0.066587892	-0.04825312	0.961514516	0.973890452	negative
PRJNA729271	stage_E14_vs_E10	ordinary_muscle_development	ENSGALG00010025938	ERP29	0.286113171	0.08904712	3.213053605	0.001313318	0.00401954	positive
PRJNA729271	stage_E14_vs_E10	ordinary_muscle_development	ENSGALG00010025939	TMEM136-1	-0.74933011	0.547857511	-1.367746348	0.171391487	0.264119834	negative
PRJNA729271	stage_E14_vs_E10	ordinary_muscle_development	ENSGALG00010025940	HECTD3	0.029363504	0.087996592	0.333689104	0.738614172	0.812122615	positive
PRJNA729271	stage_E14_vs_E10	ordinary_muscle_development	ENSGALG00010025941	ENSGALG00010025941	-0.819922335	0.539156404	-1.520750435	0.128322477	0.208677186	negative
PRJNA729271	stage_E14_vs_E10	ordinary_muscle_development	ENSGALG00010025942	ENSGALG00010025942	10.26864088	1.022034858	10.04725113	9.44657E-24	2.5642E-22	positive
PRJNA729271	stage_E14_vs_E10	ordinary_muscle_development	ENSGALG00010025943	CATH1	1.985677612	0.718118047	2.765113089	0.005690306	0.014567466	positive
PRJNA729271	stage_E14_vs_E10	ordinary_muscle_development	ENSGALG00010025946	KRT5	-0.726412709	0.963504227	-0.753927891	0.450892522	0.563956088	negative
PRJNA729271	stage_E14_vs_E10	ordinary_muscle_development	ENSGALG00010025948	WNT9B	-5.289570311	0.518488438	-10.20190601	1.94419E-24	5.56938E-23	negative
PRJNA729271	stage_E14_vs_E10	ordinary_muscle_development	ENSGALG00010025949	GPATCH1	-0.206785903	0.121750722	-1.698436765	0.089425358	0.154957629	negative
PRJNA729271	stage_E14_vs_E10	ordinary_muscle_development	ENSGALG00010025951	TBRG4	0.025468714	0.11332023	0.224749934	0.822173816	0.875507601	positive
PRJNA729271	stage_E14_vs_E10	ordinary_muscle_development	ENSGALG00010025953	POU2F3	3.819705744	0.695704147	5.490416809	4.00986E-08	2.69963E-07	positive
PRJNA729271	stage_E14_vs_E10	ordinary_muscle_development	ENSGALG00010025954	TUBA1A	-0.004272335	0.158382783	-0.026974747	0.978479876	0.985688081	negative
PRJNA729271	stage_E14_vs_E10	ordinary_muscle_development	ENSGALG00010025956	ENSGALG00010025956	0.062032628	0.526363207	0.117851376	0.906185421	0.935716909	positive
PRJNA729271	stage_E14_vs_E10	ordinary_muscle_development	ENSGALG00010025957	ENSGALG00010025957	-0.454400164	0.114683283	-3.962217953	7.42567E-05	0.000299153	negative
PRJNA729271	stage_E14_vs_E10	ordinary_muscle_development	ENSGALG00010025958	HOXC9	-2.670790305	0.329359521	-8.109042347	5.10203E-16	7.84895E-15	negative
PRJNA729271	stage_E14_vs_E10	ordinary_muscle_development	ENSGALG00010025959	UROD	0.481568755	0.101027248	4.766721478	1.87248E-06	9.91654E-06	positive
PRJNA729271	stage_E14_vs_E10	ordinary_muscle_development	ENSGALG00010025960	CMKLR2	0.392058168	0.158939418	2.466714499	0.013635903	0.031254335	positive
PRJNA729271	stage_E14_vs_E10	ordinary_muscle_development	ENSGALG00010025962	ENSGALG00010025962	-0.956427243	0.198110015	-4.827758172	1.38079E-06	7.49607E-06	negative
PRJNA729271	stage_E14_vs_E10	ordinary_muscle_development	ENSGALG00010025963	OAF	0.356713361	0.245203519	1.454764446	0.145734484	0.231853951	positive
PRJNA729271	stage_E14_vs_E10	ordinary_muscle_development	ENSGALG00010025964	HOXC10	-4.400478283	0.739700674	-5.94899861	2.69788E-09	2.10926E-08	negative
PRJNA729271	stage_E14_vs_E10	ordinary_muscle_development	ENSGALG00010025968	FADS2	-0.613976754	0.156456624	-3.924261804	8.69961E-05	0.000346146	negative
PRJNA729271	stage_E14_vs_E10	ordinary_muscle_development	ENSGALG00010025970	KLHL12	0.126549575	0.109146996	1.159441671	0.246276199	0.352931005	positive
PRJNA729271	stage_E14_vs_E10	ordinary_muscle_development	ENSGALG00010025971	TRAFD1	-0.124691988	0.198323308	-0.628730882	0.529525257	0.637074086	negative
PRJNA729271	stage_E14_vs_E10	ordinary_muscle_development	ENSGALG00010025972	WNT3	-5.103467731	1.128750963	-4.521340756	6.14492E-06	2.98895E-05	negative
PRJNA729271	stage_E14_vs_E10	ordinary_muscle_development	ENSGALG00010025974	EEF1B2	0.218194704	0.095522959	2.284212142	0.022359066	0.047942998	positive
PRJNA729271	stage_E14_vs_E10	ordinary_muscle_development	ENSGALG00010025976	NAA25	-0.30333393	0.098491867	-3.079786572	0.00207149	0.005998051	negative
PRJNA729271	stage_E14_vs_E10	ordinary_muscle_development	ENSGALG00010025978	TRIM29	-0.520153048	0.43006625	-1.209471908	0.226481597	0.329769036	negative
PRJNA729271	stage_E14_vs_E10	ordinary_muscle_development	ENSGALG00010025980	HECTD4	-0.038069984	0.063725697	-0.597403972	0.550237703	0.655249384	negative
PRJNA729271	stage_E14_vs_E10	ordinary_muscle_development	ENSGALG00010025982	SERPINE2	-0.206845547	0.198728428	-1.040845285	0.297947359	0.411270066	negative
PRJNA729271	stage_E14_vs_E10	ordinary_muscle_development	ENSGALG00010025983	RNF26	-0.225720154	0.080850237	-2.791830473	0.005241081	0.013578099	negative
PRJNA729271	stage_E14_vs_E10	ordinary_muscle_development	ENSGALG00010025984	NDUFS1	0.303832291	0.117021694	2.596375778	0.009421297	0.022620787	positive
PRJNA729271	stage_E14_vs_E10	ordinary_muscle_development	ENSGALG00010025985	CHCHD10	-0.103631878	0.321688011	-0.322150263	0.747338862	0.81892023	negative
PRJNA729271	stage_E14_vs_E10	ordinary_muscle_development	ENSGALG00010025986	RAB3IL1	0.243026306	0.107683141	2.256864956	0.024016514	0.050991559	positive
PRJNA729271	stage_E14_vs_E10	ordinary_muscle_development	ENSGALG00010025988	NSF	-0.208970154	0.058346243	-3.581552865	0.000341558	0.001207533	negative
PRJNA729271	stage_E14_vs_E10	ordinary_muscle_development	ENSGALG00010025990	FAM124B	0.008922404	0.245245712	0.036381486	0.970978176	0.980006007	positive
PRJNA729271	stage_E14_vs_E10	ordinary_muscle_development	ENSGALG00010025992	MMP11	-2.295767107	0.327983693	-6.99963796	2.56625E-12	2.82015E-11	negative
PRJNA729271	stage_E14_vs_E10	ordinary_muscle_development	ENSGALG00010025994	INO80D	0.093816631	0.10797476	0.868875568	0.384915194	0.501018834	positive
PRJNA729271	stage_E14_vs_E10	ordinary_muscle_development	ENSGALG00010025996	CUL3	0.18027623	0.096793949	1.862474178	0.062536282	0.114611633	positive
PRJNA729271	stage_E14_vs_E10	ordinary_muscle_development	ENSGALG00010025997	CCDC43	0.236027196	0.147428097	1.600964812	0.109384713	0.183425661	positive
PRJNA729271	stage_E14_vs_E10	ordinary_muscle_development	ENSGALG00010025998	SMARCB1	-0.19453423	0.142231925	-1.367725498	0.171398016	0.264119834	negative
PRJNA729271	stage_E14_vs_E10	ordinary_muscle_development	ENSGALG00010025999	DBF4B	-0.047225558	0.145566942	-0.324425021	0.745616279	0.817465242	negative
PRJNA729271	stage_E14_vs_E10	ordinary_muscle_development	ENSGALG00010026001	ANKS1A	0.169494285	0.092117569	1.83997783	0.065771492	0.119663334	positive
PRJNA729271	stage_E14_vs_E10	ordinary_muscle_development	ENSGALG00010026002	FTH1	0.370066974	0.141864554	2.608593642	0.009091513	0.021930685	positive
PRJNA729271	stage_E14_vs_E10	ordinary_muscle_development	ENSGALG00010026003	NECTIN1	0.64043596	0.154662133	4.140871123	3.45989E-05	0.000147856	positive
PRJNA729271	stage_E14_vs_E10	ordinary_muscle_development	ENSGALG00010026005	DERL3	-0.244713252	0.114226849	-2.142344416	0.032165777	0.065054129	negative
PRJNA729271	stage_E14_vs_E10	ordinary_muscle_development	ENSGALG00010026006	ITGA2B	-0.874181523	0.305912782	-2.85761686	0.004268354	0.011316956	negative
PRJNA729271	stage_E14_vs_E10	ordinary_muscle_development	ENSGALG00010026008	NRP2	0.666704755	0.100220606	6.652371991	2.88407E-11	2.81702E-10	positive
PRJNA729271	stage_E14_vs_E10	ordinary_muscle_development	ENSGALG00010026009	ENSGALG00010026009	0.440200904	0.301665419	1.459235551	0.144500287	0.230221539	positive
PRJNA729271	stage_E14_vs_E10	ordinary_muscle_development	ENSGALG00010026010	ZSWIM5	-0.298556799	0.126519384	-2.359771202	0.018286209	0.040233546	negative
PRJNA729271	stage_E14_vs_E10	ordinary_muscle_development	ENSGALG00010026011	GJC2	0.353560388	0.163369888	2.164171083	0.030451222	0.062141413	positive
PRJNA729271	stage_E14_vs_E10	ordinary_muscle_development	ENSGALG00010026013	RABIF	-0.088603202	0.108619039	-0.815724408	0.414657776	0.529369778	negative
PRJNA729271	stage_E14_vs_E10	ordinary_muscle_development	ENSGALG00010026016	SLC2A11	-1.363959623	0.388208559	-3.513471278	0.000442292	0.001516866	negative
PRJNA729271	stage_E14_vs_E10	ordinary_muscle_development	ENSGALG00010026017	PLEKHM1	0.536736394	0.143645931	3.736523492	0.000186582	0.000697757	positive
PRJNA729271	stage_E14_vs_E10	ordinary_muscle_development	ENSGALG00010026023	THY1	-2.763728337	0.306772132	-9.009059339	2.07831E-19	4.27307E-18	negative
PRJNA729271	stage_E14_vs_E10	ordinary_muscle_development	ENSGALG00010026030	IBA57	0.287648057	0.08268019	3.479044465	0.000503205	0.001704594	positive
PRJNA729271	stage_E14_vs_E10	ordinary_muscle_development	ENSGALG00010026036	TUB5A	-0.156554297	0.206600219	-0.757764428	0.448592021	0.561674536	negative
PRJNA729271	stage_E14_vs_E10	ordinary_muscle_development	ENSGALG00010026040	GUK1	-0.114737374	0.107444721	-1.06787353	0.285577564	0.39728269	negative
PRJNA729271	stage_E14_vs_E10	ordinary_muscle_development	ENSGALG00010026051	TAF11	-0.060655205	0.126481086	-0.47955949	0.631540658	0.725301602	negative
PRJNA729271	stage_E14_vs_E10	ordinary_muscle_development	ENSGALG00010026061	MPEG1	0.499075344	0.344457328	1.448874224	0.147372711	0.234023602	positive
PRJNA729271	stage_E14_vs_E10	ordinary_muscle_development	ENSGALG00010026062	ITGA4	-0.406098548	0.159773808	-2.541709143	0.011031193	0.025961542	negative
PRJNA729271	stage_E14_vs_E10	ordinary_muscle_development	ENSGALG00010026064	ENSGALG00010026064	0.31797921	0.147585331	2.154544818	0.031197471	0.06342319	positive
PRJNA729271	stage_E14_vs_E10	ordinary_muscle_development	ENSGALG00010026066	SELENOP2	-0.765256262	0.585470048	-1.307080123	0.191185501	0.288519921	negative
PRJNA729271	stage_E14_vs_E10	ordinary_muscle_development	ENSGALG00010026068	BLTP3A	0.746505639	0.110405988	6.761459736	1.36608E-11	1.38278E-10	positive
PRJNA729271	stage_E14_vs_E10	ordinary_muscle_development	ENSGALG00010026073	HPDL	-0.87047414	0.232498642	-3.743996659	0.000181116	0.000678893	negative
PRJNA729271	stage_E14_vs_E10	ordinary_muscle_development	ENSGALG00010026076	C1orf35	0.413714716	0.128743682	3.213475868	0.001311388	0.004015302	positive
PRJNA729271	stage_E14_vs_E10	ordinary_muscle_development	ENSGALG00010026078	UBE2E3	0.057887135	0.106179166	0.545183554	0.585627281	0.686174719	positive
PRJNA729271	stage_E14_vs_E10	ordinary_muscle_development	ENSGALG00010026081	KPNA1	-0.299727271	0.108023743	-2.774642525	0.005526241	0.014207674	negative
PRJNA729271	stage_E14_vs_E10	ordinary_muscle_development	ENSGALG00010026083	MUTYH	-0.132645847	0.106100875	-1.250186175	0.211231546	0.31230123	negative
PRJNA729271	stage_E14_vs_E10	ordinary_muscle_development	ENSGALG00010026088	SNRPC	0.400840765	0.080232466	4.995992072	5.85341E-07	3.3338E-06	positive
PRJNA729271	stage_E14_vs_E10	ordinary_muscle_development	ENSGALG00010026090	USP2	1.832277463	0.234991998	7.79719086	6.33005E-15	8.8395E-14	positive
PRJNA729271	stage_E14_vs_E10	ordinary_muscle_development	ENSGALG00010026091	DTX4	0.074284747	0.16248752	0.457172014	0.647547412	0.739420178	positive
PRJNA729271	stage_E14_vs_E10	ordinary_muscle_development	ENSGALG00010026093	TOE1	-0.161402783	0.122807628	-1.314273272	0.188754258	0.285415506	negative
PRJNA729271	stage_E14_vs_E10	ordinary_muscle_development	ENSGALG00010026094	C6orf106	0.448235034	0.07788826	5.754847195	8.67202E-09	6.35495E-08	positive
PRJNA729271	stage_E14_vs_E10	ordinary_muscle_development	ENSGALG00010026095	MIF	-0.919382748	0.195586297	-4.700650109	2.59335E-06	1.34108E-05	negative
PRJNA729271	stage_E14_vs_E10	ordinary_muscle_development	ENSGALG00010026097	ARF1	0.00588849	0.055689518	0.105737849	0.915790349	0.941378705	positive
PRJNA729271	stage_E14_vs_E10	ordinary_muscle_development	ENSGALG00010026098	STAT3	0.266899959	0.10812627	2.468409933	0.01357148	0.031136215	positive
PRJNA729271	stage_E14_vs_E10	ordinary_muscle_development	ENSGALG00010026102	IPO9	-0.378545289	0.057108299	-6.628551249	3.38997E-11	3.29341E-10	negative
PRJNA729271	stage_E14_vs_E10	ordinary_muscle_development	ENSGALG00010026104	DDX51	-0.160851295	0.100462453	-1.601108575	0.109352873	0.183393486	negative
PRJNA729271	stage_E14_vs_E10	ordinary_muscle_development	ENSGALG00010026105	CNTF	0.286456492	0.20149426	1.421660803	0.155124744	0.243928528	positive
PRJNA729271	stage_E14_vs_E10	ordinary_muscle_development	ENSGALG00010026106	TESK2	0.150538792	0.11863595	1.268913781	0.204471817	0.304356026	positive
PRJNA729271	stage_E14_vs_E10	ordinary_muscle_development	ENSGALG00010026111	ENSGALG00010026111	-0.496920576	0.157414152	-3.15677193	0.001595261	0.004766709	negative
PRJNA729271	stage_E14_vs_E10	ordinary_muscle_development	ENSGALG00010026112	ZFP91	0.247418231	0.12059688	2.051613866	0.040207204	0.078644497	positive
PRJNA729271	stage_E14_vs_E10	ordinary_muscle_development	ENSGALG00010026113	MFRP	0.83444577	0.50712009	1.645459893	0.099875007	0.169916459	positive
PRJNA729271	stage_E14_vs_E10	ordinary_muscle_development	ENSGALG00010026114	CWC22	-0.208392433	0.082290152	-2.532410341	0.011328134	0.026586998	negative
PRJNA729271	stage_E14_vs_E10	ordinary_muscle_development	ENSGALG00010026115	MMACHC	0.086452537	0.177961936	0.485792291	0.627114441	0.721544993	positive
PRJNA729271	stage_E14_vs_E10	ordinary_muscle_development	ENSGALG00010026116	DLX3	-1.073823172	0.540284997	-1.987512478	0.046865635	0.089821152	negative
PRJNA729271	stage_E14_vs_E10	ordinary_muscle_development	ENSGALG00010026117	LZTR1	-0.052967399	0.069605964	-0.76096064	0.446680577	0.56008953	negative
PRJNA729271	stage_E14_vs_E10	ordinary_muscle_development	ENSGALG00010026120	C1QTNF5	-0.431847173	0.197389423	-2.187792877	0.028684693	0.05919485	negative
PRJNA729271	stage_E14_vs_E10	ordinary_muscle_development	ENSGALG00010026121	ENSGALG00010026121	-0.324366797	0.259422829	-1.25034022	0.211175292	0.312249909	negative
PRJNA729271	stage_E14_vs_E10	ordinary_muscle_development	ENSGALG00010026124	PACSIN1	0.10860811	0.270891941	0.400927799	0.688473282	0.772488332	positive
PRJNA729271	stage_E14_vs_E10	ordinary_muscle_development	ENSGALG00010026127	ENSGALG00010026127	0.116487531	0.643678272	0.180971669	0.856389814	0.900825134	positive
PRJNA729271	stage_E14_vs_E10	ordinary_muscle_development	ENSGALG00010026128	PRDX1	-0.43416185	0.141894192	-3.059757726	0.002215161	0.006366996	negative
PRJNA729271	stage_E14_vs_E10	ordinary_muscle_development	ENSGALG00010026132	DDTL	0.114275112	0.185114619	0.617320837	0.537023126	0.643584143	positive

PRJNA729271	stage_E14_vs_E10	ordinary_muscle_development	ENSGALG00010026137	LPXN	-0.274920889	0.377085715	-0.729067366	0.465960456	0.578311226	negative
PRJNA729271	stage_E14_vs_E10	ordinary_muscle_development	ENSGALG00010026141	WNT3A	-3.908687131	0.560963077	-6.967815342	3.219E-12	3.49247E-11	negative
PRJNA729271	stage_E14_vs_E10	ordinary_muscle_development	ENSGALG00010026143	RNPEP	0.439719589	0.13896047	3.164350191	0.001554297	0.004658711	positive
PRJNA729271	stage_E14_vs_E10	ordinary_muscle_development	ENSGALG00010026148	RPS10	0.416856079	0.13405078	3.109687826	0.001872852	0.005503472	positive
PRJNA729271	stage_E14_vs_E10	ordinary_muscle_development	ENSGALG00010026149	LASP1	0.367036486	0.128537976	2.855471169	0.004297302	0.011389541	positive
PRJNA729271	stage_E14_vs_E10	ordinary_muscle_development	ENSGALG00010026154	ZNF385B	0.957342287	0.170436715	5.61699564	1.94306E-08	1.36921E-07	positive
PRJNA729271	stage_E14_vs_E10	ordinary_muscle_development	ENSGALG00010026162	MED1	-0.236586993	0.151210055	-1.564624739	0.117670925	0.194663325	negative
PRJNA729271	stage_E14_vs_E10	ordinary_muscle_development	ENSGALG00010026163	CTNND1	0.289249425	0.081031159	3.569607387	0.000357517	0.001258122	positive
PRJNA729271	stage_E14_vs_E10	ordinary_muscle_development	ENSGALG00010026166	WNT9A	-1.573792964	0.242717317	-6.484057174	8.92884E-11	8.22781E-10	negative
PRJNA729271	stage_E14_vs_E10	ordinary_muscle_development	ENSGALG00010026167	CABIN1	-0.395912042	0.058475634	-6.770547206	1.28296E-11	1.3041E-10	negative
PRJNA729271	stage_E14_vs_E10	ordinary_muscle_development	ENSGALG00010026173	CDK12	-0.054683854	0.161863959	-0.337838356	0.735485004	0.809972278	negative
PRJNA729271	stage_E14_vs_E10	ordinary_muscle_development	ENSGALG00010026183	SESTD1	0.093419714	0.168221667	0.55533699	0.578664183	0.680217507	positive
PRJNA729271	stage_E14_vs_E10	ordinary_muscle_development	ENSGALG00010026184	NUDT3	0.629743365	0.103811375	6.066226994	1.3095E-09	1.0551E-08	positive
PRJNA729271	stage_E14_vs_E10	ordinary_muscle_development	ENSGALG00010026192	SMIM29	0.740799183	0.126800263	5.842252746	5.14996E-09	3.89404E-08	positive
PRJNA729271	stage_E14_vs_E10	ordinary_muscle_development	ENSGALG00010026199	TBX6	-1.036086186	0.374336975	-2.767790131	0.005643779	0.014467023	negative
PRJNA729271	stage_E14_vs_E10	ordinary_muscle_development	ENSGALG00010026203	TMX2	-0.004074243	0.103172817	-0.039489505	0.968500121	0.978580476	negative
PRJNA729271	stage_E14_vs_E10	ordinary_muscle_development	ENSGALG00010026205	ENSGALG00010026205	-0.026721205	1.336903904	-0.019987379	0.984053441	0.989377445	negative
PRJNA729271	stage_E14_vs_E10	ordinary_muscle_development	ENSGALG00010026209	MED19	0.517857782	0.171324435	3.022673232	0.002505526	0.007072562	positive
PRJNA729271	stage_E14_vs_E10	ordinary_muscle_development	ENSGALG00010026213	AKR1A1	0.025361993	0.121920604	0.208020564	0.835212911	0.885041185	positive
PRJNA729271	stage_E14_vs_E10	ordinary_muscle_development	ENSGALG00010026218	FTSJ3	-0.229137595	0.16651052	-1.37611482	0.168786088	0.260882314	negative
PRJNA729271	stage_E14_vs_E10	ordinary_muscle_development	ENSGALG00010026220	ENSGALG00010026220	-0.046481953	0.095501973	-0.486711965	0.626462465	0.72114807	negative
PRJNA729271	stage_E14_vs_E10	ordinary_muscle_development	ENSGALG00010026221	CRKL	-0.414577529	0.146465404	-2.830549183	0.004646817	0.012206525	negative
PRJNA729271	stage_E14_vs_E10	ordinary_muscle_development	ENSGALG00010026229	CRHR2	1.618967399	0.209838845	7.715289316	1.20708E-14	1.64752E-13	positive
PRJNA729271	stage_E14_vs_E10	ordinary_muscle_development	ENSGALG00010026232	PSMC5	0.172043309	0.13081205	1.315194653	0.188444492	0.285095805	positive
PRJNA729271	stage_E14_vs_E10	ordinary_muscle_development	ENSGALG00010026234	ZDHHC5	0.299939216	0.159600957	1.879307128	0.060202569	0.110881352	positive
PRJNA729271	stage_E14_vs_E10	ordinary_muscle_development	ENSGALG00010026237	KLHL22	-0.208588641	0.14014073	-1.488422683	0.136639458	0.219943248	negative
PRJNA729271	stage_E14_vs_E10	ordinary_muscle_development	ENSGALG00010026239	GIP	-2.558810101	0.930788598	-2.749077618	0.005976323	0.015219045	negative
PRJNA729271	stage_E14_vs_E10	ordinary_muscle_development	ENSGALG00010026242	CCDC141	1.161896465	0.25344794	4.584359478	4.5538E-06	2.26363E-05	positive
PRJNA729271	stage_E14_vs_E10	ordinary_muscle_development	ENSGALG00010026244	CLP1	-0.062500124	0.124837928	-0.500650125	0.616617379	0.712691884	negative
PRJNA729271	stage_E14_vs_E10	ordinary_muscle_development	ENSGALG00010026249	YPEL4	-0.618965654	0.567605224	-1.090486183	0.275499037	0.385980914	negative
PRJNA729271	stage_E14_vs_E10	ordinary_muscle_development	ENSGALG00010026252	ENO2	-0.40415868	0.339380537	-1.190871709	0.233703952	0.338151267	negative
PRJNA729271	stage_E14_vs_E10	ordinary_muscle_development	ENSGALG00010026254	ENSGALG00010026254	-1.317543263	0.557738617	-2.362295211	0.018162173	0.040003145	negative
PRJNA729271	stage_E14_vs_E10	ordinary_muscle_development	ENSGALG00010026259	MED15	-0.078979816	0.086908505	-0.908769701	0.363471703	0.479566934	negative
PRJNA729271	stage_E14_vs_E10	ordinary_muscle_development	ENSGALG00010026260	ENSGALG00010026260	-20.06936967	3.258908464	-6.158310335	7.35252E-10	6.10044E-09	negative
PRJNA729271	stage_E14_vs_E10	ordinary_muscle_development	ENSGALG00010026261	IFT70A	-0.421622021	0.104793759	-4.023350469	5.7376E-05	0.00023647	negative
PRJNA729271	stage_E14_vs_E10	ordinary_muscle_development	ENSGALG00010026262	NAV1	0.038937559	0.1596229	0.243934666	0.807281421	0.864603531	positive
PRJNA729271	stage_E14_vs_E10	ordinary_muscle_development	ENSGALG00010026264	FZD7	-0.308635759	0.138122258	-2.234511383	0.025449444	0.053563191	negative
PRJNA729271	stage_E14_vs_E10	ordinary_muscle_development	ENSGALG00010026266	ENSGALG00010026266	0.247522128	0.276648058	0.894718474	0.3709376	0.48724427	positive
PRJNA729271	stage_E14_vs_E10	ordinary_muscle_development	ENSGALG00010026268	GARS1	-0.150461832	0.150612047	-0.999002635	0.317793415	0.431676089	negative
PRJNA729271	stage_E14_vs_E10	ordinary_muscle_development	ENSGALG00010026272	LRRC58	0.660543751	0.09495634	6.956289074	3.49353E-12	3.77619E-11	positive
PRJNA729271	stage_E14_vs_E10	ordinary_muscle_development	ENSGALG00010026277	SMARCD2	-0.201078717	0.124123392	-1.619990514	0.105234315	0.17769937	negative
PRJNA729271	stage_E14_vs_E10	ordinary_muscle_development	ENSGALG00010026287	NASP	-0.385909001	0.087027817	-4.434317827	9.23643E-06	4.35814E-05	negative
PRJNA729271	stage_E14_vs_E10	ordinary_muscle_development	ENSGALG00010026291	SERPING1	-0.340305502	0.175768359	-1.936102175	0.052855188	0.099214705	negative
PRJNA729271	stage_E14_vs_E10	ordinary_muscle_development	ENSGALG00010026295	SMPD4	-0.484043488	0.128506638	-3.766680833	0.000165432	0.000625604	negative
PRJNA729271	stage_E14_vs_E10	ordinary_muscle_development	ENSGALG00010026298	SNF8	0.260350743	0.114015295	2.283472072	0.022402576	0.048029188	positive
PRJNA729271	stage_E14_vs_E10	ordinary_muscle_development	ENSGALG00010026300	HMGA1	-0.118565805	0.104826035	-1.131072106	0.258024743	0.366242524	negative
PRJNA729271	stage_E14_vs_E10	ordinary_muscle_development	ENSGALG00010026305	ATP5MC1	0.621945937	0.167044847	3.723227321	0.000196692	0.000732542	positive
PRJNA729271	stage_E14_vs_E10	ordinary_muscle_development	ENSGALG00010026315	SYT2	-1.094048831	0.268576281	-4.073512475	4.63094E-05	0.000193612	negative
PRJNA729271	stage_E14_vs_E10	ordinary_muscle_development	ENSGALG00010026316	ENSGALG00010026316	0.287226794	0.349108702	0.822743153	0.41065407	0.525415372	positive
PRJNA729271	stage_E14_vs_E10	ordinary_muscle_development	ENSGALG00010026320	ENSGALG00010026320	-0.566571326	0.175562989	-3.227168377	0.001250219	0.003853268	negative
PRJNA729271	stage_E14_vs_E10	ordinary_muscle_development	ENSGALG00010026321	GPBP1L1	0.273552841	0.047022658	5.817468676	5.97455E-09	4.47782E-08	positive
PRJNA729271	stage_E14_vs_E10	ordinary_muscle_development	ENSGALG00010026325	TMEM69	-0.127718723	0.13756801	-0.928404238	0.353197921	0.469430026	negative
PRJNA729271	stage_E14_vs_E10	ordinary_muscle_development	ENSGALG00010026329	ENSGALG00010026329	1.80447469	0.270853722	6.662174253	2.69806E-11	2.63889E-10	positive
PRJNA729271	stage_E14_vs_E10	ordinary_muscle_development	ENSGALG00010026334	ENSGALG00010026334	0.061802268	0.127280086	0.485561176	0.627278328	0.721618998	positive
PRJNA729271	stage_E14_vs_E10	ordinary_muscle_development	ENSGALG00010026335	IPP	-0.407163267	0.097815219	-4.162575842	3.14678E-05	0.000135752	negative
PRJNA729271	stage_E14_vs_E10	ordinary_muscle_development	ENSGALG00010026344	TIMM10	0.066874693	0.17145254	0.390047838	0.696501173	0.778875521	positive
PRJNA729271	stage_E14_vs_E10	ordinary_muscle_development	ENSGALG00010026346	FZD2	0.137986443	0.163009147	0.846495098	0.397276608	0.51273479	positive
PRJNA729271	stage_E14_vs_E10	ordinary_muscle_development	ENSGALG00010026354	ENSGALG00010026354	1.101490571	0.24517859	4.492605053	7.03572E-06	3.38926E-05	positive
PRJNA729271	stage_E14_vs_E10	ordinary_muscle_development	ENSGALG00010026355	PPP1R12B	1.273230273	0.137186745	9.281000683	1.67894E-20	3.71718E-19	positive
PRJNA729271	stage_E14_vs_E10	ordinary_muscle_development	ENSGALG00010026360	CTDSPL	-0.253397775	0.126789306	-1.998573729	0.045654495	0.087918415	negative
PRJNA729271	stage_E14_vs_E10	ordinary_muscle_development	ENSGALG00010026362	ENSGALG00010026362	-1.151648639	0.393099377	-2.929662846	0.0033933	0.009252422	negative
PRJNA729271	stage_E14_vs_E10	ordinary_muscle_development	ENSGALG00010026367	RBP	-0.264812893	0.623143047	-0.424963247	0.670863467	0.757848548	negative
PRJNA729271	stage_E14_vs_E10	ordinary_muscle_development	ENSGALG00010026377	ENSGALG00010026377	-0.766238958	0.162079342	-4.727554716	2.2724E-06	1.18569E-05	negative
PRJNA729271	stage_E14_vs_E10	ordinary_muscle_development	ENSGALG00010026388	SNRPD3	0.091046352	0.079202942	1.149532454	0.250336492	0.357570699	positive
PRJNA729271	stage_E14_vs_E10	ordinary_muscle_development	ENSGALG00010026393	SLC43A3	0.012646535	0.119162094	0.106128842	0.915480128	0.941362463	positive
PRJNA729271	stage_E14_vs_E10	ordinary_muscle_development	ENSGALG00010026402	TFEB	1.1082079	0.165056793	6.714100505	1.8923E-11	1.88256E-10	positive
PRJNA729271	stage_E14_vs_E10	ordinary_muscle_development	ENSGALG00010026403	GUCD1	0.31797266	0.116945954	2.718971021	0.006548535	0.016436194	positive
PRJNA729271	stage_E14_vs_E10	ordinary_muscle_development	ENSGALG00010026409	VILL	0.358548619	0.261726831	1.369934514	0.170707345	0.263262364	positive
PRJNA729271	stage_E14_vs_E10	ordinary_muscle_development	ENSGALG00010026410	ENSGALG00010026410	0.710905517	0.317274123	2.24066656	0.025047682	0.052817304	positive
PRJNA729271	stage_E14_vs_E10	ordinary_muscle_development	ENSGALG00010026416	ENSGALG00010026416	-0.471250795	1.440119709	-0.327230293	0.74349371	0.815878668	negative
PRJNA729271	stage_E14_vs_E10	ordinary_muscle_development	ENSGALG00010026422	P2RX3	-2.01506241	0.275649047	-7.310246246	2.66653E-13	3.24802E-12	negative
PRJNA729271	stage_E14_vs_E10	ordinary_muscle_development	ENSGALG00010026427	PGC	0.116796274	1.081355959	0.108009091	0.913988481	0.940755838	positive
PRJNA729271	stage_E14_vs_E10	ordinary_muscle_development	ENSGALG00010026433	VPS11	-0.142545672	0.070797666	-2.013423316	0.044070115	0.085138786	negative
PRJNA729271	stage_E14_vs_E10	ordinary_muscle_development	ENSGALG00010026434	ENSGALG00010026434	-0.085266662	0.141743595	-0.601555656	0.547469954	0.652757834	negative
PRJNA729271	stage_E14_vs_E10	ordinary_muscle_development	ENSGALG00010026436	PLCD1	-0.401848569	0.075466114	-5.324887517	1.01016E-07	6.4333E-07	negative
PRJNA729271	stage_E14_vs_E10	ordinary_muscle_development	ENSGALG00010026438	TOMM6	0.426871613	0.120758111	3.534931198	0.000407881	0.001410699	positive
PRJNA729271	stage_E14_vs_E10	ordinary_muscle_development	ENSGALG00010026443	UPB1	0.998724026	0.356403804	2.802226059	0.00507513	0.013195337	positive
PRJNA729271	stage_E14_vs_E10	ordinary_muscle_development	ENSGALG00010026448	ADORA2A	0.878257262	0.10787845	8.141174302	3.91462E-16	6.06871E-15	positive
PRJNA729271	stage_E14_vs_E10	ordinary_muscle_development	ENSGALG00010026457	SSRP1	-0.15847429	0.097628111	-1.623244461	0.104537157	0.176645616	negative
PRJNA729271	stage_E14_vs_E10	ordinary_muscle_development	ENSGALG00010026458	DLEC1	1.579281086	0.176815663	8.931794044	4.19155E-19	8.36867E-18	positive
PRJNA729271	stage_E14_vs_E10	ordinary_muscle_development	ENSGALG00010026480	USP49	-0.479391628	0.213433794	-2.246090556	0.024698208	0.05217906	negative
PRJNA729271	stage_E14_vs_E10	ordinary_muscle_development	ENSGALG00010026481	DIXDC1	-0.996511745	0.134526642	-7.407541955	1.28662E-13	1.6091E-12	negative
PRJNA729271	stage_E14_vs_E10	ordinary_muscle_development	ENSGALG00010026484	TNKS1BP1	-0.17754724	0.111090602	-1.598220162	0.109993988	0.184279839	negative
PRJNA729271	stage_E14_vs_E10	ordinary_muscle_development	ENSGALG00010026485	ACAA1	0.808916445	0.104340302	7.752674923	8.99767E-15	1.24093E-13	positive
PRJNA729271	stage_E14_vs_E10	ordinary_muscle_development	ENSGALG00010026492	SPECC1L	-0.36831822	0.074976707	-4.91243524	8.99521E-07	4.9931E-06	negative
PRJNA729271	stage_E14_vs_E10	ordinary_muscle_development	ENSGALG00010026498	ENSGALG00010026498	-0.415058743	0.087504007	-4.743311285	2.10253E-06	1.10341E-05	negative
PRJNA729271	stage_E14_vs_E10	ordinary_muscle_development	ENSGALG00010026507	APLNR	0.554948293	0.28565796	1.942702012	0.052052173	0.098113946	positive
PRJNA729271	stage_E14_vs_E10	ordinary_muscle_development	ENSGALG00010026508	CSRP1	-0.461395404	0.156466962	-2.948835958	0.003189732	0.008777401	negative
PRJNA729271	stage_E14_vs_E10	ordinary_muscle_development	ENSGALG00010026516	ENSGALG00010026516	0.076640296	0.167328303	0.458023509	0.646935552	0.738954359	positive
PRJNA729271	stage_E14_vs_E10	ordinary_muscle_development	ENSGALG00010026522	MED20	0.314560236	0.12789276	2.459562506	0.013910647	0.031783548	positive
PRJNA729271	stage_E14_vs_E10	ordinary_muscle_development	ENSGALG00010026536	BCR	0.133392865	0.095916133	1.390723975	0.164309145	0.255214429	positive
PRJNA729271	stage_E14_vs_E10	ordinary_muscle_development	ENSGALG00010026537	FJX1	-2.017888895	0.172054581	-11.72819043	9.13916E-32	4.2459E-30	negative
PRJNA729271	stage_E14_vs_E10	ordinary_muscle_development	ENSGALG00010026539	ENSGALG00010026539	-0.030052127	0.350409705	-0.085762828	0.931654956	0.953157721	negative
PRJNA729271	stage_E14_vs_E10	ordinary_muscle_development	ENSGALG00010026550	MYD88	0.239213682	0.104948895	2.279334934	0.022647163	0.048489014	positive
PRJNA729271	stage_E14_vs_E10	ordinary_muscle_development	ENSGALG00010026553	PLPP3	0.620312689	0.12749537	4.865374245	1.1424E-06	6.27241E-06	positive
PRJNA729271	stage_E14_vs_E10	ordinary_muscle_development	ENSGALG00010026559	RAB36	-1.569743458	0.449974581	-3.488515852	0.00048571	0.00165228	negative
PRJNA729271	stage_E14_vs_E10	ordinary_muscle_development	ENSGALG00010026560	TNNI1	1.389063597	0.588004445	2.362335197	0.018160214	0.040003145	positive
PRJNA729271	stage_E14_vs_E10	ordinary_muscle_development	ENSGALG00010026565	RSPH14	-0.127570725	0.365215103	-0.349302984	0.726861858	0.802610089	negative
PRJNA729271	stage_E14_vs_E10	ordinary_muscle_development	ENSGALG00010026566	ENSGALG00010026566	0.405465918	0.121772668	3.329695603	0.00086941	0.002779465	positive
PRJNA729271	stage_E14_vs_E10	ordinary_muscle_development	ENSGALG00010026568	BYSL	0.233893149	0.110004746	2.126209616	0.03348581	0.067338626	positive
PRJNA729271	stage_E14_vs_E10	ordinary_muscle_development	ENSGALG00010026569	GNAZ	-0.660718938	0.201905865	-3.272410817	0.001066345	0.003339095	negative
PRJNA729271	stage_E14_vs_E10	ordinary_muscle_development	ENSGALG00010026581	DNER	-2.633358401	0.180990542	-14.5497017	5.86583E-48	7.52435E-46	negative
PRJNA729271	stage_E14_vs_E10	ordinary_muscle_development	ENSGALG00010026584	ENSGALG00010026584	0.014925191	0.11020262	0.135434085	0.892268676	0.92599946	positive
PRJNA729271	stage_E14_vs_E10	ordinary_muscle_development	ENSGALG00010026588	CCND3	-0.175714276	0.144142544	-1.219031322	0.222832304	0.325633053	negative
PRJNA729271	stage_E14_vs_E10	ordinary_muscle_development	ENSGALG00010026595	PRKAA2	1.548377037	0.261378797	5.923881562	3.1443E-09	2.43595E-08	positive
PRJNA729271	stage_E14_vs_E10	ordinary_muscle_development	ENSGALG00010026601	ENSGALG00010026601	0.05750459	0.479054193	0.120037755	0.90445324	0.934672582	positive
PRJNA729271	stage_E14_vs_E10	ordinary_muscle_development	ENSGALG00010026612	MFF	0.105923191	0.080082795	1.322671018	0.185944805	0.282020504	positive
PRJNA729271	stage_E14_vs_E10	ordinary_muscle_development	ENSGALG00010026625	ENSGALG00010026625	0.491688356	0.791350609	0.621328081	0.534383781	0.641467785	positive
PRJNA729271	stage_E14_vs_E10	ordinary_muscle_development	ENSGALG00010026628	FYB2	-3.184651037	0.855898762	-3.720826781	0.000198572	0.000738593	negative
PRJNA729271	stage_E14_vs_E10	ordinary_muscle_development	ENSGALG00010026630	XIRP1	1.692537167	0.243738155	6.944079665	3.80936E-12	4.09619E-11	positive
PRJNA729271	stage_E14_vs_E10	ordinary_muscle_development	ENSGALG00010026633	SLC5A1	0.852658987	0.501830019	1.699099205	0.089300493	0.154778267	positive
PRJNA729271	stage_E14_vs_E10	ordinary_muscle_development	ENSGALG00010026635	C8A	-0.98956202	2.653507162	-0.372926078	0.709203462	0.789062341	negative
PRJNA729271	stage_E14_vs_E10	ordinary_muscle_development	ENSGALG00010026638	LAD1	0.261027343	0.385559733	0.677008827	0.498400354	0.607645584	positive
PRJNA729271	stage_E14_vs_E10	ordinary_muscle_development	ENSGALG00010026643	YWHAH	-0.170446794	0.111557509	-1.527882762	0.126541649	0.206208117	negative
PRJNA729271	stage_E14_vs_E10	ordinary_muscle_development	ENSGALG00010026659	LIPH	-0.348558221	0.246056743	-1.416576584	0.156606759	0.245699206	negative
PRJNA729271	stage_E14_vs_E10	ordinary_muscle_development	ENSGALG00010026666	ENSGALG00010026666	2.227945538	0.418787409	5.319991689	1.03772E-07	6.60015E-07	positive
PRJNA729271	stage_E14_vs_E10	ordinary_muscle_development	ENSGALG00010026671	TAF8	0.185181184	0.154793478	1.196311279	0.231575109	0.335869642	positive
PRJNA729271	stage_E14_vs_E10	ordinary_muscle_development	ENSGALG00010026673	DEPDC5	0.314678717	0.121046391	2.599653861	0.009331783	0.022428155	positive
PRJNA729271	stage_E14_vs_E10	ordinary_muscle_development	ENSGALG00010026681	PLXDC1	1.20420535	0.258535042	4.657803224	3.19601E-06	1.62662E-05	positive
PRJNA729271	stage_E14_vs_E10	ordinary_muscle_development	ENSGALG00010026686	ENSGALG00010026686	0.724467218	0.385754539	1.878052352	0.060374004	0.111154729	positive
PRJNA729271	stage_E14_vs_E10	ordinary_muscle_development	ENSGALG00010026690	KRT14	-2.040804935	0.42651539	-4.784833051	1.7113E-06	9.12292E-06	negative
PRJNA729271	stage_E14_vs_E10	ordinary_muscle_development	ENSGALG00010026692	NMNAT3	0.038516112	0.608903923	0.063254827	0.949563587	0.965113501	positive
PRJNA729271	stage_E14_vs_E10	ordinary_muscle_development	ENSGALG00010026696	CSRNP1	-1.030157079	0.150901679	-6.826677401	8.69038E-12	8.95288E-11	negative
PRJNA729271	stage_E14_vs_E10	ordinary_muscle_development	ENSGALG00010026697	CHN1	0.777538125	0.114290824	6.803154427	1.02353E-11	1.04848E-10	positive
PRJNA729271	stage_E14_vs_E10	ordinary_muscle_development	ENSGALG00010026700	C8B	-1.131606881	1.026630825	-1.102252975	0.270351707	0.380159817	negative
PRJNA729271	stage_E14_vs_E10	ordinary_muscle_development	ENSGALG00010026704	WDFY1	-0.020209673	0.068705646	-0.294148643	0.768644323	0.835508021	negative
PRJNA729271	stage_E14_vs_E10	ordinary_muscle_development	ENSGALG00010026706	KRT24	-3.497310405	0.545685762	-6.409018976	1.46459E-10	1.32269E-09	negative
PRJNA729271	stage_E14_vs_E10	ordinary_muscle_development	ENSGALG00010026707	GORASP1	-0.462959561	0.081380748	-5.688809326	1.27928E-08	9.19801E-08	negative
PRJNA729271	stage_E14_vs_E10	ordinary_muscle_development	ENSGALG00010026715	WDR48	-0.03999495	0.065196562	-0.613451832	0.539577623	0.645684607	negative
PRJNA729271	stage_E14_vs_E10	ordinary_muscle_development	ENSGALG00010026719	ENSGALG00010026719	1.876467449	0.693911462	2.704188575	0.006847142	0.017106053	positive
PRJNA729271	stage_E14_vs_E10	ordinary_muscle_development	ENSGALG00010026724	OLA1	0.120370176	0.097866058	1.22994814	0.218716526	0.320945135	positive

PRJNA729271	stage_E14_vs_E10	ordinary_muscle_development	ENSGALG00010026731	ENSGALG00010026731	6.338527495	0.437489475	14.48841139	1.43422E-47	1.79215E-45	positive
PRJNA729271	stage_E14_vs_E10	ordinary_muscle_development	ENSGALG00010026737	SCN11A	-4.915661577	0.91878518	-5.350175084	8.78692E-08	5.64067E-07	negative
PRJNA729271	stage_E14_vs_E10	ordinary_muscle_development	ENSGALG00010026740	DAB1	-1.160357041	0.127680696	-9.087959876	1.00915E-19	2.11076E-18	negative
PRJNA729271	stage_E14_vs_E10	ordinary_muscle_development	ENSGALG00010026743	TNNT2	2.011982026	0.586389917	3.431133393	0.000601065	0.001993693	positive
PRJNA729271	stage_E14_vs_E10	ordinary_muscle_development	ENSGALG00010026746	HOXD10	-2.450463986	0.42223067	-5.803614378	6.49004E-09	4.83667E-08	negative
PRJNA729271	stage_E14_vs_E10	ordinary_muscle_development	ENSGALG00010026755	ENSGALG00010026755	-0.499446941	0.184310624	-2.709810924	0.006732157	0.016850737	negative
PRJNA729271	stage_E14_vs_E10	ordinary_muscle_development	ENSGALG00010026759	OMA1	-0.032732343	0.107169468	-0.305426014	0.760041711	0.829016826	negative
PRJNA729271	stage_E14_vs_E10	ordinary_muscle_development	ENSGALG00010026767	ENSGALG00010026767	15.23235816	2.598211678	5.862631706	4.55588E-09	3.46472E-08	positive
PRJNA729271	stage_E14_vs_E10	ordinary_muscle_development	ENSGALG00010026770	ENSGALG00010026770	-1.817431495	0.602147444	-3.018249954	0.002542391	0.007166853	negative
PRJNA729271	stage_E14_vs_E10	ordinary_muscle_development	ENSGALG00010026775	BTG2	0.719389482	0.366221678	1.964355267	0.049488907	0.094071221	positive
PRJNA729271	stage_E14_vs_E10	ordinary_muscle_development	ENSGALG00010026780	MYSM1	0.014717128	0.078146631	0.188327094	0.850620247	0.896512796	positive
PRJNA729271	stage_E14_vs_E10	ordinary_muscle_development	ENSGALG00010026782	HOXB9	-3.067874952	0.521543543	-5.882298791	4.04607E-09	3.10306E-08	negative
PRJNA729271	stage_E14_vs_E10	ordinary_muscle_development	ENSGALG00010026786	ENSGALG00010026786	-0.053423707	0.641076304	-0.083334397	0.93358565	0.954324683	negative
PRJNA729271	stage_E14_vs_E10	ordinary_muscle_development	ENSGALG00010026796	DCAKD	0.804646856	0.201258861	3.998069213	6.38613E-05	0.000260972	positive
PRJNA729271	stage_E14_vs_E10	ordinary_muscle_development	ENSGALG00010026801	FMOD	0.539884914	0.491395624	1.098676683	0.271909117	0.381874106	positive
PRJNA729271	stage_E14_vs_E10	ordinary_muscle_development	ENSGALG00010026822	PRELP	0.733094884	0.199524072	3.674217745	0.000238579	0.000869522	positive
PRJNA729271	stage_E14_vs_E10	ordinary_muscle_development	ENSGALG00010026825	ADAM23	-0.440049907	0.134294642	-3.276749539	0.001050095	0.003295329	negative
PRJNA729271	stage_E14_vs_E10	ordinary_muscle_development	ENSGALG00010026827	FAM117A	0.640750568	0.128638705	4.981009158	6.32535E-07	3.58849E-06	positive
PRJNA729271	stage_E14_vs_E10	ordinary_muscle_development	ENSGALG00010026834	ENSGALG00010026834	9.144563585	1.927369503	4.744582484	2.08937E-06	1.0969E-05	positive
PRJNA729271	stage_E14_vs_E10	ordinary_muscle_development	ENSGALG00010026836	ENSGALG00010026836	1.786143823	0.60755597	2.939883584	0.003283356	0.009003451	positive
PRJNA729271	stage_E14_vs_E10	ordinary_muscle_development	ENSGALG00010026839	FGGY	0.025750654	0.116202042	0.221602422	0.824623397	0.877278249	positive
PRJNA729271	stage_E14_vs_E10	ordinary_muscle_development	ENSGALG00010026846	MICAL1	-0.9592921	0.228471137	-4.198745254	2.68398E-05	0.000117041	negative
PRJNA729271	stage_E14_vs_E10	ordinary_muscle_development	ENSGALG00010026851	ENSGALG00010026851	10.08368057	1.099591699	9.17038622	4.71327E-20	1.02121E-18	positive
PRJNA729271	stage_E14_vs_E10	ordinary_muscle_development	ENSGALG00010026854	SMPD2	0.383688802	0.144039907	2.663767355	0.007727098	0.019038635	positive
PRJNA729271	stage_E14_vs_E10	ordinary_muscle_development	ENSGALG00010026856	UBE2Z	0.205313199	0.075303517	2.726475562	0.006401469	0.016120448	positive
PRJNA729271	stage_E14_vs_E10	ordinary_muscle_development	ENSGALG00010026874	TEAD3	1.036013314	0.159925091	6.478116142	9.28749E-11	8.54201E-10	positive
PRJNA729271	stage_E14_vs_E10	ordinary_muscle_development	ENSGALG00010026885	TULP1	0.259211756	0.719228939	0.360402289	0.718546315	0.796461638	positive
PRJNA729271	stage_E14_vs_E10	ordinary_muscle_development	ENSGALG00010026886	JUN	0.222968017	0.193081472	1.15478722	0.248177572	0.355011734	positive
PRJNA729271	stage_E14_vs_E10	ordinary_muscle_development	ENSGALG00010026889	ENSGALG00010026889	0.18484084	0.251597399	0.734669121	0.462541031	0.575150746	positive
PRJNA729271	stage_E14_vs_E10	ordinary_muscle_development	ENSGALG00010026893	FKBP5	-0.9386456	0.388364062	-2.416921882	0.015652376	0.035202667	negative
PRJNA729271	stage_E14_vs_E10	ordinary_muscle_development	ENSGALG00010026894	IP6K1	0.051816113	0.064194125	0.807178435	0.419563688	0.534062099	positive
PRJNA729271	stage_E14_vs_E10	ordinary_muscle_development	ENSGALG00010026897	ENSGALG00010026897	-0.500535897	0.214626713	-2.332123012	0.019694221	0.042856588	negative
PRJNA729271	stage_E14_vs_E10	ordinary_muscle_development	ENSGALG00010026898	PNPLA2	0.240971025	0.272153807	0.885422208	0.375928959	0.492150493	positive
PRJNA729271	stage_E14_vs_E10	ordinary_muscle_development	ENSGALG00010026899	HOOK1	-0.554874993	0.166444689	-3.333689985	0.000857021	0.002745916	negative
PRJNA729271	stage_E14_vs_E10	ordinary_muscle_development	ENSGALG00010026901	FAM212A	-0.228179901	0.17035421	-1.339443864	0.180426218	0.275292424	negative
PRJNA729271	stage_E14_vs_E10	ordinary_muscle_development	ENSGALG00010026907	ENSGALG00010026907	0.216355411	0.129868625	1.665955969	0.095722185	0.164139069	positive
PRJNA729271	stage_E14_vs_E10	ordinary_muscle_development	ENSGALG00010026909	ENSGALG00010026909	-0.506516504	0.156260281	-3.241492348	0.001189056	0.003681198	negative
PRJNA729271	stage_E14_vs_E10	ordinary_muscle_development	ENSGALG00010026916	ENSGALG00010026916	0.618812073	0.210788231	2.935705041	0.003327906	0.009100208	positive
PRJNA729271	stage_E14_vs_E10	ordinary_muscle_development	ENSGALG00010026917	AMT	-0.161454505	0.135552432	-1.191085268	0.233620112	0.338091407	negative
PRJNA729271	stage_E14_vs_E10	ordinary_muscle_development	ENSGALG00010026919	NICN1	0.298020672	0.158474108	1.88056381	0.060031279	0.110608031	positive
PRJNA729271	stage_E14_vs_E10	ordinary_muscle_development	ENSGALG00010026920	GATA2	0.205302804	0.211585157	0.970308155	0.331892913	0.446808561	positive
PRJNA729271	stage_E14_vs_E10	ordinary_muscle_development	ENSGALG00010026924	TPH1	0.281475974	0.346077091	0.81333316	0.41602707	0.530697208	positive
PRJNA729271	stage_E14_vs_E10	ordinary_muscle_development	ENSGALG00010026942	CDK18	1.472856219	0.169124703	8.708699542	3.07381E-18	5.72684E-17	positive
PRJNA729271	stage_E14_vs_E10	ordinary_muscle_development	ENSGALG00010026943	EEFSEC	0.105544985	0.067683066	1.559400179	0.118901694	0.196184411	positive
PRJNA729271	stage_E14_vs_E10	ordinary_muscle_development	ENSGALG00010026950	LHFPL5	-1.55476302	0.293038768	-5.305656419	1.12268E-07	7.09724E-07	negative
PRJNA729271	stage_E14_vs_E10	ordinary_muscle_development	ENSGALG00010026951	RUVBL1	-0.039968992	0.104938176	-0.380881325	0.70329131	0.784441623	negative
PRJNA729271	stage_E14_vs_E10	ordinary_muscle_development	ENSGALG00010026954	MFSD4A	0.567773018	0.175857006	3.228606194	0.001243951	0.003836397	positive
PRJNA729271	stage_E14_vs_E10	ordinary_muscle_development	ENSGALG00010026958	CLTCL1	0.101801686	0.083976123	1.21226942	0.225409266	0.328570727	positive
PRJNA729271	stage_E14_vs_E10	ordinary_muscle_development	ENSGALG00010026960	SEC61A1	-0.099339157	0.101975259	-0.974149592	0.329982269	0.44473203	negative
PRJNA729271	stage_E14_vs_E10	ordinary_muscle_development	ENSGALG00010026964	SRPK1	-0.18299763	0.059253633	-3.088378226	0.002012522	0.005847433	negative
PRJNA729271	stage_E14_vs_E10	ordinary_muscle_development	ENSGALG00010026972	TNNT3	2.733033727	0.158387112	17.25540473	1.01905E-66	3.07732E-64	positive
PRJNA729271	stage_E14_vs_E10	ordinary_muscle_development	ENSGALG00010026973	MGLL	1.138250921	0.124286446	9.158286841	5.27277E-20	1.12893E-18	positive
PRJNA729271	stage_E14_vs_E10	ordinary_muscle_development	ENSGALG00010026977	ELK4	0.431764304	0.095615344	4.515638227	6.31264E-06	3.06641E-05	positive
PRJNA729271	stage_E14_vs_E10	ordinary_muscle_development	ENSGALG00010026980	LIMK2	-0.500541903	0.113705164	-4.402103539	1.07206E-05	4.99986E-05	negative
PRJNA729271	stage_E14_vs_E10	ordinary_muscle_development	ENSGALG00010026982	ABTB1	1.095724933	0.084714581	12.93431334	2.88186E-38	2.0083E-36	positive
PRJNA729271	stage_E14_vs_E10	ordinary_muscle_development	ENSGALG00010026984	PGR2/3	1.472040352	0.932884708	1.577944561	0.114578346	0.190722684	positive
PRJNA729271	stage_E14_vs_E10	ordinary_muscle_development	ENSGALG00010026999	MLEC	-0.085756169	0.08187243	-1.047436476	0.294898316	0.407993805	negative
PRJNA729271	stage_E14_vs_E10	ordinary_muscle_development	ENSGALG00010027001	SLC26A8	-0.398677819	0.253756196	-1.571105753	0.116158086	0.192630469	negative
PRJNA729271	stage_E14_vs_E10	ordinary_muscle_development	ENSGALG00010027003	SEPTIN2L	0.212502097	0.081697224	2.601093227	0.00929272	0.022349091	positive
PRJNA729271	stage_E14_vs_E10	ordinary_muscle_development	ENSGALG00010027012	RILPL1	0.344715838	0.192114615	1.794323859	0.072761492	0.130384204	positive
PRJNA729271	stage_E14_vs_E10	ordinary_muscle_development	ENSGALG00010027015	ENSGALG00010027015	1.096363684	0.519179259	2.111724736	0.034710066	0.069434503	positive
PRJNA729271	stage_E14_vs_E10	ordinary_muscle_development	ENSGALG00010027025	ORAI1	0.090996425	0.130663504	0.696418069	0.48616705	0.597483256	positive
PRJNA729271	stage_E14_vs_E10	ordinary_muscle_development	ENSGALG00010027026	SLC45A3	0.999652854	0.198049472	5.047490616	4.47651E-07	2.60277E-06	positive
PRJNA729271	stage_E14_vs_E10	ordinary_muscle_development	ENSGALG00010027027	ARHGEF3	-0.085690324	0.102067115	-0.839548808	0.401161412	0.516369299	negative
PRJNA729271	stage_E14_vs_E10	ordinary_muscle_development	ENSGALG00010027036	NUCKS1	-0.056178047	0.065356671	-0.859561027	0.390031066	0.505589367	negative
PRJNA729271	stage_E14_vs_E10	ordinary_muscle_development	ENSGALG00010027040	CLIP1	-0.206988944	0.108681604	-1.904544429	0.056839316	0.105639939	negative
PRJNA729271	stage_E14_vs_E10	ordinary_muscle_development	ENSGALG00010027041	MAPK14	0.893695712	0.073357382	12.18276456	3.8404E-34	2.0169E-32	positive
PRJNA729271	stage_E14_vs_E10	ordinary_muscle_development	ENSGALG00010027043	IL17RD	-0.544074256	0.174905014	-3.110684152	0.001866545	0.005490172	negative
PRJNA729271	stage_E14_vs_E10	ordinary_muscle_development	ENSGALG00010027045	MAPK13	1.164945929	0.179644089	6.484744013	8.88826E-11	8.20085E-10	positive
PRJNA729271	stage_E14_vs_E10	ordinary_muscle_development	ENSGALG00010027046	HESX1	-0.268156134	0.296014795	-0.905887606	0.364995345	0.481050801	negative
PRJNA729271	stage_E14_vs_E10	ordinary_muscle_development	ENSGALG00010027050	GSTT1	1.430287704	0.151962163	9.412130483	4.86181E-21	1.12395E-19	positive
PRJNA729271	stage_E14_vs_E10	ordinary_muscle_development	ENSGALG00010027052	APPL1	-0.063833586	0.084196597	-0.758149239	0.448361645	0.561517371	negative
PRJNA729271	stage_E14_vs_E10	ordinary_muscle_development	ENSGALG00010027059	ASB14	3.394630225	0.217036643	15.64081612	3.83778E-55	7.22449E-53	positive
PRJNA729271	stage_E14_vs_E10	ordinary_muscle_development	ENSGALG00010027063	RAB7L1	0.35993886	0.086864083	4.14370185	3.41744E-05	0.00014621	positive
PRJNA729271	stage_E14_vs_E10	ordinary_muscle_development	ENSGALG00010027073	DNAH12	-1.130900511	0.301829943	-3.746813526	0.000179095	0.000672186	negative
PRJNA729271	stage_E14_vs_E10	ordinary_muscle_development	ENSGALG00010027074	BRPF3	0.251138452	0.078726771	3.19000066	0.001422725	0.004312504	positive
PRJNA729271	stage_E14_vs_E10	ordinary_muscle_development	ENSGALG00010027075	SLC41A1	0.510988629	0.095127781	5.371602523	7.804E-08	5.05673E-07	positive
PRJNA729271	stage_E14_vs_E10	ordinary_muscle_development	ENSGALG00010027076	VDHAP	1.317261552	0.171484097	7.681537696	1.5719E-14	2.13369E-13	positive
PRJNA729271	stage_E14_vs_E10	ordinary_muscle_development	ENSGALG00010027077	ARF4	-0.247000477	0.103713423	-2.381567099	0.01723915	0.038266689	negative
PRJNA729271	stage_E14_vs_E10	ordinary_muscle_development	ENSGALG00010027078	PDE12	-0.459802659	0.118736972	-3.872447239	0.000107748	0.00042211	negative
PRJNA729271	stage_E14_vs_E10	ordinary_muscle_development	ENSGALG00010027079	PM20D1	0.236819738	0.094125743	2.51599329	0.011869744	0.027705627	positive
PRJNA729271	stage_E14_vs_E10	ordinary_muscle_development	ENSGALG00010027080	TBC1D22B	0.212531168	0.155608995	1.365802589	0.172000933	0.264862799	positive
PRJNA729271	stage_E14_vs_E10	ordinary_muscle_development	ENSGALG00010027081	PDE4B	-0.098038793	0.292222484	-0.335493668	0.73725272	0.811241037	negative
PRJNA729271	stage_E14_vs_E10	ordinary_muscle_development	ENSGALG00010027082	SLC26A9	-3.494389801	0.649022053	-5.384084846	7.28142E-08	4.74569E-07	negative
PRJNA729271	stage_E14_vs_E10	ordinary_muscle_development	ENSGALG00010027083	DENND6A	-0.369855315	0.085939294	-4.303681104	1.67983E-05	7.57834E-05	negative
PRJNA729271	stage_E14_vs_E10	ordinary_muscle_development	ENSGALG00010027084	OSBPL9	0.181019996	0.093412023	1.937866127	0.052639558	0.098902366	positive
PRJNA729271	stage_E14_vs_E10	ordinary_muscle_development	ENSGALG00010027085	PIM1	-0.610327779	0.152879176	-3.99222311	6.54567E-05	0.00026689	negative
PRJNA729271	stage_E14_vs_E10	ordinary_muscle_development	ENSGALG00010027086	CACNA1S	2.83898275	0.201279483	14.10468023	3.55397E-45	3.78785E-43	positive
PRJNA729271	stage_E14_vs_E10	ordinary_muscle_development	ENSGALG00010027087	RAB7B	-0.422894674	0.337972632	-1.251268988	0.210836356	0.311876006	negative
PRJNA729271	stage_E14_vs_E10	ordinary_muscle_development	ENSGALG00010027088	AGBL4	0.697044113	0.248352004	2.806678029	0.005005524	0.01303775	positive
PRJNA729271	stage_E14_vs_E10	ordinary_muscle_development	ENSGALG00010027092	ENSGALG00010027092	-0.198095749	0.193613501	-1.023150493	0.306236696	0.419833625	negative
PRJNA729271	stage_E14_vs_E10	ordinary_muscle_development	ENSGALG00010027093	SLMAP	0.199105002	0.076047086	2.618180552	0.008840002	0.021405918	positive
PRJNA729271	stage_E14_vs_E10	ordinary_muscle_development	ENSGALG00010027095	SCN5A	1.046762377	0.39622125	2.641863298	0.008245133	0.020174409	positive
PRJNA729271	stage_E14_vs_E10	ordinary_muscle_development	ENSGALG00010027096	YIPF1	0.474071811	0.109810386	4.317185562	1.58031E-05	7.16953E-05	positive
PRJNA729271	stage_E14_vs_E10	ordinary_muscle_development	ENSGALG00010027097	ENSGALG00010027097	-0.328169787	0.147567437	-2.223863168	0.026157652	0.054767465	negative
PRJNA729271	stage_E14_vs_E10	ordinary_muscle_development	ENSGALG00010027098	EXOG	-0.075659538	0.145546579	-0.519830411	0.603181781	0.701449533	negative
PRJNA729271	stage_E14_vs_E10	ordinary_muscle_development	ENSGALG00010027099	GNA11	0.246995733	0.084277193	2.93075414	0.003381403	0.009223454	positive
PRJNA729271	stage_E14_vs_E10	ordinary_muscle_development	ENSGALG00010027100	MIER2	0.0652154	0.085483655	0.762899054	0.4455236	0.558961786	positive
PRJNA729271	stage_E14_vs_E10	ordinary_muscle_development	ENSGALG00010027101	RNF220	0.192809481	0.077176762	2.498284158	0.012479611	0.02893883	positive
PRJNA729271	stage_E14_vs_E10	ordinary_muscle_development	ENSGALG00010027102	DUS3L	-0.158104861	0.138630419	-1.140477406	0.254087461	0.361574489	negative
PRJNA729271	stage_E14_vs_E10	ordinary_muscle_development	ENSGALG00010027103	ACVR2B	-0.120969259	0.17621847	-0.686473208	0.492414754	0.602579304	negative
PRJNA729271	stage_E14_vs_E10	ordinary_muscle_development	ENSGALG00010027105	CHERP	-0.201881066	0.127378294	-1.584893781	0.11299047	0.188490834	negative
PRJNA729271	stage_E14_vs_E10	ordinary_muscle_development	ENSGALG00010027106	CYP2J23	-0.255927791	0.147224189	-1.738354225	0.082148417	0.144665449	negative
PRJNA729271	stage_E14_vs_E10	ordinary_muscle_development	ENSGALG00010027107	KIF21B	-0.332087725	0.196529896	-1.689756785	0.091074495	0.157363786	negative
PRJNA729271	stage_E14_vs_E10	ordinary_muscle_development	ENSGALG00010027108	ENSGALG00010027108	-0.662951837	0.143285555	-4.626787645	3.71381E-06	1.86656E-05	negative
PRJNA729271	stage_E14_vs_E10	ordinary_muscle_development	ENSGALG00010027109	C19orf44	0.063773115	0.121991174	0.522768266	0.601135526	0.69993248	positive
PRJNA729271	stage_E14_vs_E10	ordinary_muscle_development	ENSGALG00010027110	IKBKE	-0.233002134	0.206562514	-1.127998151	0.2593207	0.367614039	negative
PRJNA729271	stage_E14_vs_E10	ordinary_muscle_development	ENSGALG00010027111	CALR3	-0.895926696	0.601392649	-1.48975332	0.136289109	0.219452415	negative
PRJNA729271	stage_E14_vs_E10	ordinary_muscle_development	ENSGALG00010027112	XYLB	-0.715399612	0.179370804	-3.988383817	6.6525E-05	0.000271017	negative
PRJNA729271	stage_E14_vs_E10	ordinary_muscle_development	ENSGALG00010027113	PIAS4	-0.252103947	0.130862946	-1.926473111	0.054045326	0.101225869	negative
PRJNA729271	stage_E14_vs_E10	ordinary_muscle_development	ENSGALG00010027114	FLNB	-1.175061539	0.11364382	-10.33986311	4.65202E-25	1.40189E-23	negative
PRJNA729271	stage_E14_vs_E10	ordinary_muscle_development	ENSGALG00010027115	C9ORF152	-1.177150339	0.618797142	-1.902320257	0.057129299	0.106110865	negative
PRJNA729271	stage_E14_vs_E10	ordinary_muscle_development	ENSGALG00010027116	CCDC94	0.256050065	0.100283485	2.553262527	0.010671902	0.025201579	positive
PRJNA729271	stage_E14_vs_E10	ordinary_muscle_development	ENSGALG00010027117	RASSF5	0.180568389	0.246267454	0.733220676	0.463423847	0.576018449	positive
PRJNA729271	stage_E14_vs_E10	ordinary_muscle_development	ENSGALG00010027118	ABHD6	-0.213883827	0.16394151	-1.30463497	0.192017175	0.289353254	negative
PRJNA729271	stage_E14_vs_E10	ordinary_muscle_development	ENSGALG00010027119	NUB1	0.238087981	0.098442842	2.418540302	0.015582919	0.035073667	positive
PRJNA729271	stage_E14_vs_E10	ordinary_muscle_development	ENSGALG00010027121	ELF3	-1.7646492	0.930069432	-1.897330608	0.057784318	0.107135289	negative
PRJNA729271	stage_E14_vs_E10	ordinary_muscle_development	ENSGALG00010027122	DNASE1L3	0.664967371	0.256270161	2.594790469	0.00946486	0.022706578	positive
PRJNA729271	stage_E14_vs_E10	ordinary_muscle_development	ENSGALG00010027123	WDR86	-0.347426955	0.217639563	-1.596340989	0.110412684	0.184807373	negative
PRJNA729271	stage_E14_vs_E10	ordinary_muscle_development	ENSGALG00010027124	IP6K2	-0.047079583	0.173744089	-0.270970846	0.786413456	0.849788507	negative
PRJNA729271	stage_E14_vs_E10	ordinary_muscle_development	ENSGALG00010027125	CRYGN	0.765176404	0.405371298	1.887593936	0.059080485	0.109078032	positive
PRJNA729271	stage_E14_vs_E10	ordinary_muscle_development	ENSGALG00010027126	ENSGALG00010027126	3.096160452	0.563666753	5.492891741	3.95405E-08	2.66701E-07	positive
PRJNA729271	stage_E14_vs_E10	ordinary_muscle_development	ENSGALG00010027127	S1PR4	-0.053388657	0.702826557	-0.075962777	0.939448712	0.957549334	negative
PRJNA729271	stage_E14_vs_E10	ordinary_muscle_development	ENSGALG00010027128	EIF2D	0.326280739	0.089378337	3.650557268	0.000261672	0.000944927	positive
PRJNA729271	stage_E14_vs_E10	ordinary_muscle_development	ENSGALG00010027129	NCLN	-0.015676143	0.128729985	-0.121775383	0.90307691	0.933739911	negative
PRJNA729271	stage_E14_vs_E10	ordinary_muscle_development	ENSGALG00010027130	NCKIPSD	0.386332537	0.141737274	2.725694699	0.006416632	0.016155824	positive
PRJNA729271	stage_E14_vs_E10	ordinary_muscle_development	ENSGALG00010027131	UBA52	0.515479797	0.152161397	3.3877173	0.000704769	0.002304449	positive
PRJNA729271	stage_E14_vs_E10	ordinary_muscle_development	ENSGALG00010027133	CELF5	-0.70694765	0.214980365	-3.288428925	0.001007482	0.003173974	negative
PRJNA729271	stage_E14_vs_E10	ordinary_muscle_development	ENSGALG00010027134	DYRK3	0.935345245	0.148339771	6.305424633	2.87405E-10	2.5096E-09	positive
PRJNA729271	stage_E14_vs_E10	ordinary_muscle_development	ENSGALG00010027135	PRKAG2	-0.321544876	0.13901181	-2.313075952	0.020718464	0.044836389	negative
PRJNA729271	stage_E14_vs_E10	ordinary_muscle_development	ENSGALG00010027136	MAPKAPK2	0.827382211	0.143992318	5.746016339	9.13705E-09	6.67548E-08	positive

PRJNA729271	stage_E14_vs_E10	ordinary_muscle_development	ENSGALG00010027137	FKBP8	0.268496296	0.089554837	2.998121667	0.002716492	0.007614688	positive
PRJNA729271	stage_E14_vs_E10	ordinary_muscle_development	ENSGALG00010027139	HSD11B1L	0.35116674	0.133315857	2.634095802	0.008436168	0.020572385	positive
PRJNA729271	stage_E14_vs_E10	ordinary_muscle_development	ENSGALG00010027141	PIGR	1.86472622	1.607485972	1.160026434	0.246038044	0.352751874	positive
PRJNA729271	stage_E14_vs_E10	ordinary_muscle_development	ENSGALG00010027143	GALNT11	-0.46168233	0.118184981	-3.906438237	9.36666E-05	0.000370449	negative
PRJNA729271	stage_E14_vs_E10	ordinary_muscle_development	ENSGALG00010027144	ENSGALG00010027144	-0.991295255	0.562766474	-1.761468214	0.078159188	0.138600127	negative
PRJNA729271	stage_E14_vs_E10	ordinary_muscle_development	ENSGALG00010027145	CELSR3	-0.201802493	0.158129339	-1.276186278	0.201889678	0.301261638	negative
PRJNA729271	stage_E14_vs_E10	ordinary_muscle_development	ENSGALG00010027146	KXD1	0.277833879	0.123625989	2.247374367	0.024616112	0.052035955	positive
PRJNA729271	stage_E14_vs_E10	ordinary_muscle_development	ENSGALG00010027147	KMT2C	-0.155789094	0.103637343	-1.503213903	0.132783893	0.214882498	negative
PRJNA729271	stage_E14_vs_E10	ordinary_muscle_development	ENSGALG00010027148	ELL	0.207979798	0.16582016	1.254249175	0.209751451	0.310556413	positive
PRJNA729271	stage_E14_vs_E10	ordinary_muscle_development	ENSGALG00010027149	C1orf116	1.554730728	0.223373585	6.960226428	3.39726E-12	3.68037E-11	positive
PRJNA729271	stage_E14_vs_E10	ordinary_muscle_development	ENSGALG00010027150	YOD1	0.115447955	0.088395044	1.306045571	0.191537061	0.288930138	positive
PRJNA729271	stage_E14_vs_E10	ordinary_muscle_development	ENSGALG00010027151	MICOS13	0.546804539	0.143513467	3.810127018	0.000138895	0.000533463	positive
PRJNA729271	stage_E14_vs_E10	ordinary_muscle_development	ENSGALG00010027152	C4BPS	-0.390804683	0.464929036	-0.840568457	0.400589736	0.515918187	negative
PRJNA729271	stage_E14_vs_E10	ordinary_muscle_development	ENSGALG00010027153	ENSGALG00010027153	-0.222556452	0.229779423	-0.968565629	0.332761959	0.447729007	negative
PRJNA729271	stage_E14_vs_E10	ordinary_muscle_development	ENSGALG00010027154	PLPP2	-0.376580612	0.137360666	-2.74154619	0.006115076	0.015509717	negative
PRJNA729271	stage_E14_vs_E10	ordinary_muscle_development	ENSGALG00010027155	ENSGALG00010027155	0.600429872	0.292363009	2.053713542	0.040003425	0.078294578	positive
PRJNA729271	stage_E14_vs_E10	ordinary_muscle_development	ENSGALG00010027156	ISYNA1	-0.997184715	0.146235247	-6.819044897	9.16478E-12	9.42152E-11	negative
PRJNA729271	stage_E14_vs_E10	ordinary_muscle_development	ENSGALG00010027157	ENSGALG00010027157	0.745791569	0.250625476	2.975721307	0.002923004	0.008121342	positive
PRJNA729271	stage_E14_vs_E10	ordinary_muscle_development	ENSGALG00010027158	XRCC2	-0.087088681	0.130690477	-0.666373579	0.505172346	0.614043871	negative
PRJNA729271	stage_E14_vs_E10	ordinary_muscle_development	ENSGALG00010027159	TMUB1	0.265888109	0.163916215	1.622097663	0.104782438	0.177018816	positive
PRJNA729271	stage_E14_vs_E10	ordinary_muscle_development	ENSGALG00010027160	FASTK	0.314294153	0.166371668	1.889108623	0.058877275	0.108730552	positive
PRJNA729271	stage_E14_vs_E10	ordinary_muscle_development	ENSGALG00010027161	C4BPG	0.00290818	0.203605464	0.014283408	0.988603877	0.992300616	positive
PRJNA729271	stage_E14_vs_E10	ordinary_muscle_development	ENSGALG00010027162	SLC4A2	-0.098823444	0.13012426	-0.759454416	0.447580773	0.560713659	negative
PRJNA729271	stage_E14_vs_E10	ordinary_muscle_development	ENSGALG00010027163	ENSGALG00010027163	-0.275875877	0.077971761	-3.538151155	0.000402939	0.001395271	negative
PRJNA729271	stage_E14_vs_E10	ordinary_muscle_development	ENSGALG00010027164	ENSGALG00010027164	1.06961342	0.4628173	2.311092129	0.020827767	0.045039307	positive
PRJNA729271	stage_E14_vs_E10	ordinary_muscle_development	ENSGALG00010027165	ENSGALG00010027165	-1.067059173	0.209496063	-5.093456938	3.51593E-07	2.08099E-06	negative
PRJNA729271	stage_E14_vs_E10	ordinary_muscle_development	ENSGALG00010027166	CDK5	0.323684549	0.096902274	3.340319453	0.000836821	0.00268892	positive
PRJNA729271	stage_E14_vs_E10	ordinary_muscle_development	ENSGALG00010027167	ENSGALG00010027167	-0.355201541	0.336230266	-1.056423457	0.290774794	0.403135403	negative
PRJNA729271	stage_E14_vs_E10	ordinary_muscle_development	ENSGALG00010027168	SSBP4	-1.212587448	0.180808147	-6.706486784	1.99366E-11	1.97932E-10	negative
PRJNA729271	stage_E14_vs_E10	ordinary_muscle_development	ENSGALG00010027169	ABCB8	0.301955728	0.110885371	2.723134048	0.006466581	0.016258993	positive
PRJNA729271	stage_E14_vs_E10	ordinary_muscle_development	ENSGALG00010027170	TLE2	0.47366791	0.159795471	2.964213607	0.003034576	0.008402325	positive
PRJNA729271	stage_E14_vs_E10	ordinary_muscle_development	ENSGALG00010027171	ATG9B	0.330522702	0.276848208	1.193876978	0.232526094	0.336709864	positive
PRJNA729271	stage_E14_vs_E10	ordinary_muscle_development	ENSGALG00010027172	ENSGALG00010027172	-0.408489913	0.457372041	-0.893123928	0.37179081	0.487922843	negative
PRJNA729271	stage_E14_vs_E10	ordinary_muscle_development	ENSGALG00010027173	ENSGALG00010027173	0.670964221	0.096553713	6.949129137	3.67548E-12	3.9581E-11	positive
PRJNA729271	stage_E14_vs_E10	ordinary_muscle_development	ENSGALG00010027174	SHISA5	-0.410346222	0.132491593	-3.097149126	0.001953916	0.005703603	negative
PRJNA729271	stage_E14_vs_E10	ordinary_muscle_development	ENSGALG00010027175	NOS3	0.298513341	0.164497445	1.814698948	0.069570187	0.125409757	positive
PRJNA729271	stage_E14_vs_E10	ordinary_muscle_development	ENSGALG00010027176	ENSGALG00010027176	0.031516529	0.170463128	0.184887663	0.853317148	0.898440623	positive
PRJNA729271	stage_E14_vs_E10	ordinary_muscle_development	ENSGALG00010027178	C4BPM	0.197024653	0.080986573	2.432806399	0.014982309	0.033916691	positive
PRJNA729271	stage_E14_vs_E10	ordinary_muscle_development	ENSGALG00010027183	CD34	1.446566365	0.155748042	9.287862297	1.57418E-20	3.49965E-19	positive
PRJNA729271	stage_E14_vs_E10	ordinary_muscle_development	ENSGALG00010027184	MPND	-1.667569587	0.375668747	-4.438936162	9.04046E-06	4.27124E-05	negative
PRJNA729271	stage_E14_vs_E10	ordinary_muscle_development	ENSGALG00010027185	ATRIP	-0.193305367	0.077136495	-2.506016991	0.012209975	0.028390053	negative
PRJNA729271	stage_E14_vs_E10	ordinary_muscle_development	ENSGALG00010027186	PLXNA2	1.672162044	0.12212911	13.69175652	1.13726E-42	1.04333E-40	positive
PRJNA729271	stage_E14_vs_E10	ordinary_muscle_development	ENSGALG00010027187	ENSGALG00010027187	-0.099836841	0.130686888	-0.76393923	0.444903458	0.558518557	negative
PRJNA729271	stage_E14_vs_E10	ordinary_muscle_development	ENSGALG00010027188	KCNH2	0.565122712	0.256928234	2.199535264	0.027839884	0.057755707	positive
PRJNA729271	stage_E14_vs_E10	ordinary_muscle_development	ENSGALG00010027189	ENSGALG00010027189	-1.211663804	0.280746673	-4.315861668	1.58982E-05	7.20587E-05	negative
PRJNA729271	stage_E14_vs_E10	ordinary_muscle_development	ENSGALG00010027190	IPPK	-0.441455643	0.114423905	-3.858071814	0.000114285	0.000445552	negative
PRJNA729271	stage_E14_vs_E10	ordinary_muscle_development	ENSGALG00010027192	SH3GL1	0.362130659	0.115441215	3.136926957	0.001707287	0.005068015	positive
PRJNA729271	stage_E14_vs_E10	ordinary_muscle_development	ENSGALG00010027193	CAMK1G	-1.940313702	0.480144539	-4.041103343	5.32003E-05	0.000220325	negative
PRJNA729271	stage_E14_vs_E10	ordinary_muscle_development	ENSGALG00010027194	ENSGALG00010027194	-0.920152164	0.24033944	-3.828552493	0.000128899	0.000497973	negative
PRJNA729271	stage_E14_vs_E10	ordinary_muscle_development	ENSGALG00010027195	CENPP	0.039899208	0.187180228	0.213159306	0.831202708	0.882193307	positive
PRJNA729271	stage_E14_vs_E10	ordinary_muscle_development	ENSGALG00010027196	ENSGALG00010027196	0.431729422	0.327449182	1.318462363	0.187348916	0.28370482	positive
PRJNA729271	stage_E14_vs_E10	ordinary_muscle_development	ENSGALG00010027197	ENSGALG00010027197	-0.791476522	0.503753381	-1.57115873	0.116145784	0.192630469	negative
PRJNA729271	stage_E14_vs_E10	ordinary_muscle_development	ENSGALG00010027198	ECM2	-0.270768352	0.20333339	-1.331647266	0.182976125	0.278434396	negative
PRJNA729271	stage_E14_vs_E10	ordinary_muscle_development	ENSGALG00010027199	LAMB3	0.916292041	0.286066445	3.203074177	0.00135969	0.004146581	positive
PRJNA729271	stage_E14_vs_E10	ordinary_muscle_development	ENSGALG00010027200	G0S2	3.944069031	0.317459576	12.42384645	1.94005E-35	1.1115E-33	positive
PRJNA729271	stage_E14_vs_E10	ordinary_muscle_development	ENSGALG00010027201	ASPN	-1.966362974	0.318015385	-6.183232212	6.28022E-10	5.27415E-09	negative
PRJNA729271	stage_E14_vs_E10	ordinary_muscle_development	ENSGALG00010027202	CHAF1A	-0.294078161	0.080424487	-3.656574912	0.000255608	0.000926259	negative
PRJNA729271	stage_E14_vs_E10	ordinary_muscle_development	ENSGALG00010027203	ENSGALG00010027203	-0.60138855	0.295132573	-2.037689513	0.041580994	0.080988511	negative
PRJNA729271	stage_E14_vs_E10	ordinary_muscle_development	ENSGALG00010027204	OMD	-3.662904078	0.827660532	-4.425611632	9.61694E-06	4.52296E-05	negative
PRJNA729271	stage_E14_vs_E10	ordinary_muscle_development	ENSGALG00010027205	OGN	0.566447617	0.221858759	2.55319024	0.010674118	0.025201579	positive
PRJNA729271	stage_E14_vs_E10	ordinary_muscle_development	ENSGALG00010027206	ENSGALG00010027206	4.121600067	0.993517444	4.148492906	3.34671E-05	0.00014348	positive
PRJNA729271	stage_E14_vs_E10	ordinary_muscle_development	ENSGALG00010027207	NOL8	-0.5597802	0.123560754	-4.530404513	5.88709E-06	2.87318E-05	negative
PRJNA729271	stage_E14_vs_E10	ordinary_muscle_development	ENSGALG00010027208	UBXN6	0.516637129	0.093108735	5.548750364	2.87719E-08	1.98594E-07	positive
PRJNA729271	stage_E14_vs_E10	ordinary_muscle_development	ENSGALG00010027209	TRAF3IP3	0.221114613	0.254829142	0.867697513	0.385559948	0.501722906	positive
PRJNA729271	stage_E14_vs_E10	ordinary_muscle_development	ENSGALG00010027210	ENSGALG00010027210	-0.646295521	0.258623117	-2.498985892	0.012454927	0.028894713	negative
PRJNA729271	stage_E14_vs_E10	ordinary_muscle_development	ENSGALG00010027211	HDGFRP2	-0.045630811	0.100905562	-0.452213042	0.651115513	0.742216307	negative
PRJNA729271	stage_E14_vs_E10	ordinary_muscle_development	ENSGALG00010027212	IRF6	-1.703092527	0.535024316	-3.183205837	0.00145654	0.004403035	negative
PRJNA729271	stage_E14_vs_E10	ordinary_muscle_development	ENSGALG00010027213	KCNC4	-1.316087073	0.368101293	-3.575339447	0.000349774	0.00123357	negative
PRJNA729271	stage_E14_vs_E10	ordinary_muscle_development	ENSGALG00010027214	PLIN4	0.829292009	0.266143421	3.115959077	0.001833477	0.005401677	positive
PRJNA729271	stage_E14_vs_E10	ordinary_muscle_development	ENSGALG00010027215	ENSGALG00010027215	-1.061204108	0.18310756	-5.795523169	6.81085E-09	5.06014E-08	negative
PRJNA729271	stage_E14_vs_E10	ordinary_muscle_development	ENSGALG00010027216	ENSGALG00010027216	1.126969665	0.571102477	1.97332302	0.048458772	0.092434517	positive
PRJNA729271	stage_E14_vs_E10	ordinary_muscle_development	ENSGALG00010027217	SEMA6B	2.079281202	0.219705376	9.463952301	2.96519E-21	6.98868E-20	positive
PRJNA729271	stage_E14_vs_E10	ordinary_muscle_development	ENSGALG00010027218	IARS1	-0.085586292	0.164292709	-0.520937861	0.602410059	0.700782454	negative
PRJNA729271	stage_E14_vs_E10	ordinary_muscle_development	ENSGALG00010027219	ENSGALG00010027219	0.031354665	0.137465015	0.228091964	0.819574747	0.873638473	positive
PRJNA729271	stage_E14_vs_E10	ordinary_muscle_development	ENSGALG00010027220	ANKRD24	-1.396129649	0.579199848	-2.41044547	0.015933053	0.035745179	negative
PRJNA729271	stage_E14_vs_E10	ordinary_muscle_development	ENSGALG00010027221	LRRC61	-1.223507752	0.276913187	-4.418380234	9.94434E-06	4.66634E-05	negative
PRJNA729271	stage_E14_vs_E10	ordinary_muscle_development	ENSGALG00010027222	GUCA1B	3.046707733	0.83358654	3.654938732	0.000257243	0.000931255	positive
PRJNA729271	stage_E14_vs_E10	ordinary_muscle_development	ENSGALG00010027223	SIRT6	0.101272507	0.08105143	1.24948452	0.211487912	0.312541531	positive
PRJNA729271	stage_E14_vs_E10	ordinary_muscle_development	ENSGALG00010027224	RARRES2	0.172008754	0.121163707	1.419639256	0.155712731	0.244640801	positive
PRJNA729271	stage_E14_vs_E10	ordinary_muscle_development	ENSGALG00010027225	CREB3L3	0.000492917	0.707060439	0.000697136	0.999443766	0.999503638	positive
PRJNA729271	stage_E14_vs_E10	ordinary_muscle_development	ENSGALG00010027227	ENSGALG00010027227	0.237141271	0.183712296	1.29082961	0.196762768	0.295245996	positive
PRJNA729271	stage_E14_vs_E10	ordinary_muscle_development	ENSGALG00010027229	MDM4	0.273077705	0.079328258	3.442376189	0.000576628	0.001924084	positive
PRJNA729271	stage_E14_vs_E10	ordinary_muscle_development	ENSGALG00010027230	MAP2K2	0.42073637	0.079267708	5.307790304	1.10962E-07	7.02664E-07	positive
PRJNA729271	stage_E14_vs_E10	ordinary_muscle_development	ENSGALG00010027231	ENSGALG00010027231	-0.661916422	0.155052602	-4.268979775	1.96369E-05	8.75267E-05	negative
PRJNA729271	stage_E14_vs_E10	ordinary_muscle_development	ENSGALG00010027232	ARRDC2	0.431737428	0.328402796	1.314658199	0.188624801	0.285324953	positive
PRJNA729271	stage_E14_vs_E10	ordinary_muscle_development	ENSGALG00010027233	ENSGALG00010027233	0.608253735	0.26217923	2.319992073	0.020341306	0.044138807	positive
PRJNA729271	stage_E14_vs_E10	ordinary_muscle_development	ENSGALG00010027234	SLC6A11	-4.202446521	0.472194007	-8.899830287	5.59321E-19	1.10154E-17	negative
PRJNA729271	stage_E14_vs_E10	ordinary_muscle_development	ENSGALG00010027236	PEX11G	-0.286462332	0.280909934	-1.019765756	0.307839567	0.421433182	negative
PRJNA729271	stage_E14_vs_E10	ordinary_muscle_development	ENSGALG00010027237	CTC-575C13.4	0.878868024	0.145845644	6.026014915	1.68052E-09	1.34284E-08	positive
PRJNA729271	stage_E14_vs_E10	ordinary_muscle_development	ENSGALG00010027239	ENSGALG00010027239	0.255679653	0.080587309	3.172703711	0.001510266	0.004548232	positive
PRJNA729271	stage_E14_vs_E10	ordinary_muscle_development	ENSGALG00010027240	SLC6A1	-1.855665868	0.364522339	-5.090678039	3.56785E-07	2.10914E-06	negative
PRJNA729271	stage_E14_vs_E10	ordinary_muscle_development	ENSGALG00010027241	LRRN2	-1.906359391	0.264455979	-7.208607648	5.65269E-13	6.63985E-12	negative
PRJNA729271	stage_E14_vs_E10	ordinary_muscle_development	ENSGALG00010027242	ENSGALG00010027242	0.050440089	0.187382566	0.269182399	0.787789324	0.850712098	positive
PRJNA729271	stage_E14_vs_E10	ordinary_muscle_development	ENSGALG00010027243	HRH1	0.106260593	0.197909126	0.536916084	0.59132558	0.691342498	positive
PRJNA729271	stage_E14_vs_E10	ordinary_muscle_development	ENSGALG00010027244	ARHGEF18	-0.049231647	0.089740255	-0.548601594	0.583278892	0.684252795	negative
PRJNA729271	stage_E14_vs_E10	ordinary_muscle_development	ENSGALG00010027245	LRIG2	-0.065813051	0.065269471	-1.008328242	0.313296909	0.427129298	negative
PRJNA729271	stage_E14_vs_E10	ordinary_muscle_development	ENSGALG00010027246	ZNF767	-0.232559767	0.095984351	-2.422892516	0.015397482	0.034699393	negative
PRJNA729271	stage_E14_vs_E10	ordinary_muscle_development	ENSGALG00010027247	ATG7	-0.002136225	0.077904363	-0.027421125	0.97812385	0.985397915	negative
PRJNA729271	stage_E14_vs_E10	ordinary_muscle_development	ENSGALG00010027248	ENSGALG00010027248	0.769543092	0.514295184	1.496306239	0.13457387	0.217269798	positive
PRJNA729271	stage_E14_vs_E10	ordinary_muscle_development	ENSGALG00010027249	C2CD4C	3.849826142	1.027954682	3.745132162	0.000180299	0.000676179	positive
PRJNA729271	stage_E14_vs_E10	ordinary_muscle_development	ENSGALG00010027250	ENSGALG00010027250	-0.129527204	0.166320374	-0.778781344	0.436108529	0.550103688	negative
PRJNA729271	stage_E14_vs_E10	ordinary_muscle_development	ENSGALG00010027251	ENSGALG00010027251	0.079137367	0.197201708	0.40130163	0.688198064	0.772271498	positive
PRJNA729271	stage_E14_vs_E10	ordinary_muscle_development	ENSGALG00010027252	MAGI3	0.463868995	0.070638603	6.566791736	5.14109E-11	4.91557E-10	positive
PRJNA729271	stage_E14_vs_E10	ordinary_muscle_development	ENSGALG00010027253	VGLL4	-0.203238853	0.086803971	-2.341354327	0.019213922	0.041968928	negative
PRJNA729271	stage_E14_vs_E10	ordinary_muscle_development	ENSGALG00010027254	INSR	0.182859354	0.133246453	1.372339372	0.169957814	0.262329733	positive
PRJNA729271	stage_E14_vs_E10	ordinary_muscle_development	ENSGALG00010027255	ENSGALG00010027255	0.098418041	0.176186212	0.558602402	0.5764331	0.678252925	positive
PRJNA729271	stage_E14_vs_E10	ordinary_muscle_development	ENSGALG00010027256	PHTF1	-0.466208617	0.082080821	-5.679872703	1.34795E-08	9.6582E-08	negative
PRJNA729271	stage_E14_vs_E10	ordinary_muscle_development	ENSGALG00010027257	TAFA3	0.260980793	0.284078612	0.918692159	0.358256612	0.474630253	positive
PRJNA729271	stage_E14_vs_E10	ordinary_muscle_development	ENSGALG00010027258	ENSGALG00010027258	-0.577309518	0.163513426	-3.530655135	0.000414532	0.001430628	negative
PRJNA729271	stage_E14_vs_E10	ordinary_muscle_development	ENSGALG00010027259	ENSGALG00010027259	-0.037563127	0.079625042	-0.471750169	0.637105123	0.73043097	negative
PRJNA729271	stage_E14_vs_E10	ordinary_muscle_development	ENSGALG00010027260	LEMD2	0.048872346	0.100355023	0.486994518	0.626262216	0.721096288	positive
PRJNA729271	stage_E14_vs_E10	ordinary_muscle_development	ENSGALG00010027261	ENSGALG00010027261	-0.027001236	0.107898175	-0.250247379	0.802396047	0.860897906	negative
PRJNA729271	stage_E14_vs_E10	ordinary_muscle_development	ENSGALG00010027262	USE1	-0.104072465	0.133689182	-0.778465867	0.436294421	0.550159864	negative
PRJNA729271	stage_E14_vs_E10	ordinary_muscle_development	ENSGALG00010027263	TAMM41	-0.664314938	0.12385498	-5.363651411	8.15562E-08	5.26808E-07	negative
PRJNA729271	stage_E14_vs_E10	ordinary_muscle_development	ENSGALG00010027264	ENSGALG00010027264	0.360607355	0.093902496	3.840231834	0.000122918	0.0004769	positive
PRJNA729271	stage_E14_vs_E10	ordinary_muscle_development	ENSGALG00010027265	IP6K3	2.288189278	0.20847022	10.97609661	4.97979E-28	1.88958E-26	positive
PRJNA729271	stage_E14_vs_E10	ordinary_muscle_development	ENSGALG00010027267	MYO9B	-0.539574299	0.095221042	-5.666544809	1.45706E-08	1.04091E-07	negative
PRJNA729271	stage_E14_vs_E10	ordinary_muscle_development	ENSGALG00010027268	UQCRC1	0.567043383	0.126615473	4.478468318	7.51805E-06	3.60007E-05	positive
PRJNA729271	stage_E14_vs_E10	ordinary_muscle_development	ENSGALG00010027269	CDC25A	-0.268541431	0.084590783	-3.17459447	0.00150046	0.004520718	negative
PRJNA729271	stage_E14_vs_E10	ordinary_muscle_development	ENSGALG00010027270	ENSGALG00010027270	-1.39278672	0.389869533	-3.572443096	0.000353666	0.001246085	negative
PRJNA729271	stage_E14_vs_E10	ordinary_muscle_development	ENSGALG00010027271	INKA2	1.887503807	0.139457936	13.53457438	9.77469E-42	8.33436E-40	positive
PRJNA729271	stage_E14_vs_E10	ordinary_muscle_development	ENSGALG00010027272	DDX20	-0.240234557	0.101375333	-2.369753558	0.017799945	0.03932483	negative
PRJNA729271	stage_E14_vs_E10	ordinary_muscle_development	ENSGALG00010027273	HAUS8	-0.394168327	0.127027268	-3.103021362	0.001915558	0.005611563	negative
PRJNA729271	stage_E14_vs_E10	ordinary_muscle_development	ENSGALG00010027274	COPG1	-0.129225185	0.060042108	-2.152242661	0.031378247	0.063719206	negative
PRJNA729271	stage_E14_vs_E10	ordinary_muscle_development	ENSGALG00010027275	CPAMD8	-0.680781052	0.227366309	-2.994203737	0.002751621	0.007696787	negative
PRJNA729271	stage_E14_vs_E10	ordinary_muscle_development	ENSGALG00010027276	ENSGALG00010027276	0.450492435	0.138052435	3.26319803	0.001101625	0.003433991	positive
PRJNA729271	stage_E14_vs_E10	ordinary_muscle_development	ENSGALG00010027277	KCND3	1.233672344	0.388239409	3.177607205	0.001484958	0.004480529	positive
PRJNA729271	stage_E14_vs_E10	ordinary_muscle_development	ENSGALG00010027278	H1-10	0.020332012	0.128982589	0.157633771	0.874745391	0.914162541	positive
PRJNA729271	stage_E14_vs_E10	ordinary_muscle_development	ENSGALG00010027279	F2RL3	-0.243085027	0.366438528	-0.663371914	0.507092389	0.615964484	negative
PRJNA729271	stage_E14_vs_E10	ordinary_muscle_development	ENSGALG00010027280	HMCES	-0.218347534	0.123355746	-1.770063748	0.076716522	0.136393965	negative
PRJNA729271	stage_E14_vs_E10	ordinary_muscle_development	ENSGALG00010027281	ENSGALG00010027281	0.460679937	0.237699018	1.938080943	0.052613348	0.09888011	positive

PRJNA729271	stage_E14_vs_E10	ordinary_muscle_development	ENSGALG00010027282	ENSGALG00010027282	0.162928602	0.12948629	1.258269136	0.208294439	0.308777654	positive
PRJNA729271	stage_E14_vs_E10	ordinary_muscle_development	ENSGALG00010027283	RAB7A	0.275753992	0.062468637	4.414279012	1.01347E-05	4.74952E-05	positive
PRJNA729271	stage_E14_vs_E10	ordinary_muscle_development	ENSGALG00010027284	CTTNBP2NL	0.123660255	0.090598514	1.364925864	0.172276352	0.265202392	positive
PRJNA729271	stage_E14_vs_E10	ordinary_muscle_development	ENSGALG00010027285	PTPRS	-0.445517322	0.111792108	-3.985230548	6.74146E-05	0.000274179	negative
PRJNA729271	stage_E14_vs_E10	ordinary_muscle_development	ENSGALG00010027286	RPN1	0.061457966	0.06265986	0.980818757	0.326682127	0.441515844	positive
PRJNA729271	stage_E14_vs_E10	ordinary_muscle_development	ENSGALG00010027287	DHX30	-0.173650403	0.088596914	-1.960005092	0.049995195	0.094766621	negative
PRJNA729271	stage_E14_vs_E10	ordinary_muscle_development	ENSGALG00010027288	DAG1	1.535352775	0.16764547	9.158331407	5.27059E-20	1.12893E-18	positive
PRJNA729271	stage_E14_vs_E10	ordinary_muscle_development	ENSGALG00010027289	SMARCC1	-0.083521968	0.138712965	-0.602120847	0.547093698	0.652470229	negative
PRJNA729271	stage_E14_vs_E10	ordinary_muscle_development	ENSGALG00010027290	CSPG5	-0.631839352	0.19266625	-3.279450098	0.001040096	0.003268905	negative
PRJNA729271	stage_E14_vs_E10	ordinary_muscle_development	ENSGALG00010027291	MST1	-0.546073146	0.281623823	-1.93901617	0.052499369	0.098751409	negative
PRJNA729271	stage_E14_vs_E10	ordinary_muscle_development	ENSGALG00010027292	KDM4B	0.163961903	0.059873362	2.73847829	0.006172424	0.015636443	positive
PRJNA729271	stage_E14_vs_E10	ordinary_muscle_development	ENSGALG00010027293	ELP6	0.02534191	0.146229202	0.173302662	0.862413531	0.90532065	positive
PRJNA729271	stage_E14_vs_E10	ordinary_muscle_development	ENSGALG00010027295	ENSGALG00010027295	1.102417845	0.243644898	4.524690869	6.04838E-06	2.94595E-05	positive
PRJNA729271	stage_E14_vs_E10	ordinary_muscle_development	ENSGALG00010027297	ENSGALG00010027297	2.051606707	2.077712702	0.987435224	0.323429324	0.438059059	positive
PRJNA729271	stage_E14_vs_E10	ordinary_muscle_development	ENSGALG00010027299	PGPEP1	0.146965679	0.161955605	0.90744423	0.364171929	0.480315933	positive
PRJNA729271	stage_E14_vs_E10	ordinary_muscle_development	ENSGALG00010027301	SCAP	0.072763804	0.062703644	1.160439787	0.245869796	0.3525804	positive
PRJNA729271	stage_E14_vs_E10	ordinary_muscle_development	ENSGALG00010027303	APEH	0.115378889	0.13849648	0.833081744	0.404798629	0.519666648	positive
PRJNA729271	stage_E14_vs_E10	ordinary_muscle_development	ENSGALG00010027304	PTPN23	-0.019907196	0.138583842	-0.143647309	0.885778981	0.921904669	negative
PRJNA729271	stage_E14_vs_E10	ordinary_muscle_development	ENSGALG00010027305	LSM4	-0.05763355	0.128876728	-0.447199048	0.654731349	0.745447011	negative
PRJNA729271	stage_E14_vs_E10	ordinary_muscle_development	ENSGALG00010027306	ENSGALG00010027306	0.337591719	0.337704986	0.999664599	0.317472849	0.431321488	positive
PRJNA729271	stage_E14_vs_E10	ordinary_muscle_development	ENSGALG00010027307	JUND	0.434801105	0.176057188	2.469658355	0.013524215	0.031037601	positive
PRJNA729271	stage_E14_vs_E10	ordinary_muscle_development	ENSGALG00010027309	ENSGALG00010027309	1.027802063	0.70192713	1.464257497	0.143123606	0.228427292	positive
PRJNA729271	stage_E14_vs_E10	ordinary_muscle_development	ENSGALG00010027310	ARHGEF2	0.180519655	0.130081176	1.387746182	0.165214348	0.256345748	positive
PRJNA729271	stage_E14_vs_E10	ordinary_muscle_development	ENSGALG00010027311	NFASC	0.603237087	0.241888475	2.493864528	0.012636076	0.029249429	positive
PRJNA729271	stage_E14_vs_E10	ordinary_muscle_development	ENSGALG00010027312	ENSGALG00010027312	-0.008324914	0.749114139	-0.011113011	0.991133283	0.994357484	negative
PRJNA729271	stage_E14_vs_E10	ordinary_muscle_development	ENSGALG00010027313	BSN	-0.453654794	0.226631538	-2.001728433	0.045313947	0.087321135	negative
PRJNA729271	stage_E14_vs_E10	ordinary_muscle_development	ENSGALG00010027314	ENSGALG00010027314	-0.732711553	0.382164814	-1.917265866	0.055204164	0.103036873	negative
PRJNA729271	stage_E14_vs_E10	ordinary_muscle_development	ENSGALG00010027315	RAB3A	-0.615510656	0.229561564	-2.681244393	0.007334893	0.018155613	negative
PRJNA729271	stage_E14_vs_E10	ordinary_muscle_development	ENSGALG00010027316	TRAIP	-0.072483148	0.203704268	-0.355825375	0.721971345	0.798609176	negative
PRJNA729271	stage_E14_vs_E10	ordinary_muscle_development	ENSGALG00010027317	MPV17L2	0.290811446	0.145701654	1.995937841	0.045940689	0.088422559	positive
PRJNA729271	stage_E14_vs_E10	ordinary_muscle_development	ENSGALG00010027319	MST1R	0.654396203	0.336147233	1.946754694	0.051564151	0.097333294	positive
PRJNA729271	stage_E14_vs_E10	ordinary_muscle_development	ENSGALG00010027320	MON1A	0.110964663	0.160965601	0.689368796	0.490591215	0.601060681	positive
PRJNA729271	stage_E14_vs_E10	ordinary_muscle_development	ENSGALG00010027321	IFI30	0.463220422	0.205183292	2.257593288	0.023971028	0.050932284	positive
PRJNA729271	stage_E14_vs_E10	ordinary_muscle_development	ENSGALG00010027322	CRABP2	-1.800415447	0.172775664	-10.42053845	1.99819E-25	6.22876E-24	negative
PRJNA729271	stage_E14_vs_E10	ordinary_muscle_development	ENSGALG00010027323	ISG20L2	-0.200114682	0.127415316	-1.570570078	0.116282545	0.192718441	negative
PRJNA729271	stage_E14_vs_E10	ordinary_muscle_development	ENSGALG00010027324	PIK3R2	-0.096780719	0.096712967	-1.000700547	0.316971603	0.430842403	negative
PRJNA729271	stage_E14_vs_E10	ordinary_muscle_development	ENSGALG00010027325	METTL25B	0.101232985	0.287127624	0.35257139	0.724409789	0.800390265	positive
PRJNA729271	stage_E14_vs_E10	ordinary_muscle_development	ENSGALG00010027326	MRPL24	-0.016491605	0.117628588	-0.140200653	0.888501456	0.92354454	negative
PRJNA729271	stage_E14_vs_E10	ordinary_muscle_development	ENSGALG00010027327	RBM6	-0.017181217	0.08744804	-0.196473441	0.844239618	0.891544378	negative
PRJNA729271	stage_E14_vs_E10	ordinary_muscle_development	ENSGALG00010027328	RBM15	-0.105158727	0.130444615	-0.806156137	0.420152826	0.534595788	negative
PRJNA729271	stage_E14_vs_E10	ordinary_muscle_development	ENSGALG00010027329	MINDY4	-0.514211022	0.115162087	-4.465106837	8.00288E-06	3.81334E-05	negative
PRJNA729271	stage_E14_vs_E10	ordinary_muscle_development	ENSGALG00010027330	ENSGALG00010027330	0.958602523	0.188816088	5.076911266	3.8362E-07	2.25398E-06	positive
PRJNA729271	stage_E14_vs_E10	ordinary_muscle_development	ENSGALG00010027331	SLC16A4	0.373415363	0.161200898	2.316459566	0.02053319	0.044488578	positive
PRJNA729271	stage_E14_vs_E10	ordinary_muscle_development	ENSGALG00010027332	HDGF	-0.029928478	0.176751694	-0.169324983	0.865541026	0.907553325	negative
PRJNA729271	stage_E14_vs_E10	ordinary_muscle_development	ENSGALG00010027333	PROK1	0.373040643	0.269350584	1.384963187	0.166063724	0.257326206	positive
PRJNA729271	stage_E14_vs_E10	ordinary_muscle_development	ENSGALG00010027334	PRCC	-0.14187048	0.113504067	-1.249915391	0.211330456	0.312383741	negative
PRJNA729271	stage_E14_vs_E10	ordinary_muscle_development	ENSGALG00010027335	MAST3	-0.948545495	0.179564251	-5.282485173	1.27443E-07	7.97965E-07	negative
PRJNA729271	stage_E14_vs_E10	ordinary_muscle_development	ENSGALG00010027336	RAD54L2	0.013868567	0.136994312	0.101234621	0.919364214	0.94417584	positive
PRJNA729271	stage_E14_vs_E10	ordinary_muscle_development	ENSGALG00010027337	AQP1	0.986960217	0.265178182	3.721875637	0.000197748	0.00073572	positive
PRJNA729271	stage_E14_vs_E10	ordinary_muscle_development	ENSGALG00010027339	LAMTOR5	0.459697312	0.106461606	4.317963334	1.57476E-05	7.15103E-05	positive
PRJNA729271	stage_E14_vs_E10	ordinary_muscle_development	ENSGALG00010027340	ENSGALG00010027340	-1.794709278	1.09211113	-1.643339427	0.100312725	0.17050105	negative
PRJNA729271	stage_E14_vs_E10	ordinary_muscle_development	ENSGALG00010027341	ENSGALG00010027341	0.122714801	0.69549589	0.176442165	0.859946581	0.903648376	positive
PRJNA729271	stage_E14_vs_E10	ordinary_muscle_development	ENSGALG00010027346	SIN3B	0.029426319	0.088481719	0.332569476	0.739459287	0.812658722	positive
PRJNA729271	stage_E14_vs_E10	ordinary_muscle_development	ENSGALG00010027348	ENSGALG00010027348	0.298941338	0.205060197	1.45782235	0.144889516	0.230737589	positive
PRJNA729271	stage_E14_vs_E10	ordinary_muscle_development	ENSGALG00010027350	INSRR	0.805059166	0.350080516	2.299640027	0.021468622	0.046287027	positive
PRJNA729271	stage_E14_vs_E10	ordinary_muscle_development	ENSGALG00010027351	TMEM38A	1.285974427	0.148170736	8.679004101	3.99249E-18	7.37213E-17	positive
PRJNA729271	stage_E14_vs_E10	ordinary_muscle_development	ENSGALG00010027354	ENSGALG00010027354	1.558533888	0.884224562	1.762599632	0.077968039	0.138311924	positive
PRJNA729271	stage_E14_vs_E10	ordinary_muscle_development	ENSGALG00010027355	ADCYAP1R1	-0.814328689	0.240169877	-3.390636245	0.000697306	0.002282106	negative
PRJNA729271	stage_E14_vs_E10	ordinary_muscle_development	ENSGALG00010027356	ENSGALG00010027356	-0.291609247	0.225086108	-1.295545291	0.195132188	0.293224245	negative
PRJNA729271	stage_E14_vs_E10	ordinary_muscle_development	ENSGALG00010027358	RNF126	0.219837914	0.098550273	2.23071848	0.025699783	0.054018997	positive
PRJNA729271	stage_E14_vs_E10	ordinary_muscle_development	ENSGALG00010027359	ENSGALG00010027359	0.593885158	0.116524256	5.096665541	3.45688E-07	2.04688E-06	positive
PRJNA729271	stage_E14_vs_E10	ordinary_muscle_development	ENSGALG00010027360	SMIM7	0.24919081	0.113421064	2.197041727	0.028017466	0.058043074	positive
PRJNA729271	stage_E14_vs_E10	ordinary_muscle_development	ENSGALG00010027361	FANCD2	0.216848891	0.192147961	1.128551611	0.259087033	0.367498439	positive
PRJNA729271	stage_E14_vs_E10	ordinary_muscle_development	ENSGALG00010027362	ENSGALG00010027362	0.460900056	0.144299201	3.194058268	0.001402878	0.004259471	positive
PRJNA729271	stage_E14_vs_E10	ordinary_muscle_development	ENSGALG00010027363	VIPR1	-1.129298167	0.259980028	-4.343788154	1.40047E-05	6.41788E-05	negative
PRJNA729271	stage_E14_vs_E10	ordinary_muscle_development	ENSGALG00010027364	FSTL3	0.208578253	0.239336609	0.871484949	0.38348942	0.499566743	positive
PRJNA729271	stage_E14_vs_E10	ordinary_muscle_development	ENSGALG00010027365	PLEKHA6	-0.528706173	0.181303903	-2.916132323	0.003544002	0.009608911	negative
PRJNA729271	stage_E14_vs_E10	ordinary_muscle_development	ENSGALG00010027366	USP4	0.206063194	0.082083228	2.510417774	0.01205884	0.028097233	positive
PRJNA729271	stage_E14_vs_E10	ordinary_muscle_development	ENSGALG00010027367	SEC22C	-0.262705303	0.092565071	-2.838060839	0.004538853	0.011959766	negative
PRJNA729271	stage_E14_vs_E10	ordinary_muscle_development	ENSGALG00010027368	EMC3	0.222866155	0.088593147	2.515613936	0.011882526	0.027726532	positive
PRJNA729271	stage_E14_vs_E10	ordinary_muscle_development	ENSGALG00010027369	ENSGALG00010027369	0.226151794	0.101520958	2.22763651	0.025904764	0.054335563	positive
PRJNA729271	stage_E14_vs_E10	ordinary_muscle_development	ENSGALG00010027370	NUP210L	0.154324929	0.310899885	0.496381428	0.619625325	0.715253439	positive
PRJNA729271	stage_E14_vs_E10	ordinary_muscle_development	ENSGALG00010027372	PIK3C2B	0.265009229	0.171871206	1.541905918	0.123096458	0.201643921	positive
PRJNA729271	stage_E14_vs_E10	ordinary_muscle_development	ENSGALG00010027373	MLN	2.267297811	1.187217126	1.909758342	0.056164335	0.104586593	positive
PRJNA729271	stage_E14_vs_E10	ordinary_muscle_development	ENSGALG00010027374	NKTR	-0.172927054	0.118259516	-1.462267561	0.143667904	0.229144616	negative
PRJNA729271	stage_E14_vs_E10	ordinary_muscle_development	ENSGALG00010027375	FUT7	0.575143697	0.430535098	1.335881094	0.181588142	0.276744833	positive
PRJNA729271	stage_E14_vs_E10	ordinary_muscle_development	ENSGALG00010027376	DSTYK	-0.057219885	0.079372818	-0.720900262	0.470970882	0.583224783	negative
PRJNA729271	stage_E14_vs_E10	ordinary_muscle_development	ENSGALG00010027378	ZBTB47	-0.069649688	0.164330495	-0.423839092	0.671683166	0.758333996	negative
PRJNA729271	stage_E14_vs_E10	ordinary_muscle_development	ENSGALG00010027379	OPTC	0.427546324	0.594992559	0.718574237	0.472403292	0.584468549	positive
PRJNA729271	stage_E14_vs_E10	ordinary_muscle_development	ENSGALG00010027380	GPX1	-0.190754557	0.132352562	-1.441260784	0.149511022	0.236770705	negative
PRJNA729271	stage_E14_vs_E10	ordinary_muscle_development	ENSGALG00010027381	KLHL40	1.934267941	0.196739365	9.831626414	8.22791E-23	2.14118E-21	positive
PRJNA729271	stage_E14_vs_E10	ordinary_muscle_development	ENSGALG00010027382	PALM	-0.610157589	0.201083783	-3.034345082	0.002410585	0.006835179	negative
PRJNA729271	stage_E14_vs_E10	ordinary_muscle_development	ENSGALG00010027383	ASS1	1.146346584	0.284298858	4.032188496	5.52598E-05	0.000228008	positive
PRJNA729271	stage_E14_vs_E10	ordinary_muscle_development	ENSGALG00010027384	HHATL	2.129620176	0.218351506	9.753173737	1.7879E-22	4.61131E-21	positive
PRJNA729271	stage_E14_vs_E10	ordinary_muscle_development	ENSGALG00010027385	CNTN2	-1.891863537	0.911599352	-2.075323477	0.037956565	0.074936041	negative
PRJNA729271	stage_E14_vs_E10	ordinary_muscle_development	ENSGALG00010027386	RHOA	-0.041331116	0.065850963	-0.627646346	0.530235641	0.637769946	negative
PRJNA729271	stage_E14_vs_E10	ordinary_muscle_development	ENSGALG00010027387	ENSGALG00010027387	-0.459869093	0.244639461	-1.879782967	0.060137663	0.110789962	negative
PRJNA729271	stage_E14_vs_E10	ordinary_muscle_development	ENSGALG00010027388	URM1	0.514239446	0.143683974	3.578961737	0.000344962	0.001218675	positive
PRJNA729271	stage_E14_vs_E10	ordinary_muscle_development	ENSGALG00010027391	TPM3	-0.288532517	0.114279609	-2.524794394	0.0115766	0.027104315	negative
PRJNA729271	stage_E14_vs_E10	ordinary_muscle_development	ENSGALG00010027392	RBBP5	0.019324869	0.126268137	0.153046283	0.878361777	0.916428798	positive
PRJNA729271	stage_E14_vs_E10	ordinary_muscle_development	ENSGALG00010027393	TEX264	0.070813549	0.189824613	0.373047244	0.709113282	0.789045034	positive
PRJNA729271	stage_E14_vs_E10	ordinary_muscle_development	ENSGALG00010027394	SLC27A4	0.211672334	0.085180983	2.484971719	0.012956173	0.029909177	positive
PRJNA729271	stage_E14_vs_E10	ordinary_muscle_development	ENSGALG00010027395	BCL2L15	-0.206385125	0.26116535	-0.790246964	0.429383553	0.543620806	negative
PRJNA729271	stage_E14_vs_E10	ordinary_muscle_development	ENSGALG00010027397	RFX5	0.615413788	0.123140638	4.997649826	5.80332E-07	3.30657E-06	positive
PRJNA729271	stage_E14_vs_E10	ordinary_muscle_development	ENSGALG00010027399	MAPKAP1	0.062785732	0.11041936	0.568611628	0.569619732	0.671817576	positive
PRJNA729271	stage_E14_vs_E10	ordinary_muscle_development	ENSGALG00010027401	ENSGALG00010027401	0.489043536	0.314751079	1.553746972	0.120244784	0.197904865	positive
PRJNA729271	stage_E14_vs_E10	ordinary_muscle_development	ENSGALG00010027402	AP4B1	0.089033985	0.085352919	1.043127594	0.296889202	0.410200075	positive
PRJNA729271	stage_E14_vs_E10	ordinary_muscle_development	ENSGALG00010027403	ACKR2	-0.170457062	0.373661531	-0.456180387	0.648260268	0.740117564	negative
PRJNA729271	stage_E14_vs_E10	ordinary_muscle_development	ENSGALG00010027405	DCLRE1B	0.031697903	0.162096308	0.195549816	0.844962542	0.89205925	positive
PRJNA729271	stage_E14_vs_E10	ordinary_muscle_development	ENSGALG00010027407	ENSGALG00010027407	7.209161462	0.80513145	8.954017963	3.42773E-19	6.91029E-18	positive
PRJNA729271	stage_E14_vs_E10	ordinary_muscle_development	ENSGALG00010027410	ALAS1	0.021564107	0.087245428	0.247166044	0.804779723	0.862816723	positive
PRJNA729271	stage_E14_vs_E10	ordinary_muscle_development	ENSGALG00010027412	ENSGALG00010027412	1.021751304	0.270251938	3.780736273	0.000156365	0.000593484	positive
PRJNA729271	stage_E14_vs_E10	ordinary_muscle_development	ENSGALG00010027414	PBX3	-0.766144226	0.070154194	-10.92086141	9.16227E-28	3.3966E-26	negative
PRJNA729271	stage_E14_vs_E10	ordinary_muscle_development	ENSGALG00010027417	WNT2B	-1.933363911	0.363271796	-5.322086479	1.02584E-07	6.52744E-07	negative
PRJNA729271	stage_E14_vs_E10	ordinary_muscle_development	ENSGALG00010027418	ENSGALG00010027418	5.46201603	0.81671696	6.687771036	2.26595E-11	2.23892E-10	positive
PRJNA729271	stage_E14_vs_E10	ordinary_muscle_development	ENSGALG00010027419	PTBP1	-0.62478975	0.084014042	-7.436730044	1.03208E-13	1.30427E-12	negative
PRJNA729271	stage_E14_vs_E10	ordinary_muscle_development	ENSGALG00010027421	PLPPR3	0.017637865	0.225913626	0.078073488	0.937769596	0.956514076	positive
PRJNA729271	stage_E14_vs_E10	ordinary_muscle_development	ENSGALG00010027423	PEX11B	0.284376955	0.213559356	1.331606169	0.182989637	0.278434396	positive
PRJNA729271	stage_E14_vs_E10	ordinary_muscle_development	ENSGALG00010027425	CFD	2.349672008	0.238871744	9.836542289	7.83577E-23	2.0428E-21	positive
PRJNA729271	stage_E14_vs_E10	ordinary_muscle_development	ENSGALG00010027427	UBAC1	0.785402903	0.115861427	6.778812602	1.21168E-11	1.23859E-10	positive
PRJNA729271	stage_E14_vs_E10	ordinary_muscle_development	ENSGALG00010027438	ST7L	0.038688508	0.082478374	0.469074579	0.639016329	0.731890505	positive
PRJNA729271	stage_E14_vs_E10	ordinary_muscle_development	ENSGALG00010027440	TWF2	0.473383783	0.190210618	2.488734798	0.012819855	0.02963697	positive
PRJNA729271	stage_E14_vs_E10	ordinary_muscle_development	ENSGALG00010027443	MED16	-0.00101378	0.155178509	-0.006532995	0.994787461	0.997333258	negative
PRJNA729271	stage_E14_vs_E10	ordinary_muscle_development	ENSGALG00010027444	SELENBP1	0.565216056	0.120821075	4.678124716	2.8951E-06	1.48547E-05	positive
PRJNA729271	stage_E14_vs_E10	ordinary_muscle_development	ENSGALG00010027445	PPM1M	0.60610622	0.162618821	3.727159104	0.00019365	0.000721955	positive
PRJNA729271	stage_E14_vs_E10	ordinary_muscle_development	ENSGALG00010027448	ADAMTSL5	1.107418906	0.318075452	3.481623303	0.000498384	0.00169103	positive
PRJNA729271	stage_E14_vs_E10	ordinary_muscle_development	ENSGALG00010027450	CAMSAP1	-0.008758856	0.14299451	-0.061253093	0.951157647	0.966087176	negative
PRJNA729271	stage_E14_vs_E10	ordinary_muscle_development	ENSGALG00010027455	R3HDM4	0.534790044	0.146991826	3.638229807	0.000274518	0.000989345	positive
PRJNA729271	stage_E14_vs_E10	ordinary_muscle_development	ENSGALG00010027457	CAPZA1	0.024808041	0.127836875	0.194060131	0.846128799	0.892882712	positive
PRJNA729271	stage_E14_vs_E10	ordinary_muscle_development	ENSGALG00010027460	WDR82	-0.223660924	0.072465908	-3.086429593	0.002025759	0.005877378	negative
PRJNA729271	stage_E14_vs_E10	ordinary_muscle_development	ENSGALG00010027467	GLYCTK	0.162927142	0.133204674	1.223133823	0.221279156	0.323951253	positive
PRJNA729271	stage_E14_vs_E10	ordinary_muscle_development	ENSGALG00010027468	BSG	0.157609658	0.115188412	1.368277024	0.17122538	0.263920872	positive
PRJNA729271	stage_E14_vs_E10	ordinary_muscle_development	ENSGALG00010027471	ENSGALG00010027471	0.563054614	0.129346559	4.353069924	1.34244E-05	6.16758E-05	positive
PRJNA729271	stage_E14_vs_E10	ordinary_muscle_development	ENSGALG00010027473	DNAH1	-1.035708096	0.330936288	-3.129629886	0.001750267	0.005180747	negative
PRJNA729271	stage_E14_vs_E10	ordinary_muscle_development	ENSGALG00010027475	ARID3A	0.085151897	0.162850765	0.522883	0.601055676	0.69993248	positive
PRJNA729271	stage_E14_vs_E10	ordinary_muscle_development	ENSGALG00010027479	HCN2	-0.119903634	0.258479662	-0.463880342	0.642733478	0.735079853	negative
PRJNA729271	stage_E14_vs_E10	ordinary_muscle_development	ENSGALG00010027487	MPZ	-0.429176096	0.60865188	-0.705125721	0.480732001	0.59268609	negative
PRJNA729271	stage_E14_vs_E10	ordinary_muscle_development	ENSGALG00010027490	ATP2B4	-0.152355236	0.067718083	-2.249845669	0.024458744	0.051763687	negative
PRJNA729271	stage_E14_vs_E10	ordinary_muscle_development	ENSGALG00010027492	PCP4L1	0.984837292	0.500961653	1.96589357	0.049310906	0.093813044	positive
PRJNA729271	stage_E14_vs_E10	ordinary_muscle_development	ENSGALG00010027497	KCNT1	3.961800101	0.337759757	11.72963924	8.98408E-32	4.18727E-30	positive
PRJNA729271	stage_E14_vs_E10	ordinary_muscle_development	ENSGALG00010027499	MOV10	-0.798667241	0.409023248	-1.952620652	0.050864562	0.096188106	negative

PRJNA729271	stage_E14_vs_E10	ordinary_muscle_development	ENSGALG00010027501	BAP1	0.05835121	0.065034152	0.897239493	0.369591135	0.485827832	positive
PRJNA729271	stage_E14_vs_E10	ordinary_muscle_development	ENSGALG00010027502	THOP1	-0.319137683	0.138928504	-2.297136115	0.021611006	0.046524808	negative
PRJNA729271	stage_E14_vs_E10	ordinary_muscle_development	ENSGALG00010027510	RHOC	0.187521646	0.194690592	0.963177746	0.335458331	0.450753013	positive
PRJNA729271	stage_E14_vs_E10	ordinary_muscle_development	ENSGALG00010027511	POLRMT	0.026100976	0.080782937	0.32310011	0.746619427	0.818317474	positive
PRJNA729271	stage_E14_vs_E10	ordinary_muscle_development	ENSGALG00010027513	SEMA3G	0.714744113	0.181631751	3.93512758	8.31525E-05	0.00033222	positive
PRJNA729271	stage_E14_vs_E10	ordinary_muscle_development	ENSGALG00010027516	PPM1J	-0.419327447	0.237561985	-1.76512857	0.07754216	0.137691242	negative
PRJNA729271	stage_E14_vs_E10	ordinary_muscle_development	ENSGALG00010027520	FANCE	0.332547067	0.153109923	2.171949802	0.029859449	0.061183591	positive
PRJNA729271	stage_E14_vs_E10	ordinary_muscle_development	ENSGALG00010027522	PSMD5	-0.034178106	0.104287212	-0.327730553	0.7431154	0.815648775	negative
PRJNA729271	stage_E14_vs_E10	ordinary_muscle_development	ENSGALG00010027524	ENSGALG00010027524	0.849744438	0.111045334	7.652229989	1.97524E-14	2.65594E-13	positive
PRJNA729271	stage_E14_vs_E10	ordinary_muscle_development	ENSGALG00010027525	ENSGALG00010027525	2.170144923	0.268121916	8.093873705	5.77966E-16	8.84648E-15	positive
PRJNA729271	stage_E14_vs_E10	ordinary_muscle_development	ENSGALG00010027527	ENSGALG00010027527	-0.940978074	0.476971704	-1.972817392	0.048516371	0.09253221	negative
PRJNA729271	stage_E14_vs_E10	ordinary_muscle_development	ENSGALG00010027528	ENSGALG00010027528	2.037138931	2.378127957	0.856614517			positive
PRJNA729271	stage_E14_vs_E10	ordinary_muscle_development	ENSGALG00010027529	ACY1	-0.317500649	0.143468576	-2.213032694	0.026895392	0.056109485	negative
PRJNA729271	stage_E14_vs_E10	ordinary_muscle_development	ENSGALG00010027530	PHF19	0.220299171	0.136559981	1.613204458	0.106700095	0.179776576	positive
PRJNA729271	stage_E14_vs_E10	ordinary_muscle_development	ENSGALG00010027531	UFC1	0.516172161	0.1637421	3.152348485	0.001619629	0.00483653	positive
PRJNA729271	stage_E14_vs_E10	ordinary_muscle_development	ENSGALG00010027533	WDR18	-0.353549436	0.089601907	-3.94578027	7.95405E-05	0.000318756	negative
PRJNA729271	stage_E14_vs_E10	ordinary_muscle_development	ENSGALG00010027534	KCNN3	1.074426248	0.184540148	5.822181562	5.80844E-09	4.36009E-08	positive
PRJNA729271	stage_E14_vs_E10	ordinary_muscle_development	ENSGALG00010027536	USP21	-0.207284086	0.310384765	-0.667829447	0.504242467	0.613427995	negative
PRJNA729271	stage_E14_vs_E10	ordinary_muscle_development	ENSGALG00010027537	SGTA	0.059447637	0.066357779	0.895865379	0.370324667	0.486593467	positive
PRJNA729271	stage_E14_vs_E10	ordinary_muscle_development	ENSGALG00010027538	ABHD14A	0.255519258	0.134803393	1.895495747	0.058026755	0.107470871	positive
PRJNA729271	stage_E14_vs_E10	ordinary_muscle_development	ENSGALG00010027539	DDR2L	0.167856762	0.107365548	1.563413639	0.117955334	0.195017476	positive
PRJNA729271	stage_E14_vs_E10	ordinary_muscle_development	ENSGALG00010027540	PPOX	0.014232505	0.111489183	0.127658167	0.898419497	0.930195615	positive
PRJNA729271	stage_E14_vs_E10	ordinary_muscle_development	ENSGALG00010027543	TUBB4B	0.343630448	0.151845842	2.263021782	0.023634347	0.050295466	positive
PRJNA729271	stage_E14_vs_E10	ordinary_muscle_development	ENSGALG00010027545	PPARD	0.367157333	0.20153127	1.821838033	0.068479565	0.123776207	positive
PRJNA729271	stage_E14_vs_E10	ordinary_muscle_development	ENSGALG00010027547	B4GALT3	0.085896166	0.223705694	0.383969509	0.701001046	0.782820506	positive
PRJNA729271	stage_E14_vs_E10	ordinary_muscle_development	ENSGALG00010027548	PCBP4	0.202533219	0.144898064	1.397763459	0.162184111	0.252753326	positive
PRJNA729271	stage_E14_vs_E10	ordinary_muscle_development	ENSGALG00010027553	FGF22	0.071302939	0.29462973	0.242008634	0.808773473	0.86549808	positive
PRJNA729271	stage_E14_vs_E10	ordinary_muscle_development	ENSGALG00010027554	ENSGALG00010027554	0.232470113	0.143397081	1.621163493	0.10498258	0.177294943	positive
PRJNA729271	stage_E14_vs_E10	ordinary_muscle_development	ENSGALG00010027555	ENSGALG00010027555	-0.355364608	0.369104965	-0.96277385	0.335661027	0.450918126	negative
PRJNA729271	stage_E14_vs_E10	ordinary_muscle_development	ENSGALG00010027557	C6orf132	0.277361426	0.768692252	0.360822455	0.718232175	0.796296113	positive
PRJNA729271	stage_E14_vs_E10	ordinary_muscle_development	ENSGALG00010027559	ENSGALG00010027559	-0.13153755	0.280068837	-0.469661499	0.638596879	0.731562615	negative
PRJNA729271	stage_E14_vs_E10	ordinary_muscle_development	ENSGALG00010027561	ADAMTS4	-0.012744662	0.350738006	-0.036336702	0.971013885	0.980006007	negative
PRJNA729271	stage_E14_vs_E10	ordinary_muscle_development	ENSGALG00010027562	SLC39A3	0.126637865	0.143861605	0.880275629	0.378710012	0.494866141	positive
PRJNA729271	stage_E14_vs_E10	ordinary_muscle_development	ENSGALG00010027563	WDR77	-0.300689564	0.121858658	-2.467527289	0.013604985	0.031193334	negative
PRJNA729271	stage_E14_vs_E10	ordinary_muscle_development	ENSGALG00010027567	PARP3	0.371972655	0.131750581	2.823309413	0.004753068	0.012463045	positive
PRJNA729271	stage_E14_vs_E10	ordinary_muscle_development	ENSGALG00010027568	NELFB	0.021205553	0.074763006	0.283636982	0.776688593	0.841788095	positive
PRJNA729271	stage_E14_vs_E10	ordinary_muscle_development	ENSGALG00010027569	ATP5PB	0.292392259	0.125346676	2.33266863	0.019665544	0.042813466	positive
PRJNA729271	stage_E14_vs_E10	ordinary_muscle_development	ENSGALG00010027570	NDUFS2	0.416995165	0.137442377	3.033963581	0.002413636	0.00684249	positive
PRJNA729271	stage_E14_vs_E10	ordinary_muscle_development	ENSGALG00010027571	RBM10	-0.047840926	0.101524405	-0.471225869	0.637479447	0.730686746	negative
PRJNA729271	stage_E14_vs_E10	ordinary_muscle_development	ENSGALG00010027574	GNG7	0.127095898	0.094674165	1.342455972	0.179448197	0.274175357	positive
PRJNA729271	stage_E14_vs_E10	ordinary_muscle_development	ENSGALG00010027575	FCER1G	0.607207584	0.339204898	1.790090849	0.073439308	0.131517515	positive
PRJNA729271	stage_E14_vs_E10	ordinary_muscle_development	ENSGALG00010027576	GADD45B	1.768995373	0.41681805	4.24404695	2.19524E-05	9.71012E-05	positive
PRJNA729271	stage_E14_vs_E10	ordinary_muscle_development	ENSGALG00010027578	ENSGALG00010027578	0.284066088	0.244594943	1.161373511	0.245490036	0.352210044	positive
PRJNA729271	stage_E14_vs_E10	ordinary_muscle_development	ENSGALG00010027579	C26H6ORF125	-0.095116199	0.157617543	-0.603462004	0.546201385	0.651674136	negative
PRJNA729271	stage_E14_vs_E10	ordinary_muscle_development	ENSGALG00010027583	GLE1	-0.258396365	0.06904183	-3.742605951	0.000182122	0.000682309	negative
PRJNA729271	stage_E14_vs_E10	ordinary_muscle_development	ENSGALG00010027586	HJV	2.371839212	0.291126324	8.147113529	3.72714E-16	5.79045E-15	positive
PRJNA729271	stage_E14_vs_E10	ordinary_muscle_development	ENSGALG00010027590	SEMA3F	-1.86085317	0.237208333	-7.844805227	4.33626E-15	6.13809E-14	negative
PRJNA729271	stage_E14_vs_E10	ordinary_muscle_development	ENSGALG00010027592	MAP1S	0.157024899	0.168755441	0.93048792	0.352118516	0.468467911	positive
PRJNA729271	stage_E14_vs_E10	ordinary_muscle_development	ENSGALG00010027593	ENSGALG00010027593	1.861210085	0.459667226	4.049038042	5.14286E-05	0.000213476	positive
PRJNA729271	stage_E14_vs_E10	ordinary_muscle_development	ENSGALG00010027594	ITPR3	-0.228500079	0.1384415	-1.650517215	0.098837195	0.168528113	negative
PRJNA729271	stage_E14_vs_E10	ordinary_muscle_development	ENSGALG00010027595	GNAT1	-0.086498179	0.425356093	-0.203354743	0.838857771	0.887536014	negative
PRJNA729271	stage_E14_vs_E10	ordinary_muscle_development	ENSGALG00010027597	EXOSC2	0.025304556	0.11490987	0.220212204	0.825705893	0.878043204	positive
PRJNA729271	stage_E14_vs_E10	ordinary_muscle_development	ENSGALG00010027600	KIRREL1	-0.501919817	0.140989209	-3.559987457	0.000370873	0.001299443	negative
PRJNA729271	stage_E14_vs_E10	ordinary_muscle_development	ENSGALG00010027605	FCHO1	-0.02850279	0.317471503	-0.089780625	0.928461545	0.950830161	negative
PRJNA729271	stage_E14_vs_E10	ordinary_muscle_development	ENSGALG00010027607	ABL1	-0.147772741	0.078643236	-1.879026705	0.060240847	0.110923663	negative
PRJNA729271	stage_E14_vs_E10	ordinary_muscle_development	ENSGALG00010027608	GNAI2	-0.243661833	0.143783481	-1.694644143	0.090142948	0.155977322	negative
PRJNA729271	stage_E14_vs_E10	ordinary_muscle_development	ENSGALG00010027609	RPL36	0.492391906	0.18674841	2.636659164	0.008372691	0.020441664	positive
PRJNA729271	stage_E14_vs_E10	ordinary_muscle_development	ENSGALG00010027612	BAK1	-0.255975005	0.162408034	-1.576122795	0.114997507	0.1912886	negative
PRJNA729271	stage_E14_vs_E10	ordinary_muscle_development	ENSGALG00010027614	LONP1	-0.070860098	0.078608317	-0.90143258	0.367358366	0.483475899	negative
PRJNA729271	stage_E14_vs_E10	ordinary_muscle_development	ENSGALG00010027616	SLC38A3	0.147996697	0.288471001	0.513038388	0.607924468	0.70540067	positive
PRJNA729271	stage_E14_vs_E10	ordinary_muscle_development	ENSGALG00010027618	PPP1R26	-0.042955197	0.078101518	-0.549991835	0.582324974	0.683742446	negative
PRJNA729271	stage_E14_vs_E10	ordinary_muscle_development	ENSGALG00010027619	JAK3	0.847832439	0.353316554	2.399639727	0.016411215	0.036647753	positive
PRJNA729271	stage_E14_vs_E10	ordinary_muscle_development	ENSGALG00010027621	TSPO2	-0.546305616	0.391967094	-1.393753772	0.163391972	0.254143861	negative
PRJNA729271	stage_E14_vs_E10	ordinary_muscle_development	ENSGALG00010027622	FIBCD1	-2.127018518	1.011500315	-2.10283525	0.035480174	0.070731002	negative
PRJNA729271	stage_E14_vs_E10	ordinary_muscle_development	ENSGALG00010027623	APOBEC2	1.756482751	0.182694242	9.614330127	6.95606E-22	1.71185E-20	positive
PRJNA729271	stage_E14_vs_E10	ordinary_muscle_development	ENSGALG00010027624	ENSGALG00010027624	0.346057111	0.443393874	0.78047337	0.435112297	0.54919477	positive
PRJNA729271	stage_E14_vs_E10	ordinary_muscle_development	ENSGALG00010027626	SEMA3B	1.345201042	0.209438659	6.422887977	1.33713E-10	1.21287E-09	positive
PRJNA729271	stage_E14_vs_E10	ordinary_muscle_development	ENSGALG00010027627	OARD1	0.556024947	0.093424768	5.951579635	2.65567E-09	2.07963E-08	positive
PRJNA729271	stage_E14_vs_E10	ordinary_muscle_development	ENSGALG00010027628	PIERCE1	-0.613476865	0.238118433	-2.576351845	0.009984899	0.023794568	negative
PRJNA729271	stage_E14_vs_E10	ordinary_muscle_development	ENSGALG00010027632	LAMC3	-0.993191613	0.367784442	-2.700472069	0.006924115	0.01726562	negative
PRJNA729271	stage_E14_vs_E10	ordinary_muscle_development	ENSGALG00010027634	OASL	1.173601438	0.715617301	1.639984719	0.101008344	0.171442149	positive
PRJNA729271	stage_E14_vs_E10	ordinary_muscle_development	ENSGALG00010027635	CD48	0.521449454	0.230425501	2.262985007	0.023636614	0.050295466	positive
PRJNA729271	stage_E14_vs_E10	ordinary_muscle_development	ENSGALG00010027636	SLC1A6	-1.29703259	0.611953488	-2.11949538	0.034048624	0.06832823	negative
PRJNA729271	stage_E14_vs_E10	ordinary_muscle_development	ENSGALG00010027638	MRPS2	0.572903863	0.140220334	4.085740266	4.39365E-05	0.00018449	positive
PRJNA729271	stage_E14_vs_E10	ordinary_muscle_development	ENSGALG00010027641	HYAL3	0.520146575	0.163529783	3.180745208	0.001468968	0.004438473	positive
PRJNA729271	stage_E14_vs_E10	ordinary_muscle_development	ENSGALG00010027642	DIPK1B	-1.316785405	0.167923455	-7.841581188	4.44908E-15	6.29165E-14	negative
PRJNA729271	stage_E14_vs_E10	ordinary_muscle_development	ENSGALG00010027644	NAA80	0.841174363	0.145024252	5.800232373	6.62231E-09	4.93017E-08	positive
PRJNA729271	stage_E14_vs_E10	ordinary_muscle_development	ENSGALG00010027645	SLAMF1	-1.279928673	1.134516278	-1.128171273	0.259247594	0.367552598	negative
PRJNA729271	stage_E14_vs_E10	ordinary_muscle_development	ENSGALG00010027648	NFYA	-0.185175682	0.071382989	-2.594115006	0.009483476	0.022747474	negative
PRJNA729271	stage_E14_vs_E10	ordinary_muscle_development	ENSGALG00010027649	FBLN2	-0.944135648	0.191595648	-4.927751013	8.31815E-07	4.64094E-06	negative
PRJNA729271	stage_E14_vs_E10	ordinary_muscle_development	ENSGALG00010027650	CCDC124	0.086811966	0.191102766	0.454268497	0.649635588	0.741104033	positive
PRJNA729271	stage_E14_vs_E10	ordinary_muscle_development	ENSGALG00010027653	WNT7A	-5.483157126	0.483256702	-11.34626195	7.74015E-30	3.29013E-28	negative
PRJNA729271	stage_E14_vs_E10	ordinary_muscle_development	ENSGALG00010027654	AIF1L	1.198570075	0.090922529	13.1823222	1.10923E-39	8.12036E-38	positive
PRJNA729271	stage_E14_vs_E10	ordinary_muscle_development	ENSGALG00010027663	COPA	-0.164128987	0.070109468	-2.341038833	0.019230167	0.041991755	negative
PRJNA729271	stage_E14_vs_E10	ordinary_muscle_development	ENSGALG00010027664	TREMB1	0.479432296	0.325232045	1.474123793	0.140448263	0.224974868	positive
PRJNA729271	stage_E14_vs_E10	ordinary_muscle_development	ENSGALG00010027665	TRAF1	-0.253746071	0.128573202	-1.973553333	0.048432555	0.092396667	negative
PRJNA729271	stage_E14_vs_E10	ordinary_muscle_development	ENSGALG00010027667	PTPDC1	0.308467986	0.148551214	2.076509363	0.037846866	0.074738245	positive
PRJNA729271	stage_E14_vs_E10	ordinary_muscle_development	ENSGALG00010027668	RANBP3	-0.009833415	0.052627353	-0.186849884	0.851778334	0.897455057	negative
PRJNA729271	stage_E14_vs_E10	ordinary_muscle_development	ENSGALG00010027671	BARX1	-3.763670631	0.567071633	-6.637028575	3.2007E-11	3.11161E-10	negative
PRJNA729271	stage_E14_vs_E10	ordinary_muscle_development	ENSGALG00010027672	CNTRL	-0.434542868	0.173660172	-2.502259804	0.012340333	0.028656381	negative
PRJNA729271	stage_E14_vs_E10	ordinary_muscle_development	ENSGALG00010027674	TREM2	0.528874315	0.282031227	1.875233181	0.060760651	0.11180978	positive
PRJNA729271	stage_E14_vs_E10	ordinary_muscle_development	ENSGALG00010027685	ENSGALG00010027685	-0.13150867	0.330981343	-0.397329555	0.691124455	0.774536028	negative
PRJNA729271	stage_E14_vs_E10	ordinary_muscle_development	ENSGALG00010027692	PHF2	0.214609534	0.111854267	1.918653087	0.055028252	0.102787953	positive
PRJNA729271	stage_E14_vs_E10	ordinary_muscle_development	ENSGALG00010027693	RAB14	-0.056510286	0.059412614	-0.95114963	0.341528424	0.456473444	negative
PRJNA729271	stage_E14_vs_E10	ordinary_muscle_development	ENSGALG00010027695	TREMB2	0.729531153	0.300632616	2.426653382	0.015238807	0.034379224	positive
PRJNA729271	stage_E14_vs_E10	ordinary_muscle_development	ENSGALG00010027697	CATH2	2.390475658	0.889107544	2.688623749	0.007174724	0.017813912	positive
PRJNA729271	stage_E14_vs_E10	ordinary_muscle_development	ENSGALG00010027698	FAM120A	-0.111728828	0.066279785	-1.685715006	0.091850705	0.158459411	negative
PRJNA729271	stage_E14_vs_E10	ordinary_muscle_development	ENSGALG00010027699	VANGL2	-0.610765217	0.115729706	-5.277514616	1.30948E-07	8.17437E-07	negative
PRJNA729271	stage_E14_vs_E10	ordinary_muscle_development	ENSGALG00010027700	TCF3	-0.109417925	0.095640295	-1.144056751	0.252600102	0.359952663	negative
PRJNA729271	stage_E14_vs_E10	ordinary_muscle_development	ENSGALG00010027702	FOXP4	-1.858251533	0.244403629	-7.603207628	2.88879E-14	3.82054E-13	negative
PRJNA729271	stage_E14_vs_E10	ordinary_muscle_development	ENSGALG00010027703	NCSTN	-0.16976784	0.099641494	-1.703786577	0.088420967	0.153547611	negative
PRJNA729271	stage_E14_vs_E10	ordinary_muscle_development	ENSGALG00010027704	MDFI	-0.05372119	0.169546716	-0.316851846	0.751356022	0.821831085	negative
PRJNA729271	stage_E14_vs_E10	ordinary_muscle_development	ENSGALG00010027705	S100A11	-0.56339074	0.229892706	-2.450668184	0.014259133	0.032492711	negative
PRJNA729271	stage_E14_vs_E10	ordinary_muscle_development	ENSGALG00010027706	GSN	0.544553615	0.172008694	3.165849366	0.001546308	0.004638605	positive
PRJNA729271	stage_E14_vs_E10	ordinary_muscle_development	ENSGALG00010027707	HIGD1C	-0.054789605	0.211948175	-0.258504728	0.7960174	0.856682774	negative
PRJNA729271	stage_E14_vs_E10	ordinary_muscle_development	ENSGALG00010027709	PSMA5	0.395724746	0.106878475	3.702567303	0.000213429	0.000786988	positive
PRJNA729271	stage_E14_vs_E10	ordinary_muscle_development	ENSGALG00010027710	ENSGALG00010027710	-0.134437606	0.318500926	-0.422094866	0.672955775	0.759459199	negative
PRJNA729271	stage_E14_vs_E10	ordinary_muscle_development	ENSGALG00010027711	WNK2	1.022779027	0.18588566	5.502194334	3.75093E-08	2.54064E-07	positive
PRJNA729271	stage_E14_vs_E10	ordinary_muscle_development	ENSGALG00010027712	STOM	0.957880114	0.15716127	6.094886586	1.09515E-09	8.91809E-09	positive
PRJNA729271	stage_E14_vs_E10	ordinary_muscle_development	ENSGALG00010027713	ENSGALG00010027713	0.051732844	0.139671397	0.370389679	0.711092163	0.790678191	positive
PRJNA729271	stage_E14_vs_E10	ordinary_muscle_development	ENSGALG00010027714	SYPL2	4.690180479	0.162419907	28.87688196	2.33E-183	1.6887E-179	positive
PRJNA729271	stage_E14_vs_E10	ordinary_muscle_development	ENSGALG00010027715	NINJ1	-0.02430454	0.121765238	-0.199601634	0.84179215	0.889861232	negative
PRJNA729271	stage_E14_vs_E10	ordinary_muscle_development	ENSGALG00010027717	ATXN7L2	0.601520053	0.228018784	2.638028514	0.008338957	0.020369596	positive
PRJNA729271	stage_E14_vs_E10	ordinary_muscle_development	ENSGALG00010027718	ENSGALG00010027718	0.910316859	0.485804088	1.873835319	0.060953127	0.112105356	positive
PRJNA729271	stage_E14_vs_E10	ordinary_muscle_development	ENSGALG00010027719	GPR61	-2.182673892	0.816289036	-2.673898332	0.007497518	0.018510734	negative
PRJNA729271	stage_E14_vs_E10	ordinary_muscle_development	ENSGALG00010027720	DAB2IP	0.832513012	0.086425217	9.632755847	5.81487E-22	1.44079E-20	positive
PRJNA729271	stage_E14_vs_E10	ordinary_muscle_development	ENSGALG00010027721	SUSD3	0.511751607	0.165454622	3.093002781	0.001981423	0.00577185	positive
PRJNA729271	stage_E14_vs_E10	ordinary_muscle_development	ENSGALG00010027722	GNAI3	-0.021954943	0.084150913	-0.26089964	0.794169903	0.855427862	negative
PRJNA729271	stage_E14_vs_E10	ordinary_muscle_development	ENSGALG00010027723	ENSGALG00010027723	6.402555407	0.949579534	6.742516218	1.55667E-11	1.56585E-10	positive
PRJNA729271	stage_E14_vs_E10	ordinary_muscle_development	ENSGALG00010027724	TTLL11	0.277982517	0.107016232	2.597573393	0.009388505	0.022553263	positive
PRJNA729271	stage_E14_vs_E10	ordinary_muscle_development	ENSGALG00010027725	BTN1A1	-0.321676401	0.292750516	-1.098807292	0.271852131	0.381831069	negative
PRJNA729271	stage_E14_vs_E10	ordinary_muscle_development	ENSGALG00010027727	GSTM2	0.175940698	0.171484795	1.025984242	0.304899012	0.418593595	positive
PRJNA729271	stage_E14_vs_E10	ordinary_muscle_development	ENSGALG00010027728	CRNN	7.920763099	1.45996999	5.425291723	5.786E-08	3.80354E-07	positive
PRJNA729271	stage_E14_vs_E10	ordinary_muscle_development	ENSGALG00010027729	EPS8L3	0.489635669	0.246071784	1.989808262	0.046612061	0.089441597	positive
PRJNA729271	stage_E14_vs_E10	ordinary_muscle_development	ENSGALG00010027730	ENSGALG00010027730	-0.19655851	0.133562824	-1.471655841	0.141113838	0.225816414	negative
PRJNA729271	stage_E14_vs_E10	ordinary_muscle_development	ENSGALG00010027731	OLFML2A	2.176698238	0.1014095	21.46444103	3.3475E-102	4.04352E-99	positive
PRJNA729271	stage_E14_vs_E10	ordinary_muscle_development	ENSGALG00010027732	EEF2	0.602377837	0.117031585	5.147139031	2.64489E-07	1.5888E-06	positive
PRJNA729271	stage_E14_vs_E10	ordinary_muscle_development	ENSGALG00010027733	RPL35	0.564606555	0.154005911	3.666135606	0.000246243	0.000895234	positive
PRJNA729271	stage_E14_vs_E10	ordinary_muscle_development	ENSGALG00010027734	CSF1	0.824908315	0.138157734	5.970771912	2.36134E-09	1.85616E-08	positive
PRJNA729271	stage_E14_vs_E10	ordinary_muscle_development	ENSGALG00010027736	ENSGALG00010027736	8.398668113	1.437118196	5.84410394	5.09302E-09	3.85501E-08	positive
PRJNA729271	stage_E14_vs_E10	ordinary_muscle_development	ENSGALG00010027738	ENSGALG00010027738	0.368559765	0.249266487	1.478577279	0.139253329	0.223431149	positive

PRJNA729271	stage_E14_vs_E10	ordinary_muscle_development	ENSGALG00010027739	FGD3	-0.133314138	0.070674616	-1.886308626	0.059253378	0.10935545	negative
PRJNA729271	stage_E14_vs_E10	ordinary_muscle_development	ENSGALG00010027740	MARCHF2	-0.048185848	0.14345098	-0.335904627	0.736942788	0.811023135	negative
PRJNA729271	stage_E14_vs_E10	ordinary_muscle_development	ENSGALG00010027741	RAB11B	-0.321821092	0.060377268	-5.330169824	9.8121E-08	6.25996E-07	negative
PRJNA729271	stage_E14_vs_E10	ordinary_muscle_development	ENSGALG00010027743	C1orf74	0.342688173	0.189729211	1.806196168	0.070887703	0.127466326	positive
PRJNA729271	stage_E14_vs_E10	ordinary_muscle_development	ENSGALG00010027744	ENSGALG00010027744	9.028682619	1.042811636	8.658018678	4.80035E-18	8.81889E-17	positive
PRJNA729271	stage_E14_vs_E10	ordinary_muscle_development	ENSGALG00010027745	BICD2	0.811272447	0.138339669	5.864351517	4.50891E-09	3.4308E-08	positive
PRJNA729271	stage_E14_vs_E10	ordinary_muscle_development	ENSGALG00010027746	UQCR11	0.574791734	0.184871666	3.109139142	0.001876333	0.005510019	positive
PRJNA729271	stage_E14_vs_E10	ordinary_muscle_development	ENSGALG00010027748	ENSGALG00010027748	8.651700895	2.768182943	3.12540792	0.001775586	0.005250332	positive
PRJNA729271	stage_E14_vs_E10	ordinary_muscle_development	ENSGALG00010027751	ANGPTL4	0.09228905	0.208789163	0.442020306	0.658474512	0.748184374	positive
PRJNA729271	stage_E14_vs_E10	ordinary_muscle_development	ENSGALG00010027752	SCAI	-0.319689012	0.08097571	-3.947961839	7.88194E-05	0.000316128	negative
PRJNA729271	stage_E14_vs_E10	ordinary_muscle_development	ENSGALG00010027759	ENSGALG00010027759	5.425539093	0.693019906	7.828835857	4.92408E-15	6.9363E-14	positive
PRJNA729271	stage_E14_vs_E10	ordinary_muscle_development	ENSGALG00010027766	ENSGALG00010027766	0.316085668	0.557584031	0.566884362	0.570792749	0.672872552	positive
PRJNA729271	stage_E14_vs_E10	ordinary_muscle_development	ENSGALG00010027767	ENSGALG00010027767	5.3287134	0.83855545	6.354634511	2.08923E-10	1.8613E-09	positive
PRJNA729271	stage_E14_vs_E10	ordinary_muscle_development	ENSGALG00010027772	ENSGALG00010027772	3.599673815	0.829807179	4.337964175	1.43809E-05	6.57573E-05	positive
PRJNA729271	stage_E14_vs_E10	ordinary_muscle_development	ENSGALG00010027775	MVB12B	0.471015824	0.087705856	5.370403362	7.85607E-08	5.08819E-07	positive
PRJNA729271	stage_E14_vs_E10	ordinary_muscle_development	ENSGALG00010027776	TKT	-1.136894936	0.127264211	-8.933343677	4.13323E-19	8.27503E-18	negative
PRJNA729271	stage_E14_vs_E10	ordinary_muscle_development	ENSGALG00010027784	PEAR1	0.072829291	0.192909827	0.377530226	0.705779606	0.786339384	positive
PRJNA729271	stage_E14_vs_E10	ordinary_muscle_development	ENSGALG00010027787	DCP1A	-0.09602076	0.136310455	-0.704426964	0.481166919	0.592970114	negative
PRJNA729271	stage_E14_vs_E10	ordinary_muscle_development	ENSGALG00010027789	UAP1L1	-0.035449673	0.133377303	-0.265784899	0.790404874	0.852737196	negative
PRJNA729271	stage_E14_vs_E10	ordinary_muscle_development	ENSGALG00010027801	MBD3	-0.5541682	0.126143787	-4.393146987	1.11722E-05	5.19206E-05	negative
PRJNA729271	stage_E14_vs_E10	ordinary_muscle_development	ENSGALG00010027803	SAPCD2	-0.392823284	0.162437197	-2.418308689	0.015592843	0.035090553	negative
PRJNA729271	stage_E14_vs_E10	ordinary_muscle_development	ENSGALG00010027811	MEX3D	0.204326868	0.094856106	2.154071846	0.031234538	0.063471839	positive
PRJNA729271	stage_E14_vs_E10	ordinary_muscle_development	ENSGALG00010027813	LRRC71	-0.359601776	0.249833525	-1.439365577	0.150046972	0.237385975	negative
PRJNA729271	stage_E14_vs_E10	ordinary_muscle_development	ENSGALG00010027814	ENSGALG00010027814	0.194787683	0.130422166	1.493516698	0.135301987	0.218202303	positive
PRJNA729271	stage_E14_vs_E10	ordinary_muscle_development	ENSGALG00010027816	ZMYND19	-0.053436203	0.11610908	-0.460224148	0.64535534	0.73749808	negative
PRJNA729271	stage_E14_vs_E10	ordinary_muscle_development	ENSGALG00010027821	NMRK2	1.116860557	0.227343524	4.912656127	8.98508E-07	4.98999E-06	positive
PRJNA729271	stage_E14_vs_E10	ordinary_muscle_development	ENSGALG00010027822	DPH7	-0.060927953	0.089826665	-0.678283589	0.497591902	0.607065253	negative
PRJNA729271	stage_E14_vs_E10	ordinary_muscle_development	ENSGALG00010027826	ARHGEF11	-0.162592029	0.164617541	-0.987695649	0.323301726	0.437927158	negative
PRJNA729271	stage_E14_vs_E10	ordinary_muscle_development	ENSGALG00010027830	CACNA1D	-1.114322043	0.32297449	-3.450185941	0.000560201	0.001873583	negative
PRJNA729271	stage_E14_vs_E10	ordinary_muscle_development	ENSGALG00010027831	ATCAY	-1.251555923	0.197877896	-6.324889983	2.53413E-10	2.23296E-09	negative
PRJNA729271	stage_E14_vs_E10	ordinary_muscle_development	ENSGALG00010027838	ENSGALG00010027838	-1.471599684	0.671570098	-2.19128232	0.028431369	0.058737381	negative
PRJNA729271	stage_E14_vs_E10	ordinary_muscle_development	ENSGALG00010027842	SLAMF8	-0.11735534	0.192963721	-0.608173077	0.543072689	0.648849211	negative
PRJNA729271	stage_E14_vs_E10	ordinary_muscle_development	ENSGALG00010027847	NRTN	-2.504439986	0.397695862	-6.297375021	3.02728E-10	2.63546E-09	negative
PRJNA729271	stage_E14_vs_E10	ordinary_muscle_development	ENSGALG00010027860	ENSGALG00010027860	-2.318891767	0.369353192	-6.278250239	3.42405E-10	2.96131E-09	negative
PRJNA729271	stage_E14_vs_E10	ordinary_muscle_development	ENSGALG00010027861	PNPLA7	-0.079127385	0.078513854	-1.007814295	0.313543622	0.427425449	negative
PRJNA729271	stage_E14_vs_E10	ordinary_muscle_development	ENSGALG00010027863	CHDH	0.481720451	0.147738179	3.260636189	0.001111626	0.003460699	positive
PRJNA729271	stage_E14_vs_E10	ordinary_muscle_development	ENSGALG00010027864	ACTR8	-0.070435423	0.080459308	-0.875416712	0.38134721	0.497312443	negative
PRJNA729271	stage_E14_vs_E10	ordinary_muscle_development	ENSGALG00010027865	SELENOK	0.126386832	0.11957734	1.056946346	0.290536075	0.402958607	positive
PRJNA729271	stage_E14_vs_E10	ordinary_muscle_development	ENSGALG00010027866	KCNA2	-1.018861331	0.137264243	-7.422627374	1.14819E-13	1.44221E-12	negative
PRJNA729271	stage_E14_vs_E10	ordinary_muscle_development	ENSGALG00010027867	UBAP2L	-0.284875135	0.167104065	-1.70477681	0.088236058	0.153355115	negative
PRJNA729271	stage_E14_vs_E10	ordinary_muscle_development	ENSGALG00010027868	CACNA2D3	-1.244292574	0.265271065	-4.690645681	2.72344E-06	1.40435E-05	negative
PRJNA729271	stage_E14_vs_E10	ordinary_muscle_development	ENSGALG00010027869	NSMF	0.089221037	0.163309104	0.546332292	0.584837543	0.6855819	positive
PRJNA729271	stage_E14_vs_E10	ordinary_muscle_development	ENSGALG00010027870	HAX1	0.587322046	0.121622476	4.829058461	1.3718E-06	7.45287E-06	positive
PRJNA729271	stage_E14_vs_E10	ordinary_muscle_development	ENSGALG00010027872	KDM5B	0.266277152	0.091034065	2.925027601	0.003444257	0.009366698	positive
PRJNA729271	stage_E14_vs_E10	ordinary_muscle_development	ENSGALG00010027873	RFX2	0.146094114	0.197272961	0.740568364	0.458955196	0.571802501	positive
PRJNA729271	stage_E14_vs_E10	ordinary_muscle_development	ENSGALG00010027874	FRS3	0.182364646	0.131396417	1.387896646	0.165168519	0.256342728	positive
PRJNA729271	stage_E14_vs_E10	ordinary_muscle_development	ENSGALG00010027875	WNT5A	-1.728653992	0.169069301	-10.22452911	1.53974E-24	4.45481E-23	negative
PRJNA729271	stage_E14_vs_E10	ordinary_muscle_development	ENSGALG00010027876	ANAPC2	0.647610494	0.107872706	6.003469448	1.93145E-09	1.53237E-08	positive
PRJNA729271	stage_E14_vs_E10	ordinary_muscle_development	ENSGALG00010027877	OAZ1	0.179745966	0.114336572	1.572077622	0.115932551	0.192490529	positive
PRJNA729271	stage_E14_vs_E10	ordinary_muscle_development	ENSGALG00010027879	ENSGALG00010027879	1.479567103	0.424891472	3.482223585	0.000497268	0.001688034	positive
PRJNA729271	stage_E14_vs_E10	ordinary_muscle_development	ENSGALG00010027880	AMH	0.988607413	0.345599814	2.860555394	0.004228997	0.011224923	positive
PRJNA729271	stage_E14_vs_E10	ordinary_muscle_development	ENSGALG00010027881	ENSGALG00010027881	0.1553895	0.267330049	0.581264623	0.56106212	0.664156425	positive
PRJNA729271	stage_E14_vs_E10	ordinary_muscle_development	ENSGALG00010027882	CH1A	-1.327548824	1.613307855	-0.822873836	0.410579743	0.525415372	negative
PRJNA729271	stage_E14_vs_E10	ordinary_muscle_development	ENSGALG00010027883	ATP8B2	-0.065703727	0.116841383	-0.562332668	0.573889385	0.675753585	negative
PRJNA729271	stage_E14_vs_E10	ordinary_muscle_development	ENSGALG00010027884	SF3A2	-0.150089373	0.18717079	-0.801884592	0.42261972	0.536933372	negative
PRJNA729271	stage_E14_vs_E10	ordinary_muscle_development	ENSGALG00010027885	PLEKHJ1	-0.006351899	0.068967925	-0.092099313	0.926619134	0.949662939	negative
PRJNA729271	stage_E14_vs_E10	ordinary_muscle_development	ENSGALG00010027886	IL6R	0.857475671	0.193438986	4.432796562	9.30186E-06	4.38759E-05	positive
PRJNA729271	stage_E14_vs_E10	ordinary_muscle_development	ENSGALG00010027887	ERC2	-0.295092836	0.251640647	-1.172675558	0.240925937	0.346869344	negative
PRJNA729271	stage_E14_vs_E10	ordinary_muscle_development	ENSGALG00010027888	PFKFB2	-0.019234507	0.142771786	-0.134722042	0.892831645	0.926318424	negative
PRJNA729271	stage_E14_vs_E10	ordinary_muscle_development	ENSGALG00010027889	RBM8A	0.145617359	0.159259912	0.914337808	0.36053937	0.476773763	positive
PRJNA729271	stage_E14_vs_E10	ordinary_muscle_development	ENSGALG00010027890	ENSGALG00010027890	3.076947562	0.857992642	3.586216722	0.00033551	0.00118789	positive
PRJNA729271	stage_E14_vs_E10	ordinary_muscle_development	ENSGALG00010027891	CCDC66	-0.77583552	0.13973602	-5.552151251	2.82175E-08	1.95047E-07	negative
PRJNA729271	stage_E14_vs_E10	ordinary_muscle_development	ENSGALG00010027892	SHE	0.509780166	0.166672563	3.058572795	0.00222394	0.006385899	positive
PRJNA729271	stage_E14_vs_E10	ordinary_muscle_development	ENSGALG00010027893	DOT1L	-0.193689022	0.158073898	-1.2253068	0.220459647	0.32291457	negative
PRJNA729271	stage_E14_vs_E10	ordinary_muscle_development	ENSGALG00010027894	RSBN1	-0.194673454	0.066241539	-2.938842573	0.003294404	0.009021799	negative
PRJNA729271	stage_E14_vs_E10	ordinary_muscle_development	ENSGALG00010027895	LIX1L	-0.012759788	0.17633594	-0.072360679	0.942314877	0.959452654	negative
PRJNA729271	stage_E14_vs_E10	ordinary_muscle_development	ENSGALG00010027896	TOR4A	0.695894728	0.156901198	4.435241659	9.19691E-06	4.3409E-05	positive
PRJNA729271	stage_E14_vs_E10	ordinary_muscle_development	ENSGALG00010027897	AP3D1	-0.266907899	0.097805615	-2.728962953	0.006353384	0.016029992	negative
PRJNA729271	stage_E14_vs_E10	ordinary_muscle_development	ENSGALG00010027898	TASOR	-0.240483861	0.091521609	-2.627618372	0.008598492	0.020911936	negative
PRJNA729271	stage_E14_vs_E10	ordinary_muscle_development	ENSGALG00010027899	PMVK	0.287458272	0.171889423	1.672344158	0.094456477	0.162375075	positive
PRJNA729271	stage_E14_vs_E10	ordinary_muscle_development	ENSGALG00010027901	MOB3A	0.005480625	0.093561088	0.058578034	0.953288207	0.967776478	positive
PRJNA729271	stage_E14_vs_E10	ordinary_muscle_development	ENSGALG00010027902	UBE2Q1	0.203262143	0.069473932	2.925732529	0.003436463	0.009352521	positive
PRJNA729271	stage_E14_vs_E10	ordinary_muscle_development	ENSGALG00010027903	NDUFA8	0.09020647	0.125538243	0.718557693	0.472413488	0.584468549	positive
PRJNA729271	stage_E14_vs_E10	ordinary_muscle_development	ENSGALG00010027904	MORN5	-1.789210515	0.506308738	-3.533832981	0.00040958	0.001415223	negative
PRJNA729271	stage_E14_vs_E10	ordinary_muscle_development	ENSGALG00010027905	SYN2	-1.029350596	0.28951384	-3.555445215	0.00037734	0.001317961	negative
PRJNA729271	stage_E14_vs_E10	ordinary_muscle_development	ENSGALG00010027906	LHX6	0.538656453	0.176111768	3.05860568	0.002223696	0.006385899	positive
PRJNA729271	stage_E14_vs_E10	ordinary_muscle_development	ENSGALG00010027907	MKNK2	0.59760575	0.188752937	3.166073918	0.001545115	0.004635985	positive
PRJNA729271	stage_E14_vs_E10	ordinary_muscle_development	ENSGALG00010027908	PBXIP1	1.632264812	0.105791983	15.42900283	1.04472E-53	1.78155E-51	positive
PRJNA729271	stage_E14_vs_E10	ordinary_muscle_development	ENSGALG00010027909	TIMP4	-4.448066476	0.272321933	-16.33385318	5.66882E-60	1.34704E-57	negative
PRJNA729271	stage_E14_vs_E10	ordinary_muscle_development	ENSGALG00010027910	CHRNB2	-0.12643983	0.179472196	-0.704509293	0.481115665	0.592957364	negative
PRJNA729271	stage_E14_vs_E10	ordinary_muscle_development	ENSGALG00010027911	PTPN22	-1.344899383	0.415893695	-3.233757564	0.001221731	0.00377671	negative
PRJNA729271	stage_E14_vs_E10	ordinary_muscle_development	ENSGALG00010027912	RGS3	0.241237219	0.12725185	1.895746266	0.057993604	0.107440861	positive
PRJNA729271	stage_E14_vs_E10	ordinary_muscle_development	ENSGALG00010027913	PYGO2	-0.649171233	0.222505098	-2.917556677	0.003527855	0.009573411	negative
PRJNA729271	stage_E14_vs_E10	ordinary_muscle_development	ENSGALG00010027914	BTBD2	0.238819464	0.125786134	1.89861518	0.057615091	0.106861825	positive
PRJNA729271	stage_E14_vs_E10	ordinary_muscle_development	ENSGALG00010027915	GSTM3	0.213901142	0.082989853	2.577437282	0.009953594	0.023745449	positive
PRJNA729271	stage_E14_vs_E10	ordinary_muscle_development	ENSGALG00010027916	ADAR	-0.092195238	0.128142269	-0.71947562	0.471847919	0.584117822	negative
PRJNA729271	stage_E14_vs_E10	ordinary_muscle_development	ENSGALG00010027917	PPARG	-0.739332304	0.452946689	-1.632272233	0.10262215	0.173794609	negative
PRJNA729271	stage_E14_vs_E10	ordinary_muscle_development	ENSGALG00010027918	CSNK1G2	0.119079527	0.100357876	1.186548901	0.235405602	0.339996433	positive
PRJNA729271	stage_E14_vs_E10	ordinary_muscle_development	ENSGALG00010027919	TSEN2	-0.158686687	0.116162287	-1.3660775	0.17191464	0.264758043	negative
PRJNA729271	stage_E14_vs_E10	ordinary_muscle_development	ENSGALG00010027920	SCAMP4	-0.118690636	0.112622846	-1.053877079	0.291939198	0.40447894	negative
PRJNA729271	stage_E14_vs_E10	ordinary_muscle_development	ENSGALG00010027921	MKRN2OS	0.538819188	0.231253746	2.329991179	0.019806617	0.04307313	positive
PRJNA729271	stage_E14_vs_E10	ordinary_muscle_development	ENSGALG00010027922	SHC1	0.037883811	0.112702482	0.336139986	0.736765307	0.810889379	positive
PRJNA729271	stage_E14_vs_E10	ordinary_muscle_development	ENSGALG00010027923	AMPD2	-0.541123631	0.151376174	-3.574694858	0.000350637	0.001236011	negative
PRJNA729271	stage_E14_vs_E10	ordinary_muscle_development	ENSGALG00010027925	MKRN2	0.022533206	0.06778399	0.332426669	0.739567104	0.812677217	positive
PRJNA729271	stage_E14_vs_E10	ordinary_muscle_development	ENSGALG00010027926	CKS1B	-0.055273335	0.246424011	-0.224301741	0.822522522	0.875672553	negative
PRJNA729271	stage_E14_vs_E10	ordinary_muscle_development	ENSGALG00010027927	ENSGALG00010027927	0.350201283	0.19577878	1.788760165	0.073653449	0.131868422	positive
PRJNA729271	stage_E14_vs_E10	ordinary_muscle_development	ENSGALG00010027928	FLAD1	-0.167510588	0.160928729	-1.040899217	0.297922325	0.411270066	negative
PRJNA729271	stage_E14_vs_E10	ordinary_muscle_development	ENSGALG00010027929	CDK5RAP2	-0.870403749	0.136969281	-6.354736954	2.08784E-10	1.8612E-09	negative
PRJNA729271	stage_E14_vs_E10	ordinary_muscle_development	ENSGALG00010027930	RAF1	0.389056805	0.089311861	4.356160549	1.32364E-05	6.08504E-05	positive
PRJNA729271	stage_E14_vs_E10	ordinary_muscle_development	ENSGALG00010027931	ABHD17A	0.25032922	0.171861631	1.456574217	0.145233949	0.231159118	positive
PRJNA729271	stage_E14_vs_E10	ordinary_muscle_development	ENSGALG00010027932	POLE3	-0.320258668	0.072575336	-4.412775546	1.02054E-05	4.78109E-05	negative
PRJNA729271	stage_E14_vs_E10	ordinary_muscle_development	ENSGALG00010027933	ENSGALG00010027933	0.752099258	0.227548406	3.305227542	0.000948993	0.003006044	positive
PRJNA729271	stage_E14_vs_E10	ordinary_muscle_development	ENSGALG00010027934	CNBP	0.141610302	0.085004877	1.665907958	0.095731748	0.164139069	positive
PRJNA729271	stage_E14_vs_E10	ordinary_muscle_development	ENSGALG00010027935	ISY1	-0.003860243	0.083273531	-0.046356177	0.963026365	0.975067558	negative
PRJNA729271	stage_E14_vs_E10	ordinary_muscle_development	ENSGALG00010027936	ENSGALG00010027936	-0.192369176	0.08419226	-2.284879601	0.022319888	0.047873155	negative
PRJNA729271	stage_E14_vs_E10	ordinary_muscle_development	ENSGALG00010027937	HDHD3	0.537110275	0.092239955	5.822967666	5.78117E-09	4.34187E-08	positive
PRJNA729271	stage_E14_vs_E10	ordinary_muscle_development	ENSGALG00010027938	ENSGALG00010027938	-0.219122407	0.106366765	-2.060064603	0.039392365	0.077338796	negative
PRJNA729271	stage_E14_vs_E10	ordinary_muscle_development	ENSGALG00010027939	BSPRY	-0.621018914	0.362757478	-1.711939664	0.086907788	0.151508802	negative
PRJNA729271	stage_E14_vs_E10	ordinary_muscle_development	ENSGALG00010027940	ZBTB7B	-0.110815968	0.428849086	-0.258403182	0.796095761	0.856682774	negative
PRJNA729271	stage_E14_vs_E10	ordinary_muscle_development	ENSGALG00010027941	KLF2	0.313897958	0.150883657	2.080397332	0.037489104	0.074114212	positive
PRJNA729271	stage_E14_vs_E10	ordinary_muscle_development	ENSGALG00010027942	KHDC4	0.188723517	0.122928753	1.535226805	0.124728126	0.203822399	positive
PRJNA729271	stage_E14_vs_E10	ordinary_muscle_development	ENSGALG00010027943	RAB43	-0.306443093	0.093552224	-3.275636654	0.001054241	0.003306193	negative
PRJNA729271	stage_E14_vs_E10	ordinary_muscle_development	ENSGALG00010027944	WDR31	1.316865566	0.232623531	5.660930178	1.50555E-08	1.07214E-07	positive
PRJNA729271	stage_E14_vs_E10	ordinary_muscle_development	ENSGALG00010027946	AP1M1	-0.129178998	0.092036719	-1.403559355	0.160450106	0.250589838	negative
PRJNA729271	stage_E14_vs_E10	ordinary_muscle_development	ENSGALG00010027947	GP9	-0.940928331	0.420128603	-2.239619783	0.025115617	0.052937454	negative
PRJNA729271	stage_E14_vs_E10	ordinary_muscle_development	ENSGALG00010027948	PRPF4	0.137558992	0.112225983	1.225732117	0.2202995	0.322747661	positive
PRJNA729271	stage_E14_vs_E10	ordinary_muscle_development	ENSGALG00010027950	CDC26	0.401496914	0.12642664	3.175730334	0.001494598	0.004504928	positive
PRJNA729271	stage_E14_vs_E10	ordinary_muscle_development	ENSGALG00010027951	FAM32A	0.240969372	0.103989239	2.317252973	0.020489955	0.04441482	positive
PRJNA729271	stage_E14_vs_E10	ordinary_muscle_development	ENSGALG00010027952	CIB3	0.295100323	0.457568234	0.64493184	0.518971357	0.62708318	positive
PRJNA729271	stage_E14_vs_E10	ordinary_muscle_development	ENSGALG00010027953	SLC31A1	-0.495781246	0.118749567	-4.175015171	2.97966E-05	0.00012908	negative
PRJNA729271	stage_E14_vs_E10	ordinary_muscle_development	ENSGALG00010027954	CFAP92	-0.987715911	0.228945801	-4.314190992	1.60188E-05	7.25377E-05	negative
PRJNA729271	stage_E14_vs_E10	ordinary_muscle_development	ENSGALG00010027955	ENSGALG00010027955	-0.166559863	0.116393338	-1.431008564	0.152427761	0.240496396	negative
PRJNA729271	stage_E14_vs_E10	ordinary_muscle_development	ENSGALG00010027956	RAB8A	-0.287124435	0.061546313	-4.665176857	3.08352E-06	1.57656E-05	negative
PRJNA729271	stage_E14_vs_E10	ordinary_muscle_development	ENSGALG00010027957	ENTREP3	-1.169812163	0.478660053	-2.443931044	0.014528203	0.033043512	negative
PRJNA729271	stage_E14_vs_E10	ordinary_muscle_development	ENSGALG00010027958	ENSGALG00010027958	-0.063022716	0.139414962	-0.452051307	0.651232021	0.742282625	negative
PRJNA729271	stage_E14_vs_E10	ordinary_muscle_development	ENSGALG00010027959	TPM4	-0.512924497	0.168778568	-3.039038093	0.002373348	0.006749399	negative
PRJNA729271	stage_E14_vs_E10	ordinary_muscle_development	ENSGALG00010027960	PPP6C	0.216490988	0.118833274	1.821804451	0.068484662	0.123776207	positive
PRJNA729271	stage_E14_vs_E10	ordinary_muscle_development	ENSGALG00010027961	TINCR	-1.361630624	0.690789047	-1.971123645	0.048709735	0.092852131	negative
PRJNA729271	stage_E14_vs_E10	ordinary_muscle_development	ENSGALG00010027962	SCAMP3	0.233928802	0.126423431	1.850359546	0.064261746	0.117255036	positive
PRJNA729271	stage_E14_vs_E10	ordinary_muscle_development	ENSGALG00010027964	RABEPK	0.43682433	0.134482215	3.248194053	0.0011614	0.003602503	positive
PRJNA729271	stage_E14_vs_E10	ordinary_muscle_development	ENSGALG00010027965	ACAD9	0.500425907	0.109396583	4.574419915	4.77541E-06	2.36487E-05	positive

PRJNA729271	stage_E14_vs_E10	ordinary_muscle_development	ENSGALG00010027966	CLK2	0.073737521	0.097437112	0.75677039	0.449187435	0.562260093	positive
PRJNA729271	stage_E14_vs_E10	ordinary_muscle_development	ENSGALG00010027968	FKBP15	-0.003864194	0.096554971	-0.040020666	0.96807665	0.978469436	negative
PRJNA729271	stage_E14_vs_E10	ordinary_muscle_development	ENSGALG00010027969	HSPA5	-0.229360158	0.129298712	-1.773878143	0.076083309	0.135515798	negative
PRJNA729271	stage_E14_vs_E10	ordinary_muscle_development	ENSGALG00010027970	GRIN3B	-0.481299826	0.177018708	-2.718920681	0.006549531	0.016436194	negative
PRJNA729271	stage_E14_vs_E10	ordinary_muscle_development	ENSGALG00010027971	ENSGALG00010027971	0.145740152	0.408603112	0.356679007	0.721332123	0.798207329	positive
PRJNA729271	stage_E14_vs_E10	ordinary_muscle_development	ENSGALG00010027972	TMEM259	0.05036953	0.105454886	0.477640555	0.632906061	0.72653626	positive
PRJNA729271	stage_E14_vs_E10	ordinary_muscle_development	ENSGALG00010027973	GAPVD1	0.506143251	0.064202459	7.883549321	3.1821E-15	4.57585E-14	positive
PRJNA729271	stage_E14_vs_E10	ordinary_muscle_development	ENSGALG00010027974	ENSGALG00010027974	-1.133951531	0.336969649	-3.365144406	0.000765036	0.002478587	negative
PRJNA729271	stage_E14_vs_E10	ordinary_muscle_development	ENSGALG00010027975	IQSEC1	0.404302195	0.117059607	3.453814742	0.000552717	0.001851544	positive
PRJNA729271	stage_E14_vs_E10	ordinary_muscle_development	ENSGALG00010027976	LRSAM1	0.101983516	0.096905235	1.052404607	0.292613956	0.405220148	positive
PRJNA729271	stage_E14_vs_E10	ordinary_muscle_development	ENSGALG00010027977	BOLA1	0.428953457	0.156022553	2.749304179	0.005972194	0.015213874	positive
PRJNA729271	stage_E14_vs_E10	ordinary_muscle_development	ENSGALG00010027979	RPL12	0.409077148	0.119511195	3.422919084	0.000619525	0.002049296	positive
PRJNA729271	stage_E14_vs_E10	ordinary_muscle_development	ENSGALG00010027980	NUP210	1.069772291	0.141243512	7.573957022	3.62024E-14	4.75321E-13	positive
PRJNA729271	stage_E14_vs_E10	ordinary_muscle_development	ENSGALG00010027981	CNN2	-0.469577118	0.154907648	-3.03133593	0.002434742	0.006894488	negative
PRJNA729271	stage_E14_vs_E10	ordinary_muscle_development	ENSGALG00010027982	ENTPD2	1.447533891	0.249100334	5.811047565	6.20831E-09	4.64103E-08	positive
PRJNA729271	stage_E14_vs_E10	ordinary_muscle_development	ENSGALG00010027983	POGZ	0.32454477	0.145030406	2.237770539	0.025236023	0.053160318	positive
PRJNA729271	stage_E14_vs_E10	ordinary_muscle_development	ENSGALG00010027984	ARHGAP45	0.116757992	0.172521078	0.676775231	0.498548576	0.607725306	positive
PRJNA729271	stage_E14_vs_E10	ordinary_muscle_development	ENSGALG00010027985	PSMB4	0.167668972	0.094614799	1.772122054	0.076374299	0.135917184	positive
PRJNA729271	stage_E14_vs_E10	ordinary_muscle_development	ENSGALG00010027986	CGN	0.06327826	0.2403128	0.263316227	0.792306853	0.854246343	positive
PRJNA729271	stage_E14_vs_E10	ordinary_muscle_development	ENSGALG00010027987	TMEM141	0.373895327	0.137368135	2.721849045	0.006491779	0.016313858	positive
PRJNA729271	stage_E14_vs_E10	ordinary_muscle_development	ENSGALG00010027988	HDAC11	-0.17126072	0.129529571	-1.322174694	0.186109986	0.282182452	negative
PRJNA729271	stage_E14_vs_E10	ordinary_muscle_development	ENSGALG00010027989	ENSGALG00010027989	0.272067525	0.137695133	1.97586886	0.048169632	0.091991939	positive
PRJNA729271	stage_E14_vs_E10	ordinary_muscle_development	ENSGALG00010027990	ENSGALG00010027990	0.565707013	0.190752071	2.965666434	0.003020279	0.008368756	positive
PRJNA729271	stage_E14_vs_E10	ordinary_muscle_development	ENSGALG00010027991	ENSGALG00010027991	0.23840703	0.144627551	1.648420566	0.099266399	0.169103525	positive
PRJNA729271	stage_E14_vs_E10	ordinary_muscle_development	ENSGALG00010027993	CLIC3	0.882262927	0.161076504	5.477291256	4.31886E-08	2.89154E-07	positive
PRJNA729271	stage_E14_vs_E10	ordinary_muscle_development	ENSGALG00010027994	TUFT1	0.461333219	0.152557752	3.02399067	0.002494641	0.007044579	positive
PRJNA729271	stage_E14_vs_E10	ordinary_muscle_development	ENSGALG00010027995	POLR2E	0.135684419	0.081651545	1.661749556	0.096563	0.165193047	positive
PRJNA729271	stage_E14_vs_E10	ordinary_muscle_development	ENSGALG00010027996	GPX4	0.644126734	0.121410688	5.305354445	1.12454E-07	7.1056E-07	positive
PRJNA729271	stage_E14_vs_E10	ordinary_muscle_development	ENSGALG00010027998	SNX27	0.089383899	0.09193881	0.972210739	0.330945719	0.445740401	positive
PRJNA729271	stage_E14_vs_E10	ordinary_muscle_development	ENSGALG00010027999	ENSGALG00010027999	-1.61979733	0.491671915	-3.29446788	0.000986082	0.003109258	negative
PRJNA729271	stage_E14_vs_E10	ordinary_muscle_development	ENSGALG00010028000	MRPL9	0.404290636	0.166046264	2.434807174	0.014899727	0.033750828	positive
PRJNA729271	stage_E14_vs_E10	ordinary_muscle_development	ENSGALG00010028001	SBNO2	-0.000992428	0.116871193	-0.008491636	0.993224736	0.996111018	negative
PRJNA729271	stage_E14_vs_E10	ordinary_muscle_development	ENSGALG00010028002	ABCA2	0.183245497	0.119617923	1.531923412	0.125541335	0.204900535	positive
PRJNA729271	stage_E14_vs_E10	ordinary_muscle_development	ENSGALG00010028004	ENSGALG00010028004	-1.64406821	0.296443371	-5.545977318	2.92317E-08	2.01576E-07	negative
PRJNA729271	stage_E14_vs_E10	ordinary_muscle_development	ENSGALG00010028005	IER5L	0.39484799	0.142679105	2.767384828	0.005650801	0.014481676	positive
PRJNA729271	stage_E14_vs_E10	ordinary_muscle_development	ENSGALG00010028006	ENSGALG00010028006	1.405842095	0.860941596	1.632912269	0.102487449	0.173607056	positive
PRJNA729271	stage_E14_vs_E10	ordinary_muscle_development	ENSGALG00010028007	STK11	0.389396205	0.119403301	3.261184574	0.001109478	0.003455171	positive
PRJNA729271	stage_E14_vs_E10	ordinary_muscle_development	ENSGALG00010028008	NTMT1	0.444440853	0.112365818	3.955302978	7.64377E-05	0.000307341	positive
PRJNA729271	stage_E14_vs_E10	ordinary_muscle_development	ENSGALG00010028009	RRP9	-0.274326562	0.177553342	-1.545037441	0.122337207	0.200710562	negative
PRJNA729271	stage_E14_vs_E10	ordinary_muscle_development	ENSGALG00010028010	ASB6	-0.018451075	0.068235177	-0.270404155	0.786849344	0.850069407	negative
PRJNA729271	stage_E14_vs_E10	ordinary_muscle_development	ENSGALG00010028011	ENSGALG00010028011	0.242034187	0.084654237	2.859091248	0.004248566	0.011268611	positive
PRJNA729271	stage_E14_vs_E10	ordinary_muscle_development	ENSGALG00010028012	PRRX2	-2.585426585	0.249417217	-10.36587054	3.54515E-25	1.07956E-23	negative
PRJNA729271	stage_E14_vs_E10	ordinary_muscle_development	ENSGALG00010028013	CBARP	-0.113522601	0.214987691	-0.528042331	0.597469956	0.697120422	negative
PRJNA729271	stage_E14_vs_E10	ordinary_muscle_development	ENSGALG00010028014	ENSGALG00010028014	0.30944849	0.292691639	1.05725087	0.290397109	0.402804411	positive
PRJNA729271	stage_E14_vs_E10	ordinary_muscle_development	ENSGALG00010028015	DUSP7	-0.871737121	0.101321747	-8.603652687	7.72184E-18	1.39214E-16	negative
PRJNA729271	stage_E14_vs_E10	ordinary_muscle_development	ENSGALG00010028016	PRF1	-0.488960025	1.470453767	-0.332523222	0.739494208	0.812658722	negative
PRJNA729271	stage_E14_vs_E10	ordinary_muscle_development	ENSGALG00010028017	ENSGALG00010028017	0.694967819	1.329277589	0.52281617	0.601102186	0.69993248	positive
PRJNA729271	stage_E14_vs_E10	ordinary_muscle_development	ENSGALG00010028019	RPL29	0.664859698	0.227101636	2.927586556	0.00341604	0.009305675	positive
PRJNA729271	stage_E14_vs_E10	ordinary_muscle_development	ENSGALG00010028020	ENSGALG00010028020	1.283300909	0.142708706	8.99245004	2.41779E-19	4.93605E-18	positive
PRJNA729271	stage_E14_vs_E10	ordinary_muscle_development	ENSGALG00010028021	ATP5F1D	0.590913919	0.145204366	4.069532734	4.71075E-05	0.000196836	positive
PRJNA729271	stage_E14_vs_E10	ordinary_muscle_development	ENSGALG00010028022	ENSGALG00010028022	-0.097500287	0.141631205	-0.68840964	0.491194855	0.601441918	negative
PRJNA729271	stage_E14_vs_E10	ordinary_muscle_development	ENSGALG00010028023	ENSGALG00010028023	0.310351596	0.903390054	0.343541081	0.731191441	0.80646933	positive
PRJNA729271	stage_E14_vs_E10	ordinary_muscle_development	ENSGALG00010028024	RBM3	0.033702444	0.112725208	0.298978771	0.764956244	0.832935754	positive
PRJNA729271	stage_E14_vs_E10	ordinary_muscle_development	ENSGALG00010028025	TNNC1	3.146021307	0.237094678	13.2690507	3.49986E-40	2.62852E-38	positive
PRJNA729271	stage_E14_vs_E10	ordinary_muscle_development	ENSGALG00010028026	ANKMY1	-0.271583792	0.171002471	-1.58818636	0.112244204	0.18746166	negative
PRJNA729271	stage_E14_vs_E10	ordinary_muscle_development	ENSGALG00010028027	ENSGALG00010028027	0.56110593	0.231245297	2.426453364	0.01524721	0.034392827	positive
PRJNA729271	stage_E14_vs_E10	ordinary_muscle_development	ENSGALG00010028028	FAM174C	-0.073736691	0.15788369	-0.467031718	0.640477185	0.733137235	negative
PRJNA729271	stage_E14_vs_E10	ordinary_muscle_development	ENSGALG00010028029	EFNA2	-1.561426719	0.112887822	-13.83166666	1.64172E-43	1.57594E-41	negative
PRJNA729271	stage_E14_vs_E10	ordinary_muscle_development	ENSGALG00010028030	PFDN2	0.178238547	0.134411668	1.326064539	0.184818321	0.280664385	positive
PRJNA729271	stage_E14_vs_E10	ordinary_muscle_development	ENSGALG00010028031	NISCH	0.053493801	0.080997558	0.660437206	0.508973302	0.617783287	positive
PRJNA729271	stage_E14_vs_E10	ordinary_muscle_development	ENSGALG00010028032	ENSGALG00010028032	-0.664260491	0.208268482	-3.189443181	0.001425472	0.004319825	negative
PRJNA729271	stage_E14_vs_E10	ordinary_muscle_development	ENSGALG00010028033	ENSGALG00010028033	2.032779802	0.294859789	6.894055676	5.42238E-12	5.69546E-11	positive
PRJNA729271	stage_E14_vs_E10	ordinary_muscle_development	ENSGALG00010028034	MUM1L1	-0.101454785	0.072777595	-1.394038719	0.163305913	0.254037262	negative
PRJNA729271	stage_E14_vs_E10	ordinary_muscle_development	ENSGALG00010028035	ENSGALG00010028035	0.70942654	1.044522955	0.679187122	0.497019306	0.606524233	positive
PRJNA729271	stage_E14_vs_E10	ordinary_muscle_development	ENSGALG00010028036	NDUFS7	0.567872274	0.122378596	4.640290793	3.47919E-06	1.75901E-05	positive
PRJNA729271	stage_E14_vs_E10	ordinary_muscle_development	ENSGALG00010028037	ENSGALG00010028037	-0.45565089	0.276122502	-1.650176594	0.098906823	0.168605715	negative
PRJNA729271	stage_E14_vs_E10	ordinary_muscle_development	ENSGALG00010028039	STAB1	0.785581363	0.162477457	4.835017592	1.33134E-06	7.24267E-06	positive
PRJNA729271	stage_E14_vs_E10	ordinary_muscle_development	ENSGALG00010028042	DPP7	-0.195785689	0.191584304	-1.021929692	0.306814178	0.420466249	negative
PRJNA729271	stage_E14_vs_E10	ordinary_muscle_development	ENSGALG00010028043	NT5DC2	0.326157392	0.115783594	2.816956893	0.004848103	0.01267098	positive
PRJNA729271	stage_E14_vs_E10	ordinary_muscle_development	ENSGALG00010028044	GAMT	1.566447235	0.153946848	10.17524721	2.5575E-24	7.28309E-23	positive
PRJNA729271	stage_E14_vs_E10	ordinary_muscle_development	ENSGALG00010028045	ENSGALG00010028045	-1.352893107	0.596583337	-2.267735326	0.023345343	0.049741401	negative
PRJNA729271	stage_E14_vs_E10	ordinary_muscle_development	ENSGALG00010028046	SMIM4	0.631880539	0.203901979	3.098942645	0.001942126	0.005674485	positive
PRJNA729271	stage_E14_vs_E10	ordinary_muscle_development	ENSGALG00010028047	ENSGALG00010028047	0.808645216	0.288085239	2.806965117	0.005001066	0.013028477	positive
PRJNA729271	stage_E14_vs_E10	ordinary_muscle_development	ENSGALG00010028048	S100A10	1.507144793	0.218368609	6.90183814	5.13339E-12	5.39583E-11	positive
PRJNA729271	stage_E14_vs_E10	ordinary_muscle_development	ENSGALG00010028049	MAN1B1	0.006979852	0.059567517	0.11717547	0.906721005	0.935972153	positive
PRJNA729271	stage_E14_vs_E10	ordinary_muscle_development	ENSGALG00010028050	PBRM1	-0.189697431	0.086838313	-2.184490061	0.028926257	0.059562172	negative
PRJNA729271	stage_E14_vs_E10	ordinary_muscle_development	ENSGALG00010028051	GNL3	-0.303765123	0.092022208	-3.300998	0.000963416	0.003047733	negative
PRJNA729271	stage_E14_vs_E10	ordinary_muscle_development	ENSGALG00010028052	ENSGALG00010028052	-1.621603851	0.212377093	-7.635493193	2.24958E-14	3.01643E-13	negative
PRJNA729271	stage_E14_vs_E10	ordinary_muscle_development	ENSGALG00010028053	ORM1	-19.03364215	2.290178814	-8.310985164	9.49108E-17	1.56868E-15	negative
PRJNA729271	stage_E14_vs_E10	ordinary_muscle_development	ENSGALG00010028054	RPS15	0.435259528	0.140730094	3.092867465	0.001982326	0.005773322	positive
PRJNA729271	stage_E14_vs_E10	ordinary_muscle_development	ENSGALG00010028055	ENSGALG00010028055	0.125198165	0.116797419	1.071925778	0.283753371	0.395297341	positive
PRJNA729271	stage_E14_vs_E10	ordinary_muscle_development	ENSGALG00010028056	SLC25A25	0.072940686	0.237090814	0.307648721	0.758349643	0.827794117	positive
PRJNA729271	stage_E14_vs_E10	ordinary_muscle_development	ENSGALG00010028057	APC2	-0.232644703	0.339530455	-0.685195392	0.493220631	0.603463582	negative
PRJNA729271	stage_E14_vs_E10	ordinary_muscle_development	ENSGALG00010028058	GLT8D1	0.129009091	0.112951231	1.142166318	0.253384893	0.360858128	positive
PRJNA729271	stage_E14_vs_E10	ordinary_muscle_development	ENSGALG00010028059	GZMM	0.270403508	0.628863653	0.429987497	0.667204736	0.755203237	positive
PRJNA729271	stage_E14_vs_E10	ordinary_muscle_development	ENSGALG00010028060	ENSGALG00010028060	0.37779404	0.140739202	2.684355428	0.00726698	0.018009041	positive
PRJNA729271	stage_E14_vs_E10	ordinary_muscle_development	ENSGALG00010028061	NAIF1	-0.098274498	0.094107744	-1.044276424	0.296357517	0.409660711	negative
PRJNA729271	stage_E14_vs_E10	ordinary_muscle_development	ENSGALG00010028062	ENSGALG00010028062	0.405254402	0.486778102	0.832523896	0.405113296	0.519898371	positive
PRJNA729271	stage_E14_vs_E10	ordinary_muscle_development	ENSGALG00010028064	SPCS1	0.113606838	0.086746481	1.309642031	0.190316963	0.287418668	positive
PRJNA729271	stage_E14_vs_E10	ordinary_muscle_development	ENSGALG00010028065	REEP6	0.839946992	0.815809484	1.029587188	0.303203832	0.416882518	positive
PRJNA729271	stage_E14_vs_E10	ordinary_muscle_development	ENSGALG00010028066	ENSGALG00010028066	0.893819694	0.185683681	4.813668548	1.48185E-06	7.98489E-06	positive
PRJNA729271	stage_E14_vs_E10	ordinary_muscle_development	ENSGALG00010028067	FAM102A	-0.1904927	0.111166809	-1.713575322	0.086606749	0.151048589	negative
PRJNA729271	stage_E14_vs_E10	ordinary_muscle_development	ENSGALG00010028068	POLE4	0.190338974	0.105622347	1.80207105	0.071534228	0.128423165	positive
PRJNA729271	stage_E14_vs_E10	ordinary_muscle_development	ENSGALG00010028069	NEK4	-0.469334713	0.113572477	-4.13246877	3.58888E-05	0.000152957	negative
PRJNA729271	stage_E14_vs_E10	ordinary_muscle_development	ENSGALG00010028070	MEX3A	-0.391653857	0.121461655	-3.224506184	0.001261901	0.003887622	negative
PRJNA729271	stage_E14_vs_E10	ordinary_muscle_development	ENSGALG00010028071	DPM2	0.296113362	0.112491361	2.632320906	0.008480372	0.020662799	positive
PRJNA729271	stage_E14_vs_E10	ordinary_muscle_development	ENSGALG00010028072	ENSGALG00010028072	-1.361237398	0.545432979	-2.495700573	0.012570866	0.029107781	negative
PRJNA729271	stage_E14_vs_E10	ordinary_muscle_development	ENSGALG00010028073	ST6GALNAC4	0.951160381	0.124076553	7.665915573	1.7756E-14	2.39863E-13	positive
PRJNA729271	stage_E14_vs_E10	ordinary_muscle_development	ENSGALG00010028074	ENSGALG00010028074	2.977745003	0.434413977	6.854625216	7.14997E-12	7.40277E-11	positive
PRJNA729271	stage_E14_vs_E10	ordinary_muscle_development	ENSGALG00010028075	RAB25	0.431607737	0.475773765	0.907170105	0.36431685	0.480419645	positive
PRJNA729271	stage_E14_vs_E10	ordinary_muscle_development	ENSGALG00010028076	ST6GALNAC6	0.902450103	0.152486832	5.918216609	3.25451E-09	2.51461E-08	positive
PRJNA729271	stage_E14_vs_E10	ordinary_muscle_development	ENSGALG00010028078	LAMTOR2	0.439790411	0.130467943	3.370869495	0.000749313	0.002434375	positive
PRJNA729271	stage_E14_vs_E10	ordinary_muscle_development	ENSGALG00010028079	ITIH3	-0.577926916	1.718818017	-0.336235081	0.7366936	0.810889379	negative
PRJNA729271	stage_E14_vs_E10	ordinary_muscle_development	ENSGALG00010028080	MUSTN1	0.466403245	0.405770487	1.149426241	0.250380265	0.357597984	positive
PRJNA729271	stage_E14_vs_E10	ordinary_muscle_development	ENSGALG00010028081	UBQLN4	0.019058449	0.16140422	0.118078998	0.906005064	0.935716909	positive
PRJNA729271	stage_E14_vs_E10	ordinary_muscle_development	ENSGALG00010028082	AK1	1.852816003	0.137620207	13.46325547	2.57324E-41	2.14363E-39	positive
PRJNA729271	stage_E14_vs_E10	ordinary_muscle_development	ENSGALG00010028083	ELAVL1	0.034161842	0.048484765	0.704589191	0.481065927	0.592946481	positive
PRJNA729271	stage_E14_vs_E10	ordinary_muscle_development	ENSGALG00010028084	ENG	-0.253771726	0.194395595	-1.305439692	0.19174317	0.289090623	negative
PRJNA729271	stage_E14_vs_E10	ordinary_muscle_development	ENSGALG00010028085	ENSGALG00010028085	-0.335630481	0.282691332	-1.187268384	0.235121775	0.339688042	negative
PRJNA729271	stage_E14_vs_E10	ordinary_muscle_development	ENSGALG00010028086	MATK	-0.557625125	0.28002435	-1.991345127	0.046442957	0.08921391	negative
PRJNA729271	stage_E14_vs_E10	ordinary_muscle_development	ENSGALG00010028087	STIMATE	-0.627049046	0.088320615	-7.0996907	1.25036E-12	1.41043E-11	negative
PRJNA729271	stage_E14_vs_E10	ordinary_muscle_development	ENSGALG00010028088	NAXE	0.520139424	0.114489438	4.543121457	5.54273E-06	2.71884E-05	positive
PRJNA729271	stage_E14_vs_E10	ordinary_muscle_development	ENSGALG00010028091	FPGS	0.038901438	0.09348269	0.416135199	0.677311074	0.763601464	positive
PRJNA729271	stage_E14_vs_E10	ordinary_muscle_development	ENSGALG00010028093	SFMBT1	-0.700341589	0.137912587	-5.078155693	3.81116E-07	2.242E-06	negative
PRJNA729271	stage_E14_vs_E10	ordinary_muscle_development	ENSGALG00010028095	CDK9	0.142082937	0.091553155	1.551917433	0.120681981	0.198535604	positive
PRJNA729271	stage_E14_vs_E10	ordinary_muscle_development	ENSGALG00010028096	TOR2A	-0.269256526	0.146132688	-1.842548226	0.065394999	0.119038115	negative
PRJNA729271	stage_E14_vs_E10	ordinary_muscle_development	ENSGALG00010028097	TTC24	0.970962731	0.220059113	4.4122814	1.02287E-05	4.79047E-05	positive
PRJNA729271	stage_E14_vs_E10	ordinary_muscle_development	ENSGALG00010028098	RFT1	-0.149286491	0.116982289	-1.276146094	0.20190388	0.301261638	negative
PRJNA729271	stage_E14_vs_E10	ordinary_muscle_development	ENSGALG00010028099	PCASP2	0.701028232	0.265691326	2.638506278	0.008327216	0.020351204	positive
PRJNA729271	stage_E14_vs_E10	ordinary_muscle_development	ENSGALG00010028100	ENSGALG00010028100	0.543714926	0.469999398	1.156841748	0.247337022	0.353984018	positive
PRJNA729271	stage_E14_vs_E10	ordinary_muscle_development	ENSGALG00010028101	MRPL54	0.298632725	0.126845297	2.354306645	0.018557295	0.04073724	positive
PRJNA729271	stage_E14_vs_E10	ordinary_muscle_development	ENSGALG00010028102	CERCAM	0.465344621	0.17898068	2.599971249	0.009323157	0.022411138	positive
PRJNA729271	stage_E14_vs_E10	ordinary_muscle_development	ENSGALG00010028103	APBA3	0.055989655	0.1295167	0.432296796	0.665525716	0.75389147	positive
PRJNA729271	stage_E14_vs_E10	ordinary_muscle_development	ENSGALG00010028104	GOLGA1	-0.072900715	0.114639505	-0.63591268	0.524833338	0.632426572	negative
PRJNA729271	stage_E14_vs_E10	ordinary_muscle_development	ENSGALG00010028105	PRKCD	-0.873196116	0.140637351	-6.208849278	5.3374E-10	4.51638E-09	negative
PRJNA729271	stage_E14_vs_E10	ordinary_muscle_development	ENSGALG00010028106	ARPC5L	0.049479683	0.102567141	0.482412622	0.629512857	0.723328487	positive
PRJNA729271	stage_E14_vs_E10	ordinary_muscle_development	ENSGALG00010028107	NOTCH1	0.179591831	0.123746445	1.451288809	0.146699456	0.233108926	positive
PRJNA729271	stage_E14_vs_E10	ordinary_muscle_development	ENSGALG00010028108	IQGAP3	0.018560064	0.206948446	0.089684483	0.928537947	0.950841225	positive
PRJNA729271	stage_E14_vs_E10	ordinary_muscle_development	ENSGALG00010028109	TJP3	-0.184231169	0.213284022	-0.863783264	0.387706963	0.503633401	negative
PRJNA729271	stage_E14_vs_E10	ordinary_muscle_development	ENSGALG00010028110	COQ4	0.618061357	0.100415321	6.155050331	7.50537E-10	6.22014E-09	positive
PRJNA729271	stage_E14_vs_E10	ordinary_muscle_development	ENSGALG00010028111	TRUB2	-0.073028237	0.087052324	-0.838900483	0.401525157	0.5167568	negative

PRJNA729271	stage_E14_vs_E10	ordinary_muscle_development	ENSGALG00010028112	SWI5	0.126300094	0.080348494	1.571903685	0.11597289	0.192529888	positive
PRJNA729271	stage_E14_vs_E10	ordinary_muscle_development	ENSGALG00010028113	PIP5K1C	-0.223151151	0.093233847	-2.393456421	0.016690464	0.037225462	negative
PRJNA729271	stage_E14_vs_E10	ordinary_muscle_development	ENSGALG00010028114	PFKFB4	0.207868805	0.089625748	2.319297855	0.020378892	0.044207129	positive
PRJNA729271	stage_E14_vs_E10	ordinary_muscle_development	ENSGALG00010028115	CACTIN	0.18109955	0.174999967	1.034854766	0.300736742	0.41464654	positive
PRJNA729271	stage_E14_vs_E10	ordinary_muscle_development	ENSGALG00010028116	HYAL1	0.688023928	0.148004303	4.648675186	3.34074E-06	1.69433E-05	positive
PRJNA729271	stage_E14_vs_E10	ordinary_muscle_development	ENSGALG00010028117	ENSGALG00010028117	0.162311816	0.227695707	0.712845305	0.475941487	0.588186163	positive
PRJNA729271	stage_E14_vs_E10	ordinary_muscle_development	ENSGALG00010028118	ENSGALG00010028118	0.164740775	0.076968088	2.140377644	0.03232426	0.065310865	positive
PRJNA729271	stage_E14_vs_E10	ordinary_muscle_development	ENSGALG00010028119	HYAL2	0.163496886	0.126630107	1.291137549	0.196655986	0.295116318	positive
PRJNA729271	stage_E14_vs_E10	ordinary_muscle_development	ENSGALG00010028120	TBXA2R	-0.230795779	0.233971862	-0.986425364	0.323924424	0.438565713	negative
PRJNA729271	stage_E14_vs_E10	ordinary_muscle_development	ENSGALG00010028121	TUSC2	0.132899322	0.095257336	1.395161012	0.162967291	0.253619378	positive
PRJNA729271	stage_E14_vs_E10	ordinary_muscle_development	ENSGALG00010028122	SEC16A	-0.059584155	0.088307542	-0.674734616	0.499844388	0.608997596	negative
PRJNA729271	stage_E14_vs_E10	ordinary_muscle_development	ENSGALG00010028123	RASSF1	0.220324764	0.104306038	2.112291562	0.034661449	0.069396652	positive
PRJNA729271	stage_E14_vs_E10	ordinary_muscle_development	ENSGALG00010028124	SUGP2	-0.240439141	0.088438006	-2.718730902	0.00655329	0.016442779	negative
PRJNA729271	stage_E14_vs_E10	ordinary_muscle_development	ENSGALG00010028125	HMG20B	-0.101324538	0.117367528	-0.863309804	0.387967155	0.503815079	negative
PRJNA729271	stage_E14_vs_E10	ordinary_muscle_development	ENSGALG00010028126	ZMYND10	0.193578033	0.081414066	2.377697663	0.017421103	0.03862448	positive
PRJNA729271	stage_E14_vs_E10	ordinary_muscle_development	ENSGALG00010028127	MFSD12	-0.504784414	0.170879902	-2.954030345	0.003136532	0.008651575	negative
PRJNA729271	stage_E14_vs_E10	ordinary_muscle_development	ENSGALG00010028128	DNM1	-0.89166679	0.142054724	-6.276924586	3.45335E-10	2.98488E-09	negative
PRJNA729271	stage_E14_vs_E10	ordinary_muscle_development	ENSGALG00010028129	CYB561D2	0.128508086	0.10524144	1.221078757	0.222056202	0.324813332	positive
PRJNA729271	stage_E14_vs_E10	ordinary_muscle_development	ENSGALG00010028130	TMEM115	0.104352885	0.126663817	0.823857097	0.410020761	0.524883063	positive
PRJNA729271	stage_E14_vs_E10	ordinary_muscle_development	ENSGALG00010028131	TMEM161A	0.161857668	0.20410789	0.793000546	0.427777505	0.542155716	positive
PRJNA729271	stage_E14_vs_E10	ordinary_muscle_development	ENSGALG00010028132	CACNA2D2	0.02696358	0.128689676	0.20952403	0.834039178	0.884309699	positive
PRJNA729271	stage_E14_vs_E10	ordinary_muscle_development	ENSGALG00010028133	ENSGALG00010028133	0.216763279	0.305111159	0.710440349	0.477431109	0.589519033	positive
PRJNA729271	stage_E14_vs_E10	ordinary_muscle_development	ENSGALG00010028134	INPP5E	-0.260242586	0.067583928	-3.850657889	0.000117801	0.000458396	negative
PRJNA729271	stage_E14_vs_E10	ordinary_muscle_development	ENSGALG00010028136	C3orf18	0.416376594	0.16063096	2.592131637	0.009538327	0.022867689	positive
PRJNA729271	stage_E14_vs_E10	ordinary_muscle_development	ENSGALG00010028137	NUP214	-0.121687773	0.065227234	-1.865597618	0.062097683	0.113943119	negative
PRJNA729271	stage_E14_vs_E10	ordinary_muscle_development	ENSGALG00010028138	ENSGALG00010028138	-0.97776967	0.5044959	-1.938112224	0.052609532	0.09888011	negative
PRJNA729271	stage_E14_vs_E10	ordinary_muscle_development	ENSGALG00010028139	FZR1	0.000303388	0.084318863	0.003598104	0.997129134	0.998283737	positive
PRJNA729271	stage_E14_vs_E10	ordinary_muscle_development	ENSGALG00010028140	HEMK1	-0.018131742	0.099802904	-0.181675497	0.855837397	0.900374732	negative
PRJNA729271	stage_E14_vs_E10	ordinary_muscle_development	ENSGALG00010028141	CISH	0.552543597	0.187089986	2.953357416	0.003143378	0.008668809	positive
PRJNA729271	stage_E14_vs_E10	ordinary_muscle_development	ENSGALG00010028142	PMPCA	0.100856086	0.121720762	0.828585723	0.407338869	0.522141573	positive
PRJNA729271	stage_E14_vs_E10	ordinary_muscle_development	ENSGALG00010028144	FAM78A	2.019985741	0.184073588	10.97379455	5.10826E-28	1.93327E-26	positive
PRJNA729271	stage_E14_vs_E10	ordinary_muscle_development	ENSGALG00010028145	ENSGALG00010028145	0.379028089	0.129482293	2.927258097	0.00341965	0.009313758	positive
PRJNA729271	stage_E14_vs_E10	ordinary_muscle_development	ENSGALG00010028146	MAPKAPK3	1.650475676	0.104123935	15.85106897	1.3821E-56	2.74431E-54	positive
PRJNA729271	stage_E14_vs_E10	ordinary_muscle_development	ENSGALG00010028147	SLC25A42	1.763793681	0.197700532	8.921542419	4.59839E-19	9.14316E-18	positive
PRJNA729271	stage_E14_vs_E10	ordinary_muscle_development	ENSGALG00010028148	ENSGALG00010028148	3.617658294	0.260030456	13.91244067	5.32315E-44	5.32131E-42	positive
PRJNA729271	stage_E14_vs_E10	ordinary_muscle_development	ENSGALG00010028149	SNAPC4	0.012954114	0.086783308	0.149269647	0.881340865	0.918538671	positive
PRJNA729271	stage_E14_vs_E10	ordinary_muscle_development	ENSGALG00010028150	ARMC6	0.181273585	0.080355035	2.25590823	0.024076379	0.051073776	positive
PRJNA729271	stage_E14_vs_E10	ordinary_muscle_development	ENSGALG00010028151	DOCK3	-0.544675471	0.230805058	-2.359893993	0.018280158	0.040226338	negative
PRJNA729271	stage_E14_vs_E10	ordinary_muscle_development	ENSGALG00010028152	ENSGALG00010028152	-0.590357267	0.347948835	-1.696678385	0.089757481	0.155440226	negative
PRJNA729271	stage_E14_vs_E10	ordinary_muscle_development	ENSGALG00010028153	MANF	-0.014341072	0.146756261	-0.097720339	0.922154364	0.946376913	negative
PRJNA729271	stage_E14_vs_E10	ordinary_muscle_development	ENSGALG00010028154	ENSGALG00010028154	-0.031127217	0.121386811	-0.256429974	0.797618845	0.857676941	negative
PRJNA729271	stage_E14_vs_E10	ordinary_muscle_development	ENSGALG00010028155	CIZ1	0.125519284	0.077499211	1.619620144	0.1053139	0.177792333	positive
PRJNA729271	stage_E14_vs_E10	ordinary_muscle_development	ENSGALG00010028156	DOHH	0.15006067	0.09594499	1.564028204	0.117810945	0.194828254	positive
PRJNA729271	stage_E14_vs_E10	ordinary_muscle_development	ENSGALG00010028157	PRRC2B	-0.27568839	0.144916471	-1.902395139	0.057119516	0.106106291	negative
PRJNA729271	stage_E14_vs_E10	ordinary_muscle_development	ENSGALG00010028158	DDX49	-0.144752407	0.096767875	-1.495872547	0.134686871	0.217396466	negative
PRJNA729271	stage_E14_vs_E10	ordinary_muscle_development	ENSGALG00010028159	DCAF1	0.127629372	0.08945296	1.426776407	0.153644354	0.242125996	positive
PRJNA729271	stage_E14_vs_E10	ordinary_muscle_development	ENSGALG00010028160	ENSGALG00010028160	1.343134027	0.56980519	2.357181104	0.018414264	0.040456046	positive
PRJNA729271	stage_E14_vs_E10	ordinary_muscle_development	ENSGALG00010028161	ENSGALG00010028161	0.961737733	0.591090521	1.627056599	0.103725078	0.175425979	positive
PRJNA729271	stage_E14_vs_E10	ordinary_muscle_development	ENSGALG00010028162	CARD9	0.526151622	0.174184996	3.020648361	0.002522341	0.007114484	positive
PRJNA729271	stage_E14_vs_E10	ordinary_muscle_development	ENSGALG00010028163	GBP	1.336869355	0.351491991	3.803413418	0.000142716	0.000546832	positive
PRJNA729271	stage_E14_vs_E10	ordinary_muscle_development	ENSGALG00010028164	BBLN	0.314180176	0.138853892	2.262667404	0.0236562	0.05032705	positive
PRJNA729271	stage_E14_vs_E10	ordinary_muscle_development	ENSGALG00010028165	NFIC	-0.51888338	0.163758807	-3.168583058	0.00153184	0.004605687	negative
PRJNA729271	stage_E14_vs_E10	ordinary_muscle_development	ENSGALG00010028168	PTGES2	0.298337994	0.137610353	2.167990906	0.030159378	0.061702215	positive
PRJNA729271	stage_E14_vs_E10	ordinary_muscle_development	ENSGALG00010028169	DNLZ	0.600687082	0.142811916	4.20614118	2.59768E-05	0.000113551	positive
PRJNA729271	stage_E14_vs_E10	ordinary_muscle_development	ENSGALG00010028170	COPE	-0.171463258	0.093375485	-1.836277024	0.066316696	0.120443617	negative
PRJNA729271	stage_E14_vs_E10	ordinary_muscle_development	ENSGALG00010028171	RNF123	0.719335037	0.158371145	4.542083956	5.57008E-06	2.73042E-05	positive
PRJNA729271	stage_E14_vs_E10	ordinary_muscle_development	ENSGALG00010028172	CERS1	0.170348639	0.209735308	0.81220773	0.416672445	0.531380176	positive
PRJNA729271	stage_E14_vs_E10	ordinary_muscle_development	ENSGALG00010028173	GDF3	0.273312872	0.226498701	1.206686269	0.227552989	0.330963333	positive
PRJNA729271	stage_E14_vs_E10	ordinary_muscle_development	ENSGALG00010028174	GMPPB	0.269426135	0.152388935	1.768016389	0.077058164	0.136915676	positive
PRJNA729271	stage_E14_vs_E10	ordinary_muscle_development	ENSGALG00010028175	POMT1	-0.205978527	0.092447805	-2.228052101	0.025877041	0.054289725	negative
PRJNA729271	stage_E14_vs_E10	ordinary_muscle_development	ENSGALG00010028176	UPF1	-0.161724735	0.094473053	-1.711861008	0.086922286	0.151508802	negative
PRJNA729271	stage_E14_vs_E10	ordinary_muscle_development	ENSGALG00010028177	ENSGALG00010028177	0.60603468	0.389827071	1.554624407	0.120035547	0.197739708	positive
PRJNA729271	stage_E14_vs_E10	ordinary_muscle_development	ENSGALG00010028178	ENSGALG00010028178	0.726652375	0.1615162	4.498944227	6.82918E-06	3.30073E-05	positive
PRJNA729271	stage_E14_vs_E10	ordinary_muscle_development	ENSGALG00010028179	MCM2	-0.15051526	0.14252159	-1.05608743	0.290928273	0.403232484	negative
PRJNA729271	stage_E14_vs_E10	ordinary_muscle_development	ENSGALG00010028180	ODF2	0.052325122	0.10314284	0.507307365	0.611939172	0.708810796	positive
PRJNA729271	stage_E14_vs_E10	ordinary_muscle_development	ENSGALG00010028181	BGLAP	-0.690025704	0.273821081	-2.519987506	0.0117359	0.027419708	negative
PRJNA729271	stage_E14_vs_E10	ordinary_muscle_development	ENSGALG00010028182	SMG5	0.204386026	0.146066032	1.399271427	0.161731604	0.252183693	positive
PRJNA729271	stage_E14_vs_E10	ordinary_muscle_development	ENSGALG00010028183	CRTC1	0.071923058	0.095785635	0.750875203	0.452727765	0.565665801	positive
PRJNA729271	stage_E14_vs_E10	ordinary_muscle_development	ENSGALG00010028184	TMEM79	-0.354701755	0.251812961	-1.408592128	0.158955808	0.248631103	negative
PRJNA729271	stage_E14_vs_E10	ordinary_muscle_development	ENSGALG00010028185	GLMP	0.000274405	0.085912912	0.003193986	0.997451572	0.998370291	positive
PRJNA729271	stage_E14_vs_E10	ordinary_muscle_development	ENSGALG00010028186	CCT3	0.150588359	0.132033476	1.140531658	0.254064871	0.361574489	positive
PRJNA729271	stage_E14_vs_E10	ordinary_muscle_development	ENSGALG00010028187	TPRA1	0.298209891	0.069085606	4.316527113	1.58503E-05	7.18644E-05	positive
PRJNA729271	stage_E14_vs_E10	ordinary_muscle_development	ENSGALG00010028188	ENSGALG00010028188	0.293683692	0.107805452	2.724200726	0.006445732	0.016217824	positive
PRJNA729271	stage_E14_vs_E10	ordinary_muscle_development	ENSGALG00010028189	GPSM1	0.598393741	0.104800995	5.709809718	1.13103E-08	8.18075E-08	positive
PRJNA729271	stage_E14_vs_E10	ordinary_muscle_development	ENSGALG00010028190	ILF2	0.105783448	0.079755741	1.326342744	0.184726195	0.280583275	positive
PRJNA729271	stage_E14_vs_E10	ordinary_muscle_development	ENSGALG00010028191	KLHL26	0.541933709	0.096808974	5.597969775	2.16877E-08	1.51719E-07	positive
PRJNA729271	stage_E14_vs_E10	ordinary_muscle_development	ENSGALG00010028192	ENSGALG00010028192	-0.415436915	0.396371416	-1.048100086	0.294592497	0.407609607	negative
PRJNA729271	stage_E14_vs_E10	ordinary_muscle_development	ENSGALG00010028193	BRINP1	-0.585298037	0.173574614	-3.372025578	0.000746175	0.002426702	negative
PRJNA729271	stage_E14_vs_E10	ordinary_muscle_development	ENSGALG00010028194	ENSGALG00010028194	0.792222365	0.181728523	4.359372722	1.30436E-05	6.00402E-05	positive
PRJNA729271	stage_E14_vs_E10	ordinary_muscle_development	ENSGALG00010028195	ENSGALG00010028195	1.253627773	0.298019482	4.206529604	2.59322E-05	0.00011339	positive
PRJNA729271	stage_E14_vs_E10	ordinary_muscle_development	ENSGALG00010028196	ENSGALG00010028196	-0.317489519	0.092789547	-3.421608663	0.000622518	0.002058258	negative
PRJNA729271	stage_E14_vs_E10	ordinary_muscle_development	ENSGALG00010028197	INTS3	0.093795973	0.153022559	0.612955199	0.539905963	0.646024182	positive
PRJNA729271	stage_E14_vs_E10	ordinary_muscle_development	ENSGALG00010028198	UCK1	-0.433910557	0.091330579	-4.750988778	2.02424E-06	1.0658E-05	negative
PRJNA729271	stage_E14_vs_E10	ordinary_muscle_development	ENSGALG00010028199	DPP9	-0.28115932	0.106254413	-2.646095452	0.008142684	0.019954049	negative
PRJNA729271	stage_E14_vs_E10	ordinary_muscle_development	ENSGALG00010028201	ENSGALG00010028201	0.436750996	0.161587259	2.702880158	0.006874153	0.017170574	positive
PRJNA729271	stage_E14_vs_E10	ordinary_muscle_development	ENSGALG00010028202	QSOX2	-0.182764785	0.06577294	-2.77872305	0.005457303	0.014050375	negative
PRJNA729271	stage_E14_vs_E10	ordinary_muscle_development	ENSGALG00010028205	PRRT1B	0.221487435	0.421232354	0.525808223	0.599021461	0.698424716	positive
PRJNA729271	stage_E14_vs_E10	ordinary_muscle_development	ENSGALG00010028206	RBM15B	0.028581721	0.093046973	0.307175182	0.758710035	0.828125147	positive
PRJNA729271	stage_E14_vs_E10	ordinary_muscle_development	ENSGALG00010028207	ENSGALG00010028207	-0.051872992	0.270131286	-0.19202882	0.84771963	0.894132273	negative
PRJNA729271	stage_E14_vs_E10	ordinary_muscle_development	ENSGALG00010028211	GATAD2B	-0.675074929	0.14764896	-4.572161769	4.82718E-06	2.38728E-05	negative
PRJNA729271	stage_E14_vs_E10	ordinary_muscle_development	ENSGALG00010028216	NKX2-3	8.581779666	1.346965263	6.371195979	1.8756E-10	1.67923E-09	positive
PRJNA729271	stage_E14_vs_E10	ordinary_muscle_development	ENSGALG00010028221	TNFAIP8L1	-0.191301254	0.186693127	-1.024682892	0.305512838	0.419277465	negative
PRJNA729271	stage_E14_vs_E10	ordinary_muscle_development	ENSGALG00010028232	C9orf69	0.078632302	0.10831488	0.7259603	0.467863109	0.580175871	positive
PRJNA729271	stage_E14_vs_E10	ordinary_muscle_development	ENSGALG00010028239	EDMPN2	8.375952362	1.422106229	5.889821866	3.86612E-09	2.96819E-08	positive
PRJNA729271	stage_E14_vs_E10	ordinary_muscle_development	ENSGALG00010028242	ENSGALG00010028242	0.987922305	0.278106296	3.552319098	0.000381851	0.001331871	positive
PRJNA729271	stage_E14_vs_E10	ordinary_muscle_development	ENSGALG00010028245	JAC1	7.01709518	1.232244048	5.694566096	1.23686E-08	8.90183E-08	positive
PRJNA729271	stage_E14_vs_E10	ordinary_muscle_development	ENSGALG00010028247	MVP	0.535606994	0.091965811	5.823979472	5.74626E-09	4.31789E-08	positive
PRJNA729271	stage_E14_vs_E10	ordinary_muscle_development	ENSGALG00010028262	C1orf43	-0.029409448	0.143421304	-0.205056343	0.837528105	0.886679491	negative
PRJNA729271	stage_E14_vs_E10	ordinary_muscle_development	ENSGALG00010028270	RAPGEF1	-0.0042807	0.181794471	-0.023546919	0.981214013	0.987446602	negative
PRJNA729271	stage_E14_vs_E10	ordinary_muscle_development	ENSGALG00010028279	EDBETA	6.355276545	1.409074015	4.510250335	6.47512E-06	3.13902E-05	positive
PRJNA729271	stage_E14_vs_E10	ordinary_muscle_development	ENSGALG00010028283	ENSGALG00010028283	1.147784162	0.150902201	7.606145907	2.8239E-14	3.74497E-13	positive
PRJNA729271	stage_E14_vs_E10	ordinary_muscle_development	ENSGALG00010028286	KCTD12A	12.45386878	1.184312309	10.51569649	7.31384E-26	2.37168E-24	positive
PRJNA729271	stage_E14_vs_E10	ordinary_muscle_development	ENSGALG00010028292	ENSGALG00010028292	-0.26007273	0.444865378	-0.584609958	0.558810011	0.662528394	negative
PRJNA729271	stage_E14_vs_E10	ordinary_muscle_development	ENSGALG00010028301	FBN3	-1.266957547	0.184588212	-6.86369695	6.71009E-12	6.96226E-11	negative
PRJNA729271	stage_E14_vs_E10	ordinary_muscle_development	ENSGALG00010028311	ENSGALG00010028311	-0.740717647	0.200433121	-3.695585061	0.000219381	0.000806474	negative
PRJNA729271	stage_E14_vs_E10	ordinary_muscle_development	ENSGALG00010028314	MEF2D	1.143648101	0.214030805	5.343380841	9.12288E-08	5.84857E-07	positive
PRJNA729271	stage_E14_vs_E10	ordinary_muscle_development	ENSGALG00010028316	TIMM44	0.073457077	0.079165351	0.927894289	0.353462407	0.469670458	positive
PRJNA729271	stage_E14_vs_E10	ordinary_muscle_development	ENSGALG00010028318	NACC2	0.059294898	0.196417184	0.301882437	0.762741685	0.830927445	positive
PRJNA729271	stage_E14_vs_E10	ordinary_muscle_development	ENSGALG00010028321	RHBG	-0.316400702	0.193042713	-1.639019139	0.101209274	0.171702765	negative
PRJNA729271	stage_E14_vs_E10	ordinary_muscle_development	ENSGALG00010028322	STRBP	-1.149976285	0.110344175	-10.42172171	1.97348E-25	6.17831E-24	negative
PRJNA729271	stage_E14_vs_E10	ordinary_muscle_development	ENSGALG00010028326	CRB2	-0.046004532	0.227582799	-0.202144154	0.83980403	0.888213019	negative
PRJNA729271	stage_E14_vs_E10	ordinary_muscle_development	ENSGALG00010028328	HNRNPM	-0.223116338	0.165483695	-1.348267803	0.177572264	0.271738805	negative
PRJNA729271	stage_E14_vs_E10	ordinary_muscle_development	ENSGALG00010028329	HAPLN2	-0.170667818	0.621599017	-0.274562561	0.783652333	0.847373411	negative
PRJNA729271	stage_E14_vs_E10	ordinary_muscle_development	ENSGALG00010028337	ENSGALG00010028337	0.616465897	0.215591948	2.85941058	0.004244291	0.011259332	positive
PRJNA729271	stage_E14_vs_E10	ordinary_muscle_development	ENSGALG00010028339	BCAN	-0.6616675	0.392813958	-1.684429707	0.092098654	0.158849356	negative
PRJNA729271	stage_E14_vs_E10	ordinary_muscle_development	ENSGALG00010028344	LSM7	-0.397081387	0.091715618	-4.329484964	1.49458E-05	6.82116E-05	negative
PRJNA729271	stage_E14_vs_E10	ordinary_muscle_development	ENSGALG00010028346	DENND1A	0.283148964	0.072668766	3.896432791	9.76199E-05	0.000384406	positive
PRJNA729271	stage_E14_vs_E10	ordinary_muscle_development	ENSGALG00010028348	NES	1.79440321	0.276013504	6.501142816	7.97121E-11	7.41367E-10	positive
PRJNA729271	stage_E14_vs_E10	ordinary_muscle_development	ENSGALG00010028353	ENSGALG00010028353	8.424113945	1.39276537	6.048480332	1.46218E-09	1.1742E-08	positive
PRJNA729271	stage_E14_vs_E10	ordinary_muscle_development	ENSGALG00010028355	RBM18	0.081354423	0.073074189	1.113312702	0.265574153	0.374391883	positive
PRJNA729271	stage_E14_vs_E10	ordinary_muscle_development	ENSGALG00010028357	SPPL2C	-0.385711287	0.092788925	-4.156867714	3.22641E-05	0.000138856	negative
PRJNA729271	stage_E14_vs_E10	ordinary_muscle_development	ENSGALG00010028360	ENSGALG00010028360	0.329845222	0.541227751	0.609438856	0.542233589	0.648072287	positive
PRJNA729271	stage_E14_vs_E10	ordinary_muscle_development	ENSGALG00010028362	TMPRSS9	-2.929792322	0.401233885	-7.301956371	2.83613E-13	3.44015E-12	negative
PRJNA729271	stage_E14_vs_E10	ordinary_muscle_development	ENSGALG00010028364	MHM2	2.547943369	0.697210352	3.654482984	0.000257701	0.000932416	positive
PRJNA729271	stage_E14_vs_E10	ordinary_muscle_development	ENSGALG00010028366	MRRF	0.237436991	0.084777991	2.8006914	0.005099326	0.013239249	positive
PRJNA729271	stage_E14_vs_E10	ordinary_muscle_development	ENSGALG00010028368	TIMM13	0.432245793	0.120253591	3.594452252	0.000325075	0.001154609	positive
PRJNA729271	stage_E14_vs_E10	ordinary_muscle_development	ENSGALG00010028370	S100A12	3.625529842	0.657264498	5.516089567	3.46626E-08	2.36662E-07	positive
PRJNA729271	stage_E14_vs_E10	ordinary_muscle_development	ENSGALG00010028372	PTGS1	1.18137431	0.15888324	7.435487292	1.04183E-13	1.31545E-12	positive
PRJNA729271	stage_E14_vs_E10	ordinary_muscle_development	ENSGALG00010028374	FDPS	0.080274902	0.130518861	0.61504446	0.53852536	0.644798041	positive
PRJNA729271	stage_E14_vs_E10	ordinary_muscle_development	ENSGALG00010028375	LMNB2	0.143814799	0.152439178	0.94342413	0.345464006	0.461010934	positive
PRJNA729271	stage_E14_vs_E10	ordinary_muscle_development	ENSGALG00010028377	PDCL	0.474864665	0.056370017	8.424064583	3.63644E-17	6.18664E-16	positive

PRJNA729271	stage_E14_vs_E10	ordinary_muscle_development	ENSGALG00010028380	RUSC1	0.491979974	0.141019401	3.488739638	0.000485304	0.001651673	positive
PRJNA729271	stage_E14_vs_E10	ordinary_muscle_development	ENSGALG00010028384	ENSGALG00010028384	8.621059779	0.815860885	10.56682572	4.24624E-26	1.40524E-24	positive
PRJNA729271	stage_E14_vs_E10	ordinary_muscle_development	ENSGALG00010028386	NCAN	-2.917699631	0.353679505	-8.249558126	1.58981E-16	2.55764E-15	negative
PRJNA729271	stage_E14_vs_E10	ordinary_muscle_development	ENSGALG00010028391	MED26	-0.065950358	0.113015188	-0.583553055	0.559521052	0.663036106	negative
PRJNA729271	stage_E14_vs_E10	ordinary_muscle_development	ENSGALG00010028392	ASH1L	0.211922783	0.120806693	1.754230476	0.079391023	0.14054383	positive
PRJNA729271	stage_E14_vs_E10	ordinary_muscle_development	ENSGALG00010028394	ZBTB26	-0.170428384	0.103085441	-1.653273079	0.098275299	0.167686929	negative
PRJNA729271	stage_E14_vs_E10	ordinary_muscle_development	ENSGALG00010028399	SLC35E1	0.066056664	0.07850941	0.841385313	0.400132111	0.515496655	positive
PRJNA729271	stage_E14_vs_E10	ordinary_muscle_development	ENSGALG00010028405	S100A6	-0.666368074	0.280151987	-2.378594856	0.017378765	0.038547085	negative
PRJNA729271	stage_E14_vs_E10	ordinary_muscle_development	ENSGALG00010028411	LHX2	-2.211161022	0.584595928	-3.782375001	0.000155339	0.000590052	negative
PRJNA729271	stage_E14_vs_E10	ordinary_muscle_development	ENSGALG00010028412	ENSGALG00010028412	-1.566414123	0.330468979	-4.739973258	2.13746E-06	1.11931E-05	negative
PRJNA729271	stage_E14_vs_E10	ordinary_muscle_development	ENSGALG00010028414	KBTBD12	2.850365074	0.35830642	7.955104657	1.7898E-15	2.62583E-14	positive
PRJNA729271	stage_E14_vs_E10	ordinary_muscle_development	ENSGALG00010028415	FSD1	0.329029438	0.166994227	1.970304266	0.04880351	0.092994199	positive
PRJNA729271	stage_E14_vs_E10	ordinary_muscle_development	ENSGALG00010028418	ENSGALG00010028418	0.629185982	0.124338379	5.060271712	4.18659E-07	2.44696E-06	positive
PRJNA729271	stage_E14_vs_E10	ordinary_muscle_development	ENSGALG00010028419	ENSGALG00010028419	0.620459807	0.38134497	1.627030265	0.10373067	0.175425979	positive
PRJNA729271	stage_E14_vs_E10	ordinary_muscle_development	ENSGALG00010028420	ENSGALG00010028420	0.905276867	0.844540874	1.07191599	0.283757767	0.395297341	positive
PRJNA729271	stage_E14_vs_E10	ordinary_muscle_development	ENSGALG00010028421	ZBTB7A	0.142178857	0.191740672	0.74151642	0.458380379	0.571316738	positive
PRJNA729271	stage_E14_vs_E10	ordinary_muscle_development	ENSGALG00010028422	RXRA	-0.332101116	0.212135975	-1.565510596	0.117463235	0.194386299	negative
PRJNA729271	stage_E14_vs_E10	ordinary_muscle_development	ENSGALG00010028425	DAP3	0.356028836	0.129203685	2.755562552	0.00585913	0.014952128	positive
PRJNA729271	stage_E14_vs_E10	ordinary_muscle_development	ENSGALG00010028426	GPR21	-0.238369029	0.145446441	-1.638878387	0.10123859	0.1717324	negative
PRJNA729271	stage_E14_vs_E10	ordinary_muscle_development	ENSGALG00010028427	ENSGALG00010028427	0.645181931	0.202370655	3.188119993	0.001432011	0.004336118	positive
PRJNA729271	stage_E14_vs_E10	ordinary_muscle_development	ENSGALG00010028428	RABGAP1	0.161370673	0.066752071	2.417463156	0.015629116	0.035161266	positive
PRJNA729271	stage_E14_vs_E10	ordinary_muscle_development	ENSGALG00010028429	REXO1	-0.146158534	0.087483519	-1.670697926	0.094781359	0.162856306	negative
PRJNA729271	stage_E14_vs_E10	ordinary_muscle_development	ENSGALG00010028430	NR2C2AP	0.421738596	0.113551821	3.714062831	0.000203958	0.000756863	positive
PRJNA729271	stage_E14_vs_E10	ordinary_muscle_development	ENSGALG00010028431	ENSGALG00010028431	0.67717619	0.264276944	2.562373319	0.010395951	0.024598319	positive
PRJNA729271	stage_E14_vs_E10	ordinary_muscle_development	ENSGALG00010028432	RFXANK	0.145555589	0.133897458	1.087067606	0.277006933	0.387531656	positive
PRJNA729271	stage_E14_vs_E10	ordinary_muscle_development	ENSGALG00010028433	MSTO1	0.225915538	0.084895414	2.661104154	0.007788486	0.019170335	positive
PRJNA729271	stage_E14_vs_E10	ordinary_muscle_development	ENSGALG00010028434	COL5A1	0.574336916	0.195871778	2.932208617	0.003365606	0.00918901	positive
PRJNA729271	stage_E14_vs_E10	ordinary_muscle_development	ENSGALG00010028435	ATP8B3	0.171180112	0.360570205	0.474748355	0.634966347	0.728670509	positive
PRJNA729271	stage_E14_vs_E10	ordinary_muscle_development	ENSGALG00010028436	S100A1	2.68016633	0.185014436	14.4862552	1.47994E-47	1.83348E-45	positive
PRJNA729271	stage_E14_vs_E10	ordinary_muscle_development	ENSGALG00010028437	ENSGALG00010028437	0.186472177	0.066143257	2.819216725	0.0048141	0.012593464	positive
PRJNA729271	stage_E14_vs_E10	ordinary_muscle_development	ENSGALG00010028438	MED27	0.047161582	0.068304889	0.690456903	0.489906903	0.60047358	positive
PRJNA729271	stage_E14_vs_E10	ordinary_muscle_development	ENSGALG00010028439	BORCS8	0.308084453	0.117435074	2.623444964	0.008704551	0.021122882	positive
PRJNA729271	stage_E14_vs_E10	ordinary_muscle_development	ENSGALG00010028440	PSMB7	0.213832804	0.077882714	2.745574617	0.006040503	0.015358199	positive
PRJNA729271	stage_E14_vs_E10	ordinary_muscle_development	ENSGALG00010028442	CHTOP	0.286018482	0.089359097	3.200776342	0.001370579	0.004171014	positive
PRJNA729271	stage_E14_vs_E10	ordinary_muscle_development	ENSGALG00010028443	ENSGALG00010028443	0.248987435	0.32312985	0.77054916	0.440974205	0.554950608	positive
PRJNA729271	stage_E14_vs_E10	ordinary_muscle_development	ENSGALG00010028444	MEF2B	-0.243259507	0.323166534	-0.752737307	0.451607787	0.564704526	negative
PRJNA729271	stage_E14_vs_E10	ordinary_muscle_development	ENSGALG00010028445	SYT11	-0.975512476	0.202435895	-4.818871068	1.44373E-06	7.80248E-06	negative
PRJNA729271	stage_E14_vs_E10	ordinary_muscle_development	ENSGALG00010028446	ENSGALG00010028446	-0.533160697	0.219399507	-2.430090679	0.015095046	0.034113946	negative
PRJNA729271	stage_E14_vs_E10	ordinary_muscle_development	ENSGALG00010028447	OLFM1	-0.934662781	0.15743547	-5.936799266	2.9064E-09	2.26618E-08	negative
PRJNA729271	stage_E14_vs_E10	ordinary_muscle_development	ENSGALG00010028448	RIT1	0.015562804	0.056412612	0.275874544	0.782644426	0.846536151	positive
PRJNA729271	stage_E14_vs_E10	ordinary_muscle_development	ENSGALG00010028449	EFCAB12	0.064977263	0.310373817	0.209351625	0.834173753	0.884387694	positive
PRJNA729271	stage_E14_vs_E10	ordinary_muscle_development	ENSGALG00010028450	PTGDS	1.507298021	0.453688478	3.322319377	0.000892724	0.002842716	positive
PRJNA729271	stage_E14_vs_E10	ordinary_muscle_development	ENSGALG00010028451	TMEM59L	-1.075223055	0.301466723	-3.56663928	0.000361589	0.001269631	negative
PRJNA729271	stage_E14_vs_E10	ordinary_muscle_development	ENSGALG00010028452	GOLPH3L	-2.111501968	0.727172978	-2.903713465	0.003687655	0.009952565	negative
PRJNA729271	stage_E14_vs_E10	ordinary_muscle_development	ENSGALG00010028453	ENSGALG00010028453	0.134360218	0.094416226	1.423062785	0.154717955	0.243447326	positive
PRJNA729271	stage_E14_vs_E10	ordinary_muscle_development	ENSGALG00010028454	C8G	0.166005335	0.23539276	0.705227021	0.480668968	0.59268609	positive
PRJNA729271	stage_E14_vs_E10	ordinary_muscle_development	ENSGALG00010028455	ENSA	0.291082799	0.069532483	4.186285091	2.83557E-05	0.000123317	positive
PRJNA729271	stage_E14_vs_E10	ordinary_muscle_development	ENSGALG00010028456	FBXW5	0.276486827	0.092661029	2.983852336	0.002846441	0.007932929	positive
PRJNA729271	stage_E14_vs_E10	ordinary_muscle_development	ENSGALG00010028457	KANK3	0.594128344	0.104507487	5.685031373	1.30789E-08	9.38506E-08	positive
PRJNA729271	stage_E14_vs_E10	ordinary_muscle_development	ENSGALG00010028458	TRAF2	0.602979408	0.107769634	5.595077046	2.20524E-08	1.54122E-07	positive
PRJNA729271	stage_E14_vs_E10	ordinary_muscle_development	ENSGALG00010028459	MCL1	0.381968787	0.210192789	1.81723069	0.069181798	0.124804553	positive
PRJNA729271	stage_E14_vs_E10	ordinary_muscle_development	ENSGALG00010028460	CRLF1	-0.647995457	0.24451158	-2.650162647	0.008045303	0.019735432	negative
PRJNA729271	stage_E14_vs_E10	ordinary_muscle_development	ENSGALG00010028461	ENSGALG00010028461	-0.455511658	0.341322473	-1.33454927	0.182023911	0.277321483	negative
PRJNA729271	stage_E14_vs_E10	ordinary_muscle_development	ENSGALG00010028462	EDF1	0.429211061	0.105326535	4.075051565	4.60042E-05	0.000192392	positive
PRJNA729271	stage_E14_vs_E10	ordinary_muscle_development	ENSGALG00010028463	RPS28	0.967628099	0.169237479	5.717575723	1.08055E-08	7.83517E-08	positive
PRJNA729271	stage_E14_vs_E10	ordinary_muscle_development	ENSGALG00010028464	ENSGALG00010028464	0.334214383	0.110227582	3.032039496	0.002429074	0.006880874	positive
PRJNA729271	stage_E14_vs_E10	ordinary_muscle_development	ENSGALG00010028465	ENSGALG00010028465	-0.396329181	0.137066548	-2.891509186	0.003833964	0.010293259	negative
PRJNA729271	stage_E14_vs_E10	ordinary_muscle_development	ENSGALG00010028466	MAMDC4	-0.122184779	0.662112431	-0.184537811	0.853591568	0.89859901	negative
PRJNA729271	stage_E14_vs_E10	ordinary_muscle_development	ENSGALG00010028467	ENSGALG00010028467	-1.176849892	0.287241091	-4.097080566	4.18393E-05	0.000176502	negative
PRJNA729271	stage_E14_vs_E10	ordinary_muscle_development	ENSGALG00010028468	PHPT1	0.161575208	0.098080733	1.647369497	0.099482121	0.169416049	positive
PRJNA729271	stage_E14_vs_E10	ordinary_muscle_development	ENSGALG00010028470	AJM1	-0.105609805	0.498738103	-0.211754033	0.832298931	0.883110534	negative
PRJNA729271	stage_E14_vs_E10	ordinary_muscle_development	ENSGALG00010028471	TPGS1	0.020925081	0.164744124	0.127015641	0.898928019	0.930379267	positive
PRJNA729271	stage_E14_vs_E10	ordinary_muscle_development	ENSGALG00010028472	RPRD2	-0.222247423	0.138965298	-1.599301603	0.109753604	0.183959122	negative
PRJNA729271	stage_E14_vs_E10	ordinary_muscle_development	ENSGALG00010028473	CDC34	0.103399703	0.141567353	0.730392288	0.465150431	0.577553152	positive
PRJNA729271	stage_E14_vs_E10	ordinary_muscle_development	ENSGALG00010028474	PEX19	0.163012202	0.119449422	1.364696448	0.172348476	0.265258726	positive
PRJNA729271	stage_E14_vs_E10	ordinary_muscle_development	ENSGALG00010028475	AMBP	2.208100355	1.421962609	1.552854021			positive
PRJNA729271	stage_E14_vs_E10	ordinary_muscle_development	ENSGALG00010028476	ENSGALG00010028476	0.21389708	0.155973125	1.371371377	0.170259215	0.262738989	positive
PRJNA729271	stage_E14_vs_E10	ordinary_muscle_development	ENSGALG00010028477	NRARP	-0.138353826	0.211252756	-0.654920812	0.51251874	0.621358241	negative
PRJNA729271	stage_E14_vs_E10	ordinary_muscle_development	ENSGALG00010028478	PEA15	0.221430157	0.157929935	1.402078439	0.160891825	0.251144411	positive
PRJNA729271	stage_E14_vs_E10	ordinary_muscle_development	ENSGALG00010028479	CASQ1	2.714730101	0.328808282	8.256270452	1.50295E-16	2.42868E-15	positive
PRJNA729271	stage_E14_vs_E10	ordinary_muscle_development	ENSGALG00010028480	F11R	0.02267928	0.213890778	0.106032062	0.915556913	0.941362463	positive
PRJNA729271	stage_E14_vs_E10	ordinary_muscle_development	ENSGALG00010028481	ENSGALG00010028481	1.572092059	0.140246539	11.20948917	3.66301E-29	1.49987E-27	positive
PRJNA729271	stage_E14_vs_E10	ordinary_muscle_development	ENSGALG00010028482	CD320	0.484487107	0.174270682	2.780083838	0.005434487	0.013996603	positive
PRJNA729271	stage_E14_vs_E10	ordinary_muscle_development	ENSGALG00010028483	EXD3	0.160519022	0.090091152	1.781740133	0.074791619	0.133526853	positive
PRJNA729271	stage_E14_vs_E10	ordinary_muscle_development	ENSGALG00010028484	NOXA1	-0.921711785	0.511019634	-1.80367196	0.071282748	0.12806634	negative
PRJNA729271	stage_E14_vs_E10	ordinary_muscle_development	ENSGALG00010028485	USF1	-0.291040812	0.18207835	-1.598437221	0.109945707	0.184238499	negative
PRJNA729271	stage_E14_vs_E10	ordinary_muscle_development	ENSGALG00010028486	ARHGAP30	0.911439127	0.225890787	4.034866323	5.46334E-05	0.000225744	positive
PRJNA729271	stage_E14_vs_E10	ordinary_muscle_development	ENSGALG00010028488	PTGES	1.37428472	0.128864701	10.6645552	1.49111E-26	5.12171E-25	positive
PRJNA729271	stage_E14_vs_E10	ordinary_muscle_development	ENSGALG00010028489	ENSGALG00010028489	-1.839666766	1.046855251	-1.757326778	0.078862126	0.139692841	negative
PRJNA729271	stage_E14_vs_E10	ordinary_muscle_development	ENSGALG00010028490	NECTIN4	-0.572127533	0.338969614	-1.687843127	0.091441346	0.157922354	negative
PRJNA729271	stage_E14_vs_E10	ordinary_muscle_development	ENSGALG00010028491	TOR1B	-0.033161766	0.097089844	-0.34155752	0.732683911	0.80750101	negative
PRJNA729271	stage_E14_vs_E10	ordinary_muscle_development	ENSGALG00010028492	ENSGALG00010028492	0.729461206	0.262286124	2.781165834	0.005416406	0.013959958	positive
PRJNA729271	stage_E14_vs_E10	ordinary_muscle_development	ENSGALG00010028493	TOR1A	-0.131411384	0.085566664	-1.535777819	0.124592882	0.203673601	negative
PRJNA729271	stage_E14_vs_E10	ordinary_muscle_development	ENSGALG00010028494	ENSGALG00010028494	0.102530565	0.179978972	0.5696808	0.568894215	0.671273721	positive
PRJNA729271	stage_E14_vs_E10	ordinary_muscle_development	ENSGALG00010028495	LINGO3	-0.47737115	0.243742638	-1.958504893	0.050170797	0.095012504	negative
PRJNA729271	stage_E14_vs_E10	ordinary_muscle_development	ENSGALG00010028496	ENSGALG00010028496	0.174718619	0.146469185	1.192869471	0.232920497	0.337245522	positive
PRJNA729271	stage_E14_vs_E10	ordinary_muscle_development	ENSGALG00010028498	C9orf78	0.107043962	0.102776088	1.041525939	0.297631521	0.411068975	positive
PRJNA729271	stage_E14_vs_E10	ordinary_muscle_development	ENSGALG00010028500	VPS45	0.309484308	0.069005962	4.484892314	7.29508E-06	3.49792E-05	positive
PRJNA729271	stage_E14_vs_E10	ordinary_muscle_development	ENSGALG00010028501	USP20	0.190224936	0.087249869	2.180231766	0.029240286	0.060127386	positive
PRJNA729271	stage_E14_vs_E10	ordinary_muscle_development	ENSGALG00010028502	PLEKHO1	1.355272062	0.149308567	9.076987913	1.11621E-19	2.32464E-18	positive
PRJNA729271	stage_E14_vs_E10	ordinary_muscle_development	ENSGALG00010028503	NDOR1	0.19953549	0.124278157	1.605555596	0.108371588	0.182021573	positive
PRJNA729271	stage_E14_vs_E10	ordinary_muscle_development	ENSGALG00010028506	SHD	-2.054878576	0.255946329	-8.02855264	9.86293E-16	1.47537E-14	negative
PRJNA729271	stage_E14_vs_E10	ordinary_muscle_development	ENSGALG00010028507	ANP32E	-0.104110405	0.056463295	-1.843859896	0.065203561	0.118755305	negative
PRJNA729271	stage_E14_vs_E10	ordinary_muscle_development	ENSGALG00010028510	CILP2	-1.954260536	0.59595357	-3.27921609	0.001040959	0.003270908	negative
PRJNA729271	stage_E14_vs_E10	ordinary_muscle_development	ENSGALG00010028511	YJEFN3	-0.874707068	0.470774633	-1.858016569	0.063166661	0.115547799	negative
PRJNA729271	stage_E14_vs_E10	ordinary_muscle_development	ENSGALG00010028512	NDUFA13	0.31543405	0.122478129	2.575431656	0.010011506	0.023848278	positive
PRJNA729271	stage_E14_vs_E10	ordinary_muscle_development	ENSGALG00010028513	ENSGALG00010028513	6.44314893	1.02081126	6.311792572	2.75822E-10	2.41573E-09	positive
PRJNA729271	stage_E14_vs_E10	ordinary_muscle_development	ENSGALG00010028514	FNBP1	0.117795072	0.059162511	1.991042465	0.046476219	0.089240004	positive
PRJNA729271	stage_E14_vs_E10	ordinary_muscle_development	ENSGALG00010028515	ENSGALG00010028515	7.401223472	3.287262922	2.251485095	0.02435483	0.051573887	positive
PRJNA729271	stage_E14_vs_E10	ordinary_muscle_development	ENSGALG00010028516	GATAD2A	0.068860861	0.061837733	1.113573494	0.265462203	0.374270463	positive
PRJNA729271	stage_E14_vs_E10	ordinary_muscle_development	ENSGALG00010028517	RNF208	0.798883356	0.328602972	2.431150736	0.015050951	0.034054917	positive
PRJNA729271	stage_E14_vs_E10	ordinary_muscle_development	ENSGALG00010028518	MAU2	0.234031558	0.11446288	2.04460659	0.040893659	0.079767675	positive
PRJNA729271	stage_E14_vs_E10	ordinary_muscle_development	ENSGALG00010028519	ENSGALG00010028519	8.844369398	1.929900204	4.582811785	4.58765E-06	2.27889E-05	positive
PRJNA729271	stage_E14_vs_E10	ordinary_muscle_development	ENSGALG00010028523	SUGP1	0.045386336	0.110795343	0.409641193	0.682069174	0.767413854	positive
PRJNA729271	stage_E14_vs_E10	ordinary_muscle_development	ENSGALG00010028528	UHRF1	-0.158144163	0.182947969	-0.864421531	0.387356369	0.503382694	negative
PRJNA729271	stage_E14_vs_E10	ordinary_muscle_development	ENSGALG00010028534	ENSGALG00010028534	7.833582425	4.266148703	1.836218794	0.066325304	0.12044416	positive
PRJNA729271	stage_E14_vs_E10	ordinary_muscle_development	ENSGALG00010028535	TICAM1	0.323320025	0.10625621	3.042834149	0.002343615	0.006684513	positive
PRJNA729271	stage_E14_vs_E10	ordinary_muscle_development	ENSGALG00010028536	GPR107	0.005767602	0.062836123	0.091787998	0.92686648	0.94980061	positive
PRJNA729271	stage_E14_vs_E10	ordinary_muscle_development	ENSGALG00010028537	ENSGALG00010028537	10.78187594	1.953217724	5.520058418	3.38887E-08	2.31925E-07	positive
PRJNA729271	stage_E14_vs_E10	ordinary_muscle_development	ENSGALG00010028538	FEM1A	0.575691635	0.1170323	4.919083314	8.69505E-07	4.8419E-06	positive
PRJNA729271	stage_E14_vs_E10	ordinary_muscle_development	ENSGALG00010028539	ENSGALG00010028539	9.545073275	3.242390815	2.943837995	0.003241696	0.008906521	positive
PRJNA729271	stage_E14_vs_E10	ordinary_muscle_development	ENSGALG00010028541	ENSGALG00010028541	2.859097306	0.26768751	10.68072735	1.25286E-26	4.32386E-25	positive
PRJNA729271	stage_E14_vs_E10	ordinary_muscle_development	ENSGALG00010028542	ENSGALG00010028542	12.15576689	1.814560255	6.699015285	2.09829E-11	2.07892E-10	positive
PRJNA729271	stage_E14_vs_E10	ordinary_muscle_development	ENSGALG00010028543	ANP32B	-0.235035416	0.093192541	-2.522041064	0.011667609	0.027284443	negative
PRJNA729271	stage_E14_vs_E10	ordinary_muscle_development	ENSGALG00010028544	ZNF414	0.103515374	0.168571209	0.61407505	0.539165734	0.645511466	positive
PRJNA729271	stage_E14_vs_E10	ordinary_muscle_development	ENSGALG00010028545	ENSGALG00010028545	12.84576392	1.304017614	9.850912886	6.79242E-23	1.77398E-21	positive
PRJNA729271	stage_E14_vs_E10	ordinary_muscle_development	ENSGALG00010028546	ENSGALG00010028546	10.41617262	1.5173593	6.864671156	6.66446E-12	6.91987E-11	positive
PRJNA729271	stage_E14_vs_E10	ordinary_muscle_development	ENSGALG00010028547	ZNF618	-0.021046032	0.103956033	-0.202451277	0.839563945	0.88802389	negative
PRJNA729271	stage_E14_vs_E10	ordinary_muscle_development	ENSGALG00010028548	MYO1F	0.506235962	0.249751793	2.026956269	0.042666885	0.082825298	positive
PRJNA729271	stage_E14_vs_E10	ordinary_muscle_development	ENSGALG00010028549	ADAMTS10	-0.605316884	0.176050314	-3.438317545	0.000585341	0.001948671	negative
PRJNA729271	stage_E14_vs_E10	ordinary_muscle_development	ENSGALG00010028550	ZAP70	-4.113175847	1.002304866	-4.10371733	4.06564E-05	0.000171862	negative
PRJNA729271	stage_E14_vs_E10	ordinary_muscle_development	ENSGALG00010028551	NCS1	-1.39630451	0.204777543	-6.818640792	9.19059E-12	9.44136E-11	negative
PRJNA729271	stage_E14_vs_E10	ordinary_muscle_development	ENSGALG00010028552	ENSGALG00010028552	5.25078839	0.635161393	8.266856967	1.37537E-16	2.23748E-15	positive
PRJNA729271	stage_E14_vs_E10	ordinary_muscle_development	ENSGALG00010028553	NFILZ	-0.409849199	1.229979154	-0.333216378	0.738970956	0.812268447	negative
PRJNA729271	stage_E14_vs_E10	ordinary_muscle_development	ENSGALG00010028554	ENSGALG00010028554	6.04925229	0.748141951	8.085701228	6.18075E-16	9.44047E-15	positive
PRJNA729271	stage_E14_vs_E10	ordinary_muscle_development	ENSGALG00010028555	ENSGALG00010028555	10.81564733	1.157361554	9.345089514	9.18118E-21	2.07939E-19	positive
PRJNA729271	stage_E14_vs_E10	ordinary_muscle_development	ENSGALG00010028556	SPTAN1	0.765185651	0.093265714	8.2043617	2.31819E-16	3.66037E-15	positive
PRJNA729271	stage_E14_vs_E10	ordinary_muscle_development	ENSGALG00010028558	ENSGALG00010028558	10.1510308	1.353750459	7.498450496	6.45766E-14	8.36495E-13	positive
PRJNA729271	stage_E14_vs_E10	ordinary_muscle_development	ENSGALG00010028559	DYNC2I2	-0.100162966	0.115264132	-0.868986429	0.384854554	0.500984891	negative
PRJNA729271	stage_E14_vs_E10	ordinary_muscle_development	ENSGALG00010028560	ENSGALG00010028560	7.686309626	3.406566459	2.256321642	0.024050495	0.051041277	positive
PRJNA729271	stage_E14_vs_E10	ordinary_muscle_development	ENSGALG00010028561	SET	-0.24425836	0.051004257	-4.788979863	1.67631E-06	8.95619E-06	negative

PRJNA729271	stage_E14_vs_E10	ordinary_muscle_development	ENSGALG00010028562	ACSBG2	0.866340246	0.199504323	4.342463539	1.40894E-05	6.45263E-05	positive
PRJNA729271	stage_E14_vs_E10	ordinary_muscle_development	ENSGALG00010028563	ENSGALG00010028563	10.42809496	2.145285657	4.860935384	1.16832E-06	6.40986E-06	positive
PRJNA729271	stage_E14_vs_E10	ordinary_muscle_development	ENSGALG00010028565	PKN1	0.026121767	0.118811763	0.219858421	0.825981419	0.87820734	positive
PRJNA729271	stage_E14_vs_E10	ordinary_muscle_development	ENSGALG00010028566	ZDHHC12	0.60919083	0.122174084	4.986252466	6.15617E-07	3.50073E-06	positive
PRJNA729271	stage_E14_vs_E10	ordinary_muscle_development	ENSGALG00010028567	ENSGALG00010028567	4.687899667	0.867602697	5.403279267	6.54335E-08	4.28391E-07	positive
PRJNA729271	stage_E14_vs_E10	ordinary_muscle_development	ENSGALG00010028568	MLLT1	-0.401022148	0.063231904	-6.342085586	2.26675E-10	2.00835E-09	negative
PRJNA729271	stage_E14_vs_E10	ordinary_muscle_development	ENSGALG00010028569	ZER1	0.283582832	0.068201911	4.157989529	3.21061E-05	0.000138246	positive
PRJNA729271	stage_E14_vs_E10	ordinary_muscle_development	ENSGALG00010028572	TBC1D13	0.471998425	0.067230704	7.020578388	2.20952E-12	2.43922E-11	positive
PRJNA729271	stage_E14_vs_E10	ordinary_muscle_development	ENSGALG00010028573	KCNN1	1.505948318	0.261939739	5.749216695	8.96578E-09	6.55697E-08	positive
PRJNA729271	stage_E14_vs_E10	ordinary_muscle_development	ENSGALG00010028574	ENDOG	0.310113207	0.116084641	2.671440453	0.007552647	0.018634148	positive
PRJNA729271	stage_E14_vs_E10	ordinary_muscle_development	ENSGALG00010028575	SETDB1	0.039439273	0.118181761	0.333717083	0.738593058	0.812122615	positive
PRJNA729271	stage_E14_vs_E10	ordinary_muscle_development	ENSGALG00010028577	CERS2	0.219413479	0.131721691	1.665735361	0.095766136	0.164159193	positive
PRJNA729271	stage_E14_vs_E10	ordinary_muscle_development	ENSGALG00010028578	SPOUT1	0.246794094	0.119480536	2.065558977	0.038870141	0.076510415	positive
PRJNA729271	stage_E14_vs_E10	ordinary_muscle_development	ENSGALG00010028579	OCLN	-0.319468543	0.27295408	-1.170411311	0.241835486	0.347896524	negative
PRJNA729271	stage_E14_vs_E10	ordinary_muscle_development	ENSGALG00010028580	PRUNE1	0.068964206	0.152382552	0.452572853	0.65085635	0.742146224	positive
PRJNA729271	stage_E14_vs_E10	ordinary_muscle_development	ENSGALG00010028581	NR2F6	0.588634247	0.123494588	4.766478077	1.87474E-06	9.9249E-06	positive
PRJNA729271	stage_E14_vs_E10	ordinary_muscle_development	ENSGALG00010028582	KYAT1	-0.135357714	0.210825564	-0.642036532	0.520849464	0.628688357	negative
PRJNA729271	stage_E14_vs_E10	ordinary_muscle_development	ENSGALG00010028583	USHBP1	0.879007587	0.196596151	4.471133236	7.78062E-06	3.71231E-05	positive
PRJNA729271	stage_E14_vs_E10	ordinary_muscle_development	ENSGALG00010028584	LRRC8A	-0.1759533	0.075464548	-2.331602115	0.019721632	0.042903356	negative
PRJNA729271	stage_E14_vs_E10	ordinary_muscle_development	ENSGALG00010028585	MINDY1	0.390285046	0.121140431	3.221757107	0.001274071	0.003916789	positive
PRJNA729271	stage_E14_vs_E10	ordinary_muscle_development	ENSGALG00010028586	BABAM1	0.158776116	0.120810953	1.314252665	0.188761191	0.285415506	positive
PRJNA729271	stage_E14_vs_E10	ordinary_muscle_development	ENSGALG00010028587	ANKLE1	0.08465233	0.174049119	0.486370346	0.626704612	0.721245204	positive
PRJNA729271	stage_E14_vs_E10	ordinary_muscle_development	ENSGALG00010028588	ABHD8	-1.406288625	0.634109769	-2.217736886	0.026572778	0.055492352	negative
PRJNA729271	stage_E14_vs_E10	ordinary_muscle_development	ENSGALG00010028589	PHYHD1	2.506226664	0.176316685	14.2143477	7.46437E-46	8.38729E-44	positive
PRJNA729271	stage_E14_vs_E10	ordinary_muscle_development	ENSGALG00010028590	ENSGALG00010028590	0.133319859	0.228715149	0.582907864	0.559955327	0.663388023	positive
PRJNA729271	stage_E14_vs_E10	ordinary_muscle_development	ENSGALG00010028591	GABPB2	0.052161572	0.116658075	0.447132115	0.654779672	0.745447011	positive
PRJNA729271	stage_E14_vs_E10	ordinary_muscle_development	ENSGALG00010028592	SEMA6C	0.753991693	0.172967464	4.359153304	1.30567E-05	6.00814E-05	positive
PRJNA729271	stage_E14_vs_E10	ordinary_muscle_development	ENSGALG00010028593	DOLK	-0.011648486	0.112129242	-0.103884465	0.917261035	0.942355851	negative
PRJNA729271	stage_E14_vs_E10	ordinary_muscle_development	ENSGALG00010028594	ENSGALG00010028594	0.19671269	0.144370957	1.362550295	0.173024283	0.26604296	positive
PRJNA729271	stage_E14_vs_E10	ordinary_muscle_development	ENSGALG00010028595	SCNM1	0.048522721	0.112169261	0.43258483	0.665316412	0.753762875	positive
PRJNA729271	stage_E14_vs_E10	ordinary_muscle_development	ENSGALG00010028596	DDA1	0.082985167	0.088441155	0.938309393	0.348085434	0.463868563	positive
PRJNA729271	stage_E14_vs_E10	ordinary_muscle_development	ENSGALG00010028597	NUP188	-0.125626686	0.054634686	-2.299394325	0.021482558	0.046303298	negative
PRJNA729271	stage_E14_vs_E10	ordinary_muscle_development	ENSGALG00010028598	TMOD4	0.712542705	0.259310299	2.747838044	0.005998963	0.015265971	positive
PRJNA729271	stage_E14_vs_E10	ordinary_muscle_development	ENSGALG00010028599	VPS72	0.141842431	0.118126375	1.200768513	0.229841009	0.333744792	positive
PRJNA729271	stage_E14_vs_E10	ordinary_muscle_development	ENSGALG00010028600	PSMD4	0.331558363	0.132176325	2.508454998	0.012126041	0.028231122	positive
PRJNA729271	stage_E14_vs_E10	ordinary_muscle_development	ENSGALG00010028601	ANO8	0.765852783	0.223302435	3.42966607	0.000604324	0.002003587	positive
PRJNA729271	stage_E14_vs_E10	ordinary_muscle_development	ENSGALG00010028602	ZNF687	0.06717923	0.168894287	0.397759045	0.690807809	0.774300896	positive
PRJNA729271	stage_E14_vs_E10	ordinary_muscle_development	ENSGALG00010028603	SH3GLB2	-0.005432288	0.337265176	-0.016106876	0.987149128	0.991389635	negative
PRJNA729271	stage_E14_vs_E10	ordinary_muscle_development	ENSGALG00010028604	PI4KB	0.21120205	0.140112135	1.507378712	0.131713612	0.213364865	positive
PRJNA729271	stage_E14_vs_E10	ordinary_muscle_development	ENSGALG00010028605	GTPBP3	0.049734502	0.21906306	0.227032808	0.820398231	0.874259106	positive
PRJNA729271	stage_E14_vs_E10	ordinary_muscle_development	ENSGALG00010028606	PRPF3	-0.137597081	0.135674308	-1.014171972	0.3105007	0.424282892	negative
PRJNA729271	stage_E14_vs_E10	ordinary_muscle_development	ENSGALG00010028607	ENSGALG00010028607	-0.046853979	0.239627321	-0.195528536	0.8449792	0.89205925	negative
PRJNA729271	stage_E14_vs_E10	ordinary_muscle_development	ENSGALG00010028608	MIGA2	0.130939024	0.157995657	0.828750781	0.407245444	0.522141573	positive
PRJNA729271	stage_E14_vs_E10	ordinary_muscle_development	ENSGALG00010028609	MRPS21	0.317518041	0.144682126	2.194590646	0.028192975	0.058312953	positive
PRJNA729271	stage_E14_vs_E10	ordinary_muscle_development	ENSGALG00010028610	ENSGALG00010028610	0.126562143	0.532387148	0.237725767	0.812093797	0.868282038	positive
PRJNA729271	stage_E14_vs_E10	ordinary_muscle_development	ENSGALG00010028612	DIRAS1	-0.87083313	0.591579367	-1.472047842	0.141007959	0.225721741	negative
PRJNA729271	stage_E14_vs_E10	ordinary_muscle_development	ENSGALG00010028613	ENSGALG00010028613	-0.336879337	0.370458663	-0.909357426	0.363161487	0.479244879	negative
PRJNA729271	stage_E14_vs_E10	ordinary_muscle_development	ENSGALG00010028618	DOLPP1	-0.200486948	0.098557864	-2.034205479	0.041930882	0.081538522	negative
PRJNA729271	stage_E14_vs_E10	ordinary_muscle_development	ENSGALG00010028626	CRAT	0.595598302	0.173460914	3.433616764	0.000595586	0.001978687	positive
PRJNA729271	stage_E14_vs_E10	ordinary_muscle_development	ENSGALG00010028642	APH1A	0.075674084	0.138238813	0.547415612	0.584093232	0.684930944	positive
PRJNA729271	stage_E14_vs_E10	ordinary_muscle_development	ENSGALG00010028649	ENSGALG00010028649	-0.154798109	0.172518594	-0.897283621	0.369567594	0.485827832	negative
PRJNA729271	stage_E14_vs_E10	ordinary_muscle_development	ENSGALG00010028658	PTPA	0.234407549	0.081050119	2.892130865	0.003826385	0.010278624	positive
PRJNA729271	stage_E14_vs_E10	ordinary_muscle_development	ENSGALG00010028662	FCRL4	-0.297916176	0.226133781	-1.317433312	0.187693421	0.284167133	negative
PRJNA729271	stage_E14_vs_E10	ordinary_muscle_development	ENSGALG00010028669	ZBTB34	0.185457475	0.110470089	1.678802619	0.093190518	0.160389048	positive
PRJNA729271	stage_E14_vs_E10	ordinary_muscle_development	ENSGALG00010028671	CADM3	-0.187473437	0.217331786	-0.862613979	0.388349742	0.504221561	negative
PRJNA729271	stage_E14_vs_E10	ordinary_muscle_development	ENSGALG00010028675	ZBTB43	-0.107811932	0.069788936	-1.544828439	0.122387766	0.200748068	negative
PRJNA729271	stage_E14_vs_E10	ordinary_muscle_development	ENSGALG00010028678	ENSGALG00010028678	0.3939427	0.366415692	1.075125081	0.282318732	0.393670998	positive
PRJNA729271	stage_E14_vs_E10	ordinary_muscle_development	ENSGALG00010028686	TAGLN2	-0.141516921	0.119377967	-1.185452594	0.235838548	0.340519947	negative
PRJNA729271	stage_E14_vs_E10	ordinary_muscle_development	ENSGALG00010028689	CFAP45	0.304277031	0.61917518	0.491423171	0.623127179	0.718154445	positive
PRJNA729271	stage_E14_vs_E10	ordinary_muscle_development	ENSGALG00010028692	RALGPS1	0.092529622	0.110151789	0.840019235	0.400897602	0.516213089	positive
PRJNA729271	stage_E14_vs_E10	ordinary_muscle_development	ENSGALG00010028695	VSIG8	4.368959224	0.794576598	5.498474578	3.83091E-08	2.58756E-07	positive
PRJNA729271	stage_E14_vs_E10	ordinary_muscle_development	ENSGALG00010028700	ANGPTL2	0.979162507	0.197606264	4.955118755	7.22861E-07	4.06908E-06	positive
PRJNA729271	stage_E14_vs_E10	ordinary_muscle_development	ENSGALG00010028707	ENSGALG00010028707	2.600912472	0.91544295	2.841151896	0.00449509	0.011861701	positive
PRJNA729271	stage_E14_vs_E10	ordinary_muscle_development	ENSGALG00010028712	FBXW2	-0.047907102	0.084074386	-0.569818044	0.568801116	0.671234403	negative
PRJNA729271	stage_E14_vs_E10	ordinary_muscle_development	ENSGALG00010028726	SLC2A8	-0.019005415	0.111093258	-0.171076227	0.864163828	0.906502728	negative
PRJNA729271	stage_E14_vs_E10	ordinary_muscle_development	ENSGALG00010028727	ENSGALG00010028727	-0.140967019	0.169975821	-0.829335717	0.406914465	0.521782128	negative
PRJNA729271	stage_E14_vs_E10	ordinary_muscle_development	ENSGALG00010028734	ENSGALG00010028734	0.731426918	0.359434425	2.034938413	0.04185707	0.081438689	positive
PRJNA729271	stage_E14_vs_E10	ordinary_muscle_development	ENSGALG00010028735	ENSGALG00010028735	-0.13748853	0.316794172	-0.433999557	0.664288762	0.753137709	negative
PRJNA729271	stage_E14_vs_E10	ordinary_muscle_development	ENSGALG00010028738	MIDN	0.047464184	0.182280834	0.260390427	0.794562627	0.8557237	positive
PRJNA729271	stage_E14_vs_E10	ordinary_muscle_development	ENSGALG00010028739	GARNL3	-0.053282044	0.076996816	-0.692003215	0.48893531	0.599688383	negative
PRJNA729271	stage_E14_vs_E10	ordinary_muscle_development	ENSGALG00010028740	DAZAP1	0.265310052	0.105133546	2.523552786	0.011617562	0.027182657	positive
PRJNA729271	stage_E14_vs_E10	ordinary_muscle_development	ENSGALG00010028742	ENSGALG00010028742	0.495143169	0.132703711	3.731193099	0.000190575	0.000711405	positive
PRJNA729271	stage_E14_vs_E10	ordinary_muscle_development	ENSGALG00010028743	ARRDC1	0.930854686	0.185873198	5.008009197	5.49959E-07	3.14961E-06	positive
PRJNA729271	stage_E14_vs_E10	ordinary_muscle_development	ENSGALG00010028744	HOMER3	0.852555626	0.208203831	4.094812392	4.2251E-05	0.000178032	positive
PRJNA729271	stage_E14_vs_E10	ordinary_muscle_development	ENSGALG00010028745	ENSGALG00010028745	0.712721201	0.135826756	5.247281337	1.5436E-07	9.55767E-07	positive
PRJNA729271	stage_E14_vs_E10	ordinary_muscle_development	ENSGALG00010028746	MYDGF	0.096160526	0.122083035	0.787664937	0.43089272	0.545010007	positive
PRJNA729271	stage_E14_vs_E10	ordinary_muscle_development	ENSGALG00010028747	ENSGALG00010028747	-1.278620095	0.185169699	-6.905125967	5.01589E-12	5.27998E-11	negative
PRJNA729271	stage_E14_vs_E10	ordinary_muscle_development	ENSGALG00010028748	PIAS3	0.126047392	0.201204915	0.626462787	0.531011438	0.638491148	positive
PRJNA729271	stage_E14_vs_E10	ordinary_muscle_development	ENSGALG00010028750	OTUD7B	-0.102634719	0.120094507	-0.854616272	0.392763617	0.508222516	negative
PRJNA729271	stage_E14_vs_E10	ordinary_muscle_development	ENSGALG00010028751	EHMT1	-0.175862992	0.094754038	-1.85599469	0.063454315	0.116015426	negative
PRJNA729271	stage_E14_vs_E10	ordinary_muscle_development	ENSGALG00010028752	MTMR11	-0.389592933	0.151150136	-2.577522875	0.009951129	0.023743477	negative
PRJNA729271	stage_E14_vs_E10	ordinary_muscle_development	ENSGALG00010028753	SV2A	-0.449543956	0.762923471	-0.589238598	0.555701228	0.660020428	negative
PRJNA729271	stage_E14_vs_E10	ordinary_muscle_development	ENSGALG00010028756	ENSGALG00010028756	1.47088382	0.559880884	2.627137063	0.008610664	0.020934515	positive
PRJNA729271	stage_E14_vs_E10	ordinary_muscle_development	ENSGALG00010028757	MAT2A	2.05022184	1.108837686	1.848982827	0.064460292	0.117587709	positive
PRJNA729271	stage_E14_vs_E10	ordinary_muscle_development	ENSGALG00010028760	SSR2	0.171843781	0.096926911	1.772921266	0.076241754	0.13571463	positive
PRJNA729271	stage_E14_vs_E10	ordinary_muscle_development	ENSGALG00010028761	CTSS	0.550835473	0.238001685	2.314418376	0.020644784	0.044703636	positive
PRJNA729271	stage_E14_vs_E10	ordinary_muscle_development	ENSGALG00010028762	CACNA1B	-1.168538108	0.566036017	-2.064423594	0.038977571	0.076649016	negative
PRJNA729271	stage_E14_vs_E10	ordinary_muscle_development	ENSGALG00010028763	CTSK	0.638393952	0.217389467	2.936636999	0.003317922	0.00907763	positive
PRJNA729271	stage_E14_vs_E10	ordinary_muscle_development	ENSGALG00010028764	ARNT	0.291248296	0.125257782	2.325191231	0.020061737	0.043584364	positive
PRJNA729271	stage_E14_vs_E10	ordinary_muscle_development	ENSGALG00010028768	NTNG2	-1.213368994	0.350698651	-3.45986216	0.000540452	0.001818443	negative
PRJNA729271	stage_E14_vs_E10	ordinary_muscle_development	ENSGALG00010028771	SETX	-0.430150825	0.071920855	-5.98089144	2.2192E-09	1.74917E-08	negative
PRJNA729271	stage_E14_vs_E10	ordinary_muscle_development	ENSGALG00010028772	TTF1	-0.242825482	0.071232414	-3.40891835	0.00065221	0.002145174	negative
PRJNA729271	stage_E14_vs_E10	ordinary_muscle_development	ENSGALG00010028782	MLLT11	-0.321847641	0.255091711	-1.261693842	0.207058974	0.307172839	negative
PRJNA729271	stage_E14_vs_E10	ordinary_muscle_development	ENSGALG00010028783	DDX31	0.093602686	0.071324691	1.312346188	0.189403355	0.286247693	positive
PRJNA729271	stage_E14_vs_E10	ordinary_muscle_development	ENSGALG00010028786	GTF3C4	0.066910484	0.062918602	1.063445174	0.287580116	0.3998536	positive
PRJNA729271	stage_E14_vs_E10	ordinary_muscle_development	ENSGALG00010028800	ENSGALG00010028800	2.471069074	0.38828208	6.364107949	1.96428E-10	1.75646E-09	positive
PRJNA729271	stage_E14_vs_E10	ordinary_muscle_development	ENSGALG00010028806	SPACA9	1.630223435	0.363991561	4.478739649	7.5085E-06	3.59669E-05	positive
PRJNA729271	stage_E14_vs_E10	ordinary_muscle_development	ENSGALG00010028818	TSC1	1.375385572	0.116539202	11.80191338	3.81536E-32	1.81323E-30	positive
PRJNA729271	stage_E14_vs_E10	ordinary_muscle_development	ENSGALG00010028823	GFI1B	0.398638316	0.30970261	1.287164859	0.198036833	0.29663895	positive
PRJNA729271	stage_E14_vs_E10	ordinary_muscle_development	ENSGALG00010028826	CEL	-16.11845486	2.846051572	-5.663444406	1.48364E-08	1.05886E-07	negative
PRJNA729271	stage_E14_vs_E10	ordinary_muscle_development	ENSGALG00010028829	GTF3C5	-0.233307371	0.111559834	-2.091320524	0.036499341	0.072453841	negative
PRJNA729271	stage_E14_vs_E10	ordinary_muscle_development	ENSGALG00010028833	GBGT1	0.534159662	0.170090975	3.14043507	0.001686971	0.005016956	positive
PRJNA729271	stage_E14_vs_E10	ordinary_muscle_development	ENSGALG00010028849	ENSGALG00010028849	2.003615811	0.333059701	6.01578577	1.79016E-09	1.42574E-08	positive
PRJNA729271	stage_E14_vs_E10	ordinary_muscle_development	ENSGALG00010028853	ENSGALG00010028853	7.741328934	1.178487011	6.568870819	5.06982E-11	4.85384E-10	positive
PRJNA729271	stage_E14_vs_E10	ordinary_muscle_development	ENSGALG00010028854	RPL7A	0.486259698	0.140320494	3.465350532	0.000529541	0.001785045	positive
PRJNA729271	stage_E14_vs_E10	ordinary_muscle_development	ENSGALG00010028855	SURF6	0.042508166	0.16771924	0.253448359	0.799921762	0.859239708	positive
PRJNA729271	stage_E14_vs_E10	ordinary_muscle_development	ENSGALG00010028856	ETV3	-0.009035299	0.108715954	-0.083109232	0.933764685	0.954414283	negative
PRJNA729271	stage_E14_vs_E10	ordinary_muscle_development	ENSGALG00010028857	MED22	0.215094559	0.077819859	2.764006019	0.005709649	0.014612717	positive
PRJNA729271	stage_E14_vs_E10	ordinary_muscle_development	ENSGALG00010028858	ENSGALG00010028858	0.161032861	0.457108843	0.352285597	0.724624089	0.800566019	positive
PRJNA729271	stage_E14_vs_E10	ordinary_muscle_development	ENSGALG00010028859	SURF1	0.20091299	0.108887783	1.84513804	0.065017461	0.118544415	positive
PRJNA729271	stage_E14_vs_E10	ordinary_muscle_development	ENSGALG00010028860	SURF2	0.126639348	0.171411274	0.738804079	0.460025974	0.572662015	positive
PRJNA729271	stage_E14_vs_E10	ordinary_muscle_development	ENSGALG00010028861	SURF4	-0.030196334	0.074422668	-0.405741083	0.684932853	0.769560631	negative
PRJNA729271	stage_E14_vs_E10	ordinary_muscle_development	ENSGALG00010028862	ENSGALG00010028862	10.76905867	1.134090627	9.49576551	2.18599E-21	5.24602E-20	positive
PRJNA729271	stage_E14_vs_E10	ordinary_muscle_development	ENSGALG00010028865	STKLD1	0.088467615	0.169289152	0.522582898	0.601264544	0.700026471	positive
PRJNA729271	stage_E14_vs_E10	ordinary_muscle_development	ENSGALG00010028866	HORMAD1	-0.571791669	0.459335245	-1.244824287	0.213196346	0.31469257	negative
PRJNA729271	stage_E14_vs_E10	ordinary_muscle_development	ENSGALG00010028867	REXO4	-0.268414574	0.116036382	-2.313193225	0.020712018	0.044829132	negative
PRJNA729271	stage_E14_vs_E10	ordinary_muscle_development	ENSGALG00010028868	ENSGALG00010028868	11.35363887	4.15933299	2.729677787	0.006339625	0.016006422	positive
PRJNA729271	stage_E14_vs_E10	ordinary_muscle_development	ENSGALG00010028869	ADAMTS13	0.240803074	0.172436643	1.396472753	0.16257218	0.253086	positive
PRJNA729271	stage_E14_vs_E10	ordinary_muscle_development	ENSGALG00010028870	CACFD1	-0.962932635	0.222542054	-4.326969306	1.51175E-05	6.89299E-05	negative
PRJNA729271	stage_E14_vs_E10	ordinary_muscle_development	ENSGALG00010028871	PIP5K1A	0.050846602	0.107585864	0.472614154	0.636488482	0.729954945	positive
PRJNA729271	stage_E14_vs_E10	ordinary_muscle_development	ENSGALG00010028872	SLC2A6	0.466563678	0.34714541	1.34400071	0.178948157	0.273469008	positive
PRJNA729271	stage_E14_vs_E10	ordinary_muscle_development	ENSGALG00010028873	TMEM8C	1.848684239	0.272781179	6.777169315	1.22553E-11	1.25187E-10	positive
PRJNA729271	stage_E14_vs_E10	ordinary_muscle_development	ENSGALG00010028874	CRPL1	0.396880421	0.520144162	0.763020043	0.445451443	0.558961786	positive
PRJNA729271	stage_E14_vs_E10	ordinary_muscle_development	ENSGALG00010028875	DUSP23	0.581493346	0.119906968	4.849537549	1.2375E-06	6.75867E-06	positive
PRJNA729271	stage_E14_vs_E10	ordinary_muscle_development	ENSGALG00010028876	ADAMTSL2	-0.205562237	0.201768977	-1.018800017	0.308297917	0.421901276	negative
PRJNA729271	stage_E14_vs_E10	ordinary_muscle_development	ENSGALG00010028877	FAM163B	-1.291589085	0.493742921	-2.615914131	0.008898893	0.021526946	negative
PRJNA729271	stage_E14_vs_E10	ordinary_muscle_development	ENSGALG00010028878	IGSF9	-0.341288475	0.15899445	-2.146543325	0.031829656	0.06451837	negative
PRJNA729271	stage_E14_vs_E10	ordinary_muscle_development	ENSGALG00010028881	SARDH	2.311961453	0.229348976	10.08053968	6.73551E-24	1.85259E-22	positive
PRJNA729271	stage_E14_vs_E10	ordinary_muscle_development	ENSGALG00010028884	VAV2	-0.421737361	0.128625561	-3.278799005	0.001042499	0.003274737	negative
PRJNA729271	stage_E14_vs_E10	ordinary_muscle_development	ENSGALG00010028885	BRD3	-0.175129664	0.139989221	-1.251022493	0.210926271	0.311945342	negative
PRJNA729271	stage_E14_vs_E10	ordinary_muscle_development	ENSGALG00010028886	ENSGALG00010028886	6.324473736	1.66800508	3.791639373	0.000149656	0.000570536	positive

PRJNA729271	stage_E14_vs_E10	ordinary_muscle_development	ENSGALG00010028887	SF3B4	-0.148157769	0.173492285	-0.853973243	0.393119815	0.508460851	negative
PRJNA729271	stage_E14_vs_E10	ordinary_muscle_development	ENSGALG00010028888	WDR5B	-0.315942697	0.079979584	-3.950291819	7.8056E-05	0.000313326	negative
PRJNA729271	stage_E14_vs_E10	ordinary_muscle_development	ENSGALG00010028889	FUBP3	-0.063967698	0.08139317	-0.785909894	0.431920278	0.546097907	negative
PRJNA729271	stage_E14_vs_E10	ordinary_muscle_development	ENSGALG00010028890	ENSGALG00010028890	-1.221391086	0.216918591	-5.630642725	1.79539E-08	1.26762E-07	negative
PRJNA729271	stage_E14_vs_E10	ordinary_muscle_development	ENSGALG00010028891	SLC31A2	0.229613183	0.240718066	0.953867678	0.340150626	0.455135542	positive
PRJNA729271	stage_E14_vs_E10	ordinary_muscle_development	ENSGALG00010028892	NIBAN2	-0.062732085	0.140487401	-0.446531748	0.655213185	0.74576483	negative
PRJNA729271	stage_E14_vs_E10	ordinary_muscle_development	ENSGALG00010028893	STXBP1	-0.087440249	0.202118854	-0.432617971	0.665292331	0.753762875	negative
PRJNA729271	stage_E14_vs_E10	ordinary_muscle_development	ENSGALG00010028894	AKNA	-0.626669845	0.152898743	-4.09859382	4.15668E-05	0.000175454	negative
PRJNA729271	stage_E14_vs_E10	ordinary_muscle_development	ENSGALG00010028895	WHRN	-1.783145667	0.494548309	-3.605604614	0.000311427	0.001110301	negative
PRJNA729271	stage_E14_vs_E10	ordinary_muscle_development	ENSGALG00010028896	ATP6V1G1	0.363549749	0.098672567	3.684405494	0.000229237	0.000839513	positive
PRJNA729271	stage_E14_vs_E10	ordinary_muscle_development	ENSGALG00010028897	TMEM268	-0.287062445	0.281313132	-1.020437414	0.307521057	0.421076679	negative
PRJNA729271	stage_E14_vs_E10	ordinary_muscle_development	ENSGALG00010028898	TNFSF15	-1.668927877	0.249841648	-6.679942648	2.39036E-11	2.35382E-10	negative
PRJNA729271	stage_E14_vs_E10	ordinary_muscle_development	ENSGALG00010028899	TNFSF8	-1.430139271	0.816212987	-1.752164317	0.079745557	0.141050867	negative
PRJNA729271	stage_E14_vs_E10	ordinary_muscle_development	ENSGALG00010028900	TNC	-3.009302744	0.54952046	-5.476234218	4.34473E-08	2.90752E-07	negative
PRJNA729271	stage_E14_vs_E10	ordinary_muscle_development	ENSGALG00010028901	PAPPA	-0.781543475	0.111869053	-6.986234828	2.82361E-12	3.09594E-11	negative
PRJNA729271	stage_E14_vs_E10	ordinary_muscle_development	ENSGALG00010028902	ENSGALG00010028902	-1.054993107	0.421545263	-2.502680492	0.012325676	0.028626931	negative
PRJNA729271	stage_E14_vs_E10	ordinary_muscle_development	ENSGALG00010028903	ASTN2	-1.782297019	0.212849258	-8.373517643	5.59233E-17	9.43665E-16	negative
PRJNA729271	stage_E14_vs_E10	ordinary_muscle_development	ENSGALG00010028904	TRIM32	-0.113085637	0.079406713	-1.424131952	0.154408279	0.243107317	negative
PRJNA729271	stage_E14_vs_E10	ordinary_muscle_development	ENSGALG00010028905	COLGALT1	0.114473492	0.085386623	1.34064902	0.180034434	0.274926161	positive
PRJNA729271	stage_E14_vs_E10	ordinary_muscle_development	ENSGALG00010028909	ENSGALG00010028909	-1.08160438	0.132009495	-8.193383179	2.53984E-16	4.00163E-15	negative
PRJNA729271	stage_E14_vs_E10	ordinary_muscle_development	ENSGALG00010028911	LMX1B	-2.580397397	0.521653434	-4.946574164	7.5531E-07	4.24349E-06	negative
PRJNA729271	stage_E14_vs_E10	ordinary_muscle_development	ENSGALG00010028912	EXFABP	0.729225234	0.497377054	1.466141689	0.14260969	0.227805032	positive
PRJNA729271	stage_E14_vs_E10	ordinary_muscle_development	ENSGALG00010028914	TLR4	0.522914421	0.229774928	2.27576797	0.022859901	0.048865103	positive
PRJNA729271	stage_E14_vs_E10	ordinary_muscle_development	ENSGALG00010028915	ADGRD2	0.037225642	0.483813859	0.076942075	0.938669626	0.957092613	positive
PRJNA729271	stage_E14_vs_E10	ordinary_muscle_development	ENSGALG00010028917	NR6A1	0.513964475	0.123356544	4.166495424	3.09318E-05	0.000133638	positive
PRJNA729271	stage_E14_vs_E10	ordinary_muscle_development	ENSGALG00010028918	AGPAT2	0.740933784	0.184704796	4.01144853	6.03473E-05	0.00024738	positive
PRJNA729271	stage_E14_vs_E10	ordinary_muscle_development	ENSGALG00010028919	EGFL7	0.361404606	0.169411915	2.133289191	0.03290101	0.066346709	positive
PRJNA729271	stage_E14_vs_E10	ordinary_muscle_development	ENSGALG00010028921	MRPL41	0.093922248	0.119373363	0.786794019	0.431402455	0.545604973	positive
PRJNA729271	stage_E14_vs_E10	ordinary_muscle_development	ENSGALG00010028923	ENSGALG00010028923	-0.011613927	0.192903676	-0.060205833	0.9519917	0.966731098	negative
PRJNA729271	stage_E14_vs_E10	ordinary_muscle_development	ENSGALG00010028924	TMEM203	0.147562859	0.119194155	1.23800416	0.215714518	0.317407567	positive
PRJNA729271	stage_E14_vs_E10	ordinary_muscle_development	ENSGALG00010028925	ENSGALG00010028925	0.400954276	0.335747383	1.194214152	0.232394208	0.336573402	positive
PRJNA729271	stage_E14_vs_E10	ordinary_muscle_development	ENSGALG00010028926	ENSGALG00010028926	-0.588771381	0.633469072	-0.92943982	0.352661199	0.46895682	negative
PRJNA729271	stage_E14_vs_E10	ordinary_muscle_development	ENSGALG00010029140	NPDC1	0.05899043	0.190878236	0.309047438	0.757285441	0.826881542	positive
PRJNA729271	stage_E14_vs_E10	ordinary_muscle_development	ENSGALG00010029141	C5	-0.532500301	0.567383183	-0.93851971	0.347977393	0.463809867	negative
PRJNA729271	stage_E14_vs_E10	ordinary_muscle_development	ENSGALG00010029142	ENSGALG00010029142	0.12209877	0.182849226	0.667756558	0.504289001	0.613433121	positive
PRJNA729271	stage_E14_vs_E10	ordinary_muscle_development	ENSGALG00010029143	SH2D3C	0.343411039	0.107134473	3.205420527	0.001348653	0.004118122	positive
PRJNA729271	stage_E14_vs_E10	ordinary_muscle_development	ENSGALG00010029144	ENSGALG00010029144	2.864623651	0.222148181	12.8951029	4.79623E-38	3.29485E-36	positive
PRJNA729271	stage_E14_vs_E10	ordinary_muscle_development	ENSGALG00010029145	RC3H2	0.146917433	0.133652237	1.099251578	0.271658347	0.381632849	positive
PRJNA729271	stage_E14_vs_E10	ordinary_muscle_development	ENSGALG00010029146	NEK6	0.061028361	0.058045576	1.051386955	0.293080905	0.405750498	positive
PRJNA729271	stage_E14_vs_E10	ordinary_muscle_development	ENSGALG00010029147	RABL6	0.110446532	0.102179367	1.08090836	0.279737879	0.390598262	positive
PRJNA729271	stage_E14_vs_E10	ordinary_muscle_development	ENSGALG00010029148	RALGDS	0.452792562	0.118217778	3.830156257	0.000128062	0.000495266	positive
PRJNA729271	stage_E14_vs_E10	ordinary_muscle_development	ENSGALG00010029149	ENSGALG00010029149	-0.855300802	0.204856272	-4.175126255	2.97821E-05	0.000129056	negative
PRJNA729271	stage_E14_vs_E10	ordinary_muscle_development	ENSGALG00010029150	NARF	-0.052734392	0.122817986	-0.429370269	0.667653786	0.755475537	negative
PRJNA729271	stage_E14_vs_E10	ordinary_muscle_development	ENSGALG00010029151	CYBC1	-0.4228307	0.112705489	-3.751642494	0.00017568	0.000660223	negative
PRJNA729271	stage_E14_vs_E10	ordinary_muscle_development	ENSGALG00010029152	HEXD	0.097762146	0.121731643	0.803095594	0.421919484	0.536278755	positive
PRJNA729271	stage_E14_vs_E10	ordinary_muscle_development	ENSGALG00010029153	OGFOD3	0.100241765	0.074195439	1.351050228	0.176679344	0.270600918	positive
PRJNA729271	stage_E14_vs_E10	ordinary_muscle_development	ENSGALG00010029154	UTS2R	1.394323861	0.139010911	10.03031958	1.12153E-23	3.03695E-22	positive
PRJNA729271	stage_E14_vs_E10	ordinary_muscle_development	ENSGALG00010029155	ENSGALG00010029155	1.360482692	0.578276767	2.352649753	0.018640182	0.040875861	positive
PRJNA729271	stage_E14_vs_E10	ordinary_muscle_development	ENSGALG00010029156	ENSGALG00010029156	-0.531862736	0.973079452	-0.546576885	0.584669453	0.685495731	negative
PRJNA729271	stage_E14_vs_E10	ordinary_muscle_development	ENSGALG00010029158	CD7	-0.053037403	0.416423809	-0.127364002	0.898652306	0.930211158	negative
PRJNA729271	stage_E14_vs_E10	ordinary_muscle_development	ENSGALG00010029159	SSC4D	-2.577343541	0.7880074	-3.270709819	0.001072779	0.003354142	negative
PRJNA729271	stage_E14_vs_E10	ordinary_muscle_development	ENSGALG00010029160	DUS1L	0.208119817	0.09152205	2.27398553	0.022966857	0.04907202	positive
PRJNA729271	stage_E14_vs_E10	ordinary_muscle_development	ENSGALG00010029162	ELN	-1.422192336	0.311332406	-4.568083214	4.92205E-06	2.43084E-05	negative
PRJNA729271	stage_E14_vs_E10	ordinary_muscle_development	ENSGALG00010029163	ANKFY1	-0.266153196	0.094020645	-2.830795264	0.004643243	0.012199349	negative
PRJNA729271	stage_E14_vs_E10	ordinary_muscle_development	ENSGALG00010029164	CYB5D2	0.535919995	0.080578355	6.650917516	2.91271E-11	2.84117E-10	positive
PRJNA729271	stage_E14_vs_E10	ordinary_muscle_development	ENSGALG00010029165	RAC3	0.201256675	0.119657723	1.681936357	0.092581182	0.159491827	positive
PRJNA729271	stage_E14_vs_E10	ordinary_muscle_development	ENSGALG00010029166	ZZEF1	0.068852712	0.123805367	0.55613673	0.578117388	0.679682986	positive
PRJNA729271	stage_E14_vs_E10	ordinary_muscle_development	ENSGALG00010029167	ENSGALG00010029167	-0.118944929	0.127266294	-0.934614538	0.349986984	0.46597422	negative
PRJNA729271	stage_E14_vs_E10	ordinary_muscle_development	ENSGALG00010029168	RFNG	0.347975275	0.079577981	4.372758283	1.22687E-05	5.67254E-05	positive
PRJNA729271	stage_E14_vs_E10	ordinary_muscle_development	ENSGALG00010029169	SBDS	0.152984622	0.077970959	1.962071834	0.049754122	0.09442079	positive
PRJNA729271	stage_E14_vs_E10	ordinary_muscle_development	ENSGALG00010029170	DCXR	0.364332479	0.138694458	2.626871216	0.008617394	0.02094034	positive
PRJNA729271	stage_E14_vs_E10	ordinary_muscle_development	ENSGALG00010029171	ENSGALG00010029171	0.010538754	0.14560853	0.072377311	0.942301641	0.959452654	positive
PRJNA729271	stage_E14_vs_E10	ordinary_muscle_development	ENSGALG00010029172	LRRC45	-0.126122757	0.071958063	-1.752725857	0.079649075	0.140897406	negative
PRJNA729271	stage_E14_vs_E10	ordinary_muscle_development	ENSGALG00010029173	ZNHIT3	0.278730671	0.132374522	2.105621733	0.035237223	0.070314366	positive
PRJNA729271	stage_E14_vs_E10	ordinary_muscle_development	ENSGALG00010029174	CALN1	-1.965100674	0.288824735	-6.80378248	1.01907E-11	1.04466E-10	negative
PRJNA729271	stage_E14_vs_E10	ordinary_muscle_development	ENSGALG00010029175	CENPX	0.283865584	0.128603469	2.207293359	0.027293571	0.056744164	positive
PRJNA729271	stage_E14_vs_E10	ordinary_muscle_development	ENSGALG00010029176	ENSGALG00010029176	-1.750454196	0.589029223	-2.971761208	0.002960969	0.008218928	negative
PRJNA729271	stage_E14_vs_E10	ordinary_muscle_development	ENSGALG00010029177	ASPSCR1	0.570489486	0.079442096	7.181198813	6.91028E-13	8.03244E-12	positive
PRJNA729271	stage_E14_vs_E10	ordinary_muscle_development	ENSGALG00010029178	NOTUM	-0.188477609	0.205915224	-0.915316532	0.360025483	0.476406974	negative
PRJNA729271	stage_E14_vs_E10	ordinary_muscle_development	ENSGALG00010029179	PIGW	0.13943787	0.146440285	0.952182453	0.341004458	0.455899246	positive
PRJNA729271	stage_E14_vs_E10	ordinary_muscle_development	ENSGALG00010029180	CA4	1.58946143	0.470729507	3.376591875	0.000733899	0.002391605	positive
PRJNA729271	stage_E14_vs_E10	ordinary_muscle_development	ENSGALG00010029181	MYO19	-0.20473153	0.102730731	-1.99289471	0.046272978	0.08896761	negative
PRJNA729271	stage_E14_vs_E10	ordinary_muscle_development	ENSGALG00010029182	HEATR6	-0.242767175	0.064514471	-3.762987935	0.000167895	0.000633926	negative
PRJNA729271	stage_E14_vs_E10	ordinary_muscle_development	ENSGALG00010029183	ENSGALG00010029183	0.216911894	0.139221472	1.558034773	0.119225005	0.196666847	positive
PRJNA729271	stage_E14_vs_E10	ordinary_muscle_development	ENSGALG00010029184	DYNLL2	0.401401735	0.095349621	4.209788456	2.5561E-05	0.000111868	positive
PRJNA729271	stage_E14_vs_E10	ordinary_muscle_development	ENSGALG00010029186	ZNF302	-0.145179364	0.14011721	-1.036127996	0.300142433	0.413945249	negative
PRJNA729271	stage_E14_vs_E10	ordinary_muscle_development	ENSGALG00010029187	SRSF1	-0.126279276	0.051684702	-2.44326212	0.014555162	0.033085581	negative
PRJNA729271	stage_E14_vs_E10	ordinary_muscle_development	ENSGALG00010029188	ENSGALG00010029188	0.348609467	0.25108186	1.388429519	0.165006293	0.256160032	positive
PRJNA729271	stage_E14_vs_E10	ordinary_muscle_development	ENSGALG00010029189	ENSGALG00010029189	0.310538709	0.327103242	0.949359922	0.342437587	0.457562023	positive
PRJNA729271	stage_E14_vs_E10	ordinary_muscle_development	ENSGALG00010029190	DHRS7C	2.138245934	0.167703432	12.75016207	3.11098E-37	2.06851E-35	positive
PRJNA729271	stage_E14_vs_E10	ordinary_muscle_development	ENSGALG00010029192	DTX2	1.502236336	0.099591042	15.08405083	2.06219E-51	3.01934E-49	positive
PRJNA729271	stage_E14_vs_E10	ordinary_muscle_development	ENSGALG00010029194	DHRS11	0.821060686	0.11833142	6.938653212	3.95856E-12	4.23463E-11	positive
PRJNA729271	stage_E14_vs_E10	ordinary_muscle_development	ENSGALG00010029196	MRM1	-0.255445355	0.095300146	-2.680429826	0.007352768	0.018190539	negative
PRJNA729271	stage_E14_vs_E10	ordinary_muscle_development	ENSGALG00010029197	LHX1	4.810033985	0.548898597	8.763064819	1.90012E-18	3.60501E-17	positive
PRJNA729271	stage_E14_vs_E10	ordinary_muscle_development	ENSGALG00010029198	PPM1D	0.287578221	0.174141601	1.651404482	0.09865601	0.168277109	positive
PRJNA729271	stage_E14_vs_E10	ordinary_muscle_development	ENSGALG00010029199	APPBP2	0.220663738	0.089219309	2.473273334	0.01338817	0.0307546	positive
PRJNA729271	stage_E14_vs_E10	ordinary_muscle_development	ENSGALG00010029200	GAS7	0.60267214	0.124540869	4.839151563	1.30395E-06	7.11087E-06	positive
PRJNA729271	stage_E14_vs_E10	ordinary_muscle_development	ENSGALG00010029201	PIGS	-0.4486865	0.108069297	-4.151840661	3.29812E-05	0.000141648	negative
PRJNA729271	stage_E14_vs_E10	ordinary_muscle_development	ENSGALG00010029202	PSMD12	-0.008800338	0.14273728	-0.061654095	0.950838297	0.965830492	negative
PRJNA729271	stage_E14_vs_E10	ordinary_muscle_development	ENSGALG00010029203	ALDOC	0.080020914	0.172414668	0.464118949	0.642562526	0.734943882	positive
PRJNA729271	stage_E14_vs_E10	ordinary_muscle_development	ENSGALG00010029204	HELZ	0.112768298	0.097394205	1.157854291	0.246923504	0.353566747	positive
PRJNA729271	stage_E14_vs_E10	ordinary_muscle_development	ENSGALG00010029205	CACNG1	2.838942547	0.114283744	24.84117545	3.2209E-136	1.5562E-132	positive
PRJNA729271	stage_E14_vs_E10	ordinary_muscle_development	ENSGALG00010029206	CACNG4	2.951683594	0.290553469	10.15883101	3.02684E-24	8.5358E-23	positive
PRJNA729271	stage_E14_vs_E10	ordinary_muscle_development	ENSGALG00010029207	SPAG5	0.092712561	0.218283271	0.424735075	0.671029811	0.757934787	positive
PRJNA729271	stage_E14_vs_E10	ordinary_muscle_development	ENSGALG00010029208	CACNG5	2.873473563	0.227670321	12.62120396	1.61336E-36	9.90916E-35	positive
PRJNA729271	stage_E14_vs_E10	ordinary_muscle_development	ENSGALG00010029209	PRKCA	0.423869978	0.096180126	4.40704326	1.04791E-05	4.89984E-05	positive
PRJNA729271	stage_E14_vs_E10	ordinary_muscle_development	ENSGALG00010029210	RSKR	0.960357258	0.285085179	3.368667786	0.000755324	0.002450286	positive
PRJNA729271	stage_E14_vs_E10	ordinary_muscle_development	ENSGALG00010029211	SDF2	0.194277815	0.165606246	1.173130966	0.240743291	0.346703825	positive
PRJNA729271	stage_E14_vs_E10	ordinary_muscle_development	ENSGALG00010029212	SUPT6H	0.023521039	0.113431559	0.207358858	0.835729611	0.885324907	positive
PRJNA729271	stage_E14_vs_E10	ordinary_muscle_development	ENSGALG00010029213	APOH	-0.403381283	1.574924187	-0.256127429	0.797852443	0.857858154	negative
PRJNA729271	stage_E14_vs_E10	ordinary_muscle_development	ENSGALG00010029214	ENSGALG00010029214	2.450223676	0.383639728	6.386782957	1.69412E-10	1.52051E-09	positive
PRJNA729271	stage_E14_vs_E10	ordinary_muscle_development	ENSGALG00010029215	ENSGALG00010029215	4.529779858	0.240424203	18.8407814	3.49764E-79	1.58432E-76	positive
PRJNA729271	stage_E14_vs_E10	ordinary_muscle_development	ENSGALG00010029216	RAB34	-0.395816674	0.130845019	-3.02508019	0.002485672	0.007020619	negative
PRJNA729271	stage_E14_vs_E10	ordinary_muscle_development	ENSGALG00010029217	CEP112	-0.05741409	0.086058124	-0.667154796	0.504673262	0.613610182	negative
PRJNA729271	stage_E14_vs_E10	ordinary_muscle_development	ENSGALG00010029218	RPL23A	0.478565311	0.150210995	3.185953935	0.001442776	0.004365066	positive
PRJNA729271	stage_E14_vs_E10	ordinary_muscle_development	ENSGALG00010029219	TLCD1	-0.188165956	0.202789982	-0.92788386	0.35346678	0.469670458	negative
PRJNA729271	stage_E14_vs_E10	ordinary_muscle_development	ENSGALG00010029220	NEK8	0.298402149	0.122264593	2.44062603	0.014661829	0.033289977	positive
PRJNA729271	stage_E14_vs_E10	ordinary_muscle_development	ENSGALG00010029221	AXIN2	-1.407155254	0.125023759	-11.25510279	2.18571E-29	9.05197E-28	negative
PRJNA729271	stage_E14_vs_E10	ordinary_muscle_development	ENSGALG00010029222	TRAF4	0.698258014	0.224960146	3.103918736	0.001909758	0.00560023	positive
PRJNA729271	stage_E14_vs_E10	ordinary_muscle_development	ENSGALG00010029223	NME2	0.393586442	0.145759493	2.700245691	0.006928829	0.017274402	positive
PRJNA729271	stage_E14_vs_E10	ordinary_muscle_development	ENSGALG00010029224	FAM222B	-0.300260852	0.160454399	-1.871315808	0.061301321	0.112647394	negative
PRJNA729271	stage_E14_vs_E10	ordinary_muscle_development	ENSGALG00010029226	MBTD1	0.009761258	0.073768446	0.13232295	0.894728856	0.927741231	positive
PRJNA729271	stage_E14_vs_E10	ordinary_muscle_development	ENSGALG00010029227	UTP18	-0.05390977	0.077959061	-0.691513846	0.489242683	0.599862349	negative
PRJNA729271	stage_E14_vs_E10	ordinary_muscle_development	ENSGALG00010029228	ERAL1	-0.052191775	0.117750378	-0.443240829	0.65759155	0.747532704	negative
PRJNA729271	stage_E14_vs_E10	ordinary_muscle_development	ENSGALG00010029229	CBX4	0.231248992	0.220425095	1.04910465	0.294129956	0.407047328	positive
PRJNA729271	stage_E14_vs_E10	ordinary_muscle_development	ENSGALG00010029230	PPM1E	0.159266927	0.164801546	0.966416464	0.333835829	0.448965517	positive
PRJNA729271	stage_E14_vs_E10	ordinary_muscle_development	ENSGALG00010029231	CBX8	-0.16577017	0.104186765	-1.591086633	0.11159008	0.186541139	negative
PRJNA729271	stage_E14_vs_E10	ordinary_muscle_development	ENSGALG00010029232	CA10	-0.415532911	0.378602881	-1.097542919	0.27240413	0.382458142	negative
PRJNA729271	stage_E14_vs_E10	ordinary_muscle_development	ENSGALG00010029233	CBX2	-0.712109598	0.15146333	-4.701531366	2.58218E-06	1.33626E-05	negative
PRJNA729271	stage_E14_vs_E10	ordinary_muscle_development	ENSGALG00010029234	TOM1L1	-2.519169821	0.238312317	-10.57087544	4.0668E-26	1.34893E-24	negative
PRJNA729271	stage_E14_vs_E10	ordinary_muscle_development	ENSGALG00010029235	TRIM37	-0.104291225	0.072018503	-1.4481171	0.147584304	0.234282607	negative
PRJNA729271	stage_E14_vs_E10	ordinary_muscle_development	ENSGALG00010029237	COX11	-0.435014986	0.104082536	-4.179519457	2.92126E-05	0.000126664	negative
PRJNA729271	stage_E14_vs_E10	ordinary_muscle_development	ENSGALG00010029238	CAMKK1	0.13800735	0.242083213	0.570082278	0.568621895	0.671077541	positive
PRJNA729271	stage_E14_vs_E10	ordinary_muscle_development	ENSGALG00010029239	RBFOX3	0.238803501	0.352101629	0.678223221	0.497630172	0.607065253	positive
PRJNA729271	stage_E14_vs_E10	ordinary_muscle_development	ENSGALG00010029240	MIS12	-0.006054784	0.243536643	-0.024861903	0.980165115	0.986974181	negative
PRJNA729271	stage_E14_vs_E10	ordinary_muscle_development	ENSGALG00010029241	STXBP4	-0.192494866	0.089129177	-2.159728969	0.030793657	0.062743051	negative
PRJNA729271	stage_E14_vs_E10	ordinary_muscle_development	ENSGALG00010029242	DERL2	-0.067481718	0.096264433	-0.701003638	0.483300745	0.594790652	negative
PRJNA729271	stage_E14_vs_E10	ordinary_muscle_development	ENSGALG00010029243	NME1	-0.663100022	0.341241922	-1.943196247	0.051992451	0.098026871	negative
PRJNA729271	stage_E14_vs_E10	ordinary_muscle_development	ENSGALG00010029244	HLF	0.086277337	0.186739458	0.462019854	0.644067083	0.736258073	positive
PRJNA729271	stage_E14_vs_E10	ordinary_muscle_development	ENSGALG00010029245	SKA2	-0.142394918	0.113262502	-1.257211477	0.208677068	0.309201188	negative
PRJNA729271	stage_E14_vs_E10	ordinary_muscle_development	ENSGALG00010029246	DHX33	-0.264363649	0.132957143	-1.988337319	0.046774396	0.089689518	negative

PRJNA729271	stage_E14_vs_E10	ordinary_muscle_development	ENSGALG00010029247	CCDC92B	1.853874494	0.58835454	3.150947889	0.001627415	0.004855781	positive
PRJNA729271	stage_E14_vs_E10	ordinary_muscle_development	ENSGALG00010029248	C1QBP	-0.003189742	0.081060924	-0.039349937	0.968611393	0.978603342	negative
PRJNA729271	stage_E14_vs_E10	ordinary_muscle_development	ENSGALG00010029249	PRR11	-0.121189806	0.260594569	-0.465051159	0.641894821	0.734296064	negative
PRJNA729271	stage_E14_vs_E10	ordinary_muscle_development	ENSGALG00010029250	SPAG9	-0.18204476	0.087404554	-2.082783458	0.037270966	0.073773643	negative
PRJNA729271	stage_E14_vs_E10	ordinary_muscle_development	ENSGALG00010029251	ENGASE	-0.728018394	0.183562983	-3.966041413	7.30762E-05	0.000294888	negative
PRJNA729271	stage_E14_vs_E10	ordinary_muscle_development	ENSGALG00010029252	RPAIN	0.085033269	0.139847419	0.608043178	0.543158837	0.648898651	positive
PRJNA729271	stage_E14_vs_E10	ordinary_muscle_development	ENSGALG00010029253	NUP88	-0.019058057	0.084465146	-0.225632201	0.821487494	0.875033894	negative
PRJNA729271	stage_E14_vs_E10	ordinary_muscle_development	ENSGALG00010029254	MMD	-0.064383516	0.118628326	-0.542733075	0.587313598	0.687539218	negative
PRJNA729271	stage_E14_vs_E10	ordinary_muscle_development	ENSGALG00010029255	RAP1GAP2	1.313093443	0.183192409	7.167837622	7.61916E-13	8.82811E-12	positive
PRJNA729271	stage_E14_vs_E10	ordinary_muscle_development	ENSGALG00010029256	RABEP1	-0.105974956	0.096503283	-1.09814871	0.272139558	0.382160715	negative
PRJNA729271	stage_E14_vs_E10	ordinary_muscle_development	ENSGALG00010029257	YPEL2	0.194810128	0.161254103	1.208094083	0.227011076	0.33037405	positive
PRJNA729271	stage_E14_vs_E10	ordinary_muscle_development	ENSGALG00010029258	TMEM100	-0.170008786	0.234697487	-0.724374121	0.468836083	0.581133832	negative
PRJNA729271	stage_E14_vs_E10	ordinary_muscle_development	ENSGALG00010029259	PTRH2	0.112085878	0.106678384	1.050689692	0.293401132	0.406116253	positive
PRJNA729271	stage_E14_vs_E10	ordinary_muscle_development	ENSGALG00010029260	DHX40	-0.241380033	0.101957277	-2.367462533	0.017910533	0.039526976	negative
PRJNA729271	stage_E14_vs_E10	ordinary_muscle_development	ENSGALG00010029261	IFT22	-0.448220562	0.086627458	-5.174116548	2.28992E-07	1.3859E-06	negative
PRJNA729271	stage_E14_vs_E10	ordinary_muscle_development	ENSGALG00010029262	CLTC	-0.289222217	0.064924509	-4.454746293	8.39925E-06	3.98648E-05	negative
PRJNA729271	stage_E14_vs_E10	ordinary_muscle_development	ENSGALG00010029263	TOB1	0.237474207	0.188931671	1.256931701	0.208778368	0.309273627	positive
PRJNA729271	stage_E14_vs_E10	ordinary_muscle_development	ENSGALG00010029264	COL26A1	-2.117113281	0.177371888	-11.93601365	7.68174E-33	3.83954E-31	negative
PRJNA729271	stage_E14_vs_E10	ordinary_muscle_development	ENSGALG00010029266	CANT1	0.044266239	0.110120047	0.401981662	0.687697522	0.77188908	positive
PRJNA729271	stage_E14_vs_E10	ordinary_muscle_development	ENSGALG00010029267	ENSGALG00010029267	1.376119801	0.287346334	4.789063363	1.67562E-06	8.95577E-06	positive
PRJNA729271	stage_E14_vs_E10	ordinary_muscle_development	ENSGALG00010029268	PCTP	0.20683146	0.180124534	1.14826923	0.250857437	0.358032548	positive
PRJNA729271	stage_E14_vs_E10	ordinary_muscle_development	ENSGALG00010029269	WSCD1	1.288933503	0.151978678	8.481015365	2.23237E-17	3.87988E-16	positive
PRJNA729271	stage_E14_vs_E10	ordinary_muscle_development	ENSGALG00010029270	TIMP2	0.293916161	0.149453137	1.966610849	0.049228092	0.093700904	positive
PRJNA729271	stage_E14_vs_E10	ordinary_muscle_development	ENSGALG00010029271	MYL10	-1.126865754	0.799576854	-1.409327631	0.158738312	0.248398125	negative
PRJNA729271	stage_E14_vs_E10	ordinary_muscle_development	ENSGALG00010029273	SLC13A5	-1.074524375	0.434408886	-2.473532216	0.013378474	0.030737198	negative
PRJNA729271	stage_E14_vs_E10	ordinary_muscle_development	ENSGALG00010029274	VMP1	-0.295373746	0.146471485	-2.016595556	0.043737737	0.084564292	negative
PRJNA729271	stage_E14_vs_E10	ordinary_muscle_development	ENSGALG00010029275	PCYT2	0.062636352	0.070596603	0.887243139	0.374948012	0.491265609	positive
PRJNA729271	stage_E14_vs_E10	ordinary_muscle_development	ENSGALG00010029276	TUBD1	0.334524546	0.170521678	1.961771377	0.049789108	0.094474815	positive
PRJNA729271	stage_E14_vs_E10	ordinary_muscle_development	ENSGALG00010029277	WFIKKN2	2.380439339	0.738723408	3.222368906	0.001271353	0.003910922	positive
PRJNA729271	stage_E14_vs_E10	ordinary_muscle_development	ENSGALG00010029278	MED31	-0.188782709	0.068935107	-2.738556829	0.006170949	0.015636443	negative
PRJNA729271	stage_E14_vs_E10	ordinary_muscle_development	ENSGALG00010029279	ENSGALG00010029279	0.790945537	0.158220447	4.999009617	5.76255E-07	3.28463E-06	positive
PRJNA729271	stage_E14_vs_E10	ordinary_muscle_development	ENSGALG00010029280	USP36	0.226857896	0.182599472	1.242379804	0.214096463	0.315796096	positive
PRJNA729271	stage_E14_vs_E10	ordinary_muscle_development	ENSGALG00010029281	KIAA0753	-0.427254022	0.130309492	-3.278763613	0.001042629	0.003274737	negative
PRJNA729271	stage_E14_vs_E10	ordinary_muscle_development	ENSGALG00010029282	LUC7L3	-0.291641224	0.140398705	-2.077255854	0.037779796	0.074637883	negative
PRJNA729271	stage_E14_vs_E10	ordinary_muscle_development	ENSGALG00010029283	RPS6KB1	-0.080763213	0.077228803	-1.04576544	0.295669341	0.408865398	negative
PRJNA729271	stage_E14_vs_E10	ordinary_muscle_development	ENSGALG00010029284	CYTH1	0.458088944	0.092705888	4.941314461	7.75976E-07	4.34949E-06	positive
PRJNA729271	stage_E14_vs_E10	ordinary_muscle_development	ENSGALG00010029285	CUX1	0.396663192	0.068937358	5.753965698	8.71739E-09	6.38497E-08	positive
PRJNA729271	stage_E14_vs_E10	ordinary_muscle_development	ENSGALG00010029286	MAFG	0.817330176	0.182620526	4.475565776	7.62092E-06	3.64331E-05	positive
PRJNA729271	stage_E14_vs_E10	ordinary_muscle_development	ENSGALG00010029287	ANKRD40	0.326711999	0.122866299	2.659085532	0.007835307	0.01926595	positive
PRJNA729271	stage_E14_vs_E10	ordinary_muscle_development	ENSGALG00010029288	PYCR1	0.038333396	0.292888729	0.130880406	0.895869915	0.928359928	positive
PRJNA729271	stage_E14_vs_E10	ordinary_muscle_development	ENSGALG00010029289	PITPNM3	-1.612426904	0.224367388	-7.186547565	6.64501E-13	7.74895E-12	negative
PRJNA729271	stage_E14_vs_E10	ordinary_muscle_development	ENSGALG00010029290	ENSGALG00010029290	-2.051485477	0.197846483	-10.36907729	3.42819E-25	1.04614E-23	negative
PRJNA729271	stage_E14_vs_E10	ordinary_muscle_development	ENSGALG00010029291	RNFT1	-0.114091305	0.112457817	-1.014525345	0.310332142	0.42418602	negative
PRJNA729271	stage_E14_vs_E10	ordinary_muscle_development	ENSGALG00010029292	ENSGALG00010029292	0.081926729	0.250303195	0.327309962	0.743433458	0.815878668	positive
PRJNA729271	stage_E14_vs_E10	ordinary_muscle_development	ENSGALG00010029293	DNAH17	-0.669072045	0.135800853	-4.926861862	8.35608E-07	4.66031E-06	negative
PRJNA729271	stage_E14_vs_E10	ordinary_muscle_development	ENSGALG00010029294	PGS1	-0.045614948	0.073421087	-0.621278574	0.534416349	0.641467785	negative
PRJNA729271	stage_E14_vs_E10	ordinary_muscle_development	ENSGALG00010029295	SOCS3	-0.213998634	0.260859112	-0.820360969	0.412010359	0.526732242	negative
PRJNA729271	stage_E14_vs_E10	ordinary_muscle_development	ENSGALG00010029296	FBXO39	-0.255062132	0.155159689	-1.643868545	0.100203359	0.170355112	negative
PRJNA729271	stage_E14_vs_E10	ordinary_muscle_development	ENSGALG00010029297	SH2B2	0.51781744	0.254896848	2.031478397	0.042206489	0.082008452	positive
PRJNA729271	stage_E14_vs_E10	ordinary_muscle_development	ENSGALG00010029298	ENDOV	0.1407622	0.143632799	0.980014325	0.327079048	0.442011076	positive
PRJNA729271	stage_E14_vs_E10	ordinary_muscle_development	ENSGALG00010029299	PRKRIP1	0.002550543	0.08984518	0.028388203	0.977352533	0.984894672	positive
PRJNA729271	stage_E14_vs_E10	ordinary_muscle_development	ENSGALG00010029300	TEKT1	0.686623837	0.345623064	1.986626207	0.046963835	0.089961779	positive
PRJNA729271	stage_E14_vs_E10	ordinary_muscle_development	ENSGALG00010029301	RNF213	0.318266171	0.465858877	0.68318151	0.494492155	0.604509048	positive
PRJNA729271	stage_E14_vs_E10	ordinary_muscle_development	ENSGALG00010029303	SLC26A11	-0.317426102	0.150583151	-2.107978882	0.035032816	0.069983554	negative
PRJNA729271	stage_E14_vs_E10	ordinary_muscle_development	ENSGALG00010029304	ORAI3	-0.213172418	0.116057448	-1.836783609	0.066241847	0.120322753	negative
PRJNA729271	stage_E14_vs_E10	ordinary_muscle_development	ENSGALG00010029305	SMTNL2	1.923829882	0.18991057	10.13018857	4.05866E-24	1.13353E-22	positive
PRJNA729271	stage_E14_vs_E10	ordinary_muscle_development	ENSGALG00010029306	CEP95	-0.377671724	0.156643866	-2.411021478	0.015907912	0.035699826	negative
PRJNA729271	stage_E14_vs_E10	ordinary_muscle_development	ENSGALG00010029307	SGSH	-0.083436026	0.164683611	-0.50664438	0.612404363	0.709066319	negative
PRJNA729271	stage_E14_vs_E10	ordinary_muscle_development	ENSGALG00010029308	GAA	0.028373678	0.136116837	0.208450908	0.834876911	0.884874292	positive
PRJNA729271	stage_E14_vs_E10	ordinary_muscle_development	ENSGALG00010029309	ALKBH4	0.435852132	0.174467746	2.49818171	0.012483219	0.02894182	positive
PRJNA729271	stage_E14_vs_E10	ordinary_muscle_development	ENSGALG00010029310	LRWD1	0.11036737	0.089813432	1.228851493	0.219127491	0.321386447	positive
PRJNA729271	stage_E14_vs_E10	ordinary_muscle_development	ENSGALG00010029311	ENSGALG00010029311	0.333296232	0.164662832	2.024113322	0.042958493	0.083279839	positive
PRJNA729271	stage_E14_vs_E10	ordinary_muscle_development	ENSGALG00010029312	SMURF2	0.282184638	0.092858048	3.038881872	0.002374579	0.006750251	positive
PRJNA729271	stage_E14_vs_E10	ordinary_muscle_development	ENSGALG00010029313	CCDC40	-0.975315315	0.220223526	-4.428751704	9.47801E-06	4.46341E-05	negative
PRJNA729271	stage_E14_vs_E10	ordinary_muscle_development	ENSGALG00010029314	MED13	-0.233904224	0.125846738	-1.858643522	0.063077684	0.115414167	negative
PRJNA729271	stage_E14_vs_E10	ordinary_muscle_development	ENSGALG00010029315	TBC1D16	0.046058599	0.094143547	0.489237982	0.624673222	0.719650163	positive
PRJNA729271	stage_E14_vs_E10	ordinary_muscle_development	ENSGALG00010029316	INTS2	0.021871471	0.093885591	0.232958767	0.815793424	0.870952765	positive
PRJNA729271	stage_E14_vs_E10	ordinary_muscle_development	ENSGALG00010029317	KPNA2	-0.278133456	0.203842986	-1.364449481	0.172426144	0.265320271	negative
PRJNA729271	stage_E14_vs_E10	ordinary_muscle_development	ENSGALG00010029318	C17orf58	4.563156478	0.201221263	22.67730763	7.5042E-114	1.3597E-110	positive
PRJNA729271	stage_E14_vs_E10	ordinary_muscle_development	ENSGALG00010029319	BRIP1	-0.331577241	0.120814728	-2.744510104	0.006060129	0.015394597	negative
PRJNA729271	stage_E14_vs_E10	ordinary_muscle_development	ENSGALG00010029320	TBX4	-3.196815254	0.625342737	-5.112101039	3.18595E-07	1.89497E-06	negative
PRJNA729271	stage_E14_vs_E10	ordinary_muscle_development	ENSGALG00010029321	TBX2	-1.402760076	0.172161925	-8.147911178	3.70264E-16	5.76475E-15	negative
PRJNA729271	stage_E14_vs_E10	ordinary_muscle_development	ENSGALG00010029322	BPTF	-0.450679403	0.096645544	-4.663219667	3.113E-06	1.58996E-05	negative
PRJNA729271	stage_E14_vs_E10	ordinary_muscle_development	ENSGALG00010029323	NOL11	-0.135839903	0.090812244	-1.49583246	0.13469732	0.217396466	negative
PRJNA729271	stage_E14_vs_E10	ordinary_muscle_development	ENSGALG00010029324	BCAS3	0.617918009	0.126021036	4.903292558	9.42435E-07	5.21596E-06	positive
PRJNA729271	stage_E14_vs_E10	ordinary_muscle_development	ENSGALG00010029325	LRRC75A	-0.21455796	0.16422984	-1.306449302	0.191399809	0.288783203	negative
PRJNA729271	stage_E14_vs_E10	ordinary_muscle_development	ENSGALG00010029326	NCBP3	0.437320081	0.078481953	5.572237462	2.51488E-08	1.74251E-07	positive
PRJNA729271	stage_E14_vs_E10	ordinary_muscle_development	ENSGALG00010029327	CRK	-0.136157097	0.053846594	-2.528611127	0.011451483	0.026850411	negative
PRJNA729271	stage_E14_vs_E10	ordinary_muscle_development	ENSGALG00010029328	ENSGALG00010029328	0.48224035	0.080370301	6.000230709	1.97037E-09	1.56154E-08	positive
PRJNA729271	stage_E14_vs_E10	ordinary_muscle_development	ENSGALG00010029329	ENSGALG00010029329	0.586975179	0.178039297	3.296885522	0.000977633	0.003084631	positive
PRJNA729271	stage_E14_vs_E10	ordinary_muscle_development	ENSGALG00010029330	AFMID	0.352487243	0.125707622	2.804024424	0.005046908	0.013136099	positive
PRJNA729271	stage_E14_vs_E10	ordinary_muscle_development	ENSGALG00010029331	TK1	0.025420802	0.276194338	0.092039549	0.926666617	0.949662939	positive
PRJNA729271	stage_E14_vs_E10	ordinary_muscle_development	ENSGALG00010029332	YWHAE	-0.063087786	0.14847276	-0.424911519	0.670901177	0.757848548	negative
PRJNA729271	stage_E14_vs_E10	ordinary_muscle_development	ENSGALG00010029333	ENSGALG00010029333	-0.153433292	0.174101251	-0.881287704	0.378162119	0.494448847	negative
PRJNA729271	stage_E14_vs_E10	ordinary_muscle_development	ENSGALG00010029335	RNF222	4.555232344	1.268082898	3.592219683	0.000327873	0.001163693	positive
PRJNA729271	stage_E14_vs_E10	ordinary_muscle_development	ENSGALG00010029336	SYNGR2	0.917198256	0.110594337	8.293356409	1.10097E-16	1.80526E-15	positive
PRJNA729271	stage_E14_vs_E10	ordinary_muscle_development	ENSGALG00010029337	MYO1C	0.638767673	0.133702015	4.777547079	1.77447E-06	9.42847E-06	positive
PRJNA729271	stage_E14_vs_E10	ordinary_muscle_development	ENSGALG00010029338	INPP5K	-0.319218866	0.119980713	-2.660584848	0.007800507	0.019193405	negative
PRJNA729271	stage_E14_vs_E10	ordinary_muscle_development	ENSGALG00010029339	PITPNA	-0.401993891	0.1273211	-3.157323428	0.001592246	0.004759664	negative
PRJNA729271	stage_E14_vs_E10	ordinary_muscle_development	ENSGALG00010029340	NDEL1	0.160169829	0.103780213	1.543356138	0.12274439	0.201196419	positive
PRJNA729271	stage_E14_vs_E10	ordinary_muscle_development	ENSGALG00010029341	SLC43A2	1.30868447	0.326377464	4.009726817	6.0789E-05	0.000248909	positive
PRJNA729271	stage_E14_vs_E10	ordinary_muscle_development	ENSGALG00010029342	TMC6	-0.450745481	0.126620713	-3.559808412	0.000371125	0.001300015	negative
PRJNA729271	stage_E14_vs_E10	ordinary_muscle_development	ENSGALG00010029343	SCARF1	0.600861816	0.131169619	4.580800189	4.632E-06	2.29934E-05	positive
PRJNA729271	stage_E14_vs_E10	ordinary_muscle_development	ENSGALG00010029344	ENSGALG00010029344	0.469653343	0.165387208	2.83971989	0.004515316	0.01190424	positive
PRJNA729271	stage_E14_vs_E10	ordinary_muscle_development	ENSGALG00010029345	RILP	0.539836902	0.246862851	2.186788742	0.02875795	0.059295374	positive
PRJNA729271	stage_E14_vs_E10	ordinary_muscle_development	ENSGALG00010029346	ARL16	-0.033261844	0.115107912	-0.288962275	0.772610247	0.838782606	negative
PRJNA729271	stage_E14_vs_E10	ordinary_muscle_development	ENSGALG00010029347	PRPF8	-0.273244435	0.069773511	-3.916162897	8.99695E-05	0.000357388	negative
PRJNA729271	stage_E14_vs_E10	ordinary_muscle_development	ENSGALG00010029348	HGS	0.275505893	0.123053869	2.23890475	0.025162114	0.053020039	positive
PRJNA729271	stage_E14_vs_E10	ordinary_muscle_development	ENSGALG00010029349	MYH10	-0.77628627	0.106722652	-7.273866013	3.49342E-13	4.19182E-12	negative
PRJNA729271	stage_E14_vs_E10	ordinary_muscle_development	ENSGALG00010029350	MRPL12	0.230662995	0.121429159	1.899568418	0.05748978	0.106671065	positive
PRJNA729271	stage_E14_vs_E10	ordinary_muscle_development	ENSGALG00010029351	ENSGALG00010029351	-0.54579991	0.184136056	-2.964112099	0.003035577	0.008403492	negative
PRJNA729271	stage_E14_vs_E10	ordinary_muscle_development	ENSGALG00010029352	AATF	0.209373928	0.106586541	1.964356162	0.049488803	0.094071221	positive
PRJNA729271	stage_E14_vs_E10	ordinary_muscle_development	ENSGALG00010029353	GCGR	3.171742712	0.888576038	3.569466852	0.000357708	0.001258307	positive
PRJNA729271	stage_E14_vs_E10	ordinary_muscle_development	ENSGALG00010029354	TLCD2	-1.252793365	0.284739457	-4.399788412	1.08356E-05	5.047E-05	negative
PRJNA729271	stage_E14_vs_E10	ordinary_muscle_development	ENSGALG00010029355	MCRIP1	0.284202589	0.172410276	1.64840864	0.099268844	0.169103525	positive
PRJNA729271	stage_E14_vs_E10	ordinary_muscle_development	ENSGALG00010029357	ARHGDIA	0.057961305	0.096844096	0.598501173	0.549505576	0.654646447	positive
PRJNA729271	stage_E14_vs_E10	ordinary_muscle_development	ENSGALG00010029359	ENSGALG00010029359	-0.617817741	0.5091396	-1.213454506	0.224956098	0.32809458	negative
PRJNA729271	stage_E14_vs_E10	ordinary_muscle_development	ENSGALG00010029360	ACACA	-0.382482808	0.085184286	-4.490062978	7.12021E-06	3.42541E-05	negative
PRJNA729271	stage_E14_vs_E10	ordinary_muscle_development	ENSGALG00010029361	ALYREF	0.038634095	0.133007929	0.290464604	0.771460826	0.83781559	positive
PRJNA729271	stage_E14_vs_E10	ordinary_muscle_development	ENSGALG00010029362	TADA2A	0.226822957	0.108378422	2.092878339	0.036360016	0.072206938	positive
PRJNA729271	stage_E14_vs_E10	ordinary_muscle_development	ENSGALG00010029363	PIK3R6	0.690142913	0.19393258	3.558674431	0.000372731	0.001303852	positive
PRJNA729271	stage_E14_vs_E10	ordinary_muscle_development	ENSGALG00010029364	ANAPC11	0.254175083	0.126308366	2.012337676	0.044184353	0.085325367	positive
PRJNA729271	stage_E14_vs_E10	ordinary_muscle_development	ENSGALG00010029365	PIK3R5	0.976591605	0.186362308	5.240284994	1.60329E-07	9.90608E-07	positive
PRJNA729271	stage_E14_vs_E10	ordinary_muscle_development	ENSGALG00010029366	MGAT5B	0.187002085	0.349663266	0.534806208	0.59278387	0.69283827	positive
PRJNA729271	stage_E14_vs_E10	ordinary_muscle_development	ENSGALG00010029367	DUSP14	0.032103865	0.115610337	0.277690267	0.781250133	0.845532792	positive
PRJNA729271	stage_E14_vs_E10	ordinary_muscle_development	ENSGALG00010029368	DDX52	-0.110494761	0.087842349	-1.257875759	0.208436692	0.308893758	negative
PRJNA729271	stage_E14_vs_E10	ordinary_muscle_development	ENSGALG00010029369	MFSD11	0.100629253	0.084522818	1.190557248	0.233827443	0.338229309	positive
PRJNA729271	stage_E14_vs_E10	ordinary_muscle_development	ENSGALG00010029370	SRSF2	-0.255163652	0.123998149	-2.057802096	0.039609135	0.077648689	negative
PRJNA729271	stage_E14_vs_E10	ordinary_muscle_development	ENSGALG00010029371	AUTS2	-1.21183547	0.168584233	-7.188308453	6.55989E-13	7.66202E-12	negative
PRJNA729271	stage_E14_vs_E10	ordinary_muscle_development	ENSGALG00010029372	METTL23	-0.409545476	0.138097687	-2.965621551	0.00302072	0.008368756	negative
PRJNA729271	stage_E14_vs_E10	ordinary_muscle_development	ENSGALG00010029373	GATSL2	0.076412117	0.169396316	0.45108488	0.651928374	0.742842684	positive
PRJNA729271	stage_E14_vs_E10	ordinary_muscle_development	ENSGALG00010029374	RCC1L	-0.053989674	0.11553756	-0.467291104	0.64029162	0.7330406	negative
PRJNA729271	stage_E14_vs_E10	ordinary_muscle_development	ENSGALG00010029375	JMJD6	-0.172930728	0.160649983	-1.076444113	0.281728683	0.39292382	negative
PRJNA729271	stage_E14_vs_E10	ordinary_muscle_development	ENSGALG00010029376	ENSGALG00010029376	0.448012496	0.205389708	2.181280165	0.029162701	0.059976354	positive
PRJNA729271	stage_E14_vs_E10	ordinary_muscle_development	ENSGALG00010029377	NCF1C	0.804219019	0.29258526	2.748665535	0.005983841	0.015235513	positive
PRJNA729271	stage_E14_vs_E10	ordinary_muscle_development	ENSGALG00010029378	ST6GALNAC1	-2.115718749	0.337289846	-6.272702169	3.54835E-10	3.06333E-09	negative
PRJNA729271	stage_E14_vs_E10	ordinary_muscle_development	ENSGALG00010029379	GTF2I	-0.056604852	0.112067258	-0.505097146	0.613490606	0.710134536	negative
PRJNA729271	stage_E14_vs_E10	ordinary_muscle_development	ENSGALG00010029380	RGS9	-0.14345198	0.147295581	-0.973905523	0.330103451	0.444812636	negative
PRJNA729271	stage_E14_vs_E10	ordinary_muscle_development	ENSGALG00010029382	GTF2IRD1	0.3265978	0.205943264	1.585862987	0.112770394	0.188186224	positive
PRJNA729271	stage_E14_vs_E10	ordinary_muscle_development	ENSGALG00010029383	GNA13	-0.100731536	0.078548252	-1.282416015	0.199696749	0.298751613	negative
PRJNA729271	stage_E14_vs_E10	ordinary_muscle_development	ENSGALG00010029384	WDR81	-0.18151116	0.073871814	-2.457109832	0.014005986	0.031981217	negative

PRJNA729271	stage_E14_vs_E10	ordinary_muscle_development	ENSGALG00010029385	CLIP2	-0.078537777	0.121642769	-0.64564279	0.518510719	0.62678783	negative
PRJNA729271	stage_E14_vs_E10	ordinary_muscle_development	ENSGALG00010029386	SLC16A6	0.947858022	0.236825057	4.002355301	6.2715E-05	0.000256433	positive
PRJNA729271	stage_E14_vs_E10	ordinary_muscle_development	ENSGALG00010029387	SERPINF1	0.507841597	0.176034936	2.884890971	0.003915492	0.010483019	positive
PRJNA729271	stage_E14_vs_E10	ordinary_muscle_development	ENSGALG00010029388	ST6GALNAC2	-0.428002019	0.357729786	-1.196439423	0.231525125	0.33583075	negative
PRJNA729271	stage_E14_vs_E10	ordinary_muscle_development	ENSGALG00010029389	SMYD4	-0.040818898	0.094322727	-0.432757816	0.665190723	0.753762875	negative
PRJNA729271	stage_E14_vs_E10	ordinary_muscle_development	ENSGALG00010029390	RPA1	-0.403233191	0.077828357	-5.181057507	2.20631E-07	1.33922E-06	negative
PRJNA729271	stage_E14_vs_E10	ordinary_muscle_development	ENSGALG00010029391	ARSG	0.424847269	0.136970077	3.101752437	0.001923788	0.005632257	positive
PRJNA729271	stage_E14_vs_E10	ordinary_muscle_development	ENSGALG00010029392	RTN4RL1	0.641712026	0.565775436	1.134216839	0.256703602	0.364652951	positive
PRJNA729271	stage_E14_vs_E10	ordinary_muscle_development	ENSGALG00010029394	CYGB	0.550868884	0.142743404	3.859154727	0.00011378	0.000443941	positive
PRJNA729271	stage_E14_vs_E10	ordinary_muscle_development	ENSGALG00010029395	DPH1	-0.050010461	0.113043776	-0.44239907	0.658200452	0.747931605	negative
PRJNA729271	stage_E14_vs_E10	ordinary_muscle_development	ENSGALG00010029396	WIPI1	0.845508285	0.154288191	5.480058318	4.25186E-08	2.848E-07	positive
PRJNA729271	stage_E14_vs_E10	ordinary_muscle_development	ENSGALG00010029397	OVCA2	-0.062343464	0.150269845	-0.414876744	0.678232135	0.764461493	negative
PRJNA729271	stage_E14_vs_E10	ordinary_muscle_development	ENSGALG00010029398	RHBDF2	-0.096415041	0.123708755	-0.779371201	0.435761083	0.549965773	negative
PRJNA729271	stage_E14_vs_E10	ordinary_muscle_development	ENSGALG00010029399	PRKAR1A	-0.126335168	0.052928228	-2.386914733	0.016990431	0.037795627	negative
PRJNA729271	stage_E14_vs_E10	ordinary_muscle_development	ENSGALG00010029400	AANAT	0.19457699	0.657856464	0.295774232	0.767402503	0.834596285	positive
PRJNA729271	stage_E14_vs_E10	ordinary_muscle_development	ENSGALG00010029401	HIC1	-0.133670462	0.202015049	-0.661685662	0.508172695	0.617069885	negative
PRJNA729271	stage_E14_vs_E10	ordinary_muscle_development	ENSGALG00010029402	SMG6	0.644366921	0.087925626	7.328545132	2.32665E-13	2.84117E-12	positive
PRJNA729271	stage_E14_vs_E10	ordinary_muscle_development	ENSGALG00010029403	FAM20A	2.35829746	0.155669646	15.14937257	7.64934E-52	1.19223E-49	positive
PRJNA729271	stage_E14_vs_E10	ordinary_muscle_development	ENSGALG00010029404	SRR	1.129617966	0.174416462	6.476555898	9.38399E-11	8.62529E-10	positive
PRJNA729271	stage_E14_vs_E10	ordinary_muscle_development	ENSGALG00010029405	POLDIP2	0.651534388	0.078355206	8.315138478	9.16452E-17	1.51817E-15	positive
PRJNA729271	stage_E14_vs_E10	ordinary_muscle_development	ENSGALG00010029406	TMEM199	0.200084172	0.09981188	2.004612796	0.045004458	0.086816557	positive
PRJNA729271	stage_E14_vs_E10	ordinary_muscle_development	ENSGALG00010029407	UBE2O	-0.19607136	0.188721397	-1.038946101	0.298829804	0.412409598	negative
PRJNA729271	stage_E14_vs_E10	ordinary_muscle_development	ENSGALG00010029408	KIF12	0.666822459	0.486748315	1.369953296	0.170701481	0.263262364	positive
PRJNA729271	stage_E14_vs_E10	ordinary_muscle_development	ENSGALG00010029409	ENSGALG00010029409	0.703777387	0.190412401	3.696069078	0.000218963	0.000805143	positive
PRJNA729271	stage_E14_vs_E10	ordinary_muscle_development	ENSGALG00010029410	VTN	1.348244599	0.885098558	1.523270585	0.127691023	0.207870775	positive
PRJNA729271	stage_E14_vs_E10	ordinary_muscle_development	ENSGALG00010029411	SARM1	2.08236617	0.147508462	14.11692682	2.98738E-45	3.23149E-43	positive
PRJNA729271	stage_E14_vs_E10	ordinary_muscle_development	ENSGALG00010029412	SLC46A1	0.435267988	0.104662961	4.158758574	3.19982E-05	0.000137835	positive
PRJNA729271	stage_E14_vs_E10	ordinary_muscle_development	ENSGALG00010029413	B3GNTL1	-0.004403436	0.216415336	-0.020347151	0.983766442	0.989226124	negative
PRJNA729271	stage_E14_vs_E10	ordinary_muscle_development	ENSGALG00010029416	UNC119	-0.969619941	0.438366527	-2.211893203	0.026974045	0.056228371	negative
PRJNA729271	stage_E14_vs_E10	ordinary_muscle_development	ENSGALG00010029417	ENSGALG00010029417	1.099482627	0.211933749	5.187860042	2.12724E-07	1.29556E-06	positive
PRJNA729271	stage_E14_vs_E10	ordinary_muscle_development	ENSGALG00010029418	ABCA5	-0.31733923	0.132512668	-2.394784094	0.016630155	0.037108081	negative
PRJNA729271	stage_E14_vs_E10	ordinary_muscle_development	ENSGALG00010029419	RFC2	-0.216673541	0.132029024	-1.641105377	0.100775543	0.171147216	negative
PRJNA729271	stage_E14_vs_E10	ordinary_muscle_development	ENSGALG00010029420	LAT2	-0.43910633	0.324111324	-1.354800951	0.175480983	0.269078267	negative
PRJNA729271	stage_E14_vs_E10	ordinary_muscle_development	ENSGALG00010029421	TBCD	-0.2589485	0.092799278	-2.790415017	0.005264052	0.013627867	negative
PRJNA729271	stage_E14_vs_E10	ordinary_muscle_development	ENSGALG00010029422	EIF4H	0.114088484	0.087056638	1.31050872	0.190023796	0.287005827	positive
PRJNA729271	stage_E14_vs_E10	ordinary_muscle_development	ENSGALG00010029423	MYBBP1A	-0.569787008	0.120035513	-4.746820265	2.06639E-06	1.08636E-05	negative
PRJNA729271	stage_E14_vs_E10	ordinary_muscle_development	ENSGALG00010029424	MAP2K6	-0.157505837	0.217865577	-0.722949624	0.469710834	0.582023729	negative
PRJNA729271	stage_E14_vs_E10	ordinary_muscle_development	ENSGALG00010029425	SPNS2	0.492725809	0.167029179	2.949938511	0.003178372	0.008751994	positive
PRJNA729271	stage_E14_vs_E10	ordinary_muscle_development	ENSGALG00010029426	ZNF750	-0.153126589	0.745462699	-0.205411471	0.837250659	0.88648271	negative
PRJNA729271	stage_E14_vs_E10	ordinary_muscle_development	ENSGALG00010029427	SPNS3	-0.256121314	0.28349048	-0.903456491	0.36628367	0.482355028	negative
PRJNA729271	stage_E14_vs_E10	ordinary_muscle_development	ENSGALG00010029428	FN3K	-0.824060454	0.268069425	-3.074056117	0.002111697	0.006101066	negative
PRJNA729271	stage_E14_vs_E10	ordinary_muscle_development	ENSGALG00010029429	UBE2G1	0.308569032	0.089754372	3.437927578	0.000586184	0.001951032	positive
PRJNA729271	stage_E14_vs_E10	ordinary_muscle_development	ENSGALG00010029430	FN3KRP	-0.0839343	0.095089029	-0.882691741	0.377402846	0.493634204	negative
PRJNA729271	stage_E14_vs_E10	ordinary_muscle_development	ENSGALG00010029432	ENSGALG00010029432	-1.656848768	5.461315828	-0.303379043			negative
PRJNA729271	stage_E14_vs_E10	ordinary_muscle_development	ENSGALG00010029436	WDR45B	-0.015622198	0.07854707	-0.198889635	0.842349078	0.890320102	negative
PRJNA729271	stage_E14_vs_E10	ordinary_muscle_development	ENSGALG00010029437	ENSGALG00010029437	-5.587596466	2.917375961	-1.915281589	0.055456602	0.103454755	negative
PRJNA729271	stage_E14_vs_E10	ordinary_muscle_development	ENSGALG00010029438	ENSGALG00010029438	0.197566795	0.267869325	0.737549157	0.460788461	0.573364988	positive
PRJNA729271	stage_E14_vs_E10	ordinary_muscle_development	ENSGALG00010029439	ENSGALG00010029439	-0.288124229	0.205973313	-1.398842525	0.161860211	0.252357079	negative
PRJNA729271	stage_E14_vs_E10	ordinary_muscle_development	ENSGALG00010029440	FOXK2	-0.045614666	0.181577761	-0.251212844	0.801649555	0.860288021	negative
PRJNA729271	stage_E14_vs_E10	ordinary_muscle_development	ENSGALG00010029442	ENSGALG00010029442	-0.087694484	0.278789021	-0.314555011	0.753099542	0.823241166	negative
PRJNA729271	stage_E14_vs_E10	ordinary_muscle_development	ENSGALG00010029443	ENSGALG00010029443	0.211070009	0.314011336	0.672173215	0.501473431	0.6106744	positive
PRJNA729271	stage_E14_vs_E10	ordinary_muscle_development	ENSGALG00010029445	SPHK1	-0.025376129	0.134577922	-0.188560862	0.85043701	0.89638485	negative
PRJNA729271	stage_E14_vs_E10	ordinary_muscle_development	ENSGALG00010029446	PRPSAP1	0.415397432	0.078538169	5.289115328	1.22909E-07	7.71243E-07	positive
PRJNA729271	stage_E14_vs_E10	ordinary_muscle_development	ENSGALG00010029449	UBALD2	0.996701754	0.1813397	5.496324064	3.8779E-08	2.61686E-07	positive
PRJNA729271	stage_E14_vs_E10	ordinary_muscle_development	ENSGALG00010029451	ENSGALG00010029451	-0.068603895	0.221273859	-0.310040667	0.756530032	0.826430236	negative
PRJNA729271	stage_E14_vs_E10	ordinary_muscle_development	ENSGALG00010029452	ENSGALG00010029452	1.110225821	0.916775832	1.211011222	0.225891104	0.329008395	positive
PRJNA729271	stage_E14_vs_E10	ordinary_muscle_development	ENSGALG00010029453	ENSGALG00010029453	-0.663203183	1.511822086	-0.438678063	0.660894825	0.750287475	negative
PRJNA729271	stage_E14_vs_E10	ordinary_muscle_development	ENSGALG00010029454	ENSGALG00010029454	0.239238014	0.288119919	0.830341808	0.406345557	0.521283197	positive
PRJNA729271	stage_E14_vs_E10	ordinary_muscle_development	ENSGALG00010029455	ENSGALG00010029455	0.054671231	0.249813522	0.218848164	0.826768327	0.878721726	positive
PRJNA729271	stage_E14_vs_E10	ordinary_muscle_development	ENSGALG00010029456	RNF157	-2.679272156	0.49350712	-5.429044583	5.66565E-08	3.72781E-07	negative
PRJNA729271	stage_E14_vs_E10	ordinary_muscle_development	ENSGALG00010029457	ENSGALG00010029457	0.146023237	0.190051826	0.768333778	0.442288912	0.556122292	positive
PRJNA729271	stage_E14_vs_E10	ordinary_muscle_development	ENSGALG00010029460	ENSGALG00010029460	0.025799855	0.277619617	0.092932391	0.925957273	0.949137308	positive
PRJNA729271	stage_E14_vs_E10	ordinary_muscle_development	ENSGALG00010029461	ENSGALG00010029461	0.159811645	0.199257262	0.80203674	0.422531706	0.536913861	positive
PRJNA729271	stage_E14_vs_E10	ordinary_muscle_development	ENSGALG00010029462	ENSGALG00010029462	-1.658272778	1.811987224	-0.91516803	0.360103425	0.4764238	negative
PRJNA729271	stage_E14_vs_E10	ordinary_muscle_development	ENSGALG00010029463	ENSGALG00010029463	0.092995324	0.398009371	0.233651091	0.815255861	0.870571217	positive
PRJNA729271	stage_E14_vs_E10	ordinary_muscle_development	ENSGALG00010029464	ENSGALG00010029464	0.590809732	0.49208942	1.200614578	0.229900744	0.333775168	positive
PRJNA729271	stage_E14_vs_E10	ordinary_muscle_development	ENSGALG00010029465	ENSGALG00010029465	-0.313093752	2.367268883	-0.13225948	0.894779056	0.927741231	negative
PRJNA729271	stage_E14_vs_E10	ordinary_muscle_development	ENSGALG00010029466	SLC39A11	0.018811724	0.113047061	0.166406128	0.867837352	0.909369075	positive
PRJNA729271	stage_E14_vs_E10	ordinary_muscle_development	ENSGALG00010029468	ENSGALG00010029468	-0.118649481	0.239786315	-0.494813399	0.620731845	0.716248058	negative
PRJNA729271	stage_E14_vs_E10	ordinary_muscle_development	ENSGALG00010029469	ENSGALG00010029469	-0.467763782	0.393048291	-1.190092393	0.23401008	0.338453014	negative
PRJNA729271	stage_E14_vs_E10	ordinary_muscle_development	ENSGALG00010029470	SSTR2	1.274229267	0.91927484	1.38612438	0.165708927	0.256948106	positive
PRJNA729271	stage_E14_vs_E10	ordinary_muscle_development	ENSGALG00010029472	ENSGALG00010029472	-0.129542111	0.785500886	-0.164916569	0.869009651	0.91020268	negative
PRJNA729271	stage_E14_vs_E10	ordinary_muscle_development	ENSGALG00010029473	COG1	0.252457917	0.100319685	2.516534196	0.01185154	0.027676506	positive
PRJNA729271	stage_E14_vs_E10	ordinary_muscle_development	ENSGALG00010029474	ENSGALG00010029474	0.291233654	1.025971902	0.283861238	0.776516723	0.841666784	positive
PRJNA729271	stage_E14_vs_E10	ordinary_muscle_development	ENSGALG00010029477	FAM104A	-0.089303232	0.056409389	-1.5831271	0.113392498	0.189074457	negative
PRJNA729271	stage_E14_vs_E10	ordinary_muscle_development	ENSGALG00010029479	ENSGALG00010029479	0.158261215	0.545213972	0.290273587	0.771606944	0.837848727	positive
PRJNA729271	stage_E14_vs_E10	ordinary_muscle_development	ENSGALG00010029480	ENSGALG00010029480	0.129380666	0.115073076	1.124334814	0.260871015	0.369053812	positive
PRJNA729271	stage_E14_vs_E10	ordinary_muscle_development	ENSGALG00010029481	ENSGALG00010029481	-0.104848291	0.273869614	-0.382840175	0.701838271	0.783574347	negative
PRJNA729271	stage_E14_vs_E10	ordinary_muscle_development	ENSGALG00010029482	DDX25	-0.192797508	0.181948378	-1.059627512	0.289314092	0.401802028	negative
PRJNA729271	stage_E14_vs_E10	ordinary_muscle_development	ENSGALG00010029484	ENSGALG00010029484	-0.582172889	0.48430767	-1.202072411	0.229335474	0.33315471	negative
PRJNA729271	stage_E14_vs_E10	ordinary_muscle_development	ENSGALG00010029487	CDC42EP4	-0.362661788	0.13915723	-2.60612968	0.009157178	0.022059714	negative
PRJNA729271	stage_E14_vs_E10	ordinary_muscle_development	ENSGALG00010029489	ENSGALG00010029489	-1.391346883	0.901644346	-1.543121619	0.12280127	0.201248075	negative
PRJNA729271	stage_E14_vs_E10	ordinary_muscle_development	ENSGALG00010029490	ENSGALG00010029490	-0.65894804	0.546645738	-1.205438905	0.22803391	0.33159626	negative
PRJNA729271	stage_E14_vs_E10	ordinary_muscle_development	ENSGALG00010029491	ENSGALG00010029491	0.424328306	0.197953012	2.14358095	0.032066478	0.064907639	positive
PRJNA729271	stage_E14_vs_E10	ordinary_muscle_development	ENSGALG00010029492	SDK2	1.461203091	0.22351443	6.537399345	6.25977E-11	5.91881E-10	positive
PRJNA729271	stage_E14_vs_E10	ordinary_muscle_development	ENSGALG00010029494	MYH7	2.022106156	0.500753098	4.038130094	5.3879E-05	0.000222881	positive
PRJNA729271	stage_E14_vs_E10	ordinary_muscle_development	ENSGALG00010029495	RPL38	0.514645957	0.188560039	2.72934796	0.00634597	0.016014074	positive
PRJNA729271	stage_E14_vs_E10	ordinary_muscle_development	ENSGALG00010029496	TTYH2	1.042489855	0.143230618	7.278400857	3.378E-13	4.06005E-12	positive
PRJNA729271	stage_E14_vs_E10	ordinary_muscle_development	ENSGALG00010029497	ENSGALG00010029497	0.33037089	0.534330421	0.618289501	0.536384521	0.643084668	positive
PRJNA729271	stage_E14_vs_E10	ordinary_muscle_development	ENSGALG00010029498	ENSGALG00010029498	0.578551324	0.147717655	3.916602408	8.98057E-05	0.000356835	positive
PRJNA729271	stage_E14_vs_E10	ordinary_muscle_development	ENSGALG00010029501	ENSGALG00010029501	0.224367209	0.386868798	0.57995685	0.561943717	0.665037082	positive
PRJNA729271	stage_E14_vs_E10	ordinary_muscle_development	ENSGALG00010029502	BTBD17	0.51090655	0.420652787	1.214556437	0.224535312	0.327560321	positive
PRJNA729271	stage_E14_vs_E10	ordinary_muscle_development	ENSGALG00010029504	GPR142	-0.145822015	0.342170141	-0.426168148	0.669985325	0.757286127	negative
PRJNA729271	stage_E14_vs_E10	ordinary_muscle_development	ENSGALG00010029505	VEZF1	0.045288867	0.109242543	0.414571698	0.67845547	0.764653762	positive
PRJNA729271	stage_E14_vs_E10	ordinary_muscle_development	ENSGALG00010029506	OXLD1	0.297017241	0.141134203	2.104502198	0.035334663	0.070499097	positive
PRJNA729271	stage_E14_vs_E10	ordinary_muscle_development	ENSGALG00010029507	VPS37D	-0.384504695	0.169806961	-2.264363557	0.023551763	0.050144361	negative
PRJNA729271	stage_E14_vs_E10	ordinary_muscle_development	ENSGALG00010029508	ENSGALG00010029508	-0.219593143	0.132208592	-1.660959694	0.096721542	0.165386192	negative
PRJNA729271	stage_E14_vs_E10	ordinary_muscle_development	ENSGALG00010029509	DNAJC30	0.432854219	0.168559491	2.567961123	0.010229863	0.024264746	positive
PRJNA729271	stage_E14_vs_E10	ordinary_muscle_development	ENSGALG00010029510	PDE6G	-0.029405027	0.256578326	-0.114604486	0.908758624	0.937207844	negative
PRJNA729271	stage_E14_vs_E10	ordinary_muscle_development	ENSGALG00010029511	CUEDC1	0.681664103	0.070892227	9.615498471	6.87754E-22	1.69541E-20	positive
PRJNA729271	stage_E14_vs_E10	ordinary_muscle_development	ENSGALG00010029512	TSPAN10	-0.561581249	0.639181109	-0.878594879	0.379620973	0.495640967	negative
PRJNA729271	stage_E14_vs_E10	ordinary_muscle_development	ENSGALG00010029513	WBSCR22	0.223607579	0.08238036	2.714331175	0.006640974	0.016639744	positive
PRJNA729271	stage_E14_vs_E10	ordinary_muscle_development	ENSGALG00010029514	MRPS23	0.094419044	0.096831314	0.975087907	0.329516657	0.444187105	positive
PRJNA729271	stage_E14_vs_E10	ordinary_muscle_development	ENSGALG00010029515	NPLOC4	0.56264315	0.152259865	3.69528208	0.000219643	0.000807232	positive
PRJNA729271	stage_E14_vs_E10	ordinary_muscle_development	ENSGALG00010029516	ENSGALG00010029516	2.88656582	0.22687343	12.7232432	4.39243E-37	2.86794E-35	positive
PRJNA729271	stage_E14_vs_E10	ordinary_muscle_development	ENSGALG00010029517	MSI2	-0.258395724	0.112194705	-2.303100885	0.021273168	0.045913425	negative
PRJNA729271	stage_E14_vs_E10	ordinary_muscle_development	ENSGALG00010029518	FAAP100	0.188351414	0.185611561	1.014761219	0.310219664	0.42414796	positive
PRJNA729271	stage_E14_vs_E10	ordinary_muscle_development	ENSGALG00010029519	STX1A	1.122858772	0.104539559	10.74099397	6.53371E-27	2.30429E-25	positive
PRJNA729271	stage_E14_vs_E10	ordinary_muscle_development	ENSGALG00010029520	MKS1	-0.329111102	0.106743817	-3.08318655	0.002047967	0.00593587	negative
PRJNA729271	stage_E14_vs_E10	ordinary_muscle_development	ENSGALG00010029521	ACTG1	-0.038330845	0.098360015	-0.38969946	0.696758796	0.779103505	negative
PRJNA729271	stage_E14_vs_E10	ordinary_muscle_development	ENSGALG00010029522	AKAP1	0.488522062	0.112022612	4.360923706	1.29515E-05	5.9673E-05	positive
PRJNA729271	stage_E14_vs_E10	ordinary_muscle_development	ENSGALG00010029523	FSCN2	0.263102142	0.721274085	0.364774151	0.715280002	0.794068286	positive
PRJNA729271	stage_E14_vs_E10	ordinary_muscle_development	ENSGALG00010029524	ENSGALG00010029524	0.418580779	0.147940505	2.829385909	0.004663743	0.012248767	positive
PRJNA729271	stage_E14_vs_E10	ordinary_muscle_development	ENSGALG00010029525	SLC38A10	-0.283698104	0.137998364	-2.055807734	0.039801052	0.077955996	negative
PRJNA729271	stage_E14_vs_E10	ordinary_muscle_development	ENSGALG00010029526	BCL7B	0.464203866	0.111788148	4.152532026	3.28817E-05	0.000141321	positive
PRJNA729271	stage_E14_vs_E10	ordinary_muscle_development	ENSGALG00010029527	NDUFAF8	0.642711691	0.152684652	4.20940601	2.56043E-05	0.000112024	positive
PRJNA729271	stage_E14_vs_E10	ordinary_muscle_development	ENSGALG00010029528	TBL2	-0.04460922	0.094619762	-0.471457748	0.637313885	0.730554747	negative
PRJNA729271	stage_E14_vs_E10	ordinary_muscle_development	ENSGALG00010029529	TEPSIN	0.113569857	0.12841939	0.884366887	0.376498192	0.492717704	positive
PRJNA729271	stage_E14_vs_E10	ordinary_muscle_development	ENSGALG00010029530	ENSGALG00010029530	1.611549128	0.361594445	4.456786187	8.31975E-06	3.95263E-05	positive
PRJNA729271	stage_E14_vs_E10	ordinary_muscle_development	ENSGALG00010029531	CEP131	-0.064922462	0.170465338	-0.380854331	0.703311341	0.784441623	negative
PRJNA729271	stage_E14_vs_E10	ordinary_muscle_development	ENSGALG00010029532	EVI2A	0.195404274	0.476825092	0.409802834	0.681950587	0.76733999	positive
PRJNA729271	stage_E14_vs_E10	ordinary_muscle_development	ENSGALG00010029533	MLXIPL	-1.062305717	0.757895315	-1.401652307	0.161019099	0.251316016	negative
PRJNA729271	stage_E14_vs_E10	ordinary_muscle_development	ENSGALG00010029534	NF1	0.017435479	0.090667889	0.192300478	0.847506844	0.894011477	positive
PRJNA729271	stage_E14_vs_E10	ordinary_muscle_development	ENSGALG00010029536	BAZ1B	-0.162839377	0.076862374	-2.118583756	0.03412566	0.06844492	negative
PRJNA729271	stage_E14_vs_E10	ordinary_muscle_development	ENSGALG00010029537	MYH1B	1.951770887	0.206080416	9.47091881	2.77393E-21	6.55923E-20	positive
PRJNA729271	stage_E14_vs_E10	ordinary_muscle_development	ENSGALG00010029538	FZD9	-0.047708823	0.17288942	-0.275949928	0.782586525	0.846536151	negative
PRJNA729271	stage_E14_vs_E10	ordinary_muscle_development	ENSGALG00010029539	FKBP6	-1.571515896	0.35648417	-4.408374978	1.04149E-05	4.87295E-05	negative
PRJNA729271	stage_E14_vs_E10	ordinary_muscle_development	ENSGALG00010029541	ENSGALG00010029541	0.353708686	0.227918117	1.551911234	0.120683465	0.198535604	positive
PRJNA729271	stage_E14_vs_E10	ordinary_muscle_development	ENSGALG00010029542	AATK	0.188339138	0.147115258	1.280214851	0.200469584	0.299629473	positive
PRJNA729271	stage_E14_vs_E10	ordinary_muscle_development	ENSGALG00010029543	NSUN5	0.268554274	0.131582236	2.040961469	0.041254657	0.080396108	positive
PRJNA729271	stage_E14_vs_E10	ordinary_muscle_development	ENSGALG00010029544	ENSGALG00010029544	-0.554196073	0.176410111	-3.14152102	0.001680727	0.005001467	negative

PRJNA729271	stage_E14_vs_E10	ordinary_muscle_development	ENSGALG00010029545	OMG	-0.305669263	0.350435041	-0.872256559	0.383068431	0.499108036	negative
PRJNA729271	stage_E14_vs_E10	ordinary_muscle_development	ENSGALG00010029546	WSB1	-0.128610053	0.165273829	-0.77816345	0.436472661	0.550336745	negative
PRJNA729271	stage_E14_vs_E10	ordinary_muscle_development	ENSGALG00010029547	HIP1	0.112469305	0.09981794	1.126744397	0.259850565	0.368185136	positive
PRJNA729271	stage_E14_vs_E10	ordinary_muscle_development	ENSGALG00010029548	BAIAP2	-0.337058259	0.224412397	-1.501959177	0.133107652	0.21533431	negative
PRJNA729271	stage_E14_vs_E10	ordinary_muscle_development	ENSGALG00010029549	KSR1	0.489735883	0.125078267	3.915435481	9.02412E-05	0.000358369	positive
PRJNA729271	stage_E14_vs_E10	ordinary_muscle_development	ENSGALG00010029550	CHMP6	0.166686386	0.239511924	0.695941909	0.48646521	0.597670217	positive
PRJNA729271	stage_E14_vs_E10	ordinary_muscle_development	ENSGALG00010029552	NOS2	-0.053018022	0.124475357	-0.425931875	0.670157487	0.757421659	negative
PRJNA729271	stage_E14_vs_E10	ordinary_muscle_development	ENSGALG00010029553	MYH1C	2.335221649	0.194805683	11.98744109	4.13482E-33	2.10295E-31	positive
PRJNA729271	stage_E14_vs_E10	ordinary_muscle_development	ENSGALG00010029554	ENSGALG00010029554	3.391912879	0.374787719	9.050224181	1.42675E-19	2.95439E-18	positive
PRJNA729271	stage_E14_vs_E10	ordinary_muscle_development	ENSGALG00010029555	LYRM9	-0.015740115	0.156518258	-0.100564083	0.91989651	0.944525717	negative
PRJNA729271	stage_E14_vs_E10	ordinary_muscle_development	ENSGALG00010029556	NPTX1	-3.04800165	0.77257374	-3.945256603	7.97146E-05	0.000319365	negative
PRJNA729271	stage_E14_vs_E10	ordinary_muscle_development	ENSGALG00010029557	ENSGALG00010029557	3.009154287	0.552992423	5.441583218	5.28091E-08	3.49051E-07	positive
PRJNA729271	stage_E14_vs_E10	ordinary_muscle_development	ENSGALG00010029558	ENSGALG00010029558	1.113731456	0.664227898	1.676730922	0.093595111	0.161008917	positive
PRJNA729271	stage_E14_vs_E10	ordinary_muscle_development	ENSGALG00010029560	ENSGALG00010029560	2.207914373	0.172533216	12.79703945	1.70322E-37	1.15365E-35	positive
PRJNA729271	stage_E14_vs_E10	ordinary_muscle_development	ENSGALG00010029561	SCO1	0.197489304	0.103455402	1.908931778	0.056270893	0.104704312	positive
PRJNA729271	stage_E14_vs_E10	ordinary_muscle_development	ENSGALG00010029562	ENSGALG00010029562	0.775033322	0.765532818	1.012410317	0.311341908	0.425103707	positive
PRJNA729271	stage_E14_vs_E10	ordinary_muscle_development	ENSGALG00010029563	ADPRM	0.517307828	0.124341804	4.160369346	3.17733E-05	0.000136988	positive
PRJNA729271	stage_E14_vs_E10	ordinary_muscle_development	ENSGALG00010029564	NLK	0.020468446	0.086930908	0.235456488	0.813854447	0.869566943	positive
PRJNA729271	stage_E14_vs_E10	ordinary_muscle_development	ENSGALG00010029566	TMEM97	-0.369755211	0.143626118	-2.574428768	0.010040576	0.023893966	negative
PRJNA729271	stage_E14_vs_E10	ordinary_muscle_development	ENSGALG00010029567	IFT20	-0.15695723	0.118600142	-1.323415196	0.185697341	0.28170413	negative
PRJNA729271	stage_E14_vs_E10	ordinary_muscle_development	ENSGALG00010029568	ENSGALG00010029568	2.565457843	0.804027757	3.190757808	0.001419002	0.004303019	positive
PRJNA729271	stage_E14_vs_E10	ordinary_muscle_development	ENSGALG00010029569	TMEM220	0.78070017	0.200128343	3.90099752	9.57971E-05	0.00037805	positive
PRJNA729271	stage_E14_vs_E10	ordinary_muscle_development	ENSGALG00010029570	TNFAIP1	-0.161186018	0.078117898	-2.063368606	0.03907762	0.076835337	negative
PRJNA729271	stage_E14_vs_E10	ordinary_muscle_development	ENSGALG00010029571	EPX	-0.360195144	0.742973661	-0.484802037	0.627816777	0.721956943	negative
PRJNA729271	stage_E14_vs_E10	ordinary_muscle_development	ENSGALG00010029572	TSPOAP1	-0.311122593	0.116973997	-2.659758587	0.007819668	0.019234021	negative
PRJNA729271	stage_E14_vs_E10	ordinary_muscle_development	ENSGALG00010029573	MAP2K4	-0.289389847	0.058979775	-4.906594627	9.26713E-07	5.13286E-06	negative
PRJNA729271	stage_E14_vs_E10	ordinary_muscle_development	ENSGALG00010029574	SGSM2	-0.51414033	0.130613009	-3.936363866	8.27255E-05	0.000330697	negative
PRJNA729271	stage_E14_vs_E10	ordinary_muscle_development	ENSGALG00010029575	ANKFN1	-2.127440311	0.51274815	-4.149094076	3.33794E-05	0.000143188	negative
PRJNA729271	stage_E14_vs_E10	ordinary_muscle_development	ENSGALG00010029576	ATP2A3	0.654711148	0.130925938	5.000622159	5.71456E-07	3.26241E-06	positive
PRJNA729271	stage_E14_vs_E10	ordinary_muscle_development	ENSGALG00010029577	NOG	-3.648752897	0.340654375	-10.71101142	9.03691E-27	3.15638E-25	negative
PRJNA729271	stage_E14_vs_E10	ordinary_muscle_development	ENSGALG00010029578	C17orf67	-4.314287282	0.583737267	-7.390803233	1.45944E-13	1.81429E-12	negative
PRJNA729271	stage_E14_vs_E10	ordinary_muscle_development	ENSGALG00010029579	GALNT17	0.792247639	0.183236392	4.323636973	1.53478E-05	6.984E-05	positive
PRJNA729271	stage_E14_vs_E10	ordinary_muscle_development	ENSGALG00010029580	P2RX1	2.4552747	0.314912608	7.796685928	6.35542E-15	8.85895E-14	positive
PRJNA729271	stage_E14_vs_E10	ordinary_muscle_development	ENSGALG00010029581	DGKE	-0.033007847	0.104029277	-0.317293819	0.751020667	0.821712301	negative
PRJNA729271	stage_E14_vs_E10	ordinary_muscle_development	ENSGALG00010029582	SCPEP1	-0.184090277	0.149904417	-1.228051049	0.219427805	0.321695766	negative
PRJNA729271	stage_E14_vs_E10	ordinary_muscle_development	ENSGALG00010029583	LIMK1	-0.514838988	0.191385525	-2.690062309	0.007143868	0.01774338	negative
PRJNA729271	stage_E14_vs_E10	ordinary_muscle_development	ENSGALG00010029584	TRIM25	0.023304918	0.233204088	0.099933574	0.920397062	0.944907955	positive
PRJNA729271	stage_E14_vs_E10	ordinary_muscle_development	ENSGALG00010029585	ENSGALG00010029585	-1.153980822	0.408106443	-2.827646664	0.004689153	0.012311044	negative
PRJNA729271	stage_E14_vs_E10	ordinary_muscle_development	ENSGALG00010029586	COIL	-0.021960526	0.186659087	-0.11765045	0.906344629	0.935716909	negative
PRJNA729271	stage_E14_vs_E10	ordinary_muscle_development	ENSGALG00010029588	RAB11FIP4	-2.002143721	0.139318852	-14.37094617	7.8748E-47	9.35617E-45	negative
PRJNA729271	stage_E14_vs_E10	ordinary_muscle_development	ENSGALG00010029590	UTP6	-0.087549533	0.097332754	-0.89948686	0.368393392	0.484648958	negative
PRJNA729271	stage_E14_vs_E10	ordinary_muscle_development	ENSGALG00010029591	SUZ12	-0.314331189	0.077741123	-4.04330651	5.27027E-05	0.000218379	negative
PRJNA729271	stage_E14_vs_E10	ordinary_muscle_development	ENSGALG00010029592	MNT	0.208912668	0.165361699	1.263367932	0.206456983	0.306461236	positive
PRJNA729271	stage_E14_vs_E10	ordinary_muscle_development	ENSGALG00010029593	PAFAH1B1	0.292397966	0.078785827	3.711301582	0.000206196	0.000763325	positive
PRJNA729271	stage_E14_vs_E10	ordinary_muscle_development	ENSGALG00010029594	CRLF3	-0.079190691	0.066814354	-1.185234701	0.235924663	0.340610358	negative
PRJNA729271	stage_E14_vs_E10	ordinary_muscle_development	ENSGALG00010029595	CLUH	0.433576776	0.134967743	3.212447412	0.001316092	0.004025482	positive
PRJNA729271	stage_E14_vs_E10	ordinary_muscle_development	ENSGALG00010029596	ATAD5	-0.326067362	0.126847142	-2.570553486	0.010153615	0.024123364	negative
PRJNA729271	stage_E14_vs_E10	ordinary_muscle_development	ENSGALG00010029597	ADAP2	0.71881652	0.206465577	3.481532031	0.000498554	0.00169121	positive
PRJNA729271	stage_E14_vs_E10	ordinary_muscle_development	ENSGALG00010029598	SUPT4H1	0.203007868	0.148599824	1.36613801	0.17189565	0.264756928	positive
PRJNA729271	stage_E14_vs_E10	ordinary_muscle_development	ENSGALG00010029599	RNF43	-1.27161568	0.312470585	-4.069553235	4.71034E-05	0.000196836	negative
PRJNA729271	stage_E14_vs_E10	ordinary_muscle_development	ENSGALG00010029600	RHOT1	0.21596431	0.108893881	1.983254782	0.047338978	0.090588927	positive
PRJNA729271	stage_E14_vs_E10	ordinary_muscle_development	ENSGALG00010029601	HSF5	-0.392741946	0.491305069	-0.799385088	0.424067154	0.538444648	negative
PRJNA729271	stage_E14_vs_E10	ordinary_muscle_development	ENSGALG00010029602	MTMR4	-0.058041696	0.085305565	-0.680397532	0.496252783	0.605843855	negative
PRJNA729271	stage_E14_vs_E10	ordinary_muscle_development	ENSGALG00010029603	RHBDL3	-0.665862632	0.426678323	-1.56057291	0.118624555	0.195838601	negative
PRJNA729271	stage_E14_vs_E10	ordinary_muscle_development	ENSGALG00010029604	ENSGALG00010029604	0.079104546	0.116321047	0.680053594	0.496470527	0.606007603	positive
PRJNA729271	stage_E14_vs_E10	ordinary_muscle_development	ENSGALG00010029605	C17orf75	-0.527950896	0.09144449	-5.773457703	7.76611E-09	5.72292E-08	negative
PRJNA729271	stage_E14_vs_E10	ordinary_muscle_development	ENSGALG00010029606	ABHD11	0.240171728	0.209187762	1.148115577	0.250920854	0.358052548	positive
PRJNA729271	stage_E14_vs_E10	ordinary_muscle_development	ENSGALG00010029607	ZNF207	-0.08424447	0.064283337	-1.310518009	0.190020656	0.287005827	negative
PRJNA729271	stage_E14_vs_E10	ordinary_muscle_development	ENSGALG00010029608	ENSGALG00010029608	0.32441343	0.292487074	1.109154757	0.267363426	0.376474924	positive
PRJNA729271	stage_E14_vs_E10	ordinary_muscle_development	ENSGALG00010029609	METTL27	-0.240492541	0.138901032	-1.731394923	0.083381352	0.146480753	negative
PRJNA729271	stage_E14_vs_E10	ordinary_muscle_development	ENSGALG00010029610	ERN1	-0.119379202	0.078533902	-1.520097683	0.128486427	0.208884114	negative
PRJNA729271	stage_E14_vs_E10	ordinary_muscle_development	ENSGALG00010029611	YWHAG	0.399105999	0.130529573	3.057590626	0.002231242	0.006405595	positive
PRJNA729271	stage_E14_vs_E10	ordinary_muscle_development	ENSGALG00010029612	HSPB1	1.518602095	0.195717249	7.759163277	8.54915E-15	1.18244E-13	positive
PRJNA729271	stage_E14_vs_E10	ordinary_muscle_development	ENSGALG00010029614	MDH2	0.165502146	0.128634138	1.286611389	0.198229772	0.296863369	positive
PRJNA729271	stage_E14_vs_E10	ordinary_muscle_development	ENSGALG00010029615	ENSGALG00010029615	-0.938889214	0.282166115	-3.327434316	0.000876496	0.002799034	negative
PRJNA729271	stage_E14_vs_E10	ordinary_muscle_development	ENSGALG00010029616	TMEM120A	0.286253096	0.135329599	2.115229027	0.034410432	0.068968365	positive
PRJNA729271	stage_E14_vs_E10	ordinary_muscle_development	ENSGALG00010029617	POR	-0.076327929	0.094162312	-0.810599563	0.417595668	0.532184246	negative
PRJNA729271	stage_E14_vs_E10	ordinary_muscle_development	ENSGALG00010029618	TEX2	0.623216652	0.112531869	5.538134747	3.0571E-08	2.10412E-07	positive
PRJNA729271	stage_E14_vs_E10	ordinary_muscle_development	ENSGALG00010029619	ENSGALG00010029619	0.14505699	0.101757964	1.425509955	0.154009845	0.242570107	positive
PRJNA729271	stage_E14_vs_E10	ordinary_muscle_development	ENSGALG00010029620	ENSGALG00010029620	0.222386386	0.384887973	0.577795102	0.563402462	0.66616516	positive
PRJNA729271	stage_E14_vs_E10	ordinary_muscle_development	ENSGALG00010029621	TAF15	-0.254200225	0.120052934	-2.117401182	0.034225814	0.068626805	negative
PRJNA729271	stage_E14_vs_E10	ordinary_muscle_development	ENSGALG00010029622	MMP28	1.17181353	0.155284665	7.546228291	4.48044E-14	5.85081E-13	positive
PRJNA729271	stage_E14_vs_E10	ordinary_muscle_development	ENSGALG00010029623	ENSGALG00010029623	1.31993565	0.376676484	3.504162603	0.000458046	0.001566628	positive
PRJNA729271	stage_E14_vs_E10	ordinary_muscle_development	ENSGALG00010029624	RASL10B	1.920026393	0.26911102	7.134699983	9.69985E-13	1.10795E-11	positive
PRJNA729271	stage_E14_vs_E10	ordinary_muscle_development	ENSGALG00010029625	PECAM1	0.512885332	0.174509071	2.939018171	0.003292538	0.009018393	positive
PRJNA729271	stage_E14_vs_E10	ordinary_muscle_development	ENSGALG00010029626	AP2B1	-0.146416217	0.108274412	-1.352269794	0.176289024	0.270060177	negative
PRJNA729271	stage_E14_vs_E10	ordinary_muscle_development	ENSGALG00010029627	PEX12	-0.142961812	0.143103823	-0.999007636	0.317790992	0.431676089	negative
PRJNA729271	stage_E14_vs_E10	ordinary_muscle_development	ENSGALG00010029628	UNC45B	1.495570887	0.16040914	9.323476751	1.12589E-20	2.5302E-19	positive
PRJNA729271	stage_E14_vs_E10	ordinary_muscle_development	ENSGALG00010029629	ENSGALG00010029629	1.100370136	0.259224878	4.244847743	2.18742E-05	9.67846E-05	positive
PRJNA729271	stage_E14_vs_E10	ordinary_muscle_development	ENSGALG00010029630	RAD51D	0.344279817	0.115570159	2.9789681	0.002892209	0.00804347	positive
PRJNA729271	stage_E14_vs_E10	ordinary_muscle_development	ENSGALG00010029631	LIG3	-0.020870846	0.085534637	-0.244004615	0.807227246	0.864603531	negative
PRJNA729271	stage_E14_vs_E10	ordinary_muscle_development	ENSGALG00010029632	TMEM132E	0.237285774	0.22725528	1.044137565	0.296421748	0.409710426	positive
PRJNA729271	stage_E14_vs_E10	ordinary_muscle_development	ENSGALG00010029633	POLG2	0.00078691	0.174575361	0.004507568	0.996403494	0.998160417	positive
PRJNA729271	stage_E14_vs_E10	ordinary_muscle_development	ENSGALG00010029634	DDX5	-0.432447402	0.091111275	-4.746365383	2.07104E-06	1.08807E-05	negative
PRJNA729271	stage_E14_vs_E10	ordinary_muscle_development	ENSGALG00010029635	ENSGALG00010029635	1.02473578	0.577049183	1.775820518	0.075762505	0.135010758	positive
PRJNA729271	stage_E14_vs_E10	ordinary_muscle_development	ENSGALG00010029636	SPATA20	0.222576989	0.075284442	2.956480546	0.003111719	0.008591307	positive
PRJNA729271	stage_E14_vs_E10	ordinary_muscle_development	ENSGALG00010029638	EPN3	1.283304375	0.195467992	6.565291655	5.19311E-11	4.95907E-10	positive
PRJNA729271	stage_E14_vs_E10	ordinary_muscle_development	ENSGALG00010029639	ENSGALG00010029639	0.415056133	0.74943098	0.553828363	0.579696315	0.68126302	positive
PRJNA729271	stage_E14_vs_E10	ordinary_muscle_development	ENSGALG00010029641	ENSGALG00010029641	0.025437718	0.719982138	0.035331041	0.971815772	0.980555773	positive
PRJNA729271	stage_E14_vs_E10	ordinary_muscle_development	ENSGALG00010029642	NAT9	0.206417034	0.144317315	1.430299846	0.152630979	0.240764614	positive
PRJNA729271	stage_E14_vs_E10	ordinary_muscle_development	ENSGALG00010029644	MYCBPAP	0.392570829	0.161804507	2.426204541	0.015257668	0.03440571	positive
PRJNA729271	stage_E14_vs_E10	ordinary_muscle_development	ENSGALG00010029645	ENSGALG00010029645	-2.04858185	0.385757926	-5.310537294	1.09303E-07	6.93316E-07	negative
PRJNA729271	stage_E14_vs_E10	ordinary_muscle_development	ENSGALG00010029646	MRPS17	-0.081959262	0.108263728	-0.757033435	0.449029832	0.562111358	negative
PRJNA729271	stage_E14_vs_E10	ordinary_muscle_development	ENSGALG00010029647	RSAD1	0.080275357	0.15426354	0.520378026	0.602800122	0.701082225	positive
PRJNA729271	stage_E14_vs_E10	ordinary_muscle_development	ENSGALG00010029648	NIPSNAP2	0.389654797	0.12190422	3.196401219	0.001391535	0.004227687	positive
PRJNA729271	stage_E14_vs_E10	ordinary_muscle_development	ENSGALG00010029649	PSPH	0.896914261	0.260695205	3.440470878	0.000580703	0.001936344	positive
PRJNA729271	stage_E14_vs_E10	ordinary_muscle_development	ENSGALG00010029650	ACSF2	-0.059481211	0.133826831	-0.444464021	0.656707136	0.746878771	negative
PRJNA729271	stage_E14_vs_E10	ordinary_muscle_development	ENSGALG00010029651	ENSGALG00010029651	-2.280595752	0.302441703	-7.540612721	4.67768E-14	6.09739E-13	negative
PRJNA729271	stage_E14_vs_E10	ordinary_muscle_development	ENSGALG00010029652	LRRC59	0.08851632	0.098924282	0.894788604	0.370900103	0.48723917	positive
PRJNA729271	stage_E14_vs_E10	ordinary_muscle_development	ENSGALG00010029653	CCT6A	0.010109299	0.090023073	0.112296756	0.910588114	0.93836021	positive
PRJNA729271	stage_E14_vs_E10	ordinary_muscle_development	ENSGALG00010029654	EME1	-0.426669499	0.120387993	-3.544120029	0.000393926	0.001366999	negative
PRJNA729271	stage_E14_vs_E10	ordinary_muscle_development	ENSGALG00010029655	PHKG1	0.802544321	0.129165053	6.213324	5.18753E-10	4.39727E-09	positive
PRJNA729271	stage_E14_vs_E10	ordinary_muscle_development	ENSGALG00010029656	MRPL27	0.247163415	0.112349635	2.199948533	0.027810547	0.057711364	positive
PRJNA729271	stage_E14_vs_E10	ordinary_muscle_development	ENSGALG00010029657	CHCHD2	0.560199393	0.146808084	3.815862033	0.000135708	0.000522191	positive
PRJNA729271	stage_E14_vs_E10	ordinary_muscle_development	ENSGALG00010029658	XYLT2	0.081341502	0.124312306	0.654331856	0.512898026	0.621662086	positive
PRJNA729271	stage_E14_vs_E10	ordinary_muscle_development	ENSGALG00010029659	VKORC1L1	-0.377025177	0.145700114	-2.587679354	0.009662488	0.023130337	negative
PRJNA729271	stage_E14_vs_E10	ordinary_muscle_development	ENSGALG00010029660	CD300LG	0.361467842	0.307413275	1.175836803	0.239660096	0.345384082	positive
PRJNA729271	stage_E14_vs_E10	ordinary_muscle_development	ENSGALG00010029661	KRABZFP	0.46551426	0.682909214	0.681663464	0.495451775	0.605123095	positive
PRJNA729271	stage_E14_vs_E10	ordinary_muscle_development	ENSGALG00010029663	GUSB	0.032996201	0.153362759	0.215151326	0.829649338	0.881008583	positive
PRJNA729271	stage_E14_vs_E10	ordinary_muscle_development	ENSGALG00010029664	RAB37	0.490191645	0.404014825	1.213301133	0.22501471	0.32809458	positive
PRJNA729271	stage_E14_vs_E10	ordinary_muscle_development	ENSGALG00010029665	ASL1	0.027358332	0.191974201	0.142510459	0.886676818	0.922309328	positive
PRJNA729271	stage_E14_vs_E10	ordinary_muscle_development	ENSGALG00010029666	SLC9A3R1	0.205535715	0.196406127	1.046483213	0.295337991	0.408524113	positive
PRJNA729271	stage_E14_vs_E10	ordinary_muscle_development	ENSGALG00010029667	TMEM104	-0.037045079	0.091278953	-0.405844693	0.684856717	0.769534738	negative
PRJNA729271	stage_E14_vs_E10	ordinary_muscle_development	ENSGALG00010029668	CRCP	0.128218617	0.181187409	0.707657432	0.479158018	0.591248444	positive
PRJNA729271	stage_E14_vs_E10	ordinary_muscle_development	ENSGALG00010029669	HID1	0.700985884	0.072236649	9.704020025	2.89848E-22	7.38374E-21	positive
PRJNA729271	stage_E14_vs_E10	ordinary_muscle_development	ENSGALG00010029670	TPST1	-0.50084665	0.169954965	-2.946937441	0.003209381	0.008822299	negative
PRJNA729271	stage_E14_vs_E10	ordinary_muscle_development	ENSGALG00010029671	GRIN2C	3.508177011	0.196183157	17.88215184	1.62445E-71	5.88659E-69	positive
PRJNA729271	stage_E14_vs_E10	ordinary_muscle_development	ENSGALG00010029672	KCTD7	-0.609415511	0.207828322	-2.932302518	0.003364588	0.009187963	negative
PRJNA729271	stage_E14_vs_E10	ordinary_muscle_development	ENSGALG00010029673	TMEM248	-0.236160615	0.089804502	-2.62971909	0.008545545	0.020802837	negative
PRJNA729271	stage_E14_vs_E10	ordinary_muscle_development	ENSGALG00010029674	FDXR	0.254178429	0.10952617	2.320709563	0.020302524	0.044067848	positive
PRJNA729271	stage_E14_vs_E10	ordinary_muscle_development	ENSGALG00010029675	DHRS13	-0.840487819	0.231607236	-3.628935923	0.000284592	0.001023105	negative
PRJNA729271	stage_E14_vs_E10	ordinary_muscle_development	ENSGALG00010029676	FADS6	1.94449918	0.999327083	1.945808547	0.051677742	0.097496924	positive
PRJNA729271	stage_E14_vs_E10	ordinary_muscle_development	ENSGALG00010029677	PHF12	8.77204E-05	0.099886944	0.000878196	0.999299301	0.999437202	positive
PRJNA729271	stage_E14_vs_E10	ordinary_muscle_development	ENSGALG00010029682	CDR2L	-0.534679451	0.119399431	-4.47807368	7.53196E-06	3.60554E-05	negative
PRJNA729271	stage_E14_vs_E10	ordinary_muscle_development	ENSGALG00010029683	MRPL58	-0.04111826	0.109528055	-0.375413038	0.70735331	0.787547909	negative
PRJNA729271	stage_E14_vs_E10	ordinary_muscle_development	ENSGALG00010029684	ENSGALG00010029684	1.597611691	0.467070347	3.420494793	0.000625073	0.002064822	positive
PRJNA729271	stage_E14_vs_E10	ordinary_muscle_development	ENSGALG00010029685	ATP5PD	0.610814721	0.114928497	5.31473689	1.06812E-07	6.78157E-07	positive
PRJNA729271	stage_E14_vs_E10	ordinary_muscle_development	ENSGALG00010029686	KCTD2	0.615521702	0.059064799	10.42112582	1.98588E-25	6.20375E-24	positive
PRJNA729271	stage_E14_vs_E10	ordinary_muscle_development	ENSGALG00010029687	SLC16A5	2.291145058	0.395162681	5.797979342	6.71187E-09	4.99172E-08	positive
PRJNA729271	stage_E14_vs_E10	ordinary_muscle_development	ENSGALG00010029689	ENSGALG00010029689	0.34227117	0.17610974	1.943510735	0.05195448	0.097968022	positive
PRJNA729271	stage_E14_vs_E10	ordinary_muscle_development	ENSGALG00010029690	ENSGALG00010029690	0.23860417	0.161737433	1.475256314	0.140143646	0.224586197	positive

PRJNA729271	stage_E14_vs_E10	ordinary_muscle_development	ENSGALG00010029691	NUFIP2	-0.024666458	0.141158022	-0.174743581	0.861281122	0.904526146	negative
PRJNA729271	stage_E14_vs_E10	ordinary_muscle_development	ENSGALG00010029692	CRYBA1	-0.481040776	0.490041489	-0.981632752	0.326280806	0.441055701	negative
PRJNA729271	stage_E14_vs_E10	ordinary_muscle_development	ENSGALG00010029693	HN1	0.115972572	0.126259424	0.918526063	0.35834352	0.474702004	positive
PRJNA729271	stage_E14_vs_E10	ordinary_muscle_development	ENSGALG00010029694	ARMC7	1.315746924	0.086876749	15.14498341	8.17753E-52	1.26099E-49	positive
PRJNA729271	stage_E14_vs_E10	ordinary_muscle_development	ENSGALG00010029695	TAOK1	0.200692669	0.086055401	2.332133339	0.019693678	0.042856588	positive
PRJNA729271	stage_E14_vs_E10	ordinary_muscle_development	ENSGALG00010029696	SUMO2	-0.424478696	0.107479612	-3.94938806	7.83512E-05	0.000314337	negative
PRJNA729271	stage_E14_vs_E10	ordinary_muscle_development	ENSGALG00010029697	GIT1	-0.267638204	0.168480848	-1.58853785	0.112164769	0.187372171	negative
PRJNA729271	stage_E14_vs_E10	ordinary_muscle_development	ENSGALG00010029698	NUP85	0.081089034	0.084582862	0.958693431	0.337713209	0.452835612	positive
PRJNA729271	stage_E14_vs_E10	ordinary_muscle_development	ENSGALG00010029699	ANKRD13B	-1.041990919	0.171092707	-6.090212377	1.12761E-09	9.1567E-09	negative
PRJNA729271	stage_E14_vs_E10	ordinary_muscle_development	ENSGALG00010029700	GGA3	0.110578498	0.078744745	1.404265111	0.160239919	0.25037911	positive
PRJNA729271	stage_E14_vs_E10	ordinary_muscle_development	ENSGALG00010029701	MRPS7	0.046787481	0.065129691	0.718374064	0.472526674	0.584483906	positive
PRJNA729271	stage_E14_vs_E10	ordinary_muscle_development	ENSGALG00010029702	CORO6	1.695904323	0.171524344	9.887251474	4.72831E-23	1.24161E-21	positive
PRJNA729271	stage_E14_vs_E10	ordinary_muscle_development	ENSGALG00010029703	MIF4GD	-0.02012177	0.114597268	-0.175586821	0.860618556	0.904157859	negative
PRJNA729271	stage_E14_vs_E10	ordinary_muscle_development	ENSGALG00010029704	SLC25A19	0.032043248	0.101101702	0.31694074	0.751288568	0.821831085	positive
PRJNA729271	stage_E14_vs_E10	ordinary_muscle_development	ENSGALG00010029705	GRB2	0.536713074	0.066478963	8.073427295	6.83519E-16	1.03962E-14	positive
PRJNA729271	stage_E14_vs_E10	ordinary_muscle_development	ENSGALG00010029706	SSH2	0.652883032	0.217692785	2.999102762	0.00270776	0.007596978	positive
PRJNA729271	stage_E14_vs_E10	ordinary_muscle_development	ENSGALG00010029707	NSRP1	-0.113955659	0.222195721	-0.512861628	0.608048117	0.705478575	negative
PRJNA729271	stage_E14_vs_E10	ordinary_muscle_development	ENSGALG00010029708	TMEM94	0.309030506	0.084102561	3.674448226	0.000238364	0.00086902	positive
PRJNA729271	stage_E14_vs_E10	ordinary_muscle_development	ENSGALG00010029709	SLC6A4	-1.036856629	0.211022663	-4.913484725	8.94717E-07	4.97084E-06	negative
PRJNA729271	stage_E14_vs_E10	ordinary_muscle_development	ENSGALG00010029710	BLMH	-0.105432999	0.082593098	-1.276535222	0.201766384	0.30119503	negative
PRJNA729271	stage_E14_vs_E10	ordinary_muscle_development	ENSGALG00010029711	CASKIN2	1.187331944	0.110383905	10.7563865	5.52961E-27	1.95492E-25	positive
PRJNA729271	stage_E14_vs_E10	ordinary_muscle_development	ENSGALG00010029713	CPD	-0.166095528	0.116503939	-1.425664485	0.153965213	0.242526164	negative
PRJNA729271	stage_E14_vs_E10	ordinary_muscle_development	ENSGALG00010029716	GOSR1	0.168395316	0.061493788	2.738411828	0.006173671	0.015636443	positive
PRJNA729271	stage_E14_vs_E10	ordinary_muscle_development	ENSGALG00010029717	TRARG1	-0.222281053	0.406254752	-0.547146961	0.584277771	0.685091919	negative
PRJNA729271	stage_E14_vs_E10	ordinary_muscle_development	ENSGALG00010029718	ABR	-0.674179243	0.112125941	-6.0126964	1.82463E-09	1.45159E-08	negative
PRJNA729271	stage_E14_vs_E10	ordinary_muscle_development	ENSGALG00010029720	TIMM22	-0.154265757	0.106649584	-1.44647313	0.148044543	0.234884594	negative
PRJNA729271	stage_E14_vs_E10	ordinary_muscle_development	ENSGALG00010029721	EXOC7	-0.026456685	0.104807833	-0.252430418	0.800708391	0.859577582	negative
PRJNA729271	stage_E14_vs_E10	ordinary_muscle_development	ENSGALG00010029722	SPECC1	1.023061883	0.141663504	7.221774499	5.13135E-13	6.04707E-12	positive
PRJNA729271	stage_E14_vs_E10	ordinary_muscle_development	ENSGALG00010029723	ADORA2B	-0.040774743	0.182007032	-0.224028396	0.822735208	0.875783714	negative
PRJNA729271	stage_E14_vs_E10	ordinary_muscle_development	ENSGALG00010029724	CACNA1G	-1.383181309	0.21176508	-6.531677973	6.50369E-11	6.12945E-10	negative
PRJNA729271	stage_E14_vs_E10	ordinary_muscle_development	ENSGALG00010029725	TSEN54	0.221602951	0.06233709	3.55491334	0.000378104	0.001320311	positive
PRJNA729271	stage_E14_vs_E10	ordinary_muscle_development	ENSGALG00010029726	SEPTIN9	0.084147464	0.125065141	0.672829082	0.501056034	0.610217376	positive
PRJNA729271	stage_E14_vs_E10	ordinary_muscle_development	ENSGALG00010029727	ZSWIM7	0.299508398	0.132973331	2.252394495	0.024297353	0.051459693	positive
PRJNA729271	stage_E14_vs_E10	ordinary_muscle_development	ENSGALG00010029728	TNRC6C	-0.228406684	0.103448141	-2.207934158	0.027248863	0.056691836	negative
PRJNA729271	stage_E14_vs_E10	ordinary_muscle_development	ENSGALG00010029729	RASL10A	-1.629388051	1.119494816	-1.455467259	0.145539948	0.231587667	negative
PRJNA729271	stage_E14_vs_E10	ordinary_muscle_development	ENSGALG00010029730	TTC19	0.41641107	0.132713793	3.137662333	0.001703009	0.00505739	positive
PRJNA729271	stage_E14_vs_E10	ordinary_muscle_development	ENSGALG00010029731	NCOR1	-0.110558871	0.184642426	-0.598772848	0.549324371	0.654484363	negative
PRJNA729271	stage_E14_vs_E10	ordinary_muscle_development	ENSGALG00010029732	PIGL	0.069392163	0.17851802	0.388712374	0.697488928	0.779799615	positive
PRJNA729271	stage_E14_vs_E10	ordinary_muscle_development	ENSGALG00010029733	ENSGALG00010029733	0.383812568	0.165737573	2.315784898	0.020570016	0.044553364	positive
PRJNA729271	stage_E14_vs_E10	ordinary_muscle_development	ENSGALG00010029734	SEC14L1	0.281242879	0.083056535	3.386161973	0.000708775	0.002316505	positive
PRJNA729271	stage_E14_vs_E10	ordinary_muscle_development	ENSGALG00010029735	SRP68	-0.040604737	0.113616317	-0.357384734	0.720803804	0.797865684	negative
PRJNA729271	stage_E14_vs_E10	ordinary_muscle_development	ENSGALG00010029736	ENSGALG00010029736	0.838273398	0.133071965	6.299398958	2.98802E-10	2.60441E-09	positive
PRJNA729271	stage_E14_vs_E10	ordinary_muscle_development	ENSGALG00010029737	ENSGALG00010029737	-0.814407633	0.492410395	-1.653920471	0.098143673	0.16748205	negative
PRJNA729271	stage_E14_vs_E10	ordinary_muscle_development	ENSGALG00010029738	TRPV2	0.467095674	0.166769879	2.800839564	0.005096985	0.013237914	positive
PRJNA729271	stage_E14_vs_E10	ordinary_muscle_development	ENSGALG00010029739	P2RX5	1.733934324	0.20290844	8.545402648	1.28089E-17	2.26974E-16	positive
PRJNA729271	stage_E14_vs_E10	ordinary_muscle_development	ENSGALG00010029740	HASPIN	-0.10847224	0.198771414	-0.545713482	0.585262903	0.685858661	negative
PRJNA729271	stage_E14_vs_E10	ordinary_muscle_development	ENSGALG00010029741	EMC6	0.06840102	0.163081254	0.41942908	0.674902575	0.761182137	positive
PRJNA729271	stage_E14_vs_E10	ordinary_muscle_development	ENSGALG00010029742	TAX1BP3	-0.122186449	0.118285684	-1.032977492	0.301614432	0.415422006	negative
PRJNA729271	stage_E14_vs_E10	ordinary_muscle_development	ENSGALG00010029743	MYOCD	2.113354188	0.255315407	8.27742523	1.25867E-16	2.05687E-15	positive
PRJNA729271	stage_E14_vs_E10	ordinary_muscle_development	ENSGALG00010029744	CTNS	0.025885687	0.089593839	0.288922624	0.77264059	0.838782606	positive
PRJNA729271	stage_E14_vs_E10	ordinary_muscle_development	ENSGALG00010029745	SHPK	0.078730839	0.168735899	0.466592111	0.640791732	0.733323556	positive
PRJNA729271	stage_E14_vs_E10	ordinary_muscle_development	ENSGALG00010029746	TRPV1	-0.468674006	0.319767491	-1.46567121	0.142737881	0.227937158	negative
PRJNA729271	stage_E14_vs_E10	ordinary_muscle_development	ENSGALG00010029747	TRPV3	0.480954169	0.333387132	1.44262967	0.149124821	0.236230167	positive
PRJNA729271	stage_E14_vs_E10	ordinary_muscle_development	ENSGALG00010029749	ASPA	-0.221661357	0.30418	-0.728717724	0.466174348	0.578527156	negative
PRJNA729271	stage_E14_vs_E10	ordinary_muscle_development	ENSGALG00010029750	TEN1	-0.049321969	0.21500977	-0.229394084	0.818562634	0.872944775	negative
PRJNA729271	stage_E14_vs_E10	ordinary_muscle_development	ENSGALG00010029752	ENSGALG00010029752	0.152652029	0.169788889	0.899069601	0.368615589	0.484853263	positive
PRJNA729271	stage_E14_vs_E10	ordinary_muscle_development	ENSGALG00010029753	AKAP10	-0.118128994	0.073522849	-1.606697717	0.108120695	0.181705446	negative
PRJNA729271	stage_E14_vs_E10	ordinary_muscle_development	ENSGALG00010029754	NLE1	-0.219141297	0.176226243	-1.243522489	0.213675359	0.315303301	negative
PRJNA729271	stage_E14_vs_E10	ordinary_muscle_development	ENSGALG00010029755	ACOX1	-0.002480348	0.119095466	-0.020826552	0.983384017	0.988978792	negative
PRJNA729271	stage_E14_vs_E10	ordinary_muscle_development	ENSGALG00010029756	ELAC2	-0.023770985	0.081145903	-0.292941291	0.769567027	0.836260144	negative
PRJNA729271	stage_E14_vs_E10	ordinary_muscle_development	ENSGALG00010029757	ULK2	-0.114134018	0.209266556	-0.545400182	0.585478315	0.686055633	negative
PRJNA729271	stage_E14_vs_E10	ordinary_muscle_development	ENSGALG00010029758	DNAH9	0.446227194	0.302964252	1.47287078	0.140785882	0.225440937	positive
PRJNA729271	stage_E14_vs_E10	ordinary_muscle_development	ENSGALG00010029759	ALDH3A2	0.335434834	0.107382522	3.123737723	0.001785696	0.00527807	positive
PRJNA729271	stage_E14_vs_E10	ordinary_muscle_development	ENSGALG00010029760	SHISA6	0.416217639	0.243262329	1.710982712	0.087084304	0.151753666	positive
PRJNA729271	stage_E14_vs_E10	ordinary_muscle_development	ENSGALG00010029761	MRPL38	0.131248833	0.117566903	1.116375691	0.264261354	0.373012789	positive
PRJNA729271	stage_E14_vs_E10	ordinary_muscle_development	ENSGALG00010029762	SLC47A2	2.383217788	0.479047917	4.974904812	6.52797E-07	3.6991E-06	positive
PRJNA729271	stage_E14_vs_E10	ordinary_muscle_development	ENSGALG00010029763	GEMIN4	-0.023063584	0.095278512	-0.242064904	0.808729872	0.86549808	negative
PRJNA729271	stage_E14_vs_E10	ordinary_muscle_development	ENSGALG00010029764	NXN	-0.565549195	0.12386598	-4.565815378	4.97557E-06	2.45476E-05	negative
PRJNA729271	stage_E14_vs_E10	ordinary_muscle_development	ENSGALG00010029765	KCNJ16	-1.519226656	0.299812973	-5.067247887	4.03609E-07	2.36281E-06	negative
PRJNA729271	stage_E14_vs_E10	ordinary_muscle_development	ENSGALG00010029766	ENSGALG00010029766	0.197571303	0.144998753	1.362572433	0.173017302	0.26604296	positive
PRJNA729271	stage_E14_vs_E10	ordinary_muscle_development	ENSGALG00010029767	GLOD4	0.244962875	0.084965648	2.883081347	0.003938057	0.010533704	positive
PRJNA729271	stage_E14_vs_E10	ordinary_muscle_development	ENSGALG00010029768	KCNJ2	-0.093031329	0.175675331	-0.529563985	0.59641427	0.696000712	negative
PRJNA729271	stage_E14_vs_E10	ordinary_muscle_development	ENSGALG00010029770	TLCD3A	-0.470560181	0.228724642	-2.057321752	0.039655286	0.077700259	negative
PRJNA729271	stage_E14_vs_E10	ordinary_muscle_development	ENSGALG00010029771	SOX9	-4.026789156	0.259836028	-15.49742424	3.6108E-54	6.38275E-52	negative
PRJNA729271	stage_E14_vs_E10	ordinary_muscle_development	ENSGALG00010029772	VPS53	0.144767296	0.067630954	2.140547907	0.032310514	0.065292192	positive
PRJNA729271	stage_E14_vs_E10	ordinary_muscle_development	ENSGALG00010029773	TRIM65	0.351383315	0.133158783	2.638829428	0.008319283	0.020338675	positive
PRJNA729271	stage_E14_vs_E10	ordinary_muscle_development	ENSGALG00010029774	RFLNB	0.461953836	0.124087387	3.722810588	0.000197017	0.000733245	positive
PRJNA729271	stage_E14_vs_E10	ordinary_muscle_development	ENSGALG00010029775	ENSGALG00010029775	-0.78083245	0.142429355	-5.482243807	4.19965E-08	2.81824E-07	negative
PRJNA729271	stage_E14_vs_E10	ordinary_muscle_development	ENSGALG00010029776	WBP2	0.427704078	0.136395141	3.135772107	0.001714023	0.005084889	positive
PRJNA729271	stage_E14_vs_E10	ordinary_muscle_development	ENSGALG00010029777	ENSGALG00010029777	0.404216755	0.134783487	2.999008004	0.002708602	0.007596978	positive
PRJNA729271	stage_E14_vs_E10	ordinary_muscle_development	ENSGALG00010029778	DOC2B	0.865203342	0.342714538	2.524559789	0.01158433	0.027113654	positive
PRJNA729271	stage_E14_vs_E10	ordinary_muscle_development	ENSGALG00010029780	TEX14	-0.23853083	0.253361851	-0.941463085	0.346467598	0.462009921	negative
PRJNA729271	stage_E14_vs_E10	ordinary_muscle_development	ENSGALG00010029781	UNC13D	-0.309249841	0.260015863	-1.189349903	0.234302006	0.338807619	negative
PRJNA729271	stage_E14_vs_E10	ordinary_muscle_development	ENSGALG00010029782	UNK	0.022255178	0.119572288	0.186123206	0.852348144	0.897747345	positive
PRJNA729271	stage_E14_vs_E10	ordinary_muscle_development	ENSGALG00010029783	RAD51C	-0.283523808	0.120464533	-2.353587415	0.018593236	0.040791426	negative
PRJNA729271	stage_E14_vs_E10	ordinary_muscle_development	ENSGALG00010029784	CLDN3	-1.898745296	0.65633563	-2.892948682	0.003816436	0.0102557	negative
PRJNA729271	stage_E14_vs_E10	ordinary_muscle_development	ENSGALG00010029785	ENSGALG00010029785	1.354455967	0.247716383	5.467769017	4.55735E-08	3.04278E-07	positive
PRJNA729271	stage_E14_vs_E10	ordinary_muscle_development	ENSGALG00010029787	H3-3B	0.094569159	0.063753409	1.483358456	0.137979199	0.221632146	positive
PRJNA729271	stage_E14_vs_E10	ordinary_muscle_development	ENSGALG00010029791	GALK1	0.301735441	0.106185918	2.841576807	0.004489104	0.011849335	positive
PRJNA729271	stage_E14_vs_E10	ordinary_muscle_development	ENSGALG00010029804	ITGB4	-0.945121091	0.231778566	-4.077689787	4.54854E-05	0.000190387	negative
PRJNA729271	stage_E14_vs_E10	ordinary_muscle_development	ENSGALG00010029812	SAP30BP	0.27827405	0.114063246	2.439646942	0.014701622	0.033361764	positive
PRJNA729271	stage_E14_vs_E10	ordinary_muscle_development	ENSGALG00010029835	RECQL5	-0.01008761	0.105864131	-0.095288276	0.924085855	0.947855721	negative
PRJNA729271	stage_E14_vs_E10	ordinary_muscle_development	ENSGALG00010029841	SMIM5	1.619071621	0.128415043	12.60811496	1.90495E-36	1.16018E-34	positive
PRJNA729271	stage_E14_vs_E10	ordinary_muscle_development	ENSGALG00010029859	CSNK1D	-0.09082646	0.051534063	-1.762454878	0.077992474	0.138321413	negative
PRJNA729271	stage_E14_vs_E10	ordinary_muscle_development	ENSGALG00010029864	SLC16A3	-1.442806473	0.343440371	-4.201039232	2.65693E-05	0.000115965	negative
PRJNA729271	stage_E14_vs_E10	ordinary_muscle_development	ENSGALG00010029879	ENSGALG00010029879	-0.770570389	0.318147426	-2.422054447	0.015433038	0.034763308	negative
PRJNA729271	stage_E14_vs_E10	ordinary_muscle_development	ENSGALG00010029895	FASN	-0.654804259	0.168094496	-3.895453291	9.80153E-05	0.000385649	negative
PRJNA729271	stage_E14_vs_E10	ordinary_muscle_development	ENSGALG00010029907	NTN1	-2.443052647	0.295920006	-8.25578737	1.50904E-16	2.43581E-15	negative
PRJNA729271	stage_E14_vs_E10	ordinary_muscle_development	ENSGALG00010029921	STX8	0.28478869	0.102795847	2.770429912	0.005598235	0.014369868	positive
PRJNA729271	stage_E14_vs_E10	ordinary_muscle_development	ENSGALG00010029925	GGNBP2	0.093119843	0.124851885	0.745842505	0.45576256	0.568877479	positive
PRJNA729271	stage_E14_vs_E10	ordinary_muscle_development	ENSGALG00010029926	BLTP2	0.088063799	0.13193315	0.66748804	0.504460447	0.61359018	positive
PRJNA729271	stage_E14_vs_E10	ordinary_muscle_development	ENSGALG00010029927	ENSGALG00010029927	-1.367527936	0.701966726	-1.948137833	0.051398473	0.097071123	negative
PRJNA729271	stage_E14_vs_E10	ordinary_muscle_development	ENSGALG00010029928	USP43	-0.056686422	0.133556882	-0.424436549	0.67124747	0.758121558	negative
PRJNA729271	stage_E14_vs_E10	ordinary_muscle_development	ENSGALG00010029929	GDPD1	-0.148059624	0.093901653	-1.576752054	0.114852588	0.191091398	negative
PRJNA729271	stage_E14_vs_E10	ordinary_muscle_development	ENSGALG00010029930	ARHGAP44	0.337053527	0.220429372	1.5290772	0.126245307	0.205804845	positive
PRJNA729271	stage_E14_vs_E10	ordinary_muscle_development	ENSGALG00010029932	RASA4B	3.189988035	0.261810164	12.18435521	3.7662E-34	1.98513E-32	positive
PRJNA729271	stage_E14_vs_E10	ordinary_muscle_development	ENSGALG00010029933	TVP23B	0.165750471	0.093881405	1.765530358	0.077474673	0.13758826	positive
PRJNA729271	stage_E14_vs_E10	ordinary_muscle_development	ENSGALG00010029934	TEKT3	0.840458398	0.491532656	1.709872961	0.087289367	0.152037897	positive
PRJNA729271	stage_E14_vs_E10	ordinary_muscle_development	ENSGALG00010029935	SYNRG	0.227533156	0.072199546	3.151448557	0.001624628	0.004848462	positive
PRJNA729271	stage_E14_vs_E10	ordinary_muscle_development	ENSGALG00010029936	SERPINF2	-0.954705287	0.528229942	-1.807366852			negative
PRJNA729271	stage_E14_vs_E10	ordinary_muscle_development	ENSGALG00010029937	PMP22	1.558578477	0.12388239	12.58111404	2.6822E-36	1.61994E-34	positive
PRJNA729271	stage_E14_vs_E10	ordinary_muscle_development	ENSGALG00010029938	ENSGALG00010029938	-1.292668299	0.311015152	-4.156287207	3.23461E-05	0.000139168	negative
PRJNA729271	stage_E14_vs_E10	ordinary_muscle_development	ENSGALG00010029939	ENSGALG00010029939	3.875264695	1.476980899	2.623774416	0.008696136	0.021106914	positive
PRJNA729271	stage_E14_vs_E10	ordinary_muscle_development	ENSGALG00010029941	COX10	0.40548063	0.172751267	2.347193378	0.018915435	0.041416802	positive
PRJNA729271	stage_E14_vs_E10	ordinary_muscle_development	ENSGALG00010029942	ENSGALG00010029942	0.288676323	1.458520053	0.197924137	0.843104419	0.890950465	positive
PRJNA729271	stage_E14_vs_E10	ordinary_muscle_development	ENSGALG00010029943	HS3ST3A1	-3.847187274	0.245122408	-15.69496361	1.63737E-55	3.16448E-53	negative
PRJNA729271	stage_E14_vs_E10	ordinary_muscle_development	ENSGALG00010029945	CCL4	1.709232602	0.712084518	2.400322656	0.016380626	0.036590719	positive
PRJNA729271	stage_E14_vs_E10	ordinary_muscle_development	ENSGALG00010029946	METRNL	-0.133412252	0.1367	-0.975949172	0.329089653	0.443900476	negative
PRJNA729271	stage_E14_vs_E10	ordinary_muscle_development	ENSGALG00010029947	CCL5	0.9584614	1.270653808	0.754305692	0.450665685	0.56379864	positive
PRJNA729271	stage_E14_vs_E10	ordinary_muscle_development	ENSGALG00010029948	MYADML2	2.830894664	0.217567575	13.01156507	1.05166E-38	7.436E-37	positive
PRJNA729271	stage_E14_vs_E10	ordinary_muscle_development	ENSGALG00010029950	RFFL	-0.364740058	0.103852539	-3.512095727	0.000444588	0.001523475	negative
PRJNA729271	stage_E14_vs_E10	ordinary_muscle_development	ENSGALG00010029956	CCL15	4.312133221	0.181897214	23.70642809	3.0953E-124	7.4777E-121	positive
PRJNA729271	stage_E14_vs_E10	ordinary_muscle_development	ENSGALG00010029962	ENSGALG00010029962	0.527013099	0.750604202	0.702118503	0.482605264	0.594186979	positive
PRJNA729271	stage_E14_vs_E10	ordinary_muscle_development	ENSGALG00010029966	RABGEF1	-0.113287176	0.08041267	-1.408822472	0.158887669	0.248605004	negative
PRJNA729271	stage_E14_vs_E10	ordinary_muscle_development	ENSGALG00010030004	FLOT2	0.803324955	0.069667926	11.53077173	9.23125E-31	4.09196E-29	positive
PRJNA729271	stage_E14_vs_E10	ordinary_muscle_development	ENSGALG00010030014	ENSGALG00010030014	-0.300472415	0.304959199	-0.985287265	0.324482986	0.439116879	negative
PRJNA729271	stage_E14_vs_E10	ordinary_muscle_development	ENSGALG00010030102	GPS1	0.188548748	0.082522826	2.284807214	0.022324134	0.047875178	positive
PRJNA729271	stage_E14_vs_E10	ordinary_muscle_development	ENSGALG00010030103	C1QTNF1	-0.217329313	0.220674722	-0.984840093	0.324702624	0.439349231	negative
PRJNA729271	stage_E14_vs_E10	ordinary_muscle_development	ENSGALG00010030104	P4HB	-0.314203238	0.10990862	-2.858767919	0.004252898	0.011278038	negative
PRJNA729271	stage_E14_vs_E10	ordinary_muscle_development	ENSGALG00010030105	RAB40B	-0.436580562	0.196531629	-2.22142646	0.026322091	0.055079934	negative
PRJNA729271	stage_E14_vs_E10	ordinary_muscle_development	ENSGALG00010030106	GPRC5C	-0.432789403	0.167488925	-2.583988185	0.009766514	0.023352956	negative

PRJNA729271	stage_E14_vs_E10	ordinary_muscle_development	ENSGALG00010030107	RPTOR	0.24853848	0.138071178	1.80007503	0.071848792	0.128892108	positive
PRJNA729271	stage_E14_vs_E10	ordinary_muscle_development	ENSGALG00010030108	LLGL2	-0.078963	0.118929009	-0.663950709	0.50672186	0.615565987	negative
PRJNA729271	stage_E14_vs_E10	ordinary_muscle_development	ENSGALG00010030109	FBF1	0.135705321	0.088730981	1.529401789	0.126164869	0.205756051	positive
PRJNA729271	stage_E18_vs_E10	ordinary_muscle_development	ENSGALG00010000007	ND1	0.093746144	0.24368055	0.384709178	0.700452894	0.745233757	positive
PRJNA729271	stage_E18_vs_E10	ordinary_muscle_development	ENSGALG00010000011	ND2	0.170585483	0.253363843	0.673282663	0.50076748	0.557969454	positive
PRJNA729271	stage_E18_vs_E10	ordinary_muscle_development	ENSGALG00010000017	COX1	0.730155015	0.272479802	2.679666561	0.007369553	0.011880955	positive
PRJNA729271	stage_E18_vs_E10	ordinary_muscle_development	ENSGALG00010000020	COX2	0.749023637	0.239238309	3.130868291	0.001742903	0.003088433	positive
PRJNA729271	stage_E18_vs_E10	ordinary_muscle_development	ENSGALG00010000022	ATP8	0.101430657	0.279291079	0.363171846	0.716476517	0.759661116	positive
PRJNA729271	stage_E18_vs_E10	ordinary_muscle_development	ENSGALG00010000023	ATP6	0.075035961	0.199096326	0.376882703	0.706260777	0.750806744	positive
PRJNA729271	stage_E18_vs_E10	ordinary_muscle_development	ENSGALG00010000024	COX3	0.982788854	0.221537234	4.436224264	9.15505E-06	2.13898E-05	positive
PRJNA729271	stage_E18_vs_E10	ordinary_muscle_development	ENSGALG00010000026	ND3	0.409594665	0.318423544	1.286320288	0.198331305	0.246976999	positive
PRJNA729271	stage_E18_vs_E10	ordinary_muscle_development	ENSGALG00010000028	ND4L	0.142103865	0.287255035	0.49469582	0.620814853	0.671569363	positive
PRJNA729271	stage_E18_vs_E10	ordinary_muscle_development	ENSGALG00010000029	ND4	0.478282131	0.256642799	1.863610171	0.062376468	0.08599457	positive
PRJNA729271	stage_E18_vs_E10	ordinary_muscle_development	ENSGALG00010000033	ND5	0.327854336	0.232671042	1.409089563	0.158808687	0.202101134	positive
PRJNA729271	stage_E18_vs_E10	ordinary_muscle_development	ENSGALG00010000034	CYTB	0.237789401	0.219826856	1.081712241	0.279380412	0.334900684	positive
PRJNA729271	stage_E18_vs_E10	ordinary_muscle_development	ENSGALG00010000037	ND6	0.440061616	0.191043083	2.303467933	0.021252529	0.031906309	positive
PRJNA729271	stage_E18_vs_E10	ordinary_muscle_development	ENSGALG00010000039	PDIA3	-0.506684353	0.065801902	-7.700147578	1.35909E-14	6.71211E-14	negative
PRJNA729271	stage_E18_vs_E10	ordinary_muscle_development	ENSGALG00010000042	CKMT1B	3.038218225	0.459653978	6.609794256	3.84855E-11	1.44745E-10	positive
PRJNA729271	stage_E18_vs_E10	ordinary_muscle_development	ENSGALG00010000043	PPIP5K1	0.790006491	0.122515402	6.448221854	1.1317E-10	4.10305E-10	positive
PRJNA729271	stage_E18_vs_E10	ordinary_muscle_development	ENSGALG00010000044	MAP1A	0.744029619	0.132465553	5.616778112	1.94551E-08	5.8616E-08	positive
PRJNA729271	stage_E18_vs_E10	ordinary_muscle_development	ENSGALG00010000045	TP53BP1	-1.265838233	0.074633135	-16.96080758	1.60128E-64	7.41553E-63	negative
PRJNA729271	stage_E18_vs_E10	ordinary_muscle_development	ENSGALG00010000046	TUBGCP4	-1.088953631	0.065301642	-16.6757465	1.96738E-62	8.64158E-61	negative
PRJNA729271	stage_E18_vs_E10	ordinary_muscle_development	ENSGALG00010000047	IQGAP1	-0.760825722	0.097361923	-7.814407284	5.52223E-15	2.81352E-14	negative
PRJNA729271	stage_E18_vs_E10	ordinary_muscle_development	ENSGALG00010000048	MAN2A2	0.257367269	0.149980742	1.71600211	0.086161656	0.11565082	positive
PRJNA729271	stage_E18_vs_E10	ordinary_muscle_development	ENSGALG00010000049	FES	0.626423429	0.198089356	3.16232755	0.001565134	0.002784317	positive
PRJNA729271	stage_E18_vs_E10	ordinary_muscle_development	ENSGALG00010000050	CIB1	0.552263262	0.130805448	4.222020332	2.42122E-05	5.40348E-05	positive
PRJNA729271	stage_E18_vs_E10	ordinary_muscle_development	ENSGALG00010000051	GDPGP1	0.43900997	0.187065802	2.346821096	0.018934344	0.028657545	positive
PRJNA729271	stage_E18_vs_E10	ordinary_muscle_development	ENSGALG00010000052	TTLL13	1.252210961	0.131563619	9.517912096	1.76693E-21	1.36742E-20	positive
PRJNA729271	stage_E18_vs_E10	ordinary_muscle_development	ENSGALG00010000053	HDDC3	0.764469522	0.117395798	6.51189849	7.42069E-11	2.7328E-10	positive
PRJNA729271	stage_E18_vs_E10	ordinary_muscle_development	ENSGALG00010000054	VPS33B	0.281319167	0.099653571	2.822971255	0.004758084	0.00790469	positive
PRJNA729271	stage_E18_vs_E10	ordinary_muscle_development	ENSGALG00010000055	PRC1	-0.98322055	0.21236082	-4.629952685	3.65749E-06	8.88029E-06	negative
PRJNA729271	stage_E18_vs_E10	ordinary_muscle_development	ENSGALG00010000056	UNC45A	-0.081202247	0.083394692	-0.973710013	0.330200544	0.38836368	negative
PRJNA729271	stage_E18_vs_E10	ordinary_muscle_development	ENSGALG00010000058	FURIN	-0.128849814	0.175165345	-0.735589642	0.461980468	0.521283426	negative
PRJNA729271	stage_E18_vs_E10	ordinary_muscle_development	ENSGALG00010000059	MFAP1	-0.255782632	0.111193191	-2.300344373	0.021428718	0.032147512	negative
PRJNA729271	stage_E18_vs_E10	ordinary_muscle_development	ENSGALG00010000060	HYPK	-0.162975311	0.24728746	-0.659052064	0.509862336	0.566796116	negative
PRJNA729271	stage_E18_vs_E10	ordinary_muscle_development	ENSGALG00010000061	SERINC4	1.214065262	0.37297978	3.255043108	0.001133752	0.002052164	positive
PRJNA729271	stage_E18_vs_E10	ordinary_muscle_development	ENSGALG00010000063	SERF2	0.529180545	0.127415958	4.153173214	3.27896E-05	7.19912E-05	positive
PRJNA729271	stage_E18_vs_E10	ordinary_muscle_development	ENSGALG00010000069	ENSGALG00010000069	1.867946936	0.23392896	7.985103398	1.40405E-15	7.46849E-15	positive
PRJNA729271	stage_E18_vs_E10	ordinary_muscle_development	ENSGALG00010000076	ENSGALG00010000076	0.758217679	0.199219582	3.805939517	0.000141267	0.0002893	positive
PRJNA729271	stage_E18_vs_E10	ordinary_muscle_development	ENSGALG00010000078	ENSGALG00010000078	0.759892912	0.470574508	1.614819543	0.10634978	0.140299989	positive
PRJNA729271	stage_E18_vs_E10	ordinary_muscle_development	ENSGALG00010000081	ENSGALG00010000081	2.621680708	0.306467105	8.554525631	1.18354E-17	7.2386E-17	positive
PRJNA729271	stage_E18_vs_E10	ordinary_muscle_development	ENSGALG00010000084	ENSGALG00010000084	1.389501653	0.264857757	5.246218457	1.55253E-07	4.3144E-07	positive
PRJNA729271	stage_E18_vs_E10	ordinary_muscle_development	ENSGALG00010000085	ENSGALG00010000085	-0.322977047	0.150827941	-2.141360839	0.03224495	0.046860894	negative
PRJNA729271	stage_E18_vs_E10	ordinary_muscle_development	ENSGALG00010000089	ENSGALG00010000089	1.083904497	0.309263037	3.504798075	0.000456954	0.000874049	positive
PRJNA729271	stage_E18_vs_E10	ordinary_muscle_development	ENSGALG00010000091	ENSGALG00010000091	3.144155013	0.893417809	3.519243718	0.000432779	0.000830109	positive
PRJNA729271	stage_E18_vs_E10	ordinary_muscle_development	ENSGALG00010000092	INPPL1	0.986946592	0.171718086	5.747481888	9.05823E-09	2.80974E-08	positive
PRJNA729271	stage_E18_vs_E10	ordinary_muscle_development	ENSGALG00010000093	ENSGALG00010000093	1.964633187	0.328290431	5.984436339	2.1714E-09	7.11771E-09	positive
PRJNA729271	stage_E18_vs_E10	ordinary_muscle_development	ENSGALG00010000098	ENSGALG00010000098	0.640883843	0.197398471	3.246650486	0.001167717	0.002109955	positive
PRJNA729271	stage_E18_vs_E10	ordinary_muscle_development	ENSGALG00010000105	CACNA1F	0.599963322	0.291477316	2.058353392	0.039556222	0.056539537	positive
PRJNA729271	stage_E18_vs_E10	ordinary_muscle_development	ENSGALG00010000107	ENSGALG00010000107	0.060833292	0.533196191	0.11409176	0.909165054	0.924835786	positive
PRJNA729271	stage_E18_vs_E10	ordinary_muscle_development	ENSGALG00010000121	ENSGALG00010000121	0.345733956	0.13627474	2.53703625	0.011179538	0.01758136	positive
PRJNA729271	stage_E18_vs_E10	ordinary_muscle_development	ENSGALG00010000123	ENSGALG00010000123	-0.02049068	0.260792377	-0.078570856	0.937373969	0.948696808	negative
PRJNA729271	stage_E18_vs_E10	ordinary_muscle_development	ENSGALG00010000124	ENSGALG00010000124	1.273827389	0.182564692	6.977402757	3.00687E-12	1.23961E-11	positive
PRJNA729271	stage_E18_vs_E10	ordinary_muscle_development	ENSGALG00010000126	ENSGALG00010000126	0.37529133	0.205543151	1.825851787	0.06787259	0.093005596	positive
PRJNA729271	stage_E18_vs_E10	ordinary_muscle_development	ENSGALG00010000127	ENSGALG00010000127	0.909781368	0.209815985	4.336091788	1.45038E-05	3.31926E-05	positive
PRJNA729271	stage_E18_vs_E10	ordinary_muscle_development	ENSGALG00010000133	USP39	-0.440707896	0.143711637	-3.066612456	0.002164994	0.00378228	negative
PRJNA729271	stage_E18_vs_E10	ordinary_muscle_development	ENSGALG00010000134	ENSGALG00010000134	0.645283135	0.127735238	5.051723754	4.37841E-07	1.17051E-06	positive
PRJNA729271	stage_E18_vs_E10	ordinary_muscle_development	ENSGALG00010000137	ENSGALG00010000137	-0.733707931	0.547901659	-1.339123398	0.180530504	0.226856494	negative
PRJNA729271	stage_E18_vs_E10	ordinary_muscle_development	ENSGALG00010000140	SEPHS3	-0.317175846	0.127640221	-2.484920857	0.012958024	0.020170378	negative
PRJNA729271	stage_E18_vs_E10	ordinary_muscle_development	ENSGALG00010000141	ENSGALG00010000141	-0.335110521	0.139905359	-2.395265801	0.016608321	0.025418394	negative
PRJNA729271	stage_E18_vs_E10	ordinary_muscle_development	ENSGALG00010000143	SCARF2	-1.004297412	0.159528578	-6.295407519	3.06593E-10	1.07892E-09	negative
PRJNA729271	stage_E18_vs_E10	ordinary_muscle_development	ENSGALG00010000145	ENSGALG00010000145	-0.118385072	0.283350459	-0.417804412	0.676090125	0.722548577	negative
PRJNA729271	stage_E18_vs_E10	ordinary_muscle_development	ENSGALG00010000146	ACADM	2.100867517	0.161758554	12.98767494	1.43727E-38	2.60416E-37	positive
PRJNA729271	stage_E18_vs_E10	ordinary_muscle_development	ENSGALG00010000147	ENSGALG00010000147	0.224745875	0.110517461	2.033577979	0.041994164	0.059682852	positive
PRJNA729271	stage_E18_vs_E10	ordinary_muscle_development	ENSGALG00010000148	ENSGALG00010000148	1.055305073	0.291041169	3.625964927	0.000287884	0.000565432	positive
PRJNA729271	stage_E18_vs_E10	ordinary_muscle_development	ENSGALG00010000149	ENSGALG00010000149	-0.476198301	0.140127053	-3.398332382	0.00067798	0.00126494	negative
PRJNA729271	stage_E18_vs_E10	ordinary_muscle_development	ENSGALG00010000150	B3GAT3	0.581549247	0.125917005	4.618512389	3.86501E-06	9.35749E-06	positive
PRJNA729271	stage_E18_vs_E10	ordinary_muscle_development	ENSGALG00010000151	TBX5	-4.046736178	0.250926368	-16.12718589	1.6434E-58	6.28524E-57	negative
PRJNA729271	stage_E18_vs_E10	ordinary_muscle_development	ENSGALG00010000153	NF2	-0.311694627	0.09396292	-3.317208805	0.000909216	0.001665709	negative
PRJNA729271	stage_E18_vs_E10	ordinary_muscle_development	ENSGALG00010000154	ENSGALG00010000154	-1.080276998	3.978710308	-0.271514364	0.785995454	0.820585141	negative
PRJNA729271	stage_E18_vs_E10	ordinary_muscle_development	ENSGALG00010000155	MAZ	0.765131264	0.164205023	4.659609359	3.1681E-06	7.74787E-06	positive
PRJNA729271	stage_E18_vs_E10	ordinary_muscle_development	ENSGALG00010000156	ENSGALG00010000156	1.3119202	0.503447623	2.605872272	0.009164063	0.014568227	positive
PRJNA729271	stage_E18_vs_E10	ordinary_muscle_development	ENSGALG00010000157	ENSGALG00010000157	0.584368594	5.341390366	0.109403836			positive
PRJNA729271	stage_E18_vs_E10	ordinary_muscle_development	ENSGALG00010000161	CDC42EP2	0.616541738	0.339962148	1.813559958	0.0697455	0.095436705	positive
PRJNA729271	stage_E18_vs_E10	ordinary_muscle_development	ENSGALG00010000166	PFDN6	0.135103737	0.129952493	1.039639434	0.298507449	0.355418553	positive
PRJNA729271	stage_E18_vs_E10	ordinary_muscle_development	ENSGALG00010000171	DPF2	0.063799829	0.201768356	0.316203347	0.751848168	0.79166346	positive
PRJNA729271	stage_E18_vs_E10	ordinary_muscle_development	ENSGALG00010000172	ENSGALG00010000172	0.079565176	1.124105581	0.070780874	0.943572154	0.953904196	positive
PRJNA729271	stage_E18_vs_E10	ordinary_muscle_development	ENSGALG00010000173	NIPSNAP1	-0.342551959	0.125991239	-2.718855394	0.006550824	0.010651059	negative
PRJNA729271	stage_E18_vs_E10	ordinary_muscle_development	ENSGALG00010000174	ENSGALG00010000174	0.910995605	0.097145881	9.377604046	6.74889E-21	5.04513E-20	positive
PRJNA729271	stage_E18_vs_E10	ordinary_muscle_development	ENSGALG00010000175	ENSGALG00010000175	0.518135431	0.833680447	0.621503638	0.534268301	0.589990784	positive
PRJNA729271	stage_E18_vs_E10	ordinary_muscle_development	ENSGALG00010000176	ENSGALG00010000176	-0.140469715	0.511159066	-0.274806267	0.783465083	0.818473973	negative
PRJNA729271	stage_E18_vs_E10	ordinary_muscle_development	ENSGALG00010000177	ENSGALG00010000177	0.725155795	0.209740762	3.457390873	0.000545433	0.001031179	positive
PRJNA729271	stage_E18_vs_E10	ordinary_muscle_development	ENSGALG00010000184	ENSGALG00010000184	5.788418408	3.405165535	1.699893397	0.089150979	0.119309708	positive
PRJNA729271	stage_E18_vs_E10	ordinary_muscle_development	ENSGALG00010000186	THOC5	-0.308251993	0.080113658	-3.847683401	0.00011924	0.000246173	negative
PRJNA729271	stage_E18_vs_E10	ordinary_muscle_development	ENSGALG00010000188	ENSGALG00010000188	1.127630794	1.330465521	0.847546048	0.396690832	0.456678072	positive
PRJNA729271	stage_E18_vs_E10	ordinary_muscle_development	ENSGALG00010000189	ENSGALG00010000189	1.478867038	0.587578125	2.516885799	0.01183972	0.018545141	positive
PRJNA729271	stage_E18_vs_E10	ordinary_muscle_development	ENSGALG00010000191	NEFH	3.199947441	0.396415677	8.072202044	6.90415E-16	3.75518E-15	positive
PRJNA729271	stage_E18_vs_E10	ordinary_muscle_development	ENSGALG00010000192	ENSGALG00010000192	0.650571652	0.713009518	0.912430529	0.361542125	0.420961772	positive
PRJNA729271	stage_E18_vs_E10	ordinary_muscle_development	ENSGALG00010000196	KSR2	-2.769301325	0.92934595	-2.979839019	0.002883999	0.00495479	negative
PRJNA729271	stage_E18_vs_E10	ordinary_muscle_development	ENSGALG00010000197	ENSGALG00010000197	-0.842798689	0.534228609	-1.577599317	0.114657689	0.150444755	negative
PRJNA729271	stage_E18_vs_E10	ordinary_muscle_development	ENSGALG00010000200	GAL3ST1	-1.708743782	0.248228253	-6.883760241	5.8293E-12	2.3445E-11	negative
PRJNA729271	stage_E18_vs_E10	ordinary_muscle_development	ENSGALG00010000202	ENSGALG00010000202	-0.327258055	0.30189649	-1.084007485	0.27836148	0.333955442	negative
PRJNA729271	stage_E18_vs_E10	ordinary_muscle_development	ENSGALG00010000205	RNF215	0.030665568	0.116611857	0.262971267	0.792572725	0.826618337	positive
PRJNA729271	stage_E18_vs_E10	ordinary_muscle_development	ENSGALG00010000210	CCDC157	-0.035650373	0.518022706	-0.068820097	0.945132821	0.955055686	negative
PRJNA729271	stage_E18_vs_E10	ordinary_muscle_development	ENSGALG00010000215	SF3A1	-0.386170238	0.090502284	-4.266966786	1.98149E-05	4.46682E-05	negative
PRJNA729271	stage_E18_vs_E10	ordinary_muscle_development	ENSGALG00010000221	TBC1D10A	-0.198038235	0.088824873	-2.229535801	0.025778276	0.038112618	negative
PRJNA729271	stage_E18_vs_E10	ordinary_muscle_development	ENSGALG00010000223	CASTOR1	0.253106467	0.162821661	1.554501199	0.120064911	0.156928844	positive
PRJNA729271	stage_E18_vs_E10	ordinary_muscle_development	ENSGALG00010000224	MYBPC2	7.979637588	0.252086798	31.65432559	6.6121E-220	5.3245E-217	positive
PRJNA729271	stage_E18_vs_E10	ordinary_muscle_development	ENSGALG00010000225	LIF	2.913927512	0.473060814	6.159731319	7.28685E-10	2.49581E-09	positive
PRJNA729271	stage_E18_vs_E10	ordinary_muscle_development	ENSGALG00010000226	ENSGALG00010000226	0.69632299	0.447716829	1.555275443	0.119880483	0.156730189	positive
PRJNA729271	stage_E18_vs_E10	ordinary_muscle_development	ENSGALG00010000227	ENSGALG00010000227	0.252759133	0.287050762	0.880538103	0.378567873	0.438706534	positive
PRJNA729271	stage_E18_vs_E10	ordinary_muscle_development	ENSGALG00010000231	ENSGALG00010000231	2.395412954	0.454596221	5.269319989	1.3693E-07	3.82723E-07	positive
PRJNA729271	stage_E18_vs_E10	ordinary_muscle_development	ENSGALG00010000232	ENSGALG00010000232	0.274371899	0.163018435	1.683072825	0.092360994	0.12329827	positive
PRJNA729271	stage_E18_vs_E10	ordinary_muscle_development	ENSGALG00010000233	HORMAD2	0.420740361	0.439184488	0.958003691	0.338060897	0.396455721	positive
PRJNA729271	stage_E18_vs_E10	ordinary_muscle_development	ENSGALG00010000234	ENSGALG00010000234	-0.653440801	4.579964354	-0.142673774			negative
PRJNA729271	stage_E18_vs_E10	ordinary_muscle_development	ENSGALG00010000235	ENSGALG00010000235	-1.58098205	0.234614782	-6.738629322	1.59888E-11	6.19175E-11	negative
PRJNA729271	stage_E18_vs_E10	ordinary_muscle_development	ENSGALG00010000239	ENSGALG00010000239	-1.291990838	4.630382837	-0.279024626	0.780225926	0.815677952	negative
PRJNA729271	stage_E18_vs_E10	ordinary_muscle_development	ENSGALG00010000242	ENSGALG00010000242	1.935660767	0.196548007	9.848284899	6.97236E-23	5.81833E-22	positive
PRJNA729271	stage_E18_vs_E10	ordinary_muscle_development	ENSGALG00010000246	ENSGALG00010000246	-0.179171908	4.279379718	-0.041868663	0.966603398	0.972440051	negative
PRJNA729271	stage_E18_vs_E10	ordinary_muscle_development	ENSGALG00010000247	ENSGALG00010000247	3.487343317	0.224621926	15.5253914	2.33575E-54	7.72982E-53	positive
PRJNA729271	stage_E18_vs_E10	ordinary_muscle_development	ENSGALG00010000249	ENSGALG00010000249	-2.27942015	4.420421749	-0.515656713			negative
PRJNA729271	stage_E18_vs_E10	ordinary_muscle_development	ENSGALG00010000254	ENSGALG00010000254	-0.567532178	0.591816795	-0.95896599	0.337575879	0.395983035	negative
PRJNA729271	stage_E18_vs_E10	ordinary_muscle_development	ENSGALG00010000255	ENSGALG00010000255	-0.050557389	0.145979795	-0.346331413	0.729093663	0.771120952	negative
PRJNA729271	stage_E18_vs_E10	ordinary_muscle_development	ENSGALG00010000256	ENSGALG00010000256	-0.361382712	0.400398691	-0.902557177	0.366760963	0.426387565	negative
PRJNA729271	stage_E18_vs_E10	ordinary_muscle_development	ENSGALG00010000262	HNRNPKL	-1.911347215	3.47483414	-0.550054229	0.582282179	0.635268718	negative
PRJNA729271	stage_E18_vs_E10	ordinary_muscle_development	ENSGALG00010000263	MTMR3	1.358271605	0.091344762	14.86972621	5.18248E-50	1.51452E-48	positive
PRJNA729271	stage_E18_vs_E10	ordinary_muscle_development	ENSGALG00010000267	ENSGALG00010000267	0.045828951	0.124645421	0.367674567	0.713115906	0.756928461	positive
PRJNA729271	stage_E18_vs_E10	ordinary_muscle_development	ENSGALG00010000269	ENSGALG00010000269	-0.153854743	0.467119322	-0.329369255	0.741876603	0.782528116	negative
PRJNA729271	stage_E18_vs_E10	ordinary_muscle_development	ENSGALG00010000271	RTN4R	-1.944479856	0.373657022	-5.20391627	1.95132E-07	5.38033E-07	negative
PRJNA729271	stage_E18_vs_E10	ordinary_muscle_development	ENSGALG00010000272	ENSGALG00010000272	0.506747422	0.365533327	1.386323446	0.165648161	0.209792057	positive
PRJNA729271	stage_E18_vs_E10	ordinary_muscle_development	ENSGALG00010000277	ENSGALG00010000277	1.213297998	0.632968568	1.916837674	0.055258557	0.076987004	positive
PRJNA729271	stage_E18_vs_E10	ordinary_muscle_development	ENSGALG00010000279	ENSGALG00010000279	-0.779780894	0.135935253	-5.736414036	9.67021E-09	2.99316E-08	negative
PRJNA729271	stage_E18_vs_E10	ordinary_muscle_development	ENSGALG00010000281	OSBP2	2.550847122	0.203287999	12.5479474	4.07948E-36	6.62916E-35	positive
PRJNA729271	stage_E18_vs_E10	ordinary_muscle_development	ENSGALG00010000282	ENSGALG00010000282	-2.007096154	4.958079368	-0.404813236	0.685614799	0.731648863	negative
PRJNA729271	stage_E18_vs_E10	ordinary_muscle_development	ENSGALG00010000284	ENSGALG00010000284	-0.519602171	0.153580693	-3.3832519	0.000716329	0.001332716	negative
PRJNA729271	stage_E18_vs_E10	ordinary_muscle_development	ENSGALG00010000285	MYLPF	4.595738814	0.145699964	31.54248424	2.2734E-218	1.7343E-215	positive
PRJNA729271	stage_E18_vs_E10	ordinary_muscle_development	ENSGALG00010000286	TCN2	1.936244532	0.138587626	13.97126556	2.33441E-44	5.371E-43	positive
PRJNA729271	stage_E18_vs_E10	ordinary_muscle_development	ENSGALG00010000287	IQCD	-0.77307367	0.194539694	-3.973860827	7.07169E-05	0.000149926	negative
PRJNA729271	stage_E18_vs_E10	ordinary_muscle_development	ENSGALG00010000288	VARS2	0.040549615	0.158389227	0.256012456	0.797941219	0.831200716	positive
PRJNA729271	stage_E18_vs_E10	ordinary_muscle_development	ENSGALG00010000289	QPRT	-0.642042879	0.456477484	-1.406515989	0.15957096	0.202928678	negative
PRJNA729271	stage_E18_vs_E10	ordinary_muscle_development	ENSGALG00010000292	ABHD16A	0.210951552	0.144427835	1.460601771	0.14412476	0.185153629	positive

PRJNA729271	stage_E18_vs_E10	ordinary_muscle_development	ENSGALG00010000293	ENSGALG00010000293	1.525627614	0.363017345	4.202630076	2.63832E-05	5.8627E-05	positive
PRJNA729271	stage_E18_vs_E10	ordinary_muscle_development	ENSGALG00010000294	DUSP21	0.257105677	0.274813583	0.935563936	0.34949775	0.408282551	positive
PRJNA729271	stage_E18_vs_E10	ordinary_muscle_development	ENSGALG00010000298	SLC35E4	0.195707483	0.243080915	0.805112501	0.420754763	0.481208796	positive
PRJNA729271	stage_E18_vs_E10	ordinary_muscle_development	ENSGALG00010000301	UQCR10	0.755602542	0.15510053	4.871695418	1.10645E-06	2.85018E-06	positive
PRJNA729271	stage_E18_vs_E10	ordinary_muscle_development	ENSGALG00010000302	ENSGALG00010000302	0.149742826	0.235565993	0.635672511	0.524989897	0.581450683	positive
PRJNA729271	stage_E18_vs_E10	ordinary_muscle_development	ENSGALG00010000303	ENSGALG00010000303	-0.493066819	0.113583213	-4.341018401	1.41824E-05	3.24915E-05	negative
PRJNA729271	stage_E18_vs_E10	ordinary_muscle_development	ENSGALG00010000306	ENSGALG00010000306	1.18458912	0.213349279	5.552346481	2.8186E-08	8.40132E-08	positive
PRJNA729271	stage_E18_vs_E10	ordinary_muscle_development	ENSGALG00010000307	ZMAT5	0.532499189	0.113308797	4.699539693	2.60749E-06	6.43547E-06	positive
PRJNA729271	stage_E18_vs_E10	ordinary_muscle_development	ENSGALG00010000308	ENSGALG00010000308	0.089337066	0.347538655	0.257056486	0.797135168	0.830480433	positive
PRJNA729271	stage_E18_vs_E10	ordinary_muscle_development	ENSGALG00010000309	ENSGALG00010000309	-2.548419806	4.31874048	-0.590084035	0.555134312	0.609227124	negative
PRJNA729271	stage_E18_vs_E10	ordinary_muscle_development	ENSGALG00010000312	CABP7	-2.033509718	0.542199643	-3.750481475	0.000176495	0.000356508	negative
PRJNA729271	stage_E18_vs_E10	ordinary_muscle_development	ENSGALG00010000313	ENSGALG00010000313	-1.325455747	3.826830809	-0.346358596	0.729073237	0.771120952	negative
PRJNA729271	stage_E18_vs_E10	ordinary_muscle_development	ENSGALG00010000318	ENSGALG00010000318	-1.550029523	4.749630475	-0.326347393	0.744161535	0.784424511	negative
PRJNA729271	stage_E18_vs_E10	ordinary_muscle_development	ENSGALG00010000323	AP1B1	0.221477773	0.14557885	1.521359544	0.128169635	0.166635471	positive
PRJNA729271	stage_E18_vs_E10	ordinary_muscle_development	ENSGALG00010000324	ENSGALG00010000324	0.12887652	0.172851176	0.745592378	0.455913688	0.515521407	positive
PRJNA729271	stage_E18_vs_E10	ordinary_muscle_development	ENSGALG00010000326	ENSGALG00010000326	-1.402019143	1.79717666	-0.780123164	0.435318383	0.495013725	negative
PRJNA729271	stage_E18_vs_E10	ordinary_muscle_development	ENSGALG00010000331	ENSGALG00010000331	-0.576355402	0.181862921	-3.169174876	0.001528724	0.002724896	negative
PRJNA729271	stage_E18_vs_E10	ordinary_muscle_development	ENSGALG00010000338	ENSGALG00010000338	-0.105060191	0.191510613	-0.548586783	0.583289058	0.636169744	negative
PRJNA729271	stage_E18_vs_E10	ordinary_muscle_development	ENSGALG00010000340	NAA10	-0.778396041	0.135784429	-5.732586918	9.89103E-09	3.05694E-08	negative
PRJNA729271	stage_E18_vs_E10	ordinary_muscle_development	ENSGALG00010000341	EWSR1	-0.693560739	0.147888606	-4.689750999	2.73538E-06	6.73735E-06	negative
PRJNA729271	stage_E18_vs_E10	ordinary_muscle_development	ENSGALG00010000346	ENSGALG00010000346	-0.058233149	0.113806334	-0.511686358	0.608870535	0.659757674	negative
PRJNA729271	stage_E18_vs_E10	ordinary_muscle_development	ENSGALG00010000349	CHIR-IG1-5	1.40528071	0.45644778	3.078732706	0.002078831	0.003641849	positive
PRJNA729271	stage_E18_vs_E10	ordinary_muscle_development	ENSGALG00010000350	ENSGALG00010000350	0.087312356	0.165889516	0.526328357	0.598660084	0.650219735	positive
PRJNA729271	stage_E18_vs_E10	ordinary_muscle_development	ENSGALG00010000353	ENSGALG00010000353	0.927615175	0.110522307	8.39301312	4.73839E-17	2.78294E-16	positive
PRJNA729271	stage_E18_vs_E10	ordinary_muscle_development	ENSGALG00010000356	ENSGALG00010000356	1.583160112	0.119224233	13.27884497	3.07095E-40	5.93511E-39	positive
PRJNA729271	stage_E18_vs_E10	ordinary_muscle_development	ENSGALG00010000366	EMID1	-2.106096629	0.239770232	-8.783811948	1.58026E-18	1.02304E-17	negative
PRJNA729271	stage_E18_vs_E10	ordinary_muscle_development	ENSGALG00010000367	HSD17B10	0.065229854	0.139523067	0.467520213	0.640127734	0.689350038	positive
PRJNA729271	stage_E18_vs_E10	ordinary_muscle_development	ENSGALG00010000373	ENSGALG00010000373	1.185379113	0.51979625	2.280468766	0.022579901	0.0337453	positive
PRJNA729271	stage_E18_vs_E10	ordinary_muscle_development	ENSGALG00010000376	ASCC2	-0.840881488	0.087907927	-9.56547964	1.11682E-21	8.77418E-21	negative
PRJNA729271	stage_E18_vs_E10	ordinary_muscle_development	ENSGALG00010000377	ENSGALG00010000377	0.791744245	0.241475879	3.278771558	0.0010426	0.001894033	positive
PRJNA729271	stage_E18_vs_E10	ordinary_muscle_development	ENSGALG00010000381	ENSGALG00010000381	0.48961295	0.298813087	1.638525792	0.101312058	0.134160267	positive
PRJNA729271	stage_E18_vs_E10	ordinary_muscle_development	ENSGALG00010000382	ENSGALG00010000382	-0.832522641	0.562739952	-1.479409162	0.139030993	0.17932499	negative
PRJNA729271	stage_E18_vs_E10	ordinary_muscle_development	ENSGALG00010000384	ENSGALG00010000384	-0.150146345	0.148194264	-1.013172444	0.3109778	0.36857099	negative
PRJNA729271	stage_E18_vs_E10	ordinary_muscle_development	ENSGALG00010000386	ENSGALG00010000386	-0.172999206	0.383157224	-0.451509708	0.65162223	0.699752869	negative
PRJNA729271	stage_E18_vs_E10	ordinary_muscle_development	ENSGALG00010000387	DTX1	-1.798551251	0.500540738	-3.593216521	0.000326621	0.000636169	negative
PRJNA729271	stage_E18_vs_E10	ordinary_muscle_development	ENSGALG00010000388	ENSGALG00010000388	-0.48445596	0.18678738	-2.593622542	0.009497069	0.015061271	negative
PRJNA729271	stage_E18_vs_E10	ordinary_muscle_development	ENSGALG00010000391	ENSGALG00010000391	1.368713969	0.703521228	1.945519074	0.051712536	0.072401547	positive
PRJNA729271	stage_E18_vs_E10	ordinary_muscle_development	ENSGALG00010000402	PFKFB1	5.806869603	0.320687637	18.10755681	2.77807E-73	1.59163E-71	positive
PRJNA729271	stage_E18_vs_E10	ordinary_muscle_development	ENSGALG00010000405	ENSGALG00010000405	2.371587584	0.397776198	5.96211537	2.48993E-09	8.11776E-09	positive
PRJNA729271	stage_E18_vs_E10	ordinary_muscle_development	ENSGALG00010000406	NSMCE3	-0.146863007	0.0985215	-1.490669623	0.136048255	0.175774976	negative
PRJNA729271	stage_E18_vs_E10	ordinary_muscle_development	ENSGALG00010000407	WSB2	0.690951932	0.08526	8.104057346	5.3156E-16	2.90753E-15	positive
PRJNA729271	stage_E18_vs_E10	ordinary_muscle_development	ENSGALG00010000408	ENSGALG00010000408	6.888494133	0.350686121	19.64290491	6.64842E-86	5.4755E-84	positive
PRJNA729271	stage_E18_vs_E10	ordinary_muscle_development	ENSGALG00010000411	ENSGALG00010000411	3.572081051	0.912090603	3.916366467	8.98936E-05	0.000188378	positive
PRJNA729271	stage_E18_vs_E10	ordinary_muscle_development	ENSGALG00010000412	RFC5	-0.20622907	0.105494677	-1.954876537	0.050597645	0.070977728	negative
PRJNA729271	stage_E18_vs_E10	ordinary_muscle_development	ENSGALG00010000422	ITIH6	-1.729375102	0.59940855	-2.885135859	0.003912448	0.00657518	negative
PRJNA729271	stage_E18_vs_E10	ordinary_muscle_development	ENSGALG00010000423	ENSGALG00010000423	1.547664022	0.208791323	7.412492049	1.23948E-13	5.66044E-13	positive
PRJNA729271	stage_E18_vs_E10	ordinary_muscle_development	ENSGALG00010000428	GNL3L	-0.703013239	0.150295827	-4.677529998	2.90351E-06	7.12725E-06	negative
PRJNA729271	stage_E18_vs_E10	ordinary_muscle_development	ENSGALG00010000431	AIFM3	-4.02384279	1.177696049	-3.416707387	0.000633834	0.001187417	negative
PRJNA729271	stage_E18_vs_E10	ordinary_muscle_development	ENSGALG00010000432	ENSGALG00010000432	0.08822391	0.185041889	0.476778042	0.633520183	0.683097155	positive
PRJNA729271	stage_E18_vs_E10	ordinary_muscle_development	ENSGALG00010000434	TP53	-0.03422343	0.222031432	-0.154137771	0.877501107	0.898515015	negative
PRJNA729271	stage_E18_vs_E10	ordinary_muscle_development	ENSGALG00010000435	P2RX6	-0.910503222	0.333877218	-2.727060047	0.006390141	0.01041436	negative
PRJNA729271	stage_E18_vs_E10	ordinary_muscle_development	ENSGALG00010000436	ENSGALG00010000436	0.784099101	0.404682173	1.937567687	0.052675988	0.073643754	positive
PRJNA729271	stage_E18_vs_E10	ordinary_muscle_development	ENSGALG00010000440	ENSGALG00010000440	-2.142524215	0.487151196	-4.398068262	1.09219E-05	2.53259E-05	negative
PRJNA729271	stage_E18_vs_E10	ordinary_muscle_development	ENSGALG00010000441	SLC7A4	-1.052502269	0.20978221	-5.017118796	5.24521E-07	1.39299E-06	negative
PRJNA729271	stage_E18_vs_E10	ordinary_muscle_development	ENSGALG00010000442	DGCR6	-0.764878219	0.131265669	-5.826947913	5.64502E-09	1.78423E-08	negative
PRJNA729271	stage_E18_vs_E10	ordinary_muscle_development	ENSGALG00010000443	TBX3	-0.036134455	0.156757117	-0.230512375	0.817693648	0.84854449	negative
PRJNA729271	stage_E18_vs_E10	ordinary_muscle_development	ENSGALG00010000444	RBM19	-0.768335048	0.119876596	-6.409383253	1.46109E-10	5.25914E-10	negative
PRJNA729271	stage_E18_vs_E10	ordinary_muscle_development	ENSGALG00010000449	PLBD2	1.525367892	0.107493556	14.19031943	1.05179E-45	2.55371E-44	positive
PRJNA729271	stage_E18_vs_E10	ordinary_muscle_development	ENSGALG00010000450	ENSGALG00010000450	0.442445587	0.190582466	2.321544037	0.0202575	0.030519953	positive
PRJNA729271	stage_E18_vs_E10	ordinary_muscle_development	ENSGALG00010000452	SLC8B1	1.774207257	0.226320965	7.839341159	4.52916E-15	2.31898E-14	positive
PRJNA729271	stage_E18_vs_E10	ordinary_muscle_development	ENSGALG00010000453	ACHE	1.590870724	0.24194115	6.575445001	4.85079E-11	1.81498E-10	positive
PRJNA729271	stage_E18_vs_E10	ordinary_muscle_development	ENSGALG00010000457	TPCN1	-0.289022315	0.082318094	-3.511042355	0.000446353	0.000854901	negative
PRJNA729271	stage_E18_vs_E10	ordinary_muscle_development	ENSGALG00010000458	ENSGALG00010000458	0.830285337	0.199555032	4.160683536	3.17297E-05	6.9759E-05	positive
PRJNA729271	stage_E18_vs_E10	ordinary_muscle_development	ENSGALG00010000462	ENSGALG00010000462	-0.179086521	0.225528433	-0.794075137	0.427151691	0.487217797	negative
PRJNA729271	stage_E18_vs_E10	ordinary_muscle_development	ENSGALG00010000465	ENSGALG00010000465	0.979590265	0.192364999	5.092351883	3.53649E-07	9.553E-07	positive
PRJNA729271	stage_E18_vs_E10	ordinary_muscle_development	ENSGALG00010000466	FBXO21	-0.129523757	0.081430386	-1.590607182	0.111698007	0.146840433	negative
PRJNA729271	stage_E18_vs_E10	ordinary_muscle_development	ENSGALG00010000467	RNASEK	-0.0322018	0.19972469	-0.161230942	0.871911511	0.894434349	negative
PRJNA729271	stage_E18_vs_E10	ordinary_muscle_development	ENSGALG00010000468	TESC	-2.324701499	0.303749111	-7.653360675	1.95794E-14	9.52999E-14	negative
PRJNA729271	stage_E18_vs_E10	ordinary_muscle_development	ENSGALG00010000469	ENSGALG00010000469	5.048175978	0.391659457	12.88919719	5.178E-38	9.16424E-37	positive
PRJNA729271	stage_E18_vs_E10	ordinary_muscle_development	ENSGALG00010000470	FBXW8	-0.123107307	0.111088997	-1.108186326	0.267781355	0.32280054	negative
PRJNA729271	stage_E18_vs_E10	ordinary_muscle_development	ENSGALG00010000471	ENSGALG00010000471	-0.208731732	0.207529517	-1.005792983	0.314515163	0.371942342	negative
PRJNA729271	stage_E18_vs_E10	ordinary_muscle_development	ENSGALG00010000475	RNFT2	-0.230196358	0.108627859	-2.119128192	0.034079635	0.049270328	negative
PRJNA729271	stage_E18_vs_E10	ordinary_muscle_development	ENSGALG00010000476	ENSGALG00010000476	2.690104977	0.215608188	12.47682197	9.98948E-36	1.59293E-34	positive
PRJNA729271	stage_E18_vs_E10	ordinary_muscle_development	ENSGALG00010000479	C12orf49	-0.87350265	0.116111773	-7.522946475	5.35554E-14	2.51388E-13	negative
PRJNA729271	stage_E18_vs_E10	ordinary_muscle_development	ENSGALG00010000483	ENSGALG00010000483	1.057455192	0.247552479	4.271640482	1.9404E-05	4.37751E-05	positive
PRJNA729271	stage_E18_vs_E10	ordinary_muscle_development	ENSGALG00010000485	MED13L	1.186451619	0.125888329	9.424635547	4.31602E-21	3.26688E-20	positive
PRJNA729271	stage_E18_vs_E10	ordinary_muscle_development	ENSGALG00010000491	ENSGALG00010000491	0.576625334	0.158565702	3.636507297	0.00027636	0.000544049	positive
PRJNA729271	stage_E18_vs_E10	ordinary_muscle_development	ENSGALG00010000495	ENSGALG00010000495	0.520236226	0.576867982	0.901828913	0.367147758	0.426768785	positive
PRJNA729271	stage_E18_vs_E10	ordinary_muscle_development	ENSGALG00010000501	ENSGALG00010000501	-0.113265708	0.165777745	-0.68323832	0.494456262	0.551828112	negative
PRJNA729271	stage_E18_vs_E10	ordinary_muscle_development	ENSGALG00010000507	TRPT1	1.387818171	0.295722566	4.692973518	2.69262E-06	6.63543E-06	positive
PRJNA729271	stage_E18_vs_E10	ordinary_muscle_development	ENSGALG00010000509	ENSGALG00010000509	-3.10744922	1.127031704	-2.757197697	0.005829909	0.009562582	negative
PRJNA729271	stage_E18_vs_E10	ordinary_muscle_development	ENSGALG00010000511	FERMT3	0.249869319	0.162268732	1.539848838	0.123597204	0.161138827	positive
PRJNA729271	stage_E18_vs_E10	ordinary_muscle_development	ENSGALG00010000525	ENSGALG00010000525	-0.851065491	0.525435452	-1.619733667	0.105289501	0.139071561	negative
PRJNA729271	stage_E18_vs_E10	ordinary_muscle_development	ENSGALG00010000526	POLD1	-1.021821587	0.159881466	-6.391119699	1.64675E-10	5.90834E-10	negative
PRJNA729271	stage_E18_vs_E10	ordinary_muscle_development	ENSGALG00010000529	STIP1	-0.213962372	0.193084935	-1.108125664	0.267807549	0.32280054	negative
PRJNA729271	stage_E18_vs_E10	ordinary_muscle_development	ENSGALG00010000532	ENSGALG00010000532	1.780201226	0.475921678	3.740534015	0.00018363	0.000369939	positive
PRJNA729271	stage_E18_vs_E10	ordinary_muscle_development	ENSGALG00010000546	FLRT1	-2.99087312	0.597147892	-5.008596967	5.48283E-07	1.45343E-06	negative
PRJNA729271	stage_E18_vs_E10	ordinary_muscle_development	ENSGALG00010000555	OTUB1	-0.457712239	0.102382892	-4.470592977	7.8003E-06	1.83757E-05	negative
PRJNA729271	stage_E18_vs_E10	ordinary_muscle_development	ENSGALG00010000561	ENSGALG00010000561	-0.534637647	0.27766611	-1.925469578	0.054170638	0.075558449	negative
PRJNA729271	stage_E18_vs_E10	ordinary_muscle_development	ENSGALG00010000564	NAA40	-0.589623085	0.089806417	-6.565489465	5.18622E-11	1.93698E-10	negative
PRJNA729271	stage_E18_vs_E10	ordinary_muscle_development	ENSGALG00010000569	SIPA1	0.576351046	0.236484082	2.437166346	0.014802868	0.022872569	positive
PRJNA729271	stage_E18_vs_E10	ordinary_muscle_development	ENSGALG00010000574	ENSGALG00010000574	3.424517251	0.172200862	19.88676018	5.29898E-88	4.68346E-86	positive
PRJNA729271	stage_E18_vs_E10	ordinary_muscle_development	ENSGALG00010000575	ENSGALG00010000575	1.135258172	0.160209678	7.086077368	1.37967E-12	5.84009E-12	positive
PRJNA729271	stage_E18_vs_E10	ordinary_muscle_development	ENSGALG00010000576	NOS1	1.286661074	0.470231502	2.736229006	0.006214776	0.010151361	positive
PRJNA729271	stage_E18_vs_E10	ordinary_muscle_development	ENSGALG00010000577	ENSGALG00010000577	-0.145401316	0.274829085	-0.529060873	0.596763223	0.64857786	negative
PRJNA729271	stage_E18_vs_E10	ordinary_muscle_development	ENSGALG00010000578	ENSGALG00010000578	0.077685461	0.424103189	0.183175846	0.854660045	0.880021656	positive
PRJNA729271	stage_E18_vs_E10	ordinary_muscle_development	ENSGALG00010000579	ENSGALG00010000579	-0.766600522	0.899469214	-0.852281001	0.394058148	0.454043948	negative
PRJNA729271	stage_E18_vs_E10	ordinary_muscle_development	ENSGALG00010000580	ENSGALG00010000580	1.38259268	0.464552898	2.976179216	0.002918642	0.005007187	positive
PRJNA729271	stage_E18_vs_E10	ordinary_muscle_development	ENSGALG00010000581	ENSGALG00010000581	0.06816869	0.130861413	0.520922772	0.602420571	0.653697124	positive
PRJNA729271	stage_E18_vs_E10	ordinary_muscle_development	ENSGALG00010000583	CCS	-0.272147158	0.141850888	-1.918543911	0.05504208	0.076737035	negative
PRJNA729271	stage_E18_vs_E10	ordinary_muscle_development	ENSGALG00010000584	RBM14	0.353848924	0.212868959	1.66228522	0.0964556	0.12836246	positive
PRJNA729271	stage_E18_vs_E10	ordinary_muscle_development	ENSGALG00010000585	SPTBN2	-2.79756984	0.575230877	-4.863386081	1.15394E-06	2.96253E-06	negative
PRJNA729271	stage_E18_vs_E10	ordinary_muscle_development	ENSGALG00010000586	ENSGALG00010000586	3.00020836	0.26218992	11.44288217	2.55262E-30	3.16783E-29	positive
PRJNA729271	stage_E18_vs_E10	ordinary_muscle_development	ENSGALG00010000589	ENSGALG00010000589	0.443404735	0.357586224	1.239993897	0.214977651	0.265471209	positive
PRJNA729271	stage_E18_vs_E10	ordinary_muscle_development	ENSGALG00010000590	DNAH5	-0.434361735	0.458680346	-0.946981353	0.343648281	0.402064883	negative
PRJNA729271	stage_E18_vs_E10	ordinary_muscle_development	ENSGALG00010000591	SBK2	2.60944204	0.30913995	8.440973232	3.14706E-17	1.86724E-16	positive
PRJNA729271	stage_E18_vs_E10	ordinary_muscle_development	ENSGALG00010000592	CCT5	-1.088447602	0.119399563	-9.116009938	7.79411E-20	5.4263E-19	negative
PRJNA729271	stage_E18_vs_E10	ordinary_muscle_development	ENSGALG00010000593	ATPSCKMT	-0.594717413	0.146489488	-4.05979585	4.91156E-05	0.000105848	negative
PRJNA729271	stage_E18_vs_E10	ordinary_muscle_development	ENSGALG00010000595	MYO10	0.422320786	0.137778603	3.065213154	0.002175149	0.003798649	positive
PRJNA729271	stage_E18_vs_E10	ordinary_muscle_development	ENSGALG00010000596	ENSGALG00010000596	-0.229892493	0.671479511	-0.3423671	0.732074645	0.773652812	negative
PRJNA729271	stage_E18_vs_E10	ordinary_muscle_development	ENSGALG00010000597	CDH9	0.943661749	0.554924902	1.70052154	0.089032868	0.119173647	positive
PRJNA729271	stage_E18_vs_E10	ordinary_muscle_development	ENSGALG00010000598	ENSGALG00010000598	0.065111518	0.502978145	0.129451982	0.897000018	0.914540006	positive
PRJNA729271	stage_E18_vs_E10	ordinary_muscle_development	ENSGALG00010000599	RETREG1	4.656406907	0.374666242	12.42814642	1.83848E-35	2.88095E-34	positive
PRJNA729271	stage_E18_vs_E10	ordinary_muscle_development	ENSGALG00010000601	ENSGALG00010000601	-0.28219902	0.330023073	-0.855088757	0.392502013	0.452502719	negative
PRJNA729271	stage_E18_vs_E10	ordinary_muscle_development	ENSGALG00010000603	ZNF622	-0.947250978	0.111382653	-8.5044749	1.82419E-17	1.09853E-16	negative
PRJNA729271	stage_E18_vs_E10	ordinary_muscle_development	ENSGALG00010000604	ENSGALG00010000604	-0.064634516	0.131887502	-0.490073097	0.624082175	0.674526219	negative
PRJNA729271	stage_E18_vs_E10	ordinary_muscle_development	ENSGALG00010000605	MARCHF11	-2.606120893	0.815946468	-3.193985141	0.001403233	0.002511715	negative
PRJNA729271	stage_E18_vs_E10	ordinary_muscle_development	ENSGALG00010000606	UBE2QL1	-0.623286745	0.159760621	-3.90137909	9.56462E-05	0.000199826	negative
PRJNA729271	stage_E18_vs_E10	ordinary_muscle_development	ENSGALG00010000607	ENSGALG00010000607	-0.216190745	0.256413484	-0.843133294	0.3991539	0.459076076	negative
PRJNA729271	stage_E18_vs_E10	ordinary_muscle_development	ENSGALG00010000608	ENSGALG00010000608	-1.012768507	0.979555442	-1.033906264	0.301179987	0.358188702	negative
PRJNA729271	stage_E18_vs_E10	ordinary_muscle_development	ENSGALG00010000610	ENSGALG00010000610	-0.19475829	0.079951047	-2.435969233	0.014851948	0.022945958	negative
PRJNA729271	stage_E18_vs_E10	ordinary_muscle_development	ENSGALG00010000611	C7orf57	1.251198701	0.328734097	3.806111722	0.000141169	0.000289139	positive
PRJNA729271	stage_E18_vs_E10	ordinary_muscle_development	ENSGALG00010000612	ENSGALG00010000612	-0.76949727	0.40960354	-1.87863921	0.060293773	0.083385006	negative
PRJNA729271	stage_E18_vs_E10	ordinary_muscle_development	ENSGALG00010000613	ENSGALG00010000613	-0.076910412	0.177896801	-0.432331622	0.665500408	0.712702505	negative
PRJNA729271	stage_E18_vs_E10	ordinary_muscle_development	ENSGALG00010000614	KAT8	1.261614844	0.183801382	6.864011742	6.69531E-12	2.66949E-11	positive
PRJNA729271	stage_E18_vs_E10	ordinary_muscle_development	ENSGALG00010000615	ENSGALG00010000615	-0.291418157	0.591873188	-0.49236587	0.622460722	0.67312505	negative
PRJNA729271	stage_E18_vs_E10	ordinary_muscle_development	ENSGALG00010000616	PSENEN	0.091296052	0.525769823	0.173642626	0.86214633	0.886172958	positive
PRJNA729271	stage_E18_vs_E10	ordinary_muscle_development	ENSGALG00010000619	ENSGALG00010000619	0.686513564	0.296604438	2.31457617	0.020636138	0.031045233	positive
PRJNA729271	stage_E18_vs_E10	ordinary_muscle_development	ENSGALG00010000621	UPP1	-0.331554117	0.238957646	-1.387501604	0.165288862	0.2093553	negative
PRJNA729271	stage_E18_vs_E10	ordinary_muscle_development	ENSGALG00010000622	ENSGALG00010000622	0.314894972	0.430513088	0.731441113	0.464509761	0.523648233	positive
PRJNA729271	stage_E18_vs_E10	ordinary_muscle_development	ENSGALG00010000623	ENSGALG00010000623	0.241992321	0.247574049	0.977454308	0.328344288	0.386405006	positive

PRJNA729271	stage_E18_vs_E10	ordinary_muscle_development	ENSGALG00010000625	ENSGALG00010000625	2.309831999	0.364884227	6.330314734	2.44662E-10	8.66659E-10	positive
PRJNA729271	stage_E18_vs_E10	ordinary_muscle_development	ENSGALG00010000626	ABCA13	0.017025017	0.637884569	0.026689809	0.978707142	0.982443701	positive
PRJNA729271	stage_E18_vs_E10	ordinary_muscle_development	ENSGALG00010000627	ENSGALG00010000627	0.414917446	0.238213916	1.741785088	0.081546057	0.110087557	positive
PRJNA729271	stage_E18_vs_E10	ordinary_muscle_development	ENSGALG00010000630	ENSGALG00010000630	0.070573079	0.394151368	0.179050702	0.85789789	0.882431871	positive
PRJNA729271	stage_E18_vs_E10	ordinary_muscle_development	ENSGALG00010000632	ENSGALG00010000632	0.306360622	0.382918898	0.800066604	0.423672209	0.48412524	positive
PRJNA729271	stage_E18_vs_E10	ordinary_muscle_development	ENSGALG00010000633	VWC2	-0.958000764	0.410330946	-2.334702691	0.019558958	0.02954122	negative
PRJNA729271	stage_E18_vs_E10	ordinary_muscle_development	ENSGALG00010000634	ENSGALG00010000634	0.918838569	0.192434139	4.774820996	1.79867E-06	4.52007E-06	positive
PRJNA729271	stage_E18_vs_E10	ordinary_muscle_development	ENSGALG00010000635	ENSGALG00010000635	-0.398435688	0.132412595	-3.009046738	0.002620688	0.004528716	negative
PRJNA729271	stage_E18_vs_E10	ordinary_muscle_development	ENSGALG00010000636	ENSGALG00010000636	0.263174357	0.147505694	1.784164057	0.074397011	0.101190267	positive
PRJNA729271	stage_E18_vs_E10	ordinary_muscle_development	ENSGALG00010000639	SCN1B	1.365982931	0.280506703	4.869697996	1.11769E-06	2.87709E-06	positive
PRJNA729271	stage_E18_vs_E10	ordinary_muscle_development	ENSGALG00010000641	ENSGALG00010000641	1.452374126	0.405669686	3.580188945	0.000343346	0.000666416	positive
PRJNA729271	stage_E18_vs_E10	ordinary_muscle_development	ENSGALG00010000643	VKORC1	0.735230218	0.206765224	3.555869811	0.000376731	0.00072761	positive
PRJNA729271	stage_E18_vs_E10	ordinary_muscle_development	ENSGALG00010000645	ENSGALG00010000645	0.198622667	0.523110499	0.379695431	0.704171512	0.748970213	positive
PRJNA729271	stage_E18_vs_E10	ordinary_muscle_development	ENSGALG00010000646	ENSGALG00010000646	0.072693624	0.513709333	0.141507306	0.887469189	0.907119801	positive
PRJNA729271	stage_E18_vs_E10	ordinary_muscle_development	ENSGALG00010000649	PAPD7	0.415821021	0.08312548	5.002329293	5.66417E-07	1.49958E-06	positive
PRJNA729271	stage_E18_vs_E10	ordinary_muscle_development	ENSGALG00010000650	BCKDK	1.556027443	0.168687777	9.224304662	2.85407E-20	2.03892E-19	positive
PRJNA729271	stage_E18_vs_E10	ordinary_muscle_development	ENSGALG00010000651	ENSGALG00010000651	-0.350733163	0.648708202	-0.540663987	0.588739205	0.640866234	negative
PRJNA729271	stage_E18_vs_E10	ordinary_muscle_development	ENSGALG00010000652	SRD5A1	0.691114268	0.225793459	3.060825013	0.002207281	0.003848735	positive
PRJNA729271	stage_E18_vs_E10	ordinary_muscle_development	ENSGALG00010000653	NSUN2	-0.886933371	0.093784723	-9.457119869	3.16542E-21	2.41615E-20	negative
PRJNA729271	stage_E18_vs_E10	ordinary_muscle_development	ENSGALG00010000654	ENSGALG00010000654	-0.774756575	0.170592573	-4.541560988	5.58392E-06	1.33409E-05	negative
PRJNA729271	stage_E18_vs_E10	ordinary_muscle_development	ENSGALG00010000655	ENSGALG00010000655	-1.054241304	0.849234893	-1.241401305	0.214457537	0.264896634	negative
PRJNA729271	stage_E18_vs_E10	ordinary_muscle_development	ENSGALG00010000656	ENSGALG00010000656	1.104606134	0.416499063	2.652121533	0.007998774	0.012829726	positive
PRJNA729271	stage_E18_vs_E10	ordinary_muscle_development	ENSGALG00010000659	ENSGALG00010000659	2.203575962	0.548537093	4.017186783	5.8897E-05	0.000126059	positive
PRJNA729271	stage_E18_vs_E10	ordinary_muscle_development	ENSGALG00010000660	ENSGALG00010000660	1.353360541	0.181622291	7.451511236	9.2277E-14	4.25026E-13	positive
PRJNA729271	stage_E18_vs_E10	ordinary_muscle_development	ENSGALG00010000661	ENSGALG00010000661	-0.268336696	0.592240732	-0.453087201	0.650485951	0.698946914	negative
PRJNA729271	stage_E18_vs_E10	ordinary_muscle_development	ENSGALG00010000670	ENSGALG00010000670	-0.067240759	0.51771094	-0.129880893	0.896660661	0.914322637	negative
PRJNA729271	stage_E18_vs_E10	ordinary_muscle_development	ENSGALG00010000674	GRB10	0.447833865	0.169898111	2.635896666	0.008391528	0.013428483	positive
PRJNA729271	stage_E18_vs_E10	ordinary_muscle_development	ENSGALG00010000684	COBL	2.215399452	0.134908207	16.42153207	1.34137E-60	5.47693E-59	positive
PRJNA729271	stage_E18_vs_E10	ordinary_muscle_development	ENSGALG00010000686	ENSGALG00010000686	0.201877427	0.13249882	1.523616788	0.127604467	0.166019814	positive
PRJNA729271	stage_E18_vs_E10	ordinary_muscle_development	ENSGALG00010000687	IKZF1	-0.887749483	0.311097339	-2.853606798	0.0043226	0.007223436	negative
PRJNA729271	stage_E18_vs_E10	ordinary_muscle_development	ENSGALG00010000688	ENSGALG00010000688	1.52814528	0.283709897	5.386295284	7.19248E-08	2.062E-07	positive
PRJNA729271	stage_E18_vs_E10	ordinary_muscle_development	ENSGALG00010000695	FIGNL1	-1.000577628	0.142857766	-7.004012829	2.48733E-12	1.03188E-11	negative
PRJNA729271	stage_E18_vs_E10	ordinary_muscle_development	ENSGALG00010000697	ENSGALG00010000697	0.639017254	0.249266754	2.563587979	0.010359644	0.016355848	positive
PRJNA729271	stage_E18_vs_E10	ordinary_muscle_development	ENSGALG00010000702	DDC	0.319605714	0.357105854	0.894988729	0.370793113	0.43048828	positive
PRJNA729271	stage_E18_vs_E10	ordinary_muscle_development	ENSGALG00010000704	ENSGALG00010000704	-0.051292262	0.093728382	-0.547243648	0.584211353	0.636846173	negative
PRJNA729271	stage_E18_vs_E10	ordinary_muscle_development	ENSGALG00010000705	ENSGALG00010000705	5.048488085	1.008487589	5.005999221	5.5573E-07	1.47236E-06	positive
PRJNA729271	stage_E18_vs_E10	ordinary_muscle_development	ENSGALG00010000710	ENSGALG00010000710	1.163754402	0.502498761	2.315934869	0.020561825	0.030946283	positive
PRJNA729271	stage_E18_vs_E10	ordinary_muscle_development	ENSGALG00010000711	SPRY2	0.317991455	0.110773732	2.870639533	0.004096424	0.00686844	positive
PRJNA729271	stage_E18_vs_E10	ordinary_muscle_development	ENSGALG00010000714	FBXL7	-0.961123388	0.095935919	-10.01838935	1.26548E-23	1.09708E-22	negative
PRJNA729271	stage_E18_vs_E10	ordinary_muscle_development	ENSGALG00010000715	PCDH20	0.005744402	0.215316925	0.026678822	0.978715905	0.982443701	positive
PRJNA729271	stage_E18_vs_E10	ordinary_muscle_development	ENSGALG00010000717	NDFIP2	0.225389552	0.067916833	3.318611024	0.000904664	0.001657787	positive
PRJNA729271	stage_E18_vs_E10	ordinary_muscle_development	ENSGALG00010000718	EEF1G	0.337671846	0.150927138	2.23731696	0.025265632	0.037446353	positive
PRJNA729271	stage_E18_vs_E10	ordinary_muscle_development	ENSGALG00010000720	RBM26	-0.152439724	0.051660686	-2.950787857	0.003169645	0.00541027	negative
PRJNA729271	stage_E18_vs_E10	ordinary_muscle_development	ENSGALG00010000721	ENSGALG00010000721	-0.104364513	0.147917785	-0.705557567	0.48046332	0.538658506	negative
PRJNA729271	stage_E18_vs_E10	ordinary_muscle_development	ENSGALG00010000722	ANKH	-0.227174684	0.171957164	-1.321112059	0.186464003	0.233563405	negative
PRJNA729271	stage_E18_vs_E10	ordinary_muscle_development	ENSGALG00010000724	OBI1	-1.120714497	0.146808151	-7.633871053	2.27808E-14	1.10106E-13	negative
PRJNA729271	stage_E18_vs_E10	ordinary_muscle_development	ENSGALG00010000725	ENSGALG00010000725	-0.880689519	0.588269437	-1.497085288	0.134371067	0.173793934	negative
PRJNA729271	stage_E18_vs_E10	ordinary_muscle_development	ENSGALG00010000727	ENSGALG00010000727	0.010241105	0.148462127	0.068981262	0.945004535	0.955019224	positive
PRJNA729271	stage_E18_vs_E10	ordinary_muscle_development	ENSGALG00010000728	OTULIN	-0.649121818	0.295376056	-2.197611499	0.027976803	0.041103158	negative
PRJNA729271	stage_E18_vs_E10	ordinary_muscle_development	ENSGALG00010000729	EDNRB	-0.414163941	0.157232664	-2.634083344	0.008436478	0.013497433	negative
PRJNA729271	stage_E18_vs_E10	ordinary_muscle_development	ENSGALG00010000730	INTS5	0.548986625	0.225163069	2.438173483	0.014761688	0.022811371	positive
PRJNA729271	stage_E18_vs_E10	ordinary_muscle_development	ENSGALG00010000731	ENSGALG00010000731	-0.135640097	0.445522431	-0.304451779	0.760783725	0.799330248	negative
PRJNA729271	stage_E18_vs_E10	ordinary_muscle_development	ENSGALG00010000732	ENSGALG00010000732	-0.442206541	0.148459207	-2.978640066	0.002895307	0.004972449	negative
PRJNA729271	stage_E18_vs_E10	ordinary_muscle_development	ENSGALG00010000733	ENSGALG00010000733	-0.147406139	0.420926792	-0.350194242	0.726192928	0.768501606	negative
PRJNA729271	stage_E18_vs_E10	ordinary_muscle_development	ENSGALG00010000734	SLAIN1	-0.591552301	0.318853857	-1.85524587	0.063561124	0.087494634	negative
PRJNA729271	stage_E18_vs_E10	ordinary_muscle_development	ENSGALG00010000735	ENSGALG00010000735	1.612392584	0.536697947	3.004283123	0.002662075	0.00459257	positive
PRJNA729271	stage_E18_vs_E10	ordinary_muscle_development	ENSGALG00010000737	ENSGALG00010000737	-0.02331433	0.747171238	-0.031203463	0.975107278	0.979499653	negative
PRJNA729271	stage_E18_vs_E10	ordinary_muscle_development	ENSGALG00010000741	FAM105A	-1.213950474	0.199467548	-6.08595476	1.15799E-09	3.88364E-09	negative
PRJNA729271	stage_E18_vs_E10	ordinary_muscle_development	ENSGALG00010000753	TRIO	-0.062207678	0.068434671	-0.909008201	0.363345797	0.422858075	negative
PRJNA729271	stage_E18_vs_E10	ordinary_muscle_development	ENSGALG00010000757	SCEL	-3.908648483	0.502896692	-7.772269228	7.70924E-15	3.88409E-14	negative
PRJNA729271	stage_E18_vs_E10	ordinary_muscle_development	ENSGALG00010000760	CDH10	-0.603092787	0.290942463	-2.072893658	0.038182177	0.054742894	negative
PRJNA729271	stage_E18_vs_E10	ordinary_muscle_development	ENSGALG00010000762	ENSGALG00010000762	0.116440997	0.330072404	0.352774104	0.724257799	0.766621644	positive
PRJNA729271	stage_E18_vs_E10	ordinary_muscle_development	ENSGALG00010000768	ENSGALG00010000768	-0.411007545	0.349817219	-1.174920851	0.240026383	0.292959112	negative
PRJNA729271	stage_E18_vs_E10	ordinary_muscle_development	ENSGALG00010000770	ENSGALG00010000770	-1.76397093	0.617372778	-2.857221749	0.004273672	0.00714909	negative
PRJNA729271	stage_E18_vs_E10	ordinary_muscle_development	ENSGALG00010000777	MYCBP2	-0.184599902	0.082729833	-2.231358338	0.025657402	0.037949392	negative
PRJNA729271	stage_E18_vs_E10	ordinary_muscle_development	ENSGALG00010000783	DAP	0.336556537	0.162638158	2.069357771	0.038512526	0.055183775	positive
PRJNA729271	stage_E18_vs_E10	ordinary_muscle_development	ENSGALG00010000784	FBXL3	0.489751557	0.09585937	5.109062981	3.2376E-07	8.77671E-07	positive
PRJNA729271	stage_E18_vs_E10	ordinary_muscle_development	ENSGALG00010000785	CLN5	0.036547013	0.09466543	0.386065036	0.699448504	0.744329055	positive
PRJNA729271	stage_E18_vs_E10	ordinary_muscle_development	ENSGALG00010000786	ANKRD33B	3.91378179	0.30113481	12.99677638	1.2761E-38	2.31793E-37	positive
PRJNA729271	stage_E18_vs_E10	ordinary_muscle_development	ENSGALG00010000787	SNRNP48	0.585689572	0.103759878	5.644663262	1.65505E-08	5.01567E-08	positive
PRJNA729271	stage_E18_vs_E10	ordinary_muscle_development	ENSGALG00010000788	GATD3AL1	1.0122419	0.104472979	9.689030696	3.35699E-22	2.70933E-21	positive
PRJNA729271	stage_E18_vs_E10	ordinary_muscle_development	ENSGALG00010000789	ROPN1L	1.066033239	0.22820302	4.671424768	2.99118E-06	7.33375E-06	positive
PRJNA729271	stage_E18_vs_E10	ordinary_muscle_development	ENSGALG00010000790	MARCHF6	0.206046516	0.100067611	2.059073004	0.039487245	0.056452078	positive
PRJNA729271	stage_E18_vs_E10	ordinary_muscle_development	ENSGALG00010000791	ACOD1	0.297208211	0.517208617	0.574638939	0.565535511	0.619328894	positive
PRJNA729271	stage_E18_vs_E10	ordinary_muscle_development	ENSGALG00010000792	ENSGALG00010000792	-0.795836616	1.815067995	-0.438461048	0.661052101	0.708724127	negative
PRJNA729271	stage_E18_vs_E10	ordinary_muscle_development	ENSGALG00010000793	ENSGALG00010000793	5.956911586	1.153115851	5.165926373	2.39251E-07	6.55194E-07	positive
PRJNA729271	stage_E18_vs_E10	ordinary_muscle_development	ENSGALG00010000795	ENSGALG00010000795	-0.430775829	0.229322343	-1.878472997	0.060316488	0.083408461	negative
PRJNA729271	stage_E18_vs_E10	ordinary_muscle_development	ENSGALG00010000796	ENSGALG00010000796	-0.505613295	0.200859647	-2.517246762	0.011827596	0.018528818	negative
PRJNA729271	stage_E18_vs_E10	ordinary_muscle_development	ENSGALG00010000797	ENSGALG00010000797	2.643163878	0.416268784	6.349656711	2.15796E-10	7.66657E-10	positive
PRJNA729271	stage_E18_vs_E10	ordinary_muscle_development	ENSGALG00010000798	ENSGALG00010000798	-3.617384953	0.484989476	-7.458687519	8.73886E-14	4.03921E-13	negative
PRJNA729271	stage_E18_vs_E10	ordinary_muscle_development	ENSGALG00010000799	ENSGALG00010000799	-0.024499079	0.291969639	-0.083909679	0.933128244	0.94558822	negative
PRJNA729271	stage_E18_vs_E10	ordinary_muscle_development	ENSGALG00010000800	LMO7	1.548273353	0.151452938	10.22280169	1.56744E-24	1.44529E-23	positive
PRJNA729271	stage_E18_vs_E10	ordinary_muscle_development	ENSGALG00010000802	SEMA5A	-0.116308012	0.125982839	-0.923205195	0.355900282	0.415280941	negative
PRJNA729271	stage_E18_vs_E10	ordinary_muscle_development	ENSGALG00010000803	MTRR	-0.589998278	0.101750857	-5.798460039	6.69266E-09	2.10115E-08	negative
PRJNA729271	stage_E18_vs_E10	ordinary_muscle_development	ENSGALG00010000804	ENSGALG00010000804	0.441485922	0.4360461	1.012475337	0.311310833	0.368875216	positive
PRJNA729271	stage_E18_vs_E10	ordinary_muscle_development	ENSGALG00010000805	FASTKD3	0.020455195	0.132629414	0.154228197	0.87742981	0.898505482	positive
PRJNA729271	stage_E18_vs_E10	ordinary_muscle_development	ENSGALG00010000806	CFAP90	-0.237465339	0.488483344	-0.486127811	0.62687655	0.677243466	negative
PRJNA729271	stage_E18_vs_E10	ordinary_muscle_development	ENSGALG00010000807	ENSGALG00010000807	2.572421019	0.743928973	3.457885243	0.000544433	0.001029558	positive
PRJNA729271	stage_E18_vs_E10	ordinary_muscle_development	ENSGALG00010000808	ADCY2	0.983150223	0.171412882	5.735567897	9.71861E-09	3.00686E-08	positive
PRJNA729271	stage_E18_vs_E10	ordinary_muscle_development	ENSGALG00010000810	UCHL3	-0.80990477	0.138267886	-5.857504518	4.69874E-09	1.49656E-08	negative
PRJNA729271	stage_E18_vs_E10	ordinary_muscle_development	ENSGALG00010000811	ENSGALG00010000811	5.069713033	1.048827041	4.83369787	1.3402E-06	3.4189E-06	positive
PRJNA729271	stage_E18_vs_E10	ordinary_muscle_development	ENSGALG00010000812	ENSGALG00010000812	-0.608691269	0.096323613	-6.31923211	2.62866E-10	9.29102E-10	negative
PRJNA729271	stage_E18_vs_E10	ordinary_muscle_development	ENSGALG00010000813	ENSGALG00010000813	-1.183847154	0.454685599	-2.603660982	0.009223394	0.01465451	negative
PRJNA729271	stage_E18_vs_E10	ordinary_muscle_development	ENSGALG00010000814	CDH18	-0.729533813	0.284368417	-2.565453011	0.010304118	0.016275274	negative
PRJNA729271	stage_E18_vs_E10	ordinary_muscle_development	ENSGALG00010000815	ENSGALG00010000815	-1.349465324	0.172803362	-7.809253877	5.75275E-15	2.92583E-14	negative
PRJNA729271	stage_E18_vs_E10	ordinary_muscle_development	ENSGALG00010000816	TBC1D4	1.579529131	0.103853985	15.20913353	3.07587E-52	9.69234E-51	positive
PRJNA729271	stage_E18_vs_E10	ordinary_muscle_development	ENSGALG00010000817	ENSGALG00010000817	0.625567934	0.115597923	5.411584563	6.24695E-08	1.79912E-07	positive
PRJNA729271	stage_E18_vs_E10	ordinary_muscle_development	ENSGALG00010000818	KLF12	-0.884561669	0.078087256	-11.32786218	9.55074E-30	1.15365E-28	negative
PRJNA729271	stage_E18_vs_E10	ordinary_muscle_development	ENSGALG00010000820	KLF5	2.315559129	0.191764123	12.07503828	1.43098E-33	2.04758E-32	positive
PRJNA729271	stage_E18_vs_E10	ordinary_muscle_development	ENSGALG00010000821	ENSGALG00010000821	2.467056954	0.148876381	16.57117759	1.12599E-61	4.73079E-60	positive
PRJNA729271	stage_E18_vs_E10	ordinary_muscle_development	ENSGALG00010000822	BASP1	-1.628015631	0.164897415	-9.872899635	5.45641E-23	4.57171E-22	negative
PRJNA729271	stage_E18_vs_E10	ordinary_muscle_development	ENSGALG00010000823	PIBF1	1.935160132	0.158380182	12.21844873	2.47767E-34	3.71011E-33	positive
PRJNA729271	stage_E18_vs_E10	ordinary_muscle_development	ENSGALG00010000843	DIS3	-0.892929152	0.084951405	-10.51105813	7.68267E-26	7.60657E-25	negative
PRJNA729271	stage_E18_vs_E10	ordinary_muscle_development	ENSGALG00010000853	ENSGALG00010000853	0.423658802	0.305990308	1.384549743	0.166190188	0.210423373	positive
PRJNA729271	stage_E18_vs_E10	ordinary_muscle_development	ENSGALG00010000857	ENSGALG00010000857	-1.101635719	0.784711762	-1.40387308			negative
PRJNA729271	stage_E18_vs_E10	ordinary_muscle_development	ENSGALG00010000859	BORA	-2.14507536	0.193570152	-11.08164324	1.5402E-28	1.74551E-27	negative
PRJNA729271	stage_E18_vs_E10	ordinary_muscle_development	ENSGALG00010000864	MZT1	-0.207890776	0.129433986	-1.60615293	0.108240313	0.142670122	negative
PRJNA729271	stage_E18_vs_E10	ordinary_muscle_development	ENSGALG00010000870	DACH1	0.770701725	0.140374367	5.49033089	4.01181E-08	1.17906E-07	positive
PRJNA729271	stage_E18_vs_E10	ordinary_muscle_development	ENSGALG00010000901	PCDH9	-0.721671022	0.240623938	-2.999165536	0.002707202	0.00466598	negative
PRJNA729271	stage_E18_vs_E10	ordinary_muscle_development	ENSGALG00010001066	SLC33A1	-0.613645827	0.089561185	-6.851693915	7.29806E-12	2.90141E-11	negative
PRJNA729271	stage_E18_vs_E10	ordinary_muscle_development	ENSGALG00010001070	C3orf33	0.205685295	0.180609915	1.138837227	0.254771051	0.308616613	positive
PRJNA729271	stage_E18_vs_E10	ordinary_muscle_development	ENSGALG00010001082	P2RY1	3.934627639	0.179727564	21.89217701	3.0843E-106	4.2176E-104	positive
PRJNA729271	stage_E18_vs_E10	ordinary_muscle_development	ENSGALG00010001104	MBNL1	0.951881291	0.123130909	7.730644564	1.07003E-14	5.33137E-14	positive
PRJNA729271	stage_E18_vs_E10	ordinary_muscle_development	ENSGALG00010001111	SUCNR1	2.717711753	0.444826453	6.109600126	9.98811E-10	3.37006E-09	positive
PRJNA729271	stage_E18_vs_E10	ordinary_muscle_development	ENSGALG00010001112	TSC22D2	0.140833702	0.090073326	1.563545033	0.117924452	0.154381768	positive
PRJNA729271	stage_E18_vs_E10	ordinary_muscle_development	ENSGALG00010001113	IGSF10	0.764253739	0.299549977	2.551339672	0.010730968	0.016910784	positive
PRJNA729271	stage_E18_vs_E10	ordinary_muscle_development	ENSGALG00010001114	MED12L	1.098317766	0.101472718	10.823774	2.6561E-27	2.84975E-26	positive
PRJNA729271	stage_E18_vs_E10	ordinary_muscle_development	ENSGALG00010001115	PFN2	-0.882256792	0.095931717	-9.196716333	3.69055E-20	2.61331E-19	negative
PRJNA729271	stage_E18_vs_E10	ordinary_muscle_development	ENSGALG00010001116	RNF13	0.192502354	0.070667839	2.724044737	0.006448777	0.010499273	positive
PRJNA729271	stage_E18_vs_E10	ordinary_muscle_development	ENSGALG00010001117	P2RY13	0.386415694	0.31865095	1.21266136	0.225259319	0.276518786	positive
PRJNA729271	stage_E18_vs_E10	ordinary_muscle_development	ENSGALG00010001118	P2RY12	0.114681626	0.324092588	0.353854516	0.723447915	0.765944516	positive
PRJNA729271	stage_E18_vs_E10	ordinary_muscle_development	ENSGALG00010001119	COMMD2	-0.404385273	0.099244188	-4.074649403	4.60837E-05	9.95653E-05	negative
PRJNA729271	stage_E18_vs_E10	ordinary_muscle_development	ENSGALG00010001120	GPR87	-1.176204682	0.925818676	-1.270448213	0.203925019	0.253268199	negative
PRJNA729271	stage_E18_vs_E10	ordinary_muscle_development	ENSGALG00010001121	WWTR1	1.173219655	0.091432907	12.83148144	1.09255E-37	1.90114E-36	positive
PRJNA729271	stage_E18_vs_E10	ordinary_muscle_development	ENSGALG00010001122	P2RY14	4.459211939	0.421773406	10.57252989	3.99568E-26	4.02483E-25	positive
PRJNA729271	stage_E18_vs_E10	ordinary_muscle_development	ENSGALG00010001123	CP	0.159699263	0.151894163	1.051385118	0.293081748	0.349618957	positive
PRJNA729271	stage_E18_vs_E10	ordinary_muscle_development	ENSGALG00010001124	GPR171	1.210935368	0.672193838	1.801467524	0.071629222	0.097820385	positive
PRJNA729271	stage_E18_vs_E10	ordinary_muscle_development	ENSGALG00010001125	CLRN1	0.71787896	0.277855763	2.583638909	0.009776409	0.015487328	positive
PRJNA729271	stage_E18_vs_E10	ordinary_muscle_development	ENSGALG00010001126	MINDY4B	1.098918266	1.269662601	0.865519915	0.38675349	0.44693001	positive
PRJNA729271	stage_E18_vs_E10	ordinary_muscle_development	ENSGALG00010001127	SIAH2	0.23671336	0.10384478	2.279492153	0.022637826	0.033824893	positive
PRJNA729271	stage_E18_vs_E10	ordinary_muscle_development	ENSGALG00010001129	TM4SF4	0.838896832	0.979548915	0.856411373	0.391770276	0.451783088	positive

PRJNA729271	stage_E18_vs_E10	ordinary_muscle_development	ENSGALG00010001131	HPS3	0.820535495	0.099279116	8.264935556	1.3977E-16	7.9481E-16	positive
PRJNA729271	stage_E18_vs_E10	ordinary_muscle_development	ENSGALG00010001132	SELENOT	-0.021648817	0.095190094	-0.227427203	0.820091569	0.850457694	negative
PRJNA729271	stage_E18_vs_E10	ordinary_muscle_development	ENSGALG00010001133	EIF2A	-0.63808134	0.075796883	-8.418305761	3.81968E-17	2.25616E-16	negative
PRJNA729271	stage_E18_vs_E10	ordinary_muscle_development	ENSGALG00010001134	ENSGALG00010001134	-0.809023896	0.712575788	-1.135351368	0.256228127	0.31012247	negative
PRJNA729271	stage_E18_vs_E10	ordinary_muscle_development	ENSGALG00010001135	GYG1	1.527115803	0.159437617	9.578139896	9.88084E-22	7.79231E-21	positive
PRJNA729271	stage_E18_vs_E10	ordinary_muscle_development	ENSGALG00010001136	SERP1	-0.196192555	0.125946433	-1.557746021	0.119293466	0.15604718	negative
PRJNA729271	stage_E18_vs_E10	ordinary_muscle_development	ENSGALG00010001138	SPTSSB	-5.851439073	0.994755276	-5.882290062	4.04628E-09	1.29615E-08	negative
PRJNA729271	stage_E18_vs_E10	ordinary_muscle_development	ENSGALG00010001140	NMD3	-0.255662709	0.123188012	-2.075386275	0.037950749	0.054435941	negative
PRJNA729271	stage_E18_vs_E10	ordinary_muscle_development	ENSGALG00010001141	MTAP	-1.245466811	0.113693114	-10.95463713	6.31309E-28	7.00139E-27	negative
PRJNA729271	stage_E18_vs_E10	ordinary_muscle_development	ENSGALG00010001142	PPM1L	2.13002559	0.146205324	14.56872792	4.44083E-48	1.19869E-46	positive
PRJNA729271	stage_E18_vs_E10	ordinary_muscle_development	ENSGALG00010001143	CDKN2A	0.18490235	0.536201062	0.344837717	0.730216377	0.772026723	positive
PRJNA729271	stage_E18_vs_E10	ordinary_muscle_development	ENSGALG00010001144	CDKN2B	2.176666477	1.563549809	1.392131204	0.163882671	0.207864833	positive
PRJNA729271	stage_E18_vs_E10	ordinary_muscle_development	ENSGALG00010001145	TRIM36	1.003105174	0.207600686	4.83189721	1.35238E-06	3.44815E-06	positive
PRJNA729271	stage_E18_vs_E10	ordinary_muscle_development	ENSGALG00010001146	DIPK1C	4.148803682	0.239075655	17.35351798	1.85527E-67	9.20961E-66	positive
PRJNA729271	stage_E18_vs_E10	ordinary_muscle_development	ENSGALG00010001147	PGGT1B	-0.357092338	0.114555929	-3.117187749	0.001825853	0.003227528	negative
PRJNA729271	stage_E18_vs_E10	ordinary_muscle_development	ENSGALG00010001149	CIDEA	1.834978301	0.62079492	2.955852635	0.00311806	0.005327038	positive
PRJNA729271	stage_E18_vs_E10	ordinary_muscle_development	ENSGALG00010001150	ENSGALG00010001150	-1.625914577	0.70179544	-2.316792733	0.020515025	0.030879054	negative
PRJNA729271	stage_E18_vs_E10	ordinary_muscle_development	ENSGALG00010001151	CCDC112	1.293172682	0.216567293	5.971227994	2.35474E-09	7.69259E-09	positive
PRJNA729271	stage_E18_vs_E10	ordinary_muscle_development	ENSGALG00010001152	FEM1C	2.097272865	0.197587496	10.61440075	2.55428E-26	2.60551E-25	positive
PRJNA729271	stage_E18_vs_E10	ordinary_muscle_development	ENSGALG00010001153	TSHZ1	-1.207590465	0.113202468	-10.66752771	1.44418E-26	1.50228E-25	negative
PRJNA729271	stage_E18_vs_E10	ordinary_muscle_development	ENSGALG00010001154	PTGR3	-0.245919729	0.085408516	-2.879334996	0.003985148	0.006691152	negative
PRJNA729271	stage_E18_vs_E10	ordinary_muscle_development	ENSGALG00010001155	CYB5A	-0.185728207	0.094508871	-1.965193381	0.04939186	0.069407175	negative
PRJNA729271	stage_E18_vs_E10	ordinary_muscle_development	ENSGALG00010001156	ALDH7A1	0.470937137	0.181119385	2.600147622	0.009318367	0.014798918	positive
PRJNA729271	stage_E18_vs_E10	ordinary_muscle_development	ENSGALG00010001157	TIMM21	1.160426431	0.092607094	12.53064297	5.07496E-36	8.20999E-35	positive
PRJNA729271	stage_E18_vs_E10	ordinary_muscle_development	ENSGALG00010001158	FRMPD1	-0.415990697	0.378189787	-1.099952222	0.27135294	0.326492974	negative
PRJNA729271	stage_E18_vs_E10	ordinary_muscle_development	ENSGALG00010001159	RTTN	-0.902244807	0.115043235	-7.84265854	4.41106E-15	2.2617E-14	negative
PRJNA729271	stage_E18_vs_E10	ordinary_muscle_development	ENSGALG00010001160	TRMT10B	-0.219554462	0.128646302	-1.706651951	0.087886763	0.11777005	negative
PRJNA729271	stage_E18_vs_E10	ordinary_muscle_development	ENSGALG00010001161	B3GALNT1	-0.055915833	0.100831516	-0.554547181	0.579204425	0.632148795	negative
PRJNA729271	stage_E18_vs_E10	ordinary_muscle_development	ENSGALG00010001162	ENSGALG00010001162	-1.143745775	0.461865243	-2.476362514	0.013272873	0.020636094	negative
PRJNA729271	stage_E18_vs_E10	ordinary_muscle_development	ENSGALG00010001163	ARL14	0.387540344	0.454423266	0.852818005	0.393760237	0.453798372	positive
PRJNA729271	stage_E18_vs_E10	ordinary_muscle_development	ENSGALG00010001164	KPNA4	0.022971628	0.07494818	0.30650015	0.759223865	0.798038429	positive
PRJNA729271	stage_E18_vs_E10	ordinary_muscle_development	ENSGALG00010001165	ENSGALG00010001165	0.836366269	0.161056114	5.193011604	2.06919E-07	5.69558E-07	positive
PRJNA729271	stage_E18_vs_E10	ordinary_muscle_development	ENSGALG00010001166	TRIM59	-0.506216767	0.097173522	-5.209410503	1.89442E-07	5.23139E-07	negative
PRJNA729271	stage_E18_vs_E10	ordinary_muscle_development	ENSGALG00010001167	SMC4	-1.304780818	0.154090458	-8.46762893	2.50439E-17	1.49387E-16	negative
PRJNA729271	stage_E18_vs_E10	ordinary_muscle_development	ENSGALG00010001168	DOK6	-0.803269392	0.147228767	-5.455926896	4.8718E-08	1.41801E-07	negative
PRJNA729271	stage_E18_vs_E10	ordinary_muscle_development	ENSGALG00010001169	ENSGALG00010001169	-0.089000644	0.104059403	-0.855286893	0.392392342	0.452412265	negative
PRJNA729271	stage_E18_vs_E10	ordinary_muscle_development	ENSGALG00010001170	DCAF10	-0.468623823	0.071860005	-6.521344176	6.96801E-11	2.57394E-10	negative
PRJNA729271	stage_E18_vs_E10	ordinary_muscle_development	ENSGALG00010001171	POLR1E	-0.590343051	0.128248199	-4.603129351	4.1619E-06	1.00444E-05	negative
PRJNA729271	stage_E18_vs_E10	ordinary_muscle_development	ENSGALG00010001172	TMEM200C	-3.099559594	0.301873322	-10.2677493	9.84752E-25	9.19716E-24	negative
PRJNA729271	stage_E18_vs_E10	ordinary_muscle_development	ENSGALG00010001173	IFT80	-0.247336962	0.111560249	-2.217070728	0.026618259	0.039274395	negative
PRJNA729271	stage_E18_vs_E10	ordinary_muscle_development	ENSGALG00010001174	ENSGALG00010001174	-1.360266024	0.142209231	-9.56524422	1.11937E-21	8.78941E-21	negative
PRJNA729271	stage_E18_vs_E10	ordinary_muscle_development	ENSGALG00010001177	SEH1L	-0.582077433	0.072470591	-8.031912296	9.5965E-16	5.16529E-15	negative
PRJNA729271	stage_E18_vs_E10	ordinary_muscle_development	ENSGALG00010001178	ZBTB5	0.067147646	0.064789682	1.036394134	0.300018306	0.357012178	positive
PRJNA729271	stage_E18_vs_E10	ordinary_muscle_development	ENSGALG00010001179	GRHPR	0.074820396	0.189913494	0.393970927	0.693602504	0.738975986	positive
PRJNA729271	stage_E18_vs_E10	ordinary_muscle_development	ENSGALG00010001180	SPIRE1	-0.972050426	0.178736798	-5.438446023	5.37473E-08	1.55733E-07	negative
PRJNA729271	stage_E18_vs_E10	ordinary_muscle_development	ENSGALG00010001181	MFSD1	0.608109114	0.104927382	5.795523562	6.81084E-09	2.13594E-08	positive
PRJNA729271	stage_E18_vs_E10	ordinary_muscle_development	ENSGALG00010001182	ZCCHC7	-0.25007824	0.085188192	-2.935597456	0.00332906	0.005649775	negative
PRJNA729271	stage_E18_vs_E10	ordinary_muscle_development	ENSGALG00010001183	RARRES1	0.252750197	0.189866866	1.331196973	0.183124207	0.229896534	positive
PRJNA729271	stage_E18_vs_E10	ordinary_muscle_development	ENSGALG00010001185	PTPN2	-0.779214867	0.068797218	-11.32625541	9.72751E-30	1.17402E-28	negative
PRJNA729271	stage_E18_vs_E10	ordinary_muscle_development	ENSGALG00010001186	ENSGALG00010001186	-0.708045414	0.398573419	-1.776449156	0.075658915	0.102781254	negative
PRJNA729271	stage_E18_vs_E10	ordinary_muscle_development	ENSGALG00010001187	GFM1	-0.234685482	0.182378938	-1.286801453	0.1981635	0.246792175	negative
PRJNA729271	stage_E18_vs_E10	ordinary_muscle_development	ENSGALG00010001188	PSMG2	-0.72499212	0.11767344	-6.161051487	7.22635E-10	2.47743E-09	negative
PRJNA729271	stage_E18_vs_E10	ordinary_muscle_development	ENSGALG00010001190	LXN	-0.956080954	0.453168717	-2.109768214	0.034878326	0.05033466	negative
PRJNA729271	stage_E18_vs_E10	ordinary_muscle_development	ENSGALG00010001191	TOMM5	0.69089232	0.151081257	4.572984983	4.80825E-06	1.15486E-05	positive
PRJNA729271	stage_E18_vs_E10	ordinary_muscle_development	ENSGALG00010001192	ENSGALG00010001192	-0.254802084	0.221821028	-1.148683182	0.250686643	0.304355716	negative
PRJNA729271	stage_E18_vs_E10	ordinary_muscle_development	ENSGALG00010001193	PRELID3A	-2.832534505	0.266982232	-10.6094495	2.69331E-26	2.74154E-25	negative
PRJNA729271	stage_E18_vs_E10	ordinary_muscle_development	ENSGALG00010001194	POT1	-0.933502876	0.11160241	-8.364540481	6.03497E-17	3.51313E-16	negative
PRJNA729271	stage_E18_vs_E10	ordinary_muscle_development	ENSGALG00010001195	ZNF608	-0.41862366	0.142029575	-2.947440057	0.003204169	0.005460838	negative
PRJNA729271	stage_E18_vs_E10	ordinary_muscle_development	ENSGALG00010001196	LSMEM1	4.005415502	0.206583403	19.38885433	9.58481E-84	7.38999E-82	positive
PRJNA729271	stage_E18_vs_E10	ordinary_muscle_development	ENSGALG00010001197	CEP76	-1.123322184	0.149424689	-7.517647808	5.57705E-14	2.61701E-13	negative
PRJNA729271	stage_E18_vs_E10	ordinary_muscle_development	ENSGALG00010001198	IFRD1	-0.403573083	0.176901458	-2.281344018	0.022528098	0.033671353	negative
PRJNA729271	stage_E18_vs_E10	ordinary_muscle_development	ENSGALG00010001199	AFG3L2	-0.28527576	0.091149098	-3.129770509	0.001749429	0.003098861	negative
PRJNA729271	stage_E18_vs_E10	ordinary_muscle_development	ENSGALG00010001200	CTTNBP2	0.427074234	0.128879439	3.313749943	0.000920538	0.00168496	positive
PRJNA729271	stage_E18_vs_E10	ordinary_muscle_development	ENSGALG00010001201	TUBB6	-0.751943807	0.165447209	-4.544916841	5.49569E-06	1.31387E-05	negative
PRJNA729271	stage_E18_vs_E10	ordinary_muscle_development	ENSGALG00010001202	CFTR	1.995449325	0.251580907	7.931640562	2.1627E-15	1.13171E-14	positive
PRJNA729271	stage_E18_vs_E10	ordinary_muscle_development	ENSGALG00010001203	TMX3	-0.718156138	0.100616829	-7.137534985	9.50194E-13	4.08939E-12	negative
PRJNA729271	stage_E18_vs_E10	ordinary_muscle_development	ENSGALG00010001204	SNX2	-0.163781334	0.082005287	-1.997204576	0.045802964	0.064734201	negative
PRJNA729271	stage_E18_vs_E10	ordinary_muscle_development	ENSGALG00010001205	CEP192	-0.707890544	0.106185947	-6.666518155	2.61943E-11	9.98125E-11	negative
PRJNA729271	stage_E18_vs_E10	ordinary_muscle_development	ENSGALG00010001206	RSRC1	-0.039789956	0.077518746	-0.513294631	0.607745239	0.65868605	negative
PRJNA729271	stage_E18_vs_E10	ordinary_muscle_development	ENSGALG00010001208	MOV10L1	-1.846383008	0.735462387	-2.510506373	0.012055815	0.018851028	negative
PRJNA729271	stage_E18_vs_E10	ordinary_muscle_development	ENSGALG00010001209	CDH7	-0.320829908	0.242989986	-1.320342095	0.186720827	0.233804284	negative
PRJNA729271	stage_E18_vs_E10	ordinary_muscle_development	ENSGALG00010001210	PANX2	-3.169327098	0.24988085	-12.68335326	7.31348E-37	1.23123E-35	negative
PRJNA729271	stage_E18_vs_E10	ordinary_muscle_development	ENSGALG00010001213	PP1C	0.131179497	0.218306366	0.600896342	0.547909029	0.602392399	positive
PRJNA729271	stage_E18_vs_E10	ordinary_muscle_development	ENSGALG00010001214	TES	-2.083292168	0.173368779	-12.01653596	2.90913E-33	4.08208E-32	negative
PRJNA729271	stage_E18_vs_E10	ordinary_muscle_development	ENSGALG00010001215	PRDM6	-2.347410317	0.67142021	-3.496186561	0.000471959	0.000900848	negative
PRJNA729271	stage_E18_vs_E10	ordinary_muscle_development	ENSGALG00010001216	SHOX2	-4.162131687	0.483487629	-8.6085588	7.39848E-18	4.57318E-17	negative
PRJNA729271	stage_E18_vs_E10	ordinary_muscle_development	ENSGALG00010001218	ENSGALG00010001218	-0.020435122	0.109745165	-0.186205214	0.852283835	0.877965617	negative
PRJNA729271	stage_E18_vs_E10	ordinary_muscle_development	ENSGALG00010001219	TFEC	-0.66835078	0.443977852	-1.505369641	0.13222907	0.171237413	negative
PRJNA729271	stage_E18_vs_E10	ordinary_muscle_development	ENSGALG00010001220	PLXNB2	-0.243109629	0.082652833	-2.941334503	0.003268014	0.005556582	negative
PRJNA729271	stage_E18_vs_E10	ordinary_muscle_development	ENSGALG00010001221	GRAMD2B	0.300877158	0.154177806	1.951494611	0.050998237	0.07153275	positive
PRJNA729271	stage_E18_vs_E10	ordinary_muscle_development	ENSGALG00010001222	ZNF800	0.219422001	0.097861623	2.242165977	0.024950647	0.037017362	positive
PRJNA729271	stage_E18_vs_E10	ordinary_muscle_development	ENSGALG00010001223	GRM8	-1.029128204	0.501979846	-2.05013849	0.040350918	0.057578909	negative
PRJNA729271	stage_E18_vs_E10	ordinary_muscle_development	ENSGALG00010001224	DNAJB9	0.168891323	0.190155627	0.888174204	0.374447052	0.434347793	positive
PRJNA729271	stage_E18_vs_E10	ordinary_muscle_development	ENSGALG00010001225	SOCS6	-0.327947782	0.098204747	-3.339429013	0.000839508	0.001546996	negative
PRJNA729271	stage_E18_vs_E10	ordinary_muscle_development	ENSGALG00010001226	VEPH1	-1.171427982	0.553236327	-2.11740973	0.034225089	0.049465816	negative
PRJNA729271	stage_E18_vs_E10	ordinary_muscle_development	ENSGALG00010001227	ENSGALG00010001227	-2.873432735	0.924838499	-3.106956228	0.001890244	0.003330791	negative
PRJNA729271	stage_E18_vs_E10	ordinary_muscle_development	ENSGALG00010001228	THAP5	-0.205890312	0.111724369	-1.842841576	0.065352145	0.089763985	negative
PRJNA729271	stage_E18_vs_E10	ordinary_muscle_development	ENSGALG00010001229	ZNF407	-0.311181715	0.104133755	-2.988288635	0.002805445	0.00482727	negative
PRJNA729271	stage_E18_vs_E10	ordinary_muscle_development	ENSGALG00010001230	PTX3	-2.6985441	0.583951589	-4.621177769	3.81568E-06	9.24578E-06	negative
PRJNA729271	stage_E18_vs_E10	ordinary_muscle_development	ENSGALG00010001231	CNDP1	2.197486362	0.512348786	4.28904376	1.79444E-05	4.06095E-05	positive
PRJNA729271	stage_E18_vs_E10	ordinary_muscle_development	ENSGALG00010001232	TSPAN12	1.070887095	0.101416904	10.5592564	4.60294E-26	4.62367E-25	positive
PRJNA729271	stage_E18_vs_E10	ordinary_muscle_development	ENSGALG00010001233	CNDP2	-0.362292552	0.096686579	-3.747082129	0.000178904	0.00036107	negative
PRJNA729271	stage_E18_vs_E10	ordinary_muscle_development	ENSGALG00010001234	AASS	0.427107033	0.09952192	4.291587556	1.774E-05	4.01907E-05	positive
PRJNA729271	stage_E18_vs_E10	ordinary_muscle_development	ENSGALG00010001235	CCDC102B	0.619422315	0.28943224	2.140128947	0.032344347	0.046981793	positive
PRJNA729271	stage_E18_vs_E10	ordinary_muscle_development	ENSGALG00010001237	MELK	-2.123210959	0.271760056	-7.812814708	5.59248E-15	2.84731E-14	negative
PRJNA729271	stage_E18_vs_E10	ordinary_muscle_development	ENSGALG00010001238	GOLIM4	0.399009022	0.121908606	3.273017668	0.001064058	0.001931562	positive
PRJNA729271	stage_E18_vs_E10	ordinary_muscle_development	ENSGALG00010001239	ENSGALG00010001239	0.357038532	0.250973986	1.422611714	0.154848746	0.197525051	positive
PRJNA729271	stage_E18_vs_E10	ordinary_muscle_development	ENSGALG00010001242	ENSGALG00010001242	-0.484746329	0.631521265	-0.767585125	0.442733702	0.502263835	negative
PRJNA729271	stage_E18_vs_E10	ordinary_muscle_development	ENSGALG00010001244	ENSGALG00010001244	1.065329015	0.150345609	7.085867155	1.38176E-12	5.84608E-12	positive
PRJNA729271	stage_E18_vs_E10	ordinary_muscle_development	ENSGALG00010001245	SERPINI1	-0.667782568	0.224686177	-2.972067869	0.002958013	0.005070529	negative
PRJNA729271	stage_E18_vs_E10	ordinary_muscle_development	ENSGALG00010001248	PTPRZ1	0.272378241	0.240196025	1.133983136	0.256801622	0.310712814	positive
PRJNA729271	stage_E18_vs_E10	ordinary_muscle_development	ENSGALG00010001249	PDCD10	-0.059154823	0.139674147	-0.423520201	0.671915763	0.718723267	negative
PRJNA729271	stage_E18_vs_E10	ordinary_muscle_development	ENSGALG00010001250	ENSGALG00010001250	-1.019688485	0.667907666	-1.526690795	0.126837917	0.165155912	negative
PRJNA729271	stage_E18_vs_E10	ordinary_muscle_development	ENSGALG00010001251	ZNF277	-0.424778136	0.125292443	-3.390293342	0.000698179	0.001300951	negative
PRJNA729271	stage_E18_vs_E10	ordinary_muscle_development	ENSGALG00010001252	SNCAIP	-0.439016564	0.122245129	-3.591280623	0.000329057	0.00064057	negative
PRJNA729271	stage_E18_vs_E10	ordinary_muscle_development	ENSGALG00010001253	ENSGALG00010001253	-3.457269747	1.106481149	-3.12456272	0.001780696	0.003151933	negative
PRJNA729271	stage_E18_vs_E10	ordinary_muscle_development	ENSGALG00010001254	NETO1	-1.639150327	0.410915008	-3.989025216	6.63454E-05	0.000141174	negative
PRJNA729271	stage_E18_vs_E10	ordinary_muscle_development	ENSGALG00010001258	ENSGALG00010001258	-0.226192598	0.126451138	-1.788774711	0.073651105	0.100260403	negative
PRJNA729271	stage_E18_vs_E10	ordinary_muscle_development	ENSGALG00010001259	ENSGALG00010001259	0.738121375	0.170299417	4.334256615	1.46253E-05	3.3464E-05	positive
PRJNA729271	stage_E18_vs_E10	ordinary_muscle_development	ENSGALG00010001260	ENSGALG00010001260	-3.165677864	0.908614207	-3.484072602	0.000493846	0.000939408	negative
PRJNA729271	stage_E18_vs_E10	ordinary_muscle_development	ENSGALG00010001262	ENSGALG00010001262	0.617224676	0.145032914	4.255755886	2.08344E-05	4.68645E-05	positive
PRJNA729271	stage_E18_vs_E10	ordinary_muscle_development	ENSGALG00010001264	FAM3C	0.27260074	0.113448718	2.402854305	0.016267667	0.024955004	positive
PRJNA729271	stage_E18_vs_E10	ordinary_muscle_development	ENSGALG00010001265	OPN1LW	0.409102904	0.677086308	0.604210865	0.545703458	0.600422926	positive
PRJNA729271	stage_E18_vs_E10	ordinary_muscle_development	ENSGALG00010001267	BCHE	2.283281546	0.248754291	9.178862963	4.35667E-20	3.0715E-19	positive
PRJNA729271	stage_E18_vs_E10	ordinary_muscle_development	ENSGALG00010001268	CBLN2	-1.788447939	0.42228953	-4.235122612	2.28427E-05	5.10886E-05	negative
PRJNA729271	stage_E18_vs_E10	ordinary_muscle_development	ENSGALG00010001270	DRAP1	-0.540215757	0.111326602	-4.852530716	1.21896E-06	3.12556E-06	negative
PRJNA729271	stage_E18_vs_E10	ordinary_muscle_development	ENSGALG00010001271	ENSGALG00010001271	0.915171765	0.184479997	4.960818411	7.01968E-07	1.84263E-06	positive
PRJNA729271	stage_E18_vs_E10	ordinary_muscle_development	ENSGALG00010001273	ENSGALG00010001273	-0.432226514	0.567527954	-0.761595109	0.446301695	0.505796956	negative
PRJNA729271	stage_E18_vs_E10	ordinary_muscle_development	ENSGALG00010001274	DLGAP1	-2.089481503	0.474881713	-4.400004142	1.08249E-05	2.51292E-05	negative
PRJNA729271	stage_E18_vs_E10	ordinary_muscle_development	ENSGALG00010001276	ENSGALG00010001276	0.842585774	1.191476798	0.70717766			positive
PRJNA729271	stage_E18_vs_E10	ordinary_muscle_development	ENSGALG00010001277	ENSGALG00010001277	0.108288724	0.164195768	0.659509838	0.509568431	0.56651284	positive
PRJNA729271	stage_E18_vs_E10	ordinary_muscle_development	ENSGALG00010001279	LPIN2	-0.595913009	0.138005172	-4.318048391	1.57415E-05	3.59038E-05	negative
PRJNA729271	stage_E18_vs_E10	ordinary_muscle_development	ENSGALG00010001281	LRRN3	0.344746124	0.145545399	2.368650101	0.017853134	0.027151525	positive
PRJNA729271	stage_E18_vs_E10	ordinary_muscle_development	ENSGALG00010001283	ENSGALG00010001283	1.029055949	0.179666198	5.727599077	1.01862E-08	3.14347E-08	positive
PRJNA729271	stage_E18_vs_E10	ordinary_muscle_development	ENSGALG00010001285	ENSGALG00010001285	1.467108452	0.217700377	6.739117632	1.59351E-11	6.17593E-11	positive
PRJNA729271	stage_E18_vs_E10	ordinary_muscle_development	ENSGALG00010001286	SRFBP1	-0.646303249	0.113007335	-5.719126525	1.07073E-08	3.29377E-08	negative
PRJNA729271	stage_E18_vs_E10	ordinary_muscle_development	ENSGALG00010001287	PLPP4	-3.342299997	0.419441091	-7.968461057	1.60662E-15	8.49618E-15	negative
PRJNA729271	stage_E18_vs_E10	ordinary_muscle_development	ENSGALG00010001288	TGIF1	-0.212409173	0.154812752	-1.372039254	0.170051218	0.214899077	negative
PRJNA729271	stage_E18_vs_E10	ordinary_muscle_development	ENSGALG00010001289	ENSGALG00010001289	0.931686425	0.373654005	2.493446912	0.01265095	0.019726282	positive
PRJNA729271	stage_E18_vs_E10	ordinary_muscle_development	ENSGALG00010001291	ENSGALG00010001291	0.541754915	0.144884409	3.739221629	0.000184591	0.000371772	positive
PRJNA729271	stage_E18_vs_E10	ordinary_muscle_development	ENSGALG00010001292	LOX	2.019368633	0.141927014	14.22821897	6.12212E-46	1.50919E-44	positive

PRJNA729271	stage_E18_vs_E10	ordinary_muscle_development	ENSGALG00010001295	ENSGALG00010001295	0.792476935	0.127864267	6.197798295	5.72585E-10	1.97987E-09	positive
PRJNA729271	stage_E18_vs_E10	ordinary_muscle_development	ENSGALG00010001297	MYL12A	-0.322869685	0.222916225	-1.448390246	0.147507941	0.18906425	negative
PRJNA729271	stage_E18_vs_E10	ordinary_muscle_development	ENSGALG00010001298	DOCK4	0.725871353	0.131901189	5.503144881	3.73076E-08	1.10092E-07	positive
PRJNA729271	stage_E18_vs_E10	ordinary_muscle_development	ENSGALG00010001300	MRPL49	0.167727493	0.18173284	0.922934419	0.356041383	0.415391165	positive
PRJNA729271	stage_E18_vs_E10	ordinary_muscle_development	ENSGALG00010001301	GMPS	-0.810329479	0.076976484	-10.52697446	6.48866E-26	6.46858E-25	negative
PRJNA729271	stage_E18_vs_E10	ordinary_muscle_development	ENSGALG00010001302	ENSGALG00010001302	-0.158953782	0.366629562	-0.433554187	0.664612209	0.712119601	negative
PRJNA729271	stage_E18_vs_E10	ordinary_muscle_development	ENSGALG00010001304	SYVN1	-0.044570333	0.178718003	-0.249389164	0.803059767	0.835645555	negative
PRJNA729271	stage_E18_vs_E10	ordinary_muscle_development	ENSGALG00010001305	WNT16	-0.704889983	0.392920846	-1.793974514	0.072817237	0.09927444	negative
PRJNA729271	stage_E18_vs_E10	ordinary_muscle_development	ENSGALG00010001306	CEP120	-0.347173823	0.086770022	-4.001080282	6.3054E-05	0.000134506	negative
PRJNA729271	stage_E18_vs_E10	ordinary_muscle_development	ENSGALG00010001307	CALU	0.035042534	0.155277421	0.225676948	0.821452689	0.85153091	positive
PRJNA729271	stage_E18_vs_E10	ordinary_muscle_development	ENSGALG00010001308	MGMT	-0.075475269	0.139778643	-0.539962807	0.589222682	0.641296199	negative
PRJNA729271	stage_E18_vs_E10	ordinary_muscle_development	ENSGALG00010001313	EMILIN2	0.541092998	0.176735323	3.061600752	0.002201569	0.003841086	positive
PRJNA729271	stage_E18_vs_E10	ordinary_muscle_development	ENSGALG00010001320	CPED1	-0.530317127	0.198805781	-2.66751361	0.007641479	0.012292004	negative
PRJNA729271	stage_E18_vs_E10	ordinary_muscle_development	ENSGALG00010001330	ENSGALG00010001330	0.815699188	0.144252597	5.654658611	1.56156E-08	4.74724E-08	positive
PRJNA729271	stage_E18_vs_E10	ordinary_muscle_development	ENSGALG00010001333	PLCH1	-1.015150738	0.295581625	-3.434417609	0.000593829	0.001116848	negative
PRJNA729271	stage_E18_vs_E10	ordinary_muscle_development	ENSGALG00010001334	EBF3	-1.035102589	0.159464906	-6.491099597	8.52121E-11	3.12459E-10	negative
PRJNA729271	stage_E18_vs_E10	ordinary_muscle_development	ENSGALG00010001343	ENSGALG00010001343	0.223015907	0.286948537	0.777198272	0.437041801	0.496611956	positive
PRJNA729271	stage_E18_vs_E10	ordinary_muscle_development	ENSGALG00010001347	MYOM1	2.145103444	0.164816839	13.01507457	1.00444E-38	1.83137E-37	positive
PRJNA729271	stage_E18_vs_E10	ordinary_muscle_development	ENSGALG00010001349	ING3	0.891567361	0.104644542	8.519960432	1.59608E-17	9.66785E-17	positive
PRJNA729271	stage_E18_vs_E10	ordinary_muscle_development	ENSGALG00010001351	CAPN1	0.91886937	0.156664692	5.865197548	4.48598E-09	1.43139E-08	positive
PRJNA729271	stage_E18_vs_E10	ordinary_muscle_development	ENSGALG00010001353	GLRX3	-0.54908603	0.07965013	-6.893724198	5.43504E-12	2.19384E-11	negative
PRJNA729271	stage_E18_vs_E10	ordinary_muscle_development	ENSGALG00010001358	TCERG1L	-1.309459887	0.484687049	-2.701660567	0.006899416	0.011172722	negative
PRJNA729271	stage_E18_vs_E10	ordinary_muscle_development	ENSGALG00010001363	HIBADH	0.978205789	0.210995861	4.636137334	3.5498E-06	8.62894E-06	positive
PRJNA729271	stage_E18_vs_E10	ordinary_muscle_development	ENSGALG00010001364	SMCHD1	-0.662849081	0.18493612	-3.584205625	0.000338106	0.000656925	negative
PRJNA729271	stage_E18_vs_E10	ordinary_muscle_development	ENSGALG00010001365	ENSGALG00010001365	1.789527702	0.269064375	6.650927685	2.91251E-11	1.10437E-10	positive
PRJNA729271	stage_E18_vs_E10	ordinary_muscle_development	ENSGALG00010001367	ENSGALG00010001367	-0.573938218	0.445780656	-1.287490181	0.19792349	0.246532696	negative
PRJNA729271	stage_E18_vs_E10	ordinary_muscle_development	ENSGALG00010001368	WIPF3	1.212231618	0.141013404	8.596570156	8.21339E-18	5.05748E-17	positive
PRJNA729271	stage_E18_vs_E10	ordinary_muscle_development	ENSGALG00010001369	PPP2R2D	-0.436969673	0.124796996	-3.501443846	0.000462744	0.000884892	negative
PRJNA729271	stage_E18_vs_E10	ordinary_muscle_development	ENSGALG00010001372	ENSGALG00010001372	1.193058642	0.291338955	4.095087948	4.22008E-05	9.15857E-05	positive
PRJNA729271	stage_E18_vs_E10	ordinary_muscle_development	ENSGALG00010001373	IMPA2	0.472278955	0.140207522	3.368428092	0.000755981	0.001402636	positive
PRJNA729271	stage_E18_vs_E10	ordinary_muscle_development	ENSGALG00010001378	POLA2	-1.134530126	0.130746578	-8.677321747	4.05198E-18	2.55807E-17	negative
PRJNA729271	stage_E18_vs_E10	ordinary_muscle_development	ENSGALG00010001380	SCRN1	-1.909906596	0.160327158	-11.91255817	1.01805E-32	1.39609E-31	negative
PRJNA729271	stage_E18_vs_E10	ordinary_muscle_development	ENSGALG00010001381	MDFIC	-0.199021326	0.130647157	-1.523349841	0.127671204	0.166075743	negative
PRJNA729271	stage_E18_vs_E10	ordinary_muscle_development	ENSGALG00010001382	BNIP3	1.843084026	0.193087292	9.545340877	1.35661E-21	1.05948E-20	positive
PRJNA729271	stage_E18_vs_E10	ordinary_muscle_development	ENSGALG00010001385	MME	0.932024712	0.256736438	3.630278268	0.000283116	0.00055682	positive
PRJNA729271	stage_E18_vs_E10	ordinary_muscle_development	ENSGALG00010001386	FKBP14	-1.11856677	0.184993907	-6.046506021	1.48021E-09	4.92327E-09	negative
PRJNA729271	stage_E18_vs_E10	ordinary_muscle_development	ENSGALG00010001388	MPPE1	0.916255817	0.164741708	5.561772007	2.67049E-08	7.96804E-08	positive
PRJNA729271	stage_E18_vs_E10	ordinary_muscle_development	ENSGALG00010001395	GNAL	0.135946112	0.106582511	1.275501112	0.202131931	0.251278074	positive
PRJNA729271	stage_E18_vs_E10	ordinary_muscle_development	ENSGALG00010001397	KLHL7	-0.078946575	0.064294628	-1.227887581	0.219489172	0.270282521	negative
PRJNA729271	stage_E18_vs_E10	ordinary_muscle_development	ENSGALG00010001398	WDR11	-0.517187717	0.09744593	-5.307432715	1.1118E-07	3.14204E-07	negative
PRJNA729271	stage_E18_vs_E10	ordinary_muscle_development	ENSGALG00010001400	SNX24	0.593369428	0.16019551	3.704032825	0.000212199	0.000424017	positive
PRJNA729271	stage_E18_vs_E10	ordinary_muscle_development	ENSGALG00010001402	NUP42	-1.076419483	0.144111603	-7.469346425	8.05941E-14	3.74067E-13	negative
PRJNA729271	stage_E18_vs_E10	ordinary_muscle_development	ENSGALG00010001406	EML3	0.946553448	0.159909264	5.919315886	3.23284E-09	1.04388E-08	positive
PRJNA729271	stage_E18_vs_E10	ordinary_muscle_development	ENSGALG00010001407	FOXP2	-0.876709203	0.150226949	-5.835898348	5.35015E-09	1.69509E-08	negative
PRJNA729271	stage_E18_vs_E10	ordinary_muscle_development	ENSGALG00010001409	GPNMB	-3.822643812	1.008227305	-3.791450392	0.00014977	0.000305247	negative
PRJNA729271	stage_E18_vs_E10	ordinary_muscle_development	ENSGALG00010001411	FGFR2	-3.428273394	0.365475818	-9.380301582	6.57843E-21	4.92787E-20	negative
PRJNA729271	stage_E18_vs_E10	ordinary_muscle_development	ENSGALG00010001412	ENSGALG00010001412	-1.490932784	0.22409511	-6.653125025	2.86934E-11	1.08906E-10	negative
PRJNA729271	stage_E18_vs_E10	ordinary_muscle_development	ENSGALG00010001414	CCDC126	-0.056970676	0.176519651	-0.322744099	0.746889052	0.787070651	negative
PRJNA729271	stage_E18_vs_E10	ordinary_muscle_development	ENSGALG00010001415	MC5R	0.286308217	0.266133645	1.075806171	0.282013952	0.337555098	positive
PRJNA729271	stage_E18_vs_E10	ordinary_muscle_development	ENSGALG00010001416	AP4M1	0.514432359	0.092684742	5.550345703	2.85105E-08	8.49281E-08	positive
PRJNA729271	stage_E18_vs_E10	ordinary_muscle_development	ENSGALG00010001418	RNMT	-0.884672224	0.067517062	-13.10294308	3.16733E-39	5.87091E-38	negative
PRJNA729271	stage_E18_vs_E10	ordinary_muscle_development	ENSGALG00010001419	KCND2	-0.684038885	0.220664892	-3.099899024	0.001935866	0.003404147	negative
PRJNA729271	stage_E18_vs_E10	ordinary_muscle_development	ENSGALG00010001420	FAM221A	-4.004340234	0.623634152	-6.420976495	1.35403E-10	4.88468E-10	negative
PRJNA729271	stage_E18_vs_E10	ordinary_muscle_development	ENSGALG00010001422	AP1S1	-0.842991061	0.140282766	-6.009227545	1.86409E-09	6.14092E-09	negative
PRJNA729271	stage_E18_vs_E10	ordinary_muscle_development	ENSGALG00010001423	FAM210A	0.925409557	0.137183179	6.745794643	1.52192E-11	5.90793E-11	positive
PRJNA729271	stage_E18_vs_E10	ordinary_muscle_development	ENSGALG00010001426	ATE1	-0.060755823	0.091622801	-0.663108114	0.507261314	0.564294148	negative
PRJNA729271	stage_E18_vs_E10	ordinary_muscle_development	ENSGALG00010001427	ASB11	5.852113909	0.467868711	12.5080258	6.74805E-36	1.08561E-34	positive
PRJNA729271	stage_E18_vs_E10	ordinary_muscle_development	ENSGALG00010001432	NSMCE4A	-0.238181024	0.116095332	-2.051598638	0.040208685	0.057398551	negative
PRJNA729271	stage_E18_vs_E10	ordinary_muscle_development	ENSGALG00010001436	ENSGALG00010001436	0.529140698	1.231808641	0.429564042	0.667512799	0.71464643	positive
PRJNA729271	stage_E18_vs_E10	ordinary_muscle_development	ENSGALG00010001440	CDCA7L	-1.107933236	0.150037703	-7.384365502	1.53182E-13	6.94953E-13	negative
PRJNA729271	stage_E18_vs_E10	ordinary_muscle_development	ENSGALG00010001441	HMGCLL1	-1.055187857	0.305606398	-3.452767558	0.000554867	0.00104744	negative
PRJNA729271	stage_E18_vs_E10	ordinary_muscle_development	ENSGALG00010001442	LDLRAD4	0.908375458	0.113813966	7.981230125	1.44882E-15	7.68973E-15	positive
PRJNA729271	stage_E18_vs_E10	ordinary_muscle_development	ENSGALG00010001443	ANKRD26	0.089964173	0.145471619	0.618431096	0.536291205	0.591728783	positive
PRJNA729271	stage_E18_vs_E10	ordinary_muscle_development	ENSGALG00010001448	GPR37	-2.669823757	0.326998189	-8.164643874	3.22385E-16	1.7863E-15	negative
PRJNA729271	stage_E18_vs_E10	ordinary_muscle_development	ENSGALG00010001450	GPR149	0.073576927	0.442866053	0.166138106	0.868048267	0.89116507	positive
PRJNA729271	stage_E18_vs_E10	ordinary_muscle_development	ENSGALG00010001453	TAX1BP1	0.018087452	0.074080808	0.244158404	0.807108141	0.839362355	positive
PRJNA729271	stage_E18_vs_E10	ordinary_muscle_development	ENSGALG00010001454	VEGFD	0.622584678	0.169024734	3.683393927	0.000230149	0.000457803	positive
PRJNA729271	stage_E18_vs_E10	ordinary_muscle_development	ENSGALG00010001456	LSM8	-0.956450876	0.116630557	-8.200688555	2.39014E-16	1.33456E-15	negative
PRJNA729271	stage_E18_vs_E10	ordinary_muscle_development	ENSGALG00010001457	NDC80	-2.013938568	0.226332702	-8.89813337	5.67937E-19	3.77973E-18	negative
PRJNA729271	stage_E18_vs_E10	ordinary_muscle_development	ENSGALG00010001458	JAZF1	0.011458931	0.156076758	0.073418558	0.94147305	0.95217711	positive
PRJNA729271	stage_E18_vs_E10	ordinary_muscle_development	ENSGALG00010001460	MALSU1	-0.639154515	0.108247701	-5.904555088	3.536E-09	1.13949E-08	negative
PRJNA729271	stage_E18_vs_E10	ordinary_muscle_development	ENSGALG00010001461	ASB9	-2.828302102	0.232531889	-12.16307199	4.88851E-34	7.20844E-33	negative
PRJNA729271	stage_E18_vs_E10	ordinary_muscle_development	ENSGALG00010001462	DHX36	-0.728834842	0.109584127	-6.650916183	2.91274E-11	1.10437E-10	negative
PRJNA729271	stage_E18_vs_E10	ordinary_muscle_development	ENSGALG00010001463	DSEL	0.231004984	0.15841162	1.458257825	0.144769491	0.185932462	positive
PRJNA729271	stage_E18_vs_E10	ordinary_muscle_development	ENSGALG00010001465	TACC2	1.178899832	0.157604789	7.480101593	7.42652E-14	3.45356E-13	positive
PRJNA729271	stage_E18_vs_E10	ordinary_muscle_development	ENSGALG00010001466	PRKX	-0.018233028	0.130691391	-0.139512085	0.889045507	0.90841073	negative
PRJNA729271	stage_E18_vs_E10	ordinary_muscle_development	ENSGALG00010001468	ANOS1	0.061652909	0.152204435	0.40506644	0.685428674	0.731557958	positive
PRJNA729271	stage_E18_vs_E10	ordinary_muscle_development	ENSGALG00010001469	ARHGEF26	0.505474692	0.122722448	4.118844603	3.80777E-05	8.30478E-05	positive
PRJNA729271	stage_E18_vs_E10	ordinary_muscle_development	ENSGALG00010001470	GPR85	-1.040562364	0.320840773	-3.243236055	0.001181803	0.002133279	negative
PRJNA729271	stage_E18_vs_E10	ordinary_muscle_development	ENSGALG00010001471	BMP5	-2.827209786	0.371993859	-7.600151766	2.95784E-14	1.41358E-13	negative
PRJNA729271	stage_E18_vs_E10	ordinary_muscle_development	ENSGALG00010001472	ENSGALG00010001472	1.077205449	0.184672544	5.833056865	5.4421E-09	1.72347E-08	positive
PRJNA729271	stage_E18_vs_E10	ordinary_muscle_development	ENSGALG00010001475	BMT2	-0.667342333	0.137823856	-4.841994361	1.28542E-06	3.28727E-06	negative
PRJNA729271	stage_E18_vs_E10	ordinary_muscle_development	ENSGALG00010001476	IGF2BP3	-0.923617316	0.193476151	-4.773804482	1.80778E-06	4.54217E-06	negative
PRJNA729271	stage_E18_vs_E10	ordinary_muscle_development	ENSGALG00010001477	NLGN4	-0.513483596	0.215176984	-2.386331407	0.017017408	0.025984128	negative
PRJNA729271	stage_E18_vs_E10	ordinary_muscle_development	ENSGALG00010001479	TMEM168	-0.440996881	0.156074499	-2.825553724	0.004719897	0.007845747	negative
PRJNA729271	stage_E18_vs_E10	ordinary_muscle_development	ENSGALG00010001480	JAKMIP3	0.265104088	0.27812242	0.953192078	0.34049276	0.398855871	positive
PRJNA729271	stage_E18_vs_E10	ordinary_muscle_development	ENSGALG00010001482	TRA2A	-0.57655198	0.065799793	-8.762215664	1.9145E-18	1.23336E-17	negative
PRJNA729271	stage_E18_vs_E10	ordinary_muscle_development	ENSGALG00010001483	ENSGALG00010001483	-0.762892681	0.271295384	-2.812037091	0.004922883	0.008167241	negative
PRJNA729271	stage_E18_vs_E10	ordinary_muscle_development	ENSGALG00010001486	WNT2	1.811991506	0.262630617	6.899391732	5.22257E-12	2.11161E-11	positive
PRJNA729271	stage_E18_vs_E10	ordinary_muscle_development	ENSGALG00010001487	CDH19	0.775575461	0.229626706	3.377549045	0.000731349	0.001359263	positive
PRJNA729271	stage_E18_vs_E10	ordinary_muscle_development	ENSGALG00010001488	GRPR	-3.411157629	0.447014375	-7.630979714	2.32976E-14	1.12491E-13	negative
PRJNA729271	stage_E18_vs_E10	ordinary_muscle_development	ENSGALG00010001491	PHF3	0.239937483	0.088418941	2.713643482	0.006654774	0.010812796	positive
PRJNA729271	stage_E18_vs_E10	ordinary_muscle_development	ENSGALG00010001492	ENSGALG00010001492	1.366601156	0.290163399	4.709764091	2.48004E-06	6.13554E-06	positive
PRJNA729271	stage_E18_vs_E10	ordinary_muscle_development	ENSGALG00010001495	CCNL1	0.411032944	0.110475453	3.720581662	0.000198764	0.000398767	positive
PRJNA729271	stage_E18_vs_E10	ordinary_muscle_development	ENSGALG00010001496	PTP4A1	0.183512543	0.190618185	0.962723166	0.335686468	0.394117556	positive
PRJNA729271	stage_E18_vs_E10	ordinary_muscle_development	ENSGALG00010001497	LEKR1	0.48887291	0.261567371	1.86901336	0.061620956	0.085049632	positive
PRJNA729271	stage_E18_vs_E10	ordinary_muscle_development	ENSGALG00010001499	TIPARP	1.848045976	0.186340988	9.917549505	3.49225E-23	2.95851E-22	positive
PRJNA729271	stage_E18_vs_E10	ordinary_muscle_development	ENSGALG00010001501	CAPZA2	0.011055824	0.106661785	0.103653093	0.917444652	0.931845017	positive
PRJNA729271	stage_E18_vs_E10	ordinary_muscle_development	ENSGALG00010001502	LGSN	3.42494393	0.897606362	3.815641329	0.00013583	0.000278677	positive
PRJNA729271	stage_E18_vs_E10	ordinary_muscle_development	ENSGALG00010001504	RBMS3	0.356554799	0.098867943	3.606374194	0.000310505	0.000606655	positive
PRJNA729271	stage_E18_vs_E10	ordinary_muscle_development	ENSGALG00010001505	SSR3	0.099436107	0.096882182	1.026361141	0.304721387	0.361954971	positive
PRJNA729271	stage_E18_vs_E10	ordinary_muscle_development	ENSGALG00010001506	DPYSL4	1.295889838	0.246597538	5.255080199	1.4796E-07	4.11647E-07	positive
PRJNA729271	stage_E18_vs_E10	ordinary_muscle_development	ENSGALG00010001507	METTL4	-0.405495678	0.101827529	-3.982181269	6.82857E-05	0.000145047	negative
PRJNA729271	stage_E18_vs_E10	ordinary_muscle_development	ENSGALG00010001508	CD109	-1.339823441	0.291161934	-4.601643575	4.1917E-06	1.01096E-05	negative
PRJNA729271	stage_E18_vs_E10	ordinary_muscle_development	ENSGALG00010001510	AP1S2	-0.185002276	0.099120787	-1.86643268	0.061980855	0.085489817	negative
PRJNA729271	stage_E18_vs_E10	ordinary_muscle_development	ENSGALG00010001512	PLCL2	1.694366815	0.173423475	9.770112212	1.51284E-22	1.24101E-21	positive
PRJNA729271	stage_E18_vs_E10	ordinary_muscle_development	ENSGALG00010001513	ZRSR2	-0.024166825	0.093764753	-0.257738903	0.79660842	0.83005097	negative
PRJNA729271	stage_E18_vs_E10	ordinary_muscle_development	ENSGALG00010001514	MET	1.522092952	0.150741039	10.09740254	5.67247E-24	5.03506E-23	positive
PRJNA729271	stage_E18_vs_E10	ordinary_muscle_development	ENSGALG00010001516	CLTRN	-1.403958904	0.581746271	-2.413352648	0.015806518	0.024304177	negative
PRJNA729271	stage_E18_vs_E10	ordinary_muscle_development	ENSGALG00010001517	CAV2	2.304825844	0.164346729	14.02416624	1.10908E-44	2.5763E-43	positive
PRJNA729271	stage_E18_vs_E10	ordinary_muscle_development	ENSGALG00010001518	SLC17A5	-0.810894437	0.166710825	-4.864077874	1.14992E-06	2.95271E-06	negative
PRJNA729271	stage_E18_vs_E10	ordinary_muscle_development	ENSGALG00010001520	KCNAB1	-1.441971977	0.200847648	-7.17943172	7.00018E-13	3.03613E-12	negative
PRJNA729271	stage_E18_vs_E10	ordinary_muscle_development	ENSGALG00010001521	TBC1D5	-0.394702336	0.075155558	-5.251805033	1.50616E-07	4.18795E-07	negative
PRJNA729271	stage_E18_vs_E10	ordinary_muscle_development	ENSGALG00010001524	ACE2	-0.135674916	1.65901423	-0.081780441	0.93482131	0.946509841	negative
PRJNA729271	stage_E18_vs_E10	ordinary_muscle_development	ENSGALG00010001525	EEF1A1	0.217415305	0.176696387	1.230445674	0.218530259	0.269307839	positive
PRJNA729271	stage_E18_vs_E10	ordinary_muscle_development	ENSGALG00010001526	PPP1R3A	5.222830977	0.149344364	34.97173137	6.0536E-268	7.3123E-265	positive
PRJNA729271	stage_E18_vs_E10	ordinary_muscle_development	ENSGALG00010001527	STK32C	1.937053193	0.217733487	8.896441338	5.76659E-19	3.83601E-18	positive
PRJNA729271	stage_E18_vs_E10	ordinary_muscle_development	ENSGALG00010001528	NR1D2	0.645424436	0.183181573	3.523413556	0.000426026	0.000817914	positive
PRJNA729271	stage_E18_vs_E10	ordinary_muscle_development	ENSGALG00010001531	ENSGALG00010001531	30	5.461707949	5.492787289			positive
PRJNA729271	stage_E18_vs_E10	ordinary_muscle_development	ENSGALG00010001532	ENSGALG00010001532	0.229994467	0.27659849	0.831510202	0.405685468	0.465701343	positive
PRJNA729271	stage_E18_vs_E10	ordinary_muscle_development	ENSGALG00010001533	ENSGALG00010001533	0.613856161	0.150860882	4.069021414	4.7211E-05	0.000101925	positive
PRJNA729271	stage_E18_vs_E10	ordinary_muscle_development	ENSGALG00010001534	SLITRK3	-0.968152551	0.265520533	-3.646243626	0.000266102	0.000525496	negative
PRJNA729271	stage_E18_vs_E10	ordinary_muscle_development	ENSGALG00010001535	RAP2B	-0.021136092	0.104852083	-0.20158009	0.840245012	0.867846049	negative
PRJNA729271	stage_E18_vs_E10	ordinary_muscle_development	ENSGALG00010001540	TRABD	-1.234398985	0.132113405	-9.343480227	9.32185E-21	6.90446E-20	negative
PRJNA729271	stage_E18_vs_E10	ordinary_muscle_development	ENSGALG00010001542	MTO1	0.018856974	0.129184829	0.145968948	0.883945901	0.90421989	positive
PRJNA729271	stage_E18_vs_E10	ordinary_muscle_development	ENSGALG00010001545	SELENOO	0.344433016	0.141979609	2.425932973	0.01526909	0.023547767	positive
PRJNA729271	stage_E18_vs_E10	ordinary_muscle_development	ENSGALG00010001546	CGAS	-0.674445244	0.166902413	-4.040955607	5.32338E-05	0.000114332	negative
PRJNA729271	stage_E18_vs_E10	ordinary_muscle_development	ENSGALG00010001547	PWWP2B	1.143403027	0.109735641	10.4196141	2.0177E-25	1.96418E-24	positive
PRJNA729271	stage_E18_vs_E10	ordinary_muscle_development	ENSGALG00010001548	AKAP17A	-0.41423481	0.097433886	-4.25144504	2.12396E-05	4.77092E-05	negative
PRJNA729271	stage_E18_vs_E10	ordinary_muscle_development	ENSGALG00010001549	ENSGALG00010001549	-0.715638487	0.486731399	-1.470294475	0.141482017	0.182129825	negative
PRJNA729271	stage_E18_vs_E10	ordinary_muscle_development	ENSGALG00010001551	FHIP1B	0.980225181	0.161158486	6.082367758	1.18421E-09	3.96697E-09	positive

PRJNA729271	stage_E18_vs_E10	ordinary_muscle_development	ENSGALG00010001552	TUBGCP6	-0.174863068	0.109842306	-1.591946438	0.11139674	0.14651082	negative
PRJNA729271	stage_E18_vs_E10	ordinary_muscle_development	ENSGALG00010001553	CNGA4	0.789701241	0.398628836	1.981043941	0.047586346	0.067065055	positive
PRJNA729271	stage_E18_vs_E10	ordinary_muscle_development	ENSGALG00010001554	ENSGALG00010001554	0.834465391	0.182450087	4.573663977	4.79268E-06	1.1515E-05	positive
PRJNA729271	stage_E18_vs_E10	ordinary_muscle_development	ENSGALG00010001556	EPB41L3	-0.166925589	0.117158354	-1.424786053	0.154219055	0.196795951	negative
PRJNA729271	stage_E18_vs_E10	ordinary_muscle_development	ENSGALG00010001557	THRB	-1.28349699	0.322086939	-3.984939571	6.74973E-05	0.00014352	negative
PRJNA729271	stage_E18_vs_E10	ordinary_muscle_development	ENSGALG00010001558	CCKBR	-0.224269215	0.554870861	-0.404182723	0.686078358	0.731935475	negative
PRJNA729271	stage_E18_vs_E10	ordinary_muscle_development	ENSGALG00010001559	HDAC10	-0.541311983	0.124185013	-4.35891555	1.30709E-05	3.00733E-05	negative
PRJNA729271	stage_E18_vs_E10	ordinary_muscle_development	ENSGALG00010001567	INPP5A	0.003142949	0.082644192	0.038029886	0.969663854	0.974842389	positive
PRJNA729271	stage_E18_vs_E10	ordinary_muscle_development	ENSGALG00010001570	SMPD1	1.172576032	0.163228082	7.183666041	6.78665E-13	2.94529E-12	positive
PRJNA729271	stage_E18_vs_E10	ordinary_muscle_development	ENSGALG00010001585	ENSGALG00010001585	-1.305335566	0.141013533	-9.256810602	2.1063E-20	1.52349E-19	negative
PRJNA729271	stage_E18_vs_E10	ordinary_muscle_development	ENSGALG00010001586	ZBTB14	-0.054065286	0.096873845	-0.558099929	0.576776149	0.629783072	negative
PRJNA729271	stage_E18_vs_E10	ordinary_muscle_development	ENSGALG00010001588	ENSGALG00010001588	1.371104595	0.583898524	2.348189862	0.018864902	0.028555426	positive
PRJNA729271	stage_E18_vs_E10	ordinary_muscle_development	ENSGALG00010001595	AKAIN1	0.437145365	0.282243956	1.548820995	0.121424757	0.158606097	positive
PRJNA729271	stage_E18_vs_E10	ordinary_muscle_development	ENSGALG00010001599	TBLX1	-0.094129012	0.075046218	-1.254280557	0.209740048	0.259667065	negative
PRJNA729271	stage_E18_vs_E10	ordinary_muscle_development	ENSGALG00010001604	ENSGALG00010001604	4.409851126	0.428966715	10.28017087	8.6574E-25	8.12752E-24	positive
PRJNA729271	stage_E18_vs_E10	ordinary_muscle_development	ENSGALG00010001605	APBB1	0.28346387	0.113133306	2.505574009	0.012225281	0.019093357	positive
PRJNA729271	stage_E18_vs_E10	ordinary_muscle_development	ENSGALG00010001606	PIGA	-0.224029296	0.110273532	-2.031578132	0.042196382	0.059934989	negative
PRJNA729271	stage_E18_vs_E10	ordinary_muscle_development	ENSGALG00010001608	ENSGALG00010001608	0.424840048	0.477552227	0.889620075	0.373669923	0.433585137	positive
PRJNA729271	stage_E18_vs_E10	ordinary_muscle_development	ENSGALG00010001609	ENSGALG00010001609	0.890326011	0.468743	1.899390519	0.057513149	0.079890091	positive
PRJNA729271	stage_E18_vs_E10	ordinary_muscle_development	ENSGALG00010001611	MAPK12	-0.993669192	0.162578397	-6.11193869	9.8428E-10	3.32335E-09	negative
PRJNA729271	stage_E18_vs_E10	ordinary_muscle_development	ENSGALG00010001612	HPX	2.66019507	0.936038746	2.841971107	0.004483555	0.007477751	positive
PRJNA729271	stage_E18_vs_E10	ordinary_muscle_development	ENSGALG00010001616	OPNVA	-1.096307866	0.577482267	-1.898426892	0.05763987	0.080035436	negative
PRJNA729271	stage_E18_vs_E10	ordinary_muscle_development	ENSGALG00010001617	TRIM3	-0.347068517	0.09391813	-3.695436837	0.000219509	0.00043772	negative
PRJNA729271	stage_E18_vs_E10	ordinary_muscle_development	ENSGALG00010001620	MOSPD2	0.984816532	0.143195365	6.877433018	6.09407E-12	2.44353E-11	positive
PRJNA729271	stage_E18_vs_E10	ordinary_muscle_development	ENSGALG00010001621	DLGAP2	1.150489563	0.234921315	4.897340048	9.71427E-07	2.51443E-06	positive
PRJNA729271	stage_E18_vs_E10	ordinary_muscle_development	ENSGALG00010001627	ARFIP2	-0.176954069	0.120528762	-1.468148058	0.14206401	0.182781628	negative
PRJNA729271	stage_E18_vs_E10	ordinary_muscle_development	ENSGALG00010001628	MAPK11	-0.922615289	0.207408068	-4.44830955	8.65487E-06	2.02932E-05	negative
PRJNA729271	stage_E18_vs_E10	ordinary_muscle_development	ENSGALG00010001632	FANCB	1.851499122	0.269714914	6.864652356	6.66534E-12	2.66008E-11	positive
PRJNA729271	stage_E18_vs_E10	ordinary_muscle_development	ENSGALG00010001633	B3GAT2	-1.65509495	0.372096452	-4.448026689	8.66628E-06	2.03166E-05	negative
PRJNA729271	stage_E18_vs_E10	ordinary_muscle_development	ENSGALG00010001635	TIMM10B	0.183401123	0.150241848	1.220705986	0.222197361	0.273106991	positive
PRJNA729271	stage_E18_vs_E10	ordinary_muscle_development	ENSGALG00010001639	ENSGALG00010001639	-1.358365337	0.163487435	-8.308683372	9.67697E-17	5.55736E-16	negative
PRJNA729271	stage_E18_vs_E10	ordinary_muscle_development	ENSGALG00010001642	ENSGALG00010001642	1.079095441	0.121129814	8.908586625	5.16872E-19	3.45097E-18	positive
PRJNA729271	stage_E18_vs_E10	ordinary_muscle_development	ENSGALG00010001647	ARL8BL	1.110545207	0.150551005	7.376538001	1.62459E-13	7.35656E-13	positive
PRJNA729271	stage_E18_vs_E10	ordinary_muscle_development	ENSGALG00010001649	UBE2E2	0.318101486	0.214995485	1.479572866	0.138987272	0.179287494	positive
PRJNA729271	stage_E18_vs_E10	ordinary_muscle_development	ENSGALG00010001654	RPS11	0.948091908	0.169381267	5.597383506	2.17611E-08	6.53193E-08	positive
PRJNA729271	stage_E18_vs_E10	ordinary_muscle_development	ENSGALG00010001657	SMAP1	-0.415559777	0.097622002	-4.256824964	2.07351E-05	4.66627E-05	negative
PRJNA729271	stage_E18_vs_E10	ordinary_muscle_development	ENSGALG00010001661	TCTN3	0.099766832	0.104091262	0.958455392	0.337833175	0.396252781	positive
PRJNA729271	stage_E18_vs_E10	ordinary_muscle_development	ENSGALG00010001664	SPAM1	1.14092924	2.0670095	0.551970971	0.580968242	0.633978369	positive
PRJNA729271	stage_E18_vs_E10	ordinary_muscle_development	ENSGALG00010001666	PIEZO2	-1.103265857	0.182496101	-6.045421527	1.4902E-09	4.95423E-09	negative
PRJNA729271	stage_E18_vs_E10	ordinary_muscle_development	ENSGALG00010001668	UBE2E1	0.146824494	0.085593217	1.715375335	0.086276435	0.115783438	positive
PRJNA729271	stage_E18_vs_E10	ordinary_muscle_development	ENSGALG00010001670	SHOX	-1.00368044	0.162625308	-6.171735817	6.75443E-10	2.32279E-09	negative
PRJNA729271	stage_E18_vs_E10	ordinary_muscle_development	ENSGALG00010001672	ENSGALG00010001672	1.368334424	0.594279642	2.302509337	0.021306465	0.031980658	positive
PRJNA729271	stage_E18_vs_E10	ordinary_muscle_development	ENSGALG00010001674	ENSGALG00010001674	0.115573806	0.106106323	1.089226376	0.27605407	0.33156696	positive
PRJNA729271	stage_E18_vs_E10	ordinary_muscle_development	ENSGALG00010001677	SDHAF4	0.092473011	0.108252122	0.854237393	0.392973468	0.45293817	positive
PRJNA729271	stage_E18_vs_E10	ordinary_muscle_development	ENSGALG00010001678	SYAP1	0.475617709	0.059118002	8.045226385	8.60861E-16	4.64737E-15	positive
PRJNA729271	stage_E18_vs_E10	ordinary_muscle_development	ENSGALG00010001681	NAPG	-0.285592945	0.092415629	-3.09031004	0.001999477	0.003510892	negative
PRJNA729271	stage_E18_vs_E10	ordinary_muscle_development	ENSGALG00010001686	WASL	0.124134012	0.086890608	1.428624043	0.15311232	0.195590295	positive
PRJNA729271	stage_E18_vs_E10	ordinary_muscle_development	ENSGALG00010001688	ENSGALG00010001688	-0.27624982	0.165889522	-1.665263829	0.095860132	0.127616882	negative
PRJNA729271	stage_E18_vs_E10	ordinary_muscle_development	ENSGALG00010001689	ILK	0.101801372	0.068006298	1.496940357	0.134408778	0.173827198	positive
PRJNA729271	stage_E18_vs_E10	ordinary_muscle_development	ENSGALG00010001691	CTPS2	1.503492389	0.1329959	11.30480253	1.24233E-29	1.49317E-28	positive
PRJNA729271	stage_E18_vs_E10	ordinary_muscle_development	ENSGALG00010001692	ALDH18A1	-0.549472497	0.254786195	-2.156602311	0.031036664	0.045231897	negative
PRJNA729271	stage_E18_vs_E10	ordinary_muscle_development	ENSGALG00010001693	NKIRAS1	0.439986844	0.201829761	2.179989922	0.029258209	0.042825178	positive
PRJNA729271	stage_E18_vs_E10	ordinary_muscle_development	ENSGALG00010001694	ENSGALG00010001694	-0.252784998	0.217724209	-1.161033031	0.245628467	0.298890583	negative
PRJNA729271	stage_E18_vs_E10	ordinary_muscle_development	ENSGALG00010001695	LMOD2	4.984877589	0.468675662	10.63609229	2.02443E-26	2.08854E-25	positive
PRJNA729271	stage_E18_vs_E10	ordinary_muscle_development	ENSGALG00010001696	FAM135A	-0.122899855	0.091469806	-1.343611189	0.179074149	0.225143533	negative
PRJNA729271	stage_E18_vs_E10	ordinary_muscle_development	ENSGALG00010001697	TAF10	-0.096114954	0.118536692	-0.81084559	0.41745435	0.477773454	negative
PRJNA729271	stage_E18_vs_E10	ordinary_muscle_development	ENSGALG00010001698	RPL15	1.14882181	0.176417707	6.51194162	7.41856E-11	2.73271E-10	positive
PRJNA729271	stage_E18_vs_E10	ordinary_muscle_development	ENSGALG00010001699	ASB15	5.606361629	0.251513477	22.29050183	4.5684E-110	7.1978E-108	positive
PRJNA729271	stage_E18_vs_E10	ordinary_muscle_development	ENSGALG00010001700	ENSGALG00010001700	1.29679834	0.202512903	6.403534402	1.51821E-10	5.45658E-10	positive
PRJNA729271	stage_E18_vs_E10	ordinary_muscle_development	ENSGALG00010001701	NDUFA5	1.700618474	0.169262467	10.04722727	9.44886E-24	8.26561E-23	positive
PRJNA729271	stage_E18_vs_E10	ordinary_muscle_development	ENSGALG00010001702	PLEKHA1	0.322626393	0.083800766	3.84992179	0.000118156	0.000244213	positive
PRJNA729271	stage_E18_vs_E10	ordinary_muscle_development	ENSGALG00010001704	ENSGALG00010001704	-0.022805936	0.145638925	-0.156592312	0.875566162	0.897230931	negative
PRJNA729271	stage_E18_vs_E10	ordinary_muscle_development	ENSGALG00010001705	HTRA1	2.855190385	0.15451426	18.47849113	3.07666E-76	1.92225E-74	positive
PRJNA729271	stage_E18_vs_E10	ordinary_muscle_development	ENSGALG00010001706	IQUB	1.314635752	0.354868446	3.704572116	0.000211748	0.000423233	positive
PRJNA729271	stage_E18_vs_E10	ordinary_muscle_development	ENSGALG00010001707	ENSGALG00010001707	-0.818415945	1.327459156	-0.616528156	0.537545995	0.592752317	negative
PRJNA729271	stage_E18_vs_E10	ordinary_muscle_development	ENSGALG00010001708	AADAC	3.214454974	0.250946143	12.809342	1.45362E-37	2.51135E-36	positive
PRJNA729271	stage_E18_vs_E10	ordinary_muscle_development	ENSGALG00010001710	LCMT2	-0.877785894	0.082795111	-10.60190495	2.91972E-26	2.96785E-25	negative
PRJNA729271	stage_E18_vs_E10	ordinary_muscle_development	ENSGALG00010001711	CREB5	0.034619597	0.175312012	0.197474189	0.843456478	0.870604689	positive
PRJNA729271	stage_E18_vs_E10	ordinary_muscle_development	ENSGALG00010001712	ATP6AP1	1.449209658	0.159016758	9.11356559	7.9718E-20	5.54735E-19	positive
PRJNA729271	stage_E18_vs_E10	ordinary_muscle_development	ENSGALG00010001713	PNPLA4	0.397576642	0.22616188	1.757929505	0.078759505	0.106643533	positive
PRJNA729271	stage_E18_vs_E10	ordinary_muscle_development	ENSGALG00010001716	TRIL	-1.419685072	0.152242354	-9.325164998	1.10811E-20	8.16577E-20	negative
PRJNA729271	stage_E18_vs_E10	ordinary_muscle_development	ENSGALG00010001717	STARD10	1.144096674	0.47718992	2.397570916	0.016504186	0.025275032	positive
PRJNA729271	stage_E18_vs_E10	ordinary_muscle_development	ENSGALG00010001721	TM4SF1	0.626540301	0.160993337	3.891715728	9.95378E-05	0.000207567	positive
PRJNA729271	stage_E18_vs_E10	ordinary_muscle_development	ENSGALG00010001725	MTA2	-0.729388419	0.188175919	-3.876098615	0.000106145	0.0002209	negative
PRJNA729271	stage_E18_vs_E10	ordinary_muscle_development	ENSGALG00010001727	COL9A1	-6.169399144	0.774511021	-7.965540811	1.64503E-15	8.68658E-15	negative
PRJNA729271	stage_E18_vs_E10	ordinary_muscle_development	ENSGALG00010001728	STS	1.092999306	0.324150297	3.371890493	0.000746541	0.001386611	positive
PRJNA729271	stage_E18_vs_E10	ordinary_muscle_development	ENSGALG00010001729	ENSGALG00010001729	2.120708226	0.24338417	8.713418892	2.94845E-18	1.87611E-17	positive
PRJNA729271	stage_E18_vs_E10	ordinary_muscle_development	ENSGALG00010001731	ENSGALG00010001731	-0.708037498	0.240025175	-2.949846813	0.003179315	0.005423582	negative
PRJNA729271	stage_E18_vs_E10	ordinary_muscle_development	ENSGALG00010001738	PUDP	0.421469196	0.123013561	3.426201069	0.000612087	0.001149249	positive
PRJNA729271	stage_E18_vs_E10	ordinary_muscle_development	ENSGALG00010001741	SATB1	-1.586679555	0.205986601	-7.702828964	1.33086E-14	6.57943E-14	negative
PRJNA729271	stage_E18_vs_E10	ordinary_muscle_development	ENSGALG00010001745	CADPS2	0.018505307	0.102561546	0.18043124	0.856814031	0.881567247	positive
PRJNA729271	stage_E18_vs_E10	ordinary_muscle_development	ENSGALG00010001749	VAPA	0.694666076	0.106140788	6.544760872	5.95907E-11	2.21025E-10	positive
PRJNA729271	stage_E18_vs_E10	ordinary_muscle_development	ENSGALG00010001751	AVPR2C	-0.276278293	0.432218518	-0.639209755	0.522686487	0.57933069	negative
PRJNA729271	stage_E18_vs_E10	ordinary_muscle_development	ENSGALG00010001752	ARRB1	1.509185144	0.10734576	14.05910342	6.7739E-45	1.58112E-43	positive
PRJNA729271	stage_E18_vs_E10	ordinary_muscle_development	ENSGALG00010001753	PNPLA8	0.33126634	0.204799696	1.617513828	0.105767413	0.139639189	positive
PRJNA729271	stage_E18_vs_E10	ordinary_muscle_development	ENSGALG00010001754	APCDD1	-2.019067749	0.189508285	-10.65424526	1.6659E-26	1.72851E-25	negative
PRJNA729271	stage_E18_vs_E10	ordinary_muscle_development	ENSGALG00010001755	CUZD1	-2.360811199	1.61447076	-1.462281794	0.143664005	0.184610794	negative
PRJNA729271	stage_E18_vs_E10	ordinary_muscle_development	ENSGALG00010001756	ENSGALG00010001756	0.478453248	0.219007647	2.184641751	0.028915124	0.042369785	positive
PRJNA729271	stage_E18_vs_E10	ordinary_muscle_development	ENSGALG00010001758	COL19A1	2.6170107	0.276766758	9.45565399	3.21009E-21	2.44896E-20	positive
PRJNA729271	stage_E18_vs_E10	ordinary_muscle_development	ENSGALG00010001759	C10orf88	-0.581074872	0.092822244	-6.260082137	3.84775E-10	1.3449E-09	negative
PRJNA729271	stage_E18_vs_E10	ordinary_muscle_development	ENSGALG00010001760	RAB5A	0.278552415	0.096448462	2.888095978	0.003875816	0.006518152	positive
PRJNA729271	stage_E18_vs_E10	ordinary_muscle_development	ENSGALG00010001761	ELOB	0.011342255	0.150546403	0.075340588	0.939943728	0.95083288	positive
PRJNA729271	stage_E18_vs_E10	ordinary_muscle_development	ENSGALG00010001763	ENSGALG00010001763	0.482998813	0.266199642	1.814423222	0.069612593	0.09527283	positive
PRJNA729271	stage_E18_vs_E10	ordinary_muscle_development	ENSGALG00010001764	ENSGALG00010001764	-1.409743146	0.571692806	-2.465910243	0.013666558	0.021210685	negative
PRJNA729271	stage_E18_vs_E10	ordinary_muscle_development	ENSGALG00010001767	ENSGALG00010001767	0.633955349	0.174864898	3.62540084	0.000288514	0.000566514	positive
PRJNA729271	stage_E18_vs_E10	ordinary_muscle_development	ENSGALG00010001771	ENSGALG00010001771	-0.203039998	0.138251945	-1.468623085	0.14193505	0.182648131	negative
PRJNA729271	stage_E18_vs_E10	ordinary_muscle_development	ENSGALG00010001773	NRCAM	-0.44548389	0.234951131	-1.896070418	0.057950734	0.08042856	negative
PRJNA729271	stage_E18_vs_E10	ordinary_muscle_development	ENSGALG00010001776	LMBRD1	0.631137122	0.132169729	4.775201761	1.79527E-06	4.51231E-06	positive
PRJNA729271	stage_E18_vs_E10	ordinary_muscle_development	ENSGALG00010001778	KAT2B	1.743327665	0.148238613	11.7602805	6.25262E-32	8.35316E-31	positive
PRJNA729271	stage_E18_vs_E10	ordinary_muscle_development	ENSGALG00010001779	ENSGALG00010001779	1.107055674	0.602853512	1.836359333	0.06630453	0.091011758	positive
PRJNA729271	stage_E18_vs_E10	ordinary_muscle_development	ENSGALG00010001780	ENSGALG00010001780	2.214824631	0.235844269	9.391047049	5.94067E-21	4.46397E-20	positive
PRJNA729271	stage_E18_vs_E10	ordinary_muscle_development	ENSGALG00010001783	SGO1	-2.101903202	0.248127215	-8.471070782	2.43147E-17	1.45277E-16	negative
PRJNA729271	stage_E18_vs_E10	ordinary_muscle_development	ENSGALG00010001785	CNTN1	0.157739223	0.213266582	0.739634037	0.459522083	0.518913415	positive
PRJNA729271	stage_E18_vs_E10	ordinary_muscle_development	ENSGALG00010001786	P2RY8	-0.370916159	0.210864728	-1.759024195	0.078573399	0.106411419	negative
PRJNA729271	stage_E18_vs_E10	ordinary_muscle_development	ENSGALG00010001788	HOXA1	-1.867566579	0.454624753	-4.107929816	3.99221E-05	8.68876E-05	negative
PRJNA729271	stage_E18_vs_E10	ordinary_muscle_development	ENSGALG00010001790	HOXA2	-0.826524061	0.236888773	-3.489080765	0.000484685	0.000922709	negative
PRJNA729271	stage_E18_vs_E10	ordinary_muscle_development	ENSGALG00010001793	IKZF5	0.206988274	0.104724097	1.976510462	0.048096992	0.067725461	positive
PRJNA729271	stage_E18_vs_E10	ordinary_muscle_development	ENSGALG00010001794	ENSGALG00010001794	-0.678984955	0.541698064	-1.25343803	0.210046338	0.259957452	negative
PRJNA729271	stage_E18_vs_E10	ordinary_muscle_development	ENSGALG00010001795	ENSGALG00010001795	1.804729341	0.475520899	3.795268184	0.000147484	0.000300926	positive
PRJNA729271	stage_E18_vs_E10	ordinary_muscle_development	ENSGALG00010001796	SLCO2B1	1.332907305	0.158024306	8.434824609	3.317E-17	1.96485E-16	positive
PRJNA729271	stage_E18_vs_E10	ordinary_muscle_development	ENSGALG00010001798	PSTK	-0.028813215	0.132175767	-0.217991664	0.827435606	0.856507828	negative
PRJNA729271	stage_E18_vs_E10	ordinary_muscle_development	ENSGALG00010001800	ENSGALG00010001800	0.460347331	0.18991678	2.423942376	0.015353041	0.02366213	positive
PRJNA729271	stage_E18_vs_E10	ordinary_muscle_development	ENSGALG00010001801	HOXA3	-0.815176632	0.145324614	-5.60935006	2.03088E-08	6.11119E-08	negative
PRJNA729271	stage_E18_vs_E10	ordinary_muscle_development	ENSGALG00010001803	PPP4R1	-0.472725591	0.096958296	-4.875555886	1.08502E-06	2.79649E-06	negative
PRJNA729271	stage_E18_vs_E10	ordinary_muscle_development	ENSGALG00010001805	ENSGALG00010001805	0.710557687	0.265063767	2.680704709	0.007346731	0.011848116	positive
PRJNA729271	stage_E18_vs_E10	ordinary_muscle_development	ENSGALG00010001806	ENSGALG00010001806	1.153223114	0.975922838	1.181674482	0.237334887	0.289942621	positive
PRJNA729271	stage_E18_vs_E10	ordinary_muscle_development	ENSGALG00010001807	ASMTL	-0.051443084	0.105421567	-0.487974949	0.625567585	0.675930089	negative
PRJNA729271	stage_E18_vs_E10	ordinary_muscle_development	ENSGALG00010001808	HOXA4	-0.771279121	0.150371316	-5.129163884	2.91032E-07	7.91762E-07	negative
PRJNA729271	stage_E18_vs_E10	ordinary_muscle_development	ENSGALG00010001811	ACADSB	-0.119646131	0.149590973	-0.799821863	0.423814014	0.484249104	negative
PRJNA729271	stage_E18_vs_E10	ordinary_muscle_development	ENSGALG00010001813	HOXA5	-0.635414568	0.10534652	-6.03166171	1.62282E-09	5.37787E-09	negative
PRJNA729271	stage_E18_vs_E10	ordinary_muscle_development	ENSGALG00010001817	SLC25A6	-0.90703051	0.112978027	-8.028379827	9.87683E-16	5.31223E-15	negative
PRJNA729271	stage_E18_vs_E10	ordinary_muscle_development	ENSGALG00010001818	ADGRB3	-0.656625471	0.234106623	-2.804813737	0.005034566	0.008343933	negative
PRJNA729271	stage_E18_vs_E10	ordinary_muscle_development	ENSGALG00010001819	HOXA6	0.497822975	0.143885134	3.45986387	0.000540449	0.001022156	positive
PRJNA729271	stage_E18_vs_E10	ordinary_muscle_development	ENSGALG00010001820	PGM2L1	0.64799899	0.159370745	4.065984572	4.78301E-05	0.000103169	positive
PRJNA729271	stage_E18_vs_E10	ordinary_muscle_development	ENSGALG00010001822	KCNE3	0.693613227	0.327277342	2.119343868	0.034061417	0.049248902	positive
PRJNA729271	stage_E18_vs_E10	ordinary_muscle_development	ENSGALG00010001823	RALBP1	-0.445169218	0.076699622	-5.804060121	6.47281E-09	2.03654E-08	negative
PRJNA729271	stage_E18_vs_E10	ordinary_muscle_development	ENSGALG00010001825	LIPT2	1.364439215	0.145754099	9.361240785	7.88057E-21	5.86692E-20	positive
PRJNA729271	stage_E18_vs_E10	ordinary_muscle_development	ENSGALG00010001826	HOXA7	0.823020755	0.133602128	6.160236875	7.26362E-10	2.48845E-09	positive
PRJNA729271	stage_E18_vs_E10	ordinary_muscle_development	ENSGALG00010001827	PDZRN4	-1.51467511	0.181262914	-8.3562328	6.47529E-17	3.75888E-16	negative
PRJNA729271	stage_E18_vs_E10	ordinary_muscle_development	ENSGALG00010001828	ENSGALG00010001828	-2.421029522	1.100477485	-2.199980968	0.027808245	0.040866754	negative
PRJNA729271	stage_E18_vs_E10	ordinary_muscle_development	ENSGALG00010001829	POLD3	0.014140854	0.121453222	0.116430456	0.907311399	0.923440726	positive

PRJNA729271	stage_E18_vs_E10	ordinary_muscle_development	ENSGALG00010001831	CSF2RA	0.443761284	0.256815316	1.727939327	0.083999103	0.113051717	positive
PRJNA729271	stage_E18_vs_E10	ordinary_muscle_development	ENSGALG00010001832	BUB3	-1.226077214	0.087468034	-14.01743197	1.21948E-44	2.82822E-43	negative
PRJNA729271	stage_E18_vs_E10	ordinary_muscle_development	ENSGALG00010001834	ZCRB1	0.061086227	0.085272479	0.716365085	0.473765948	0.532509105	positive
PRJNA729271	stage_E18_vs_E10	ordinary_muscle_development	ENSGALG00010001835	MECP2	0.338009415	0.191413389	1.765860879	0.077419192	0.10498561	positive
PRJNA729271	stage_E18_vs_E10	ordinary_muscle_development	ENSGALG00010001836	TWSG1	-1.072940961	0.140445446	-7.639556814	2.17971E-14	1.05598E-13	negative
PRJNA729271	stage_E18_vs_E10	ordinary_muscle_development	ENSGALG00010001837	TAB3	-0.07782095	0.142057405	-0.547813398	0.583820038	0.63656322	negative
PRJNA729271	stage_E18_vs_E10	ordinary_muscle_development	ENSGALG00010001838	PPHLN1	-0.742513436	0.06617431	-11.22056934	3.23183E-29	3.78702E-28	negative
PRJNA729271	stage_E18_vs_E10	ordinary_muscle_development	ENSGALG00010001839	ENSGALG00010001839	0.701207148	0.748028951	0.93740643	0.348549535	0.407328997	positive
PRJNA729271	stage_E18_vs_E10	ordinary_muscle_development	ENSGALG00010001840	HOXA9	0.124839353	0.14502728	0.860799106	0.389348697	0.449475101	positive
PRJNA729271	stage_E18_vs_E10	ordinary_muscle_development	ENSGALG00010001842	GPR26	-0.433967368	0.530627615	-0.817837889	0.41344976	0.473751326	negative
PRJNA729271	stage_E18_vs_E10	ordinary_muscle_development	ENSGALG00010001843	PRICKLE1	-1.480629683	0.121908949	-12.14537325	6.07049E-34	8.88806E-33	negative
PRJNA729271	stage_E18_vs_E10	ordinary_muscle_development	ENSGALG00010001844	HOXA10	-1.18433442	0.169545554	-6.985346378	2.84154E-12	1.17379E-11	negative
PRJNA729271	stage_E18_vs_E10	ordinary_muscle_development	ENSGALG00010001845	ANKRD12	0.722743093	0.134706195	5.365329291	8.08017E-08	2.30828E-07	positive
PRJNA729271	stage_E18_vs_E10	ordinary_muscle_development	ENSGALG00010001848	HOXA11	-1.348611565	0.372880859	-3.616735839	0.000298341	0.000584308	negative
PRJNA729271	stage_E18_vs_E10	ordinary_muscle_development	ENSGALG00010001849	NDUFV2	0.67557379	0.094828329	7.124176861	1.04705E-12	4.48491E-12	positive
PRJNA729271	stage_E18_vs_E10	ordinary_muscle_development	ENSGALG00010001850	MYOM2	3.762575839	0.187803977	20.03459086	2.75091E-89	2.52369E-87	positive
PRJNA729271	stage_E18_vs_E10	ordinary_muscle_development	ENSGALG00010001852	CPXM2	-0.34974284	0.380392411	-0.91942644	0.357872565	0.417292481	negative
PRJNA729271	stage_E18_vs_E10	ordinary_muscle_development	ENSGALG00010001853	ENSGALG00010001853	0.421215749	0.193469975	2.177163397	0.029468378	0.043097986	positive
PRJNA729271	stage_E18_vs_E10	ordinary_muscle_development	ENSGALG00010001854	CHRDL2	0.994742552	0.299317057	3.323374093	0.000889356	0.001632832	positive
PRJNA729271	stage_E18_vs_E10	ordinary_muscle_development	ENSGALG00010001856	CHST15	-0.989846617	0.119342165	-8.294190183	1.09327E-16	6.26364E-16	negative
PRJNA729271	stage_E18_vs_E10	ordinary_muscle_development	ENSGALG00010001857	HNRNPA2B1	-0.920762875	0.187881187	-4.900772081	9.54608E-07	2.47355E-06	negative
PRJNA729271	stage_E18_vs_E10	ordinary_muscle_development	ENSGALG00010001858	ADAMTS20	1.289579657	0.160676029	8.0259617	1.00734E-15	5.41392E-15	positive
PRJNA729271	stage_E18_vs_E10	ordinary_muscle_development	ENSGALG00010001860	RNF169	0.619675776	0.128963466	4.805049034	1.54714E-06	3.91647E-06	positive
PRJNA729271	stage_E18_vs_E10	ordinary_muscle_development	ENSGALG00010001861	CBX3	-0.533982676	0.074303537	-7.186504103	6.64713E-13	2.88646E-12	negative
PRJNA729271	stage_E18_vs_E10	ordinary_muscle_development	ENSGALG00010001864	ENSGALG00010001864	0.268933865	0.196987219	1.365235099	0.17217917	0.217360832	positive
PRJNA729271	stage_E18_vs_E10	ordinary_muscle_development	ENSGALG00010001865	CYBB	0.194744363	0.323409135	0.602160986	0.547066983	0.601763678	positive
PRJNA729271	stage_E18_vs_E10	ordinary_muscle_development	ENSGALG00010001866	PUS7L	-0.133618605	0.120555926	-1.108353687	0.267709098	0.322778521	negative
PRJNA729271	stage_E18_vs_E10	ordinary_muscle_development	ENSGALG00010001868	ENSGALG00010001868	-0.198744205	0.124856929	-1.591775534	0.111435149	0.146539702	negative
PRJNA729271	stage_E18_vs_E10	ordinary_muscle_development	ENSGALG00010001869	CRLF2	6.293253622	0.936650829	6.718889714	1.83114E-11	7.05166E-11	positive
PRJNA729271	stage_E18_vs_E10	ordinary_muscle_development	ENSGALG00010001870	PLP2	2.846993826	0.16330046	17.43408337	4.54793E-68	2.30498E-66	positive
PRJNA729271	stage_E18_vs_E10	ordinary_muscle_development	ENSGALG00010001871	ENSGALG00010001871	-0.202344793	0.123055014	-1.644344152	0.100105134	0.132671109	negative
PRJNA729271	stage_E18_vs_E10	ordinary_muscle_development	ENSGALG00010001873	ENSGALG00010001873	-0.487603523	0.226794581	-2.149978722	0.031556898	0.045930037	negative
PRJNA729271	stage_E18_vs_E10	ordinary_muscle_development	ENSGALG00010001877	OAT	0.466627601	0.136809227	3.410790417	0.000647749	0.001212124	positive
PRJNA729271	stage_E18_vs_E10	ordinary_muscle_development	ENSGALG00010001878	XK	-0.364320127	0.203287765	-1.792139959	0.073110549	0.099608743	negative
PRJNA729271	stage_E18_vs_E10	ordinary_muscle_development	ENSGALG00010001879	IRAK4	0.006167115	0.169078809	0.036474797	0.970903774	0.975899666	positive
PRJNA729271	stage_E18_vs_E10	ordinary_muscle_development	ENSGALG00010001880	MTCL1	0.912786152	0.145190758	6.286806169	3.24064E-10	1.13791E-09	positive
PRJNA729271	stage_E18_vs_E10	ordinary_muscle_development	ENSGALG00010001883	KBTBD11	-0.951498301	0.089146977	-10.6733659	1.35623E-26	1.41326E-25	negative
PRJNA729271	stage_E18_vs_E10	ordinary_muscle_development	ENSGALG00010001885	SMPX	3.678617083	0.371306051	9.907237086	3.87206E-23	3.27072E-22	positive
PRJNA729271	stage_E18_vs_E10	ordinary_muscle_development	ENSGALG00010001886	NEU3	-0.372797247	0.085236863	-4.373662205	1.22179E-05	2.8196E-05	negative
PRJNA729271	stage_E18_vs_E10	ordinary_muscle_development	ENSGALG00010001888	SNX10	0.921935573	0.201698152	4.57086772	4.85709E-06	1.16639E-05	positive
PRJNA729271	stage_E18_vs_E10	ordinary_muscle_development	ENSGALG00010001890	KLHL34	4.193225071	0.25263343	16.59806094	7.19789E-62	3.07768E-60	positive
PRJNA729271	stage_E18_vs_E10	ordinary_muscle_development	ENSGALG00010001892	RAB12	0.867892998	0.071055991	12.21421291	2.61015E-34	3.89641E-33	positive
PRJNA729271	stage_E18_vs_E10	ordinary_muscle_development	ENSGALG00010001895	P4HA3	-0.646409515	0.23987039	-2.694828305	0.007042491	0.011383076	negative
PRJNA729271	stage_E18_vs_E10	ordinary_muscle_development	ENSGALG00010001896	ARHGEF10	1.225432214	0.133358708	9.188992869	3.96536E-20	2.8038E-19	positive
PRJNA729271	stage_E18_vs_E10	ordinary_muscle_development	ENSGALG00010001897	TLR7	0.569036488	0.185179879	3.072885085	0.002120001	0.003709938	positive
PRJNA729271	stage_E18_vs_E10	ordinary_muscle_development	ENSGALG00010001898	LHPP	0.036381559	0.134642934	0.27020771	0.787000461	0.821516037	positive
PRJNA729271	stage_E18_vs_E10	ordinary_muscle_development	ENSGALG00010001899	TWF1	-0.471622083	0.112940202	-4.175856569	2.96867E-05	6.54945E-05	negative
PRJNA729271	stage_E18_vs_E10	ordinary_muscle_development	ENSGALG00010001901	PRPS2	3.123707608	0.181265015	17.23282124	1.50622E-66	7.27757E-65	positive
PRJNA729271	stage_E18_vs_E10	ordinary_muscle_development	ENSGALG00010001902	WDR45	0.231192712	0.142946497	1.617337376	0.105805476	0.139676718	positive
PRJNA729271	stage_E18_vs_E10	ordinary_muscle_development	ENSGALG00010001903	NARS2	-0.045581996	0.117561128	-0.387730173	0.698215724	0.743235436	negative
PRJNA729271	stage_E18_vs_E10	ordinary_muscle_development	ENSGALG00010001906	SKAP2	0.306326481	0.127664902	2.399457303	0.016419395	0.025155811	positive
PRJNA729271	stage_E18_vs_E10	ordinary_muscle_development	ENSGALG00010001908	FAM83B	-0.769291398	0.364151342	-2.112559556	0.034638483	0.050018412	negative
PRJNA729271	stage_E18_vs_E10	ordinary_muscle_development	ENSGALG00010001910	FAM53B	0.298512945	0.095963412	3.110695401	0.001866474	0.003292908	positive
PRJNA729271	stage_E18_vs_E10	ordinary_muscle_development	ENSGALG00010001911	USP35	-3.727293593	0.577965879	-6.448985531	1.12601E-10	4.08447E-10	negative
PRJNA729271	stage_E18_vs_E10	ordinary_muscle_development	ENSGALG00010001912	TMEM117	3.058175049	0.208071873	14.69768596	6.67003E-49	1.8557E-47	positive
PRJNA729271	stage_E18_vs_E10	ordinary_muscle_development	ENSGALG00010001914	ENSGALG00010001914	0.016209718	0.152187217	0.106511694	0.915176377	0.929939387	positive
PRJNA729271	stage_E18_vs_E10	ordinary_muscle_development	ENSGALG00010001915	EEF1AKMT2	-0.64751203	0.17164246	-3.772446681	0.000161655	0.000328085	negative
PRJNA729271	stage_E18_vs_E10	ordinary_muscle_development	ENSGALG00010001918	CNKSR2	2.287396243	0.141507658	16.16446966	8.98074E-59	3.47136E-57	positive
PRJNA729271	stage_E18_vs_E10	ordinary_muscle_development	ENSGALG00010001921	ENSGALG00010001921	-0.024129553	0.290183004	-0.083152881	0.933729978	0.94583334	negative
PRJNA729271	stage_E18_vs_E10	ordinary_muscle_development	ENSGALG00010001923	RAPGEF5	1.510534581	0.101600789	14.86735088	5.36964E-50	1.56605E-48	positive
PRJNA729271	stage_E18_vs_E10	ordinary_muscle_development	ENSGALG00010001925	FRMPD4	-0.461553743	0.177144867	-2.605515756	0.009173605	0.014581797	negative
PRJNA729271	stage_E18_vs_E10	ordinary_muscle_development	ENSGALG00010001928	KCTD21	-0.213210886	0.172310469	-1.237364672	0.215951728	0.266492448	negative
PRJNA729271	stage_E18_vs_E10	ordinary_muscle_development	ENSGALG00010001929	POLR2I	0.03839609	0.123913562	0.309861888	0.756665986	0.795753716	positive
PRJNA729271	stage_E18_vs_E10	ordinary_muscle_development	ENSGALG00010001935	ABRAXAS2	-0.199131305	0.131450383	-1.514878086	0.129803281	0.168442127	negative
PRJNA729271	stage_E18_vs_E10	ordinary_muscle_development	ENSGALG00010001938	ALG8	-0.726934155	0.104969904	-6.925167367	4.3546E-12	1.77503E-11	negative
PRJNA729271	stage_E18_vs_E10	ordinary_muscle_development	ENSGALG00010001940	PTPRM	1.143833226	0.110552225	10.34654184	4.33875E-25	4.12936E-24	positive
PRJNA729271	stage_E18_vs_E10	ordinary_muscle_development	ENSGALG00010001947	TINAG	5.585159647	0.303169056	18.42259141	8.65585E-76	5.29395E-74	positive
PRJNA729271	stage_E18_vs_E10	ordinary_muscle_development	ENSGALG00010001948	EMX2	-2.207404825	0.265067312	-8.327714218	8.24182E-17	4.75578E-16	negative
PRJNA729271	stage_E18_vs_E10	ordinary_muscle_development	ENSGALG00010001952	ENSGALG00010001952	0.198076149	0.072891392	2.717414839	0.006579408	0.010695136	positive
PRJNA729271	stage_E18_vs_E10	ordinary_muscle_development	ENSGALG00010001957	NDUFC2	0.771106569	0.112051975	6.881686549	5.91481E-12	2.37625E-11	positive
PRJNA729271	stage_E18_vs_E10	ordinary_muscle_development	ENSGALG00010001958	RAB11FIP2	-0.263056509	0.112257851	-2.343323942	0.01911278	0.028912518	negative
PRJNA729271	stage_E18_vs_E10	ordinary_muscle_development	ENSGALG00010001966	ARHGAP6	1.001716525	0.141909347	7.05884811	1.67888E-12	7.0654E-12	positive
PRJNA729271	stage_E18_vs_E10	ordinary_muscle_development	ENSGALG00010001967	LAMA1	-3.412066864	0.23362562	-14.60484882	2.61562E-48	7.11321E-47	negative
PRJNA729271	stage_E18_vs_E10	ordinary_muscle_development	ENSGALG00010001969	PLEKHA8	-1.608484757	0.122627128	-13.11687536	2.6358E-39	4.91711E-38	negative
PRJNA729271	stage_E18_vs_E10	ordinary_muscle_development	ENSGALG00010001971	MLIP	4.664269314	0.204511533	22.80687672	3.9185E-115	6.7617E-113	positive
PRJNA729271	stage_E18_vs_E10	ordinary_muscle_development	ENSGALG00010001972	FAM204A	0.1149388	0.158712418	0.724195382	0.468945793	0.527910009	positive
PRJNA729271	stage_E18_vs_E10	ordinary_muscle_development	ENSGALG00010001974	ENSGALG00010001974	1.372906076	0.394987108	3.475825026	0.000509284	0.000966747	positive
PRJNA729271	stage_E18_vs_E10	ordinary_muscle_development	ENSGALG00010001976	THRSP	8.564369807	1.424415759	6.012549184	1.82628E-09	6.02321E-09	positive
PRJNA729271	stage_E18_vs_E10	ordinary_muscle_development	ENSGALG00010001982	DMD	2.863794207	0.121807087	23.51089981	3.1554E-122	5.8638E-120	positive
PRJNA729271	stage_E18_vs_E10	ordinary_muscle_development	ENSGALG00010001987	KCTD14	-4.383762881	0.669876655	-6.544134428	5.9841E-11	2.21897E-10	negative
PRJNA729271	stage_E18_vs_E10	ordinary_muscle_development	ENSGALG00010001991	TOMM7	0.378709069	0.130744164	2.896565753	0.003772716	0.006355086	positive
PRJNA729271	stage_E18_vs_E10	ordinary_muscle_development	ENSGALG00010001995	LRRC1	-0.834026261	0.136433732	-6.113050243	9.77446E-10	3.30258E-09	negative
PRJNA729271	stage_E18_vs_E10	ordinary_muscle_development	ENSGALG00010001997	INTS4	-0.220478496	0.07809668	-2.823148121	0.00475546	0.007901236	negative
PRJNA729271	stage_E18_vs_E10	ordinary_muscle_development	ENSGALG00010002005	COLQ	2.572746501	0.209212455	12.29729127	9.36627E-35	1.4291E-33	positive
PRJNA729271	stage_E18_vs_E10	ordinary_muscle_development	ENSGALG00010002006	ENSGALG00010002006	1.936209198	0.419647538	4.613893856	3.95194E-06	9.55679E-06	positive
PRJNA729271	stage_E18_vs_E10	ordinary_muscle_development	ENSGALG00010002012	MID1	-2.340196682	0.143371304	-16.32262951	6.81364E-60	2.73584E-58	negative
PRJNA729271	stage_E18_vs_E10	ordinary_muscle_development	ENSGALG00010002017	ARHGAP28	-0.933890098	0.118300083	-7.894247203	2.92073E-15	1.51851E-14	negative
PRJNA729271	stage_E18_vs_E10	ordinary_muscle_development	ENSGALG00010002022	ERNI	-1.008137306	0.684538241	-1.472726058	0.140824917	0.181386103	negative
PRJNA729271	stage_E18_vs_E10	ordinary_muscle_development	ENSGALG00010002023	CLCN4	-0.26253401	0.244458945	-1.07393906	0.282849995	0.338471946	negative
PRJNA729271	stage_E18_vs_E10	ordinary_muscle_development	ENSGALG00010002024	KLHL31	2.902449102	0.165825156	17.5030687	1.35754E-68	6.95319E-67	positive
PRJNA729271	stage_E18_vs_E10	ordinary_muscle_development	ENSGALG00010002027	ENSGALG00010002027	3.945806697	0.558428603	7.065910801	1.59566E-12	6.72552E-12	positive
PRJNA729271	stage_E18_vs_E10	ordinary_muscle_development	ENSGALG00010002034	WWC3	0.044863648	0.099313836	0.451736129	0.651459088	0.699694333	positive
PRJNA729271	stage_E18_vs_E10	ordinary_muscle_development	ENSGALG00010002039	AAMDC	0.976471287	0.128486822	7.599777714	2.9664E-14	1.41674E-13	positive
PRJNA729271	stage_E18_vs_E10	ordinary_muscle_development	ENSGALG00010002041	GCLC	1.016624924	0.138345143	7.348468479	2.00491E-13	9.00562E-13	positive
PRJNA729271	stage_E18_vs_E10	ordinary_muscle_development	ENSGALG00010002046	FAM126A	-0.315845114	0.088216868	-3.580325635	0.000343166	0.000666157	negative
PRJNA729271	stage_E18_vs_E10	ordinary_muscle_development	ENSGALG00010002050	ENSGALG00010002050	-2.732162237	1.102232344	-2.478753461	0.01318424	0.020502689	negative
PRJNA729271	stage_E18_vs_E10	ordinary_muscle_development	ENSGALG00010002054	RSF1	-0.274420051	0.100496713	-2.730637087	0.006321204	0.010315903	negative
PRJNA729271	stage_E18_vs_E10	ordinary_muscle_development	ENSGALG00010002056	PRLHR	-2.81467348	0.555270526	-5.069012936	3.99884E-07	1.07439E-06	negative
PRJNA729271	stage_E18_vs_E10	ordinary_muscle_development	ENSGALG00010002062	GSTA4	-1.017560953	0.369306392	-2.755329923	0.005863298	0.009611909	negative
PRJNA729271	stage_E18_vs_E10	ordinary_muscle_development	ENSGALG00010002065	CLNS1A	-0.70618741	0.063888585	-11.0534207	2.11018E-28	2.37846E-27	negative
PRJNA729271	stage_E18_vs_E10	ordinary_muscle_development	ENSGALG00010002072	NEURL4	-0.110781115	0.162609964	-0.681268924	0.495701343	0.55296221	negative
PRJNA729271	stage_E18_vs_E10	ordinary_muscle_development	ENSGALG00010002079	ARX	2.818856741	0.189258566	14.89420956	3.5941E-50	1.05458E-48	positive
PRJNA729271	stage_E18_vs_E10	ordinary_muscle_development	ENSGALG00010002084	HACL1	-0.774515295	0.093040915	-8.324459138	8.47146E-17	4.8844E-16	negative
PRJNA729271	stage_E18_vs_E10	ordinary_muscle_development	ENSGALG00010002087	POLA1	-0.664960633	0.073217308	-9.082014174	1.06584E-19	7.36384E-19	negative
PRJNA729271	stage_E18_vs_E10	ordinary_muscle_development	ENSGALG00010002088	OZFL1	-0.240538856	0.139370067	-1.725900417	0.08436533	0.113460332	negative
PRJNA729271	stage_E18_vs_E10	ordinary_muscle_development	ENSGALG00010002090	AQP11	-0.610120485	0.311166317	-1.960753628	0.04990777	0.070098171	negative
PRJNA729271	stage_E18_vs_E10	ordinary_muscle_development	ENSGALG00010002097	PCYT1B	-2.551570102	0.49132323	-5.193261681	2.06641E-07	5.68902E-07	negative
PRJNA729271	stage_E18_vs_E10	ordinary_muscle_development	ENSGALG00010002098	ENSGALG00010002098	0.192589781	0.458414896	0.420121123	0.674396972	0.720898533	positive
PRJNA729271	stage_E18_vs_E10	ordinary_muscle_development	ENSGALG00010002100	BTD	0.391943815	0.119271286	3.286154019	0.001015655	0.00184879	positive
PRJNA729271	stage_E18_vs_E10	ordinary_muscle_development	ENSGALG00010002102	PAK1	-3.712455346	0.3024977	-12.27267297	1.26991E-34	1.92344E-33	negative
PRJNA729271	stage_E18_vs_E10	ordinary_muscle_development	ENSGALG00010002106	ENSGALG00010002106	1.263723204	0.88150326	1.433600149	0.151686401	0.193922595	positive
PRJNA729271	stage_E18_vs_E10	ordinary_muscle_development	ENSGALG00010002113	GDPD4	-5.780192089	0.286601762	-20.16802703	1.86949E-90	1.77113E-88	negative
PRJNA729271	stage_E18_vs_E10	ordinary_muscle_development	ENSGALG00010002118	ENSGALG00010002118	1.368715465	0.268304758	5.10134629	3.37246E-07	9.13204E-07	positive
PRJNA729271	stage_E18_vs_E10	ordinary_muscle_development	ENSGALG00010002122	ENSGALG00010002122	-0.010000647	0.544652842	-0.018361508	0.98535046	0.987598874	negative
PRJNA729271	stage_E18_vs_E10	ordinary_muscle_development	ENSGALG00010002126	ENSGALG00010002126	1.965255223	0.299571662	6.560217376	5.37294E-11	2.00259E-10	positive
PRJNA729271	stage_E18_vs_E10	ordinary_muscle_development	ENSGALG00010002134	ANKRD28	0.068735719	0.121737864	0.564620706	0.572331778	0.625778768	positive
PRJNA729271	stage_E18_vs_E10	ordinary_muscle_development	ENSGALG00010002135	ELOVL5	-0.575727968	0.168842557	-3.409851034	0.000649984	0.001215993	negative
PRJNA729271	stage_E18_vs_E10	ordinary_muscle_development	ENSGALG00010002136	ACAP1	-1.193673282	0.269887191	-4.422860077	9.74028E-06	2.27059E-05	negative
PRJNA729271	stage_E18_vs_E10	ordinary_muscle_development	ENSGALG00010002137	PDK3	1.306120131	0.131451504	9.936136819	2.89851E-23	2.46127E-22	positive
PRJNA729271	stage_E18_vs_E10	ordinary_muscle_development	ENSGALG00010002138	MYO7A	-0.372397004	0.170606098	-2.182788354	0.0290514	0.042561152	negative
PRJNA729271	stage_E18_vs_E10	ordinary_muscle_development	ENSGALG00010002140	ENSGALG00010002140	0.343868576	0.435505424	0.789585059	0.429770137	0.489857524	positive
PRJNA729271	stage_E18_vs_E10	ordinary_muscle_development	ENSGALG00010002142	CLDN34	0.43501727	0.256883375	1.693442681	0.090371237	0.120831203	positive
PRJNA729271	stage_E18_vs_E10	ordinary_muscle_development	ENSGALG00010002145	ENSGALG00010002145	0.225375145	0.472227002	0.477260182	0.633176861	0.682828554	positive
PRJNA729271	stage_E18_vs_E10	ordinary_muscle_development	ENSGALG00010002146	CACUL1	-0.011946002	0.089981383	-0.132760812	0.894382547	0.912541595	negative
PRJNA729271	stage_E18_vs_E10	ordinary_muscle_development	ENSGALG00010002148	POLR2A	0.526201141	0.1786003	2.946250043	0.003216523	0.005477384	positive
PRJNA729271	stage_E18_vs_E10	ordinary_muscle_development	ENSGALG00010002149	ENSGALG00010002149	-1.635479946	0.342170842	-4.779717462	1.75542E-06	4.4198E-06	negative
PRJNA729271	stage_E18_vs_E10	ordinary_muscle_development	ENSGALG00010002150	EIF3A	-0.130447138	0.132526231	-0.984311838	0.324962211	0.382953434	negative
PRJNA729271	stage_E18_vs_E10	ordinary_muscle_development	ENSGALG00010002159	GALNT15	1.487396125	0.189404529	7.85301247	4.06161E-15	2.08992E-14	positive
PRJNA729271	stage_E18_vs_E10	ordinary_muscle_development	ENSGALG00010002160	CAPN5	0.89071666	0.16540008	5.385225089	7.2354E-08	2.0739E-07	positive
PRJNA729271	stage_E18_vs_E10	ordinary_muscle_development	ENSGALG00010002161	CDKL5	0.52447559	0.173578295	3.021550541	0.002514837	0.004352024	positive
PRJNA729271	stage_E18_vs_E10	ordinary_muscle_development	ENSGALG00010002173	GCM1	-1.090556408	0.928873874	-1.174062957	0.240369809	0.293279494	negative

PRJNA729271	stage_E18_vs_E10	ordinary_muscle_development	ENSGALG00010002174	ENSGALG00010002174	0.287465282	0.457399174	0.628477921	0.529690906	0.585783908	positive
PRJNA729271	stage_E18_vs_E10	ordinary_muscle_development	ENSGALG00010002177	FBXO9	-0.104949067	0.156107095	-0.672288898	0.501399796	0.558588121	negative
PRJNA729271	stage_E18_vs_E10	ordinary_muscle_development	ENSGALG00010002181	FIS1	0.497708335	0.258402129	1.92609998	0.054091891	0.075463134	positive
PRJNA729271	stage_E18_vs_E10	ordinary_muscle_development	ENSGALG00010002185	ENSGALG00010002185	0.397913981	0.331168626	1.201544925	0.229539891	0.281178122	positive
PRJNA729271	stage_E18_vs_E10	ordinary_muscle_development	ENSGALG00010002186	CILK1	0.176668442	0.073977134	2.388149306	0.016933461	0.025869574	positive
PRJNA729271	stage_E18_vs_E10	ordinary_muscle_development	ENSGALG00010002192	ENSGALG00010002192	0.956158144	0.194961164	4.904351852	9.37363E-07	2.4306E-06	positive
PRJNA729271	stage_E18_vs_E10	ordinary_muscle_development	ENSGALG00010002194	SHROOM2	-1.796828584	0.132580742	-13.55271177	7.63563E-42	1.57662E-40	negative
PRJNA729271	stage_E18_vs_E10	ordinary_muscle_development	ENSGALG00010002196	ENSGALG00010002196	-0.940167521	0.10188596	-9.227645541	2.76646E-20	1.98122E-19	negative
PRJNA729271	stage_E18_vs_E10	ordinary_muscle_development	ENSGALG00010002197	GSTA2	-0.622335393	0.189128401	-3.290544367	0.000999937	0.001821344	negative
PRJNA729271	stage_E18_vs_E10	ordinary_muscle_development	ENSGALG00010002202	SFXN4	0.383613791	0.101878981	3.765387012	0.000166291	0.000336694	positive
PRJNA729271	stage_E18_vs_E10	ordinary_muscle_development	ENSGALG00010002206	DPH3P1	0.418390941	0.111877955	3.739708501	0.000184234	0.000371105	positive
PRJNA729271	stage_E18_vs_E10	ordinary_muscle_development	ENSGALG00010002210	GPR143	-2.856005513	1.059656486	-2.695218263	0.007034254	0.011374555	negative
PRJNA729271	stage_E18_vs_E10	ordinary_muscle_development	ENSGALG00010002211	ACER3	-0.603290838	0.174727584	-3.452750975	0.000554901	0.00104744	negative
PRJNA729271	stage_E18_vs_E10	ordinary_muscle_development	ENSGALG00010002215	YLEC18	0.340388197	0.355587864	0.95725482	0.338438653	0.396770324	positive
PRJNA729271	stage_E18_vs_E10	ordinary_muscle_development	ENSGALG00010002225	PRDX3	0.071439223	0.105838624	0.674982536	0.499686861	0.556893822	positive
PRJNA729271	stage_E18_vs_E10	ordinary_muscle_development	ENSGALG00010002226	OXNAD1	-0.702665417	0.138443379	-5.075471451	3.86536E-07	1.0403E-06	negative
PRJNA729271	stage_E18_vs_E10	ordinary_muscle_development	ENSGALG00010002227	ENSGALG00010002227	-0.53939514	1.217116456	-0.443174634	0.657639425	0.705743945	negative
PRJNA729271	stage_E18_vs_E10	ordinary_muscle_development	ENSGALG00010002231	TMEM14A	-0.104399085	0.134065157	-0.778719	0.436145261	0.495837299	negative
PRJNA729271	stage_E18_vs_E10	ordinary_muscle_development	ENSGALG00010002235	MXRA5	0.586476385	0.201218135	2.914629858	0.003561106	0.006016813	positive
PRJNA729271	stage_E18_vs_E10	ordinary_muscle_development	ENSGALG00010002238	ENSGALG00010002238	-0.369152243	0.44580881	-0.828050579	0.407641856	0.46776193	negative
PRJNA729271	stage_E18_vs_E10	ordinary_muscle_development	ENSGALG00010002239	AGPAT5	-0.155805492	0.128932634	-1.208425579	0.226883606	0.278324125	negative
PRJNA729271	stage_E18_vs_E10	ordinary_muscle_development	ENSGALG00010002243	ARSH	-1.01578838	0.182875561	-5.554533223	2.78355E-08	8.29854E-08	negative
PRJNA729271	stage_E18_vs_E10	ordinary_muscle_development	ENSGALG00010002247	RFTN1	0.102273491	0.184905852	0.553111164	0.580187293	0.63317383	positive
PRJNA729271	stage_E18_vs_E10	ordinary_muscle_development	ENSGALG00010002251	GRK5	-0.526775217	0.152932973	-3.44448426	0.00057215	0.001079016	negative
PRJNA729271	stage_E18_vs_E10	ordinary_muscle_development	ENSGALG00010002253	ENSGALG00010002253	-0.746554208	0.323160859	-2.310162841	0.02087914	0.031388005	negative
PRJNA729271	stage_E18_vs_E10	ordinary_muscle_development	ENSGALG00010002254	ENSGALG00010002254	4.001723017	0.460789359	8.684495284	3.80428E-18	2.40904E-17	positive
PRJNA729271	stage_E18_vs_E10	ordinary_muscle_development	ENSGALG00010002259	TASL	0.712335084	0.539064884	1.321427354	0.18635891	0.233472118	positive
PRJNA729271	stage_E18_vs_E10	ordinary_muscle_development	ENSGALG00010002261	NR0B1	-0.396196637	0.354264287	-1.1183646	0.263411307	0.318019898	negative
PRJNA729271	stage_E18_vs_E10	ordinary_muscle_development	ENSGALG00010002269	MCPH1	-1.197189264	0.140324456	-8.531579584	1.44363E-17	8.7664E-17	negative
PRJNA729271	stage_E18_vs_E10	ordinary_muscle_development	ENSGALG00010002270	IMMP2L	-0.420151037	0.258018368	-1.628376464	0.103445085	0.136772463	negative
PRJNA729271	stage_E18_vs_E10	ordinary_muscle_development	ENSGALG00010002271	ENSGALG00010002271	1.326190776	0.791595402	1.675339159	0.093867706	0.125171334	positive
PRJNA729271	stage_E18_vs_E10	ordinary_muscle_development	ENSGALG00010002274	ENSGALG00010002274	3.529116561	0.780655316	4.520710345	6.16325E-06	1.46525E-05	positive
PRJNA729271	stage_E18_vs_E10	ordinary_muscle_development	ENSGALG00010002275	IL1RAPL1	-1.83354786	0.257099476	-7.13166705	9.91605E-13	4.25876E-12	negative
PRJNA729271	stage_E18_vs_E10	ordinary_muscle_development	ENSGALG00010002277	RGS10	-1.870296319	0.180116848	-10.38379442	2.93858E-25	2.82271E-24	negative
PRJNA729271	stage_E18_vs_E10	ordinary_muscle_development	ENSGALG00010002279	ENSGALG00010002279	7.359841995	0.692064158	10.63462385	2.05657E-26	2.1202E-25	positive
PRJNA729271	stage_E18_vs_E10	ordinary_muscle_development	ENSGALG00010002280	ST7	0.823977843	0.145188531	5.675226809	1.38505E-08	4.2257E-08	positive
PRJNA729271	stage_E18_vs_E10	ordinary_muscle_development	ENSGALG00010002281	TGFBR2	2.142495583	0.154841447	13.83670603	1.53061E-43	3.38721E-42	positive
PRJNA729271	stage_E18_vs_E10	ordinary_muscle_development	ENSGALG00010002282	TSKU	-1.082495493	0.165717044	-6.532191647	6.48141E-11	2.39786E-10	negative
PRJNA729271	stage_E18_vs_E10	ordinary_muscle_development	ENSGALG00010002283	ENSGALG00010002283	5.440373851	0.650488104	8.36352551	6.08714E-17	3.54065E-16	positive
PRJNA729271	stage_E18_vs_E10	ordinary_muscle_development	ENSGALG00010002285	CAV1	2.902914778	0.15740901	18.44185908	6.06206E-76	3.73913E-74	positive
PRJNA729271	stage_E18_vs_E10	ordinary_muscle_development	ENSGALG00010002288	ANGPT2	-0.185019218	0.17792399	-1.039877856	0.298396652	0.355345011	negative
PRJNA729271	stage_E18_vs_E10	ordinary_muscle_development	ENSGALG00010002289	ENSGALG00010002289	1.643460839	0.937427517	1.75316044	0.079574471	0.107656521	positive
PRJNA729271	stage_E18_vs_E10	ordinary_muscle_development	ENSGALG00010002291	RNASEH1	-0.065026402	0.118332614	-0.549522236	0.582647109	0.635619015	negative
PRJNA729271	stage_E18_vs_E10	ordinary_muscle_development	ENSGALG00010002293	GYG2	-1.172310042	0.366453561	-3.199068496	0.001378724	0.002471507	negative
PRJNA729271	stage_E18_vs_E10	ordinary_muscle_development	ENSGALG00010002294	DAZL	1.241762331	0.832840028	1.490997418	0.135962173	0.175679417	positive
PRJNA729271	stage_E18_vs_E10	ordinary_muscle_development	ENSGALG00010002295	YAF2	-0.150745793	0.11527634	-1.307690655	0.190978254	0.238558238	negative
PRJNA729271	stage_E18_vs_E10	ordinary_muscle_development	ENSGALG00010002298	ENSGALG00010002298	-0.145108569	0.400019985	-0.362753298	0.716789182	0.759881459	negative
PRJNA729271	stage_E18_vs_E10	ordinary_muscle_development	ENSGALG00010002303	ENSGALG00010002303	1.383585081	0.838288768	1.650487439	0.09884328	0.131226722	positive
PRJNA729271	stage_E18_vs_E10	ordinary_muscle_development	ENSGALG00010002308	GXYLT1	-1.236994801	0.104939244	-11.78772364	4.51582E-32	6.07206E-31	negative
PRJNA729271	stage_E18_vs_E10	ordinary_muscle_development	ENSGALG00010002309	GADL1	4.23973259	0.326315669	12.99273369	1.34534E-38	2.44064E-37	positive
PRJNA729271	stage_E18_vs_E10	ordinary_muscle_development	ENSGALG00010002310	PRRG1	0.003381561	0.105132263	0.032164822	0.97434061	0.978865202	positive
PRJNA729271	stage_E18_vs_E10	ordinary_muscle_development	ENSGALG00010002312	ENSGALG00010002312	-1.017348002	0.194738081	-5.224186245	1.74923E-07	4.84429E-07	negative
PRJNA729271	stage_E18_vs_E10	ordinary_muscle_development	ENSGALG00010002315	ENSGALG00010002315	-0.615530053	0.177740626	-3.463080265	0.000534029	0.001010674	negative
PRJNA729271	stage_E18_vs_E10	ordinary_muscle_development	ENSGALG00010002324	SNTG2	-0.851193146	0.323347593	-2.632440022	0.008477399	0.013558413	negative
PRJNA729271	stage_E18_vs_E10	ordinary_muscle_development	ENSGALG00010002328	ENSGALG00010002328	-0.139668852	0.262240607	-0.53259811	0.59431181	0.64610738	negative
PRJNA729271	stage_E18_vs_E10	ordinary_muscle_development	ENSGALG00010002329	STT3B	-0.283385947	0.072769391	-3.894301468	9.84821E-05	0.000205484	negative
PRJNA729271	stage_E18_vs_E10	ordinary_muscle_development	ENSGALG00010002330	KHDRBS2	-0.56264892	0.283495735	-1.984682135	0.047179848	0.066518032	negative
PRJNA729271	stage_E18_vs_E10	ordinary_muscle_development	ENSGALG00010002331	ENSGALG00010002331	-1.186018664	0.285949936	-4.147644447	3.35914E-05	7.36733E-05	negative
PRJNA729271	stage_E18_vs_E10	ordinary_muscle_development	ENSGALG00010002332	ENSGALG00010002332	-2.57596185	0.641142848	-4.017765869	5.87525E-05	0.000125774	negative
PRJNA729271	stage_E18_vs_E10	ordinary_muscle_development	ENSGALG00010002333	ENSGALG00010002333	-0.045640512	0.704352672	-0.064797812	0.948334984	0.957450414	negative
PRJNA729271	stage_E18_vs_E10	ordinary_muscle_development	ENSGALG00010002334	OSBPL10	-0.344104801	0.168078593	-2.047285117	0.040630098	0.057943061	negative
PRJNA729271	stage_E18_vs_E10	ordinary_muscle_development	ENSGALG00010002335	TMEM47	0.944195771	0.134042457	7.044005273	1.86791E-12	7.82299E-12	positive
PRJNA729271	stage_E18_vs_E10	ordinary_muscle_development	ENSGALG00010002336	PRIM2	-1.202575729	0.119914028	-10.0286493	1.14066E-23	9.91836E-23	negative
PRJNA729271	stage_E18_vs_E10	ordinary_muscle_development	ENSGALG00010002338	CD99	0.388939357	0.148301672	2.622622882	0.00872558	0.013935354	positive
PRJNA729271	stage_E18_vs_E10	ordinary_muscle_development	ENSGALG00010002339	ZNFY4	-0.079064963	0.31314684	-0.252485265	0.800666002	0.833619717	negative
PRJNA729271	stage_E18_vs_E10	ordinary_muscle_development	ENSGALG00010002340	RAB23	-0.583355481	0.137232417	-4.25085772	2.12953E-05	4.78197E-05	negative
PRJNA729271	stage_E18_vs_E10	ordinary_muscle_development	ENSGALG00010002341	FAM49A	-0.420940392	0.111107721	-3.788579118	0.000151511	0.000308709	negative
PRJNA729271	stage_E18_vs_E10	ordinary_muscle_development	ENSGALG00010002342	BAG2	2.066110573	0.124006891	16.66125613	2.50703E-62	1.09787E-60	positive
PRJNA729271	stage_E18_vs_E10	ordinary_muscle_development	ENSGALG00010002343	GPD1L	-0.267225648	0.082221886	-3.250054956	0.001153827	0.002086938	negative
PRJNA729271	stage_E18_vs_E10	ordinary_muscle_development	ENSGALG00010002345	SCML2	-1.070995339	0.102643469	-10.4341304	1.73192E-25	1.6928E-24	negative
PRJNA729271	stage_E18_vs_E10	ordinary_muscle_development	ENSGALG00010002346	FBXO15	-0.01044224	0.354541145	-0.029452829	0.976503439	0.980562338	negative
PRJNA729271	stage_E18_vs_E10	ordinary_muscle_development	ENSGALG00010002347	ZNF451	-0.300400727	0.06256165	-4.801675245	1.57344E-06	3.9775E-06	negative
PRJNA729271	stage_E18_vs_E10	ordinary_muscle_development	ENSGALG00010002348	THAP12	-1.168534477	0.160256166	-7.291666242	3.06145E-13	1.36122E-12	negative
PRJNA729271	stage_E18_vs_E10	ordinary_muscle_development	ENSGALG00010002349	ZBED1	0.301001373	0.130542196	2.305778383	0.021123019	0.031731595	positive
PRJNA729271	stage_E18_vs_E10	ordinary_muscle_development	ENSGALG00010002350	BEND6	0.490288736	0.248199364	1.975382727	0.048224731	0.067878955	positive
PRJNA729271	stage_E18_vs_E10	ordinary_muscle_development	ENSGALG00010002352	ENSGALG00010002352	0.325191148	0.106946543	3.04068874	0.002360377	0.004097936	positive
PRJNA729271	stage_E18_vs_E10	ordinary_muscle_development	ENSGALG00010002353	CMTM8	0.600082624	0.13866857	4.327459537	1.50839E-05	3.44751E-05	positive
PRJNA729271	stage_E18_vs_E10	ordinary_muscle_development	ENSGALG00010002355	UFM1	-0.621164957	0.100985223	-6.151047997	7.69726E-10	2.62992E-09	negative
PRJNA729271	stage_E18_vs_E10	ordinary_muscle_development	ENSGALG00010002356	WNT11	-3.020573361	0.32735635	-9.227172033	2.77871E-20	1.98803E-19	negative
PRJNA729271	stage_E18_vs_E10	ordinary_muscle_development	ENSGALG00010002357	RAB31	-0.036124388	0.141321121	-0.255619176	0.79824491	0.831397569	negative
PRJNA729271	stage_E18_vs_E10	ordinary_muscle_development	ENSGALG00010002358	FREM2	-0.360816011	0.233840903	-1.542997852	0.122831297	0.160183504	negative
PRJNA729271	stage_E18_vs_E10	ordinary_muscle_development	ENSGALG00010002359	CMTM6	-0.266845453	0.117107518	-2.278636402	0.022688688	0.033897396	negative
PRJNA729271	stage_E18_vs_E10	ordinary_muscle_development	ENSGALG00010002360	DHRSX	1.368098253	0.163069824	8.389646957	4.87604E-17	2.86031E-16	positive
PRJNA729271	stage_E18_vs_E10	ordinary_muscle_development	ENSGALG00010002361	ENSGALG00010002361	-0.268940774	0.099820625	-2.694240526	0.007054923	0.011400347	negative
PRJNA729271	stage_E18_vs_E10	ordinary_muscle_development	ENSGALG00010002362	TRPC4	-1.815468409	0.426644014	-4.255229999	2.08834E-05	4.69564E-05	negative
PRJNA729271	stage_E18_vs_E10	ordinary_muscle_development	ENSGALG00010002364	VSNL1	0.34776721	0.391221982	0.888925535	0.374043101	0.433913946	positive
PRJNA729271	stage_E18_vs_E10	ordinary_muscle_development	ENSGALG00010002365	RAI2	0.732570972	0.138730805	5.280521317	1.28817E-07	3.6137E-07	positive
PRJNA729271	stage_E18_vs_E10	ordinary_muscle_development	ENSGALG00010002366	ENSGALG00010002366	1.064557202	0.756704595	1.406833272	0.159476834	0.202844569	positive
PRJNA729271	stage_E18_vs_E10	ordinary_muscle_development	ENSGALG00010002367	STK31	-6.44128198	1.023871724	-6.291102515	3.15219E-10	1.10793E-09	negative
PRJNA729271	stage_E18_vs_E10	ordinary_muscle_development	ENSGALG00010002368	UVRAG	0.428315911	0.096272727	4.448984925	8.62771E-06	2.02327E-05	positive
PRJNA729271	stage_E18_vs_E10	ordinary_muscle_development	ENSGALG00010002369	GEMIN8	-0.915202357	0.122728026	-7.457158623	8.84082E-14	4.08374E-13	negative
PRJNA729271	stage_E18_vs_E10	ordinary_muscle_development	ENSGALG00010002370	ENSGALG00010002370	0.420706457	0.153909331	2.733469469	0.006267093	0.010232205	positive
PRJNA729271	stage_E18_vs_E10	ordinary_muscle_development	ENSGALG00010002371	BAG3	1.962303222	0.1550679	12.65447733	1.05677E-36	1.77289E-35	positive
PRJNA729271	stage_E18_vs_E10	ordinary_muscle_development	ENSGALG00010002372	SMC6	-0.412372712	0.127698945	-3.229256991	0.001241123	0.002235626	negative
PRJNA729271	stage_E18_vs_E10	ordinary_muscle_development	ENSGALG00010002373	POSTN	0.495481083	0.255607188	1.938447374	0.052568665	0.073500801	positive
PRJNA729271	stage_E18_vs_E10	ordinary_muscle_development	ENSGALG00010002374	DST	0.594097867	0.114294085	5.197975635	2.01471E-07	5.55193E-07	positive
PRJNA729271	stage_E18_vs_E10	ordinary_muscle_development	ENSGALG00010002375	NPY	-5.183293025	0.580560599	-8.928082678	4.33456E-19	2.90473E-18	negative
PRJNA729271	stage_E18_vs_E10	ordinary_muscle_development	ENSGALG00010002376	GEN1	-1.020217932	0.192607202	-5.29688362	1.17796E-07	3.31543E-07	negative
PRJNA729271	stage_E18_vs_E10	ordinary_muscle_development	ENSGALG00010002377	ART1A	3.633645682	0.248660165	14.61289824	2.32417E-48	6.33248E-47	positive
PRJNA729271	stage_E18_vs_E10	ordinary_muscle_development	ENSGALG00010002378	ENSGALG00010002378	0.80184559	0.447527204	1.791724802	0.07317706	0.09968062	positive
PRJNA729271	stage_E18_vs_E10	ordinary_muscle_development	ENSGALG00010002379	NHS	-1.19390303	0.133557025	-8.939275421	3.9173E-19	2.62755E-18	negative
PRJNA729271	stage_E18_vs_E10	ordinary_muscle_development	ENSGALG00010002380	CMPK2	-0.759412157	0.760107824	-0.999084779	0.317753624	0.375312808	negative
PRJNA729271	stage_E18_vs_E10	ordinary_muscle_development	ENSGALG00010002381	TDRD3	0.096503941	0.096941337	0.995488037	0.31949896	0.377159168	positive
PRJNA729271	stage_E18_vs_E10	ordinary_muscle_development	ENSGALG00010002382	ART1D	-1.094072882	0.774569176	-1.412492151	0.1578051	0.200912159	negative
PRJNA729271	stage_E18_vs_E10	ordinary_muscle_development	ENSGALG00010002383	INPP5F	-0.031241088	0.125995917	-0.247953177	0.804170635	0.836607576	negative
PRJNA729271	stage_E18_vs_E10	ordinary_muscle_development	ENSGALG00010002384	ENSGALG00010002384	-1.016437403	0.309737319	-3.281611038	0.001032159	0.001876499	negative
PRJNA729271	stage_E18_vs_E10	ordinary_muscle_development	ENSGALG00010002385	PALS2	0.023264996	0.123636102	0.188173163	0.85074091	0.876625399	positive
PRJNA729271	stage_E18_vs_E10	ordinary_muscle_development	ENSGALG00010002386	MHCY2B2	-0.708716439	0.3839796	-1.845713784	0.064933775	0.089257	negative
PRJNA729271	stage_E18_vs_E10	ordinary_muscle_development	ENSGALG00010002387	MCMBP	-0.361454078	0.10942397	-3.30324406	0.000955732	0.001745411	negative
PRJNA729271	stage_E18_vs_E10	ordinary_muscle_development	ENSGALG00010002388	ENSGALG00010002388	2.081434131	0.820542032	2.536657538	0.011191638	0.017594662	positive
PRJNA729271	stage_E18_vs_E10	ordinary_muscle_development	ENSGALG00010002389	COL21A1	-0.292882632	0.322225383	-0.908937184	0.363383284	0.422867751	negative
PRJNA729271	stage_E18_vs_E10	ordinary_muscle_development	ENSGALG00010002391	REPS2	0.367832906	0.159885904	2.300596215	0.021414465	0.032129456	positive
PRJNA729271	stage_E18_vs_E10	ordinary_muscle_development	ENSGALG00010002392	DFNA5	-1.409187709	0.207758322	-6.782821972	1.17851E-11	4.60568E-11	negative
PRJNA729271	stage_E18_vs_E10	ordinary_muscle_development	ENSGALG00010002393	GPM6B	-0.296662747	0.107069288	-2.770754842	0.005592652	0.009201531	negative
PRJNA729271	stage_E18_vs_E10	ordinary_muscle_development	ENSGALG00010002395	CLN8	0.409444736	0.090651976	4.516666441	6.28207E-06	1.49179E-05	positive
PRJNA729271	stage_E18_vs_E10	ordinary_muscle_development	ENSGALG00010002396	SEC23IP	-0.278140633	0.089319653	-3.113991412	0.001845749	0.003260313	negative
PRJNA729271	stage_E18_vs_E10	ordinary_muscle_development	ENSGALG00010002398	ENSGALG00010002398	-4.046736862	0.543190916	-7.449934716	9.33864E-14	4.29999E-13	negative
PRJNA729271	stage_E18_vs_E10	ordinary_muscle_development	ENSGALG00010002399	CSMD1	0.216435418	0.390039542	0.554906349	0.578958719	0.631975798	positive
PRJNA729271	stage_E18_vs_E10	ordinary_muscle_development	ENSGALG00010002400	RBBP7	-0.849236602	0.154974703	-5.47984016	4.2571E-08	1.24715E-07	negative
PRJNA729271	stage_E18_vs_E10	ordinary_muscle_development	ENSGALG00010002401	ZRANB1	0.136160664	0.161749223	0.841801037	0.399899331	0.459787484	positive
PRJNA729271	stage_E18_vs_E10	ordinary_muscle_development	ENSGALG00010002402	TXLNG	0.927215378	0.125566192	7.384275697	1.53286E-13	6.95205E-13	positive
PRJNA729271	stage_E18_vs_E10	ordinary_muscle_development	ENSGALG00010002403	ZNFY3	-0.701810363	0.27031437	-2.596274713	0.009424068	0.014958593	negative
PRJNA729271	stage_E18_vs_E10	ordinary_muscle_development	ENSGALG00010002404	OSBPL3	-1.787138553	0.259542897	-6.88571551	5.74978E-12	2.31444E-11	negative
PRJNA729271	stage_E18_vs_E10	ordinary_muscle_development	ENSGALG00010002405	KCNQ5	0.90319997	0.198135853	4.558488305	5.15231E-06	1.23483E-05	positive
PRJNA729271	stage_E18_vs_E10	ordinary_muscle_development	ENSGALG00010002406	DIAPH3	-0.858429473	0.246448086	-3.48320608	0.000495447	0.000942331	negative
PRJNA729271	stage_E18_vs_E10	ordinary_muscle_development	ENSGALG00010002408	OFD1	-0.218304565	0.116494844	-1.873941865	0.060938438	0.084212286	negative
PRJNA729271	stage_E18_vs_E10	ordinary_muscle_development	ENSGALG00010002409	CYCS	-0.162723653	0.16713418	-0.973610862	0.330249791	0.38836368	negative
PRJNA729271	stage_E18_vs_E10	ordinary_muscle_development	ENSGALG00010002410	RNF121	0.336631732	0.105094063	3.203147006	0.001359346	0.002438579	positive
PRJNA729271	stage_E18_vs_E10	ordinary_muscle_development	ENSGALG00010002411	ENSGALG00010002411	-0.716558009	0.180210433	-3.976229328	7.00166E-05	0.000148593	negative
PRJNA729271	stage_E18_vs_E10	ordinary_muscle_development	ENSGALG00010002413	TSSC1	-0.926379497	0.087128988	-10.63227654	2.109E-26	2.17117E-25	negative
PRJNA729271	stage_E18_vs_E10	ordinary_muscle_development	ENSGALG00010002414	ENSGALG00010002414	-0.113864501	0.374591974	-0.303969409	0.761151197	0.79960042	negative

PRJNA729271	stage_E18_vs_E10	ordinary_muscle_development	ENSGALG00010002415	PCDH17	-3.8357026	0.340238062	-11.27358466	1.77205E-29	2.10886E-28	negative
PRJNA729271	stage_E18_vs_E10	ordinary_muscle_development	ENSGALG00010002416	SH3BGRL2	-0.464322499	0.266811099	-1.740266806	0.08181218	0.110415973	negative
PRJNA729271	stage_E18_vs_E10	ordinary_muscle_development	ENSGALG00010002418	TRAPPC2	-0.848117799	0.085204	-9.953966895	2.42327E-23	2.06619E-22	negative
PRJNA729271	stage_E18_vs_E10	ordinary_muscle_development	ENSGALG00010002419	CTBP2	-0.910806569	0.114579624	-7.949114633	1.87849E-15	9.88339E-15	negative
PRJNA729271	stage_E18_vs_E10	ordinary_muscle_development	ENSGALG00010002420	TMEM272	0.251368198	0.702252055	0.357945834	0.72038385	0.76312253	positive
PRJNA729271	stage_E18_vs_E10	ordinary_muscle_development	ENSGALG00010002421	ENSGALG00010002421	-0.160182762	0.188956784	-0.847721679	0.39659299	0.456601699	negative
PRJNA729271	stage_E18_vs_E10	ordinary_muscle_development	ENSGALG00010002422	TRAPPC12	0.083391143	0.104374852	0.798958191	0.424314655	0.484744714	positive
PRJNA729271	stage_E18_vs_E10	ordinary_muscle_development	ENSGALG00010002423	ENSGALG00010002423	0.350902302	0.58408056	0.600777232	0.547988371	0.602433935	positive
PRJNA729271	stage_E18_vs_E10	ordinary_muscle_development	ENSGALG00010002424	CASK	-0.347574167	0.057137848	-6.083081144	1.17895E-09	3.95209E-09	negative
PRJNA729271	stage_E18_vs_E10	ordinary_muscle_development	ENSGALG00010002425	FDX2	0.403975936	0.134472925	3.004143299	0.002663299	0.004594134	positive
PRJNA729271	stage_E18_vs_E10	ordinary_muscle_development	ENSGALG00010002427	CYB5R4	-0.338259653	0.114891029	-2.944178104	0.003238136	0.005511599	negative
PRJNA729271	stage_E18_vs_E10	ordinary_muscle_development	ENSGALG00010002428	IBSP	-1.482832848	0.702301754	-2.111389925	0.03473881	0.050153292	negative
PRJNA729271	stage_E18_vs_E10	ordinary_muscle_development	ENSGALG00010002429	ENSGALG00010002429	0.637336028	0.545047853	1.169321235	0.242274231	0.295279995	positive
PRJNA729271	stage_E18_vs_E10	ordinary_muscle_development	ENSGALG00010002430	NUMA1	0.65776479	0.174509773	3.769214627	0.000163762	0.000331944	positive
PRJNA729271	stage_E18_vs_E10	ordinary_muscle_development	ENSGALG00010002431	BCKDHB	1.205421333	0.147521533	8.171155165	3.0545E-16	1.69441E-15	positive
PRJNA729271	stage_E18_vs_E10	ordinary_muscle_development	ENSGALG00010002432	RAVER1	-0.151161187	0.176347084	-0.857179964	0.391345433	0.451420664	negative
PRJNA729271	stage_E18_vs_E10	ordinary_muscle_development	ENSGALG00010002433	GRHL1	-0.891068829	0.323492197	-2.754529592	0.005877657	0.00963436	negative
PRJNA729271	stage_E18_vs_E10	ordinary_muscle_development	ENSGALG00010002434	GPR34	2.136174461	0.247273289	8.638921208	5.67463E-18	3.54237E-17	positive
PRJNA729271	stage_E18_vs_E10	ordinary_muscle_development	ENSGALG00010002435	ATP7B	0.57229908	0.63304077	0.904047744	0.365970086	0.425570509	positive
PRJNA729271	stage_E18_vs_E10	ordinary_muscle_development	ENSGALG00010002436	ENSGALG00010002436	2.686231485	0.875459347	3.068368045	0.002152314	0.00376285	positive
PRJNA729271	stage_E18_vs_E10	ordinary_muscle_development	ENSGALG00010002437	TTK	-1.813847386	0.196040763	-9.252399123	2.19509E-20	1.58535E-19	negative
PRJNA729271	stage_E18_vs_E10	ordinary_muscle_development	ENSGALG00010002438	RARB	-0.783170807	0.091184707	-8.588839442	8.78522E-18	5.405E-17	negative
PRJNA729271	stage_E18_vs_E10	ordinary_muscle_development	ENSGALG00010002439	GPR82	1.218255105	1.051895579	1.15815213	0.24680196	0.300041466	positive
PRJNA729271	stage_E18_vs_E10	ordinary_muscle_development	ENSGALG00010002440	LRRC51	0.825977421	0.266084208	3.104195579	0.001907972	0.003360122	positive
PRJNA729271	stage_E18_vs_E10	ordinary_muscle_development	ENSGALG00010002441	USO1	-0.093122098	0.068021117	-1.369017481	0.170993811	0.215977282	negative
PRJNA729271	stage_E18_vs_E10	ordinary_muscle_development	ENSGALG00010002442	ENSGALG00010002442	0.531427511	0.450453936	1.179759947	0.238095704	0.290749556	positive
PRJNA729271	stage_E18_vs_E10	ordinary_muscle_development	ENSGALG00010002443	TYK2	0.598039374	0.138119932	4.329855682	1.49207E-05	3.41129E-05	positive
PRJNA729271	stage_E18_vs_E10	ordinary_muscle_development	ENSGALG00010002444	KLF11	1.535045732	0.181020783	8.479941962	2.25306E-17	1.34895E-16	positive
PRJNA729271	stage_E18_vs_E10	ordinary_muscle_development	ENSGALG00010002445	EDRF1	0.120857413	0.112078989	1.078323551	0.280889391	0.33651473	positive
PRJNA729271	stage_E18_vs_E10	ordinary_muscle_development	ENSGALG00010002446	RIMS1	-2.675371763	0.26356296	-10.15078814	3.28698E-24	2.96483E-23	negative
PRJNA729271	stage_E18_vs_E10	ordinary_muscle_development	ENSGALG00010002447	YLEC12	-0.039295063	0.568573777	-0.069111635	0.94490076	0.954980931	negative
PRJNA729271	stage_E18_vs_E10	ordinary_muscle_development	ENSGALG00010002448	RAB9A	-0.445633062	0.13669208	-3.26012349	0.001113637	0.002017267	negative
PRJNA729271	stage_E18_vs_E10	ordinary_muscle_development	ENSGALG00010002449	ALG11	0.182639627	0.136179955	1.34116381	0.179867273	0.226062267	positive
PRJNA729271	stage_E18_vs_E10	ordinary_muscle_development	ENSGALG00010002453	TOP2B	-0.59517765	0.063141027	-9.426163609	4.25363E-21	3.22302E-20	negative
PRJNA729271	stage_E18_vs_E10	ordinary_muscle_development	ENSGALG00010002454	NEK5	-1.83175524	0.584182347	-3.135588143	0.001715099	0.00304065	negative
PRJNA729271	stage_E18_vs_E10	ordinary_muscle_development	ENSGALG00010002455	NYX	0.868651237	0.367582807	2.363144357	0.01812061	0.027523655	positive
PRJNA729271	stage_E18_vs_E10	ordinary_muscle_development	ENSGALG00010002456	ELOVL4	-0.746552171	0.481247336	-1.551285827	0.120833202	0.157890314	negative
PRJNA729271	stage_E18_vs_E10	ordinary_muscle_development	ENSGALG00010002457	ENSGALG00010002457	0.598980174	0.446435262	1.341695482	0.179694752	0.22588461	positive
PRJNA729271	stage_E18_vs_E10	ordinary_muscle_development	ENSGALG00010002458	ENSGALG00010002458	1.062747949	0.197600664	5.378261028	7.52087E-08	2.15232E-07	positive
PRJNA729271	stage_E18_vs_E10	ordinary_muscle_development	ENSGALG00010002459	G3BP2	-0.788918778	0.074968359	-10.52335663	6.74278E-26	6.71267E-25	negative
PRJNA729271	stage_E18_vs_E10	ordinary_muscle_development	ENSGALG00010002460	LAMTOR1	0.199734713	0.184430905	1.082978545	0.278817946	0.334392366	positive
PRJNA729271	stage_E18_vs_E10	ordinary_muscle_development	ENSGALG00010002461	TOMT	0.348487033	0.332153097	1.049175924	0.294097158	0.350685941	positive
PRJNA729271	stage_E18_vs_E10	ordinary_muscle_development	ENSGALG00010002462	RRM2	-2.463455324	0.275575255	-8.939319761	3.91573E-19	2.62755E-18	negative
PRJNA729271	stage_E18_vs_E10	ordinary_muscle_development	ENSGALG00010002463	ASRGL1	0.085109133	0.103452303	0.822689597	0.410684533	0.470880581	positive
PRJNA729271	stage_E18_vs_E10	ordinary_muscle_development	ENSGALG00010002464	NGLY1	0.299980861	0.083653334	3.586000065	0.000335789	0.000652973	positive
PRJNA729271	stage_E18_vs_E10	ordinary_muscle_development	ENSGALG00010002466	UROS	-0.232789841	0.181726231	-1.280991961	0.20019649	0.249064297	negative
PRJNA729271	stage_E18_vs_E10	ordinary_muscle_development	ENSGALG00010002467	NEK3	-1.158428302	0.219519847	-5.277100546	1.31244E-07	3.6761E-07	negative
PRJNA729271	stage_E18_vs_E10	ordinary_muscle_development	ENSGALG00010002468	ENSGALG00010002468	0.165063269	0.554641471	0.297603547	0.766005767	0.80360291	positive
PRJNA729271	stage_E18_vs_E10	ordinary_muscle_development	ENSGALG00010002469	MEPE	-0.970688642	1.179197501	-0.823177323	0.410407162	0.470637011	negative
PRJNA729271	stage_E18_vs_E10	ordinary_muscle_development	ENSGALG00010002470	OXSM	0.030065513	0.165644212	0.181506571	0.855969976	0.880827232	positive
PRJNA729271	stage_E18_vs_E10	ordinary_muscle_development	ENSGALG00010002471	OGFRL1	-0.186791856	0.153655166	-1.215656203	0.224115915	0.275231736	negative
PRJNA729271	stage_E18_vs_E10	ordinary_muscle_development	ENSGALG00010002472	CDC37	-0.277903752	0.21499118	-1.29262862	0.196139536	0.244457659	negative
PRJNA729271	stage_E18_vs_E10	ordinary_muscle_development	ENSGALG00010002473	ENSGALG00010002473	0.496870455	0.644458701	0.770988822	0.440713557	0.500285301	positive
PRJNA729271	stage_E18_vs_E10	ordinary_muscle_development	ENSGALG00010002474	HPCAL1	-0.156018998	0.14206359	-1.098233525	0.272102531	0.327259059	negative
PRJNA729271	stage_E18_vs_E10	ordinary_muscle_development	ENSGALG00010002475	ENSGALG00010002475	0.25957486	0.185479334	1.399481301	0.161668702	0.205380178	positive
PRJNA729271	stage_E18_vs_E10	ordinary_muscle_development	ENSGALG00010002476	DDX3X	0.510603033	0.144973904	3.522034105	0.000428249	0.000822073	positive
PRJNA729271	stage_E18_vs_E10	ordinary_muscle_development	ENSGALG00010002478	PPME1	-0.690236628	0.056727626	-12.16755706	4.62725E-34	6.83014E-33	negative
PRJNA729271	stage_E18_vs_E10	ordinary_muscle_development	ENSGALG00010002481	CKAP2	-1.566412031	0.245456662	-6.381623614	1.7522E-10	6.27408E-10	negative
PRJNA729271	stage_E18_vs_E10	ordinary_muscle_development	ENSGALG00010002483	COQ2	0.618554059	0.142807594	4.331380702	1.48177E-05	3.38828E-05	positive
PRJNA729271	stage_E18_vs_E10	ordinary_muscle_development	ENSGALG00010002487	BCCIP	-1.078613012	0.119832772	-9.000985261	2.23701E-19	1.51805E-18	negative
PRJNA729271	stage_E18_vs_E10	ordinary_muscle_development	ENSGALG00010002492	RPL8	1.137295988	0.129552395	8.778656579	1.65439E-18	1.0696E-17	positive
PRJNA729271	stage_E18_vs_E10	ordinary_muscle_development	ENSGALG00010002500	OSR1	-0.935969001	0.197503396	-4.739002056	2.14773E-06	5.34536E-06	negative
PRJNA729271	stage_E18_vs_E10	ordinary_muscle_development	ENSGALG00010002501	TCEANC	-0.552422604	0.09986075	-5.53192927	3.16728E-08	9.39809E-08	negative
PRJNA729271	stage_E18_vs_E10	ordinary_muscle_development	ENSGALG00010002504	ENSGALG00010002504	-0.221603163	0.065966116	-3.359348363	0.000781265	0.001446473	negative
PRJNA729271	stage_E18_vs_E10	ordinary_muscle_development	ENSGALG00010002507	C2CD3	-0.155215525	0.110270185	-1.40759286	0.159251663	0.202611503	negative
PRJNA729271	stage_E18_vs_E10	ordinary_muscle_development	ENSGALG00010002511	VPS36	-0.290961695	0.077935528	-3.733364007	0.000188939	0.000379949	negative
PRJNA729271	stage_E18_vs_E10	ordinary_muscle_development	ENSGALG00010002512	DAXX	0.208579145	0.23462766	0.888979351	0.374014177	0.433913946	positive
PRJNA729271	stage_E18_vs_E10	ordinary_muscle_development	ENSGALG00010002518	MAST1	0.193057352	0.372254692	0.518616302	0.604028339	0.655098449	positive
PRJNA729271	stage_E18_vs_E10	ordinary_muscle_development	ENSGALG00010002520	TTC32	0.9353591	0.163947654	5.705230157	1.16186E-08	3.56653E-08	positive
PRJNA729271	stage_E18_vs_E10	ordinary_muscle_development	ENSGALG00010002522	HPSE	-0.607688912	0.334893265	-1.814574895	0.069589263	0.095249894	negative
PRJNA729271	stage_E18_vs_E10	ordinary_muscle_development	ENSGALG00010002523	DNAJB13	0.96703494	0.447276494	2.162051782	0.030614185	0.044670084	positive
PRJNA729271	stage_E18_vs_E10	ordinary_muscle_development	ENSGALG00010002526	ZBTB22	0.773034121	0.10417755	7.420352318	1.16809E-13	5.34454E-13	positive
PRJNA729271	stage_E18_vs_E10	ordinary_muscle_development	ENSGALG00010002527	LRRC3B	-0.054270247	0.164327509	-0.330256616	0.741206071	0.781919382	negative
PRJNA729271	stage_E18_vs_E10	ordinary_muscle_development	ENSGALG00010002528	MED14	-0.456442172	0.118176637	-3.86237231	0.000112291	0.000232889	negative
PRJNA729271	stage_E18_vs_E10	ordinary_muscle_development	ENSGALG00010002530	STAG3	0.433910157	0.121614706	3.567908632	0.000359842	0.000697221	positive
PRJNA729271	stage_E18_vs_E10	ordinary_muscle_development	ENSGALG00010002533	LPAR2	-0.580577579	0.145802525	-3.981944603	6.83537E-05	0.00014517	negative
PRJNA729271	stage_E18_vs_E10	ordinary_muscle_development	ENSGALG00010002536	RGL2	1.297984743	0.14468364	8.97119218	2.93322E-19	1.97938E-18	positive
PRJNA729271	stage_E18_vs_E10	ordinary_muscle_development	ENSGALG00010002539	SNAP91	-0.449777299	0.201180642	-2.235688757	0.025372166	0.037600403	negative
PRJNA729271	stage_E18_vs_E10	ordinary_muscle_development	ENSGALG00010002543	UCP3	2.335836238	0.535437334	4.362482945	1.28595E-05	2.96152E-05	positive
PRJNA729271	stage_E18_vs_E10	ordinary_muscle_development	ENSGALG00010002544	THSD1	1.214117532	0.124579503	9.745724649	1.92401E-22	1.57473E-21	positive
PRJNA729271	stage_E18_vs_E10	ordinary_muscle_development	ENSGALG00010002546	ATP6AP2	0.379821127	0.064054123	5.929690585	3.03506E-09	9.82869E-09	positive
PRJNA729271	stage_E18_vs_E10	ordinary_muscle_development	ENSGALG00010002547	PBX4	-0.567416774	0.103110847	-5.502978474	3.73428E-08	1.10174E-07	negative
PRJNA729271	stage_E18_vs_E10	ordinary_muscle_development	ENSGALG00010002551	WDR35	-0.168688619	0.109612358	-1.538956207	0.123814988	0.161408242	negative
PRJNA729271	stage_E18_vs_E10	ordinary_muscle_development	ENSGALG00010002553	ENSGALG00010002553	0.460674393	0.225439807	2.043447423	0.041008167	0.058436235	positive
PRJNA729271	stage_E18_vs_E10	ordinary_muscle_development	ENSGALG00010002554	EGFL6	-1.119455397	0.292843332	-3.8227109	0.000131993	0.00027138	negative
PRJNA729271	stage_E18_vs_E10	ordinary_muscle_development	ENSGALG00010002556	ENSGALG00010002556	4.917723568	0.22958335	21.42021003	8.6598E-102	1.06376E-99	positive
PRJNA729271	stage_E18_vs_E10	ordinary_muscle_development	ENSGALG00010002557	ENSGALG00010002557	0.611807876	0.409494151	1.494057668	0.135160547	0.174737079	positive
PRJNA729271	stage_E18_vs_E10	ordinary_muscle_development	ENSGALG00010002559	NEK10	-0.316127449	0.53990229	-0.58552715	0.558193316	0.612167067	negative
PRJNA729271	stage_E18_vs_E10	ordinary_muscle_development	ENSGALG00010002561	PAAF1	-0.108575044	0.083006334	-1.308033227	0.19086204	0.238433618	negative
PRJNA729271	stage_E18_vs_E10	ordinary_muscle_development	ENSGALG00010002565	MRPS18C	-0.05820075	0.118716421	-0.490250207	0.623956857	0.674441062	negative
PRJNA729271	stage_E18_vs_E10	ordinary_muscle_development	ENSGALG00010002566	TMSB4X	-1.030498847	0.115731212	-8.90424314	5.37515E-19	3.58055E-18	negative
PRJNA729271	stage_E18_vs_E10	ordinary_muscle_development	ENSGALG00010002567	COA4	0.356586026	0.225050857	1.584468646	0.113087111	0.148545326	positive
PRJNA729271	stage_E18_vs_E10	ordinary_muscle_development	ENSGALG00010002569	GTPBP6	-0.016127019	0.1196527	-0.134781905	0.892784313	0.911223097	negative
PRJNA729271	stage_E18_vs_E10	ordinary_muscle_development	ENSGALG00010002570	ADAM12	-0.47120568	0.267050137	-1.764483944	0.077650536	0.105257785	negative
PRJNA729271	stage_E18_vs_E10	ordinary_muscle_development	ENSGALG00010002571	BCOR	0.807407946	0.141091948	5.722565727	1.04927E-08	3.23394E-08	positive
PRJNA729271	stage_E18_vs_E10	ordinary_muscle_development	ENSGALG00010002573	MVB12A	1.226928446	0.094835053	12.9374995	2.76483E-38	4.94767E-37	positive
PRJNA729271	stage_E18_vs_E10	ordinary_muscle_development	ENSGALG00010002575	BGV	0.770641256	0.399749897	1.927808517	0.053878948	0.075195008	positive
PRJNA729271	stage_E18_vs_E10	ordinary_muscle_development	ENSGALG00010002576	HELQ	-0.514759078	0.109808862	-4.687773555	2.76193E-06	6.79583E-06	negative
PRJNA729271	stage_E18_vs_E10	ordinary_muscle_development	ENSGALG00010002577	MRPL48	-0.174717724	0.104040322	-1.679327028	0.093088326	0.124212031	negative
PRJNA729271	stage_E18_vs_E10	ordinary_muscle_development	ENSGALG00010002579	MATN3	0.215378041	0.565385414	0.380940214	0.703247612	0.748042425	positive
PRJNA729271	stage_E18_vs_E10	ordinary_muscle_development	ENSGALG00010002581	ATP13A1	-0.305570592	0.078439084	-3.895642049	9.79389E-05	0.00020441	negative
PRJNA729271	stage_E18_vs_E10	ordinary_muscle_development	ENSGALG00010002582	ENSGALG00010002582	-0.832255043	0.391790703	-2.124233774	0.0336506	0.048719762	negative
PRJNA729271	stage_E18_vs_E10	ordinary_muscle_development	ENSGALG00010002584	ENSGALG00010002584	1.069055806	0.441174876	2.42320192	0.015384373	0.023702858	positive
PRJNA729271	stage_E18_vs_E10	ordinary_muscle_development	ENSGALG00010002586	GPR63	-0.297408503	0.148319128	-2.005193174	0.0449424	0.063592355	negative
PRJNA729271	stage_E18_vs_E10	ordinary_muscle_development	ENSGALG00010002587	DOCK1	-0.353240943	0.066857964	-5.283453478	1.26771E-07	3.55976E-07	negative
PRJNA729271	stage_E18_vs_E10	ordinary_muscle_development	ENSGALG00010002588	FAM175A	-0.361482839	0.086001953	-4.203193379	2.63176E-05	5.84902E-05	negative
PRJNA729271	stage_E18_vs_E10	ordinary_muscle_development	ENSGALG00010002589	SLC4A7	0.822386599	0.144229796	5.701918915	1.18466E-08	3.63422E-08	positive
PRJNA729271	stage_E18_vs_E10	ordinary_muscle_development	ENSGALG00010002590	ATP6V1C2	-0.945674131	0.256356618	-3.688900791	0.000225225	0.000448562	negative
PRJNA729271	stage_E18_vs_E10	ordinary_muscle_development	ENSGALG00010002591	ENSGALG00010002591	1.073635418	0.211785071	5.069457518	3.98951E-07	1.07248E-06	positive
PRJNA729271	stage_E18_vs_E10	ordinary_muscle_development	ENSGALG00010002592	RPS6KA3	0.499251353	0.101618582	4.912992704	8.96966E-07	2.33379E-06	positive
PRJNA729271	stage_E18_vs_E10	ordinary_muscle_development	ENSGALG00010002597	ENSGALG00010002597	-0.840427302	0.120936164	-6.949346456	3.66982E-12	1.50351E-11	negative
PRJNA729271	stage_E18_vs_E10	ordinary_muscle_development	ENSGALG00010002598	NMT2	0.007965659	0.1029473	0.077376083	0.938324367	0.949526089	positive
PRJNA729271	stage_E18_vs_E10	ordinary_muscle_development	ENSGALG00010002599	KIFC1	-1.172269221	0.219722229	-5.335232696	9.54221E-08	2.71098E-07	negative
PRJNA729271	stage_E18_vs_E10	ordinary_muscle_development	ENSGALG00010002600	TRPA1	0.647845918	0.529841716	1.22271595	0.221437	0.272357176	positive
PRJNA729271	stage_E18_vs_E10	ordinary_muscle_development	ENSGALG00010002601	PLCXD1	2.740173661	0.186356897	14.70390262	6.08494E-49	1.69618E-47	positive
PRJNA729271	stage_E18_vs_E10	ordinary_muscle_development	ENSGALG00010002603	INSYN2A	-0.934484737	0.26926775	-3.470466623	0.000519555	0.000985339	negative
PRJNA729271	stage_E18_vs_E10	ordinary_muscle_development	ENSGALG00010002604	BLEC3	-0.152613434	0.394720491	-0.386636714	0.699025174	0.743987803	negative
PRJNA729271	stage_E18_vs_E10	ordinary_muscle_development	ENSGALG00010002605	EOMES	-1.076360173	0.971321267	-1.10814023	0.267801259	0.32280054	negative
PRJNA729271	stage_E18_vs_E10	ordinary_muscle_development	ENSGALG00010002608	RPP38	-0.401857145	0.131009671	-3.067385352	0.002159403	0.003774333	negative
PRJNA729271	stage_E18_vs_E10	ordinary_muscle_development	ENSGALG00010002609	PDIA6	-0.55727596	0.125250207	-4.449301705	8.61499E-06	2.02127E-05	negative
PRJNA729271	stage_E18_vs_E10	ordinary_muscle_development	ENSGALG00010002611	NPS	-1.075390583	0.48103732	-2.235565806	0.025380227	0.037608504	negative
PRJNA729271	stage_E18_vs_E10	ordinary_muscle_development	ENSGALG00010002612	JPH1	2.463718073	0.214577877	11.48169656	1.63049E-30	2.04093E-29	positive
PRJNA729271	stage_E18_vs_E10	ordinary_muscle_development	ENSGALG00010002613	ENSGALG00010002613	-0.259214137	0.106228979	-2.440145236	0.014681358	0.022698747	negative
PRJNA729271	stage_E18_vs_E10	ordinary_muscle_development	ENSGALG00010002614	GPAT3	0.836648226	0.14445667	5.791689819	6.96817E-09	2.18245E-08	positive
PRJNA729271	stage_E18_vs_E10	ordinary_muscle_development	ENSGALG00010002616	ZNFB3	-0.24394124	0.168212373	-1.45019796	0.147003324	0.188526676	negative
PRJNA729271	stage_E18_vs_E10	ordinary_muscle_development	ENSGALG00010002619	RAB6A	0.221447307	0.067411952	3.284985811	0.001019875	0.00185554	positive
PRJNA729271	stage_E18_vs_E10	ordinary_muscle_development	ENSGALG00010002621	SLC25A15	-1.305878909	0.165461533	-7.892341417	2.96569E-15	1.54078E-14	negative
PRJNA729271	stage_E18_vs_E10	ordinary_muscle_development	ENSGALG00010002622	AZI2	-0.20562807	0.081623998	-2.519210962	0.011761816	0.018431084	negative
PRJNA729271	stage_E18_vs_E10	ordinary_muscle_development	ENSGALG00010002623	MID1IP1	0.060178487	0.138141037	0.435630772	0.66310464	0.710714416	positive
PRJNA729271	stage_E18_vs_E10	ordinary_muscle_development	ENSGALG00010002625	FHL5	0.50750168	0.219634468	2.310665013	0.020851365	0.031349501	positive
PRJNA729271	stage_E18_vs_E10	ordinary_muscle_development	ENSGALG00010002626	CHD7	0.222550936	0.136494886	1.630470879	0.103002015	0.136223924	positive

PRJNA729271	stage_E18_vs_E10	ordinary_muscle_development	ENSGALG00010002627	MRPS31	-0.150885767	0.146789999	-1.027902231	0.30399582	0.361152316	negative
PRJNA729271	stage_E18_vs_E10	ordinary_muscle_development	ENSGALG00010002628	TRIM7.2	-0.017859452	0.192210978	-0.092915878	0.925970392	0.939254082	negative
PRJNA729271	stage_E18_vs_E10	ordinary_muscle_development	ENSGALG00010002632	HPGD	2.148950127	0.273186425	7.866240532	3.65458E-15	1.88715E-14	positive
PRJNA729271	stage_E18_vs_E10	ordinary_muscle_development	ENSGALG00010002633	ENSGALG00010002633	-0.024942571	0.123388042	-0.202147391	0.8398015	0.867449778	negative
PRJNA729271	stage_E18_vs_E10	ordinary_muscle_development	ENSGALG00010002634	RGN	1.215764989	0.183242958	6.634716018	3.25128E-11	1.22855E-10	positive
PRJNA729271	stage_E18_vs_E10	ordinary_muscle_development	ENSGALG00010002638	FABP9	6.204380014	0.497355816	12.474731	1.02552E-35	1.63351E-34	positive
PRJNA729271	stage_E18_vs_E10	ordinary_muscle_development	ENSGALG00010002644	KCNF1	-0.34846468	0.495316582	-0.703519109	0.481732302	0.539705497	negative
PRJNA729271	stage_E18_vs_E10	ordinary_muscle_development	ENSGALG00010002646	FOXO1	0.389507449	0.170178316	2.288819504	0.022089842	0.033057222	positive
PRJNA729271	stage_E18_vs_E10	ordinary_muscle_development	ENSGALG00010002647	FAM168A	-0.362343463	0.098605321	-3.674684687	0.000238143	0.000472991	negative
PRJNA729271	stage_E18_vs_E10	ordinary_muscle_development	ENSGALG00010002649	UFL1	0.085825005	0.079760761	1.07603042	0.281913652	0.337490781	positive
PRJNA729271	stage_E18_vs_E10	ordinary_muscle_development	ENSGALG00010002653	EIF1AX	-0.722730947	0.064087605	-11.27723447	1.70007E-29	2.02819E-28	negative
PRJNA729271	stage_E18_vs_E10	ordinary_muscle_development	ENSGALG00010002655	FABP4	7.376002491	0.946292103	7.794635997	6.45944E-15	3.2772E-14	positive
PRJNA729271	stage_E18_vs_E10	ordinary_muscle_development	ENSGALG00010002656	GLRA3	3.773040441	0.530116369	7.117381509	1.09997E-12	4.70048E-12	positive
PRJNA729271	stage_E18_vs_E10	ordinary_muscle_development	ENSGALG00010002658	FBXL12	-0.206919791	0.147956504	-1.398517704	0.16195766	0.205693182	negative
PRJNA729271	stage_E18_vs_E10	ordinary_muscle_development	ENSGALG00010002661	ACBD7	-0.663675872	0.1961914	-3.382797989	0.000717514	0.001334749	negative
PRJNA729271	stage_E18_vs_E10	ordinary_muscle_development	ENSGALG00010002664	ZNFB1	0.283615122	0.139069564	2.03937594	0.041412522	0.058931328	positive
PRJNA729271	stage_E18_vs_E10	ordinary_muscle_development	ENSGALG00010002667	ENSGALG00010002667	5.847677442	1.554062363	3.762833194			positive
PRJNA729271	stage_E18_vs_E10	ordinary_muscle_development	ENSGALG00010002669	IL4I1	3.354141954	1.142248557	2.9364379	0.003320053	0.005637202	positive
PRJNA729271	stage_E18_vs_E10	ordinary_muscle_development	ENSGALG00010002673	SLC66A3	-0.26128711	0.150819254	-1.732451946	0.083193126	0.11205021	negative
PRJNA729271	stage_E18_vs_E10	ordinary_muscle_development	ENSGALG00010002678	CCNI	1.872201247	0.17270582	10.84040621	2.21485E-27	2.38871E-26	positive
PRJNA729271	stage_E18_vs_E10	ordinary_muscle_development	ENSGALG00010002681	PLEKHB1	-0.669045266	0.16297032	-4.105319703	4.03756E-05	8.77954E-05	negative
PRJNA729271	stage_E18_vs_E10	ordinary_muscle_development	ENSGALG00010002682	UBL5	-0.201788741	0.166647514	-1.21087159	0.225944623	0.277336549	negative
PRJNA729271	stage_E18_vs_E10	ordinary_muscle_development	ENSGALG00010002685	CA8	0.604469997	0.179397248	3.36944966	0.000753184	0.001398055	positive
PRJNA729271	stage_E18_vs_E10	ordinary_muscle_development	ENSGALG00010002689	COG6	-0.480834871	0.062198822	-7.730610631	1.07032E-14	5.33137E-14	negative
PRJNA729271	stage_E18_vs_E10	ordinary_muscle_development	ENSGALG00010002693	MSC	0.106469393	0.168739713	0.630968201	0.528061317	0.584249201	positive
PRJNA729271	stage_E18_vs_E10	ordinary_muscle_development	ENSGALG00010002700	LHFPL6	0.454209804	0.170209582	2.668532515	0.00761834	0.012260225	positive
PRJNA729271	stage_E18_vs_E10	ordinary_muscle_development	ENSGALG00010002701	TMEM196	1.440463266	0.419050487	3.437445635	0.000587229	0.001105295	positive
PRJNA729271	stage_E18_vs_E10	ordinary_muscle_development	ENSGALG00010002703	PIN1	-0.236629535	0.09764869	-2.423274028	0.015381319	0.023700672	negative
PRJNA729271	stage_E18_vs_E10	ordinary_muscle_development	ENSGALG00010002704	KCNB2	2.281708396	0.250625939	9.104039303	8.70333E-20	6.04479E-19	positive
PRJNA729271	stage_E18_vs_E10	ordinary_muscle_development	ENSGALG00010002705	JADE3	-0.466810427	0.053946757	-8.653169403	5.00886E-18	3.13893E-17	negative
PRJNA729271	stage_E18_vs_E10	ordinary_muscle_development	ENSGALG00010002706	ROCK2	0.116477086	0.144618499	0.805409309	0.420583522	0.481090891	positive
PRJNA729271	stage_E18_vs_E10	ordinary_muscle_development	ENSGALG00010002707	RELT	-2.363925211	0.237029282	-9.973135774	1.99818E-23	1.71484E-22	negative
PRJNA729271	stage_E18_vs_E10	ordinary_muscle_development	ENSGALG00010002712	PTPRE	1.408174165	0.124028077	11.35367249	7.11118E-30	8.64011E-29	positive
PRJNA729271	stage_E18_vs_E10	ordinary_muscle_development	ENSGALG00010002713	OLFM2	0.021803004	0.182056281	0.119759692	0.904673513	0.921326675	positive
PRJNA729271	stage_E18_vs_E10	ordinary_muscle_development	ENSGALG00010002716	NHLRC3	-0.5425774	0.110820521	-4.896001148	9.78065E-07	2.53026E-06	negative
PRJNA729271	stage_E18_vs_E10	ordinary_muscle_development	ENSGALG00010002722	TRIM7.1	2.072829835	0.150124438	13.80741108	2.29948E-43	5.04251E-42	positive
PRJNA729271	stage_E18_vs_E10	ordinary_muscle_development	ENSGALG00010002725	TSPAN7	-0.52399503	0.179651396	-2.916732298	0.003537192	0.00598059	negative
PRJNA729271	stage_E18_vs_E10	ordinary_muscle_development	ENSGALG00010002728	RP2	-0.283285047	0.111972536	-2.529951158	0.011407841	0.017911249	negative
PRJNA729271	stage_E18_vs_E10	ordinary_muscle_development	ENSGALG00010002730	E2F6	-0.061023598	0.103098027	-0.591898793	0.553918362	0.608122901	negative
PRJNA729271	stage_E18_vs_E10	ordinary_muscle_development	ENSGALG00010002733	RDH8	1.395268789	0.355201726	3.928102501	8.56187E-05	0.000179783	positive
PRJNA729271	stage_E18_vs_E10	ordinary_muscle_development	ENSGALG00010002739	HEP21	1.352981878	0.662722206	2.041552051	0.041195986	0.058677201	positive
PRJNA729271	stage_E18_vs_E10	ordinary_muscle_development	ENSGALG00010002741	PDK4	7.170041702	0.574119125	12.48877	8.59725E-36	1.37698E-34	positive
PRJNA729271	stage_E18_vs_E10	ordinary_muscle_development	ENSGALG00010002742	PROSER1	0.527588001	0.119615415	4.410702419	1.03036E-05	2.39728E-05	positive
PRJNA729271	stage_E18_vs_E10	ordinary_muscle_development	ENSGALG00010002747	FUT9	0.377182164	0.504093182	0.748238971	0.454316029	0.513995538	positive
PRJNA729271	stage_E18_vs_E10	ordinary_muscle_development	ENSGALG00010002750	ENSGALG00010002750	-1.520140716	0.234781891	-6.474693203	9.50048E-11	3.4705E-10	negative
PRJNA729271	stage_E18_vs_E10	ordinary_muscle_development	ENSGALG00010002752	NDUFA4	1.166619126	0.154249477	7.563196664	3.93282E-14	1.86173E-13	positive
PRJNA729271	stage_E18_vs_E10	ordinary_muscle_development	ENSGALG00010002753	SLC9A7	0.221710647	0.153632515	1.443123198	0.14898577	0.190840291	positive
PRJNA729271	stage_E18_vs_E10	ordinary_muscle_development	ENSGALG00010002758	GREB1	-3.797594919	0.325096012	-11.68145648	1.58555E-31	2.09887E-30	negative
PRJNA729271	stage_E18_vs_E10	ordinary_muscle_development	ENSGALG00010002760	RAB2A	0.215427023	0.092715076	2.323538235	0.020150256	0.030367848	positive
PRJNA729271	stage_E18_vs_E10	ordinary_muscle_development	ENSGALG00010002762	PPAN	-0.855567392	0.148663224	-5.755070869	8.66055E-09	2.69099E-08	negative
PRJNA729271	stage_E18_vs_E10	ordinary_muscle_development	ENSGALG00010002763	SEPTIN11	-1.053891103	0.115676379	-9.110685461	8.18631E-20	5.69115E-19	negative
PRJNA729271	stage_E18_vs_E10	ordinary_muscle_development	ENSGALG00010002765	MAP7D2	-3.279602442	0.426431835	-7.690801143	1.46217E-14	7.19908E-14	negative
PRJNA729271	stage_E18_vs_E10	ordinary_muscle_development	ENSGALG00010002766	NANOS1	-1.380071466	0.336417481	-4.102258487	4.09137E-05	8.89255E-05	negative
PRJNA729271	stage_E18_vs_E10	ordinary_muscle_development	ENSGALG00010002768	TRIM39.2	1.245619944	1.197160641	1.040478529	0.298117634	0.355113737	positive
PRJNA729271	stage_E18_vs_E10	ordinary_muscle_development	ENSGALG00010002774	TBX18	-1.105457013	0.119115777	-9.280525582	1.68645E-20	1.22839E-19	negative
PRJNA729271	stage_E18_vs_E10	ordinary_muscle_development	ENSGALG00010002779	FBXO8	0.313222893	0.088566061	3.536601839	0.00040531	0.000780002	positive
PRJNA729271	stage_E18_vs_E10	ordinary_muscle_development	ENSGALG00010002781	EIF3G	1.130723927	0.137600372	8.217448162	2.07879E-16	1.16655E-15	positive
PRJNA729271	stage_E18_vs_E10	ordinary_muscle_development	ENSGALG00010002782	GSTA3	-0.160524249	0.14759886	-1.087571062	0.276784512	0.332256024	negative
PRJNA729271	stage_E18_vs_E10	ordinary_muscle_development	ENSGALG00010002784	TRIM27.2	1.109242623	0.515839337	2.150364551	0.03152639	0.045890241	positive
PRJNA729271	stage_E18_vs_E10	ordinary_muscle_development	ENSGALG00010002787	CEP44	-0.565195555	0.132836041	-4.254835898	2.09202E-05	4.70284E-05	negative
PRJNA729271	stage_E18_vs_E10	ordinary_muscle_development	ENSGALG00010002788	ENSGALG00010002788	3.725548821	0.631329621	5.901115193	3.61053E-09	1.16222E-08	positive
PRJNA729271	stage_E18_vs_E10	ordinary_muscle_development	ENSGALG00010002790	BCLAF3	0.428720907	0.102450811	4.184651187	2.85604E-05	6.31843E-05	positive
PRJNA729271	stage_E18_vs_E10	ordinary_muscle_development	ENSGALG00010002792	ENSGALG00010002792	0.449523774	0.229520191	1.958536944	0.05016704	0.070421388	positive
PRJNA729271	stage_E18_vs_E10	ordinary_muscle_development	ENSGALG00010002796	PRDM8	1.946577679	0.73320273	2.654896932	0.007933264	0.012730285	positive
PRJNA729271	stage_E18_vs_E10	ordinary_muscle_development	ENSGALG00010002800	TUBGCP5	0.0122255	0.075749929	0.161392888	0.871783968	0.894430111	positive
PRJNA729271	stage_E18_vs_E10	ordinary_muscle_development	ENSGALG00010002801	FGF5	-2.179239817	0.449165004	-4.851757806	1.22372E-06	3.13666E-06	negative
PRJNA729271	stage_E18_vs_E10	ordinary_muscle_development	ENSGALG00010002805	FAM171A1	-0.42173624	0.099179481	-4.252252943	2.11631E-05	4.75668E-05	negative
PRJNA729271	stage_E18_vs_E10	ordinary_muscle_development	ENSGALG00010002807	P2RY6	1.577250668	0.223326371	7.062536607	1.6349E-12	6.88492E-12	positive
PRJNA729271	stage_E18_vs_E10	ordinary_muscle_development	ENSGALG00010002809	SH3KBP1	-0.156575749	0.110129756	-1.421738813	0.155102088	0.197818281	negative
PRJNA729271	stage_E18_vs_E10	ordinary_muscle_development	ENSGALG00010002810	TRIM27.1	1.612539763	0.304338609	5.298505409	1.16754E-07	3.28794E-07	positive
PRJNA729271	stage_E18_vs_E10	ordinary_muscle_development	ENSGALG00010002812	TRIM13	-0.238744534	0.101848971	-2.344103546	0.019072876	0.028855164	negative
PRJNA729271	stage_E18_vs_E10	ordinary_muscle_development	ENSGALG00010002814	TERF1	-1.745499095	0.153134956	-11.39843666	4.25695E-30	5.22476E-29	negative
PRJNA729271	stage_E18_vs_E10	ordinary_muscle_development	ENSGALG00010002817	SHFM1	-0.259977397	0.092442155	-2.812325163	0.004918475	0.008161798	negative
PRJNA729271	stage_E18_vs_E10	ordinary_muscle_development	ENSGALG00010002819	BMP3	-0.950950496	0.345856434	-2.749552711	0.005967666	0.009770849	negative
PRJNA729271	stage_E18_vs_E10	ordinary_muscle_development	ENSGALG00010002824	NXPH1	-3.197593084	0.597275829	-5.353628807	8.62076E-08	2.45642E-07	negative
PRJNA729271	stage_E18_vs_E10	ordinary_muscle_development	ENSGALG00010002825	TRIM41	0.450326295	0.122681415	3.670696943	0.00024189	0.000480038	positive
PRJNA729271	stage_E18_vs_E10	ordinary_muscle_development	ENSGALG00010002827	SBSPON	-0.205781907	0.359912145	-0.571755941	0.56748733	0.62118478	negative
PRJNA729271	stage_E18_vs_E10	ordinary_muscle_development	ENSGALG00010002832	LPIN1	-0.2510494	0.207965373	-1.207169235	0.227366976	0.278846291	negative
PRJNA729271	stage_E18_vs_E10	ordinary_muscle_development	ENSGALG00010002834	MRPL53	-0.257568005	0.114355295	-2.252348752	0.024300242	0.036115247	negative
PRJNA729271	stage_E18_vs_E10	ordinary_muscle_development	ENSGALG00010002835	CYFIP1	-0.533796087	0.084576848	-6.311373639	2.7657E-10	9.76108E-10	negative
PRJNA729271	stage_E18_vs_E10	ordinary_muscle_development	ENSGALG00010002837	DNMT1	-0.285195297	0.13031614	-2.188487914	0.028634081	0.041979468	negative
PRJNA729271	stage_E18_vs_E10	ordinary_muscle_development	ENSGALG00010002838	ENSGALG00010002838	0.35478963	0.273876279	1.295437601	0.195169314	0.243290265	positive
PRJNA729271	stage_E18_vs_E10	ordinary_muscle_development	ENSGALG00010002840	SRPX	0.454606159	0.170246399	2.670283548	0.007578722	0.012199175	positive
PRJNA729271	stage_E18_vs_E10	ordinary_muscle_development	ENSGALG00010002848	TRIB2	-1.166829006	0.107511523	-10.85305999	1.92859E-27	2.09714E-26	negative
PRJNA729271	stage_E18_vs_E10	ordinary_muscle_development	ENSGALG00010002853	CEP162	-0.358372406	0.169188054	-2.118189777	0.034158999	0.049375218	negative
PRJNA729271	stage_E18_vs_E10	ordinary_muscle_development	ENSGALG00010002855	RACK1	1.052783034	0.125271878	8.40398542	4.31576E-17	2.53884E-16	positive
PRJNA729271	stage_E18_vs_E10	ordinary_muscle_development	ENSGALG00010002857	DCAF15	-0.122851846	0.14499566	-0.847279474	0.396839365	0.45677651	negative
PRJNA729271	stage_E18_vs_E10	ordinary_muscle_development	ENSGALG00010002860	NIPA2	0.331885188	0.108333548	3.063549517	0.00218728	0.003818454	positive
PRJNA729271	stage_E18_vs_E10	ordinary_muscle_development	ENSGALG00010002864	RNASEH2B	-0.320522705	0.093503706	-3.427914447	0.000608237	0.001142316	negative
PRJNA729271	stage_E18_vs_E10	ordinary_muscle_development	ENSGALG00010002866	ENSGALG00010002866	2.907612689	0.252345374	11.52235386	1.01792E-30	1.2875E-29	positive
PRJNA729271	stage_E18_vs_E10	ordinary_muscle_development	ENSGALG00010002868	PPP1R9A	0.811070096	0.06740044	12.03360243	2.36607E-33	3.3362E-32	positive
PRJNA729271	stage_E18_vs_E10	ordinary_muscle_development	ENSGALG00010002869	ENSGALG00010002869	0.744402961	0.301141368	2.471938565	0.013438261	0.020875318	positive
PRJNA729271	stage_E18_vs_E10	ordinary_muscle_development	ENSGALG00010002870	FABP5	3.795216024	0.216173774	17.55632035	5.3215E-69	2.74502E-67	positive
PRJNA729271	stage_E18_vs_E10	ordinary_muscle_development	ENSGALG00010002878	P2RY2	3.735432025	0.252269702	14.80729552	1.31423E-49	3.7797E-48	positive
PRJNA729271	stage_E18_vs_E10	ordinary_muscle_development	ENSGALG00010002880	RASGEF1B	0.174131209	0.207416245	0.839525413	0.401174535	0.460997691	positive
PRJNA729271	stage_E18_vs_E10	ordinary_muscle_development	ENSGALG00010002881	ENSGALG00010002881	3.401579938	0.320004448	10.62978955	2.16601E-26	2.22827E-25	positive
PRJNA729271	stage_E18_vs_E10	ordinary_muscle_development	ENSGALG00010002883	MAP3K15	4.952040669	0.412911934	11.99297056	3.86781E-33	5.40115E-32	positive
PRJNA729271	stage_E18_vs_E10	ordinary_muscle_development	ENSGALG00010002884	ADHFE1	3.177104812	0.255126366	12.4530634	1.34579E-35	2.12265E-34	positive
PRJNA729271	stage_E18_vs_E10	ordinary_muscle_development	ENSGALG00010002886	HERC2	0.32026543	0.10586921	3.025104554	0.002485472	0.004304805	positive
PRJNA729271	stage_E18_vs_E10	ordinary_muscle_development	ENSGALG00010002888	ENSGALG00010002888	-3.613549337	0.387752352	-9.31921964	1.17199E-20	8.61461E-20	negative
PRJNA729271	stage_E18_vs_E10	ordinary_muscle_development	ENSGALG00010002890	VXN	0.706987305	0.164476481	4.29840971	1.72028E-05	3.9096E-05	positive
PRJNA729271	stage_E18_vs_E10	ordinary_muscle_development	ENSGALG00010002894	FCHSD2	-0.068938571	0.071354115	-0.966147086	0.333970586	0.39229365	negative
PRJNA729271	stage_E18_vs_E10	ordinary_muscle_development	ENSGALG00010002896	OCA2	-1.354515683	0.254355318	-5.325289419	1.00792E-07	2.85851E-07	negative
PRJNA729271	stage_E18_vs_E10	ordinary_muscle_development	ENSGALG00010002904	PON1	1.918202175	0.17950764	10.68590828	1.18484E-26	1.23733E-25	positive
PRJNA729271	stage_E18_vs_E10	ordinary_muscle_development	ENSGALG00010002907	GABRG3	-1.766149143	0.373282013	-4.731407036	2.22969E-06	5.54458E-06	negative
PRJNA729271	stage_E18_vs_E10	ordinary_muscle_development	ENSGALG00010002908	S1PR2	-0.05005839	0.18269572	-0.2739987	0.784085621	0.819004185	negative
PRJNA729271	stage_E18_vs_E10	ordinary_muscle_development	ENSGALG00010002911	MYBL1	-0.35732829	0.22904671	-1.560067333	0.11874397	0.155384477	negative
PRJNA729271	stage_E18_vs_E10	ordinary_muscle_development	ENSGALG00010002912	RPS3	1.089867761	0.192267791	5.668488484	1.44063E-08	4.39157E-08	positive
PRJNA729271	stage_E18_vs_E10	ordinary_muscle_development	ENSGALG00010002913	NXNL1	0.424418708	0.584266186	0.726413266	0.467585461	0.526583114	positive
PRJNA729271	stage_E18_vs_E10	ordinary_muscle_development	ENSGALG00010002914	SLC27A1	0.427409122	0.30789136	1.388181605	0.165081752	0.209147802	positive
PRJNA729271	stage_E18_vs_E10	ordinary_muscle_development	ENSGALG00010002916	SDCBP	-0.757836779	0.122855492	-6.168521785	6.89314E-10	2.36824E-09	negative
PRJNA729271	stage_E18_vs_E10	ordinary_muscle_development	ENSGALG00010002917	PGLS	0.677068646	0.095698508	7.075017779	1.4943E-12	6.31482E-12	positive
PRJNA729271	stage_E18_vs_E10	ordinary_muscle_development	ENSGALG00010002918	LRATD1	-3.122310954	0.403878174	-7.73082369	1.06853E-14	5.32611E-14	negative
PRJNA729271	stage_E18_vs_E10	ordinary_muscle_development	ENSGALG00010002919	ENSGALG00010002919	0.735431595	0.204113944	3.603044363	0.000314512	0.000613727	positive
PRJNA729271	stage_E18_vs_E10	ordinary_muscle_development	ENSGALG00010002920	VCPIP1	0.163160659	0.104510649	1.561186918	0.118479655	0.155080603	positive
PRJNA729271	stage_E18_vs_E10	ordinary_muscle_development	ENSGALG00010002922	TMEM106B	0.185132964	0.091351254	2.026605604	0.042702763	0.060626731	positive
PRJNA729271	stage_E18_vs_E10	ordinary_muscle_development	ENSGALG00010002923	SERPINH1	0.606437845	0.158447215	3.82738091	0.000129514	0.000266511	positive
PRJNA729271	stage_E18_vs_E10	ordinary_muscle_development	ENSGALG00010002925	INTS6	0.123237023	0.122444868	1.006469482	0.314189785	0.371678848	positive
PRJNA729271	stage_E18_vs_E10	ordinary_muscle_development	ENSGALG00010002926	GALNTL6	-2.214291825	0.370706076	-5.973173813	2.32682E-09	7.6065E-09	negative
PRJNA729271	stage_E18_vs_E10	ordinary_muscle_development	ENSGALG00010002927	GABRA5	-4.239947767	0.624568414	-6.788604217	1.13224E-11	4.43321E-11	negative
PRJNA729271	stage_E18_vs_E10	ordinary_muscle_development	ENSGALG00010002928	ASB4	4.661276014	0.564935045	8.250994614	1.57082E-16	8.89633E-16	positive
PRJNA729271	stage_E18_vs_E10	ordinary_muscle_development	ENSGALG00010002929	PDHA2	0.226892993	0.113448185	1.999970229	0.045503479	0.064342301	positive
PRJNA729271	stage_E18_vs_E10	ordinary_muscle_development	ENSGALG00010002930	SERAF	-0.282934037	0.277628257	-1.019111093	0.308150228	0.365698179	negative
PRJNA729271	stage_E18_vs_E10	ordinary_muscle_development	ENSGALG00010002934	VWDE	-5.248813109	0.782136298	-6.710867562	1.93471E-11	7.43467E-11	negative
PRJNA729271	stage_E18_vs_E10	ordinary_muscle_development	ENSGALG00010002935	SYTL5	-3.114246501	0.66929066	-4.653055372	3.27052E-06	7.98354E-06	negative
PRJNA729271	stage_E18_vs_E10	ordinary_muscle_development	ENSGALG00010002937	GALNT7	-0.408444489	0.109141704	-3.742331988	0.00018232	0.000367506	negative
PRJNA729271	stage_E18_vs_E10	ordinary_muscle_development	ENSGALG00010002939	HMGB2	-1.683814904	0.171420455	-9.822718684	8.98861E-23	7.45366E-22	negative
PRJNA729271	stage_E18_vs_E10	ordinary_muscle_development	ENSGALG00010002940	BG1	1.649000591	0.416331477	3.960787693	7.47029E-05	0.000157868	positive
PRJNA729271	stage_E18_vs_E10	ordinary_muscle_development	ENSGALG00010002942	GABRB3	-3.607877769	0.559856012	-6.444295837	1.16138E-10	4.20856E-10	negative
PRJNA729271	stage_E18_vs_E10	ordinary_muscle_development	ENSGALG00010002943	SAP30	-0.771239044	0.152313144	-5.063509451	4.11608E-07	1.10404E-06	negative
PRJNA729271	stage_E18_vs_E10	ordinary_muscle_development	ENSGALG00010002946	GDPD5	1.042969245	0.115253703	9.049333926	1.43843E-19	9.88623E-19	positive

PRJNA729271	stage_E18_vs_E10	ordinary_muscle_development	ENSGALG00010002947	ENSGALG00010002947	-0.660976291	0.682767354	-0.968084206	0.333002317	0.391267509	negative
PRJNA729271	stage_E18_vs_E10	ordinary_muscle_development	ENSGALG00010002948	DLX6	-3.680294903	1.563480161	-2.353912121	0.018577002	0.028149033	negative
PRJNA729271	stage_E18_vs_E10	ordinary_muscle_development	ENSGALG00010002949	ENSGALG00010002949	2.080635055	1.182982833	1.758804098	0.078610789	0.10645211	positive
PRJNA729271	stage_E18_vs_E10	ordinary_muscle_development	ENSGALG00010002951	ENSGALG00010002951	-1.441171519	4.280680577	-0.336668783	0.736366599	0.777621583	negative
PRJNA729271	stage_E18_vs_E10	ordinary_muscle_development	ENSGALG00010002952	NBAS	0.21883121	0.064543728	3.390433395	0.000697822	0.001300454	positive
PRJNA729271	stage_E18_vs_E10	ordinary_muscle_development	ENSGALG00010002953	WDFY2	0.104706953	0.13188807	0.79390769	0.427249173	0.487290641	positive
PRJNA729271	stage_E18_vs_E10	ordinary_muscle_development	ENSGALG00010002954	CHST7	1.714266864	0.120630256	14.21091961	7.83893E-46	1.91288E-44	positive
PRJNA729271	stage_E18_vs_E10	ordinary_muscle_development	ENSGALG00010002955	BLEC1	0.436081507	0.475459167	0.917179723	0.35904847	0.418562617	positive
PRJNA729271	stage_E18_vs_E10	ordinary_muscle_development	ENSGALG00010002956	SCRG1	0.720510925	0.372895175	1.932207693	0.053333876	0.074491668	positive
PRJNA729271	stage_E18_vs_E10	ordinary_muscle_development	ENSGALG00010002957	ADGRG2	-0.94845543	0.293611611	-3.230306276	0.001236577	0.002228267	negative
PRJNA729271	stage_E18_vs_E10	ordinary_muscle_development	ENSGALG00010002958	ENSGALG00010002958	1.364982579	0.170624375	7.99992722	1.24493E-15	6.64894E-15	positive
PRJNA729271	stage_E18_vs_E10	ordinary_muscle_development	ENSGALG00010002959	DLX5	-4.170103263	0.625679266	-6.664921615	2.64807E-11	1.00745E-10	negative
PRJNA729271	stage_E18_vs_E10	ordinary_muscle_development	ENSGALG00010002960	BLEC2	0.172844864	0.805592877	0.214556097	0.830113426	0.8588504	positive
PRJNA729271	stage_E18_vs_E10	ordinary_muscle_development	ENSGALG00010002965	PRSS35	0.441564551	0.211543633	2.087345029	0.036856957	0.052963378	positive
PRJNA729271	stage_E18_vs_E10	ordinary_muscle_development	ENSGALG00010002967	OLFM4	-2.05142967	0.866399562	-2.367763974	0.017895949	0.027210928	negative
PRJNA729271	stage_E18_vs_E10	ordinary_muscle_development	ENSGALG00010002972	ME1	-0.405686063	0.155558807	-2.607927321	0.009109229	0.014492183	negative
PRJNA729271	stage_E18_vs_E10	ordinary_muscle_development	ENSGALG00010002978	DYNLT3	0.649994533	0.102546014	6.338564617	2.31916E-10	8.22716E-10	positive
PRJNA729271	stage_E18_vs_E10	ordinary_muscle_development	ENSGALG00010002981	KLHL35	-4.079719443	0.298200659	-13.68112151	1.31646E-42	2.81033E-41	negative
PRJNA729271	stage_E18_vs_E10	ordinary_muscle_development	ENSGALG00010002982	HAND2	-2.508745295	0.300052443	-8.36102274	6.21769E-17	3.61369E-16	negative
PRJNA729271	stage_E18_vs_E10	ordinary_muscle_development	ENSGALG00010002983	NSMAF	0.427112965	0.112135661	3.808895045	0.000139589	0.000286066	positive
PRJNA729271	stage_E18_vs_E10	ordinary_muscle_development	ENSGALG00010002985	ENSGALG00010002985	0.107069857	0.217361496	0.492588884	0.622303104	0.673004812	positive
PRJNA729271	stage_E18_vs_E10	ordinary_muscle_development	ENSGALG00010002987	PCDH8	-1.552819539	0.367080013	-4.230193644	2.3349E-05	5.21727E-05	negative
PRJNA729271	stage_E18_vs_E10	ordinary_muscle_development	ENSGALG00010002991	BLB1	1.830088061	0.310497876	5.894043745	3.76857E-09	1.21175E-08	positive
PRJNA729271	stage_E18_vs_E10	ordinary_muscle_development	ENSGALG00010002992	ENSGALG00010002992	-0.729705016	0.254503958	-2.867165688	0.004141661	0.006939473	negative
PRJNA729271	stage_E18_vs_E10	ordinary_muscle_development	ENSGALG00010002994	DUSP4	-3.379410574	0.172792596	-19.55761219	3.55332E-85	2.8145E-83	negative
PRJNA729271	stage_E18_vs_E10	ordinary_muscle_development	ENSGALG00010002995	RWDD2A	-0.514913566	0.127778702	-4.029729202	5.58411E-05	0.000119736	negative
PRJNA729271	stage_E18_vs_E10	ordinary_muscle_development	ENSGALG00010002998	LECT1	-2.806172121	0.280553922	-10.00225589	1.48964E-23	1.28679E-22	negative
PRJNA729271	stage_E18_vs_E10	ordinary_muscle_development	ENSGALG00010002999	CMTM7	-0.547999329	0.217300024	-2.521855812	0.011673755	0.018306943	negative
PRJNA729271	stage_E18_vs_E10	ordinary_muscle_development	ENSGALG00010003001	MAP6	-2.5076183	0.32072218	-7.81866193	5.33878E-15	2.72293E-14	negative
PRJNA729271	stage_E18_vs_E10	ordinary_muscle_development	ENSGALG00010003002	PHKA2	1.816914726	0.18372695	9.889211826	4.63664E-23	3.90291E-22	positive
PRJNA729271	stage_E18_vs_E10	ordinary_muscle_development	ENSGALG00010003006	CYP7B1	0.801971337	0.200320518	4.003440822	6.24278E-05	0.000133268	positive
PRJNA729271	stage_E18_vs_E10	ordinary_muscle_development	ENSGALG00010003009	TNKS	0.215152015	0.057934613	3.713704212	0.000204248	0.000408918	positive
PRJNA729271	stage_E18_vs_E10	ordinary_muscle_development	ENSGALG00010003017	PGM3	-0.582474475	0.154146662	-3.778703143	0.000157647	0.000320535	negative
PRJNA729271	stage_E18_vs_E10	ordinary_muscle_development	ENSGALG00010003018	MOGAT2	-1.246777526	0.958714189	-1.300468419	0.193440474	0.241259651	negative
PRJNA729271	stage_E18_vs_E10	ordinary_muscle_development	ENSGALG00010003019	TAPBP	1.180719463	0.255336469	4.62417087	3.761E-06	9.11938E-06	positive
PRJNA729271	stage_E18_vs_E10	ordinary_muscle_development	ENSGALG00010003022	BLB2	2.283205887	0.269036827	8.486592381	2.12785E-17	1.27609E-16	positive
PRJNA729271	stage_E18_vs_E10	ordinary_muscle_development	ENSGALG00010003024	SUGT1	-0.369240275	0.073699583	-5.010072764	5.44095E-07	1.44312E-06	negative
PRJNA729271	stage_E18_vs_E10	ordinary_muscle_development	ENSGALG00010003025	MFHAS1	1.189669787	0.122932616	9.677413752	3.7611E-22	3.02034E-21	positive
PRJNA729271	stage_E18_vs_E10	ordinary_muscle_development	ENSGALG00010003027	SCIN	-2.363140855	0.256587929	-9.20986759	3.26527E-20	2.32124E-19	negative
PRJNA729271	stage_E18_vs_E10	ordinary_muscle_development	ENSGALG00010003030	PPEF1	-0.968991694	0.69188107	-1.400517713	0.161358343	0.20506652	negative
PRJNA729271	stage_E18_vs_E10	ordinary_muscle_development	ENSGALG00010003034	ERI1	-0.364384039	0.062451288	-5.834692116	5.389E-09	1.70703E-08	negative
PRJNA729271	stage_E18_vs_E10	ordinary_muscle_development	ENSGALG00010003035	DGAT2	2.41442804	0.457280516	5.27997139	1.29204E-07	3.62246E-07	positive
PRJNA729271	stage_E18_vs_E10	ordinary_muscle_development	ENSGALG00010003042	TOX	-1.938781836	0.292720568	-6.62331947	3.51221E-11	1.32439E-10	negative
PRJNA729271	stage_E18_vs_E10	ordinary_muscle_development	ENSGALG00010003052	ENSGALG00010003052	-0.023307582	0.094517323	-0.246595876	0.805220996	0.837519973	negative
PRJNA729271	stage_E18_vs_E10	ordinary_muscle_development	ENSGALG00010003057	DDX1	-0.582227084	0.090087952	-6.462873996	1.02733E-10	3.74149E-10	negative
PRJNA729271	stage_E18_vs_E10	ordinary_muscle_development	ENSGALG00010003061	ENSGALG00010003061	-0.589956088	0.322695586	-1.828212452	0.067517673	0.092580519	negative
PRJNA729271	stage_E18_vs_E10	ordinary_muscle_development	ENSGALG00010003063	GGH	0.324166605	0.088409478	3.666649936	0.000245749	0.000486962	positive
PRJNA729271	stage_E18_vs_E10	ordinary_muscle_development	ENSGALG00010003064	VAV1	2.015104724	0.252470324	7.981550813	1.44506E-15	7.67258E-15	positive
PRJNA729271	stage_E18_vs_E10	ordinary_muscle_development	ENSGALG00010003068	BRD2	0.098081232	0.126632673	0.774533376	0.438615439	0.49817668	positive
PRJNA729271	stage_E18_vs_E10	ordinary_muscle_development	ENSGALG00010003072	ELF1	0.594173702	0.075469252	7.873056748	3.4608E-15	1.79094E-14	positive
PRJNA729271	stage_E18_vs_E10	ordinary_muscle_development	ENSGALG00010003074	TTPA	2.991402474	0.347832716	8.600118215	7.9634E-18	4.90563E-17	positive
PRJNA729271	stage_E18_vs_E10	ordinary_muscle_development	ENSGALG00010003077	EIF2S3	0.388774945	0.081776156	4.75413572	1.99297E-06	4.97985E-06	positive
PRJNA729271	stage_E18_vs_E10	ordinary_muscle_development	ENSGALG00010003081	DOP1A	0.007738651	0.104965441	0.073725705	0.941228645	0.952066239	positive
PRJNA729271	stage_E18_vs_E10	ordinary_muscle_development	ENSGALG00010003087	TWISTNB	-0.526272747	0.182379768	-2.885587326	0.003906841	0.00656728	negative
PRJNA729271	stage_E18_vs_E10	ordinary_muscle_development	ENSGALG00010003090	DMA	-0.589322919	0.211750941	-2.783094684	0.005384309	0.008882946	negative
PRJNA729271	stage_E18_vs_E10	ordinary_muscle_development	ENSGALG00010003092	ART1F	0.764883401	1.565978392	0.48843803	0.62523961	0.675626073	positive
PRJNA729271	stage_E18_vs_E10	ordinary_muscle_development	ENSGALG00010003097	TFPI2	0.248047766	0.136927165	1.811530717	0.070058739	0.095829142	positive
PRJNA729271	stage_E18_vs_E10	ordinary_muscle_development	ENSGALG00010003101	KLHL15	-0.152530085	0.072771444	-2.096015654	0.036080802	0.051940732	negative
PRJNA729271	stage_E18_vs_E10	ordinary_muscle_development	ENSGALG00010003103	YTHDF3	0.852754115	0.086239202	9.888242151	4.68176E-23	3.93632E-22	positive
PRJNA729271	stage_E18_vs_E10	ordinary_muscle_development	ENSGALG00010003107	GNG11	1.925220856	0.180475519	10.66749037	1.44476E-26	1.50228E-25	positive
PRJNA729271	stage_E18_vs_E10	ordinary_muscle_development	ENSGALG00010003108	PPP1R3B	-0.318953885	0.182969442	-1.743208494	0.081297202	0.109782276	negative
PRJNA729271	stage_E18_vs_E10	ordinary_muscle_development	ENSGALG00010003109	ART5	1.564373901	0.666850144	2.34591522	0.018980424	0.028721291	positive
PRJNA729271	stage_E18_vs_E10	ordinary_muscle_development	ENSGALG00010003110	BF1	2.264241548	0.258925809	8.744750299	2.23509E-18	1.43353E-17	positive
PRJNA729271	stage_E18_vs_E10	ordinary_muscle_development	ENSGALG00010003113	WBP4	0.208315712	0.116724316	1.784681362	0.074313016	0.101085507	positive
PRJNA729271	stage_E18_vs_E10	ordinary_muscle_development	ENSGALG00010003115	UBE3D	-0.502294986	0.158779839	-3.163468283	0.001559013	0.00277445	negative
PRJNA729271	stage_E18_vs_E10	ordinary_muscle_development	ENSGALG00010003117	ID2	-1.796339148	0.126809643	-14.16563528	1.49509E-45	3.57612E-44	negative
PRJNA729271	stage_E18_vs_E10	ordinary_muscle_development	ENSGALG00010003121	ARMC1	-0.084453797	0.083147877	-1.015705994	0.309769413	0.367409184	negative
PRJNA729271	stage_E18_vs_E10	ordinary_muscle_development	ENSGALG00010003125	APOO	-0.31269129	0.109170485	-2.864247505	0.004180013	0.007000495	negative
PRJNA729271	stage_E18_vs_E10	ordinary_muscle_development	ENSGALG00010003126	ENSGALG00010003126	-1.024800246	0.480562663	-2.132500762	0.032965702	0.047793344	negative
PRJNA729271	stage_E18_vs_E10	ordinary_muscle_development	ENSGALG00010003131	TAP1	0.984766264	0.367176291	2.681998503	0.007318379	0.011806333	positive
PRJNA729271	stage_E18_vs_E10	ordinary_muscle_development	ENSGALG00010003132	TPBG	-1.220600323	0.135510586	-9.007416776	2.10967E-19	1.43365E-18	negative
PRJNA729271	stage_E18_vs_E10	ordinary_muscle_development	ENSGALG00010003133	SAT1	1.528810283	0.16756751	9.123548373	7.2704E-20	5.07632E-19	positive
PRJNA729271	stage_E18_vs_E10	ordinary_muscle_development	ENSGALG00010003136	NUP98	-0.164803736	0.105209642	-1.566431863	0.117247548	0.153557873	negative
PRJNA729271	stage_E18_vs_E10	ordinary_muscle_development	ENSGALG00010003152	ENSGALG00010003152	1.550512549	0.126914611	12.21697439	2.52301E-34	3.77021E-33	positive
PRJNA729271	stage_E18_vs_E10	ordinary_muscle_development	ENSGALG00010003154	MTFR1	-0.788029495	0.082378761	-9.565930403	1.11197E-21	8.74076E-21	negative
PRJNA729271	stage_E18_vs_E10	ordinary_muscle_development	ENSGALG00010003159	TAP2	1.074115483	0.27804311	3.863125701	0.000111945	0.000232205	positive
PRJNA729271	stage_E18_vs_E10	ordinary_muscle_development	ENSGALG00010003160	KIDINS220	-0.370714909	0.15167139	-2.444198007	0.014517456	0.022462695	negative
PRJNA729271	stage_E18_vs_E10	ordinary_muscle_development	ENSGALG00010003164	C3	3.233399066	0.459417726	7.038037245	1.94967E-12	8.15126E-12	positive
PRJNA729271	stage_E18_vs_E10	ordinary_muscle_development	ENSGALG00010003165	BMP2K	0.516397078	0.109703006	4.707228142	2.51108E-06	6.21022E-06	positive
PRJNA729271	stage_E18_vs_E10	ordinary_muscle_development	ENSGALG00010003166	PGAP2	0.211570321	0.112925594	1.873537383	0.060994216	0.08428133	positive
PRJNA729271	stage_E18_vs_E10	ordinary_muscle_development	ENSGALG00010003169	MTRF1	0.505569802	0.108648929	4.6532424	3.26756E-06	7.97764E-06	positive
PRJNA729271	stage_E18_vs_E10	ordinary_muscle_development	ENSGALG00010003170	C4	0.879666657	0.407248297	2.160025381	0.030770705	0.044880395	positive
PRJNA729271	stage_E18_vs_E10	ordinary_muscle_development	ENSGALG00010003171	PAQR3	-0.856635579	0.110029489	-7.785508987	6.94331E-15	3.51163E-14	negative
PRJNA729271	stage_E18_vs_E10	ordinary_muscle_development	ENSGALG00010003176	PRAG1	0.526741711	0.252198404	2.088600491	0.036743701	0.052805864	positive
PRJNA729271	stage_E18_vs_E10	ordinary_muscle_development	ENSGALG00010003178	CENPA	-1.200389359	0.221370384	-5.422538191	5.87587E-08	1.69561E-07	negative
PRJNA729271	stage_E18_vs_E10	ordinary_muscle_development	ENSGALG00010003180	IBTK	0.373439265	0.126915899	2.942415163	0.00325663	0.005540476	positive
PRJNA729271	stage_E18_vs_E10	ordinary_muscle_development	ENSGALG00010003183	NAA16	0.588628111	0.088260514	6.66921232	2.5718E-11	9.8049E-11	positive
PRJNA729271	stage_E18_vs_E10	ordinary_muscle_development	ENSGALG00010003184	BET1	0.064762911	0.081239704	0.797182999	0.425344758	0.485615333	positive
PRJNA729271	stage_E18_vs_E10	ordinary_muscle_development	ENSGALG00010003186	CYP21A1	-1.323546286	0.256858806	-5.152816476	2.56603E-07	7.00859E-07	negative
PRJNA729271	stage_E18_vs_E10	ordinary_muscle_development	ENSGALG00010003187	RHOG	0.451253833	0.255367371	1.767077102	0.077215317	0.104728737	positive
PRJNA729271	stage_E18_vs_E10	ordinary_muscle_development	ENSGALG00010003189	ANTXR2	1.12149533	0.12663792	8.855920347	8.29961E-19	5.45839E-18	positive
PRJNA729271	stage_E18_vs_E10	ordinary_muscle_development	ENSGALG00010003191	CC2D1A	1.182557749	0.163708071	7.223576363	5.06378E-13	2.21483E-12	positive
PRJNA729271	stage_E18_vs_E10	ordinary_muscle_development	ENSGALG00010003192	RGCC	1.272666961	0.308603191	4.123959169	3.72415E-05	8.1322E-05	positive
PRJNA729271	stage_E18_vs_E10	ordinary_muscle_development	ENSGALG00010003193	ACOT9	0.558078093	0.136735992	4.081427898	4.47599E-05	9.67772E-05	positive
PRJNA729271	stage_E18_vs_E10	ordinary_muscle_development	ENSGALG00010003198	PRKG2	-0.605719617	0.332630032	-1.82100099	0.068606706	0.093949382	negative
PRJNA729271	stage_E18_vs_E10	ordinary_muscle_development	ENSGALG00010003201	MBOAT2	-0.248300577	0.127906841	-1.941261113	0.052226614	0.073057785	negative
PRJNA729271	stage_E18_vs_E10	ordinary_muscle_development	ENSGALG00010003202	FAM46A	1.509019953	0.102462114	14.72758945	4.28728E-49	1.20434E-47	positive
PRJNA729271	stage_E18_vs_E10	ordinary_muscle_development	ENSGALG00010003204	PKD2	0.406744903	0.143189839	2.840598922	0.00450289	0.007508271	positive
PRJNA729271	stage_E18_vs_E10	ordinary_muscle_development	ENSGALG00010003205	ENSGALG00010003205	5.878636377	0.264021112	22.26578144	7.9327E-110	1.2232E-107	positive
PRJNA729271	stage_E18_vs_E10	ordinary_muscle_development	ENSGALG00010003206	ENSGALG00010003206	-0.967211018	0.574341931	-1.684033441	0.092175206	0.123072919	negative
PRJNA729271	stage_E18_vs_E10	ordinary_muscle_development	ENSGALG00010003210	EYA1	2.275934164	0.199765168	11.39304811	4.52865E-30	5.54884E-29	positive
PRJNA729271	stage_E18_vs_E10	ordinary_muscle_development	ENSGALG00010003212	LTB4R	2.728446089	0.728838938	3.743551486	0.000181437	0.000365879	positive
PRJNA729271	stage_E18_vs_E10	ordinary_muscle_development	ENSGALG00010003214	PRDX4	-0.221980255	0.094735587	-2.343155951	0.019121389	0.028922522	negative
PRJNA729271	stage_E18_vs_E10	ordinary_muscle_development	ENSGALG00010003218	MAP3K7	-0.210177839	0.061002264	-3.445410479	0.000570193	0.001075464	negative
PRJNA729271	stage_E18_vs_E10	ordinary_muscle_development	ENSGALG00010003220	SPP1	-0.543416499	0.941792277	-0.577002501	0.563937766	0.617765865	negative
PRJNA729271	stage_E18_vs_E10	ordinary_muscle_development	ENSGALG00010003222	ITGA8	-2.121867895	0.310161286	-6.841175827	7.85458E-12	3.11593E-11	negative
PRJNA729271	stage_E18_vs_E10	ordinary_muscle_development	ENSGALG00010003223	AREG	-1.186372846	0.845428428	-1.403280048	0.160533345	0.204080937	negative
PRJNA729271	stage_E18_vs_E10	ordinary_muscle_development	ENSGALG00010003229	STIM1	0.622722617	0.106780047	5.831825649	5.48242E-09	1.73548E-08	positive
PRJNA729271	stage_E18_vs_E10	ordinary_muscle_development	ENSGALG00010003239	RRM1	-1.40710296	0.11885055	-11.83926332	2.44592E-32	3.31962E-31	negative
PRJNA729271	stage_E18_vs_E10	ordinary_muscle_development	ENSGALG00010003240	PLACL2	0.266107037	1.032289127	0.257783435	0.79657405	0.83005097	positive
PRJNA729271	stage_E18_vs_E10	ordinary_muscle_development	ENSGALG00010003242	ASAP2	-1.35876317	0.127857817	-10.62714191	2.22837E-26	2.28755E-25	negative
PRJNA729271	stage_E18_vs_E10	ordinary_muscle_development	ENSGALG00010003243	VPS50	-0.38983086	0.09352604	-4.168153147	3.07078E-05	6.76252E-05	negative
PRJNA729271	stage_E18_vs_E10	ordinary_muscle_development	ENSGALG00010003245	ENSGALG00010003245	0.01944114	0.4217932	0.046091639	0.96323721	0.96979394	positive
PRJNA729271	stage_E18_vs_E10	ordinary_muscle_development	ENSGALG00010003246	VWA8	0.654124183	0.109694598	5.963139422	2.47437E-09	8.07066E-09	positive
PRJNA729271	stage_E18_vs_E10	ordinary_muscle_development	ENSGALG00010003247	ENSGALG00010003247	0.307548828	0.219335453	1.402184749	0.160860084	0.204478376	positive
PRJNA729271	stage_E18_vs_E10	ordinary_muscle_development	ENSGALG00010003248	MRI1	-0.128838037	0.097042155	-1.327650201	0.184293692	0.231244552	negative
PRJNA729271	stage_E18_vs_E10	ordinary_muscle_development	ENSGALG00010003249	YJU2B	1.390718561	0.332591497	4.181461565	2.89641E-05	6.40285E-05	positive
PRJNA729271	stage_E18_vs_E10	ordinary_muscle_development	ENSGALG00010003250	CD1G	0.675466134	0.766565513	0.881159043	0.378231744	0.438387104	positive
PRJNA729271	stage_E18_vs_E10	ordinary_muscle_development	ENSGALG00010003251	ELOF1	-0.461930873	0.156239256	-2.956560878	0.003110908	0.005316271	negative
PRJNA729271	stage_E18_vs_E10	ordinary_muscle_development	ENSGALG00010003252	PHEX	-0.629414042	0.195255331	-3.223543442	0.001266151	0.002279292	negative
PRJNA729271	stage_E18_vs_E10	ordinary_muscle_development	ENSGALG00010003253	ITGB1BP1	0.042442829	0.114259227	0.371460843	0.710294316	0.754320178	positive
PRJNA729271	stage_E18_vs_E10	ordinary_muscle_development	ENSGALG00010003254	DGKH	1.281920211	0.167531263	7.651826802	1.98144E-14	9.63468E-14	positive
PRJNA729271	stage_E18_vs_E10	ordinary_muscle_development	ENSGALG00010003255	CALCR	-2.743687511	0.362522779	-7.568317558	3.78089E-14	1.79391E-13	negative
PRJNA729271	stage_E18_vs_E10	ordinary_muscle_development	ENSGALG00010003256	ENSGALG00010003256	-0.888360476	1.413023753	-0.628694652	0.52954898	0.585671637	negative
PRJNA729271	stage_E18_vs_E10	ordinary_muscle_development	ENSGALG00010003257	ENSGALG00010003257	0.363235458	0.797060603	0.455718746	0.64859224	0.697170524	positive
PRJNA729271	stage_E18_vs_E10	ordinary_muscle_development	ENSGALG00010003259	VEGFC	1.011496096	0.141636652	7.14148548	9.23276E-13	3.9759E-12	positive
PRJNA729271	stage_E18_vs_E10	ordinary_muscle_development	ENSGALG00010003260	ACP5	0.473745039	0.270808259	1.749374416	0.08022632	0.10848778	positive
PRJNA729271	stage_E18_vs_E10	ordinary_muscle_development	ENSGALG00010003261	PEX2	-0.514944429	0.173655317	-2.965324863	0.003023635	0.00517506	negative
PRJNA729271	stage_E18_vs_E10	ordinary_muscle_development	ENSGALG00010003262	ENSGALG00010003262	-0.699808651	0.582479457	-1.201430612	0.229584208	0.281208644	negative
PRJNA729271	stage_E18_vs_E10	ordinary_muscle_development	ENSGALG00010003263	ENSGALG00010003263	1.724642152	0.761319972	2.265331551	0.023492341	0.034989877	positive
PRJNA729271	stage_E18_vs_E10	ordinary_muscle_development	ENSGALG00010003264	ENSGALG00010003264	-0.261924112	0.390400123	-0.670911962	0.502276616	0.559521945	negative

PRJNA729271	stage_E18_vs_E10	ordinary_muscle_development	ENSGALG00010003268	SMS	-0.496459735	0.064192858	-7.733878077	1.04319E-14	5.21056E-14	negative
PRJNA729271	stage_E18_vs_E10	ordinary_muscle_development	ENSGALG00010003269	ENSGALG00010003269	2.765828398	0.404600357	6.835951451	8.14625E-12	3.22535E-11	positive
PRJNA729271	stage_E18_vs_E10	ordinary_muscle_development	ENSGALG00010003271	AKAP11	0.185698591	0.084237092	2.204475332	0.027490933	0.040454931	positive
PRJNA729271	stage_E18_vs_E10	ordinary_muscle_development	ENSGALG00010003272	CPSF3	-0.508914156	0.067716464	-7.515368082	5.6751E-14	2.65871E-13	negative
PRJNA729271	stage_E18_vs_E10	ordinary_muscle_development	ENSGALG00010003273	NFIX	1.793943167	0.197168971	9.098506501	9.15825E-20	6.34858E-19	positive
PRJNA729271	stage_E18_vs_E10	ordinary_muscle_development	ENSGALG00010003274	BACH2	0.348358085	0.147691107	2.358693711	0.018339385	0.027818061	positive
PRJNA729271	stage_E18_vs_E10	ordinary_muscle_development	ENSGALG00010003275	MBTPS2	0.573108646	0.127454714	4.496566894	6.90595E-06	1.63351E-05	positive
PRJNA729271	stage_E18_vs_E10	ordinary_muscle_development	ENSGALG00010003276	TNFSF11	-0.370844982	0.383081858	-0.96805676	0.333016023	0.391267509	negative
PRJNA729271	stage_E18_vs_E10	ordinary_muscle_development	ENSGALG00010003278	ENSGALG00010003278	0.443004988	0.513836313	0.862151968	0.388603897	0.448794	positive
PRJNA729271	stage_E18_vs_E10	ordinary_muscle_development	ENSGALG00010003279	ENSGALG00010003279	2.261477476	0.361868928	6.249438118	4.11932E-10	1.43878E-09	positive
PRJNA729271	stage_E18_vs_E10	ordinary_muscle_development	ENSGALG00010003280	ENSGALG00010003280	-0.128504539	0.166698009	-0.770882266	0.44077672	0.500317819	negative
PRJNA729271	stage_E18_vs_E10	ordinary_muscle_development	ENSGALG00010003287	ENSGALG00010003287	2.041219339	1.926738343	1.059416992	0.289409915	0.345637037	positive
PRJNA729271	stage_E18_vs_E10	ordinary_muscle_development	ENSGALG00010003292	GADD45GIP1	0.427620093	0.125315698	3.412342582	0.000644071	0.001205554	positive
PRJNA729271	stage_E18_vs_E10	ordinary_muscle_development	ENSGALG00010003294	ENSGALG00010003294	0.733837737	4.422067577	0.16594901	0.868197078	0.89125472	positive
PRJNA729271	stage_E18_vs_E10	ordinary_muscle_development	ENSGALG00010003295	IAH1	-0.502914395	0.135603539	-3.708711436	0.000208317	0.000416777	negative
PRJNA729271	stage_E18_vs_E10	ordinary_muscle_development	ENSGALG00010003296	ENSGALG00010003296	2.676268163	0.553861274	4.832018938	1.35155E-06	3.44665E-06	positive
PRJNA729271	stage_E18_vs_E10	ordinary_muscle_development	ENSGALG00010003298	IL7	4.269250709	1.045101762	4.085009578	4.4075E-05	9.54103E-05	positive
PRJNA729271	stage_E18_vs_E10	ordinary_muscle_development	ENSGALG00010003299	RAD23A	0.742822225	0.120794154	6.149488227	7.77334E-10	2.65304E-09	positive
PRJNA729271	stage_E18_vs_E10	ordinary_muscle_development	ENSGALG00010003300	CASP8AP2	-0.334620518	0.112271632	-2.980454731	0.002878208	0.004947186	negative
PRJNA729271	stage_E18_vs_E10	ordinary_muscle_development	ENSGALG00010003303	MTHFD2L	0.017042667	0.195060144	0.087371345	0.930376345	0.943063295	positive
PRJNA729271	stage_E18_vs_E10	ordinary_muscle_development	ENSGALG00010003304	ENSGALG00010003304	0.013835029	0.645681518	0.021427017	0.982905022	0.985829525	positive
PRJNA729271	stage_E18_vs_E10	ordinary_muscle_development	ENSGALG00010003305	CALR	-0.203872248	0.078146486	-2.608847277	0.009084778	0.01445963	negative
PRJNA729271	stage_E18_vs_E10	ordinary_muscle_development	ENSGALG00010003307	COL1A2	2.269887673	0.220378405	10.29995507	7.04942E-25	6.6481E-24	positive
PRJNA729271	stage_E18_vs_E10	ordinary_muscle_development	ENSGALG00010003308	MDN1	-1.066317908	0.099659091	-10.69965515	1.02158E-26	1.06761E-25	negative
PRJNA729271	stage_E18_vs_E10	ordinary_muscle_development	ENSGALG00010003311	ENSGALG00010003311	0.821200506	1.169544541	0.70215411	0.482583061	0.540616853	positive
PRJNA729271	stage_E18_vs_E10	ordinary_muscle_development	ENSGALG00010003315	EPGN	0.075810001	0.33902791	0.22360991	0.823060852	0.85295396	positive
PRJNA729271	stage_E18_vs_E10	ordinary_muscle_development	ENSGALG00010003317	LYRM2	1.020183933	0.176882427	5.767582178	8.04169E-09	2.5073E-08	positive
PRJNA729271	stage_E18_vs_E10	ordinary_muscle_development	ENSGALG00010003320	OR51E2	1.129640429	0.470804562	2.399382927	0.01642273	0.025158262	positive
PRJNA729271	stage_E18_vs_E10	ordinary_muscle_development	ENSGALG00010003323	STMN2	-3.093448572	0.519664046	-5.952785452	2.63617E-09	8.57716E-09	negative
PRJNA729271	stage_E18_vs_E10	ordinary_muscle_development	ENSGALG00010003324	ENSGALG00010003324	0.619652231	0.587063571	1.055511296	0.291191546	0.347535732	positive
PRJNA729271	stage_E18_vs_E10	ordinary_muscle_development	ENSGALG00010003330	HEY1	1.732141054	0.119240152	14.52649145	8.23306E-48	2.19776E-46	positive
PRJNA729271	stage_E18_vs_E10	ordinary_muscle_development	ENSGALG00010003331	EPSTI1	0.303090754	0.637009187	0.475802799	0.634214875	0.683744486	positive
PRJNA729271	stage_E18_vs_E10	ordinary_muscle_development	ENSGALG00010003333	ENSGALG00010003333	-24.95271153	2.810126854	-8.879567659	6.71207E-19	4.44863E-18	negative
PRJNA729271	stage_E18_vs_E10	ordinary_muscle_development	ENSGALG00010003334	TAC1	-3.331642372	0.896079618	-3.718020479	0.00020079	0.000402496	negative
PRJNA729271	stage_E18_vs_E10	ordinary_muscle_development	ENSGALG00010003339	ENSGALG00010003339	0.766889078	0.455061482	1.685242783	0.09194174	0.122783814	positive
PRJNA729271	stage_E18_vs_E10	ordinary_muscle_development	ENSGALG00010003344	ZFAND1	-0.025923535	0.190682864	-0.135951048	0.891859979	0.910452173	negative
PRJNA729271	stage_E18_vs_E10	ordinary_muscle_development	ENSGALG00010003346	ENSGALG00010003346	-2.000952669	5.47463579	-0.365495121	0.714741848	0.758154635	negative
PRJNA729271	stage_E18_vs_E10	ordinary_muscle_development	ENSGALG00010003347	GPM6A	1.456535946	0.35018157	4.15937351	3.19122E-05	7.01497E-05	positive
PRJNA729271	stage_E18_vs_E10	ordinary_muscle_development	ENSGALG00010003348	ADAM17	-0.284608704	0.083942301	-3.390527783	0.000697582	0.001300341	negative
PRJNA729271	stage_E18_vs_E10	ordinary_muscle_development	ENSGALG00010003351	ENSGALG00010003351	-0.183725133	1.578175366	-0.116416171	0.90732272	0.923440726	negative
PRJNA729271	stage_E18_vs_E10	ordinary_muscle_development	ENSGALG00010003352	FARSA	-0.320796698	0.102877727	-3.118232758	0.001819391	0.003217283	negative
PRJNA729271	stage_E18_vs_E10	ordinary_muscle_development	ENSGALG00010003356	ENSGALG00010003356	0.741683431	1.114379214	0.665557488	0.505693987	0.562817583	positive
PRJNA729271	stage_E18_vs_E10	ordinary_muscle_development	ENSGALG00010003358	DNAJC15	-0.255014617	0.13631582	-1.870763186	0.061377912	0.08474195	negative
PRJNA729271	stage_E18_vs_E10	ordinary_muscle_development	ENSGALG00010003363	HBE1	-0.648016455	0.502621388	-1.289273536	0.197303008	0.245780947	negative
PRJNA729271	stage_E18_vs_E10	ordinary_muscle_development	ENSGALG00010003364	ANKRD6	-1.431232367	0.153079181	-9.349621281	8.79621E-21	6.53182E-20	negative
PRJNA729271	stage_E18_vs_E10	ordinary_muscle_development	ENSGALG00010003366	CASD1	0.123716633	0.108881203	1.136253356	0.255850546	0.309768933	positive
PRJNA729271	stage_E18_vs_E10	ordinary_muscle_development	ENSGALG00010003371	ENSGALG00010003371	-0.539704897	0.368826203	-1.463304103	0.143384186	0.184316581	negative
PRJNA729271	stage_E18_vs_E10	ordinary_muscle_development	ENSGALG00010003372	ENOX1	-0.355317605	0.140747124	-2.524510599	0.011585951	0.018179082	negative
PRJNA729271	stage_E18_vs_E10	ordinary_muscle_development	ENSGALG00010003374	SYCE2	-0.392401039	0.471041787	-0.833049316	0.404816917	0.464789583	negative
PRJNA729271	stage_E18_vs_E10	ordinary_muscle_development	ENSGALG00010003375	YWHAQ	-0.774306667	0.086117108	-8.99132224	2.44274E-19	1.65301E-18	negative
PRJNA729271	stage_E18_vs_E10	ordinary_muscle_development	ENSGALG00010003379	ENSGALG00010003379	-0.438052803	0.442194451	-0.990633876	0.32186439	0.379673204	negative
PRJNA729271	stage_E18_vs_E10	ordinary_muscle_development	ENSGALG00010003382	ASNS	-1.138151233	0.600102122	-1.896595916	0.05788129	0.080339874	negative
PRJNA729271	stage_E18_vs_E10	ordinary_muscle_development	ENSGALG00010003383	UBE2J1	0.306261079	0.088228908	3.471210117	0.000518118	0.000982872	positive
PRJNA729271	stage_E18_vs_E10	ordinary_muscle_development	ENSGALG00010003388	PXDN	-0.082214098	0.186019287	-0.441965447	0.65851421	0.706368939	negative
PRJNA729271	stage_E18_vs_E10	ordinary_muscle_development	ENSGALG00010003389	ENSGALG00010003389	-0.260503722	0.368874671	-0.706212009	0.480056303	0.538319464	negative
PRJNA729271	stage_E18_vs_E10	ordinary_muscle_development	ENSGALG00010003395	ENSGALG00010003395	0.56766902	1.01018259	0.561946945	0.574152168	0.627343259	positive
PRJNA729271	stage_E18_vs_E10	ordinary_muscle_development	ENSGALG00010003397	SPATA4	2.169374091	0.399530446	5.429809196	5.64143E-08	1.63121E-07	positive
PRJNA729271	stage_E18_vs_E10	ordinary_muscle_development	ENSGALG00010003400	PKIA	1.954204842	0.11438771	17.08404552	1.9511E-65	9.2121E-64	positive
PRJNA729271	stage_E18_vs_E10	ordinary_muscle_development	ENSGALG00010003401	ENSGALG00010003401	-3.664787263	1.741155746	-2.104801522	0.035308589	0.050920107	negative
PRJNA729271	stage_E18_vs_E10	ordinary_muscle_development	ENSGALG00010003403	GCDH	-0.019840145	0.149883175	-0.132370731	0.894691065	0.912699485	negative
PRJNA729271	stage_E18_vs_E10	ordinary_muscle_development	ENSGALG00010003405	GABRR2	-0.722421426	0.234253132	-3.083934971	0.002042822	0.003583097	negative
PRJNA729271	stage_E18_vs_E10	ordinary_muscle_development	ENSGALG00010003406	C1GALT1	-0.782363663	0.106333224	-7.357659572	1.87163E-13	8.42784E-13	negative
PRJNA729271	stage_E18_vs_E10	ordinary_muscle_development	ENSGALG00010003407	CHMP4C	0.156709894	0.215427662	0.727436264	0.466958746	0.525959051	positive
PRJNA729271	stage_E18_vs_E10	ordinary_muscle_development	ENSGALG00010003408	ENSGALG00010003408	7.672099802	3.663444757	2.094231062	0.036239402	0.052153508	positive
PRJNA729271	stage_E18_vs_E10	ordinary_muscle_development	ENSGALG00010003412	ASB5	1.937605293	0.146593757	13.21751575	6.95224E-40	1.3277E-38	positive
PRJNA729271	stage_E18_vs_E10	ordinary_muscle_development	ENSGALG00010003419	ENSGALG00010003419	-2.377217885	0.951709452	-2.497839945	0.01249526	0.019489808	negative
PRJNA729271	stage_E18_vs_E10	ordinary_muscle_development	ENSGALG00010003420	ENSGALG00010003420	0.540023082	0.631596177	0.855013222	0.392543828	0.452514934	positive
PRJNA729271	stage_E18_vs_E10	ordinary_muscle_development	ENSGALG00010003428	SNX16	-0.263103072	0.159920684	-1.64520977	0.099926559	0.132507134	negative
PRJNA729271	stage_E18_vs_E10	ordinary_muscle_development	ENSGALG00010003431	PM20D2	1.599342868	0.286092091	5.590307871	2.26667E-08	6.78831E-08	positive
PRJNA729271	stage_E18_vs_E10	ordinary_muscle_development	ENSGALG00010003432	LACC1	2.677142548	0.306010764	8.74852412	2.16161E-18	1.38824E-17	positive
PRJNA729271	stage_E18_vs_E10	ordinary_muscle_development	ENSGALG00010003437	PNRC1	1.90257055	0.178934341	10.63278598	2.09751E-26	2.16087E-25	positive
PRJNA729271	stage_E18_vs_E10	ordinary_muscle_development	ENSGALG00010003438	KCNS3	-0.408037799	0.169212118	-2.411398207	0.015891487	0.024424463	negative
PRJNA729271	stage_E18_vs_E10	ordinary_muscle_development	ENSGALG00010003439	GMIP	-1.094520663	0.244444454	-4.477584348	7.54924E-06	1.77987E-05	negative
PRJNA729271	stage_E18_vs_E10	ordinary_muscle_development	ENSGALG00010003442	SPCS3	0.184942408	0.08983381	2.05871717	0.03952134	0.056495249	positive
PRJNA729271	stage_E18_vs_E10	ordinary_muscle_development	ENSGALG00010003447	ZC2HC1A	1.01325339	0.124657827	8.128277368	4.35434E-16	2.39621E-15	positive
PRJNA729271	stage_E18_vs_E10	ordinary_muscle_development	ENSGALG00010003449	BORCS6	0.99165181	0.110387891	8.983338641	2.62673E-19	1.77503E-18	positive
PRJNA729271	stage_E18_vs_E10	ordinary_muscle_development	ENSGALG00010003454	BPNT2	-2.037468854	0.12279789	-16.59205096	7.95566E-62	3.39168E-60	negative
PRJNA729271	stage_E18_vs_E10	ordinary_muscle_development	ENSGALG00010003456	CDS1	-2.237418638	0.364585995	-6.136874884	8.41606E-10	2.86431E-09	negative
PRJNA729271	stage_E18_vs_E10	ordinary_muscle_development	ENSGALG00010003466	TSC22D1	0.201194889	0.100390484	2.004123114	0.045056874	0.06374811	positive
PRJNA729271	stage_E18_vs_E10	ordinary_muscle_development	ENSGALG00010003468	ILVBL	0.999893793	0.135631569	7.372131739	1.67921E-13	7.5968E-13	positive
PRJNA729271	stage_E18_vs_E10	ordinary_muscle_development	ENSGALG00010003470	METTL3	0.463169165	0.18727526	2.473199959	0.01339092	0.020806236	positive
PRJNA729271	stage_E18_vs_E10	ordinary_muscle_development	ENSGALG00010003471	ADI1	-0.228234682	0.13516502	-1.688563224	0.091303163	0.12198722	negative
PRJNA729271	stage_E18_vs_E10	ordinary_muscle_development	ENSGALG00010003472	FAM110B	-0.356430951	0.128639242	-2.770779313	0.005592231	0.009201531	negative
PRJNA729271	stage_E18_vs_E10	ordinary_muscle_development	ENSGALG00010003473	ENSGALG00010003473	0.001776945	0.247925842	0.007167243	0.994281416	0.995036532	positive
PRJNA729271	stage_E18_vs_E10	ordinary_muscle_development	ENSGALG00010003474	RPS7	0.710416696	0.166095524	4.277157371	1.89295E-05	4.27521E-05	positive
PRJNA729271	stage_E18_vs_E10	ordinary_muscle_development	ENSGALG00010003475	ENSGALG00010003475	1.243619714	2.232576104	0.557033515	0.577504531	0.630483406	positive
PRJNA729271	stage_E18_vs_E10	ordinary_muscle_development	ENSGALG00010003478	COLEC11	1.092699627	0.17904712	6.102860667	1.04187E-09	3.51124E-09	positive
PRJNA729271	stage_E18_vs_E10	ordinary_muscle_development	ENSGALG00010003479	SYDE1	0.037097406	0.203936719	0.181906458	0.855656136	0.880688468	positive
PRJNA729271	stage_E18_vs_E10	ordinary_muscle_development	ENSGALG00010003485	RNGTT	-0.734053033	0.103444404	-7.096111603	1.28316E-12	5.44797E-12	negative
PRJNA729271	stage_E18_vs_E10	ordinary_muscle_development	ENSGALG00010003489	SGCE	-0.185440816	0.119552138	-1.551129234	0.120870717	0.157925098	negative
PRJNA729271	stage_E18_vs_E10	ordinary_muscle_development	ENSGALG00010003503	NUFIP1	-0.994363439	0.090044785	-11.0429875	2.37019E-28	2.66739E-27	negative
PRJNA729271	stage_E18_vs_E10	ordinary_muscle_development	ENSGALG00010003506	DYNC1I1	-1.066714224	0.163871445	-6.509457597	7.54226E-11	2.77686E-10	negative
PRJNA729271	stage_E18_vs_E10	ordinary_muscle_development	ENSGALG00010003507	WDFY3	0.077165147	0.097721661	0.789642191	0.429736761	0.489857524	positive
PRJNA729271	stage_E18_vs_E10	ordinary_muscle_development	ENSGALG00010003509	GPALPP1	-0.490216444	0.074651871	-6.566700044	5.14425E-11	1.9218E-10	negative
PRJNA729271	stage_E18_vs_E10	ordinary_muscle_development	ENSGALG00010003511	UBXN2B	-0.282132369	0.141620843	-1.992166995	0.046352738	0.065441019	negative
PRJNA729271	stage_E18_vs_E10	ordinary_muscle_development	ENSGALG00010003512	ENSGALG00010003512	-1.460517977	0.558397227	-2.615553777	0.008908289	0.014197433	negative
PRJNA729271	stage_E18_vs_E10	ordinary_muscle_development	ENSGALG00010003517	GTF2F2	-1.138978976	0.090696675	-12.55811175	3.58793E-36	5.86326E-35	negative
PRJNA729271	stage_E18_vs_E10	ordinary_muscle_development	ENSGALG00010003519	KCTD4	-1.144736495	0.652446598	-1.754529026	0.079339901	0.10735921	negative
PRJNA729271	stage_E18_vs_E10	ordinary_muscle_development	ENSGALG00010003520	ENSGALG00010003520	1.243645094	1.808471231	0.687677566	0.491655851	0.5492102	positive
PRJNA729271	stage_E18_vs_E10	ordinary_muscle_development	ENSGALG00010003522	ENSGALG00010003522	2.240328166	0.641416366	3.49278298	0.000478015	0.000910968	positive
PRJNA729271	stage_E18_vs_E10	ordinary_muscle_development	ENSGALG00010003525	NOL10	-0.818575421	0.068477327	-11.95396283	6.19016E-33	8.59448E-32	negative
PRJNA729271	stage_E18_vs_E10	ordinary_muscle_development	ENSGALG00010003528	GDAP1	-1.496153905	0.209758599	-7.132741682	9.83891E-13	4.22814E-12	negative
PRJNA729271	stage_E18_vs_E10	ordinary_muscle_development	ENSGALG00010003531	PPM1K	0.313631785	0.149905152	2.092201501	0.036420494	0.052377722	positive
PRJNA729271	stage_E18_vs_E10	ordinary_muscle_development	ENSGALG00010003539	ENSGALG00010003539	0.871275092	0.212002232	4.109744902	3.96096E-05	8.62204E-05	positive
PRJNA729271	stage_E18_vs_E10	ordinary_muscle_development	ENSGALG00010003541	CNR1	1.521332405	0.21818271	6.972745036	3.10816E-12	1.27918E-11	positive
PRJNA729271	stage_E18_vs_E10	ordinary_muscle_development	ENSGALG00010003543	SOX11	-1.103811746	0.079725119	-13.84521916	1.35965E-43	3.0181E-42	negative
PRJNA729271	stage_E18_vs_E10	ordinary_muscle_development	ENSGALG00010003552	PI15	-0.058516887	0.388821732	-0.150497985	0.88037174	0.900945239	negative
PRJNA729271	stage_E18_vs_E10	ordinary_muscle_development	ENSGALG00010003556	ENSGALG00010003556	1.243628856	2.007218613	0.619578181	0.535535536	0.591119981	positive
PRJNA729271	stage_E18_vs_E10	ordinary_muscle_development	ENSGALG00010003558	TPT1	2.020637462	0.184651415	10.94298393	7.17972E-28	7.95035E-27	positive
PRJNA729271	stage_E18_vs_E10	ordinary_muscle_development	ENSGALG00010003560	ADGRL3	-0.334545345	0.137521524	-2.432676242	0.014987696	0.023143352	negative
PRJNA729271	stage_E18_vs_E10	ordinary_muscle_development	ENSGALG00010003571	RPL7	0.940301042	0.157433423	5.972690067	2.33373E-09	7.62566E-09	positive
PRJNA729271	stage_E18_vs_E10	ordinary_muscle_development	ENSGALG00010003577	ENSGALG00010003577	1.243643128	1.755449981	0.708446917	0.478667765	0.537144016	positive
PRJNA729271	stage_E18_vs_E10	ordinary_muscle_development	ENSGALG00010003582	RDH10	-0.969075243	0.141887123	-6.829902699	8.49722E-12	3.35697E-11	negative
PRJNA729271	stage_E18_vs_E10	ordinary_muscle_development	ENSGALG00010003583	RSAD2	-0.099637067	0.835937475	-0.119192009	0.905123238	0.921590428	negative
PRJNA729271	stage_E18_vs_E10	ordinary_muscle_development	ENSGALG00010003585	PNPLA6	1.157376365	0.142536696	8.119848392	4.66766E-16	2.56377E-15	positive
PRJNA729271	stage_E18_vs_E10	ordinary_muscle_development	ENSGALG00010003589	SLC25A30	-0.106253471	0.389573666	-0.27274295	0.785050817	0.819776067	negative
PRJNA729271	stage_E18_vs_E10	ordinary_muscle_development	ENSGALG00010003595	SNX13	0.482545824	0.094318249	5.116144874	3.11844E-07	8.46474E-07	positive
PRJNA729271	stage_E18_vs_E10	ordinary_muscle_development	ENSGALG00010003596	CRISPLD1	-0.490632002	0.150848423	-3.252483475	0.001144012	0.002069703	negative
PRJNA729271	stage_E18_vs_E10	ordinary_muscle_development	ENSGALG00010003597	RNF144A	-0.042107414	0.143244437	-0.293954967	0.768792315	0.805847549	negative
PRJNA729271	stage_E18_vs_E10	ordinary_muscle_development	ENSGALG00010003604	ODC	-0.751990602	0.216144567	-3.479109432	0.000503083	0.00095535	negative
PRJNA729271	stage_E18_vs_E10	ordinary_muscle_development	ENSGALG00010003617	COG3	-0.145627528	0.08389717	-1.735785934	0.082601692	0.111357099	negative
PRJNA729271	stage_E18_vs_E10	ordinary_muscle_development	ENSGALG00010003619	HNF4G	-0.594641873	0.526131098	-1.130216166	0.25838515	0.312420149	negative
PRJNA729271	stage_E18_vs_E10	ordinary_muscle_development	ENSGALG00010003627	MCOLN1	0.082284465	0.121478418	0.677358714	0.498178386	0.555383457	positive
PRJNA729271	stage_E18_vs_E10	ordinary_muscle_development	ENSGALG00010003632	TECRL	0.602012315	0.334256672	1.801048012	0.071695313	0.097892197	positive
PRJNA729271	stage_E18_vs_E10	ordinary_muscle_development	ENSGALG00010003634	CYP4F11	0.80495154	0.320665081	2.510256296	0.012064356	0.018860316	positive
PRJNA729271	stage_E18_vs_E10	ordinary_muscle_development	ENSGALG00010003639	ENSGALG00010003639	-0.546423785	0.803003232	-0.680475201	0.496203619	0.553479912	negative
PRJNA729271	stage_E18_vs_E10	ordinary_muscle_development	ENSGALG00010003641	ENSGALG00010003641	0.715517094	2.578076982	0.277539072	0.781366208	0.816693336	positive
PRJNA729271	stage_E18_vs_E10	ordinary_muscle_development	ENSGALG00010003650	SPACA1	4.02534568	0.479559177	8.393845585	4.70494E-17	2.76441E-16	positive
PRJNA729271	stage_E18_vs_E10	ordinary_muscle_development	ENSGALG00010003652	MYCN	-0.793750371	0.142032279	-5.588520992	2.29012E-08	6.85569E-08	negative
PRJNA729271	stage_E18_vs_E10	ordinary_muscle_development	ENSGALG00010003660	IGFBP7	2.6808207	0.1649256	16.25472754	2.06786E-59	8.1895E-58	positive
PRJNA729271	stage_E18_vs_E10	ordinary_muscle_development	ENSGALG00010003665	RDH14	0.20178495	0.097423827	2.071207392	0.038339419	0.05495203	positive

PRJNA729271	stage_E18_vs_E10	ordinary_muscle_development	ENSGALG00010003666	SLC25A13	0.072964663	0.076346915	0.955698905	0.339224376	0.397601192	positive
PRJNA729271	stage_E18_vs_E10	ordinary_muscle_development	ENSGALG00010003669	ENSGALG00010003669	1.243648642	1.676252672	0.741921945	0.458134629	0.517632523	positive
PRJNA729271	stage_E18_vs_E10	ordinary_muscle_development	ENSGALG00010003671	ENSGALG00010003671	0.533790537	0.390285362	1.367692949	0.171408208	0.21646297	positive
PRJNA729271	stage_E18_vs_E10	ordinary_muscle_development	ENSGALG00010003674	AKIRIN2	-0.309513754	0.134670762	-2.298299562	0.021544745	0.032294838	negative
PRJNA729271	stage_E18_vs_E10	ordinary_muscle_development	ENSGALG00010003676	POLR2B	-0.365664823	0.079424313	-4.60394066	4.14571E-06	1.00087E-05	negative
PRJNA729271	stage_E18_vs_E10	ordinary_muscle_development	ENSGALG00010003678	ZNFY1	-0.60160522	0.446588075	-1.347114384	0.177943396	0.22389666	negative
PRJNA729271	stage_E18_vs_E10	ordinary_muscle_development	ENSGALG00010003680	TRAPPC5	0.074387502	0.108299945	0.686865558	0.492167453	0.549654613	positive
PRJNA729271	stage_E18_vs_E10	ordinary_muscle_development	ENSGALG00010003681	NT5C1B	-1.008196569	0.133923771	-7.528137537	5.14692E-14	2.41752E-13	negative
PRJNA729271	stage_E18_vs_E10	ordinary_muscle_development	ENSGALG00010003683	ENSGALG00010003683	0.765032296	1.755426205	0.435810001	0.662974587	0.710627571	positive
PRJNA729271	stage_E18_vs_E10	ordinary_muscle_development	ENSGALG00010003691	ERICH1	0.295425948	0.148152487	1.994066747	0.046144761	0.065179137	positive
PRJNA729271	stage_E18_vs_E10	ordinary_muscle_development	ENSGALG00010003692	ZNFY2	-0.680378327	0.263710079	-2.580023973	0.009879346	0.015641848	negative
PRJNA729271	stage_E18_vs_E10	ordinary_muscle_development	ENSGALG00010003695	NOA1	-0.235165804	0.102534719	-2.293523674	0.021817874	0.032673839	negative
PRJNA729271	stage_E18_vs_E10	ordinary_muscle_development	ENSGALG00010003698	MACC1	-5.057569739	1.238858153	-4.082444568	4.45644E-05	9.63834E-05	negative
PRJNA729271	stage_E18_vs_E10	ordinary_muscle_development	ENSGALG00010003700	REST	-0.466359846	0.067740062	-6.884549986	5.79705E-12	2.33217E-11	negative
PRJNA729271	stage_E18_vs_E10	ordinary_muscle_development	ENSGALG00010003701	SARAF	0.986405466	0.082272154	11.98954228	4.03127E-33	5.61858E-32	positive
PRJNA729271	stage_E18_vs_E10	ordinary_muscle_development	ENSGALG00010003703	TDRP	-0.172984331	0.225521998	-0.767039722	0.443057899	0.502513634	negative
PRJNA729271	stage_E18_vs_E10	ordinary_muscle_development	ENSGALG00010003713	SPERT	0.838729125	0.260208692	3.223294032	0.001267254	0.002280994	positive
PRJNA729271	stage_E18_vs_E10	ordinary_muscle_development	ENSGALG00010003714	ORC3	-0.98483991	0.11436807	-8.611143891	7.23351E-18	4.47502E-17	negative
PRJNA729271	stage_E18_vs_E10	ordinary_muscle_development	ENSGALG00010003716	YLEC8	-0.369197369	0.689699488	-0.535301787	0.592441191	0.644412056	negative
PRJNA729271	stage_E18_vs_E10	ordinary_muscle_development	ENSGALG00010003717	LEPROTL1	-0.164144317	0.09819838	-1.671558303	0.094611453	0.126093509	negative
PRJNA729271	stage_E18_vs_E10	ordinary_muscle_development	ENSGALG00010003719	RPGR	0.040975583	0.11224464	0.365056031	0.715069581	0.758446772	positive
PRJNA729271	stage_E18_vs_E10	ordinary_muscle_development	ENSGALG00010003720	ENSGALG00010003720	1.243598929	2.731725995	0.455242924	0.648934483	0.697486678	positive
PRJNA729271	stage_E18_vs_E10	ordinary_muscle_development	ENSGALG00010003722	MHCY11	1.141932864	0.65868575	1.733653512	0.08297958	0.111804146	positive
PRJNA729271	stage_E18_vs_E10	ordinary_muscle_development	ENSGALG00010003724	ENSGALG00010003724	0.111314005	0.21773731	0.511230737	0.609189498	0.660053952	positive
PRJNA729271	stage_E18_vs_E10	ordinary_muscle_development	ENSGALG00010003726	ENPEP	-1.757186211	0.450306459	-3.902200772	9.5322E-05	0.000199206	negative
PRJNA729271	stage_E18_vs_E10	ordinary_muscle_development	ENSGALG00010003727	FBXO25	0.498246486	0.153700976	3.241661181	0.001188352	0.002144033	positive
PRJNA729271	stage_E18_vs_E10	ordinary_muscle_development	ENSGALG00010003729	RARS2	0.101210572	0.101918801	0.993051054	0.320685075	0.378374453	positive
PRJNA729271	stage_E18_vs_E10	ordinary_muscle_development	ENSGALG00010003731	ENSGALG00010003731	0.845358559	0.591556478	1.429041165	0.152992402	0.195454333	positive
PRJNA729271	stage_E18_vs_E10	ordinary_muscle_development	ENSGALG00010003733	STAU2	0.18679622	0.070825161	2.6374274	0.00835375	0.013369506	positive
PRJNA729271	stage_E18_vs_E10	ordinary_muscle_development	ENSGALG00010003734	ZC3H13	-0.665131736	0.142186557	-4.6778806	2.89855E-06	7.11749E-06	negative
PRJNA729271	stage_E18_vs_E10	ordinary_muscle_development	ENSGALG00010003736	ENSGALG00010003736	1.24357711	3.115575725	0.399148414	0.689783851	0.735394007	positive
PRJNA729271	stage_E18_vs_E10	ordinary_muscle_development	ENSGALG00010003737	SRP72	0.074462961	0.056121216	1.326823711	0.184567005	0.231527368	positive
PRJNA729271	stage_E18_vs_E10	ordinary_muscle_development	ENSGALG00010003740	PITX2	-1.348467021	0.354516715	-3.80367685	0.000142564	0.000291668	negative
PRJNA729271	stage_E18_vs_E10	ordinary_muscle_development	ENSGALG00010003742	ZFHX4	-1.114849385	0.166433429	-6.698470327	2.10612E-11	8.07626E-11	negative
PRJNA729271	stage_E18_vs_E10	ordinary_muscle_development	ENSGALG00010003743	ARL9	-0.183806008	0.178261375	-1.031103948	0.302492077	0.359601628	negative
PRJNA729271	stage_E18_vs_E10	ordinary_muscle_development	ENSGALG00010003746	HOPX	2.473685982	0.407398961	6.07190057	1.26405E-09	4.22273E-09	positive
PRJNA729271	stage_E18_vs_E10	ordinary_muscle_development	ENSGALG00010003749	MHCY15	3.473768164	0.945379456	3.674469697	0.000238344	0.000473324	positive
PRJNA729271	stage_E18_vs_E10	ordinary_muscle_development	ENSGALG00010003753	SLCO5A1	-1.149279683	0.166077572	-6.920137815	4.51204E-12	1.83662E-11	negative
PRJNA729271	stage_E18_vs_E10	ordinary_muscle_development	ENSGALG00010003754	FAM110C	1.958804196	0.243609132	8.040766688	8.92781E-16	4.8161E-15	positive
PRJNA729271	stage_E18_vs_E10	ordinary_muscle_development	ENSGALG00010003755	SLC35A1	-0.8639723	0.093762373	-9.214488426	3.12766E-20	2.22669E-19	negative
PRJNA729271	stage_E18_vs_E10	ordinary_muscle_development	ENSGALG00010003756	MHCY7	-0.238334795	0.384597484	-0.619699307	0.535455772	0.591076949	negative
PRJNA729271	stage_E18_vs_E10	ordinary_muscle_development	ENSGALG00010003761	UGT8	2.811606356	0.327507354	8.584864813	9.09433E-18	5.59043E-17	positive
PRJNA729271	stage_E18_vs_E10	ordinary_muscle_development	ENSGALG00010003762	EVI5L	0.256580598	0.099338271	2.582897754	0.009797435	0.015517245	positive
PRJNA729271	stage_E18_vs_E10	ordinary_muscle_development	ENSGALG00010003764	HDAC9	1.271106571	0.192947822	6.587825448	4.46315E-11	1.67383E-10	positive
PRJNA729271	stage_E18_vs_E10	ordinary_muscle_development	ENSGALG00010003765	ENSGALG00010003765	1.888212509	0.870102229	2.17010421	0.029998952	0.043838574	positive
PRJNA729271	stage_E18_vs_E10	ordinary_muscle_development	ENSGALG00010003767	ENSGALG00010003767	-0.009317543	0.413892402	-0.022511995	0.982039544	0.985234163	negative
PRJNA729271	stage_E18_vs_E10	ordinary_muscle_development	ENSGALG00010003768	ARSJ	-0.90125595	0.129816923	-6.942515136	3.8518E-12	1.57672E-11	negative
PRJNA729271	stage_E18_vs_E10	ordinary_muscle_development	ENSGALG00010003769	C6orf163	1.178449663	0.471405421	2.499864472	0.012424083	0.019391308	positive
PRJNA729271	stage_E18_vs_E10	ordinary_muscle_development	ENSGALG00010003772	ENSGALG00010003772	0.624258349	0.446335986	1.398628764	0.161924336	0.205668879	positive
PRJNA729271	stage_E18_vs_E10	ordinary_muscle_development	ENSGALG00010003775	NCOA2	0.571501112	0.114393708	4.99591387	5.85578E-07	1.5472E-06	positive
PRJNA729271	stage_E18_vs_E10	ordinary_muscle_development	ENSGALG00010003777	ENSGALG00010003777	2.785511959	0.825420437	3.374658337	0.000739074	0.001373094	positive
PRJNA729271	stage_E18_vs_E10	ordinary_muscle_development	ENSGALG00010003778	LCA5	-0.051981148	0.127192325	-0.408681487	0.682773422	0.729099804	negative
PRJNA729271	stage_E18_vs_E10	ordinary_muscle_development	ENSGALG00010003785	ENSGALG00010003785	0.246880287	1.32497777	0.186327871	0.852187652	0.877928928	positive
PRJNA729271	stage_E18_vs_E10	ordinary_muscle_development	ENSGALG00010003786	ENSGALG00010003786	-0.31951536	0.608720697	-0.524896495	0.599655146	0.651135017	negative
PRJNA729271	stage_E18_vs_E10	ordinary_muscle_development	ENSGALG00010003787	SMIM8	1.228238781	0.188479402	6.516567677	7.19344E-11	2.65315E-10	positive
PRJNA729271	stage_E18_vs_E10	ordinary_muscle_development	ENSGALG00010003788	NUDT9	-0.147822343	0.127407573	-1.160232005	0.24595436	0.299161571	negative
PRJNA729271	stage_E18_vs_E10	ordinary_muscle_development	ENSGALG00010003790	ELOVL6	-0.371410791	0.182106639	-2.039523613	0.041397797	0.058916158	negative
PRJNA729271	stage_E18_vs_E10	ordinary_muscle_development	ENSGALG00010003791	ITGB8	-0.633444247	0.17014507	-3.722965623	0.000196896	0.000395293	negative
PRJNA729271	stage_E18_vs_E10	ordinary_muscle_development	ENSGALG00010003793	TRAM1L1	-0.267169088	0.084970965	-3.144239781	0.001665189	0.002956869	negative
PRJNA729271	stage_E18_vs_E10	ordinary_muscle_development	ENSGALG00010003795	LCP1	0.223407242	0.201940606	1.106301733	0.268595942	0.323632434	positive
PRJNA729271	stage_E18_vs_E10	ordinary_muscle_development	ENSGALG00010003796	ENSGALG00010003796	1.396576039	0.309474123	4.512739306	6.39957E-06	1.51795E-05	positive
PRJNA729271	stage_E18_vs_E10	ordinary_muscle_development	ENSGALG00010003797	ENSGALG00010003797	0.489526439	0.17411999	2.811431582	0.004932158	0.008181692	positive
PRJNA729271	stage_E18_vs_E10	ordinary_muscle_development	ENSGALG00010003803	LACTB2	1.163116757	0.132499264	8.77828842	1.65981E-18	1.07262E-17	positive
PRJNA729271	stage_E18_vs_E10	ordinary_muscle_development	ENSGALG00010003804	PSME1	2.379967449	0.258103478	9.220981703	2.94392E-20	2.10104E-19	positive
PRJNA729271	stage_E18_vs_E10	ordinary_muscle_development	ENSGALG00010003806	EGF	4.500935905	0.315295128	14.27531068	3.11895E-46	7.79468E-45	positive
PRJNA729271	stage_E18_vs_E10	ordinary_muscle_development	ENSGALG00010003808	TWIST1	-2.241491291	0.164442143	-13.63088101	2.62393E-42	5.50418E-41	negative
PRJNA729271	stage_E18_vs_E10	ordinary_muscle_development	ENSGALG00010003810	HMGN2P46	0.820802983	0.090918578	9.027890376	1.75013E-19	1.19492E-18	positive
PRJNA729271	stage_E18_vs_E10	ordinary_muscle_development	ENSGALG00010003812	BG8	0.663029333	0.402006503	1.649300018	0.099086186	0.131488993	positive
PRJNA729271	stage_E18_vs_E10	ordinary_muscle_development	ENSGALG00010003813	LRIT3	-1.373004679	0.511117522	-2.686279806	0.007225255	0.011663891	negative
PRJNA729271	stage_E18_vs_E10	ordinary_muscle_development	ENSGALG00010003814	TPP1	1.339813998	0.132353034	10.123032	4.36675E-24	3.90475E-23	positive
PRJNA729271	stage_E18_vs_E10	ordinary_muscle_development	ENSGALG00010003815	RASAL3	1.044346383	0.687302317	1.519486196	0.12864016	0.167160868	positive
PRJNA729271	stage_E18_vs_E10	ordinary_muscle_development	ENSGALG00010003816	KLHL8	-0.377091145	0.179618263	-2.099403134	0.035781379	0.051540404	negative
PRJNA729271	stage_E18_vs_E10	ordinary_muscle_development	ENSGALG00010003817	BF2	2.628685928	0.283681676	9.266322603	1.92674E-20	1.39641E-19	positive
PRJNA729271	stage_E18_vs_E10	ordinary_muscle_development	ENSGALG00010003818	GAB2	1.299655868	0.155861189	8.338547126	7.52089E-17	4.35017E-16	positive
PRJNA729271	stage_E18_vs_E10	ordinary_muscle_development	ENSGALG00010003819	PHIP	-0.427848007	0.094634986	-4.521034162	6.15383E-06	1.46325E-05	negative
PRJNA729271	stage_E18_vs_E10	ordinary_muscle_development	ENSGALG00010003820	SH3YL1	-0.690460181	0.301963562	-2.286567882	0.022221058	0.033239859	negative
PRJNA729271	stage_E18_vs_E10	ordinary_muscle_development	ENSGALG00010003821	RUBCNL	0.479040921	0.157284659	3.045693859	0.002321441	0.004033719	positive
PRJNA729271	stage_E18_vs_E10	ordinary_muscle_development	ENSGALG00010003822	ENSGALG00010003822	-0.505784896	0.165358897	-3.058709912	0.002222923	0.003874145	negative
PRJNA729271	stage_E18_vs_E10	ordinary_muscle_development	ENSGALG00010003823	PTPN13	0.56212689	0.1487953	3.777853797	0.000158186	0.00032154	positive
PRJNA729271	stage_E18_vs_E10	ordinary_muscle_development	ENSGALG00010003824	SP4	-0.109312474	0.159580968	-0.684996366	0.493345855	0.550758484	negative
PRJNA729271	stage_E18_vs_E10	ordinary_muscle_development	ENSGALG00010003826	ENSGALG00010003826	0.126354441	0.106581465	1.185519834	0.235811978	0.288179295	positive
PRJNA729271	stage_E18_vs_E10	ordinary_muscle_development	ENSGALG00010003827	RRH	-0.733640797	0.765014586	-0.958989294	0.337564139	0.395983035	negative
PRJNA729271	stage_E18_vs_E10	ordinary_muscle_development	ENSGALG00010003829	ENSGALG00010003829	-0.171277473	0.133561656	-1.282385068	0.1997076	0.248541397	negative
PRJNA729271	stage_E18_vs_E10	ordinary_muscle_development	ENSGALG00010003830	DMB2	-0.23452054	0.240057532	-0.976934729	0.328601467	0.386644879	negative
PRJNA729271	stage_E18_vs_E10	ordinary_muscle_development	ENSGALG00010003831	IRAK1BP1	-0.195344508	0.169918473	-1.149636673	0.250293547	0.303980307	negative
PRJNA729271	stage_E18_vs_E10	ordinary_muscle_development	ENSGALG00010003832	ART1B	4.677097551	0.955352802	4.895675753	9.79685E-07	2.534E-06	positive
PRJNA729271	stage_E18_vs_E10	ordinary_muscle_development	ENSGALG00010003833	ACP1	0.006137583	0.157852646	0.038881721	0.96898469	0.974294747	positive
PRJNA729271	stage_E18_vs_E10	ordinary_muscle_development	ENSGALG00010003834	MAPK10	-0.017737934	0.420148593	-0.042218239	0.966324723	0.972362156	negative
PRJNA729271	stage_E18_vs_E10	ordinary_muscle_development	ENSGALG00010003835	ENSGALG00010003835	1.306810654	0.16739194	7.806891135	5.86159E-15	2.97909E-14	positive
PRJNA729271	stage_E18_vs_E10	ordinary_muscle_development	ENSGALG00010003836	CD1F	2.011492583	0.368140408	5.46392773	4.65713E-08	1.3588E-07	positive
PRJNA729271	stage_E18_vs_E10	ordinary_muscle_development	ENSGALG00010003837	ALKAL2	2.072128815	0.237440409	8.726942593	2.6165E-18	1.67149E-17	positive
PRJNA729271	stage_E18_vs_E10	ordinary_muscle_development	ENSGALG00010003838	GAR1	-1.48510508	0.184502739	-8.049230532	8.33162E-16	4.50454E-15	negative
PRJNA729271	stage_E18_vs_E10	ordinary_muscle_development	ENSGALG00010003839	ENSGALG00010003839	2.18420989	0.761663218	2.867684611	0.004134875	0.006928903	positive
PRJNA729271	stage_E18_vs_E10	ordinary_muscle_development	ENSGALG00010003841	UBE2W	0.98265397	0.14385111	6.831048899	8.4296E-12	3.33298E-11	positive
PRJNA729271	stage_E18_vs_E10	ordinary_muscle_development	ENSGALG00010003842	ENSGALG00010003842	0.23333437	0.144560439	1.614095612	0.106506689	0.140487256	positive
PRJNA729271	stage_E18_vs_E10	ordinary_muscle_development	ENSGALG00010003843	LRCH1	1.239891331	0.10723616	11.56225036	6.4009E-31	8.19621E-30	positive
PRJNA729271	stage_E18_vs_E10	ordinary_muscle_development	ENSGALG00010003844	TMEM18	-0.968844678	0.115770422	-8.368671901	5.82712E-17	3.40033E-16	negative
PRJNA729271	stage_E18_vs_E10	ordinary_muscle_development	ENSGALG00010003845	CFI	3.502006687	0.845413086	4.14236158			positive
PRJNA729271	stage_E18_vs_E10	ordinary_muscle_development	ENSGALG00010003848	ESD	1.307171903	0.123404094	10.59261377	3.2246E-26	3.26857E-25	positive
PRJNA729271	stage_E18_vs_E10	ordinary_muscle_development	ENSGALG00010003850	PLA2G12A	-0.449584253	0.100350812	-4.480125693	7.45991E-06	1.75966E-05	negative
PRJNA729271	stage_E18_vs_E10	ordinary_muscle_development	ENSGALG00010003851	PHF14	-0.702230027	0.063279616	-11.09725492	1.29356E-28	1.47872E-27	negative
PRJNA729271	stage_E18_vs_E10	ordinary_muscle_development	ENSGALG00010003852	TCEB1	-0.343379514	0.107783162	-3.185836329	0.001443362	0.002580357	negative
PRJNA729271	stage_E18_vs_E10	ordinary_muscle_development	ENSGALG00010003853	ENSGALG00010003853	0.580979935	0.230518496	2.520318088	0.011724883	0.018375194	positive
PRJNA729271	stage_E18_vs_E10	ordinary_muscle_development	ENSGALG00010003858	SUCLA2	0.599231418	0.124937982	4.796230963	1.61679E-06	4.0821E-06	positive
PRJNA729271	stage_E18_vs_E10	ordinary_muscle_development	ENSGALG00010003860	TMEM70	-0.010388104	0.114782252	-0.090502706	0.927887744	0.941001389	negative
PRJNA729271	stage_E18_vs_E10	ordinary_muscle_development	ENSGALG00010003861	NUDT15	-0.668491791	0.105895195	-6.312767953	2.74088E-10	9.68058E-10	negative
PRJNA729271	stage_E18_vs_E10	ordinary_muscle_development	ENSGALG00010003862	PCK2	0.085784053	0.144989804	0.591655763	0.554081125	0.608255522	positive
PRJNA729271	stage_E18_vs_E10	ordinary_muscle_development	ENSGALG00010003863	HTR1B	-4.058262416	0.357836533	-11.34110703	8.20993E-30	9.9584E-29	negative
PRJNA729271	stage_E18_vs_E10	ordinary_muscle_development	ENSGALG00010003865	AKAP8L	-0.755902387	0.190788874	-3.961983592	7.43296E-05	0.000157125	negative
PRJNA729271	stage_E18_vs_E10	ordinary_muscle_development	ENSGALG00010003868	HBBA	-0.334808218	0.398843293	-0.839448033	0.401217939	0.461011021	negative
PRJNA729271	stage_E18_vs_E10	ordinary_muscle_development	ENSGALG00010003869	LY96	0.203017638	0.293028712	0.692825069	0.488419338	0.546352701	positive
PRJNA729271	stage_E18_vs_E10	ordinary_muscle_development	ENSGALG00010003870	MED4	-0.677942204	0.096380518	-7.034017048	2.00671E-12	8.37765E-12	negative
PRJNA729271	stage_E18_vs_E10	ordinary_muscle_development	ENSGALG00010003871	ENSGALG00010003871	-0.125293971	0.197935955	-0.633002583	0.526731957	0.58295638	negative
PRJNA729271	stage_E18_vs_E10	ordinary_muscle_development	ENSGALG00010003874	YES1	-0.29638506	0.097746576	-3.032178435	0.002427956	0.00421022	negative
PRJNA729271	stage_E18_vs_E10	ordinary_muscle_development	ENSGALG00010003875	AKAP8	-0.087866396	0.110360929	-0.796173037	0.425931467	0.486092978	negative
PRJNA729271	stage_E18_vs_E10	ordinary_muscle_development	ENSGALG00010003878	ARHGAP24	0.921849648	0.088695397	10.39343276	2.65618E-25	2.55992E-24	positive
PRJNA729271	stage_E18_vs_E10	ordinary_muscle_development	ENSGALG00010003879	CASP6	-1.016385945	0.119357994	-8.515440901	1.65958E-17	1.00357E-16	negative
PRJNA729271	stage_E18_vs_E10	ordinary_muscle_development	ENSGALG00010003882	ENSGALG00010003882	-0.210711874	0.121999243	-1.727157226	0.08413943	0.113209045	negative
PRJNA729271	stage_E18_vs_E10	ordinary_muscle_development	ENSGALG00010003884	TYMS	-1.931067599	0.192023165	-10.05643043	8.60624E-24	7.54217E-23	negative
PRJNA729271	stage_E18_vs_E10	ordinary_muscle_development	ENSGALG00010003887	GIPC1	-0.863633533	0.145663867	-5.928948259	3.04881E-09	9.86881E-09	negative
PRJNA729271	stage_E18_vs_E10	ordinary_muscle_development	ENSGALG00010003889	ITM2B	0.915627917	0.087484746	10.46614361	1.23575E-25	1.21439E-24	positive
PRJNA729271	stage_E18_vs_E10	ordinary_muscle_development	ENSGALG00010003892	OPRK1	-4.681745348	2.563370122	-1.826402402	0.06778967	0.092900754	negative
PRJNA729271	stage_E18_vs_E10	ordinary_muscle_development	ENSGALG00010003894	COPS4	0.065934112	0.124716046	0.528673853	0.597031718	0.648821019	positive
PRJNA729271	stage_E18_vs_E10	ordinary_muscle_development	ENSGALG00010003895	THSD7A	1.031684431	0.159133555	6.483135691	8.98356E-11	3.2883E-10	positive
PRJNA729271	stage_E18_vs_E10	ordinary_muscle_development	ENSGALG00010003896	MCUB	-0.614414774	0.133999954	-4.585186466	4.53581E-06	1.09177E-05	negative
PRJNA729271	stage_E18_vs_E10	ordinary_muscle_development	ENSGALG00010003898	NDST4	-4.61766912	0.622862327	-7.413627245	1.22891E-13	5.61572E-13	negative
PRJNA729271	stage_E18_vs_E10	ordinary_muscle_development	ENSGALG00010003899	LIN54	0.370049993	0.165607331	2.234502484	0.025450029	0.037692671	positive
PRJNA729271	stage_E18_vs_E10	ordinary_muscle_development	ENSGALG00010003900	RB1	-0.931386481	0.089858341	-10.36505314	3.5756E-25	3.4165E-24	negative
PRJNA729271	stage_E18_vs_E10	ordinary_muscle_development	ENSGALG00010003902	ENSGALG00010003902	0.747699657	0.125720965	5.947294918	2.7261E-09	8.86181E-09	positive
PRJNA729271	stage_E18_vs_E10	ordinary_muscle_development	ENSGALG00010003903	COL28A1	3.950240818	0.280363153	14.08972888	4.39245E-45	1.03358E-43	positive
PRJNA729271	stage_E18_vs_E10	ordinary_muscle_development	ENSGALG00010003909	DNAJB1	-0.733122114	0.158792057	-4.616868941	3.89573E-06	9.42715E-06	negative

PRJNA729271	stage_E18_vs_E10	ordinary_muscle_development	ENSGALG00010003910	THAP9	1.086186692	0.147982211	7.339981493	2.13623E-13	9.57474E-13	positive
PRJNA729271	stage_E18_vs_E10	ordinary_muscle_development	ENSGALG00010003913	PSMB5	-0.032016226	0.117369898	-0.272780554	0.785021908	0.819776067	negative
PRJNA729271	stage_E18_vs_E10	ordinary_muscle_development	ENSGALG00010003914	CAMK2D	-0.083604755	0.106291595	-0.78656036	0.431539272	0.491602444	negative
PRJNA729271	stage_E18_vs_E10	ordinary_muscle_development	ENSGALG00010003915	MIOS	-0.019427048	0.092968366	-0.208964065	0.834476291	0.862563919	negative
PRJNA729271	stage_E18_vs_E10	ordinary_muscle_development	ENSGALG00010003918	HTR1E	-1.940047364	0.690767236	-2.808539929	0.004976671	0.008253644	negative
PRJNA729271	stage_E18_vs_E10	ordinary_muscle_development	ENSGALG00010003920	LPAR6	0.525873486	0.155092758	3.390703041	0.000697136	0.001299677	positive
PRJNA729271	stage_E18_vs_E10	ordinary_muscle_development	ENSGALG00010003921	COLEC12	0.334702505	0.168264756	1.989142072	0.046685524	0.065859529	positive
PRJNA729271	stage_E18_vs_E10	ordinary_muscle_development	ENSGALG00010003923	ENSGALG00010003923	-0.99520498	0.122064555	-8.153103753	3.54701E-16	1.96012E-15	negative
PRJNA729271	stage_E18_vs_E10	ordinary_muscle_development	ENSGALG00010003925	SEC31A	-0.591862576	0.085945907	-6.886454506	5.72E-12	2.30374E-11	negative
PRJNA729271	stage_E18_vs_E10	ordinary_muscle_development	ENSGALG00010003928	ENSGALG00010003928	0.545734023	0.156163488	3.494632637	0.000474715	0.000905393	positive
PRJNA729271	stage_E18_vs_E10	ordinary_muscle_development	ENSGALG00010003929	RCBTB2	-0.105097526	0.099370707	-1.05763086	0.290223767	0.346523353	negative
PRJNA729271	stage_E18_vs_E10	ordinary_muscle_development	ENSGALG00010003933	TECR	0.86397533	0.208417457	4.145407704	3.3921E-05	7.43512E-05	positive
PRJNA729271	stage_E18_vs_E10	ordinary_muscle_development	ENSGALG00010003937	ENSGALG00010003937	1.696608267	0.205808472	8.243626935	1.67068E-16	9.43743E-16	positive
PRJNA729271	stage_E18_vs_E10	ordinary_muscle_development	ENSGALG00010003948	SCD5	-2.278487053	0.141430813	-16.11025915	2.16116E-58	8.22206E-57	negative
PRJNA729271	stage_E18_vs_E10	ordinary_muscle_development	ENSGALG00010003958	CYSLTR2	1.31499427	0.394865321	3.330234894	0.000867727	0.001595144	positive
PRJNA729271	stage_E18_vs_E10	ordinary_muscle_development	ENSGALG00010003960	ASPH	1.183048361	0.19600778	6.035721432	1.58254E-09	5.24918E-09	positive
PRJNA729271	stage_E18_vs_E10	ordinary_muscle_development	ENSGALG00010003962	UMAD1	0.219803892	0.205444351	1.069895035	0.284666558	0.340364741	positive
PRJNA729271	stage_E18_vs_E10	ordinary_muscle_development	ENSGALG00010003965	SNTG1	-0.393891243	0.494801703	-0.796058786	0.425997868	0.486092978	negative
PRJNA729271	stage_E18_vs_E10	ordinary_muscle_development	ENSGALG00010003968	DSN1	-0.113390021	0.174086677	-0.651342326	0.514825532	0.571568328	negative
PRJNA729271	stage_E18_vs_E10	ordinary_muscle_development	ENSGALG00010003972	PENK	-1.894916537	0.240924704	-7.865181562	3.68562E-15	1.90186E-14	negative
PRJNA729271	stage_E18_vs_E10	ordinary_muscle_development	ENSGALG00010003977	MYO6	-1.143911223	0.119722229	-9.554710353	1.2393E-21	9.69437E-21	negative
PRJNA729271	stage_E18_vs_E10	ordinary_muscle_development	ENSGALG00010003978	ANK2	1.087216482	0.119694097	9.083292421	1.05339E-19	7.28131E-19	positive
PRJNA729271	stage_E18_vs_E10	ordinary_muscle_development	ENSGALG00010003980	CHCHD7	0.21780466	0.120282365	1.810778003	0.070175223	0.095961307	positive
PRJNA729271	stage_E18_vs_E10	ordinary_muscle_development	ENSGALG00010003981	TMEM150C	-0.594067324	0.170142534	-3.491586212	0.000480161	0.000914939	negative
PRJNA729271	stage_E18_vs_E10	ordinary_muscle_development	ENSGALG00010003982	GLCCI1	-0.402151344	0.1216286	-3.306388017	0.000945071	0.001726813	negative
PRJNA729271	stage_E18_vs_E10	ordinary_muscle_development	ENSGALG00010003985	PRMT5	-0.242813934	0.135089426	-1.797431092	0.072267208	0.098570922	negative
PRJNA729271	stage_E18_vs_E10	ordinary_muscle_development	ENSGALG00010003986	FNDC3A	-0.023350918	0.064614535	-0.361388001	0.717809416	0.760851736	negative
PRJNA729271	stage_E18_vs_E10	ordinary_muscle_development	ENSGALG00010003988	ENOPH1	-0.96892923	0.090412379	-10.71677617	8.49115E-27	8.93173E-26	negative
PRJNA729271	stage_E18_vs_E10	ordinary_muscle_development	ENSGALG00010003990	ICA1	-0.927056333	0.176875676	-5.241287864	1.5946E-07	4.42537E-07	negative
PRJNA729271	stage_E18_vs_E10	ordinary_muscle_development	ENSGALG00010003993	ENSGALG00010003993	0.710516355	0.134319972	5.289729774	1.22497E-07	3.44375E-07	positive
PRJNA729271	stage_E18_vs_E10	ordinary_muscle_development	ENSGALG00010003994	SDR16C5	1.123565167	0.085943632	13.07327999	4.68031E-39	8.64218E-38	positive
PRJNA729271	stage_E18_vs_E10	ordinary_muscle_development	ENSGALG00010003995	MLNR	-2.74592573	0.26534421	-10.34854211	4.24907E-25	4.04666E-24	negative
PRJNA729271	stage_E18_vs_E10	ordinary_muscle_development	ENSGALG00010004000	ENSGALG00010004000	1.072239326	0.177957747	6.025246707	1.68852E-09	5.58027E-09	positive
PRJNA729271	stage_E18_vs_E10	ordinary_muscle_development	ENSGALG00010004001	LARP7	-0.707166048	0.114308136	-6.186489167	6.15189E-10	2.12061E-09	negative
PRJNA729271	stage_E18_vs_E10	ordinary_muscle_development	ENSGALG00010004002	CDADC1	0.466399524	0.129216258	3.609449229	0.000306848	0.000600156	positive
PRJNA729271	stage_E18_vs_E10	ordinary_muscle_development	ENSGALG00010004003	HNRNPDL	-0.535139778	0.068488975	-7.813517066	5.56139E-15	2.83248E-14	negative
PRJNA729271	stage_E18_vs_E10	ordinary_muscle_development	ENSGALG00010004006	NKAIN3	-2.31546576	0.343636605	-6.73812314	1.60446E-11	6.21004E-11	negative
PRJNA729271	stage_E18_vs_E10	ordinary_muscle_development	ENSGALG00010004007	ENSGALG00010004007	-1.179293207	0.162589822	-7.25317976	4.07099E-13	1.79577E-12	negative
PRJNA729271	stage_E18_vs_E10	ordinary_muscle_development	ENSGALG00010004016	ENSGALG00010004016	1.124166108	0.386223495	2.910662148	0.003606638	0.006089483	positive
PRJNA729271	stage_E18_vs_E10	ordinary_muscle_development	ENSGALG00010004018	HNRNPD	-0.676083852	0.162194053	-4.168364013	3.06794E-05	6.75729E-05	negative
PRJNA729271	stage_E18_vs_E10	ordinary_muscle_development	ENSGALG00010004029	ARL4A	-0.285285286	0.19492947	-1.463530816	0.143322188	0.18426737	negative
PRJNA729271	stage_E18_vs_E10	ordinary_muscle_development	ENSGALG00010004034	NEDD8	0.072702585	0.113586574	0.64006319	0.522131519	0.578846561	positive
PRJNA729271	stage_E18_vs_E10	ordinary_muscle_development	ENSGALG00010004036	ADCYAP1	0.860224463	0.476882588	1.803849594	0.071254889	0.097355039	positive
PRJNA729271	stage_E18_vs_E10	ordinary_muscle_development	ENSGALG00010004038	SENP6	-0.009349139	0.088006058	-0.106232899	0.915397569	0.930039892	negative
PRJNA729271	stage_E18_vs_E10	ordinary_muscle_development	ENSGALG00010004039	ALPK1	1.593554816	0.250320789	6.366050637	1.93957E-10	6.91785E-10	positive
PRJNA729271	stage_E18_vs_E10	ordinary_muscle_development	ENSGALG00010004040	LGALS1B	-0.702757746	0.224823265	-3.125823063	0.001773082	0.00313884	negative
PRJNA729271	stage_E18_vs_E10	ordinary_muscle_development	ENSGALG00010004042	PLAG1	0.201950305	0.120290071	1.678860965	0.093179144	0.124321768	positive
PRJNA729271	stage_E18_vs_E10	ordinary_muscle_development	ENSGALG00010004043	CAB39L	0.569097401	0.201225011	2.828164338	0.004681577	0.007786512	positive
PRJNA729271	stage_E18_vs_E10	ordinary_muscle_development	ENSGALG00010004044	RNF138	-0.804540947	0.136547678	-5.892014868	3.81515E-09	1.22563E-08	negative
PRJNA729271	stage_E18_vs_E10	ordinary_muscle_development	ENSGALG00010004046	ENSGALG00010004046	1.208169744	0.120433883	10.03180925	1.10473E-23	9.61172E-23	positive
PRJNA729271	stage_E18_vs_E10	ordinary_muscle_development	ENSGALG00010004047	PDE7A	-1.024530283	0.120026995	-8.535832149	1.3915E-17	8.4605E-17	negative
PRJNA729271	stage_E18_vs_E10	ordinary_muscle_development	ENSGALG00010004049	SETDB2	-0.456280043	0.139898036	-3.26151857	0.001108172	0.002007618	negative
PRJNA729271	stage_E18_vs_E10	ordinary_muscle_development	ENSGALG00010004055	FILIP1	1.944757546	0.175347399	11.09088332	1.38909E-28	1.58045E-27	positive
PRJNA729271	stage_E18_vs_E10	ordinary_muscle_development	ENSGALG00010004056	SHROOM3	0.708133789	0.181071329	3.910800203	9.19908E-05	0.000192605	positive
PRJNA729271	stage_E18_vs_E10	ordinary_muscle_development	ENSGALG00010004057	ENSGALG00010004057	0.768195111	0.381718638	2.012464245	0.044171022	0.062598647	positive
PRJNA729271	stage_E18_vs_E10	ordinary_muscle_development	ENSGALG00010004058	TMEM30A	-0.053854147	0.062087276	-0.867394274	0.385726018	0.446074568	negative
PRJNA729271	stage_E18_vs_E10	ordinary_muscle_development	ENSGALG00010004060	ETV1	-1.06000937	0.092677892	-11.43756447	2.71398E-30	3.36519E-29	negative
PRJNA729271	stage_E18_vs_E10	ordinary_muscle_development	ENSGALG00010004063	ENSGALG00010004063	2.330910846	0.40884691	5.701182496	1.18979E-08	3.64841E-08	positive
PRJNA729271	stage_E18_vs_E10	ordinary_muscle_development	ENSGALG00010004065	TIFA	-0.712062111	0.318070531	-2.238692492	0.025175931	0.03732104	negative
PRJNA729271	stage_E18_vs_E10	ordinary_muscle_development	ENSGALG00010004070	ARL11	-1.040537637	0.414098282	-2.512779411	0.011978421	0.018738097	negative
PRJNA729271	stage_E18_vs_E10	ordinary_muscle_development	ENSGALG00010004072	SGK3	-0.5427989	0.136471985	-3.977365017	6.96832E-05	0.000147929	negative
PRJNA729271	stage_E18_vs_E10	ordinary_muscle_development	ENSGALG00010004076	COX7A2	-0.527855833	0.136894541	-3.855930468	0.00011529	0.00023871	negative
PRJNA729271	stage_E18_vs_E10	ordinary_muscle_development	ENSGALG00010004079	AP1AR	-0.626798676	0.151141675	-4.147093617	3.36722E-05	7.38395E-05	negative
PRJNA729271	stage_E18_vs_E10	ordinary_muscle_development	ENSGALG00010004081	RPS20	1.178644565	0.167919586	7.019101182	2.233E-12	9.29028E-12	positive
PRJNA729271	stage_E18_vs_E10	ordinary_muscle_development	ENSGALG00010004082	FAM241A	0.648303146	0.123342376	5.256126612	1.47121E-07	4.09549E-07	positive
PRJNA729271	stage_E18_vs_E10	ordinary_muscle_development	ENSGALG00010004083	PHF11	-1.029947821	0.189642879	-5.430986005	5.60435E-08	1.62146E-07	negative
PRJNA729271	stage_E18_vs_E10	ordinary_muscle_development	ENSGALG00010004085	RB1CC1	0.463420603	0.141172384	3.282657639	0.001028335	0.001870227	positive
PRJNA729271	stage_E18_vs_E10	ordinary_muscle_development	ENSGALG00010004086	UTP3	-0.436916238	0.258780453	-1.688366458	0.091340905	0.122015153	negative
PRJNA729271	stage_E18_vs_E10	ordinary_muscle_development	ENSGALG00010004087	NCOA7	-0.088133794	0.109522767	-0.804707515	0.420988482	0.48143811	negative
PRJNA729271	stage_E18_vs_E10	ordinary_muscle_development	ENSGALG00010004094	DGKB	-0.620101713	0.177337466	-3.496732683	0.000470994	0.000899361	negative
PRJNA729271	stage_E18_vs_E10	ordinary_muscle_development	ENSGALG00010004095	ANKRD50	0.009459609	0.113345416	0.083458243	0.933487179	0.945819702	positive
PRJNA729271	stage_E18_vs_E10	ordinary_muscle_development	ENSGALG00010004096	COL12A1	-1.24581191	0.414070715	-3.008693602	0.002623736	0.004533443	negative
PRJNA729271	stage_E18_vs_E10	ordinary_muscle_development	ENSGALG00010004097	ENSGALG00010004097	-0.824758546	0.12115283	-6.807587959	9.92486E-12	3.90608E-11	negative
PRJNA729271	stage_E18_vs_E10	ordinary_muscle_development	ENSGALG00010004101	ARID1B	0.434772726	0.166441217	2.612169838	0.008996955	0.014332438	positive
PRJNA729271	stage_E18_vs_E10	ordinary_muscle_development	ENSGALG00010004102	PCMTD1	1.305452133	0.188353497	6.930862217	4.18283E-12	1.70741E-11	positive
PRJNA729271	stage_E18_vs_E10	ordinary_muscle_development	ENSGALG00010004104	SCARB2	0.655571729	0.095199419	6.886299657	5.72623E-12	2.3056E-11	positive
PRJNA729271	stage_E18_vs_E10	ordinary_muscle_development	ENSGALG00010004108	PERPB	0.467149983	0.242447778	1.926806618	0.054003734	0.075361907	positive
PRJNA729271	stage_E18_vs_E10	ordinary_muscle_development	ENSGALG00010004109	AGMO	-1.175734744	0.539304357	-2.180095023	0.029250419	0.042822424	negative
PRJNA729271	stage_E18_vs_E10	ordinary_muscle_development	ENSGALG00010004113	ECHDC1	0.930812319	0.183997099	5.058842363	4.21809E-07	1.12994E-06	positive
PRJNA729271	stage_E18_vs_E10	ordinary_muscle_development	ENSGALG00010004116	SPRY1	0.197805508	0.095421884	2.072957479	0.038176237	0.054739792	positive
PRJNA729271	stage_E18_vs_E10	ordinary_muscle_development	ENSGALG00010004117	STBD1	2.369248922	0.191257828	12.38772261	3.0457E-35	4.74194E-34	positive
PRJNA729271	stage_E18_vs_E10	ordinary_muscle_development	ENSGALG00010004122	TRIM55	1.739358258	0.194845089	8.926877588	4.38202E-19	2.93518E-18	positive
PRJNA729271	stage_E18_vs_E10	ordinary_muscle_development	ENSGALG00010004123	NDUFAF4	-0.189106721	0.111998049	-1.68848227	0.09131869	0.121996719	negative
PRJNA729271	stage_E18_vs_E10	ordinary_muscle_development	ENSGALG00010004125	MANEA	0.112291301	0.093568446	1.200097963	0.230101296	0.281722974	positive
PRJNA729271	stage_E18_vs_E10	ordinary_muscle_development	ENSGALG00010004127	EPHA7	-2.645413036	0.179433647	-14.74312693	3.4064E-49	9.66258E-48	negative
PRJNA729271	stage_E18_vs_E10	ordinary_muscle_development	ENSGALG00010004131	NUDT6	0.866738503	0.159798925	5.423931993	5.83021E-08	1.68311E-07	positive
PRJNA729271	stage_E18_vs_E10	ordinary_muscle_development	ENSGALG00010004133	CRH	3.249348368	0.974137525	3.335615644	0.000851107	0.001566777	positive
PRJNA729271	stage_E18_vs_E10	ordinary_muscle_development	ENSGALG00010004135	NUP54	-0.747441308	0.066810581	-11.18746908	4.69648E-29	5.4504E-28	negative
PRJNA729271	stage_E18_vs_E10	ordinary_muscle_development	ENSGALG00010004137	CROT	-0.546636796	0.084584277	-6.462628953	1.02899E-10	3.74567E-10	negative
PRJNA729271	stage_E18_vs_E10	ordinary_muscle_development	ENSGALG00010004139	PPEF2	-0.011376068	0.694036231	-0.016391173	0.986922321	0.988900805	negative
PRJNA729271	stage_E18_vs_E10	ordinary_muscle_development	ENSGALG00010004141	KPNA3	-0.266499165	0.142124796	-1.875106749	0.060778039	0.083998635	negative
PRJNA729271	stage_E18_vs_E10	ordinary_muscle_development	ENSGALG00010004142	RBBP8	-1.281282323	0.115571697	-11.08647148	1.45931E-28	1.65644E-27	negative
PRJNA729271	stage_E18_vs_E10	ordinary_muscle_development	ENSGALG00010004143	TCF24	-1.638709178	0.454816192	-3.603014158	0.000314548	0.000613727	negative
PRJNA729271	stage_E18_vs_E10	ordinary_muscle_development	ENSGALG00010004146	NAAA	1.364260747	0.178085113	7.660723138	1.84889E-14	9.01738E-14	positive
PRJNA729271	stage_E18_vs_E10	ordinary_muscle_development	ENSGALG00010004147	SPRYD7	-0.189756073	0.093943681	-2.019891828	0.043394609	0.061552486	negative
PRJNA729271	stage_E18_vs_E10	ordinary_muscle_development	ENSGALG00010004148	ENSGALG00010004148	0.580668842	0.154098816	3.768159014	0.000164456	0.000333211	positive
PRJNA729271	stage_E18_vs_E10	ordinary_muscle_development	ENSGALG00010004149	SDAD1	-1.230471861	0.124832789	-9.856960427	6.39562E-23	5.34628E-22	negative
PRJNA729271	stage_E18_vs_E10	ordinary_muscle_development	ENSGALG00010004154	COPS5	-0.512841558	0.131489875	-3.900236101	9.60989E-05	0.000200714	negative
PRJNA729271	stage_E18_vs_E10	ordinary_muscle_development	ENSGALG00010004158	JCHAIN	1.690618296	0.48426832	3.491077624	0.000481076	0.000916321	positive
PRJNA729271	stage_E18_vs_E10	ordinary_muscle_development	ENSGALG00010004172	ST8SIA6	0.493555673	0.124676903	3.958677692	7.53659E-05	0.0001592	positive
PRJNA729271	stage_E18_vs_E10	ordinary_muscle_development	ENSGALG00010004177	CSPP1	0.04273848	0.128807423	0.33180137	0.74003925	0.780987909	positive
PRJNA729271	stage_E18_vs_E10	ordinary_muscle_development	ENSGALG00010004181	BBS12	-0.720070262	0.132152062	-5.44880082	5.07106E-08	1.47305E-07	negative
PRJNA729271	stage_E18_vs_E10	ordinary_muscle_development	ENSGALG00010004187	ARFGEF1	0.575990212	0.094406429	6.101175712	1.05291E-09	3.54599E-09	positive
PRJNA729271	stage_E18_vs_E10	ordinary_muscle_development	ENSGALG00010004188	BMI1	0.275963301	0.115322272	2.392974884	0.016712385	0.025556074	positive
PRJNA729271	stage_E18_vs_E10	ordinary_muscle_development	ENSGALG00010004191	CABLES1	0.279740387	0.12770649	2.190494679	0.02848838	0.041786994	positive
PRJNA729271	stage_E18_vs_E10	ordinary_muscle_development	ENSGALG00010004200	ABCB1	1.08438909	0.187804962	5.774017235	7.74035E-09	2.41542E-08	positive
PRJNA729271	stage_E18_vs_E10	ordinary_muscle_development	ENSGALG00010004201	CPA6	-4.745527207	0.482194015	-9.841530714	7.45676E-23	6.21182E-22	negative
PRJNA729271	stage_E18_vs_E10	ordinary_muscle_development	ENSGALG00010004210	SYNCRIP	-0.922891551	0.070941601	-13.00917293	1.0851E-38	1.97595E-37	negative
PRJNA729271	stage_E18_vs_E10	ordinary_muscle_development	ENSGALG00010004211	RNF146	-0.110192391	0.076581565	-1.438889251	0.150181904	0.192251864	negative
PRJNA729271	stage_E18_vs_E10	ordinary_muscle_development	ENSGALG00010004212	RSPO3	-0.709782108	0.380443892	-1.865668297	0.062087788	0.085621015	negative
PRJNA729271	stage_E18_vs_E10	ordinary_muscle_development	ENSGALG00010004214	REPS1	-0.201003442	0.126808268	-1.585097287	0.112944232	0.148371093	negative
PRJNA729271	stage_E18_vs_E10	ordinary_muscle_development	ENSGALG00010004216	SNX14	-0.101696975	0.074875627	-1.358212008	0.174396422	0.219834432	negative
PRJNA729271	stage_E18_vs_E10	ordinary_muscle_development	ENSGALG00010004217	CCDC28A	-0.718177432	0.115837943	-6.199846235	5.65184E-10	1.95708E-09	negative
PRJNA729271	stage_E18_vs_E10	ordinary_muscle_development	ENSGALG00010004219	FGF2	1.411208905	0.181274126	7.784943907	6.97441E-15	3.5249E-14	positive
PRJNA729271	stage_E18_vs_E10	ordinary_muscle_development	ENSGALG00010004222	SPAG6	-1.906672723	0.252769139	-7.543138897	4.58792E-14	2.16126E-13	negative
PRJNA729271	stage_E18_vs_E10	ordinary_muscle_development	ENSGALG00010004224	NT5E	1.758790727	0.257578534	6.828172755	8.6003E-12	3.39492E-11	positive
PRJNA729271	stage_E18_vs_E10	ordinary_muscle_development	ENSGALG00010004225	CETN4	-1.208491466	0.126803822	-9.530402507	1.56675E-21	1.21966E-20	negative
PRJNA729271	stage_E18_vs_E10	ordinary_muscle_development	ENSGALG00010004226	RUNDC3B	-1.402933587	0.206254948	-6.801939073	1.0322E-11	4.05688E-11	negative
PRJNA729271	stage_E18_vs_E10	ordinary_muscle_development	ENSGALG00010004230	TAF1B	-0.350237606	0.119626621	-2.927756398	0.003414174	0.005781362	negative
PRJNA729271	stage_E18_vs_E10	ordinary_muscle_development	ENSGALG00010004231	ARMC3	2.159970004	0.347798905	6.210399093	5.28502E-10	1.83357E-09	positive
PRJNA729271	stage_E18_vs_E10	ordinary_muscle_development	ENSGALG00010004233	NHSL1	-1.129840325	0.120073713	-9.40955595	4.98237E-21	3.75706E-20	negative
PRJNA729271	stage_E18_vs_E10	ordinary_muscle_development	ENSGALG00010004235	ADAD1	-2.211601252	0.492803307	-4.487797097	7.19634E-06	1.69943E-05	negative
PRJNA729271	stage_E18_vs_E10	ordinary_muscle_development	ENSGALG00010004236	DBF4	-1.49946007	0.127216284	-11.78669913	4.57108E-32	6.14066E-31	negative
PRJNA729271	stage_E18_vs_E10	ordinary_muscle_development	ENSGALG00010004237	RUFY3	-0.143219562	0.092148889	-1.554219093	0.120132165	0.15700259	negative
PRJNA729271	stage_E18_vs_E10	ordinary_muscle_development	ENSGALG00010004238	CCNC	-0.342192139	0.124241194	-2.754256686	0.005882561	0.009641308	negative
PRJNA729271	stage_E18_vs_E10	ordinary_muscle_development	ENSGALG00010004240	ENSGALG00010004240	0.695682042	0.17725401	3.924774638	8.6811E-05	0.000182208	positive
PRJNA729271	stage_E18_vs_E10	ordinary_muscle_development	ENSGALG00010004241	PREX2	0.093850752	0.115387427	0.813353369	0.416015487	0.47631473	positive
PRJNA729271	stage_E18_vs_E10	ordinary_muscle_development	ENSGALG00010004242	ADAM22	-1.01843108	0.173621477	-5.865812771	4.46938E-09	1.42695E-08	negative
PRJNA729271	stage_E18_vs_E10	ordinary_muscle_development	ENSGALG00010004243	BLTP1	-0.539783279	0.113470308	-4.757044289	1.96448E-06	4.9129E-06	negative
PRJNA729271	stage_E18_vs_E10	ordinary_muscle_development	ENSGALG00010004244	TMEM241	0.144281791	0.081497927	1.770373745	0.0766649	0.104011466	positive
PRJNA729271	stage_E18_vs_E10	ordinary_muscle_development	ENSGALG00010004245	ARFGEF3	-0.904697983	0.226287683	-3.997999234	6.38802E-05	0.000136188	negative

PRJNA729271	stage_E18_vs_E10	ordinary_muscle_development	ENSGALG00010004246	C8orf34	-2.035472084	0.319444006	-6.371921354	1.86675E-10	6.66629E-10	negative
PRJNA729271	stage_E18_vs_E10	ordinary_muscle_development	ENSGALG00010004247	MEOX2	-0.69358043	0.104613239	-6.629948891	3.35803E-11	1.2679E-10	negative
PRJNA729271	stage_E18_vs_E10	ordinary_muscle_development	ENSGALG00010004248	GRSF1	-0.161960608	0.159763922	-1.013749575	0.310702262	0.368364902	negative
PRJNA729271	stage_E18_vs_E10	ordinary_muscle_development	ENSGALG00010004249	USP45	0.833091299	0.101830412	8.181164023	2.81115E-16	1.56241E-15	positive
PRJNA729271	stage_E18_vs_E10	ordinary_muscle_development	ENSGALG00010004250	MOB1B	-0.376320556	0.088344142	-4.259711489	2.04691E-05	4.60714E-05	negative
PRJNA729271	stage_E18_vs_E10	ordinary_muscle_development	ENSGALG00010004251	CCN2	-1.312609643	0.267527522	-4.906447131	9.2741E-07	2.40695E-06	negative
PRJNA729271	stage_E18_vs_E10	ordinary_muscle_development	ENSGALG00010004255	RPS12	0.953437513	0.151061279	6.311594325	2.76175E-10	9.74954E-10	positive
PRJNA729271	stage_E18_vs_E10	ordinary_muscle_development	ENSGALG00010004256	ENSGALG00010004256	-6.200437027	0.64958107	-9.545285898	1.35733E-21	1.05948E-20	negative
PRJNA729271	stage_E18_vs_E10	ordinary_muscle_development	ENSGALG00010004257	ENSGALG00010004257	-0.66008217	0.636040563	-1.037798859	0.299363708	0.356350247	negative
PRJNA729271	stage_E18_vs_E10	ordinary_muscle_development	ENSGALG00010004265	PIP4K2A	1.613282887	0.091598568	17.61253397	1.97406E-69	1.03674E-67	positive
PRJNA729271	stage_E18_vs_E10	ordinary_muscle_development	ENSGALG00010004275	ADARB2	-1.071267099	0.597245028	-1.793681067	0.072864089	0.099328973	negative
PRJNA729271	stage_E18_vs_E10	ordinary_muscle_development	ENSGALG00010004281	VIM	-0.47398166	0.171627022	-2.76169599	0.005750199	0.009440383	negative
PRJNA729271	stage_E18_vs_E10	ordinary_muscle_development	ENSGALG00010004283	DCK	-1.352246423	0.129885193	-10.41108989	2.20681E-25	2.14107E-24	negative
PRJNA729271	stage_E18_vs_E10	ordinary_muscle_development	ENSGALG00010004285	SLC18B1	-0.705935255	0.123835339	-5.700596117	1.19389E-08	3.66021E-08	negative
PRJNA729271	stage_E18_vs_E10	ordinary_muscle_development	ENSGALG00010004296	ENSGALG00010004296	-4.160312655	0.646874839	-6.431402803	1.26432E-10	4.57585E-10	negative
PRJNA729271	stage_E18_vs_E10	ordinary_muscle_development	ENSGALG00010004297	SULF1	-1.140548598	0.183355963	-6.22040634	4.95869E-10	1.72324E-09	negative
PRJNA729271	stage_E18_vs_E10	ordinary_muscle_development	ENSGALG00010004300	VNN2	3.599522212	0.244759571	14.70635935	5.86806E-49	1.63887E-47	positive
PRJNA729271	stage_E18_vs_E10	ordinary_muscle_development	ENSGALG00010004302	CRPPA	-0.037081983	0.128943352	-0.287583518	0.773665563	0.81016344	negative
PRJNA729271	stage_E18_vs_E10	ordinary_muscle_development	ENSGALG00010004306	PNISR	0.409078097	0.144026293	2.8403015	0.004507091	0.007513547	positive
PRJNA729271	stage_E18_vs_E10	ordinary_muscle_development	ENSGALG00010004307	NPC2	1.081431269	0.141028298	7.668186368	1.74445E-14	8.52697E-14	positive
PRJNA729271	stage_E18_vs_E10	ordinary_muscle_development	ENSGALG00010004308	DSG2	-3.979718416	0.494495894	-8.048031272	8.41365E-16	4.5455E-15	negative
PRJNA729271	stage_E18_vs_E10	ordinary_muscle_development	ENSGALG00010004309	MRPS28	-0.139495177	0.136976868	-1.018384926	0.308495063	0.366047474	negative
PRJNA729271	stage_E18_vs_E10	ordinary_muscle_development	ENSGALG00010004312	PDLIM5	3.812393077	0.181631002	20.98977064	8.13373E-98	8.99988E-96	positive
PRJNA729271	stage_E18_vs_E10	ordinary_muscle_development	ENSGALG00010004319	TAAR1	3.156276067	0.890781692	3.543265532	0.000395205	0.000761261	positive
PRJNA729271	stage_E18_vs_E10	ordinary_muscle_development	ENSGALG00010004323	TRDMT1	0.190451736	0.158695316	1.200109376	0.230096865	0.281722974	positive
PRJNA729271	stage_E18_vs_E10	ordinary_muscle_development	ENSGALG00010004325	LTBP2	2.125141627	0.112768355	18.84519486	3.21778E-79	2.18975E-77	positive
PRJNA729271	stage_E18_vs_E10	ordinary_muscle_development	ENSGALG00010004326	ENSGALG00010004326	-0.636117389	0.958387207	-0.663737354	0.506858427	0.56388924	negative
PRJNA729271	stage_E18_vs_E10	ordinary_muscle_development	ENSGALG00010004328	ENSGALG00010004328	-0.996914527	0.701614508	-1.420886421	0.155349781	0.198009329	negative
PRJNA729271	stage_E18_vs_E10	ordinary_muscle_development	ENSGALG00010004329	COQ3	0.681748698	0.121688198	5.602422486	2.11377E-08	6.35269E-08	positive
PRJNA729271	stage_E18_vs_E10	ordinary_muscle_development	ENSGALG00010004330	STX7	-0.114980193	0.103813481	-1.107565139	0.268049666	0.323054786	negative
PRJNA729271	stage_E18_vs_E10	ordinary_muscle_development	ENSGALG00010004332	ENSGALG00010004332	-0.689415683	0.8969792	-0.76859718	0.44213248	0.501660298	negative
PRJNA729271	stage_E18_vs_E10	ordinary_muscle_development	ENSGALG00010004333	DIO2	-1.13730351	0.328099884	-3.466333165	0.000527609	0.000999191	negative
PRJNA729271	stage_E18_vs_E10	ordinary_muscle_development	ENSGALG00010004334	ENSGALG00010004334	0.752718398	0.230514212	3.26538825	0.001093141	0.001981626	positive
PRJNA729271	stage_E18_vs_E10	ordinary_muscle_development	ENSGALG00010004336	ENSGALG00010004336	-3.759647999	0.573927331	-6.55073874	5.72531E-11	2.12791E-10	negative
PRJNA729271	stage_E18_vs_E10	ordinary_muscle_development	ENSGALG00010004337	BMPR1B	1.050754293	0.228261538	4.603291049	4.15866E-06	1.00383E-05	positive
PRJNA729271	stage_E18_vs_E10	ordinary_muscle_development	ENSGALG00010004338	ENSGALG00010004338	1.227288521	0.166585841	7.367303925	1.74113E-13	7.85977E-13	positive
PRJNA729271	stage_E18_vs_E10	ordinary_muscle_development	ENSGALG00010004339	B4GALT6	-2.559077007	0.123507642	-20.71998921	2.28737E-95	2.40256E-93	negative
PRJNA729271	stage_E18_vs_E10	ordinary_muscle_development	ENSGALG00010004340	SLC4A4	1.733627785	0.169197389	10.24618521	1.23107E-24	1.1424E-23	positive
PRJNA729271	stage_E18_vs_E10	ordinary_muscle_development	ENSGALG00010004341	ENSGALG00010004341	-0.226248621	0.167188708	-1.353253006	0.175974817	0.221650588	negative
PRJNA729271	stage_E18_vs_E10	ordinary_muscle_development	ENSGALG00010004342	ZNF438	0.386359345	0.130268008	2.965880492	0.003018177	0.00516633	positive
PRJNA729271	stage_E18_vs_E10	ordinary_muscle_development	ENSGALG00010004344	SOSTDC1	-5.604139958	0.406858319	-13.77418057	3.64495E-43	7.90922E-42	negative
PRJNA729271	stage_E18_vs_E10	ordinary_muscle_development	ENSGALG00010004345	FAXC	-1.962733101	0.189447834	-10.36028264	3.7585E-25	3.58417E-24	negative
PRJNA729271	stage_E18_vs_E10	ordinary_muscle_development	ENSGALG00010004346	CEP128	0.366855113	0.09977101	3.676971013	0.00023602	0.00046903	positive
PRJNA729271	stage_E18_vs_E10	ordinary_muscle_development	ENSGALG00010004350	MOXD1	-1.575613728	0.244254269	-6.450711127	1.11327E-10	4.04026E-10	negative
PRJNA729271	stage_E18_vs_E10	ordinary_muscle_development	ENSGALG00010004352	CHMP2A	0.361202263	0.056835934	6.355174176	2.08191E-10	7.40727E-10	positive
PRJNA729271	stage_E18_vs_E10	ordinary_muscle_development	ENSGALG00010004355	ANKMY2	0.18025078	0.123504672	1.459465273	0.144437092	0.18553843	positive
PRJNA729271	stage_E18_vs_E10	ordinary_muscle_development	ENSGALG00010004356	TRAPPC8	-0.109180242	0.078267502	-1.394962642	0.163027105	0.20694263	negative
PRJNA729271	stage_E18_vs_E10	ordinary_muscle_development	ENSGALG00010004358	GC	9.412917087	2.103157292	4.47561251	7.61926E-06	1.79608E-05	positive
PRJNA729271	stage_E18_vs_E10	ordinary_muscle_development	ENSGALG00010004362	FBXL4	0.966870064	0.122738037	7.877590609	3.33971E-15	1.73075E-14	positive
PRJNA729271	stage_E18_vs_E10	ordinary_muscle_development	ENSGALG00010004364	TSHR	1.677095799	0.449985913	3.726996223	0.000193775	0.000389189	positive
PRJNA729271	stage_E18_vs_E10	ordinary_muscle_development	ENSGALG00010004365	TPD52	-1.290230684	0.156467397	-8.246003398	1.6378E-16	9.25894E-16	negative
PRJNA729271	stage_E18_vs_E10	ordinary_muscle_development	ENSGALG00010004366	UNC5C	-4.019515572	0.356455604	-11.27634277	1.71738E-29	2.04716E-28	negative
PRJNA729271	stage_E18_vs_E10	ordinary_muscle_development	ENSGALG00010004367	TSPAN13	-0.978741308	0.18127872	-5.399096529	6.69773E-08	1.92435E-07	negative
PRJNA729271	stage_E18_vs_E10	ordinary_muscle_development	ENSGALG00010004368	GJD4	-4.732013701	0.375506198	-12.60169267	2.06661E-36	3.43527E-35	negative
PRJNA729271	stage_E18_vs_E10	ordinary_muscle_development	ENSGALG00010004369	UBE2M	-0.30791442	0.239586033	-1.285193535	0.198724662	0.247424324	negative
PRJNA729271	stage_E18_vs_E10	ordinary_muscle_development	ENSGALG00010004370	ATP6V1H	-0.199192065	0.07768766	-2.564011659	0.010347007	0.016337676	negative
PRJNA729271	stage_E18_vs_E10	ordinary_muscle_development	ENSGALG00010004372	SYNJ2	1.489780024	0.184506185	8.074417805	6.77994E-16	3.68912E-15	positive
PRJNA729271	stage_E18_vs_E10	ordinary_muscle_development	ENSGALG00010004373	MMS22L	-1.097680757	0.12534424	-8.757329072	1.99931E-18	1.28629E-17	negative
PRJNA729271	stage_E18_vs_E10	ordinary_muscle_development	ENSGALG00010004374	ILDR1	-4.363169799	0.762665973	-5.720944623	1.05933E-08	3.26079E-08	negative
PRJNA729271	stage_E18_vs_E10	ordinary_muscle_development	ENSGALG00010004375	ENSGALG00010004375	-0.223678708	0.220796399	-1.013054149	0.311034297	0.368607811	negative
PRJNA729271	stage_E18_vs_E10	ordinary_muscle_development	ENSGALG00010004377	AGR2	-5.849682416	1.004877019	-5.821291864	5.83945E-09	1.84407E-08	negative
PRJNA729271	stage_E18_vs_E10	ordinary_muscle_development	ENSGALG00010004378	TRIM28	-0.247216737	0.156356523	-1.581109202	0.113853071	0.149490223	negative
PRJNA729271	stage_E18_vs_E10	ordinary_muscle_development	ENSGALG00010004379	ZBTB10	0.366189671	0.059355253	6.169456814	6.8525E-10	2.35484E-09	positive
PRJNA729271	stage_E18_vs_E10	ordinary_muscle_development	ENSGALG00010004380	ISCA2	0.308141596	0.084957728	3.626999004	0.000286734	0.000563478	positive
PRJNA729271	stage_E18_vs_E10	ordinary_muscle_development	ENSGALG00010004381	ENSGALG00010004381	0.040706241	0.454368454	0.089588615	0.928614132	0.94154042	positive
PRJNA729271	stage_E18_vs_E10	ordinary_muscle_development	ENSGALG00010004383	ENSGALG00010004383	-1.260223344	0.560006685	-2.250371965	0.024425343	0.036282573	negative
PRJNA729271	stage_E18_vs_E10	ordinary_muscle_development	ENSGALG00010004384	ENSGALG00010004384	1.550389862	0.553560987	2.800757095	0.005098288	0.008443748	positive
PRJNA729271	stage_E18_vs_E10	ordinary_muscle_development	ENSGALG00010004385	ADAMTS3	-2.592843716	0.206427425	-12.56055837	3.47869E-36	5.70403E-35	negative
PRJNA729271	stage_E18_vs_E10	ordinary_muscle_development	ENSGALG00010004386	CFAP44	-0.662212336	0.401315646	-1.650103461	0.098921777	0.131318908	negative
PRJNA729271	stage_E18_vs_E10	ordinary_muscle_development	ENSGALG00010004389	ENSGALG00010004389	0.358343144	0.132265046	2.709280758	0.006742925	0.010936411	positive
PRJNA729271	stage_E18_vs_E10	ordinary_muscle_development	ENSGALG00010004390	PNLDC1	1.10567442	0.480508344	2.301051447	0.021388723	0.032097478	positive
PRJNA729271	stage_E18_vs_E10	ordinary_muscle_development	ENSGALG00010004393	ZBTB45	0.251223277	0.215799594	1.164150834	0.244362891	0.29757541	positive
PRJNA729271	stage_E18_vs_E10	ordinary_muscle_development	ENSGALG00010004394	CCNY	-0.707034509	0.088436322	-7.99484295	1.29739E-15	6.91131E-15	negative
PRJNA729271	stage_E18_vs_E10	ordinary_muscle_development	ENSGALG00010004395	BOC	-0.335224112	0.190277008	-1.761768881	0.078108355	0.105831052	negative
PRJNA729271	stage_E18_vs_E10	ordinary_muscle_development	ENSGALG00010004397	ENSGALG00010004397	0.601133984	0.168948553	3.55808898	0.000373563	0.000722039	positive
PRJNA729271	stage_E18_vs_E10	ordinary_muscle_development	ENSGALG00010004398	COX18	-0.560896078	0.125943388	-4.453557167	8.44592E-06	1.98321E-05	negative
PRJNA729271	stage_E18_vs_E10	ordinary_muscle_development	ENSGALG00010004399	POU3F2	-1.150376356	0.612743303	-1.877419713	0.060460592	0.083591785	negative
PRJNA729271	stage_E18_vs_E10	ordinary_muscle_development	ENSGALG00010004404	RGS20	-0.953202614	0.42646743	-2.235112335	0.025409975	0.037641041	negative
PRJNA729271	stage_E18_vs_E10	ordinary_muscle_development	ENSGALG00010004406	ENSGALG00010004406	-0.259875691	0.140829241	-1.845324793	0.064990306	0.089317767	negative
PRJNA729271	stage_E18_vs_E10	ordinary_muscle_development	ENSGALG00010004408	SERAC1	-0.56837108	0.11366397	-5.00045071	5.71964E-07	1.51344E-06	negative
PRJNA729271	stage_E18_vs_E10	ordinary_muscle_development	ENSGALG00010004416	STXBP6	-0.490268503	0.214160189	-2.289260697	0.02206421	0.033029094	negative
PRJNA729271	stage_E18_vs_E10	ordinary_muscle_development	ENSGALG00010004420	ENSGALG00010004420	1.316767281	0.290746887	4.528912736	5.9288E-06	1.41299E-05	positive
PRJNA729271	stage_E18_vs_E10	ordinary_muscle_development	ENSGALG00010004421	RAP1GDS1	-1.142041917	0.075998449	-15.0271741	4.87357E-51	1.48097E-49	negative
PRJNA729271	stage_E18_vs_E10	ordinary_muscle_development	ENSGALG00010004424	MRPL18	0.266514308	0.145988482	1.825584489	0.067912874	0.093052	positive
PRJNA729271	stage_E18_vs_E10	ordinary_muscle_development	ENSGALG00010004425	CREM	1.615198746	0.242155253	6.670095827	2.55636E-11	9.75119E-11	positive
PRJNA729271	stage_E18_vs_E10	ordinary_muscle_development	ENSGALG00010004429	ENSGALG00010004429	1.167194182	0.346512254	3.368406655	0.00075604	0.001402636	positive
PRJNA729271	stage_E18_vs_E10	ordinary_muscle_development	ENSGALG00010004430	TCEA1	-0.947716462	0.095327807	-9.941658045	2.74224E-23	2.33131E-22	negative
PRJNA729271	stage_E18_vs_E10	ordinary_muscle_development	ENSGALG00010004433	AHR1A	-1.327830893	0.159767958	-8.310996197	9.4902E-17	5.45226E-16	negative
PRJNA729271	stage_E18_vs_E10	ordinary_muscle_development	ENSGALG00010004434	TCP1	-1.011921347	0.086918678	-11.64216222	2.51556E-31	3.28792E-30	negative
PRJNA729271	stage_E18_vs_E10	ordinary_muscle_development	ENSGALG00010004436	NOVA1	0.262782985	0.151873932	1.730270498	0.083581958	0.112521638	positive
PRJNA729271	stage_E18_vs_E10	ordinary_muscle_development	ENSGALG00010004440	GTF2H5	-0.348187508	0.10498476	-3.316552863	0.000911353	0.001668781	negative
PRJNA729271	stage_E18_vs_E10	ordinary_muscle_development	ENSGALG00010004443	ANKRD17	-0.550344626	0.094213561	-5.84145869	5.17457E-09	1.6409E-08	negative
PRJNA729271	stage_E18_vs_E10	ordinary_muscle_development	ENSGALG00010004445	EPHA6	-2.023325961	0.510434624	-3.963927729	7.37266E-05	0.000155987	negative
PRJNA729271	stage_E18_vs_E10	ordinary_muscle_development	ENSGALG00010004446	ACAT2	-2.065099098	0.130327557	-15.84545242	1.51129E-56	5.38236E-55	negative
PRJNA729271	stage_E18_vs_E10	ordinary_muscle_development	ENSGALG00010004451	CLDND1	0.208227035	0.084607201	2.461102977	0.01385106	0.021482037	positive
PRJNA729271	stage_E18_vs_E10	ordinary_muscle_development	ENSGALG00010004454	SRI	-0.319660105	0.164184979	-1.946950978	0.051540612	0.072209663	negative
PRJNA729271	stage_E18_vs_E10	ordinary_muscle_development	ENSGALG00010004457	AGA	0.486977805	0.126411532	3.85232104	0.000117003	0.000242108	positive
PRJNA729271	stage_E18_vs_E10	ordinary_muscle_development	ENSGALG00010004460	ENSGALG00010004460	-0.225240316	0.604869788	-0.372378189	0.709611291	0.753705258	negative
PRJNA729271	stage_E18_vs_E10	ordinary_muscle_development	ENSGALG00010004468	LYPLA1	-0.395363816	0.123313132	-3.206177704	0.001345109	0.002413935	negative
PRJNA729271	stage_E18_vs_E10	ordinary_muscle_development	ENSGALG00010004471	C1H3ORF52	-0.389180985	0.278057803	-1.399640581	0.161620975	0.205355543	negative
PRJNA729271	stage_E18_vs_E10	ordinary_muscle_development	ENSGALG00010004472	NEIL3	-1.495103104	0.212847894	-7.024279529	2.15174E-12	8.97279E-12	negative
PRJNA729271	stage_E18_vs_E10	ordinary_muscle_development	ENSGALG00010004473	JDP2	1.272956532	0.130821276	9.730500819	2.2349E-22	1.81994E-21	positive
PRJNA729271	stage_E18_vs_E10	ordinary_muscle_development	ENSGALG00010004477	TULP4	-0.376697315	0.126484722	-2.978204086	0.002899429	0.004978348	negative
PRJNA729271	stage_E18_vs_E10	ordinary_muscle_development	ENSGALG00010004481	ZNF704	0.489465075	0.078779078	6.213135358	5.19377E-10	1.80277E-09	positive
PRJNA729271	stage_E18_vs_E10	ordinary_muscle_development	ENSGALG00010004483	STEAP4	-0.872081935	0.721526663	-1.208662105	0.226792686	0.278236139	negative
PRJNA729271	stage_E18_vs_E10	ordinary_muscle_development	ENSGALG00010004487	ABHD10	0.200526769	0.099559726	2.014135401	0.04399532	0.062374037	positive
PRJNA729271	stage_E18_vs_E10	ordinary_muscle_development	ENSGALG00010004489	LIG1	-1.92285895	0.261732925	-7.346645236	2.03243E-13	9.12643E-13	negative
PRJNA729271	stage_E18_vs_E10	ordinary_muscle_development	ENSGALG00010004494	WTAP	-0.129202239	0.057437629	-2.249435479	0.024484803	0.036367172	negative
PRJNA729271	stage_E18_vs_E10	ordinary_muscle_development	ENSGALG00010004495	MRPL15	0.278592534	0.114065008	2.442401384	0.014589915	0.022565175	positive
PRJNA729271	stage_E18_vs_E10	ordinary_muscle_development	ENSGALG00010004501	TAGLN3	-1.848278544	0.33363773	-5.539776763	3.02857E-08	9.00127E-08	negative
PRJNA729271	stage_E18_vs_E10	ordinary_muscle_development	ENSGALG00010004503	ENSGALG00010004503	0.783727331	0.181224498	4.32462134	1.52794E-05	3.49054E-05	positive
PRJNA729271	stage_E18_vs_E10	ordinary_muscle_development	ENSGALG00010004504	ZNF804B	-2.197699782	0.693812429	-3.167570499	0.001537184	0.002738629	negative
PRJNA729271	stage_E18_vs_E10	ordinary_muscle_development	ENSGALG00010004505	ENSGALG00010004505	1.25339309	0.131927649	9.500609634	2.08665E-21	1.60797E-20	positive
PRJNA729271	stage_E18_vs_E10	ordinary_muscle_development	ENSGALG00010004507	CUL2	-0.090586198	0.090888633	-0.996672458	0.318923523	0.376602563	negative
PRJNA729271	stage_E18_vs_E10	ordinary_muscle_development	ENSGALG00010004515	ENSGALG00010004515	-0.765057248	0.25857199	-2.958778514	0.00308861	0.005280033	negative
PRJNA729271	stage_E18_vs_E10	ordinary_muscle_development	ENSGALG00010004516	ENSGALG00010004516	0.779622798	0.284375644	2.741524511	0.00611548	0.00999931	positive
PRJNA729271	stage_E18_vs_E10	ordinary_muscle_development	ENSGALG00010004522	TSPAN5	-0.438860193	0.063443664	-6.917321063	4.60264E-12	1.8714E-11	negative
PRJNA729271	stage_E18_vs_E10	ordinary_muscle_development	ENSGALG00010004533	PAG1	1.567171649	0.111701779	14.02996137	1.02207E-44	2.378E-43	positive
PRJNA729271	stage_E18_vs_E10	ordinary_muscle_development	ENSGALG00010004534	CUBN	1.928508749	0.21322765	9.044365245	1.50537E-19	1.03365E-18	positive
PRJNA729271	stage_E18_vs_E10	ordinary_muscle_development	ENSGALG00010004542	ERMARD	-0.094776918	0.130632172	-0.725525087	0.46812996	0.527104651	negative
PRJNA729271	stage_E18_vs_E10	ordinary_muscle_development	ENSGALG00010004545	TMPRSS7	1.591778332	0.550973776	2.889027392	0.003864354	0.006500384	positive
PRJNA729271	stage_E18_vs_E10	ordinary_muscle_development	ENSGALG00010004553	BATF	-0.171426211	0.370126198	-0.463156113	0.643252471	0.692354984	negative
PRJNA729271	stage_E18_vs_E10	ordinary_muscle_development	ENSGALG00010004565	ENSGALG00010004565	2.154856229	0.396627863	5.432942139	5.54324E-08	1.60442E-07	positive
PRJNA729271	stage_E18_vs_E10	ordinary_muscle_development	ENSGALG00010004568	RSU1	0.052393518	0.068379738	0.766214075	0.443548935	0.502991849	positive
PRJNA729271	stage_E18_vs_E10	ordinary_muscle_development	ENSGALG00010004570	ENSGALG00010004570	-1.213809713	0.661793239	-1.834122262	0.066635847	0.091431902	negative
PRJNA729271	stage_E18_vs_E10	ordinary_muscle_development	ENSGALG00010004576	STEAP2	0.814022204	0.210658877	3.864172323	0.000111467	0.000231344	positive
PRJNA729271	stage_E18_vs_E10	ordinary_muscle_development	ENSGALG00010004577	ZPLD1	-4.718553746	0.471814011	-10.00087668	1.51054E-23	1.30406E-22	negative
PRJNA729271	stage_E18_vs_E10	ordinary_muscle_development	ENSGALG00010004583	XKR4	-0.094201243	0.116939647	-0.805554365	0.420499848	0.481069084	negative
PRJNA729271	stage_E18_vs_E10	ordinary_muscle_development	ENSGALG00010004584	EIF4E	-0.911350227	0.067424862	-13.51653085	1.2493E-41	2.5541E-40	negative
PRJNA729271	stage_E18_vs_E10	ordinary_muscle_development	ENSGALG00010004586	ENSGALG00010004586	-2.828687216	0.345089679	-8.196962667	2.46537E-16	1.3755E-15	negative
PRJNA729271	stage_E18_vs_E10	ordinary_muscle_development	ENSGALG00010004588	MINDY3	-0.720637405	0.1542506	-4.671861268	2.98483E-06	7.32065E-06	negative

PRJNA729271	stage_E18_vs_E10	ordinary_muscle_development	ENSGALG00010004591	RRS1	-0.479303134	0.163212264	-2.936685776	0.003317401	0.005633945	negative
PRJNA729271	stage_E18_vs_E10	ordinary_muscle_development	ENSGALG00010004602	DYNLT2	-1.69469716	0.393248933	-4.309476812	1.63641E-05	3.72484E-05	negative
PRJNA729271	stage_E18_vs_E10	ordinary_muscle_development	ENSGALG00010004609	CFAP69	-2.322697385	0.62725014	-3.702984243	0.000213078	0.00042554	negative
PRJNA729271	stage_E18_vs_E10	ordinary_muscle_development	ENSGALG00010004610	TGS1	-0.609587325	0.124211156	-4.907669668	9.21649E-07	2.39285E-06	negative
PRJNA729271	stage_E18_vs_E10	ordinary_muscle_development	ENSGALG00010004622	ENSGALG00010004622	1.060994783	0.227773692	4.658109422	3.19127E-06	7.80057E-06	positive
PRJNA729271	stage_E18_vs_E10	ordinary_muscle_development	ENSGALG00010004623	MBIP	-0.249843745	0.126143732	-1.980627505	0.047633062	0.067117841	negative
PRJNA729271	stage_E18_vs_E10	ordinary_muscle_development	ENSGALG00010004624	PHF10	-0.234315479	0.076829757	-3.049801115	0.00228993	0.003982307	negative
PRJNA729271	stage_E18_vs_E10	ordinary_muscle_development	ENSGALG00010004630	ENSGALG00010004630	1.338091353	0.329875791	4.056349059	4.98457E-05	0.000107373	positive
PRJNA729271	stage_E18_vs_E10	ordinary_muscle_development	ENSGALG00010004639	HBS1L	0.2377486	0.080020509	2.971095835	0.002967392	0.005084201	positive
PRJNA729271	stage_E18_vs_E10	ordinary_muscle_development	ENSGALG00010004641	METAP1	-1.027215267	0.083239043	-12.34054631	5.47829E-35	8.43866E-34	negative
PRJNA729271	stage_E18_vs_E10	ordinary_muscle_development	ENSGALG00010004650	GTPBP10	-0.569137304	0.129161876	-4.406387718	1.05109E-05	2.44394E-05	negative
PRJNA729271	stage_E18_vs_E10	ordinary_muscle_development	ENSGALG00010004666	C6orf120	-0.130881726	0.117881375	-1.110283333	0.266876953	0.321882296	negative
PRJNA729271	stage_E18_vs_E10	ordinary_muscle_development	ENSGALG00010004669	ELMOD1	-3.568267439	0.412657234	-8.647049273	5.28482E-18	3.30757E-17	negative
PRJNA729271	stage_E18_vs_E10	ordinary_muscle_development	ENSGALG00010004673	RAB18	0.062615339	0.085155182	0.735308614	0.462151564	0.521395308	positive
PRJNA729271	stage_E18_vs_E10	ordinary_muscle_development	ENSGALG00010004689	BBX	-0.15328797	0.084814836	-1.807324957	0.070711627	0.096676574	negative
PRJNA729271	stage_E18_vs_E10	ordinary_muscle_development	ENSGALG00010004705	ENSGALG00010004705	2.624746361	0.228192358	11.50234119	1.28385E-30	1.6126E-29	positive
PRJNA729271	stage_E18_vs_E10	ordinary_muscle_development	ENSGALG00010004707	OTUD1	2.60929648	0.296734215	8.793379234	1.45128E-18	9.42062E-18	positive
PRJNA729271	stage_E18_vs_E10	ordinary_muscle_development	ENSGALG00010004715	WDR27	-0.395316165	0.340251318	-1.161835809	0.245302165	0.298593793	negative
PRJNA729271	stage_E18_vs_E10	ordinary_muscle_development	ENSGALG00010004716	ADH6	2.529082122	0.951854887	2.657003874	0.007883854	0.012658004	positive
PRJNA729271	stage_E18_vs_E10	ordinary_muscle_development	ENSGALG00010004717	ALKBH8	-0.220892899	0.177329215	-1.245665574	0.212887197	0.263203679	negative
PRJNA729271	stage_E18_vs_E10	ordinary_muscle_development	ENSGALG00010004721	LAMA2	3.536468265	0.158822752	22.26676108	7.7612E-110	1.2097E-107	positive
PRJNA729271	stage_E18_vs_E10	ordinary_muscle_development	ENSGALG00010004728	ENSGALG00010004728	0.473452679	0.216119274	2.190700859	0.028473447	0.041773544	positive
PRJNA729271	stage_E18_vs_E10	ordinary_muscle_development	ENSGALG00010004729	ENSGALG00010004729	3.407151826	1.248604039	2.728768864	0.006357124	0.010369854	positive
PRJNA729271	stage_E18_vs_E10	ordinary_muscle_development	ENSGALG00010004730	CLDN12	0.173787517	0.062604297	2.775967866	0.005503765	0.009062487	positive
PRJNA729271	stage_E18_vs_E10	ordinary_muscle_development	ENSGALG00010004735	ENSGALG00010004735	2.469449153	0.165324182	14.93701121	1.89276E-50	5.62204E-49	positive
PRJNA729271	stage_E18_vs_E10	ordinary_muscle_development	ENSGALG00010004736	CD47	-0.043130009	0.101390045	-0.425387023	0.670554564	0.717373121	negative
PRJNA729271	stage_E18_vs_E10	ordinary_muscle_development	ENSGALG00010004739	ENSGALG00010004739	0.37159301	0.696034683	0.533871399	0.593430511	0.645246063	positive
PRJNA729271	stage_E18_vs_E10	ordinary_muscle_development	ENSGALG00010004746	LYN	-0.362482201	0.142797645	-2.538432627	0.011135024	0.017515157	negative
PRJNA729271	stage_E18_vs_E10	ordinary_muscle_development	ENSGALG00010004751	PTER	0.857034745	0.125318303	6.838863311	7.9824E-12	3.16393E-11	positive
PRJNA729271	stage_E18_vs_E10	ordinary_muscle_development	ENSGALG00010004755	PAX9	-3.664984438	0.814159203	-4.501557464	6.74573E-06	1.59718E-05	negative
PRJNA729271	stage_E18_vs_E10	ordinary_muscle_development	ENSGALG00010004757	TRMT10A	1.155113437	0.072797827	15.86741649	1.06537E-56	3.81296E-55	positive
PRJNA729271	stage_E18_vs_E10	ordinary_muscle_development	ENSGALG00010004758	FLT3	2.326349351	0.858355445	2.710240106	0.006723452	0.010910862	positive
PRJNA729271	stage_E18_vs_E10	ordinary_muscle_development	ENSGALG00010004760	THBS2	0.077505595	0.240316503	0.32251466	0.747062834	0.787196552	positive
PRJNA729271	stage_E18_vs_E10	ordinary_muscle_development	ENSGALG00010004762	IFT57	-0.82781197	0.111522009	-7.422857394	1.1462E-13	5.25101E-13	negative
PRJNA729271	stage_E18_vs_E10	ordinary_muscle_development	ENSGALG00010004765	ENSGALG00010004765	2.096378584	0.201309625	10.4137027	2.14705E-25	2.08729E-24	positive
PRJNA729271	stage_E18_vs_E10	ordinary_muscle_development	ENSGALG00010004769	HTR2A	0.293662355	0.365368466	0.803743023	0.421545402	0.481998943	positive
PRJNA729271	stage_E18_vs_E10	ordinary_muscle_development	ENSGALG00010004777	CDK14	-0.854381297	0.124237522	-6.876998843	6.11266E-12	2.45014E-11	negative
PRJNA729271	stage_E18_vs_E10	ordinary_muscle_development	ENSGALG00010004778	ENSGALG00010004778	-1.035674359	0.461484407	-2.244223952	0.024817996	0.036835639	negative
PRJNA729271	stage_E18_vs_E10	ordinary_muscle_development	ENSGALG00010004780	ENSGALG00010004780	0.137368616	0.260989949	0.526336803	0.598654217	0.650219735	positive
PRJNA729271	stage_E18_vs_E10	ordinary_muscle_development	ENSGALG00010004781	ENSGALG00010004781	-0.878378972	0.083347913	-10.53870391	5.72829E-26	5.71843E-25	negative
PRJNA729271	stage_E18_vs_E10	ordinary_muscle_development	ENSGALG00010004787	RIOK3	-0.099804262	0.116236702	-0.85862951	0.390544952	0.450604878	negative
PRJNA729271	stage_E18_vs_E10	ordinary_muscle_development	ENSGALG00010004791	FZD1	-0.51084369	0.118340467	-4.316728701	1.58359E-05	3.61027E-05	negative
PRJNA729271	stage_E18_vs_E10	ordinary_muscle_development	ENSGALG00010004798	PTPRK	-2.283756282	0.114016957	-20.02997038	3.01837E-89	2.75166E-87	negative
PRJNA729271	stage_E18_vs_E10	ordinary_muscle_development	ENSGALG00010004800	ZC3H12C	0.12048829	0.278460721	0.432694025	0.665237071	0.712525777	positive
PRJNA729271	stage_E18_vs_E10	ordinary_muscle_development	ENSGALG00010004803	RMC1	0.154604958	0.142123272	1.087822957	0.276673273	0.332177511	positive
PRJNA729271	stage_E18_vs_E10	ordinary_muscle_development	ENSGALG00010004808	THEMIS	-1.671434223	1.060281702	-1.576405798	0.114932313	0.150764152	negative
PRJNA729271	stage_E18_vs_E10	ordinary_muscle_development	ENSGALG00010004824	ATM	-0.188502075	0.111581436	-1.689367701	0.091148987	0.121814915	negative
PRJNA729271	stage_E18_vs_E10	ordinary_muscle_development	ENSGALG00010004832	C6orf58	2.997133365	1.938731677	1.545924793	0.122122732	0.159374179	positive
PRJNA729271	stage_E18_vs_E10	ordinary_muscle_development	ENSGALG00010004835	HHLA2	-1.075260203	0.41220147	-2.60857925	0.009091896	0.014466194	negative
PRJNA729271	stage_E18_vs_E10	ordinary_muscle_development	ENSGALG00010004837	CENPC	-1.401501723	0.137272717	-10.20961597	1.79574E-24	1.64742E-23	negative
PRJNA729271	stage_E18_vs_E10	ordinary_muscle_development	ENSGALG00010004841	AKAP9	0.000110723	0.194669681	0.000568775	0.999546183	0.999546183	positive
PRJNA729271	stage_E18_vs_E10	ordinary_muscle_development	ENSGALG00010004846	TTLL2	0.633438162	0.353234205	1.793252613	0.072932541	0.099394244	positive
PRJNA729271	stage_E18_vs_E10	ordinary_muscle_development	ENSGALG00010004851	MTCL3	-3.037129594	0.478972536	-6.340926394	2.28388E-10	8.10597E-10	negative
PRJNA729271	stage_E18_vs_E10	ordinary_muscle_development	ENSGALG00010004852	ENSGALG00010004852	3.462554363	0.222521788	15.56051832	1.35001E-54	4.49849E-53	positive
PRJNA729271	stage_E18_vs_E10	ordinary_muscle_development	ENSGALG00010004855	ZEB1	0.95461229	0.091684934	10.41187737	2.18862E-25	2.12486E-24	positive
PRJNA729271	stage_E18_vs_E10	ordinary_muscle_development	ENSGALG00010004859	NPC1	0.751722545	0.191719639	3.920947002	8.82017E-05	0.000184966	positive
PRJNA729271	stage_E18_vs_E10	ordinary_muscle_development	ENSGALG00010004866	SLC25A21	-0.753491993	0.305915829	-2.463069645	0.013775315	0.021366848	negative
PRJNA729271	stage_E18_vs_E10	ordinary_muscle_development	ENSGALG00010004868	NPAT	-0.261494836	0.115092377	-2.272043053	0.02308391	0.034420458	negative
PRJNA729271	stage_E18_vs_E10	ordinary_muscle_development	ENSGALG00010004871	C11orf87	-1.371263818	0.323378013	-4.24043616	2.23086E-05	4.99557E-05	negative
PRJNA729271	stage_E18_vs_E10	ordinary_muscle_development	ENSGALG00010004872	KIAA0408	-0.507585478	0.254407929	-1.995163748	0.046025023	0.065035359	negative
PRJNA729271	stage_E18_vs_E10	ordinary_muscle_development	ENSGALG00010004876	ENSGALG00010004876	-1.851307281	0.798694936	-2.317915386	0.02045392	0.030793474	negative
PRJNA729271	stage_E18_vs_E10	ordinary_muscle_development	ENSGALG00010004879	ENSGALG00010004879	-1.364836158	0.588397102	-2.319583413	0.020363424	0.030663602	negative
PRJNA729271	stage_E18_vs_E10	ordinary_muscle_development	ENSGALG00010004880	STAP1	-0.862880616	0.909854	-0.948372613	0.342939797	0.401397961	negative
PRJNA729271	stage_E18_vs_E10	ordinary_muscle_development	ENSGALG00010004881	MIPOL1	-0.282118026	0.151056436	-1.867633272	0.061813205	0.085282925	negative
PRJNA729271	stage_E18_vs_E10	ordinary_muscle_development	ENSGALG00010004883	KRIT1	-0.21227215	0.074621225	-2.844661806	0.00444586	0.007418296	negative
PRJNA729271	stage_E18_vs_E10	ordinary_muscle_development	ENSGALG00010004887	MYH15	-5.853781027	0.726105915	-8.061883127	7.5128E-16	4.07553E-15	negative
PRJNA729271	stage_E18_vs_E10	ordinary_muscle_development	ENSGALG00010004890	ACAT1	-0.251213563	0.098714608	-2.544846887	0.010932566	0.017206032	negative
PRJNA729271	stage_E18_vs_E10	ordinary_muscle_development	ENSGALG00010004891	ANKIB1	-0.480480482	0.077970767	-6.162315668	7.16888E-10	2.45889E-09	negative
PRJNA729271	stage_E18_vs_E10	ordinary_muscle_development	ENSGALG00010004892	HEY2	-1.09193784	0.268760034	-4.062872835	4.84724E-05	0.000104493	negative
PRJNA729271	stage_E18_vs_E10	ordinary_muscle_development	ENSGALG00010004896	UBA6	-0.97797607	0.085003615	-11.50511159	1.24328E-30	1.563E-29	negative
PRJNA729271	stage_E18_vs_E10	ordinary_muscle_development	ENSGALG00010004899	GATAD1	0.442430597	0.105079891	4.210421183	2.54895E-05	5.67368E-05	positive
PRJNA729271	stage_E18_vs_E10	ordinary_muscle_development	ENSGALG00010004901	CUL5	-0.071829593	0.093783481	-0.765908792	0.443730574	0.503158466	negative
PRJNA729271	stage_E18_vs_E10	ordinary_muscle_development	ENSGALG00010004902	ANKRD29	0.724185012	0.319899947	2.26378597	0.023587282	0.035120457	positive
PRJNA729271	stage_E18_vs_E10	ordinary_muscle_development	ENSGALG00010004903	ENSGALG00010004903	2.137671075	0.496205264	4.308037881	1.64709E-05	3.74797E-05	positive
PRJNA729271	stage_E18_vs_E10	ordinary_muscle_development	ENSGALG00010004907	ENSGALG00010004907	0.611096684	0.114626662	5.331191488	9.75705E-08	2.77093E-07	positive
PRJNA729271	stage_E18_vs_E10	ordinary_muscle_development	ENSGALG00010004917	ENSGALG00010004917	0.972601672	0.087607301	11.10183352	1.22896E-28	1.40599E-27	positive
PRJNA729271	stage_E18_vs_E10	ordinary_muscle_development	ENSGALG00010004921	RRAGD	1.224416825	0.133740876	9.155142879	5.42859E-20	3.80908E-19	positive
PRJNA729271	stage_E18_vs_E10	ordinary_muscle_development	ENSGALG00010004924	ENSGALG00010004924	1.462883848	0.345289044	4.236693505	2.26835E-05	5.07404E-05	positive
PRJNA729271	stage_E18_vs_E10	ordinary_muscle_development	ENSGALG00010004927	KIF25	-0.286882156	0.220032743	-1.303815748	0.192296412	0.240055118	negative
PRJNA729271	stage_E18_vs_E10	ordinary_muscle_development	ENSGALG00010004928	HDDC2	-0.456802083	0.122748299	-3.721453458	0.000198079	0.000397447	negative
PRJNA729271	stage_E18_vs_E10	ordinary_muscle_development	ENSGALG00010004929	ENSGALG00010004929	2.698319711	0.157830726	17.09628906	1.5816E-65	7.51648E-64	positive
PRJNA729271	stage_E18_vs_E10	ordinary_muscle_development	ENSGALG00010004933	SPTLC2	-0.352540208	0.091193002	-3.865869096	0.000110694	0.00022984	negative
PRJNA729271	stage_E18_vs_E10	ordinary_muscle_development	ENSGALG00010004934	CIP2A	-1.790812841	0.163170435	-10.97510615	5.03467E-28	5.59696E-27	negative
PRJNA729271	stage_E18_vs_E10	ordinary_muscle_development	ENSGALG00010004935	SVIL	0.731477763	0.069058409	10.59216073	3.24025E-26	3.28214E-25	positive
PRJNA729271	stage_E18_vs_E10	ordinary_muscle_development	ENSGALG00010004939	DDX10	-0.560207917	0.111609725	-5.019346803	5.18475E-07	1.37794E-06	negative
PRJNA729271	stage_E18_vs_E10	ordinary_muscle_development	ENSGALG00010004942	GABRR1	0.00435371	0.464735665	0.009368142	0.992525413	0.993690832	positive
PRJNA729271	stage_E18_vs_E10	ordinary_muscle_development	ENSGALG00010004944	ALKBH1	-0.020987219	0.099603486	-0.210707673	0.833115387	0.861402919	negative
PRJNA729271	stage_E18_vs_E10	ordinary_muscle_development	ENSGALG00010004946	SH2D1B	0.695447023	0.503826787	1.380329591	0.167485188	0.211840995	positive
PRJNA729271	stage_E18_vs_E10	ordinary_muscle_development	ENSGALG00010004949	RAB39A	-1.455648654	0.339816699	-4.28362896	1.83869E-05	4.15835E-05	negative
PRJNA729271	stage_E18_vs_E10	ordinary_muscle_development	ENSGALG00010004950	SLIRP	0.081010894	0.111095997	0.729197234	0.465881023	0.525030744	positive
PRJNA729271	stage_E18_vs_E10	ordinary_muscle_development	ENSGALG00010004951	AFDN	-0.865188082	0.063411296	-13.64406872	2.18993E-42	4.62053E-41	negative
PRJNA729271	stage_E18_vs_E10	ordinary_muscle_development	ENSGALG00010004952	PEX1	0.011883669	0.125225275	0.094898323	0.924395589	0.937787938	positive
PRJNA729271	stage_E18_vs_E10	ordinary_muscle_development	ENSGALG00010004957	ENSGALG00010004957	0.47684773	0.140175449	3.401792062	0.000669456	0.001250968	positive
PRJNA729271	stage_E18_vs_E10	ordinary_muscle_development	ENSGALG00010004958	TPD52L1	-1.155048855	0.408447158	-2.827902785	0.004685403	0.007791983	negative
PRJNA729271	stage_E18_vs_E10	ordinary_muscle_development	ENSGALG00010004962	ENSGALG00010004962	-0.22229006	0.096001738	-2.315479535	0.020586703	0.030977293	negative
PRJNA729271	stage_E18_vs_E10	ordinary_muscle_development	ENSGALG00010004963	ENSGALG00010004963	0.224973372	0.252356558	0.891490092	0.372666305	0.432524468	positive
PRJNA729271	stage_E18_vs_E10	ordinary_muscle_development	ENSGALG00010004965	JCAD	0.806736836	0.100655084	8.014864252	1.10259E-15	5.90177E-15	positive
PRJNA729271	stage_E18_vs_E10	ordinary_muscle_development	ENSGALG00010004966	LAMA3	1.217122074	0.211311054	5.759859948	8.41838E-09	2.61967E-08	positive
PRJNA729271	stage_E18_vs_E10	ordinary_muscle_development	ENSGALG00010004967	SLC35F2	-3.216394779	0.344245204	-9.34332488	9.33555E-21	6.91107E-20	negative
PRJNA729271	stage_E18_vs_E10	ordinary_muscle_development	ENSGALG00010004968	TRAT1	0.763072928	1.240826633	0.614971429	0.538573589	0.593704781	positive
PRJNA729271	stage_E18_vs_E10	ordinary_muscle_development	ENSGALG00010004969	YTHDC1	0.375454674	0.077464799	4.846777867	1.25483E-06	3.21242E-06	positive
PRJNA729271	stage_E18_vs_E10	ordinary_muscle_development	ENSGALG00010004970	RBM48	-0.02015232	0.170271257	-0.118354206	0.905787009	0.922201496	negative
PRJNA729271	stage_E18_vs_E10	ordinary_muscle_development	ENSGALG00010004973	SLN	8.921807534	0.195024889	45.74702029	0	0	positive
PRJNA729271	stage_E18_vs_E10	ordinary_muscle_development	ENSGALG00010004974	MTPAP	-0.15978648	0.061746893	-2.58776551	0.009660072	0.015311398	negative
PRJNA729271	stage_E18_vs_E10	ordinary_muscle_development	ENSGALG00010004975	SNW1	-0.033418205	0.069798697	-0.478779789	0.632095297	0.681865098	negative
PRJNA729271	stage_E18_vs_E10	ordinary_muscle_development	ENSGALG00010004976	ENSGALG00010004976	1.738893383	0.229939689	7.562389034	3.95733E-14	1.87272E-13	positive
PRJNA729271	stage_E18_vs_E10	ordinary_muscle_development	ENSGALG00010004977	RNF217	1.454024351	0.131038014	11.09620257	1.30888E-28	1.49505E-27	positive
PRJNA729271	stage_E18_vs_E10	ordinary_muscle_development	ENSGALG00010004980	FNDC1	2.181429798	0.377360824	5.780753216	7.43669E-09	2.32316E-08	positive
PRJNA729271	stage_E18_vs_E10	ordinary_muscle_development	ENSGALG00010004981	ENSGALG00010004981	0.203230158	0.206141115	0.98587881	0.324192587	0.382232922	positive
PRJNA729271	stage_E18_vs_E10	ordinary_muscle_development	ENSGALG00010004983	CLXN	-0.136087395	0.262973385	-0.517494937	0.604810702	0.655750702	negative
PRJNA729271	stage_E18_vs_E10	ordinary_muscle_development	ENSGALG00010004987	MAP3K8	3.715563297	0.257530381	14.4276698	3.46594E-47	9.11775E-46	positive
PRJNA729271	stage_E18_vs_E10	ordinary_muscle_development	ENSGALG00010004988	HJURP	-1.38536622	0.217794945	-6.36087409	2.00609E-10	7.14805E-10	negative
PRJNA729271	stage_E18_vs_E10	ordinary_muscle_development	ENSGALG00010004990	ENSGALG00010004990	1.831514102	2.458566885	0.744951912	0.456300791	0.51586298	positive
PRJNA729271	stage_E18_vs_E10	ordinary_muscle_development	ENSGALG00010004991	FAM133B	-0.392635725	0.063699288	-6.163895073	7.0977E-10	2.43506E-09	negative
PRJNA729271	stage_E18_vs_E10	ordinary_muscle_development	ENSGALG00010004993	SULT1B	2.510056792	0.248780611	10.08943896	6.15201E-24	5.44404E-23	positive
PRJNA729271	stage_E18_vs_E10	ordinary_muscle_development	ENSGALG00010004995	PIT54	10.58305642	2.194272113	4.823037379	1.41388E-06	3.59476E-06	positive
PRJNA729271	stage_E18_vs_E10	ordinary_muscle_development	ENSGALG00010004996	GPR158	-0.858176107	0.231312884	-3.71002294	0.00020724	0.000414738	negative
PRJNA729271	stage_E18_vs_E10	ordinary_muscle_development	ENSGALG00010004999	ADCK1	-0.908371084	0.200015382	-4.54150613	5.58538E-06	1.33411E-05	negative
PRJNA729271	stage_E18_vs_E10	ordinary_muscle_development	ENSGALG00010005003	EXPH5	-0.040289602	0.432625735	-0.093128075	0.925801814	0.939148806	negative
PRJNA729271	stage_E18_vs_E10	ordinary_muscle_development	ENSGALG00010005004	CDK6	-0.110778382	0.09681265	-1.144255238	0.252517801	0.306168593	negative
PRJNA729271	stage_E18_vs_E10	ordinary_muscle_development	ENSGALG00010005010	NKAIN2	-2.015601058	0.329068952	-6.125163264	9.05907E-10	3.07592E-09	negative
PRJNA729271	stage_E18_vs_E10	ordinary_muscle_development	ENSGALG00010005012	ENSGALG00010005012	1.427221074	1.179155	1.210376137	0.226134594	0.277546227	positive
PRJNA729271	stage_E18_vs_E10	ordinary_muscle_development	ENSGALG00010005014	SULT1B1	1.005571997	0.1525645	6.59112702	4.365E-11	1.63744E-10	positive
PRJNA729271	stage_E18_vs_E10	ordinary_muscle_development	ENSGALG00010005015	NRXN3	-0.558118086	0.389765824	-1.431931821	0.152163334	0.194446578	negative
PRJNA729271	stage_E18_vs_E10	ordinary_muscle_development	ENSGALG00010005016	STX11	-0.180290444	0.172943092	-1.042484216	0.29718724	0.354079322	negative
PRJNA729271	stage_E18_vs_E10	ordinary_muscle_development	ENSGALG00010005017	ENSGALG00010005017	2.182276701	0.169575135	12.86908428	6.71941E-38	1.18633E-36	positive
PRJNA729271	stage_E18_vs_E10	ordinary_muscle_development	ENSGALG00010005023	POGLUT3	0.279313814	0.180593396	1.546644676	0.121948951	0.15917605	positive
PRJNA729271	stage_E18_vs_E10	ordinary_muscle_development	ENSGALG00010005025	THOC1	-0.78177625	0.059089181	-13.23044663	5.85385E-40	1.12237E-38	negative
PRJNA729271	stage_E18_vs_E10	ordinary_muscle_development	ENSGALG00010005029	FAT4	0.067624883	0.197313447	0.342728202	0.731802944	0.773422069	positive
PRJNA729271	stage_E18_vs_E10	ordinary_muscle_development	ENSGALG00010005033	USP14	-0.939206056	0.136872794	-6.861890016	6.79554E-12	2.70757E-11	negative
PRJNA729271	stage_E18_vs_E10	ordinary_muscle_development	ENSGALG00010005035	NECTIN3	0.703027454	0.097182474	7.234097139	4.68636E-13	2.05658E-12	positive

PRJNA729271	stage_E18_vs_E10	ordinary_muscle_development	ENSGALG00010005037	THNSL1	1.463613447	0.140790085	10.3957139	2.59338E-25	2.50273E-24	positive
PRJNA729271	stage_E18_vs_E10	ordinary_muscle_development	ENSGALG00010005042	SLC46A3	0.833697529	0.214564864	3.885526799	0.000102108	0.000212805	positive
PRJNA729271	stage_E18_vs_E10	ordinary_muscle_development	ENSGALG00010005045	UTRN	0.151399478	0.099388661	1.523307358	0.127681827	0.166075743	positive
PRJNA729271	stage_E18_vs_E10	ordinary_muscle_development	ENSGALG00010005048	ROCK1	-0.166151999	0.114843428	-1.446769759	0.147961419	0.189595188	negative
PRJNA729271	stage_E18_vs_E10	ordinary_muscle_development	ENSGALG00010005057	FLVCR2	-0.960955635	0.290191995	-3.311447775	0.000928146	0.001698027	negative
PRJNA729271	stage_E18_vs_E10	ordinary_muscle_development	ENSGALG00010005060	POMP	-0.316818026	0.138692365	-2.284322042	0.022352611	0.033422849	negative
PRJNA729271	stage_E18_vs_E10	ordinary_muscle_development	ENSGALG00010005065	ENKUR	-0.794440969	0.424825323	-1.870041463	0.06147806	0.084868998	negative
PRJNA729271	stage_E18_vs_E10	ordinary_muscle_development	ENSGALG00010005068	PLCXD2	1.092039012	0.266123188	4.103509438	4.0693E-05	8.84723E-05	positive
PRJNA729271	stage_E18_vs_E10	ordinary_muscle_development	ENSGALG00010005070	ALB	8.18471507	1.40354879	5.831443216	5.495E-09	1.73908E-08	positive
PRJNA729271	stage_E18_vs_E10	ordinary_muscle_development	ENSGALG00010005075	TRDN	4.950350705	0.167240473	29.60019561	1.4856E-192	7.4256E-190	positive
PRJNA729271	stage_E18_vs_E10	ordinary_muscle_development	ENSGALG00010005077	ERG28	-0.690155971	0.106100238	-6.504754209	7.78205E-11	2.86297E-10	negative
PRJNA729271	stage_E18_vs_E10	ordinary_muscle_development	ENSGALG00010005081	GREB1L	0.452139922	0.179736723	2.515567851	0.01188408	0.018610603	positive
PRJNA729271	stage_E18_vs_E10	ordinary_muscle_development	ENSGALG00010005082	ENSGALG00010005082	-0.963431122	0.090287386	-10.67071678	1.39546E-26	1.4531E-25	negative
PRJNA729271	stage_E18_vs_E10	ordinary_muscle_development	ENSGALG00010005087	ENPP1	1.979141877	0.214167625	9.24108802	2.44003E-20	1.75525E-19	positive
PRJNA729271	stage_E18_vs_E10	ordinary_muscle_development	ENSGALG00010005092	ESCO1	0.540434562	0.142012499	3.805542228	0.000141494	0.000289724	positive
PRJNA729271	stage_E18_vs_E10	ordinary_muscle_development	ENSGALG00010005104	ENPP3	0.348069114	0.199621191	1.743648119	0.081220467	0.109688873	positive
PRJNA729271	stage_E18_vs_E10	ordinary_muscle_development	ENSGALG00010005107	TTLL5	-0.414677967	0.074041659	-5.600603367	2.13607E-08	6.41839E-08	negative
PRJNA729271	stage_E18_vs_E10	ordinary_muscle_development	ENSGALG00010005109	ABHD3	-0.018888791	0.375191877	-0.05034435	0.959847983	0.966918932	negative
PRJNA729271	stage_E18_vs_E10	ordinary_muscle_development	ENSGALG00010005111	RASSF6	-0.863057173	0.413739695	-2.085990739	0.036979462	0.053118353	negative
PRJNA729271	stage_E18_vs_E10	ordinary_muscle_development	ENSGALG00010005114	PHLDB2	-0.010123495	0.099755137	-0.101483449	0.919166696	0.933397874	negative
PRJNA729271	stage_E18_vs_E10	ordinary_muscle_development	ENSGALG00010005118	PRTFDC1	-0.524616706	0.13744678	-3.816871569	0.000135154	0.000277449	negative
PRJNA729271	stage_E18_vs_E10	ordinary_muscle_development	ENSGALG00010005119	MIB1	-1.154932793	0.057083295	-20.23241299	5.07573E-91	4.93777E-89	negative
PRJNA729271	stage_E18_vs_E10	ordinary_muscle_development	ENSGALG00010005122	IL8L1	0.438529978	1.025373765	0.427678173	0.668885443	0.715851632	positive
PRJNA729271	stage_E18_vs_E10	ordinary_muscle_development	ENSGALG00010005123	MTIF3	0.827651141	0.106463441	7.774040846	7.60212E-15	3.83412E-14	positive
PRJNA729271	stage_E18_vs_E10	ordinary_muscle_development	ENSGALG00010005126	MED23	-0.67659941	0.058760796	-11.51446986	1.11543E-30	1.40593E-29	negative
PRJNA729271	stage_E18_vs_E10	ordinary_muscle_development	ENSGALG00010005127	GATA6	-0.668022966	0.313684753	-2.129599732	0.033204673	0.04812055	negative
PRJNA729271	stage_E18_vs_E10	ordinary_muscle_development	ENSGALG00010005131	IL8L2	3.062413414	0.658275757	4.652174078	3.28454E-06	8.01504E-06	positive
PRJNA729271	stage_E18_vs_E10	ordinary_muscle_development	ENSGALG00010005132	LNX2	-0.099313095	0.095516974	-1.039742888	0.298459369	0.3553905	negative
PRJNA729271	stage_E18_vs_E10	ordinary_muscle_development	ENSGALG00010005138	ENSGALG00010005138	5.31406183	0.505476855	10.51296767	7.52864E-26	7.46938E-25	positive
PRJNA729271	stage_E18_vs_E10	ordinary_muscle_development	ENSGALG00010005140	DCT	-0.754366119	0.911520318	-0.827591118	0.4079021	0.468023505	negative
PRJNA729271	stage_E18_vs_E10	ordinary_muscle_development	ENSGALG00010005141	CCDC170	-0.469667661	0.311752925	-1.506538106	0.131929093	0.170877652	negative
PRJNA729271	stage_E18_vs_E10	ordinary_muscle_development	ENSGALG00010005143	GTF3A	-0.747931035	0.138627053	-5.395274729	6.84188E-08	1.96343E-07	negative
PRJNA729271	stage_E18_vs_E10	ordinary_muscle_development	ENSGALG00010005145	AKAP7	3.302213401	0.174284859	18.94721903	4.65545E-80	3.25994E-78	positive
PRJNA729271	stage_E18_vs_E10	ordinary_muscle_development	ENSGALG00010005146	TGFB3	0.695799615	0.204546453	3.401670403	0.000669754	0.001251318	positive
PRJNA729271	stage_E18_vs_E10	ordinary_muscle_development	ENSGALG00010005148	TTC39C	0.075265945	0.08267367	0.910398014	0.362612645	0.422072616	positive
PRJNA729271	stage_E18_vs_E10	ordinary_muscle_development	ENSGALG00010005150	ALCAM	-2.256267845	0.189063214	-11.93393365	7.87617E-33	1.08522E-31	negative
PRJNA729271	stage_E18_vs_E10	ordinary_muscle_development	ENSGALG00010005154	CWF19L2	-0.28276127	0.149830487	-1.887207835	0.059132377	0.08193528	negative
PRJNA729271	stage_E18_vs_E10	ordinary_muscle_development	ENSGALG00010005157	IMPA1	-0.475031751	0.110531132	-4.297719055	1.72565E-05	3.92119E-05	negative
PRJNA729271	stage_E18_vs_E10	ordinary_muscle_development	ENSGALG00010005158	IFT43	-0.615210659	0.19788518	-3.108927401	0.001877679	0.003310662	negative
PRJNA729271	stage_E18_vs_E10	ordinary_muscle_development	ENSGALG00010005159	ARHGAP21	0.626107348	0.098238687	6.373327734	1.8497E-10	6.60868E-10	positive
PRJNA729271	stage_E18_vs_E10	ordinary_muscle_development	ENSGALG00010005163	GPC6	-2.981299675	0.387743587	-7.688843287	1.48471E-14	7.30512E-14	negative
PRJNA729271	stage_E18_vs_E10	ordinary_muscle_development	ENSGALG00010005165	METTL21C	1.34261257	0.591841824	2.268532764	0.023296754	0.034719973	positive
PRJNA729271	stage_E18_vs_E10	ordinary_muscle_development	ENSGALG00010005166	CBLB	-0.243989509	0.125236521	-1.948229684	0.051387486	0.072018433	negative
PRJNA729271	stage_E18_vs_E10	ordinary_muscle_development	ENSGALG00010005167	GUCY1A2	0.179935578	0.178459366	1.008271982	0.31332391	0.370866411	positive
PRJNA729271	stage_E18_vs_E10	ordinary_muscle_development	ENSGALG00010005170	GPATCH2L	0.21185939	0.060862375	3.480958326	0.000499623	0.000949526	positive
PRJNA729271	stage_E18_vs_E10	ordinary_muscle_development	ENSGALG00010005172	OSBPL1A	1.23592439	0.080596102	15.33479122	4.4771E-53	1.43574E-51	positive
PRJNA729271	stage_E18_vs_E10	ordinary_muscle_development	ENSGALG00010005174	TPP2	-0.351122973	0.185448881	-1.893367982	0.05830895	0.080902482	negative
PRJNA729271	stage_E18_vs_E10	ordinary_muscle_development	ENSGALG00010005176	BAMBI	-2.431805849	0.308120855	-7.892376668	2.96485E-15	1.54078E-14	negative
PRJNA729271	stage_E18_vs_E10	ordinary_muscle_development	ENSGALG00010005177	AASDHPPT	0.474751261	0.129177557	3.675183774	0.000237678	0.000472132	positive
PRJNA729271	stage_E18_vs_E10	ordinary_muscle_development	ENSGALG00010005178	EPHA5	0.573303222	0.197645678	2.900661564	0.003723758	0.006276994	positive
PRJNA729271	stage_E18_vs_E10	ordinary_muscle_development	ENSGALG00010005180	WAC	0.29165116	0.115956964	2.515167272	0.011897592	0.018625726	positive
PRJNA729271	stage_E18_vs_E10	ordinary_muscle_development	ENSGALG00010005181	IMPACT	0.607028082	0.134081971	4.527290848	5.97447E-06	1.4234E-05	positive
PRJNA729271	stage_E18_vs_E10	ordinary_muscle_development	ENSGALG00010005182	NEPRO	-0.908120395	0.092338367	-9.834702742	7.98029E-23	6.64032E-22	negative
PRJNA729271	stage_E18_vs_E10	ordinary_muscle_development	ENSGALG00010005184	FGF14	-2.289840444	0.308903649	-7.412798292	1.23662E-13	5.64916E-13	negative
PRJNA729271	stage_E18_vs_E10	ordinary_muscle_development	ENSGALG00010005186	ESR1	0.634337225	0.191951669	3.304671575	0.000950878	0.001736984	positive
PRJNA729271	stage_E18_vs_E10	ordinary_muscle_development	ENSGALG00010005187	KBTBD3	0.92822308	0.260607797	3.561762503	0.000368374	0.000712608	positive
PRJNA729271	stage_E18_vs_E10	ordinary_muscle_development	ENSGALG00010005190	ENSGALG00010005190	1.521215879	0.288657147	5.26997476	1.36443E-07	3.81434E-07	positive
PRJNA729271	stage_E18_vs_E10	ordinary_muscle_development	ENSGALG00010005191	MSANTD4	-0.407185772	0.105979365	-3.842123166	0.000121975	0.000251532	negative
PRJNA729271	stage_E18_vs_E10	ordinary_muscle_development	ENSGALG00010005193	EPB41L2	0.972616566	0.098325358	9.891818179	4.51748E-23	3.80702E-22	positive
PRJNA729271	stage_E18_vs_E10	ordinary_muscle_development	ENSGALG00010005194	ENSGALG00010005194	2.249035615	1.112835414	2.020995726	0.043280208	0.061402232	positive
PRJNA729271	stage_E18_vs_E10	ordinary_muscle_development	ENSGALG00010005198	TRPC3	-1.58614558	0.534303548	-2.968622582	0.002991378	0.005123481	negative
PRJNA729271	stage_E18_vs_E10	ordinary_muscle_development	ENSGALG00010005203	ENSGALG00010005203	0.994865476	0.105443885	9.435022991	3.90909E-21	2.96661E-20	positive
PRJNA729271	stage_E18_vs_E10	ordinary_muscle_development	ENSGALG00010005204	ITGBL1	1.710045179	0.310024694	5.515835385	3.47127E-08	1.02686E-07	positive
PRJNA729271	stage_E18_vs_E10	ordinary_muscle_development	ENSGALG00010005208	ENSGALG00010005208	-0.620953579	0.112105026	-5.539034252	3.04144E-08	9.03766E-08	negative
PRJNA729271	stage_E18_vs_E10	ordinary_muscle_development	ENSGALG00010005211	ESRRB	2.846636091	0.337162835	8.442911849	3.09529E-17	1.83878E-16	positive
PRJNA729271	stage_E18_vs_E10	ordinary_muscle_development	ENSGALG00010005212	TAF13	-0.074500359	0.10280329	-0.72468847	0.46864317	0.527610311	negative
PRJNA729271	stage_E18_vs_E10	ordinary_muscle_development	ENSGALG00010005213	ENSGALG00010005213	-0.032483474	0.162330899	-0.200106537	0.841397261	0.868850416	negative
PRJNA729271	stage_E18_vs_E10	ordinary_muscle_development	ENSGALG00010005214	BBS7	-0.411462407	0.120450318	-3.4160342	0.000635403	0.001190097	negative
PRJNA729271	stage_E18_vs_E10	ordinary_muscle_development	ENSGALG00010005215	VASH1	-1.003274875	0.14234211	-7.048334998	1.81071E-12	7.59441E-12	negative
PRJNA729271	stage_E18_vs_E10	ordinary_muscle_development	ENSGALG00010005216	ENSGALG00010005216	-0.242648218	0.166115083	-1.460723576	0.144091316	0.185140069	negative
PRJNA729271	stage_E18_vs_E10	ordinary_muscle_development	ENSGALG00010005217	CCNA2	-1.740281578	0.144709435	-12.0260409	2.593E-33	3.642E-32	negative
PRJNA729271	stage_E18_vs_E10	ordinary_muscle_development	ENSGALG00010005218	GTPBP8	-0.055073199	0.121724394	-0.452441761	0.650950768	0.699342676	negative
PRJNA729271	stage_E18_vs_E10	ordinary_muscle_development	ENSGALG00010005219	NALCN	-0.444073434	0.184020022	-2.41317998	0.015814008	0.024313115	negative
PRJNA729271	stage_E18_vs_E10	ordinary_muscle_development	ENSGALG00010005220	ANGEL1	0.207698785	0.085746392	2.422245181	0.015424939	0.023755259	positive
PRJNA729271	stage_E18_vs_E10	ordinary_muscle_development	ENSGALG00010005221	ENSGALG00010005221	2.242591763	0.298315712	7.517511389	5.58287E-14	2.61889E-13	positive
PRJNA729271	stage_E18_vs_E10	ordinary_muscle_development	ENSGALG00010005222	ABCB1	3.618977	1.000066715	3.618735576	0.000296046	0.000580282	positive
PRJNA729271	stage_E18_vs_E10	ordinary_muscle_development	ENSGALG00010005223	GRIA4	-2.623030942	0.443057373	-5.920296336	3.21362E-09	1.03791E-08	negative
PRJNA729271	stage_E18_vs_E10	ordinary_muscle_development	ENSGALG00010005224	ENSGALG00010005224	0.81261	0.425058773	1.911759153	0.055907093	0.077823232	positive
PRJNA729271	stage_E18_vs_E10	ordinary_muscle_development	ENSGALG00010005226	MYCT1	1.075901623	0.168382808	6.389616816	1.66302E-10	5.96279E-10	positive
PRJNA729271	stage_E18_vs_E10	ordinary_muscle_development	ENSGALG00010005227	EXOSC9	-0.378043493	0.105434161	-3.585588288	0.000336319	0.000653741	negative
PRJNA729271	stage_E18_vs_E10	ordinary_muscle_development	ENSGALG00010005228	ZNF521	-0.980879657	0.131918847	-7.435477791	1.04191E-13	4.78228E-13	negative
PRJNA729271	stage_E18_vs_E10	ordinary_muscle_development	ENSGALG00010005229	CIPC	-0.398868852	0.075192057	-5.304667379	1.12879E-07	3.18694E-07	negative
PRJNA729271	stage_E18_vs_E10	ordinary_muscle_development	ENSGALG00010005231	ENSGALG00010005231	1.126735252	0.430544441	2.617001046	0.008870607	0.014145154	positive
PRJNA729271	stage_E18_vs_E10	ordinary_muscle_development	ENSGALG00010005232	GATA3	-3.895487077	0.546532783	-7.127636618	1.02107E-12	4.37752E-12	negative
PRJNA729271	stage_E18_vs_E10	ordinary_muscle_development	ENSGALG00010005236	TMTC4	-1.812488486	0.092825469	-19.52576715	6.63136E-85	5.19576E-83	negative
PRJNA729271	stage_E18_vs_E10	ordinary_muscle_development	ENSGALG00010005237	RAP2A	-1.47801281	0.111070327	-13.30699978	2.10777E-40	4.09546E-39	negative
PRJNA729271	stage_E18_vs_E10	ordinary_muscle_development	ENSGALG00010005238	TMEM63C	-0.900678442	0.17040265	-5.285589415	1.25301E-07	3.51983E-07	negative
PRJNA729271	stage_E18_vs_E10	ordinary_muscle_development	ENSGALG00010005239	ENSGALG00010005239	-1.117811854	0.184655169	-6.05350969	1.41724E-09	4.71815E-09	negative
PRJNA729271	stage_E18_vs_E10	ordinary_muscle_development	ENSGALG00010005240	VIP	-2.311196701	0.387296584	-5.967511203	2.409E-09	7.86805E-09	negative
PRJNA729271	stage_E18_vs_E10	ordinary_muscle_development	ENSGALG00010005241	C4orf54	3.779354566	0.187433218	20.16373944	2.03876E-90	1.91895E-88	positive
PRJNA729271	stage_E18_vs_E10	ordinary_muscle_development	ENSGALG00010005242	CCDC80	0.5627141	0.233909742	2.405689027	0.016141999	0.024785834	positive
PRJNA729271	stage_E18_vs_E10	ordinary_muscle_development	ENSGALG00010005243	NGB	-2.156852883	0.517845488	-4.165051027	3.11283E-05	6.84889E-05	negative
PRJNA729271	stage_E18_vs_E10	ordinary_muscle_development	ENSGALG00010005244	FBXO5	-1.175961392	0.097132759	-12.10674338	9.72803E-34	1.40306E-32	negative
PRJNA729271	stage_E18_vs_E10	ordinary_muscle_development	ENSGALG00010005245	KIAA1217	1.525919994	0.135354838	11.27347955	1.77417E-29	2.10965E-28	positive
PRJNA729271	stage_E18_vs_E10	ordinary_muscle_development	ENSGALG00010005246	ENSGALG00010005246	-0.784531569	0.198350166	-3.955285673	7.64433E-05	0.000161396	negative
PRJNA729271	stage_E18_vs_E10	ordinary_muscle_development	ENSGALG00010005247	THAP7	0.395030082	0.118237356	3.34099219	0.000834796	0.001538703	positive
PRJNA729271	stage_E18_vs_E10	ordinary_muscle_development	ENSGALG00010005248	TMEM200A	-0.903626372	0.240327121	-3.759985003	0.000169924	0.000343856	negative
PRJNA729271	stage_E18_vs_E10	ordinary_muscle_development	ENSGALG00010005249	PDGFD	-0.132106261	0.228890502	-0.577159207	0.563831911	0.617696588	negative
PRJNA729271	stage_E18_vs_E10	ordinary_muscle_development	ENSGALG00010005250	SLC35A5	-0.150170234	0.089864186	-1.671079892	0.094705899	0.126207778	negative
PRJNA729271	stage_E18_vs_E10	ordinary_muscle_development	ENSGALG00010005251	MTRF1L	0.178352697	0.103897428	1.716622823	0.08604811	0.115509108	positive
PRJNA729271	stage_E18_vs_E10	ordinary_muscle_development	ENSGALG00010005252	KLHL14	-1.997400927	0.220104474	-9.074785675	1.13901E-19	7.86191E-19	negative
PRJNA729271	stage_E18_vs_E10	ordinary_muscle_development	ENSGALG00010005253	POMT2	-0.652009683	0.097998243	-6.653279316	2.86633E-11	1.0882E-10	negative
PRJNA729271	stage_E18_vs_E10	ordinary_muscle_development	ENSGALG00010005254	ENSGALG00010005254	-0.853624788	0.750039484	-1.138106469	0.255076027	0.30893441	negative
PRJNA729271	stage_E18_vs_E10	ordinary_muscle_development	ENSGALG00010005255	BZW2	-0.441222295	0.093354684	-4.726300584	2.28647E-06	5.68285E-06	negative
PRJNA729271	stage_E18_vs_E10	ordinary_muscle_development	ENSGALG00010005256	OPRM1	-4.505829592	0.531030736	-8.485063641	2.15601E-17	1.29245E-16	negative
PRJNA729271	stage_E18_vs_E10	ordinary_muscle_development	ENSGALG00010005258	ITIH5	3.73856756	0.278759275	13.41145532	5.1812E-41	1.03446E-39	positive
PRJNA729271	stage_E18_vs_E10	ordinary_muscle_development	ENSGALG00010005259	SLC2A5	1.834955357	0.673656473	2.723874009	0.006452111	0.010503522	positive
PRJNA729271	stage_E18_vs_E10	ordinary_muscle_development	ENSGALG00010005260	ANXA5	0.393129457	0.149924105	2.622189795	0.008736676	0.013951539	positive
PRJNA729271	stage_E18_vs_E10	ordinary_muscle_development	ENSGALG00010005261	ATG3	-0.439779477	0.111068185	-3.959545006	7.50927E-05	0.000158669	negative
PRJNA729271	stage_E18_vs_E10	ordinary_muscle_development	ENSGALG00010005262	ENSGALG00010005262	0.15654067	0.124727622	1.255060169	0.209456918	0.259360843	positive
PRJNA729271	stage_E18_vs_E10	ordinary_muscle_development	ENSGALG00010005263	ENSGALG00010005263	1.478550195	0.330505486	4.473602574	7.69126E-06	1.81247E-05	positive
PRJNA729271	stage_E18_vs_E10	ordinary_muscle_development	ENSGALG00010005264	ITIH2	6.661395877	1.035950174	6.430228059			positive
PRJNA729271	stage_E18_vs_E10	ordinary_muscle_development	ENSGALG00010005265	DYNC2H1	-0.480966123	0.094953115	-5.065301129	4.07755E-07	1.09452E-06	negative
PRJNA729271	stage_E18_vs_E10	ordinary_muscle_development	ENSGALG00010005266	SLC19A2	-0.301503953	0.273585537	-1.102046387	0.270441505	0.325512251	negative
PRJNA729271	stage_E18_vs_E10	ordinary_muscle_development	ENSGALG00010005267	CYP51A1	-2.678907663	0.189861039	-14.10983358	3.30357E-45	7.81161E-44	negative
PRJNA729271	stage_E18_vs_E10	ordinary_muscle_development	ENSGALG00010005268	MTTP	4.661575002	0.883146992	5.278368204	1.30339E-07	3.65147E-07	positive
PRJNA729271	stage_E18_vs_E10	ordinary_muscle_development	ENSGALG00010005269	KIN	0.243346592	0.106721488	2.280202381	0.022595688	0.033765412	positive
PRJNA729271	stage_E18_vs_E10	ordinary_muscle_development	ENSGALG00010005270	GSTZ1	-0.060280096	0.215730628	-0.279422986	0.779920233	0.815475999	negative
PRJNA729271	stage_E18_vs_E10	ordinary_muscle_development	ENSGALG00010005272	MBNL2	0.730639057	0.094140605	7.761146796	8.41648E-15	4.22866E-14	positive
PRJNA729271	stage_E18_vs_E10	ordinary_muscle_development	ENSGALG00010005273	ENSGALG00010005273	2.320577269	0.653183052	3.552721187	0.000381268	0.000735688	positive
PRJNA729271	stage_E18_vs_E10	ordinary_muscle_development	ENSGALG00010005275	L3MBTL3	0.51007098	0.171653142	2.971521378	0.002963282	0.005078962	positive
PRJNA729271	stage_E18_vs_E10	ordinary_muscle_development	ENSGALG00010005277	ENSGALG00010005277	1.006264536	0.206662849	4.869111898	1.12101E-06	2.88359E-06	positive
PRJNA729271	stage_E18_vs_E10	ordinary_muscle_development	ENSGALG00010005278	DAPP1	0.308793016	0.130144312	2.372696979	0.017658744	0.026884098	positive
PRJNA729271	stage_E18_vs_E10	ordinary_muscle_development	ENSGALG00010005279	CCDC181	-0.316459623	0.345609998	-0.915655291	0.359847722	0.41939316	negative
PRJNA729271	stage_E18_vs_E10	ordinary_muscle_development	ENSGALG00010005280	TMEM244	-1.820422019	0.592432035	-3.072794702	0.002120643	0.003710614	negative
PRJNA729271	stage_E18_vs_E10	ordinary_muscle_development	ENSGALG00010005281	TMED8	-1.056946881	0.069700283	-15.16416925	6.10667E-52	1.90768E-50	negative
PRJNA729271	stage_E18_vs_E10	ordinary_muscle_development	ENSGALG00010005282	FLT1	1.531907689	0.126137805	12.14471494	6.11955E-34	8.94182E-33	positive
PRJNA729271	stage_E18_vs_E10	ordinary_muscle_development	ENSGALG00010005283	ATP5C1	0.865876819	0.132150087	6.552222864	5.66869E-11	2.10848E-10	positive
PRJNA729271	stage_E18_vs_E10	ordinary_muscle_development	ENSGALG00010005284	ENSGALG00010005284	0.574968217	0.217468672	2.643912853	0.008195375	0.013127635	positive
PRJNA729271	stage_E18_vs_E10	ordinary_muscle_development	ENSGALG00010005285	MPP7	0.450503514	0.128679405	3.500976032	0.000463558	0.00088633	positive
PRJNA729271	stage_E18_vs_E10	ordinary_muscle_development	ENSGALG00010005286	ENSGALG00010005286	1.172771984	0.620744431	1.889299244	0.058851743	0.081593115	positive

PRJNA729271	stage_E18_vs_E10	ordinary_muscle_development	ENSGALG00010005287	ENSGALG00010005287	0.164494569	0.380020389	0.432857221	0.6651185	0.712498729	positive
PRJNA729271	stage_E18_vs_E10	ordinary_muscle_development	ENSGALG00010005288	ENSGALG00010005288	0.091029808	0.094702353	0.961220128	0.336441498	0.394844103	positive
PRJNA729271	stage_E18_vs_E10	ordinary_muscle_development	ENSGALG00010005291	IFNGR1	0.644087495	0.095999372	6.709288632	1.95575E-11	7.51356E-11	positive
PRJNA729271	stage_E18_vs_E10	ordinary_muscle_development	ENSGALG00010005292	TAF3	-0.220376131	0.099301843	-2.2192552	0.026469368	0.039066641	negative
PRJNA729271	stage_E18_vs_E10	ordinary_muscle_development	ENSGALG00010005293	BLZF1	-0.428907198	0.098478609	-4.355333629	1.32864E-05	3.05354E-05	negative
PRJNA729271	stage_E18_vs_E10	ordinary_muscle_development	ENSGALG00010005295	ENSGALG00010005295	-1.026740183	0.570813323	-1.798731987	0.072061086	0.098299026	negative
PRJNA729271	stage_E18_vs_E10	ordinary_muscle_development	ENSGALG00010005296	PAN3	0.817386111	0.101090307	8.085702093	6.18071E-16	3.37056E-15	positive
PRJNA729271	stage_E18_vs_E10	ordinary_muscle_development	ENSGALG00010005297	IL22RA2	-0.991432539	0.637739264	-1.554604828	0.120040213	0.156910712	negative
PRJNA729271	stage_E18_vs_E10	ordinary_muscle_development	ENSGALG00010005299	ODAD2	-3.410866653	0.627717383	-5.433761668	5.51783E-08	1.5977E-07	negative
PRJNA729271	stage_E18_vs_E10	ordinary_muscle_development	ENSGALG00010005300	QRFPR	-2.365894882	0.974020542	-2.428998959	0.015140576	0.023357029	negative
PRJNA729271	stage_E18_vs_E10	ordinary_muscle_development	ENSGALG00010005301	ENSGALG00010005301	0.02817534	0.41019537	0.068687611	0.94523828	0.955055686	positive
PRJNA729271	stage_E18_vs_E10	ordinary_muscle_development	ENSGALG00010005302	PSPC1	-0.59155763	0.079875019	-7.406040564	1.30126E-13	5.93489E-13	negative
PRJNA729271	stage_E18_vs_E10	ordinary_muscle_development	ENSGALG00010005303	ENSGALG00010005303	1.64292784	0.291773762	5.630827908	1.79347E-08	5.41477E-08	positive
PRJNA729271	stage_E18_vs_E10	ordinary_muscle_development	ENSGALG00010005304	ENSGALG00010005304	0.295259228	0.212059623	1.392340625	0.163819275	0.207802607	positive
PRJNA729271	stage_E18_vs_E10	ordinary_muscle_development	ENSGALG00010005305	SCAF8	-0.1187326	0.08675876	-1.368537309	0.171143952	0.216148086	negative
PRJNA729271	stage_E18_vs_E10	ordinary_muscle_development	ENSGALG00010005307	TIAM2	-1.163075751	0.156183855	-7.446837255	9.56044E-14	4.39653E-13	negative
PRJNA729271	stage_E18_vs_E10	ordinary_muscle_development	ENSGALG00010005308	ENSGALG00010005308	0.591642296	0.654801633	0.90354432	0.366237077	0.425846818	positive
PRJNA729271	stage_E18_vs_E10	ordinary_muscle_development	ENSGALG00010005309	ASXL3	-1.080750888	0.139168996	-7.765744686	8.11669E-15	4.08513E-14	negative
PRJNA729271	stage_E18_vs_E10	ordinary_muscle_development	ENSGALG00010005310	LAMTOR3	0.540891603	0.076684437	7.053472984	1.74506E-12	7.33392E-12	positive
PRJNA729271	stage_E18_vs_E10	ordinary_muscle_development	ENSGALG00010005311	YME1L1	-0.525296039	0.078805536	-6.66572507	2.63362E-11	1.00248E-10	negative
PRJNA729271	stage_E18_vs_E10	ordinary_muscle_development	ENSGALG00010005313	MPHOSPH8	0.073408311	0.141777342	0.517771811	0.604617488	0.655639298	positive
PRJNA729271	stage_E18_vs_E10	ordinary_muscle_development	ENSGALG00010005314	IL20RA	-0.083529404	0.629716623	-0.132646021	0.894473334	0.912541595	negative
PRJNA729271	stage_E18_vs_E10	ordinary_muscle_development	ENSGALG00010005315	ENSGALG00010005315	0.905492661	0.560454549	1.615639774	0.106172219	0.14009707	positive
PRJNA729271	stage_E18_vs_E10	ordinary_muscle_development	ENSGALG00010005316	PARP4	0.572749605	0.107880478	5.309112619	1.1016E-07	3.11383E-07	positive
PRJNA729271	stage_E18_vs_E10	ordinary_muscle_development	ENSGALG00010005317	CHCHD3	0.311010008	0.109786489	2.832862324	0.004613325	0.007681781	positive
PRJNA729271	stage_E18_vs_E10	ordinary_muscle_development	ENSGALG00010005318	SLC35D3	1.104715645	0.31152528	3.54615088	0.000390902	0.000753575	positive
PRJNA729271	stage_E18_vs_E10	ordinary_muscle_development	ENSGALG00010005319	NOL4	0.340880084	0.64172393	0.531194286	0.595284152	0.647115928	positive
PRJNA729271	stage_E18_vs_E10	ordinary_muscle_development	ENSGALG00010005321	HS6ST3	-4.375801938	0.348793295	-12.54554487	4.20512E-36	6.82567E-35	negative
PRJNA729271	stage_E18_vs_E10	ordinary_muscle_development	ENSGALG00010005322	TFB1M	0.262260297	0.123280116	2.127352779	0.033390783	0.048370917	positive
PRJNA729271	stage_E18_vs_E10	ordinary_muscle_development	ENSGALG00010005323	DNAJB14	0.08661696	0.064784836	1.336994364	0.181224476	0.227649604	positive
PRJNA729271	stage_E18_vs_E10	ordinary_muscle_development	ENSGALG00010005324	CENPJ	-1.350443408	0.186425218	-7.243888029	4.36001E-13	1.91801E-12	negative
PRJNA729271	stage_E18_vs_E10	ordinary_muscle_development	ENSGALG00010005326	MASTL	-1.589960917	0.184610238	-8.612528391	7.14666E-18	4.42507E-17	negative
PRJNA729271	stage_E18_vs_E10	ordinary_muscle_development	ENSGALG00010005329	VIPAS39	-0.231818495	0.119678283	-1.937013878	0.052743647	0.073731234	negative
PRJNA729271	stage_E18_vs_E10	ordinary_muscle_development	ENSGALG00010005331	ENSGALG00010005331	0.546987992	0.875628898	0.624680151	0.532181003	0.588089017	positive
PRJNA729271	stage_E18_vs_E10	ordinary_muscle_development	ENSGALG00010005332	ENSGALG00010005332	1.252963526	0.731749585	1.712284574	0.086844238	0.116469951	positive
PRJNA729271	stage_E18_vs_E10	ordinary_muscle_development	ENSGALG00010005333	PLXNA4	1.499607948	0.151998441	9.865942951	5.84823E-23	4.89153E-22	positive
PRJNA729271	stage_E18_vs_E10	ordinary_muscle_development	ENSGALG00010005334	NME7	-0.288593506	0.134485064	-2.145914929	0.031879766	0.046372024	negative
PRJNA729271	stage_E18_vs_E10	ordinary_muscle_development	ENSGALG00010005335	AHSA1	-0.90110383	0.135335529	-6.658294684	2.77023E-11	1.05282E-10	negative
PRJNA729271	stage_E18_vs_E10	ordinary_muscle_development	ENSGALG00010005336	PEX7	-0.556202727	0.128995298	-4.311806195	1.61926E-05	3.6887E-05	negative
PRJNA729271	stage_E18_vs_E10	ordinary_muscle_development	ENSGALG00010005337	DCTN1	-0.247298896	0.155017716	-1.595294411	0.110646415	0.145629691	negative
PRJNA729271	stage_E18_vs_E10	ordinary_muscle_development	ENSGALG00010005338	PCGF1	1.259331365	0.113809371	11.06526954	1.84906E-28	2.08902E-27	positive
PRJNA729271	stage_E18_vs_E10	ordinary_muscle_development	ENSGALG00010005341	UGGT2	0.195689898	0.118708529	1.648490639	0.09925203	0.131697013	positive
PRJNA729271	stage_E18_vs_E10	ordinary_muscle_development	ENSGALG00010005342	MAP3K5	0.597171437	0.145384747	4.107524669	3.99922E-05	8.7014E-05	positive
PRJNA729271	stage_E18_vs_E10	ordinary_muscle_development	ENSGALG00010005343	RNF17	-4.854048967	1.784097237	-2.720731173	0.006513771	0.010595568	negative
PRJNA729271	stage_E18_vs_E10	ordinary_muscle_development	ENSGALG00010005344	ENSGALG00010005344	1.170074782	0.715923727	1.634356759	0.102183962	0.135216043	positive
PRJNA729271	stage_E18_vs_E10	ordinary_muscle_development	ENSGALG00010005345	NOX3	-0.199330775	0.56730523	-0.351364247	0.725315101	0.76762868	negative
PRJNA729271	stage_E18_vs_E10	ordinary_muscle_development	ENSGALG00010005346	FBXO30	0.472202231	0.220749461	2.139086676	0.032428649	0.047090089	positive
PRJNA729271	stage_E18_vs_E10	ordinary_muscle_development	ENSGALG00010005347	DOK1	-0.02407277	0.151611415	-0.158779403	0.873842677	0.895971536	negative
PRJNA729271	stage_E18_vs_E10	ordinary_muscle_development	ENSGALG00010005349	ACBD5	0.625525579	0.104296942	5.997544775	2.00323E-09	6.5873E-09	positive
PRJNA729271	stage_E18_vs_E10	ordinary_muscle_development	ENSGALG00010005350	ARHGAP20	-0.706623027	0.277424797	-2.547079546	0.010862867	0.017103764	negative
PRJNA729271	stage_E18_vs_E10	ordinary_muscle_development	ENSGALG00010005351	DTNA	3.765590481	0.162793835	23.1310386	2.2563E-118	4.0377E-116	positive
PRJNA729271	stage_E18_vs_E10	ordinary_muscle_development	ENSGALG00010005352	ENSGALG00010005352	1.086995205	0.667895986	1.627491747	0.1036327	0.136983037	positive
PRJNA729271	stage_E18_vs_E10	ordinary_muscle_development	ENSGALG00010005353	ENSGALG00010005353	0.179134376	0.104540344	1.713543001	0.08661269	0.116191665	positive
PRJNA729271	stage_E18_vs_E10	ordinary_muscle_development	ENSGALG00010005354	M1AP	0.401154868	0.337703321	1.187891391	0.234876202	0.28715676	positive
PRJNA729271	stage_E18_vs_E10	ordinary_muscle_development	ENSGALG00010005355	FDX1	-0.236522281	0.105193594	-2.248447563	0.024547665	0.036449335	negative
PRJNA729271	stage_E18_vs_E10	ordinary_muscle_development	ENSGALG00010005356	DNAJC3	0.247056757	0.084293935	2.930896	0.003379859	0.005726599	positive
PRJNA729271	stage_E18_vs_E10	ordinary_muscle_development	ENSGALG00010005357	TNIP3	-2.227909494	0.579288622	-3.845940367	0.000120091	0.000247824	negative
PRJNA729271	stage_E18_vs_E10	ordinary_muscle_development	ENSGALG00010005358	SHPRH	0.488569621	0.089384489	5.46593289	4.60479E-08	1.34434E-07	positive
PRJNA729271	stage_E18_vs_E10	ordinary_muscle_development	ENSGALG00010005359	MEIS2	-2.139677322	0.133375963	-16.04245074	6.45558E-58	2.40549E-56	negative
PRJNA729271	stage_E18_vs_E10	ordinary_muscle_development	ENSGALG00010005360	CAMK1D	0.104319596	0.151158293	0.690134781	0.490109432	0.547862745	positive
PRJNA729271	stage_E18_vs_E10	ordinary_muscle_development	ENSGALG00010005361	ENSGALG00010005361	1.356534898	0.44513167	3.047491317	0.002307602	0.004010634	positive
PRJNA729271	stage_E18_vs_E10	ordinary_muscle_development	ENSGALG00010005362	ATP1B1	1.229103398	0.174352047	7.049549567	1.79498E-12	7.53933E-12	positive
PRJNA729271	stage_E18_vs_E10	ordinary_muscle_development	ENSGALG00010005363	RDX	0.020273107	0.085561593	0.236941678	0.812702036	0.84433171	positive
PRJNA729271	stage_E18_vs_E10	ordinary_muscle_development	ENSGALG00010005364	MAP7	-2.124472431	0.348964991	-6.087924246	1.14384E-09	3.83884E-09	negative
PRJNA729271	stage_E18_vs_E10	ordinary_muscle_development	ENSGALG00010005366	AUP1	0.351626668	0.114860242	3.061343603	0.002203461	0.003843461	positive
PRJNA729271	stage_E18_vs_E10	ordinary_muscle_development	ENSGALG00010005367	H2AZ1	-1.511193351	0.096166358	-15.71436599	1.20581E-55	4.19143E-54	negative
PRJNA729271	stage_E18_vs_E10	ordinary_muscle_development	ENSGALG00010005368	CCDC3	-1.530834811	0.233260548	-6.562767782	5.28181E-11	1.97014E-10	negative
PRJNA729271	stage_E18_vs_E10	ordinary_muscle_development	ENSGALG00010005369	DPT	4.179714958	0.312769086	13.3635808	9.86995E-41	1.94911E-39	positive
PRJNA729271	stage_E18_vs_E10	ordinary_muscle_development	ENSGALG00010005371	GRM1	-3.8173899	1.325544304	-2.879865946	0.003978443	0.006682217	negative
PRJNA729271	stage_E18_vs_E10	ordinary_muscle_development	ENSGALG00010005372	CDIN1	-0.320930746	0.108328809	-2.962561379	0.00305091	0.00522051	negative
PRJNA729271	stage_E18_vs_E10	ordinary_muscle_development	ENSGALG00010005376	HTRA2	-0.077387144	0.096295082	-0.803645858	0.421601531	0.482025098	negative
PRJNA729271	stage_E18_vs_E10	ordinary_muscle_development	ENSGALG00010005377	EMCN	1.926900166	0.140868173	13.67874745	1.36015E-42	2.89933E-41	positive
PRJNA729271	stage_E18_vs_E10	ordinary_muscle_development	ENSGALG00010005378	ENSGALG00010005378	-5.174118977	0.818602709	-6.320671702	2.60429E-10	9.20936E-10	negative
PRJNA729271	stage_E18_vs_E10	ordinary_muscle_development	ENSGALG00010005379	DZIP1	-0.070734784	0.167104589	-0.423296477	0.672078964	0.718796207	negative
PRJNA729271	stage_E18_vs_E10	ordinary_muscle_development	ENSGALG00010005380	XCL1	0.861205889	1.309490183	0.657665021	0.510753403	0.567482031	positive
PRJNA729271	stage_E18_vs_E10	ordinary_muscle_development	ENSGALG00010005381	FGF9	-2.56394924	0.205882722	-12.45344543	1.33936E-35	2.11482E-34	negative
PRJNA729271	stage_E18_vs_E10	ordinary_muscle_development	ENSGALG00010005382	RAB32	-0.481652105	0.128626084	-3.744591236	0.000180688	0.000364519	negative
PRJNA729271	stage_E18_vs_E10	ordinary_muscle_development	ENSGALG00010005383	OPTN	0.288255922	0.140279997	2.05486119	0.039892414	0.056975125	positive
PRJNA729271	stage_E18_vs_E10	ordinary_muscle_development	ENSGALG00010005384	MAPRE2	-0.186411545	0.173257206	-1.075923761	0.281961354	0.337520012	negative
PRJNA729271	stage_E18_vs_E10	ordinary_muscle_development	ENSGALG00010005385	PDSS1	-0.045597628	0.10072161	-0.452709489	0.650757946	0.699187342	negative
PRJNA729271	stage_E18_vs_E10	ordinary_muscle_development	ENSGALG00010005386	CPOX	-0.524705179	0.141287787	-3.713733448	0.000204224	0.000408918	negative
PRJNA729271	stage_E18_vs_E10	ordinary_muscle_development	ENSGALG00010005387	CLDN10	-5.589777954	0.951121753	-5.87703723	4.17674E-09	1.33558E-08	negative
PRJNA729271	stage_E18_vs_E10	ordinary_muscle_development	ENSGALG00010005388	ENSGALG00010005388	1.172299105	0.489330706	2.395719482	0.01658778	0.025389638	positive
PRJNA729271	stage_E18_vs_E10	ordinary_muscle_development	ENSGALG00010005389	MCM10	-2.173586076	0.188490955	-11.53151392	9.152E-31	1.16163E-29	negative
PRJNA729271	stage_E18_vs_E10	ordinary_muscle_development	ENSGALG00010005391	ADGB	-0.947381882	0.413696872	-2.290038784	0.022019068	0.032968329	negative
PRJNA729271	stage_E18_vs_E10	ordinary_muscle_development	ENSGALG00010005392	ENSGALG00010005392	0.463769477	0.108437406	4.276840379	1.89565E-05	4.28063E-05	positive
PRJNA729271	stage_E18_vs_E10	ordinary_muscle_development	ENSGALG00010005394	PHYH	2.247876563	0.135620426	16.57476407	1.06078E-61	4.46979E-60	positive
PRJNA729271	stage_E18_vs_E10	ordinary_muscle_development	ENSGALG00010005395	ENSGALG00010005395	-1.163275565	0.30077053	-3.867651413	0.000109889	0.000228233	negative
PRJNA729271	stage_E18_vs_E10	ordinary_muscle_development	ENSGALG00010005396	MICU2	1.151986002	0.122353788	9.415205058	4.7216E-21	3.56828E-20	positive
PRJNA729271	stage_E18_vs_E10	ordinary_muscle_development	ENSGALG00010005398	SEPHS1	0.440582986	0.08508734	5.178008674	2.24267E-07	6.15206E-07	positive
PRJNA729271	stage_E18_vs_E10	ordinary_muscle_development	ENSGALG00010005399	NDNF	-4.027574817	0.238061521	-16.91821008	3.30317E-64	1.51998E-62	negative
PRJNA729271	stage_E18_vs_E10	ordinary_muscle_development	ENSGALG00010005401	BCLAF1	-0.888493544	0.086767638	-10.23991851	1.31347E-24	1.21653E-23	negative
PRJNA729271	stage_E18_vs_E10	ordinary_muscle_development	ENSGALG00010005402	ABCC4	-1.198793632	0.177486248	-6.754290245	1.43536E-11	5.58689E-11	negative
PRJNA729271	stage_E18_vs_E10	ordinary_muscle_development	ENSGALG00010005403	ENSGALG00010005403	-0.133206553	0.14045149	-0.948416806	0.342917307	0.401397961	negative
PRJNA729271	stage_E18_vs_E10	ordinary_muscle_development	ENSGALG00010005404	BEND7	1.114805184	0.187616554	5.941934028	2.81679E-09	9.14431E-09	positive
PRJNA729271	stage_E18_vs_E10	ordinary_muscle_development	ENSGALG00010005405	CEBPD	2.454334366	0.475820178	5.158113257	2.49451E-07	6.82095E-07	positive
PRJNA729271	stage_E18_vs_E10	ordinary_muscle_development	ENSGALG00010005407	DPH6	-0.725328175	0.122421478	-5.924844114	3.12594E-09	1.01027E-08	negative
PRJNA729271	stage_E18_vs_E10	ordinary_muscle_development	ENSGALG00010005408	APBB1IP	0.031748786	0.208684672	0.152137606	0.879078404	0.89987581	positive
PRJNA729271	stage_E18_vs_E10	ordinary_muscle_development	ENSGALG00010005409	PRKCQ	0.272415048	0.218984945	1.243989847	0.2135033	0.263807887	positive
PRJNA729271	stage_E18_vs_E10	ordinary_muscle_development	ENSGALG00010005411	STXBP5	0.267157693	0.098623254	2.708871213	0.006751254	0.010948694	positive
PRJNA729271	stage_E18_vs_E10	ordinary_muscle_development	ENSGALG00010005412	MTFR2	-1.727718067	0.165660357	-10.42927893	1.82266E-25	1.77909E-24	negative
PRJNA729271	stage_E18_vs_E10	ordinary_muscle_development	ENSGALG00010005413	GAD2	-0.217984574	0.251168491	-0.867881848	0.385459017	0.445836934	negative
PRJNA729271	stage_E18_vs_E10	ordinary_muscle_development	ENSGALG00010005414	ENSGALG00010005414	-0.580360326	0.217112176	-2.673089726	0.007515615	0.012107005	negative
PRJNA729271	stage_E18_vs_E10	ordinary_muscle_development	ENSGALG00010005415	PRKDC	-0.69038522	0.088179421	-7.829323622	4.90502E-15	2.50699E-14	negative
PRJNA729271	stage_E18_vs_E10	ordinary_muscle_development	ENSGALG00010005416	PPP3CA	0.2119661	0.162578883	1.303773873	0.192310693	0.240055118	positive
PRJNA729271	stage_E18_vs_E10	ordinary_muscle_development	ENSGALG00010005417	LOXL3	0.171980093	0.165835049	1.03705516	0.299710151	0.356704051	positive
PRJNA729271	stage_E18_vs_E10	ordinary_muscle_development	ENSGALG00010005420	DHRS4	0.713506375	0.117652474	6.064525022	1.32344E-09	4.41401E-09	positive
PRJNA729271	stage_E18_vs_E10	ordinary_muscle_development	ENSGALG00010005421	ENSGALG00010005421	1.1886659	0.331696327	3.58359681	0.000338895	0.000658307	positive
PRJNA729271	stage_E18_vs_E10	ordinary_muscle_development	ENSGALG00010005423	ST3GAL6	0.809921024	0.150077992	5.396667509	6.789E-08	1.94942E-07	positive
PRJNA729271	stage_E18_vs_E10	ordinary_muscle_development	ENSGALG00010005424	ENSGALG00010005424	-0.151258585	0.145406834	-1.040243985	0.298226561	0.355171638	negative
PRJNA729271	stage_E18_vs_E10	ordinary_muscle_development	ENSGALG00010005425	ELAPOR2	-1.40704127	0.259570397	-5.420653842	5.93814E-08	1.71256E-07	negative
PRJNA729271	stage_E18_vs_E10	ordinary_muscle_development	ENSGALG00010005426	ENSGALG00010005426	1.44152988	1.009464675	1.42801419	0.153287774	0.195779918	positive
PRJNA729271	stage_E18_vs_E10	ordinary_muscle_development	ENSGALG00010005427	ENSGALG00010005427	0.121009378	0.145142903	0.833725767	0.404435535	0.464413616	positive
PRJNA729271	stage_E18_vs_E10	ordinary_muscle_development	ENSGALG00010005428	ZNF770	0.151457514	0.102887842	1.472064247	0.141003529	0.181578372	positive
PRJNA729271	stage_E18_vs_E10	ordinary_muscle_development	ENSGALG00010005429	ZDHHC20	-0.996473576	0.117455497	-8.483839409	2.17882E-17	1.30558E-16	negative
PRJNA729271	stage_E18_vs_E10	ordinary_muscle_development	ENSGALG00010005430	MCM4	-1.570016756	0.135513701	-11.58566806	4.87154E-31	6.29349E-30	negative
PRJNA729271	stage_E18_vs_E10	ordinary_muscle_development	ENSGALG00010005431	GRM3	-6.495070327	1.035005469	-6.27539711	3.48743E-10	1.2222E-09	negative
PRJNA729271	stage_E18_vs_E10	ordinary_muscle_development	ENSGALG00010005433	GPC5	-0.297673908	0.139329455	-2.136475077	0.03264071	0.047374296	negative
PRJNA729271	stage_E18_vs_E10	ordinary_muscle_development	ENSGALG00010005434	PRDM5	-0.045792703	0.218927818	-0.209168041	0.83431706	0.862522344	negative
PRJNA729271	stage_E18_vs_E10	ordinary_muscle_development	ENSGALG00010005435	MAD2L1	-1.622983808	0.169793954	-9.558548859	1.19419E-21	9.35161E-21	negative
PRJNA729271	stage_E18_vs_E10	ordinary_muscle_development	ENSGALG00010005436	AQR	-1.25141053	0.056455985	-22.16612712	7.2921E-109	1.0786E-106	negative
PRJNA729271	stage_E18_vs_E10	ordinary_muscle_development	ENSGALG00010005437	PDE7B	2.51650631	0.158102951	15.91688384	4.83884E-57	1.7491E-55	positive
PRJNA729271	stage_E18_vs_E10	ordinary_muscle_development	ENSGALG00010005438	GPR180	-0.577117265	0.140805368	-4.098687955	4.15499E-05	9.02406E-05	negative
PRJNA729271	stage_E18_vs_E10	ordinary_muscle_development	ENSGALG00010005439	TBX19	-0.280127638	0.596327685	-0.46975454	0.638530397	0.687834282	negative
PRJNA729271	stage_E18_vs_E10	ordinary_muscle_development	ENSGALG00010005440	ENSGALG00010005440	2.081729354	0.518651477	4.013734557	5.97656E-05	0.000127811	positive
PRJNA729271	stage_E18_vs_E10	ordinary_muscle_development	ENSGALG00010005441	UBE2V2	-0.545266569	0.096774682	-5.634392803	1.75676E-08	5.30948E-08	negative
PRJNA729271	stage_E18_vs_E10	ordinary_muscle_development	ENSGALG00010005442	SASH1	0.372886251	0.097754134	3.814531802	0.000136441	0.000279852	positive
PRJNA729271	stage_E18_vs_E10	ordinary_muscle_development	ENSGALG00010005443	BANK1	-0.613294181	0.449020105	-1.365850158	0.171986	0.217135882	negative
PRJNA729271	stage_E18_vs_E10	ordinary_muscle_development	ENSGALG00010005444	ACTC1	1.249874665	0.426552284	2.930179284	0.003387665	0.005737813	positive
PRJNA729271	stage_E18_vs_E10	ordinary_muscle_development	ENSGALG00010005445	SFT2D2	-1.262703307	0.106142418	-11.89631183	1.23694E-32	1.69146E-31	negative
PRJNA729271	stage_E18_vs_E10	ordinary_muscle_development	ENSGALG00010005446	ENSGALG00010005446	1.028762244	0.403137745	2.551887675	0.010714105	0.016887881	positive

PRJNA729271	stage_E18_vs_E10	ordinary_muscle_development	ENSGALG00010005447	MYO3A	-1.071005671	0.323260752	-3.313132402	0.000922573	0.001688258	negative
PRJNA729271	stage_E18_vs_E10	ordinary_muscle_development	ENSGALG00010005448	ENSGALG00010005448	-0.026598466	0.148004032	-0.179714465	0.857376737	0.882020994	negative
PRJNA729271	stage_E18_vs_E10	ordinary_muscle_development	ENSGALG00010005449	TIPRL	-0.250591672	0.086827152	-2.886098015	0.003900507	0.006557393	negative
PRJNA729271	stage_E18_vs_E10	ordinary_muscle_development	ENSGALG00010005450	ENSGALG00010005450	1.779221604	0.449915259	3.954570488	7.66723E-05	0.000161841	positive
PRJNA729271	stage_E18_vs_E10	ordinary_muscle_development	ENSGALG00010005453	UST	-0.196666712	0.114895586	-1.711699447	0.086952071	0.116592994	negative
PRJNA729271	stage_E18_vs_E10	ordinary_muscle_development	ENSGALG00010005454	GPR161	-0.847510834	0.096894173	-8.746767784	2.19551E-18	1.40938E-17	negative
PRJNA729271	stage_E18_vs_E10	ordinary_muscle_development	ENSGALG00010005455	ENSGALG00010005455	1.153739875	0.721538224	1.599000354	0.109820524	0.144595194	positive
PRJNA729271	stage_E18_vs_E10	ordinary_muscle_development	ENSGALG00010005456	RPL7L1	-0.700190178	0.070848212	-9.882961862	4.93522E-23	4.14702E-22	negative
PRJNA729271	stage_E18_vs_E10	ordinary_muscle_development	ENSGALG00010005457	SEMA3D	0.531999973	0.159520063	3.335003532	0.000852983	0.001569831	positive
PRJNA729271	stage_E18_vs_E10	ordinary_muscle_development	ENSGALG00010005458	PRKD1	-0.015490806	0.175309271	-0.088362733	0.92958838	0.942396389	negative
PRJNA729271	stage_E18_vs_E10	ordinary_muscle_development	ENSGALG00010005459	ZC3H12D	2.177965473	0.551271692	3.950802306	7.78896E-05	0.000164196	positive
PRJNA729271	stage_E18_vs_E10	ordinary_muscle_development	ENSGALG00010005460	TGDS	-0.874316941	0.10232373	-8.544615649	1.28965E-17	7.86099E-17	negative
PRJNA729271	stage_E18_vs_E10	ordinary_muscle_development	ENSGALG00010005462	AHI1	0.59735469	0.150874591	3.959279603	7.51762E-05	0.000158822	positive
PRJNA729271	stage_E18_vs_E10	ordinary_muscle_development	ENSGALG00010005463	SKA3	-1.800964689	0.248533907	-7.246354081	4.2814E-13	1.88514E-12	negative
PRJNA729271	stage_E18_vs_E10	ordinary_muscle_development	ENSGALG00010005464	SLC39A8	-3.142593415	0.30025117	-10.46654844	1.23048E-25	1.21003E-24	negative
PRJNA729271	stage_E18_vs_E10	ordinary_muscle_development	ENSGALG00010005465	ENSGALG00010005465	-1.287910286	0.094215861	-13.66978198	1.53855E-42	3.27479E-41	negative
PRJNA729271	stage_E18_vs_E10	ordinary_muscle_development	ENSGALG00010005466	LATS2	0.295475003	0.090040954	3.281562329	0.001032337	0.001876565	positive
PRJNA729271	stage_E18_vs_E10	ordinary_muscle_development	ENSGALG00010005467	ENSGALG00010005467	1.373031339	0.266659751	5.149001048	2.61877E-07	7.14592E-07	positive
PRJNA729271	stage_E18_vs_E10	ordinary_muscle_development	ENSGALG00010005468	PPIL4	-0.413880831	0.105228842	-3.933150122	8.38398E-05	0.000176252	negative
PRJNA729271	stage_E18_vs_E10	ordinary_muscle_development	ENSGALG00010005470	SEMA3A	-1.149458812	0.141185619	-8.141472338	3.905E-16	2.15139E-15	negative
PRJNA729271	stage_E18_vs_E10	ordinary_muscle_development	ENSGALG00010005472	PFKP	-1.332568112	0.115568014	-11.53059624	9.25009E-31	1.17305E-29	negative
PRJNA729271	stage_E18_vs_E10	ordinary_muscle_development	ENSGALG00010005473	ENSGALG00010005473	0.893367648	0.767235292	1.164398532	0.244262542	0.297478201	positive
PRJNA729271	stage_E18_vs_E10	ordinary_muscle_development	ENSGALG00010005474	COL8A1	-0.210124172	0.345774941	-0.607690572	0.543392718	0.598289209	negative
PRJNA729271	stage_E18_vs_E10	ordinary_muscle_development	ENSGALG00010005475	TMEM181	-0.371704384	0.092529867	-4.017128713	5.89116E-05	0.000126059	negative
PRJNA729271	stage_E18_vs_E10	ordinary_muscle_development	ENSGALG00010005476	NFKB1	0.285841688	0.117433107	2.434080943	0.014929656	0.023058643	positive
PRJNA729271	stage_E18_vs_E10	ordinary_muscle_development	ENSGALG00010005477	TMEM161B	-0.319663615	0.163199318	-1.958731313	0.050144261	0.070396229	negative
PRJNA729271	stage_E18_vs_E10	ordinary_muscle_development	ENSGALG00010005479	ENSGALG00010005479	1.558064594	0.604127633	2.579032159	0.009907756	0.015681691	positive
PRJNA729271	stage_E18_vs_E10	ordinary_muscle_development	ENSGALG00010005481	ENSGALG00010005481	-0.542895866	0.551200654	-0.984933277	0.324656847	0.382655797	negative
PRJNA729271	stage_E18_vs_E10	ordinary_muscle_development	ENSGALG00010005482	XPO4	-1.139136832	0.075649402	-15.05810753	3.05405E-51	9.30009E-50	negative
PRJNA729271	stage_E18_vs_E10	ordinary_muscle_development	ENSGALG00010005484	GINM1	0.97845032	0.122864146	7.96367656	1.67001E-15	8.81532E-15	positive
PRJNA729271	stage_E18_vs_E10	ordinary_muscle_development	ENSGALG00010005485	PITRM1	-1.020282894	0.098416074	-10.3670351	3.50223E-25	3.3486E-24	negative
PRJNA729271	stage_E18_vs_E10	ordinary_muscle_development	ENSGALG00010005486	IRF5	0.744753749	0.333616414	2.232365428	0.025590821	0.037866369	positive
PRJNA729271	stage_E18_vs_E10	ordinary_muscle_development	ENSGALG00010005487	ENSGALG00010005487	-1.260268509	0.212060638	-5.942962926	2.79916E-09	9.09115E-09	negative
PRJNA729271	stage_E18_vs_E10	ordinary_muscle_development	ENSGALG00010005489	G2E3	-1.097934286	0.086210665	-12.73548099	3.75525E-37	6.38128E-36	negative
PRJNA729271	stage_E18_vs_E10	ordinary_muscle_development	ENSGALG00010005490	DCBLD2	0.390397263	0.114805998	3.400495358	0.000672639	0.001256107	positive
PRJNA729271	stage_E18_vs_E10	ordinary_muscle_development	ENSGALG00010005491	GGACT	-0.038426359	0.176780415	-0.217367738	0.827921772	0.856949877	negative
PRJNA729271	stage_E18_vs_E10	ordinary_muscle_development	ENSGALG00010005492	TNPO3	-0.498504299	0.097658554	-5.104563587	3.31559E-07	8.98307E-07	negative
PRJNA729271	stage_E18_vs_E10	ordinary_muscle_development	ENSGALG00010005493	KLF6	0.640370986	0.181209421	3.533872483	0.000409519	0.000787682	positive
PRJNA729271	stage_E18_vs_E10	ordinary_muscle_development	ENSGALG00010005494	PPDPFL	4.870693812	0.433289685	11.24119494	2.55895E-29	3.02298E-28	positive
PRJNA729271	stage_E18_vs_E10	ordinary_muscle_development	ENSGALG00010005495	TXN	-0.983410151	0.146623773	-6.707030737	1.98624E-11	7.62867E-11	negative
PRJNA729271	stage_E18_vs_E10	ordinary_muscle_development	ENSGALG00010005497	MKX	-1.431042773	0.201790166	-7.091736917	1.32439E-12	5.61646E-12	negative
PRJNA729271	stage_E18_vs_E10	ordinary_muscle_development	ENSGALG00010005498	SMO	0.252770458	0.137189496	1.842491334	0.065403313	0.089825755	positive
PRJNA729271	stage_E18_vs_E10	ordinary_muscle_development	ENSGALG00010005499	PDE5A	0.225293509	0.112459373	2.003332432	0.045141619	0.063855544	positive
PRJNA729271	stage_E18_vs_E10	ordinary_muscle_development	ENSGALG00010005500	KATNA1	0.496006211	0.129636198	3.826139748	0.000130168	0.000267706	positive
PRJNA729271	stage_E18_vs_E10	ordinary_muscle_development	ENSGALG00010005502	SCFD1	-0.405730431	0.072231664	-5.617071658	1.94221E-08	5.85287E-08	negative
PRJNA729271	stage_E18_vs_E10	ordinary_muscle_development	ENSGALG00010005503	DYNLT1	-1.360374332	0.148616747	-9.153573631	5.50806E-20	3.86257E-19	negative
PRJNA729271	stage_E18_vs_E10	ordinary_muscle_development	ENSGALG00010005504	LATS1	0.00946915	0.07938788	0.119277024	0.905055886	0.921590428	positive
PRJNA729271	stage_E18_vs_E10	ordinary_muscle_development	ENSGALG00010005505	FABP2	-3.51588386	1.252539662	-2.807004015	0.005000462	0.008291203	negative
PRJNA729271	stage_E18_vs_E10	ordinary_muscle_development	ENSGALG00010005507	USP53	0.386874213	0.121369759	3.187566788	0.001434753	0.002565599	positive
PRJNA729271	stage_E18_vs_E10	ordinary_muscle_development	ENSGALG00010005508	SS18	-0.779810778	0.064643964	-12.06316457	1.65307E-33	2.34914E-32	negative
PRJNA729271	stage_E18_vs_E10	ordinary_muscle_development	ENSGALG00010005509	COCH	1.765113569	0.446595177	3.952375837	7.73791E-05	0.000163286	positive
PRJNA729271	stage_E18_vs_E10	ordinary_muscle_development	ENSGALG00010005510	MYOZ2	1.568888712	0.317413029	4.942735709	7.70339E-07	2.01553E-06	positive
PRJNA729271	stage_E18_vs_E10	ordinary_muscle_development	ENSGALG00010005511	AHCYL2	0.012903116	0.100562268	0.12830972	0.897903873	0.915332769	positive
PRJNA729271	stage_E18_vs_E10	ordinary_muscle_development	ENSGALG00010005512	SMARCAD1	-1.552390294	0.11133277	-13.94369598	3.43672E-44	7.83259E-43	negative
PRJNA729271	stage_E18_vs_E10	ordinary_muscle_development	ENSGALG00010005513	TMEM30CP	-0.101679346	0.266326568	-0.381784465	0.702621243	0.747431008	negative
PRJNA729271	stage_E18_vs_E10	ordinary_muscle_development	ENSGALG00010005514	EEF1AKMT1	0.180699338	0.14408057	1.254154792	0.209785748	0.259701462	positive
PRJNA729271	stage_E18_vs_E10	ordinary_muscle_development	ENSGALG00010005515	PCCA	0.328112981	0.096492801	3.400388188	0.000672902	0.001256437	positive
PRJNA729271	stage_E18_vs_E10	ordinary_muscle_development	ENSGALG00010005516	LARP4B	1.210295835	0.131636601	9.194219711	3.77726E-20	2.67341E-19	positive
PRJNA729271	stage_E18_vs_E10	ordinary_muscle_development	ENSGALG00010005518	HPGDS	-1.705379641	0.657455754	-2.593907851	0.009489192	0.015050425	negative
PRJNA729271	stage_E18_vs_E10	ordinary_muscle_development	ENSGALG00010005519	PTBP3	-2.05625106	0.267030867	-7.700424598	1.35615E-14	6.69985E-14	negative
PRJNA729271	stage_E18_vs_E10	ordinary_muscle_development	ENSGALG00010005521	SYTL3	0.462172485	0.422879798	1.092916917	0.27443028	0.329812363	positive
PRJNA729271	stage_E18_vs_E10	ordinary_muscle_development	ENSGALG00010005522	SYNPO2	1.571680733	0.17825418	8.817076453	1.17487E-18	7.6607E-18	positive
PRJNA729271	stage_E18_vs_E10	ordinary_muscle_development	ENSGALG00010005523	ENSGALG00010005523	0.567148073	0.74869691	0.757513575	0.448742236	0.508284654	positive
PRJNA729271	stage_E18_vs_E10	ordinary_muscle_development	ENSGALG00010005524	STRIP2	2.291815248	0.273969501	8.365220349	6.00027E-17	3.49574E-16	positive
PRJNA729271	stage_E18_vs_E10	ordinary_muscle_development	ENSGALG00010005526	STRN3	-0.09851951	0.070300269	-1.401410136	0.161091463	0.204754538	negative
PRJNA729271	stage_E18_vs_E10	ordinary_muscle_development	ENSGALG00010005527	NUP43	-0.735406384	0.096221419	-7.642855343	2.12457E-14	1.02995E-13	negative
PRJNA729271	stage_E18_vs_E10	ordinary_muscle_development	ENSGALG00010005529	ENSGALG00010005529	1.133165028	0.379657232	2.984705494	0.002838515	0.004881275	positive
PRJNA729271	stage_E18_vs_E10	ordinary_muscle_development	ENSGALG00010005530	IL17D	1.113987208	0.210946011	5.280911477	1.28543E-07	3.60671E-07	positive
PRJNA729271	stage_E18_vs_E10	ordinary_muscle_development	ENSGALG00010005531	SUSD1	-0.329971389	0.256985549	-1.284007569	0.199139307	0.247876707	negative
PRJNA729271	stage_E18_vs_E10	ordinary_muscle_development	ENSGALG00010005532	GTPBP4	-0.114437786	0.097802123	-1.170095112	0.241962695	0.294999518	negative
PRJNA729271	stage_E18_vs_E10	ordinary_muscle_development	ENSGALG00010005533	OPN4-1	-3.940499469	1.038439984	-3.794633806	0.000147862	0.000301654	negative
PRJNA729271	stage_E18_vs_E10	ordinary_muscle_development	ENSGALG00010005534	ENSGALG00010005534	-1.950289279	0.167382716	-11.65167663	2.24988E-31	2.94865E-30	negative
PRJNA729271	stage_E18_vs_E10	ordinary_muscle_development	ENSGALG00010005535	AP4S1	-0.191684447	0.161952179	-1.18358671	0.236576704	0.289065103	negative
PRJNA729271	stage_E18_vs_E10	ordinary_muscle_development	ENSGALG00010005536	TAF4B	-1.956049789	0.219142261	-8.925935948	4.41946E-19	2.95753E-18	negative
PRJNA729271	stage_E18_vs_E10	ordinary_muscle_development	ENSGALG00010005537	GRIN3A	-2.21910161	0.302488483	-7.336152393	2.19822E-13	9.84951E-13	negative
PRJNA729271	stage_E18_vs_E10	ordinary_muscle_development	ENSGALG00010005538	ENSGALG00010005538	1.743958436	0.329145626	5.298440255	1.16796E-07	3.28794E-07	positive
PRJNA729271	stage_E18_vs_E10	ordinary_muscle_development	ENSGALG00010005539	IFT88	-0.5845246	0.132897231	-4.398320368	1.09092E-05	2.53006E-05	negative
PRJNA729271	stage_E18_vs_E10	ordinary_muscle_development	ENSGALG00010005540	PCMT1	0.672415972	0.137453098	4.891966645	9.98334E-07	2.57947E-06	positive
PRJNA729271	stage_E18_vs_E10	ordinary_muscle_development	ENSGALG00010005542	KCTD1	-0.019688523	0.134795595	-0.146062065	0.883872393	0.904208507	negative
PRJNA729271	stage_E18_vs_E10	ordinary_muscle_development	ENSGALG00010005543	SEC24D	0.527295233	0.201716922	2.614035684	0.008947969	0.014259104	positive
PRJNA729271	stage_E18_vs_E10	ordinary_muscle_development	ENSGALG00010005544	AQP4	8.061249901	0.308691694	26.11424301	2.5123E-150	8.2762E-148	positive
PRJNA729271	stage_E18_vs_E10	ordinary_muscle_development	ENSGALG00010005545	RPL34	1.078541403	0.166901699	6.462135547	1.03236E-10	3.75602E-10	positive
PRJNA729271	stage_E18_vs_E10	ordinary_muscle_development	ENSGALG00010005546	NRF1	0.210257113	0.155491069	1.352213437	0.176307046	0.222011175	positive
PRJNA729271	stage_E18_vs_E10	ordinary_muscle_development	ENSGALG00010005547	LRP11	1.136267395	0.215039334	5.283997929	1.26395E-07	3.54988E-07	positive
PRJNA729271	stage_E18_vs_E10	ordinary_muscle_development	ENSGALG00010005548	EZR	-0.488369853	0.179119938	-2.726496322	0.006401067	0.010429795	negative
PRJNA729271	stage_E18_vs_E10	ordinary_muscle_development	ENSGALG00010005549	RNF20	-0.374664523	0.113861067	-3.290541123	0.000999949	0.001821344	negative
PRJNA729271	stage_E18_vs_E10	ordinary_muscle_development	ENSGALG00010005550	ENSGALG00010005550	2.028642436	0.561990566	3.609744648	0.000306499	0.000599554	positive
PRJNA729271	stage_E18_vs_E10	ordinary_muscle_development	ENSGALG00010005551	METTL14	-1.394802377	0.084552441	-16.49629942	3.90077E-61	1.59722E-59	negative
PRJNA729271	stage_E18_vs_E10	ordinary_muscle_development	ENSGALG00010005552	WDR37	0.062916272	0.087346174	0.720309429	0.4713345	0.530187303	positive
PRJNA729271	stage_E18_vs_E10	ordinary_muscle_development	ENSGALG00010005553	PPP1R14C	1.351454233	0.163430349	8.269297847	1.3475E-16	7.67468E-16	positive
PRJNA729271	stage_E18_vs_E10	ordinary_muscle_development	ENSGALG00010005554	CRYL1	-0.013762888	0.164189404	-0.083823244	0.933196967	0.945591754	negative
PRJNA729271	stage_E18_vs_E10	ordinary_muscle_development	ENSGALG00010005555	PRSS12	-2.664114394	0.159002518	-16.75517114	5.19113E-63	2.32958E-61	negative
PRJNA729271	stage_E18_vs_E10	ordinary_muscle_development	ENSGALG00010005556	IYD	-0.86320582	0.790482712	-1.091998353	0.274833825	0.330242584	negative
PRJNA729271	stage_E18_vs_E10	ordinary_muscle_development	ENSGALG00010005557	CMSS1	-1.126625297	0.171893492	-6.55420567	5.59388E-11	2.0828E-10	negative
PRJNA729271	stage_E18_vs_E10	ordinary_muscle_development	ENSGALG00010005558	TAGAP	0.992121364	0.215852969	4.596283151	4.30094E-06	1.03679E-05	positive
PRJNA729271	stage_E18_vs_E10	ordinary_muscle_development	ENSGALG00010005559	FILIP1L	0.547695209	0.130834811	4.186158153	2.83716E-05	6.28048E-05	positive
PRJNA729271	stage_E18_vs_E10	ordinary_muscle_development	ENSGALG00010005560	RSPH3	-0.394349801	0.369371401	-1.067624077	0.285690118	0.341504063	negative
PRJNA729271	stage_E18_vs_E10	ordinary_muscle_development	ENSGALG00010005561	CLYBL	0.974335756	0.198614882	4.905653322	9.31169E-07	2.41541E-06	positive
PRJNA729271	stage_E18_vs_E10	ordinary_muscle_development	ENSGALG00010005562	HECTD1	-0.157451386	0.085689272	-1.837469068	0.066140679	0.090795448	negative
PRJNA729271	stage_E18_vs_E10	ordinary_muscle_development	ENSGALG00010005564	TM9SF2	0.071426042	0.058162913	1.228034119	0.21943416	0.270237736	positive
PRJNA729271	stage_E18_vs_E10	ordinary_muscle_development	ENSGALG00010005565	PGAP4	-0.491606973	0.176500297	-2.785303945	0.005347757	0.008826661	negative
PRJNA729271	stage_E18_vs_E10	ordinary_muscle_development	ENSGALG00010005567	NDST3	-0.009981063	0.237141993	-0.042088972	0.966427771	0.972373158	negative
PRJNA729271	stage_E18_vs_E10	ordinary_muscle_development	ENSGALG00010005568	PLEKHG1	1.099284921	0.13167314	8.348588968	6.90833E-17	4.00545E-16	positive
PRJNA729271	stage_E18_vs_E10	ordinary_muscle_development	ENSGALG00010005572	ENSGALG00010005572	0.956311669	0.155835225	6.136684877	8.42613E-10	2.86706E-09	positive
PRJNA729271	stage_E18_vs_E10	ordinary_muscle_development	ENSGALG00010005573	CHST9	0.791832346	0.282443234	2.803509698	0.005054971	0.008373921	positive
PRJNA729271	stage_E18_vs_E10	ordinary_muscle_development	ENSGALG00010005575	ENSGALG00010005575	2.603660913	0.449659025	5.790300583	7.02606E-09	2.19963E-08	positive
PRJNA729271	stage_E18_vs_E10	ordinary_muscle_development	ENSGALG00010005578	HEATR5A	0.314405297	0.120484785	2.609502087	0.009067409	0.014439914	positive
PRJNA729271	stage_E18_vs_E10	ordinary_muscle_development	ENSGALG00010005581	GR42L5	1.391755873	0.835553324	1.665669722	0.095779216	0.127520872	positive
PRJNA729271	stage_E18_vs_E10	ordinary_muscle_development	ENSGALG00010005583	ALDOB	1.568267055	1.010570471	1.551863132			positive
PRJNA729271	stage_E18_vs_E10	ordinary_muscle_development	ENSGALG00010005587	LEF1	-4.5639688	0.215164933	-21.21148993	7.48059E-100	8.60565E-98	negative
PRJNA729271	stage_E18_vs_E10	ordinary_muscle_development	ENSGALG00010005588	PLAGL1	0.345593037	0.126943603	2.722413964	0.006480691	0.010547677	positive
PRJNA729271	stage_E18_vs_E10	ordinary_muscle_development	ENSGALG00010005590	TBC1D23	-0.003309958	0.057027816	-0.058041117	0.953715877	0.961800323	negative
PRJNA729271	stage_E18_vs_E10	ordinary_muscle_development	ENSGALG00010005593	ENSGALG00010005593	1.073923981	1.181611019	0.908864223	0.363421801	0.422878623	positive
PRJNA729271	stage_E18_vs_E10	ordinary_muscle_development	ENSGALG00010005594	MRPL50	0.191552446	0.15102201	1.268374362	0.204664293	0.253968746	positive
PRJNA729271	stage_E18_vs_E10	ordinary_muscle_development	ENSGALG00010005596	PARD3	0.101081079	0.053823125	1.878023215	0.060377989	0.083485544	positive
PRJNA729271	stage_E18_vs_E10	ordinary_muscle_development	ENSGALG00010005597	ENSGALG00010005597	1.803016794	0.493021088	3.657078446	0.000255106	0.000505021	positive
PRJNA729271	stage_E18_vs_E10	ordinary_muscle_development	ENSGALG00010005601	TMEM215	-1.410665966	0.395734832	-3.564674759	0.000364308	0.000705025	negative
PRJNA729271	stage_E18_vs_E10	ordinary_muscle_development	ENSGALG00010005609	GJB6	-4.615544957	0.599579659	-7.697967875	1.38247E-14	6.81828E-14	negative
PRJNA729271	stage_E18_vs_E10	ordinary_muscle_development	ENSGALG00010005610	MTHFD1L	-0.98993903	0.191029085	-5.182137746	2.19357E-07	6.02193E-07	negative
PRJNA729271	stage_E18_vs_E10	ordinary_muscle_development	ENSGALG00010005617	DTD2	1.686472707	0.208057335	8.105807493	5.23963E-16	2.86706E-15	positive
PRJNA729271	stage_E18_vs_E10	ordinary_muscle_development	ENSGALG00010005618	PHACTR2	-0.126753834	0.068210363	-1.858278238	0.063129513	0.086941785	negative
PRJNA729271	stage_E18_vs_E10	ordinary_muscle_development	ENSGALG00010005619	CELF2	0.536548984	0.077040704	6.964487064	3.29602E-12	1.35534E-11	positive
PRJNA729271	stage_E18_vs_E10	ordinary_muscle_development	ENSGALG00010005621	CDH2	-1.060541004	0.140494568	-7.548626401	4.39873E-14	2.07551E-13	negative
PRJNA729271	stage_E18_vs_E10	ordinary_muscle_development	ENSGALG00010005629	NRP1	0.952430725	0.173202976	5.498928185	3.82107E-08	1.12574E-07	positive
PRJNA729271	stage_E18_vs_E10	ordinary_muscle_development	ENSGALG00010005630	NIT2	0.784433946	0.163584751	4.795275483	1.62451E-06	4.10089E-06	positive
PRJNA729271	stage_E18_vs_E10	ordinary_muscle_development	ENSGALG00010005632	ITGB1	0.364105185	0.09345598	3.896007332	9.77914E-05	0.000204131	positive
PRJNA729271	stage_E18_vs_E10	ordinary_muscle_development	ENSGALG00010005634	HADH	0.372557852	0.08708521	4.278084098	1.88509E-05	4.25878E-05	positive
PRJNA729271	stage_E18_vs_E10	ordinary_muscle_development	ENSGALG00010005636	ENSGALG00010005636	2.11465347	0.606493067	3.48669026	0.000489037	0.000930628	positive
PRJNA729271	stage_E18_vs_E10	ordinary_muscle_development	ENSGALG00010005637	TOPORS	-0.240957186	0.072560849	-3.320760306	0.000897726	0.001645953	negative
PRJNA729271	stage_E18_vs_E10	ordinary_muscle_development	ENSGALG00010005643	ENSGALG00010005643	-2.757577118	1.266068633	-2.1780629	0.029401354	0.043017324	negative
PRJNA729271	stage_E18_vs_E10	ordinary_muscle_development	ENSGALG00010005645	FUCA2	0.234358728	0.157568052	1.487349274	0.136922587	0.176810058	positive

PRJNA729271	stage_E18_vs_E10	ordinary_muscle_development	ENSGALG00010005649	TOMM70	0.474537345	0.078523845	6.043226028	1.51063E-09	5.01984E-09	positive
PRJNA729271	stage_E18_vs_E10	ordinary_muscle_development	ENSGALG00010005651	USP6NL	0.067896128	0.121437198	0.559104865	0.576090155	0.62917622	positive
PRJNA729271	stage_E18_vs_E10	ordinary_muscle_development	ENSGALG00010005656	CYP2U1	0.243510085	0.136182056	1.788121668	0.07375638	0.100375433	positive
PRJNA729271	stage_E18_vs_E10	ordinary_muscle_development	ENSGALG00010005660	UBAC2	0.476764632	0.101872679	4.680004862	2.86868E-06	7.04772E-06	positive
PRJNA729271	stage_E18_vs_E10	ordinary_muscle_development	ENSGALG00010005668	LNP1	1.633694796	0.289595644	5.641296167	1.68775E-08	5.11369E-08	positive
PRJNA729271	stage_E18_vs_E10	ordinary_muscle_development	ENSGALG00010005670	SGMS2	-1.070588969	0.208839146	-5.126380714	2.95365E-07	8.03249E-07	negative
PRJNA729271	stage_E18_vs_E10	ordinary_muscle_development	ENSGALG00010005675	GJA3	-0.624240756	0.296608021	-2.104598367	0.035326284	0.050940558	negative
PRJNA729271	stage_E18_vs_E10	ordinary_muscle_development	ENSGALG00010005679	TMEM45A	-0.859794269	0.204607085	-4.202172525	2.64366E-05	5.87276E-05	negative
PRJNA729271	stage_E18_vs_E10	ordinary_muscle_development	ENSGALG00010005680	ENSGALG00010005680	2.000957903	0.465908671	4.294742789	1.74896E-05	3.96918E-05	positive
PRJNA729271	stage_E18_vs_E10	ordinary_muscle_development	ENSGALG00010005681	EPC1	0.401188167	0.066163944	6.063546721	1.33152E-09	4.43993E-09	positive
PRJNA729271	stage_E18_vs_E10	ordinary_muscle_development	ENSGALG00010005688	ENSGALG00010005688	1.284579139	0.547951114	2.344331649	0.019061214	0.028840531	positive
PRJNA729271	stage_E18_vs_E10	ordinary_muscle_development	ENSGALG00010005692	SEC24B	0.100548168	0.144715162	0.694800503	0.487180333	0.545219188	positive
PRJNA729271	stage_E18_vs_E10	ordinary_muscle_development	ENSGALG00010005694	ENSGALG00010005694	-0.274990542	0.075426203	-3.645822398	0.000266538	0.000526286	negative
PRJNA729271	stage_E18_vs_E10	ordinary_muscle_development	ENSGALG00010005695	KIF5B	-0.801534152	0.084132041	-9.527097384	1.61742E-21	1.25506E-20	negative
PRJNA729271	stage_E18_vs_E10	ordinary_muscle_development	ENSGALG00010005696	NUBPL	-0.906181621	0.142107133	-6.376749707	1.80886E-10	6.46753E-10	negative
PRJNA729271	stage_E18_vs_E10	ordinary_muscle_development	ENSGALG00010005699	PRPF18	0.284175666	0.093799882	3.029595135	0.002448818	0.004243856	positive
PRJNA729271	stage_E18_vs_E10	ordinary_muscle_development	ENSGALG00010005704	AKAP12	-0.203841974	0.093617943	-2.17738146	0.029452118	0.043078552	negative
PRJNA729271	stage_E18_vs_E10	ordinary_muscle_development	ENSGALG00010005705	ECHDC3	0.5293221	0.125655984	4.212470297	2.52593E-05	5.6259E-05	positive
PRJNA729271	stage_E18_vs_E10	ordinary_muscle_development	ENSGALG00010005712	ENSGALG00010005712	0.158491834	0.111263892	1.424467826	0.154311092	0.196878733	positive
PRJNA729271	stage_E18_vs_E10	ordinary_muscle_development	ENSGALG00010005714	ARHGAP5	-0.476631841	0.068231788	-6.985480742	2.83882E-12	1.173E-11	negative
PRJNA729271	stage_E18_vs_E10	ordinary_muscle_development	ENSGALG00010005716	ZBTB2	-0.71139878	0.113243308	-6.282038143	3.34163E-10	1.17224E-09	negative
PRJNA729271	stage_E18_vs_E10	ordinary_muscle_development	ENSGALG00010005720	COL25A1	-1.195884331	0.181705843	-6.58143023	4.65944E-11	1.74428E-10	negative
PRJNA729271	stage_E18_vs_E10	ordinary_muscle_development	ENSGALG00010005724	PROSER2	-0.237136016	0.12328351	-1.923501498	0.054417099	0.075894914	negative
PRJNA729271	stage_E18_vs_E10	ordinary_muscle_development	ENSGALG00010005738	ELAVL2	-1.710873512	0.346675722	-4.935083144	8.01166E-07	2.09355E-06	negative
PRJNA729271	stage_E18_vs_E10	ordinary_muscle_development	ENSGALG00010005740	ENSGALG00010005740	1.611878002	0.332711486	4.844671953	1.26821E-06	3.24496E-06	positive
PRJNA729271	stage_E18_vs_E10	ordinary_muscle_development	ENSGALG00010005742	GPR183	0.153887839	0.208734216	0.737242995	0.460974591	0.520310442	positive
PRJNA729271	stage_E18_vs_E10	ordinary_muscle_development	ENSGALG00010005743	ETNPPL	5.540357357	0.223329927	24.80794868	7.3584E-136	1.7777E-133	positive
PRJNA729271	stage_E18_vs_E10	ordinary_muscle_development	ENSGALG00010005748	AIG1	0.807935523	0.179062432	4.512032572	6.42093E-06	1.52227E-05	positive
PRJNA729271	stage_E18_vs_E10	ordinary_muscle_development	ENSGALG00010005752	RMND1	0.029344587	0.099659993	0.294447012	0.768416348	0.805656055	positive
PRJNA729271	stage_E18_vs_E10	ordinary_muscle_development	ENSGALG00010005755	ZMYM2	-0.207888167	0.098037341	-2.120499858	0.033963913	0.049122623	negative
PRJNA729271	stage_E18_vs_E10	ordinary_muscle_development	ENSGALG00010005756	TET2	0.526088895	0.164098687	3.205929951	0.001346268	0.002415715	positive
PRJNA729271	stage_E18_vs_E10	ordinary_muscle_development	ENSGALG00010005757	ARHGAP12	0.794088802	0.133322408	5.956154056	2.58243E-09	8.40609E-09	positive
PRJNA729271	stage_E18_vs_E10	ordinary_muscle_development	ENSGALG00010005758	HIVEP2	0.512714684	0.106174408	4.828985548	1.3723E-06	3.49526E-06	positive
PRJNA729271	stage_E18_vs_E10	ordinary_muscle_development	ENSGALG00010005760	GPR18	-0.722111121	0.32885767	-2.195816572	0.028105075	0.04127488	negative
PRJNA729271	stage_E18_vs_E10	ordinary_muscle_development	ENSGALG00010005763	AKAP6	2.268703731	0.142531585	15.91719992	4.81446E-57	1.74464E-55	positive
PRJNA729271	stage_E18_vs_E10	ordinary_muscle_development	ENSGALG00010005768	CXXC4	-3.753425657	0.71429507	-5.254727096	1.48244E-07	4.12279E-07	negative
PRJNA729271	stage_E18_vs_E10	ordinary_muscle_development	ENSGALG00010005769	TFG	0.146210321	0.105274438	1.388849217	0.164878605	0.208963486	positive
PRJNA729271	stage_E18_vs_E10	ordinary_muscle_development	ENSGALG00010005772	SVEP1	2.047892228	0.14076114	14.54870443	5.95196E-48	1.60063E-46	positive
PRJNA729271	stage_E18_vs_E10	ordinary_muscle_development	ENSGALG00010005777	ENSGALG00010005777	1.549199049	0.93800519	1.651588994	0.098618365	0.130952107	positive
PRJNA729271	stage_E18_vs_E10	ordinary_muscle_development	ENSGALG00010005779	ARMT1	-0.482887287	0.113045718	-4.271610601	1.94066E-05	4.37751E-05	negative
PRJNA729271	stage_E18_vs_E10	ordinary_muscle_development	ENSGALG00010005784	RASL11A	-1.390383041	0.48200059	-2.884608586	0.003919006	0.006585438	negative
PRJNA729271	stage_E18_vs_E10	ordinary_muscle_development	ENSGALG00010005785	RPL21	0.617879863	0.139807121	4.419516395	9.8922E-06	2.30415E-05	positive
PRJNA729271	stage_E18_vs_E10	ordinary_muscle_development	ENSGALG00010005789	RNASET2	0.432810652	0.155316814	2.786631023	0.005325908	0.008792601	positive
PRJNA729271	stage_E18_vs_E10	ordinary_muscle_development	ENSGALG00010005790	MUSK	1.8243234	0.146282713	12.47121658	1.07177E-35	1.69971E-34	positive
PRJNA729271	stage_E18_vs_E10	ordinary_muscle_development	ENSGALG00010005792	ADGRG6	-1.505513499	0.160909543	-9.356272272	8.25995E-21	6.14305E-20	negative
PRJNA729271	stage_E18_vs_E10	ordinary_muscle_development	ENSGALG00010005799	UFSP2	-0.593238871	0.099167336	-5.982200316	2.20143E-09	7.21288E-09	negative
PRJNA729271	stage_E18_vs_E10	ordinary_muscle_development	ENSGALG00010005800	FRMD4A	-0.189489626	0.108608925	-1.744696633	0.08103769	0.109462428	negative
PRJNA729271	stage_E18_vs_E10	ordinary_muscle_development	ENSGALG00010005814	UPF2	-0.455500749	0.12471262	-3.652403009	0.000259798	0.000513607	negative
PRJNA729271	stage_E18_vs_E10	ordinary_muscle_development	ENSGALG00010005817	VTA1	-0.233823845	0.070678001	-3.308297386	0.000938651	0.001715731	negative
PRJNA729271	stage_E18_vs_E10	ordinary_muscle_development	ENSGALG00010005821	DOCK9	1.0607427	0.153992946	6.888255136	5.64808E-12	2.27604E-11	positive
PRJNA729271	stage_E18_vs_E10	ordinary_muscle_development	ENSGALG00010005824	NMBR	-5.203212394	1.332630883	-3.904466314	9.44335E-05	0.000197435	negative
PRJNA729271	stage_E18_vs_E10	ordinary_muscle_development	ENSGALG00010005826	LRP2BP	1.007283892	0.201510148	4.998675754	5.77254E-07	1.5266E-06	positive
PRJNA729271	stage_E18_vs_E10	ordinary_muscle_development	ENSGALG00010005828	LPAR1	-1.44748975	0.154810295	-9.350087144	8.75755E-21	6.50645E-20	negative
PRJNA729271	stage_E18_vs_E10	ordinary_muscle_development	ENSGALG00010005829	NPAS3	0.177371711	0.133717814	1.32646284	0.184686436	0.231637094	positive
PRJNA729271	stage_E18_vs_E10	ordinary_muscle_development	ENSGALG00010005830	USP12	-0.397493428	0.188434265	-2.109454072	0.034905406	0.050368727	negative
PRJNA729271	stage_E18_vs_E10	ordinary_muscle_development	ENSGALG00010005831	SEC61A2	-0.13471624	0.106032214	-1.270521803	0.203898822	0.253257362	negative
PRJNA729271	stage_E18_vs_E10	ordinary_muscle_development	ENSGALG00010005836	NEBL	-0.084814474	0.246456073	-0.344136271	0.730743806	0.772415334	negative
PRJNA729271	stage_E18_vs_E10	ordinary_muscle_development	ENSGALG00010005839	RPS6KA2	0.242317197	0.182558884	1.327337196	0.184397165	0.231354359	positive
PRJNA729271	stage_E18_vs_E10	ordinary_muscle_development	ENSGALG00010005841	ENSGALG00010005841	1.248474558	0.314856568	3.965216811	7.33293E-05	0.000155192	positive
PRJNA729271	stage_E18_vs_E10	ordinary_muscle_development	ENSGALG00010005843	GPR12	0.855861535	0.434477901	1.969862067	0.048854181	0.068700925	positive
PRJNA729271	stage_E18_vs_E10	ordinary_muscle_development	ENSGALG00010005848	WASF3	0.448826042	0.193121431	2.324061289	0.020122209	0.030328733	positive
PRJNA729271	stage_E18_vs_E10	ordinary_muscle_development	ENSGALG00010005849	SNX25	-0.062197788	0.098724185	-0.630015717	0.528684301	0.58484919	negative
PRJNA729271	stage_E18_vs_E10	ordinary_muscle_development	ENSGALG00010005851	SLC15A1	-2.238336742	1.123089286	-1.993017626	0.046259517	0.065322133	negative
PRJNA729271	stage_E18_vs_E10	ordinary_muscle_development	ENSGALG00010005853	COMMD3	-0.671118741	0.142109973	-4.722530925	2.32928E-06	5.78428E-06	negative
PRJNA729271	stage_E18_vs_E10	ordinary_muscle_development	ENSGALG00010005858	CFAP97	-0.572571816	0.112590556	-5.085433763	3.66787E-07	9.88946E-07	negative
PRJNA729271	stage_E18_vs_E10	ordinary_muscle_development	ENSGALG00010005859	ENSGALG00010005859	1.064296968	0.547618476	1.943500839	0.051955674	0.072706845	positive
PRJNA729271	stage_E18_vs_E10	ordinary_muscle_development	ENSGALG00010005860	ABI3BP	0.92558894	0.212351671	4.358755155	1.30804E-05	3.00906E-05	positive
PRJNA729271	stage_E18_vs_E10	ordinary_muscle_development	ENSGALG00010005861	MPC1L	0.711927563	0.132063097	5.390813788	7.01393E-08	2.01161E-07	positive
PRJNA729271	stage_E18_vs_E10	ordinary_muscle_development	ENSGALG00010005866	DNAJC1	-0.15477505	0.149980027	-1.031971077	0.302085667	0.359236339	negative
PRJNA729271	stage_E18_vs_E10	ordinary_muscle_development	ENSGALG00010005871	EGLN3	3.606020035	0.194817625	18.50972182	1.72389E-76	1.08642E-74	positive
PRJNA729271	stage_E18_vs_E10	ordinary_muscle_development	ENSGALG00010005872	STK24	0.079216925	0.136181886	0.581699426	0.560769158	0.614666436	positive
PRJNA729271	stage_E18_vs_E10	ordinary_muscle_development	ENSGALG00010005875	CDK8	-1.332980457	0.155936974	-8.548200109	1.25023E-17	7.63677E-17	negative
PRJNA729271	stage_E18_vs_E10	ordinary_muscle_development	ENSGALG00010005879	FAM107B	-1.177597397	0.122646373	-9.601567219	7.8738E-22	6.24347E-21	negative
PRJNA729271	stage_E18_vs_E10	ordinary_muscle_development	ENSGALG00010005880	SLC25A4	1.111167198	0.180199016	6.166333329	6.98917E-10	2.40066E-09	positive
PRJNA729271	stage_E18_vs_E10	ordinary_muscle_development	ENSGALG00010005881	CORO2A	-0.340160234	0.285451705	-1.191655991	0.233396161	0.285515854	negative
PRJNA729271	stage_E18_vs_E10	ordinary_muscle_development	ENSGALG00010005884	MYB	-2.022810337	0.292019737	-6.92696444	4.29967E-12	1.75411E-11	negative
PRJNA729271	stage_E18_vs_E10	ordinary_muscle_development	ENSGALG00010005885	SLITRK5	2.314730603	0.334013252	6.930056189	4.20673E-12	1.71668E-11	positive
PRJNA729271	stage_E18_vs_E10	ordinary_muscle_development	ENSGALG00010005887	CCNH	-0.545975644	0.086878376	-6.284367489	3.29191E-10	1.15536E-09	negative
PRJNA729271	stage_E18_vs_E10	ordinary_muscle_development	ENSGALG00010005888	IMPG2	0.483725648	0.227683598	2.124552016	0.033624011	0.048699045	positive
PRJNA729271	stage_E18_vs_E10	ordinary_muscle_development	ENSGALG00010005890	SNX32	-0.204331536	0.089282867	-2.288586185	0.022103407	0.033074109	negative
PRJNA729271	stage_E18_vs_E10	ordinary_muscle_development	ENSGALG00010005891	RNF6	-0.026280944	0.118239754	-0.222268254	0.824105063	0.85373091	negative
PRJNA729271	stage_E18_vs_E10	ordinary_muscle_development	ENSGALG00010005892	ENSGALG00010005892	0.385232331	0.882203523	0.436670588	0.662350263	0.710010875	positive
PRJNA729271	stage_E18_vs_E10	ordinary_muscle_development	ENSGALG00010005893	SFT2D1	0.1442424	0.067156387	2.147858239	0.031725019	0.046160826	positive
PRJNA729271	stage_E18_vs_E10	ordinary_muscle_development	ENSGALG00010005895	MLLT10	0.219455034	0.100097199	2.192419339	0.028349241	0.041616595	positive
PRJNA729271	stage_E18_vs_E10	ordinary_muscle_development	ENSGALG00010005899	ENSGALG00010005899	-0.552303484	0.088699784	-6.226660961	4.76481E-10	1.65745E-09	negative
PRJNA729271	stage_E18_vs_E10	ordinary_muscle_development	ENSGALG00010005900	SPTSSA	0.012309138	0.087225407	0.141118726	0.887776151	0.907305599	positive
PRJNA729271	stage_E18_vs_E10	ordinary_muscle_development	ENSGALG00010005901	ENSGALG00010005901	1.264466589	0.237949389	5.314014853	1.07236E-07	3.03294E-07	positive
PRJNA729271	stage_E18_vs_E10	ordinary_muscle_development	ENSGALG00010005902	EAPP	-0.372190995	0.102023398	-3.648094466	0.000264193	0.00052194	negative
PRJNA729271	stage_E18_vs_E10	ordinary_muscle_development	ENSGALG00010005903	PRR18	-1.373696276	0.267091936	-5.14315894	2.70157E-07	7.3663E-07	negative
PRJNA729271	stage_E18_vs_E10	ordinary_muscle_development	ENSGALG00010005905	CITED2	-0.187258479	0.203334425	-0.920938396	0.357082591	0.41647185	negative
PRJNA729271	stage_E18_vs_E10	ordinary_muscle_development	ENSGALG00010005909	BRMS1L	-0.699055584	0.102968373	-6.789032039	1.12888E-11	4.42248E-11	negative
PRJNA729271	stage_E18_vs_E10	ordinary_muscle_development	ENSGALG00010005910	ENSGALG00010005910	1.392458192	0.46358134	3.003697671	0.002667202	0.004599839	positive
PRJNA729271	stage_E18_vs_E10	ordinary_muscle_development	ENSGALG00010005911	TBXT	-1.191935063	0.428039498	-2.784638029	0.005358751	0.008842793	negative
PRJNA729271	stage_E18_vs_E10	ordinary_muscle_development	ENSGALG00010005913	NFKBIA	2.869620067	0.142332014	20.16145194	2.13524E-90	1.99679E-88	positive
PRJNA729271	stage_E18_vs_E10	ordinary_muscle_development	ENSGALG00010005915	PAPSS1	-1.957773367	0.100707375	-19.44021838	3.52661E-84	2.74829E-82	negative
PRJNA729271	stage_E18_vs_E10	ordinary_muscle_development	ENSGALG00010005926	NUDT5	0.60211788	0.229737781	2.620891857	0.008770008	0.014000139	positive
PRJNA729271	stage_E18_vs_E10	ordinary_muscle_development	ENSGALG00010005934	SLITRK6	-3.414524697	0.27586781	-12.37739444	3.46407E-35	5.37599E-34	negative
PRJNA729271	stage_E18_vs_E10	ordinary_muscle_development	ENSGALG00010005935	ATP8A2	0.524361242	0.162774509	3.221396558	0.001275675	0.002295297	positive
PRJNA729271	stage_E18_vs_E10	ordinary_muscle_development	ENSGALG00010005941	TXLNB	4.755978446	0.181429575	26.2139094	1.8447E-151	6.3663E-149	positive
PRJNA729271	stage_E18_vs_E10	ordinary_muscle_development	ENSGALG00010005943	PROX2	-4.379014482	1.046262583	-4.185387638	2.8468E-05	6.29894E-05	negative
PRJNA729271	stage_E18_vs_E10	ordinary_muscle_development	ENSGALG00010005949	SLITRK1	1.368533886	0.342456738	3.996224144	6.43608E-05	0.000137115	positive
PRJNA729271	stage_E18_vs_E10	ordinary_muscle_development	ENSGALG00010005956	TXNL4B	0.107719831	0.112771985	0.95520027	0.33947643	0.397826085	positive
PRJNA729271	stage_E18_vs_E10	ordinary_muscle_development	ENSGALG00010005963	UGCG	1.163953749	0.103715021	11.22261501	3.15792E-29	3.7034E-28	positive
PRJNA729271	stage_E18_vs_E10	ordinary_muscle_development	ENSGALG00010005964	HSPA14	-0.913145252	0.116282674	-7.852805776	4.06831E-15	2.09263E-14	negative
PRJNA729271	stage_E18_vs_E10	ordinary_muscle_development	ENSGALG00010005966	PLXDC2	0.683580165	0.153703642	4.447390813	8.69196E-06	2.03702E-05	positive
PRJNA729271	stage_E18_vs_E10	ordinary_muscle_development	ENSGALG00010005967	GJD2	-2.722000878	0.284540176	-9.56631474	1.10784E-21	8.71307E-21	negative
PRJNA729271	stage_E18_vs_E10	ordinary_muscle_development	ENSGALG00010005969	HECA	0.207482679	0.066372129	3.126051266	0.001771707	0.003136789	positive
PRJNA729271	stage_E18_vs_E10	ordinary_muscle_development	ENSGALG00010005970	SHISA2	0.930602948	0.118555718	7.849498654	4.17704E-15	2.14703E-14	positive
PRJNA729271	stage_E18_vs_E10	ordinary_muscle_development	ENSGALG00010005975	ABRACL	-0.836075792	0.109328013	-7.647406814	2.05073E-14	9.95489E-14	negative
PRJNA729271	stage_E18_vs_E10	ordinary_muscle_development	ENSGALG00010005978	VPS16	0.303056275	0.114652921	2.6432495	0.00821145	0.013150477	positive
PRJNA729271	stage_E18_vs_E10	ordinary_muscle_development	ENSGALG00010005979	SUV39H2	-0.235031749	0.09347415	-2.514403691	0.011923386	0.018662076	negative
PRJNA729271	stage_E18_vs_E10	ordinary_muscle_development	ENSGALG00010005981	AREL1	-0.199576437	0.101547511	-1.965350349	0.049373702	0.069393187	negative
PRJNA729271	stage_E18_vs_E10	ordinary_muscle_development	ENSGALG00010005984	IDH3B	1.340453963	0.117862683	11.3730142	5.69851E-30	6.94701E-29	positive
PRJNA729271	stage_E18_vs_E10	ordinary_muscle_development	ENSGALG00010005985	ENSGALG00010005985	-3.912182053	0.324500189	-12.05602397	1.80278E-33	2.55688E-32	negative
PRJNA729271	stage_E18_vs_E10	ordinary_muscle_development	ENSGALG00010005988	NUP58	-0.419500848	0.09024449	-4.648492661	3.3437E-06	8.15391E-06	negative
PRJNA729271	stage_E18_vs_E10	ordinary_muscle_development	ENSGALG00010005989	FCF1	-0.546320794	0.102100995	-5.350788172	8.7572E-08	2.49333E-07	negative
PRJNA729271	stage_E18_vs_E10	ordinary_muscle_development	ENSGALG00010005990	SLC2A12	1.291590615	0.15257487	8.465290591	2.55515E-17	1.52352E-16	positive
PRJNA729271	stage_E18_vs_E10	ordinary_muscle_development	ENSGALG00010005991	FARP1	-0.844443861	0.090320146	-9.349451956	8.8103E-21	6.53893E-20	negative
PRJNA729271	stage_E18_vs_E10	ordinary_muscle_development	ENSGALG00010005997	ENSGALG00010005997	1.63377476	0.231451533	7.058820227	1.67922E-12	7.0654E-12	positive
PRJNA729271	stage_E18_vs_E10	ordinary_muscle_development	ENSGALG00010005998	YLPM1	-0.079709219	0.133921569	-0.595193286	0.551714278	0.605840792	negative
PRJNA729271	stage_E18_vs_E10	ordinary_muscle_development	ENSGALG00010005999	PDE10A	-0.332475846	0.294198385	-1.130107652	0.258430866	0.312446855	negative
PRJNA729271	stage_E18_vs_E10	ordinary_muscle_development	ENSGALG00010006001	NOP56	-0.649098642	0.140444585	-4.621742044	3.80531E-06	9.22374E-06	negative
PRJNA729271	stage_E18_vs_E10	ordinary_muscle_development	ENSGALG00010006004	DCLRE1C	-0.739060155	0.14949202	-4.943810086	7.66103E-07	2.00518E-06	negative
PRJNA729271	stage_E18_vs_E10	ordinary_muscle_development	ENSGALG00010006009	MALRD1	-0.082967723	0.682186313	-0.121620328	0.903199714	0.919955017	negative
PRJNA729271	stage_E18_vs_E10	ordinary_muscle_development	ENSGALG00010006010	IPO5	-0.906645473	0.081157575	-11.17142145	5.6273E-29	6.52542E-28	negative
PRJNA729271	stage_E18_vs_E10	ordinary_muscle_development	ENSGALG00010006013	TRMT10C	-0.55315901	0.107788774	-5.131879615	2.86863E-07	7.80567E-07	negative
PRJNA729271	stage_E18_vs_E10	ordinary_muscle_development	ENSGALG00010006015	CDC123	-0.61918272	0.11276611	-5.490858184	3.99985E-08	1.17602E-07	negative
PRJNA729271	stage_E18_vs_E10	ordinary_muscle_development	ENSGALG00010006016	MTMR6	-0.19703743	0.077925935	-2.528521846	0.011454396	0.017976556	negative
PRJNA729271	stage_E18_vs_E10	ordinary_muscle_development	ENSGALG00010006019	SLC10A2	-2.783097377	1.109548319	-2.508315617	0.012130826	0.01895605	negative
PRJNA729271	stage_E18_vs_E10	ordinary_muscle_development	ENSGALG00010006021	ENSGALG00010006021	0.068551337	0.12614621	0.543427637	0.586835402	0.639171177	positive
PRJNA729271	stage_E18_vs_E10	ordinary_muscle_development	ENSGALG00010006022	AFG2A	-0.899635799	0.092922064	-9.681616667	3.60962E-22	2.90675E-21	negative

PRJNA729271	stage_E18_vs_E10	ordinary_muscle_development	ENSGALG00010006024	AMER2	-3.385134143	0.483123034	-7.006774473	2.43875E-12	1.0123E-11	negative
PRJNA729271	stage_E18_vs_E10	ordinary_muscle_development	ENSGALG00010006025	TNFAIP3	0.536971606	0.15159646	3.542111782	0.000396937	0.000764497	positive
PRJNA729271	stage_E18_vs_E10	ordinary_muscle_development	ENSGALG00010006028	ARL5B	-0.322437669	0.12845093	-2.510201119	0.012066242	0.018861229	negative
PRJNA729271	stage_E18_vs_E10	ordinary_muscle_development	ENSGALG00010006029	HSDL2	0.401261789	0.134710804	2.978690469	0.00289483	0.00497222	positive
PRJNA729271	stage_E18_vs_E10	ordinary_muscle_development	ENSGALG00010006031	ERCC5	-0.035298429	0.09905441	-0.356353941	0.721575518	0.76422893	negative
PRJNA729271	stage_E18_vs_E10	ordinary_muscle_development	ENSGALG00010006033	NSUN6	-0.943878353	0.186771768	-5.053645747	4.33455E-07	1.15964E-06	negative
PRJNA729271	stage_E18_vs_E10	ordinary_muscle_development	ENSGALG00010006035	ADH1C	1.33453119	0.695867768	1.917794231	0.055137108	0.076832568	positive
PRJNA729271	stage_E18_vs_E10	ordinary_muscle_development	ENSGALG00010006037	METTL21EP	1.537047432	0.772616931	1.989404284	0.046656597	0.065825129	positive
PRJNA729271	stage_E18_vs_E10	ordinary_muscle_development	ENSGALG00010006038	ENSGALG00010006038	-0.437715527	0.206268714	-2.122064551	0.033832318	0.048956718	negative
PRJNA729271	stage_E18_vs_E10	ordinary_muscle_development	ENSGALG00010006039	PCNP	-0.535651435	0.125865141	-4.255756843	2.08343E-05	4.68645E-05	negative
PRJNA729271	stage_E18_vs_E10	ordinary_muscle_development	ENSGALG00010006041	QKI	0.335677595	0.08586748	3.909251721	9.25825E-05	0.000193816	positive
PRJNA729271	stage_E18_vs_E10	ordinary_muscle_development	ENSGALG00010006047	ZBTB11	-0.44384137	0.136595683	-3.249307457	0.001156864	0.002091909	negative
PRJNA729271	stage_E18_vs_E10	ordinary_muscle_development	ENSGALG00010006049	AIMP1	-0.612695003	0.124874958	-4.906468134	9.2731E-07	2.40695E-06	negative
PRJNA729271	stage_E18_vs_E10	ordinary_muscle_development	ENSGALG00010006052	RALGAPA1	-0.362311788	0.081845598	-4.426771827	9.56538E-06	2.23161E-05	negative
PRJNA729271	stage_E18_vs_E10	ordinary_muscle_development	ENSGALG00010006053	TMEM243	-0.275318237	0.234858984	-1.172270412	0.241088507	0.294106877	negative
PRJNA729271	stage_E18_vs_E10	ordinary_muscle_development	ENSGALG00010006054	ENSGALG00010006054	0.79934591	2.296061289	0.34813788	0.727736637	0.769910412	positive
PRJNA729271	stage_E18_vs_E10	ordinary_muscle_development	ENSGALG00010006055	ENSGALG00010006055	1.447972714	0.610650094	2.37119871	0.017730496	0.026981999	positive
PRJNA729271	stage_E18_vs_E10	ordinary_muscle_development	ENSGALG00010006059	BIVM	-0.503164171	0.056732822	-8.869013672	7.37966E-19	4.87105E-18	negative
PRJNA729271	stage_E18_vs_E10	ordinary_muscle_development	ENSGALG00010006060	ENSGALG00010006060	-0.042613709	0.407653812	-0.104534064	0.916745538	0.93120018	negative
PRJNA729271	stage_E18_vs_E10	ordinary_muscle_development	ENSGALG00010006061	RPL24	0.952890613	0.159002527	5.992927469	2.06097E-09	6.76795E-09	positive
PRJNA729271	stage_E18_vs_E10	ordinary_muscle_development	ENSGALG00010006062	ENSGALG00010006062	1.365501417	0.654297314	2.086973901	0.036890494	0.053001061	positive
PRJNA729271	stage_E18_vs_E10	ordinary_muscle_development	ENSGALG00010006063	POGLUT2	-0.747405046	0.101761997	-7.344638168	2.06316E-13	9.25869E-13	negative
PRJNA729271	stage_E18_vs_E10	ordinary_muscle_development	ENSGALG00010006064	DMTF1	-0.049011741	0.083002568	-0.590484636	0.554865783	0.608978536	negative
PRJNA729271	stage_E18_vs_E10	ordinary_muscle_development	ENSGALG00010006065	WDR54	-0.223147738	0.16729671	-1.333844156	0.182254936	0.228864706	negative
PRJNA729271	stage_E18_vs_E10	ordinary_muscle_development	ENSGALG00010006066	ARHGAP18	0.037537672	0.120861344	0.310584596	0.756116439	0.795291182	positive
PRJNA729271	stage_E18_vs_E10	ordinary_muscle_development	ENSGALG00010006068	PACRG	2.993010698	0.255503832	11.71415189	1.07865E-31	1.43178E-30	positive
PRJNA729271	stage_E18_vs_E10	ordinary_muscle_development	ENSGALG00010006069	TTR	6.140383764	1.389007928	4.42069742	9.83829E-06	2.29196E-05	positive
PRJNA729271	stage_E18_vs_E10	ordinary_muscle_development	ENSGALG00010006070	TMEM242	0.674716989	0.114286683	5.903723596	3.55387E-09	1.14474E-08	positive
PRJNA729271	stage_E18_vs_E10	ordinary_muscle_development	ENSGALG00010006071	TEX30	0.167082519	0.240813979	0.693824004	0.487792587	0.545693722	positive
PRJNA729271	stage_E18_vs_E10	ordinary_muscle_development	ENSGALG00010006072	ENSGALG00010006072	0.136432505	0.233614127	0.584007938	0.559214971	0.613241111	positive
PRJNA729271	stage_E18_vs_E10	ordinary_muscle_development	ENSGALG00010006073	CEP97	-0.608950945	0.109787659	-5.546624738	2.91237E-08	8.66478E-08	negative
PRJNA729271	stage_E18_vs_E10	ordinary_muscle_development	ENSGALG00010006075	SOX21	-4.159961464	0.412738323	-10.07893193	6.84663E-24	6.04765E-23	negative
PRJNA729271	stage_E18_vs_E10	ordinary_muscle_development	ENSGALG00010006076	PODXL	1.781877216	0.164669692	10.82091791	2.74019E-27	2.9378E-26	positive
PRJNA729271	stage_E18_vs_E10	ordinary_muscle_development	ENSGALG00010006077	TBCK	-0.5069609	0.101223095	-5.008352114	5.4898E-07	1.45501E-06	negative
PRJNA729271	stage_E18_vs_E10	ordinary_muscle_development	ENSGALG00010006078	SPATA13	-1.521553047	0.166983986	-9.111969849	8.08995E-20	5.62687E-19	negative
PRJNA729271	stage_E18_vs_E10	ordinary_muscle_development	ENSGALG00010006079	ENSGALG00010006079	0.447890681	0.791772812	0.565680804	0.571610787	0.625131911	positive
PRJNA729271	stage_E18_vs_E10	ordinary_muscle_development	ENSGALG00010006080	ENSGALG00010006080	-0.233740669	0.206492874	-1.131955135	0.257653297	0.311639231	negative
PRJNA729271	stage_E18_vs_E10	ordinary_muscle_development	ENSGALG00010006081	ZDHHC14	-0.081605136	0.080108259	-1.018685682	0.308352212	0.365907926	negative
PRJNA729271	stage_E18_vs_E10	ordinary_muscle_development	ENSGALG00010006083	CACNB2	0.936930875	0.163785803	5.720464525	1.06233E-08	3.26932E-08	positive
PRJNA729271	stage_E18_vs_E10	ordinary_muscle_development	ENSGALG00010006084	TMEM68	-0.608090596	0.150416168	-4.042720966	5.28345E-05	0.000113508	negative
PRJNA729271	stage_E18_vs_E10	ordinary_muscle_development	ENSGALG00010006085	DLST	0.307215135	0.090392125	3.398693577	0.000677085	0.001263433	positive
PRJNA729271	stage_E18_vs_E10	ordinary_muscle_development	ENSGALG00010006086	ENSGALG00010006086	-0.060684155	0.311639435	-0.194725532	0.845607823	0.872142123	negative
PRJNA729271	stage_E18_vs_E10	ordinary_muscle_development	ENSGALG00010006087	ENSGALG00010006087	-0.433440905	0.595604379	-0.727732904	0.466777104	0.525836179	negative
PRJNA729271	stage_E18_vs_E10	ordinary_muscle_development	ENSGALG00010006091	RTKN	1.002624237	0.100372098	9.989073237	1.70165E-23	1.46556E-22	positive
PRJNA729271	stage_E18_vs_E10	ordinary_muscle_development	ENSGALG00010006096	EDIL3	1.308372007	0.235704905	5.550890035	2.84219E-08	8.46814E-08	positive
PRJNA729271	stage_E18_vs_E10	ordinary_muscle_development	ENSGALG00010006100	RPS6KL1	-0.613428636	0.202162682	-3.034331707	0.002410692	0.004180783	negative
PRJNA729271	stage_E18_vs_E10	ordinary_muscle_development	ENSGALG00010006101	ENSGALG00010006101	0.007545365	0.118360506	0.063749013	0.949170078	0.958093334	positive
PRJNA729271	stage_E18_vs_E10	ordinary_muscle_development	ENSGALG00010006103	NXPE3	-0.35287908	0.120710771	-2.923343769	0.00346294	0.005859147	negative
PRJNA729271	stage_E18_vs_E10	ordinary_muscle_development	ENSGALG00010006106	SHOC1	1.844005394	0.373332403	4.939312464	7.83985E-07	2.05013E-06	positive
PRJNA729271	stage_E18_vs_E10	ordinary_muscle_development	ENSGALG00010006107	PGF	2.558821109	0.192325421	13.30464324	2.17529E-40	4.22099E-39	positive
PRJNA729271	stage_E18_vs_E10	ordinary_muscle_development	ENSGALG00010006108	MKLN1	0.178405585	0.061917408	2.88134777	0.003959785	0.006651649	positive
PRJNA729271	stage_E18_vs_E10	ordinary_muscle_development	ENSGALG00010006111	WBP1	0.011208167	0.084976595	0.131897102	0.895065681	0.913017385	positive
PRJNA729271	stage_E18_vs_E10	ordinary_muscle_development	ENSGALG00010006113	EIF2B2	-0.722055664	0.170053711	-4.246044726	2.17577E-05	4.87824E-05	negative
PRJNA729271	stage_E18_vs_E10	ordinary_muscle_development	ENSGALG00010006118	NPNT	-1.822859339	0.208205699	-8.755088592	2.03943E-18	1.31093E-17	negative
PRJNA729271	stage_E18_vs_E10	ordinary_muscle_development	ENSGALG00010006120	MOGS	0.335571455	0.136061328	2.466325	0.013650741	0.021189494	positive
PRJNA729271	stage_E18_vs_E10	ordinary_muscle_development	ENSGALG00010006121	ENSGALG00010006121	0.120977832	0.323708685	0.373724394	0.708609375	0.752861752	positive
PRJNA729271	stage_E18_vs_E10	ordinary_muscle_development	ENSGALG00010006123	MLH3	-0.467727896	0.104729098	-4.46607394	7.96681E-06	1.87466E-05	negative
PRJNA729271	stage_E18_vs_E10	ordinary_muscle_development	ENSGALG00010006126	ENSGALG00010006126	3.000097769	0.284730931	10.53660648	5.85745E-26	5.84334E-25	positive
PRJNA729271	stage_E18_vs_E10	ordinary_muscle_development	ENSGALG00010006128	ACYP1	0.23163066	0.131181442	1.765727349	0.077441602	0.105006176	positive
PRJNA729271	stage_E18_vs_E10	ordinary_muscle_development	ENSGALG00010006131	ENSGALG00010006131	1.817284588	0.124147346	14.63812682	1.60427E-48	4.40416E-47	positive
PRJNA729271	stage_E18_vs_E10	ordinary_muscle_development	ENSGALG00010006132	MIPEP	0.13765782	0.133111354	1.034155365	0.301063537	0.358079591	positive
PRJNA729271	stage_E18_vs_E10	ordinary_muscle_development	ENSGALG00010006134	K123	0.369881619	0.83169633	0.444731576	0.656513748	0.704588092	positive
PRJNA729271	stage_E18_vs_E10	ordinary_muscle_development	ENSGALG00010006135	ZC2HC1C	-0.172766547	0.148726618	-1.161638381	0.245382385	0.298641282	negative
PRJNA729271	stage_E18_vs_E10	ordinary_muscle_development	ENSGALG00010006139	GAREM1	-0.111654014	0.13262316	-0.841889261	0.399849942	0.459767167	negative
PRJNA729271	stage_E18_vs_E10	ordinary_muscle_development	ENSGALG00010006146	CCDC142	0.286388972	0.187416399	1.528089178	0.126490398	0.164747805	positive
PRJNA729271	stage_E18_vs_E10	ordinary_muscle_development	ENSGALG00010006147	ENSGALG00010006147	1.601823106	0.217773409	7.355457742	1.90274E-13	8.55731E-13	positive
PRJNA729271	stage_E18_vs_E10	ordinary_muscle_development	ENSGALG00010006151	ENSGALG00010006151	-0.338213553	0.161446745	-2.094892359	0.036180562	0.052079171	negative
PRJNA729271	stage_E18_vs_E10	ordinary_muscle_development	ENSGALG00010006155	TNFRSF19	0.035832863	0.193057382	0.18560732	0.852752713	0.878323781	positive
PRJNA729271	stage_E18_vs_E10	ordinary_muscle_development	ENSGALG00010006156	NFKBIZ	0.444375558	0.203968767	2.178645113	0.029358042	0.04295829	positive
PRJNA729271	stage_E18_vs_E10	ordinary_muscle_development	ENSGALG00010006157	SNX9	0.452864306	0.07384556	6.132586812	8.64615E-10	2.93985E-09	positive
PRJNA729271	stage_E18_vs_E10	ordinary_muscle_development	ENSGALG00010006163	ENSGALG00010006163	1.223357865	0.514516199	2.377685809	0.017421663	0.026551047	positive
PRJNA729271	stage_E18_vs_E10	ordinary_muscle_development	ENSGALG00010006172	ENSGALG00010006172	0.438342629	0.19396888	2.2598606	0.023829904	0.035452577	positive
PRJNA729271	stage_E18_vs_E10	ordinary_muscle_development	ENSGALG00010006173	TBPL1	-0.528322472	0.085189311	-6.20174603	5.58401E-10	1.93406E-09	negative
PRJNA729271	stage_E18_vs_E10	ordinary_muscle_development	ENSGALG00010006175	IL2RA	2.87227521	0.523241491	5.489387326	4.0333E-08	1.18466E-07	positive
PRJNA729271	stage_E18_vs_E10	ordinary_muscle_development	ENSGALG00010006176	MMR1L3	0.674374226	0.224299149	3.006583969	0.002642011	0.004561756	positive
PRJNA729271	stage_E18_vs_E10	ordinary_muscle_development	ENSGALG00010006177	NEK9	-0.575565447	0.114103244	-5.044251379	4.553E-07	1.21517E-06	negative
PRJNA729271	stage_E18_vs_E10	ordinary_muscle_development	ENSGALG00010006178	RPS23	0.721983543	0.140860096	5.125536338	2.96692E-07	8.06555E-07	positive
PRJNA729271	stage_E18_vs_E10	ordinary_muscle_development	ENSGALG00010006180	CD200L	3.146603888	1.104059962	2.850029887			positive
PRJNA729271	stage_E18_vs_E10	ordinary_muscle_development	ENSGALG00010006183	PRKN	1.716075035	0.13932017	12.31749172	7.29274E-35	1.11624E-33	positive
PRJNA729271	stage_E18_vs_E10	ordinary_muscle_development	ENSGALG00010006184	LBX2	1.023760185	0.27907191	3.668445829	0.000244029	0.00048382	positive
PRJNA729271	stage_E18_vs_E10	ordinary_muscle_development	ENSGALG00010006187	TCF21	0.728778819	0.591277327	1.232549915	0.217743733	0.268475282	positive
PRJNA729271	stage_E18_vs_E10	ordinary_muscle_development	ENSGALG00010006188	TMED10	0.205591524	0.074835369	2.747250737	0.006009717	0.009836365	positive
PRJNA729271	stage_E18_vs_E10	ordinary_muscle_development	ENSGALG00010006193	RBM17	-0.570662473	0.099770364	-5.719759363	1.06675E-08	3.28222E-08	negative
PRJNA729271	stage_E18_vs_E10	ordinary_muscle_development	ENSGALG00010006197	ENSGALG00010006197	2.836020091	0.364151262	7.78802763	6.80633E-15	3.44837E-14	positive
PRJNA729271	stage_E18_vs_E10	ordinary_muscle_development	ENSGALG00010006198	ENSGALG00010006198	1.248434545	0.698167119	1.788160042	0.073750191	0.100375433	positive
PRJNA729271	stage_E18_vs_E10	ordinary_muscle_development	ENSGALG00010006200	GSTCD	-0.984917549	0.101232973	-9.729216915	2.26329E-22	1.84098E-21	negative
PRJNA729271	stage_E18_vs_E10	ordinary_muscle_development	ENSGALG00010006201	SSTR1	-2.96051072	0.387348575	-7.643014348	2.12194E-14	1.02903E-13	negative
PRJNA729271	stage_E18_vs_E10	ordinary_muscle_development	ENSGALG00010006202	SACS	-0.330360973	0.200290541	-1.649408756	0.099063922	0.131471484	negative
PRJNA729271	stage_E18_vs_E10	ordinary_muscle_development	ENSGALG00010006203	MMR1L4	1.773013224	0.175292749	10.11458393	4.76035E-24	4.24362E-23	positive
PRJNA729271	stage_E18_vs_E10	ordinary_muscle_development	ENSGALG00010006205	CGGBP1	-0.431230773	0.074398649	-5.796217791	6.78272E-09	2.12758E-08	negative
PRJNA729271	stage_E18_vs_E10	ordinary_muscle_development	ENSGALG00010006207	ENSGALG00010006207	0.732165825	0.202130591	3.622241546	0.000292061	0.000573014	positive
PRJNA729271	stage_E18_vs_E10	ordinary_muscle_development	ENSGALG00010006212	FOS	2.445354495	0.368154566	6.642195213	3.09045E-11	1.16931E-10	positive
PRJNA729271	stage_E18_vs_E10	ordinary_muscle_development	ENSGALG00010006219	CLEC14A	0.884110146	0.190044967	4.652110291	3.28555E-06	8.01617E-06	positive
PRJNA729271	stage_E18_vs_E10	ordinary_muscle_development	ENSGALG00010006220	SGCG	1.875985374	0.170918817	10.97588738	4.99143E-28	5.5569E-27	positive
PRJNA729271	stage_E18_vs_E10	ordinary_muscle_development	ENSGALG00010006223	ATG10	0.060597646	0.112975574	0.536378293	0.591697131	0.643651025	positive
PRJNA729271	stage_E18_vs_E10	ordinary_muscle_development	ENSGALG00010006227	AGPAT4	-1.381109407	0.148244036	-9.316458513	1.20288E-20	8.82378E-20	negative
PRJNA729271	stage_E18_vs_E10	ordinary_muscle_development	ENSGALG00010006229	INTS12	-0.782212929	0.137588838	-5.685148168	1.30699E-08	3.99428E-08	negative
PRJNA729271	stage_E18_vs_E10	ordinary_muscle_development	ENSGALG00010006232	ZNF654	-0.207595832	0.104388882	-1.988677617	0.046736798	0.065919032	negative
PRJNA729271	stage_E18_vs_E10	ordinary_muscle_development	ENSGALG00010006236	C3orf38	0.106461908	0.087495023	1.216776732	0.223689175	0.274730943	positive
PRJNA729271	stage_E18_vs_E10	ordinary_muscle_development	ENSGALG00010006237	ENSGALG00010006237	1.273809045	0.312996762	4.069719561	4.70698E-05	0.000101635	positive
PRJNA729271	stage_E18_vs_E10	ordinary_muscle_development	ENSGALG00010006239	EYA4	0.76810562	0.162984682	4.712747312	2.44399E-06	6.05567E-06	positive
PRJNA729271	stage_E18_vs_E10	ordinary_muscle_development	ENSGALG00010006241	MRC1	0.974001506	0.246882666	3.945200052	7.97334E-05	0.000167887	positive
PRJNA729271	stage_E18_vs_E10	ordinary_muscle_development	ENSGALG00010006242	ENSGALG00010006242	0.545551935	0.569742538	0.957541168	0.338294178	0.396697201	positive
PRJNA729271	stage_E18_vs_E10	ordinary_muscle_development	ENSGALG00010006244	EPHA3	-0.666007546	0.162038252	-4.11018718	3.95339E-05	8.60684E-05	negative
PRJNA729271	stage_E18_vs_E10	ordinary_muscle_development	ENSGALG00010006245	PFKFB3	-0.379375793	0.510679871	-0.742883781	0.457552046	0.517091603	negative
PRJNA729271	stage_E18_vs_E10	ordinary_muscle_development	ENSGALG00010006246	SUPT20H	-0.246410138	0.100499937	-2.451843719	0.014212637	0.022016905	negative
PRJNA729271	stage_E18_vs_E10	ordinary_muscle_development	ENSGALG00010006249	C9orf72	0.098195902	0.105992325	0.926443511	0.354215541	0.413593867	positive
PRJNA729271	stage_E18_vs_E10	ordinary_muscle_development	ENSGALG00010006251	SEMA3E	-3.86997852	0.385704873	-10.0335225	1.08573E-23	9.45201E-23	negative
PRJNA729271	stage_E18_vs_E10	ordinary_muscle_development	ENSGALG00010006252	COX7C	0.763970765	0.136707879	5.5883448	2.29244E-08	6.86123E-08	positive
PRJNA729271	stage_E18_vs_E10	ordinary_muscle_development	ENSGALG00010006254	PROS1	2.239587783	0.137018031	16.34520481	4.70587E-60	1.89476E-58	positive
PRJNA729271	stage_E18_vs_E10	ordinary_muscle_development	ENSGALG00010006259	PPA2	0.513417732	0.087264188	5.88348719	4.01711E-09	1.28738E-08	positive
PRJNA729271	stage_E18_vs_E10	ordinary_muscle_development	ENSGALG00010006260	MAP3K4	-0.415201131	0.161917611	-2.564274074	0.010339187	0.016328885	negative
PRJNA729271	stage_E18_vs_E10	ordinary_muscle_development	ENSGALG00010006261	PCLO	-1.560262483	0.500206885	-3.119234319	0.001813217	0.003207148	negative
PRJNA729271	stage_E18_vs_E10	ordinary_muscle_development	ENSGALG00010006263	EXOSC8	-0.568341428	0.150057574	-3.787489118	0.000152177	0.000309892	negative
PRJNA729271	stage_E18_vs_E10	ordinary_muscle_development	ENSGALG00010006266	CENPW	-1.490247882	0.219317387	-6.794937256	1.0836E-11	4.24851E-11	negative
PRJNA729271	stage_E18_vs_E10	ordinary_muscle_development	ENSGALG00010006269	ENSGALG00010006269	4.879482678	1.213781623	4.020066368	5.81817E-05	0.000124589	positive
PRJNA729271	stage_E18_vs_E10	ordinary_muscle_development	ENSGALG00010006270	STAM	0.39282817	0.092548097	4.244584001	2.18999E-05	4.90785E-05	positive
PRJNA729271	stage_E18_vs_E10	ordinary_muscle_development	ENSGALG00010006273	ARL13B	-0.309582558	0.099287027	-3.118056469	0.001820479	0.003218815	negative
PRJNA729271	stage_E18_vs_E10	ordinary_muscle_development	ENSGALG00010006277	GNG10	-1.656160076	0.16607809	-9.972176825	2.01758E-23	1.73046E-22	negative
PRJNA729271	stage_E18_vs_E10	ordinary_muscle_development	ENSGALG00010006279	HACD1	3.429685571	0.141390259	24.25687305	5.5953E-130	1.2289E-127	positive
PRJNA729271	stage_E18_vs_E10	ordinary_muscle_development	ENSGALG00010006281	TRMT11	0.223562306	0.08088214	2.764050321	0.005708873	0.009377847	positive
PRJNA729271	stage_E18_vs_E10	ordinary_muscle_development	ENSGALG00010006283	DNAJC25	-0.521406397	0.109878721	-4.745290011	2.08208E-06	5.19266E-06	negative
PRJNA729271	stage_E18_vs_E10	ordinary_muscle_development	ENSGALG00010006285	FZD8	-0.838918726	0.155009525	-5.412046296	6.23086E-08	1.79484E-07	negative
PRJNA729271	stage_E18_vs_E10	ordinary_muscle_development	ENSGALG00010006287	ECPAS	0.14001953	0.122998058	1.138388133	0.254958447	0.308817807	positive
PRJNA729271	stage_E18_vs_E10	ordinary_muscle_development	ENSGALG00010006288	ARF5	-1.972875932	0.17184079	-11.480836	1.6468E-30	2.05957E-29	negative
PRJNA729271	stage_E18_vs_E10	ordinary_muscle_development	ENSGALG00010006289	SKIDA1	-1.479393136	0.147313327	-10.04249356	9.91358E-24	8.66691E-23	negative
PRJNA729271	stage_E18_vs_E10	ordinary_muscle_development	ENSGALG00010006291	ENSGALG00010006291	3.399062408	0.191333141	17.76515236	1.31582E-70	7.1434E-69	positive
PRJNA729271	stage_E18_vs_E10	ordinary_muscle_development	ENSGALG00010006293	SLC22A3	-1.383781117	0.449453578	-3.078807658	0.002078308	0.003641373	negative
PRJNA729271	stage_E18_vs_E10	ordinary_muscle_development	ENSGALG00010006300	GCC1	-0.173451687	0.121164601	-1.431537644	0.152276186	0.194573635	negative
PRJNA729271	stage_E18_vs_E10	ordinary_muscle_development	ENSGALG00010006301	ENSGALG00010006301	0.246570674	0.150372856	1.639728604	0.101061609	0.133840842	positive
PRJNA729271	stage_E18_vs_E10	ordinary_muscle_development	ENSGALG00010006310	ALG5	-0.017473252	0.110338742	-0.15836008	0.874173068	0.896246896	negative

PRJNA729271	stage_E18_vs_E10	ordinary_muscle_development	ENSGALG00010006311	ABCD4	-0.177142817	0.116322743	-1.522856263	0.12779467	0.166197708	negative
PRJNA729271	stage_E18_vs_E10	ordinary_muscle_development	ENSGALG00010006323	ANAPC10	-0.242798929	0.108407692	-2.239683581	0.025111472	0.0372331	negative
PRJNA729271	stage_E18_vs_E10	ordinary_muscle_development	ENSGALG00010006327	DENND6B	0.439762975	0.110642358	3.974634882	7.04873E-05	0.000149504	positive
PRJNA729271	stage_E18_vs_E10	ordinary_muscle_development	ENSGALG00010006328	SYNDIG1L	-2.061041345	0.45308282	-4.548928488	5.39198E-06	1.2895E-05	negative
PRJNA729271	stage_E18_vs_E10	ordinary_muscle_development	ENSGALG00010006330	STX19	1.274612152	0.412049409	3.093347845	0.00197912	0.003476833	positive
PRJNA729271	stage_E18_vs_E10	ordinary_muscle_development	ENSGALG00010006331	SMAD9	-1.585503546	0.163501752	-9.697165484	3.09991E-22	2.50743E-21	negative
PRJNA729271	stage_E18_vs_E10	ordinary_muscle_development	ENSGALG00010006336	ALDH8A1	3.026694431	0.838619564	3.60913883	0.000307215	0.000600794	positive
PRJNA729271	stage_E18_vs_E10	ordinary_muscle_development	ENSGALG00010006339	ENSGALG00010006339	1.869480449	0.754824954	2.476707269	0.013260061	0.020618385	positive
PRJNA729271	stage_E18_vs_E10	ordinary_muscle_development	ENSGALG00010006341	HHIP	-1.133906523	0.221729078	-5.113927916	3.15528E-07	8.56154E-07	negative
PRJNA729271	stage_E18_vs_E10	ordinary_muscle_development	ENSGALG00010006342	RFXAP	-0.35528567	0.085805047	-4.140615085	3.46376E-05	7.58187E-05	negative
PRJNA729271	stage_E18_vs_E10	ordinary_muscle_development	ENSGALG00010006343	SEC23A	-0.066635051	0.094925907	-0.701969077	0.482698448	0.540704328	negative
PRJNA729271	stage_E18_vs_E10	ordinary_muscle_development	ENSGALG00010006344	HINT3	0.195910891	0.106195289	1.844817157	0.065064141	0.089402287	positive
PRJNA729271	stage_E18_vs_E10	ordinary_muscle_development	ENSGALG00010006345	SMC2	-1.834603399	0.249868785	-7.342267271	2.10005E-13	9.42132E-13	negative
PRJNA729271	stage_E18_vs_E10	ordinary_muscle_development	ENSGALG00010006346	NSUN3	0.305134492	0.131261602	2.324628737	0.020091821	0.03028608	positive
PRJNA729271	stage_E18_vs_E10	ordinary_muscle_development	ENSGALG00010006347	PPP6R2	0.947044123	0.135986278	6.964262386	3.30129E-12	1.35712E-11	positive
PRJNA729271	stage_E18_vs_E10	ordinary_muscle_development	ENSGALG00010006351	IGF2R	0.194307046	0.1744591	1.113768477	0.265378524	0.320181597	positive
PRJNA729271	stage_E18_vs_E10	ordinary_muscle_development	ENSGALG00010006352	GEMIN2	-0.442888932	0.09983722	-4.436110428	9.15989E-06	2.13977E-05	negative
PRJNA729271	stage_E18_vs_E10	ordinary_muscle_development	ENSGALG00010006353	ABCE1	-1.00660907	0.081276709	-12.38496342	3.15229E-35	4.90262E-34	negative
PRJNA729271	stage_E18_vs_E10	ordinary_muscle_development	ENSGALG00010006355	PTGR1	-0.621965749	0.152332549	-4.082947168	4.44681E-05	9.62326E-05	negative
PRJNA729271	stage_E18_vs_E10	ordinary_muscle_development	ENSGALG00010006359	SGK1	0.957782453	0.164266931	5.830646796	5.52129E-09	1.74702E-08	positive
PRJNA729271	stage_E18_vs_E10	ordinary_muscle_development	ENSGALG00010006361	SERTM1	-1.653636705	0.264070249	-6.262109086	3.79805E-10	1.32817E-09	negative
PRJNA729271	stage_E18_vs_E10	ordinary_muscle_development	ENSGALG00010006363	TRAPPC6A	-0.176837115	0.102878255	-1.718896915	0.085633144	0.11500532	negative
PRJNA729271	stage_E18_vs_E10	ordinary_muscle_development	ENSGALG00010006372	SBF1	0.034974377	0.113790467	0.30735771	0.758571114	0.797583657	positive
PRJNA729271	stage_E18_vs_E10	ordinary_muscle_development	ENSGALG00010006378	CAAP1	-1.366209076	0.106281395	-12.8546401	8.1002E-38	1.42146E-36	negative
PRJNA729271	stage_E18_vs_E10	ordinary_muscle_development	ENSGALG00010006381	SPART	0.990210999	0.157278724	6.295899236	3.05623E-10	1.07577E-09	positive
PRJNA729271	stage_E18_vs_E10	ordinary_muscle_development	ENSGALG00010006382	PNN	-0.93738899	0.197544004	-4.745216103	2.08284E-06	5.19366E-06	negative
PRJNA729271	stage_E18_vs_E10	ordinary_muscle_development	ENSGALG00010006387	OTUD4	-0.723797321	0.137015329	-5.282601033	1.27363E-07	3.57567E-07	negative
PRJNA729271	stage_E18_vs_E10	ordinary_muscle_development	ENSGALG00010006404	SMAD1	0.335390463	0.109233661	3.070394797	0.00213776	0.003738307	positive
PRJNA729271	stage_E18_vs_E10	ordinary_muscle_development	ENSGALG00010006408	ENSGALG00010006408	-0.363948936	0.670209435	-0.543037619	0.587103901	0.639374233	negative
PRJNA729271	stage_E18_vs_E10	ordinary_muscle_development	ENSGALG00010006411	RGS17	-1.250967051	0.388867934	-3.216945756	0.001295631	0.00232889	negative
PRJNA729271	stage_E18_vs_E10	ordinary_muscle_development	ENSGALG00010006421	LMF2	-0.640673485	0.113193616	-5.659978944	1.51392E-08	4.60916E-08	negative
PRJNA729271	stage_E18_vs_E10	ordinary_muscle_development	ENSGALG00010006434	MMAA	0.396894576	0.11638824	3.410091748	0.00064941	0.001215077	positive
PRJNA729271	stage_E18_vs_E10	ordinary_muscle_development	ENSGALG00010006446	LSAMP	0.004644315	0.183941464	0.025248875	0.979856453	0.983496825	positive
PRJNA729271	stage_E18_vs_E10	ordinary_muscle_development	ENSGALG00010006454	SOD2	0.379998762	0.105981926	3.585505365	0.000336426	0.000653861	positive
PRJNA729271	stage_E18_vs_E10	ordinary_muscle_development	ENSGALG00010006457	NCAPH2	-1.545841726	0.202427521	-7.636519573	2.23172E-14	1.08046E-13	negative
PRJNA729271	stage_E18_vs_E10	ordinary_muscle_development	ENSGALG00010006463	SCO2	0.386513436	0.134459749	2.874566104	0.004045831	0.006789894	positive
PRJNA729271	stage_E18_vs_E10	ordinary_muscle_development	ENSGALG00010006465	PLAA	-0.795092926	0.117950903	-6.740880361	1.5743E-11	6.10472E-11	negative
PRJNA729271	stage_E18_vs_E10	ordinary_muscle_development	ENSGALG00010006472	DCLK1	-2.410808085	0.325697743	-7.401979708	1.34169E-13	6.1099E-13	negative
PRJNA729271	stage_E18_vs_E10	ordinary_muscle_development	ENSGALG00010006478	ENSGALG00010006478	-0.539508794	0.135238655	-3.989309084	6.6266E-05	0.000141028	negative
PRJNA729271	stage_E18_vs_E10	ordinary_muscle_development	ENSGALG00010006480	IFT74	-0.785153698	0.121048416	-6.486278146	8.79827E-11	3.22292E-10	negative
PRJNA729271	stage_E18_vs_E10	ordinary_muscle_development	ENSGALG00010006486	CEP43	-0.493819916	0.114362642	-4.318017723	1.57437E-05	3.59038E-05	negative
PRJNA729271	stage_E18_vs_E10	ordinary_muscle_development	ENSGALG00010006492	GAP43	-2.48272813	0.464659067	-5.343117799	9.13613E-08	2.59663E-07	negative
PRJNA729271	stage_E18_vs_E10	ordinary_muscle_development	ENSGALG00010006496	UBE2H	0.303890093	0.114055023	2.664416563	0.0077122	0.012401634	positive
PRJNA729271	stage_E18_vs_E10	ordinary_muscle_development	ENSGALG00010006499	CCR6	-2.61222149	1.05935862	-2.465851922	0.013668783	0.021210685	negative
PRJNA729271	stage_E18_vs_E10	ordinary_muscle_development	ENSGALG00010006503	LRRC19	1.243657217	1.27540687	0.975106255	0.329507556	0.387679548	positive
PRJNA729271	stage_E18_vs_E10	ordinary_muscle_development	ENSGALG00010006506	ZNF827	0.22881181	0.104631386	2.186837221	0.028754409	0.042151614	positive
PRJNA729271	stage_E18_vs_E10	ordinary_muscle_development	ENSGALG00010006515	FBXO33	-0.59292096	0.105947781	-5.596350906	2.1891E-08	6.56958E-08	negative
PRJNA729271	stage_E18_vs_E10	ordinary_muscle_development	ENSGALG00010006516	NBEA	-0.303861941	0.126368078	-2.404578328	0.016191136	0.024853385	negative
PRJNA729271	stage_E18_vs_E10	ordinary_muscle_development	ENSGALG00010006517	ZBTB20	2.277800874	0.150245839	15.16049224	6.45835E-52	2.0132E-50	positive
PRJNA729271	stage_E18_vs_E10	ordinary_muscle_development	ENSGALG00010006521	ZNF410	-1.134972991	0.107153641	-10.59201519	3.24529E-26	3.28495E-25	negative
PRJNA729271	stage_E18_vs_E10	ordinary_muscle_development	ENSGALG00010006524	TAB2	-0.131218704	0.092085302	-1.424969028	0.154166154	0.196745765	negative
PRJNA729271	stage_E18_vs_E10	ordinary_muscle_development	ENSGALG00010006526	LSM6	-0.858737871	0.161309643	-5.323537122	1.01769E-07	2.88507E-07	negative
PRJNA729271	stage_E18_vs_E10	ordinary_muscle_development	ENSGALG00010006528	ZC3HC1	-0.694628593	0.08561856	-8.113060963	4.93603E-16	2.70604E-15	negative
PRJNA729271	stage_E18_vs_E10	ordinary_muscle_development	ENSGALG00010006531	FAM161B	-0.421341794	0.138591637	-3.04016753	0.002364466	0.004104051	negative
PRJNA729271	stage_E18_vs_E10	ordinary_muscle_development	ENSGALG00010006532	KLHDC10	-0.018723766	0.10525435	-0.17789066	0.858808846	0.883114194	negative
PRJNA729271	stage_E18_vs_E10	ordinary_muscle_development	ENSGALG00010006537	DRD3	-0.575737064	0.254061395	-2.266133599	0.023443205	0.03492387	negative
PRJNA729271	stage_E18_vs_E10	ordinary_muscle_development	ENSGALG00010006538	TEK	0.837200087	0.106291634	7.876443862	3.3683E-15	1.74432E-14	positive
PRJNA729271	stage_E18_vs_E10	ordinary_muscle_development	ENSGALG00010006542	RFC3	-1.407129939	0.141536976	-9.94178324	2.7388E-23	2.32975E-22	negative
PRJNA729271	stage_E18_vs_E10	ordinary_muscle_development	ENSGALG00010006544	MOB3B	-2.235841893	0.151353462	-14.77232076	2.20971E-49	6.32999E-48	negative
PRJNA729271	stage_E18_vs_E10	ordinary_muscle_development	ENSGALG00010006546	SMOC2	-0.669523145	0.208563478	-3.210164849	0.001326589	0.002382173	negative
PRJNA729271	stage_E18_vs_E10	ordinary_muscle_development	ENSGALG00010006548	TMEM209	-0.475106208	0.080138797	-5.928541779	3.05637E-09	9.89105E-09	negative
PRJNA729271	stage_E18_vs_E10	ordinary_muscle_development	ENSGALG00010006549	ZDHHC23	-0.762421826	0.189420346	-4.025026042	5.6969E-05	0.000122028	negative
PRJNA729271	stage_E18_vs_E10	ordinary_muscle_development	ENSGALG00010006552	CPA2	1.751246212	1.815022373	0.964862053	0.33461391	0.393017473	positive
PRJNA729271	stage_E18_vs_E10	ordinary_muscle_development	ENSGALG00010006557	COQ6	0.475127286	0.109033509	4.357626311	1.31481E-05	3.02222E-05	positive
PRJNA729271	stage_E18_vs_E10	ordinary_muscle_development	ENSGALG00010006558	USP10	-0.532777005	0.088951567	-5.989517904	2.10464E-09	6.90667E-09	negative
PRJNA729271	stage_E18_vs_E10	ordinary_muscle_development	ENSGALG00010006564	SLC10A7	-0.682952412	0.144046831	-4.74118318	2.12474E-06	5.29449E-06	negative
PRJNA729271	stage_E18_vs_E10	ordinary_muscle_development	ENSGALG00010006566	CPA5	1.243535908	2.919585631	0.425928904	0.670159652	0.717056482	positive
PRJNA729271	stage_E18_vs_E10	ordinary_muscle_development	ENSGALG00010006568	ENTPD5	-1.520243152	0.201263584	-7.553493402	4.23736E-14	2.00132E-13	negative
PRJNA729271	stage_E18_vs_E10	ordinary_muscle_development	ENSGALG00010006570	ENSGALG00010006570	2.625500167	0.479173528	5.479226241	4.2719E-08	1.25018E-07	positive
PRJNA729271	stage_E18_vs_E10	ordinary_muscle_development	ENSGALG00010006574	CPA1	1.243545203	2.682463718	0.463583233	0.642946369	0.692128305	positive
PRJNA729271	stage_E18_vs_E10	ordinary_muscle_development	ENSGALG00010006575	SSBP2	-0.408030464	0.116697796	-3.496471033	0.000471456	0.000900125	negative
PRJNA729271	stage_E18_vs_E10	ordinary_muscle_development	ENSGALG00010006576	CCDC191	0.361072759	0.110602181	3.264607952	0.001096157	0.001986844	positive
PRJNA729271	stage_E18_vs_E10	ordinary_muscle_development	ENSGALG00010006580	ALDH6A1	0.712199737	0.138991874	5.124038671	2.9906E-07	8.12686E-07	positive
PRJNA729271	stage_E18_vs_E10	ordinary_muscle_development	ENSGALG00010006581	CEP41	-1.068069037	0.106570185	-10.02221251	1.21747E-23	1.05672E-22	negative
PRJNA729271	stage_E18_vs_E10	ordinary_muscle_development	ENSGALG00010006585	TTC29	-0.127383015	0.184913802	-0.688877812	0.490900163	0.548535142	negative
PRJNA729271	stage_E18_vs_E10	ordinary_muscle_development	ENSGALG00010006588	ACOT12	-2.162809015	0.798924794	-2.707149699	0.006786365	0.010999481	negative
PRJNA729271	stage_E18_vs_E10	ordinary_muscle_development	ENSGALG00010006592	STARD13	0.463400059	0.11437531	4.051574224	5.08742E-05	0.000109491	positive
PRJNA729271	stage_E18_vs_E10	ordinary_muscle_development	ENSGALG00010006598	ENSGALG00010006598	0.783409032	0.151580739	5.168262393	2.3628E-07	6.47182E-07	positive
PRJNA729271	stage_E18_vs_E10	ordinary_muscle_development	ENSGALG00010006602	EDNRA	-0.517429718	0.118467076	-4.367709016	1.25557E-05	2.89385E-05	negative
PRJNA729271	stage_E18_vs_E10	ordinary_muscle_development	ENSGALG00010006603	MEST	-0.571656393	0.225690833	-2.532918087	0.011311738	0.017773837	negative
PRJNA729271	stage_E18_vs_E10	ordinary_muscle_development	ENSGALG00010006606	SULT	3.961188886	1.183546345	3.346881095			positive
PRJNA729271	stage_E18_vs_E10	ordinary_muscle_development	ENSGALG00010006608	KL	3.282359924	0.409200868	8.021390427	1.04555E-15	5.60889E-15	positive
PRJNA729271	stage_E18_vs_E10	ordinary_muscle_development	ENSGALG00010006614	TMEM184C	-0.574551801	0.056418373	-10.18377111	2.34301E-24	2.13329E-23	negative
PRJNA729271	stage_E18_vs_E10	ordinary_muscle_development	ENSGALG00010006615	GRAMD1C	-0.129246794	0.102449013	-1.261571883	0.207102879	0.256709101	negative
PRJNA729271	stage_E18_vs_E10	ordinary_muscle_development	ENSGALG00010006616	COPG2	-0.533233607	0.069001019	-7.72790912	1.09327E-14	5.44196E-14	negative
PRJNA729271	stage_E18_vs_E10	ordinary_muscle_development	ENSGALG00010006619	LIN52	-0.60270142	0.102877811	-5.858419963	4.67292E-09	1.48866E-08	negative
PRJNA729271	stage_E18_vs_E10	ordinary_muscle_development	ENSGALG00010006621	DACT2	-2.068874719	0.210760614	-9.816230254	9.58615E-23	7.94007E-22	negative
PRJNA729271	stage_E18_vs_E10	ordinary_muscle_development	ENSGALG00010006623	ENSGALG00010006623	0.613536588	0.186813731	3.284215693	0.001022666	0.001860385	positive
PRJNA729271	stage_E18_vs_E10	ordinary_muscle_development	ENSGALG00010006627	SF3B5	-0.371515309	0.11085658	-3.351314914	0.000804288	0.00148644	negative
PRJNA729271	stage_E18_vs_E10	ordinary_muscle_development	ENSGALG00010006633	FBXO18	0.510116724	0.09329565	5.467743908	4.558E-08	1.33148E-07	positive
PRJNA729271	stage_E18_vs_E10	ordinary_muscle_development	ENSGALG00010006634	PRMT9	-0.184812298	0.097055935	-1.904183378	0.056886306	0.07908757	negative
PRJNA729271	stage_E18_vs_E10	ordinary_muscle_development	ENSGALG00010006635	ENSGALG00010006635	-1.199514373	0.472868387	-2.536677026	0.011191015	0.017594662	negative
PRJNA729271	stage_E18_vs_E10	ordinary_muscle_development	ENSGALG00010006641	ENSGALG00010006641	-3.071086999	0.496260843	-6.188453187	6.07574E-10	2.09636E-09	negative
PRJNA729271	stage_E18_vs_E10	ordinary_muscle_development	ENSGALG00010006651	ENSGALG00010006651	0.34560204	0.114346544	3.022409146	0.002507713	0.004340216	positive
PRJNA729271	stage_E18_vs_E10	ordinary_muscle_development	ENSGALG00010006653	CFL2	0.744547945	0.104636269	7.115581898	1.11442E-12	4.75942E-12	positive
PRJNA729271	stage_E18_vs_E10	ordinary_muscle_development	ENSGALG00010006657	PDS5B	-0.644321652	0.088358431	-7.29213355	3.0508E-13	1.35773E-12	negative
PRJNA729271	stage_E18_vs_E10	ordinary_muscle_development	ENSGALG00010006664	ENSGALG00010006664	1.752179522	1.407314365	1.245051969	0.213112648	0.263437475	positive
PRJNA729271	stage_E18_vs_E10	ordinary_muscle_development	ENSGALG00010006670	ANKRD16	0.284563091	0.186778552	1.523531945	0.127625675	0.166032504	positive
PRJNA729271	stage_E18_vs_E10	ordinary_muscle_development	ENSGALG00010006673	BAZ1A	-1.023623918	0.082968901	-12.3374409	5.69369E-35	8.76116E-34	negative
PRJNA729271	stage_E18_vs_E10	ordinary_muscle_development	ENSGALG00010006678	ENSGALG00010006678	-5.3482642	1.390465911	-3.846382827	0.000119874	0.000247448	negative
PRJNA729271	stage_E18_vs_E10	ordinary_muscle_development	ENSGALG00010006681	CRISPLD2	-1.140055989	0.209677091	-5.437198612	5.41248E-08	1.56782E-07	negative
PRJNA729271	stage_E18_vs_E10	ordinary_muscle_development	ENSGALG00010006682	ARHGAP10	-0.42284316	0.12105876	-3.492875372	0.000477849	0.000910773	negative
PRJNA729271	stage_E18_vs_E10	ordinary_muscle_development	ENSGALG00010006685	ENSGALG00010006685	-0.498814058	0.120968383	-4.123507699	3.73146E-05	8.14694E-05	negative
PRJNA729271	stage_E18_vs_E10	ordinary_muscle_development	ENSGALG00010006694	ENSGALG00010006694	-2.799910193	0.698326954	-4.009454562	6.08592E-05	0.000129996	negative
PRJNA729271	stage_E18_vs_E10	ordinary_muscle_development	ENSGALG00010006701	LINGO2	-2.470040155	0.324651023	-7.608293146	2.77739E-14	1.33129E-13	negative
PRJNA729271	stage_E18_vs_E10	ordinary_muscle_development	ENSGALG00010006715	IRF8	0.579604521	0.162568141	3.565302027	0.000363437	0.000703529	positive
PRJNA729271	stage_E18_vs_E10	ordinary_muscle_development	ENSGALG00010006716	ENSGALG00010006716	0.719364967	0.098206046	7.325057843	2.38796E-13	1.06766E-12	positive
PRJNA729271	stage_E18_vs_E10	ordinary_muscle_development	ENSGALG00010006720	SRP54	-0.545700169	0.092084143	-5.926103566	3.10207E-09	1.003E-08	negative
PRJNA729271	stage_E18_vs_E10	ordinary_muscle_development	ENSGALG00010006728	ENSGALG00010006728	0.750395095	0.135909579	5.521281866	3.36535E-08	9.9634E-08	positive
PRJNA729271	stage_E18_vs_E10	ordinary_muscle_development	ENSGALG00010006730	HAPLN1	-6.38162491	0.493200445	-12.93921159	2.7039E-38	4.84463E-37	negative
PRJNA729271	stage_E18_vs_E10	ordinary_muscle_development	ENSGALG00010006735	NR3C2	-0.987074826	0.115327801	-8.558862785	1.13986E-17	6.98028E-17	negative
PRJNA729271	stage_E18_vs_E10	ordinary_muscle_development	ENSGALG00010006739	VCAN	-2.302142468	0.253697957	-9.074343732	1.14365E-19	7.89012E-19	negative
PRJNA729271	stage_E18_vs_E10	ordinary_muscle_development	ENSGALG00010006740	BRCA2	-0.472568451	0.152482229	-3.099170661	0.001940632	0.003411699	negative
PRJNA729271	stage_E18_vs_E10	ordinary_muscle_development	ENSGALG00010006748	FAM177A1	0.563599257	0.077669441	7.256383555	3.97577E-13	1.75483E-12	positive
PRJNA729271	stage_E18_vs_E10	ordinary_muscle_development	ENSGALG00010006752	ZAR1L	-1.037844903	0.471405669	-2.201596142	0.027693849	0.040716334	negative
PRJNA729271	stage_E18_vs_E10	ordinary_muscle_development	ENSGALG00010006760	TAS0R2	0.138807032	0.103116919	1.346113069	0.178266055	0.224224788	positive
PRJNA729271	stage_E18_vs_E10	ordinary_muscle_development	ENSGALG00010006761	PPP2R3C	-0.332391231	0.096232766	-3.454033828	0.000552268	0.001043013	negative
PRJNA729271	stage_E18_vs_E10	ordinary_muscle_development	ENSGALG00010006767	ENSGALG00010006767	7.052985536	0.330757498	21.3237359	6.8376E-101	8.05782E-99	positive
PRJNA729271	stage_E18_vs_E10	ordinary_muscle_development	ENSGALG00010006794	PERPA	-5.295690203	0.590956408	-8.961219697	3.21107E-19	2.16385E-18	negative
PRJNA729271	stage_E18_vs_E10	ordinary_muscle_development	ENSGALG00010006803	COX4I1	1.31886886	0.103916714	12.69159507	6.58313E-37	1.11085E-35	positive
PRJNA729271	stage_E18_vs_E10	ordinary_muscle_development	ENSGALG00010006804	PSMA6	-0.158568305	0.102618183	-1.545226198	0.12229156	0.159580137	negative
PRJNA729271	stage_E18_vs_E10	ordinary_muscle_development	ENSGALG00010006814	ENSGALG00010006814	-0.60112769	0.401954865	-1.495510421	0.134781282	0.174293397	negative
PRJNA729271	stage_E18_vs_E10	ordinary_muscle_development	ENSGALG00010006816	ENSGALG00010006816	3.49040289	0.518496527	6.731776792	1.67604E-11	6.47154E-11	positive
PRJNA729271	stage_E18_vs_E10	ordinary_muscle_development	ENSGALG00010006819	EMC8	0.084935789	0.132929146	0.638955353	0.522851977	0.579414328	positive
PRJNA729271	stage_E18_vs_E10	ordinary_muscle_development	ENSGALG00010006821	XRCC4	-0.418947522	0.126681785	-3.307085717	0.00094272	0.001722952	negative
PRJNA729271	stage_E18_vs_E10	ordinary_muscle_development	ENSGALG00010006828	GTF2A1	-0.362070578	0.106465782	-3.400816419	0.000671849	0.001254794	negative
PRJNA729271	stage_E18_vs_E10	ordinary_muscle_development	ENSGALG00010006833	LTV1	-0.611325402	0.087939248	-6.951678768	3.60965E-12	1.47969E-11	negative
PRJNA729271	stage_E18_vs_E10	ordinary_muscle_development	ENSGALG00010006835	FRY	1.746392315	0.152267351	11.46925002	1.88284E-30	2.34869E-29	positive
PRJNA729271	stage_E18_vs_E10	ordinary_muscle_development	ENSGALG00010006838	ASB13	0.601850531	0.12818117	4.69531158	2.66201E-06	6.56332E-06	positive
PRJNA729271	stage_E18_vs_E10	ordinary_muscle_development	ENSGALG00010006840	ENSGALG00010006840	-2.171852029	0.281588064	-7.71286963	1.2302E-14	6.09631E-14	negative
PRJNA729271	stage_E18_vs_E10	ordinary_muscle_development	ENSGALG00010006843	STON2	0.373788208	0.195574183	1.911234918	0.055974398	0.07790944	positive

PRJNA729271	stage_E18_vs_E10	ordinary_muscle_development	ENSGALG00010006845	CALML3	-6.856417552	0.652316955	-10.51086822	7.69815E-26	7.61671E-25	negative
PRJNA729271	stage_E18_vs_E10	ordinary_muscle_development	ENSGALG00010006847	SEL1L	-0.114237134	0.087137698	-1.310995542	0.18985927	0.237303623	negative
PRJNA729271	stage_E18_vs_E10	ordinary_muscle_development	ENSGALG00010006850	IRF2BPL	0.130154645	0.159763752	0.814669433	0.415261555	0.47563937	positive
PRJNA729271	stage_E18_vs_E10	ordinary_muscle_development	ENSGALG00010006851	TMEM167A	-0.169217993	0.104701323	-1.616197276	0.106051668	0.139973344	negative
PRJNA729271	stage_E18_vs_E10	ordinary_muscle_development	ENSGALG00010006853	NET1	0.457561498	0.083505777	5.479399324	4.26772E-08	1.24946E-07	positive
PRJNA729271	stage_E18_vs_E10	ordinary_muscle_development	ENSGALG00010006855	DCLK2	-0.611389973	0.126391699	-4.837263647	1.31639E-06	3.3623E-06	negative
PRJNA729271	stage_E18_vs_E10	ordinary_muscle_development	ENSGALG00010006865	EFCAB2	0.216392316	0.320673228	0.674806304	0.499798835	0.556975791	positive
PRJNA729271	stage_E18_vs_E10	ordinary_muscle_development	ENSGALG00010006877	GSE1	0.909099993	0.241352376	3.766691709	0.000165425	0.000335034	positive
PRJNA729271	stage_E18_vs_E10	ordinary_muscle_development	ENSGALG00010006883	LRBA	-0.72118511	0.071635492	-10.06742733	7.69649E-24	6.77768E-23	negative
PRJNA729271	stage_E18_vs_E10	ordinary_muscle_development	ENSGALG00010006885	RHOU	-0.084960137	0.153143787	-0.554773643	0.579049498	0.632027294	negative
PRJNA729271	stage_E18_vs_E10	ordinary_muscle_development	ENSGALG00010006901	TUBAL3	-0.638371413	0.261174793	-2.444230572	0.014516146	0.022462695	negative
PRJNA729271	stage_E18_vs_E10	ordinary_muscle_development	ENSGALG00010006903	B3GLCT	-0.882088083	0.146289997	-6.02972247	1.64241E-09	5.43907E-09	negative
PRJNA729271	stage_E18_vs_E10	ordinary_muscle_development	ENSGALG00010006912	ENSGALG00010006912	-0.994913837	0.183287588	-5.428157193	5.69389E-08	1.64506E-07	negative
PRJNA729271	stage_E18_vs_E10	ordinary_muscle_development	ENSGALG00010006913	HSPH1	-2.503066264	0.190707677	-13.12514686	2.36324E-39	4.42574E-38	negative
PRJNA729271	stage_E18_vs_E10	ordinary_muscle_development	ENSGALG00010006922	SMYD3	-0.087294587	0.15846299	-0.550883125	0.581713796	0.634744164	negative
PRJNA729271	stage_E18_vs_E10	ordinary_muscle_development	ENSGALG00010006923	ENSGALG00010006923	0.416008267	0.226206239	1.839066285	0.065905437	0.090489657	positive
PRJNA729271	stage_E18_vs_E10	ordinary_muscle_development	ENSGALG00010006926	ID4	-1.75829635	0.226185177	-7.77370284	7.62244E-15	3.8417E-14	negative
PRJNA729271	stage_E18_vs_E10	ordinary_muscle_development	ENSGALG00010006927	UCN3	-1.603818383	0.766002349	-2.093751258	0.036282144	0.052209836	negative
PRJNA729271	stage_E18_vs_E10	ordinary_muscle_development	ENSGALG00010006939	FBXO31	0.381339854	0.128435571	2.969114015	0.002986598	0.005115898	positive
PRJNA729271	stage_E18_vs_E10	ordinary_muscle_development	ENSGALG00010006942	ALOX5AP	-1.20297402	0.199351343	-6.034441502	1.59513E-09	5.28974E-09	negative
PRJNA729271	stage_E18_vs_E10	ordinary_muscle_development	ENSGALG00010006944	MAB21L2	-4.466020821	0.491608352	-9.084509659	1.04167E-19	7.20373E-19	negative
PRJNA729271	stage_E18_vs_E10	ordinary_muscle_development	ENSGALG00010006946	PLPPR1	-3.978641549	0.304429843	-13.06915746	4.94097E-39	9.11189E-38	negative
PRJNA729271	stage_E18_vs_E10	ordinary_muscle_development	ENSGALG00010006948	ENSGALG00010006948	-4.903872791	0.249046925	-19.6905575	2.59814E-86	2.17688E-84	negative
PRJNA729271	stage_E18_vs_E10	ordinary_muscle_development	ENSGALG00010006953	MAP1LC3B	0.148680727	0.128640897	1.155781181	0.247770672	0.301042405	positive
PRJNA729271	stage_E18_vs_E10	ordinary_muscle_development	ENSGALG00010006957	PALM2AKAP2	0.013190376	0.092707755	0.142279105	0.88685955	0.90670072	positive
PRJNA729271	stage_E18_vs_E10	ordinary_muscle_development	ENSGALG00010006958	KIF26B	-3.785789039	0.23684457	-15.98427625	1.64466E-57	6.00487E-56	negative
PRJNA729271	stage_E18_vs_E10	ordinary_muscle_development	ENSGALG00010006961	ZCCHC14	1.108554699	0.116425169	9.521606958	1.70521E-21	1.32177E-20	positive
PRJNA729271	stage_E18_vs_E10	ordinary_muscle_development	ENSGALG00010006963	ENSGALG00010006963	-0.392609816	0.455701187	-0.861551004	0.388934643	0.449104409	negative
PRJNA729271	stage_E18_vs_E10	ordinary_muscle_development	ENSGALG00010006966	PGBD5	-1.395512854	0.223277371	-6.250131148	4.10108E-10	1.43276E-09	negative
PRJNA729271	stage_E18_vs_E10	ordinary_muscle_development	ENSGALG00010006983	GTPBP2	0.890672591	0.124257848	7.167938339	7.61356E-13	3.29527E-12	positive
PRJNA729271	stage_E18_vs_E10	ordinary_muscle_development	ENSGALG00010006988	USPL1	-0.090129259	0.104811339	-0.85991897	0.389833711	0.449891691	negative
PRJNA729271	stage_E18_vs_E10	ordinary_muscle_development	ENSGALG00010006992	SLC35B4	-1.323354573	0.104647607	-12.64581787	1.1799E-36	1.97719E-35	negative
PRJNA729271	stage_E18_vs_E10	ordinary_muscle_development	ENSGALG00010006998	POLH	0.206587248	0.123755309	1.669320281	0.095053923	0.126636637	positive
PRJNA729271	stage_E18_vs_E10	ordinary_muscle_development	ENSGALG00010007000	LRGUK	-1.578168409	0.532997034	-2.960932815	0.003067088	0.005245716	negative
PRJNA729271	stage_E18_vs_E10	ordinary_muscle_development	ENSGALG00010007001	SNX30	-1.074024482	0.094013829	-11.42411163	3.16879E-30	3.92578E-29	negative
PRJNA729271	stage_E18_vs_E10	ordinary_muscle_development	ENSGALG00010007002	EXOC4	-0.081672152	0.12017881	-0.679588624	0.496764975	0.554063428	negative
PRJNA729271	stage_E18_vs_E10	ordinary_muscle_development	ENSGALG00010007004	ENSGALG00010007004	1.259770789	0.570892609	2.206668591	0.027337221	0.040240989	positive
PRJNA729271	stage_E18_vs_E10	ordinary_muscle_development	ENSGALG00010007007	ACSL1	0.819244164	0.217207261	3.771716293	0.000162129	0.000328955	positive
PRJNA729271	stage_E18_vs_E10	ordinary_muscle_development	ENSGALG00010007010	ENSGALG00010007010	0.361824675	1.02506771	0.352976366	0.724106159	0.766517109	positive
PRJNA729271	stage_E18_vs_E10	ordinary_muscle_development	ENSGALG00010007011	HMGB1	-1.286795693	0.101842447	-12.63516079	1.35118E-36	2.25897E-35	negative
PRJNA729271	stage_E18_vs_E10	ordinary_muscle_development	ENSGALG00010007014	ENSGALG00010007014	0.611230107	0.571022658	1.070413054	0.284433426	0.340142109	positive
PRJNA729271	stage_E18_vs_E10	ordinary_muscle_development	ENSGALG00010007017	SRD5A2	0.247788428	0.350769735	0.706413363	0.479931113	0.538228354	positive
PRJNA729271	stage_E18_vs_E10	ordinary_muscle_development	ENSGALG00010007021	SULT6B1	-0.107267693	0.253617318	-0.422950979	0.672331027	0.718902599	negative
PRJNA729271	stage_E18_vs_E10	ordinary_muscle_development	ENSGALG00010007025	ENSGALG00010007025	1.224981503	0.762445125	1.606648745	0.108131443	0.142548544	positive
PRJNA729271	stage_E18_vs_E10	ordinary_muscle_development	ENSGALG00010007028	KATNAL1	-0.872033419	0.14505993	-6.011538955	1.8377E-09	6.05811E-09	negative
PRJNA729271	stage_E18_vs_E10	ordinary_muscle_development	ENSGALG00010007033	CEBPZOS	1.496162712	0.185443006	8.068046034	7.14321E-16	3.87939E-15	positive
PRJNA729271	stage_E18_vs_E10	ordinary_muscle_development	ENSGALG00010007038	CENPU	-1.389835395	0.203292438	-6.836631067	8.10772E-12	3.21097E-11	negative
PRJNA729271	stage_E18_vs_E10	ordinary_muscle_development	ENSGALG00010007041	NAE1	-1.014662282	0.082517188	-12.29637489	9.47311E-35	1.44388E-33	negative
PRJNA729271	stage_E18_vs_E10	ordinary_muscle_development	ENSGALG00010007049	ENSGALG00010007049	0.260978508	0.504895022	0.516896575	0.605228359	0.656056316	positive
PRJNA729271	stage_E18_vs_E10	ordinary_muscle_development	ENSGALG00010007051	E2F3	-2.157151253	0.1598331	-13.49627361	1.64488E-41	3.34397E-40	negative
PRJNA729271	stage_E18_vs_E10	ordinary_muscle_development	ENSGALG00010007066	INIP	-0.789166166	0.154843656	-5.096535335	3.45926E-07	9.35659E-07	negative
PRJNA729271	stage_E18_vs_E10	ordinary_muscle_development	ENSGALG00010007067	CASP3	-0.548478881	0.107123346	-5.120068602	3.05425E-07	8.29361E-07	negative
PRJNA729271	stage_E18_vs_E10	ordinary_muscle_development	ENSGALG00010007068	NCAM2	-2.640305802	0.235649296	-11.20438655	3.88033E-29	4.52497E-28	negative
PRJNA729271	stage_E18_vs_E10	ordinary_muscle_development	ENSGALG00010007070	UBL3	0.700550684	0.052930615	13.23526446	5.49031E-40	1.05407E-38	positive
PRJNA729271	stage_E18_vs_E10	ordinary_muscle_development	ENSGALG00010007085	PRIMPOL	1.029227454	0.157944398	6.516391001	7.20191E-11	2.6556E-10	positive
PRJNA729271	stage_E18_vs_E10	ordinary_muscle_development	ENSGALG00010007090	CEBPZ	-0.979936082	0.096105947	-10.19641459	2.05727E-24	1.87903E-23	negative
PRJNA729271	stage_E18_vs_E10	ordinary_muscle_development	ENSGALG00010007092	DYNC1LI2	-0.047237476	0.061911409	-0.76298499	0.445472348	0.504936009	negative
PRJNA729271	stage_E18_vs_E10	ordinary_muscle_development	ENSGALG00010007097	ZCCHC9	-0.215623351	0.169197298	-1.274390035	0.202525222	0.251704741	negative
PRJNA729271	stage_E18_vs_E10	ordinary_muscle_development	ENSGALG00010007098	NDUFAF7	-0.402853415	0.134075891	-3.004667061	0.002658717	0.004587869	negative
PRJNA729271	stage_E18_vs_E10	ordinary_muscle_development	ENSGALG00010007102	CMTM3	-0.720367478	0.165471916	-4.353412323	1.34035E-05	3.07897E-05	negative
PRJNA729271	stage_E18_vs_E10	ordinary_muscle_development	ENSGALG00010007106	SLC7A1	0.481106332	0.239110924	2.012063371	0.044213257	0.062640129	positive
PRJNA729271	stage_E18_vs_E10	ordinary_muscle_development	ENSGALG00010007108	MBOAT1	-1.502823512	0.196451762	-7.649834717	2.01238E-14	9.77855E-14	negative
PRJNA729271	stage_E18_vs_E10	ordinary_muscle_development	ENSGALG00010007110	ARL6	0.098058118	0.1027446	0.954387069	0.33988775	0.398275904	positive
PRJNA729271	stage_E18_vs_E10	ordinary_muscle_development	ENSGALG00010007114	CKLF	-0.634000616	0.157279491	-4.031044419	5.55295E-05	0.000119086	negative
PRJNA729271	stage_E18_vs_E10	ordinary_muscle_development	ENSGALG00010007115	ENSGALG00010007115	1.2055925	0.277984438	4.336906446	1.44502E-05	3.30789E-05	positive
PRJNA729271	stage_E18_vs_E10	ordinary_muscle_development	ENSGALG00010007118	ENSGALG00010007118	-0.595848845	0.127881546	-4.659380978	3.17162E-06	7.75516E-06	negative
PRJNA729271	stage_E18_vs_E10	ordinary_muscle_development	ENSGALG00010007123	ENSGALG00010007123	1.744930161	0.247202391	7.058710701	1.68055E-12	7.06892E-12	positive
PRJNA729271	stage_E18_vs_E10	ordinary_muscle_development	ENSGALG00010007126	ENSGALG00010007126	1.134303281	1.090200139	1.04045417	0.298128946	0.355113737	positive
PRJNA729271	stage_E18_vs_E10	ordinary_muscle_development	ENSGALG00010007130	IRF2	1.26515508	0.122597667	10.319569	5.74806E-25	5.43852E-24	positive
PRJNA729271	stage_E18_vs_E10	ordinary_muscle_development	ENSGALG00010007133	BLVRA	1.559771094	0.186920742	8.344558662	7.14803E-17	4.13946E-16	positive
PRJNA729271	stage_E18_vs_E10	ordinary_muscle_development	ENSGALG00010007135	TK2	-0.605870682	0.117898167	-5.138932154	2.76304E-07	7.52542E-07	negative
PRJNA729271	stage_E18_vs_E10	ordinary_muscle_development	ENSGALG00010007137	ENSGALG00010007137	1.372969043	0.359774119	3.816197355	0.000135524	0.000278129	positive
PRJNA729271	stage_E18_vs_E10	ordinary_muscle_development	ENSGALG00010007141	PRKD3	-0.542668762	0.084379568	-6.431281583	1.26533E-10	4.57835E-10	negative
PRJNA729271	stage_E18_vs_E10	ordinary_muscle_development	ENSGALG00010007143	CKMT2	5.715892399	0.318332397	17.95573575	4.32808E-72	2.40366E-70	positive
PRJNA729271	stage_E18_vs_E10	ordinary_muscle_development	ENSGALG00010007146	ENSGALG00010007146	0.043616927	0.1453978	0.299983403	0.764189816	0.802185238	positive
PRJNA729271	stage_E18_vs_E10	ordinary_muscle_development	ENSGALG00010007155	FOXL1	-0.713812688	0.607805549	-1.174409626	0.240230991	0.293134794	negative
PRJNA729271	stage_E18_vs_E10	ordinary_muscle_development	ENSGALG00010007160	ENSGALG00010007160	1.105677981	0.29426218	3.75745868	0.000171648	0.000347103	positive
PRJNA729271	stage_E18_vs_E10	ordinary_muscle_development	ENSGALG00010007162	ENSGALG00010007162	0.324953984	0.419242422	0.775098051	0.438281723	0.497836656	positive
PRJNA729271	stage_E18_vs_E10	ordinary_muscle_development	ENSGALG00010007165	ENSGALG00010007165	0.337174414	0.329132742	1.024432914	0.305630842	0.362961577	positive
PRJNA729271	stage_E18_vs_E10	ordinary_muscle_development	ENSGALG00010007166	RASGRF2	3.522652866	0.180099901	19.55943811	3.42834E-85	2.73043E-83	positive
PRJNA729271	stage_E18_vs_E10	ordinary_muscle_development	ENSGALG00010007168	MINA	-0.238970942	0.108167445	-2.209268618	0.027155963	0.039998545	negative
PRJNA729271	stage_E18_vs_E10	ordinary_muscle_development	ENSGALG00010007169	QPCT	-0.327546816	0.219053073	-1.495285191	0.134840028	0.17435381	negative
PRJNA729271	stage_E18_vs_E10	ordinary_muscle_development	ENSGALG00010007175	FOXC2	-3.206101968	0.439676512	-7.291956423	3.05486E-13	1.35912E-12	negative
PRJNA729271	stage_E18_vs_E10	ordinary_muscle_development	ENSGALG00010007181	GJB2	-0.8159334	0.659074752	-1.237998267	0.215716703	0.266247754	negative
PRJNA729271	stage_E18_vs_E10	ordinary_muscle_development	ENSGALG00010007182	ENSGALG00010007182	0.884376359	0.295338711	2.994447818	0.002749421	0.004735368	positive
PRJNA729271	stage_E18_vs_E10	ordinary_muscle_development	ENSGALG00010007183	VOPP1	0.174701735	0.337692211	0.517340137	0.60491874	0.655769736	positive
PRJNA729271	stage_E18_vs_E10	ordinary_muscle_development	ENSGALG00010007184	VGLL3	0.441310025	0.093470787	4.721368451	2.34263E-06	5.81645E-06	positive
PRJNA729271	stage_E18_vs_E10	ordinary_muscle_development	ENSGALG00010007187	ENPP6	2.576751907	0.276224002	9.328486627	1.07393E-20	7.92196E-20	positive
PRJNA729271	stage_E18_vs_E10	ordinary_muscle_development	ENSGALG00010007188	MAB21L1	-1.246098614	0.214416291	-5.811585534	6.18839E-09	1.95086E-08	negative
PRJNA729271	stage_E18_vs_E10	ordinary_muscle_development	ENSGALG00010007190	ENSGALG00010007190	-0.201735011	0.149683886	-1.347740339	0.177741913	0.22366256	negative
PRJNA729271	stage_E18_vs_E10	ordinary_muscle_development	ENSGALG00010007201	ENSGALG00010007201	0.71435412	0.268948831	2.656096764	0.007905093	0.012690699	positive
PRJNA729271	stage_E18_vs_E10	ordinary_muscle_development	ENSGALG00010007203	SLC30A6	0.251098748	0.104577829	2.401070568	0.016347184	0.025063722	positive
PRJNA729271	stage_E18_vs_E10	ordinary_muscle_development	ENSGALG00010007204	LANCL2	-0.587260103	0.087186343	-6.735689112	1.63155E-11	6.30984E-11	negative
PRJNA729271	stage_E18_vs_E10	ordinary_muscle_development	ENSGALG00010007207	EPM2A	0.755837462	0.105483288	7.165471226	7.75194E-13	3.35416E-12	positive
PRJNA729271	stage_E18_vs_E10	ordinary_muscle_development	ENSGALG00010007214	MTHFSD	-0.201422714	0.144848906	-1.390571178	0.164355501	0.208373392	negative
PRJNA729271	stage_E18_vs_E10	ordinary_muscle_development	ENSGALG00010007217	MSH3	0.058443402	0.143343639	0.407715352	0.683482665	0.7296959	positive
PRJNA729271	stage_E18_vs_E10	ordinary_muscle_development	ENSGALG00010007223	FOXF1	-0.49497863	0.477571228	-1.036449855	0.299992322	0.357010567	negative
PRJNA729271	stage_E18_vs_E10	ordinary_muscle_development	ENSGALG00010007224	DHFR	-2.078080729	0.216107108	-9.615975831	6.84571E-22	5.44315E-21	negative
PRJNA729271	stage_E18_vs_E10	ordinary_muscle_development	ENSGALG00010007226	ENSGALG00010007226	5.584385647	1.481650365	3.769030655	0.000163883	0.000332142	positive
PRJNA729271	stage_E18_vs_E10	ordinary_muscle_development	ENSGALG00010007230	ENSGALG00010007230	-0.244735061	0.347563961	-0.704143953	0.48134313	0.539311175	negative
PRJNA729271	stage_E18_vs_E10	ordinary_muscle_development	ENSGALG00010007243	BEAN1	-0.024933042	0.187929124	-0.132672579	0.89445233	0.912541595	negative
PRJNA729271	stage_E18_vs_E10	ordinary_muscle_development	ENSGALG00010007251	SPAST	-0.433573272	0.064234716	-6.749827808	1.48021E-11	5.75063E-11	negative
PRJNA729271	stage_E18_vs_E10	ordinary_muscle_development	ENSGALG00010007252	NFATC1	0.242897449	0.153834226	1.578955832	0.114346188	0.150117562	positive
PRJNA729271	stage_E18_vs_E10	ordinary_muscle_development	ENSGALG00010007259	ANKRD34B	-0.561616783	0.096292251	-5.83241929	5.46294E-09	1.72969E-08	negative
PRJNA729271	stage_E18_vs_E10	ordinary_muscle_development	ENSGALG00010007265	CD200R1A	-0.737482432	0.29868812	-2.469071862	0.013546401	0.021036542	negative
PRJNA729271	stage_E18_vs_E10	ordinary_muscle_development	ENSGALG00010007270	CDH5	0.939147273	0.10833726	8.668737541	4.36938E-18	2.75007E-17	positive
PRJNA729271	stage_E18_vs_E10	ordinary_muscle_development	ENSGALG00010007271	CADM2	-3.029252453	0.316233461	-9.579164844	9.78328E-22	7.72378E-21	negative
PRJNA729271	stage_E18_vs_E10	ordinary_muscle_development	ENSGALG00010007275	DPY30	-0.618405606	0.103687322	-5.964139037	2.45927E-09	8.02502E-09	negative
PRJNA729271	stage_E18_vs_E10	ordinary_muscle_development	ENSGALG00010007277	FAM151B	-0.097352856	0.187177267	-0.520110468	0.602986583	0.654262334	negative
PRJNA729271	stage_E18_vs_E10	ordinary_muscle_development	ENSGALG00010007283	STOX2	-0.005027996	0.120393418	-0.041763051	0.966687591	0.972444579	negative
PRJNA729271	stage_E18_vs_E10	ordinary_muscle_development	ENSGALG00010007284	ENSGALG00010007284	-0.064615352	0.082695388	-0.781365847	0.434587352	0.494376367	negative
PRJNA729271	stage_E18_vs_E10	ordinary_muscle_development	ENSGALG00010007287	ENSGALG00010007287	1.828950846	0.62032908	2.948355808	0.003194691	0.005445966	positive
PRJNA729271	stage_E18_vs_E10	ordinary_muscle_development	ENSGALG00010007294	TRAPPC11	-0.361142649	0.066718401	-5.41293921	6.19985E-08	1.78626E-07	negative
PRJNA729271	stage_E18_vs_E10	ordinary_muscle_development	ENSGALG00010007300	CCDC125	-1.19006098	0.313246596	-3.799118634	0.000145212	0.000296749	negative
PRJNA729271	stage_E18_vs_E10	ordinary_muscle_development	ENSGALG00010007302	CDH11	-1.889497576	0.174972709	-10.79881305	3.48682E-27	3.7245E-26	negative
PRJNA729271	stage_E18_vs_E10	ordinary_muscle_development	ENSGALG00010007304	F5	1.477887757	0.355632795	4.155656563	3.24355E-05	7.12676E-05	positive
PRJNA729271	stage_E18_vs_E10	ordinary_muscle_development	ENSGALG00010007318	RMDN2	1.004130857	0.124997367	8.03321607	9.49502E-16	5.11257E-15	positive
PRJNA729271	stage_E18_vs_E10	ordinary_muscle_development	ENSGALG00010007319	ATP9B	0.046560057	0.102330145	0.454998445	0.649110358	0.697623981	positive
PRJNA729271	stage_E18_vs_E10	ordinary_muscle_development	ENSGALG00010007320	ENSGALG00010007320	1.262507504	0.303068326	4.165752066	3.10328E-05	6.82995E-05	positive
PRJNA729271	stage_E18_vs_E10	ordinary_muscle_development	ENSGALG00010007321	CD200	2.009193216	0.149767438	13.41542091	4.91137E-41	9.81935E-40	positive
PRJNA729271	stage_E18_vs_E10	ordinary_muscle_development	ENSGALG00010007322	AK6	-0.493346948	0.10990574	-4.488818783	7.16192E-06	1.69185E-05	negative
PRJNA729271	stage_E18_vs_E10	ordinary_muscle_development	ENSGALG00010007324	ENSGALG00010007324	-0.320772373	0.142874264	-2.245137533	0.024759305	0.036752291	negative
PRJNA729271	stage_E18_vs_E10	ordinary_muscle_development	ENSGALG00010007326	RWDD4	0.080406307	0.116579314	0.689713328	0.490374482	0.548074495	positive
PRJNA729271	stage_E18_vs_E10	ordinary_muscle_development	ENSGALG00010007328	NOVA1	0.274921635	0.137491047	1.999560267	0.045547768	0.064398644	positive
PRJNA729271	stage_E18_vs_E10	ordinary_muscle_development	ENSGALG00010007330	MMP13	-0.437031448	0.70837076	-0.616952976	0.537265743	0.592488355	negative
PRJNA729271	stage_E18_vs_E10	ordinary_muscle_development	ENSGALG00010007333	QTRT2	-1.426201245	0.075443511	-18.90422685	1.05271E-79	7.23179E-78	negative
PRJNA729271	stage_E18_vs_E10	ordinary_muscle_development	ENSGALG00010007337	PRORP	-0.488986975	0.100562478	-4.862519154	1.15901E-06	2.97501E-06	negative
PRJNA729271	stage_E18_vs_E10	ordinary_muscle_development	ENSGALG00010007339	ENSGALG00010007339	0.844593972	0.224031243	3.769982967	0.000163259	0.000331016	positive
PRJNA729271	stage_E18_vs_E10	ordinary_muscle_development	ENSGALG00010007344	RAD17	-0.077463171	0.109409674	-0.708010257	0.478938888	0.537365058	negative
PRJNA729271	stage_E18_vs_E10	ordinary_muscle_development	ENSGALG00010007353	ING2	-0.90028112	0.130331297	-6.907635685	4.92798E-12	1.99975E-11	negative
PRJNA729271	stage_E18_vs_E10	ordinary_muscle_development	ENSGALG00010007357	NOX4	0.721852845	0.118538206	6.089621822	1.13178E-09	3.80088E-09	positive

PRJNA729271	stage_E18_vs_E10	ordinary_muscle_development	ENSGALG00010007360	ROBO1	-2.051688806	0.185860102	-11.03888777	2.48085E-28	2.78975E-27	negative
PRJNA729271	stage_E18_vs_E10	ordinary_muscle_development	ENSGALG00010007365	MEMO1	0.291136636	0.088217623	3.30020948	0.000966127	0.001763285	positive
PRJNA729271	stage_E18_vs_E10	ordinary_muscle_development	ENSGALG00010007369	ENSGALG00010007369	0.012047698	0.212547187	0.056682461	0.954798145	0.96256775	positive
PRJNA729271	stage_E18_vs_E10	ordinary_muscle_development	ENSGALG00010007374	CMIP	-0.05322188	0.134352407	-0.396136406	0.692004402	0.737435952	negative
PRJNA729271	stage_E18_vs_E10	ordinary_muscle_development	ENSGALG00010007375	CHORDC1	-0.715521856	0.076067479	-9.406409496	5.13374E-21	3.86765E-20	negative
PRJNA729271	stage_E18_vs_E10	ordinary_muscle_development	ENSGALG00010007376	CDKN2AIP	-0.46434679	0.061091465	-7.600845548	2.94202E-14	1.40695E-13	negative
PRJNA729271	stage_E18_vs_E10	ordinary_muscle_development	ENSGALG00010007381	EGFR	0.07115543	0.098080496	0.725479919	0.46815766	0.527104651	positive
PRJNA729271	stage_E18_vs_E10	ordinary_muscle_development	ENSGALG00010007382	EIF2AK2	0.160199726	0.258917139	0.618729711	0.536094434	0.591601737	positive
PRJNA729271	stage_E18_vs_E10	ordinary_muscle_development	ENSGALG00010007387	ENSGALG00010007387	-1.397506561	0.503896834	-2.773398179	0.00554742	0.009131257	negative
PRJNA729271	stage_E18_vs_E10	ordinary_muscle_development	ENSGALG00010007388	CYP1B1	-2.497376728	0.200238799	-12.47199213	1.06139E-35	1.68509E-34	negative
PRJNA729271	stage_E18_vs_E10	ordinary_muscle_development	ENSGALG00010007391	NANP	0.205778974	0.139281535	1.477431838	0.139559917	0.179943159	positive
PRJNA729271	stage_E18_vs_E10	ordinary_muscle_development	ENSGALG00010007392	NAALAD2	2.032207959	0.20052141	10.13461835	3.87885E-24	3.48136E-23	positive
PRJNA729271	stage_E18_vs_E10	ordinary_muscle_development	ENSGALG00010007393	PLCG2	0.087064257	0.132739471	0.655903299	0.511886345	0.568653631	positive
PRJNA729271	stage_E18_vs_E10	ordinary_muscle_development	ENSGALG00010007399	WWC2	-0.736003956	0.110126573	-6.683254879	2.33693E-11	8.93766E-11	negative
PRJNA729271	stage_E18_vs_E10	ordinary_muscle_development	ENSGALG00010007401	SEC61G	-0.13599716	0.220209319	-0.617581311	0.536851367	0.592121486	negative
PRJNA729271	stage_E18_vs_E10	ordinary_muscle_development	ENSGALG00010007403	SDR42E1	0.457377951	0.220669937	2.072679026	0.038202161	0.054766128	positive
PRJNA729271	stage_E18_vs_E10	ordinary_muscle_development	ENSGALG00010007404	FOLH1	1.597185915	0.171086043	9.335571062	1.00448E-20	7.41721E-20	positive
PRJNA729271	stage_E18_vs_E10	ordinary_muscle_development	ENSGALG00010007408	HCLS1	0.495413317	0.254828759	1.944102857	0.051883049	0.072612223	positive
PRJNA729271	stage_E18_vs_E10	ordinary_muscle_development	ENSGALG00010007411	ENSGALG00010007411	0.979734194	0.586891384	1.669362034	0.095045653	0.126636637	positive
PRJNA729271	stage_E18_vs_E10	ordinary_muscle_development	ENSGALG00010007413	GPATCH11	-0.94791659	0.102501524	-9.247829194	2.29098E-20	1.65213E-19	negative
PRJNA729271	stage_E18_vs_E10	ordinary_muscle_development	ENSGALG00010007414	ROBO2	-3.31990175	0.229989827	-14.43499389	3.11673E-47	8.21401E-46	negative
PRJNA729271	stage_E18_vs_E10	ordinary_muscle_development	ENSGALG00010007416	DCTD	-1.459494415	0.251069298	-5.813113868	6.13213E-09	1.93397E-08	negative
PRJNA729271	stage_E18_vs_E10	ordinary_muscle_development	ENSGALG00010007418	NME6	0.610268507	0.146476996	4.16630954	3.0957E-05	6.81431E-05	positive
PRJNA729271	stage_E18_vs_E10	ordinary_muscle_development	ENSGALG00010007419	RABL2A	-0.145815357	0.151327133	-0.963577079	0.335258003	0.393646396	negative
PRJNA729271	stage_E18_vs_E10	ordinary_muscle_development	ENSGALG00010007423	HEATR5B	-0.573467511	0.067104253	-8.545918957	1.27517E-17	7.77932E-17	negative
PRJNA729271	stage_E18_vs_E10	ordinary_muscle_development	ENSGALG00010007425	ARSA	-0.260978256	0.132520217	-1.969346731	0.048913288	0.068774674	negative
PRJNA729271	stage_E18_vs_E10	ordinary_muscle_development	ENSGALG00010007429	C21orf91	0.129461424	0.209625468	0.617584427	0.536849313	0.592121486	positive
PRJNA729271	stage_E18_vs_E10	ordinary_muscle_development	ENSGALG00010007430	MEF2C	0.959054412	0.146925801	6.527474467	6.68879E-11	2.47332E-10	positive
PRJNA729271	stage_E18_vs_E10	ordinary_muscle_development	ENSGALG00010007432	NAT	1.061908106	0.193485598	5.488305672	4.05807E-08	1.19169E-07	positive
PRJNA729271	stage_E18_vs_E10	ordinary_muscle_development	ENSGALG00010007433	RNF144B	1.43353657	0.276029035	5.193426731	2.06458E-07	5.68505E-07	positive
PRJNA729271	stage_E18_vs_E10	ordinary_muscle_development	ENSGALG00010007434	ENSGALG00010007434	0.126258544	0.252082687	0.500861625	0.616468512	0.667142831	positive
PRJNA729271	stage_E18_vs_E10	ordinary_muscle_development	ENSGALG00010007436	PNAT3	0.337992543	0.274691266	1.230445175	0.218530445	0.269307839	positive
PRJNA729271	stage_E18_vs_E10	ordinary_muscle_development	ENSGALG00010007437	TUSC1	0.481234318	0.138469146	3.475390247	0.000510111	0.000968188	positive
PRJNA729271	stage_E18_vs_E10	ordinary_muscle_development	ENSGALG00010007439	HSD17B2	-1.565322901	0.332341269	-4.709986528	2.47733E-06	6.12989E-06	negative
PRJNA729271	stage_E18_vs_E10	ordinary_muscle_development	ENSGALG00010007441	STRN	0.704144054	0.136627937	5.153734054	2.5535E-07	6.977E-07	positive
PRJNA729271	stage_E18_vs_E10	ordinary_muscle_development	ENSGALG00010007455	TRPC6	1.46786923	0.207060719	7.089076277	1.3501E-12	5.71712E-12	positive
PRJNA729271	stage_E18_vs_E10	ordinary_muscle_development	ENSGALG00010007457	SHANK3	0.689837411	0.177857067	3.878605577	0.000105057	0.000218699	positive
PRJNA729271	stage_E18_vs_E10	ordinary_muscle_development	ENSGALG00010007465	PNAT10	0.921899168	0.202644133	4.549350402	5.38118E-06	1.28713E-05	positive
PRJNA729271	stage_E18_vs_E10	ordinary_muscle_development	ENSGALG00010007467	CHODL	-0.691477262	0.330844036	-2.09003998	0.036614208	0.052635421	negative
PRJNA729271	stage_E18_vs_E10	ordinary_muscle_development	ENSGALG00010007479	NFX1	-0.141435828	0.097075907	-1.456961172	0.145127099	0.18630921	negative
PRJNA729271	stage_E18_vs_E10	ordinary_muscle_development	ENSGALG00010007480	TENM3	-0.168932586	0.105915682	-1.594972373	0.110718414	0.145711223	negative
PRJNA729271	stage_E18_vs_E10	ordinary_muscle_development	ENSGALG00010007485	CTSC	-0.048684965	0.1561825	-0.311718435	0.755254516	0.794653629	negative
PRJNA729271	stage_E18_vs_E10	ordinary_muscle_development	ENSGALG00010007500	MPHOSPH6	0.342809406	0.116587442	2.94036304	0.003278279	0.005572075	positive
PRJNA729271	stage_E18_vs_E10	ordinary_muscle_development	ENSGALG00010007506	RAB38	-3.027674408	0.650966648	-4.651043827	3.30259E-06	8.05639E-06	negative
PRJNA729271	stage_E18_vs_E10	ordinary_muscle_development	ENSGALG00010007513	GALNT12	-0.126809373	0.129323033	-0.980562939	0.326808318	0.384847394	negative
PRJNA729271	stage_E18_vs_E10	ordinary_muscle_development	ENSGALG00010007514	VIT	-6.215660919	0.436563929	-14.23768777	5.3467E-46	1.32479E-44	negative
PRJNA729271	stage_E18_vs_E10	ordinary_muscle_development	ENSGALG00010007521	SLC12A7	-0.752419959	0.137924217	-5.455314323	4.88863E-08	1.42233E-07	negative
PRJNA729271	stage_E18_vs_E10	ordinary_muscle_development	ENSGALG00010007529	CDH13	1.214345723	0.13473046	9.013149075	2.00223E-19	1.36319E-18	positive
PRJNA729271	stage_E18_vs_E10	ordinary_muscle_development	ENSGALG00010007532	DCUN1D5	-0.479551149	0.267192376	-1.794778561	0.072688988	0.099108915	negative
PRJNA729271	stage_E18_vs_E10	ordinary_muscle_development	ENSGALG00010007536	GABBR2	0.586587531	0.195790835	2.99599075	0.002735547	0.004712033	positive
PRJNA729271	stage_E18_vs_E10	ordinary_muscle_development	ENSGALG00010007539	RPS3A	1.258383718	0.17533006	7.177227454	7.11393E-13	3.08455E-12	positive
PRJNA729271	stage_E18_vs_E10	ordinary_muscle_development	ENSGALG00010007542	MRPL36	0.472595254	0.110457527	4.278524691	1.88136E-05	4.25103E-05	positive
PRJNA729271	stage_E18_vs_E10	ordinary_muscle_development	ENSGALG00010007544	MLYCD	0.279929926	0.10631148	2.633110984	0.00846067	0.013534644	positive
PRJNA729271	stage_E18_vs_E10	ordinary_muscle_development	ENSGALG00010007547	ENSGALG00010007547	-1.222053266	0.730495465	-1.672910134	0.094344988	0.125749941	negative
PRJNA729271	stage_E18_vs_E10	ordinary_muscle_development	ENSGALG00010007548	HSBP1	-0.0634945	0.086950211	-0.730239745	0.465243652	0.524393992	negative
PRJNA729271	stage_E18_vs_E10	ordinary_muscle_development	ENSGALG00010007549	SH3D19	1.283522559	0.15758979	8.144706341	3.80203E-16	2.09625E-15	positive
PRJNA729271	stage_E18_vs_E10	ordinary_muscle_development	ENSGALG00010007551	FEZ2	0.106870273	0.111634374	0.957324073	0.338403709	0.396761446	positive
PRJNA729271	stage_E18_vs_E10	ordinary_muscle_development	ENSGALG00010007555	NDUFS6	1.111116994	0.130296953	8.52757464	1.49448E-17	9.0638E-17	positive
PRJNA729271	stage_E18_vs_E10	ordinary_muscle_development	ENSGALG00010007564	IRX4	-8.777973777	0.947294196	-9.266364998	1.92598E-20	1.39641E-19	negative
PRJNA729271	stage_E18_vs_E10	ordinary_muscle_development	ENSGALG00010007566	FHIP1A	1.737711762	0.170686593	10.1807162	2.41775E-24	2.1972E-23	positive
PRJNA729271	stage_E18_vs_E10	ordinary_muscle_development	ENSGALG00010007567	TRIP13	-1.852292906	0.133538536	-13.8708493	9.51435E-44	2.1217E-42	negative
PRJNA729271	stage_E18_vs_E10	ordinary_muscle_development	ENSGALG00010007569	GPR89A	0.048798343	0.082037226	0.5948317	0.551955975	0.606060288	positive
PRJNA729271	stage_E18_vs_E10	ordinary_muscle_development	ENSGALG00010007574	ENSGALG00010007574	0.598323153	0.187705244	3.187567599	0.001434749	0.002565599	positive
PRJNA729271	stage_E18_vs_E10	ordinary_muscle_development	ENSGALG00010007575	MMP27	-2.699790524	0.34340094	-7.861919437	3.78291E-15	1.94928E-14	negative
PRJNA729271	stage_E18_vs_E10	ordinary_muscle_development	ENSGALG00010007577	WFDC1	0.975448824	0.118999202	8.197103921	2.46248E-16	1.37442E-15	positive
PRJNA729271	stage_E18_vs_E10	ordinary_muscle_development	ENSGALG00010007579	GATB	1.48023253	0.178103002	8.311103773	9.4816E-17	5.44947E-16	positive
PRJNA729271	stage_E18_vs_E10	ordinary_muscle_development	ENSGALG00010007580	BRD9	-0.472082088	0.102027087	-4.627027008	3.70952E-06	9.00059E-06	negative
PRJNA729271	stage_E18_vs_E10	ordinary_muscle_development	ENSGALG00010007581	LPCAT1	-0.589808584	0.116895068	-5.045624205	4.52043E-07	1.20692E-06	negative
PRJNA729271	stage_E18_vs_E10	ordinary_muscle_development	ENSGALG00010007584	ELP2	-0.81312399	0.118544409	-6.859235275	6.92301E-12	2.75684E-11	negative
PRJNA729271	stage_E18_vs_E10	ordinary_muscle_development	ENSGALG00010007585	KCNG4	-2.65461283	0.801834948	-3.310672398	0.000930721	0.001702095	negative
PRJNA729271	stage_E18_vs_E10	ordinary_muscle_development	ENSGALG00010007586	MMP7	-1.883206385	1.697970734	-1.10909237	0.267390336	0.32244783	negative
PRJNA729271	stage_E18_vs_E10	ordinary_muscle_development	ENSGALG00010007588	TTC27	-1.215216063	0.104886142	-11.58604978	4.84988E-31	6.2711E-30	negative
PRJNA729271	stage_E18_vs_E10	ordinary_muscle_development	ENSGALG00010007590	FBXW7	1.641072168	0.137299695	11.95248223	6.30146E-33	8.74063E-32	positive
PRJNA729271	stage_E18_vs_E10	ordinary_muscle_development	ENSGALG00010007593	SEC61B	-0.786614264	0.118946336	-6.613186169	3.76135E-11	1.41576E-10	negative
PRJNA729271	stage_E18_vs_E10	ordinary_muscle_development	ENSGALG00010007595	ATP2C2	1.758701508	0.153507722	11.45676244	2.17494E-30	2.70607E-29	positive
PRJNA729271	stage_E18_vs_E10	ordinary_muscle_development	ENSGALG00010007596	ENSGALG00010007596	-1.164500402	0.496316302	-2.346286829	0.018961509	0.028695665	negative
PRJNA729271	stage_E18_vs_E10	ordinary_muscle_development	ENSGALG00010007597	SMKR1	-1.658340835	0.777148083	-2.133880108	0.032852596	0.047648427	negative
PRJNA729271	stage_E18_vs_E10	ordinary_muscle_development	ENSGALG00010007601	TMEM154	-0.32606091	0.326930089	-0.997341393	0.318598828	0.376280454	negative
PRJNA729271	stage_E18_vs_E10	ordinary_muscle_development	ENSGALG00010007603	ALG2	-0.420822084	0.10666482	-3.945275339	7.97083E-05	0.000167859	negative
PRJNA729271	stage_E18_vs_E10	ordinary_muscle_development	ENSGALG00010007604	ENSGALG00010007604	-0.071233557	0.504191564	-0.141282722	0.887646598	0.907237163	negative
PRJNA729271	stage_E18_vs_E10	ordinary_muscle_development	ENSGALG00010007610	TLDC1	-0.059425587	0.158707088	-0.37443562	0.708080246	0.752465042	negative
PRJNA729271	stage_E18_vs_E10	ordinary_muscle_development	ENSGALG00010007614	BIRC6	-0.174466383	0.08817455	-1.978647848	0.047855669	0.067411848	negative
PRJNA729271	stage_E18_vs_E10	ordinary_muscle_development	ENSGALG00010007624	TPPP	1.231591241	0.247662418	4.972862855	6.59713E-07	1.73486E-06	positive
PRJNA729271	stage_E18_vs_E10	ordinary_muscle_development	ENSGALG00010007626	DLK2	-0.491540949	0.355900902	-1.381117458	0.167242849	0.211589866	negative
PRJNA729271	stage_E18_vs_E10	ordinary_muscle_development	ENSGALG00010007633	ENSGALG00010007633	0.432604721	0.103695109	4.171891299	3.02082E-05	6.65857E-05	positive
PRJNA729271	stage_E18_vs_E10	ordinary_muscle_development	ENSGALG00010007640	TMEM123	-2.080257913	0.220139237	-9.449737079	3.39683E-21	2.58598E-20	negative
PRJNA729271	stage_E18_vs_E10	ordinary_muscle_development	ENSGALG00010007642	ZNF318	0.231874116	0.107622267	2.154518035	0.031199569	0.045455599	positive
PRJNA729271	stage_E18_vs_E10	ordinary_muscle_development	ENSGALG00010007643	COTL1	0.408463521	0.163934781	2.491622093	0.012716125	0.019815119	positive
PRJNA729271	stage_E18_vs_E10	ordinary_muscle_development	ENSGALG00010007650	KLHL36	0.066891055	0.088153894	0.758798635	0.447973022	0.507532356	positive
PRJNA729271	stage_E18_vs_E10	ordinary_muscle_development	ENSGALG00010007651	CTNNAL1	-0.655209359	0.137659894	-4.759624168	1.93954E-06	4.85303E-06	negative
PRJNA729271	stage_E18_vs_E10	ordinary_muscle_development	ENSGALG00010007658	BIRC2	0.087877259	0.120033655	0.73210517	0.464104379	0.523231933	positive
PRJNA729271	stage_E18_vs_E10	ordinary_muscle_development	ENSGALG00010007660	FAM98A	-0.775222235	0.098993275	-7.831059592	4.83775E-15	2.47348E-14	negative
PRJNA729271	stage_E18_vs_E10	ordinary_muscle_development	ENSGALG00010007663	ARFIP1	0.006093681	0.110249487	0.055271741	0.955921975	0.963499689	positive
PRJNA729271	stage_E18_vs_E10	ordinary_muscle_development	ENSGALG00010007671	FAM206A	0.076917038	0.10438441	0.736863267	0.461205504	0.520490017	positive
PRJNA729271	stage_E18_vs_E10	ordinary_muscle_development	ENSGALG00010007673	FHDC1	0.171192235	0.322998112	0.530010019	0.596104984	0.647959636	positive
PRJNA729271	stage_E18_vs_E10	ordinary_muscle_development	ENSGALG00010007678	CDH8	-0.132709595	0.29006849	-0.457511242	0.647303624	0.695888603	negative
PRJNA729271	stage_E18_vs_E10	ordinary_muscle_development	ENSGALG00010007679	TMEM135	1.269950597	0.141669824	8.964157378	3.12662E-19	2.10793E-18	positive
PRJNA729271	stage_E18_vs_E10	ordinary_muscle_development	ENSGALG00010007680	CHMP2B	-0.224826798	0.12618398	-1.78173805	0.074791959	0.101698822	negative
PRJNA729271	stage_E18_vs_E10	ordinary_muscle_development	ENSGALG00010007695	RASGRP3	1.02954354	0.143246306	7.187225741	6.6121E-13	2.87211E-12	positive
PRJNA729271	stage_E18_vs_E10	ordinary_muscle_development	ENSGALG00010007698	ADAMTS18	-0.761722326	0.399036836	-1.908902283	0.056274699	0.078282318	negative
PRJNA729271	stage_E18_vs_E10	ordinary_muscle_development	ENSGALG00010007699	SLC39A6	-0.936494835	0.105949632	-8.839057001	9.65299E-19	6.32836E-18	negative
PRJNA729271	stage_E18_vs_E10	ordinary_muscle_development	ENSGALG00010007707	ENSGALG00010007707	2.88303586	0.760955137	3.788706744	0.000151434	0.000308594	positive
PRJNA729271	stage_E18_vs_E10	ordinary_muscle_development	ENSGALG00010007713	SCLT1	-0.53792228	0.214061098	-2.512938064	0.011973035	0.018733716	negative
PRJNA729271	stage_E18_vs_E10	ordinary_muscle_development	ENSGALG00010007719	LTBP1	-2.156729126	0.191128131	-11.28420561	1.57054E-29	1.87675E-28	negative
PRJNA729271	stage_E18_vs_E10	ordinary_muscle_development	ENSGALG00010007724	RBM11	0.32915657	0.375175028	0.877341362	0.380301253	0.440644817	positive
PRJNA729271	stage_E18_vs_E10	ordinary_muscle_development	ENSGALG00010007730	COG2	-0.44340618	0.065905478	-6.727910845	1.72116E-11	6.63787E-11	negative
PRJNA729271	stage_E18_vs_E10	ordinary_muscle_development	ENSGALG00010007731	ENSGALG00010007731	-0.855801912	0.274569673	-3.116884336	0.001827733	0.003230458	negative
PRJNA729271	stage_E18_vs_E10	ordinary_muscle_development	ENSGALG00010007741	ENSGALG00010007741	-0.600324832	0.209024678	-2.872028497	0.004078462	0.006839906	negative
PRJNA729271	stage_E18_vs_E10	ordinary_muscle_development	ENSGALG00010007748	CSGALNACT1	-2.831771447	0.272038738	-10.40944195	2.24534E-25	2.17555E-24	negative
PRJNA729271	stage_E18_vs_E10	ordinary_muscle_development	ENSGALG00010007751	HSPA13	-0.860942673	0.100637426	-8.554895634	1.17975E-17	7.21846E-17	negative
PRJNA729271	stage_E18_vs_E10	ordinary_muscle_development	ENSGALG00010007753	PRSS23	1.572227692	0.227649768	6.906344364	4.97302E-12	2.01577E-11	positive
PRJNA729271	stage_E18_vs_E10	ordinary_muscle_development	ENSGALG00010007758	DEK	-0.62316418	0.077153155	-8.076975997	6.63926E-16	3.61654E-15	negative
PRJNA729271	stage_E18_vs_E10	ordinary_muscle_development	ENSGALG00010007759	AGT	1.953075944	1.270009916	1.537843067			positive
PRJNA729271	stage_E18_vs_E10	ordinary_muscle_development	ENSGALG00010007761	ENSGALG00010007761	0.380836465	0.122111401	3.118762561	0.001816122	0.003211896	positive
PRJNA729271	stage_E18_vs_E10	ordinary_muscle_development	ENSGALG00010007762	SH2D4A	-1.156473172	0.350896932	-3.295763131	0.000981547	0.001789176	negative
PRJNA729271	stage_E18_vs_E10	ordinary_muscle_development	ENSGALG00010007763	FZD4	1.106986127	0.099564628	11.1182671	1.02238E-28	1.17335E-27	positive
PRJNA729271	stage_E18_vs_E10	ordinary_muscle_development	ENSGALG00010007765	NUDT7	0.035897995	0.097837277	0.366915313	0.713682189	0.757418606	positive
PRJNA729271	stage_E18_vs_E10	ordinary_muscle_development	ENSGALG00010007766	ENSGALG00010007766	0.3835519	0.138721062	2.764914673	0.005693769	0.009356216	positive
PRJNA729271	stage_E18_vs_E10	ordinary_muscle_development	ENSGALG00010007767	VAT1L	-0.990066059	0.321417564	-3.080311	0.002067845	0.003624794	negative
PRJNA729271	stage_E18_vs_E10	ordinary_muscle_development	ENSGALG00010007770	ENSGALG00010007770	-1.160900478	0.452760635	-2.564049054	0.010345892	0.016337676	negative
PRJNA729271	stage_E18_vs_E10	ordinary_muscle_development	ENSGALG00010007771	CAPN9	-1.290089543	0.458316457	-2.814844468	0.004880085	0.008102729	negative
PRJNA729271	stage_E18_vs_E10	ordinary_muscle_development	ENSGALG00010007772	CLEC3A	-1.497477469	0.564072649	-2.654759935	0.007936487	0.012734046	negative
PRJNA729271	stage_E18_vs_E10	ordinary_muscle_development	ENSGALG00010007774	ENSGALG00010007774	0.729990987	0.280317694	2.604155941	0.009210084	0.014636572	positive
PRJNA729271	stage_E18_vs_E10	ordinary_muscle_development	ENSGALG00010007777	KIF13A	0.315274874	0.125065104	2.520886031	0.011705976	0.018349532	positive
PRJNA729271	stage_E18_vs_E10	ordinary_muscle_development	ENSGALG00010007780	SIRT5	0.297554086	0.14339214	2.075107372	0.037976585	0.054464292	positive
PRJNA729271	stage_E18_vs_E10	ordinary_muscle_development	ENSGALG00010007781	MBP	4.172892133	0.235151959	17.74551294	1.86688E-70	1.00972E-68	positive
PRJNA729271	stage_E18_vs_E10	ordinary_muscle_development	ENSGALG00010007783	NOL7	-0.360675237	0.110563528	-3.262153835	0.001105691	0.002003625	negative
PRJNA729271	stage_E18_vs_E10	ordinary_muscle_development	ENSGALG00010007784	INTS10	-0.948327012	0.096033329	-9.874977995	5.34447E-23	4.48051E-22	negative
PRJNA729271	stage_E18_vs_E10	ordinary_muscle_development	ENSGALG00010007786	SAMSN1	1.420554951	0.228790921	6.208965583	5.33345E-10	1.84948E-09	positive
PRJNA729271	stage_E18_vs_E10	ordinary_muscle_development	ENSGALG00010007787	C1orf198	-1.227866148	0.119608342	-10.26572331	1.00564E-24	9.38623E-24	negative
PRJNA729271	stage_E18_vs_E10	ordinary_muscle_development	ENSGALG00010007788	PALM2AKAP2	2.369137729	0.164564112	14.39644223	5.44765E-47	1.42021E-45	positive

PRJNA729271	stage_E18_vs_E10	ordinary_muscle_development	ENSGALG00010007789	WWOX	-0.091628421	0.111798976	-0.819581933	0.412454477	0.472685614	negative
PRJNA729271	stage_E18_vs_E10	ordinary_muscle_development	ENSGALG00010007792	ENSGALG00010007792	0.313717975	0.240263981	1.305722037	0.191647101	0.239311228	positive
PRJNA729271	stage_E18_vs_E10	ordinary_muscle_development	ENSGALG00010007795	ZNF236	-0.055063225	0.116670461	-0.471955152	0.6369588	0.686345362	negative
PRJNA729271	stage_E18_vs_E10	ordinary_muscle_development	ENSGALG00010007796	MCUR1	-0.83407309	0.104768853	-7.961078761	1.70546E-15	8.99586E-15	negative
PRJNA729271	stage_E18_vs_E10	ordinary_muscle_development	ENSGALG00010007798	HGSNAT	0.486579317	0.097975257	4.966348968	6.82251E-07	1.79185E-06	positive
PRJNA729271	stage_E18_vs_E10	ordinary_muscle_development	ENSGALG00010007800	MAF	0.890912581	0.11404379	7.81202186	5.62778E-15	2.86327E-14	positive
PRJNA729271	stage_E18_vs_E10	ordinary_muscle_development	ENSGALG00010007801	TTC13	-0.420967788	0.163317089	-2.577610166	0.009948616	0.015742925	negative
PRJNA729271	stage_E18_vs_E10	ordinary_muscle_development	ENSGALG00010007802	ENSGALG00010007802	6.838403155	0.715206981	9.561432333	1.16138E-21	9.1045E-21	positive
PRJNA729271	stage_E18_vs_E10	ordinary_muscle_development	ENSGALG00010007803	ME3	1.348175589	0.176767388	7.626834372	2.40588E-14	1.15974E-13	positive
PRJNA729271	stage_E18_vs_E10	ordinary_muscle_development	ENSGALG00010007805	ENSGALG00010007805	9.811498018	1.517179559	6.466932643	1.00012E-10	3.64607E-10	positive
PRJNA729271	stage_E18_vs_E10	ordinary_muscle_development	ENSGALG00010007806	DYNLRB2	0.542769162	0.357316343	1.519015777	0.128758525	0.167296057	positive
PRJNA729271	stage_E18_vs_E10	ordinary_muscle_development	ENSGALG00010007809	ARV1	-0.287585558	0.120968715	-2.377354817	0.017437306	0.026566507	negative
PRJNA729271	stage_E18_vs_E10	ordinary_muscle_development	ENSGALG00010007811	PRCP	1.044806506	0.182403328	5.728001332	1.01621E-08	3.1367E-08	positive
PRJNA729271	stage_E18_vs_E10	ordinary_muscle_development	ENSGALG00010007812	POMK	0.136278184	0.121620281	1.120521869	0.262491439	0.31696213	positive
PRJNA729271	stage_E18_vs_E10	ordinary_muscle_development	ENSGALG00010007817	CDYL2	-0.961159538	0.137677907	-6.981218378	2.92631E-12	1.20811E-11	negative
PRJNA729271	stage_E18_vs_E10	ordinary_muscle_development	ENSGALG00010007818	ENSGALG00010007818	0.72692566	0.222824145	3.26232896	0.001105008	0.002002638	positive
PRJNA729271	stage_E18_vs_E10	ordinary_muscle_development	ENSGALG00010007819	NRIP1	0.80503817	0.166619248	4.831603682	1.35438E-06	3.45264E-06	positive
PRJNA729271	stage_E18_vs_E10	ordinary_muscle_development	ENSGALG00010007821	FAM89A	-1.461894042	0.125748149	-11.62557107	3.05543E-31	3.97563E-30	negative
PRJNA729271	stage_E18_vs_E10	ordinary_muscle_development	ENSGALG00010007826	ENSGALG00010007826	9.453905786	1.374078288	6.880179876	5.9777E-12	2.39952E-11	positive
PRJNA729271	stage_E18_vs_E10	ordinary_muscle_development	ENSGALG00010007830	SMIM19	1.154485101	0.130270522	8.862212938	7.84416E-19	5.16823E-18	positive
PRJNA729271	stage_E18_vs_E10	ordinary_muscle_development	ENSGALG00010007835	GUCY1A1	2.071334979	0.185860797	11.14455021	7.6128E-29	8.77864E-28	positive
PRJNA729271	stage_E18_vs_E10	ordinary_muscle_development	ENSGALG00010007845	TFAP2B	-7.675260668	1.094265546	-7.014075051	2.31475E-12	9.61936E-12	negative
PRJNA729271	stage_E18_vs_E10	ordinary_muscle_development	ENSGALG00010007846	ENSGALG00010007846	0.43658763	0.149189289	2.92640063	0.00342909	0.005805263	positive
PRJNA729271	stage_E18_vs_E10	ordinary_muscle_development	ENSGALG00010007847	CMC2	-0.425030878	0.121133037	-3.508794031	0.000450143	0.000861477	negative
PRJNA729271	stage_E18_vs_E10	ordinary_muscle_development	ENSGALG00010007849	CXADR	-4.640792289	0.324761452	-14.28984954	2.53156E-46	6.39285E-45	negative
PRJNA729271	stage_E18_vs_E10	ordinary_muscle_development	ENSGALG00010007850	ENSGALG00010007850	-1.130149492	0.276812757	-4.082721851	4.45113E-05	9.62972E-05	negative
PRJNA729271	stage_E18_vs_E10	ordinary_muscle_development	ENSGALG00010007851	AvBD7	5.436679036	0.893954403	6.08160664	1.18984E-09	3.98493E-09	positive
PRJNA729271	stage_E18_vs_E10	ordinary_muscle_development	ENSGALG00010007852	SLC20A2	1.34896175	0.20753314	6.499982363	8.03294E-11	2.95377E-10	positive
PRJNA729271	stage_E18_vs_E10	ordinary_muscle_development	ENSGALG00010007853	C1orf131	-0.515972206	0.116085879	-4.444745638	8.79959E-06	2.06158E-05	negative
PRJNA729271	stage_E18_vs_E10	ordinary_muscle_development	ENSGALG00010007854	FAM181B	0.607012625	0.118053329	5.141850982	2.72045E-07	7.41359E-07	positive
PRJNA729271	stage_E18_vs_E10	ordinary_muscle_development	ENSGALG00010007855	RNF182	-1.737843527	0.228755463	-7.596948737	3.03195E-14	1.44661E-13	negative
PRJNA729271	stage_E18_vs_E10	ordinary_muscle_development	ENSGALG00010007856	PDCD2	-0.106799654	0.134437029	-0.794421413	0.426950143	0.487064561	negative
PRJNA729271	stage_E18_vs_E10	ordinary_muscle_development	ENSGALG00010007857	ENSGALG00010007857	-0.2306871	0.340421407	-0.677651568	0.497992641	0.555261795	negative
PRJNA729271	stage_E18_vs_E10	ordinary_muscle_development	ENSGALG00010007859	CENPN	-1.546648202	0.161116506	-9.599563939	8.02834E-22	6.35906E-21	negative
PRJNA729271	stage_E18_vs_E10	ordinary_muscle_development	ENSGALG00010007860	DEFB4A	5.163477043	0.886104377	5.82716571	5.63766E-09	1.78229E-08	positive
PRJNA729271	stage_E18_vs_E10	ordinary_muscle_development	ENSGALG00010007861	INTU	-1.591829724	0.176981787	-8.994313781	2.37713E-19	1.60936E-18	negative
PRJNA729271	stage_E18_vs_E10	ordinary_muscle_development	ENSGALG00010007862	ATMIN	0.303827311	0.086578697	3.509261768	0.000449352	0.000860077	positive
PRJNA729271	stage_E18_vs_E10	ordinary_muscle_development	ENSGALG00010007863	AvBD1	4.983587312	0.820801222	6.071612932	1.26632E-09	4.22933E-09	positive
PRJNA729271	stage_E18_vs_E10	ordinary_muscle_development	ENSGALG00010007864	TYR	-1.476674328	0.985681276	-1.498125574	0.134100628	0.173475109	negative
PRJNA729271	stage_E18_vs_E10	ordinary_muscle_development	ENSGALG00010007865	GABRR3	1.048237013	0.424015043	2.472169394	0.013429586	0.020864079	positive
PRJNA729271	stage_E18_vs_E10	ordinary_muscle_development	ENSGALG00010007866	TBP	-0.392955153	0.086887662	-4.522565611	6.10945E-06	1.45341E-05	negative
PRJNA729271	stage_E18_vs_E10	ordinary_muscle_development	ENSGALG00010007867	ZNF516	-0.060821356	0.105142518	-0.578465847	0.562949649	0.616869909	negative
PRJNA729271	stage_E18_vs_E10	ordinary_muscle_development	ENSGALG00010007868	INHBA	-0.166095428	0.292094573	-0.56863579	0.569603331	0.623218621	negative
PRJNA729271	stage_E18_vs_E10	ordinary_muscle_development	ENSGALG00010007869	GCSH	-1.563150773	0.126275614	-12.37888083	3.40052E-35	5.28301E-34	negative
PRJNA729271	stage_E18_vs_E10	ordinary_muscle_development	ENSGALG00010007870	AvBD3	0.058191264	0.528521875	0.110101902	0.91232856	0.92762363	positive
PRJNA729271	stage_E18_vs_E10	ordinary_muscle_development	ENSGALG00010007871	HSPA4L	-0.814983569	0.088842205	-9.173382986	4.58402E-20	3.22707E-19	negative
PRJNA729271	stage_E18_vs_E10	ordinary_muscle_development	ENSGALG00010007872	KLHL2	-0.861195677	0.078849971	-10.92195299	9.0528E-28	9.97115E-27	negative
PRJNA729271	stage_E18_vs_E10	ordinary_muscle_development	ENSGALG00010007873	ENSGALG00010007873	1.432425779	0.767081463	1.867371132	0.061849778	0.085325263	positive
PRJNA729271	stage_E18_vs_E10	ordinary_muscle_development	ENSGALG00010007874	ENSGALG00010007874	-0.136774694	0.570636729	-0.239687856	0.81057225	0.842420934	negative
PRJNA729271	stage_E18_vs_E10	ordinary_muscle_development	ENSGALG00010007875	AvBD4	4.94175217	1.393457845	3.546395169	0.00039054	0.000753077	positive
PRJNA729271	stage_E18_vs_E10	ordinary_muscle_development	ENSGALG00010007877	MSMO1	-1.67683132	0.190578584	-8.798634581	1.38491E-18	8.99384E-18	negative
PRJNA729271	stage_E18_vs_E10	ordinary_muscle_development	ENSGALG00010007878	PSMB1	-0.622774423	0.122815832	-5.070799206	3.96149E-07	1.06514E-06	negative
PRJNA729271	stage_E18_vs_E10	ordinary_muscle_development	ENSGALG00010007879	PLK4	-1.445455937	0.191152922	-7.561777873	3.97597E-14	1.88032E-13	negative
PRJNA729271	stage_E18_vs_E10	ordinary_muscle_development	ENSGALG00010007880	BCO1	0.455670403	0.288868126	1.577434002	0.114695697	0.150481004	positive
PRJNA729271	stage_E18_vs_E10	ordinary_muscle_development	ENSGALG00010007881	AvBD14	-1.743236323	0.718657863	-2.425683226	0.015279601	0.02355863	negative
PRJNA729271	stage_E18_vs_E10	ordinary_muscle_development	ENSGALG00010007882	CPE	0.361910058	0.226937443	1.594756922	0.110766604	0.145761409	positive
PRJNA729271	stage_E18_vs_E10	ordinary_muscle_development	ENSGALG00010007883	DHTKD1	1.063439118	0.1201537	8.850656418	8.7006E-19	5.71174E-18	positive
PRJNA729271	stage_E18_vs_E10	ordinary_muscle_development	ENSGALG00010007884	GLI3	-1.220816729	0.112453922	-10.85615078	1.86443E-27	2.0289E-26	negative
PRJNA729271	stage_E18_vs_E10	ordinary_muscle_development	ENSGALG00010007885	USP38	0.244770589	0.104980809	2.331574622	0.01972308	0.029767394	positive
PRJNA729271	stage_E18_vs_E10	ordinary_muscle_development	ENSGALG00010007886	GRM5	-0.379450064	0.704274861	-0.538781213	0.590037828	0.642086961	negative
PRJNA729271	stage_E18_vs_E10	ordinary_muscle_development	ENSGALG00010007887	CDT1	-0.809446836	0.125247478	-6.462779515	1.02797E-10	3.74289E-10	negative
PRJNA729271	stage_E18_vs_E10	ordinary_muscle_development	ENSGALG00010007889	DCAF6	1.566782973	0.097200723	16.11904652	1.8748E-58	7.15136E-57	positive
PRJNA729271	stage_E18_vs_E10	ordinary_muscle_development	ENSGALG00010007890	ENSGALG00010007890	-0.339493609	0.125613942	-2.702674576	0.006878405	0.011141187	negative
PRJNA729271	stage_E18_vs_E10	ordinary_muscle_development	ENSGALG00010007891	GDI1	-0.703466405	0.056015443	-12.55843686	3.57322E-36	5.84581E-35	negative
PRJNA729271	stage_E18_vs_E10	ordinary_muscle_development	ENSGALG00010007892	ENSGALG00010007892	0.991803304	0.440487987	2.251601255	0.024347482	0.036177212	positive
PRJNA729271	stage_E18_vs_E10	ordinary_muscle_development	ENSGALG00010007893	MFSD8	-0.005017041	0.152371498	-0.032926371	0.973733303	0.978322882	negative
PRJNA729271	stage_E18_vs_E10	ordinary_muscle_development	ENSGALG00010007894	CD88	0.976223127	0.263935291	3.698721463	0.000216688	0.000432333	positive
PRJNA729271	stage_E18_vs_E10	ordinary_muscle_development	ENSGALG00010007895	GOLGB1	-0.351197066	0.111990352	-3.135958221	0.001712936	0.003037188	negative
PRJNA729271	stage_E18_vs_E10	ordinary_muscle_development	ENSGALG00010007896	TIMMDC1	0.001673753	0.106107107	0.015774182	0.987414546	0.989325627	positive
PRJNA729271	stage_E18_vs_E10	ordinary_muscle_development	ENSGALG00010007897	EFHC1	-0.678026214	0.378941372	-1.789264156	0.073572284	0.100171919	negative
PRJNA729271	stage_E18_vs_E10	ordinary_muscle_development	ENSGALG00010007898	GAB1	0.681382722	0.089077745	7.649303672	2.02071E-14	9.81573E-14	positive
PRJNA729271	stage_E18_vs_E10	ordinary_muscle_development	ENSGALG00010007899	APRT	-0.52096036	0.15324718	-3.399477638	0.000675147	0.001260303	negative
PRJNA729271	stage_E18_vs_E10	ordinary_muscle_development	ENSGALG00010007900	FHOD3	1.33017852	0.199782808	6.65812306	2.77347E-11	1.05377E-10	positive
PRJNA729271	stage_E18_vs_E10	ordinary_muscle_development	ENSGALG00010007901	FSTL5	0.519496355	0.292627191	1.775283953	0.075851014	0.103013253	positive
PRJNA729271	stage_E18_vs_E10	ordinary_muscle_development	ENSGALG00010007902	C7orf25	-0.264434119	0.148692911	-1.778390899	0.075339676	0.102385956	negative
PRJNA729271	stage_E18_vs_E10	ordinary_muscle_development	ENSGALG00010007903	PSMA2	-0.435993498	0.104221856	-4.183321195	2.87281E-05	6.35455E-05	negative
PRJNA729271	stage_E18_vs_E10	ordinary_muscle_development	ENSGALG00010007904	ENSGALG00010007904	-0.337036472	0.126118067	-2.672388502	0.00753134	0.012128294	negative
PRJNA729271	stage_E18_vs_E10	ordinary_muscle_development	ENSGALG00010007905	MRPL32	0.112708609	0.156925264	0.718231127	0.472614789	0.531421252	positive
PRJNA729271	stage_E18_vs_E10	ordinary_muscle_development	ENSGALG00010007906	NAF1	-0.684132537	0.112724441	-6.069070137	1.28653E-09	4.29287E-09	negative
PRJNA729271	stage_E18_vs_E10	ordinary_muscle_development	ENSGALG00010007907	IRX2	-6.158621863	0.803156582	-7.668021403	1.7467E-14	8.53333E-14	negative
PRJNA729271	stage_E18_vs_E10	ordinary_muscle_development	ENSGALG00010007908	IRX1	-5.956598678	0.808460907	-7.367825244	1.73434E-13	7.83642E-13	negative
PRJNA729271	stage_E18_vs_E10	ordinary_muscle_development	ENSGALG00010007909	GALNS	0.210986695	0.106852097	1.97456766	0.048317232	0.067995949	positive
PRJNA729271	stage_E18_vs_E10	ordinary_muscle_development	ENSGALG00010007910	ANKS6	0.458004055	0.147091458	3.113736583	0.001847343	0.003262732	positive
PRJNA729271	stage_E18_vs_E10	ordinary_muscle_development	ENSGALG00010007911	IFI6	0.574241716	0.824905516	0.696130289	0.486347239	0.544328873	positive
PRJNA729271	stage_E18_vs_E10	ordinary_muscle_development	ENSGALG00010007912	TRAPPC2L	0.086150521	0.113645374	0.758064472	0.448412387	0.50799043	positive
PRJNA729271	stage_E18_vs_E10	ordinary_muscle_development	ENSGALG00010007914	PAQR8	-0.145453986	0.128612348	-1.130948841	0.258076625	0.312099172	negative
PRJNA729271	stage_E18_vs_E10	ordinary_muscle_development	ENSGALG00010007915	HECW1	1.129962156	0.13189463	8.567158164	1.0607E-17	6.50926E-17	positive
PRJNA729271	stage_E18_vs_E10	ordinary_muscle_development	ENSGALG00010007916	MCM3	-1.869175035	0.203080656	-9.204101824	3.44541E-20	2.44211E-19	negative
PRJNA729271	stage_E18_vs_E10	ordinary_muscle_development	ENSGALG00010007917	POGLUT1	-1.549178844	0.080002363	-19.36416363	1.54849E-83	1.17515E-81	negative
PRJNA729271	stage_E18_vs_E10	ordinary_muscle_development	ENSGALG00010007918	NPY1R	-1.435063952	0.705044556	-2.035423066	0.041808322	0.059447874	negative
PRJNA729271	stage_E18_vs_E10	ordinary_muscle_development	ENSGALG00010007920	FAT3	-1.004475083	0.178386207	-5.630901054	1.79271E-08	5.4136E-08	negative
PRJNA729271	stage_E18_vs_E10	ordinary_muscle_development	ENSGALG00010007921	FAM120B	-0.298240336	0.101919675	-2.926229267	0.00343098	0.005807784	negative
PRJNA729271	stage_E18_vs_E10	ordinary_muscle_development	ENSGALG00010007922	IL17A	-0.475021511	0.500331665	-0.949413247	0.342410476	0.400875522	negative
PRJNA729271	stage_E18_vs_E10	ordinary_muscle_development	ENSGALG00010007923	YAP1	-1.366987722	0.091655793	-14.91436257	2.65803E-50	7.8629E-49	negative
PRJNA729271	stage_E18_vs_E10	ordinary_muscle_development	ENSGALG00010007924	ABHD18	0.947125877	0.115076806	8.230380326	1.8662E-16	1.05092E-15	positive
PRJNA729271	stage_E18_vs_E10	ordinary_muscle_development	ENSGALG00010007925	STK17A	1.701082385	0.23540801	7.226102411	4.97053E-13	2.17601E-12	positive
PRJNA729271	stage_E18_vs_E10	ordinary_muscle_development	ENSGALG00010007926	EIF3E	0.382837488	0.131410286	2.913299251	0.003576317	0.006041104	positive
PRJNA729271	stage_E18_vs_E10	ordinary_muscle_development	ENSGALG00010007927	DLL1	-1.030406235	0.177334771	-5.810514352	6.22812E-09	1.96296E-08	negative
PRJNA729271	stage_E18_vs_E10	ordinary_muscle_development	ENSGALG00010007928	C11orf70	-1.975145383	0.64629788	-3.056091387	0.002242429	0.003906732	negative
PRJNA729271	stage_E18_vs_E10	ordinary_muscle_development	ENSGALG00010007929	LRRC14B	7.950484899	0.329540629	24.12596261	1.3352E-128	2.8461E-126	positive
PRJNA729271	stage_E18_vs_E10	ordinary_muscle_development	ENSGALG00010007930	CBFA2T3	1.04911684	0.196317459	5.343981342	9.09269E-08	2.5853E-07	positive
PRJNA729271	stage_E18_vs_E10	ordinary_muscle_development	ENSGALG00010007931	GBE1	0.855801276	0.189515255	4.515738207	6.30966E-06	1.49785E-05	positive
PRJNA729271	stage_E18_vs_E10	ordinary_muscle_development	ENSGALG00010007932	NPY5R	0.75700803	0.180444231	4.195246499	2.72575E-05	6.04402E-05	positive
PRJNA729271	stage_E18_vs_E10	ordinary_muscle_development	ENSGALG00010007933	CEP126	-0.189844694	0.472969195	-0.401389131	0.68813365	0.733904588	negative
PRJNA729271	stage_E18_vs_E10	ordinary_muscle_development	ENSGALG00010007934	PKHD1	0.211457611	0.340299279	0.621387186	0.534344901	0.590030421	positive
PRJNA729271	stage_E18_vs_E10	ordinary_muscle_development	ENSGALG00010007935	LARP1B	0.365477944	0.124651342	2.932001674	0.003367849	0.00570892	positive
PRJNA729271	stage_E18_vs_E10	ordinary_muscle_development	ENSGALG00010007936	ENSGALG00010007936	3.549267934	0.138961257	25.54142068	6.8384E-144	2.0229E-141	positive
PRJNA729271	stage_E18_vs_E10	ordinary_muscle_development	ENSGALG00010007937	ANGPTL5	1.673207162	0.408275931	4.098226305	4.16328E-05	9.03936E-05	positive
PRJNA729271	stage_E18_vs_E10	ordinary_muscle_development	ENSGALG00010007938	BCL9	-0.26778036	0.103428215	-2.589045549	0.009624236	0.015257936	negative
PRJNA729271	stage_E18_vs_E10	ordinary_muscle_development	ENSGALG00010007940	GLIPR2	-2.769632345	0.273249226	-10.1359202	3.82753E-24	3.43742E-23	negative
PRJNA729271	stage_E18_vs_E10	ordinary_muscle_development	ENSGALG00010007941	MRPL19	-0.714571211	0.075215958	-9.500260694	2.09366E-21	1.61251E-20	negative
PRJNA729271	stage_E18_vs_E10	ordinary_muscle_development	ENSGALG00010007942	PGRMC2	-0.067857578	0.085593868	-0.792785509	0.427902801	0.487959334	negative
PRJNA729271	stage_E18_vs_E10	ordinary_muscle_development	ENSGALG00010007943	BLOC1S4	-0.022755682	0.101141966	-0.224987541	0.821988966	0.852025891	negative
PRJNA729271	stage_E18_vs_E10	ordinary_muscle_development	ENSGALG00010007944	ACSF3	1.070533989	0.132850319	8.058196594	7.74282E-16	4.19874E-15	positive
PRJNA729271	stage_E18_vs_E10	ordinary_muscle_development	ENSGALG00010007945	ENSGALG00010007945	-0.864340155	0.119730202	-7.219065365	5.23461E-13	2.28541E-12	negative
PRJNA729271	stage_E18_vs_E10	ordinary_muscle_development	ENSGALG00010007946	ENSGALG00010007946	-0.606899622	0.164454973	-3.690369531	0.000223929	0.000446041	negative
PRJNA729271	stage_E18_vs_E10	ordinary_muscle_development	ENSGALG00010007948	ENSGALG00010007948	-2.503547068	0.491436823	-5.094341636	3.49955E-07	9.45851E-07	negative
PRJNA729271	stage_E18_vs_E10	ordinary_muscle_development	ENSGALG00010007950	ARC	-1.22209563	0.580279065	-2.106048114	0.035200173	0.050778912	negative
PRJNA729271	stage_E18_vs_E10	ordinary_muscle_development	ENSGALG00010007951	TGFBR1	-0.031476228	0.113863992	-0.276437068	0.782212389	0.817518825	negative
PRJNA729271	stage_E18_vs_E10	ordinary_muscle_development	ENSGALG00010007952	EMC2	0.082587967	0.109289367	0.755681629	0.449840101	0.509408771	positive
PRJNA729271	stage_E18_vs_E10	ordinary_muscle_development	ENSGALG00010007953	SCGN	-1.009814854	0.45651649	-2.212000828	0.026966608	0.039735791	negative
PRJNA729271	stage_E18_vs_E10	ordinary_muscle_development	ENSGALG00010007954	PSCA	-2.657787616	0.739171434	-3.595630855	0.000323606	0.000630552	negative
PRJNA729271	stage_E18_vs_E10	ordinary_muscle_development	ENSGALG00010007955	TMA16	-0.47513907	0.123023584	-3.862178717	0.00011238	0.00023304	negative
PRJNA729271	stage_E18_vs_E10	ordinary_muscle_development	ENSGALG00010007956	HS1BP3	0.178327296	0.132651191	1.344332412	0.178840919	0.224908825	positive
PRJNA729271	stage_E18_vs_E10	ordinary_muscle_development	ENSGALG00010007957	CDH15	1.597731152	0.162831495	9.812175182	9.97938E-23	8.24693E-22	positive
PRJNA729271	stage_E18_vs_E10	ordinary_muscle_development	ENSGALG00010007958	ENSGALG00010007958	-2.808666223	0.883677531	-3.178383659	0.001480986	0.002644032	negative
PRJNA729271	stage_E18_vs_E10	ordinary_muscle_development	ENSGALG00010007960	TSNAX	-0.733353432	0.06380605	-11.49347798	1.42266E-30	1.78233E-29	negative
PRJNA729271	stage_E18_vs_E10	ordinary_muscle_development	ENSGALG00010007962	ADCY8	-1.243956128	0.462381166	-2.690326119	0.007138222	0.011528527	negative
PRJNA729271	stage_E18_vs_E10	ordinary_muscle_development	ENSGALG00010007963	LDAH	-0.010821283	0.085177163	-0.127044423	0.898905239	0.916160275	negative
PRJNA729271	stage_E18_vs_E10	ordinary_muscle_development	ENSGALG00010007964	RECQL4	-1.487196008	0.158708733	-9.370599707	7.21216E-21	5.37758E-20	negative
PRJNA729271	stage_E18_vs_E10	ordinary_muscle_development	ENSGALG00010007965	EGLN1	0.427213455	0.157741998	2.708305083	0.006762782	0.010966163	positive

PRJNA729271	stage_E18_vs_E10	ordinary_muscle_development	ENSGALG00010007966	TMEM74	-1.57241907	0.229946997	-6.838180502	8.02053E-12	3.1773E-11	negative
PRJNA729271	stage_E18_vs_E10	ordinary_muscle_development	ENSGALG00010007968	SLC22A31	0.753249345	0.202710244	3.715891858	0.000202488	0.000405732	positive
PRJNA729271	stage_E18_vs_E10	ordinary_muscle_development	ENSGALG00010007970	SPRTN	-0.103966842	0.108992478	-0.953890064	0.340139293	0.398506228	negative
PRJNA729271	stage_E18_vs_E10	ordinary_muscle_development	ENSGALG00010007972	EXOC8	-0.710969061	0.114363947	-6.216723705	5.07642E-10	1.76288E-09	negative
PRJNA729271	stage_E18_vs_E10	ordinary_muscle_development	ENSGALG00010007973	CARMIL1	-0.129116249	0.134545617	-0.95964664	0.337233087	0.395676995	negative
PRJNA729271	stage_E18_vs_E10	ordinary_muscle_development	ENSGALG00010007975	ANKRD11	0.518057477	0.176247	2.939383235	0.003288662	0.005587756	positive
PRJNA729271	stage_E18_vs_E10	ordinary_muscle_development	ENSGALG00010007976	ENSGALG00010007976	0.545312358	0.360317518	1.513421719	0.130172572	0.168830657	positive
PRJNA729271	stage_E18_vs_E10	ordinary_muscle_development	ENSGALG00010007977	APOB	4.961049055	1.376904576	3.603044934	0.000314511	0.000613727	positive
PRJNA729271	stage_E18_vs_E10	ordinary_muscle_development	ENSGALG00010007978	JADE1	0.315248959	0.118726854	2.655245617	0.007925068	0.012719949	positive
PRJNA729271	stage_E18_vs_E10	ordinary_muscle_development	ENSGALG00010007979	RAB30	0.267051924	0.088885999	3.004431832	0.002660774	0.004590872	positive
PRJNA729271	stage_E18_vs_E10	ordinary_muscle_development	ENSGALG00010007980	MARCHF1	-0.60256207	0.230254217	-2.616942607	0.008872126	0.014146019	negative
PRJNA729271	stage_E18_vs_E10	ordinary_muscle_development	ENSGALG00010007981	TDRD15	0.373581192	0.519526125	0.719080666	0.472091219	0.530873718	positive
PRJNA729271	stage_E18_vs_E10	ordinary_muscle_development	ENSGALG00010007982	RECK	0.011032087	0.146252875	0.075431589	0.939871326	0.950825996	positive
PRJNA729271	stage_E18_vs_E10	ordinary_muscle_development	ENSGALG00010007983	RCHY1	-0.017319139	0.087829678	-0.197190053	0.843678815	0.870772174	negative
PRJNA729271	stage_E18_vs_E10	ordinary_muscle_development	ENSGALG00010007984	EIF3H	0.609797923	0.122044573	4.996518158	5.83747E-07	1.54293E-06	positive
PRJNA729271	stage_E18_vs_E10	ordinary_muscle_development	ENSGALG00010007985	APELA	-0.047386413	0.29476588	-0.16075949	0.872282832	0.894734959	negative
PRJNA729271	stage_E18_vs_E10	ordinary_muscle_development	ENSGALG00010007986	MYC	-1.065211972	0.194602304	-5.473789096	4.40513E-08	1.28839E-07	negative
PRJNA729271	stage_E18_vs_E10	ordinary_muscle_development	ENSGALG00010007987	ENSGALG00010007987	1.044816701	0.293357594	3.561580547	0.000368629	0.000713007	positive
PRJNA729271	stage_E18_vs_E10	ordinary_muscle_development	ENSGALG00010007988	TARBP1	0.177650446	0.123467222	1.438847032	0.150193868	0.192251864	positive
PRJNA729271	stage_E18_vs_E10	ordinary_muscle_development	ENSGALG00010007989	SPG7	0.146961338	0.097551655	1.506497634	0.131939474	0.170877652	positive
PRJNA729271	stage_E18_vs_E10	ordinary_muscle_development	ENSGALG00010007990	TRHR	-3.524546821	1.154799176	-3.052086366	0.002272567	0.00395401	negative
PRJNA729271	stage_E18_vs_E10	ordinary_muscle_development	ENSGALG00010007991	PCF11	0.265090048	0.110368565	2.401861876	0.016311866	0.025017512	positive
PRJNA729271	stage_E18_vs_E10	ordinary_muscle_development	ENSGALG00010007992	LAPTM4A	0.119646342	0.09914155	1.206823397	0.227500163	0.278938832	positive
PRJNA729271	stage_E18_vs_E10	ordinary_muscle_development	ENSGALG00010007993	LRRC14	0.579563727	0.175556571	3.301293279	0.000962402	0.00175693	positive
PRJNA729271	stage_E18_vs_E10	ordinary_muscle_development	ENSGALG00010007994	FAM65B	1.066430328	0.166375024	6.409798194	1.45712E-10	5.24615E-10	positive
PRJNA729271	stage_E18_vs_E10	ordinary_muscle_development	ENSGALG00010007995	KIAA0319	-0.98483739	0.720695379	-1.366509927	0.171778967	0.216912286	negative
PRJNA729271	stage_E18_vs_E10	ordinary_muscle_development	ENSGALG00010007996	NUDCD1	-0.980907676	0.076432021	-12.83372685	1.06133E-37	1.85127E-36	negative
PRJNA729271	stage_E18_vs_E10	ordinary_muscle_development	ENSGALG00010007997	TMEM192	-0.0717106	0.137583091	-0.521216664	0.602215847	0.653572828	negative
PRJNA729271	stage_E18_vs_E10	ordinary_muscle_development	ENSGALG00010007998	RPL13	0.987296121	0.144791731	6.818732793	9.18471E-12	3.62068E-11	positive
PRJNA729271	stage_E18_vs_E10	ordinary_muscle_development	ENSGALG00010007999	SDC1	-1.102961723	0.208088744	-5.300439144	1.15524E-07	3.25593E-07	negative
PRJNA729271	stage_E18_vs_E10	ordinary_muscle_development	ENSGALG00010008000	C8orf82	0.293341371	0.17027996	1.722700495	0.084942701	0.114173261	positive
PRJNA729271	stage_E18_vs_E10	ordinary_muscle_development	ENSGALG00010008001	ALDH5A1	-0.702199628	0.137708751	-5.099164902	3.41155E-07	9.23445E-07	negative
PRJNA729271	stage_E18_vs_E10	ordinary_muscle_development	ENSGALG00010008002	GJA8	-0.727717624	0.166575502	-4.36869537	1.24991E-05	2.88265E-05	negative
PRJNA729271	stage_E18_vs_E10	ordinary_muscle_development	ENSGALG00010008004	ENY2	-0.844906347	0.171344607	-4.931035543	8.17948E-07	2.13394E-06	negative
PRJNA729271	stage_E18_vs_E10	ordinary_muscle_development	ENSGALG00010008005	ANKRD42	0.201547889	0.332749204	0.605705096	0.5447106	0.599467022	positive
PRJNA729271	stage_E18_vs_E10	ordinary_muscle_development	ENSGALG00010008006	CPNE7	1.155649695	0.485411705	2.380761905	0.017276875	0.026349779	positive
PRJNA729271	stage_E18_vs_E10	ordinary_muscle_development	ENSGALG00010008007	CBR4	0.334457398	0.122947693	2.720322674	0.006521824	0.010606287	positive
PRJNA729271	stage_E18_vs_E10	ordinary_muscle_development	ENSGALG00010008008	BTC	2.855590226	0.204518384	13.96251121	2.63966E-44	6.03501E-43	positive
PRJNA729271	stage_E18_vs_E10	ordinary_muscle_development	ENSGALG00010008009	GPLD1	1.378256437	1.253934818	1.099145201	0.271704737	0.326861994	positive
PRJNA729271	stage_E18_vs_E10	ordinary_muscle_development	ENSGALG00010008010	CERK	-0.027634958	0.092401648	-0.299074298	0.764883357	0.80270665	negative
PRJNA729271	stage_E18_vs_E10	ordinary_muscle_development	ENSGALG00010008011	ADNP2	-0.242008527	0.101070207	-2.394459602	0.016644877	0.0254609	negative
PRJNA729271	stage_E18_vs_E10	ordinary_muscle_development	ENSGALG00010008012	CCDC90B	-0.086274139	0.080023602	-1.07810867	0.280985263	0.336601767	negative
PRJNA729271	stage_E18_vs_E10	ordinary_muscle_development	ENSGALG00010008013	ENSGALG00010008013	1.651860421	0.226028086	7.308208675	2.70727E-13	1.20781E-12	positive
PRJNA729271	stage_E18_vs_E10	ordinary_muscle_development	ENSGALG00010008015	ARHGAP39	0.949356089	0.148125342	6.409140232	1.46343E-10	5.26622E-10	positive
PRJNA729271	stage_E18_vs_E10	ordinary_muscle_development	ENSGALG00010008016	GSR	-0.335590566	0.143369465	-2.34073948	0.019245591	0.029101277	negative
PRJNA729271	stage_E18_vs_E10	ordinary_muscle_development	ENSGALG00010008017	GMNN	-1.010628358	0.133700584	-7.558892635	4.06515E-14	1.92124E-13	negative
PRJNA729271	stage_E18_vs_E10	ordinary_muscle_development	ENSGALG00010008018	ENSGALG00010008018	1.090764613	0.204614469	5.330828357	9.77658E-08	2.77539E-07	positive
PRJNA729271	stage_E18_vs_E10	ordinary_muscle_development	ENSGALG00010008019	PPP2CB	-0.630112073	0.100526313	-6.268130768	3.65408E-10	1.27937E-09	negative
PRJNA729271	stage_E18_vs_E10	ordinary_muscle_development	ENSGALG00010008020	COA6	0.200294268	0.133380442	1.501676444	0.13318069	0.172377365	positive
PRJNA729271	stage_E18_vs_E10	ordinary_muscle_development	ENSGALG00010008022	SLC35F3	-0.833802327	0.396935643	-2.100598277	0.035676245	0.051405489	negative
PRJNA729271	stage_E18_vs_E10	ordinary_muscle_development	ENSGALG00010008023	ATAD2	-0.89262309	0.079159519	-11.27625713	1.71905E-29	2.04747E-28	negative
PRJNA729271	stage_E18_vs_E10	ordinary_muscle_development	ENSGALG00010008024	MRS2	0.510059361	0.096182015	5.303063785	1.13875E-07	3.21257E-07	positive
PRJNA729271	stage_E18_vs_E10	ordinary_muscle_development	ENSGALG00010008025	TBC1D22A	0.032334692	0.09721361	0.332614865	0.739425021	0.780510171	positive
PRJNA729271	stage_E18_vs_E10	ordinary_muscle_development	ENSGALG00010008026	PURG	-0.002698978	0.123140338	-0.021917907	0.982513441	0.98550497	negative
PRJNA729271	stage_E18_vs_E10	ordinary_muscle_development	ENSGALG00010008027	C6orf62	1.84282976	0.106846661	17.24742493	1.16999E-66	5.69094E-65	positive
PRJNA729271	stage_E18_vs_E10	ordinary_muscle_development	ENSGALG00010008028	KCNK1	-3.271493362	0.171953253	-19.02548109	1.0492E-80	7.52879E-79	negative
PRJNA729271	stage_E18_vs_E10	ordinary_muscle_development	ENSGALG00010008029	LY6E	-0.62726853	0.314524536	-1.99433894	0.046115026	0.065156186	negative
PRJNA729271	stage_E18_vs_E10	ordinary_muscle_development	ENSGALG00010008030	GJA5	-1.626206245	0.147940837	-10.99227423	4.16302E-28	4.64534E-27	negative
PRJNA729271	stage_E18_vs_E10	ordinary_muscle_development	ENSGALG00010008031	MAP3K21	-2.145188609	0.185738851	-11.54948787	7.42616E-31	9.46721E-30	negative
PRJNA729271	stage_E18_vs_E10	ordinary_muscle_development	ENSGALG00010008032	PUM2	-0.363881626	0.05442221	-6.686270635	2.28929E-11	8.76243E-11	negative
PRJNA729271	stage_E18_vs_E10	ordinary_muscle_development	ENSGALG00010008033	ACOT13	0.916741719	0.119404154	7.67763672	1.6205E-14	7.94627E-14	positive
PRJNA729271	stage_E18_vs_E10	ordinary_muscle_development	ENSGALG00010008034	DPEP1	1.54844847	0.313904446	4.932865677	8.10319E-07	2.1167E-06	positive
PRJNA729271	stage_E18_vs_E10	ordinary_muscle_development	ENSGALG00010008035	TDP2	-0.047864473	0.103515272	-0.46239045	0.643801347	0.692894307	negative
PRJNA729271	stage_E18_vs_E10	ordinary_muscle_development	ENSGALG00010008036	SPOCK3	-2.511852428	0.169195498	-14.8458585	7.40013E-50	2.14102E-48	negative
PRJNA729271	stage_E18_vs_E10	ordinary_muscle_development	ENSGALG00010008037	ENSGALG00010008037	2.144734288	0.907601191	2.363080072	0.018123753	0.027525545	positive
PRJNA729271	stage_E18_vs_E10	ordinary_muscle_development	ENSGALG00010008038	WRN	-0.239983839	0.07794619	-3.078839877	0.002078083	0.003641373	negative
PRJNA729271	stage_E18_vs_E10	ordinary_muscle_development	ENSGALG00010008039	CHMP1A	-0.081700899	0.090682784	-0.900952706	0.367613466	0.427207343	negative
PRJNA729271	stage_E18_vs_E10	ordinary_muscle_development	ENSGALG00010008040	ENSGALG00010008040	0.52999247	0.272113743	1.947687259	0.051452396	0.072100007	positive
PRJNA729271	stage_E18_vs_E10	ordinary_muscle_development	ENSGALG00010008041	ENSGALG00010008041	-0.681288317	0.198131702	-3.438562893	0.000584811	0.001101316	negative
PRJNA729271	stage_E18_vs_E10	ordinary_muscle_development	ENSGALG00010008042	ENSGALG00010008042	-0.049921208	0.876467263	-0.056957299	0.95457921	0.962413971	negative
PRJNA729271	stage_E18_vs_E10	ordinary_muscle_development	ENSGALG00010008043	IL17REL	-3.171065751	0.380051103	-8.343787777	7.19481E-17	4.16488E-16	negative
PRJNA729271	stage_E18_vs_E10	ordinary_muscle_development	ENSGALG00010008045	DCDC2	-0.849416774	0.838098251	-1.013505007	0.310819006	0.36844304	negative
PRJNA729271	stage_E18_vs_E10	ordinary_muscle_development	ENSGALG00010008046	RHOB	0.371681091	0.168036451	2.211907526	0.026973055	0.039741252	positive
PRJNA729271	stage_E18_vs_E10	ordinary_muscle_development	ENSGALG00010008047	ASAP1	-0.983451647	0.129088369	-7.618437323	2.56765E-14	1.23361E-13	negative
PRJNA729271	stage_E18_vs_E10	ordinary_muscle_development	ENSGALG00010008048	ENSGALG00010008048	2.037524365	0.763023062	2.670331298	0.007577644	0.012198795	positive
PRJNA729271	stage_E18_vs_E10	ordinary_muscle_development	ENSGALG00010008050	CDK10	-0.244595647	0.155321197	-1.57477313	0.115308821	0.151216987	negative
PRJNA729271	stage_E18_vs_E10	ordinary_muscle_development	ENSGALG00010008051	ENSGALG00010008051	-0.144264281	0.079292616	-1.819391108	0.068851782	0.094267175	negative
PRJNA729271	stage_E18_vs_E10	ordinary_muscle_development	ENSGALG00010008052	RBFA	-0.224677491	0.125387809	-1.791860726	0.073155279	0.099660316	negative
PRJNA729271	stage_E18_vs_E10	ordinary_muscle_development	ENSGALG00010008054	CRISP3	-5.862885301	0.978118125	-5.994046274	2.04683E-09	6.72305E-09	negative
PRJNA729271	stage_E18_vs_E10	ordinary_muscle_development	ENSGALG00010008056	GTF2E2	-0.80416034	0.073120642	-10.99771993	3.91916E-28	4.3766E-27	negative
PRJNA729271	stage_E18_vs_E10	ordinary_muscle_development	ENSGALG00010008057	BAG1	-0.163224648	0.132648523	-1.230504831	0.218508119	0.269307839	negative
PRJNA729271	stage_E18_vs_E10	ordinary_muscle_development	ENSGALG00010008058	USP25	1.175427245	0.138853719	8.465219779	2.5567E-17	1.52382E-16	positive
PRJNA729271	stage_E18_vs_E10	ordinary_muscle_development	ENSGALG00010008059	ENSGALG00010008059	0.233729248	0.094229599	2.480422836	0.013122666	0.020411315	positive
PRJNA729271	stage_E18_vs_E10	ordinary_muscle_development	ENSGALG00010008062	VPS9D1	-0.022791549	0.142968595	-0.159416471	0.873340763	0.895646976	negative
PRJNA729271	stage_E18_vs_E10	ordinary_muscle_development	ENSGALG00010008063	KCNV1	1.380843057	0.259029375	5.33083577	9.77618E-08	2.77539E-07	positive
PRJNA729271	stage_E18_vs_E10	ordinary_muscle_development	ENSGALG00010008064	DDX60	-1.464377119	0.671717994	-2.180047478	0.029253942	0.042823257	negative
PRJNA729271	stage_E18_vs_E10	ordinary_muscle_development	ENSGALG00010008065	CHMP5	-0.585595232	0.088117342	-6.645629782	3.01923E-11	1.14325E-10	negative
PRJNA729271	stage_E18_vs_E10	ordinary_muscle_development	ENSGALG00010008066	PCNX2	-3.45943404	0.185978003	-18.60130761	3.13565E-77	2.02907E-75	negative
PRJNA729271	stage_E18_vs_E10	ordinary_muscle_development	ENSGALG00010008067	MPC2	0.022411566	0.147779522	0.151655427	0.879458714	0.900201544	positive
PRJNA729271	stage_E18_vs_E10	ordinary_muscle_development	ENSGALG00010008069	DLG2	-1.723497628	0.221018372	-7.79798355	6.29042E-15	3.19256E-14	negative
PRJNA729271	stage_E18_vs_E10	ordinary_muscle_development	ENSGALG00010008071	SMIM18	-1.390879212	0.368803992	-3.771323639	0.000162384	0.000329427	negative
PRJNA729271	stage_E18_vs_E10	ordinary_muscle_development	ENSGALG00010008072	NRSN1	0.432065877	0.200662129	2.153200901	0.031302893	0.045596968	positive
PRJNA729271	stage_E18_vs_E10	ordinary_muscle_development	ENSGALG00010008073	ZNF276	0.013749807	0.085101572	0.16156937	0.871644979	0.894350816	positive
PRJNA729271	stage_E18_vs_E10	ordinary_muscle_development	ENSGALG00010008074	RHAG	-0.041984404	0.431354931	-0.097331457	0.922463175	0.936220678	negative
PRJNA729271	stage_E18_vs_E10	ordinary_muscle_development	ENSGALG00010008075	SDHA	0.501006815	0.136706444	3.664836873	0.000247496	0.000490358	positive
PRJNA729271	stage_E18_vs_E10	ordinary_muscle_development	ENSGALG00010008076	ENSGALG00010008076	-2.535976002	1.927717772	-1.315532823	0.188330894	0.23559647	negative
PRJNA729271	stage_E18_vs_E10	ordinary_muscle_development	ENSGALG00010008077	ENSGALG00010008077	2.312074285	1.460226111	1.583367306	0.11333777	0.148847602	positive
PRJNA729271	stage_E18_vs_E10	ordinary_muscle_development	ENSGALG00010008078	GUCY1B1	0.10351349	0.141645715	0.730791536	0.464906494	0.524054719	positive
PRJNA729271	stage_E18_vs_E10	ordinary_muscle_development	ENSGALG00010008079	CCDC127	-0.439016413	0.110361817	-3.977973772	6.9505E-05	0.000147572	negative
PRJNA729271	stage_E18_vs_E10	ordinary_muscle_development	ENSGALG00010008081	TOP1MT	-0.549907337	0.141816866	-3.877587709	0.000105497	0.000219584	negative
PRJNA729271	stage_E18_vs_E10	ordinary_muscle_development	ENSGALG00010008083	FANCA	-1.953514753	0.257556899	-7.584789071	3.33028E-14	1.5853E-13	negative
PRJNA729271	stage_E18_vs_E10	ordinary_muscle_development	ENSGALG00010008084	CYP2AC1	5.954213612	1.320638876	4.508585747			positive
PRJNA729271	stage_E18_vs_E10	ordinary_muscle_development	ENSGALG00010008086	RHPN1	-1.045171325	0.214107444	-4.881527253	1.05267E-06	2.71612E-06	negative
PRJNA729271	stage_E18_vs_E10	ordinary_muscle_development	ENSGALG00010008087	CPNE8	-0.777852059	0.184688077	-4.211706961	2.53448E-05	5.64408E-05	negative
PRJNA729271	stage_E18_vs_E10	ordinary_muscle_development	ENSGALG00010008088	SLC35A4	0.557542423	0.071061128	7.845955172	4.2967E-15	2.20619E-14	positive
PRJNA729271	stage_E18_vs_E10	ordinary_muscle_development	ENSGALG00010008091	ENSGALG00010008091	0.199068839	0.275306606	0.723080503	0.469630427	0.52847551	positive
PRJNA729271	stage_E18_vs_E10	ordinary_muscle_development	ENSGALG00010008092	SPIRE2	-0.624641076	0.695164579	-0.898551357	0.368891678	0.428520987	negative
PRJNA729271	stage_E18_vs_E10	ordinary_muscle_development	ENSGALG00010008093	EFR3A	0.277253508	0.092465638	2.998449098	0.002713575	0.004675852	positive
PRJNA729271	stage_E18_vs_E10	ordinary_muscle_development	ENSGALG00010008094	PIM3	-0.030918928	0.143507015	-0.215452382	0.829414633	0.858249937	negative
PRJNA729271	stage_E18_vs_E10	ordinary_muscle_development	ENSGALG00010008096	CPSF1	-0.791188888	0.150939219	-5.241771436	1.59042E-07	4.41463E-07	negative
PRJNA729271	stage_E18_vs_E10	ordinary_muscle_development	ENSGALG00010008098	ENSGALG00010008098	-0.119483525	0.169433401	-0.705194631	0.480689122	0.538799405	negative
PRJNA729271	stage_E18_vs_E10	ordinary_muscle_development	ENSGALG00010008099	PANK3	-0.534061798	0.077435624	-6.896848852	5.31687E-12	2.14794E-11	negative
PRJNA729271	stage_E18_vs_E10	ordinary_muscle_development	ENSGALG00010008100	ENSGALG00010008100	1.618225348	0.235509712	6.871161836	6.36811E-12	2.54847E-11	positive
PRJNA729271	stage_E18_vs_E10	ordinary_muscle_development	ENSGALG00010008101	CENPQ	-1.964652507	0.206654077	-9.506962232	1.96311E-21	1.516E-20	negative
PRJNA729271	stage_E18_vs_E10	ordinary_muscle_development	ENSGALG00010008103	RBPMS	-0.110991457	0.110042819	-1.008620629	0.31315661	0.370698658	negative
PRJNA729271	stage_E18_vs_E10	ordinary_muscle_development	ENSGALG00010008104	TCF25	-0.095978173	0.095508004	-1.004922819	0.314934015	0.372346538	negative
PRJNA729271	stage_E18_vs_E10	ordinary_muscle_development	ENSGALG00010008105	MMUT	0.804259871	0.095506977	8.420954111	3.73431E-17	2.20753E-16	positive
PRJNA729271	stage_E18_vs_E10	ordinary_muscle_development	ENSGALG00010008106	ENSGALG00010008106	0.851609729	0.370770537	2.29686462	0.021626494	0.032410673	positive
PRJNA729271	stage_E18_vs_E10	ordinary_muscle_development	ENSGALG00010008107	TUBB3	-1.606673104	0.208869372	-7.692238874	1.44582E-14	7.12104E-14	negative
PRJNA729271	stage_E18_vs_E10	ordinary_muscle_development	ENSGALG00010008108	ENSGALG00010008108	1.077995771	0.337320018	3.19576578	0.001394603	0.0024975	positive
PRJNA729271	stage_E18_vs_E10	ordinary_muscle_development	ENSGALG00010008110	DEF8	-0.468686188	0.158857654	-2.950353206	0.003174108	0.005415337	negative
PRJNA729271	stage_E18_vs_E10	ordinary_muscle_development	ENSGALG00010008111	SYTL2	-1.066852393	0.261777813	-4.075411812	4.5933E-05	9.92544E-05	negative
PRJNA729271	stage_E18_vs_E10	ordinary_muscle_development	ENSGALG00010008113	DBNDD1	-0.298356292	0.169613808	-1.75903304	0.078571897	0.106411419	negative
PRJNA729271	stage_E18_vs_E10	ordinary_muscle_development	ENSGALG00010008115	ENSGALG00010008115	-0.484404515	0.20447149	-2.369056511	0.017833528	0.027124553	negative
PRJNA729271	stage_E18_vs_E10	ordinary_muscle_development	ENSGALG00010008116	URAH	2.468276696	0.370773119	6.657107991	2.79268E-11	1.06079E-10	positive
PRJNA729271	stage_E18_vs_E10	ordinary_muscle_development	ENSGALG00010008117	ENSGALG00010008117	2.164469907	0.178945092	12.09572099	1.11261E-33	1.59676E-32	positive
PRJNA729271	stage_E18_vs_E10	ordinary_muscle_development	ENSGALG00010008118	ENSGALG00010008118	0.201088328	0.628880628	0.319755958	0.749153336	0.789112536	positive
PRJNA729271	stage_E18_vs_E10	ordinary_muscle_development	ENSGALG00010008119	CCDC83	-0.922423913	0.544089094	-1.695354535	0.090008185	0.120390204	negative
PRJNA729271	stage_E18_vs_E10	ordinary_muscle_development	ENSGALG00010008120	CDC5L	-0.532561278	0.065089821	-8.181944112	2.79301E-16	1.55292E-15	negative
PRJNA729271	stage_E18_vs_E10	ordinary_muscle_development	ENSGALG00010008121	MPZL1	0.015020788	0.13534101	0.110984746	0.911628443	0.9269768	positive
PRJNA729271	stage_E18_vs_E10	ordinary_muscle_development	ENSGALG00010008123	ENSGALG00010008123	-0.763993287	0.16472834	-4.637898288	3.5197E-06	8.55864E-06	negative

PRJNA729271	stage_E18_vs_E10	ordinary_muscle_development	ENSGALG00010008124	ENSGALG00010008124	-0.695777371	0.228229644	-3.048584567	0.002299222	0.003997508	negative
PRJNA729271	stage_E18_vs_E10	ordinary_muscle_development	ENSGALG00010008126	CDH3	-4.138205225	0.33101757	-12.50146701	7.32863E-36	1.1777E-34	negative
PRJNA729271	stage_E18_vs_E10	ordinary_muscle_development	ENSGALG00010008127	TBC1D2	-0.008033783	0.13297677	-0.060414932	0.951825167	0.960305268	negative
PRJNA729271	stage_E18_vs_E10	ordinary_muscle_development	ENSGALG00010008128	RARS	-0.409686575	0.070418615	-5.817873253	5.96011E-09	1.88135E-08	negative
PRJNA729271	stage_E18_vs_E10	ordinary_muscle_development	ENSGALG00010008129	TANGO6	0.432324543	0.117452878	3.680833951	0.000232472	0.000462234	positive
PRJNA729271	stage_E18_vs_E10	ordinary_muscle_development	ENSGALG00010008130	RCSD1	2.632124469	0.119186618	22.08406032	4.498E-108	6.4554E-106	positive
PRJNA729271	stage_E18_vs_E10	ordinary_muscle_development	ENSGALG00010008131	OC90	0.676407773	0.525653221	1.286794688	0.198165859	0.246792175	positive
PRJNA729271	stage_E18_vs_E10	ordinary_muscle_development	ENSGALG00010008132	TDO2	2.252580125	0.634444031	3.550478869	0.000384531	0.000741786	positive
PRJNA729271	stage_E18_vs_E10	ordinary_muscle_development	ENSGALG00010008133	CREG1	1.376815593	0.16806611	8.192107226	2.56692E-16	1.42996E-15	positive
PRJNA729271	stage_E18_vs_E10	ordinary_muscle_development	ENSGALG00010008134	SLC2A13	-0.217075948	0.077333274	-2.807018743	0.005000233	0.008291203	negative
PRJNA729271	stage_E18_vs_E10	ordinary_muscle_development	ENSGALG00010008135	WWC1	0.404926006	0.130031463	3.114061744	0.001845309	0.003259933	positive
PRJNA729271	stage_E18_vs_E10	ordinary_muscle_development	ENSGALG00010008136	ELP1	-0.658938672	0.114046622	-5.777800867	7.56833E-09	2.36276E-08	negative
PRJNA729271	stage_E18_vs_E10	ordinary_muscle_development	ENSGALG00010008137	HHLA1	4.131252103	0.440447695	9.379665623	6.61823E-21	4.95512E-20	positive
PRJNA729271	stage_E18_vs_E10	ordinary_muscle_development	ENSGALG00010008139	ENSGALG00010008139	1.685063251	0.392246659	4.295927602	1.73964E-05	3.95051E-05	positive
PRJNA729271	stage_E18_vs_E10	ordinary_muscle_development	ENSGALG00010008141	APTX	-0.912635261	0.159521286	-5.721087656	1.05844E-08	3.25874E-08	negative
PRJNA729271	stage_E18_vs_E10	ordinary_muscle_development	ENSGALG00010008142	CD247	1.418812532	0.341164527	4.158734037	3.20016E-05	7.03356E-05	positive
PRJNA729271	stage_E18_vs_E10	ordinary_muscle_development	ENSGALG00010008143	DCTN6	0.374385945	0.115843934	3.231813102	0.001230075	0.002217102	positive
PRJNA729271	stage_E18_vs_E10	ordinary_muscle_development	ENSGALG00010008144	NTPCR	0.115825176	0.133477572	0.867750098	0.385531154	0.44588479	positive
PRJNA729271	stage_E18_vs_E10	ordinary_muscle_development	ENSGALG00010008145	ENSGALG00010008145	1.002073303	0.211397857	4.740224512	2.13482E-06	5.31869E-06	positive
PRJNA729271	stage_E18_vs_E10	ordinary_muscle_development	ENSGALG00010008146	SOX4	-1.052419794	0.118694373	-8.866635969	7.53889E-19	4.97388E-18	negative
PRJNA729271	stage_E18_vs_E10	ordinary_muscle_development	ENSGALG00010008147	CDKAL1	0.372754407	0.112524482	3.312651622	0.00092416	0.001690949	positive
PRJNA729271	stage_E18_vs_E10	ordinary_muscle_development	ENSGALG00010008148	ABCD2	-0.007117431	0.140155441	-0.050782412	0.959498906	0.966634462	negative
PRJNA729271	stage_E18_vs_E10	ordinary_muscle_development	ENSGALG00010008149	DNAJA1	-0.642073974	0.282027968	-2.276632273	0.022808194	0.03405488	negative
PRJNA729271	stage_E18_vs_E10	ordinary_muscle_development	ENSGALG00010008150	TERT	-3.240788737	0.705902392	-4.590987046	4.41155E-06	1.06275E-05	negative
PRJNA729271	stage_E18_vs_E10	ordinary_muscle_development	ENSGALG00010008151	SIPA1L2	1.500502623	0.120193354	12.48407313	9.11992E-36	1.45748E-34	positive
PRJNA729271	stage_E18_vs_E10	ordinary_muscle_development	ENSGALG00010008152	ENSGALG00010008152	0.004567521	0.16586889	0.02756937	0.97803148	0.981960677	positive
PRJNA729271	stage_E18_vs_E10	ordinary_muscle_development	ENSGALG00010008153	SMU1	-0.452084892	0.062080062	-7.282288018	3.28205E-13	1.45723E-12	negative
PRJNA729271	stage_E18_vs_E10	ordinary_muscle_development	ENSGALG00010008154	KCNQ3	-0.080515579	0.226880255	-0.354881383	0.722678456	0.765229325	negative
PRJNA729271	stage_E18_vs_E10	ordinary_muscle_development	ENSGALG00010008155	MAP10	0.768971744	0.222407636	3.457488055	0.000545236	0.001030942	positive
PRJNA729271	stage_E18_vs_E10	ordinary_muscle_development	ENSGALG00010008156	HAS3	-2.913124502	1.070893815	-2.720273907	0.006522786	0.010606662	negative
PRJNA729271	stage_E18_vs_E10	ordinary_muscle_development	ENSGALG00010008157	CTSO	-0.012348173	0.155199929	-0.079563005	0.936584819	0.948045553	negative
PRJNA729271	stage_E18_vs_E10	ordinary_muscle_development	ENSGALG00010008158	CLPTM1L	-0.567570529	0.076162857	-7.452064566	9.18907E-14	4.23381E-13	negative
PRJNA729271	stage_E18_vs_E10	ordinary_muscle_development	ENSGALG00010008159	SUPT3H	-2.45114212	0.148368915	-16.52059071	2.60831E-61	1.07714E-59	negative
PRJNA729271	stage_E18_vs_E10	ordinary_muscle_development	ENSGALG00010008160	TXNL4A	-0.441034775	0.094207397	-4.681530204	2.84741E-06	6.99666E-06	negative
PRJNA729271	stage_E18_vs_E10	ordinary_muscle_development	ENSGALG00010008161	TMEFF1	-4.086768115	0.395530013	-10.33238436	5.02946E-25	4.7742E-24	negative
PRJNA729271	stage_E18_vs_E10	ordinary_muscle_development	ENSGALG00010008162	HSBP1L1	0.889112948	0.204776379	4.341872606	1.41273E-05	3.23807E-05	positive
PRJNA729271	stage_E18_vs_E10	ordinary_muscle_development	ENSGALG00010008163	CHTF8	-1.725384783	0.223389578	-7.723658373	1.13038E-14	5.61893E-14	negative
PRJNA729271	stage_E18_vs_E10	ordinary_muscle_development	ENSGALG00010008164	TG	1.271262419	0.291708117	4.35799467	1.3126E-05	3.01905E-05	positive
PRJNA729271	stage_E18_vs_E10	ordinary_muscle_development	ENSGALG00010008165	MRPL1	0.395368946	0.085148231	4.643301927	3.42885E-06	8.34612E-06	positive
PRJNA729271	stage_E18_vs_E10	ordinary_muscle_development	ENSGALG00010008166	RUNX2	-3.87518486	0.415209156	-9.333091057	1.02827E-20	7.58902E-20	negative
PRJNA729271	stage_E18_vs_E10	ordinary_muscle_development	ENSGALG00010008167	UTP4	-0.507042708	0.177221466	-2.861068236	0.004222162	0.007067818	negative
PRJNA729271	stage_E18_vs_E10	ordinary_muscle_development	ENSGALG00010008168	CAVIN4	4.762464533	0.124915989	38.12533986	4.8741E-318	1.1775E-314	positive
PRJNA729271	stage_E18_vs_E10	ordinary_muscle_development	ENSGALG00010008169	HTR4	0.880722605	0.31584764	2.788441305	0.005296234	0.008748595	positive
PRJNA729271	stage_E18_vs_E10	ordinary_muscle_development	ENSGALG00010008170	PICALM	-0.381813909	0.071340271	-5.352010903	8.69822E-08	2.47703E-07	negative
PRJNA729271	stage_E18_vs_E10	ordinary_muscle_development	ENSGALG00010008171	KIF21A	-2.474219	0.185155727	-13.36290831	9.95955E-41	1.96413E-39	negative
PRJNA729271	stage_E18_vs_E10	ordinary_muscle_development	ENSGALG00010008172	SNTB2	0.067715536	0.130829839	0.517584801	0.604747988	0.655731754	positive
PRJNA729271	stage_E18_vs_E10	ordinary_muscle_development	ENSGALG00010008174	SLC26A2	-1.071854772	0.125945151	-8.510489958	1.732E-17	1.04649E-16	negative
PRJNA729271	stage_E18_vs_E10	ordinary_muscle_development	ENSGALG00010008175	ENSGALG00010008175	-0.943914634	0.449053831	-2.102007754	0.035552597	0.051251605	negative
PRJNA729271	stage_E18_vs_E10	ordinary_muscle_development	ENSGALG00010008176	SLA	0.14868325	0.317508509	0.468281152	0.639583545	0.688815179	positive
PRJNA729271	stage_E18_vs_E10	ordinary_muscle_development	ENSGALG00010008177	NREP	-0.956666966	0.184628595	-5.181575279	2.2002E-07	6.03898E-07	negative
PRJNA729271	stage_E18_vs_E10	ordinary_muscle_development	ENSGALG00010008178	PGR	-0.820888724	0.311979668	-2.631225073	0.008507767	0.01360248	negative
PRJNA729271	stage_E18_vs_E10	ordinary_muscle_development	ENSGALG00010008179	SEMA6A	-0.437330582	0.096368355	-4.538114035	5.67596E-06	1.35451E-05	negative
PRJNA729271	stage_E18_vs_E10	ordinary_muscle_development	ENSGALG00010008180	POU2F1	0.78469052	0.1582229	4.959399192	7.07115E-07	1.85547E-06	positive
PRJNA729271	stage_E18_vs_E10	ordinary_muscle_development	ENSGALG00010008181	DISC1	0.185966057	0.130246159	1.427804541	0.153348125	0.195839743	positive
PRJNA729271	stage_E18_vs_E10	ordinary_muscle_development	ENSGALG00010008182	CLIC5	3.177250955	0.291087218	10.9151167	9.76039E-28	1.07424E-26	positive
PRJNA729271	stage_E18_vs_E10	ordinary_muscle_development	ENSGALG00010008183	ALG10	-0.824789988	0.089377285	-9.228183503	2.7526E-20	1.97227E-19	negative
PRJNA729271	stage_E18_vs_E10	ordinary_muscle_development	ENSGALG00010008184	SLC66A2	-0.1763705	0.086337535	-2.042802137	0.041072029	0.058515732	negative
PRJNA729271	stage_E18_vs_E10	ordinary_muscle_development	ENSGALG00010008185	PDGFC	-0.851521345	0.088769866	-9.592459529	8.60098E-22	6.79407E-21	negative
PRJNA729271	stage_E18_vs_E10	ordinary_muscle_development	ENSGALG00010008186	NOP53	0.404165835	0.121650653	3.322348256	0.000892632	0.001637603	positive
PRJNA729271	stage_E18_vs_E10	ordinary_muscle_development	ENSGALG00010008187	ENSGALG00010008187	1.307736029	0.383312981	3.411666425	0.000645671	0.001208392	positive
PRJNA729271	stage_E18_vs_E10	ordinary_muscle_development	ENSGALG00010008188	VPS4A	0.313183147	0.113372733	2.762420367	0.005737456	0.009420528	positive
PRJNA729271	stage_E18_vs_E10	ordinary_muscle_development	ENSGALG00010008189	EXOC3	-0.135485203	0.115325897	-1.174802949	0.24007356	0.292992023	negative
PRJNA729271	stage_E18_vs_E10	ordinary_muscle_development	ENSGALG00010008190	TSNARE1	0.240401026	0.16853407	1.426423902	0.153746018	0.19626143	positive
PRJNA729271	stage_E18_vs_E10	ordinary_muscle_development	ENSGALG00010008192	HNRNPU	-0.393029482	0.082917777	-4.739900589	2.13728E-06	5.32301E-06	negative
PRJNA729271	stage_E18_vs_E10	ordinary_muscle_development	ENSGALG00010008193	CNOT6L	0.922227237	0.130672641	7.057538799	1.69477E-12	7.12671E-12	positive
PRJNA729271	stage_E18_vs_E10	ordinary_muscle_development	ENSGALG00010008194	PDF	-0.121945989	0.171802335	-0.709804028	0.477825671	0.536282083	negative
PRJNA729271	stage_E18_vs_E10	ordinary_muscle_development	ENSGALG00010008195	ENSGALG00010008195	2.864468231	0.423339115	6.766367973	1.32055E-11	5.14831E-11	positive
PRJNA729271	stage_E18_vs_E10	ordinary_muscle_development	ENSGALG00010008196	PDCD6	-0.042023925	0.147425014	-0.285052883	0.775603627	0.811899659	negative
PRJNA729271	stage_E18_vs_E10	ordinary_muscle_development	ENSGALG00010008197	STARD4	-1.671959497	0.162768482	-10.27201016	9.42206E-25	8.81684E-24	negative
PRJNA729271	stage_E18_vs_E10	ordinary_muscle_development	ENSGALG00010008198	GLRB	-2.984547924	0.386054466	-7.730898589	1.0679E-14	5.32481E-14	negative
PRJNA729271	stage_E18_vs_E10	ordinary_muscle_development	ENSGALG00010008199	TPRKB	-0.484629984	0.115988403	-4.178262399	2.93745E-05	6.48664E-05	negative
PRJNA729271	stage_E18_vs_E10	ordinary_muscle_development	ENSGALG00010008200	COG8	-0.624220964	0.131704816	-4.739545485	2.14198E-06	5.33337E-06	negative
PRJNA729271	stage_E18_vs_E10	ordinary_muscle_development	ENSGALG00010008201	ENPP4	0.733543149	0.243287445	3.015129481	0.002568696	0.004441518	positive
PRJNA729271	stage_E18_vs_E10	ordinary_muscle_development	ENSGALG00010008202	KCNG2	2.634642602	0.191194969	13.77987413	3.36859E-43	7.35357E-42	positive
PRJNA729271	stage_E18_vs_E10	ordinary_muscle_development	ENSGALG00010008203	CXCL13	-0.469853018	1.499324004	-0.313376573	0.753994581	0.793577654	negative
PRJNA729271	stage_E18_vs_E10	ordinary_muscle_development	ENSGALG00010008204	ENSGALG00010008204	0.696500812	0.153364002	4.541488246	5.58585E-06	1.33411E-05	positive
PRJNA729271	stage_E18_vs_E10	ordinary_muscle_development	ENSGALG00010008205	ENSGALG00010008205	-0.439769617	0.181621289	-2.421355005	0.015462768	0.023808458	negative
PRJNA729271	stage_E18_vs_E10	ordinary_muscle_development	ENSGALG00010008206	CXCL13L2	-3.405134053	1.046558104	-3.253650265	0.001139325	0.002061736	negative
PRJNA729271	stage_E18_vs_E10	ordinary_muscle_development	ENSGALG00010008207	CAMK4	0.036458248	0.155681332	0.234185096	0.814841285	0.846190316	positive
PRJNA729271	stage_E18_vs_E10	ordinary_muscle_development	ENSGALG00010008208	ILDR2	1.998959333	0.151512383	13.19337263	9.58014E-40	1.82236E-38	positive
PRJNA729271	stage_E18_vs_E10	ordinary_muscle_development	ENSGALG00010008209	PPARGC1B	2.604311581	0.235075535	11.07861597	1.59316E-28	1.80413E-27	positive
PRJNA729271	stage_E18_vs_E10	ordinary_muscle_development	ENSGALG00010008210	CTDP1	0.013727094	0.085912856	0.159779278	0.873054948	0.895417213	positive
PRJNA729271	stage_E18_vs_E10	ordinary_muscle_development	ENSGALG00010008211	ADGRB1	0.567613809	0.233657847	2.429252074	0.015130009	0.023343212	positive
PRJNA729271	stage_E18_vs_E10	ordinary_muscle_development	ENSGALG00010008212	GTSE1	-1.811003035	0.226251784	-8.004370178	1.2008E-15	6.41797E-15	negative
PRJNA729271	stage_E18_vs_E10	ordinary_muscle_development	ENSGALG00010008213	ISCA1	0.434567817	0.117393268	3.701812063	0.000214065	0.000427334	positive
PRJNA729271	stage_E18_vs_E10	ordinary_muscle_development	ENSGALG00010008214	CCNG2	-0.131485304	0.135891415	-0.967576235	0.333256051	0.391517787	negative
PRJNA729271	stage_E18_vs_E10	ordinary_muscle_development	ENSGALG00010008215	SELENOW	1.30333772	0.128113208	10.17332829	2.6084E-24	2.36749E-23	positive
PRJNA729271	stage_E18_vs_E10	ordinary_muscle_development	ENSGALG00010008217	ARHGEF37	3.320233023	0.163734903	20.27810172	2.0073E-91	1.96593E-89	positive
PRJNA729271	stage_E18_vs_E10	ordinary_muscle_development	ENSGALG00010008218	ARHGAP42	-0.145847727	0.102333766	-1.425216066	0.154094754	0.19667196	negative
PRJNA729271	stage_E18_vs_E10	ordinary_muscle_development	ENSGALG00010008219	NIP7	-0.215190358	0.119528544	-1.800326107	0.071809161	0.097983036	negative
PRJNA729271	stage_E18_vs_E10	ordinary_muscle_development	ENSGALG00010008221	ENSGALG00010008221	-0.421938874	0.523279282	-0.806335905	0.420049193	0.480629386	negative
PRJNA729271	stage_E18_vs_E10	ordinary_muscle_development	ENSGALG00010008222	STYXL2	1.671438353	0.275186344	6.073841924	1.24886E-09	4.17389E-09	positive
PRJNA729271	stage_E18_vs_E10	ordinary_muscle_development	ENSGALG00010008223	ENSGALG00010008223	-1.28372025	0.153435297	-8.366525002	5.93423E-17	3.46004E-16	negative
PRJNA729271	stage_E18_vs_E10	ordinary_muscle_development	ENSGALG00010008224	ENSGALG00010008224	-0.159481528	0.212385599	-0.750905564	0.452709491	0.512377924	negative
PRJNA729271	stage_E18_vs_E10	ordinary_muscle_development	ENSGALG00010008226	COX20	-0.842408386	0.166890619	-5.047667701	4.47236E-07	1.19519E-06	negative
PRJNA729271	stage_E18_vs_E10	ordinary_muscle_development	ENSGALG00010008227	DUSP1	1.929007901	0.178293396	10.81928966	2.78931E-27	2.98825E-26	positive
PRJNA729271	stage_E18_vs_E10	ordinary_muscle_development	ENSGALG00010008228	TMED6	2.352895768	0.696944598	3.376015501	0.000735438	0.001366513	positive
PRJNA729271	stage_E18_vs_E10	ordinary_muscle_development	ENSGALG00010008229	PIGY	0.45407087	0.123459959	3.677879644	0.000235181	0.000467491	positive
PRJNA729271	stage_E18_vs_E10	ordinary_muscle_development	ENSGALG00010008230	TRMU	0.276997034	0.114608275	2.416902563	0.015653207	0.024091445	positive
PRJNA729271	stage_E18_vs_E10	ordinary_muscle_development	ENSGALG00010008231	ZCCHC6	0.018885095	0.110444334	0.17099198	0.864230071	0.887999921	positive
PRJNA729271	stage_E18_vs_E10	ordinary_muscle_development	ENSGALG00010008232	PYURF	0.197490751	0.128344125	1.538759579	0.123863001	0.161456313	positive
PRJNA729271	stage_E18_vs_E10	ordinary_muscle_development	ENSGALG00010008233	CYRIB	-0.251889983	0.071309136	-3.532366232	0.000411859	0.000791868	negative
PRJNA729271	stage_E18_vs_E10	ordinary_muscle_development	ENSGALG00010008234	COMMD10	-1.039085603	0.120751482	-8.605158165	7.62116E-18	4.7048E-17	negative
PRJNA729271	stage_E18_vs_E10	ordinary_muscle_development	ENSGALG00010008236	ENSGALG00010008236	1.759529012	0.661146319	2.661330724	0.007783247	0.012508943	positive
PRJNA729271	stage_E18_vs_E10	ordinary_muscle_development	ENSGALG00010008237	GRIA2	-2.985860448	0.395909531	-7.541774606	4.63619E-14	2.18258E-13	negative
PRJNA729271	stage_E18_vs_E10	ordinary_muscle_development	ENSGALG00010008238	NTAQ1	-0.630868396	0.168796593	-3.737447443	0.000185898	0.000374301	negative
PRJNA729271	stage_E18_vs_E10	ordinary_muscle_development	ENSGALG00010008240	RCAN2	-0.742237025	0.140562449	-5.280478754	1.28847E-07	3.61384E-07	negative
PRJNA729271	stage_E18_vs_E10	ordinary_muscle_development	ENSGALG00010008241	HERC3	0.118804873	0.084221252	1.410628195	0.158354273	0.201558236	positive
PRJNA729271	stage_E18_vs_E10	ordinary_muscle_development	ENSGALG00010008242	CNTN5	-4.120634658	1.220653772	-3.375760392			negative
PRJNA729271	stage_E18_vs_E10	ordinary_muscle_development	ENSGALG00010008243	YTHDC2	-0.353192488	0.10447227	-3.380729513	0.000722937	0.001344319	negative
PRJNA729271	stage_E18_vs_E10	ordinary_muscle_development	ENSGALG00010008245	FBXO32	1.684437894	0.492259214	3.421851428	0.000621963	0.001166885	positive
PRJNA729271	stage_E18_vs_E10	ordinary_muscle_development	ENSGALG00010008246	TERF2	-0.413988975	0.058727258	-7.049349635	1.79756E-12	7.54579E-12	negative
PRJNA729271	stage_E18_vs_E10	ordinary_muscle_development	ENSGALG00010008247	GASK1B	0.964204856	0.160849113	5.994467988	2.04153E-09	6.70715E-09	positive
PRJNA729271	stage_E18_vs_E10	ordinary_muscle_development	ENSGALG00010008248	TPK1	-0.198514165	0.162512036	-1.221535155	0.221883463	0.272859999	negative
PRJNA729271	stage_E18_vs_E10	ordinary_muscle_development	ENSGALG00010008250	KLHL38	1.586074363	0.605921978	2.617621442	0.008854497	0.014124127	positive
PRJNA729271	stage_E18_vs_E10	ordinary_muscle_development	ENSGALG00010008251	AHRR	-1.499257435	0.26202012	-5.721917213	1.05329E-08	3.24424E-08	negative
PRJNA729271	stage_E18_vs_E10	ordinary_muscle_development	ENSGALG00010008252	ZFP92	-0.864905117	0.121858414	-7.097623273	1.26921E-12	5.39347E-12	negative
PRJNA729271	stage_E18_vs_E10	ordinary_muscle_development	ENSGALG00010008253	ENSGALG00010008253	0.954352265	0.674241586	1.415445569	0.156937897	0.199907092	positive
PRJNA729271	stage_E18_vs_E10	ordinary_muscle_development	ENSGALG00010008255	MSANTD3	-2.31463368	0.135469785	-17.08597733	1.88754E-65	8.94114E-64	negative
PRJNA729271	stage_E18_vs_E10	ordinary_muscle_development	ENSGALG00010008257	DESI2	-0.58088981	0.060092183	-9.666645204	4.17851E-22	3.34258E-21	negative
PRJNA729271	stage_E18_vs_E10	ordinary_muscle_development	ENSGALG00010008258	CCDC82	0.909916369	0.131774978	6.905076981	5.01762E-12	2.03214E-11	positive
PRJNA729271	stage_E18_vs_E10	ordinary_muscle_development	ENSGALG00010008259	MCC	0.093834623	0.108633779	0.863770219	0.387714131	0.447909168	positive
PRJNA729271	stage_E18_vs_E10	ordinary_muscle_development	ENSGALG00010008261	CRX	-0.977144529	0.413805454	-2.36136213	0.018207941	0.027636032	negative
PRJNA729271	stage_E18_vs_E10	ordinary_muscle_development	ENSGALG00010008262	CYB5B	-0.75531473	0.072299919	-10.44696516	1.51289E-25	1.48271E-24	negative
PRJNA729271	stage_E18_vs_E10	ordinary_muscle_development	ENSGALG00010008263	C1orf101	-1.077071823	0.534670719	-2.014458215	0.043961448	0.062332112	negative
PRJNA729271	stage_E18_vs_E10	ordinary_muscle_development	ENSGALG00010008264	CNTNAP2	-1.464115029	0.328330252	-4.459275436	8.22372E-06	1.9326E-05	negative
PRJNA729271	stage_E18_vs_E10	ordinary_muscle_development	ENSGALG00010008265	ENSGALG00010008265	-5.175153168	1.047020867	-4.942741189	7.70317E-07	2.01553E-06	negative
PRJNA729271	stage_E18_vs_E10	ordinary_muscle_development	ENSGALG00010008266	ECSCR	0.656358066	0.188228852	3.487021559	0.000488432	0.000929598	positive
PRJNA729271	stage_E18_vs_E10	ordinary_muscle_development	ENSGALG00010008267	MAML2	-0.35446338	0.118997973	-2.9787346	0.002894414	0.004972094	negative
PRJNA729271	stage_E18_vs_E10	ordinary_muscle_development	ENSGALG00010008268	ENSGALG00010008268	1.682941534	0.238644296	7.052092016	1.76248E-12	7.40495E-12	positive
PRJNA729271	stage_E18_vs_E10	ordinary_muscle_development	ENSGALG00010008269	ENSGALG00010008269	0.818902733	0.314251948	2.605879573	0.009163867	0.014568227	positive

PRJNA729271	stage_E18_vs_E10	ordinary_muscle_development	ENSGALG00010008270	ENSGALG00010008270	1.292331929	0.160980112	8.027898041	9.91568E-16	5.33115E-15	positive
PRJNA729271	stage_E18_vs_E10	ordinary_muscle_development	ENSGALG00010008272	DCP2	-1.07478006	0.086393262	-12.44055429	1.57408E-35	2.47734E-34	negative
PRJNA729271	stage_E18_vs_E10	ordinary_muscle_development	ENSGALG00010008273	TMEM144	0.545735524	0.119836951	4.553983725	5.26394E-06	1.26034E-05	positive
PRJNA729271	stage_E18_vs_E10	ordinary_muscle_development	ENSGALG00010008274	NFAT5	-0.262675367	0.10985588	-2.391090639	0.016798405	0.025679486	negative
PRJNA729271	stage_E18_vs_E10	ordinary_muscle_development	ENSGALG00010008275	CELSR1	0.08897119	0.170211528	0.522709542	0.601176397	0.652571011	positive
PRJNA729271	stage_E18_vs_E10	ordinary_muscle_development	ENSGALG00010008276	DNAJC18	0.76002004	0.093789038	8.103506094	5.33976E-16	2.91964E-15	positive
PRJNA729271	stage_E18_vs_E10	ordinary_muscle_development	ENSGALG00010008277	MZB1	2.360438294	1.213923373	1.944470588	0.051838729	0.072557201	positive
PRJNA729271	stage_E18_vs_E10	ordinary_muscle_development	ENSGALG00010008278	CYP39A1	0.278401204	0.163800373	1.699637181	0.089199192	0.119352191	positive
PRJNA729271	stage_E18_vs_E10	ordinary_muscle_development	ENSGALG00010008279	MAPK15	-0.145846304	0.168189093	-0.867156732	0.38585614	0.446189449	negative
PRJNA729271	stage_E18_vs_E10	ordinary_muscle_development	ENSGALG00010008282	FAM83H	-1.48875638	0.273531116	-5.442731344	5.24698E-08	1.52201E-07	negative
PRJNA729271	stage_E18_vs_E10	ordinary_muscle_development	ENSGALG00010008283	NQO1	-0.717771455	0.198056615	-3.624072104	0.000290001	0.00056928	negative
PRJNA729271	stage_E18_vs_E10	ordinary_muscle_development	ENSGALG00010008284	BCKDHA	1.325414381	0.129433311	10.24013348	1.31055E-24	1.21461E-23	positive
PRJNA729271	stage_E18_vs_E10	ordinary_muscle_development	ENSGALG00010008285	ADSS2	-2.049171919	0.091900289	-22.29777458	3.8834E-110	6.1857E-108	negative
PRJNA729271	stage_E18_vs_E10	ordinary_muscle_development	ENSGALG00010008289	MTMR2	-0.726861674	0.07273522	-9.993256052	1.63133E-23	1.40584E-22	negative
PRJNA729271	stage_E18_vs_E10	ordinary_muscle_development	ENSGALG00010008290	FAM13A	1.783234941	0.163041578	10.93730177	7.64411E-28	8.44523E-27	positive
PRJNA729271	stage_E18_vs_E10	ordinary_muscle_development	ENSGALG00010008291	PTGFRN	0.715304298	0.160825268	4.447710906	8.67902E-06	2.03432E-05	positive
PRJNA729271	stage_E18_vs_E10	ordinary_muscle_development	ENSGALG00010008292	REEP5	0.376434593	0.113711823	3.310426164	0.00093154	0.001703378	positive
PRJNA729271	stage_E18_vs_E10	ordinary_muscle_development	ENSGALG00010008293	PLA2G7	-0.292345054	0.119777854	-2.440727097	0.014657727	0.022665218	negative
PRJNA729271	stage_E18_vs_E10	ordinary_muscle_development	ENSGALG00010008294	NOB1	-0.11257321	0.155676317	-0.723123544	0.469603986	0.52847551	negative
PRJNA729271	stage_E18_vs_E10	ordinary_muscle_development	ENSGALG00010008295	DMAC2	0.269167463	0.133116431	2.022045361	0.043171666	0.061272231	positive
PRJNA729271	stage_E18_vs_E10	ordinary_muscle_development	ENSGALG00010008296	PALLD	-0.192098386	0.153120293	-1.254558637	0.209639027	0.259564166	negative
PRJNA729271	stage_E18_vs_E10	ordinary_muscle_development	ENSGALG00010008297	ENSGALG00010008297	-0.431039175	0.307776356	-1.400494762	0.161365211	0.20506652	negative
PRJNA729271	stage_E18_vs_E10	ordinary_muscle_development	ENSGALG00010008298	ENSGALG00010008298	-1.373132858	0.132486667	-10.36430975	3.60351E-25	3.4409E-24	negative
PRJNA729271	stage_E18_vs_E10	ordinary_muscle_development	ENSGALG00010008299	ANKRD66	-0.494106399	0.337621852	-1.463490577	0.14333319	0.18426737	negative
PRJNA729271	stage_E18_vs_E10	ordinary_muscle_development	ENSGALG00010008300	CUL1	-0.355240616	0.085792337	-4.14070334	3.46243E-05	7.5801E-05	negative
PRJNA729271	stage_E18_vs_E10	ordinary_muscle_development	ENSGALG00010008301	FAM91A1	-0.674677001	0.106030905	-6.363022162	1.97822E-10	7.05222E-10	negative
PRJNA729271	stage_E18_vs_E10	ordinary_muscle_development	ENSGALG00010008302	ENSGALG00010008302	0.389672275	0.145299485	2.68185585	0.0073215	0.011810054	positive
PRJNA729271	stage_E18_vs_E10	ordinary_muscle_development	ENSGALG00010008303	TEX10	-1.169095583	0.072421665	-16.14289852	1.27415E-58	4.9119E-57	negative
PRJNA729271	stage_E18_vs_E10	ordinary_muscle_development	ENSGALG00010008306	ENSGALG00010008306	0.138052589	0.314454723	0.439022153	0.660645485	0.708445388	positive
PRJNA729271	stage_E18_vs_E10	ordinary_muscle_development	ENSGALG00010008307	ENSGALG00010008307	-1.476991733	1.058365295	-1.395540595	0.162852882	0.20673958	negative
PRJNA729271	stage_E18_vs_E10	ordinary_muscle_development	ENSGALG00010008308	WWP2	-2.042204333	0.126922002	-16.09023096	2.98721E-58	1.13054E-56	negative
PRJNA729271	stage_E18_vs_E10	ordinary_muscle_development	ENSGALG00010008309	ENSGALG00010008309	1.675257939	0.270225186	6.199488514	5.6647E-10	1.96106E-09	positive
PRJNA729271	stage_E18_vs_E10	ordinary_muscle_development	ENSGALG00010008310	EXOSC5	0.104789484	0.183775706	0.570203137	0.568539928	0.622289984	positive
PRJNA729271	stage_E18_vs_E10	ordinary_muscle_development	ENSGALG00010008311	SRP19	-0.892376051	0.116267032	-7.675228602	1.65124E-14	8.08605E-14	negative
PRJNA729271	stage_E18_vs_E10	ordinary_muscle_development	ENSGALG00010008312	ZBTB18	0.430800829	0.218038364	1.975802884	0.048177107	0.067818506	positive
PRJNA729271	stage_E18_vs_E10	ordinary_muscle_development	ENSGALG00010008313	PSMD7	-0.108557654	0.17897357	-0.606556902	0.544145009	0.598981008	negative
PRJNA729271	stage_E18_vs_E10	ordinary_muscle_development	ENSGALG00010008314	INVS	0.414414025	0.105685712	3.921192525	8.81118E-05	0.000184804	positive
PRJNA729271	stage_E18_vs_E10	ordinary_muscle_development	ENSGALG00010008316	ADGRF5	1.334343516	0.137874211	9.677977522	3.74042E-22	3.0054E-21	positive
PRJNA729271	stage_E18_vs_E10	ordinary_muscle_development	ENSGALG00010008317	CEP57	-0.536369579	0.114969774	-4.665309479	3.08153E-06	7.54634E-06	negative
PRJNA729271	stage_E18_vs_E10	ordinary_muscle_development	ENSGALG00010008318	ADPRH	1.391177562	0.133803015	10.39720635	2.55309E-25	2.46549E-24	positive
PRJNA729271	stage_E18_vs_E10	ordinary_muscle_development	ENSGALG00010008319	ERP44	-0.04092758	0.065178894	-0.627926888	0.530051836	0.586048922	negative
PRJNA729271	stage_E18_vs_E10	ordinary_muscle_development	ENSGALG00010008320	GPRIN3	0.480285168	0.114915141	4.179476808	2.92181E-05	6.45505E-05	positive
PRJNA729271	stage_E18_vs_E10	ordinary_muscle_development	ENSGALG00010008321	ZFHX3	0.136140194	0.102239023	1.331587393	0.18299581	0.229775144	positive
PRJNA729271	stage_E18_vs_E10	ordinary_muscle_development	ENSGALG00010008323	AKT3	0.605152646	0.104326683	5.800554827	6.60959E-09	2.07777E-08	positive
PRJNA729271	stage_E18_vs_E10	ordinary_muscle_development	ENSGALG00010008324	SCRIB	0.489020363	0.103797158	4.711307809	2.46132E-06	6.09444E-06	positive
PRJNA729271	stage_E18_vs_E10	ordinary_muscle_development	ENSGALG00010008325	AADAT	0.427908896	0.131874686	3.244814531	0.001175271	0.002122282	positive
PRJNA729271	stage_E18_vs_E10	ordinary_muscle_development	ENSGALG00010008327	TGFB1	0.47011958	0.128634235	3.65470033	0.000257483	0.000509378	positive
PRJNA729271	stage_E18_vs_E10	ordinary_muscle_development	ENSGALG00010008328	ENSGALG00010008328	-0.264193288	0.474636317	-0.556622573	0.577785328	0.630742455	negative
PRJNA729271	stage_E18_vs_E10	ordinary_muscle_development	ENSGALG00010008330	EZH2	-1.310481235	0.078126993	-16.77373189	3.79882E-63	1.71006E-61	negative
PRJNA729271	stage_E18_vs_E10	ordinary_muscle_development	ENSGALG00010008331	CCDC97	-0.33480889	0.270212412	-1.239058143	0.215323966	0.265830925	negative
PRJNA729271	stage_E18_vs_E10	ordinary_muscle_development	ENSGALG00010008332	DHX38	-0.456754928	0.094140989	-4.851817822	1.22335E-06	3.13627E-06	negative
PRJNA729271	stage_E18_vs_E10	ordinary_muscle_development	ENSGALG00010008333	APC	-0.438972502	0.092343834	-4.753674216	1.99753E-06	4.99038E-06	negative
PRJNA729271	stage_E18_vs_E10	ordinary_muscle_development	ENSGALG00010008334	STX17	-0.109628207	0.136125141	-0.805348712	0.420618479	0.481090891	negative
PRJNA729271	stage_E18_vs_E10	ordinary_muscle_development	ENSGALG00010008335	HNRNPUL1	-0.577702549	0.191123679	-3.02266339	0.002505608	0.004337089	negative
PRJNA729271	stage_E18_vs_E10	ordinary_muscle_development	ENSGALG00010008336	PAIP2B	0.381524087	0.109170912	3.494741232	0.000474522	0.000905144	positive
PRJNA729271	stage_E18_vs_E10	ordinary_muscle_development	ENSGALG00010008339	FAM76B	-0.927101313	0.114247323	-8.114862441	4.86335E-16	2.66721E-15	negative
PRJNA729271	stage_E18_vs_E10	ordinary_muscle_development	ENSGALG00010008340	PDIA4	-0.370574239	0.105660225	-3.507225522	0.000452805	0.000866343	negative
PRJNA729271	stage_E18_vs_E10	ordinary_muscle_development	ENSGALG00010008341	DHODH	-1.463452668	0.111299062	-13.14883204	1.72823E-39	3.24072E-38	negative
PRJNA729271	stage_E18_vs_E10	ordinary_muscle_development	ENSGALG00010008342	PLD3	1.36635188	0.146781479	9.30874856	1.29347E-20	9.47392E-20	positive
PRJNA729271	stage_E18_vs_E10	ordinary_muscle_development	ENSGALG00010008343	TNFRSF21	0.082273297	0.226674289	0.362958223	0.716636092	0.759774733	positive
PRJNA729271	stage_E18_vs_E10	ordinary_muscle_development	ENSGALG00010008344	MFAP3L	1.163311983	0.29028493	4.007483207	6.13692E-05	0.000131066	positive
PRJNA729271	stage_E18_vs_E10	ordinary_muscle_development	ENSGALG00010008346	NR4A3	1.201086156	0.531366412	2.260372747	0.023798126	0.035408935	positive
PRJNA729271	stage_E18_vs_E10	ordinary_muscle_development	ENSGALG00010008347	PUF60	-0.416275355	0.055019712	-7.56593113	3.85096E-14	1.82537E-13	negative
PRJNA729271	stage_E18_vs_E10	ordinary_muscle_development	ENSGALG00010008348	ENSGALG00010008348	-0.796307035	0.101183356	-7.869940924	3.54809E-15	1.83415E-14	negative
PRJNA729271	stage_E18_vs_E10	ordinary_muscle_development	ENSGALG00010008350	LRRC6	0.263886856	0.174597488	1.51140122	0.130686259	0.169405949	positive
PRJNA729271	stage_E18_vs_E10	ordinary_muscle_development	ENSGALG00010008351	IST1	-0.412484675	0.068924657	-5.984573493	2.16958E-09	7.11332E-09	negative
PRJNA729271	stage_E18_vs_E10	ordinary_muscle_development	ENSGALG00010008352	ENSGALG00010008352	-0.078195214	0.156430997	-0.499870328	0.617166386	0.66784821	negative
PRJNA729271	stage_E18_vs_E10	ordinary_muscle_development	ENSGALG00010008354	TMEM71	1.615594536	0.230064842	7.022344307	2.18176E-12	9.09276E-12	positive
PRJNA729271	stage_E18_vs_E10	ordinary_muscle_development	ENSGALG00010008355	MAFA	3.927983587	0.177760513	22.09705361	3.3738E-108	4.8903E-106	positive
PRJNA729271	stage_E18_vs_E10	ordinary_muscle_development	ENSGALG00010008356	ENSGALG00010008356	-0.442121876	0.12805157	-3.452686109	0.000555034	0.001047555	negative
PRJNA729271	stage_E18_vs_E10	ordinary_muscle_development	ENSGALG00010008358	HPF1	-1.141962871	0.114824293	-9.945307245	2.64357E-23	2.25006E-22	negative
PRJNA729271	stage_E18_vs_E10	ordinary_muscle_development	ENSGALG00010008360	ZC3H3	0.170554988	0.062180334	2.74290887	0.006089758	0.0099595	positive
PRJNA729271	stage_E18_vs_E10	ordinary_muscle_development	ENSGALG00010008361	MATR3	-0.947364305	0.057036769	-16.60971198	5.92771E-62	2.54208E-60	negative
PRJNA729271	stage_E18_vs_E10	ordinary_muscle_development	ENSGALG00010008362	RAMP3	1.508274548	0.260141055	5.797910467	6.71463E-09	2.10713E-08	positive
PRJNA729271	stage_E18_vs_E10	ordinary_muscle_development	ENSGALG00010008364	ENSGALG00010008364	-1.044852144	0.597784706	-1.747873666	0.080485906	0.10879821	negative
PRJNA729271	stage_E18_vs_E10	ordinary_muscle_development	ENSGALG00010008366	RPRD1A	0.024687272	0.107463888	0.229726217	0.818304522	0.848996066	positive
PRJNA729271	stage_E18_vs_E10	ordinary_muscle_development	ENSGALG00010008367	ENSGALG00010008367	-0.228102061	0.618583736	-0.368748881	0.71231491	0.756133623	negative
PRJNA729271	stage_E18_vs_E10	ordinary_muscle_development	ENSGALG00010008368	SNCA	1.719610093	0.317512037	5.415889455	6.09848E-08	1.7581E-07	positive
PRJNA729271	stage_E18_vs_E10	ordinary_muscle_development	ENSGALG00010008372	CLCN3	-0.299095963	0.070120361	-4.265465269	1.99486E-05	4.49487E-05	negative
PRJNA729271	stage_E18_vs_E10	ordinary_muscle_development	ENSGALG00010008373	MAC1R	0.349112184	0.163897608	2.130062713	0.033166436	0.048069942	positive
PRJNA729271	stage_E18_vs_E10	ordinary_muscle_development	ENSGALG00010008374	ZNF821	-0.033779879	0.086290109	-0.39146873	0.695450798	0.740564158	negative
PRJNA729271	stage_E18_vs_E10	ordinary_muscle_development	ENSGALG00010008375	ENSGALG00010008375	-0.499323102	0.098637128	-5.062222649	4.14397E-07	1.11111E-06	negative
PRJNA729271	stage_E18_vs_E10	ordinary_muscle_development	ENSGALG00010008376	MMRN1	-0.618395461	0.261490385	-2.364887953	0.018035527	0.027408781	negative
PRJNA729271	stage_E18_vs_E10	ordinary_muscle_development	ENSGALG00010008377	AKT2	0.033900695	0.117051984	0.289620851	0.772106314	0.808939719	positive
PRJNA729271	stage_E18_vs_E10	ordinary_muscle_development	ENSGALG00010008380	S1L1	1.104891083	0.170867532	6.466360627	1.00391E-10	3.65897E-10	positive
PRJNA729271	stage_E18_vs_E10	ordinary_muscle_development	ENSGALG00010008381	C18orf21	-0.03413801	0.114919989	-0.29705894	0.766421512	0.803971618	negative
PRJNA729271	stage_E18_vs_E10	ordinary_muscle_development	ENSGALG00010008382	ENSGALG00010008382	1.817530535	0.127744311	14.22787849	6.15199E-46	1.51398E-44	positive
PRJNA729271	stage_E18_vs_E10	ordinary_muscle_development	ENSGALG00010008383	PLD5	-1.940604503	0.421659145	-4.60230621	4.17838E-06	1.00799E-05	negative
PRJNA729271	stage_E18_vs_E10	ordinary_muscle_development	ENSGALG00010008385	CTNNA1	-0.005913996	0.091214666	-0.064836019	0.948304563	0.957450414	negative
PRJNA729271	stage_E18_vs_E10	ordinary_muscle_development	ENSGALG00010008386	SESN3	0.4442761	0.227892632	1.949497424	0.05123605	0.071832644	positive
PRJNA729271	stage_E18_vs_E10	ordinary_muscle_development	ENSGALG00010008387	PHF20L1	-0.006822652	0.112732038	-0.060520969	0.951740716	0.960286905	negative
PRJNA729271	stage_E18_vs_E10	ordinary_muscle_development	ENSGALG00010008388	ADCY1	-0.060125559	0.384799228	-0.156251765	0.875834575	0.89744254	negative
PRJNA729271	stage_E18_vs_E10	ordinary_muscle_development	ENSGALG00010008389	GALNT1	-0.913230185	0.088894207	-10.27322492	9.30414E-25	8.71211E-24	negative
PRJNA729271	stage_E18_vs_E10	ordinary_muscle_development	ENSGALG00010008391	ENSGALG00010008391	0.038560593	0.071296817	0.54084593	0.588613782	0.640777827	positive
PRJNA729271	stage_E18_vs_E10	ordinary_muscle_development	ENSGALG00010008392	CD36	2.817731154	0.369455327	7.626716814	2.40808E-14	1.16041E-13	positive
PRJNA729271	stage_E18_vs_E10	ordinary_muscle_development	ENSGALG00010008394	CD2AP	0.688597395	0.11128358	6.187771786	6.10206E-10	2.10443E-09	positive
PRJNA729271	stage_E18_vs_E10	ordinary_muscle_development	ENSGALG00010008395	ENDOD1	1.691520667	0.144134395	11.73571834	8.36134E-32	1.11293E-30	positive
PRJNA729271	stage_E18_vs_E10	ordinary_muscle_development	ENSGALG00010008396	LRRTM2	-1.124829701	0.344301964	-3.26698601	0.001086991	0.001971956	negative
PRJNA729271	stage_E18_vs_E10	ordinary_muscle_development	ENSGALG00010008397	ATXN1L	-0.141064893	0.072350334	-1.949747631	0.051206206	0.071803614	negative
PRJNA729271	stage_E18_vs_E10	ordinary_muscle_development	ENSGALG00010008399	INO80C	-0.761581611	0.114953772	-6.625111989	3.46985E-11	1.3091E-10	negative
PRJNA729271	stage_E18_vs_E10	ordinary_muscle_development	ENSGALG00010008400	MAP1LC3C	-1.932730106	0.265517435	-7.279108079	3.36034E-13	1.49137E-12	negative
PRJNA729271	stage_E18_vs_E10	ordinary_muscle_development	ENSGALG00010008401	PSMD8	-0.569506119	0.157885949	-3.607072839	0.000309671	0.000605188	negative
PRJNA729271	stage_E18_vs_E10	ordinary_muscle_development	ENSGALG00010008403	GNAT3	-0.936075442	0.437296297	-2.140597688	0.032306495	0.046936218	negative
PRJNA729271	stage_E18_vs_E10	ordinary_muscle_development	ENSGALG00010008405	LRATD2	-0.687261767	0.147559008	-4.657538529	3.20013E-06	7.82091E-06	negative
PRJNA729271	stage_E18_vs_E10	ordinary_muscle_development	ENSGALG00010008407	GNAI1	-0.576843009	0.194489388	-2.965935647	0.003017636	0.005166014	negative
PRJNA729271	stage_E18_vs_E10	ordinary_muscle_development	ENSGALG00010008408	SMARCA4	-0.734721485	0.154286587	-4.762056775	1.9163E-06	4.79985E-06	negative
PRJNA729271	stage_E18_vs_E10	ordinary_muscle_development	ENSGALG00010008409	CWC15	0.139957341	0.085075716	1.645091539	0.099950935	0.132527333	positive
PRJNA729271	stage_E18_vs_E10	ordinary_muscle_development	ENSGALG00010008410	NAPRT	0.217509684	0.131653373	1.652139092	0.098506199	0.13081515	positive
PRJNA729271	stage_E18_vs_E10	ordinary_muscle_development	ENSGALG00010008411	ENSGALG00010008411	0.708680379	0.387814822	1.827367956	0.067644464	0.09272806	positive
PRJNA729271	stage_E18_vs_E10	ordinary_muscle_development	ENSGALG00010008412	NEK1	-0.629888399	0.104749213	-6.013299565	1.81785E-09	5.99811E-09	negative
PRJNA729271	stage_E18_vs_E10	ordinary_muscle_development	ENSGALG00010008415	ENSGALG00010008415	1.493177914	0.350905542	4.255213255	2.0885E-05	4.69564E-05	positive
PRJNA729271	stage_E18_vs_E10	ordinary_muscle_development	ENSGALG00010008416	MSX2	-4.704328518	0.458924913	-10.25075864	1.17418E-24	1.09171E-23	negative
PRJNA729271	stage_E18_vs_E10	ordinary_muscle_development	ENSGALG00010008417	IGFBP1	1.655680868	0.85084115	1.94593417	0.051662648	0.072345675	positive
PRJNA729271	stage_E18_vs_E10	ordinary_muscle_development	ENSGALG00010008418	TMEM245	0.630640022	0.090841201	6.942224625	3.85973E-12	1.57952E-11	positive
PRJNA729271	stage_E18_vs_E10	ordinary_muscle_development	ENSGALG00010008422	EXO1	-2.590607034	0.230730244	-11.22786066	2.97597E-29	3.5042E-28	negative
PRJNA729271	stage_E18_vs_E10	ordinary_muscle_development	ENSGALG00010008424	PPIP5K2	-1.129173432	0.122190035	-9.241125394	2.43918E-20	1.75525E-19	negative
PRJNA729271	stage_E18_vs_E10	ordinary_muscle_development	ENSGALG00010008425	MAGI2	0.057937307	0.10591853	0.546998785	0.584379567	0.636981638	positive
PRJNA729271	stage_E18_vs_E10	ordinary_muscle_development	ENSGALG00010008426	AP1G1	-0.562630186	0.056401659	-9.975419019	1.95275E-23	1.67784E-22	negative
PRJNA729271	stage_E18_vs_E10	ordinary_muscle_development	ENSGALG00010008429	IGFBP3	1.132559374	0.11886849	9.527835164	1.60597E-21	1.24751E-20	positive
PRJNA729271	stage_E18_vs_E10	ordinary_muscle_development	ENSGALG00010008430	ENSGALG00010008430	-0.83255461	0.452689	-1.839131524	0.065895843	0.090485056	negative
PRJNA729271	stage_E18_vs_E10	ordinary_muscle_development	ENSGALG00010008431	SH3RF1	-0.265660481	0.126492835	-2.100201803	0.035711093	0.051444275	negative
PRJNA729271	stage_E18_vs_E10	ordinary_muscle_development	ENSGALG00010008433	EBAG9	0.228254536	0.096679021	2.360952077	0.018228086	0.027659774	positive
PRJNA729271	stage_E18_vs_E10	ordinary_muscle_development	ENSGALG00010008434	PTCHD4	-5.938532863	0.920876715	-6.448781652	1.12753E-10	4.08895E-10	negative
PRJNA729271	stage_E18_vs_E10	ordinary_muscle_development	ENSGALG00010008437	SHKBP1	0.109543517	0.163955022	0.66813151	0.50404965	0.561195151	positive
PRJNA729271	stage_E18_vs_E10	ordinary_muscle_development	ENSGALG00010008438	SYBU	0.119963789	0.265542799	0.451768188	0.65143599	0.699694333	positive
PRJNA729271	stage_E18_vs_E10	ordinary_muscle_development	ENSGALG00010008440	ENSGALG00010008440	0.553757948	0.314536711	1.76055109	0.078314413	0.106100329	positive
PRJNA729271	stage_E18_vs_E10	ordinary_muscle_development	ENSGALG00010008441	PHLPP2	-0.245490046	0.088512172	-2.773517372	0.005545388	0.009128949	negative
PRJNA729271	stage_E18_vs_E10	ordinary_muscle_development	ENSGALG00010008443	RSFR	2.431217081	0.441811632	5.502836283	3.73729E-08	1.1024E-07	positive
PRJNA729271	stage_E18_vs_E10	ordinary_muscle_development	ENSGALG00010008444	SLC7A11	-3.986926984	0.519375951	-7.676379661	1.63648E-14	8.01647E-14	negative
PRJNA729271	stage_E18_vs_E10	ordinary_muscle_development	ENSGALG00010008447	FRRS1L	-0.131883355	0.156100422	-0.844862262	0.398187747	0.458141888	negative
PRJNA729271	stage_E18_vs_E10	ordinary_muscle_development	ENSGALG00010008448	TNS3	-0.250103435	0.08076494	-3.096683238	0.001956989	0.003438787	negative

PRJNA729271	stage_E18_vs_E10	ordinary_muscle_development	ENSGALG00010008449	SRA1	0.190066329	0.109549351	1.734983617	0.082743707	0.111517437	positive
PRJNA729271	stage_E18_vs_E10	ordinary_muscle_development	ENSGALG00010008450	GIN1	-0.692134078	0.181570449	-3.811931291	0.000137885	0.000282734	negative
PRJNA729271	stage_E18_vs_E10	ordinary_muscle_development	ENSGALG00010008451	OPN3	0.437032944	0.314314545	1.390431819	0.16439779	0.208408778	positive
PRJNA729271	stage_E18_vs_E10	ordinary_muscle_development	ENSGALG00010008452	ENSGALG00010008452	0.523763212	0.424429834	1.234039574	0.217188158	0.267858619	positive
PRJNA729271	stage_E18_vs_E10	ordinary_muscle_development	ENSGALG00010008453	AMOTL1	1.860311664	0.119774217	15.53182065	2.11295E-54	7.02458E-53	positive
PRJNA729271	stage_E18_vs_E10	ordinary_muscle_development	ENSGALG00010008455	FKBP1B	0.575545926	0.152493709	3.774227346	0.000160504	0.000325887	positive
PRJNA729271	stage_E18_vs_E10	ordinary_muscle_development	ENSGALG00010008456	NHLRC1	-0.030106236	0.130792644	-0.230182949	0.817949611	0.848749346	negative
PRJNA729271	stage_E18_vs_E10	ordinary_muscle_development	ENSGALG00010008457	MARVELD3	0.724260717	0.119446223	6.063487829	1.33201E-09	4.44054E-09	positive
PRJNA729271	stage_E18_vs_E10	ordinary_muscle_development	ENSGALG00010008459	EPB41L4B	-1.758146762	0.219908219	-7.994911578	1.29667E-15	6.91001E-15	negative
PRJNA729271	stage_E18_vs_E10	ordinary_muscle_development	ENSGALG00010008464	SF3B6	-0.65479252	0.111989903	-5.846888913	5.00852E-09	1.59137E-08	negative
PRJNA729271	stage_E18_vs_E10	ordinary_muscle_development	ENSGALG00010008466	C11orf97	0.126066675	0.299804248	0.420496626	0.674122694	0.720711642	positive
PRJNA729271	stage_E18_vs_E10	ordinary_muscle_development	ENSGALG00010008468	EPPK1	-0.432562739	0.286725326	-1.508631081	0.131393087	0.17026129	negative
PRJNA729271	stage_E18_vs_E10	ordinary_muscle_development	ENSGALG00010008469	NOCT	-0.127736977	0.282387342	-0.452346681	0.651019252	0.699364415	negative
PRJNA729271	stage_E18_vs_E10	ordinary_muscle_development	ENSGALG00010008473	CSMD3	-3.064226682	0.383876444	-7.98232539	1.43602E-15	7.62737E-15	negative
PRJNA729271	stage_E18_vs_E10	ordinary_muscle_development	ENSGALG00010008474	ENSGALG00010008474	2.62401389	0.137829771	19.0380777	8.25004E-81	5.97922E-79	positive
PRJNA729271	stage_E18_vs_E10	ordinary_muscle_development	ENSGALG00010008476	RNF166	0.242672106	0.134762062	1.800744981	0.071743084	0.097938972	positive
PRJNA729271	stage_E18_vs_E10	ordinary_muscle_development	ENSGALG00010008486	CTU2	-0.613848134	0.139423666	-4.402754221	1.06885E-05	2.48445E-05	negative
PRJNA729271	stage_E18_vs_E10	ordinary_muscle_development	ENSGALG00010008488	CCSER1	-0.546976687	0.15386239	-3.5549733	0.000378017	0.000729711	negative
PRJNA729271	stage_E18_vs_E10	ordinary_muscle_development	ENSGALG00010008492	ANKRD49	-0.489000918	0.127586936	-3.832687997	0.000126751	0.000261122	negative
PRJNA729271	stage_E18_vs_E10	ordinary_muscle_development	ENSGALG00010008497	PAM	-0.350596461	0.129540611	-2.706459845	0.006800481	0.011019896	negative
PRJNA729271	stage_E18_vs_E10	ordinary_muscle_development	ENSGALG00010008498	ENSGALG00010008498	2.003063845	0.147816265	13.5510381	7.81175E-42	1.61069E-40	positive
PRJNA729271	stage_E18_vs_E10	ordinary_muscle_development	ENSGALG00010008500	ENSGALG00010008500	0.666092881	0.152326471	4.372797952	1.22664E-05	2.82989E-05	positive
PRJNA729271	stage_E18_vs_E10	ordinary_muscle_development	ENSGALG00010008501	ENSGALG00010008501	-1.595943106	0.436075551	-3.659785792	0.000252426	0.000499784	negative
PRJNA729271	stage_E18_vs_E10	ordinary_muscle_development	ENSGALG00010008504	RNF139	0.03035272	0.088792962	0.341837005	0.732473559	0.774017951	positive
PRJNA729271	stage_E18_vs_E10	ordinary_muscle_development	ENSGALG00010008505	PTPN3	-0.446664577	0.271567731	-1.644763075	0.10001868	0.132588713	negative
PRJNA729271	stage_E18_vs_E10	ordinary_muscle_development	ENSGALG00010008509	MGARP	1.726973831	0.355272132	4.860988733	1.16801E-06	2.99704E-06	positive
PRJNA729271	stage_E18_vs_E10	ordinary_muscle_development	ENSGALG00010008514	PIEZO1	1.562483831	0.16656067	9.380869002	6.54312E-21	4.90396E-20	positive
PRJNA729271	stage_E18_vs_E10	ordinary_muscle_development	ENSGALG00010008515	TAFA5	0.081087771	0.207909994	0.39001382	0.696526328	0.741600494	positive
PRJNA729271	stage_E18_vs_E10	ordinary_muscle_development	ENSGALG00010008516	MRE11	-1.026889573	0.079236887	-12.95974155	2.06944E-38	3.71704E-37	negative
PRJNA729271	stage_E18_vs_E10	ordinary_muscle_development	ENSGALG00010008522	NUDT12	1.043230189	0.149221348	6.991159139	2.72624E-12	1.12712E-11	positive
PRJNA729271	stage_E18_vs_E10	ordinary_muscle_development	ENSGALG00010008527	CHUK	-0.03872638	0.056130374	-0.689936251	0.490234278	0.547960044	negative
PRJNA729271	stage_E18_vs_E10	ordinary_muscle_development	ENSGALG00010008529	GPR83	-0.246052505	0.519113099	-0.473986315	0.635509666	0.684987553	negative
PRJNA729271	stage_E18_vs_E10	ordinary_muscle_development	ENSGALG00010008533	ENSGALG00010008533	-0.722575375	0.622762281	-1.160274791	0.245936945	0.299161571	negative
PRJNA729271	stage_E18_vs_E10	ordinary_muscle_development	ENSGALG00010008534	RAB33B	0.065715691	0.073176668	0.898041593	0.369163376	0.428802238	positive
PRJNA729271	stage_E18_vs_E10	ordinary_muscle_development	ENSGALG00010008542	MOCOS	1.679007264	0.15859461	10.58678643	3.43171E-26	3.47123E-25	positive
PRJNA729271	stage_E18_vs_E10	ordinary_muscle_development	ENSGALG00010008561	EEF1D	1.592637393	0.114697109	13.88559318	7.74565E-44	1.73529E-42	positive
PRJNA729271	stage_E18_vs_E10	ordinary_muscle_development	ENSGALG00010008580	PANX1	-0.824399144	0.247478129	-3.331199998	0.000864725	0.001590027	negative
PRJNA729271	stage_E18_vs_E10	ordinary_muscle_development	ENSGALG00010008588	PYCR3	-0.752487215	0.179950624	-4.18163159	2.89425E-05	6.39904E-05	negative
PRJNA729271	stage_E18_vs_E10	ordinary_muscle_development	ENSGALG00010008592	KIAA0513	1.680021614	0.138877843	12.09711768	1.09385E-33	1.57139E-32	positive
PRJNA729271	stage_E18_vs_E10	ordinary_muscle_development	ENSGALG00010008596	ENSGALG00010008596	-1.292302706	0.162715797	-7.9420851	1.9881E-15	1.04336E-14	negative
PRJNA729271	stage_E18_vs_E10	ordinary_muscle_development	ENSGALG00010008618	EIF4EBP3	0.074359181	0.116924161	0.635960779	0.524801986	0.581308634	positive
PRJNA729271	stage_E18_vs_E10	ordinary_muscle_development	ENSGALG00010008623	TRPS1	-1.472316693	0.132314988	-11.1273614	9.23282E-29	1.06214E-27	negative
PRJNA729271	stage_E18_vs_E10	ordinary_muscle_development	ENSGALG00010008636	AGTPBP1	1.455961691	0.24271082	5.998750656	1.98841E-09	6.54303E-09	positive
PRJNA729271	stage_E18_vs_E10	ordinary_muscle_development	ENSGALG00010008637	RGS7	-4.271645096	0.805235235	-5.304841259	1.12771E-07	3.18453E-07	negative
PRJNA729271	stage_E18_vs_E10	ordinary_muscle_development	ENSGALG00010008653	ENSGALG00010008653	-0.468470436	0.155789145	-3.007080094	0.002637703	0.00455486	negative
PRJNA729271	stage_E18_vs_E10	ordinary_muscle_development	ENSGALG00010008656	CCDC6	-0.236463526	0.072410455	-3.265599237	0.001092327	0.001980646	negative
PRJNA729271	stage_E18_vs_E10	ordinary_muscle_development	ENSGALG00010008658	TSTA3	-0.654445514	0.115676645	-5.657542298	1.53556E-08	4.67113E-08	negative
PRJNA729271	stage_E18_vs_E10	ordinary_muscle_development	ENSGALG00010008666	SLC16A9	0.666357148	0.189273321	3.520607894	0.000430559	0.000826178	positive
PRJNA729271	stage_E18_vs_E10	ordinary_muscle_development	ENSGALG00010008668	ENSGALG00010008668	-0.368608178	0.339251567	-1.086533456	0.277243047	0.332723793	negative
PRJNA729271	stage_E18_vs_E10	ordinary_muscle_development	ENSGALG00010008674	RXFP1	0.59707166	0.279257502	2.13806847	0.032511187	0.047195758	positive
PRJNA729271	stage_E18_vs_E10	ordinary_muscle_development	ENSGALG00010008686	ENSGALG00010008686	-2.573898329	0.440306637	-5.845695054	5.04457E-09	1.60248E-08	negative
PRJNA729271	stage_E18_vs_E10	ordinary_muscle_development	ENSGALG00010008688	HBEGF	0.97286394	0.23915517	4.06791933	4.74348E-05	0.000102379	positive
PRJNA729271	stage_E18_vs_E10	ordinary_muscle_development	ENSGALG00010008690	YIF1B	-0.164365914	0.124154755	-1.323879333	0.185543123	0.232530483	negative
PRJNA729271	stage_E18_vs_E10	ordinary_muscle_development	ENSGALG00010008693	C4orf46	-0.224405466	0.104682587	-2.143675205	0.03205892	0.046618584	negative
PRJNA729271	stage_E18_vs_E10	ordinary_muscle_development	ENSGALG00010008696	PFN4	0.253303101	0.208790141	1.21319474	0.225055375	0.276291832	positive
PRJNA729271	stage_E18_vs_E10	ordinary_muscle_development	ENSGALG00010008699	NAA35	-0.283182114	0.101261456	-2.796543976	0.005165238	0.008546818	negative
PRJNA729271	stage_E18_vs_E10	ordinary_muscle_development	ENSGALG00010008700	ENSGALG00010008700	-0.430615591	0.266151226	-1.617935781	0.105676438	0.139531788	negative
PRJNA729271	stage_E18_vs_E10	ordinary_muscle_development	ENSGALG00010008708	ZC3H4	0.440858059	0.248860935	1.771503668	0.076476983	0.103805026	positive
PRJNA729271	stage_E18_vs_E10	ordinary_muscle_development	ENSGALG00010008716	TATDN1	-0.715941623	0.114311038	-6.263101407	3.77395E-10	1.32038E-09	negative
PRJNA729271	stage_E18_vs_E10	ordinary_muscle_development	ENSGALG00010008729	TMEM254	-0.446332221	0.119418766	-3.737538388	0.000185831	0.000374217	negative
PRJNA729271	stage_E18_vs_E10	ordinary_muscle_development	ENSGALG00010008731	NDUFB9	0.502810236	0.088009273	5.713150649	1.10903E-08	3.4087E-08	positive
PRJNA729271	stage_E18_vs_E10	ordinary_muscle_development	ENSGALG00010008739	VSTM5	-1.656485395	0.283411021	-5.844816436	5.07127E-09	1.61025E-08	negative
PRJNA729271	stage_E18_vs_E10	ordinary_muscle_development	ENSGALG00010008745	GRAMD4	-0.10766581	0.154427714	-0.697192282	0.485682469	0.543710796	negative
PRJNA729271	stage_E18_vs_E10	ordinary_muscle_development	ENSGALG00010008750	PFDN1	1.043186621	0.16586187	6.289490308	3.1851E-10	1.11922E-09	positive
PRJNA729271	stage_E18_vs_E10	ordinary_muscle_development	ENSGALG00010008752	GOLM1	0.137317142	0.172728663	0.794987577	0.426620729	0.486765386	positive
PRJNA729271	stage_E18_vs_E10	ordinary_muscle_development	ENSGALG00010008756	ITSN2	3.011479785	0.071675627	42.01539503	0	0	positive
PRJNA729271	stage_E18_vs_E10	ordinary_muscle_development	ENSGALG00010008762	SAE1	-0.664266113	0.102988271	-6.449920013	1.11909E-10	4.06039E-10	negative
PRJNA729271	stage_E18_vs_E10	ordinary_muscle_development	ENSGALG00010008764	ETFDH	1.137779221	0.123659312	9.20091828	3.54904E-20	2.51433E-19	positive
PRJNA729271	stage_E18_vs_E10	ordinary_muscle_development	ENSGALG00010008768	PRXL2C	1.994990382	0.14847566	13.43648099	3.69599E-41	7.42014E-40	positive
PRJNA729271	stage_E18_vs_E10	ordinary_muscle_development	ENSGALG00010008775	MED17	-0.386412951	0.090930362	-4.249548157	2.14202E-05	4.80852E-05	negative
PRJNA729271	stage_E18_vs_E10	ordinary_muscle_development	ENSGALG00010008780	KARS1	0.02326	0.057549586	0.404173199	0.68608536	0.731935475	positive
PRJNA729271	stage_E18_vs_E10	ordinary_muscle_development	ENSGALG00010008782	CCDC9	0.331267158	0.285394064	1.160735975	0.245749287	0.2990125	positive
PRJNA729271	stage_E18_vs_E10	ordinary_muscle_development	ENSGALG00010008790	GPN1	-0.140227877	0.097333941	-1.440688373	0.149672741	0.191652507	negative
PRJNA729271	stage_E18_vs_E10	ordinary_muscle_development	ENSGALG00010008793	C11orf54	0.203180869	0.118118974	1.72013744	0.085407462	0.114723488	positive
PRJNA729271	stage_E18_vs_E10	ordinary_muscle_development	ENSGALG00010008799	ENSGALG00010008799	0.741329026	0.172572331	4.295758305	1.74097E-05	3.95291E-05	positive
PRJNA729271	stage_E18_vs_E10	ordinary_muscle_development	ENSGALG00010008807	BRD1	0.143706771	0.06214118	2.312585167	0.020745458	0.03119998	positive
PRJNA729271	stage_E18_vs_E10	ordinary_muscle_development	ENSGALG00010008811	TERF2IP	0.272811439	0.095040576	2.870473334	0.004098578	0.006871256	positive
PRJNA729271	stage_E18_vs_E10	ordinary_muscle_development	ENSGALG00010008814	ENSGALG00010008814	0.286642799	0.24454699	1.172137917	0.241141689	0.294146998	positive
PRJNA729271	stage_E18_vs_E10	ordinary_muscle_development	ENSGALG00010008821	ENSGALG00010008821	0.105823107	0.138108241	0.766233106	0.443537613	0.502991849	positive
PRJNA729271	stage_E18_vs_E10	ordinary_muscle_development	ENSGALG00010008824	GREM2	-1.867305531	0.313967477	-5.947448908	2.72354E-09	8.85547E-09	negative
PRJNA729271	stage_E18_vs_E10	ordinary_muscle_development	ENSGALG00010008825	PPID	-0.202892308	0.109310234	-1.856114475	0.063437243	0.087340695	negative
PRJNA729271	stage_E18_vs_E10	ordinary_muscle_development	ENSGALG00010008830	DHX34	0.092259049	0.200696123	0.459695222	0.645734999	0.694717496	positive
PRJNA729271	stage_E18_vs_E10	ordinary_muscle_development	ENSGALG00010008832	MTSS1	1.924853446	0.123255605	15.61676198	5.59771E-55	1.90467E-53	positive
PRJNA729271	stage_E18_vs_E10	ordinary_muscle_development	ENSGALG00010008837	ENSGALG00010008837	-0.762663377	0.099538239	-7.662013945	1.8304E-14	8.9332E-14	negative
PRJNA729271	stage_E18_vs_E10	ordinary_muscle_development	ENSGALG00010008839	ZNF512	-0.301028592	0.074626157	-4.033821431	5.4877E-05	0.000117739	negative
PRJNA729271	stage_E18_vs_E10	ordinary_muscle_development	ENSGALG00010008846	SLC35D2	0.561101536	0.284592167	1.971598668	0.048655439	0.068458609	positive
PRJNA729271	stage_E18_vs_E10	ordinary_muscle_development	ENSGALG00010008850	CYSTM1	2.259233732	0.124083346	18.20738887	4.50947E-74	2.62509E-72	positive
PRJNA729271	stage_E18_vs_E10	ordinary_muscle_development	ENSGALG00010008852	KHDRBS3	0.278792309	0.083794201	3.327107417	0.000877525	0.00161152	positive
PRJNA729271	stage_E18_vs_E10	ordinary_muscle_development	ENSGALG00010008859	ENSGALG00010008859	-0.138631205	0.204638188	-0.677445427	0.498123385	0.555364853	negative
PRJNA729271	stage_E18_vs_E10	ordinary_muscle_development	ENSGALG00010008860	SQLE	-3.28092612	0.255389366	-12.84676091	8.96876E-38	1.57007E-36	negative
PRJNA729271	stage_E18_vs_E10	ordinary_muscle_development	ENSGALG00010008862	ZNF367	-0.238760503	0.143252416	-1.66671188	0.09557171	0.127267977	negative
PRJNA729271	stage_E18_vs_E10	ordinary_muscle_development	ENSGALG00010008864	ADAT1	0.415058251	0.138436361	2.998188113	0.0027159	0.004679302	positive
PRJNA729271	stage_E18_vs_E10	ordinary_muscle_development	ENSGALG00010008866	WASHC5	-0.703416934	0.075135736	-9.361949032	7.82792E-21	5.83071E-20	negative
PRJNA729271	stage_E18_vs_E10	ordinary_muscle_development	ENSGALG00010008871	PRR7	-1.27429893	0.195417729	-6.520897256	6.9888E-11	2.58096E-10	negative
PRJNA729271	stage_E18_vs_E10	ordinary_muscle_development	ENSGALG00010008877	FNDC4	1.056096789	0.246727896	4.280410952	1.86549E-05	4.21647E-05	positive
PRJNA729271	stage_E18_vs_E10	ordinary_muscle_development	ENSGALG00010008879	HABP4	-0.86001169	0.172894719	-4.974192944	6.552E-07	1.72362E-06	negative
PRJNA729271	stage_E18_vs_E10	ordinary_muscle_development	ENSGALG00010008880	FMN2	0.486211203	0.279597656	1.738967379	0.082040501	0.110703506	positive
PRJNA729271	stage_E18_vs_E10	ordinary_muscle_development	ENSGALG00010008884	FAM135B	-0.095002096	0.522769881	-0.181728328	0.855795934	0.880769814	negative
PRJNA729271	stage_E18_vs_E10	ordinary_muscle_development	ENSGALG00010008888	FBXO46	0.679276446	0.142169494	4.777933901	1.77106E-06	4.45685E-06	positive
PRJNA729271	stage_E18_vs_E10	ordinary_muscle_development	ENSGALG00010008892	ENSGALG00010008892	0.381785681	0.119993252	3.181726272	0.001464001	0.002614352	positive
PRJNA729271	stage_E18_vs_E10	ordinary_muscle_development	ENSGALG00010008893	ENSGALG00010008893	3.919810963	0.138686664	28.26379163	9.6349E-176	4.3643E-173	positive
PRJNA729271	stage_E18_vs_E10	ordinary_muscle_development	ENSGALG00010008897	NSMCE2	0.143486347	0.163929712	0.87529189	0.381415106	0.441723551	positive
PRJNA729271	stage_E18_vs_E10	ordinary_muscle_development	ENSGALG00010008903	ENSGALG00010008903	3.195234167	0.408653264	7.818937096	5.32712E-15	2.71794E-14	positive
PRJNA729271	stage_E18_vs_E10	ordinary_muscle_development	ENSGALG00010008905	GABARAPL2	0.170783045	0.095460119	1.789051238	0.073606564	0.100209181	positive
PRJNA729271	stage_E18_vs_E10	ordinary_muscle_development	ENSGALG00010008906	DBN1	-2.503210049	0.211666231	-11.82621355	2.85743E-32	3.86366E-31	negative
PRJNA729271	stage_E18_vs_E10	ordinary_muscle_development	ENSGALG00010008910	TRIB1	1.046828693	0.261880864	3.997347035	6.40564E-05	0.000136524	positive
PRJNA729271	stage_E18_vs_E10	ordinary_muscle_development	ENSGALG00010008918	PLAC9	0.817504746	0.245642578	3.328025428	0.000874639	0.001606627	positive
PRJNA729271	stage_E18_vs_E10	ordinary_muscle_development	ENSGALG00010008921	OPA3	0.287440178	0.12981231	2.214275191	0.026809854	0.039532944	positive
PRJNA729271	stage_E18_vs_E10	ordinary_muscle_development	ENSGALG00010008922	ENSGALG00010008922	4.894386186	1.188054391	4.119665079	3.79424E-05	8.27709E-05	positive
PRJNA729271	stage_E18_vs_E10	ordinary_muscle_development	ENSGALG00010008928	CFC1	-2.977542338	0.552095477	-5.393165609	6.92271E-08	1.98624E-07	negative
PRJNA729271	stage_E18_vs_E10	ordinary_muscle_development	ENSGALG00010008931	FNIP2	0.563135108	0.131681617	4.276489925	1.89863E-05	4.28671E-05	positive
PRJNA729271	stage_E18_vs_E10	ordinary_muscle_development	ENSGALG00010008932	VASP	1.333878243	0.120542058	11.06566677	1.84089E-28	2.08141E-27	positive
PRJNA729271	stage_E18_vs_E10	ordinary_muscle_development	ENSGALG00010008936	IFT172	-0.009376919	0.094059223	-0.099691652	0.920589128	0.934645893	negative
PRJNA729271	stage_E18_vs_E10	ordinary_muscle_development	ENSGALG00010008938	ZBED4	-0.867168168	0.09171471	-9.455060873	3.22834E-21	2.46159E-20	negative
PRJNA729271	stage_E18_vs_E10	ordinary_muscle_development	ENSGALG00010008939	RANBP9	0.183273213	0.104922598	1.746746799	0.080681269	0.109031791	positive
PRJNA729271	stage_E18_vs_E10	ordinary_muscle_development	ENSGALG00010008943	ENSGALG00010008943	3.07809464	0.465768261	6.608639739	3.87867E-11	1.4584E-10	positive
PRJNA729271	stage_E18_vs_E10	ordinary_muscle_development	ENSGALG00010008945	FER1L6	0.572416171	0.590666516	0.969102117	0.332494242	0.390749476	positive
PRJNA729271	stage_E18_vs_E10	ordinary_muscle_development	ENSGALG00010008946	COL22A1	-0.300765656	0.619589242	-0.485427499	0.62737313	0.677628429	negative
PRJNA729271	stage_E18_vs_E10	ordinary_muscle_development	ENSGALG00010008955	KRTCAP3	-0.509871441	0.34534811	-1.476398527	0.139836938	0.18023621	negative
PRJNA729271	stage_E18_vs_E10	ordinary_muscle_development	ENSGALG00010008956	LVRN	-0.405133613	0.484914252	-0.83547475	0.403450458	0.463429304	negative
PRJNA729271	stage_E18_vs_E10	ordinary_muscle_development	ENSGALG00010008960	LRRC2	10.10127565	0.397379235	25.41973701	1.5261E-142	4.3374E-140	positive
PRJNA729271	stage_E18_vs_E10	ordinary_muscle_development	ENSGALG00010008966	NRBP1	0.134824935	0.116610034	1.156203549	0.247597909	0.300882938	positive
PRJNA729271	stage_E18_vs_E10	ordinary_muscle_development	ENSGALG00010008969	PPP1R37	0.128938406	0.188745448	0.683133857	0.494522263	0.551859281	positive
PRJNA729271	stage_E18_vs_E10	ordinary_muscle_development	ENSGALG00010008971	ANXA11	0.6112754	0.103857675	5.885702727	3.96366E-09	1.27109E-08	positive
PRJNA729271	stage_E18_vs_E10	ordinary_muscle_development	ENSGALG00010008973	KCNK9	0.613051877	0.477422387	1.284086993	0.199111519	0.247863403	positive
PRJNA729271	stage_E18_vs_E10	ordinary_muscle_development	ENSGALG00010008974	SMCO4	-0.605591082	0.104638537	-5.787457476	7.14598E-09	2.23524E-08	negative
PRJNA729271	stage_E18_vs_E10	ordinary_muscle_development	ENSGALG00010008980	ENSGALG00010008980	-0.826019057	0.286125624	-2.886910465	0.003890449	0.006541244	negative
PRJNA729271	stage_E18_vs_E10	ordinary_muscle_development	ENSGALG00010008983	ENSGALG00010008983	-0.353506967	0.350680404	-1.008060224	0.313425552	0.370956428	negative
PRJNA729271	stage_E18_vs_E10	ordinary_muscle_development	ENSGALG00010008988	PPM1G	-0.673859461	0.080938608	-8.325562767	8.3929E-17	4.84103E-16	negative
PRJNA729271	stage_E18_vs_E10	ordinary_muscle_development	ENSGALG00010008991	ENSGALG00010008991	-0.215499105	0.136222957	-1.581958789	0.113658979	0.149255925	negative
PRJNA729271	stage_E18_vs_E10	ordinary_muscle_development	ENSGALG00010008995	ECD	-0.898847343	0.101825734	-8.827310265	1.07223E-18	7.00405E-18	negative

PRJNA729271	stage_E18_vs_E10	ordinary_muscle_development	ENSGALG00010008996	CHRM3	-0.649756947	0.239763893	-2.709986641	0.006728592	0.010914821	negative
PRJNA729271	stage_E18_vs_E10	ordinary_muscle_development	ENSGALG00010008997	CHST6	-0.528365978	0.129927871	-4.066609995	4.7702E-05	0.000102924	negative
PRJNA729271	stage_E18_vs_E10	ordinary_muscle_development	ENSGALG00010008999	TRAPPC9	-0.256271374	0.064859376	-3.951184676	7.77653E-05	0.000164005	negative
PRJNA729271	stage_E18_vs_E10	ordinary_muscle_development	ENSGALG00010009000	FAM149B1	-0.633549789	0.104325509	-6.072817601	1.25685E-09	4.19965E-09	negative
PRJNA729271	stage_E18_vs_E10	ordinary_muscle_development	ENSGALG00010009002	ENSGALG00010009002	1.190103521	0.0971083	12.25542531	1.57123E-34	2.36992E-33	positive
PRJNA729271	stage_E18_vs_E10	ordinary_muscle_development	ENSGALG00010009003	ALG12	-0.655679609	0.13588114	-4.825390849	1.39729E-06	3.55515E-06	negative
PRJNA729271	stage_E18_vs_E10	ordinary_muscle_development	ENSGALG00010009007	TMEM65	0.373415965	0.099441893	3.755117213	0.00017326	0.000350266	positive
PRJNA729271	stage_E18_vs_E10	ordinary_muscle_development	ENSGALG00010009011	ACP6	-0.936489524	0.132027495	-7.093140156	1.31103E-12	5.56303E-12	negative
PRJNA729271	stage_E18_vs_E10	ordinary_muscle_development	ENSGALG00010009012	DEUP1	-1.813642622	0.808710395	-2.242635477	0.024920331	0.036976169	negative
PRJNA729271	stage_E18_vs_E10	ordinary_muscle_development	ENSGALG00010009013	LIPG	0.035005446	0.22337759	0.156709748	0.875473604	0.897199512	positive
PRJNA729271	stage_E18_vs_E10	ordinary_muscle_development	ENSGALG00010009015	TMEM231	-0.149565576	0.092621063	-1.614811696	0.106351479	0.140299989	negative
PRJNA729271	stage_E18_vs_E10	ordinary_muscle_development	ENSGALG00010009016	DNAJC9	-1.389916068	0.112129859	-12.39559271	2.76097E-35	4.30325E-34	negative
PRJNA729271	stage_E18_vs_E10	ordinary_muscle_development	ENSGALG00010009019	ZNF513	-0.032692953	0.137359158	-0.238010726	0.811872775	0.843591102	negative
PRJNA729271	stage_E18_vs_E10	ordinary_muscle_development	ENSGALG00010009020	ARHGAP35	-0.395418351	0.183149258	-2.158995107	0.030850547	0.04499232	negative
PRJNA729271	stage_E18_vs_E10	ordinary_muscle_development	ENSGALG00010009021	HNF4beta	0.273802396	0.423424578	0.646637937	0.517866299	0.574724141	positive
PRJNA729271	stage_E18_vs_E10	ordinary_muscle_development	ENSGALG00010009024	PMAIP1	-0.214078316	0.26295229	-0.814133685	0.415568371	0.475877985	negative
PRJNA729271	stage_E18_vs_E10	ordinary_muscle_development	ENSGALG00010009025	UTP23	-0.206782172	0.098597665	-2.097231936	0.035973049	0.051795902	negative
PRJNA729271	stage_E18_vs_E10	ordinary_muscle_development	ENSGALG00010009026	RAPGEF2	-0.244010787	0.137247541	-1.777888223	0.075422215	0.102488516	negative
PRJNA729271	stage_E18_vs_E10	ordinary_muscle_development	ENSGALG00010009028	PDLIM7	0.384045981	0.109808507	3.497415572	0.000469789	0.000897298	positive
PRJNA729271	stage_E18_vs_E10	ordinary_muscle_development	ENSGALG00010009029	IGSF3	-0.428764744	0.163211146	-2.627055522	0.008612728	0.013764222	negative
PRJNA729271	stage_E18_vs_E10	ordinary_muscle_development	ENSGALG00010009030	ZDHHC7	-0.343186336	0.091307471	-3.758578915	0.000170881	0.000345649	negative
PRJNA729271	stage_E18_vs_E10	ordinary_muscle_development	ENSGALG00010009031	SPIN1	-0.322277054	0.079505373	-4.053525473	5.04515E-05	0.00010863	negative
PRJNA729271	stage_E18_vs_E10	ordinary_muscle_development	ENSGALG00010009034	SNX17	0.30378478	0.120002141	2.531494669	0.011357754	0.017842273	positive
PRJNA729271	stage_E18_vs_E10	ordinary_muscle_development	ENSGALG00010009035	ENSGALG00010009035	-0.981366836	0.080932936	-12.12567948	7.72171E-34	1.12489E-32	negative
PRJNA729271	stage_E18_vs_E10	ordinary_muscle_development	ENSGALG00010009037	ENSGALG00010009037	1.377234969	0.186893154	7.369103375	1.71779E-13	7.76409E-13	positive
PRJNA729271	stage_E18_vs_E10	ordinary_muscle_development	ENSGALG00010009038	RAD21	-0.527625703	0.124251275	-4.246440943	2.17193E-05	4.87038E-05	negative
PRJNA729271	stage_E18_vs_E10	ordinary_muscle_development	ENSGALG00010009039	SLC36A4	-0.666110329	0.175608283	-3.79316009	0.000148742	0.000303323	negative
PRJNA729271	stage_E18_vs_E10	ordinary_muscle_development	ENSGALG00010009040	CRELD2	-0.972227808	0.142971758	-6.800138882	1.04518E-11	4.10528E-11	negative
PRJNA729271	stage_E18_vs_E10	ordinary_muscle_development	ENSGALG00010009043	TFAM	-0.217132938	0.083212718	-2.609372035	0.009070856	0.014443817	negative
PRJNA729271	stage_E18_vs_E10	ordinary_muscle_development	ENSGALG00010009047	SUPT5H	-0.113146351	0.110735454	-1.021771681	0.306888975	0.364320696	negative
PRJNA729271	stage_E18_vs_E10	ordinary_muscle_development	ENSGALG00010009050	PIK3CG	0.718168652	0.094175661	7.625841399	2.42448E-14	1.16754E-13	positive
PRJNA729271	stage_E18_vs_E10	ordinary_muscle_development	ENSGALG00010009051	TROJANZ	0.871525993	0.262768552	3.316705851	0.000910855	0.001668113	positive
PRJNA729271	stage_E18_vs_E10	ordinary_muscle_development	ENSGALG00010009054	RYR2	-2.717989598	0.521390359	-5.212964822	1.85846E-07	5.13797E-07	negative
PRJNA729271	stage_E18_vs_E10	ordinary_muscle_development	ENSGALG00010009057	NXNL2	0.440444581	0.371080276	1.186925334	0.235257073	0.287573898	positive
PRJNA729271	stage_E18_vs_E10	ordinary_muscle_development	ENSGALG00010009059	MED29	1.137696772	0.125873715	9.03839833	1.58984E-19	1.0901E-18	positive
PRJNA729271	stage_E18_vs_E10	ordinary_muscle_development	ENSGALG00010009065	EIF2B4	-0.258762522	0.097280434	-2.659964712	0.007814884	0.012556907	negative
PRJNA729271	stage_E18_vs_E10	ordinary_muscle_development	ENSGALG00010009067	ENSGALG00010009067	2.05806721	1.619627011	1.270704425	0.203833821	0.253198323	positive
PRJNA729271	stage_E18_vs_E10	ordinary_muscle_development	ENSGALG00010009068	AGO2	0.835169489	0.079477056	10.50830933	7.90988E-26	7.82085E-25	positive
PRJNA729271	stage_E18_vs_E10	ordinary_muscle_development	ENSGALG00010009069	GRID2	-2.294065638	0.287796598	-7.971135352	1.57223E-15	8.32037E-15	negative
PRJNA729271	stage_E18_vs_E10	ordinary_muscle_development	ENSGALG00010009070	ENSGALG00010009070	-5.919026022	1.274366987	-4.644679344	3.40605E-06	8.29481E-06	negative
PRJNA729271	stage_E18_vs_E10	ordinary_muscle_development	ENSGALG00010009073	PRKAR2B	1.702885866	0.402948834	4.226059788	2.37819E-05	5.31071E-05	positive
PRJNA729271	stage_E18_vs_E10	ordinary_muscle_development	ENSGALG00010009076	TMEM214	-0.471384945	0.119175902	-3.955371327	7.64159E-05	0.000161371	negative
PRJNA729271	stage_E18_vs_E10	ordinary_muscle_development	ENSGALG00010009078	PTTG2	-1.347608742	0.168813137	-7.982842854	1.43001E-15	7.60102E-15	negative
PRJNA729271	stage_E18_vs_E10	ordinary_muscle_development	ENSGALG00010009079	KCTD15	0.296497002	0.095401624	3.107882121	0.001884332	0.003321181	positive
PRJNA729271	stage_E18_vs_E10	ordinary_muscle_development	ENSGALG00010009080	TLL1	-1.302905973	0.173630591	-7.503896429	6.19483E-14	2.89565E-13	negative
PRJNA729271	stage_E18_vs_E10	ordinary_muscle_development	ENSGALG00010009081	HBP1	0.800618011	0.122441408	6.538784731	6.20207E-11	2.29745E-10	positive
PRJNA729271	stage_E18_vs_E10	ordinary_muscle_development	ENSGALG00010009083	MED30	0.060425487	0.128687489	0.46955215	0.638675018	0.687938945	positive
PRJNA729271	stage_E18_vs_E10	ordinary_muscle_development	ENSGALG00010009085	PAF1	0.122498312	0.088674258	1.381441636	0.167143211	0.211500729	positive
PRJNA729271	stage_E18_vs_E10	ordinary_muscle_development	ENSGALG00010009086	UBE2D1	0.605875549	0.171778356	3.527077344	0.000420174	0.00080732	positive
PRJNA729271	stage_E18_vs_E10	ordinary_muscle_development	ENSGALG00010009088	ENSGALG00010009088	-0.132453999	0.130802026	-1.01262957	0.31123713	0.368818035	negative
PRJNA729271	stage_E18_vs_E10	ordinary_muscle_development	ENSGALG00010009089	AGBL5	0.138493852	0.153681633	0.901173739	0.367495951	0.42710502	positive
PRJNA729271	stage_E18_vs_E10	ordinary_muscle_development	ENSGALG00010009091	EXT1	0.194584618	0.111445702	1.746003786	0.080810294	0.109175619	positive
PRJNA729271	stage_E18_vs_E10	ordinary_muscle_development	ENSGALG00010009092	EED	-1.43071388	0.12394528	-11.543109	7.99812E-31	1.01874E-29	negative
PRJNA729271	stage_E18_vs_E10	ordinary_muscle_development	ENSGALG00010009093	SLU7	0.15175401	0.132349464	1.146615975	0.251540371	0.305111103	positive
PRJNA729271	stage_E18_vs_E10	ordinary_muscle_development	ENSGALG00010009094	DARS	-0.196376004	0.095572734	-2.054728335	0.039905252	0.056987844	negative
PRJNA729271	stage_E18_vs_E10	ordinary_muscle_development	ENSGALG00010009096	ENSGALG00010009096	5.993297604	1.109946815	5.399625929	6.678E-08	1.91906E-07	positive
PRJNA729271	stage_E18_vs_E10	ordinary_muscle_development	ENSGALG00010009097	C1QTNF2	1.313317449	0.181160421	7.249472266	4.18399E-13	1.84337E-12	positive
PRJNA729271	stage_E18_vs_E10	ordinary_muscle_development	ENSGALG00010009098	CAD	-0.250883352	0.197671186	-1.26919536	0.204371396	0.253648718	negative
PRJNA729271	stage_E18_vs_E10	ordinary_muscle_development	ENSGALG00010009099	CXCR4	0.214650668	0.190148437	1.128858444	0.258957553	0.313007817	positive
PRJNA729271	stage_E18_vs_E10	ordinary_muscle_development	ENSGALG00010009100	ENSGALG00010009100	-0.196091836	0.154555663	-1.268745722	0.20453177	0.253826027	negative
PRJNA729271	stage_E18_vs_E10	ordinary_muscle_development	ENSGALG00010009101	HIKESHI	0.349064241	0.09812304	3.557413633	0.000374524	0.000723637	positive
PRJNA729271	stage_E18_vs_E10	ordinary_muscle_development	ENSGALG00010009102	HTRA3	2.010044902	0.216384475	9.289228836	1.5541E-20	1.13428E-19	positive
PRJNA729271	stage_E18_vs_E10	ordinary_muscle_development	ENSGALG00010009103	AP3S1	-0.984465167	0.123857187	-7.948389527	1.88952E-15	9.93284E-15	negative
PRJNA729271	stage_E18_vs_E10	ordinary_muscle_development	ENSGALG00010009104	MTR	0.097313838	0.123769108	0.786253039	0.431719259	0.491692516	positive
PRJNA729271	stage_E18_vs_E10	ordinary_muscle_development	ENSGALG00010009106	THSD7B	-0.30270538	0.237530683	-1.274384329	0.202527243	0.251704741	negative
PRJNA729271	stage_E18_vs_E10	ordinary_muscle_development	ENSGALG00010009109	ENSGALG00010009109	-0.998808433	0.231465973	-4.315141529	1.59501E-05	3.63516E-05	negative
PRJNA729271	stage_E18_vs_E10	ordinary_muscle_development	ENSGALG00010009111	LRFN1	0.0309115	0.249080966	0.124102217	0.901234343	0.918082212	positive
PRJNA729271	stage_E18_vs_E10	ordinary_muscle_development	ENSGALG00010009112	ENSGALG00010009112	4.370103582	0.266599535	16.39201504	2.18094E-60	8.85508E-59	positive
PRJNA729271	stage_E18_vs_E10	ordinary_muscle_development	ENSGALG00010009113	RPLP1	0.775875521	0.176355993	4.399484856	1.08508E-05	2.51733E-05	positive
PRJNA729271	stage_E18_vs_E10	ordinary_muscle_development	ENSGALG00010009114	SETD7	0.079091404	0.150266036	0.52634252	0.598650245	0.650219735	positive
PRJNA729271	stage_E18_vs_E10	ordinary_muscle_development	ENSGALG00010009115	COG5	-0.389310013	0.115676608	-3.365503364	0.000764041	0.001416756	negative
PRJNA729271	stage_E18_vs_E10	ordinary_muscle_development	ENSGALG00010009116	S1PR3	0.05459722	0.18401156	0.296705379	0.76669145	0.804196582	positive
PRJNA729271	stage_E18_vs_E10	ordinary_muscle_development	ENSGALG00010009117	ERGIC1	-0.654583787	0.067534371	-9.692602072	3.24163E-22	2.61768E-21	negative
PRJNA729271	stage_E18_vs_E10	ordinary_muscle_development	ENSGALG00010009118	ACTN2	3.615132372	0.143913642	25.12015076	2.9961E-139	8.0422E-137	positive
PRJNA729271	stage_E18_vs_E10	ordinary_muscle_development	ENSGALG00010009119	CISD1	0.260791246	0.153407059	1.699995089	0.089131849	0.119295121	positive
PRJNA729271	stage_E18_vs_E10	ordinary_muscle_development	ENSGALG00010009120	ATG12	-0.246948461	0.091194237	-2.707939315	0.00677024	0.010977028	negative
PRJNA729271	stage_E18_vs_E10	ordinary_muscle_development	ENSGALG00010009121	SYCN	-0.376720053	0.81734012	-0.460909778	0.644863336	0.69383121	negative
PRJNA729271	stage_E18_vs_E10	ordinary_muscle_development	ENSGALG00010009123	ENSGALG00010009123	0.102258794	0.13231076	0.772868318	0.439600316	0.499256177	positive
PRJNA729271	stage_E18_vs_E10	ordinary_muscle_development	ENSGALG00010009125	CD72L1	4.252661335	0.986310308	4.311687001	1.62014E-05	3.69011E-05	positive
PRJNA729271	stage_E18_vs_E10	ordinary_muscle_development	ENSGALG00010009126	JPH3	-1.149666362	0.14326652	-8.024668705	1.018E-15	5.46517E-15	negative
PRJNA729271	stage_E18_vs_E10	ordinary_muscle_development	ENSGALG00010009127	SCOC	-0.669325608	0.076305979	-8.771601079	1.76143E-18	1.13577E-17	negative
PRJNA729271	stage_E18_vs_E10	ordinary_muscle_development	ENSGALG00010009129	ENSGALG00010009129	2.778676796	0.355688612	7.812105034	5.62407E-15	2.86239E-14	positive
PRJNA729271	stage_E18_vs_E10	ordinary_muscle_development	ENSGALG00010009130	HEATR1	-1.416162496	0.074912021	-18.90434245	1.05041E-79	7.23179E-78	negative
PRJNA729271	stage_E18_vs_E10	ordinary_muscle_development	ENSGALG00010009131	CDKL2	-2.375460243	0.315090448	-7.538978923	4.73666E-14	2.22915E-13	negative
PRJNA729271	stage_E18_vs_E10	ordinary_muscle_development	ENSGALG00010009133	BOD1L1	0.072947453	0.129390835	0.563776048	0.572906555	0.626265745	positive
PRJNA729271	stage_E18_vs_E10	ordinary_muscle_development	ENSGALG00010009135	TENM4	-1.132439194	0.185347791	-6.109806787	9.97518E-10	3.36648E-09	negative
PRJNA729271	stage_E18_vs_E10	ordinary_muscle_development	ENSGALG00010009136	KHK	2.488095821	0.340501122	7.307158958	2.7285E-13	1.21691E-12	positive
PRJNA729271	stage_E18_vs_E10	ordinary_muscle_development	ENSGALG00010009137	IPMK	0.506704556	0.091762341	5.521922739	3.3531E-08	9.93118E-08	positive
PRJNA729271	stage_E18_vs_E10	ordinary_muscle_development	ENSGALG00010009138	ENSGALG00010009138	0.649574594	0.104780154	6.199404868	5.66771E-10	1.96164E-09	positive
PRJNA729271	stage_E18_vs_E10	ordinary_muscle_development	ENSGALG00010009139	CKS2	-2.007367084	0.2907789	-6.903413837	5.07675E-12	2.05437E-11	negative
PRJNA729271	stage_E18_vs_E10	ordinary_muscle_development	ENSGALG00010009141	MAML3	0.905907229	0.115084255	7.87168694	3.49891E-15	1.81002E-14	positive
PRJNA729271	stage_E18_vs_E10	ordinary_muscle_development	ENSGALG00010009142	LGALS8	-0.840852344	0.137924999	-6.096446235	1.08452E-09	3.64907E-09	negative
PRJNA729271	stage_E18_vs_E10	ordinary_muscle_development	ENSGALG00010009143	MCCC2	0.52095352	0.135501864	3.84462254	0.000120738	0.000249124	positive
PRJNA729271	stage_E18_vs_E10	ordinary_muscle_development	ENSGALG00010009144	CREBZF	0.777251126	0.153598963	5.06026284	4.18679E-07	1.12197E-06	positive
PRJNA729271	stage_E18_vs_E10	ordinary_muscle_development	ENSGALG00010009147	HNMT	5.434323469	0.226292423	24.01460644	1.9571E-127	4.1113E-125	positive
PRJNA729271	stage_E18_vs_E10	ordinary_muscle_development	ENSGALG00010009149	FUT4	-0.308342368	0.189449495	-1.62757028	0.103616035	0.1369735	negative
PRJNA729271	stage_E18_vs_E10	ordinary_muscle_development	ENSGALG00010009150	ENSGALG00010009150	2.198581137	0.383046241	5.739727744	9.48289E-09	2.93832E-08	positive
PRJNA729271	stage_E18_vs_E10	ordinary_muscle_development	ENSGALG00010009151	CCNJL	0.637384335	0.3644605	1.748843384	0.080318095	0.108601753	positive
PRJNA729271	stage_E18_vs_E10	ordinary_muscle_development	ENSGALG00010009152	RPP25L	-0.186092405	0.12459391	-1.493591498	0.135282423	0.174879044	negative
PRJNA729271	stage_E18_vs_E10	ordinary_muscle_development	ENSGALG00010009153	SECISBP2	0.013246354	0.124711171	0.106216258	0.915410772	0.930039892	positive
PRJNA729271	stage_E18_vs_E10	ordinary_muscle_development	ENSGALG00010009155	PAK4	-0.265232892	0.222511924	-1.191994064	0.233263573	0.285377742	negative
PRJNA729271	stage_E18_vs_E10	ordinary_muscle_development	ENSGALG00010009157	ABHD1	0.778177817	0.140267583	5.547809394	2.89271E-08	8.61336E-08	positive
PRJNA729271	stage_E18_vs_E10	ordinary_muscle_development	ENSGALG00010009159	PTK2	-0.037266043	0.099162019	-0.375809646	0.707058416	0.751544455	negative
PRJNA729271	stage_E18_vs_E10	ordinary_muscle_development	ENSGALG00010009160	ENSGALG00010009160	-0.530736435	0.391204279	-1.356673388	0.174885012	0.220354507	negative
PRJNA729271	stage_E18_vs_E10	ordinary_muscle_development	ENSGALG00010009163	SAMD12	-0.733258439	0.180308761	-4.066682247	4.76872E-05	0.000102907	negative
PRJNA729271	stage_E18_vs_E10	ordinary_muscle_development	ENSGALG00010009164	TMEM128	0.127543907	0.10200073	1.250421506	0.211145613	0.261184132	positive
PRJNA729271	stage_E18_vs_E10	ordinary_muscle_development	ENSGALG00010009166	CLGN	-1.22612514	0.240947373	-5.088767406	3.60398E-07	9.72626E-07	negative
PRJNA729271	stage_E18_vs_E10	ordinary_muscle_development	ENSGALG00010009167	DCTN3	-0.128798542	0.110985864	-1.160495011	0.245847324	0.299106678	negative
PRJNA729271	stage_E18_vs_E10	ordinary_muscle_development	ENSGALG00010009168	MRPS12	0.172110407	0.176037767	0.977690245	0.328227548	0.386298986	positive
PRJNA729271	stage_E18_vs_E10	ordinary_muscle_development	ENSGALG00010009170	RPL26L1	1.286448102	0.191264838	6.726004176	1.74386E-11	6.72087E-11	positive
PRJNA729271	stage_E18_vs_E10	ordinary_muscle_development	ENSGALG00010009172	SPOPL	0.247728397	0.133027698	1.8622317	0.062570438	0.086237377	positive
PRJNA729271	stage_E18_vs_E10	ordinary_muscle_development	ENSGALG00010009173	LYAR	-1.08582808	0.144783481	-7.499668284	6.39795E-14	2.9877E-13	negative
PRJNA729271	stage_E18_vs_E10	ordinary_muscle_development	ENSGALG00010009178	ENSGALG00010009178	0.960530797	0.251491999	3.819329441	0.000133815	0.000274815	positive
PRJNA729271	stage_E18_vs_E10	ordinary_muscle_development	ENSGALG00010009180	NFKBIB	0.345805773	0.147151017	2.350005998	0.018773109	0.028426819	positive
PRJNA729271	stage_E18_vs_E10	ordinary_muscle_development	ENSGALG00010009181	ARID3C	-2.788127023	0.554929893	-5.024286953	5.05306E-07	1.34393E-06	negative
PRJNA729271	stage_E18_vs_E10	ordinary_muscle_development	ENSGALG00010009186	PREB	0.172699175	0.097391748	1.773242377	0.076188553	0.1034329	positive
PRJNA729271	stage_E18_vs_E10	ordinary_muscle_development	ENSGALG00010009188	ENSGALG00010009188	-0.278046229	0.440808068	-0.630764837	0.528194298	0.584351729	negative
PRJNA729271	stage_E18_vs_E10	ordinary_muscle_development	ENSGALG00010009189	ENSGALG00010009189	-0.530124327	0.220542595	-2.403727617	0.01622886	0.02490338	negative
PRJNA729271	stage_E18_vs_E10	ordinary_muscle_development	ENSGALG00010009190	ZBTB49	0.111719627	0.13955135	0.800562853	0.423384763	0.483834921	positive
PRJNA729271	stage_E18_vs_E10	ordinary_muscle_development	ENSGALG00010009192	LSM14A	-0.111991555	0.059530459	-1.881247989	0.059938193	0.082956565	negative
PRJNA729271	stage_E18_vs_E10	ordinary_muscle_development	ENSGALG00010009193	EDARADD	-1.266865537	0.221927682	-5.70846108	1.14002E-08	3.50098E-08	negative
PRJNA729271	stage_E18_vs_E10	ordinary_muscle_development	ENSGALG00010009195	ENSGALG00010009195	-0.98218776	1.018142731	-0.964685727	0.334702247	0.393089375	negative
PRJNA729271	stage_E18_vs_E10	ordinary_muscle_development	ENSGALG00010009197	FH	0.745327667	0.102654713	7.260530423	3.85575E-13	1.70446E-12	positive
PRJNA729271	stage_E18_vs_E10	ordinary_muscle_development	ENSGALG00010009198	KCNN2	0.10935189	0.177432827	0.616300216	0.537696396	0.592827966	positive
PRJNA729271	stage_E18_vs_E10	ordinary_muscle_development	ENSGALG00010009199	SEMA4D	-1.523662465	0.148879476	-10.23420091	1.39341E-24	1.28892E-23	negative
PRJNA729271	stage_E18_vs_E10	ordinary_muscle_development	ENSGALG00010009200	ADGRA3	-0.852047403	0.09065918	-9.398357696	5.54213E-21	4.17099E-20	negative
PRJNA729271	stage_E18_vs_E10	ordinary_muscle_development	ENSGALG00010009202	FABP6	0.578595334	0.297476747	1.945010289	0.05177374	0.072473236	positive
PRJNA729271	stage_E18_vs_E10	ordinary_muscle_development	ENSGALG00010009205	SIRT2	0.226283315	0.098411894	2.29934925	0.021485115	0.032222115	positive
PRJNA729271	stage_E18_vs_E10	ordinary_muscle_development	ENSGALG00010009208	SLC5A6	-1.20087814	0.201929023	-5.947030911	2.7305E-09	8.87412E-09	negative
PRJNA729271	stage_E18_vs_E10	ordinary_muscle_development	ENSGALG00010009209	SLC6A19	1.243629494	1.868583938	0.665546497	0.505701014	0.562817583	positive
PRJNA729271	stage_E18_vs_E10	ordinary_muscle_development	ENSGALG00010009210	PCDH15	0.363163542	0.284234102	1.27769166	0.201358167	0.250402079	positive

PRJNA729271	stage_E18_vs_E10	ordinary_muscle_development	ENSGALG00010009212	NXPH2	1.105555181	0.251218508	4.400771221	1.07867E-05	2.50521E-05	positive
PRJNA729271	stage_E18_vs_E10	ordinary_muscle_development	ENSGALG00010009213	DUS4L	-0.873644797	0.097013544	-9.005389954	2.14901E-19	1.45969E-18	negative
PRJNA729271	stage_E18_vs_E10	ordinary_muscle_development	ENSGALG00010009214	PCDH10	-3.394990977	0.254785376	-13.32490516	1.65843E-40	3.23976E-39	negative
PRJNA729271	stage_E18_vs_E10	ordinary_muscle_development	ENSGALG00010009216	NSG1	-1.890565362	0.211021888	-8.959096062	3.27351E-19	2.20388E-18	negative
PRJNA729271	stage_E18_vs_E10	ordinary_muscle_development	ENSGALG00010009219	TNFRSF11B	0.145521725	0.182731138	0.796370702	0.425816602	0.486000917	positive
PRJNA729271	stage_E18_vs_E10	ordinary_muscle_development	ENSGALG00010009220	GADD45G	1.897456119	0.189098079	10.03424324	1.07783E-23	9.38888E-23	positive
PRJNA729271	stage_E18_vs_E10	ordinary_muscle_development	ENSGALG00010009223	ENSGALG00010009223	0.490390216	0.108587933	4.516065497	6.29992E-06	1.49578E-05	positive
PRJNA729271	stage_E18_vs_E10	ordinary_muscle_development	ENSGALG00010009224	ENSGALG00010009224	5.186311794	0.447511019	11.5892382	4.6727E-31	6.04739E-30	positive
PRJNA729271	stage_E18_vs_E10	ordinary_muscle_development	ENSGALG00010009225	HNRNPL	-0.13498192	0.124854708	-1.081111979	0.279647305	0.335192896	negative
PRJNA729271	stage_E18_vs_E10	ordinary_muscle_development	ENSGALG00010009227	ENSGALG00010009227	0.628258249	0.173157778	3.628241565	0.000285358	0.000561002	positive
PRJNA729271	stage_E18_vs_E10	ordinary_muscle_development	ENSGALG00010009228	ATP6V0E1	0.182652279	0.101865933	1.793065385	0.07296247	0.099425684	positive
PRJNA729271	stage_E18_vs_E10	ordinary_muscle_development	ENSGALG00010009232	ECH1	0.951334643	0.164094957	5.797464232	6.73251E-09	2.11229E-08	positive
PRJNA729271	stage_E18_vs_E10	ordinary_muscle_development	ENSGALG00010009234	PCDH18	-0.599403246	0.128083677	-4.679778557	2.87185E-06	7.05311E-06	negative
PRJNA729271	stage_E18_vs_E10	ordinary_muscle_development	ENSGALG00010009235	KLHDC4	-0.487791552	0.131884356	-3.698630898	0.000216766	0.000432405	negative
PRJNA729271	stage_E18_vs_E10	ordinary_muscle_development	ENSGALG00010009236	DTWD2	-0.538956902	0.119221565	-4.520632679	6.16551E-06	1.46555E-05	negative
PRJNA729271	stage_E18_vs_E10	ordinary_muscle_development	ENSGALG00010009237	DENND3	-0.825272492	0.179054843	-4.609048714	4.04516E-06	9.77082E-06	negative
PRJNA729271	stage_E18_vs_E10	ordinary_muscle_development	ENSGALG00010009239	STX18	0.075197102	0.082651027	0.909814482	0.362920356	0.422396865	positive
PRJNA729271	stage_E18_vs_E10	ordinary_muscle_development	ENSGALG00010009243	BCAP29	-0.304203956	0.100330438	-3.032020605	0.002429226	0.004211918	negative
PRJNA729271	stage_E18_vs_E10	ordinary_muscle_development	ENSGALG00010009246	RUSC2	2.801112645	0.141467817	19.80035246	2.95618E-87	2.52058E-85	positive
PRJNA729271	stage_E18_vs_E10	ordinary_muscle_development	ENSGALG00010009250	ERO1B	-0.274687836	0.108954352	-2.521127697	0.011697939	0.018342885	negative
PRJNA729271	stage_E18_vs_E10	ordinary_muscle_development	ENSGALG00010009252	SIRT1	-0.347489429	0.09699852	-3.582419901	0.000340426	0.000661192	negative
PRJNA729271	stage_E18_vs_E10	ordinary_muscle_development	ENSGALG00010009253	SLC45A4	0.020244932	0.140498334	0.144093754	0.885426437	0.905478778	positive
PRJNA729271	stage_E18_vs_E10	ordinary_muscle_development	ENSGALG00010009254	SLC26A4	-0.796445335	0.812041421	-0.980793977	0.326694349	0.384744442	negative
PRJNA729271	stage_E18_vs_E10	ordinary_muscle_development	ENSGALG00010009256	CREBRF	1.204700959	0.174090635	6.919964168	4.51758E-12	1.83836E-11	positive
PRJNA729271	stage_E18_vs_E10	ordinary_muscle_development	ENSGALG00010009257	ENSGALG00010009257	3.993245027	0.185838073	21.48776605	2.0263E-102	2.53199E-100	positive
PRJNA729271	stage_E18_vs_E10	ordinary_muscle_development	ENSGALG00010009258	DNAJC12	-0.532961633	0.169256792	-3.148834546	0.00163923	0.002912556	negative
PRJNA729271	stage_E18_vs_E10	ordinary_muscle_development	ENSGALG00010009259	MGAT4D	2.238182799	0.791283052	2.828548891	0.004675956	0.007778949	positive
PRJNA729271	stage_E18_vs_E10	ordinary_muscle_development	ENSGALG00010009263	COLEC10	0.185524436	0.220600243	0.84099833	0.40034887	0.460194835	positive
PRJNA729271	stage_E18_vs_E10	ordinary_muscle_development	ENSGALG00010009266	PCDH7	-1.254928585	0.23815955	-5.269276782	1.36962E-07	3.82739E-07	negative
PRJNA729271	stage_E18_vs_E10	ordinary_muscle_development	ENSGALG00010009268	PWWP2A	0.09634211	0.13379262	0.720085379	0.471472429	0.5303013	positive
PRJNA729271	stage_E18_vs_E10	ordinary_muscle_development	ENSGALG00010009269	ENSGALG00010009269	-3.164041323	0.49602881	-6.378745068	1.78545E-10	6.38857E-10	negative
PRJNA729271	stage_E18_vs_E10	ordinary_muscle_development	ENSGALG00010009273	MAL2	-1.76492933	0.525351006	-3.359524035	0.000780769	0.001445738	negative
PRJNA729271	stage_E18_vs_E10	ordinary_muscle_development	ENSGALG00010009275	CBLL1	0.141751775	0.143590008	0.987198048	0.323545559	0.381532124	positive
PRJNA729271	stage_E18_vs_E10	ordinary_muscle_development	ENSGALG00010009276	ENSGALG00010009276	-0.556102062	0.335016153	-1.659926115	0.096929317	0.128910033	negative
PRJNA729271	stage_E18_vs_E10	ordinary_muscle_development	ENSGALG00010009277	ACTN4	-0.165899662	0.128459566	-1.291454319	0.196546185	0.244901312	negative
PRJNA729271	stage_E18_vs_E10	ordinary_muscle_development	ENSGALG00010009279	ELMOD2	-1.04738269	0.112996717	-9.269142644	1.87648E-20	1.36407E-19	negative
PRJNA729271	stage_E18_vs_E10	ordinary_muscle_development	ENSGALG00010009280	HSD17B4	-0.084566379	0.083636374	-1.01111963	0.311959176	0.369552816	negative
PRJNA729271	stage_E18_vs_E10	ordinary_muscle_development	ENSGALG00010009284	CCN3	-2.948046526	0.67908088	-4.341230351	1.41687E-05	3.24653E-05	negative
PRJNA729271	stage_E18_vs_E10	ordinary_muscle_development	ENSGALG00010009286	ENSGALG00010009286	0.703955037	0.182858903	3.849717055	0.000118254	0.000244367	positive
PRJNA729271	stage_E18_vs_E10	ordinary_muscle_development	ENSGALG00010009289	GPR137B	0.326018715	0.145935313	2.233994696	0.025483422	0.037738272	positive
PRJNA729271	stage_E18_vs_E10	ordinary_muscle_development	ENSGALG00010009294	ACOX3	0.354401397	0.141576432	2.503251373	0.012305811	0.019210848	positive
PRJNA729271	stage_E18_vs_E10	ordinary_muscle_development	ENSGALG00010009297	CTNNA3	4.510018903	0.204443704	22.05995497	7.6659E-108	1.0894E-105	positive
PRJNA729271	stage_E18_vs_E10	ordinary_muscle_development	ENSGALG00010009299	MAP4K1	1.165034867	0.322902325	3.608010155	0.000308554	0.000603169	positive
PRJNA729271	stage_E18_vs_E10	ordinary_muscle_development	ENSGALG00010009303	TTC1	-0.155216186	0.097533902	-1.591407528	0.111517891	0.146630246	negative
PRJNA729271	stage_E18_vs_E10	ordinary_muscle_development	ENSGALG00010009306	TBC1D9	-0.721953683	0.107465393	-6.718010942	1.84222E-11	7.09242E-11	negative
PRJNA729271	stage_E18_vs_E10	ordinary_muscle_development	ENSGALG00010009307	DIRAS2	2.197239383	0.313865842	7.000568688	2.54926E-12	1.05636E-11	positive
PRJNA729271	stage_E18_vs_E10	ordinary_muscle_development	ENSGALG00010009309	TRIM54	2.555001896	0.149060737	17.14067662	7.3787E-66	3.54153E-64	positive
PRJNA729271	stage_E18_vs_E10	ordinary_muscle_development	ENSGALG00010009311	BNIP1	0.067478328	0.112106758	0.601911331	0.547233161	0.601840227	positive
PRJNA729271	stage_E18_vs_E10	ordinary_muscle_development	ENSGALG00010009313	UNC13B	1.706883119	0.169699816	10.05824967	8.4487E-24	7.40859E-23	positive
PRJNA729271	stage_E18_vs_E10	ordinary_muscle_development	ENSGALG00010009317	LRRTM3	-3.264690097	0.538797746	-6.059212608	1.36789E-09	4.55913E-09	negative
PRJNA729271	stage_E18_vs_E10	ordinary_muscle_development	ENSGALG00010009320	GPR20	2.215354132	0.204215237	10.84813341	2.03539E-27	2.20008E-26	positive
PRJNA729271	stage_E18_vs_E10	ordinary_muscle_development	ENSGALG00010009322	ENSGALG00010009322	1.405215087	0.171144758	8.210681459	2.19937E-16	1.23183E-15	positive
PRJNA729271	stage_E18_vs_E10	ordinary_muscle_development	ENSGALG00010009323	RNF150	0.506268222	0.107588296	4.705606866	2.53112E-06	6.25658E-06	positive
PRJNA729271	stage_E18_vs_E10	ordinary_muscle_development	ENSGALG00010009326	DLD	0.178548074	0.132012104	1.352512906	0.176211293	0.221909877	positive
PRJNA729271	stage_E18_vs_E10	ordinary_muscle_development	ENSGALG00010009327	NID1	2.395903058	0.163060811	14.69331007	7.11515E-49	1.97575E-47	positive
PRJNA729271	stage_E18_vs_E10	ordinary_muscle_development	ENSGALG00010009328	ENSGALG00010009328	-0.013714791	0.127528036	-0.107543343	0.914357942	0.929426253	negative
PRJNA729271	stage_E18_vs_E10	ordinary_muscle_development	ENSGALG00010009330	EIF3K	0.665408804	0.141478697	4.703243788	2.5606E-06	6.3273E-06	positive
PRJNA729271	stage_E18_vs_E10	ordinary_muscle_development	ENSGALG00010009332	ENSGALG00010009332	0.297083667	0.163972722	1.811787131	0.070019094	0.095783953	positive
PRJNA729271	stage_E18_vs_E10	ordinary_muscle_development	ENSGALG00010009333	ADRA1B	1.828513791	0.319345441	5.725817737	1.02937E-08	3.17461E-08	positive
PRJNA729271	stage_E18_vs_E10	ordinary_muscle_development	ENSGALG00010009334	SS18L2	0.255903705	0.166586976	1.536156738	0.124499944	0.162271979	positive
PRJNA729271	stage_E18_vs_E10	ordinary_muscle_development	ENSGALG00010009335	PTP4A3	1.326119675	0.19533925	6.788802952	1.13068E-11	4.42831E-11	positive
PRJNA729271	stage_E18_vs_E10	ordinary_muscle_development	ENSGALG00010009336	LAMB1	2.05694641	0.109855744	18.72406798	3.15139E-78	2.06694E-76	positive
PRJNA729271	stage_E18_vs_E10	ordinary_muscle_development	ENSGALG00010009337	ZNF330	-0.247664547	0.103456367	-2.393903379	0.01667014	0.025496853	negative
PRJNA729271	stage_E18_vs_E10	ordinary_muscle_development	ENSGALG00010009338	KYNU	1.506098487	0.555674267	2.710398117	0.006720249	0.010908176	positive
PRJNA729271	stage_E18_vs_E10	ordinary_muscle_development	ENSGALG00010009340	SYK	0.570556236	0.143942577	3.963776736	7.37733E-05	0.000156063	positive
PRJNA729271	stage_E18_vs_E10	ordinary_muscle_development	ENSGALG00010009344	ENPP2	-0.230363471	0.257678169	-0.89399685	0.371323576	0.431035096	negative
PRJNA729271	stage_E18_vs_E10	ordinary_muscle_development	ENSGALG00010009345	ZFAT	-0.03312692	0.126957398	-0.260929417	0.794146939	0.827962302	negative
PRJNA729271	stage_E18_vs_E10	ordinary_muscle_development	ENSGALG00010009346	STC2	-1.097342948	0.307626518	-3.567127289	0.000360916	0.000699115	negative
PRJNA729271	stage_E18_vs_E10	ordinary_muscle_development	ENSGALG00010009347	TESK1	2.829341342	0.141378699	20.01250092	4.28597E-89	3.88282E-87	positive
PRJNA729271	stage_E18_vs_E10	ordinary_muscle_development	ENSGALG00010009348	ERGIC2	-0.562176266	0.083373867	-6.742835477	1.55325E-11	6.02633E-11	negative
PRJNA729271	stage_E18_vs_E10	ordinary_muscle_development	ENSGALG00010009351	GTF3C2	0.150995167	0.1226538	1.231067987	0.218297438	0.269089324	positive
PRJNA729271	stage_E18_vs_E10	ordinary_muscle_development	ENSGALG00010009352	HSF1	0.883208981	0.182921366	4.828353306	1.37667E-06	3.50576E-06	positive
PRJNA729271	stage_E18_vs_E10	ordinary_muscle_development	ENSGALG00010009355	GARRE1	0.365319109	0.07230667	5.052356979	4.36391E-07	1.16706E-06	positive
PRJNA729271	stage_E18_vs_E10	ordinary_muscle_development	ENSGALG00010009357	LYST	0.196303689	0.116792906	1.680784356	0.092804808	0.123856523	positive
PRJNA729271	stage_E18_vs_E10	ordinary_muscle_development	ENSGALG00010009359	REEP3	-0.164614987	0.110385445	-1.491274395	0.135889469	0.175601127	negative
PRJNA729271	stage_E18_vs_E10	ordinary_muscle_development	ENSGALG00010009360	TNFAIP8	-0.530702322	0.130900388	-4.054245597	5.02964E-05	0.000108328	negative
PRJNA729271	stage_E18_vs_E10	ordinary_muscle_development	ENSGALG00010009361	LRRK2	0.640360398	0.089140067	7.183754926	6.78224E-13	2.94425E-12	positive
PRJNA729271	stage_E18_vs_E10	ordinary_muscle_development	ENSGALG00010009362	IL15	1.57935578	0.322381578	4.899026146	9.63128E-07	2.49518E-06	positive
PRJNA729271	stage_E18_vs_E10	ordinary_muscle_development	ENSGALG00010009363	TAF2	-0.341720686	0.077667063	-4.3998147	1.08343E-05	2.51431E-05	negative
PRJNA729271	stage_E18_vs_E10	ordinary_muscle_development	ENSGALG00010009365	CA5B	0.318567769	0.16169133	1.970221706	0.048812967	0.068660258	positive
PRJNA729271	stage_E18_vs_E10	ordinary_muscle_development	ENSGALG00010009367	DMXL1	0.272767646	0.119456046	2.283414312	0.022405975	0.033492277	positive
PRJNA729271	stage_E18_vs_E10	ordinary_muscle_development	ENSGALG00010009368	DSCC1	-2.133852598	0.198517685	-10.74892947	5.99534E-27	6.34788E-26	negative
PRJNA729271	stage_E18_vs_E10	ordinary_muscle_development	ENSGALG00010009370	ENSGALG00010009370	1.220225505	0.454024019	2.687579188	0.007197203	0.011621195	positive
PRJNA729271	stage_E18_vs_E10	ordinary_muscle_development	ENSGALG00010009371	PHTF2	2.422576509	0.154530796	15.67698199	2.17338E-55	7.50076E-54	positive
PRJNA729271	stage_E18_vs_E10	ordinary_muscle_development	ENSGALG00010009372	RAB28	-0.093898792	0.098075834	-0.957410079	0.338360313	0.396742658	negative
PRJNA729271	stage_E18_vs_E10	ordinary_muscle_development	ENSGALG00010009373	ARHGAP15	-0.18414195	0.220597487	-0.83474183	0.403863084	0.46382976	negative
PRJNA729271	stage_E18_vs_E10	ordinary_muscle_development	ENSGALG00010009376	CDO1	0.305905145	0.249808366	1.22455925	0.22074133	0.271654741	positive
PRJNA729271	stage_E18_vs_E10	ordinary_muscle_development	ENSGALG00010009378	ENSGALG00010009378	-0.917241502	0.084527589	-10.8513861	1.96424E-27	2.13271E-26	negative
PRJNA729271	stage_E18_vs_E10	ordinary_muscle_development	ENSGALG00010009379	RYR1	2.939448144	0.219518669	13.3904244	6.87866E-41	1.36397E-39	positive
PRJNA729271	stage_E18_vs_E10	ordinary_muscle_development	ENSGALG00010009381	INPP4B	-1.362471356	0.117750499	-11.57083292	5.79177E-31	7.43593E-30	negative
PRJNA729271	stage_E18_vs_E10	ordinary_muscle_development	ENSGALG00010009383	ASXL2	0.514288481	0.112001424	4.591803055	4.39433E-06	1.05895E-05	positive
PRJNA729271	stage_E18_vs_E10	ordinary_muscle_development	ENSGALG00010009385	GPI	-0.069598948	0.178137909	-0.390702619	0.696017063	0.74111271	negative
PRJNA729271	stage_E18_vs_E10	ordinary_muscle_development	ENSGALG00010009386	TMEM60	0.071864955	0.11243054	0.639194255	0.522696569	0.57933069	positive
PRJNA729271	stage_E18_vs_E10	ordinary_muscle_development	ENSGALG00010009387	DEPTOR	2.313272116	0.138673284	16.68145473	1.78811E-62	7.87801E-61	positive
PRJNA729271	stage_E18_vs_E10	ordinary_muscle_development	ENSGALG00010009388	TMED7	-0.537878799	0.074003668	-7.268272201	3.64115E-13	1.61205E-12	negative
PRJNA729271	stage_E18_vs_E10	ordinary_muscle_development	ENSGALG00010009389	CPEB4	2.167100823	0.125329093	17.29128306	5.47206E-67	2.68873E-65	positive
PRJNA729271	stage_E18_vs_E10	ordinary_muscle_development	ENSGALG00010009391	RSBN1L	0.513023165	0.106790801	4.80400148	1.55526E-06	3.93497E-06	positive
PRJNA729271	stage_E18_vs_E10	ordinary_muscle_development	ENSGALG00010009392	MAPRE3	0.054947717	0.109490876	0.50184745	0.615774833	0.666459553	positive
PRJNA729271	stage_E18_vs_E10	ordinary_muscle_development	ENSGALG00010009393	MARVELD2	-0.009423966	0.130959406	-0.071960968	0.942632971	0.953087675	negative
PRJNA729271	stage_E18_vs_E10	ordinary_muscle_development	ENSGALG00010009394	BANP	0.656862001	0.091566707	7.173589874	7.30563E-13	3.16388E-12	positive
PRJNA729271	stage_E18_vs_E10	ordinary_muscle_development	ENSGALG00010009396	AUH	1.257824583	0.111280172	11.30322284	1.26489E-29	1.51902E-28	positive
PRJNA729271	stage_E18_vs_E10	ordinary_muscle_development	ENSGALG00010009398	WTIP	-0.058770266	0.13943932	-0.421475566	0.673407849	0.7200005	negative
PRJNA729271	stage_E18_vs_E10	ordinary_muscle_development	ENSGALG00010009400	CNTFR	0.217003761	0.282399464	0.768428375	0.442232728	0.501734768	positive
PRJNA729271	stage_E18_vs_E10	ordinary_muscle_development	ENSGALG00010009404	GNG4	-0.311859522	0.399645037	-0.780341285	0.435190018	0.494932658	negative
PRJNA729271	stage_E18_vs_E10	ordinary_muscle_development	ENSGALG00010009407	PTPN12	0.272538629	0.087052055	3.130754685	0.001743577	0.00308925	positive
PRJNA729271	stage_E18_vs_E10	ordinary_muscle_development	ENSGALG00010009408	ENSGALG00010009408	-0.086609123	0.091943107	-0.941986036	0.346199789	0.404886715	negative
PRJNA729271	stage_E18_vs_E10	ordinary_muscle_development	ENSGALG00010009410	JMJD1C	0.567806424	0.108002098	5.257364776	1.46134E-07	4.06958E-07	positive
PRJNA729271	stage_E18_vs_E10	ordinary_muscle_development	ENSGALG00010009411	SMARCA5	-2.275734739	0.100010461	-22.75496704	1.2814E-114	2.1349E-112	negative
PRJNA729271	stage_E18_vs_E10	ordinary_muscle_development	ENSGALG00010009415	HMP19	-2.854423487	0.426665494	-6.690073429	2.23059E-11	8.55127E-11	negative
PRJNA729271	stage_E18_vs_E10	ordinary_muscle_development	ENSGALG00010009416	COL14A1	1.160285956	0.473462555	2.450639325	0.014260276	0.022083622	positive
PRJNA729271	stage_E18_vs_E10	ordinary_muscle_development	ENSGALG00010009424	ENSGALG00010009424	5.33902193	0.98778961	5.405019325	6.48014E-08	1.86294E-07	positive
PRJNA729271	stage_E18_vs_E10	ordinary_muscle_development	ENSGALG00010009431	SMN	-0.538892095	0.151523452	-3.556492991	0.000375839	0.000725983	negative
PRJNA729271	stage_E18_vs_E10	ordinary_muscle_development	ENSGALG00010009432	BOP1	-0.508313917	0.185788744	-2.735977995	0.006219518	0.010157963	negative
PRJNA729271	stage_E18_vs_E10	ordinary_muscle_development	ENSGALG00010009435	GTDC1	-0.267840029	0.090669126	-2.954037827	0.003136456	0.005357403	negative
PRJNA729271	stage_E18_vs_E10	ordinary_muscle_development	ENSGALG00010009437	DPYSL5	-1.936628094	0.160466155	-12.06876365	1.54437E-33	2.20331E-32	negative
PRJNA729271	stage_E18_vs_E10	ordinary_muscle_development	ENSGALG00010009439	B3GALNT2	-0.438118892	0.103465219	-4.23445577	2.29106E-05	5.12325E-05	negative
PRJNA729271	stage_E18_vs_E10	ordinary_muscle_development	ENSGALG00010009440	DOK3	-0.410789426	0.228106665	-1.800865513	0.07172408	0.097922251	negative
PRJNA729271	stage_E18_vs_E10	ordinary_muscle_development	ENSGALG00010009441	ZNF518B	-0.656853543	0.098892798	-6.642076628	3.09294E-11	1.16994E-10	negative
PRJNA729271	stage_E18_vs_E10	ordinary_muscle_development	ENSGALG00010009444	NFIL3	1.710455313	0.221401419	7.725584235	1.11341E-14	5.54032E-14	positive
PRJNA729271	stage_E18_vs_E10	ordinary_muscle_development	ENSGALG00010009446	ENSGALG00010009446	0.240893206	0.249798291	0.964350899	0.33487003	0.393254564	positive
PRJNA729271	stage_E18_vs_E10	ordinary_muscle_development	ENSGALG00010009447	DDX41	-0.564921136	0.107342066	-5.262812199	1.41868E-07	3.95763E-07	negative
PRJNA729271	stage_E18_vs_E10	ordinary_muscle_development	ENSGALG00010009451	ROR2	-1.976599323	0.142986141	-13.82371265	1.83363E-43	4.03316E-42	negative
PRJNA729271	stage_E18_vs_E10	ordinary_muscle_development	ENSGALG00010009452	TBCE	-0.95618682	0.092258342	-10.36423156	3.60646E-25	3.44145E-24	negative
PRJNA729271	stage_E18_vs_E10	ordinary_muscle_development	ENSGALG00010009453	GSAP	0.632402939	0.11344215	5.574673427	2.47995E-08	7.4163E-08	positive
PRJNA729271	stage_E18_vs_E10	ordinary_muscle_development	ENSGALG00010009457	BDP1	-0.604169193	0.130531121	-4.628545206	3.68243E-06	8.93786E-06	negative
PRJNA729271	stage_E18_vs_E10	ordinary_muscle_development	ENSGALG00010009458	SCX	-2.963872588	0.451547859	-6.563806094	5.24514E-11	1.95697E-10	negative
PRJNA729271	stage_E18_vs_E10	ordinary_muscle_development	ENSGALG00010009461	ZEB2	0.300092067	0.125592367	2.38941325	0.016875309	0.025791607	positive
PRJNA729271	stage_E18_vs_E10	ordinary_muscle_development	ENSGALG00010009462	NRBF2	0.231647583	0.121708825	1.903293238	0.057002293	0.079241223	positive
PRJNA729271	stage_E18_vs_E10	ordinary_muscle_development	ENSGALG00010009463	MRPL13	0.24110878	0.10910039	2.209971751	0.027107123	0.039930665	positive
PRJNA729271	stage_E18_vs_E10	ordinary_muscle_development	ENSGALG00010009464	SPTLC1	-0.126469733	0.148509143	-0.851595599	0.394438583	0.454410051	negative
PRJNA729271	stage_E18_vs_E10	ordinary_muscle_development	ENSGALG00010009465	GGPS1	-0.809938969	0.10677239	-7.585659291	3.30801E-14	1.57522E-13	negative

PRJNA729271	stage_E18_vs_E10	ordinary_muscle_development	ENSGALG00010009466	FAM193B	-0.207046921	0.110588503	-1.872228272	0.061175029	0.084507009	negative
PRJNA729271	stage_E18_vs_E10	ordinary_muscle_development	ENSGALG00010009467	EGR2	0.773495202	0.318072567	2.431819914	0.015023175	0.023193196	positive
PRJNA729271	stage_E18_vs_E10	ordinary_muscle_development	ENSGALG00010009469	DNMT3A	0.307033242	0.210859462	1.456103702	0.145363954	0.186580228	positive
PRJNA729271	stage_E18_vs_E10	ordinary_muscle_development	ENSGALG00010009470	ZNF469	0.178993169	0.210638931	0.849762998	0.395456865	0.455438365	positive
PRJNA729271	stage_E18_vs_E10	ordinary_muscle_development	ENSGALG00010009472	ADO	-0.299910556	0.086194711	-3.479454262	0.000502436	0.000954497	negative
PRJNA729271	stage_E18_vs_E10	ordinary_muscle_development	ENSGALG00010009474	ACVR2A	-0.285811803	0.095750638	-2.984959761	0.002836156	0.004877798	negative
PRJNA729271	stage_E18_vs_E10	ordinary_muscle_development	ENSGALG00010009477	ZNF365	-0.281497207	0.246494165	-1.142003533	0.253452551	0.307173472	negative
PRJNA729271	stage_E18_vs_E10	ordinary_muscle_development	ENSGALG00010009479	MTBP	-1.905052621	0.132374552	-14.39138109	5.86136E-47	1.52532E-45	negative
PRJNA729271	stage_E18_vs_E10	ordinary_muscle_development	ENSGALG00010009482	ARID4B	0.235304478	0.108116218	2.176403153	0.029525128	0.043176628	positive
PRJNA729271	stage_E18_vs_E10	ordinary_muscle_development	ENSGALG00010009484	RTKN2	-1.01224395	0.132565984	-7.635774402	2.24467E-14	1.08567E-13	negative
PRJNA729271	stage_E18_vs_E10	ordinary_muscle_development	ENSGALG00010009486	B4GALT1	0.261174317	0.128227717	2.036800811	0.041670007	0.059268644	positive
PRJNA729271	stage_E18_vs_E10	ordinary_muscle_development	ENSGALG00010009489	MROH1	0.017293866	0.13847206	0.124890651	0.90061012	0.91776882	positive
PRJNA729271	stage_E18_vs_E10	ordinary_muscle_development	ENSGALG00010009490	SNTB1	0.36339626	0.159898968	2.272661706	0.023046574	0.034369627	positive
PRJNA729271	stage_E18_vs_E10	ordinary_muscle_development	ENSGALG00010009491	RBM34	0.088415326	0.102411616	0.86333298	0.387954416	0.448151041	positive
PRJNA729271	stage_E18_vs_E10	ordinary_muscle_development	ENSGALG00010009492	CDC42SE2	-1.596835621	0.128517652	-12.42502949	1.91156E-35	2.99223E-34	negative
PRJNA729271	stage_E18_vs_E10	ordinary_muscle_development	ENSGALG00010009493	ENSGALG00010009493	0.765370164	0.399007315	1.918185797	0.055087457	0.076785526	positive
PRJNA729271	stage_E18_vs_E10	ordinary_muscle_development	ENSGALG00010009494	UBA2	-0.998138583	0.075666884	-13.19122087	9.85758E-40	1.87268E-38	negative
PRJNA729271	stage_E18_vs_E10	ordinary_muscle_development	ENSGALG00010009497	HS3ST1	1.669676031	0.188841927	8.841659571	9.43072E-19	6.18544E-18	positive
PRJNA729271	stage_E18_vs_E10	ordinary_muscle_development	ENSGALG00010009498	ORC4	-1.459607318	0.117191929	-12.45484507	1.31607E-35	2.08031E-34	negative
PRJNA729271	stage_E18_vs_E10	ordinary_muscle_development	ENSGALG00010009499	MXD3	0.175492054	0.240558819	0.72951827	0.465684696	0.524850297	positive
PRJNA729271	stage_E18_vs_E10	ordinary_muscle_development	ENSGALG00010009500	CCDC146	0.705044519	0.599951741	1.175168719	0.239927222	0.292862744	positive
PRJNA729271	stage_E18_vs_E10	ordinary_muscle_development	ENSGALG00010009501	HGH1	0.642647613	0.109569829	5.865187712	4.48625E-09	1.43139E-08	positive
PRJNA729271	stage_E18_vs_E10	ordinary_muscle_development	ENSGALG00010009502	ARID5B	-0.466713601	0.09534374	-4.895062879	9.82743E-07	2.54146E-06	negative
PRJNA729271	stage_E18_vs_E10	ordinary_muscle_development	ENSGALG00010009503	IL11RA	-1.761070371	0.135714743	-12.97626424	1.66821E-38	3.01129E-37	negative
PRJNA729271	stage_E18_vs_E10	ordinary_muscle_development	ENSGALG00010009506	HAS2	-1.421751796	0.258825944	-5.493080686	3.94982E-08	1.16205E-07	negative
PRJNA729271	stage_E18_vs_E10	ordinary_muscle_development	ENSGALG00010009508	TOMM20	0.814187734	0.090121981	9.034285783	1.65077E-19	1.13081E-18	positive
PRJNA729271	stage_E18_vs_E10	ordinary_muscle_development	ENSGALG00010009511	ZFPM1	-0.793159407	0.258613567	-3.066967508	0.002162424	0.003778246	negative
PRJNA729271	stage_E18_vs_E10	ordinary_muscle_development	ENSGALG00010009514	PRELID1	-0.309137479	0.09075192	-3.406401516	0.000658253	0.001231304	negative
PRJNA729271	stage_E18_vs_E10	ordinary_muscle_development	ENSGALG00010009515	MBD5	0.581568321	0.096198482	6.045504144	1.48944E-09	4.95282E-09	positive
PRJNA729271	stage_E18_vs_E10	ordinary_muscle_development	ENSGALG00010009518	FGL2	3.790361834	0.290747699	13.03660132	7.57596E-39	1.38829E-37	positive
PRJNA729271	stage_E18_vs_E10	ordinary_muscle_development	ENSGALG00010009520	SPINK4	0.765095231	0.591559718	1.293352483	0.195889176	0.244166619	positive
PRJNA729271	stage_E18_vs_E10	ordinary_muscle_development	ENSGALG00010009522	TAF5L	-0.093948441	0.082908333	-1.133160418	0.257146893	0.311078636	negative
PRJNA729271	stage_E18_vs_E10	ordinary_muscle_development	ENSGALG00010009524	FAM185A	0.646329943	0.162109372	3.986999244	6.69142E-05	0.000142363	positive
PRJNA729271	stage_E18_vs_E10	ordinary_muscle_development	ENSGALG00010009525	ENSGALG00010009525	2.252029328	0.154353882	14.5900401	3.25007E-48	8.82204E-47	positive
PRJNA729271	stage_E18_vs_E10	ordinary_muscle_development	ENSGALG00010009526	ENSGALG00010009526	-0.307688843	0.235896271	-1.304339581	0.192117826	0.23987836	negative
PRJNA729271	stage_E18_vs_E10	ordinary_muscle_development	ENSGALG00010009527	ENSGALG00010009527	-0.553830699	0.474094044	-1.168187422	0.242731173	0.295787167	negative
PRJNA729271	stage_E18_vs_E10	ordinary_muscle_development	ENSGALG00010009530	KIF3C	-0.645104513	0.15280945	-4.221627084	2.42545E-05	5.41209E-05	negative
PRJNA729271	stage_E18_vs_E10	ordinary_muscle_development	ENSGALG00010009531	FBXL13	-0.532926664	0.447415178	-1.191123346	0.233605166	0.285747416	negative
PRJNA729271	stage_E18_vs_E10	ordinary_muscle_development	ENSGALG00010009534	XPA	-0.36708675	0.131726022	-2.78674437	0.005324046	0.008790528	negative
PRJNA729271	stage_E18_vs_E10	ordinary_muscle_development	ENSGALG00010009538	CRIM1	1.248297254	0.074749875	16.69965674	1.31821E-62	5.84326E-61	positive
PRJNA729271	stage_E18_vs_E10	ordinary_muscle_development	ENSGALG00010009540	RAB10	-0.140725395	0.090365055	-1.557298839	0.119399551	0.156171855	negative
PRJNA729271	stage_E18_vs_E10	ordinary_muscle_development	ENSGALG00010009541	PMPCB	-0.072735072	0.092925555	-0.782724109	0.433789141	0.493584552	negative
PRJNA729271	stage_E18_vs_E10	ordinary_muscle_development	ENSGALG00010009542	WDR1	-0.687800599	0.143364463	-4.79756689	1.60605E-06	4.0571E-06	negative
PRJNA729271	stage_E18_vs_E10	ordinary_muscle_development	ENSGALG00010009543	MAF1	1.377444517	0.129830227	10.60958258	2.68948E-26	2.73956E-25	positive
PRJNA729271	stage_E18_vs_E10	ordinary_muscle_development	ENSGALG00010009546	GAREM2	-3.427364815	0.836242861	-4.098528042	4.15786E-05	9.02894E-05	negative
PRJNA729271	stage_E18_vs_E10	ordinary_muscle_development	ENSGALG00010009548	PIK3C3	-0.153975036	0.082306721	-1.870746824	0.061380181	0.08474195	negative
PRJNA729271	stage_E18_vs_E10	ordinary_muscle_development	ENSGALG00010009551	HADHA	0.970924133	0.130140682	7.460573576	8.61466E-14	3.98435E-13	positive
PRJNA729271	stage_E18_vs_E10	ordinary_muscle_development	ENSGALG00010009554	ABCB10	-0.0145793	0.105011632	-0.138835099	0.889580457	0.908829203	negative
PRJNA729271	stage_E18_vs_E10	ordinary_muscle_development	ENSGALG00010009555	EPC2	0.115368425	0.076969089	1.498892945	0.133901407	0.173232854	positive
PRJNA729271	stage_E18_vs_E10	ordinary_muscle_development	ENSGALG00010009557	CIDEC	3.247505876	1.057488605	3.070960634	0.002133713	0.00373168	positive
PRJNA729271	stage_E18_vs_E10	ordinary_muscle_development	ENSGALG00010009558	TMEM26	-1.909483071	0.198906172	-9.599918667	8.00075E-22	6.34067E-21	negative
PRJNA729271	stage_E18_vs_E10	ordinary_muscle_development	ENSGALG00010009560	RAB24	0.378811694	0.146036904	2.593944977	0.009488167	0.015050425	positive
PRJNA729271	stage_E18_vs_E10	ordinary_muscle_development	ENSGALG00010009562	HADHB	0.861162673	0.18960551	4.541865239	5.57587E-06	1.3326E-05	positive
PRJNA729271	stage_E18_vs_E10	ordinary_muscle_development	ENSGALG00010009565	TMEM8B	1.005466026	0.21677571	4.638278092	3.51324E-06	8.54436E-06	positive
PRJNA729271	stage_E18_vs_E10	ordinary_muscle_development	ENSGALG00010009567	NSD1	0.127080499	0.106608955	1.192024611	0.233251595	0.285377742	positive
PRJNA729271	stage_E18_vs_E10	ordinary_muscle_development	ENSGALG00010009569	ZC3H18	-0.473817657	0.145101906	-3.265413055	0.001093046	0.001981626	negative
PRJNA729271	stage_E18_vs_E10	ordinary_muscle_development	ENSGALG00010009570	SHARPIN	1.156421307	0.137308737	8.422051859	3.69947E-17	2.18873E-16	positive
PRJNA729271	stage_E18_vs_E10	ordinary_muscle_development	ENSGALG00010009571	DNAJC2	-1.446280877	0.188260649	-7.682332371	1.56218E-14	7.67846E-14	negative
PRJNA729271	stage_E18_vs_E10	ordinary_muscle_development	ENSGALG00010009574	TAF1C	-0.42870613	0.15012712	-2.85562082	0.004295278	0.007181918	negative
PRJNA729271	stage_E18_vs_E10	ordinary_muscle_development	ENSGALG00010009575	RHOBTB1	-1.997628413	0.245389844	-8.140631969	3.9322E-16	2.16555E-15	negative
PRJNA729271	stage_E18_vs_E10	ordinary_muscle_development	ENSGALG00010009578	KIF5C	-2.286349867	0.161203167	-14.18303317	1.16692E-45	2.80971E-44	negative
PRJNA729271	stage_E18_vs_E10	ordinary_muscle_development	ENSGALG00010009582	TIGD5	-0.12980963	0.13225747	-0.981491856	0.326350248	0.384464146	negative
PRJNA729271	stage_E18_vs_E10	ordinary_muscle_development	ENSGALG00010009583	LYPD6B	-2.806603519	0.31672046	-8.861453161	7.89781E-19	5.20122E-18	negative
PRJNA729271	stage_E18_vs_E10	ordinary_muscle_development	ENSGALG00010009591	LYPD6	-0.824040011	0.441046674	-1.868373712	0.061709999	0.085156743	negative
PRJNA729271	stage_E18_vs_E10	ordinary_muscle_development	ENSGALG00010009593	NCBP1	-1.033475469	0.063537768	-16.26552985	1.73361E-59	6.88457E-58	negative
PRJNA729271	stage_E18_vs_E10	ordinary_muscle_development	ENSGALG00010009594	HINT2	1.849739011	0.159780892	11.57672227	5.40743E-31	6.96098E-30	positive
PRJNA729271	stage_E18_vs_E10	ordinary_muscle_development	ENSGALG00010009595	ENSGALG00010009595	0.801315745	0.186600197	4.294292069	1.75252E-05	3.97601E-05	positive
PRJNA729271	stage_E18_vs_E10	ordinary_muscle_development	ENSGALG00010009596	URB2	-1.755355733	0.104559426	-16.78811568	2.98162E-63	1.35481E-61	negative
PRJNA729271	stage_E18_vs_E10	ordinary_muscle_development	ENSGALG00010009597	CDK1	-2.030035114	0.259462919	-7.823989341	5.11751E-15	2.61376E-14	negative
PRJNA729271	stage_E18_vs_E10	ordinary_muscle_development	ENSGALG00010009600	LRRC17	-0.522557665	0.217221124	-2.405648476	0.01614379	0.024785959	negative
PRJNA729271	stage_E18_vs_E10	ordinary_muscle_development	ENSGALG00010009601	ZHX2	-0.074628282	0.137187836	-0.543986146	0.586451007	0.63885196	negative
PRJNA729271	stage_E18_vs_E10	ordinary_muscle_development	ENSGALG00010009602	TSTD2	-0.198636542	0.0822715	-2.414402821	0.015761027	0.024236801	negative
PRJNA729271	stage_E18_vs_E10	ordinary_muscle_development	ENSGALG00010009603	HINT1	0.209578616	0.177500679	1.180720084	0.23771394	0.290332286	positive
PRJNA729271	stage_E18_vs_E10	ordinary_muscle_development	ENSGALG00010009605	ENSGALG00010009605	1.572113268	0.155456352	10.11289182	4.84332E-24	4.31493E-23	positive
PRJNA729271	stage_E18_vs_E10	ordinary_muscle_development	ENSGALG00010009606	CHSY3	0.443702998	0.211062861	2.102231516	0.035533	0.05122845	positive
PRJNA729271	stage_E18_vs_E10	ordinary_muscle_development	ENSGALG00010009608	AFAP1	-0.607780231	0.106798888	-5.690885362	1.26382E-08	3.86724E-08	negative
PRJNA729271	stage_E18_vs_E10	ordinary_muscle_development	ENSGALG00010009609	MMADHC	-0.109851853	0.072136135	-1.522840848	0.127798528	0.166197708	negative
PRJNA729271	stage_E18_vs_E10	ordinary_muscle_development	ENSGALG00010009612	DERL1	-0.234446536	0.06378523	-3.675561512	0.000237327	0.000471498	negative
PRJNA729271	stage_E18_vs_E10	ordinary_muscle_development	ENSGALG00010009615	ENSGALG00010009615	0.538950817	0.397237164	1.356748225	0.174861224	0.220346531	positive
PRJNA729271	stage_E18_vs_E10	ordinary_muscle_development	ENSGALG00010009616	ARMC10	-0.593196943	0.183486479	-3.232919108	0.001225323	0.002209086	negative
PRJNA729271	stage_E18_vs_E10	ordinary_muscle_development	ENSGALG00010009617	SLC25A46	0.446118185	0.098800131	4.515360262	6.32092E-06	1.50027E-05	positive
PRJNA729271	stage_E18_vs_E10	ordinary_muscle_development	ENSGALG00010009619	ENSGALG00010009619	1.439588298	0.186448958	7.721085254	1.15343E-14	5.72765E-14	positive
PRJNA729271	stage_E18_vs_E10	ordinary_muscle_development	ENSGALG00010009620	TMOD1	3.059956192	0.231768612	13.20263417	8.47198E-40	1.61581E-38	positive
PRJNA729271	stage_E18_vs_E10	ordinary_muscle_development	ENSGALG00010009623	XPO5	-0.84964846	0.111791576	-7.600290554	2.95467E-14	1.41253E-13	negative
PRJNA729271	stage_E18_vs_E10	ordinary_muscle_development	ENSGALG00010009629	ENSGALG00010009629	1.619241616	0.524990917	3.084323106	0.002040159	0.003578858	positive
PRJNA729271	stage_E18_vs_E10	ordinary_muscle_development	ENSGALG00010009634	TBC1D31	-0.501085074	0.105078958	-4.768652851	1.85462E-06	4.6526E-06	negative
PRJNA729271	stage_E18_vs_E10	ordinary_muscle_development	ENSGALG00010009635	TDRD7	0.440282974	0.140711935	3.128966805	0.001754221	0.00310659	positive
PRJNA729271	stage_E18_vs_E10	ordinary_muscle_development	ENSGALG00010009637	CYBA	0.91302403	0.135531816	6.73660292	1.62132E-11	6.27365E-11	positive
PRJNA729271	stage_E18_vs_E10	ordinary_muscle_development	ENSGALG00010009638	RND3	-0.684409385	0.150144456	-4.55833938	5.15597E-06	1.23551E-05	negative
PRJNA729271	stage_E18_vs_E10	ordinary_muscle_development	ENSGALG00010009640	NPR2	2.266984943	0.186678516	12.1437913	6.18904E-34	9.03426E-33	positive
PRJNA729271	stage_E18_vs_E10	ordinary_muscle_development	ENSGALG00010009643	PSMC2	-0.142585486	0.108956784	-1.30864258	0.19065545	0.238216598	negative
PRJNA729271	stage_E18_vs_E10	ordinary_muscle_development	ENSGALG00010009644	SLC27A6	-1.418663336	0.244592325	-5.800113869	6.62699E-09	2.08279E-08	negative
PRJNA729271	stage_E18_vs_E10	ordinary_muscle_development	ENSGALG00010009646	MVD	-0.143172316	0.170027737	-0.842052705	0.399758453	0.459698435	negative
PRJNA729271	stage_E18_vs_E10	ordinary_muscle_development	ENSGALG00010009647	GALNT2	-1.086362522	0.078074915	-13.91436052	5.18213E-44	1.17367E-42	negative
PRJNA729271	stage_E18_vs_E10	ordinary_muscle_development	ENSGALG00010009649	TENM2	-0.383004393	0.311789466	-1.228407097	0.219294185	0.270121907	negative
PRJNA729271	stage_E18_vs_E10	ordinary_muscle_development	ENSGALG00010009650	KLHL29	-0.472826329	0.194324509	-2.433179068	0.014966897	0.023113698	negative
PRJNA729271	stage_E18_vs_E10	ordinary_muscle_development	ENSGALG00010009652	CIAO2B	0.096699816	0.082369738	1.173972607	0.240405998	0.29329896	positive
PRJNA729271	stage_E18_vs_E10	ordinary_muscle_development	ENSGALG00010009653	ABLIM2	4.556458058	0.182767589	24.93033956	3.4895E-137	8.7208E-135	positive
PRJNA729271	stage_E18_vs_E10	ordinary_muscle_development	ENSGALG00010009654	NDUFA12	0.920388372	0.140046384	6.572025243	4.96354E-11	1.85525E-10	positive
PRJNA729271	stage_E18_vs_E10	ordinary_muscle_development	ENSGALG00010009657	MSMP	4.047772064	0.637067386	6.3537581	2.10117E-10	7.47215E-10	positive
PRJNA729271	stage_E18_vs_E10	ordinary_muscle_development	ENSGALG00010009658	NAPEPLD	0.845126048	0.123887991	6.821694645	8.99728E-12	3.54776E-11	positive
PRJNA729271	stage_E18_vs_E10	ordinary_muscle_development	ENSGALG00010009659	APOLD1	1.088461733	0.253271557	4.297607456	1.72652E-05	3.92255E-05	positive
PRJNA729271	stage_E18_vs_E10	ordinary_muscle_development	ENSGALG00010009663	FER	0.079069267	0.122422386	0.645872624	0.51836185	0.575230058	positive
PRJNA729271	stage_E18_vs_E10	ordinary_muscle_development	ENSGALG00010009665	DGKQ	-0.05875059	0.174489022	-0.336700783	0.736342473	0.777621583	negative
PRJNA729271	stage_E18_vs_E10	ordinary_muscle_development	ENSGALG00010009666	RRAD	1.300336904	0.364740137	3.565105045	0.00036371	0.000703964	positive
PRJNA729271	stage_E18_vs_E10	ordinary_muscle_development	ENSGALG00010009668	TFAP2A	-6.165379122	0.936761024	-6.581592281	4.65436E-11	1.74283E-10	negative
PRJNA729271	stage_E18_vs_E10	ordinary_muscle_development	ENSGALG00010009671	MINAR2	3.110351391	0.817766644	3.803470605	0.000142683	0.00029187	positive
PRJNA729271	stage_E18_vs_E10	ordinary_muscle_development	ENSGALG00010009672	DDX47	-0.555743769	0.121297258	-4.58166803	4.61282E-06	1.10939E-05	negative
PRJNA729271	stage_E18_vs_E10	ordinary_muscle_development	ENSGALG00010009676	ACO1	-0.432204603	0.096317035	-4.487312166	7.21274E-06	1.70302E-05	negative
PRJNA729271	stage_E18_vs_E10	ordinary_muscle_development	ENSGALG00010009677	SLC26A5	-0.60753569	0.167111557	-3.635509725	0.000277432	0.000546085	negative
PRJNA729271	stage_E18_vs_E10	ordinary_muscle_development	ENSGALG00010009678	JARID2	-0.334962552	0.100935916	-3.318566523	0.000904808	0.001657842	negative
PRJNA729271	stage_E18_vs_E10	ordinary_muscle_development	ENSGALG00010009679	ANK3	1.607801895	0.102979147	15.612888	5.94824E-55	2.0192E-53	positive
PRJNA729271	stage_E18_vs_E10	ordinary_muscle_development	ENSGALG00010009683	ADAMTS19	2.356245251	0.238448395	9.88157311	5.00411E-23	4.20003E-22	positive
PRJNA729271	stage_E18_vs_E10	ordinary_muscle_development	ENSGALG00010009686	MAT2B	0.314967767	0.087834861	3.585908431	0.000335907	0.000653115	positive
PRJNA729271	stage_E18_vs_E10	ordinary_muscle_development	ENSGALG00010009689	ENSGALG00010009689	-0.955070517	0.46642319	-2.047648012	0.040594501	0.05790369	negative
PRJNA729271	stage_E18_vs_E10	ordinary_muscle_development	ENSGALG00010009690	RBM43	0.066077677	0.186835717	0.353667264	0.723588259	0.766024818	positive
PRJNA729271	stage_E18_vs_E10	ordinary_muscle_development	ENSGALG00010009695	ATAD2B	-0.041476929	0.077790961	-0.533184424	0.593905923	0.645714548	negative
PRJNA729271	stage_E18_vs_E10	ordinary_muscle_development	ENSGALG00010009697	ENSGALG00010009697	0.281453312	0.261205458	1.077516963	0.281249377	0.336862478	positive
PRJNA729271	stage_E18_vs_E10	ordinary_muscle_development	ENSGALG00010009699	POLR1C	-0.690047075	0.110747307	-6.230824859	4.63986E-10	1.6167E-09	negative
PRJNA729271	stage_E18_vs_E10	ordinary_muscle_development	ENSGALG00010009700	SH3TC1	1.478229828	0.205332369	7.199205035	6.05646E-13	2.63757E-12	positive
PRJNA729271	stage_E18_vs_E10	ordinary_muscle_development	ENSGALG00010009702	ENSGALG00010009702	-0.231231021	0.122344739	-1.889995619	0.058758546	0.081487287	negative
PRJNA729271	stage_E18_vs_E10	ordinary_muscle_development	ENSGALG00010009704	RGP1	-0.039729324	0.131752629	-0.301544828	0.762999072	0.801193245	negative
PRJNA729271	stage_E18_vs_E10	ordinary_muscle_development	ENSGALG00010009705	DTNBP1	-0.219877635	0.072212108	-3.04488597	0.002327685	0.004044085	negative
PRJNA729271	stage_E18_vs_E10	ordinary_muscle_development	ENSGALG00010009710	ENSGALG00010009710	-0.538819385	0.111972628	-4.81206339	1.4938E-06	3.79205E-06	negative
PRJNA729271	stage_E18_vs_E10	ordinary_muscle_development	ENSGALG00010009712	UBXN2A	1.280577379	0.179227634	7.144977306	9.00108E-13	3.88074E-12	positive
PRJNA729271	stage_E18_vs_E10	ordinary_muscle_development	ENSGALG00010009715	NUDCD2	-0.512528512	0.094204659	-5.440585605	5.31057E-08	1.53984E-07	negative
PRJNA729271	stage_E18_vs_E10	ordinary_muscle_development	ENSGALG00010009717	EDN1	1.029098843	0.274237333	3.752584784	0.000175021	0.000353628	positive
PRJNA729271	stage_E18_vs_E10	ordinary_muscle_development	ENSGALG00010009718	ENSGALG00010009718	-0.360341524	0.181099253	-1.989746048	0.046618917	0.065778371	negative
PRJNA729271	stage_E18_vs_E10	ordinary_muscle_development	ENSGALG00010009719	YIPF3	-0.020432116	0.082855614	-0.246599054	0.805218537	0.837519973	negative
PRJNA729271	stage_E18_vs_E10	ordinary_muscle_development	ENSGALG00010009720	RELN	-0.724689793	0.169025191	-4.28746619	1.80723E-05	4.08925E-05	negative
PRJNA729271	stage_E18_vs_E10	ordinary_muscle_development	ENSGALG00010009721	ZHX1	0.759503566	0.156128555	4.864603819	1.14686E-06	2.94622E-06	positive

PRJNA729271	stage_E18_vs_E10	ordinary_muscle_development	ENSGALG00010009722	GBA2	-0.072260202	0.098528628	-0.733392957	0.463318794	0.522468174	negative
PRJNA729271	stage_E18_vs_E10	ordinary_muscle_development	ENSGALG00010009724	ISOC1	-0.522367685	0.146463026	-3.566549849	0.000361712	0.00070047	negative
PRJNA729271	stage_E18_vs_E10	ordinary_muscle_development	ENSGALG00010009726	PRR16	-0.044524651	0.147833338	-0.301181397	0.763276174	0.801426161	negative
PRJNA729271	stage_E18_vs_E10	ordinary_muscle_development	ENSGALG00010009727	CDH20	4.683933518	0.211670123	22.12845841	1.6823E-108	2.4631E-106	positive
PRJNA729271	stage_E18_vs_E10	ordinary_muscle_development	ENSGALG00010009728	CCDC149	-0.63226673	0.102153637	-6.189370727	6.04049E-10	2.08568E-09	negative
PRJNA729271	stage_E18_vs_E10	ordinary_muscle_development	ENSGALG00010009730	C8orf76	-0.082111809	0.116490099	-0.704882302	0.480883485	0.538879483	negative
PRJNA729271	stage_E18_vs_E10	ordinary_muscle_development	ENSGALG00010009731	NMI	-0.891960553	0.244506172	-3.648008328	0.000264281	0.000522043	negative
PRJNA729271	stage_E18_vs_E10	ordinary_muscle_development	ENSGALG00010009732	HMMR	-1.930749195	0.243471894	-7.930070127	2.19022E-15	1.14487E-14	negative
PRJNA729271	stage_E18_vs_E10	ordinary_muscle_development	ENSGALG00010009736	WISP1	-0.386548006	0.328314484	-1.177371163	0.239047394	0.291813372	negative
PRJNA729271	stage_E18_vs_E10	ordinary_muscle_development	ENSGALG00010009738	ENSGALG00010009738	-0.724055559	0.259969552	-2.785155236	0.00535021	0.008829705	negative
PRJNA729271	stage_E18_vs_E10	ordinary_muscle_development	ENSGALG00010009740	LRRC73	-0.313334627	0.304446918	-1.029192966	0.303389007	0.360549615	negative
PRJNA729271	stage_E18_vs_E10	ordinary_muscle_development	ENSGALG00010009741	C2H6orf52	-0.23497918	0.072302509	-3.249945049	0.001154273	0.002087485	negative
PRJNA729271	stage_E18_vs_E10	ordinary_muscle_development	ENSGALG00010009743	CCNG1	0.646127445	0.173004759	3.734737977	0.000187911	0.000378038	positive
PRJNA729271	stage_E18_vs_E10	ordinary_muscle_development	ENSGALG00010009744	DAPK1	-0.679326432	0.10605521	-6.40540369	1.49972E-10	5.39148E-10	negative
PRJNA729271	stage_E18_vs_E10	ordinary_muscle_development	ENSGALG00010009749	NDRG1	1.169334429	0.167161682	6.995230104	2.64824E-12	1.09675E-11	positive
PRJNA729271	stage_E18_vs_E10	ordinary_muscle_development	ENSGALG00010009751	ENSGALG00010009751	0.745414522	0.201084739	3.706967141	0.000209756	0.000419425	positive
PRJNA729271	stage_E18_vs_E10	ordinary_muscle_development	ENSGALG00010009761	PDP2	0.2010031	0.11674984	1.72165631	0.085131796	0.114385	positive
PRJNA729271	stage_E18_vs_E10	ordinary_muscle_development	ENSGALG00010009762	MAN2A1	0.208324712	0.102795661	2.026590522	0.042704307	0.060626731	positive
PRJNA729271	stage_E18_vs_E10	ordinary_muscle_development	ENSGALG00010009765	STX10	-0.335195296	0.098428515	-3.405469404	0.000660504	0.001235196	negative
PRJNA729271	stage_E18_vs_E10	ordinary_muscle_development	ENSGALG00010009769	TMCC3	-0.276727223	0.249898589	-1.107358083	0.268139141	0.323108891	negative
PRJNA729271	stage_E18_vs_E10	ordinary_muscle_development	ENSGALG00010009772	ENSGALG00010009772	0.57574076	0.559933168	1.028231212	0.30384108	0.361027662	positive
PRJNA729271	stage_E18_vs_E10	ordinary_muscle_development	ENSGALG00010009774	PIAS2	-0.537809592	0.061661242	-8.722003862	2.73321E-18	1.74528E-17	negative
PRJNA729271	stage_E18_vs_E10	ordinary_muscle_development	ENSGALG00010009775	KIAA1614	-0.09109791	0.139199043	-0.654443511	0.51282611	0.569610303	negative
PRJNA729271	stage_E18_vs_E10	ordinary_muscle_development	ENSGALG00010009777	ST3GAL1	-2.296250974	0.192279065	-11.9422828	7.12407E-33	9.84399E-32	negative
PRJNA729271	stage_E18_vs_E10	ordinary_muscle_development	ENSGALG00010009778	ENSGALG00010009778	0.066002189	0.112075334	0.588909144	0.555922223	0.609999442	positive
PRJNA729271	stage_E18_vs_E10	ordinary_muscle_development	ENSGALG00010009779	PPP1R3E	1.971542009	0.357686252	5.511931189	3.54918E-08	1.04905E-07	positive
PRJNA729271	stage_E18_vs_E10	ordinary_muscle_development	ENSGALG00010009780	MFSD2B	-0.399328329	0.254863209	-1.566833951	0.117153509	0.153455641	negative
PRJNA729271	stage_E18_vs_E10	ordinary_muscle_development	ENSGALG00010009784	ENSGALG00010009784	-0.597382438	0.179361378	-3.330607978	0.000866565	0.00159321	negative
PRJNA729271	stage_E18_vs_E10	ordinary_muscle_development	ENSGALG00010009786	DIPK2B	0.719830112	0.16018049	4.493868822	6.99407E-06	1.65328E-05	positive
PRJNA729271	stage_E18_vs_E10	ordinary_muscle_development	ENSGALG00010009788	PAK1IP1	-0.335509121	0.093463941	-3.589717259	0.000331037	0.000644337	negative
PRJNA729271	stage_E18_vs_E10	ordinary_muscle_development	ENSGALG00010009791	GABRG2	1.149913366	0.280075484	4.105726605	4.03046E-05	8.76542E-05	positive
PRJNA729271	stage_E18_vs_E10	ordinary_muscle_development	ENSGALG00010009793	TNFAIP6	-0.893507325	0.290473398	-3.076038395	0.002097708	0.0036727	negative
PRJNA729271	stage_E18_vs_E10	ordinary_muscle_development	ENSGALG00010009795	NDUFA6	0.93028562	0.114884797	8.097552009	5.60761E-16	3.06379E-15	positive
PRJNA729271	stage_E18_vs_E10	ordinary_muscle_development	ENSGALG00010009796	ENSGALG00010009796	-0.437336858	0.19616151	-2.229473344	0.025782427	0.038114868	negative
PRJNA729271	stage_E18_vs_E10	ordinary_muscle_development	ENSGALG00010009798	YIPF4	0.260847429	0.078270979	3.332619978	0.000860324	0.001582336	positive
PRJNA729271	stage_E18_vs_E10	ordinary_muscle_development	ENSGALG00010009801	ENSGALG00010009801	-1.1733397	0.636539152	-1.843311125	0.065283598	0.08969125	negative
PRJNA729271	stage_E18_vs_E10	ordinary_muscle_development	ENSGALG00010009804	XPR1	-0.279528468	0.118266626	-2.363544794	0.018101038	0.027499691	negative
PRJNA729271	stage_E18_vs_E10	ordinary_muscle_development	ENSGALG00010009806	WDCP	-0.506334433	0.174348813	-2.904146147	0.003682562	0.006210441	negative
PRJNA729271	stage_E18_vs_E10	ordinary_muscle_development	ENSGALG00010009809	SMDT1	0.101826938	0.091892854	1.108105074	0.26781644	0.32280054	positive
PRJNA729271	stage_E18_vs_E10	ordinary_muscle_development	ENSGALG00010009811	ENSGALG00010009811	0.39458301	0.087198501	4.525112298	6.03634E-06	1.437E-05	positive
PRJNA729271	stage_E18_vs_E10	ordinary_muscle_development	ENSGALG00010009822	ENSGALG00010009822	-0.335101845	0.202603887	-1.653975401	0.098132511	0.130330836	negative
PRJNA729271	stage_E18_vs_E10	ordinary_muscle_development	ENSGALG00010009830	RNASEL	-0.632983644	0.195960038	-3.230166981	0.001237179	0.002228799	negative
PRJNA729271	stage_E18_vs_E10	ordinary_muscle_development	ENSGALG00010009831	N6AMT1	-0.429367675	0.121436213	-3.535746577	0.000406625	0.000782219	negative
PRJNA729271	stage_E18_vs_E10	ordinary_muscle_development	ENSGALG00010009833	SOD3	2.403795538	0.313070937	7.678117817	1.61443E-14	7.92186E-14	positive
PRJNA729271	stage_E18_vs_E10	ordinary_muscle_development	ENSGALG00010009840	ENSGALG00010009840	0.537172867	0.176142116	3.049656025	0.002291036	0.003983753	positive
PRJNA729271	stage_E18_vs_E10	ordinary_muscle_development	ENSGALG00010009841	GABRA1	3.08314421	0.228343065	13.5022459	1.51681E-41	3.08794E-40	positive
PRJNA729271	stage_E18_vs_E10	ordinary_muscle_development	ENSGALG00010009842	PHETA2	0.617675116	0.08997563	6.864915745	6.65305E-12	2.65664E-11	positive
PRJNA729271	stage_E18_vs_E10	ordinary_muscle_development	ENSGALG00010009847	ENSGALG00010009847	-0.788577443	0.119611344	-6.59283157	4.31516E-11	1.61916E-10	negative
PRJNA729271	stage_E18_vs_E10	ordinary_muscle_development	ENSGALG00010009850	RIF1	-1.468912527	0.105717803	-13.89465619	6.82504E-44	1.53141E-42	negative
PRJNA729271	stage_E18_vs_E10	ordinary_muscle_development	ENSGALG00010009852	ENSGALG00010009852	-0.26023277	0.25131169	-1.03549807	0.300436367	0.35742163	negative
PRJNA729271	stage_E18_vs_E10	ordinary_muscle_development	ENSGALG00010009857	ORC5	-1.04474538	0.140728948	-7.423812923	1.13796E-13	5.21489E-13	negative
PRJNA729271	stage_E18_vs_E10	ordinary_muscle_development	ENSGALG00010009858	ST8SIA5	-0.468765546	0.249410521	-1.87949387	0.06017709	0.083263356	negative
PRJNA729271	stage_E18_vs_E10	ordinary_muscle_development	ENSGALG00010009866	NAGA	0.607112168	0.115745976	5.245211864	1.56103E-07	4.33699E-07	positive
PRJNA729271	stage_E18_vs_E10	ordinary_muscle_development	ENSGALG00010009868	LTN1	0.204729889	0.095714811	2.138957244	0.032439131	0.047095874	positive
PRJNA729271	stage_E18_vs_E10	ordinary_muscle_development	ENSGALG00010009876	SELENOI	-0.75128736	0.111933239	-6.711923677	1.92075E-11	7.383E-11	negative
PRJNA729271	stage_E18_vs_E10	ordinary_muscle_development	ENSGALG00010009883	ENSGALG00010009883	0.097354961	0.2013566	0.483495256	0.628744128	0.678957471	positive
PRJNA729271	stage_E18_vs_E10	ordinary_muscle_development	ENSGALG00010009884	GFOD1	0.806943779	0.116423274	6.931120838	4.17519E-12	1.70477E-11	positive
PRJNA729271	stage_E18_vs_E10	ordinary_muscle_development	ENSGALG00010009910	ENSGALG00010009910	0.568922279	0.175641279	3.239114871	0.001199013	0.002162998	positive
PRJNA729271	stage_E18_vs_E10	ordinary_muscle_development	ENSGALG00010009914	RWDD2B	-0.295119914	0.12532281	-2.354877876	0.018528794	0.02808773	negative
PRJNA729271	stage_E18_vs_E10	ordinary_muscle_development	ENSGALG00010009941	CACNA1E	-2.019498473	0.419800675	-4.810612732	1.50468E-06	3.81634E-06	negative
PRJNA729271	stage_E18_vs_E10	ordinary_muscle_development	ENSGALG00010009943	DHX15	-0.894701889	0.069538964	-12.86619528	6.97544E-38	1.22854E-36	negative
PRJNA729271	stage_E18_vs_E10	ordinary_muscle_development	ENSGALG00010009948	WBP2NL	0.457907777	0.06592242	6.94616156	3.75359E-12	1.53696E-11	positive
PRJNA729271	stage_E18_vs_E10	ordinary_muscle_development	ENSGALG00010009972	PTK2B	0.093322492	0.288650538	0.323306143	0.746463402	0.786793704	positive
PRJNA729271	stage_E18_vs_E10	ordinary_muscle_development	ENSGALG00010009985	USP16	-0.908707363	0.117344723	-7.743913299	9.64028E-15	4.82513E-14	negative
PRJNA729271	stage_E18_vs_E10	ordinary_muscle_development	ENSGALG00010009989	TBC1D7	-0.477672957	0.135317585	-3.530013915	0.000415538	0.000798624	negative
PRJNA729271	stage_E18_vs_E10	ordinary_muscle_development	ENSGALG00010009991	LHFPL3	-2.292222036	0.515009255	-4.450836589	8.55364E-06	2.0072E-05	negative
PRJNA729271	stage_E18_vs_E10	ordinary_muscle_development	ENSGALG00010009993	ENSGALG00010009993	-0.3943078	0.364068609	-1.083059045	0.278782216	0.33437718	negative
PRJNA729271	stage_E18_vs_E10	ordinary_muscle_development	ENSGALG00010009995	SEPTIN3	1.990524876	0.337592142	5.896241733	3.71874E-09	1.19625E-08	positive
PRJNA729271	stage_E18_vs_E10	ordinary_muscle_development	ENSGALG00010009999	GABRB2	0.353075122	0.22617244	1.561088177	0.118502947	0.155097085	positive
PRJNA729271	stage_E18_vs_E10	ordinary_muscle_development	ENSGALG00010010006	PPARGC1A	2.856612641	0.265245681	10.76968579	4.78632E-27	5.08634E-26	positive
PRJNA729271	stage_E18_vs_E10	ordinary_muscle_development	ENSGALG00010010007	CTIF	-0.025544811	0.096773342	-0.26396537	0.791806603	0.826057055	negative
PRJNA729271	stage_E18_vs_E10	ordinary_muscle_development	ENSGALG00010010009	ELF2	-0.370079053	0.088882216	-4.163701918	3.13129E-05	6.88845E-05	negative
PRJNA729271	stage_E18_vs_E10	ordinary_muscle_development	ENSGALG00010010011	EPB41L4A	0.166428519	0.126687906	1.313689074	0.188950859	0.236249694	positive
PRJNA729271	stage_E18_vs_E10	ordinary_muscle_development	ENSGALG00010010014	GLUL	0.738876835	0.392747302	1.8813034	0.059930659	0.082956565	positive
PRJNA729271	stage_E18_vs_E10	ordinary_muscle_development	ENSGALG00010010017	CCDC59	-1.029302902	0.109213561	-9.424680307	4.31418E-21	3.26688E-20	negative
PRJNA729271	stage_E18_vs_E10	ordinary_muscle_development	ENSGALG00010010022	KMT2E	0.47875066	0.101420517	4.720451762	2.35321E-06	5.84073E-06	positive
PRJNA729271	stage_E18_vs_E10	ordinary_muscle_development	ENSGALG00010010035	ATP10B	-1.43315708	0.335206747	-4.275442223	1.90758E-05	4.30625E-05	negative
PRJNA729271	stage_E18_vs_E10	ordinary_muscle_development	ENSGALG00010010037	NAA15	-0.633520738	0.138648276	-4.569265166	4.89437E-06	1.17476E-05	negative
PRJNA729271	stage_E18_vs_E10	ordinary_muscle_development	ENSGALG00010010042	METTL25	-1.252578799	0.122727936	-10.20614246	1.86118E-24	1.70637E-23	negative
PRJNA729271	stage_E18_vs_E10	ordinary_muscle_development	ENSGALG00010010049	ZBTB7C	0.413777687	0.272476815	1.518579433	0.128868391	0.167363796	positive
PRJNA729271	stage_E18_vs_E10	ordinary_muscle_development	ENSGALG00010010052	ZNF346	-0.434357775	0.115015909	-3.776501693	0.000159046	0.000323154	negative
PRJNA729271	stage_E18_vs_E10	ordinary_muscle_development	ENSGALG00010010053	CHRNA2	-5.537841738	0.90032727	-6.15092081	7.70344E-10	2.63104E-09	negative
PRJNA729271	stage_E18_vs_E10	ordinary_muscle_development	ENSGALG00010010059	ENSGALG00010010059	-1.005926804	0.131663465	-7.640136184	2.16992E-14	1.05159E-13	negative
PRJNA729271	stage_E18_vs_E10	ordinary_muscle_development	ENSGALG00010010061	CYP2D6	2.765495293	0.202343021	13.6673619	1.59058E-42	3.38056E-41	positive
PRJNA729271	stage_E18_vs_E10	ordinary_muscle_development	ENSGALG00010010062	RGS7BP	-0.239668737	0.172947455	-1.385789324	0.165811243	0.209980253	negative
PRJNA729271	stage_E18_vs_E10	ordinary_muscle_development	ENSGALG00010010064	ENSGALG00010010064	-0.345486605	0.089532939	-3.858765361	0.000113961	0.000236082	negative
PRJNA729271	stage_E18_vs_E10	ordinary_muscle_development	ENSGALG00010010071	GLT8D2	-0.79989897	0.26866972	-2.977257616	0.002908395	0.00499197	negative
PRJNA729271	stage_E18_vs_E10	ordinary_muscle_development	ENSGALG00010010077	CCT8	-1.09028421	0.115623609	-9.429598503	4.11662E-21	3.12083E-20	negative
PRJNA729271	stage_E18_vs_E10	ordinary_muscle_development	ENSGALG00010010080	CDC42EP3	-0.377208856	0.119909705	-3.145774208	0.001656478	0.002941761	negative
PRJNA729271	stage_E18_vs_E10	ordinary_muscle_development	ENSGALG00010010084	HK3	0.869845689	0.220671493	3.941812678	8.08681E-05	0.000170178	positive
PRJNA729271	stage_E18_vs_E10	ordinary_muscle_development	ENSGALG00010010085	TDG	-1.264528316	0.104420969	-12.10990792	9.35992E-34	1.35131E-32	negative
PRJNA729271	stage_E18_vs_E10	ordinary_muscle_development	ENSGALG00010010094	SLC2A9	1.927350895	0.244008674	7.898698286	2.81831E-15	1.46631E-14	positive
PRJNA729271	stage_E18_vs_E10	ordinary_muscle_development	ENSGALG00010010095	C12orf73	-0.208583744	0.101498307	-2.055046539	0.03987451	0.056960779	negative
PRJNA729271	stage_E18_vs_E10	ordinary_muscle_development	ENSGALG00010010100	IRF2BP2	1.334525136	0.162225836	8.226341564	1.93018E-16	1.0861E-15	positive
PRJNA729271	stage_E18_vs_E10	ordinary_muscle_development	ENSGALG00010010106	C7orf73	0.144031057	0.206331161	0.698057707	0.485141106	0.5431467	positive
PRJNA729271	stage_E18_vs_E10	ordinary_muscle_development	ENSGALG00010010107	MGST2	-0.329971237	0.327907954	-1.006292263	0.314275001	0.371688654	negative
PRJNA729271	stage_E18_vs_E10	ordinary_muscle_development	ENSGALG00010010111	CNOT4	0.542692211	0.136272827	3.982394905	6.82243E-05	0.000144938	positive
PRJNA729271	stage_E18_vs_E10	ordinary_muscle_development	ENSGALG00010010115	NEB	3.364558285	0.171698292	19.59575865	1.68062E-85	1.36093E-83	positive
PRJNA729271	stage_E18_vs_E10	ordinary_muscle_development	ENSGALG00010010116	DRD5	-3.099226094	0.550347406	-5.631399478	1.78753E-08	5.40022E-08	negative
PRJNA729271	stage_E18_vs_E10	ordinary_muscle_development	ENSGALG00010010119	SRPK2	-0.685460328	0.125195804	-5.475106256	4.37249E-08	1.27936E-07	negative
PRJNA729271	stage_E18_vs_E10	ordinary_muscle_development	ENSGALG00010010122	THBS4	-0.583358802	0.245911588	-2.372229818	0.017681089	0.026912463	negative
PRJNA729271	stage_E18_vs_E10	ordinary_muscle_development	ENSGALG00010010124	ENSGALG00010010124	0.120039063	0.149614779	0.80232089	0.422367361	0.482786445	positive
PRJNA729271	stage_E18_vs_E10	ordinary_muscle_development	ENSGALG00010010125	AHCTF1	-0.349210375	0.067213805	-5.195515636	2.04153E-07	5.62479E-07	negative
PRJNA729271	stage_E18_vs_E10	ordinary_muscle_development	ENSGALG00010010127	FAM13C	1.496593571	0.341884736	4.377479931	1.20059E-05	2.772E-05	positive
PRJNA729271	stage_E18_vs_E10	ordinary_muscle_development	ENSGALG00010010136	EPHX2	0.634261297	0.115712905	5.481335884	4.22126E-08	1.23735E-07	positive
PRJNA729271	stage_E18_vs_E10	ordinary_muscle_development	ENSGALG00010010142	ENSGALG00010010142	0.981903546	0.295054002	3.3278774	0.000875104	0.001607277	positive
PRJNA729271	stage_E18_vs_E10	ordinary_muscle_development	ENSGALG00010010146	PHACTR1	-3.892999951	0.346718918	-11.2281152	2.96741E-29	3.49696E-28	negative
PRJNA729271	stage_E18_vs_E10	ordinary_muscle_development	ENSGALG00010010150	NTRK2	2.288375486	0.296499179	7.717982537	1.18185E-14	5.86276E-14	positive
PRJNA729271	stage_E18_vs_E10	ordinary_muscle_development	ENSGALG00010010153	ENSGALG00010010153	-0.168327959	0.468448707	-0.359330609	0.719347779	0.76219278	negative
PRJNA729271	stage_E18_vs_E10	ordinary_muscle_development	ENSGALG00010010154	IQSEC3	-0.350046004	0.255671963	-1.36912159	0.170961271	0.215954999	negative
PRJNA729271	stage_E18_vs_E10	ordinary_muscle_development	ENSGALG00010010155	BRINP3	-1.192815079	0.352468207	-3.384177796	0.000713918	0.001328741	negative
PRJNA729271	stage_E18_vs_E10	ordinary_muscle_development	ENSGALG00010010156	ENSGALG00010010156	-0.136664881	0.098024256	-1.394194523	0.163258871	0.207200538	negative
PRJNA729271	stage_E18_vs_E10	ordinary_muscle_development	ENSGALG00010010161	SEPSECS	0.505830885	0.063094803	8.01699758	1.08361E-15	5.8045E-15	positive
PRJNA729271	stage_E18_vs_E10	ordinary_muscle_development	ENSGALG00010010164	ENSGALG00010010164	0.634398209	0.433114397	1.464735906	0.142992985	0.183878931	positive
PRJNA729271	stage_E18_vs_E10	ordinary_muscle_development	ENSGALG00010010167	PUS7	-1.061933275	0.074178527	-14.31591208	1.74065E-46	4.42645E-45	negative
PRJNA729271	stage_E18_vs_E10	ordinary_muscle_development	ENSGALG00010010168	SNCB	-1.880745499	0.267242038	-7.037610985	1.95564E-12	8.17151E-12	negative
PRJNA729271	stage_E18_vs_E10	ordinary_muscle_development	ENSGALG00010010171	ENSGALG00010010171	0.619142905	0.400113176	1.547419436	0.121762137	0.158975156	positive
PRJNA729271	stage_E18_vs_E10	ordinary_muscle_development	ENSGALG00010010178	LGI2	-1.503029006	0.178492513	-8.420683764	3.74293E-17	2.21173E-16	negative
PRJNA729271	stage_E18_vs_E10	ordinary_muscle_development	ENSGALG00010010179	CDHR2	-0.958419429	0.954280034	-1.004337716	0.31521586	0.372618977	negative
PRJNA729271	stage_E18_vs_E10	ordinary_muscle_development	ENSGALG00010010183	NKX3-2	-5.013305995	0.485669055	-10.3224736	5.57676E-25	5.27989E-24	negative
PRJNA729271	stage_E18_vs_E10	ordinary_muscle_development	ENSGALG00010010185	PHYHIPL	-1.118616927	0.336624957	-3.323036226	0.000890434	0.001634162	negative
PRJNA729271	stage_E18_vs_E10	ordinary_muscle_development	ENSGALG00010010186	TNPO1	0.291610191	0.072377466	4.029019064	5.60101E-05	0.000120081	positive
PRJNA729271	stage_E18_vs_E10	ordinary_muscle_development	ENSGALG00010010188	DSP	-5.128655768	0.356080598	-14.40307559	4.94912E-47	1.2949E-45	negative
PRJNA729271	stage_E18_vs_E10	ordinary_muscle_development	ENSGALG00010010190	RNF44	-0.026501735	0.185102058	-0.143173639	0.886153048	0.906157914	negative
PRJNA729271	stage_E18_vs_E10	ordinary_muscle_development	ENSGALG00010010191	RINT1	-0.204253333	0.093801165	-2.177513824	0.029442251	0.043068467	negative
PRJNA729271	stage_E18_vs_E10	ordinary_muscle_development	ENSGALG00010010195	ENSGALG00010010195	4.546641709	0.277685	16.37337884	2.96298E-60	1.19967E-58	positive
PRJNA729271	stage_E18_vs_E10	ordinary_muscle_development	ENSGALG00010010196	EFCAB10	1.34967449	0.325289532	4.149148249	3.33715E-05	7.32312E-05	positive
PRJNA729271	stage_E18_vs_E10	ordinary_muscle_development	ENSGALG00010010197	SCCPDH	-0.431557681	0.121740369	-3.544902021	0.000392759	0.000756852	negative
PRJNA729271	stage_E18_vs_E10	ordinary_muscle_development	ENSGALG00010010201	RIOK1	-1.357826463	0.104049329	-13.04983392	6.36861E-39	1.16852E-37	negative
PRJNA729271	stage_E18_vs_E10	ordinary_muscle_development	ENSGALG00010010205	ARL5A	-0.598925005	0.084715212	-7.069863691	1.55086E-12	6.54812E-12	negative
PRJNA729271	stage_E18_vs_E10	ordinary_muscle_development	ENSGALG00010010206	SCARA5	1.901408895	0.158083626	12.02786744	2.53627E-33	3.56579E-32	positive

PRJNA729271	stage_E18_vs_E10	ordinary_muscle_development	ENSGALG00010010214	FAF2	-0.757838014	0.07843402	-9.662108543	4.3678E-22	3.49207E-21	negative
PRJNA729271	stage_E18_vs_E10	ordinary_muscle_development	ENSGALG00010010215	ENSGALG00010010215	0.165035978	0.127142787	1.298036502	0.194274792	0.242258526	positive
PRJNA729271	stage_E18_vs_E10	ordinary_muscle_development	ENSGALG00010010216	ATXN7L1	0.610395817	0.128717683	4.742128685	2.11484E-06	5.27165E-06	positive
PRJNA729271	stage_E18_vs_E10	ordinary_muscle_development	ENSGALG00010010218	PBK	-1.847717749	0.220251906	-8.389111285	4.89831E-17	2.87221E-16	negative
PRJNA729271	stage_E18_vs_E10	ordinary_muscle_development	ENSGALG00010010220	LHX9	-8.997785847	0.902676048	-9.967901406	2.10632E-23	1.80551E-22	negative
PRJNA729271	stage_E18_vs_E10	ordinary_muscle_development	ENSGALG00010010222	PAH	2.488774026	1.056987636	2.354591427	0.018543082	0.028100572	positive
PRJNA729271	stage_E18_vs_E10	ordinary_muscle_development	ENSGALG00010010223	ENSGALG00010010223	0.620105399	0.214623453	2.889271371	0.003861357	0.006496097	positive
PRJNA729271	stage_E18_vs_E10	ordinary_muscle_development	ENSGALG00010010226	BICC1	-0.002165115	0.164102299	-0.013193692	0.989473262	0.991093869	negative
PRJNA729271	stage_E18_vs_E10	ordinary_muscle_development	ENSGALG00010010231	C1orf53	0.82913559	0.228659306	3.626074106	0.000287763	0.000565269	positive
PRJNA729271	stage_E18_vs_E10	ordinary_muscle_development	ENSGALG00010010234	ENSGALG00010010234	1.265066863	0.268805016	4.70626211	2.523E-06	6.23859E-06	positive
PRJNA729271	stage_E18_vs_E10	ordinary_muscle_development	ENSGALG00010010243	ESCO2	-0.775072038	0.113468163	-6.830744584	8.4475E-12	3.33915E-11	negative
PRJNA729271	stage_E18_vs_E10	ordinary_muscle_development	ENSGALG00010010246	CLTB	0.58832889	0.066645407	8.827748455	1.06804E-18	6.97981E-18	positive
PRJNA729271	stage_E18_vs_E10	ordinary_muscle_development	ENSGALG00010010252	WDR7	0.532087137	0.097050881	5.482558547	4.19218E-08	1.22933E-07	positive
PRJNA729271	stage_E18_vs_E10	ordinary_muscle_development	ENSGALG00010010253	IL10RB	0.660645891	0.124310596	5.314477707	1.06964E-07	3.02643E-07	positive
PRJNA729271	stage_E18_vs_E10	ordinary_muscle_development	ENSGALG00010010254	WDR41	-0.178943558	0.118252129	-1.513237508	0.13021934	0.168864546	negative
PRJNA729271	stage_E18_vs_E10	ordinary_muscle_development	ENSGALG00010010255	CACNB4	0.518244403	0.175823986	2.947518227	0.003203359	0.005460099	positive
PRJNA729271	stage_E18_vs_E10	ordinary_muscle_development	ENSGALG00010010257	ENSGALG00010010257	0.567731844	0.139374209	4.073435441	4.63247E-05	0.000100056	positive
PRJNA729271	stage_E18_vs_E10	ordinary_muscle_development	ENSGALG00010010259	IGF1	0.873817397	0.39585825	2.207399741	0.027286144	0.040173963	positive
PRJNA729271	stage_E18_vs_E10	ordinary_muscle_development	ENSGALG00010010262	HSPA9	-0.239478109	0.145791336	-1.642608642	0.100463932	0.133110118	negative
PRJNA729271	stage_E18_vs_E10	ordinary_muscle_development	ENSGALG00010010263	RAB4A	-0.805506637	0.137930311	-5.839953733	5.22153E-09	1.65543E-08	negative
PRJNA729271	stage_E18_vs_E10	ordinary_muscle_development	ENSGALG00010010266	EFNA5	-1.327529225	0.215458583	-6.1614126	7.20989E-10	2.47238E-09	negative
PRJNA729271	stage_E18_vs_E10	ordinary_muscle_development	ENSGALG00010010267	CCSER2	0.338233229	0.107796583	3.13769897	0.001702797	0.00302066	positive
PRJNA729271	stage_E18_vs_E10	ordinary_muscle_development	ENSGALG00010010270	SSR1	-0.592749731	0.077775989	-7.621243284	2.51244E-14	1.20869E-13	negative
PRJNA729271	stage_E18_vs_E10	ordinary_muscle_development	ENSGALG00010010271	HUNK	-1.137348827	0.108884659	-10.44544604	1.53731E-25	1.50563E-24	negative
PRJNA729271	stage_E18_vs_E10	ordinary_muscle_development	ENSGALG00010010274	MIS18A	-1.045008007	0.093900641	-11.12886977	9.07792E-29	1.04515E-27	negative
PRJNA729271	stage_E18_vs_E10	ordinary_muscle_development	ENSGALG00010010275	PDE8B	-0.169956708	0.852795128	-0.199293714	0.842032996	0.869321103	negative
PRJNA729271	stage_E18_vs_E10	ordinary_muscle_development	ENSGALG00010010279	CCSAP	0.437463723	0.241353741	1.812541715	0.069902534	0.095639465	positive
PRJNA729271	stage_E18_vs_E10	ordinary_muscle_development	ENSGALG00010010280	HSP90B1	-0.696846605	0.135516	-5.142172169	2.7158E-07	7.40371E-07	negative
PRJNA729271	stage_E18_vs_E10	ordinary_muscle_development	ENSGALG00010010284	CCDC25	0.140897118	0.095348678	1.477703944	0.139487038	0.179865192	positive
PRJNA729271	stage_E18_vs_E10	ordinary_muscle_development	ENSGALG00010010285	ENSGALG00010010285	0.874398392	0.609476673	1.434670809	0.151380926	0.193583284	positive
PRJNA729271	stage_E18_vs_E10	ordinary_muscle_development	ENSGALG00010010287	STAM2	-0.292466199	0.097789422	-2.990775417	0.002782701	0.004790978	negative
PRJNA729271	stage_E18_vs_E10	ordinary_muscle_development	ENSGALG00010010288	ENSGALG00010010288	-0.519959028	0.164147438	-3.16763414	0.001536848	0.002738366	negative
PRJNA729271	stage_E18_vs_E10	ordinary_muscle_development	ENSGALG00010010290	ENSGALG00010010290	1.928469036	0.161441643	11.94530107	6.87011E-33	9.5112E-32	positive
PRJNA729271	stage_E18_vs_E10	ordinary_muscle_development	ENSGALG00010010291	TCF20	-0.274720241	0.117536244	-2.33732364	0.019422362	0.029353263	negative
PRJNA729271	stage_E18_vs_E10	ordinary_muscle_development	ENSGALG00010010293	ENSGALG00010010293	-2.541219242	0.861157441	-2.950934547	0.00316814	0.005408338	negative
PRJNA729271	stage_E18_vs_E10	ordinary_muscle_development	ENSGALG00010010295	ENSGALG00010010295	6.324872342	0.181419567	34.86323131	2.6836E-266	2.9922E-263	positive
PRJNA729271	stage_E18_vs_E10	ordinary_muscle_development	ENSGALG00010010298	KCNIP4	-1.180290714	0.412095635	-2.864118458	0.004181716	0.007002538	negative
PRJNA729271	stage_E18_vs_E10	ordinary_muscle_development	ENSGALG00010010300	DENND1B	0.950827643	0.093995909	10.11562792	4.70987E-24	4.20638E-23	positive
PRJNA729271	stage_E18_vs_E10	ordinary_muscle_development	ENSGALG00010010301	ENSGALG00010010301	-1.358804891	0.551615259	-2.463319982	0.0137657	0.021354219	negative
PRJNA729271	stage_E18_vs_E10	ordinary_muscle_development	ENSGALG00010010302	WDR36	-0.928368632	0.105158542	-8.828275978	1.06302E-18	6.95011E-18	negative
PRJNA729271	stage_E18_vs_E10	ordinary_muscle_development	ENSGALG00010010303	ETF1	-0.518534126	0.184863432	-2.804957804	0.005032317	0.008341158	negative
PRJNA729271	stage_E18_vs_E10	ordinary_muscle_development	ENSGALG00010010305	URB1	-1.091567884	0.103496644	-10.54689162	5.25055E-26	5.25962E-25	negative
PRJNA729271	stage_E18_vs_E10	ordinary_muscle_development	ENSGALG00010010308	PACRGL	-0.738822153	0.224584862	-3.289723736	0.001002858	0.001826184	negative
PRJNA729271	stage_E18_vs_E10	ordinary_muscle_development	ENSGALG00010010310	NT5DC3	1.381369062	0.118679153	11.6395258	2.59454E-31	3.3881E-30	positive
PRJNA729271	stage_E18_vs_E10	ordinary_muscle_development	ENSGALG00010010311	CTSV	0.49968715	0.189584639	2.635694282	0.008396534	0.013435011	positive
PRJNA729271	stage_E18_vs_E10	ordinary_muscle_development	ENSGALG00010010313	GRID1	-2.044543513	0.198406651	-10.30481337	6.70216E-25	6.32885E-24	negative
PRJNA729271	stage_E18_vs_E10	ordinary_muscle_development	ENSGALG00010010314	CRB1	-0.023453257	0.281496307	-0.083316393	0.933599966	0.94583334	negative
PRJNA729271	stage_E18_vs_E10	ordinary_muscle_development	ENSGALG00010010317	ENSGALG00010010317	0.061464858	0.107823981	0.570048124	0.568645058	0.622358058	positive
PRJNA729271	stage_E18_vs_E10	ordinary_muscle_development	ENSGALG00010010320	SYPL1	-1.878703402	0.701084796	-2.679709235	0.007368614	0.011880762	negative
PRJNA729271	stage_E18_vs_E10	ordinary_muscle_development	ENSGALG00010010321	NUP133	-0.891798848	0.07298861	-12.21832895	2.48133E-34	3.71175E-33	negative
PRJNA729271	stage_E18_vs_E10	ordinary_muscle_development	ENSGALG00010010322	ENSGALG00010010322	0.343676186	0.067302616	5.10643129	3.283E-07	8.89811E-07	positive
PRJNA729271	stage_E18_vs_E10	ordinary_muscle_development	ENSGALG00010010323	FBP2	5.013832173	0.242409882	20.68328294	4.89907E-95	5.07229E-93	positive
PRJNA729271	stage_E18_vs_E10	ordinary_muscle_development	ENSGALG00010010327	FBP1	1.030430292	0.576623842	1.787006046	0.073936511	0.100592241	positive
PRJNA729271	stage_E18_vs_E10	ordinary_muscle_development	ENSGALG00010010330	ALPK2	2.16485159	0.153284334	14.12311049	2.73641E-45	6.50235E-44	positive
PRJNA729271	stage_E18_vs_E10	ordinary_muscle_development	ENSGALG00010010333	WAPL	-0.078924872	0.063779915	-1.237456513	0.21591765	0.26647308	negative
PRJNA729271	stage_E18_vs_E10	ordinary_muscle_development	ENSGALG00010010334	ZBTB41	0.343677654	0.086988827	3.95082524	7.78822E-05	0.000164196	positive
PRJNA729271	stage_E18_vs_E10	ordinary_muscle_development	ENSGALG00010010336	FMNL2	-0.43397552	0.088093304	-4.926316757	8.37941E-07	2.18531E-06	negative
PRJNA729271	stage_E18_vs_E10	ordinary_muscle_development	ENSGALG00010010337	SCARA3	0.193338062	0.154190359	1.253892021	0.209881256	0.259797506	positive
PRJNA729271	stage_E18_vs_E10	ordinary_muscle_development	ENSGALG00010010338	CNST	0.182384986	0.158991691	1.147135332	0.251325693	0.304952781	positive
PRJNA729271	stage_E18_vs_E10	ordinary_muscle_development	ENSGALG00010010345	ENSGALG00010010345	0.008905058	0.138306802	0.064386259	0.948662671	0.957647846	positive
PRJNA729271	stage_E18_vs_E10	ordinary_muscle_development	ENSGALG00010010346	TFB2M	-0.405749931	0.142401328	-2.849340928	0.004380991	0.007315952	negative
PRJNA729271	stage_E18_vs_E10	ordinary_muscle_development	ENSGALG00010010347	FBXW11	-0.43107586	0.084635561	-5.093318406	3.5185E-07	9.50795E-07	negative
PRJNA729271	stage_E18_vs_E10	ordinary_muscle_development	ENSGALG00010010349	RREB1	1.314164091	0.081846747	16.05639981	5.15624E-58	1.92628E-56	positive
PRJNA729271	stage_E18_vs_E10	ordinary_muscle_development	ENSGALG00010010351	NAMPTP1	0.577920871	0.213229103	2.710328291	0.006721664	0.010909251	positive
PRJNA729271	stage_E18_vs_E10	ordinary_muscle_development	ENSGALG00010010352	MALT1	-0.08560347	0.115781149	-0.739355846	0.459690946	0.519023233	negative
PRJNA729271	stage_E18_vs_E10	ordinary_muscle_development	ENSGALG00010010356	STAB2	0.639306366	0.281927333	2.267628185	0.023351878	0.034798548	positive
PRJNA729271	stage_E18_vs_E10	ordinary_muscle_development	ENSGALG00010010358	ASPM	-1.190738661	0.181375927	-6.56503141	5.20219E-11	1.94245E-10	negative
PRJNA729271	stage_E18_vs_E10	ordinary_muscle_development	ENSGALG00010010361	PIGM	-0.334692776	0.162244095	-2.062896501	0.039122463	0.055991321	negative
PRJNA729271	stage_E18_vs_E10	ordinary_muscle_development	ENSGALG00010010366	CLU	1.08379243	0.236078835	4.590807255	4.41535E-06	1.06348E-05	positive
PRJNA729271	stage_E18_vs_E10	ordinary_muscle_development	ENSGALG00010010368	ENSGALG00010010368	0.190612554	0.079822705	2.38794906	0.01694269	0.025880945	positive
PRJNA729271	stage_E18_vs_E10	ordinary_muscle_development	ENSGALG00010010369	ENSGALG00010010369	0.116346556	0.24422424	0.476392337	0.633794893	0.68334253	positive
PRJNA729271	stage_E18_vs_E10	ordinary_muscle_development	ENSGALG00010010371	SOCS1L	-1.660211701	0.209861765	-7.910977509	2.55376E-15	1.33154E-14	negative
PRJNA729271	stage_E18_vs_E10	ordinary_muscle_development	ENSGALG00010010374	CMTM4	-0.310242879	0.112663345	-2.7537162	0.005892284	0.009653968	negative
PRJNA729271	stage_E18_vs_E10	ordinary_muscle_development	ENSGALG00010010379	SLC35F6	0.147279985	0.189382371	0.777685819	0.436754254	0.496412837	positive
PRJNA729271	stage_E18_vs_E10	ordinary_muscle_development	ENSGALG00010010381	MAST4	0.744083847	0.131984498	5.637660915	1.72376E-08	5.21516E-08	positive
PRJNA729271	stage_E18_vs_E10	ordinary_muscle_development	ENSGALG00010010382	SEC11C	0.24351924	0.154580209	1.575358464	0.115173726	0.151053489	positive
PRJNA729271	stage_E18_vs_E10	ordinary_muscle_development	ENSGALG00010010383	SLIT2	-0.917132337	0.160855502	-5.701591346	1.18694E-08	3.64044E-08	negative
PRJNA729271	stage_E18_vs_E10	ordinary_muscle_development	ENSGALG00010010386	PRPF40A	-0.407180062	0.147182243	-2.766502631	0.005666113	0.009313938	negative
PRJNA729271	stage_E18_vs_E10	ordinary_muscle_development	ENSGALG00010010390	KCNK3	2.723478703	0.411725726	6.614788759	3.72083E-11	1.40123E-10	positive
PRJNA729271	stage_E18_vs_E10	ordinary_muscle_development	ENSGALG00010010393	FANCC	-1.62229532	0.144441221	-11.23152595	2.85505E-29	3.37002E-28	negative
PRJNA729271	stage_E18_vs_E10	ordinary_muscle_development	ENSGALG00010010394	MRPL42	0.128637574	0.124712705	1.031471287	0.302319866	0.359426383	positive
PRJNA729271	stage_E18_vs_E10	ordinary_muscle_development	ENSGALG00010010395	GRP	-0.784626402	0.900349984	-0.871468225	0.383498548	0.443817282	negative
PRJNA729271	stage_E18_vs_E10	ordinary_muscle_development	ENSGALG00010010397	PICK1	-0.566270799	0.093677317	-6.044908379	1.49495E-09	4.96888E-09	negative
PRJNA729271	stage_E18_vs_E10	ordinary_muscle_development	ENSGALG00010010399	FGF18	0.78676254	0.180255952	4.364696595	1.27299E-05	2.93308E-05	positive
PRJNA729271	stage_E18_vs_E10	ordinary_muscle_development	ENSGALG00010010403	ENSGALG00010010403	-1.185546765	0.460940282	-2.572018138	0.010110759	0.0159838	negative
PRJNA729271	stage_E18_vs_E10	ordinary_muscle_development	ENSGALG00010010404	COP1	0.287196899	0.116469428	2.465856523	0.013668607	0.021210685	positive
PRJNA729271	stage_E18_vs_E10	ordinary_muscle_development	ENSGALG00010010405	EVA1C	1.217315501	0.244602036	4.976718591	6.46712E-07	1.7016E-06	positive
PRJNA729271	stage_E18_vs_E10	ordinary_muscle_development	ENSGALG00010010406	PTCH1	-1.272088401	0.095799358	-13.27867358	3.07798E-40	5.94079E-39	negative
PRJNA729271	stage_E18_vs_E10	ordinary_muscle_development	ENSGALG00010010407	SOX10	0.450845082	0.288778343	1.561215005	0.11847303	0.155080603	positive
PRJNA729271	stage_E18_vs_E10	ordinary_muscle_development	ENSGALG00010010408	GAS8	-0.282323075	0.118314831	-2.38620191	0.017023402	0.025990542	negative
PRJNA729271	stage_E18_vs_E10	ordinary_muscle_development	ENSGALG00010010410	CACNA2D1	1.703210981	0.158205052	10.76584443	4.9902E-27	5.29911E-26	positive
PRJNA729271	stage_E18_vs_E10	ordinary_muscle_development	ENSGALG00010010411	CD180	-0.037967944	0.477318079	-0.079544325	0.936599677	0.948045553	negative
PRJNA729271	stage_E18_vs_E10	ordinary_muscle_development	ENSGALG00010010416	SLC35B3	-0.544124375	0.128287688	-4.241438776	2.22091E-05	4.97484E-05	negative
PRJNA729271	stage_E18_vs_E10	ordinary_muscle_development	ENSGALG00010010417	OTOF	0.565619826	0.656101578	0.862091855	0.388636973	0.448796441	positive
PRJNA729271	stage_E18_vs_E10	ordinary_muscle_development	ENSGALG00010010418	BRINP2	-2.761542071	0.438584446	-6.296488849	3.04463E-10	1.0722E-09	negative
PRJNA729271	stage_E18_vs_E10	ordinary_muscle_development	ENSGALG00010010420	NPM1	0.131377902	0.11224662	1.170439718	0.24182406	0.2948801	positive
PRJNA729271	stage_E18_vs_E10	ordinary_muscle_development	ENSGALG00010010421	POLR2F	0.214782346	0.091299708	2.352497627	0.018647809	0.028241562	positive
PRJNA729271	stage_E18_vs_E10	ordinary_muscle_development	ENSGALG00010010422	CDH1	-3.350575167	0.906802683	-3.69493301	0.000219945	0.000438468	negative
PRJNA729271	stage_E18_vs_E10	ordinary_muscle_development	ENSGALG00010010423	C21orf59	-0.079595524	0.100600288	-0.791205727	0.42882395	0.488894381	negative
PRJNA729271	stage_E18_vs_E10	ordinary_muscle_development	ENSGALG00010010425	BLOC1S5	0.634792079	0.079017831	8.033529563	9.47078E-16	5.10141E-15	positive
PRJNA729271	stage_E18_vs_E10	ordinary_muscle_development	ENSGALG00010010430	ASTN1	-4.534571117	0.530901126	-8.541272367	1.32752E-17	8.08504E-17	negative
PRJNA729271	stage_E18_vs_E10	ordinary_muscle_development	ENSGALG00010010431	SOCS2	-2.25954403	0.266103104	-8.491235158	2.04452E-17	1.22714E-16	negative
PRJNA729271	stage_E18_vs_E10	ordinary_muscle_development	ENSGALG00010010433	ENSGALG00010010433	-0.477132164	1.614098565	-0.295602867	0.767533384	0.804788513	negative
PRJNA729271	stage_E18_vs_E10	ordinary_muscle_development	ENSGALG00010010434	PROM1	-1.029760403	0.146822286	-7.013651876	2.32177E-12	9.64575E-12	negative
PRJNA729271	stage_E18_vs_E10	ordinary_muscle_development	ENSGALG00010010435	GAS1	-0.693825979	0.165752823	-4.185907451	2.84029E-05	6.28581E-05	negative
PRJNA729271	stage_E18_vs_E10	ordinary_muscle_development	ENSGALG00010010438	LDB3	3.56233212	0.18524021	19.23087934	2.04137E-82	1.51742E-80	positive
PRJNA729271	stage_E18_vs_E10	ordinary_muscle_development	ENSGALG00010010439	HGF	1.646811784	0.238851015	6.894723827	5.39695E-12	2.17968E-11	positive
PRJNA729271	stage_E18_vs_E10	ordinary_muscle_development	ENSGALG00010010444	TXNDC5	-0.523534179	0.139512909	-3.752585925	0.00017502	0.000353628	negative
PRJNA729271	stage_E18_vs_E10	ordinary_muscle_development	ENSGALG00010010445	LMAN1L	-0.409932437	0.083128644	-4.931301857	8.16834E-07	2.1318E-06	negative
PRJNA729271	stage_E18_vs_E10	ordinary_muscle_development	ENSGALG00010010446	C22orf23	-0.028075489	0.601055495	-0.046710311	0.962744112	0.969432158	negative
PRJNA729271	stage_E18_vs_E10	ordinary_muscle_development	ENSGALG00010010447	BTF3	-0.031758087	0.096837142	-0.327953573	0.742946766	0.783314909	negative
PRJNA729271	stage_E18_vs_E10	ordinary_muscle_development	ENSGALG00010010448	GALNT13	-0.304445726	0.363516575	-0.837501635	0.402310658	0.462229946	negative
PRJNA729271	stage_E18_vs_E10	ordinary_muscle_development	ENSGALG00010010449	PAPPA2	-5.270902396	0.863437224	-6.104557748	1.03086E-09	3.47495E-09	negative
PRJNA729271	stage_E18_vs_E10	ordinary_muscle_development	ENSGALG00010010451	RANBP17	0.139940335	0.467738949	0.299184695	0.764799126	0.802676369	positive
PRJNA729271	stage_E18_vs_E10	ordinary_muscle_development	ENSGALG00010010453	DRC1	-0.45006174	0.410019043	-1.097660579	0.272352729	0.327532798	negative
PRJNA729271	stage_E18_vs_E10	ordinary_muscle_development	ENSGALG00010010456	BMP6	-0.158192677	0.176581213	-0.895863574	0.370325631	0.430014421	negative
PRJNA729271	stage_E18_vs_E10	ordinary_muscle_development	ENSGALG00010010457	AGGF1	-0.514690902	0.092065988	-5.590456512	2.26473E-08	6.78391E-08	negative
PRJNA729271	stage_E18_vs_E10	ordinary_muscle_development	ENSGALG00010010460	SEMA3C	1.428113084	0.141977514	10.05872718	8.40783E-24	7.37794E-23	positive
PRJNA729271	stage_E18_vs_E10	ordinary_muscle_development	ENSGALG00010010461	SLC7A5	-0.769468337	0.262656569	-2.929560604	0.003394416	0.005748576	negative
PRJNA729271	stage_E18_vs_E10	ordinary_muscle_development	ENSGALG00010010463	ACAA2	0.93448276	0.12823328	7.287365322	3.16075E-13	1.40451E-12	positive
PRJNA729271	stage_E18_vs_E10	ordinary_muscle_development	ENSGALG00010010465	BMPR1A	-0.673328837	0.098043811	-6.86763224	6.52762E-12	2.60943E-11	negative
PRJNA729271	stage_E18_vs_E10	ordinary_muscle_development	ENSGALG00010010466	CRADD	-0.18134594	0.128871073	-1.407188875	0.15937139	0.20272824	negative
PRJNA729271	stage_E18_vs_E10	ordinary_muscle_development	ENSGALG00010010467	FGFBP2	-8.868964538	0.582880759	-15.21574422	2.7804E-52	8.78035E-51	negative
PRJNA729271	stage_E18_vs_E10	ordinary_muscle_development	ENSGALG00010010468	ENSGALG00010010468	3.736342694	1.33429959	2.800227717	0.005106657	0.008456642	positive
PRJNA729271	stage_E18_vs_E10	ordinary_muscle_development	ENSGALG00010010471	KCNJ3	0.563454359	0.149385962	3.771802597	0.000162072	0.000328887	positive
PRJNA729271	stage_E18_vs_E10	ordinary_muscle_development	ENSGALG00010010472	MMRN2	1.188714215	0.136971698	8.678538943	4.00885E-18	2.53306E-17	positive
PRJNA729271	stage_E18_vs_E10	ordinary_muscle_development	ENSGALG00010010473	TRIM35	-0.197688576	0.186226084	-1.06155149	0.288439346	0.344563073	negative
PRJNA729271	stage_E18_vs_E10	ordinary_muscle_development	ENSGALG00010010475	CCDC71L	0.215268572	0.11079376	1.942966577	0.052020197	0.07279011	positive
PRJNA729271	stage_E18_vs_E10	ordinary_muscle_development	ENSGALG00010010478	ENSGALG00010010478	1.041006778	0.208969352	4.981624179	6.30528E-07	1.65992E-06	positive
PRJNA729271	stage_E18_vs_E10	ordinary_muscle_development	ENSGALG00010010483	FGFBP1	-3.484008556	0.955994965	-3.644379607	0.000268038	0.000528959	negative
PRJNA729271	stage_E18_vs_E10	ordinary_muscle_development	ENSGALG00010010486	ENSGALG00010010486	-1.363319379	0.472176252	-2.887310347	0.003885508	0.006533693	negative

PRJNA729271	stage_E18_vs_E10	ordinary_muscle_development	ENSGALG00000010494	ENSGALG00000010494	2.447180345	0.261111391	9.37217001	7.10563E-21	5.30361E-20	positive
PRJNA729271	stage_E18_vs_E10	ordinary_muscle_development	ENSGALG00000010496	MRPL17	0.45114664	0.142591862	3.16390173	0.001556693	0.00277178	positive
PRJNA729271	stage_E18_vs_E10	ordinary_muscle_development	ENSGALG00000010499	NR4A2	1.680106112	0.473995115	3.544564191	0.000393263	0.000757722	positive
PRJNA729271	stage_E18_vs_E10	ordinary_muscle_development	ENSGALG00000010505	CD38	-0.06858897	0.217353756	-0.315563766	0.752333644	0.792117104	negative
PRJNA729271	stage_E18_vs_E10	ordinary_muscle_development	ENSGALG00000010511	GABRP	1.442427517	0.341799637	4.220096695	2.44198E-05	5.44728E-05	positive
PRJNA729271	stage_E18_vs_E10	ordinary_muscle_development	ENSGALG00000010512	PLXNC1	-0.636900313	0.200092148	-3.183035017	0.0014574	0.002603847	negative
PRJNA729271	stage_E18_vs_E10	ordinary_muscle_development	ENSGALG00000010515	RXRG	-0.727036516	0.380602411	-1.910225724	0.056104156	0.078060064	negative
PRJNA729271	stage_E18_vs_E10	ordinary_muscle_development	ENSGALG00000010517	STMN4	-5.748587388	0.368980485	-15.57965157	1.00099E-54	3.36643E-53	negative
PRJNA729271	stage_E18_vs_E10	ordinary_muscle_development	ENSGALG00000010521	SNCG	3.234231974	0.378681557	8.540769721	1.33331E-17	8.11688E-17	positive
PRJNA729271	stage_E18_vs_E10	ordinary_muscle_development	ENSGALG00000010526	ENSGALG00000010526	0.726727481	0.252390266	2.879379992	0.003984579	0.006690973	positive
PRJNA729271	stage_E18_vs_E10	ordinary_muscle_development	ENSGALG00000010528	GPD2	1.432281713	0.124855229	11.4715397	1.83367E-30	2.28933E-29	positive
PRJNA729271	stage_E18_vs_E10	ordinary_muscle_development	ENSGALG00000010534	SYNJ1	-0.022247517	0.124219862	-0.179097904	0.857860828	0.882431871	negative
PRJNA729271	stage_E18_vs_E10	ordinary_muscle_development	ENSGALG00000010535	LMX1A	-0.826852959	0.419755266	-1.969845348	0.048856098	0.068700925	negative
PRJNA729271	stage_E18_vs_E10	ordinary_muscle_development	ENSGALG00000010537	ENSGALG00000010537	0.074854562	0.223459394	0.334980598	0.737639722	0.778795817	positive
PRJNA729271	stage_E18_vs_E10	ordinary_muscle_development	ENSGALG00000010544	ENSGALG00000010544	0.773738951	0.156905998	4.931226073	8.17151E-07	2.13224E-06	positive
PRJNA729271	stage_E18_vs_E10	ordinary_muscle_development	ENSGALG00000010546	MICALL1	0.03794623	0.168075701	0.225768683	0.821381336	0.85153091	positive
PRJNA729271	stage_E18_vs_E10	ordinary_muscle_development	ENSGALG00000010547	BST1	0.907842347	0.165160346	5.496733127	3.86892E-08	1.13937E-07	positive
PRJNA729271	stage_E18_vs_E10	ordinary_muscle_development	ENSGALG00000010567	ERMN	-2.514801652	0.762651443	-3.297445609	0.000975686	0.001779163	negative
PRJNA729271	stage_E18_vs_E10	ordinary_muscle_development	ENSGALG00000010572	AQP3	1.762609892	0.146988498	11.99148177	3.93797E-33	5.49383E-32	positive
PRJNA729271	stage_E18_vs_E10	ordinary_muscle_development	ENSGALG00000010573	CEP83	-0.625467962	0.157726637	-3.965518916	7.32364E-05	0.000155018	negative
PRJNA729271	stage_E18_vs_E10	ordinary_muscle_development	ENSGALG00000010575	PAXBP1	-0.331833444	0.12383906	-2.679553952	0.007372032	0.011883631	negative
PRJNA729271	stage_E18_vs_E10	ordinary_muscle_development	ENSGALG00000010577	PBX1	-0.253209818	0.145800558	-1.736686206	0.082442574	0.111183951	negative
PRJNA729271	stage_E18_vs_E10	ordinary_muscle_development	ENSGALG00000010578	GLUD2	0.611124514	0.111532541	5.479338214	4.2692E-08	1.24964E-07	positive
PRJNA729271	stage_E18_vs_E10	ordinary_muscle_development	ENSGALG00000010585	FBXL5	-0.010684166	0.164083113	-0.065114356	0.94808295	0.957329318	negative
PRJNA729271	stage_E18_vs_E10	ordinary_muscle_development	ENSGALG00000010586	CYTIP	-0.769657191	0.282001982	-2.729261633	0.006347632	0.010355535	negative
PRJNA729271	stage_E18_vs_E10	ordinary_muscle_development	ENSGALG00000010588	SLC25A3	0.186929184	0.101077586	1.84936335	0.064405364	0.088589462	positive
PRJNA729271	stage_E18_vs_E10	ordinary_muscle_development	ENSGALG00000010593	EIF3L	0.984777965	0.103686948	9.49760782	2.14767E-21	1.65236E-20	positive
PRJNA729271	stage_E18_vs_E10	ordinary_muscle_development	ENSGALG00000010596	SHLD2	0.206353193	0.1345715	1.533409331	0.125175031	0.163137212	positive
PRJNA729271	stage_E18_vs_E10	ordinary_muscle_development	ENSGALG00000010599	F2RL1	-2.050921494	0.205375744	-9.986191426	1.75184E-23	1.50789E-22	negative
PRJNA729271	stage_E18_vs_E10	ordinary_muscle_development	ENSGALG00000010604	HIVEP1	-0.029057919	0.100611916	-0.288811901	0.772725324	0.809295778	negative
PRJNA729271	stage_E18_vs_E10	ordinary_muscle_development	ENSGALG00000010605	NUF2	-1.3429855	0.266876142	-5.032242638	4.84775E-07	1.29051E-06	negative
PRJNA729271	stage_E18_vs_E10	ordinary_muscle_development	ENSGALG00000010614	IKBIP	0.185375505	0.141428095	1.310740306	0.189945516	0.237390952	positive
PRJNA729271	stage_E18_vs_E10	ordinary_muscle_development	ENSGALG00000010618	APAF1	-0.569797768	0.137615	-4.140520783	3.46518E-05	7.58385E-05	negative
PRJNA729271	stage_E18_vs_E10	ordinary_muscle_development	ENSGALG00000010619	LRRC52	-0.035856443	0.263026418	-0.136322592	0.891566265	0.910344675	negative
PRJNA729271	stage_E18_vs_E10	ordinary_muscle_development	ENSGALG00000010620	ANKRD54	0.227815926	0.11122691	2.048208712	0.040539553	0.057831003	positive
PRJNA729271	stage_E18_vs_E10	ordinary_muscle_development	ENSGALG00000010623	SMAD2	-0.314109011	0.07759142	-4.048244164	5.16033E-05	0.000110928	negative
PRJNA729271	stage_E18_vs_E10	ordinary_muscle_development	ENSGALG00000010625	FAM169A	-3.545222077	0.266956142	-13.28016673	3.01722E-40	5.83906E-39	negative
PRJNA729271	stage_E18_vs_E10	ordinary_muscle_development	ENSGALG00000010626	ADTRP	0.794952115	0.63457157	1.252738307	0.21030096	0.260250356	positive
PRJNA729271	stage_E18_vs_E10	ordinary_muscle_development	ENSGALG00000010629	ENSGALG00000010629	0.09197291	0.317316934	0.289845578	0.771934377	0.808818043	positive
PRJNA729271	stage_E18_vs_E10	ordinary_muscle_development	ENSGALG00000010633	EPCIP	0.985706918	0.335247015	2.940240696	0.003279574	0.005572969	positive
PRJNA729271	stage_E18_vs_E10	ordinary_muscle_development	ENSGALG00000010635	ALDH9A1	-0.31377969	0.076808378	-4.085227399	4.40337E-05	9.5335E-05	negative
PRJNA729271	stage_E18_vs_E10	ordinary_muscle_development	ENSGALG00000010638	ACVR1C	1.275130512	0.434353211	2.935699517	0.003327965	0.005648578	positive
PRJNA729271	stage_E18_vs_E10	ordinary_muscle_development	ENSGALG00000010642	CC2D2A	0.027069056	0.119195755	0.227097484	0.820347941	0.850629043	positive
PRJNA729271	stage_E18_vs_E10	ordinary_muscle_development	ENSGALG00000010645	FAM219A	0.086844255	0.086607176	1.002737406	0.315987577	0.373439864	positive
PRJNA729271	stage_E18_vs_E10	ordinary_muscle_development	ENSGALG00000010646	MCM3AP	-0.742890018	0.058972749	-12.59717456	2.18844E-36	3.62531E-35	negative
PRJNA729271	stage_E18_vs_E10	ordinary_muscle_development	ENSGALG00000010648	CENPM	-1.452828937	0.250102524	-5.808933536	6.2872E-09	1.98115E-08	negative
PRJNA729271	stage_E18_vs_E10	ordinary_muscle_development	ENSGALG00000010651	NUAK1	-0.191508265	0.134461739	-1.424258432	0.154371676	0.196938694	negative
PRJNA729271	stage_E18_vs_E10	ordinary_muscle_development	ENSGALG00000010655	C1QTNF7	0.885205905	0.249486098	3.548117157	0.000387996	0.00074827	positive
PRJNA729271	stage_E18_vs_E10	ordinary_muscle_development	ENSGALG00000010657	TMEM170B	-0.977489794	0.179078317	-5.458448622	4.80313E-08	1.39942E-07	negative
PRJNA729271	stage_E18_vs_E10	ordinary_muscle_development	ENSGALG00000010659	ADAMTS1	1.553428182	0.151454406	10.25673816	1.10372E-24	1.0295E-23	positive
PRJNA729271	stage_E18_vs_E10	ordinary_muscle_development	ENSGALG00000010661	CPEB2	-0.204814283	0.187610252	-1.091700907	0.274964586	0.330372321	negative
PRJNA729271	stage_E18_vs_E10	ordinary_muscle_development	ENSGALG00000010662	MGST3	-0.592550415	0.152661898	-3.881455829	0.000103833	0.000216213	negative
PRJNA729271	stage_E18_vs_E10	ordinary_muscle_development	ENSGALG00000010663	EFR3B	-0.133606441	0.16733788	-0.798423175	0.424624959	0.485022757	negative
PRJNA729271	stage_E18_vs_E10	ordinary_muscle_development	ENSGALG00000010666	C12orf75	2.091248019	0.376432726	5.555436265	2.76919E-08	8.25745E-08	positive
PRJNA729271	stage_E18_vs_E10	ordinary_muscle_development	ENSGALG00000010671	SIGMAR1	-0.399673266	0.140995252	-2.834657627	0.004587482	0.007642288	negative
PRJNA729271	stage_E18_vs_E10	ordinary_muscle_development	ENSGALG00000010678	KCNIP1	4.209226214	0.341450784	12.32747561	6.44338E-35	9.88326E-34	positive
PRJNA729271	stage_E18_vs_E10	ordinary_muscle_development	ENSGALG00000010680	ENSGALG00000010680	-0.977014187	0.638645027	-1.529823525	0.126060417	0.164217286	negative
PRJNA729271	stage_E18_vs_E10	ordinary_muscle_development	ENSGALG00000010682	SMIM13	-0.590084594	0.105912872	-5.571415303	2.52678E-08	7.54702E-08	negative
PRJNA729271	stage_E18_vs_E10	ordinary_muscle_development	ENSGALG00000010685	STIM2	-0.386499883	0.109750208	-3.52163234	0.000428899	0.00082321	negative
PRJNA729271	stage_E18_vs_E10	ordinary_muscle_development	ENSGALG00000010688	IQGAP2	-1.277498662	0.170035377	-7.513134547	5.77281E-14	2.70362E-13	negative
PRJNA729271	stage_E18_vs_E10	ordinary_muscle_development	ENSGALG00000010689	POFUT2	0.129192945	0.149714868	0.862926618	0.388177814	0.44837337	positive
PRJNA729271	stage_E18_vs_E10	ordinary_muscle_development	ENSGALG00000010690	MTPN	-0.413118349	0.135329271	-3.052690276	0.002267999	0.00394701	negative
PRJNA729271	stage_E18_vs_E10	ordinary_muscle_development	ENSGALG00000010693	KCNMB1	0.076206453	0.34491998	0.220939516	0.825139528	0.854619326	positive
PRJNA729271	stage_E18_vs_E10	ordinary_muscle_development	ENSGALG00000010697	NEDD9	-0.399743355	0.139190787	-2.871909578	0.004079997	0.006841688	negative
PRJNA729271	stage_E18_vs_E10	ordinary_muscle_development	ENSGALG00000010698	GPRIN2	-1.552332466	0.185409084	-8.372472546	5.64217E-17	3.29505E-16	negative
PRJNA729271	stage_E18_vs_E10	ordinary_muscle_development	ENSGALG00000010699	DNAJC27	0.106802797	0.151050553	0.707066573	0.479525108	0.537897882	positive
PRJNA729271	stage_E18_vs_E10	ordinary_muscle_development	ENSGALG00000010706	SREBF2	-0.804069197	0.140276567	-5.732027926	9.92369E-09	3.06638E-08	negative
PRJNA729271	stage_E18_vs_E10	ordinary_muscle_development	ENSGALG00000010710	UCK2	-0.445216462	0.101525553	-4.385265099	1.15845E-05	2.6781E-05	negative
PRJNA729271	stage_E18_vs_E10	ordinary_muscle_development	ENSGALG00000010711	ENSGALG00000010711	-2.210255351	0.448207998	-4.93131618	8.16774E-07	2.1318E-06	negative
PRJNA729271	stage_E18_vs_E10	ordinary_muscle_development	ENSGALG00000010712	ADAMTS5	0.770323084	0.145241669	5.303733359	1.13458E-07	3.20268E-07	positive
PRJNA729271	stage_E18_vs_E10	ordinary_muscle_development	ENSGALG00000010713	NSA2	0.239833987	0.09082603	2.640586476	0.008276267	0.013251352	positive
PRJNA729271	stage_E18_vs_E10	ordinary_muscle_development	ENSGALG00000010719	SH3BP4	0.631104017	0.164923578	3.826645202	0.000129902	0.000267233	positive
PRJNA729271	stage_E18_vs_E10	ordinary_muscle_development	ENSGALG00000010722	TBC1D19	-0.337043769	0.165877809	-2.031879803	0.042165826	0.059909199	negative
PRJNA729271	stage_E18_vs_E10	ordinary_muscle_development	ENSGALG00000010724	ADCY3	0.493012274	0.144778579	3.405284671	0.000660951	0.001235873	positive
PRJNA729271	stage_E18_vs_E10	ordinary_muscle_development	ENSGALG00000010728	ELOVL2	-0.429871085	0.951675379	-0.451699282	0.651485636	0.699694333	negative
PRJNA729271	stage_E18_vs_E10	ordinary_muscle_development	ENSGALG00000010732	CCKAR	-6.583188259	1.165005816	-5.650777162	1.59724E-08	4.85163E-08	negative
PRJNA729271	stage_E18_vs_E10	ordinary_muscle_development	ENSGALG00000010733	GFM2	-0.78587824	0.128923064	-6.095714866	1.08949E-09	3.66409E-09	negative
PRJNA729271	stage_E18_vs_E10	ordinary_muscle_development	ENSGALG00000010734	AMN1	1.004587038	0.143612746	6.995110563	2.6505E-12	1.09737E-11	positive
PRJNA729271	stage_E18_vs_E10	ordinary_muscle_development	ENSGALG00000010740	CCDC134	0.885845515	0.096209017	9.207510257	3.33777E-20	2.36929E-19	positive
PRJNA729271	stage_E18_vs_E10	ordinary_muscle_development	ENSGALG00000010742	LCP2	0.846394611	0.266832195	3.172010827	0.001513874	0.002699754	positive
PRJNA729271	stage_E18_vs_E10	ordinary_muscle_development	ENSGALG00000010743	KLHL20	-0.124358775	0.084662228	-1.468881442	0.141864949	0.182588183	negative
PRJNA729271	stage_E18_vs_E10	ordinary_muscle_development	ENSGALG00000010745	ATVR1	-0.07037096	0.063701305	-1.10470201	0.269288732	0.324332378	negative
PRJNA729271	stage_E18_vs_E10	ordinary_muscle_development	ENSGALG00000010748	SF3B1	-0.109185798	0.071857575	-1.519475126	0.128642945	0.167160868	negative
PRJNA729271	stage_E18_vs_E10	ordinary_muscle_development	ENSGALG00000010749	NCOA4	-0.302396643	0.098515783	-3.069524825	0.002143996	0.00374876	negative
PRJNA729271	stage_E18_vs_E10	ordinary_muscle_development	ENSGALG00000010751	SLC30A5	-0.296813288	0.07919315	-3.747966703	0.000178274	0.00035985	negative
PRJNA729271	stage_E18_vs_E10	ordinary_muscle_development	ENSGALG00000010752	ENSGALG00000010752	-0.285175599	0.256142447	-1.113347682	0.265559135	0.320372839	negative
PRJNA729271	stage_E18_vs_E10	ordinary_muscle_development	ENSGALG00000010753	ENSGALG00000010753	1.394400077	1.161120848	1.200908656			positive
PRJNA729271	stage_E18_vs_E10	ordinary_muscle_development	ENSGALG00000010754	DNAI1	-1.616805452	0.272604185	-5.930963436	3.01162E-09	9.75497E-09	negative
PRJNA729271	stage_E18_vs_E10	ordinary_muscle_development	ENSGALG00000010756	CENPL	-1.799727801	0.197386036	-9.117807099	7.66597E-20	5.34994E-19	negative
PRJNA729271	stage_E18_vs_E10	ordinary_muscle_development	ENSGALG00000010759	ENSGALG00000010759	-0.080550284	0.278862809	-0.28885273	0.772694079	0.809295778	negative
PRJNA729271	stage_E18_vs_E10	ordinary_muscle_development	ENSGALG00000010760	FOXI1	-0.882738687	1.029542281	-0.857408874	0.391218957	0.451310687	negative
PRJNA729271	stage_E18_vs_E10	ordinary_muscle_development	ENSGALG00000010761	MEI1	1.170381054	0.257172824	4.55095152	5.34039E-06	1.27801E-05	positive
PRJNA729271	stage_E18_vs_E10	ordinary_muscle_development	ENSGALG00000010762	MRPL39	-0.042041933	0.111791231	-0.376075408	0.706860836	0.751389544	negative
PRJNA729271	stage_E18_vs_E10	ordinary_muscle_development	ENSGALG00000010765	ENSGALG00000010765	2.804160194	0.820932436	3.415823364	0.000635895	0.001190865	positive
PRJNA729271	stage_E18_vs_E10	ordinary_muscle_development	ENSGALG00000010766	CENPO	-1.658260865	0.237802003	-6.973283826	3.09627E-12	1.27465E-11	negative
PRJNA729271	stage_E18_vs_E10	ordinary_muscle_development	ENSGALG00000010768	ENSGALG00000010768	-0.159944157	0.092731907	-1.724801773	0.084563202	0.113705345	negative
PRJNA729271	stage_E18_vs_E10	ordinary_muscle_development	ENSGALG00000010769	HEXB	-0.442949493	0.12313828	-3.597171364	0.000321697	0.000627083	negative
PRJNA729271	stage_E18_vs_E10	ordinary_muscle_development	ENSGALG00000010771	ENSGALG00000010771	-0.399617407	0.072169503	-5.537206008	3.07335E-08	9.12686E-08	negative
PRJNA729271	stage_E18_vs_E10	ordinary_muscle_development	ENSGALG00000010774	KPTN	-0.279161825	0.13728722	-2.033414503	0.042010663	0.059700447	negative
PRJNA729271	stage_E18_vs_E10	ordinary_muscle_development	ENSGALG00000010776	GCAT	-0.31324841	0.195457477	-1.60264225	0.10901366	0.14362416	negative
PRJNA729271	stage_E18_vs_E10	ordinary_muscle_development	ENSGALG00000010778	UPP2	1.150810734	0.39484452	2.914592138	0.003561537	0.006016838	positive
PRJNA729271	stage_E18_vs_E10	ordinary_muscle_development	ENSGALG00000010779	DOCK2	-0.697312635	0.192624995	-3.62005272	0.000294543	0.00057757	negative
PRJNA729271	stage_E18_vs_E10	ordinary_muscle_development	ENSGALG00000010780	PARG	-0.778789911	0.050008634	-15.57310897	1.10884E-54	3.71193E-53	negative
PRJNA729271	stage_E18_vs_E10	ordinary_muscle_development	ENSGALG00000010781	NAPA	0.052803933	0.098303691	0.537151069	0.591163267	0.64311855	positive
PRJNA729271	stage_E18_vs_E10	ordinary_muscle_development	ENSGALG00000010783	F2RL2	0.010100217	0.143706933	0.070283436	0.943968066	0.954171347	positive
PRJNA729271	stage_E18_vs_E10	ordinary_muscle_development	ENSGALG00000010784	CCDC148	2.148942712	0.28608647	7.511514663	5.84471E-14	2.73552E-13	positive
PRJNA729271	stage_E18_vs_E10	ordinary_muscle_development	ENSGALG00000010786	NABP1	-0.440058888	0.155839116	-2.823802513	0.004745762	0.007886027	negative
PRJNA729271	stage_E18_vs_E10	ordinary_muscle_development	ENSGALG00000010789	ENSGALG00000010789	-0.15878702	0.272876786	-0.581900067	0.560633995	0.614564755	negative
PRJNA729271	stage_E18_vs_E10	ordinary_muscle_development	ENSGALG00000010791	H-10	0.854622452	0.293115548	2.915650362	0.00354948	0.005999267	positive
PRJNA729271	stage_E18_vs_E10	ordinary_muscle_development	ENSGALG00000010792	XRCC6	-1.009786186	0.149579564	-6.750829859	1.47002E-11	5.71369E-11	negative
PRJNA729271	stage_E18_vs_E10	ordinary_muscle_development	ENSGALG00000010796	CAVIN2	2.574760377	0.145973291	17.63857181	1.2457E-69	6.58992E-68	positive
PRJNA729271	stage_E18_vs_E10	ordinary_muscle_development	ENSGALG00000010799	DARS2	0.156263118	0.105957984	1.474764923	0.140275755	0.180753584	positive
PRJNA729271	stage_E18_vs_E10	ordinary_muscle_development	ENSGALG00000010800	BICRA	0.629385347	0.19158138	3.285211469	0.001019059	0.001854287	positive
PRJNA729271	stage_E18_vs_E10	ordinary_muscle_development	ENSGALG00000010805	PTRHD1	-1.078332953	0.177049195	-6.090583782	1.125E-09	3.77998E-09	negative
PRJNA729271	stage_E18_vs_E10	ordinary_muscle_development	ENSGALG00000010806	INSYN2B	-1.420170156	0.938532808	-1.513181153	0.13023365	0.168864546	negative
PRJNA729271	stage_E18_vs_E10	ordinary_muscle_development	ENSGALG00000010809	CENPH	-1.63114211	0.154768281	-10.53925326	5.69493E-26	5.68904E-25	negative
PRJNA729271	stage_E18_vs_E10	ordinary_muscle_development	ENSGALG00000010810	GALT	0.694999326	0.111788616	6.217084975	5.06476E-10	1.75925E-09	positive
PRJNA729271	stage_E18_vs_E10	ordinary_muscle_development	ENSGALG00000010813	EHD2	1.818506159	0.149381329	12.17358404	4.29788E-34	6.35691E-33	positive
PRJNA729271	stage_E18_vs_E10	ordinary_muscle_development	ENSGALG00000010816	AP2S1	-0.57744383	0.112119876	-5.150236084	2.60159E-07	7.1017E-07	negative
PRJNA729271	stage_E18_vs_E10	ordinary_muscle_development	ENSGALG00000010821	RGS8	-2.64733803	0.48791788	-5.425786058	5.77E-08	1.66672E-07	negative
PRJNA729271	stage_E18_vs_E10	ordinary_muscle_development	ENSGALG00000010822	OGDHL	-1.993326133	0.426419586	-4.674565146	2.94577E-06	7.22853E-06	negative
PRJNA729271	stage_E18_vs_E10	ordinary_muscle_development	ENSGALG00000010823	TMEFF2	-0.432709324	0.163613059	-2.644711405	0.008176062	0.013101041	negative
PRJNA729271	stage_E18_vs_E10	ordinary_muscle_development	ENSGALG00000010824	PKP4	-0.474489337	0.110459489	-4.295595981	1.74225E-05	3.95519E-05	negative
PRJNA729271	stage_E18_vs_E10	ordinary_muscle_development	ENSGALG00000010825	MAK	0.833135453	0.4076318	2.043843129	0.040969047	0.058386229	positive
PRJNA729271	stage_E18_vs_E10	ordinary_muscle_development	ENSGALG00000010826	NCOA1	0.68163317	0.161788749	4.213106115	2.51883E-05	5.6118E-05	positive
PRJNA729271	stage_E18_vs_E10	ordinary_muscle_development	ENSGALG00000010828	SPDL1	-1.147462471	0.12664167	-9.060702284	1.29613E-19	8.91245E-19	negative
PRJNA729271	stage_E18_vs_E10	ordinary_muscle_development	ENSGALG00000010829	LAMB4	-1.082822921	0.245142251	-4.417120739	1.00024E-05	2.32946E-05	negative
PRJNA729271	stage_E18_vs_E10	ordinary_muscle_development	ENSGALG00000010831	DESI1	-0.228362544	0.093199202	-2.450262869	0.014275196	0.022104365	negative
PRJNA729271	stage_E18_vs_E10	ordinary_muscle_development	ENSGALG00000010834	KGD4	0.561509789	0.126203124	4.44925426	8.6169E-06	2.02139E-05	positive
PRJNA729271	stage_E18_vs_E10	ordinary_muscle_development	ENSGALG00000010836	DIP2A	0.081494995	0.105749319	0.770643214	0.440918439	0.500439494	positive
PRJNA729271	stage_E18_vs_E10	ordinary_muscle_development	ENSGALG00000010837	RBPJ	0.039124423	0.122900445	0.318342402	0.750225224	0.790069356	positive

PRJNA729271	stage_E18_vs_E10	ordinary_muscle_development	ENSGALG00010010838	JAM2	0.736450358	0.175521173	4.195792151	2.7192E-05	6.03133E-05	positive
PRJNA729271	stage_E18_vs_E10	ordinary_muscle_development	ENSGALG00010010840	RGS16	-0.8312866	0.714980267	-1.16267069	0.244963138	0.298206155	negative
PRJNA729271	stage_E18_vs_E10	ordinary_muscle_development	ENSGALG00010010841	PMM1	0.945657133	0.132271671	7.149354995	8.71866E-13	3.76234E-12	positive
PRJNA729271	stage_E18_vs_E10	ordinary_muscle_development	ENSGALG00010010843	ZNF395	2.50311674	0.177968636	14.06493185	6.23829E-45	1.46081E-43	positive
PRJNA729271	stage_E18_vs_E10	ordinary_muscle_development	ENSGALG00010010844	CDC14B	0.41969202	0.17485165	2.400274863	0.016382765	0.025104998	positive
PRJNA729271	stage_E18_vs_E10	ordinary_muscle_development	ENSGALG00010010845	FKRP	0.665340511	0.2553141	2.605968538	0.009161488	0.014567742	positive
PRJNA729271	stage_E18_vs_E10	ordinary_muscle_development	ENSGALG00010010848	RGR	0.409779679	0.592704187	0.691373012	0.489331159	0.547245981	positive
PRJNA729271	stage_E18_vs_E10	ordinary_muscle_development	ENSGALG00010010849	ACBD6	-0.52873035	0.110850693	-4.769752327	1.84453E-06	4.62888E-06	negative
PRJNA729271	stage_E18_vs_E10	ordinary_muscle_development	ENSGALG00010010850	ENSGALG00010010850	1.194590207	0.106679837	11.19790058	4.1751E-29	4.85699E-28	positive
PRJNA729271	stage_E18_vs_E10	ordinary_muscle_development	ENSGALG00010010851	PNOC	11.90459353	0.741355254	16.05787977	5.03469E-58	1.88573E-56	positive
PRJNA729271	stage_E18_vs_E10	ordinary_muscle_development	ENSGALG00010010853	SMIM20	0.510976416	0.146151194	3.496217874	0.000471903	0.000900848	positive
PRJNA729271	stage_E18_vs_E10	ordinary_muscle_development	ENSGALG00010010854	LRIT1	0.756731992	0.591102176	1.280205052	0.20047303	0.249365534	positive
PRJNA729271	stage_E18_vs_E10	ordinary_muscle_development	ENSGALG00010010855	ATP5PF	0.735980834	0.148605467	4.952582499	7.3235E-07	1.9196E-06	positive
PRJNA729271	stage_E18_vs_E10	ordinary_muscle_development	ENSGALG00010010856	CSDC2	-3.24302734	0.572472417	-5.664949518	1.47068E-08	4.48129E-08	negative
PRJNA729271	stage_E18_vs_E10	ordinary_muscle_development	ENSGALG00010010857	SEL1L3	-0.267513978	0.062058562	-4.310669921	1.62761E-05	3.70596E-05	negative
PRJNA729271	stage_E18_vs_E10	ordinary_muscle_development	ENSGALG00010010859	ENSGALG00010010859	-0.099141252	0.265608806	-0.373260411	0.708954638	0.753118174	negative
PRJNA729271	stage_E18_vs_E10	ordinary_muscle_development	ENSGALG00010010861	ENSGALG00010010861	0.209496277	0.241698179	0.866768125	0.38606907	0.446390611	positive
PRJNA729271	stage_E18_vs_E10	ordinary_muscle_development	ENSGALG00010010862	STRN4	0.285471096	0.154250603	1.850696789	0.064213186	0.088341888	positive
PRJNA729271	stage_E18_vs_E10	ordinary_muscle_development	ENSGALG00010010865	ENSGALG00010010865	0.472459638	0.157096266	3.007453009	0.002634469	0.00455036	positive
PRJNA729271	stage_E18_vs_E10	ordinary_muscle_development	ENSGALG00010010867	QSOX1	0.445178894	0.161457749	2.757246992	0.00582903	0.009562222	positive
PRJNA729271	stage_E18_vs_E10	ordinary_muscle_development	ENSGALG00010010868	SLC39A10	-1.670902011	0.109609267	-15.24416733	1.80013E-52	5.70961E-51	negative
PRJNA729271	stage_E18_vs_E10	ordinary_muscle_development	ENSGALG00010010869	ENSGALG00010010869	3.747769375	0.173836552	21.5591562	4.3446E-103	5.6734E-101	positive
PRJNA729271	stage_E18_vs_E10	ordinary_muscle_development	ENSGALG00010010870	NOL6	-1.149271789	0.124476099	-9.232871215	2.63473E-20	1.89155E-19	negative
PRJNA729271	stage_E18_vs_E10	ordinary_muscle_development	ENSGALG00010010871	GCNT2	-1.068722291	0.919921194	-1.161754179	0.245335331	0.298609088	negative
PRJNA729271	stage_E18_vs_E10	ordinary_muscle_development	ENSGALG00010010874	ENSGALG00010010874	0.016451687	0.282370986	0.058262667	0.953539405	0.961767008	positive
PRJNA729271	stage_E18_vs_E10	ordinary_muscle_development	ENSGALG00010010876	ENSGALG00010010876	-0.229740434	0.164848257	-1.393647945	0.163423943	0.207355572	negative
PRJNA729271	stage_E18_vs_E10	ordinary_muscle_development	ENSGALG00010010880	ELP3	-0.15451754	0.107547457	-1.436738193	0.150792398	0.192932811	negative
PRJNA729271	stage_E18_vs_E10	ordinary_muscle_development	ENSGALG00010010882	CEP350	1.593080018	0.098157513	16.22983278	3.10304E-59	1.21564E-57	positive
PRJNA729271	stage_E18_vs_E10	ordinary_muscle_development	ENSGALG00010010884	DNAH7	-2.473231814	0.311269306	-7.945633461	1.93201E-15	1.01502E-14	negative
PRJNA729271	stage_E18_vs_E10	ordinary_muscle_development	ENSGALG00010010885	SNRPD2	-1.164552522	0.277116811	-4.202388585	2.64113E-05	5.86806E-05	negative
PRJNA729271	stage_E18_vs_E10	ordinary_muscle_development	ENSGALG00010010887	GABPA	-0.336671131	0.066694166	-5.047984694	4.46495E-07	1.19342E-06	negative
PRJNA729271	stage_E18_vs_E10	ordinary_muscle_development	ENSGALG00010010888	GNPAT	0.146394315	0.119647679	1.223544961	0.221123933	0.272021911	positive
PRJNA729271	stage_E18_vs_E10	ordinary_muscle_development	ENSGALG00010010889	CCL19	-0.864191505	0.681532003	-1.268013095	0.204793274	0.254107046	negative
PRJNA729271	stage_E18_vs_E10	ordinary_muscle_development	ENSGALG00010010890	NOL12	-0.676344996	0.136872986	-4.941406012	7.75612E-07	2.02897E-06	negative
PRJNA729271	stage_E18_vs_E10	ordinary_muscle_development	ENSGALG00010010891	POLR3H	-0.080864728	0.105698549	-0.765050506	0.44424147	0.503698382	negative
PRJNA729271	stage_E18_vs_E10	ordinary_muscle_development	ENSGALG00010010893	CCL21	0.103151611	0.488357265	0.211221616	0.832714344	0.86104968	positive
PRJNA729271	stage_E18_vs_E10	ordinary_muscle_development	ENSGALG00010010894	SPAG1	-0.024864396	0.145493859	-0.170896531	0.864305124	0.88801409	negative
PRJNA729271	stage_E18_vs_E10	ordinary_muscle_development	ENSGALG00010010896	LY86	2.991518294	0.292709675	10.22008684	1.61196E-24	1.4854E-23	positive
PRJNA729271	stage_E18_vs_E10	ordinary_muscle_development	ENSGALG00010010897	CEP170	-0.734636497	0.091392726	-8.038238129	9.11394E-16	4.91285E-15	negative
PRJNA729271	stage_E18_vs_E10	ordinary_muscle_development	ENSGALG00010010898	PHF24	-4.53274355	3.569493153	-1.269856351	0.204135804	0.253443124	negative
PRJNA729271	stage_E18_vs_E10	ordinary_muscle_development	ENSGALG00010010900	SLC34A2	-1.113261415	1.052226036	-1.058005959	0.290052726	0.346376196	negative
PRJNA729271	stage_E18_vs_E10	ordinary_muscle_development	ENSGALG00010010904	TOR1AIP1	-0.421595854	0.082096095	-5.135394728	2.81552E-07	7.66404E-07	negative
PRJNA729271	stage_E18_vs_E10	ordinary_muscle_development	ENSGALG00010010905	ENC1	0.309314061	0.147867766	2.091828866	0.036453827	0.052420459	positive
PRJNA729271	stage_E18_vs_E10	ordinary_muscle_development	ENSGALG00010010906	ATP6V1C1	-0.2334698	0.141086098	-1.654803728	0.097964318	0.130131303	negative
PRJNA729271	stage_E18_vs_E10	ordinary_muscle_development	ENSGALG00010010908	STK17B	-0.473416437	0.127998516	-3.698608788	0.000216784	0.000432405	negative
PRJNA729271	stage_E18_vs_E10	ordinary_muscle_development	ENSGALG00010010909	SLIT3	-1.895197383	0.329234855	-5.756369209	8.59423E-09	2.67096E-08	negative
PRJNA729271	stage_E18_vs_E10	ordinary_muscle_development	ENSGALG00010010911	ENSGALG00010010911	-2.047938142	0.388278557	-5.274404435	1.33188E-07	3.72767E-07	negative
PRJNA729271	stage_E18_vs_E10	ordinary_muscle_development	ENSGALG00010010912	BAALC	-1.271915882	0.364862688	-3.486012471	0.000490278	0.000932867	negative
PRJNA729271	stage_E18_vs_E10	ordinary_muscle_development	ENSGALG00010010913	IFT56	-1.171052488	0.145672296	-8.038951267	9.06106E-16	4.88616E-15	negative
PRJNA729271	stage_E18_vs_E10	ordinary_muscle_development	ENSGALG00010010915	APP	-0.446037052	0.110151977	-4.049287771	5.13737E-05	0.000110484	negative
PRJNA729271	stage_E18_vs_E10	ordinary_muscle_development	ENSGALG00010010916	ENSGALG00010010916	-1.47022564	0.565098389	-2.601716211	0.009275857	0.014733021	negative
PRJNA729271	stage_E18_vs_E10	ordinary_muscle_development	ENSGALG00010010917	TANC1	-0.212677094	0.06937356	-3.065679398	0.00217176	0.003793188	negative
PRJNA729271	stage_E18_vs_E10	ordinary_muscle_development	ENSGALG00010010918	TOR1AIP2	0.592800229	0.121027562	4.89805974	9.67876E-07	2.50614E-06	positive
PRJNA729271	stage_E18_vs_E10	ordinary_muscle_development	ENSGALG00010010919	CDHR1	-2.200811173	0.579541942	-3.797501118	0.000146162	0.000298523	negative
PRJNA729271	stage_E18_vs_E10	ordinary_muscle_development	ENSGALG00010010920	UBN2	0.411793636	0.114769711	3.587999249	0.000333225	0.000648249	positive
PRJNA729271	stage_E18_vs_E10	ordinary_muscle_development	ENSGALG00010010921	ENSGALG00010010921	-0.130644477	0.130415036	-1.001759314	0.316459852	0.373967516	negative
PRJNA729271	stage_E18_vs_E10	ordinary_muscle_development	ENSGALG00010010922	DYM	0.36443861	0.102515193	3.554971721	0.00037802	0.000729711	positive
PRJNA729271	stage_E18_vs_E10	ordinary_muscle_development	ENSGALG00010010923	F13A1	1.706071988	0.231116055	7.38188433	1.56065E-13	7.07588E-13	positive
PRJNA729271	stage_E18_vs_E10	ordinary_muscle_development	ENSGALG00010010924	ENSGALG00010010924	-0.774758125	0.202482366	-3.826299249	0.000130084	0.000267571	negative
PRJNA729271	stage_E18_vs_E10	ordinary_muscle_development	ENSGALG00010010928	HECW2	-0.140872967	0.116631587	-1.207845751	0.2271066	0.27855053	negative
PRJNA729271	stage_E18_vs_E10	ordinary_muscle_development	ENSGALG00010010929	FMC1	0.840475303	0.147733743	5.689122122	1.27694E-08	3.90408E-08	positive
PRJNA729271	stage_E18_vs_E10	ordinary_muscle_development	ENSGALG00010010930	FAM163A	-2.319590514	0.45650796	-5.081161161	3.75135E-07	1.01034E-06	negative
PRJNA729271	stage_E18_vs_E10	ordinary_muscle_development	ENSGALG00010010931	CYYR1	-1.61882442	0.193193571	-8.379287201	5.32492E-17	3.11354E-16	negative
PRJNA729271	stage_E18_vs_E10	ordinary_muscle_development	ENSGALG00010010932	GHITM	0.582485483	0.088316076	6.595463837	4.23928E-11	1.59193E-10	positive
PRJNA729271	stage_E18_vs_E10	ordinary_muscle_development	ENSGALG00010010933	ANAPC4	-0.879262091	0.112013591	-7.849601855	4.1736E-15	2.14602E-14	negative
PRJNA729271	stage_E18_vs_E10	ordinary_muscle_development	ENSGALG00010010934	ENSGALG00010010934	-0.277859193	0.124642821	-2.229243446	0.025797711	0.038133574	negative
PRJNA729271	stage_E18_vs_E10	ordinary_muscle_development	ENSGALG00010010935	LGALS1A	1.116264914	0.227578301	4.904970773	9.34413E-07	2.42339E-06	positive
PRJNA729271	stage_E18_vs_E10	ordinary_muscle_development	ENSGALG00010010937	CDK7	-0.068059227	0.116687426	-0.583261019	0.5597176	0.613745866	negative
PRJNA729271	stage_E18_vs_E10	ordinary_muscle_development	ENSGALG00010010938	FZD6	-0.400299318	0.122042832	-3.279990404	0.001038106	0.001886578	negative
PRJNA729271	stage_E18_vs_E10	ordinary_muscle_development	ENSGALG00010010939	ZCCHC4	0.204284588	0.137841591	1.482024305	0.138333828	0.178552879	positive
PRJNA729271	stage_E18_vs_E10	ordinary_muscle_development	ENSGALG00010010940	NRG3	2.259612262	0.315810378	7.154965196	8.36942E-13	3.61486E-12	positive
PRJNA729271	stage_E18_vs_E10	ordinary_muscle_development	ENSGALG00010010941	RIPK4	-4.818911469	0.728993177	-6.610365669	3.83372E-11	1.44225E-10	negative
PRJNA729271	stage_E18_vs_E10	ordinary_muscle_development	ENSGALG00010010942	NRN1	1.916228906	0.339207348	5.649137366	1.61255E-08	4.89506E-08	positive
PRJNA729271	stage_E18_vs_E10	ordinary_muscle_development	ENSGALG00010010943	WDSUB1	0.384159807	0.103402499	3.715188806	0.000203052	0.000406806	positive
PRJNA729271	stage_E18_vs_E10	ordinary_muscle_development	ENSGALG00010010945	PI4K2B	-0.756395777	0.10100736	-7.488521392	6.96538E-14	3.24432E-13	negative
PRJNA729271	stage_E18_vs_E10	ordinary_muscle_development	ENSGALG00010010946	SH2D4B	1.79732753	0.300040619	5.990280702	2.09479E-09	6.87591E-09	positive
PRJNA729271	stage_E18_vs_E10	ordinary_muscle_development	ENSGALG00010010947	LUC7L2	-0.567668925	0.070941524	-8.001927371	1.22487E-15	6.54421E-15	negative
PRJNA729271	stage_E18_vs_E10	ordinary_muscle_development	ENSGALG00010010948	PRDM15	-0.555184161	0.086997781	-6.381589901	1.75259E-10	6.27408E-10	negative
PRJNA729271	stage_E18_vs_E10	ordinary_muscle_development	ENSGALG00010010949	FZD3	-1.947389183	0.229032756	-8.502666677	1.85284E-17	1.11486E-16	negative
PRJNA729271	stage_E18_vs_E10	ordinary_muscle_development	ENSGALG00010010951	ENSGALG00010010951	-1.028733892	0.136494707	-7.536804275	4.81628E-14	2.26589E-13	negative
PRJNA729271	stage_E18_vs_E10	ordinary_muscle_development	ENSGALG00010010952	ENSGALG00010010952	-0.885740768	0.550892424	-1.607828916	0.107872654	0.142224314	negative
PRJNA729271	stage_E18_vs_E10	ordinary_muscle_development	ENSGALG00010010953	EXTL3	-0.196414919	0.110917071	-1.77082677	0.076589513	0.103948033	negative
PRJNA729271	stage_E18_vs_E10	ordinary_muscle_development	ENSGALG00010010954	MTERF2	0.965527681	0.227108223	4.251399048	2.12439E-05	4.77116E-05	positive
PRJNA729271	stage_E18_vs_E10	ordinary_muscle_development	ENSGALG00010010955	GTF3C3	-0.158976142	0.084238837	-1.887207239	0.059132457	0.08193528	negative
PRJNA729271	stage_E18_vs_E10	ordinary_muscle_development	ENSGALG00010010956	AXDND1	4.381962304	0.54966277	7.972092242	1.5601E-15	8.2592E-15	positive
PRJNA729271	stage_E18_vs_E10	ordinary_muscle_development	ENSGALG00010010957	ZMAT2	-0.04855435	0.075630431	-0.641994883	0.520876506	0.5777552	negative
PRJNA729271	stage_E18_vs_E10	ordinary_muscle_development	ENSGALG00010010958	LCORL	0.357044452	0.160417007	2.225726923	0.026032478	0.038468832	positive
PRJNA729271	stage_E18_vs_E10	ordinary_muscle_development	ENSGALG00010010959	TMEM263	-0.228973673	0.089594233	-2.55567424	0.010598229	0.01671614	negative
PRJNA729271	stage_E18_vs_E10	ordinary_muscle_development	ENSGALG00010010960	POLDIP3	-0.213157229	0.070079115	-3.041665555	0.002352731	0.00408613	negative
PRJNA729271	stage_E18_vs_E10	ordinary_muscle_development	ENSGALG00010010961	C2orf66	0.388580938	0.385719684	1.00741796	0.313733964	0.371260822	positive
PRJNA729271	stage_E18_vs_E10	ordinary_muscle_development	ENSGALG00010010962	ANKDD1B	0.370590225	0.281411132	1.316899667	0.187872258	0.235083597	positive
PRJNA729271	stage_E18_vs_E10	ordinary_muscle_development	ENSGALG00010010963	TSPAN14	0.050899938	0.073749016	0.690177856	0.490082347	0.547862745	positive
PRJNA729271	stage_E18_vs_E10	ordinary_muscle_development	ENSGALG00010010964	INTS9	-0.575931881	0.093029096	-6.190879031	5.98296E-10	2.0668E-09	negative
PRJNA729271	stage_E18_vs_E10	ordinary_muscle_development	ENSGALG00010010965	BAZ2B	0.01520381	0.132419708	0.11481531	0.908591514	0.924407524	positive
PRJNA729271	stage_E18_vs_E10	ordinary_muscle_development	ENSGALG00010010966	ENSGALG00010010966	2.878960202	0.520470144	5.53146081	3.17575E-08	9.4213E-08	positive
PRJNA729271	stage_E18_vs_E10	ordinary_muscle_development	ENSGALG00010010967	PRXL2A	-0.064258877	0.390641958	-0.164495584	0.869341023	0.892302657	negative
PRJNA729271	stage_E18_vs_E10	ordinary_muscle_development	ENSGALG00010010968	RRP7A	-0.063442544	0.246919143	-0.256936515	0.797227782	0.830517227	negative
PRJNA729271	stage_E18_vs_E10	ordinary_muscle_development	ENSGALG00010010970	TPM2	2.158198688	0.352421325	6.123916278	9.13029E-10	3.0972E-09	positive
PRJNA729271	stage_E18_vs_E10	ordinary_muscle_development	ENSGALG00010010971	LCORL	0.409370264	0.124831789	3.279375114	0.001040372	0.001890223	positive
PRJNA729271	stage_E18_vs_E10	ordinary_muscle_development	ENSGALG00010010972	EXOSC3	0.093929358	0.162733306	0.577198119	0.563805628	0.617696588	positive
PRJNA729271	stage_E18_vs_E10	ordinary_muscle_development	ENSGALG00010010973	PGAP1	-0.381457892	0.113448026	-3.362402185	0.000772675	0.001431483	negative
PRJNA729271	stage_E18_vs_E10	ordinary_muscle_development	ENSGALG00010010974	NCAPG	-1.848754574	0.192229405	-9.617438997	6.74906E-22	5.36924E-21	negative
PRJNA729271	stage_E18_vs_E10	ordinary_muscle_development	ENSGALG00010010975	ATP8B1	-4.160600557	0.958374883	-4.34130801	1.41637E-05	3.24589E-05	negative
PRJNA729271	stage_E18_vs_E10	ordinary_muscle_development	ENSGALG00010010976	DYDC2	3.649188716	0.605009424	6.031622931	1.62321E-09	5.37793E-09	positive
PRJNA729271	stage_E18_vs_E10	ordinary_muscle_development	ENSGALG00010010977	FARS2	0.106951536	0.082056375	1.30339095	0.192441325	0.240178836	positive
PRJNA729271	stage_E18_vs_E10	ordinary_muscle_development	ENSGALG00010010978	SERHL	0.030208738	0.135553993	0.222853913	0.823649206	0.85344165	positive
PRJNA729271	stage_E18_vs_E10	ordinary_muscle_development	ENSGALG00010010979	ENSGALG00010010979	-1.270349736	0.366527216	-3.46590834	0.000528443	0.001000495	negative
PRJNA729271	stage_E18_vs_E10	ordinary_muscle_development	ENSGALG00010010980	POLK	0.457354977	0.126000661	3.629782359	0.00028366	0.000557815	positive
PRJNA729271	stage_E18_vs_E10	ordinary_muscle_development	ENSGALG00010010981	RIC8B	-0.834216561	0.065851362	-12.66817483	8.87555E-37	1.49247E-35	negative
PRJNA729271	stage_E18_vs_E10	ordinary_muscle_development	ENSGALG00010010982	ZBTB21	0.773340218	0.119550276	6.468744734	9.88204E-11	3.60443E-10	positive
PRJNA729271	stage_E18_vs_E10	ordinary_muscle_development	ENSGALG00010010983	MED28	-0.344995415	0.090663895	-3.805212833	0.000141682	0.000289998	negative
PRJNA729271	stage_E18_vs_E10	ordinary_muscle_development	ENSGALG00010010984	MAT1A	2.886097219	0.389913115	7.401898288	1.34251E-13	6.11172E-13	positive
PRJNA729271	stage_E18_vs_E10	ordinary_muscle_development	ENSGALG00010010985	CERT1	0.278910712	0.109059301	2.557422521	0.010545105	0.016634159	positive
PRJNA729271	stage_E18_vs_E10	ordinary_muscle_development	ENSGALG00010010986	NARS	-0.382933795	0.130415268	-2.936265068	0.003321903	0.00563961	negative
PRJNA729271	stage_E18_vs_E10	ordinary_muscle_development	ENSGALG00010010987	MARCHF7	0.120303891	0.080872278	1.487578862	0.136861991	0.176747556	positive
PRJNA729271	stage_E18_vs_E10	ordinary_muscle_development	ENSGALG00010010988	ANKRD44	-0.893786043	0.087047455	-10.26780208	9.84214E-25	9.19716E-24	negative
PRJNA729271	stage_E18_vs_E10	ordinary_muscle_development	ENSGALG00010010989	TRIQK	-0.778671461	0.17885768	-4.353581341	1.33931E-05	3.07709E-05	negative
PRJNA729271	stage_E18_vs_E10	ordinary_muscle_development	ENSGALG00010010990	MED18	0.137767209	0.119911335	1.148908974	0.250593517	0.304293627	positive
PRJNA729271	stage_E18_vs_E10	ordinary_muscle_development	ENSGALG00010010991	C2CD2	0.990999376	0.161309813	6.143453756	8.07462E-10	2.75198E-09	positive
PRJNA729271	stage_E18_vs_E10	ordinary_muscle_development	ENSGALG00010010992	PCDHA6	1.11457073	0.293135284	3.802240097	0.000143394	0.000293241	positive
PRJNA729271	stage_E18_vs_E10	ordinary_muscle_development	ENSGALG00010010993	ENSGALG00010010993	5.686117199	0.328426816	17.31319404	3.74076E-67	1.85059E-65	positive
PRJNA729271	stage_E18_vs_E10	ordinary_muscle_development	ENSGALG00010010994	NAA20	0.215041597	0.14975467	1.435959202	0.151013951	0.193148082	positive
PRJNA729271	stage_E18_vs_E10	ordinary_muscle_development	ENSGALG00010010995	HEBP1	0.275170053	0.183198981	1.502028298	0.133089801	0.172275108	positive
PRJNA729271	stage_E18_vs_E10	ordinary_muscle_development	ENSGALG00010010996	RFX4	1.049510369	0.582011687	1.803246211	0.071349556	0.097466008	positive
PRJNA729271	stage_E18_vs_E10	ordinary_muscle_development	ENSGALG00010010997	LAP3	-0.440258987	0.149412629	-2.946598224	0.003212904	0.005473151	negative
PRJNA729271	stage_E18_vs_E10	ordinary_muscle_development	ENSGALG00010010998	SOAT1	1.366956221	0.165873739	8.240944182	1.70857E-16	9.64021E-16	positive
PRJNA729271	stage_E18_vs_E10	ordinary_muscle_development	ENSGALG00010011001	ANGPT1	-1.680662485	0.25303607	-6.641987788	3.09481E-11	1.17034E-10	negative
PRJNA729271	stage_E18_vs_E10	ordinary_muscle_development	ENSGALG00010011002	RPRM	1.237464132	0.53913909	2.295259525	0.021718256	0.0325381	positive
PRJNA729271	stage_E18_vs_E10	ordinary_muscle_development	ENSGALG00010011003	FECH	-1.816196594	0.114893537	-15.80764801	2.75564E-56	9.74219E-55	negative
PRJNA729271	stage_E18_vs_E10	ordinary_muscle_development	ENSGALG00010011004	UMODL1	1.983546379	0.37526695	5.285694297	1.25229E-07	3.5185E-07	positive
PRJNA729271	stage_E18_vs_E10	ordinary_muscle_development	ENSGALG00010011006	TMEM64	0.243012803	0.100454162	2.419141205	0.0155572	0.023951313	positive

PRJNA729271	stage_E18_vs_E10	ordinary_muscle_development	ENSGALG00010011007	ENSGALG00010011007	0.825886495	0.164155658	5.031118055	4.87628E-07	1.29786E-06	positive
PRJNA729271	stage_E18_vs_E10	ordinary_muscle_development	ENSGALG00010011009	SFTPA1	3.177398265	1.149632924	2.76383722	0.005712603	0.00938291	positive
PRJNA729271	stage_E18_vs_E10	ordinary_muscle_development	ENSGALG00010011013	ENSGALG00010011013	3.418279838	0.341613427	10.00628068	1.43029E-23	1.23847E-22	positive
PRJNA729271	stage_E18_vs_E10	ordinary_muscle_development	ENSGALG00010011015	LYRM4	0.188519626	0.183591161	1.026844785	0.304493557	0.361713991	positive
PRJNA729271	stage_E18_vs_E10	ordinary_muscle_development	ENSGALG00010011018	QDPR	0.022971799	0.10381309	0.221280367	0.824874136	0.854405503	positive
PRJNA729271	stage_E18_vs_E10	ordinary_muscle_development	ENSGALG00010011019	NECAB1	-1.808929602	0.218474891	-8.279805503	1.23377E-16	7.04073E-16	negative
PRJNA729271	stage_E18_vs_E10	ordinary_muscle_development	ENSGALG00010011020	SFTPA2	1.427484505	0.868065122	1.644444027	0.100084517	0.132655914	positive
PRJNA729271	stage_E18_vs_E10	ordinary_muscle_development	ENSGALG00010011021	PPP1R3G	1.828557763	0.18803387	9.724619086	2.3679E-22	1.925E-21	positive
PRJNA729271	stage_E18_vs_E10	ordinary_muscle_development	ENSGALG00010011022	C5orf22	-0.37442215	0.195814938	-1.912122505	0.055860483	0.077765818	negative
PRJNA729271	stage_E18_vs_E10	ordinary_muscle_development	ENSGALG00010011025	POLR3B	-0.645185433	0.092788349	-6.953302192	3.56833E-12	1.46358E-11	negative
PRJNA729271	stage_E18_vs_E10	ordinary_muscle_development	ENSGALG00010011027	FAM234B	0.08792686	0.097917373	0.89796996	0.369201565	0.428812234	positive
PRJNA729271	stage_E18_vs_E10	ordinary_muscle_development	ENSGALG00010011028	HMBOX1	-0.24415224	0.084499847	-2.889380864	0.003860012	0.006494588	negative
PRJNA729271	stage_E18_vs_E10	ordinary_muscle_development	ENSGALG00010011029	HMGCR	-1.437287948	0.1268458	-11.33098573	9.21617E-30	1.11416E-28	negative
PRJNA729271	stage_E18_vs_E10	ordinary_muscle_development	ENSGALG00010011033	DROSHA	-0.730268775	0.101400868	-7.201800064	5.94228E-13	2.58892E-12	negative
PRJNA729271	stage_E18_vs_E10	ordinary_muscle_development	ENSGALG00010011034	FAM180A	1.441365294	0.396861512	3.631910002	0.000281331	0.00055346	positive
PRJNA729271	stage_E18_vs_E10	ordinary_muscle_development	ENSGALG00010011038	GSG1	-2.230996719	0.487384008	-4.577492657	4.70582E-06	1.13138E-05	negative
PRJNA729271	stage_E18_vs_E10	ordinary_muscle_development	ENSGALG00010011040	CDH6	-2.238743638	0.221331331	-10.11489709	4.74515E-24	4.23268E-23	negative
PRJNA729271	stage_E18_vs_E10	ordinary_muscle_development	ENSGALG00010011042	SLC13A4	0.426352749	0.52222134	0.816421536	0.414259083	0.474603653	positive
PRJNA729271	stage_E18_vs_E10	ordinary_muscle_development	ENSGALG00010011044	MBL2	3.43582	0.602163779	5.705789884	1.15805E-08	3.55558E-08	positive
PRJNA729271	stage_E18_vs_E10	ordinary_muscle_development	ENSGALG00010011045	ENSGALG00010011045	-0.50325026	0.401924014	-1.252102991	0.210532338	0.260502553	negative
PRJNA729271	stage_E18_vs_E10	ordinary_muscle_development	ENSGALG00010011049	ABL2	-0.088934875	0.129417053	-0.687195952	0.491959255	0.549492181	negative
PRJNA729271	stage_E18_vs_E10	ordinary_muscle_development	ENSGALG00010011050	SYT10	3.107265621	0.402358642	7.72262677	1.13956E-14	5.66266E-14	positive
PRJNA729271	stage_E18_vs_E10	ordinary_muscle_development	ENSGALG00010011055	TOR3A	0.751675477	0.13091022	5.741915946	9.36113E-09	2.90121E-08	positive
PRJNA729271	stage_E18_vs_E10	ordinary_muscle_development	ENSGALG00010011056	ENSGALG00010011056	0.262567271	0.51928396	0.505633317	0.613114089	0.663810032	positive
PRJNA729271	stage_E18_vs_E10	ordinary_muscle_development	ENSGALG00010011059	ENSGALG00010011059	-0.277548442	0.20692933	-1.341271642	0.179832273	0.226037877	negative
PRJNA729271	stage_E18_vs_E10	ordinary_muscle_development	ENSGALG00010011061	KIF13B	0.633238462	0.154790973	4.090926286	4.29654E-05	9.31752E-05	positive
PRJNA729271	stage_E18_vs_E10	ordinary_muscle_development	ENSGALG00010011062	TMEM55A	-0.309552198	0.122359567	-2.529856922	0.011410905	0.017914119	negative
PRJNA729271	stage_E18_vs_E10	ordinary_muscle_development	ENSGALG00010011064	MYLIP	0.136311421	0.09805244	1.390188971	0.164471502	0.208483989	positive
PRJNA729271	stage_E18_vs_E10	ordinary_muscle_development	ENSGALG00010011067	TMTC2	-0.263309756	0.102836465	-2.560470705	0.010453048	0.016494331	negative
PRJNA729271	stage_E18_vs_E10	ordinary_muscle_development	ENSGALG00010011069	ENSGALG00010011069	-0.746503972	0.528310613	-1.413002037	0.157655125	0.200756483	negative
PRJNA729271	stage_E18_vs_E10	ordinary_muscle_development	ENSGALG00010011076	PKP2	-0.037403744	0.288600239	-0.129603996	0.896879741	0.9144817	negative
PRJNA729271	stage_E18_vs_E10	ordinary_muscle_development	ENSGALG00010011077	RASGEF1A	1.609698619	0.143523447	11.21557939	3.4194E-29	3.99711E-28	positive
PRJNA729271	stage_E18_vs_E10	ordinary_muscle_development	ENSGALG00010011080	EMP1	0.078560758	0.255893559	0.307005608	0.758839104	0.797797919	positive
PRJNA729271	stage_E18_vs_E10	ordinary_muscle_development	ENSGALG00010011085	UGT1A1	2.365273503	1.34218082	1.762261438	0.078025136	0.10572818	positive
PRJNA729271	stage_E18_vs_E10	ordinary_muscle_development	ENSGALG00010011086	OTUD6A	-0.475514543	0.1138052	-4.178319988	2.9367E-05	6.48598E-05	negative
PRJNA729271	stage_E18_vs_E10	ordinary_muscle_development	ENSGALG00010011090	GRIN2B	0.208404945	0.307158624	0.678492899	0.497459225	0.554709706	positive
PRJNA729271	stage_E18_vs_E10	ordinary_muscle_development	ENSGALG00010011091	YARS2	-0.459547352	0.103568674	-4.437126938	9.11675E-06	2.13113E-05	negative
PRJNA729271	stage_E18_vs_E10	ordinary_muscle_development	ENSGALG00010011094	CSGALNACT2	-0.017891093	0.090342412	-0.198036481	0.843016521	0.870212539	negative
PRJNA729271	stage_E18_vs_E10	ordinary_muscle_development	ENSGALG00010011095	TNN	-1.412821171	0.46547699	-3.035211628	0.00240367	0.004169103	negative
PRJNA729271	stage_E18_vs_E10	ordinary_muscle_development	ENSGALG00010011096	LDB2	-0.527544297	0.115119352	-4.58258572	4.59261E-06	1.10508E-05	negative
PRJNA729271	stage_E18_vs_E10	ordinary_muscle_development	ENSGALG00010011099	CCBE1	-0.222015607	0.199323943	-1.113843146	0.265346484	0.320181597	negative
PRJNA729271	stage_E18_vs_E10	ordinary_muscle_development	ENSGALG00010011101	ENSGALG00010011101	0.067858156	0.822579119	0.082494382	0.934253586	0.946199394	positive
PRJNA729271	stage_E18_vs_E10	ordinary_muscle_development	ENSGALG00010011104	ABCG1	0.814921686	0.248882914	3.274317517	0.001059175	0.001923662	positive
PRJNA729271	stage_E18_vs_E10	ordinary_muscle_development	ENSGALG00010011106	USP40	0.22848902	0.116634836	1.959011806	0.050111404	0.070363732	positive
PRJNA729271	stage_E18_vs_E10	ordinary_muscle_development	ENSGALG00010011107	ENSGALG00010011107	1.064245458	0.190274197	5.593220081	2.22896E-08	6.68367E-08	positive
PRJNA729271	stage_E18_vs_E10	ordinary_muscle_development	ENSGALG00010011109	ENSGALG00010011109	-0.921156891	1.25321566	-0.735034616	0.462318413	0.521542952	negative
PRJNA729271	stage_E18_vs_E10	ordinary_muscle_development	ENSGALG00010011111	MRPS14	0.47522634	0.117518157	4.043854621	5.25795E-05	0.000112993	positive
PRJNA729271	stage_E18_vs_E10	ordinary_muscle_development	ENSGALG00010011116	TAPT1	0.403391797	0.1285643	3.137665714	0.00170299	0.00302066	positive
PRJNA729271	stage_E18_vs_E10	ordinary_muscle_development	ENSGALG00010011117	SLC6A15	-6.156549704	0.544967273	-11.29709987	1.35623E-29	1.62737E-28	negative
PRJNA729271	stage_E18_vs_E10	ordinary_muscle_development	ENSGALG00010011118	GMPR2	2.446503924	0.135851869	18.00861435	1.66751E-72	9.33225E-71	positive
PRJNA729271	stage_E18_vs_E10	ordinary_muscle_development	ENSGALG00010011119	ENSGALG00010011119	-1.015793156	0.296062727	-3.431006552	0.000601346	0.0011304	negative
PRJNA729271	stage_E18_vs_E10	ordinary_muscle_development	ENSGALG00010011120	ENSGALG00010011120	1.785355146	3.005774113	0.593975155	0.55252873	0.606643232	positive
PRJNA729271	stage_E18_vs_E10	ordinary_muscle_development	ENSGALG00010011121	NUP35	-1.041817908	0.112446265	-9.265029051	1.95024E-20	1.41273E-19	negative
PRJNA729271	stage_E18_vs_E10	ordinary_muscle_development	ENSGALG00010011124	CACYBP	-0.114714677	0.068713183	-1.669471158	0.095024042	0.126620103	negative
PRJNA729271	stage_E18_vs_E10	ordinary_muscle_development	ENSGALG00010011126	ATXN1	0.98789514	0.140333073	7.039645903	1.92729E-12	8.06236E-12	positive
PRJNA729271	stage_E18_vs_E10	ordinary_muscle_development	ENSGALG00010011128	GCNT4	-0.462108835	0.252494321	-1.830175161	0.067223751	0.092203659	negative
PRJNA729271	stage_E18_vs_E10	ordinary_muscle_development	ENSGALG00010011129	ENSGALG00010011129	0.196980911	0.118506565	1.662194078	0.096473867	0.128374984	positive
PRJNA729271	stage_E18_vs_E10	ordinary_muscle_development	ENSGALG00010011130	DNM1L	-0.50288876	0.115678674	-4.347290146	1.3783E-05	3.16415E-05	negative
PRJNA729271	stage_E18_vs_E10	ordinary_muscle_development	ENSGALG00010011131	RET	-0.100960269	0.495618219	-0.203705726	0.838583467	0.86639009	negative
PRJNA729271	stage_E18_vs_E10	ordinary_muscle_development	ENSGALG00010011133	CDC37L1	-0.286121589	0.122799482	-2.329990201	0.019806669	0.029877996	negative
PRJNA729271	stage_E18_vs_E10	ordinary_muscle_development	ENSGALG00010011136	ENSGALG00010011136	0.403644407	0.385332995	1.047521007	0.294859349	0.35150804	positive
PRJNA729271	stage_E18_vs_E10	ordinary_muscle_development	ENSGALG00010011139	UBASH3A	-1.784897464	1.003186779	-1.77922746	0.075202478	0.102209088	negative
PRJNA729271	stage_E18_vs_E10	ordinary_muscle_development	ENSGALG00010011141	MSX1	-4.105735099	0.816491906	-5.028506794	4.94314E-07	1.31518E-06	negative
PRJNA729271	stage_E18_vs_E10	ordinary_muscle_development	ENSGALG00010011142	DUSP19	-0.330044543	0.152247389	-2.167817431	0.03017258	0.044070087	negative
PRJNA729271	stage_E18_vs_E10	ordinary_muscle_development	ENSGALG00010011143	AK3	0.552977288	0.169740986	3.257771161	0.001122909	0.0020333	positive
PRJNA729271	stage_E18_vs_E10	ordinary_muscle_development	ENSGALG00010011144	ATF7IP	0.397869857	0.061516313	6.467712956	9.94973E-11	3.62821E-10	positive
PRJNA729271	stage_E18_vs_E10	ordinary_muscle_development	ENSGALG00010011146	BMS1	-1.140614029	0.129735804	-8.791821462	1.47154E-18	9.54792E-18	negative
PRJNA729271	stage_E18_vs_E10	ordinary_muscle_development	ENSGALG00010011148	ENSGALG00010011148	-0.607427175	0.185061789	-3.282293862	0.001029662	0.001872407	negative
PRJNA729271	stage_E18_vs_E10	ordinary_muscle_development	ENSGALG00010011150	FGD4	0.648366928	0.170138395	3.810820757	0.000138506	0.000283927	positive
PRJNA729271	stage_E18_vs_E10	ordinary_muscle_development	ENSGALG00010011152	SLC18A3	0.20218563	0.557795932	0.3624724	0.716999045	0.760048351	positive
PRJNA729271	stage_E18_vs_E10	ordinary_muscle_development	ENSGALG00010011154	SLC26A7	-0.065270892	0.452224756	-0.144332859	0.885237632	0.905349571	negative
PRJNA729271	stage_E18_vs_E10	ordinary_muscle_development	ENSGALG00010011157	ENSGALG00010011157	-4.848628154	1.574356484	-3.079752396	0.002071727	0.003630721	negative
PRJNA729271	stage_E18_vs_E10	ordinary_muscle_development	ENSGALG00010011161	ENSGALG00010011161	0.077235924	0.094848839	0.814305421	0.415470005	0.475802933	positive
PRJNA729271	stage_E18_vs_E10	ordinary_muscle_development	ENSGALG00010011166	SOX7	1.272934585	0.140917808	9.033170508	1.66769E-19	1.14186E-18	positive
PRJNA729271	stage_E18_vs_E10	ordinary_muscle_development	ENSGALG00010011170	RBM24	1.740078946	0.142733809	12.19107765	3.46805E-34	5.15583E-33	positive
PRJNA729271	stage_E18_vs_E10	ordinary_muscle_development	ENSGALG00010011175	GPR52	-0.017371945	0.192224012	-0.090373437	0.927990464	0.941039722	negative
PRJNA729271	stage_E18_vs_E10	ordinary_muscle_development	ENSGALG00010011178	RSPO2	-2.278915257	0.320339248	-7.114068201	1.12671E-12	4.80486E-12	negative
PRJNA729271	stage_E18_vs_E10	ordinary_muscle_development	ENSGALG00010011181	ENSGALG00010011181	0.637583049	0.266914987	2.388712061	0.016907548	0.025832708	positive
PRJNA729271	stage_E18_vs_E10	ordinary_muscle_development	ENSGALG00010011184	FAM8A1	0.075596977	0.137974757	0.547904404	0.583757545	0.63656322	positive
PRJNA729271	stage_E18_vs_E10	ordinary_muscle_development	ENSGALG00010011187	STK32B	-5.622062231	0.591069642	-9.511674827	1.87617E-21	1.44963E-20	negative
PRJNA729271	stage_E18_vs_E10	ordinary_muscle_development	ENSGALG00010011190	ENSGALG00010011190	-0.108888882	0.213373016	-0.510321707	0.609826098	0.660595567	negative
PRJNA729271	stage_E18_vs_E10	ordinary_muscle_development	ENSGALG00010011192	RPL17	1.027302312	0.123473461	8.320025257	8.79446E-17	5.0666E-16	positive
PRJNA729271	stage_E18_vs_E10	ordinary_muscle_development	ENSGALG00010011193	FAM20B	-1.025874698	0.081621156	-12.56873526	3.13702E-36	5.16131E-35	negative
PRJNA729271	stage_E18_vs_E10	ordinary_muscle_development	ENSGALG00010011198	PINX1	-0.476294575	0.066597625	-7.151825195	8.56316E-13	3.69634E-12	negative
PRJNA729271	stage_E18_vs_E10	ordinary_muscle_development	ENSGALG00010011199	ENSGALG00010011199	-4.678639243	1.718897299	-2.721884109	0.00649109	0.010562231	negative
PRJNA729271	stage_E18_vs_E10	ordinary_muscle_development	ENSGALG00010011200	PCDHB4	-0.519664365	0.592919907	-0.876449516	0.38078571	0.441154855	negative
PRJNA729271	stage_E18_vs_E10	ordinary_muscle_development	ENSGALG00010011203	ENSGALG00010011203	-0.256330102	0.104491219	-2.453125774	0.014162081	0.021943278	negative
PRJNA729271	stage_E18_vs_E10	ordinary_muscle_development	ENSGALG00010011205	CYTL1	-7.840197527	0.880177678	-8.907516884	5.21883E-19	3.48122E-18	negative
PRJNA729271	stage_E18_vs_E10	ordinary_muscle_development	ENSGALG00010011210	NUP153	-0.685499096	0.099066478	-6.919586834	4.52963E-12	1.84223E-11	negative
PRJNA729271	stage_E18_vs_E10	ordinary_muscle_development	ENSGALG00010011211	SLC37A1	1.382605162	0.14894562	9.282617124	1.65366E-20	1.20512E-19	positive
PRJNA729271	stage_E18_vs_E10	ordinary_muscle_development	ENSGALG00010011212	NCKAP1	-0.411113084	0.06326361	-6.498413324	8.11715E-11	2.98246E-10	negative
PRJNA729271	stage_E18_vs_E10	ordinary_muscle_development	ENSGALG00010011213	XKR6	0.447578508	0.180927109	2.473805669	0.013368239	0.02077545	positive
PRJNA729271	stage_E18_vs_E10	ordinary_muscle_development	ENSGALG00010011218	ENSGALG00010011218	-0.214412138	0.581413421	-0.368777415	0.71229364	0.756133623	negative
PRJNA729271	stage_E18_vs_E10	ordinary_muscle_development	ENSGALG00010011222	RCL1	-0.421869283	0.090243534	-4.674786838	2.94259E-06	7.22195E-06	negative
PRJNA729271	stage_E18_vs_E10	ordinary_muscle_development	ENSGALG00010011231	PLBD1	1.236749843	0.20519258	6.027263969	1.66759E-09	5.51738E-09	positive
PRJNA729271	stage_E18_vs_E10	ordinary_muscle_development	ENSGALG00010011235	ENSGALG00010011235	-0.56902261	0.093489276	-6.086501425	1.15405E-09	3.8713E-09	negative
PRJNA729271	stage_E18_vs_E10	ordinary_muscle_development	ENSGALG00010011237	ENSGALG00010011237	-0.288696908	0.261159868	-1.105441317	0.268968409	0.324000423	negative
PRJNA729271	stage_E18_vs_E10	ordinary_muscle_development	ENSGALG00010011239	RALGPS2	-0.777918617	0.074876777	-10.38931761	2.7733E-25	2.66749E-24	negative
PRJNA729271	stage_E18_vs_E10	ordinary_muscle_development	ENSGALG00010011243	ALX1	-4.630201032	0.54064659	-8.564191685	1.08837E-17	6.66774E-17	negative
PRJNA729271	stage_E18_vs_E10	ordinary_muscle_development	ENSGALG00010011244	PDE9A	-0.692540594	0.170910217	-4.05207252	5.07659E-05	0.000109274	negative
PRJNA729271	stage_E18_vs_E10	ordinary_muscle_development	ENSGALG00010011246	DNAJB5	1.665739005	0.2334993	7.133807262	9.763E-13	4.19676E-12	positive
PRJNA729271	stage_E18_vs_E10	ordinary_muscle_development	ENSGALG00010011247	ENSGALG00010011247	0.123823532	0.095188116	1.300829737	0.193316741	0.241126078	positive
PRJNA729271	stage_E18_vs_E10	ordinary_muscle_development	ENSGALG00010011250	ENSGALG00010011250	-0.171742573	0.099770892	-1.721369526	0.085183791	0.114444253	negative
PRJNA729271	stage_E18_vs_E10	ordinary_muscle_development	ENSGALG00010011251	FRZB	0.396701085	0.175387135	2.26185966	0.023706075	0.035282838	positive
PRJNA729271	stage_E18_vs_E10	ordinary_muscle_development	ENSGALG00010011253	KDM4C	1.320440662	0.107867172	12.24135794	1.8688E-34	2.80416E-33	positive
PRJNA729271	stage_E18_vs_E10	ordinary_muscle_development	ENSGALG00010011262	ENSGALG00010011262	-0.224793724	0.200916112	-1.118843688	0.26320683	0.3177995	negative
PRJNA729271	stage_E18_vs_E10	ordinary_muscle_development	ENSGALG00010011267	EVC2	-0.91997315	0.165840407	-5.54734016	2.90048E-08	8.63118E-08	negative
PRJNA729271	stage_E18_vs_E10	ordinary_muscle_development	ENSGALG00010011271	ANGPTL1	-2.150607187	0.161585887	-13.30937512	2.04182E-40	3.97265E-39	negative
PRJNA729271	stage_E18_vs_E10	ordinary_muscle_development	ENSGALG00010011273	RUNX1T1	-0.697972179	0.098872466	-7.059318006	1.67322E-12	7.04423E-12	negative
PRJNA729271	stage_E18_vs_E10	ordinary_muscle_development	ENSGALG00010011274	ENSGALG00010011274	0.122755938	0.522589232	0.234899479	0.814286754	0.845675038	positive
PRJNA729271	stage_E18_vs_E10	ordinary_muscle_development	ENSGALG00010011275	WDR4	-0.865918775	0.104763182	-8.265487537	1.39125E-16	7.91762E-16	negative
PRJNA729271	stage_E18_vs_E10	ordinary_muscle_development	ENSGALG00010011278	ENSGALG00010011278	-1.767043989	0.524891013	-3.366496939	0.000761294	0.001412075	negative
PRJNA729271	stage_E18_vs_E10	ordinary_muscle_development	ENSGALG00010011280	FAM199X	-0.245787519	0.08201409	-2.996893804	0.002727457	0.004698656	negative
PRJNA729271	stage_E18_vs_E10	ordinary_muscle_development	ENSGALG00010011281	ENSGALG00010011281	-0.329763729	0.609376087	-0.541149769	0.588404356	0.640597953	negative
PRJNA729271	stage_E18_vs_E10	ordinary_muscle_development	ENSGALG00010011282	CRY1	0.69783612	0.127199093	5.486172151	4.10737E-08	1.20549E-07	positive
PRJNA729271	stage_E18_vs_E10	ordinary_muscle_development	ENSGALG00010011283	LONRF3	-0.893988267	0.155910639	-5.733978609	9.81017E-09	3.03324E-08	negative
PRJNA729271	stage_E18_vs_E10	ordinary_muscle_development	ENSGALG00010011285	ENSGALG00010011285	-0.272779138	0.312918541	-0.871725712	0.38335803	0.443690086	negative
PRJNA729271	stage_E18_vs_E10	ordinary_muscle_development	ENSGALG00010011287	ENSGALG00010011287	-0.810391988	0.120377648	-6.732080245	1.67254E-11	6.46149E-11	negative
PRJNA729271	stage_E18_vs_E10	ordinary_muscle_development	ENSGALG00010011288	ENSGALG00010011288	-0.002027506	0.294743009	-0.006878894	0.99451148	0.995060668	negative
PRJNA729271	stage_E18_vs_E10	ordinary_muscle_development	ENSGALG00010011293	NDUFV3	1.012823716	0.167703881	6.039357636	1.54729E-09	5.13696E-09	positive
PRJNA729271	stage_E18_vs_E10	ordinary_muscle_development	ENSGALG00010011295	FAM167A	-0.884892186	0.384803526	-2.2995948	0.021471187	0.032207891	negative
PRJNA729271	stage_E18_vs_E10	ordinary_muscle_development	ENSGALG00010011297	OSR2	-0.214292624	0.184823181	-1.159446681	0.246274158	0.299500287	negative
PRJNA729271	stage_E18_vs_E10	ordinary_muscle_development	ENSGALG00010011300	EVC	-0.502659307	0.161876334	-3.105205652	0.001901468	0.003349754	negative
PRJNA729271	stage_E18_vs_E10	ordinary_muscle_development	ENSGALG00010011304	COMMD5	-0.497179285	0.092884037	-5.352688182	8.66571E-08	2.46825E-07	negative
PRJNA729271	stage_E18_vs_E10	ordinary_muscle_development	ENSGALG00010011306	ECI2	0.093062693	0.13884875	0.670245094	0.502701562	0.559823228	positive
PRJNA729271	stage_E18_vs_E10	ordinary_muscle_development	ENSGALG00010011307	ENSGALG00010011307	-0.934707784	0.462445735	-2.021226953	0.043256277	0.061380297	negative
PRJNA729271	stage_E18_vs_E10	ordinary_muscle_development	ENSGALG00010011308	SPIC	1.125892294	1.591362866	0.707501927	0.479254616	0.537677682	positive
PRJNA729271	stage_E18_vs_E10	ordinary_muscle_development	ENSGALG00010011311	DOCK8	-0.364084007	0.193413425	-1.882413319	0.059779919	0.082777028	negative
PRJNA729271	stage_E18_vs_E10	ordinary_muscle_development	ENSGALG00010011312	VPS13B	0.192337397	0.052687367	3.650541084	0.000261688	0.000517275	positive
PRJNA729271	stage_E18_vs_E10	ordinary_muscle_development	ENSGALG00010011313	RASSF9	-1.484303974	0.256274906	-5.791842816	6.96183E-09	2.18093E-08	negative
PRJNA729271	stage_E18_vs_E10	ordinary_muscle_development	ENSGALG00010011316	PARPBP	-1.661509379	0.201924675	-8.228362286	1.8979E-16	1.06835E-15	negative

PRJNA729271	stage_E18_vs_E10	ordinary_muscle_development	ENSGALG00010011317	BLK	-1.647659286	0.459538383	-3.585466084	0.000336477	0.000653872	negative
PRJNA729271	stage_E18_vs_E10	ordinary_muscle_development	ENSGALG00010011318	PRP4K	-0.500155435	0.098271549	-5.089524296	3.58963E-07	9.68932E-07	negative
PRJNA729271	stage_E18_vs_E10	ordinary_muscle_development	ENSGALG00010011321	MAP2	-0.900422558	0.149000539	-6.043082548	1.51197E-09	5.02316E-09	negative
PRJNA729271	stage_E18_vs_E10	ordinary_muscle_development	ENSGALG00010011322	GATA4	-2.247186016	1.247706565	-1.801053291	0.071694481	0.097892197	negative
PRJNA729271	stage_E18_vs_E10	ordinary_muscle_development	ENSGALG00010011323	GUCY2C	-0.722530564	0.855312269	-0.844756459	0.39824683	0.458141888	negative
PRJNA729271	stage_E18_vs_E10	ordinary_muscle_development	ENSGALG00010011326	COX6C	0.465293304	0.135714439	3.428473111	0.000606987	0.001140116	positive
PRJNA729271	stage_E18_vs_E10	ordinary_muscle_development	ENSGALG00010011329	PCDHGC5	0.046309151	0.116881545	0.396205844	0.69195318	0.737435582	positive
PRJNA729271	stage_E18_vs_E10	ordinary_muscle_development	ENSGALG00010011333	ENSGALG00010011333	1.590313827	0.129823888	12.24977813	1.68456E-34	2.53822E-33	positive
PRJNA729271	stage_E18_vs_E10	ordinary_muscle_development	ENSGALG00010011335	VCP	-0.74670215	0.173993421	-4.291553935	1.77427E-05	4.01907E-05	negative
PRJNA729271	stage_E18_vs_E10	ordinary_muscle_development	ENSGALG00010011339	ENSGALG00010011339	-0.28004945	0.303356086	-0.923170698	0.355918256	0.415280941	negative
PRJNA729271	stage_E18_vs_E10	ordinary_muscle_development	ENSGALG00010011340	PKNOX1	-0.27655726	0.109300832	-2.530239298	0.011398476	0.017898484	negative
PRJNA729271	stage_E18_vs_E10	ordinary_muscle_development	ENSGALG00010011342	RASAL2	0.435306203	0.066898798	6.50693605	7.6699E-11	2.82314E-10	positive
PRJNA729271	stage_E18_vs_E10	ordinary_muscle_development	ENSGALG00010011343	PXDC1	-0.668824654	0.141792964	-4.716910013	2.39454E-06	5.93922E-06	negative
PRJNA729271	stage_E18_vs_E10	ordinary_muscle_development	ENSGALG00010011345	CRMP1	-1.111230755	0.225362184	-4.930866104	8.18658E-07	2.13541E-06	negative
PRJNA729271	stage_E18_vs_E10	ordinary_muscle_development	ENSGALG00010011347	RGS22	-0.111633656	0.390481798	-0.285886964	0.774964699	0.811348018	negative
PRJNA729271	stage_E18_vs_E10	ordinary_muscle_development	ENSGALG00010011348	SLC22A23	-0.31642998	0.164991303	-1.917858547	0.05512895	0.076832568	negative
PRJNA729271	stage_E18_vs_E10	ordinary_muscle_development	ENSGALG00010011349	ENSGALG00010011349	-0.527448516	0.091779484	-5.746910892	9.08886E-09	2.81864E-08	negative
PRJNA729271	stage_E18_vs_E10	ordinary_muscle_development	ENSGALG00010011351	KANK1	-0.585189068	0.109152278	-5.361217188	8.26631E-08	2.35866E-07	negative
PRJNA729271	stage_E18_vs_E10	ordinary_muscle_development	ENSGALG00010011352	NTS	-3.376490994	0.672447335	-5.021197674	5.13503E-07	1.36523E-06	negative
PRJNA729271	stage_E18_vs_E10	ordinary_muscle_development	ENSGALG00010011353	ENSGALG00010011353	-3.318389688	0.217124866	-15.28332403	9.87714E-53	3.14658E-51	negative
PRJNA729271	stage_E18_vs_E10	ordinary_muscle_development	ENSGALG00010011357	UNC80	-0.141306742	0.415610471	-0.339998031	0.733858011	0.775254855	negative
PRJNA729271	stage_E18_vs_E10	ordinary_muscle_development	ENSGALG00010011359	RNF19A	0.223028833	0.086645599	2.574035331	0.010052001	0.01589958	positive
PRJNA729271	stage_E18_vs_E10	ordinary_muscle_development	ENSGALG00010011360	TCF4	0.039229141	0.175561189	0.223449961	0.823185324	0.853021966	positive
PRJNA729271	stage_E18_vs_E10	ordinary_muscle_development	ENSGALG00010011361	NUP37	-1.179439385	0.091717854	-12.85943068	7.61354E-38	1.33768E-36	negative
PRJNA729271	stage_E18_vs_E10	ordinary_muscle_development	ENSGALG00010011363	FDFT1	-0.105274934	0.147789132	-0.712332041	0.476259188	0.534730978	negative
PRJNA729271	stage_E18_vs_E10	ordinary_muscle_development	ENSGALG00010011365	PSMG4	1.347299868	0.17738823	7.595204404	3.07307E-14	1.46575E-13	positive
PRJNA729271	stage_E18_vs_E10	ordinary_muscle_development	ENSGALG00010011367	IER3IP1	0.649793121	0.112606563	5.770472889	7.90494E-09	2.46519E-08	positive
PRJNA729271	stage_E18_vs_E10	ordinary_muscle_development	ENSGALG00010011368	ENSGALG00010011368	-0.75155394	0.269213806	-2.791661954	0.005243811	0.008668914	negative
PRJNA729271	stage_E18_vs_E10	ordinary_muscle_development	ENSGALG00010011370	MGAT4C	-5.301631925	1.574826278	-3.366486831	0.000761322	0.001412075	negative
PRJNA729271	stage_E18_vs_E10	ordinary_muscle_development	ENSGALG00010011371	ENSGALG00010011371	0.184360098	0.20858175	0.883874538	0.376763944	0.436790639	positive
PRJNA729271	stage_E18_vs_E10	ordinary_muscle_development	ENSGALG00010011372	VMA21	-0.147872978	0.092745408	-1.594396762	0.110847197	0.145854223	negative
PRJNA729271	stage_E18_vs_E10	ordinary_muscle_development	ENSGALG00010011374	ENSGALG00010011374	-0.099565003	0.09303862	-1.070147033	0.284553131	0.340257189	negative
PRJNA729271	stage_E18_vs_E10	ordinary_muscle_development	ENSGALG00010011375	C12orf50	0.478198884	0.137917335	3.467286284	0.000525742	0.00099603	positive
PRJNA729271	stage_E18_vs_E10	ordinary_muscle_development	ENSGALG00010011377	SEC16B	0.938028835	0.322766129	2.906218311	0.003658261	0.00617305	positive
PRJNA729271	stage_E18_vs_E10	ordinary_muscle_development	ENSGALG00010011378	TUBB2B	-1.522590825	0.191796611	-7.938570001	2.04525E-15	1.07257E-14	negative
PRJNA729271	stage_E18_vs_E10	ordinary_muscle_development	ENSGALG00010011379	CELF4	-1.954309969	0.579873836	-3.370233051	0.000751046	0.001394622	negative
PRJNA729271	stage_E18_vs_E10	ordinary_muscle_development	ENSGALG00010011381	RLIG1	-0.59061125	0.124433437	-4.7464031	2.07066E-06	5.16507E-06	negative
PRJNA729271	stage_E18_vs_E10	ordinary_muscle_development	ENSGALG00010011382	ENSGALG00010011382	0.379511309	0.195083734	1.945376489	0.051729682	0.072418558	positive
PRJNA729271	stage_E18_vs_E10	ordinary_muscle_development	ENSGALG00010011384	PUM3	-1.19277428	0.082832998	-14.39974777	5.19327E-47	1.35633E-45	negative
PRJNA729271	stage_E18_vs_E10	ordinary_muscle_development	ENSGALG00010011386	CTSB	1.201022328	0.126197834	9.516980532	1.78284E-21	1.37899E-20	positive
PRJNA729271	stage_E18_vs_E10	ordinary_muscle_development	ENSGALG00010011388	GPR50	1.162813443	0.160862268	7.228627659	4.87899E-13	2.13723E-12	positive
PRJNA729271	stage_E18_vs_E10	ordinary_muscle_development	ENSGALG00010011389	RC3H1	0.437100619	0.086223361	5.069398962	3.99074E-07	1.07261E-06	positive
PRJNA729271	stage_E18_vs_E10	ordinary_muscle_development	ENSGALG00010011394	SERPINC1	5.485159018	0.900815744	6.089102078	1.13546E-09	3.81159E-09	positive
PRJNA729271	stage_E18_vs_E10	ordinary_muscle_development	ENSGALG00010011398	ENSGALG00010011398	0.690607526	0.185457746	3.723799856	0.000196247	0.000394043	positive
PRJNA729271	stage_E18_vs_E10	ordinary_muscle_development	ENSGALG00010011399	JAKMIP1	-0.870749001	0.247986352	-3.51127792	0.000445958	0.000854257	negative
PRJNA729271	stage_E18_vs_E10	ordinary_muscle_development	ENSGALG00010011400	BPHL	0.751487883	0.200722802	3.743908887	0.000181179	0.000365409	positive
PRJNA729271	stage_E18_vs_E10	ordinary_muscle_development	ENSGALG00010011401	ANKRD46	-0.250947131	0.068378257	-3.669984314	0.000242565	0.000481115	negative
PRJNA729271	stage_E18_vs_E10	ordinary_muscle_development	ENSGALG00010011403	SV2C	-3.048635049	0.518277469	-5.882244994	4.04739E-09	1.29622E-08	negative
PRJNA729271	stage_E18_vs_E10	ordinary_muscle_development	ENSGALG00010011406	UBE2R2	-0.438987054	0.116301687	-3.774554498	0.000160294	0.000325551	negative
PRJNA729271	stage_E18_vs_E10	ordinary_muscle_development	ENSGALG00010011407	CEP290	-0.729764454	0.181240481	-4.026498113	5.66137E-05	0.000121321	negative
PRJNA729271	stage_E18_vs_E10	ordinary_muscle_development	ENSGALG00010011411	ENSGALG00010011411	0.0587082	0.214733162	0.273400715	0.784545203	0.819366145	positive
PRJNA729271	stage_E18_vs_E10	ordinary_muscle_development	ENSGALG00010011412	KANSL1L	0.413034031	0.120720401	3.421410362	0.000622973	0.001168628	positive
PRJNA729271	stage_E18_vs_E10	ordinary_muscle_development	ENSGALG00010011413	ZBTB37	1.027966434	0.103661014	9.916615664	3.52506E-23	2.98457E-22	positive
PRJNA729271	stage_E18_vs_E10	ordinary_muscle_development	ENSGALG00010011414	PABPC1	0.490215221	0.114128754	4.295282323	1.74471E-05	3.96016E-05	positive
PRJNA729271	stage_E18_vs_E10	ordinary_muscle_development	ENSGALG00010011416	ENSGALG00010011416	0.445260455	0.43809656	1.016352321	0.309461642	0.367104223	positive
PRJNA729271	stage_E18_vs_E10	ordinary_muscle_development	ENSGALG00010011417	WFS1	0.438075207	0.144850093	3.024335002	0.002491804	0.004315256	positive
PRJNA729271	stage_E18_vs_E10	ordinary_muscle_development	ENSGALG00010011418	RIPK1	-0.758316186	0.08829234	-8.588697331	8.79609E-18	5.40939E-17	negative
PRJNA729271	stage_E18_vs_E10	ordinary_muscle_development	ENSGALG00010011419	RNF180	1.04840258	0.146764458	7.143436448	9.1026E-13	3.92218E-12	positive
PRJNA729271	stage_E18_vs_E10	ordinary_muscle_development	ENSGALG00010011420	HMGB3	-1.311923193	0.099655897	-13.16453153	1.40406E-39	2.64998E-38	negative
PRJNA729271	stage_E18_vs_E10	ordinary_muscle_development	ENSGALG00010011421	ENSGALG00010011421	0.917550331	0.116953776	7.845410059	4.31541E-15	2.21344E-14	positive
PRJNA729271	stage_E18_vs_E10	ordinary_muscle_development	ENSGALG00010011422	KATNAL2	-0.742389762	0.221043644	-3.358566429	0.000783479	0.001450201	negative
PRJNA729271	stage_E18_vs_E10	ordinary_muscle_development	ENSGALG00010011423	GDF6	-1.786129807	0.407535763	-4.382755995	1.17187E-05	2.70784E-05	negative
PRJNA729271	stage_E18_vs_E10	ordinary_muscle_development	ENSGALG00010011424	ACADL	1.29596192	0.097176531	13.33616161	1.42616E-40	2.78977E-39	positive
PRJNA729271	stage_E18_vs_E10	ordinary_muscle_development	ENSGALG00010011425	WASHC3	-0.279645328	0.080717181	-3.464508117	0.000531202	0.001005586	negative
PRJNA729271	stage_E18_vs_E10	ordinary_muscle_development	ENSGALG00010011426	NQO2	0.657742475	0.127393264	5.163086743	2.42911E-07	6.64839E-07	positive
PRJNA729271	stage_E18_vs_E10	ordinary_muscle_development	ENSGALG00010011427	TMTC3	-0.30690182	0.087732746	-3.498144469	0.000468507	0.000895203	negative
PRJNA729271	stage_E18_vs_E10	ordinary_muscle_development	ENSGALG00010011428	UQCRB	0.810569216	0.163078996	4.97040842	6.6812E-07	1.75601E-06	positive
PRJNA729271	stage_E18_vs_E10	ordinary_muscle_development	ENSGALG00010011429	UBAP2	0.184526346	0.107369574	1.718609277	0.085685541	0.115065029	positive
PRJNA729271	stage_E18_vs_E10	ordinary_muscle_development	ENSGALG00010011430	ENSGALG00010011430	0.180173289	0.332448349	0.54195874	0.58784693	0.640039153	positive
PRJNA729271	stage_E18_vs_E10	ordinary_muscle_development	ENSGALG00010011431	DRAM1	-0.323783103	0.459191862	-0.705115073	0.480738627	0.538800464	negative
PRJNA729271	stage_E18_vs_E10	ordinary_muscle_development	ENSGALG00010011433	RNASE6	2.286343604	0.340933093	6.706135748	1.99846E-11	7.67355E-11	positive
PRJNA729271	stage_E18_vs_E10	ordinary_muscle_development	ENSGALG00010011434	NPL	0.886795571	0.180083722	4.924351636	8.46405E-07	2.20699E-06	positive
PRJNA729271	stage_E18_vs_E10	ordinary_muscle_development	ENSGALG00010011436	UBAP2L2	-0.640679614	2.254218716	-0.284213599			negative
PRJNA729271	stage_E18_vs_E10	ordinary_muscle_development	ENSGALG00010011437	DMRT1	-1.461374786	0.546757277	-2.67280354	0.007522029	0.012115992	negative
PRJNA729271	stage_E18_vs_E10	ordinary_muscle_development	ENSGALG00010011438	ENSGALG00010011438	3.398566979	0.442528533	7.67988214	1.59235E-14	7.81616E-14	positive
PRJNA729271	stage_E18_vs_E10	ordinary_muscle_development	ENSGALG00010011439	ENSGALG00010011439	-0.643944663	0.338972731	-1.899694588	0.057473211	0.079849918	negative
PRJNA729271	stage_E18_vs_E10	ordinary_muscle_development	ENSGALG00010011440	FUNDC1	-0.211565704	0.112586042	-1.879146829	0.060224447	0.083310044	negative
PRJNA729271	stage_E18_vs_E10	ordinary_muscle_development	ENSGALG00010011441	CD99L2	0.501793892	0.080117799	6.263201192	3.77153E-10	1.31986E-09	positive
PRJNA729271	stage_E18_vs_E10	ordinary_muscle_development	ENSGALG00010011442	ENSGALG00010011442	-1.010285686	2.057744595	-0.490967484	0.623449446	0.673993117	negative
PRJNA729271	stage_E18_vs_E10	ordinary_muscle_development	ENSGALG00010011443	ENSGALG00010011443	-0.620731992	0.168155478	-3.691417	0.000223008	0.00044433	negative
PRJNA729271	stage_E18_vs_E10	ordinary_muscle_development	ENSGALG00010011445	MTERF3	-0.289248489	0.082636668	-3.500243861	0.000464833	0.000888651	negative
PRJNA729271	stage_E18_vs_E10	ordinary_muscle_development	ENSGALG00010011446	ENSGALG00010011446	0.32076056	0.223996176	1.431991232	0.152146331	0.194441991	positive
PRJNA729271	stage_E18_vs_E10	ordinary_muscle_development	ENSGALG00010011447	LAMC1	2.626085804	0.130282513	20.1568556	2.34307E-90	2.1771E-88	positive
PRJNA729271	stage_E18_vs_E10	ordinary_muscle_development	ENSGALG00010011448	AvBD10	7.038620465	2.605362143	2.701590058	0.006900879	0.011173843	positive
PRJNA729271	stage_E18_vs_E10	ordinary_muscle_development	ENSGALG00010011449	DUSP22	0.529610786	0.144674946	3.660694559	0.000251532	0.000498082	positive
PRJNA729271	stage_E18_vs_E10	ordinary_muscle_development	ENSGALG00010011450	PTDSS1	-0.794198401	0.06980418	-11.3775193	5.41173E-30	6.60294E-29	negative
PRJNA729271	stage_E18_vs_E10	ordinary_muscle_development	ENSGALG00010011451	CRHBP	-7.385230313	0.602558315	-12.25645739	1.55135E-34	2.34238E-33	negative
PRJNA729271	stage_E18_vs_E10	ordinary_muscle_development	ENSGALG00010011452	KITLG	0.625717987	0.189608878	3.300045827	0.00096669	0.001764091	positive
PRJNA729271	stage_E18_vs_E10	ordinary_muscle_development	ENSGALG00010011453	GNPTAB	-0.585087134	0.124772215	-4.689242184	2.74219E-06	6.75068E-06	negative
PRJNA729271	stage_E18_vs_E10	ordinary_muscle_development	ENSGALG00010011456	KIAA1328	-0.410439872	0.096609975	-4.248421281	2.15282E-05	4.83052E-05	negative
PRJNA729271	stage_E18_vs_E10	ordinary_muscle_development	ENSGALG00010011457	ENSGALG00010011457	0.227852938	0.28402964	0.802215353	0.422428397	0.482818137	positive
PRJNA729271	stage_E18_vs_E10	ordinary_muscle_development	ENSGALG00010011458	TPGS2	-1.016735966	0.13297365	-7.646146192	2.07092E-14	1.00462E-13	negative
PRJNA729271	stage_E18_vs_E10	ordinary_muscle_development	ENSGALG00010011459	ENSGALG00010011459	0.317555845	0.278371017	1.140764754	0.253967831	0.307720782	positive
PRJNA729271	stage_E18_vs_E10	ordinary_muscle_development	ENSGALG00010011460	LAMC2	0.507170941	0.575925402	0.880619155	0.378523987	0.438690749	positive
PRJNA729271	stage_E18_vs_E10	ordinary_muscle_development	ENSGALG00010011461	IRF4	0.740376702	0.359522769	2.059331887	0.039462456	0.056433336	positive
PRJNA729271	stage_E18_vs_E10	ordinary_muscle_development	ENSGALG00010011463	IMP3	-1.123169717	0.136672791	-8.217946724	2.07017E-16	1.16216E-15	negative
PRJNA729271	stage_E18_vs_E10	ordinary_muscle_development	ENSGALG00010011464	FANCG	-1.270476559	0.159841318	-7.948361392	1.88995E-15	9.93284E-15	negative
PRJNA729271	stage_E18_vs_E10	ordinary_muscle_development	ENSGALG00010011465	PPP2R2C	1.442193293	0.333597308	4.323156264	1.53813E-05	3.51271E-05	positive
PRJNA729271	stage_E18_vs_E10	ordinary_muscle_development	ENSGALG00010011466	MTMR1	-0.301533938	0.067470241	-4.469139779	7.85348E-06	1.84949E-05	negative
PRJNA729271	stage_E18_vs_E10	ordinary_muscle_development	ENSGALG00010011467	ENSGALG00010011467	-0.881310551	3.984134098	-0.221205042			negative
PRJNA729271	stage_E18_vs_E10	ordinary_muscle_development	ENSGALG00010011468	EFHC2	-0.181620102	0.266573392	-0.681313693	0.495673021	0.55296221	negative
PRJNA729271	stage_E18_vs_E10	ordinary_muscle_development	ENSGALG00010011469	CPS1	2.114317704	0.330042361	6.406200999	1.4919E-10	5.36604E-10	positive
PRJNA729271	stage_E18_vs_E10	ordinary_muscle_development	ENSGALG00010011471	NMNAT2	-1.064923857	0.375959063	-2.832552699	0.004617795	0.007687486	negative
PRJNA729271	stage_E18_vs_E10	ordinary_muscle_development	ENSGALG00010011475	NDP	-1.252630959	0.299044456	-4.188778409	2.8046E-05	6.21316E-05	negative
PRJNA729271	stage_E18_vs_E10	ordinary_muscle_development	ENSGALG00010011477	SDC2	0.584933674	0.167245273	3.497460117	0.000469711	0.000897267	positive
PRJNA729271	stage_E18_vs_E10	ordinary_muscle_development	ENSGALG00010011478	HDAC3	-0.593895386	0.077546424	-7.658578608	1.88002E-14	9.15689E-14	negative
PRJNA729271	stage_E18_vs_E10	ordinary_muscle_development	ENSGALG00010011480	BLTP3B	-0.419155287	0.066680884	-6.285988798	3.25773E-10	1.14364E-09	negative
PRJNA729271	stage_E18_vs_E10	ordinary_muscle_development	ENSGALG00010011482	DMRT2	0.695264687	0.150021705	4.634427297	3.57927E-06	8.69328E-06	positive
PRJNA729271	stage_E18_vs_E10	ordinary_muscle_development	ENSGALG00010011484	CHPT1	0.680400925	0.078648201	8.651195038	5.09629E-18	3.19234E-17	positive
PRJNA729271	stage_E18_vs_E10	ordinary_muscle_development	ENSGALG00010011485	MTM1	0.177790054	0.078322497	2.269974278	0.023209144	0.03459296	positive
PRJNA729271	stage_E18_vs_E10	ordinary_muscle_development	ENSGALG00010011487	MAN2B2	-0.709990363	0.159084976	-4.46296302	8.0834E-06	1.90086E-05	negative
PRJNA729271	stage_E18_vs_E10	ordinary_muscle_development	ENSGALG00010011493	ENSGALG00010011493	0.717152784	0.268811673	2.667863256	0.007633532	0.012281945	positive
PRJNA729271	stage_E18_vs_E10	ordinary_muscle_development	ENSGALG00010011496	ACTR6	-1.50691974	0.089243354	-16.88551223	5.7515E-64	2.6299E-62	negative
PRJNA729271	stage_E18_vs_E10	ordinary_muscle_development	ENSGALG00010011497	CPQ	1.649213335	0.247961558	6.651084749	2.90941E-11	1.10369E-10	positive
PRJNA729271	stage_E18_vs_E10	ordinary_muscle_development	ENSGALG00010011499	SMG7	-0.126745744	0.147588923	-0.858775454	0.390464412	0.450547816	negative
PRJNA729271	stage_E18_vs_E10	ordinary_muscle_development	ENSGALG00010011503	NCF2	0.769116331	0.387152774	1.986596462	0.046967134	0.066224572	positive
PRJNA729271	stage_E18_vs_E10	ordinary_muscle_development	ENSGALG00010011505	MYL1	4.293017702	0.152243045	28.19844867	6.1094E-175	2.6835E-172	positive
PRJNA729271	stage_E18_vs_E10	ordinary_muscle_development	ENSGALG00010011509	EXOC2	-0.253130288	0.068697878	-3.684688574	0.000228982	0.000455545	negative
PRJNA729271	stage_E18_vs_E10	ordinary_muscle_development	ENSGALG00010011510	ENSGALG00010011510	0.938272517	0.263796759	3.556800775	0.000375399	0.00072523	positive
PRJNA729271	stage_E18_vs_E10	ordinary_muscle_development	ENSGALG00010011515	SERINC5	0.79351416	0.085207322	9.312746199	1.24569E-20	9.13317E-20	positive
PRJNA729271	stage_E18_vs_E10	ordinary_muscle_development	ENSGALG00010011523	PIGO	-0.261193958	0.122544902	-2.131414306	0.033055025	0.047913259	negative
PRJNA729271	stage_E18_vs_E10	ordinary_muscle_development	ENSGALG00010011525	ENSGALG00010011525	-2.134015033	0.251793201	-8.4752687	2.34538E-17	1.40248E-16	negative
PRJNA729271	stage_E18_vs_E10	ordinary_muscle_development	ENSGALG00010011529	SCYL2	-0.125106715	0.083002142	-1.507270916	0.131741229	0.170651396	negative
PRJNA729271	stage_E18_vs_E10	ordinary_muscle_development	ENSGALG00010011530	ARPC5	-0.598922542	0.119848963	-4.99731099	5.81353E-07	1.53688E-06	negative
PRJNA729271	stage_E18_vs_E10	ordinary_muscle_development	ENSGALG00010011531	MTDH	-0.076721087	0.072105339	-1.064013946	0.287322382	0.343341956	negative
PRJNA729271	stage_E18_vs_E10	ordinary_muscle_development	ENSGALG00010011533	ENSGALG00010011533	-0.777227945	0.275452478	-2.821640785	0.004777866	0.007936646	negative
PRJNA729271	stage_E18_vs_E10	ordinary_muscle_development	ENSGALG00010011536	FCHSD1	-1.348999493	0.192185652	-7.01925183	2.23059E-12	9.28294E-12	negative
PRJNA729271	stage_E18_vs_E10	ordinary_muscle_development	ENSGALG00010011538	ENSGALG00010011538	1.220207668	0.258924235	4.712605083	2.4457E-06	6.05886E-06	positive
PRJNA729271	stage_E18_vs_E10	ordinary_muscle_development	ENSGALG00010011539	LANCL1	-0.317177835	0.107192453	-2.958956779	0.003086824	0.005277602	negative
PRJNA729271	stage_E18_vs_E10	ordinary_muscle_development	ENSGALG00010011544	STOML2	-0.646214784	0.093380274	-6.920249368	4.50849E-12	1.83569E-11	negative
PRJNA729271	stage_E18_vs_E10	ordinary_muscle_development	ENSGALG00010011545	USP12-like	-0.370005124	0.076906687	-4.811091701	1.50108E-06	3.8092E-06	negative

PRJNA729271	stage_E18_vs_E10	ordinary_muscle_development	ENSGALG00010011546	MAOA	1.733316764	0.126580292	13.6934173	1.11156E-42	2.37641E-41	positive
PRJNA729271	stage_E18_vs_E10	ordinary_muscle_development	ENSGALG00010011549	KIAA0232	0.723333091	0.143938125	5.025305763	5.02631E-07	1.33706E-06	positive
PRJNA729271	stage_E18_vs_E10	ordinary_muscle_development	ENSGALG00010011551	GMDS	-1.134984031	0.155274538	-7.309530898	2.68077E-13	1.19636E-12	negative
PRJNA729271	stage_E18_vs_E10	ordinary_muscle_development	ENSGALG00010011555	ENSGALG00010011555	-0.476196652	4.224155429	-0.112731802	0.910243188	0.925698099	negative
PRJNA729271	stage_E18_vs_E10	ordinary_muscle_development	ENSGALG00010011556	LAPTM4B	0.752890522	0.143259852	5.255418826	1.47688E-07	4.10969E-07	positive
PRJNA729271	stage_E18_vs_E10	ordinary_muscle_development	ENSGALG00010011558	MYBPC1	4.679399203	0.559837887	8.358489687			positive
PRJNA729271	stage_E18_vs_E10	ordinary_muscle_development	ENSGALG00010011559	MTX3	-0.108095233	0.128137273	-0.84358931	0.39889894	0.458819246	negative
PRJNA729271	stage_E18_vs_E10	ordinary_muscle_development	ENSGALG00010011562	ENSGALG00010011562	-6.627975808	0.722847629	-9.169257181	4.7629E-20	3.35137E-19	negative
PRJNA729271	stage_E18_vs_E10	ordinary_muscle_development	ENSGALG00010011564	ARAP3	1.288113481	0.121762008	10.57894412	3.73139E-26	3.7691E-25	positive
PRJNA729271	stage_E18_vs_E10	ordinary_muscle_development	ENSGALG00010011570	RGL1	0.035609546	0.136521917	0.260833913	0.794220591	0.827979536	positive
PRJNA729271	stage_E18_vs_E10	ordinary_muscle_development	ENSGALG00010011571	ATOSB	0.750651147	0.116859027	6.42356149	1.33122E-10	4.8072E-10	positive
PRJNA729271	stage_E18_vs_E10	ordinary_muscle_development	ENSGALG00010011572	PTCD2	0.416637228	0.103813385	4.013328627	5.98685E-05	0.000127993	positive
PRJNA729271	stage_E18_vs_E10	ordinary_muscle_development	ENSGALG00010011573	CMAS	-0.755168038	0.180749404	-4.177983553	2.94105E-05	6.49261E-05	negative
PRJNA729271	stage_E18_vs_E10	ordinary_muscle_development	ENSGALG00010011574	GAS2L3	-0.040542217	0.142655194	-0.284197272	0.776259206	0.812527238	negative
PRJNA729271	stage_E18_vs_E10	ordinary_muscle_development	ENSGALG00010011575	ENSGALG00010011575	-2.545329012	0.497844689	-5.112696928	3.17592E-07	8.61432E-07	negative
PRJNA729271	stage_E18_vs_E10	ordinary_muscle_development	ENSGALG00010011577	ENSGALG00010011577	1.476493269	0.159568267	9.253050749	2.18175E-20	1.57649E-19	positive
PRJNA729271	stage_E18_vs_E10	ordinary_muscle_development	ENSGALG00010011578	MYLK4	0.014265469	0.329978394	0.043231524	0.965516976	0.971751741	positive
PRJNA729271	stage_E18_vs_E10	ordinary_muscle_development	ENSGALG00010011584	ENSGALG00010011584	-0.800672276	0.957161278	-0.836507174	0.402869642	0.462798817	negative
PRJNA729271	stage_E18_vs_E10	ordinary_muscle_development	ENSGALG00010011586	ENSGALG00010011586	0.480402304	0.252912796	1.899478049	0.05750165	0.079881773	positive
PRJNA729271	stage_E18_vs_E10	ordinary_muscle_development	ENSGALG00010011588	COLGALT2	-0.101055882	0.199004263	-0.507807625	0.611588262	0.662306452	negative
PRJNA729271	stage_E18_vs_E10	ordinary_muscle_development	ENSGALG00010011589	RERG	0.446245508	0.223959757	1.992525417	0.046313439	0.065391906	positive
PRJNA729271	stage_E18_vs_E10	ordinary_muscle_development	ENSGALG00010011593	WRNIP1	-0.501279056	0.101572521	-4.935183762	8.00753E-07	2.09285E-06	negative
PRJNA729271	stage_E18_vs_E10	ordinary_muscle_development	ENSGALG00010011595	ENSGALG00010011595	0.523278133	0.163857276	3.193499524	0.001405596	0.0025155	positive
PRJNA729271	stage_E18_vs_E10	ordinary_muscle_development	ENSGALG00010011597	TBC1D14	-0.177726976	0.130504388	-1.361846745	0.173246256	0.218441586	negative
PRJNA729271	stage_E18_vs_E10	ordinary_muscle_development	ENSGALG00010011601	ANO4	-3.032562505	0.639757767	-4.740173015	2.13536E-06	5.31913E-06	negative
PRJNA729271	stage_E18_vs_E10	ordinary_muscle_development	ENSGALG00010011603	ENSGALG00010011603	0.673211432	0.279266137	2.410644694	0.015924353	0.024472381	positive
PRJNA729271	stage_E18_vs_E10	ordinary_muscle_development	ENSGALG00010011605	APPL2	0.215742819	0.141100923	1.528996505	0.12626531	0.164469417	positive
PRJNA729271	stage_E18_vs_E10	ordinary_muscle_development	ENSGALG00010011607	TSEN15	-0.803873523	0.179891066	-4.468668417	7.8708E-06	1.85297E-05	negative
PRJNA729271	stage_E18_vs_E10	ordinary_muscle_development	ENSGALG00010011609	ENSGALG00010011609	2.623141503	0.801707701	3.271942504	0.001068113	0.001938437	positive
PRJNA729271	stage_E18_vs_E10	ordinary_muscle_development	ENSGALG00010011612	ENSGALG00010011612	-0.398994791	0.468114844	-0.852343812	0.394023296	0.454039882	negative
PRJNA729271	stage_E18_vs_E10	ordinary_muscle_development	ENSGALG00010011617	MAMLD1	1.439944608	0.096203754	14.96765508	1.1946E-50	3.57024E-49	positive
PRJNA729271	stage_E18_vs_E10	ordinary_muscle_development	ENSGALG00010011619	PCDH1	1.187214651	0.158618245	7.484729476	7.16947E-14	3.33616E-13	positive
PRJNA729271	stage_E18_vs_E10	ordinary_muscle_development	ENSGALG00010011621	WASHC4	0.276206501	0.088402599	3.124416089	0.001781583	0.00315312	positive
PRJNA729271	stage_E18_vs_E10	ordinary_muscle_development	ENSGALG00010011622	BACH1	0.177000414	0.160265789	1.104417954	0.269411876	0.324453734	positive
PRJNA729271	stage_E18_vs_E10	ordinary_muscle_development	ENSGALG00010011625	ERBB4	1.649209226	0.217000317	7.600031416	2.96059E-14	1.41443E-13	positive
PRJNA729271	stage_E18_vs_E10	ordinary_muscle_development	ENSGALG00010011626	SERPINB1	-0.488227162	0.660833679	-0.738804903	0.460025474	0.519360483	negative
PRJNA729271	stage_E18_vs_E10	ordinary_muscle_development	ENSGALG00010011628	MATN2	4.251734632	0.191497728	22.20253306	3.2463E-109	4.8511E-107	positive
PRJNA729271	stage_E18_vs_E10	ordinary_muscle_development	ENSGALG00010011630	DELE1	0.661118938	0.101011704	6.54497362	5.95059E-11	2.20824E-10	positive
PRJNA729271	stage_E18_vs_E10	ordinary_muscle_development	ENSGALG00010011633	TADA2B	0.019300726	0.086109779	0.224140934	0.822647642	0.852586699	positive
PRJNA729271	stage_E18_vs_E10	ordinary_muscle_development	ENSGALG00010011634	AVD	2.379180757	0.897978185	2.64948614	0.008061428	0.012927359	positive
PRJNA729271	stage_E18_vs_E10	ordinary_muscle_development	ENSGALG00010011635	PCDH12	1.014596451	0.194313537	5.221439866	1.77537E-07	4.91295E-07	positive
PRJNA729271	stage_E18_vs_E10	ordinary_muscle_development	ENSGALG00010011636	GRIK1	-0.365998841	0.400226544	-0.914479178	0.360465114	0.419943886	negative
PRJNA729271	stage_E18_vs_E10	ordinary_muscle_development	ENSGALG00010011637	CECR2	0.0177358	0.155518797	0.114042807	0.90920386	0.924835786	positive
PRJNA729271	stage_E18_vs_E10	ordinary_muscle_development	ENSGALG00010011638	GRPEL1	-0.192887226	0.082801163	-2.329523154	0.019831368	0.029905917	negative
PRJNA729271	stage_E18_vs_E10	ordinary_muscle_development	ENSGALG00010011639	ENSGALG00010011639	0.372973024	0.144061067	2.58899252	0.009625719	0.015258617	positive
PRJNA729271	stage_E18_vs_E10	ordinary_muscle_development	ENSGALG00010011641	ENSGALG00010011641	-1.912957388	0.609853004	-3.136751602	0.001708308	0.003029723	negative
PRJNA729271	stage_E18_vs_E10	ordinary_muscle_development	ENSGALG00010011643	C1orf21	0.796494103	0.08053702	9.889788665	4.61E-23	3.88274E-22	positive
PRJNA729271	stage_E18_vs_E10	ordinary_muscle_development	ENSGALG00010011647	FBXO38	0.123844307	0.083875641	1.476522932	0.139803564	0.180225245	positive
PRJNA729271	stage_E18_vs_E10	ordinary_muscle_development	ENSGALG00010011648	ENSGALG00010011648	-0.312964477	0.225394984	-1.388515712	0.164980064	0.209073791	negative
PRJNA729271	stage_E18_vs_E10	ordinary_muscle_development	ENSGALG00010011649	MRPS27	0.104540991	0.137506307	0.760263248	0.447097245	0.506658945	positive
PRJNA729271	stage_E18_vs_E10	ordinary_muscle_development	ENSGALG00010011650	RPL30	0.709435504	0.155325157	4.567421773	4.9376E-06	1.18435E-05	positive
PRJNA729271	stage_E18_vs_E10	ordinary_muscle_development	ENSGALG00010011651	SERPINB6	0.13851636	0.128266243	1.079912818	0.280181005	0.33577707	positive
PRJNA729271	stage_E18_vs_E10	ordinary_muscle_development	ENSGALG00010011653	NR2C1	-0.397142814	0.089762928	-4.424352266	9.67321E-06	2.25604E-05	negative
PRJNA729271	stage_E18_vs_E10	ordinary_muscle_development	ENSGALG00010011654	CLDN8	-3.504514134	1.364434345	-2.568473997	0.010214738	0.016142894	negative
PRJNA729271	stage_E18_vs_E10	ordinary_muscle_development	ENSGALG00010011655	SORCS2	-0.013495012	0.184557392	-0.073120949	0.941709872	0.952287191	negative
PRJNA729271	stage_E18_vs_E10	ordinary_muscle_development	ENSGALG00010011657	ALDH1L2	1.218195446	0.142222955	8.565392589	1.07708E-17	6.60419E-17	positive
PRJNA729271	stage_E18_vs_E10	ordinary_muscle_development	ENSGALG00010011658	TENM1	-2.117806871	0.324826219	-6.519815038	7.03941E-11	2.59833E-10	negative
PRJNA729271	stage_E18_vs_E10	ordinary_muscle_development	ENSGALG00010011659	CD14	0.666686741	0.157993103	4.219720528	2.44605E-05	5.45554E-05	positive
PRJNA729271	stage_E18_vs_E10	ordinary_muscle_development	ENSGALG00010011660	ENSGALG00010011660	-0.98968226	0.089786795	-11.02258146	2.97406E-28	3.33402E-27	negative
PRJNA729271	stage_E18_vs_E10	ordinary_muscle_development	ENSGALG00010011662	RIDA	-0.560511501	0.16930331	-3.310694278	0.000930648	0.001702095	negative
PRJNA729271	stage_E18_vs_E10	ordinary_muscle_development	ENSGALG00010011663	UTP20	-1.010555527	0.137987468	-7.323531186	2.41529E-13	1.07921E-12	negative
PRJNA729271	stage_E18_vs_E10	ordinary_muscle_development	ENSGALG00010011664	SERPINB10B	4.208323667	0.538940208	7.808516799	5.78649E-15	2.94196E-14	positive
PRJNA729271	stage_E18_vs_E10	ordinary_muscle_development	ENSGALG00010011667	ENSGALG00010011667	-0.194115355	0.410299508	-0.473106478	0.636137215	0.685562003	negative
PRJNA729271	stage_E18_vs_E10	ordinary_muscle_development	ENSGALG00010011669	ENSGALG00010011669	-0.588023812	0.749169434	-0.784900965	0.432511636	0.49236285	negative
PRJNA729271	stage_E18_vs_E10	ordinary_muscle_development	ENSGALG00010011670	TMCO6	0.156807202	0.171569576	0.913956923	0.360739481	0.42022975	positive
PRJNA729271	stage_E18_vs_E10	ordinary_muscle_development	ENSGALG00010011671	SLC41A2	-1.645261784	0.195960026	-8.395905119	4.62319E-17	2.71748E-16	negative
PRJNA729271	stage_E18_vs_E10	ordinary_muscle_development	ENSGALG00010011673	POP1	-1.277367961	0.126546911	-10.09402723	5.87103E-24	5.20493E-23	negative
PRJNA729271	stage_E18_vs_E10	ordinary_muscle_development	ENSGALG00010011674	ENSGALG00010011674	-2.342275779	0.532356785	-4.399823287	1.08339E-05	2.51431E-05	negative
PRJNA729271	stage_E18_vs_E10	ordinary_muscle_development	ENSGALG00010011677	EDEM3	-0.387213583	0.080784846	-4.793146254	1.64186E-06	4.14396E-06	negative
PRJNA729271	stage_E18_vs_E10	ordinary_muscle_development	ENSGALG00010011679	OVAL	-7.786174533	1.214507677	-6.410971856	1.44595E-10	5.20851E-10	negative
PRJNA729271	stage_E18_vs_E10	ordinary_muscle_development	ENSGALG00010011681	MAP1B	-1.627317867	0.358824924	-4.535130523	5.75679E-06	1.3729E-05	negative
PRJNA729271	stage_E18_vs_E10	ordinary_muscle_development	ENSGALG00010011682	USP15	0.369693554	0.123001849	3.005593466	0.002650631	0.004575551	positive
PRJNA729271	stage_E18_vs_E10	ordinary_muscle_development	ENSGALG00010011683	NIPAL2	-0.171790807	0.273056058	-0.629141167	0.529256641	0.585392981	negative
PRJNA729271	stage_E18_vs_E10	ordinary_muscle_development	ENSGALG00010011685	NDUFA2	0.829041191	0.112366896	7.377984288	1.60704E-13	7.27939E-13	positive
PRJNA729271	stage_E18_vs_E10	ordinary_muscle_development	ENSGALG00010011688	PDE3A	0.094109164	0.148062629	0.635603761	0.525034718	0.581450683	positive
PRJNA729271	stage_E18_vs_E10	ordinary_muscle_development	ENSGALG00010011692	ARL1	-0.23098658	0.089310061	-2.586344445	0.009699994	0.015372996	negative
PRJNA729271	stage_E18_vs_E10	ordinary_muscle_development	ENSGALG00010011696	ZFC3H1	0.2397931	0.109402325	2.191846475	0.028390594	0.041660423	positive
PRJNA729271	stage_E18_vs_E10	ordinary_muscle_development	ENSGALG00010011699	TIAM1	-1.409745442	0.145705454	-9.675310035	3.83926E-22	3.0797E-21	negative
PRJNA729271	stage_E18_vs_E10	ordinary_muscle_development	ENSGALG00010011702	NUP205	-0.60710997	0.083997897	-7.227680578	4.91313E-13	2.15153E-12	negative
PRJNA729271	stage_E18_vs_E10	ordinary_muscle_development	ENSGALG00010011703	YWHAZ	-1.024666805	0.094437409	-10.85022148	1.98943E-27	2.15521E-26	negative
PRJNA729271	stage_E18_vs_E10	ordinary_muscle_development	ENSGALG00010011708	IK	0.004499924	0.092024728	0.048899071	0.960999729	0.968011889	positive
PRJNA729271	stage_E18_vs_E10	ordinary_muscle_development	ENSGALG00010011712	DCAF12L2	-0.058891957	0.076267152	-0.772179839	0.440007921	0.4996408	negative
PRJNA729271	stage_E18_vs_E10	ordinary_muscle_development	ENSGALG00010011713	AVPR1A	-2.208828454	0.687367103	-3.213462566	0.001311449	0.002356446	negative
PRJNA729271	stage_E18_vs_E10	ordinary_muscle_development	ENSGALG00010011715	SLCO1B3	3.761244807	1.739303262	2.162500864	0.030579591	0.044633085	positive
PRJNA729271	stage_E18_vs_E10	ordinary_muscle_development	ENSGALG00010011720	IKZF2	-0.556734845	0.188135477	-2.959223074	0.003084158	0.005273666	negative
PRJNA729271	stage_E18_vs_E10	ordinary_muscle_development	ENSGALG00010011723	SPRY3	1.371217171	0.156900113	8.739427552	2.34295E-18	1.50137E-17	positive
PRJNA729271	stage_E18_vs_E10	ordinary_muscle_development	ENSGALG00010011724	ARHGEF28	-0.829694866	0.154845674	-5.358205005	8.40528E-08	2.39596E-07	negative
PRJNA729271	stage_E18_vs_E10	ordinary_muscle_development	ENSGALG00010011726	WDR55	-0.658802758	0.089187764	-7.386694378	1.50524E-13	6.83108E-13	negative
PRJNA729271	stage_E18_vs_E10	ordinary_muscle_development	ENSGALG00010011729	FAM129A	0.191250215	0.232446398	0.822771256	0.410638085	0.470864571	positive
PRJNA729271	stage_E18_vs_E10	ordinary_muscle_development	ENSGALG00010011734	ENSGALG00010011734	-0.662133392	0.291348957	-2.272647199	0.023047449	0.034369627	negative
PRJNA729271	stage_E18_vs_E10	ordinary_muscle_development	ENSGALG00010011738	FGD6	0.447633829	0.117853919	3.798209127	0.000145745	0.000297756	positive
PRJNA729271	stage_E18_vs_E10	ordinary_muscle_development	ENSGALG00010011745	AFAP1L1	2.853228538	0.190130937	15.00665062	6.64179E-51	1.99736E-49	positive
PRJNA729271	stage_E18_vs_E10	ordinary_muscle_development	ENSGALG00010011746	SOD1	-0.826214356	0.143276458	-5.766574394	8.0899E-09	2.52125E-08	negative
PRJNA729271	stage_E18_vs_E10	ordinary_muscle_development	ENSGALG00010011747	UTP15	-1.125406328	0.118511311	-9.496193392	2.17703E-21	1.67406E-20	negative
PRJNA729271	stage_E18_vs_E10	ordinary_muscle_development	ENSGALG00010011751	ZNF706	0.603652815	0.109645044	5.505518455	3.68083E-08	1.08685E-07	positive
PRJNA729271	stage_E18_vs_E10	ordinary_muscle_development	ENSGALG00010011752	RNF2	-1.626677558	0.124513885	-13.06422616	5.2718E-39	9.6973E-38	negative
PRJNA729271	stage_E18_vs_E10	ordinary_muscle_development	ENSGALG00010011758	SERPINB11	-0.151379396	0.542153496	-0.279218703	0.780076991	0.815581073	negative
PRJNA729271	stage_E18_vs_E10	ordinary_muscle_development	ENSGALG00010011759	CREB3	-0.165685769	0.131548622	-1.259502123	0.207849024	0.257545871	negative
PRJNA729271	stage_E18_vs_E10	ordinary_muscle_development	ENSGALG00010011764	PCYOX1L	0.270729794	0.127684449	2.120303578	0.033980452	0.04914164	positive
PRJNA729271	stage_E18_vs_E10	ordinary_muscle_development	ENSGALG00010011770	CHST11	-3.781644323	0.313265567	-12.07168842	1.49045E-33	2.12847E-32	negative
PRJNA729271	stage_E18_vs_E10	ordinary_muscle_development	ENSGALG00010011772	GRHL2	-2.265108863	0.458646057	-4.938686001	7.86507E-07	2.05635E-06	negative
PRJNA729271	stage_E18_vs_E10	ordinary_muscle_development	ENSGALG00010011774	ANKRA2	-0.395124716	0.094040087	-4.201662599	2.64962E-05	5.88331E-05	negative
PRJNA729271	stage_E18_vs_E10	ordinary_muscle_development	ENSGALG00010011775	VEZT	-0.234691574	0.081858539	-2.867038382	0.004143328	0.00694066	negative
PRJNA729271	stage_E18_vs_E10	ordinary_muscle_development	ENSGALG00010011776	IL17RA	-1.110603519	0.139275725	-7.974135593	1.53451E-15	8.12965E-15	negative
PRJNA729271	stage_E18_vs_E10	ordinary_muscle_development	ENSGALG00010011780	SERPINB5	-6.947732666	1.038713458	-6.688786605	2.25029E-11	8.62223E-11	negative
PRJNA729271	stage_E18_vs_E10	ordinary_muscle_development	ENSGALG00010011784	GRPEL2	-0.24807666	0.13443142	-1.845377072	0.064982706	0.08931579	negative
PRJNA729271	stage_E18_vs_E10	ordinary_muscle_development	ENSGALG00010011789	TXNRD1	-1.278630558	0.138102748	-9.25854534	2.07236E-20	1.49969E-19	negative
PRJNA729271	stage_E18_vs_E10	ordinary_muscle_development	ENSGALG00010011790	AMY2A	-0.233005909	1.121458091	-0.207770501	0.835408167	0.863465586	negative
PRJNA729271	stage_E18_vs_E10	ordinary_muscle_development	ENSGALG00010011792	HDHD5	0.346323722	0.142764516	2.425838941	0.015273047	0.023551363	positive
PRJNA729271	stage_E18_vs_E10	ordinary_muscle_development	ENSGALG00010011793	TLN1	0.199397637	0.11414498	1.746880482	0.080658072	0.109010607	positive
PRJNA729271	stage_E18_vs_E10	ordinary_muscle_development	ENSGALG00010011796	METAP2	-0.244957887	0.089949787	-2.72327368	0.006463849	0.010521447	negative
PRJNA729271	stage_E18_vs_E10	ordinary_muscle_development	ENSGALG00010011797	SCAF4	-0.070294199	0.157072169	-0.447528034	0.654493851	0.702576344	negative
PRJNA729271	stage_E18_vs_E10	ordinary_muscle_development	ENSGALG00010011802	VPS4B	-0.717395203	0.094159413	-7.618943059	2.55761E-14	1.22961E-13	negative
PRJNA729271	stage_E18_vs_E10	ordinary_muscle_development	ENSGALG00010011808	SMARCA2	0.365461584	0.122708453	2.978291837	0.002898598	0.004977513	positive
PRJNA729271	stage_E18_vs_E10	ordinary_muscle_development	ENSGALG00010011810	FGFR4	0.077189587	0.125855861	0.613317384	0.539666501	0.59472865	positive
PRJNA729271	stage_E18_vs_E10	ordinary_muscle_development	ENSGALG00010011814	ENSGALG00010011814	1.891003731	0.530299852	3.565914123	0.00036259	0.000702076	positive
PRJNA729271	stage_E18_vs_E10	ordinary_muscle_development	ENSGALG00010011817	KDSR	0.214842186	0.091023988	2.360280966	0.018261099	0.02770511	positive
PRJNA729271	stage_E18_vs_E10	ordinary_muscle_development	ENSGALG00010011819	USP44	0.168588624	0.141943679	1.187714913	0.234945746	0.287217559	positive
PRJNA729271	stage_E18_vs_E10	ordinary_muscle_development	ENSGALG00010011823	ENSGALG00010011823	-3.679779702	0.683957712	-5.380127512	7.44331E-08	2.13097E-07	negative
PRJNA729271	stage_E18_vs_E10	ordinary_muscle_development	ENSGALG00010011825	TAFA2	-4.790419611	0.405000269	-11.82818871	2.79099E-32	3.77735E-31	negative
PRJNA729271	stage_E18_vs_E10	ordinary_muscle_development	ENSGALG00010011837	VLDLR	1.183462441	0.134731561	8.783854626	1.57966E-18	1.02304E-17	positive
PRJNA729271	stage_E18_vs_E10	ordinary_muscle_development	ENSGALG00010011839	CBS	0.033731698	0.459934009	0.0733403	0.941535324	0.95217711	positive
PRJNA729271	stage_E18_vs_E10	ordinary_muscle_development	ENSGALG00010011841	KCNMB4	-1.229978261	0.242066531	-5.081157876	3.75141E-07	1.01034E-06	negative
PRJNA729271	stage_E18_vs_E10	ordinary_muscle_development	ENSGALG00010011843	BCL2	1.636201499	0.111060122	14.73257425	3.98242E-49	1.12088E-47	positive
PRJNA729271	stage_E18_vs_E10	ordinary_muscle_development	ENSGALG00010011846	UBAP1	0.090941407	0.106756963	0.851854571	0.394294814	0.454280528	positive
PRJNA729271	stage_E18_vs_E10	ordinary_muscle_development	ENSGALG00010011850	SPAG16	-0.886148818	0.351821436	-2.51874595	0.01177736	0.018453446	negative
PRJNA729271	stage_E18_vs_E10	ordinary_muscle_development	ENSGALG00010011856	NFYB	-0.330267583	0.129490862	-2.550508812	0.01075658	0.01694746	negative
PRJNA729271	stage_E18_vs_E10	ordinary_muscle_development	ENSGALG00010011857	ENSGALG00010011857	0.707177698	0.244572704	2.891482512	0.003834289	0.006452058	positive
PRJNA729271	stage_E18_vs_E10	ordinary_muscle_development	ENSGALG00010011859	NTN4	1.575258985	0.20320543	7.752051617	9.04196E-15	4.53349E-14	positive
PRJNA729271	stage_E18_vs_E10	ordinary_muscle_development	ENSGALG00010011860	CECR1	0.805086056	0.155922876	5.163360734	2.42555E-07	6.63992E-07	positive
PRJNA729271	stage_E18_vs_E10	ordinary_muscle_development	ENSGALG00010011861	ENSGALG00010011861	0.02661837	0.390718883	0.068126653	0.945684813	0.955272409	positive

PRJNA729271	stage_E18_vs_E10	ordinary_muscle_development	ENSGALG00010011866	KLF8	0.285955563	0.189000915	1.512985068	0.130283452	0.16891401	positive
PRJNA729271	stage_E18_vs_E10	ordinary_muscle_development	ENSGALG00010011867	ENSGALG00010011867	0.406602629	0.275092866	1.478055883	0.139392821	0.179759692	positive
PRJNA729271	stage_E18_vs_E10	ordinary_muscle_development	ENSGALG00010011868	SNRPF	-1.191255973	0.125125259	-9.520507562	1.72335E-21	1.33511E-20	negative
PRJNA729271	stage_E18_vs_E10	ordinary_muscle_development	ENSGALG00010011870	AvBD6	4.76793682	1.349600888	3.532849499	0.000411106	0.000790526	positive
PRJNA729271	stage_E18_vs_E10	ordinary_muscle_development	ENSGALG00010011871	CCT2	-1.155996944	0.098282721	-11.76195501	6.12983E-32	8.20423E-31	negative
PRJNA729271	stage_E18_vs_E10	ordinary_muscle_development	ENSGALG00010011873	RFX3	-0.13704734	0.193814354	-0.707106245	0.479500455	0.537897882	negative
PRJNA729271	stage_E18_vs_E10	ordinary_muscle_development	ENSGALG00010011874	KCNS2	-5.210024493	0.786210749	-6.626753064	3.43151E-11	1.29531E-10	negative
PRJNA729271	stage_E18_vs_E10	ordinary_muscle_development	ENSGALG00010011875	PSD2	-0.615714055	0.288606856	-2.13340065	0.032891874	0.047700621	negative
PRJNA729271	stage_E18_vs_E10	ordinary_muscle_development	ENSGALG00010011876	AMDHD1	0.829002591	0.440437452	1.882225473	0.059805408	0.082804413	positive
PRJNA729271	stage_E18_vs_E10	ordinary_muscle_development	ENSGALG00010011880	U2AF1	-0.429750783	0.156660781	-2.743193166	0.006084488	0.009953127	negative
PRJNA729271	stage_E18_vs_E10	ordinary_muscle_development	ENSGALG00010011881	RIOK2	-0.951542014	0.098152774	-9.694499454	3.18194E-22	2.57092E-21	negative
PRJNA729271	stage_E18_vs_E10	ordinary_muscle_development	ENSGALG00010011882	ENSGALG00010011882	0.090808496	0.291695812	0.311312305	0.755563214	0.794882333	positive
PRJNA729271	stage_E18_vs_E10	ordinary_muscle_development	ENSGALG00010011883	HCFC2	0.661579545	0.107292156	6.166150175	6.99726E-10	2.40287E-09	positive
PRJNA729271	stage_E18_vs_E10	ordinary_muscle_development	ENSGALG00010011885	NCALD	-0.072945478	0.211534524	-0.344839586	0.730214971	0.772026723	negative
PRJNA729271	stage_E18_vs_E10	ordinary_muscle_development	ENSGALG00010011886	NTNG1	-2.443744111	0.17247131	-14.16898911	1.42538E-45	3.41501E-44	negative
PRJNA729271	stage_E18_vs_E10	ordinary_muscle_development	ENSGALG00010011887	PGM5	1.63461981	0.132502644	12.33650709	5.7601E-35	8.85393E-34	positive
PRJNA729271	stage_E18_vs_E10	ordinary_muscle_development	ENSGALG00010011888	CAND1	-0.97012452	0.070211122	-13.81724845	2.00592E-43	4.40542E-42	negative
PRJNA729271	stage_E18_vs_E10	ordinary_muscle_development	ENSGALG00010011891	HAL	0.400227978	0.561112706	0.713275557	0.47567526	0.53432369	positive
PRJNA729271	stage_E18_vs_E10	ordinary_muscle_development	ENSGALG00010011893	LIX1	-6.243928718	0.908321312	-6.874140942	6.23645E-12	2.49786E-11	negative
PRJNA729271	stage_E18_vs_E10	ordinary_muscle_development	ENSGALG00010011894	VWC2L	-1.038681515	0.334347353	-3.106594102	0.001892561	0.003334468	negative
PRJNA729271	stage_E18_vs_E10	ordinary_muscle_development	ENSGALG00010011895	PHF21B	-1.498069198	0.71316632	-2.10058882	0.035677076	0.051405489	negative
PRJNA729271	stage_E18_vs_E10	ordinary_muscle_development	ENSGALG00010011896	SIK1	2.306874316	0.298453687	7.729421412	1.08036E-14	5.37956E-14	positive
PRJNA729271	stage_E18_vs_E10	ordinary_muscle_development	ENSGALG00010011898	ENSGALG00010011898	-0.383880008	0.111796039	-3.433753196	0.000595286	0.001119298	negative
PRJNA729271	stage_E18_vs_E10	ordinary_muscle_development	ENSGALG00010011899	PHLPP1	0.063135872	0.085348715	0.739740165	0.459457672	0.518913415	positive
PRJNA729271	stage_E18_vs_E10	ordinary_muscle_development	ENSGALG00010011902	TP53I3	-1.088784113	0.254174844	-4.283602952	1.83891E-05	4.15835E-05	negative
PRJNA729271	stage_E18_vs_E10	ordinary_muscle_development	ENSGALG00010011903	LTA4H	-0.537163006	0.116275709	-4.619735373	3.8423E-06	9.30873E-06	negative
PRJNA729271	stage_E18_vs_E10	ordinary_muscle_development	ENSGALG00010011904	KIF24	-0.95525195	0.334116711	-2.859036736	0.004249296	0.007110776	negative
PRJNA729271	stage_E18_vs_E10	ordinary_muscle_development	ENSGALG00010011906	ENSGALG00010011906	0.2902208	0.934121046	0.310688643	0.756037332	0.795265684	positive
PRJNA729271	stage_E18_vs_E10	ordinary_muscle_development	ENSGALG00010011907	BIK	-0.165171458	0.223980508	-0.737436749	0.460856794	0.520252013	negative
PRJNA729271	stage_E18_vs_E10	ordinary_muscle_development	ENSGALG00010011908	ENSGALG00010011908	-0.278292781	0.189294232	-1.470159858	0.141518464	0.182160566	negative
PRJNA729271	stage_E18_vs_E10	ordinary_muscle_development	ENSGALG00010011909	C8orf48	-0.412658228	0.204234319	-2.020513645	0.043330136	0.061467051	negative
PRJNA729271	stage_E18_vs_E10	ordinary_muscle_development	ENSGALG00010011911	NUDT2	0.63574294	0.137643385	4.618768561	3.86024E-06	9.34751E-06	positive
PRJNA729271	stage_E18_vs_E10	ordinary_muscle_development	ENSGALG00010011912	TCP11X2	1.013965153	0.125835322	8.057873889	7.76328E-16	4.20826E-15	positive
PRJNA729271	stage_E18_vs_E10	ordinary_muscle_development	ENSGALG00010011913	CXXC5	1.066002526	0.110905841	9.611779852	7.13056E-22	5.66653E-21	positive
PRJNA729271	stage_E18_vs_E10	ordinary_muscle_development	ENSGALG00010011915	RRM2B	-0.174855148	0.12393016	-1.410916829	0.158269139	0.201467566	negative
PRJNA729271	stage_E18_vs_E10	ordinary_muscle_development	ENSGALG00010011916	MCAT	0.136719711	0.098267043	1.391307867	0.164132089	0.208126553	positive
PRJNA729271	stage_E18_vs_E10	ordinary_muscle_development	ENSGALG00010011917	HSF2BP	-0.408693871	0.179586054	-2.275755063	0.022860674	0.034126207	negative
PRJNA729271	stage_E18_vs_E10	ordinary_muscle_development	ENSGALG00010011921	MEGF10	-0.071872184	0.167571041	-0.428905755	0.667991812	0.715053638	negative
PRJNA729271	stage_E18_vs_E10	ordinary_muscle_development	ENSGALG00010011922	PIK3R1	0.517128334	0.173080992	2.987782346	0.002810096	0.004834699	positive
PRJNA729271	stage_E18_vs_E10	ordinary_muscle_development	ENSGALG00010011923	BARD1	-1.808811377	0.158418861	-11.41790419	3.40342E-30	4.2021E-29	negative
PRJNA729271	stage_E18_vs_E10	ordinary_muscle_development	ENSGALG00010011925	RRP1B	-0.703305676	0.446924387	-1.573656969	0.115566775	0.15154156	negative
PRJNA729271	stage_E18_vs_E10	ordinary_muscle_development	ENSGALG00010011926	DYRK2	1.60742419	0.194789055	8.252127879	1.55599E-16	8.81706E-16	positive
PRJNA729271	stage_E18_vs_E10	ordinary_muscle_development	ENSGALG00010011927	ENSGALG00010011927	-0.722122887	0.480214023	-1.503752186	0.132645187	0.171730259	negative
PRJNA729271	stage_E18_vs_E10	ordinary_muscle_development	ENSGALG00010011929	ELK3	-0.551399314	0.134834238	-4.089460687	4.32377E-05	9.37378E-05	negative
PRJNA729271	stage_E18_vs_E10	ordinary_muscle_development	ENSGALG00010011930	TSPO	0.148762516	0.151457388	0.982207064	0.325997848	0.384080209	positive
PRJNA729271	stage_E18_vs_E10	ordinary_muscle_development	ENSGALG00010011931	ENSGALG00010011931	-0.363417996	1.298714324	-0.27982905			negative
PRJNA729271	stage_E18_vs_E10	ordinary_muscle_development	ENSGALG00010011932	VAV3	-0.47869982	0.168490469	-2.841109197	0.004495692	0.007497129	negative
PRJNA729271	stage_E18_vs_E10	ordinary_muscle_development	ENSGALG00010011935	ST8SIA4	-0.301776803	0.126637441	-2.38299826	0.017172276	0.026204036	negative
PRJNA729271	stage_E18_vs_E10	ordinary_muscle_development	ENSGALG00010011936	CKAP4	-0.687949351	0.175438015	-3.921324302	8.80636E-05	0.00018473	negative
PRJNA729271	stage_E18_vs_E10	ordinary_muscle_development	ENSGALG00010011937	SLC1A1	-0.419646174	0.303355716	-1.383346849	0.166558539	0.210816104	negative
PRJNA729271	stage_E18_vs_E10	ordinary_muscle_development	ENSGALG00010011941	ENSGALG00010011941	-2.760503014	0.244042955	-11.31154562	1.15045E-29	1.38503E-28	negative
PRJNA729271	stage_E18_vs_E10	ordinary_muscle_development	ENSGALG00010011943	TTLL12	-0.752235401	0.056156789	-13.39527086	6.44405E-41	1.27954E-39	negative
PRJNA729271	stage_E18_vs_E10	ordinary_muscle_development	ENSGALG00010011945	ENSGALG00010011945	-0.281713618	0.516975445	-0.544926496	0.585804072	0.638246394	negative
PRJNA729271	stage_E18_vs_E10	ordinary_muscle_development	ENSGALG00010011947	THAP1	-0.557748629	0.12728256	-4.381972101	1.1761E-05	2.71717E-05	negative
PRJNA729271	stage_E18_vs_E10	ordinary_muscle_development	ENSGALG00010011949	ABCA12	-0.364801636	0.42139807	-0.865693656	0.38665818	0.446902983	negative
PRJNA729271	stage_E18_vs_E10	ordinary_muscle_development	ENSGALG00010011950	PDXK	1.875231222	0.164889849	11.37262988	5.72366E-30	6.97181E-29	positive
PRJNA729271	stage_E18_vs_E10	ordinary_muscle_development	ENSGALG00010011952	UBE2D2	0.75852869	0.17379238	4.364568177	1.27374E-05	2.93434E-05	positive
PRJNA729271	stage_E18_vs_E10	ordinary_muscle_development	ENSGALG00010011953	CDK17	-0.115525855	0.059817547	-1.931303803	0.053445493	0.074640373	negative
PRJNA729271	stage_E18_vs_E10	ordinary_muscle_development	ENSGALG00010011954	ENSGALG00010011954	1.509271	0.454738466	3.318986878	0.000903447	0.001655767	positive
PRJNA729271	stage_E18_vs_E10	ordinary_muscle_development	ENSGALG00010011957	ENSGALG00010011957	2.907559599	0.21028816	13.82654922	1.76276E-43	3.88316E-42	positive
PRJNA729271	stage_E18_vs_E10	ordinary_muscle_development	ENSGALG00010011960	CFAP54	-0.497828019	0.293992746	-1.69333436	0.090391841	0.120847606	negative
PRJNA729271	stage_E18_vs_E10	ordinary_muscle_development	ENSGALG00010011961	AGPAT3	0.687707006	0.103167849	6.665904254	2.63041E-11	1.00152E-10	positive
PRJNA729271	stage_E18_vs_E10	ordinary_muscle_development	ENSGALG00010011962	CPSF6	-0.628660335	0.059542349	-10.55820516	4.65477E-26	4.6725E-25	negative
PRJNA729271	stage_E18_vs_E10	ordinary_muscle_development	ENSGALG00010011963	ZCCHC2	0.084268662	0.101208372	0.832625411	0.405056023	0.465015607	positive
PRJNA729271	stage_E18_vs_E10	ordinary_muscle_development	ENSGALG00010011964	ENSGALG00010011964	-0.934447491	4.330150397	-0.215800239	0.829143462	0.858096172	negative
PRJNA729271	stage_E18_vs_E10	ordinary_muscle_development	ENSGALG00010011965	ATIC	0.750636378	0.14677027	5.114362586	3.14802E-07	8.54345E-07	positive
PRJNA729271	stage_E18_vs_E10	ordinary_muscle_development	ENSGALG00010011966	SLC25A24	-0.265396266	0.138349112	-1.91830842	0.055071915	0.076771246	negative
PRJNA729271	stage_E18_vs_E10	ordinary_muscle_development	ENSGALG00010011967	MTMR9	-0.113180866	0.137772445	-0.821505824	0.411358208	0.471541102	negative
PRJNA729271	stage_E18_vs_E10	ordinary_muscle_development	ENSGALG00010011968	ENSGALG00010011968	3.842076095	0.231774364	16.57679493	1.02554E-61	4.3339E-60	positive
PRJNA729271	stage_E18_vs_E10	ordinary_muscle_development	ENSGALG00010011969	TMEM173	1.166808364	0.301292906	3.87267121	0.000107649	0.000223798	positive
PRJNA729271	stage_E18_vs_E10	ordinary_muscle_development	ENSGALG00010011971	RNF170	-0.411227478	0.091269191	-4.505654918	6.61685E-06	1.56743E-05	negative
PRJNA729271	stage_E18_vs_E10	ordinary_muscle_development	ENSGALG00010011972	IPO11	-0.555931067	0.082763422	-6.717110675	1.85363E-11	7.13257E-11	negative
PRJNA729271	stage_E18_vs_E10	ordinary_muscle_development	ENSGALG00010011973	TRAPPC10	-0.190991801	0.051223056	-3.728629555	0.000192524	0.000386836	negative
PRJNA729271	stage_E18_vs_E10	ordinary_muscle_development	ENSGALG00010011976	TNFRSF11A	-0.468445849	0.092262633	-5.077308503	3.82819E-07	1.03064E-06	negative
PRJNA729271	stage_E18_vs_E10	ordinary_muscle_development	ENSGALG00010011977	GATD3A	0.079202716	0.119166653	0.664638252	0.506281897	0.563291073	positive
PRJNA729271	stage_E18_vs_E10	ordinary_muscle_development	ENSGALG00010011978	ENSGALG00010011978	-0.430682822	5.460732126	-0.078869062	0.937136771	0.948522973	negative
PRJNA729271	stage_E18_vs_E10	ordinary_muscle_development	ENSGALG00010011979	NEDD1	-0.648993777	0.078968295	-8.21840938	2.0622E-16	1.15814E-15	negative
PRJNA729271	stage_E18_vs_E10	ordinary_muscle_development	ENSGALG00010011980	AvBD9	23.02417435	2.272990739	10.12946246	4.08891E-24	3.66309E-23	positive
PRJNA729271	stage_E18_vs_E10	ordinary_muscle_development	ENSGALG00010011981	SCUBE1	-3.652215101	0.348838365	-10.46964861	1.19084E-25	1.17264E-24	negative
PRJNA729271	stage_E18_vs_E10	ordinary_muscle_development	ENSGALG00010011982	STK3	0.063495601	0.105013963	0.604639603	0.545418487	0.600154936	positive
PRJNA729271	stage_E18_vs_E10	ordinary_muscle_development	ENSGALG00010011983	LYZ	2.040462797	0.531057807	3.842261184	0.000121906	0.000251427	positive
PRJNA729271	stage_E18_vs_E10	ordinary_muscle_development	ENSGALG00010011984	FN1	0.403985068	0.240232757	1.681640229	0.092638625	0.12364612	positive
PRJNA729271	stage_E18_vs_E10	ordinary_muscle_development	ENSGALG00010011987	H2BC32	-1.219949082	0.37513318	-3.252042598	0.001145788	0.002072657	negative
PRJNA729271	stage_E18_vs_E10	ordinary_muscle_development	ENSGALG00010011988	HINTW	-2.908681286	3.141879118	-0.925777593	0.354561574	0.413897884	negative
PRJNA729271	stage_E18_vs_E10	ordinary_muscle_development	ENSGALG00010011989	H1-010	-0.00095323	0.136571197	-0.006979729	0.994431027	0.995052002	negative
PRJNA729271	stage_E18_vs_E10	ordinary_muscle_development	ENSGALG00010011991	CSNK1A1	0.139413939	0.056368834	2.473245039	0.01338923	0.020805842	positive
PRJNA729271	stage_E18_vs_E10	ordinary_muscle_development	ENSGALG00010011992	YEATS4	-1.238677962	0.087087925	-14.2233032	6.56781E-46	1.61357E-44	negative
PRJNA729271	stage_E18_vs_E10	ordinary_muscle_development	ENSGALG00010011993	RELCH	-0.071038372	0.052290556	-1.35853159	0.174295066	0.219725776	negative
PRJNA729271	stage_E18_vs_E10	ordinary_muscle_development	ENSGALG00010011996	LRRC70	0.612307657	0.221227915	2.76776851	0.005644153	0.009278893	positive
PRJNA729271	stage_E18_vs_E10	ordinary_muscle_development	ENSGALG00010011997	DENND5B	0.356876451	0.131140359	2.721331973	0.006501943	0.010578703	positive
PRJNA729271	stage_E18_vs_E10	ordinary_muscle_development	ENSGALG00010011998	MPPED1	-0.349920666	0.075317179	-4.645960839	3.38497E-06	8.24902E-06	negative
PRJNA729271	stage_E18_vs_E10	ordinary_muscle_development	ENSGALG00010012001	EEIG2	-0.259500628	0.254382496	-1.020119826	0.307671635	0.365160104	negative
PRJNA729271	stage_E18_vs_E10	ordinary_muscle_development	ENSGALG00010012004	LGR5	-2.27198815	0.19533216	-11.63140854	2.8535E-31	3.71621E-30	negative
PRJNA729271	stage_E18_vs_E10	ordinary_muscle_development	ENSGALG00010012007	MREG	0.134003346	0.193008471	0.694287382	0.487502002	0.545452839	positive
PRJNA729271	stage_E18_vs_E10	ordinary_muscle_development	ENSGALG00010012008	ZNF366	2.585619124	0.182331423	14.180875	1.20337E-45	2.89267E-44	positive
PRJNA729271	stage_E18_vs_E10	ordinary_muscle_development	ENSGALG00010012010	HENMT1	-0.613516148	0.166177185	-3.691939703	0.00022255	0.000443479	negative
PRJNA729271	stage_E18_vs_E10	ordinary_muscle_development	ENSGALG00010012011	HOOK3	0.079025852	0.068917573	1.146672003	0.251517206	0.305111103	positive
PRJNA729271	stage_E18_vs_E10	ordinary_muscle_development	ENSGALG00010012013	FOXD1	-4.240842452	0.823990388	-5.146713498	2.6509E-07	7.23221E-07	negative
PRJNA729271	stage_E18_vs_E10	ordinary_muscle_development	ENSGALG00010012015	ENSGALG00010012015	0.286196114	0.215933654	1.325389116	0.185042125	0.231942719	positive
PRJNA729271	stage_E18_vs_E10	ordinary_muscle_development	ENSGALG00010012016	WDR91	-0.220579596	0.064073137	-3.442622084	0.000576104	0.001085483	negative
PRJNA729271	stage_E18_vs_E10	ordinary_muscle_development	ENSGALG00010012017	N4BP3	-0.113044869	0.129459581	-0.873205893	0.382550863	0.442932723	negative
PRJNA729271	stage_E18_vs_E10	ordinary_muscle_development	ENSGALG00010012019	ENSGALG00010012019	5.444273533	0.302133379	18.01943747	1.37131E-72	7.7043E-71	positive
PRJNA729271	stage_E18_vs_E10	ordinary_muscle_development	ENSGALG00010012021	ENSGALG00010012021	-0.441959411	0.131549136	-3.359652708	0.000780405	0.00144525	negative
PRJNA729271	stage_E18_vs_E10	ordinary_muscle_development	ENSGALG00010012024	UBR5	-0.022486443	0.075981392	-0.295946716	0.767270774	0.80462427	negative
PRJNA729271	stage_E18_vs_E10	ordinary_muscle_development	ENSGALG00010012026	SYT1	-1.109500437	0.238336965	-4.655175654	3.23705E-06	7.90848E-06	negative
PRJNA729271	stage_E18_vs_E10	ordinary_muscle_development	ENSGALG00010012028	TMEM140	2.471736401	0.177234387	13.94614463	3.32077E-44	7.58025E-43	positive
PRJNA729271	stage_E18_vs_E10	ordinary_muscle_development	ENSGALG00010012031	GRK6	-0.322928615	0.110875197	-2.912541523	0.003585005	0.006054369	negative
PRJNA729271	stage_E18_vs_E10	ordinary_muscle_development	ENSGALG00010012034	ARHGEF9	1.560619765	0.150464004	10.3720473	3.32327E-25	3.1859E-24	positive
PRJNA729271	stage_E18_vs_E10	ordinary_muscle_development	ENSGALG00010012035	ENSGALG00010012035	1.177891557	0.448006116	2.629186332	0.008558945	0.013681287	positive
PRJNA729271	stage_E18_vs_E10	ordinary_muscle_development	ENSGALG00010012039	ODF1	0.826181735	0.567903864	1.454791536	0.145726982	0.187013068	positive
PRJNA729271	stage_E18_vs_E10	ordinary_muscle_development	ENSGALG00010012040	B4GALT7	-0.419563547	0.15384226	-2.727232084	0.00638681	0.010411247	negative
PRJNA729271	stage_E18_vs_E10	ordinary_muscle_development	ENSGALG00010012041	ICOSLG	-0.638448903	0.15440869	-4.134799043	3.55265E-05	7.76942E-05	negative
PRJNA729271	stage_E18_vs_E10	ordinary_muscle_development	ENSGALG00010012042	FNDC7	-2.821436441	0.351374781	-8.029706715	9.7706E-16	5.25704E-15	negative
PRJNA729271	stage_E18_vs_E10	ordinary_muscle_development	ENSGALG00010012043	NUP50	-1.295507551	0.096871382	-13.37348061	8.64012E-41	1.70858E-39	negative
PRJNA729271	stage_E18_vs_E10	ordinary_muscle_development	ENSGALG00010012044	ENSGALG00010012044	2.142296874	0.644669012	3.323095781	0.000890243	0.001634048	positive
PRJNA729271	stage_E18_vs_E10	ordinary_muscle_development	ENSGALG00010012045	ENSGALG00010012045	0.189775562	0.30130331	0.629848913	0.52879344	0.584925284	positive
PRJNA729271	stage_E18_vs_E10	ordinary_muscle_development	ENSGALG00010012046	LOXHD1	0.415229909	0.27208996	1.526075822	0.126990983	0.165295824	positive
PRJNA729271	stage_E18_vs_E10	ordinary_muscle_development	ENSGALG00010012047	LMAN2	0.051618292	0.090998425	0.567243797	0.570548556	0.624017303	positive
PRJNA729271	stage_E18_vs_E10	ordinary_muscle_development	ENSGALG00010012048	PDE6H	4.615423535	0.443546796	10.40571948	2.33486E-25	2.25775E-24	positive
PRJNA729271	stage_E18_vs_E10	ordinary_muscle_development	ENSGALG00010012049	IFNAR1	0.282813707	0.145745249	1.940466039	0.052323078	0.0731786	positive
PRJNA729271	stage_E18_vs_E10	ordinary_muscle_development	ENSGALG00010012051	ARK2C	-1.212002364	0.196429429	-6.170166902	6.82179E-10	2.34484E-09	negative
PRJNA729271	stage_E18_vs_E10	ordinary_muscle_development	ENSGALG00010012052	KLF10	1.48095961	0.123683758	11.97375976	4.87676E-33	6.78393E-32	positive
PRJNA729271	stage_E18_vs_E10	ordinary_muscle_development	ENSGALG00010012054	FAM60A	-1.638090372	0.090808891	-18.03887645	9.64857E-73	5.44187E-71	negative
PRJNA729271	stage_E18_vs_E10	ordinary_muscle_development	ENSGALG00010012055	ENSGALG00010012055	1.500590492	0.156349741	9.597652581	8.17858E-22	6.47452E-21	positive
PRJNA729271	stage_E18_vs_E10	ordinary_muscle_development	ENSGALG00010012058	ENSGALG00010012058	-1.287244893	0.300953177	-4.277226466	1.89236E-05	4.27455E-05	negative
PRJNA729271	stage_E18_vs_E10	ordinary_muscle_development	ENSGALG00010012059	ARHGDIB	0.128518504	0.144882389	0.88705401	0.375049823	0.435012177	positive
PRJNA729271	stage_E18_vs_E10	ordinary_muscle_development	ENSGALG00010012060	ENSGALG00010012060	0.469519718	0.125146842	3.751750434	0.000175604	0.000354757	positive
PRJNA729271	stage_E18_vs_E10	ordinary_muscle_development	ENSGALG00010012061	AZIN1	1.34771311	0.120304862	11.20248248	3.96465E-29	4.61959E-28	positive
PRJNA729271	stage_E18_vs_E10	ordinary_muscle_development	ENSGALG00010012062	HMGA2	-2.774394346	0.152292735	-18.21750948	3.74831E-74	2.19967E-72	negative
PRJNA729271	stage_E18_vs_E10	ordinary_muscle_development	ENSGALG00010012063	PTPRD	-0.435749633	0.19655135	-2.216976038	0.026624729	0.039279944	negative
PRJNA729271	stage_E18_vs_E10	ordinary_muscle_development	ENSGALG00010012064	RGS14	-1.70478917	0.185795746	-9.175609281	4.49028E-20	3.16415E-19	negative

PRJNA729271	stage_E18_vs_E10	ordinary_muscle_development	ENSGALG00010012066	STXBP3	-0.639675456	0.123897177	-5.16295425	2.43083E-07	6.65184E-07	negative
PRJNA729271	stage_E18_vs_E10	ordinary_muscle_development	ENSGALG00010012067	ENSGALG00010012067	-0.044610323	0.08484843	-0.525764854	0.599051597	0.650528386	negative
PRJNA729271	stage_E18_vs_E10	ordinary_muscle_development	ENSGALG00010012069	ERP27	1.966074691	0.410790911	4.786071545	1.70077E-06	4.28818E-06	positive
PRJNA729271	stage_E18_vs_E10	ordinary_muscle_development	ENSGALG00010012070	DNAJC28	0.610562557	0.162659802	3.753616747	0.000174301	0.000352272	positive
PRJNA729271	stage_E18_vs_E10	ordinary_muscle_development	ENSGALG00010012071	ENSGALG00010012071	-1.002193281	0.154255942	-6.496950889	8.19642E-11	3.01082E-10	negative
PRJNA729271	stage_E18_vs_E10	ordinary_muscle_development	ENSGALG00010012072	CAPRIN2	-0.746843238	0.154445187	-4.835652384	1.3271E-06	3.38726E-06	negative
PRJNA729271	stage_E18_vs_E10	ordinary_muscle_development	ENSGALG00010012074	KIAA0930	-0.454191685	0.089006527	-5.102903122	3.34482E-07	9.05889E-07	negative
PRJNA729271	stage_E18_vs_E10	ordinary_muscle_development	ENSGALG00010012075	PIGN	-0.128309023	0.096446864	-1.330359713	0.183399782	0.230202618	negative
PRJNA729271	stage_E18_vs_E10	ordinary_muscle_development	ENSGALG00010012077	IFNGR2	0.336696389	0.069578651	4.839076102	1.30444E-06	3.33413E-06	positive
PRJNA729271	stage_E18_vs_E10	ordinary_muscle_development	ENSGALG00010012078	TMBIM4	-0.289407512	0.083713752	-3.457108368	0.000546005	0.001032126	negative
PRJNA729271	stage_E18_vs_E10	ordinary_muscle_development	ENSGALG00010012079	ENSGALG00010012079	-1.241891925	0.379898057	-3.269013628	0.001079231	0.001958369	negative
PRJNA729271	stage_E18_vs_E10	ordinary_muscle_development	ENSGALG00010012081	CHIC1	-0.077343837	0.125962102	-0.614024656	0.539199034	0.59434905	negative
PRJNA729271	stage_E18_vs_E10	ordinary_muscle_development	ENSGALG00010012082	HAUS1	-1.926459685	0.208459724	-9.241399954	2.43293E-20	1.75188E-19	negative
PRJNA729271	stage_E18_vs_E10	ordinary_muscle_development	ENSGALG00010012083	MGP	4.229333119	0.277663972	15.23183972	2.17387E-52	6.87998E-51	positive
PRJNA729271	stage_E18_vs_E10	ordinary_muscle_development	ENSGALG00010012084	TMEM50B	0.268155441	0.106372552	2.520908225	0.011705238	0.018349532	positive
PRJNA729271	stage_E18_vs_E10	ordinary_muscle_development	ENSGALG00010012085	IPO8	-0.613223514	0.097192471	-6.309372582	2.80169E-10	9.8833E-10	negative
PRJNA729271	stage_E18_vs_E10	ordinary_muscle_development	ENSGALG00010012086	CIBAR1	-1.270637806	0.13305982	-9.549372644	1.30484E-21	1.02015E-20	negative
PRJNA729271	stage_E18_vs_E10	ordinary_muscle_development	ENSGALG00010012087	TMEM171	-0.522684085	0.183099853	-2.85463957	0.00430857	0.007203312	negative
PRJNA729271	stage_E18_vs_E10	ordinary_muscle_development	ENSGALG00010012088	ATP5F1AZ	0.394744166	0.169926031	2.323035282	0.020177257	0.030405379	positive
PRJNA729271	stage_E18_vs_E10	ordinary_muscle_development	ENSGALG00010012089	ENSGALG00010012089	-1.054050988	0.253254148	-4.162028521	3.15433E-05	6.93704E-05	negative
PRJNA729271	stage_E18_vs_E10	ordinary_muscle_development	ENSGALG00010012090	HELB	0.162225244	0.22420133	0.723569498	0.469330073	0.528260554	positive
PRJNA729271	stage_E18_vs_E10	ordinary_muscle_development	ENSGALG00010012091	ENSGALG00010012091	-0.657725559	0.445470377	-1.47647429	0.139816612	0.180226038	negative
PRJNA729271	stage_E18_vs_E10	ordinary_muscle_development	ENSGALG00010012092	OC3	4.306998467	0.714634524	6.026854736	1.67181E-09	5.5301E-09	positive
PRJNA729271	stage_E18_vs_E10	ordinary_muscle_development	ENSGALG00010012093	ENSGALG00010012093	-0.943959479	0.068448143	-13.79087052	2.89248E-43	6.32376E-42	negative
PRJNA729271	stage_E18_vs_E10	ordinary_muscle_development	ENSGALG00010012094	ENSGALG00010012094	-1.071351855	0.376442511	-2.845990621	0.00442735	0.007389963	negative
PRJNA729271	stage_E18_vs_E10	ordinary_muscle_development	ENSGALG00010012095	RNF38	0.209248341	0.087162675	2.400664519	0.016365332	0.025086241	positive
PRJNA729271	stage_E18_vs_E10	ordinary_muscle_development	ENSGALG00010012096	ENSGALG00010012096	-0.721976828	0.163277689	-4.421772689	9.78944E-06	2.28132E-05	negative
PRJNA729271	stage_E18_vs_E10	ordinary_muscle_development	ENSGALG00010012097	GART	-0.909415715	0.193072837	-4.710220914	2.47448E-06	6.12494E-06	negative
PRJNA729271	stage_E18_vs_E10	ordinary_muscle_development	ENSGALG00010012098	ART4	1.408711572	0.553205212	2.546453907	0.010882358	0.017130732	positive
PRJNA729271	stage_E18_vs_E10	ordinary_muscle_development	ENSGALG00010012099	UPK3A	-1.922483169	0.476229749	-4.036881721	5.41664E-05	0.000116248	negative
PRJNA729271	stage_E18_vs_E10	ordinary_muscle_development	ENSGALG00010012100	UBE2F	0.404679946	0.111787573	3.620079896	0.000294512	0.00057757	positive
PRJNA729271	stage_E18_vs_E10	ordinary_muscle_development	ENSGALG00010012101	ENSGALG00010012101	-1.374252869	0.136623071	-10.05871742	8.40866E-24	7.37794E-23	negative
PRJNA729271	stage_E18_vs_E10	ordinary_muscle_development	ENSGALG00010012102	SON	-0.127981476	0.104983762	-1.21905973	0.222821523	0.273827721	negative
PRJNA729271	stage_E18_vs_E10	ordinary_muscle_development	ENSGALG00010012103	ENSGALG00010012103	1.134891161	0.207988066	5.456520561	4.85555E-08	1.41356E-07	positive
PRJNA729271	stage_E18_vs_E10	ordinary_muscle_development	ENSGALG00010012105	DONSON	-1.400885613	0.206009147	-6.800113648	1.04537E-11	4.10528E-11	negative
PRJNA729271	stage_E18_vs_E10	ordinary_muscle_development	ENSGALG00010012106	TMTC1	0.337150675	0.138448158	2.435212432	0.014883049	0.022991559	positive
PRJNA729271	stage_E18_vs_E10	ordinary_muscle_development	ENSGALG00010012107	CLCC1	-0.117511105	0.085824009	-1.369210167	0.170933589	0.21593885	negative
PRJNA729271	stage_E18_vs_E10	ordinary_muscle_development	ENSGALG00010012108	PSTPIP2	-0.805888077	0.354571193	-2.272852654	0.023035061	0.034361758	negative
PRJNA729271	stage_E18_vs_E10	ordinary_muscle_development	ENSGALG00010012110	ENSGALG00010012110	0.770610634	0.113137873	6.811252629	9.67526E-12	3.81302E-11	positive
PRJNA729271	stage_E18_vs_E10	ordinary_muscle_development	ENSGALG00010012111	DRD1	-1.306571444	0.370806437	-3.523594292	0.000425736	0.000817464	negative
PRJNA729271	stage_E18_vs_E10	ordinary_muscle_development	ENSGALG00010012112	MBD2	1.687007891	0.190109466	8.873876339	7.0643E-19	4.6714E-18	positive
PRJNA729271	stage_E18_vs_E10	ordinary_muscle_development	ENSGALG00010012113	WDR47	-0.498485591	0.09602662	-5.191118789	2.09034E-07	5.74871E-07	negative
PRJNA729271	stage_E18_vs_E10	ordinary_muscle_development	ENSGALG00010012114	TAL2	-0.961617515	0.329094148	-2.922013411	0.003477766	0.005882172	negative
PRJNA729271	stage_E18_vs_E10	ordinary_muscle_development	ENSGALG00010012116	RBM12B	-0.375283743	0.078196696	-4.799227642	1.59279E-06	4.02501E-06	negative
PRJNA729271	stage_E18_vs_E10	ordinary_muscle_development	ENSGALG00010012118	ENSGALG00010012118	0.249915981	0.735379711	0.339846174	0.733972373	0.775319163	positive
PRJNA729271	stage_E18_vs_E10	ordinary_muscle_development	ENSGALG00010012119	EPG5	0.27720053	0.123275405	2.248628028	0.024536172	0.036436001	positive
PRJNA729271	stage_E18_vs_E10	ordinary_muscle_development	ENSGALG00010012120	ENSGALG00010012120	-0.818553096	0.192129323	-4.260427738	2.04036E-05	4.59311E-05	negative
PRJNA729271	stage_E18_vs_E10	ordinary_muscle_development	ENSGALG00010012121	SREK1	-0.377303619	0.100641755	-3.748976957	0.000177557	0.000358553	negative
PRJNA729271	stage_E18_vs_E10	ordinary_muscle_development	ENSGALG00010012122	GRIP1	-3.213546365	0.262412538	-12.24616165	1.76137E-34	2.64845E-33	negative
PRJNA729271	stage_E18_vs_E10	ordinary_muscle_development	ENSGALG00010012123	ENSGALG00010012123	-0.732256744	0.208050859	-3.519604513	0.000432191	0.00082909	negative
PRJNA729271	stage_E18_vs_E10	ordinary_muscle_development	ENSGALG00010012124	ERAP1	1.074110033	0.148544944	7.230875732	4.79889E-13	2.10278E-12	positive
PRJNA729271	stage_E18_vs_E10	ordinary_muscle_development	ENSGALG00010012125	RNF152	0.974166656	0.147244207	6.615993107	3.69066E-11	1.39023E-10	positive
PRJNA729271	stage_E18_vs_E10	ordinary_muscle_development	ENSGALG00010012127	ENSGALG00010012127	-0.233829692	0.116587272	-2.005619374	0.044896873	0.063540342	negative
PRJNA729271	stage_E18_vs_E10	ordinary_muscle_development	ENSGALG00010012128	ENSGALG00010012128	0.912731297	0.857989761	1.063802085	0.287418367	0.343428343	positive
PRJNA729271	stage_E18_vs_E10	ordinary_muscle_development	ENSGALG00010012129	CRYZL1	-0.681562961	0.098993569	-6.884921599	5.78194E-12	2.32674E-11	negative
PRJNA729271	stage_E18_vs_E10	ordinary_muscle_development	ENSGALG00010012130	TMEM67	-0.775539031	0.166929312	-4.64591282	3.38576E-06	8.24955E-06	negative
PRJNA729271	stage_E18_vs_E10	ordinary_muscle_development	ENSGALG00010012131	SLC14A1	-1.602648212	0.270305339	-5.929029061	3.04731E-09	9.86616E-09	negative
PRJNA729271	stage_E18_vs_E10	ordinary_muscle_development	ENSGALG00010012132	MC4R	-4.91138013	1.155122634	-4.251825723	2.12035E-05	4.76429E-05	negative
PRJNA729271	stage_E18_vs_E10	ordinary_muscle_development	ENSGALG00010012133	RAB3IP	-2.080710997	0.236349663	-8.803528502	1.3258E-18	8.6216E-18	negative
PRJNA729271	stage_E18_vs_E10	ordinary_muscle_development	ENSGALG00010012135	CAMSAP2	-0.260168722	0.092457129	-2.813939019	0.004893851	0.008124656	negative
PRJNA729271	stage_E18_vs_E10	ordinary_muscle_development	ENSGALG00010012136	ENSGALG00010012136	0.967620359	0.174808395	5.535319734	3.10661E-08	9.22375E-08	positive
PRJNA729271	stage_E18_vs_E10	ordinary_muscle_development	ENSGALG00010012137	ENSGALG00010012137	-3.378011596	1.226603588	-2.753955416	0.005887979	0.009648876	negative
PRJNA729271	stage_E18_vs_E10	ordinary_muscle_development	ENSGALG00010012138	DDX11	-1.785699754	0.171849576	-10.3910629	2.72301E-25	2.62086E-24	negative
PRJNA729271	stage_E18_vs_E10	ordinary_muscle_development	ENSGALG00010012140	GLOD5	2.48745084	0.561763199	4.427934839	9.51396E-06	2.21998E-05	positive
PRJNA729271	stage_E18_vs_E10	ordinary_muscle_development	ENSGALG00010012141	ENSGALG00010012141	-0.023137119	0.095217374	-0.242992619	0.808011115	0.840120588	negative
PRJNA729271	stage_E18_vs_E10	ordinary_muscle_development	ENSGALG00010012144	H1-01	-0.096687006	0.15756816	-0.613620204	0.539466329	0.594553258	negative
PRJNA729271	stage_E18_vs_E10	ordinary_muscle_development	ENSGALG00010012145	ENSGALG00010012145	0.761997114	1.197883845	0.636119368	0.524698623	0.581238559	positive
PRJNA729271	stage_E18_vs_E10	ordinary_muscle_development	ENSGALG00010012147	ARFGAP3	-0.334191822	0.12298517	-2.717334298	0.00658101	0.010696539	negative
PRJNA729271	stage_E18_vs_E10	ordinary_muscle_development	ENSGALG00010012148	SETBP1	0.262287883	0.109134266	2.403350403	0.016245612	0.024926448	positive
PRJNA729271	stage_E18_vs_E10	ordinary_muscle_development	ENSGALG00010012149	SFXN1	-0.340693911	0.173921047	-1.958899843	0.050124517	0.070375327	negative
PRJNA729271	stage_E18_vs_E10	ordinary_muscle_development	ENSGALG00010012151	MYRFL	-1.379201409	0.578378911	-2.38459837	0.017097776	0.026098596	negative
PRJNA729271	stage_E18_vs_E10	ordinary_muscle_development	ENSGALG00010012154	CWC27	-0.187876788	0.114210198	-1.645008872	0.099967981	0.132537811	negative
PRJNA729271	stage_E18_vs_E10	ordinary_muscle_development	ENSGALG00010012156	ENSGALG00010012156	0.226790261	0.512749732	0.442302056	0.658270643	0.706212195	positive
PRJNA729271	stage_E18_vs_E10	ordinary_muscle_development	ENSGALG00010012157	H2BC32	-1.126904408	0.198464769	-5.678108081	1.36193E-08	4.15865E-08	negative
PRJNA729271	stage_E18_vs_E10	ordinary_muscle_development	ENSGALG00010012159	WASHC1	0.471958172	0.10085039	4.679785309	2.87175E-06	7.05311E-06	positive
PRJNA729271	stage_E18_vs_E10	ordinary_muscle_development	ENSGALG00010012160	SYT4	-4.799043087	0.478158147	-10.0365185	1.05326E-23	9.197E-23	negative
PRJNA729271	stage_E18_vs_E10	ordinary_muscle_development	ENSGALG00010012163	PDP1	0.239553044	0.221395884	1.082012184	0.279247114	0.334768581	positive
PRJNA729271	stage_E18_vs_E10	ordinary_muscle_development	ENSGALG00010012168	ENSGALG00010012168	-1.527024773	0.572417478	-2.667676706	0.007637771	0.012287402	negative
PRJNA729271	stage_E18_vs_E10	ordinary_muscle_development	ENSGALG00010012169	H3C33	-0.937966967	0.227966279	-4.114498742	3.88022E-05	8.45643E-05	negative
PRJNA729271	stage_E18_vs_E10	ordinary_muscle_development	ENSGALG00010012171	CDH17	1.505847822	0.384931087	3.911993274	9.15375E-05	0.000191712	positive
PRJNA729271	stage_E18_vs_E10	ordinary_muscle_development	ENSGALG00010012172	CNOT2	-0.096998019	0.08909734	-1.088674697	0.276297364	0.33180864	negative
PRJNA729271	stage_E18_vs_E10	ordinary_muscle_development	ENSGALG00010012175	PACSIN2	0.155719486	0.154744423	1.00630112	0.314270742	0.371688654	positive
PRJNA729271	stage_E18_vs_E10	ordinary_muscle_development	ENSGALG00010012179	ENSGALG00010012179	-0.254584898	0.180225636	-1.412589819	0.157776364	0.200893218	negative
PRJNA729271	stage_E18_vs_E10	ordinary_muscle_development	ENSGALG00010012180	DDX59	-0.401574409	0.108551183	-3.699401493	0.000216108	0.000431235	negative
PRJNA729271	stage_E18_vs_E10	ordinary_muscle_development	ENSGALG00010012186	KIF20A	-1.659941212	0.247694785	-6.701558976	2.06208E-11	7.91154E-11	negative
PRJNA729271	stage_E18_vs_E10	ordinary_muscle_development	ENSGALG00010012187	ENSGALG00010012187	-0.636749068	0.838642402	-0.759261715	0.447696015	0.507258167	negative
PRJNA729271	stage_E18_vs_E10	ordinary_muscle_development	ENSGALG00010012188	ADAMTS6	-0.878446288	0.185200893	-4.743207635	2.1036E-06	5.24454E-06	negative
PRJNA729271	stage_E18_vs_E10	ordinary_muscle_development	ENSGALG00010012190	CAST	0.775751234	0.132581811	5.851113589	4.88293E-09	1.55351E-08	positive
PRJNA729271	stage_E18_vs_E10	ordinary_muscle_development	ENSGALG00010012191	BEST3	2.632591845	0.385487144	6.829259771	8.53539E-12	3.37021E-11	positive
PRJNA729271	stage_E18_vs_E10	ordinary_muscle_development	ENSGALG00010012193	TTLL1	-2.191320708	0.254338704	-8.615757951	6.94804E-18	4.30577E-17	negative
PRJNA729271	stage_E18_vs_E10	ordinary_muscle_development	ENSGALG00010012194	LRRFIP1	1.936257971	0.159868902	12.11153603	9.17595E-34	1.32608E-32	positive
PRJNA729271	stage_E18_vs_E10	ordinary_muscle_development	ENSGALG00010012195	ITSN1	0.214110888	0.072095663	2.969816475	0.002979777	0.005104818	positive
PRJNA729271	stage_E18_vs_E10	ordinary_muscle_development	ENSGALG00010012196	CENPK	-1.893285057	0.261654443	-7.235822325	4.62717E-13	2.03245E-12	negative
PRJNA729271	stage_E18_vs_E10	ordinary_muscle_development	ENSGALG00010012198	TMEM5	0.350292416	0.103353767	3.389256384	0.000700825	0.001305546	positive
PRJNA729271	stage_E18_vs_E10	ordinary_muscle_development	ENSGALG00010012199	ENSGALG00010012199	1.087431185	0.288176912	3.773484755	0.000160983	0.000326814	positive
PRJNA729271	stage_E18_vs_E10	ordinary_muscle_development	ENSGALG00010012200	GEM	1.82947146	0.238580234	7.668160212	1.74481E-14	8.52697E-14	positive
PRJNA729271	stage_E18_vs_E10	ordinary_muscle_development	ENSGALG00010012205	SLC6A13	-4.139536079	0.373374344	-11.08682518	1.45355E-28	1.6512E-27	negative
PRJNA729271	stage_E18_vs_E10	ordinary_muscle_development	ENSGALG00010012206	RIBC2	-3.746445718	0.823252463	-4.550785921	5.34459E-06	1.2788E-05	negative
PRJNA729271	stage_E18_vs_E10	ordinary_muscle_development	ENSGALG00010012207	GRIA3	-1.778209098	0.263066401	-6.759544696	1.38426E-11	5.39233E-11	negative
PRJNA729271	stage_E18_vs_E10	ordinary_muscle_development	ENSGALG00010012209	KIF14	-0.947278939	0.228130136	-4.15236214	3.29061E-05	7.22359E-05	negative
PRJNA729271	stage_E18_vs_E10	ordinary_muscle_development	ENSGALG00010012210	PPWD1	-1.094294239	0.077091526	-14.194741	9.87506E-46	2.40166E-44	negative
PRJNA729271	stage_E18_vs_E10	ordinary_muscle_development	ENSGALG00010012211	ENSGALG00010012211	-1.19983894	4.002438348	-0.299776995	0.764347264	0.802260217	negative
PRJNA729271	stage_E18_vs_E10	ordinary_muscle_development	ENSGALG00010012212	RAD54B	-1.445507497	0.185611344	-7.787818711	6.81759E-15	3.45286E-14	negative
PRJNA729271	stage_E18_vs_E10	ordinary_muscle_development	ENSGALG00010012215	RAB17	3.4045122	0.798510352	4.263579288	2.01178E-05	4.53159E-05	positive
PRJNA729271	stage_E18_vs_E10	ordinary_muscle_development	ENSGALG00010012216	BRD8	0.030964292	0.057921805	0.534587829	0.592934901	0.644755544	positive
PRJNA729271	stage_E18_vs_E10	ordinary_muscle_development	ENSGALG00010012219	KDM5A	-0.408819017	0.077610844	-5.267550208	1.38256E-07	3.86207E-07	negative
PRJNA729271	stage_E18_vs_E10	ordinary_muscle_development	ENSGALG00010012226	SLCO4C1	-1.397158363	0.606494772	-2.303661017	0.02124168	0.031898189	negative
PRJNA729271	stage_E18_vs_E10	ordinary_muscle_development	ENSGALG00010012228	NHP2	-1.378411279	0.119475043	-11.53723189	8.56369E-31	1.08982E-29	negative
PRJNA729271	stage_E18_vs_E10	ordinary_muscle_development	ENSGALG00010012229	FSBP	0.423535867	0.202365626	2.092923962	0.036355943	0.052310839	positive
PRJNA729271	stage_E18_vs_E10	ordinary_muscle_development	ENSGALG00010012230	PTPRQ	0.215533308	0.396254525	0.543926427	0.586492103	0.63885196	positive
PRJNA729271	stage_E18_vs_E10	ordinary_muscle_development	ENSGALG00010012232	PCSK1	3.721805676	0.412521635	9.022086017	1.84541E-19	1.25938E-18	positive
PRJNA729271	stage_E18_vs_E10	ordinary_muscle_development	ENSGALG00010012234	C1GALT1C1	-0.287103326	0.065826255	-4.361532119	1.29155E-05	2.97347E-05	negative
PRJNA729271	stage_E18_vs_E10	ordinary_muscle_development	ENSGALG00010012236	PRLH	-1.078584713	0.5535021	-1.948655141	0.051336622	0.071958644	negative
PRJNA729271	stage_E18_vs_E10	ordinary_muscle_development	ENSGALG00010012240	TRIM14	-1.282165195	0.274483857	-4.671186167	2.99465E-06	7.33979E-06	negative
PRJNA729271	stage_E18_vs_E10	ordinary_muscle_development	ENSGALG00010012242	CCDC77	-1.290136263	0.133699342	-9.649533352	4.93799E-22	3.94577E-21	negative
PRJNA729271	stage_E18_vs_E10	ordinary_muscle_development	ENSGALG00010012243	ENSGALG00010012243	-0.404757751	1.511353591	-0.26781142	0.78884448	0.823263067	negative
PRJNA729271	stage_E18_vs_E10	ordinary_muscle_development	ENSGALG00010012244	ENSGALG00010012244	-0.622918856	0.082880501	-7.515867416	5.65348E-14	2.64944E-13	negative
PRJNA729271	stage_E18_vs_E10	ordinary_muscle_development	ENSGALG00010012245	ENSGALG00010012245	-0.283220037	0.078986402	-3.585680958	0.0003362	0.000653597	negative
PRJNA729271	stage_E18_vs_E10	ordinary_muscle_development	ENSGALG00010012247	TRIM23	0.64063319	0.126222179	5.075440724	3.86599E-07	1.0403E-06	positive
PRJNA729271	stage_E18_vs_E10	ordinary_muscle_development	ENSGALG00010012248	H2AC39	-0.721910001	0.209817945	-3.440649468	0.00058032	0.001093143	negative
PRJNA729271	stage_E18_vs_E10	ordinary_muscle_development	ENSGALG00010012250	NANS	-1.461295513	0.11305718	-12.9252783	3.24122E-38	5.77878E-37	negative
PRJNA729271	stage_E18_vs_E10	ordinary_muscle_development	ENSGALG00010012253	MYF6	1.76191251	0.290039082	6.074741723	1.24187E-09	4.15151E-09	positive
PRJNA729271	stage_E18_vs_E10	ordinary_muscle_development	ENSGALG00010012259	IL1RAPL2	-0.219174449	0.22023371	-0.995190286	0.319643725	0.377299332	negative
PRJNA729271	stage_E18_vs_E10	ordinary_muscle_development	ENSGALG00010012260	ENSGALG00010012260	1.953591798	0.302638001	6.455209826	1.08069E-10	3.92498E-10	positive
PRJNA729271	stage_E18_vs_E10	ordinary_muscle_development	ENSGALG00010012263	SHLD3	0.059833547	0.131766425	0.454087957	0.649765521	0.69822457	positive
PRJNA729271	stage_E18_vs_E10	ordinary_muscle_development	ENSGALG00010012264	MYF5	2.449792982	0.154118886	15.89547556	6.81096E-57	2.44975E-55	positive
PRJNA729271	stage_E18_vs_E10	ordinary_muscle_development	ENSGALG00010012266	H3C33	-0.963711969	0.202861766	-4.75058454	2.02829E-06	5.06221E-06	negative
PRJNA729271	stage_E18_vs_E10	ordinary_muscle_development	ENSGALG00010012268	ENSGALG00010012268	-0.43922019	0.167667781	-2.619586112	0.008803654	0.014052304	negative
PRJNA729271	stage_E18_vs_E10	ordinary_muscle_development	ENSGALG00010012271	ENSGALG00010012271	0.807673256	0.270897196	2.981475147	0.002868633	0.004931314	positive
PRJNA729271	stage_E18_vs_E10	ordinary_muscle_development	ENSGALG00010012274	B4GALNT3	0.913609829	0.276880035	3.299659475	0.000968022	0.001766077	positive
PRJNA729271	stage_E18_vs_E10	ordinary_muscle_development	ENSGALG00010012275	VIRMA	-0.282010507	0.074220128	-3.799649987	0.000144901	0.000296197	negative

PRJNA729271	stage_E18_vs_E10	ordinary_muscle_development	ENSGALG00010012277	H3C33	-0.945643522	0.209942495	-4.504297822	6.65928E-06	1.57723E-05	negative
PRJNA729271	stage_E18_vs_E10	ordinary_muscle_development	ENSGALG00010012282	ENSGALG00010012282	-1.186985207	1.498404337	-0.792166158			negative
PRJNA729271	stage_E18_vs_E10	ordinary_muscle_development	ENSGALG00010012283	CUPIN1A	1.112481427	0.751460497	1.480425692	0.138759678	0.179054707	positive
PRJNA729271	stage_E18_vs_E10	ordinary_muscle_development	ENSGALG00010012285	FBLN1	-1.640147562	0.199055383	-8.239654399	1.72709E-16	9.74091E-16	negative
PRJNA729271	stage_E18_vs_E10	ordinary_muscle_development	ENSGALG00010012286	ENSGALG00010012286	-1.233185557	2.670832691	-0.461723252	0.644279794	0.693357756	negative
PRJNA729271	stage_E18_vs_E10	ordinary_muscle_development	ENSGALG00010012288	MCTS1	-0.38874715	0.115400569	-3.368676212	0.000755301	0.001401624	negative
PRJNA729271	stage_E18_vs_E10	ordinary_muscle_development	ENSGALG00010012290	ENSGALG00010012290	0.077964109	0.097723666	0.797801724	0.424985561	0.485314806	positive
PRJNA729271	stage_E18_vs_E10	ordinary_muscle_development	ENSGALG00010012291	NR5A2	0.910861765	0.188250248	4.838568749	1.30777E-06	3.34207E-06	positive
PRJNA729271	stage_E18_vs_E10	ordinary_muscle_development	ENSGALG00010012293	SRGAP1	-0.643855903	0.105687444	-6.092075618	1.11456E-09	3.74665E-09	negative
PRJNA729271	stage_E18_vs_E10	ordinary_muscle_development	ENSGALG00010012294	ENSGALG00010012294	0.010495202	0.522688477	0.020079268	0.983980139	0.986498279	positive
PRJNA729271	stage_E18_vs_E10	ordinary_muscle_development	ENSGALG00010012295	CUL4B	-0.597051934	0.066238556	-9.013661693	1.99289E-19	1.35747E-18	negative
PRJNA729271	stage_E18_vs_E10	ordinary_muscle_development	ENSGALG00010012297	ABLIM3	1.67922069	0.186291162	9.013957915	1.98751E-19	1.35444E-18	positive
PRJNA729271	stage_E18_vs_E10	ordinary_muscle_development	ENSGALG00010012300	NINJ2	-1.153286596	0.20966814	-5.500533357	3.78644E-08	1.11622E-07	negative
PRJNA729271	stage_E18_vs_E10	ordinary_muscle_development	ENSGALG00010012303	CA9	2.046496323	0.210962631	9.700752745	2.99282E-22	2.42352E-21	positive
PRJNA729271	stage_E18_vs_E10	ordinary_muscle_development	ENSGALG00010012304	ESRP1	-4.704510788	0.705791388	-6.665582594	2.63618E-11	1.00319E-10	negative
PRJNA729271	stage_E18_vs_E10	ordinary_muscle_development	ENSGALG00010012307	ENSGALG00010012307	-0.695918297	0.172855731	-4.026006491	5.67321E-05	0.000121539	negative
PRJNA729271	stage_E18_vs_E10	ordinary_muscle_development	ENSGALG00010012308	TRABD2A	2.023921183	0.342980852	5.900974275	3.61361E-09	1.16295E-08	positive
PRJNA729271	stage_E18_vs_E10	ordinary_muscle_development	ENSGALG00010012309	TRAPPC13	0.13596405	0.124831239	1.089182892	0.276073242	0.33156696	positive
PRJNA729271	stage_E18_vs_E10	ordinary_muscle_development	ENSGALG00010012311	ENSGALG00010012311	-2.323343685	0.519180579	-4.475020402	7.6404E-06	1.80077E-05	negative
PRJNA729271	stage_E18_vs_E10	ordinary_muscle_development	ENSGALG00010012312	SH3TC2	0.88631002	0.181729387	4.877086934	1.07664E-06	2.77537E-06	positive
PRJNA729271	stage_E18_vs_E10	ordinary_muscle_development	ENSGALG00010012314	ADRB2	2.534397554	0.268054333	9.45479046	3.2367E-21	2.46666E-20	positive
PRJNA729271	stage_E18_vs_E10	ordinary_muscle_development	ENSGALG00010012315	ATP6V1E1	-0.690133477	0.10382108	-6.647334807	2.98448E-11	1.13039E-10	negative
PRJNA729271	stage_E18_vs_E10	ordinary_muscle_development	ENSGALG00010012316	ENSGALG00010012316	-1.487437826	0.86145872	-1.726650148	0.084230514	0.113300046	negative
PRJNA729271	stage_E18_vs_E10	ordinary_muscle_development	ENSGALG00010012317	ENSGALG00010012317	-0.884209418	1.148267688	-0.770037707	0.441277525	0.500768632	negative
PRJNA729271	stage_E18_vs_E10	ordinary_muscle_development	ENSGALG00010012318	POLR1D	-0.667658072	0.143850956	-4.641318295	3.46193E-06	8.421E-06	negative
PRJNA729271	stage_E18_vs_E10	ordinary_muscle_development	ENSGALG00010012320	DPY19L4	-0.061227917	0.061151144	-1.001255467	0.316703317	0.374163711	negative
PRJNA729271	stage_E18_vs_E10	ordinary_muscle_development	ENSGALG00010012322	ENSGALG00010012322	-0.850787624	0.267860433	-3.176234786	0.001492001	0.002663042	negative
PRJNA729271	stage_E18_vs_E10	ordinary_muscle_development	ENSGALG00010012323	SMAD7B	-0.14859735	3.563938135	-0.041694705	0.966742075	0.972444579	negative
PRJNA729271	stage_E18_vs_E10	ordinary_muscle_development	ENSGALG00010012324	ENSGALG00010012324	-1.107912724	0.163559446	-6.773761771	1.25476E-11	4.89709E-11	negative
PRJNA729271	stage_E18_vs_E10	ordinary_muscle_development	ENSGALG00010012325	MLPH	-1.365778329	0.529956887	-2.577149882	0.009961874	0.015762184	negative
PRJNA729271	stage_E18_vs_E10	ordinary_muscle_development	ENSGALG00010012326	SGTB	-0.899587696	0.120508114	-7.46495538	8.33278E-14	3.86137E-13	negative
PRJNA729271	stage_E18_vs_E10	ordinary_muscle_development	ENSGALG00010012327	UBTD2	-0.093641483	0.08629056	-1.085188035	0.277838382	0.333410659	negative
PRJNA729271	stage_E18_vs_E10	ordinary_muscle_development	ENSGALG00010012329	ATXN10	-0.607348359	0.109539618	-5.544554311	2.94704E-08	8.76433E-08	negative
PRJNA729271	stage_E18_vs_E10	ordinary_muscle_development	ENSGALG00010012331	ENSGALG00010012331	-0.722543823	0.264835421	-2.728274863	0.006366653	0.010384229	negative
PRJNA729271	stage_E18_vs_E10	ordinary_muscle_development	ENSGALG00010012334	ARHGEF39	-1.063111436	0.225352436	-4.717550228	2.38701E-06	5.92158E-06	negative
PRJNA729271	stage_E18_vs_E10	ordinary_muscle_development	ENSGALG00010012336	ENSGALG00010012336	-0.909612418	4.133185306	-0.220075402	0.825812432	0.855071876	negative
PRJNA729271	stage_E18_vs_E10	ordinary_muscle_development	ENSGALG00010012337	ACSS3	1.583845361	0.150547601	10.52056196	6.94581E-26	6.91005E-25	positive
PRJNA729271	stage_E18_vs_E10	ordinary_muscle_development	ENSGALG00010012338	H3C33	-0.800035366	0.225093909	-3.5542293	0.000379089	0.000731676	negative
PRJNA729271	stage_E18_vs_E10	ordinary_muscle_development	ENSGALG00010012339	WNK1	0.207702444	0.155169815	1.338549278	0.180717448	0.227052042	positive
PRJNA729271	stage_E18_vs_E10	ordinary_muscle_development	ENSGALG00010012340	ENHO	0.533090294	0.158853336	3.355864641	0.000791173	0.001463508	positive
PRJNA729271	stage_E18_vs_E10	ordinary_muscle_development	ENSGALG00010012341	GOPC	0.0335631	0.080506049	0.41690159	0.676750391	0.723094273	positive
PRJNA729271	stage_E18_vs_E10	ordinary_muscle_development	ENSGALG00010012342	ENSGALG00010012342	1.206306116	1.53619854	0.785254044	0.432304634	0.492167695	positive
PRJNA729271	stage_E18_vs_E10	ordinary_muscle_development	ENSGALG00010012343	BCL2L13	-0.124818535	0.171337676	-0.728494383	0.466311006	0.525433615	negative
PRJNA729271	stage_E18_vs_E10	ordinary_muscle_development	ENSGALG00010012345	H2BC32	-1.079642562	0.165159175	-6.536982046	6.27726E-11	2.32352E-10	negative
PRJNA729271	stage_E18_vs_E10	ordinary_muscle_development	ENSGALG00010012346	NLN	-0.60247127	0.141231928	-4.265829126	1.99161E-05	4.48825E-05	negative
PRJNA729271	stage_E18_vs_E10	ordinary_muscle_development	ENSGALG00010012347	ENSGALG00010012347	-1.514188644	0.487995648	-3.102873252	0.001916517	0.003373396	negative
PRJNA729271	stage_E18_vs_E10	ordinary_muscle_development	ENSGALG00010012350	H2AC32	-0.988882416	0.192172372	-5.145809494	2.6637E-07	7.26577E-07	negative
PRJNA729271	stage_E18_vs_E10	ordinary_muscle_development	ENSGALG00010012352	PTPRC	0.286102338	0.197432005	1.449118335	0.147304539	0.188820241	positive
PRJNA729271	stage_E18_vs_E10	ordinary_muscle_development	ENSGALG00010012353	PIGG	-0.037594262	0.153056149	-0.245623991	0.805973316	0.83824232	negative
PRJNA729271	stage_E18_vs_E10	ordinary_muscle_development	ENSGALG00010012354	DCBLD1	0.953008142	0.149439318	6.377224927	1.80326E-10	6.45069E-10	positive
PRJNA729271	stage_E18_vs_E10	ordinary_muscle_development	ENSGALG00010012358	STK10	0.319105281	0.149390918	2.136042043	0.032675987	0.047411255	positive
PRJNA729271	stage_E18_vs_E10	ordinary_muscle_development	ENSGALG00010012360	ROS1	2.346333973	0.377122214	6.221680626	4.91857E-10	1.70971E-09	positive
PRJNA729271	stage_E18_vs_E10	ordinary_muscle_development	ENSGALG00010012361	WIF1	-4.988688564	0.36835232	-13.54325275	8.6856E-42	1.78073E-40	negative
PRJNA729271	stage_E18_vs_E10	ordinary_muscle_development	ENSGALG00010012362	COL6A3	3.994367574	0.292449917	13.65829615	1.8015E-42	3.81765E-41	positive
PRJNA729271	stage_E18_vs_E10	ordinary_muscle_development	ENSGALG00010012366	INTS8	0.053776431	0.06745485	0.797221111	0.425322627	0.485615333	positive
PRJNA729271	stage_E18_vs_E10	ordinary_muscle_development	ENSGALG00010012367	RAD52	-1.497093495	0.140841453	-10.62963685	2.16956E-26	2.23033E-25	negative
PRJNA729271	stage_E18_vs_E10	ordinary_muscle_development	ENSGALG00010012371	ATP6V1G3	-2.028876307	0.543009766	-3.736353253	0.000186708	0.000375724	negative
PRJNA729271	stage_E18_vs_E10	ordinary_muscle_development	ENSGALG00010012372	LEMD3	0.382684642	0.073026384	5.240361365	1.60262E-07	4.44424E-07	positive
PRJNA729271	stage_E18_vs_E10	ordinary_muscle_development	ENSGALG00010012373	WNT7B	-5.666628287	0.652957516	-8.678402732	4.01366E-18	2.53499E-17	negative
PRJNA729271	stage_E18_vs_E10	ordinary_muscle_development	ENSGALG00010012376	VGLL2	1.328071999	0.186419639	7.124099195	1.04764E-12	4.48611E-12	positive
PRJNA729271	stage_E18_vs_E10	ordinary_muscle_development	ENSGALG00010012378	RPS19BP1	-0.093532247	0.092127867	-1.015243819	0.309989618	0.367614073	negative
PRJNA729271	stage_E18_vs_E10	ordinary_muscle_development	ENSGALG00010012382	SH3PXD2B	-0.585018764	0.133071807	-4.396263766	1.1013E-05	2.5521E-05	negative
PRJNA729271	stage_E18_vs_E10	ordinary_muscle_development	ENSGALG00010012384	NRK	0.204775626	0.121990213	1.678623395	0.093225464	0.124372122	positive
PRJNA729271	stage_E18_vs_E10	ordinary_muscle_development	ENSGALG00010012387	ERBIN	-0.027244864	0.089104711	-0.305762326	0.759785615	0.798455194	negative
PRJNA729271	stage_E18_vs_E10	ordinary_muscle_development	ENSGALG00010012390	MMP16	-0.264875628	0.174301845	-1.519637547	0.128602096	0.167159507	negative
PRJNA729271	stage_E18_vs_E10	ordinary_muscle_development	ENSGALG00010012391	NEURL1B	0.560242703	0.156443994	3.58110713	0.000342141	0.000664345	positive
PRJNA729271	stage_E18_vs_E10	ordinary_muscle_development	ENSGALG00010012393	ENSGALG00010012393	-0.933420329	1.608477375	-0.580312998	0.561703563	0.615597547	negative
PRJNA729271	stage_E18_vs_E10	ordinary_muscle_development	ENSGALG00010012398	ENSGALG00010012398	-3.671232714	0.767904035	-4.780848315	1.74557E-06	4.39577E-06	negative
PRJNA729271	stage_E18_vs_E10	ordinary_muscle_development	ENSGALG00010012399	ERC1	-0.714085179	0.086162728	-8.287634284	1.15523E-16	6.61078E-16	negative
PRJNA729271	stage_E18_vs_E10	ordinary_muscle_development	ENSGALG00010012401	RIPK2	0.487769513	0.132248182	3.688288977	0.000225767	0.000449519	positive
PRJNA729271	stage_E18_vs_E10	ordinary_muscle_development	ENSGALG00010012404	VAMP7	-0.033911776	0.0535551	-0.633212823	0.526594673	0.582891116	negative
PRJNA729271	stage_E18_vs_E10	ordinary_muscle_development	ENSGALG00010012405	MSRB3	2.465259525	0.125707253	19.61111605	1.24275E-85	1.012E-83	positive
PRJNA729271	stage_E18_vs_E10	ordinary_muscle_development	ENSGALG00010012406	ENSGALG00010012406	2.972782458	0.243627938	12.20214102	3.02757E-34	4.50561E-33	positive
PRJNA729271	stage_E18_vs_E10	ordinary_muscle_development	ENSGALG00010012407	ATF4	0.930643115	0.128865455	7.221819985	5.12963E-13	2.24296E-12	positive
PRJNA729271	stage_E18_vs_E10	ordinary_muscle_development	ENSGALG00010012410	CACNA1I	-1.816238101	0.402017714	-4.517806149	6.24836E-06	1.48427E-05	negative
PRJNA729271	stage_E18_vs_E10	ordinary_muscle_development	ENSGALG00010012412	PURA	0.814057233	0.135062455	6.027265206	1.66757E-09	5.51738E-09	positive
PRJNA729271	stage_E18_vs_E10	ordinary_muscle_development	ENSGALG00010012413	NEK7	0.070693816	0.073660296	0.95972756	0.337192349	0.395661224	positive
PRJNA729271	stage_E18_vs_E10	ordinary_muscle_development	ENSGALG00010012414	ENSGALG00010012414	6.123245727	0.203339401	30.11342468	3.233E-199	1.8024E-196	positive
PRJNA729271	stage_E18_vs_E10	ordinary_muscle_development	ENSGALG00010012415	BID	-1.345214181	0.115188113	-11.6784115	1.64338E-31	2.17145E-30	negative
PRJNA729271	stage_E18_vs_E10	ordinary_muscle_development	ENSGALG00010012418	ENSGALG00010012418	0.451083341	0.792980618	0.568845354	0.569461093	0.623157069	positive
PRJNA729271	stage_E18_vs_E10	ordinary_muscle_development	ENSGALG00010012419	HACE1	-0.151204432	0.098975128	-1.527701304	0.126586716	0.164858441	negative
PRJNA729271	stage_E18_vs_E10	ordinary_muscle_development	ENSGALG00010012426	MIEF1	-0.304704675	0.081553247	-3.736266629	0.000186773	0.000375801	negative
PRJNA729271	stage_E18_vs_E10	ordinary_muscle_development	ENSGALG00010012427	C7	0.070006428	0.453682791	0.154306994	0.877367683	0.898505339	positive
PRJNA729271	stage_E18_vs_E10	ordinary_muscle_development	ENSGALG00010012428	CCNE2	-1.62807917	0.215294755	-7.562093977	3.96632E-14	1.87636E-13	negative
PRJNA729271	stage_E18_vs_E10	ordinary_muscle_development	ENSGALG00010012429	CXCR7	1.997114094	0.100733621	19.82569553	1.78694E-87	1.54177E-85	positive
PRJNA729271	stage_E18_vs_E10	ordinary_muscle_development	ENSGALG00010012431	LAMP2	0.44475361	0.079562032	5.59002326	2.27039E-08	6.79804E-08	positive
PRJNA729271	stage_E18_vs_E10	ordinary_muscle_development	ENSGALG00010012433	ENSGALG00010012433	-0.532392837	0.789613705	-0.674244676	0.500155771	0.557330711	negative
PRJNA729271	stage_E18_vs_E10	ordinary_muscle_development	ENSGALG00010012436	ENSGALG00010012436	3.720321569	0.199837534	18.61673081	2.35138E-77	1.5284E-75	positive
PRJNA729271	stage_E18_vs_E10	ordinary_muscle_development	ENSGALG00010012437	ENSGALG00010012437	-1.135423138	3.812466377	-0.297818532	0.76584167	0.803537821	negative
PRJNA729271	stage_E18_vs_E10	ordinary_muscle_development	ENSGALG00010012439	TENT2	0.12594699	0.103134667	1.221189664	0.222014217	0.272940118	positive
PRJNA729271	stage_E18_vs_E10	ordinary_muscle_development	ENSGALG00010012441	FST	-0.027025659	0.239480394	-0.112851239	0.910148495	0.925672487	negative
PRJNA729271	stage_E18_vs_E10	ordinary_muscle_development	ENSGALG00010012442	CPLX1	-5.005543991	0.390679812	-12.81239479	1.39754E-37	2.41734E-36	negative
PRJNA729271	stage_E18_vs_E10	ordinary_muscle_development	ENSGALG00010012445	SOBP	-0.545839273	0.16614801	-3.285259165	0.001018886	0.001854206	negative
PRJNA729271	stage_E18_vs_E10	ordinary_muscle_development	ENSGALG00010012447	COPS8	-0.54118932	0.117806481	-4.593884106	4.35071E-06	1.04861E-05	negative
PRJNA729271	stage_E18_vs_E10	ordinary_muscle_development	ENSGALG00010012449	SUB1	-1.235222292	0.109725951	-11.25733963	2.13095E-29	2.52148E-28	negative
PRJNA729271	stage_E18_vs_E10	ordinary_muscle_development	ENSGALG00010012450	PPFIA2	-1.06547232	0.255121247	-4.176337056	2.96241E-05	6.53777E-05	negative
PRJNA729271	stage_E18_vs_E10	ordinary_muscle_development	ENSGALG00010012454	IQCA1	2.745117252	0.407774906	6.731942583	1.67413E-11	6.46589E-11	positive
PRJNA729271	stage_E18_vs_E10	ordinary_muscle_development	ENSGALG00010012456	ENSGALG00010012456	-0.640039129	0.088466245	-7.234840036	4.66078E-13	2.04659E-12	negative
PRJNA729271	stage_E18_vs_E10	ordinary_muscle_development	ENSGALG00010012458	MRPS30	0.113665567	0.108703104	1.045651524	0.295721951	0.352478389	positive
PRJNA729271	stage_E18_vs_E10	ordinary_muscle_development	ENSGALG00010012464	ENSGALG00010012464	0.664727346	0.241001857	2.758183498	0.005812356	0.009539187	positive
PRJNA729271	stage_E18_vs_E10	ordinary_muscle_development	ENSGALG00010012466	PDSS2	-0.155036363	0.121906116	-1.271768542	0.203455369	0.252759838	negative
PRJNA729271	stage_E18_vs_E10	ordinary_muscle_development	ENSGALG00010012469	CETN2	-0.780204384	0.131484606	-5.93380781	2.95988E-09	9.59381E-09	negative
PRJNA729271	stage_E18_vs_E10	ordinary_muscle_development	ENSGALG00010012470	CHD1	-0.604435614	0.106502693	-5.675308253	1.38439E-08	4.22458E-08	negative
PRJNA729271	stage_E18_vs_E10	ordinary_muscle_development	ENSGALG00010012474	ASB18	5.538487244	0.925687284	5.983108271	2.18919E-09	7.17439E-09	positive
PRJNA729271	stage_E18_vs_E10	ordinary_muscle_development	ENSGALG00010012477	ENSGALG00010012477	-0.024920418	0.257706094	-0.096700928	0.922963903	0.93666329	negative
PRJNA729271	stage_E18_vs_E10	ordinary_muscle_development	ENSGALG00010012483	ENSGALG00010012483	0.339252148	0.253925713	1.336029124	0.181539754	0.228025887	positive
PRJNA729271	stage_E18_vs_E10	ordinary_muscle_development	ENSGALG00010012485	C6	3.105108331	1.280612392	2.424705828	0.015320796	0.023614944	positive
PRJNA729271	stage_E18_vs_E10	ordinary_muscle_development	ENSGALG00010012486	RADX	0.262773096	0.108748422	2.416339405	0.01567744	0.02412362	positive
PRJNA729271	stage_E18_vs_E10	ordinary_muscle_development	ENSGALG00010012489	GBX2	-1.28733721	0.791614942	-1.626216412	0.103903626	0.137328628	negative
PRJNA729271	stage_E18_vs_E10	ordinary_muscle_development	ENSGALG00010012497	PLCXD3	-0.756556799	0.44025987	-1.718432341	0.085717786	0.115087005	negative
PRJNA729271	stage_E18_vs_E10	ordinary_muscle_development	ENSGALG00010012498	KCNT2	-0.087990837	0.220675957	-0.39873323	0.69008978	0.735611955	negative
PRJNA729271	stage_E18_vs_E10	ordinary_muscle_development	ENSGALG00010012506	TP53INP1	-0.484464825	0.126670244	-3.824614297	0.000130977	0.000269331	negative
PRJNA729271	stage_E18_vs_E10	ordinary_muscle_development	ENSGALG00010012508	MDM1	-0.441193883	0.06871265	-6.42085383	1.35512E-10	4.88619E-10	negative
PRJNA729271	stage_E18_vs_E10	ordinary_muscle_development	ENSGALG00010012509	MICAL3	0.22826489	0.093941031	2.429874222	0.015104063	0.023307303	positive
PRJNA729271	stage_E18_vs_E10	ordinary_muscle_development	ENSGALG00010012511	OXCT1	-1.048237495	0.122364202	-8.566537224	1.06643E-17	6.54167E-17	negative
PRJNA729271	stage_E18_vs_E10	ordinary_muscle_development	ENSGALG00010012517	MGAT3	0.675336908	0.175510856	3.847835525	0.000119166	0.000246056	positive
PRJNA729271	stage_E18_vs_E10	ordinary_muscle_development	ENSGALG00010012519	ENSGALG00010012519	-0.69625373	1.684052067	-0.41343955	0.679284603	0.725641559	negative
PRJNA729271	stage_E18_vs_E10	ordinary_muscle_development	ENSGALG00010012521	HOMER1	2.350908681	0.128382875	18.31169987	6.6753E-75	3.98183E-73	positive
PRJNA729271	stage_E18_vs_E10	ordinary_muscle_development	ENSGALG00010012529	BEND3	-0.816877741	0.125981694	-6.484098711	8.92638E-11	3.26819E-10	negative
PRJNA729271	stage_E18_vs_E10	ordinary_muscle_development	ENSGALG00010012532	FAM174A	0.164789263	0.154328665	1.067781306	0.285619173	0.341447415	positive
PRJNA729271	stage_E18_vs_E10	ordinary_muscle_development	ENSGALG00010012535	JMY	-0.183978307	0.104007171	-1.768900201	0.076910531	0.104325112	negative
PRJNA729271	stage_E18_vs_E10	ordinary_muscle_development	ENSGALG00010012536	FBXO4	0.155328879	0.134884565	1.151568966	0.24949824	0.303065197	positive
PRJNA729271	stage_E18_vs_E10	ordinary_muscle_development	ENSGALG00010012538	RAP1B	-0.61907895	0.088349494	-7.00715895	2.43206E-12	1.00982E-11	negative
PRJNA729271	stage_E18_vs_E10	ordinary_muscle_development	ENSGALG00010012547	ENSGALG00010012547	0.058510938	0.360568615	0.162274074	0.87109003	0.893861462	positive
PRJNA729271	stage_E18_vs_E10	ordinary_muscle_development	ENSGALG00010012550	TAB1	0.489263145	0.094714629	5.165655511	2.39598E-07	6.5602E-07	positive
PRJNA729271	stage_E18_vs_E10	ordinary_muscle_development	ENSGALG00010012551	PCGF3	0.109286498	0.156137626	0.699936978	0.483966663	0.541999288	positive
PRJNA729271	stage_E18_vs_E10	ordinary_muscle_development	ENSGALG00010012553	ENSGALG00010012553	0.390568936	0.224803101	1.737382328	0.082319708	0.111049248	positive
PRJNA729271	stage_E18_vs_E10	ordinary_muscle_development	ENSGALG00010012555	ENSGALG00010012555	1.65914895	0.212888434	7.793513812	6.5171E-15	3.30529E-14	positive
PRJNA729271	stage_E18_vs_E10	ordinary_muscle_development	ENSGALG00010012561	OSGIN2	-0.719297755	0.127667256	-5.634160054	1.75914E-08	5.31555E-08	negative
PRJNA729271	stage_E18_vs_E10	ordinary_muscle_development	ENSGALG00010012565	SYNGR1	0.408722242	0.122529626	3.335701387	0.000850845	0.001566493	positive
PRJNA729271	stage_E18_vs_E10	ordinary_muscle_development	ENSGALG00010012569	C6orf203	0.435505313	0.122399486	3.558064874	0.000373597	0.000722039	positive

PRJNA729271	stage_E18_vs_E10	ordinary_muscle_development	ENSGALG00010012571	AGAP1	0.695081301	0.08172558	8.505064201	1.81495E-17	1.09433E-16	positive
PRJNA729271	stage_E18_vs_E10	ordinary_muscle_development	ENSGALG00010012574	PHAX	-0.523607091	0.105595712	-4.95860184	7.10023E-07	1.86277E-06	negative
PRJNA729271	stage_E18_vs_E10	ordinary_muscle_development	ENSGALG00010012578	NDUFS4	0.327650019	0.153177742	2.139018467	0.032434173	0.047093392	positive
PRJNA729271	stage_E18_vs_E10	ordinary_muscle_development	ENSGALG00010012582	CD24	-3.151275426	0.227852986	-13.83030117	1.67319E-43	3.69145E-42	negative
PRJNA729271	stage_E18_vs_E10	ordinary_muscle_development	ENSGALG00010012583	NDUFAF6	0.166475545	0.103700401	1.605351025	0.108416576	0.142889459	positive
PRJNA729271	stage_E18_vs_E10	ordinary_muscle_development	ENSGALG00010012586	CDC73	-0.078280927	0.134344766	-0.58268684	0.560104134	0.614123254	negative
PRJNA729271	stage_E18_vs_E10	ordinary_muscle_development	ENSGALG00010012587	MBLAC2	0.354797242	0.14966488	2.370611204	0.017758701	0.027016411	positive
PRJNA729271	stage_E18_vs_E10	ordinary_muscle_development	ENSGALG00010012588	PEX26	-0.108629743	0.107941038	-1.006380376	0.31423263	0.371688654	negative
PRJNA729271	stage_E18_vs_E10	ordinary_muscle_development	ENSGALG00010012593	TUBA8B	2.084758548	0.970168435	2.148862479	0.031645303	0.046049459	positive
PRJNA729271	stage_E18_vs_E10	ordinary_muscle_development	ENSGALG00010012599	DMGDH	1.350707165	0.196713547	6.866365767	6.58581E-12	2.63196E-11	positive
PRJNA729271	stage_E18_vs_E10	ordinary_muscle_development	ENSGALG00010012600	NUP107	-1.512104913	0.101803399	-14.85318684	6.63386E-50	1.92316E-48	negative
PRJNA729271	stage_E18_vs_E10	ordinary_muscle_development	ENSGALG00010012601	TUBA8A	0.471007281	0.25218344	1.867716932	0.061801537	0.085274944	positive
PRJNA729271	stage_E18_vs_E10	ordinary_muscle_development	ENSGALG00010012604	ENSGALG00010012604	-2.254230651	2.137506767	-1.054607492			negative
PRJNA729271	stage_E18_vs_E10	ordinary_muscle_development	ENSGALG00010012606	ATP5PO	0.52446231	0.114268446	4.589738705	4.43801E-06	1.06877E-05	positive
PRJNA729271	stage_E18_vs_E10	ordinary_muscle_development	ENSGALG00010012607	LMNB1	-2.258692227	0.164256708	-13.75098924	5.02379E-43	1.08041E-41	negative
PRJNA729271	stage_E18_vs_E10	ordinary_muscle_development	ENSGALG00010012610	RPL3	0.578635538	0.154958529	3.734131588	0.000188364	0.000378898	positive
PRJNA729271	stage_E18_vs_E10	ordinary_muscle_development	ENSGALG00010012614	LHFPL2	-0.347967039	0.144800806	-2.403073914	0.016257901	0.024942662	negative
PRJNA729271	stage_E18_vs_E10	ordinary_muscle_development	ENSGALG00010012618	B3GALT2	2.598057466	0.324476918	8.006909958	1.17627E-15	6.28918E-15	positive
PRJNA729271	stage_E18_vs_E10	ordinary_muscle_development	ENSGALG00010012624	PDGFB	0.87392503	0.183622541	4.759355927	1.94212E-06	4.85864E-06	positive
PRJNA729271	stage_E18_vs_E10	ordinary_muscle_development	ENSGALG00010012629	NUS1	0.156568971	0.110550481	1.416266754	0.156697418	0.199694837	positive
PRJNA729271	stage_E18_vs_E10	ordinary_muscle_development	ENSGALG00010012631	GLRX2	0.180536532	0.155587251	1.160355562	0.245904072	0.29915061	positive
PRJNA729271	stage_E18_vs_E10	ordinary_muscle_development	ENSGALG00010012633	USP18	-1.319727299	0.546947761	-2.412894599	0.015826396	0.024329579	negative
PRJNA729271	stage_E18_vs_E10	ordinary_muscle_development	ENSGALG00010012640	PRRC1	-0.238919332	0.07377605	-3.238440285	0.001201852	0.00216758	negative
PRJNA729271	stage_E18_vs_E10	ordinary_muscle_development	ENSGALG00010012648	MRPS6	0.120583861	0.131522758	0.916828869	0.359232322	0.418743266	positive
PRJNA729271	stage_E18_vs_E10	ordinary_muscle_development	ENSGALG00010012650	DSE	-0.402590639	0.105049481	-3.832390543	0.000126904	0.000261363	negative
PRJNA729271	stage_E18_vs_E10	ordinary_muscle_development	ENSGALG00010012653	CBX7	2.186951012	0.250248555	8.739115456	2.34943E-18	1.50486E-17	positive
PRJNA729271	stage_E18_vs_E10	ordinary_muscle_development	ENSGALG00010012654	TENT5B	1.769728717	0.309483549	5.718328882	1.07577E-08	3.30856E-08	positive
PRJNA729271	stage_E18_vs_E10	ordinary_muscle_development	ENSGALG00010012662	SLC35E3	-0.842665662	0.1081983	-7.788159939	6.79921E-15	3.44596E-14	negative
PRJNA729271	stage_E18_vs_E10	ordinary_muscle_development	ENSGALG00010012667	SCAMP1	-0.48427262	0.089546017	-5.408086672	6.37016E-08	1.83351E-07	negative
PRJNA729271	stage_E18_vs_E10	ordinary_muscle_development	ENSGALG00010012669	ERCC8	0.237646615	0.148074595	1.60491146	0.108513291	0.143003923	positive
PRJNA729271	stage_E18_vs_E10	ordinary_muscle_development	ENSGALG00010012670	ENSGALG00010012670	-0.185549622	0.216665872	-0.856386012	0.391784299	0.451783088	negative
PRJNA729271	stage_E18_vs_E10	ordinary_muscle_development	ENSGALG00010012671	NBN	-1.886766591	0.189371647	-9.96330032	2.20615E-23	1.8855E-22	negative
PRJNA729271	stage_E18_vs_E10	ordinary_muscle_development	ENSGALG00010012672	RO60	-0.600278877	0.09090809	-6.603140327	4.02538E-11	1.512E-10	negative
PRJNA729271	stage_E18_vs_E10	ordinary_muscle_development	ENSGALG00010012673	ENSGALG00010012673	-1.017305612	0.326906037	-3.111920539	0.001858745	0.003280471	negative
PRJNA729271	stage_E18_vs_E10	ordinary_muscle_development	ENSGALG00010012674	ENSGALG00010012674	1.335236072	0.126269425	10.5745003	3.91258E-26	3.94387E-25	positive
PRJNA729271	stage_E18_vs_E10	ordinary_muscle_development	ENSGALG00010012676	ENSGALG00010012676	1.094175288	0.207960585	5.261455137	1.4292E-07	3.98465E-07	positive
PRJNA729271	stage_E18_vs_E10	ordinary_muscle_development	ENSGALG00010012680	TRPM8	0.662605824	0.278108518	2.382544157	0.01719347	0.026225334	positive
PRJNA729271	stage_E18_vs_E10	ordinary_muscle_development	ENSGALG00010012682	ENSGALG00010012682	-0.10586276	0.111045529	-0.953327534	0.340424145	0.398807725	negative
PRJNA729271	stage_E18_vs_E10	ordinary_muscle_development	ENSGALG00010012683	MDM2	0.48651109	0.086456744	5.627219675	1.83137E-08	5.52576E-08	positive
PRJNA729271	stage_E18_vs_E10	ordinary_muscle_development	ENSGALG00010012685	NPTXR	1.450259712	0.243618929	5.952984514	2.63296E-09	8.56865E-09	positive
PRJNA729271	stage_E18_vs_E10	ordinary_muscle_development	ENSGALG00010012688	NDUFAF2	-0.054578524	0.114888787	-0.475055271	0.634747578	0.684217011	negative
PRJNA729271	stage_E18_vs_E10	ordinary_muscle_development	ENSGALG00010012690	NT5DC1	-0.84150237	0.338263605	-2.487711825	0.012856786	0.020025695	negative
PRJNA729271	stage_E18_vs_E10	ordinary_muscle_development	ENSGALG00010012693	UCHL5	-0.557954901	0.078298082	-7.126035361	1.03301E-12	4.42741E-12	negative
PRJNA729271	stage_E18_vs_E10	ordinary_muscle_development	ENSGALG00010012696	DNAL4	-0.477078001	0.091603252	-5.208090221	1.90794E-07	5.26774E-07	negative
PRJNA729271	stage_E18_vs_E10	ordinary_muscle_development	ENSGALG00010012699	RNF128	-0.367507628	0.146489077	-2.508771547	0.012115181	0.018933644	negative
PRJNA729271	stage_E18_vs_E10	ordinary_muscle_development	ENSGALG00010012702	PTPRO	-2.997163705	0.367875159	-8.147230483	3.72354E-16	2.05454E-15	negative
PRJNA729271	stage_E18_vs_E10	ordinary_muscle_development	ENSGALG00010012704	SMIM15	-0.106521914	0.068292274	-1.559794523	0.118808446	0.155440782	negative
PRJNA729271	stage_E18_vs_E10	ordinary_muscle_development	ENSGALG00010012705	CPM	2.861961949	0.183891956	15.56327975	1.293E-54	4.31844E-53	positive
PRJNA729271	stage_E18_vs_E10	ordinary_muscle_development	ENSGALG00010012706	TBC1D8B	0.643232507	0.105188988	6.115017526	9.65464E-10	3.26438E-09	positive
PRJNA729271	stage_E18_vs_E10	ordinary_muscle_development	ENSGALG00010012708	SPP2	3.641522514	1.094134279	3.328222673	0.00087402	0.001605693	positive
PRJNA729271	stage_E18_vs_E10	ordinary_muscle_development	ENSGALG00010012709	PPP1R8	-0.864479071	0.057566894	-15.01694828	5.68661E-51	1.72082E-49	negative
PRJNA729271	stage_E18_vs_E10	ordinary_muscle_development	ENSGALG00010012711	ELL2	-0.114630581	0.127101642	-0.901881198	0.36711998	0.426768785	negative
PRJNA729271	stage_E18_vs_E10	ordinary_muscle_development	ENSGALG00010012712	EEF1E1	-0.103122354	0.133878968	-0.770265528	0.4411424	0.500654486	negative
PRJNA729271	stage_E18_vs_E10	ordinary_muscle_development	ENSGALG00010012714	RGS2	1.504564858	0.172061682	8.744334223	2.24334E-18	1.43818E-17	positive
PRJNA729271	stage_E18_vs_E10	ordinary_muscle_development	ENSGALG00010012719	COL10A1	-1.230115065	0.769992395	-1.597567811	0.110139195	0.144988433	negative
PRJNA729271	stage_E18_vs_E10	ordinary_muscle_development	ENSGALG00010012720	ACO2	0.855823085	0.119952891	7.134659913	9.70268E-13	4.17206E-12	positive
PRJNA729271	stage_E18_vs_E10	ordinary_muscle_development	ENSGALG00010012722	ENSGALG00010012722	-0.293519872	0.102873749	-2.853204774	0.004328072	0.007230914	negative
PRJNA729271	stage_E18_vs_E10	ordinary_muscle_development	ENSGALG00010012725	ENSGALG00010012725	1.591778616	0.25836708	6.160918853	7.2324E-10	2.47892E-09	positive
PRJNA729271	stage_E18_vs_E10	ordinary_muscle_development	ENSGALG00010012726	IL7R	3.660542801	0.485733186	7.536118401	4.84167E-14	2.27709E-13	positive
PRJNA729271	stage_E18_vs_E10	ordinary_muscle_development	ENSGALG00010012730	CAP2	0.731636926	0.168404515	4.344520848	1.3958E-05	3.20179E-05	positive
PRJNA729271	stage_E18_vs_E10	ordinary_muscle_development	ENSGALG00010012732	MGST1	-0.708025383	0.175273296	-4.039550807	5.35537E-05	0.000114967	negative
PRJNA729271	stage_E18_vs_E10	ordinary_muscle_development	ENSGALG00010012733	ENSGALG00010012733	-1.545816222	1.060617605	-1.457468003	0.144987238	0.186162637	negative
PRJNA729271	stage_E18_vs_E10	ordinary_muscle_development	ENSGALG00010012734	TMEM19	-0.957223486	0.14740224	-6.493954806	8.36118E-11	3.06746E-10	negative
PRJNA729271	stage_E18_vs_E10	ordinary_muscle_development	ENSGALG00010012738	RAB21	0.169706926	0.069419651	2.444652533	0.014499175	0.0224392	positive
PRJNA729271	stage_E18_vs_E10	ordinary_muscle_development	ENSGALG00010012741	ENSGALG00010012741	0.517727816	0.150904305	3.430835301	0.000601726	0.001130967	positive
PRJNA729271	stage_E18_vs_E10	ordinary_muscle_development	ENSGALG00010012742	ENSGALG00010012742	0.315086744	0.173839657	1.812513608	0.069906873	0.095639465	positive
PRJNA729271	stage_E18_vs_E10	ordinary_muscle_development	ENSGALG00010012743	ENSGALG00010012743	-0.090340134	0.525780062	-0.171821149	0.863578138	0.887455875	negative
PRJNA729271	stage_E18_vs_E10	ordinary_muscle_development	ENSGALG00010012744	ARL4C	-2.021635636	0.233209308	-8.668760509	4.3685E-18	2.75007E-17	negative
PRJNA729271	stage_E18_vs_E10	ordinary_muscle_development	ENSGALG00010012745	MARCHF3	-0.385547141	0.319628155	-1.206236479	0.227726322	0.279168897	negative
PRJNA729271	stage_E18_vs_E10	ordinary_muscle_development	ENSGALG00010012747	TOB2	0.387936897	0.145845522	2.659916409	0.007816005	0.012556907	positive
PRJNA729271	stage_E18_vs_E10	ordinary_muscle_development	ENSGALG00010012748	ENSGALG00010012748	0.708957412	0.211905712	3.345626722	0.000820968	0.001515336	positive
PRJNA729271	stage_E18_vs_E10	ordinary_muscle_development	ENSGALG00010012749	RGS4	1.735187047	0.178305371	9.731546726	2.21204E-22	1.80233E-21	positive
PRJNA729271	stage_E18_vs_E10	ordinary_muscle_development	ENSGALG00010012751	AP3B1	-0.481104299	0.075568519	-6.366464556	1.93435E-10	6.90091E-10	negative
PRJNA729271	stage_E18_vs_E10	ordinary_muscle_development	ENSGALG00010012753	GTPBP1	-0.179068763	0.052853336	-3.388031438	0.000703962	0.00131079	negative
PRJNA729271	stage_E18_vs_E10	ordinary_muscle_development	ENSGALG00010012755	CLVS2	-2.604968492	0.399430309	-6.521709634	6.95105E-11	2.56898E-10	negative
PRJNA729271	stage_E18_vs_E10	ordinary_muscle_development	ENSGALG00010012756	RPA2	-1.459771633	0.105404433	-13.84924328	1.28559E-43	2.85808E-42	negative
PRJNA729271	stage_E18_vs_E10	ordinary_muscle_development	ENSGALG00010012758	NAB1	-0.175714193	0.128855982	-1.363647928	0.1726784	0.217915149	negative
PRJNA729271	stage_E18_vs_E10	ordinary_muscle_development	ENSGALG00010012759	ATP1B4	5.810947564	0.197767277	29.38275564	9.1216E-190	4.4073E-187	positive
PRJNA729271	stage_E18_vs_E10	ordinary_muscle_development	ENSGALG00010012760	PLEKHF2	0.80585705	0.105683803	7.625170783	2.43712E-14	1.17323E-13	positive
PRJNA729271	stage_E18_vs_E10	ordinary_muscle_development	ENSGALG00010012761	SMPDL3A	0.340846242	0.229470872	1.485357334	0.137449192	0.177458451	positive
PRJNA729271	stage_E18_vs_E10	ordinary_muscle_development	ENSGALG00010012763	SMPDL3B	-0.448721531	0.138226215	-3.246283868	0.001169222	0.002112147	negative
PRJNA729271	stage_E18_vs_E10	ordinary_muscle_development	ENSGALG00010012765	PHF5A	-0.253195808	0.107610176	-2.352898368	0.018627725	0.028217041	negative
PRJNA729271	stage_E18_vs_E10	ordinary_muscle_development	ENSGALG00010012767	JOSD1	0.153789316	0.069826394	2.202452508	0.02763336	0.040639768	positive
PRJNA729271	stage_E18_vs_E10	ordinary_muscle_development	ENSGALG00010012768	EPS8	-0.489682282	0.116499899	-4.203285052	2.63069E-05	5.84755E-05	negative
PRJNA729271	stage_E18_vs_E10	ordinary_muscle_development	ENSGALG00010012769	NEMP2	-1.118359047	0.086570364	-12.91849773	3.53985E-38	6.28801E-37	negative
PRJNA729271	stage_E18_vs_E10	ordinary_muscle_development	ENSGALG00010012771	ITGA2	-1.14120932	0.188097326	-6.067121447	1.30223E-09	4.34427E-09	negative
PRJNA729271	stage_E18_vs_E10	ordinary_muscle_development	ENSGALG00010012772	TOMM22	-0.412197102	0.074613496	-5.524430897	3.30555E-08	9.79436E-08	negative
PRJNA729271	stage_E18_vs_E10	ordinary_muscle_development	ENSGALG00010012773	XKR8	-0.608075699	0.131990547	-4.606963996	4.08591E-06	9.86594E-06	negative
PRJNA729271	stage_E18_vs_E10	ordinary_muscle_development	ENSGALG00010012775	KCNE2	1.140755077	0.146546873	7.784233482	7.01371E-15	3.54353E-14	positive
PRJNA729271	stage_E18_vs_E10	ordinary_muscle_development	ENSGALG00010012778	TBC1D15	-0.10191725	0.151964317	-0.670665663	0.502433542	0.559653745	negative
PRJNA729271	stage_E18_vs_E10	ordinary_muscle_development	ENSGALG00010012780	FABP7	-1.686818786	0.24681349	-6.834386517	8.23567E-12	3.25897E-11	negative
PRJNA729271	stage_E18_vs_E10	ordinary_muscle_development	ENSGALG00010012781	SPIN1L	-1.296345585	3.188394749	-0.406582524	0.684314635	0.730476517	negative
PRJNA729271	stage_E18_vs_E10	ordinary_muscle_development	ENSGALG00010012786	TEF	0.629851738	0.088256915	7.136570977	9.56879E-13	4.11572E-12	positive
PRJNA729271	stage_E18_vs_E10	ordinary_muscle_development	ENSGALG00010012788	RGS1	0.183132842	0.71605674	0.255751859	0.798142449	0.831350589	positive
PRJNA729271	stage_E18_vs_E10	ordinary_muscle_development	ENSGALG00010012790	PITX1	-4.420794714	0.483367515	-9.14582502	5.91764E-20	4.14178E-19	negative
PRJNA729271	stage_E18_vs_E10	ordinary_muscle_development	ENSGALG00010012791	CFAP418	0.123255335	0.192344159	0.640806231	0.521648584	0.578434534	positive
PRJNA729271	stage_E18_vs_E10	ordinary_muscle_development	ENSGALG00010012792	CBY1	-1.032890084	0.149268332	-6.919686658	4.52644E-12	1.84145E-11	negative
PRJNA729271	stage_E18_vs_E10	ordinary_muscle_development	ENSGALG00010012795	TBCA	-0.627734526	0.137022656	-4.581246234	4.62213E-06	1.11144E-05	negative
PRJNA729271	stage_E18_vs_E10	ordinary_muscle_development	ENSGALG00010012796	MOCS2	0.038120529	0.123254236	0.309283725	0.75710571	0.796158391	positive
PRJNA729271	stage_E18_vs_E10	ordinary_muscle_development	ENSGALG00010012797	ZC3H7B	0.215682936	0.070418885	3.062856448	0.002192352	0.003826387	positive
PRJNA729271	stage_E18_vs_E10	ordinary_muscle_development	ENSGALG00010012798	MFSD6	-0.177481302	0.100291971	-1.769646161	0.076786104	0.104166081	negative
PRJNA729271	stage_E18_vs_E10	ordinary_muscle_development	ENSGALG00010012801	DMC1	1.153577373	0.468006611	2.464874097	0.01370614	0.021266073	positive
PRJNA729271	stage_E18_vs_E10	ordinary_muscle_development	ENSGALG00010012803	NUP155	-1.067041877	0.090806887	-11.75067121	7.00601E-32	9.35103E-31	negative
PRJNA729271	stage_E18_vs_E10	ordinary_muscle_development	ENSGALG00010012804	ENSGALG00010012804	0.700610076	0.16022893	4.372556667	1.228E-05	2.83257E-05	positive
PRJNA729271	stage_E18_vs_E10	ordinary_muscle_development	ENSGALG00010012808	EYA3	-0.779776557	0.094089162	-8.287634167	1.15523E-16	6.61078E-16	negative
PRJNA729271	stage_E18_vs_E10	ordinary_muscle_development	ENSGALG00010012809	RHOBTB3	1.821613665	0.248784839	7.322044504	2.44221E-13	1.0909E-12	positive
PRJNA729271	stage_E18_vs_E10	ordinary_muscle_development	ENSGALG00010012812	DECR1	1.62147354	0.239605185	6.767272349	1.31233E-11	5.11762E-11	positive
PRJNA729271	stage_E18_vs_E10	ordinary_muscle_development	ENSGALG00010012818	RGS18	0.38376992	0.198376358	1.934554718	0.053044961	0.07410242	positive
PRJNA729271	stage_E18_vs_E10	ordinary_muscle_development	ENSGALG00010012819	RANGAP1	-1.586164527	0.072427305	-21.90009048	2.5927E-106	3.5791E-104	negative
PRJNA729271	stage_E18_vs_E10	ordinary_muscle_development	ENSGALG00010012821	ZBTB33	-0.097705898	0.052256308	-1.869743603	0.061519431	0.084918023	negative
PRJNA729271	stage_E18_vs_E10	ordinary_muscle_development	ENSGALG00010012824	HIBCH	0.333344812	0.117829032	2.829055011	0.004668567	0.00776893	positive
PRJNA729271	stage_E18_vs_E10	ordinary_muscle_development	ENSGALG00010012827	ENSGALG00010012827	-0.055718147	0.190184421	-0.292969039	0.769545817	0.806490248	negative
PRJNA729271	stage_E18_vs_E10	ordinary_muscle_development	ENSGALG00010012829	RGS5	2.860465213	0.181499535	15.76017927	5.84669E-56	2.05699E-54	positive
PRJNA729271	stage_E18_vs_E10	ordinary_muscle_development	ENSGALG00010012830	ARRDC3	0.89234141	0.149270451	5.978017793	2.25869E-09	7.38879E-09	positive
PRJNA729271	stage_E18_vs_E10	ordinary_muscle_development	ENSGALG00010012831	ENSGALG00010012831	0.243518027	0.380467076	0.640050198	0.522139965	0.578846561	positive
PRJNA729271	stage_E18_vs_E10	ordinary_muscle_development	ENSGALG00010012832	CHADL	-2.233876949	0.35017633	-6.379291674	1.77909E-10	6.36738E-10	negative
PRJNA729271	stage_E18_vs_E10	ordinary_muscle_development	ENSGALG00010012833	JAK2	0.290434851	0.105878142	2.743104909	0.006086124	0.009954679	positive
PRJNA729271	stage_E18_vs_E10	ordinary_muscle_development	ENSGALG00010012834	TPH2	-5.035245489	0.916988256	-5.491068679	3.99509E-08	1.17486E-07	negative
PRJNA729271	stage_E18_vs_E10	ordinary_muscle_development	ENSGALG00010012836	ENSGALG00010012836	0.704910655	1.152172735	0.611809873			positive
PRJNA729271	stage_E18_vs_E10	ordinary_muscle_development	ENSGALG00010012837	PKIB	0.74474437	0.273688958	2.721134153	0.006505835	0.010583848	positive
PRJNA729271	stage_E18_vs_E10	ordinary_muscle_development	ENSGALG00010012838	C2orf88	0.243422117	0.23178367	1.050212543	0.293620404	0.35020388	positive
PRJNA729271	stage_E18_vs_E10	ordinary_muscle_development	ENSGALG00010012839	TGFBI	-0.341509422	0.179943991	-1.897865111	0.057713853	0.080130489	negative
PRJNA729271	stage_E18_vs_E10	ordinary_muscle_development	ENSGALG00010012841	L3MBTL2	-0.573620013	0.094095631	-6.096138644	1.08661E-09	3.65524E-09	negative
PRJNA729271	stage_E18_vs_E10	ordinary_muscle_development	ENSGALG00010012842	DDX17	0.16671228	0.049036394	3.399766312	0.000674435	0.001259136	positive
PRJNA729271	stage_E18_vs_E10	ordinary_muscle_development	ENSGALG00010012843	ENSGALG00010012843	0.318913595	0.158368757	2.013740595	0.044036776	0.062426705	positive
PRJNA729271	stage_E18_vs_E10	ordinary_muscle_development	ENSGALG00010012845	HDHD2	0.098395322	0.102581963	0.959187363	0.337464366	0.395916307	positive
PRJNA729271	stage_E18_vs_E10	ordinary_muscle_development	ENSGALG00010012847	RLN3	-1.246002627	0.841529668	-1.480640166	0.138702486	0.178996843	negative
PRJNA729271	stage_E18_vs_E10	ordinary_muscle_development	ENSGALG00010012848	SERINC1	0.136178226	0.092711334	1.468841195	0.141875868	0.182588183	positive
PRJNA729271	stage_E18_vs_E10	ordinary_muscle_development	ENSGALG00010012849	TRHDE	-1.873069886	0.325616834	-5.752374233	8.79987E-09	2.73252E-08	negative
PRJNA729271	stage_E18_vs_E10	ordinary_muscle_development	ENSGALG00010012850	CALB1	2.086439026	1.102338075	1.89273969	0.058392495	0.081010645	positive
PRJNA729271	stage_E18_vs_E10	ordinary_muscle_development	ENSGALG00010012851	ENSGALG00010012851	-0.921816546	0.263460174	-3.498883842	0.00046721	0.00089296	negative

PRJNA729271	stage_E18_vs_E10	ordinary_muscle_development	ENSGALG00010012852	MSTN	2.647882491	0.418686023	6.324267698	2.54436E-10	9.00624E-10	positive
PRJNA729271	stage_E18_vs_E10	ordinary_muscle_development	ENSGALG00010012854	LECT2	5.131367581	0.744913396	6.888542496	5.63669E-12	2.27207E-11	positive
PRJNA729271	stage_E18_vs_E10	ordinary_muscle_development	ENSGALG00010012856	AVR2	-0.20890311	1.161142473	-0.179911695	0.857221894	0.881924292	negative
PRJNA729271	stage_E18_vs_E10	ordinary_muscle_development	ENSGALG00010012857	NSDHL	-2.292034763	0.230125402	-9.959938099	2.28205E-23	1.94693E-22	negative
PRJNA729271	stage_E18_vs_E10	ordinary_muscle_development	ENSGALG00010012859	FBXL21	0.795209449	0.11163117	7.123543082	1.05187E-12	4.50293E-12	positive
PRJNA729271	stage_E18_vs_E10	ordinary_muscle_development	ENSGALG00010012860	ENSGALG00010012860	0.343152523	0.272900697	1.257426333	0.208599299	0.258387185	positive
PRJNA729271	stage_E18_vs_E10	ordinary_muscle_development	ENSGALG00010012861	WDR70	-0.571124953	0.083916468	-6.805874518	1.00437E-11	3.95071E-11	negative
PRJNA729271	stage_E18_vs_E10	ordinary_muscle_development	ENSGALG00010012863	KCNE1	0.131116984	0.492014738	0.266489952	0.789861897	0.82420619	positive
PRJNA729271	stage_E18_vs_E10	ordinary_muscle_development	ENSGALG00010012865	EP300	0.146074965	0.123595117	1.181882977	0.237252138	0.289865959	positive
PRJNA729271	stage_E18_vs_E10	ordinary_muscle_development	ENSGALG00010012867	A4GALT	0.346988529	0.102284773	3.392377176	0.00069289	0.001292093	positive
PRJNA729271	stage_E18_vs_E10	ordinary_muscle_development	ENSGALG00010012868	CTHRC1	-3.115722994	0.596592321	-5.222532846	1.76492E-07	4.88683E-07	negative
PRJNA729271	stage_E18_vs_E10	ordinary_muscle_development	ENSGALG00010012869	ENSGALG00010012869	-0.690705253	0.227492281	-3.036170057	0.002396042	0.00415637	negative
PRJNA729271	stage_E18_vs_E10	ordinary_muscle_development	ENSGALG00010012870	KCNC2	-0.390990628	0.187478529	-2.085522167	0.037021928	0.053174083	negative
PRJNA729271	stage_E18_vs_E10	ordinary_muscle_development	ENSGALG00010012871	ENSGALG00010012871	-0.699619063	0.369231757	-1.894796562	0.058119359	0.080647148	negative
PRJNA729271	stage_E18_vs_E10	ordinary_muscle_development	ENSGALG00010012873	PTAFR	0.635850344	0.227539872	2.794456808	0.005198698	0.008599135	positive
PRJNA729271	stage_E18_vs_E10	ordinary_muscle_development	ENSGALG00010012875	HSF2	-0.924014212	0.134617632	-6.863990965	6.69629E-12	2.66949E-11	negative
PRJNA729271	stage_E18_vs_E10	ordinary_muscle_development	ENSGALG00010012876	KDELR3	0.592939384	0.159650851	3.71397573	0.000204028	0.000408592	positive
PRJNA729271	stage_E18_vs_E10	ordinary_muscle_development	ENSGALG00010012877	SLC25A32	-0.68564771	0.096667974	-7.092811433	1.31414E-12	5.57463E-12	negative
PRJNA729271	stage_E18_vs_E10	ordinary_muscle_development	ENSGALG00010012878	GDNF	0.019208764	0.321336121	0.059777794	0.952332612	0.9606835	positive
PRJNA729271	stage_E18_vs_E10	ordinary_muscle_development	ENSGALG00010012879	RCAN1	0.671627224	0.173406917	3.873128224	0.000107447	0.000223418	positive
PRJNA729271	stage_E18_vs_E10	ordinary_muscle_development	ENSGALG00010012880	CLK4	0.473383218	0.141191926	3.352764067	0.000800089	0.001479246	positive
PRJNA729271	stage_E18_vs_E10	ordinary_muscle_development	ENSGALG00010012882	ENSGALG00010012882	2.102505087	0.546104494	3.850005102	0.000118115	0.000244187	positive
PRJNA729271	stage_E18_vs_E10	ordinary_muscle_development	ENSGALG00010012883	DCAF13	-0.672029189	0.067437179	-9.965262532	2.16302E-23	1.85192E-22	negative
PRJNA729271	stage_E18_vs_E10	ordinary_muscle_development	ENSGALG00010012884	ENSGALG00010012884	-0.162498658	0.215488374	-0.754094784	0.450792309	0.510367455	negative
PRJNA729271	stage_E18_vs_E10	ordinary_muscle_development	ENSGALG00010012885	PMS1	-0.016364658	0.130326106	-0.125567001	0.900074686	0.917287673	negative
PRJNA729271	stage_E18_vs_E10	ordinary_muscle_development	ENSGALG00010012887	KCNJ4	0.937797122	0.396586589	2.364671797	0.018046055	0.027421907	positive
PRJNA729271	stage_E18_vs_E10	ordinary_muscle_development	ENSGALG00010012888	ENSGALG00010012888	-0.624370613	3.616066201	-0.172665703	0.862914204	0.886836456	negative
PRJNA729271	stage_E18_vs_E10	ordinary_muscle_development	ENSGALG00010012889	RIC1	0.357999578	0.10525763	3.401174603	0.00067097	0.001253312	positive
PRJNA729271	stage_E18_vs_E10	ordinary_muscle_development	ENSGALG00010012890	MFAP3	-0.198659816	0.07010252	-2.833846993	0.004599135	0.00766082	negative
PRJNA729271	stage_E18_vs_E10	ordinary_muscle_development	ENSGALG00010012891	CAPS2	-2.724902984	0.327655804	-8.316358059	9.07075E-17	5.22163E-16	negative
PRJNA729271	stage_E18_vs_E10	ordinary_muscle_development	ENSGALG00010012892	ORMDL1	0.243359978	0.16279551	1.494881383	0.134945402	0.174474498	positive
PRJNA729271	stage_E18_vs_E10	ordinary_muscle_development	ENSGALG00010012893	ENSGALG00010012893	0.401103544	0.189921942	2.111938935	0.034691687	0.050090249	positive
PRJNA729271	stage_E18_vs_E10	ordinary_muscle_development	ENSGALG00010012894	CLIC6	0.273045269	0.661970904	0.412473218	0.679992607	0.72634435	positive
PRJNA729271	stage_E18_vs_E10	ordinary_muscle_development	ENSGALG00010012896	STK38L	-0.114944847	0.100361952	-1.14530302	0.252083655	0.305744505	negative
PRJNA729271	stage_E18_vs_E10	ordinary_muscle_development	ENSGALG00010012897	ENSGALG00010012897	0.334634499	0.318569085	1.050429922	0.293520494	0.350113526	positive
PRJNA729271	stage_E18_vs_E10	ordinary_muscle_development	ENSGALG00010012898	FAM114A2	-0.024362171	0.116401582	-0.20929416	0.834218611	0.86248208	negative
PRJNA729271	stage_E18_vs_E10	ordinary_muscle_development	ENSGALG00010012899	ENSGALG00010012899	0.116904775	0.135849001	0.860549392	0.389486269	0.449582975	positive
PRJNA729271	stage_E18_vs_E10	ordinary_muscle_development	ENSGALG00010012900	RBX1	-0.386658691	0.173313751	-2.230975259	0.025682768	0.037979159	negative
PRJNA729271	stage_E18_vs_E10	ordinary_muscle_development	ENSGALG00010012903	RGMB	0.472544551	0.092201947	5.125103831	2.97374E-07	8.08257E-07	positive
PRJNA729271	stage_E18_vs_E10	ordinary_muscle_development	ENSGALG00010012904	ERMP1	-0.750142103	0.138909681	-5.400214707	6.65612E-08	1.91316E-07	negative
PRJNA729271	stage_E18_vs_E10	ordinary_muscle_development	ENSGALG00010012905	NKAIN1	-1.092008362	0.20530756	-5.318890168	1.04402E-07	2.95626E-07	negative
PRJNA729271	stage_E18_vs_E10	ordinary_muscle_development	ENSGALG00010012908	STRAP	-0.669319725	0.083687371	-7.997858146	1.26602E-15	6.75662E-15	negative
PRJNA729271	stage_E18_vs_E10	ordinary_muscle_development	ENSGALG00010012910	XPNPEP3	-0.130420625	0.100948688	-1.291949685	0.19637457	0.244729549	negative
PRJNA729271	stage_E18_vs_E10	ordinary_muscle_development	ENSGALG00010012911	CSNK1E	-0.505775753	0.084851547	-5.960713381	2.51139E-09	8.18404E-09	negative
PRJNA729271	stage_E18_vs_E10	ordinary_muscle_development	ENSGALG00010012912	NKAPL	0.016968843	0.100968954	0.168060003	0.866536075	0.889990817	positive
PRJNA729271	stage_E18_vs_E10	ordinary_muscle_development	ENSGALG00010012913	EGFLAM	0.231623028	0.152948	1.514390692	0.129926779	0.168557116	positive
PRJNA729271	stage_E18_vs_E10	ordinary_muscle_development	ENSGALG00010012915	ENSGALG00010012915	1.944756472	0.295448728	6.582382288	4.62969E-11	1.73404E-10	positive
PRJNA729271	stage_E18_vs_E10	ordinary_muscle_development	ENSGALG00010012917	RUNX1	-1.509417246	0.267672626	-5.639042252	1.70999E-08	5.17782E-08	negative
PRJNA729271	stage_E18_vs_E10	ordinary_muscle_development	ENSGALG00010012918	GLRX	0.243118311	0.283477258	0.85762898	0.391097368	0.451206331	positive
PRJNA729271	stage_E18_vs_E10	ordinary_muscle_development	ENSGALG00010012921	EBF1	-0.064360069	0.14050555	-0.458060686	0.646908843	0.695567367	negative
PRJNA729271	stage_E18_vs_E10	ordinary_muscle_development	ENSGALG00010012922	SNRNP40	-1.031631077	0.130030988	-7.933732506	2.12656E-15	1.1136E-14	negative
PRJNA729271	stage_E18_vs_E10	ordinary_muscle_development	ENSGALG00010012923	MLANA	-3.6740505	0.88624677	-4.145629216	3.38882E-05	7.42906E-05	negative
PRJNA729271	stage_E18_vs_E10	ordinary_muscle_development	ENSGALG00010012924	GLIPR1	0.229615912	0.20236419	1.134666721	0.256514986	0.310443744	positive
PRJNA729271	stage_E18_vs_E10	ordinary_muscle_development	ENSGALG00010012925	SETD4	-1.068218829	0.19200748	-5.563422996	2.64534E-08	7.89624E-08	negative
PRJNA729271	stage_E18_vs_E10	ordinary_muscle_development	ENSGALG00010012926	OSGEPL1	0.00380621	0.166348023	0.022881004	0.981745193	0.985075216	positive
PRJNA729271	stage_E18_vs_E10	ordinary_muscle_development	ENSGALG00010012927	ENSGALG00010012927	-1.568356603	3.702348435	-0.423611292			negative
PRJNA729271	stage_E18_vs_E10	ordinary_muscle_development	ENSGALG00010012930	IL6ST	0.330144876	0.091067071	3.625293678	0.000288633	0.000566672	positive
PRJNA729271	stage_E18_vs_E10	ordinary_muscle_development	ENSGALG00010012932	ATF6	0.528255534	0.118350194	4.463495274	8.06334E-06	1.89645E-05	positive
PRJNA729271	stage_E18_vs_E10	ordinary_muscle_development	ENSGALG00010012933	ZCCHC17	-0.029083908	0.145448638	-0.199959986	0.841511875	0.868906869	negative
PRJNA729271	stage_E18_vs_E10	ordinary_muscle_development	ENSGALG00010012934	NDUFA1	0.703817746	0.152068882	4.628282516	3.68711E-06	8.9477E-06	positive
PRJNA729271	stage_E18_vs_E10	ordinary_muscle_development	ENSGALG00010012938	UPF3B	-0.189699768	0.151299492	-1.253803071	0.209913593	0.259815347	negative
PRJNA729271	stage_E18_vs_E10	ordinary_muscle_development	ENSGALG00010012939	GJA1	-3.363111435	0.207573924	-16.20199379	4.88183E-59	1.89711E-57	negative
PRJNA729271	stage_E18_vs_E10	ordinary_muscle_development	ENSGALG00010012940	FBN2	-0.717232076	0.223427909	-3.210127508	0.001326761	0.002382188	negative
PRJNA729271	stage_E18_vs_E10	ordinary_muscle_development	ENSGALG00010012943	ASNSD1	-0.665331247	0.124784907	-5.331824686	9.72308E-08	2.76183E-07	negative
PRJNA729271	stage_E18_vs_E10	ordinary_muscle_development	ENSGALG00010012944	NCK1	-0.130783755	0.052466813	-2.492694831	0.012677776	0.019763859	negative
PRJNA729271	stage_E18_vs_E10	ordinary_muscle_development	ENSGALG00010012945	CLINT1	-0.016837911	0.063082991	-0.266916819	0.789533207	0.823922522	negative
PRJNA729271	stage_E18_vs_E10	ordinary_muscle_development	ENSGALG00010012948	ST13P5	-0.24623308	0.095220124	-2.585935294	0.009711516	0.015389573	negative
PRJNA729271	stage_E18_vs_E10	ordinary_muscle_development	ENSGALG00010012949	ENSGALG00010012949	-0.896369178	0.180812386	-4.957454508	7.14228E-07	1.87346E-06	negative
PRJNA729271	stage_E18_vs_E10	ordinary_muscle_development	ENSGALG00010012952	BRD10	0.119966965	0.187304231	0.640492554	0.521852429	0.578616305	positive
PRJNA729271	stage_E18_vs_E10	ordinary_muscle_development	ENSGALG00010012954	RIMS2	-1.055223437	0.340002363	-3.103576772	0.001911966	0.003366203	negative
PRJNA729271	stage_E18_vs_E10	ordinary_muscle_development	ENSGALG00010012955	RPL39L	0.74470206	0.155162172	4.79950783	1.59056E-06	4.02008E-06	positive
PRJNA729271	stage_E18_vs_E10	ordinary_muscle_development	ENSGALG00010012956	FABP3	3.59870182	0.168222089	21.39256408	1.567E-101	1.90876E-99	positive
PRJNA729271	stage_E18_vs_E10	ordinary_muscle_development	ENSGALG00010012957	DERA	-0.617501747	0.229480687	-2.690865865	0.007126684	0.011511175	negative
PRJNA729271	stage_E18_vs_E10	ordinary_muscle_development	ENSGALG00010012958	OLFML2B	1.970701913	0.204625038	9.630795596	5.92688E-22	4.72034E-21	positive
PRJNA729271	stage_E18_vs_E10	ordinary_muscle_development	ENSGALG00010012959	TBC1D32	0.2023842	0.146692202	1.379652073	0.167693796	0.212084037	positive
PRJNA729271	stage_E18_vs_E10	ordinary_muscle_development	ENSGALG00010012962	SLC25A17	-1.243387157	0.211154456	-5.888519623	3.8967E-09	1.251E-08	negative
PRJNA729271	stage_E18_vs_E10	ordinary_muscle_development	ENSGALG00010012963	UIMC1	-0.09921763	0.091692327	-1.082071238	0.279220875	0.334764812	negative
PRJNA729271	stage_E18_vs_E10	ordinary_muscle_development	ENSGALG00010012964	CBR3	-0.03579438	0.07236089	-0.494664728	0.620836804	0.671569363	negative
PRJNA729271	stage_E18_vs_E10	ordinary_muscle_development	ENSGALG00010012965	ENSGALG00010012965	-0.413590453	0.085434712	-4.841011821	1.2918E-06	3.30298E-06	negative
PRJNA729271	stage_E18_vs_E10	ordinary_muscle_development	ENSGALG00010012966	GLIPR1L	-0.365058029	0.276519425	-1.320189455	0.186771772	0.233847874	negative
PRJNA729271	stage_E18_vs_E10	ordinary_muscle_development	ENSGALG00010012967	ECRG4	0.522464784	0.208858416	2.501526121	0.012365932	0.019302626	positive
PRJNA729271	stage_E18_vs_E10	ordinary_muscle_development	ENSGALG00010012968	LSM11	-0.379108582	0.076866827	-4.932018121	8.13844E-07	2.12514E-06	negative
PRJNA729271	stage_E18_vs_E10	ordinary_muscle_development	ENSGALG00010012969	SLC12A2	-0.736160974	0.143313865	-5.136704477	2.79598E-07	7.61227E-07	negative
PRJNA729271	stage_E18_vs_E10	ordinary_muscle_development	ENSGALG00010012970	UNC5A	0.210494342	0.17135874	1.228384042	0.219302835	0.270121907	positive
PRJNA729271	stage_E18_vs_E10	ordinary_muscle_development	ENSGALG00010012971	ENSGALG00010012971	3.132303011	0.390500127	8.021259904	1.04666E-15	5.61278E-15	positive
PRJNA729271	stage_E18_vs_E10	ordinary_muscle_development	ENSGALG00010012973	GLDC	-4.365339319	0.337894141	-12.91925128	3.50536E-38	6.23438E-37	negative
PRJNA729271	stage_E18_vs_E10	ordinary_muscle_development	ENSGALG00010012974	SLC49A3	-1.333203214	0.223569623	-5.96325742	2.47258E-09	8.06665E-09	negative
PRJNA729271	stage_E18_vs_E10	ordinary_muscle_development	ENSGALG00010012975	KRR1	-0.421867224	0.093847205	-4.495256135	6.94863E-06	1.6428E-05	negative
PRJNA729271	stage_E18_vs_E10	ordinary_muscle_development	ENSGALG00010012976	SLC9A2	3.737245985	0.226169269	16.5241105	2.46043E-61	1.02189E-59	positive
PRJNA729271	stage_E18_vs_E10	ordinary_muscle_development	ENSGALG00010012977	NKRF	-0.887210978	0.101829176	-8.712738477	2.9662E-18	1.88658E-17	negative
PRJNA729271	stage_E18_vs_E10	ordinary_muscle_development	ENSGALG00010012978	HSD17B7	-0.784746733	0.214599761	-3.656792198	0.000255391	0.000505378	negative
PRJNA729271	stage_E18_vs_E10	ordinary_muscle_development	ENSGALG00010012979	HTR1A	3.214070019	0.514924618	6.241826287	4.32491E-10	1.50986E-09	positive
PRJNA729271	stage_E18_vs_E10	ordinary_muscle_development	ENSGALG00010012980	KICS2	-0.08152065	0.077097915	-1.057365177	0.290344958	0.346639499	negative
PRJNA729271	stage_E18_vs_E10	ordinary_muscle_development	ENSGALG00010012982	DDR2	-0.441985524	0.24363578	-1.814124032	0.069658632	0.095326839	negative
PRJNA729271	stage_E18_vs_E10	ordinary_muscle_development	ENSGALG00010012985	ARNTL2	0.102050996	0.170505182	0.598521373	0.549492102	0.603675005	positive
PRJNA729271	stage_E18_vs_E10	ordinary_muscle_development	ENSGALG00010012986	TMEM184B	1.320750781	0.139789903	9.448112864	3.44994E-21	2.62366E-20	positive
PRJNA729271	stage_E18_vs_E10	ordinary_muscle_development	ENSGALG00010012988	RPL18A	0.734494352	0.134872288	5.445850763	5.15584E-08	1.49617E-07	positive
PRJNA729271	stage_E18_vs_E10	ordinary_muscle_development	ENSGALG00010012991	MFSD9	0.062737198	0.102410374	0.612605885	0.540136966	0.595156619	positive
PRJNA729271	stage_E18_vs_E10	ordinary_muscle_development	ENSGALG00010012992	HARS1	-0.520491325	0.12775879	-4.07401576	4.62094E-05	9.98218E-05	negative
PRJNA729271	stage_E18_vs_E10	ordinary_muscle_development	ENSGALG00010012993	DOP1B	-0.391233007	0.100170509	-3.905670559	9.39645E-05	0.000196539	negative
PRJNA729271	stage_E18_vs_E10	ordinary_muscle_development	ENSGALG00010012995	SERINC2	1.495267003	0.147877281	10.11153971	4.91064E-24	4.36953E-23	positive
PRJNA729271	stage_E18_vs_E10	ordinary_muscle_development	ENSGALG00010012997	UBE2A	-0.072225278	0.058831784	-1.227657443	0.219575588	0.270343001	negative
PRJNA729271	stage_E18_vs_E10	ordinary_muscle_development	ENSGALG00010012998	MAFF	0.290091514	0.200071559	1.449938787	0.14707559	0.188576796	positive
PRJNA729271	stage_E18_vs_E10	ordinary_muscle_development	ENSGALG00010013001	ADGRV1	-1.465652981	0.33500274	-4.375047738	1.21406E-05	2.8022E-05	negative
PRJNA729271	stage_E18_vs_E10	ordinary_muscle_development	ENSGALG00010013004	UAP1	0.04696606	0.153413957	0.306139423	0.759498493	0.798211329	positive
PRJNA729271	stage_E18_vs_E10	ordinary_muscle_development	ENSGALG00010013005	SLC40A1	0.229544083	0.091279391	2.51474163	0.011911964	0.018646212	positive
PRJNA729271	stage_E18_vs_E10	ordinary_muscle_development	ENSGALG00010013006	GRIK2	-4.560731082	1.098075546	-4.153385531	3.27592E-05	7.19353E-05	negative
PRJNA729271	stage_E18_vs_E10	ordinary_muscle_development	ENSGALG00010013008	STEEP1	-0.579054761	0.111531905	-5.191830644	2.08236E-07	5.72966E-07	negative
PRJNA729271	stage_E18_vs_E10	ordinary_muscle_development	ENSGALG00010013012	TMEM182	2.137719674	0.151494494	14.11087372	3.2552E-45	7.72244E-44	positive
PRJNA729271	stage_E18_vs_E10	ordinary_muscle_development	ENSGALG00010013013	DCSTAMP	1.192269505	1.319764487	0.903395656	0.366315945	0.425904357	positive
PRJNA729271	stage_E18_vs_E10	ordinary_muscle_development	ENSGALG00010013015	PLA2G6	0.206170525	0.15267185	1.350416104	0.176882548	0.222658492	positive
PRJNA729271	stage_E18_vs_E10	ordinary_muscle_development	ENSGALG00010013016	QRSL1	-0.054246626	0.125339816	-0.432796435	0.665162663	0.712498729	negative
PRJNA729271	stage_E18_vs_E10	ordinary_muscle_development	ENSGALG00010013017	SCMH1	1.070574216	0.157940479	6.778339662	1.21565E-11	4.74827E-11	positive
PRJNA729271	stage_E18_vs_E10	ordinary_muscle_development	ENSGALG00010013018	ID1	-0.068906016	0.192883119	-0.357242334	0.720910396	0.763580284	negative
PRJNA729271	stage_E18_vs_E10	ordinary_muscle_development	ENSGALG00010013019	DPYS	1.837525513	0.929988774	1.975857736	0.048170892	0.067816344	positive
PRJNA729271	stage_E18_vs_E10	ordinary_muscle_development	ENSGALG00010013021	SLC25A5	-1.01150899	0.163462631	-6.188013645	6.09271E-10	2.10171E-09	negative
PRJNA729271	stage_E18_vs_E10	ordinary_muscle_development	ENSGALG00010013027	UHMK1	0.141909173	0.126586289	1.12104695	0.262267878	0.316718562	positive
PRJNA729271	stage_E18_vs_E10	ordinary_muscle_development	ENSGALG00010013029	MYLK2	4.840474697	0.255565835	18.94022613	5.31683E-80	3.70516E-78	positive
PRJNA729271	stage_E18_vs_E10	ordinary_muscle_development	ENSGALG00010013032	BAIAP2L2	0.515321978	0.436385264	1.180887673	0.237647349	0.290275413	positive
PRJNA729271	stage_E18_vs_E10	ordinary_muscle_development	ENSGALG00010013033	MAP3K1	0.063868306	0.125434883	0.509174992	0.610629571	0.66136708	positive
PRJNA729271	stage_E18_vs_E10	ordinary_muscle_development	ENSGALG00010013036	ENSGALG00010013036	0.858773603	0.434908956	1.974605471	0.048312938	0.067995949	positive
PRJNA729271	stage_E18_vs_E10	ordinary_muscle_development	ENSGALG00010013037	MORC4	0.816248548	0.089696632	9.100102494	9.02466E-20	6.26197E-19	positive
PRJNA729271	stage_E18_vs_E10	ordinary_muscle_development	ENSGALG00010013040	SLC25A43	1.190818395	0.205221051	5.802613277	6.52893E-09	2.0533E-08	positive
PRJNA729271	stage_E18_vs_E10	ordinary_muscle_development	ENSGALG00010013041	LRP12	-0.356142481	0.075814302	-4.697563248	2.63284E-06	6.49582E-06	negative
PRJNA729271	stage_E18_vs_E10	ordinary_muscle_development	ENSGALG00010013042	SLC25A48	1.172849811	0.297176334	3.946646065	7.92536E-05	0.000166974	positive
PRJNA729271	stage_E18_vs_E10	ordinary_muscle_development	ENSGALG00010013044	ENSGALG00010013044	2.278680812	0.124030572	18.37192858	2.20438E-75	1.33692E-73	positive
PRJNA729271	stage_E18_vs_E10	ordinary_muscle_development	ENSGALG00010013045	FSD1L	0.6563831	0.146370022	4.484409376	7.31162E-06	1.72525E-05	positive
PRJNA729271	stage_E18_vs_E10	ordinary_muscle_development	ENSGALG00010013046	SLC12A5	0.108226947	0.365779071	0.295880644	0.767321233	0.80462427	positive
PRJNA729271	stage_E18_vs_E10	ordinary_muscle_development	ENSGALG00010013047	CTPS1	-0.872998077	0.102623805	-8.506779454	1.78831E-17	1.07917E-16	negative
PRJNA729271	stage_E18_vs_E10	ordinary_muscle_development	ENSGALG00010013048	WDR75	-1.580066112	0.089961034	-17.56389444	4.65678E-69	2.41072E-67	negative
PRJNA729271	stage_E18_vs_E10	ordinary_muscle_development	ENSGALG00010013051	XPOT	-0.517091994	0.079291865	-6.521375111	6.96657E-11	2.57394E-10	negative

PRJNA729271	stage_E18_vs_E10	ordinary_muscle_development	ENSGALG00010013053	MRTFA	0.878635149	0.174467232	5.036104149	4.75102E-07	1.26569E-06	positive
PRJNA729271	stage_E18_vs_E10	ordinary_muscle_development	ENSGALG00010013056	ENSGALG00010013056	3.662829805	0.535241433	6.843322621	7.73771E-12	3.07283E-11	positive
PRJNA729271	stage_E18_vs_E10	ordinary_muscle_development	ENSGALG00010013058	ENSGALG00010013058	0.221066747	0.277422328	0.796859969	0.425532362	0.485745201	positive
PRJNA729271	stage_E18_vs_E10	ordinary_muscle_development	ENSGALG00010013062	ENSGALG00010013062	-0.313812458	0.112927952	-2.778873191	0.005454781	0.008986935	negative
PRJNA729271	stage_E18_vs_E10	ordinary_muscle_development	ENSGALG00010013063	PARP12	-0.243828208	0.220519192	-1.105700621	0.268856121	0.323892078	negative
PRJNA729271	stage_E18_vs_E10	ordinary_muscle_development	ENSGALG00010013065	FKTN	-0.147212613	0.104337467	-1.410927607	0.158265961	0.201467566	negative
PRJNA729271	stage_E18_vs_E10	ordinary_muscle_development	ENSGALG00010013071	ENSGALG00010013071	0.431855769	0.225765499	1.912851038	0.055767125	0.077643308	positive
PRJNA729271	stage_E18_vs_E10	ordinary_muscle_development	ENSGALG00010013076	RTN4IP1	-0.257697255	0.084013432	-3.067333997	0.002159774	0.003774526	negative
PRJNA729271	stage_E18_vs_E10	ordinary_muscle_development	ENSGALG00010013078	COL5A2	0.386169031	0.241979245	1.595876667	0.110516332	0.145471688	positive
PRJNA729271	stage_E18_vs_E10	ordinary_muscle_development	ENSGALG00010013079	ENSGALG00010013079	-0.065512741	0.205217509	-0.319235632	0.749547839	0.789470711	negative
PRJNA729271	stage_E18_vs_E10	ordinary_muscle_development	ENSGALG00010013080	ARHGEF7	0.392197295	0.099158293	3.955264672	7.645E-05	0.000161396	positive
PRJNA729271	stage_E18_vs_E10	ordinary_muscle_development	ENSGALG00010013081	NCOA5	-0.607103476	0.101389179	-5.987852741	2.12629E-09	6.97615E-09	negative
PRJNA729271	stage_E18_vs_E10	ordinary_muscle_development	ENSGALG00010013082	CHAF1B	-1.619992707	0.114841776	-14.10630144	3.47322E-45	8.1994E-44	negative
PRJNA729271	stage_E18_vs_E10	ordinary_muscle_development	ENSGALG00010013084	SGSM3	0.262343291	0.119190883	2.201034869	0.027733555	0.040770577	positive
PRJNA729271	stage_E18_vs_E10	ordinary_muscle_development	ENSGALG00010013085	LYSMD3	0.317108962	0.088742606	3.573356444	0.000352434	0.000683234	positive
PRJNA729271	stage_E18_vs_E10	ordinary_muscle_development	ENSGALG00010013086	CXCL14	-0.073436914	0.516489406	-0.142184744	0.886934082	0.90670072	negative
PRJNA729271	stage_E18_vs_E10	ordinary_muscle_development	ENSGALG00010013087	GPN2	-0.272551266	0.092934759	-2.932716107	0.00336011	0.005698466	negative
PRJNA729271	stage_E18_vs_E10	ordinary_muscle_development	ENSGALG00010013089	TBK1	0.034313207	0.078067838	0.439530636	0.660277088	0.708102722	positive
PRJNA729271	stage_E18_vs_E10	ordinary_muscle_development	ENSGALG00010013093	GPATCH3	0.13282267	0.161657714	0.821629022	0.411288066	0.47152845	positive
PRJNA729271	stage_E18_vs_E10	ordinary_muscle_development	ENSGALG00010013095	ADSL	3.046734313	0.127574176	23.88206146	4.705E-126	9.6055E-124	positive
PRJNA729271	stage_E18_vs_E10	ordinary_muscle_development	ENSGALG00010013096	CLDN14	-1.091710353	0.435383637	-2.507467575	0.012159974	0.018997502	negative
PRJNA729271	stage_E18_vs_E10	ordinary_muscle_development	ENSGALG00010013097	ENSGALG00010013097	0.326443461	0.410786391	0.794679348	0.426800049	0.486931657	positive
PRJNA729271	stage_E18_vs_E10	ordinary_muscle_development	ENSGALG00010013099	SIM2	0.791876781	0.202002608	3.920131478	8.85007E-05	0.000185566	positive
PRJNA729271	stage_E18_vs_E10	ordinary_muscle_development	ENSGALG00010013100	ZFPM2	0.111420736	0.150496377	0.740354938	0.459084654	0.518581052	positive
PRJNA729271	stage_E18_vs_E10	ordinary_muscle_development	ENSGALG00010013103	AIRIM	0.38079079	0.145137212	2.623660635	0.008699041	0.013894502	positive
PRJNA729271	stage_E18_vs_E10	ordinary_muscle_development	ENSGALG00010013104	HLCS	-0.488712634	0.110141854	-4.437120087	9.11704E-06	2.13113E-05	negative
PRJNA729271	stage_E18_vs_E10	ordinary_muscle_development	ENSGALG00010013106	SETD9	-0.796047079	0.185531896	-4.290621164	1.78174E-05	4.0341E-05	negative
PRJNA729271	stage_E18_vs_E10	ordinary_muscle_development	ENSGALG00010013109	ENSGALG00010013109	1.984047278	1.473299672	1.346669191	0.178086799	0.224038201	positive
PRJNA729271	stage_E18_vs_E10	ordinary_muscle_development	ENSGALG00010013110	ANKRD10	0.198076469	0.156835523	1.262956667	0.206604753	0.256201206	positive
PRJNA729271	stage_E18_vs_E10	ordinary_muscle_development	ENSGALG00010013111	RFESD	2.258744201	0.276281394	8.175520505	2.94591E-16	1.63543E-15	positive
PRJNA729271	stage_E18_vs_E10	ordinary_muscle_development	ENSGALG00010013112	CD40	0.394483657	0.163786898	2.408517783	0.016017446	0.02460501	positive
PRJNA729271	stage_E18_vs_E10	ordinary_muscle_development	ENSGALG00010013114	PIGP	-0.158681236	0.124774317	-1.271745985	0.203463386	0.252759838	negative
PRJNA729271	stage_E18_vs_E10	ordinary_muscle_development	ENSGALG00010013115	ENSGALG00010013115	-1.782134067	0.382929257	-4.653951181	3.25634E-06	7.95159E-06	negative
PRJNA729271	stage_E18_vs_E10	ordinary_muscle_development	ENSGALG00010013116	TARS	-0.782169242	0.168407497	-4.644503706	3.40895E-06	8.30047E-06	negative
PRJNA729271	stage_E18_vs_E10	ordinary_muscle_development	ENSGALG00010013117	ARSK	0.200387563	0.131841313	1.519914798	0.128532391	0.167092108	positive
PRJNA729271	stage_E18_vs_E10	ordinary_muscle_development	ENSGALG00010013119	RNF114	0.327252528	0.079336695	4.124857072	3.70965E-05	8.10176E-05	positive
PRJNA729271	stage_E18_vs_E10	ordinary_muscle_development	ENSGALG00010013121	PCBD2	-0.439325819	0.166934975	-2.631718242	0.008495428	0.013584251	negative
PRJNA729271	stage_E18_vs_E10	ordinary_muscle_development	ENSGALG00010013123	PPFIBP1	0.316033398	0.10822413	2.920174989	0.003498349	0.005915605	positive
PRJNA729271	stage_E18_vs_E10	ordinary_muscle_development	ENSGALG00010013125	POU3F3	-4.306335684	0.456128036	-9.441067733	3.69E-21	2.80328E-20	negative
PRJNA729271	stage_E18_vs_E10	ordinary_muscle_development	ENSGALG00010013129	RASSF3	0.503443549	0.110928807	4.538438345	5.66724E-06	1.35265E-05	positive
PRJNA729271	stage_E18_vs_E10	ordinary_muscle_development	ENSGALG00010013131	SPATA2	0.294781277	0.117290766	2.513252207	0.011962378	0.018719062	positive
PRJNA729271	stage_E18_vs_E10	ordinary_muscle_development	ENSGALG00010013132	SKIC3	-0.934759217	0.090377269	-10.34285748	4.50889E-25	4.28566E-24	negative
PRJNA729271	stage_E18_vs_E10	ordinary_muscle_development	ENSGALG00010013133	TXNDC15	-0.177297699	0.121668791	-1.457215917	0.145056788	0.186235442	negative
PRJNA729271	stage_E18_vs_E10	ordinary_muscle_development	ENSGALG00010013134	ENSGALG00010013134	-0.925023701	5.393403066	-0.171510212	0.863822602	0.887644167	negative
PRJNA729271	stage_E18_vs_E10	ordinary_muscle_development	ENSGALG00010013135	TTC3	-0.288453259	0.085324655	-3.38065544	0.000723132	0.001344337	negative
PRJNA729271	stage_E18_vs_E10	ordinary_muscle_development	ENSGALG00010013137	TMEM121	-1.636042651	0.157004366	-10.42036402	2.00186E-25	1.95006E-24	negative
PRJNA729271	stage_E18_vs_E10	ordinary_muscle_development	ENSGALG00010013139	TMEM185A	-0.129215588	0.085305913	-1.514731901	0.129840312	0.168460019	negative
PRJNA729271	stage_E18_vs_E10	ordinary_muscle_development	ENSGALG00010013143	ENSGALG00010013143	0.647082507	0.420140343	1.540157992	0.123521847	0.161055066	positive
PRJNA729271	stage_E18_vs_E10	ordinary_muscle_development	ENSGALG00010013144	TEDC1	-1.512088556	0.17383248	-8.698538705	3.36185E-18	2.13354E-17	negative
PRJNA729271	stage_E18_vs_E10	ordinary_muscle_development	ENSGALG00010013145	ENSGALG00010013145	-0.777128747	0.305343522	-2.545096561	0.010924752	0.017195601	negative
PRJNA729271	stage_E18_vs_E10	ordinary_muscle_development	ENSGALG00010013146	TNRC6B	0.323656227	0.119793742	2.701779081	0.006896957	0.011169988	positive
PRJNA729271	stage_E18_vs_E10	ordinary_muscle_development	ENSGALG00010013149	MCTP1	-0.239140775	0.204075394	-1.171825618	0.241267075	0.294275181	negative
PRJNA729271	stage_E18_vs_E10	ordinary_muscle_development	ENSGALG00010013150	GNS	0.811500277	0.103139371	7.867997159	3.60364E-15	1.8622E-14	positive
PRJNA729271	stage_E18_vs_E10	ordinary_muscle_development	ENSGALG00010013152	TBXAS1	-0.454506067	0.22954762	-1.980007754	0.047702657	0.067209372	negative
PRJNA729271	stage_E18_vs_E10	ordinary_muscle_development	ENSGALG00010013154	ENSGALG00010013154	0.039570184	0.362816439	0.109063923	0.91315179	0.928330425	positive
PRJNA729271	stage_E18_vs_E10	ordinary_muscle_development	ENSGALG00010013156	ABCC9	1.378156049	0.129991368	10.60190431	2.91974E-26	2.96785E-25	positive
PRJNA729271	stage_E18_vs_E10	ordinary_muscle_development	ENSGALG00010013158	CRIP1	2.032888047	0.185906089	10.93502667	7.83829E-28	8.65317E-27	positive
PRJNA729271	stage_E18_vs_E10	ordinary_muscle_development	ENSGALG00010013159	ENSGALG00010013159	-2.147094417	1.516631592	-1.415699388	0.156863538	0.199836261	negative
PRJNA729271	stage_E18_vs_E10	ordinary_muscle_development	ENSGALG00010013160	CRYBG1	-3.189811304	0.56497361	-5.645947431	1.64274E-08	4.98149E-08	negative
PRJNA729271	stage_E18_vs_E10	ordinary_muscle_development	ENSGALG00010013162	IDS	0.468776331	0.110510967	4.241898725	2.21637E-05	4.96542E-05	positive
PRJNA729271	stage_E18_vs_E10	ordinary_muscle_development	ENSGALG00010013163	ENSGALG00010013163	-0.422130402	0.473782169	-0.890979926	0.372939939	0.4328074	negative
PRJNA729271	stage_E18_vs_E10	ordinary_muscle_development	ENSGALG00010013164	FAM83F	-2.744331928	0.51172707	-5.362882063	8.19045E-08	2.33794E-07	negative
PRJNA729271	stage_E18_vs_E10	ordinary_muscle_development	ENSGALG00010013165	NUDC	-0.815200952	0.081925896	-9.950467318	2.51002E-23	2.13764E-22	negative
PRJNA729271	stage_E18_vs_E10	ordinary_muscle_development	ENSGALG00010013168	VPS26C	0.103131934	0.138155721	0.746490505	0.455371165	0.5149883	positive
PRJNA729271	stage_E18_vs_E10	ordinary_muscle_development	ENSGALG00010013170	COL3A1	3.193026545	0.23490505	13.59283907	4.41612E-42	9.18388E-41	positive
PRJNA729271	stage_E18_vs_E10	ordinary_muscle_development	ENSGALG00010013171	SLC9A8	0.302079325	0.136747972	2.209022337	0.027173087	0.040019701	positive
PRJNA729271	stage_E18_vs_E10	ordinary_muscle_development	ENSGALG00010013172	TNFSF13B	-0.036878183	0.501243836	-0.073573339	0.941349886	0.952122434	negative
PRJNA729271	stage_E18_vs_E10	ordinary_muscle_development	ENSGALG00010013173	KCNJ8	0.133630826	0.156237723	0.855304491	0.392382602	0.452412265	positive
PRJNA729271	stage_E18_vs_E10	ordinary_muscle_development	ENSGALG00010013174	ADAMTS12	0.732763769	0.165260377	4.433995512	9.25026E-06	2.16018E-05	positive
PRJNA729271	stage_E18_vs_E10	ordinary_muscle_development	ENSGALG00010013175	HIPK2	0.684967153	0.164162441	4.172496151	3.01281E-05	6.64192E-05	positive
PRJNA729271	stage_E18_vs_E10	ordinary_muscle_development	ENSGALG00010013177	OXR1	0.580100432	0.069914065	8.297335162	1.06473E-16	6.10491E-16	positive
PRJNA729271	stage_E18_vs_E10	ordinary_muscle_development	ENSGALG00010013179	TBC1D30	-0.704915065	0.194314708	-3.627697931	0.00028596	0.000562108	negative
PRJNA729271	stage_E18_vs_E10	ordinary_muscle_development	ENSGALG00010013180	C5orf24	-0.015659193	0.059570848	-0.262866714	0.792653313	0.826642907	negative
PRJNA729271	stage_E18_vs_E10	ordinary_muscle_development	ENSGALG00010013181	DYRK1A	1.147291087	0.078904759	14.54020146	6.7394E-48	1.80235E-46	positive
PRJNA729271	stage_E18_vs_E10	ordinary_muscle_development	ENSGALG00010013183	CLTA	-0.923264108	0.137731046	-6.703384122	2.03647E-11	7.81744E-11	negative
PRJNA729271	stage_E18_vs_E10	ordinary_muscle_development	ENSGALG00010013184	LDHB	-0.730434285	0.114386444	-6.385671774	1.70647E-10	6.1135E-10	negative
PRJNA729271	stage_E18_vs_E10	ordinary_muscle_development	ENSGALG00010013185	ENSGALG00010013185	-0.387115886	0.32117497	-1.205311502	0.228083071	0.279558947	negative
PRJNA729271	stage_E18_vs_E10	ordinary_muscle_development	ENSGALG00010013186	DDX46	-0.722778363	0.080732954	-8.952705564	3.46874E-19	2.33207E-18	negative
PRJNA729271	stage_E18_vs_E10	ordinary_muscle_development	ENSGALG00010013187	KDF1	-3.681912723	0.291408872	-12.63486831	1.35621E-36	2.26478E-35	negative
PRJNA729271	stage_E18_vs_E10	ordinary_muscle_development	ENSGALG00010013188	ENSGALG00010013188	-0.084006989	0.366465168	-0.229235944	0.818685537	0.849148255	negative
PRJNA729271	stage_E18_vs_E10	ordinary_muscle_development	ENSGALG00010013190	SLC45A2	-3.112787226	0.558633486	-5.572145784	2.51621E-08	7.51854E-08	negative
PRJNA729271	stage_E18_vs_E10	ordinary_muscle_development	ENSGALG00010013191	ABRA	4.723255953	0.419541602	11.25813489	2.11181E-29	2.50087E-28	positive
PRJNA729271	stage_E18_vs_E10	ordinary_muscle_development	ENSGALG00010013192	B4GALT5	0.297303355	0.082860158	3.588013378	0.000333207	0.000648249	positive
PRJNA729271	stage_E18_vs_E10	ordinary_muscle_development	ENSGALG00010013194	GRAP2	-3.182107204	0.431974654	-7.366421094	1.7527E-13	7.9095E-13	negative
PRJNA729271	stage_E18_vs_E10	ordinary_muscle_development	ENSGALG00010013196	MRPS9	-0.255416049	0.114890807	-2.223119972	0.026207711	0.038708047	negative
PRJNA729271	stage_E18_vs_E10	ordinary_muscle_development	ENSGALG00010013198	CAMLG	-0.918855727	0.088304148	-10.40557835	2.33832E-25	2.25959E-24	negative
PRJNA729271	stage_E18_vs_E10	ordinary_muscle_development	ENSGALG00010013199	RALYL	-4.602586426	0.838944459	-5.486163448	4.10757E-08	1.20549E-07	negative
PRJNA729271	stage_E18_vs_E10	ordinary_muscle_development	ENSGALG00010013202	ENSGALG00010013202	-0.079884258	0.68962068	-0.115837967	0.90778096	0.923777381	negative
PRJNA729271	stage_E18_vs_E10	ordinary_muscle_development	ENSGALG00010013203	AMACR	0.315658322	0.121401986	2.600108384	0.009319432	0.014798989	positive
PRJNA729271	stage_E18_vs_E10	ordinary_muscle_development	ENSGALG00010013204	ENSGALG00010013204	2.366844216	0.317538945	7.453713161	9.07492E-14	4.18654E-13	positive
PRJNA729271	stage_E18_vs_E10	ordinary_muscle_development	ENSGALG00010013205	KCNJ6	2.492944951	0.328287478	7.593786295	3.10691E-14	1.4814E-13	positive
PRJNA729271	stage_E18_vs_E10	ordinary_muscle_development	ENSGALG00010013206	AFF2	-0.291174487	0.112157215	-2.596128005	0.009428093	0.014963343	negative
PRJNA729271	stage_E18_vs_E10	ordinary_muscle_development	ENSGALG00010013207	C1QTNF3	-1.872575548	0.199177265	-9.401552658	5.37638E-21	4.04834E-20	negative
PRJNA729271	stage_E18_vs_E10	ordinary_muscle_development	ENSGALG00010013208	ENSGALG00010013208	0.099345575	0.326617461	0.304164924	0.761002246	0.799501888	positive
PRJNA729271	stage_E18_vs_E10	ordinary_muscle_development	ENSGALG00010013209	KIAA1549	-0.489960054	0.103011607	-4.756357736	1.97117E-06	4.92708E-06	negative
PRJNA729271	stage_E18_vs_E10	ordinary_muscle_development	ENSGALG00010013212	ENSGALG00010013212	0.959071055	0.152509805	6.288586193	3.2037E-10	1.12549E-09	positive
PRJNA729271	stage_E18_vs_E10	ordinary_muscle_development	ENSGALG00010013213	SH2D1A	1.178050348	0.380606192	3.095194909	0.001966836	0.003455671	positive
PRJNA729271	stage_E18_vs_E10	ordinary_muscle_development	ENSGALG00010013214	RAI14	-1.044390779	0.169640848	-6.156481704	7.43788E-10	2.54514E-09	negative
PRJNA729271	stage_E18_vs_E10	ordinary_muscle_development	ENSGALG00010013215	SF3A3	-0.665205918	0.082610058	-8.052359927	8.12127E-16	4.39409E-15	negative
PRJNA729271	stage_E18_vs_E10	ordinary_muscle_development	ENSGALG00010013217	TGFBRAP1	0.239607099	0.083969681	2.853495417	0.004324115	0.007225135	positive
PRJNA729271	stage_E18_vs_E10	ordinary_muscle_development	ENSGALG00010013220	CSTF1	-0.045579239	0.08720433	-0.522671737	0.60120271	0.652571011	negative
PRJNA729271	stage_E18_vs_E10	ordinary_muscle_development	ENSGALG00010013221	C2orf49	0.010154925	0.119628757	0.084886986	0.932351238	0.944866895	positive
PRJNA729271	stage_E18_vs_E10	ordinary_muscle_development	ENSGALG00010013224	ATP6V0A4	1.147604784	0.71068129	1.614795269	0.106355038	0.140299989	positive
PRJNA729271	stage_E18_vs_E10	ordinary_muscle_development	ENSGALG00010013225	GNE	-0.987412065	0.097397305	-10.1379814	3.74763E-24	3.36776E-23	negative
PRJNA729271	stage_E18_vs_E10	ordinary_muscle_development	ENSGALG00010013226	DUSP6	-0.02630037	0.112960691	-0.232827634	0.815895254	0.84698143	negative
PRJNA729271	stage_E18_vs_E10	ordinary_muscle_development	ENSGALG00010013227	CITED4	1.510125777	0.162552066	9.290105082	1.54136E-20	1.12554E-19	positive
PRJNA729271	stage_E18_vs_E10	ordinary_muscle_development	ENSGALG00010013228	RTFDC1	0.159863991	0.085173842	1.876914183	0.060529857	0.083679568	positive
PRJNA729271	stage_E18_vs_E10	ordinary_muscle_development	ENSGALG00010013230	ENSGALG00010013230	1.175789174	0.347717044	3.381453956	0.000721033	0.001340951	positive
PRJNA729271	stage_E18_vs_E10	ordinary_muscle_development	ENSGALG00010013232	FHL2	-0.550455786	0.422138244	-1.303970425	0.192243666	0.24001481	negative
PRJNA729271	stage_E18_vs_E10	ordinary_muscle_development	ENSGALG00010013233	LRRCC1	-0.053225968	0.126599973	-0.420426373	0.674174005	0.720713342	negative
PRJNA729271	stage_E18_vs_E10	ordinary_muscle_development	ENSGALG00010013235	GCNT7	0.4470456	0.254682729	1.75530395	0.079207331	0.107209848	positive
PRJNA729271	stage_E18_vs_E10	ordinary_muscle_development	ENSGALG00010013236	MON2	0.183885148	0.08281283	2.220491068	0.026385451	0.038962624	positive
PRJNA729271	stage_E18_vs_E10	ordinary_muscle_development	ENSGALG00010013238	RAD1	-0.434705729	0.138060349	-3.148664561	0.001640183	0.002913894	negative
PRJNA729271	stage_E18_vs_E10	ordinary_muscle_development	ENSGALG00010013241	SEC24A	0.030009026	0.135904254	0.220810055	0.825240334	0.854662663	positive
PRJNA729271	stage_E18_vs_E10	ordinary_muscle_development	ENSGALG00010013242	SULT4A1	-0.457474869	0.160787783	-2.845209125	0.004438227	0.007406413	negative
PRJNA729271	stage_E18_vs_E10	ordinary_muscle_development	ENSGALG00010013244	TRIM24	-0.107993886	0.148749302	-0.726012724	0.46783097	0.52681867	negative
PRJNA729271	stage_E18_vs_E10	ordinary_muscle_development	ENSGALG00010013246	LNPEP	1.325756172	0.175047019	7.573714664	3.62701E-14	1.72203E-13	positive
PRJNA729271	stage_E18_vs_E10	ordinary_muscle_development	ENSGALG00010013247	ATG5	-0.152326699	0.093443832	-1.630141815	0.103071528	0.136303421	negative
PRJNA729271	stage_E18_vs_E10	ordinary_muscle_development	ENSGALG00010013248	ENSGALG00010013248	-0.50712871	0.687737362	-0.737387173	0.460886933	0.520252013	negative
PRJNA729271	stage_E18_vs_E10	ordinary_muscle_development	ENSGALG00010013249	AURKA	-2.557161164	0.228358498	-11.19801184	4.16987E-29	4.8548E-28	negative
PRJNA729271	stage_E18_vs_E10	ordinary_muscle_development	ENSGALG00010013250	E2F5	-0.377328196	0.098772001	-3.820193918	0.000133347	0.00027397	negative
PRJNA729271	stage_E18_vs_E10	ordinary_muscle_development	ENSGALG00010013251	MTA1	-0.041698032	0.081833478	-0.509547354	0.610368615	0.661133842	negative
PRJNA729271	stage_E18_vs_E10	ordinary_muscle_development	ENSGALG00010013252	PPM1H	-1.787193644	0.189905267	-9.410974625	4.91557E-21	3.711E-20	negative
PRJNA729271	stage_E18_vs_E10	ordinary_muscle_development	ENSGALG00010013253	SHB	0.378682632	0.100178406	3.780082419	0.000156776	0.000318899	positive
PRJNA729271	stage_E18_vs_E10	ordinary_muscle_development	ENSGALG00010013254	SAR1B	-0.165209562	0.142401064	-1.160170841	0.245979256	0.299166749	negative
PRJNA729271	stage_E18_vs_E10	ordinary_muscle_development	ENSGALG00010013256	BRIX1	-0.732603782	0.118379807	-6.188587428	6.07057E-10	2.09507E-09	negative
PRJNA729271	stage_E18_vs_E10	ordinary_muscle_development	ENSGALG00010013257	STX12	-0.389904519	0.090257914	-4.31989287	1.56105E-05	3.56337E-05	negative
PRJNA729271	stage_E18_vs_E10	ordinary_muscle_development	ENSGALG00010013258	RBIS	0.003047558	0.146984411	0.020733889	0.983457935	0.986179368	positive
PRJNA729271	stage_E18_vs_E10	ordinary_muscle_development	ENSGALG00010013259	PTPRB	1.429847518	0.139950263	10.21682624	1.6671E-24	1.53329E-23	positive
PRJNA729271	stage_E18_vs_E10	ordinary_muscle_development	ENSGALG00010013260	FAM210B	0.257747799	0.081359971	3.167992762	0.001534953	0.002735663	positive
PRJNA729271	stage_E18_vs_E10	ordinary_muscle_development	ENSGALG00010013261	JADE2	0.9345216	0.198730412	4.702458924	2.57047E-06	6.3506E-06	positive

PRJNA729271	stage_E18_vs_E10	ordinary_muscle_development	ENSGALG00010013262	CA13	0.765113616	0.262339922	2.916497077	0.00353986	0.005984404	positive
PRJNA729271	stage_E18_vs_E10	ordinary_muscle_development	ENSGALG00010013263	ATP11A	-0.503668865	0.113522053	-4.4367491	9.13276E-06	2.13446E-05	negative
PRJNA729271	stage_E18_vs_E10	ordinary_muscle_development	ENSGALG00010013264	DNAJC21	-0.955311178	0.125589134	-7.60663877	2.81316E-14	1.34755E-13	negative
PRJNA729271	stage_E18_vs_E10	ordinary_muscle_development	ENSGALG00010013265	PACS2	1.731480446	0.121755609	14.2209501	6.79244E-46	1.66593E-44	positive
PRJNA729271	stage_E18_vs_E10	ordinary_muscle_development	ENSGALG00010013266	KIAA1755	2.590345015	0.187219511	13.83587109	1.54849E-43	3.42155E-42	positive
PRJNA729271	stage_E18_vs_E10	ordinary_muscle_development	ENSGALG00010013267	PNPLA3	-0.168722015	0.179566134	-0.939609334	0.347417987	0.40614757	negative
PRJNA729271	stage_E18_vs_E10	ordinary_muscle_development	ENSGALG00010013268	PTPRR	0.58714295	0.276571384	2.122934562	0.033759336	0.048855988	positive
PRJNA729271	stage_E18_vs_E10	ordinary_muscle_development	ENSGALG00010013269	INPP5B	-0.009181887	0.10390053	-0.088371901	0.929581093	0.942396389	negative
PRJNA729271	stage_E18_vs_E10	ordinary_muscle_development	ENSGALG00010013271	GULP1	0.860239269	0.139027296	6.18755663	6.11039E-10	2.10681E-09	positive
PRJNA729271	stage_E18_vs_E10	ordinary_muscle_development	ENSGALG00010013273	STAG2	0.305000373	0.116493197	2.61818184	0.008839968	0.014105608	positive
PRJNA729271	stage_E18_vs_E10	ordinary_muscle_development	ENSGALG00010013274	MTF1	0.324576508	0.121722725	2.666523517	0.007664024	0.012325534	positive
PRJNA729271	stage_E18_vs_E10	ordinary_muscle_development	ENSGALG00010013275	MAPRE1	-0.815390701	0.076089942	-10.71614298	8.54946E-27	8.98003E-26	negative
PRJNA729271	stage_E18_vs_E10	ordinary_muscle_development	ENSGALG00010013276	POC1B	-1.40157049	0.110867357	-12.64186793	1.24071E-36	2.07668E-35	negative
PRJNA729271	stage_E18_vs_E10	ordinary_muscle_development	ENSGALG00010013277	YRDC	-0.815453586	0.094389646	-8.639227078	5.65946E-18	3.53594E-17	negative
PRJNA729271	stage_E18_vs_E10	ordinary_muscle_development	ENSGALG00010013278	SAMM50	0.399948797	0.103185842	3.876004577	0.000106186	0.000220953	positive
PRJNA729271	stage_E18_vs_E10	ordinary_muscle_development	ENSGALG00010013280	FRS2	-0.222250472	0.112452327	-1.976397274	0.0481098	0.067736916	negative
PRJNA729271	stage_E18_vs_E10	ordinary_muscle_development	ENSGALG00010013281	BIRC8	1.924629659	0.138734597	13.8727448	9.26619E-44	2.06954E-42	positive
PRJNA729271	stage_E18_vs_E10	ordinary_muscle_development	ENSGALG00010013282	SEC22B	-0.603140568	0.089792219	-6.717069391	1.85416E-11	7.1327E-11	negative
PRJNA729271	stage_E18_vs_E10	ordinary_muscle_development	ENSGALG00010013283	PAWR	-1.635981001	0.285124409	-5.737779535	9.59259E-09	2.97104E-08	negative
PRJNA729271	stage_E18_vs_E10	ordinary_muscle_development	ENSGALG00010013284	MANEAL	-1.567593047	0.338954417	-4.624790148	3.74978E-06	9.09522E-06	negative
PRJNA729271	stage_E18_vs_E10	ordinary_muscle_development	ENSGALG00010013285	ATP2B1	-0.798923701	0.108611367	-7.355801939	1.89784E-13	8.53793E-13	negative
PRJNA729271	stage_E18_vs_E10	ordinary_muscle_development	ENSGALG00010013286	RNF14	0.031961395	0.121306657	0.26347602	0.792183704	0.826390989	positive
PRJNA729271	stage_E18_vs_E10	ordinary_muscle_development	ENSGALG00010013288	CA3A	4.412845848	0.77662664	5.682068606	1.33075E-08	4.06518E-08	positive
PRJNA729271	stage_E18_vs_E10	ordinary_muscle_development	ENSGALG00010013289	TM9SF4	-0.815771046	0.074821342	-10.90291921	1.11618E-27	1.22661E-26	negative
PRJNA729271	stage_E18_vs_E10	ordinary_muscle_development	ENSGALG00010013290	AKR1D1	-1.987620234	0.338144221	-5.87802515	4.1519E-09	1.32822E-08	negative
PRJNA729271	stage_E18_vs_E10	ordinary_muscle_development	ENSGALG00010013292	PRDM1	-1.595501581	0.289835415	-5.504853791	3.69475E-08	1.09074E-07	negative
PRJNA729271	stage_E18_vs_E10	ordinary_muscle_development	ENSGALG00010013293	TUBGCP3	-0.864702108	0.05914374	-14.62034868	2.08331E-48	5.69765E-47	negative
PRJNA729271	stage_E18_vs_E10	ordinary_muscle_development	ENSGALG00010013295	CA3B	-0.789206106	0.406923302	-1.939446824	0.052446954	0.073344775	negative
PRJNA729271	stage_E18_vs_E10	ordinary_muscle_development	ENSGALG00010013296	AGXT2	-1.882674389	0.484309545	-3.887336948	0.00010135	0.000211255	negative
PRJNA729271	stage_E18_vs_E10	ordinary_muscle_development	ENSGALG00010013297	FCHO2	0.6446839	0.125581278	5.133598803	2.84254E-07	7.73612E-07	positive
PRJNA729271	stage_E18_vs_E10	ordinary_muscle_development	ENSGALG00010013298	EPYC	-5.673912406	0.37759971	-15.02626262	4.94108E-51	1.49835E-49	negative
PRJNA729271	stage_E18_vs_E10	ordinary_muscle_development	ENSGALG00010013299	PLAGL2	-0.830981511	0.111566212	-7.448325929	9.45321E-14	4.34859E-13	negative
PRJNA729271	stage_E18_vs_E10	ordinary_muscle_development	ENSGALG00010013300	GNPDA1	-0.715163259	0.098591904	-7.253772659	4.0532E-13	1.78847E-12	negative
PRJNA729271	stage_E18_vs_E10	ordinary_muscle_development	ENSGALG00010013301	PARVB	-0.482104052	0.140976156	-3.419755971	0.000626773	0.001174997	negative
PRJNA729271	stage_E18_vs_E10	ordinary_muscle_development	ENSGALG00010013302	ENSGALG00010013302	-0.552042779	0.802033038	-0.688304287	0.491261183	0.548853926	negative
PRJNA729271	stage_E18_vs_E10	ordinary_muscle_development	ENSGALG00010013303	TFPI	-0.661750331	0.235862164	-2.805665472	0.005021279	0.008323815	negative
PRJNA729271	stage_E18_vs_E10	ordinary_muscle_development	ENSGALG00010013304	KCNJ15	-0.039554804	0.238046301	-0.166164332	0.868027628	0.89116507	negative
PRJNA729271	stage_E18_vs_E10	ordinary_muscle_development	ENSGALG00010013305	THOC2	-0.25050392	0.09122592	-2.745973079	0.006033171	0.00987141	negative
PRJNA729271	stage_E18_vs_E10	ordinary_muscle_development	ENSGALG00010013306	ARSB	0.340296493	0.14968566	2.273407433	0.023001639	0.034315434	positive
PRJNA729271	stage_E18_vs_E10	ordinary_muscle_development	ENSGALG00010013307	POFUT1	-0.558912104	0.088125548	-6.342225544	2.26469E-10	8.04183E-10	negative
PRJNA729271	stage_E18_vs_E10	ordinary_muscle_development	ENSGALG00010013309	ENSGALG00010013309	4.586089998	0.285172621	16.0818033	3.42269E-58	1.29198E-56	positive
PRJNA729271	stage_E18_vs_E10	ordinary_muscle_development	ENSGALG00010013310	MYO16	0.526813216	0.294339603	1.789814252	0.073483777	0.100060813	positive
PRJNA729271	stage_E18_vs_E10	ordinary_muscle_development	ENSGALG00010013311	RSPO1	-4.60314731	0.287911072	-15.98808712	1.54709E-57	5.6629E-56	negative
PRJNA729271	stage_E18_vs_E10	ordinary_muscle_development	ENSGALG00010013312	ENSGALG00010013312	-2.48936539	0.753339739	-3.304439232	0.000951666	0.001738206	negative
PRJNA729271	stage_E18_vs_E10	ordinary_muscle_development	ENSGALG00010013313	GAK	-0.155737806	0.094759338	-1.643508806	0.100277705	0.13288767	negative
PRJNA729271	stage_E18_vs_E10	ordinary_muscle_development	ENSGALG00010013314	KIT	0.469033607	0.205257118	2.285102754	0.022306803	0.033357796	positive
PRJNA729271	stage_E18_vs_E10	ordinary_muscle_development	ENSGALG00010013316	KDM7A	0.728736781	0.130805617	5.57114287	2.53074E-08	7.55728E-08	positive
PRJNA729271	stage_E18_vs_E10	ordinary_muscle_development	ENSGALG00010013317	PREP	-0.73393569	0.10998159	-6.67325952	2.50183E-11	9.55072E-11	negative
PRJNA729271	stage_E18_vs_E10	ordinary_muscle_development	ENSGALG00010013318	KERA	-1.845426587	0.48582615	-3.798532847	0.000145555	0.000297409	negative
PRJNA729271	stage_E18_vs_E10	ordinary_muscle_development	ENSGALG00010013319	ENSGALG00010013319	-0.821934942	1.5626175	-0.525998808			negative
PRJNA729271	stage_E18_vs_E10	ordinary_muscle_development	ENSGALG00010013320	CALCRL	0.506756618	0.123720909	4.09596586	4.20412E-05	9.12666E-05	positive
PRJNA729271	stage_E18_vs_E10	ordinary_muscle_development	ENSGALG00010013322	IRS2	0.106263678	0.515377969	0.206185915	0.83664569	0.864498094	positive
PRJNA729271	stage_E18_vs_E10	ordinary_muscle_development	ENSGALG00010013323	NOTCH2	-0.584396171	0.108769778	-5.372780744	7.75316E-08	2.21661E-07	negative
PRJNA729271	stage_E18_vs_E10	ordinary_muscle_development	ENSGALG00010013324	PARVG	1.457085722	0.147350939	9.888540431	4.66784E-23	3.92689E-22	positive
PRJNA729271	stage_E18_vs_E10	ordinary_muscle_development	ENSGALG00010013325	CA2	3.259867166	0.316865605	10.28785426	7.99379E-25	7.51914E-24	positive
PRJNA729271	stage_E18_vs_E10	ordinary_muscle_development	ENSGALG00010013326	FGF20	-7.390155828	1.03478446	-7.141734454	9.21605E-13	3.96989E-12	negative
PRJNA729271	stage_E18_vs_E10	ordinary_muscle_development	ENSGALG00010013327	SLF1	-0.686211119	0.097493806	-7.038509876	1.94307E-12	8.126E-12	negative
PRJNA729271	stage_E18_vs_E10	ordinary_muscle_development	ENSGALG00010013328	SLC37A3	-0.085426122	0.078150582	-1.09309642	0.274351468	0.32977233	negative
PRJNA729271	stage_E18_vs_E10	ordinary_muscle_development	ENSGALG00010013329	LIFR	0.097085756	0.122604112	0.791863785	0.428440105	0.488495188	positive
PRJNA729271	stage_E18_vs_E10	ordinary_muscle_development	ENSGALG00010013330	ERG	-0.036242985	0.128908896	-0.281151927	0.778593875	0.814324137	negative
PRJNA729271	stage_E18_vs_E10	ordinary_muscle_development	ENSGALG00010013331	KIF3B	-0.725469088	0.151219936	-4.797434434	1.60704E-06	4.05889E-06	negative
PRJNA729271	stage_E18_vs_E10	ordinary_muscle_development	ENSGALG00010013332	LUM	0.806612889	0.320670683	2.515393307	0.011889966	0.018615798	positive
PRJNA729271	stage_E18_vs_E10	ordinary_muscle_development	ENSGALG00010013333	PPP1R12A	0.134738193	0.098187939	1.372247901	0.169986278	0.214854474	positive
PRJNA729271	stage_E18_vs_E10	ordinary_muscle_development	ENSGALG00010013334	SH3BGRL3	0.889501339	0.120899593	7.357355949	1.87589E-13	8.4444E-13	positive
PRJNA729271	stage_E18_vs_E10	ordinary_muscle_development	ENSGALG00010013335	POPDC3	1.533242803	0.176948907	8.66488995	4.51949E-18	2.83962E-17	positive
PRJNA729271	stage_E18_vs_E10	ordinary_muscle_development	ENSGALG00010013336	MTNR1A	2.024462842	0.673107438	3.007637011	0.002632874	0.004548148	positive
PRJNA729271	stage_E18_vs_E10	ordinary_muscle_development	ENSGALG00010013337	RAB19	0.322489351	0.256031904	1.259567057	0.207825586	0.257538845	positive
PRJNA729271	stage_E18_vs_E10	ordinary_muscle_development	ENSGALG00010013339	ATP6V0D2	0.355659717	0.569574457	0.624430594	0.532344838	0.588225219	positive
PRJNA729271	stage_E18_vs_E10	ordinary_muscle_development	ENSGALG00010013340	LIN28B	-3.806861436	0.383909056	-9.916050119	3.54509E-23	2.99977E-22	negative
PRJNA729271	stage_E18_vs_E10	ordinary_muscle_development	ENSGALG00010013343	SHISAL1	-4.729622124	0.403093963	-11.73329934	8.60383E-32	1.14415E-30	negative
PRJNA729271	stage_E18_vs_E10	ordinary_muscle_development	ENSGALG00010013344	OSMR	0.638661203	0.123882248	5.155389183	2.53105E-07	6.91695E-07	positive
PRJNA729271	stage_E18_vs_E10	ordinary_muscle_development	ENSGALG00010013345	DCN	1.220734381	0.213766791	5.71058945	1.12586E-08	3.45894E-08	positive
PRJNA729271	stage_E18_vs_E10	ordinary_muscle_development	ENSGALG00010013346	HMGCS2	2.474957101	0.684618617	3.61508881	0.000300245	0.000587876	positive
PRJNA729271	stage_E18_vs_E10	ordinary_muscle_development	ENSGALG00010013347	ENSGALG00010013347	0.251646434	0.31544454	0.797751749	0.425014567	0.485314806	positive
PRJNA729271	stage_E18_vs_E10	ordinary_muscle_development	ENSGALG00010013348	MKRN4P	-0.053373687	0.07057284	-0.756292169	0.449474042	0.509034006	negative
PRJNA729271	stage_E18_vs_E10	ordinary_muscle_development	ENSGALG00010013349	ETS2	0.617580401	0.100540695	6.142591324	8.1186E-10	2.76632E-09	positive
PRJNA729271	stage_E18_vs_E10	ordinary_muscle_development	ENSGALG00010013351	EDAR	-3.193105586	0.404201007	-7.89979622	2.7936E-15	1.4545E-14	negative
PRJNA729271	stage_E18_vs_E10	ordinary_muscle_development	ENSGALG00010013352	ASXL1	-0.234114599	0.088370163	-2.649249366	0.008067078	0.012934989	negative
PRJNA729271	stage_E18_vs_E10	ordinary_muscle_development	ENSGALG00010013353	NDFIP1	0.206473889	0.084658221	2.438911259	0.014731585	0.022769709	positive
PRJNA729271	stage_E18_vs_E10	ordinary_muscle_development	ENSGALG00010013354	WWP1	1.644069878	0.145867279	11.27099845	1.82489E-29	2.1664E-28	positive
PRJNA729271	stage_E18_vs_E10	ordinary_muscle_development	ENSGALG00010013355	FAM171B	-2.184743381	0.15246605	-14.32937614	1.43403E-46	3.67077E-45	negative
PRJNA729271	stage_E18_vs_E10	ordinary_muscle_development	ENSGALG00010013356	AMER1	-0.535260807	0.065568936	-8.163329205	3.25915E-16	1.80448E-15	negative
PRJNA729271	stage_E18_vs_E10	ordinary_muscle_development	ENSGALG00010013357	RICTOR	0.45077351	0.109786042	4.10592732	4.02696E-05	8.75912E-05	positive
PRJNA729271	stage_E18_vs_E10	ordinary_muscle_development	ENSGALG00010013358	RPS16	1.064166158	0.174132155	6.111255908	9.88501E-10	3.33682E-09	positive
PRJNA729271	stage_E18_vs_E10	ordinary_muscle_development	ENSGALG00010013359	BTG1	0.542716035	0.132551959	4.094364493	4.23328E-05	9.18584E-05	positive
PRJNA729271	stage_E18_vs_E10	ordinary_muscle_development	ENSGALG00010013360	PRR5	0.019244957	0.113093802	0.170168094	0.864877943	0.888518422	positive
PRJNA729271	stage_E18_vs_E10	ordinary_muscle_development	ENSGALG00010013361	ENSGALG00010013361	-0.037834961	0.23275411	-0.162553355	0.870870116	0.893745563	negative
PRJNA729271	stage_E18_vs_E10	ordinary_muscle_development	ENSGALG00010013362	ENSGALG00010013362	5.289464823	0.35592055	14.86136393	5.87175E-50	1.70906E-48	positive
PRJNA729271	stage_E18_vs_E10	ordinary_muscle_development	ENSGALG00010013363	HS3ST5	1.535174143	0.236357882	6.495125656	8.29641E-11	3.04524E-10	positive
PRJNA729271	stage_E18_vs_E10	ordinary_muscle_development	ENSGALG00010013364	OTOGL	3.083841724	0.357972665	8.614740805	7.01E-18	4.34231E-17	positive
PRJNA729271	stage_E18_vs_E10	ordinary_muscle_development	ENSGALG00010013365	CEP85	1.488507412	0.227569201	6.540900097	6.11497E-11	2.26692E-10	positive
PRJNA729271	stage_E18_vs_E10	ordinary_muscle_development	ENSGALG00010013367	NOL4L	0.86866923	0.166513124	5.216821398	1.8202E-07	5.03507E-07	positive
PRJNA729271	stage_E18_vs_E10	ordinary_muscle_development	ENSGALG00010013369	MTMR8	-0.471754113	0.063305127	-7.452068031	9.18883E-14	4.23381E-13	negative
PRJNA729271	stage_E18_vs_E10	ordinary_muscle_development	ENSGALG00010013370	CNKSR1	3.359136506	0.234424557	14.32928595	1.43589E-46	3.67077E-45	positive
PRJNA729271	stage_E18_vs_E10	ordinary_muscle_development	ENSGALG00010013371	FAT1	-0.369556185	0.156518016	-2.361109567	0.018220346	0.027651965	negative
PRJNA729271	stage_E18_vs_E10	ordinary_muscle_development	ENSGALG00010013372	SPRY4	-0.921893519	0.297546661	-3.098315798	0.001946239	0.003421142	negative
PRJNA729271	stage_E18_vs_E10	ordinary_muscle_development	ENSGALG00010013373	ENSGALG00010013373	0.322927994	0.569134108	0.567402286	0.570440897	0.62397844	positive
PRJNA729271	stage_E18_vs_E10	ordinary_muscle_development	ENSGALG00010013374	RMDN1	1.814875092	0.163563075	11.09587292	1.31371E-28	1.49939E-27	positive
PRJNA729271	stage_E18_vs_E10	ordinary_muscle_development	ENSGALG00010013375	PSMG1	-0.498066804	0.089345696	-5.574603243	2.48095E-08	7.41776E-08	negative
PRJNA729271	stage_E18_vs_E10	ordinary_muscle_development	ENSGALG00010013376	ARHGAP8	-2.092744872	0.738221931	-2.834845165	0.00458479	0.007638682	negative
PRJNA729271	stage_E18_vs_E10	ordinary_muscle_development	ENSGALG00010013378	SH3RF3	0.641621575	0.142155103	4.513531762	6.37569E-06	1.51253E-05	positive
PRJNA729271	stage_E18_vs_E10	ordinary_muscle_development	ENSGALG00010013379	ZNF593	1.104778775	0.131637384	8.392591354	4.75542E-17	2.79181E-16	positive
PRJNA729271	stage_E18_vs_E10	ordinary_muscle_development	ENSGALG00010013380	PHGDH	1.103098231	0.609524326	1.809769003	0.070331617	0.096166096	positive
PRJNA729271	stage_E18_vs_E10	ordinary_muscle_development	ENSGALG00010013381	DENND2A	0.016136128	0.088470613	0.182389696	0.855276912	0.880423183	positive
PRJNA729271	stage_E18_vs_E10	ordinary_muscle_development	ENSGALG00010013382	ASB12	2.213963603	0.220596383	10.0362643	1.05598E-23	9.21517E-23	positive
PRJNA729271	stage_E18_vs_E10	ordinary_muscle_development	ENSGALG00010013383	SEPTIN10	0.7357925	0.139394238	5.278500103	1.30246E-07	3.64955E-07	positive
PRJNA729271	stage_E18_vs_E10	ordinary_muscle_development	ENSGALG00010013385	PGRMC1	-0.456366384	0.155922371	-2.926882016	0.003423787	0.005796963	negative
PRJNA729271	stage_E18_vs_E10	ordinary_muscle_development	ENSGALG00010013386	ITGAV	0.263438629	0.121865993	2.161707479	0.030640731	0.044699818	positive
PRJNA729271	stage_E18_vs_E10	ordinary_muscle_development	ENSGALG00010013387	NDST1	-0.025234669	0.092609417	-0.272484912	0.785249191	0.819924148	negative
PRJNA729271	stage_E18_vs_E10	ordinary_muscle_development	ENSGALG00010013388	SOWAHC	0.331022498	0.216715101	1.527454692	0.126647986	0.164923417	positive
PRJNA729271	stage_E18_vs_E10	ordinary_muscle_development	ENSGALG00010013389	NAP1L1	-1.299753011	0.062457705	-20.81013097	3.50386E-96	3.73445E-94	negative
PRJNA729271	stage_E18_vs_E10	ordinary_muscle_development	ENSGALG00010013390	SLC16A2	-1.762125936	0.134876833	-13.06470429	5.23878E-39	9.64881E-38	negative
PRJNA729271	stage_E18_vs_E10	ordinary_muscle_development	ENSGALG00010013391	HMGN1	-0.207931837	0.088000023	-2.362861159	0.018134462	0.027538924	negative
PRJNA729271	stage_E18_vs_E10	ordinary_muscle_development	ENSGALG00010013392	HDAC2	-0.93627296	0.069300273	-13.51037905	1.3582E-41	2.77284E-40	negative
PRJNA729271	stage_E18_vs_E10	ordinary_muscle_development	ENSGALG00010013393	ADCK2	-0.069639592	0.161767458	-0.430491973	0.666837805	0.714029252	negative
PRJNA729271	stage_E18_vs_E10	ordinary_muscle_development	ENSGALG00010013394	DNMT3B	-2.978599642	0.13354526	-22.30404609	3.3756E-110	5.4365E-108	negative
PRJNA729271	stage_E18_vs_E10	ordinary_muscle_development	ENSGALG00010013396	FYB1	0.913660226	0.187289688	4.878326381	1.0699E-06	2.75848E-06	positive
PRJNA729271	stage_E18_vs_E10	ordinary_muscle_development	ENSGALG00010013397	MATN1	-7.913477619	0.462014743	-17.12819286	9.14515E-66	4.36049E-64	negative
PRJNA729271	stage_E18_vs_E10	ordinary_muscle_development	ENSGALG00010013398	CHAMP1	0.402756065	0.156296088	2.576878738	0.009969692	0.015772832	positive
PRJNA729271	stage_E18_vs_E10	ordinary_muscle_development	ENSGALG00010013399	FGF1	3.987154137	0.322407839	12.3668027	3.95243E-35	6.11425E-34	positive
PRJNA729271	stage_E18_vs_E10	ordinary_muscle_development	ENSGALG00010013400	BBS10	0.656617162	0.163453284	4.017154912	5.8905E-05	0.000126059	positive
PRJNA729271	stage_E18_vs_E10	ordinary_muscle_development	ENSGALG00010013402	MARCKS	0.147065041	0.154973693	0.948967775	0.342637002	0.40110833	positive
PRJNA729271	stage_E18_vs_E10	ordinary_muscle_development	ENSGALG00010013403	CPNE3	1.432618884	0.102771562	13.93983763	3.62765E-44	8.24181E-43	positive
PRJNA729271	stage_E18_vs_E10	ordinary_muscle_development	ENSGALG00010013404	ENSGALG00010013404	0.382884257	0.224478801	1.705658864	0.088071613	0.11798503	positive
PRJNA729271	stage_E18_vs_E10	ordinary_muscle_development	ENSGALG00010013405	RPS14	0.997385604	0.175327455	5.688701777	1.28009E-08	3.91288E-08	positive
PRJNA729271	stage_E18_vs_E10	ordinary_muscle_development	ENSGALG00010013406	ENSGALG00010013406	0.58238975	0.277283178	2.100342883	0.03569869	0.051431518	positive
PRJNA729271	stage_E18_vs_E10	ordinary_muscle_development	ENSGALG00010013407	ZC3H15	-0.064876279	0.11327718	-0.572721521	0.566833263	0.620515682	negative
PRJNA729271	stage_E18_vs_E10	ordinary_muscle_development	ENSGALG00010013408	RLIM	-0.242726541	0.065538766	-3.703556763	0.000212598	0.000424639	negative
PRJNA729271	stage_E18_vs_E10	ordinary_muscle_development	ENSGALG00010013409	DAB2	-0.257015407	0.148085366	-1.735589508	0.082636443	0.111393586	negative
PRJNA729271	stage_E18_vs_E10	ordinary_muscle_development	ENSGALG00010013410	KIAA0825	-0.267185966	0.102474503	-2.607340935	0.009124846	0.014513841	negative
PRJNA729271	stage_E18_vs_E10	ordinary_muscle_development	ENSGALG00010013411	ENSGALG00010013411	1.182887945	0.447283629	2.64460371	0.008178664	0.013103762	positive

PRJNA729271	stage_E18_vs_E10	ordinary_muscle_development	ENSGALG00010013412	COMMD7	-0.365877924	0.100498358	-3.64063586	0.000271966	0.000535981	negative
PRJNA729271	stage_E18_vs_E10	ordinary_muscle_development	ENSGALG00010013413	WRB	-0.397964151	0.085001323	-4.681858311	2.84286E-06	6.98783E-06	negative
PRJNA729271	stage_E18_vs_E10	ordinary_muscle_development	ENSGALG00010013414	LAPTM5	0.335681896	0.175436541	1.913409227	0.055695684	0.077571602	positive
PRJNA729271	stage_E18_vs_E10	ordinary_muscle_development	ENSGALG00010013416	NDUFB2	0.673470558	0.110641988	6.086934697	1.15093E-09	3.86174E-09	positive
PRJNA729271	stage_E18_vs_E10	ordinary_muscle_development	ENSGALG00010013417	AKR1B1	-0.163827955	0.088424279	-1.852748553	0.063918407	0.087961389	negative
PRJNA729271	stage_E18_vs_E10	ordinary_muscle_development	ENSGALG00010013418	HCN1	-0.54176491	0.720343918	-0.752092017	0.451995725	0.511610029	negative
PRJNA729271	stage_E18_vs_E10	ordinary_muscle_development	ENSGALG00010013419	ENSGALG00010013419	1.794145028	0.338615917	5.298466312	1.16779E-07	3.28794E-07	positive
PRJNA729271	stage_E18_vs_E10	ordinary_muscle_development	ENSGALG00010013420	ENSGALG00010013420	-1.541658921	4.212068456	-0.36600994			negative
PRJNA729271	stage_E18_vs_E10	ordinary_muscle_development	ENSGALG00010013423	ZNF804A	1.397638396	0.372268805	3.754379568	0.000173771	0.00035125	positive
PRJNA729271	stage_E18_vs_E10	ordinary_muscle_development	ENSGALG00010013425	ENSGALG00010013425	0.46163182	0.223921535	2.061578491	0.039247884	0.056143104	positive
PRJNA729271	stage_E18_vs_E10	ordinary_muscle_development	ENSGALG00010013427	NEXMIF	-1.500536497	0.176907238	-8.48205261	2.21255E-17	1.32525E-16	negative
PRJNA729271	stage_E18_vs_E10	ordinary_muscle_development	ENSGALG00010013428	BPGM	0.71749406	0.153244789	4.682012789	2.84072E-06	6.98375E-06	positive
PRJNA729271	stage_E18_vs_E10	ordinary_muscle_development	ENSGALG00010013429	PDE4DIP	2.554372633	0.187517199	13.62207117	2.96051E-42	6.18337E-41	positive
PRJNA729271	stage_E18_vs_E10	ordinary_muscle_development	ENSGALG00010013431	TMEM175	-0.487226101	0.088159488	-5.526643927	3.26414E-08	9.67364E-08	negative
PRJNA729271	stage_E18_vs_E10	ordinary_muscle_development	ENSGALG00010013434	CORIN	-2.773500643	0.412092248	-6.730290739	1.69324E-11	6.53276E-11	negative
PRJNA729271	stage_E18_vs_E10	ordinary_muscle_development	ENSGALG00010013435	SDC3	-2.327371458	0.18122047	-12.84276248	9.44432E-38	1.65133E-36	negative
PRJNA729271	stage_E18_vs_E10	ordinary_muscle_development	ENSGALG00010013436	NPAS2	-0.004454238	0.214600256	-0.020755976	0.983440316	0.986179368	negative
PRJNA729271	stage_E18_vs_E10	ordinary_muscle_development	ENSGALG00010013437	ENSGALG00010013437	0.527914629	0.124882687	4.227284367	2.36529E-05	5.28272E-05	positive
PRJNA729271	stage_E18_vs_E10	ordinary_muscle_development	ENSGALG00010013438	RPL37	0.908564987	0.187489602	4.845948671	1.26008E-06	3.2253E-06	positive
PRJNA729271	stage_E18_vs_E10	ordinary_muscle_development	ENSGALG00010013440	EEA1	-0.083869141	0.098819423	-0.848711098	0.39604207	0.456076094	negative
PRJNA729271	stage_E18_vs_E10	ordinary_muscle_development	ENSGALG00010013442	ENSGALG00010013442	0.929909778	0.104803895	8.87285514	7.1294E-19	4.71015E-18	positive
PRJNA729271	stage_E18_vs_E10	ordinary_muscle_development	ENSGALG00010013444	OSBPL8	-0.719663026	0.103179713	-6.974850065	3.06197E-12	1.26125E-11	negative
PRJNA729271	stage_E18_vs_E10	ordinary_muscle_development	ENSGALG00010013446	ATP5I	1.453725327	0.145305994	10.00457919	1.45509E-23	1.25844E-22	positive
PRJNA729271	stage_E18_vs_E10	ordinary_muscle_development	ENSGALG00010013449	LCA5L	-0.31088696	0.225123748	-1.380960303	0.167291167	0.211632524	negative
PRJNA729271	stage_E18_vs_E10	ordinary_muscle_development	ENSGALG00010013450	CD74	2.036829688	0.190781967	10.67621705	1.31523E-26	1.37247E-25	positive
PRJNA729271	stage_E18_vs_E10	ordinary_muscle_development	ENSGALG00010013451	PRKAB2	1.986151824	0.169061281	11.74811768	7.22094E-32	9.62903E-31	positive
PRJNA729271	stage_E18_vs_E10	ordinary_muscle_development	ENSGALG00010013452	LSS	-1.889176832	0.230507619	-8.195724044	2.49089E-16	1.38921E-15	negative
PRJNA729271	stage_E18_vs_E10	ordinary_muscle_development	ENSGALG00010013453	PUM1	-0.135474234	0.089436126	-1.514759633	0.129833287	0.168460019	negative
PRJNA729271	stage_E18_vs_E10	ordinary_muscle_development	ENSGALG00010013457	SH3BGR	2.287491885	0.147818593	15.47499429	5.11778E-54	1.67077E-52	positive
PRJNA729271	stage_E18_vs_E10	ordinary_muscle_development	ENSGALG00010013458	LAMA4	2.282137304	0.143610503	15.89115878	7.29662E-57	2.61793E-55	positive
PRJNA729271	stage_E18_vs_E10	ordinary_muscle_development	ENSGALG00010013459	ATP10D	-0.935109954	0.145917278	-6.408493692	1.46964E-10	5.28729E-10	negative
PRJNA729271	stage_E18_vs_E10	ordinary_muscle_development	ENSGALG00010013461	S100B	1.774198578	0.601205684	2.951067536	0.003166777	0.005406646	positive
PRJNA729271	stage_E18_vs_E10	ordinary_muscle_development	ENSGALG00010013463	ENSGALG00010013463	1.533014914	0.210241823	7.291674376	3.06126E-13	1.36122E-12	positive
PRJNA729271	stage_E18_vs_E10	ordinary_muscle_development	ENSGALG00010013464	ABCB7	-0.485126337	0.075910502	-6.390767092	1.65056E-10	5.92052E-10	negative
PRJNA729271	stage_E18_vs_E10	ordinary_muscle_development	ENSGALG00010013465	CALD1	-0.727105647	0.157336926	-4.62132868	3.8129E-06	9.2406E-06	negative
PRJNA729271	stage_E18_vs_E10	ordinary_muscle_development	ENSGALG00010013466	BRAF	0.873379652	0.060732488	14.38076517	6.83348E-47	1.76877E-45	positive
PRJNA729271	stage_E18_vs_E10	ordinary_muscle_development	ENSGALG00010013468	DEPDC1B	-1.886776465	0.272848866	-6.915097318	4.67542E-12	1.89992E-11	negative
PRJNA729271	stage_E18_vs_E10	ordinary_muscle_development	ENSGALG00010013470	RPL31	0.771990413	0.146314159	5.276252252	1.31853E-07	3.69243E-07	positive
PRJNA729271	stage_E18_vs_E10	ordinary_muscle_development	ENSGALG00010013472	ENSGALG00010013472	1.589942932	0.226054075	7.033462833	2.0147E-12	8.40859E-12	positive
PRJNA729271	stage_E18_vs_E10	ordinary_muscle_development	ENSGALG00010013476	ZDHHC17	-0.67930418	0.130859215	-5.191106948	2.09048E-07	5.74871E-07	negative
PRJNA729271	stage_E18_vs_E10	ordinary_muscle_development	ENSGALG00010013477	RFC1	-0.585877581	0.090669538	-6.461680462	1.03547E-10	3.76639E-10	negative
PRJNA729271	stage_E18_vs_E10	ordinary_muscle_development	ENSGALG00010013480	TBC1D8	1.422693439	0.152298532	9.341478361	9.49983E-21	7.02551E-20	positive
PRJNA729271	stage_E18_vs_E10	ordinary_muscle_development	ENSGALG00010013482	ENSGALG00010013482	-0.334146047	0.235047312	-1.421611864	0.155138959	0.197847898	negative
PRJNA729271	stage_E18_vs_E10	ordinary_muscle_development	ENSGALG00010013483	ENSGALG00010013483	-0.193249324	0.206320807	-0.936644866	0.348941265	0.407698181	negative
PRJNA729271	stage_E18_vs_E10	ordinary_muscle_development	ENSGALG00010013484	TCOF1	-0.237585969	0.10203152	-2.32855463	0.019882672	0.029980165	negative
PRJNA729271	stage_E18_vs_E10	ordinary_muscle_development	ENSGALG00010013486	PLPP6	0.240080406	0.16715982	1.436232736	0.150936127	0.193065581	positive
PRJNA729271	stage_E18_vs_E10	ordinary_muscle_development	ENSGALG00010013487	ENSGALG00010013487	-0.398610672	0.094061992	-4.23774433	2.25777E-05	5.05192E-05	negative
PRJNA729271	stage_E18_vs_E10	ordinary_muscle_development	ENSGALG00010013488	UPRT	-0.390494375	0.120034796	-3.253176468	0.001141226	0.00206492	negative
PRJNA729271	stage_E18_vs_E10	ordinary_muscle_development	ENSGALG00010013491	CNOT11	-0.278821431	0.112682832	-2.474391398	0.013346339	0.020745864	negative
PRJNA729271	stage_E18_vs_E10	ordinary_muscle_development	ENSGALG00010013492	B3GALT5	-1.078181262	0.611057908	-1.764450221	0.077656209	0.105257785	negative
PRJNA729271	stage_E18_vs_E10	ordinary_muscle_development	ENSGALG00010013493	ENSGALG00010013493	1.072128703	0.127125835	8.433602074	3.35185E-17	1.98468E-16	positive
PRJNA729271	stage_E18_vs_E10	ordinary_muscle_development	ENSGALG00010013494	ASB1	-1.383265089	0.14606867	-9.469964297	2.79939E-21	2.14467E-20	negative
PRJNA729271	stage_E18_vs_E10	ordinary_muscle_development	ENSGALG00010013497	FAM172A	0.446178796	0.107169792	4.163288805	3.13696E-05	6.89989E-05	positive
PRJNA729271	stage_E18_vs_E10	ordinary_muscle_development	ENSGALG00010013498	KDR	0.718048373	0.125485329	5.722169902	1.05172E-08	3.2401E-08	positive
PRJNA729271	stage_E18_vs_E10	ordinary_muscle_development	ENSGALG00010013501	AKR1B10	-0.589146534	0.134503934	-4.380143505	1.18601E-05	2.73877E-05	negative
PRJNA729271	stage_E18_vs_E10	ordinary_muscle_development	ENSGALG00010013502	PAFAH2	1.525225518	0.204995876	7.440274137	1.00477E-13	4.61473E-13	positive
PRJNA729271	stage_E18_vs_E10	ordinary_muscle_development	ENSGALG00010013504	IGSF5	0.094718426	0.75909761	0.124777663	0.900699571	0.917795451	positive
PRJNA729271	stage_E18_vs_E10	ordinary_muscle_development	ENSGALG00010013506	RNF149	-0.806403736	0.106728348	-7.555665877	4.16722E-14	1.96884E-13	negative
PRJNA729271	stage_E18_vs_E10	ordinary_muscle_development	ENSGALG00010013508	TUBE1	-0.037128039	0.194038096	-0.191344072	0.848256034	0.874500086	negative
PRJNA729271	stage_E18_vs_E10	ordinary_muscle_development	ENSGALG00010013509	ENSGALG00010013509	0.269831937	0.193586193	1.393859413	0.163360062	0.207310813	positive
PRJNA729271	stage_E18_vs_E10	ordinary_muscle_development	ENSGALG00010013510	MRPS33	-0.119722679	0.088408324	-1.354201435	0.175672121	0.221288555	negative
PRJNA729271	stage_E18_vs_E10	ordinary_muscle_development	ENSGALG00010013511	FAM78B	0.244368018	0.229341614	1.065519742	0.286640797	0.342583967	positive
PRJNA729271	stage_E18_vs_E10	ordinary_muscle_development	ENSGALG00010013512	ENSGALG00010013512	-1.313008906	3.450860753	-0.380487362			negative
PRJNA729271	stage_E18_vs_E10	ordinary_muscle_development	ENSGALG00010013514	LMBRD2	-0.260709203	0.140465331	-1.856039511	0.063447926	0.087347107	negative
PRJNA729271	stage_E18_vs_E10	ordinary_muscle_development	ENSGALG00010013516	CSRP2	0.195292409	0.133185082	1.466323442	0.142560192	0.183387468	positive
PRJNA729271	stage_E18_vs_E10	ordinary_muscle_development	ENSGALG00010013517	STMN1	-1.06901152	0.21761856	-4.912317784	9.0006E-07	2.34058E-06	negative
PRJNA729271	stage_E18_vs_E10	ordinary_muscle_development	ENSGALG00010013520	CREG2	-0.111529459	0.186853148	-0.596882956	0.550585527	0.604692924	negative
PRJNA729271	stage_E18_vs_E10	ordinary_muscle_development	ENSGALG00010013523	TMEM178B	-1.53973887	0.189854512	-8.110098896	5.05786E-16	2.77178E-15	negative
PRJNA729271	stage_E18_vs_E10	ordinary_muscle_development	ENSGALG00010013524	ZDHHC15	0.242795929	0.12335911	1.968204285	0.049044537	0.068945841	positive
PRJNA729271	stage_E18_vs_E10	ordinary_muscle_development	ENSGALG00010013526	SIRPA	0.084796471	0.245410335	0.34552934	0.729696457	0.771590093	positive
PRJNA729271	stage_E18_vs_E10	ordinary_muscle_development	ENSGALG00010013527	ADARB1	0.5871797	0.098882451	5.938158831	2.88241E-09	9.35105E-09	positive
PRJNA729271	stage_E18_vs_E10	ordinary_muscle_development	ENSGALG00010013528	PRDX6	0.515485979	0.131791653	3.911370472	9.17739E-05	0.000192179	positive
PRJNA729271	stage_E18_vs_E10	ordinary_muscle_development	ENSGALG00010013531	ARSI	-0.729867804	0.230170335	-3.170989884	0.001519204	0.002708927	negative
PRJNA729271	stage_E18_vs_E10	ordinary_muscle_development	ENSGALG00010013532	WDR19	-0.086585663	0.074817725	-1.157288107	0.247154671	0.300419871	negative
PRJNA729271	stage_E18_vs_E10	ordinary_muscle_development	ENSGALG00010013533	SKP2	-0.889896734	0.082103983	-10.8386548	2.25766E-27	2.43126E-26	negative
PRJNA729271	stage_E18_vs_E10	ordinary_muscle_development	ENSGALG00010013537	LMO3	-0.798637067	0.363647842	-2.196182609	0.028078876	0.041240582	negative
PRJNA729271	stage_E18_vs_E10	ordinary_muscle_development	ENSGALG00010013538	E2F7	-1.406689578	0.265340369	-5.301453316	1.14884E-07	3.23853E-07	negative
PRJNA729271	stage_E18_vs_E10	ordinary_muscle_development	ENSGALG00010013539	NSFL1C	-0.203217803	0.092617409	-2.194164197	0.028223607	0.041444756	negative
PRJNA729271	stage_E18_vs_E10	ordinary_muscle_development	ENSGALG00010013544	ENSGALG00010013544	-0.039086575	0.287441202	-0.135981115	0.89183621	0.910452173	negative
PRJNA729271	stage_E18_vs_E10	ordinary_muscle_development	ENSGALG00010013546	IDUA	-0.005555407	0.132157612	-0.042036221	0.966469823	0.972373158	negative
PRJNA729271	stage_E18_vs_E10	ordinary_muscle_development	ENSGALG00010013549	PCP4	-2.110252242	0.282671223	-7.465394673	8.30503E-14	3.84974E-13	negative
PRJNA729271	stage_E18_vs_E10	ordinary_muscle_development	ENSGALG00010013550	KLHL5	0.004870917	0.1247672	0.039040042	0.968858464	0.97423541	positive
PRJNA729271	stage_E18_vs_E10	ordinary_muscle_development	ENSGALG00010013551	UBE2N	-0.46392147	0.112102606	-4.138364728	3.4979E-05	7.65199E-05	negative
PRJNA729271	stage_E18_vs_E10	ordinary_muscle_development	ENSGALG00010013552	FGF16	0.4129686	0.404503594	1.020926898	0.307289074	0.364735926	positive
PRJNA729271	stage_E18_vs_E10	ordinary_muscle_development	ENSGALG00010013553	TADA1	-0.072942311	0.113607781	-0.642053832	0.520838231	0.5777552	negative
PRJNA729271	stage_E18_vs_E10	ordinary_muscle_development	ENSGALG00010013554	ENSGALG00010013554	-1.042295909	5.425338598	-0.192116287	0.847651117	0.873938612	negative
PRJNA729271	stage_E18_vs_E10	ordinary_muscle_development	ENSGALG00010013556	RASL11B	-2.370301953	0.169711034	-13.96669323	2.48918E-44	5.69996E-43	negative
PRJNA729271	stage_E18_vs_E10	ordinary_muscle_development	ENSGALG00010013558	SNAPC3	-0.074547999	0.085181897	-0.875162457	0.381485517	0.441769799	negative
PRJNA729271	stage_E18_vs_E10	ordinary_muscle_development	ENSGALG00010013561	AGK	-0.1851667	0.109733253	-1.687425591	0.091521546	0.122245191	negative
PRJNA729271	stage_E18_vs_E10	ordinary_muscle_development	ENSGALG00010013562	RERGL	0.571443326	0.158879039	3.596719418	0.000322256	0.000628089	positive
PRJNA729271	stage_E18_vs_E10	ordinary_muscle_development	ENSGALG00010013563	FKBP1A	1.32501076	0.098522756	13.44877885	3.12999E-41	6.30127E-40	positive
PRJNA729271	stage_E18_vs_E10	ordinary_muscle_development	ENSGALG00010013565	ENSGALG00010013565	3.175391192	5.275612717	0.601899981	0.547240717	0.601840227	positive
PRJNA729271	stage_E18_vs_E10	ordinary_muscle_development	ENSGALG00010013569	ENSGALG00010013569	0.537730585	0.534203256	1.006602971	0.314125606	0.371633257	positive
PRJNA729271	stage_E18_vs_E10	ordinary_muscle_development	ENSGALG00010013572	DENND11	-0.057102389	0.106721644	-0.535059122	0.592608976	0.644546192	negative
PRJNA729271	stage_E18_vs_E10	ordinary_muscle_development	ENSGALG00010013573	ENSGALG00010013573	-0.41925573	0.47298218	-0.886409146	0.375397093	0.435380129	negative
PRJNA729271	stage_E18_vs_E10	ordinary_muscle_development	ENSGALG00010013574	NR2F1	-0.395539806	0.148513746	-2.663321183	0.007737352	0.012439321	negative
PRJNA729271	stage_E18_vs_E10	ordinary_muscle_development	ENSGALG00010013576	CAMK2A	0.939846905	0.185935069	5.054704902	4.31057E-07	1.15386E-06	positive
PRJNA729271	stage_E18_vs_E10	ordinary_muscle_development	ENSGALG00010013579	H2AC32	-0.974588302	0.176785177	-5.512839476	3.5309E-08	1.04386E-07	negative
PRJNA729271	stage_E18_vs_E10	ordinary_muscle_development	ENSGALG00010013580	RANBP3L	1.914201928	0.300946842	6.360598154	2.0097E-10	7.15914E-10	positive
PRJNA729271	stage_E18_vs_E10	ordinary_muscle_development	ENSGALG00010013582	MAP4K4	-0.7138065	0.107055724	-6.667616413	2.59991E-11	9.90947E-11	negative
PRJNA729271	stage_E18_vs_E10	ordinary_muscle_development	ENSGALG00010013583	FASLG	-0.440966639	0.407472035	-1.082200989	0.279163229	0.334723385	negative
PRJNA729271	stage_E18_vs_E10	ordinary_muscle_development	ENSGALG00010013587	SLC26A1	0.357855727	0.469448375	0.762289841	0.445887036	0.505366533	positive
PRJNA729271	stage_E18_vs_E10	ordinary_muscle_development	ENSGALG00010013589	DSCAM	-2.51771943	0.528367914	-4.765087663	1.88772E-06	4.73071E-06	negative
PRJNA729271	stage_E18_vs_E10	ordinary_muscle_development	ENSGALG00010013592	H3C33	-0.96106852	0.183833207	-5.227937519	1.71411E-07	4.74978E-07	negative
PRJNA729271	stage_E18_vs_E10	ordinary_muscle_development	ENSGALG00010013596	SDCBP2	0.745851629	0.120068793	6.211869153	5.2358E-10	1.81692E-09	positive
PRJNA729271	stage_E18_vs_E10	ordinary_muscle_development	ENSGALG00010013598	PAQR7	0.415943402	0.23772655	1.749671637	0.080174991	0.108428484	positive
PRJNA729271	stage_E18_vs_E10	ordinary_muscle_development	ENSGALG00010013604	FYN	-0.122688201	0.111422135	-1.101111563	0.270848105	0.325946	negative
PRJNA729271	stage_E18_vs_E10	ordinary_muscle_development	ENSGALG00010013609	PIGC	1.214349719	0.16288867	7.455090131	8.98064E-14	4.14436E-13	positive
PRJNA729271	stage_E18_vs_E10	ordinary_muscle_development	ENSGALG00010013612	TWIST2	-3.122740166	0.288241822	-10.83375114	2.38193E-27	2.55939E-26	negative
PRJNA729271	stage_E18_vs_E10	ordinary_muscle_development	ENSGALG00010013614	ATRX	-0.767675426	0.086877933	-8.836253304	9.89822E-19	6.4862E-18	negative
PRJNA729271	stage_E18_vs_E10	ordinary_muscle_development	ENSGALG00010013617	PIK3C2G	-0.30325082	0.16814138	-1.803546632	0.071302409	0.097410784	negative
PRJNA729271	stage_E18_vs_E10	ordinary_muscle_development	ENSGALG00010013619	IL1R2	0.795906359	0.19814022	4.016884393	5.89726E-05	0.000126171	positive
PRJNA729271	stage_E18_vs_E10	ordinary_muscle_development	ENSGALG00010013621	PDGFRA	0.533964745	0.164629908	3.24342491	0.001181019	0.00213213	positive
PRJNA729271	stage_E18_vs_E10	ordinary_muscle_development	ENSGALG00010013624	SST1	-8.292861281	1.740043611	-4.765892779	1.88019E-06	4.71349E-06	negative
PRJNA729271	stage_E18_vs_E10	ordinary_muscle_development	ENSGALG00010013625	SSBP1	-0.306451103	0.112733134	-2.718376502	0.006560314	0.010665293	negative
PRJNA729271	stage_E18_vs_E10	ordinary_muscle_development	ENSGALG00010013627	MYCBP	0.521932035	0.147182123	3.546164606	0.000390882	0.000753575	positive
PRJNA729271	stage_E18_vs_E10	ordinary_muscle_development	ENSGALG00010013632	SLC1A3	-3.777884171	0.514318574	-7.345416558	2.05119E-13	9.20782E-13	negative
PRJNA729271	stage_E18_vs_E10	ordinary_muscle_development	ENSGALG00010013635	BCL6	2.344975993	0.156379485	14.99541959	7.8664E-51	2.36073E-49	positive
PRJNA729271	stage_E18_vs_E10	ordinary_muscle_development	ENSGALG00010013638	COMMD8	-0.831901242	0.120101433	-6.926655396	4.30907E-12	1.75745E-11	negative
PRJNA729271	stage_E18_vs_E10	ordinary_muscle_development	ENSGALG00010013639	ENSGALG00010013639	1.008729241	0.458531445	2.199912901	0.027813075	0.040866754	positive
PRJNA729271	stage_E18_vs_E10	ordinary_muscle_development	ENSGALG00010013640	SNPH	-3.28301692	0.798532988	-4.111310328	3.9342E-05	8.56764E-05	negative
PRJNA729271	stage_E18_vs_E10	ordinary_muscle_development	ENSGALG00010013645	RRAGC	-0.731658098	0.124173397	-5.892229065	3.8102E-09	1.22432E-08	negative
PRJNA729271	stage_E18_vs_E10	ordinary_muscle_development	ENSGALG00010013646	CLDN1	0.054241812	0.33636716	0.161257753	0.871890396	0.894434349	positive
PRJNA729271	stage_E18_vs_E10	ordinary_muscle_development	ENSGALG00010013648	NAV3	-0.101749162	0.118257121	-0.860406214	0.389565162	0.449617566	negative
PRJNA729271	stage_E18_vs_E10	ordinary_muscle_development	ENSGALG00010013650	BACE2	-0.823299219	0.161656502	-5.092892695	3.52641E-07	9.52756E-07	negative
PRJNA729271	stage_E18_vs_E10	ordinary_muscle_development	ENSGALG00010013651	ENSGALG00010013651	-0.191370439	0.163628089	-1.169545156	0.242184059	0.29524457	negative
PRJNA729271	stage_E18_vs_E10	ordinary_muscle_development	ENSGALG00010013654	SLC6A7	-2.410711597	0.472846435	-5.098297073	3.42723E-07	9.27341E-07	negative
PRJNA729271	stage_E18_vs_E10	ordinary_muscle_development	ENSGALG00010013657	MAGT1	-0.613361786	0.104931286	-5.845366133	5.05455E-09	1.6053E-08	negative
PRJNA729271	stage_E18_vs_E10	ordinary_muscle_development	ENSGALG00010013661	GABRA4	-0.799968692	0.363067774	-2.203359124	0.027569447	0.040554002	negative
PRJNA729271	stage_E18_vs_E10	ordinary_muscle_development	ENSGALG00010013663	OPA1	-0.186343367	0.106916888	-1.742880574	0.081354478	0.109849386	negative
PRJNA729271	stage_E18_vs_E10	ordinary_muscle_development	ENSGALG00010013664	REV3L	0.413003308	0.096262974	4.29036513	1.7838E-05	4.03813E-05	positive

PRJNA729271	stage_E18_vs_E10	ordinary_muscle_development	ENSGALG00010013666	COX7B	0.665558289	0.143574005	4.635646198	3.55824E-06	8.64801E-06	positive
PRJNA729271	stage_E18_vs_E10	ordinary_muscle_development	ENSGALG00010013667	SOWAHA	-3.60017906	0.757100877	-4.755217128	1.98233E-06	4.95412E-06	negative
PRJNA729271	stage_E18_vs_E10	ordinary_muscle_development	ENSGALG00010013669	NPR3	-1.840409775	0.266153652	-6.914839469	4.68393E-12	1.90285E-11	negative
PRJNA729271	stage_E18_vs_E10	ordinary_muscle_development	ENSGALG00010013672	ENSGALG00010013672	-2.822384249	0.473057174	-5.96626455	2.42747E-09	7.9248E-09	negative
PRJNA729271	stage_E18_vs_E10	ordinary_muscle_development	ENSGALG00010013675	CHRNA6	1.618783428	0.368153066	4.39703911	1.09738E-05	2.54341E-05	positive
PRJNA729271	stage_E18_vs_E10	ordinary_muscle_development	ENSGALG00010013677	GABRG1	-4.352726879	0.535314626	-8.131156268	4.25215E-16	2.34086E-15	negative
PRJNA729271	stage_E18_vs_E10	ordinary_muscle_development	ENSGALG00010013678	ATP7A	-0.170037108	0.102345867	-1.661396921	0.096633756	0.128540542	negative
PRJNA729271	stage_E18_vs_E10	ordinary_muscle_development	ENSGALG00010013679	MFSD4B	0.127278135	0.21655127	0.587750585	0.556699717	0.610713872	positive
PRJNA729271	stage_E18_vs_E10	ordinary_muscle_development	ENSGALG00010013686	UQCRQ	1.786994516	0.138160169	12.93422357	2.88523E-38	5.15677E-37	positive
PRJNA729271	stage_E18_vs_E10	ordinary_muscle_development	ENSGALG00010013690	CREB3L2	-0.377199622	0.10164979	-3.7107762	0.000206625	0.000413563	negative
PRJNA729271	stage_E18_vs_E10	ordinary_muscle_development	ENSGALG00010013696	CETN3	-0.357348184	0.11133207	-3.209750649	0.001328502	0.002385017	negative
PRJNA729271	stage_E18_vs_E10	ordinary_muscle_development	ENSGALG00010013697	HDAC4	0.292088784	0.067077883	4.354472317	1.33388E-05	3.06509E-05	positive
PRJNA729271	stage_E18_vs_E10	ordinary_muscle_development	ENSGALG00010013698	TFAP2E	-6.871927816	0.955685501	-7.190574526	6.45192E-13	2.8059E-12	negative
PRJNA729271	stage_E18_vs_E10	ordinary_muscle_development	ENSGALG00010013709	PSMF1	0.472909561	0.100848167	4.689322313	2.74111E-06	6.74918E-06	positive
PRJNA729271	stage_E18_vs_E10	ordinary_muscle_development	ENSGALG00010013715	IL1R1	0.603543704	0.233740331	2.58211196	0.009819772	0.015550923	positive
PRJNA729271	stage_E18_vs_E10	ordinary_muscle_development	ENSGALG00010013719	NCDN	-0.643055228	0.142223407	-4.521444417	6.14191E-06	1.46066E-05	negative
PRJNA729271	stage_E18_vs_E10	ordinary_muscle_development	ENSGALG00010013720	SLC16A10	-0.454992544	0.191473151	-2.376273346	0.017488502	0.026641706	negative
PRJNA729271	stage_E18_vs_E10	ordinary_muscle_development	ENSGALG00010013732	FNTA	-0.296578025	0.078186228	-3.793225889	0.000148703	0.000303285	negative
PRJNA729271	stage_E18_vs_E10	ordinary_muscle_development	ENSGALG00010013737	AFF4	0.396078828	0.085095249	4.65453518	3.24712E-06	7.93176E-06	positive
PRJNA729271	stage_E18_vs_E10	ordinary_muscle_development	ENSGALG00010013740	GNPDA2	-1.006601928	0.120966401	-8.321334831	8.69782E-17	5.01292E-16	negative
PRJNA729271	stage_E18_vs_E10	ordinary_muscle_development	ENSGALG00010013742	RSPO4	0.836800385	1.110750761	0.753364675	0.451230804	0.510824001	positive
PRJNA729271	stage_E18_vs_E10	ordinary_muscle_development	ENSGALG00010013744	ENSGALG00010013744	0.158577447	0.309782527	0.511899261	0.608721515	0.659645512	positive
PRJNA729271	stage_E18_vs_E10	ordinary_muscle_development	ENSGALG00010013746	ENSGALG00010013746	0.052350068	0.086902042	0.602403201	0.54690578	0.601654469	positive
PRJNA729271	stage_E18_vs_E10	ordinary_muscle_development	ENSGALG00010013749	NELL2	-3.319801404	0.465640758	-7.129533549	1.0071E-12	4.32017E-12	negative
PRJNA729271	stage_E18_vs_E10	ordinary_muscle_development	ENSGALG00010013751	FUT10	0.018260778	0.171208902	0.106657876	0.915060402	0.929939387	positive
PRJNA729271	stage_E18_vs_E10	ordinary_muscle_development	ENSGALG00010013753	PLEKHA5	0.946479806	0.12367704	7.652833572	1.96598E-14	9.56594E-14	positive
PRJNA729271	stage_E18_vs_E10	ordinary_muscle_development	ENSGALG00010013755	ANGPT4	1.870747268	0.153663612	12.17430232	4.26021E-34	6.30764E-33	positive
PRJNA729271	stage_E18_vs_E10	ordinary_muscle_development	ENSGALG00010013757	DGKI	0.448706482	0.22012292	2.038435987	0.04150635	0.059053254	positive
PRJNA729271	stage_E18_vs_E10	ordinary_muscle_development	ENSGALG00010013758	FAM3B	-3.195620435	0.69311326	-4.61053138	4.01641E-06	9.70621E-06	negative
PRJNA729271	stage_E18_vs_E10	ordinary_muscle_development	ENSGALG00010013760	RPF2	-0.775993979	0.116166492	-6.680015603	2.38917E-11	9.13505E-11	negative
PRJNA729271	stage_E18_vs_E10	ordinary_muscle_development	ENSGALG00010013766	DNM3	0.294552449	0.107990493	2.727577588	0.006380125	0.01040386	positive
PRJNA729271	stage_E18_vs_E10	ordinary_muscle_development	ENSGALG00010013767	PGK2	0.269964108	0.172206004	1.567681161	0.11695556	0.153224046	positive
PRJNA729271	stage_E18_vs_E10	ordinary_muscle_development	ENSGALG00010013768	FAM110A	0.585572186	0.220218888	2.659046148	0.007836223	0.012584318	positive
PRJNA729271	stage_E18_vs_E10	ordinary_muscle_development	ENSGALG00010013771	PELO	-0.58194345	0.105397155	-5.521434117	3.36244E-08	9.9568E-08	negative
PRJNA729271	stage_E18_vs_E10	ordinary_muscle_development	ENSGALG00010013772	KIAA0319L	-0.009575528	0.144282992	-0.066366301	0.947086199	0.95638947	negative
PRJNA729271	stage_E18_vs_E10	ordinary_muscle_development	ENSGALG00010013775	DBX2	-1.339162742	0.370111254	-3.618270794	0.000296578	0.000581089	negative
PRJNA729271	stage_E18_vs_E10	ordinary_muscle_development	ENSGALG00010013777	CPLANE1	-0.88051592	0.095527148	-9.217441734	3.04273E-20	2.16836E-19	negative
PRJNA729271	stage_E18_vs_E10	ordinary_muscle_development	ENSGALG00010013778	ENSGALG00010013778	-0.877192632	0.664856233	-1.3193719	0.187044811	0.234108845	negative
PRJNA729271	stage_E18_vs_E10	ordinary_muscle_development	ENSGALG00010013779	SLC52A3	-1.509828896	0.287049165	-5.259826825	1.44191E-07	4.01778E-07	negative
PRJNA729271	stage_E18_vs_E10	ordinary_muscle_development	ENSGALG00010013783	NDUFA10	0.369467086	0.095856887	3.854361414	0.000116032	0.000240166	positive
PRJNA729271	stage_E18_vs_E10	ordinary_muscle_development	ENSGALG00010013784	ANO6	2.227503624	0.094146339	23.66001319	9.3099E-124	1.7698E-121	positive
PRJNA729271	stage_E18_vs_E10	ordinary_muscle_development	ENSGALG00010013787	ZCCHC10	-0.361400561	0.104934867	-3.444046487	0.000573077	0.001080624	negative
PRJNA729271	stage_E18_vs_E10	ordinary_muscle_development	ENSGALG00010013791	AEBP2	0.247767743	0.064328026	3.851629829	0.000117334	0.000242758	positive
PRJNA729271	stage_E18_vs_E10	ordinary_muscle_development	ENSGALG00010013792	FGF10	0.104767516	0.231964951	0.451652352	0.651519449	0.699694333	positive
PRJNA729271	stage_E18_vs_E10	ordinary_muscle_development	ENSGALG00010013793	METTL13	-0.935398818	0.132333137	-7.068515406	1.566E-12	6.6082E-12	negative
PRJNA729271	stage_E18_vs_E10	ordinary_muscle_development	ENSGALG00010013794	GTF3C6	-0.416340004	0.178713213	-2.329654287	0.01982443	0.029901677	negative
PRJNA729271	stage_E18_vs_E10	ordinary_muscle_development	ENSGALG00010013797	IL1RL1	0.828268274	0.318107041	2.603740779	0.009221247	0.014652705	positive
PRJNA729271	stage_E18_vs_E10	ordinary_muscle_development	ENSGALG00010013802	PYROXD1	-0.495304925	0.14695545	-3.370442711	0.000750475	0.001393739	negative
PRJNA729271	stage_E18_vs_E10	ordinary_muscle_development	ENSGALG00010013805	HSPA4	-0.592690043	0.145807755	-4.064873244	4.80586E-05	0.000103647	negative
PRJNA729271	stage_E18_vs_E10	ordinary_muscle_development	ENSGALG00010013808	PLAAT1	1.326562168	0.1370619	9.67856253	3.71908E-22	2.98991E-21	positive
PRJNA729271	stage_E18_vs_E10	ordinary_muscle_development	ENSGALG00010013809	MX1	-1.593351727	1.320234082	-1.206870621	0.227481974	0.278938832	negative
PRJNA729271	stage_E18_vs_E10	ordinary_muscle_development	ENSGALG00010013814	AMD1	0.629964463	0.109815878	5.736551718	9.66236E-09	2.99201E-08	positive
PRJNA729271	stage_E18_vs_E10	ordinary_muscle_development	ENSGALG00010013815	SRXN1	0.329917103	0.251111549	1.313826879	0.18890447	0.236212068	positive
PRJNA729271	stage_E18_vs_E10	ordinary_muscle_development	ENSGALG00010013820	MASP1	-1.712635793	0.469926969	-3.644472241	0.000267941	0.000528841	negative
PRJNA729271	stage_E18_vs_E10	ordinary_muscle_development	ENSGALG00010013823	GUF1	0.137479815	0.112851643	1.218234944	0.223134704	0.274142866	positive
PRJNA729271	stage_E18_vs_E10	ordinary_muscle_development	ENSGALG00010013828	PTN	-5.022526419	0.273992338	-18.33090102	4.6907E-75	2.82123E-73	negative
PRJNA729271	stage_E18_vs_E10	ordinary_muscle_development	ENSGALG00010013831	TCERG1	-0.477461133	0.11363426	-4.201735764	2.64876E-05	5.88231E-05	negative
PRJNA729271	stage_E18_vs_E10	ordinary_muscle_development	ENSGALG00010013833	TMPRSS2	-2.208873385	1.254833922	-1.760291418	0.078358408	0.106140093	negative
PRJNA729271	stage_E18_vs_E10	ordinary_muscle_development	ENSGALG00010013836	IL18R1	-0.434223978	0.268590339	-1.616677572	0.105947898	0.139851997	negative
PRJNA729271	stage_E18_vs_E10	ordinary_muscle_development	ENSGALG00010013837	IFI27L2	1.121879535	0.336959234	3.329422148	0.000870264	0.001599401	positive
PRJNA729271	stage_E18_vs_E10	ordinary_muscle_development	ENSGALG00010013842	CDK19	0.704476814	0.111133083	6.339037796	2.31205E-10	8.20394E-10	positive
PRJNA729271	stage_E18_vs_E10	ordinary_muscle_development	ENSGALG00010013846	YIPF7	7.286840249	0.319690094	22.79345022	5.325E-115	9.0807E-113	positive
PRJNA729271	stage_E18_vs_E10	ordinary_muscle_development	ENSGALG00010013848	ITPA	-1.327545895	0.10570944	-12.55844226	3.57298E-36	5.84581E-35	negative
PRJNA729271	stage_E18_vs_E10	ordinary_muscle_development	ENSGALG00010013857	IL18RAP	-0.77670348	0.776613472	-1.000115898	0.317254423	0.37478426	negative
PRJNA729271	stage_E18_vs_E10	ordinary_muscle_development	ENSGALG00010013859	MYOC	1.929077396	0.245296849	7.864256742	3.71295E-15	1.91459E-14	positive
PRJNA729271	stage_E18_vs_E10	ordinary_muscle_development	ENSGALG00010013861	TCF15	2.148650566	0.155987141	13.77453651	3.62703E-43	7.88214E-42	positive
PRJNA729271	stage_E18_vs_E10	ordinary_muscle_development	ENSGALG00010013866	KCTD8	-4.110492277	0.491773646	-8.358504589	6.35182E-17	3.68869E-16	negative
PRJNA729271	stage_E18_vs_E10	ordinary_muscle_development	ENSGALG00010013867	RECQL	-0.693483116	0.126687675	-5.473958805	4.40091E-08	1.28741E-07	negative
PRJNA729271	stage_E18_vs_E10	ordinary_muscle_development	ENSGALG00010013871	ENSGALG00010013871	1.245640238	0.222436511	5.599981022	2.14375E-08	6.44014E-08	positive
PRJNA729271	stage_E18_vs_E10	ordinary_muscle_development	ENSGALG00010013872	GLS2	-0.217311778	0.197805235	-1.098614901	0.271936075	0.32710321	negative
PRJNA729271	stage_E18_vs_E10	ordinary_muscle_development	ENSGALG00010013878	ARID2	0.227995487	0.081626149	2.793167267	0.005219469	0.008631627	positive
PRJNA729271	stage_E18_vs_E10	ordinary_muscle_development	ENSGALG00010013880	ZMYM4	-0.386740454	0.090004942	-4.296880224	1.73219E-05	3.9342E-05	negative
PRJNA729271	stage_E18_vs_E10	ordinary_muscle_development	ENSGALG00010013889	ENSGALG00010013889	0.327984795	0.420893301	0.779258767	0.435827298	0.495514683	positive
PRJNA729271	stage_E18_vs_E10	ordinary_muscle_development	ENSGALG00010013890	SFPQ	-1.020919938	0.072353448	-14.11017682	3.28753E-45	7.78639E-44	negative
PRJNA729271	stage_E18_vs_E10	ordinary_muscle_development	ENSGALG00010013893	CSNK2A1	-0.596637892	0.079623224	-7.493264656	6.71812E-14	3.13419E-13	negative
PRJNA729271	stage_E18_vs_E10	ordinary_muscle_development	ENSGALG00010013897	AMOT	-0.542115133	0.074864724	-7.241262703	4.44526E-13	1.95433E-12	negative
PRJNA729271	stage_E18_vs_E10	ordinary_muscle_development	ENSGALG00010013898	EMB	-2.125578565	0.298354398	-7.12434132	1.0458E-12	4.48088E-12	negative
PRJNA729271	stage_E18_vs_E10	ordinary_muscle_development	ENSGALG00010013899	WNT5B	-0.162398032	0.14232089	-1.141069532	0.253840989	0.307592805	negative
PRJNA729271	stage_E18_vs_E10	ordinary_muscle_development	ENSGALG00010013901	NFXL1	-0.808804657	0.12466644	-6.487749698	8.71279E-11	3.19241E-10	negative
PRJNA729271	stage_E18_vs_E10	ordinary_muscle_development	ENSGALG00010013908	TBC1D20	0.849294674	0.115934095	7.32566786	2.37712E-13	1.06314E-12	positive
PRJNA729271	stage_E18_vs_E10	ordinary_muscle_development	ENSGALG00010013910	ADIPOR2	0.274867778	0.208753635	1.316708943	0.187936205	0.235143313	positive
PRJNA729271	stage_E18_vs_E10	ordinary_muscle_development	ENSGALG00010013911	TNIK	0.399828721	0.148024532	2.701097688	0.006911104	0.011187899	positive
PRJNA729271	stage_E18_vs_E10	ordinary_muscle_development	ENSGALG00010013914	NRG1	2.168717838	0.226650253	9.568565688	1.08399E-21	8.53473E-21	positive
PRJNA729271	stage_E18_vs_E10	ordinary_muscle_development	ENSGALG00010013915	ENSGALG00010013915	1.814278276	0.189701721	9.563847222	1.13459E-21	8.89927E-21	positive
PRJNA729271	stage_E18_vs_E10	ordinary_muscle_development	ENSGALG00010013917	TRPC5	-0.784967811	0.506218423	-1.550650421	0.120985482	0.158060799	negative
PRJNA729271	stage_E18_vs_E10	ordinary_muscle_development	ENSGALG00010013921	PPP2R2B	1.477122563	0.104874515	14.08466649	4.71881E-45	1.10678E-43	positive
PRJNA729271	stage_E18_vs_E10	ordinary_muscle_development	ENSGALG00010013922	ENSGALG00010013922	-3.70956704	1.085721654	-3.416683295	0.00063389	0.001187417	negative
PRJNA729271	stage_E18_vs_E10	ordinary_muscle_development	ENSGALG00010013923	FMR1NB	1.139550163	0.532172136	2.141318732	0.032248344	0.046861127	positive
PRJNA729271	stage_E18_vs_E10	ordinary_muscle_development	ENSGALG00010013928	CCNO	4.748870435	0.453610693	10.4690443	1.19847E-25	1.17935E-24	positive
PRJNA729271	stage_E18_vs_E10	ordinary_muscle_development	ENSGALG00010013930	SLC22A16	4.169050992	0.302986677	13.75984924	4.44453E-43	9.60111E-42	positive
PRJNA729271	stage_E18_vs_E10	ordinary_muscle_development	ENSGALG00010013934	STAT1	0.067816096	0.28320733	0.239457419	0.81075091	0.842546204	positive
PRJNA729271	stage_E18_vs_E10	ordinary_muscle_development	ENSGALG00010013936	DLGAP3	-0.658376065	0.353885806	-1.860419531	0.062826194	0.086548724	negative
PRJNA729271	stage_E18_vs_E10	ordinary_muscle_development	ENSGALG00010013938	ENSGALG00010013938	1.119668701	0.40238374	2.78258933	0.005392702	0.008895779	positive
PRJNA729271	stage_E18_vs_E10	ordinary_muscle_development	ENSGALG00010013940	UPF3A	-0.29938799	0.240167643	-1.246579209	0.212551829	0.262833882	negative
PRJNA729271	stage_E18_vs_E10	ordinary_muscle_development	ENSGALG00010013942	CACNA2D4	1.839241458	0.482073667	3.81527054	0.000136034	0.000279056	positive
PRJNA729271	stage_E18_vs_E10	ordinary_muscle_development	ENSGALG00010013944	METTL24	-0.96761002	0.214534046	-4.510286532	6.47401E-06	1.5346E-05	negative
PRJNA729271	stage_E18_vs_E10	ordinary_muscle_development	ENSGALG00010013945	SCAF11	-0.376807287	0.077222943	-4.879473299	1.0637E-06	2.74346E-06	negative
PRJNA729271	stage_E18_vs_E10	ordinary_muscle_development	ENSGALG00010013947	ENSGALG00010013947	0.213928011	0.176915003	1.209213508	0.22658083	0.27802329	positive
PRJNA729271	stage_E18_vs_E10	ordinary_muscle_development	ENSGALG00010013949	PRRC2C	-0.927310116	0.126501351	-7.33043645	2.29404E-13	1.02662E-12	negative
PRJNA729271	stage_E18_vs_E10	ordinary_muscle_development	ENSGALG00010013953	DHX29	-0.481018493	0.082103285	-5.858699742	4.66505E-09	1.48648E-08	negative
PRJNA729271	stage_E18_vs_E10	ordinary_muscle_development	ENSGALG00010013954	LPL	1.573535494	0.334051313	4.710460443	2.47158E-06	6.11879E-06	positive
PRJNA729271	stage_E18_vs_E10	ordinary_muscle_development	ENSGALG00010013958	TUSC3	0.024046531	0.180647278	0.133113164	0.894103884	0.912421557	positive
PRJNA729271	stage_E18_vs_E10	ordinary_muscle_development	ENSGALG00010013959	STK32A	-1.934245526	0.499775084	-3.870232003	0.000108732	0.000225895	negative
PRJNA729271	stage_E18_vs_E10	ordinary_muscle_development	ENSGALG00010013964	SLC38A1	-1.3709547	0.16153208	-8.487197707	2.11679E-17	1.26999E-16	negative
PRJNA729271	stage_E18_vs_E10	ordinary_muscle_development	ENSGALG00010013965	SLC2A2	3.57280774	0.994976301	3.590847074			positive
PRJNA729271	stage_E18_vs_E10	ordinary_muscle_development	ENSGALG00010013967	MLLT3	-0.828559851	0.15637147	-5.298663827	1.16653E-07	3.28583E-07	negative
PRJNA729271	stage_E18_vs_E10	ordinary_muscle_development	ENSGALG00010013971	ENSGALG00010013971	-0.51840689	0.372266004	-1.39257113	0.163749518	0.207750483	negative
PRJNA729271	stage_E18_vs_E10	ordinary_muscle_development	ENSGALG00010013973	ENSGALG00010013973	1.010548806	0.24078367	4.196915874	2.70574E-05	6.00241E-05	positive
PRJNA729271	stage_E18_vs_E10	ordinary_muscle_development	ENSGALG00010013976	CDC40	-0.410791465	0.076541975	-5.366878312	8.01111E-08	2.289E-07	negative
PRJNA729271	stage_E18_vs_E10	ordinary_muscle_development	ENSGALG00010013977	SLC38A2	0.149852969	0.192758097	0.777414656	0.436914169	0.496555659	positive
PRJNA729271	stage_E18_vs_E10	ordinary_muscle_development	ENSGALG00010013980	FMO4	-0.106574534	0.427371517	-0.249372103	0.803072962	0.835645555	negative
PRJNA729271	stage_E18_vs_E10	ordinary_muscle_development	ENSGALG00010013981	RLF	-0.044454098	0.107937617	-0.411849909	0.680449438	0.726778763	negative
PRJNA729271	stage_E18_vs_E10	ordinary_muscle_development	ENSGALG00010013989	ENSGALG00010013989	1.532400089	0.205187712	7.468283904	8.12474E-14	3.76979E-13	positive
PRJNA729271	stage_E18_vs_E10	ordinary_muscle_development	ENSGALG00010013994	CHRM2	-3.265587816	0.452370909	-7.218828064	5.24375E-13	2.28871E-12	negative
PRJNA729271	stage_E18_vs_E10	ordinary_muscle_development	ENSGALG00010013995	REM1	2.024300883	0.207142816	9.772488941	1.47776E-22	1.21292E-21	positive
PRJNA729271	stage_E18_vs_E10	ordinary_muscle_development	ENSGALG00010014000	STAT4	3.770266315	1.034673262	3.643919732	0.000268517	0.000529833	positive
PRJNA729271	stage_E18_vs_E10	ordinary_muscle_development	ENSGALG00010014001	CDC16	-0.973622368	0.087549183	-11.12086185	9.9308E-29	1.14153E-27	negative
PRJNA729271	stage_E18_vs_E10	ordinary_muscle_development	ENSGALG00010014002	ZDHHC18	-0.467160128	0.281360278	-1.660362759	0.096841497	0.128805056	negative
PRJNA729271	stage_E18_vs_E10	ordinary_muscle_development	ENSGALG00010014010	EIF5A2	-0.760667851	0.102693118	-7.40719402	1.29E-13	5.88723E-13	negative
PRJNA729271	stage_E18_vs_E10	ordinary_muscle_development	ENSGALG00010014011	SFN	-4.163932981	0.52289097	-7.96329105	1.67523E-15	8.83962E-15	negative
PRJNA729271	stage_E18_vs_E10	ordinary_muscle_development	ENSGALG00010014014	PARP8	-0.14649845	0.205619881	-0.712472204	0.476172418	0.53467497	negative
PRJNA729271	stage_E18_vs_E10	ordinary_muscle_development	ENSGALG00010014018	DPYSL3	-1.133774391	0.238778383	-4.748228783	2.05206E-06	5.12043E-06	negative
PRJNA729271	stage_E18_vs_E10	ordinary_muscle_development	ENSGALG00010014019	IRAG2	-0.567015145	0.261065732	-2.17192483	0.029861333	0.043646267	negative
PRJNA729271	stage_E18_vs_E10	ordinary_muscle_development	ENSGALG00010014020	PDXP	-0.607511232	0.10571499	-5.746689571	9.10076E-09	2.82112E-08	negative
PRJNA729271	stage_E18_vs_E10	ordinary_muscle_development	ENSGALG00010014026	SH3BP1	0.533308213	0.391364725	1.362688508	0.172980701	0.21822065	positive
PRJNA729271	stage_E18_vs_E10	ordinary_muscle_development	ENSGALG00010014030	WASF1	-1.873661558	0.346512225	-5.407201895	6.4017E-08	1.84213E-07	negative
PRJNA729271	stage_E18_vs_E10	ordinary_muscle_development	ENSGALG00010014031	RPL22L1	0.200154992	0.18843962	1.062170432	0.288158321	0.344255737	positive
PRJNA729271	stage_E18_vs_E10	ordinary_muscle_development	ENSGALG00010014032	PSD3	-0.280034618	0.07762616	-3.607477383	0.000309188	0.000604327	negative
PRJNA729271	stage_E18_vs_E10	ordinary_muscle_development	ENSGALG00010014033	SLC38A4	0.740174472	0.478207638	1.547809807	0.121668095	0.158866682	positive
PRJNA729271	stage_E18_vs_E10	ordinary_muscle_development	ENSGALG00010014035	PSIP1	-1.642008095	0.120584388	-13.61708699	3.1696E-42	6.60106E-41	negative
PRJNA729271	stage_E18_vs_E10	ordinary_muscle_development	ENSGALG00010014041	SKIV2L2	-0.061393649	0.098708188	-0.621971186	0.533960815	0.589696153	negative

PRJNA729271	stage_E18_vs_E10	ordinary_muscle_development	ENSGALG00010014042	SGCZ	0.062593785	0.320231299	0.195464293	0.845029488	0.871731722	positive
PRJNA729271	stage_E18_vs_E10	ordinary_muscle_development	ENSGALG00010014045	GOLT1B	-1.033352338	0.110906926	-9.317293134	1.19346E-20	8.76354E-20	negative
PRJNA729271	stage_E18_vs_E10	ordinary_muscle_development	ENSGALG00010014047	HM13	-0.087457113	0.167507825	-0.522107627	0.601595396	0.652948354	negative
PRJNA729271	stage_E18_vs_E10	ordinary_muscle_development	ENSGALG00010014056	FMO3	6.039071366	0.253444171	23.82801444	1.7119E-125	3.4464E-123	positive
PRJNA729271	stage_E18_vs_E10	ordinary_muscle_development	ENSGALG00010014062	GGA1	-0.064994278	0.104857896	-0.619831993	0.535368403	0.591025514	negative
PRJNA729271	stage_E18_vs_E10	ordinary_muscle_development	ENSGALG00010014065	CHUNK-1	-1.020692128	0.678152534	-1.505107002	0.132296569	0.17130952	negative
PRJNA729271	stage_E18_vs_E10	ordinary_muscle_development	ENSGALG00010014066	BCL2L1	-0.26104712	0.117846615	-2.215143131	0.026750242	0.039457083	negative
PRJNA729271	stage_E18_vs_E10	ordinary_muscle_development	ENSGALG00010014076	PRRX1	-0.860972375	0.244764793	-3.517549914	0.00043555	0.000834983	negative
PRJNA729271	stage_E18_vs_E10	ordinary_muscle_development	ENSGALG00010014077	SLITRK2	0.942908762	0.098978859	9.526365273	1.62887E-21	1.26326E-20	positive
PRJNA729271	stage_E18_vs_E10	ordinary_muscle_development	ENSGALG00010014080	MYO1B	-0.448129601	0.160968486	-2.783958601	0.005369989	0.008860329	negative
PRJNA729271	stage_E18_vs_E10	ordinary_muscle_development	ENSGALG00010014082	HNRNPR	-0.676575114	0.04308184	-15.70441536	1.41072E-55	4.89197E-54	negative
PRJNA729271	stage_E18_vs_E10	ordinary_muscle_development	ENSGALG00010014083	ENSGALG00010014083	-0.440850359	0.145783781	-3.024001399	0.002494553	0.004318985	negative
PRJNA729271	stage_E18_vs_E10	ordinary_muscle_development	ENSGALG00010014089	FIG4	-0.047937284	0.115697945	-0.414331336	0.678631468	0.724997282	negative
PRJNA729271	stage_E18_vs_E10	ordinary_muscle_development	ENSGALG00010014091	CHIC2	0.776680069	0.098205142	7.908751572	2.59983E-15	1.35507E-14	positive
PRJNA729271	stage_E18_vs_E10	ordinary_muscle_development	ENSGALG00010014092	TYRP1	0.65630404	0.914828068	0.717406978	0.473123015	0.531868939	positive
PRJNA729271	stage_E18_vs_E10	ordinary_muscle_development	ENSGALG00010014093	PLD1	1.233280498	0.092464352	13.33790231	1.39325E-40	2.72908E-39	positive
PRJNA729271	stage_E18_vs_E10	ordinary_muscle_development	ENSGALG00010014096	JAKMIP2	-2.455955578	0.158633937	-15.48190519	4.59656E-54	1.5074E-52	negative
PRJNA729271	stage_E18_vs_E10	ordinary_muscle_development	ENSGALG00010014099	TPX2	-1.76070497	0.14383011	-12.24156035	1.86414E-34	2.80007E-33	negative
PRJNA729271	stage_E18_vs_E10	ordinary_muscle_development	ENSGALG00010014102	TRIM2	-0.748340931	0.139073123	-5.380916988	7.41074E-08	2.12206E-07	negative
PRJNA729271	stage_E18_vs_E10	ordinary_muscle_development	ENSGALG00010014104	ALDH1A1	-0.322182322	0.555532056	-0.579952711	0.561946508	0.61581724	negative
PRJNA729271	stage_E18_vs_E10	ordinary_muscle_development	ENSGALG00010014105	AHR1B	-1.570531005	0.519989379	-3.02031362	0.002525131	0.004368796	negative
PRJNA729271	stage_E18_vs_E10	ordinary_muscle_development	ENSGALG00010014109	AMIGO2	-0.885493391	0.165042531	-5.365243632	8.084E-08	2.30892E-07	negative
PRJNA729271	stage_E18_vs_E10	ordinary_muscle_development	ENSGALG00010014111	GORAB	0.281791818	0.080934349	3.481733279	0.00049818	0.000947031	positive
PRJNA729271	stage_E18_vs_E10	ordinary_muscle_development	ENSGALG00010014114	ANXA1	1.891246971	0.332296159	5.691449997	1.25965E-08	3.85691E-08	positive
PRJNA729271	stage_E18_vs_E10	ordinary_muscle_development	ENSGALG00010014120	SOX5	-1.988403391	0.135027152	-14.72595223	4.39239E-49	1.23148E-47	negative
PRJNA729271	stage_E18_vs_E10	ordinary_muscle_development	ENSGALG00010014122	ABCA1	0.298744657	0.1408426	2.12112427	0.033911346	0.049051488	positive
PRJNA729271	stage_E18_vs_E10	ordinary_muscle_development	ENSGALG00010014125	PLPP1	3.097082832	0.138785179	22.31565969	2.6037E-110	4.2406E-108	positive
PRJNA729271	stage_E18_vs_E10	ordinary_muscle_development	ENSGALG00010014128	COL4A1	2.957726958	0.144549144	20.46173973	4.72229E-93	4.75345E-91	positive
PRJNA729271	stage_E18_vs_E10	ordinary_muscle_development	ENSGALG00010014131	CDC23	-0.768303294	0.118530933	-6.481880114	9.05866E-11	3.31328E-10	negative
PRJNA729271	stage_E18_vs_E10	ordinary_muscle_development	ENSGALG00010014134	RPAP3	-0.549411024	0.115185568	-4.769790469	1.84418E-06	4.62888E-06	negative
PRJNA729271	stage_E18_vs_E10	ordinary_muscle_development	ENSGALG00010014135	ZBTB24	-0.370791824	0.158979297	-2.33232774	0.019683456	0.029713778	negative
PRJNA729271	stage_E18_vs_E10	ordinary_muscle_development	ENSGALG00010014142	PPIL6	1.39804676	0.492190278	2.840459921	0.004504853	0.00751068	positive
PRJNA729271	stage_E18_vs_E10	ordinary_muscle_development	ENSGALG00010014143	NFATC2	0.208355822	0.152717412	1.364322634	0.172466045	0.217666115	positive
PRJNA729271	stage_E18_vs_E10	ordinary_muscle_development	ENSGALG00010014145	NTMT2	4.974939708	0.251900717	19.74960522	8.08473E-87	6.85311E-85	positive
PRJNA729271	stage_E18_vs_E10	ordinary_muscle_development	ENSGALG00010014147	ENSGALG00010014147	0.016085533	0.342438066	0.046973553	0.962534306	0.969303122	positive
PRJNA729271	stage_E18_vs_E10	ordinary_muscle_development	ENSGALG00010014148	KCNG1	1.747419056	0.346600056	5.041600612	4.61654E-07	1.23122E-06	positive
PRJNA729271	stage_E18_vs_E10	ordinary_muscle_development	ENSGALG00010014149	LGALS2	1.887250763	0.539712538	3.496770278	0.000470927	0.000899353	positive
PRJNA729271	stage_E18_vs_E10	ordinary_muscle_development	ENSGALG00010014151	ATP23	-0.255408392	0.092246396	-2.768762833	0.005626958	0.009254823	negative
PRJNA729271	stage_E18_vs_E10	ordinary_muscle_development	ENSGALG00010014153	FOCAD	-0.143981561	0.132967451	-1.082833128	0.278882498	0.334442112	negative
PRJNA729271	stage_E18_vs_E10	ordinary_muscle_development	ENSGALG00010014154	DNAI7	-0.763009331	0.413329944	-1.846005455	0.064891413	0.08920723	negative
PRJNA729271	stage_E18_vs_E10	ordinary_muscle_development	ENSGALG00010014155	ENSGALG00010014155	-4.977152718	1.40846176	-3.533750691	0.000409707	0.000787941	negative
PRJNA729271	stage_E18_vs_E10	ordinary_muscle_development	ENSGALG00010014156	CD164	0.194739435	0.092303613	2.109770456	0.034878132	0.05033466	positive
PRJNA729271	stage_E18_vs_E10	ordinary_muscle_development	ENSGALG00010014159	CDC42EP1	-0.037336895	0.149617424	-0.24954911	0.802936058	0.835623073	negative
PRJNA729271	stage_E18_vs_E10	ordinary_muscle_development	ENSGALG00010014161	GFRA3	1.042611152	0.248947672	4.18807351	2.81332E-05	6.2314E-05	positive
PRJNA729271	stage_E18_vs_E10	ordinary_muscle_development	ENSGALG00010014162	COL18A1	2.568423128	0.188102776	13.65436058	1.90151E-42	4.02371E-41	positive
PRJNA729271	stage_E18_vs_E10	ordinary_muscle_development	ENSGALG00010014165	LUZP1	-0.873452346	0.118602409	-7.364541327	1.77757E-13	8.01427E-13	negative
PRJNA729271	stage_E18_vs_E10	ordinary_muscle_development	ENSGALG00010014166	FGF12	0.12654139	0.192981281	0.655718472	0.512005281	0.568742168	positive
PRJNA729271	stage_E18_vs_E10	ordinary_muscle_development	ENSGALG00010014167	SLC38A9	-0.14902422	0.110253076	-1.35165589	0.176485422	0.222177887	negative
PRJNA729271	stage_E18_vs_E10	ordinary_muscle_development	ENSGALG00010014170	CEMIP2	-1.225557122	0.108406326	-11.30521775	1.23647E-29	1.48736E-28	negative
PRJNA729271	stage_E18_vs_E10	ordinary_muscle_development	ENSGALG00010014171	DLC1	0.496754804	0.132758849	3.741783009	0.000182719	0.000368259	positive
PRJNA729271	stage_E18_vs_E10	ordinary_muscle_development	ENSGALG00010014173	MND1	-1.419560937	0.202737426	-7.001967841	2.52392E-12	1.04646E-11	negative
PRJNA729271	stage_E18_vs_E10	ordinary_muscle_development	ENSGALG00010014176	ABHD17B	-0.832252005	0.09453975	-8.803196567	1.32973E-18	8.64327E-18	negative
PRJNA729271	stage_E18_vs_E10	ordinary_muscle_development	ENSGALG00010014178	LRTM2	2.181043459	0.333701055	6.535920179	6.32196E-11	2.33947E-10	positive
PRJNA729271	stage_E18_vs_E10	ordinary_muscle_development	ENSGALG00010014179	CTBPL	-0.872650495	0.043132805	-20.23171213	5.14839E-91	4.97506E-89	negative
PRJNA729271	stage_E18_vs_E10	ordinary_muscle_development	ENSGALG00010014180	SLC19A1	-0.869130191	0.146292382	-5.941048867	2.83204E-09	9.19177E-09	negative
PRJNA729271	stage_E18_vs_E10	ordinary_muscle_development	ENSGALG00010014184	SLC44A1	0.558862554	0.13244832	4.219476353	2.4487E-05	5.46061E-05	positive
PRJNA729271	stage_E18_vs_E10	ordinary_muscle_development	ENSGALG00010014186	MOCS3	-0.368351658	0.10945218	-3.365411807	0.000764295	0.001417045	negative
PRJNA729271	stage_E18_vs_E10	ordinary_muscle_development	ENSGALG00010014189	DDX4	-0.142753217	0.494025705	-0.288959088	0.772612685	0.809295778	negative
PRJNA729271	stage_E18_vs_E10	ordinary_muscle_development	ENSGALG00010014190	HACD4	1.68495216	0.222447514	7.574605471	3.60221E-14	1.71171E-13	positive
PRJNA729271	stage_E18_vs_E10	ordinary_muscle_development	ENSGALG00010014192	KDM1A	-1.031164471	0.061445814	-16.78168795	3.32256E-63	1.50033E-61	negative
PRJNA729271	stage_E18_vs_E10	ordinary_muscle_development	ENSGALG00010014193	CARD10	-2.211299912	0.398549079	-5.548375422	2.88336E-08	8.58729E-08	negative
PRJNA729271	stage_E18_vs_E10	ordinary_muscle_development	ENSGALG00010014194	LRIG3	-0.75618156	0.09164765	-8.25096511	1.5712E-16	8.89633E-16	negative
PRJNA729271	stage_E18_vs_E10	ordinary_muscle_development	ENSGALG00010014197	SH3GL2	-1.758790797	0.341437148	-5.151140725	2.58907E-07	7.07019E-07	negative
PRJNA729271	stage_E18_vs_E10	ordinary_muscle_development	ENSGALG00010014198	KDM3B	-0.225450501	0.045420648	-4.963612645	6.91939E-07	1.81697E-06	negative
PRJNA729271	stage_E18_vs_E10	ordinary_muscle_development	ENSGALG00010014199	LNX1	1.038706977	0.221174639	4.696320433	2.6489E-06	6.53323E-06	positive
PRJNA729271	stage_E18_vs_E10	ordinary_muscle_development	ENSGALG00010014200	DCP1B	0.546217842	0.088801099	6.151025671	7.69835E-10	2.62992E-09	positive
PRJNA729271	stage_E18_vs_E10	ordinary_muscle_development	ENSGALG00010014203	TMEM38B	1.01193293	0.169384144	5.974189248	2.31237E-09	7.56098E-09	positive
PRJNA729271	stage_E18_vs_E10	ordinary_muscle_development	ENSGALG00010014204	KIFAP3	-0.3153265	0.084180827	-3.745823249	0.000179803	0.000362785	negative
PRJNA729271	stage_E18_vs_E10	ordinary_muscle_development	ENSGALG00010014205	DPM1	-0.670552626	0.086846931	-7.721085997	1.15343E-14	5.72765E-14	negative
PRJNA729271	stage_E18_vs_E10	ordinary_muscle_development	ENSGALG00010014209	CCDC50	0.210287261	0.107452203	1.957030714	0.050343857	0.070649067	positive
PRJNA729271	stage_E18_vs_E10	ordinary_muscle_development	ENSGALG00010014210	ENSGALG00010014210	0.177668884	0.25200637	0.705017432	0.480799388	0.5388269	positive
PRJNA729271	stage_E18_vs_E10	ordinary_muscle_development	ENSGALG00010014213	ETFRF1	1.170374871	0.151040538	7.748746723	9.28039E-15	4.65143E-14	positive
PRJNA729271	stage_E18_vs_E10	ordinary_muscle_development	ENSGALG00010014214	REEP2	-0.156688698	0.136062023	-1.15159759	0.249486472	0.303065197	negative
PRJNA729271	stage_E18_vs_E10	ordinary_muscle_development	ENSGALG00010014217	RASA3	0.809157903	0.109510565	7.38885699	1.48096E-13	6.72301E-13	positive
PRJNA729271	stage_E18_vs_E10	ordinary_muscle_development	ENSGALG00010014218	IL31RA	1.375411593	0.167239793	8.224188556	1.96517E-16	1.10493E-15	positive
PRJNA729271	stage_E18_vs_E10	ordinary_muscle_development	ENSGALG00010014220	C9orf85	-0.892623641	0.134956612	-6.614152694	3.73686E-11	1.4069E-10	negative
PRJNA729271	stage_E18_vs_E10	ordinary_muscle_development	ENSGALG00010014221	SCYL3	0.142670436	0.132560735	1.076264676	0.281808902	0.337421116	positive
PRJNA729271	stage_E18_vs_E10	ordinary_muscle_development	ENSGALG00010014222	TBX22	-1.104556822	0.405001107	-2.727293341	0.006385624	0.010410485	negative
PRJNA729271	stage_E18_vs_E10	ordinary_muscle_development	ENSGALG00010014224	NMUR2	-3.546267142	0.722188118	-4.91044792	9.08686E-07	2.36047E-06	negative
PRJNA729271	stage_E18_vs_E10	ordinary_muscle_development	ENSGALG00010014225	ZMPSTE24	-0.471731365	0.076946751	-6.130620959	8.75367E-10	2.97502E-09	negative
PRJNA729271	stage_E18_vs_E10	ordinary_muscle_development	ENSGALG00010014226	SLC16A7	-0.10460068	0.262445781	-0.398561102	0.690216628	0.735693067	negative
PRJNA729271	stage_E18_vs_E10	ordinary_muscle_development	ENSGALG00010014234	GLRA1	1.351424129	0.625610344	2.160169093	0.030759582	0.044868687	positive
PRJNA729271	stage_E18_vs_E10	ordinary_muscle_development	ENSGALG00010014236	WDR44	-0.052209246	0.082438234	-0.633313501	0.526528939	0.582891116	negative
PRJNA729271	stage_E18_vs_E10	ordinary_muscle_development	ENSGALG00010014238	PHLDA1	-2.406922507	0.122408422	-19.66304654	4.47057E-86	3.72419E-84	negative
PRJNA729271	stage_E18_vs_E10	ordinary_muscle_development	ENSGALG00010014240	PIGV	0.290266353	0.131435714	2.208428326	0.027214429	0.040076516	positive
PRJNA729271	stage_E18_vs_E10	ordinary_muscle_development	ENSGALG00010014241	ENSGALG00010014241	2.085474136	0.860786371	2.422754595	0.015403328	0.02372954	positive
PRJNA729271	stage_E18_vs_E10	ordinary_muscle_development	ENSGALG00010014243	ENSGALG00010014243	-0.05939365	0.145131402	-0.409240517	0.682363163	0.728715395	negative
PRJNA729271	stage_E18_vs_E10	ordinary_muscle_development	ENSGALG00010014244	SESN1	2.54903444	0.369929182	6.89060113	5.55571E-12	2.2413E-11	positive
PRJNA729271	stage_E18_vs_E10	ordinary_muscle_development	ENSGALG00010014246	KRAS	-0.992334403	0.114938803	-8.633589177	5.94563E-18	3.70515E-17	negative
PRJNA729271	stage_E18_vs_E10	ordinary_muscle_development	ENSGALG00010014249	ENSGALG00010014249	0.214698388	0.101571162	2.113773076	0.034534654	0.049878418	positive
PRJNA729271	stage_E18_vs_E10	ordinary_muscle_development	ENSGALG00010014250	ENSGALG00010014250	5.059354738	0.253120529	19.98792729	7.01498E-89	6.31566E-87	positive
PRJNA729271	stage_E18_vs_E10	ordinary_muscle_development	ENSGALG00010014257	NOP16	-1.550886899	0.131285356	-11.81309893	3.34019E-32	4.49963E-31	negative
PRJNA729271	stage_E18_vs_E10	ordinary_muscle_development	ENSGALG00010014258	ENSGALG00010014258	-0.954361422	0.562347203	-1.697103528	0.089677089	0.119969489	negative
PRJNA729271	stage_E18_vs_E10	ordinary_muscle_development	ENSGALG00010014260	ADNP	-0.370525212	0.075634754	-4.898875076	9.63869E-07	2.49665E-06	negative
PRJNA729271	stage_E18_vs_E10	ordinary_muscle_development	ENSGALG00010014263	ZNF131	-0.467572296	0.121094156	-3.861229226	0.000112818	0.000233881	negative
PRJNA729271	stage_E18_vs_E10	ordinary_muscle_development	ENSGALG00010014265	ZNF462	-0.384337959	0.13973798	-2.750418738	0.005951915	0.009749462	negative
PRJNA729271	stage_E18_vs_E10	ordinary_muscle_development	ENSGALG00010014267	SCFD2	-1.111429393	0.078020752	-14.24530486	4.79451E-46	1.19001E-44	negative
PRJNA729271	stage_E18_vs_E10	ordinary_muscle_development	ENSGALG00010014272	PARD6B	-1.034486833	0.255467904	-4.049380832	5.13533E-05	0.000110457	negative
PRJNA729271	stage_E18_vs_E10	ordinary_muscle_development	ENSGALG00010014274	FIRRM	-1.066375866	0.153073289	-6.96643991	3.25062E-12	1.33705E-11	negative
PRJNA729271	stage_E18_vs_E10	ordinary_muscle_development	ENSGALG00010014278	G3BP1	-1.191436574	0.139714031	-8.527680177	1.49312E-17	9.05932E-17	negative
PRJNA729271	stage_E18_vs_E10	ordinary_muscle_development	ENSGALG00010014284	TMEM131L	-0.537029528	0.084918441	-6.324062509	2.54775E-10	9.01601E-10	negative
PRJNA729271	stage_E18_vs_E10	ordinary_muscle_development	ENSGALG00010014288	ARMC2	-1.572984799	0.250604321	-6.276766457	3.45687E-10	1.21237E-09	negative
PRJNA729271	stage_E18_vs_E10	ordinary_muscle_development	ENSGALG00010014291	OSTN	-5.014118895	0.582694097	-8.605062104	7.62755E-18	4.70674E-17	negative
PRJNA729271	stage_E18_vs_E10	ordinary_muscle_development	ENSGALG00010014292	NIM1K	2.252524396	0.45179248	4.985750081	6.17219E-07	1.62695E-06	positive
PRJNA729271	stage_E18_vs_E10	ordinary_muscle_development	ENSGALG00010014294	TMPO	0.323010019	0.101906384	3.16967403	0.0015261	0.002720889	positive
PRJNA729271	stage_E18_vs_E10	ordinary_muscle_development	ENSGALG00010014295	CACNA1C	2.6871522	0.173048	15.52836324	2.22999E-54	7.39674E-53	positive
PRJNA729271	stage_E18_vs_E10	ordinary_muscle_development	ENSGALG00010014298	METTL18	-0.921844834	0.228231947	-4.039070096	5.36635E-05	0.000115186	negative
PRJNA729271	stage_E18_vs_E10	ordinary_muscle_development	ENSGALG00010014300	ELFN2	-2.183591926	0.585790564	-3.727598325	0.000193313	0.000388368	negative
PRJNA729271	stage_E18_vs_E10	ordinary_muscle_development	ENSGALG00010014301	ARID1A	-0.213843894	0.119897859	-1.783550563	0.074496725	0.101306879	negative
PRJNA729271	stage_E18_vs_E10	ordinary_muscle_development	ENSGALG00010014302	FOXO3	1.594732604	0.233467622	6.830637103	8.45383E-12	3.34074E-11	positive
PRJNA729271	stage_E18_vs_E10	ordinary_muscle_development	ENSGALG00010014303	GABRA2	-1.479358806	0.412224615	-3.588720211	0.000332305	0.000646719	negative
PRJNA729271	stage_E18_vs_E10	ordinary_muscle_development	ENSGALG00010014305	ARL10	-1.351343345	0.307414416	-4.395835966	1.10347E-05	2.55672E-05	negative
PRJNA729271	stage_E18_vs_E10	ordinary_muscle_development	ENSGALG00010014306	ENSGALG00010014306	-0.862001614	0.33664084	-2.560597267	0.010449241	0.016490119	negative
PRJNA729271	stage_E18_vs_E10	ordinary_muscle_development	ENSGALG00010014308	ENSGALG00010014308	0.333668276	0.204941914	1.628111448	0.103501257	0.13683425	positive
PRJNA729271	stage_E18_vs_E10	ordinary_muscle_development	ENSGALG00010014310	COL6A1	2.928496434	0.237713344	12.31944486	7.11826E-35	1.09069E-33	positive
PRJNA729271	stage_E18_vs_E10	ordinary_muscle_development	ENSGALG00010014312	KIAA1191	-0.175302312	0.084138952	-2.083485804	0.037206964	0.053423967	negative
PRJNA729271	stage_E18_vs_E10	ordinary_muscle_development	ENSGALG00010014314	ENSGALG00010014314	1.637018965	0.156204996	10.47993989	1.06812E-25	1.0525E-24	positive
PRJNA729271	stage_E18_vs_E10	ordinary_muscle_development	ENSGALG00010014317	RAD23B	-0.032349032	0.146616773	-0.220636637	0.825375372	0.854741446	negative
PRJNA729271	stage_E18_vs_E10	ordinary_muscle_development	ENSGALG00010014318	ENSGALG00010014318	-0.050385129	0.475421306	-0.105979956	0.915598255	0.930165175	negative
PRJNA729271	stage_E18_vs_E10	ordinary_muscle_development	ENSGALG00010014319	ENSGALG00010014319	0.73713552	0.11507583	6.405650234	1.4973E-10	5.38411E-10	positive
PRJNA729271	stage_E18_vs_E10	ordinary_muscle_development	ENSGALG00010014320	UTS2B	-2.010985111	0.942184107	-2.134386576	0.03281115	0.047593076	negative
PRJNA729271	stage_E18_vs_E10	ordinary_muscle_development	ENSGALG00010014323	KLF4	0.26695588	0.17763116	1.502866277	0.132873531	0.172010523	positive
PRJNA729271	stage_E18_vs_E10	ordinary_muscle_development	ENSGALG00010014324	COL4A2	2.826245245	0.142231533	19.87073599	7.29258E-88	6.40642E-86	positive
PRJNA729271	stage_E18_vs_E10	ordinary_muscle_development	ENSGALG00010014328	RASSF8	-0.647686692	0.091691681	-7.06374544	1.62074E-12	6.82725E-12	negative
PRJNA729271	stage_E18_vs_E10	ordinary_muscle_development	ENSGALG00010014329	IER5	0.434710172	0.157253185	2.764396608	0.005702818	0.009370023	positive
PRJNA729271	stage_E18_vs_E10	ordinary_muscle_development	ENSGALG00010014334	HMGCS1	-1.08433781	0.159444332	-6.800729742	1.04091E-11	4.08998E-11	negative
PRJNA729271	stage_E18_vs_E10	ordinary_muscle_development	ENSGALG00010014338	BHLHE41	2.180579226	0.218642576	9.973259865	1.99569E-23	1.71372E-22	positive
PRJNA729271	stage_E18_vs_E10	ordinary_muscle_development	ENSGALG00010014340	EFNB1	-0.767871973	0.137163985	-5.598204024	2.16584E-08	6.50379E-08	negative
PRJNA729271	stage_E18_vs_E10	ordinary_muscle_development	ENSGALG00010014344	LSG1	-0.676071585	0.088580673	-7.63226967	2.30656E-14	1.11408E-13	negative
PRJNA729271	stage_E18_vs_E10	ordinary_muscle_development	ENSGALG00010014346	AFG1L	0.471249725	0.160778845	2.931043102	0.003378259	0.005724558	positive
PRJNA729271	stage_E18_vs_E10	ordinary_muscle_development	ENSGALG00010014347	KIAA0040	0.721160344	0.38135036	1.891070309	0.058614959	0.081303716	positive

PRJNA729271	stage_E18_vs_E10	ordinary_muscle_development	ENSGALG00010014350	PTGER4	-0.899421465	0.180263683	-4.989476814	6.0543E-07	1.59733E-06	negative
PRJNA729271	stage_E18_vs_E10	ordinary_muscle_development	ENSGALG00010014355	RAB20	1.117263023	0.165593549	6.747020208	1.50912E-11	5.85983E-11	positive
PRJNA729271	stage_E18_vs_E10	ordinary_muscle_development	ENSGALG00010014359	TMEM271	-0.35677294	0.438404579	-0.813798389	0.415760458	0.47606034	negative
PRJNA729271	stage_E18_vs_E10	ordinary_muscle_development	ENSGALG00010014361	SSPN	3.230140512	0.148944706	21.68684334	2.731E-104	3.6317E-102	positive
PRJNA729271	stage_E18_vs_E10	ordinary_muscle_development	ENSGALG00010014362	NAXD	0.456363114	0.108429527	4.208845378	2.56679E-05	5.71252E-05	positive
PRJNA729271	stage_E18_vs_E10	ordinary_muscle_development	ENSGALG00010014367	OPHN1	0.99676157	0.108509756	9.18591659	4.08037E-20	2.88372E-19	positive
PRJNA729271	stage_E18_vs_E10	ordinary_muscle_development	ENSGALG00010014371	TTC33	-0.4931083	0.103799682	-4.750576214	2.02838E-06	5.06221E-06	negative
PRJNA729271	stage_E18_vs_E10	ordinary_muscle_development	ENSGALG00010014372	CCDC171	-0.421191246	0.126261719	-3.33585863	0.000850364	0.001565806	negative
PRJNA729271	stage_E18_vs_E10	ordinary_muscle_development	ENSGALG00010014375	H2BC35	-1.300168935	0.246933955	-5.265249707	1.39999E-07	3.90924E-07	negative
PRJNA729271	stage_E18_vs_E10	ordinary_muscle_development	ENSGALG00010014379	CARS2	-0.269032774	0.09050612	-2.972536834	0.002953498	0.005063987	negative
PRJNA729271	stage_E18_vs_E10	ordinary_muscle_development	ENSGALG00010014380	SNX3	0.056313464	0.117778203	0.478131459	0.632556643	0.68226122	positive
PRJNA729271	stage_E18_vs_E10	ordinary_muscle_development	ENSGALG00010014388	MFNG	1.014510619	0.244511852	4.149126552	3.33746E-05	7.32312E-05	positive
PRJNA729271	stage_E18_vs_E10	ordinary_muscle_development	ENSGALG00010014389	COL6A2	3.012295034	0.264456541	11.3905106	4.66249E-30	5.7032E-29	positive
PRJNA729271	stage_E18_vs_E10	ordinary_muscle_development	ENSGALG00010014390	SPARC	2.546162053	0.159017905	16.01179474	1.05716E-57	3.91906E-56	positive
PRJNA729271	stage_E18_vs_E10	ordinary_muscle_development	ENSGALG00010014393	ATP8A1	0.429463871	0.14589693	2.943611423	0.00324407	0.005520647	positive
PRJNA729271	stage_E18_vs_E10	ordinary_muscle_development	ENSGALG00010014396	ING1	0.349512918	0.117784007	2.967405563	0.003003245	0.005142592	positive
PRJNA729271	stage_E18_vs_E10	ordinary_muscle_development	ENSGALG00010014397	PRKAA1	-0.463453307	0.077242421	-5.99998421	1.97337E-09	6.49499E-09	negative
PRJNA729271	stage_E18_vs_E10	ordinary_muscle_development	ENSGALG00010014399	GAS6	1.614677629	0.191112739	8.448822606	2.94255E-17	1.7502E-16	positive
PRJNA729271	stage_E18_vs_E10	ordinary_muscle_development	ENSGALG00010014400	RIPOR3	-0.457611321	0.101505895	-4.508224098	6.53725E-06	1.54908E-05	negative
PRJNA729271	stage_E18_vs_E10	ordinary_muscle_development	ENSGALG00010014403	H2BC37	0.119450714	0.187939863	0.635579448	0.525050569	0.581450683	positive
PRJNA729271	stage_E18_vs_E10	ordinary_muscle_development	ENSGALG00010014407	YIPF6	-0.154603998	0.09061531	-1.706157568	0.087978747	0.117871516	negative
PRJNA729271	stage_E18_vs_E10	ordinary_muscle_development	ENSGALG00010014408	ENSGALG00010014408	-0.386683179	0.327943692	-1.179114552	0.238352565	0.291038702	negative
PRJNA729271	stage_E18_vs_E10	ordinary_muscle_development	ENSGALG00010014411	ATP13A3	-0.866953688	0.082014775	-10.57070123	4.07437E-26	4.10125E-25	negative
PRJNA729271	stage_E18_vs_E10	ordinary_muscle_development	ENSGALG00010014414	TMEM255B	2.304919728	0.479810002	4.803817591	1.55669E-06	3.9379E-06	positive
PRJNA729271	stage_E18_vs_E10	ordinary_muscle_development	ENSGALG00010014416	H2BC32	-0.863188524	0.26980199	-3.199340841	0.001377422	0.002469479	negative
PRJNA729271	stage_E18_vs_E10	ordinary_muscle_development	ENSGALG00010014418	RAC2	0.741400242	0.311687724	2.378663595	0.017375525	0.026483516	positive
PRJNA729271	stage_E18_vs_E10	ordinary_muscle_development	ENSGALG00010014427	TMEM267	-0.682810684	0.136959933	-4.985477633	6.18089E-07	1.62895E-06	negative
PRJNA729271	stage_E18_vs_E10	ordinary_muscle_development	ENSGALG00010014431	ENSGALG00010014431	0.0944414	0.157093875	0.601178118	0.547721357	0.602231744	positive
PRJNA729271	stage_E18_vs_E10	ordinary_muscle_development	ENSGALG00010014439	ENSGALG00010014439	1.910382652	0.839414855	2.275850421			positive
PRJNA729271	stage_E18_vs_E10	ordinary_muscle_development	ENSGALG00010014442	SHISA3	-2.476979863	0.72879208	-3.398746957	0.000676953	0.00126335	negative
PRJNA729271	stage_E18_vs_E10	ordinary_muscle_development	ENSGALG00010014449	ENSGALG00010014449	-0.222008274	0.154533004	-1.436639865	0.15082035	0.192951542	negative
PRJNA729271	stage_E18_vs_E10	ordinary_muscle_development	ENSGALG00010014453	NR2E1	-0.207258468	0.371087277	-0.558516771	0.576491556	0.629519745	negative
PRJNA729271	stage_E18_vs_E10	ordinary_muscle_development	ENSGALG00010014455	C4orf19	-1.989312688	0.260614734	-7.633155103	2.29077E-14	1.10682E-13	negative
PRJNA729271	stage_E18_vs_E10	ordinary_muscle_development	ENSGALG00010014465	SIMC1	0.13321038	0.161519197	0.824734037	0.409522603	0.469734125	positive
PRJNA729271	stage_E18_vs_E10	ordinary_muscle_development	ENSGALG00010014469	TMCO3	-0.54057819	0.128236684	-4.215472299	2.49256E-05	5.55584E-05	negative
PRJNA729271	stage_E18_vs_E10	ordinary_muscle_development	ENSGALG00010014470	RPS6KA1	-1.054495997	0.13039751	-8.086780134	6.12627E-16	3.34213E-15	negative
PRJNA729271	stage_E18_vs_E10	ordinary_muscle_development	ENSGALG00010014474	PTPN1	-0.289352749	0.078148147	-3.702618193	0.000213386	0.000426096	negative
PRJNA729271	stage_E18_vs_E10	ordinary_muscle_development	ENSGALG00010014476	C5orf34	0.563941734	0.175098952	3.220703072	0.001278766	0.002300001	positive
PRJNA729271	stage_E18_vs_E10	ordinary_muscle_development	ENSGALG00010014477	ITPR2	-0.347085305	0.09921542	-3.498299999	0.000468234	0.000894799	negative
PRJNA729271	stage_E18_vs_E10	ordinary_muscle_development	ENSGALG00010014482	THOC3	-1.067979148	0.085312258	-12.51847243	5.91628E-36	9.54972E-35	negative
PRJNA729271	stage_E18_vs_E10	ordinary_muscle_development	ENSGALG00010014487	OSTM1	-0.225648478	0.086208163	-2.617483892	0.008858067	0.014128266	negative
PRJNA729271	stage_E18_vs_E10	ordinary_muscle_development	ENSGALG00010014488	ENSGALG00010014488	1.870479355	0.409380838	4.569044717	4.89952E-06	1.1758E-05	positive
PRJNA729271	stage_E18_vs_E10	ordinary_muscle_development	ENSGALG00010014492	CPLX2	-2.717433762	0.386907144	-7.023477864	2.16412E-12	9.02185E-12	negative
PRJNA729271	stage_E18_vs_E10	ordinary_muscle_development	ENSGALG00010014494	KLHL9	-0.476989396	0.066989353	-7.12037621	1.07633E-12	4.60218E-12	negative
PRJNA729271	stage_E18_vs_E10	ordinary_muscle_development	ENSGALG00010014504	SEC63	-0.735799551	0.046547932	-15.80735195	2.76862E-56	9.76426E-55	negative
PRJNA729271	stage_E18_vs_E10	ordinary_muscle_development	ENSGALG00010014505	CYTH4	1.207410335	0.219677416	5.496287946	3.87869E-08	1.14202E-07	positive
PRJNA729271	stage_E18_vs_E10	ordinary_muscle_development	ENSGALG00010014512	HMGN4	-0.561496443	0.11077012	-5.06902444	3.9986E-07	1.07439E-06	negative
PRJNA729271	stage_E18_vs_E10	ordinary_muscle_development	ENSGALG00010014515	ETNK1	-1.476643689	0.113437842	-13.01720541	9.76808E-39	1.78323E-37	negative
PRJNA729271	stage_E18_vs_E10	ordinary_muscle_development	ENSGALG00010014517	DCUN1D2	1.668822979	0.127773327	13.06080871	5.51389E-39	1.01298E-37	positive
PRJNA729271	stage_E18_vs_E10	ordinary_muscle_development	ENSGALG00010014522	FAT2	-3.637760127	0.427618126	-8.507029774	1.78446E-17	1.07729E-16	negative
PRJNA729271	stage_E18_vs_E10	ordinary_muscle_development	ENSGALG00010014537	GKAP1	0.798882769	0.124111106	6.436835489	1.2199E-10	4.4173E-10	positive
PRJNA729271	stage_E18_vs_E10	ordinary_muscle_development	ENSGALG00010014539	PAIP1	-0.398260324	0.085711587	-4.646516725	3.37587E-06	8.23098E-06	negative
PRJNA729271	stage_E18_vs_E10	ordinary_muscle_development	ENSGALG00010014547	DHDDS	-0.002397195	0.107530363	-0.02229319	0.982214081	0.985319306	negative
PRJNA729271	stage_E18_vs_E10	ordinary_muscle_development	ENSGALG00010014550	SCML4	-5.019840784	0.968001012	-5.185780514	2.15112E-07	5.90987E-07	negative
PRJNA729271	stage_E18_vs_E10	ordinary_muscle_development	ENSGALG00010014555	VGLL1	-5.553925818	0.804188214	-6.906251204	4.97628E-12	2.01653E-11	negative
PRJNA729271	stage_E18_vs_E10	ordinary_muscle_development	ENSGALG00010014567	UTP11	-0.691115671	0.118391063	-5.837566229	5.29688E-09	1.67858E-08	negative
PRJNA729271	stage_E18_vs_E10	ordinary_muscle_development	ENSGALG00010014570	KIF27	-1.445806164	0.169643168	-8.52263125	1.55969E-17	9.45532E-17	negative
PRJNA729271	stage_E18_vs_E10	ordinary_muscle_development	ENSGALG00010014572	IL1RAP	0.382485087	0.124710052	3.066994848	0.002162226	0.003778246	positive
PRJNA729271	stage_E18_vs_E10	ordinary_muscle_development	ENSGALG00010014573	CEBPB	2.773975185	0.34894005	7.949718537	1.86936E-15	9.84248E-15	positive
PRJNA729271	stage_E18_vs_E10	ordinary_muscle_development	ENSGALG00010014576	HRH2	-0.013537361	0.277674623	-0.048752604	0.961116454	0.968062192	negative
PRJNA729271	stage_E18_vs_E10	ordinary_muscle_development	ENSGALG00010014578	ENSGALG00010014578	5.849543558	0.301761976	19.38462768	1.04055E-83	7.98032E-82	positive
PRJNA729271	stage_E18_vs_E10	ordinary_muscle_development	ENSGALG00010014579	NNT	0.135739646	0.144737958	0.937830323	0.348331615	0.407149969	positive
PRJNA729271	stage_E18_vs_E10	ordinary_muscle_development	ENSGALG00010014592	QNG1	-0.358166039	0.094442325	-3.792431401	0.000149179	0.000304086	negative
PRJNA729271	stage_E18_vs_E10	ordinary_muscle_development	ENSGALG00010014599	SSTR3	-2.00893241	0.442870503	-4.536162142	5.72871E-06	1.36665E-05	negative
PRJNA729271	stage_E18_vs_E10	ordinary_muscle_development	ENSGALG00010014602	FRK	-2.877552956	1.3068138	-2.201960949	0.027668067	0.040682556	negative
PRJNA729271	stage_E18_vs_E10	ordinary_muscle_development	ENSGALG00010014603	ENSGALG00010014603	3.960301853	0.221810643	17.85442664	2.67009E-71	1.46602E-69	positive
PRJNA729271	stage_E18_vs_E10	ordinary_muscle_development	ENSGALG00010014611	TMEM189	0.227171173	0.10484578	2.166717368	0.030256411	0.044188079	positive
PRJNA729271	stage_E18_vs_E10	ordinary_muscle_development	ENSGALG00010014614	GHR	1.734360948	0.09010443	19.24834277	1.45751E-82	1.089E-80	positive
PRJNA729271	stage_E18_vs_E10	ordinary_muscle_development	ENSGALG00010014617	GRTP1	1.799044093	0.202390657	8.88896808	6.1679E-19	4.09358E-18	positive
PRJNA729271	stage_E18_vs_E10	ordinary_muscle_development	ENSGALG00010014624	GPRC6A	-0.092612147	0.461901119	-0.200502106	0.841087911	0.86865474	negative
PRJNA729271	stage_E18_vs_E10	ordinary_muscle_development	ENSGALG00010014626	C2CD5	-0.368495708	0.092002183	-4.005293073	6.19406E-05	0.000132248	negative
PRJNA729271	stage_E18_vs_E10	ordinary_muscle_development	ENSGALG00010014632	NWD2	-1.071846363	0.171336015	-6.25581472	3.95446E-10	1.38187E-09	negative
PRJNA729271	stage_E18_vs_E10	ordinary_muscle_development	ENSGALG00010014634	CCDC152	2.621470809	0.452631524	5.791622253	6.97098E-09	2.18285E-08	positive
PRJNA729271	stage_E18_vs_E10	ordinary_muscle_development	ENSGALG00010014641	DOCK11	0.279338922	0.096458907	2.895937057	0.003780282	0.006366352	positive
PRJNA729271	stage_E18_vs_E10	ordinary_muscle_development	ENSGALG00010014645	SLC36A1	-0.815226427	0.132567912	-6.149500407	7.77274E-10	2.65304E-09	negative
PRJNA729271	stage_E18_vs_E10	ordinary_muscle_development	ENSGALG00010014648	C1QTNF6	0.646377203	0.274606396	2.353831569	0.018581028	0.02815219	positive
PRJNA729271	stage_E18_vs_E10	ordinary_muscle_development	ENSGALG00010014653	FAM162B	0.480529692	0.519745543	0.92454798	0.355201083	0.414577639	positive
PRJNA729271	stage_E18_vs_E10	ordinary_muscle_development	ENSGALG00010014654	LAMP1	0.617878638	0.104668851	5.903175891	3.5657E-09	1.1483E-08	positive
PRJNA729271	stage_E18_vs_E10	ordinary_muscle_development	ENSGALG00010014655	FHL3	0.296679173	0.134057751	2.213069902	0.026892827	0.039635132	positive
PRJNA729271	stage_E18_vs_E10	ordinary_muscle_development	ENSGALG00010014658	SELENOP1	2.232816987	0.173520694	12.86772736	6.83848E-38	1.20588E-36	positive
PRJNA729271	stage_E18_vs_E10	ordinary_muscle_development	ENSGALG00010014668	GP5	-0.593877495	0.162593764	-3.652523201	0.000259676	0.000513437	negative
PRJNA729271	stage_E18_vs_E10	ordinary_muscle_development	ENSGALG00010014672	ISL1	-4.405630154	1.231354105	-3.577874257	0.0003464	0.000672164	negative
PRJNA729271	stage_E18_vs_E10	ordinary_muscle_development	ENSGALG00010014676	UBE2V1	-0.665036818	0.059293877	-11.21594422	3.40533E-29	3.98387E-28	negative
PRJNA729271	stage_E18_vs_E10	ordinary_muscle_development	ENSGALG00010014677	ENSGALG00010014677	-1.008111052	0.090082457	-11.19098084	4.51412E-29	5.24717E-28	negative
PRJNA729271	stage_E18_vs_E10	ordinary_muscle_development	ENSGALG00010014682	IL13RA1	0.452921586	0.12663406	3.576617434	0.000348069	0.000675132	positive
PRJNA729271	stage_E18_vs_E10	ordinary_muscle_development	ENSGALG00010014687	CUL4A	0.564636297	0.116922649	4.829143876	1.37121E-06	3.49371E-06	positive
PRJNA729271	stage_E18_vs_E10	ordinary_muscle_development	ENSGALG00010014690	CSF3R	2.574735724	0.370541894	6.948568468	3.69011E-12	1.51139E-11	positive
PRJNA729271	stage_E18_vs_E10	ordinary_muscle_development	ENSGALG00010014691	PABIR2	0.241732578	0.054131917	4.465620104	7.98372E-06	1.87803E-05	positive
PRJNA729271	stage_E18_vs_E10	ordinary_muscle_development	ENSGALG00010014696	SPINK5	0.439398562	0.632699403	0.694482341	0.487379771	0.545358174	positive
PRJNA729271	stage_E18_vs_E10	ordinary_muscle_development	ENSGALG00010014697	ENSGALG00010014697	0.318322772	0.718756666	0.44287975	0.657852715	0.705868318	positive
PRJNA729271	stage_E18_vs_E10	ordinary_muscle_development	ENSGALG00010014702	ENSGALG00010014702	0.707066214	0.269349727	2.625086064	0.008662707	0.013839517	positive
PRJNA729271	stage_E18_vs_E10	ordinary_muscle_development	ENSGALG00010014703	PCID2	-0.540447662	0.080329438	-6.72789052	1.7214E-11	6.63787E-11	negative
PRJNA729271	stage_E18_vs_E10	ordinary_muscle_development	ENSGALG00010014705	ARAP2	-0.980762754	0.222116899	-4.415525151	1.00765E-05	2.3452E-05	negative
PRJNA729271	stage_E18_vs_E10	ordinary_muscle_development	ENSGALG00010014707	KPNA5	0.371543935	0.13017315	2.854228657	0.004314147	0.007211805	positive
PRJNA729271	stage_E18_vs_E10	ordinary_muscle_development	ENSGALG00010014710	NPEPL1	-0.009171157	0.100720153	-0.091055831	0.927448229	0.940687291	negative
PRJNA729271	stage_E18_vs_E10	ordinary_muscle_development	ENSGALG00010014713	ST8SIA1	-2.592772664	0.138136042	-18.76970425	1.33627E-78	8.88499E-77	negative
PRJNA729271	stage_E18_vs_E10	ordinary_muscle_development	ENSGALG00010014716	TMEM35A	-1.695147786	0.128414325	-13.20061287	8.70241E-40	1.65758E-38	negative
PRJNA729271	stage_E18_vs_E10	ordinary_muscle_development	ENSGALG00010014717	ELOVL7	1.134347645	0.18569841	6.108547966	1.00542E-09	3.39077E-09	positive
PRJNA729271	stage_E18_vs_E10	ordinary_muscle_development	ENSGALG00010014719	ENSGALG00010014719	4.281237334	0.869556056	4.923474805	8.50208E-07	2.21611E-06	positive
PRJNA729271	stage_E18_vs_E10	ordinary_muscle_development	ENSGALG00010014722	KCTD17	-1.620353364	0.385681631	-4.20127181	2.6542E-05	5.89257E-05	negative
PRJNA729271	stage_E18_vs_E10	ordinary_muscle_development	ENSGALG00010014726	PWP2	-1.637836423	0.064347739	-25.4528976	6.5569E-143	1.9008E-140	negative
PRJNA729271	stage_E18_vs_E10	ordinary_muscle_development	ENSGALG00010014728	INTS13	-0.641376076	0.112661887	-5.692928556	1.24879E-08	3.82527E-08	negative
PRJNA729271	stage_E18_vs_E10	ordinary_muscle_development	ENSGALG00010014729	MRPS15	0.057829392	0.096154377	0.601422357	0.547558711	0.602098583	positive
PRJNA729271	stage_E18_vs_E10	ordinary_muscle_development	ENSGALG00010014734	ZUP1	-0.697550347	0.101481076	-6.873698746	6.25582E-12	2.50492E-11	negative
PRJNA729271	stage_E18_vs_E10	ordinary_muscle_development	ENSGALG00010014735	STX16	-0.330657979	0.106224026	-3.112836051	0.001852989	0.003271906	negative
PRJNA729271	stage_E18_vs_E10	ordinary_muscle_development	ENSGALG00010014736	KIF2A	-1.158595528	0.090089786	-12.86045382	7.51343E-38	1.32169E-36	negative
PRJNA729271	stage_E18_vs_E10	ordinary_muscle_development	ENSGALG00010014737	RMI1	-1.22183284	0.119669888	-10.21002744	1.78814E-24	1.64149E-23	negative
PRJNA729271	stage_E18_vs_E10	ordinary_muscle_development	ENSGALG00010014738	BRWD1	0.028806346	0.087122591	0.330641524	0.740915277	0.781741661	positive
PRJNA729271	stage_E18_vs_E10	ordinary_muscle_development	ENSGALG00010014739	TRMT2B	0.180422749	0.180196191	1.001257285	0.316702438	0.374163711	positive
PRJNA729271	stage_E18_vs_E10	ordinary_muscle_development	ENSGALG00010014740	TRMT9B	-2.396449374	0.487635712	-4.914425492	8.90432E-07	2.31803E-06	negative
PRJNA729271	stage_E18_vs_E10	ordinary_muscle_development	ENSGALG00010014742	PROZ	20.31001537	2.849239682	7.128222837	1.01673E-12	4.36021E-12	positive
PRJNA729271	stage_E18_vs_E10	ordinary_muscle_development	ENSGALG00010014744	IL2RB	1.05322813	0.50351509	2.091750874	0.036460807	0.052425295	positive
PRJNA729271	stage_E18_vs_E10	ordinary_muscle_development	ENSGALG00010014745	LSM10	1.022030235	0.185848584	5.499262952	3.81382E-08	1.12406E-07	positive
PRJNA729271	stage_E18_vs_E10	ordinary_muscle_development	ENSGALG00010014748	FGFR1OP2	-0.496681695	0.11090628	-4.478391083	7.52077E-06	1.77344E-05	negative
PRJNA729271	stage_E18_vs_E10	ordinary_muscle_development	ENSGALG00010014750	LONRF1	-0.607991314	0.186164284	-3.265885915	0.001091222	0.001979137	negative
PRJNA729271	stage_E18_vs_E10	ordinary_muscle_development	ENSGALG00010014753	ENSGALG00010014753	-2.829383212	0.491430394	-5.757444489	8.53968E-09	2.65571E-08	negative
PRJNA729271	stage_E18_vs_E10	ordinary_muscle_development	ENSGALG00010014754	DIMT1	-0.934880582	0.145553662	-6.42292725	1.33678E-10	4.82607E-10	negative
PRJNA729271	stage_E18_vs_E10	ordinary_muscle_development	ENSGALG00010014758	FXN	-0.750123573	0.10287861	-7.29134634	3.06873E-13	1.36404E-12	negative
PRJNA729271	stage_E18_vs_E10	ordinary_muscle_development	ENSGALG00010014762	STK40	-0.668557925	0.106753263	-6.262646296	3.78498E-10	1.32392E-09	negative
PRJNA729271	stage_E18_vs_E10	ordinary_muscle_development	ENSGALG00010014764	ENSGALG00010014764	10.01677958	2.254989263	4.442052008	8.9105E-06	2.08487E-05	positive
PRJNA729271	stage_E18_vs_E10	ordinary_muscle_development	ENSGALG00010014766	F10	0.76337258	0.212983877	3.584180131	0.000338139	0.000656925	positive
PRJNA729271	stage_E18_vs_E10	ordinary_muscle_development	ENSGALG00010014767	LRRC15	2.481732027	0.243680442	10.18437099	2.32861E-24	2.12151E-23	positive
PRJNA729271	stage_E18_vs_E10	ordinary_muscle_development	ENSGALG00010014769	CCDC69	2.687063327	0.321238127	8.364708605	6.02637E-17	3.50953E-16	positive
PRJNA729271	stage_E18_vs_E10	ordinary_muscle_development	ENSGALG00010014777	APCDD1L	-2.026660196	0.226744763	-8.938068371	3.96032E-19	2.65517E-18	negative
PRJNA729271	stage_E18_vs_E10	ordinary_muscle_development	ENSGALG00010014780	EVA1B	0.261999155	0.125984115	2.079620558	0.03756035	0.05390468	positive
PRJNA729271	stage_E18_vs_E10	ordinary_muscle_development	ENSGALG00010014783	ENSGALG00010014783	-0.205242547	0.11998475	-1.710571944	0.087160162	0.116850402	negative
PRJNA729271	stage_E18_vs_E10	ordinary_muscle_development	ENSGALG00010014789	F7	0.021735868	0.925963967	0.023473773	0.981272359	0.984737111	positive
PRJNA729271	stage_E18_vs_E10	ordinary_muscle_development	ENSGALG00010014792	ENSGALG00010014792	1.351320749	0.270973309	4.986914599	6.13512E-07	1.61776E-06	positive
PRJNA729271	stage_E18_vs_E10	ordinary_muscle_development	ENSGALG00010014793	TLR2A	0.49002113	0.176132661	2.782113927	0.005400608	0.008907807	positive
PRJNA729271	stage_E18_vs_E10	ordinary_muscle_development	ENSGALG00010014795	THRAP3	-0.299473373	0.095432285	-3.138071916	0.001700631	0.003017216	negative
PRJNA729271	stage_E18_vs_E10	ordinary_muscle_development	ENSGALG00010014796	PRLR	-0.418259339	0.248500553	-1.68313243	0.092349458	0.123294224	negative

PRJNA729271	stage_E18_vs_E10	ordinary_muscle_development	ENSGALG00010014797	ZDHHC9	0.587405722	0.091931482	6.389603565	1.66316E-10	5.96279E-10	positive
PRJNA729271	stage_E18_vs_E10	ordinary_muscle_development	ENSGALG00010014801	MPST	-0.131269373	0.087082058	-1.507421576	0.131702632	0.170616646	negative
PRJNA729271	stage_E18_vs_E10	ordinary_muscle_development	ENSGALG00010014802	ENSGALG00010014802	0.545060016	0.394608919	1.381266336	0.167197085	0.211550432	positive
PRJNA729271	stage_E18_vs_E10	ordinary_muscle_development	ENSGALG00010014805	SPEF2	-0.85749012	0.363117332	-2.361468443	0.018202721	0.027631002	negative
PRJNA729271	stage_E18_vs_E10	ordinary_muscle_development	ENSGALG00010014806	MAP7D1	0.809559854	0.2274778	3.558852132	0.000372479	0.000720166	positive
PRJNA729271	stage_E18_vs_E10	ordinary_muscle_development	ENSGALG00010014807	HES1	-0.225232355	0.225899065	-0.997048639	0.318740902	0.376417579	negative
PRJNA729271	stage_E18_vs_E10	ordinary_muscle_development	ENSGALG00010014810	ENTREP1	1.305427872	0.227576593	5.736213258	9.68167E-09	2.99607E-08	positive
PRJNA729271	stage_E18_vs_E10	ordinary_muscle_development	ENSGALG00010014812	RWDD1	-1.180558065	0.086997359	-13.57004483	6.02852E-42	1.24655E-40	negative
PRJNA729271	stage_E18_vs_E10	ordinary_muscle_development	ENSGALG00010014813	SASH3	0.17526995	0.287837054	0.608920731	0.542576982	0.597572628	positive
PRJNA729271	stage_E18_vs_E10	ordinary_muscle_development	ENSGALG00010014816	TST	0.237981212	0.109285446	2.177611204	0.029434995	0.043062197	positive
PRJNA729271	stage_E18_vs_E10	ordinary_muscle_development	ENSGALG00010014818	ENSGALG00010014818	0.593794243	0.440443941	1.348172124	0.177603028	0.223526604	positive
PRJNA729271	stage_E18_vs_E10	ordinary_muscle_development	ENSGALG00010014821	C3AR1L	1.594340213	0.230850662	6.906370554	4.9721E-12	2.01577E-11	positive
PRJNA729271	stage_E18_vs_E10	ordinary_muscle_development	ENSGALG00010014824	ANXA6	0.845808201	0.09058892	9.336773241	9.93147E-21	7.33724E-20	positive
PRJNA729271	stage_E18_vs_E10	ordinary_muscle_development	ENSGALG00010014829	ENSGALG00010014829	1.2366974	0.419647354	2.946992013	0.003208815	0.005468074	positive
PRJNA729271	stage_E18_vs_E10	ordinary_muscle_development	ENSGALG00010014830	TRAPPC3L	-1.274299139	0.434729766	-2.931244279	0.003376072	0.005722189	negative
PRJNA729271	stage_E18_vs_E10	ordinary_muscle_development	ENSGALG00010014831	VAPB	0.424795098	0.060562618	7.014146918	2.31356E-12	9.61717E-12	positive
PRJNA729271	stage_E18_vs_E10	ordinary_muscle_development	ENSGALG00010014837	APBA1	0.247211583	0.111678003	2.213610344	0.026855595	0.03958831	positive
PRJNA729271	stage_E18_vs_E10	ordinary_muscle_development	ENSGALG00010014839	TM7SF3	0.367769302	0.110030749	3.342422951	0.000830504	0.001531182	positive
PRJNA729271	stage_E18_vs_E10	ordinary_muscle_development	ENSGALG00010014840	NCF4	0.167487357	0.224701626	0.745376702	0.456044024	0.515628559	positive
PRJNA729271	stage_E18_vs_E10	ordinary_muscle_development	ENSGALG00010014847	PAICS	-1.432217131	0.092417954	-15.49717414	3.62488E-54	1.19144E-52	negative
PRJNA729271	stage_E18_vs_E10	ordinary_muscle_development	ENSGALG00010014851	CALHM4	0.87722371	0.443409878	1.978358519	0.047888276	0.067451225	positive
PRJNA729271	stage_E18_vs_E10	ordinary_muscle_development	ENSGALG00010014856	MMGT1	0.10149752	0.072491292	1.400133974	0.161473203	0.20518577	positive
PRJNA729271	stage_E18_vs_E10	ordinary_muscle_development	ENSGALG00010014859	TRAPPC3	-0.461717733	0.129171032	-3.574468093	0.000350941	0.00068052	negative
PRJNA729271	stage_E18_vs_E10	ordinary_muscle_development	ENSGALG00010014861	MED21	-0.287164128	0.097447243	-2.946867656	0.003210106	0.005469027	negative
PRJNA729271	stage_E18_vs_E10	ordinary_muscle_development	ENSGALG00010014862	ENSGALG00010014862	0.747513422	0.306490621	2.438943875	0.014730256	0.022769709	positive
PRJNA729271	stage_E18_vs_E10	ordinary_muscle_development	ENSGALG00010014865	TLR2	1.201842173	0.380024145	3.162541614	0.001563984	0.002782613	positive
PRJNA729271	stage_E18_vs_E10	ordinary_muscle_development	ENSGALG00010014866	FAM26E	0.467344163	0.149472847	3.126615797	0.001768309	0.003131155	positive
PRJNA729271	stage_E18_vs_E10	ordinary_muscle_development	ENSGALG00010014867	TP63	-0.28322223	0.272650929	-1.038772289	0.298910652	0.355869396	negative
PRJNA729271	stage_E18_vs_E10	ordinary_muscle_development	ENSGALG00010014870	ENSGALG00010014870	-0.286626012	2.0675433	-0.138631202	0.889741586	0.908929755	negative
PRJNA729271	stage_E18_vs_E10	ordinary_muscle_development	ENSGALG00010014873	CALHM6	-1.353529667	0.820324185	-1.649993614	0.098944242	0.131336702	negative
PRJNA729271	stage_E18_vs_E10	ordinary_muscle_development	ENSGALG00010014876	PCNT	-0.259341712	0.103550274	-2.504500482	0.012262444	0.019149335	negative
PRJNA729271	stage_E18_vs_E10	ordinary_muscle_development	ENSGALG00010014877	ENSGALG00010014877	-0.19372656	0.091233986	-2.123403438	0.033720059	0.04880402	negative
PRJNA729271	stage_E18_vs_E10	ordinary_muscle_development	ENSGALG00010014878	TMCO1	0.156744365	0.131460689	1.192328792	0.233132349	0.285265356	positive
PRJNA729271	stage_E18_vs_E10	ordinary_muscle_development	ENSGALG00010014880	SFRP2	-0.506530092	0.292584121	-1.731228923	0.083410943	0.112322707	negative
PRJNA729271	stage_E18_vs_E10	ordinary_muscle_development	ENSGALG00010014881	ENSGALG00010014881	1.164690461	0.407144947	2.860628557	0.004228021	0.007076809	positive
PRJNA729271	stage_E18_vs_E10	ordinary_muscle_development	ENSGALG00010014882	XPNPEP2	1.986136396	0.583767372	3.402273734	0.000668277	0.001248926	positive
PRJNA729271	stage_E18_vs_E10	ordinary_muscle_development	ENSGALG00010014883	PPAT	-1.622797189	0.1250111	-12.98122482	1.5636E-38	2.82951E-37	negative
PRJNA729271	stage_E18_vs_E10	ordinary_muscle_development	ENSGALG00010014886	RABGAP1L	0.384357364	0.07032365	5.465549135	4.61476E-08	1.34698E-07	positive
PRJNA729271	stage_E18_vs_E10	ordinary_muscle_development	ENSGALG00010014887	MTMR12	0.001577905	0.13199377	0.011954392	0.990462002	0.991693495	positive
PRJNA729271	stage_E18_vs_E10	ordinary_muscle_development	ENSGALG00010014889	PVALB	-1.175949193	0.47172803	-2.492854183	0.012672088	0.019757116	negative
PRJNA729271	stage_E18_vs_E10	ordinary_muscle_development	ENSGALG00010014893	SLC9A6	-0.209991378	0.090045741	-2.332052305	0.01969794	0.029732546	negative
PRJNA729271	stage_E18_vs_E10	ordinary_muscle_development	ENSGALG00010014894	RAB22A	-0.291286199	0.080866112	-3.602079923	0.000315681	0.000615855	negative
PRJNA729271	stage_E18_vs_E10	ordinary_muscle_development	ENSGALG00010014897	COL8A2	-1.924088793	0.249498023	-7.71183984	1.24017E-14	6.14362E-14	negative
PRJNA729271	stage_E18_vs_E10	ordinary_muscle_development	ENSGALG00010014898	ENSGALG00010014898	3.442623087	0.632996437	5.438613686	5.36967E-08	1.55636E-07	positive
PRJNA729271	stage_E18_vs_E10	ordinary_muscle_development	ENSGALG00010014900	UBLCP1	-0.736202803	0.086126377	-8.547936543	1.25309E-17	7.651E-17	negative
PRJNA729271	stage_E18_vs_E10	ordinary_muscle_development	ENSGALG00010014902	AASDH	0.273739942	0.126631177	2.161710468	0.0306405	0.044699818	positive
PRJNA729271	stage_E18_vs_E10	ordinary_muscle_development	ENSGALG00010014903	MCF2L	0.840026632	0.154746289	5.428412136	5.68576E-08	1.64304E-07	positive
PRJNA729271	stage_E18_vs_E10	ordinary_muscle_development	ENSGALG00010014906	IFT27	-0.021352247	0.108881962	-0.196104541	0.844528342	0.871462826	negative
PRJNA729271	stage_E18_vs_E10	ordinary_muscle_development	ENSGALG00010014907	ENSGALG00010014907	-0.464418541	0.071018434	-6.539408366	6.17627E-11	2.28847E-10	negative
PRJNA729271	stage_E18_vs_E10	ordinary_muscle_development	ENSGALG00010014908	ENSGALG00010014908	-0.100008731	0.101912041	-0.981323991	0.326432996	0.384530376	negative
PRJNA729271	stage_E18_vs_E10	ordinary_muscle_development	ENSGALG00010014909	FBXL14	-0.429551324	0.099621651	-4.311827016	1.61911E-05	3.6887E-05	negative
PRJNA729271	stage_E18_vs_E10	ordinary_muscle_development	ENSGALG00010014912	ENSGALG00010014912	1.938915243	0.326276602	5.942550677	2.80621E-09	9.11201E-09	positive
PRJNA729271	stage_E18_vs_E10	ordinary_muscle_development	ENSGALG00010014914	PTAR1	-0.050203176	0.182153697	-0.275608877	0.782848491	0.817947731	negative
PRJNA729271	stage_E18_vs_E10	ordinary_muscle_development	ENSGALG00010014916	DYNLT2B	-0.714781221	0.162115486	-4.409086629	1.03807E-05	2.41446E-05	negative
PRJNA729271	stage_E18_vs_E10	ordinary_muscle_development	ENSGALG00010014917	EIF3D	0.8673117	0.104962393	8.263070924	1.41972E-16	8.06696E-16	positive
PRJNA729271	stage_E18_vs_E10	ordinary_muscle_development	ENSGALG00010014918	PLRG1	-0.581102976	0.084579078	-6.870528648	6.39644E-12	2.55911E-11	negative
PRJNA729271	stage_E18_vs_E10	ordinary_muscle_development	ENSGALG00010014922	MAN1A1	-0.709148225	0.166092541	-4.269597055	1.95826E-05	4.41584E-05	negative
PRJNA729271	stage_E18_vs_E10	ordinary_muscle_development	ENSGALG00010014923	CIMIP1	-1.813453329	0.390591478	-4.64283895	3.43654E-06	8.36345E-06	negative
PRJNA729271	stage_E18_vs_E10	ordinary_muscle_development	ENSGALG00010014928	SUCO	0.390723137	0.080257355	4.868377971	1.12518E-06	2.89242E-06	positive
PRJNA729271	stage_E18_vs_E10	ordinary_muscle_development	ENSGALG00010014929	FHL1	4.408113487	0.284758353	15.48019028	4.72074E-54	1.54463E-52	positive
PRJNA729271	stage_E18_vs_E10	ordinary_muscle_development	ENSGALG00010014931	RNF145	-0.327736137	0.109008502	-3.006519054	0.002642575	0.004562187	negative
PRJNA729271	stage_E18_vs_E10	ordinary_muscle_development	ENSGALG00010014934	MAMDC2	-0.055571998	0.205837565	-0.269979863	0.787175746	0.821639838	negative
PRJNA729271	stage_E18_vs_E10	ordinary_muscle_development	ENSGALG00010014935	PCYT1A	0.613745957	0.131534354	4.666050645	3.07044E-06	7.52045E-06	positive
PRJNA729271	stage_E18_vs_E10	ordinary_muscle_development	ENSGALG00010014936	FOXRED2	-0.239209036	0.170991382	-1.398953755	0.161826851	0.205563071	negative
PRJNA729271	stage_E18_vs_E10	ordinary_muscle_development	ENSGALG00010014940	VAMP4	-0.177280877	0.086373854	-2.052483124	0.040122733	0.057287138	negative
PRJNA729271	stage_E18_vs_E10	ordinary_muscle_development	ENSGALG00010014942	PMEPA1	-0.821086115	0.145598204	-5.639397259	1.70646E-08	5.16824E-08	negative
PRJNA729271	stage_E18_vs_E10	ordinary_muscle_development	ENSGALG00010014943	APLN	0.876431291	0.301608682	2.905855648	0.003662504	0.006179489	positive
PRJNA729271	stage_E18_vs_E10	ordinary_muscle_development	ENSGALG00010014944	FGB	3.356731193	0.914600576	3.670160812			positive
PRJNA729271	stage_E18_vs_E10	ordinary_muscle_development	ENSGALG00010014945	TXN2	0.489470767	0.104783035	4.671278786	2.9933E-06	7.33772E-06	positive
PRJNA729271	stage_E18_vs_E10	ordinary_muscle_development	ENSGALG00010014946	PCK1	4.345260174	1.040574757	4.175827009	2.96905E-05	6.54945E-05	positive
PRJNA729271	stage_E18_vs_E10	ordinary_muscle_development	ENSGALG00010014948	RBM38	2.220866081	0.259297577	8.564931863	1.0814E-17	6.62785E-17	positive
PRJNA729271	stage_E18_vs_E10	ordinary_muscle_development	ENSGALG00010014949	AGO3	-0.105271463	0.109473673	-0.961614424	0.336243324	0.39464348	negative
PRJNA729271	stage_E18_vs_E10	ordinary_muscle_development	ENSGALG00010014952	C21orf58	-0.504558229	0.58598549	-0.861042189	0.389214807	0.449356323	negative
PRJNA729271	stage_E18_vs_E10	ordinary_muscle_development	ENSGALG00010014953	ENSGALG00010014953	0.141604291	0.400194159	0.353838974	0.723459563	0.765944516	positive
PRJNA729271	stage_E18_vs_E10	ordinary_muscle_development	ENSGALG00010014956	FGG	6.472816511	1.523206604	4.249467204	2.1428E-05	4.80951E-05	positive
PRJNA729271	stage_E18_vs_E10	ordinary_muscle_development	ENSGALG00010014965	ABHD13	-0.100283478	0.099554642	-1.007320974	0.313780554	0.371285643	negative
PRJNA729271	stage_E18_vs_E10	ordinary_muscle_development	ENSGALG00010014967	HNRNPH1	-0.778187861	0.107770453	-7.22079047	5.16862E-13	2.25864E-12	negative
PRJNA729271	stage_E18_vs_E10	ordinary_muscle_development	ENSGALG00010014970	SLC51A	0.115963227	0.54361931	0.213316976	0.831079733	0.859666077	positive
PRJNA729271	stage_E18_vs_E10	ordinary_muscle_development	ENSGALG00010014971	SMC5	-0.349717123	0.125328519	-2.790403373	0.005264241	0.008699712	negative
PRJNA729271	stage_E18_vs_E10	ordinary_muscle_development	ENSGALG00010014972	FGA	5.726756945	1.656595336	3.45694378	0.000546339	0.001032622	positive
PRJNA729271	stage_E18_vs_E10	ordinary_muscle_development	ENSGALG00010014974	LIG4	-0.083684879	0.114034878	-0.733853368	0.463038111	0.522273552	negative
PRJNA729271	stage_E18_vs_E10	ordinary_muscle_development	ENSGALG00010014976	ITGB2	0.942368494	0.214759144	4.388025001	1.14385E-05	2.64603E-05	positive
PRJNA729271	stage_E18_vs_E10	ordinary_muscle_development	ENSGALG00010014980	ENSGALG00010014980	2.286621572	0.727287232	3.144041956	0.001666315	0.002958144	positive
PRJNA729271	stage_E18_vs_E10	ordinary_muscle_development	ENSGALG00010014982	ENSGALG00010014982	-0.028593383	0.373169615	-0.076623021	0.938923445	0.949999674	negative
PRJNA729271	stage_E18_vs_E10	ordinary_muscle_development	ENSGALG00010014984	LPP	0.926521012	0.092178511	10.05137754	9.05921E-24	7.93433E-23	positive
PRJNA729271	stage_E18_vs_E10	ordinary_muscle_development	ENSGALG00010014985	ENSGALG00010014985	-0.030560381	0.108559023	-0.281509364	0.778319747	0.81409617	negative
PRJNA729271	stage_E18_vs_E10	ordinary_muscle_development	ENSGALG00010014987	KLF9	3.877340204	0.296296649	13.08600763	3.95875E-39	7.3285E-38	positive
PRJNA729271	stage_E18_vs_E10	ordinary_muscle_development	ENSGALG00010014990	AGO1	-0.856491194	0.048128974	-17.7957501	7.62397E-71	4.15449E-69	negative
PRJNA729271	stage_E18_vs_E10	ordinary_muscle_development	ENSGALG00010014992	ARL15	-0.141573019	0.096771986	-1.462954569	0.143479811	0.184406797	negative
PRJNA729271	stage_E18_vs_E10	ordinary_muscle_development	ENSGALG00010014994	ENSGALG00010014994	0.600205372	0.118960874	5.045401517	4.5257E-07	1.2081E-06	positive
PRJNA729271	stage_E18_vs_E10	ordinary_muscle_development	ENSGALG00010014995	LRAT	-0.726058745	0.247523259	-2.933295034	0.00335385	0.005688516	negative
PRJNA729271	stage_E18_vs_E10	ordinary_muscle_development	ENSGALG00010014997	RAE1	-0.752803877	0.090672225	-8.302474956	1.01965E-16	5.85106E-16	negative
PRJNA729271	stage_E18_vs_E10	ordinary_muscle_development	ENSGALG00010014998	NALF1	-0.25967844	0.506596288	-0.51259444	0.608235043	0.659167622	negative
PRJNA729271	stage_E18_vs_E10	ordinary_muscle_development	ENSGALG00010015002	ADAM19	0.861011822	0.199762011	4.310187988	1.63116E-05	3.71347E-05	positive
PRJNA729271	stage_E18_vs_E10	ordinary_muscle_development	ENSGALG00010015006	HSPB3	4.704318184	0.329387604	14.28201345	2.83297E-46	7.10447E-45	positive
PRJNA729271	stage_E18_vs_E10	ordinary_muscle_development	ENSGALG00010015009	AGO4	0.053666718	0.105005165	0.511086454	0.609290521	0.660114067	positive
PRJNA729271	stage_E18_vs_E10	ordinary_muscle_development	ENSGALG00010015013	SNX18	-0.67175642	0.10986003	-6.11465717	9.67648E-10	3.271E-09	negative
PRJNA729271	stage_E18_vs_E10	ordinary_muscle_development	ENSGALG00010015016	ARGLU1	0.006975595	0.08003959	0.087151804	0.930550849	0.943174222	positive
PRJNA729271	stage_E18_vs_E10	ordinary_muscle_development	ENSGALG00010015017	FAM184A	-0.125066244	0.192693871	-0.649041113	0.516311802	0.573130615	negative
PRJNA729271	stage_E18_vs_E10	ordinary_muscle_development	ENSGALG00010015018	MAP7D3	-0.211716716	0.069225151	-3.058378556	0.002225383	0.0038775	negative
PRJNA729271	stage_E18_vs_E10	ordinary_muscle_development	ENSGALG00010015020	NIPAL4	2.865748889	0.722617304	3.965790567	7.31531E-05	0.000154864	positive
PRJNA729271	stage_E18_vs_E10	ordinary_muscle_development	ENSGALG00010015021	RBM46	-3.698180391	1.07088971	-3.453371861	0.000553625	0.00104544	negative
PRJNA729271	stage_E18_vs_E10	ordinary_muscle_development	ENSGALG00010015024	CRACD	-2.047126596	0.392538201	-5.215101586	1.83717E-07	5.08104E-07	negative
PRJNA729271	stage_E18_vs_E10	ordinary_muscle_development	ENSGALG00010015025	TRAF3IP1	-0.97117328	0.193621853	-5.015824727	5.28065E-07	1.40189E-06	negative
PRJNA729271	stage_E18_vs_E10	ordinary_muscle_development	ENSGALG00010015027	CLSPN	-1.688319052	0.17978423	-9.390807254	5.95421E-21	4.47183E-20	negative
PRJNA729271	stage_E18_vs_E10	ordinary_muscle_development	ENSGALG00010015028	MCM9	-1.301208995	0.268435716	-4.847376545	1.25105E-06	3.20388E-06	negative
PRJNA729271	stage_E18_vs_E10	ordinary_muscle_development	ENSGALG00010015029	TFRC	-0.704367595	0.182470016	-3.860182686	0.000113302	0.000234818	negative
PRJNA729271	stage_E18_vs_E10	ordinary_muscle_development	ENSGALG00010015031	MYH9	0.355737752	0.109580322	3.246365267	0.001168888	0.002111807	positive
PRJNA729271	stage_E18_vs_E10	ordinary_muscle_development	ENSGALG00010015032	C1orf216	-2.041249598	0.268666895	-7.597696749	3.01448E-14	1.43875E-13	negative
PRJNA729271	stage_E18_vs_E10	ordinary_muscle_development	ENSGALG00010015033	BMP7	-2.168954804	0.327381946	-6.625150921	3.46894E-11	1.30909E-10	negative
PRJNA729271	stage_E18_vs_E10	ordinary_muscle_development	ENSGALG00010015035	SLC35F1	-1.091697152	0.114564701	-9.5290883	1.58671E-21	1.23321E-20	negative
PRJNA729271	stage_E18_vs_E10	ordinary_muscle_development	ENSGALG00010015037	CYFIP2	1.257459104	0.189573479	6.633096081	3.28718E-11	1.24179E-10	positive
PRJNA729271	stage_E18_vs_E10	ordinary_muscle_development	ENSGALG00010015038	PCSK5	-0.300771182	0.206760597	-1.454683275	0.145756965	0.187034986	negative
PRJNA729271	stage_E18_vs_E10	ordinary_muscle_development	ENSGALG00010015039	FAM114A1	0.419785972	0.126567366	3.316699923	0.000910874	0.001668113	positive
PRJNA729271	stage_E18_vs_E10	ordinary_muscle_development	ENSGALG00010015040	ZC4H2	-0.86717271	0.088473928	-9.801449223	1.10982E-22	9.14543E-22	negative
PRJNA729271	stage_E18_vs_E10	ordinary_muscle_development	ENSGALG00010015041	TFAP2C	-3.897230682	0.503247848	-7.744157673	9.62176E-15	4.81752E-14	negative
PRJNA729271	stage_E18_vs_E10	ordinary_muscle_development	ENSGALG00010015042	EFNB2	0.065409378	0.132957653	0.491956469	0.622750117	0.673337532	positive
PRJNA729271	stage_E18_vs_E10	ordinary_muscle_development	ENSGALG00010015043	COQ10B	0.307870865	0.223311941	1.378658317	0.168000127	0.212436695	positive
PRJNA729271	stage_E18_vs_E10	ordinary_muscle_development	ENSGALG00010015044	ZC3H12B	-0.602678778	0.132459613	-4.549905936	5.36699E-06	1.28395E-05	negative
PRJNA729271	stage_E18_vs_E10	ordinary_muscle_development	ENSGALG00010015046	PSMB2	-0.490663425	0.094536296	-5.190212071	2.10055E-07	5.77421E-07	negative
PRJNA729271	stage_E18_vs_E10	ordinary_muscle_development	ENSGALG00010015047	NPY2R	-3.494946665	0.225180819	-15.52062329	2.51595E-54	8.30723E-53	negative
PRJNA729271	stage_E18_vs_E10	ordinary_muscle_development	ENSGALG00010015049	DCK2	-1.374506068	0.249324844	-5.512912568	3.52944E-08	1.04364E-07	negative
PRJNA729271	stage_E18_vs_E10	ordinary_muscle_development	ENSGALG00010015050	HIVEP3	-0.54848065	0.179747497	-3.051395199	0.002277806	0.003962173	negative
PRJNA729271	stage_E18_vs_E10	ordinary_muscle_development	ENSGALG00010015052	ENSGALG00010015052	-0.394326692	0.108851099	-3.622624828	0.000291629	0.00057232	negative
PRJNA729271	stage_E18_vs_E10	ordinary_muscle_development	ENSGALG00010015054	ENSGALG00010015054	-0.143764405	0.174193896	-0.825312526	0.409194182	0.469394561	negative
PRJNA729271	stage_E18_vs_E10	ordinary_muscle_development	ENSGALG00010015055	ZNF335	-0.185822916	0.182882755	-1.016076753	0.309592838	0.367229803	negative
PRJNA729271	stage_E18_vs_E10	ordinary_muscle_development	ENSGALG00010015056	ENSGALG00010015056	-0.894903989	0.481159353	-1.85989108	0.062900938	0.086635223	negative
PRJNA729271	stage_E18_vs_E10	ordinary_muscle_development	ENSGALG00010015057	LAS1L	-0.633586112	0.113499575	-5.582277408	2.37389E-08	7.10355E-08	negative
PRJNA729271	stage_E18_vs_E10	ordinary_muscle_development	ENSGALG00010015059	ENSGALG00010015059	0.07751855	0.168962244	0.458792144	0.646383437	0.695311905	positive
PRJNA729271	stage_E18_vs_E10	ordinary_muscle_development	ENSGALG00010015061	MMP9	0.917402325	0.460137134	1.993758507	0.046178452	0.065220371	positive
PRJNA729271	stage_E18_vs_E10	ordinary_muscle_development	ENSGALG00010015062	ENSGALG00010015062	-0.168206273	0.245219094	-0.685942803	0.492749177	0.550177116	negative

PRJNA729271	stage_E18_vs_E10	ordinary_muscle_development	ENSGALG00010015063	EEF1AKMT4	-0.97548599	0.221832079	-4.397407237	1.09552E-05	2.5395E-05	negative
PRJNA729271	stage_E18_vs_E10	ordinary_muscle_development	ENSGALG00010015064	RFK	-0.729093572	0.153443536	-4.751543081	2.0187E-06	5.04154E-06	negative
PRJNA729271	stage_E18_vs_E10	ordinary_muscle_development	ENSGALG00010015065	F11	4.485394057	1.234793603	3.632505096	0.000280683	0.00055226	positive
PRJNA729271	stage_E18_vs_E10	ordinary_muscle_development	ENSGALG00010015066	RUFY1	-0.134580772	0.070814547	-1.900467862	0.057371749	0.079731879	negative
PRJNA729271	stage_E18_vs_E10	ordinary_muscle_development	ENSGALG00010015068	ESM1	-1.104582064	0.328461087	-3.362900831	0.000771281	0.001429265	negative
PRJNA729271	stage_E18_vs_E10	ordinary_muscle_development	ENSGALG00010015069	HSPD1	-1.041549403	0.102700647	-10.14160506	3.61117E-24	3.24915E-23	negative
PRJNA729271	stage_E18_vs_E10	ordinary_muscle_development	ENSGALG00010015070	EDN2	0.488009261	0.442176488	1.103652668	0.269743835	0.324799542	positive
PRJNA729271	stage_E18_vs_E10	ordinary_muscle_development	ENSGALG00010015071	SNAI1	-1.72388773	0.127969236	-13.47111064	2.31359E-41	4.68374E-40	negative
PRJNA729271	stage_E18_vs_E10	ordinary_muscle_development	ENSGALG00010015072	MAP9	-0.156500975	0.138753787	-1.127904171	0.259360392	0.313442462	negative
PRJNA729271	stage_E18_vs_E10	ordinary_muscle_development	ENSGALG00010015075	ENSGALG00010015075	1.106727642	0.169301426	6.537024941	6.27546E-11	2.32344E-10	positive
PRJNA729271	stage_E18_vs_E10	ordinary_muscle_development	ENSGALG00010015076	ITK	-1.532684214	0.975870881	-1.570580948	0.116280018	0.152421673	negative
PRJNA729271	stage_E18_vs_E10	ordinary_muscle_development	ENSGALG00010015080	ASF1A	-1.011866335	0.079389688	-12.74556385	3.29996E-37	5.62079E-36	negative
PRJNA729271	stage_E18_vs_E10	ordinary_muscle_development	ENSGALG00010015083	CEP135	-1.534835987	0.190566657	-8.054063638	8.00895E-16	4.33656E-15	negative
PRJNA729271	stage_E18_vs_E10	ordinary_muscle_development	ENSGALG00010015084	FOXO6	2.06320653	0.201681969	10.22999994	1.45519E-24	1.34351E-23	positive
PRJNA729271	stage_E18_vs_E10	ordinary_muscle_development	ENSGALG00010015085	PSMD2	-0.888793877	0.164397442	-5.406372909	6.43138E-08	1.84966E-07	negative
PRJNA729271	stage_E18_vs_E10	ordinary_muscle_development	ENSGALG00010015086	FOXN3	0.11750912	0.096573613	1.216782892	0.223686831	0.274730943	positive
PRJNA729271	stage_E18_vs_E10	ordinary_muscle_development	ENSGALG00010015087	GABRA3	-2.814803279	0.485348035	-5.799556353	6.64906E-09	2.08927E-08	negative
PRJNA729271	stage_E18_vs_E10	ordinary_muscle_development	ENSGALG00010015088	ENSGALG00010015088	4.17105872	1.079050354	3.86549034			positive
PRJNA729271	stage_E18_vs_E10	ordinary_muscle_development	ENSGALG00010015090	GZMA	-2.547635017	0.808821318	-3.149811905	0.001633756	0.002903543	negative
PRJNA729271	stage_E18_vs_E10	ordinary_muscle_development	ENSGALG00010015091	MSN	-0.410863824	0.121310234	-3.386885106	0.00070691	0.001315867	negative
PRJNA729271	stage_E18_vs_E10	ordinary_muscle_development	ENSGALG00010015092	HSPE1	-0.444072488	0.122766926	-3.6171997	0.000297808	0.000583341	negative
PRJNA729271	stage_E18_vs_E10	ordinary_muscle_development	ENSGALG00010015094	CDK5RAP1	0.626505679	0.213791931	2.930445855	0.00338476	0.005733562	positive
PRJNA729271	stage_E18_vs_E10	ordinary_muscle_development	ENSGALG00010015095	RBFOX2	0.509242386	0.133065595	3.827002653	0.000129713	0.000266883	positive
PRJNA729271	stage_E18_vs_E10	ordinary_muscle_development	ENSGALG00010015096	GABRE	-3.251304069	1.143752049	-2.842665131	0.004473805	0.007462348	negative
PRJNA729271	stage_E18_vs_E10	ordinary_muscle_development	ENSGALG00010015097	MFSD2A	-0.190511373	0.161380395	-1.180511255	0.237796937	0.290409183	negative
PRJNA729271	stage_E18_vs_E10	ordinary_muscle_development	ENSGALG00010015098	HAVCR1	1.316509526	0.216350199	6.085085811	1.16429E-09	3.90386E-09	positive
PRJNA729271	stage_E18_vs_E10	ordinary_muscle_development	ENSGALG00010015100	MOB4	-0.023152696	0.089473053	-0.258767254	0.795814825	0.829343295	negative
PRJNA729271	stage_E18_vs_E10	ordinary_muscle_development	ENSGALG00010015101	CAP1	-0.892530184	0.09964424	-8.957167824	3.33124E-19	2.24067E-18	negative
PRJNA729271	stage_E18_vs_E10	ordinary_muscle_development	ENSGALG00010015103	ITPK1	-0.942379852	0.093505092	-10.07838007	6.88519E-24	6.0743E-23	negative
PRJNA729271	stage_E18_vs_E10	ordinary_muscle_development	ENSGALG00010015104	CNGA2	0.485971329	0.314120491	1.547085733	0.121842574	0.159065848	positive
PRJNA729271	stage_E18_vs_E10	ordinary_muscle_development	ENSGALG00010015105	ENSGALG00010015105	2.689879215	1.105810528	2.432495573	0.014995175	0.023152435	positive
PRJNA729271	stage_E18_vs_E10	ordinary_muscle_development	ENSGALG00010015107	MB	-1.605534212	0.577748253	-2.778951218	0.005453471	0.008985798	negative
PRJNA729271	stage_E18_vs_E10	ordinary_muscle_development	ENSGALG00010015108	ENSGALG00010015108	-0.258182858	0.093555468	-2.75967683	0.005785857	0.009496772	negative
PRJNA729271	stage_E18_vs_E10	ordinary_muscle_development	ENSGALG00010015109	PPT1	0.132495934	0.110247292	1.201806702	0.229438428	0.281077587	positive
PRJNA729271	stage_E18_vs_E10	ordinary_muscle_development	ENSGALG00010015110	ENSGALG00010015110	1.226689098	0.853102244	1.437915685	0.150457979	0.192555925	positive
PRJNA729271	stage_E18_vs_E10	ordinary_muscle_development	ENSGALG00010015111	ADAMTS2	3.041681971	0.141459683	21.50211216	1.4876E-102	1.87504E-100	positive
PRJNA729271	stage_E18_vs_E10	ordinary_muscle_development	ENSGALG00010015112	CBLN4	-2.438014324	0.393305244	-6.198784177	5.6901E-10	1.96845E-09	negative
PRJNA729271	stage_E18_vs_E10	ordinary_muscle_development	ENSGALG00010015115	CLDN2	0.203434872	0.409580548	0.496690756	0.619407142	0.670172914	positive
PRJNA729271	stage_E18_vs_E10	ordinary_muscle_development	ENSGALG00010015117	RASD2	1.0372381	0.202576858	5.120220089	3.05179E-07	8.2885E-07	positive
PRJNA729271	stage_E18_vs_E10	ordinary_muscle_development	ENSGALG00010015118	GPX8	0.059042919	0.18503491	0.3190907	0.749657736	0.789529091	positive
PRJNA729271	stage_E18_vs_E10	ordinary_muscle_development	ENSGALG00010015119	TLR1A	0.709203606	0.2208006	3.211964125	0.001318308	0.002367598	positive
PRJNA729271	stage_E18_vs_E10	ordinary_muscle_development	ENSGALG00010015121	UNC79	-4.438448934	0.30448969	-14.57668052	3.95275E-48	1.06894E-46	negative
PRJNA729271	stage_E18_vs_E10	ordinary_muscle_development	ENSGALG00010015122	ENSGALG00010015122	-1.071838221	0.20304902	-5.278716544	1.30092E-07	3.64594E-07	negative
PRJNA729271	stage_E18_vs_E10	ordinary_muscle_development	ENSGALG00010015123	CEP85L	0.613087436	0.251384611	2.438842351	0.014734395	0.022771623	positive
PRJNA729271	stage_E18_vs_E10	ordinary_muscle_development	ENSGALG00010015124	UXS1	0.802640761	0.095123954	8.437840629	3.23254E-17	1.9156E-16	positive
PRJNA729271	stage_E18_vs_E10	ordinary_muscle_development	ENSGALG00010015126	MCM5	-1.875217466	0.180092564	-10.41252021	2.17389E-25	2.11197E-24	negative
PRJNA729271	stage_E18_vs_E10	ordinary_muscle_development	ENSGALG00010015127	RPL11	0.965601173	0.151547549	6.371605339	1.8706E-10	6.6784E-10	positive
PRJNA729271	stage_E18_vs_E10	ordinary_muscle_development	ENSGALG00010015128	OCRL	0.069058148	0.107656324	0.641468567	0.521218297	0.578045847	positive
PRJNA729271	stage_E18_vs_E10	ordinary_muscle_development	ENSGALG00010015129	PRRG3	-0.187187415	0.13001776	-1.43970651	0.149950451	0.19197419	negative
PRJNA729271	stage_E18_vs_E10	ordinary_muscle_development	ENSGALG00010015131	DOK5	4.455802518	0.239576083	18.59869509	3.29224E-77	2.12094E-75	positive
PRJNA729271	stage_E18_vs_E10	ordinary_muscle_development	ENSGALG00010015132	GCNT1	-1.702748612	0.295716274	-5.758048371	8.5092E-09	2.6468E-08	negative
PRJNA729271	stage_E18_vs_E10	ordinary_muscle_development	ENSGALG00010015133	RFTN2	1.181943591	0.127415831	9.276269542	1.75516E-20	1.2778E-19	positive
PRJNA729271	stage_E18_vs_E10	ordinary_muscle_development	ENSGALG00010015134	PLN	2.979856574	0.523112709	5.696394908	1.22367E-08	3.74913E-08	positive
PRJNA729271	stage_E18_vs_E10	ordinary_muscle_development	ENSGALG00010015136	TCEB3	0.065583013	0.092039952	0.712549397	0.476124634	0.534662734	positive
PRJNA729271	stage_E18_vs_E10	ordinary_muscle_development	ENSGALG00010015137	AMN	3.344531505	0.394796871	8.47152485	2.42201E-17	1.44771E-16	positive
PRJNA729271	stage_E18_vs_E10	ordinary_muscle_development	ENSGALG00010015138	ENSGALG00010015138	-2.118480728	0.503425566	-4.208130999	2.57492E-05	5.72972E-05	negative
PRJNA729271	stage_E18_vs_E10	ordinary_muscle_development	ENSGALG00010015140	ASCC3	-0.554293818	0.074911125	-7.399352447	1.3685E-13	6.22613E-13	negative
PRJNA729271	stage_E18_vs_E10	ordinary_muscle_development	ENSGALG00010015141	PFDN4	0.222110686	0.136157719	1.631275027	0.1028323	0.13601188	positive
PRJNA729271	stage_E18_vs_E10	ordinary_muscle_development	ENSGALG00010015142	HNRNPAB	-0.696225381	0.128694892	-5.409891321	6.3063E-08	1.81549E-07	negative
PRJNA729271	stage_E18_vs_E10	ordinary_muscle_development	ENSGALG00010015143	EXOC1	-0.357640032	0.08151042	-4.38766025	1.14577E-05	2.64963E-05	negative
PRJNA729271	stage_E18_vs_E10	ordinary_muscle_development	ENSGALG00010015145	CLIC2	1.480451649	0.125286771	11.81650411	3.20756E-32	4.32499E-31	positive
PRJNA729271	stage_E18_vs_E10	ordinary_muscle_development	ENSGALG00010015146	ENSGALG00010015146	0.577288944	0.130124121	4.436448377	9.14553E-06	2.1371E-05	positive
PRJNA729271	stage_E18_vs_E10	ordinary_muscle_development	ENSGALG00010015148	ENSGALG00010015148	-0.032971916	0.07609837	-0.433280183	0.664811233	0.7122802	negative
PRJNA729271	stage_E18_vs_E10	ordinary_muscle_development	ENSGALG00010015149	ENSGALG00010015149	1.489391221	0.138473161	10.75581153	5.56421E-27	5.8957E-26	positive
PRJNA729271	stage_E18_vs_E10	ordinary_muscle_development	ENSGALG00010015151	ENSGALG00010015151	-1.897536706	0.374123145	-5.071957538	3.93744E-07	1.05887E-06	negative
PRJNA729271	stage_E18_vs_E10	ordinary_muscle_development	ENSGALG00010015152	BOLL	2.86896299	0.279400614	10.26827733	9.79378E-25	9.15877E-24	positive
PRJNA729271	stage_E18_vs_E10	ordinary_muscle_development	ENSGALG00010015153	ERCC6L	-2.2044341	0.24445816	-9.01763353	1.92196E-19	1.31039E-18	negative
PRJNA729271	stage_E18_vs_E10	ordinary_muscle_development	ENSGALG00010015154	CYP24A1	-2.938883655	0.799373849	-3.67648211	0.000236472	0.000469865	negative
PRJNA729271	stage_E18_vs_E10	ordinary_muscle_development	ENSGALG00010015155	HMOX1	-0.714308124	0.179666409	-3.975746646	7.01588E-05	0.000148851	negative
PRJNA729271	stage_E18_vs_E10	ordinary_muscle_development	ENSGALG00010015156	CNOT6	-0.076530561	0.073934113	-1.035118413	0.300613615	0.357573801	negative
PRJNA729271	stage_E18_vs_E10	ordinary_muscle_development	ENSGALG00010015157	CDC42BPB	-0.279523458	0.070068212	-3.98930484	6.62672E-05	0.000141028	negative
PRJNA729271	stage_E18_vs_E10	ordinary_muscle_development	ENSGALG00010015158	PIN4	0.076924926	0.165413987	0.465044872	0.641899323	0.691103817	positive
PRJNA729271	stage_E18_vs_E10	ordinary_muscle_development	ENSGALG00010015160	SIM1	-6.875564498	0.594369664	-11.56782541	5.99837E-31	7.69437E-30	negative
PRJNA729271	stage_E18_vs_E10	ordinary_muscle_development	ENSGALG00010015161	GFPT2	-0.98998696	0.201299508	-4.917980016	8.74418E-07	2.27716E-06	negative
PRJNA729271	stage_E18_vs_E10	ordinary_muscle_development	ENSGALG00010015163	PLK2	-0.279349697	0.209389471	-1.334115298	0.182166073	0.228772936	negative
PRJNA729271	stage_E18_vs_E10	ordinary_muscle_development	ENSGALG00010015165	KLF3	0.31666637	0.119602253	2.647662244	0.008105046	0.012992992	positive
PRJNA729271	stage_E18_vs_E10	ordinary_muscle_development	ENSGALG00010015166	YTHDF2	-0.675925747	0.182709558	-3.69945478	0.000216063	0.000431204	negative
PRJNA729271	stage_E18_vs_E10	ordinary_muscle_development	ENSGALG00010015167	RAB3C	-2.810749791	0.436605186	-6.437737985	1.21267E-10	4.39222E-10	negative
PRJNA729271	stage_E18_vs_E10	ordinary_muscle_development	ENSGALG00010015169	ENSGALG00010015169	0.409143597	0.571411329	0.716022899	0.473977209	0.532681843	positive
PRJNA729271	stage_E18_vs_E10	ordinary_muscle_development	ENSGALG00010015170	EXOC1L	-0.104083272	0.669299706	-0.15551071	0.876418711	0.897850677	negative
PRJNA729271	stage_E18_vs_E10	ordinary_muscle_development	ENSGALG00010015171	NHSL2	2.522818889	0.198179488	12.72996974	4.02995E-37	6.83207E-36	positive
PRJNA729271	stage_E18_vs_E10	ordinary_muscle_development	ENSGALG00010015172	PPP4R3A	-0.175348448	0.077545094	-2.261244899	0.023744096	0.035335798	negative
PRJNA729271	stage_E18_vs_E10	ordinary_muscle_development	ENSGALG00010015173	ENS-1	-0.362750449	0.686969297	-0.528044632	0.597468359	0.649221178	negative
PRJNA729271	stage_E18_vs_E10	ordinary_muscle_development	ENSGALG00010015174	ST6GAL2	-3.976911214	0.218413781	-18.20815154	4.4471E-74	2.59922E-72	negative
PRJNA729271	stage_E18_vs_E10	ordinary_muscle_development	ENSGALG00010015175	MAPK9	0.389432279	0.06878046	5.661960936	1.49653E-08	4.55814E-08	positive
PRJNA729271	stage_E18_vs_E10	ordinary_muscle_development	ENSGALG00010015177	TC2N	0.921469961	0.156768673	5.877896042	4.15514E-09	1.32897E-08	positive
PRJNA729271	stage_E18_vs_E10	ordinary_muscle_development	ENSGALG00010015180	PITHD1	1.049372499	0.125149307	8.384964543	5.0741E-17	2.97048E-16	positive
PRJNA729271	stage_E18_vs_E10	ordinary_muscle_development	ENSGALG00010015181	VPS13A	0.667274869	0.120312562	5.546177884	2.91982E-08	8.68515E-08	positive
PRJNA729271	stage_E18_vs_E10	ordinary_muscle_development	ENSGALG00010015182	PHYKPL	-0.73886395	0.326507256	-2.262932709	0.023639838	0.035187867	negative
PRJNA729271	stage_E18_vs_E10	ordinary_muscle_development	ENSGALG00010015183	SLC5A7	-7.060021788	1.036872695	-6.808957187	9.83087E-12	3.87119E-11	negative
PRJNA729271	stage_E18_vs_E10	ordinary_muscle_development	ENSGALG00010015186	ENSGALG00010015186	3.950881971	0.284060413	13.90859757	5.617E-44	1.2682E-42	positive
PRJNA729271	stage_E18_vs_E10	ordinary_muscle_development	ENSGALG00010015188	SULT1C3	0.943955451	1.162215845	0.812203219	0.416675032	0.47695685	positive
PRJNA729271	stage_E18_vs_E10	ordinary_muscle_development	ENSGALG00010015190	FBLN5	1.304925146	0.192066348	6.794137337	1.08963E-11	4.27099E-11	positive
PRJNA729271	stage_E18_vs_E10	ordinary_muscle_development	ENSGALG00010015191	TOM1	1.240420213	0.189498007	6.54582196	5.9169E-11	2.1963E-10	positive
PRJNA729271	stage_E18_vs_E10	ordinary_muscle_development	ENSGALG00010015192	ENSGALG00010015192	1.761133881	0.224613571	7.840727851	4.47942E-15	2.29513E-14	positive
PRJNA729271	stage_E18_vs_E10	ordinary_muscle_development	ENSGALG00010015193	LYPLA2	-0.520923709	0.106974079	-4.869625564	1.1181E-06	2.87729E-06	negative
PRJNA729271	stage_E18_vs_E10	ordinary_muscle_development	ENSGALG00010015194	TMLHE	-0.037890505	0.113819582	-0.332899704	0.739209993	0.780340022	negative
PRJNA729271	stage_E18_vs_E10	ordinary_muscle_development	ENSGALG00010015196	PDE4D	1.655397996	0.217565496	7.608734056	2.76794E-14	1.32731E-13	positive
PRJNA729271	stage_E18_vs_E10	ordinary_muscle_development	ENSGALG00010015198	NME5	0.659332782	0.220436129	2.991037746	0.002780311	0.004787433	positive
PRJNA729271	stage_E18_vs_E10	ordinary_muscle_development	ENSGALG00010015199	ENSGALG00010015199	-0.455086227	0.17150202	-2.653532756	0.007965404	0.012777615	negative
PRJNA729271	stage_E18_vs_E10	ordinary_muscle_development	ENSGALG00010015200	TBC1D1	-0.53230852	0.086328218	-6.166101111	6.99943E-10	2.40305E-09	negative
PRJNA729271	stage_E18_vs_E10	ordinary_muscle_development	ENSGALG00010015201	GCC2	0.081036158	0.109273861	0.741587762	0.458337141	0.517777013	positive
PRJNA729271	stage_E18_vs_E10	ordinary_muscle_development	ENSGALG00010015203	HMGXB4	-0.300612071	0.065866341	-4.563971032	5.0195E-06	1.2038E-05	negative
PRJNA729271	stage_E18_vs_E10	ordinary_muscle_development	ENSGALG00010015204	ENSGALG00010015204	0.753766737	0.138037912	5.460577651	4.74588E-08	1.3833E-07	positive
PRJNA729271	stage_E18_vs_E10	ordinary_muscle_development	ENSGALG00010015207	AKR1E2	-0.60008918	0.179488641	-3.343326781	0.000827804	0.001526398	negative
PRJNA729271	stage_E18_vs_E10	ordinary_muscle_development	ENSGALG00010015208	GNA14	-0.355019212	0.730880572	-0.485741756	0.627150274	0.677488689	negative
PRJNA729271	stage_E18_vs_E10	ordinary_muscle_development	ENSGALG00010015212	GNAQ	0.160669621	0.098239996	1.635480736	0.10194831	0.134928849	positive
PRJNA729271	stage_E18_vs_E10	ordinary_muscle_development	ENSGALG00010015213	RUBCN	0.499717172	0.119970804	4.165323182	3.10912E-05	6.84176E-05	positive
PRJNA729271	stage_E18_vs_E10	ordinary_muscle_development	ENSGALG00010015218	PLCL1	-1.216951163	0.15845179	-7.680261378	1.58764E-14	7.79569E-14	negative
PRJNA729271	stage_E18_vs_E10	ordinary_muscle_development	ENSGALG00010015227	FYTTD1	-0.627133815	0.075852967	-8.267755884	1.36504E-16	7.77151E-16	negative
PRJNA729271	stage_E18_vs_E10	ordinary_muscle_development	ENSGALG00010015233	ENSGALG00010015233	0.452969393	0.72757599	0.622573311	0.533564954	0.589393691	positive
PRJNA729271	stage_E18_vs_E10	ordinary_muscle_development	ENSGALG00010015234	TRIP11	-0.06931999	0.14402147	-0.481317059	0.630291175	0.680374634	negative
PRJNA729271	stage_E18_vs_E10	ordinary_muscle_development	ENSGALG00010015241	ENSGALG00010015241	0.825565785	0.155690649	5.302603523	1.14163E-07	3.21943E-07	positive
PRJNA729271	stage_E18_vs_E10	ordinary_muscle_development	ENSGALG00010015248	ENSGALG00010015248	-0.02155943	0.141291013	-0.15258883	0.878722534	0.899638588	negative
PRJNA729271	stage_E18_vs_E10	ordinary_muscle_development	ENSGALG00010015250	RORB	-0.476892845	0.442472642	-1.077790579	0.281127225	0.336743998	negative
PRJNA729271	stage_E18_vs_E10	ordinary_muscle_development	ENSGALG00010015253	ENSGALG00010015253	0.438146229	0.07189574	6.09418905	1.09994E-09	3.69835E-09	positive
PRJNA729271	stage_E18_vs_E10	ordinary_muscle_development	ENSGALG00010015260	RASGEF1C	1.76840425	0.139250773	12.69942141	5.95685E-37	1.00752E-35	positive
PRJNA729271	stage_E18_vs_E10	ordinary_muscle_development	ENSGALG00010015261	IQCG	0.135120485	0.408230195	0.330990913	0.740651349	0.781520078	positive
PRJNA729271	stage_E18_vs_E10	ordinary_muscle_development	ENSGALG00010015283	ENSGALG00010015283	1.114050906	0.504951601	2.206252845	0.027366301	0.040279705	positive
PRJNA729271	stage_E18_vs_E10	ordinary_muscle_development	ENSGALG00010015285	ALG13	1.152130869	0.403243261	2.85716088	0.004274491	0.007149637	positive
PRJNA729271	stage_E18_vs_E10	ordinary_muscle_development	ENSGALG00010015291	ZFP64	-0.205240815	0.093627998	-2.192088043	0.02837315	0.041643257	negative
PRJNA729271	stage_E18_vs_E10	ordinary_muscle_development	ENSGALG00010015296	RPL35A	0.860430416	0.195363318	4.404257791	1.06147E-05	2.46768E-05	positive
PRJNA729271	stage_E18_vs_E10	ordinary_muscle_development	ENSGALG00010015298	ENSGALG00010015298	2.222905894	0.243135142	9.142676261	6.09256E-20	4.26214E-19	positive
PRJNA729271	stage_E18_vs_E10	ordinary_muscle_development	ENSGALG00010015299	GALE	0.252454888	0.094074842	2.683553675	0.007284428	0.011755487	positive
PRJNA729271	stage_E18_vs_E10	ordinary_muscle_development	ENSGALG00010015300	ATXN3	0.679642255	0.126951638	5.353552464	8.6244E-08	2.45697E-07	positive
PRJNA729271	stage_E18_vs_E10	ordinary_muscle_development	ENSGALG00010015306	BCAT1	0.80144388	0.090912456	8.815556387	1.19092E-18	7.75838E-18	positive
PRJNA729271	stage_E18_vs_E10	ordinary_muscle_development	ENSGALG00010015310	PGM2	-1.580333577	0.095451622	-16.55638266	1.43995E-61	6.03239E-60	negative
PRJNA729271	stage_E18_vs_E10	ordinary_muscle_development	ENSGALG00010015314	GMEB1	-0.060390571	0.069717078	-0.866223488	0.386367615	0.446602758	negative
PRJNA729271	stage_E18_vs_E10	ordinary_muscle_development	ENSGALG00010015318	SALL4	1.108481547	0.163835878	6.765804643	1.3257E-11	5.16561E-11	positive
PRJNA729271	stage_E18_vs_E10	ordinary_muscle_development	ENSGALG00010015320	TRPM6	-2.29376795	0.41616584	-5.511668015	3.55449E-08	1.0504E-07	negative
PRJNA729271	stage_E18_vs_E10	ordinary_muscle_development	ENSGALG00010015327	LARGE1	0.184057561	0.126264459	1.457714722	0.144919192	0.186091752	positive

PRJNA729271	stage_E18_vs_E10	ordinary_muscle_development	ENSGALG00010015328	ENSGALG00010015328	-0.081654608	0.43975327	-0.185682776	0.852693536	0.878323781	negative
PRJNA729271	stage_E18_vs_E10	ordinary_muscle_development	ENSGALG00010015333	MED7	0.073782386	0.068875935	1.07123607	0.284063297	0.339755548	positive
PRJNA729271	stage_E18_vs_E10	ordinary_muscle_development	ENSGALG00010015339	ENSGALG00010015339	0.153501773	0.804672242	0.190763101	0.848711198	0.874844889	positive
PRJNA729271	stage_E18_vs_E10	ordinary_muscle_development	ENSGALG00010015348	CPSF2	-0.727597071	0.061866353	-11.76078807	6.21515E-32	8.31075E-31	negative
PRJNA729271	stage_E18_vs_E10	ordinary_muscle_development	ENSGALG00010015353	RELL1	0.192268841	0.091877548	2.092664051	0.036379154	0.052339042	positive
PRJNA729271	stage_E18_vs_E10	ordinary_muscle_development	ENSGALG00010015356	ENSGALG00010015356	0.360279719	0.08357316	4.310950041	1.62555E-05	3.70185E-05	positive
PRJNA729271	stage_E18_vs_E10	ordinary_muscle_development	ENSGALG00010015362	ATP9A	1.467229333	0.12576239	11.66667819	1.88646E-31	2.49037E-30	positive
PRJNA729271	stage_E18_vs_E10	ordinary_muscle_development	ENSGALG00010015364	CLOCK	1.31292683	0.202923417	6.47006073	9.79635E-11	3.57588E-10	positive
PRJNA729271	stage_E18_vs_E10	ordinary_muscle_development	ENSGALG00010015370	ENSGALG00010015370	1.601257756	0.142803034	11.21305139	3.5185E-29	4.10965E-28	positive
PRJNA729271	stage_E18_vs_E10	ordinary_muscle_development	ENSGALG00010015374	LIMS1	-1.249193209	0.151440911	-8.248716956	1.60104E-16	9.06173E-16	negative
PRJNA729271	stage_E18_vs_E10	ordinary_muscle_development	ENSGALG00010015384	TAF12	0.119596715	0.116938215	1.022734232	0.306433521	0.363809804	positive
PRJNA729271	stage_E18_vs_E10	ordinary_muscle_development	ENSGALG00010015386	SLC24A4	0.311947691	0.256044659	1.218333131	0.223097405	0.274120275	positive
PRJNA729271	stage_E18_vs_E10	ordinary_muscle_development	ENSGALG00010015388	PSMD1	-0.982644635	0.12294201	-7.992749036	1.31963E-15	7.02718E-15	negative
PRJNA729271	stage_E18_vs_E10	ordinary_muscle_development	ENSGALG00010015390	CCNB3	-1.714142302	0.205303739	-8.34929901	6.86693E-17	3.98304E-16	negative
PRJNA729271	stage_E18_vs_E10	ordinary_muscle_development	ENSGALG00010015391	SYN3	-0.264785184	0.37002561	-0.715586102	0.474246958	0.532884469	negative
PRJNA729271	stage_E18_vs_E10	ordinary_muscle_development	ENSGALG00010015392	TMEM165	-0.566783463	0.082383896	-6.879784687	5.99431E-12	2.40552E-11	negative
PRJNA729271	stage_E18_vs_E10	ordinary_muscle_development	ENSGALG00010015393	DCX	-0.527658132	0.37752023	-1.397694985	0.162204681	0.205954086	negative
PRJNA729271	stage_E18_vs_E10	ordinary_muscle_development	ENSGALG00010015397	SRD5A3	-0.599726731	0.088729501	-6.75904546	1.38904E-11	5.40949E-11	negative
PRJNA729271	stage_E18_vs_E10	ordinary_muscle_development	ENSGALG00010015404	BEND4	-2.02451203	0.273641405	-7.398412636	1.37822E-13	6.26837E-13	negative
PRJNA729271	stage_E18_vs_E10	ordinary_muscle_development	ENSGALG00010015407	TIMP3	0.652873341	0.175517986	3.719694811	0.000199464	0.000400114	positive
PRJNA729271	stage_E18_vs_E10	ordinary_muscle_development	ENSGALG00010015409	FAM13B	0.952557577	0.131836791	7.225278819	5.00075E-13	2.18858E-12	positive
PRJNA729271	stage_E18_vs_E10	ordinary_muscle_development	ENSGALG00010015410	HTR2B	-1.053028757	0.208653391	-5.046784773	4.49307E-07	1.2005E-06	negative
PRJNA729271	stage_E18_vs_E10	ordinary_muscle_development	ENSGALG00010015413	CARNMT1	-0.507124731	0.108580138	-4.670511016	3.00451E-06	7.36146E-06	negative
PRJNA729271	stage_E18_vs_E10	ordinary_muscle_development	ENSGALG00010015416	CAPN6	2.415009934	0.162325408	14.87758425	4.60841E-50	1.34947E-48	positive
PRJNA729271	stage_E18_vs_E10	ordinary_muscle_development	ENSGALG00010015422	ENSGALG00010015422	-1.601032868	0.54961125	-2.913027829	0.003579427	0.006045653	negative
PRJNA729271	stage_E18_vs_E10	ordinary_muscle_development	ENSGALG00010015430	HMGCL	0.745704119	0.131045182	5.690435238	1.26716E-08	3.87663E-08	positive
PRJNA729271	stage_E18_vs_E10	ordinary_muscle_development	ENSGALG00010015431	RIN3	-0.43786445	0.159210636	-2.750221105	0.005955506	0.009753143	negative
PRJNA729271	stage_E18_vs_E10	ordinary_muscle_development	ENSGALG00010015436	SLC30A9	-0.063878347	0.099099774	-0.644586199	0.519195381	0.576110927	negative
PRJNA729271	stage_E18_vs_E10	ordinary_muscle_development	ENSGALG00010015440	ENSGALG00010015440	-0.102974315	0.423522937	-0.243137515	0.80789887	0.840064141	negative
PRJNA729271	stage_E18_vs_E10	ordinary_muscle_development	ENSGALG00010015443	PAK3	-0.704249684	0.10294286	-6.841170769	7.85486E-12	3.11593E-11	negative
PRJNA729271	stage_E18_vs_E10	ordinary_muscle_development	ENSGALG00010015449	HDX	-0.396727787	0.092918573	-4.269628476	1.95799E-05	4.41584E-05	negative
PRJNA729271	stage_E18_vs_E10	ordinary_muscle_development	ENSGALG00010015466	NMRK1	0.215556401	0.126221358	1.707764871	0.08767998	0.117503819	positive
PRJNA729271	stage_E18_vs_E10	ordinary_muscle_development	ENSGALG00010015469	TMEM33	-0.733941591	0.075325834	-9.74355748	1.9655E-22	1.60597E-21	negative
PRJNA729271	stage_E18_vs_E10	ordinary_muscle_development	ENSGALG00010015472	LGMN	-0.165178112	0.134664564	-1.226589295	0.219976995	0.270768218	negative
PRJNA729271	stage_E18_vs_E10	ordinary_muscle_development	ENSGALG00010015477	ARMC9	-0.601815617	0.102970832	-5.844525167	5.08015E-09	1.61237E-08	negative
PRJNA729271	stage_E18_vs_E10	ordinary_muscle_development	ENSGALG00010015478	FBXO7	0.337476972	0.062313218	5.415816823	6.10095E-08	1.75847E-07	positive
PRJNA729271	stage_E18_vs_E10	ordinary_muscle_development	ENSGALG00010015481	GOLGA5	0.389589546	0.070970227	5.489478656	4.03122E-08	1.18428E-07	positive
PRJNA729271	stage_E18_vs_E10	ordinary_muscle_development	ENSGALG00010015483	SGCD	1.649047374	0.162177457	10.16816644	2.75037E-24	2.48855E-23	positive
PRJNA729271	stage_E18_vs_E10	ordinary_muscle_development	ENSGALG00010015485	BCAS1	2.040091045	0.184831118	11.03759512	2.51679E-28	2.82798E-27	positive
PRJNA729271	stage_E18_vs_E10	ordinary_muscle_development	ENSGALG00010015486	MTFR1L	0.301319596	0.121332437	2.483421608	0.013012697	0.020251132	positive
PRJNA729271	stage_E18_vs_E10	ordinary_muscle_development	ENSGALG00010015487	RPS6KA6	-0.570107351	0.120350807	-4.737046335	2.16856E-06	5.39441E-06	negative
PRJNA729271	stage_E18_vs_E10	ordinary_muscle_development	ENSGALG00010015490	RANBP2	-0.575650492	0.083330718	-6.908022721	4.91455E-12	1.99486E-11	negative
PRJNA729271	stage_E18_vs_E10	ordinary_muscle_development	ENSGALG00010015493	OSTF1	-0.58694447	0.142600752	-4.115998417	3.85507E-05	8.40416E-05	negative
PRJNA729271	stage_E18_vs_E10	ordinary_muscle_development	ENSGALG00010015494	CHRDL1	-0.9366374	0.241194436	-3.88332921	0.000103036	0.000214646	negative
PRJNA729271	stage_E18_vs_E10	ordinary_muscle_development	ENSGALG00010015498	MID2	1.908150458	0.132162353	14.43792744	2.98689E-47	7.88616E-46	positive
PRJNA729271	stage_E18_vs_E10	ordinary_muscle_development	ENSGALG00010015502	BPIFB6	3.593916454	1.254179445	2.86555203	0.004162829	0.006972521	positive
PRJNA729271	stage_E18_vs_E10	ordinary_muscle_development	ENSGALG00010015505	CHGA	-0.948673439	0.486944881	-1.948215242	0.051389214	0.072018433	negative
PRJNA729271	stage_E18_vs_E10	ordinary_muscle_development	ENSGALG00010015507	AMMECR1	-0.458186171	0.069698383	-6.573842161	4.90332E-11	1.83369E-10	negative
PRJNA729271	stage_E18_vs_E10	ordinary_muscle_development	ENSGALG00010015508	FUCA1	0.792842513	0.104608918	7.579110127	3.47933E-14	1.65462E-13	positive
PRJNA729271	stage_E18_vs_E10	ordinary_muscle_development	ENSGALG00010015511	BPIFB4	-1.641422863	0.408899111	-4.014249043	5.96354E-05	0.000127551	negative
PRJNA729271	stage_E18_vs_E10	ordinary_muscle_development	ENSGALG00010015516	SELENON	-0.179550242	0.112799908	-1.591758762	0.111438919	0.146539702	negative
PRJNA729271	stage_E18_vs_E10	ordinary_muscle_development	ENSGALG00010015520	UBE3A	-0.026060862	0.079150266	-0.329258041	0.741960656	0.782559827	negative
PRJNA729271	stage_E18_vs_E10	ordinary_muscle_development	ENSGALG00010015521	DKC1	-0.896768227	0.082294	-10.89712764	1.18953E-27	1.30327E-26	negative
PRJNA729271	stage_E18_vs_E10	ordinary_muscle_development	ENSGALG00010015523	MRPL22	-0.58681826	0.146698014	-4.00017862	6.32947E-05	0.00013498	negative
PRJNA729271	stage_E18_vs_E10	ordinary_muscle_development	ENSGALG00010015532	NXT2	-0.876929499	0.112020423	-7.828300166	4.9451E-15	2.52659E-14	negative
PRJNA729271	stage_E18_vs_E10	ordinary_muscle_development	ENSGALG00010015533	PSAT1	-0.92925726	0.277798525	-3.345076292	0.000822599	0.00151796	negative
PRJNA729271	stage_E18_vs_E10	ordinary_muscle_development	ENSGALG00010015540	ACSL4	-0.748102995	0.155637296	-4.806707748	1.53436E-06	3.8872E-06	negative
PRJNA729271	stage_E18_vs_E10	ordinary_muscle_development	ENSGALG00010015541	RASEF	-1.096320238	0.333518232	-3.287137355	0.001012115	0.001842577	negative
PRJNA729271	stage_E18_vs_E10	ordinary_muscle_development	ENSGALG00010015542	CNGA3	-0.102305835	0.276952322	-0.369398726	0.711830548	0.755858439	negative
PRJNA729271	stage_E18_vs_E10	ordinary_muscle_development	ENSGALG00010015544	RTCB	-0.712814648	0.074418242	-9.578493551	9.84707E-22	7.76991E-21	negative
PRJNA729271	stage_E18_vs_E10	ordinary_muscle_development	ENSGALG00010015551	VWA3B	0.061123774	0.365048057	0.167440348	0.867023581	0.890302267	positive
PRJNA729271	stage_E18_vs_E10	ordinary_muscle_development	ENSGALG00010015553	ASCL4	-6.736112766	1.113444173	-6.04979839	1.45027E-09	4.82481E-09	negative
PRJNA729271	stage_E18_vs_E10	ordinary_muscle_development	ENSGALG00010015555	PNRC2	-0.286753983	0.102498135	-2.797650742	0.005147574	0.008519535	negative
PRJNA729271	stage_E18_vs_E10	ordinary_muscle_development	ENSGALG00010015557	IRS4	-0.212315678	0.192658465	-1.102031403	0.27044802	0.325512251	negative
PRJNA729271	stage_E18_vs_E10	ordinary_muscle_development	ENSGALG00010015558	MPP1	-0.476164835	0.260696899	-1.826507473	0.067773857	0.092887864	negative
PRJNA729271	stage_E18_vs_E10	ordinary_muscle_development	ENSGALG00010015562	IRF9	0.597677224	0.284443062	2.101219205	0.035621728	0.051341051	positive
PRJNA729271	stage_E18_vs_E10	ordinary_muscle_development	ENSGALG00010015563	B3GNT7	-2.95877533	0.326410056	-9.064596138	1.25067E-19	8.61617E-19	negative
PRJNA729271	stage_E18_vs_E10	ordinary_muscle_development	ENSGALG00010015564	PKD2L2	-0.633420792	0.306481817	-2.066748357	0.038757872	0.05550789	negative
PRJNA729271	stage_E18_vs_E10	ordinary_muscle_development	ENSGALG00010015565	ENSGALG00010015565	-0.690307099	0.454288414	-1.5195349	0.12862791	0.167160868	negative
PRJNA729271	stage_E18_vs_E10	ordinary_muscle_development	ENSGALG00010015567	ENSGALG00010015567	2.006436476	0.491062081	4.085912054	4.3904E-05	9.50969E-05	positive
PRJNA729271	stage_E18_vs_E10	ordinary_muscle_development	ENSGALG00010015570	LIMCH1	1.238835186	0.127881005	9.687405807	3.41082E-22	2.75124E-21	positive
PRJNA729271	stage_E18_vs_E10	ordinary_muscle_development	ENSGALG00010015571	DUSP15	0.385014206	0.306051483	1.258004708	0.208390054	0.258150058	positive
PRJNA729271	stage_E18_vs_E10	ordinary_muscle_development	ENSGALG00010015573	MAN1C1	0.480106278	0.125016797	3.84033418	0.000122867	0.000253337	positive
PRJNA729271	stage_E18_vs_E10	ordinary_muscle_development	ENSGALG00010015574	C14orf132	-0.338125334	0.264128419	-1.280155066	0.200490605	0.249365997	negative
PRJNA729271	stage_E18_vs_E10	ordinary_muscle_development	ENSGALG00010015580	GEMIN5	-1.16635709	0.073726308	-15.82009365	2.26156E-56	8.01499E-55	negative
PRJNA729271	stage_E18_vs_E10	ordinary_muscle_development	ENSGALG00010015583	PRDM4	-0.415939067	0.138603067	-3.000936974	0.002691503	0.004640025	negative
PRJNA729271	stage_E18_vs_E10	ordinary_muscle_development	ENSGALG00010015584	TTLL9	0.644557112	0.256197595	2.515859336	0.011874256	0.018597228	positive
PRJNA729271	stage_E18_vs_E10	ordinary_muscle_development	ENSGALG00010015585	BDKRB1	0.862780809	0.306728679	2.81284689	0.004910503	0.0081495	positive
PRJNA729271	stage_E18_vs_E10	ordinary_muscle_development	ENSGALG00010015588	BDKRB2	-0.929543077	0.466100633	-1.994296964	0.046119611	0.065156312	negative
PRJNA729271	stage_E18_vs_E10	ordinary_muscle_development	ENSGALG00010015589	FRMD3	3.136040065	0.178639752	17.55510761	5.43639E-69	2.79434E-67	positive
PRJNA729271	stage_E18_vs_E10	ordinary_muscle_development	ENSGALG00010015596	UCHL1	-3.577067671	0.505933014	-7.070239681	1.54666E-12	6.53231E-12	negative
PRJNA729271	stage_E18_vs_E10	ordinary_muscle_development	ENSGALG00010015605	LDLRAP1	-0.358281155	0.059618974	-6.009515596	1.86078E-09	6.13141E-09	negative
PRJNA729271	stage_E18_vs_E10	ordinary_muscle_development	ENSGALG00010015606	TMEM131	0.178422467	0.108691089	1.641555604	0.100682135	0.133362656	positive
PRJNA729271	stage_E18_vs_E10	ordinary_muscle_development	ENSGALG00010015610	ENSGALG00010015610	1.842418774	0.286481595	6.431194216	1.26605E-10	4.57914E-10	positive
PRJNA729271	stage_E18_vs_E10	ordinary_muscle_development	ENSGALG00010015613	PWP1	-0.984497242	0.082659975	-11.91020498	1.0472E-32	1.4347E-31	negative
PRJNA729271	stage_E18_vs_E10	ordinary_muscle_development	ENSGALG00010015614	ATG2B	-0.110714872	0.10803074	-1.024846001	0.305435857	0.362773906	negative
PRJNA729271	stage_E18_vs_E10	ordinary_muscle_development	ENSGALG00010015616	NADK2	-0.368911545	0.118632419	-3.109702633	0.001872758	0.003303191	negative
PRJNA729271	stage_E18_vs_E10	ordinary_muscle_development	ENSGALG00010015617	SRSF10	-0.236038301	0.056388953	-4.185896175	2.84043E-05	6.28581E-05	negative
PRJNA729271	stage_E18_vs_E10	ordinary_muscle_development	ENSGALG00010015618	NCL	-1.116965878	0.10110779	-11.0472781	2.25962E-28	2.54493E-27	negative
PRJNA729271	stage_E18_vs_E10	ordinary_muscle_development	ENSGALG00010015620	PDRG1	0.431699413	0.144996196	2.977315442	0.002907847	0.00499162	positive
PRJNA729271	stage_E18_vs_E10	ordinary_muscle_development	ENSGALG00010015621	IDNK	-0.863741553	0.179087741	-4.823007698	1.4141E-06	3.59476E-06	negative
PRJNA729271	stage_E18_vs_E10	ordinary_muscle_development	ENSGALG00010015624	ITGA1	1.100540844	0.129521168	8.496995969	1.9456E-17	1.16873E-16	positive
PRJNA729271	stage_E18_vs_E10	ordinary_muscle_development	ENSGALG00010015626	XKR7	0.549575072	0.507629412	1.082630475	0.278972475	0.334522338	positive
PRJNA729271	stage_E18_vs_E10	ordinary_muscle_development	ENSGALG00010015628	UBQLN1	-0.160494567	0.119209133	-1.346327782	0.17819683	0.224157168	negative
PRJNA729271	stage_E18_vs_E10	ordinary_muscle_development	ENSGALG00010015631	GSKIP	-0.494468793	0.108030561	-4.577119557	4.71422E-06	1.13321E-05	negative
PRJNA729271	stage_E18_vs_E10	ordinary_muscle_development	ENSGALG00010015632	COL4A5	2.947693658	0.161399512	18.26333688	1.62076E-74	9.54998E-73	positive
PRJNA729271	stage_E18_vs_E10	ordinary_muscle_development	ENSGALG00010015633	CCM2L	1.050694523	0.186042376	5.647608611	1.62695E-08	4.93671E-08	positive
PRJNA729271	stage_E18_vs_E10	ordinary_muscle_development	ENSGALG00010015634	ENSGALG00010015634	1.913946455	1.077956739	1.775531786	0.075810122	0.102977014	positive
PRJNA729271	stage_E18_vs_E10	ordinary_muscle_development	ENSGALG00010015635	ABTB3	0.089737994	0.124277199	0.722079312	0.470245717	0.529126818	positive
PRJNA729271	stage_E18_vs_E10	ordinary_muscle_development	ENSGALG00010015636	APBB2	0.281751695	0.124341901	2.265943283	0.023454856	0.034937636	positive
PRJNA729271	stage_E18_vs_E10	ordinary_muscle_development	ENSGALG00010015642	ENSGALG00010015642	-0.265437106	0.765695259	-0.346661551	0.728845599	0.770971098	negative
PRJNA729271	stage_E18_vs_E10	ordinary_muscle_development	ENSGALG00010015643	ENSGALG00010015643	-2.545581356	0.122333326	-20.80856815	3.61996E-96	3.83002E-94	negative
PRJNA729271	stage_E18_vs_E10	ordinary_muscle_development	ENSGALG00010015645	MACO1	0.799817268	0.09388639	8.51899054	1.6095E-17	9.74507E-17	positive
PRJNA729271	stage_E18_vs_E10	ordinary_muscle_development	ENSGALG00010015647	PDZD2	-0.344529752	0.138225903	-2.492512219	0.012684297	0.019771898	negative
PRJNA729271	stage_E18_vs_E10	ordinary_muscle_development	ENSGALG00010015650	ENSGALG00010015650	1.548432027	1.236736446	1.252030723	0.210558669	0.260502553	positive
PRJNA729271	stage_E18_vs_E10	ordinary_muscle_development	ENSGALG00010015653	HCK	0.800363301	0.547018684	1.463137046	0.143429883	0.184358974	positive
PRJNA729271	stage_E18_vs_E10	ordinary_muscle_development	ENSGALG00010015655	ENSGALG00010015655	3.244946683	0.380952619	8.517979719	1.62361E-17	9.82638E-17	positive
PRJNA729271	stage_E18_vs_E10	ordinary_muscle_development	ENSGALG00010015657	NMUR1	-2.148720446	1.044000491	-2.058160379	0.039574741	0.056560428	negative
PRJNA729271	stage_E18_vs_E10	ordinary_muscle_development	ENSGALG00010015660	KLHL3	0.451923195	0.123603758	3.656225347	0.000255956	0.000506427	positive
PRJNA729271	stage_E18_vs_E10	ordinary_muscle_development	ENSGALG00010015668	ZNF217	0.179431517	0.135407387	1.325123555	0.185130175	0.23203302	positive
PRJNA729271	stage_E18_vs_E10	ordinary_muscle_development	ENSGALG00010015669	INPP4A	0.378350599	0.130423604	2.900936542	0.003720492	0.006272218	positive
PRJNA729271	stage_E18_vs_E10	ordinary_muscle_development	ENSGALG00010015670	ENSGALG00010015670	-1.309517414	0.131395198	-9.966250202	2.14162E-23	1.83468E-22	negative
PRJNA729271	stage_E18_vs_E10	ordinary_muscle_development	ENSGALG00010015674	RFKL	-0.45588529	0.196105171	-2.324697959	0.020088116	0.030283645	negative
PRJNA729271	stage_E18_vs_E10	ordinary_muscle_development	ENSGALG00010015676	TGM2	2.576096718	0.192266493	13.39857336	6.16366E-41	1.22554E-39	positive
PRJNA729271	stage_E18_vs_E10	ordinary_muscle_development	ENSGALG00010015677	GRHL3	-1.504586323	0.24574494	-6.122552614	9.2088E-10	3.12238E-09	negative
PRJNA729271	stage_E18_vs_E10	ordinary_muscle_development	ENSGALG00010015680	ENSGALG00010015680	-0.193524194	0.104180763	-1.857580892	0.063228554	0.087061641	negative
PRJNA729271	stage_E18_vs_E10	ordinary_muscle_development	ENSGALG00010015684	PDE6D	-0.485793722	0.099784846	-4.868411794	1.12499E-06	2.89242E-06	negative
PRJNA729271	stage_E18_vs_E10	ordinary_muscle_development	ENSGALG00010015686	CNOT8	-0.516810669	0.053417842	-9.674869754	3.85582E-22	3.09127E-21	negative
PRJNA729271	stage_E18_vs_E10	ordinary_muscle_development	ENSGALG00010015691	EXOC3L4	-1.210796044	0.829659404	-1.459389285	0.144457993	0.185548836	negative
PRJNA729271	stage_E18_vs_E10	ordinary_muscle_development	ENSGALG00010015693	COA5	-0.373819771	0.109214577	-3.42280107	0.000619794	0.001162967	negative
PRJNA729271	stage_E18_vs_E10	ordinary_muscle_development	ENSGALG00010015695	ASAH1	0.202378104	0.094738349	2.136179348	0.032664798	0.047399765	positive
PRJNA729271	stage_E18_vs_E10	ordinary_muscle_development	ENSGALG00010015697	FAXDC2	0.844144376	0.342035205	2.46800436	0.013586866	0.021097122	positive
PRJNA729271	stage_E18_vs_E10	ordinary_muscle_development	ENSGALG00010015698	TNFAIP2	-1.560774294	0.203967855	-7.652060132	1.97785E-14	9.62043E-14	negative
PRJNA729271	stage_E18_vs_E10	ordinary_muscle_development	ENSGALG00010015701	UNC50	-0.027844364	0.08700496	-0.320031919	0.748944134	0.788949508	negative
PRJNA729271	stage_E18_vs_E10	ordinary_muscle_development	ENSGALG00010015703	GK5	0.024378683	0.188831112	0.129103105	0.897276065	0.914757108	positive
PRJNA729271	stage_E18_vs_E10	ordinary_muscle_development	ENSGALG00010015705	NPY6R	-3.614874213	1.022077672	-3.536790121	0.000405021	0.000779653	negative
PRJNA729271	stage_E18_vs_E10	ordinary_muscle_development	ENSGALG00010015706	ENSGALG00010015706	-0.149981636	0.174593707	-0.859032314	0.390322688	0.450420139	negative
PRJNA729271	stage_E18_vs_E10	ordinary_muscle_development	ENSGALG00010015709	HNRNPA0	-0.779417762	0.112718146	-6.914749646	4.6869E-12	1.90352E-11	negative
PRJNA729271	stage_E18_vs_E10	ordinary_muscle_development	ENSGALG00010015711	LBFABP	2.086898383	0.670973306	3.110255453	0.001869256	0.003297416	positive
PRJNA729271	stage_E18_vs_E10	ordinary_muscle_development	ENSGALG00010015716	MGAT4A	-0.314703472	0.132759885	-2.370471112	0.017765432	0.027023816	negative
PRJNA729271	stage_E18_vs_E10	ordinary_muscle_development	ENSGALG00010015719	XRN1	0.165458016	0.099734608	1.65898296	0.097119226	0.129127058	positive

PRJNA729271	stage_E18_vs_E10	ordinary_muscle_development	ENSGALG00010015729	RHCE	0.566146808	0.196977555	2.87416913	0.00405092	0.006797648	positive
PRJNA729271	stage_E18_vs_E10	ordinary_muscle_development	ENSGALG00010015731	PHF6	-1.106197911	0.069788419	-15.85073742	1.38941E-56	4.96046E-55	negative
PRJNA729271	stage_E18_vs_E10	ordinary_muscle_development	ENSGALG00010015733	COL4A6	2.307374268	0.152421536	15.13811186	9.07829E-52	2.80575E-50	positive
PRJNA729271	stage_E18_vs_E10	ordinary_muscle_development	ENSGALG00010015734	TSHZ2	-0.926036177	0.166367798	-5.566198435	2.60357E-08	7.77316E-08	negative
PRJNA729271	stage_E18_vs_E10	ordinary_muscle_development	ENSGALG00010015738	CRACDL	-3.034025181	0.449431831	-6.750801722	1.4703E-11	5.71369E-11	negative
PRJNA729271	stage_E18_vs_E10	ordinary_muscle_development	ENSGALG00010015742	HPRT1	0.728505186	0.086673182	8.405197146	4.27143E-17	2.5148E-16	positive
PRJNA729271	stage_E18_vs_E10	ordinary_muscle_development	ENSGALG00010015746	PAPOLB	-0.429864993	0.072462002	-5.932281484	2.98754E-09	9.68128E-09	negative
PRJNA729271	stage_E18_vs_E10	ordinary_muscle_development	ENSGALG00010015749	ATR	-1.135355891	0.13506386	-8.406067269	4.23988E-17	2.49927E-16	negative
PRJNA729271	stage_E18_vs_E10	ordinary_muscle_development	ENSGALG00010015751	MYOM3	1.762479703	0.538079568	3.275500147	0.001054751	0.001915866	positive
PRJNA729271	stage_E18_vs_E10	ordinary_muscle_development	ENSGALG00010015753	RPRD1B	-0.398821966	0.078833099	-5.059067484	4.21312E-07	1.12882E-06	negative
PRJNA729271	stage_E18_vs_E10	ordinary_muscle_development	ENSGALG00010015756	PCM1	-0.35618399	0.103437572	-3.443468212	0.000574304	0.001082515	negative
PRJNA729271	stage_E18_vs_E10	ordinary_muscle_development	ENSGALG00010015758	PCDH19	0.75653352	0.169618474	4.46020708	8.18805E-06	1.92484E-05	positive
PRJNA729271	stage_E18_vs_E10	ordinary_muscle_development	ENSGALG00010015759	EIF5	0.099538186	0.09178669	1.084451196	0.278164795	0.333747099	positive
PRJNA729271	stage_E18_vs_E10	ordinary_muscle_development	ENSGALG00010015764	TTI1	0.357727998	0.097046189	3.686162243	0.000227661	0.000453103	positive
PRJNA729271	stage_E18_vs_E10	ordinary_muscle_development	ENSGALG00010015765	ENSGALG00010015765	0.686123248	0.376645832	1.821666909	0.068505542	0.093828577	positive
PRJNA729271	stage_E18_vs_E10	ordinary_muscle_development	ENSGALG00010015766	TLE1	-0.444571142	0.100727196	-4.413615789	1.01658E-05	2.36561E-05	negative
PRJNA729271	stage_E18_vs_E10	ordinary_muscle_development	ENSGALG00010015769	TMEM50A	0.238741565	0.099933503	2.389004272	0.016894106	0.025814892	positive
PRJNA729271	stage_E18_vs_E10	ordinary_muscle_development	ENSGALG00010015774	PLS1	-2.142324542	0.383124327	-5.591721514	2.24829E-08	6.73744E-08	negative
PRJNA729271	stage_E18_vs_E10	ordinary_muscle_development	ENSGALG00010015776	SPOCK1	-3.943513466	0.203563372	-19.37241182	1.3193E-83	1.00649E-81	negative
PRJNA729271	stage_E18_vs_E10	ordinary_muscle_development	ENSGALG00010015778	PABIR3	-0.622984486	0.08698312	-7.162130849	7.94325E-13	3.43489E-12	negative
PRJNA729271	stage_E18_vs_E10	ordinary_muscle_development	ENSGALG00010015782	ATG4A	0.17214277	0.083170482	2.069757995	0.038475013	0.055135473	positive
PRJNA729271	stage_E18_vs_E10	ordinary_muscle_development	ENSGALG00010015785	FGL1	2.068472583	0.190669465	10.84847318	2.02784E-27	2.19355E-26	positive
PRJNA729271	stage_E18_vs_E10	ordinary_muscle_development	ENSGALG00010015788	TRPC1	-0.193124895	0.128721308	-1.50033354	0.133528026	0.172796065	negative
PRJNA729271	stage_E18_vs_E10	ordinary_muscle_development	ENSGALG00010015792	MARK3	0.051790864	0.081840837	0.632824222	0.526848437	0.582996266	positive
PRJNA729271	stage_E18_vs_E10	ordinary_muscle_development	ENSGALG00010015793	RSRP1	1.347120251	0.233076177	5.779742355	7.48151E-09	2.33666E-08	positive
PRJNA729271	stage_E18_vs_E10	ordinary_muscle_development	ENSGALG00010015795	PSMD10	-0.085383206	0.134543646	-0.634613442	0.525680559	0.582059408	negative
PRJNA729271	stage_E18_vs_E10	ordinary_muscle_development	ENSGALG00010015796	LARP1	-0.235027245	0.141428183	-1.661813368	0.096550201	0.128464766	negative
PRJNA729271	stage_E18_vs_E10	ordinary_muscle_development	ENSGALG00010015797	PIP5K1B	-0.469154687	0.164631958	-2.849718196	0.004375798	0.007308122	negative
PRJNA729271	stage_E18_vs_E10	ordinary_muscle_development	ENSGALG00010015798	IL22RA1	0.532061644	0.261259749	2.036523596	0.041697806	0.059302365	positive
PRJNA729271	stage_E18_vs_E10	ordinary_muscle_development	ENSGALG00010015800	BVES	1.952750447	0.181876227	10.73669979	6.84476E-27	7.23667E-26	positive
PRJNA729271	stage_E18_vs_E10	ordinary_muscle_development	ENSGALG00010015801	VSTM2L	-0.278203256	0.22362311	-1.24407203	0.213473054	0.263800761	negative
PRJNA729271	stage_E18_vs_E10	ordinary_muscle_development	ENSGALG00010015802	TRPC7	-2.633454492	0.884761358	-2.976457401	0.002915996	0.005003831	negative
PRJNA729271	stage_E18_vs_E10	ordinary_muscle_development	ENSGALG00010015803	SYF2	0.028808959	0.161007849	0.178928909	0.857993523	0.882467616	positive
PRJNA729271	stage_E18_vs_E10	ordinary_muscle_development	ENSGALG00010015804	CKB	-3.349559318	0.161744424	-20.70896311	2.8758E-95	2.9989E-93	negative
PRJNA729271	stage_E18_vs_E10	ordinary_muscle_development	ENSGALG00010015807	ENSGALG00010015807	1.246137364	0.93933134	1.326621726	0.184633845	0.231591172	positive
PRJNA729271	stage_E18_vs_E10	ordinary_muscle_development	ENSGALG00010015809	PCOLCE2	0.918942224	0.235297321	3.905451285	9.40497E-05	0.000196689	positive
PRJNA729271	stage_E18_vs_E10	ordinary_muscle_development	ENSGALG00010015810	LIPT1	-0.238541014	0.106393011	-2.242074095	0.024956584	0.037022381	negative
PRJNA729271	stage_E18_vs_E10	ordinary_muscle_development	ENSGALG00010015811	CTNNBL1	-0.605506983	0.056869149	-10.64737202	1.79357E-26	1.85831E-25	negative
PRJNA729271	stage_E18_vs_E10	ordinary_muscle_development	ENSGALG00010015813	ENSGALG00010015813	0.056410725	0.139556041	0.404215575	0.686054201	0.731935475	positive
PRJNA729271	stage_E18_vs_E10	ordinary_muscle_development	ENSGALG00010015814	PAQR9	0.057721863	0.453439584	0.127297804	0.898704698	0.916020294	positive
PRJNA729271	stage_E18_vs_E10	ordinary_muscle_development	ENSGALG00010015816	MITD1	-0.919026353	0.219824781	-4.180722241	2.90585E-05	6.42273E-05	negative
PRJNA729271	stage_E18_vs_E10	ordinary_muscle_development	ENSGALG00010015820	ENSGALG00010015820	-0.634192223	0.542313357	-1.169420252	0.242234354	0.29525622	negative
PRJNA729271	stage_E18_vs_E10	ordinary_muscle_development	ENSGALG00010015822	WFDC2	-1.489790219	0.584181417	-2.550218437	0.010765544	0.01695974	negative
PRJNA729271	stage_E18_vs_E10	ordinary_muscle_development	ENSGALG00010015825	RUNX3	-6.298719794	0.942017927	-6.686411813	2.28709E-11	8.7563E-11	negative
PRJNA729271	stage_E18_vs_E10	ordinary_muscle_development	ENSGALG00010015827	ENSGALG00010015827	1.22654699	0.152994652	8.016927237	1.08423E-15	5.80567E-15	positive
PRJNA729271	stage_E18_vs_E10	ordinary_muscle_development	ENSGALG00010015829	SMAD5	0.043074658	0.047835367	0.900477218	0.367866344	0.427432678	positive
PRJNA729271	stage_E18_vs_E10	ordinary_muscle_development	ENSGALG00010015831	LYG2	4.759454234	0.621349026	7.65987236	1.86118E-14	9.07427E-14	positive
PRJNA729271	stage_E18_vs_E10	ordinary_muscle_development	ENSGALG00010015832	RAB39B	-0.553856558	0.085257721	-6.496262775	8.23398E-11	3.02385E-10	negative
PRJNA729271	stage_E18_vs_E10	ordinary_muscle_development	ENSGALG00010015834	STARD8	1.653183919	0.132855942	12.44343225	1.51836E-35	2.39225E-34	positive
PRJNA729271	stage_E18_vs_E10	ordinary_muscle_development	ENSGALG00010015837	GDA	1.153938903	0.169513475	6.807357943	9.94074E-12	3.91127E-11	positive
PRJNA729271	stage_E18_vs_E10	ordinary_muscle_development	ENSGALG00010015838	IFNLR1	-2.182703027	1.282523281	-1.701881797	0.088777529	0.118853817	negative
PRJNA729271	stage_E18_vs_E10	ordinary_muscle_development	ENSGALG00010015839	U2SURP	-0.239729484	0.071145055	-3.369587453	0.000752808	0.001397535	negative
PRJNA729271	stage_E18_vs_E10	ordinary_muscle_development	ENSGALG00010015840	MTUS1	2.17862973	0.145671598	14.95576185	1.42838E-50	4.26015E-49	positive
PRJNA729271	stage_E18_vs_E10	ordinary_muscle_development	ENSGALG00010015843	ENSGALG00010015843	-0.426787534	0.392517781	-1.087307518	0.276900927	0.332368246	negative
PRJNA729271	stage_E18_vs_E10	ordinary_muscle_development	ENSGALG00010015845	SAP30L	0.30616147	0.125761624	2.434458616	0.014914085	0.023037049	positive
PRJNA729271	stage_E18_vs_E10	ordinary_muscle_development	ENSGALG00010015848	UBE2B	0.997809827	0.162277714	6.148779173	7.80816E-10	2.6643E-09	positive
PRJNA729271	stage_E18_vs_E10	ordinary_muscle_development	ENSGALG00010015852	ZFAND5	0.261255064	0.286132204	0.91305718	0.361212469	0.42071433	positive
PRJNA729271	stage_E18_vs_E10	ordinary_muscle_development	ENSGALG00010015855	NIPAL3	-0.488317731	0.080812903	-6.042571335	1.51677E-09	5.03795E-09	negative
PRJNA729271	stage_E18_vs_E10	ordinary_muscle_development	ENSGALG00010015856	TXNDC9	-0.189866183	0.133748368	-1.419577567	0.1557307	0.198532674	negative
PRJNA729271	stage_E18_vs_E10	ordinary_muscle_development	ENSGALG00010015858	CHST2	-0.274724224	0.091094322	-3.015821597	0.00256284	0.00443245	negative
PRJNA729271	stage_E18_vs_E10	ordinary_muscle_development	ENSGALG00010015861	PPP2CA	-0.462609766	0.067424058	-6.861197348	6.82857E-12	2.71998E-11	negative
PRJNA729271	stage_E18_vs_E10	ordinary_muscle_development	ENSGALG00010015862	PDGFRL	0.828824497	0.209052916	3.964663648	7.34995E-05	0.000155529	positive
PRJNA729271	stage_E18_vs_E10	ordinary_muscle_development	ENSGALG00010015863	ENSGALG00010015863	-0.811329388	0.535848556	-1.514102033	0.129999963	0.168621876	negative
PRJNA729271	stage_E18_vs_E10	ordinary_muscle_development	ENSGALG00010015865	VRK1	-1.136278753	0.13997065	-8.117978666	4.74012E-16	2.60159E-15	negative
PRJNA729271	stage_E18_vs_E10	ordinary_muscle_development	ENSGALG00010015866	ARHGEF6	-0.230743593	0.072930402	-3.163887584	0.001556769	0.00277178	negative
PRJNA729271	stage_E18_vs_E10	ordinary_muscle_development	ENSGALG00010015867	PICALML	-0.097871485	0.05712033	-1.713426468	0.08663411	0.116209645	negative
PRJNA729271	stage_E18_vs_E10	ordinary_muscle_development	ENSGALG00010015869	SLC7A2	2.516485188	0.194088334	12.96566948	1.91551E-38	3.4491E-37	positive
PRJNA729271	stage_E18_vs_E10	ordinary_muscle_development	ENSGALG00010015870	EIF5B	-0.73942768	0.15687588	-4.71345679	2.43549E-06	6.03564E-06	negative
PRJNA729271	stage_E18_vs_E10	ordinary_muscle_development	ENSGALG00010015872	TMC1	0.109604919	0.342095649	0.320392612	0.748670725	0.788718814	positive
PRJNA729271	stage_E18_vs_E10	ordinary_muscle_development	ENSGALG00010015874	CLIC4	-0.372255248	0.114602571	-3.248227722	0.001161263	0.00209934	negative
PRJNA729271	stage_E18_vs_E10	ordinary_muscle_development	ENSGALG00010015875	DNTTIP1	-0.026115435	0.14098793	-0.185231706	0.853047301	0.878564774	negative
PRJNA729271	stage_E18_vs_E10	ordinary_muscle_development	ENSGALG00010015876	SKP1	-0.563042766	0.077633612	-7.252564342	4.08954E-13	1.8034E-12	negative
PRJNA729271	stage_E18_vs_E10	ordinary_muscle_development	ENSGALG00010015877	REV1	0.49901941	0.103911644	4.802343495	1.56819E-06	3.96563E-06	positive
PRJNA729271	stage_E18_vs_E10	ordinary_muscle_development	ENSGALG00010015879	TRMT61A	-1.294927658	0.124090814	-10.43532243	1.71032E-25	1.67281E-24	negative
PRJNA729271	stage_E18_vs_E10	ordinary_muscle_development	ENSGALG00010015881	GAB3	1.276222121	0.179988361	7.090581376	1.3355E-12	5.66024E-12	positive
PRJNA729271	stage_E18_vs_E10	ordinary_muscle_development	ENSGALG00010015886	AFF3	0.411250146	0.207615108	1.98082957	0.047610389	0.067092416	positive
PRJNA729271	stage_E18_vs_E10	ordinary_muscle_development	ENSGALG00010015887	SLC9A9	1.036501656	0.180173477	5.752798222	8.77782E-09	2.72626E-08	positive
PRJNA729271	stage_E18_vs_E10	ordinary_muscle_development	ENSGALG00010015889	SH3BP5	1.332947843	0.165054172	8.075820366	6.70245E-16	3.64959E-15	positive
PRJNA729271	stage_E18_vs_E10	ordinary_muscle_development	ENSGALG00010015892	UBE2C	-1.522544492	0.222502218	-6.84282839	7.76446E-12	3.08261E-11	negative
PRJNA729271	stage_E18_vs_E10	ordinary_muscle_development	ENSGALG00010015893	TCF7	-2.28304232	0.460654338	-4.956085574	7.19275E-07	1.88602E-06	negative
PRJNA729271	stage_E18_vs_E10	ordinary_muscle_development	ENSGALG00010015894	CEP78	-1.326654899	0.11507862	-11.5282482	9.50583E-31	1.20443E-29	negative
PRJNA729271	stage_E18_vs_E10	ordinary_muscle_development	ENSGALG00010015895	RBMXL1	-0.608035399	0.085076101	-7.146958931	8.87214E-13	3.82743E-12	negative
PRJNA729271	stage_E18_vs_E10	ordinary_muscle_development	ENSGALG00010015896	LONRF2	1.952685473	0.077401874	25.22788352	1.9809E-140	5.4176E-138	positive
PRJNA729271	stage_E18_vs_E10	ordinary_muscle_development	ENSGALG00010015898	ENSGALG00010015898	0.763198826	0.221090213	3.451979244	0.000556491	0.001050167	positive
PRJNA729271	stage_E18_vs_E10	ordinary_muscle_development	ENSGALG00010015899	SMARCA1	-0.148420998	0.115605334	-1.283859425	0.199191146	0.247919944	negative
PRJNA729271	stage_E18_vs_E10	ordinary_muscle_development	ENSGALG00010015900	ENSGALG00010015900	0.286429812	0.167205717	1.713038392	0.086705477	0.116294613	positive
PRJNA729271	stage_E18_vs_E10	ordinary_muscle_development	ENSGALG00010015902	ENSGALG00010015902	0.103766761	0.566902814	0.183041535	0.854765429	0.880021656	positive
PRJNA729271	stage_E18_vs_E10	ordinary_muscle_development	ENSGALG00010015904	MTMR7	0.829306578	0.261299397	3.173779153	0.001504681	0.002684351	positive
PRJNA729271	stage_E18_vs_E10	ordinary_muscle_development	ENSGALG00010015905	ANPEP	1.386310757	0.217050931	6.387029759	1.69139E-10	6.06248E-10	positive
PRJNA729271	stage_E18_vs_E10	ordinary_muscle_development	ENSGALG00010015906	BAG5	0.748530977	0.101117832	7.402561567	1.33582E-13	6.087E-13	positive
PRJNA729271	stage_E18_vs_E10	ordinary_muscle_development	ENSGALG00010015908	CHRD	0.945718212	0.137815737	6.86219319	6.78113E-12	2.70257E-11	positive
PRJNA729271	stage_E18_vs_E10	ordinary_muscle_development	ENSGALG00010015909	TNNC2	3.949394474	0.106585357	37.05381848	1.5588E-300	3.2278E-297	positive
PRJNA729271	stage_E18_vs_E10	ordinary_muscle_development	ENSGALG00010015910	C5orf15	-0.191705719	0.093491916	-2.050505822	0.040315096	0.057533457	negative
PRJNA729271	stage_E18_vs_E10	ordinary_muscle_development	ENSGALG00010015911	TM9SF2L	0.104515098	0.078667616	1.328565725	0.183991286	0.230905082	positive
PRJNA729271	stage_E18_vs_E10	ordinary_muscle_development	ENSGALG00010015912	ENSGALG00010015912	0.319363238	0.268138926	1.191036462	0.233639271	0.285765019	positive
PRJNA729271	stage_E18_vs_E10	ordinary_muscle_development	ENSGALG00010015913	CHST10	0.497199443	0.103307167	4.812826215	1.48811E-06	3.77959E-06	positive
PRJNA729271	stage_E18_vs_E10	ordinary_muscle_development	ENSGALG00010015914	SRRM1	-0.388594701	0.117899435	-3.295984415	0.000980775	0.001787992	negative
PRJNA729271	stage_E18_vs_E10	ordinary_muscle_development	ENSGALG00010015916	COA8	0.463778811	0.146604328	3.163472839	0.001558989	0.00277445	positive
PRJNA729271	stage_E18_vs_E10	ordinary_muscle_development	ENSGALG00010015917	TMEM63A	-0.084006423	0.113562103	-0.739739939	0.459457809	0.518913415	negative
PRJNA729271	stage_E18_vs_E10	ordinary_muscle_development	ENSGALG00010015918	BICD1	-0.620787034	0.188698665	-3.289832679	0.00100247	0.001825707	negative
PRJNA729271	stage_E18_vs_E10	ordinary_muscle_development	ENSGALG00010015919	ENSGALG00010015919	2.55694403	0.221954068	11.52014941	1.0443E-30	1.31971E-29	positive
PRJNA729271	stage_E18_vs_E10	ordinary_muscle_development	ENSGALG00010015921	SNX21	2.077758554	0.109534781	18.96893869	3.08063E-80	2.16766E-78	positive
PRJNA729271	stage_E18_vs_E10	ordinary_muscle_development	ENSGALG00010015923	RCAN3	-0.349930729	0.121010047	-2.89174939	0.003831034	0.006448077	negative
PRJNA729271	stage_E18_vs_E10	ordinary_muscle_development	ENSGALG00010015927	GLA	0.749510823	0.174521562	4.294660288	1.74961E-05	3.97004E-05	positive
PRJNA729271	stage_E18_vs_E10	ordinary_muscle_development	ENSGALG00010015928	CAPN7	0.422957711	0.122830274	3.443432125	0.000574381	0.001082518	positive
PRJNA729271	stage_E18_vs_E10	ordinary_muscle_development	ENSGALG00010015929	SLC6A12	-2.570688207	0.857204262	-2.998921401	0.002709372	0.004669165	negative
PRJNA729271	stage_E18_vs_E10	ordinary_muscle_development	ENSGALG00010015931	RBM47	0.180826296	0.283457217	0.637931532	0.523518251	0.580019647	positive
PRJNA729271	stage_E18_vs_E10	ordinary_muscle_development	ENSGALG00010015932	ACOT8	-1.038224799	0.163796767	-6.33849383	2.32022E-10	8.22892E-10	negative
PRJNA729271	stage_E18_vs_E10	ordinary_muscle_development	ENSGALG00010015933	ENSGALG00010015933	9.153345624	0.647604636	14.13415704	2.33918E-45	5.57672E-44	positive
PRJNA729271	stage_E18_vs_E10	ordinary_muscle_development	ENSGALG00010015934	TMC2	4.675405268	0.994547938	4.701035605	2.58845E-06	6.39067E-06	positive
PRJNA729271	stage_E18_vs_E10	ordinary_muscle_development	ENSGALG00010015935	EPHX1	0.586896405	0.110597919	5.306577284	1.11703E-07	3.15559E-07	positive
PRJNA729271	stage_E18_vs_E10	ordinary_muscle_development	ENSGALG00010015936	RPL36AL	0.683963575	0.139507798	4.902690633	9.45328E-07	2.45038E-06	positive
PRJNA729271	stage_E18_vs_E10	ordinary_muscle_development	ENSGALG00010015937	ENSGALG00010015937	0.800416043	0.193174831	4.143479978	3.42075E-05	7.49566E-05	positive
PRJNA729271	stage_E18_vs_E10	ordinary_muscle_development	ENSGALG00010015939	SUN2	0.263120552	0.081022585	3.247496388	0.001164251	0.002104217	positive
PRJNA729271	stage_E18_vs_E10	ordinary_muscle_development	ENSGALG00010015942	AP3S2	0.561143274	0.13051698	4.299389044	1.7127E-05	3.89298E-05	positive
PRJNA729271	stage_E18_vs_E10	ordinary_muscle_development	ENSGALG00010015946	TLE4	0.368156623	0.099283978	3.708117179	0.000208806	0.000417583	positive
PRJNA729271	stage_E18_vs_E10	ordinary_muscle_development	ENSGALG00010015954	CTSA	0.473263514	0.097139397	4.872003853	1.10472E-06	2.84624E-06	positive
PRJNA729271	stage_E18_vs_E10	ordinary_muscle_development	ENSGALG00010015957	ENSGALG00010015957	1.120169131	0.220352531	5.083531955	3.7048E-07	9.98347E-07	positive
PRJNA729271	stage_E18_vs_E10	ordinary_muscle_development	ENSGALG00010015959	TRNAU1AP	0.780411536	0.130536321	5.978501095	2.252E-09	7.37023E-09	positive
PRJNA729271	stage_E18_vs_E10	ordinary_muscle_development	ENSGALG00010015960	CHRNA9	-1.967087985	0.944214733	-2.083305752	0.037223363	0.053442219	negative
PRJNA729271	stage_E18_vs_E10	ordinary_muscle_development	ENSGALG00010015963	DIPK2A	0.489232682	0.108380059	4.514047027	6.36021E-06	1.50911E-05	positive
PRJNA729271	stage_E18_vs_E10	ordinary_muscle_development	ENSGALG00010015964	KLC1	-0.249965498	0.088358312	-2.828998115	0.004669397	0.00776893	negative
PRJNA729271	stage_E18_vs_E10	ordinary_muscle_development	ENSGALG00010015967	ZSWIM1	-0.518558957	0.219807077	-2.359154969	0.018316605	0.027786414	negative
PRJNA729271	stage_E18_vs_E10	ordinary_muscle_development	ENSGALG00010015970	THPO	0.569238505	0.387069275	1.470637279	0.141389237	0.182026555	positive
PRJNA729271	stage_E18_vs_E10	ordinary_muscle_development	ENSGALG00010015971	ENSGALG00010015971	-1.406334338	0.161392688	-8.713742598	2.94004E-18	1.8724E-17	negative
PRJNA729271	stage_E18_vs_E10	ordinary_muscle_development	ENSGALG00010015972	FSTL4	-1.592358839	0.303584872	-5.245185076	1.56126E-07	4.33699E-07	negative
PRJNA729271	stage_E18_vs_E10	ordinary_muscle_development	ENSGALG00010015974	VPS37A	-0.045851917	0.168759799	-0.271699284	0.785853252	0.820495778	negative
PRJNA729271	stage_E18_vs_E10	ordinary_muscle_development	ENSGALG00010015975	BTK	0.639105233	0.183631529	3.480367641	0.000500726	0.000951498	positive
PRJNA729271	stage_E18_vs_E10	ordinary_muscle_development	ENSGALG00010015979	ENSGALG00010015979	3.39929606	0.20303046	16.74278855	6.39222E-63	2.85973E-61	positive
PRJNA729271	stage_E18_vs_E10	ordinary_muscle_development	ENSGALG00010015980	EXOSC7	-0.873870071	0.092248462	-9.473004183	2.71909E-21	2.08425E-20	negative
PRJNA729271	stage_E18_vs_E10	ordinary_muscle_development	ENSGALG00010015983	CNOT7	-0.535031728	0.065303211	-8.193038626	2.54712E-16	1.42002E-15	negative

PRJNA729271	stage_E18_vs_E10	ordinary_muscle_development	ENSGALG00010015984	PDCL3	0.53026219	0.107816637	4.918185197	8.73503E-07	2.27519E-06	positive
PRJNA729271	stage_E18_vs_E10	ordinary_muscle_development	ENSGALG00010015985	TIMM8A	0.114128524	0.110967741	1.0284838	0.303722307	0.360916121	positive
PRJNA729271	stage_E18_vs_E10	ordinary_muscle_development	ENSGALG00010015986	NEURL2	1.386932811	0.249583114	5.556997791	2.74454E-08	8.18563E-08	positive
PRJNA729271	stage_E18_vs_E10	ordinary_muscle_development	ENSGALG00010015987	POLR2H	-0.65151792	0.098258826	-6.630629983	3.34257E-11	1.26239E-10	negative
PRJNA729271	stage_E18_vs_E10	ordinary_muscle_development	ENSGALG00010015989	ENAH	-0.605099504	0.163162432	-3.708571256	0.000208432	0.00041695	negative
PRJNA729271	stage_E18_vs_E10	ordinary_muscle_development	ENSGALG00010015992	ATP10A	-1.703582169	0.160471544	-10.61610133	2.50819E-26	2.5603E-25	negative
PRJNA729271	stage_E18_vs_E10	ordinary_muscle_development	ENSGALG00010015994	ENSGALG00010015994	-0.228675676	0.11125603	-2.055400296	0.039840357	0.0569176	negative
PRJNA729271	stage_E18_vs_E10	ordinary_muscle_development	ENSGALG00010016004	XRCC3	-0.312740101	0.169677212	-1.843147335	0.065307502	0.089711167	negative
PRJNA729271	stage_E18_vs_E10	ordinary_muscle_development	ENSGALG00010016006	PLTP	-0.564117793	0.319334902	-1.766539735	0.077305341	0.104841029	negative
PRJNA729271	stage_E18_vs_E10	ordinary_muscle_development	ENSGALG00010016008	TAF7	-0.409447194	0.095132781	-4.303954842	1.67776E-05	3.81476E-05	negative
PRJNA729271	stage_E18_vs_E10	ordinary_muscle_development	ENSGALG00010016011	ADD1	-0.120243882	0.080739786	-1.489276702	0.13641452	0.176216778	negative
PRJNA729271	stage_E18_vs_E10	ordinary_muscle_development	ENSGALG00010016015	ZDHHC3	-0.392846193	0.084346988	-4.657501122	3.20071E-06	7.82101E-06	negative
PRJNA729271	stage_E18_vs_E10	ordinary_muscle_development	ENSGALG00010016016	ZDHHC2	-0.10146883	0.279191285	-0.363438385	0.716277432	0.759599366	negative
PRJNA729271	stage_E18_vs_E10	ordinary_muscle_development	ENSGALG00010016017	RAPGEF6	1.005371368	0.071034736	14.15323576	1.78358E-45	4.25914E-44	positive
PRJNA729271	stage_E18_vs_E10	ordinary_muscle_development	ENSGALG00010016022	EPHB3	-0.448533379	0.165294269	-2.713544654	0.00665676	0.01081481	negative
PRJNA729271	stage_E18_vs_E10	ordinary_muscle_development	ENSGALG00010016025	PCIF1	0.11807425	0.108310723	1.090143676	0.27564986	0.331140785	positive
PRJNA729271	stage_E18_vs_E10	ordinary_muscle_development	ENSGALG00010016027	COPS2	-0.514837664	0.150644127	-3.417575419	0.000631816	0.001183838	negative
PRJNA729271	stage_E18_vs_E10	ordinary_muscle_development	ENSGALG00010016030	TGM4	0.949725199	0.716101146	1.326244491	0.184758727	0.231660373	positive
PRJNA729271	stage_E18_vs_E10	ordinary_muscle_development	ENSGALG00010016040	RPSA2	0.428108184	0.10591353	4.042053787	5.29851E-05	0.000113814	positive
PRJNA729271	stage_E18_vs_E10	ordinary_muscle_development	ENSGALG00010016041	ENSGALG00010016041	0.11704841	0.094889856	1.233518677	0.217382313	0.268075262	positive
PRJNA729271	stage_E18_vs_E10	ordinary_muscle_development	ENSGALG00010016043	ADAMTSL1	1.077647151	0.191815776	5.618136176	1.93028E-08	5.81935E-08	positive
PRJNA729271	stage_E18_vs_E10	ordinary_muscle_development	ENSGALG00010016047	SRP9	-0.536269163	0.090014665	-5.957575522	2.56007E-09	8.33894E-09	negative
PRJNA729271	stage_E18_vs_E10	ordinary_muscle_development	ENSGALG00010016049	ZFYVE21	0.742687943	0.122097171	6.08276128	1.1813E-09	3.95907E-09	positive
PRJNA729271	stage_E18_vs_E10	ordinary_muscle_development	ENSGALG00010016053	DRP2	1.339357225	0.472238209	2.836189868	0.00456553	0.007608341	positive
PRJNA729271	stage_E18_vs_E10	ordinary_muscle_development	ENSGALG00010016057	SOHO1	-2.26844775	0.597362809	-3.797437197	0.0001462	0.000298558	negative
PRJNA729271	stage_E18_vs_E10	ordinary_muscle_development	ENSGALG00010016059	EPDR1	-0.488175532	0.202160643	-2.414790165	0.015744277	0.024213612	negative
PRJNA729271	stage_E18_vs_E10	ordinary_muscle_development	ENSGALG00010016060	ENSGALG00010016060	2.453651375	1.362018814	1.801481264	0.071627058	0.097820385	positive
PRJNA729271	stage_E18_vs_E10	ordinary_muscle_development	ENSGALG00010016066	C14orf2	0.89938513	0.149591541	6.012272653	1.8294E-09	6.03212E-09	positive
PRJNA729271	stage_E18_vs_E10	ordinary_muscle_development	ENSGALG00010016067	AHSG	9.601063541	0.84506376	11.3613481	6.51317E-30	7.92682E-29	positive
PRJNA729271	stage_E18_vs_E10	ordinary_muscle_development	ENSGALG00010016069	GALK2	0.587201092	0.145001033	4.049633866	5.12978E-05	0.000110355	positive
PRJNA729271	stage_E18_vs_E10	ordinary_muscle_development	ENSGALG00010016073	SAXO1	0.471209925	1.268507039	0.37146812	0.710288896	0.754320178	positive
PRJNA729271	stage_E18_vs_E10	ordinary_muscle_development	ENSGALG00010016076	RAB3GAP2	-0.12450339	0.091221352	-1.364849207	0.172300449	0.217494994	negative
PRJNA729271	stage_E18_vs_E10	ordinary_muscle_development	ENSGALG00010016083	CDH22	-0.711093138	0.379962264	-1.871483581	0.061278084	0.084625174	negative
PRJNA729271	stage_E18_vs_E10	ordinary_muscle_development	ENSGALG00010016086	TDRD9	-0.558711551	0.335106774	-1.667264267	0.095461869	0.127133387	negative
PRJNA729271	stage_E18_vs_E10	ordinary_muscle_development	ENSGALG00010016087	IARS2	0.01650351	0.090713242	0.181930546	0.855637233	0.880688468	positive
PRJNA729271	stage_E18_vs_E10	ordinary_muscle_development	ENSGALG00010016091	HMX1	-3.776108063	0.99559196	-3.792826995	0.000148942	0.000303687	negative
PRJNA729271	stage_E18_vs_E10	ordinary_muscle_development	ENSGALG00010016094	FGF7	0.356393413	0.185382261	1.922478513	0.054545575	0.07606678	positive
PRJNA729271	stage_E18_vs_E10	ordinary_muscle_development	ENSGALG00010016097	FNIP1	1.15991977	0.110405093	10.50603503	8.1029E-26	8.00624E-25	positive
PRJNA729271	stage_E18_vs_E10	ordinary_muscle_development	ENSGALG00010016100	SFRP4	-3.570889281	0.458635047	-7.785905813	6.92155E-15	3.50185E-14	negative
PRJNA729271	stage_E18_vs_E10	ordinary_muscle_development	ENSGALG00010016103	BPNT1	-0.694772733	0.089838719	-7.733555648	1.04584E-14	5.22198E-14	negative
PRJNA729271	stage_E18_vs_E10	ordinary_muscle_development	ENSGALG00010016107	UVSSA	-0.089973736	0.093902871	-0.95815746	0.337983364	0.396396866	negative
PRJNA729271	stage_E18_vs_E10	ordinary_muscle_development	ENSGALG00010016109	MLH1	-0.263646414	0.103285066	-2.552609229	0.010691938	0.01685844	negative
PRJNA729271	stage_E18_vs_E10	ordinary_muscle_development	ENSGALG00010016111	EPB41	-0.112952621	0.102710529	-1.099718031	0.271454996	0.326588659	negative
PRJNA729271	stage_E18_vs_E10	ordinary_muscle_development	ENSGALG00010016113	DTWD1	0.149854169	0.148728575	1.007568112	0.313661844	0.371205783	positive
PRJNA729271	stage_E18_vs_E10	ordinary_muscle_development	ENSGALG00010016114	HAUS6	-1.282227487	0.151908436	-8.440791845	3.15195E-17	1.86937E-16	negative
PRJNA729271	stage_E18_vs_E10	ordinary_muscle_development	ENSGALG00010016115	RD3L	1.178216026	0.456158078	2.582911678	0.00979704	0.015517245	positive
PRJNA729271	stage_E18_vs_E10	ordinary_muscle_development	ENSGALG00010016117	ENSGALG00010016117	0.93163863	0.233417577	3.991295947	6.57132E-05	0.000139911	positive
PRJNA729271	stage_E18_vs_E10	ordinary_muscle_development	ENSGALG00010016118	EPRS	-0.233129769	0.087663634	-2.659366926	0.007828765	0.012575127	negative
PRJNA729271	stage_E18_vs_E10	ordinary_muscle_development	ENSGALG00010016119	FETUB	4.077542933	1.389399641	2.934751683			positive
PRJNA729271	stage_E18_vs_E10	ordinary_muscle_development	ENSGALG00010016121	SLC35C2	-0.095359688	0.099070231	-0.962546338	0.335775238	0.39418985	negative
PRJNA729271	stage_E18_vs_E10	ordinary_muscle_development	ENSGALG00010016122	AFG2B	-0.077312652	0.134280565	-0.575754594	0.564781068	0.61859616	negative
PRJNA729271	stage_E18_vs_E10	ordinary_muscle_development	ENSGALG00010016127	ENSGALG00010016127	0.133465378	0.172883898	0.771994268	0.440117823	0.499710899	positive
PRJNA729271	stage_E18_vs_E10	ordinary_muscle_development	ENSGALG00010016129	CENPI	-1.453450974	0.234140861	-6.207592164	5.38026E-10	1.86527E-09	negative
PRJNA729271	stage_E18_vs_E10	ordinary_muscle_development	ENSGALG00010016131	SMYD1	0.854832865	0.217519195	3.929919211	8.49744E-05	0.000178508	positive
PRJNA729271	stage_E18_vs_E10	ordinary_muscle_development	ENSGALG00010016137	STARD3NL	-0.016327291	0.141111227	-0.115705115	0.907886252	0.923819672	negative
PRJNA729271	stage_E18_vs_E10	ordinary_muscle_development	ENSGALG00010016139	LURAP1L	-0.372276274	0.197036596	-1.889376295	0.058841425	0.081586613	negative
PRJNA729271	stage_E18_vs_E10	ordinary_muscle_development	ENSGALG00010016141	RRP15	-0.979033098	0.178672972	-5.479469496	4.26603E-08	1.24921E-07	negative
PRJNA729271	stage_E18_vs_E10	ordinary_muscle_development	ENSGALG00010016147	FABP1	0.935411014	0.847956989	1.103134977	0.269968553	0.325043125	positive
PRJNA729271	stage_E18_vs_E10	ordinary_muscle_development	ENSGALG00010016150	POLG	0.208972936	0.095831383	2.180631526	0.029210681	0.042768568	positive
PRJNA729271	stage_E18_vs_E10	ordinary_muscle_development	ENSGALG00010016159	ASPG	-2.544179273	0.199221475	-12.77060757	2.39279E-37	4.10456E-36	negative
PRJNA729271	stage_E18_vs_E10	ordinary_muscle_development	ENSGALG00010016165	KK34	0.458523228	1.169128803	0.392192226	0.694916188	0.740157994	positive
PRJNA729271	stage_E18_vs_E10	ordinary_muscle_development	ENSGALG00010016168	PLIN2	0.551110426	0.229468305	2.401684299	0.016319786	0.025025551	positive
PRJNA729271	stage_E18_vs_E10	ordinary_muscle_development	ENSGALG00010016169	RHOH	-0.353755506	0.124746978	-2.835784186	0.004571332	0.007617136	negative
PRJNA729271	stage_E18_vs_E10	ordinary_muscle_development	ENSGALG00010016173	ENSGALG00010016173	-0.278021482	0.30393321	-0.914745323	0.360325345	0.419914048	negative
PRJNA729271	stage_E18_vs_E10	ordinary_muscle_development	ENSGALG00010016177	ENSGALG00010016177	3.220459536	1.560034992	2.064350833	0.038984465	0.055815865	positive
PRJNA729271	stage_E18_vs_E10	ordinary_muscle_development	ENSGALG00010016181	FANCI	-1.708969847	0.175604122	-9.731946102	2.20337E-22	1.79628E-21	negative
PRJNA729271	stage_E18_vs_E10	ordinary_muscle_development	ENSGALG00010016182	ELMO2	-0.423712946	0.062832384	-6.74354402	1.54569E-11	5.99861E-11	negative
PRJNA729271	stage_E18_vs_E10	ordinary_muscle_development	ENSGALG00010016190	THNSL2	0.19610109	0.153922767	1.274022635	0.202655394	0.251842416	positive
PRJNA729271	stage_E18_vs_E10	ordinary_muscle_development	ENSGALG00010016193	TCAIM	-0.083578038	0.135635011	-0.616198111	0.537763775	0.592857159	negative
PRJNA729271	stage_E18_vs_E10	ordinary_muscle_development	ENSGALG00010016197	SNRPN	-1.456598945	0.116788747	-12.47208302	1.06018E-35	1.68501E-34	negative
PRJNA729271	stage_E18_vs_E10	ordinary_muscle_development	ENSGALG00010016199	RLBP1	-2.594659091	0.369623535	-7.019734537	2.2229E-12	9.2589E-12	negative
PRJNA729271	stage_E18_vs_E10	ordinary_muscle_development	ENSGALG00010016200	MICU3	0.482505923	0.118204457	4.081960476	4.46574E-05	9.657E-05	positive
PRJNA729271	stage_E18_vs_E10	ordinary_muscle_development	ENSGALG00010016205	CD40LG	3.207476921	1.230077337	2.607540862	0.009119519	0.01450696	positive
PRJNA729271	stage_E18_vs_E10	ordinary_muscle_development	ENSGALG00010016206	KNG1	4.356957055	1.147916213	3.795535778	0.000147325	0.000300686	positive
PRJNA729271	stage_E18_vs_E10	ordinary_muscle_development	ENSGALG00010016209	ADAMTS7	0.075717912	0.165238236	0.458234815	0.64678375	0.695536053	positive
PRJNA729271	stage_E18_vs_E10	ordinary_muscle_development	ENSGALG00010016211	TACC3	-1.50624741	0.161783441	-9.310269345	1.27509E-20	9.34397E-20	negative
PRJNA729271	stage_E18_vs_E10	ordinary_muscle_development	ENSGALG00010016215	SPATA17	-1.712596467	0.359090177	-4.769265714	1.84899E-06	4.63927E-06	negative
PRJNA729271	stage_E18_vs_E10	ordinary_muscle_development	ENSGALG00010016218	N4BP2	0.263097959	0.075686019	3.476176467	0.000508617	0.000965607	positive
PRJNA729271	stage_E18_vs_E10	ordinary_muscle_development	ENSGALG00010016221	TMEM200B	0.538846146	0.200906418	2.682075327	0.007316698	0.011804936	positive
PRJNA729271	stage_E18_vs_E10	ordinary_muscle_development	ENSGALG00010016224	MOSPD1	0.410491517	0.116351352	3.528033918	0.000418659	0.000804515	positive
PRJNA729271	stage_E18_vs_E10	ordinary_muscle_development	ENSGALG00010016225	P4HA2	1.346384968	0.156442634	8.606253519	7.54872E-18	4.66405E-17	positive
PRJNA729271	stage_E18_vs_E10	ordinary_muscle_development	ENSGALG00010016229	ENSGALG00010016229	1.124953421	0.219984511	5.113784679	3.15767E-07	8.56644E-07	positive
PRJNA729271	stage_E18_vs_E10	ordinary_muscle_development	ENSGALG00010016235	DDX27	-1.028111651	0.125133199	-8.216138128	2.10161E-16	1.17845E-15	negative
PRJNA729271	stage_E18_vs_E10	ordinary_muscle_development	ENSGALG00010016236	GPATCH2	-0.086228996	0.065170928	-1.323120575	0.185795283	0.232826371	negative
PRJNA729271	stage_E18_vs_E10	ordinary_muscle_development	ENSGALG00010016240	EIF4A2	0.269038555	0.100360276	2.680727539	0.00734623	0.011848116	positive
PRJNA729271	stage_E18_vs_E10	ordinary_muscle_development	ENSGALG00010016241	TOPAZ1	0.966626785	0.801457331	1.206086398	0.227784179	0.279216209	positive
PRJNA729271	stage_E18_vs_E10	ordinary_muscle_development	ENSGALG00010016242	PDS5A	-0.29744855	0.092770233	-3.206293032	0.00134457	0.002413267	negative
PRJNA729271	stage_E18_vs_E10	ordinary_muscle_development	ENSGALG00010016245	FAM96A	-0.134345786	0.105236443	-1.276608956	0.201740339	0.250834295	negative
PRJNA729271	stage_E18_vs_E10	ordinary_muscle_development	ENSGALG00010016247	KIF26A	-1.987136501	0.16715751	-11.88780867	1.36953E-32	1.86924E-31	negative
PRJNA729271	stage_E18_vs_E10	ordinary_muscle_development	ENSGALG00010016251	MPDZ	-0.348659124	0.07329545	-4.756899937	1.96589E-06	4.91556E-06	negative
PRJNA729271	stage_E18_vs_E10	ordinary_muscle_development	ENSGALG00010016253	EDN3	0.175208905	0.203608907	0.860516896	0.389504174	0.449582975	positive
PRJNA729271	stage_E18_vs_E10	ordinary_muscle_development	ENSGALG00010016257	ENSGALG00010016257	-0.55723897	0.111900302	-4.979780756	6.36563E-07	1.6752E-06	negative
PRJNA729271	stage_E18_vs_E10	ordinary_muscle_development	ENSGALG00010016258	CTNNB1	-1.060745735	0.077096647	-13.75864939	4.5189E-43	9.74724E-42	negative
PRJNA729271	stage_E18_vs_E10	ordinary_muscle_development	ENSGALG00010016260	INTS6L	0.035110962	0.145543609	0.241240148	0.809368996	0.841351377	positive
PRJNA729271	stage_E18_vs_E10	ordinary_muscle_development	ENSGALG00010016262	SPG11	0.319241479	0.061338624	5.20457514	1.94441E-07	5.36331E-07	positive
PRJNA729271	stage_E18_vs_E10	ordinary_muscle_development	ENSGALG00010016263	HGFAC	3.738870747	1.325304452	2.821141016			positive
PRJNA729271	stage_E18_vs_E10	ordinary_muscle_development	ENSGALG00010016266	PDLIM4	0.194909761	0.120605218	1.616097244	0.10607329	0.139979273	positive
PRJNA729271	stage_E18_vs_E10	ordinary_muscle_development	ENSGALG00010016270	RFC4	-1.129129937	0.111168258	-10.15694545	3.08593E-24	2.78695E-23	negative
PRJNA729271	stage_E18_vs_E10	ordinary_muscle_development	ENSGALG00010016274	ZNF831	0.087838345	0.566619323	0.155021795	0.876804135	0.898070225	positive
PRJNA729271	stage_E18_vs_E10	ordinary_muscle_development	ENSGALG00010016276	ACTR2	-1.186178193	0.108197127	-10.96312093	5.74822E-28	6.37982E-27	negative
PRJNA729271	stage_E18_vs_E10	ordinary_muscle_development	ENSGALG00010016279	PRELID3B	-0.768524014	0.10689361	-7.18961608	6.49738E-13	2.82482E-12	negative
PRJNA729271	stage_E18_vs_E10	ordinary_muscle_development	ENSGALG00010016281	SLC22A4	-0.60249322	0.210935156	-2.856295892	0.004286155	0.007168318	negative
PRJNA729271	stage_E18_vs_E10	ordinary_muscle_development	ENSGALG00010016286	ULK4	-0.568468516	0.136014141	-4.179480994	2.92175E-05	6.45505E-05	negative
PRJNA729271	stage_E18_vs_E10	ordinary_muscle_development	ENSGALG00010016287	BCL11B	-6.678498764	0.387456462	-17.23677217	1.40675E-66	6.8197E-65	negative
PRJNA729271	stage_E18_vs_E10	ordinary_muscle_development	ENSGALG00010016289	IRF1	1.430023621	0.266956211	5.356772247	8.47218E-08	2.41455E-07	positive
PRJNA729271	stage_E18_vs_E10	ordinary_muscle_development	ENSGALG00010016292	PHKA1	0.831973176	0.150333118	5.534197552	3.12656E-08	9.28109E-08	positive
PRJNA729271	stage_E18_vs_E10	ordinary_muscle_development	ENSGALG00010016295	UBE2K	-0.779843397	0.094625444	-8.241371037	1.70248E-16	9.60962E-16	negative
PRJNA729271	stage_E18_vs_E10	ordinary_muscle_development	ENSGALG00010016299	SRSF4	-0.201038054	0.107693896	-1.866754393	0.061935894	0.085435933	negative
PRJNA729271	stage_E18_vs_E10	ordinary_muscle_development	ENSGALG00010016303	ENSGALG00010016303	0.954591829	0.203388073	4.693450377	2.68635E-06	6.6211E-06	positive
PRJNA729271	stage_E18_vs_E10	ordinary_muscle_development	ENSGALG00010016304	EIF3J	0.165151514	0.100264156	1.647164062	0.099524328	0.132022068	positive
PRJNA729271	stage_E18_vs_E10	ordinary_muscle_development	ENSGALG00010016305	DENND4C	0.880309292	0.096343548	9.137189921	6.40961E-20	4.48178E-19	positive
PRJNA729271	stage_E18_vs_E10	ordinary_muscle_development	ENSGALG00010016307	RAD50	-0.263971986	0.12845717	-2.054941626	0.039884643	0.05696964	negative
PRJNA729271	stage_E18_vs_E10	ordinary_muscle_development	ENSGALG00010016308	RPS6	0.58788009	0.140705013	4.17810336	2.9395E-05	6.49018E-05	positive
PRJNA729271	stage_E18_vs_E10	ordinary_muscle_development	ENSGALG00010016310	HDAC8	-0.451082192	0.093242988	-4.837706308	1.31346E-06	3.35541E-06	negative
PRJNA729271	stage_E18_vs_E10	ordinary_muscle_development	ENSGALG00010016312	MECR	-1.178292642	0.15878799	-7.420540073	1.16644E-13	5.34034E-13	negative
PRJNA729271	stage_E18_vs_E10	ordinary_muscle_development	ENSGALG00010016314	ACER2	0.369661906	0.157125674	2.352651202	0.01864011	0.028232852	positive
PRJNA729271	stage_E18_vs_E10	ordinary_muscle_development	ENSGALG00010016315	RPS4Y1	1.119995932	0.136405299	8.210794906	2.19729E-16	1.23114E-15	positive
PRJNA729271	stage_E18_vs_E10	ordinary_muscle_development	ENSGALG00010016317	CTDSPL2	0.163491515	0.05644588	2.896429552	0.003774354	0.006357106	positive
PRJNA729271	stage_E18_vs_E10	ordinary_muscle_development	ENSGALG00010016319	RBM41	0.155275132	0.19067843	0.814329822	0.41545603	0.475802933	positive
PRJNA729271	stage_E18_vs_E10	ordinary_muscle_development	ENSGALG00010016322	EBF4	0.288729827	0.131453699	2.196437442	0.028060648	0.041217987	positive
PRJNA729271	stage_E18_vs_E10	ordinary_muscle_development	ENSGALG00010016324	TUBB1	-0.843880459	0.212521622	-3.970798132	7.16323E-05	0.000151777	negative
PRJNA729271	stage_E18_vs_E10	ordinary_muscle_development	ENSGALG00010016328	USH2A	0.314585557	0.642691094	0.489481744	0.624500676	0.674928221	positive
PRJNA729271	stage_E18_vs_E10	ordinary_muscle_development	ENSGALG00010016332	ENSGALG00010016332	-1.373774045	0.252827353	-5.433644843	5.52144E-08	1.59843E-07	negative
PRJNA729271	stage_E18_vs_E10	ordinary_muscle_development	ENSGALG00010016335	ABHD4	1.408589986	0.184613636	7.62993471	2.34873E-14	1.13369E-13	positive
PRJNA729271	stage_E18_vs_E10	ordinary_muscle_development	ENSGALG00010016336	SLC24A2	-0.616334449	0.37773881	-1.631641846	0.102754957	0.13593439	negative
PRJNA729271	stage_E18_vs_E10	ordinary_muscle_development	ENSGALG00010016338	PTPRU	-1.253314592	0.151974979	-8.246848283	1.62627E-16	9.19732E-16	negative
PRJNA729271	stage_E18_vs_E10	ordinary_muscle_development	ENSGALG00010016339	SETD3	1.740607943	0.121624019	14.3113818	1.85781E-46	4.71611E-45	positive
PRJNA729271	stage_E18_vs_E10	ordinary_muscle_development	ENSGALG00010016340	RGS12	-1.735581935	0.101600889	-17.08234993	2.00864E-65	9.42239E-64	negative
PRJNA729271	stage_E18_vs_E10	ordinary_muscle_development	ENSGALG00010016341	SLX4	-0.575879533	0.064700393	-8.900711534	5.54898E-19	3.69465E-18	negative
PRJNA729271	stage_E18_vs_E10	ordinary_muscle_development	ENSGALG00010016344	SMIM14	0.153630543	0.112132292	1.370082962	0.170661006	0.215632085	positive
PRJNA729271	stage_E18_vs_E10	ordinary_muscle_development	ENSGALG00010016345	CTSZ	-0.783286508	0.112982304	-6.932824693	4.12519E-12	1.68531E-11	negative

PRJNA729271	stage_E18_vs_E10	ordinary_muscle_development	ENSGALG00010016346	TRAP1	-0.475185196	0.104693481	-4.538823172	5.6569E-06	1.35041E-05	negative
PRJNA729271	stage_E18_vs_E10	ordinary_muscle_development	ENSGALG00010016347	ANO10	0.299909137	0.090313608	3.320752463	0.000897751	0.001645953	positive
PRJNA729271	stage_E18_vs_E10	ordinary_muscle_development	ENSGALG00010016350	CASC4	0.09894385	0.063017614	1.570098313	0.116392241	0.152554981	positive
PRJNA729271	stage_E18_vs_E10	ordinary_muscle_development	ENSGALG00010016353	C1orf232	0.623765781	0.14172034	4.401385001	1.07562E-05	2.49858E-05	positive
PRJNA729271	stage_E18_vs_E10	ordinary_muscle_development	ENSGALG00010016355	MCF2L2	0.18105611	0.230570917	0.785251288	0.43230625	0.492167695	positive
PRJNA729271	stage_E18_vs_E10	ordinary_muscle_development	ENSGALG00010016361	NELFCD	-0.776527387	0.083264585	-9.32602242	1.09919E-20	8.10412E-20	negative
PRJNA729271	stage_E18_vs_E10	ordinary_muscle_development	ENSGALG00010016362	NUP62	-0.260402588	0.170083638	-1.53102668	0.125762799	0.163873766	negative
PRJNA729271	stage_E18_vs_E10	ordinary_muscle_development	ENSGALG00010016366	KIF3A	-0.475989298	0.099005154	-4.80772241	1.5266E-06	3.86921E-06	negative
PRJNA729271	stage_E18_vs_E10	ordinary_muscle_development	ENSGALG00010016368	CCNK	-0.301159771	0.151224881	-1.991469721	0.046429271	0.065536302	negative
PRJNA729271	stage_E18_vs_E10	ordinary_muscle_development	ENSGALG00010016369	MSANTD1	-2.051195554	0.273306489	-7.505111064	6.13765E-14	2.86985E-13	negative
PRJNA729271	stage_E18_vs_E10	ordinary_muscle_development	ENSGALG00010016370	TMEM255A	2.746899843	0.185888109	14.77716812	2.05632E-49	5.90225E-48	positive
PRJNA729271	stage_E18_vs_E10	ordinary_muscle_development	ENSGALG00010016372	DNAAF6	-0.524017198	0.201897974	-2.595455449	0.009446565	0.014991018	negative
PRJNA729271	stage_E18_vs_E10	ordinary_muscle_development	ENSGALG00010016373	SRL	2.812597819	0.13168327	21.358809	3.2294E-101	3.86861E-99	positive
PRJNA729271	stage_E18_vs_E10	ordinary_muscle_development	ENSGALG00010016374	FRMD5	2.581482213	0.348047909	7.417031237	1.19775E-13	5.47676E-13	positive
PRJNA729271	stage_E18_vs_E10	ordinary_muscle_development	ENSGALG00010016375	CCNI2	-1.313920156	0.138905844	-9.459070359	3.10693E-21	2.37276E-20	negative
PRJNA729271	stage_E18_vs_E10	ordinary_muscle_development	ENSGALG00010016377	FRMPD3	4.495594291	0.276921267	16.23419661	2.89007E-59	1.13836E-57	positive
PRJNA729271	stage_E18_vs_E10	ordinary_muscle_development	ENSGALG00010016378	WDR76	-0.023339174	0.172148993	-0.13557543	0.89215693	0.910691176	negative
PRJNA729271	stage_E18_vs_E10	ordinary_muscle_development	ENSGALG00010016379	TFAP4	-1.016899282	0.155974324	-6.519658211	7.04678E-11	2.60038E-10	negative
PRJNA729271	stage_E18_vs_E10	ordinary_muscle_development	ENSGALG00010016382	UGDH	-0.497358664	0.186091804	-2.67265217	0.007525423	0.012120113	negative
PRJNA729271	stage_E18_vs_E10	ordinary_muscle_development	ENSGALG00010016383	FAM110D	1.001638086	0.175802065	5.697533102	1.21553E-08	3.72498E-08	positive
PRJNA729271	stage_E18_vs_E10	ordinary_muscle_development	ENSGALG00010016384	GNAS	0.124330827	0.074517153	1.668485991	0.095219291	0.126845291	positive
PRJNA729271	stage_E18_vs_E10	ordinary_muscle_development	ENSGALG00010016385	HTT	-0.075524178	0.107091645	-0.70522941	0.480667482	0.538799405	negative
PRJNA729271	stage_E18_vs_E10	ordinary_muscle_development	ENSGALG00010016387	CCDC85C	-0.507245675	0.159255934	-3.185097481	0.001447053	0.002586316	negative
PRJNA729271	stage_E18_vs_E10	ordinary_muscle_development	ENSGALG00010016390	ENSGALG00010016390	1.091976249	0.354124919	3.08359054	0.002045189	0.003586813	positive
PRJNA729271	stage_E18_vs_E10	ordinary_muscle_development	ENSGALG00010016392	STMN3	1.118886316	0.310375472	3.604944385	0.00031222	0.000609676	positive
PRJNA729271	stage_E18_vs_E10	ordinary_muscle_development	ENSGALG00010016393	BLM	-1.447268697	0.124210125	-11.65177712	2.24723E-31	2.94784E-30	negative
PRJNA729271	stage_E18_vs_E10	ordinary_muscle_development	ENSGALG00010016394	PDIK1L	0.58120523	0.095998144	6.054338172	1.40996E-09	4.69501E-09	positive
PRJNA729271	stage_E18_vs_E10	ordinary_muscle_development	ENSGALG00010016395	ENSGALG00010016395	-0.437095125	0.161671422	-2.703601656	0.006859246	0.011112638	negative
PRJNA729271	stage_E18_vs_E10	ordinary_muscle_development	ENSGALG00010016397	LIAS	0.245001391	0.104712437	2.339754455	0.019296422	0.029172052	positive
PRJNA729271	stage_E18_vs_E10	ordinary_muscle_development	ENSGALG00010016398	SEPTIN6	-0.531975149	0.06891113	-7.719727571	1.16579E-14	5.78701E-14	negative
PRJNA729271	stage_E18_vs_E10	ordinary_muscle_development	ENSGALG00010016399	ACSM5	5.753681291	0.329651234	17.45384422	3.21826E-68	1.64256E-66	positive
PRJNA729271	stage_E18_vs_E10	ordinary_muscle_development	ENSGALG00010016400	ENSGALG00010016400	0.20698725	0.086768247	2.3855184	0.017055069	0.026036148	positive
PRJNA729271	stage_E18_vs_E10	ordinary_muscle_development	ENSGALG00010016402	SEPTIN8	-0.693626613	0.077881168	-8.90621737	5.28033E-19	3.52063E-18	negative
PRJNA729271	stage_E18_vs_E10	ordinary_muscle_development	ENSGALG00010016403	GRK4	-1.159608063	0.135798158	-8.539203156	1.35151E-17	8.22421E-17	negative
PRJNA729271	stage_E18_vs_E10	ordinary_muscle_development	ENSGALG00010016404	ESRRG	1.269635237	0.14948757	8.493249554	2.00938E-17	1.20654E-16	positive
PRJNA729271	stage_E18_vs_E10	ordinary_muscle_development	ENSGALG00010016407	RPL9	0.951412196	0.180707806	5.264920306	1.4025E-07	3.9155E-07	positive
PRJNA729271	stage_E18_vs_E10	ordinary_muscle_development	ENSGALG00010016409	GMEB2	1.246891752	0.206914169	6.026130332	1.67932E-09	5.5524E-09	positive
PRJNA729271	stage_E18_vs_E10	ordinary_muscle_development	ENSGALG00010016410	TRAK1	0.338669335	0.097667748	3.467565721	0.000525195	0.000995255	positive
PRJNA729271	stage_E18_vs_E10	ordinary_muscle_development	ENSGALG00010016411	CTSH	0.213004836	0.124719427	1.707872152	0.087660067	0.117487996	positive
PRJNA729271	stage_E18_vs_E10	ordinary_muscle_development	ENSGALG00010016412	TMSB15B	-1.664277775	0.215054512	-7.738864727	1.00308E-14	5.01369E-14	negative
PRJNA729271	stage_E18_vs_E10	ordinary_muscle_development	ENSGALG00010016415	RASGRF1	2.048107961	0.548706013	3.732614392	0.000189503	0.000381029	positive
PRJNA729271	stage_E18_vs_E10	ordinary_muscle_development	ENSGALG00010016416	CYP46A1	0.356298429	0.114506876	3.111589798	0.001860829	0.003283349	positive
PRJNA729271	stage_E18_vs_E10	ordinary_muscle_development	ENSGALG00010016417	NOP14	-1.500052393	0.110761993	-13.54302461	8.71263E-42	1.78375E-40	negative
PRJNA729271	stage_E18_vs_E10	ordinary_muscle_development	ENSGALG00010016418	NFIB	0.299319743	0.098011872	3.053913144	0.002258775	0.003932846	positive
PRJNA729271	stage_E18_vs_E10	ordinary_muscle_development	ENSGALG00010016421	ENSGALG00010016421	-0.88162663	0.393560318	-2.240130905	0.025082426	0.037197643	negative
PRJNA729271	stage_E18_vs_E10	ordinary_muscle_development	ENSGALG00010016422	KCTD3	-0.25865347	0.090905556	-2.845298801	0.004436978	0.007405181	negative
PRJNA729271	stage_E18_vs_E10	ordinary_muscle_development	ENSGALG00010016423	TRMT44	-0.762282018	0.113194884	-6.734244419	1.64784E-11	6.36775E-11	negative
PRJNA729271	stage_E18_vs_E10	ordinary_muscle_development	ENSGALG00010016425	TRIM63	3.329977778	0.414024928	8.042940308	8.77081E-16	4.73317E-15	positive
PRJNA729271	stage_E18_vs_E10	ordinary_muscle_development	ENSGALG00010016426	HELZ2	-0.24653569	0.37418924	-0.658852964	0.509990193	0.566894773	negative
PRJNA729271	stage_E18_vs_E10	ordinary_muscle_development	ENSGALG00010016428	ADRA1D	1.06976262	0.201485446	5.309379125	1.09999E-07	3.10989E-07	positive
PRJNA729271	stage_E18_vs_E10	ordinary_muscle_development	ENSGALG00010016429	ENSGALG00010016429	-0.398770764	0.393388146	-1.013682715	0.310734174	0.368372606	negative
PRJNA729271	stage_E18_vs_E10	ordinary_muscle_development	ENSGALG00010016431	B3GNT5	-3.346653451	0.160793445	-20.81336986	3.27496E-96	3.51633E-94	negative
PRJNA729271	stage_E18_vs_E10	ordinary_muscle_development	ENSGALG00010016433	TMED3	0.669352169	0.14210753	4.710180861	2.47497E-06	6.1251E-06	positive
PRJNA729271	stage_E18_vs_E10	ordinary_muscle_development	ENSGALG00010016434	GPR139	-2.084826923	0.568118357	-3.66970526	0.00024283	0.000481574	negative
PRJNA729271	stage_E18_vs_E10	ordinary_muscle_development	ENSGALG00010016436	SLC30A2	0.795721218	0.612700101	1.298712399	0.194042647	0.241989863	positive
PRJNA729271	stage_E18_vs_E10	ordinary_muscle_development	ENSGALG00010016441	ENSGALG00010016441	0.962727002	0.568671659	1.692940007	0.090466887	0.120936782	positive
PRJNA729271	stage_E18_vs_E10	ordinary_muscle_development	ENSGALG00010016445	ZDHHC21	-0.285874664	0.142066023	-2.012266254	0.044191878	0.062622081	negative
PRJNA729271	stage_E18_vs_E10	ordinary_muscle_development	ENSGALG00010016447	ID3	-1.149299498	0.1132622	-10.14724684	3.40843E-24	3.07056E-23	negative
PRJNA729271	stage_E18_vs_E10	ordinary_muscle_development	ENSGALG00010016449	FMR1	-0.180736753	0.107127209	-1.687122764	0.091579749	0.122311661	negative
PRJNA729271	stage_E18_vs_E10	ordinary_muscle_development	ENSGALG00010016450	ENSGALG00010016450	-0.113511857	0.268269875	-0.423125621	0.67220361	0.718872018	negative
PRJNA729271	stage_E18_vs_E10	ordinary_muscle_development	ENSGALG00010016451	USP46	-0.722528426	0.148966006	-4.85029065	1.23281E-06	3.15939E-06	negative
PRJNA729271	stage_E18_vs_E10	ordinary_muscle_development	ENSGALG00010016452	GLCE	-0.121857773	0.114465338	-1.064582305	0.287064991	0.343062663	negative
PRJNA729271	stage_E18_vs_E10	ordinary_muscle_development	ENSGALG00010016453	MYCL	-0.903399429	0.178395403	-5.06402863	4.10488E-07	1.10124E-06	negative
PRJNA729271	stage_E18_vs_E10	ordinary_muscle_development	ENSGALG00010016454	ANKS3	0.10420257	0.094941914	1.097540226	0.272405306	0.32756885	positive
PRJNA729271	stage_E18_vs_E10	ordinary_muscle_development	ENSGALG00010016455	RBM27	-0.616390756	0.092227702	-6.683358036	2.33528E-11	8.93373E-11	negative
PRJNA729271	stage_E18_vs_E10	ordinary_muscle_development	ENSGALG00010016456	LAMP3	-0.943693738	0.370051516	-2.550168551	0.010767085	0.016960324	negative
PRJNA729271	stage_E18_vs_E10	ordinary_muscle_development	ENSGALG00010016457	SMOX	0.825264674	0.17804783	4.635072908	3.56812E-06	8.6691E-06	positive
PRJNA729271	stage_E18_vs_E10	ordinary_muscle_development	ENSGALG00010016458	ZC3H12A	0.471018766	0.091031001	5.174267655	2.28807E-07	6.27303E-07	positive
PRJNA729271	stage_E18_vs_E10	ordinary_muscle_development	ENSGALG00010016459	PAQR5	1.540739933	0.292983182	5.258799909	1.44999E-07	4.0395E-07	positive
PRJNA729271	stage_E18_vs_E10	ordinary_muscle_development	ENSGALG00010016460	LBR	-1.265079406	0.069656623	-18.16165288	1.0385E-73	6.02125E-72	negative
PRJNA729271	stage_E18_vs_E10	ordinary_muscle_development	ENSGALG00010016461	HPCAL4	-1.528802626	0.266865988	-5.728727884	1.01187E-08	3.12396E-08	negative
PRJNA729271	stage_E18_vs_E10	ordinary_muscle_development	ENSGALG00010016462	RNF24	-0.287255656	0.154934529	-1.854045435	0.06373266	0.08772243	negative
PRJNA729271	stage_E18_vs_E10	ordinary_muscle_development	ENSGALG00010016463	FREM1	1.028767305	0.142207449	7.234271576	4.68034E-13	2.05456E-12	positive
PRJNA729271	stage_E18_vs_E10	ordinary_muscle_development	ENSGALG00010016464	PANK2	-0.182565927	0.088808292	-2.055730653	0.039808486	0.056883281	negative
PRJNA729271	stage_E18_vs_E10	ordinary_muscle_development	ENSGALG00010016465	STK35	-1.465984748	0.21351554	-6.865939355	6.60551E-12	2.63838E-11	negative
PRJNA729271	stage_E18_vs_E10	ordinary_muscle_development	ENSGALG00010016466	TMEM164	-0.756770055	0.23665726	-3.197747054	0.001385057	0.002481632	negative
PRJNA729271	stage_E18_vs_E10	ordinary_muscle_development	ENSGALG00010016468	TTC39B	-0.225852122	0.167540309	-1.348046472	0.177643436	0.223558049	negative
PRJNA729271	stage_E18_vs_E10	ordinary_muscle_development	ENSGALG00010016469	MAVS	0.835292142	0.102265541	8.167874853	3.13869E-16	1.73978E-15	positive
PRJNA729271	stage_E18_vs_E10	ordinary_muscle_development	ENSGALG00010016470	TNIP1	0.709278128	0.158712393	4.468952377	7.86036E-06	1.85081E-05	positive
PRJNA729271	stage_E18_vs_E10	ordinary_muscle_development	ENSGALG00010016471	POMGNT2	0.1085254	0.138768711	0.782059586	0.434179556	0.493951234	positive
PRJNA729271	stage_E18_vs_E10	ordinary_muscle_development	ENSGALG00010016472	TFDP1	-1.041201979	0.122074423	-8.529239396	1.47313E-17	8.9418E-17	negative
PRJNA729271	stage_E18_vs_E10	ordinary_muscle_development	ENSGALG00010016473	RAB9B	-1.126516432	0.162720455	-6.923016732	4.42126E-12	1.80118E-11	negative
PRJNA729271	stage_E18_vs_E10	ordinary_muscle_development	ENSGALG00010016474	MAPK8IP3	0.460403284	0.109858276	4.190883949	2.7787E-05	6.15766E-05	positive
PRJNA729271	stage_E18_vs_E10	ordinary_muscle_development	ENSGALG00010016475	MCCC1	0.689595237	0.08404479	8.205092046	2.30414E-16	1.28952E-15	positive
PRJNA729271	stage_E18_vs_E10	ordinary_muscle_development	ENSGALG00010016477	CNIH3	0.199955184	0.195307734	1.023795522	0.305931866	0.363257429	positive
PRJNA729271	stage_E18_vs_E10	ordinary_muscle_development	ENSGALG00010016479	ENSGALG00010016479	-1.85115926	0.407489736	-4.542836531	5.55023E-06	1.32669E-05	negative
PRJNA729271	stage_E18_vs_E10	ordinary_muscle_development	ENSGALG00010016480	GPX3	4.048782994	0.133268088	30.38073905	9.8694E-203	5.9607E-200	positive
PRJNA729271	stage_E18_vs_E10	ordinary_muscle_development	ENSGALG00010016482	EML1	1.152281647	0.140458564	8.2037123	2.33076E-16	1.30341E-15	positive
PRJNA729271	stage_E18_vs_E10	ordinary_muscle_development	ENSGALG00010016483	WDR26	0.068088131	0.103450802	0.65816919	0.51042942	0.567252507	positive
PRJNA729271	stage_E18_vs_E10	ordinary_muscle_development	ENSGALG00010016489	CCK	-4.327540849	0.546015177	-7.925678677	2.26904E-15	1.18564E-14	negative
PRJNA729271	stage_E18_vs_E10	ordinary_muscle_development	ENSGALG00010016491	DNAJC8	-0.308335653	0.054391936	-5.668775102	1.43822E-08	4.38515E-08	negative
PRJNA729271	stage_E18_vs_E10	ordinary_muscle_development	ENSGALG00010016496	PLP1	3.810857731	0.327201766	11.64681284	2.38202E-31	3.11619E-30	positive
PRJNA729271	stage_E18_vs_E10	ordinary_muscle_development	ENSGALG00010016498	KIF23	-1.771577927	0.207111924	-8.553722528	1.19181E-17	7.28609E-17	negative
PRJNA729271	stage_E18_vs_E10	ordinary_muscle_development	ENSGALG00010016500	ENSGALG00010016500	0.881807747	0.106763156	8.259476183	1.46313E-16	8.30387E-16	positive
PRJNA729271	stage_E18_vs_E10	ordinary_muscle_development	ENSGALG00010016502	YIPF5	-0.510274124	0.132136966	-3.861706074	0.000112598	0.000233458	negative
PRJNA729271	stage_E18_vs_E10	ordinary_muscle_development	ENSGALG00010016504	SNRK	0.766156582	0.085019844	9.011503067	2.03251E-19	1.38251E-18	positive
PRJNA729271	stage_E18_vs_E10	ordinary_muscle_development	ENSGALG00010016505	KCTD16	-0.983036643	0.294949361	-3.332899719	0.000859459	0.001580947	negative
PRJNA729271	stage_E18_vs_E10	ordinary_muscle_development	ENSGALG00010016507	SPATA18	0.610790985	0.140424251	4.349611857	1.36379E-05	3.13183E-05	positive
PRJNA729271	stage_E18_vs_E10	ordinary_muscle_development	ENSGALG00010016508	CREBBP	0.541548292	0.119667539	4.525440197	6.02699E-06	1.43545E-05	positive
PRJNA729271	stage_E18_vs_E10	ordinary_muscle_development	ENSGALG00010016510	THG1L	-0.340564171	0.11204179	-3.039617368	0.002368789	0.004111063	negative
PRJNA729271	stage_E18_vs_E10	ordinary_muscle_development	ENSGALG00010016514	GASK1A	-0.240989877	0.238225834	-1.011602616	0.311728094	0.369309254	negative
PRJNA729271	stage_E18_vs_E10	ordinary_muscle_development	ENSGALG00010016518	TMEM42	-1.119788485	0.154689921	-7.238923365	4.5226E-13	1.98772E-12	negative
PRJNA729271	stage_E18_vs_E10	ordinary_muscle_development	ENSGALG00010016519	MEX3B	-1.576461514	0.177272155	-8.892888529	5.95405E-19	3.95527E-18	negative
PRJNA729271	stage_E18_vs_E10	ordinary_muscle_development	ENSGALG00010016520	CHMP1B	-0.12052898	0.083371487	-1.445685854	0.148265334	0.189967826	negative
PRJNA729271	stage_E18_vs_E10	ordinary_muscle_development	ENSGALG00010016526	EIF1B	0.266832843	0.119603249	2.230983232	0.02568224	0.037979159	positive
PRJNA729271	stage_E18_vs_E10	ordinary_muscle_development	ENSGALG00010016527	MRPS34	-0.145526252	0.098519781	-1.477127238	0.139641533	0.180032378	negative
PRJNA729271	stage_E18_vs_E10	ordinary_muscle_development	ENSGALG00010016529	DCUN1D1	-0.539257552	0.098650923	-5.466320402	4.59474E-08	1.34167E-07	negative
PRJNA729271	stage_E18_vs_E10	ordinary_muscle_development	ENSGALG00010016533	CNIH4	-0.039937238	0.110875623	-0.360198542	0.718698665	0.761571544	negative
PRJNA729271	stage_E18_vs_E10	ordinary_muscle_development	ENSGALG00010016534	TRIT1	-0.215292377	0.145625677	-1.478395713	0.139301892	0.179658415	negative
PRJNA729271	stage_E18_vs_E10	ordinary_muscle_development	ENSGALG00010016535	BNC2	-0.778256368	0.131116815	-5.935595438	2.92781E-09	9.49409E-09	negative
PRJNA729271	stage_E18_vs_E10	ordinary_muscle_development	ENSGALG00010016541	UBE2J2	-0.523943124	0.079886058	-6.558630322	5.43042E-11	2.0235E-10	negative
PRJNA729271	stage_E18_vs_E10	ordinary_muscle_development	ENSGALG00010016542	ENOX2	-0.510155007	0.078497706	-6.498979796	8.08665E-11	2.97201E-10	negative
PRJNA729271	stage_E18_vs_E10	ordinary_muscle_development	ENSGALG00010016543	TCEA3	5.666373203	0.173869282	32.58984645	5.7119E-233	5.1746E-230	positive
PRJNA729271	stage_E18_vs_E10	ordinary_muscle_development	ENSGALG00010016544	ADCY9	1.225712444	0.135629108	9.037237344	1.60681E-19	1.10122E-18	positive
PRJNA729271	stage_E18_vs_E10	ordinary_muscle_development	ENSGALG00010016546	C1QTNF12	-1.812337497	0.439640646	-4.122315609	3.75083E-05	8.18676E-05	negative
PRJNA729271	stage_E18_vs_E10	ordinary_muscle_development	ENSGALG00010016547	SGCB	3.294385176	0.143099546	23.02163263	2.8308E-117	5.004E-115	positive
PRJNA729271	stage_E18_vs_E10	ordinary_muscle_development	ENSGALG00010016548	EVL	0.250243011	0.108836337	2.299259762	0.021490193	0.032226397	positive
PRJNA729271	stage_E18_vs_E10	ordinary_muscle_development	ENSGALG00010016549	PTPRA	-0.457271274	0.103203145	-4.430788162	9.38893E-06	2.19151E-05	negative
PRJNA729271	stage_E18_vs_E10	ordinary_muscle_development	ENSGALG00010016550	FBLIM1	-1.728965266	0.144495712	-11.9655126	5.38639E-33	7.48569E-32	negative
PRJNA729271	stage_E18_vs_E10	ordinary_muscle_development	ENSGALG00010016552	ENSGALG00010016552	4.532224338	0.438137284	10.3443019	4.44141E-25	4.2243E-24	positive
PRJNA729271	stage_E18_vs_E10	ordinary_muscle_development	ENSGALG00010016553	SLC25A14	-0.401271132	0.097402499	-4.119721106	3.79331E-05	8.27699E-05	negative
PRJNA729271	stage_E18_vs_E10	ordinary_muscle_development	ENSGALG00010016556	ASAP3	0.144135201	0.195981447	0.735453294	0.462063475	0.521336504	positive
PRJNA729271	stage_E18_vs_E10	ordinary_muscle_development	ENSGALG00010016558	NR2F2	-0.298320827	0.109463975	-2.725287727	0.006424547	0.010464526	negative
PRJNA729271	stage_E18_vs_E10	ordinary_muscle_development	ENSGALG00010016559	ROGDI	-0.352251125	0.115821979	-3.041315025	0.002355472	0.004089911	negative
PRJNA729271	stage_E18_vs_E10	ordinary_muscle_development	ENSGALG00010016560	DCUN1D4	1.182391314	0.169377565	6.980802412	2.93499E-12	1.21135E-11	positive
PRJNA729271	stage_E18_vs_E10	ordinary_muscle_development	ENSGALG00010016561	DEGS2	1.138392348	0.603167176	1.887357921	0.059112201	0.081922875	positive
PRJNA729271	stage_E18_vs_E10	ordinary_muscle_development	ENSGALG00010016562	GLIS2	0.830670579	0.221038616	3.758033757	0.000171254	0.000346355	positive
PRJNA729271	stage_E18_vs_E10	ordinary_muscle_development	ENSGALG00010016564	SPEN	0.67942268	0.166062109	4.091376915	4.2882E-05	9.30082E-05	positive
PRJNA729271	stage_E18_vs_E10	ordinary_muscle_development	ENSGALG00010016565	NVL	-0.790319082	0.099335125	-7.95608889	1.77563E-15	9.35918E-15	negative
PRJNA729271	stage_E18_vs_E10	ordinary_muscle_development	ENSGALG00010016566	CNTLN	-0.735019029	0.206137712	-3.565669867	0.000362928	0.000702637	negative
PRJNA729271	stage_E18_vs_E10	ordinary_muscle_development	ENSGALG00010016567	E2F2	-0.929309027	0.270389043	-3.43693301	0.000588341	0.001107102	negative

PRJNA729271	stage_E18_vs_E10	ordinary_muscle_development	ENSGALG00010016568	PAM16	1.133978049	0.136022333	8.336704921	7.63894E-17	4.41493E-16	positive
PRJNA729271	stage_E18_vs_E10	ordinary_muscle_development	ENSGALG00010016569	YY1	-0.184476374	0.087609915	-2.105656356	0.035234213	0.050820785	negative
PRJNA729271	stage_E18_vs_E10	ordinary_muscle_development	ENSGALG00010016572	RAB33A	-0.039830855	0.115259946	-0.345574124	0.729662795	0.771590093	negative
PRJNA729271	stage_E18_vs_E10	ordinary_muscle_development	ENSGALG00010016573	DEGS1	-0.419598244	0.128752951	-3.258940791	0.00111829	0.002025189	negative
PRJNA729271	stage_E18_vs_E10	ordinary_muscle_development	ENSGALG00010016575	CWH43	1.569976278	0.371772167	4.222952701	2.41122E-05	5.382E-05	positive
PRJNA729271	stage_E18_vs_E10	ordinary_muscle_development	ENSGALG00010016576	MRPS26	-0.113038826	0.107321373	-1.053274127	0.292215373	0.348614143	negative
PRJNA729271	stage_E18_vs_E10	ordinary_muscle_development	ENSGALG00010016577	ZBTB17	0.276520335	0.187649511	1.473600089	0.140589298	0.181109302	positive
PRJNA729271	stage_E18_vs_E10	ordinary_muscle_development	ENSGALG00010016580	MARS2	-0.237387482	0.110191258	-2.154322283	0.031214907	0.045473374	negative
PRJNA729271	stage_E18_vs_E10	ordinary_muscle_development	ENSGALG00010016581	H2AFYMACROH2A1	-0.459269151	0.078267296	-5.867957274	4.41197E-09	1.40924E-08	negative
PRJNA729271	stage_E18_vs_E10	ordinary_muscle_development	ENSGALG00010016583	VPS41	-0.220047954	0.083829514	-2.624946075	0.008666269	0.013843682	negative
PRJNA729271	stage_E18_vs_E10	ordinary_muscle_development	ENSGALG00010016584	EHBP1	0.22936225	0.160652758	1.427689461	0.153381261	0.195864803	positive
PRJNA729271	stage_E18_vs_E10	ordinary_muscle_development	ENSGALG00010016588	ATP11B	-0.25289544	0.100465601	-2.517234123	0.01182802	0.018528818	negative
PRJNA729271	stage_E18_vs_E10	ordinary_muscle_development	ENSGALG00010016589	FBXO28	-0.130560777	0.079509189	-1.642084124	0.100572571	0.133229704	negative
PRJNA729271	stage_E18_vs_E10	ordinary_muscle_development	ENSGALG00010016591	HOMER2	-2.068201139	0.212233834	-9.74491721	1.93937E-22	1.58551E-21	negative
PRJNA729271	stage_E18_vs_E10	ordinary_muscle_development	ENSGALG00010016592	TMEM129	-0.246008899	0.106509951	-2.3097269	0.020903279	0.031414518	negative
PRJNA729271	stage_E18_vs_E10	ordinary_muscle_development	ENSGALG00010016597	FRYL	0.08023182	0.125726129	0.638147541	0.523377642	0.579908188	positive
PRJNA729271	stage_E18_vs_E10	ordinary_muscle_development	ENSGALG00010016598	AIFM1	-0.212362453	0.089072063	-2.384164512	0.017117948	0.026126635	negative
PRJNA729271	stage_E18_vs_E10	ordinary_muscle_development	ENSGALG00010016599	ENSGALG00010016599	1.778599016	0.267908793	6.638822838	3.16198E-11	1.19512E-10	positive
PRJNA729271	stage_E18_vs_E10	ordinary_muscle_development	ENSGALG00010016600	GNL2	-0.576627174	0.110676402	-5.210028187	1.88812E-07	5.21536E-07	negative
PRJNA729271	stage_E18_vs_E10	ordinary_muscle_development	ENSGALG00010016602	HSPB7	1.912017234	0.518353296	3.688637167	0.000225458	0.000448966	positive
PRJNA729271	stage_E18_vs_E10	ordinary_muscle_development	ENSGALG00010016603	ENSGALG00010016603	-0.221651801	0.128887949	-1.719724786	0.08548248	0.114813617	negative
PRJNA729271	stage_E18_vs_E10	ordinary_muscle_development	ENSGALG00010016604	TP53BP2	-0.598560278	0.062813395	-9.529182134	1.58527E-21	1.23275E-20	negative
PRJNA729271	stage_E18_vs_E10	ordinary_muscle_development	ENSGALG00010016605	WDPCP	0.078886877	0.164520186	0.479496645	0.631585354	0.681467151	positive
PRJNA729271	stage_E18_vs_E10	ordinary_muscle_development	ENSGALG00010016606	VASN	-0.054080956	0.178881057	-0.302329137	0.762401169	0.800623411	negative
PRJNA729271	stage_E18_vs_E10	ordinary_muscle_development	ENSGALG00010016607	FAM103A1	-0.647151046	0.11559159	-5.598599751	2.1609E-08	6.49031E-08	negative
PRJNA729271	stage_E18_vs_E10	ordinary_muscle_development	ENSGALG00010016609	SOX2	-0.506219422	0.484679529	-1.044441515	0.296281165	0.353056688	negative
PRJNA729271	stage_E18_vs_E10	ordinary_muscle_development	ENSGALG00010016611	DNALI1	0.389542956	0.201283821	1.935291936	0.052954481	0.073983152	positive
PRJNA729271	stage_E18_vs_E10	ordinary_muscle_development	ENSGALG00010016612	GRIA1	-0.512638164	0.331960489	-1.544274638	0.122521814	0.159837431	negative
PRJNA729271	stage_E18_vs_E10	ordinary_muscle_development	ENSGALG00010016613	SNIP1	-0.425011264	0.078052514	-5.445196327	5.17483E-08	1.50138E-07	negative
PRJNA729271	stage_E18_vs_E10	ordinary_muscle_development	ENSGALG00010016614	DNAJC19	0.246126679	0.140208376	1.755434918	0.079184944	0.107189555	positive
PRJNA729271	stage_E18_vs_E10	ordinary_muscle_development	ENSGALG00010016615	BCORL1	1.336151458	0.124476846	10.73413652	7.03738E-27	7.4349E-26	positive
PRJNA729271	stage_E18_vs_E10	ordinary_muscle_development	ENSGALG00010016616	SLBP	-1.989000281	0.23775811	-8.365646431	5.97862E-17	3.48453E-16	negative
PRJNA729271	stage_E18_vs_E10	ordinary_muscle_development	ENSGALG00010016618	TRPM3	-1.731143412	0.485285653	-3.56726683	0.000360724	0.000698837	negative
PRJNA729271	stage_E18_vs_E10	ordinary_muscle_development	ENSGALG00010016619	SLC25A47	1.936326065	0.323766004	5.98063428	2.2227E-09	7.27764E-09	positive
PRJNA729271	stage_E18_vs_E10	ordinary_muscle_development	ENSGALG00010016620	ENSGALG00010016620	0.172633336	0.122097617	1.413896022	0.157392434	0.200439583	positive
PRJNA729271	stage_E18_vs_E10	ordinary_muscle_development	ENSGALG00010016622	KIAA1143	-0.477291751	0.134679127	-3.543917754	0.000394228	0.000759481	negative
PRJNA729271	stage_E18_vs_E10	ordinary_muscle_development	ENSGALG00010016623	DNAJA3	0.466030718	0.141694025	3.288993432	0.001005464	0.001830699	positive
PRJNA729271	stage_E18_vs_E10	ordinary_muscle_development	ENSGALG00010016624	MDH1	0.444130798	0.154797658	2.86910541	0.004116346	0.006898651	positive
PRJNA729271	stage_E18_vs_E10	ordinary_muscle_development	ENSGALG00010016626	CAPN2	-0.260758482	0.230747178	-1.130061413	0.258450348	0.312446855	negative
PRJNA729271	stage_E18_vs_E10	ordinary_muscle_development	ENSGALG00010016629	LARS1	-0.636184232	0.071861182	-8.852960834	8.52276E-19	5.59875E-18	negative
PRJNA729271	stage_E18_vs_E10	ordinary_muscle_development	ENSGALG00010016630	ENSGALG00010016630	1.202979731	0.358307817	3.357391815	0.000786815	0.001456005	positive
PRJNA729271	stage_E18_vs_E10	ordinary_muscle_development	ENSGALG00010016631	FAM53A	1.385671893	0.091476087	15.14791393	7.82099E-52	2.42233E-50	positive
PRJNA729271	stage_E18_vs_E10	ordinary_muscle_development	ENSGALG00010016637	POU6F2	-2.051936959	0.695787285	-2.9490866	0.003187147	0.005434383	negative
PRJNA729271	stage_E18_vs_E10	ordinary_muscle_development	ENSGALG00010016640	OCIAD1	0.568309986	0.104135111	5.457429105	4.83078E-08	1.40691E-07	positive
PRJNA729271	stage_E18_vs_E10	ordinary_muscle_development	ENSGALG00010016643	BTBD1	0.143426586	0.173739515	0.825526572	0.409072703	0.469292349	positive
PRJNA729271	stage_E18_vs_E10	ordinary_muscle_development	ENSGALG00010016644	NMRAL1	-0.471130027	0.197737126	-2.382607845	0.017190496	0.026224301	negative
PRJNA729271	stage_E18_vs_E10	ordinary_muscle_development	ENSGALG00010016647	EGR1	2.08530847	0.204433439	10.20042748	1.97402E-24	1.8064E-23	positive
PRJNA729271	stage_E18_vs_E10	ordinary_muscle_development	ENSGALG00010016648	ADRA2C	-0.400872745	0.252243375	-1.58923002	0.112008471	0.147221871	negative
PRJNA729271	stage_E18_vs_E10	ordinary_muscle_development	ENSGALG00010016651	ENSGALG00010016651	4.331597951	0.177664649	24.38075316	2.7369E-131	6.1986E-129	positive
PRJNA729271	stage_E18_vs_E10	ordinary_muscle_development	ENSGALG00010016652	FXR1	1.147297349	0.099057484	11.58213702	5.07645E-31	6.54655E-30	positive
PRJNA729271	stage_E18_vs_E10	ordinary_muscle_development	ENSGALG00010016653	EPHA2	-0.431552844	0.211461043	-2.040814882	0.041269231	0.058756262	negative
PRJNA729271	stage_E18_vs_E10	ordinary_muscle_development	ENSGALG00010016654	SMIM3	1.602197401	0.181939758	8.806197267	1.29463E-18	8.42644E-18	positive
PRJNA729271	stage_E18_vs_E10	ordinary_muscle_development	ENSGALG00010016655	TRANK1	-0.369275985	0.324117859	-1.139326251	0.254567103	0.308395333	negative
PRJNA729271	stage_E18_vs_E10	ordinary_muscle_development	ENSGALG00010016656	CAPN8	-3.700239175	1.104596065	-3.349857284	0.000808532	0.001493713	negative
PRJNA729271	stage_E18_vs_E10	ordinary_muscle_development	ENSGALG00010016657	SLC25A29	1.505734886	0.279391364	5.38933939	7.07171E-08	2.02778E-07	positive
PRJNA729271	stage_E18_vs_E10	ordinary_muscle_development	ENSGALG00010016658	ZAR1	-0.393217689	0.498328089	-0.789073901	0.430068815	0.490082349	negative
PRJNA729271	stage_E18_vs_E10	ordinary_muscle_development	ENSGALG00010016661	UBE4B	-0.905356232	0.129313335	-7.001259613	2.53671E-12	1.05146E-11	negative
PRJNA729271	stage_E18_vs_E10	ordinary_muscle_development	ENSGALG00010016664	TM6SF1	3.238398499	0.248420029	13.03597986	7.63794E-39	1.39788E-37	positive
PRJNA729271	stage_E18_vs_E10	ordinary_muscle_development	ENSGALG00010016665	ENSGALG00010016665	5.053458783	1.017435467	4.966859274	6.80459E-07	1.78747E-06	positive
PRJNA729271	stage_E18_vs_E10	ordinary_muscle_development	ENSGALG00010016667	WARS1	-0.492253735	0.144180819	-3.414141633	0.000639833	0.001197931	negative
PRJNA729271	stage_E18_vs_E10	ordinary_muscle_development	ENSGALG00010016671	CASS4	-0.899847606	0.250223387	-3.596177074	0.000322928	0.000629315	negative
PRJNA729271	stage_E18_vs_E10	ordinary_muscle_development	ENSGALG00010016672	DCTN4	-0.182446817	0.081298583	-2.244157392	0.024822276	0.03683822	negative
PRJNA729271	stage_E18_vs_E10	ordinary_muscle_development	ENSGALG00010016673	LRPAP1	-0.015625408	0.080211659	-0.194802205	0.845547796	0.872142123	negative
PRJNA729271	stage_E18_vs_E10	ordinary_muscle_development	ENSGALG00010016674	SLC10A4	0.696078986	0.327808819	2.123429713	0.03371786	0.04880402	positive
PRJNA729271	stage_E18_vs_E10	ordinary_muscle_development	ENSGALG00010016675	SKIL	-0.19856304	0.139984451	-1.418464967	0.15605506	0.198928686	negative
PRJNA729271	stage_E18_vs_E10	ordinary_muscle_development	ENSGALG00010016676	NLRC3	-1.410761811	0.674921359	-2.090261024	0.036594358	0.052612103	negative
PRJNA729271	stage_E18_vs_E10	ordinary_muscle_development	ENSGALG00010016677	MEAF6	0.324229523	0.088244227	3.674229279	0.000238568	0.000473705	positive
PRJNA729271	stage_E18_vs_E10	ordinary_muscle_development	ENSGALG00010016678	NOX1	2.930704679	0.316269932	9.266466326	1.92415E-20	1.39592E-19	positive
PRJNA729271	stage_E18_vs_E10	ordinary_muscle_development	ENSGALG00010016680	BPIFB3	0.233835972	0.883966747	0.264530281	0.791371335	0.825662384	positive
PRJNA729271	stage_E18_vs_E10	ordinary_muscle_development	ENSGALG00010016681	SUSD4	1.672457328	0.288698711	5.793088998	6.91035E-09	2.16527E-08	positive
PRJNA729271	stage_E18_vs_E10	ordinary_muscle_development	ENSGALG00010016682	GPR141	-0.435004283	0.47878602	-0.908556777	0.363584131	0.422999597	negative
PRJNA729271	stage_E18_vs_E10	ordinary_muscle_development	ENSGALG00010016683	RBM22	-0.359035827	0.074112595	-4.84446435	1.26954E-06	3.24778E-06	negative
PRJNA729271	stage_E18_vs_E10	ordinary_muscle_development	ENSGALG00010016691	ENSGALG00010016691	0.426276527	0.329981065	1.291821176	0.19641908	0.244763976	positive
PRJNA729271	stage_E18_vs_E10	ordinary_muscle_development	ENSGALG00010016692	PRKCI	-0.821589874	0.074059172	-11.09369511	1.3461E-28	1.53394E-27	negative
PRJNA729271	stage_E18_vs_E10	ordinary_muscle_development	ENSGALG00010016696	DOK7	1.385672883	0.129222474	10.72315701	7.92512E-27	8.34238E-26	positive
PRJNA729271	stage_E18_vs_E10	ordinary_muscle_development	ENSGALG00010016699	SYNPO	-0.898323974	0.148284814	-6.058098251	1.3774E-09	4.5887E-09	negative
PRJNA729271	stage_E18_vs_E10	ordinary_muscle_development	ENSGALG00010016700	CLUAP1	-0.139167643	0.123104172	-1.130486811	0.258271153	0.312308364	negative
PRJNA729271	stage_E18_vs_E10	ordinary_muscle_development	ENSGALG00010016702	WDR25	0.40638288	0.097344949	4.174668359	2.9842E-05	6.58087E-05	positive
PRJNA729271	stage_E18_vs_E10	ordinary_muscle_development	ENSGALG00010016704	CSTF2	-0.379452654	0.072552841	-5.230017886	1.69494E-07	4.69753E-07	negative
PRJNA729271	stage_E18_vs_E10	ordinary_muscle_development	ENSGALG00010016706	RTN4	1.787876954	0.12549337	14.24678416	4.69405E-46	1.16707E-44	positive
PRJNA729271	stage_E18_vs_E10	ordinary_muscle_development	ENSGALG00010016707	KIF1B	1.160616769	0.130482716	8.894793139	5.85282E-19	3.88981E-18	positive
PRJNA729271	stage_E18_vs_E10	ordinary_muscle_development	ENSGALG00010016712	ENSGALG00010016712	-0.008814901	0.162560337	-0.054225413	0.956755574	0.964272844	negative
PRJNA729271	stage_E18_vs_E10	ordinary_muscle_development	ENSGALG00010016720	INTS15	-0.534000084	0.103854501	-5.141809715	2.72105E-07	7.41383E-07	negative
PRJNA729271	stage_E18_vs_E10	ordinary_muscle_development	ENSGALG00010016724	PGD	-1.532203011	0.111209655	-13.77760786	3.476E-43	7.573E-42	negative
PRJNA729271	stage_E18_vs_E10	ordinary_muscle_development	ENSGALG00010016729	ELMO1	0.981261711	0.094296699	10.40610878	2.32533E-25	2.25155E-24	positive
PRJNA729271	stage_E18_vs_E10	ordinary_muscle_development	ENSGALG00010016731	C4orf48	-0.517895162	0.271455994	-1.907842055	0.056411633	0.078450218	negative
PRJNA729271	stage_E18_vs_E10	ordinary_muscle_development	ENSGALG00010016732	ARHGAP26	0.377036703	0.076069555	4.95647312	7.17843E-07	1.8826E-06	positive
PRJNA729271	stage_E18_vs_E10	ordinary_muscle_development	ENSGALG00010016733	PHC3	0.695167708	0.156338909	4.446543161	8.72631E-06	2.04474E-05	positive
PRJNA729271	stage_E18_vs_E10	ordinary_muscle_development	ENSGALG00010016735	ENSGALG00010016735	0.541466461	0.325649445	1.662728031	0.096366888	0.128256179	positive
PRJNA729271	stage_E18_vs_E10	ordinary_muscle_development	ENSGALG00010016736	CENPS	-1.523880988	0.267389512	-5.699105317	1.20438E-08	3.69158E-08	negative
PRJNA729271	stage_E18_vs_E10	ordinary_muscle_development	ENSGALG00010016738	NAA60	1.264555921	0.149783235	8.442573149	3.10427E-17	1.84336E-16	positive
PRJNA729271	stage_E18_vs_E10	ordinary_muscle_development	ENSGALG00010016741	NELFA	-0.090214236	0.089777357	-1.004866252	0.314961257	0.372348374	negative
PRJNA729271	stage_E18_vs_E10	ordinary_muscle_development	ENSGALG00010016742	TRMT12	0.158938609	0.136600963	1.163524805	0.24461664	0.297834372	positive
PRJNA729271	stage_E18_vs_E10	ordinary_muscle_development	ENSGALG00010016744	CRYBG2	-5.458373901	0.898311191	-6.076261717	1.23017E-09	4.11522E-09	negative
PRJNA729271	stage_E18_vs_E10	ordinary_muscle_development	ENSGALG00010016745	BEGAIN	1.32057925	0.36188776	3.649140409	0.000263119	0.00051989	positive
PRJNA729271	stage_E18_vs_E10	ordinary_muscle_development	ENSGALG00010016746	HDGFL3	-0.944930775	0.123214242	-7.669006106	1.73334E-14	8.47665E-14	negative
PRJNA729271	stage_E18_vs_E10	ordinary_muscle_development	ENSGALG00010016750	SYTL4	0.461050986	0.128510657	3.587647884	0.000333674	0.000649035	positive
PRJNA729271	stage_E18_vs_E10	ordinary_muscle_development	ENSGALG00010016751	DFFA	-0.621904071	0.088618517	-7.017766627	2.25443E-12	9.37673E-12	negative
PRJNA729271	stage_E18_vs_E10	ordinary_muscle_development	ENSGALG00010016752	ENSGALG00010016752	1.461262824	0.172522648	8.469976771	2.45442E-17	1.46588E-16	positive
PRJNA729271	stage_E18_vs_E10	ordinary_muscle_development	ENSGALG00010016754	ENSGALG00010016754	-0.086446744	0.185634672	-0.465682102	0.641443065	0.690663886	negative
PRJNA729271	stage_E18_vs_E10	ordinary_muscle_development	ENSGALG00010016756	NUDT16L1	-0.800027243	0.107516392	-7.440979243	9.99417E-14	4.59161E-13	negative
PRJNA729271	stage_E18_vs_E10	ordinary_muscle_development	ENSGALG00010016757	SRPX2	-0.443203925	0.279002068	-1.588532758	0.11216592	0.147402086	negative
PRJNA729271	stage_E18_vs_E10	ordinary_muscle_development	ENSGALG00010016758	NR3C1	0.785742693	0.068175231	11.52533964	9.83237E-31	1.24472E-29	positive
PRJNA729271	stage_E18_vs_E10	ordinary_muscle_development	ENSGALG00010016760	DLK1	1.792448026	0.259579441	6.905200276	5.01326E-12	2.03095E-11	positive
PRJNA729271	stage_E18_vs_E10	ordinary_muscle_development	ENSGALG00010016761	GPR160	-1.371348674	0.389775288	-3.51830584	0.000434312	0.000832938	negative
PRJNA729271	stage_E18_vs_E10	ordinary_muscle_development	ENSGALG00010016762	EML6	0.419330892	0.122510896	3.422804882	0.000619785	0.001162967	positive
PRJNA729271	stage_E18_vs_E10	ordinary_muscle_development	ENSGALG00010016763	UBALD1	0.943307571	0.172704171	5.46198487	4.7084E-08	1.37321E-07	positive
PRJNA729271	stage_E18_vs_E10	ordinary_muscle_development	ENSGALG00010016766	AOAH	-0.252904556	0.365257493	-0.692400733	0.488685706	0.546566304	negative
PRJNA729271	stage_E18_vs_E10	ordinary_muscle_development	ENSGALG00010016767	SLAIN2	0.080202068	0.094953025	0.844649957	0.398306307	0.458173949	positive
PRJNA729271	stage_E18_vs_E10	ordinary_muscle_development	ENSGALG00010016768	GALNT10	0.447506861	0.099636062	4.491414588	7.07517E-06	1.6719E-05	positive
PRJNA729271	stage_E18_vs_E10	ordinary_muscle_development	ENSGALG00010016769	BNC1	-5.801009283	0.284003427	-20.42584258	9.85438E-93	9.85098E-91	negative
PRJNA729271	stage_E18_vs_E10	ordinary_muscle_development	ENSGALG00010016772	TSPAN6	-0.556083464	0.126119794	-4.409168839	1.03768E-05	2.41393E-05	negative
PRJNA729271	stage_E18_vs_E10	ordinary_muscle_development	ENSGALG00010016773	RPS27A	0.986441266	0.161251147	6.117421686	9.51015E-10	3.21853E-09	positive
PRJNA729271	stage_E18_vs_E10	ordinary_muscle_development	ENSGALG00010016774	PEX14	-0.36680276	0.123238533	-2.97636421	0.002916882	0.005004759	negative
PRJNA729271	stage_E18_vs_E10	ordinary_muscle_development	ENSGALG00010016777	TNMD	0.341290406	0.487301438	0.700368148	0.483697423	0.541781481	positive
PRJNA729271	stage_E18_vs_E10	ordinary_muscle_development	ENSGALG00010016778	MTIF2	-0.56070341	0.110336847	-5.08174217	3.73989E-07	1.00761E-06	negative
PRJNA729271	stage_E18_vs_E10	ordinary_muscle_development	ENSGALG00010016779	CTNNA2	-1.841181503	0.152104519	-12.10471268	9.97179E-34	1.43679E-32	negative
PRJNA729271	stage_E18_vs_E10	ordinary_muscle_development	ENSGALG00010016780	PRORSD1P	-0.732605929	0.139619727	-5.247151983	1.54468E-07	4.29342E-07	negative
PRJNA729271	stage_E18_vs_E10	ordinary_muscle_development	ENSGALG00010016781	CDIP1	1.706045727	0.143587435	11.88158088	1.4755E-32	2.01198E-31	positive
PRJNA729271	stage_E18_vs_E10	ordinary_muscle_development	ENSGALG00010016783	CLBA1	-0.231273256	0.308043813	-0.750780396	0.452784829	0.512423181	negative
PRJNA729271	stage_E18_vs_E10	ordinary_muscle_development	ENSGALG00010016785	PDGFRB	0.359718113	0.194514177	1.849315658	0.064412246	0.088590522	positive
PRJNA729271	stage_E18_vs_E10	ordinary_muscle_development	ENSGALG00010016786	DISP1	0.078354643	0.11104761	0.705595045	0.480440007	0.538658506	positive
PRJNA729271	stage_E18_vs_E10	ordinary_muscle_development	ENSGALG00010016787	PPP2R5C	-0.426829524	0.075799201	-5.631055718	1.7911E-08	5.40987E-08	negative
PRJNA729271	stage_E18_vs_E10	ordinary_muscle_development	ENSGALG00010016788	ANLN	-1.734476895	0.28855732	-6.010857374	1.84545E-09	6.08225E-09	negative
PRJNA729271	stage_E18_vs_E10	ordinary_muscle_development	ENSGALG00010016789	DISP3	0.621446767	0.114421153	5.431222714	5.59692E-08	1.61963E-07	positive
PRJNA729271	stage_E18_vs_E10	ordinary_muscle_development	ENSGALG00010016791	LRRTM1	-2.075998315	0.449550816	-4.617939153	3.8757E-06	9.38024E-06	negative
PRJNA729271	stage_E18_vs_E10	ordinary_muscle_development	ENSGALG00010016792	HMOX2	-0.480476798	0.122391835	-3.925725917	8.64686E-05	0.000181515	negative
PRJNA729271	stage_E18_vs_E10	ordinary_muscle_development	ENSGALG00010016793	SLC9A1	0.143970414	0.157426214	0.914526306	0.360440362	0.419943886	positive
PRJNA729271	stage_E18_vs_E10	ordinary_muscle_development	ENSGALG00010016795	GUCA2B	-1.860404977	1.429040645	-1.301855888	0.192965655	0.240729724	negative
PRJNA729271	stage_E18_vs_E10	ordinary_muscle_development	ENSGALG00010016796	BROX	-0.010983492	0.088411962	-0.124230835	0.901132509	0.918082212	negative
PRJNA729271	stage_E18_vs_E10	ordinary_muscle_development	ENSGALG00010016797	SEC62	-0.023412189	0.10932449	-0.214153198	0.830427592	0.859114121	negative

PRJNA729271	stage_E18_vs_E10	ordinary_muscle_development	ENSGALG00010016799	WDTC1	0.649490634	0.132632573	4.896916479	9.73522E-07	2.51941E-06	positive
PRJNA729271	stage_E18_vs_E10	ordinary_muscle_development	ENSGALG00010016800	RPL14	0.653048298	0.169027036	3.863573021	0.00011174	0.000231846	positive
PRJNA729271	stage_E18_vs_E10	ordinary_muscle_development	ENSGALG00010016802	TMEM222	-0.238314613	0.085455844	-2.788745653	0.00529126	0.008741374	negative
PRJNA729271	stage_E18_vs_E10	ordinary_muscle_development	ENSGALG00010016804	CSF1R	1.694108812	0.136184302	12.43982447	1.58853E-35	2.49736E-34	positive
PRJNA729271	stage_E18_vs_E10	ordinary_muscle_development	ENSGALG00010016805	ENSGALG00010016805	-6.264165715	1.069024506	-5.859702634	4.63697E-09	1.47785E-08	negative
PRJNA729271	stage_E18_vs_E10	ordinary_muscle_development	ENSGALG00010016807	VSIG4	1.460299343	0.256539437	5.692299629	1.2534E-08	3.83858E-08	positive
PRJNA729271	stage_E18_vs_E10	ordinary_muscle_development	ENSGALG00010016808	ENTPD3	-1.528829821	0.566036035	-2.7009408	0.006914364	0.011191928	negative
PRJNA729271	stage_E18_vs_E10	ordinary_muscle_development	ENSGALG00010016809	SYTL1	-2.222643513	0.565019927	-3.933743588	8.3633E-05	0.000175843	negative
PRJNA729271	stage_E18_vs_E10	ordinary_muscle_development	ENSGALG00010016810	SH3GL3	-0.743206674	0.196783804	-3.776767487	0.000158877	0.000322854	negative
PRJNA729271	stage_E18_vs_E10	ordinary_muscle_development	ENSGALG00010016812	LRRC31	-0.40172562	0.128319682	-3.13066252	0.001744125	0.003089842	negative
PRJNA729271	stage_E18_vs_E10	ordinary_muscle_development	ENSGALG00010016813	ENSGALG00010016813	-1.530233987	0.494867558	-3.092209144	0.001986728	0.003489352	negative
PRJNA729271	stage_E18_vs_E10	ordinary_muscle_development	ENSGALG00010016814	HMGXB3	-0.008461613	0.10180847	-0.083113051	0.933761648	0.94583334	negative
PRJNA729271	stage_E18_vs_E10	ordinary_muscle_development	ENSGALG00010016816	MAP3K6	0.914560152	0.299772426	3.050848157	0.002281959	0.003968923	positive
PRJNA729271	stage_E18_vs_E10	ordinary_muscle_development	ENSGALG00010016817	GGCT	-1.818548183	0.118434406	-15.35489759	3.28428E-53	1.05556E-51	negative
PRJNA729271	stage_E18_vs_E10	ordinary_muscle_development	ENSGALG00010016820	EIF2AK3	-0.070269199	0.10855344	-0.64732356	0.517422557	0.574275648	negative
PRJNA729271	stage_E18_vs_E10	ordinary_muscle_development	ENSGALG00010016823	HSF3	0.642796809	0.080122304	8.022695047	1.0345E-15	5.55167E-15	positive
PRJNA729271	stage_E18_vs_E10	ordinary_muscle_development	ENSGALG00010016825	ENSGALG00010016825	0.296227781	0.300694868	0.98514412	0.324553284	0.382564841	positive
PRJNA729271	stage_E18_vs_E10	ordinary_muscle_development	ENSGALG00010016828	LACTBL1	3.424847935	0.386860112	8.852936327	8.52463E-19	5.59875E-18	positive
PRJNA729271	stage_E18_vs_E10	ordinary_muscle_development	ENSGALG00010016831	AIDA	-0.670227869	0.067650818	-9.907165832	3.87482E-23	3.27114E-22	negative
PRJNA729271	stage_E18_vs_E10	ordinary_muscle_development	ENSGALG00010016832	DYNC1H1	-0.479421112	0.094242002	-5.087127859	3.63527E-07	9.80521E-07	negative
PRJNA729271	stage_E18_vs_E10	ordinary_muscle_development	ENSGALG00010016834	ENSGALG00010016834	1.207685805	0.074608286	16.18701991	6.22723E-59	2.41347E-57	positive
PRJNA729271	stage_E18_vs_E10	ordinary_muscle_development	ENSGALG00010016835	CCDC88A	-1.199795326	0.151919876	-7.897553342	2.84431E-15	1.47931E-14	negative
PRJNA729271	stage_E18_vs_E10	ordinary_muscle_development	ENSGALG00010016836	HEPH	1.060507189	0.15087127	7.029218934	2.07693E-12	8.66583E-12	positive
PRJNA729271	stage_E18_vs_E10	ordinary_muscle_development	ENSGALG00010016840	LRRC34	-0.731683193	0.216975941	-3.372185825	0.000745741	0.001385303	negative
PRJNA729271	stage_E18_vs_E10	ordinary_muscle_development	ENSGALG00010016842	NPY7R	-0.068142422	0.436790631	-0.15600706	0.876027457	0.897576729	negative
PRJNA729271	stage_E18_vs_E10	ordinary_muscle_development	ENSGALG00010016845	MIA3	0.428495872	0.138076965	3.103311787	0.001913679	0.00336881	positive
PRJNA729271	stage_E18_vs_E10	ordinary_muscle_development	ENSGALG00010016847	EPHB2	-0.628247702	0.106299658	-5.910157306	3.41781E-09	1.10189E-08	negative
PRJNA729271	stage_E18_vs_E10	ordinary_muscle_development	ENSGALG00010016848	ENSGALG00010016848	0.668041183	0.169048671	3.951768336	7.75758E-05	0.000163677	positive
PRJNA729271	stage_E18_vs_E10	ordinary_muscle_development	ENSGALG00010016849	WASF2	0.333955389	0.153088082	2.181459096	0.029149477	0.042691893	positive
PRJNA729271	stage_E18_vs_E10	ordinary_muscle_development	ENSGALG00010016851	TEC	1.246396378	0.140572558	8.866569645	7.54338E-19	4.97458E-18	positive
PRJNA729271	stage_E18_vs_E10	ordinary_muscle_development	ENSGALG00010016852	AHDC1	0.357777332	0.169467132	2.111190101	0.034755975	0.050173077	positive
PRJNA729271	stage_E18_vs_E10	ordinary_muscle_development	ENSGALG00010016854	TAF1A	-0.059991042	0.10573727	-0.567359478	0.570469975	0.62397844	negative
PRJNA729271	stage_E18_vs_E10	ordinary_muscle_development	ENSGALG00010016856	NOD1	-0.789116355	0.142249342	-5.547416546	2.89921E-08	8.62918E-08	negative
PRJNA729271	stage_E18_vs_E10	ordinary_muscle_development	ENSGALG00010016858	ENSGALG00010016858	-1.10497343	0.17397191	-6.351447384	2.13298E-10	7.5797E-10	negative
PRJNA729271	stage_E18_vs_E10	ordinary_muscle_development	ENSGALG00010016859	RPIA	0.769789524	0.130409562	5.902861032	3.57251E-09	1.15023E-08	positive
PRJNA729271	stage_E18_vs_E10	ordinary_muscle_development	ENSGALG00010016860	VSX1	-0.02290241	0.436129137	-0.052512909	0.95812001	0.965446614	negative
PRJNA729271	stage_E18_vs_E10	ordinary_muscle_development	ENSGALG00010016861	FGR	0.987775523	0.121964379	8.09888538	5.5465E-16	3.03154E-15	positive
PRJNA729271	stage_E18_vs_E10	ordinary_muscle_development	ENSGALG00010016862	TXK	2.845646008	0.553639163	5.139892906	2.74895E-07	7.48845E-07	positive
PRJNA729271	stage_E18_vs_E10	ordinary_muscle_development	ENSGALG00010016863	MYNN	-0.42826067	0.083979821	-5.099566373	3.40432E-07	9.2166E-07	negative
PRJNA729271	stage_E18_vs_E10	ordinary_muscle_development	ENSGALG00010016864	ENSGALG00010016864	0.696421233	0.267312227	2.605272646	0.009180118	0.014590549	positive
PRJNA729271	stage_E18_vs_E10	ordinary_muscle_development	ENSGALG00010016865	CFAP36	-0.90618331	0.138414877	-6.546863512	5.8758E-11	2.1816E-10	negative
PRJNA729271	stage_E18_vs_E10	ordinary_muscle_development	ENSGALG00010016866	ZNRF2	1.328590619	0.099300549	13.37948913	7.96932E-41	1.57808E-39	positive
PRJNA729271	stage_E18_vs_E10	ordinary_muscle_development	ENSGALG00010016867	FLT4	0.158107601	0.11977147	1.320077323	0.186809203	0.233874538	positive
PRJNA729271	stage_E18_vs_E10	ordinary_muscle_development	ENSGALG00010016868	NIPAL1	0.462999173	0.591185028	0.783171344	0.4335265	0.493324432	positive
PRJNA729271	stage_E18_vs_E10	ordinary_muscle_development	ENSGALG00010016869	HSP90AA1	-0.916313458	0.156435133	-5.857465904	4.69983E-09	1.49657E-08	negative
PRJNA729271	stage_E18_vs_E10	ordinary_muscle_development	ENSGALG00010016872	EDA2R	1.914740075	0.280353591	6.829732641	8.5073E-12	3.36004E-11	positive
PRJNA729271	stage_E18_vs_E10	ordinary_muscle_development	ENSGALG00010016873	ENTPD6	-0.71297755	0.14259779	-4.999920056	5.73541E-07	1.51706E-06	negative
PRJNA729271	stage_E18_vs_E10	ordinary_muscle_development	ENSGALG00010016874	ADAMTSL3	0.888702189	0.144931731	6.13186763	8.68534E-10	2.95248E-09	positive
PRJNA729271	stage_E18_vs_E10	ordinary_muscle_development	ENSGALG00010016875	SESN2	2.422369039	0.218387028	11.09209215	1.37045E-28	1.56046E-27	positive
PRJNA729271	stage_E18_vs_E10	ordinary_muscle_development	ENSGALG00010016876	MTURN	-0.382235294	0.189964989	-2.012135478	0.044205658	0.062635485	negative
PRJNA729271	stage_E18_vs_E10	ordinary_muscle_development	ENSGALG00010016877	UBE2D3	-0.062321268	0.135884499	-0.458634119	0.646496931	0.695382384	negative
PRJNA729271	stage_E18_vs_E10	ordinary_muscle_development	ENSGALG00010016878	SUCLG1	0.661386341	0.125654809	5.263517955	1.41325E-07	3.94322E-07	positive
PRJNA729271	stage_E18_vs_E10	ordinary_muscle_development	ENSGALG00010016879	RNF130	-0.246186173	0.10131749	-2.429848722	0.015105126	0.023307303	negative
PRJNA729271	stage_E18_vs_E10	ordinary_muscle_development	ENSGALG00010016880	AR	2.427349248	0.201034955	12.07426463	1.4445E-33	2.06489E-32	positive
PRJNA729271	stage_E18_vs_E10	ordinary_muscle_development	ENSGALG00010016881	C1QB	1.131166879	0.293066298	3.859764454	0.000113496	0.000235153	positive
PRJNA729271	stage_E18_vs_E10	ordinary_muscle_development	ENSGALG00010016882	ENSGALG00010016882	-0.623065326	0.049925602	-12.47987598	9.61363E-36	1.53469E-34	negative
PRJNA729271	stage_E18_vs_E10	ordinary_muscle_development	ENSGALG00010016883	TMEM251	-0.20167705	0.078856875	-2.557507521	0.010542528	0.016631905	negative
PRJNA729271	stage_E18_vs_E10	ordinary_muscle_development	ENSGALG00010016884	SBK1	0.101460022	0.256481441	0.395584265	0.692411747	0.737815796	positive
PRJNA729271	stage_E18_vs_E10	ordinary_muscle_development	ENSGALG00010016885	CDCA4	-0.169752046	0.15345067	-1.106232029	0.268626103	0.323641872	negative
PRJNA729271	stage_E18_vs_E10	ordinary_muscle_development	ENSGALG00010016887	TJP2	-0.050195536	0.139724196	-0.35924727	0.719410117	0.76219278	negative
PRJNA729271	stage_E18_vs_E10	ordinary_muscle_development	ENSGALG00010016888	ENSGALG00010016888	-0.107656512	0.085427286	-1.260212252	0.207592804	0.257272375	negative
PRJNA729271	stage_E18_vs_E10	ordinary_muscle_development	ENSGALG00010016889	FUNDC2	0.503232529	0.087411361	5.757060904	8.5591E-09	2.66118E-08	positive
PRJNA729271	stage_E18_vs_E10	ordinary_muscle_development	ENSGALG00010016890	UBR7	-1.144658884	0.076396295	-14.98317276	9.45914E-51	2.83286E-49	negative
PRJNA729271	stage_E18_vs_E10	ordinary_muscle_development	ENSGALG00010016891	ENSGALG00010016891	1.480103896	1.164794499	1.270699593			positive
PRJNA729271	stage_E18_vs_E10	ordinary_muscle_development	ENSGALG00010016892	MAL	0.51777917	0.452203349	1.145014012	0.252203353	0.30586409	positive
PRJNA729271	stage_E18_vs_E10	ordinary_muscle_development	ENSGALG00010016893	TBC1D9B	-0.222251467	0.051425842	-4.321785686	1.54771E-05	3.53349E-05	negative
PRJNA729271	stage_E18_vs_E10	ordinary_muscle_development	ENSGALG00010016894	BTBD7	0.34588622	0.091993711	3.759889833	0.000169988	0.000343939	positive
PRJNA729271	stage_E18_vs_E10	ordinary_muscle_development	ENSGALG00010016895	ENSGALG00010016895	0.657541912	0.160999414	4.084126111	4.4243E-05	9.57596E-05	positive
PRJNA729271	stage_E18_vs_E10	ordinary_muscle_development	ENSGALG00010016896	SPTBN1	0.08193186	0.098764122	0.829571087	0.406781329	0.466848406	positive
PRJNA729271	stage_E18_vs_E10	ordinary_muscle_development	ENSGALG00010016898	ENSGALG00010016898	-1.224701317	0.732055459	-1.672962481	0.094334682	0.125747767	negative
PRJNA729271	stage_E18_vs_E10	ordinary_muscle_development	ENSGALG00010016899	SAXO2	-0.728572583	0.549406522	-1.326108364	0.184803806	0.231684066	negative
PRJNA729271	stage_E18_vs_E10	ordinary_muscle_development	ENSGALG00010016900	CISD2	-0.336549879	0.091416294	-3.681508669	0.000231858	0.000461076	negative
PRJNA729271	stage_E18_vs_E10	ordinary_muscle_development	ENSGALG00010016901	MECOM	-0.219988269	0.192206393	-1.144541897	0.252398973	0.306075722	negative
PRJNA729271	stage_E18_vs_E10	ordinary_muscle_development	ENSGALG00010016902	MRNIP	-1.028229592	0.144371809	-7.122093975	1.063E-12	4.54919E-12	negative
PRJNA729271	stage_E18_vs_E10	ordinary_muscle_development	ENSGALG00010016903	PPM1A	2.724263747	0.146882798	18.54719402	8.59219E-77	5.51078E-75	positive
PRJNA729271	stage_E18_vs_E10	ordinary_muscle_development	ENSGALG00010016904	EAF1	0.431424646	0.070820875	6.091772293	1.11667E-09	3.75289E-09	positive
PRJNA729271	stage_E18_vs_E10	ordinary_muscle_development	ENSGALG00010016905	C1QC	1.581536372	0.375425928	4.212645572	2.52397E-05	5.6224E-05	positive
PRJNA729271	stage_E18_vs_E10	ordinary_muscle_development	ENSGALG00010016907	SQSTM1	2.159058051	0.160255551	13.47259449	2.26756E-41	4.59696E-40	positive
PRJNA729271	stage_E18_vs_E10	ordinary_muscle_development	ENSGALG00010016908	CMC4	0.658019279	0.162891757	4.039610669	5.354E-05	0.000114955	positive
PRJNA729271	stage_E18_vs_E10	ordinary_muscle_development	ENSGALG00010016909	MRPS5	-0.34268013	0.134857667	-2.541050405	0.011051999	0.017388334	negative
PRJNA729271	stage_E18_vs_E10	ordinary_muscle_development	ENSGALG00010016910	NSD2	-0.964639743	0.111210219	-8.674020708	4.17124E-18	2.62993E-17	negative
PRJNA729271	stage_E18_vs_E10	ordinary_muscle_development	ENSGALG00010016911	AXIN1	-0.330116438	0.100630063	-3.28049518	0.00103625	0.001883442	negative
PRJNA729271	stage_E18_vs_E10	ordinary_muscle_development	ENSGALG00010016912	CCDC88C	0.594493898	0.160036875	3.714730746	0.00020342	0.000407487	positive
PRJNA729271	stage_E18_vs_E10	ordinary_muscle_development	ENSGALG00010016913	BRCC3	-0.335143073	0.112705457	-2.973618858	0.002943104	0.005047361	negative
PRJNA729271	stage_E18_vs_E10	ordinary_muscle_development	ENSGALG00010016915	PLOD2	-0.62025229	0.167834912	-3.695609475	0.00021936	0.000437482	negative
PRJNA729271	stage_E18_vs_E10	ordinary_muscle_development	ENSGALG00010016916	ACYP2	1.506695707	0.159104959	9.469822415	2.8032E-21	2.14645E-20	positive
PRJNA729271	stage_E18_vs_E10	ordinary_muscle_development	ENSGALG00010016917	METTL6	0.189038655	0.171681724	1.10109947	0.270853368	0.325946	positive
PRJNA729271	stage_E18_vs_E10	ordinary_muscle_development	ENSGALG00010016918	RCOR1	-0.630386272	0.075569395	-8.341819712	7.31559E-17	4.23311E-16	negative
PRJNA729271	stage_E18_vs_E10	ordinary_muscle_development	ENSGALG00010016919	ARHGDIG	0.953697506	0.196187594	4.861150942	1.16705E-06	2.99512E-06	positive
PRJNA729271	stage_E18_vs_E10	ordinary_muscle_development	ENSGALG00010016920	C1QA	1.415607856	0.3217506	4.399705415	1.08398E-05	2.51517E-05	positive
PRJNA729271	stage_E18_vs_E10	ordinary_muscle_development	ENSGALG00010016921	EFL1	-0.706893195	0.076574182	-9.231482119	2.66913E-20	1.9153E-19	negative
PRJNA729271	stage_E18_vs_E10	ordinary_muscle_development	ENSGALG00010016922	ENSGALG00010016922	0.834914867	0.191589913	4.357822681	1.31363E-05	3.02047E-05	positive
PRJNA729271	stage_E18_vs_E10	ordinary_muscle_development	ENSGALG00010016923	ENSGALG00010016923	-1.91046392	0.16767887	-11.39358777	4.50068E-30	5.51924E-29	negative
PRJNA729271	stage_E18_vs_E10	ordinary_muscle_development	ENSGALG00010016924	LETM1	-0.596665053	0.117822997	-5.064079739	4.10378E-07	1.10115E-06	negative
PRJNA729271	stage_E18_vs_E10	ordinary_muscle_development	ENSGALG00010016925	PHACTR4	-0.312641498	0.136154973	-2.29621799	0.02166342	0.032459301	negative
PRJNA729271	stage_E18_vs_E10	ordinary_muscle_development	ENSGALG00010016926	ENSGALG00010016926	1.026173492	0.180335538	5.690356459	1.26774E-08	3.8776E-08	positive
PRJNA729271	stage_E18_vs_E10	ordinary_muscle_development	ENSGALG00010016927	MGAT4B	0.004378217	0.118050934	0.037087523	0.970415221	0.975530108	positive
PRJNA729271	stage_E18_vs_E10	ordinary_muscle_development	ENSGALG00010016928	RGS11	1.637397917	0.427476222	3.830383617	0.000127944	0.000263429	positive
PRJNA729271	stage_E18_vs_E10	ordinary_muscle_development	ENSGALG00010016930	C10H15ORF48	1.265436718	0.453536612	2.790153398	0.005268307	0.00870544	positive
PRJNA729271	stage_E18_vs_E10	ordinary_muscle_development	ENSGALG00010016931	SLC30A4	0.033613177	0.082821255	0.40585206	0.684851304	0.730995556	positive
PRJNA729271	stage_E18_vs_E10	ordinary_muscle_development	ENSGALG00010016932	OGT	0.888962898	0.092194284	9.642277852	5.29987E-22	4.22561E-21	positive
PRJNA729271	stage_E18_vs_E10	ordinary_muscle_development	ENSGALG00010016934	SLC25A38	-0.680169292	0.142858266	-4.761147625	1.92495E-06	4.81986E-06	negative
PRJNA729271	stage_E18_vs_E10	ordinary_muscle_development	ENSGALG00010016935	PRIMA1	-0.270827442	0.249955595	-1.08350222	0.278585566	0.334168965	negative
PRJNA729271	stage_E18_vs_E10	ordinary_muscle_development	ENSGALG00010016936	RCC1	-0.863763074	0.164802596	-5.241198233	1.59537E-07	4.42667E-07	negative
PRJNA729271	stage_E18_vs_E10	ordinary_muscle_development	ENSGALG00010016937	DHRS7	0.579891364	0.171036396	3.390455938	0.000697765	0.001300454	positive
PRJNA729271	stage_E18_vs_E10	ordinary_muscle_development	ENSGALG00010016938	PSME4	-0.152541448	0.167134323	-0.912687742	0.361406792	0.420871813	negative
PRJNA729271	stage_E18_vs_E10	ordinary_muscle_development	ENSGALG00010016939	FAM234A	0.073446495	0.080536534	0.911964941	0.361787173	0.421197866	positive
PRJNA729271	stage_E18_vs_E10	ordinary_muscle_development	ENSGALG00010016940	FGFR3	-2.584482846	0.213690778	-12.09449875	1.12929E-33	1.6191E-32	negative
PRJNA729271	stage_E18_vs_E10	ordinary_muscle_development	ENSGALG00010016941	SLC8A1	0.546304471	0.17596561	3.104609314	0.001905305	0.003356106	positive
PRJNA729271	stage_E18_vs_E10	ordinary_muscle_development	ENSGALG00010016942	ZBTB40	0.589699838	0.155088584	3.802342021	0.000143335	0.000293162	positive
PRJNA729271	stage_E18_vs_E10	ordinary_muscle_development	ENSGALG00010016943	PCNX4	0.271723796	0.108519952	2.503906349	0.012283054	0.019177388	positive
PRJNA729271	stage_E18_vs_E10	ordinary_muscle_development	ENSGALG00010016944	OPRD1	-2.914882463	0.500867914	-5.819662993	5.89664E-09	1.86172E-08	negative
PRJNA729271	stage_E18_vs_E10	ordinary_muscle_development	ENSGALG00010016945	BDH2	0.292415515	0.145817401	2.00535404	0.044925211	0.06357424	positive
PRJNA729271	stage_E18_vs_E10	ordinary_muscle_development	ENSGALG00010016946	TAF1	-0.403343996	0.107006706	-3.769333824	0.000163684	0.000331832	negative
PRJNA729271	stage_E18_vs_E10	ordinary_muscle_development	ENSGALG00010016947	CANX	-0.214704472	0.071255706	-3.013154794	0.00258547	0.004469456	negative
PRJNA729271	stage_E18_vs_E10	ordinary_muscle_development	ENSGALG00010016948	CCR8	-1.891801369	0.929016769	-2.036347923	0.041715431	0.05932161	negative
PRJNA729271	stage_E18_vs_E10	ordinary_muscle_development	ENSGALG00010016949	LUC7L	-0.086404823	0.095826759	-0.901677406	0.367228258	0.42682813	negative
PRJNA729271	stage_E18_vs_E10	ordinary_muscle_development	ENSGALG00010016950	ENSGALG00010016950	1.824797523	1.066049342	1.711738332	0.086944901	0.116592994	positive
PRJNA729271	stage_E18_vs_E10	ordinary_muscle_development	ENSGALG00010016952	GINS1	-1.345115845	0.207220182	-6.491239558	8.5133E-11	3.12248E-10	negative
PRJNA729271	stage_E18_vs_E10	ordinary_muscle_development	ENSGALG00010016953	GJA4	1.869960509	0.160337749	11.66263415	1.97826E-31	2.60444E-30	positive
PRJNA729271	stage_E18_vs_E10	ordinary_muscle_development	ENSGALG00010016955	ENSGALG00010016955	-4.116120194	0.226758379	-18.15200923	1.23789E-73	7.14869E-72	negative
PRJNA729271	stage_E18_vs_E10	ordinary_muscle_development	ENSGALG00010016956	CENPE	-1.744244939	0.25618193	-6.808618166	9.85406E-12	3.87927E-11	negative
PRJNA729271	stage_E18_vs_E10	ordinary_muscle_development	ENSGALG00010016957	WNT4	1.881446584	0.198580049	9.474499553	2.68042E-21	2.05679E-20	positive
PRJNA729271	stage_E18_vs_E10	ordinary_muscle_development	ENSGALG00010016958	TLR15	-0.504585859	0.61249237	-0.82382391	0.41003962	0.470252733	negative
PRJNA729271	stage_E18_vs_E10	ordinary_muscle_development	ENSGALG00010016959	PERCC1	2.756501053	1.103161912	2.498727542	0.01246401	0.019447343	positive
PRJNA729271	stage_E18_vs_E10	ordinary_muscle_development	ENSGALG00010016960	GJB3	-3.858239359	0.888562198	-4.342115123	1.41118E-05	3.23501E-05	negative
PRJNA729271	stage_E18_vs_E10	ordinary_muscle_development	ENSGALG00010016961	EIF4A3	-0.45890227	0.074776178	-6.137011562	8.40883E-10	2.86252E-09	negative
PRJNA729271	stage_E18_vs_E10	ordinary_muscle_development	ENSGALG00010016962	SQOR	1.40013439	0.138207645	10.13065805	4.03922E-24	3.62081E-23	positive
PRJNA729271	stage_E18_vs_E10	ordinary_muscle_development	ENSGALG00010016963	CD8A	-1.400687438	1.052574292	-1.330725487	0.183279354	0.23007138	negative
PRJNA729271	stage_E18_vs_E10	ordinary_muscle_development	ENSGALG00010016964	MAML1	0.250481668	0.08576141	2.920680398	0.003492679	0.005906707	positive
PRJNA729271	stage_E18_vs_E10	ordinary_muscle_development	ENSGALG00010016965	SLC25A33	-0.841187988	0.341274763	-2.464840883	0.013707411	0.021266073	negative

PRJNA729271	stage_E18_vs_E10	ordinary_muscle_development	ENSGALG00010016966	KDM3A	0.030299279	0.158535157	0.191120253	0.848431381	0.874618652	positive
PRJNA729271	stage_E18_vs_E10	ordinary_muscle_development	ENSGALG00010016967	ERLEC1	0.198848133	0.075176329	2.645089687	0.008166927	0.01308785	positive
PRJNA729271	stage_E18_vs_E10	ordinary_muscle_development	ENSGALG00010016968	C15orf61	-0.147519703	0.151473313	-0.973898967	0.330106707	0.388289922	negative
PRJNA729271	stage_E18_vs_E10	ordinary_muscle_development	ENSGALG00010016969	CHMP3	0.203510512	0.085978741	2.366986407	0.017933591	0.027262444	positive
PRJNA729271	stage_E18_vs_E10	ordinary_muscle_development	ENSGALG00010016970	SYNE3	1.159182228	0.099452027	11.65569231	2.14629E-31	2.82309E-30	positive
PRJNA729271	stage_E18_vs_E10	ordinary_muscle_development	ENSGALG00010016972	ASB3	-0.840260414	0.109028714	-7.706780953	1.29031E-14	6.38547E-14	negative
PRJNA729271	stage_E18_vs_E10	ordinary_muscle_development	ENSGALG00010016973	PLSCR1	1.313423068	0.124564712	10.54410234	5.40869E-26	5.41055E-25	positive
PRJNA729271	stage_E18_vs_E10	ordinary_muscle_development	ENSGALG00010016974	CFAP96	-0.582569732	0.612374914	-0.951328539	0.341437624	0.399865748	negative
PRJNA729271	stage_E18_vs_E10	ordinary_muscle_development	ENSGALG00010016975	MALL	-0.061108975	0.253997406	-0.240588974	0.809873697	0.84181565	negative
PRJNA729271	stage_E18_vs_E10	ordinary_muscle_development	ENSGALG00010016976	ENSGALG00010016976	3.499630274	1.005154984	3.481682259	0.000498275	0.000947088	positive
PRJNA729271	stage_E18_vs_E10	ordinary_muscle_development	ENSGALG00010016977	CLCN7	-0.409402298	0.123997444	-3.301699478	0.00096101	0.001754608	negative
PRJNA729271	stage_E18_vs_E10	ordinary_muscle_development	ENSGALG00010016978	FAM181A	2.234927249	0.362541321	6.164613845	7.06553E-10	2.2446E-09	positive
PRJNA729271	stage_E18_vs_E10	ordinary_muscle_development	ENSGALG00010016979	PLSCR5	3.798812527	0.428551846	8.864300935	7.69856E-19	5.07461E-18	positive
PRJNA729271	stage_E18_vs_E10	ordinary_muscle_development	ENSGALG00010016980	NPHP1	-0.296790886	0.101984005	-2.91017092	0.003612312	0.006098353	negative
PRJNA729271	stage_E18_vs_E10	ordinary_muscle_development	ENSGALG00010016981	ENSGALG00010016981	-1.598289963	0.666981477	-2.396303371	0.016561377	0.025351901	negative
PRJNA729271	stage_E18_vs_E10	ordinary_muscle_development	ENSGALG00010016982	GLRX5	-0.270508173	0.141392907	-1.913166497	0.055726741	0.07760199	negative
PRJNA729271	stage_E18_vs_E10	ordinary_muscle_development	ENSGALG00010016983	ZFAND2A	-1.076454126	0.29204368	-3.685935353	0.000227864	0.000453382	negative
PRJNA729271	stage_E18_vs_E10	ordinary_muscle_development	ENSGALG00010016984	RNF103	0.613731626	0.116935542	5.248460929	1.53375E-07	4.26385E-07	positive
PRJNA729271	stage_E18_vs_E10	ordinary_muscle_development	ENSGALG00010016985	ENSGALG00010016985	1.245300952	0.192593156	6.465966786	1.00653E-10	3.66759E-10	positive
PRJNA729271	stage_E18_vs_E10	ordinary_muscle_development	ENSGALG00010016986	GFER	-0.083549612	0.08889251	-0.939894843	0.347271503	0.406009068	negative
PRJNA729271	stage_E18_vs_E10	ordinary_muscle_development	ENSGALG00010016987	ASB2	3.092596883	0.31829194	9.716227431	2.57132E-22	2.08803E-21	positive
PRJNA729271	stage_E18_vs_E10	ordinary_muscle_development	ENSGALG00010016988	ECI1	1.16594259	0.109787421	10.62000166	2.40557E-26	2.46248E-25	positive
PRJNA729271	stage_E18_vs_E10	ordinary_muscle_development	ENSGALG00010016989	RHOG2	-0.064332186	0.093436905	-0.68850938	0.491132065	0.548751969	negative
PRJNA729271	stage_E18_vs_E10	ordinary_muscle_development	ENSGALG00010016991	PDLIM3	2.826686657	0.199260779	14.18586575	1.12074E-45	2.70302E-44	positive
PRJNA729271	stage_E18_vs_E10	ordinary_muscle_development	ENSGALG00010016993	ENSGALG00010016993	0.784477317	0.180020112	4.357720409	1.31424E-05	3.0214E-05	positive
PRJNA729271	stage_E18_vs_E10	ordinary_muscle_development	ENSGALG00010016994	ATL2	0.275431863	0.116076776	2.372842118	0.017651807	0.02687636	positive
PRJNA729271	stage_E18_vs_E10	ordinary_muscle_development	ENSGALG00010016998	DNASE1L2	1.045910535	0.345074169	3.030973134	0.002437669	0.004225801	positive
PRJNA729271	stage_E18_vs_E10	ordinary_muscle_development	ENSGALG00010016999	ITGB1BP2	2.696103312	0.172777498	15.60448174	6.78585E-55	2.2928E-53	positive
PRJNA729271	stage_E18_vs_E10	ordinary_muscle_development	ENSGALG00010017001	TPCN3	-0.866602402	0.12028501	-7.204575201	5.82251E-13	2.5375E-12	negative
PRJNA729271	stage_E18_vs_E10	ordinary_muscle_development	ENSGALG00010017003	CPZ	3.352983198	0.396787262	8.450329737	2.90481E-17	1.72846E-16	positive
PRJNA729271	stage_E18_vs_E10	ordinary_muscle_development	ENSGALG00010017004	CX3CR1	-4.323365158	0.607126906	-7.121023812	1.07128E-12	4.58195E-12	negative
PRJNA729271	stage_E18_vs_E10	ordinary_muscle_development	ENSGALG00010017006	DNAH3	0.680699219	0.244888804	2.779625723	0.005442158	0.008969197	positive
PRJNA729271	stage_E18_vs_E10	ordinary_muscle_development	ENSGALG00010017008	RTN1	-2.30745335	0.180916082	-12.75427439	2.9511E-37	5.03249E-36	negative
PRJNA729271	stage_E18_vs_E10	ordinary_muscle_development	ENSGALG00010017010	NONO	-1.139341811	0.05525255	-20.62061952	1.79249E-94	1.8427E-92	negative
PRJNA729271	stage_E18_vs_E10	ordinary_muscle_development	ENSGALG00010017012	SYNGR3	2.991581591	0.177043649	16.89742392	4.69996E-64	2.15588E-62	positive
PRJNA729271	stage_E18_vs_E10	ordinary_muscle_development	ENSGALG00010017016	ZIC1	-5.386112395	0.847869187	-6.352527583	2.11805E-10	7.52849E-10	negative
PRJNA729271	stage_E18_vs_E10	ordinary_muscle_development	ENSGALG00010017017	ENSGALG00010017017	0.205790172	0.284350541	0.723719994	0.469237656	0.528197548	positive
PRJNA729271	stage_E18_vs_E10	ordinary_muscle_development	ENSGALG00010017018	HP1BP3	-0.564821505	0.08364627	-6.752500843	1.45318E-11	5.6517E-11	negative
PRJNA729271	stage_E18_vs_E10	ordinary_muscle_development	ENSGALG00010017020	GJB5	-0.60052554	0.34074877	-1.762370382	0.078006739	0.105713135	negative
PRJNA729271	stage_E18_vs_E10	ordinary_muscle_development	ENSGALG00010017022	CCR4	-1.438822416	0.820228748	-1.754172137	0.079401016	0.10743188	negative
PRJNA729271	stage_E18_vs_E10	ordinary_muscle_development	ENSGALG00010017024	BUB1	-2.183095128	0.21752887	-10.03588684	1.06003E-23	9.24492E-23	negative
PRJNA729271	stage_E18_vs_E10	ordinary_muscle_development	ENSGALG00010017033	LDAF1	0.586536545	0.11122612	5.273370567	1.33941E-07	3.74729E-07	positive
PRJNA729271	stage_E18_vs_E10	ordinary_muscle_development	ENSGALG00010017034	ZMYM3	0.429400061	0.089873474	4.777828665	1.77198E-06	4.45841E-06	positive
PRJNA729271	stage_E18_vs_E10	ordinary_muscle_development	ENSGALG00010017035	PIK3CD	0.100349347	0.207947887	0.482569691	0.629401304	0.679565877	positive
PRJNA729271	stage_E18_vs_E10	ordinary_muscle_development	ENSGALG00010017038	ZNF598	-0.674979251	0.132397086	-5.098142793	3.43002E-07	9.27924E-07	negative
PRJNA729271	stage_E18_vs_E10	ordinary_muscle_development	ENSGALG00010017039	MAP2K5	-0.189720287	0.077677056	-2.44242377	0.01458901	0.022565175	negative
PRJNA729271	stage_E18_vs_E10	ordinary_muscle_development	ENSGALG00010017041	JKAMP	-0.129022273	0.092846832	-1.389624928	0.164642802	0.20868288	negative
PRJNA729271	stage_E18_vs_E10	ordinary_muscle_development	ENSGALG00010017042	HNRNPLL	-0.763564011	0.070356656	-10.85276147	1.9349E-27	2.10242E-26	negative
PRJNA729271	stage_E18_vs_E10	ordinary_muscle_development	ENSGALG00010017045	GLB1	0.347620234	0.098908541	3.514562359	0.000440479	0.000844208	positive
PRJNA729271	stage_E18_vs_E10	ordinary_muscle_development	ENSGALG00010017049	TRAF3	0.09813995	0.09145315	1.07311722	0.283218526	0.338856998	positive
PRJNA729271	stage_E18_vs_E10	ordinary_muscle_development	ENSGALG00010017050	L3HYPDH	0.023411126	0.153682584	0.152334279	0.87892329	0.899780569	positive
PRJNA729271	stage_E18_vs_E10	ordinary_muscle_development	ENSGALG00010017054	GALM	-0.145750049	0.150473157	-0.968611628	0.332738999	0.391005415	negative
PRJNA729271	stage_E18_vs_E10	ordinary_muscle_development	ENSGALG00010017055	GPR135	-0.597793361	0.302918313	-1.973447411	0.048444611	0.068168589	negative
PRJNA729271	stage_E18_vs_E10	ordinary_muscle_development	ENSGALG00010017056	AGTR1	4.154164092	0.202312558	20.5333971	1.0833E-93	1.10581E-91	positive
PRJNA729271	stage_E18_vs_E10	ordinary_muscle_development	ENSGALG00010017057	NDUFAB1	0.683238476	0.121899088	5.604951494	2.08313E-08	6.26453E-08	positive
PRJNA729271	stage_E18_vs_E10	ordinary_muscle_development	ENSGALG00010017060	CLSTN1	-1.185333017	0.102438014	-11.57122221	5.76554E-31	7.40883E-30	negative
PRJNA729271	stage_E18_vs_E10	ordinary_muscle_development	ENSGALG00010017063	TMPPE	0.398567293	0.109481603	3.640495591	0.000272114	0.0005362	positive
PRJNA729271	stage_E18_vs_E10	ordinary_muscle_development	ENSGALG00010017065	VRK2	-0.320919471	0.135143968	-2.374648874	0.017565651	0.0267508	negative
PRJNA729271	stage_E18_vs_E10	ordinary_muscle_development	ENSGALG00010017068	ZP2	-4.677338679	1.197543657	-3.905777172	9.3923E-05	0.000196481	negative
PRJNA729271	stage_E18_vs_E10	ordinary_muscle_development	ENSGALG00010017070	BLOC1S6	-0.186899169	0.107092169	-1.745217886	0.080946949	0.109350049	negative
PRJNA729271	stage_E18_vs_E10	ordinary_muscle_development	ENSGALG00010017077	CTNNBIP1	-0.327807898	0.105039911	-3.120793749	0.001803643	0.003191383	negative
PRJNA729271	stage_E18_vs_E10	ordinary_muscle_development	ENSGALG00010017080	PALB2	-0.827068546	0.103430314	-7.996384343	1.28126E-15	6.83292E-15	negative
PRJNA729271	stage_E18_vs_E10	ordinary_muscle_development	ENSGALG00010017083	ENSGALG00010017083	-0.330536565	0.425731735	-0.776396351	0.437514999	0.497004695	negative
PRJNA729271	stage_E18_vs_E10	ordinary_muscle_development	ENSGALG00010017085	ENSGALG00010017085	-0.603699421	0.120455433	-5.011807317	5.39212E-07	1.43043E-06	negative
PRJNA729271	stage_E18_vs_E10	ordinary_muscle_development	ENSGALG00010017089	CRTAP	1.275586254	0.156596495	8.145688425	3.7713E-16	2.0801E-15	positive
PRJNA729271	stage_E18_vs_E10	ordinary_muscle_development	ENSGALG00010017094	SORBS2	-0.189650171	0.149311617	-1.270163527	0.204026387	0.253350679	negative
PRJNA729271	stage_E18_vs_E10	ordinary_muscle_development	ENSGALG00010017096	DCTN5	-0.386039235	0.091474352	-4.220190997	2.44095E-05	5.44584E-05	negative
PRJNA729271	stage_E18_vs_E10	ordinary_muscle_development	ENSGALG00010017098	DAAM1	-0.68513396	0.123900234	-5.529722888	3.20737E-08	9.51122E-08	negative
PRJNA729271	stage_E18_vs_E10	ordinary_muscle_development	ENSGALG00010017100	SUSD5	-5.935269412	0.583276409	-10.17574056	2.54457E-24	2.311E-23	negative
PRJNA729271	stage_E18_vs_E10	ordinary_muscle_development	ENSGALG00010017101	CSMD2	-0.191059686	0.235070985	-0.812774432	0.416347397	0.476619454	negative
PRJNA729271	stage_E18_vs_E10	ordinary_muscle_development	ENSGALG00010017104	FANCL	-1.847517296	0.178132448	-10.37159323	3.3391E-25	3.19896E-24	negative
PRJNA729271	stage_E18_vs_E10	ordinary_muscle_development	ENSGALG00010017105	PLK1	-2.140133248	0.23427383	-9.135178469	6.5299E-20	4.56369E-19	negative
PRJNA729271	stage_E18_vs_E10	ordinary_muscle_development	ENSGALG00010017106	CRYM	0.131102442	0.11406031	1.149413337	0.250385583	0.304066608	positive
PRJNA729271	stage_E18_vs_E10	ordinary_muscle_development	ENSGALG00010017109	ITGB1BP3	0.740542303	0.127517144	5.807394055	6.34527E-09	1.99858E-08	positive
PRJNA729271	stage_E18_vs_E10	ordinary_muscle_development	ENSGALG00010017110	BRF1	-0.302556989	0.073184813	-4.134149925	3.56271E-05	7.79023E-05	negative
PRJNA729271	stage_E18_vs_E10	ordinary_muscle_development	ENSGALG00010017111	GJB1	-0.18943676	0.45362234	-0.417608973	0.676233035	0.722648027	negative
PRJNA729271	stage_E18_vs_E10	ordinary_muscle_development	ENSGALG00010017112	LZIC	-0.55432429	0.063834242	-8.683807859	3.82735E-18	2.4226E-17	negative
PRJNA729271	stage_E18_vs_E10	ordinary_muscle_development	ENSGALG00010017115	LGALSL	2.351097341	0.191949479	12.24852158	1.71086E-34	2.57518E-33	positive
PRJNA729271	stage_E18_vs_E10	ordinary_muscle_development	ENSGALG00010017116	ENSGALG00010017116	-0.095754976	0.259420837	-0.369110582	0.712045302	0.756013525	negative
PRJNA729271	stage_E18_vs_E10	ordinary_muscle_development	ENSGALG00010017119	BTBD6	-0.0985812	0.149883438	-0.657719097	0.510718649	0.567482031	negative
PRJNA729271	stage_E18_vs_E10	ordinary_muscle_development	ENSGALG00010017120	ERN2	0.84395849	0.145868032	5.785767288	7.21821E-09	2.25734E-08	positive
PRJNA729271	stage_E18_vs_E10	ordinary_muscle_development	ENSGALG00010017122	NCBP2	-1.443179804	0.193581874	-7.455139149	8.9773E-14	4.14414E-13	negative
PRJNA729271	stage_E18_vs_E10	ordinary_muscle_development	ENSGALG00010017127	SOX8	-0.335015198	0.133782643	-2.50417535	0.012273719	0.019164877	negative
PRJNA729271	stage_E18_vs_E10	ordinary_muscle_development	ENSGALG00010017131	TLR3	0.368138445	0.403418301	0.912547706	0.361480469	0.420923794	positive
PRJNA729271	stage_E18_vs_E10	ordinary_muscle_development	ENSGALG00010017132	TMEM108	0.148060281	0.263793336	0.561273773	0.574610918	0.627655257	positive
PRJNA729271	stage_E18_vs_E10	ordinary_muscle_development	ENSGALG00010017133	WDR20	0.347733715	0.112840251	3.081646056	0.002058595	0.003609451	positive
PRJNA729271	stage_E18_vs_E10	ordinary_muscle_development	ENSGALG00010017135	ENSGALG00010017135	0.894388035	0.132732053	6.738297302	1.60253E-11	6.20426E-11	positive
PRJNA729271	stage_E18_vs_E10	ordinary_muscle_development	ENSGALG00010017136	ENSGALG00010017136	0.862248931	0.386160917	2.23287467	0.02555721	0.037828539	positive
PRJNA729271	stage_E18_vs_E10	ordinary_muscle_development	ENSGALG00010017139	COL9A2	-5.465894591	0.561795771	-9.729326689	2.26084E-22	1.84003E-21	negative
PRJNA729271	stage_E18_vs_E10	ordinary_muscle_development	ENSGALG00010017140	UQCRC2	0.872563792	0.115090707	7.581531242	3.415E-14	1.62456E-13	positive
PRJNA729271	stage_E18_vs_E10	ordinary_muscle_development	ENSGALG00010017141	AFTPH	0.326913056	0.076800184	4.25667021	2.07494E-05	4.66878E-05	positive
PRJNA729271	stage_E18_vs_E10	ordinary_muscle_development	ENSGALG00010017142	RBP7	1.915000198	0.69462192	2.75689572	0.005835296	0.009570334	positive
PRJNA729271	stage_E18_vs_E10	ordinary_muscle_development	ENSGALG00010017143	FAM149A	-0.117508098	0.274807787	-0.427601047	0.668941604	0.715858881	negative
PRJNA729271	stage_E18_vs_E10	ordinary_muscle_development	ENSGALG00010017144	CPB1	1.467559667	0.737476203	1.989975623	0.046593621	0.065749078	positive
PRJNA729271	stage_E18_vs_E10	ordinary_muscle_development	ENSGALG00010017147	SRSF7L	-0.609853251	0.070870282	-8.605204185	7.6181E-18	4.7048E-17	negative
PRJNA729271	stage_E18_vs_E10	ordinary_muscle_development	ENSGALG00010017148	MOK	-0.363076318	0.487948964	-0.744086665	0.456824046	0.516389655	negative
PRJNA729271	stage_E18_vs_E10	ordinary_muscle_development	ENSGALG00010017152	CYP4V2	2.552003997	0.199116365	12.81664617	1.323E-37	2.29115E-36	positive
PRJNA729271	stage_E18_vs_E10	ordinary_muscle_development	ENSGALG00010017153	SLC35F4	0.231290183	0.571171032	0.404940324	0.685521377	0.73160303	positive
PRJNA729271	stage_E18_vs_E10	ordinary_muscle_development	ENSGALG00010017154	PIGZ	0.047485322	0.197909576	0.239934435	0.810381086	0.84228265	positive
PRJNA729271	stage_E18_vs_E10	ordinary_muscle_development	ENSGALG00010017162	BCL11A	-1.566147683	0.201070766	-7.789037232	6.75217E-15	3.42332E-14	negative
PRJNA729271	stage_E18_vs_E10	ordinary_muscle_development	ENSGALG00010017163	CDV3	-1.09889278	0.12524449	-8.773981039	1.72458E-18	1.1125E-17	negative
PRJNA729271	stage_E18_vs_E10	ordinary_muscle_development	ENSGALG00010017167	SMAP2	-0.206986894	0.097261931	-2.128138856	0.033325573	0.048286103	negative
PRJNA729271	stage_E18_vs_E10	ordinary_muscle_development	ENSGALG00010017169	CCDC198	1.024903287	0.233587261	4.387667732	1.14573E-05	2.64963E-05	positive
PRJNA729271	stage_E18_vs_E10	ordinary_muscle_development	ENSGALG00010017174	DHX57	-0.696623138	0.098834444	-7.048384246	1.81007E-12	7.59392E-12	negative
PRJNA729271	stage_E18_vs_E10	ordinary_muscle_development	ENSGALG00010017180	ENSGALG00010017180	-1.568000342	0.477554662	-3.28339448	0.00102565	0.001865579	negative
PRJNA729271	stage_E18_vs_E10	ordinary_muscle_development	ENSGALG00010017183	NFYC	0.130169017	0.093765453	1.388240678	0.165063769	0.209143299	positive
PRJNA729271	stage_E18_vs_E10	ordinary_muscle_development	ENSGALG00010017184	CARD11	0.843694881	0.213939344	3.943617213	8.02617E-05	0.000168975	positive
PRJNA729271	stage_E18_vs_E10	ordinary_muscle_development	ENSGALG00010017185	NAA30	-0.197151609	0.065250824	-3.021442468	0.002515734	0.004353058	negative
PRJNA729271	stage_E18_vs_E10	ordinary_muscle_development	ENSGALG00010017187	ZNF839	0.46326261	0.139750119	3.314935359	0.000916643	0.001678043	positive
PRJNA729271	stage_E18_vs_E10	ordinary_muscle_development	ENSGALG00010017188	ENSGALG00010017188	0.661641381	0.160163711	4.131031779	3.61139E-05	7.8931E-05	positive
PRJNA729271	stage_E18_vs_E10	ordinary_muscle_development	ENSGALG00010017190	STK26	-0.756335074	0.068711891	-11.007339	3.52254E-28	3.93976E-27	negative
PRJNA729271	stage_E18_vs_E10	ordinary_muscle_development	ENSGALG00010017198	TOPBP1	-0.689300448	0.068907765	-10.00323333	1.47501E-23	1.27491E-22	negative
PRJNA729271	stage_E18_vs_E10	ordinary_muscle_development	ENSGALG00010017200	KCNQ4	1.027397139	0.309181996	3.322952666	0.0008907	0.001634266	positive
PRJNA729271	stage_E18_vs_E10	ordinary_muscle_development	ENSGALG00010017201	AP5M1	-1.52983983	0.156332529	-9.785806212	1.29559E-22	1.0652E-21	negative
PRJNA729271	stage_E18_vs_E10	ordinary_muscle_development	ENSGALG00010017207	ENSGALG00010017207	3.823679542	0.286343265	13.35348169	1.1304E-40	2.22623E-39	positive
PRJNA729271	stage_E18_vs_E10	ordinary_muscle_development	ENSGALG00010017208	CINP	-0.84874925	0.176663989	-4.804313867	1.55283E-06	3.92952E-06	negative
PRJNA729271	stage_E18_vs_E10	ordinary_muscle_development	ENSGALG00010017211	UBFD1	0.053419285	0.130760083	0.408528994	0.682885349	0.729111898	positive
PRJNA729271	stage_E18_vs_E10	ordinary_muscle_development	ENSGALG00010017212	TINAGL1	2.152882472	0.136989137	15.71571675	1.18039E-55	4.11291E-54	positive
PRJNA729271	stage_E18_vs_E10	ordinary_muscle_development	ENSGALG00010017215	FRMD7	-0.611973349	0.862266274	-0.709726645	0.477873666	0.536294425	negative
PRJNA729271	stage_E18_vs_E10	ordinary_muscle_development	ENSGALG00010017219	EARS2	-0.772570214	0.141730243	-5.450990541	5.009E-08	1.4559E-07	negative
PRJNA729271	stage_E18_vs_E10	ordinary_muscle_development	ENSGALG00010017221	SPPL2A	0.014345415	0.075738914	0.189406138	0.849774514	0.875691851	positive
PRJNA729271	stage_E18_vs_E10	ordinary_muscle_development	ENSGALG00010017223	NOC2L	-1.27324239	0.078006068	-16.32235052	6.84485E-60	2.74078E-58	negative
PRJNA729271	stage_E18_vs_E10	ordinary_muscle_development	ENSGALG00010017226	PEF1	0.024758118	0.075387318	0.328412237	0.742599992	0.78312018	positive
PRJNA729271	stage_E18_vs_E10	ordinary_muscle_development	ENSGALG00010017228	RAP2C	-0.471238738	0.072082916	-6.537453891	6.25749E-11	2.31738E-10	negative
PRJNA729271	stage_E18_vs_E10	ordinary_muscle_development	ENSGALG00010017240	MELTF	0.968525681	0.233888252	4.140976183	3.45831E-05	7.57223E-05	positive
PRJNA729271	stage_E18_vs_E10	ordinary_muscle_development	ENSGALG00010017246	GGA2	0.162133009	0.134841902	1.202393367	0.229211158	0.2808229	positive
PRJNA729271	stage_E18_vs_E10	ordinary_muscle_development	ENSGALG00010017248	TECPR2	0.027979005	0.085432938	0.327496693	0.743292244	0.78362216	positive
PRJNA729271	stage_E18_vs_E10	ordinary_muscle_development	ENSGALG00010017250	COL16A1	0.670378487	0.169654478	3.951434091	7.76843E-05	0.000163858	positive
PRJNA729271	stage_E18_vs_E10	ordinary_muscle_development	ENSGALG00010017252	EXOC5	-0.786927749	0.088708949	-8.870894759	7.25605E-19	4.79164E-18	negative
PRJNA729271	stage_E18_vs_E10	ordinary_muscle_development	ENSGALG00010017256	CARHSP1	-0.068050874	0.12928753	-0.52635296	0.598642993	0.650219735	negative
PRJNA729271	stage_E18_vs_E10	ordinary_muscle_development	ENSGALG00010017263	COG7	0.080013576	0.071265003	1.122761141	0.261538953	0.315917261	positive

PRJNA729271	stage_E18_vs_E10	ordinary_muscle_development	ENSGALG00010017264	PIAS1	0.439615016	0.086435583	5.086042144	3.65613E-07	9.85964E-07	positive
PRJNA729271	stage_E18_vs_E10	ordinary_muscle_development	ENSGALG00010017266	PMM2	-0.782409124	0.109061583	-7.174012151	7.28311E-13	3.15507E-12	negative
PRJNA729271	stage_E18_vs_E10	ordinary_muscle_development	ENSGALG00010017272	NRXN1	0.955181741	0.134361609	7.109037661	1.16855E-12	4.97302E-12	positive
PRJNA729271	stage_E18_vs_E10	ordinary_muscle_development	ENSGALG00010017278	CCR5	0.291316959	0.234240931	1.243663769	0.213623336	0.263933707	positive
PRJNA729271	stage_E18_vs_E10	ordinary_muscle_development	ENSGALG00010017280	SOS1	0.027956364	0.157218933	0.177818051	0.858865871	0.883114194	positive
PRJNA729271	stage_E18_vs_E10	ordinary_muscle_development	ENSGALG00010017283	INF2	0.320712386	0.138537504	2.314986031	0.020613697	0.031014691	positive
PRJNA729271	stage_E18_vs_E10	ordinary_muscle_development	ENSGALG00010017284	ENSGALG00010017284	-0.692140368	0.206616423	-3.34988071	0.000808464	0.001493713	negative
PRJNA729271	stage_E18_vs_E10	ordinary_muscle_development	ENSGALG00010017285	IL21R	0.586422125	1.091531344	0.537247169	0.591096893	0.643100642	positive
PRJNA729271	stage_E18_vs_E10	ordinary_muscle_development	ENSGALG00010017289	ADGRB2	-1.530151275	0.329349042	-4.64598672	3.38455E-06	8.24902E-06	negative
PRJNA729271	stage_E18_vs_E10	ordinary_muscle_development	ENSGALG00010017290	DACT1	-0.237068447	0.156368777	-1.516085576	0.129497715	0.168105802	negative
PRJNA729271	stage_E18_vs_E10	ordinary_muscle_development	ENSGALG00010017296	SCNN1G	1.351245874	0.228291603	5.918946905	3.2401E-09	1.046E-08	positive
PRJNA729271	stage_E18_vs_E10	ordinary_muscle_development	ENSGALG00010017299	USP31	0.6272793	0.112587333	5.571490881	2.52569E-08	7.5453E-08	positive
PRJNA729271	stage_E18_vs_E10	ordinary_muscle_development	ENSGALG00010017300	TRPM7	-0.887090956	0.105758197	-8.387916806	4.94832E-17	2.89919E-16	negative
PRJNA729271	stage_E18_vs_E10	ordinary_muscle_development	ENSGALG00010017301	LHCGR	0.043506543	0.44510268	0.097744958	0.922134815	0.935952954	positive
PRJNA729271	stage_E18_vs_E10	ordinary_muscle_development	ENSGALG00010017304	ANKRD9	0.551314637	0.304411916	1.811081	0.070128314	0.095915259	positive
PRJNA729271	stage_E18_vs_E10	ordinary_muscle_development	ENSGALG00010017305	HS3ST2	2.096678107	0.520116125	4.031173053	5.54992E-05	0.000119038	positive
PRJNA729271	stage_E18_vs_E10	ordinary_muscle_development	ENSGALG00010017307	TMEM186	-0.23823507	0.159094798	-1.497440983	0.134278551	0.173689773	negative
PRJNA729271	stage_E18_vs_E10	ordinary_muscle_development	ENSGALG00010017310	SAMD11	-0.752619449	0.177012398	-4.251789462	2.12069E-05	4.76433E-05	negative
PRJNA729271	stage_E18_vs_E10	ordinary_muscle_development	ENSGALG00010017312	PTP4A2	0.605733661	0.154953322	3.909136338	9.26267E-05	0.000193881	positive
PRJNA729271	stage_E18_vs_E10	ordinary_muscle_development	ENSGALG00010017313	MAP4K3	0.006786563	0.083390569	0.081382854	0.935137484	0.946763835	positive
PRJNA729271	stage_E18_vs_E10	ordinary_muscle_development	ENSGALG00010017314	CALML4	-0.000864402	0.221865023	-0.003896074	0.996891391	0.997304211	negative
PRJNA729271	stage_E18_vs_E10	ordinary_muscle_development	ENSGALG00010017315	HS3ST4	-2.440562023	0.968059664	-2.52108637	0.011699313	0.018343055	negative
PRJNA729271	stage_E18_vs_E10	ordinary_muscle_development	ENSGALG00010017318	ADSS1	2.630880081	0.123933498	21.2281596	5.24777E-100	6.08531E-98	positive
PRJNA729271	stage_E18_vs_E10	ordinary_muscle_development	ENSGALG00010017322	KHDRBS1	-0.713937201	0.070007713	-10.19797921	2.02441E-24	1.85018E-23	negative
PRJNA729271	stage_E18_vs_E10	ordinary_muscle_development	ENSGALG00010017323	ENSGALG00010017323	1.294309833	0.104679617	12.3644877	4.06797E-35	6.27957E-34	positive
PRJNA729271	stage_E18_vs_E10	ordinary_muscle_development	ENSGALG00010017326	STON1	0.300953397	0.121782673	2.471233294	0.013464795	0.020914295	positive
PRJNA729271	stage_E18_vs_E10	ordinary_muscle_development	ENSGALG00010017327	CLN6	-0.793344714	0.138049567	-5.746810606	9.09425E-09	2.8197E-08	negative
PRJNA729271	stage_E18_vs_E10	ordinary_muscle_development	ENSGALG00010017332	FGFR1	0.40825106	0.158694379	2.57256157	0.0100949	0.015963951	positive
PRJNA729271	stage_E18_vs_E10	ordinary_muscle_development	ENSGALG00010017334	KDM8	-0.563330544	0.159061476	-3.541590084	0.000397723	0.000765909	negative
PRJNA729271	stage_E18_vs_E10	ordinary_muscle_development	ENSGALG00010017335	THUMPD2	-0.410383011	0.13948699	-2.942088072	0.003260072	0.00554568	negative
PRJNA729271	stage_E18_vs_E10	ordinary_muscle_development	ENSGALG00010017337	ABAT	-1.600870929	0.20800512	-7.696305405	1.40057E-14	6.90283E-14	negative
PRJNA729271	stage_E18_vs_E10	ordinary_muscle_development	ENSGALG00010017338	MBNL3	-0.046979107	0.075176029	-0.624921374	0.532022663	0.588003699	negative
PRJNA729271	stage_E18_vs_E10	ordinary_muscle_development	ENSGALG00010017340	SIVA1	-0.810534684	0.108256227	-7.487187638	7.03651E-14	3.27639E-13	negative
PRJNA729271	stage_E18_vs_E10	ordinary_muscle_development	ENSGALG00010017341	TA3	-0.2025249	0.08061759	-2.512167633	0.011999208	0.018766564	negative
PRJNA729271	stage_E18_vs_E10	ordinary_muscle_development	ENSGALG00010017343	DLG1	0.309224427	0.09936079	3.112137376	0.00185738	0.003278462	positive
PRJNA729271	stage_E18_vs_E10	ordinary_muscle_development	ENSGALG00010017344	RERE	1.427223361	0.153369549	9.305780519	1.33012E-20	9.7374E-20	positive
PRJNA729271	stage_E18_vs_E10	ordinary_muscle_development	ENSGALG00010017346	GM2A	0.644262454	0.149531944	4.308527244	1.64345E-05	3.74028E-05	positive
PRJNA729271	stage_E18_vs_E10	ordinary_muscle_development	ENSGALG00010017347	TMEM39B	-0.710956494	0.121704624	-5.841655569	5.16846E-09	1.63932E-08	negative
PRJNA729271	stage_E18_vs_E10	ordinary_muscle_development	ENSGALG00010017349	ENSGALG00010017349	1.274280202	0.112589228	11.31795844	1.06933E-29	1.28951E-28	positive
PRJNA729271	stage_E18_vs_E10	ordinary_muscle_development	ENSGALG00010017351	CCR2	1.580779209	0.208028157	7.598871377	2.98724E-14	1.42622E-13	positive
PRJNA729271	stage_E18_vs_E10	ordinary_muscle_development	ENSGALG00010017352	GPCPD1	0.543515876	0.238579464	2.278133528	0.022718623	0.033938621	positive
PRJNA729271	stage_E18_vs_E10	ordinary_muscle_development	ENSGALG00010017354	FEM1B	-0.223852147	0.105527618	-2.121265994	0.033899424	0.049044032	negative
PRJNA729271	stage_E18_vs_E10	ordinary_muscle_development	ENSGALG00010017356	NSMCE1	-0.707233163	0.120881661	-5.850624146	4.89732E-09	1.55775E-08	negative
PRJNA729271	stage_E18_vs_E10	ordinary_muscle_development	ENSGALG00010017359	EHD3	-0.166155093	0.095860605	-1.733299023	0.083042534	0.11187857	negative
PRJNA729271	stage_E18_vs_E10	ordinary_muscle_development	ENSGALG00010017361	METTL22	-0.127340178	0.152108071	-0.837169107	0.40249752	0.462407985	negative
PRJNA729271	stage_E18_vs_E10	ordinary_muscle_development	ENSGALG00010017362	ENO1	1.19774236	0.130126138	9.204471763	3.43356E-20	2.4349E-19	positive
PRJNA729271	stage_E18_vs_E10	ordinary_muscle_development	ENSGALG00010017363	TIMM9	-0.124852745	0.143193905	-0.871913821	0.383255393	0.443606718	negative
PRJNA729271	stage_E18_vs_E10	ordinary_muscle_development	ENSGALG00010017364	AKT1	-0.699822423	0.074617376	-9.378813058	6.67196E-21	4.99277E-20	negative
PRJNA729271	stage_E18_vs_E10	ordinary_muscle_development	ENSGALG00010017367	TIMD4	0.853233116	0.30734071	2.776179946	0.005500176	0.009057606	positive
PRJNA729271	stage_E18_vs_E10	ordinary_muscle_development	ENSGALG00010017368	ERLIN2	-0.429920463	0.050762498	-8.469253558	2.46971E-17	1.4744E-16	negative
PRJNA729271	stage_E18_vs_E10	ordinary_muscle_development	ENSGALG00010017369	CAPN14	0.681106583	0.492080731	1.384135854	0.16631686	0.210565367	positive
PRJNA729271	stage_E18_vs_E10	ordinary_muscle_development	ENSGALG00010017371	TMEM114	-1.31894268	1.259227053	-1.047422446	0.294904784	0.351533293	negative
PRJNA729271	stage_E18_vs_E10	ordinary_muscle_development	ENSGALG00010017372	ENSGALG00010017372	0.414638631	0.11147931	3.719422302	0.000199679	0.000400435	positive
PRJNA729271	stage_E18_vs_E10	ordinary_muscle_development	ENSGALG00010017373	RP1-2705.3	0.081135307	0.107051493	0.757909159	0.448505367	0.508056056	positive
PRJNA729271	stage_E18_vs_E10	ordinary_muscle_development	ENSGALG00010017375	BDH1A	-1.673912936	0.162666076	-10.29048577	7.7783E-25	7.32119E-24	negative
PRJNA729271	stage_E18_vs_E10	ordinary_muscle_development	ENSGALG00010017376	GALNT14	-0.009986185	0.275532006	-0.036243285	0.971088372	0.976003741	negative
PRJNA729271	stage_E18_vs_E10	ordinary_muscle_development	ENSGALG00010017377	USP8	0.245190752	0.075738491	3.237333462	0.001206523	0.002175735	positive
PRJNA729271	stage_E18_vs_E10	ordinary_muscle_development	ENSGALG00010017378	ENSGALG00010017378	2.082933114	0.533368718	3.905240492	9.41317E-05	0.000196832	positive
PRJNA729271	stage_E18_vs_E10	ordinary_muscle_development	ENSGALG00010017380	MARCKSL1	-0.343087214	0.13430528	-2.55453259	0.010633048	0.016767409	negative
PRJNA729271	stage_E18_vs_E10	ordinary_muscle_development	ENSGALG00010017381	CA6	0.250484941	0.667362512	0.375335648	0.707410857	0.751863937	positive
PRJNA729271	stage_E18_vs_E10	ordinary_muscle_development	ENSGALG00010017383	ENSGALG00010017383	-0.382967153	0.153537442	-2.494291605	0.012620881	0.019681514	negative
PRJNA729271	stage_E18_vs_E10	ordinary_muscle_development	ENSGALG00010017384	ARID4A	0.107821329	0.093025045	1.159056995	0.246432954	0.299643123	positive
PRJNA729271	stage_E18_vs_E10	ordinary_muscle_development	ENSGALG00010017386	ENSGALG00010017386	1.696696258	0.511536489	3.31686262	0.000910344	0.001667564	positive
PRJNA729271	stage_E18_vs_E10	ordinary_muscle_development	ENSGALG00010017387	HS6ST2	-0.59729617	0.114643841	-5.210015325	1.88825E-07	5.21536E-07	negative
PRJNA729271	stage_E18_vs_E10	ordinary_muscle_development	ENSGALG00010017388	PLPBP	0.137903045	0.083211807	1.657253333	0.097468268	0.129555483	positive
PRJNA729271	stage_E18_vs_E10	ordinary_muscle_development	ENSGALG00010017390	DDX24	-0.891383732	0.103323091	-8.627149321	6.29E-18	3.90967E-17	negative
PRJNA729271	stage_E18_vs_E10	ordinary_muscle_development	ENSGALG00010017391	CRAMP1	0.154274477	0.117421086	1.313856664	0.188894445	0.236212068	positive
PRJNA729271	stage_E18_vs_E10	ordinary_muscle_development	ENSGALG00010017393	VDAC1	2.097857477	0.163893795	12.80010309	1.63734E-37	2.82538E-36	positive
PRJNA729271	stage_E18_vs_E10	ordinary_muscle_development	ENSGALG00010017395	BRF2	-0.614489078	0.13136277	-4.677802362	2.89966E-06	7.11899E-06	negative
PRJNA729271	stage_E18_vs_E10	ordinary_muscle_development	ENSGALG00010017396	CCR8L	3.234464415	0.271959783	11.89317178	1.28435E-32	1.75463E-31	positive
PRJNA729271	stage_E18_vs_E10	ordinary_muscle_development	ENSGALG00010017398	SLC22A5	-1.374377372	0.186575594	-7.366329876	1.7539E-13	7.91245E-13	negative
PRJNA729271	stage_E18_vs_E10	ordinary_muscle_development	ENSGALG00010017399	CPXM1	1.382671264	0.289978928	4.768178409	1.85899E-06	4.66276E-06	positive
PRJNA729271	stage_E18_vs_E10	ordinary_muscle_development	ENSGALG00010017400	GPC4	-1.15671862	0.119128937	-9.7098039	2.73863E-22	2.22141E-21	negative
PRJNA729271	stage_E18_vs_E10	ordinary_muscle_development	ENSGALG00010017402	SLC2A5	2.12936083	0.227783409	9.348182297	8.91669E-21	6.6145E-20	positive
PRJNA729271	stage_E18_vs_E10	ordinary_muscle_development	ENSGALG00010017403	ITGA11	-0.546934212	0.293175572	-1.865551786	0.0621041	0.085635363	negative
PRJNA729271	stage_E18_vs_E10	ordinary_muscle_development	ENSGALG00010017405	XCR1	0.33393561	1.219872021	0.273746429	0.784279495	0.819147664	positive
PRJNA729271	stage_E18_vs_E10	ordinary_muscle_development	ENSGALG00010017406	IFT140	-0.538267387	0.123833569	-4.346700091	1.38201E-05	3.17166E-05	negative
PRJNA729271	stage_E18_vs_E10	ordinary_muscle_development	ENSGALG00010017407	EPN2	-0.251904003	0.085660588	-2.940722332	0.003274479	0.005566269	negative
PRJNA729271	stage_E18_vs_E10	ordinary_muscle_development	ENSGALG00010017409	GPC3	-1.589189116	0.343659493	-4.624313155	3.75842E-06	9.11465E-06	negative
PRJNA729271	stage_E18_vs_E10	ordinary_muscle_development	ENSGALG00010017411	RAB11FIP1	0.257987066	0.138036776	1.868973427	0.061626512	0.085049632	positive
PRJNA729271	stage_E18_vs_E10	ordinary_muscle_development	ENSGALG00010017413	ZIC3	-5.433064698	0.995874432	-5.455572031	4.88154E-08	1.42056E-07	negative
PRJNA729271	stage_E18_vs_E10	ordinary_muscle_development	ENSGALG00010017414	ENSGALG00010017414	1.202417363	0.19669917	6.11297629	9.77899E-10	3.30334E-09	positive
PRJNA729271	stage_E18_vs_E10	ordinary_muscle_development	ENSGALG00010017415	HDAC1	-1.079728791	0.063721307	-16.94454883	2.11148E-64	9.74709E-63	negative
PRJNA729271	stage_E18_vs_E10	ordinary_muscle_development	ENSGALG00010017418	CHGB	-1.483658884	0.289420327	-5.126311966	2.95473E-07	8.03391E-07	negative
PRJNA729271	stage_E18_vs_E10	ordinary_muscle_development	ENSGALG00010017419	B9D1	-0.853445408	0.102377548	-8.336255566	7.66801E-17	4.42996E-16	negative
PRJNA729271	stage_E18_vs_E10	ordinary_muscle_development	ENSGALG00010017421	GPR157	2.039818122	0.262093697	7.782782062	7.09468E-15	3.58318E-14	positive
PRJNA729271	stage_E18_vs_E10	ordinary_muscle_development	ENSGALG00010017422	ENSGALG00010017422	1.072522255	0.220163924	4.871471387	1.1077E-06	2.8529E-06	positive
PRJNA729271	stage_E18_vs_E10	ordinary_muscle_development	ENSGALG00010017423	TMEM204	0.845422284	0.132395693	6.385572422	1.70758E-10	6.11596E-10	positive
PRJNA729271	stage_E18_vs_E10	ordinary_muscle_development	ENSGALG00010017424	APOD	0.837915978	0.537779598	1.55810295	0.119208845	0.155950561	positive
PRJNA729271	stage_E18_vs_E10	ordinary_muscle_development	ENSGALG00010017425	H6PD	1.148072264	0.124884522	9.19307088	3.81783E-20	2.7008E-19	positive
PRJNA729271	stage_E18_vs_E10	ordinary_muscle_development	ENSGALG00010017428	PSMA3	-0.59487381	0.108778001	-5.468695895	4.53359E-08	1.32462E-07	negative
PRJNA729271	stage_E18_vs_E10	ordinary_muscle_development	ENSGALG00010017429	TELO2	-0.40688678	0.105673258	-3.850423368	0.000117914	0.000243818	negative
PRJNA729271	stage_E18_vs_E10	ordinary_muscle_development	ENSGALG00010017430	CACNA1H	0.535619788	0.198864349	2.693392716	0.00707289	0.01142556	positive
PRJNA729271	stage_E18_vs_E10	ordinary_muscle_development	ENSGALG00010017431	FYCO1	2.222671119	0.117845172	18.86094335	2.38919E-79	1.63355E-77	positive
PRJNA729271	stage_E18_vs_E10	ordinary_muscle_development	ENSGALG00010017433	RNPS1	-0.635118371	0.079564324	-7.982451638	1.43455E-15	7.62236E-15	negative
PRJNA729271	stage_E18_vs_E10	ordinary_muscle_development	ENSGALG00010017434	FGF13	3.374998552	0.148096545	22.78917824	5.8707E-115	9.8948E-113	positive
PRJNA729271	stage_E18_vs_E10	ordinary_muscle_development	ENSGALG00010017435	ACTR10	-0.544076516	0.090651058	-6.001877191	1.95049E-09	6.42262E-09	negative
PRJNA729271	stage_E18_vs_E10	ordinary_muscle_development	ENSGALG00010017437	DRG2	0.019662561	0.140147822	0.140298729	0.888423968	0.907903654	positive
PRJNA729271	stage_E18_vs_E10	ordinary_muscle_development	ENSGALG00010017438	ADRB3	3.414601538	0.790504204	4.319523563	1.56366E-05	3.56765E-05	positive
PRJNA729271	stage_E18_vs_E10	ordinary_muscle_development	ENSGALG00010017439	PPP4R4	-2.350743166	0.284409952	-8.265333712	1.39304E-16	7.92472E-16	negative
PRJNA729271	stage_E18_vs_E10	ordinary_muscle_development	ENSGALG00010017441	SPSB1	0.28715681	0.256980709	1.117425546	0.263812417	0.318451115	positive
PRJNA729271	stage_E18_vs_E10	ordinary_muscle_development	ENSGALG00010017442	LCK	1.164778362	0.259453327	4.489356043	7.14388E-06	1.68787E-05	positive
PRJNA729271	stage_E18_vs_E10	ordinary_muscle_development	ENSGALG00010017445	F9	5.522625697	1.1587622	4.765969838	1.87947E-06	4.7125E-06	positive
PRJNA729271	stage_E18_vs_E10	ordinary_muscle_development	ENSGALG00010017447	CAPN13	-0.360432191	0.5439059	-0.662673803	0.507539491	0.564516952	negative
PRJNA729271	stage_E18_vs_E10	ordinary_muscle_development	ENSGALG00010017449	TRMT6	-0.870077592	0.087028916	-9.997568972	1.56184E-23	1.34755E-22	negative
PRJNA729271	stage_E18_vs_E10	ordinary_muscle_development	ENSGALG00010017450	ENSGALG00010017450	0.985320745	0.212134715	4.644787839	3.40426E-06	8.29184E-06	positive
PRJNA729271	stage_E18_vs_E10	ordinary_muscle_development	ENSGALG00010017451	GABPB1	-0.58923633	0.077899048	-7.564101786	3.90554E-14	1.85003E-13	negative
PRJNA729271	stage_E18_vs_E10	ordinary_muscle_development	ENSGALG00010017452	ARMH4	-3.594584744	0.453087894	-7.93352635	2.13009E-15	1.11505E-14	negative
PRJNA729271	stage_E18_vs_E10	ordinary_muscle_development	ENSGALG00010017453	PPP1R2	-0.648025388	0.139749641	-4.637045091	3.53425E-06	8.59259E-06	negative
PRJNA729271	stage_E18_vs_E10	ordinary_muscle_development	ENSGALG00010017454	ENSGALG00010017454	0.977399949	0.159507774	6.127600714	8.92141E-10	3.02989E-09	positive
PRJNA729271	stage_E18_vs_E10	ordinary_muscle_development	ENSGALG00010017455	CCR9	1.548850976	0.760244078	2.037307519	0.041619235	0.059208049	positive
PRJNA729271	stage_E18_vs_E10	ordinary_muscle_development	ENSGALG00010017457	ZMAT4	-2.073947174	0.270159787	-7.676742694	1.63185E-14	7.99649E-14	negative
PRJNA729271	stage_E18_vs_E10	ordinary_muscle_development	ENSGALG00010017458	ENSGALG00010017458	-0.128264165	0.173996163	-0.737166633	0.461021022	0.520322332	negative
PRJNA729271	stage_E18_vs_E10	ordinary_muscle_development	ENSGALG00010017459	FAM167B	1.76114403	0.291017436	6.051678744	1.43344E-09	4.76991E-09	positive
PRJNA729271	stage_E18_vs_E10	ordinary_muscle_development	ENSGALG00010017462	MCM8	0.221162438	0.11765512	1.879751927	0.060141895	0.083222603	positive
PRJNA729271	stage_E18_vs_E10	ordinary_muscle_development	ENSGALG00010017463	PPP1R36	1.32324512	0.32642759	4.053717149	5.04102E-05	0.000108557	positive
PRJNA729271	stage_E18_vs_E10	ordinary_muscle_development	ENSGALG00010017464	SH3BP2	0.310947206	0.130628553	2.380392327	0.017294214	0.026370675	positive
PRJNA729271	stage_E18_vs_E10	ordinary_muscle_development	ENSGALG00010017465	TMEM234	0.36573035	0.231972947	1.576607769	0.114885804	0.150716782	positive
PRJNA729271	stage_E18_vs_E10	ordinary_muscle_development	ENSGALG00010017466	SPIA4	9.421297209	2.715619603	3.469299308			positive
PRJNA729271	stage_E18_vs_E10	ordinary_muscle_development	ENSGALG00010017467	SFRP1	-2.99162579	0.109246064	-27.38428901	4.2199E-165	1.799E-162	negative
PRJNA729271	stage_E18_vs_E10	ordinary_muscle_development	ENSGALG00010017468	AMDHD2	-0.035601212	0.128971662	-0.276039026	0.782518092	0.817779361	negative
PRJNA729271	stage_E18_vs_E10	ordinary_muscle_development	ENSGALG00010017470	HSPA2	2.346141599	0.169982568	13.80224824	2.47027E-43	5.40884E-42	positive
PRJNA729271	stage_E18_vs_E10	ordinary_muscle_development	ENSGALG00010017471	CRLS1	-0.002133949	0.091368563	-0.0233554	0.981366781	0.984763689	negative
PRJNA729271	stage_E18_vs_E10	ordinary_muscle_development	ENSGALG00010017472	LZTFL1	-0.730787501	0.169709693	-4.306103484	1.66155E-05	3.78029E-05	negative
PRJNA729271	stage_E18_vs_E10	ordinary_muscle_development	ENSGALG00010017473	EIF3I	0.385443153	0.1045451	3.68686004	0.000227038	0.000451925	positive
PRJNA729271	stage_E18_vs_E10	ordinary_muscle_development	ENSGALG00010017475	STUM	4.817082867	0.246087269	19.57469353	2.54161E-85	2.0467E-83	positive
PRJNA729271	stage_E18_vs_E10	ordinary_muscle_development	ENSGALG00010017476	ATP6V0C	0.181824097	0.113564762	1.601060866	0.109363439	0.144032623	positive
PRJNA729271	stage_E18_vs_E10	ordinary_muscle_development	ENSGALG00010017477	MCF2	2.641194614	0.181831946	14.52547074	8.35662E-48	2.22664E-46	positive
PRJNA729271	stage_E18_vs_E10	ordinary_muscle_development	ENSGALG00010017479	SSTR5	3.091346245	0.464954544	6.648706383	2.9568E-11	1.12049E-10	positive
PRJNA729271	stage_E18_vs_E10	ordinary_muscle_development	ENSGALG00010017480	DCDC2B	0.874330451	0.196960418	4.439117574	9.03285E-06	2.11213E-05	positive
PRJNA729271	stage_E18_vs_E10	ordinary_muscle_development	ENSGALG00010017481	SPIA1	8.91106261	2.479207251	3.594319356			positive

PRJNA729271	stage_E18_vs_E10	ordinary_muscle_development	ENSGALG00010017482	CORO2B	0.192451957	0.14158203	1.359296493	0.174052655	0.219439265	positive
PRJNA729271	stage_E18_vs_E10	ordinary_muscle_development	ENSGALG00010017484	IQCC	0.257808762	0.133679022	1.928565594	0.053784814	0.075070859	positive
PRJNA729271	stage_E18_vs_E10	ordinary_muscle_development	ENSGALG00010017485	LBH	-0.767776868	0.108185443	-7.096859274	1.27624E-12	5.42177E-12	negative
PRJNA729271	stage_E18_vs_E10	ordinary_muscle_development	ENSGALG00010017487	C1QTNF8	-1.168953465	0.51434403	-2.272707367	0.02304382	0.034369627	negative
PRJNA729271	stage_E18_vs_E10	ordinary_muscle_development	ENSGALG00010017489	TNFRSF1B	0.801702768	0.178821683	4.483252573	7.35138E-06	1.73435E-05	positive
PRJNA729271	stage_E18_vs_E10	ordinary_muscle_development	ENSGALG00010017491	CCDC28B	0.349189603	0.216818783	1.610513619	0.107285775	0.141489155	positive
PRJNA729271	stage_E18_vs_E10	ordinary_muscle_development	ENSGALG00010017493	YPEL5	-0.17218938	0.134912014	-1.276308724	0.201846407	0.25094465	negative
PRJNA729271	stage_E18_vs_E10	ordinary_muscle_development	ENSGALG00010017494	TBC1D24	-0.638846817	0.09389571	-6.803791319	1.01901E-11	4.00721E-11	negative
PRJNA729271	stage_E18_vs_E10	ordinary_muscle_development	ENSGALG00010017495	NT5M	-0.101461339	0.135521567	-0.748673008	0.454054316	0.513739546	negative
PRJNA729271	stage_E18_vs_E10	ordinary_muscle_development	ENSGALG00010017496	PARP1	-1.530096689	0.104001714	-14.71222572	5.38081E-49	1.50569E-47	negative
PRJNA729271	stage_E18_vs_E10	ordinary_muscle_development	ENSGALG00010017499	SNRPD1	-0.853627063	0.098481016	-8.667935206	4.40027E-18	2.76711E-17	negative
PRJNA729271	stage_E18_vs_E10	ordinary_muscle_development	ENSGALG00010017500	ACAP2	-0.102354027	0.101109147	-1.012312233	0.311388788	0.368937427	negative
PRJNA729271	stage_E18_vs_E10	ordinary_muscle_development	ENSGALG00010017502	NAT8B	0.915717167	0.117747865	7.77693221	7.43044E-15	3.74884E-14	positive
PRJNA729271	stage_E18_vs_E10	ordinary_muscle_development	ENSGALG00010017503	MED9	0.202294696	0.126282633	1.601920158	0.109173266	0.143821366	positive
PRJNA729271	stage_E18_vs_E10	ordinary_muscle_development	ENSGALG00010017505	TNFRSF8	-0.088894094	0.341020149	-0.260671091	0.794346161	0.828050889	negative
PRJNA729271	stage_E18_vs_E10	ordinary_muscle_development	ENSGALG00010017509	RASD1	1.660362694	0.294886302	5.63051821	1.79669E-08	5.42337E-08	positive
PRJNA729271	stage_E18_vs_E10	ordinary_muscle_development	ENSGALG00010017510	HDC	3.919383855	0.383544918	10.21883922	1.63284E-24	1.50369E-23	positive
PRJNA729271	stage_E18_vs_E10	ordinary_muscle_development	ENSGALG00010017513	TNIP2	1.086589494	0.142619984	7.618774465	2.56095E-14	1.2308E-13	positive
PRJNA729271	stage_E18_vs_E10	ordinary_muscle_development	ENSGALG00010017514	TXLNA	-0.475548118	0.097465698	-4.879133163	1.06553E-06	2.74771E-06	negative
PRJNA729271	stage_E18_vs_E10	ordinary_muscle_development	ENSGALG00010017516	AAK1	0.05708412	0.08033001	0.7106201	0.477319684	0.535801844	positive
PRJNA729271	stage_E18_vs_E10	ordinary_muscle_development	ENSGALG00010017517	LIN9	-0.465318659	0.102635205	-4.533713945	5.79555E-06	1.38191E-05	negative
PRJNA729271	stage_E18_vs_E10	ordinary_muscle_development	ENSGALG00010017518	PEMT	0.71365262	0.147708957	4.831478282	1.35523E-06	3.4542E-06	positive
PRJNA729271	stage_E18_vs_E10	ordinary_muscle_development	ENSGALG00010017519	NTN3	-0.507954303	0.191009758	-2.65931075	0.007830071	0.012575831	negative
PRJNA729271	stage_E18_vs_E10	ordinary_muscle_development	ENSGALG00010017520	ZBTB1	-0.459082494	0.081246876	-5.650463303	1.60016E-08	4.85948E-08	negative
PRJNA729271	stage_E18_vs_E10	ordinary_muscle_development	ENSGALG00010017521	MIIP	0.152694137	0.119933512	1.273156554	0.202962494	0.25220243	positive
PRJNA729271	stage_E18_vs_E10	ordinary_muscle_development	ENSGALG00010017522	XXYLT1	0.244615283	0.106608532	2.294518813	0.021760716	0.032594975	positive
PRJNA729271	stage_E18_vs_E10	ordinary_muscle_development	ENSGALG00010017524	ACBD3	-0.164258721	0.060105984	-2.732818093	0.0062795	0.010251307	negative
PRJNA729271	stage_E18_vs_E10	ordinary_muscle_development	ENSGALG00010017525	ENSGALG00010017525	-1.594924886	0.553969136	-2.879086183	0.003988293	0.006695658	negative
PRJNA729271	stage_E18_vs_E10	ordinary_muscle_development	ENSGALG00010017526	PLEKHA2	1.322933786	0.172313261	7.677492591	1.62233E-14	7.95253E-14	positive
PRJNA729271	stage_E18_vs_E10	ordinary_muscle_development	ENSGALG00010017528	RAI1	0.807270016	0.176452474	4.574999712	4.76221E-06	1.14456E-05	positive
PRJNA729271	stage_E18_vs_E10	ordinary_muscle_development	ENSGALG00010017529	MFN2	-0.242607081	0.119224794	-2.034871051	0.04186385	0.059509316	negative
PRJNA729271	stage_E18_vs_E10	ordinary_muscle_development	ENSGALG00010017530	TLR1B	0.533622811	0.214248661	2.49067046	0.012750232	0.01986613	positive
PRJNA729271	stage_E18_vs_E10	ordinary_muscle_development	ENSGALG00010017531	SACM1L	-0.767416425	0.113298315	-6.773414275	1.25778E-11	4.90755E-11	negative
PRJNA729271	stage_E18_vs_E10	ordinary_muscle_development	ENSGALG00010017532	MIXL1	1.144227344	0.311920093	3.668334834	0.000244135	0.000483964	positive
PRJNA729271	stage_E18_vs_E10	ordinary_muscle_development	ENSGALG00010017533	FAM193A	0.063948875	0.078481369	0.814828736	0.415170349	0.475572484	positive
PRJNA729271	stage_E18_vs_E10	ordinary_muscle_development	ENSGALG00010017534	ATP11C	-0.842467005	0.084647235	-9.952681902	2.45477E-23	2.09182E-22	negative
PRJNA729271	stage_E18_vs_E10	ordinary_muscle_development	ENSGALG00010017535	LIMD1	-0.01517069	0.090220775	-0.168150736	0.866464696	0.889980568	negative
PRJNA729271	stage_E18_vs_E10	ordinary_muscle_development	ENSGALG00010017536	GSC	-4.834559674	0.544132106	-8.884900597	6.39779E-19	4.24227E-18	negative
PRJNA729271	stage_E18_vs_E10	ordinary_muscle_development	ENSGALG00010017537	MANBA	1.440175015	0.187049027	7.699452072	1.36651E-14	6.74644E-14	positive
PRJNA729271	stage_E18_vs_E10	ordinary_muscle_development	ENSGALG00010017538	H3-3A	-0.151034385	0.076023517	-1.986679792	0.046957893	0.066217984	negative
PRJNA729271	stage_E18_vs_E10	ordinary_muscle_development	ENSGALG00010017539	KPNA6	0.22762734	0.139231531	1.634883557	0.10207346	0.135082151	positive
PRJNA729271	stage_E18_vs_E10	ordinary_muscle_development	ENSGALG00010017540	EIF4G1	-0.128935189	0.165079673	-0.781048247	0.434774119	0.494550016	negative
PRJNA729271	stage_E18_vs_E10	ordinary_muscle_development	ENSGALG00010017541	SREBF1	0.671347497	0.229748854	2.922092911	0.003476878	0.005881357	positive
PRJNA729271	stage_E18_vs_E10	ordinary_muscle_development	ENSGALG00010017542	PLOD1	0.671001233	0.125127321	5.362547727	8.20563E-08	2.34181E-07	positive
PRJNA729271	stage_E18_vs_E10	ordinary_muscle_development	ENSGALG00010017543	ZBTB25	0.262040396	0.177012432	1.480350239	0.138779802	0.179064735	positive
PRJNA729271	stage_E18_vs_E10	ordinary_muscle_development	ENSGALG00010017544	SDE2	0.114731185	0.06913844	1.659441342	0.097026891	0.129016126	positive
PRJNA729271	stage_E18_vs_E10	ordinary_muscle_development	ENSGALG00010017545	DICER1	-0.557167029	0.121599768	-4.581974435	4.60606E-06	1.10795E-05	negative
PRJNA729271	stage_E18_vs_E10	ordinary_muscle_development	ENSGALG00010017546	NFU1	1.148638181	0.108187702	10.61708643	2.48187E-26	2.537E-25	positive
PRJNA729271	stage_E18_vs_E10	ordinary_muscle_development	ENSGALG00010017547	RNF4	-0.494014475	0.067054103	-7.367401152	1.73986E-13	7.85649E-13	negative
PRJNA729271	stage_E18_vs_E10	ordinary_muscle_development	ENSGALG00010017550	TM2D2	0.529524938	0.076756738	6.898742041	5.2465E-12	2.1201E-11	positive
PRJNA729271	stage_E18_vs_E10	ordinary_muscle_development	ENSGALG00010017551	NALF2	0.300736753	0.276406254	1.088024417	0.276584329	0.332098231	positive
PRJNA729271	stage_E18_vs_E10	ordinary_muscle_development	ENSGALG00010017552	LARS2	-0.200114646	0.103862633	-1.926724176	0.054014013	0.075368995	negative
PRJNA729271	stage_E18_vs_E10	ordinary_muscle_development	ENSGALG00010017553	EDA	0.733360674	0.10900533	6.727750598	1.72306E-11	6.64248E-11	positive
PRJNA729271	stage_E18_vs_E10	ordinary_muscle_development	ENSGALG00010017554	FAM131A	-2.34647245	0.345834894	-6.784949967	1.16127E-11	4.54075E-11	negative
PRJNA729271	stage_E18_vs_E10	ordinary_muscle_development	ENSGALG00010017555	CELA2A	-0.520289752	1.304595997	-0.39881293	0.690031049	0.735603446	negative
PRJNA729271	stage_E18_vs_E10	ordinary_muscle_development	ENSGALG00010017556	TOM1L2	0.718182269	0.114430511	6.276143189	3.47075E-10	1.21694E-09	positive
PRJNA729271	stage_E18_vs_E10	ordinary_muscle_development	ENSGALG00010017558	ENSGALG00010017558	-0.548236529	0.074330338	-7.375676485	1.63513E-13	7.40199E-13	negative
PRJNA729271	stage_E18_vs_E10	ordinary_muscle_development	ENSGALG00010017559	DUSP10	1.181522396	0.150395993	7.856076323	3.96354E-15	2.0409E-14	positive
PRJNA729271	stage_E18_vs_E10	ordinary_muscle_development	ENSGALG00010017561	CFAP99	-2.428508479	0.825949018	-2.940264383	0.003279323	0.005572969	negative
PRJNA729271	stage_E18_vs_E10	ordinary_muscle_development	ENSGALG00010017562	SLC30A10	-1.540518479	0.575370431	-2.677437692	0.007418765	0.011957634	negative
PRJNA729271	stage_E18_vs_E10	ordinary_muscle_development	ENSGALG00010017563	ALK	1.891499154	0.152821236	12.37720101	3.47243E-35	5.38319E-34	positive
PRJNA729271	stage_E18_vs_E10	ordinary_muscle_development	ENSGALG00010017565	ENSGALG00010017565	0.669834598	0.185306604	3.614736793	0.000300653	0.000588596	positive
PRJNA729271	stage_E18_vs_E10	ordinary_muscle_development	ENSGALG00010017568	CDCP1	-2.375129604	0.249856936	-9.50595824	1.98215E-21	1.52988E-20	negative
PRJNA729271	stage_E18_vs_E10	ordinary_muscle_development	ENSGALG00010017569	CLMN	1.035419948	0.243910868	4.245075083	2.1852E-05	4.89787E-05	positive
PRJNA729271	stage_E18_vs_E10	ordinary_muscle_development	ENSGALG00010017570	DRC3	-0.102545529	0.362352758	-0.282999169	0.777177474	0.81315076	negative
PRJNA729271	stage_E18_vs_E10	ordinary_muscle_development	ENSGALG00010017571	ADAM9	0.564414332	0.150557104	3.748838937	0.000177655	0.000358651	positive
PRJNA729271	stage_E18_vs_E10	ordinary_muscle_development	ENSGALG00010017573	ENSGALG00010017573	0.591043834	0.226557208	2.608806132	0.00908587	0.014459781	positive
PRJNA729271	stage_E18_vs_E10	ordinary_muscle_development	ENSGALG00010017574	DYNC1LI1	-0.437514733	0.056956844	-7.681512911	1.57221E-14	7.72251E-14	negative
PRJNA729271	stage_E18_vs_E10	ordinary_muscle_development	ENSGALG00010017575	CLCN2	0.476099152	0.890294171	0.534766111	0.5928116	0.644669828	positive
PRJNA729271	stage_E18_vs_E10	ordinary_muscle_development	ENSGALG00010017576	ENSGALG00010017576	-1.86915998	0.288580036	-6.477093868	9.3506E-11	3.41747E-10	negative
PRJNA729271	stage_E18_vs_E10	ordinary_muscle_development	ENSGALG00010017577	CLPX	0.676769088	0.137869086	4.908780548	9.16445E-07	2.37977E-06	positive
PRJNA729271	stage_E18_vs_E10	ordinary_muscle_development	ENSGALG00010017578	BSDC1	0.288114027	0.10063311	2.863014252	0.004196317	0.007026177	positive
PRJNA729271	stage_E18_vs_E10	ordinary_muscle_development	ENSGALG00010017579	P3H2	1.515115982	0.140743431	10.76509201	5.03113E-27	5.33867E-26	positive
PRJNA729271	stage_E18_vs_E10	ordinary_muscle_development	ENSGALG00010017580	LYPLAL1	0.314715987	0.154111665	2.042129565	0.041138681	0.058604932	positive
PRJNA729271	stage_E18_vs_E10	ordinary_muscle_development	ENSGALG00010017581	EFCAB11	-2.104999897	0.450654064	-4.670988384	2.99754E-06	7.34562E-06	negative
PRJNA729271	stage_E18_vs_E10	ordinary_muscle_development	ENSGALG00010017582	CLCN6	-0.20017135	0.104130177	-1.922318345	0.054565713	0.076087543	negative
PRJNA729271	stage_E18_vs_E10	ordinary_muscle_development	ENSGALG00010017583	MTHFD1	-1.040251236	0.106544718	-9.763517627	1.61456E-22	1.3222E-21	negative
PRJNA729271	stage_E18_vs_E10	ordinary_muscle_development	ENSGALG00010017584	WNT11B	-2.905670309	0.922126166	-3.151055047	0.001626818	0.002891567	negative
PRJNA729271	stage_E18_vs_E10	ordinary_muscle_development	ENSGALG00010017585	ENSGALG00010017585	0.017710737	0.117672362	0.15050889	0.880363138	0.900945239	positive
PRJNA729271	stage_E18_vs_E10	ordinary_muscle_development	ENSGALG00010017586	ZFYVE28	0.502880318	0.161459806	3.114585166	0.001842038	0.003254948	positive
PRJNA729271	stage_E18_vs_E10	ordinary_muscle_development	ENSGALG00010017587	MB21D2	-0.740644334	0.148936208	-4.972896404	6.59599E-07	1.73486E-06	negative
PRJNA729271	stage_E18_vs_E10	ordinary_muscle_development	ENSGALG00010017588	NOX5	-1.507061248	0.523473294	-2.878964913	0.003989827	0.006697457	negative
PRJNA729271	stage_E18_vs_E10	ordinary_muscle_development	ENSGALG00010017589	ATPAF2	-0.182770545	0.149880759	-1.219439679	0.222677357	0.273673757	negative
PRJNA729271	stage_E18_vs_E10	ordinary_muscle_development	ENSGALG00010017590	ENSGALG00010017590	0.142184823	0.190141043	0.747786069	0.454589206	0.514264461	positive
PRJNA729271	stage_E18_vs_E10	ordinary_muscle_development	ENSGALG00010017591	ESR2	-3.268546583	0.321923822	-10.15316781	3.20779E-24	2.8952E-23	negative
PRJNA729271	stage_E18_vs_E10	ordinary_muscle_development	ENSGALG00010017592	GID4	0.187954835	0.11867395	1.583791847	0.113241095	0.148734113	positive
PRJNA729271	stage_E18_vs_E10	ordinary_muscle_development	ENSGALG00010017593	MTHFR	-0.742361945	0.137669061	-5.392365854	6.9536E-08	1.99471E-07	negative
PRJNA729271	stage_E18_vs_E10	ordinary_muscle_development	ENSGALG00010017594	CEP68	1.010483067	0.099930032	10.11190579	4.89232E-24	4.35591E-23	positive
PRJNA729271	stage_E18_vs_E10	ordinary_muscle_development	ENSGALG00010017595	CCNF	-2.034133252	0.127857881	-15.90933023	5.45944E-57	1.96852E-55	negative
PRJNA729271	stage_E18_vs_E10	ordinary_muscle_development	ENSGALG00010017596	TDP1	-1.217143915	0.096657068	-12.59239433	2.32512E-36	3.84294E-35	negative
PRJNA729271	stage_E18_vs_E10	ordinary_muscle_development	ENSGALG00010017597	CNOT10	-0.341339342	0.137279018	-2.48646404	0.012901961	0.020091741	negative
PRJNA729271	stage_E18_vs_E10	ordinary_muscle_development	ENSGALG00010017598	ENSGALG00010017598	-0.061136463	0.11967928	-0.510835823	0.609466023	0.660254858	negative
PRJNA729271	stage_E18_vs_E10	ordinary_muscle_development	ENSGALG00010017599	KCNK13	-0.363931766	0.238649974	-1.524960426	0.127268969	0.165627914	negative
PRJNA729271	stage_E18_vs_E10	ordinary_muscle_development	ENSGALG00010017600	MXD4	1.097223968	0.103041159	10.64840477	1.77379E-26	1.83913E-25	positive
PRJNA729271	stage_E18_vs_E10	ordinary_muscle_development	ENSGALG00010017601	ENSGALG00010017601	1.45807586	0.114435575	12.74145611	3.47841E-37	5.9178E-36	positive
PRJNA729271	stage_E18_vs_E10	ordinary_muscle_development	ENSGALG00010017603	CYP3A5	1.540130375	1.356162366	1.13565338	0.256101659	0.309995286	positive
PRJNA729271	stage_E18_vs_E10	ordinary_muscle_development	ENSGALG00010017604	GATM	5.444986665	0.241138004	22.5803754	6.7572E-113	1.113E-110	positive
PRJNA729271	stage_E18_vs_E10	ordinary_muscle_development	ENSGALG00010017605	GFPT1	-0.939568613	0.071680848	-13.10766605	2.97617E-39	5.53782E-38	negative
PRJNA729271	stage_E18_vs_E10	ordinary_muscle_development	ENSGALG00010017606	ENSGALG00010017606	0.705569977	0.25686776	2.746821856	0.006017581	0.009848124	positive
PRJNA729271	stage_E18_vs_E10	ordinary_muscle_development	ENSGALG00010017608	RBBP4	-1.046143346	0.08114886	-12.8916579	5.01539E-38	8.88729E-37	negative
PRJNA729271	stage_E18_vs_E10	ordinary_muscle_development	ENSGALG00010017609	TRIM71	2.047926743	0.192439011	10.64195212	1.90104E-26	1.96545E-25	positive
PRJNA729271	stage_E18_vs_E10	ordinary_muscle_development	ENSGALG00010017610	PSMC1	-0.591625604	0.119939207	-4.932712324	8.10955E-07	2.11798E-06	negative
PRJNA729271	stage_E18_vs_E10	ordinary_muscle_development	ENSGALG00010017612	CLDN11	0.756417497	0.401828206	1.88244002	0.059776297	0.082777028	positive
PRJNA729271	stage_E18_vs_E10	ordinary_muscle_development	ENSGALG00010017613	HAUS3	-1.298256546	0.121044335	-10.72546305	7.72989E-27	8.14871E-26	negative
PRJNA729271	stage_E18_vs_E10	ordinary_muscle_development	ENSGALG00010017615	AGTRAP	0.706953962	0.134791493	5.244796583	1.56455E-07	4.34447E-07	positive
PRJNA729271	stage_E18_vs_E10	ordinary_muscle_development	ENSGALG00010017616	PDCD7	0.629809906	0.156690214	4.019459905	5.83319E-05	0.000124892	positive
PRJNA729271	stage_E18_vs_E10	ordinary_muscle_development	ENSGALG00010017619	POLN	0.619972106	0.328239232	1.888781249	0.058921146	0.081681524	positive
PRJNA729271	stage_E18_vs_E10	ordinary_muscle_development	ENSGALG00010017620	UBAP1L	0.354420154	0.335018974	1.057910692	0.29009616	0.346399526	positive
PRJNA729271	stage_E18_vs_E10	ordinary_muscle_development	ENSGALG00010017621	SYNE2	0.874699567	0.155971395	5.608076819	2.04587E-08	6.15503E-08	positive
PRJNA729271	stage_E18_vs_E10	ordinary_muscle_development	ENSGALG00010017622	RAB1B	-0.428991337	0.112703076	-3.806385348	0.000141013	0.000288901	negative
PRJNA729271	stage_E18_vs_E10	ordinary_muscle_development	ENSGALG00010017625	YBX1	0.084520739	0.091262781	0.926124955	0.354381047	0.413720463	positive
PRJNA729271	stage_E18_vs_E10	ordinary_muscle_development	ENSGALG00010017626	COPS7B	-1.04562699	0.141813685	-7.373244597	1.66525E-13	7.53598E-13	negative
PRJNA729271	stage_E18_vs_E10	ordinary_muscle_development	ENSGALG00010017628	NRDE2	-0.338843835	0.105984126	-3.19711875	0.001388078	0.002486737	negative
PRJNA729271	stage_E18_vs_E10	ordinary_muscle_development	ENSGALG00010017629	ENSGALG00010017629	-0.223777543	0.14345416	-1.559923699	0.118777913	0.155414863	negative
PRJNA729271	stage_E18_vs_E10	ordinary_muscle_development	ENSGALG00010017631	NAT8L	-1.445273458	0.253941082	-5.691373164	1.26022E-08	3.85784E-08	negative
PRJNA729271	stage_E18_vs_E10	ordinary_muscle_development	ENSGALG00010017632	AWAT1	1.793914206	0.583844722	3.072587861	0.002122114	0.00371229	positive
PRJNA729271	stage_E18_vs_E10	ordinary_muscle_development	ENSGALG00010017633	HLX	0.562993607	0.153361386	3.671025806	0.000241579	0.000479486	positive
PRJNA729271	stage_E18_vs_E10	ordinary_muscle_development	ENSGALG00010017634	PPIH	-0.466225651	0.09808091	-4.753480061	1.99945E-06	4.99431E-06	negative
PRJNA729271	stage_E18_vs_E10	ordinary_muscle_development	ENSGALG00010017636	ZBTB8OS	-0.394803236	0.159043514	-2.48235987	0.013051539	0.02030504	negative
PRJNA729271	stage_E18_vs_E10	ordinary_muscle_development	ENSGALG00010017638	GFRA2	0.160316374	0.258893675	0.61923635	0.535760668	0.591323451	positive
PRJNA729271	stage_E18_vs_E10	ordinary_muscle_development	ENSGALG00010017639	ENSGALG00010017639	-0.671083631	0.134313193	-4.996408902	5.84077E-07	1.54352E-06	negative
PRJNA729271	stage_E18_vs_E10	ordinary_muscle_development	ENSGALG00010017641	GPRC5B	1.620745232	0.159178295	10.18194867	2.38732E-24	2.1709E-23	positive
PRJNA729271	stage_E18_vs_E10	ordinary_muscle_development	ENSGALG00010017642	MTARC1	1.272615716	0.122298029	10.40585626	2.3315E-25	2.25602E-24	positive
PRJNA729271	stage_E18_vs_E10	ordinary_muscle_development	ENSGALG00010017643	CALM1	-0.701137024	0.061235811	-11.4497875	2.35723E-30	2.92785E-29	negative
PRJNA729271	stage_E18_vs_E10	ordinary_muscle_development	ENSGALG00010017644	RBBP6	0.316438664	0.097576691	3.242973932	0.00118289	0.002134711	positive
PRJNA729271	stage_E18_vs_E10	ordinary_muscle_development	ENSGALG00010017645	SYNC	2.517787322	0.268566546	9.37491047	6.92345E-21	5.17029E-20	positive
PRJNA729271	stage_E18_vs_E10	ordinary_muscle_development	ENSGALG00010017646	MAEA	-0.565236203	0.087310314	-6.473876623	9.55199E-11	3.48844E-10	negative
PRJNA729271	stage_E18_vs_E10	ordinary_muscle_development	ENSGALG00010017647	ITGA9	0.317006749	0.166923814	1.899110385	0.057549964	0.079925911	positive
PRJNA729271	stage_E18_vs_E10	ordinary_muscle_development	ENSGALG00010017648	ENSGALG00010017648	-0.507227308	0.152641119	-3.323005696	0.000890531	0.001634162	negative
PRJNA729271	stage_E18_vs_E10	ordinary_muscle_development	ENSGALG00010017649	ENSGALG00010017649	0.379069029	0.107373905	3.530364571	0.000414987	0.000797672	positive
PRJNA729271	stage_E18_vs_E10	ordinary_muscle_development	ENSGALG00010017651	PPCS	-0.7308257	0.126614196	-5.772067604	7.83047E-09	2.44249E-08	negative

PRJNA729271	stage_E18_vs_E10	ordinary_muscle_development	ENSGALG00010017652	C1orf115	1.707698669	0.172022005	9.927210582	3.16999E-23	2.69023E-22	positive
PRJNA729271	stage_E18_vs_E10	ordinary_muscle_development	ENSGALG00010017653	ENSGALG00010017653	1.521000791	0.17259821	8.812378697	1.22518E-18	7.97798E-18	positive
PRJNA729271	stage_E18_vs_E10	ordinary_muscle_development	ENSGALG00010017654	KBTBD13	5.953143574	0.313563919	18.98542277	2.25117E-80	1.59174E-78	positive
PRJNA729271	stage_E18_vs_E10	ordinary_muscle_development	ENSGALG00010017655	ENSGALG00010017655	-0.813608916	0.110261732	-7.378887511	1.59618E-13	7.23244E-13	negative
PRJNA729271	stage_E18_vs_E10	ordinary_muscle_development	ENSGALG00010017656	FSD2	6.642412159	0.208401174	31.87319934	6.2809E-223	5.3554E-220	positive
PRJNA729271	stage_E18_vs_E10	ordinary_muscle_development	ENSGALG00010017657	DIS3L2	0.291595898	0.094680689	3.079782186	0.00207152	0.003630721	positive
PRJNA729271	stage_E18_vs_E10	ordinary_muscle_development	ENSGALG00010017658	WHAMM	1.131407296	0.16318307	6.933361992	4.10955E-12	1.67939E-11	positive
PRJNA729271	stage_E18_vs_E10	ordinary_muscle_development	ENSGALG00010017659	TNRC6A	0.230638096	0.078526169	2.937085793	0.003313124	0.005628002	positive
PRJNA729271	stage_E18_vs_E10	ordinary_muscle_development	ENSGALG00010017660	CLIP4	2.258272414	0.247683818	9.117561391	7.68337E-20	5.3595E-19	positive
PRJNA729271	stage_E18_vs_E10	ordinary_muscle_development	ENSGALG00010017661	NHSL3	0.731081024	0.168950861	4.327181416	1.5103E-05	3.45132E-05	positive
PRJNA729271	stage_E18_vs_E10	ordinary_muscle_development	ENSGALG00010017662	GOLGA4	0.528215596	0.165659571	3.188560692	0.00142983	0.002557427	positive
PRJNA729271	stage_E18_vs_E10	ordinary_muscle_development	ENSGALG00010017663	ZMYND12	0.214331768	0.214498617	0.999222145	0.31768709	0.375264801	positive
PRJNA729271	stage_E18_vs_E10	ordinary_muscle_development	ENSGALG00010017664	ENSGALG00010017664	0.257817752	0.074864174	3.443806793	0.000573585	0.001081441	positive
PRJNA729271	stage_E18_vs_E10	ordinary_muscle_development	ENSGALG00010017665	CTBP1	-0.264279174	0.063266179	-4.177258344	2.95044E-05	6.51235E-05	negative
PRJNA729271	stage_E18_vs_E10	ordinary_muscle_development	ENSGALG00010017667	SLC2A1	-0.765052032	0.112825395	-6.780849569	1.19471E-11	4.66775E-11	negative
PRJNA729271	stage_E18_vs_E10	ordinary_muscle_development	ENSGALG00010017668	ENSGALG00010017668	-1.487096848	1.014526674	-1.465803597	0.142701801	0.183553341	negative
PRJNA729271	stage_E18_vs_E10	ordinary_muscle_development	ENSGALG00010017669	ENSGALG00010017669	1.512414907	0.172641082	8.760457742	1.94459E-18	1.25219E-17	positive
PRJNA729271	stage_E18_vs_E10	ordinary_muscle_development	ENSGALG00010017670	FAM179A	1.90269443	0.337974582	5.629696812	1.80527E-08	5.44812E-08	positive
PRJNA729271	stage_E18_vs_E10	ordinary_muscle_development	ENSGALG00010017671	ANTXR1	0.348941979	0.184383937	1.892474931	0.058427731	0.081051771	positive
PRJNA729271	stage_E18_vs_E10	ordinary_muscle_development	ENSGALG00010017672	SPRED2	-0.245984864	0.077961033	-3.155228379	0.001603725	0.002852269	negative
PRJNA729271	stage_E18_vs_E10	ordinary_muscle_development	ENSGALG00010017673	WDR89	-0.164104264	0.091141812	-1.800537653	0.071775784	0.097955935	negative
PRJNA729271	stage_E18_vs_E10	ordinary_muscle_development	ENSGALG00010017675	ENSGALG00010017675	0.515104288	0.50314379	1.023771531	0.3059432	0.363257429	positive
PRJNA729271	stage_E18_vs_E10	ordinary_muscle_development	ENSGALG00010017676	SPON2	3.670106409	0.26259079	13.97652374	2.16823E-44	4.99658E-43	positive
PRJNA729271	stage_E18_vs_E10	ordinary_muscle_development	ENSGALG00010017677	WDR43	-0.673012024	0.110518451	-6.08958973	1.132E-09	3.80088E-09	negative
PRJNA729271	stage_E18_vs_E10	ordinary_muscle_development	ENSGALG00010017678	YARS	-0.734144809	0.138837296	-5.287806862	1.23792E-07	3.47879E-07	negative
PRJNA729271	stage_E18_vs_E10	ordinary_muscle_development	ENSGALG00010017679	RASL12	4.363166829	0.17540337	24.87504559	1.3859E-136	3.4049E-134	positive
PRJNA729271	stage_E18_vs_E10	ordinary_muscle_development	ENSGALG00010017681	ENSGALG00010017681	1.132277134	1.409163222	0.80351028	0.421679858	0.482076626	positive
PRJNA729271	stage_E18_vs_E10	ordinary_muscle_development	ENSGALG00010017684	TRMT61B	0.338022587	0.097149767	3.479396787	0.000502544	0.000954577	positive
PRJNA729271	stage_E18_vs_E10	ordinary_muscle_development	ENSGALG00010017685	PHC2	-0.184076209	0.18512502	-0.99433458	0.320060006	0.37772918	negative
PRJNA729271	stage_E18_vs_E10	ordinary_muscle_development	ENSGALG00010017687	TTC7B	0.493439813	0.098625967	5.00314295	5.64031E-07	1.49354E-06	positive
PRJNA729271	stage_E18_vs_E10	ordinary_muscle_development	ENSGALG00010017688	SPDYA	-0.455257057	0.547616604	-0.831342684	0.405780068	0.465773051	negative
PRJNA729271	stage_E18_vs_E10	ordinary_muscle_development	ENSGALG00010017691	LZTS1	0.635794881	0.225980662	2.813492428	0.004900654	0.008135018	positive
PRJNA729271	stage_E18_vs_E10	ordinary_muscle_development	ENSGALG00010017692	EPCAM	-5.390407308	1.005091365	-5.363101798	8.18049E-08	2.33556E-07	negative
PRJNA729271	stage_E18_vs_E10	ordinary_muscle_development	ENSGALG00010017693	MTFMT	0.370631814	0.117001011	3.167765921	0.001536152	0.002737462	positive
PRJNA729271	stage_E18_vs_E10	ordinary_muscle_development	ENSGALG00010017694	ATP6V1B2	-0.447729809	0.071861726	-6.230434958	4.65142E-10	1.62034E-09	negative
PRJNA729271	stage_E18_vs_E10	ordinary_muscle_development	ENSGALG00010017695	FNDC5	1.001050733	0.204628223	4.89204627	9.9793E-07	2.57889E-06	positive
PRJNA729271	stage_E18_vs_E10	ordinary_muscle_development	ENSGALG00010017698	ENSGALG00010017698	1.329604248	0.76067049	1.747937203	0.080474902	0.108793481	positive
PRJNA729271	stage_E18_vs_E10	ordinary_muscle_development	ENSGALG00010017699	RNF19B	0.348365861	0.246003363	1.41610203	0.156745634	0.199738722	positive
PRJNA729271	stage_E18_vs_E10	ordinary_muscle_development	ENSGALG00010017700	VPS35L	-0.087673897	0.07907079	-1.10880259	0.267515353	0.322571753	negative
PRJNA729271	stage_E18_vs_E10	ordinary_muscle_development	ENSGALG00010017701	PPP2R5E	0.00427794	0.100013206	0.042773751	0.965881888	0.97198403	positive
PRJNA729271	stage_E18_vs_E10	ordinary_muscle_development	ENSGALG00010017703	RPS6KA5	0.850507848	0.140124873	6.069642244	1.28196E-09	4.27958E-09	positive
PRJNA729271	stage_E18_vs_E10	ordinary_muscle_development	ENSGALG00010017704	ZNF362	-0.306640804	0.201438307	-1.52225666	0.127944783	0.166358058	negative
PRJNA729271	stage_E18_vs_E10	ordinary_muscle_development	ENSGALG00010017705	MAK16	-0.131155806	0.104753337	-1.252044174	0.210553768	0.260502553	negative
PRJNA729271	stage_E18_vs_E10	ordinary_muscle_development	ENSGALG00010017706	FGFRL1	-0.167255074	0.143301164	-1.167157819	0.243146642	0.296243639	negative
PRJNA729271	stage_E18_vs_E10	ordinary_muscle_development	ENSGALG00010017707	AK2	-0.254029725	0.095716244	-2.65398761	0.007954675	0.012761816	negative
PRJNA729271	stage_E18_vs_E10	ordinary_muscle_development	ENSGALG00010017708	MEIS1	-1.368832485	0.104431922	-13.10741445	2.98606E-39	5.54909E-38	negative
PRJNA729271	stage_E18_vs_E10	ordinary_muscle_development	ENSGALG00010017710	RAB41	-0.26421275	0.11767641	-2.245248212	0.024752203	0.036745512	negative
PRJNA729271	stage_E18_vs_E10	ordinary_muscle_development	ENSGALG00010017711	LRRFIP2	0.241693114	0.134991357	1.790433999	0.073384169	0.099934566	positive
PRJNA729271	stage_E18_vs_E10	ordinary_muscle_development	ENSGALG00010017712	MSH2	-1.360632625	0.073667397	-18.46994301	3.60465E-76	2.24246E-74	negative
PRJNA729271	stage_E18_vs_E10	ordinary_muscle_development	ENSGALG00010017713	ALKBH5	0.460127272	0.150048654	3.066520479	0.00216566	0.003782988	positive
PRJNA729271	stage_E18_vs_E10	ordinary_muscle_development	ENSGALG00010017714	ATAD3A	-1.137828543	0.156346461	-7.277609831	3.39786E-13	1.50756E-12	negative
PRJNA729271	stage_E18_vs_E10	ordinary_muscle_development	ENSGALG00010017715	CILP	1.981874857	0.168800947	11.7408989	7.86461E-32	1.04777E-30	positive
PRJNA729271	stage_E18_vs_E10	ordinary_muscle_development	ENSGALG00010017717	LLGL1	-0.328236919	0.150250038	-2.184604565	0.028917853	0.042369785	negative
PRJNA729271	stage_E18_vs_E10	ordinary_muscle_development	ENSGALG00010017718	ATOH8	-0.100587117	0.299980531	-0.335312152	0.737389628	0.77858848	negative
PRJNA729271	stage_E18_vs_E10	ordinary_muscle_development	ENSGALG00010017719	ENSGALG00010017719	-0.44503736	1.940268166	-0.229368995	0.818582132	0.849148255	negative
PRJNA729271	stage_E18_vs_E10	ordinary_muscle_development	ENSGALG00010017720	ENSGALG00010017720	-1.267501278	0.342013401	-3.705998873	0.000210559	0.000420915	negative
PRJNA729271	stage_E18_vs_E10	ordinary_muscle_development	ENSGALG00010017721	VWA1	1.994268894	0.119852739	16.63932682	3.61661E-62	1.5602E-60	positive
PRJNA729271	stage_E18_vs_E10	ordinary_muscle_development	ENSGALG00010017723	KCNK12	-2.184839674	0.397744289	-5.493076169	3.94992E-08	1.16205E-07	negative
PRJNA729271	stage_E18_vs_E10	ordinary_muscle_development	ENSGALG00010017724	FLII	0.221829264	0.128782377	1.722512578	0.084976706	0.114195325	positive
PRJNA729271	stage_E18_vs_E10	ordinary_muscle_development	ENSGALG00010017725	ENSGALG00010017725	0.372818761	0.430764109	0.865482415	0.386774064	0.44693001	positive
PRJNA729271	stage_E18_vs_E10	ordinary_muscle_development	ENSGALG00010017726	TTI2	0.578516731	0.121229634	4.772073577	1.82339E-06	4.57901E-06	positive
PRJNA729271	stage_E18_vs_E10	ordinary_muscle_development	ENSGALG00010017727	AZIN2	1.027124916	0.149906384	6.851775685	7.29389E-12	2.90055E-11	positive
PRJNA729271	stage_E18_vs_E10	ordinary_muscle_development	ENSGALG00010017729	MIEF2	1.211177693	0.141894413	8.535767297	1.39228E-17	8.4617E-17	positive
PRJNA729271	stage_E18_vs_E10	ordinary_muscle_development	ENSGALG00010017730	TARP	2.200549407	0.583508172	3.771240081	0.000162438	0.000329491	positive
PRJNA729271	stage_E18_vs_E10	ordinary_muscle_development	ENSGALG00010017732	RHOJ	0.376900475	0.077193756	4.882525435	1.04736E-06	2.70325E-06	positive
PRJNA729271	stage_E18_vs_E10	ordinary_muscle_development	ENSGALG00010017733	TRIM62	0.407070503	0.129989577	3.131562638	0.001738787	0.003081516	positive
PRJNA729271	stage_E18_vs_E10	ordinary_muscle_development	ENSGALG00010017734	TOP3A	-0.350310194	0.118294763	-2.961333063	0.003063105	0.005239522	negative
PRJNA729271	stage_E18_vs_E10	ordinary_muscle_development	ENSGALG00010017736	ENSGALG00010017736	1.243359001	4.395975581	0.282840288	0.777299269	0.813204829	positive
PRJNA729271	stage_E18_vs_E10	ordinary_muscle_development	ENSGALG00010017737	ENSGALG00010017737	-0.559996838	0.208276904	-2.688713083	0.007172804	0.011583088	negative
PRJNA729271	stage_E18_vs_E10	ordinary_muscle_development	ENSGALG00010017738	NDUFS5	0.627669249	0.112020857	5.603146266	2.10495E-08	6.32752E-08	positive
PRJNA729271	stage_E18_vs_E10	ordinary_muscle_development	ENSGALG00010017739	MRPL20	-0.473765907	0.10034684	-4.721283775	2.34361E-06	5.81788E-06	negative
PRJNA729271	stage_E18_vs_E10	ordinary_muscle_development	ENSGALG00010017741	SMCR8	0.238510723	0.140137364	1.701978095	0.088759475	0.118840623	positive
PRJNA729271	stage_E18_vs_E10	ordinary_muscle_development	ENSGALG00010017742	DGLUCY	1.559060507	0.237955905	6.55188828	5.6814E-11	2.11267E-10	positive
PRJNA729271	stage_E18_vs_E10	ordinary_muscle_development	ENSGALG00010017743	KCNH5	-1.368517709	0.317306059	-4.312926499	1.61108E-05	3.67121E-05	negative
PRJNA729271	stage_E18_vs_E10	ordinary_muscle_development	ENSGALG00010017744	ETAA1	-0.690177393	0.099510364	-6.935733801	4.04118E-12	1.65191E-11	negative
PRJNA729271	stage_E18_vs_E10	ordinary_muscle_development	ENSGALG00010017745	SHMT1	-0.459934241	0.187669177	-2.450771345	0.014255047	0.022077884	negative
PRJNA729271	stage_E18_vs_E10	ordinary_muscle_development	ENSGALG00010017746	MSH6	-0.997126966	0.089875361	-11.09455317	1.33325E-28	1.52049E-27	negative
PRJNA729271	stage_E18_vs_E10	ordinary_muscle_development	ENSGALG00010017747	DUSP26	3.640749596	0.117286472	31.04151333	1.4852E-211	1.0251E-208	positive
PRJNA729271	stage_E18_vs_E10	ordinary_muscle_development	ENSGALG00010017748	ENSGALG00010017748	0.991711084	0.423395154	2.342282558	0.019166199	0.028987275	positive
PRJNA729271	stage_E18_vs_E10	ordinary_muscle_development	ENSGALG00010017751	ST3GAL5	-0.570751412	0.129227513	-4.416640067	1.00247E-05	2.33389E-05	negative
PRJNA729271	stage_E18_vs_E10	ordinary_muscle_development	ENSGALG00010017753	GNRHR	0.589454638	0.358002856	1.646508198	0.099659174	0.132188847	positive
PRJNA729271	stage_E18_vs_E10	ordinary_muscle_development	ENSGALG00010017754	PDZD11	-0.781032067	0.161138427	-4.84696346	1.25366E-06	3.20999E-06	negative
PRJNA729271	stage_E18_vs_E10	ordinary_muscle_development	ENSGALG00010017755	C1D	-0.936284181	0.141456976	-6.618861855	3.61975E-11	1.36423E-10	negative
PRJNA729271	stage_E18_vs_E10	ordinary_muscle_development	ENSGALG00010017756	EIF2B5	-0.309491962	0.119283603	-2.594589323	0.0094704	0.015025553	negative
PRJNA729271	stage_E18_vs_E10	ordinary_muscle_development	ENSGALG00010017757	ENSGALG00010017757	1.217762045	0.138570527	8.788030681	1.52204E-18	9.86674E-18	positive
PRJNA729271	stage_E18_vs_E10	ordinary_muscle_development	ENSGALG00010017759	ENSGALG00010017759	2.590087948	0.893894664	2.897531502	0.00376112	0.006336289	positive
PRJNA729271	stage_E18_vs_E10	ordinary_muscle_development	ENSGALG00010017761	PARP16	1.156460148	0.1063892	10.8700897	1.60043E-27	1.74423E-26	positive
PRJNA729271	stage_E18_vs_E10	ordinary_muscle_development	ENSGALG00010017762	FBXO11	-0.154572652	0.055829672	-2.768646949	0.005628959	0.009256015	negative
PRJNA729271	stage_E18_vs_E10	ordinary_muscle_development	ENSGALG00010017763	POLR1A	-1.024849834	0.088630552	-11.56316656	6.33296E-31	8.11638E-30	negative
PRJNA729271	stage_E18_vs_E10	ordinary_muscle_development	ENSGALG00010017764	IGDCC3	-1.08522637	0.233256859	-4.652495002	3.27943E-06	8.00392E-06	negative
PRJNA729271	stage_E18_vs_E10	ordinary_muscle_development	ENSGALG00010017765	SYT16	-0.894869251	0.292591316	-3.058427244	0.002225021	0.003877336	negative
PRJNA729271	stage_E18_vs_E10	ordinary_muscle_development	ENSGALG00010017766	DNAAF10	-0.006900751	0.155055654	-0.044504997	0.964501869	0.970932328	negative
PRJNA729271	stage_E18_vs_E10	ordinary_muscle_development	ENSGALG00010017767	KIF4B	-2.191718995	0.221269871	-9.905184965	3.95238E-23	3.33274E-22	negative
PRJNA729271	stage_E18_vs_E10	ordinary_muscle_development	ENSGALG00010017768	FOXN2	-0.670083427	0.090464321	-7.407157017	1.29036E-13	5.88723E-13	negative
PRJNA729271	stage_E18_vs_E10	ordinary_muscle_development	ENSGALG00010017770	PTCD3	-0.22324276	0.07090364	-3.14853736	0.001640897	0.002914805	negative
PRJNA729271	stage_E18_vs_E10	ordinary_muscle_development	ENSGALG00010017771	PNO1	-0.5413394	0.160002545	-3.383317423	0.000716158	0.001332569	negative
PRJNA729271	stage_E18_vs_E10	ordinary_muscle_development	ENSGALG00010017772	ENSGALG00010017772	0.800960444	0.403947996	1.982830593	0.047386355	0.06679619	positive
PRJNA729271	stage_E18_vs_E10	ordinary_muscle_development	ENSGALG00010017773	DVL3	-0.7674607	0.081220828	-9.449062775	3.41878E-21	2.60132E-20	negative
PRJNA729271	stage_E18_vs_E10	ordinary_muscle_development	ENSGALG00010017774	ASH2L	-0.20450159	0.164730944	-1.241427899	0.214447718	0.264896634	negative
PRJNA729271	stage_E18_vs_E10	ordinary_muscle_development	ENSGALG00010017775	PRPSAP2	-0.953928758	0.086500787	-11.02797775	2.80092E-28	3.1448E-27	negative
PRJNA729271	stage_E18_vs_E10	ordinary_muscle_development	ENSGALG00010017776	GPR68	0.189440841	0.367109112	0.516034158	0.605830553	0.656659976	positive
PRJNA729271	stage_E18_vs_E10	ordinary_muscle_development	ENSGALG00010017778	PPP3R1	0.916728239	0.078688753	11.65005428	2.29313E-31	3.00261E-30	positive
PRJNA729271	stage_E18_vs_E10	ordinary_muscle_development	ENSGALG00010017779	SNAPC1	-0.000626292	0.125052644	-0.005008229	0.996004028	0.996485256	negative
PRJNA729271	stage_E18_vs_E10	ordinary_muscle_development	ENSGALG00010017780	TAF9	-0.269839541	0.139584924	-1.933156774	0.053216888	0.074335433	negative
PRJNA729271	stage_E18_vs_E10	ordinary_muscle_development	ENSGALG00010017781	CYP26C1	1.11666715	0.314087435	3.555274828	0.000377584	0.000729064	positive
PRJNA729271	stage_E18_vs_E10	ordinary_muscle_development	ENSGALG00010017782	IGDCC4	0.824449139	0.11391934	7.237130567	4.58277E-13	2.01356E-12	positive
PRJNA729271	stage_E18_vs_E10	ordinary_muscle_development	ENSGALG00010017783	STAR	0.083308326	0.40980568	0.203287386	0.838910414	0.866652872	positive
PRJNA729271	stage_E18_vs_E10	ordinary_muscle_development	ENSGALG00010017786	KAZALD1	-0.029565367	0.16547889	-0.178665493	0.858200366	0.882617731	negative
PRJNA729271	stage_E18_vs_E10	ordinary_muscle_development	ENSGALG00010017787	CNRIP1	1.587321312	0.237490475	6.683726208	2.32942E-11	8.91365E-11	positive
PRJNA729271	stage_E18_vs_E10	ordinary_muscle_development	ENSGALG00010017789	IMMT	0.287113329	0.130476073	2.20050559	0.027771044	0.040817408	positive
PRJNA729271	stage_E18_vs_E10	ordinary_muscle_development	ENSGALG00010017790	R3HCC1L	0.000273368	0.179726911	0.001521018	0.998786404	0.998855314	positive
PRJNA729271	stage_E18_vs_E10	ordinary_muscle_development	ENSGALG00010017791	CCNL2	0.290145446	0.111796101	2.595309155	0.009450587	0.014995759	positive
PRJNA729271	stage_E18_vs_E10	ordinary_muscle_development	ENSGALG00010017792	LSM1	-0.950441947	0.132325259	-7.182619187	6.83884E-13	2.96705E-12	negative
PRJNA729271	stage_E18_vs_E10	ordinary_muscle_development	ENSGALG00010017793	FAM83G	-4.132196125	0.475970515	-8.681622071	3.90164E-18	2.46746E-17	negative
PRJNA729271	stage_E18_vs_E10	ordinary_muscle_development	ENSGALG00010017795	MACF1	-0.133050591	0.114255774	-1.164497745	0.244222357	0.297454253	negative
PRJNA729271	stage_E18_vs_E10	ordinary_muscle_development	ENSGALG00010017796	MXI1	1.145739807	0.133158264	8.604346228	7.6753E-18	4.73419E-17	positive
PRJNA729271	stage_E18_vs_E10	ordinary_muscle_development	ENSGALG00010017797	PPP1R21	-0.324655014	0.093075104	-3.488097259	0.000486471	0.000925988	negative
PRJNA729271	stage_E18_vs_E10	ordinary_muscle_development	ENSGALG00010017798	BAG4	-0.556799345	0.162556537	-3.425265785	0.000614198	0.001153064	negative
PRJNA729271	stage_E18_vs_E10	ordinary_muscle_development	ENSGALG00010017799	GRAPL	-0.44455517	0.396439349	-1.121369942	0.262130425	0.316578946	negative
PRJNA729271	stage_E18_vs_E10	ordinary_muscle_development	ENSGALG00010017801	SMNDC1	-0.394526768	0.07228524	-5.457915998	4.81755E-08	1.40335E-07	negative
PRJNA729271	stage_E18_vs_E10	ordinary_muscle_development	ENSGALG00010017803	ENSGALG00010017803	1.022989751	0.118187675	8.65563814	4.90161E-18	3.07438E-17	positive
PRJNA729271	stage_E18_vs_E10	ordinary_muscle_development	ENSGALG00010017804	DUSP5	0.841908972	0.239743729	3.511703836	0.000445244	0.000853002	positive
PRJNA729271	stage_E18_vs_E10	ordinary_muscle_development	ENSGALG00010017805	DPP8	-0.542439826	0.10888076	-4.981962144	6.29428E-07	1.65732E-06	negative
PRJNA729271	stage_E18_vs_E10	ordinary_muscle_development	ENSGALG00010017806	ENSGALG00010017806	1.872283211	0.762549351	2.455294478	0.014076922	0.021815993	positive
PRJNA729271	stage_E18_vs_E10	ordinary_muscle_development	ENSGALG00010017807	MRPL35	-0.043698758	0.147528986	-0.296204556	0.76707387	0.804481278	negative
PRJNA729271	stage_E18_vs_E10	ordinary_muscle_development	ENSGALG00010017808	SFRP5	2.352119634	0.330849544	7.109333161	1.16605E-12	4.96405E-12	positive
PRJNA729271	stage_E18_vs_E10	ordinary_muscle_development	ENSGALG00010017809	MOSMO	0.287135056	0.101208328	2.837069447	0.004552971	0.007588285	positive
PRJNA729271	stage_E18_vs_E10	ordinary_muscle_development	ENSGALG00010017810	HIF1A	-1.18753164	0.079404888	-14.95539722	1.43622E-50	4.27475E-49	negative
PRJNA729271	stage_E18_vs_E10	ordinary_muscle_development	ENSGALG00010017811	HACD3	-0.749734023	0.066668581	-11.24568749	2.43194E-29	2.87529E-28	negative
PRJNA729271	stage_E18_vs_E10	ordinary_muscle_development	ENSGALG00010017812	ENSGALG00010017812	1.160445818	1.864336871	0.622444278	0.533649773	0.589423304	positive
PRJNA729271	stage_E18_vs_E10	ordinary_muscle_development	ENSGALG00010017813	DDHD2	-0.478574264	0.165062238	-2.899356446	0.003739295	0.006300986	negative

PRJNA729271	stage_E18_vs_E10	ordinary_muscle_development	ENSGALG00010017814	ENSGALG00010017814	-1.402602309	0.245094008	-5.722711545	1.04837E-08	3.23185E-08	negative
PRJNA729271	stage_E18_vs_E10	ordinary_muscle_development	ENSGALG00010017815	VWA9	-0.807438307	0.087471161	-9.23090881	2.68345E-20	1.92462E-19	negative
PRJNA729271	stage_E18_vs_E10	ordinary_muscle_development	ENSGALG00010017816	PRKCH	-2.092557506	0.325559319	-6.427576743	1.29654E-10	4.68779E-10	negative
PRJNA729271	stage_E18_vs_E10	ordinary_muscle_development	ENSGALG00010017817	REEP1	2.724739158	0.116493268	23.38967055	5.4442E-121	9.989E-119	positive
PRJNA729271	stage_E18_vs_E10	ordinary_muscle_development	ENSGALG00010017818	SLC38A6	-0.082510731	0.137828545	-0.598647621	0.549407893	0.603674	negative
PRJNA729271	stage_E18_vs_E10	ordinary_muscle_development	ENSGALG00010017820	FLRT2	-0.452938209	0.135121657	-3.352077088	0.000802077	0.001482543	negative
PRJNA729271	stage_E18_vs_E10	ordinary_muscle_development	ENSGALG00010017821	ENSGALG00010017821	-2.833101819	0.368011571	-7.698404179	1.37776E-14	6.79967E-14	negative
PRJNA729271	stage_E18_vs_E10	ordinary_muscle_development	ENSGALG00010017822	VWA3A	3.116260722	0.258791724	12.04157796	2.14807E-33	3.03177E-32	positive
PRJNA729271	stage_E18_vs_E10	ordinary_muscle_development	ENSGALG00010017823	PPIE	-0.025280041	0.090156878	-0.280400583	0.77917019	0.814750534	negative
PRJNA729271	stage_E18_vs_E10	ordinary_muscle_development	ENSGALG00010017824	YAE1	-0.214029479	0.126706009	-1.689181753	0.091184605	0.121848037	negative
PRJNA729271	stage_E18_vs_E10	ordinary_muscle_development	ENSGALG00010017825	PLPP5	-0.976741923	0.147719074	-6.612158427	3.78756E-11	1.42525E-10	negative
PRJNA729271	stage_E18_vs_E10	ordinary_muscle_development	ENSGALG00010017826	PABPC4	2.835630855	0.140196408	20.22613054	5.76537E-91	5.49796E-89	positive
PRJNA729271	stage_E18_vs_E10	ordinary_muscle_development	ENSGALG00010017828	ENSGALG00010017828	0.36903043	0.237078315	1.556576063	0.119571171	0.156368109	positive
PRJNA729271	stage_E18_vs_E10	ordinary_muscle_development	ENSGALG00010017830	DENND4A	0.100058637	0.128538595	0.778432632	0.436314008	0.495990239	positive
PRJNA729271	stage_E18_vs_E10	ordinary_muscle_development	ENSGALG00010017831	PLEK	0.861760238	0.185625161	4.642475357	3.4426E-06	8.37677E-06	positive
PRJNA729271	stage_E18_vs_E10	ordinary_muscle_development	ENSGALG00010017832	POU3F1	-0.654148621	0.416839203	-1.569306861	0.116576454	0.152782614	negative
PRJNA729271	stage_E18_vs_E10	ordinary_muscle_development	ENSGALG00010017834	ENSGALG00010017834	2.846695418	0.300930656	9.459639161	3.09008E-21	2.36113E-20	positive
PRJNA729271	stage_E18_vs_E10	ordinary_muscle_development	ENSGALG00010017838	B3GNT2	-0.654276599	0.12627372	-5.181415401	2.20208E-07	6.04302E-07	negative
PRJNA729271	stage_E18_vs_E10	ordinary_muscle_development	ENSGALG00010017841	GALC	-0.663554843	0.266534679	-2.489562882	0.012790029	0.019923855	negative
PRJNA729271	stage_E18_vs_E10	ordinary_muscle_development	ENSGALG00010017843	NSD3	-0.050892035	0.071528564	-0.711492481	0.476779108	0.535273269	negative
PRJNA729271	stage_E18_vs_E10	ordinary_muscle_development	ENSGALG00010017848	CYP3A4	1.583197286	0.195679838	8.090753243	5.92969E-16	3.2361E-15	positive
PRJNA729271	stage_E18_vs_E10	ordinary_muscle_development	ENSGALG00010017849	FBXO48	1.352012954	0.184315349	7.335324819	2.21184E-13	9.9075E-13	positive
PRJNA729271	stage_E18_vs_E10	ordinary_muscle_development	ENSGALG00010017850	TRMT5	-0.987714439	0.098143367	-10.06399582	7.96965E-24	7.00546E-23	negative
PRJNA729271	stage_E18_vs_E10	ordinary_muscle_development	ENSGALG00010017852	LETM2	0.146352018	0.11752631	1.245270254	0.213032426	0.263360769	positive
PRJNA729271	stage_E18_vs_E10	ordinary_muscle_development	ENSGALG00010017855	SDR42E2	3.728923806	0.232115876	16.06492359	4.49418E-58	1.68765E-56	positive
PRJNA729271	stage_E18_vs_E10	ordinary_muscle_development	ENSGALG00010017856	FASTKD5	-0.081430176	0.118597095	-0.686611892	0.492327333	0.549790808	negative
PRJNA729271	stage_E18_vs_E10	ordinary_muscle_development	ENSGALG00010017858	GPR65	1.422623261	0.289768462	4.909517252	9.13009E-07	2.37127E-06	positive
PRJNA729271	stage_E18_vs_E10	ordinary_muscle_development	ENSGALG00010017859	EXOC6	1.258665006	0.134590821	9.351789357	8.61773E-21	6.40584E-20	positive
PRJNA729271	stage_E18_vs_E10	ordinary_muscle_development	ENSGALG00010017860	CYSLTR1	-0.285737213	0.210605552	-1.356741126	0.17486348	0.220346531	negative
PRJNA729271	stage_E18_vs_E10	ordinary_muscle_development	ENSGALG00010017861	PRSS56	0.920131058	0.502476746	1.831191323	0.067071991	0.092004213	positive
PRJNA729271	stage_E18_vs_E10	ordinary_muscle_development	ENSGALG00010017865	UBOX5	-0.191008893	0.140193129	-1.362469715	0.173049695	0.218269695	negative
PRJNA729271	stage_E18_vs_E10	ordinary_muscle_development	ENSGALG00010017866	RALA	-0.082721511	0.095232891	-0.868623327	0.385053191	0.445438628	negative
PRJNA729271	stage_E18_vs_E10	ordinary_muscle_development	ENSGALG00010017867	APLF	0.65335328	0.110757367	5.898960019	3.658E-09	1.17697E-08	positive
PRJNA729271	stage_E18_vs_E10	ordinary_muscle_development	ENSGALG00010017868	CHRND	1.792301379	0.240761126	7.444313822	9.74496E-14	4.47854E-13	positive
PRJNA729271	stage_E18_vs_E10	ordinary_muscle_development	ENSGALG00010017870	RAB11A	0.131574267	0.074118144	1.775196463	0.075865453	0.10302321	positive
PRJNA729271	stage_E18_vs_E10	ordinary_muscle_development	ENSGALG00010017873	HSPG2	1.575986432	0.10965104	14.37274498	7.67288E-47	1.9825E-45	positive
PRJNA729271	stage_E18_vs_E10	ordinary_muscle_development	ENSGALG00010017876	COMMD1	0.24569511	0.137005443	1.793323706	0.072921179	0.099388104	positive
PRJNA729271	stage_E18_vs_E10	ordinary_muscle_development	ENSGALG00010017878	CHRNG	1.936128646	0.245976383	7.871197307	3.51263E-15	1.81647E-14	positive
PRJNA729271	stage_E18_vs_E10	ordinary_muscle_development	ENSGALG00010017880	CDK13	0.015504357	0.084970466	0.182467599	0.85521578	0.880422779	positive
PRJNA729271	stage_E18_vs_E10	ordinary_muscle_development	ENSGALG00010017881	KCNK10	0.16427774	0.448254812	0.366482936	0.714004743	0.757594521	positive
PRJNA729271	stage_E18_vs_E10	ordinary_muscle_development	ENSGALG00010017882	LZTS3	2.422964632	0.19938782	12.15201927	5.5966E-34	8.2025E-33	positive
PRJNA729271	stage_E18_vs_E10	ordinary_muscle_development	ENSGALG00010017886	DDRGK1	-0.116487722	0.10314566	-1.129351651	0.25874952	0.312782445	negative
PRJNA729271	stage_E18_vs_E10	ordinary_muscle_development	ENSGALG00010017887	PROKR1	5.726264169	0.388526236	14.73842342	3.65211E-49	1.03192E-47	positive
PRJNA729271	stage_E18_vs_E10	ordinary_muscle_development	ENSGALG00010017888	MPLKIP	-0.432837989	0.113359028	-3.818293054	0.000134378	0.000275894	negative
PRJNA729271	stage_E18_vs_E10	ordinary_muscle_development	ENSGALG00010017890	ANAPC1	-1.049291068	0.06716438	-15.62273151	5.09743E-55	1.73852E-53	negative
PRJNA729271	stage_E18_vs_E10	ordinary_muscle_development	ENSGALG00010017891	GOLGA7B	2.62969553	0.341306689	7.704787545	1.31061E-14	6.48372E-14	positive
PRJNA729271	stage_E18_vs_E10	ordinary_muscle_development	ENSGALG00010017894	KAT6A	-0.074245698	0.081170035	-0.914693432	0.360352593	0.419914048	negative
PRJNA729271	stage_E18_vs_E10	ordinary_muscle_development	ENSGALG00010017895	MNAT1	0.32352408	0.081637729	3.962923552	7.40375E-05	0.000156576	positive
PRJNA729271	stage_E18_vs_E10	ordinary_muscle_development	ENSGALG00010017898	NHERF2	0.785835162	0.114776467	6.84665756	7.55954E-12	3.00371E-11	positive
PRJNA729271	stage_E18_vs_E10	ordinary_muscle_development	ENSGALG00010017901	HTR7L	-2.459362225	0.743995013	-3.305616546	0.000947677	0.001731356	negative
PRJNA729271	stage_E18_vs_E10	ordinary_muscle_development	ENSGALG00010017903	ENSGALG00010017903	-2.301767011	0.351403571	-6.550209498	5.74564E-11	2.13492E-10	negative
PRJNA729271	stage_E18_vs_E10	ordinary_muscle_development	ENSGALG00010017904	MEGF11	-1.179282964	0.265384147	-4.443682779	8.8432E-06	2.07012E-05	negative
PRJNA729271	stage_E18_vs_E10	ordinary_muscle_development	ENSGALG00010017905	EIF4E2	-0.208279449	0.087973187	-2.367533287	0.017907109	0.027225042	negative
PRJNA729271	stage_E18_vs_E10	ordinary_muscle_development	ENSGALG00010017909	NTHL1	-0.712100717	0.142287238	-5.00467032	5.59577E-07	1.48229E-06	negative
PRJNA729271	stage_E18_vs_E10	ordinary_muscle_development	ENSGALG00010017915	BFSP1	2.739520674	0.443522221	6.176738271	6.54394E-10	2.252E-09	positive
PRJNA729271	stage_E18_vs_E10	ordinary_muscle_development	ENSGALG00010017918	SIX4	0.186767818	0.202170105	0.923815206	0.355582537	0.414956032	positive
PRJNA729271	stage_E18_vs_E10	ordinary_muscle_development	ENSGALG00010017920	AP3M2	-0.993158503	0.082661804	-12.01472091	2.97372E-33	4.16867E-32	negative
PRJNA729271	stage_E18_vs_E10	ordinary_muscle_development	ENSGALG00010017921	SPATA7	2.007042568	0.197835174	10.14502388	3.48693E-24	3.13932E-23	positive
PRJNA729271	stage_E18_vs_E10	ordinary_muscle_development	ENSGALG00010017922	MERTK	0.518208261	0.131506587	3.940549848	8.12951E-05	0.000171026	positive
PRJNA729271	stage_E18_vs_E10	ordinary_muscle_development	ENSGALG00010017927	PCSK2	0.010513582	0.128363778	0.081904585	0.934722589	0.946475999	positive
PRJNA729271	stage_E18_vs_E10	ordinary_muscle_development	ENSGALG00010017930	TSC2	0.010863332	0.077912	0.139430791	0.889109742	0.908412329	positive
PRJNA729271	stage_E18_vs_E10	ordinary_muscle_development	ENSGALG00010017933	SIX1	0.045784002	0.196404087	0.233111249	0.81567502	0.846813452	positive
PRJNA729271	stage_E18_vs_E10	ordinary_muscle_development	ENSGALG00010017937	PTPN21	2.818258727	0.118893206	23.70411923	3.2697E-124	6.3193E-122	positive
PRJNA729271	stage_E18_vs_E10	ordinary_muscle_development	ENSGALG00010017941	DIS3L	-0.169972605	0.138214173	-1.229776959	0.218780639	0.269580897	negative
PRJNA729271	stage_E18_vs_E10	ordinary_muscle_development	ENSGALG00010017946	PTGDR	0.605329778	0.256212486	2.362608423	0.018146832	0.027554822	positive
PRJNA729271	stage_E18_vs_E10	ordinary_muscle_development	ENSGALG00010017948	PLAT	0.653850631	0.160320692	4.078392017	4.53483E-05	9.80201E-05	positive
PRJNA729271	stage_E18_vs_E10	ordinary_muscle_development	ENSGALG00010017949	SFXN3	0.606347568	0.139625295	4.342677065	1.40757E-05	3.22777E-05	positive
PRJNA729271	stage_E18_vs_E10	ordinary_muscle_development	ENSGALG00010017950	FBLN7	-3.083437588	0.336076035	-9.174821377	4.52324E-20	3.18583E-19	negative
PRJNA729271	stage_E18_vs_E10	ordinary_muscle_development	ENSGALG00010017954	PTGER2	0.750324657	0.267659404	2.803281503	0.00505855	0.008378891	positive
PRJNA729271	stage_E18_vs_E10	ordinary_muscle_development	ENSGALG00010017955	IKBKB	-0.018385291	0.066152542	-0.277922676	0.781071716	0.816494478	negative
PRJNA729271	stage_E18_vs_E10	ordinary_muscle_development	ENSGALG00010017956	TIPIN	-0.727177335	0.081745625	-8.89561162	5.80984E-19	3.86301E-18	negative
PRJNA729271	stage_E18_vs_E10	ordinary_muscle_development	ENSGALG00010017957	THUMPD1	-0.429492554	0.099770861	-4.304789488	1.67145E-05	3.80101E-05	negative
PRJNA729271	stage_E18_vs_E10	ordinary_muscle_development	ENSGALG00010017958	CAPN10	-1.185410568	0.094745872	-12.51147457	6.46135E-36	1.04179E-34	negative
PRJNA729271	stage_E18_vs_E10	ordinary_muscle_development	ENSGALG00010017959	PDZD7	0.603573575	0.293125105	2.059098877	0.039484767	0.056452078	positive
PRJNA729271	stage_E18_vs_E10	ordinary_muscle_development	ENSGALG00010017962	TXNDC16	-0.103925442	0.150418205	-0.690910002	0.4896221	0.547529111	negative
PRJNA729271	stage_E18_vs_E10	ordinary_muscle_development	ENSGALG00010017963	LPAR4	-0.817623074	0.169427063	-4.825811544	1.39434E-06	3.54828E-06	negative
PRJNA729271	stage_E18_vs_E10	ordinary_muscle_development	ENSGALG00010017970	AMPH	-0.747163268	0.799214951	-0.934871485	0.349854534	0.408666409	negative
PRJNA729271	stage_E18_vs_E10	ordinary_muscle_development	ENSGALG00010017972	RNPEPL1	0.546124823	0.174252989	3.134091556	0.001723871	0.003055828	positive
PRJNA729271	stage_E18_vs_E10	ordinary_muscle_development	ENSGALG00010017973	PKD1	1.568903308	0.129079688	12.15453287	5.42711E-34	7.96214E-33	positive
PRJNA729271	stage_E18_vs_E10	ordinary_muscle_development	ENSGALG00010017974	MXRA8	-0.034314091	0.184151504	-0.186336197	0.852181124	0.877928928	negative
PRJNA729271	stage_E18_vs_E10	ordinary_muscle_development	ENSGALG00010017976	GPR137C	-0.917441328	0.109160819	-8.40449291	4.29714E-17	2.52891E-16	negative
PRJNA729271	stage_E18_vs_E10	ordinary_muscle_development	ENSGALG00010017979	ENSGALG00010017979	0.236927896	0.160878832	1.472710195	0.140829196	0.181386103	positive
PRJNA729271	stage_E18_vs_E10	ordinary_muscle_development	ENSGALG00010017983	SLC4A11	1.152058679	0.204283015	5.639522608	1.70522E-08	5.16556E-08	positive
PRJNA729271	stage_E18_vs_E10	ordinary_muscle_development	ENSGALG00010017985	ABCC5	0.861202688	0.267814999	3.215662651	0.001301437	0.002338747	positive
PRJNA729271	stage_E18_vs_E10	ordinary_muscle_development	ENSGALG00010017987	ERI2	-1.286549784	0.224277135	-5.736428656	9.66938E-09	2.99316E-08	negative
PRJNA729271	stage_E18_vs_E10	ordinary_muscle_development	ENSGALG00010017991	RAB26	0.468343976	0.24184565	1.936540831	0.052801497	0.073804985	positive
PRJNA729271	stage_E18_vs_E10	ordinary_muscle_development	ENSGALG00010017992	ENSGALG00010017992	0.21937465	0.809100211	0.271134091	0.786287902	0.820831339	positive
PRJNA729271	stage_E18_vs_E10	ordinary_muscle_development	ENSGALG00010017993	REXO5	-0.720672569	0.094920163	-7.592407616	3.14015E-14	1.49627E-13	negative
PRJNA729271	stage_E18_vs_E10	ordinary_muscle_development	ENSGALG00010017996	ZC3H14	-0.51139993	0.093708024	-5.457376096	4.83222E-08	1.40705E-07	negative
PRJNA729271	stage_E18_vs_E10	ordinary_muscle_development	ENSGALG00010017997	ENSGALG00010017997	-0.09109061	0.347381419	-0.262220732	0.793151271	0.82710271	negative
PRJNA729271	stage_E18_vs_E10	ordinary_muscle_development	ENSGALG00010017999	GPR174	-0.200062544	0.790553498	-0.25306642	0.800216887	0.833211966	negative
PRJNA729271	stage_E18_vs_E10	ordinary_muscle_development	ENSGALG00010018000	MAP2K1	-0.481961028	0.074125495	-6.501960371	7.928E-11	2.91592E-10	negative
PRJNA729271	stage_E18_vs_E10	ordinary_muscle_development	ENSGALG00010018001	TRAF7	0.116345348	0.085694359	1.357678022	0.174565873	0.220028899	positive
PRJNA729271	stage_E18_vs_E10	ordinary_muscle_development	ENSGALG00010018002	ZC3H8	0.089367809	0.080376494	1.111864989	0.266196204	0.321087957	positive
PRJNA729271	stage_E18_vs_E10	ordinary_muscle_development	ENSGALG00010018004	CDC42BPA	0.836941364	0.061764189	13.5505926	7.85931E-42	1.61819E-40	positive
PRJNA729271	stage_E18_vs_E10	ordinary_muscle_development	ENSGALG00010018006	DCUN1D3	-0.182004472	0.105912162	-1.718447331	0.085715053	0.115087005	negative
PRJNA729271	stage_E18_vs_E10	ordinary_muscle_development	ENSGALG00010018007	DNAAF9	0.001252686	0.179576236	0.006975789	0.994434171	0.995052002	positive
PRJNA729271	stage_E18_vs_E10	ordinary_muscle_development	ENSGALG00010018008	ABCF3	0.217631052	0.133010501	1.636194508	0.101798885	0.134743388	positive
PRJNA729271	stage_E18_vs_E10	ordinary_muscle_development	ENSGALG00010018010	ENSGALG00010018010	-0.734001803	0.119958735	-6.118785808	9.4291E-10	3.19259E-09	negative
PRJNA729271	stage_E18_vs_E10	ordinary_muscle_development	ENSGALG00010018011	LIPA	-0.74274714	0.186814709	-3.975849354	7.01285E-05	0.000148809	negative
PRJNA729271	stage_E18_vs_E10	ordinary_muscle_development	ENSGALG00010018012	DVL1	1.150904678	0.138439666	8.313402568	9.29964E-17	5.34914E-16	positive
PRJNA729271	stage_E18_vs_E10	ordinary_muscle_development	ENSGALG00010018014	LYRM1	0.169995987	0.097747971	1.739125484	0.082012693	0.110676286	positive
PRJNA729271	stage_E18_vs_E10	ordinary_muscle_development	ENSGALG00010018016	IFIT5	1.340015207	0.868033274	1.543737144	0.122652024	0.159978502	positive
PRJNA729271	stage_E18_vs_E10	ordinary_muscle_development	ENSGALG00010018019	NAPB	0.509413052	0.353180771	1.442357833	0.149201453	0.191099678	positive
PRJNA729271	stage_E18_vs_E10	ordinary_muscle_development	ENSGALG00010018020	SNAPC5	-0.459670404	0.103954042	-4.421861751	9.78541E-06	2.28074E-05	negative
PRJNA729271	stage_E18_vs_E10	ordinary_muscle_development	ENSGALG00010018021	ATRN	0.594153699	0.07128905	8.334431382	7.78715E-17	4.49521E-16	positive
PRJNA729271	stage_E18_vs_E10	ordinary_muscle_development	ENSGALG00010018022	XPO6	-0.79244165	0.118688288	-6.676662559	2.44445E-11	9.34149E-11	negative
PRJNA729271	stage_E18_vs_E10	ordinary_muscle_development	ENSGALG00010018025	CPTP	-0.087574276	0.134090906	-0.653096311	0.51369419	0.570487074	negative
PRJNA729271	stage_E18_vs_E10	ordinary_muscle_development	ENSGALG00010018026	CCDC39	-1.496604237	0.716422261	-2.088997395	0.036707958	0.052759727	negative
PRJNA729271	stage_E18_vs_E10	ordinary_muscle_development	ENSGALG00010018027	EML5	1.262770252	0.257130089	4.911017049	9.06052E-07	2.3546E-06	positive
PRJNA729271	stage_E18_vs_E10	ordinary_muscle_development	ENSGALG00010018030	POLB	-0.234315034	0.103212661	-2.270215993	0.023194481	0.034574661	negative
PRJNA729271	stage_E18_vs_E10	ordinary_muscle_development	ENSGALG00010018032	ERO1A	0.268280004	0.123658618	2.169521289	0.03004313	0.043894282	positive
PRJNA729271	stage_E18_vs_E10	ordinary_muscle_development	ENSGALG00010018033	COQ8A	2.32155916	0.213201342	10.88904573	1.29995E-27	1.4221E-26	positive
PRJNA729271	stage_E18_vs_E10	ordinary_muscle_development	ENSGALG00010018035	ITM2A	1.005217578	0.086401506	11.63425988	2.75974E-31	3.59733E-30	positive
PRJNA729271	stage_E18_vs_E10	ordinary_muscle_development	ENSGALG00010018036	RPL4	0.855804175	0.107027486	7.996115822	1.28406E-15	6.84531E-15	positive
PRJNA729271	stage_E18_vs_E10	ordinary_muscle_development	ENSGALG00010018037	GZF1	0.26074265	0.082414948	3.163778635	0.001557352	0.002772175	positive
PRJNA729271	stage_E18_vs_E10	ordinary_muscle_development	ENSGALG00010018039	TTC14	0.438598905	0.131587559	3.333133538	0.000858737	0.00158002	positive
PRJNA729271	stage_E18_vs_E10	ordinary_muscle_development	ENSGALG00010018042	ZWILCH	-0.907082635	0.138430045	-6.552642757	5.65276E-11	2.1031E-10	negative
PRJNA729271	stage_E18_vs_E10	ordinary_muscle_development	ENSGALG00010018043	PSMC6	-0.012940721	0.09119459	-0.14190229	0.887157186	0.906864838	negative
PRJNA729271	stage_E18_vs_E10	ordinary_muscle_development	ENSGALG00010018044	INTS11	-0.656500078	0.060506554	-10.8500655	1.99282E-27	2.15728E-26	negative
PRJNA729271	stage_E18_vs_E10	ordinary_muscle_development	ENSGALG00010018045	PSEN2	0.208197825	0.074607746	2.790565805	0.0052616	0.008696339	positive
PRJNA729271	stage_E18_vs_E10	ordinary_muscle_development	ENSGALG00010018046	CAPN15	0.128175542	0.102837639	1.246387448	0.212622187	0.262898456	positive
PRJNA729271	stage_E18_vs_E10	ordinary_muscle_development	ENSGALG00010018048	GFRA4	-3.145782357	0.164136084	-19.16569636	7.15922E-82	5.26766E-80	negative
PRJNA729271	stage_E18_vs_E10	ordinary_muscle_development	ENSGALG00010018049	SUGCT	-0.271385978	0.163113444	-1.663786695	0.096155064	0.12798601	negative
PRJNA729271	stage_E18_vs_E10	ordinary_muscle_development	ENSGALG00010018051	GNPNAT1	-1.242281203	0.138494863	-8.96987207	2.96859E-19	2.00231E-18	negative
PRJNA729271	stage_E18_vs_E10	ordinary_muscle_development	ENSGALG00010018052	PPP1RL	-0.240856795	0.131687528	-1.829002329	0.067399259	0.092435639	negative
PRJNA729271	stage_E18_vs_E10	ordinary_muscle_development	ENSGALG00010018053	DIAPH2	0.859647086	0.092044033	9.339520025	9.67718E-21	7.15302E-20	positive
PRJNA729271	stage_E18_vs_E10	ordinary_muscle_development	ENSGALG00010018054	SMAD6	-0.932404786	0.16123209	-5.782997598	7.33811E-09	2.29286E-08	negative
PRJNA729271	stage_E18_vs_E10	ordinary_muscle_development	ENSGALG00010018055	PEX5L	0.512441737	0.476088414	1.076358344	0.281767025	0.337398845	positive
PRJNA729271	stage_E18_vs_E10	ordinary_muscle_development	ENSGALG00010018057	SSTR4	0.30208539	0.176194573	1.714498833	0.086437151	0.115988382	positive
PRJNA729271	stage_E18_vs_E10	ordinary_muscle_development	ENSGALG00010018058	ITPKB	-0.218874343	0.093204649	-2.348320013	0.018858311	0.02854843	negative

PRJNA729271	stage_E18_vs_E10	ordinary_muscle_development	ENSGALG00010018060	CASKIN1	-1.982056798	0.342056415	-5.794531867	6.8512E-09	2.14767E-08	negative
PRJNA729271	stage_E18_vs_E10	ordinary_muscle_development	ENSGALG00010018061	NDUFAF5	0.335570654	0.137001782	2.449388966	0.014309882	0.022155709	positive
PRJNA729271	stage_E18_vs_E10	ordinary_muscle_development	ENSGALG00010018062	GOLGA7	-0.824964013	0.094785063	-8.703523421	3.21736E-18	2.04453E-17	negative
PRJNA729271	stage_E18_vs_E10	ordinary_muscle_development	ENSGALG00010018063	DECR2	0.170499915	0.09878512	1.725967583	0.084353245	0.113454605	positive
PRJNA729271	stage_E18_vs_E10	ordinary_muscle_development	ENSGALG00010018064	TTC8	-0.643667702	0.160371945	-4.013592921	5.98014E-05	0.000127869	negative
PRJNA729271	stage_E18_vs_E10	ordinary_muscle_development	ENSGALG00010018067	ENSGALG00010018067	0.900167746	0.177971608	5.057928947	4.23834E-07	1.13495E-06	positive
PRJNA729271	stage_E18_vs_E10	ordinary_muscle_development	ENSGALG00010018068	ADAM33	0.867907969	0.137786323	6.298941374	2.99685E-10	1.05564E-09	positive
PRJNA729271	stage_E18_vs_E10	ordinary_muscle_development	ENSGALG00010018069	ENSGALG00010018069	-1.245261094	0.397929457	-3.129351375	0.001751927	0.003102905	negative
PRJNA729271	stage_E18_vs_E10	ordinary_muscle_development	ENSGALG00010018071	GPAT4	0.047852128	0.079678012	0.600568796	0.548127227	0.602510975	positive
PRJNA729271	stage_E18_vs_E10	ordinary_muscle_development	ENSGALG00010018075	MLST8	0.490903298	0.103870723	4.726098794	2.28874E-06	5.68655E-06	positive
PRJNA729271	stage_E18_vs_E10	ordinary_muscle_development	ENSGALG00010018076	FOXA2	-3.800177127	1.420849389	-2.674581244	0.007482264	0.012054621	negative
PRJNA729271	stage_E18_vs_E10	ordinary_muscle_development	ENSGALG00010018078	NKX6-3	4.744633797	0.708303766	6.698586153	2.10446E-11	8.072E-11	positive
PRJNA729271	stage_E18_vs_E10	ordinary_muscle_development	ENSGALG00010018080	BRICD5	0.61062977	0.343516288	1.777586075	0.075471862	0.102546367	positive
PRJNA729271	stage_E18_vs_E10	ordinary_muscle_development	ENSGALG00010018081	DIO3	-3.015749881	0.246942337	-12.21236473	2.67014E-34	3.98186E-33	negative
PRJNA729271	stage_E18_vs_E10	ordinary_muscle_development	ENSGALG00010018082	RAB11FIP3	-0.807278451	0.178253541	-4.528821409	5.93136E-06	1.41337E-05	negative
PRJNA729271	stage_E18_vs_E10	ordinary_muscle_development	ENSGALG00010018083	PAX1	-5.097508919	0.98283229	-5.186550109	2.14225E-07	5.88663E-07	negative
PRJNA729271	stage_E18_vs_E10	ordinary_muscle_development	ENSGALG00010018085	PGP	0.599036838	0.161051357	3.719539212	0.000199587	0.000400305	positive
PRJNA729271	stage_E18_vs_E10	ordinary_muscle_development	ENSGALG00010018087	ENSGALG00010018087	-0.5946371	0.055979951	-10.62232266	2.34649E-26	2.4037E-25	negative
PRJNA729271	stage_E18_vs_E10	ordinary_muscle_development	ENSGALG00010018089	TASP1	0.178361151	0.095733442	1.86310183	0.062447941	0.086084917	positive
PRJNA729271	stage_E18_vs_E10	ordinary_muscle_development	ENSGALG00010018092	ENSGALG00010018092	-2.75153106	0.213032499	-12.91601548	3.65589E-38	6.48618E-37	negative
PRJNA729271	stage_E18_vs_E10	ordinary_muscle_development	ENSGALG00010018095	E4F1	0.109578399	0.069433904	1.578168499	0.114526905	0.150286754	positive
PRJNA729271	stage_E18_vs_E10	ordinary_muscle_development	ENSGALG00010018099	SMAD3	-0.787155393	0.13072001	-6.021690135	1.72605E-09	5.70171E-09	negative
PRJNA729271	stage_E18_vs_E10	ordinary_muscle_development	ENSGALG00010018102	ZDHHC4	0.340372596	0.172718079	1.970683079	0.048760137	0.068592604	positive
PRJNA729271	stage_E18_vs_E10	ordinary_muscle_development	ENSGALG00010018103	BTRC	-0.38962127	0.108223148	-3.600165752	0.000318014	0.000620072	negative
PRJNA729271	stage_E18_vs_E10	ordinary_muscle_development	ENSGALG00010018111	NKX2-2	1.910660291	0.592369841	3.225451667	0.001257741	0.00226478	positive
PRJNA729271	stage_E18_vs_E10	ordinary_muscle_development	ENSGALG00010018115	KLHL4	-0.446675284	0.188394756	-2.370953918	0.017742243	0.026994207	negative
PRJNA729271	stage_E18_vs_E10	ordinary_muscle_development	ENSGALG00010018119	GRID2IP	0.034216449	0.505275571	0.067718392	0.946009809	0.95549056	positive
PRJNA729271	stage_E18_vs_E10	ordinary_muscle_development	ENSGALG00010018121	FERMT2	1.400830207	0.159633975	8.775263556	1.70504E-18	1.10087E-17	positive
PRJNA729271	stage_E18_vs_E10	ordinary_muscle_development	ENSGALG00010018123	USP13	2.160663207	0.141371749	15.28355717	9.84186E-53	3.14224E-51	positive
PRJNA729271	stage_E18_vs_E10	ordinary_muscle_development	ENSGALG00010018128	SEPTIN12	-0.133414796	0.192230684	-0.694034861	0.487660347	0.545587892	negative
PRJNA729271	stage_E18_vs_E10	ordinary_muscle_development	ENSGALG00010018137	NDUFB5	0.910347171	0.127682354	7.129780626	1.00529E-12	4.31498E-12	positive
PRJNA729271	stage_E18_vs_E10	ordinary_muscle_development	ENSGALG00010018140	CHPF	0.300780112	0.101192636	2.972351775	0.002955279	0.005066442	positive
PRJNA729271	stage_E18_vs_E10	ordinary_muscle_development	ENSGALG00010018142	DDHD1	-0.655384483	0.142819229	-4.588909266	4.45568E-06	1.07284E-05	negative
PRJNA729271	stage_E18_vs_E10	ordinary_muscle_development	ENSGALG00010018143	MRPL47	0.017249692	0.124649085	0.138386031	0.889935337	0.909063616	positive
PRJNA729271	stage_E18_vs_E10	ordinary_muscle_development	ENSGALG00010018145	ENSGALG00010018145	0.436989421	0.351070123	1.244735431	0.213229018	0.263536375	positive
PRJNA729271	stage_E18_vs_E10	ordinary_muscle_development	ENSGALG00010018147	ISM1	1.681283871	0.283643498	5.927454292	3.07667E-09	9.95454E-09	positive
PRJNA729271	stage_E18_vs_E10	ordinary_muscle_development	ENSGALG00010018148	XRN2	-0.3503955	0.055346589	-6.330932099	2.43685E-10	8.63409E-10	negative
PRJNA729271	stage_E18_vs_E10	ordinary_muscle_development	ENSGALG00010018149	ENSGALG00010018149	-0.021386167	0.295740386	-0.072313989	0.942352033	0.952870088	negative
PRJNA729271	stage_E18_vs_E10	ordinary_muscle_development	ENSGALG00010018150	AAGAB	-1.033217279	0.080228467	-12.87843731	5.95287E-38	1.05228E-36	negative
PRJNA729271	stage_E18_vs_E10	ordinary_muscle_development	ENSGALG00010018154	SLC16A12	2.533042448	0.20787494	12.18541518	3.71755E-34	5.5211E-33	positive
PRJNA729271	stage_E18_vs_E10	ordinary_muscle_development	ENSGALG00010018157	FBXL2	1.122649692	0.150701701	7.449482536	9.3707E-14	4.31338E-13	positive
PRJNA729271	stage_E18_vs_E10	ordinary_muscle_development	ENSGALG00010018160	ENSGALG00010018160	0.87660594	0.164917712	5.315414146	1.06415E-07	3.01149E-07	positive
PRJNA729271	stage_E18_vs_E10	ordinary_muscle_development	ENSGALG00010018162	ENSGALG00010018162	-1.095495069	0.396449551	-2.763264747	0.005722633	0.009398319	negative
PRJNA729271	stage_E18_vs_E10	ordinary_muscle_development	ENSGALG00010018163	CCT7	-0.762016373	0.081254744	-9.378115538	6.71623E-21	5.02331E-20	negative
PRJNA729271	stage_E18_vs_E10	ordinary_muscle_development	ENSGALG00010018165	DACH2	1.169201641	0.182068242	6.421776971	1.34693E-10	4.86027E-10	positive
PRJNA729271	stage_E18_vs_E10	ordinary_muscle_development	ENSGALG00010018166	MSS51	4.970620881	0.237852112	20.89794725	5.58975E-97	6.13814E-95	positive
PRJNA729271	stage_E18_vs_E10	ordinary_muscle_development	ENSGALG00010018168	ENSGALG00010018168	0.646538178	0.200939821	3.217571183	0.001292809	0.002324107	positive
PRJNA729271	stage_E18_vs_E10	ordinary_muscle_development	ENSGALG00010018172	ACTL6A	-1.915096226	0.095408236	-20.07265104	1.27992E-89	1.18168E-87	negative
PRJNA729271	stage_E18_vs_E10	ordinary_muscle_development	ENSGALG00010018174	BMP4	0.295003811	0.205359197	1.436525927	0.150852744	0.192975953	positive
PRJNA729271	stage_E18_vs_E10	ordinary_muscle_development	ENSGALG00010018177	KIF20B	-1.95931688	0.187543096	-10.44728879	1.50774E-25	1.47867E-24	negative
PRJNA729271	stage_E18_vs_E10	ordinary_muscle_development	ENSGALG00010018179	PRADC1	-0.059100797	0.12136115	-0.486982839	0.626270492	0.676639146	negative
PRJNA729271	stage_E18_vs_E10	ordinary_muscle_development	ENSGALG00010018183	CYP17A1	2.638318042	0.286001356	9.224844517	2.83973E-20	2.02968E-19	positive
PRJNA729271	stage_E18_vs_E10	ordinary_muscle_development	ENSGALG00010018185	GNB4	-0.479433975	0.069660237	-6.88246257	5.88266E-12	2.36465E-11	negative
PRJNA729271	stage_E18_vs_E10	ordinary_muscle_development	ENSGALG00010018186	SPTLC3	-2.694652127	0.596412397	-4.518102142	6.23964E-06	1.48268E-05	negative
PRJNA729271	stage_E18_vs_E10	ordinary_muscle_development	ENSGALG00010018188	UBP1	-0.23726391	0.052752767	-4.497658078	6.87061E-06	1.62542E-05	negative
PRJNA729271	stage_E18_vs_E10	ordinary_muscle_development	ENSGALG00010018189	MIS18BP1	-0.840752122	0.119772241	-7.019590816	2.22519E-12	9.26576E-12	negative
PRJNA729271	stage_E18_vs_E10	ordinary_muscle_development	ENSGALG00010018192	IQCH	-0.420273676	0.2052608	-2.047510657	0.040607972	0.057917204	negative
PRJNA729271	stage_E18_vs_E10	ordinary_muscle_development	ENSGALG00010018193	ANK1	4.642000521	0.246481624	18.83304909	4.0477E-79	2.7289E-77	positive
PRJNA729271	stage_E18_vs_E10	ordinary_muscle_development	ENSGALG00010018198	BTBD3	0.391787103	0.101366773	3.865044647	0.000111069	0.000230585	positive
PRJNA729271	stage_E18_vs_E10	ordinary_muscle_development	ENSGALG00010018199	BORCS7	0.082931765	0.119375409	0.694713978	0.487234566	0.545237786	positive
PRJNA729271	stage_E18_vs_E10	ordinary_muscle_development	ENSGALG00010018206	KIZ	0.262497	0.125119956	2.097962692	0.035908441	0.051708013	positive
PRJNA729271	stage_E18_vs_E10	ordinary_muscle_development	ENSGALG00010018209	GMPPA	0.030644973	0.131921344	0.232297311	0.816307101	0.847348283	positive
PRJNA729271	stage_E18_vs_E10	ordinary_muscle_development	ENSGALG00010018215	CFAP70	3.072781422	0.266817401	11.51642062	1.09047E-30	1.37567E-29	positive
PRJNA729271	stage_E18_vs_E10	ordinary_muscle_development	ENSGALG00010018216	FANCM	0.926291452	0.083558557	11.08553666	1.47463E-28	1.67252E-27	positive
PRJNA729271	stage_E18_vs_E10	ordinary_muscle_development	ENSGALG00010018217	ENSGALG00010018217	0.557205203	0.38409767	1.450686233	0.146867251	0.188392991	positive
PRJNA729271	stage_E18_vs_E10	ordinary_muscle_development	ENSGALG00010018219	SLC24A5	-2.453897823	0.564279787	-4.348725364	1.36931E-05	3.14401E-05	negative
PRJNA729271	stage_E18_vs_E10	ordinary_muscle_development	ENSGALG00010018226	JAG1	-0.047544284	0.102421835	-0.464200665	0.642503985	0.69170345	negative
PRJNA729271	stage_E18_vs_E10	ordinary_muscle_development	ENSGALG00010018229	AS3MT	1.471898522	0.187732843	7.840389017	4.49153E-15	2.30052E-14	positive
PRJNA729271	stage_E18_vs_E10	ordinary_muscle_development	ENSGALG00010018231	SMYD5	-0.177447294	0.078111956	-2.271704666	0.023104354	0.034447399	negative
PRJNA729271	stage_E18_vs_E10	ordinary_muscle_development	ENSGALG00010018233	RASSF2	-0.183238817	0.187193441	-0.978874132	0.327642175	0.385703998	negative
PRJNA729271	stage_E18_vs_E10	ordinary_muscle_development	ENSGALG00010018240	SOUL	-0.41677312	0.685723905	-0.607785607	0.543329677	0.598265243	negative
PRJNA729271	stage_E18_vs_E10	ordinary_muscle_development	ENSGALG00010018241	RALGAPA2	0.241249219	0.092477346	2.608738571	0.009087664	0.014461048	positive
PRJNA729271	stage_E18_vs_E10	ordinary_muscle_development	ENSGALG00010018244	CHM	0.156857756	0.082099636	1.910577968	0.056058838	0.078011987	positive
PRJNA729271	stage_E18_vs_E10	ordinary_muscle_development	ENSGALG00010018251	USP48	0.169255707	0.166715468	1.015236971	0.309992881	0.367614073	positive
PRJNA729271	stage_E18_vs_E10	ordinary_muscle_development	ENSGALG00010018255	MFN1	0.290656745	0.11322699	2.567027036	0.010257461	0.016206879	positive
PRJNA729271	stage_E18_vs_E10	ordinary_muscle_development	ENSGALG00010018258	OSCP1	-0.624332534	0.283496041	-2.202261912	0.027646813	0.040655428	negative
PRJNA729271	stage_E18_vs_E10	ordinary_muscle_development	ENSGALG00010018259	WDR90	-0.029722282	0.129266156	-0.229930885	0.818145477	0.848891817	negative
PRJNA729271	stage_E18_vs_E10	ordinary_muscle_development	ENSGALG00010018261	PRNP	0.695038092	0.195046287	3.563452057	0.00036601	0.00070813	positive
PRJNA729271	stage_E18_vs_E10	ordinary_muscle_development	ENSGALG00010018263	SLX4IP	-0.81973282	0.109845548	-7.462594825	8.48349E-14	3.92619E-13	negative
PRJNA729271	stage_E18_vs_E10	ordinary_muscle_development	ENSGALG00010018264	CFAP61	-0.835790328	0.341447468	-2.447785988	0.0143737	0.022249764	negative
PRJNA729271	stage_E18_vs_E10	ordinary_muscle_development	ENSGALG00010018265	PDCD4	-0.638366303	0.066517084	-9.597027706	8.2283E-22	6.51033E-21	negative
PRJNA729271	stage_E18_vs_E10	ordinary_muscle_development	ENSGALG00010018267	ADPRHL2	-0.281626435	0.077068232	-3.654248047	0.000257937	0.000510207	negative
PRJNA729271	stage_E18_vs_E10	ordinary_muscle_development	ENSGALG00010018273	PROM2	-3.468068823	0.914396536	-3.792740554	0.000148994	0.00030375	negative
PRJNA729271	stage_E18_vs_E10	ordinary_muscle_development	ENSGALG00010018274	ZNF711	0.068909845	0.097478479	0.706923675	0.479613911	0.537955864	positive
PRJNA729271	stage_E18_vs_E10	ordinary_muscle_development	ENSGALG00010018275	ZNF639	-0.09512383	0.084065066	-1.131550066	0.257823643	0.31181925	negative
PRJNA729271	stage_E18_vs_E10	ordinary_muscle_development	ENSGALG00010018276	ARHGAP17	0.247390299	0.071952624	3.43823875	0.000585511	0.001102492	positive
PRJNA729271	stage_E18_vs_E10	ordinary_muscle_development	ENSGALG00010018278	PUSL1	0.552550289	0.153495088	3.599791349	0.000318473	0.000620882	positive
PRJNA729271	stage_E18_vs_E10	ordinary_muscle_development	ENSGALG00010018283	POF1B	2.427311447	0.171088186	14.18748717	1.09513E-45	2.65007E-44	positive
PRJNA729271	stage_E18_vs_E10	ordinary_muscle_development	ENSGALG00010018284	ENSGALG00010018284	0.918544508	0.167221102	5.49299399	3.95176E-08	1.16235E-07	positive
PRJNA729271	stage_E18_vs_E10	ordinary_muscle_development	ENSGALG00010018285	BBIP1	0.116146778	0.191568383	0.606294089	0.544319483	0.599127566	positive
PRJNA729271	stage_E18_vs_E10	ordinary_muscle_development	ENSGALG00010018287	MKKS	-0.284950903	0.103951686	-2.741185965	0.006121785	0.010006233	negative
PRJNA729271	stage_E18_vs_E10	ordinary_muscle_development	ENSGALG00010018288	CRNKL1	-0.484222886	0.062739389	-7.718004489	1.18165E-14	5.86276E-14	negative
PRJNA729271	stage_E18_vs_E10	ordinary_muscle_development	ENSGALG00010018289	HEYL	-0.13399683	0.206313695	-0.649481027	0.516027506	0.572858904	negative
PRJNA729271	stage_E18_vs_E10	ordinary_muscle_development	ENSGALG00010018290	RHOT2	-0.70627502	0.138902328	-5.084688146	3.68231E-07	9.92654E-07	negative
PRJNA729271	stage_E18_vs_E10	ordinary_muscle_development	ENSGALG00010018294	FKBP3	0.931553555	0.177744454	5.240971145	1.59734E-07	4.43127E-07	positive
PRJNA729271	stage_E18_vs_E10	ordinary_muscle_development	ENSGALG00010018296	PIK3CA	0.483919558	0.096946528	4.9916131	5.98771E-07	1.58062E-06	positive
PRJNA729271	stage_E18_vs_E10	ordinary_muscle_development	ENSGALG00010018298	ENSGALG00010018298	0.142135831	0.198753897	0.715134815	0.474525743	0.533156395	positive
PRJNA729271	stage_E18_vs_E10	ordinary_muscle_development	ENSGALG00010018299	SHOC2	-0.34645804	0.086791969	-3.991821408	6.55677E-05	0.000139622	negative
PRJNA729271	stage_E18_vs_E10	ordinary_muscle_development	ENSGALG00010018301	ENSGALG00010018301	-0.018072706	0.312656815	-0.057803652	0.953905029	0.961868063	negative
PRJNA729271	stage_E18_vs_E10	ordinary_muscle_development	ENSGALG00010018302	RAB11FIP5	0.644980347	0.101706306	6.341596484	2.27396E-10	8.07276E-10	positive
PRJNA729271	stage_E18_vs_E10	ordinary_muscle_development	ENSGALG00010018306	DES	2.564184167	0.150989527	16.98252996	1.10613E-64	5.13891E-63	positive
PRJNA729271	stage_E18_vs_E10	ordinary_muscle_development	ENSGALG00010018308	ACAP3	0.055302318	0.175621569	0.314894793	0.752841535	0.792594281	positive
PRJNA729271	stage_E18_vs_E10	ordinary_muscle_development	ENSGALG00010018309	DPYSL2	-1.043350999	0.124264675	-8.396199495	4.61162E-17	2.71178E-16	negative
PRJNA729271	stage_E18_vs_E10	ordinary_muscle_development	ENSGALG00010018310	APOOL	0.201336662	0.112254204	1.793577925	0.072880563	0.099342087	positive
PRJNA729271	stage_E18_vs_E10	ordinary_muscle_development	ENSGALG00010018311	NKX2-3	2.553556081	0.736073649	3.469158395	0.000522092	0.000989632	positive
PRJNA729271	stage_E18_vs_E10	ordinary_muscle_development	ENSGALG00010018312	PRPF39	-0.402634523	0.079443862	-5.068164034	4.01671E-07	1.07859E-06	negative
PRJNA729271	stage_E18_vs_E10	ordinary_muscle_development	ENSGALG00010018313	MYEF2	-1.254782613	0.082154479	-15.27345348	1.14922E-52	3.65306E-51	negative
PRJNA729271	stage_E18_vs_E10	ordinary_muscle_development	ENSGALG00010018314	SLC24A3	0.14875885	0.124343791	1.196351251	0.231559517	0.283388652	positive
PRJNA729271	stage_E18_vs_E10	ordinary_muscle_development	ENSGALG00010018315	CLASP2	2.300957813	0.117056257	19.65685443	5.0509E-86	4.18359E-84	positive
PRJNA729271	stage_E18_vs_E10	ordinary_muscle_development	ENSGALG00010018317	ZMAT3	-0.771350342	0.143382905	-5.37965348	7.46293E-08	2.13616E-07	negative
PRJNA729271	stage_E18_vs_E10	ordinary_muscle_development	ENSGALG00010018319	STUB1	0.393910327	0.202075018	1.949327189	0.051256364	0.071853094	positive
PRJNA729271	stage_E18_vs_E10	ordinary_muscle_development	ENSGALG00010018322	NT5C1A	5.081004981	0.24025927	21.14800811	2.87842E-99	3.25958E-97	positive
PRJNA729271	stage_E18_vs_E10	ordinary_muscle_development	ENSGALG00010018323	GOT1	0.91872238	0.142941967	6.427240372	1.29941E-10	4.697E-10	positive
PRJNA729271	stage_E18_vs_E10	ordinary_muscle_development	ENSGALG00010018325	ENSGALG00010018325	1.570823979	0.189815777	8.275518543	1.27898E-16	7.29299E-16	positive
PRJNA729271	stage_E18_vs_E10	ordinary_muscle_development	ENSGALG00010018326	LCMT1	-0.400608075	0.110045127	-3.640398125	0.000272217	0.00053633	negative
PRJNA729271	stage_E18_vs_E10	ordinary_muscle_development	ENSGALG00010018327	SNAP25	0.535006009	0.456674362	1.171526263	0.241387308	0.294397057	positive
PRJNA729271	stage_E18_vs_E10	ordinary_muscle_development	ENSGALG00010018328	ENSGALG00010018328	1.365666385	0.592577559	2.30462049	0.021187837	0.031825669	positive
PRJNA729271	stage_E18_vs_E10	ordinary_muscle_development	ENSGALG00010018329	ENSGALG00010018329	0.39166791	0.243784125	1.60661778	0.10813824	0.142548544	positive
PRJNA729271	stage_E18_vs_E10	ordinary_muscle_development	ENSGALG00010018333	ENSGALG00010018333	0.681373555	0.226286503	3.01111001	0.002602945	0.004499129	positive
PRJNA729271	stage_E18_vs_E10	ordinary_muscle_development	ENSGALG00010018336	JMJD8	0.057794635	0.147105437	0.392878988	0.694408862	0.739671991	positive
PRJNA729271	stage_E18_vs_E10	ordinary_muscle_development	ENSGALG00010018337	TMEM151B	-1.121770065	0.323541899	-3.467155471	0.000525998	0.000996385	negative
PRJNA729271	stage_E18_vs_E10	ordinary_muscle_development	ENSGALG00010018338	PDCD6IP	0.632816706	0.084658616	7.474923805	7.72486E-14	3.58999E-13	positive
PRJNA729271	stage_E18_vs_E10	ordinary_muscle_development	ENSGALG00010018339	FAM76A	-0.032691373	0.116526448	-0.280548952	0.779056375	0.814690293	negative
PRJNA729271	stage_E18_vs_E10	ordinary_muscle_development	ENSGALG00010018340	DNPEP	0.081002442	0.098253175	0.824425695	0.40969772	0.469897804	positive
PRJNA729271	stage_E18_vs_E10	ordinary_muscle_development	ENSGALG00010018342	BNIP3L	0.014090754	0.106153462	0.132739466	0.89439943	0.912541595	positive
PRJNA729271	stage_E18_vs_E10	ordinary_muscle_development	ENSGALG00010018344	SFXN5	-1.843639505	0.211257565	-8.726975079	2.61575E-18	1.67149E-17	negative
PRJNA729271	stage_E18_vs_E10	ordinary_muscle_development	ENSGALG00010018345	WDR24	-0.312946533	0.173841741	-1.800180619	0.071832123	0.098005142	negative
PRJNA729271	stage_E18_vs_E10	ordinary_muscle_development	ENSGALG00010018347	CTXN2	-2.605807698	0.664942428	-3.918847088	8.89735E-05	0.000186503	negative
PRJNA729271	stage_E18_vs_E10	ordinary_muscle_development	ENSGALG00010018348	TOGARAM1	-0.068546418	0.109122309	-0.628161358	0.529898241	0.585923793	negative
PRJNA729271	stage_E18_vs_E10	ordinary_muscle_development	ENSGALG00010018349	KCNMB2	0.261008194	0.198655357	1.313874432	0.188888464	0.236212068	positive
PRJNA729271	stage_E18_vs_E10	ordinary_muscle_development	ENSGALG00010018350	CNNM1	-2.357842216	0.305902305	-7.707827563	1.27978E-14	6.33766E-14	negative

PRJNA729271	stage_E18_vs_E10	ordinary_muscle_development	ENSGALG00010018351	RIN2	0.881582027	0.086720228	10.16581772	2.81748E-24	2.54768E-23	positive
PRJNA729271	stage_E18_vs_E10	ordinary_muscle_development	ENSGALG00010018354	AARS2	0.314889128	0.117854834	2.671838896	0.007543686	0.012145476	positive
PRJNA729271	stage_E18_vs_E10	ordinary_muscle_development	ENSGALG00010018355	PRKCB	-0.683912252	0.156654966	-4.365723421	1.26703E-05	2.9198E-05	negative
PRJNA729271	stage_E18_vs_E10	ordinary_muscle_development	ENSGALG00010018356	ADRA1A	0.597265549	0.326749048	1.827902951	0.067564118	0.09262668	positive
PRJNA729271	stage_E18_vs_E10	ordinary_muscle_development	ENSGALG00010018359	CACNG3	-0.7085193	0.519721202	-1.36326803	0.172798054	0.218028185	negative
PRJNA729271	stage_E18_vs_E10	ordinary_muscle_development	ENSGALG00010018361	ASIC4	1.060619529	0.205003477	5.17366605	2.29545E-07	6.29208E-07	positive
PRJNA729271	stage_E18_vs_E10	ordinary_muscle_development	ENSGALG00010018363	TCTE1	-0.427089217	0.40437338	-1.056175403	0.290888086	0.347230735	negative
PRJNA729271	stage_E18_vs_E10	ordinary_muscle_development	ENSGALG00010018365	HPSE2	-2.147530953	0.267968015	-8.014131649	1.10918E-15	5.93486E-15	negative
PRJNA729271	stage_E18_vs_E10	ordinary_muscle_development	ENSGALG00010018367	PAK5	-0.146307278	0.210032969	-0.696591961	0.486058188	0.544089384	negative
PRJNA729271	stage_E18_vs_E10	ordinary_muscle_development	ENSGALG00010018370	ZBTB8A	-0.276606277	0.113211179	-2.443277058	0.014554559	0.022516778	negative
PRJNA729271	stage_E18_vs_E10	ordinary_muscle_development	ENSGALG00010018371	ENSGALG00010018371	0.097475662	0.137171161	0.710613378	0.477323851	0.535801844	positive
PRJNA729271	stage_E18_vs_E10	ordinary_muscle_development	ENSGALG00010018372	NLGN3	-0.4315744	0.467162754	-0.923820224	0.355579924	0.414956032	negative
PRJNA729271	stage_E18_vs_E10	ordinary_muscle_development	ENSGALG00010018374	HPS1	0.594620026	0.130296859	4.56357912	5.02888E-06	1.20585E-05	positive
PRJNA729271	stage_E18_vs_E10	ordinary_muscle_development	ENSGALG00010018376	PYGB	-0.897728967	0.145467856	-6.171321908	6.77214E-10	2.32832E-09	negative
PRJNA729271	stage_E18_vs_E10	ordinary_muscle_development	ENSGALG00010018377	RAP1GAP	3.073002618	0.254714676	12.06448983	1.62667E-33	2.3139E-32	positive
PRJNA729271	stage_E18_vs_E10	ordinary_muscle_development	ENSGALG00010018378	ARPP21	0.748396403	0.144994175	5.161561852	2.44898E-07	6.70026E-07	positive
PRJNA729271	stage_E18_vs_E10	ordinary_muscle_development	ENSGALG00010018379	SRBD1	0.114924548	0.126469749	0.90871176	0.363502295	0.422938334	positive
PRJNA729271	stage_E18_vs_E10	ordinary_muscle_development	ENSGALG00010018380	SLC20A1	-1.49397222	0.146562904	-10.19338576	2.12241E-24	1.93608E-23	negative
PRJNA729271	stage_E18_vs_E10	ordinary_muscle_development	ENSGALG00010018381	HAPSTR1	0.361729355	0.067613578	5.349951411	8.79778E-08	2.5044E-07	positive
PRJNA729271	stage_E18_vs_E10	ordinary_muscle_development	ENSGALG00010018383	METRN	-0.123790108	0.159424795	-0.776479645	0.437465835	0.496987795	negative
PRJNA729271	stage_E18_vs_E10	ordinary_muscle_development	ENSGALG00010018384	LAMP5	0.154047657	0.239263471	0.643841102	0.519678478	0.576602842	positive
PRJNA729271	stage_E18_vs_E10	ordinary_muscle_development	ENSGALG00010018385	SPR	-1.191277921	0.144248309	-8.258522604	1.47487E-16	8.36065E-16	negative
PRJNA729271	stage_E18_vs_E10	ordinary_muscle_development	ENSGALG00010018387	NT5DC4	0.245256996	0.311564201	0.787179641	0.431176714	0.491228992	positive
PRJNA729271	stage_E18_vs_E10	ordinary_muscle_development	ENSGALG00010018388	SMC3	-0.648129109	0.06588895	-9.83668899	7.82435E-23	6.5143E-22	negative
PRJNA729271	stage_E18_vs_E10	ordinary_muscle_development	ENSGALG00010018391	STAC	-1.539228849	0.223648071	-6.882370342	5.88647E-12	2.36552E-11	negative
PRJNA729271	stage_E18_vs_E10	ordinary_muscle_development	ENSGALG00010018393	PRKCE	-0.173762279	0.077950305	-2.22914173	0.025804476	0.038139684	negative
PRJNA729271	stage_E18_vs_E10	ordinary_muscle_development	ENSGALG00010018394	KLHL28	-0.470271805	0.099244502	-4.738517469	2.15287E-06	5.35724E-06	negative
PRJNA729271	stage_E18_vs_E10	ordinary_muscle_development	ENSGALG00010018395	MED12	-0.255001846	0.079923582	-3.19057077	0.001419921	0.002540643	negative
PRJNA729271	stage_E18_vs_E10	ordinary_muscle_development	ENSGALG00010018396	TBL1XR1	-0.23436132	0.075218899	-3.115723869	0.00183494	0.003242801	negative
PRJNA729271	stage_E18_vs_E10	ordinary_muscle_development	ENSGALG00010018397	FAM173A	1.703976323	0.160182751	10.63770171	1.98977E-26	2.05572E-25	positive
PRJNA729271	stage_E18_vs_E10	ordinary_muscle_development	ENSGALG00010018398	LRFN5	0.900975705	0.181648741	4.959988714	7.04973E-07	1.85019E-06	positive
PRJNA729271	stage_E18_vs_E10	ordinary_muscle_development	ENSGALG00010018399	ABHD12	0.820162585	0.162621517	5.04338294	4.57373E-07	1.22047E-06	positive
PRJNA729271	stage_E18_vs_E10	ordinary_muscle_development	ENSGALG00010018401	ENSGALG00010018401	-0.518701187	0.103015055	-5.035197877	4.77355E-07	1.27122E-06	negative
PRJNA729271	stage_E18_vs_E10	ordinary_muscle_development	ENSGALG00010018404	IL2RG	0.886101361	0.262968848	3.369605821	0.000752758	0.001397535	positive
PRJNA729271	stage_E18_vs_E10	ordinary_muscle_development	ENSGALG00010018405	DCLK3	-1.047561479	0.191445122	-5.471862986	4.45329E-08	1.30168E-07	negative
PRJNA729271	stage_E18_vs_E10	ordinary_muscle_development	ENSGALG00010018406	TMEM260	-0.405863681	0.128281347	-3.163855778	0.001556939	0.00277178	negative
PRJNA729271	stage_E18_vs_E10	ordinary_muscle_development	ENSGALG00010018410	EXOC6B	-0.028768825	0.101588587	-0.283189537	0.777031548	0.813100801	negative
PRJNA729271	stage_E18_vs_E10	ordinary_muscle_development	ENSGALG00010018413	SLC15A2	-0.124299052	0.355508955	-0.349636908	0.726611209	0.76888812	negative
PRJNA729271	stage_E18_vs_E10	ordinary_muscle_development	ENSGALG00010018414	EPAS1	0.248975687	0.138276036	1.800570031	0.071770676	0.097955935	positive
PRJNA729271	stage_E18_vs_E10	ordinary_muscle_development	ENSGALG00010018415	PELI2	-0.914036831	0.128473208	-7.114610494	1.12229E-12	4.78883E-12	negative
PRJNA729271	stage_E18_vs_E10	ordinary_muscle_development	ENSGALG00010018416	DUT	-1.400313332	0.134247769	-10.43081267	1.79348E-25	1.75178E-24	negative
PRJNA729271	stage_E18_vs_E10	ordinary_muscle_development	ENSGALG00010018418	PLCB4	-0.226692527	0.119184938	-1.902023285	0.05716811	0.079464112	negative
PRJNA729271	stage_E18_vs_E10	ordinary_muscle_development	ENSGALG00010018419	USP7	-0.06966218	0.1341483	-0.519292303	0.60355692	0.654636154	negative
PRJNA729271	stage_E18_vs_E10	ordinary_muscle_development	ENSGALG00010018420	RHOQ	0.452782112	0.13255646	3.415767989	0.000636024	0.001190953	positive
PRJNA729271	stage_E18_vs_E10	ordinary_muscle_development	ENSGALG00010018422	ENSGALG00010018422	1.177597223	0.212905421	5.531081431	3.18263E-08	9.43977E-08	positive
PRJNA729271	stage_E18_vs_E10	ordinary_muscle_development	ENSGALG00010018425	CYP26B1	-1.030519904	0.228884146	-4.502364714	6.72015E-06	1.59138E-05	negative
PRJNA729271	stage_E18_vs_E10	ordinary_muscle_development	ENSGALG00010018426	ENSGALG00010018426	2.71102543	0.350098887	7.743599102	9.66414E-15	4.83541E-14	positive
PRJNA729271	stage_E18_vs_E10	ordinary_muscle_development	ENSGALG00010018427	PIGF	-0.753416712	0.14961543	-5.035688581	4.76134E-07	1.2682E-06	negative
PRJNA729271	stage_E18_vs_E10	ordinary_muscle_development	ENSGALG00010018428	PPP2R5D	-0.086899995	0.063792252	-1.36223432	0.173123948	0.218314662	negative
PRJNA729271	stage_E18_vs_E10	ordinary_muscle_development	ENSGALG00010018430	LOXL4	-0.533347387	0.245945384	-2.168560262	0.030116085	0.043992003	negative
PRJNA729271	stage_E18_vs_E10	ordinary_muscle_development	ENSGALG00010018434	HSPBAP1	1.044473701	0.161744867	6.457538486	1.0642E-10	3.86799E-10	positive
PRJNA729271	stage_E18_vs_E10	ordinary_muscle_development	ENSGALG00010018436	FOXO4	1.06947167	0.14531566	7.359645018	1.844E-13	8.31118E-13	positive
PRJNA729271	stage_E18_vs_E10	ordinary_muscle_development	ENSGALG00010018438	PPP2R2A	-0.198679204	0.098959315	-2.007685733	0.044676698	0.063253441	negative
PRJNA729271	stage_E18_vs_E10	ordinary_muscle_development	ENSGALG00010018440	MEA1	0.481910521	0.10174214	4.736587248	2.17347E-06	5.40571E-06	positive
PRJNA729271	stage_E18_vs_E10	ordinary_muscle_development	ENSGALG00010018441	CRIPT	-0.025573569	0.094754499	-0.269892924	0.787242631	0.821650485	negative
PRJNA729271	stage_E18_vs_E10	ordinary_muscle_development	ENSGALG00010018443	PLCB1	1.376634396	0.146903327	9.371022587	7.18332E-21	5.35883E-20	positive
PRJNA729271	stage_E18_vs_E10	ordinary_muscle_development	ENSGALG00010018444	NUBP2	-1.249623169	0.107118573	-11.66579361	1.90617E-31	2.51182E-30	negative
PRJNA729271	stage_E18_vs_E10	ordinary_muscle_development	ENSGALG00010018446	FBN1	2.137388018	0.23207175	9.210031033	3.26031E-20	2.31885E-19	positive
PRJNA729271	stage_E18_vs_E10	ordinary_muscle_development	ENSGALG00010018448	KLHDC3	3.090913927	0.164190861	18.8251277	4.70074E-79	3.1545E-77	positive
PRJNA729271	stage_E18_vs_E10	ordinary_muscle_development	ENSGALG00010018451	PIK3R4	-1.104399538	0.114778649	-9.621994578	6.45668E-22	5.13946E-21	negative
PRJNA729271	stage_E18_vs_E10	ordinary_muscle_development	ENSGALG00010018452	SPSB3	0.352728276	0.120634401	2.923944365	0.003456265	0.005849219	positive
PRJNA729271	stage_E18_vs_E10	ordinary_muscle_development	ENSGALG00010018453	TMX4	0.111848428	0.09250135	1.209154539	0.226603479	0.278027547	positive
PRJNA729271	stage_E18_vs_E10	ordinary_muscle_development	ENSGALG00010018455	RBM20	2.581755014	0.246944966	10.45477886	1.39323E-25	1.36729E-24	positive
PRJNA729271	stage_E18_vs_E10	ordinary_muscle_development	ENSGALG00010018458	SEMA5B	-0.293724647	0.1061932	-2.765945915	0.005675795	0.009327737	negative
PRJNA729271	stage_E18_vs_E10	ordinary_muscle_development	ENSGALG00010018459	SNX12	-0.037508107	0.103008032	-0.364127988	0.715762438	0.75912611	negative
PRJNA729271	stage_E18_vs_E10	ordinary_muscle_development	ENSGALG00010018462	KTN1	-0.142622305	0.116777765	-1.221313881	0.2219672	0.272916665	negative
PRJNA729271	stage_E18_vs_E10	ordinary_muscle_development	ENSGALG00010018464	ENSGALG00010018464	-2.773469015	1.16405273	-2.382597405	0.017190983	0.026224301	negative
PRJNA729271	stage_E18_vs_E10	ordinary_muscle_development	ENSGALG00010018465	NAALADL2	-1.099254473	0.199215944	-5.517904096	3.43067E-08	1.01505E-07	negative
PRJNA729271	stage_E18_vs_E10	ordinary_muscle_development	ENSGALG00010018466	DYSF	2.213158469	0.182520555	12.12553	7.73581E-34	1.12581E-32	positive
PRJNA729271	stage_E18_vs_E10	ordinary_muscle_development	ENSGALG00010018467	TLX1	-0.940655074	0.847325426	-1.110146167	0.266936046	0.321926782	negative
PRJNA729271	stage_E18_vs_E10	ordinary_muscle_development	ENSGALG00010018469	KLHDC7A	0.450130599	0.200126776	2.249227255	0.024498041	0.036383105	positive
PRJNA729271	stage_E18_vs_E10	ordinary_muscle_development	ENSGALG00010018470	IQCB1	1.178955979	0.15801277	7.461143662	8.57747E-14	3.96841E-13	positive
PRJNA729271	stage_E18_vs_E10	ordinary_muscle_development	ENSGALG00010018471	HHEX	1.147410925	0.181825832	6.310494554	2.78145E-10	9.8143E-10	positive
PRJNA729271	stage_E18_vs_E10	ordinary_muscle_development	ENSGALG00010018472	LMF1	0.531013675	0.14043114	3.781310004	0.000156005	0.000317419	positive
PRJNA729271	stage_E18_vs_E10	ordinary_muscle_development	ENSGALG00010018476	ENSGALG00010018476	-0.089105399	0.376468896	-0.23668728	0.812899404	0.844415713	negative
PRJNA729271	stage_E18_vs_E10	ordinary_muscle_development	ENSGALG00010018480	EME2	0.233027754	0.24419751	0.954259334	0.339952387	0.398319445	positive
PRJNA729271	stage_E18_vs_E10	ordinary_muscle_development	ENSGALG00010018482	TWNK	-0.677644665	0.129265632	-5.242264747	1.58618E-07	4.40368E-07	negative
PRJNA729271	stage_E18_vs_E10	ordinary_muscle_development	ENSGALG00010018484	NLGN1	-0.478999996	0.238576495	-2.007741777	0.044670739	0.063253441	negative
PRJNA729271	stage_E18_vs_E10	ordinary_muscle_development	ENSGALG00010018485	ATP2C1	-0.249302002	0.067369357	-3.700525179	0.000215154	0.000429448	negative
PRJNA729271	stage_E18_vs_E10	ordinary_muscle_development	ENSGALG00010018486	ENSGALG00010018486	0.467144352	0.101154648	4.618120481	3.87231E-06	9.37361E-06	positive
PRJNA729271	stage_E18_vs_E10	ordinary_muscle_development	ENSGALG00010018487	ENSGALG00010018487	-1.56045572	0.130030759	-12.00066612	3.52448E-33	4.93121E-32	negative
PRJNA729271	stage_E18_vs_E10	ordinary_muscle_development	ENSGALG00010018488	ENSGALG00010018488	1.0657846	0.154830815	6.883543164	5.83819E-12	2.34742E-11	positive
PRJNA729271	stage_E18_vs_E10	ordinary_muscle_development	ENSGALG00010018489	SLC7A3	-0.859260004	0.207197751	-4.147052749	3.36782E-05	7.38415E-05	negative
PRJNA729271	stage_E18_vs_E10	ordinary_muscle_development	ENSGALG00010018490	NME3	-0.486375176	0.121750904	-3.994838315	6.47384E-05	0.000137876	negative
PRJNA729271	stage_E18_vs_E10	ordinary_muscle_development	ENSGALG00010018491	TIA1	0.56416533	0.119807957	4.708913694	2.4904E-06	6.16014E-06	positive
PRJNA729271	stage_E18_vs_E10	ordinary_muscle_development	ENSGALG00010018492	ENSGALG00010018492	0.662010802	0.172502809	3.837681286	0.000124202	0.000256016	positive
PRJNA729271	stage_E18_vs_E10	ordinary_muscle_development	ENSGALG00010018494	HAO1	1.051262716	0.284743354	3.691965774	0.000222527	0.000443479	positive
PRJNA729271	stage_E18_vs_E10	ordinary_muscle_development	ENSGALG00010018495	ENSGALG00010018495	1.790212467	0.628462953	2.848556879	0.0043918	0.007333158	positive
PRJNA729271	stage_E18_vs_E10	ordinary_muscle_development	ENSGALG00010018496	GNG13	-1.563416098	0.525317783	-2.976133969	0.002919073	0.005007333	negative
PRJNA729271	stage_E18_vs_E10	ordinary_muscle_development	ENSGALG00010018497	ASTE1	-0.64571903	0.109581397	-5.892597167	3.80172E-09	1.22186E-08	negative
PRJNA729271	stage_E18_vs_E10	ordinary_muscle_development	ENSGALG00010018498	ENSGALG00010018498	5.647678748	0.378591767	14.91759526	2.53236E-50	7.50647E-49	positive
PRJNA729271	stage_E18_vs_E10	ordinary_muscle_development	ENSGALG00010018501	ATG14	-0.078679053	0.137149636	-0.573673073	0.566189053	0.619904096	negative
PRJNA729271	stage_E18_vs_E10	ordinary_muscle_development	ENSGALG00010018502	ARHGEF10L	0.86609378	0.122470749	7.071841958	1.5289E-12	6.45919E-12	positive
PRJNA729271	stage_E18_vs_E10	ordinary_muscle_development	ENSGALG00010018503	BMP2	-2.625647829	0.188534517	-13.92661606	4.36554E-44	9.90274E-43	negative
PRJNA729271	stage_E18_vs_E10	ordinary_muscle_development	ENSGALG00010018504	SLCO3A1	0.366218296	0.180652492	2.027197592	0.042642209	0.060550433	positive
PRJNA729271	stage_E18_vs_E10	ordinary_muscle_development	ENSGALG00010018505	CHTF18	-0.794826622	0.143648544	-5.533133874	3.14559E-08	9.33566E-08	negative
PRJNA729271	stage_E18_vs_E10	ordinary_muscle_development	ENSGALG00010018506	ENSGALG00010018506	0.841247225	0.143279229	5.871382953	4.32175E-09	1.38073E-08	positive
PRJNA729271	stage_E18_vs_E10	ordinary_muscle_development	ENSGALG00010018508	ENSGALG00010018508	-0.706342379	0.103269432	-6.839801143	7.93032E-12	3.14415E-11	negative
PRJNA729271	stage_E18_vs_E10	ordinary_muscle_development	ENSGALG00010018509	ENSGALG00010018509	-0.497927084	0.110491871	-4.506458966	6.59184E-06	1.56176E-05	negative
PRJNA729271	stage_E18_vs_E10	ordinary_muscle_development	ENSGALG00010018510	ENSGALG00010018510	0.692103767	0.126205967	5.483922694	4.15997E-08	1.22037E-07	positive
PRJNA729271	stage_E18_vs_E10	ordinary_muscle_development	ENSGALG00010018511	SOCS5	-0.122782605	0.101066885	-1.214864843	0.224417644	0.275578936	negative
PRJNA729271	stage_E18_vs_E10	ordinary_muscle_development	ENSGALG00010018512	FERMT1	-0.617642432	0.178751816	-3.455307167	0.000549666	0.001038505	negative
PRJNA729271	stage_E18_vs_E10	ordinary_muscle_development	ENSGALG00010018513	RPUSD1	0.269006003	0.154872517	1.736951191	0.082395787	0.111131193	positive
PRJNA729271	stage_E18_vs_E10	ordinary_muscle_development	ENSGALG00010018514	EAF2	-1.190679309	0.247718992	-4.806572561	1.5354E-06	3.8888E-06	negative
PRJNA729271	stage_E18_vs_E10	ordinary_muscle_development	ENSGALG00010018515	SEMA4G	0.537500232	0.173329148	3.101037755	0.001928437	0.003392729	positive
PRJNA729271	stage_E18_vs_E10	ordinary_muscle_development	ENSGALG00010018516	LRRN4	1.353881788	0.153087004	8.84387146	9.2458E-19	6.0669E-18	positive
PRJNA729271	stage_E18_vs_E10	ordinary_muscle_development	ENSGALG00010018517	ECT2	-1.620883869	0.198149764	-8.180094881	2.8362E-16	1.57573E-15	negative
PRJNA729271	stage_E18_vs_E10	ordinary_muscle_development	ENSGALG00010018518	ENSGALG00010018518	-0.842684882	0.899606312	-0.936726289	0.34889937	0.407682093	negative
PRJNA729271	stage_E18_vs_E10	ordinary_muscle_development	ENSGALG00010018519	OGDH	0.762196174	0.136718499	5.57493083	2.47628E-08	7.40687E-08	positive
PRJNA729271	stage_E18_vs_E10	ordinary_muscle_development	ENSGALG00010018520	ENSGALG00010018520	0.098899113	0.126891554	0.779398701	0.435744889	0.495459849	positive
PRJNA729271	stage_E18_vs_E10	ordinary_muscle_development	ENSGALG00010018521	ENSGALG00010018521	0.795910572	0.158948493	5.00734898	5.51848E-07	1.46235E-06	positive
PRJNA729271	stage_E18_vs_E10	ordinary_muscle_development	ENSGALG00010018522	NEK11	0.072639793	0.254254808	0.285696832	0.775110331	0.811441878	positive
PRJNA729271	stage_E18_vs_E10	ordinary_muscle_development	ENSGALG00010018523	TEX11	-0.027380721	0.173617583	-0.157707077	0.874687624	0.896604716	negative
PRJNA729271	stage_E18_vs_E10	ordinary_muscle_development	ENSGALG00010018524	SEC22A	-0.213245666	0.117132882	-1.820544854	0.068676072	0.094035487	negative
PRJNA729271	stage_E18_vs_E10	ordinary_muscle_development	ENSGALG00010018525	ST8SIA2	-1.792065331	0.291322242	-6.151488189	7.67592E-10	2.62412E-09	negative
PRJNA729271	stage_E18_vs_E10	ordinary_muscle_development	ENSGALG00010018526	ENSGALG00010018526	0.204499405	0.101683121	2.011144066	0.044310242	0.062771399	positive
PRJNA729271	stage_E18_vs_E10	ordinary_muscle_development	ENSGALG00010018527	CCT4	-1.099167661	0.081848825	-13.42924179	4.07563E-41	8.17099E-40	negative
PRJNA729271	stage_E18_vs_E10	ordinary_muscle_development	ENSGALG00010018528	ENSGALG00010018528	0.63862935	0.662626117	0.963785359	0.335153548	0.393555629	positive
PRJNA729271	stage_E18_vs_E10	ordinary_muscle_development	ENSGALG00010018529	UBR2	-0.173473516	0.093695462	-1.851461245	0.064103225	0.08819898	negative
PRJNA729271	stage_E18_vs_E10	ordinary_muscle_development	ENSGALG00010018530	FAM161A	-0.903463961	0.236762181	-3.815913323	0.00013568	0.000278409	negative
PRJNA729271	stage_E18_vs_E10	ordinary_muscle_development	ENSGALG00010018531	ADCY5	0.978101416	0.134979848	7.246277362	4.28382E-13	1.88564E-12	positive
PRJNA729271	stage_E18_vs_E10	ordinary_muscle_development	ENSGALG00010018532	MFSD13A	-0.332223414	0.129645442	-2.562553753	0.01039055	0.01640107	negative
PRJNA729271	stage_E18_vs_E10	ordinary_muscle_development	ENSGALG00010018533	PCYOX1	0.78520757	0.173583646	4.523511228	6.08221E-06	1.44717E-05	positive
PRJNA729271	stage_E18_vs_E10	ordinary_muscle_development	ENSGALG00010018534	NCEH1	-0.547261878	0.198243932	-2.760547933	0.005770449	0.009472555	negative
PRJNA729271	stage_E18_vs_E10	ordinary_muscle_development	ENSGALG00010018535	FAM174B	-0.258114537	0.104029918	-2.481156793	0.013095675	0.020371519	negative
PRJNA729271	stage_E18_vs_E10	ordinary_muscle_development	ENSGALG00010018536	ENSGALG00010018536	0.139430297	0.25508529	0.546602655	0.584651744	0.637230396	positive
PRJNA729271	stage_E18_vs_E10	ordinary_muscle_development	ENSGALG00010018537	ENSGALG00010018537	-0.793993219	0.697802109	-1.137848696	0.255183667	0.309038955	negative
PRJNA729271	stage_E18_vs_E10	ordinary_muscle_development	ENSGALG00010018539	CHD2	0.279136447	0.088286368	3.161716283	0.001568423	0.002789826	positive
PRJNA729271	stage_E18_vs_E10	ordinary_muscle_development	ENSGALG00010018540	TNFSF10	0.926401148	0.109014962	8.497926642	1.93007E-17	1.15988E-16	positive
PRJNA729271	stage_E18_vs_E10	ordinary_muscle_development	ENSGALG00010018542	ZMIZ2	3.226641095	0.261967148	12.31696845	7.3402E-35	1.12114E-33	positive
PRJNA729271	stage_E18_vs_E10	ordinary_muscle_development	ENSGALG00010018543	MRPL3	-0.754266532	0.078181846	-9.647591783	5.03236E-22	4.01674E-21	negative

PRJNA729271	stage_E18_vs_E10	ordinary_muscle_development	ENSGALG00010018544	HACD2	-0.834180127	0.102644328	-8.126899407	4.40411E-16	2.42176E-15	negative
PRJNA729271	stage_E18_vs_E10	ordinary_muscle_development	ENSGALG00010018546	SNRPGP15	-0.857981089	0.136265714	-6.296382722	3.04672E-10	1.07268E-09	negative
PRJNA729271	stage_E18_vs_E10	ordinary_muscle_development	ENSGALG00010018548	RGMA	-0.098451353	0.123665236	-0.796111789	0.425967062	0.486092978	negative
PRJNA729271	stage_E18_vs_E10	ordinary_muscle_development	ENSGALG00010018549	GHSR	-1.114963334	0.442013966	-2.52246178	0.011653661	0.018279388	negative
PRJNA729271	stage_E18_vs_E10	ordinary_muscle_development	ENSGALG00010018551	FBXO34	0.863654439	0.111706454	7.731464073	1.06317E-14	5.30303E-14	positive
PRJNA729271	stage_E18_vs_E10	ordinary_muscle_development	ENSGALG00010018552	DTD1	-0.175929107	0.104418766	-1.684841849	0.092019088	0.122875789	negative
PRJNA729271	stage_E18_vs_E10	ordinary_muscle_development	ENSGALG00010018553	ENSGALG00010018553	-1.040125765	0.497706665	-2.089836923	0.036632451	0.052656424	negative
PRJNA729271	stage_E18_vs_E10	ordinary_muscle_development	ENSGALG00010018554	PPIA	-0.445036723	0.123567988	-3.601553525	0.000316321	0.00061702	negative
PRJNA729271	stage_E18_vs_E10	ordinary_muscle_development	ENSGALG00010018555	UBN1	-0.604766192	0.076911482	-7.863145736	3.74605E-15	1.93097E-14	negative
PRJNA729271	stage_E18_vs_E10	ordinary_muscle_development	ENSGALG00010018556	MRPL2	1.411463505	0.12612731	11.19078421	4.52414E-29	5.25461E-28	positive
PRJNA729271	stage_E18_vs_E10	ordinary_muscle_development	ENSGALG00010018557	TBCC	0.124133025	0.103620462	1.197958613	0.23093313	0.282669795	positive
PRJNA729271	stage_E18_vs_E10	ordinary_muscle_development	ENSGALG00010018558	ENSGALG00010018558	1.813338251	0.222481773	8.1505025	3.62415E-16	2.00122E-15	positive
PRJNA729271	stage_E18_vs_E10	ordinary_muscle_development	ENSGALG00010018559	DLG3	-1.789135899	0.134537766	-13.29839159	2.36501E-40	4.583E-39	negative
PRJNA729271	stage_E18_vs_E10	ordinary_muscle_development	ENSGALG00010018560	ENSGALG00010018560	0.385341694	0.080788518	4.769758177	1.84447E-06	4.62888E-06	positive
PRJNA729271	stage_E18_vs_E10	ordinary_muscle_development	ENSGALG00010018561	ACTR1A	0.014854027	0.059317059	0.250417464	0.802264526	0.835044112	positive
PRJNA729271	stage_E18_vs_E10	ordinary_muscle_development	ENSGALG00010018562	ENSGALG00010018562	0.372171258	0.756620815	0.491886095	0.622799869	0.673341098	positive
PRJNA729271	stage_E18_vs_E10	ordinary_muscle_development	ENSGALG00010018564	DLGAP5	-1.639726603	0.153587778	-10.67615292	1.31613E-26	1.37247E-25	negative
PRJNA729271	stage_E18_vs_E10	ordinary_muscle_development	ENSGALG00010018566	ENSGALG00010018566	1.136224871	0.146318384	7.765427985	8.137E-15	4.09392E-14	positive
PRJNA729271	stage_E18_vs_E10	ordinary_muscle_development	ENSGALG00010018568	FAM136A	-0.031548509	0.193432462	-0.163098317	0.870441025	0.893368453	negative
PRJNA729271	stage_E18_vs_E10	ordinary_muscle_development	ENSGALG00010018573	SEC23B	-0.662495159	0.080070155	-8.27393376	1.2961E-16	7.38773E-16	negative
PRJNA729271	stage_E18_vs_E10	ordinary_muscle_development	ENSGALG00010018574	ENSGALG00010018574	-0.622983484	0.225924721	-2.757482582	0.005824832	0.00955858	negative
PRJNA729271	stage_E18_vs_E10	ordinary_muscle_development	ENSGALG00010018576	PTK7	-1.04919996	0.113259015	-9.263721398	1.97428E-20	1.42943E-19	negative
PRJNA729271	stage_E18_vs_E10	ordinary_muscle_development	ENSGALG00010018577	NARFL	2.105738012	0.07876434	26.73466206	1.862E-157	7.1024E-155	positive
PRJNA729271	stage_E18_vs_E10	ordinary_muscle_development	ENSGALG00010018579	FNDC3B	1.126996599	0.101106289	11.14665176	7.43517E-29	8.58748E-28	positive
PRJNA729271	stage_E18_vs_E10	ordinary_muscle_development	ENSGALG00010018581	SUFU	-0.055743216	0.091615889	-0.608444852	0.542892471	0.597874667	negative
PRJNA729271	stage_E18_vs_E10	ordinary_muscle_development	ENSGALG00010018582	RCC2	-1.20541096	0.111236187	-10.83650017	2.31145E-27	2.48734E-26	negative
PRJNA729271	stage_E18_vs_E10	ordinary_muscle_development	ENSGALG00010018583	PPL	2.468110704	0.213068632	11.58364178	4.9881E-31	6.43834E-30	positive
PRJNA729271	stage_E18_vs_E10	ordinary_muscle_development	ENSGALG00010018585	GDPD2	1.243535814	0.407026072	3.055174837	0.002249294	0.003916808	positive
PRJNA729271	stage_E18_vs_E10	ordinary_muscle_development	ENSGALG00010018587	SRF	0.627097812	0.173525198	3.61387175	0.000301658	0.000590325	positive
PRJNA729271	stage_E18_vs_E10	ordinary_muscle_development	ENSGALG00010018588	POLR3F	0.036991229	0.103867112	0.356139957	0.721735755	0.76434279	positive
PRJNA729271	stage_E18_vs_E10	ordinary_muscle_development	ENSGALG00010018590	TGFA	-0.996600743	0.569071166	-1.751276118	0.079898359	0.108074535	negative
PRJNA729271	stage_E18_vs_E10	ordinary_muscle_development	ENSGALG00010018593	LGALS3	-2.153724081	0.372646498	-5.779536615	7.49067E-09	2.33902E-08	negative
PRJNA729271	stage_E18_vs_E10	ordinary_muscle_development	ENSGALG00010018594	BICRAL	0.388075302	0.104410173	3.716834188	0.000201735	0.000404278	positive
PRJNA729271	stage_E18_vs_E10	ordinary_muscle_development	ENSGALG00010018598	ENSGALG00010018598	0.175043548	0.079311455	2.207039934	0.02731127	0.040206871	positive
PRJNA729271	stage_E18_vs_E10	ordinary_muscle_development	ENSGALG00010018601	CPNE4	-2.216680091	0.295958568	-7.489832479	6.89616E-14	3.21518E-13	negative
PRJNA729271	stage_E18_vs_E10	ordinary_muscle_development	ENSGALG00010018602	MCTP2	-0.51213017	0.222527115	-2.301428165	0.021367441	0.032068861	negative
PRJNA729271	stage_E18_vs_E10	ordinary_muscle_development	ENSGALG00010018604	FAM43A	1.413561894	0.123778166	11.42012309	3.31764E-30	4.10667E-29	positive
PRJNA729271	stage_E18_vs_E10	ordinary_muscle_development	ENSGALG00010018606	DZANK1	-0.573507277	0.250862134	-2.286145258	0.022245762	0.03327338	negative
PRJNA729271	stage_E18_vs_E10	ordinary_muscle_development	ENSGALG00010018607	HAGHL	1.081401531	0.1153283	9.376723079	6.8055E-21	5.08483E-20	positive
PRJNA729271	stage_E18_vs_E10	ordinary_muscle_development	ENSGALG00010018608	TRIM8	0.587218875	0.126171978	4.654114842	3.25375E-06	7.94662E-06	positive
PRJNA729271	stage_E18_vs_E10	ordinary_muscle_development	ENSGALG00010018609	ENSGALG00010018609	-0.339677579	0.127340912	-2.6674662	0.007642558	0.012292374	negative
PRJNA729271	stage_E18_vs_E10	ordinary_muscle_development	ENSGALG00010018611	NUDCD3	0.035850028	0.113917631	0.314701316	0.752988445	0.792691373	positive
PRJNA729271	stage_E18_vs_E10	ordinary_muscle_development	ENSGALG00010018613	PADI3	2.653025614	0.363631427	7.295919491	2.96626E-13	1.32092E-12	positive
PRJNA729271	stage_E18_vs_E10	ordinary_muscle_development	ENSGALG00010018615	HTR2C	1.594326685	0.233235121	6.835705868	8.16022E-12	3.23E-11	positive
PRJNA729271	stage_E18_vs_E10	ordinary_muscle_development	ENSGALG00010018616	ENSGALG00010018616	1.237150504	0.849430753	1.456446567	0.145269211	0.186475133	positive
PRJNA729271	stage_E18_vs_E10	ordinary_muscle_development	ENSGALG00010018618	FAHD1	1.079540716	0.169759281	6.359244145	2.02749E-10	7.22075E-10	positive
PRJNA729271	stage_E18_vs_E10	ordinary_muscle_development	ENSGALG00010018619	MAPK1IP1L	-0.163860901	0.076756198	-2.13482306	0.032775466	0.047546075	negative
PRJNA729271	stage_E18_vs_E10	ordinary_muscle_development	ENSGALG00010018623	ARRDC4	0.639171452	0.132810791	4.812646968	1.48944E-06	3.78232E-06	positive
PRJNA729271	stage_E18_vs_E10	ordinary_muscle_development	ENSGALG00010018624	ENSGALG00010018624	1.054671817	0.392584084	2.686486439	0.007220788	0.011657977	positive
PRJNA729271	stage_E18_vs_E10	ordinary_muscle_development	ENSGALG00010018627	MYLK	-0.529659015	0.180065447	-2.941480586	0.003266473	0.005555266	negative
PRJNA729271	stage_E18_vs_E10	ordinary_muscle_development	ENSGALG00010018628	XDH	-0.070400507	0.610391358	-0.115336671	0.908178273	0.924051949	negative
PRJNA729271	stage_E18_vs_E10	ordinary_muscle_development	ENSGALG00010018631	KAT14	-0.436263695	0.053674336	-8.12797555	4.36519E-16	2.40127E-15	negative
PRJNA729271	stage_E18_vs_E10	ordinary_muscle_development	ENSGALG00010018633	ENSGALG00010018633	0.559855932	0.186582955	3.000573826	0.002694714	0.004645009	positive
PRJNA729271	stage_E18_vs_E10	ordinary_muscle_development	ENSGALG00010018638	SEC14L5	0.20006567	0.171244743	1.168302551	0.242684747	0.295755457	positive
PRJNA729271	stage_E18_vs_E10	ordinary_muscle_development	ENSGALG00010018641	ENSGALG00010018641	-3.719406068	0.774135001	-4.80459618	1.55064E-06	3.92466E-06	negative
PRJNA729271	stage_E18_vs_E10	ordinary_muscle_development	ENSGALG00010018644	ESF1	-0.680337896	0.098740882	-6.890133894	5.57399E-12	2.24805E-11	negative
PRJNA729271	stage_E18_vs_E10	ordinary_muscle_development	ENSGALG00010018649	HAGH	1.326529542	0.107491017	12.34084092	5.45828E-35	8.41678E-34	positive
PRJNA729271	stage_E18_vs_E10	ordinary_muscle_development	ENSGALG00010018654	ENSGALG00010018654	-0.411696641	0.23970637	-1.717503962	0.085887131	0.115303692	negative
PRJNA729271	stage_E18_vs_E10	ordinary_muscle_development	ENSGALG00010018655	BIRC5	-1.622323856	0.209402805	-7.747383601	9.38053E-15	4.69999E-14	negative
PRJNA729271	stage_E18_vs_E10	ordinary_muscle_development	ENSGALG00010018662	PADI1	1.636185091	0.844969577	1.936383434	0.052820757	0.073819027	positive
PRJNA729271	stage_E18_vs_E10	ordinary_muscle_development	ENSGALG00010018665	IL13RA2	-0.332588929	0.330213389	-1.007193953	0.313841579	0.371327539	negative
PRJNA729271	stage_E18_vs_E10	ordinary_muscle_development	ENSGALG00010018668	OVOL2	-4.993353722	1.001997985	-4.983396971	6.24776E-07	1.64537E-06	negative
PRJNA729271	stage_E18_vs_E10	ordinary_muscle_development	ENSGALG00010018670	MRAS	0.891175307	0.213254347	4.178931499	2.92882E-05	6.46956E-05	positive
PRJNA729271	stage_E18_vs_E10	ordinary_muscle_development	ENSGALG00010018672	PKDCC	-0.893073574	0.098266597	-9.088272102	1.00625E-19	6.96213E-19	negative
PRJNA729271	stage_E18_vs_E10	ordinary_muscle_development	ENSGALG00010018673	NAGPA	-0.027081394	0.092121182	-0.29397576	0.768776426	0.805847549	negative
PRJNA729271	stage_E18_vs_E10	ordinary_muscle_development	ENSGALG00010018684	PADI2	4.837240915	0.994414562	4.864410783	1.14798E-06	2.94827E-06	positive
PRJNA729271	stage_E18_vs_E10	ordinary_muscle_development	ENSGALG00010018685	ARL3	-1.678150002	0.104794023	-16.01379496	1.02371E-57	3.80478E-56	negative
PRJNA729271	stage_E18_vs_E10	ordinary_muscle_development	ENSGALG00010018693	ENSGALG00010018693	0.06770616	0.169166877	0.400232962	0.688984939	0.734704384	positive
PRJNA729271	stage_E18_vs_E10	ordinary_muscle_development	ENSGALG00010018694	MGME1	-1.014947971	0.103099657	-9.844338972	7.25143E-23	6.04425E-22	negative
PRJNA729271	stage_E18_vs_E10	ordinary_muscle_development	ENSGALG00010018698	MRPS16	0.510388005	0.116297908	4.388625841	1.14069E-05	2.63958E-05	positive
PRJNA729271	stage_E18_vs_E10	ordinary_muscle_development	ENSGALG00010018701	SFXN2	-1.047691952	0.12272144	-8.537154974	1.37567E-17	8.36774E-17	negative
PRJNA729271	stage_E18_vs_E10	ordinary_muscle_development	ENSGALG00010018704	PGPEP1L	0.592278885	0.453226839	1.306804526	0.191279108	0.238913457	positive
PRJNA729271	stage_E18_vs_E10	ordinary_muscle_development	ENSGALG00010018707	SLC45A1	0.768941455	0.099083472	7.760542125	8.45671E-15	4.2474E-14	positive
PRJNA729271	stage_E18_vs_E10	ordinary_muscle_development	ENSGALG00010018709	SNX5	0.006955501	0.113643969	0.061204309	0.951196498	0.959871432	positive
PRJNA729271	stage_E18_vs_E10	ordinary_muscle_development	ENSGALG00010018713	ENSGALG00010018713	-0.325128734	0.13807121	-2.354790207	0.018533166	0.028091419	negative
PRJNA729271	stage_E18_vs_E10	ordinary_muscle_development	ENSGALG00010018715	DNAJC13	-0.38744505	0.103572908	-3.740795319	0.000183439	0.000369606	negative
PRJNA729271	stage_E18_vs_E10	ordinary_muscle_development	ENSGALG00010018717	MEIOB	0.146689634	0.566262296	0.259048915	0.795597499	0.82923605	positive
PRJNA729271	stage_E18_vs_E10	ordinary_muscle_development	ENSGALG00010018719	ERRFI1	-0.8070697	0.092175937	-8.755752605	2.02746E-18	1.30382E-17	negative
PRJNA729271	stage_E18_vs_E10	ordinary_muscle_development	ENSGALG00010018721	LRCH2	1.030645478	0.188303074	5.47333326	4.41648E-08	1.29145E-07	positive
PRJNA729271	stage_E18_vs_E10	ordinary_muscle_development	ENSGALG00010018724	RRBP1	0.159242064	0.141141762	1.128242001	0.25921773	0.313296173	positive
PRJNA729271	stage_E18_vs_E10	ordinary_muscle_development	ENSGALG00010018726	EBF2	-0.345470132	0.155839248	-2.2168365	0.026634266	0.039290015	negative
PRJNA729271	stage_E18_vs_E10	ordinary_muscle_development	ENSGALG00010018727	PARK7	-0.333132739	0.133302526	-2.49907296	0.012451867	0.019432581	negative
PRJNA729271	stage_E18_vs_E10	ordinary_muscle_development	ENSGALG00010018729	HS3ST6	-5.0971897	0.475696794	-10.71520718	8.63637E-27	9.06475E-26	negative
PRJNA729271	stage_E18_vs_E10	ordinary_muscle_development	ENSGALG00010018730	IGF1R	0.047762214	0.087238817	0.547488104	0.584043441	0.636711017	positive
PRJNA729271	stage_E18_vs_E10	ordinary_muscle_development	ENSGALG00010018732	ENSGALG00010018732	2.08809031	0.220078497	9.487934251	2.35658E-21	1.81021E-20	positive
PRJNA729271	stage_E18_vs_E10	ordinary_muscle_development	ENSGALG00010018736	EEF2KMT	0.188358829	0.132640944	1.4200655	0.155588613	0.198378289	positive
PRJNA729271	stage_E18_vs_E10	ordinary_muscle_development	ENSGALG00010018747	FLRT3	-1.573863745	0.122585907	-12.83886362	9.93217E-38	1.73454E-36	negative
PRJNA729271	stage_E18_vs_E10	ordinary_muscle_development	ENSGALG00010018750	SOCS4	0.548429656	0.125537299	4.368659041	1.25012E-05	2.88267E-05	positive
PRJNA729271	stage_E18_vs_E10	ordinary_muscle_development	ENSGALG00010018752	PLS3	-0.764859949	0.141030405	-5.423369155	5.8486E-08	1.68808E-07	negative
PRJNA729271	stage_E18_vs_E10	ordinary_muscle_development	ENSGALG00010018753	SYNM	0.758224492	0.154335363	4.912837066	8.97679E-07	2.33522E-06	positive
PRJNA729271	stage_E18_vs_E10	ordinary_muscle_development	ENSGALG00010018756	EML4	-1.229231916	0.062359475	-19.71203128	1.70004E-86	1.43268E-84	negative
PRJNA729271	stage_E18_vs_E10	ordinary_muscle_development	ENSGALG00010018758	ENSGALG00010018758	-1.458156548	0.141695304	-10.29078954	7.7538E-25	7.30288E-24	negative
PRJNA729271	stage_E18_vs_E10	ordinary_muscle_development	ENSGALG00010018764	TMEM177	-0.096320329	0.092180417	-1.044910966	0.296064121	0.352828203	negative
PRJNA729271	stage_E18_vs_E10	ordinary_muscle_development	ENSGALG00010018767	NDUFB6	1.281112995	0.169181907	7.572399521	3.66393E-14	1.73899E-13	positive
PRJNA729271	stage_E18_vs_E10	ordinary_muscle_development	ENSGALG00010018774	MSRB1	0.094594359	0.12519805	0.755557767	0.449914385	0.509453091	positive
PRJNA729271	stage_E18_vs_E10	ordinary_muscle_development	ENSGALG00010018780	WDHD1	-1.837348267	0.205369009	-8.946570256	3.66699E-19	2.46421E-18	negative
PRJNA729271	stage_E18_vs_E10	ordinary_muscle_development	ENSGALG00010018783	ENSGALG00010018783	-0.240399029	0.178890367	-1.343834398	0.179001944	0.225072274	negative
PRJNA729271	stage_E18_vs_E10	ordinary_muscle_development	ENSGALG00010018786	DSTN	-1.091567038	0.114916633	-9.498773269	2.12377E-21	1.63484E-20	negative
PRJNA729271	stage_E18_vs_E10	ordinary_muscle_development	ENSGALG00010018796	PER3	-0.466793164	0.314222506	-1.485549748	0.137398256	0.177408491	negative
PRJNA729271	stage_E18_vs_E10	ordinary_muscle_development	ENSGALG00010018799	ENSGALG00010018799	-2.053672157	0.369421059	-5.559163739	2.7107E-08	8.08636E-08	negative
PRJNA729271	stage_E18_vs_E10	ordinary_muscle_development	ENSGALG00010018806	GCH1	-0.876518371	0.323240189	-2.711662725	0.006694667	0.01087274	negative
PRJNA729271	stage_E18_vs_E10	ordinary_muscle_development	ENSGALG00010018807	RD3	-2.993909316	0.267179826	-11.20559647	3.82767E-29	4.46715E-28	negative
PRJNA729271	stage_E18_vs_E10	ordinary_muscle_development	ENSGALG00010018808	MATCAP2	-0.988911399	0.091063311	-10.85960299	1.79528E-27	1.95512E-26	negative
PRJNA729271	stage_E18_vs_E10	ordinary_muscle_development	ENSGALG00010018811	VAMP3	0.731092443	0.124085456	5.891846347	3.81904E-09	1.22661E-08	positive
PRJNA729271	stage_E18_vs_E10	ordinary_muscle_development	ENSGALG00010018815	ENSGALG00010018815	1.146691481	0.17034277	6.731670971	1.67726E-11	6.47452E-11	positive
PRJNA729271	stage_E18_vs_E10	ordinary_muscle_development	ENSGALG00010018819	LRRC28	0.139163678	0.079053023	1.760384008	0.078342719	0.106128758	positive
PRJNA729271	stage_E18_vs_E10	ordinary_muscle_development	ENSGALG00010018822	TRAF5	1.261860305	0.195403666	6.457710502	1.06299E-10	3.86456E-10	positive
PRJNA729271	stage_E18_vs_E10	ordinary_muscle_development	ENSGALG00010018823	RAB40C	-0.173947298	0.075991848	-2.289025757	0.022077856	0.033042697	negative
PRJNA729271	stage_E18_vs_E10	ordinary_muscle_development	ENSGALG00010018829	PTPN4	0.968203312	0.139228201	6.954074711	3.54884E-12	1.456E-11	positive
PRJNA729271	stage_E18_vs_E10	ordinary_muscle_development	ENSGALG00010018831	WBP1L	1.937436947	0.240065763	8.070442547	7.00438E-16	3.8072E-15	positive
PRJNA729271	stage_E18_vs_E10	ordinary_muscle_development	ENSGALG00010018832	EEPD1	2.098068551	0.183919673	11.40752654	3.83479E-30	4.72262E-29	positive
PRJNA729271	stage_E18_vs_E10	ordinary_muscle_development	ENSGALG00010018834	SAMD4A	0.865718129	0.1719466	5.034808068	4.78328E-07	1.27358E-06	positive
PRJNA729271	stage_E18_vs_E10	ordinary_muscle_development	ENSGALG00010018835	CAMK2B	1.910040847	0.171363815	11.14611537	7.48011E-29	8.6325E-28	positive
PRJNA729271	stage_E18_vs_E10	ordinary_muscle_development	ENSGALG00010018837	RCOR3	-0.618135916	0.094803698	-6.520166726	7.02293E-11	2.5929E-10	negative
PRJNA729271	stage_E18_vs_E10	ordinary_muscle_development	ENSGALG00010018838	WFIKKN1	0.713694773	0.208053193	3.430347613	0.000602808	0.001132855	positive
PRJNA729271	stage_E18_vs_E10	ordinary_muscle_development	ENSGALG00010018839	RPL3L	1.647987885	0.271456791	6.070903132	1.27193E-09	4.24709E-09	positive
PRJNA729271	stage_E18_vs_E10	ordinary_muscle_development	ENSGALG00010018843	MCRIP2	1.922664137	0.236092673	8.143684054	3.83429E-16	2.11323E-15	positive
PRJNA729271	stage_E18_vs_E10	ordinary_muscle_development	ENSGALG00010018847	CGRRF1	-0.982763087	0.167265314	-5.875474503	4.21634E-09	1.34765E-08	negative
PRJNA729271	stage_E18_vs_E10	ordinary_muscle_development	ENSGALG00010018848	MEF2A	1.856675191	0.114511514	16.21387342	4.02388E-59	1.56791E-57	positive
PRJNA729271	stage_E18_vs_E10	ordinary_muscle_development	ENSGALG00010018849	KCNH1	-0.468901043	0.300446786	-1.560679175	0.118599467	0.155209397	negative
PRJNA729271	stage_E18_vs_E10	ordinary_muscle_development	ENSGALG00010018852	NDUFB10	1.05249595	0.116159687	9.060767804	1.29535E-19	8.91133E-19	positive
PRJNA729271	stage_E18_vs_E10	ordinary_muscle_development	ENSGALG00010018854	LYSMD4	0.250782535	0.172315138	1.455371465	0.145566452	0.186823598	positive
PRJNA729271	stage_E18_vs_E10	ordinary_muscle_development	ENSGALG00010018855	SYNDIG1	0.238709158	0.557517514	0.428164411	0.668531425	0.715525585	positive
PRJNA729271	stage_E18_vs_E10	ordinary_muscle_development	ENSGALG00010018857	ENSGALG00010018857	0.243974941	0.082725715	2.949203157	0.003185945	0.005432973	positive
PRJNA729271	stage_E18_vs_E10	ordinary_muscle_development	ENSGALG00010018858	EPB41L5	-0.251036995	0.114670127	-2.18921006	0.028581576	0.04191097	negative
PRJNA729271	stage_E18_vs_E10	ordinary_muscle_development	ENSGALG00010018860	AGTR2	2.139819582	0.214748399	9.964309828	2.18385E-23	1.86865E-22	positive
PRJNA729271	stage_E18_vs_E10	ordinary_muscle_development	ENSGALG00010018864	ABCA3	0.080304579	0.120147189	0.668384995	0.503887871	0.561058126	positive
PRJNA729271	stage_E18_vs_E10	ordinary_muscle_development	ENSGALG00010018865	ENSGALG00010018865	-1.676612563	0.232176055	-7.221298354	5.14935E-13	2.2509E-12	negative
PRJNA729271	stage_E18_vs_E10	ordinary_muscle_development	ENSGALG00010018867	RPS2	0.699748282	0.155921438	4.487825992	7.19537E-06	1.69943E-05	positive
PRJNA729271	stage_E18_vs_E10	ordinary_muscle_development	ENSGALG00010018872	KCTD9	0.003702353	0.101552329	0.03645759	0.970917494	0.975899666	positive
PRJNA729271	stage_E18_vs_E10	ordinary_muscle_development	ENSGALG00010018874	ENSGALG00010018874	-0.323518412	0.11654816	-2.775834584	0.005506021	0.009065173	negative

PRJNA729271	stage_E18_vs_E10	ordinary_muscle_development	ENSGALG00010018876	GMFB	-0.989533259	0.098299738	-10.06648927	7.77023E-24	6.83846E-23	negative
PRJNA729271	stage_E18_vs_E10	ordinary_muscle_development	ENSGALG00010018877	CST7	2.770211571	0.866284512	3.197808033	0.001384764	0.002481414	positive
PRJNA729271	stage_E18_vs_E10	ordinary_muscle_development	ENSGALG00010018880	TMEM17	-0.0375745	0.256123218	-0.14670478	0.883365046	0.90375327	negative
PRJNA729271	stage_E18_vs_E10	ordinary_muscle_development	ENSGALG00010018881	ENSGALG00010018881	0.523594349	0.644082725	0.812930278	0.416258034	0.47655479	positive
PRJNA729271	stage_E18_vs_E10	ordinary_muscle_development	ENSGALG00010018882	HHAT	-0.154972262	0.157220919	-0.985697472	0.324281591	0.382306763	negative
PRJNA729271	stage_E18_vs_E10	ordinary_muscle_development	ENSGALG00010018883	TBL3	-1.008299919	0.076006918	-13.2658967	3.65028E-40	7.03602E-39	negative
PRJNA729271	stage_E18_vs_E10	ordinary_muscle_development	ENSGALG00010018885	EFEMP1	2.219483506	0.207150287	10.71436367	8.71545E-27	9.14113E-26	positive
PRJNA729271	stage_E18_vs_E10	ordinary_muscle_development	ENSGALG00010018887	ADAMTS17	0.026241183	0.199149609	0.131766178	0.895169239	0.913058766	positive
PRJNA729271	stage_E18_vs_E10	ordinary_muscle_development	ENSGALG00010018891	GNRH1	-3.69433848	0.461140108	-8.011314598	1.13489E-15	6.07018E-15	negative
PRJNA729271	stage_E18_vs_E10	ordinary_muscle_development	ENSGALG00010018892	APMAP	-0.034936452	0.1234587	-0.282980886	0.777191489	0.81315076	negative
PRJNA729271	stage_E18_vs_E10	ordinary_muscle_development	ENSGALG00010018894	SERTAD4	-0.133120352	0.091564742	-1.453838561	0.14599107	0.187304798	negative
PRJNA729271	stage_E18_vs_E10	ordinary_muscle_development	ENSGALG00010018895	SEPTIN7	-0.639638342	0.089814866	-7.121742402	1.06571E-12	4.55947E-12	negative
PRJNA729271	stage_E18_vs_E10	ordinary_muscle_development	ENSGALG00010018897	CCDC85A	-0.290465144	0.167270116	-1.736503511	0.082474844	0.11121625	negative
PRJNA729271	stage_E18_vs_E10	ordinary_muscle_development	ENSGALG00010018899	ACSS1	0.019039834	0.100158029	0.190097926	0.849232395	0.875257649	positive
PRJNA729271	stage_E18_vs_E10	ordinary_muscle_development	ENSGALG00010018901	TCF7L2	-0.184555174	0.147812273	-1.248578145	0.21181941	0.261972896	negative
PRJNA729271	stage_E18_vs_E10	ordinary_muscle_development	ENSGALG00010018902	NOXO1	-3.391918267	0.896249082	-3.784570981	0.000153974	0.000313506	negative
PRJNA729271	stage_E18_vs_E10	ordinary_muscle_development	ENSGALG00010018903	CAMTA1	-0.248833649	0.097783905	-2.544730112	0.010936223	0.017209917	negative
PRJNA729271	stage_E18_vs_E10	ordinary_muscle_development	ENSGALG00010018904	UGP2	1.016011881	0.271351444	3.744265621	0.000180922	0.000364941	positive
PRJNA729271	stage_E18_vs_E10	ordinary_muscle_development	ENSGALG00010018908	ENSGALG00010018908	0.412418245	0.234641167	1.757655101	0.078806212	0.106696809	positive
PRJNA729271	stage_E18_vs_E10	ordinary_muscle_development	ENSGALG00010018909	TTBK1	0.173680897	0.132330964	1.312473601	0.189360387	0.236700484	positive
PRJNA729271	stage_E18_vs_E10	ordinary_muscle_development	ENSGALG00010018911	CNIH1	-0.326276524	0.094770102	-3.442821298	0.00057568	0.001084825	negative
PRJNA729271	stage_E18_vs_E10	ordinary_muscle_development	ENSGALG00010018913	ENSGALG00010018913	0.568155834	0.224531252	2.530408704	0.011392973	0.017891782	positive
PRJNA729271	stage_E18_vs_E10	ordinary_muscle_development	ENSGALG00010018915	DOCK5	-1.071091885	0.192535126	-5.563098583	2.65026E-08	7.90931E-08	negative
PRJNA729271	stage_E18_vs_E10	ordinary_muscle_development	ENSGALG00010018916	CERS3	-1.300208558	0.339237708	-3.832735944	0.000126726	0.000261122	negative
PRJNA729271	stage_E18_vs_E10	ordinary_muscle_development	ENSGALG00010018917	DNAJC11	-0.524368455	0.106640204	-4.917174139	8.78024E-07	2.28614E-06	negative
PRJNA729271	stage_E18_vs_E10	ordinary_muscle_development	ENSGALG00010018919	RALB	-0.076299904	0.107765185	-0.708019975	0.478932853	0.537365058	negative
PRJNA729271	stage_E18_vs_E10	ordinary_muscle_development	ENSGALG00010018920	ACAD11	-0.494220018	0.117532463	-4.204966064	2.61121E-05	5.80603E-05	negative
PRJNA729271	stage_E18_vs_E10	ordinary_muscle_development	ENSGALG00010018922	EEF2K	2.491352983	0.113912605	21.87074019	4.935E-106	6.6854E-104	positive
PRJNA729271	stage_E18_vs_E10	ordinary_muscle_development	ENSGALG00010018923	VPS54	-0.122397871	0.092663583	-1.320884294	0.186539947	0.233618156	negative
PRJNA729271	stage_E18_vs_E10	ordinary_muscle_development	ENSGALG00010018925	LINS1	-0.417082367	0.13454223	-3.100010798	0.001935136	0.003403537	negative
PRJNA729271	stage_E18_vs_E10	ordinary_muscle_development	ENSGALG00010018926	SLC22A7	4.544078474	0.853625079	5.323271991	1.01917E-07	2.88872E-07	positive
PRJNA729271	stage_E18_vs_E10	ordinary_muscle_development	ENSGALG00010018932	CDKN3	-1.298385042	0.232228501	-5.590980589	2.25791E-08	6.76486E-08	negative
PRJNA729271	stage_E18_vs_E10	ordinary_muscle_development	ENSGALG00010018933	PHF13	0.292537688	0.087890938	3.328416961	0.00087341	0.00160498	positive
PRJNA729271	stage_E18_vs_E10	ordinary_muscle_development	ENSGALG00010018936	SYT14	4.489719378	0.212887323	21.08965119	9.89887E-99	1.11228E-96	positive
PRJNA729271	stage_E18_vs_E10	ordinary_muscle_development	ENSGALG00010018937	INHBB	-2.296372025	0.333731288	-6.880901205	5.94751E-12	2.38873E-11	negative
PRJNA729271	stage_E18_vs_E10	ordinary_muscle_development	ENSGALG00010018941	KLHL21	1.960472493	0.143700516	13.64276582	2.22942E-42	4.69019E-41	positive
PRJNA729271	stage_E18_vs_E10	ordinary_muscle_development	ENSGALG00010018943	ACKR4	2.030252916	0.352144596	5.765395632	8.14665E-09	2.53839E-08	positive
PRJNA729271	stage_E18_vs_E10	ordinary_muscle_development	ENSGALG00010018944	NEFL	-2.120756706	0.154811195	-13.69898802	1.02949E-42	2.20421E-41	negative
PRJNA729271	stage_E18_vs_E10	ordinary_muscle_development	ENSGALG00010018945	HABP2	0.783813482	0.674828263	1.161500674	0.245438349	0.298684315	positive
PRJNA729271	stage_E18_vs_E10	ordinary_muscle_development	ENSGALG00010018947	ENSGALG00010018947	0.190084281	0.224916435	0.845132908	0.398036636	0.458045494	positive
PRJNA729271	stage_E18_vs_E10	ordinary_muscle_development	ENSGALG00010018948	POLR3E	-0.506387821	0.111160193	-4.555478052	5.22666E-06	1.25224E-05	negative
PRJNA729271	stage_E18_vs_E10	ordinary_muscle_development	ENSGALG00010018950	TTL	-0.217007689	0.097184315	-2.232949731	0.02555226	0.037828539	negative
PRJNA729271	stage_E18_vs_E10	ordinary_muscle_development	ENSGALG00010018951	YKT6	-0.194093965	0.088549156	-2.191934678	0.028384224	0.041655292	negative
PRJNA729271	stage_E18_vs_E10	ordinary_muscle_development	ENSGALG00010018955	ZBTB48	0.460728801	0.14400098	3.199483789	0.001376739	0.00246856	positive
PRJNA729271	stage_E18_vs_E10	ordinary_muscle_development	ENSGALG00010018958	PELI3	0.116828278	0.117772498	0.991982679	0.321205975	0.378927371	positive
PRJNA729271	stage_E18_vs_E10	ordinary_muscle_development	ENSGALG00010018959	UBA5	-0.297044328	0.117232851	-2.533797705	0.011283385	0.017733131	negative
PRJNA729271	stage_E18_vs_E10	ordinary_muscle_development	ENSGALG00010018960	RBFOX1	-0.24523098	0.219829114	-1.115552781	0.264613614	0.319365057	negative
PRJNA729271	stage_E18_vs_E10	ordinary_muscle_development	ENSGALG00010018961	ASB7	0.188626055	0.098398669	1.916957387	0.055243346	0.076973209	positive
PRJNA729271	stage_E18_vs_E10	ordinary_muscle_development	ENSGALG00010018968	GLI2	-0.83370874	0.196525632	-4.2422392	2.21301E-05	4.95865E-05	negative
PRJNA729271	stage_E18_vs_E10	ordinary_muscle_development	ENSGALG00010018969	DIEXF	-0.686276292	0.091305411	-7.516271838	5.63603E-14	2.64212E-13	negative
PRJNA729271	stage_E18_vs_E10	ordinary_muscle_development	ENSGALG00010018971	POLR1B	-0.431494779	0.100224325	-4.305289958	1.66767E-05	3.79302E-05	negative
PRJNA729271	stage_E18_vs_E10	ordinary_muscle_development	ENSGALG00010018973	NEFM	-5.652056229	0.628841007	-8.988052892	2.51649E-19	1.70212E-18	negative
PRJNA729271	stage_E18_vs_E10	ordinary_muscle_development	ENSGALG00010018974	TAS1R1	0.772502385	0.297259564	2.598746952	0.009356472	0.014856179	positive
PRJNA729271	stage_E18_vs_E10	ordinary_muscle_development	ENSGALG00010018977	NPHP3	0.187246855	0.110852674	1.689150547	0.091190583	0.121848037	positive
PRJNA729271	stage_E18_vs_E10	ordinary_muscle_development	ENSGALG00010018983	MDGA2	-0.920568456	0.170261969	-5.40677675	6.4169E-08	1.84586E-07	negative
PRJNA729271	stage_E18_vs_E10	ordinary_muscle_development	ENSGALG00010018985	NOL9	-0.388759492	0.089807017	-4.328832037	1.49902E-05	3.42664E-05	negative
PRJNA729271	stage_E18_vs_E10	ordinary_muscle_development	ENSGALG00010018986	CTDSP1	1.12901132	0.192191065	5.874421467	4.24323E-09	1.35594E-08	positive
PRJNA729271	stage_E18_vs_E10	ordinary_muscle_development	ENSGALG00010018987	MRPS10	-0.235661274	0.109729064	-2.147665027	0.031740376	0.046178535	negative
PRJNA729271	stage_E18_vs_E10	ordinary_muscle_development	ENSGALG00010018990	GCK	-1.36492639	0.41364476	-3.299755058	0.000967692	0.001765698	negative
PRJNA729271	stage_E18_vs_E10	ordinary_muscle_development	ENSGALG00010018991	RYBP	-1.409950783	0.131774632	-10.69971333	1.02094E-26	1.06761E-25	negative
PRJNA729271	stage_E18_vs_E10	ordinary_muscle_development	ENSGALG00010018993	ENSGALG00010018993	0.849279666	0.462591973	1.835915268	0.066370189	0.091091709	positive
PRJNA729271	stage_E18_vs_E10	ordinary_muscle_development	ENSGALG00010018998	RPS29	2.745781662	0.439935913	6.241321936	4.33888E-10	1.51401E-09	positive
PRJNA729271	stage_E18_vs_E10	ordinary_muscle_development	ENSGALG00010018999	THUMPD3	-1.078265693	0.07376827	-14.61693079	2.19055E-48	5.97967E-47	negative
PRJNA729271	stage_E18_vs_E10	ordinary_muscle_development	ENSGALG00010019000	TRERF1	0.285961835	0.1195933	2.391119203	0.016797098	0.025679486	positive
PRJNA729271	stage_E18_vs_E10	ordinary_muscle_development	ENSGALG00010019001	ALDH1A3	1.379294076	0.159739957	8.634621582	5.89218E-18	3.67342E-17	positive
PRJNA729271	stage_E18_vs_E10	ordinary_muscle_development	ENSGALG00010019004	ECE2	-0.089312698	0.209158105	-0.427010456	0.669371712	0.716266275	negative
PRJNA729271	stage_E18_vs_E10	ordinary_muscle_development	ENSGALG00010019005	ENSGALG00010019005	-0.065036051	0.21772633	-0.298705495	0.765164764	0.80294384	negative
PRJNA729271	stage_E18_vs_E10	ordinary_muscle_development	ENSGALG00010019006	ENSGALG00010019006	-0.430594014	0.078753948	-5.467586412	4.56205E-08	1.3324E-07	negative
PRJNA729271	stage_E18_vs_E10	ordinary_muscle_development	ENSGALG00010019008	HERPUD2	0.170855716	0.045413903	3.762189694	0.000168432	0.000340934	positive
PRJNA729271	stage_E18_vs_E10	ordinary_muscle_development	ENSGALG00010019009	NRAP	3.932261347	0.218378547	18.00662844	1.72841E-72	9.63588E-71	positive
PRJNA729271	stage_E18_vs_E10	ordinary_muscle_development	ENSGALG00010019011	VIL1	1.360123658	0.411295885	3.306922603	0.00094327	0.001723738	positive
PRJNA729271	stage_E18_vs_E10	ordinary_muscle_development	ENSGALG00010019012	ADAM28	0.683916552	0.357116823	1.915105948	0.055478993	0.077279263	positive
PRJNA729271	stage_E18_vs_E10	ordinary_muscle_development	ENSGALG00010019013	LRRK1	-0.68304091	0.105746757	-6.459213749	1.05248E-10	3.82733E-10	negative
PRJNA729271	stage_E18_vs_E10	ordinary_muscle_development	ENSGALG00010019015	GSG1L	-3.583587518	1.005620015	-3.563560258	0.000365859	0.000707932	negative
PRJNA729271	stage_E18_vs_E10	ordinary_muscle_development	ENSGALG00010019016	PLEKHG5	0.592727075	0.145051777	4.086313774	4.38281E-05	9.49467E-05	positive
PRJNA729271	stage_E18_vs_E10	ordinary_muscle_development	ENSGALG00010019017	IRAK2	-0.424002446	0.083914059	-5.052817723	4.35339E-07	1.16447E-06	negative
PRJNA729271	stage_E18_vs_E10	ordinary_muscle_development	ENSGALG00010019018	SH3BGRL	-1.367627562	0.110572071	-12.36865288	3.86245E-35	5.98143E-34	negative
PRJNA729271	stage_E18_vs_E10	ordinary_muscle_development	ENSGALG00010019019	TBX20	0.033907266	0.318370097	0.106502672	0.915183536	0.929939387	positive
PRJNA729271	stage_E18_vs_E10	ordinary_muscle_development	ENSGALG00010019020	KCNK2	2.226558208	0.439924965	5.061222671	4.16576E-07	1.11654E-06	positive
PRJNA729271	stage_E18_vs_E10	ordinary_muscle_development	ENSGALG00010019021	LRR1	-1.241685014	0.154556413	-8.033862788	9.44508E-16	5.08946E-15	negative
PRJNA729271	stage_E18_vs_E10	ordinary_muscle_development	ENSGALG00010019023	AP2M1	-0.153084514	0.071656208	-2.136374778	0.032648878	0.047381407	negative
PRJNA729271	stage_E18_vs_E10	ordinary_muscle_development	ENSGALG00010019024	CNPY3	0.370205527	0.101445774	3.6492947	0.000262961	0.000519699	positive
PRJNA729271	stage_E18_vs_E10	ordinary_muscle_development	ENSGALG00010019025	DNAAF2	-0.683262868	0.249957428	-2.733516952	0.00626619	0.010231882	negative
PRJNA729271	stage_E18_vs_E10	ordinary_muscle_development	ENSGALG00010019026	CHSY1	-1.012361623	0.124655061	-8.121303803	4.61202E-16	2.53512E-15	negative
PRJNA729271	stage_E18_vs_E10	ordinary_muscle_development	ENSGALG00010019027	GNLY	-0.011010329	0.329356989	-0.033429771	0.973331869	0.978055143	negative
PRJNA729271	stage_E18_vs_E10	ordinary_muscle_development	ENSGALG00010019028	TNFRSF25	4.634950545	0.724989558	6.393127315	1.62527E-10	5.8327E-10	positive
PRJNA729271	stage_E18_vs_E10	ordinary_muscle_development	ENSGALG00010019029	CENPF	-1.598957044	0.259933046	-6.151418877	7.67928E-10	2.62464E-09	negative
PRJNA729271	stage_E18_vs_E10	ordinary_muscle_development	ENSGALG00010019030	CDR2	-1.344872113	0.151519629	-8.875893651	6.93741E-19	4.59378E-18	negative
PRJNA729271	stage_E18_vs_E10	ordinary_muscle_development	ENSGALG00010019032	GNMT	1.13220472	0.187479211	6.039094743	1.54981E-09	5.14416E-09	positive
PRJNA729271	stage_E18_vs_E10	ordinary_muscle_development	ENSGALG00010019033	CYP2AB4	-2.572136493	1.158639498	-2.219962721	0.026421298	0.039003638	negative
PRJNA729271	stage_E18_vs_E10	ordinary_muscle_development	ENSGALG00010019034	CASP7	-0.290022033	0.176333696	-1.644734043	0.100024669	0.132588713	negative
PRJNA729271	stage_E18_vs_E10	ordinary_muscle_development	ENSGALG00010019035	ENSGALG00010019035	-1.26425349	0.495922961	-2.549294125	0.010794122	0.017001065	negative
PRJNA729271	stage_E18_vs_E10	ordinary_muscle_development	ENSGALG00010019036	REL	0.223082012	0.111538524	2.0000445	0.045495459	0.064337237	positive
PRJNA729271	stage_E18_vs_E10	ordinary_muscle_development	ENSGALG00010019038	PLEKHS1	-4.420916172	0.594525286	-7.436043974	1.03745E-13	4.76335E-13	negative
PRJNA729271	stage_E18_vs_E10	ordinary_muscle_development	ENSGALG00010019040	HMGN5	-1.984561326	0.08270339	-23.99613035	3.0519E-127	6.3196E-125	negative
PRJNA729271	stage_E18_vs_E10	ordinary_muscle_development	ENSGALG00010019041	KATNIP	0.044612695	0.08850395	0.504075752	0.614208146	0.664845573	positive
PRJNA729271	stage_E18_vs_E10	ordinary_muscle_development	ENSGALG00010019042	PEX6	0.693593005	0.105923787	6.548038209	5.82978E-11	2.16562E-10	positive
PRJNA729271	stage_E18_vs_E10	ordinary_muscle_development	ENSGALG00010019043	METTL9	-1.180712311	0.105165396	-11.22719408	2.9985E-29	3.52786E-28	negative
PRJNA729271	stage_E18_vs_E10	ordinary_muscle_development	ENSGALG00010019045	LBX1	2.045439465	0.2640278	7.747060962	9.40438E-15	4.71031E-14	positive
PRJNA729271	stage_E18_vs_E10	ordinary_muscle_development	ENSGALG00010019046	SELENOS	0.181821881	0.081758583	2.223887378	0.026156023	0.038635641	positive
PRJNA729271	stage_E18_vs_E10	ordinary_muscle_development	ENSGALG00010019047	GHRL	-0.454125025	0.447774503	-1.01418241	0.31049572	0.36815014	negative
PRJNA729271	stage_E18_vs_E10	ordinary_muscle_development	ENSGALG00010019049	POLD2	-0.651091003	0.163866383	-3.973304292	7.08824E-05	0.000150211	negative
PRJNA729271	stage_E18_vs_E10	ordinary_muscle_development	ENSGALG00010019050	TNS1	1.124909198	0.133696596	8.413895576	3.96614E-17	2.34172E-16	positive
PRJNA729271	stage_E18_vs_E10	ordinary_muscle_development	ENSGALG00010019051	IGSF6	0.97118368	0.26659797	3.642877246	0.000269607	0.000531768	positive
PRJNA729271	stage_E18_vs_E10	ordinary_muscle_development	ENSGALG00010019059	BRWD3	0.116337865	0.078859918	1.475247084	0.140146127	0.180602606	positive
PRJNA729271	stage_E18_vs_E10	ordinary_muscle_development	ENSGALG00010019062	PUS10	-0.825005639	0.100579485	-8.202523999	2.35392E-16	1.31534E-15	negative
PRJNA729271	stage_E18_vs_E10	ordinary_muscle_development	ENSGALG00010019064	ENSGALG00010019064	-0.422075691	0.172077255	-2.452826728	0.014173859	0.02195918	negative
PRJNA729271	stage_E18_vs_E10	ordinary_muscle_development	ENSGALG00010019065	CAND2	1.63180475	0.121704501	13.4079244	5.43384E-41	1.0834E-39	positive
PRJNA729271	stage_E18_vs_E10	ordinary_muscle_development	ENSGALG00010019067	GTF3C1	-0.447661432	0.058511074	-7.650883878	1.99603E-14	9.70234E-14	negative
PRJNA729271	stage_E18_vs_E10	ordinary_muscle_development	ENSGALG00010019068	SNRPA1	-0.493266986	0.164130452	-3.005334972	0.002652885	0.004578896	negative
PRJNA729271	stage_E18_vs_E10	ordinary_muscle_development	ENSGALG00010019072	AEBP1	1.894437561	0.211160271	8.971562455	2.92338E-19	1.97365E-18	positive
PRJNA729271	stage_E18_vs_E10	ordinary_muscle_development	ENSGALG00010019076	ESPN	0.07382905	0.388966933	0.189808037	0.849459558	0.875429527	positive
PRJNA729271	stage_E18_vs_E10	ordinary_muscle_development	ENSGALG00010019079	CAPG	0.743034879	0.249353164	2.979849412	0.002883901	0.00495479	positive
PRJNA729271	stage_E18_vs_E10	ordinary_muscle_development	ENSGALG00010019081	PCSK6	0.798216719	0.137991453	5.784537401	7.27121E-09	2.27245E-08	positive
PRJNA729271	stage_E18_vs_E10	ordinary_muscle_development	ENSGALG00010019082	POLR3K	0.364331575	0.18163708	2.005821581	0.044875287	0.063515994	positive
PRJNA729271	stage_E18_vs_E10	ordinary_muscle_development	ENSGALG00010019083	OTOA	-0.678366128	0.235243881	-2.883671722	0.003930683	0.006604294	negative
PRJNA729271	stage_E18_vs_E10	ordinary_muscle_development	ENSGALG00010019087	PEX13	0.053761452	0.088268973	0.609063984	0.542482029	0.597513451	positive
PRJNA729271	stage_E18_vs_E10	ordinary_muscle_development	ENSGALG00010019093	RPL32	0.726367479	0.15175334	4.786500773	1.69714E-06	4.27976E-06	positive
PRJNA729271	stage_E18_vs_E10	ordinary_muscle_development	ENSGALG00010019094	SNRNP25	0.787527997	0.167395717	4.704588692	2.54378E-06	6.28681E-06	positive
PRJNA729271	stage_E18_vs_E10	ordinary_muscle_development	ENSGALG00010019097	STC1	-3.260959599	0.294568841	-11.07028018	1.74853E-28	1.97853E-27	negative
PRJNA729271	stage_E18_vs_E10	ordinary_muscle_development	ENSGALG00010019101	POLE2	-1.129865862	0.121229205	-9.320079766	1.16253E-20	8.54939E-20	negative
PRJNA729271	stage_E18_vs_E10	ordinary_muscle_development	ENSGALG00010019104	POLL	0.073265504	0.121792547	0.60155983	0.547467175	0.6020436	positive
PRJNA729271	stage_E18_vs_E10	ordinary_muscle_development	ENSGALG00010019108	KDELR2	-0.012364064	0.099531712	-0.124222354	0.901139223	0.918082212	negative
PRJNA729271	stage_E18_vs_E10	ordinary_muscle_development	ENSGALG00010019110	MBD4	-0.285725856	0.116774614	-2.446814811	0.014412487	0.022307421	negative
PRJNA729271	stage_E18_vs_E10	ordinary_muscle_development	ENSGALG00010019113	KLHDC1	1.50126391	0.133297642	11.26249415	2.00988E-29	2.38406E-28	positive
PRJNA729271	stage_E18_vs_E10	ordinary_muscle_development	ENSGALG00010019114	TENT5D	-1.291677338	0.374395191	-3.450037203	0.000560509	0.001057613	negative
PRJNA729271	stage_E18_vs_E10	ordinary_muscle_development	ENSGALG00010019115	DBNL	-0.735365997	0.102821252	-7.151887181	8.55929E-13	3.69577E-12	negative
PRJNA729271	stage_E18_vs_E10	ordinary_muscle_development	ENSGALG00010019120	DAGLB	-0.201564806	0.088050564	-2.289193818	0.022068094	0.033031497	negative
PRJNA729271	stage_E18_vs_E10	ordinary_muscle_development	ENSGALG00010019123	PLEKHO2	0.910903221	0.118462508	7.689379834	1.4785E-14	7.27703E-14	positive
PRJNA729271	stage_E18_vs_E10	ordinary_muscle_development	ENSGALG00010019124	BMPER	-0.976278732	0.250199395	-3.902002767	9.54001E-05	0.00019934	negative
PRJNA729271	stage_E18_vs_E10	ordinary_muscle_development	ENSGALG00010019125	ENSGALG00010019125	0.974076804	0.203914293	4.776893213	1.78024E-06	4.47686E-06	positive

PRJNA729271	stage_E18_vs_E10	ordinary_muscle_development	ENSGALG00010019127	TMBIM1	0.923997921	0.122415785	7.548029239	4.41894E-14	2.08436E-13	positive
PRJNA729271	stage_E18_vs_E10	ordinary_muscle_development	ENSGALG00010019130	RPS6KC1	0.253224218	0.082042751	3.086491057	0.00202534	0.003554154	positive
PRJNA729271	stage_E18_vs_E10	ordinary_muscle_development	ENSGALG00010019132	ENSGALG00010019132	-0.427874313	0.153510203	-2.787269547	0.005315425	0.008778293	negative
PRJNA729271	stage_E18_vs_E10	ordinary_muscle_development	ENSGALG00010019135	KIAA1841	-1.995634496	0.210850418	-9.464693087	2.94424E-21	2.25089E-20	negative
PRJNA729271	stage_E18_vs_E10	ordinary_muscle_development	ENSGALG00010019139	DPCD	-0.446628588	0.130610116	-3.419555858	0.000627235	0.00117571	negative
PRJNA729271	stage_E18_vs_E10	ordinary_muscle_development	ENSGALG00010019141	ACOT7	-1.004193893	0.157627283	-6.370685813	1.88185E-10	6.71691E-10	negative
PRJNA729271	stage_E18_vs_E10	ordinary_muscle_development	ENSGALG00010019144	IFT122	-1.102673392	0.074747108	-14.75205426	2.9844E-49	8.49881E-48	negative
PRJNA729271	stage_E18_vs_E10	ordinary_muscle_development	ENSGALG00010019145	RAC1	-0.527201227	0.077540382	-6.799053756	1.05309E-11	4.13335E-11	negative
PRJNA729271	stage_E18_vs_E10	ordinary_muscle_development	ENSGALG00010019146	KLHDC2	0.277205178	0.086134419	3.218285786	0.001289593	0.002318899	positive
PRJNA729271	stage_E18_vs_E10	ordinary_muscle_development	ENSGALG00010019148	ENSGALG00010019148	0.685808043	0.216699465	3.164788813	0.001551956	0.002763926	positive
PRJNA729271	stage_E18_vs_E10	ordinary_muscle_development	ENSGALG00010019149	COX5B	0.785324312	0.180827317	4.342951744	1.40581E-05	3.22424E-05	positive
PRJNA729271	stage_E18_vs_E10	ordinary_muscle_development	ENSGALG00010019158	ENSGALG00010019158	2.340317435	0.254135649	9.20893014	3.29391E-20	2.34046E-19	positive
PRJNA729271	stage_E18_vs_E10	ordinary_muscle_development	ENSGALG00010019166	PNPT1	-0.847151679	0.111339529	-7.608723425	2.76816E-14	1.32731E-13	negative
PRJNA729271	stage_E18_vs_E10	ordinary_muscle_development	ENSGALG00010019169	GPR153	-1.551124948	0.200401591	-7.740083016	9.93519E-15	4.9676E-14	negative
PRJNA729271	stage_E18_vs_E10	ordinary_muscle_development	ENSGALG00010019172	NEMF	-0.258401981	0.088091864	-2.933324032	0.003353537	0.005688516	negative
PRJNA729271	stage_E18_vs_E10	ordinary_muscle_development	ENSGALG00010019173	RHO	-3.204570212	1.033736315	-3.099988039	0.001935285	0.003403537	negative
PRJNA729271	stage_E18_vs_E10	ordinary_muscle_development	ENSGALG00010019174	ENSGALG00010019174	-0.690540456	0.506477492	-1.363417854	0.172750857	0.21798761	negative
PRJNA729271	stage_E18_vs_E10	ordinary_muscle_development	ENSGALG00010019183	ANGEL2	-0.351647518	0.082507108	-4.262026942	2.02581E-05	4.56248E-05	negative
PRJNA729271	stage_E18_vs_E10	ordinary_muscle_development	ENSGALG00010019184	PNKD	-0.429669653	0.188624186	-2.27791389	0.022731709	0.03395467	negative
PRJNA729271	stage_E18_vs_E10	ordinary_muscle_development	ENSGALG00010019185	ANKDD1A	-0.066210181	0.2183453	-0.30323612	0.761709925	0.800071403	negative
PRJNA729271	stage_E18_vs_E10	ordinary_muscle_development	ENSGALG00010019188	ENSGALG00010019188	2.793883413	0.372442004	7.501526089	6.30791E-14	2.9466E-13	positive
PRJNA729271	stage_E18_vs_E10	ordinary_muscle_development	ENSGALG00010019189	ICMT	-0.303630999	0.095896591	-3.166233502	0.001544268	0.002750573	negative
PRJNA729271	stage_E18_vs_E10	ordinary_muscle_development	ENSGALG00010019193	ENSGALG00010019193	3.000231097	1.0831172	2.769996725	0.005605686	0.009221929	positive
PRJNA729271	stage_E18_vs_E10	ordinary_muscle_development	ENSGALG00010019194	ENSGALG00010019194	0.551016467	0.187983248	2.931199848	0.003376555	0.005722339	positive
PRJNA729271	stage_E18_vs_E10	ordinary_muscle_development	ENSGALG00010019204	ARF6	-0.888803576	0.23080711	-3.850850077	0.000117709	0.000243428	negative
PRJNA729271	stage_E18_vs_E10	ordinary_muscle_development	ENSGALG00010019205	ENSGALG00010019205	-0.577509422	0.266165514	-2.169737971	0.030026702	0.043874702	negative
PRJNA729271	stage_E18_vs_E10	ordinary_muscle_development	ENSGALG00010019206	RNF207	0.555580156	0.283872199	1.957148876	0.050329967	0.070637128	positive
PRJNA729271	stage_E18_vs_E10	ordinary_muscle_development	ENSGALG00010019208	PLXND1	1.065036368	0.157965041	6.74222829	1.55976E-11	6.04996E-11	positive
PRJNA729271	stage_E18_vs_E10	ordinary_muscle_development	ENSGALG00010019209	VCPKMT	0.309826214	0.085533433	3.62228201	0.000292016	0.000573002	positive
PRJNA729271	stage_E18_vs_E10	ordinary_muscle_development	ENSGALG00010019212	RPL22	0.493819815	0.113836901	4.337959063	1.43812E-05	3.29261E-05	positive
PRJNA729271	stage_E18_vs_E10	ordinary_muscle_development	ENSGALG00010019213	RHBDF1	0.731226811	0.10950396	6.677628919	2.42839E-11	9.28257E-11	positive
PRJNA729271	stage_E18_vs_E10	ordinary_muscle_development	ENSGALG00010019215	ENSGALG00010019215	0.09580893	0.101809163	0.941063918	0.346672105	0.405373681	positive
PRJNA729271	stage_E18_vs_E10	ordinary_muscle_development	ENSGALG00010019218	RMND5A	0.260250794	0.13870849	1.876242719	0.060621959	0.083798902	positive
PRJNA729271	stage_E18_vs_E10	ordinary_muscle_development	ENSGALG00010019221	VASH2	0.51278792	0.231601375	2.214097046	0.026822104	0.039546983	positive
PRJNA729271	stage_E18_vs_E10	ordinary_muscle_development	ENSGALG00010019223	AAMP	-0.04338477	0.12606835	-0.344136889	0.730743342	0.772415334	negative
PRJNA729271	stage_E18_vs_E10	ordinary_muscle_development	ENSGALG00010019225	PROK2	-4.76612272	0.741643426	-6.426434256	1.30632E-10	4.72079E-10	negative
PRJNA729271	stage_E18_vs_E10	ordinary_muscle_development	ENSGALG00010019227	CLCN5	0.031422444	0.07407004	0.424226096	0.671400931	0.718225571	positive
PRJNA729271	stage_E18_vs_E10	ordinary_muscle_development	ENSGALG00010019230	SPG21	-0.439622131	0.098685232	-4.454791471	8.39748E-06	1.97216E-05	negative
PRJNA729271	stage_E18_vs_E10	ordinary_muscle_development	ENSGALG00010019231	ENSGALG00010019231	1.809948725	0.311863897	5.803649414	6.48869E-09	2.04109E-08	positive
PRJNA729271	stage_E18_vs_E10	ordinary_muscle_development	ENSGALG00010019232	SOS2	0.147101571	0.095270912	1.544034463	0.122579984	0.159898926	positive
PRJNA729271	stage_E18_vs_E10	ordinary_muscle_development	ENSGALG00010019233	ARPC2	-0.817591711	0.054268557	-15.06566149	2.72425E-51	8.34842E-50	negative
PRJNA729271	stage_E18_vs_E10	ordinary_muscle_development	ENSGALG00010019236	MPG	0.299984821	0.207725159	1.444142934	0.148698773	0.190489502	positive
PRJNA729271	stage_E18_vs_E10	ordinary_muscle_development	ENSGALG00010019237	SORCS3	-1.913462794	0.339527419	-5.635665013	1.74384E-08	5.27373E-08	negative
PRJNA729271	stage_E18_vs_E10	ordinary_muscle_development	ENSGALG00010019239	CYTH3	0.146237664	0.081283756	1.799100712	0.072002751	0.098228694	positive
PRJNA729271	stage_E18_vs_E10	ordinary_muscle_development	ENSGALG00010019240	ENSGALG00010019240	0.715847213	0.198356461	3.608892854	0.000307507	0.000601283	positive
PRJNA729271	stage_E18_vs_E10	ordinary_muscle_development	ENSGALG00010019243	SERTAD2	1.319443527	0.153097036	8.618347951	6.7927E-18	4.2131E-17	positive
PRJNA729271	stage_E18_vs_E10	ordinary_muscle_development	ENSGALG00010019246	IL17RC	1.050553754	0.134941231	7.785268805	6.95651E-15	3.51708E-14	positive
PRJNA729271	stage_E18_vs_E10	ordinary_muscle_development	ENSGALG00010019247	HBZ	-1.788147964	0.434436252	-4.116019225	3.85472E-05	8.40416E-05	negative
PRJNA729271	stage_E18_vs_E10	ordinary_muscle_development	ENSGALG00010019248	RMI2	0.011422436	0.166606762	0.06855926	0.945340448	0.95509234	positive
PRJNA729271	stage_E18_vs_E10	ordinary_muscle_development	ENSGALG00010019250	CXCR1	0.432153303	0.249686153	1.73078602	0.083489936	0.112408195	positive
PRJNA729271	stage_E18_vs_E10	ordinary_muscle_development	ENSGALG00010019252	TATDN3	0.024396136	0.117807274	0.207085139	0.835943368	0.863932857	positive
PRJNA729271	stage_E18_vs_E10	ordinary_muscle_development	ENSGALG00010019253	HBAD	-0.467357243	0.426323564	-1.096250085	0.272969347	0.328165438	negative
PRJNA729271	stage_E18_vs_E10	ordinary_muscle_development	ENSGALG00010019255	ENSGALG00010019255	2.814288979	0.198340479	14.1891811	1.069E-45	2.59116E-44	positive
PRJNA729271	stage_E18_vs_E10	ordinary_muscle_development	ENSGALG00010019256	BBS9	-0.599935805	0.104128149	-5.761514142	8.33627E-09	2.59468E-08	negative
PRJNA729271	stage_E18_vs_E10	ordinary_muscle_development	ENSGALG00010019257	SLC1A4	-0.783050009	0.158679344	-4.934794835	8.02351E-07	2.09626E-06	negative
PRJNA729271	stage_E18_vs_E10	ordinary_muscle_development	ENSGALG00010019259	L2HGDH	0.153122771	0.109407227	1.399567243	0.161642949	0.205365461	positive
PRJNA729271	stage_E18_vs_E10	ordinary_muscle_development	ENSGALG00010019260	TRAIL-LIKE	0.175354895	0.175986	0.99641389	0.319049087	0.376720146	positive
PRJNA729271	stage_E18_vs_E10	ordinary_muscle_development	ENSGALG00010019262	TMC3	-3.108991384	0.489310164	-6.353825479	2.10025E-10	7.47071E-10	negative
PRJNA729271	stage_E18_vs_E10	ordinary_muscle_development	ENSGALG00010019263	SNRPB2	-1.044246596	0.103179624	-10.12066684	4.47357E-24	3.99781E-23	negative
PRJNA729271	stage_E18_vs_E10	ordinary_muscle_development	ENSGALG00010019264	HK2	0.507821159	0.214891367	2.363152909	0.018120191	0.027523655	positive
PRJNA729271	stage_E18_vs_E10	ordinary_muscle_development	ENSGALG00010019265	RUFY4	0.618888341	0.907349035	0.68208409	0.49518578	0.552472128	positive
PRJNA729271	stage_E18_vs_E10	ordinary_muscle_development	ENSGALG00010019266	ATP5S	-0.297390793	0.161007232	-1.847064816	0.064737747	0.089004424	negative
PRJNA729271	stage_E18_vs_E10	ordinary_muscle_development	ENSGALG00010019267	STARD5	-1.759266128	0.208698038	-8.429720499	3.46492E-17	2.0508E-16	negative
PRJNA729271	stage_E18_vs_E10	ordinary_muscle_development	ENSGALG00010019268	NSL1	-0.83611944	0.094412576	-8.856017696	8.29237E-19	5.4561E-18	negative
PRJNA729271	stage_E18_vs_E10	ordinary_muscle_development	ENSGALG00010019269	SOCS1	-0.187633891	0.259918415	-0.721895334	0.470358831	0.529213013	negative
PRJNA729271	stage_E18_vs_E10	ordinary_muscle_development	ENSGALG00010019270	ENSGALG00010019270	-2.725093446	0.242758622	-11.22552693	3.0556E-29	3.59212E-28	negative
PRJNA729271	stage_E18_vs_E10	ordinary_muscle_development	ENSGALG00010019271	CDKL1	-0.323243037	0.194774976	-1.659571693	0.097000647	0.128993062	negative
PRJNA729271	stage_E18_vs_E10	ordinary_muscle_development	ENSGALG00010019274	ENSGALG00010019274	0.436975878	0.584687147	0.747366998	0.45484206	0.514470204	positive
PRJNA729271	stage_E18_vs_E10	ordinary_muscle_development	ENSGALG00010019275	CTAGE1	1.406163328	0.589131045	2.38684303	0.016993745	0.025950731	positive
PRJNA729271	stage_E18_vs_E10	ordinary_muscle_development	ENSGALG00010019277	IGFBP5	0.876439912	0.132704429	6.604451118	3.98993E-11	1.49907E-10	positive
PRJNA729271	stage_E18_vs_E10	ordinary_muscle_development	ENSGALG00010019278	JPT2	-1.420575777	0.073398146	-19.35438219	1.87226E-83	1.41346E-81	negative
PRJNA729271	stage_E18_vs_E10	ordinary_muscle_development	ENSGALG00010019280	TLNRD1	-0.158385703	0.117175084	-1.351701207	0.176470919	0.222177887	negative
PRJNA729271	stage_E18_vs_E10	ordinary_muscle_development	ENSGALG00010019281	ENSGALG00010019281	-3.126048674	1.359848722	-2.298820908	0.021515111	0.032260426	negative
PRJNA729271	stage_E18_vs_E10	ordinary_muscle_development	ENSGALG00010019282	IGFBP2	0.556619819	0.152695729	3.645287414	0.000267093	0.000527239	positive
PRJNA729271	stage_E18_vs_E10	ordinary_muscle_development	ENSGALG00010019283	NT5C3A	0.296399565	0.256211548	1.156854822	0.247331679	0.300609818	positive
PRJNA729271	stage_E18_vs_E10	ordinary_muscle_development	ENSGALG00010019284	CHD5	0.670594699	0.133679699	5.016428864	5.26408E-07	1.39774E-06	positive
PRJNA729271	stage_E18_vs_E10	ordinary_muscle_development	ENSGALG00010019285	BATF3	0.811330848	0.265493313	3.055937037	0.002243584	0.003907597	positive
PRJNA729271	stage_E18_vs_E10	ordinary_muscle_development	ENSGALG00010019287	AHSA2	-0.605301612	0.098755396	-6.129301634	8.82656E-10	2.99909E-09	negative
PRJNA729271	stage_E18_vs_E10	ordinary_muscle_development	ENSGALG00010019288	RPL37A	0.83297016	0.156701886	5.315635834	1.06286E-07	3.00841E-07	positive
PRJNA729271	stage_E18_vs_E10	ordinary_muscle_development	ENSGALG00010019289	FKBP9	-0.457491265	0.166380358	-2.749671115	0.005965511	0.009768423	negative
PRJNA729271	stage_E18_vs_E10	ordinary_muscle_development	ENSGALG00010019291	ADD2	-0.286350978	0.271488068	-1.05474609	0.291541466	0.347838798	negative
PRJNA729271	stage_E18_vs_E10	ordinary_muscle_development	ENSGALG00010019292	ENSGALG00010019292	-0.276910016	0.095874234	-2.888263134	0.003873757	0.006515445	negative
PRJNA729271	stage_E18_vs_E10	ordinary_muscle_development	ENSGALG00010019294	SMARCAL1	-0.626437748	0.099301738	-6.30842682	2.81886E-10	9.94145E-10	negative
PRJNA729271	stage_E18_vs_E10	ordinary_muscle_development	ENSGALG00010019295	MAP4K5	-0.110952117	0.130534493	-0.84998313	0.395334466	0.455333578	negative
PRJNA729271	stage_E18_vs_E10	ordinary_muscle_development	ENSGALG00010019296	ENSGALG00010019296	-0.121850049	0.104789615	-1.162806538	0.244908004	0.298164078	negative
PRJNA729271	stage_E18_vs_E10	ordinary_muscle_development	ENSGALG00010019297	SLC25A26	0.578220741	0.148699409	3.88852076	0.000100857	0.000210288	positive
PRJNA729271	stage_E18_vs_E10	ordinary_muscle_development	ENSGALG00010019298	PGAP6	0.420221335	0.093705124	4.484507543	7.30825E-06	1.72473E-05	positive
PRJNA729271	stage_E18_vs_E10	ordinary_muscle_development	ENSGALG00010019300	MESDC2	-0.383162721	0.066498721	-5.761956285	8.31445E-09	2.589E-08	negative
PRJNA729271	stage_E18_vs_E10	ordinary_muscle_development	ENSGALG00010019302	USP34	-0.325020699	0.095035577	-3.419989751	0.000626235	0.001174292	negative
PRJNA729271	stage_E18_vs_E10	ordinary_muscle_development	ENSGALG00010019305	SNRNP27	-0.233483289	0.120610297	-1.935848723	0.052886232	0.073894923	negative
PRJNA729271	stage_E18_vs_E10	ordinary_muscle_development	ENSGALG00010019307	ATF3	1.952187036	0.486738605	4.010750361	6.05261E-05	0.000129361	positive
PRJNA729271	stage_E18_vs_E10	ordinary_muscle_development	ENSGALG00010019310	CRELD1	0.772288244	0.09732115	7.935461556	2.09714E-15	1.09859E-14	positive
PRJNA729271	stage_E18_vs_E10	ordinary_muscle_development	ENSGALG00010019312	KBTBD2	-0.289605407	0.074552248	-3.884596583	0.0001025	0.000213591	negative
PRJNA729271	stage_E18_vs_E10	ordinary_muscle_development	ENSGALG00010019313	MRPL28	-0.274898847	0.100275255	-2.741442525	0.006117006	0.010000677	negative
PRJNA729271	stage_E18_vs_E10	ordinary_muscle_development	ENSGALG00010019314	GKN2	-3.371645488	1.265975235	-2.663279181	0.007738318	0.012439495	negative
PRJNA729271	stage_E18_vs_E10	ordinary_muscle_development	ENSGALG00010019316	CLEC16A	-0.080466645	0.088873751	-0.905403951	0.365251423	0.424802967	negative
PRJNA729271	stage_E18_vs_E10	ordinary_muscle_development	ENSGALG00010019318	NOG2	0.485760533	0.27797449	1.747500404	0.080550575	0.108875474	positive
PRJNA729271	stage_E18_vs_E10	ordinary_muscle_development	ENSGALG00010019319	ACTR3	-0.991754359	0.067179433	-14.76276762	2.54614E-49	7.26502E-48	negative
PRJNA729271	stage_E18_vs_E10	ordinary_muscle_development	ENSGALG00010019322	EGR3	0.688371355	0.33380896	2.062171595	0.039191402	0.056078911	positive
PRJNA729271	stage_E18_vs_E10	ordinary_muscle_development	ENSGALG00010019323	LRIG1	0.414341902	0.119261802	3.474221377	0.000512338	0.000972289	positive
PRJNA729271	stage_E18_vs_E10	ordinary_muscle_development	ENSGALG00010019325	SLITRK4	-2.963102079	0.218695376	-13.54899283	8.03247E-42	1.6515E-40	negative
PRJNA729271	stage_E18_vs_E10	ordinary_muscle_development	ENSGALG00010019331	CEMIP	1.164270089	0.301574056	3.860644068	0.000113089	0.000234408	positive
PRJNA729271	stage_E18_vs_E10	ordinary_muscle_development	ENSGALG00010019334	ATL1	-0.302242224	0.12950764	-2.333779107	0.019607291	0.029608052	negative
PRJNA729271	stage_E18_vs_E10	ordinary_muscle_development	ENSGALG00010019335	HIPK3	1.237209986	0.115262356	10.73385992	7.05848E-27	7.45176E-26	positive
PRJNA729271	stage_E18_vs_E10	ordinary_muscle_development	ENSGALG00010019336	DEXI	1.204672394	0.239549896	5.028899666	4.93302E-07	1.31273E-06	positive
PRJNA729271	stage_E18_vs_E10	ordinary_muscle_development	ENSGALG00010019337	XPO1	-0.024698372	0.084141598	-0.293533429	0.769114452	0.806096455	negative
PRJNA729271	stage_E18_vs_E10	ordinary_muscle_development	ENSGALG00010019344	DNAJC24	-0.277153416	0.08674852	-3.194906562	0.001398761	0.002504328	negative
PRJNA729271	stage_E18_vs_E10	ordinary_muscle_development	ENSGALG00010019345	ENSGALG00010019345	-0.098992786	0.18809084	-0.526303066	0.598677653	0.650219735	negative
PRJNA729271	stage_E18_vs_E10	ordinary_muscle_development	ENSGALG00010019346	POU3F4	0.231939821	0.491082768	0.472302911	0.636710593	0.686128915	positive
PRJNA729271	stage_E18_vs_E10	ordinary_muscle_development	ENSGALG00010019350	SAV1	0.013693349	0.088655863	0.154455091	0.877250919	0.898449238	positive
PRJNA729271	stage_E18_vs_E10	ordinary_muscle_development	ENSGALG00010019355	ENTPD4	0.18603503	0.100013778	1.860094022	0.062872225	0.086603906	positive
PRJNA729271	stage_E18_vs_E10	ordinary_muscle_development	ENSGALG00010019356	AVL9	-0.069942796	0.096048596	-0.728202168	0.466489839	0.525594265	negative
PRJNA729271	stage_E18_vs_E10	ordinary_muscle_development	ENSGALG00010019365	ENSGALG00010019365	0.482303743	0.176858549	2.727059249	0.006390156	0.01041436	positive
PRJNA729271	stage_E18_vs_E10	ordinary_muscle_development	ENSGALG00010019371	TMEM87A	0.471344003	0.077074891	6.115402752	9.63134E-10	3.25726E-09	positive
PRJNA729271	stage_E18_vs_E10	ordinary_muscle_development	ENSGALG00010019373	DENND5A	-0.012357239	0.062086731	-0.199031891	0.842237798	0.869470614	negative
PRJNA729271	stage_E18_vs_E10	ordinary_muscle_development	ENSGALG00010019376	ENSGALG00010019376	-0.596969842	0.417376096	-1.430292361	0.152633126	0.195012531	negative
PRJNA729271	stage_E18_vs_E10	ordinary_muscle_development	ENSGALG00010019388	GPR75	-0.16883644	0.136032655	-1.241146396	0.214551673	0.264990329	negative
PRJNA729271	stage_E18_vs_E10	ordinary_muscle_development	ENSGALG00010019390	TVP23A	-0.59812756	0.223550601	-2.675580191	0.007460002	0.012021427	negative
PRJNA729271	stage_E18_vs_E10	ordinary_muscle_development	ENSGALG00010019391	GANC	0.281995782	0.136077965	2.072310401	0.038236503	0.05480994	positive
PRJNA729271	stage_E18_vs_E10	ordinary_muscle_development	ENSGALG00010019392	NIN	-1.021009042	0.105258575	-9.700008257	3.01474E-22	2.4399E-21	negative
PRJNA729271	stage_E18_vs_E10	ordinary_muscle_development	ENSGALG00010019398	SLC35F5	0.789507268	0.120473623	6.553362053	5.62559E-11	2.09353E-10	positive
PRJNA729271	stage_E18_vs_E10	ordinary_muscle_development	ENSGALG00010019399	CD93	1.285656405	0.151159688	8.505286178	1.81148E-17	1.09269E-16	positive
PRJNA729271	stage_E18_vs_E10	ordinary_muscle_development	ENSGALG00010019403	PACC1	-0.173013383	0.106586123	-1.62322616	0.104541068	0.138133343	negative
PRJNA729271	stage_E18_vs_E10	ordinary_muscle_development	ENSGALG00010019411	LSM5	-1.136265855	0.159584713	-7.120142247	1.07816E-12	4.60864E-12	negative
PRJNA729271	stage_E18_vs_E10	ordinary_muscle_development	ENSGALG00010019416	GPR39	3.555387561	0.416224427	8.54199641	1.31923E-17	8.0379E-17	positive
PRJNA729271	stage_E18_vs_E10	ordinary_muscle_development	ENSGALG00010019418	ZNF703	0.584138883	0.229332053	2.547131438	0.010861252	0.017103079	positive
PRJNA729271	stage_E18_vs_E10	ordinary_muscle_development	ENSGALG00010019422	IMMP1L	1.05944698	0.13923285	7.609173985	2.75853E-14	1.32357E-13	positive
PRJNA729271	stage_E18_vs_E10	ordinary_muscle_development	ENSGALG00010019423	LYPD1	0.220674802	0.178589462	1.23565411	0.216587162	0.267231096	positive
PRJNA729271	stage_E18_vs_E10	ordinary_muscle_development	ENSGALG00010019426	GRM7	-0.740321271	0.251215422	-2.946957891	0.003209169	0.005468074	negative
PRJNA729271	stage_E18_vs_E10	ordinary_muscle_development	ENSGALG00010019430	ENSGALG00010019430	0.461499897	0.404336827	1.14137488	0.253713954	0.307464573	positive
PRJNA729271	stage_E18_vs_E10	ordinary_muscle_development	ENSGALG00010019441	LMCD1	1.879685929	0.138743482	13.54792239	8.15045E-42	1.67338E-40	positive

PRJNA729271	stage_E18_vs_E10	ordinary_muscle_development	ENSGALG00010019443	NCKAP5	0.453336121	0.131970637	3.43512868	0.000592273	0.00111421	positive
PRJNA729271	stage_E18_vs_E10	ordinary_muscle_development	ENSGALG00010019451	CAV3	4.847562588	0.142664781	33.97869155	4.5993E-253	4.7619E-250	positive
PRJNA729271	stage_E18_vs_E10	ordinary_muscle_development	ENSGALG00010019458	ELP4	-0.370958318	0.1042275	-3.559121318	0.000372098	0.000719524	negative
PRJNA729271	stage_E18_vs_E10	ordinary_muscle_development	ENSGALG00010019461	MGAT5	0.168333048	0.070726759	2.380047528	0.017310405	0.026392587	positive
PRJNA729271	stage_E18_vs_E10	ordinary_muscle_development	ENSGALG00010019470	TGFB2	-0.269385783	0.20014705	-1.345939313	0.178322089	0.224275807	negative
PRJNA729271	stage_E18_vs_E10	ordinary_muscle_development	ENSGALG00010019471	NUBP1	0.056785546	0.094307895	0.602129286	0.547088082	0.601763678	positive
PRJNA729271	stage_E18_vs_E10	ordinary_muscle_development	ENSGALG00010019472	CAPN3	6.957414096	0.255749065	27.20406467	5.8142E-163	2.4079E-160	positive
PRJNA729271	stage_E18_vs_E10	ordinary_muscle_development	ENSGALG00010019473	OXTR	-0.090935248	0.464445665	-0.195793081	0.844772126	0.871528253	negative
PRJNA729271	stage_E18_vs_E10	ordinary_muscle_development	ENSGALG00010019474	TMEM163	-1.711253327	0.597892931	-2.862140088	0.004207909	0.007044772	negative
PRJNA729271	stage_E18_vs_E10	ordinary_muscle_development	ENSGALG00010019477	NCAPH	-1.832495324	0.177440847	-10.32735899	5.29997E-25	5.0244E-24	negative
PRJNA729271	stage_E18_vs_E10	ordinary_muscle_development	ENSGALG00010019481	PYGL	0.463392165	0.09817263	4.720176756	2.3564E-06	5.84763E-06	positive
PRJNA729271	stage_E18_vs_E10	ordinary_muscle_development	ENSGALG00010019483	SCUBE2	-2.280385646	0.262821258	-8.676564679	4.07903E-18	2.57403E-17	negative
PRJNA729271	stage_E18_vs_E10	ordinary_muscle_development	ENSGALG00010019488	TEKT5	-3.884629163	1.317367126	-2.948782528	0.003190284	0.005439092	negative
PRJNA729271	stage_E18_vs_E10	ordinary_muscle_development	ENSGALG00010019495	EMP2	0.602961859	0.196846698	3.063103754	0.002190541	0.003823686	positive
PRJNA729271	stage_E18_vs_E10	ordinary_muscle_development	ENSGALG00010019498	ENSGALG00010019498	1.605197845	0.257859237	6.225093447	4.81269E-10	1.6737E-09	positive
PRJNA729271	stage_E18_vs_E10	ordinary_muscle_development	ENSGALG00010019509	ZNF106	2.306717368	0.22216133	10.38307326	2.96087E-25	2.84224E-24	positive
PRJNA729271	stage_E18_vs_E10	ordinary_muscle_development	ENSGALG00010019515	ENSGALG00010019515	-0.191067075	0.109396249	-1.746559648	0.080713752	0.109055354	negative
PRJNA729271	stage_E18_vs_E10	ordinary_muscle_development	ENSGALG00010019520	CD6	1.578817262	1.196594591	1.319425371	0.187026944	0.234106698	positive
PRJNA729271	stage_E18_vs_E10	ordinary_muscle_development	ENSGALG00010019524	HHIPL2	-0.116899306	0.406883148	-0.287304369	0.773879278	0.810314074	negative
PRJNA729271	stage_E18_vs_E10	ordinary_muscle_development	ENSGALG00010019529	SNAP23	-0.226886008	0.068001947	-3.336463427	0.000848516	0.001562602	negative
PRJNA729271	stage_E18_vs_E10	ordinary_muscle_development	ENSGALG00010019534	PPP2R5A	0.821758193	0.1046272	7.8541545	4.02478E-15	2.0717E-14	positive
PRJNA729271	stage_E18_vs_E10	ordinary_muscle_development	ENSGALG00010019540	UNC5D	0.001279903	0.35859075	0.003569259	0.99715215	0.997496232	positive
PRJNA729271	stage_E18_vs_E10	ordinary_muscle_development	ENSGALG00010019546	HSD17B12	-1.14820171	0.078942212	-14.5448383	6.29795E-48	1.68741E-46	negative
PRJNA729271	stage_E18_vs_E10	ordinary_muscle_development	ENSGALG00010019557	TRIM9	-1.337244258	0.353691048	-3.780825858	0.000156309	0.000317993	negative
PRJNA729271	stage_E18_vs_E10	ordinary_muscle_development	ENSGALG00010019572	NFKBIE	-0.248422813	0.190841032	-1.30172642	0.193009925	0.240764101	negative
PRJNA729271	stage_E18_vs_E10	ordinary_muscle_development	ENSGALG00010019577	MXD1	-0.436681166	0.1929391	-2.263310888	0.023616532	0.035156786	negative
PRJNA729271	stage_E18_vs_E10	ordinary_muscle_development	ENSGALG00010019580	CD5	1.397014891	0.493955794	2.828218448	0.004680785	0.00778609	positive
PRJNA729271	stage_E18_vs_E10	ordinary_muscle_development	ENSGALG00010019581	SLC35B2	-0.434333713	0.134854631	-3.220754896	0.001278534	0.002300001	negative
PRJNA729271	stage_E18_vs_E10	ordinary_muscle_development	ENSGALG00010019584	RAD18	-1.18133164	0.118035352	-10.00828667	1.40159E-23	1.21435E-22	negative
PRJNA729271	stage_E18_vs_E10	ordinary_muscle_development	ENSGALG00010019585	PDE1C	-0.464168532	0.211285486	-2.196878458	0.028029128	0.04117586	negative
PRJNA729271	stage_E18_vs_E10	ordinary_muscle_development	ENSGALG00010019586	GLYR1	-0.512166237	0.090700439	-5.646788921	1.63472E-08	4.95822E-08	negative
PRJNA729271	stage_E18_vs_E10	ordinary_muscle_development	ENSGALG00010019587	CCNT2	0.210394065	0.127694989	1.647629763	0.099428669	0.131919319	positive
PRJNA729271	stage_E18_vs_E10	ordinary_muscle_development	ENSGALG00010019588	CHMP7	0.19029688	0.093248705	2.040745547	0.041276126	0.058760306	positive
PRJNA729271	stage_E18_vs_E10	ordinary_muscle_development	ENSGALG00010019591	ENSGALG00010019591	-1.402691152	0.227223044	-6.173190555	6.69255E-10	2.3026E-09	negative
PRJNA729271	stage_E18_vs_E10	ordinary_muscle_development	ENSGALG00010019592	ALKBH3	0.143619953	0.134105468	1.070947779	0.284192911	0.339882528	positive
PRJNA729271	stage_E18_vs_E10	ordinary_muscle_development	ENSGALG00010019594	TMX1	0.805870571	0.313100157	2.573842753	0.010057597	0.015906696	positive
PRJNA729271	stage_E18_vs_E10	ordinary_muscle_development	ENSGALG00010019595	GRIN2A	-0.993865192	0.222966563	-4.457462943	8.29354E-06	1.94869E-05	negative
PRJNA729271	stage_E18_vs_E10	ordinary_muscle_development	ENSGALG00010019598	KCNAB2	-1.350843159	0.139717756	-9.668371451	4.10864E-22	3.2885E-21	negative
PRJNA729271	stage_E18_vs_E10	ordinary_muscle_development	ENSGALG00010019600	MAP3K19	-0.180244858	0.214363229	-0.840838505	0.400438412	0.460261263	negative
PRJNA729271	stage_E18_vs_E10	ordinary_muscle_development	ENSGALG00010019602	SLC29A1	0.779102537	0.194960361	3.996209963	6.43647E-05	0.000137115	positive
PRJNA729271	stage_E18_vs_E10	ordinary_muscle_development	ENSGALG00010019610	CORO7	0.387331972	0.101180895	3.828113729	0.000129129	0.000265757	positive
PRJNA729271	stage_E18_vs_E10	ordinary_muscle_development	ENSGALG00010019613	FRMD6	-0.120810749	0.123931771	-0.974816611	0.329651236	0.387785641	negative
PRJNA729271	stage_E18_vs_E10	ordinary_muscle_development	ENSGALG00010019614	MICALL2	0.614431034	0.157002801	3.913503639	9.09665E-05	0.000190543	positive
PRJNA729271	stage_E18_vs_E10	ordinary_muscle_development	ENSGALG00010019617	ANO3	-0.954795906	0.144149486	-6.623651123	3.50433E-11	1.32176E-10	negative
PRJNA729271	stage_E18_vs_E10	ordinary_muscle_development	ENSGALG00010019618	NPHP4	-0.391695137	0.107568094	-3.641369146	0.000271192	0.000534602	negative
PRJNA729271	stage_E18_vs_E10	ordinary_muscle_development	ENSGALG00010019621	ENSGALG00010019621	-0.031323092	0.151290833	-0.207038926	0.835979459	0.863932857	negative
PRJNA729271	stage_E18_vs_E10	ordinary_muscle_development	ENSGALG00010019622	C2orf42	0.83460911	0.142799143	5.844636678	5.07675E-09	1.61164E-08	positive
PRJNA729271	stage_E18_vs_E10	ordinary_muscle_development	ENSGALG00010019624	SRGAP3	0.5409033	0.129658986	4.171737838	3.02285E-05	6.66155E-05	positive
PRJNA729271	stage_E18_vs_E10	ordinary_muscle_development	ENSGALG00010019625	INTS1	-0.061141991	0.076498652	-0.799255789	0.424142109	0.484585786	negative
PRJNA729271	stage_E18_vs_E10	ordinary_muscle_development	ENSGALG00010019630	AJAP1	-0.223993041	0.369575549	-0.606081874	0.544460387	0.599237153	negative
PRJNA729271	stage_E18_vs_E10	ordinary_muscle_development	ENSGALG00010019634	GNG2	-1.42830599	0.277354562	-5.14974761	2.60837E-07	7.11888E-07	negative
PRJNA729271	stage_E18_vs_E10	ordinary_muscle_development	ENSGALG00010019635	LRRC57	0.21546728	0.081552964	2.642053337	0.008240508	0.013195555	positive
PRJNA729271	stage_E18_vs_E10	ordinary_muscle_development	ENSGALG00010019640	MGRN1	0.133811082	0.116567889	1.147924042	0.250999922	0.304608496	positive
PRJNA729271	stage_E18_vs_E10	ordinary_muscle_development	ENSGALG00010019641	CAPN11	0.3258552	0.082148384	3.966665973	7.2885E-05	0.000154319	positive
PRJNA729271	stage_E18_vs_E10	ordinary_muscle_development	ENSGALG00010019643	RAB3GAP1	-0.015116568	0.070674962	-0.21388859	0.830633938	0.859266267	negative
PRJNA729271	stage_E18_vs_E10	ordinary_muscle_development	ENSGALG00010019645	ENSGALG00010019645	1.319498058	0.165564599	7.969687132	1.59077E-15	8.41538E-15	positive
PRJNA729271	stage_E18_vs_E10	ordinary_muscle_development	ENSGALG00010019646	VPS37C	-0.169648755	0.129112784	-1.313957846	0.18886039	0.236212068	negative
PRJNA729271	stage_E18_vs_E10	ordinary_muscle_development	ENSGALG00010019648	PRDM16	-1.058840433	0.190730747	-5.551493126	2.8324E-08	8.44071E-08	negative
PRJNA729271	stage_E18_vs_E10	ordinary_muscle_development	ENSGALG00010019649	TMEM63B	-0.479036869	0.0783173	-6.116616221	9.55832E-10	3.23408E-09	negative
PRJNA729271	stage_E18_vs_E10	ordinary_muscle_development	ENSGALG00010019650	KBTBD8	0.190142884	0.087937823	2.162242331	0.030599502	0.044657651	positive
PRJNA729271	stage_E18_vs_E10	ordinary_muscle_development	ENSGALG00010019652	TNFRSF10B	0.197147557	0.328468802	0.600201771	0.548371773	0.602626903	positive
PRJNA729271	stage_E18_vs_E10	ordinary_muscle_development	ENSGALG00010019655	RTRAF	-0.284467102	0.099141743	-2.869296955	0.004113854	0.006895272	negative
PRJNA729271	stage_E18_vs_E10	ordinary_muscle_development	ENSGALG00010019657	PPP1R17	-2.126013932	0.274949566	-7.732377848	1.05556E-14	5.26872E-14	negative
PRJNA729271	stage_E18_vs_E10	ordinary_muscle_development	ENSGALG00010019659	MRPL14	-0.086060514	0.126713623	-0.67917334	0.497028037	0.554271534	negative
PRJNA729271	stage_E18_vs_E10	ordinary_muscle_development	ENSGALG00010019660	ENSGALG00010019660	0.301343995	0.548991346	0.548904817	0.583070772	0.636033326	positive
PRJNA729271	stage_E18_vs_E10	ordinary_muscle_development	ENSGALG00010019667	ITPRID1	0.48206829	0.620266268	0.777195722	0.437043306	0.496611956	positive
PRJNA729271	stage_E18_vs_E10	ordinary_muscle_development	ENSGALG00010019670	ENSGALG00010019670	-0.6113862	0.121267147	-5.041647436	4.61541E-07	1.23114E-06	negative
PRJNA729271	stage_E18_vs_E10	ordinary_muscle_development	ENSGALG00010019673	NID2	1.190718703	0.132503439	8.986323026	2.5564E-19	1.72831E-18	positive
PRJNA729271	stage_E18_vs_E10	ordinary_muscle_development	ENSGALG00010019675	MAFK	1.348910968	0.137491999	9.810832488	1.01131E-22	8.35265E-22	positive
PRJNA729271	stage_E18_vs_E10	ordinary_muscle_development	ENSGALG00010019679	RHOBTB2	-1.510642634	0.172064468	-8.779515347	1.64181E-18	1.06194E-17	negative
PRJNA729271	stage_E18_vs_E10	ordinary_muscle_development	ENSGALG00010019680	CCDC34	-0.050320593	0.201089667	-0.250239577	0.802402081	0.835127318	negative
PRJNA729271	stage_E18_vs_E10	ordinary_muscle_development	ENSGALG00010019682	TMEM158	-1.149310828	0.36600371	-3.14016169	0.001688546	0.002996508	negative
PRJNA729271	stage_E18_vs_E10	ordinary_muscle_development	ENSGALG00010019683	TMEM184A	0.718641125	0.290872708	2.470637864	0.013487232	0.020946901	positive
PRJNA729271	stage_E18_vs_E10	ordinary_muscle_development	ENSGALG00010019684	PIGQ	0.254292473	0.127102411	2.000689607	0.04542585	0.064245068	positive
PRJNA729271	stage_E18_vs_E10	ordinary_muscle_development	ENSGALG00010019690	ENSGALG00010019690	0.26578677	0.090208097	2.946373768	0.003215236	0.005475837	positive
PRJNA729271	stage_E18_vs_E10	ordinary_muscle_development	ENSGALG00010019692	MMEL1	-1.042547256	0.307782505	-3.387285625	0.000705879	0.001314116	negative
PRJNA729271	stage_E18_vs_E10	ordinary_muscle_development	ENSGALG00010019693	HAUS2	-0.589315581	0.120642595	-4.884805255	1.03531E-06	2.67263E-06	negative
PRJNA729271	stage_E18_vs_E10	ordinary_muscle_development	ENSGALG00010019695	PGA5	-14.87073456	2.525620648	-5.887952561	3.91009E-09	1.25447E-08	negative
PRJNA729271	stage_E18_vs_E10	ordinary_muscle_development	ENSGALG00010019697	EIF4EBP1	1.768441836	0.145278378	12.1727807	4.3404E-34	6.41326E-33	positive
PRJNA729271	stage_E18_vs_E10	ordinary_muscle_development	ENSGALG00010019700	HES5B	-2.002151555	0.258216983	-7.753756298	8.92135E-15	4.47456E-14	negative
PRJNA729271	stage_E18_vs_E10	ordinary_muscle_development	ENSGALG00010019706	NPRL3	0.404310525	0.09981269	4.050692603	5.10663E-05	0.000109888	positive
PRJNA729271	stage_E18_vs_E10	ordinary_muscle_development	ENSGALG00010019713	NUDT14	0.610989343	0.113994289	5.359824152	8.3303E-08	2.37646E-07	positive
PRJNA729271	stage_E18_vs_E10	ordinary_muscle_development	ENSGALG00010019716	VEGFA	1.879830962	0.129158734	14.55442389	5.47452E-48	1.47497E-46	positive
PRJNA729271	stage_E18_vs_E10	ordinary_muscle_development	ENSGALG00010019717	ZRANB3	-0.736578975	0.142732947	-5.160539242	2.4624E-07	6.73442E-07	negative
PRJNA729271	stage_E18_vs_E10	ordinary_muscle_development	ENSGALG00010019724	HES5A	-3.381642955	0.283820838	-11.91470991	9.92109E-33	1.3618E-31	negative
PRJNA729271	stage_E18_vs_E10	ordinary_muscle_development	ENSGALG00010019726	PEBP4	2.336226242	0.237796386	9.824481684	8.83272E-23	7.32858E-22	positive
PRJNA729271	stage_E18_vs_E10	ordinary_muscle_development	ENSGALG00010019731	PANK4	0.056847051	0.136224426	0.417304392	0.676455776	0.722832767	positive
PRJNA729271	stage_E18_vs_E10	ordinary_muscle_development	ENSGALG00010019732	HBA1	0.074612658	0.40348259	0.184921629	0.853290506	0.878752816	positive
PRJNA729271	stage_E18_vs_E10	ordinary_muscle_development	ENSGALG00010019735	ENSGALG00010019735	2.014333988	0.171084596	11.77390623	5.32027E-32	7.13389E-31	positive
PRJNA729271	stage_E18_vs_E10	ordinary_muscle_development	ENSGALG00010019738	ADRA2A	-1.784739853	0.358009788	-4.985170547	6.19072E-07	1.63094E-06	negative
PRJNA729271	stage_E18_vs_E10	ordinary_muscle_development	ENSGALG00010019743	KCNU1	0.02700825	0.614812563	0.043929243	0.964960805	0.971326866	positive
PRJNA729271	stage_E18_vs_E10	ordinary_muscle_development	ENSGALG00010019744	KBP	-0.539956471	0.485382359	-1.112435302	0.265951033	0.320818927	negative
PRJNA729271	stage_E18_vs_E10	ordinary_muscle_development	ENSGALG00010019752	GPAM	-0.335787887	0.168389357	-1.994115851	0.046139395	0.06517791	negative
PRJNA729271	stage_E18_vs_E10	ordinary_muscle_development	ENSGALG00010019761	MRPS18A	-0.010063579	0.089181246	-0.112844116	0.910154142	0.925672487	negative
PRJNA729271	stage_E18_vs_E10	ordinary_muscle_development	ENSGALG00010019767	SUCLG2	-0.349971147	0.077757161	-4.500822085	6.76911E-06	1.60245E-05	negative
PRJNA729271	stage_E18_vs_E10	ordinary_muscle_development	ENSGALG00010019771	VWCE	-1.197988844	0.358567282	-3.341043377	0.000834642	0.001538615	negative
PRJNA729271	stage_E18_vs_E10	ordinary_muscle_development	ENSGALG00010019772	ENSGALG00010019772	-0.412946799	0.168129369	-2.456125315	0.014044417	0.021770274	negative
PRJNA729271	stage_E18_vs_E10	ordinary_muscle_development	ENSGALG00010019778	ABHD17C	-0.010343757	0.134385877	-0.076970568	0.938646958	0.949786224	negative
PRJNA729271	stage_E18_vs_E10	ordinary_muscle_development	ENSGALG00010019785	ACSL5	-0.162968923	0.238281005	-0.683935857	0.494015671	0.551378852	negative
PRJNA729271	stage_E18_vs_E10	ordinary_muscle_development	ENSGALG00010019786	R3HDM1	-0.208384943	0.088674926	-2.349987221	0.018774056	0.028426819	negative
PRJNA729271	stage_E18_vs_E10	ordinary_muscle_development	ENSGALG00010019789	ARNT2	-1.41562463	0.17318035	-8.174279776	2.97638E-16	1.65171E-15	negative
PRJNA729271	stage_E18_vs_E10	ordinary_muscle_development	ENSGALG00010019794	RSPH9	-0.856539684	0.496051551	-1.726715061	0.08421885	0.11329487	negative
PRJNA729271	stage_E18_vs_E10	ordinary_muscle_development	ENSGALG00010019799	DOK2	0.217489607	0.281602929	0.772327218	0.439920649	0.499580837	positive
PRJNA729271	stage_E18_vs_E10	ordinary_muscle_development	ENSGALG00010019800	EXT2	-0.292550181	0.060465382	-4.838308645	1.30949E-06	3.34585E-06	negative
PRJNA729271	stage_E18_vs_E10	ordinary_muscle_development	ENSGALG00010019801	FIBIN	-3.375261409	0.18704978	-18.04472266	8.67991E-73	4.91466E-71	negative
PRJNA729271	stage_E18_vs_E10	ordinary_muscle_development	ENSGALG00010019803	FAH	-0.647103936	0.489346652	-1.322383495	0.186040482	0.233113484	negative
PRJNA729271	stage_E18_vs_E10	ordinary_muscle_development	ENSGALG00010019804	ELFN1	0.901782189	0.262411196	3.436523299	0.000589232	0.001108634	positive
PRJNA729271	stage_E18_vs_E10	ordinary_muscle_development	ENSGALG00010019807	ZDHHC6	-0.332646972	0.080557798	-4.129295715	3.63876E-05	7.94934E-05	negative
PRJNA729271	stage_E18_vs_E10	ordinary_muscle_development	ENSGALG00010019810	ZFAND6	0.165221291	0.125234098	1.319299568	0.187068982	0.234118882	positive
PRJNA729271	stage_E18_vs_E10	ordinary_muscle_development	ENSGALG00010019811	UBXN4	0.309598694	0.110706895	2.796561975	0.00516495	0.008546818	positive
PRJNA729271	stage_E18_vs_E10	ordinary_muscle_development	ENSGALG00010019813	ENSGALG00010019813	2.955137801	0.219736614	13.44854529	3.13989E-41	6.31244E-40	positive
PRJNA729271	stage_E18_vs_E10	ordinary_muscle_development	ENSGALG00010019816	ENSGALG00010019816	3.421022834	0.707351943	4.836380061	1.32225E-06	3.37549E-06	positive
PRJNA729271	stage_E18_vs_E10	ordinary_muscle_development	ENSGALG00010019820	BBOX1	-0.683725343	0.155611232	-4.393804589	1.11384E-05	2.5795E-05	negative
PRJNA729271	stage_E18_vs_E10	ordinary_muscle_development	ENSGALG00010019823	LCT	-0.31392411	0.203821645	-1.540190249	0.123513986	0.161055066	negative
PRJNA729271	stage_E18_vs_E10	ordinary_muscle_development	ENSGALG00010019825	BCL2A1	1.301588015	0.289202085	4.50061767	6.77563E-06	1.60374E-05	positive
PRJNA729271	stage_E18_vs_E10	ordinary_muscle_development	ENSGALG00010019830	JAG2	1.433344496	0.097676543	14.67439833	9.40461E-49	2.60152E-47	positive
PRJNA729271	stage_E18_vs_E10	ordinary_muscle_development	ENSGALG00010019832	MAD2L1BP	-1.588628444	0.139258503	-11.40776621	3.82424E-30	4.71364E-29	negative
PRJNA729271	stage_E18_vs_E10	ordinary_muscle_development	ENSGALG00010019833	MTHFS	0.100874303	0.127204403	0.793009525	0.427772273	0.487848867	positive
PRJNA729271	stage_E18_vs_E10	ordinary_muscle_development	ENSGALG00010019834	PLCH2	-1.12536298	0.239970564	-4.689587596	2.73756E-06	6.74158E-06	negative
PRJNA729271	stage_E18_vs_E10	ordinary_muscle_development	ENSGALG00010019836	MINAR1	2.072754462	0.482971373	4.291671465	1.77333E-05	4.0182E-05	positive
PRJNA729271	stage_E18_vs_E10	ordinary_muscle_development	ENSGALG00010019838	ALX4	-3.151904842	0.202381156	-15.57410239	1.09175E-54	3.66317E-53	negative
PRJNA729271	stage_E18_vs_E10	ordinary_muscle_development	ENSGALG00010019843	MCM6	-1.256381922	0.110020331	-11.41954315	3.33985E-30	4.13064E-29	negative
PRJNA729271	stage_E18_vs_E10	ordinary_muscle_development	ENSGALG00010019846	ENSGALG00010019846	-0.089429732	0.073930598	-1.209644384	0.226415379	0.277867321	negative
PRJNA729271	stage_E18_vs_E10	ordinary_muscle_development	ENSGALG00010019848	MAD1L1	-0.461266394	0.126399638	-3.649269895	0.000262987	0.000519699	negative
PRJNA729271	stage_E18_vs_E10	ordinary_muscle_development	ENSGALG00010019854	CCDC14	-0.881512355	0.151375277	-5.8233575	5.7677E-09	1.82181E-08	negative
PRJNA729271	stage_E18_vs_E10	ordinary_muscle_development	ENSGALG00010019855	TTC7A	0.023329841	0.127405538	0.183114813	0.854707933	0.880021656	positive
PRJNA729271	stage_E18_vs_E10	ordinary_muscle_development	ENSGALG00010019859	BIN3	0.371672729	0.13524277	2.748189273	0.00599254	0.009810467	positive
PRJNA729271	stage_E18_vs_E10	ordinary_muscle_development	ENSGALG00010019865	ENSGALG00010019865	0.141524129	0.136664694	1.035557353	0.300408697	0.357418046	positive
PRJNA729271	stage_E18_vs_E10	ordinary_muscle_development	ENSGALG00010019867	PEX11A	-0.677023554	0.378857277	-1.787014781	0.0739351	0.100592241	negative
PRJNA729271	stage_E18_vs_E10	ordinary_muscle_development	ENSGALG00010019871	TAFA1	-3.132985341	0.60172792	-5.206647784	1.92283E-07	5.30681E-07	negative
PRJNA729271	stage_E18_vs_E10	ordinary_muscle_development	ENSGALG00010019872	VTI1A	-0.210423765	0.110269759	-1.908263587	0.056357157	0.078381979	negative

PRJNA729271	stage_E18_vs_E10	ordinary_muscle_development	ENSGALG00010019873	LGR4	-0.633217526	0.09772928	-6.479302045	9.21479E-11	3.36868E-10	negative
PRJNA729271	stage_E18_vs_E10	ordinary_muscle_development	ENSGALG00010019875	PLEKHH1	-3.53060318	0.346143194	-10.19983416	1.98611E-24	1.81632E-23	negative
PRJNA729271	stage_E18_vs_E10	ordinary_muscle_development	ENSGALG00010019876	MRM2	0.218043423	0.224992984	0.969112101	0.332489261	0.390749476	positive
PRJNA729271	stage_E18_vs_E10	ordinary_muscle_development	ENSGALG00010019878	MCFD2	0.713905309	0.149910783	4.762201199	1.91493E-06	4.79724E-06	positive
PRJNA729271	stage_E18_vs_E10	ordinary_muscle_development	ENSGALG00010019891	PIGH	-0.588985363	0.104875301	-5.616054108	1.95368E-08	5.88498E-08	negative
PRJNA729271	stage_E18_vs_E10	ordinary_muscle_development	ENSGALG00010019896	DDB1	-0.272264251	0.091211338	-2.984982554	0.002835945	0.004877798	negative
PRJNA729271	stage_E18_vs_E10	ordinary_muscle_development	ENSGALG00010019898	NUDT1	-1.472791785	0.18969175	-7.764132005	8.22062E-15	4.13169E-14	negative
PRJNA729271	stage_E18_vs_E10	ordinary_muscle_development	ENSGALG00010019899	ENSGALG00010019899	-0.013925053	0.451669425	-0.030830188	0.975404965	0.9796629	negative
PRJNA729271	stage_E18_vs_E10	ordinary_muscle_development	ENSGALG00010019905	ENSGALG00010019905	0.038811377	0.124526978	0.311670434	0.755291	0.794653629	positive
PRJNA729271	stage_E18_vs_E10	ordinary_muscle_development	ENSGALG00010019906	ENSGALG00010019906	1.394317738	0.2331316	5.980818281	2.22019E-09	7.27106E-09	positive
PRJNA729271	stage_E18_vs_E10	ordinary_muscle_development	ENSGALG00010019910	ENSGALG00010019910	0.565393798	0.242024167	2.336104716	0.019485785	0.029442974	positive
PRJNA729271	stage_E18_vs_E10	ordinary_muscle_development	ENSGALG00010019912	ARG2	-4.156438983	0.221238955	-18.7871028	9.6295E-79	6.43224E-77	negative
PRJNA729271	stage_E18_vs_E10	ordinary_muscle_development	ENSGALG00010019916	NOC3L	-0.604125746	0.114934338	-5.256268567	1.47008E-07	4.09311E-07	negative
PRJNA729271	stage_E18_vs_E10	ordinary_muscle_development	ENSGALG00010019919	LIN7C	-0.417039571	0.10813152	-3.856780796	0.00011489	0.000237973	negative
PRJNA729271	stage_E18_vs_E10	ordinary_muscle_development	ENSGALG00010019921	CALM2	-0.180804525	0.112723021	-1.603971612	0.10872031	0.143263718	negative
PRJNA729271	stage_E18_vs_E10	ordinary_muscle_development	ENSGALG00010019925	RDH11	-2.110036191	0.151046612	-13.96943739	2.39511E-44	5.50193E-43	negative
PRJNA729271	stage_E18_vs_E10	ordinary_muscle_development	ENSGALG00010019926	KIF7	-0.586525912	0.100819877	-5.817562274	5.9712E-09	1.88445E-08	negative
PRJNA729271	stage_E18_vs_E10	ordinary_muscle_development	ENSGALG00010019931	ZFYVE26	0.502133908	0.135215104	3.713593322	0.000204337	0.000409041	positive
PRJNA729271	stage_E18_vs_E10	ordinary_muscle_development	ENSGALG00010019932	KALRN	-0.413749495	0.11733234	-3.526303942	0.000421403	0.000809574	negative
PRJNA729271	stage_E18_vs_E10	ordinary_muscle_development	ENSGALG00010019934	TKFC	-0.740346493	0.157118063	-4.712039325	2.4525E-06	6.07467E-06	negative
PRJNA729271	stage_E18_vs_E10	ordinary_muscle_development	ENSGALG00010019935	ENSGALG00010019935	1.722527798	1.42780973	1.206412705	0.2276584	0.279109237	positive
PRJNA729271	stage_E18_vs_E10	ordinary_muscle_development	ENSGALG00010019938	TAFA4	-3.037394384	0.387152565	-7.845471428	4.3133E-15	2.21314E-14	negative
PRJNA729271	stage_E18_vs_E10	ordinary_muscle_development	ENSGALG00010019947	ENSGALG00010019947	-1.144040422	0.298509908	-3.832504024	0.000126846	0.00026128	negative
PRJNA729271	stage_E18_vs_E10	ordinary_muscle_development	ENSGALG00010019948	TICRR	-1.162991455	0.129158347	-9.004384792	2.16878E-19	1.47244E-18	negative
PRJNA729271	stage_E18_vs_E10	ordinary_muscle_development	ENSGALG00010019954	CYB561A3	0.698205378	0.132635237	5.264101713	1.40876E-07	3.93222E-07	positive
PRJNA729271	stage_E18_vs_E10	ordinary_muscle_development	ENSGALG00010019956	SLC29A3	-1.117988999	0.182622278	-6.121865366	9.24862E-10	3.13441E-09	negative
PRJNA729271	stage_E18_vs_E10	ordinary_muscle_development	ENSGALG00010019961	HTATSF1	-0.38614309	0.070060985	-5.511528115	3.55732E-08	1.05103E-07	negative
PRJNA729271	stage_E18_vs_E10	ordinary_muscle_development	ENSGALG00010019962	SNX8	0.277722902	0.143436501	1.936208006	0.05284223	0.07384056	positive
PRJNA729271	stage_E18_vs_E10	ordinary_muscle_development	ENSGALG00010019967	CEP170B	-0.901989018	0.109441621	-8.241736612	1.69729E-16	9.58403E-16	negative
PRJNA729271	stage_E18_vs_E10	ordinary_muscle_development	ENSGALG00010019968	RHCG	3.902287465	0.800622276	4.874068063	1.09323E-06	2.81714E-06	positive
PRJNA729271	stage_E18_vs_E10	ordinary_muscle_development	ENSGALG00010019972	PPP1CB	0.199425803	0.091486746	2.179832725	0.029269863	0.042837911	positive
PRJNA729271	stage_E18_vs_E10	ordinary_muscle_development	ENSGALG00010019975	ENSGALG00010019975	-0.211156285	0.201546765	-1.047678861	0.29478659	0.351450208	negative
PRJNA729271	stage_E18_vs_E10	ordinary_muscle_development	ENSGALG00010019977	ZMIZ1	-0.066554418	0.088434919	-0.752580755	0.451701886	0.511317363	negative
PRJNA729271	stage_E18_vs_E10	ordinary_muscle_development	ENSGALG00010019979	ENSGALG00010019979	1.358005556	0.259832693	5.226461453	1.72785E-07	4.786E-07	positive
PRJNA729271	stage_E18_vs_E10	ordinary_muscle_development	ENSGALG00010019982	PLCE1	0.692323825	0.11229586	6.165176763	7.04044E-10	2.41656E-09	positive
PRJNA729271	stage_E18_vs_E10	ordinary_muscle_development	ENSGALG00010019985	UMPS	-1.408494032	0.087297279	-16.13445513	1.46091E-58	5.61695E-57	negative
PRJNA729271	stage_E18_vs_E10	ordinary_muscle_development	ENSGALG00010019989	ENSGALG00010019989	2.030288093	0.27307943	7.434789546	1.04735E-13	4.80572E-13	positive
PRJNA729271	stage_E18_vs_E10	ordinary_muscle_development	ENSGALG00010019990	MARCHF8	0.897078549	0.106526745	8.421157968	3.72781E-17	2.2046E-16	positive
PRJNA729271	stage_E18_vs_E10	ordinary_muscle_development	ENSGALG00010019991	EIF3B	-0.075412502	0.075306731	-1.001404533	0.316631273	0.374139586	negative
PRJNA729271	stage_E18_vs_E10	ordinary_muscle_development	ENSGALG00010019992	CHAC2	-0.545716536	0.149861552	-3.641471278	0.000271084	0.000534462	negative
PRJNA729271	stage_E18_vs_E10	ordinary_muscle_development	ENSGALG00010019994	PLD4	-1.304917446	0.966380413	-1.350314461	0.176915136	0.222680175	negative
PRJNA729271	stage_E18_vs_E10	ordinary_muscle_development	ENSGALG00010019996	ITGB5	0.229638854	0.124664561	1.842054008	0.06546725	0.089905049	positive
PRJNA729271	stage_E18_vs_E10	ordinary_muscle_development	ENSGALG00010019997	FSHR	2.918111335	0.401591826	7.266361386	3.693E-13	1.63401E-12	positive
PRJNA729271	stage_E18_vs_E10	ordinary_muscle_development	ENSGALG00010019998	PLEK2	-6.862772039	1.005256479	-6.826886654	8.67772E-12	3.42455E-11	negative
PRJNA729271	stage_E18_vs_E10	ordinary_muscle_development	ENSGALG00010019999	ERLIN1	-0.554707769	0.064032081	-8.662966494	4.59644E-18	2.88671E-17	negative
PRJNA729271	stage_E18_vs_E10	ordinary_muscle_development	ENSGALG00010020003	ENSGALG00010020003	1.974271611	0.186914641	10.56242356	4.45021E-26	4.47646E-25	positive
PRJNA729271	stage_E18_vs_E10	ordinary_muscle_development	ENSGALG00010020005	EOGT	0.066281518	0.164246629	0.403548725	0.686544598	0.7323715	positive
PRJNA729271	stage_E18_vs_E10	ordinary_muscle_development	ENSGALG00010020006	PLB1	-0.007434811	0.369618012	-0.020114851	0.983951753	0.986498279	negative
PRJNA729271	stage_E18_vs_E10	ordinary_muscle_development	ENSGALG00010020008	EIF2S1	-0.713240291	0.079753437	-8.943066479	3.78518E-19	2.54128E-18	negative
PRJNA729271	stage_E18_vs_E10	ordinary_muscle_development	ENSGALG00010020009	MACROD2	-1.106004912	0.12007662	-9.210826468	3.23623E-20	2.30286E-19	negative
PRJNA729271	stage_E18_vs_E10	ordinary_muscle_development	ENSGALG00010020010	SORBS3	2.513424768	0.206421656	12.17616802	4.1639E-34	6.17135E-33	positive
PRJNA729271	stage_E18_vs_E10	ordinary_muscle_development	ENSGALG00010020011	TLE3	0.204977147	0.202861183	1.0104306	0.312289033	0.369792463	positive
PRJNA729271	stage_E18_vs_E10	ordinary_muscle_development	ENSGALG00010020013	ADAM8	-2.377521653	0.680345515	-3.494579741	0.000474809	0.000905454	negative
PRJNA729271	stage_E18_vs_E10	ordinary_muscle_development	ENSGALG00010020015	ATP6V1D	0.49058511	0.078844931	6.222151543	4.90383E-10	1.70499E-09	positive
PRJNA729271	stage_E18_vs_E10	ordinary_muscle_development	ENSGALG00010020017	ENSGALG00010020017	1.336667147	0.320137438	4.175291576	2.97605E-05	6.56388E-05	positive
PRJNA729271	stage_E18_vs_E10	ordinary_muscle_development	ENSGALG00010020018	ENSGALG00010020018	1.999200622	0.204035231	9.798310868	1.14484E-22	9.42332E-22	positive
PRJNA729271	stage_E18_vs_E10	ordinary_muscle_development	ENSGALG00010020019	PEX10	-0.336362476	0.102146174	-3.292952282	0.000991413	0.001806705	negative
PRJNA729271	stage_E18_vs_E10	ordinary_muscle_development	ENSGALG00010020023	FOSL2	-0.395302002	0.210359716	-1.879171588	0.060221068	0.083310044	negative
PRJNA729271	stage_E18_vs_E10	ordinary_muscle_development	ENSGALG00010020025	LOXL2	-0.863607247	0.140662792	-6.139557143	8.27519E-10	2.81835E-09	negative
PRJNA729271	stage_E18_vs_E10	ordinary_muscle_development	ENSGALG00010020027	RER1	-0.230024569	0.071210607	-3.230200934	0.001237032	0.002228799	negative
PRJNA729271	stage_E18_vs_E10	ordinary_muscle_development	ENSGALG00010020030	MRPL33	-0.129666953	0.136810857	-0.947782624	0.343240128	0.401652188	negative
PRJNA729271	stage_E18_vs_E10	ordinary_muscle_development	ENSGALG00010020032	CHST12	0.271623178	0.122861317	2.210811225	0.027048912	0.039848967	positive
PRJNA729271	stage_E18_vs_E10	ordinary_muscle_development	ENSGALG00010020036	TMF1	-0.097720983	0.084288067	-1.159369127	0.246305755	0.299513584	negative
PRJNA729271	stage_E18_vs_E10	ordinary_muscle_development	ENSGALG00010020037	MPP5	-0.060528643	0.089577835	-0.675710046	0.499224757	0.556507179	negative
PRJNA729271	stage_E18_vs_E10	ordinary_muscle_development	ENSGALG00010020038	SKI	-0.044023641	0.090881911	-0.484404871	0.628098562	0.678310882	negative
PRJNA729271	stage_E18_vs_E10	ordinary_muscle_development	ENSGALG00010020041	CDH23	0.034083056	0.153300603	0.222328255	0.824058358	0.85373091	positive
PRJNA729271	stage_E18_vs_E10	ordinary_muscle_development	ENSGALG00010020046	BDNF	0.962570998	0.224422397	4.289103985	1.79395E-05	4.06049E-05	positive
PRJNA729271	stage_E18_vs_E10	ordinary_muscle_development	ENSGALG00010020048	ENSGALG00010020048	-0.452205883	0.26573769	-1.701700213	0.088811581	0.118888424	negative
PRJNA729271	stage_E18_vs_E10	ordinary_muscle_development	ENSGALG00010020053	LFNG	0.761537774	0.134722802	5.652627178	1.58014E-08	4.80271E-08	positive
PRJNA729271	stage_E18_vs_E10	ordinary_muscle_development	ENSGALG00010020055	R3HCC1	-0.193278872	0.08417047	-2.296278868	0.021659942	0.032457444	negative
PRJNA729271	stage_E18_vs_E10	ordinary_muscle_development	ENSGALG00010020056	UACA	-0.753889831	0.165498512	-4.555266512	5.23192E-06	1.25329E-05	negative
PRJNA729271	stage_E18_vs_E10	ordinary_muscle_development	ENSGALG00010020057	RBKS	-0.190551233	0.096035111	-1.984182992	0.047235444	0.06658994	negative
PRJNA729271	stage_E18_vs_E10	ordinary_muscle_development	ENSGALG00010020059	PRKCZ	-0.517111571	0.142836698	-3.620299124	0.000294263	0.000577177	negative
PRJNA729271	stage_E18_vs_E10	ordinary_muscle_development	ENSGALG00010020061	UBA3	-0.147725426	0.073021666	-2.023035557	0.043069482	0.06113319	negative
PRJNA729271	stage_E18_vs_E10	ordinary_muscle_development	ENSGALG00010020063	ENSGALG00010020063	1.489455642	0.128958802	11.54985638	7.39439E-31	9.44332E-30	positive
PRJNA729271	stage_E18_vs_E10	ordinary_muscle_development	ENSGALG00010020064	TMEM138	0.202379706	0.132117584	1.531815064	0.125568078	0.163634746	positive
PRJNA729271	stage_E18_vs_E10	ordinary_muscle_development	ENSGALG00010020065	TTYH3	-1.232361481	0.111790252	-11.02387244	2.93169E-28	3.28908E-27	negative
PRJNA729271	stage_E18_vs_E10	ordinary_muscle_development	ENSGALG00010020066	ENSGALG00010020066	2.357554779	0.383156446	6.152982172	7.60394E-10	2.60012E-09	positive
PRJNA729271	stage_E18_vs_E10	ordinary_muscle_development	ENSGALG00010020069	TMEM216	-0.712782021	0.206203647	-3.456689695	0.000546854	0.001033462	negative
PRJNA729271	stage_E18_vs_E10	ordinary_muscle_development	ENSGALG00010020070	ARL6IP5	0.564039942	0.114545058	4.92417528	8.47169E-07	2.20858E-06	positive
PRJNA729271	stage_E18_vs_E10	ordinary_muscle_development	ENSGALG00010020071	ENSGALG00010020071	-0.438710446	0.145356892	-3.018160618	0.002543141	0.00439943	negative
PRJNA729271	stage_E18_vs_E10	ordinary_muscle_development	ENSGALG00010020074	ENSGALG00010020074	-0.548708766	0.208396908	-2.632998598	0.00846347	0.013537629	negative
PRJNA729271	stage_E18_vs_E10	ordinary_muscle_development	ENSGALG00010020075	CPSF7	-0.019376717	0.141925225	-0.136527643	0.891404173	0.910307418	negative
PRJNA729271	stage_E18_vs_E10	ordinary_muscle_development	ENSGALG00010020080	SLC4A1AP	-0.35120935	0.104885038	-3.348517161	0.000812452	0.001500573	negative
PRJNA729271	stage_E18_vs_E10	ordinary_muscle_development	ENSGALG00010020083	CFAP74	-0.942190138	0.436666416	-2.157688579	0.030952052	0.045122197	negative
PRJNA729271	stage_E18_vs_E10	ordinary_muscle_development	ENSGALG00010020084	ENSGALG00010020084	0.271930505	0.086709778	3.13609965	0.00171211	0.003036095	positive
PRJNA729271	stage_E18_vs_E10	ordinary_muscle_development	ENSGALG00010020087	ENSGALG00010020087	-1.000573324	0.624975561	-1.60097992	0.109381367	0.144043146	negative
PRJNA729271	stage_E18_vs_E10	ordinary_muscle_development	ENSGALG00010020088	LMOD3	3.509989919	0.194128881	18.08071993	4.5214E-73	2.57011E-71	positive
PRJNA729271	stage_E18_vs_E10	ordinary_muscle_development	ENSGALG00010020090	MORF4L2	-0.449891783	0.053692033	-8.379116248	5.33266E-17	3.11681E-16	negative
PRJNA729271	stage_E18_vs_E10	ordinary_muscle_development	ENSGALG00010020091	SUPT7L	-0.042671318	0.08396329	-0.508213981	0.611303287	0.662047306	negative
PRJNA729271	stage_E18_vs_E10	ordinary_muscle_development	ENSGALG00010020092	SDHAF2	0.719922962	0.137879381	5.221396812	1.77579E-07	4.91315E-07	positive
PRJNA729271	stage_E18_vs_E10	ordinary_muscle_development	ENSGALG00010020094	CLEC3B	3.511427326	0.227720693	15.41988685	1.20315E-53	3.91024E-52	positive
PRJNA729271	stage_E18_vs_E10	ordinary_muscle_development	ENSGALG00010020095	LRFN2	-0.760096732	0.327360803	-2.321892926	0.020238702	0.030494801	negative
PRJNA729271	stage_E18_vs_E10	ordinary_muscle_development	ENSGALG00010020096	GPHN	0.509031103	0.117220424	4.342512035	1.40863E-05	3.22968E-05	positive
PRJNA729271	stage_E18_vs_E10	ordinary_muscle_development	ENSGALG00010020097	RETSAT	0.660805537	0.229193943	2.883171902	0.003936925	0.006614016	positive
PRJNA729271	stage_E18_vs_E10	ordinary_muscle_development	ENSGALG00010020098	GABRD	-0.513124679	0.356817823	-1.43805787	0.150417635	0.192521291	negative
PRJNA729271	stage_E18_vs_E10	ordinary_muscle_development	ENSGALG00010020100	MOCS1	-0.035267	0.159975652	-0.220452296	0.825518921	0.85482903	negative
PRJNA729271	stage_E18_vs_E10	ordinary_muscle_development	ENSGALG00010020101	KIF15	-2.067438164	0.206267438	-10.02309521	1.20664E-23	1.04795E-22	negative
PRJNA729271	stage_E18_vs_E10	ordinary_muscle_development	ENSGALG00010020102	TMEM127	-0.196955249	0.215735322	-0.912948551	0.361269601	0.420745851	negative
PRJNA729271	stage_E18_vs_E10	ordinary_muscle_development	ENSGALG00010020103	PSAP	0.087279642	0.105110326	0.830362205	0.406334028	0.46637198	positive
PRJNA729271	stage_E18_vs_E10	ordinary_muscle_development	ENSGALG00010020104	BRAT1	0.026968876	0.115205404	0.234093848	0.814912122	0.846203253	positive
PRJNA729271	stage_E18_vs_E10	ordinary_muscle_development	ENSGALG00010020106	ENSGALG00010020106	1.594811477	0.163004018	9.78378337	1.32051E-22	1.08508E-21	positive
PRJNA729271	stage_E18_vs_E10	ordinary_muscle_development	ENSGALG00010020108	RPAP1	-0.542184353	0.11749368	-4.614583109	3.93885E-06	9.52831E-06	negative
PRJNA729271	stage_E18_vs_E10	ordinary_muscle_development	ENSGALG00010020109	WDFY4	-1.144793031	0.367695414	-3.113427549	0.001849279	0.003265753	negative
PRJNA729271	stage_E18_vs_E10	ordinary_muscle_development	ENSGALG00010020111	ADRA2B	1.322409472	0.122669435	10.78026876	4.26637E-27	4.54046E-26	positive
PRJNA729271	stage_E18_vs_E10	ordinary_muscle_development	ENSGALG00010020113	CIAO1	-0.268004182	0.105800512	-2.533108541	0.011305594	0.017766108	negative
PRJNA729271	stage_E18_vs_E10	ordinary_muscle_development	ENSGALG00010020116	GNA12	-0.219077264	0.072900218	-3.005166116	0.002654358	0.004580894	negative
PRJNA729271	stage_E18_vs_E10	ordinary_muscle_development	ENSGALG00010020117	AKAP13	0.095917954	0.102232166	0.938236545	0.348122862	0.406938781	positive
PRJNA729271	stage_E18_vs_E10	ordinary_muscle_development	ENSGALG00010020118	GNB1	-0.742212415	0.093513063	-7.936991815	2.07144E-15	1.08591E-14	negative
PRJNA729271	stage_E18_vs_E10	ordinary_muscle_development	ENSGALG00010020120	DAAM2	-0.532198358	0.102352749	-5.199648886	1.99665E-07	5.50428E-07	negative
PRJNA729271	stage_E18_vs_E10	ordinary_muscle_development	ENSGALG00010020121	ENSGALG00010020121	-0.841827411	0.231289445	-3.639713919	0.000272941	0.000537611	negative
PRJNA729271	stage_E18_vs_E10	ordinary_muscle_development	ENSGALG00010020123	ENSGALG00010020123	0.859683315	0.143474787	5.991877282	2.07432E-09	6.81027E-09	positive
PRJNA729271	stage_E18_vs_E10	ordinary_muscle_development	ENSGALG00010020125	NAV2	0.999482416	0.129688122	7.70681538	1.28996E-14	6.38547E-14	positive
PRJNA729271	stage_E18_vs_E10	ordinary_muscle_development	ENSGALG00010020126	NADK	-0.352271463	0.105497678	-3.339139483	0.000840384	0.001548412	negative
PRJNA729271	stage_E18_vs_E10	ordinary_muscle_development	ENSGALG00010020127	FRMD4B	-0.488894616	0.140033719	-3.49126354	0.000480742	0.000915924	negative
PRJNA729271	stage_E18_vs_E10	ordinary_muscle_development	ENSGALG00010020128	KIF6	-0.145477313	0.528585444	-0.275220052	0.783147182	0.818200837	negative
PRJNA729271	stage_E18_vs_E10	ordinary_muscle_development	ENSGALG00010020129	MGAT2	-0.207695235	0.116984159	-1.775413318	0.075829667	0.102993911	negative
PRJNA729271	stage_E18_vs_E10	ordinary_muscle_development	ENSGALG00010020130	PDXDC1	-0.395482645	0.074887223	-5.281042973	1.28451E-07	3.60482E-07	negative
PRJNA729271	stage_E18_vs_E10	ordinary_muscle_development	ENSGALG00010020131	SNRNP200	-0.685920895	0.124992183	-5.487710341	4.07177E-08	1.19547E-07	negative
PRJNA729271	stage_E18_vs_E10	ordinary_muscle_development	ENSGALG00010020132	SHC4	-0.485877492	0.232211741	-2.092389859	0.036403655	0.052368347	negative
PRJNA729271	stage_E18_vs_E10	ordinary_muscle_development	ENSGALG00010020133	ENSGALG00010020133	2.642179452	0.366198407	7.215158237	5.38713E-13	2.34988E-12	positive
PRJNA729271	stage_E18_vs_E10	ordinary_muscle_development	ENSGALG00010020136	KCNK17	-0.826882232	0.332505118	-2.486825576	0.012888857	0.020073492	negative
PRJNA729271	stage_E18_vs_E10	ordinary_muscle_development	ENSGALG00010020137	NTAN1	-0.148316671	0.083291935	-1.780684649	0.074963982	0.101904054	negative
PRJNA729271	stage_E18_vs_E10	ordinary_muscle_development	ENSGALG00010020138	ENSGALG00010020138	-0.176816542	0.086617908	-2.041339336	0.04121711	0.058699352	negative
PRJNA729271	stage_E18_vs_E10	ordinary_muscle_development	ENSGALG00010020140	SECISBP2L	0.325774342	0.095220616	3.42125851	0.000623321	0.001169129	positive
PRJNA729271	stage_E18_vs_E10	ordinary_muscle_development	ENSGALG00010020141	THOC7	-0.346770523	0.128116014	-2.706691484	0.006795738	0.011013441	negative
PRJNA729271	stage_E18_vs_E10	ordinary_muscle_development	ENSGALG00010020142	SLC35E2B	-0.880879241	0.06995021	-12.59294641	2.30891E-36	3.82051E-35	negative
PRJNA729271	stage_E18_vs_E10	ordinary_muscle_development	ENSGALG00010020143	KCNK5	-1.607076919	0.188072207	-8.544999506	1.28537E-17	7.8382E-17	negative
PRJNA729271	stage_E18_vs_E10	ordinary_muscle_development	ENSGALG00010020147	ABHD2	0.585019891	0.147642677	3.962403707	7.41989E-05	0.000156895	positive
PRJNA729271	stage_E18_vs_E10	ordinary_muscle_development	ENSGALG00010020149	FAHD2A	0.338166168	0.173703382	1.946802443	0.051558424	0.072227637	positive
PRJNA729271	stage_E18_vs_E10	ordinary_muscle_development	ENSGALG00010020152	ATXN7	0.641569286	0.108252682	5.926590197	3.0929E-09	1.00048E-08	positive

PRJNA729271	stage_E18_vs_E10	ordinary_muscle_development	ENSGALG00010020153	AXL	0.053121967	0.121061215	0.43880253	0.660804627	0.708563624	positive
PRJNA729271	stage_E18_vs_E10	ordinary_muscle_development	ENSGALG00010020155	MFGE8	0.537137629	0.135408249	3.966801372	7.28437E-05	0.000154254	positive
PRJNA729271	stage_E18_vs_E10	ordinary_muscle_development	ENSGALG00010020160	SYT7	-2.330677073	0.281700222	-8.273607512	1.29966E-16	7.40507E-16	negative
PRJNA729271	stage_E18_vs_E10	ordinary_muscle_development	ENSGALG00010020163	CDC2L1	-0.373598738	0.096676124	-3.864436472	0.000111346	0.000231127	negative
PRJNA729271	stage_E18_vs_E10	ordinary_muscle_development	ENSGALG00010020164	C10orf71	3.6921763	0.147987156	24.94930236	2.1729E-137	5.5257E-135	positive
PRJNA729271	stage_E18_vs_E10	ordinary_muscle_development	ENSGALG00010020166	HAPLN3	-0.338643163	0.177810572	-1.904516465	0.056842954	0.07903488	negative
PRJNA729271	stage_E18_vs_E10	ordinary_muscle_development	ENSGALG00010020168	COX7A2L	0.430835229	0.115538568	3.728929985	0.000192295	0.000386429	positive
PRJNA729271	stage_E18_vs_E10	ordinary_muscle_development	ENSGALG00010020170	SORCS1	-2.193700555	0.313219607	-7.003714017	2.49265E-12	1.03379E-11	negative
PRJNA729271	stage_E18_vs_E10	ordinary_muscle_development	ENSGALG00010020171	RRN3	-0.293486938	0.076984333	-3.81229438	0.000137683	0.000282359	negative
PRJNA729271	stage_E18_vs_E10	ordinary_muscle_development	ENSGALG00010020175	ACAN	-5.336189656	0.502572889	-10.61774277	2.46448E-26	2.52101E-25	negative
PRJNA729271	stage_E18_vs_E10	ordinary_muscle_development	ENSGALG00010020176	ENSGALG00010020176	0.503562936	0.247615207	2.033651096	0.041986786	0.059678218	positive
PRJNA729271	stage_E18_vs_E10	ordinary_muscle_development	ENSGALG00010020177	KCNG3	-1.155910588	0.240650717	-4.803270905	1.56094E-06	3.94798E-06	negative
PRJNA729271	stage_E18_vs_E10	ordinary_muscle_development	ENSGALG00010020179	PSMD6	-0.456747345	0.136229829	-3.352770457	0.00080007	0.001479246	negative
PRJNA729271	stage_E18_vs_E10	ordinary_muscle_development	ENSGALG00010020180	TCF7L1	-0.260248712	0.208214805	-1.249904935	0.211334276	0.261395199	negative
PRJNA729271	stage_E18_vs_E10	ordinary_muscle_development	ENSGALG00010020181	LEUTX	2.607458341	0.21552565	12.0981347	1.08038E-33	1.55358E-32	positive
PRJNA729271	stage_E18_vs_E10	ordinary_muscle_development	ENSGALG00010020183	XPNPEP1	-0.320120087	0.068657749	-4.662582499	3.12266E-06	7.6406E-06	negative
PRJNA729271	stage_E18_vs_E10	ordinary_muscle_development	ENSGALG00010020184	RSL1D1	-1.004948446	0.071332375	-14.08825162	4.48529E-45	1.05372E-43	negative
PRJNA729271	stage_E18_vs_E10	ordinary_muscle_development	ENSGALG00010020186	MGA	0.119728502	0.094985794	1.26048851	0.20749319	0.25717091	positive
PRJNA729271	stage_E18_vs_E10	ordinary_muscle_development	ENSGALG00010020187	MMP23B	0.238287748	0.207528075	1.148219334	0.25087803	0.304486062	positive
PRJNA729271	stage_E18_vs_E10	ordinary_muscle_development	ENSGALG00010020193	VSTM4	0.244702736	0.176836578	1.383778958	0.166426148	0.21066693	positive
PRJNA729271	stage_E18_vs_E10	ordinary_muscle_development	ENSGALG00010020194	PRICKLE2	-0.00314812	0.153554504	-0.020501642	0.983643202	0.986296916	negative
PRJNA729271	stage_E18_vs_E10	ordinary_muscle_development	ENSGALG00010020199	DET1	-0.195213759	0.084746914	-2.30349105	0.02125123	0.031906309	negative
PRJNA729271	stage_E18_vs_E10	ordinary_muscle_development	ENSGALG00010020202	MAPKBP1	2.050294909	0.089832481	22.82353647	2.6775E-115	4.6759E-113	positive
PRJNA729271	stage_E18_vs_E10	ordinary_muscle_development	ENSGALG00010020204	GSPT2	-0.403771899	0.087110105	-4.635190158	3.5661E-06	8.66564E-06	negative
PRJNA729271	stage_E18_vs_E10	ordinary_muscle_development	ENSGALG00010020206	MRPS11	0.011323467	0.082516259	0.137227099	0.890851292	0.90980691	positive
PRJNA729271	stage_E18_vs_E10	ordinary_muscle_development	ENSGALG00010020211	ADD3	0.534018972	0.083145909	6.422672885	1.33902E-10	4.83294E-10	positive
PRJNA729271	stage_E18_vs_E10	ordinary_muscle_development	ENSGALG00010020212	JMJD7	0.081939268	0.140725626	0.582262598	0.560389815	0.614390013	positive
PRJNA729271	stage_E18_vs_E10	ordinary_muscle_development	ENSGALG00010020215	PRMT3	-1.248411008	0.085160072	-14.65958149	1.16992E-48	3.22395E-47	negative
PRJNA729271	stage_E18_vs_E10	ordinary_muscle_development	ENSGALG00010020224	MRPL46	0.132555511	0.087598095	1.513223679	0.130222852	0.168864546	positive
PRJNA729271	stage_E18_vs_E10	ordinary_muscle_development	ENSGALG00010020226	TCIM	1.642687282	0.198323249	8.282878042	1.20234E-16	6.87489E-16	positive
PRJNA729271	stage_E18_vs_E10	ordinary_muscle_development	ENSGALG00010020228	TM9SF3	-0.482104475	0.083188579	-5.795320471	6.81908E-09	2.13806E-08	negative
PRJNA729271	stage_E18_vs_E10	ordinary_muscle_development	ENSGALG00010020230	ENSGALG00010020230	0.324727456	0.217862829	1.490513356	0.136089307	0.175812345	positive
PRJNA729271	stage_E18_vs_E10	ordinary_muscle_development	ENSGALG00010020236	SLC35G1	-1.37821413	0.34736996	-3.967568557	7.26096E-05	0.000153803	negative
PRJNA729271	stage_E18_vs_E10	ordinary_muscle_development	ENSGALG00010020237	MIB2	-0.246215345	0.077066911	-3.194825675	0.001399153	0.002504721	negative
PRJNA729271	stage_E18_vs_E10	ordinary_muscle_development	ENSGALG00010020240	SV2B	0.978511011	0.338236646	2.892977513	0.003816086	0.006423664	positive
PRJNA729271	stage_E18_vs_E10	ordinary_muscle_development	ENSGALG00010020244	SLC25A37	-1.269481792	0.215668968	-5.886251516	3.95053E-09	1.26716E-08	negative
PRJNA729271	stage_E18_vs_E10	ordinary_muscle_development	ENSGALG00010020246	ADAMTS9	-0.757489774	0.12994477	-5.82932098	5.56534E-09	1.76019E-08	negative
PRJNA729271	stage_E18_vs_E10	ordinary_muscle_development	ENSGALG00010020248	NOLC1	-1.187884315	0.081665041	-14.54581174	6.20899E-48	1.66665E-46	negative
PRJNA729271	stage_E18_vs_E10	ordinary_muscle_development	ENSGALG00010020250	ENSGALG00010020250	0.370905555	0.244965871	1.514111141	0.129997653	0.168621876	positive
PRJNA729271	stage_E18_vs_E10	ordinary_muscle_development	ENSGALG00010020252	DMTN	1.167450476	0.188482325	6.193952012	5.86741E-10	2.02785E-09	positive
PRJNA729271	stage_E18_vs_E10	ordinary_muscle_development	ENSGALG00010020254	ENSGALG00010020254	-1.332309856	0.135501245	-9.832454694	8.1605E-23	6.77859E-22	negative
PRJNA729271	stage_E18_vs_E10	ordinary_muscle_development	ENSGALG00010020257	ZNF710	0.064869384	0.163485538	0.396789739	0.691522518	0.737030802	positive
PRJNA729271	stage_E18_vs_E10	ordinary_muscle_development	ENSGALG00010020259	ENSGALG00010020259	-0.664124386	0.190230004	-3.491165291	0.000480919	0.000916141	negative
PRJNA729271	stage_E18_vs_E10	ordinary_muscle_development	ENSGALG00010020261	SSU72	-0.183218653	0.09269742	-1.976523766	0.048095487	0.067725461	negative
PRJNA729271	stage_E18_vs_E10	ordinary_muscle_development	ENSGALG00010020265	ENSGALG00010020265	0.196558541	0.226782569	0.866726846	0.386091693	0.446390611	positive
PRJNA729271	stage_E18_vs_E10	ordinary_muscle_development	ENSGALG00010020268	CPLANE2	-0.071810945	0.100004934	-0.718074019	0.472711649	0.531488935	negative
PRJNA729271	stage_E18_vs_E10	ordinary_muscle_development	ENSGALG00010020269	ENSGALG00010020269	-2.553549694	0.828248073	-3.08307351	0.002048745	0.003592616	negative
PRJNA729271	stage_E18_vs_E10	ordinary_muscle_development	ENSGALG00010020272	ENSGALG00010020272	-0.389490748	0.09009973	-4.322884738	1.54002E-05	3.51648E-05	negative
PRJNA729271	stage_E18_vs_E10	ordinary_muscle_development	ENSGALG00010020275	ELOVL3	-5.36753054	0.907820277	-5.912547534	3.36857E-09	1.0865E-08	negative
PRJNA729271	stage_E18_vs_E10	ordinary_muscle_development	ENSGALG00010020277	IDH2	1.41934219	0.124678007	11.38406219	5.02054E-30	6.13082E-29	positive
PRJNA729271	stage_E18_vs_E10	ordinary_muscle_development	ENSGALG00010020282	MAGI1	-0.750108442	0.138130212	-5.430444443	5.62139E-08	1.62574E-07	negative
PRJNA729271	stage_E18_vs_E10	ordinary_muscle_development	ENSGALG00010020283	FBXO42	-0.507989173	0.225577203	-2.251952614	0.024325267	0.036148733	negative
PRJNA729271	stage_E18_vs_E10	ordinary_muscle_development	ENSGALG00010020284	HEG1	0.471297473	0.119836907	3.932824084	8.39537E-05	0.000176454	positive
PRJNA729271	stage_E18_vs_E10	ordinary_muscle_development	ENSGALG00010020285	ADAMTS14	-1.880043886	0.262732774	-7.155726555	8.32309E-13	3.59592E-12	negative
PRJNA729271	stage_E18_vs_E10	ordinary_muscle_development	ENSGALG00010020287	GALNT16	1.566660409	0.159775429	9.805390123	1.06734E-22	8.80538E-22	positive
PRJNA729271	stage_E18_vs_E10	ordinary_muscle_development	ENSGALG00010020290	NELL1	-3.598673602	0.582396923	-6.179073863	6.44787E-10	2.22E-09	negative
PRJNA729271	stage_E18_vs_E10	ordinary_muscle_development	ENSGALG00010020291	SNX29	0.036104364	0.146176938	0.246990839	0.804915314	0.837322196	positive
PRJNA729271	stage_E18_vs_E10	ordinary_muscle_development	ENSGALG00010020292	NPM2	-0.782178491	0.248196431	-3.151449394	0.001624623	0.00288802	negative
PRJNA729271	stage_E18_vs_E10	ordinary_muscle_development	ENSGALG00010020293	SEMA4B	0.259556942	0.111344557	2.331114766	0.019747309	0.029798772	positive
PRJNA729271	stage_E18_vs_E10	ordinary_muscle_development	ENSGALG00010020295	LGI1	-0.415579972	0.152757662	-2.720518011	0.006517972	0.010601212	negative
PRJNA729271	stage_E18_vs_E10	ordinary_muscle_development	ENSGALG00010020298	ENSGALG00010020298	0.643131215	0.173645379	3.703704748	0.000212474	0.000424508	positive
PRJNA729271	stage_E18_vs_E10	ordinary_muscle_development	ENSGALG00010020300	EHD4	0.392838382	0.129307972	3.038005889	0.002381493	0.004132616	positive
PRJNA729271	stage_E18_vs_E10	ordinary_muscle_development	ENSGALG00010020302	HAAO	2.601814897	1.447843363	1.797027886	0.072331193	0.098648912	positive
PRJNA729271	stage_E18_vs_E10	ordinary_muscle_development	ENSGALG00010020303	EXD2	-0.268133496	0.074079863	-3.619519342	0.000295151	0.000578606	negative
PRJNA729271	stage_E18_vs_E10	ordinary_muscle_development	ENSGALG00010020304	ZFP36L2	1.382600217	0.155176032	8.909882556	5.10867E-19	3.41245E-18	positive
PRJNA729271	stage_E18_vs_E10	ordinary_muscle_development	ENSGALG00010020305	XPO7	-0.335618721	0.138826764	-2.41753615	0.015625982	0.024052098	negative
PRJNA729271	stage_E18_vs_E10	ordinary_muscle_development	ENSGALG00010020307	SHQ1	-0.710314143	0.227857301	-3.117363981	0.001824761	0.003225993	negative
PRJNA729271	stage_E18_vs_E10	ordinary_muscle_development	ENSGALG00010020309	GXYLT2	0.04748648	0.159569078	0.297591995	0.766014585	0.80360291	positive
PRJNA729271	stage_E18_vs_E10	ordinary_muscle_development	ENSGALG00010020311	SGPL1	-0.651969623	0.135138855	-4.824442411	1.40395E-06	3.57149E-06	negative
PRJNA729271	stage_E18_vs_E10	ordinary_muscle_development	ENSGALG00010020313	FRA10AC1	0.239921273	0.115066233	2.08507106	0.037062851	0.053222312	positive
PRJNA729271	stage_E18_vs_E10	ordinary_muscle_development	ENSGALG00010020314	THADA	-0.825423006	0.081601178	-10.11533204	4.72412E-24	4.21651E-23	negative
PRJNA729271	stage_E18_vs_E10	ordinary_muscle_development	ENSGALG00010020315	ANO5	-0.336755848	0.108726333	-3.09727956	0.001953056	0.003432708	negative
PRJNA729271	stage_E18_vs_E10	ordinary_muscle_development	ENSGALG00010020316	PDPK1	-0.180989558	0.11328036	-1.597713474	0.110106759	0.144958898	negative
PRJNA729271	stage_E18_vs_E10	ordinary_muscle_development	ENSGALG00010020319	NTRK3	1.148047728	0.157948465	7.268495612	3.63513E-13	1.60987E-12	positive
PRJNA729271	stage_E18_vs_E10	ordinary_muscle_development	ENSGALG00010020321	SZRD1	0.278107312	0.169917632	1.636718384	0.101689326	0.134610665	positive
PRJNA729271	stage_E18_vs_E10	ordinary_muscle_development	ENSGALG00010020323	ARHGAP25	2.258671855	0.150433754	15.01439538	5.90983E-51	1.78465E-49	positive
PRJNA729271	stage_E18_vs_E10	ordinary_muscle_development	ENSGALG00010020324	PLEKHH2	0.221122192	0.185914409	1.189376302	0.234291622	0.286490388	positive
PRJNA729271	stage_E18_vs_E10	ordinary_muscle_development	ENSGALG00010020329	PPP4R2	-0.405514856	0.10666624	-3.801716987	0.000143697	0.000293819	negative
PRJNA729271	stage_E18_vs_E10	ordinary_muscle_development	ENSGALG00010020334	CABP4	-0.36231278	0.543253399	-0.666931455	0.504815918	0.562005125	negative
PRJNA729271	stage_E18_vs_E10	ordinary_muscle_development	ENSGALG00010020336	OSBP	-0.199084672	0.135097156	-1.473640734	0.140578348	0.181109302	negative
PRJNA729271	stage_E18_vs_E10	ordinary_muscle_development	ENSGALG00010020338	NECAP2	-0.23048444	0.167392844	-1.376907361	0.168540891	0.213064732	negative
PRJNA729271	stage_E18_vs_E10	ordinary_muscle_development	ENSGALG00010020340	DCAF5	0.747591235	0.097072416	7.701376591	1.34608E-14	6.65239E-14	positive
PRJNA729271	stage_E18_vs_E10	ordinary_muscle_development	ENSGALG00010020341	DYNC2LI1	-0.785169619	0.130245865	-6.028365034	1.65627E-09	5.48369E-09	negative
PRJNA729271	stage_E18_vs_E10	ordinary_muscle_development	ENSGALG00010020342	CPPED1	-0.114736567	0.106628479	-1.076040549	0.281909122	0.337490781	negative
PRJNA729271	stage_E18_vs_E10	ordinary_muscle_development	ENSGALG00010020343	PATL1	0.373671178	0.177863924	2.100882351	0.035651295	0.051378557	positive
PRJNA729271	stage_E18_vs_E10	ordinary_muscle_development	ENSGALG00010020345	AGBL1	1.30370317	0.452750463	2.879518139	0.003982834	0.006688817	positive
PRJNA729271	stage_E18_vs_E10	ordinary_muscle_development	ENSGALG00010020346	PCBD1	-1.07725036	0.263658862	-4.085773377	4.39302E-05	9.51395E-05	negative
PRJNA729271	stage_E18_vs_E10	ordinary_muscle_development	ENSGALG00010020350	PDZRN3	-0.040653327	0.10835089	-0.375200671	0.707511231	0.751915484	negative
PRJNA729271	stage_E18_vs_E10	ordinary_muscle_development	ENSGALG00010020351	CROCC	0.098112933	0.148001628	0.662917929	0.507383119	0.564386333	positive
PRJNA729271	stage_E18_vs_E10	ordinary_muscle_development	ENSGALG00010020352	ABCG5	0.40208586	0.599796793	0.67037014	0.502621865	0.559777482	positive
PRJNA729271	stage_E18_vs_E10	ordinary_muscle_development	ENSGALG00010020356	STX3	0.222414146	0.378513397	0.58759914	0.556801388	0.610779183	positive
PRJNA729271	stage_E18_vs_E10	ordinary_muscle_development	ENSGALG00010020357	RBP4	2.320635179	0.725095719	3.200453565	0.001372115	0.002460572	positive
PRJNA729271	stage_E18_vs_E10	ordinary_muscle_development	ENSGALG00010020362	CDS2	-0.173268035	0.127184958	-1.362331189	0.173093389	0.21830581	negative
PRJNA729271	stage_E18_vs_E10	ordinary_muscle_development	ENSGALG00010020364	MRPL16	0.461378748	0.111731774	4.129342349	3.63802E-05	7.94892E-05	positive
PRJNA729271	stage_E18_vs_E10	ordinary_muscle_development	ENSGALG00010020365	UNC5B	-0.005413199	0.138234577	-0.039159513	0.968763213	0.974207213	negative
PRJNA729271	stage_E18_vs_E10	ordinary_muscle_development	ENSGALG00010020369	CNTN3	-2.523620277	0.289515907	-8.716689524	2.86454E-18	1.82512E-17	negative
PRJNA729271	stage_E18_vs_E10	ordinary_muscle_development	ENSGALG00010020370	CEP55	-0.875234036	0.22162348	-3.949193637	7.84149E-05	0.000165279	negative
PRJNA729271	stage_E18_vs_E10	ordinary_muscle_development	ENSGALG00010020373	MS4A4A	-0.355190656	0.215166256	-1.650773048	0.098784925	0.131161262	negative
PRJNA729271	stage_E18_vs_E10	ordinary_muscle_development	ENSGALG00010020374	PCNA	-1.589742166	0.096338608	-16.50161031	3.57238E-61	1.47107E-59	negative
PRJNA729271	stage_E18_vs_E10	ordinary_muscle_development	ENSGALG00010020375	LRPPRC	-0.421752448	0.102041134	-4.133161103	3.57808E-05	7.82266E-05	negative
PRJNA729271	stage_E18_vs_E10	ordinary_muscle_development	ENSGALG00010020377	TMEM230	-0.441863381	0.101250129	-4.364077203	1.2766E-05	2.94047E-05	negative
PRJNA729271	stage_E18_vs_E10	ordinary_muscle_development	ENSGALG00010020379	COL13A1	-0.016059046	0.194141059	-0.08271844	0.934075422	0.946085057	negative
PRJNA729271	stage_E18_vs_E10	ordinary_muscle_development	ENSGALG00010020382	MFAP2	-1.460880035	0.146671675	-9.960205584	2.27592E-23	1.94284E-22	negative
PRJNA729271	stage_E18_vs_E10	ordinary_muscle_development	ENSGALG00010020383	BAAT	-0.622349232	0.107316227	-5.799209032	6.66284E-09	2.09279E-08	negative
PRJNA729271	stage_E18_vs_E10	ordinary_muscle_development	ENSGALG00010020385	SLC12A8	-0.673205599	0.357844145	-1.881281583	0.059933625	0.082956565	negative
PRJNA729271	stage_E18_vs_E10	ordinary_muscle_development	ENSGALG00010020386	PPM1B	1.085845712	0.071886021	15.10510241	1.49873E-51	4.61235E-50	positive
PRJNA729271	stage_E18_vs_E10	ordinary_muscle_development	ENSGALG00010020388	MYOF	0.138496346	0.167647312	0.826117305	0.408737552	0.468944975	positive
PRJNA729271	stage_E18_vs_E10	ordinary_muscle_development	ENSGALG00010020389	ACTN1	-1.2246577	0.157699974	-7.765744438	8.11671E-15	4.08513E-14	negative
PRJNA729271	stage_E18_vs_E10	ordinary_muscle_development	ENSGALG00010020390	KLHL25	1.320489599	0.221888371	5.951143769	2.66275E-09	8.66171E-09	positive
PRJNA729271	stage_E18_vs_E10	ordinary_muscle_development	ENSGALG00010020392	SLC23A2	-0.133694245	0.08195456	-1.63132161	0.102822476	0.136011296	negative
PRJNA729271	stage_E18_vs_E10	ordinary_muscle_development	ENSGALG00010020393	SHISA9	-1.76969104	0.313847011	-5.638706044	1.71333E-08	5.18578E-08	negative
PRJNA729271	stage_E18_vs_E10	ordinary_muscle_development	ENSGALG00010020396	CHL1	-1.594217652	0.203940542	-7.817070765	5.40667E-15	2.75562E-14	negative
PRJNA729271	stage_E18_vs_E10	ordinary_muscle_development	ENSGALG00010020399	ATP13A2	-0.020650861	0.132950189	-0.155327802	0.876562899	0.897934927	negative
PRJNA729271	stage_E18_vs_E10	ordinary_muscle_development	ENSGALG00010020402	SLC3A1	2.481784628	0.320974161	7.732038674	1.05838E-14	5.28096E-14	positive
PRJNA729271	stage_E18_vs_E10	ordinary_muscle_development	ENSGALG00010020405	UBE2I	-0.620134222	0.081976297	-7.564799169	3.88464E-14	1.84073E-13	negative
PRJNA729271	stage_E18_vs_E10	ordinary_muscle_development	ENSGALG00010020413	CYP26A1	0.735792895	0.313222269	2.349107863	0.018818455	0.028491069	positive
PRJNA729271	stage_E18_vs_E10	ordinary_muscle_development	ENSGALG00010020415	TSR3	-0.182301194	0.095423817	-1.910437039	0.056076966	0.078029722	negative
PRJNA729271	stage_E18_vs_E10	ordinary_muscle_development	ENSGALG00010020416	ENSGALG00010020416	-2.123803426	0.807764726	-2.62923517	0.008557716	0.013680831	negative
PRJNA729271	stage_E18_vs_E10	ordinary_muscle_development	ENSGALG00010020417	ENSGALG00010020417	0.559140402	0.847003593	0.660139351	0.509164408	0.566107087	positive
PRJNA729271	stage_E18_vs_E10	ordinary_muscle_development	ENSGALG00010020422	ERCC4	-0.642895096	0.091718992	-7.009399923	2.39342E-12	9.9406E-12	negative
PRJNA729271	stage_E18_vs_E10	ordinary_muscle_development	ENSGALG00010020428	SEMA4C	0.126788008	0.150512178	0.842374046	0.399578616	0.459528089	positive
PRJNA729271	stage_E18_vs_E10	ordinary_muscle_development	ENSGALG00010020429	ZNF148	-0.049296681	0.102883485	-0.479150575	0.631831512	0.681631272	negative
PRJNA729271	stage_E18_vs_E10	ordinary_muscle_development	ENSGALG00010020433	GNPTG	-0.351507253	0.073095899	-4.808850499	1.51801E-06	3.84811E-06	negative
PRJNA729271	stage_E18_vs_E10	ordinary_muscle_development	ENSGALG00010020436	CNTN4	-2.199513025	0.266303419	-8.259424664	1.46376E-16	8.3042E-16	negative
PRJNA729271	stage_E18_vs_E10	ordinary_muscle_development	ENSGALG00010020437	SNX4	-0.711519117	0.084781204	-8.392415789	4.76253E-17	2.79485E-16	negative
PRJNA729271	stage_E18_vs_E10	ordinary_muscle_development	ENSGALG00010020438	HTR7	-0.161922689	0.287697598	-0.562822528	0.573555738	0.626871073	negative
PRJNA729271	stage_E18_vs_E10	ordinary_muscle_development	ENSGALG00010020440	PAX7	0.947836383	0.185698503	5.10416814	3.32253E-07	9.00019E-07	positive
PRJNA729271	stage_E18_vs_E10	ordinary_muscle_development	ENSGALG00010020443	PREPL	0.129592553	0.137135663	0.944995269	0.344661291	0.403185007	positive
PRJNA729271	stage_E18_vs_E10	ordinary_muscle_development	ENSGALG00010020444	ENSGALG00010020444	2.789294421	0.879339661	3.172032997	0.001513758	0.002699754	positive
PRJNA729271	stage_E18_vs_E10	ordinary_muscle_development	ENSGALG00010020449	PRPF19	-0.18925994	0.097525051	-1.940628984	0.052303296	0.073157992	negative
PRJNA729271	stage_E18_vs_E10	ordinary_muscle_development	ENSGALG00010020450	ANKRD39	1.166589873	0.179973383	6.482013345	9.05066E-11	3.31119E-10	positive

PRJNA729271	stage_E18_vs_E10	ordinary_muscle_development	ENSGALG00010020453	ENSGALG00010020453	0.503345443	0.138600981	3.631615296	0.000281653	0.000554018	positive
PRJNA729271	stage_E18_vs_E10	ordinary_muscle_development	ENSGALG00010020455	ENSGALG00010020455	-0.152825997	0.699860201	-0.218366464	0.827143593	0.856266703	negative
PRJNA729271	stage_E18_vs_E10	ordinary_muscle_development	ENSGALG00010020457	IL5RA	-0.697980918	1.102197495	-0.633263023	0.526561896	0.582891116	negative
PRJNA729271	stage_E18_vs_E10	ordinary_muscle_development	ENSGALG00010020460	MAPK8	0.079085782	0.141242911	0.559927446	0.575528927	0.62861064	positive
PRJNA729271	stage_E18_vs_E10	ordinary_muscle_development	ENSGALG00010020462	CNNM4	-0.08696791	0.184988043	-0.470127196	0.638264145	0.687700794	negative
PRJNA729271	stage_E18_vs_E10	ordinary_muscle_development	ENSGALG00010020467	ALDH4A1	-0.443595198	0.134071722	-3.308641006	0.0009375	0.001713842	negative
PRJNA729271	stage_E18_vs_E10	ordinary_muscle_development	ENSGALG00010020468	ENSGALG00010020468	-0.670793699	0.508231889	-1.319857556	0.186882581	0.233946196	negative
PRJNA729271	stage_E18_vs_E10	ordinary_muscle_development	ENSGALG00010020473	TMEM109	2.113587934	0.161298478	13.10358261	3.14075E-39	5.82909E-38	positive
PRJNA729271	stage_E18_vs_E10	ordinary_muscle_development	ENSGALG00010020476	TMEM132A	-0.488605749	0.111451695	-4.384013634	1.16512E-05	2.69311E-05	negative
PRJNA729271	stage_E18_vs_E10	ordinary_muscle_development	ENSGALG00010020478	ENSGALG00010020478	-0.339117331	0.907306121	-0.373762861	0.708580753	0.752861752	negative
PRJNA729271	stage_E18_vs_E10	ordinary_muscle_development	ENSGALG00010020481	RPP30	-0.515505472	0.12411072	-4.15359343	3.27294E-05	7.18808E-05	negative
PRJNA729271	stage_E18_vs_E10	ordinary_muscle_development	ENSGALG00010020484	TRNT1	-0.753756633	0.127424323	-5.91532773	3.31215E-09	1.06902E-08	negative
PRJNA729271	stage_E18_vs_E10	ordinary_muscle_development	ENSGALG00010020485	LMAN2L	-0.613246942	0.13305284	-4.609048123	4.04517E-06	9.77082E-06	negative
PRJNA729271	stage_E18_vs_E10	ordinary_muscle_development	ENSGALG00010020486	MRTFB	0.659409541	0.113124107	5.829080634	5.57336E-09	1.76234E-08	positive
PRJNA729271	stage_E18_vs_E10	ordinary_muscle_development	ENSGALG00010020489	IFFO2	-1.697301235	0.193396946	-8.77625665	1.69006E-18	1.09168E-17	negative
PRJNA729271	stage_E18_vs_E10	ordinary_muscle_development	ENSGALG00010020493	DEPDC7	0.260442229	0.147097076	1.770546616	0.076636126	0.103991822	positive
PRJNA729271	stage_E18_vs_E10	ordinary_muscle_development	ENSGALG00010020497	OSBPL11	-0.550145382	0.070655134	-7.786346836	6.89744E-15	3.49087E-14	negative
PRJNA729271	stage_E18_vs_E10	ordinary_muscle_development	ENSGALG00010020498	FRMPD2	-1.643051154	0.628117776	-2.615832917	0.00890101	0.014188952	negative
PRJNA729271	stage_E18_vs_E10	ordinary_muscle_development	ENSGALG00010020507	ANKRD1	2.413221352	0.585766258	4.119768459	3.79253E-05	8.27654E-05	positive
PRJNA729271	stage_E18_vs_E10	ordinary_muscle_development	ENSGALG00010020509	CRBN	0.221791163	0.103665322	2.139492341	0.032395816	0.047047124	positive
PRJNA729271	stage_E18_vs_E10	ordinary_muscle_development	ENSGALG00010020514	CAMKMT	-0.837602968	0.13895822	-6.027732438	1.66276E-09	5.50393E-09	negative
PRJNA729271	stage_E18_vs_E10	ordinary_muscle_development	ENSGALG00010020517	UBR4	-0.243892489	0.094359778	-2.584708179	0.009746145	0.01544276	negative
PRJNA729271	stage_E18_vs_E10	ordinary_muscle_development	ENSGALG00010020520	ARHGAP22	-0.459478913	0.201982716	-2.274842735	0.022915366	0.034200806	negative
PRJNA729271	stage_E18_vs_E10	ordinary_muscle_development	ENSGALG00010020521	ENSGALG00010020521	1.019962695	0.715035415	1.426450597	0.153738318	0.19626143	positive
PRJNA729271	stage_E18_vs_E10	ordinary_muscle_development	ENSGALG00010020524	ENSGALG00010020524	0.102868793	0.408833608	0.251615304	0.801338428	0.834140073	positive
PRJNA729271	stage_E18_vs_E10	ordinary_muscle_development	ENSGALG00010020525	ENSGALG00010020525	1.554114362	0.722579977	2.150785258	0.031493153	0.045846465	positive
PRJNA729271	stage_E18_vs_E10	ordinary_muscle_development	ENSGALG00010020530	SRSF5A	-0.343250193	0.06941102	-4.94518293	7.60724E-07	1.99146E-06	negative
PRJNA729271	stage_E18_vs_E10	ordinary_muscle_development	ENSGALG00010020533	ENSGALG00010020533	0.372922506	0.096830266	3.851301054	0.000117492	0.000243049	positive
PRJNA729271	stage_E18_vs_E10	ordinary_muscle_development	ENSGALG00010020535	LMLN	-0.62829404	0.154570082	-4.06478427	4.80769E-05	0.000103671	negative
PRJNA729271	stage_E18_vs_E10	ordinary_muscle_development	ENSGALG00010020537	CCDC167	-0.226092305	0.097718223	-2.313716902	0.020683256	0.03110966	negative
PRJNA729271	stage_E18_vs_E10	ordinary_muscle_development	ENSGALG00010020540	PCGF5	0.595557732	0.163367435	3.645510691	0.000266861	0.000526853	positive
PRJNA729271	stage_E18_vs_E10	ordinary_muscle_development	ENSGALG00010020544	BMERB1	-1.499465257	0.165137265	-9.080114393	1.08461E-19	7.48994E-19	negative
PRJNA729271	stage_E18_vs_E10	ordinary_muscle_development	ENSGALG00010020545	STYX	1.943843443	0.141087906	13.77753413	3.47956E-43	7.573E-42	positive
PRJNA729271	stage_E18_vs_E10	ordinary_muscle_development	ENSGALG00010020546	LRRC20	2.062391397	0.238533765	8.646119339	5.32804E-18	3.33319E-17	positive
PRJNA729271	stage_E18_vs_E10	ordinary_muscle_development	ENSGALG00010020548	CMTR1	-0.763612835	0.185931043	-4.106967957	4.00887E-05	8.72108E-05	negative
PRJNA729271	stage_E18_vs_E10	ordinary_muscle_development	ENSGALG00010020550	HECTD2	0.365468789	0.215018794	1.699706249	0.089186193	0.119345815	positive
PRJNA729271	stage_E18_vs_E10	ordinary_muscle_development	ENSGALG00010020554	ZFP36L1	0.654274078	0.210891424	3.102421455	0.001919445	0.003377729	positive
PRJNA729271	stage_E18_vs_E10	ordinary_muscle_development	ENSGALG00010020556	EMC1	-0.37946652	0.086418397	-4.391038643	1.1281E-05	2.61129E-05	negative
PRJNA729271	stage_E18_vs_E10	ordinary_muscle_development	ENSGALG00010020557	ENSGALG00010020557	0.214729858	0.269111053	0.797922848	0.424915264	0.485277872	positive
PRJNA729271	stage_E18_vs_E10	ordinary_muscle_development	ENSGALG00010020558	EIF4EBP2	0.825769591	0.099940453	8.262616065	1.42514E-16	8.0946E-16	positive
PRJNA729271	stage_E18_vs_E10	ordinary_muscle_development	ENSGALG00010020562	LRRN1	-2.045103811	0.227307963	-8.997061892	2.31839E-19	1.57106E-18	negative
PRJNA729271	stage_E18_vs_E10	ordinary_muscle_development	ENSGALG00010020568	PLA2G4F	-1.592379679	0.454218977	-3.505753301	0.000455317	0.000871033	negative
PRJNA729271	stage_E18_vs_E10	ordinary_muscle_development	ENSGALG00010020569	DTX3L	0.607517366	0.260613808	2.331101991	0.019747982	0.029798772	positive
PRJNA729271	stage_E18_vs_E10	ordinary_muscle_development	ENSGALG00010020571	MRTO4	-0.287797285	0.081809899	-3.517878498	0.000435011	0.00083417	negative
PRJNA729271	stage_E18_vs_E10	ordinary_muscle_development	ENSGALG00010020572	PPP1R3C	0.476636516	0.273535187	1.742505309	0.081420063	0.109927703	positive
PRJNA729271	stage_E18_vs_E10	ordinary_muscle_development	ENSGALG00010020574	DTL	-1.588158279	0.180655769	-8.791074267	1.48137E-18	9.60734E-18	negative
PRJNA729271	stage_E18_vs_E10	ordinary_muscle_development	ENSGALG00010020576	AKR7L	-0.241679333	0.106997658	-2.25873479	0.023899887	0.035542101	negative
PRJNA729271	stage_E18_vs_E10	ordinary_muscle_development	ENSGALG00010020578	TNKS2	-0.054630905	0.063767348	-0.856722227	0.391598416	0.45165388	negative
PRJNA729271	stage_E18_vs_E10	ordinary_muscle_development	ENSGALG00010020580	ENSGALG00010020580	-0.016423235	0.115412091	-0.142300818	0.8868424	0.90670072	negative
PRJNA729271	stage_E18_vs_E10	ordinary_muscle_development	ENSGALG00010020582	KANSL3	-0.106726085	0.14965292	-0.713157387	0.475748372	0.534364405	negative
PRJNA729271	stage_E18_vs_E10	ordinary_muscle_development	ENSGALG00010020584	PARP9	0.438170933	0.295542416	1.482599141	0.138180946	0.178371432	positive
PRJNA729271	stage_E18_vs_E10	ordinary_muscle_development	ENSGALG00010020586	C11orf16	0.364597015	0.098430211	3.70411696	0.000212128	0.000423935	positive
PRJNA729271	stage_E18_vs_E10	ordinary_muscle_development	ENSGALG00010020588	PALD1	-0.37292804	0.109705733	-3.39934869	0.000675465	0.001260735	negative
PRJNA729271	stage_E18_vs_E10	ordinary_muscle_development	ENSGALG00010020590	INTS7	-0.547137927	0.078210515	-6.995708068	2.63923E-12	1.09333E-11	negative
PRJNA729271	stage_E18_vs_E10	ordinary_muscle_development	ENSGALG00010020594	PQLC2	-0.844794133	0.159287122	-5.303593415	1.13545E-07	3.20451E-07	negative
PRJNA729271	stage_E18_vs_E10	ordinary_muscle_development	ENSGALG00010020595	ZFYVE27	0.603158684	0.133527749	4.517103668	6.26912E-06	1.48895E-05	positive
PRJNA729271	stage_E18_vs_E10	ordinary_muscle_development	ENSGALG00010020596	SUMF1	0.4869292	0.121698006	4.001127186	6.30415E-05	0.000134499	positive
PRJNA729271	stage_E18_vs_E10	ordinary_muscle_development	ENSGALG00010020597	ARID5A	0.125162504	0.246871829	0.506993871	0.612159119	0.662875126	positive
PRJNA729271	stage_E18_vs_E10	ordinary_muscle_development	ENSGALG00010020599	PARN	-0.935982827	0.066900777	-13.9906122	1.77874E-44	4.10555E-43	negative
PRJNA729271	stage_E18_vs_E10	ordinary_muscle_development	ENSGALG00010020601	AKIP1	-1.094263352	0.177021193	-6.181538688	6.34798E-10	2.18664E-09	negative
PRJNA729271	stage_E18_vs_E10	ordinary_muscle_development	ENSGALG00010020602	DENND2B	-0.567374866	0.097003969	-5.848986122	4.94578E-09	1.57213E-08	negative
PRJNA729271	stage_E18_vs_E10	ordinary_muscle_development	ENSGALG00010020603	VPS39	-0.067556448	0.09435296	-0.715997116	0.473993129	0.532681843	negative
PRJNA729271	stage_E18_vs_E10	ordinary_muscle_development	ENSGALG00010020605	STAMBP	0.03830262	0.088440177	0.433090718	0.664948866	0.712375005	positive
PRJNA729271	stage_E18_vs_E10	ordinary_muscle_development	ENSGALG00010020607	ENSGALG00010020607	2.590790338	0.238868268	10.84610508	2.08106E-27	2.24776E-26	positive
PRJNA729271	stage_E18_vs_E10	ordinary_muscle_development	ENSGALG00010020608	CAPZB	0.476629237	0.099630714	4.783958839	1.71876E-06	4.33126E-06	positive
PRJNA729271	stage_E18_vs_E10	ordinary_muscle_development	ENSGALG00010020609	AVPI1	-0.075305716	0.233175697	-0.322956967	0.746727832	0.786957971	negative
PRJNA729271	stage_E18_vs_E10	ordinary_muscle_development	ENSGALG00010020611	LPGAT1	-0.607818735	0.100608977	-6.041396618	1.52786E-09	5.07361E-09	negative
PRJNA729271	stage_E18_vs_E10	ordinary_muscle_development	ENSGALG00010020612	RPL27A	0.974728769	0.192775179	5.056298098	4.27473E-07	1.14448E-06	positive
PRJNA729271	stage_E18_vs_E10	ordinary_muscle_development	ENSGALG00010020613	ACTG2	1.165764952	0.24191728	4.818857713	1.44382E-06	3.6684E-06	positive
PRJNA729271	stage_E18_vs_E10	ordinary_muscle_development	ENSGALG00010020614	FAIM	-0.187191559	0.109979919	-1.702052158	0.088745591	0.118833011	negative
PRJNA729271	stage_E18_vs_E10	ordinary_muscle_development	ENSGALG00010020616	PI4K2A	0.317505082	0.132784057	2.391138586	0.016796212	0.025679486	positive
PRJNA729271	stage_E18_vs_E10	ordinary_muscle_development	ENSGALG00010020617	DGUOK	0.748399477	0.11029726	6.785295289	1.15849E-11	4.53235E-11	positive
PRJNA729271	stage_E18_vs_E10	ordinary_muscle_development	ENSGALG00010020618	ENSGALG00010020618	0.005622606	0.320651476	0.017534945	0.986009855	0.988191443	positive
PRJNA729271	stage_E18_vs_E10	ordinary_muscle_development	ENSGALG00010020619	QARS	-0.778561614	0.101192516	-7.693865553	1.42755E-14	7.03343E-14	negative
PRJNA729271	stage_E18_vs_E10	ordinary_muscle_development	ENSGALG00010020620	MARF1	-0.123370563	0.103992325	-1.186342954	0.23548689	0.287830548	negative
PRJNA729271	stage_E18_vs_E10	ordinary_muscle_development	ENSGALG00010020622	ABCG2	0.84457898	0.117990825	7.158005565	8.18592E-13	3.53877E-12	positive
PRJNA729271	stage_E18_vs_E10	ordinary_muscle_development	ENSGALG00010020623	NEK2	-1.374868267	0.2211778	-6.216122349	5.09591E-10	1.76923E-09	negative
PRJNA729271	stage_E18_vs_E10	ordinary_muscle_development	ENSGALG00010020624	MINOS1	1.602143909	0.135506691	11.82335649	2.95632E-32	3.98993E-31	positive
PRJNA729271	stage_E18_vs_E10	ordinary_muscle_development	ENSGALG00010020625	SLC30A1	-0.362124825	0.077038846	-4.700548378	2.59464E-06	6.40485E-06	negative
PRJNA729271	stage_E18_vs_E10	ordinary_muscle_development	ENSGALG00010020626	MORN4	-0.475098515	0.119426492	-3.978166873	6.94486E-05	0.000147474	negative
PRJNA729271	stage_E18_vs_E10	ordinary_muscle_development	ENSGALG00010020629	TRIM66	-3.256410842	0.254605	-12.79005062	1.86352E-37	3.20425E-36	negative
PRJNA729271	stage_E18_vs_E10	ordinary_muscle_development	ENSGALG00010020630	QRICH1	-0.179733392	0.126569208	-1.420040422	0.155595913	0.198378289	negative
PRJNA729271	stage_E18_vs_E10	ordinary_muscle_development	ENSGALG00010020632	BLOC1S2	-0.038339505	0.113119432	-0.338929431	0.734662893	0.77599203	negative
PRJNA729271	stage_E18_vs_E10	ordinary_muscle_development	ENSGALG00010020633	HOGA1	-0.531826512	0.369393327	-1.439729614	0.149943912	0.19197419	negative
PRJNA729271	stage_E18_vs_E10	ordinary_muscle_development	ENSGALG00010020635	NDE1	-1.139445747	0.135564383	-8.40519999	4.27133E-17	2.5148E-16	negative
PRJNA729271	stage_E18_vs_E10	ordinary_muscle_development	ENSGALG00010020636	NBL1	1.880456044	0.17807955	10.55964058	4.58414E-26	4.60798E-25	positive
PRJNA729271	stage_E18_vs_E10	ordinary_muscle_development	ENSGALG00010020638	IMPDH2	-0.223364637	0.11216593	-1.991376864	0.046439471	0.065544316	negative
PRJNA729271	stage_E18_vs_E10	ordinary_muscle_development	ENSGALG00010020640	PTPN14	-0.556617838	0.093443169	-5.956752583	2.57299E-09	8.37725E-09	negative
PRJNA729271	stage_E18_vs_E10	ordinary_muscle_development	ENSGALG00010020642	ANKRD2	2.710167358	0.637790649	4.249305572	2.14434E-05	4.81224E-05	positive
PRJNA729271	stage_E18_vs_E10	ordinary_muscle_development	ENSGALG00010020646	CEP70	-0.443256976	0.116836846	-3.793811555	0.000148352	0.000302613	negative
PRJNA729271	stage_E18_vs_E10	ordinary_muscle_development	ENSGALG00010020648	TET3	-0.331440324	0.149284331	-2.220194999	0.026405534	0.038984336	negative
PRJNA729271	stage_E18_vs_E10	ordinary_muscle_development	ENSGALG00010020649	MYH11	2.829467474	0.212484193	13.31613157	1.86525E-40	3.63887E-39	positive
PRJNA729271	stage_E18_vs_E10	ordinary_muscle_development	ENSGALG00010020651	LMO1	0.11721766	0.173532506	0.675479558	0.499371134	0.556627545	positive
PRJNA729271	stage_E18_vs_E10	ordinary_muscle_development	ENSGALG00010020653	NDUFAF3	1.021292442	0.118454451	8.62181568	6.59007E-18	4.09092E-17	positive
PRJNA729271	stage_E18_vs_E10	ordinary_muscle_development	ENSGALG00010020654	SAYSD1	0.460179753	0.147846027	3.112560824	0.001854718	0.00327456	positive
PRJNA729271	stage_E18_vs_E10	ordinary_muscle_development	ENSGALG00010020655	RIC3	-1.009632512	0.136398455	-7.40208175	1.34066E-13	6.10712E-13	negative
PRJNA729271	stage_E18_vs_E10	ordinary_muscle_development	ENSGALG00010020657	DALRD3	0.397621035	0.094971727	4.18673058	2.83001E-05	6.26563E-05	positive
PRJNA729271	stage_E18_vs_E10	ordinary_muscle_development	ENSGALG00010020659	RAD51B	-0.892171113	0.136636954	-6.52950089	6.59892E-11	2.44071E-10	negative
PRJNA729271	stage_E18_vs_E10	ordinary_muscle_development	ENSGALG00010020662	BFAR	-0.01580716	0.087311687	-0.181042887	0.856333913	0.881135804	negative
PRJNA729271	stage_E18_vs_E10	ordinary_muscle_development	ENSGALG00010020664	ESYT3	0.590431881	0.455491979	1.296250887	0.194889063	0.242982708	positive
PRJNA729271	stage_E18_vs_E10	ordinary_muscle_development	ENSGALG00010020666	TMCO4	-0.174890117	0.219094103	-0.798242008	0.424730064	0.485104584	negative
PRJNA729271	stage_E18_vs_E10	ordinary_muscle_development	ENSGALG00010020667	UBTD1	-0.405631512	0.163642264	-2.478769861	0.013183634	0.020502689	negative
PRJNA729271	stage_E18_vs_E10	ordinary_muscle_development	ENSGALG00010020670	ENSGALG00010020670	0.104876071	0.133380003	0.786295312	0.431694498	0.491692516	positive
PRJNA729271	stage_E18_vs_E10	ordinary_muscle_development	ENSGALG00010020673	MMS19	-0.381193528	0.129620338	-2.940846591	0.003273166	0.005564689	negative
PRJNA729271	stage_E18_vs_E10	ordinary_muscle_development	ENSGALG00010020674	TRPV4	-4.962662079	0.558212476	-8.890274394	6.09582E-19	4.04759E-18	negative
PRJNA729271	stage_E18_vs_E10	ordinary_muscle_development	ENSGALG00010020675	SMYD2	-1.246448677	0.181380886	-6.871995763	6.33098E-12	2.53432E-11	negative
PRJNA729271	stage_E18_vs_E10	ordinary_muscle_development	ENSGALG00010020676	CYP2C23a	5.074283133	1.404657523	3.612469979	0.000303294	0.000593446	positive
PRJNA729271	stage_E18_vs_E10	ordinary_muscle_development	ENSGALG00010020677	CYR61	-1.023188869	0.239029167	-4.280602578	1.86388E-05	4.21415E-05	negative
PRJNA729271	stage_E18_vs_E10	ordinary_muscle_development	ENSGALG00010020679	OTUD3	-0.448449047	0.112838928	-3.974240584	7.06042E-05	0.000149709	negative
PRJNA729271	stage_E18_vs_E10	ordinary_muscle_development	ENSGALG00010020680	CFAP221	-0.283144904	0.579345288	-0.488732556	0.625031052	0.675451062	negative
PRJNA729271	stage_E18_vs_E10	ordinary_muscle_development	ENSGALG00010020681	BCL10	-0.507537048	0.090014407	-5.638397948	1.71639E-08	5.19398E-08	negative
PRJNA729271	stage_E18_vs_E10	ordinary_muscle_development	ENSGALG00010020682	ZDHHC16	-0.058595118	0.17219037	-0.340292655	0.733636148	0.775076965	negative
PRJNA729271	stage_E18_vs_E10	ordinary_muscle_development	ENSGALG00010020684	P4HTM	1.171251	0.122764415	9.540639265	1.41955E-21	1.10744E-20	positive
PRJNA729271	stage_E18_vs_E10	ordinary_muscle_development	ENSGALG00010020685	RPL6	0.732609197	0.116016224	6.314713338	2.70663E-10	9.56192E-10	positive
PRJNA729271	stage_E18_vs_E10	ordinary_muscle_development	ENSGALG00010020686	C1orf52	-0.433653603	0.126883003	-3.417743851	0.000631425	0.001183259	negative
PRJNA729271	stage_E18_vs_E10	ordinary_muscle_development	ENSGALG00010020691	ENSGALG00010020691	0.284490386	0.165096967	1.723171491	0.084857518	0.114069342	positive
PRJNA729271	stage_E18_vs_E10	ordinary_muscle_development	ENSGALG00010020692	TUB	0.400031249	0.153109635	2.612711136	0.008982719	0.014311333	positive
PRJNA729271	stage_E18_vs_E10	ordinary_muscle_development	ENSGALG00010020693	PTGFR	-1.216142482	0.193264179	-6.292642979	3.12106E-10	1.09725E-09	negative
PRJNA729271	stage_E18_vs_E10	ordinary_muscle_development	ENSGALG00010020696	MOB1A	-0.778290609	0.102029165	-7.628119002	2.38204E-14	1.14862E-13	negative
PRJNA729271	stage_E18_vs_E10	ordinary_muscle_development	ENSGALG00010020699	PLA2G10	0.379485141	0.416352755	0.911451014	0.362057785	0.421460616	positive
PRJNA729271	stage_E18_vs_E10	ordinary_muscle_development	ENSGALG00010020700	MTHFD2	2.811429689	0.130578668	21.5305435	8.0583E-103	1.02461E-100	positive
PRJNA729271	stage_E18_vs_E10	ordinary_muscle_development	ENSGALG00010020701	PTPN11	-0.585353668	0.085418891	-6.852742513	7.24474E-12	2.88179E-11	negative
PRJNA729271	stage_E18_vs_E10	ordinary_muscle_development	ENSGALG00010020704	PROX1	-0.004245032	0.243537261	-0.017430731	0.986092993	0.988206439	negative
PRJNA729271	stage_E18_vs_E10	ordinary_muscle_development	ENSGALG00010020705	EXOSC1	-0.046394186	0.177476622	-0.261410127	0.793776249	0.827635356	negative
PRJNA729271	stage_E18_vs_E10	ordinary_muscle_development	ENSGALG00010020707	SLC4A5	-1.450589817	0.452853092	-3.203223836	0.001358983	0.00243823	negative
PRJNA729271	stage_E18_vs_E10	ordinary_muscle_development	ENSGALG00010020708	FAM214A	-0.63898003	0.135198338	-4.72624174	2.28713E-06	5.68352E-06	negative
PRJNA729271	stage_E18_vs_E10	ordinary_muscle_development	ENSGALG00010020712	ZC3H7A	-0.473750942	0.070960641	-6.676249509	2.45134E-11	9.36538E-11	negative
PRJNA729271	stage_E18_vs_E10	ordinary_muscle_development	ENSGALG00010020713	SCTR	-1.071836454	1.130252809	-0.948315674	0.342968774	0.401399465	negative
PRJNA729271	stage_E18_vs_E10	ordinary_muscle_development	ENSGALG00010020714	PGAM1	1.083875899	0.144606953	7.495323522	6.6135E-14	3.08637E-13	positive
PRJNA729271	stage_E18_vs_E10	ordinary_muscle_development	ENSGALG00010020715	ENSGALG00010020715	1.965379321	0.718741942	2.73447145	0.006248051	0.010203414	positive
PRJNA729271	stage_E18_vs_E10	ordinary_muscle_development	ENSGALG00010020716	RRAS	-0.253459892	0.180024662	-1.407917617	0.159155466	0.202506889	negative

PRJNA729271	stage_E18_vs_E10	ordinary_muscle_development	ENSGALG00010020719	RPH3A	0.655067476	0.637246498	1.027965596	0.303966011	0.361146503	positive
PRJNA729271	stage_E18_vs_E10	ordinary_muscle_development	ENSGALG00010020720	ENSGALG00010020720	-3.16217424	0.32220066	-9.814300939	9.77129E-23	8.08418E-22	negative
PRJNA729271	stage_E18_vs_E10	ordinary_muscle_development	ENSGALG00010020721	PTDSS2	-0.303028855	0.170568808	-1.776578368	0.075637637	0.102761979	negative
PRJNA729271	stage_E18_vs_E10	ordinary_muscle_development	ENSGALG00010020723	RPS24	0.377856559	0.19011759	1.987488688	0.046868268	0.06609803	positive
PRJNA729271	stage_E18_vs_E10	ordinary_muscle_development	ENSGALG00010020724	TACR1	-1.798049181	0.265663276	-6.76815106	1.30439E-11	5.08802E-11	negative
PRJNA729271	stage_E18_vs_E10	ordinary_muscle_development	ENSGALG00010020725	FOPNL	0.227099465	0.107583036	2.110922639	0.034778962	0.05020126	positive
PRJNA729271	stage_E18_vs_E10	ordinary_muscle_development	ENSGALG00010020728	TMEM37	1.52015804	0.209089552	7.270368243	3.58509E-13	1.58868E-12	positive
PRJNA729271	stage_E18_vs_E10	ordinary_muscle_development	ENSGALG00010020730	RRP12	-0.801360428	0.462509154	-1.732636903	0.083160226	0.112016306	negative
PRJNA729271	stage_E18_vs_E10	ordinary_muscle_development	ENSGALG00010020732	ARIH2	-0.383458722	0.145057745	-2.643490164	0.008205615	0.013142584	negative
PRJNA729271	stage_E18_vs_E10	ordinary_muscle_development	ENSGALG00010020733	TMEM229B	-1.854951776	0.113856402	-16.29202876	1.12437E-59	4.48972E-58	negative
PRJNA729271	stage_E18_vs_E10	ordinary_muscle_development	ENSGALG00010020736	LRRTM4	0.73690877	0.220304747	3.344951839	0.000822969	0.001518448	positive
PRJNA729271	stage_E18_vs_E10	ordinary_muscle_development	ENSGALG00010020737	POLR3A	-1.088135011	0.117924324	-9.22740088	2.77278E-20	1.98476E-19	negative
PRJNA729271	stage_E18_vs_E10	ordinary_muscle_development	ENSGALG00010020739	PKP3	-4.396019629	0.759867643	-5.785243877	7.24072E-09	2.2639E-08	negative
PRJNA729271	stage_E18_vs_E10	ordinary_muscle_development	ENSGALG00010020742	THAP10	-1.414222065	0.211980139	-6.671483799	2.5323E-11	9.66448E-11	negative
PRJNA729271	stage_E18_vs_E10	ordinary_muscle_development	ENSGALG00010020743	PURB	0.638826148	0.171190612	3.731665776	0.000190218	0.000382308	positive
PRJNA729271	stage_E18_vs_E10	ordinary_muscle_development	ENSGALG00010020744	CHST3	-0.914642246	0.115726835	-7.903458582	2.71269E-15	1.41288E-14	negative
PRJNA729271	stage_E18_vs_E10	ordinary_muscle_development	ENSGALG00010020745	ABCC1	-0.791272806	0.167803396	-4.715475524	2.41147E-06	5.97917E-06	negative
PRJNA729271	stage_E18_vs_E10	ordinary_muscle_development	ENSGALG00010020747	DBI	0.080484735	0.120923439	0.665584235	0.505676886	0.562817583	positive
PRJNA729271	stage_E18_vs_E10	ordinary_muscle_development	ENSGALG00010020750	AIFM2	-1.494775637	0.306999296	-4.868987183	1.12172E-06	2.8849E-06	negative
PRJNA729271	stage_E18_vs_E10	ordinary_muscle_development	ENSGALG00010020753	SYDE2	0.818252023	0.099013024	8.264084762	1.40771E-16	8.00184E-16	positive
PRJNA729271	stage_E18_vs_E10	ordinary_muscle_development	ENSGALG00010020754	SLC25A20	-0.046077724	0.114869399	-0.401131405	0.68832338	0.734052928	negative
PRJNA729271	stage_E18_vs_E10	ordinary_muscle_development	ENSGALG00010020756	MACROH2A2	-2.274853971	0.152638674	-14.90352288	3.12651E-50	9.22988E-49	negative
PRJNA729271	stage_E18_vs_E10	ordinary_muscle_development	ENSGALG00010020759	ARHGAP19	-1.598365071	0.169287376	-9.441726304	3.66688E-21	2.78717E-20	negative
PRJNA729271	stage_E18_vs_E10	ordinary_muscle_development	ENSGALG00010020760	TXNDC11	-0.383309514	0.096442627	-3.974482297	7.05325E-05	0.000149578	negative
PRJNA729271	stage_E18_vs_E10	ordinary_muscle_development	ENSGALG00010020762	TTBK2	-0.406112875	0.144086216	-2.81854078	0.004824248	0.008010938	negative
PRJNA729271	stage_E18_vs_E10	ordinary_muscle_development	ENSGALG00010020764	PRKAR2A	-0.844174623	0.100744336	-8.37937551	5.32093E-17	3.11246E-16	negative
PRJNA729271	stage_E18_vs_E10	ordinary_muscle_development	ENSGALG00010020765	GLT1D1	-2.636067097	0.219572019	-12.00547821	3.32536E-33	4.65711E-32	negative
PRJNA729271	stage_E18_vs_E10	ordinary_muscle_development	ENSGALG00010020766	C2orf76	0.517867665	0.120621994	4.293310427	1.76029E-05	3.99239E-05	positive
PRJNA729271	stage_E18_vs_E10	ordinary_muscle_development	ENSGALG00010020767	UBXN10	-0.153194214	0.178818681	-0.856701395	0.391609932	0.45165388	negative
PRJNA729271	stage_E18_vs_E10	ordinary_muscle_development	ENSGALG00010020768	DNAH8	0.35230012	0.61942407	0.568754327	0.569522874	0.62317763	positive
PRJNA729271	stage_E18_vs_E10	ordinary_muscle_development	ENSGALG00010020770	B4GALNT4	-2.501480492	0.198160848	-12.623485	1.56729E-36	2.61124E-35	negative
PRJNA729271	stage_E18_vs_E10	ordinary_muscle_development	ENSGALG00010020771	LINGO1	1.936821609	0.200739787	9.648419176	4.99193E-22	3.98667E-21	positive
PRJNA729271	stage_E18_vs_E10	ordinary_muscle_development	ENSGALG00010020773	ZCCHC24	1.07350184	0.089463119	11.99937861	3.57974E-33	5.00369E-32	positive
PRJNA729271	stage_E18_vs_E10	ordinary_muscle_development	ENSGALG00010020775	UBR1	0.029225427	0.081660343	0.35789008	0.720425575	0.76312253	positive
PRJNA729271	stage_E18_vs_E10	ordinary_muscle_development	ENSGALG00010020776	MCOLN3	-0.297632778	0.450310318	-0.660950386	0.508644132	0.565615396	negative
PRJNA729271	stage_E18_vs_E10	ordinary_muscle_development	ENSGALG00010020778	SLIT1	-0.821245063	0.218590036	-3.757010504	0.000171955	0.000347676	negative
PRJNA729271	stage_E18_vs_E10	ordinary_muscle_development	ENSGALG00010020779	SLC15A4	-0.31511967	0.067173312	-4.691143863	2.71682E-06	6.69277E-06	negative
PRJNA729271	stage_E18_vs_E10	ordinary_muscle_development	ENSGALG00010020781	SNN	0.02662015	0.137524683	0.193566344	0.846515453	0.872954005	positive
PRJNA729271	stage_E18_vs_E10	ordinary_muscle_development	ENSGALG00010020782	PPIF	0.151586099	0.157866596	0.960216428	0.3369463	0.395404519	positive
PRJNA729271	stage_E18_vs_E10	ordinary_muscle_development	ENSGALG00010020786	DDX19	-0.908629535	0.132126455	-6.876969012	6.11394E-12	2.45014E-11	negative
PRJNA729271	stage_E18_vs_E10	ordinary_muscle_development	ENSGALG00010020787	TMEM132C	-2.872724872	0.296699905	-9.682257466	3.58706E-22	2.8918E-21	negative
PRJNA729271	stage_E18_vs_E10	ordinary_muscle_development	ENSGALG00010020790	PRPH2L	0.290189712	0.360200647	0.80563351	0.420454198	0.481054827	positive
PRJNA729271	stage_E18_vs_E10	ordinary_muscle_development	ENSGALG00010020794	STEAP3	2.787391158	0.129444321	21.53351452	7.5579E-103	9.7814E-101	positive
PRJNA729271	stage_E18_vs_E10	ordinary_muscle_development	ENSGALG00010020797	DGCR8	-0.840453728	0.066929278	-12.55734034	3.62308E-36	5.90737E-35	negative
PRJNA729271	stage_E18_vs_E10	ordinary_muscle_development	ENSGALG00010020798	C1QL2	-1.273836134	0.574181549	-2.218525023	0.026519055	0.039135991	negative
PRJNA729271	stage_E18_vs_E10	ordinary_muscle_development	ENSGALG00010020803	ABCC6	1.816915439	0.159124911	11.41817094	3.393E-30	4.19279E-29	positive
PRJNA729271	stage_E18_vs_E10	ordinary_muscle_development	ENSGALG00010020806	HTD2	-0.401435488	0.175326602	-2.289643917	0.022041967	0.032999206	negative
PRJNA729271	stage_E18_vs_E10	ordinary_muscle_development	ENSGALG00010020809	ENSGALG00010020809	-0.548371045	0.223175941	-2.457124373	0.014005419	0.021714467	negative
PRJNA729271	stage_E18_vs_E10	ordinary_muscle_development	ENSGALG00010020810	IFITM5	1.100588256	0.242324931	4.54178714	5.57793E-06	1.33287E-05	positive
PRJNA729271	stage_E18_vs_E10	ordinary_muscle_development	ENSGALG00010020811	MCOLN2	0.531718693	0.649478309	0.818685837	0.412965679	0.473234052	positive
PRJNA729271	stage_E18_vs_E10	ordinary_muscle_development	ENSGALG00010020813	NPY4R	-0.657833949	0.555040585	-1.185199727	0.235938488	0.288309592	negative
PRJNA729271	stage_E18_vs_E10	ordinary_muscle_development	ENSGALG00010020816	TRMT2A	0.198627896	0.090845187	2.186443791	0.028783153	0.042189483	positive
PRJNA729271	stage_E18_vs_E10	ordinary_muscle_development	ENSGALG00010020818	TMEM62	-1.234794475	0.203058518	-6.080978466	1.19451E-09	3.99965E-09	negative
PRJNA729271	stage_E18_vs_E10	ordinary_muscle_development	ENSGALG00010020820	VWA5B1	-1.089789394	0.520362864	-2.094287409	0.036234385	0.052151466	negative
PRJNA729271	stage_E18_vs_E10	ordinary_muscle_development	ENSGALG00010020827	ENSGALG00010020827	-0.452779663	0.092203528	-4.910654439	9.07729E-07	2.3584E-06	negative
PRJNA729271	stage_E18_vs_E10	ordinary_muscle_development	ENSGALG00010020828	CAMK2N1	0.594652192	0.210377395	2.826597366	0.004704544	0.007822019	positive
PRJNA729271	stage_E18_vs_E10	ordinary_muscle_development	ENSGALG00010020831	MUL1	0.438911681	0.14595088	3.007256139	0.002636175	0.004552766	positive
PRJNA729271	stage_E18_vs_E10	ordinary_muscle_development	ENSGALG00010020833	RANBP1	-1.527258388	0.082850104	-18.43399484	7.01096E-76	4.3061E-74	negative
PRJNA729271	stage_E18_vs_E10	ordinary_muscle_development	ENSGALG00010020835	PTGR2	0.317872261	0.11477322	2.769568204	0.005613065	0.009233021	positive
PRJNA729271	stage_E18_vs_E10	ordinary_muscle_development	ENSGALG00010020839	PGGHG	0.07876417	0.139446596	0.564833942	0.572186718	0.625667356	positive
PRJNA729271	stage_E18_vs_E10	ordinary_muscle_development	ENSGALG00010020840	LPAR3	0.777828136	0.241212627	3.224657627	0.001261234	0.002270723	positive
PRJNA729271	stage_E18_vs_E10	ordinary_muscle_development	ENSGALG00010020843	HMG20A	-0.63231719	0.083478849	-7.574579637	3.60292E-14	1.71171E-13	negative
PRJNA729271	stage_E18_vs_E10	ordinary_muscle_development	ENSGALG00010020845	RAB35	-0.161813324	0.093652785	-1.727800461	0.084024005	0.113074733	negative
PRJNA729271	stage_E18_vs_E10	ordinary_muscle_development	ENSGALG00010020847	MIDEAS	0.001244369	0.090709194	0.013718219	0.989054788	0.990763591	positive
PRJNA729271	stage_E18_vs_E10	ordinary_muscle_development	ENSGALG00010020854	ENSGALG00010020854	10.25023934	0.381146032	26.89320756	2.6371E-159	1.0618E-156	positive
PRJNA729271	stage_E18_vs_E10	ordinary_muscle_development	ENSGALG00010020855	DNAL1	-0.553065162	0.103833302	-5.32647189	1.00139E-07	2.84053E-07	negative
PRJNA729271	stage_E18_vs_E10	ordinary_muscle_development	ENSGALG00010020856	VTG2	-1.769417124	0.686249085	-2.578389046	0.009926217	0.015709195	negative
PRJNA729271	stage_E18_vs_E10	ordinary_muscle_development	ENSGALG00010020861	CDA	0.396613742	0.188074394	2.108813079	0.034960719	0.050443522	positive
PRJNA729271	stage_E18_vs_E10	ordinary_muscle_development	ENSGALG00010020865	BICDL1	-1.529698054	0.80042037	-1.911118348	0.055989374	0.077922801	negative
PRJNA729271	stage_E18_vs_E10	ordinary_muscle_development	ENSGALG00010020866	LCOR	-0.057133911	0.097099745	-0.588404339	0.556260926	0.610278695	negative
PRJNA729271	stage_E18_vs_E10	ordinary_muscle_development	ENSGALG00010020870	RIOX1	-0.783232396	0.150674548	-5.19817319	2.01257E-07	5.54709E-07	negative
PRJNA729271	stage_E18_vs_E10	ordinary_muscle_development	ENSGALG00010020873	PINK1	0.929193982	0.13816041	6.725472093	1.75024E-11	6.74368E-11	positive
PRJNA729271	stage_E18_vs_E10	ordinary_muscle_development	ENSGALG00010020875	ENSGALG00010020875	0.090707662	0.174121018	0.520946082	0.602404333	0.653697124	positive
PRJNA729271	stage_E18_vs_E10	ordinary_muscle_development	ENSGALG00010020878	XYLT1	-0.107995561	0.124429127	-0.867928304	0.385433583	0.445836934	negative
PRJNA729271	stage_E18_vs_E10	ordinary_muscle_development	ENSGALG00010020880	PXK	0.614689435	0.169360862	3.62946568	0.000284008	0.000558424	positive
PRJNA729271	stage_E18_vs_E10	ordinary_muscle_development	ENSGALG00010020886	GBF1	-0.734877408	0.086894055	-8.457165582	2.73955E-17	1.63146E-16	negative
PRJNA729271	stage_E18_vs_E10	ordinary_muscle_development	ENSGALG00010020887	ARPP19	-0.936294439	0.071357331	-13.12120886	2.48932E-39	4.65583E-38	negative
PRJNA729271	stage_E18_vs_E10	ordinary_muscle_development	ENSGALG00010020889	PPRC1	-1.035220617	0.285991436	-3.619760903	0.000294875	0.000578144	negative
PRJNA729271	stage_E18_vs_E10	ordinary_muscle_development	ENSGALG00010020890	RPS15A	0.923949503	0.188343664	4.905657482	9.31149E-07	2.41541E-06	positive
PRJNA729271	stage_E18_vs_E10	ordinary_muscle_development	ENSGALG00010020894	PSMD13	-0.118945157	0.121071973	-0.982433459	0.325886349	0.383980055	negative
PRJNA729271	stage_E18_vs_E10	ordinary_muscle_development	ENSGALG00010020896	MARCO	1.966370744	0.393964712	4.991235724	5.99942E-07	1.58342E-06	positive
PRJNA729271	stage_E18_vs_E10	ordinary_muscle_development	ENSGALG00010020898	ENSGALG00010020898	0.430020205	0.12795928	3.360601955	0.000777728	0.001440477	positive
PRJNA729271	stage_E18_vs_E10	ordinary_muscle_development	ENSGALG00010020900	PDHB	0.29850099	0.11627733	2.56714692	0.010253916	0.016203042	positive
PRJNA729271	stage_E18_vs_E10	ordinary_muscle_development	ENSGALG00010020903	AGMAT	0.633508686	0.39605474	1.599548302	0.109698825	0.14444808	positive
PRJNA729271	stage_E18_vs_E10	ordinary_muscle_development	ENSGALG00010020905	KCTD6	-0.362826307	0.088865951	-4.082849529	4.44868E-05	9.62586E-05	negative
PRJNA729271	stage_E18_vs_E10	ordinary_muscle_development	ENSGALG00010020906	SIRT3	0.63806135	0.112416508	5.67586879	1.37987E-08	4.21165E-08	positive
PRJNA729271	stage_E18_vs_E10	ordinary_muscle_development	ENSGALG00010020912	PDE8A	1.619113099	0.143344975	11.29522049	1.38556E-29	1.66118E-28	positive
PRJNA729271	stage_E18_vs_E10	ordinary_muscle_development	ENSGALG00010020913	TMEM132B	-1.384204081	0.246762684	-5.609454642	2.02965E-08	6.10876E-08	negative
PRJNA729271	stage_E18_vs_E10	ordinary_muscle_development	ENSGALG00010020915	EN1	-3.422450536	0.36461472	-9.386484835	6.20361E-21	4.65672E-20	negative
PRJNA729271	stage_E18_vs_E10	ordinary_muscle_development	ENSGALG00010020916	ARL6IP1	-0.306512326	0.098105991	-3.124297738	0.0017823	0.003154003	negative
PRJNA729271	stage_E18_vs_E10	ordinary_muscle_development	ENSGALG00010020918	ENSGALG00010020918	-1.462146939	0.356374929	-4.102833336	4.08121E-05	8.8718E-05	negative
PRJNA729271	stage_E18_vs_E10	ordinary_muscle_development	ENSGALG00010020920	ACOX2	1.409128502	0.207310596	6.797185144	1.06683E-11	4.18503E-11	positive
PRJNA729271	stage_E18_vs_E10	ordinary_muscle_development	ENSGALG00010020921	LDB1	-0.194166329	0.152021351	-1.277230649	0.20152083	0.250582862	negative
PRJNA729271	stage_E18_vs_E10	ordinary_muscle_development	ENSGALG00010020922	INSIG2	0.166563804	0.080559569	2.067585584	0.038679009	0.055400418	positive
PRJNA729271	stage_E18_vs_E10	ordinary_muscle_development	ENSGALG00010020924	DNAJC16	-0.259740753	0.122275831	-2.124219899	0.03365176	0.048719762	negative
PRJNA729271	stage_E18_vs_E10	ordinary_muscle_development	ENSGALG00010020925	RIC8A	0.183404726	0.10394809	1.764387646	0.077666737	0.105262211	positive
PRJNA729271	stage_E18_vs_E10	ordinary_muscle_development	ENSGALG00010020927	ENSGALG00010020927	0.022149455	0.116341256	0.190383496	0.849008629	0.875089247	positive
PRJNA729271	stage_E18_vs_E10	ordinary_muscle_development	ENSGALG00010020928	AACS	-1.437717752	0.152794048	-9.409514135	4.98436E-21	3.75706E-20	negative
PRJNA729271	stage_E18_vs_E10	ordinary_muscle_development	ENSGALG00010020932	CASP9	0.379215595	0.119152361	3.182610828	0.001459536	0.002607343	positive
PRJNA729271	stage_E18_vs_E10	ordinary_muscle_development	ENSGALG00010020934	ABCD3	-0.408322975	0.154181598	-2.648324962	0.008089173	0.012968981	negative
PRJNA729271	stage_E18_vs_E10	ordinary_muscle_development	ENSGALG00010020935	BRI3BP	-0.344295768	0.066068783	-5.211171652	1.87652E-07	5.18591E-07	negative
PRJNA729271	stage_E18_vs_E10	ordinary_muscle_development	ENSGALG00010020936	BET1L	-0.299024306	0.126927155	-2.355873392	0.018479215	0.028018433	negative
PRJNA729271	stage_E18_vs_E10	ordinary_muscle_development	ENSGALG00010020938	SMG1	-0.024889574	0.080303555	-0.309943614	0.756603836	0.795746089	negative
PRJNA729271	stage_E18_vs_E10	ordinary_muscle_development	ENSGALG00010020939	CTRC	3.566296167	2.193330779	1.62597279	0.103955444	0.137384588	positive
PRJNA729271	stage_E18_vs_E10	ordinary_muscle_development	ENSGALG00010020940	ENSGALG00010020940	-1.357873044	0.136807776	-9.925408354	3.22779E-23	2.73767E-22	negative
PRJNA729271	stage_E18_vs_E10	ordinary_muscle_development	ENSGALG00010020941	ENSGALG00010020941	2.462123249	0.186274083	13.21774459	6.93112E-40	1.32542E-38	positive
PRJNA729271	stage_E18_vs_E10	ordinary_muscle_development	ENSGALG00010020943	FAM107A	0.994422628	0.37171434	2.675233425	0.007467724	0.012032531	positive
PRJNA729271	stage_E18_vs_E10	ordinary_muscle_development	ENSGALG00010020944	MYO5A	-1.723255229	0.158071362	-10.9017548	1.13056E-27	1.24147E-26	negative
PRJNA729271	stage_E18_vs_E10	ordinary_muscle_development	ENSGALG00010020945	NUMB	1.401977218	0.084708274	16.55065255	1.58377E-61	6.61576E-60	positive
PRJNA729271	stage_E18_vs_E10	ordinary_muscle_development	ENSGALG00010020946	CTBS	1.313453608	0.215079096	6.106839926	1.01623E-09	3.42644E-09	positive
PRJNA729271	stage_E18_vs_E10	ordinary_muscle_development	ENSGALG00010020949	HPS6	-0.123525834	0.112054884	-1.102369034	0.270301268	0.325389659	negative
PRJNA729271	stage_E18_vs_E10	ordinary_muscle_development	ENSGALG00010020950	EFHD2	1.794033699	0.234906465	7.637225746	2.21952E-14	1.07491E-13	positive
PRJNA729271	stage_E18_vs_E10	ordinary_muscle_development	ENSGALG00010020951	ENSGALG00010020951	-2.311012347	0.305699332	-7.559755954	4.03826E-14	1.90915E-13	negative
PRJNA729271	stage_E18_vs_E10	ordinary_muscle_development	ENSGALG00010020952	GNG5	-0.150752623	0.098493658	-1.530582032	0.125872725	0.163987521	negative
PRJNA729271	stage_E18_vs_E10	ordinary_muscle_development	ENSGALG00010020954	PLIN1	10.77209281	1.405755163	7.662851321	1.8185E-14	8.87812E-14	positive
PRJNA729271	stage_E18_vs_E10	ordinary_muscle_development	ENSGALG00010020955	FHAD1	-0.355429211	0.929167088	-0.382524538	0.702072331	0.746901904	negative
PRJNA729271	stage_E18_vs_E10	ordinary_muscle_development	ENSGALG00010020957	RPF1	-0.38799413	0.103144011	-3.761673858	0.00016878	0.00034159	negative
PRJNA729271	stage_E18_vs_E10	ordinary_muscle_development	ENSGALG00010020959	ENSGALG00010020959	1.011724606	0.347467603	2.911709173	0.003594571	0.006069817	positive
PRJNA729271	stage_E18_vs_E10	ordinary_muscle_development	ENSGALG00010020960	CLEC19A	6.987134402	0.365062999	19.13953051	1.18331E-81	8.6627E-80	positive
PRJNA729271	stage_E18_vs_E10	ordinary_muscle_development	ENSGALG00010020962	MYO5C	-2.055063846	0.340618361	-6.033332555	1.60612E-09	5.32497E-09	negative
PRJNA729271	stage_E18_vs_E10	ordinary_muscle_development	ENSGALG00010020963	CCDC93	0.905933533	0.099841677	9.073701072	1.15041E-19	7.93304E-19	positive
PRJNA729271	stage_E18_vs_E10	ordinary_muscle_development	ENSGALG00010020965	DHX37	-1.050609449	0.09640306	-10.89809232	1.17699E-27	1.29051E-26	negative
PRJNA729271	stage_E18_vs_E10	ordinary_muscle_development	ENSGALG00010020966	PAPLN	3.487085107	0.245366251	14.21175528	7.74593E-46	1.89338E-44	positive
PRJNA729271	stage_E18_vs_E10	ordinary_muscle_development	ENSGALG00010020967	VPS51	-0.08217331	0.077545789	-1.059674703	0.289292615	0.345525417	negative
PRJNA729271	stage_E18_vs_E10	ordinary_muscle_development	ENSGALG00010020969	RBP3	-1.88356306	0.36733947	-5.127581477	2.93488E-07	7.98294E-07	negative
PRJNA729271	stage_E18_vs_E10	ordinary_muscle_development	ENSGALG00010020970	ARMH3	0.194646802	0.079289599	2.454884413	0.014092989	0.021838559	positive
PRJNA729271	stage_E18_vs_E10	ordinary_muscle_development	ENSGALG00010020971	TMEM51	-0.427673596	0.283256634	-1.509844942	0.131082994	0.169889843	negative
PRJNA729271	stage_E18_vs_E10	ordinary_muscle_development	ENSGALG00010020973	GNB5	-0.572477985	0.163158955	-3.508713233	0.00045028	0.000861625	negative
PRJNA729271	stage_E18_vs_E10	ordinary_muscle_development	ENSGALG00010020975	HCCS	-0.247013734	0.131213694	-1.88253015	0.05976407	0.082770896	negative
PRJNA729271	stage_E18_vs_E10	ordinary_muscle_development	ENSGALG00010020976	GDF10	-1.948549977	0.318256318	-6.122580656	9.20718E-10	3.12238E-09	negative
PRJNA729271	stage_E18_vs_E10	ordinary_muscle_development	ENSGALG00010020978	BCL2L10	3.583447976	0.11505063	31.1467045	5.6209E-213	4.0738E-210	positive

PRJNA729271	stage_E18_vs_E10	ordinary_muscle_development	ENSGALG00010020979	ENSGALG00010020979	0.896519364	0.244673537	3.664145191	0.000248166	0.00049155	positive
PRJNA729271	stage_E18_vs_E10	ordinary_muscle_development	ENSGALG00010020981	CIT	-1.642022965	0.236037227	-6.956627075	3.48516E-12	1.43028E-11	negative
PRJNA729271	stage_E18_vs_E10	ordinary_muscle_development	ENSGALG00010020982	TLCD4	0.505981431	0.151556783	3.338560122	0.000842138	0.001551393	positive
PRJNA729271	stage_E18_vs_E10	ordinary_muscle_development	ENSGALG00010020984	PSEN1	-1.018418111	0.060686247	-16.78169544	3.32214E-63	1.50033E-61	negative
PRJNA729271	stage_E18_vs_E10	ordinary_muscle_development	ENSGALG00010020986	CFAP20DC	-1.636458535	0.321104718	-5.096339117	3.46285E-07	9.36454E-07	negative
PRJNA729271	stage_E18_vs_E10	ordinary_muscle_development	ENSGALG00010020987	NADSYN1	0.005382822	0.093400878	0.057631383	0.954042251	0.961939512	positive
PRJNA729271	stage_E18_vs_E10	ordinary_muscle_development	ENSGALG00010020989	MAPK4	-0.40573814	0.153261439	-2.647359597	0.008112304	0.01300319	negative
PRJNA729271	stage_E18_vs_E10	ordinary_muscle_development	ENSGALG00010020990	UBB	-1.216672818	0.106825822	-11.38931392	4.72697E-30	5.77718E-29	negative
PRJNA729271	stage_E18_vs_E10	ordinary_muscle_development	ENSGALG00010020991	ALG14	0.322493021	0.143043398	2.254511757	0.024163992	0.035927486	positive
PRJNA729271	stage_E18_vs_E10	ordinary_muscle_development	ENSGALG00010020992	KCNH7	-0.166422382	0.488792474	-0.340476565	0.733497668	0.774987149	negative
PRJNA729271	stage_E18_vs_E10	ordinary_muscle_development	ENSGALG00010020994	DHCR7	-1.66577056	0.147491655	-11.29399867	1.40496E-29	1.68166E-28	negative
PRJNA729271	stage_E18_vs_E10	ordinary_muscle_development	ENSGALG00010020998	SYT17	0.790457193	0.273367877	2.89155113	0.003833452	0.006451398	positive
PRJNA729271	stage_E18_vs_E10	ordinary_muscle_development	ENSGALG00010020999	IFIH1	-0.27128472	0.343745699	-0.789201788	0.429994077	0.490035706	negative
PRJNA729271	stage_E18_vs_E10	ordinary_muscle_development	ENSGALG00010021000	NFKB2	0.462333624	0.116047723	3.983995639	6.77661E-05	0.000144028	positive
PRJNA729271	stage_E18_vs_E10	ordinary_muscle_development	ENSGALG00010021002	SLC44A3	-0.14400359	0.160570483	-0.896824792	0.369812416	0.429473792	negative
PRJNA729271	stage_E18_vs_E10	ordinary_muscle_development	ENSGALG00010021003	KAZN	-0.031341997	0.118289716	-0.264959606	0.79104058	0.825376706	negative
PRJNA729271	stage_E18_vs_E10	ordinary_muscle_development	ENSGALG00010021004	PSD	1.041805588	0.166964506	6.239682975	4.38459E-10	1.52959E-09	positive
PRJNA729271	stage_E18_vs_E10	ordinary_muscle_development	ENSGALG00010021010	LEO1	-0.761213329	0.113582868	-6.701832266	2.05822E-11	7.89884E-11	negative
PRJNA729271	stage_E18_vs_E10	ordinary_muscle_development	ENSGALG00010021011	SCARB1	0.408634259	0.109061117	3.746837263	0.000179078	0.000361372	positive
PRJNA729271	stage_E18_vs_E10	ordinary_muscle_development	ENSGALG00010021012	KCNIP2	-2.037544838	0.820448126	-2.483453581	0.013011529	0.020251132	negative
PRJNA729271	stage_E18_vs_E10	ordinary_muscle_development	ENSGALG00010021013	FAP	0.301001265	0.296683562	1.014553225	0.310318846	0.367970523	positive
PRJNA729271	stage_E18_vs_E10	ordinary_muscle_development	ENSGALG00010021014	FBXL15	0.00232706	0.093999412	0.024756116	0.980249495	0.983778746	positive
PRJNA729271	stage_E18_vs_E10	ordinary_muscle_development	ENSGALG00010021015	RBM25	-0.685680496	0.136084187	-5.038649306	4.68829E-07	1.24943E-06	negative
PRJNA729271	stage_E18_vs_E10	ordinary_muscle_development	ENSGALG00010021016	FHIT	0.344572	0.206793937	1.666257747	0.095662089	0.127376628	positive
PRJNA729271	stage_E18_vs_E10	ordinary_muscle_development	ENSGALG00010021017	COQ7	0.710355018	0.156980839	4.525106528	6.03651E-06	1.437E-05	positive
PRJNA729271	stage_E18_vs_E10	ordinary_muscle_development	ENSGALG00010021018	F3	-0.503838499	0.224645124	-2.242819648	0.024908447	0.03696232	negative
PRJNA729271	stage_E18_vs_E10	ordinary_muscle_development	ENSGALG00010021019	VSIG10	0.253548268	0.09195063	2.757439162	0.005825605	0.009558767	positive
PRJNA729271	stage_E18_vs_E10	ordinary_muscle_development	ENSGALG00010021020	ZDHHC13	0.829991657	0.11695965	7.096393117	1.28055E-12	5.43849E-12	positive
PRJNA729271	stage_E18_vs_E10	ordinary_muscle_development	ENSGALG00010021021	GCG	-0.441236439	0.378218674	-1.166617276	0.243364963	0.296434886	negative
PRJNA729271	stage_E18_vs_E10	ordinary_muscle_development	ENSGALG00010021022	ZFYVE1	0.607458952	0.079559822	7.635247713	2.25387E-14	1.08972E-13	positive
PRJNA729271	stage_E18_vs_E10	ordinary_muscle_development	ENSGALG00010021023	MGEA5	0.468756396	0.097345284	4.815399125	1.46906E-06	3.73186E-06	positive
PRJNA729271	stage_E18_vs_E10	ordinary_muscle_development	ENSGALG00010021024	ENSGALG00010021024	-0.380399382	0.191276844	-1.988737235	0.046730214	0.065916159	negative
PRJNA729271	stage_E18_vs_E10	ordinary_muscle_development	ENSGALG00010021025	CUEDC2	-0.50995036	0.119669336	-4.26132856	2.03215E-05	4.57534E-05	negative
PRJNA729271	stage_E18_vs_E10	ordinary_muscle_development	ENSGALG00010021026	ADGRA2	0.00167905	0.133873522	0.012542063	0.989993144	0.99136101	positive
PRJNA729271	stage_E18_vs_E10	ordinary_muscle_development	ENSGALG00010021027	PLA2G4A	1.814686761	0.165871769	10.94029907	7.39555E-28	8.17685E-27	positive
PRJNA729271	stage_E18_vs_E10	ordinary_muscle_development	ENSGALG00010021028	ENSGALG00010021028	1.0802284	0.320928151	3.365950904	0.000762803	0.00141464	positive
PRJNA729271	stage_E18_vs_E10	ordinary_muscle_development	ENSGALG00010021029	PRDM2	-0.330013887	0.075407003	-4.376435552	1.20636E-05	2.78486E-05	negative
PRJNA729271	stage_E18_vs_E10	ordinary_muscle_development	ENSGALG00010021030	CSRP3	5.354217682	0.546514046	9.79703582	1.15938E-22	9.53758E-22	positive
PRJNA729271	stage_E18_vs_E10	ordinary_muscle_development	ENSGALG00010021031	DDX54	-0.66594966	0.111692219	-5.962363961	2.48615E-09	8.10724E-09	negative
PRJNA729271	stage_E18_vs_E10	ordinary_muscle_development	ENSGALG00010021032	TMC7	0.186341094	0.119001926	1.565866196	0.117379945	0.15371057	positive
PRJNA729271	stage_E18_vs_E10	ordinary_muscle_development	ENSGALG00010021033	RNF122	1.465034887	0.230195375	6.364310706	1.96169E-10	6.99499E-10	positive
PRJNA729271	stage_E18_vs_E10	ordinary_muscle_development	ENSGALG00010021034	NPM3	-2.762083282	0.285323975	-9.680515902	3.6487E-22	2.93496E-21	negative
PRJNA729271	stage_E18_vs_E10	ordinary_muscle_development	ENSGALG00010021036	E2F8	-0.763427529	0.195547949	-3.904042636	9.45991E-05	0.000197724	negative
PRJNA729271	stage_E18_vs_E10	ordinary_muscle_development	ENSGALG00010021038	PTGS2	0.67657823	0.299445325	2.259438283	0.023856135	0.03548796	positive
PRJNA729271	stage_E18_vs_E10	ordinary_muscle_development	ENSGALG00010021040	FGF8	-2.348462326	0.529961763	-4.431380698	9.36316E-06	2.18584E-05	negative
PRJNA729271	stage_E18_vs_E10	ordinary_muscle_development	ENSGALG00010021041	GLTP	-1.98599488	0.21839175	-9.093726646	9.57013E-20	6.62776E-19	negative
PRJNA729271	stage_E18_vs_E10	ordinary_muscle_development	ENSGALG00010021042	LRRC38	1.507968355	0.094474415	15.96165855	2.36366E-57	8.58677E-56	positive
PRJNA729271	stage_E18_vs_E10	ordinary_muscle_development	ENSGALG00010021043	PTPRG	0.205956256	0.090117321	2.285423629	0.022288	0.033333116	positive
PRJNA729271	stage_E18_vs_E10	ordinary_muscle_development	ENSGALG00010021044	EIF4G2	-0.440140926	0.068097182	-6.463423448	1.0236E-10	3.72886E-10	negative
PRJNA729271	stage_E18_vs_E10	ordinary_muscle_development	ENSGALG00010021045	DPP4	1.367527541	0.261878503	5.221992355	1.77008E-07	4.90018E-07	positive
PRJNA729271	stage_E18_vs_E10	ordinary_muscle_development	ENSGALG00010021046	ENSGALG00010021046	0.566560028	0.25014872	2.264892768	0.023519261	0.035026372	positive
PRJNA729271	stage_E18_vs_E10	ordinary_muscle_development	ENSGALG00010021047	TMC5	-0.280943724	0.48950398	-0.573935526	0.566011431	0.619756436	negative
PRJNA729271	stage_E18_vs_E10	ordinary_muscle_development	ENSGALG00010021048	API5	-0.343172054	0.075863455	-4.523548985	6.08112E-06	1.44715E-05	negative
PRJNA729271	stage_E18_vs_E10	ordinary_muscle_development	ENSGALG00010021050	CTR9	-0.246493421	0.095082964	-2.592403626	0.009530788	0.015113092	negative
PRJNA729271	stage_E18_vs_E10	ordinary_muscle_development	ENSGALG00010021051	GDE1	-0.284213792	0.137714026	-2.063796985	0.039036969	0.055880258	negative
PRJNA729271	stage_E18_vs_E10	ordinary_muscle_development	ENSGALG00010021052	CEP15	-0.778746504	0.147636119	-5.274769551	1.32923E-07	3.72097E-07	negative
PRJNA729271	stage_E18_vs_E10	ordinary_muscle_development	ENSGALG00010021053	ENSGALG00010021053	-1.144621286	0.34898492	-3.279858873	0.00103859	0.001887221	negative
PRJNA729271	stage_E18_vs_E10	ordinary_muscle_development	ENSGALG00010021055	IRAG1	-0.871944102	0.225114933	-3.873328567	0.000107359	0.000223267	negative
PRJNA729271	stage_E18_vs_E10	ordinary_muscle_development	ENSGALG00010021057	ZDHHC8	0.882573084	0.077065891	11.45218812	2.29284E-30	2.85031E-29	positive
PRJNA729271	stage_E18_vs_E10	ordinary_muscle_development	ENSGALG00010021058	CCP110	-0.406993979	0.095869825	-4.245277161	2.18323E-05	4.89421E-05	negative
PRJNA729271	stage_E18_vs_E10	ordinary_muscle_development	ENSGALG00010021059	ENSGALG00010021059	-0.260535233	1.285414935	-0.202685706	0.839380697	0.86707691	negative
PRJNA729271	stage_E18_vs_E10	ordinary_muscle_development	ENSGALG00010021062	MYO9A	0.122993459	0.10060877	1.222492424	0.221521467	0.272437949	positive
PRJNA729271	stage_E18_vs_E10	ordinary_muscle_development	ENSGALG00010021064	LYVE1	1.084548314	0.292925549	3.702470868	0.00021351	0.000426285	positive
PRJNA729271	stage_E18_vs_E10	ordinary_muscle_development	ENSGALG00010021065	CNNM2	-0.245343152	0.112472294	-2.181365232	0.029156413	0.042697738	negative
PRJNA729271	stage_E18_vs_E10	ordinary_muscle_development	ENSGALG00010021067	SLC4A10	0.504092607	0.209892805	2.401666919	0.016320561	0.025025551	positive
PRJNA729271	stage_E18_vs_E10	ordinary_muscle_development	ENSGALG00010021069	PGAM5	0.128451849	0.071519025	1.796051449	0.072486335	0.098851202	positive
PRJNA729271	stage_E18_vs_E10	ordinary_muscle_development	ENSGALG00010021070	RNF141	0.032575257	0.08622983	0.377772477	0.705599621	0.750268963	positive
PRJNA729271	stage_E18_vs_E10	ordinary_muscle_development	ENSGALG00010021071	HIF1AN	0.033467981	0.096860203	0.345528711	0.72969693	0.771590093	positive
PRJNA729271	stage_E18_vs_E10	ordinary_muscle_development	ENSGALG00010021072	ENSGALG00010021072	0.387593203	0.090125916	4.300574341	1.70356E-05	3.87282E-05	positive
PRJNA729271	stage_E18_vs_E10	ordinary_muscle_development	ENSGALG00010021073	NDUFB8	0.313666247	0.107948911	2.90569163	0.003664424	0.006182009	positive
PRJNA729271	stage_E18_vs_E10	ordinary_muscle_development	ENSGALG00010021074	TTC17	0.568871023	0.069362047	8.201474014	2.37457E-16	1.32637E-15	positive
PRJNA729271	stage_E18_vs_E10	ordinary_muscle_development	ENSGALG00010021075	DHRS3	-0.342448262	0.132153001	-2.591301448	0.00956137	0.015159927	negative
PRJNA729271	stage_E18_vs_E10	ordinary_muscle_development	ENSGALG00010021077	SEC31B	-0.199171561	0.08132879	-2.448967487	0.014326638	0.022179282	negative
PRJNA729271	stage_E18_vs_E10	ordinary_muscle_development	ENSGALG00010021078	ENSGALG00010021078	1.996564263	0.192576406	10.36764733	3.47987E-25	3.32942E-24	positive
PRJNA729271	stage_E18_vs_E10	ordinary_muscle_development	ENSGALG00010021080	TTLL7	1.308812294	0.175549174	7.455530898	8.95067E-14	4.13316E-13	positive
PRJNA729271	stage_E18_vs_E10	ordinary_muscle_development	ENSGALG00010021081	KCNH6	0.487459597	0.203638184	2.393753403	0.016676957	0.025504589	positive
PRJNA729271	stage_E18_vs_E10	ordinary_muscle_development	ENSGALG00010021082	SENP8	-1.097935601	0.165883831	-6.618701737	3.62367E-11	1.36535E-10	negative
PRJNA729271	stage_E18_vs_E10	ordinary_muscle_development	ENSGALG00010021083	SCD	-3.243613017	0.574520204	-5.645777108			negative
PRJNA729271	stage_E18_vs_E10	ordinary_muscle_development	ENSGALG00010021084	ENSGALG00010021084	-3.643698138	0.946489503	-3.849697358	0.000118264	0.000244367	negative
PRJNA729271	stage_E18_vs_E10	ordinary_muscle_development	ENSGALG00010021085	AMPD3	2.628174939	0.183926629	14.28925737	2.55318E-46	6.42505E-45	positive
PRJNA729271	stage_E18_vs_E10	ordinary_muscle_development	ENSGALG00010021086	PSMD14	-0.791382004	0.110348882	-7.171635925	7.41068E-13	3.20842E-12	negative
PRJNA729271	stage_E18_vs_E10	ordinary_muscle_development	ENSGALG00010021087	THBS1	-3.385977543	0.357074729	-9.482546012	2.48153E-21	1.90518E-20	negative
PRJNA729271	stage_E18_vs_E10	ordinary_muscle_development	ENSGALG00010021088	ANKLE2	-0.439929283	0.087956528	-5.001667212	5.68366E-07	1.50419E-06	negative
PRJNA729271	stage_E18_vs_E10	ordinary_muscle_development	ENSGALG00010021090	ADM	1.995620111	0.265658303	7.511980954	5.82393E-14	2.72667E-13	positive
PRJNA729271	stage_E18_vs_E10	ordinary_muscle_development	ENSGALG00010021091	TANK	-0.153473905	0.083469637	-1.838679436	0.06596235	0.090559221	negative
PRJNA729271	stage_E18_vs_E10	ordinary_muscle_development	ENSGALG00010021092	VPS13D	0.608987369	0.094993236	6.410849788	1.44711E-10	5.21138E-10	positive
PRJNA729271	stage_E18_vs_E10	ordinary_muscle_development	ENSGALG00010021093	GRAMD2	0.295865589	0.227052278	1.303072541	0.192549997	0.240293776	positive
PRJNA729271	stage_E18_vs_E10	ordinary_muscle_development	ENSGALG00010021094	ENSGALG00010021094	2.232725608	0.316859537	7.046420719	1.83579E-12	7.69512E-12	positive
PRJNA729271	stage_E18_vs_E10	ordinary_muscle_development	ENSGALG00010021095	NT5C2	1.966360326	0.10485606	18.75294886	1.83148E-78	1.2067E-76	positive
PRJNA729271	stage_E18_vs_E10	ordinary_muscle_development	ENSGALG00010021096	ZFAND4	-0.302324032	0.102190311	-2.958441251	0.003091992	0.005285191	negative
PRJNA729271	stage_E18_vs_E10	ordinary_muscle_development	ENSGALG00010021097	KATNBL1	0.309665514	0.129096611	2.398711404	0.016452876	0.025199117	positive
PRJNA729271	stage_E18_vs_E10	ordinary_muscle_development	ENSGALG00010021098	SDK1	-0.283794469	0.142200788	-1.995730644	0.04596325	0.064954402	negative
PRJNA729271	stage_E18_vs_E10	ordinary_muscle_development	ENSGALG00010021099	HMCN1	-0.007294857	0.264014191	-0.027630549	0.977956816	0.98195373	negative
PRJNA729271	stage_E18_vs_E10	ordinary_muscle_development	ENSGALG00010021100	EMC7	0.176587369	0.086778161	2.034928686	0.041858049	0.059506907	positive
PRJNA729271	stage_E18_vs_E10	ordinary_muscle_development	ENSGALG00010021101	TMEM240	-1.501695749	0.329719237	-4.554468099	5.25183E-06	1.25785E-05	negative
PRJNA729271	stage_E18_vs_E10	ordinary_muscle_development	ENSGALG00010021102	GOLGA3	-0.128646172	0.113268101	-1.135767007	0.256054089	0.309963589	negative
PRJNA729271	stage_E18_vs_E10	ordinary_muscle_development	ENSGALG00010021103	RBMS1	0.25489161	0.074139485	3.438000834	0.000586026	0.001103318	positive
PRJNA729271	stage_E18_vs_E10	ordinary_muscle_development	ENSGALG00010021104	INA	2.068973636	0.260697306	7.936306147	2.08292E-15	1.09153E-14	positive
PRJNA729271	stage_E18_vs_E10	ordinary_muscle_development	ENSGALG00010021105	ENSGALG00010021105	0.939052571	0.487654249	1.92565239	0.054147792	0.075533851	positive
PRJNA729271	stage_E18_vs_E10	ordinary_muscle_development	ENSGALG00010021106	ITGB6	-1.41589841	0.201786328	-7.016820345	2.26974E-12	9.43772E-12	negative
PRJNA729271	stage_E18_vs_E10	ordinary_muscle_development	ENSGALG00010021108	PCGF6	-0.291344293	0.107957665	-2.698690207	0.006961294	0.01126286	negative
PRJNA729271	stage_E18_vs_E10	ordinary_muscle_development	ENSGALG00010021110	PKLR	0.658206524	0.168896162	3.89710764	9.73484E-05	0.000203265	positive
PRJNA729271	stage_E18_vs_E10	ordinary_muscle_development	ENSGALG00010021111	AVEN	0.235976938	0.080345278	2.93703557	0.003313661	0.005628253	positive
PRJNA729271	stage_E18_vs_E10	ordinary_muscle_development	ENSGALG00010021112	TAF5	-1.055807347	0.073235521	-14.41660182	4.06888E-47	1.06845E-45	negative
PRJNA729271	stage_E18_vs_E10	ordinary_muscle_development	ENSGALG00010021113	SYP	-0.576588159	0.303894228	-1.897331723	0.057784171	0.080212752	negative
PRJNA729271	stage_E18_vs_E10	ordinary_muscle_development	ENSGALG00010021114	SBF2	-0.096670035	0.098526854	-0.98115418	0.326516716	0.38456649	negative
PRJNA729271	stage_E18_vs_E10	ordinary_muscle_development	ENSGALG00010021115	ALPL	-0.78770753	0.246390423	-3.196989236	0.001388701	0.002487546	negative
PRJNA729271	stage_E18_vs_E10	ordinary_muscle_development	ENSGALG00010021116	FAM21C	0.28847161	0.06477777	4.453250093	8.45801E-06	1.98573E-05	positive
PRJNA729271	stage_E18_vs_E10	ordinary_muscle_development	ENSGALG00010021118	USMG5	0.840800898	0.179911914	4.673403099	2.9625E-06	7.26711E-06	positive
PRJNA729271	stage_E18_vs_E10	ordinary_muscle_development	ENSGALG00010021120	PDCD11	-1.32655946	0.088380588	-15.00962482	6.35059E-51	1.91376E-49	negative
PRJNA729271	stage_E18_vs_E10	ordinary_muscle_development	ENSGALG00010021121	PLA2R1	0.936812733	0.091685352	10.2176925	1.65227E-24	1.52061E-23	positive
PRJNA729271	stage_E18_vs_E10	ordinary_muscle_development	ENSGALG00010021122	CYP1A1	1.603938587	0.961636843	1.667925474	0.095330522	0.126970129	positive
PRJNA729271	stage_E18_vs_E10	ordinary_muscle_development	ENSGALG00010021123	RYR3	5.409945812	0.216182684	25.0248804	3.2778E-138	8.6385E-136	positive
PRJNA729271	stage_E18_vs_E10	ordinary_muscle_development	ENSGALG00010021124	CALHM2	0.642544274	0.131045507	4.903214853	9.42808E-07	2.44428E-06	positive
PRJNA729271	stage_E18_vs_E10	ordinary_muscle_development	ENSGALG00010021125	LY75	0.597731066	0.12494598	4.783915953	1.71913E-06	4.33143E-06	positive
PRJNA729271	stage_E18_vs_E10	ordinary_muscle_development	ENSGALG00010021126	ECE1	0.794536882	0.104181678	7.626455019	2.41297E-14	1.16238E-13	positive
PRJNA729271	stage_E18_vs_E10	ordinary_muscle_development	ENSGALG00010021127	SWAP70	-0.591796266	0.078731373	-7.516651202	5.61971E-14	2.63532E-13	negative
PRJNA729271	stage_E18_vs_E10	ordinary_muscle_development	ENSGALG00010021129	TXNRD3	-0.41032122	0.086419769	-4.7480018	2.05436E-06	5.1253E-06	negative
PRJNA729271	stage_E18_vs_E10	ordinary_muscle_development	ENSGALG00010021130	ENSGALG00010021130	4.185199298	1.102722144	3.79533441	0.000147445	0.000300888	positive
PRJNA729271	stage_E18_vs_E10	ordinary_muscle_development	ENSGALG00010021131	EDC3	-0.207827496	0.116202544	-1.788493505	0.073696423	0.100312672	negative
PRJNA729271	stage_E18_vs_E10	ordinary_muscle_development	ENSGALG00010021133	ENSGALG00010021133	-0.004383722	0.101851493	-0.043040335	0.965669381	0.97183765	negative
PRJNA729271	stage_E18_vs_E10	ordinary_muscle_development	ENSGALG00010021134	CHFR	-0.561218428	0.093731116	-5.987535992	2.13044E-09	6.98816E-09	negative
PRJNA729271	stage_E18_vs_E10	ordinary_muscle_development	ENSGALG00010021135	CLK3	0.768459247	0.106465634	7.217908901	5.27931E-13	2.30354E-12	positive
PRJNA729271	stage_E18_vs_E10	ordinary_muscle_development	ENSGALG00010021136	WEE1	-1.647710529	0.149681462	-11.00811355	3.4924E-28	3.90906E-27	negative
PRJNA729271	stage_E18_vs_E10	ordinary_muscle_development	ENSGALG00010021137	PLAU	-0.481419844	0.198233788	-2.428545854	0.015159508	0.023383747	negative
PRJNA729271	stage_E18_vs_E10	ordinary_muscle_development	ENSGALG00010021138	DDX18	-1.279567611	0.101862176	-12.56175417	3.42651E-36	5.62483E-35	negative
PRJNA729271	stage_E18_vs_E10	ordinary_muscle_development	ENSGALG00010021139	ARHGAP29	0.182018459	0.096741342	1.881496107	0.059904464	0.08293364	positive
PRJNA729271	stage_E18_vs_E10	ordinary_muscle_development	ENSGALG00010021140	SLC11A1	-1.385406879	0.497125931	-2.786832859	0.005322592	0.008789129	negative
PRJNA729271	stage_E18_vs_E10	ordinary_muscle_development	ENSGALG00010021141	SGSM1	0.726151162	0.144103936	5.039079292	4.67777E-07	1.24686E-06	positive
PRJNA729271	stage_E18_vs_E10	ordinary_muscle_development	ENSGALG00010021142	WNT6	-3.591914076	0.499744568	-7.187499984	6.59884E-13	2.86721E-12	negative
PRJNA729271	stage_E18_vs_E10	ordinary_muscle_development	ENSGALG00010021143	ABCA4	-1.953888554	0.402861546	-4.850024964	1.23446E-06	3.16307E-06	negative

PRJNA729271	stage_E18_vs_E10	ordinary_muscle_development	ENSGALG00010021144	FOXK1	0.031240356	0.110071278	0.283819327	0.776548842	0.812771714	positive
PRJNA729271	stage_E18_vs_E10	ordinary_muscle_development	ENSGALG00010021145	EIF4G3	-0.558288578	0.158711925	-3.517622108	0.000435432	0.000834866	negative
PRJNA729271	stage_E18_vs_E10	ordinary_muscle_development	ENSGALG00010021146	AP5Z1	-0.310609101	0.087944164	-3.531889841	0.000412601	0.00079319	negative
PRJNA729271	stage_E18_vs_E10	ordinary_muscle_development	ENSGALG00010021147	ARID3B	0.300953208	0.083010141	3.62549932	0.000288404	0.000566375	positive
PRJNA729271	stage_E18_vs_E10	ordinary_muscle_development	ENSGALG00010021148	GPATCH8	0.487316868	0.10488858	4.646043151	3.38362E-06	8.2485E-06	positive
PRJNA729271	stage_E18_vs_E10	ordinary_muscle_development	ENSGALG00010021149	ENSGALG00010021149	-2.603009704	1.344269078	-1.936375497	0.052821728	0.073819027	negative
PRJNA729271	stage_E18_vs_E10	ordinary_muscle_development	ENSGALG00010021150	ENSGALG00010021150	1.761641286	0.247954797	7.10468725	1.20595E-12	5.1307E-12	positive
PRJNA729271	stage_E18_vs_E10	ordinary_muscle_development	ENSGALG00010021151	WNT10A	-3.817439711	0.665666336	-5.734764556	9.76479E-09	3.0205E-08	negative
PRJNA729271	stage_E18_vs_E10	ordinary_muscle_development	ENSGALG00010021152	STK11IP	0.532092104	0.130033584	4.091959088	4.27744E-05	9.27889E-05	positive
PRJNA729271	stage_E18_vs_E10	ordinary_muscle_development	ENSGALG00010021153	PTPRF	-1.234006236	0.157761908	-7.821953053	5.201E-15	2.65546E-14	negative
PRJNA729271	stage_E18_vs_E10	ordinary_muscle_development	ENSGALG00010021154	CHST13	-1.284927775	0.209461389	-6.134437381	8.54611E-10	2.90652E-09	negative
PRJNA729271	stage_E18_vs_E10	ordinary_muscle_development	ENSGALG00010021155	RADIL	-1.737631637	0.221408109	-7.848093947	4.22408E-15	2.16967E-14	negative
PRJNA729271	stage_E18_vs_E10	ordinary_muscle_development	ENSGALG00010021156	DDOST	-0.479003846	0.080687904	-5.936501289	2.91169E-09	9.44392E-09	negative
PRJNA729271	stage_E18_vs_E10	ordinary_muscle_development	ENSGALG00010021157	ENSGALG00010021157	2.067673197	0.950612704	2.175095272	0.029622978	0.043310981	positive
PRJNA729271	stage_E18_vs_E10	ordinary_muscle_development	ENSGALG00010021158	ACTG1L	-1.577660239	0.128609711	-12.26703825	1.36143E-34	2.05991E-33	negative
PRJNA729271	stage_E18_vs_E10	ordinary_muscle_development	ENSGALG00010021159	MMD2	0.914776955	0.240419121	3.804925961	0.000141847	0.000290282	positive
PRJNA729271	stage_E18_vs_E10	ordinary_muscle_development	ENSGALG00010021160	ZNF143	-0.264237055	0.166073248	-1.591087411	0.111589905	0.146711626	negative
PRJNA729271	stage_E18_vs_E10	ordinary_muscle_development	ENSGALG00010021161	ENSGALG00010021161	-0.982749151	0.106727676	-9.208006653	3.32237E-20	2.35952E-19	negative
PRJNA729271	stage_E18_vs_E10	ordinary_muscle_development	ENSGALG00010021162	WIPI2	-0.220105449	0.095235732	-2.311164565	0.020823767	0.031314504	negative
PRJNA729271	stage_E18_vs_E10	ordinary_muscle_development	ENSGALG00010021163	INHA	0.121681026	0.315634809	0.385512061	0.699858072	0.744655565	positive
PRJNA729271	stage_E18_vs_E10	ordinary_muscle_development	ENSGALG00010021164	SLC41A3	-1.110923735	0.218915826	-5.07466159	3.88186E-07	1.04431E-06	negative
PRJNA729271	stage_E18_vs_E10	ordinary_muscle_development	ENSGALG00010021165	PTRF	3.773931872	0.105566055	35.74948284	6.7393E-280	8.8806E-277	positive
PRJNA729271	stage_E18_vs_E10	ordinary_muscle_development	ENSGALG00010021166	NEURL1	-0.329673827	0.31015089	-1.062946577	0.287806179	0.343863383	negative
PRJNA729271	stage_E18_vs_E10	ordinary_muscle_development	ENSGALG00010021167	KIAA1671	0.640558551	0.125734509	5.094532563	3.49603E-07	9.45075E-07	positive
PRJNA729271	stage_E18_vs_E10	ordinary_muscle_development	ENSGALG00010021168	IVNS1ABP	0.062426046	0.106492351	0.586202159	0.557739671	0.611715839	positive
PRJNA729271	stage_E18_vs_E10	ordinary_muscle_development	ENSGALG00010021169	CAMK2G	1.268690702	0.140481741	9.031000709	1.70109E-19	1.16308E-18	positive
PRJNA729271	stage_E18_vs_E10	ordinary_muscle_development	ENSGALG00010021170	UBL7	-0.614040682	0.140824996	-4.360310296	1.29878E-05	2.98918E-05	negative
PRJNA729271	stage_E18_vs_E10	ordinary_muscle_development	ENSGALG00010021171	ENSGALG00010021171	0.482128742	0.211752705	2.276848092	0.022795299	0.034039132	positive
PRJNA729271	stage_E18_vs_E10	ordinary_muscle_development	ENSGALG00010021172	SLC29A4	-0.523469147	0.272640094	-1.920000613	0.054857822	0.076487508	negative
PRJNA729271	stage_E18_vs_E10	ordinary_muscle_development	ENSGALG00010021173	KLF15	1.940489265	0.28739063	6.752096506	1.45724E-11	5.66595E-11	positive
PRJNA729271	stage_E18_vs_E10	ordinary_muscle_development	ENSGALG00010021175	IPO7	-0.135939603	0.097677627	-1.391716886	0.164008147	0.208005782	negative
PRJNA729271	stage_E18_vs_E10	ordinary_muscle_development	ENSGALG00010021176	ENSGALG00010021176	-0.421766628	0.118527732	-3.558379286	0.00037315	0.000721367	negative
PRJNA729271	stage_E18_vs_E10	ordinary_muscle_development	ENSGALG00010021177	OBSL1	0.438137305	0.18843187	2.325176233	0.020062539	0.030248232	positive
PRJNA729271	stage_E18_vs_E10	ordinary_muscle_development	ENSGALG00010021178	NDST2	-0.154666649	0.0973472	-1.588814558	0.112102266	0.147331792	negative
PRJNA729271	stage_E18_vs_E10	ordinary_muscle_development	ENSGALG00010021180	TMEM198	-0.119025556	0.142708399	-0.834047304	0.404254328	0.464242314	negative
PRJNA729271	stage_E18_vs_E10	ordinary_muscle_development	ENSGALG00010021181	DAGLA	0.113236724	0.129203873	0.876418961	0.380802314	0.441154855	positive
PRJNA729271	stage_E18_vs_E10	ordinary_muscle_development	ENSGALG00010021182	ZSWIM8	0.638237165	0.068655865	9.296178329	1.45585E-20	1.06417E-19	positive
PRJNA729271	stage_E18_vs_E10	ordinary_muscle_development	ENSGALG00010021183	CFAP100	-0.340125975	0.531544422	-0.639882502	0.52224899	0.578923151	negative
PRJNA729271	stage_E18_vs_E10	ordinary_muscle_development	ENSGALG00010021184	DRAXIN	-2.664120917	0.552409697	-4.822726562	1.41609E-06	3.5992E-06	negative
PRJNA729271	stage_E18_vs_E10	ordinary_muscle_development	ENSGALG00010021186	SWT1	0.456845691	0.090619451	5.041364605	4.62224E-07	1.23251E-06	positive
PRJNA729271	stage_E18_vs_E10	ordinary_muscle_development	ENSGALG00010021187	SEMA7A	2.897036243	0.277811187	10.42807628	1.84587E-25	1.80054E-24	positive
PRJNA729271	stage_E18_vs_E10	ordinary_muscle_development	ENSGALG00010021188	TNRC18	0.292384114	0.133464763	2.190721412	0.028471959	0.041773544	positive
PRJNA729271	stage_E18_vs_E10	ordinary_muscle_development	ENSGALG00010021189	ENSGALG00010021189	2.613837057	0.382106025	6.840606759	7.88585E-12	3.12737E-11	positive
PRJNA729271	stage_E18_vs_E10	ordinary_muscle_development	ENSGALG00010021190	ENSGALG00010021190	0.156765278	0.104176167	1.504809419	0.132373082	0.171393284	positive
PRJNA729271	stage_E18_vs_E10	ordinary_muscle_development	ENSGALG00010021191	MYRF	-0.881905884	0.20929003	-4.213797871	2.51112E-05	5.59579E-05	negative
PRJNA729271	stage_E18_vs_E10	ordinary_muscle_development	ENSGALG00010021192	ENSGALG00010021192	-0.265017681	0.17113287	-1.548607708	0.121476052	0.158658801	negative
PRJNA729271	stage_E18_vs_E10	ordinary_muscle_development	ENSGALG00010021193	USP37	-0.553431813	0.158548225	-3.490621313	0.000481899	0.000917767	negative
PRJNA729271	stage_E18_vs_E10	ordinary_muscle_development	ENSGALG00010021194	CYP11A1	-1.487645421	0.404987855	-3.673308721	0.00023943	0.000475351	negative
PRJNA729271	stage_E18_vs_E10	ordinary_muscle_development	ENSGALG00010021195	GCLM	-1.041239202	0.091822987	-11.3396355	8.34915E-30	1.01188E-28	negative
PRJNA729271	stage_E18_vs_E10	ordinary_muscle_development	ENSGALG00010021196	ATP6V0A1	1.59227741	0.12045747	13.21858588	6.85404E-40	1.31241E-38	positive
PRJNA729271	stage_E18_vs_E10	ordinary_muscle_development	ENSGALG00010021197	CHCHD1	-0.13631636	0.107962569	-1.262626114	0.206723579	0.256304702	negative
PRJNA729271	stage_E18_vs_E10	ordinary_muscle_development	ENSGALG00010021199	TMEM258	-0.375741978	0.136438668	-2.753925869	0.00588851	0.009648876	negative
PRJNA729271	stage_E18_vs_E10	ordinary_muscle_development	ENSGALG00010021200	FTO	-0.309266467	0.166141719	-1.86146182	0.062678987	0.08636235	negative
PRJNA729271	stage_E18_vs_E10	ordinary_muscle_development	ENSGALG00010021201	TRMT1L	-0.174267083	0.089379058	-1.949752964	0.05120557	0.071803614	negative
PRJNA729271	stage_E18_vs_E10	ordinary_muscle_development	ENSGALG00010021202	UROC1	0.50208014	0.510121133	0.984237091	0.324998952	0.382965598	positive
PRJNA729271	stage_E18_vs_E10	ordinary_muscle_development	ENSGALG00010021203	FUT11	0.351091781	0.081310466	4.317916248	1.57509E-05	3.59147E-05	positive
PRJNA729271	stage_E18_vs_E10	ordinary_muscle_development	ENSGALG00010021204	NAGLU	0.540536838	0.095101762	5.683773095	1.31755E-08	4.02569E-08	positive
PRJNA729271	stage_E18_vs_E10	ordinary_muscle_development	ENSGALG00010021206	HSD17B1	0.189701276	0.436906378	0.43419205	0.664148986	0.711675874	positive
PRJNA729271	stage_E18_vs_E10	ordinary_muscle_development	ENSGALG00010021207	MAD2L2	-0.396808637	0.124128556	-3.19675544	0.001389827	0.002489255	negative
PRJNA729271	stage_E18_vs_E10	ordinary_muscle_development	ENSGALG00010021210	ENSGALG00010021210	-0.345486827	0.063911953	-5.405668439	6.45671E-08	1.85658E-07	negative
PRJNA729271	stage_E18_vs_E10	ordinary_muscle_development	ENSGALG00010021211	FEN1	-1.70385047	0.155682243	-10.94441114	7.06755E-28	7.83212E-27	negative
PRJNA729271	stage_E18_vs_E10	ordinary_muscle_development	ENSGALG00010021212	MYO18B	1.961456005	0.161273431	12.16230097	4.93488E-34	7.26942E-33	positive
PRJNA729271	stage_E18_vs_E10	ordinary_muscle_development	ENSGALG00010021213	FBXO44	-0.68511588	0.23440157	-2.922829736	0.003468661	0.005868143	negative
PRJNA729271	stage_E18_vs_E10	ordinary_muscle_development	ENSGALG00010021214	FBXL18	-0.533007253	0.117428166	-4.539006888	5.65198E-06	1.34946E-05	negative
PRJNA729271	stage_E18_vs_E10	ordinary_muscle_development	ENSGALG00010021215	SH3PXD2A	0.610866146	0.111475297	5.479834202	4.25725E-08	1.24715E-07	positive
PRJNA729271	stage_E18_vs_E10	ordinary_muscle_development	ENSGALG00010021217	FBXO2	-1.264990576	0.211503503	-5.980943866	2.21848E-09	7.2671E-09	negative
PRJNA729271	stage_E18_vs_E10	ordinary_muscle_development	ENSGALG00010021218	DPF3	-0.404949607	0.177822488	-2.277268818	0.022770178	0.034008627	negative
PRJNA729271	stage_E18_vs_E10	ordinary_muscle_development	ENSGALG00010021219	STRA6	-1.98475799	0.228512138	-8.685569212	3.76851E-18	2.38743E-17	negative
PRJNA729271	stage_E18_vs_E10	ordinary_muscle_development	ENSGALG00010021220	SYNPO2L	3.413536495	0.205768712	16.58919111	8.34365E-62	3.54666E-60	positive
PRJNA729271	stage_E18_vs_E10	ordinary_muscle_development	ENSGALG00010021222	ENSGALG00010021222	2.441160694	0.314411972	7.764210367	8.21554E-15	4.13057E-14	positive
PRJNA729271	stage_E18_vs_E10	ordinary_muscle_development	ENSGALG00010021223	DFFB	0.53642466	0.110392164	4.859263925	1.17823E-06	3.02273E-06	positive
PRJNA729271	stage_E18_vs_E10	ordinary_muscle_development	ENSGALG00010021224	MYOZ1	6.556772035	0.141061974	46.48149921	0	0	positive
PRJNA729271	stage_E18_vs_E10	ordinary_muscle_development	ENSGALG00010021227	CHCHD4	-0.494212449	0.095850861	-5.156056451	2.52205E-07	6.89496E-07	negative
PRJNA729271	stage_E18_vs_E10	ordinary_muscle_development	ENSGALG00010021228	ESRP2	-5.088837037	0.926813817	-5.490678865	4.00392E-08	1.17698E-07	negative
PRJNA729271	stage_E18_vs_E10	ordinary_muscle_development	ENSGALG00010021229	GLO1	0.368241097	0.1784291	2.063795073	0.03903715	0.055880258	positive
PRJNA729271	stage_E18_vs_E10	ordinary_muscle_development	ENSGALG00010021230	C1orf174	-0.398579674	0.061196053	-6.51315983	7.35861E-11	2.712E-10	negative
PRJNA729271	stage_E18_vs_E10	ordinary_muscle_development	ENSGALG00010021231	STN1	-0.787977831	0.116659919	-6.754486349	1.43342E-11	5.58083E-11	negative
PRJNA729271	stage_E18_vs_E10	ordinary_muscle_development	ENSGALG00010021232	ACTB	-1.473009679	0.109440424	-13.45946612	2.70866E-41	5.46065E-40	negative
PRJNA729271	stage_E18_vs_E10	ordinary_muscle_development	ENSGALG00010021233	COASY	0.008178298	0.062677704	0.130481778	0.89618527	0.913902173	positive
PRJNA729271	stage_E18_vs_E10	ordinary_muscle_development	ENSGALG00010021234	ISLR2	5.058376229	0.321453824	15.73593422	8.57813E-56	3.01065E-54	positive
PRJNA729271	stage_E18_vs_E10	ordinary_muscle_development	ENSGALG00010021235	XRCC5	-1.147177413	0.139838626	-8.20358043	2.33331E-16	1.30433E-15	negative
PRJNA729271	stage_E18_vs_E10	ordinary_muscle_development	ENSGALG00010021236	BTBD9	-0.334079382	0.075048838	-4.451493067	8.52753E-06	2.0014E-05	negative
PRJNA729271	stage_E18_vs_E10	ordinary_muscle_development	ENSGALG00010021237	FSCN1	-2.07545565	0.222717358	-9.318787142	1.17678E-20	8.64541E-20	negative
PRJNA729271	stage_E18_vs_E10	ordinary_muscle_development	ENSGALG00010021238	NDUFS8	0.565450112	0.148032356	3.81977379	0.000133574	0.00027436	positive
PRJNA729271	stage_E18_vs_E10	ordinary_muscle_development	ENSGALG00010021239	USP54	0.583934952	0.06961118	8.388522541	4.9229E-17	2.88546E-16	positive
PRJNA729271	stage_E18_vs_E10	ordinary_muscle_development	ENSGALG00010021240	SLK	-0.512896187	0.079281559	-6.469300006	9.84579E-11	3.59302E-10	negative
PRJNA729271	stage_E18_vs_E10	ordinary_muscle_development	ENSGALG00010021241	TMEM43	0.984755653	0.171170573	5.753066275	8.76391E-09	2.72252E-08	positive
PRJNA729271	stage_E18_vs_E10	ordinary_muscle_development	ENSGALG00010021242	BCAR3	1.846968256	0.227526876	8.117582798	4.7556E-16	2.6091E-15	positive
PRJNA729271	stage_E18_vs_E10	ordinary_muscle_development	ENSGALG00010021243	DYL1	-0.767156524	0.094913802	-8.082665624	6.33662E-16	3.45428E-15	negative
PRJNA729271	stage_E18_vs_E10	ordinary_muscle_development	ENSGALG00010021244	PPP3CB	0.696453646	0.08741763	7.966970123	1.62612E-15	8.593E-15	positive
PRJNA729271	stage_E18_vs_E10	ordinary_muscle_development	ENSGALG00010021245	ZFAND3	0.615695151	0.070758955	8.70130364	3.28093E-18	2.08401E-17	positive
PRJNA729271	stage_E18_vs_E10	ordinary_muscle_development	ENSGALG00010021246	PECR	0.478946409	0.115634688	4.141892189	3.44452E-05	7.54433E-05	positive
PRJNA729271	stage_E18_vs_E10	ordinary_muscle_development	ENSGALG00010021247	GNAO1	-0.982836925	0.221838477	-4.430416853	9.40511E-06	2.19493E-05	negative
PRJNA729271	stage_E18_vs_E10	ordinary_muscle_development	ENSGALG00010021248	ANXA7	0.538148723	0.16851487	3.193479151	0.001405695	0.0025155	positive
PRJNA729271	stage_E18_vs_E10	ordinary_muscle_development	ENSGALG00010021249	ENSGALG00010021249	-0.685397181	0.394798997	-1.736066164	0.082552136	0.111300643	negative
PRJNA729271	stage_E18_vs_E10	ordinary_muscle_development	ENSGALG00010021250	ISLR	1.545018257	0.144855937	10.66589527	1.46977E-26	1.52719E-25	positive
PRJNA729271	stage_E18_vs_E10	ordinary_muscle_development	ENSGALG00010021251	RNF216	-0.35733576	0.086866467	-4.113621426	3.895E-05	8.4861E-05	negative
PRJNA729271	stage_E18_vs_E10	ordinary_muscle_development	ENSGALG00010021252	XPC	0.585540377	0.131765238	4.443815283	8.83775E-06	2.06918E-05	positive
PRJNA729271	stage_E18_vs_E10	ordinary_muscle_development	ENSGALG00010021253	TMEM169	-1.453140588	0.115317834	-12.60117831	2.08014E-36	3.45379E-35	negative
PRJNA729271	stage_E18_vs_E10	ordinary_muscle_development	ENSGALG00010021254	FNBP1L	-0.110909562	0.108267282	-1.024405157	0.305643947	0.362961577	negative
PRJNA729271	stage_E18_vs_E10	ordinary_muscle_development	ENSGALG00010021255	EIF3F	0.700725943	0.091924793	7.622817754	2.48197E-14	1.19443E-13	positive
PRJNA729271	stage_E18_vs_E10	ordinary_muscle_development	ENSGALG00010021256	ENSGALG00010021256	-0.324713089	0.296297842	-1.09590096	0.273122119	0.32832187	negative
PRJNA729271	stage_E18_vs_E10	ordinary_muscle_development	ENSGALG00010021257	RGS6	2.922750234	0.286262025	10.21005226	1.78769E-24	1.64149E-23	positive
PRJNA729271	stage_E18_vs_E10	ordinary_muscle_development	ENSGALG00010021258	LSM3	-0.515683765	0.111834482	-4.611133848	4.00479E-06	9.68135E-06	negative
PRJNA729271	stage_E18_vs_E10	ordinary_muscle_development	ENSGALG00010021259	CEP104	-0.841334539	0.135104139	-6.227303935	4.7453E-10	1.65176E-09	negative
PRJNA729271	stage_E18_vs_E10	ordinary_muscle_development	ENSGALG00010021260	KDM4A	0.019910888	0.06805504	0.292570374	0.769850561	0.806751293	positive
PRJNA729271	stage_E18_vs_E10	ordinary_muscle_development	ENSGALG00010021261	JUP	0.701354267	0.128229543	5.469521713	4.51252E-08	1.31873E-07	positive
PRJNA729271	stage_E18_vs_E10	ordinary_muscle_development	ENSGALG00010021262	MDGA1	-0.525066326	0.314799159	-1.667940687	0.095327502	0.126970129	negative
PRJNA729271	stage_E18_vs_E10	ordinary_muscle_development	ENSGALG00010021263	STOML1	-0.914017538	0.251187372	-3.638787769	0.000273924	0.000539402	negative
PRJNA729271	stage_E18_vs_E10	ordinary_muscle_development	ENSGALG00010021264	CBFB	-0.267905651	0.169694124	-1.578756206	0.114391987	0.15015049	negative
PRJNA729271	stage_E18_vs_E10	ordinary_muscle_development	ENSGALG00010021265	SIPA1L1	0.454422207	0.08889106	5.112124985	3.18555E-07	8.63883E-07	positive
PRJNA729271	stage_E18_vs_E10	ordinary_muscle_development	ENSGALG00010021266	COQ5	0.331312868	0.099401865	3.333064897	0.000858949	0.001580209	positive
PRJNA729271	stage_E18_vs_E10	ordinary_muscle_development	ENSGALG00010021267	LRRC47	0.198188704	0.079059299	2.506836093	0.012181719	0.019029422	positive
PRJNA729271	stage_E18_vs_E10	ordinary_muscle_development	ENSGALG00010021269	NUDT13	1.269186671	0.161346901	7.866198026	3.65582E-15	1.88715E-14	positive
PRJNA729271	stage_E18_vs_E10	ordinary_muscle_development	ENSGALG00010021271	ENSGALG00010021271	-0.809853578	0.467485128	-1.732362228	0.083209089	0.112061297	negative
PRJNA729271	stage_E18_vs_E10	ordinary_muscle_development	ENSGALG00010021272	ENSGALG00010021272	-0.425216581	0.367199395	-1.157999133	0.24686439	0.300092195	negative
PRJNA729271	stage_E18_vs_E10	ordinary_muscle_development	ENSGALG00010021275	MAP3K2	0.064197458	0.092941971	0.690726237	0.489737598	0.547616022	positive
PRJNA729271	stage_E18_vs_E10	ordinary_muscle_development	ENSGALG00010021277	ENSGALG00010021277	0.337765465	0.23503246	1.43710135	0.150689198	0.19281779	positive
PRJNA729271	stage_E18_vs_E10	ordinary_muscle_development	ENSGALG00010021287	COL17A1	-5.874735053	0.446288217	-13.16354507	1.42252E-39	2.67784E-38	negative
PRJNA729271	stage_E18_vs_E10	ordinary_muscle_development	ENSGALG00010021290	ERCC3	-0.431118951	0.069693579	-6.185920624	6.17411E-10	2.12776E-09	negative
PRJNA729271	stage_E18_vs_E10	ordinary_muscle_development	ENSGALG00010021293	PCNX1	0.021737927	0.069201269	0.314126127	0.753425247	0.793093606	positive
PRJNA729271	stage_E18_vs_E10	ordinary_muscle_development	ENSGALG00010021296	GATC	-0.023938927	0.123058787	-0.194532448	0.845758991	0.872235971	negative
PRJNA729271	stage_E18_vs_E10	ordinary_muscle_development	ENSGALG00010021298	RPGRIP1L	-1.283741417	0.148774435	-8.62877696	6.20115E-18	3.8561E-17	negative
PRJNA729271	stage_E18_vs_E10	ordinary_muscle_development	ENSGALG00010021299	CYP27C1	-3.219578987	0.16573338	-19.4262555	4.62926E-84	3.5883E-82	negative
PRJNA729271	stage_E18_vs_E10	ordinary_muscle_development	ENSGALG00010021301	SMURF1	-0.022927196	0.081261619	-0.282140528	0.777835758	0.813700158	negative
PRJNA729271	stage_E18_vs_E10	ordinary_muscle_development	ENSGALG00010021302	P4HA1	0.139735195	0.059786591	2.337233035	0.01942707	0.029357317	positive
PRJNA729271	stage_E18_vs_E10	ordinary_muscle_development	ENSGALG00010021307	MAP3K9	-0.628741685	0.141318081	-4.44912414	8.62212E-06	2.02229E-05	negative
PRJNA729271	stage_E18_vs_E10	ordinary_muscle_development	ENSGALG00010021309	ST3GAL3	0.121391868	0.127020336	0.955688446	0.339229662	0.397601192	positive
PRJNA729271	stage_E18_vs_E10	ordinary_muscle_development	ENSGALG00010021311	TRIAP1	0.031472726	0.121192969	0.259691024	0.795102117	0.828779318	positive
PRJNA729271	stage_E18_vs_E10	ordinary_muscle_development	ENSGALG00010021313	KPNA7	0.220504871	1.437638784	0.153379885	0.878098707	0.899063414	positive
PRJNA729271	stage_E18_vs_E10	ordinary_muscle_development	ENSGALG00010021315	COX6A1	0.847508529	0.135039202	6.276018507	3.47353E-10	1.21763E-09	positive

PRJNA729271	stage_E18_vs_E10	ordinary_muscle_development	ENSGALG00010021318	TP73	-3.594127104	0.296589609	-12.11818284	8.46144E-34	1.22526E-32	negative
PRJNA729271	stage_E18_vs_E10	ordinary_muscle_development	ENSGALG00010021320	SLC6A6	1.472345802	0.195152717	7.544582648	4.53738E-14	2.13884E-13	positive
PRJNA729271	stage_E18_vs_E10	ordinary_muscle_development	ENSGALG00010021325	ARPC1A	-0.479925186	0.065641605	-7.31129571	2.64579E-13	1.18111E-12	negative
PRJNA729271	stage_E18_vs_E10	ordinary_muscle_development	ENSGALG00010021327	IGSF1	1.482603745	0.46568534	3.183702853	0.001454042	0.002598168	positive
PRJNA729271	stage_E18_vs_E10	ordinary_muscle_development	ENSGALG00010021329	PSMC3IP	-0.947620142	0.158464377	-5.980019978	2.2311E-09	7.30349E-09	negative
PRJNA729271	stage_E18_vs_E10	ordinary_muscle_development	ENSGALG00010021330	COMMD4	-0.131686308	0.130532787	-1.008837022	0.313052803	0.370630289	negative
PRJNA729271	stage_E18_vs_E10	ordinary_muscle_development	ENSGALG00010021332	OIT3	2.326330077	0.123475601	18.84040289	3.52274E-79	2.38608E-77	positive
PRJNA729271	stage_E18_vs_E10	ordinary_muscle_development	ENSGALG00010021335	NFATC3	0.138053996	0.102026783	1.353115242	0.176018817	0.221686745	positive
PRJNA729271	stage_E18_vs_E10	ordinary_muscle_development	ENSGALG00010021338	PDIA5	0.315809109	0.157590287	2.003988416	0.045071301	0.063762299	positive
PRJNA729271	stage_E18_vs_E10	ordinary_muscle_development	ENSGALG00010021341	L3MBTL1	-0.166584465	0.154444289	-1.078605533	0.280763614	0.336391848	negative
PRJNA729271	stage_E18_vs_E10	ordinary_muscle_development	ENSGALG00010021342	SFR1	-1.97184019	0.180402973	-10.93019787	8.2668E-28	9.11927E-27	negative
PRJNA729271	stage_E18_vs_E10	ordinary_muscle_development	ENSGALG00010021345	NEIL1	0.36296119	0.157119286	2.310099529	0.020882645	0.031390017	positive
PRJNA729271	stage_E18_vs_E10	ordinary_muscle_development	ENSGALG00010021346	RPS21	1.41730551	0.278427653	5.090390609	3.57327E-07	9.64875E-07	positive
PRJNA729271	stage_E18_vs_E10	ordinary_muscle_development	ENSGALG00010021348	SLC4A3	1.132835084	0.114341109	9.907504754	3.8617E-23	3.26387E-22	positive
PRJNA729271	stage_E18_vs_E10	ordinary_muscle_development	ENSGALG00010021350	CABLES2	-0.346643567	0.136592831	-2.537787402	0.011155573	0.017545576	negative
PRJNA729271	stage_E18_vs_E10	ordinary_muscle_development	ENSGALG00010021352	CFAP43	-0.480798904	0.22732402	-2.115037843	0.034426722	0.049732443	negative
PRJNA729271	stage_E18_vs_E10	ordinary_muscle_development	ENSGALG00010021353	IPO13	1.77911634	0.178024183	9.993677881	1.6244E-23	1.4007E-22	positive
PRJNA729271	stage_E18_vs_E10	ordinary_muscle_development	ENSGALG00010021354	ENSGALG00010021354	0.306991781	0.11237267	2.731907874	0.006296875	0.010278513	positive
PRJNA729271	stage_E18_vs_E10	ordinary_muscle_development	ENSGALG00010021358	MMP15	0.007505944	0.129476926	0.05797129	0.953771497	0.961800323	positive
PRJNA729271	stage_E18_vs_E10	ordinary_muscle_development	ENSGALG00010021360	PTPRN	-0.124596605	0.265583297	-0.469143228	0.638967262	0.688202591	negative
PRJNA729271	stage_E18_vs_E10	ordinary_muscle_development	ENSGALG00010021361	WRAP73	-0.402520336	0.091441064	-4.401964722	1.07275E-05	2.49271E-05	negative
PRJNA729271	stage_E18_vs_E10	ordinary_muscle_development	ENSGALG00010021362	ARPC1B	0.14353682	0.121820342	1.178266429	0.238690407	0.291402127	positive
PRJNA729271	stage_E18_vs_E10	ordinary_muscle_development	ENSGALG00010021363	ENSGALG00010021363	-0.43603228	0.075602201	-5.767454823	8.04776E-09	2.50865E-08	negative
PRJNA729271	stage_E18_vs_E10	ordinary_muscle_development	ENSGALG00010021367	GRIP2	1.805776483	0.176173531	10.24998747	1.18359E-24	1.09975E-23	positive
PRJNA729271	stage_E18_vs_E10	ordinary_muscle_development	ENSGALG00010021372	CD86	1.061222422	0.675447692	1.571139312	0.116150293	0.152265397	positive
PRJNA729271	stage_E18_vs_E10	ordinary_muscle_development	ENSGALG00010021373	CEP250	-0.514374583	0.154316197	-3.333250778	0.000858375	0.001579554	negative
PRJNA729271	stage_E18_vs_E10	ordinary_muscle_development	ENSGALG00010021375	TUBG1	-1.219600104	0.119548947	-10.20168005	1.94872E-24	1.78438E-23	negative
PRJNA729271	stage_E18_vs_E10	ordinary_muscle_development	ENSGALG00010021378	KIF11	-1.582538577	0.185884113	-8.513576309	1.68649E-17	1.01942E-16	negative
PRJNA729271	stage_E18_vs_E10	ordinary_muscle_development	ENSGALG00010021381	DNAJB2	0.048442329	0.148435194	0.326353394	0.744156995	0.784424511	positive
PRJNA729271	stage_E18_vs_E10	ordinary_muscle_development	ENSGALG00010021382	TPRG1L	0.334509565	0.086918127	3.848559272	0.000118815	0.000245371	positive
PRJNA729271	stage_E18_vs_E10	ordinary_muscle_development	ENSGALG00010021383	MSI1	-1.625339834	0.121844061	-13.33950804	1.36356E-40	2.67454E-39	negative
PRJNA729271	stage_E18_vs_E10	ordinary_muscle_development	ENSGALG00010021384	ARTN	2.642044037	0.353985109	7.463715187	8.41163E-14	3.89542E-13	positive
PRJNA729271	stage_E18_vs_E10	ordinary_muscle_development	ENSGALG00010021385	GDF5	-4.990247929	0.411777072	-12.11880959	8.39699E-34	1.21836E-32	negative
PRJNA729271	stage_E18_vs_E10	ordinary_muscle_development	ENSGALG00010021387	SLC2A14	0.67387663	0.094683543	7.117146346	1.10184E-12	4.70711E-12	positive
PRJNA729271	stage_E18_vs_E10	ordinary_muscle_development	ENSGALG00010021388	DPH2	-0.590723472	0.150572956	-3.923171112	8.7391E-05	0.000183398	negative
PRJNA729271	stage_E18_vs_E10	ordinary_muscle_development	ENSGALG00010021390	GSTO2	-1.805520071	0.219174581	-8.237816915	1.75381E-16	9.88779E-16	negative
PRJNA729271	stage_E18_vs_E10	ordinary_muscle_development	ENSGALG00010021391	ENSGALG00010021391	-0.027993745	0.38950661	-0.071869756	0.942705559	0.953094586	negative
PRJNA729271	stage_E18_vs_E10	ordinary_muscle_development	ENSGALG00010021393	CCDC174	-0.412928096	0.116188509	-3.553949517	0.000379492	0.000732358	negative
PRJNA729271	stage_E18_vs_E10	ordinary_muscle_development	ENSGALG00010021394	MAN2C1	-0.234882465	0.098309454	-2.389215421	0.016884399	0.025802779	negative
PRJNA729271	stage_E18_vs_E10	ordinary_muscle_development	ENSGALG00010021396	MED6	-0.584170664	0.080864168	-7.224097864	5.04439E-13	2.20702E-12	negative
PRJNA729271	stage_E18_vs_E10	ordinary_muscle_development	ENSGALG00010021397	ENSGALG00010021397	0.762299027	1.870696737	0.407494711	0.683644678	0.729815113	positive
PRJNA729271	stage_E18_vs_E10	ordinary_muscle_development	ENSGALG00010021400	B4GALT2	0.638367066	0.091836669	6.951113027	3.62415E-12	1.48522E-11	positive
PRJNA729271	stage_E18_vs_E10	ordinary_muscle_development	ENSGALG00010021401	PDAP1	-0.431406958	0.094911451	-4.545362578	5.48408E-06	1.31131E-05	negative
PRJNA729271	stage_E18_vs_E10	ordinary_muscle_development	ENSGALG00010021402	ENSGALG00010021402	0.146264152	0.089012457	1.643187443	0.100344157	0.132963576	positive
PRJNA729271	stage_E18_vs_E10	ordinary_muscle_development	ENSGALG00010021404	UQCC1	-0.042573387	0.095020309	-0.448045134	0.654120624	0.702227702	negative
PRJNA729271	stage_E18_vs_E10	ordinary_muscle_development	ENSGALG00010021405	IDE	-0.027756579	0.114756449	-0.241873805	0.808877948	0.840961545	negative
PRJNA729271	stage_E18_vs_E10	ordinary_muscle_development	ENSGALG00010021407	SIN3A	-0.190171231	0.111953757	-1.698658769	0.089383496	0.119587759	negative
PRJNA729271	stage_E18_vs_E10	ordinary_muscle_development	ENSGALG00010021408	TTC9	1.080115104	0.205203946	5.263617618	1.41248E-07	3.94184E-07	positive
PRJNA729271	stage_E18_vs_E10	ordinary_muscle_development	ENSGALG00010021409	MEGF6	-0.065539778	0.239045464	-0.274172858	0.783951785	0.818923402	negative
PRJNA729271	stage_E18_vs_E10	ordinary_muscle_development	ENSGALG00010021411	MCU	1.075284652	0.128996758	8.335749424	7.70089E-17	4.44718E-16	positive
PRJNA729271	stage_E18_vs_E10	ordinary_muscle_development	ENSGALG00010021412	ZNF319	-0.046149015	0.125571623	-0.367511493	0.71323752	0.757002113	negative
PRJNA729271	stage_E18_vs_E10	ordinary_muscle_development	ENSGALG00010021413	SYNJ2BP	0.393351901	0.160995136	2.44325332	0.014555516	0.022516778	positive
PRJNA729271	stage_E18_vs_E10	ordinary_muscle_development	ENSGALG00010021414	BUD31	-0.049829086	0.111877921	-0.445388022	0.656039367	0.704131109	negative
PRJNA729271	stage_E18_vs_E10	ordinary_muscle_development	ENSGALG00010021415	SIRT4	0.751651612	0.150321935	5.000278992	5.72474E-07	1.51451E-06	positive
PRJNA729271	stage_E18_vs_E10	ordinary_muscle_development	ENSGALG00010021416	PLEKHH3	0.194880021	0.128318816	1.518717424	0.128833639	0.167346975	positive
PRJNA729271	stage_E18_vs_E10	ordinary_muscle_development	ENSGALG00010021417	STK16	-1.079895449	0.164597984	-6.560806066	5.35177E-11	1.99521E-10	negative
PRJNA729271	stage_E18_vs_E10	ordinary_muscle_development	ENSGALG00010021419	MANSC1	-1.183966768	0.201227443	-5.883724154	4.01136E-09	1.28582E-08	negative
PRJNA729271	stage_E18_vs_E10	ordinary_muscle_development	ENSGALG00010021420	PLXNB3	-1.345733164	0.191014921	-7.045172994	1.85231E-12	7.7599E-12	negative
PRJNA729271	stage_E18_vs_E10	ordinary_muscle_development	ENSGALG00010021421	GLB1L	0.87720968	0.163230807	5.374044864	7.69897E-08	2.20155E-07	positive
PRJNA729271	stage_E18_vs_E10	ordinary_muscle_development	ENSGALG00010021424	PTCD1	-0.476461808	0.110883359	-4.296964052	1.73153E-05	3.93333E-05	negative
PRJNA729271	stage_E18_vs_E10	ordinary_muscle_development	ENSGALG00010021425	PTPN9	-1.11099455	0.125789797	-8.83215152	1.02681E-18	6.72251E-18	negative
PRJNA729271	stage_E18_vs_E10	ordinary_muscle_development	ENSGALG00010021426	SLC38A7	-0.298916651	0.146460109	-2.040942437	0.041256549	0.058743976	negative
PRJNA729271	stage_E18_vs_E10	ordinary_muscle_development	ENSGALG00010021427	RPLP0	0.693468679	0.135391868	5.121937469	3.02412E-07	8.21489E-07	positive
PRJNA729271	stage_E18_vs_E10	ordinary_muscle_development	ENSGALG00010021429	MED8	0.427309155	0.116046965	3.682208791	0.000231222	0.000459874	positive
PRJNA729271	stage_E18_vs_E10	ordinary_muscle_development	ENSGALG00010021431	CPSF4	-0.923579613	0.108212807	-8.534845733	1.40343E-17	8.52584E-17	negative
PRJNA729271	stage_E18_vs_E10	ordinary_muscle_development	ENSGALG00010021432	SYT9	-1.106278931	0.306920741	-3.604445	0.000312821	0.000610767	negative
PRJNA729271	stage_E18_vs_E10	ordinary_muscle_development	ENSGALG00010021433	ENSGALG00010021433	5.888503223	0.269593997	21.84211553	9.2377E-106	1.2398E-103	positive
PRJNA729271	stage_E18_vs_E10	ordinary_muscle_development	ENSGALG00010021434	ANKZF1	-0.175561522	0.179633397	-0.97733231	0.328404663	0.386444681	negative
PRJNA729271	stage_E18_vs_E10	ordinary_muscle_development	ENSGALG00010021435	GCN1	-0.415662414	0.089472158	-4.645717975	3.38896E-06	8.25596E-06	negative
PRJNA729271	stage_E18_vs_E10	ordinary_muscle_development	ENSGALG00010021438	GOT2	-0.015098971	0.110700004	-0.136395399	0.891508711	0.910344675	negative
PRJNA729271	stage_E18_vs_E10	ordinary_muscle_development	ENSGALG00010021439	ITPRIP	-0.571047891	0.187097786	-3.052136012	0.002272191	0.003953831	negative
PRJNA729271	stage_E18_vs_E10	ordinary_muscle_development	ENSGALG00010021441	ENSGALG00010021441	-0.436253575	0.328549121	-1.327818421	0.1842381	0.231194811	negative
PRJNA729271	stage_E18_vs_E10	ordinary_muscle_development	ENSGALG00010021443	SNUPN	-0.245550533	0.137819172	-1.781686317	0.074800399	0.101700759	negative
PRJNA729271	stage_E18_vs_E10	ordinary_muscle_development	ENSGALG00010021444	ATG9A	0.261658189	0.24794066	1.055325853	0.291276321	0.347579672	positive
PRJNA729271	stage_E18_vs_E10	ordinary_muscle_development	ENSGALG00010021446	HOXB8	-1.746564695	0.646564242	-2.701301097	0.006906878	0.011182307	negative
PRJNA729271	stage_E18_vs_E10	ordinary_muscle_development	ENSGALG00010021447	OCSTAMP	-0.410431468	0.761452183	-0.53901148	0.589878933	0.641962245	negative
PRJNA729271	stage_E18_vs_E10	ordinary_muscle_development	ENSGALG00010021448	SZT2	0.188067676	0.101025961	1.861577698	0.062662639	0.086348033	positive
PRJNA729271	stage_E18_vs_E10	ordinary_muscle_development	ENSGALG00010021450	OLFML1	1.499884425	0.236742677	6.335505053	2.36565E-10	8.38799E-10	positive
PRJNA729271	stage_E18_vs_E10	ordinary_muscle_development	ENSGALG00010021452	SLC25A1	-0.189265481	0.115875589	-1.63335075	0.102395249	0.135470896	negative
PRJNA729271	stage_E18_vs_E10	ordinary_muscle_development	ENSGALG00010021454	ATP5J2	1.032734277	0.174263031	5.926295841	3.09844E-09	1.00205E-08	positive
PRJNA729271	stage_E18_vs_E10	ordinary_muscle_development	ENSGALG00010021455	CFAP58	-0.29655492	0.521843644	-0.568283093	0.569842758	0.623433525	negative
PRJNA729271	stage_E18_vs_E10	ordinary_muscle_development	ENSGALG00010021457	USB1	0.868472133	0.113154626	7.675091688	1.653E-14	8.09196E-14	positive
PRJNA729271	stage_E18_vs_E10	ordinary_muscle_development	ENSGALG00010021458	USP30	-0.139110919	0.08313298	-1.673354183	0.094257592	0.125656562	negative
PRJNA729271	stage_E18_vs_E10	ordinary_muscle_development	ENSGALG00010021459	ARHGEF16	0.395482053	0.12490574	3.166244031	0.001544212	0.002750573	positive
PRJNA729271	stage_E18_vs_E10	ordinary_muscle_development	ENSGALG00010021461	SNX33	0.172025702	0.111445045	1.543592195	0.122687157	0.160009928	positive
PRJNA729271	stage_E18_vs_E10	ordinary_muscle_development	ENSGALG00010021462	SETD5	-0.207130188	0.094923937	-2.182064861	0.029104746	0.042634997	negative
PRJNA729271	stage_E18_vs_E10	ordinary_muscle_development	ENSGALG00010021464	ENSGALG00010021464	0.56208198	0.438486049	1.281869701	0.199888358	0.248742852	positive
PRJNA729271	stage_E18_vs_E10	ordinary_muscle_development	ENSGALG00010021465	ABCB6	-0.302709535	0.217409115	-1.392349788	0.163816502	0.207802607	negative
PRJNA729271	stage_E18_vs_E10	ordinary_muscle_development	ENSGALG00010021466	HES4	-0.166691125	0.179984543	-0.926141333	0.354372536	0.413720463	negative
PRJNA729271	stage_E18_vs_E10	ordinary_muscle_development	ENSGALG00010021467	LHFPL4	0.326355127	0.261792882	1.246615738	0.212538428	0.262833882	positive
PRJNA729271	stage_E18_vs_E10	ordinary_muscle_development	ENSGALG00010021469	CSPG4	0.567230789	0.16158685	3.510377167	0.000447471	0.000856816	positive
PRJNA729271	stage_E18_vs_E10	ordinary_muscle_development	ENSGALG00010021470	ENSGALG00010021470	-1.300903099	0.63565882	-2.046542985	0.040702978	0.058035576	negative
PRJNA729271	stage_E18_vs_E10	ordinary_muscle_development	ENSGALG00010021473	SLC8A3	1.52423678	0.131447457	11.59578747	4.32866E-31	5.61216E-30	positive
PRJNA729271	stage_E18_vs_E10	ordinary_muscle_development	ENSGALG00010021474	PPFIBP2	2.455633052	0.180296067	13.62000341	3.04554E-42	6.3518E-41	positive
PRJNA729271	stage_E18_vs_E10	ordinary_muscle_development	ENSGALG00010021476	ALKBH2	0.219690369	0.121616296	1.806422133	0.070852427	0.096850804	positive
PRJNA729271	stage_E18_vs_E10	ordinary_muscle_development	ENSGALG00010021477	ZFAND2B	0.214538131	0.121158494	1.770722995	0.076606777	0.103961729	positive
PRJNA729271	stage_E18_vs_E10	ordinary_muscle_development	ENSGALG00010021478	SLC13A3	-0.752170367	0.198583897	-3.787670496	0.000152066	0.000309753	negative
PRJNA729271	stage_E18_vs_E10	ordinary_muscle_development	ENSGALG00010021479	SKAP1	-1.842849448	0.622088333	-2.962359766	0.003052908	0.005223313	negative
PRJNA729271	stage_E18_vs_E10	ordinary_muscle_development	ENSGALG00010021480	ENSGALG00010021480	-0.350647734	0.076189814	-4.602291508	4.17868E-06	1.00799E-05	negative
PRJNA729271	stage_E18_vs_E10	ordinary_muscle_development	ENSGALG00010021481	MICU1	1.087981844	0.174311105	6.241609471	4.33091E-10	1.5116E-09	positive
PRJNA729271	stage_E18_vs_E10	ordinary_muscle_development	ENSGALG00010021483	UNG	-1.257486186	0.156094923	-8.055907005	7.88916E-16	4.27329E-15	negative
PRJNA729271	stage_E18_vs_E10	ordinary_muscle_development	ENSGALG00010021484	MARCHF5	0.115660565	0.071826849	1.610269238	0.107339092	0.141546592	positive
PRJNA729271	stage_E18_vs_E10	ordinary_muscle_development	ENSGALG00010021485	SMOC1	-3.304828261	0.230522479	-14.3362516	1.29884E-46	3.33806E-45	negative
PRJNA729271	stage_E18_vs_E10	ordinary_muscle_development	ENSGALG00010021487	KLHL17	-0.466405862	0.137060877	-3.402910234	0.000666722	0.001246181	negative
PRJNA729271	stage_E18_vs_E10	ordinary_muscle_development	ENSGALG00010021488	HYI	-1.141067362	0.1446985	-7.885827178	3.12459E-15	1.62043E-14	negative
PRJNA729271	stage_E18_vs_E10	ordinary_muscle_development	ENSGALG00010021489	CNPPD1	0.002895897	0.087823865	0.032973919	0.973695386	0.978322882	positive
PRJNA729271	stage_E18_vs_E10	ordinary_muscle_development	ENSGALG00010021490	ACACB	4.219119961	0.330529124	12.76474492	2.57994E-37	4.40994E-36	positive
PRJNA729271	stage_E18_vs_E10	ordinary_muscle_development	ENSGALG00010021492	PARP6	0.287317539	0.159203227	1.804721832	0.071118222	0.097196936	positive
PRJNA729271	stage_E18_vs_E10	ordinary_muscle_development	ENSGALG00010021493	UNC93B1	0.312701292	0.13531884	2.310848149	0.020841244	0.031337534	positive
PRJNA729271	stage_E18_vs_E10	ordinary_muscle_development	ENSGALG00010021494	SLC2A10	-0.084860543	0.235225798	-0.360762059	0.718277328	0.761236372	negative
PRJNA729271	stage_E18_vs_E10	ordinary_muscle_development	ENSGALG00010021496	MTMR14	0.035670757	0.117462075	0.303678931	0.761372511	0.799774951	positive
PRJNA729271	stage_E18_vs_E10	ordinary_muscle_development	ENSGALG00010021498	B3GALT6	-0.528635036	0.106857465	-4.94710439	7.53256E-07	1.97226E-06	negative
PRJNA729271	stage_E18_vs_E10	ordinary_muscle_development	ENSGALG00010021501	SLC23A3	0.80155656	0.693375442	1.156020983	0.247672574	0.300948442	positive
PRJNA729271	stage_E18_vs_E10	ordinary_muscle_development	ENSGALG00010021502	DNAJB12	-0.10522168	0.147386884	-0.713914814	0.475279857	0.533920912	negative
PRJNA729271	stage_E18_vs_E10	ordinary_muscle_development	ENSGALG00010021503	KCNS1	-1.344165073	0.526048059	-2.555213446	0.01061227	0.016736465	negative
PRJNA729271	stage_E18_vs_E10	ordinary_muscle_development	ENSGALG00010021504	ODR4	-0.440086023	0.078259731	-5.623403182	1.87232E-08	5.64577E-08	negative
PRJNA729271	stage_E18_vs_E10	ordinary_muscle_development	ENSGALG00010021505	IHH	-3.456353739	0.530354582	-6.51706209	7.16978E-11	2.6451E-10	negative
PRJNA729271	stage_E18_vs_E10	ordinary_muscle_development	ENSGALG00010021506	MAX	-0.060032598	0.26167024	-0.229420808	0.818541865	0.849148255	negative
PRJNA729271	stage_E18_vs_E10	ordinary_muscle_development	ENSGALG00010021509	DDIT4	3.546219124	0.277700854	12.76992515	2.41386E-37	4.13581E-36	positive
PRJNA729271	stage_E18_vs_E10	ordinary_muscle_development	ENSGALG00010021510	NTSR1	-1.129502868	0.420500696	-2.686090363	0.007229353	0.011669207	negative
PRJNA729271	stage_E18_vs_E10	ordinary_muscle_development	ENSGALG00010021512	CPNE9	3.669840854	0.392593483	9.347686638	8.95856E-21	6.64217E-20	positive
PRJNA729271	stage_E18_vs_E10	ordinary_muscle_development	ENSGALG00010021513	SDF4	0.35037514	0.097097975	3.608470105	0.000308008	0.000602182	positive
PRJNA729271	stage_E18_vs_E10	ordinary_muscle_development	ENSGALG00010021514	TPR	-0.593733994	0.079471897	-7.470993122	7.95918E-14	3.69533E-13	negative
PRJNA729271	stage_E18_vs_E10	ordinary_muscle_development	ENSGALG00010021516	SRSF5	0.439901429	0.151409418	2.90537693	0.003668111	0.006187509	positive
PRJNA729271	stage_E18_vs_E10	ordinary_muscle_development	ENSGALG00010021518	CPEB3	2.142334971	0.146144683	14.65900043	1.17997E-48	3.24548E-47	positive
PRJNA729271	stage_E18_vs_E10	ordinary_muscle_development	ENSGALG00010021519	FNTB	-0.323520681	0.10155866	-3.185554845	0.001444767	0.00258255	negative
PRJNA729271	stage_E18_vs_E10	ordinary_muscle_development	ENSGALG00010021521	ENSGALG00010021521	0.819527756	0.269767685	3.037901871	0.002382315	0.004133548	positive
PRJNA729271	stage_E18_vs_E10	ordinary_muscle_development	ENSGALG00010021523	CELF6	-0.382061679	0.151994088	-2.513661437	0.011948508	0.018699376	negative
PRJNA729271	stage_E18_vs_E10	ordinary_muscle_development	ENSGALG00010021525	ENSGALG00010021525	-0.271533952	0.818055964	-0.331925887	0.739945222	0.780945536	negative
PRJNA729271	stage_E18_vs_E10	ordinary_muscle_development	ENSGALG00010021526	KCNJ12	0.849200101	0.219396466	3.870618867	0.000108559	0.000225569	positive
PRJNA729271	stage_E18_vs_E10	ordinary_muscle_development	ENSGALG00010021528	BRPF1	-0.063508448	0.167977773	-0.378076494	0.70537377	0.750083838	negative

PRJNA729271	stage_E18_vs_E10	ordinary_muscle_development	ENSGALG00010021529	BTAF1	-0.022147921	0.076516865	-0.28945149	0.772235897	0.809017009	negative
PRJNA729271	stage_E18_vs_E10	ordinary_muscle_development	ENSGALG00010021530	MLF2	-0.507981209	0.061191173	-8.301543858	1.02767E-16	5.89477E-16	negative
PRJNA729271	stage_E18_vs_E10	ordinary_muscle_development	ENSGALG00010021531	RAB15	0.247457292	0.244833518	1.010716566	0.312152106	0.369690724	positive
PRJNA729271	stage_E18_vs_E10	ordinary_muscle_development	ENSGALG00010021532	DPP10	-0.26656026	0.181885934	-1.465535313	0.142774925	0.183631104	negative
PRJNA729271	stage_E18_vs_E10	ordinary_muscle_development	ENSGALG00010021533	SSX2IP	-2.853953812	0.313655992	-9.098993433	9.11729E-20	6.32321E-19	negative
PRJNA729271	stage_E18_vs_E10	ordinary_muscle_development	ENSGALG00010021534	KIFC3	1.549643277	0.169264989	9.155131778	5.42915E-20	3.80908E-19	positive
PRJNA729271	stage_E18_vs_E10	ordinary_muscle_development	ENSGALG00010021535	SUSD6	-0.177163838	0.086817195	-2.040653781	0.041285252	0.058767528	negative
PRJNA729271	stage_E18_vs_E10	ordinary_muscle_development	ENSGALG00010021538	TNFRSF18	0.723690028	0.4459698	1.622733261	0.104646438	0.138259969	positive
PRJNA729271	stage_E18_vs_E10	ordinary_muscle_development	ENSGALG00010021539	HEXA	-0.144180449	0.11979492	-1.20356063	0.228759444	0.28031686	negative
PRJNA729271	stage_E18_vs_E10	ordinary_muscle_development	ENSGALG00010021540	STK4	-0.649186956	0.078033225	-8.319366	8.84351E-17	5.09283E-16	negative
PRJNA729271	stage_E18_vs_E10	ordinary_muscle_development	ENSGALG00010021541	CYP27A1	2.110375074	0.277589335	7.602507755	2.90447E-14	1.38945E-13	positive
PRJNA729271	stage_E18_vs_E10	ordinary_muscle_development	ENSGALG00010021543	PLEKHD1	0.917490535	0.261424506	3.509581217	0.000448813	0.000859271	positive
PRJNA729271	stage_E18_vs_E10	ordinary_muscle_development	ENSGALG00010021545	CYB5R2	-1.376364232	0.143902348	-9.564571025	1.12668E-21	8.84201E-21	negative
PRJNA729271	stage_E18_vs_E10	ordinary_muscle_development	ENSGALG00010021546	OGG1	-0.671132218	0.177989042	-3.77063784	0.000162831	0.000330195	negative
PRJNA729271	stage_E18_vs_E10	ordinary_muscle_development	ENSGALG00010021549	ANAPC16	-0.187580191	0.092328272	-2.031665787	0.042187502	0.059928248	negative
PRJNA729271	stage_E18_vs_E10	ordinary_muscle_development	ENSGALG00010021550	MAP2K3	0.238189304	0.174312942	1.366446472	0.171798871	0.216918522	positive
PRJNA729271	stage_E18_vs_E10	ordinary_muscle_development	ENSGALG00010021552	ENSGALG00010021552	1.800070372	0.171436648	10.49991584	8.64572E-26	8.53676E-25	positive
PRJNA729271	stage_E18_vs_E10	ordinary_muscle_development	ENSGALG00010021553	SLC39A9	-0.26426285	0.086808659	-3.044199199	0.002333006	0.004052843	negative
PRJNA729271	stage_E18_vs_E10	ordinary_muscle_development	ENSGALG00010021554	SNX11	0.098669017	0.149155098	0.661519571	0.508279168	0.565252918	positive
PRJNA729271	stage_E18_vs_E10	ordinary_muscle_development	ENSGALG00010021556	CAMK1	-0.162784166	0.143470895	-1.134614559	0.256536851	0.310444285	negative
PRJNA729271	stage_E18_vs_E10	ordinary_muscle_development	ENSGALG00010021558	CHURC1	1.040920752	0.179801877	5.789265227	7.0695E-09	2.21227E-08	positive
PRJNA729271	stage_E18_vs_E10	ordinary_muscle_development	ENSGALG00010021559	MYO1H	0.451086749	0.571530145	0.789261517	0.429959174	0.490034457	positive
PRJNA729271	stage_E18_vs_E10	ordinary_muscle_development	ENSGALG00010021561	TGIF2	-0.812254458	0.097728427	-8.311342779	9.46252E-17	5.44066E-16	negative
PRJNA729271	stage_E18_vs_E10	ordinary_muscle_development	ENSGALG00010021562	LRP6	0.326574768	0.082155068	3.975101927	7.03491E-05	0.000149233	positive
PRJNA729271	stage_E18_vs_E10	ordinary_muscle_development	ENSGALG00010021564	PRKAG3	6.475910626	0.636741286	10.17039537	2.68815E-24	2.4353E-23	positive
PRJNA729271	stage_E18_vs_E10	ordinary_muscle_development	ENSGALG00010021565	CBX1	-0.899827152	0.104748126	-8.590389052	8.66753E-18	5.33486E-17	negative
PRJNA729271	stage_E18_vs_E10	ordinary_muscle_development	ENSGALG00010021566	ENSGALG00010021566	0.201250952	0.09647688	2.086001855	0.036978455	0.053118353	positive
PRJNA729271	stage_E18_vs_E10	ordinary_muscle_development	ENSGALG00010021568	TADA3	0.265941503	0.143149668	1.857786379	0.063199356	0.087029704	positive
PRJNA729271	stage_E18_vs_E10	ordinary_muscle_development	ENSGALG00010021570	KATNB1	-0.147864051	0.100471757	-1.471697671	0.141102537	0.181689728	negative
PRJNA729271	stage_E18_vs_E10	ordinary_muscle_development	ENSGALG00010021572	MYL9	0.24149172	0.112747172	2.141887162	0.032202563	0.046803986	positive
PRJNA729271	stage_E18_vs_E10	ordinary_muscle_development	ENSGALG00010021573	NATD1	0.363828788	0.199013109	1.828164936	0.067524802	0.092581536	positive
PRJNA729271	stage_E18_vs_E10	ordinary_muscle_development	ENSGALG00010021574	TTLL4	-0.145402416	0.232231873	-0.626108785	0.53124359	0.587236659	negative
PRJNA729271	stage_E18_vs_E10	ordinary_muscle_development	ENSGALG00010021575	ADGRL2	-0.094851134	0.08293476	-1.143683709	0.252754832	0.306379101	negative
PRJNA729271	stage_E18_vs_E10	ordinary_muscle_development	ENSGALG00010021576	ERH	-0.92097113	0.122229827	-7.534749494	4.89273E-14	2.30036E-13	negative
PRJNA729271	stage_E18_vs_E10	ordinary_muscle_development	ENSGALG00010021577	ARIH1	-0.220513179	0.077820732	-2.833604532	0.004602625	0.007665753	negative
PRJNA729271	stage_E18_vs_E10	ordinary_muscle_development	ENSGALG00010021578	ENSGALG00010021578	2.246854224	1.080820576	2.07884109	0.037631959	0.053991413	positive
PRJNA729271	stage_E18_vs_E10	ordinary_muscle_development	ENSGALG00010021579	C20H20ORF24	-0.662576808	0.112960889	-5.865541732	4.47669E-09	1.42897E-08	negative
PRJNA729271	stage_E18_vs_E10	ordinary_muscle_development	ENSGALG00010021580	KCTD10	-1.148106144	0.110673241	-10.37383686	3.2616E-25	3.12885E-24	negative
PRJNA729271	stage_E18_vs_E10	ordinary_muscle_development	ENSGALG00010021582	STK36	0.423833274	0.141796908	2.989016337	0.002798772	0.004817503	positive
PRJNA729271	stage_E18_vs_E10	ordinary_muscle_development	ENSGALG00010021583	NFE2L1	0.525975886	0.221362547	2.376083452	0.017497505	0.02665262	positive
PRJNA729271	stage_E18_vs_E10	ordinary_muscle_development	ENSGALG00010021584	OVCH2	-0.076864407	0.392459821	-0.195852934	0.844725277	0.871528253	negative
PRJNA729271	stage_E18_vs_E10	ordinary_muscle_development	ENSGALG00010021586	BBS4	-0.036187628	0.123121954	-0.293916944	0.76882137	0.805847549	negative
PRJNA729271	stage_E18_vs_E10	ordinary_muscle_development	ENSGALG00010021588	RNF25	0.102452022	0.139692629	0.733410364	0.46330818	0.522468174	positive
PRJNA729271	stage_E18_vs_E10	ordinary_muscle_development	ENSGALG00010021593	DCAF7	-0.647597088	0.056419284	-11.47829335	1.69595E-30	2.11921E-29	negative
PRJNA729271	stage_E18_vs_E10	ordinary_muscle_development	ENSGALG00010021594	ADPGK	-0.611956053	0.090208914	-6.783764765	1.17084E-11	4.57695E-11	negative
PRJNA729271	stage_E18_vs_E10	ordinary_muscle_development	ENSGALG00010021595	UBE3B	-0.375980563	0.086065799	-4.36852461	1.25089E-05	2.88398E-05	negative
PRJNA729271	stage_E18_vs_E10	ordinary_muscle_development	ENSGALG00010021597	GGT7	0.500742944	0.185031114	2.706263462	0.006804504	0.011025183	positive
PRJNA729271	stage_E18_vs_E10	ordinary_muscle_development	ENSGALG00010021599	ARPC4	-0.25883638	0.103717072	-2.495600526	0.012574412	0.019611158	negative
PRJNA729271	stage_E18_vs_E10	ordinary_muscle_development	ENSGALG00010021600	FMN1	-0.229386324	0.13251846	-1.730976379	0.083455977	0.112372912	negative
PRJNA729271	stage_E18_vs_E10	ordinary_muscle_development	ENSGALG00010021601	LPAR5	-1.8525425	0.450398598	-4.113117819	3.90351E-05	8.50336E-05	negative
PRJNA729271	stage_E18_vs_E10	ordinary_muscle_development	ENSGALG00010021602	PLEKHG4	0.348469636	0.0979552	3.557438883	0.000374488	0.000723637	positive
PRJNA729271	stage_E18_vs_E10	ordinary_muscle_development	ENSGALG00010021604	SPTB	2.37729022	0.189313758	12.55740865	3.61995E-36	5.90737E-35	positive
PRJNA729271	stage_E18_vs_E10	ordinary_muscle_development	ENSGALG00010021605	MAP3K3	-0.06419576	0.112991021	-0.568149215	0.569933652	0.623485909	negative
PRJNA729271	stage_E18_vs_E10	ordinary_muscle_development	ENSGALG00010021606	RNF8	-0.642900804	0.09631266	-6.67514324	2.46991E-11	9.43133E-11	negative
PRJNA729271	stage_E18_vs_E10	ordinary_muscle_development	ENSGALG00010021607	SLC9A5	-0.192818268	0.203401754	-0.947967576	0.343145961	0.401574415	negative
PRJNA729271	stage_E18_vs_E10	ordinary_muscle_development	ENSGALG00010021609	ENSGALG00010021609	1.898507181	0.203607815	9.324333568	1.11683E-20	8.22586E-20	positive
PRJNA729271	stage_E18_vs_E10	ordinary_muscle_development	ENSGALG00010021610	TMEM11	0.109149185	0.091013845	1.199259124	0.230427204	0.282074345	positive
PRJNA729271	stage_E18_vs_E10	ordinary_muscle_development	ENSGALG00010021611	GREM1	-1.996161121	0.604512731	-3.302099391	0.000959641	0.001752329	negative
PRJNA729271	stage_E18_vs_E10	ordinary_muscle_development	ENSGALG00010021612	BCS1L	-0.096658278	0.134265538	-0.719903853	0.471584196	0.530385856	negative
PRJNA729271	stage_E18_vs_E10	ordinary_muscle_development	ENSGALG00010021613	ADGRL4	1.926574455	0.149918176	12.85083977	8.50816E-38	1.49124E-36	positive
PRJNA729271	stage_E18_vs_E10	ordinary_muscle_development	ENSGALG00010021615	FLVCR1	-2.387063152	0.186663559	-12.78805124	1.91209E-37	3.28385E-36	negative
PRJNA729271	stage_E18_vs_E10	ordinary_muscle_development	ENSGALG00010021617	SORBS1	0.721456591	0.111753026	6.45581259	1.0764E-10	3.91037E-10	positive
PRJNA729271	stage_E18_vs_E10	ordinary_muscle_development	ENSGALG00010021618	NEO1	-0.653230127	0.10640247	-6.139238394	8.29181E-10	2.82334E-09	negative
PRJNA729271	stage_E18_vs_E10	ordinary_muscle_development	ENSGALG00010021619	FHOD1	2.489172585	0.179836667	13.84129627	1.43594E-43	3.18255E-42	positive
PRJNA729271	stage_E18_vs_E10	ordinary_muscle_development	ENSGALG00010021621	ZNF518A	0.278981972	0.137242287	2.032769763	0.042075789	0.059787135	positive
PRJNA729271	stage_E18_vs_E10	ordinary_muscle_development	ENSGALG00010021622	PTHLH	-1.8192793	0.26222989	-6.937726666	3.9846E-12	1.62924E-11	negative
PRJNA729271	stage_E18_vs_E10	ordinary_muscle_development	ENSGALG00010021623	FAR1	-0.27953915	0.130487594	-2.14226611	0.032172074	0.046764362	negative
PRJNA729271	stage_E18_vs_E10	ordinary_muscle_development	ENSGALG00010021624	HSD11B2	-0.773530777	0.445463246	-1.736463748	0.082481869	0.11121625	negative
PRJNA729271	stage_E18_vs_E10	ordinary_muscle_development	ENSGALG00010021625	HCN4	-2.407920137	0.960890692	-2.505925134	0.012213148	0.019076463	negative
PRJNA729271	stage_E18_vs_E10	ordinary_muscle_development	ENSGALG00010021626	ZNF142	-0.308646746	0.216299742	-1.426939964	0.1535972	0.196106	negative
PRJNA729271	stage_E18_vs_E10	ordinary_muscle_development	ENSGALG00010021627	NCOA6	0.176871089	0.104441311	1.693497397	0.09036083	0.120828434	positive
PRJNA729271	stage_E18_vs_E10	ordinary_muscle_development	ENSGALG00010021628	REC114	-1.37873675	0.651608567	-2.115897213	0.034353551	0.049641583	negative
PRJNA729271	stage_E18_vs_E10	ordinary_muscle_development	ENSGALG00010021629	DHRS7B	1.187866081	0.101621635	11.68910618	1.44904E-31	1.91991E-30	positive
PRJNA729271	stage_E18_vs_E10	ordinary_muscle_development	ENSGALG00010021630	VTG1	-1.585982209	0.307282257	-5.161320484	2.45214E-07	6.70764E-07	negative
PRJNA729271	stage_E18_vs_E10	ordinary_muscle_development	ENSGALG00010021632	LIMD2	-3.028986665	0.165077263	-18.34890292	3.36845E-75	2.0344E-73	negative
PRJNA729271	stage_E18_vs_E10	ordinary_muscle_development	ENSGALG00010021633	CCNJ	-0.544776213	0.105255201	-5.175765276	2.26979E-07	6.22527E-07	negative
PRJNA729271	stage_E18_vs_E10	ordinary_muscle_development	ENSGALG00010021634	PLCD4	3.584375931	0.244972371	14.6317559	1.76181E-48	4.82749E-47	positive
PRJNA729271	stage_E18_vs_E10	ordinary_muscle_development	ENSGALG00010021635	FOXJ2	0.385404592	0.114108947	3.377514213	0.000731442	0.001359263	positive
PRJNA729271	stage_E18_vs_E10	ordinary_muscle_development	ENSGALG00010021636	C1orf159	0.416973577	0.194421428	2.144689408	0.031977688	0.046505126	positive
PRJNA729271	stage_E18_vs_E10	ordinary_muscle_development	ENSGALG00010021637	ATP6V0D1	-0.546661858	0.105756594	-5.169056939	2.35278E-07	6.44558E-07	negative
PRJNA729271	stage_E18_vs_E10	ordinary_muscle_development	ENSGALG00010021638	SRC	-1.079879713	0.177634997	-6.079205851	1.20779E-09	4.04181E-09	negative
PRJNA729271	stage_E18_vs_E10	ordinary_muscle_development	ENSGALG00010021639	SCG5	-0.759493797	0.351920856	-2.158138073	0.030917098	0.045075774	negative
PRJNA729271	stage_E18_vs_E10	ordinary_muscle_development	ENSGALG00010021640	PLEKHG3	0.331143653	0.106676568	3.104183607	0.001908049	0.003360122	positive
PRJNA729271	stage_E18_vs_E10	ordinary_muscle_development	ENSGALG00010021642	NPTN	-0.031530316	0.090494905	-0.348420894	0.727524113	0.769741753	negative
PRJNA729271	stage_E18_vs_E10	ordinary_muscle_development	ENSGALG00010021643	ENSGALG00010021643	-1.326690948	0.138039838	-9.610928056	7.1898E-22	5.71047E-21	negative
PRJNA729271	stage_E18_vs_E10	ordinary_muscle_development	ENSGALG00010021644	TARDBP	-0.584638014	0.154334846	-3.788114147	0.000151795	0.000309244	negative
PRJNA729271	stage_E18_vs_E10	ordinary_muscle_development	ENSGALG00010021645	ASCC1	-0.193625386	0.139207616	-1.390910867	0.164252457	0.208260966	negative
PRJNA729271	stage_E18_vs_E10	ordinary_muscle_development	ENSGALG00010021646	RPUSD3	0.015230197	0.168087332	0.090608831	0.927803414	0.940981702	positive
PRJNA729271	stage_E18_vs_E10	ordinary_muscle_development	ENSGALG00010021647	ARHGAP11B	-1.842569471	0.138966066	-13.25913241	3.99491E-40	7.69006E-39	negative
PRJNA729271	stage_E18_vs_E10	ordinary_muscle_development	ENSGALG00010021648	BRE	-0.500164995	0.068200945	-7.333695963	2.23891E-13	1.00256E-12	negative
PRJNA729271	stage_E18_vs_E10	ordinary_muscle_development	ENSGALG00010021649	PXN	0.539985696	0.096466708	5.597637849	2.17292E-08	6.52371E-08	positive
PRJNA729271	stage_E18_vs_E10	ordinary_muscle_development	ENSGALG00010021650	CD276	-0.88442308	0.109930916	-8.045262531	8.60607E-16	4.64737E-15	negative
PRJNA729271	stage_E18_vs_E10	ordinary_muscle_development	ENSGALG00010021651	RNF113B	-0.121553865	0.157461519	-0.771959183	0.440138603	0.499710899	negative
PRJNA729271	stage_E18_vs_E10	ordinary_muscle_development	ENSGALG00010021652	BORCS5	-0.476102967	0.097760137	-4.870113549	1.11534E-06	2.87207E-06	negative
PRJNA729271	stage_E18_vs_E10	ordinary_muscle_development	ENSGALG00010021655	USP22	-0.711839391	0.0943604	-7.543836076	4.56345E-14	2.15043E-13	negative
PRJNA729271	stage_E18_vs_E10	ordinary_muscle_development	ENSGALG00010021656	SETD6	-1.006619282	0.084815895	-11.86828578	1.72976E-32	2.35426E-31	negative
PRJNA729271	stage_E18_vs_E10	ordinary_muscle_development	ENSGALG00010021657	JAGN1	-0.13021378	0.144657967	-0.90014938	0.368040761	0.42760106	negative
PRJNA729271	stage_E18_vs_E10	ordinary_muscle_development	ENSGALG00010021658	STRADA	-0.947357405	0.092436309	-10.24875851	1.19873E-24	1.11311E-23	negative
PRJNA729271	stage_E18_vs_E10	ordinary_muscle_development	ENSGALG00010021659	SPOCK2	-0.549521326	0.147658532	-3.721568392	0.000197989	0.000397322	negative
PRJNA729271	stage_E18_vs_E10	ordinary_muscle_development	ENSGALG00010021660	RNF223	1.980802512	0.456818448	4.336082571	1.45044E-05	3.31926E-05	positive
PRJNA729271	stage_E18_vs_E10	ordinary_muscle_development	ENSGALG00010021661	DUSP16	0.134715116	0.121641471	1.107476873	0.268087806	0.32307389	positive
PRJNA729271	stage_E18_vs_E10	ordinary_muscle_development	ENSGALG00010021662	RASGRP1	0.121152261	0.168121907	0.720621499	0.471142423	0.530012372	positive
PRJNA729271	stage_E18_vs_E10	ordinary_muscle_development	ENSGALG00010021663	CNOT9	-0.370979081	0.140495769	-2.640500011	0.008278379	0.01325327	negative
PRJNA729271	stage_E18_vs_E10	ordinary_muscle_development	ENSGALG00010021664	TYSND1	-0.417040156	0.167794531	-2.485421621	0.012939808	0.02014635	negative
PRJNA729271	stage_E18_vs_E10	ordinary_muscle_development	ENSGALG00010021665	CNOT1	-0.253491707	0.091639162	-2.766194092	0.005671477	0.009321698	negative
PRJNA729271	stage_E18_vs_E10	ordinary_muscle_development	ENSGALG00010021666	CCDC47	0.522668897	0.092548683	5.647502249	1.62796E-08	4.93873E-08	positive
PRJNA729271	stage_E18_vs_E10	ordinary_muscle_development	ENSGALG00010021667	TBX15	0.271320722	0.191689811	1.415415457	0.15694672	0.199907092	positive
PRJNA729271	stage_E18_vs_E10	ordinary_muscle_development	ENSGALG00010021668	PYGO1	-0.641657863	0.131091287	-4.894740741	9.84354E-07	2.54517E-06	negative
PRJNA729271	stage_E18_vs_E10	ordinary_muscle_development	ENSGALG00010021669	ENSGALG00010021669	-0.355475692	0.074029636	-4.801802521	1.57244E-06	3.97566E-06	negative
PRJNA729271	stage_E18_vs_E10	ordinary_muscle_development	ENSGALG00010021670	BLCAP	0.510016228	0.109126231	4.673635511	2.95914E-06	7.26012E-06	positive
PRJNA729271	stage_E18_vs_E10	ordinary_muscle_development	ENSGALG00010021671	TNFRSF13B	2.346912425	0.753602647	3.114257142	0.001844087	0.003258172	positive
PRJNA729271	stage_E18_vs_E10	ordinary_muscle_development	ENSGALG00010021674	ING4	-0.436150085	0.121835293	-3.579833667	0.000343813	0.000667234	negative
PRJNA729271	stage_E18_vs_E10	ordinary_muscle_development	ENSGALG00010021675	DDX42	-0.04072274	0.058968436	-0.690585386	0.489826134	0.547672772	negative
PRJNA729271	stage_E18_vs_E10	ordinary_muscle_development	ENSGALG00010021676	TP53INP2	0.429278825	0.287427598	1.493519856	0.135301161	0.174887669	positive
PRJNA729271	stage_E18_vs_E10	ordinary_muscle_development	ENSGALG00010021677	ENSGALG00010021677	-0.641096028	0.154964354	-4.137054834	3.51792E-05	7.69462E-05	negative
PRJNA729271	stage_E18_vs_E10	ordinary_muscle_development	ENSGALG00010021678	DNAAF4	1.537775503	0.151667151	10.13914679	3.7032E-24	3.32989E-23	positive
PRJNA729271	stage_E18_vs_E10	ordinary_muscle_development	ENSGALG00010021679	MASP2	0.774713776	1.006306336	0.769858788	0.441383663	0.500849866	positive
PRJNA729271	stage_E18_vs_E10	ordinary_muscle_development	ENSGALG00010021680	PIGT	0.003276382	0.120641858	0.027157919	0.978333779	0.982196158	positive
PRJNA729271	stage_E18_vs_E10	ordinary_muscle_development	ENSGALG00010021681	BTBD10	-0.731685444	0.083108489	-8.803979585	1.32048E-18	8.59085E-18	negative
PRJNA729271	stage_E18_vs_E10	ordinary_muscle_development	ENSGALG00010021682	MYL4	-3.62696047	0.20472511	-17.71624627	3.14191E-70	1.68052E-68	negative
PRJNA729271	stage_E18_vs_E10	ordinary_muscle_development	ENSGALG00010021683	HFM1	-0.388202304	0.428463027	-0.906034544	0.364917569	0.424448737	negative
PRJNA729271	stage_E18_vs_E10	ordinary_muscle_development	ENSGALG00010021684	BIN1	3.137899748	0.121174759	25.89565503	7.4541E-148	2.2989E-145	positive
PRJNA729271	stage_E18_vs_E10	ordinary_muscle_development	ENSGALG00010021685	DBNDD2	1.38022544	0.139895153	9.866141963	5.83665E-23	4.88465E-22	positive
PRJNA729271	stage_E18_vs_E10	ordinary_muscle_development	ENSGALG00010021686	CCPG1	0.970461804	0.165575793	5.861133347	4.59719E-09	1.4655E-08	positive
PRJNA729271	stage_E18_vs_E10	ordinary_muscle_development	ENSGALG00010021687	ZNF384	-0.067616168	0.106919935	-0.632400014	0.527125525	0.583258357	negative
PRJNA729271	stage_E18_vs_E10	ordinary_muscle_development	ENSGALG00010021688	ITPR1	-0.10387765	0.117351678	-0.88518249	0.376058215	0.436077106	negative
PRJNA729271	stage_E18_vs_E10	ordinary_muscle_development	ENSGALG00010021689	MPRIP	0.309144399	0.109463477	2.824178517	0.004740198	0.007877684	positive
PRJNA729271	stage_E18_vs_E10	ordinary_muscle_development	ENSGALG00010021691	PIGB	-0.579823044	0.149120828	-3.888276712	0.000100958	0.000210469	negative
PRJNA729271	stage_E18_vs_E10	ordinary_muscle_development	ENSGALG00010021693	PPA1	-0.744139628	0.0931167	-7.991473342	1.33336E-15	7.09768E-15	negative
PRJNA729271	stage_E18_vs_E10	ordinary_muscle_development	ENSGALG00010021694	ENSGALG00010021694	-0.080630381	1.356017962	-0.059461145	0.952584812	0.96087104	negative
PRJNA729271	stage_E18_vs_E10	ordinary_muscle_development	ENSGALG00010021695	FNBP4	0.085974572	0.071616856	1.200479559	0.229953147	0.281636744	positive

PRJNA729271	stage_E18_vs_E10	ordinary_muscle_development	ENSGALG00010021697	PIANP	-1.02888751	0.227731894	-4.51797722	6.24332E-06	1.48331E-05	negative
PRJNA729271	stage_E18_vs_E10	ordinary_muscle_development	ENSGALG00010021698	GYPC	0.696220538	0.134654483	5.170422267	2.33566E-07	6.40108E-07	positive
PRJNA729271	stage_E18_vs_E10	ordinary_muscle_development	ENSGALG00010021699	SYS1	-0.415403662	0.086921369	-4.77907406	1.76104E-06	4.43319E-06	negative
PRJNA729271	stage_E18_vs_E10	ordinary_muscle_development	ENSGALG00010021700	FLCN	-0.26623695	0.080712596	-3.298579943	0.000971752	0.001772436	negative
PRJNA729271	stage_E18_vs_E10	ordinary_muscle_development	ENSGALG00010021701	DR1	-0.653563047	0.077756253	-8.405279621	4.26843E-17	2.5148E-16	negative
PRJNA729271	stage_E18_vs_E10	ordinary_muscle_development	ENSGALG00010021702	PLD6	0.265964674	0.303491496	0.876349676	0.380839967	0.441163216	positive
PRJNA729271	stage_E18_vs_E10	ordinary_muscle_development	ENSGALG00010021703	CD82	0.926394144	0.173162826	5.349844235	8.803E-08	2.50539E-07	positive
PRJNA729271	stage_E18_vs_E10	ordinary_muscle_development	ENSGALG00010021704	COPS7A	0.782835357	0.102728132	7.620457424	2.52778E-14	1.21567E-13	positive
PRJNA729271	stage_E18_vs_E10	ordinary_muscle_development	ENSGALG00010021705	CCDC18	-0.347011071	0.191611682	-1.811012081	0.070138982	0.095920798	negative
PRJNA729271	stage_E18_vs_E10	ordinary_muscle_development	ENSGALG00010021706	ENSGALG00010021706	2.211279308	0.159032881	13.90454163	5.94465E-44	1.34009E-42	positive
PRJNA729271	stage_E18_vs_E10	ordinary_muscle_development	ENSGALG00010021707	DLX2	-4.534799283	1.134199094	-3.998239203	6.38154E-05	0.00013607	negative
PRJNA729271	stage_E18_vs_E10	ordinary_muscle_development	ENSGALG00010021708	CNTNAP5	-5.347833666	0.394082203	-13.57035061	6.00342E-42	1.24314E-40	negative
PRJNA729271	stage_E18_vs_E10	ordinary_muscle_development	ENSGALG00010021710	ENSGALG00010021710	0.311310874	0.317729402	0.97979876	0.327185464	0.385221485	positive
PRJNA729271	stage_E18_vs_E10	ordinary_muscle_development	ENSGALG00010021711	ENSGALG00010021711	0.948238362	0.220860794	4.293375692	1.75977E-05	3.99184E-05	positive
PRJNA729271	stage_E18_vs_E10	ordinary_muscle_development	ENSGALG00010021713	RAB27A	0.036005269	0.081310442	0.442812365	0.657901459	0.705868368	positive
PRJNA729271	stage_E18_vs_E10	ordinary_muscle_development	ENSGALG00010021714	TSPAN18	1.262066459	0.114856412	10.98821074	4.35473E-28	4.85553E-27	positive
PRJNA729271	stage_E18_vs_E10	ordinary_muscle_development	ENSGALG00010021715	MMAB	0.269895902	0.129661046	2.081549626	0.037383627	0.053661682	positive
PRJNA729271	stage_E18_vs_E10	ordinary_muscle_development	ENSGALG00010021716	TFCP2L1	2.316010409	0.384080601	6.030011415	1.63948E-09	5.43059E-09	positive
PRJNA729271	stage_E18_vs_E10	ordinary_muscle_development	ENSGALG00010021717	SDC4	1.281072166	0.197565835	6.484279865	8.91566E-11	3.2651E-10	positive
PRJNA729271	stage_E18_vs_E10	ordinary_muscle_development	ENSGALG00010021718	SST7	-3.376274461	0.479490037	-7.041386055	1.90337E-12	7.96919E-12	negative
PRJNA729271	stage_E18_vs_E10	ordinary_muscle_development	ENSGALG00010021719	BHLHE40	3.227923013	0.214360261	15.05840217	3.04047E-51	9.27823E-50	positive
PRJNA729271	stage_E18_vs_E10	ordinary_muscle_development	ENSGALG00010021720	CACNB1	0.667277625	0.262015223	2.546713189	0.010874277	0.01711987	positive
PRJNA729271	stage_E18_vs_E10	ordinary_muscle_development	ENSGALG00010021721	TP53I11	0.312414436	0.142739306	2.188706423	0.028618185	0.041960408	positive
PRJNA729271	stage_E18_vs_E10	ordinary_muscle_development	ENSGALG00010021722	AARS1	-0.468245773	0.159343209	-2.938598868	0.003296995	0.005601259	negative
PRJNA729271	stage_E18_vs_E10	ordinary_muscle_development	ENSGALG00010021723	COPS3	-0.749606279	0.108609214	-6.901866344	5.13237E-12	2.0763E-11	negative
PRJNA729271	stage_E18_vs_E10	ordinary_muscle_development	ENSGALG00010021725	SH2D6	0.771683905	0.216130596	3.570451931	0.000356366	0.000690763	positive
PRJNA729271	stage_E18_vs_E10	ordinary_muscle_development	ENSGALG00010021726	ARNTL	-0.017259048	0.147304016	-0.117166178	0.906728369	0.923095077	negative
PRJNA729271	stage_E18_vs_E10	ordinary_muscle_development	ENSGALG00010021727	MVK	-0.094034067	0.102201787	-0.920082415	0.357529693	0.416959764	negative
PRJNA729271	stage_E18_vs_E10	ordinary_muscle_development	ENSGALG00010021728	TMED5	-0.746750368	0.065482686	-11.40378348	4.00335E-30	4.91767E-29	negative
PRJNA729271	stage_E18_vs_E10	ordinary_muscle_development	ENSGALG00010021729	RBPJL	-3.438333555	0.66235684	-5.191059178	2.09101E-07	5.74909E-07	negative
PRJNA729271	stage_E18_vs_E10	ordinary_muscle_development	ENSGALG00010021730	ARL8B	-0.355372188	0.072680348	-4.889522396	1.01081E-06	2.60985E-06	negative
PRJNA729271	stage_E18_vs_E10	ordinary_muscle_development	ENSGALG00010021731	SRM	-0.883964629	0.1213858	-7.28227378	3.2824E-13	1.45723E-12	negative
PRJNA729271	stage_E18_vs_E10	ordinary_muscle_development	ENSGALG00010021732	CASR	1.86695799	0.252435639	7.395778163	1.40583E-13	6.38791E-13	positive
PRJNA729271	stage_E18_vs_E10	ordinary_muscle_development	ENSGALG00010021733	ENSGALG00010021733	0.658169702	0.656186647	1.00302209	0.315850203	0.37330795	positive
PRJNA729271	stage_E18_vs_E10	ordinary_muscle_development	ENSGALG00010021734	PARD3B	0.679278909	0.063466231	10.70299749	9.85381E-27	1.03127E-25	positive
PRJNA729271	stage_E18_vs_E10	ordinary_muscle_development	ENSGALG00010021735	PIGBOS1	1.181675497	0.144928485	8.153507564	3.53518E-16	1.95433E-15	positive
PRJNA729271	stage_E18_vs_E10	ordinary_muscle_development	ENSGALG00010021736	EDEM1	0.545455853	0.082835518	6.584806432	4.55478E-11	1.70731E-10	positive
PRJNA729271	stage_E18_vs_E10	ordinary_muscle_development	ENSGALG00010021738	PRDM11	0.577480166	0.071716513	8.052262221	8.12775E-16	4.39596E-15	positive
PRJNA729271	stage_E18_vs_E10	ordinary_muscle_development	ENSGALG00010021739	EXOSC10	0.237453866	0.110609885	2.146768953	0.03181168	0.04627763	positive
PRJNA729271	stage_E18_vs_E10	ordinary_muscle_development	ENSGALG00010021740	PDLIM1	0.241283498	0.160034053	1.507700972	0.131631077	0.17053919	positive
PRJNA729271	stage_E18_vs_E10	ordinary_muscle_development	ENSGALG00010021741	RPL19	0.818442618	0.146011074	5.605346208	2.07839E-08	6.25156E-08	positive
PRJNA729271	stage_E18_vs_E10	ordinary_muscle_development	ENSGALG00010021742	MATN4	-3.943074173	0.481661854	-8.186394951	2.69167E-16	1.49773E-15	negative
PRJNA729271	stage_E18_vs_E10	ordinary_muscle_development	ENSGALG00010021743	SYT13	-2.971802245	0.36275171	-8.192386589	2.56097E-16	1.42719E-15	negative
PRJNA729271	stage_E18_vs_E10	ordinary_muscle_development	ENSGALG00010021744	LITAF	2.326662062	0.349974735	6.648085788	2.96929E-11	1.12493E-10	positive
PRJNA729271	stage_E18_vs_E10	ordinary_muscle_development	ENSGALG00010021745	RSL24D1	-0.35769796	0.126596809	-2.825489528	0.004720843	0.007846419	negative
PRJNA729271	stage_E18_vs_E10	ordinary_muscle_development	ENSGALG00010021746	MTOR	0.150429119	0.111050716	1.354598363	0.175545555	0.221167564	positive
PRJNA729271	stage_E18_vs_E10	ordinary_muscle_development	ENSGALG00010021747	AKTIP	0.445864313	0.117831196	3.783924203	0.000154375	0.000314279	positive
PRJNA729271	stage_E18_vs_E10	ordinary_muscle_development	ENSGALG00010021748	CDCA7	-1.19376598	0.17714849	-6.738787205	1.59714E-11	6.18668E-11	negative
PRJNA729271	stage_E18_vs_E10	ordinary_muscle_development	ENSGALG00010021749	CLASP1	0.391727612	0.09098415	4.305448927	1.66647E-05	3.79089E-05	positive
PRJNA729271	stage_E18_vs_E10	ordinary_muscle_development	ENSGALG00010021750	HELLS	-2.107401213	0.149549411	-14.09167175	4.27325E-45	1.00717E-43	negative
PRJNA729271	stage_E18_vs_E10	ordinary_muscle_development	ENSGALG00010021751	MTCH2	-0.322184818	0.092918331	-3.4673978	0.000525524	0.000995747	negative
PRJNA729271	stage_E18_vs_E10	ordinary_muscle_development	ENSGALG00010021752	CSTB	-0.13563157	0.110440539	-1.228095873	0.21941098	0.270232149	negative
PRJNA729271	stage_E18_vs_E10	ordinary_muscle_development	ENSGALG00010021753	MTF2	-0.962458047	0.097494177	-9.871954148	5.50809E-23	4.61235E-22	negative
PRJNA729271	stage_E18_vs_E10	ordinary_muscle_development	ENSGALG00010021754	SLC17A9	-2.397365769	0.545417137	-4.395472028	1.10532E-05	2.56019E-05	negative
PRJNA729271	stage_E18_vs_E10	ordinary_muscle_development	ENSGALG00010021755	ENSGALG00010021755	2.204191656	0.153753813	14.33585036	1.30637E-46	3.35146E-45	positive
PRJNA729271	stage_E18_vs_E10	ordinary_muscle_development	ENSGALG00010021756	USP42	-1.699466702	0.131127281	-12.96043579	2.05079E-38	3.68812E-37	negative
PRJNA729271	stage_E18_vs_E10	ordinary_muscle_development	ENSGALG00010021757	ZP4	0.432412584	0.351625254	1.229754062	0.218789215	0.269580897	positive
PRJNA729271	stage_E18_vs_E10	ordinary_muscle_development	ENSGALG00010021758	RASSF10	-4.645488823	0.459688385	-10.1057346	5.21036E-24	4.63054E-23	negative
PRJNA729271	stage_E18_vs_E10	ordinary_muscle_development	ENSGALG00010021759	NIFK	-0.725320998	0.094616506	-7.665903407	1.77577E-14	8.67242E-14	negative
PRJNA729271	stage_E18_vs_E10	ordinary_muscle_development	ENSGALG00010021760	CSTA	-0.040170547	0.403470114	-0.099562632	0.920691561	0.934684422	negative
PRJNA729271	stage_E18_vs_E10	ordinary_muscle_development	ENSGALG00010021761	MAFB	-0.124773541	0.170120645	-0.733441498	0.463289197	0.522468174	negative
PRJNA729271	stage_E18_vs_E10	ordinary_muscle_development	ENSGALG00010021762	LAMB2	3.040465023	0.19873938	15.29875469	7.79336E-53	2.4937E-51	positive
PRJNA729271	stage_E18_vs_E10	ordinary_muscle_development	ENSGALG00010021764	FAM222A	-1.510988825	0.831571222	-1.817028758	0.06921271	0.094752383	negative
PRJNA729271	stage_E18_vs_E10	ordinary_muscle_development	ENSGALG00010021765	EIF2AK1	-0.134516702	0.10596121	-1.269490049	0.204266338	0.253583467	negative
PRJNA729271	stage_E18_vs_E10	ordinary_muscle_development	ENSGALG00010021766	AGRN	0.869841793	0.148335222	5.86402733	4.51773E-09	1.44112E-08	positive
PRJNA729271	stage_E18_vs_E10	ordinary_muscle_development	ENSGALG00010021767	CCDC58	0.161752619	0.213611692	0.757227365	0.448913658	0.50843909	positive
PRJNA729271	stage_E18_vs_E10	ordinary_muscle_development	ENSGALG00010021768	DIPK1A	0.586573207	0.15210085	3.85647553	0.000115034	0.000238236	positive
PRJNA729271	stage_E18_vs_E10	ordinary_muscle_development	ENSGALG00010021769	ENSGALG00010021769	-0.412853472	0.176588729	-2.33793784	0.019390473	0.029308124	negative
PRJNA729271	stage_E18_vs_E10	ordinary_muscle_development	ENSGALG00010021770	RALGAPB	0.064599251	0.098161449	0.658091861	0.510479105	0.567264231	positive
PRJNA729271	stage_E18_vs_E10	ordinary_muscle_development	ENSGALG00010021771	ANKRD61	-0.617376841	0.146450189	-4.215609736	2.49104E-05	5.55332E-05	negative
PRJNA729271	stage_E18_vs_E10	ordinary_muscle_development	ENSGALG00010021772	TSN	-1.123860217	0.084413762	-13.31370854	1.92675E-40	3.7538E-39	negative
PRJNA729271	stage_E18_vs_E10	ordinary_muscle_development	ENSGALG00010021773	ENSGALG00010021773	-2.056265147	0.966741381	-2.127006444	0.033419548	0.048407749	negative
PRJNA729271	stage_E18_vs_E10	ordinary_muscle_development	ENSGALG00010021774	FBXL20	0.838210053	0.072545614	11.55424844	7.02594E-31	8.98069E-30	positive
PRJNA729271	stage_E18_vs_E10	ordinary_muscle_development	ENSGALG00010021775	CCDC36	1.520820237	0.338135109	4.497670305	6.87021E-06	1.62542E-05	positive
PRJNA729271	stage_E18_vs_E10	ordinary_muscle_development	ENSGALG00010021776	PMS2	-0.51820184	0.09483474	-5.464261694	4.64837E-08	1.35652E-07	negative
PRJNA729271	stage_E18_vs_E10	ordinary_muscle_development	ENSGALG00010021778	RBL2	-0.054942827	0.104942002	-0.523554208	0.600588638	0.65200212	negative
PRJNA729271	stage_E18_vs_E10	ordinary_muscle_development	ENSGALG00010021779	UNC13C	-0.432304556	0.247869265	-1.744082936	0.081144629	0.109596664	negative
PRJNA729271	stage_E18_vs_E10	ordinary_muscle_development	ENSGALG00010021781	RAPGEF4	-1.065024602	0.273660126	-3.891778525	9.95121E-05	0.000207567	negative
PRJNA729271	stage_E18_vs_E10	ordinary_muscle_development	ENSGALG00010021782	ADAM11	2.124485467	0.280002169	7.587389312	3.26415E-14	1.55484E-13	positive
PRJNA729271	stage_E18_vs_E10	ordinary_muscle_development	ENSGALG00010021783	GIT2	-0.589461905	0.048469122	-12.16159639	4.97763E-34	7.32496E-33	negative
PRJNA729271	stage_E18_vs_E10	ordinary_muscle_development	ENSGALG00010021784	ANGPTL7	-0.12733483	0.955904222	-0.133208775	0.894028272	0.912408632	negative
PRJNA729271	stage_E18_vs_E10	ordinary_muscle_development	ENSGALG00010021785	CCDC71	-0.111440477	0.09029933	-1.23412297	0.217157085	0.267843086	negative
PRJNA729271	stage_E18_vs_E10	ordinary_muscle_development	ENSGALG00010021786	FAM162A	-0.07232479	0.173923273	-0.415843084	0.67752483	0.723868387	negative
PRJNA729271	stage_E18_vs_E10	ordinary_muscle_development	ENSGALG00010021788	RPL5	0.899326226	0.12389664	7.258681301	3.90882E-13	1.72686E-12	positive
PRJNA729271	stage_E18_vs_E10	ordinary_muscle_development	ENSGALG00010021789	CHD9	-0.002295533	0.091116596	-0.025193359	0.979900734	0.983496825	negative
PRJNA729271	stage_E18_vs_E10	ordinary_muscle_development	ENSGALG00010021790	PHF21A	-0.067408669	0.128740184	-0.523602398	0.600555114	0.65200212	negative
PRJNA729271	stage_E18_vs_E10	ordinary_muscle_development	ENSGALG00010021791	PLEKHM2	1.089937221	0.184288145	5.914310004	3.3327E-09	1.07517E-08	positive
PRJNA729271	stage_E18_vs_E10	ordinary_muscle_development	ENSGALG00010021792	KLHDC8B	3.412026664	0.260172782	13.1144643	2.72098E-39	5.06949E-38	positive
PRJNA729271	stage_E18_vs_E10	ordinary_muscle_development	ENSGALG00010021793	ENSGALG00010021793	0.667946937	0.158801774	4.206167973	2.59737E-05	5.77703E-05	positive
PRJNA729271	stage_E18_vs_E10	ordinary_muscle_development	ENSGALG00010021794	ENSGALG00010021794	-1.052087072	0.200896808	-5.236952663	1.6325E-07	4.52621E-07	negative
PRJNA729271	stage_E18_vs_E10	ordinary_muscle_development	ENSGALG00010021795	NDRG3	-0.805121185	0.108132728	-7.445675358	9.64497E-14	4.434E-13	negative
PRJNA729271	stage_E18_vs_E10	ordinary_muscle_development	ENSGALG00010021797	SLC25A34	2.664217074	0.160046028	16.64656794	3.20461E-62	1.39492E-60	positive
PRJNA729271	stage_E18_vs_E10	ordinary_muscle_development	ENSGALG00010021798	RANBP10	0.011607038	0.183886051	0.063120817	0.949670298	0.958531507	positive
PRJNA729271	stage_E18_vs_E10	ordinary_muscle_development	ENSGALG00010021799	ENSGALG00010021799	-1.17478632	0.240044133	-4.894043051	9.87852E-07	2.55376E-06	negative
PRJNA729271	stage_E18_vs_E10	ordinary_muscle_development	ENSGALG00010021800	TMEM82	2.253672921	0.328299552	6.864684734	6.66383E-12	2.66008E-11	positive
PRJNA729271	stage_E18_vs_E10	ordinary_muscle_development	ENSGALG00010021801	VAMP1	0.696721437	0.252046159	2.764261275	0.005705184	0.009372848	positive
PRJNA729271	stage_E18_vs_E10	ordinary_muscle_development	ENSGALG00010021803	TLL2	-1.838697731	0.163104804	-11.27310594	1.78172E-29	2.11689E-28	negative
PRJNA729271	stage_E18_vs_E10	ordinary_muscle_development	ENSGALG00010021805	FUT8	-0.770099266	0.110301974	-6.981736057	2.91555E-12	1.20401E-11	negative
PRJNA729271	stage_E18_vs_E10	ordinary_muscle_development	ENSGALG00010021807	CDC42	-0.051312425	0.082998209	-0.618235329	0.536420225	0.591781046	negative
PRJNA729271	stage_E18_vs_E10	ordinary_muscle_development	ENSGALG00010021810	ANKRD13A	-0.920010444	0.097181007	-9.466977837	2.88057E-21	2.20453E-20	negative
PRJNA729271	stage_E18_vs_E10	ordinary_muscle_development	ENSGALG00010021812	TEAD1	0.440361767	0.083173544	5.294493253	1.19347E-07	3.35844E-07	positive
PRJNA729271	stage_E18_vs_E10	ordinary_muscle_development	ENSGALG00010021817	EVI5	-0.005459309	0.101037186	-0.05403267	0.956909135	0.964360559	negative
PRJNA729271	stage_E18_vs_E10	ordinary_muscle_development	ENSGALG00010021821	PPP1R19L	-0.796487908	0.119899817	-6.642945133	3.07476E-11	1.16398E-10	negative
PRJNA729271	stage_E18_vs_E10	ordinary_muscle_development	ENSGALG00010021823	ENSGALG00010021823	0.085598126	0.259227724	0.330204365	0.741245549	0.781919382	positive
PRJNA729271	stage_E18_vs_E10	ordinary_muscle_development	ENSGALG00010021826	FBXO40	3.44335329	0.210445038	16.3622451	3.55766E-60	1.43644E-58	positive
PRJNA729271	stage_E18_vs_E10	ordinary_muscle_development	ENSGALG00010021827	CCZ1	-0.463761821	0.096679035	-4.796922294	1.61122E-06	4.06875E-06	negative
PRJNA729271	stage_E18_vs_E10	ordinary_muscle_development	ENSGALG00010021829	ERCC6	-0.21220863	0.097689082	-2.172286055	0.029834093	0.043610849	negative
PRJNA729271	stage_E18_vs_E10	ordinary_muscle_development	ENSGALG00010021831	ENSGALG00010021831	1.533109093	0.357595288	4.287274315	1.80879E-05	4.09215E-05	positive
PRJNA729271	stage_E18_vs_E10	ordinary_muscle_development	ENSGALG00010021835	NUDT8	0.876352084	0.1704307	5.141984878	2.71851E-07	7.4097E-07	positive
PRJNA729271	stage_E18_vs_E10	ordinary_muscle_development	ENSGALG00010021837	ENSGALG00010021837	0.297885755	0.328733727	0.90616122	0.364850526	0.424438874	positive
PRJNA729271	stage_E18_vs_E10	ordinary_muscle_development	ENSGALG00010021839	C1orf50	0.036041873	0.122569399	0.294052787	0.768717567	0.805847549	positive
PRJNA729271	stage_E18_vs_E10	ordinary_muscle_development	ENSGALG00010021840	ENSGALG00010021840	0.697082422	0.217728818	3.201608442	0.001366626	0.002451033	positive
PRJNA729271	stage_E18_vs_E10	ordinary_muscle_development	ENSGALG00010021841	CENPT	-1.170964064	0.091717984	-12.76700607	2.5061E-37	4.28877E-36	negative
PRJNA729271	stage_E18_vs_E10	ordinary_muscle_development	ENSGALG00010021842	ENSGALG00010021842	-0.974271629	0.18115947	-5.37797792	7.53271E-08	2.15486E-07	negative
PRJNA729271	stage_E18_vs_E10	ordinary_muscle_development	ENSGALG00010021843	ENSGALG00010021843	0.285128079	0.400031196	0.712764611	0.475991428	0.534554563	positive
PRJNA729271	stage_E18_vs_E10	ordinary_muscle_development	ENSGALG00010021847	P3H1	0.229857838	0.15059398	1.526341474	0.126924845	0.165254256	positive
PRJNA729271	stage_E18_vs_E10	ordinary_muscle_development	ENSGALG00010021848	LARGE2	2.04886968	0.136035609	15.06127477	2.91119E-51	8.90247E-50	positive
PRJNA729271	stage_E18_vs_E10	ordinary_muscle_development	ENSGALG00010021851	ENSGALG00010021851	0.697949161	0.229086174	3.046666454	0.002313943	0.004021173	positive
PRJNA729271	stage_E18_vs_E10	ordinary_muscle_development	ENSGALG00010021853	ENSGALG00010021853	-0.626773358	0.669831612	-0.935717794	0.349418506	0.408222878	negative
PRJNA729271	stage_E18_vs_E10	ordinary_muscle_development	ENSGALG00010021854	TAPBPL	0.872418178	0.156540826	5.57310319	2.50241E-08	7.47886E-08	positive
PRJNA729271	stage_E18_vs_E10	ordinary_muscle_development	ENSGALG00010021860	ENSGALG00010021860	0.379703191	0.27969281	1.35757223	0.17459946	0.220052097	positive
PRJNA729271	stage_E18_vs_E10	ordinary_muscle_development	ENSGALG00010021862	PDK1	0.253788283	0.106324828	2.386914581	0.016990438	0.025948415	positive
PRJNA729271	stage_E18_vs_E10	ordinary_muscle_development	ENSGALG00010021863	CLDN19	3.139787836	0.285148104	11.01107736	3.37938E-28	3.78548E-27	positive
PRJNA729271	stage_E18_vs_E10	ordinary_muscle_development	ENSGALG00010021864	GFI1	1.31245391	0.522334536	2.51266922	0.011982162	0.018741928	positive
PRJNA729271	stage_E18_vs_E10	ordinary_muscle_development	ENSGALG00010021866	SCNN1A	2.150973012	0.357290827	6.020230155	1.74169E-09	5.75207E-09	positive
PRJNA729271	stage_E18_vs_E10	ordinary_muscle_development	ENSGALG00010021867	CXCL12	1.335000946	0.182068011	7.332429988	2.26017E-13	1.01177E-12	positive
PRJNA729271	stage_E18_vs_E10	ordinary_muscle_development	ENSGALG00010021869	GJC3	0.312702127	0.121877331	2.565711969	0.010296429	0.016264902	positive
PRJNA729271	stage_E18_vs_E10	ordinary_muscle_development	ENSGALG00010021870	ENSGALG00010021870	-0.219585593	0.169396589	-1.296281084	0.194878663	0.242982708	negative
PRJNA729271	stage_E18_vs_E10	ordinary_muscle_development	ENSGALG00010021871	ENSGALG00010021871	4.957967728	0.250321959	19.80636355	2.62362E-87	2.25026E-85	positive
PRJNA729271	stage_E18_vs_E10	ordinary_muscle_development	ENSGALG00010021874	TMCC1	0.894989387	0.078780368	11.36056372	6.57192E-30	7.99161E-29	positive
PRJNA729271	stage_E18_vs_E10	ordinary_muscle_development	ENSGALG00010021877	PLEKHN1	1.775555169	0.144842659	12.2585099	1.51256E-34	2.28618E-33	positive

PRJNA729271	stage_E18_vs_E10	ordinary_muscle_development	ENSGALG00010021879	CREBL2	0.832909973	0.156050687	5.337432279	9.42721E-08	2.67884E-07	positive
PRJNA729271	stage_E18_vs_E10	ordinary_muscle_development	ENSGALG00010021881	PHF20	-0.269249339	0.048873894	-5.509062588	3.6075E-08	1.06563E-07	negative
PRJNA729271	stage_E18_vs_E10	ordinary_muscle_development	ENSGALG00010021887	NUTF2	-0.913636708	0.111067709	-8.225943587	1.9366E-16	1.08929E-15	negative
PRJNA729271	stage_E18_vs_E10	ordinary_muscle_development	ENSGALG00010021892	NDUFV1	0.782945572	0.122040157	6.415474992	1.40384E-10	5.0581E-10	positive
PRJNA729271	stage_E18_vs_E10	ordinary_muscle_development	ENSGALG00010021897	ENSGALG00010021897	-0.204369035	0.221813853	-0.921353796	0.356865743	0.41625243	negative
PRJNA729271	stage_E18_vs_E10	ordinary_muscle_development	ENSGALG00010021902	LMTK2	0.059784753	0.119977509	0.498299672	0.618272839	0.668995581	positive
PRJNA729271	stage_E18_vs_E10	ordinary_muscle_development	ENSGALG00010021903	MORC2	-0.284298023	0.078942526	-3.601329211	0.000316594	0.00061747	negative
PRJNA729271	stage_E18_vs_E10	ordinary_muscle_development	ENSGALG00010021905	RBSN	-0.328089769	0.073904188	-4.439393457	9.02128E-06	2.10977E-05	negative
PRJNA729271	stage_E18_vs_E10	ordinary_muscle_development	ENSGALG00010021908	EDC4	-0.512748782	0.10945639	-4.684502965	2.8064E-06	6.9029E-06	negative
PRJNA729271	stage_E18_vs_E10	ordinary_muscle_development	ENSGALG00010021913	ENSGALG00010021913	1.377044315	0.176108334	7.81930236	5.31169E-15	2.71102E-14	positive
PRJNA729271	stage_E18_vs_E10	ordinary_muscle_development	ENSGALG00010021916	MRPS25	-0.13776463	0.149975885	-0.91857854	0.358316061	0.417742404	negative
PRJNA729271	stage_E18_vs_E10	ordinary_muscle_development	ENSGALG00010021919	PARVA	0.474293622	0.124643442	3.805203168	0.000141688	0.000289998	positive
PRJNA729271	stage_E18_vs_E10	ordinary_muscle_development	ENSGALG00010021922	MRPL51	0.069883983	0.138283033	0.505369182	0.61329956	0.663961246	positive
PRJNA729271	stage_E18_vs_E10	ordinary_muscle_development	ENSGALG00010021924	ENSGALG00010021924	-1.638818016	0.526640543	-3.111834128	0.001859289	0.003281032	negative
PRJNA729271	stage_E18_vs_E10	ordinary_muscle_development	ENSGALG00010021928	ENSGALG00010021928	0.561096595	1.02423311	0.547821185	0.583814691	0.63656322	positive
PRJNA729271	stage_E18_vs_E10	ordinary_muscle_development	ENSGALG00010021931	ITGA6	1.161181994	0.121638928	9.546137974	1.34621E-21	1.05193E-20	positive
PRJNA729271	stage_E18_vs_E10	ordinary_muscle_development	ENSGALG00010021932	RPAP2	-0.092486835	0.164372877	-0.562664817	0.573663146	0.626903446	negative
PRJNA729271	stage_E18_vs_E10	ordinary_muscle_development	ENSGALG00010021933	PRR14L	-0.176358944	0.089701501	-1.966064586	0.049291151	0.069285806	negative
PRJNA729271	stage_E18_vs_E10	ordinary_muscle_development	ENSGALG00010021934	EFTUD2	-0.756066418	0.06714464	-11.26026474	2.06139E-29	2.44315E-28	negative
PRJNA729271	stage_E18_vs_E10	ordinary_muscle_development	ENSGALG00010021935	CETP	3.687078171	0.301639686	12.22345182	2.32978E-34	3.49226E-33	positive
PRJNA729271	stage_E18_vs_E10	ordinary_muscle_development	ENSGALG00010021938	CABP2	-0.996075042	0.193376788	-5.150954542	2.59164E-07	7.07588E-07	negative
PRJNA729271	stage_E18_vs_E10	ordinary_muscle_development	ENSGALG00010021943	OCM3	-1.090445285	0.568434189	-1.918331631			negative
PRJNA729271	stage_E18_vs_E10	ordinary_muscle_development	ENSGALG00010021945	C1QTNF4	-0.581612144	0.209128822	-2.781119023	0.005417187	0.008932104	negative
PRJNA729271	stage_E18_vs_E10	ordinary_muscle_development	ENSGALG00010021947	CDK2AP2	0.307848347	0.252104459	1.221114252	0.222042764	0.272940118	positive
PRJNA729271	stage_E18_vs_E10	ordinary_muscle_development	ENSGALG00010021949	ENSGALG00010021949	-0.446021713	0.244783	-1.822110657	0.068438197	0.093745196	negative
PRJNA729271	stage_E18_vs_E10	ordinary_muscle_development	ENSGALG00010021951	PSKH1	-0.08810774	0.144405372	-0.610141705	0.541767942	0.596817626	negative
PRJNA729271	stage_E18_vs_E10	ordinary_muscle_development	ENSGALG00010021953	PITPNM1	-0.230523844	0.181476436	-1.270268743	0.203988918	0.253325854	negative
PRJNA729271	stage_E18_vs_E10	ordinary_muscle_development	ENSGALG00010021958	BHLHA15	0.084019123	0.395019142	0.212696332	0.831563828	0.860105443	positive
PRJNA729271	stage_E18_vs_E10	ordinary_muscle_development	ENSGALG00010021960	CDKN1B	0.347848849	0.144959853	2.399621977	0.016412011	0.025147156	positive
PRJNA729271	stage_E18_vs_E10	ordinary_muscle_development	ENSGALG00010021965	PEX16	0.633178086	0.101555013	6.234828495	4.52273E-10	1.57665E-09	positive
PRJNA729271	stage_E18_vs_E10	ordinary_muscle_development	ENSGALG00010021966	ADGRA1	0.744250839	0.158456415	4.696880465	2.64165E-06	6.51646E-06	positive
PRJNA729271	stage_E18_vs_E10	ordinary_muscle_development	ENSGALG00010021969	CCDC103	-0.414199409	0.264686921	-1.564865415	0.11761447	0.15400377	negative
PRJNA729271	stage_E18_vs_E10	ordinary_muscle_development	ENSGALG00010021970	ERGIC3	0.105656326	0.090218284	1.171118774	0.241551038	0.294571959	positive
PRJNA729271	stage_E18_vs_E10	ordinary_muscle_development	ENSGALG00010021971	ICOS	-3.42953541	0.564426447	-6.076142304	1.23108E-09	4.11733E-09	negative
PRJNA729271	stage_E18_vs_E10	ordinary_muscle_development	ENSGALG00010021972	PISD	-0.802094313	0.185689471	-4.319546554	1.5635E-05	3.56765E-05	negative
PRJNA729271	stage_E18_vs_E10	ordinary_muscle_development	ENSGALG00010021974	GFAP	3.965748431	0.809559512	4.898649665	9.64975E-07	2.49907E-06	positive
PRJNA729271	stage_E18_vs_E10	ordinary_muscle_development	ENSGALG00010021978	NR2C2	0.564813219	0.073748805	7.658608376	1.87959E-14	9.15689E-14	positive
PRJNA729271	stage_E18_vs_E10	ordinary_muscle_development	ENSGALG00010021980	GLMN	-0.806106834	0.137686304	-5.854662445	4.7798E-09	1.52104E-08	negative
PRJNA729271	stage_E18_vs_E10	ordinary_muscle_development	ENSGALG00010021983	CTLA4	-5.648837748	0.962771854	-5.867265149	4.43042E-09	1.41483E-08	negative
PRJNA729271	stage_E18_vs_E10	ordinary_muscle_development	ENSGALG00010021988	FAM180B	4.614700416	0.262450174	17.58314863	3.31638E-69	1.72297E-67	positive
PRJNA729271	stage_E18_vs_E10	ordinary_muscle_development	ENSGALG00010021989	NCAPD2	-1.554382704	0.151900897	-10.23287377	1.41264E-24	1.30588E-23	negative
PRJNA729271	stage_E18_vs_E10	ordinary_muscle_development	ENSGALG00010021994	ENSGALG00010021994	0.06274289	0.32473819	0.193210691	0.846793966	0.873179095	positive
PRJNA729271	stage_E18_vs_E10	ordinary_muscle_development	ENSGALG00010021995	CD28	1.58623014	0.523847436	3.02803838	0.002461468	0.00426476	positive
PRJNA729271	stage_E18_vs_E10	ordinary_muscle_development	ENSGALG00010021999	ANKRD22	-2.263658695	0.481523003	-4.701039576	2.5884E-06	6.39067E-06	negative
PRJNA729271	stage_E18_vs_E10	ordinary_muscle_development	ENSGALG00010022002	KIF18B	-1.522883692	0.215464712	-7.067903024	1.57292E-12	6.63549E-12	negative
PRJNA729271	stage_E18_vs_E10	ordinary_muscle_development	ENSGALG00010022005	NLRC5	2.44515252	0.18546304	13.18404205	1.08422E-39	2.05436E-38	positive
PRJNA729271	stage_E18_vs_E10	ordinary_muscle_development	ENSGALG00010022006	SFI1	0.523903162	0.106665974	4.911624025	9.03251E-07	2.34845E-06	positive
PRJNA729271	stage_E18_vs_E10	ordinary_muscle_development	ENSGALG00010022012	ENSGALG00010022012	-0.104107182	2.998349023	-0.034721502	0.972301815	0.977155572	negative
PRJNA729271	stage_E18_vs_E10	ordinary_muscle_development	ENSGALG00010022017	CNP1	0.916039215	0.690717658	1.326213692	0.184768925	0.231660373	positive
PRJNA729271	stage_E18_vs_E10	ordinary_muscle_development	ENSGALG00010022019	BTBD8	-0.527004948	0.15754223	-3.345166233	0.000822333	0.001517661	negative
PRJNA729271	stage_E18_vs_E10	ordinary_muscle_development	ENSGALG00010022021	TECPR1	0.741167614	0.080418016	9.216437404	3.07135E-20	2.18768E-19	positive
PRJNA729271	stage_E18_vs_E10	ordinary_muscle_development	ENSGALG00010022022	STAMBPL1	-1.782646947	0.267560799	-6.662586432	2.6905E-11	1.02305E-10	negative
PRJNA729271	stage_E18_vs_E10	ordinary_muscle_development	ENSGALG00010022024	MICAL2	1.556846859	0.183605605	8.479299211	2.26554E-17	1.35587E-16	positive
PRJNA729271	stage_E18_vs_E10	ordinary_muscle_development	ENSGALG00010022027	EIF4ENIF1	-0.49058682	0.068219867	-7.1912603	6.4196E-13	2.79268E-12	negative
PRJNA729271	stage_E18_vs_E10	ordinary_muscle_development	ENSGALG00010022028	FGD5	0.092405036	0.115690839	0.79872388	0.424450537	0.484861733	positive
PRJNA729271	stage_E18_vs_E10	ordinary_muscle_development	ENSGALG00010022029	ENSGALG00010022029	0.396322265	0.648960032	0.610703658	0.541395784	0.596452982	positive
PRJNA729271	stage_E18_vs_E10	ordinary_muscle_development	ENSGALG00010022030	ZNF341	-0.049234452	0.159664972	-0.30836101	0.757807647	0.796838726	negative
PRJNA729271	stage_E18_vs_E10	ordinary_muscle_development	ENSGALG00010022032	KBTBD4	-0.061057456	0.083784478	-0.728744238	0.466158127	0.525302188	negative
PRJNA729271	stage_E18_vs_E10	ordinary_muscle_development	ENSGALG00010022033	RAPH1	0.548413294	0.11484443	4.775271146	1.79465E-06	4.51153E-06	positive
PRJNA729271	stage_E18_vs_E10	ordinary_muscle_development	ENSGALG00010022034	ENSGALG00010022034	1.600366177	0.105491329	15.17059448	5.5373E-52	1.73355E-50	positive
PRJNA729271	stage_E18_vs_E10	ordinary_muscle_development	ENSGALG00010022037	DRG1	-0.347034883	0.096317584	-3.603027291	0.000314532	0.000613727	negative
PRJNA729271	stage_E18_vs_E10	ordinary_muscle_development	ENSGALG00010022038	GAPDH	1.872798719	0.137189751	13.65115619	1.98701E-42	4.19849E-41	positive
PRJNA729271	stage_E18_vs_E10	ordinary_muscle_development	ENSGALG00010022041	MAPK8IP1	-1.833524713	0.162331826	-11.29491827	1.39033E-29	1.66553E-28	negative
PRJNA729271	stage_E18_vs_E10	ordinary_muscle_development	ENSGALG00010022042	AIP	0.059176691	0.086722222	0.68237056	0.495004666	0.552312573	positive
PRJNA729271	stage_E18_vs_E10	ordinary_muscle_development	ENSGALG00010022044	PXMP4	-0.314312956	0.062599071	-5.021048208	5.13903E-07	1.36604E-06	negative
PRJNA729271	stage_E18_vs_E10	ordinary_muscle_development	ENSGALG00010022046	CRY2	0.511523511	0.104152368	4.91129984	9.04746E-07	2.35192E-06	positive
PRJNA729271	stage_E18_vs_E10	ordinary_muscle_development	ENSGALG00010022049	EPHX4	-2.245151993	0.636969081	-3.524742503	0.000423895	0.000814145	negative
PRJNA729271	stage_E18_vs_E10	ordinary_muscle_development	ENSGALG00010022050	ENSGALG00010022050	0.697673821	0.198740551	3.510475436	0.000447306	0.000856613	positive
PRJNA729271	stage_E18_vs_E10	ordinary_muscle_development	ENSGALG00010022051	BAIAP2L1	0.444347595	0.111065325	4.000776966	6.31348E-05	0.000134659	positive
PRJNA729271	stage_E18_vs_E10	ordinary_muscle_development	ENSGALG00010022052	IFFO1	0.821636803	0.104008878	7.899679492	2.79622E-15	1.45534E-14	positive
PRJNA729271	stage_E18_vs_E10	ordinary_muscle_development	ENSGALG00010022054	ENSGALG00010022054	2.321938441	0.422693872	5.493191633	3.94734E-08	1.16176E-07	positive
PRJNA729271	stage_E18_vs_E10	ordinary_muscle_development	ENSGALG00010022055	E2F1	-0.548297499	0.161773808	-3.38928474	0.000700752	0.001305546	negative
PRJNA729271	stage_E18_vs_E10	ordinary_muscle_development	ENSGALG00010022058	NLRP3	-0.431010685	0.128824782	-3.345712499	0.000820714	0.001515061	negative
PRJNA729271	stage_E18_vs_E10	ordinary_muscle_development	ENSGALG00010022059	NPTX2	0.312536037	0.247446267	1.263046075	0.206572622	0.256183278	positive
PRJNA729271	stage_E18_vs_E10	ordinary_muscle_development	ENSGALG00010022062	MITF	0.519139384	0.176005274	2.949567204	0.003182194	0.005427853	positive
PRJNA729271	stage_E18_vs_E10	ordinary_muscle_development	ENSGALG00010022065	PABPC1L	0.589865104	0.244892881	2.408665791	0.016010952	0.024597642	positive
PRJNA729271	stage_E18_vs_E10	ordinary_muscle_development	ENSGALG00010022066	DAD1	-0.161717118	0.138944552	-1.16389679	0.244465841	0.297675769	negative
PRJNA729271	stage_E18_vs_E10	ordinary_muscle_development	ENSGALG00010022067	TMEM130	-0.563128504	0.235890918	-2.387241135	0.016975353	0.025928108	negative
PRJNA729271	stage_E18_vs_E10	ordinary_muscle_development	ENSGALG00010022069	NOP2	-1.088808277	0.136483465	-7.977583798	1.49226E-15	7.91447E-15	negative
PRJNA729271	stage_E18_vs_E10	ordinary_muscle_development	ENSGALG00010022071	ACTA2	-0.764926826	0.198396409	-3.855547741	0.000115471	0.000239039	negative
PRJNA729271	stage_E18_vs_E10	ordinary_muscle_development	ENSGALG00010022072	SMTN	1.29759514	0.312587613	4.151140627	3.30822E-05	7.26116E-05	positive
PRJNA729271	stage_E18_vs_E10	ordinary_muscle_development	ENSGALG00010022073	PTPMT1	-0.107233992	0.138961711	-0.771680137	0.440303898	0.499859415	negative
PRJNA729271	stage_E18_vs_E10	ordinary_muscle_development	ENSGALG00010022076	YWHAB	-1.291310645	0.070245128	-18.38292121	1.80009E-75	1.09631E-73	negative
PRJNA729271	stage_E18_vs_E10	ordinary_muscle_development	ENSGALG00010022079	ABI2	-0.66561249	0.077241811	-8.617256361	6.85775E-18	4.25163E-17	negative
PRJNA729271	stage_E18_vs_E10	ordinary_muscle_development	ENSGALG00010022081	FAS	0.44528759	0.185748954	2.397254893	0.016518428	0.025294171	positive
PRJNA729271	stage_E18_vs_E10	ordinary_muscle_development	ENSGALG00010022082	TGFBR3	1.513979209	0.144395677	10.48493443	1.01316E-25	9.9903E-25	positive
PRJNA729271	stage_E18_vs_E10	ordinary_muscle_development	ENSGALG00010022084	SELENOM	0.227195675	0.200683482	1.132109491	0.257588405	0.311586742	positive
PRJNA729271	stage_E18_vs_E10	ordinary_muscle_development	ENSGALG00010022085	MLLT6	0.952673727	0.177136249	5.378197469	7.52353E-08	2.15266E-07	positive
PRJNA729271	stage_E18_vs_E10	ordinary_muscle_development	ENSGALG00010022087	SLC35C1	0.070025579	0.074425173	0.940885669	0.346763453	0.405447789	positive
PRJNA729271	stage_E18_vs_E10	ordinary_muscle_development	ENSGALG00010022088	DKK3	0.995302436	0.269646387	3.691139521	0.000223252	0.000444754	positive
PRJNA729271	stage_E18_vs_E10	ordinary_muscle_development	ENSGALG00010022090	TRRAP	-0.914217456	0.091165801	-10.02807467	1.14732E-23	9.97026E-23	negative
PRJNA729271	stage_E18_vs_E10	ordinary_muscle_development	ENSGALG00010022092	CTRL	1.721947239	1.945796587	0.884957477	0.376179566	0.436148041	positive
PRJNA729271	stage_E18_vs_E10	ordinary_muscle_development	ENSGALG00010022093	CHD4	-1.288604705	0.136793888	-9.420045917	4.50891E-21	3.4111E-20	negative
PRJNA729271	stage_E18_vs_E10	ordinary_muscle_development	ENSGALG00010022097	INPP5J	-0.111417994	0.282505643	-0.394392101	0.693291575	0.73869913	negative
PRJNA729271	stage_E18_vs_E10	ordinary_muscle_development	ENSGALG00010022098	CDC7	-2.182296991	0.165957205	-13.14975745	1.70721E-39	3.20545E-38	negative
PRJNA729271	stage_E18_vs_E10	ordinary_muscle_development	ENSGALG00010022100	ENTPD1	-0.70716582	0.166743477	-4.241040391	2.22486E-05	4.9829E-05	negative
PRJNA729271	stage_E18_vs_E10	ordinary_muscle_development	ENSGALG00010022101	ENSGALG00010022101	1.428684669	0.181723834	7.861845301	3.78515E-15	1.94974E-14	positive
PRJNA729271	stage_E18_vs_E10	ordinary_muscle_development	ENSGALG00010022104	EXOC3L1	0.581214117	0.141492966	4.107724557	3.99576E-05	8.69518E-05	positive
PRJNA729271	stage_E18_vs_E10	ordinary_muscle_development	ENSGALG00010022105	DLG5	-0.819116242	0.084471956	-9.696901591	3.10793E-22	2.51252E-21	negative
PRJNA729271	stage_E18_vs_E10	ordinary_muscle_development	ENSGALG00010022108	USP47	0.140982903	0.088450429	1.593919946	0.110953966	0.14598146	positive
PRJNA729271	stage_E18_vs_E10	ordinary_muscle_development	ENSGALG00010022111	PCGF2	-0.190410005	0.161526915	-1.178812861	0.238472702	0.291160867	negative
PRJNA729271	stage_E18_vs_E10	ordinary_muscle_development	ENSGALG00010022115	E2F4	-0.625253118	0.187780227	-3.329706894	0.000869374	0.001597969	negative
PRJNA729271	stage_E18_vs_E10	ordinary_muscle_development	ENSGALG00010022116	CYP20A1	-0.406842015	0.102645146	-3.963577734	7.38348E-05	0.00015617	negative
PRJNA729271	stage_E18_vs_E10	ordinary_muscle_development	ENSGALG00010022117	FAM20C	0.639964551	0.120680066	5.302984764	1.13924E-07	3.21334E-07	positive
PRJNA729271	stage_E18_vs_E10	ordinary_muscle_development	ENSGALG00010022120	FOXL3	-1.307192361	0.479855328	-2.724138475	0.006446947	0.010498651	negative
PRJNA729271	stage_E18_vs_E10	ordinary_muscle_development	ENSGALG00010022122	PSMB3	-0.49258132	0.158415633	-3.109423677	0.001874527	0.003305909	negative
PRJNA729271	stage_E18_vs_E10	ordinary_muscle_development	ENSGALG00010022124	ENSGALG00010022124	-0.050627004	0.157796629	-0.320837045	0.748333884	0.788421257	negative
PRJNA729271	stage_E18_vs_E10	ordinary_muscle_development	ENSGALG00010022125	CBFA2T2	0.295188691	0.187281232	1.576178716	0.114984622	0.15081912	positive
PRJNA729271	stage_E18_vs_E10	ordinary_muscle_development	ENSGALG00010022129	ELMO3	0.333756312	0.244942403	1.362590993	0.173011449	0.218240445	positive
PRJNA729271	stage_E18_vs_E10	ordinary_muscle_development	ENSGALG00010022134	CH25H	-0.297019819	0.239133963	-1.242064553	0.214212746	0.264639372	negative
PRJNA729271	stage_E18_vs_E10	ordinary_muscle_development	ENSGALG00010022135	CHST1	-1.441174941	0.248236328	-5.805656869	6.41141E-09	2.01854E-08	negative
PRJNA729271	stage_E18_vs_E10	ordinary_muscle_development	ENSGALG00010022137	PDDC1	-0.789624192	0.129889787	-6.079186112	1.20794E-09	4.04181E-09	negative
PRJNA729271	stage_E18_vs_E10	ordinary_muscle_development	ENSGALG00010022138	SPAG17	1.439517094	0.206804097	6.960776465	3.38402E-12	1.38995E-11	positive
PRJNA729271	stage_E18_vs_E10	ordinary_muscle_development	ENSGALG00010022142	TMEM208	0.362077895	0.107044083	3.382511993	0.000718261	0.001335968	positive
PRJNA729271	stage_E18_vs_E10	ordinary_muscle_development	ENSGALG00010022143	MATCAP1	-0.325580025	0.198217827	-1.642536541	0.10047886	0.133117729	negative
PRJNA729271	stage_E18_vs_E10	ordinary_muscle_development	ENSGALG00010022144	SNTA1	2.985948827	0.186746655	15.98930284	1.51719E-57	5.56753E-56	positive
PRJNA729271	stage_E18_vs_E10	ordinary_muscle_development	ENSGALG00010022148	TALDO1	-1.148146556	0.088150756	-13.02480669	8.84247E-39	1.61629E-37	negative
PRJNA729271	stage_E18_vs_E10	ordinary_muscle_development	ENSGALG00010022149	NBEAL1	-0.118840858	0.106698368	-1.113802021	0.26536413	0.320181597	negative
PRJNA729271	stage_E18_vs_E10	ordinary_muscle_development	ENSGALG00010022150	CPNE2	-0.5530346	0.130908737	-4.2245813	2.39385E-05	5.34487E-05	negative
PRJNA729271	stage_E18_vs_E10	ordinary_muscle_development	ENSGALG00010022151	PIP4K2B	0.307181262	0.154088692	1.993535398	0.046202851	0.065248473	positive
PRJNA729271	stage_E18_vs_E10	ordinary_muscle_development	ENSGALG00010022152	ZNF644	-0.258920565	0.100744566	-2.570069773	0.010167803	0.016070473	negative
PRJNA729271	stage_E18_vs_E10	ordinary_muscle_development	ENSGALG00010022154	CPN1	0.398017503	0.196685046	2.023628693	0.04300837	0.061052427	positive
PRJNA729271	stage_E18_vs_E10	ordinary_muscle_development	ENSGALG00010022157	EPS8L2	-0.533356198	0.14115484	-3.778518656	0.000157764	0.000320728	negative
PRJNA729271	stage_E18_vs_E10	ordinary_muscle_development	ENSGALG00010022158	CWC25	-0.379185753	0.081384072	-4.659213306	3.1742E-06	7.76017E-06	negative
PRJNA729271	stage_E18_vs_E10	ordinary_muscle_development	ENSGALG00010022159	KIAA2013	-0.240250395	0.090402292	-2.657569725	0.007870631	0.012638174	negative
PRJNA729271	stage_E18_vs_E10	ordinary_muscle_development	ENSGALG00010022161	CELF1	0.043265942	0.09444069	0.458128184	0.646860351	0.695566824	positive
PRJNA729271	stage_E18_vs_E10	ordinary_muscle_development	ENSGALG00010022162	POLE	-1.280224051	0.155231771	-8.247178027	1.62179E-16	9.17557E-16	negative
PRJNA729271	stage_E18_vs_E10	ordinary_muscle_development	ENSGALG00010022164	PDGFA	0.431333235	0.107568858	4.009833712	6.07615E-05	0.000129807	positive
PRJNA729271	stage_E18_vs_E10	ordinary_muscle_development	ENSGALG00010022165	FAM192A	-0.530535343	0.090223051	-5.880263811	4.09613E-09	1.31125E-08	negative
PRJNA729271	stage_E18_vs_E10	ordinary_muscle_development	ENSGALG00010022166	SDHB	1.00223782	0.108130767	9.268757189	1.88327E-20	1.36832E-19	positive
PRJNA729271	stage_E18_vs_E10	ordinary_muscle_development	ENSGALG00010022167	WDR3	-1.362955021	0.075342815	-18.09004641	3.81765E-73	2.17862E-71	negative

PRJNA729271	stage_E18_vs_E10	ordinary_muscle_development	ENSGALG00010022169	TOX2	-0.02382845	0.230963429	-0.103169795	0.917828212	0.932169278	negative
PRJNA729271	stage_E18_vs_E10	ordinary_muscle_development	ENSGALG00010022170	RSPRY1	-0.673485618	0.14283066	-4.715273458	2.41386E-06	5.98409E-06	negative
PRJNA729271	stage_E18_vs_E10	ordinary_muscle_development	ENSGALG00010022171	FOXP1	-0.417228144	0.094982207	-4.392697921	1.11953E-05	2.59226E-05	negative
PRJNA729271	stage_E18_vs_E10	ordinary_muscle_development	ENSGALG00010022172	GIPC2	0.892324101	0.184143373	4.845811653	1.26095E-06	3.22696E-06	positive
PRJNA729271	stage_E18_vs_E10	ordinary_muscle_development	ENSGALG00010022173	RPL23	0.667700617	0.104030023	6.418345366	1.37763E-10	4.96613E-10	positive
PRJNA729271	stage_E18_vs_E10	ordinary_muscle_development	ENSGALG00010022174	KCNMA1	2.515231157	0.123647735	20.34191043	5.47554E-92	5.39918E-90	positive
PRJNA729271	stage_E18_vs_E10	ordinary_muscle_development	ENSGALG00010022176	ARL2BPL	-0.326243974	0.13449756	-2.425649755	0.01528101	0.02355863	negative
PRJNA729271	stage_E18_vs_E10	ordinary_muscle_development	ENSGALG00010022178	ENSGALG00010022178	1.073463795	0.323967968	3.313487448	0.000921402	0.001686329	positive
PRJNA729271	stage_E18_vs_E10	ordinary_muscle_development	ENSGALG00010022180	PLLP	1.351622527	0.387896516	3.48449257	0.000493071	0.000938058	positive
PRJNA729271	stage_E18_vs_E10	ordinary_muscle_development	ENSGALG00010022181	ENSGALG00010022181	-1.881937041	0.236764852	-7.948549072	1.88709E-15	9.925E-15	negative
PRJNA729271	stage_E18_vs_E10	ordinary_muscle_development	ENSGALG00010022182	CNTNAP1	0.670305098	0.301382469	2.224101153	0.026141639	0.038618331	positive
PRJNA729271	stage_E18_vs_E10	ordinary_muscle_development	ENSGALG00010022184	PRKAR1B	-0.09886953	0.0919405	-1.075364283	0.282211666	0.337763859	negative
PRJNA729271	stage_E18_vs_E10	ordinary_muscle_development	ENSGALG00010022186	DNAJB4	0.941785305	0.11273361	8.354077429	6.59461E-17	3.82662E-16	positive
PRJNA729271	stage_E18_vs_E10	ordinary_muscle_development	ENSGALG00010022190	ACVR1B	0.392836907	0.165500167	2.373634502	0.017613977	0.026821577	positive
PRJNA729271	stage_E18_vs_E10	ordinary_muscle_development	ENSGALG00010022193	GDAP2	-0.562518326	0.083451552	-6.740657479	1.57672E-11	6.11246E-11	negative
PRJNA729271	stage_E18_vs_E10	ordinary_muscle_development	ENSGALG00010022194	ENSGALG00010022194	-0.138990136	0.089453947	-1.553761924	0.120241216	0.157130944	negative
PRJNA729271	stage_E18_vs_E10	ordinary_muscle_development	ENSGALG00010022196	PLXNA1	-0.106261222	0.108666819	-0.977862633	0.328142268	0.386261343	negative
PRJNA729271	stage_E18_vs_E10	ordinary_muscle_development	ENSGALG00010022199	RBM39	-0.18218936	0.061751558	-2.950360517	0.003174033	0.005415337	negative
PRJNA729271	stage_E18_vs_E10	ordinary_muscle_development	ENSGALG00010022200	DNMBP	0.589282614	0.1091926	5.396726654	6.78677E-08	1.94916E-07	positive
PRJNA729271	stage_E18_vs_E10	ordinary_muscle_development	ENSGALG00010022202	CX3CL1	0.624813769	0.168008286	3.718946151	0.000200056	0.00040108	positive
PRJNA729271	stage_E18_vs_E10	ordinary_muscle_development	ENSGALG00010022203	DNAAF5	-0.810386045	0.136840888	-5.922104561	3.17847E-09	1.02679E-08	negative
PRJNA729271	stage_E18_vs_E10	ordinary_muscle_development	ENSGALG00010022204	ENSGALG00010022204	0.213169378	0.341193812	0.624775041	0.532118714	0.588065017	positive
PRJNA729271	stage_E18_vs_E10	ordinary_muscle_development	ENSGALG00010022205	GALNT18	-0.504989351	0.108788114	-4.641953345	3.45131E-06	8.39656E-06	negative
PRJNA729271	stage_E18_vs_E10	ordinary_muscle_development	ENSGALG00010022207	ENSGALG00010022207	0.656081472	0.308828703	2.124418703	0.033635147	0.048705441	positive
PRJNA729271	stage_E18_vs_E10	ordinary_muscle_development	ENSGALG00010022208	CCL17	-2.414174727	1.064769749	-2.26732092	0.023370628	0.034820161	negative
PRJNA729271	stage_E18_vs_E10	ordinary_muscle_development	ENSGALG00010022209	ZFYVE19	-0.697457919	0.121706617	-5.730649144	1.00047E-08	3.09075E-08	negative
PRJNA729271	stage_E18_vs_E10	ordinary_muscle_development	ENSGALG00010022210	EZH1	0.607485233	0.08438256	7.199179922	6.05758E-13	2.63757E-12	positive
PRJNA729271	stage_E18_vs_E10	ordinary_muscle_development	ENSGALG00010022211	ENSGALG00010022211	0.817299245	0.488010018	1.674759152	0.093981496	0.125304163	positive
PRJNA729271	stage_E18_vs_E10	ordinary_muscle_development	ENSGALG00010022213	IDH1	-0.340456557	0.080348515	-4.23724767	2.26276E-05	5.06232E-05	negative
PRJNA729271	stage_E18_vs_E10	ordinary_muscle_development	ENSGALG00010022214	ENSGALG00010022214	-1.71872542	0.270986654	-6.34247258	2.26106E-10	8.03091E-10	negative
PRJNA729271	stage_E18_vs_E10	ordinary_muscle_development	ENSGALG00010022215	ENSGALG00010022215	1.199761195	0.130637003	9.183930797	4.15636E-20	2.93455E-19	positive
PRJNA729271	stage_E18_vs_E10	ordinary_muscle_development	ENSGALG00010022216	ROMO1	0.283702423	0.144808595	1.959154591	0.050094685	0.070347071	positive
PRJNA729271	stage_E18_vs_E10	ordinary_muscle_development	ENSGALG00010022218	ENSGALG00010022218	2.525133742	0.248311847	10.16920364	2.72124E-24	2.46374E-23	positive
PRJNA729271	stage_E18_vs_E10	ordinary_muscle_development	ENSGALG00010022219	EIF4E3	-0.151644717	0.139179985	-1.089558364	0.275907732	0.331423103	negative
PRJNA729271	stage_E18_vs_E10	ordinary_muscle_development	ENSGALG00010022221	CUTC	0.248830477	0.085351376	2.915365727	0.003552719	0.006004042	positive
PRJNA729271	stage_E18_vs_E10	ordinary_muscle_development	ENSGALG00010022222	ENSGALG00010022222	0.291501036	0.106388039	2.7399794	0.006144304	0.010040776	positive
PRJNA729271	stage_E18_vs_E10	ordinary_muscle_development	ENSGALG00010022223	DOK4	-0.91879454	0.174147201	-5.27596501	1.32059E-07	3.69751E-07	negative
PRJNA729271	stage_E18_vs_E10	ordinary_muscle_development	ENSGALG00010022224	NHEJ1	-0.73133783	0.182347097	-4.010690834	6.05413E-05	0.000129374	negative
PRJNA729271	stage_E18_vs_E10	ordinary_muscle_development	ENSGALG00010022225	ENSGALG00010022225	-0.351524359	0.573565008	-0.612876228	0.539958182	0.595004854	negative
PRJNA729271	stage_E18_vs_E10	ordinary_muscle_development	ENSGALG00010022226	FBRSL1	0.296603615	0.132537969	2.237876572	0.025229105	0.037396041	positive
PRJNA729271	stage_E18_vs_E10	ordinary_muscle_development	ENSGALG00010022227	TMEM80	0.672731006	0.157487212	4.271654807	1.94028E-05	4.37751E-05	positive
PRJNA729271	stage_E18_vs_E10	ordinary_muscle_development	ENSGALG00010022228	FUBP1	-1.467773962	0.055602844	-26.39746223	1.4652E-153	5.1802E-151	negative
PRJNA729271	stage_E18_vs_E10	ordinary_muscle_development	ENSGALG00010022229	RAPSN	1.561749499	0.17785128	8.781210352	1.61725E-18	1.04652E-17	positive
PRJNA729271	stage_E18_vs_E10	ordinary_muscle_development	ENSGALG00010022230	NFS1	-0.041912906	0.087201675	-0.48064336	0.630769993	0.680739413	negative
PRJNA729271	stage_E18_vs_E10	ordinary_muscle_development	ENSGALG00010022231	ZNF503	-1.56328491	0.205479203	-7.607995774	2.78379E-14	1.33392E-13	negative
PRJNA729271	stage_E18_vs_E10	ordinary_muscle_development	ENSGALG00010022232	GPR27	-1.503704028	0.201173102	-7.474677346	7.73935E-14	3.59557E-13	negative
PRJNA729271	stage_E18_vs_E10	ordinary_muscle_development	ENSGALG00010022233	FAM46C	3.89459378	0.16389838	23.76224689	8.2095E-125	1.6081E-122	positive
PRJNA729271	stage_E18_vs_E10	ordinary_muscle_development	ENSGALG00010022234	COX15	-0.371867578	0.098827781	-3.762783852	0.000168032	0.000340172	negative
PRJNA729271	stage_E18_vs_E10	ordinary_muscle_development	ENSGALG00010022235	RAMP2	0.592713508	0.114097698	5.194789363	2.04952E-07	5.64572E-07	positive
PRJNA729271	stage_E18_vs_E10	ordinary_muscle_development	ENSGALG00010022236	SHF	2.017265658	0.181436055	11.11832848	1.02168E-28	1.17335E-27	positive
PRJNA729271	stage_E18_vs_E10	ordinary_muscle_development	ENSGALG00010022237	SUN1	0.823327383	0.091069388	9.04066007	1.55729E-19	1.06879E-18	positive
PRJNA729271	stage_E18_vs_E10	ordinary_muscle_development	ENSGALG00010022238	COMTD1	-1.094496691	0.174658094	-6.266510005	3.6923E-10	1.29244E-09	negative
PRJNA729271	stage_E18_vs_E10	ordinary_muscle_development	ENSGALG00010022239	DEAF1	0.395208127	0.116087657	3.404394049	0.00066311	0.00123975	positive
PRJNA729271	stage_E18_vs_E10	ordinary_muscle_development	ENSGALG00010022240	ENSGALG00010022240	-0.702674233	0.133559803	-5.261120619	1.4318E-07	3.99065E-07	negative
PRJNA729271	stage_E18_vs_E10	ordinary_muscle_development	ENSGALG00010022241	POLR2C	-0.085049017	0.09555619	-0.890041942	0.373443366	0.433356944	negative
PRJNA729271	stage_E18_vs_E10	ordinary_muscle_development	ENSGALG00010022242	USP19	-0.028710354	0.127207298	-0.225697381	0.821436795	0.85153091	negative
PRJNA729271	stage_E18_vs_E10	ordinary_muscle_development	ENSGALG00010022243	ENSGALG00010022243	1.136712303	0.615350785	1.847259046	0.064709605	0.088974173	positive
PRJNA729271	stage_E18_vs_E10	ordinary_muscle_development	ENSGALG00010022244	ENTPD7	-0.949540308	0.175014324	-5.42550052	5.77924E-08	1.66873E-07	negative
PRJNA729271	stage_E18_vs_E10	ordinary_muscle_development	ENSGALG00010022245	PIKFYVE	0.313967901	0.100985712	3.109032895	0.001877008	0.003309882	positive
PRJNA729271	stage_E18_vs_E10	ordinary_muscle_development	ENSGALG00010022246	RHOV	-3.091099831	0.447700971	-6.90438492	5.04214E-12	2.04093E-11	negative
PRJNA729271	stage_E18_vs_E10	ordinary_muscle_development	ENSGALG00010022247	GALNT9	-1.550564927	0.208028635	-7.453612934	9.08182E-14	4.18839E-13	negative
PRJNA729271	stage_E18_vs_E10	ordinary_muscle_development	ENSGALG00010022248	FIGNL2	-0.021464091	0.217689093	-0.098599756	0.921456064	0.935395031	negative
PRJNA729271	stage_E18_vs_E10	ordinary_muscle_development	ENSGALG00010022250	SLC25A28	0.593905546	0.12986044	4.57341393	4.79841E-06	1.15268E-05	positive
PRJNA729271	stage_E18_vs_E10	ordinary_muscle_development	ENSGALG00010022251	WNK4	-0.296840171	0.152513088	-1.946325889	0.051615606	0.072293768	negative
PRJNA729271	stage_E18_vs_E10	ordinary_muscle_development	ENSGALG00010022252	COQ9	1.954769978	0.132576136	14.74450856	3.3374E-49	9.48542E-48	positive
PRJNA729271	stage_E18_vs_E10	ordinary_muscle_development	ENSGALG00010022253	ALPK3	1.940938036	0.217430798	8.926693224	4.38932E-19	2.93872E-18	positive
PRJNA729271	stage_E18_vs_E10	ordinary_muscle_development	ENSGALG00010022255	NOC4L	-1.151187239	0.092103037	-12.49890639	7.56853E-36	1.2149E-34	negative
PRJNA729271	stage_E18_vs_E10	ordinary_muscle_development	ENSGALG00010022256	PSMC3	-0.411120805	0.092508942	-4.444119644	8.82525E-06	2.06659E-05	negative
PRJNA729271	stage_E18_vs_E10	ordinary_muscle_development	ENSGALG00010022257	KCNK18	4.139949826	0.836426198	4.949569773	7.43777E-07	1.9478E-06	positive
PRJNA729271	stage_E18_vs_E10	ordinary_muscle_development	ENSGALG00010022258	ENSGALG00010022258	3.007916381	0.723873129	4.155308799	3.24849E-05	7.13652E-05	positive
PRJNA729271	stage_E18_vs_E10	ordinary_muscle_development	ENSGALG00010022259	GET4	-0.083591448	0.085896705	-0.973162448	0.330472571	0.388594136	negative
PRJNA729271	stage_E18_vs_E10	ordinary_muscle_development	ENSGALG00010022264	SCN8A	-0.310507014	0.251066688	-1.236751143	0.216179485	0.266750799	negative
PRJNA729271	stage_E18_vs_E10	ordinary_muscle_development	ENSGALG00010022266	COA3	0.441189743	0.145650823	3.029092004	0.0024529	0.004250422	positive
PRJNA729271	stage_E18_vs_E10	ordinary_muscle_development	ENSGALG00010022269	CNTD1	0.087335023	0.378331217	0.230842761	0.81743696	0.848338851	positive
PRJNA729271	stage_E18_vs_E10	ordinary_muscle_development	ENSGALG00010022271	SLC18A2	-1.916914641	0.26064004	-7.354643737	1.91437E-13	8.60695E-13	negative
PRJNA729271	stage_E18_vs_E10	ordinary_muscle_development	ENSGALG00010022274	CCDC91	3.000443588	0.173409077	17.3026905	4.48925E-67	2.21332E-65	positive
PRJNA729271	stage_E18_vs_E10	ordinary_muscle_development	ENSGALG00010022275	VDAC2	1.376112767	0.124101466	11.08861005	1.42484E-28	1.61985E-27	positive
PRJNA729271	stage_E18_vs_E10	ordinary_muscle_development	ENSGALG00010022276	BECN1	-0.661980834	0.091955713	-7.198909229	6.06961E-13	2.64201E-12	negative
PRJNA729271	stage_E18_vs_E10	ordinary_muscle_development	ENSGALG00010022279	EP400	-0.0067096	0.149473116	-0.044888343	0.964196309	0.970692144	negative
PRJNA729271	stage_E18_vs_E10	ordinary_muscle_development	ENSGALG00010022280	PDZD8	-0.371421345	0.069731031	-5.326485802	1.00131E-07	2.84053E-07	negative
PRJNA729271	stage_E18_vs_E10	ordinary_muscle_development	ENSGALG00010022282	TMT1A	1.44742965	0.226363399	6.394274232	1.61312E-10	5.79196E-10	positive
PRJNA729271	stage_E18_vs_E10	ordinary_muscle_development	ENSGALG00010022290	NEXN	1.929489238	0.220071163	8.767569607	1.82564E-18	1.17664E-17	positive
PRJNA729271	stage_E18_vs_E10	ordinary_muscle_development	ENSGALG00010022293	DNAJC10	-1.160606673	0.065490479	-17.72176196	2.84849E-70	1.52922E-68	negative
PRJNA729271	stage_E18_vs_E10	ordinary_muscle_development	ENSGALG00010022294	ENSGALG00010022294	0.609250687	0.720587971	0.84549106	0.397836722	0.45785179	positive
PRJNA729271	stage_E18_vs_E10	ordinary_muscle_development	ENSGALG00010022296	CIAPIN1	-0.232679659	0.097862843	-2.377609836	0.017425253	0.026552388	negative
PRJNA729271	stage_E18_vs_E10	ordinary_muscle_development	ENSGALG00010022297	VPS18	-0.740061329	0.060084647	-12.31697887	7.33926E-35	1.12114E-33	negative
PRJNA729271	stage_E18_vs_E10	ordinary_muscle_development	ENSGALG00010022299	MRGBP	0.314261594	0.079646821	3.945689115	7.95708E-05	0.000167594	positive
PRJNA729271	stage_E18_vs_E10	ordinary_muscle_development	ENSGALG00010022302	ENSGALG00010022302	-1.161012367	0.158153358	-7.341054145	2.11918E-13	9.50124E-13	negative
PRJNA729271	stage_E18_vs_E10	ordinary_muscle_development	ENSGALG00010022303	DUSP13A	3.253641525	0.209854706	15.50425808	3.24645E-54	1.06948E-52	positive
PRJNA729271	stage_E18_vs_E10	ordinary_muscle_development	ENSGALG00010022304	ADAP1	-1.991471037	0.196624717	-10.12828429	4.13847E-24	3.70291E-23	negative
PRJNA729271	stage_E18_vs_E10	ordinary_muscle_development	ENSGALG00010022307	ENSGALG00010022307	4.743876866	0.653429682	7.259965371	3.87189E-13	1.71107E-12	positive
PRJNA729271	stage_E18_vs_E10	ordinary_muscle_development	ENSGALG00010022308	OGFR	-0.908996515	0.185659806	-4.896032877	9.77907E-07	2.53026E-06	negative
PRJNA729271	stage_E18_vs_E10	ordinary_muscle_development	ENSGALG00010022309	WSCD2	0.03106358	0.322731469	0.096252096	0.923320356	0.936865526	positive
PRJNA729271	stage_E18_vs_E10	ordinary_muscle_development	ENSGALG00010022310	DCLRE1A	-1.568242763	0.101737889	-15.41453999	1.30698E-53	4.23819E-52	negative
PRJNA729271	stage_E18_vs_E10	ordinary_muscle_development	ENSGALG00010022311	DLL4	0.845925288	0.141999514	5.957240723	2.56532E-09	8.35416E-09	positive
PRJNA729271	stage_E18_vs_E10	ordinary_muscle_development	ENSGALG00010022312	ZNF592	-0.049906447	0.056130818	-0.889109566	0.373944198	0.433868659	negative
PRJNA729271	stage_E18_vs_E10	ordinary_muscle_development	ENSGALG00010022313	ENSGALG00010022313	-2.191314959	0.452427559	-4.843460383	1.27597E-06	3.26367E-06	negative
PRJNA729271	stage_E18_vs_E10	ordinary_muscle_development	ENSGALG00010022314	DUSP13B	4.702105298	0.561978689	8.367052677	5.90773E-17	3.44598E-16	positive
PRJNA729271	stage_E18_vs_E10	ordinary_muscle_development	ENSGALG00010022316	INO80	-0.064518224	0.070225039	-0.918735321	0.35823403	0.417680363	negative
PRJNA729271	stage_E18_vs_E10	ordinary_muscle_development	ENSGALG00010022317	PSME3	-1.438978609	0.074385193	-19.3449603	2.24777E-83	1.68816E-81	negative
PRJNA729271	stage_E18_vs_E10	ordinary_muscle_development	ENSGALG00010022320	COL9A3	-5.773046568	0.544755162	-10.59750687	3.0603E-26	3.10637E-25	negative
PRJNA729271	stage_E18_vs_E10	ordinary_muscle_development	ENSGALG00010022322	ENSGALG00010022322	0.190611938	0.415064142	0.459234896	0.646065494	0.695021474	positive
PRJNA729271	stage_E18_vs_E10	ordinary_muscle_development	ENSGALG00010022323	DUSP29	0.779172956	0.410389268	1.898619231	0.057614558	0.080007953	positive
PRJNA729271	stage_E18_vs_E10	ordinary_muscle_development	ENSGALG00010022325	MIGA1	-0.651099001	0.114612086	-5.68089303	1.33993E-08	4.09236E-08	negative
PRJNA729271	stage_E18_vs_E10	ordinary_muscle_development	ENSGALG00010022326	COX19	0.54284797	0.096476303	5.626749312	1.83637E-08	5.53969E-08	positive
PRJNA729271	stage_E18_vs_E10	ordinary_muscle_development	ENSGALG00010022328	ENSGALG00010022328	-1.70525453	0.293391532	-5.812214547	6.16518E-09	1.94397E-08	negative
PRJNA729271	stage_E18_vs_E10	ordinary_muscle_development	ENSGALG00010022332	KAT6B	-0.615219287	0.061396576	-10.02041694	1.23979E-23	1.07545E-22	negative
PRJNA729271	stage_E18_vs_E10	ordinary_muscle_development	ENSGALG00010022333	ENSGALG00010022333	1.779902977	0.130621085	13.6264599	2.78779E-42	5.83103E-41	positive
PRJNA729271	stage_E18_vs_E10	ordinary_muscle_development	ENSGALG00010022334	PDE1A	-0.327235673	0.119884669	-2.729587318	0.006341365	0.010346476	negative
PRJNA729271	stage_E18_vs_E10	ordinary_muscle_development	ENSGALG00010022340	GFOD2	-0.282704013	0.094660118	-2.986516594	0.002821755	0.004854183	negative
PRJNA729271	stage_E18_vs_E10	ordinary_muscle_development	ENSGALG00010022343	GATA5	-3.165379258	0.624474773	-5.068866506	4.00192E-07	1.07501E-06	negative
PRJNA729271	stage_E18_vs_E10	ordinary_muscle_development	ENSGALG00010022345	USP33	-0.126739086	0.123099983	-1.029562172	0.30321558	0.36037306	negative
PRJNA729271	stage_E18_vs_E10	ordinary_muscle_development	ENSGALG00010022347	SEC11A	-0.726319341	0.104047861	-6.980627322	2.93865E-12	1.21252E-11	negative
PRJNA729271	stage_E18_vs_E10	ordinary_muscle_development	ENSGALG00010022348	G6PC1	3.719093182	0.612759245	6.069419939	1.28373E-09	4.28452E-09	positive
PRJNA729271	stage_E18_vs_E10	ordinary_muscle_development	ENSGALG00010022353	SLC39A13	-0.180278804	0.095682941	-1.884126916	0.05954781	0.082487147	negative
PRJNA729271	stage_E18_vs_E10	ordinary_muscle_development	ENSGALG00010022354	FAR2	-0.200573197	0.137314314	-1.460686731	0.144101432	0.185140069	negative
PRJNA729271	stage_E18_vs_E10	ordinary_muscle_development	ENSGALG00010022356	TCFL5	0.683662251	0.188931719	3.618567895	0.000296238	0.00058058	positive
PRJNA729271	stage_E18_vs_E10	ordinary_muscle_development	ENSGALG00010022357	ENSGALG00010022357	-6.163753904	0.953822021	-6.462163556	1.03216E-10	3.75602E-10	negative
PRJNA729271	stage_E18_vs_E10	ordinary_muscle_development	ENSGALG00010022359	ZZZ3	-0.319261764	0.079613384	-4.010151934	6.06797E-05	0.000129651	negative
PRJNA729271	stage_E18_vs_E10	ordinary_muscle_development	ENSGALG00010022363	KIF18A	-1.645050103	0.202847325	-8.109794406	5.07055E-16	2.77664E-15	negative
PRJNA729271	stage_E18_vs_E10	ordinary_muscle_development	ENSGALG00010022365	PLCG1	-0.99023932	0.109634297	-9.032203849	1.68249E-19	1.1509E-18	negative
PRJNA729271	stage_E18_vs_E10	ordinary_muscle_development	ENSGALG00010022366	AARSD1	-0.293555823	0.116388837	-2.522199125	0.011662367	0.018291064	negative
PRJNA729271	stage_E18_vs_E10	ordinary_muscle_development	ENSGALG00010022373	AK5	-3.667245706	0.225699204	-16.24837675	2.29357E-59	9.05867E-58	negative
PRJNA729271	stage_E18_vs_E10	ordinary_muscle_development	ENSGALG00010022375	NMB	1.101613517	0.397921146	2.768421656	0.005632853	0.009261366	positive
PRJNA729271	stage_E18_vs_E10	ordinary_muscle_development	ENSGALG00010022377	ZHX3	1.268232519	0.089754419	14.13002866	2.48043E-45	5.90376E-44	positive
PRJNA729271	stage_E18_vs_E10	ordinary_muscle_development	ENSGALG00010022379	METTL15	-0.249200327	0.116321877	-2.142334136	0.032166604	0.046761099	negative
PRJNA729271	stage_E18_vs_E10	ordinary_muscle_development	ENSGALG00010022382	CALB2	-5.28342873	1.036732459	-5.096231609	3.46481E-07	9.36811E-07	negative
PRJNA729271	stage_E18_vs_E10	ordinary_muscle_development	ENSGALG00010022385	ENSGALG00010022385	4.311142228	0.604682838	7.129592502	1.00667E-12	4.3196E-12	positive
PRJNA729271	stage_E18_vs_E10	ordinary_muscle_development	ENSGALG00010022386	SPI1	0.569113963	0.247049601	2.303642513	0.021242719	0.031898189	positive
PRJNA729271	stage_E18_vs_E10	ordinary_muscle_development	ENSGALG00010022388	CYP2W1	-0.610219436	0.441391769	-1.382489388	0.166821487	0.211112053	negative
PRJNA729271	stage_E18_vs_E10	ordinary_muscle_development	ENSGALG00010022391	ENSGALG00010022391	2.013417521	0.175149497	11.49542281	1.39098E-30	1.74414E-29	positive

PRJNA729271	stage_E18_vs_E10	ordinary_muscle_development	ENSGALG00010022392	EMILIN3	-0.928710502	0.185210638	-5.014347506	5.32137E-07	1.41192E-06	negative
PRJNA729271	stage_E18_vs_E10	ordinary_muscle_development	ENSGALG00010022395	PTGES3L	1.557997726	0.133700767	11.65287051	2.21857E-31	2.91288E-30	positive
PRJNA729271	stage_E18_vs_E10	ordinary_muscle_development	ENSGALG00010022396	PPP1R1C	1.128087626	0.322824669	3.494428191	0.000475078	0.000905849	positive
PRJNA729271	stage_E18_vs_E10	ordinary_muscle_development	ENSGALG00010022397	ATF1	-0.345492413	0.047944841	-7.206039351	5.76028E-13	2.51189E-12	negative
PRJNA729271	stage_E18_vs_E10	ordinary_muscle_development	ENSGALG00010022400	NHLRC2	0.135832696	0.07223406	1.880452181	0.060046478	0.0830985	positive
PRJNA729271	stage_E18_vs_E10	ordinary_muscle_development	ENSGALG00010022401	LCAT	2.017495174	0.437578126	4.61059421	4.0152E-06	9.7049E-06	positive
PRJNA729271	stage_E18_vs_E10	ordinary_muscle_development	ENSGALG00010022406	ITPRID2	0.970881165	0.10435895	9.303286089	1.36171E-20	9.9586E-20	positive
PRJNA729271	stage_E18_vs_E10	ordinary_muscle_development	ENSGALG00010022407	RUNDC1	0.0862532	0.096355925	0.895152006	0.370705836	0.430421427	positive
PRJNA729271	stage_E18_vs_E10	ordinary_muscle_development	ENSGALG00010022408	RAD51AP1	-0.252873733	0.137793309	-1.835166999	0.066480949	0.091228	negative
PRJNA729271	stage_E18_vs_E10	ordinary_muscle_development	ENSGALG00010022409	CARNS1	6.462141431	0.339828835	19.01587143	1.26025E-80	8.95459E-79	positive
PRJNA729271	stage_E18_vs_E10	ordinary_muscle_development	ENSGALG00010022411	ADRB1	0.548423101	0.294758509	1.860584457	0.062802881	0.086524833	positive
PRJNA729271	stage_E18_vs_E10	ordinary_muscle_development	ENSGALG00010022412	CHAC1	0.358611615	0.657661613	0.545282874	0.585558981	0.638027319	positive
PRJNA729271	stage_E18_vs_E10	ordinary_muscle_development	ENSGALG00010022415	DIP2B	-0.17692415	0.109471744	-1.616162704	0.10605914	0.139973344	negative
PRJNA729271	stage_E18_vs_E10	ordinary_muscle_development	ENSGALG00010022416	CCDC186	0.352775481	0.086257894	4.089776174	4.3179E-05	9.36244E-05	positive
PRJNA729271	stage_E18_vs_E10	ordinary_muscle_development	ENSGALG00010022419	DUOX2	4.184669704	0.662992094	6.311794277	2.75819E-10	9.73932E-10	positive
PRJNA729271	stage_E18_vs_E10	ordinary_muscle_development	ENSGALG00010022421	CMKLR1	1.405772505	0.17684188	7.94931894	1.8754E-15	9.87069E-15	positive
PRJNA729271	stage_E18_vs_E10	ordinary_muscle_development	ENSGALG00010022422	RPS6KB2	0.548837243	0.15822824	3.468642788	0.000523094	0.000991404	positive
PRJNA729271	stage_E18_vs_E10	ordinary_muscle_development	ENSGALG00010022425	VWA2	-1.483467362	0.334414615	-4.436012353	9.16406E-06	2.1404E-05	negative
PRJNA729271	stage_E18_vs_E10	ordinary_muscle_development	ENSGALG00010022426	EXD1	-1.34495424	0.24499065	-5.489818654	4.02347E-08	1.18224E-07	negative
PRJNA729271	stage_E18_vs_E10	ordinary_muscle_development	ENSGALG00010022427	RPL27	1.069606745	0.138626429	7.715749105	1.20274E-14	5.96431E-14	positive
PRJNA729271	stage_E18_vs_E10	ordinary_muscle_development	ENSGALG00010022429	ADK	0.8185088	0.130062749	6.293183921	3.11019E-10	1.0937E-09	positive
PRJNA729271	stage_E18_vs_E10	ordinary_muscle_development	ENSGALG00010022431	LARP4	-0.595181234	0.133277476	-4.46573007	7.97962E-06	1.87737E-05	negative
PRJNA729271	stage_E18_vs_E10	ordinary_muscle_development	ENSGALG00010022433	LNPK	0.844743852	0.108061032	7.817284668	5.39749E-15	2.75191E-14	positive
PRJNA729271	stage_E18_vs_E10	ordinary_muscle_development	ENSGALG00010022435	PIGK	0.196912353	0.164058611	1.200256129	0.230039882	0.28169537	positive
PRJNA729271	stage_E18_vs_E10	ordinary_muscle_development	ENSGALG00010022436	AFAP1L2	0.748300595	0.154890505	4.831158609	1.35741E-06	3.45915E-06	positive
PRJNA729271	stage_E18_vs_E10	ordinary_muscle_development	ENSGALG00010022438	RPN2	-0.789224595	0.101463873	-7.778380318	7.3459E-15	3.70748E-14	negative
PRJNA729271	stage_E18_vs_E10	ordinary_muscle_development	ENSGALG00010022439	IFI35	0.248200462	0.208491427	1.19045884	0.233866098	0.286018317	positive
PRJNA729271	stage_E18_vs_E10	ordinary_muscle_development	ENSGALG00010022440	SORD	1.420734269	0.123163146	11.53538465	8.74952E-31	1.11249E-29	positive
PRJNA729271	stage_E18_vs_E10	ordinary_muscle_development	ENSGALG00010022441	FICD	-0.018839788	0.121122959	-0.155542666	0.876393521	0.897850677	negative
PRJNA729271	stage_E18_vs_E10	ordinary_muscle_development	ENSGALG00010022448	FERRY3	-0.495444223	0.121274274	-4.08532006	4.40161E-05	9.53112E-05	negative
PRJNA729271	stage_E18_vs_E10	ordinary_muscle_development	ENSGALG00010022449	ENSGALG00010022449	0.043543879	0.101507515	0.428971971	0.667943623	0.715053638	positive
PRJNA729271	stage_E18_vs_E10	ordinary_muscle_development	ENSGALG00010022451	CHP1	-0.590095909	0.121915773	-4.840193297	1.29713E-06	3.31603E-06	negative
PRJNA729271	stage_E18_vs_E10	ordinary_muscle_development	ENSGALG00010022452	AP3M1	0.744528499	0.081063305	9.18453178	4.13321E-20	2.91964E-19	positive
PRJNA729271	stage_E18_vs_E10	ordinary_muscle_development	ENSGALG00010022453	ENSGALG00010022453	1.296374915	0.23880036	5.428697486	5.67668E-08	1.64075E-07	positive
PRJNA729271	stage_E18_vs_E10	ordinary_muscle_development	ENSGALG00010022454	ABLIM1	2.85359404	0.140116409	20.36588051	3.35763E-92	3.33348E-90	positive
PRJNA729271	stage_E18_vs_E10	ordinary_muscle_development	ENSGALG00010022455	C7orf50	-1.607920439	0.158280194	-10.15869638	3.03102E-24	2.73907E-23	negative
PRJNA729271	stage_E18_vs_E10	ordinary_muscle_development	ENSGALG00010022456	ATP5G3	-0.015502321	0.158331843	-0.097910317	0.922003507	0.935885213	negative
PRJNA729271	stage_E18_vs_E10	ordinary_muscle_development	ENSGALG00010022459	FGF6	3.429669517	0.731885724	4.686072436	2.78498E-06	6.85137E-06	positive
PRJNA729271	stage_E18_vs_E10	ordinary_muscle_development	ENSGALG00010022460	VAT1	-0.285003055	0.116798974	-2.440116092	0.014682543	0.022698747	negative
PRJNA729271	stage_E18_vs_E10	ordinary_muscle_development	ENSGALG00010022462	ITGA5	0.588709906	0.129117033	4.559506137	5.12741E-06	1.22907E-05	positive
PRJNA729271	stage_E18_vs_E10	ordinary_muscle_development	ENSGALG00010022463	DUSP12	0.280962009	0.116933225	2.402756011	0.01627204	0.024959071	positive
PRJNA729271	stage_E18_vs_E10	ordinary_muscle_development	ENSGALG00010022466	NEDD4	-0.478750384	0.138583565	-3.454597113	0.000551116	0.001041108	negative
PRJNA729271	stage_E18_vs_E10	ordinary_muscle_development	ENSGALG00010022467	SART3	-0.925908145	0.117341385	-7.890721107	3.00445E-15	1.56036E-14	negative
PRJNA729271	stage_E18_vs_E10	ordinary_muscle_development	ENSGALG00010022468	MYBPC3	0.225200998	0.42925673	0.524630092	0.599840364	0.651287346	positive
PRJNA729271	stage_E18_vs_E10	ordinary_muscle_development	ENSGALG00010022470	ENSGALG00010022470	-0.098104525	0.174013393	-0.563775716	0.57290678	0.626265745	negative
PRJNA729271	stage_E18_vs_E10	ordinary_muscle_development	ENSGALG00010022471	ST6GALNAC5	-0.293281403	0.13784673	-2.12759057	0.033371045	0.048347156	negative
PRJNA729271	stage_E18_vs_E10	ordinary_muscle_development	ENSGALG00010022472	QSER1	-0.067518985	0.088274109	-0.764878689	0.444343785	0.503774983	negative
PRJNA729271	stage_E18_vs_E10	ordinary_muscle_development	ENSGALG00010022473	ZNF385A	1.584004647	0.423825711	3.737396304	0.000185936	0.000374325	positive
PRJNA729271	stage_E18_vs_E10	ordinary_muscle_development	ENSGALG00010022474	VCL	-0.016137108	0.119749399	-0.134757316	0.892803755	0.911223097	negative
PRJNA729271	stage_E18_vs_E10	ordinary_muscle_development	ENSGALG00010022476	OIP5	-1.12182886	0.115146561	-9.742617164	1.98378E-22	1.62E-21	negative
PRJNA729271	stage_E18_vs_E10	ordinary_muscle_development	ENSGALG00010022478	ATP6V1A	0.329011536	0.096375208	3.413860706	0.000640493	0.001199012	positive
PRJNA729271	stage_E18_vs_E10	ordinary_muscle_development	ENSGALG00010022479	ENSGALG00010022479	2.982316762	0.635078904	4.695978319	2.65334E-06	6.54306E-06	positive
PRJNA729271	stage_E18_vs_E10	ordinary_muscle_development	ENSGALG00010022480	PIF1	-2.191006824	0.251253579	-8.720300951	2.77463E-18	1.77017E-17	negative
PRJNA729271	stage_E18_vs_E10	ordinary_muscle_development	ENSGALG00010022481	CORO1B	-0.894614979	0.152628836	-5.861375895	4.59047E-09	1.46368E-08	negative
PRJNA729271	stage_E18_vs_E10	ordinary_muscle_development	ENSGALG00010022482	PRTG	-1.269028995	0.327690526	-3.872644754	0.000107661	0.000223798	negative
PRJNA729271	stage_E18_vs_E10	ordinary_muscle_development	ENSGALG00010022483	TOP2A	-2.155659728	0.238751177	-9.028896751	1.73411E-19	1.18454E-18	negative
PRJNA729271	stage_E18_vs_E10	ordinary_muscle_development	ENSGALG00010022485	ATF2	-0.350855399	0.058311617	-6.016903962	1.77784E-09	5.87013E-09	negative
PRJNA729271	stage_E18_vs_E10	ordinary_muscle_development	ENSGALG00010022486	FAM183A	-1.424365789	0.661588558	-2.152948038	0.031322763	0.045616743	negative
PRJNA729271	stage_E18_vs_E10	ordinary_muscle_development	ENSGALG00010022489	NUSAP1	-1.238959877	0.23886154	-5.186937483	2.1378E-07	5.87552E-07	negative
PRJNA729271	stage_E18_vs_E10	ordinary_muscle_development	ENSGALG00010022493	ENSGALG00010022493	0.185580401	0.161575553	1.148567323	0.250734438	0.304381169	positive
PRJNA729271	stage_E18_vs_E10	ordinary_muscle_development	ENSGALG00010022494	FHIP2A	-0.18805528	0.06652656	-2.826769931	0.00470201	0.007818703	negative
PRJNA729271	stage_E18_vs_E10	ordinary_muscle_development	ENSGALG00010022496	TUBGCP2	0.252522228	0.072155287	3.499705112	0.000465773	0.000890331	positive
PRJNA729271	stage_E18_vs_E10	ordinary_muscle_development	ENSGALG00010022497	CORO1C	-0.653666358	0.149950667	-4.359209409	1.30533E-05	3.00378E-05	negative
PRJNA729271	stage_E18_vs_E10	ordinary_muscle_development	ENSGALG00010022498	NAA50	0.004549502	0.113936416	0.039930185	0.968148786	0.973656883	positive
PRJNA729271	stage_E18_vs_E10	ordinary_muscle_development	ENSGALG00010022500	ENSGALG00010022500	1.688278137	0.404288952	4.175919548	2.96785E-05	6.54878E-05	positive
PRJNA729271	stage_E18_vs_E10	ordinary_muscle_development	ENSGALG00010022502	ENSGALG00010022502	-0.572883964	0.452621491	-1.265702082	0.205619763	0.255088879	negative
PRJNA729271	stage_E18_vs_E10	ordinary_muscle_development	ENSGALG00010022504	RAD9A	0.258301815	0.148931591	1.734365513	0.08285325	0.111644312	positive
PRJNA729271	stage_E18_vs_E10	ordinary_muscle_development	ENSGALG00010022505	ZNF511	0.500147736	0.12427404	4.024555209	5.70831E-05	0.000122255	positive
PRJNA729271	stage_E18_vs_E10	ordinary_muscle_development	ENSGALG00010022506	EBNA1BP2	-1.026580229	0.086926849	-11.8097026	3.47789E-32	4.68079E-31	negative
PRJNA729271	stage_E18_vs_E10	ordinary_muscle_development	ENSGALG00010022507	ENSGALG00010022507	-0.24511929	0.216140157	-1.134075657	0.256762814	0.310691793	negative
PRJNA729271	stage_E18_vs_E10	ordinary_muscle_development	ENSGALG00010022508	USF3	0.58650745	0.122777625	4.776989687	1.77939E-06	4.47549E-06	positive
PRJNA729271	stage_E18_vs_E10	ordinary_muscle_development	ENSGALG00010022511	IGFBP4	0.376591705	0.271383302	1.387674562	0.165236166	0.209306844	positive
PRJNA729271	stage_E18_vs_E10	ordinary_muscle_development	ENSGALG00010022512	PRAP1	1.28437636	0.876543667	1.465273674	0.142846266	0.183706559	positive
PRJNA729271	stage_E18_vs_E10	ordinary_muscle_development	ENSGALG00010022513	NDUFAF1	-0.21267311	0.085671275	-2.482431947	0.013048899	0.020303112	negative
PRJNA729271	stage_E18_vs_E10	ordinary_muscle_development	ENSGALG00010022515	CHRNA1	-1.402596314	0.170952619	-8.20459099	2.31377E-16	1.29441E-15	negative
PRJNA729271	stage_E18_vs_E10	ordinary_muscle_development	ENSGALG00010022516	TRUB1	-0.596753685	0.153133641	-3.896947014	9.74129E-05	0.00020337	negative
PRJNA729271	stage_E18_vs_E10	ordinary_muscle_development	ENSGALG00010022517	HNRNPA1	0.009430245	0.181380303	0.051991562	0.958535417	0.965798058	positive
PRJNA729271	stage_E18_vs_E10	ordinary_muscle_development	ENSGALG00010022518	ECHS1	0.987487004	0.111896527	8.825001385	1.09458E-18	7.14363E-18	positive
PRJNA729271	stage_E18_vs_E10	ordinary_muscle_development	ENSGALG00010022520	TMOD2	-1.416838565	0.202561172	-6.994620694	2.65978E-12	1.1009E-11	negative
PRJNA729271	stage_E18_vs_E10	ordinary_muscle_development	ENSGALG00010022521	RTF1	-0.455761856	0.091434075	-4.984595241	6.20917E-07	1.63551E-06	negative
PRJNA729271	stage_E18_vs_E10	ordinary_muscle_development	ENSGALG00010022522	TNS4	-0.854727092	0.353443348	-2.418285976	0.015593816	0.024005136	negative
PRJNA729271	stage_E18_vs_E10	ordinary_muscle_development	ENSGALG00010022524	SOX18	1.099545974	0.228753279	4.806689457	1.5345E-06	3.8872E-06	positive
PRJNA729271	stage_E18_vs_E10	ordinary_muscle_development	ENSGALG00010022525	ISCU	-0.067982721	0.195010809	-0.348610013	0.72738211	0.769647688	negative
PRJNA729271	stage_E18_vs_E10	ordinary_muscle_development	ENSGALG00010022526	PPP1CA	0.262962268	0.137800565	1.9082815	0.056354843	0.078381979	positive
PRJNA729271	stage_E18_vs_E10	ordinary_muscle_development	ENSGALG00010022528	SIDT1	-1.728421891	0.458640413	-3.768577394	0.000164181	0.000332699	negative
PRJNA729271	stage_E18_vs_E10	ordinary_muscle_development	ENSGALG00010022529	CCR7	-0.810623456	0.36179184	-2.240579708	0.025053312	0.037158269	negative
PRJNA729271	stage_E18_vs_E10	ordinary_muscle_development	ENSGALG00010022531	ENSGALG00010022531	-2.048900948	0.485006633	-4.224480261	2.39493E-05	5.34645E-05	negative
PRJNA729271	stage_E18_vs_E10	ordinary_muscle_development	ENSGALG00010022532	CBX5	-2.207007859	0.175794932	-12.55444533	3.75805E-36	6.12056E-35	negative
PRJNA729271	stage_E18_vs_E10	ordinary_muscle_development	ENSGALG00010022533	TMEM119	-0.371285378	0.132770785	-2.796438823	0.005166919	0.008548624	negative
PRJNA729271	stage_E18_vs_E10	ordinary_muscle_development	ENSGALG00010022534	CFAP57	-0.436760299	0.518970088	-0.841590506	0.400017206	0.459886532	negative
PRJNA729271	stage_E18_vs_E10	ordinary_muscle_development	ENSGALG00010022536	SMUG1	0.369462911	0.099981295	3.695320331	0.00021961	0.00043786	positive
PRJNA729271	stage_E18_vs_E10	ordinary_muscle_development	ENSGALG00010022538	WIPF1	-0.437681609	0.159356018	-2.746564675	0.006022301	0.009854736	negative
PRJNA729271	stage_E18_vs_E10	ordinary_muscle_development	ENSGALG00010022539	LYSMD2	0.698607745	0.102028346	6.847192722	7.53132E-12	2.99332E-11	positive
PRJNA729271	stage_E18_vs_E10	ordinary_muscle_development	ENSGALG00010022540	GPR146	1.162564055	0.238844948	4.867442524	1.13052E-06	2.90547E-06	positive
PRJNA729271	stage_E18_vs_E10	ordinary_muscle_development	ENSGALG00010022541	ITPKA	-1.363184381	0.126687135	-10.76024317	5.303E-27	5.62305E-26	negative
PRJNA729271	stage_E18_vs_E10	ordinary_muscle_development	ENSGALG00010022542	ENSGALG00010022542	1.090992097	0.722016421	1.511035021	0.130779528	0.169511693	positive
PRJNA729271	stage_E18_vs_E10	ordinary_muscle_development	ENSGALG00010022544	SPICE1	0.458146349	0.068628121	6.675781664	2.45918E-11	9.39283E-11	positive
PRJNA729271	stage_E18_vs_E10	ordinary_muscle_development	ENSGALG00010022545	CSTF3	-0.472789526	0.067200368	-7.035519921	1.98519E-12	8.29262E-12	negative
PRJNA729271	stage_E18_vs_E10	ordinary_muscle_development	ENSGALG00010022546	MTG1	0.745350006	0.158511158	4.702192675	2.57382E-06	6.3578E-06	positive
PRJNA729271	stage_E18_vs_E10	ordinary_muscle_development	ENSGALG00010022547	SMARCE1	-0.715475444	0.061948034	-11.549607	7.41588E-31	9.46242E-30	negative
PRJNA729271	stage_E18_vs_E10	ordinary_muscle_development	ENSGALG00010022548	ENSGALG00010022548	1.258204406	0.11284141	11.150201	7.14448E-29	8.2649E-28	positive
PRJNA729271	stage_E18_vs_E10	ordinary_muscle_development	ENSGALG00010022550	TRIM45	0.532631591	0.160402329	3.32059762	0.000898249	0.001646658	positive
PRJNA729271	stage_E18_vs_E10	ordinary_muscle_development	ENSGALG00010022551	GPER1	-1.352081748	0.632813376	-2.13662005	0.032628908	0.047361908	negative
PRJNA729271	stage_E18_vs_E10	ordinary_muscle_development	ENSGALG00010022552	GPR155	-0.356805205	0.107447062	-3.320753473	0.000897748	0.001645953	negative
PRJNA729271	stage_E18_vs_E10	ordinary_muscle_development	ENSGALG00010022553	PRPF6	-0.391377432	0.169923672	-2.30325432	0.021264538	0.031921032	negative
PRJNA729271	stage_E18_vs_E10	ordinary_muscle_development	ENSGALG00010022554	SCG3	-0.974797844	0.348408894	-2.797855797	0.005144307	0.008515101	negative
PRJNA729271	stage_E18_vs_E10	ordinary_muscle_development	ENSGALG00010022557	WNT1	-0.02777287	0.400322577	-0.069376227	0.944690152	0.954834652	negative
PRJNA729271	stage_E18_vs_E10	ordinary_muscle_development	ENSGALG00010022558	NYAP2	-0.367214815	0.512576564	-0.716409687	0.473738415	0.532509105	negative
PRJNA729271	stage_E18_vs_E10	ordinary_muscle_development	ENSGALG00010022559	SSH1	0.017220359	0.088171289	0.195305742	0.845153602	0.871797713	positive
PRJNA729271	stage_E18_vs_E10	ordinary_muscle_development	ENSGALG00010022560	SAMD10	-3.361293661	1.448819092	-2.320023031	0.020339631	0.030634139	negative
PRJNA729271	stage_E18_vs_E10	ordinary_muscle_development	ENSGALG00010022562	GHRH	-4.70985688	1.216494837	-3.871662036	0.000108096	0.000224664	negative
PRJNA729271	stage_E18_vs_E10	ordinary_muscle_development	ENSGALG00010022565	ARF3	-0.768537545	0.168181676	-4.569686561	4.88454E-06	1.1726E-05	negative
PRJNA729271	stage_E18_vs_E10	ordinary_muscle_development	ENSGALG00010022567	PAOX	0.612073356	0.125151967	4.890641123	1.00508E-06	2.59598E-06	positive
PRJNA729271	stage_E18_vs_E10	ordinary_muscle_development	ENSGALG00010022568	SCRN3	0.799977614	0.16803807	4.760692702	1.9293E-06	4.8299E-06	positive
PRJNA729271	stage_E18_vs_E10	ordinary_muscle_development	ENSGALG00010022569	KIAA1549L	-2.926870973	0.252929184	-11.57189898	5.72024E-31	7.35713E-30	negative
PRJNA729271	stage_E18_vs_E10	ordinary_muscle_development	ENSGALG00010022571	RIPOR1	0.803519127	0.107734059	7.458357488	8.76077E-14	4.04805E-13	positive
PRJNA729271	stage_E18_vs_E10	ordinary_muscle_development	ENSGALG00010022572	ENSGALG00010022572	-3.349624583	0.362740095	-9.23422757	2.60156E-20	1.86866E-19	negative
PRJNA729271	stage_E18_vs_E10	ordinary_muscle_development	ENSGALG00010022573	AP4E1	-0.001825987	0.082121594	-0.022235168	0.982260364	0.985319306	negative
PRJNA729271	stage_E18_vs_E10	ordinary_muscle_development	ENSGALG00010022574	ITPRIPL2	0.387991578	0.107574303	3.606731051	0.000310079	0.000605903	positive
PRJNA729271	stage_E18_vs_E10	ordinary_muscle_development	ENSGALG00010022575	TMEM41B	-1.151389632	0.11191984	-10.28762754	8.01263E-25	7.53197E-24	negative
PRJNA729271	stage_E18_vs_E10	ordinary_muscle_development	ENSGALG00010022578	ZNF512B	0.307542534	0.134682082	2.28347029	0.022402681	0.033490807	positive
PRJNA729271	stage_E18_vs_E10	ordinary_muscle_development	ENSGALG00010022579	TNFAIP8L3	-0.90826501	0.148598585	-6.11220499	9.82638E-10	3.31858E-09	negative
PRJNA729271	stage_E18_vs_E10	ordinary_muscle_development	ENSGALG00010022581	TMEM125	-1.379860967	0.621622165	-2.219774399	0.026434086	0.039018541	negative
PRJNA729271	stage_E18_vs_E10	ordinary_muscle_development	ENSGALG00010022582	ENSGALG00010022582	-0.512644988	0.223035343	-2.298492163	0.021533793	0.03228176	negative
PRJNA729271	stage_E18_vs_E10	ordinary_muscle_development	ENSGALG00010022583	FKBP11	0.839097709	0.116027559	7.231882826	4.76343E-13	2.08787E-12	positive
PRJNA729271	stage_E18_vs_E10	ordinary_muscle_development	ENSGALG00010022585	CIR1	-0.643731079	0.096226044	-6.689780125	2.23506E-11	8.56616E-11	negative
PRJNA729271	stage_E18_vs_E10	ordinary_muscle_development	ENSGALG00010022587	ENSGALG00010022587	-3.234483206	1.003801419	-3.222234144	0.001271951	0.002288926	negative
PRJNA729271	stage_E18_vs_E10	ordinary_muscle_development	ENSGALG00010022588	TCP11L1	-0.589811459	0.080182586	-7.355854747	1.89709E-13	8.53721E-13	negative
PRJNA729271	stage_E18_vs_E10	ordinary_muscle_development	ENSGALG00010022590	ENSGALG00010022590	-0.357562875	0.159219878	-2.245717552	0.024722105	0.03670459	negative
PRJNA729271	stage_E18_vs_E10	ordinary_muscle_development	ENSGALG00010022592	CCDC65	0.708688143	0.380212314	1.863927384	0.062331903	0.085941304	positive
PRJNA729271	stage_E18_vs_E10	ordinary_muscle_development	ENSGALG00010022593	MANBAL	-1.261238347	0.139630307	-9.03269765	1.67491E-19	1.14626E-18	negative

PRJNA729271	stage_E18_vs_E10	ordinary_muscle_development	ENSGALG00010022597	SLCO4A1	0.297709057	0.257617698	1.155623464	0.247835206	0.301095576	positive
PRJNA729271	stage_E18_vs_E10	ordinary_muscle_development	ENSGALG00010022599	RNLS	-1.372176677	0.283030677	-4.848155297	1.24615E-06	3.19189E-06	negative
PRJNA729271	stage_E18_vs_E10	ordinary_muscle_development	ENSGALG00010022603	ATRNL1	0.172354141	0.16927176	1.018209659	0.30857833	0.366116305	positive
PRJNA729271	stage_E18_vs_E10	ordinary_muscle_development	ENSGALG00010022608	P3H4	-0.367256083	0.205593433	-1.786322054	0.074047128	0.100733282	negative
PRJNA729271	stage_E18_vs_E10	ordinary_muscle_development	ENSGALG00010022609	RBBP8NL	-7.819256307	1.061065745	-7.369247705	1.71594E-13	7.75811E-13	negative
PRJNA729271	stage_E18_vs_E10	ordinary_muscle_development	ENSGALG00010022614	DAO	0.478368088	0.199723794	2.395148206	0.016613648	0.025423863	positive
PRJNA729271	stage_E18_vs_E10	ordinary_muscle_development	ENSGALG00010022616	GLDN	3.136850614	0.332265203	9.440803869	3.6993E-21	2.80887E-20	positive
PRJNA729271	stage_E18_vs_E10	ordinary_muscle_development	ENSGALG00010022619	MADD	-0.647223408	0.133823422	-4.836398585	1.32213E-06	3.37549E-06	negative
PRJNA729271	stage_E18_vs_E10	ordinary_muscle_development	ENSGALG00010022622	MPPED2	-0.21938538	0.134607204	-1.629819008	0.103139755	0.136381204	negative
PRJNA729271	stage_E18_vs_E10	ordinary_muscle_development	ENSGALG00010022623	TOP1	-0.397682369	0.126185019	-3.151581483	0.001623888	0.002887068	negative
PRJNA729271	stage_E18_vs_E10	ordinary_muscle_development	ENSGALG00010022625	ENSGALG00010022625	-0.001144085	0.125648669	-0.009105425	0.992735023	0.993763408	negative
PRJNA729271	stage_E18_vs_E10	ordinary_muscle_development	ENSGALG00010022627	CTCF	-0.61487396	0.098738854	-6.227274651	4.74619E-10	1.65176E-09	negative
PRJNA729271	stage_E18_vs_E10	ordinary_muscle_development	ENSGALG00010022628	SVOP	0.09339169	0.591256484	0.157954614	0.874492562	0.896511047	positive
PRJNA729271	stage_E18_vs_E10	ordinary_muscle_development	ENSGALG00010022630	ENSGALG00010022630	-2.184116315	0.29188566	-7.482780456	7.27664E-14	3.38494E-13	negative
PRJNA729271	stage_E18_vs_E10	ordinary_muscle_development	ENSGALG00010022637	DMXL2	-0.907232533	0.11133499	-8.148673936	3.67937E-16	2.03094E-15	negative
PRJNA729271	stage_E18_vs_E10	ordinary_muscle_development	ENSGALG00010022642	SEC13	-0.114877441	0.068524316	-1.676447832	0.093650506	0.124893191	negative
PRJNA729271	stage_E18_vs_E10	ordinary_muscle_development	ENSGALG00010022643	SP3	0.141149653	0.076883904	1.835880412	0.066375345	0.091091709	positive
PRJNA729271	stage_E18_vs_E10	ordinary_muscle_development	ENSGALG00010022644	ARMC8	0.503190169	0.085625207	5.876659279	4.18629E-09	1.33834E-08	positive
PRJNA729271	stage_E18_vs_E10	ordinary_muscle_development	ENSGALG00010022645	ENSGALG00010022645	0.19517874	0.637152552	0.306329684	0.75935364	0.798116961	positive
PRJNA729271	stage_E18_vs_E10	ordinary_muscle_development	ENSGALG00010022647	SVIP	-0.259775253	0.133996882	-1.93866641	0.052541971	0.073470565	negative
PRJNA729271	stage_E18_vs_E10	ordinary_muscle_development	ENSGALG00010022649	PATZ1	-1.166067138	0.08205998	-14.2099369	7.94971E-46	1.93666E-44	negative
PRJNA729271	stage_E18_vs_E10	ordinary_muscle_development	ENSGALG00010022650	NR1H3	0.267730407	0.099757574	2.683810333	0.007278839	0.011747775	positive
PRJNA729271	stage_E18_vs_E10	ordinary_muscle_development	ENSGALG00010022652	CARMIL2	-0.834389968	0.279189776	-2.988612196	0.002802476	0.004822734	negative
PRJNA729271	stage_E18_vs_E10	ordinary_muscle_development	ENSGALG00010022655	NRIP3	-7.771749549	0.582270112	-13.34732694	1.22776E-40	2.41471E-39	negative
PRJNA729271	stage_E18_vs_E10	ordinary_muscle_development	ENSGALG00010022656	FKBP10	-0.126037597	0.210669774	-0.59827091	0.549659184	0.603812798	negative
PRJNA729271	stage_E18_vs_E10	ordinary_muscle_development	ENSGALG00010022657	MRPS35	0.135738237	0.112235384	1.209406803	0.226506596	0.277955733	positive
PRJNA729271	stage_E18_vs_E10	ordinary_muscle_development	ENSGALG00010022659	TIE1	1.145081517	0.151176046	7.574490443	3.6054E-14	1.71233E-13	positive
PRJNA729271	stage_E18_vs_E10	ordinary_muscle_development	ENSGALG00010022660	METAP1D	0.417694475	0.189802167	2.200683382	0.027758446	0.04080303	positive
PRJNA729271	stage_E18_vs_E10	ordinary_muscle_development	ENSGALG00010022661	ENSGALG00010022661	-0.488619391	0.382147306	-1.278615299	0.20103256	0.250018615	negative
PRJNA729271	stage_E18_vs_E10	ordinary_muscle_development	ENSGALG00010022662	GFRA1	-2.081619624	0.149789745	-13.89694349	6.61045E-44	1.48556E-42	negative
PRJNA729271	stage_E18_vs_E10	ordinary_muscle_development	ENSGALG00010022664	TRPC4AP	0.169025868	0.097869626	1.727051325	0.084158446	0.113224121	positive
PRJNA729271	stage_E18_vs_E10	ordinary_muscle_development	ENSGALG00010022666	ENSGALG00010022666	-0.734768699	0.10588719	-6.939165174	3.94424E-12	1.6132E-11	negative
PRJNA729271	stage_E18_vs_E10	ordinary_muscle_development	ENSGALG00010022667	PTEN	0.035254452	0.042927673	0.821252333	0.411502553	0.471669263	positive
PRJNA729271	stage_E18_vs_E10	ordinary_muscle_development	ENSGALG00010022669	MANSC4	-5.900435941	1.138165471	-5.184163543	2.16986E-07	5.96024E-07	negative
PRJNA729271	stage_E18_vs_E10	ordinary_muscle_development	ENSGALG00010022671	DBR1	-0.711453962	0.057886299	-12.29054159	1.01822E-34	1.54707E-33	negative
PRJNA729271	stage_E18_vs_E10	ordinary_muscle_development	ENSGALG00010022673	KLHL42	0.54583473	0.148724698	3.670101458	0.000242454	0.000481009	positive
PRJNA729271	stage_E18_vs_E10	ordinary_muscle_development	ENSGALG00010022674	ENSGALG00010022674	2.230908893	0.338854603	6.58367593	4.58957E-11	1.71946E-10	positive
PRJNA729271	stage_E18_vs_E10	ordinary_muscle_development	ENSGALG00010022681	PPP2R3A	0.895073823	0.093293945	9.594125561	8.46317E-22	6.6925E-21	positive
PRJNA729271	stage_E18_vs_E10	ordinary_muscle_development	ENSGALG00010022682	EIF1	0.295583524	0.156317385	1.890919065	0.058635149	0.081323939	positive
PRJNA729271	stage_E18_vs_E10	ordinary_muscle_development	ENSGALG00010022684	ENSGALG00010022684	-0.705005238	0.536570313	-1.313910257	0.188876407	0.236212068	negative
PRJNA729271	stage_E18_vs_E10	ordinary_muscle_development	ENSGALG00010022685	ATAD1	-0.339144779	0.109950295	-3.084528148	0.002038753	0.003576825	negative
PRJNA729271	stage_E18_vs_E10	ordinary_muscle_development	ENSGALG00010022689	MPL	0.307573084	0.429723165	0.715747042	0.474147558	0.532814083	positive
PRJNA729271	stage_E18_vs_E10	ordinary_muscle_development	ENSGALG00010022691	ASIC2	-4.805094074	0.728647655	-6.594537212	4.26584E-11	1.60107E-10	negative
PRJNA729271	stage_E18_vs_E10	ordinary_muscle_development	ENSGALG00010022695	PIK3IP1	1.5646852	0.202837754	7.713974202	1.21959E-14	6.04582E-14	positive
PRJNA729271	stage_E18_vs_E10	ordinary_muscle_development	ENSGALG00010022698	SEMA6D	1.098539646	0.116910759	9.396394773	5.64647E-21	4.2473E-20	positive
PRJNA729271	stage_E18_vs_E10	ordinary_muscle_development	ENSGALG00010022702	CDC20	-1.900154594	0.23302004	-8.154468561	3.50719E-16	1.93959E-15	negative
PRJNA729271	stage_E18_vs_E10	ordinary_muscle_development	ENSGALG00010022707	SALL1	-2.903651671	0.471334446	-6.160491126	7.25197E-10	2.48504E-09	negative
PRJNA729271	stage_E18_vs_E10	ordinary_muscle_development	ENSGALG00010022708	SPRED1	-0.571780967	0.131112214	-4.361004597	1.29467E-05	2.98018E-05	negative
PRJNA729271	stage_E18_vs_E10	ordinary_muscle_development	ENSGALG00010022709	RNF5	-0.297721266	0.085164349	-3.495843858	0.000472565	0.000901659	negative
PRJNA729271	stage_E18_vs_E10	ordinary_muscle_development	ENSGALG00010022711	LRRC49	0.474173053	0.18981408	2.498092094	0.012486375	0.019478046	positive
PRJNA729271	stage_E18_vs_E10	ordinary_muscle_development	ENSGALG00010022712	DDX23	-0.189380468	0.201992478	-0.937561986	0.348469555	0.407278358	negative
PRJNA729271	stage_E18_vs_E10	ordinary_muscle_development	ENSGALG00010022713	PNLIPRP1	1.777787585	2.897046189	0.613655243	0.53944317	0.594553258	positive
PRJNA729271	stage_E18_vs_E10	ordinary_muscle_development	ENSGALG00010022717	ELOVL1	0.096573107	0.171602601	0.562771815	0.573590275	0.626871073	positive
PRJNA729271	stage_E18_vs_E10	ordinary_muscle_development	ENSGALG00010022718	C12orf43	-0.201848672	0.13562826	-1.488249362	0.136685144	0.176534898	negative
PRJNA729271	stage_E18_vs_E10	ordinary_muscle_development	ENSGALG00010022719	MSL2	-0.078100196	0.098336138	-0.794216628	0.42706933	0.487162189	negative
PRJNA729271	stage_E18_vs_E10	ordinary_muscle_development	ENSGALG00010022724	LUZP2	-1.726019776	0.410887347	-4.200712888	2.66076E-05	5.90623E-05	negative
PRJNA729271	stage_E18_vs_E10	ordinary_muscle_development	ENSGALG00010022725	KRT222	-5.925463988	0.91234631	-6.49475306	8.31697E-11	3.05201E-10	negative
PRJNA729271	stage_E18_vs_E10	ordinary_muscle_development	ENSGALG00010022727	ACP2	-0.120573087	0.085076512	-1.417231203	0.156415342	0.199352886	negative
PRJNA729271	stage_E18_vs_E10	ordinary_muscle_development	ENSGALG00010022729	ENSGALG00010022729	-0.830462752	0.135587532	-6.12491977	9.07293E-10	3.07991E-09	negative
PRJNA729271	stage_E18_vs_E10	ordinary_muscle_development	ENSGALG00010022731	LRRC8B	-1.543400127	0.182736606	-8.446036947	3.01358E-17	1.79098E-16	negative
PRJNA729271	stage_E18_vs_E10	ordinary_muscle_development	ENSGALG00010022733	PCCB	0.36552651	0.115323228	3.169582728	0.00152658	0.002721409	positive
PRJNA729271	stage_E18_vs_E10	ordinary_muscle_development	ENSGALG00010022734	ENSGALG00010022734	0.453228166	0.119404895	3.795725179	0.000147213	0.000300499	positive
PRJNA729271	stage_E18_vs_E10	ordinary_muscle_development	ENSGALG00010022737	CYLD	0.251859949	0.080194216	3.140624849	0.001685878	0.002992506	positive
PRJNA729271	stage_E18_vs_E10	ordinary_muscle_development	ENSGALG00010022742	IFT46	-0.31343658	0.123927971	-2.529183512	0.011432823	0.017944642	negative
PRJNA729271	stage_E18_vs_E10	ordinary_muscle_development	ENSGALG00010022743	ZNF326	-1.456136011	0.153240849	-9.502270557	2.05363E-21	1.58421E-20	negative
PRJNA729271	stage_E18_vs_E10	ordinary_muscle_development	ENSGALG00010022745	PAPSS2	-0.839085579	0.238613388	-3.516506709	0.000437266	0.00083816	negative
PRJNA729271	stage_E18_vs_E10	ordinary_muscle_development	ENSGALG00010022747	NT5C3B	-0.950215319	0.141545339	-6.713151598	1.90465E-11	7.325E-11	negative
PRJNA729271	stage_E18_vs_E10	ordinary_muscle_development	ENSGALG00010022748	ENSGALG00010022748	-0.502646859	0.330976868	-1.518676703	0.128843894	0.167346975	negative
PRJNA729271	stage_E18_vs_E10	ordinary_muscle_development	ENSGALG00010022749	HSPA12A	-0.265757799	0.129288125	-2.055546857	0.039826215	0.056903005	negative
PRJNA729271	stage_E18_vs_E10	ordinary_muscle_development	ENSGALG00010022751	SLC25A12	0.330329865	0.192564069	1.715428361	0.086266719	0.11578112	positive
PRJNA729271	stage_E18_vs_E10	ordinary_muscle_development	ENSGALG00010022754	SNX20	0.620791012	0.182291006	3.405494465	0.000660444	0.001235196	positive
PRJNA729271	stage_E18_vs_E10	ordinary_muscle_development	ENSGALG00010022755	MINPP1	-0.320527268	0.115635595	-2.771873722	0.005573465	0.009172046	negative
PRJNA729271	stage_E18_vs_E10	ordinary_muscle_development	ENSGALG00010022759	THSD4	0.827125232	0.119965962	6.894665954	5.39915E-12	2.17996E-11	positive
PRJNA729271	stage_E18_vs_E10	ordinary_muscle_development	ENSGALG00010022760	ELAVL4	-2.60456055	0.135844898	-19.17304652	6.21597E-82	4.59697E-80	negative
PRJNA729271	stage_E18_vs_E10	ordinary_muscle_development	ENSGALG00010022764	MFAP5	3.184219344	0.222839719	14.28928093	2.55231E-46	6.42505E-45	positive
PRJNA729271	stage_E18_vs_E10	ordinary_muscle_development	ENSGALG00010022767	CACNB3	0.360640869	0.460827864	0.782593453	0.433865887	0.493633127	positive
PRJNA729271	stage_E18_vs_E10	ordinary_muscle_development	ENSGALG00010022768	ENSGALG00010022768	-3.334503646	0.973901128	-3.423862597	0.000617378	0.001158883	negative
PRJNA729271	stage_E18_vs_E10	ordinary_muscle_development	ENSGALG00010022769	FXYD6	2.820102723	0.096933475	29.09317664	4.3793E-186	2.0477E-183	positive
PRJNA729271	stage_E18_vs_E10	ordinary_muscle_development	ENSGALG00010022770	HAT1	-1.470536119	0.09398325	-15.64678933	3.49408E-55	1.19732E-53	negative
PRJNA729271	stage_E18_vs_E10	ordinary_muscle_development	ENSGALG00010022771	SGMS1	0.74493304	0.220146454	3.383806676	0.000714883	0.001330367	positive
PRJNA729271	stage_E18_vs_E10	ordinary_muscle_development	ENSGALG00010022776	CDH4	0.137912131	0.177231397	0.778147285	0.436482189	0.496142514	positive
PRJNA729271	stage_E18_vs_E10	ordinary_muscle_development	ENSGALG00010022780	DYNC1I2	-0.535222373	0.057998434	-9.228221139	2.75163E-20	1.97227E-19	negative
PRJNA729271	stage_E18_vs_E10	ordinary_muscle_development	ENSGALG00010022782	TMPRSS13	0.02613194	0.243792898	0.107189093	0.914638968	0.929581534	positive
PRJNA729271	stage_E18_vs_E10	ordinary_muscle_development	ENSGALG00010022783	STAG1	0.261302405	0.055003067	4.750687935	2.02726E-06	5.06116E-06	positive
PRJNA729271	stage_E18_vs_E10	ordinary_muscle_development	ENSGALG00010022784	DMRTA2	-2.75701177	0.902175761	-3.055958593	0.002243422	0.003907597	negative
PRJNA729271	stage_E18_vs_E10	ordinary_muscle_development	ENSGALG00010022786	KRT40	1.942382864	0.520432134	3.73225006	0.000189777	0.000381528	positive
PRJNA729271	stage_E18_vs_E10	ordinary_muscle_development	ENSGALG00010022787	ENO4	-0.156408716	0.29622069	-0.528014151	0.597489514	0.649221178	negative
PRJNA729271	stage_E18_vs_E10	ordinary_muscle_development	ENSGALG00010022788	CYBRD1	2.434728227	0.173269898	14.05165153	7.52583E-45	1.75381E-43	positive
PRJNA729271	stage_E18_vs_E10	ordinary_muscle_development	ENSGALG00010022789	ENSGALG00010022789	-4.405469384	0.434919018	-10.12940157	4.09146E-24	3.66311E-23	negative
PRJNA729271	stage_E18_vs_E10	ordinary_muscle_development	ENSGALG00010022792	DDB2	-0.738472712	0.126958863	-5.816629836	6.00459E-09	1.89416E-08	negative
PRJNA729271	stage_E18_vs_E10	ordinary_muscle_development	ENSGALG00010022795	TAF4	0.291143855	0.111385477	2.61384036	0.008953086	0.01426569	positive
PRJNA729271	stage_E18_vs_E10	ordinary_muscle_development	ENSGALG00010022796	RIMKLB	-0.508351198	0.135872157	-3.741393444	0.000183003	0.000368779	negative
PRJNA729271	stage_E18_vs_E10	ordinary_muscle_development	ENSGALG00010022797	EFHD1	0.705565572	0.132609971	5.320607232	1.03421E-07	2.93007E-07	positive
PRJNA729271	stage_E18_vs_E10	ordinary_muscle_development	ENSGALG00010022799	DCAF17	-0.157414127	0.154862611	-1.016475998	0.309402772	0.367064428	negative
PRJNA729271	stage_E18_vs_E10	ordinary_muscle_development	ENSGALG00010022800	KLHL10	-0.986563314	0.473070105	-2.085448445	0.037028613	0.053178416	negative
PRJNA729271	stage_E18_vs_E10	ordinary_muscle_development	ENSGALG00010022801	LSM14B	0.619091699	0.102247483	6.054835599	1.40561E-09	4.6816E-09	positive
PRJNA729271	stage_E18_vs_E10	ordinary_muscle_development	ENSGALG00010022803	ENSGALG00010022803	-0.929437865	0.180661331	-5.144641956	2.68032E-07	7.30972E-07	negative
PRJNA729271	stage_E18_vs_E10	ordinary_muscle_development	ENSGALG00010022804	SNX19	-0.440171601	0.117891362	-3.733705272	0.000188683	0.000379487	negative
PRJNA729271	stage_E18_vs_E10	ordinary_muscle_development	ENSGALG00010022805	ENSGALG00010022805	0.540641096	0.269283076	2.007705435	0.044674603	0.063253441	positive
PRJNA729271	stage_E18_vs_E10	ordinary_muscle_development	ENSGALG00010022806	SPPL3	-0.264354328	0.063630425	-4.154527118	3.25961E-05	7.15988E-05	negative
PRJNA729271	stage_E18_vs_E10	ordinary_muscle_development	ENSGALG00010022807	ADCY6	-0.303015812	0.148903304	-2.034983797	0.041852503	0.059504859	negative
PRJNA729271	stage_E18_vs_E10	ordinary_muscle_development	ENSGALG00010022808	PSMA7	-0.824099785	0.137458299	-5.995271225	2.03146E-09	6.6771E-09	negative
PRJNA729271	stage_E18_vs_E10	ordinary_muscle_development	ENSGALG00010022809	MYO1E	0.122276982	0.155469012	0.786503888	0.431572342	0.491602444	positive
PRJNA729271	stage_E18_vs_E10	ordinary_muscle_development	ENSGALG00010022811	GIGYF2	0.300933916	0.097924031	3.073136524	0.002118216	0.003707261	positive
PRJNA729271	stage_E18_vs_E10	ordinary_muscle_development	ENSGALG00010022813	USP24	0.332827912	0.122145583	2.72484606	0.006433147	0.010477356	positive
PRJNA729271	stage_E18_vs_E10	ordinary_muscle_development	ENSGALG00010022815	LRRC8D	-1.207101539	0.242801453	-4.971558126	6.64169E-07	1.74626E-06	negative
PRJNA729271	stage_E18_vs_E10	ordinary_muscle_development	ENSGALG00010022816	METTL8	-0.786260227	0.155582646	-5.053649922	4.33446E-07	1.15964E-06	negative
PRJNA729271	stage_E18_vs_E10	ordinary_muscle_development	ENSGALG00010022817	KRT23	-2.314714604	0.804578131	-2.876929555	0.004015652	0.006740028	negative
PRJNA729271	stage_E18_vs_E10	ordinary_muscle_development	ENSGALG00010022819	PACSIN3	3.933400734	0.107954273	36.43580407	1.1545E-290	2.0918E-287	positive
PRJNA729271	stage_E18_vs_E10	ordinary_muscle_development	ENSGALG00010022820	SHTN1	-0.25411681	0.167388253	-1.518128103	0.128982108	0.167496475	negative
PRJNA729271	stage_E18_vs_E10	ordinary_muscle_development	ENSGALG00010022821	ENSGALG00010022821	6.420939711	1.36847203	4.69205038			positive
PRJNA729271	stage_E18_vs_E10	ordinary_muscle_development	ENSGALG00010022823	IGHMBP2	0.257383137	0.115140539	2.235382427	0.025392253	0.037622478	positive
PRJNA729271	stage_E18_vs_E10	ordinary_muscle_development	ENSGALG00010022824	PUS1	-1.058598239	0.085349202	-12.40314161	2.51275E-35	3.92482E-34	negative
PRJNA729271	stage_E18_vs_E10	ordinary_muscle_development	ENSGALG00010022825	SS18L1	-0.275833673	0.302099669	-0.9130552	0.361213511	0.42071433	negative
PRJNA729271	stage_E18_vs_E10	ordinary_muscle_development	ENSGALG00010022826	SNORC	-4.988506499	0.536076324	-9.305590043	1.3325E-20	9.74995E-20	negative
PRJNA729271	stage_E18_vs_E10	ordinary_muscle_development	ENSGALG00010022827	CMTR2	0.04362903	0.094157915	0.4633602	0.643106202	0.692248952	positive
PRJNA729271	stage_E18_vs_E10	ordinary_muscle_development	ENSGALG00010022828	PRKG1	0.349053346	0.169276418	2.06203174	0.039204714	0.056092423	positive
PRJNA729271	stage_E18_vs_E10	ordinary_muscle_development	ENSGALG00010022830	MTG2	-0.609913551	0.132027866	-4.619581973	3.84514E-06	9.31405E-06	negative
PRJNA729271	stage_E18_vs_E10	ordinary_muscle_development	ENSGALG00010022831	LRRC8C	0.609406779	0.09024518	6.752790322	1.45028E-11	5.64194E-11	positive
PRJNA729271	stage_E18_vs_E10	ordinary_muscle_development	ENSGALG00010022833	HRH3	0.29720261	0.503845947	0.589868018	0.555279137	0.609339927	positive
PRJNA729271	stage_E18_vs_E10	ordinary_muscle_development	ENSGALG00010022834	CHRNA7	-2.469390334	0.248407796	-9.940872947	2.76394E-23	2.34838E-22	negative
PRJNA729271	stage_E18_vs_E10	ordinary_muscle_development	ENSGALG00010022837	ITGB3	-0.968074946	0.151413986	-6.39356357	1.62064E-10	5.81751E-10	negative
PRJNA729271	stage_E18_vs_E10	ordinary_muscle_development	ENSGALG00010022838	ARFGAP2	0.114078895	0.080073757	1.424672702	0.154251834	0.196820452	positive
PRJNA729271	stage_E18_vs_E10	ordinary_muscle_development	ENSGALG00010022839	SYT12	-2.334840196	0.338165657	-6.904427302	5.04064E-12	2.04089E-11	negative
PRJNA729271	stage_E18_vs_E10	ordinary_muscle_development	ENSGALG00010022840	MARVELD1	0.6311335	0.185873365	3.395502639	0.000685027	0.001277924	positive
PRJNA729271	stage_E18_vs_E10	ordinary_muscle_development	ENSGALG00010022841	ENSGALG00010022841	-3.457293079	0.324762754	-10.64559602	1.82811E-26	1.89139E-25	negative
PRJNA729271	stage_E18_vs_E10	ordinary_muscle_development	ENSGALG00010022842	PHC1	-0.537542945	0.14149074	-3.799198702	0.0001452	0.000296749	negative
PRJNA729271	stage_E18_vs_E10	ordinary_muscle_development	ENSGALG00010022845	HYDIN	1.694569955	0.331579672	5.110596636	3.21143E-07	8.70738E-07	positive
PRJNA729271	stage_E18_vs_E10	ordinary_muscle_development	ENSGALG00010022848	SGIP1	-0.783410937	0.717293543	-1.0921762	0.274755661	0.330176033	negative
PRJNA729271	stage_E18_vs_E10	ordinary_muscle_development	ENSGALG00010022850	ULK1	0.999167174	0.250690882	3.985654226	6.72945E-05	0.000143151	positive
PRJNA729271	stage_E18_vs_E10	ordinary_muscle_development	ENSGALG00010022858	HERC4	-0.126560745	0.127776211	-0.990487544	0.321935874	0.379726625	negative

PRJNA729271	stage_E18_vs_E10	ordinary_muscle_development	ENSGALG00010022860	MOB3C	-0.143727128	0.128119723	-1.121818908	0.261939445	0.316374657	negative
PRJNA729271	stage_E18_vs_E10	ordinary_muscle_development	ENSGALG00010022862	ACADS	0.847547974	0.122828324	6.900264937	5.19056E-12	2.09925E-11	positive
PRJNA729271	stage_E18_vs_E10	ordinary_muscle_development	ENSGALG00010022863	FAM81A	4.778033105	0.273991619	17.43861044	4.20166E-68	2.13695E-66	positive
PRJNA729271	stage_E18_vs_E10	ordinary_muscle_development	ENSGALG00010022864	CSTPP1	-0.453355511	0.201206237	-2.253188156	0.024247288	0.036043938	negative
PRJNA729271	stage_E18_vs_E10	ordinary_muscle_development	ENSGALG00010022866	KLHL11	-0.121584705	0.098900334	-1.229365971	0.218934623	0.269737132	negative
PRJNA729271	stage_E18_vs_E10	ordinary_muscle_development	ENSGALG00010022867	ATPAF1	-0.799666963	0.135661353	-5.894581949	3.75631E-09	1.20807E-08	negative
PRJNA729271	stage_E18_vs_E10	ordinary_muscle_development	ENSGALG00010022868	VAC14	-0.381803315	0.102170182	-3.736934842	0.000186277	0.00037496	negative
PRJNA729271	stage_E18_vs_E10	ordinary_muscle_development	ENSGALG00010022872	KRT13	-0.607700154	0.466795366	-1.301855585	0.192965758	0.240729724	negative
PRJNA729271	stage_E18_vs_E10	ordinary_muscle_development	ENSGALG00010022873	NGEF	-0.434468912	0.38014978	-1.142888764	0.253084778	0.306753396	negative
PRJNA729271	stage_E18_vs_E10	ordinary_muscle_development	ENSGALG00010022874	EFCAB14	0.525773636	0.07745758	6.787891306	1.13784E-11	4.45397E-11	positive
PRJNA729271	stage_E18_vs_E10	ordinary_muscle_development	ENSGALG00010022875	SPON1	-1.314643486	0.245043262	-5.364944434	8.09741E-08	2.31229E-07	negative
PRJNA729271	stage_E18_vs_E10	ordinary_muscle_development	ENSGALG00010022877	KYAT3	0.091928978	0.102057566	0.900756128	0.367717998	0.427294564	positive
PRJNA729271	stage_E18_vs_E10	ordinary_muscle_development	ENSGALG00010022879	ENSGALG00010022879	1.096105204	0.280033078	3.914199029	9.07048E-05	0.000190023	positive
PRJNA729271	stage_E18_vs_E10	ordinary_muscle_development	ENSGALG00010022881	CCNB1	-1.716914185	0.211450658	-8.119691867	4.67369E-16	2.5661E-15	negative
PRJNA729271	stage_E18_vs_E10	ordinary_muscle_development	ENSGALG00010022885	M6PR	0.16824315	0.099431477	1.692051195	0.090636213	0.121140793	positive
PRJNA729271	stage_E18_vs_E10	ordinary_muscle_development	ENSGALG00010022886	PLA2G15	0.875002967	0.281494577	3.108418558	0.001880915	0.003315964	positive
PRJNA729271	stage_E18_vs_E10	ordinary_muscle_development	ENSGALG00010022888	OSBPL2	0.597366035	0.091271791	6.544914111	5.95296E-11	2.20855E-10	positive
PRJNA729271	stage_E18_vs_E10	ordinary_muscle_development	ENSGALG00010022890	MYPN	4.473066691	0.166340746	26.89098616	2.7997E-159	1.0968E-156	positive
PRJNA729271	stage_E18_vs_E10	ordinary_muscle_development	ENSGALG00010022891	NTM	2.092264514	0.201731607	10.37152554	3.34147E-25	3.19912E-24	positive
PRJNA729271	stage_E18_vs_E10	ordinary_muscle_development	ENSGALG00010022900	MMP17	1.35916004	0.198279005	6.854785442	7.14196E-12	2.84247E-11	positive
PRJNA729271	stage_E18_vs_E10	ordinary_muscle_development	ENSGALG00010022911	SMPD3	-2.319749621	0.339043481	-6.842041653	7.80724E-12	3.09874E-11	negative
PRJNA729271	stage_E18_vs_E10	ordinary_muscle_development	ENSGALG00010022917	CYP4B7	-2.033895169	0.508044073	-4.003383321	6.2443E-05	0.000133281	negative
PRJNA729271	stage_E18_vs_E10	ordinary_muscle_development	ENSGALG00010022919	ACLY	-1.557555412	0.125242991	-12.43626811	1.66084E-35	2.60822E-34	negative
PRJNA729271	stage_E18_vs_E10	ordinary_muscle_development	ENSGALG00010022921	ENSGALG00010022921	1.456800029	0.19355204	7.52665811	5.20554E-14	2.44426E-13	positive
PRJNA729271	stage_E18_vs_E10	ordinary_muscle_development	ENSGALG00010022922	ATOH7	-0.187130558	0.465122175	-0.4023256	0.687444416	0.73322346	negative
PRJNA729271	stage_E18_vs_E10	ordinary_muscle_development	ENSGALG00010022923	CD63	0.985433583	0.126651257	7.780685346	7.21327E-15	3.64181E-14	positive
PRJNA729271	stage_E18_vs_E10	ordinary_muscle_development	ENSGALG00010022928	RNF111	0.073262026	0.083998843	0.872178991	0.383110739	0.443510116	positive
PRJNA729271	stage_E18_vs_E10	ordinary_muscle_development	ENSGALG00010022929	LRP4	0.325686835	0.11496885	2.832826765	0.004613838	0.007681781	positive
PRJNA729271	stage_E18_vs_E10	ordinary_muscle_development	ENSGALG00010022930	GTF2B	-0.729471765	0.120075956	-6.075086055	1.23921E-09	4.14357E-09	negative
PRJNA729271	stage_E18_vs_E10	ordinary_muscle_development	ENSGALG00010022931	RDH5	0.604864569	0.126346883	4.787332742	1.69012E-06	4.26281E-06	positive
PRJNA729271	stage_E18_vs_E10	ordinary_muscle_development	ENSGALG00010022932	OPCML	0.683923642	0.18085744	3.781562115	0.000155847	0.000317143	positive
PRJNA729271	stage_E18_vs_E10	ordinary_muscle_development	ENSGALG00010022933	UNC119B	-0.66667744	0.076807382	-8.67986152	3.96251E-18	2.50487E-17	negative
PRJNA729271	stage_E18_vs_E10	ordinary_muscle_development	ENSGALG00010022937	BLOC1S1	0.685369652	0.161666284	4.239409957	2.24108E-05	5.01768E-05	positive
PRJNA729271	stage_E18_vs_E10	ordinary_muscle_development	ENSGALG00010022942	NEU2	1.03732473	0.473555671	2.190502179	0.028487837	0.041786994	positive
PRJNA729271	stage_E18_vs_E10	ordinary_muscle_development	ENSGALG00010022945	KCNA4	-2.139821475	0.369067513	-5.797913391	6.71451E-09	2.10713E-08	negative
PRJNA729271	stage_E18_vs_E10	ordinary_muscle_development	ENSGALG00010022946	KRT17	-4.152874042	0.490854806	-8.460493796	2.66248E-17	1.58621E-16	negative
PRJNA729271	stage_E18_vs_E10	ordinary_muscle_development	ENSGALG00010022949	INSC	-0.765635905	0.259430971	-2.951212426	0.003165291	0.005405384	negative
PRJNA729271	stage_E18_vs_E10	ordinary_muscle_development	ENSGALG00010022950	ITGA7	1.958715466	0.155736489	12.57711327	2.82153E-36	4.64751E-35	positive
PRJNA729271	stage_E18_vs_E10	ordinary_muscle_development	ENSGALG00010022953	ENSGALG00010022953	-1.108258185	1.166613399	-0.949978961	0.342122943	0.400571249	negative
PRJNA729271	stage_E18_vs_E10	ordinary_muscle_development	ENSGALG00010022954	ADRM1	-0.603370347	0.144652343	-4.17117575	3.03032E-05	6.67647E-05	negative
PRJNA729271	stage_E18_vs_E10	ordinary_muscle_development	ENSGALG00010022956	PBLD	0.389598053	0.126189311	3.087409294	0.002019094	0.00354405	positive
PRJNA729271	stage_E18_vs_E10	ordinary_muscle_development	ENSGALG00010022957	SLTM	-0.657215669	0.143857314	-4.56852454	4.9117E-06	1.17853E-05	negative
PRJNA729271	stage_E18_vs_E10	ordinary_muscle_development	ENSGALG00010022958	SFSWAP	0.247816622	0.097599186	2.539125917	0.011112982	0.017482382	positive
PRJNA729271	stage_E18_vs_E10	ordinary_muscle_development	ENSGALG00010022959	ADAMTS8	0.649829901	0.264208981	2.459529951	0.013911909	0.021571793	positive
PRJNA729271	stage_E18_vs_E10	ordinary_muscle_development	ENSGALG00010022967	ADAMTS15	0.254798271	0.161921853	1.573587917	0.115582749	0.151548796	positive
PRJNA729271	stage_E18_vs_E10	ordinary_muscle_development	ENSGALG00010022969	TTN	4.01395163	0.575497673	6.974748677	3.06418E-12	1.2618E-11	positive
PRJNA729271	stage_E18_vs_E10	ordinary_muscle_development	ENSGALG00010022971	CKAP5	-1.08574655	0.058578291	-18.53496444	1.07861E-76	6.85722E-75	negative
PRJNA729271	stage_E18_vs_E10	ordinary_muscle_development	ENSGALG00010022972	ENSGALG00010022972	2.828959092	1.089483343	2.596606097			positive
PRJNA729271	stage_E18_vs_E10	ordinary_muscle_development	ENSGALG00010022974	INPP5D	1.110943978	0.156155398	7.114348874	1.12442E-12	4.79651E-12	positive
PRJNA729271	stage_E18_vs_E10	ordinary_muscle_development	ENSGALG00010022976	MINDY2	0.195830876	0.065755914	2.978148471	0.002899955	0.004978662	positive
PRJNA729271	stage_E18_vs_E10	ordinary_muscle_development	ENSGALG00010022978	ARL14EP	-0.271123413	0.087379398	-3.102829955	0.001916797	0.00337348	negative
PRJNA729271	stage_E18_vs_E10	ordinary_muscle_development	ENSGALG00010022979	LAMA5	0.69481266	0.148795036	4.669595681	3.01793E-06	7.39309E-06	positive
PRJNA729271	stage_E18_vs_E10	ordinary_muscle_development	ENSGALG00010022981	HNRNPH3	-1.116792253	0.12972252	-8.609085394	7.36458E-18	4.55416E-17	negative
PRJNA729271	stage_E18_vs_E10	ordinary_muscle_development	ENSGALG00010022983	PKN2	-0.147235091	0.062347953	-2.361506425	0.018200857	0.027631002	negative
PRJNA729271	stage_E18_vs_E10	ordinary_muscle_development	ENSGALG00010022986	ALG9	0.37154729	0.102463285	3.626150488	0.000287678	0.000565179	positive
PRJNA729271	stage_E18_vs_E10	ordinary_muscle_development	ENSGALG00010022987	F2	6.173397822	1.375881093	4.486868706			positive
PRJNA729271	stage_E18_vs_E10	ordinary_muscle_development	ENSGALG00010022990	SLC25A22	-0.562145629	0.099324654	-5.659678709	1.51657E-08	4.61529E-08	negative
PRJNA729271	stage_E18_vs_E10	ordinary_muscle_development	ENSGALG00010022991	TAL1	0.655613853	0.177454097	3.694554605	0.000220273	0.000439061	positive
PRJNA729271	stage_E18_vs_E10	ordinary_muscle_development	ENSGALG00010022992	SLC7A6	-0.582036545	0.108138736	-5.382313196	7.35347E-08	2.10622E-07	negative
PRJNA729271	stage_E18_vs_E10	ordinary_muscle_development	ENSGALG00010022994	LIPC	1.098480011	0.838562333	1.3099563	0.19021062	0.237701779	positive
PRJNA729271	stage_E18_vs_E10	ordinary_muscle_development	ENSGALG00010022997	ARHGAP1	-0.505188949	0.094810196	-5.328424267	9.90685E-08	2.81182E-07	negative
PRJNA729271	stage_E18_vs_E10	ordinary_muscle_development	ENSGALG00010022999	ENSGALG00010022999	1.684269818	0.501964584	3.355355878	0.000792629	0.001466016	positive
PRJNA729271	stage_E18_vs_E10	ordinary_muscle_development	ENSGALG00010023002	ATG16L1	-0.163470978	0.135829618	-1.203500247	0.228782796	0.280321777	negative
PRJNA729271	stage_E18_vs_E10	ordinary_muscle_development	ENSGALG00010023003	ENSGALG00010023003	-4.397124039	0.619447504	-7.098461151	1.26154E-12	5.36403E-12	negative
PRJNA729271	stage_E18_vs_E10	ordinary_muscle_development	ENSGALG00010023005	IRF7	0.188880171	0.363509648	0.519601536	0.603341327	0.654500265	positive
PRJNA729271	stage_E18_vs_E10	ordinary_muscle_development	ENSGALG00010023007	LMO4	-0.721840284	0.143050649	-5.046046895	4.51045E-07	1.20448E-06	negative
PRJNA729271	stage_E18_vs_E10	ordinary_muscle_development	ENSGALG00010023008	CDHR5	-0.595485723	0.928602631	-0.641270769	0.521346777	0.578144101	negative
PRJNA729271	stage_E18_vs_E10	ordinary_muscle_development	ENSGALG00010023012	ENSGALG00010023012	0.61838915	0.529939851	1.166904411	0.243248974	0.296343409	positive
PRJNA729271	stage_E18_vs_E10	ordinary_muscle_development	ENSGALG00010023013	ENSGALG00010023013	0.255358829	0.444783322	0.574119613	0.565886862	0.619666848	positive
PRJNA729271	stage_E18_vs_E10	ordinary_muscle_development	ENSGALG00010023015	METTL2B	-0.17639197	0.103115874	-1.71061897	0.087151475	0.116849563	negative
PRJNA729271	stage_E18_vs_E10	ordinary_muscle_development	ENSGALG00010023017	BUB1B	-1.908986351	0.20054014	-9.519223207	1.74478E-21	1.351E-20	negative
PRJNA729271	stage_E18_vs_E10	ordinary_muscle_development	ENSGALG00010023019	CABP1	0.483575989	0.318839632	1.516674658	0.129348846	0.167933643	positive
PRJNA729271	stage_E18_vs_E10	ordinary_muscle_development	ENSGALG00010023024	PRMT7	-0.881614772	0.116753641	-7.551068782	4.31701E-14	2.03827E-13	negative
PRJNA729271	stage_E18_vs_E10	ordinary_muscle_development	ENSGALG00010023026	PHRF1	-0.112229126	0.091725148	-1.223537145	0.221126883	0.272021911	negative
PRJNA729271	stage_E18_vs_E10	ordinary_muscle_development	ENSGALG00010023028	TMEM61	-1.484848353	0.303650778	-4.889986981	1.00843E-06	2.60416E-06	negative
PRJNA729271	stage_E18_vs_E10	ordinary_muscle_development	ENSGALG00010023030	HS2ST1	-0.507277485	0.085418838	-5.938707393	2.87278E-09	9.32191E-09	negative
PRJNA729271	stage_E18_vs_E10	ordinary_muscle_development	ENSGALG00010023041	CD151	1.278598356	0.119423786	10.70639608	9.49879E-27	9.94834E-26	positive
PRJNA729271	stage_E18_vs_E10	ordinary_muscle_development	ENSGALG00010023042	ENSGALG00010023042	-3.506056047	0.827052573	-4.239217869	2.243E-05	5.0212E-05	negative
PRJNA729271	stage_E18_vs_E10	ordinary_muscle_development	ENSGALG00010023043	CNP	1.476485087	0.145159926	10.17143733	2.65955E-24	2.41089E-23	positive
PRJNA729271	stage_E18_vs_E10	ordinary_muscle_development	ENSGALG00010023046	SLC7A6OS	-0.69887472	0.076311388	-9.158196951	5.27716E-20	3.70783E-19	negative
PRJNA729271	stage_E18_vs_E10	ordinary_muscle_development	ENSGALG00010023048	ARFGAP1	-0.907902872	0.0841033	-10.79509219	3.63098E-27	3.87278E-26	negative
PRJNA729271	stage_E18_vs_E10	ordinary_muscle_development	ENSGALG00010023050	RUFY2	0.023633442	0.093337126	0.253205158	0.800109681	0.833211966	positive
PRJNA729271	stage_E18_vs_E10	ordinary_muscle_development	ENSGALG00010023052	RAD54L	-1.30389718	0.132426829	-9.846170807	7.12053E-23	5.93855E-22	negative
PRJNA729271	stage_E18_vs_E10	ordinary_muscle_development	ENSGALG00010023053	MAN1A2	-0.702174447	0.048128719	-14.58951044	3.2754E-48	8.87418E-47	negative
PRJNA729271	stage_E18_vs_E10	ordinary_muscle_development	ENSGALG00010023054	AQP9	1.463354896	0.232014155	6.307179399	2.84166E-10	1.00194E-09	positive
PRJNA729271	stage_E18_vs_E10	ordinary_muscle_development	ENSGALG00010023056	SAG	0.583483558	0.45967938	1.269327239	0.204324375	0.253633795	positive
PRJNA729271	stage_E18_vs_E10	ordinary_muscle_development	ENSGALG00010023057	PAFAH1B2	-0.787974153	0.074963738	-10.5114042	7.65452E-26	7.58389E-25	negative
PRJNA729271	stage_E18_vs_E10	ordinary_muscle_development	ENSGALG00010023058	PPARA	0.606349409	0.205997609	2.9434779	0.00324547	0.005522137	positive
PRJNA729271	stage_E18_vs_E10	ordinary_muscle_development	ENSGALG00010023059	SELENOF	-0.284043496	0.093390568	-3.041458048	0.002354354	0.004088458	negative
PRJNA729271	stage_E18_vs_E10	ordinary_muscle_development	ENSGALG00010023060	ALDH1A2	-1.31664604	0.167816263	-7.845759487	4.30341E-15	2.20885E-14	negative
PRJNA729271	stage_E18_vs_E10	ordinary_muscle_development	ENSGALG00010023062	STX2	0.127987137	0.103647129	1.234835335	0.216891794	0.267561409	positive
PRJNA729271	stage_E18_vs_E10	ordinary_muscle_development	ENSGALG00010023063	ENSGALG00010023063	-0.577555096	0.104647664	-5.519044335	3.40848E-08	1.0087E-07	negative
PRJNA729271	stage_E18_vs_E10	ordinary_muscle_development	ENSGALG00010023064	ATG13	0.580900838	0.068824425	8.440329666	3.16444E-17	1.87601E-16	positive
PRJNA729271	stage_E18_vs_E10	ordinary_muscle_development	ENSGALG00010023065	DNA2	-1.981261235	0.227172446	-8.721397634	2.74789E-18	1.75388E-17	negative
PRJNA729271	stage_E18_vs_E10	ordinary_muscle_development	ENSGALG00010023066	TLR21	0.727059217	0.189443421	3.837869965	0.000124106	0.000255855	positive
PRJNA729271	stage_E18_vs_E10	ordinary_muscle_development	ENSGALG00010023068	CDPF1	-0.199660318	0.125778513	-1.587396075	0.112422968	0.147726491	negative
PRJNA729271	stage_E18_vs_E10	ordinary_muscle_development	ENSGALG00010023069	ENSGALG00010023069	0.596367979	0.274689865	2.171059272	0.029926692	0.043737387	positive
PRJNA729271	stage_E18_vs_E10	ordinary_muscle_development	ENSGALG00010023070	SIDT2	0.120586345	0.205365474	0.58717925	0.557083326	0.611042211	positive
PRJNA729271	stage_E18_vs_E10	ordinary_muscle_development	ENSGALG00010023071	POLR2M	-0.284550889	0.084737784	-3.35801663	0.000785039	0.001452903	negative
PRJNA729271	stage_E18_vs_E10	ordinary_muscle_development	ENSGALG00010023073	POMGNT1	0.075567746	0.086310327	0.875535395	0.38128266	0.441605446	positive
PRJNA729271	stage_E18_vs_E10	ordinary_muscle_development	ENSGALG00010023074	CCNT1	0.119353909	0.168086653	0.710073687	0.477658443	0.536135909	positive
PRJNA729271	stage_E18_vs_E10	ordinary_muscle_development	ENSGALG00010023075	PKDREJ	-1.494837727	0.28865326	-5.178662197	2.23483E-07	6.13171E-07	negative
PRJNA729271	stage_E18_vs_E10	ordinary_muscle_development	ENSGALG00010023076	DNAJC7	0.437922931	0.080354866	5.449862016	5.04089E-08	1.46458E-07	positive
PRJNA729271	stage_E18_vs_E10	ordinary_muscle_development	ENSGALG00010023077	KANSL2	-0.244373688	0.153766287	-1.589254005	0.112003058	0.147221871	negative
PRJNA729271	stage_E18_vs_E10	ordinary_muscle_development	ENSGALG00010023078	TAGLN	0.844802769	0.201508974	4.192382852	2.7604E-05	6.11991E-05	positive
PRJNA729271	stage_E18_vs_E10	ordinary_muscle_development	ENSGALG00010023079	SLC25A16	-0.343312677	0.099540867	-3.448962102	0.000562746	0.001061694	negative
PRJNA729271	stage_E18_vs_E10	ordinary_muscle_development	ENSGALG00010023080	MCRS1	-0.087538118	0.112184874	-0.780302322	0.435212947	0.494932658	negative
PRJNA729271	stage_E18_vs_E10	ordinary_muscle_development	ENSGALG00010023081	PRLHR2	-8.126227727	0.827349882	-9.821996605	9.05324E-23	7.50296E-22	negative
PRJNA729271	stage_E18_vs_E10	ordinary_muscle_development	ENSGALG00010023083	HARBI1	0.335316813	0.158588421	2.114383954	0.034482487	0.049808037	positive
PRJNA729271	stage_E18_vs_E10	ordinary_muscle_development	ENSGALG00010023084	ENSGALG00010023084	0.106473002	0.242169331	0.439663442	0.660180884	0.708074465	positive
PRJNA729271	stage_E18_vs_E10	ordinary_muscle_development	ENSGALG00010023086	PCSK7	-0.527964059	0.09563257	-5.520755759	3.37545E-08	9.99124E-08	negative
PRJNA729271	stage_E18_vs_E10	ordinary_muscle_development	ENSGALG00010023087	SH3GLB1	0.386787387	0.118137452	3.274045455	0.001060196	0.001925274	positive
PRJNA729271	stage_E18_vs_E10	ordinary_muscle_development	ENSGALG00010023089	ENSGALG00010023089	4.097652598	0.676336699	6.058598633	1.37313E-09	4.57551E-09	positive
PRJNA729271	stage_E18_vs_E10	ordinary_muscle_development	ENSGALG00010023090	LRRC41	0.205399838	0.091905317	2.234907011	0.025423454	0.03765716	positive
PRJNA729271	stage_E18_vs_E10	ordinary_muscle_development	ENSGALG00010023091	ENSGALG00010023091	0.237799666	0.132078444	1.800442664	0.071790769	0.097967163	positive
PRJNA729271	stage_E18_vs_E10	ordinary_muscle_development	ENSGALG00010023093	POP5	-0.484903626	0.132211742	-3.667629042	0.00024481	0.000485235	negative
PRJNA729271	stage_E18_vs_E10	ordinary_muscle_development	ENSGALG00010023094	CCDC32	0.072745471	0.10202787	0.712996078	0.475848184	0.534435102	positive
PRJNA729271	stage_E18_vs_E10	ordinary_muscle_development	ENSGALG00010023096	SPATS2	-0.042749299	0.172051518	-0.248468016	0.803772314	0.836253208	negative
PRJNA729271	stage_E18_vs_E10	ordinary_muscle_development	ENSGALG00010023097	LURAP1	1.196668149	0.211574168	5.656021994	1.54922E-08	4.71168E-08	positive
PRJNA729271	stage_E18_vs_E10	ordinary_muscle_development	ENSGALG00010023101	CLCA2	-1.861433683	1.365903183	-1.362785961	0.172949977	0.21820088	negative
PRJNA729271	stage_E18_vs_E10	ordinary_muscle_development	ENSGALG00010023102	RNF10	-0.085589544	0.101758679	-0.841103134	0.400290159	0.46016384	negative
PRJNA729271	stage_E18_vs_E10	ordinary_muscle_development	ENSGALG00010023103	DGKD	0.581915791	0.125556427	4.634695383	3.57463E-06	8.68348E-06	positive
PRJNA729271	stage_E18_vs_E10	ordinary_muscle_development	ENSGALG00010023104	ENSGALG00010023104	0.824585062	0.158410322	5.205374587	1.93606E-07	5.34129E-07	positive
PRJNA729271	stage_E18_vs_E10	ordinary_muscle_development	ENSGALG00010023107	ZC3HAV1	-0.46102676	0.293785924	-1.569260887	0.116587162	0.152782833	negative
PRJNA729271	stage_E18_vs_E10	ordinary_muscle_development	ENSGALG00010023108	TET1	0.486340233	0.061242307	7.941246176	2.0016E-15	1.05006E-14	positive
PRJNA729271	stage_E18_vs_E10	ordinary_muscle_development	ENSGALG00010023110	LIMA1	-1.437405437	0.155117996	-9.266529164	1.92302E-20	1.3958E-19	negative
PRJNA729271	stage_E18_vs_E10	ordinary_muscle_development	ENSGALG00010023112	PCMT1L	1.06110678	0.133071966	7.973931809	1.53704E-15	8.1401E-15	positive
PRJNA729271	stage_E18_vs_E10	ordinary_muscle_development	ENSGALG00010023113	MYZAP	-0.195906201	0.133413008	-1.468419033	0.141990435	0.182703182	negative
PRJNA729271	stage_E18_vs_E10	ordinary_muscle_development	ENSGALG00010023114	UQCRHL	1.229252233	0.167445194	7.341221338	2.11653E-13	9.49232E-13	positive
PRJNA729271	stage_E18_vs_E10	ordinary_muscle_development	ENSGALG00010023115	RPLP2	0.680831907	0.136343809	4.993493386	5.92968E-07	1.56587E-06	positive
PRJNA729271	stage_E18_vs_E10	ordinary_muscle_development	ENSGALG00010023116	PROC	4.958835161	1.003552616	4.94128069	7.76111E-07	2.0299E-06	positive

PRJNA729271	stage_E18_vs_E10	ordinary_muscle_development	ENSGALG00010023117	RPUSD2	-0.622003289	0.106804201	-5.823771759	5.75341E-09	1.81769E-08	negative
PRJNA729271	stage_E18_vs_E10	ordinary_muscle_development	ENSGALG00010023118	PIDD1	0.09105565	0.14568952	0.624997939	0.531972411	0.587992992	positive
PRJNA729271	stage_E18_vs_E10	ordinary_muscle_development	ENSGALG00010023119	NDRG4	-0.353267731	0.172781706	-2.044589902	0.040895306	0.058292601	negative
PRJNA729271	stage_E18_vs_E10	ordinary_muscle_development	ENSGALG00010023120	CHRM4	0.194948285	0.242740376	0.803114374	0.42190863	0.482300126	positive
PRJNA729271	stage_E18_vs_E10	ordinary_muscle_development	ENSGALG00010023122	ENSGALG00010023122	2.523541895	0.662068645	3.81160158	0.000138069	0.000283071	positive
PRJNA729271	stage_E18_vs_E10	ordinary_muscle_development	ENSGALG00010023123	RNF214	0.210247934	0.145531618	1.444689047	0.148545248	0.190309649	positive
PRJNA729271	stage_E18_vs_E10	ordinary_muscle_development	ENSGALG00010023124	NSUN4	0.44954168	0.137994091	3.257687895	0.001123239	0.002033643	positive
PRJNA729271	stage_E18_vs_E10	ordinary_muscle_development	ENSGALG00010023125	COL20A1	3.523093364	0.282195692	12.48457529	9.06256E-36	1.44991E-34	positive
PRJNA729271	stage_E18_vs_E10	ordinary_muscle_development	ENSGALG00010023126	CGNL1	0.48670669	0.137436744	3.541314189	0.000398139	0.000766608	positive
PRJNA729271	stage_E18_vs_E10	ordinary_muscle_development	ENSGALG00010023127	CERS5	-0.694801335	0.097726124	-7.109678604	1.16313E-12	4.95435E-12	negative
PRJNA729271	stage_E18_vs_E10	ordinary_muscle_development	ENSGALG00010023128	IWS1	-0.180223274	0.137699881	-1.308812127	0.190597998	0.238165343	negative
PRJNA729271	stage_E18_vs_E10	ordinary_muscle_development	ENSGALG00010023130	DYNLT5	0.736273991	0.486766099	1.512582723	0.130385686	0.169031438	positive
PRJNA729271	stage_E18_vs_E10	ordinary_muscle_development	ENSGALG00010023131	SOX6	1.022432378	0.137050686	7.460250002	8.63585E-14	3.99287E-13	positive
PRJNA729271	stage_E18_vs_E10	ordinary_muscle_development	ENSGALG00010023134	KNL1	-1.846812822	0.215961159	-8.5515971	1.21397E-17	7.41841E-17	negative
PRJNA729271	stage_E18_vs_E10	ordinary_muscle_development	ENSGALG00010023135	GINS3	-0.960622752	0.069298242	-13.86215174	1.07406E-43	2.39147E-42	negative
PRJNA729271	stage_E18_vs_E10	ordinary_muscle_development	ENSGALG00010023136	MDK	-2.862464722	0.40629947	-7.045209103	1.85183E-12	7.7599E-12	negative
PRJNA729271	stage_E18_vs_E10	ordinary_muscle_development	ENSGALG00010023137	ETV6	-0.093694478	0.114262269	-0.8199949	0.412219015	0.472453121	negative
PRJNA729271	stage_E18_vs_E10	ordinary_muscle_development	ENSGALG00010023140	CCAR1	-0.917624303	0.084653667	-10.83974671	2.23088E-27	2.40421E-26	negative
PRJNA729271	stage_E18_vs_E10	ordinary_muscle_development	ENSGALG00010023142	TLK2	-0.039133979	0.169192562	-0.23129846	0.817082943	0.848092894	negative
PRJNA729271	stage_E18_vs_E10	ordinary_muscle_development	ENSGALG00010023144	C11orf58	0.013957583	0.10649984	0.131057314	0.895729968	0.91350625	positive
PRJNA729271	stage_E18_vs_E10	ordinary_muscle_development	ENSGALG00010023147	RAD51	-2.309411481	0.19192559	-12.03284817	2.38779E-33	3.36356E-32	negative
PRJNA729271	stage_E18_vs_E10	ordinary_muscle_development	ENSGALG00010023149	ADGRD1	-0.456408265	0.185048165	-2.466429571	0.013646756	0.021187155	negative
PRJNA729271	stage_E18_vs_E10	ordinary_muscle_development	ENSGALG00010023151	ENSGALG00010023151	-1.960490943	0.538150115	-3.643018718	0.000269459	0.000531548	negative
PRJNA729271	stage_E18_vs_E10	ordinary_muscle_development	ENSGALG00010023155	CFAP263	0.438523991	0.321344277	1.36465474	0.172361591	0.217553227	positive
PRJNA729271	stage_E18_vs_E10	ordinary_muscle_development	ENSGALG00010023157	STOX1	-1.021242549	0.34353014	-2.972788791	0.002951074	0.005060431	negative
PRJNA729271	stage_E18_vs_E10	ordinary_muscle_development	ENSGALG00010023158	ENSGALG00010023158	-2.38700613	0.938477491	-2.543487888	0.010975186	0.017269358	negative
PRJNA729271	stage_E18_vs_E10	ordinary_muscle_development	ENSGALG00010023159	DAW1	-0.317802233	0.515570396	-0.616409001	0.537624614	0.592793913	negative
PRJNA729271	stage_E18_vs_E10	ordinary_muscle_development	ENSGALG00010023160	ENSGALG00010023160	-1.311947007	0.111539761	-11.76214645	6.11594E-32	8.19321E-31	negative
PRJNA729271	stage_E18_vs_E10	ordinary_muscle_development	ENSGALG00010023161	ODF2L	-0.939773128	0.423247125	-2.220388689	0.026392394	0.038968906	negative
PRJNA729271	stage_E18_vs_E10	ordinary_muscle_development	ENSGALG00010023162	CHRNA4	-1.438276979	0.271763803	-5.292378765	1.20736E-07	3.3962E-07	negative
PRJNA729271	stage_E18_vs_E10	ordinary_muscle_development	ENSGALG00010023163	CRTAC1	-0.171517833	0.280496071	-0.611480339	0.540881631	0.595931836	negative
PRJNA729271	stage_E18_vs_E10	ordinary_muscle_development	ENSGALG00010023164	NKIRAS2	0.721066163	0.13302567	5.420503878	5.94313E-08	1.71366E-07	positive
PRJNA729271	stage_E18_vs_E10	ordinary_muscle_development	ENSGALG00010023165	CSNK2A2	-0.026976859	0.130357328	-0.206945474	0.836052442	0.863946685	negative
PRJNA729271	stage_E18_vs_E10	ordinary_muscle_development	ENSGALG00010023166	KDM2A	-0.065088514	0.114227378	-0.569815356	0.568802939	0.622483851	negative
PRJNA729271	stage_E18_vs_E10	ordinary_muscle_development	ENSGALG00010023167	GPD1	5.288973408	0.211583475	24.99710055	6.5737E-138	1.7015E-135	positive
PRJNA729271	stage_E18_vs_E10	ordinary_muscle_development	ENSGALG00010023168	SPHKAP	-1.630107404	0.299061624	-5.450740828	5.01604E-08	1.45765E-07	negative
PRJNA729271	stage_E18_vs_E10	ordinary_muscle_development	ENSGALG00010023169	RAN	-1.271729422	0.09723775	-13.0785566	4.36653E-39	8.07307E-38	negative
PRJNA729271	stage_E18_vs_E10	ordinary_muscle_development	ENSGALG00010023170	TCF12	-0.707297526	0.064839822	-10.90838173	1.05111E-27	1.15598E-26	negative
PRJNA729271	stage_E18_vs_E10	ordinary_muscle_development	ENSGALG00010023171	ENSGALG00010023171	-1.445221911	0.116272666	-12.42959301	1.80551E-35	2.83235E-34	negative
PRJNA729271	stage_E18_vs_E10	ordinary_muscle_development	ENSGALG00010023172	DHX58	0.775796922	0.342625815	2.264268739	0.023557591	0.035079852	positive
PRJNA729271	stage_E18_vs_E10	ordinary_muscle_development	ENSGALG00010023174	KCNQ2	-0.660184939	0.319080007	-2.069026341	0.038543615	0.055222862	negative
PRJNA729271	stage_E18_vs_E10	ordinary_muscle_development	ENSGALG00010023175	GRK2	-0.235030243	0.141722122	-1.658387828	0.097239212	0.129274729	negative
PRJNA729271	stage_E18_vs_E10	ordinary_muscle_development	ENSGALG00010023177	LZTS2	0.023703772	0.172287495	0.13758266	0.89057026	0.909583986	positive
PRJNA729271	stage_E18_vs_E10	ordinary_muscle_development	ENSGALG00010023178	CFAP20	-0.209163076	0.098687285	-2.11945315	0.034052189	0.049240471	negative
PRJNA729271	stage_E18_vs_E10	ordinary_muscle_development	ENSGALG00010023179	KAT2A	-1.176567335	0.137186435	-8.576411622	9.78788E-18	6.01167E-17	negative
PRJNA729271	stage_E18_vs_E10	ordinary_muscle_development	ENSGALG00010023180	EEF1A2	5.554833846	0.121668498	45.6554815	0	0	positive
PRJNA729271	stage_E18_vs_E10	ordinary_muscle_development	ENSGALG00010023181	PLEKHA7	-0.091595948	0.166360148	-0.550588283	0.581915943	0.634916944	negative
PRJNA729271	stage_E18_vs_E10	ordinary_muscle_development	ENSGALG00010023182	EPHB6	-1.353810964	0.142093456	-9.527609507	1.60947E-21	1.24956E-20	negative
PRJNA729271	stage_E18_vs_E10	ordinary_muscle_development	ENSGALG00010023183	ENSGALG00010023183	0.92902228	0.442669871	2.098679719	0.035845144	0.051627122	positive
PRJNA729271	stage_E18_vs_E10	ordinary_muscle_development	ENSGALG00010023184	SMARCD1	-0.44518303	0.053672392	-8.294451084	1.09088E-16	6.25238E-16	negative
PRJNA729271	stage_E18_vs_E10	ordinary_muscle_development	ENSGALG00010023185	FBXW4	1.876475433	0.11722898	16.00692458	1.14323E-57	4.21656E-56	positive
PRJNA729271	stage_E18_vs_E10	ordinary_muscle_development	ENSGALG00010023186	MIER1	-0.332268805	0.100097519	-3.319450961	0.000901946	0.001653226	negative
PRJNA729271	stage_E18_vs_E10	ordinary_muscle_development	ENSGALG00010023187	ENSGALG00010023187	3.292732645	0.230468362	14.28713517	2.63217E-46	6.61235E-45	positive
PRJNA729271	stage_E18_vs_E10	ordinary_muscle_development	ENSGALG00010023188	KIF1BP	0.122672834	0.059109146	2.075361313	0.037953061	0.054435941	positive
PRJNA729271	stage_E18_vs_E10	ordinary_muscle_development	ENSGALG00010023189	RMDN3	-0.760278258	0.087091917	-8.72960754	2.55558E-18	1.63474E-17	negative
PRJNA729271	stage_E18_vs_E10	ordinary_muscle_development	ENSGALG00010023190	SF3B3	-0.943366967	0.056451835	-16.7110061	1.08981E-62	4.84565E-61	negative
PRJNA729271	stage_E18_vs_E10	ordinary_muscle_development	ENSGALG00010023193	PPDPF	2.613660922	0.313093838	8.347851686	6.95158E-17	4.02891E-16	positive
PRJNA729271	stage_E18_vs_E10	ordinary_muscle_development	ENSGALG00010023194	MRC2	0.235176564	0.193262538	1.216876105	0.223651358	0.274730943	positive
PRJNA729271	stage_E18_vs_E10	ordinary_muscle_development	ENSGALG00010023195	ENSGALG00010023195	-2.336938957	0.186603866	-12.52352914	5.55109E-36	8.97024E-35	negative
PRJNA729271	stage_E18_vs_E10	ordinary_muscle_development	ENSGALG00010023196	DGKZ	0.664409599	0.128381539	5.175273692	2.27577E-07	6.2405E-07	positive
PRJNA729271	stage_E18_vs_E10	ordinary_muscle_development	ENSGALG00010023198	PTK6	-0.480480997	0.483007473	-0.994769282	0.31984849	0.377510289	negative
PRJNA729271	stage_E18_vs_E10	ordinary_muscle_development	ENSGALG00010023199	RPS13	0.953746751	0.196680965	4.849207193	1.23956E-06	3.17558E-06	positive
PRJNA729271	stage_E18_vs_E10	ordinary_muscle_development	ENSGALG00010023200	VPS26A	-0.313069084	0.08377775	-3.736900105	0.000186303	0.00037496	negative
PRJNA729271	stage_E18_vs_E10	ordinary_muscle_development	ENSGALG00010023201	ZNF280D	-0.142566592	0.118294447	-1.20518415	0.22813222	0.279595546	negative
PRJNA729271	stage_E18_vs_E10	ordinary_muscle_development	ENSGALG00010023202	PID1	1.003800391	0.155923757	6.437764287	1.21246E-10	4.39222E-10	positive
PRJNA729271	stage_E18_vs_E10	ordinary_muscle_development	ENSGALG00010023203	COG4	-0.163541076	0.064463667	-2.536949629	0.011182304	0.017583803	negative
PRJNA729271	stage_E18_vs_E10	ordinary_muscle_development	ENSGALG00010023204	SRGN	3.776075952	0.313273144	12.05362165	1.85612E-33	2.62995E-32	positive
PRJNA729271	stage_E18_vs_E10	ordinary_muscle_development	ENSGALG00010023205	DNAJC17	-0.267581724	0.113071954	-2.366472972	0.017958485	0.027297425	negative
PRJNA729271	stage_E18_vs_E10	ordinary_muscle_development	ENSGALG00010023206	HSPB9	0.91769279	0.316331289	2.901049691	0.003719149	0.006270683	positive
PRJNA729271	stage_E18_vs_E10	ordinary_muscle_development	ENSGALG00010023207	MNS1	-0.291788899	0.154407081	-1.889737813	0.058793034	0.081527316	negative
PRJNA729271	stage_E18_vs_E10	ordinary_muscle_development	ENSGALG00010023208	BACE1	-0.2967407	0.0787931	-3.766074674	0.000165834	0.000335815	negative
PRJNA729271	stage_E18_vs_E10	ordinary_muscle_development	ENSGALG00010023209	PIK3C2A	-0.142785502	0.106751895	-1.337545361	0.181044686	0.227443467	negative
PRJNA729271	stage_E18_vs_E10	ordinary_muscle_development	ENSGALG00010023210	ZNHIT6	-0.285191804	0.142689389	-1.998689645	0.045641944	0.064525503	negative
PRJNA729271	stage_E18_vs_E10	ordinary_muscle_development	ENSGALG00010023211	TLK1	-0.128767527	0.076747974	-1.677797085	0.093386719	0.124575786	negative
PRJNA729271	stage_E18_vs_E10	ordinary_muscle_development	ENSGALG00010023212	RIMBP2	3.97957796	0.213387033	18.6495773	1.2728E-77	8.31044E-76	positive
PRJNA729271	stage_E18_vs_E10	ordinary_muscle_development	ENSGALG00010023213	FCSK	0.357228875	0.116209785	3.073999963	0.002112095	0.003696994	positive
PRJNA729271	stage_E18_vs_E10	ordinary_muscle_development	ENSGALG00010023214	SUPV3L1	-0.746935578	0.092145681	-8.106029128	5.23009E-16	2.86292E-15	negative
PRJNA729271	stage_E18_vs_E10	ordinary_muscle_development	ENSGALG00010023215	TRIP12	-0.167334835	0.0565056	-2.961384958	0.003062589	0.005239257	negative
PRJNA729271	stage_E18_vs_E10	ordinary_muscle_development	ENSGALG00010023216	SLC35D1	-0.846399897	0.125435915	-6.747667916	1.5024E-11	5.8353E-11	negative
PRJNA729271	stage_E18_vs_E10	ordinary_muscle_development	ENSGALG00010023217	CREB3L1	0.986594994	0.205600253	4.79860789	1.59772E-06	4.03678E-06	positive
PRJNA729271	stage_E18_vs_E10	ordinary_muscle_development	ENSGALG00010023218	SSH3	0.74128304	0.12953865	5.722485465	1.04977E-08	3.23478E-08	positive
PRJNA729271	stage_E18_vs_E10	ordinary_muscle_development	ENSGALG00010023219	TEX9	0.414302127	0.149170522	2.777372647	0.005480031	0.009026483	positive
PRJNA729271	stage_E18_vs_E10	ordinary_muscle_development	ENSGALG00010023220	ST3GAL2	-0.594526794	0.087287406	-6.811140616	9.68279E-12	3.81495E-11	negative
PRJNA729271	stage_E18_vs_E10	ordinary_muscle_development	ENSGALG00010023221	AMY1A	2.403420061	0.136186794	17.64796709	1.05485E-69	5.60077E-68	positive
PRJNA729271	stage_E18_vs_E10	ordinary_muscle_development	ENSGALG00010023222	ENSGALG00010023222	-1.197637597	0.620862262	-1.928990809	0.053732003	0.07501882	negative
PRJNA729271	stage_E18_vs_E10	ordinary_muscle_development	ENSGALG00010023223	CEP164	-0.871393871	0.09841155	-8.854589445	8.39924E-19	5.5214E-18	negative
PRJNA729271	stage_E18_vs_E10	ordinary_muscle_development	ENSGALG00010023225	PDPR	0.290028011	0.120987342	2.397176477	0.016521964	0.025296912	positive
PRJNA729271	stage_E18_vs_E10	ordinary_muscle_development	ENSGALG00010023226	ENSGALG00010023226	0.394958898	0.205004951	1.926582238	0.054031714	0.075386437	positive
PRJNA729271	stage_E18_vs_E10	ordinary_muscle_development	ENSGALG00010023227	RNPC3	0.376928069	0.123457548	3.053098627	0.002264915	0.003942116	positive
PRJNA729271	stage_E18_vs_E10	ordinary_muscle_development	ENSGALG00010023228	GORASP2	0.267338172	0.10220491	2.615707724	0.008904274	0.014192594	positive
PRJNA729271	stage_E18_vs_E10	ordinary_muscle_development	ENSGALG00010023229	HKDC1	-1.985649743	0.524254851	-3.787565799	0.00015213	0.00030984	negative
PRJNA729271	stage_E18_vs_E10	ordinary_muscle_development	ENSGALG00010023230	ENSGALG00010023230	0.42749511	0.083551302	5.116558333	3.11161E-07	8.4478E-07	positive
PRJNA729271	stage_E18_vs_E10	ordinary_muscle_development	ENSGALG00010023231	ASIC1	-1.698587266	0.239602058	-7.089201493	1.34888E-12	5.71529E-12	negative
PRJNA729271	stage_E18_vs_E10	ordinary_muscle_development	ENSGALG00010023233	SEZ6L	3.850363293	0.741596255	5.19199398	2.08054E-07	5.72572E-07	positive
PRJNA729271	stage_E18_vs_E10	ordinary_muscle_development	ENSGALG00010023234	ENSGALG00010023234	-0.004099796	0.094269189	-0.043490311	0.965310688	0.971611584	negative
PRJNA729271	stage_E18_vs_E10	ordinary_muscle_development	ENSGALG00010023235	ENSGALG00010023235	-1.769015239	0.383416365	-4.613822987	3.95329E-06	9.55846E-06	negative
PRJNA729271	stage_E18_vs_E10	ordinary_muscle_development	ENSGALG00010023237	GAD1	-3.518261262	0.59804649	-5.882922689	4.03084E-09	1.29149E-08	negative
PRJNA729271	stage_E18_vs_E10	ordinary_muscle_development	ENSGALG00010023238	NUCB2	-0.029209706	0.078186965	-0.373587924	0.708710919	0.752914452	negative
PRJNA729271	stage_E18_vs_E10	ordinary_muscle_development	ENSGALG00010023239	RBPMS2	1.074668161	0.200059539	5.371741675	7.79798E-08	2.22898E-07	positive
PRJNA729271	stage_E18_vs_E10	ordinary_muscle_development	ENSGALG00010023240	PIWIL1	-1.336938248	0.419842827	-3.184377964	0.001450655	0.002592435	negative
PRJNA729271	stage_E18_vs_E10	ordinary_muscle_development	ENSGALG00010023243	HK1	0.581294875	0.11453119	5.075428597	3.86624E-07	1.0403E-06	positive
PRJNA729271	stage_E18_vs_E10	ordinary_muscle_development	ENSGALG00010023244	PPIB	-0.205903032	0.118533308	-1.737090075	0.082371273	0.111108468	negative
PRJNA729271	stage_E18_vs_E10	ordinary_muscle_development	ENSGALG00010023245	TANC2	0.449246454	0.143159695	3.138079144	0.00170059	0.003017216	positive
PRJNA729271	stage_E18_vs_E10	ordinary_muscle_development	ENSGALG00010023246	SP5	-2.635018978	0.607288376	-4.338991299	1.43138E-05	3.27807E-05	negative
PRJNA729271	stage_E18_vs_E10	ordinary_muscle_development	ENSGALG00010023248	PRDM10	-0.246354573	0.12189671	-2.021010845	0.043278642	0.061402232	negative
PRJNA729271	stage_E18_vs_E10	ordinary_muscle_development	ENSGALG00010023249	IL12RB2	-0.826855935	0.603291551	-1.370574366	0.170507678	0.215457135	negative
PRJNA729271	stage_E18_vs_E10	ordinary_muscle_development	ENSGALG00010023250	FZD10	-5.134611139	0.461215682	-11.13277656	8.68862E-29	1.00112E-27	negative
PRJNA729271	stage_E18_vs_E10	ordinary_muscle_development	ENSGALG00010023251	ENSGALG00010023251	1.243647576	1.237512286	1.00495776	0.31491719	0.372346538	positive
PRJNA729271	stage_E18_vs_E10	ordinary_muscle_development	ENSGALG00010023252	GLG1	0.106815559	0.079792144	1.338672616	0.180677275	0.227021246	positive
PRJNA729271	stage_E18_vs_E10	ordinary_muscle_development	ENSGALG00010023254	MYO3B	-1.113571441	0.317014835	-3.512679278	0.000443613	0.000850101	negative
PRJNA729271	stage_E18_vs_E10	ordinary_muscle_development	ENSGALG00010023255	SNX22	1.729198981	0.318707687	5.425658216	5.77414E-08	1.66759E-07	positive
PRJNA729271	stage_E18_vs_E10	ordinary_muscle_development	ENSGALG00010023257	PAK6	-0.098188862	0.303951743	-0.323040956	0.746664225	0.786948153	negative
PRJNA729271	stage_E18_vs_E10	ordinary_muscle_development	ENSGALG00010023258	ENSGALG00010023258	-0.910182872	0.386787314	-2.353186982	0.018613272	0.028198095	negative
PRJNA729271	stage_E18_vs_E10	ordinary_muscle_development	ENSGALG00010023259	TSPAN15	1.400548673	0.290207253	4.826029189	1.39282E-06	3.54503E-06	positive
PRJNA729271	stage_E18_vs_E10	ordinary_muscle_development	ENSGALG00010023260	ENSGALG00010023260	-1.568223738	0.225577724	-6.9520328	3.6006E-12	1.4764E-11	negative
PRJNA729271	stage_E18_vs_E10	ordinary_muscle_development	ENSGALG00010023261	SNX1	-0.238092354	0.096159977	-2.476002607	0.013286261	0.020654692	negative
PRJNA729271	stage_E18_vs_E10	ordinary_muscle_development	ENSGALG00010023262	POLD4	0.898248693	0.216990747	4.139571422	3.47955E-05	7.61415E-05	positive
PRJNA729271	stage_E18_vs_E10	ordinary_muscle_development	ENSGALG00010023263	SRP14	-0.776436551	0.073249595	-10.59987499	2.98379E-26	3.03083E-25	negative
PRJNA729271	stage_E18_vs_E10	ordinary_muscle_development	ENSGALG00010023264	RACGAP1	-1.426139831	0.22875156	-6.234448541	4.53372E-10	1.5801E-09	negative
PRJNA729271	stage_E18_vs_E10	ordinary_muscle_development	ENSGALG00010023265	RAB5C	-0.253207265	0.071313424	-3.550625523	0.000384317	0.000741471	negative
PRJNA729271	stage_E18_vs_E10	ordinary_muscle_development	ENSGALG00010023266	RFWD3	-1.009061267	0.141920427	-7.110049521	1.16001E-12	4.9425E-12	negative
PRJNA729271	stage_E18_vs_E10	ordinary_muscle_development	ENSGALG00010023267	DSCAML1	-1.647827813	0.747530884	-2.204360847	0.027498977	0.040462661	negative
PRJNA729271	stage_E18_vs_E10	ordinary_muscle_development	ENSGALG00010023268	KCNJ11	3.287679931	0.179990083	18.26589486	1.54657E-74	9.14999E-73	positive
PRJNA729271	stage_E18_vs_E10	ordinary_muscle_development	ENSGALG00010023269	OPRL1	-1.716600814	0.521418638	-3.292173864	0.000994161	0.001811258	negative
PRJNA729271	stage_E18_vs_E10	ordinary_muscle_development	ENSGALG00010023271	PATL2	-0.26332183	0.420572946	-0.626102635	0.531247624	0.587236659	negative
PRJNA729271	stage_E18_vs_E10	ordinary_muscle_development	ENSGALG00010023273	HDAC7	0.979093887	0.113343291	8.638304704	5.70533E-18	3.56E-17	positive
PRJNA729271	stage_E18_vs_E10	ordinary_muscle_development	ENSGALG00010023274	ZBTB38	0.717775229	0.115067228	6.237877126	4.43549E-10	1.54698E-09	positive
PRJNA729271	stage_E18_vs_E10	ordinary_muscle_development	ENSGALG00010023275	TWIST3	-7.478675344	0.596548991	-12.53656524	4.7097E-36	7.63614E-35	negative
PRJNA729271	stage_E18_vs_E10	ordinary_muscle_development	ENSGALG00010023276	ABCC8	2.689882102	0.161570499	16.64834927	3.11065E-62	1.3581E-60	positive
PRJNA729271	stage_E18_vs_E10	ordinary_muscle_development	ENSGALG00010023278	ENSGALG00010023278	0.930709107	0.242858415	3.832311544	0.000126945	0.00026141	positive
PRJNA729271	stage_E18_vs_E10	ordinary_muscle_development	ENSGALG00010023279	FA2H	-0.407235733	0.44143437	-0.922528376	0.356253036	0.415604648	negative

PRJNA729271	stage_E18_vs_E10	ordinary_muscle_development	ENSGALG00010023281	B2M	1.720237172	0.199952395	8.603233637	7.7501E-18	4.77829E-17	positive
PRJNA729271	stage_E18_vs_E10	ordinary_muscle_development	ENSGALG00010023282	SERBP1	-0.107220389	0.087284774	-1.228397392	0.219297827	0.270121907	negative
PRJNA729271	stage_E18_vs_E10	ordinary_muscle_development	ENSGALG00010023285	SLC48A1	2.015188128	0.111279446	18.10925742	2.69357E-73	1.54934E-71	positive
PRJNA729271	stage_E18_vs_E10	ordinary_muscle_development	ENSGALG00010023286	HCRT	-0.613138732	0.568086698	-1.079304856	0.280451849	0.336073872	negative
PRJNA729271	stage_E18_vs_E10	ordinary_muscle_development	ENSGALG00010023287	RGS19	-0.30943191	0.203883794	-1.517687617	0.129093167	0.167625679	negative
PRJNA729271	stage_E18_vs_E10	ordinary_muscle_development	ENSGALG00010023289	NFRKB	-0.362813597	0.087588434	-4.142254642	3.43908E-05	7.53355E-05	negative
PRJNA729271	stage_E18_vs_E10	ordinary_muscle_development	ENSGALG00010023290	SLC16A14	-1.610843317	0.23371687	-6.892285164	5.49032E-12	2.21554E-11	negative
PRJNA729271	stage_E18_vs_E10	ordinary_muscle_development	ENSGALG00010023293	COL11A1	-2.01099362	0.489112005	-4.111519649	3.93063E-05	8.56116E-05	negative
PRJNA729271	stage_E18_vs_E10	ordinary_muscle_development	ENSGALG00010023295	ENSGALG00010023295	0.873401083	0.51604601	1.692486844	0.090553186	0.121040985	positive
PRJNA729271	stage_E18_vs_E10	ordinary_muscle_development	ENSGALG00010023299	PASK	-1.034658658	0.139957393	-7.392668813	1.43911E-13	6.53709E-13	negative
PRJNA729271	stage_E18_vs_E10	ordinary_muscle_development	ENSGALG00010023304	ENSGALG00010023304	-0.334970754	0.175444722	-1.909266633	0.056227704	0.078224453	negative
PRJNA729271	stage_E18_vs_E10	ordinary_muscle_development	ENSGALG00010023305	PLCB2	0.043782722	0.166415861	0.263092241	0.792479484	0.826618337	positive
PRJNA729271	stage_E18_vs_E10	ordinary_muscle_development	ENSGALG00010023309	PIK3R3	-0.917607569	0.201135293	-4.562141003	5.06346E-06	1.21394E-05	negative
PRJNA729271	stage_E18_vs_E10	ordinary_muscle_development	ENSGALG00010023311	ETS1	0.203686035	0.133563372	1.525014166	0.127255564	0.165625339	positive
PRJNA729271	stage_E18_vs_E10	ordinary_muscle_development	ENSGALG00010023312	UBR3	0.471614273	0.114715442	4.111166438	3.93665E-05	8.5717E-05	positive
PRJNA729271	stage_E18_vs_E10	ordinary_muscle_development	ENSGALG00010023317	RAPGEF3	1.122230428	0.148998399	7.531828791	5.00345E-14	2.35166E-13	positive
PRJNA729271	stage_E18_vs_E10	ordinary_muscle_development	ENSGALG00010023319	EIF2AK4	-0.239899484	0.063065469	-3.803975257	0.000142392	0.000291358	negative
PRJNA729271	stage_E18_vs_E10	ordinary_muscle_development	ENSGALG00010023320	ENSGALG00010023320	-0.864030986	0.140586971	-6.14588237	7.95202E-10	2.71083E-09	negative
PRJNA729271	stage_E18_vs_E10	ordinary_muscle_development	ENSGALG00010023322	PRKAB1	0.007854071	0.091630828	0.085714289	0.931693543	0.94426639	positive
PRJNA729271	stage_E18_vs_E10	ordinary_muscle_development	ENSGALG00010023324	LAG3	-0.616884157	1.25175392	-0.492815838	0.622142719	0.672881563	negative
PRJNA729271	stage_E18_vs_E10	ordinary_muscle_development	ENSGALG00010023328	TCEA2	-0.55751692	0.108824815	-5.123067928	3.00604E-07	8.1673E-07	negative
PRJNA729271	stage_E18_vs_E10	ordinary_muscle_development	ENSGALG00010023330	TSPAN1	0.515695304	1.449874909	0.355682618	0.722078264	0.764649652	positive
PRJNA729271	stage_E18_vs_E10	ordinary_muscle_development	ENSGALG00010023331	CD4	-3.573969004	0.36560778	-9.775418359	1.43563E-22	1.17901E-21	negative
PRJNA729271	stage_E18_vs_E10	ordinary_muscle_development	ENSGALG00010023332	PPP1R7	-0.103232951	0.075783241	-1.362213456	0.17313053	0.218314662	negative
PRJNA729271	stage_E18_vs_E10	ordinary_muscle_development	ENSGALG00010023333	ENDOU	-1.478491801	0.597535893	-2.47431463	0.013349208	0.020748098	negative
PRJNA729271	stage_E18_vs_E10	ordinary_muscle_development	ENSGALG00010023334	RPS17	1.226959946	0.151606057	8.093080014	5.81746E-16	3.17605E-15	positive
PRJNA729271	stage_E18_vs_E10	ordinary_muscle_development	ENSGALG00010023335	WDR59	0.579884379	0.067854044	8.546054853	1.27367E-17	7.77344E-17	positive
PRJNA729271	stage_E18_vs_E10	ordinary_muscle_development	ENSGALG00010023336	GPR162	-0.799843705	0.151653091	-5.274166862	1.33361E-07	3.73178E-07	negative
PRJNA729271	stage_E18_vs_E10	ordinary_muscle_development	ENSGALG00010023337	FLI1	-0.050284833	0.117320087	-0.428612306	0.668205388	0.715229442	negative
PRJNA729271	stage_E18_vs_E10	ordinary_muscle_development	ENSGALG00010023338	HDLBP	0.058303359	0.124092087	0.469839461	0.638469719	0.687820036	positive
PRJNA729271	stage_E18_vs_E10	ordinary_muscle_development	ENSGALG00010023340	P3H3	0.631476708	0.157404055	4.011819832	6.02525E-05	0.000128795	positive
PRJNA729271	stage_E18_vs_E10	ordinary_muscle_development	ENSGALG00010023342	FITM2	1.509207631	0.134408924	11.22847786	2.95526E-29	3.48547E-28	positive
PRJNA729271	stage_E18_vs_E10	ordinary_muscle_development	ENSGALG00010023343	TMEM233	5.063464832	0.33425284	15.14860678	7.73898E-52	2.40207E-50	positive
PRJNA729271	stage_E18_vs_E10	ordinary_muscle_development	ENSGALG00010023347	STAT5A	1.529343433	0.169369273	9.029639222	1.72239E-19	1.17709E-18	positive
PRJNA729271	stage_E18_vs_E10	ordinary_muscle_development	ENSGALG00010023349	ENSGALG00010023349	3.054685126	0.571482984	5.34518999	9.03223E-08	2.56912E-07	positive
PRJNA729271	stage_E18_vs_E10	ordinary_muscle_development	ENSGALG00010023350	ZNRF1	-0.193825689	0.232071268	-0.835198992	0.403605677	0.463570863	negative
PRJNA729271	stage_E18_vs_E10	ordinary_muscle_development	ENSGALG00010023351	GNB3	0.975097537	0.365376025	2.66875074	0.007613393	0.012253623	positive
PRJNA729271	stage_E18_vs_E10	ordinary_muscle_development	ENSGALG00010023354	SEPTIN2	-2.14936749	0.172084631	-12.49017693	8.44656E-36	1.35435E-34	negative
PRJNA729271	stage_E18_vs_E10	ordinary_muscle_development	ENSGALG00010023356	ENSGALG00010023356	-0.711536547	0.203887203	-3.489853883	0.000483285	0.000920164	negative
PRJNA729271	stage_E18_vs_E10	ordinary_muscle_development	ENSGALG00010023359	CDCA3	-1.699024413	0.270893627	-6.271924632	3.56612E-10	1.24918E-09	negative
PRJNA729271	stage_E18_vs_E10	ordinary_muscle_development	ENSGALG00010023362	GADD45A	1.196373268	0.148635235	8.049055577	8.34354E-16	4.5093E-15	positive
PRJNA729271	stage_E18_vs_E10	ordinary_muscle_development	ENSGALG00010023363	CPEB1	-1.784585984	0.253497568	-7.039854469	1.92441E-12	8.05262E-12	negative
PRJNA729271	stage_E18_vs_E10	ordinary_muscle_development	ENSGALG00010023364	CTRB1	4.963071134	2.26294406	2.193192144	0.028293538	0.041539282	positive
PRJNA729271	stage_E18_vs_E10	ordinary_muscle_development	ENSGALG00010023366	GDAP1L1	-2.137468705	0.440000587	-4.857876944	1.18651E-06	3.04344E-06	negative
PRJNA729271	stage_E18_vs_E10	ordinary_muscle_development	ENSGALG00010023368	ENSGALG00010023368	-0.669069508	0.213049536	-3.140441052	0.001686937	0.002994018	negative
PRJNA729271	stage_E18_vs_E10	ordinary_muscle_development	ENSGALG00010023369	ACE	3.232698711	0.476354289	6.786332749	1.1502E-11	4.50111E-11	positive
PRJNA729271	stage_E18_vs_E10	ordinary_muscle_development	ENSGALG00010023374	ENSGALG00010023374	-0.926352074	0.208623154	-4.44031285	8.98282E-06	2.10111E-05	negative
PRJNA729271	stage_E18_vs_E10	ordinary_muscle_development	ENSGALG00010023375	ST14	0.354014976	0.146963234	2.408867626	0.016002101	0.02458665	positive
PRJNA729271	stage_E18_vs_E10	ordinary_muscle_development	ENSGALG00010023377	OSER1	0.211672171	0.152460228	1.388376319	0.165022483	0.209109266	positive
PRJNA729271	stage_E18_vs_E10	ordinary_muscle_development	ENSGALG00010023379	CCDC60	1.69195273	0.482794762	3.504496868	0.000457471	0.000874923	positive
PRJNA729271	stage_E18_vs_E10	ordinary_muscle_development	ENSGALG00010023380	USP5	-1.023795329	0.097635351	-10.48590819	1.00278E-25	9.89464E-25	negative
PRJNA729271	stage_E18_vs_E10	ordinary_muscle_development	ENSGALG00010023381	HOXB7	-0.394754593	0.165582134	-2.38404098	0.017123695	0.026132656	negative
PRJNA729271	stage_E18_vs_E10	ordinary_muscle_development	ENSGALG00010023383	HOXB6	-0.475871451	0.174217663	-2.731476494	0.006305124	0.01029082	negative
PRJNA729271	stage_E18_vs_E10	ordinary_muscle_development	ENSGALG00010023384	AP3B2	-2.093343287	0.2369068	-8.836146896	9.90765E-19	6.48944E-18	negative
PRJNA729271	stage_E18_vs_E10	ordinary_muscle_development	ENSGALG00010023387	ENSGALG00010023387	2.118216358	0.182586293	11.60117946	4.06438E-31	5.27423E-30	positive
PRJNA729271	stage_E18_vs_E10	ordinary_muscle_development	ENSGALG00010023388	FARP2	-0.552658235	0.08432235	-6.554113256	5.59735E-11	2.08355E-10	negative
PRJNA729271	stage_E18_vs_E10	ordinary_muscle_development	ENSGALG00010023389	GNG12	0.276966288	0.109501966	2.529327091	0.011428147	0.017939245	positive
PRJNA729271	stage_E18_vs_E10	ordinary_muscle_development	ENSGALG00010023390	HSPB8	2.244242631	0.250867972	8.945911321	3.68893E-19	2.47781E-18	positive
PRJNA729271	stage_E18_vs_E10	ordinary_muscle_development	ENSGALG00010023392	TPI1	1.722075592	0.149577211	11.51295425	1.13522E-30	1.42962E-29	positive
PRJNA729271	stage_E18_vs_E10	ordinary_muscle_development	ENSGALG00010023394	HOXB5	-1.667844288	0.182931093	-9.117336261	7.69934E-20	5.36806E-19	negative
PRJNA729271	stage_E18_vs_E10	ordinary_muscle_development	ENSGALG00010023396	ENSGALG00010023396	0.590074076	0.140856629	4.189182146	2.79962E-05	6.20306E-05	positive
PRJNA729271	stage_E18_vs_E10	ordinary_muscle_development	ENSGALG00010023397	ENSGALG00010023397	0.254183853	0.245535047	1.035224323	0.300564162	0.357544319	positive
PRJNA729271	stage_E18_vs_E10	ordinary_muscle_development	ENSGALG00010023401	LRRC23	2.344616941	0.355509305	6.595093031	4.24989E-11	1.5955E-10	positive
PRJNA729271	stage_E18_vs_E10	ordinary_muscle_development	ENSGALG00010023405	DNAJA2	-0.442201524	0.07745664	-5.709020198	1.13628E-08	3.49024E-08	negative
PRJNA729271	stage_E18_vs_E10	ordinary_muscle_development	ENSGALG00010023408	ZBTB44	0.124807803	0.127643571	0.977783698	0.328181316	0.386275937	positive
PRJNA729271	stage_E18_vs_E10	ordinary_muscle_development	ENSGALG00010023411	ENSGALG00010023411	3.119341218	0.181144022	17.22022717	1.87252E-66	9.01732E-65	positive
PRJNA729271	stage_E18_vs_E10	ordinary_muscle_development	ENSGALG00010023412	GSS	-0.17254231	0.106583053	-1.618853134	0.105478867	0.139296298	negative
PRJNA729271	stage_E18_vs_E10	ordinary_muscle_development	ENSGALG00010023414	SUDS3	-0.484083657	0.076560132	-6.322920844	2.56665E-10	9.07847E-10	negative
PRJNA729271	stage_E18_vs_E10	ordinary_muscle_development	ENSGALG00010023415	HOXB4	-0.034899415	0.166927199	-0.209069672	0.83439385	0.862540212	negative
PRJNA729271	stage_E18_vs_E10	ordinary_muscle_development	ENSGALG00010023421	OLFM3	-0.099322131	0.252798746	-0.392890127	0.694400634	0.739671991	negative
PRJNA729271	stage_E18_vs_E10	ordinary_muscle_development	ENSGALG00010023422	WLS	-1.472660674	0.15556268	-9.466670736	2.88905E-21	2.20986E-20	negative
PRJNA729271	stage_E18_vs_E10	ordinary_muscle_development	ENSGALG00010023426	KCNJ1	-0.333935297	0.494425493	-0.675400645	0.499421256	0.556640608	negative
PRJNA729271	stage_E18_vs_E10	ordinary_muscle_development	ENSGALG00010023428	HOXB3	-0.803869723	0.18092161	-4.443193505	8.86334E-06	2.07417E-05	negative
PRJNA729271	stage_E18_vs_E10	ordinary_muscle_development	ENSGALG00010023429	BMF	1.428372366	0.145625898	9.808505127	1.0349E-22	8.54266E-22	positive
PRJNA729271	stage_E18_vs_E10	ordinary_muscle_development	ENSGALG00010023431	BCAR1	0.125126808	0.174004069	0.719102766	0.472077603	0.530873718	positive
PRJNA729271	stage_E18_vs_E10	ordinary_muscle_development	ENSGALG00010023432	TMOD3	-0.405946759	0.174985176	-2.319892284	0.020346705	0.030641609	negative
PRJNA729271	stage_E18_vs_E10	ordinary_muscle_development	ENSGALG00010023434	ATN1	0.308224109	0.185113113	1.665058213	0.095901142	0.127659754	positive
PRJNA729271	stage_E18_vs_E10	ordinary_muscle_development	ENSGALG00010023437	KCNJ5	1.50342026	0.245619372	6.12093519	9.30277E-10	3.15129E-09	positive
PRJNA729271	stage_E18_vs_E10	ordinary_muscle_development	ENSGALG00010023438	HOXB2	-0.389281017	0.245444734	-1.586023097	0.11273407	0.148108434	negative
PRJNA729271	stage_E18_vs_E10	ordinary_muscle_development	ENSGALG00010023439	ACSS2	1.600287073	0.132220133	12.10320269	1.0157E-33	1.46202E-32	positive
PRJNA729271	stage_E18_vs_E10	ordinary_muscle_development	ENSGALG00010023441	ENSGALG00010023441	1.217949763	0.15185883	8.0202762	1.05508E-15	5.65582E-15	positive
PRJNA729271	stage_E18_vs_E10	ordinary_muscle_development	ENSGALG00010023442	STK25	-0.804706305	0.113172073	-7.110467127	1.15651E-12	4.92902E-12	negative
PRJNA729271	stage_E18_vs_E10	ordinary_muscle_development	ENSGALG00010023443	ENSGALG00010023443	0.26195092	0.184994011	1.415996758	0.156776454	0.199760434	positive
PRJNA729271	stage_E18_vs_E10	ordinary_muscle_development	ENSGALG00010023447	C12orf57	0.745465803	0.113562583	6.564361097	5.22564E-11	1.9502E-10	positive
PRJNA729271	stage_E18_vs_E10	ordinary_muscle_development	ENSGALG00010023448	FAM219B	0.681049782	0.115853738	5.878530944	4.13923E-09	1.32446E-08	positive
PRJNA729271	stage_E18_vs_E10	ordinary_muscle_development	ENSGALG00010023453	ARHGAP32	0.097285384	0.173200979	0.561690726	0.574326754	0.627439425	positive
PRJNA729271	stage_E18_vs_E10	ordinary_muscle_development	ENSGALG00010023454	HOXB1	1.062598906	0.405290471	2.6218206	0.008746146	0.013965122	positive
PRJNA729271	stage_E18_vs_E10	ordinary_muscle_development	ENSGALG00010023455	CFDP1	-0.719913322	0.097323571	-7.397111684	1.39178E-13	6.3261E-13	negative
PRJNA729271	stage_E18_vs_E10	ordinary_muscle_development	ENSGALG00010023456	MPI	-0.398558251	0.14707091	-2.709973374	0.006728861	0.010914821	negative
PRJNA729271	stage_E18_vs_E10	ordinary_muscle_development	ENSGALG00010023469	ENSGALG00010023469	0.929839016	0.213899717	4.34707923	1.37962E-05	3.16669E-05	positive
PRJNA729271	stage_E18_vs_E10	ordinary_muscle_development	ENSGALG00010023472	PTPN6	0.073267739	0.198355154	0.36937653	0.711847089	0.755858439	positive
PRJNA729271	stage_E18_vs_E10	ordinary_muscle_development	ENSGALG00010023473	BOK	-0.304493211	0.200498806	-1.518678428	0.128843459	0.167346975	negative
PRJNA729271	stage_E18_vs_E10	ordinary_muscle_development	ENSGALG00010023475	ENSGALG00010023475	-2.85583851	0.566675196	-5.03963916	4.6641E-07	1.24345E-06	negative
PRJNA729271	stage_E18_vs_E10	ordinary_muscle_development	ENSGALG00010023476	SCAMP2	-0.012482455	0.151549567	-0.082365497	0.934356072	0.946237076	negative
PRJNA729271	stage_E18_vs_E10	ordinary_muscle_development	ENSGALG00010023478	TAOK3	0.62611579	0.123885557	5.053985329	4.32685E-07	1.15801E-06	positive
PRJNA729271	stage_E18_vs_E10	ordinary_muscle_development	ENSGALG00010023479	MCCC2L	0.751516795	0.156833011	4.791827867	1.65269E-06	4.17056E-06	positive
PRJNA729271	stage_E18_vs_E10	ordinary_muscle_development	ENSGALG00010023482	MTCL2	1.247362615	0.097503881	12.79295345	1.7952E-37	3.09042E-36	positive
PRJNA729271	stage_E18_vs_E10	ordinary_muscle_development	ENSGALG00010023485	BARX2	-2.606142495	0.30153832	-8.642823558	5.48406E-18	3.42783E-17	negative
PRJNA729271	stage_E18_vs_E10	ordinary_muscle_development	ENSGALG00010023487	GRN	-0.085601587	0.147038285	-0.582172096	0.560450767	0.614410366	negative
PRJNA729271	stage_E18_vs_E10	ordinary_muscle_development	ENSGALG00010023489	ETV5	-0.694851499	0.218362033	-3.182107667	0.001462075	0.002611555	negative
PRJNA729271	stage_E18_vs_E10	ordinary_muscle_development	ENSGALG00010023490	PHB2	0.115973673	0.103013637	1.125808933	0.260246402	0.314487003	positive
PRJNA729271	stage_E18_vs_E10	ordinary_muscle_development	ENSGALG00010023496	PLEKHA3	-0.279978781	0.10731534	-2.608935318	0.009082441	0.014457498	negative
PRJNA729271	stage_E18_vs_E10	ordinary_muscle_development	ENSGALG00010023499	PEBP1	-0.008133706	0.139853555	-0.058158733	0.953622192	0.961783584	negative
PRJNA729271	stage_E18_vs_E10	ordinary_muscle_development	ENSGALG00010023501	ENSGALG00010023501	1.243527409	3.076410128	0.404213794	0.686055511	0.731935475	positive
PRJNA729271	stage_E18_vs_E10	ordinary_muscle_development	ENSGALG00010023506	RPE65	-0.721836043	0.571032054	-1.264090235	0.206197641	0.255762006	negative
PRJNA729271	stage_E18_vs_E10	ordinary_muscle_development	ENSGALG00010023510	ASPHD2	0.692165394	0.245353784	2.821091173	0.00478606	0.007949345	positive
PRJNA729271	stage_E18_vs_E10	ordinary_muscle_development	ENSGALG00010023513	CDCA9	-0.364772923	0.212840426	-1.713832894	0.08655942	0.116130951	negative
PRJNA729271	stage_E18_vs_E10	ordinary_muscle_development	ENSGALG00010023514	ULK3	-0.087403795	0.111492298	-0.783944692	0.433072564	0.492885271	negative
PRJNA729271	stage_E18_vs_E10	ordinary_muscle_development	ENSGALG00010023518	METTL5	-0.206293418	0.120734907	-1.708647664	0.087516234	0.117306067	negative
PRJNA729271	stage_E18_vs_E10	ordinary_muscle_development	ENSGALG00010023519	DGKG	0.343609797	0.242812731	1.415122656	0.157032536	0.199998824	positive
PRJNA729271	stage_E18_vs_E10	ordinary_muscle_development	ENSGALG00010023522	EMG1	-1.29132302	0.16369199	-7.888736771	3.05261E-15	1.5848E-14	negative
PRJNA729271	stage_E18_vs_E10	ordinary_muscle_development	ENSGALG00010023526	KNSTRN	-1.947404951	0.188902571	-10.30904419	6.4136E-25	6.06031E-24	negative
PRJNA729271	stage_E18_vs_E10	ordinary_muscle_development	ENSGALG00010023527	TMEM98	0.176852858	0.225061441	0.785798125	0.431985765	0.491880099	positive
PRJNA729271	stage_E18_vs_E10	ordinary_muscle_development	ENSGALG00010023533	FKBP7	0.386128456	0.162260085	2.379688484	0.017327279	0.026415535	positive
PRJNA729271	stage_E18_vs_E10	ordinary_muscle_development	ENSGALG00010023536	ENSGALG00010023536	0.264685361	0.192197896	1.377150149	0.168465831	0.212988418	positive
PRJNA729271	stage_E18_vs_E10	ordinary_muscle_development	ENSGALG00010023541	PRSS2	1.243558569	2.614160467	0.475700932	0.634287457	0.68377188	positive
PRJNA729271	stage_E18_vs_E10	ordinary_muscle_development	ENSGALG00010023543	ENSGALG00010023543	-0.152463717	0.638156521	-0.238912731	0.811173253	0.842924676	negative
PRJNA729271	stage_E18_vs_E10	ordinary_muscle_development	ENSGALG00010023544	SSB	-1.29400705	0.103779085	-12.46886163	1.10392E-35	1.74877E-34	negative
PRJNA729271	stage_E18_vs_E10	ordinary_muscle_development	ENSGALG00010023545	PTPRT	2.160298393	0.126744948	17.0444537	3.84318E-65	1.797E-63	positive
PRJNA729271	stage_E18_vs_E10	ordinary_muscle_development	ENSGALG00010023546	SLC11A2	0.191624913	0.156579591	1.223817945	0.221020913	0.271937708	positive
PRJNA729271	stage_E18_vs_E10	ordinary_muscle_development	ENSGALG00010023551	ENSGALG00010023551	0.508648461	0.175655278	2.895719766	0.0037829	0.006370021	positive
PRJNA729271	stage_E18_vs_E10	ordinary_muscle_development	ENSGALG00010023552	SGPP2	0.783336512	0.3445256	2.273667072	0.022986011	0.03429565	positive
PRJNA729271	stage_E18_vs_E10	ordinary_muscle_development	ENSGALG00010023555	HPS4	-0.821035143	0.170830047	-4.806151827	1.53863E-06	3.89631E-06	negative
PRJNA729271	stage_E18_vs_E10	ordinary_muscle_development	ENSGALG00010023556	IVD	0.251603496	0.12166263	2.068042554	0.038636022	0.055349786	positive
PRJNA729271	stage_E18_vs_E10	ordinary_muscle_development	ENSGALG00010023559	PJVK	0.086502032	0.74483568	0.116135725	0.907544976	0.92360208	positive
PRJNA729271	stage_E18_vs_E10	ordinary_muscle_development	ENSGALG00010023569	LPCAT3	-1.005307171	0.108744614	-9.244661704	2.35986E-20	1.70011E-19	negative
PRJNA729271	stage_E18_vs_E10	ordinary_muscle_development	ENSGALG00010023571	APLP2	1.577823858	0.098558383	16.00902747	1.10524E-57	4.08685E-56	positive
PRJNA729271	stage_E18_vs_E10	ordinary_muscle_development	ENSGALG00010023572	MYBL2	-0.985866019	0.128481264	-7.67322787	1.67721E-14	8.20769E-14	negative
PRJNA729271	stage_E18_vs_E10	ordinary_muscle_development	ENSGALG00010023574	DEPDC1	-1.779730825	0.205737138	-8.650508317	5.12705E-18	3.21022E-17	negative
PRJNA729271	stage_E18_vs_E10	ordinary_muscle_development	ENSGALG00010023578	SRRD	-0.352805234	0.104126835	-3.388225845	0.000703463	0.001310293	negative
PRJNA729271	stage_E18_vs_E10	ordinary_muscle_development	ENSGALG00010023580	RDM1	-0.949519235	0.163752832	-5.798490467	6.69145E-09	2.10115E-08	negative

PRJNA729271	stage_E18_vs_E10	ordinary_muscle_development	ENSGALG00010023585	TFIP11	-0.471446437	0.097734261	-4.823758159	1.40878E-06	3.58314E-06	negative
PRJNA729271	stage_E18_vs_E10	ordinary_muscle_development	ENSGALG00010023588	FARSB	-1.009105194	0.135971562	-7.421442988	1.15851E-13	5.30573E-13	negative
PRJNA729271	stage_E18_vs_E10	ordinary_muscle_development	ENSGALG00010023590	S1PR1	0.48281297	0.11568898	4.173370433	3.00126E-05	6.61748E-05	positive
PRJNA729271	stage_E18_vs_E10	ordinary_muscle_development	ENSGALG00010023592	BAHD1	-0.554935218	0.105449748	-5.262556127	1.42066E-07	3.96238E-07	negative
PRJNA729271	stage_E18_vs_E10	ordinary_muscle_development	ENSGALG00010023593	CSK	-0.71187063	0.083546055	-8.520697164	1.58596E-17	9.61056E-17	negative
PRJNA729271	stage_E18_vs_E10	ordinary_muscle_development	ENSGALG00010023597	MOGAT1	0.429187269	0.33973799	1.263289009	0.206485335	0.256096939	positive
PRJNA729271	stage_E18_vs_E10	ordinary_muscle_development	ENSGALG00010023598	IL10RA	1.510660652	0.421727408	3.582078435	0.000340871	0.000661968	positive
PRJNA729271	stage_E18_vs_E10	ordinary_muscle_development	ENSGALG00010023600	CHST14	-1.988815523	0.08511883	-23.36516505	9.6646E-121	1.7511E-118	negative
PRJNA729271	stage_E18_vs_E10	ordinary_muscle_development	ENSGALG00010023601	TPST2	1.403290922	0.104179905	13.46988104	2.35245E-41	4.75575E-40	positive
PRJNA729271	stage_E18_vs_E10	ordinary_muscle_development	ENSGALG00010023603	C1S	0.032508473	0.395683128	0.082157845	0.934521195	0.946338181	positive
PRJNA729271	stage_E18_vs_E10	ordinary_muscle_development	ENSGALG00010023604	ENSGALG00010023604	0.470057761	0.412178307	1.14042334	0.254109974	0.307867274	positive
PRJNA729271	stage_E18_vs_E10	ordinary_muscle_development	ENSGALG00010023605	INSYN1	1.859447318	0.313459951	5.93200922	2.9925E-09	9.69519E-09	positive
PRJNA729271	stage_E18_vs_E10	ordinary_muscle_development	ENSGALG00010023606	LRRC7	-0.764602969	0.262885339	-2.908503655	0.003631629	0.006128824	negative
PRJNA729271	stage_E18_vs_E10	ordinary_muscle_development	ENSGALG00010023612	GPT2	1.375949118	0.186005943	7.397339531	1.3894E-13	6.31724E-13	positive
PRJNA729271	stage_E18_vs_E10	ordinary_muscle_development	ENSGALG00010023613	COPB1	-0.572666302	0.051767691	-11.06223376	1.91274E-28	2.15928E-27	negative
PRJNA729271	stage_E18_vs_E10	ordinary_muscle_development	ENSGALG00010023615	PRSS3	1.243396642	5.150527732	0.241411503	0.809236198	0.841273664	positive
PRJNA729271	stage_E18_vs_E10	ordinary_muscle_development	ENSGALG00010023617	ENSGALG00010023617	1.040061955	0.298842351	3.480303086	0.000500847	0.000951602	positive
PRJNA729271	stage_E18_vs_E10	ordinary_muscle_development	ENSGALG00010023620	IRS1	0.563303419	0.141757596	3.97370889	7.0762E-05	0.000149999	positive
PRJNA729271	stage_E18_vs_E10	ordinary_muscle_development	ENSGALG00010023621	CD79B	-0.009286979	1.194433752	-0.007775214	0.993796339	0.994757125	negative
PRJNA729271	stage_E18_vs_E10	ordinary_muscle_development	ENSGALG00010023623	OSBPL6	-1.101215	0.224671803	-4.901438395	9.51375E-07	2.46561E-06	negative
PRJNA729271	stage_E18_vs_E10	ordinary_muscle_development	ENSGALG00010023624	PSMA1	-0.146443062	0.071478329	-2.048775662	0.040484057	0.05775752	negative
PRJNA729271	stage_E18_vs_E10	ordinary_muscle_development	ENSGALG00010023626	LOXL1	0.978834732	0.19515566	5.015661506	5.28513E-07	1.40282E-06	positive
PRJNA729271	stage_E18_vs_E10	ordinary_muscle_development	ENSGALG00010023627	IFT52	-0.34518442	0.10267775	-3.361822971	0.000774297	0.001434306	negative
PRJNA729271	stage_E18_vs_E10	ordinary_muscle_development	ENSGALG00010023631	ENSGALG00010023631	3.1255397	0.451604013	6.920974156	4.48548E-12	1.82684E-11	positive
PRJNA729271	stage_E18_vs_E10	ordinary_muscle_development	ENSGALG00010023635	SCN4A	7.76021632	0.317930917	24.40849855	1.3894E-131	3.1967E-129	positive
PRJNA729271	stage_E18_vs_E10	ordinary_muscle_development	ENSGALG00010023636	PDE3B	-0.101097068	0.082032602	-1.232401086	0.217799296	0.268520948	negative
PRJNA729271	stage_E18_vs_E10	ordinary_muscle_development	ENSGALG00010023637	RHBDD1	0.394800973	0.106449737	3.708801768	0.000208242	0.000416686	positive
PRJNA729271	stage_E18_vs_E10	ordinary_muscle_development	ENSGALG00010023638	DCTN2	0.041051808	0.13156656	0.312023113	0.755022958	0.794486953	positive
PRJNA729271	stage_E18_vs_E10	ordinary_muscle_development	ENSGALG00010023639	KLHL23	0.821327599	0.217999632	3.767564154	0.000164848	0.000333959	positive
PRJNA729271	stage_E18_vs_E10	ordinary_muscle_development	ENSGALG00010023641	LRRC40	-0.945973596	0.111195453	-8.50730467	1.78023E-17	1.07519E-16	negative
PRJNA729271	stage_E18_vs_E10	ordinary_muscle_development	ENSGALG00010023642	PAX6	3.298180674	0.517434707	6.374100208	1.8404E-10	6.57708E-10	positive
PRJNA729271	stage_E18_vs_E10	ordinary_muscle_development	ENSGALG00010023643	SCN4B	3.565818154	0.149661044	23.82596066	1.7979E-125	3.5699E-123	positive
PRJNA729271	stage_E18_vs_E10	ordinary_muscle_development	ENSGALG00010023646	DPH5	-0.777990618	0.146593171	-5.307140933	1.11358E-07	3.14646E-07	negative
PRJNA729271	stage_E18_vs_E10	ordinary_muscle_development	ENSGALG00010023648	SCN2B	2.475150977	0.359737603	6.880434388	5.96703E-12	2.3959E-11	positive
PRJNA729271	stage_E18_vs_E10	ordinary_muscle_development	ENSGALG00010023652	SLC30A7	-0.380554792	0.095771079	-3.973587796	7.0798E-05	0.000150054	negative
PRJNA729271	stage_E18_vs_E10	ordinary_muscle_development	ENSGALG00010023655	C1R	0.42788805	0.186149016	2.298631815	0.021525855	0.032273197	positive
PRJNA729271	stage_E18_vs_E10	ordinary_muscle_development	ENSGALG00010023658	ENSGALG00010023658	0.649112271	0.280107106	2.317371668	0.020483494	0.030834796	positive
PRJNA729271	stage_E18_vs_E10	ordinary_muscle_development	ENSGALG00010023660	CRYBB1	1.037830629	0.464875715	2.232490526	0.025582561	0.037858011	positive
PRJNA729271	stage_E18_vs_E10	ordinary_muscle_development	ENSGALG00010023663	PHOSPHO2	-0.104568776	0.097116446	-1.076736022	0.281598215	0.337224564	negative
PRJNA729271	stage_E18_vs_E10	ordinary_muscle_development	ENSGALG00010023664	SLC6A9	0.176223409	0.324847843	0.542479849	0.587487986	0.639744449	positive
PRJNA729271	stage_E18_vs_E10	ordinary_muscle_development	ENSGALG00010023665	C16orf87	0.376887735	0.136687234	2.75730018	0.005828082	0.009561749	positive
PRJNA729271	stage_E18_vs_E10	ordinary_muscle_development	ENSGALG00010023666	ENSGALG00010023666	0.537751402	0.263092447	2.043963665	0.040957137	0.058374995	positive
PRJNA729271	stage_E18_vs_E10	ordinary_muscle_development	ENSGALG00010023668	RCN1	0.720714032	0.160926272	4.478535553	7.51569E-06	1.77253E-05	positive
PRJNA729271	stage_E18_vs_E10	ordinary_muscle_development	ENSGALG00010023670	EXTL2	0.604190358	0.126940193	4.759645826	1.93933E-06	4.85303E-06	positive
PRJNA729271	stage_E18_vs_E10	ordinary_muscle_development	ENSGALG00010023671	ENSGALG00010023671	1.051511836	0.806996756	1.302993882	0.19257685	0.240306598	positive
PRJNA729271	stage_E18_vs_E10	ordinary_muscle_development	ENSGALG00010023676	ENSGALG00010023676	-0.678026041	0.299527743	-2.263650219	0.023595637	0.035129288	negative
PRJNA729271	stage_E18_vs_E10	ordinary_muscle_development	ENSGALG00010023677	MN1	0.934179567	0.161002288	5.802275108	6.54211E-09	2.057E-08	positive
PRJNA729271	stage_E18_vs_E10	ordinary_muscle_development	ENSGALG00010023680	MPZL3	-7.35427829	0.797588415	-9.220643319	2.95323E-20	2.10665E-19	negative
PRJNA729271	stage_E18_vs_E10	ordinary_muscle_development	ENSGALG00010023686	MYLK3	1.94289989	0.218030932	8.911120401	5.05194E-19	3.37611E-18	positive
PRJNA729271	stage_E18_vs_E10	ordinary_muscle_development	ENSGALG00010023689	WT1	-1.523045223	0.237313158	-6.417870942	1.38193E-10	4.98039E-10	negative
PRJNA729271	stage_E18_vs_E10	ordinary_muscle_development	ENSGALG00010023693	SRSF11	-0.422066239	0.06901559	-6.115520258	9.62425E-10	3.25562E-09	negative
PRJNA729271	stage_E18_vs_E10	ordinary_muscle_development	ENSGALG00010023694	PPCDC	1.551392515	0.152273448	10.18820116	2.23868E-24	2.04086E-23	positive
PRJNA729271	stage_E18_vs_E10	ordinary_muscle_development	ENSGALG00010023695	CLSTN3	-0.449099165	0.130647475	-3.437488281	0.000587136	0.001105265	negative
PRJNA729271	stage_E18_vs_E10	ordinary_muscle_development	ENSGALG00010023696	LRRC4C	-0.180915644	0.530508516	-0.341023072	0.733086213	0.774608883	negative
PRJNA729271	stage_E18_vs_E10	ordinary_muscle_development	ENSGALG00010023698	ENSGALG00010023698	-1.176329974	0.144851725	-8.120924843	4.62644E-16	2.54209E-15	negative
PRJNA729271	stage_E18_vs_E10	ordinary_muscle_development	ENSGALG00010023699	PP1G	-0.675907987	0.204135532	-3.311074667	0.000929384	0.001700078	negative
PRJNA729271	stage_E18_vs_E10	ordinary_muscle_development	ENSGALG00010023702	COL4A4	1.79563039	0.250272617	7.174697772	7.24671E-13	3.14118E-12	positive
PRJNA729271	stage_E18_vs_E10	ordinary_muscle_development	ENSGALG00010023703	PITPNB	-0.793747366	0.080983156	-9.801388431	1.11049E-22	9.14573E-22	negative
PRJNA729271	stage_E18_vs_E10	ordinary_muscle_development	ENSGALG00010023704	ANKRD13C	-0.51396389	0.069415316	-7.404185684	1.31958E-13	6.01486E-13	negative
PRJNA729271	stage_E18_vs_E10	ordinary_muscle_development	ENSGALG00010023706	CDK5RAP3	-0.611977898	0.130162856	-4.701632376	2.5809E-06	6.37419E-06	negative
PRJNA729271	stage_E18_vs_E10	ordinary_muscle_development	ENSGALG00010023707	MPZL2	-4.327818979	0.375720232	-11.51872752	1.06167E-30	1.3405E-29	negative
PRJNA729271	stage_E18_vs_E10	ordinary_muscle_development	ENSGALG00010023710	CDC14A	0.343574825	0.121279067	2.83292767	0.004612382	0.007681121	positive
PRJNA729271	stage_E18_vs_E10	ordinary_muscle_development	ENSGALG00010023711	ENSGALG00010023711	-0.231999668	0.350175202	-0.662524548	0.507635108	0.564579981	negative
PRJNA729271	stage_E18_vs_E10	ordinary_muscle_development	ENSGALG00010023712	ARHGAP40	-0.004991062	0.164565461	-0.030328732	0.975804882	0.979928763	negative
PRJNA729271	stage_E18_vs_E10	ordinary_muscle_development	ENSGALG00010023714	EIF3M	0.420049842	0.110383868	3.805355345	0.000141601	0.000289902	positive
PRJNA729271	stage_E18_vs_E10	ordinary_muscle_development	ENSGALG00010023715	CD3E	-1.377573199	0.39219824	-3.51244105	0.00044401	0.000850751	negative
PRJNA729271	stage_E18_vs_E10	ordinary_muscle_development	ENSGALG00010023716	FASTKD1	-0.539923268	0.108127899	-4.993376112	5.93329E-07	1.56654E-06	negative
PRJNA729271	stage_E18_vs_E10	ordinary_muscle_development	ENSGALG00010023720	SCAMP5	-0.678463569	0.097248429	-6.97660182	3.02405E-12	1.24634E-11	negative
PRJNA729271	stage_E18_vs_E10	ordinary_muscle_development	ENSGALG00010023724	CYP2R1	-0.530514326	0.128510548	-4.128177307	3.6565E-05	7.98689E-05	negative
PRJNA729271	stage_E18_vs_E10	ordinary_muscle_development	ENSGALG00010023726	PNPO	-0.102713766	0.192065618	-0.534784764	0.5927987	0.644669828	negative
PRJNA729271	stage_E18_vs_E10	ordinary_muscle_development	ENSGALG00010023730	SP2	0.832740687	0.188125793	4.426509916	9.577E-06	2.23397E-05	positive
PRJNA729271	stage_E18_vs_E10	ordinary_muscle_development	ENSGALG00010023731	ACTR5	0.098778728	0.098472938	1.00310532	0.315810048	0.373290928	positive
PRJNA729271	stage_E18_vs_E10	ordinary_muscle_development	ENSGALG00010023732	TM2D1	-0.519717961	0.080172269	-6.482515314	9.02059E-11	3.30102E-10	negative
PRJNA729271	stage_E18_vs_E10	ordinary_muscle_development	ENSGALG00010023733	ORC6	-1.612114356	0.192224646	-8.386616334	5.00335E-17	2.93024E-16	negative
PRJNA729271	stage_E18_vs_E10	ordinary_muscle_development	ENSGALG00010023736	KIF5A	-2.751094853	0.689289265	-3.991205132	6.57384E-05	0.000139944	negative
PRJNA729271	stage_E18_vs_E10	ordinary_muscle_development	ENSGALG00010023738	ENSGALG00010023738	0.125134757	0.407677652	0.306945343	0.758884975	0.797797919	positive
PRJNA729271	stage_E18_vs_E10	ordinary_muscle_development	ENSGALG00010023740	PPP1R16B	0.617397991	0.102465366	6.025431013	1.6866E-09	5.57519E-09	positive
PRJNA729271	stage_E18_vs_E10	ordinary_muscle_development	ENSGALG00010023743	CD3D	0.511472958	0.237064682	2.157524915	0.030964788	0.045131684	positive
PRJNA729271	stage_E18_vs_E10	ordinary_muscle_development	ENSGALG00010023744	MT4	2.472667635	0.315668941	7.833103975	4.7597E-15	2.43529E-14	positive
PRJNA729271	stage_E18_vs_E10	ordinary_muscle_development	ENSGALG00010023746	TTC28	-0.737370285	0.088906482	-8.29377417	1.09711E-16	6.28311E-16	negative
PRJNA729271	stage_E18_vs_E10	ordinary_muscle_development	ENSGALG00010023748	ENSGALG00010023748	-1.088016569	0.176069652	-6.179466807	6.43185E-10	2.21501E-09	negative
PRJNA729271	stage_E18_vs_E10	ordinary_muscle_development	ENSGALG00010023749	RPP25	1.142471588	0.2431524	4.698582494	2.61973E-06	6.4646E-06	positive
PRJNA729271	stage_E18_vs_E10	ordinary_muscle_development	ENSGALG00010023750	ENSGALG00010023750	-1.370253365	0.164351943	-8.337311622	7.59986E-17	4.39409E-16	negative
PRJNA729271	stage_E18_vs_E10	ordinary_muscle_development	ENSGALG00010023751	PRRG4	-1.573999741	0.309208052	-5.090422877	3.57266E-07	9.64875E-07	negative
PRJNA729271	stage_E18_vs_E10	ordinary_muscle_development	ENSGALG00010023752	C1QL1	-0.231146152	0.263822126	-0.876143922	0.380951798	0.441257496	negative
PRJNA729271	stage_E18_vs_E10	ordinary_muscle_development	ENSGALG00010023759	CALCA	-1.610841617	0.293667302	-5.485260379	4.12861E-08	1.21142E-07	negative
PRJNA729271	stage_E18_vs_E10	ordinary_muscle_development	ENSGALG00010023760	ZW10	-0.882121478	0.094614429	-9.3233293	1.12746E-20	8.2999E-20	negative
PRJNA729271	stage_E18_vs_E10	ordinary_muscle_development	ENSGALG00010023762	BBS5	2.901996841	0.152089542	19.08084413	3.64327E-81	2.65373E-79	positive
PRJNA729271	stage_E18_vs_E10	ordinary_muscle_development	ENSGALG00010023764	COX5A	0.643272907	0.178389641	3.605999216	0.000310954	0.000607368	positive
PRJNA729271	stage_E18_vs_E10	ordinary_muscle_development	ENSGALG00010023766	ENSGALG00010023766	0.766793596	0.331102913	2.315876928	0.020564989	0.030947832	positive
PRJNA729271	stage_E18_vs_E10	ordinary_muscle_development	ENSGALG00010023767	ENSGALG00010023767	0.275562352	0.213622647	1.289949152	0.197068313	0.245509686	positive
PRJNA729271	stage_E18_vs_E10	ordinary_muscle_development	ENSGALG00010023770	SCRN2	0.211266676	0.139380035	1.51575996	0.12958006	0.168197633	positive
PRJNA729271	stage_E18_vs_E10	ordinary_muscle_development	ENSGALG00010023773	OVOA	1.228594285	0.527385551	2.329594132	0.019827613	0.029903365	positive
PRJNA729271	stage_E18_vs_E10	ordinary_muscle_development	ENSGALG00010023775	ENSGALG00010023775	0.860610227	0.143500347	5.997269303	2.00663E-09	6.59699E-09	positive
PRJNA729271	stage_E18_vs_E10	ordinary_muscle_development	ENSGALG00010023777	OAZ2	0.304346109	0.132496747	2.29700815	0.021618305	0.03240175	positive
PRJNA729271	stage_E18_vs_E10	ordinary_muscle_development	ENSGALG00010023778	FANCF	-1.531063673	0.209194207	-7.318862685	2.50081E-13	1.11674E-12	negative
PRJNA729271	stage_E18_vs_E10	ordinary_muscle_development	ENSGALG00010023781	COL4A3	2.167934746	0.234432878	9.24757127	2.29651E-20	1.65529E-19	positive
PRJNA729271	stage_E18_vs_E10	ordinary_muscle_development	ENSGALG00010023783	VPS35	-0.078156041	0.063072508	-1.239145927	0.215291461	0.265813436	negative
PRJNA729271	stage_E18_vs_E10	ordinary_muscle_development	ENSGALG00010023786	ENSGALG00010023786	-1.776039595	0.3572821	-4.970972787	6.66178E-07	1.75122E-06	negative
PRJNA729271	stage_E18_vs_E10	ordinary_muscle_development	ENSGALG00010023788	RTCA	-0.788140672	0.107872155	-7.306247577	2.74706E-13	1.22481E-12	negative
PRJNA729271	stage_E18_vs_E10	ordinary_muscle_development	ENSGALG00010023791	VTI1B	-0.112148454	0.083273943	-1.346741251	0.178063582	0.224028436	negative
PRJNA729271	stage_E18_vs_E10	ordinary_muscle_development	ENSGALG00010023792	ZNF609	-0.326580988	0.177172963	-1.84328908	0.065286815	0.08969125	negative
PRJNA729271	stage_E18_vs_E10	ordinary_muscle_development	ENSGALG00010023793	RBM45	0.005226242	0.06529049	0.080045993	0.936200676	0.947774046	positive
PRJNA729271	stage_E18_vs_E10	ordinary_muscle_development	ENSGALG00010023794	MT3	0.472581035	0.479576141	0.985413982	0.324420764	0.382439735	positive
PRJNA729271	stage_E18_vs_E10	ordinary_muscle_development	ENSGALG00010023798	ARCN1	-0.219947803	0.056751339	-3.875640784	0.000106344	0.000221227	negative
PRJNA729271	stage_E18_vs_E10	ordinary_muscle_development	ENSGALG00010023799	NMT1	0.097538091	0.12715571	0.767075985	0.44303634	0.502513634	positive
PRJNA729271	stage_E18_vs_E10	ordinary_muscle_development	ENSGALG00010023800	PEX5	0.217513864	0.073326619	2.966369744	0.00301338	0.005159336	positive
PRJNA729271	stage_E18_vs_E10	ordinary_muscle_development	ENSGALG00010023802	ENSGALG00010023802	0.250039736	0.313795125	0.796824796	0.425552792	0.485745201	positive
PRJNA729271	stage_E18_vs_E10	ordinary_muscle_development	ENSGALG00010023805	GALNT6	-1.059361241	0.350449956	-3.022860246	0.002503979	0.004334787	negative
PRJNA729271	stage_E18_vs_E10	ordinary_muscle_development	ENSGALG00010023806	PATJ	-1.119489136	0.173489814	-6.452765792	1.09827E-10	3.98783E-10	negative
PRJNA729271	stage_E18_vs_E10	ordinary_muscle_development	ENSGALG00010023807	MRPL10	0.492772582	0.126123985	3.907048949	9.34302E-05	0.000195478	positive
PRJNA729271	stage_E18_vs_E10	ordinary_muscle_development	ENSGALG00010023808	DHX35	-0.575041793	0.119670645	-4.805203407	1.54594E-06	3.91414E-06	negative
PRJNA729271	stage_E18_vs_E10	ordinary_muscle_development	ENSGALG00010023809	CDON	-0.364370304	0.09717351	-3.749687596	0.000177055	0.000357588	negative
PRJNA729271	stage_E18_vs_E10	ordinary_muscle_development	ENSGALG00010023811	MRPL44	0.492575116	0.084860926	5.804498491	6.45589E-09	2.03166E-08	positive
PRJNA729271	stage_E18_vs_E10	ordinary_muscle_development	ENSGALG00010023813	CELA1	1.243341577	5.322023468	0.233621964	0.815278474	0.846523029	positive
PRJNA729271	stage_E18_vs_E10	ordinary_muscle_development	ENSGALG00010023815	PLCD3	1.248700936	0.134673168	9.272084077	1.82544E-20	1.3283E-19	positive
PRJNA729271	stage_E18_vs_E10	ordinary_muscle_development	ENSGALG00010023817	BBS2	-0.357762477	0.120052457	-2.980051255	0.002882002	0.00495312	negative
PRJNA729271	stage_E18_vs_E10	ordinary_muscle_development	ENSGALG00010023818	ALOX5	2.243354973	0.184879035	12.13417722	6.96061E-34	1.01503E-32	positive
PRJNA729271	stage_E18_vs_E10	ordinary_muscle_development	ENSGALG00010023819	BIN2	0.034626742	0.251305342	0.137787527	0.890408342	0.909482695	positive
PRJNA729271	stage_E18_vs_E10	ordinary_muscle_development	ENSGALG00010023820	CYP1C1	-2.516644707	1.128462548	-2.230153506	0.025737254	0.038055849	negative
PRJNA729271	stage_E18_vs_E10	ordinary_muscle_development	ENSGALG00010023821	KANK4	-0.415794907	0.124462408	-3.340726836	0.000835594	0.001539979	negative
PRJNA729271	stage_E18_vs_E10	ordinary_muscle_development	ENSGALG00010023822	CHEK2	-1.189943842	0.131247504	-9.066411181	1.23002E-19	8.47793E-19	negative
PRJNA729271	stage_E18_vs_E10	ordinary_muscle_development	ENSGALG00010023823	MUC2	1.334612443	0.549948475	2.426795424	0.015232843	0.023494366	positive
PRJNA729271	stage_E18_vs_E10	ordinary_muscle_development	ENSGALG00010023826	DBT	0.926019849	0.127389964	7.269174281	3.61692E-13	1.6023E-12	positive
PRJNA729271	stage_E18_vs_E10	ordinary_muscle_development	ENSGALG00010023828	CHEK1	0.059683591	0.160040056	0.372929079	0.709201229	0.753324917	positive
PRJNA729271	stage_E18_vs_E10	ordinary_muscle_development	ENSGALG00010023829	USP1	-1.041579341	0.101201479	-10.29213552	7.64615E-25	7.20617E-24	negative
PRJNA729271	stage_E18_vs_E10	ordinary_muscle_development	ENSGALG00010023830	AGFG1	0.317038957	0.114116691	2.778199695	0.005466101	0.009004562	positive
PRJNA729271	stage_E18_vs_E10	ordinary_muscle_development	ENSGALG00010023831	SATB2	-2.1838581	0.510182006	-4.28054709	1.86434E-05	4.21455E-05	negative
PRJNA729271	stage_E18_vs_E10	ordinary_muscle_development	ENSGALG00010023832	ENSGALG00010023832	-0.124903006	0.495870762	-0.251886209	0.80112902	0.833981984	negative
PRJNA729271	stage_E18_vs_E10	ordinary_muscle_development	ENSGALG00010023833	PIP4K2C	0.134719672	0.108229771	1.244756146	0.213221401	0.263536375	positive
PRJNA729271	stage_E18_vs_E10	ordinary_muscle_development	ENSGALG00010023835	INAFM2	0.458371012	0.12168608	3.766831922	0.000165332	0.000334892	positive

PRJNA729271	stage_E18_vs_E10	ordinary_muscle_development	ENSGALG00010023836	NDUFS3	1.05188097	0.147957872	7.109327501	1.1661E-12	4.96405E-12	positive
PRJNA729271	stage_E18_vs_E10	ordinary_muscle_development	ENSGALG00010023837	STT3A	-0.364192683	0.09265281	-3.930724624	8.46902E-05	0.000177937	negative
PRJNA729271	stage_E18_vs_E10	ordinary_muscle_development	ENSGALG00010023840	SMAGP	-4.288260331	0.782599211	-5.479510166	4.26505E-08	1.24918E-07	negative
PRJNA729271	stage_E18_vs_E10	ordinary_muscle_development	ENSGALG00010023841	OSBPL7	0.224461421	0.151707781	1.479564326	0.138989553	0.179287494	positive
PRJNA729271	stage_E18_vs_E10	ordinary_muscle_development	ENSGALG00010023847	PIK3AP1	0.032185144	0.189194822	0.170116411	0.864918588	0.888518422	positive
PRJNA729271	stage_E18_vs_E10	ordinary_muscle_development	ENSGALG00010023850	DAZAP2	0.489518116	0.219669394	2.228431128	0.025851779	0.038205704	positive
PRJNA729271	stage_E18_vs_E10	ordinary_muscle_development	ENSGALG00010023851	HSCB	0.045837738	0.150472231	0.304625892	0.760651098	0.799248834	positive
PRJNA729271	stage_E18_vs_E10	ordinary_muscle_development	ENSGALG00010023852	LRRC39	3.161302186	0.190209913	16.62007062	4.98738E-62	2.14517E-60	positive
PRJNA729271	stage_E18_vs_E10	ordinary_muscle_development	ENSGALG00010023854	SHCBP1	0.700431215	0.215815966	3.245502304	0.001172436	0.002117426	positive
PRJNA729271	stage_E18_vs_E10	ordinary_muscle_development	ENSGALG00010023857	ACBD4	0.516859837	0.154571776	3.343817668	0.00082634	0.001523893	positive
PRJNA729271	stage_E18_vs_E10	ordinary_muscle_development	ENSGALG00010023859	ENSGALG00010023859	2.920829795	0.766981926	3.808212027	0.000139975	0.000286817	positive
PRJNA729271	stage_E18_vs_E10	ordinary_muscle_development	ENSGALG00010023860	TRIP4	-0.086253028	0.115646711	-0.745832087	0.455768854	0.515397842	negative
PRJNA729271	stage_E18_vs_E10	ordinary_muscle_development	ENSGALG00010023861	ENSGALG00010023861	-0.03353773	0.151499665	-0.221371644	0.82480307	0.854392946	negative
PRJNA729271	stage_E18_vs_E10	ordinary_muscle_development	ENSGALG00010023862	TRMT13	-0.377560913	0.132735834	-2.844453537	0.004448767	0.007422293	negative
PRJNA729271	stage_E18_vs_E10	ordinary_muscle_development	ENSGALG00010023863	EYA2	-2.066542584	0.232046861	-8.905712303	5.30443E-19	3.53507E-18	negative
PRJNA729271	stage_E18_vs_E10	ordinary_muscle_development	ENSGALG00010023864	EPHA1	-5.765742394	0.727852622	-7.921579475	2.34512E-15	1.22452E-14	negative
PRJNA729271	stage_E18_vs_E10	ordinary_muscle_development	ENSGALG00010023866	AMBRA1	-0.06989768	0.075029505	-0.931602564	0.351541957	0.410571322	negative
PRJNA729271	stage_E18_vs_E10	ordinary_muscle_development	ENSGALG00010023867	ENSGALG00010023867	1.473246058	0.477207465	3.087223414	0.002020357	0.003545838	positive
PRJNA729271	stage_E18_vs_E10	ordinary_muscle_development	ENSGALG00010023869	DOCK7	-0.700432539	0.047215315	-14.83485909	8.71868E-50	2.51748E-48	negative
PRJNA729271	stage_E18_vs_E10	ordinary_muscle_development	ENSGALG00010023871	UQCRFS1	0.559766433	0.140502565	3.984030008	6.77563E-05	0.000144028	positive
PRJNA729271	stage_E18_vs_E10	ordinary_muscle_development	ENSGALG00010023874	HEXIM1	0.588236921	0.225246495	2.611525298	0.009013932	0.014356328	positive
PRJNA729271	stage_E18_vs_E10	ordinary_muscle_development	ENSGALG00010023878	ENSGALG00010023878	1.304190679	1.565596252	0.833031299	0.404827078	0.464789583	positive
PRJNA729271	stage_E18_vs_E10	ordinary_muscle_development	ENSGALG00010023879	EI24	-0.188920498	0.117820376	-1.603462029	0.108832686	0.143398762	negative
PRJNA729271	stage_E18_vs_E10	ordinary_muscle_development	ENSGALG00010023880	ENSGALG00010023880	-1.388290817	0.195675939	-7.094846847	1.29495E-12	5.49642E-12	negative
PRJNA729271	stage_E18_vs_E10	ordinary_muscle_development	ENSGALG00010023881	VSTM2B	-0.161566908	0.216096465	-0.74766104	0.454664637	0.514309654	negative
PRJNA729271	stage_E18_vs_E10	ordinary_muscle_development	ENSGALG00010023883	TBX21	6.034615851	0.342741246	17.60691459	2.18009E-69	1.14081E-67	positive
PRJNA729271	stage_E18_vs_E10	ordinary_muscle_development	ENSGALG00010023884	C2orf69	-0.980379745	0.128176015	-7.648698875	2.03023E-14	9.85869E-14	negative
PRJNA729271	stage_E18_vs_E10	ordinary_muscle_development	ENSGALG00010023887	RASA2	-0.281089554	0.128825259	-2.181944407	0.029113636	0.04264371	negative
PRJNA729271	stage_E18_vs_E10	ordinary_muscle_development	ENSGALG00010023888	CTH	-1.026885478	0.259221254	-3.961424702	7.45039E-05	0.000157471	negative
PRJNA729271	stage_E18_vs_E10	ordinary_muscle_development	ENSGALG00010023889	SAMD8	0.844596525	0.192357189	4.390771831	1.12949E-05	2.61408E-05	positive
PRJNA729271	stage_E18_vs_E10	ordinary_muscle_development	ENSGALG00010023890	SASS6	-0.933243353	0.120820224	-7.724231263	1.1253E-14	5.59756E-14	negative
PRJNA729271	stage_E18_vs_E10	ordinary_muscle_development	ENSGALG00010023891	ENSGALG00010023891	2.578150924	0.280798403	9.181501395	4.25122E-20	2.99861E-19	positive
PRJNA729271	stage_E18_vs_E10	ordinary_muscle_development	ENSGALG00010023896	POP4	-0.701994195	0.130998011	-5.358815676	8.37693E-08	2.38929E-07	negative
PRJNA729271	stage_E18_vs_E10	ordinary_muscle_development	ENSGALG00010023902	PTGER3	2.653880254	0.232527195	11.41320377	3.59249E-30	4.43176E-29	positive
PRJNA729271	stage_E18_vs_E10	ordinary_muscle_development	ENSGALG00010023903	TBKBP1	-0.377108166	0.412322878	-0.914594329	0.360404637	0.419940934	negative
PRJNA729271	stage_E18_vs_E10	ordinary_muscle_development	ENSGALG00010023909	FOXRED1	0.035134477	0.155580701	0.225827988	0.821335208	0.85153091	positive
PRJNA729271	stage_E18_vs_E10	ordinary_muscle_development	ENSGALG00010023910	RNF7	0.310620519	0.100965529	3.076500668	0.002094458	0.003667453	positive
PRJNA729271	stage_E18_vs_E10	ordinary_muscle_development	ENSGALG00010023911	TFCP2	-0.468345552	0.122274679	-3.830274224	0.000128001	0.000263509	negative
PRJNA729271	stage_E18_vs_E10	ordinary_muscle_development	ENSGALG00010023913	ZMYND8	-0.438309046	0.083311165	-5.26110814	1.4319E-07	3.99065E-07	negative
PRJNA729271	stage_E18_vs_E10	ordinary_muscle_development	ENSGALG00010023914	CCDC117	-0.334807867	0.066342308	-5.046671963	4.49572E-07	1.20099E-06	negative
PRJNA729271	stage_E18_vs_E10	ordinary_muscle_development	ENSGALG00010023917	ZRANB2	-0.186616645	0.079996068	-2.332822707	0.019657452	0.029677614	negative
PRJNA729271	stage_E18_vs_E10	ordinary_muscle_development	ENSGALG00010023918	MFSD14A	-0.505641124	0.080638561	-6.270463113	3.59976E-10	1.26065E-09	negative
PRJNA729271	stage_E18_vs_E10	ordinary_muscle_development	ENSGALG00010023920	ENSGALG00010023920	0.387488585	0.390447194	0.992422511	0.320991462	0.378705131	positive
PRJNA729271	stage_E18_vs_E10	ordinary_muscle_development	ENSGALG00010023921	GRK7	0.711934189	0.181113415	3.930874964	8.46373E-05	0.000177851	positive
PRJNA729271	stage_E18_vs_E10	ordinary_muscle_development	ENSGALG00010023923	CSNK1G1	-0.060738086	0.095929333	-0.633154472	0.526632774	0.582891116	negative
PRJNA729271	stage_E18_vs_E10	ordinary_muscle_development	ENSGALG00010023925	NCOA3	-0.055709508	0.077020487	-0.723307657	0.469490889	0.528400531	negative
PRJNA729271	stage_E18_vs_E10	ordinary_muscle_development	ENSGALG00010023926	ZYX	-0.318693547	0.1395464	-2.283781923	0.022384351	0.033466855	negative
PRJNA729271	stage_E18_vs_E10	ordinary_muscle_development	ENSGALG00010023927	KPNB1	-1.259776317	0.131308628	-9.594010216	8.47264E-22	6.69634E-21	negative
PRJNA729271	stage_E18_vs_E10	ordinary_muscle_development	ENSGALG00010023930	SRPRA	-0.333364725	0.131729484	-2.530676608	0.011384276	0.01788006	negative
PRJNA729271	stage_E18_vs_E10	ordinary_muscle_development	ENSGALG00010023931	CSRNP2	-0.947711795	0.14759802	-6.420897756	1.35473E-10	4.88599E-10	negative
PRJNA729271	stage_E18_vs_E10	ordinary_muscle_development	ENSGALG00010023937	XBP1	-0.442084968	0.110624439	-3.996268563	6.43487E-05	0.000137115	negative
PRJNA729271	stage_E18_vs_E10	ordinary_muscle_development	ENSGALG00010023940	ATP1B3	-0.125663874	0.071947015	-1.746616915	0.080703811	0.109052087	negative
PRJNA729271	stage_E18_vs_E10	ordinary_muscle_development	ENSGALG00010023942	NEGR1	3.262557012	0.220066974	14.82529139	1.00543E-49	2.89735E-48	positive
PRJNA729271	stage_E18_vs_E10	ordinary_muscle_development	ENSGALG00010023945	LETMD1	0.620612617	0.213964333	2.900542391	0.003725174	0.006278652	positive
PRJNA729271	stage_E18_vs_E10	ordinary_muscle_development	ENSGALG00010023946	ZNRF3	-0.387159518	0.126789736	-3.053555682	0.002261467	0.003936588	negative
PRJNA729271	stage_E18_vs_E10	ordinary_muscle_development	ENSGALG00010023947	ENSGALG00010023947	-3.212301336	0.459363911	-6.992933618	2.69197E-12	1.1139E-11	negative
PRJNA729271	stage_E18_vs_E10	ordinary_muscle_development	ENSGALG00010023948	SULF2	-1.579974361	0.221086726	-7.146400828	8.90827E-13	3.84187E-12	negative
PRJNA729271	stage_E18_vs_E10	ordinary_muscle_development	ENSGALG00010023953	SLC35A3	-0.639168533	0.156389656	-4.087025624	4.36939E-05	9.46842E-05	negative
PRJNA729271	stage_E18_vs_E10	ordinary_muscle_development	ENSGALG00010023954	PLEKHF1	1.480349477	0.094332039	15.69296592	1.68973E-55	5.84548E-54	positive
PRJNA729271	stage_E18_vs_E10	ordinary_muscle_development	ENSGALG00010023956	KREMEN1	-2.149290694	0.133231699	-16.13197693	1.52074E-58	5.8315E-57	negative
PRJNA729271	stage_E18_vs_E10	ordinary_muscle_development	ENSGALG00010023957	YBX3	2.011155898	0.138848326	14.48455273	1.51707E-47	4.03485E-46	positive
PRJNA729271	stage_E18_vs_E10	ordinary_muscle_development	ENSGALG00010023962	FPGT	-0.280822079	0.114368814	-2.455407813	0.014072484	0.021811448	negative
PRJNA729271	stage_E18_vs_E10	ordinary_muscle_development	ENSGALG00010023966	PEAK1	0.686035672	0.098341415	6.976060602	3.03572E-12	1.25079E-11	positive
PRJNA729271	stage_E18_vs_E10	ordinary_muscle_development	ENSGALG00010023967	PRPH	-3.021230026	0.387326485	-7.80021544	6.18016E-15	3.1377E-14	negative
PRJNA729271	stage_E18_vs_E10	ordinary_muscle_development	ENSGALG00010023968	TYW5	-0.152081344	0.11975899	-1.269895014	0.20412203	0.253443124	negative
PRJNA729271	stage_E18_vs_E10	ordinary_muscle_development	ENSGALG00010023972	ENSGALG00010023972	0.483117586	0.525443516	0.91944723	0.357861695	0.417292481	positive
PRJNA729271	stage_E18_vs_E10	ordinary_muscle_development	ENSGALG00010023974	PREX1	-0.773129447	0.104322807	-7.410934075	1.25413E-13	5.72554E-13	negative
PRJNA729271	stage_E18_vs_E10	ordinary_muscle_development	ENSGALG00010023978	TFDP2	-0.450847272	0.140101866	-3.217996202	0.001290895	0.002320953	negative
PRJNA729271	stage_E18_vs_E10	ordinary_muscle_development	ENSGALG00010023979	FAM118B	-0.316674228	0.115486759	-2.742082572	0.006105099	0.009983463	negative
PRJNA729271	stage_E18_vs_E10	ordinary_muscle_development	ENSGALG00010023981	ENSGALG00010023981	-0.162636471	0.377150737	-0.431224056	0.666305464	0.713511946	negative
PRJNA729271	stage_E18_vs_E10	ordinary_muscle_development	ENSGALG00010023984	FMNL1	1.306045251	0.210706486	6.198410251	5.70363E-10	1.97266E-09	positive
PRJNA729271	stage_E18_vs_E10	ordinary_muscle_development	ENSGALG00010023986	SYCE3	0.397747037	0.582592027	0.682719671	0.494783998	0.552108856	positive
PRJNA729271	stage_E18_vs_E10	ordinary_muscle_development	ENSGALG00010023987	TNNI3K	-0.77126015	0.446169619	-1.728625434	0.083876155	0.112907212	negative
PRJNA729271	stage_E18_vs_E10	ordinary_muscle_development	ENSGALG00010023989	ENSGALG00010023989	-0.288903568	0.102234775	-2.825883533	0.00471504	0.007838572	negative
PRJNA729271	stage_E18_vs_E10	ordinary_muscle_development	ENSGALG00010023991	TSPAN3	0.173182946	0.105995769	1.633866594	0.102286866	0.135339855	positive
PRJNA729271	stage_E18_vs_E10	ordinary_muscle_development	ENSGALG00010023992	FAM131B	-0.823498607	0.136639634	-6.026791655	1.67246E-09	5.531E-09	negative
PRJNA729271	stage_E18_vs_E10	ordinary_muscle_development	ENSGALG00010023993	C19orf12	0.170113656	0.175179913	0.971079689	0.331508597	0.389717527	positive
PRJNA729271	stage_E18_vs_E10	ordinary_muscle_development	ENSGALG00010023996	MYO7B	4.159403752	0.470945412	8.832029458	1.02793E-18	6.72682E-18	positive
PRJNA729271	stage_E18_vs_E10	ordinary_muscle_development	ENSGALG00010023997	ARFGEF2	-0.169270896	0.063440291	-2.668192292	0.00762606	0.012271285	negative
PRJNA729271	stage_E18_vs_E10	ordinary_muscle_development	ENSGALG00010023999	DGCR14	0.111787919	0.092531732	1.208103608	0.227007412	0.278452436	positive
PRJNA729271	stage_E18_vs_E10	ordinary_muscle_development	ENSGALG00010024001	ENSGALG00010024001	2.239260834	0.463801672	4.828056842	1.37872E-06	3.51037E-06	positive
PRJNA729271	stage_E18_vs_E10	ordinary_muscle_development	ENSGALG00010024002	DGCR2	-0.468479965	0.060984796	-7.681914161	1.56729E-14	7.70096E-14	negative
PRJNA729271	stage_E18_vs_E10	ordinary_muscle_development	ENSGALG00010024003	ERICH3	-1.276443779	0.307100418	-4.156437775	3.23248E-05	7.10352E-05	negative
PRJNA729271	stage_E18_vs_E10	ordinary_muscle_development	ENSGALG00010024004	EIF4B	0.796449064	0.125060977	6.368485872	1.90903E-10	6.81227E-10	positive
PRJNA729271	stage_E18_vs_E10	ordinary_muscle_development	ENSGALG00010024005	STYK1	4.15244288	0.24951159	16.64228456	3.44231E-62	1.4939E-60	positive
PRJNA729271	stage_E18_vs_E10	ordinary_muscle_development	ENSGALG00010024008	CCNE1	-0.882966255	0.110959065	-7.957585602	1.75429E-15	9.25005E-15	negative
PRJNA729271	stage_E18_vs_E10	ordinary_muscle_development	ENSGALG00010024009	CSE1L	-1.034508377	0.061816185	-16.73523494	7.25702E-63	3.23663E-61	negative
PRJNA729271	stage_E18_vs_E10	ordinary_muscle_development	ENSGALG00010024010	ENSGALG00010024010	0.875768309	0.088850613	9.856637752	6.4162E-23	5.36039E-22	positive
PRJNA729271	stage_E18_vs_E10	ordinary_muscle_development	ENSGALG00010024011	AGPS	0.037367416	0.070865918	0.52729743	0.597987059	0.649713096	positive
PRJNA729271	stage_E18_vs_E10	ordinary_muscle_development	ENSGALG00010024013	PTPRJ	-0.03178572	0.1215409	-0.261522832	0.793689345	0.827604277	negative
PRJNA729271	stage_E18_vs_E10	ordinary_muscle_development	ENSGALG00010024016	CRYZ	-0.064552015	0.104498123	-0.617733727	0.536750876	0.592100757	negative
PRJNA729271	stage_E18_vs_E10	ordinary_muscle_development	ENSGALG00010024017	TYW3	-1.153659917	0.130706933	-8.826310061	1.08186E-18	7.06376E-18	negative
PRJNA729271	stage_E18_vs_E10	ordinary_muscle_development	ENSGALG00010024018	KRT18	-4.003133256	0.964881756	-4.148832984	3.34174E-05	7.33141E-05	negative
PRJNA729271	stage_E18_vs_E10	ordinary_muscle_development	ENSGALG00010024019	MAP3K14	0.317615323	0.109759133	2.893748466	0.00380673	0.006408658	positive
PRJNA729271	stage_E18_vs_E10	ordinary_muscle_development	ENSGALG00010024020	MAIP1	-0.468959415	0.122873666	-3.816598207	0.000135304	0.000277717	negative
PRJNA729271	stage_E18_vs_E10	ordinary_muscle_development	ENSGALG00010024021	IGLL1	1.243627914	1.466554754	0.847992828	0.396441965	0.456464078	positive
PRJNA729271	stage_E18_vs_E10	ordinary_muscle_development	ENSGALG00010024023	LHX8	-3.002091397	0.488079987	-6.150818465	7.70841E-10	2.63212E-09	negative
PRJNA729271	stage_E18_vs_E10	ordinary_muscle_development	ENSGALG00010024024	AGL	1.93017454	0.122807502	15.71707353	1.15538E-55	4.03549E-54	positive
PRJNA729271	stage_E18_vs_E10	ordinary_muscle_development	ENSGALG00010024025	CLCN1	3.023652963	0.351131093	8.611179775	7.23125E-18	4.47502E-17	positive
PRJNA729271	stage_E18_vs_E10	ordinary_muscle_development	ENSGALG00010024026	TIRAP	0.3597396	0.105177398	3.420312781	0.000625492	0.00117305	positive
PRJNA729271	stage_E18_vs_E10	ordinary_muscle_development	ENSGALG00010024029	SCAPER	0.843192164	0.146467022	5.75687381	8.56859E-09	2.66356E-08	positive
PRJNA729271	stage_E18_vs_E10	ordinary_muscle_development	ENSGALG00010024030	STAU1	-0.145993345	0.089933047	-1.623355938	0.104513338	0.138109293	negative
PRJNA729271	stage_E18_vs_E10	ordinary_muscle_development	ENSGALG00010024031	LIMS2	1.11433551	0.11510337	9.681171909	3.62536E-22	2.9178E-21	positive
PRJNA729271	stage_E18_vs_E10	ordinary_muscle_development	ENSGALG00010024034	LRP2	0.6255036	0.24318194	2.572163046	0.010106528	0.015978853	positive
PRJNA729271	stage_E18_vs_E10	ordinary_muscle_development	ENSGALG00010024035	OSBPL5	-0.298663463	0.136931328	-2.18111857	0.029174648	0.042720125	negative
PRJNA729271	stage_E18_vs_E10	ordinary_muscle_development	ENSGALG00010024038	NKD1	-0.493272313	0.16212351	-3.042571141	0.002345664	0.004074343	negative
PRJNA729271	stage_E18_vs_E10	ordinary_muscle_development	ENSGALG00010024039	MRPL45	-0.348049446	0.107907475	-3.225443335	0.001257777	0.00226478	negative
PRJNA729271	stage_E18_vs_E10	ordinary_muscle_development	ENSGALG00010024040	ENSGALG00010024040	-4.079497433	0.813502857	-5.014730306	5.31079E-07	1.40937E-06	negative
PRJNA729271	stage_E18_vs_E10	ordinary_muscle_development	ENSGALG00010024042	DCPS	-0.225623871	0.130781548	-1.725196514	0.084492064	0.11362023	negative
PRJNA729271	stage_E18_vs_E10	ordinary_muscle_development	ENSGALG00010024045	PSTPIP1	1.178247296	0.249362817	4.725032017	2.30079E-06	5.71452E-06	positive
PRJNA729271	stage_E18_vs_E10	ordinary_muscle_development	ENSGALG00010024046	ENSGALG00010024046	-0.969428177	0.537163688	-1.80471651	0.071119055	0.097196936	negative
PRJNA729271	stage_E18_vs_E10	ordinary_muscle_development	ENSGALG00010024049	RCN2	0.151803687	0.143755828	1.055982838	0.290976055	0.347307141	positive
PRJNA729271	stage_E18_vs_E10	ordinary_muscle_development	ENSGALG00010024051	ENSGALG00010024051	-4.334335466	0.66165506	-6.550747856	5.72497E-11	2.12791E-10	negative
PRJNA729271	stage_E18_vs_E10	ordinary_muscle_development	ENSGALG00010024052	ENSGALG00010024052	-0.283563588	0.173074301	-1.638392213	0.101339903	0.134184881	negative
PRJNA729271	stage_E18_vs_E10	ordinary_muscle_development	ENSGALG00010024053	ISL2	-0.080747869	0.513818741	-0.157152439	0.87512471	0.896968793	negative
PRJNA729271	stage_E18_vs_E10	ordinary_muscle_development	ENSGALG00010024054	ENSGALG00010024054	-4.052608398	0.848119063	-4.778348433	1.76741E-06	4.44845E-06	negative
PRJNA729271	stage_E18_vs_E10	ordinary_muscle_development	ENSGALG00010024057	HSD3B1	-2.864444976	0.868379984	-3.298607784	0.000971656	0.001772436	negative
PRJNA729271	stage_E18_vs_E10	ordinary_muscle_development	ENSGALG00010024058	SLC44A5	0.46345635	0.105318151	4.400536326	1.07984E-05	2.50716E-05	positive
PRJNA729271	stage_E18_vs_E10	ordinary_muscle_development	ENSGALG00010024060	FRRS1	0.22852062	0.162173504	1.409111934	0.158802073	0.202101134	positive
PRJNA729271	stage_E18_vs_E10	ordinary_muscle_development	ENSGALG00010024061	ENSGALG00010024061	4.203481938	1.204284281	3.490439927	0.000482226	0.000918269	positive
PRJNA729271	stage_E18_vs_E10	ordinary_muscle_development	ENSGALG00010024062	ST3GAL4	0.316522502	0.132400507	2.390644182	0.016818844	0.025708019	positive
PRJNA729271	stage_E18_vs_E10	ordinary_muscle_development	ENSGALG00010024063	BRD7	-0.722671	0.105050197	-6.879292171	6.01507E-12	2.41319E-11	negative
PRJNA729271	stage_E18_vs_E10	ordinary_muscle_development	ENSGALG00010024064	GPR17	0.385764542	0.211437718	1.824483095	0.06807907	0.093270899	positive
PRJNA729271	stage_E18_vs_E10	ordinary_muscle_development	ENSGALG00010024066	ENSGALG00010024066	1.243618982	2.266538334	0.548686498	0.583220613	0.636148905	positive
PRJNA729271	stage_E18_vs_E10	ordinary_muscle_development	ENSGALG00010024068	CASP2	-1.178435802	0.086401884	-13.6390058	2.34739E-42	4.93122E-41	negative
PRJNA729271	stage_E18_vs_E10	ordinary_muscle_development	ENSGALG00010024069	ENSGALG00010024069	1.243607339	2.364835876	0.525874692	0.598975274	0.650494238	positive
PRJNA729271	stage_E18_vs_E10	ordinary_muscle_development	ENSGALG00010024073	RABGGTB	-0.515388657	0.126056831	-4.088542062	4.34093E-05	9.40956E-05	negative
PRJNA729271	stage_E18_vs_E10	ordinary_muscle_development	ENSGALG00010024074	ADCY7	-1.169431733	0.231041493	-5.061565866	4.15827E-07	1.11474E-06	negative
PRJNA729271	stage_E18_vs_E10	ordinary_muscle_development	ENSGALG00010024075	SPATS2L	0.191670358	0.136541522	1.403751439	0.160392879	0.203938139	positive
PRJNA729271	stage_E18_vs_E10	ordinary_muscle_development	ENSGALG00010024077	SFT2D3	0.181480794	0.118911939	1.526178075	0.126965522	0.16529237	positive
PRJNA729271	stage_E18_vs_E10	ordinary_muscle_development	ENSGALG00010024078	KIRREL3	0.037903747	0.230369905	0.164534282	0.869310561	0.892302657	positive

PRJNA729271	stage_E18_vs_E10	ordinary_muscle_development	ENSGALG00010024080	TENT4B	0.110834132	0.087828303	1.261940956	0.206970033	0.256566376	positive
PRJNA729271	stage_E18_vs_E10	ordinary_muscle_development	ENSGALG00010024081	CARS	-0.444108289	0.099542862	-4.461478024	8.13963E-06	1.91377E-05	negative
PRJNA729271	stage_E18_vs_E10	ordinary_muscle_development	ENSGALG00010024083	PALMD	0.696507519	0.18981874	3.669329598	0.000243187	0.000482216	positive
PRJNA729271	stage_E18_vs_E10	ordinary_muscle_development	ENSGALG00010024086	GABARAPL1	-0.137527515	0.215204896	-0.63905384	0.522787907	0.579387623	negative
PRJNA729271	stage_E18_vs_E10	ordinary_muscle_development	ENSGALG00010024089	ENSGALG00010024089	1.243628609	1.936858367	0.642085467	0.520817692	0.5777552	positive
PRJNA729271	stage_E18_vs_E10	ordinary_muscle_development	ENSGALG00010024091	ETFA	1.01957661	0.116065678	8.784479878	1.5709E-18	1.01789E-17	positive
PRJNA729271	stage_E18_vs_E10	ordinary_muscle_development	ENSGALG00010024095	RBM12	-0.689042726	0.05313485	-12.96781164	1.86273E-38	3.35824E-37	negative
PRJNA729271	stage_E18_vs_E10	ordinary_muscle_development	ENSGALG00010024096	MYO1D	-0.083778049	0.220739573	-0.379533438	0.704291779	0.749043168	negative
PRJNA729271	stage_E18_vs_E10	ordinary_muscle_development	ENSGALG00010024099	ENSGALG00010024099	1.243626269	2.081202727	0.59755172	0.550139087	0.604294185	positive
PRJNA729271	stage_E18_vs_E10	ordinary_muscle_development	ENSGALG00010024101	CNEP1R1	-0.032669038	0.090611389	-0.360540083	0.718443287	0.761356591	negative
PRJNA729271	stage_E18_vs_E10	ordinary_muscle_development	ENSGALG00010024102	SGF29	0.50828271	0.090031561	5.645605871	1.64601E-08	4.99035E-08	positive
PRJNA729271	stage_E18_vs_E10	ordinary_muscle_development	ENSGALG00010024104	PLPPR5	0.756827766	0.16035383	4.719736146	2.36151E-06	5.8593E-06	positive
PRJNA729271	stage_E18_vs_E10	ordinary_muscle_development	ENSGALG00010024106	WDR33	-0.138874267	0.064144856	-2.165010192	0.030386905	0.044374187	negative
PRJNA729271	stage_E18_vs_E10	ordinary_muscle_development	ENSGALG00010024107	NFE2L2	0.527523434	0.063861182	8.260470861	1.45099E-16	8.23819E-16	positive
PRJNA729271	stage_E18_vs_E10	ordinary_muscle_development	ENSGALG00010024110	CPNE1	-0.546393777	0.126321286	-4.32542917	1.52235E-05	3.47832E-05	negative
PRJNA729271	stage_E18_vs_E10	ordinary_muscle_development	ENSGALG00010024112	NAP1L4	-0.150916703	0.090823983	-1.661639339	0.096585111	0.12849942	negative
PRJNA729271	stage_E18_vs_E10	ordinary_muscle_development	ENSGALG00010024113	ENSGALG00010024113	0.934233462	0.166456911	5.612464247	1.99465E-08	6.00592E-08	positive
PRJNA729271	stage_E18_vs_E10	ordinary_muscle_development	ENSGALG00010024115	POLR2D	-0.318386387	0.073371663	-4.33936449	1.42895E-05	3.27318E-05	negative
PRJNA729271	stage_E18_vs_E10	ordinary_muscle_development	ENSGALG00010024116	MCAM	2.266069962	0.181849045	12.46126952	1.21422E-35	1.92141E-34	positive
PRJNA729271	stage_E18_vs_E10	ordinary_muscle_development	ENSGALG00010024118	TMEM266	0.045389583	0.124957527	0.363240083	0.716425548	0.759661116	positive
PRJNA729271	stage_E18_vs_E10	ordinary_muscle_development	ENSGALG00010024119	AMMECR1L	-0.281370295	0.07446102	-3.77875959	0.000157611	0.000320535	negative
PRJNA729271	stage_E18_vs_E10	ordinary_muscle_development	ENSGALG00010024121	NRG4	0.056100995	0.170944342	0.328182814	0.742773442	0.783243944	positive
PRJNA729271	stage_E18_vs_E10	ordinary_muscle_development	ENSGALG00010024122	SOCS7	0.428324436	0.108684936	3.940973341	8.11516E-05	0.000170749	positive
PRJNA729271	stage_E18_vs_E10	ordinary_muscle_development	ENSGALG00010024123	PLPPR4	0.088306922	0.388357807	0.227385468	0.820124019	0.850457694	positive
PRJNA729271	stage_E18_vs_E10	ordinary_muscle_development	ENSGALG00010024124	DIDO1	0.03751452	0.067319027	0.557264741	0.577346562	0.630358423	positive
PRJNA729271	stage_E18_vs_E10	ordinary_muscle_development	ENSGALG00010024125	ENSGALG00010024125	-0.203788957	1.555021055	-0.131052217	0.895734	0.91350625	negative
PRJNA729271	stage_E18_vs_E10	ordinary_muscle_development	ENSGALG00010024126	CDK5R1	0.22464102	0.27114974	0.828475885	0.407401046	0.467522616	positive
PRJNA729271	stage_E18_vs_E10	ordinary_muscle_development	ENSGALG00010024128	PHLDA2	-2.245434547	0.235963405	-9.516028754	1.79924E-21	1.39093E-20	negative
PRJNA729271	stage_E18_vs_E10	ordinary_muscle_development	ENSGALG00010024129	GID8	-0.098667968	0.101566706	-0.97145976	0.331319382	0.389526681	negative
PRJNA729271	stage_E18_vs_E10	ordinary_muscle_development	ENSGALG00010024131	ENSGALG00010024131	1.266570697	1.271602597	0.996042867	0.319229317	0.376871555	positive
PRJNA729271	stage_E18_vs_E10	ordinary_muscle_development	ENSGALG00010024132	ENSGALG00010024132	-0.781723343	0.148086617	-5.278825031	1.30015E-07	3.64449E-07	negative
PRJNA729271	stage_E18_vs_E10	ordinary_muscle_development	ENSGALG00010024133	PSMD11	-0.188221232	0.141842609	-1.326972439	0.1845178	0.231485677	negative
PRJNA729271	stage_E18_vs_E10	ordinary_muscle_development	ENSGALG00010024135	FBXO22	-0.244676803	0.088567596	-2.76259958	0.005734307	0.009416424	negative
PRJNA729271	stage_E18_vs_E10	ordinary_muscle_development	ENSGALG00010024136	SAP130	-0.130582265	0.17570537	-0.743188811	0.457367376	0.516923206	negative
PRJNA729271	stage_E18_vs_E10	ordinary_muscle_development	ENSGALG00010024137	ENSGALG00010024137	1.368924092	0.727182299	1.882504695			positive
PRJNA729271	stage_E18_vs_E10	ordinary_muscle_development	ENSGALG00010024138	CBL	0.414210612	0.150255233	2.756713377	0.005838551	0.009574589	positive
PRJNA729271	stage_E18_vs_E10	ordinary_muscle_development	ENSGALG00010024139	SLC22A18	-2.80390588	0.853272883	-3.286059988	0.001015994	0.001849175	negative
PRJNA729271	stage_E18_vs_E10	ordinary_muscle_development	ENSGALG00010024140	KRT6A	-1.961040699	0.742833746	-2.639945628	0.008291933	0.013273503	negative
PRJNA729271	stage_E18_vs_E10	ordinary_muscle_development	ENSGALG00010024141	YTHDF1	-0.347492178	0.09639064	-3.605040662	0.000312104	0.000609532	negative
PRJNA729271	stage_E18_vs_E10	ordinary_muscle_development	ENSGALG00010024142	DNAJC6	-2.175016296	0.281208662	-7.734528089	1.03787E-14	5.18579E-14	negative
PRJNA729271	stage_E18_vs_E10	ordinary_muscle_development	ENSGALG00010024143	UBE2Q2	1.798875448	0.116707283	15.41356632	1.32683E-53	4.29294E-52	positive
PRJNA729271	stage_E18_vs_E10	ordinary_muscle_development	ENSGALG00010024144	HNRNPA3	-0.745282907	0.12673106	-5.880822779	4.08232E-09	1.30712E-08	negative
PRJNA729271	stage_E18_vs_E10	ordinary_muscle_development	ENSGALG00010024145	RAP1GAP3	0.653788187	0.179474664	3.642788191	0.000269701	0.000531879	positive
PRJNA729271	stage_E18_vs_E10	ordinary_muscle_development	ENSGALG00010024146	CDKN1C	-0.387596028	0.239321028	-1.619565281	0.105325693	0.139106689	negative
PRJNA729271	stage_E18_vs_E10	ordinary_muscle_development	ENSGALG00010024147	ENSGALG00010024147	-0.082563204	0.270886859	-0.304788516	0.760527229	0.799176612	negative
PRJNA729271	stage_E18_vs_E10	ordinary_muscle_development	ENSGALG00010024148	UGGT1	-0.639303726	0.059197949	-10.79942365	3.46371E-27	3.70254E-26	negative
PRJNA729271	stage_E18_vs_E10	ordinary_muscle_development	ENSGALG00010024149	TCIRG1	0.444850976	0.155648371	2.858050951	0.004262519	0.007132081	positive
PRJNA729271	stage_E18_vs_E10	ordinary_muscle_development	ENSGALG00010024150	NHERF4	1.349067815	0.357015516	3.778737208	0.000157626	0.000320535	positive
PRJNA729271	stage_E18_vs_E10	ordinary_muscle_development	ENSGALG00010024151	LEPROT	0.33982711	0.148137911	2.293991503	0.021790987	0.032636945	positive
PRJNA729271	stage_E18_vs_E10	ordinary_muscle_development	ENSGALG00010024152	ZNF423	-0.269599124	0.107420179	-2.509762373	0.012081243	0.018882641	negative
PRJNA729271	stage_E18_vs_E10	ordinary_muscle_development	ENSGALG00010024153	KCNQ1	0.023138354	0.211733738	0.10928043	0.912980068	0.92822095	positive
PRJNA729271	stage_E18_vs_E10	ordinary_muscle_development	ENSGALG00010024154	VPS29L	0.868420897	0.48228045	1.800655402	0.071757211	0.097949032	positive
PRJNA729271	stage_E18_vs_E10	ordinary_muscle_development	ENSGALG00010024155	SNX7	-0.321338444	0.165411569	-1.94266003	0.052057249	0.07283384	negative
PRJNA729271	stage_E18_vs_E10	ordinary_muscle_development	ENSGALG00010024157	CHRNB4	-0.215828674	0.365045807	-0.591237234	0.554361478	0.608471121	negative
PRJNA729271	stage_E18_vs_E10	ordinary_muscle_development	ENSGALG00010024158	ENSGALG00010024158	0.979401976	0.33272276	2.943597778	0.003244213	0.005520647	positive
PRJNA729271	stage_E18_vs_E10	ordinary_muscle_development	ENSGALG00010024159	NLRX1	0.498915496	0.162944272	3.06187808	0.00219953	0.003838453	positive
PRJNA729271	stage_E18_vs_E10	ordinary_muscle_development	ENSGALG00010024162	SRCIN1	1.443646454	0.2738289	5.272074832	1.3489E-07	3.77239E-07	positive
PRJNA729271	stage_E18_vs_E10	ordinary_muscle_development	ENSGALG00010024163	HS6ST1	-0.122451645	0.096068587	-1.274627314	0.202441185	0.251640938	negative
PRJNA729271	stage_E18_vs_E10	ordinary_muscle_development	ENSGALG00010024164	SNAP29	0.272620217	0.113161266	2.409130143	0.015990595	0.024571575	positive
PRJNA729271	stage_E18_vs_E10	ordinary_muscle_development	ENSGALG00010024165	LEPR	1.260666946	0.256626653	4.912455247	8.99429E-07	2.33936E-06	positive
PRJNA729271	stage_E18_vs_E10	ordinary_muscle_development	ENSGALG00010024166	ENSGALG00010024166	3.201045718	0.962884517	3.32443368	0.000885983	0.001626846	positive
PRJNA729271	stage_E18_vs_E10	ordinary_muscle_development	ENSGALG00010024167	CHRNA3	1.85532822	0.41092262	4.515030639	6.33076E-06	1.50236E-05	positive
PRJNA729271	stage_E18_vs_E10	ordinary_muscle_development	ENSGALG00010024168	BIRC7	-1.813399454	0.39498606	-4.591046722	4.41029E-06	1.06262E-05	negative
PRJNA729271	stage_E18_vs_E10	ordinary_muscle_development	ENSGALG00010024169	FAM168B	-0.517027096	0.072489398	-7.132451241	9.8597E-13	4.23581E-12	negative
PRJNA729271	stage_E18_vs_E10	ordinary_muscle_development	ENSGALG00010024170	NUP160	-0.639474007	0.08606697	-7.429958432	1.08632E-13	4.98139E-13	negative
PRJNA729271	stage_E18_vs_E10	ordinary_muscle_development	ENSGALG00010024171	ENSGALG00010024171	0.051223671	0.417419835	0.12271499	0.902332794	0.919136602	positive
PRJNA729271	stage_E18_vs_E10	ordinary_muscle_development	ENSGALG00010024172	ABCG4	-1.803796477	0.380681597	-4.738333793	2.15483E-06	5.36118E-06	negative
PRJNA729271	stage_E18_vs_E10	ordinary_muscle_development	ENSGALG00010024173	CBLN1	2.386846539	0.388682773	6.140860121	8.20758E-10	2.79598E-09	positive
PRJNA729271	stage_E18_vs_E10	ordinary_muscle_development	ENSGALG00010024174	PLEKHB2	0.485458162	0.101813603	4.768107085	1.85965E-06	4.6636E-06	positive
PRJNA729271	stage_E18_vs_E10	ordinary_muscle_development	ENSGALG00010024175	DHRS9	2.422564373	0.440557446	5.498861484	3.82251E-08	1.12594E-07	positive
PRJNA729271	stage_E18_vs_E10	ordinary_muscle_development	ENSGALG00010024177	ENSGALG00010024177	-0.133326782	0.462618315	-0.288200397	0.773193345	0.809727443	negative
PRJNA729271	stage_E18_vs_E10	ordinary_muscle_development	ENSGALG00010024178	N4BP1	0.21826736	0.121890168	1.790688803	0.073343248	0.099888225	positive
PRJNA729271	stage_E18_vs_E10	ordinary_muscle_development	ENSGALG00010024179	ATG4C	-0.347900091	0.117232468	-2.967608699	0.003001261	0.005139802	negative
PRJNA729271	stage_E18_vs_E10	ordinary_muscle_development	ENSGALG00010024180	HINFP	0.03179431	0.142986621	0.222358638	0.824034708	0.85373091	positive
PRJNA729271	stage_E18_vs_E10	ordinary_muscle_development	ENSGALG00010024181	FOXD3	3.28482537	0.312351788	10.51642891	7.25723E-26	7.20503E-25	positive
PRJNA729271	stage_E18_vs_E10	ordinary_muscle_development	ENSGALG00010024183	MTX2	0.252385661	0.106662694	2.36620369	0.017971554	0.027314425	positive
PRJNA729271	stage_E18_vs_E10	ordinary_muscle_development	ENSGALG00010024184	ENSGALG00010024184	-2.298082853	0.221086877	-10.39447879	2.62719E-25	2.53368E-24	negative
PRJNA729271	stage_E18_vs_E10	ordinary_muscle_development	ENSGALG00010024185	CHRNA5	0.538884771	0.155182495	3.472587369	0.000515467	0.000977971	positive
PRJNA729271	stage_E18_vs_E10	ordinary_muscle_development	ENSGALG00010024186	HAO2	0.126730399	0.689580252	0.183779043	0.854186797	0.879613358	positive
PRJNA729271	stage_E18_vs_E10	ordinary_muscle_development	ENSGALG00010024188	TSSC4	-0.558822429	0.083559181	-6.68774424	2.26637E-11	8.67926E-11	negative
PRJNA729271	stage_E18_vs_E10	ordinary_muscle_development	ENSGALG00010024189	KAT7	-0.581275193	0.141244417	-4.115385258	3.86533E-05	8.42526E-05	negative
PRJNA729271	stage_E18_vs_E10	ordinary_muscle_development	ENSGALG00010024190	WARS2	-0.704805983	0.108951223	-6.469004774	9.86505E-11	3.59914E-10	negative
PRJNA729271	stage_E18_vs_E10	ordinary_muscle_development	ENSGALG00010024191	DPYD	-0.638168556	0.111884252	-5.703828261	1.17146E-08	3.59448E-08	negative
PRJNA729271	stage_E18_vs_E10	ordinary_muscle_development	ENSGALG00010024192	ALG6	0.111811971	0.09056621	1.234588167	0.216983815	0.267652149	positive
PRJNA729271	stage_E18_vs_E10	ordinary_muscle_development	ENSGALG00010024193	GSTK1	1.502490846	0.12580817	11.94271288	7.08732E-33	9.80255E-32	positive
PRJNA729271	stage_E18_vs_E10	ordinary_muscle_development	ENSGALG00010024194	NKAIN4	0.51251013	0.085104353	6.02213769	1.72128E-09	5.68726E-09	positive
PRJNA729271	stage_E18_vs_E10	ordinary_muscle_development	ENSGALG00010024195	PSMA4	0.05321047	0.121033442	0.439634444	0.66020189	0.708074465	positive
PRJNA729271	stage_E18_vs_E10	ordinary_muscle_development	ENSGALG00010024196	MRPL21	0.076617475	0.109629969	0.698873454	0.484631119	0.542659565	positive
PRJNA729271	stage_E18_vs_E10	ordinary_muscle_development	ENSGALG00010024197	KRT7	-3.959913456	0.308660279	-12.82935878	1.1229E-37	1.9516E-36	negative
PRJNA729271	stage_E18_vs_E10	ordinary_muscle_development	ENSGALG00010024198	PI4KA	-0.22723042	0.064964339	-3.497771588	0.000469163	0.000896338	negative
PRJNA729271	stage_E18_vs_E10	ordinary_muscle_development	ENSGALG00010024199	SLC35B1	-0.806649136	0.103364377	-7.80393751	6.00049E-15	3.04862E-14	negative
PRJNA729271	stage_E18_vs_E10	ordinary_muscle_development	ENSGALG00010024200	GCHFR	-1.754919187	0.620033828	-2.83036039	0.00464956	0.007739477	negative
PRJNA729271	stage_E18_vs_E10	ordinary_muscle_development	ENSGALG00010024201	IREB2	-0.489689934	0.070171096	-6.978513441	2.9832E-12	1.2302E-11	negative
PRJNA729271	stage_E18_vs_E10	ordinary_muscle_development	ENSGALG00010024203	SPOP	-0.063479244	0.073408879	-0.864735233	0.387184127	0.447368188	negative
PRJNA729271	stage_E18_vs_E10	ordinary_muscle_development	ENSGALG00010024204	PTBP2	0.486488714	0.108308447	4.491696868	7.06579E-06	1.66996E-05	positive
PRJNA729271	stage_E18_vs_E10	ordinary_muscle_development	ENSGALG00010024205	ANKRD13D	0.053379488	0.170077141	0.313854571	0.753631495	0.793253106	positive
PRJNA729271	stage_E18_vs_E10	ordinary_muscle_development	ENSGALG00010024206	KRT80	-1.616858696	0.590563367	-2.737824231	0.006184712	0.010103393	negative
PRJNA729271	stage_E18_vs_E10	ordinary_muscle_development	ENSGALG00010024207	KCTD18	-0.000284451	0.12196754	-0.002332185	0.998139187	0.998345813	negative
PRJNA729271	stage_E18_vs_E10	ordinary_muscle_development	ENSGALG00010024208	SERPIND1	4.175448387	0.57608884	7.24792445	4.23207E-13	1.86399E-12	positive
PRJNA729271	stage_E18_vs_E10	ordinary_muscle_development	ENSGALG00010024209	RWDD3	0.136623298	0.143765149	0.950322794	0.34194826	0.400399065	positive
PRJNA729271	stage_E18_vs_E10	ordinary_muscle_development	ENSGALG00010024210	NXPH3	-4.328190666	1.247172595	-3.470402319	0.000519679	0.000985446	negative
PRJNA729271	stage_E18_vs_E10	ordinary_muscle_development	ENSGALG00010024211	TRPM5	2.689898567	0.661830448	4.064331845	4.81703E-05	0.000103857	positive
PRJNA729271	stage_E18_vs_E10	ordinary_muscle_development	ENSGALG00010024212	RIMS4	-0.067279408	0.200569282	-0.335442236	0.737291512	0.778541595	negative
PRJNA729271	stage_E18_vs_E10	ordinary_muscle_development	ENSGALG00010024213	ENSGALG00010024213	0.225310135	0.187790102	1.199797714	0.230217912	0.281841946	positive
PRJNA729271	stage_E18_vs_E10	ordinary_muscle_development	ENSGALG00010024214	CRABP1	-7.056826644	0.42291987	-16.68596619	1.65802E-62	7.32712E-61	negative
PRJNA729271	stage_E18_vs_E10	ordinary_muscle_development	ENSGALG00010024215	ENSGALG00010024215	0.776935714	0.159535929	4.869973297	1.11613E-06	2.8736E-06	positive
PRJNA729271	stage_E18_vs_E10	ordinary_muscle_development	ENSGALG00010024218	NGFR	-1.4315502	0.229837809	-6.228523537	4.70851E-10	1.63944E-09	negative
PRJNA729271	stage_E18_vs_E10	ordinary_muscle_development	ENSGALG00010024220	SIAH1	1.874313435	0.200625842	9.34233305	9.42344E-21	6.97257E-20	positive
PRJNA729271	stage_E18_vs_E10	ordinary_muscle_development	ENSGALG00010024222	ENSGALG00010024222	-0.159281562	0.306379355	-0.51988347	0.603144798	0.654385018	negative
PRJNA729271	stage_E18_vs_E10	ordinary_muscle_development	ENSGALG00010024223	ENSGALG00010024223	-3.051747933	0.384206912	-7.942980292	1.9738E-15	1.03623E-14	negative
PRJNA729271	stage_E18_vs_E10	ordinary_muscle_development	ENSGALG00010024225	BCDIN3D	-0.009621178	0.182112024	-0.052831097	0.957866484	0.965258251	negative
PRJNA729271	stage_E18_vs_E10	ordinary_muscle_development	ENSGALG00010024226	POU2AF1	-1.50561108	1.489806763	-1.0106083	0.312203942	0.369721907	negative
PRJNA729271	stage_E18_vs_E10	ordinary_muscle_development	ENSGALG00010024227	MUC6	2.555712899	0.415302346	6.153860977	7.5619E-10	2.58697E-09	positive
PRJNA729271	stage_E18_vs_E10	ordinary_muscle_development	ENSGALG00010024230	LONP2	0.218599201	0.150808639	1.449513786	0.147194153	0.188695432	positive
PRJNA729271	stage_E18_vs_E10	ordinary_muscle_development	ENSGALG00010024233	FAIM2	-3.133547436	0.253405799	-12.36572898	4.00561E-35	6.1899E-34	negative
PRJNA729271	stage_E18_vs_E10	ordinary_muscle_development	ENSGALG00010024234	ENSGALG00010024234	1.970943087	0.270022758	7.299173978	2.8954E-13	1.28976E-12	positive
PRJNA729271	stage_E18_vs_E10	ordinary_muscle_development	ENSGALG00010024238	ATF7	1.764410136	0.202406866	8.717145652	2.85303E-18	1.81859E-17	positive
PRJNA729271	stage_E18_vs_E10	ordinary_muscle_development	ENSGALG00010024240	WDR61	-0.672697986	0.126932745	-5.299641042	1.16031E-07	3.26956E-07	negative
PRJNA729271	stage_E18_vs_E10	ordinary_muscle_development	ENSGALG00010024242	KMT2A	-0.14434258	0.125642479	-1.148835818	0.250623687	0.30430477	negative
PRJNA729271	stage_E18_vs_E10	ordinary_muscle_development	ENSGALG00010024243	MPP3	0.320715479	0.20741123	1.54627828	0.122037375	0.159277125	positive
PRJNA729271	stage_E18_vs_E10	ordinary_muscle_development	ENSGALG00010024245	TARBP2	-0.311617045	0.167374991	-1.861789765	0.06263273	0.086315024	negative
PRJNA729271	stage_E18_vs_E10	ordinary_muscle_development	ENSGALG00010024250	AP2A2	-0.238719492	0.053725541	-4.443314784	8.85834E-06	2.07334E-05	negative
PRJNA729271	stage_E18_vs_E10	ordinary_muscle_development	ENSGALG00010024251	ENSGALG00010024251	0.457961794	0.209515507	2.185813355	0.028829263	0.042252798	positive
PRJNA729271	stage_E18_vs_E10	ordinary_muscle_development	ENSGALG00010024254	ENSGALG00010024254	2.51389523	0.310469309	8.097081276	5.62935E-16	3.07451E-15	positive
PRJNA729271	stage_E18_vs_E10	ordinary_muscle_development	ENSGALG00010024255	CFAP97D1	0.672741955	0.681978274	0.986456578	0.323909113	0.381929765	positive
PRJNA729271	stage_E18_vs_E10	ordinary_muscle_development	ENSGALG00010024258	DNAJA4	0.091314087	0.152560138	0.598544861	0.549476435	0.603675005	positive
PRJNA729271	stage_E18_vs_E10	ordinary_muscle_development	ENSGALG00010024259	ENSGALG00010024259	0.951731394	0.134883682	7.055941684	1.71436E-12	7.20696E-12	positive
PRJNA729271	stage_E18_vs_E10	ordinary_muscle_development	ENSGALG00010024263	PHKB	1.702217283	0.131701848	12.92477902	3.26233E-38	5.80926E-37	positive
PRJNA729271	stage_E18_vs_E10	ordinary_muscle_development	ENSGALG00010024264	AMER3	-0.193227361	0.305041743	-0.633445635	0.526442672	0.582858733	negative
PRJNA729271	stage_E18_vs_E10	ordinary_muscle_development	ENSGALG00010024266	ENSGALG00010024266	-0.441191498	0.255970448	-1.723603261	0.084779489	0.113975023	negative
PRJNA729271	stage_E18_vs_E10	ordinary_muscle_development	ENSGALG00010024267	MAP3K12	-1.526756165	0.205493257	-7.429714184	1.08833E-13	4.98902E-13	negative

PRJNA729271	stage_E18_vs_E10	ordinary_muscle_development	ENSGALG00010024270	RAB18L	1.1389487	0.774263615	1.47100894	0.141288699	0.181913279	positive
PRJNA729271	stage_E18_vs_E10	ordinary_muscle_development	ENSGALG00010024274	ACSBG1	1.003143105	0.311566242	3.219678413	0.001283345	0.002307951	positive
PRJNA729271	stage_E18_vs_E10	ordinary_muscle_development	ENSGALG00010024277	ITGB3BP	-1.266685045	0.188719907	-6.711984257	1.91995E-11	7.38189E-11	negative
PRJNA729271	stage_E18_vs_E10	ordinary_muscle_development	ENSGALG00010024280	ENSGALG00010024280	1.608934893	0.125491603	12.82105619	1.24986E-37	2.16708E-36	positive
PRJNA729271	stage_E18_vs_E10	ordinary_muscle_development	ENSGALG00010024282	HIC2	-1.281096754	0.151755683	-8.441837065	3.12389E-17	1.85425E-16	negative
PRJNA729271	stage_E18_vs_E10	ordinary_muscle_development	ENSGALG00010024283	UBE4A	-0.040519439	0.110737468	-0.365905413	0.714435657	0.757940778	negative
PRJNA729271	stage_E18_vs_E10	ordinary_muscle_development	ENSGALG00010024285	CCND2	-0.177973322	0.097113151	-1.832638736	0.066856314	0.09172251	negative
PRJNA729271	stage_E18_vs_E10	ordinary_muscle_development	ENSGALG00010024286	ADA	0.380875572	0.181508221	2.098392959	0.035870447	0.051658433	positive
PRJNA729271	stage_E18_vs_E10	ordinary_muscle_development	ENSGALG00010024288	CHID1	0.525430145	0.133946113	3.92269795	8.75629E-05	0.000183733	positive
PRJNA729271	stage_E18_vs_E10	ordinary_muscle_development	ENSGALG00010024290	SOST	0.354117271	0.451914041	0.783594311	0.433278195	0.493080587	positive
PRJNA729271	stage_E18_vs_E10	ordinary_muscle_development	ENSGALG00010024291	ENSGALG00010024291	4.026974982	0.493603373	8.158321443	3.39712E-16	1.88016E-15	positive
PRJNA729271	stage_E18_vs_E10	ordinary_muscle_development	ENSGALG00010024300	MEOX1	-3.94410994	0.291987489	-13.50780457	1.40654E-41	2.86747E-40	negative
PRJNA729271	stage_E18_vs_E10	ordinary_muscle_development	ENSGALG00010024304	PKIG	-0.787989985	0.134027611	-5.879310814	4.11978E-09	1.31853E-08	negative
PRJNA729271	stage_E18_vs_E10	ordinary_muscle_development	ENSGALG00010024305	ENSGALG00010024305	5.421143252	0.72261072	7.50216279	6.27733E-14	2.93327E-13	positive
PRJNA729271	stage_E18_vs_E10	ordinary_muscle_development	ENSGALG00010024309	ABCB11	0.573851218	0.400310145	1.433516552	0.151710272	0.193936008	positive
PRJNA729271	stage_E18_vs_E10	ordinary_muscle_development	ENSGALG00010024311	KLHL6	1.212318675	0.131200723	9.240182895	2.46076E-20	1.76928E-19	positive
PRJNA729271	stage_E18_vs_E10	ordinary_muscle_development	ENSGALG00010024313	SERINC3	1.414401866	0.109284232	12.94241479	2.59348E-38	4.65253E-37	positive
PRJNA729271	stage_E18_vs_E10	ordinary_muscle_development	ENSGALG00010024314	ITFG1	-0.036583151	0.147100226	-0.248695412	0.803596398	0.836130189	negative
PRJNA729271	stage_E18_vs_E10	ordinary_muscle_development	ENSGALG00010024321	UBE2L3	-0.656735114	0.082677289	-7.943355722	1.96784E-15	1.03347E-14	negative
PRJNA729271	stage_E18_vs_E10	ordinary_muscle_development	ENSGALG00010024322	IDH3A	-0.220823937	0.15948789	-1.384581216	0.166180558	0.210423373	negative
PRJNA729271	stage_E18_vs_E10	ordinary_muscle_development	ENSGALG00010024323	TTPAL	-1.047881629	0.103265623	-10.14743919	3.40172E-24	3.06642E-23	negative
PRJNA729271	stage_E18_vs_E10	ordinary_muscle_development	ENSGALG00010024325	PCBP2	0.006872351	0.170845931	0.040225428	0.967913405	0.973487705	positive
PRJNA729271	stage_E18_vs_E10	ordinary_muscle_development	ENSGALG00010024326	KLHL24	1.669729675	0.178671068	9.345271699	9.16538E-21	6.79204E-20	positive
PRJNA729271	stage_E18_vs_E10	ordinary_muscle_development	ENSGALG00010024327	PPP1R1B	3.611386293	0.41803391	8.638979286	5.67175E-18	3.54209E-17	positive
PRJNA729271	stage_E18_vs_E10	ordinary_muscle_development	ENSGALG00010024328	YDJC	-1.644070054	0.152456544	-10.78386018	4.10298E-27	4.37299E-26	negative
PRJNA729271	stage_E18_vs_E10	ordinary_muscle_development	ENSGALG00010024330	SDF2L1	-1.189152131	0.102704727	-11.57835831	5.30523E-31	6.83549E-30	negative
PRJNA729271	stage_E18_vs_E10	ordinary_muscle_development	ENSGALG00010024332	EFCAB7	-0.330443652	0.16591788	-1.991609654	0.046413903	0.06552099	negative
PRJNA729271	stage_E18_vs_E10	ordinary_muscle_development	ENSGALG00010024333	ETV4	-4.246030741	0.244530039	-17.36404561	1.54447E-67	7.69314E-66	negative
PRJNA729271	stage_E18_vs_E10	ordinary_muscle_development	ENSGALG00010024334	USP28	2.038262286	0.12249575	16.63945315	3.60899E-62	1.5602E-60	positive
PRJNA729271	stage_E18_vs_E10	ordinary_muscle_development	ENSGALG00010024336	TSPAN4	0.138988891	0.116839887	1.18956715	0.234216564	0.28642277	positive
PRJNA729271	stage_E18_vs_E10	ordinary_muscle_development	ENSGALG00010024338	NDUFA9	0.14049482	0.114435653	1.227718953	0.219552489	0.270337524	positive
PRJNA729271	stage_E18_vs_E10	ordinary_muscle_development	ENSGALG00010024339	TOP3B	-0.568558662	0.089412746	-6.358809977	2.03323E-10	7.23941E-10	negative
PRJNA729271	stage_E18_vs_E10	ordinary_muscle_development	ENSGALG00010024340	NETO2	-1.231985693	0.379812991	-3.24366391	0.001180029	0.002130608	negative
PRJNA729271	stage_E18_vs_E10	ordinary_muscle_development	ENSGALG00010024342	HNF4A	2.294902749	1.346781573	1.703990309	0.088382898	0.118380161	positive
PRJNA729271	stage_E18_vs_E10	ordinary_muscle_development	ENSGALG00010024343	POLR2L	-0.071502448	0.127370853	-0.561372134	0.574543876	0.627629323	negative
PRJNA729271	stage_E18_vs_E10	ordinary_muscle_development	ENSGALG00010024344	YEATS2	0.150970105	0.133163896	1.13371649	0.25691349	0.310822222	positive
PRJNA729271	stage_E18_vs_E10	ordinary_muscle_development	ENSGALG00010024345	STARD3	0.469342918	0.092233672	5.088628779	3.60662E-07	9.73156E-07	positive
PRJNA729271	stage_E18_vs_E10	ordinary_muscle_development	ENSGALG00010024346	PPM1F	0.304175291	0.091931381	3.308721019	0.000937232	0.001713569	positive
PRJNA729271	stage_E18_vs_E10	ordinary_muscle_development	ENSGALG00010024347	OTOG	-1.352253402	0.28842867	-4.688345998	2.75422E-06	6.778E-06	negative
PRJNA729271	stage_E18_vs_E10	ordinary_muscle_development	ENSGALG00010024349	R3HDML	-1.822939721	0.430043797	-4.238962943	2.24555E-05	5.02613E-05	negative
PRJNA729271	stage_E18_vs_E10	ordinary_muscle_development	ENSGALG00010024350	PGM1	2.516506537	0.136330157	18.45891327	4.42148E-76	2.73886E-74	positive
PRJNA729271	stage_E18_vs_E10	ordinary_muscle_development	ENSGALG00010024351	SP1	0.625310049	0.154437094	4.04896281	5.14451E-05	0.000110605	positive
PRJNA729271	stage_E18_vs_E10	ordinary_muscle_development	ENSGALG00010024354	C2CD2L	-0.363905085	0.080481515	-4.521598344	6.13744E-06	1.45983E-05	negative
PRJNA729271	stage_E18_vs_E10	ordinary_muscle_development	ENSGALG00010024356	PARL	0.166074954	0.118054518	1.406764916	0.159497109	0.202852557	positive
PRJNA729271	stage_E18_vs_E10	ordinary_muscle_development	ENSGALG00010024358	MAPK3	-0.021922472	0.062213466	-0.352375031	0.724557025	0.766882372	negative
PRJNA729271	stage_E18_vs_E10	ordinary_muscle_development	ENSGALG00010024360	FTL	-1.333413901	0.246306679	-5.413632749	6.17588E-08	1.77971E-07	negative
PRJNA729271	stage_E18_vs_E10	ordinary_muscle_development	ENSGALG00010024361	H2AX	-1.102876395	0.214660483	-5.137770962	2.78016E-07	7.57063E-07	negative
PRJNA729271	stage_E18_vs_E10	ordinary_muscle_development	ENSGALG00010024362	DHX8	-0.388964841	0.059252837	-6.56449315	5.22101E-11	1.94897E-10	negative
PRJNA729271	stage_E18_vs_E10	ordinary_muscle_development	ENSGALG00010024363	EDEM2	-0.755593672	0.117236407	-6.445042906	1.15568E-10	4.18893E-10	negative
PRJNA729271	stage_E18_vs_E10	ordinary_muscle_development	ENSGALG00010024365	ENSGALG00010024365	0.095269054	0.108927666	0.874608419	0.381787005	0.442083611	positive
PRJNA729271	stage_E18_vs_E10	ordinary_muscle_development	ENSGALG00010024366	PARD6A	1.13223743	0.410761758	2.756433401	0.005843551	0.009581705	positive
PRJNA729271	stage_E18_vs_E10	ordinary_muscle_development	ENSGALG00010024367	ENSGALG00010024367	-1.663067634	0.23523987	-7.069667371	1.55306E-12	6.55548E-12	negative
PRJNA729271	stage_E18_vs_E10	ordinary_muscle_development	ENSGALG00010024368	DPAGT1	-0.035073135	0.079987786	-0.43848113	0.661037546	0.708724127	negative
PRJNA729271	stage_E18_vs_E10	ordinary_muscle_development	ENSGALG00010024369	MYOD1	0.310037759	0.275771032	1.124257891	0.260903637	0.315202385	positive
PRJNA729271	stage_E18_vs_E10	ordinary_muscle_development	ENSGALG00010024370	HERPUD1	0.446153235	0.092388818	4.829082647	1.37163E-06	3.49417E-06	positive
PRJNA729271	stage_E18_vs_E10	ordinary_muscle_development	ENSGALG00010024371	YPEL1	1.602756289	0.104309951	15.36532495	2.79633E-53	9.00729E-52	positive
PRJNA729271	stage_E18_vs_E10	ordinary_muscle_development	ENSGALG00010024373	TCAP	6.187384061	0.483585743	12.79480248	1.75298E-37	3.02133E-36	positive
PRJNA729271	stage_E18_vs_E10	ordinary_muscle_development	ENSGALG00010024374	ENSGALG00010024374	2.158343139	0.579189677	3.726487584	0.000194167	0.00038992	positive
PRJNA729271	stage_E18_vs_E10	ordinary_muscle_development	ENSGALG00010024377	CIB2	-0.409015329	0.172190632	-2.375363422	0.017531679	0.026701869	negative
PRJNA729271	stage_E18_vs_E10	ordinary_muscle_development	ENSGALG00010024378	ZDHHC1	-0.469273023	0.180620792	-2.598111864	0.009373795	0.014882055	negative
PRJNA729271	stage_E18_vs_E10	ordinary_muscle_development	ENSGALG00010024382	ENSGALG00010024382	1.201031045	0.430103291	2.792424682	0.005231465	0.008650477	positive
PRJNA729271	stage_E18_vs_E10	ordinary_muscle_development	ENSGALG00010024383	DUSP28	1.449561115	0.189999298	7.629297198	2.36037E-14	1.13893E-13	positive
PRJNA729271	stage_E18_vs_E10	ordinary_muscle_development	ENSGALG00010024386	TPPP3	1.224291113	0.268827688	4.55418533	5.2589E-06	1.25934E-05	positive
PRJNA729271	stage_E18_vs_E10	ordinary_muscle_development	ENSGALG00010024387	PPIL2	0.502073592	0.064470096	7.787697296	6.82414E-15	3.45498E-14	positive
PRJNA729271	stage_E18_vs_E10	ordinary_muscle_development	ENSGALG00010024389	AAAS	-0.310560844	0.108318295	-2.867113486	0.004142344	0.006939815	negative
PRJNA729271	stage_E18_vs_E10	ordinary_muscle_development	ENSGALG00010024391	TBC1D2B	-0.194527763	0.110842294	-1.75499583	0.079260021	0.107271149	negative
PRJNA729271	stage_E18_vs_E10	ordinary_muscle_development	ENSGALG00010024392	CHMP4B	-0.327740322	0.085579109	-3.829676741	0.000128312	0.000264112	negative
PRJNA729271	stage_E18_vs_E10	ordinary_muscle_development	ENSGALG00010024393	OGFOD1	-0.779136463	0.105272119	-7.401166304	1.34994E-13	6.14358E-13	negative
PRJNA729271	stage_E18_vs_E10	ordinary_muscle_development	ENSGALG00010024396	ENSGALG00010024396	-0.140261252	0.343317579	-0.408546666	0.682872377	0.729111898	negative
PRJNA729271	stage_E18_vs_E10	ordinary_muscle_development	ENSGALG00010024398	PHB	-0.04682644	0.088422948	-0.529573383	0.596407753	0.648240131	negative
PRJNA729271	stage_E18_vs_E10	ordinary_muscle_development	ENSGALG00010024399	ROR1	-0.453689632	0.168356187	-2.694820071	0.007042665	0.011383076	negative
PRJNA729271	stage_E18_vs_E10	ordinary_muscle_development	ENSGALG00010024400	KCNA1	-2.528900132	0.395334848	-6.396856094	1.58609E-10	5.69773E-10	negative
PRJNA729271	stage_E18_vs_E10	ordinary_muscle_development	ENSGALG00010024401	KCNC1	2.57801601	0.212732865	12.11856008	8.42259E-34	1.22085E-32	positive
PRJNA729271	stage_E18_vs_E10	ordinary_muscle_development	ENSGALG00010024402	GPC1	-0.505581228	0.119523113	-4.229987099	2.33705E-05	5.22125E-05	negative
PRJNA729271	stage_E18_vs_E10	ordinary_muscle_development	ENSGALG00010024403	HMBS	-0.902345149	0.078067674	-11.55849929	6.68671E-31	8.55463E-30	negative
PRJNA729271	stage_E18_vs_E10	ordinary_muscle_development	ENSGALG00010024406	NUDT21	-0.983969181	0.066222644	-14.8585003	6.12817E-50	1.78012E-48	negative
PRJNA729271	stage_E18_vs_E10	ordinary_muscle_development	ENSGALG00010024407	HIRA	0.100080584	0.080816639	1.238366075	0.215580354	0.266124785	positive
PRJNA729271	stage_E18_vs_E10	ordinary_muscle_development	ENSGALG00010024408	ENSGALG00010024408	2.008859515	1.146000619	1.752930567	0.079613926	0.107699847	positive
PRJNA729271	stage_E18_vs_E10	ordinary_muscle_development	ENSGALG00010024410	MYG1	-0.320742745	0.110271797	-2.90865618	0.003629858	0.006126548	negative
PRJNA729271	stage_E18_vs_E10	ordinary_muscle_development	ENSGALG00010024412	PIGU	-0.711401005	0.103611842	-6.866020258	6.60177E-12	2.63761E-11	negative
PRJNA729271	stage_E18_vs_E10	ordinary_muscle_development	ENSGALG00010024413	AMFR	0.394515249	0.092876473	4.247741499	2.15936E-05	4.8437E-05	positive
PRJNA729271	stage_E18_vs_E10	ordinary_muscle_development	ENSGALG00010024415	SIK2	0.466211992	0.060903992	7.654867528	1.93512E-14	9.42207E-14	positive
PRJNA729271	stage_E18_vs_E10	ordinary_muscle_development	ENSGALG00010024416	VDR	-1.365143674	0.337103247	-4.049630746	5.12985E-05	0.000110355	negative
PRJNA729271	stage_E18_vs_E10	ordinary_muscle_development	ENSGALG00010024419	PGAP3	0.503091103	0.127599568	3.942733602	8.05581E-05	0.000169574	positive
PRJNA729271	stage_E18_vs_E10	ordinary_muscle_development	ENSGALG00010024420	TMEM106C	0.027625613	0.164573841	0.167861505	0.866692235	0.890025076	positive
PRJNA729271	stage_E18_vs_E10	ordinary_muscle_development	ENSGALG00010024421	DAPK2	-0.736448869	0.261879252	-2.812169593	0.004920855	0.008164812	negative
PRJNA729271	stage_E18_vs_E10	ordinary_muscle_development	ENSGALG00010024423	SERGEF	0.011547078	0.120024692	0.096205854	0.923357082	0.936865526	positive
PRJNA729271	stage_E18_vs_E10	ordinary_muscle_development	ENSGALG00010024424	SAMHD1	-0.409811284	0.182039407	-2.251222913	0.024371422	0.036206187	negative
PRJNA729271	stage_E18_vs_E10	ordinary_muscle_development	ENSGALG00010024425	MRPL40	0.787112051	0.088684483	8.875420151	6.96699E-19	4.61126E-18	positive
PRJNA729271	stage_E18_vs_E10	ordinary_muscle_development	ENSGALG00010024426	PHAF1	0.83358959	0.105317928	7.914982789	2.47287E-15	1.29029E-14	positive
PRJNA729271	stage_E18_vs_E10	ordinary_muscle_development	ENSGALG00010024427	C22orf39	0.473979464	0.135583819	3.495840924	0.00047257	0.000901659	positive
PRJNA729271	stage_E18_vs_E10	ordinary_muscle_development	ENSGALG00010024428	SAAL1	-1.04318884	0.06426545	-16.2324989	2.97113E-59	1.16712E-57	negative
PRJNA729271	stage_E18_vs_E10	ordinary_muscle_development	ENSGALG00010024430	ZNF652	0.023227586	0.064664099	0.359203741	0.719442678	0.76219278	positive
PRJNA729271	stage_E18_vs_E10	ordinary_muscle_development	ENSGALG00010024431	FEZ1	-1.827096449	0.153163427	-11.92906479	8.35064E-33	1.14841E-31	negative
PRJNA729271	stage_E18_vs_E10	ordinary_muscle_development	ENSGALG00010024433	ENSGALG00010024433	0.073079867	0.168935365	0.432590692	0.665312152	0.712553543	positive
PRJNA729271	stage_E18_vs_E10	ordinary_muscle_development	ENSGALG00010024434	B3GNT9	-0.41872444	0.158358083	-2.644162086	0.008189343	0.013119422	negative
PRJNA729271	stage_E18_vs_E10	ordinary_muscle_development	ENSGALG00010024437	HERC1	-0.240898393	0.081758554	-2.946461014	0.003214329	0.005474936	negative
PRJNA729271	stage_E18_vs_E10	ordinary_muscle_development	ENSGALG00010024438	RBL1	-1.327677956	0.121336315	-10.94213184	7.24754E-28	8.01932E-27	negative
PRJNA729271	stage_E18_vs_E10	ordinary_muscle_development	ENSGALG00010024439	UFD1	0.430438211	0.112680713	3.819981241	0.000133462	0.000274168	positive
PRJNA729271	stage_E18_vs_E10	ordinary_muscle_development	ENSGALG00010024440	PKNOX2	2.445569762	0.205487257	11.90132078	1.16489E-32	1.59443E-31	positive
PRJNA729271	stage_E18_vs_E10	ordinary_muscle_development	ENSGALG00010024441	SAA	3.815888642	0.79303613	4.811746272	1.49617E-06	3.79741E-06	positive
PRJNA729271	stage_E18_vs_E10	ordinary_muscle_development	ENSGALG00010024446	GRM4	-2.989895643	0.525513085	-5.68947897	1.27428E-08	3.89676E-08	negative
PRJNA729271	stage_E18_vs_E10	ordinary_muscle_development	ENSGALG00010024449	CACHD1	0.585458771	0.083072235	7.04758665	1.82047E-12	7.63314E-12	positive
PRJNA729271	stage_E18_vs_E10	ordinary_muscle_development	ENSGALG00010024451	PHOSPHO1	0.047610806	0.274769383	0.173275515	0.862434869	0.886406681	positive
PRJNA729271	stage_E18_vs_E10	ordinary_muscle_development	ENSGALG00010024452	SLC16A1	-0.394007846	0.253329949	-1.555314911	0.119871088	0.156730189	negative
PRJNA729271	stage_E18_vs_E10	ordinary_muscle_development	ENSGALG00010024453	HTR3A	4.348538339	0.560009573	7.765114287	8.15716E-15	4.10264E-14	positive
PRJNA729271	stage_E18_vs_E10	ordinary_muscle_development	ENSGALG00010024454	HPS5	-0.18741483	0.121307285	-1.544959396	0.122356085	0.159649964	negative
PRJNA729271	stage_E18_vs_E10	ordinary_muscle_development	ENSGALG00010024457	CHD6	-0.039685052	0.100794834	-0.393721092	0.693786967	0.73911819	negative
PRJNA729271	stage_E18_vs_E10	ordinary_muscle_development	ENSGALG00010024459	AHCYL1	-0.294259411	0.093329203	-3.152918931	0.001616467	0.002874578	negative
PRJNA729271	stage_E18_vs_E10	ordinary_muscle_development	ENSGALG00010024460	IMP4	-0.608368513	0.157309563	-3.867333311	0.000110032	0.000228498	negative
PRJNA729271	stage_E18_vs_E10	ordinary_muscle_development	ENSGALG00010024462	ZBTB32	6.487487314	0.428740342	15.1315066	1.00371E-51	3.09548E-50	positive
PRJNA729271	stage_E18_vs_E10	ordinary_muscle_development	ENSGALG00010024463	FBXL22	1.888840196	0.228064538	8.28204248	1.2108E-16	6.91786E-16	positive
PRJNA729271	stage_E18_vs_E10	ordinary_muscle_development	ENSGALG00010024465	SENP1	-0.979194118	0.163086531	-6.004138507	1.9235E-09	6.3352E-09	negative
PRJNA729271	stage_E18_vs_E10	ordinary_muscle_development	ENSGALG00010024466	ADGRG3	1.609950152	0.817669083	1.968950747	0.048958747	0.068831915	positive
PRJNA729271	stage_E18_vs_E10	ordinary_muscle_development	ENSGALG00010024467	ENSGALG00010024467	0.590957166	0.301255303	1.961649009	0.049803362	0.069965084	positive
PRJNA729271	stage_E18_vs_E10	ordinary_muscle_development	ENSGALG00010024469	GTF2H1	0.058758943	0.128193279	0.45836212	0.646692301	0.695489309	positive
PRJNA729271	stage_E18_vs_E10	ordinary_muscle_development	ENSGALG00010024471	CCDC61	-0.134633524	0.116236642	-1.158270935	0.246753489	0.300007701	negative
PRJNA729271	stage_E18_vs_E10	ordinary_muscle_development	ENSGALG00010024472	LDHA	1.740922342	0.201414245	8.643491637	5.45207E-18	3.40931E-17	positive
PRJNA729271	stage_E18_vs_E10	ordinary_muscle_development	ENSGALG00010024473	RAVER2	-0.758654606	0.059803782	-12.68572953	7.09501E-37	1.19584E-35	negative
PRJNA729271	stage_E18_vs_E10	ordinary_muscle_development	ENSGALG00010024474	STRIP1	-0.503766303	0.105604536	-4.770309334	1.83943E-06	4.61849E-06	negative
PRJNA729271	stage_E18_vs_E10	ordinary_muscle_development	ENSGALG00010024475	ADGRG1	0.484515892	0.159370971	3.040176563	0.002364395	0.004104051	positive
PRJNA729271	stage_E18_vs_E10	ordinary_muscle_development	ENSGALG00010024476	KCNB1	0.31180463	0.165111847	1.888444929	0.058966244	0.081736226	positive
PRJNA729271	stage_E18_vs_E10	ordinary_muscle_development	ENSGALG00010024479	ERBB2	0.369794449	0.128669104	2.87399568	0.004053145	0.006800595	positive
PRJNA729271	stage_E18_vs_E10	ordinary_muscle_development	ENSGALG00010024480	CDC45	-1.858028501	0.216707695	-8.573892589	1.00045E-17	6.14209E-17	negative
PRJNA729271	stage_E18_vs_E10	ordinary_muscle_development	ENSGALG00010024482	USP3	-0.870727789	0.058444643	-14.89833365	3.37902E-50	9.93486E-49	negative
PRJNA729271	stage_E18_vs_E10	ordinary_muscle_development	ENSGALG00010024486	GPR37L1	-0.000543404	0.298299384	-0.001821674	0.998546516	0.998684313	negative
PRJNA729271	stage_E18_vs_E10	ordinary_muscle_development	ENSGALG00010024488	ADGRG5	1.824943094	0.437710973	4.169287973	3.05553E-05	6.73098E-05	positive
PRJNA729271	stage_E18_vs_E10	ordinary_muscle_development	ENSGALG00010024490	TGM3	4.537732084	0.8109993	5.595235511	2.20322E-08	6.60922E-08	positive
PRJNA729271	stage_E18_vs_E10	ordinary_muscle_development	ENSGALG00010024492	NTF3	-2.005490048	0.252199046	-7.952012829	1.83505E-15	9.66537E-15	negative
PRJNA729271	stage_E18_vs_E10	ordinary_muscle_development	ENSGALG00010024493	JAK1	0.681043916	0.060085355	11.33460757	8.84276E-30	1.07081E-28	positive
PRJNA729271	stage_E18_vs_E10	ordinary_muscle_development	ENSGALG00010024494	DDAH1	-1.95586821	0.224357299	-8.717649136	2.84038E-18	1.81132E-17	negative

PRJNA729271	stage_E18_vs_E10	ordinary_muscle_development	ENSGALG00010024495	CLDN5	1.659733819	0.165392284	10.03513453	1.06814E-23	9.31007E-23	positive
PRJNA729271	stage_E18_vs_E10	ordinary_muscle_development	ENSGALG00010024496	UEVLD	0.002726593	0.208396173	0.013083698	0.989561017	0.991093869	positive
PRJNA729271	stage_E18_vs_E10	ordinary_muscle_development	ENSGALG00010024497	ABI3	1.145433162	0.185022562	6.190775582	5.98689E-10	2.06767E-09	positive
PRJNA729271	stage_E18_vs_E10	ordinary_muscle_development	ENSGALG00010024498	CCDC102A	-0.137565484	0.117857453	-1.167219216	0.243121853	0.296238337	negative
PRJNA729271	stage_E18_vs_E10	ordinary_muscle_development	ENSGALG00010024499	CA12	-3.54101277	0.800571001	-4.423108965	9.72906E-06	2.26834E-05	negative
PRJNA729271	stage_E18_vs_E10	ordinary_muscle_development	ENSGALG00010024501	ARL8A	0.280380889	0.090089734	3.112240188	0.001856734	0.003277719	positive
PRJNA729271	stage_E18_vs_E10	ordinary_muscle_development	ENSGALG00010024502	VPS8	-0.150720315	0.073984014	-2.037201119	0.041629892	0.059217398	negative
PRJNA729271	stage_E18_vs_E10	ordinary_muscle_development	ENSGALG00010024504	CASC3	0.007156949	0.187419564	0.03818677 8	0.969538763	0.974784238	positive
PRJNA729271	stage_E18_vs_E10	ordinary_muscle_development	ENSGALG00010024505	DNASE2B	0.687411366	0.211676793	3.247457383	0.001164411	0.002104244	positive
PRJNA729271	stage_E18_vs_E10	ordinary_muscle_development	ENSGALG00010024506	RAB8B	-0.049735867	0.081600252	-0.609506291	0.542188904	0.597235973	negative
PRJNA729271	stage_E18_vs_E10	ordinary_muscle_development	ENSGALG00010024508	RPS27L	1.113369914	0.190162075	5.854847312	4.77448E-09	1.51968E-08	positive
PRJNA729271	stage_E18_vs_E10	ordinary_muscle_development	ENSGALG00010024509	SEPTIN5	1.469375903	0.106759837	13.76337714	4.23283E-43	9.15744E-42	positive
PRJNA729271	stage_E18_vs_E10	ordinary_muscle_development	ENSGALG00010024510	CNN3	-0.371744659	0.101078563	-3.677779417	0.000235273	0.000467611	negative
PRJNA729271	stage_E18_vs_E10	ordinary_muscle_development	ENSGALG00010024511	ENSGALG00010024511	-0.213475408	0.169661365	-1.258244077	0.208303499	0.25806489	negative
PRJNA729271	stage_E18_vs_E10	ordinary_muscle_development	ENSGALG00010024512	CCNDBP1	0.242609424	0.115293621	2.104274473	0.035354511	0.050976191	positive
PRJNA729271	stage_E18_vs_E10	ordinary_muscle_development	ENSGALG00010024513	SPTY2D1	-0.132459213	0.139810685	-0.947418381	0.343425629	0.401836817	negative
PRJNA729271	stage_E18_vs_E10	ordinary_muscle_development	ENSGALG00010024514	FAF1	0.1288994	0.068888114	1.871141376	0.061325488	0.084682571	positive
PRJNA729271	stage_E18_vs_E10	ordinary_muscle_development	ENSGALG00010024515	C3orf70	-0.198629937	0.154958615	-1.281825709	0.199903793	0.248742852	negative
PRJNA729271	stage_E18_vs_E10	ordinary_muscle_development	ENSGALG00010024516	EHHADH	0.323783567	0.240893008	1.344096991	0.178917025	0.224985016	positive
PRJNA729271	stage_E18_vs_E10	ordinary_muscle_development	ENSGALG00010024517	LACTB	-0.521592724	0.102301286	-5.098594037	3.42186E-07	9.2606E-07	negative
PRJNA729271	stage_E18_vs_E10	ordinary_muscle_development	ENSGALG00010024518	ENSGALG00010024518	2.968724552	1.151096144	2.579041349	0.009907493	0.015681691	positive
PRJNA729271	stage_E18_vs_E10	ordinary_muscle_development	ENSGALG00010024519	MIEN1	0.119974778	0.142002226	0.844879555	0.39817809	0.458141888	positive
PRJNA729271	stage_E18_vs_E10	ordinary_muscle_development	ENSGALG00010024520	MAP3K13	0.012669236	0.133394509	0.094975691	0.924334136	0.937787938	positive
PRJNA729271	stage_E18_vs_E10	ordinary_muscle_development	ENSGALG00010024521	HEPACAM	-0.263006236	0.444700917	-0.591422742	0.554237206	0.608380788	negative
PRJNA729271	stage_E18_vs_E10	ordinary_muscle_development	ENSGALG00010024522	DNTTIP2	-0.092692227	0.075764457	-1.223426261	0.221168739	0.272050312	negative
PRJNA729271	stage_E18_vs_E10	ordinary_muscle_development	ENSGALG00010024523	TOX3	-1.830423632	0.279246888	-6.554857762	5.56949E-11	2.07425E-10	negative
PRJNA729271	stage_E18_vs_E10	ordinary_muscle_development	ENSGALG00010024524	TMEM41A	0.084481901	0.135578978	0.62311947	0.533206016	0.589082337	positive
PRJNA729271	stage_E18_vs_E10	ordinary_muscle_development	ENSGALG00010024525	GP1BB	1.088624022	0.185922701	5.855250698	4.76291E-09	1.51633E-08	positive
PRJNA729271	stage_E18_vs_E10	ordinary_muscle_development	ENSGALG00010024526	TGM6	3.330562396	1.429251658	2.330284088	0.019791141	0.029860788	positive
PRJNA729271	stage_E18_vs_E10	ordinary_muscle_development	ENSGALG00010024527	ATP6V0B	0.020679299	0.101523715	0.203689341	0.838596272	0.86639009	positive
PRJNA729271	stage_E18_vs_E10	ordinary_muscle_development	ENSGALG00010024528	TBX1	-0.964126907	0.216517425	-4.452883673	8.47247E-06	1.9888E-05	negative
PRJNA729271	stage_E18_vs_E10	ordinary_muscle_development	ENSGALG00010024529	GRB7	-3.423794518	1.016343164	-3.368738668	0.00075513	0.001401486	negative
PRJNA729271	stage_E18_vs_E10	ordinary_muscle_development	ENSGALG00010024530	ENSGALG00010024530	-0.317978509	0.579691273	-0.548530785	0.583327497	0.636169744	negative
PRJNA729271	stage_E18_vs_E10	ordinary_muscle_development	ENSGALG00010024531	CEP63	-0.684303374	0.129597547	-5.280218567	1.2903E-07	3.61828E-07	negative
PRJNA729271	stage_E18_vs_E10	ordinary_muscle_development	ENSGALG00010024532	TMEM86A	0.571691327	0.214930223	2.659892677	0.007816555	0.012556907	positive
PRJNA729271	stage_E18_vs_E10	ordinary_muscle_development	ENSGALG00010024533	CDKN2C	2.088100871	0.224735099	9.291387427	1.5229E-20	1.11262E-19	positive
PRJNA729271	stage_E18_vs_E10	ordinary_muscle_development	ENSGALG00010024534	VWF	1.663156614	0.126121834	13.18690479	1.04384E-39	1.98042E-38	positive
PRJNA729271	stage_E18_vs_E10	ordinary_muscle_development	ENSGALG00010024535	ENSGALG00010024535	1.604457321	0.154120816	10.41038685	2.22316E-25	2.1555E-24	positive
PRJNA729271	stage_E18_vs_E10	ordinary_muscle_development	ENSGALG00010024536	URI1	-0.131950335	0.062191564	-2.12167579	0.033864973	0.04899908	negative
PRJNA729271	stage_E18_vs_E10	ordinary_muscle_development	ENSGALG00010024537	EPB42	-0.126276945	0.178821183	-0.706163235	0.480086631	0.538319464	negative
PRJNA729271	stage_E18_vs_E10	ordinary_muscle_development	ENSGALG00010024540	PTPN5	-1.523648104	0.286050129	-5.326507312	1.00119E-07	2.84053E-07	negative
PRJNA729271	stage_E18_vs_E10	ordinary_muscle_development	ENSGALG00010024541	WIPF2	0.591194691	0.203679187	2.902577828	0.003701052	0.006240897	positive
PRJNA729271	stage_E18_vs_E10	ordinary_muscle_development	ENSGALG00010024542	ZNFX1	0.457256324	0.426732234	1.071529844	0.283931259	0.339625648	positive
PRJNA729271	stage_E18_vs_E10	ordinary_muscle_development	ENSGALG00010024544	ZNF536	-0.537122313	0.24372114	-2.20383966	0.027535622	0.040512469	negative
PRJNA729271	stage_E18_vs_E10	ordinary_muscle_development	ENSGALG00010024545	PHACTR3	-1.873209779	0.306020023	-6.121199972	9.28733E-10	3.14679E-09	negative
PRJNA729271	stage_E18_vs_E10	ordinary_muscle_development	ENSGALG00010024546	KY	3.6148466	0.403541168	8.957813681	3.31179E-19	2.22862E-18	positive
PRJNA729271	stage_E18_vs_E10	ordinary_muscle_development	ENSGALG00010024547	PFKM	5.165920573	0.197997765	26.09080246	4.6364E-150	1.4934E-147	positive
PRJNA729271	stage_E18_vs_E10	ordinary_muscle_development	ENSGALG00010024548	CDC6	-1.451146988	0.287811132	-5.042011323	4.60664E-07	1.22903E-06	negative
PRJNA729271	stage_E18_vs_E10	ordinary_muscle_development	ENSGALG00010024549	GNB1L	-0.64280698	0.137252237	-4.683398927	2.82157E-06	6.93902E-06	negative
PRJNA729271	stage_E18_vs_E10	ordinary_muscle_development	ENSGALG00010024551	RNF11	1.101085493	0.126927344	8.674927405	4.13814E-18	2.6102E-17	positive
PRJNA729271	stage_E18_vs_E10	ordinary_muscle_development	ENSGALG00010024552	CD81	1.67147236	0.066147198	25.26898212	7.0065E-141	1.9531E-138	positive
PRJNA729271	stage_E18_vs_E10	ordinary_muscle_development	ENSGALG00010024553	ASB8	-0.032731934	0.113949454	-0.287249592	0.773921217	0.810314074	negative
PRJNA729271	stage_E18_vs_E10	ordinary_muscle_development	ENSGALG00010024554	ST6GALNAC3	-0.815268537	0.210163161	-3.879217137	0.000104793	0.000218181	negative
PRJNA729271	stage_E18_vs_E10	ordinary_muscle_development	ENSGALG00010024555	EPHB1	1.378270591	0.162128328	8.501109007	1.87788E-17	1.12945E-16	positive
PRJNA729271	stage_E18_vs_E10	ordinary_muscle_development	ENSGALG00010024556	ENSGALG00010024556	-0.38689119	0.209431584	-1.847339273	0.064697984	0.088966633	negative
PRJNA729271	stage_E18_vs_E10	ordinary_muscle_development	ENSGALG00010024557	TSPAN32	1.555807334	0.281504913	5.526750191	3.26217E-08	9.66976E-08	positive
PRJNA729271	stage_E18_vs_E10	ordinary_muscle_development	ENSGALG00010024558	PFDN5	0.293070329	0.108257241	2.707166058	0.00678603	0.010999481	positive
PRJNA729271	stage_E18_vs_E10	ordinary_muscle_development	ENSGALG00010024559	ENSGALG00010024559	-0.422357958	0.281777525	-1.498905772	0.133898079	0.173232854	negative
PRJNA729271	stage_E18_vs_E10	ordinary_muscle_development	ENSGALG00010024560	A4GNT	2.385016371	0.410021924	5.816802052	5.99841E-09	1.89262E-08	positive
PRJNA729271	stage_E18_vs_E10	ordinary_muscle_development	ENSGALG00010024561	ROBO3	0.410754141	0.512721047	0.80112596	0.423058729	0.483500455	positive
PRJNA729271	stage_E18_vs_E10	ordinary_muscle_development	ENSGALG00010024563	ESPL1	-1.123786936	0.164332329	-6.838501851	8.00256E-12	3.17105E-11	negative
PRJNA729271	stage_E18_vs_E10	ordinary_muscle_development	ENSGALG00010024564	PPP1R3D	1.518914705	0.202799445	7.489737985	6.90112E-14	3.21646E-13	positive
PRJNA729271	stage_E18_vs_E10	ordinary_muscle_development	ENSGALG00010024565	FAM217B	0.726326018	0.138591868	5.240754937	1.59921E-07	4.43562E-07	positive
PRJNA729271	stage_E18_vs_E10	ordinary_muscle_development	ENSGALG00010024566	MTSS2	1.51997145	0.21628803	7.027533831	2.10216E-12	8.76857E-12	positive
PRJNA729271	stage_E18_vs_E10	ordinary_muscle_development	ENSGALG00010024567	IKZF3	-0.263723959	0.535881561	-0.492131057	0.622626698	0.673254307	negative
PRJNA729271	stage_E18_vs_E10	ordinary_muscle_development	ENSGALG00010024568	DZIP1L	1.3323417	0.258555401	5.153022126	2.56322E-07	7.00223E-07	positive
PRJNA729271	stage_E18_vs_E10	ordinary_muscle_development	ENSGALG00010024569	ASCL2	-0.436683949	0.269462067	-1.620576709	0.105108451	0.138845074	negative
PRJNA729271	stage_E18_vs_E10	ordinary_muscle_development	ENSGALG00010024570	TPM1	3.610574257	0.16416185	21.99399098	3.2876E-107	4.5821E-105	positive
PRJNA729271	stage_E18_vs_E10	ordinary_muscle_development	ENSGALG00010024571	KCNA3	-3.477443707	0.424801012	-8.186053256	2.69932E-16	1.50141E-15	negative
PRJNA729271	stage_E18_vs_E10	ordinary_muscle_development	ENSGALG00010024573	ENSGALG00010024573	0.731210896	0.285300251	2.562952161	0.010378635	0.016384046	positive
PRJNA729271	stage_E18_vs_E10	ordinary_muscle_development	ENSGALG00010024575	TXNRD2	0.276017129	0.128195586	2.153093865	0.031311302	0.045604635	positive
PRJNA729271	stage_E18_vs_E10	ordinary_muscle_development	ENSGALG00010024576	CSAD	0.168037236	0.156693649	1.072393402	0.28354337	0.339189663	positive
PRJNA729271	stage_E18_vs_E10	ordinary_muscle_development	ENSGALG00010024578	ENSGALG00010024578	2.133493999	0.586475638	3.637822035	0.000274953	0.000541354	positive
PRJNA729271	stage_E18_vs_E10	ordinary_muscle_development	ENSGALG00010024579	ZNF740	-0.416903148	0.106652113	-3.909000359	9.26788E-05	0.000193962	negative
PRJNA729271	stage_E18_vs_E10	ordinary_muscle_development	ENSGALG00010024580	IGF2	0.013201297	0.193888695	0.068086987	0.945716389	0.955272409	positive
PRJNA729271	stage_E18_vs_E10	ordinary_muscle_development	ENSGALG00010024581	RARA	0.495704579	0.172559182	2.872664166	0.004070265	0.00682853	positive
PRJNA729271	stage_E18_vs_E10	ordinary_muscle_development	ENSGALG00010024582	TLN2	-0.262632635	0.108000859	-2.431764316	0.015025481	0.023196286	negative
PRJNA729271	stage_E18_vs_E10	ordinary_muscle_development	ENSGALG00010024583	ITGB7	-0.566907991	0.66848265	-0.848051914	0.39640906	0.45646245	negative
PRJNA729271	stage_E18_vs_E10	ordinary_muscle_development	ENSGALG00010024585	TBCCD1	-0.934500952	0.127954064	-7.303409678	2.80565E-13	1.25055E-12	negative
PRJNA729271	stage_E18_vs_E10	ordinary_muscle_development	ENSGALG00010024586	DLGAP4	-0.176945503	0.096775248	-1.828416946	0.067487	0.092547215	negative
PRJNA729271	stage_E18_vs_E10	ordinary_muscle_development	ENSGALG00010024587	ENSGALG00010024587	1.991688347	0.183006567	10.88315234	1.38682E-27	1.51371E-26	positive
PRJNA729271	stage_E18_vs_E10	ordinary_muscle_development	ENSGALG00010024588	TTC39A	-4.220599746	0.405271076	-10.41426344	2.13443E-25	2.07642E-24	negative
PRJNA729271	stage_E18_vs_E10	ordinary_muscle_development	ENSGALG00010024589	ADORA1	0.835841983	0.480969859	1.737826119	0.082241457	0.11096164	positive
PRJNA729271	stage_E18_vs_E10	ordinary_muscle_development	ENSGALG00010024590	MRPL23	-0.578727684	0.105761311	-5.47201693	4.44942E-08	1.30081E-07	negative
PRJNA729271	stage_E18_vs_E10	ordinary_muscle_development	ENSGALG00010024592	COMT	1.994513482	0.197620367	10.09265145	5.95392E-24	5.27519E-23	positive
PRJNA729271	stage_E18_vs_E10	ordinary_muscle_development	ENSGALG00010024593	SRSF6	0.179830839	0.074415272	2.416585132	0.015666862	0.024109902	positive
PRJNA729271	stage_E18_vs_E10	ordinary_muscle_development	ENSGALG00010024594	TNNI2	4.841076974	0.183210213	26.42361957	7.336E-154	2.6584E-151	positive
PRJNA729271	stage_E18_vs_E10	ordinary_muscle_development	ENSGALG00010024595	MYBPHL	2.922462068	0.316870006	9.222905331	2.89157E-20	2.0647E-19	positive
PRJNA729271	stage_E18_vs_E10	ordinary_muscle_development	ENSGALG00010024596	EPS15	0.513153318	0.067198213	7.636413197	2.23357E-14	1.08099E-13	positive
PRJNA729271	stage_E18_vs_E10	ordinary_muscle_development	ENSGALG00010024597	DNAJB11	-1.404255464	0.102377053	-13.71650609	8.08695E-43	1.73403E-41	negative
PRJNA729271	stage_E18_vs_E10	ordinary_muscle_development	ENSGALG00010024598	LRRC3C	0.698771432	0.238215637	2.933356691	0.003353184	0.005688516	positive
PRJNA729271	stage_E18_vs_E10	ordinary_muscle_development	ENSGALG00010024599	ENSGALG00010024599	0.182998844	0.216121126	0.846742048	0.397138916	0.457085008	positive
PRJNA729271	stage_E18_vs_E10	ordinary_muscle_development	ENSGALG00010024600	CD9	1.729089413	0.333064265	5.191458813	2.08653E-07	5.74003E-07	positive
PRJNA729271	stage_E18_vs_E10	ordinary_muscle_development	ENSGALG00010024601	ENSGALG00010024601	-1.387993258	0.514019991	-2.700270966	0.006928302	0.011210732	negative
PRJNA729271	stage_E18_vs_E10	ordinary_muscle_development	ENSGALG00010024602	PROCR	0.253674168	0.25240108	1.005043906	0.314875709	0.372338342	positive
PRJNA729271	stage_E18_vs_E10	ordinary_muscle_development	ENSGALG00010024603	ENSGALG00010024603	1.567050263	1.011672233	1.548970321	0.121388855	0.158573491	positive
PRJNA729271	stage_E18_vs_E10	ordinary_muscle_development	ENSGALG00010024604	MFSD5	-0.177184859	0.173517519	-1.021135275	0.307190352	0.364648608	negative
PRJNA729271	stage_E18_vs_E10	ordinary_muscle_development	ENSGALG00010024605	LSP1P1	-0.452485016	0.195903277	-2.309736843	0.020902728	0.031414518	negative
PRJNA729271	stage_E18_vs_E10	ordinary_muscle_development	ENSGALG00010024607	RARG	-0.273063412	0.141579183	-1.928697471	0.05376843	0.075055219	negative
PRJNA729271	stage_E18_vs_E10	ordinary_muscle_development	ENSGALG00010024608	DENND2D	0.235810604	0.342043402	0.689417199	0.490560763	0.548198155	positive
PRJNA729271	stage_E18_vs_E10	ordinary_muscle_development	ENSGALG00010024609	PRR33	9.448578811	0.837772996	11.27820885	1.68134E-29	2.0075E-28	positive
PRJNA729271	stage_E18_vs_E10	ordinary_muscle_development	ENSGALG00010024610	PFKL	-1.040097574	0.139906842	-7.434215199	1.05191E-13	4.82512E-13	negative
PRJNA729271	stage_E18_vs_E10	ordinary_muscle_development	ENSGALG00010024611	ENSGALG00010024611	-1.411736734	0.257488069	-5.482726793	4.1882E-08	1.22841E-07	negative
PRJNA729271	stage_E18_vs_E10	ordinary_muscle_development	ENSGALG00010024612	ENSGALG00010024612	-0.902944698	0.551163975	-1.638250573	0.101369434	0.134211723	negative
PRJNA729271	stage_E18_vs_E10	ordinary_muscle_development	ENSGALG00010024613	ENSGALG00010024613	-0.274827495	0.096327599	-2.853050405	0.004330175	0.007233593	negative
PRJNA729271	stage_E18_vs_E10	ordinary_muscle_development	ENSGALG00010024614	ENSGALG00010024614	-7.197030819	0.992704511	-7.249922551	4.1701E-13	1.83781E-12	negative
PRJNA729271	stage_E18_vs_E10	ordinary_muscle_development	ENSGALG00010024615	SYT8	-3.170235334	0.733900896	-4.319704954	1.56238E-05	3.56585E-05	negative
PRJNA729271	stage_E18_vs_E10	ordinary_muscle_development	ENSGALG00010024616	ARVCF	-0.332523029	0.098406665	-3.379070218	0.000727314	0.001351939	negative
PRJNA729271	stage_E18_vs_E10	ordinary_muscle_development	ENSGALG00010024618	VPS13C	-0.298679622	0.123137572	-2.425576676	0.015284087	0.023560867	negative
PRJNA729271	stage_E18_vs_E10	ordinary_muscle_development	ENSGALG00010024619	ENSGALG00010024619	-1.464129231	0.233845648	-6.261092482	3.8229E-10	1.33654E-09	negative
PRJNA729271	stage_E18_vs_E10	ordinary_muscle_development	ENSGALG00010024620	MSANTD2	-0.492567692	0.130558015	-3.772787853	0.000161434	0.000327682	negative
PRJNA729271	stage_E18_vs_E10	ordinary_muscle_development	ENSGALG00010024621	THRA	1.422969305	0.184876318	7.696871723	1.39438E-14	6.87466E-14	positive
PRJNA729271	stage_E18_vs_E10	ordinary_muscle_development	ENSGALG00010024622	PRMT8	0.395965226	0.21024615	1.88334115	0.05965415	0.082626556	positive
PRJNA729271	stage_E18_vs_E10	ordinary_muscle_development	ENSGALG00010024623	NRDC	-0.206074122	0.109867771	-1.8756558	0.060702559	0.083902316	negative
PRJNA729271	stage_E18_vs_E10	ordinary_muscle_development	ENSGALG00010024624	CTSD	-0.453989817	0.128968545	-3.520159261	0.000431288	0.000827467	negative
PRJNA729271	stage_E18_vs_E10	ordinary_muscle_development	ENSGALG00010024625	SPRYD3	0.008140194	0.08093649	0.100575082	0.919887778	0.934031161	positive
PRJNA729271	stage_E18_vs_E10	ordinary_muscle_development	ENSGALG00010024626	TSHZ3	-1.358578502	0.097669251	-13.90999209	5.50855E-44	1.24566E-42	negative
PRJNA729271	stage_E18_vs_E10	ordinary_muscle_development	ENSGALG00010024627	CEPT1	0.138510895	0.079818443	1.735324445	0.082683354	0.111446459	positive
PRJNA729271	stage_E18_vs_E10	ordinary_muscle_development	ENSGALG00010024628	TANGO2	0.760721422	0.110662104	6.874272175	6.23071E-12	2.49625E-11	positive
PRJNA729271	stage_E18_vs_E10	ordinary_muscle_development	ENSGALG00010024629	ENSGALG00010024629	1.286609938	0.172022829	7.479297655	7.47209E-14	3.47363E-13	positive
PRJNA729271	stage_E18_vs_E10	ordinary_muscle_development	ENSGALG00010024630	CFAP410	0.056750745	0.09644476	0.588427461	0.55624541	0.610278695	positive
PRJNA729271	stage_E18_vs_E10	ordinary_muscle_development	ENSGALG00010024631	EPB41L1	0.193230133	0.149695005	1.290825521	0.196764186	0.245151872	positive
PRJNA729271	stage_E18_vs_E10	ordinary_muscle_development	ENSGALG00010024632	IFITM10	1.116592369	0.33912482	3.292570479	0.00099276	0.001808932	positive
PRJNA729271	stage_E18_vs_E10	ordinary_muscle_development	ENSGALG00010024633	NRGN	0.133102279	0.303975557	0.43787165	0.661479328	0.709129714	positive
PRJNA729271	stage_E18_vs_E10	ordinary_muscle_development	ENSGALG00010024634	ENSGALG00010024634	1.744030277	0.164620862	10.59422394	3.16959E-26	3.21506E-25	positive
PRJNA729271	stage_E18_vs_E10	ordinary_muscle_development	ENSGALG00010024635	ENSGALG00010024635	-0.148510255	0.175244437	-0.84744633	0.39674639	0.45670576	negative
PRJNA729271	stage_E18_vs_E10	ordinary_muscle_development	ENSGALG00010024636	PANX3	-1.487978584	0.305642231	-4.868367107	1.12524E-06	2.89242E-06	negative
PRJNA729271	stage_E18_vs_E10	ordinary_muscle_development	ENSGALG00010024637	DUSP8	1.597728846	0.277306695	5.761594927	8.33228E-09	2.59399E-08	positive
PRJNA729271	stage_E18_vs_E10	ordinary_muscle_development	ENSGALG00010024638	ZNF507	-0.420586227	0.13217997	-3.18192104	0.001463017	0.002612917	negative
PRJNA729271	stage_E18_vs_E10	ordinary_muscle_development	ENSGALG00010024639	AAR2	-0.311976789	0.076788988	-4.062780312	4.84917E-05	0.000104518	negative
PRJNA729271	stage_E18_vs_E10	ordinary_muscle_development	ENSGALG00010024640	GSDMA	-1.609596656	0.307892837	-5.227782082	1.71556E-07	4.75286E-07	negative

PRJNA729271	stage_E18_vs_E10	ordinary_muscle_development	ENSGALG00010024641	TRA2B	-1.000272045	0.087805599	-11.39189363	4.58907E-30	5.61812E-29	negative
PRJNA729271	stage_E18_vs_E10	ordinary_muscle_development	ENSGALG00010024642	RAB3B	0.245677748	0.228992197	1.072865151	0.283331624	0.338964336	positive
PRJNA729271	stage_E18_vs_E10	ordinary_muscle_development	ENSGALG00010024643	ETV7	1.112652209	0.408206857	2.725706809	0.006416397	0.010453599	positive
PRJNA729271	stage_E18_vs_E10	ordinary_muscle_development	ENSGALG00010024644	EIF6	-0.733795376	0.076020441	-9.646261656	5.09804E-22	4.06693E-21	negative
PRJNA729271	stage_E18_vs_E10	ordinary_muscle_development	ENSGALG00010024645	PARP11	0.159166119	0.119724651	1.329434808	0.183704561	0.23056521	positive
PRJNA729271	stage_E18_vs_E10	ordinary_muscle_development	ENSGALG00010024646	RORA	1.480638338	0.192842437	7.677969427	1.6163E-14	7.92835E-14	positive
PRJNA729271	stage_E18_vs_E10	ordinary_muscle_development	ENSGALG00010024647	RPUSD4	-0.357039519	0.136849215	-2.608999383	0.009080741	0.014456379	negative
PRJNA729271	stage_E18_vs_E10	ordinary_muscle_development	ENSGALG00010024648	MMP24	-0.688541893	0.121653595	-5.659856513	1.515E-08	4.61148E-08	negative
PRJNA729271	stage_E18_vs_E10	ordinary_muscle_development	ENSGALG00010024649	NCOR2	0.204791062	0.129737301	1.578505649	0.114449491	0.150200316	positive
PRJNA729271	stage_E18_vs_E10	ordinary_muscle_development	ENSGALG00010024650	PRKAG1	0.827227795	0.121453042	6.811091589	9.68609E-12	3.81522E-11	positive
PRJNA729271	stage_E18_vs_E10	ordinary_muscle_development	ENSGALG00010024651	PSMD3	-0.872393991	0.128384467	-6.795167765	1.08187E-11	4.24287E-11	negative
PRJNA729271	stage_E18_vs_E10	ordinary_muscle_development	ENSGALG00010024652	PUS3	-0.929792513	0.131214513	-7.086049338	1.37995E-12	5.84009E-12	negative
PRJNA729271	stage_E18_vs_E10	ordinary_muscle_development	ENSGALG00010024653	RFLNA	-2.617125275	0.254502853	-10.2832846	8.38213E-25	7.87421E-24	negative
PRJNA729271	stage_E18_vs_E10	ordinary_muscle_development	ENSGALG00010024654	CCDC92	-1.650963009	0.280391672	-5.888060071	3.90755E-09	1.25393E-08	negative
PRJNA729271	stage_E18_vs_E10	ordinary_muscle_development	ENSGALG00010024655	IGF2BP2	-0.050024718	0.165381369	-0.302480977	0.762285433	0.800559872	negative
PRJNA729271	stage_E18_vs_E10	ordinary_muscle_development	ENSGALG00010024656	HYLS1	-0.846008942	0.188185544	-4.495610683	6.93706E-06	1.64034E-05	negative
PRJNA729271	stage_E18_vs_E10	ordinary_muscle_development	ENSGALG00010024657	DPY19L3	-0.04937117	0.127964119	-0.385820421	0.699629671	0.744467191	negative
PRJNA729271	stage_E18_vs_E10	ordinary_muscle_development	ENSGALG00010024659	BEND5	0.882298055	0.107323011	8.220958808	2.01883E-16	1.13422E-15	positive
PRJNA729271	stage_E18_vs_E10	ordinary_muscle_development	ENSGALG00010024660	KMT2D	0.354474479	0.15833849	2.23871328	0.025174578	0.03732104	positive
PRJNA729271	stage_E18_vs_E10	ordinary_muscle_development	ENSGALG00010024662	MOB2	-0.107717162	0.072187254	-1.492190876	0.135649113	0.175321792	negative
PRJNA729271	stage_E18_vs_E10	ordinary_muscle_development	ENSGALG00010024663	ICE2	-0.557227219	0.123927443	-4.496398917	6.9114E-06	1.63454E-05	negative
PRJNA729271	stage_E18_vs_E10	ordinary_muscle_development	ENSGALG00010024664	DNAH10	-1.384370376	0.218940313	-6.323049222	2.56452E-10	9.07314E-10	negative
PRJNA729271	stage_E18_vs_E10	ordinary_muscle_development	ENSGALG00010024665	MAP1LC3A	1.18742472	0.148883888	7.975508541	1.51755E-15	8.04566E-15	positive
PRJNA729271	stage_E18_vs_E10	ordinary_muscle_development	ENSGALG00010024666	MED24	-0.83898848	0.115321588	-7.275207515	3.45888E-13	1.5337E-12	negative
PRJNA729271	stage_E18_vs_E10	ordinary_muscle_development	ENSGALG00010024667	RBM7	0.499243693	0.136019221	3.670390766	0.00024218	0.000480547	positive
PRJNA729271	stage_E18_vs_E10	ordinary_muscle_development	ENSGALG00010024668	ENSGALG00010024668	2.107852775	0.18392671	11.46028641	2.08823E-30	2.60266E-29	positive
PRJNA729271	stage_E18_vs_E10	ordinary_muscle_development	ENSGALG00010024669	STIL	-1.129065443	0.182362595	-6.191321447	5.96619E-10	2.0615E-09	negative
PRJNA729271	stage_E18_vs_E10	ordinary_muscle_development	ENSGALG00010024670	SENP2	0.313115171	0.096925496	3.230472717	0.001235857	0.002227247	positive
PRJNA729271	stage_E18_vs_E10	ordinary_muscle_development	ENSGALG00010024671	KCTD20	-0.005393123	0.280528676	-0.019224855	0.98466173	0.986976818	negative
PRJNA729271	stage_E18_vs_E10	ordinary_muscle_development	ENSGALG00010024672	DYNLRB1	-0.563596203	0.105927584	-5.320580145	1.03437E-07	2.93007E-07	negative
PRJNA729271	stage_E18_vs_E10	ordinary_muscle_development	ENSGALG00010024673	REXO2	-0.118338275	0.099612309	-1.187988479	0.234837949	0.287134211	negative
PRJNA729271	stage_E18_vs_E10	ordinary_muscle_development	ENSGALG00010024674	DHH	2.109742354	0.333027808	6.335033611	2.3729E-10	8.41162E-10	positive
PRJNA729271	stage_E18_vs_E10	ordinary_muscle_development	ENSGALG00010024675	NR1D1	1.768916306	0.465539015	3.79971656	0.000144862	0.000296159	positive
PRJNA729271	stage_E18_vs_E10	ordinary_muscle_development	ENSGALG00010024676	ENSGALG00010024676	0.833433933	0.166375832	5.00934496	5.46156E-07	1.44806E-06	positive
PRJNA729271	stage_E18_vs_E10	ordinary_muscle_development	ENSGALG00010024677	LMBR1L	-1.939826017	0.346805569	-5.593410798	2.22652E-08	6.67771E-08	negative
PRJNA729271	stage_E18_vs_E10	ordinary_muscle_development	ENSGALG00010024678	AMOTL2	0.709011563	0.09137239	7.759582124	8.52097E-15	4.27819E-14	positive
PRJNA729271	stage_E18_vs_E10	ordinary_muscle_development	ENSGALG00010024679	MSL1	0.141804351	0.111727249	1.269201129	0.204369339	0.253648718	positive
PRJNA729271	stage_E18_vs_E10	ordinary_muscle_development	ENSGALG00010024680	BRSK1	-0.458715343	0.25995535	-1.764592813	0.077632224	0.105244958	negative
PRJNA729271	stage_E18_vs_E10	ordinary_muscle_development	ENSGALG00010024681	ATP6V0A2	-0.169905625	0.087461881	-1.942624882	0.052061498	0.07283384	negative
PRJNA729271	stage_E18_vs_E10	ordinary_muscle_development	ENSGALG00010024682	ENSGALG00010024682	-1.27054131	0.179224558	-7.089102767	1.34984E-12	5.71712E-12	negative
PRJNA729271	stage_E18_vs_E10	ordinary_muscle_development	ENSGALG00010024683	SHISA4	1.647267199	0.181073873	9.097210846	9.26813E-20	6.42168E-19	positive
PRJNA729271	stage_E18_vs_E10	ordinary_muscle_development	ENSGALG00010024684	ENSGALG00010024684	-1.820119121	0.390355445	-4.662722507	3.12053E-06	7.63669E-06	negative
PRJNA729271	stage_E18_vs_E10	ordinary_muscle_development	ENSGALG00010024685	ENSGALG00010024685	-0.738011711	0.219841276	-3.357020693	0.000787872	0.001457775	negative
PRJNA729271	stage_E18_vs_E10	ordinary_muscle_development	ENSGALG00010024686	CMPK1	-0.989137148	0.13382866	-7.39107113	1.45651E-13	6.61406E-13	negative
PRJNA729271	stage_E18_vs_E10	ordinary_muscle_development	ENSGALG00010024687	RYK	0.198160694	0.106942796	1.852959721	0.063888132	0.087928074	positive
PRJNA729271	stage_E18_vs_E10	ordinary_muscle_development	ENSGALG00010024688	CADM1	1.553037218	0.128211124	12.11312377	9.00001E-34	1.30195E-32	positive
PRJNA729271	stage_E18_vs_E10	ordinary_muscle_development	ENSGALG00010024689	FOXD2	0.018730456	0.311446771	0.060140151	0.952044012	0.960459212	positive
PRJNA729271	stage_E18_vs_E10	ordinary_muscle_development	ENSGALG00010024690	TUBA1C	-2.629973463	0.649523333	-4.049082351	5.14189E-05	0.000110565	negative
PRJNA729271	stage_E18_vs_E10	ordinary_muscle_development	ENSGALG00010024691	TCTN2	-0.213856023	0.150835353	-1.417810998	0.156245953	0.199154509	negative
PRJNA729271	stage_E18_vs_E10	ordinary_muscle_development	ENSGALG00010024692	DRAM2	1.695032046	0.105448727	16.07446666	3.85295E-58	1.45061E-56	positive
PRJNA729271	stage_E18_vs_E10	ordinary_muscle_development	ENSGALG00010024693	ANXA2	0.930044913	0.201450435	4.616743135	3.89809E-06	9.43129E-06	positive
PRJNA729271	stage_E18_vs_E10	ordinary_muscle_development	ENSGALG00010024694	GTF2H3	-0.514424087	0.097894055	-5.254906284	1.481E-07	4.11957E-07	negative
PRJNA729271	stage_E18_vs_E10	ordinary_muscle_development	ENSGALG00010024695	TRABD2B	-0.314182489	0.194927701	-1.611789841	0.10700768	0.141135243	negative
PRJNA729271	stage_E18_vs_E10	ordinary_muscle_development	ENSGALG00010024696	TRADD	0.079007929	0.115174815	0.685982691	0.492724024	0.550177116	positive
PRJNA729271	stage_E18_vs_E10	ordinary_muscle_development	ENSGALG00010024697	IGF2BP1	-1.542205078	0.122483114	-12.59116483	2.36162E-36	3.89883E-35	negative
PRJNA729271	stage_E18_vs_E10	ordinary_muscle_development	ENSGALG00010024698	ENSGALG00010024698	-0.276408484	0.320356783	-0.86281452	0.388239455	0.448408836	negative
PRJNA729271	stage_E18_vs_E10	ordinary_muscle_development	ENSGALG00010024699	FBXL8	-0.295909394	0.133671463	-2.213706551	0.026848972	0.039582572	negative
PRJNA729271	stage_E18_vs_E10	ordinary_muscle_development	ENSGALG00010024700	TOLLIP	0.042142147	0.08529424	0.494079641	0.621249936	0.671966108	positive
PRJNA729271	stage_E18_vs_E10	ordinary_muscle_development	ENSGALG00010024701	EIF2B1	-0.198684792	0.102084632	-1.946275242	0.051621686	0.072295298	negative
PRJNA729271	stage_E18_vs_E10	ordinary_muscle_development	ENSGALG00010024702	INAVA	-1.860564475	0.767598186	-2.423878156	0.015355757	0.023663799	negative
PRJNA729271	stage_E18_vs_E10	ordinary_muscle_development	ENSGALG00010024703	HSF4	-0.616743596	0.623115891	-0.989773499	0.322284841	0.380107304	negative
PRJNA729271	stage_E18_vs_E10	ordinary_muscle_development	ENSGALG00010024705	SLCO2A1	1.445952919	0.243648026	5.934597302	2.94568E-09	9.54989E-09	positive
PRJNA729271	stage_E18_vs_E10	ordinary_muscle_development	ENSGALG00010024706	ENSGALG00010024706	0.367284657	1.066590746	0.344353875	0.730580172	0.772355014	positive
PRJNA729271	stage_E18_vs_E10	ordinary_muscle_development	ENSGALG00010024707	ENSGALG00010024707	-0.781625605	0.375916088	-2.079255528	0.03759387	0.053947446	negative
PRJNA729271	stage_E18_vs_E10	ordinary_muscle_development	ENSGALG00010024708	BNIP2	0.729857075	0.06294859	11.59449438	4.39453E-31	5.69247E-30	positive
PRJNA729271	stage_E18_vs_E10	ordinary_muscle_development	ENSGALG00010024709	TEAD4	1.878008938	0.096141141	19.53387401	5.658E-85	4.45721E-83	positive
PRJNA729271	stage_E18_vs_E10	ordinary_muscle_development	ENSGALG00010024710	HSDL1	-0.533539075	0.128840613	-4.141078375	3.45677E-05	7.57E-05	negative
PRJNA729271	stage_E18_vs_E10	ordinary_muscle_development	ENSGALG00010024711	DDX55	-0.121705254	0.091759924	-1.326344322	0.184725672	0.231660373	negative
PRJNA729271	stage_E18_vs_E10	ordinary_muscle_development	ENSGALG00010024712	LRIF1	0.745553646	0.098179995	7.593742961	3.10795E-14	1.48141E-13	positive
PRJNA729271	stage_E18_vs_E10	ordinary_muscle_development	ENSGALG00010024713	RND2	-0.813253079	0.404395788	-2.011032515	0.044322022	0.062781952	negative
PRJNA729271	stage_E18_vs_E10	ordinary_muscle_development	ENSGALG00010024714	SRPRB	-0.635455073	0.175186262	-3.627311105	0.000286388	0.000562874	negative
PRJNA729271	stage_E18_vs_E10	ordinary_muscle_development	ENSGALG00010024715	DNAAF1	0.677277222	0.169020508	4.007071274	6.14763E-05	0.000131276	positive
PRJNA729271	stage_E18_vs_E10	ordinary_muscle_development	ENSGALG00010024716	GTF2A2	-0.284627318	0.077741228	-3.661214605	0.000251022	0.00049714	negative
PRJNA729271	stage_E18_vs_E10	ordinary_muscle_development	ENSGALG00010024717	TF	4.456229353	0.652215859	6.832445568	8.34791E-12	3.30248E-11	positive
PRJNA729271	stage_E18_vs_E10	ordinary_muscle_development	ENSGALG00010024718	ENSGALG00010024718	1.523724449	0.192254207	7.92557142	2.271E-15	1.18624E-14	positive
PRJNA729271	stage_E18_vs_E10	ordinary_muscle_development	ENSGALG00010024719	PYM1	-0.365811134	0.134120001	-2.727491279	0.006381794	0.010405412	negative
PRJNA729271	stage_E18_vs_E10	ordinary_muscle_development	ENSGALG00010024720	BUD13	0.188292634	0.106666649	1.76524373	0.077522812	0.10510646	positive
PRJNA729271	stage_E18_vs_E10	ordinary_muscle_development	ENSGALG00010024721	ENSGALG00010024721	0.338250761	0.799097902	0.423290764	0.672083132	0.718796207	positive
PRJNA729271	stage_E18_vs_E10	ordinary_muscle_development	ENSGALG00010024722	BRCA1	-1.898247522	0.261563228	-7.257317995	3.94841E-13	1.74329E-12	negative
PRJNA729271	stage_E18_vs_E10	ordinary_muscle_development	ENSGALG00010024723	ITCH	0.295808087	0.100737188	2.936433837	0.003320096	0.005637202	positive
PRJNA729271	stage_E18_vs_E10	ordinary_muscle_development	ENSGALG00010024724	HOXD3	-0.337233809	0.235342971	-1.432946172	0.151873221	0.194110073	negative
PRJNA729271	stage_E18_vs_E10	ordinary_muscle_development	ENSGALG00010024726	TMED2	-0.046095513	0.058632653	-0.786174785	0.431765096	0.491706087	negative
PRJNA729271	stage_E18_vs_E10	ordinary_muscle_development	ENSGALG00010024728	SLC5A9	-0.864334496	0.728756501	-1.186040186	0.23560643	0.287952378	negative
PRJNA729271	stage_E18_vs_E10	ordinary_muscle_development	ENSGALG00010024729	FOXM1	-1.549942403	0.168010155	-9.22529002	2.82795E-20	2.02225E-19	negative
PRJNA729271	stage_E18_vs_E10	ordinary_muscle_development	ENSGALG00010024731	AOX1	2.87031308	0.198243309	14.47873877	1.65099E-47	4.37497E-46	positive
PRJNA729271	stage_E18_vs_E10	ordinary_muscle_development	ENSGALG00010024732	AHCY	1.439692094	0.224833213	6.403378203	1.51976E-10	5.46082E-10	positive
PRJNA729271	stage_E18_vs_E10	ordinary_muscle_development	ENSGALG00010024733	MBTPS1	0.231916343	0.087862933	2.639524268	0.008302248	0.013288547	positive
PRJNA729271	stage_E18_vs_E10	ordinary_muscle_development	ENSGALG00010024734	ZPR1	-0.315883427	0.11644977	-2.712615301	0.006675455	0.010843968	negative
PRJNA729271	stage_E18_vs_E10	ordinary_muscle_development	ENSGALG00010024735	OTUD7A	3.083782667	0.177586322	17.36497855	1.51957E-67	7.59521E-66	positive
PRJNA729271	stage_E18_vs_E10	ordinary_muscle_development	ENSGALG00010024736	DGKA	-0.066234348	0.149789985	-0.442181421	0.658357929	0.706253566	negative
PRJNA729271	stage_E18_vs_E10	ordinary_muscle_development	ENSGALG00010024737	STK38	-0.617561934	0.062704759	-9.848725162	6.94189E-23	5.79624E-22	negative
PRJNA729271	stage_E18_vs_E10	ordinary_muscle_development	ENSGALG00010024738	NBR1	-0.138641125	0.078905364	-1.757055773	0.078908303	0.106825054	negative
PRJNA729271	stage_E18_vs_E10	ordinary_muscle_development	ENSGALG00010024739	ASIP	-7.483746107	0.617385894	-12.12166682	8.10929E-34	1.1778E-32	negative
PRJNA729271	stage_E18_vs_E10	ordinary_muscle_development	ENSGALG00010024741	RILPL2	-0.657761733	0.157274709	-4.18224735	2.88642E-05	6.3827E-05	negative
PRJNA729271	stage_E18_vs_E10	ordinary_muscle_development	ENSGALG00010024742	PAK2	-0.344102009	0.070506902	-4.880401748	1.0587E-06	2.73106E-06	negative
PRJNA729271	stage_E18_vs_E10	ordinary_muscle_development	ENSGALG00010024744	KLF13	2.899512156	0.130586972	22.20368626	3.1641E-109	4.7774E-107	positive
PRJNA729271	stage_E18_vs_E10	ordinary_muscle_development	ENSGALG00010024745	PMEL	-2.401888303	0.559702284	-4.2913677	1.77576E-05	4.02119E-05	negative
PRJNA729271	stage_E18_vs_E10	ordinary_muscle_development	ENSGALG00010024746	HOXD4	-0.40369121	0.294220913	-1.372068376	0.170042153	0.214899077	negative
PRJNA729271	stage_E18_vs_E10	ordinary_muscle_development	ENSGALG00010024747	TULP3	-0.206215893	0.087345299	-2.360927216	0.018229308	0.027659774	negative
PRJNA729271	stage_E18_vs_E10	ordinary_muscle_development	ENSGALG00010024748	TRPM1	1.705868613	0.384823942	4.432854684	9.29935E-06	2.1713E-05	positive
PRJNA729271	stage_E18_vs_E10	ordinary_muscle_development	ENSGALG00010024749	APOA5	2.312676033	0.674438809	3.429037598	0.000605726	0.001137894	positive
PRJNA729271	stage_E18_vs_E10	ordinary_muscle_development	ENSGALG00010024750	APOA4	3.396874445	1.570345979	2.163137609	0.030530597	0.044566063	positive
PRJNA729271	stage_E18_vs_E10	ordinary_muscle_development	ENSGALG00010024751	MTMR10	0.169624116	0.058498033	2.899655021	0.003735736	0.00629572	positive
PRJNA729271	stage_E18_vs_E10	ordinary_muscle_development	ENSGALG00010024752	MPP2	0.681807998	0.208690964	3.267070052	0.001086668	0.001971617	positive
PRJNA729271	stage_E18_vs_E10	ordinary_muscle_development	ENSGALG00010024753	HOXD8	0.073574059	0.169326123	0.434510975	0.663917427	0.711480342	positive
PRJNA729271	stage_E18_vs_E10	ordinary_muscle_development	ENSGALG00010024754	EIF2S2	-0.629735708	0.145316187	-4.333555124	1.46721E-05	3.35656E-05	negative
PRJNA729271	stage_E18_vs_E10	ordinary_muscle_development	ENSGALG00010024755	PIGX	0.692378381	0.157091876	4.407474135	1.04583E-05	2.43211E-05	positive
PRJNA729271	stage_E18_vs_E10	ordinary_muscle_development	ENSGALG00010024756	CDK2	-1.515492664	0.165629339	-9.149904696	5.69838E-20	3.9941E-19	negative
PRJNA729271	stage_E18_vs_E10	ordinary_muscle_development	ENSGALG00010024757	PPFIA1	-0.273627397	0.07576345	-3.611601616	0.000304312	0.000595357	negative
PRJNA729271	stage_E18_vs_E10	ordinary_muscle_development	ENSGALG00010024758	SPATA6	0.271289728	0.10568699	2.566916963	0.010260718	0.016210257	positive
PRJNA729271	stage_E18_vs_E10	ordinary_muscle_development	ENSGALG00010024759	APOC3	5.263585758	0.958444834	5.491798349			positive
PRJNA729271	stage_E18_vs_E10	ordinary_muscle_development	ENSGALG00010024760	ENSGALG00010024760	2.643444996	0.185679737	14.23658302	5.43188E-46	1.3436E-44	positive
PRJNA729271	stage_E18_vs_E10	ordinary_muscle_development	ENSGALG00010024761	NECAB2	0.1461241	0.235498238	0.620489141	0.534935801	0.590637906	positive
PRJNA729271	stage_E18_vs_E10	ordinary_muscle_development	ENSGALG00010024762	FAN1	-0.525557824	0.091959237	-5.715117257	1.09628E-08	3.37023E-08	negative
PRJNA729271	stage_E18_vs_E10	ordinary_muscle_development	ENSGALG00010024763	HOXD9	-0.76024817	0.305080071	-2.491962743	0.012703936	0.019798254	negative
PRJNA729271	stage_E18_vs_E10	ordinary_muscle_development	ENSGALG00010024764	ENSGALG00010024764	0.6830616	0.259521915	2.631999686	0.008488394	0.013574501	positive
PRJNA729271	stage_E18_vs_E10	ordinary_muscle_development	ENSGALG00010024765	SNRNP35	-0.081896156	0.136407927	-0.600376813	0.548255137	0.602544409	negative
PRJNA729271	stage_E18_vs_E10	ordinary_muscle_development	ENSGALG00010024767	CTTN	-0.733471465	0.069313326	-10.58196892	3.61285E-26	3.6519E-25	negative
PRJNA729271	stage_E18_vs_E10	ordinary_muscle_development	ENSGALG00010024769	RALY	2.832170319	0.204150826	13.87293097	9.24217E-44	2.06736E-42	positive
PRJNA729271	stage_E18_vs_E10	ordinary_muscle_development	ENSGALG00010024770	CDC27	-0.371560128	0.073234539	-5.073564108	3.90433E-07	1.05016E-06	negative
PRJNA729271	stage_E18_vs_E10	ordinary_muscle_development	ENSGALG00010024771	AOX2	2.917603871	0.268876127	10.85110793	1.97022E-27	2.13761E-26	positive
PRJNA729271	stage_E18_vs_E10	ordinary_muscle_development	ENSGALG00010024772	ELAPOR1	-0.464973992	0.237277103	-1.959624363	0.050039712	0.070276681	negative
PRJNA729271	stage_E18_vs_E10	ordinary_muscle_development	ENSGALG00010024773	APOA1	5.869318029	0.264073044	22.22611568	1.9205E-109	2.9303E-107	positive
PRJNA729271	stage_E18_vs_E10	ordinary_muscle_development	ENSGALG00010024775	ITFG2	1.059717624	0.093840737	11.29272485	1.42548E-29	1.70481E-28	positive
PRJNA729271	stage_E18_vs_E10	ordinary_muscle_development	ENSGALG00010024776	MPHOSPH10	-0.733875411	0.108462652	-6.766157761	1.32247E-11	5.15441E-11	negative
PRJNA729271	stage_E18_vs_E10	ordinary_muscle_development	ENSGALG00010024778	RAB5B	-0.006632178	0.141245412	-0.046954997	0.962549095	0.969303122	negative
PRJNA729271	stage_E18_vs_E10	ordinary_muscle_development	ENSGALG00010024779	ENSGALG00010024779	-2.024363506	0.93539276	-2.164185561	0.030450111	0.04445753	negative
PRJNA729271	stage_E18_vs_E10	ordinary_muscle_development	ENSGALG00010024780	HOXD11	-3.610448069	0.844626793	-4.274607553	1.91474E-05	4.32174E-05	negative
PRJNA729271	stage_E18_vs_E10	ordinary_muscle_development	ENSGALG00010024781	CEP19	-0.064908167	0.152554436	-0.425475446	0.670490118	0.717357119	negative
PRJNA729271	stage_E18_vs_E10	ordinary_muscle_development	ENSGALG00010024782	HOXD12	-4.769575268	2.178365665	-2.189520036	0.028559064	0.041886434	negative
PRJNA729271	stage_E18_vs_E10	ordinary_muscle_development	ENSGALG00010024783	UBE2T	-1.177059382	0.234516545	-5.019088876	5.19171E-07	1.37929E-06	negative

PRJNA729271	stage_E18_vs_E10	ordinary_muscle_development	ENSGALG00010024784	ENSGALG00010024784	0.827026289	0.138179123	5.985175396	2.16157E-09	7.08867E-09	positive
PRJNA729271	stage_E18_vs_E10	ordinary_muscle_development	ENSGALG00010024785	MCEE	0.927622999	0.200305088	4.631050606	3.63815E-06	8.83481E-06	positive
PRJNA729271	stage_E18_vs_E10	ordinary_muscle_development	ENSGALG00010024786	PDCD5	-0.226507479	0.098320835	-2.303758685	0.021236194	0.031894998	negative
PRJNA729271	stage_E18_vs_E10	ordinary_muscle_development	ENSGALG00010024787	SARS1	0.103209643	0.10004332	1.031649516	0.302236335	0.359356548	positive
PRJNA729271	stage_E18_vs_E10	ordinary_muscle_development	ENSGALG00010024788	SIK3	0.540432811	0.166405139	3.247693028	0.001163447	0.002103026	positive
PRJNA729271	stage_E18_vs_E10	ordinary_muscle_development	ENSGALG00010024789	SUOX	0.574398721	0.177110743	3.243161373	0.001182112	0.002133572	positive
PRJNA729271	stage_E18_vs_E10	ordinary_muscle_development	ENSGALG00010024791	SHANK2	1.070443194	0.333133826	3.213252786	0.001312407	0.002357876	positive
PRJNA729271	stage_E18_vs_E10	ordinary_muscle_development	ENSGALG00010024792	BZW1	-0.62679363	0.06917495	-9.060991376	1.2927E-19	8.8973E-19	negative
PRJNA729271	stage_E18_vs_E10	ordinary_muscle_development	ENSGALG00010024793	FKBP4	-1.202393756	0.117352169	-10.24602929	1.23305E-24	1.14351E-23	negative
PRJNA729271	stage_E18_vs_E10	ordinary_muscle_development	ENSGALG00010024794	DDX6	-0.180647571	0.064410984	-2.804608162	0.005037778	0.008347347	negative
PRJNA729271	stage_E18_vs_E10	ordinary_muscle_development	ENSGALG00010024795	NRROS	0.516806579	0.214709127	2.407007964	0.016083819	0.024699116	positive
PRJNA729271	stage_E18_vs_E10	ordinary_muscle_development	ENSGALG00010024797	IKZF4	0.654175153	0.240013224	2.725579629	0.006418869	0.010456452	positive
PRJNA729271	stage_E18_vs_E10	ordinary_muscle_development	ENSGALG00010024798	FBXO45	0.275619473	0.084493192	3.262031709	0.001106168	0.002004238	positive
PRJNA729271	stage_E18_vs_E10	ordinary_muscle_development	ENSGALG00010024799	ENSGALG00010024799	0.577107372	0.185668155	3.108273314	0.001881839	0.003317191	positive
PRJNA729271	stage_E18_vs_E10	ordinary_muscle_development	ENSGALG00010024800	ENSGALG00010024800	-0.408911124	0.132045911	-3.096734477	0.00195665	0.003438609	negative
PRJNA729271	stage_E18_vs_E10	ordinary_muscle_development	ENSGALG00010024801	CXCR5	4.586720453	1.01104381	4.536618894	5.71633E-06	1.36392E-05	positive
PRJNA729271	stage_E18_vs_E10	ordinary_muscle_development	ENSGALG00010024802	CD59	0.45478756	0.164259665	2.768711104	0.005627851	0.009255242	positive
PRJNA729271	stage_E18_vs_E10	ordinary_muscle_development	ENSGALG00010024803	CLK1	-0.015903076	0.138921867	-0.11447497	0.908861287	0.924617094	negative
PRJNA729271	stage_E18_vs_E10	ordinary_muscle_development	ENSGALG00010024804	WDR53	0.226042496	0.10557555	2.141049656	0.032270034	0.046887945	positive
PRJNA729271	stage_E18_vs_E10	ordinary_muscle_development	ENSGALG00010024805	ENSGALG00010024805	1.135015683	0.37069787	3.061834923	0.002199847	0.003838544	positive
PRJNA729271	stage_E18_vs_E10	ordinary_muscle_development	ENSGALG00010024806	ANKRD27	-0.042289989	0.077828723	-0.543372511	0.586873349	0.639171177	negative
PRJNA729271	stage_E18_vs_E10	ordinary_muscle_development	ENSGALG00010024807	PPIL3	-0.785653864	0.197137925	-3.98530046	6.73948E-05	0.000143323	negative
PRJNA729271	stage_E18_vs_E10	ordinary_muscle_development	ENSGALG00010024808	CELSR2	0.855615219	0.236470529	3.618274213	0.000296574	0.000581089	positive
PRJNA729271	stage_E18_vs_E10	ordinary_muscle_development	ENSGALG00010024809	KANSL1	0.177201197	0.171752693	1.031722959	0.302201919	0.359345103	positive
PRJNA729271	stage_E18_vs_E10	ordinary_muscle_development	ENSGALG00010024810	ADIPOR1	-0.120022286	0.115631798	-1.03796956	0.299284227	0.356284894	negative
PRJNA729271	stage_E18_vs_E10	ordinary_muscle_development	ENSGALG00010024811	NIF3L1	-1.362690575	0.146728661	-9.287146508	1.5848E-20	1.1561E-19	negative
PRJNA729271	stage_E18_vs_E10	ordinary_muscle_development	ENSGALG00010024812	SBNO1	-0.345866672	0.112564732	-3.072602444	0.00212201	0.00371229	negative
PRJNA729271	stage_E18_vs_E10	ordinary_muscle_development	ENSGALG00010024813	RNF168	0.125363619	0.095502351	1.312675744	0.189292234	0.236635699	positive
PRJNA729271	stage_E18_vs_E10	ordinary_muscle_development	ENSGALG00010024814	APBA2	-2.390732893	0.153159982	-15.60938344	6.28413E-55	2.12824E-53	negative
PRJNA729271	stage_E18_vs_E10	ordinary_muscle_development	ENSGALG00010024816	NFIA	-0.215174828	0.122629949	-1.754667841	0.07931614	0.107337078	negative
PRJNA729271	stage_E18_vs_E10	ordinary_muscle_development	ENSGALG00010024817	UCKL1	1.640579796	0.222937297	7.358929242	1.85391E-13	8.35327E-13	positive
PRJNA729271	stage_E18_vs_E10	ordinary_muscle_development	ENSGALG00010024818	FBXO3	-0.312835209	0.077305727	-4.046727477	5.19387E-05	0.000111633	negative
PRJNA729271	stage_E18_vs_E10	ordinary_muscle_development	ENSGALG00010024820	NUDT19	0.123510594	0.143439692	0.861062878	0.389203413	0.449356323	positive
PRJNA729271	stage_E18_vs_E10	ordinary_muscle_development	ENSGALG00010024821	NECAP1	0.077673399	0.105989845	0.732838121	0.463657168	0.522768408	positive
PRJNA729271	stage_E18_vs_E10	ordinary_muscle_development	ENSGALG00010024822	RPS26	0.754033231	0.137122728	5.498966079	3.82025E-08	1.12573E-07	positive
PRJNA729271	stage_E18_vs_E10	ordinary_muscle_development	ENSGALG00010024823	ASB10	7.630276829	0.877910698	8.69140432	3.57987E-18	2.26892E-17	positive
PRJNA729271	stage_E18_vs_E10	ordinary_muscle_development	ENSGALG00010024824	DNAJC5	-0.837766727	0.109356547	-7.660873991	1.84672E-14	9.00983E-14	negative
PRJNA729271	stage_E18_vs_E10	ordinary_muscle_development	ENSGALG00010024825	UBXN7	-0.231450983	0.075608013	-3.061196486	0.002204544	0.003844888	negative
PRJNA729271	stage_E18_vs_E10	ordinary_muscle_development	ENSGALG00010024826	RBM33	0.237317898	0.112176858	2.115569131	0.03438147	0.049676974	positive
PRJNA729271	stage_E18_vs_E10	ordinary_muscle_development	ENSGALG00010024827	ENSGALG00010024827	0.446168236	0.359663057	1.240517276	0.214784128	0.26525483	positive
PRJNA729271	stage_E18_vs_E10	ordinary_muscle_development	ENSGALG00010024828	TIMM17B	0.333009118	0.133623454	2.492145705	0.012697394	0.019790185	positive
PRJNA729271	stage_E18_vs_E10	ordinary_muscle_development	ENSGALG00010024829	ERBB3	0.918622526	0.199705122	4.599894662	4.22705E-06	1.01932E-05	positive
PRJNA729271	stage_E18_vs_E10	ordinary_muscle_development	ENSGALG00010024830	ORC2	-1.090756618	0.111703727	-9.764728949	1.59538E-22	1.30724E-21	negative
PRJNA729271	stage_E18_vs_E10	ordinary_muscle_development	ENSGALG00010024833	MAPT	1.010131126	0.205594566	4.913218982	8.95931E-07	2.33151E-06	positive
PRJNA729271	stage_E18_vs_E10	ordinary_muscle_development	ENSGALG00010024834	INSIG1	-1.302995584	0.271025648	-4.807646772	1.52717E-06	3.87E-06	negative
PRJNA729271	stage_E18_vs_E10	ordinary_muscle_development	ENSGALG00010024835	ENTREP2	-0.773470871	0.141114398	-5.481161984	4.22541E-08	1.23832E-07	negative
PRJNA729271	stage_E18_vs_E10	ordinary_muscle_development	ENSGALG00010024836	TXNDC12	-0.396168853	0.106434359	-3.722189494	0.000197503	0.000396455	negative
PRJNA729271	stage_E18_vs_E10	ordinary_muscle_development	ENSGALG00010024837	TNFRSF1A	1.660186046	0.15581028	10.6551766	1.64931E-26	1.71252E-25	positive
PRJNA729271	stage_E18_vs_E10	ordinary_muscle_development	ENSGALG00010024838	EN2	-5.31544281	0.974393774	-5.455128054	4.89376E-08	1.42354E-07	negative
PRJNA729271	stage_E18_vs_E10	ordinary_muscle_development	ENSGALG00010024839	PA2G4	-0.863972884	0.099358283	-8.695529494	3.45216E-18	2.18894E-17	negative
PRJNA729271	stage_E18_vs_E10	ordinary_muscle_development	ENSGALG00010024840	BTF3L4	-0.583198466	0.101771058	-5.730494301	1.00138E-08	3.09292E-08	negative
PRJNA729271	stage_E18_vs_E10	ordinary_muscle_development	ENSGALG00010024841	ENSGALG00010024841	-1.421767315	0.207427954	-6.854270534	7.16773E-12	2.85194E-11	negative
PRJNA729271	stage_E18_vs_E10	ordinary_muscle_development	ENSGALG00010024842	ZFYVE9	-0.140331641	0.076574662	-1.832611954	0.066860299	0.09172251	negative
PRJNA729271	stage_E18_vs_E10	ordinary_muscle_development	ENSGALG00010024843	BCL9L	0.214271237	0.134591817	1.592007905	0.111382928	0.146505948	positive
PRJNA729271	stage_E18_vs_E10	ordinary_muscle_development	ENSGALG00010024844	TM4SF19	0.303324745	0.4292879	0.706576509	0.479829692	0.53815625	positive
PRJNA729271	stage_E18_vs_E10	ordinary_muscle_development	ENSGALG00010024845	HYCC2	0.474859537	0.111285306	4.267046151	1.98078E-05	4.46593E-05	positive
PRJNA729271	stage_E18_vs_E10	ordinary_muscle_development	ENSGALG00010024846	LMO2	0.409038763	0.143332311	2.853779175	0.004320255	0.007221183	positive
PRJNA729271	stage_E18_vs_E10	ordinary_muscle_development	ENSGALG00010024847	PPIL1	-0.794500487	0.152604885	-5.206258538	1.92686E-07	5.31693E-07	negative
PRJNA729271	stage_E18_vs_E10	ordinary_muscle_development	ENSGALG00010024848	ESYT1	0.213614137	0.085945755	2.485453029	0.012938666	0.02014635	positive
PRJNA729271	stage_E18_vs_E10	ordinary_muscle_development	ENSGALG00010024849	CENATAC	-0.196841909	0.202519721	-0.971964155	0.331068381	0.389263156	negative
PRJNA729271	stage_E18_vs_E10	ordinary_muscle_development	ENSGALG00010024850	CDK2AP1	-0.394332778	0.117385094	-3.35930879	0.000781377	0.001446496	negative
PRJNA729271	stage_E18_vs_E10	ordinary_muscle_development	ENSGALG00010024851	CPNE5	-3.637358111	0.967736611	-3.758624059	0.00017085	0.000345635	negative
PRJNA729271	stage_E18_vs_E10	ordinary_muscle_development	ENSGALG00010024852	CEP89	-0.278216849	0.093077401	-2.989091288	0.002798085	0.004816894	negative
PRJNA729271	stage_E18_vs_E10	ordinary_muscle_development	ENSGALG00010024853	TJP1	0.258291049	0.067588187	3.821541339	0.00013262	0.000272555	positive
PRJNA729271	stage_E18_vs_E10	ordinary_muscle_development	ENSGALG00010024854	C12orf65	0.747639784	0.151049532	4.949633233	7.43535E-07	1.94751E-06	positive
PRJNA729271	stage_E18_vs_E10	ordinary_muscle_development	ENSGALG00010024855	CAPRIN1	-0.766003514	0.072018348	-10.63622726	2.0215E-26	2.08701E-25	negative
PRJNA729271	stage_E18_vs_E10	ordinary_muscle_development	ENSGALG00010024856	SORT1	1.81286768	0.133409471	13.58874796	4.67001E-42	9.68409E-41	positive
PRJNA729271	stage_E18_vs_E10	ordinary_muscle_development	ENSGALG00010024857	SLC37A4	1.050858658	0.141616165	7.420471102	1.16704E-13	5.34143E-13	positive
PRJNA729271	stage_E18_vs_E10	ordinary_muscle_development	ENSGALG00010024858	SLC25A39	-0.803355672	0.124438998	-6.455819223	1.07635E-10	3.91037E-10	negative
PRJNA729271	stage_E18_vs_E10	ordinary_muscle_development	ENSGALG00010024859	TARSL2	-0.349929823	0.104379907	-3.352463462	0.000800958	0.001480664	negative
PRJNA729271	stage_E18_vs_E10	ordinary_muscle_development	ENSGALG00010024860	NEU4	-0.841568248	0.443094712	-1.899296527	0.057525499	0.079899589	negative
PRJNA729271	stage_E18_vs_E10	ordinary_muscle_development	ENSGALG00010024861	ORC1	-0.413237711	0.065001834	-6.357323851	2.05299E-10	7.30797E-10	negative
PRJNA729271	stage_E18_vs_E10	ordinary_muscle_development	ENSGALG00010024862	METTL16	0.225407137	0.120405987	1.872059211	0.061198412	0.084531255	positive
PRJNA729271	stage_E18_vs_E10	ordinary_muscle_development	ENSGALG00010024863	LMOD1	0.6018526	0.298075644	2.019127067	0.043474014	0.061653051	positive
PRJNA729271	stage_E18_vs_E10	ordinary_muscle_development	ENSGALG00010024864	MPHOSPH9	-0.768869619	0.131587641	-5.843023041	5.12619E-09	1.62662E-08	negative
PRJNA729271	stage_E18_vs_E10	ordinary_muscle_development	ENSGALG00010024865	SNAP47	0.224096434	0.144856891	1.547019496	0.121858545	0.159072371	positive
PRJNA729271	stage_E18_vs_E10	ordinary_muscle_development	ENSGALG00010024866	TRAPPC4	-0.115417237	0.087899948	-1.313052397	0.189165293	0.236497405	negative
PRJNA729271	stage_E18_vs_E10	ordinary_muscle_development	ENSGALG00010024867	TM2D3	-0.151876023	0.109153283	-1.391401325	0.164103763	0.208108841	negative
PRJNA729271	stage_E18_vs_E10	ordinary_muscle_development	ENSGALG00010024868	FAAP24	-1.689962802	0.212119324	-7.967038396	1.62522E-15	8.59139E-15	negative
PRJNA729271	stage_E18_vs_E10	ordinary_muscle_development	ENSGALG00010024869	NDUFB3	1.058760185	0.122231307	8.661939515	4.63804E-18	2.91158E-17	positive
PRJNA729271	stage_E18_vs_E10	ordinary_muscle_development	ENSGALG00010024870	TPD52L2	0.22767585	0.082866506	2.747501515	0.006005123	0.009829956	positive
PRJNA729271	stage_E18_vs_E10	ordinary_muscle_development	ENSGALG00010024871	POLQ	-0.870407314	0.135460626	-6.425537355	1.31404E-10	4.74753E-10	negative
PRJNA729271	stage_E18_vs_E10	ordinary_muscle_development	ENSGALG00010024872	RUNDC3A	-1.263989969	0.212565639	-5.946351334	2.74185E-09	8.90903E-09	negative
PRJNA729271	stage_E18_vs_E10	ordinary_muscle_development	ENSGALG00010024873	HYOU1	-1.054893509	0.109056315	-9.672924602	3.92984E-22	3.14887E-21	negative
PRJNA729271	stage_E18_vs_E10	ordinary_muscle_development	ENSGALG00010024874	JMJD4	0.1003404	0.081942163	1.224527111	0.220753446	0.271654741	positive
PRJNA729271	stage_E18_vs_E10	ordinary_muscle_development	ENSGALG00010024876	CFLAR	-0.094718056	0.096534954	-0.981178859	0.326504547	0.38456649	negative
PRJNA729271	stage_E18_vs_E10	ordinary_muscle_development	ENSGALG00010024877	PRPF38A	-0.880935016	0.081307655	-10.83458888	2.36023E-27	2.53795E-26	negative
PRJNA729271	stage_E18_vs_E10	ordinary_muscle_development	ENSGALG00010024878	RHPN2	-1.312401578	0.274748878	-4.776731354	1.78168E-06	4.47969E-06	negative
PRJNA729271	stage_E18_vs_E10	ordinary_muscle_development	ENSGALG00010024879	D2HGDH	-0.043053178	0.151893181	-0.283443781	0.776836671	0.812955567	negative
PRJNA729271	stage_E18_vs_E10	ordinary_muscle_development	ENSGALG00010024880	NAT10	-1.481702025	0.140446761	-10.54991949	5.08406E-26	5.0999E-25	negative
PRJNA729271	stage_E18_vs_E10	ordinary_muscle_development	ENSGALG00010024881	CASP10	-1.621534492	0.321841116	-5.038307446	4.69667E-07	1.25144E-06	negative
PRJNA729271	stage_E18_vs_E10	ordinary_muscle_development	ENSGALG00010024882	MYL6	0.072740287	0.164191738	0.443020386	0.657750989	0.705811415	positive
PRJNA729271	stage_E18_vs_E10	ordinary_muscle_development	ENSGALG00010024883	ING5	0.164754458	0.126087736	1.306665202	0.191326442	0.238951988	positive
PRJNA729271	stage_E18_vs_E10	ordinary_muscle_development	ENSGALG00010024884	ZBTB46	0.816155977	0.133771283	6.101129925	1.05321E-09	3.54618E-09	positive
PRJNA729271	stage_E18_vs_E10	ordinary_muscle_development	ENSGALG00010024885	SLC4A1	0.173850552	0.233804879	0.743571104	0.45713599	0.516701979	positive
PRJNA729271	stage_E18_vs_E10	ordinary_muscle_development	ENSGALG00010024886	ABTB2	-1.628489449	0.142800063	-11.40398272	3.99419E-30	4.91058E-29	negative
PRJNA729271	stage_E18_vs_E10	ordinary_muscle_development	ENSGALG00010024887	CASP18	0.447316431	0.269240757	1.661399398	0.096633259	0.128540542	positive
PRJNA729271	stage_E18_vs_E10	ordinary_muscle_development	ENSGALG00010024888	PITPNM2	0.975984971	0.126061969	7.742104737	9.77844E-15	4.89091E-14	positive
PRJNA729271	stage_E18_vs_E10	ordinary_muscle_development	ENSGALG00010024889	SLC2A4RG	-0.282974956	0.517759317	-0.546537641	0.584696421	0.637231175	negative
PRJNA729271	stage_E18_vs_E10	ordinary_muscle_development	ENSGALG00010024890	SMARCC2	0.55967986	0.145577619	3.844546037	0.000120776	0.000249167	positive
PRJNA729271	stage_E18_vs_E10	ordinary_muscle_development	ENSGALG00010024891	PKP1	-1.03150895	0.465230564	-2.217199449	0.026609465	0.039265418	negative
PRJNA729271	stage_E18_vs_E10	ordinary_muscle_development	ENSGALG00010024893	ARL6IP4	0.102914989	0.139805956	0.736127362	0.461653193	0.520954693	positive
PRJNA729271	stage_E18_vs_E10	ordinary_muscle_development	ENSGALG00010024894	TUT4	0.236882927	0.122696629	1.930639245	0.053527681	0.074747952	positive
PRJNA729271	stage_E18_vs_E10	ordinary_muscle_development	ENSGALG00010024895	ENSGALG00010024895	0.574084432	0.448174596	1.280939251	0.200215005	0.249065954	positive
PRJNA729271	stage_E18_vs_E10	ordinary_muscle_development	ENSGALG00010024896	RNF41	-0.184711879	0.102863335	-1.795701831	0.072541951	0.09891774	negative
PRJNA729271	stage_E18_vs_E10	ordinary_muscle_development	ENSGALG00010024897	ADAL	0.483298143	0.19213588	2.51539766	0.011889819	0.018615798	positive
PRJNA729271	stage_E18_vs_E10	ordinary_muscle_development	ENSGALG00010024898	NABP2	-0.152122085	0.313312026	-0.485529033	0.627301123	0.677601146	negative
PRJNA729271	stage_E18_vs_E10	ordinary_muscle_development	ENSGALG00010024899	OGFOD2	-0.291995601	0.12836289	-2.274766483	0.022919942	0.034204114	negative
PRJNA729271	stage_E18_vs_E10	ordinary_muscle_development	ENSGALG00010024900	DTYMK	-0.575591267	0.101856463	-5.651003865	1.59514E-08	4.84625E-08	negative
PRJNA729271	stage_E18_vs_E10	ordinary_muscle_development	ENSGALG00010024901	ZGPAT	-0.517655717	0.132124532	-3.917937944	8.93097E-05	0.000187181	negative
PRJNA729271	stage_E18_vs_E10	ordinary_muscle_development	ENSGALG00010024902	LARP6	0.57444504	0.118809223	4.835020586	1.33132E-06	3.39684E-06	positive
PRJNA729271	stage_E18_vs_E10	ordinary_muscle_development	ENSGALG00010024903	CASP8	-1.437482935	0.119501281	-12.02901692	2.50121E-33	3.51991E-32	negative
PRJNA729271	stage_E18_vs_E10	ordinary_muscle_development	ENSGALG00010024904	ALS2CL	0.627709532	0.163077001	3.849160385	0.000118523	0.000244833	positive
PRJNA729271	stage_E18_vs_E10	ordinary_muscle_development	ENSGALG00010024905	GPX7	0.094942561	0.207449393	0.45766613	0.647192325	0.695820557	positive
PRJNA729271	stage_E18_vs_E10	ordinary_muscle_development	ENSGALG00010024907	PHLDB1	0.873479256	0.133403408	6.54765323	5.84482E-11	2.17066E-10	positive
PRJNA729271	stage_E18_vs_E10	ordinary_muscle_development	ENSGALG00010024908	ENSGALG00010024908	0.094631116	0.129070612	0.733173218	0.463452788	0.52257862	positive
PRJNA729271	stage_E18_vs_E10	ordinary_muscle_development	ENSGALG00010024910	UBTF	-0.336852674	0.123526557	-2.726965619	0.00639197	0.010416144	negative
PRJNA729271	stage_E18_vs_E10	ordinary_muscle_development	ENSGALG00010024911	CAT	1.06719522	0.15326006	6.963296386	3.32401E-12	1.36608E-11	positive
PRJNA729271	stage_E18_vs_E10	ordinary_muscle_development	ENSGALG00010024915	STXBP5L	-0.539799573	0.176321845	-3.061444666	0.002202717	0.003842626	negative
PRJNA729271	stage_E18_vs_E10	ordinary_muscle_development	ENSGALG00010024917	TRAK2	0.530642393	0.123620986	4.292494413	1.76677E-05	4.00584E-05	positive
PRJNA729271	stage_E18_vs_E10	ordinary_muscle_development	ENSGALG00010024918	ATG4B	-0.480120251	0.075828518	-6.331658088	2.4254E-10	8.59566E-10	negative
PRJNA729271	stage_E18_vs_E10	ordinary_muscle_development	ENSGALG00010024919	ANKRD52	-0.703667726	0.124569811	-5.648782154	1.61588E-08	4.90416E-08	negative
PRJNA729271	stage_E18_vs_E10	ordinary_muscle_development	ENSGALG00010024920	VIPR2	-0.293041467	0.301877035	-0.970731233	0.331682134	0.389889915	negative
PRJNA729271	stage_E18_vs_E10	ordinary_muscle_development	ENSGALG00010024921	STRADB	-1.175230122	0.113492698	-10.35511664	3.96703E-25	3.78054E-24	negative
PRJNA729271	stage_E18_vs_E10	ordinary_muscle_development	ENSGALG00010024924	MNX1	-1.83497405	0.548318835	-3.34654572	0.000818252	0.001510707	negative
PRJNA729271	stage_E18_vs_E10	ordinary_muscle_development	ENSGALG00010024925	GTF2E1	-0.380041898	0.083794795	-4.535387882	5.74977E-06	1.37145E-05	negative
PRJNA729271	stage_E18_vs_E10	ordinary_muscle_development	ENSGALG00010024926	ELF5	-4.961312794	0.904996792	-5.482133018	4.20228E-08	1.23204E-07	negative
PRJNA729271	stage_E18_vs_E10	ordinary_muscle_development	ENSGALG00010024927	NOM1	-0.830305162	0.117509454	-7.06585837	1.59626E-12	6.72611E-12	negative
PRJNA729271	stage_E18_vs_E10	ordinary_muscle_development	ENSGALG00010024928	THAP4	0.38134308	0.086638744	4.40153055	1.0749E-05	2.4973E-05	positive
PRJNA729271	stage_E18_vs_E10	ordinary_muscle_development	ENSGALG00010024930	TMEM237	0.242334814	0.09971033	2.430388246	0.015082657	0.023280067	positive
PRJNA729271	stage_E18_vs_E10	ordinary_muscle_development	ENSGALG00010024931	UBE3C	-0.154290475	0.089462321	-1.724641989	0.084592012	0.113733532	negative

PRJNA729271	stage_E18_vs_E10	ordinary_muscle_development	ENSGALG00010024932	ANKK1	-1.181412244	1.757202222	-0.672325717	0.501376361	0.558588121	negative
PRJNA729271	stage_E18_vs_E10	ordinary_muscle_development	ENSGALG00010024933	CS	0.341937837	0.157369216	2.172838149	0.029792501	0.043554443	positive
PRJNA729271	stage_E18_vs_E10	ordinary_muscle_development	ENSGALG00010024934	G6PC3	-0.842549612	0.231531073	-3.639034709	0.000273662	0.000538958	negative
PRJNA729271	stage_E18_vs_E10	ordinary_muscle_development	ENSGALG00010024935	KLHL30	4.0772659	0.387602257	10.51920062	7.04689E-26	7.00581E-25	positive
PRJNA729271	stage_E18_vs_E10	ordinary_muscle_development	ENSGALG00010024936	EHF	-0.257240398	0.563246647	-0.456710039	0.647879474	0.69645602	negative
PRJNA729271	stage_E18_vs_E10	ordinary_muscle_development	ENSGALG00010024937	ILKAP	-0.77307509	0.127093852	-6.082710348	1.18168E-09	3.95941E-09	negative
PRJNA729271	stage_E18_vs_E10	ordinary_muscle_development	ENSGALG00010024938	ENSGALG00010024938	0.314168368	0.179513881	1.750106268	0.080099978	0.108337145	positive
PRJNA729271	stage_E18_vs_E10	ordinary_muscle_development	ENSGALG00010024939	COQ10A	0.396615966	0.209014314	1.897554084	0.057754848	0.080179726	positive
PRJNA729271	stage_E18_vs_E10	ordinary_muscle_development	ENSGALG00010024940	RABL3	-0.159258596	0.091891298	-1.733119458	0.083074439	0.111911151	negative
PRJNA729271	stage_E18_vs_E10	ordinary_muscle_development	ENSGALG00010024941	PDHX	-0.140341495	0.119477792	-1.174624107	0.240145134	0.2930547	negative
PRJNA729271	stage_E18_vs_E10	ordinary_muscle_development	ENSGALG00010024942	DNAJB6	0.065634146	0.066644394	0.984841227	0.324702067	0.382677978	positive
PRJNA729271	stage_E18_vs_E10	ordinary_muscle_development	ENSGALG00010024943	ABCB9	-0.236553099	0.192714206	-1.227481376	0.219641717	0.270385739	negative
PRJNA729271	stage_E18_vs_E10	ordinary_muscle_development	ENSGALG00010024944	ENSGALG00010024944	0.125547511	0.112341824	1.117549158	0.263759594	0.318413868	positive
PRJNA729271	stage_E18_vs_E10	ordinary_muscle_development	ENSGALG00010024945	APIP	-0.357823724	0.08511152	-4.204174992	2.62036E-05	5.82548E-05	negative
PRJNA729271	stage_E18_vs_E10	ordinary_muscle_development	ENSGALG00010024946	KIF1A	-2.772127612	0.217674665	-12.73518723	3.76941E-37	6.39784E-36	negative
PRJNA729271	stage_E18_vs_E10	ordinary_muscle_development	ENSGALG00010024947	MPP4	1.750422158	0.310495401	5.637513951	1.72523E-08	5.21852E-08	positive
PRJNA729271	stage_E18_vs_E10	ordinary_muscle_development	ENSGALG00010024949	PAN2	-0.24777972	0.115618636	-2.143077693	0.03210686	0.046683613	negative
PRJNA729271	stage_E18_vs_E10	ordinary_muscle_development	ENSGALG00010024950	NCAM1	0.857217657	0.154607127	5.544489921	2.94812E-08	8.76575E-08	positive
PRJNA729271	stage_E18_vs_E10	ordinary_muscle_development	ENSGALG00010024953	NCAPG2	-1.487513839	0.220835405	-6.735848548	1.62976E-11	6.30461E-11	negative
PRJNA729271	stage_E18_vs_E10	ordinary_muscle_development	ENSGALG00010024956	VPS37B	0.055338302	0.138463761	0.399659098	0.689407622	0.735101036	positive
PRJNA729271	stage_E18_vs_E10	ordinary_muscle_development	ENSGALG00010024960	ARFRP1	-0.558778141	0.102552807	-5.448686935	5.0743E-08	1.47369E-07	negative
PRJNA729271	stage_E18_vs_E10	ordinary_muscle_development	ENSGALG00010024961	LSM12	-0.50081921	0.207373283	-2.415061392	0.015732558	0.024198452	negative
PRJNA729271	stage_E18_vs_E10	ordinary_muscle_development	ENSGALG00010024963	TNFRSF6B	2.073866476	0.575078769	3.606230288	0.000310677	0.00060691	positive
PRJNA729271	stage_E18_vs_E10	ordinary_muscle_development	ENSGALG00010024964	ITM2C	-0.706550969	0.170972766	-4.132535168	3.58784E-05	7.84282E-05	negative
PRJNA729271	stage_E18_vs_E10	ordinary_muscle_development	ENSGALG00010024965	STAT2	-0.401596204	0.224650919	-1.787645498	0.07383322	0.100470571	negative
PRJNA729271	stage_E18_vs_E10	ordinary_muscle_development	ENSGALG00010024966	AGAP3	-0.256066066	0.115712629	-2.212948306	0.02690121	0.039643456	negative
PRJNA729271	stage_E18_vs_E10	ordinary_muscle_development	ENSGALG00010024968	TMEM101	0.17177115	0.136021637	1.262822247	0.206653068	0.256239197	positive
PRJNA729271	stage_E18_vs_E10	ordinary_muscle_development	ENSGALG00010024970	CAB39	-0.24638303	0.078362943	-3.144126819	0.001665832	0.002957648	negative
PRJNA729271	stage_E18_vs_E10	ordinary_muscle_development	ENSGALG00010024973	RTEL1	-1.167592624	0.085577391	-13.64370445	2.2009E-42	4.63693E-41	negative
PRJNA729271	stage_E18_vs_E10	ordinary_muscle_development	ENSGALG00010024974	CD44	-0.432845356	0.166859432	-2.594071855	0.009484666	0.01504654	negative
PRJNA729271	stage_E18_vs_E10	ordinary_muscle_development	ENSGALG00010024977	LRP3	1.058908743	0.105194796	10.06617037	7.79546E-24	6.8565E-23	positive
PRJNA729271	stage_E18_vs_E10	ordinary_muscle_development	ENSGALG00010024983	APOF	2.461980678	0.493842297	4.985358066	6.18472E-07	1.62966E-06	positive
PRJNA729271	stage_E18_vs_E10	ordinary_muscle_development	ENSGALG00010024984	SHISAL2A	-0.516144615	0.509346974	-1.013345797	0.31089502	0.36850301	negative
PRJNA729271	stage_E18_vs_E10	ordinary_muscle_development	ENSGALG00010024986	PTS	-0.177177085	0.162985754	-1.087070988	0.277005438	0.332466161	negative
PRJNA729271	stage_E18_vs_E10	ordinary_muscle_development	ENSGALG00010024989	TIMELESS	-1.329709434	0.164681846	-8.074414195	6.78014E-16	3.68912E-15	negative
PRJNA729271	stage_E18_vs_E10	ordinary_muscle_development	ENSGALG00010024994	NDUFB4	0.533076611	0.104772574	5.087940411	3.61973E-07	9.76512E-07	positive
PRJNA729271	stage_E18_vs_E10	ordinary_muscle_development	ENSGALG00010024996	COA7	-0.25879591	0.126241287	-2.050010064	0.040363449	0.05759112	negative
PRJNA729271	stage_E18_vs_E10	ordinary_muscle_development	ENSGALG00010024997	HES6	0.684321537	0.248291953	2.756116453	0.005849217	0.00958991	positive
PRJNA729271	stage_E18_vs_E10	ordinary_muscle_development	ENSGALG00010025003	ENSGALG00010025003	6.377611401	0.243671359	26.17300379	5.3941E-151	1.8183E-148	positive
PRJNA729271	stage_E18_vs_E10	ordinary_muscle_development	ENSGALG00010025005	HIP1R	-0.471187209	0.124118383	-3.796272552	0.000146888	0.000299921	negative
PRJNA729271	stage_E18_vs_E10	ordinary_muscle_development	ENSGALG00010025006	ENSGALG00010025006	1.921050249	0.257786983	7.452084005	9.18772E-14	4.23381E-13	positive
PRJNA729271	stage_E18_vs_E10	ordinary_muscle_development	ENSGALG00010025007	ZYG11B	-0.544066667	0.104753188	-5.193795805	2.06049E-07	5.67487E-07	negative
PRJNA729271	stage_E18_vs_E10	ordinary_muscle_development	ENSGALG00010025008	ENSGALG00010025008	-0.946591286	0.599570882	-1.578781282	0.114386234	0.15015049	negative
PRJNA729271	stage_E18_vs_E10	ordinary_muscle_development	ENSGALG00010025012	ENSGALG00010025012	-1.271172419	1.105489551	-1.149872849	0.250196245	0.303887596	negative
PRJNA729271	stage_E18_vs_E10	ordinary_muscle_development	ENSGALG00010025026	DENR	-0.528839788	0.117294241	-4.508659443	6.52385E-06	1.54616E-05	negative
PRJNA729271	stage_E18_vs_E10	ordinary_muscle_development	ENSGALG00010025028	HES6	-0.568038078	0.134192295	-4.2330156	2.30579E-05	5.15538E-05	negative
PRJNA729271	stage_E18_vs_E10	ordinary_muscle_development	ENSGALG00010025031	ECHDC2	0.69893168	0.121888556	5.734186235	9.79816E-09	3.03018E-08	positive
PRJNA729271	stage_E18_vs_E10	ordinary_muscle_development	ENSGALG00010025032	FSTL1	0.052591625	0.181244201	0.290169972	0.771686207	0.808616466	positive
PRJNA729271	stage_E18_vs_E10	ordinary_muscle_development	ENSGALG00010025039	CEBPA	0.096041212	0.309071427	0.310741156	0.755997407	0.795265684	positive
PRJNA729271	stage_E18_vs_E10	ordinary_muscle_development	ENSGALG00010025042	BCO2	-5.012808335	0.941349665	-5.325128933	1.00881E-07	2.86048E-07	negative
PRJNA729271	stage_E18_vs_E10	ordinary_muscle_development	ENSGALG00010025044	TMEM9	1.702781171	0.211024583	8.069112841	7.08108E-16	3.84709E-15	positive
PRJNA729271	stage_E18_vs_E10	ordinary_muscle_development	ENSGALG00010025047	PER2	-0.421303021	0.293966976	-1.433164458	0.151810844	0.194047459	negative
PRJNA729271	stage_E18_vs_E10	ordinary_muscle_development	ENSGALG00010025048	ESYT2	0.704894781	0.082893257	8.503644389	1.8373E-17	1.10596E-16	positive
PRJNA729271	stage_E18_vs_E10	ordinary_muscle_development	ENSGALG00010025055	ALS2	0.406340255	0.154619081	2.628008464	0.008588638	0.013727236	positive
PRJNA729271	stage_E18_vs_E10	ordinary_muscle_development	ENSGALG00010025056	RBMS2	0.151374178	0.175068741	0.864655659	0.387227813	0.447383003	positive
PRJNA729271	stage_E18_vs_E10	ordinary_muscle_development	ENSGALG00010025057	SCP2	-0.393504961	0.067558985	-5.824613312	5.7245E-09	1.80895E-08	negative
PRJNA729271	stage_E18_vs_E10	ordinary_muscle_development	ENSGALG00010025060	GPR156	1.488922594	0.151683957	9.815953024	9.61253E-23	7.95738E-22	positive
PRJNA729271	stage_E18_vs_E10	ordinary_muscle_development	ENSGALG00010025064	GPN3	-0.335746222	0.08442983	-3.976630325	6.98987E-05	0.000148365	negative
PRJNA729271	stage_E18_vs_E10	ordinary_muscle_development	ENSGALG00010025067	SLC1A2	-1.34397274	0.341122771	-3.939850561	8.15324E-05	0.000171476	negative
PRJNA729271	stage_E18_vs_E10	ordinary_muscle_development	ENSGALG00010025069	PODN	2.205820537	0.197733644	11.15551454	6.73023E-29	7.79191E-28	positive
PRJNA729271	stage_E18_vs_E10	ordinary_muscle_development	ENSGALG00010025071	CDK15	0.828055538	0.320410153	2.58436111	0.009755959	0.015456621	positive
PRJNA729271	stage_E18_vs_E10	ordinary_muscle_development	ENSGALG00010025072	ENSGALG00010025072	-3.041767974	1.010596235	-3.009874634	0.002613555	0.004516929	negative
PRJNA729271	stage_E18_vs_E10	ordinary_muscle_development	ENSGALG00010025073	IL18	0.369621827	0.202950669	1.82123976	0.068570419	0.093908562	positive
PRJNA729271	stage_E18_vs_E10	ordinary_muscle_development	ENSGALG00010025080	BAZ2A	0.784338146	0.206510045	3.798062931	0.000145831	0.00029789	positive
PRJNA729271	stage_E18_vs_E10	ordinary_muscle_development	ENSGALG00010025092	COL1A1	3.818068681	0.220960409	17.27942441	6.72154E-67	3.29151E-65	positive
PRJNA729271	stage_E18_vs_E10	ordinary_muscle_development	ENSGALG00010025097	LMBR1	0.604310972	0.120977756	4.995223851	5.87676E-07	1.55218E-06	positive
PRJNA729271	stage_E18_vs_E10	ordinary_muscle_development	ENSGALG00010025098	ATP5B	0.732558989	0.138251113	5.298756543	1.16594E-07	3.2848E-07	positive
PRJNA729271	stage_E18_vs_E10	ordinary_muscle_development	ENSGALG00010025102	ARPC3	-0.644434599	0.086297173	-7.467621176	8.16575E-14	3.7876E-13	negative
PRJNA729271	stage_E18_vs_E10	ordinary_muscle_development	ENSGALG00010025103	GSK3B	0.058365158	0.100388245	0.581394348	0.560974706	0.614845245	positive
PRJNA729271	stage_E18_vs_E10	ordinary_muscle_development	ENSGALG00010025113	SDHD	0.758270388	0.120249703	6.305798407	2.86712E-10	1.01067E-09	positive
PRJNA729271	stage_E18_vs_E10	ordinary_muscle_development	ENSGALG00010025114	CEBPG	-0.092482765	0.098103314	-0.942707867	0.345830348	0.404487283	negative
PRJNA729271	stage_E18_vs_E10	ordinary_muscle_development	ENSGALG00010025115	MGAT4F	-0.40396674	0.261577476	-1.544348336	0.122503969	0.159828535	negative
PRJNA729271	stage_E18_vs_E10	ordinary_muscle_development	ENSGALG00010025116	ANAPC7	-0.478623929	0.071024558	-6.738851199	1.59644E-11	6.18561E-11	negative
PRJNA729271	stage_E18_vs_E10	ordinary_muscle_development	ENSGALG00010025121	DYNC2I1	-0.095583992	0.153409584	-0.62306402	0.533242452	0.589082337	negative
PRJNA729271	stage_E18_vs_E10	ordinary_muscle_development	ENSGALG00010025125	PIK3CB	0.583429336	0.132732557	4.395525487	1.10505E-05	2.55997E-05	positive
PRJNA729271	stage_E18_vs_E10	ordinary_muscle_development	ENSGALG00010025126	ENSGALG00010025126	-0.917015527	0.139526951	-6.572318264	4.95378E-11	1.85208E-10	negative
PRJNA729271	stage_E18_vs_E10	ordinary_muscle_development	ENSGALG00010025127	PAMR1	-0.593444028	0.159454954	-3.721703287	0.000197883	0.000397164	negative
PRJNA729271	stage_E18_vs_E10	ordinary_muscle_development	ENSGALG00010025130	ATP2A2	-0.812056505	0.125400732	-6.47569191	9.43784E-11	3.44849E-10	negative
PRJNA729271	stage_E18_vs_E10	ordinary_muscle_development	ENSGALG00010025131	SUMO1	-0.248027366	0.0810776	-3.059135531	0.002219767	0.003869111	negative
PRJNA729271	stage_E18_vs_E10	ordinary_muscle_development	ENSGALG00010025138	CFAP91	-0.793980763	0.281490381	-2.820631952	0.004792916	0.007959821	negative
PRJNA729271	stage_E18_vs_E10	ordinary_muscle_development	ENSGALG00010025139	COPB2	-0.5123076	0.046902291	-10.92286941	8.9619E-28	9.87853E-27	negative
PRJNA729271	stage_E18_vs_E10	ordinary_muscle_development	ENSGALG00010025144	PIH1D2	0.594983899	0.153677727	3.871633899	0.000108108	0.000224664	positive
PRJNA729271	stage_E18_vs_E10	ordinary_muscle_development	ENSGALG00010025145	CPT2	0.320037362	0.094457704	3.388155196	0.000703644	0.001310462	positive
PRJNA729271	stage_E18_vs_E10	ordinary_muscle_development	ENSGALG00010025147	COX17	1.435678304	0.113963341	12.59772043	2.17335E-36	3.60443E-35	positive
PRJNA729271	stage_E18_vs_E10	ordinary_muscle_development	ENSGALG00010025149	IFT81	-0.473639779	0.140848025	-3.362771898	0.000771641	0.00142975	negative
PRJNA729271	stage_E18_vs_E10	ordinary_muscle_development	ENSGALG00010025154	TMEM183A	0.16212476	0.123009988	1.31798046	0.187510189	0.234650798	positive
PRJNA729271	stage_E18_vs_E10	ordinary_muscle_development	ENSGALG00010025155	CZIB	0.133124399	0.092780135	1.434837319	0.151333461	0.193539661	positive
PRJNA729271	stage_E18_vs_E10	ordinary_muscle_development	ENSGALG00010025156	DLAT	0.747763061	0.115399282	6.479789534	9.18507E-11	3.35867E-10	positive
PRJNA729271	stage_E18_vs_E10	ordinary_muscle_development	ENSGALG00010025157	NOP58	-1.303787422	0.14088402	-9.254331496	2.15575E-20	1.55848E-19	negative
PRJNA729271	stage_E18_vs_E10	ordinary_muscle_development	ENSGALG00010025160	MRPS22	-0.282236273	0.132852563	-2.124432271	0.033634014	0.048705441	negative
PRJNA729271	stage_E18_vs_E10	ordinary_muscle_development	ENSGALG00010025161	ENSGALG00010025161	-0.625737837	0.104670249	-5.97818238	2.25641E-09	7.383E-09	negative
PRJNA729271	stage_E18_vs_E10	ordinary_muscle_development	ENSGALG00010025163	LDLRAD3	-0.773551289	0.135330583	-5.716012404	1.09053E-08	3.35325E-08	negative
PRJNA729271	stage_E18_vs_E10	ordinary_muscle_development	ENSGALG00010025164	C11orf52	1.643789341	0.124730417	13.17873686	1.16322E-39	2.19829E-38	positive
PRJNA729271	stage_E18_vs_E10	ordinary_muscle_development	ENSGALG00010025167	PTGES3	-0.914622296	0.189152499	-4.835369873	1.32898E-06	3.39148E-06	negative
PRJNA729271	stage_E18_vs_E10	ordinary_muscle_development	ENSGALG00010025168	POPDC2	2.662704774	0.211057637	12.61600772	1.72339E-36	2.86803E-35	positive
PRJNA729271	stage_E18_vs_E10	ordinary_muscle_development	ENSGALG00010025169	RBP2	1.721183547	2.24302561	0.767349039	0.442874019	0.502383699	positive
PRJNA729271	stage_E18_vs_E10	ordinary_muscle_development	ENSGALG00010025170	COMMD9	-0.191163819	0.098058325	-1.949490971	0.05123682	0.071832644	negative
PRJNA729271	stage_E18_vs_E10	ordinary_muscle_development	ENSGALG00010025172	RBP1	3.505920138	0.969017665	3.618014682	0.000296872	0.000581586	positive
PRJNA729271	stage_E18_vs_E10	ordinary_muscle_development	ENSGALG00010025182	PLA1A	2.060554943	0.200444006	10.27995292	8.677E-25	8.14065E-24	positive
PRJNA729271	stage_E18_vs_E10	ordinary_muscle_development	ENSGALG00010025186	TMEM120B	-0.628007397	0.207446305	-3.027325054	0.002467285	0.004274327	negative
PRJNA729271	stage_E18_vs_E10	ordinary_muscle_development	ENSGALG00010025188	PPFIA4	-3.070784166	0.403434625	-7.611602912	2.70717E-14	1.29978E-13	negative
PRJNA729271	stage_E18_vs_E10	ordinary_muscle_development	ENSGALG00010025190	PRR5L	-0.095297676	0.243267535	-0.391740215	0.695250173	0.740404912	negative
PRJNA729271	stage_E18_vs_E10	ordinary_muscle_development	ENSGALG00010025192	CRYAB	0.241575806	0.182949477	1.320450923	0.186684511	0.233779006	positive
PRJNA729271	stage_E18_vs_E10	ordinary_muscle_development	ENSGALG00010025195	P2RX7	2.381424454	0.425599127	5.59546367	2.20033E-08	6.6019E-08	positive
PRJNA729271	stage_E18_vs_E10	ordinary_muscle_development	ENSGALG00010025200	PEPD	-0.248236175	0.080713969	-3.075504503	0.002101468	0.003678837	negative
PRJNA729271	stage_E18_vs_E10	ordinary_muscle_development	ENSGALG00010025204	TRAF6	0.361609971	0.119888783	3.016211868	0.002559543	0.004427277	positive
PRJNA729271	stage_E18_vs_E10	ordinary_muscle_development	ENSGALG00010025207	BMPR2	0.201682186	0.097633574	2.065705245	0.03885632	0.055637889	positive
PRJNA729271	stage_E18_vs_E10	ordinary_muscle_development	ENSGALG00010025210	HSPB2	0.230532844	0.296869658	0.77654566	0.437426872	0.496982483	positive
PRJNA729271	stage_E18_vs_E10	ordinary_muscle_development	ENSGALG00010025212	P2RX4	0.950410271	0.180327941	5.270454838	1.36086E-07	3.80511E-07	positive
PRJNA729271	stage_E18_vs_E10	ordinary_muscle_development	ENSGALG00010025214	LRP8	-3.067106593	0.245878089	-12.4740948	1.03374E-35	1.6448E-34	negative
PRJNA729271	stage_E18_vs_E10	ordinary_muscle_development	ENSGALG00010025215	MYOG	-0.82963582	0.213608261	-3.883912623	0.000102789	0.000214162	negative
PRJNA729271	stage_E18_vs_E10	ordinary_muscle_development	ENSGALG00010025216	DPP6	-1.034768248	0.24971943	-4.14372341	3.41712E-05	7.48883E-05	negative
PRJNA729271	stage_E18_vs_E10	ordinary_muscle_development	ENSGALG00010025219	NACA	-0.045871949	0.125478919	-0.365574946	0.714682273	0.758146923	negative
PRJNA729271	stage_E18_vs_E10	ordinary_muscle_development	ENSGALG00010025222	FAM117B	-0.037204884	0.134911861	-0.275771777	0.782723362	0.81788099	negative
PRJNA729271	stage_E18_vs_E10	ordinary_muscle_development	ENSGALG00010025225	DMRTB1	-2.064713601	1.048003532	-1.970139927	0.048822336	0.068666773	negative
PRJNA729271	stage_E18_vs_E10	ordinary_muscle_development	ENSGALG00010025228	FDXACB1	0.667805397	0.10573428	6.315883515	2.68622E-10	9.49215E-10	positive
PRJNA729271	stage_E18_vs_E10	ordinary_muscle_development	ENSGALG00010025229	ENSGALG00010025229	-1.313882287	0.166856595	-7.874320379	3.426E-15	1.77357E-14	negative
PRJNA729271	stage_E18_vs_E10	ordinary_muscle_development	ENSGALG00010025230	CHST8	-1.053789877	0.249905266	-4.216757383	2.4784E-05	5.52599E-05	negative
PRJNA729271	stage_E18_vs_E10	ordinary_muscle_development	ENSGALG00010025232	PRIM1	-0.861728104	0.15180665	-5.676484564	1.37491E-08	4.19741E-08	negative
PRJNA729271	stage_E18_vs_E10	ordinary_muscle_development	ENSGALG00010025233	WDR12	-0.21651232	0.074810989	-2.89412453	0.003802173	0.006401731	negative
PRJNA729271	stage_E18_vs_E10	ordinary_muscle_development	ENSGALG00010025236	ENSGALG00010025236	0.9793066	0.255513665	3.832697554	0.000126746	0.000261122	positive
PRJNA729271	stage_E18_vs_E10	ordinary_muscle_development	ENSGALG00010025238	IGSF9B	0.489471592	0.295584441	1.655945054	0.097732947	0.129871568	positive
PRJNA729271	stage_E18_vs_E10	ordinary_muscle_development	ENSGALG00010025239	RAG2	-3.372180539	0.778466909	-4.331822585	1.4788E-05	3.38202E-05	negative
PRJNA729271	stage_E18_vs_E10	ordinary_muscle_development	ENSGALG00010025242	CLSTN2	-1.969564529	0.302452568	-6.51197819	7.41675E-11	2.73271E-10	negative
PRJNA729271	stage_E18_vs_E10	ordinary_muscle_development	ENSGALG00010025243	PAXIP1	-0.52511802	0.090400915	-5.808768876	6.29339E-09	1.98267E-08	negative
PRJNA729271	stage_E18_vs_E10	ordinary_muscle_development	ENSGALG00010025245	ENSGALG00010025245	0.750454869	0.068858111	10.89856886	1.17084E-27	1.28474E-26	positive
PRJNA729271	stage_E18_vs_E10	ordinary_muscle_development	ENSGALG00010025247	SGCA	2.947122468	0.140372889	20.9949549	7.29321E-98	8.13193E-96	positive
PRJNA729271	stage_E18_vs_E10	ordinary_muscle_development	ENSGALG00010025248	C11orf74	-0.459750909	0.21760065	-2.112819561	0.034616215	0.049991236	negative
PRJNA729271	stage_E18_vs_E10	ordinary_muscle_development	ENSGALG00010025250	GLIS1	-4.356785597	0.484211661	-8.997688292	2.3052E-19	1.56286E-18	negative
PRJNA729271	stage_E18_vs_E10	ordinary_muscle_development	ENSGALG00010025252	PPP1R9B	-0.233516629	0.145842427	-1.601157047	0.10934214	0.144017657	negative
PRJNA729271	stage_E18_vs_E10	ordinary_muscle_development	ENSGALG00010025256	ANAPC5	-1.26709049	0.125723333	-10.07840356	6.88354E-24	6.0743E-23	negative

PRJNA729271	stage_E18_vs_E10	ordinary_muscle_development	ENSGALG00010025257	LPCAT2	-1.055914253	0.170054105	-6.209284117	5.32265E-10	1.84618E-09	negative
PRJNA729271	stage_E18_vs_E10	ordinary_muscle_development	ENSGALG00010025259	SHH	-6.623676455	1.053579774	-6.286829548	3.24015E-10	1.13791E-09	negative
PRJNA729271	stage_E18_vs_E10	ordinary_muscle_development	ENSGALG00010025262	CARF	-0.002305271	0.110857773	-0.020794856	0.983409301	0.986179368	negative
PRJNA729271	stage_E18_vs_E10	ordinary_muscle_development	ENSGALG00010025263	SAMD14	-2.45558719	0.883497175	-2.779394501	0.005446034	0.008974564	negative
PRJNA729271	stage_E18_vs_E10	ordinary_muscle_development	ENSGALG00010025268	ZBTB39	-0.456686594	0.107508454	-4.247913333	2.15771E-05	4.84073E-05	negative
PRJNA729271	stage_E18_vs_E10	ordinary_muscle_development	ENSGALG00010025270	NDC1	-1.815811783	0.122941071	-14.76977357	2.29483E-49	6.56085E-48	negative
PRJNA729271	stage_E18_vs_E10	ordinary_muscle_development	ENSGALG00010025271	RNF34	0.446229794	0.118148798	3.776845834	0.000158827	0.000322798	positive
PRJNA729271	stage_E18_vs_E10	ordinary_muscle_development	ENSGALG00010025272	NCAPD3	-1.680130566	0.184610853	-9.100930612	8.95611E-20	6.21738E-19	negative
PRJNA729271	stage_E18_vs_E10	ordinary_muscle_development	ENSGALG00010025274	PDK2	2.334915949	0.270677812	8.626181549	6.34343E-18	3.94119E-17	positive
PRJNA729271	stage_E18_vs_E10	ordinary_muscle_development	ENSGALG00010025280	HSPB11	0.029831333	0.152303587	0.19586757	0.844713821	0.871528253	positive
PRJNA729271	stage_E18_vs_E10	ordinary_muscle_development	ENSGALG00010025286	ITGA3	-0.391167315	0.177172663	-2.207831092	0.02725605	0.04013373	negative
PRJNA729271	stage_E18_vs_E10	ordinary_muscle_development	ENSGALG00010025289	KDM2B	-0.906722813	0.074989236	-12.09137286	1.1731E-33	1.68024E-32	negative
PRJNA729271	stage_E18_vs_E10	ordinary_muscle_development	ENSGALG00010025291	DUS2	-1.553856908	0.103068225	-15.07600341	2.3295E-51	7.15383E-50	negative
PRJNA729271	stage_E18_vs_E10	ordinary_muscle_development	ENSGALG00010025292	ATP1A1	-0.061093752	0.127665365	-0.47854602	0.632261628	0.681993771	negative
PRJNA729271	stage_E18_vs_E10	ordinary_muscle_development	ENSGALG00010025297	SPSB4	-1.113858841	0.148331104	-7.509273591	5.94564E-14	2.78186E-13	negative
PRJNA729271	stage_E18_vs_E10	ordinary_muscle_development	ENSGALG00010025298	ENSGALG00010025298	-3.989700329	0.343231062	-11.62394892	3.11402E-31	4.04823E-30	negative
PRJNA729271	stage_E18_vs_E10	ordinary_muscle_development	ENSGALG00010025301	MORN3	2.533350886	0.228047249	11.10888597	1.13567E-28	1.30131E-27	positive
PRJNA729271	stage_E18_vs_E10	ordinary_muscle_development	ENSGALG00010025303	TMCC2	1.95968181	0.18867456	10.38657154	2.85429E-25	2.74357E-24	positive
PRJNA729271	stage_E18_vs_E10	ordinary_muscle_development	ENSGALG00010025304	DDX28	-0.284743373	0.094867863	-3.001473476	0.002686764	0.004632408	negative
PRJNA729271	stage_E18_vs_E10	ordinary_muscle_development	ENSGALG00010025305	GPR182	3.387448734	0.39058214	8.672820362	4.21547E-18	2.65666E-17	positive
PRJNA729271	stage_E18_vs_E10	ordinary_muscle_development	ENSGALG00010025311	ENSGALG00010025311	2.443134273	0.563068176	4.33896707	1.43154E-05	3.27807E-05	positive
PRJNA729271	stage_E18_vs_E10	ordinary_muscle_development	ENSGALG00010025312	PLEKHM3	0.185756507	0.114794943	1.618159321	0.105628267	0.13948089	positive
PRJNA729271	stage_E18_vs_E10	ordinary_muscle_development	ENSGALG00010025313	DIO1	0.138410414	0.870227311	0.159050873	0.873628793	0.895815602	positive
PRJNA729271	stage_E18_vs_E10	ordinary_muscle_development	ENSGALG00010025315	LRRC42	-0.126899549	0.12089081	-1.049703846	0.293854295	0.350454008	negative
PRJNA729271	stage_E18_vs_E10	ordinary_muscle_development	ENSGALG00010025316	SLC25A36	-0.494170192	0.110594125	-4.468322285	7.88355E-06	1.85567E-05	negative
PRJNA729271	stage_E18_vs_E10	ordinary_muscle_development	ENSGALG00010025318	NUAK2	-2.10920312	0.456615694	-4.619208557	3.85207E-06	9.32927E-06	negative
PRJNA729271	stage_E18_vs_E10	ordinary_muscle_development	ENSGALG00010025319	LDLRAD1	-0.456792652	0.375042756	-1.217974871	0.223233522	0.274241029	negative
PRJNA729271	stage_E18_vs_E10	ordinary_muscle_development	ENSGALG00010025320	JAM3	-1.211773797	0.14470305	-8.374210482	5.55953E-17	3.2481E-16	negative
PRJNA729271	stage_E18_vs_E10	ordinary_muscle_development	ENSGALG00010025323	KLHDC8A	0.968484405	0.166814204	5.805767023	6.4072E-09	2.01765E-08	positive
PRJNA729271	stage_E18_vs_E10	ordinary_muscle_development	ENSGALG00010025325	DPEP2	-0.083399347	0.251517281	-0.331584959	0.740202678	0.781103511	negative
PRJNA729271	stage_E18_vs_E10	ordinary_muscle_development	ENSGALG00010025327	TMEM59	0.328783425	0.071876418	4.574287827	4.77843E-06	1.14826E-05	positive
PRJNA729271	stage_E18_vs_E10	ordinary_muscle_development	ENSGALG00010025329	MYO1A	0.718252393	0.20311689	3.536152972	0.000406	0.000781121	positive
PRJNA729271	stage_E18_vs_E10	ordinary_muscle_development	ENSGALG00010025330	USH1C	-1.328539755	0.377296968	-3.521204425	0.000429591	0.000824431	negative
PRJNA729271	stage_E18_vs_E10	ordinary_muscle_development	ENSGALG00010025331	ENSGALG00010025331	0.447151318	0.361146	1.238145564	0.215662092	0.266203016	positive
PRJNA729271	stage_E18_vs_E10	ordinary_muscle_development	ENSGALG00010025333	FZD5	-0.131184392	0.207277743	-0.632891841	0.526804276	0.582991906	negative
PRJNA729271	stage_E18_vs_E10	ordinary_muscle_development	ENSGALG00010025334	RHOF	0.403427972	0.233533417	1.727495693	0.084078678	0.113137805	positive
PRJNA729271	stage_E18_vs_E10	ordinary_muscle_development	ENSGALG00010025336	ADAM10	-0.393638836	0.063711076	-6.178499242	6.47138E-10	2.22756E-09	negative
PRJNA729271	stage_E18_vs_E10	ordinary_muscle_development	ENSGALG00010025337	NEMP1	-0.524330153	0.15442635	-3.395341227	0.000685431	0.001278513	negative
PRJNA729271	stage_E18_vs_E10	ordinary_muscle_development	ENSGALG00010025338	FADD	-0.579162421	0.09632114	-6.012827755	1.82315E-09	6.01423E-09	negative
PRJNA729271	stage_E18_vs_E10	ordinary_muscle_development	ENSGALG00010025340	METTL21A	0.035874687	0.12032437	0.2981498	0.765588834	0.80333069	positive
PRJNA729271	stage_E18_vs_E10	ordinary_muscle_development	ENSGALG00010025341	VPS26B	-1.28857352	0.132591698	-9.718357459	2.51811E-22	2.04596E-21	negative
PRJNA729271	stage_E18_vs_E10	ordinary_muscle_development	ENSGALG00010025342	TCEANC2	-1.072151509	0.123495276	-8.681720851	3.89825E-18	2.4664E-17	negative
PRJNA729271	stage_E18_vs_E10	ordinary_muscle_development	ENSGALG00010025343	NAB2	0.363672643	0.30128927	1.207054745	0.227411062	0.278876763	positive
PRJNA729271	stage_E18_vs_E10	ordinary_muscle_development	ENSGALG00010025344	ENSGALG00010025344	-0.251935831	0.546041543	-0.461385832	0.644521815	0.693515233	negative
PRJNA729271	stage_E18_vs_E10	ordinary_muscle_development	ENSGALG00010025345	CCM2	-0.398924161	0.090344263	-4.415600355	1.0073E-05	2.34476E-05	negative
PRJNA729271	stage_E18_vs_E10	ordinary_muscle_development	ENSGALG00010025346	PXYLP1	-2.56463894	0.13076266	-19.61293038	1.19919E-85	9.82053E-84	negative
PRJNA729271	stage_E18_vs_E10	ordinary_muscle_development	ENSGALG00010025348	HIPK1	0.006285079	0.093836797	0.066978836	0.946598552	0.955963632	positive
PRJNA729271	stage_E18_vs_E10	ordinary_muscle_development	ENSGALG00010025349	SETD1A	0.008760207	0.127392718	0.06876537	0.945176384	0.955055686	positive
PRJNA729271	stage_E18_vs_E10	ordinary_muscle_development	ENSGALG00010025351	CREB1	-0.516556793	0.067446007	-7.658819478	1.8765E-14	9.14588E-14	negative
PRJNA729271	stage_E18_vs_E10	ordinary_muscle_development	ENSGALG00010025353	SLC12A4	-0.603021835	0.078266857	-7.704689588	1.31162E-14	6.48649E-14	negative
PRJNA729271	stage_E18_vs_E10	ordinary_muscle_development	ENSGALG00010025354	ENSGALG00010025354	0.18879254	1.52031273	0.124180069	0.901172703	0.918082212	positive
PRJNA729271	stage_E18_vs_E10	ordinary_muscle_development	ENSGALG00010025355	STAT6	0.878587897	0.141292608	6.218215598	5.0284E-10	1.74704E-09	positive
PRJNA729271	stage_E18_vs_E10	ordinary_muscle_development	ENSGALG00010025357	OLFML3	0.289595954	0.120281632	2.407649034	0.016055608	0.024661019	positive
PRJNA729271	stage_E18_vs_E10	ordinary_muscle_development	ENSGALG00010025358	INCENP	-1.43820199	0.204354367	-7.037784475	1.9532E-12	8.1637E-12	negative
PRJNA729271	stage_E18_vs_E10	ordinary_muscle_development	ENSGALG00010025359	CDCP2	1.083396614	0.386949463	2.799840077	0.005112793	0.008465837	positive
PRJNA729271	stage_E18_vs_E10	ordinary_muscle_development	ENSGALG00010025360	THYN1	0.387761392	0.111450475	3.479226016	0.000502864	0.00095506	positive
PRJNA729271	stage_E18_vs_E10	ordinary_muscle_development	ENSGALG00010025362	G6PC2	-0.052280723	0.239308748	-0.218465573	0.827066379	0.856247923	negative
PRJNA729271	stage_E18_vs_E10	ordinary_muscle_development	ENSGALG00010025364	EPHA4	-2.624190325	0.183409299	-14.30783683	1.955E-46	4.95415E-45	negative
PRJNA729271	stage_E18_vs_E10	ordinary_muscle_development	ENSGALG00010025365	SIKE1	-0.768754023	0.09246622	-8.313890433	9.26147E-17	5.3293E-16	negative
PRJNA729271	stage_E18_vs_E10	ordinary_muscle_development	ENSGALG00010025368	LRP1	1.00811389	0.13876319	7.264995048	3.73052E-13	1.6496E-12	positive
PRJNA729271	stage_E18_vs_E10	ordinary_muscle_development	ENSGALG00010025370	HPD	4.61221958	0.487869051	9.453806439	3.26728E-21	2.48866E-20	positive
PRJNA729271	stage_E18_vs_E10	ordinary_muscle_development	ENSGALG00010025373	CYB5RL	-0.034276015	0.088751697	-0.386201234	0.69934764	0.744276362	negative
PRJNA729271	stage_E18_vs_E10	ordinary_muscle_development	ENSGALG00010025377	MYO1G	0.81410982	0.303768619	2.680032659	0.007361498	0.01187061	positive
PRJNA729271	stage_E18_vs_E10	ordinary_muscle_development	ENSGALG00010025378	PAX3	-1.333801139	0.262584366	-5.079514672	3.784E-07	1.01893E-06	negative
PRJNA729271	stage_E18_vs_E10	ordinary_muscle_development	ENSGALG00010025379	ACAD8	1.620080209	0.18548454	8.734313975	2.45139E-18	1.56948E-17	positive
PRJNA729271	stage_E18_vs_E10	ordinary_muscle_development	ENSGALG00010025380	SPC25	-1.738897209	0.225125413	-7.724126686	1.12623E-14	5.60023E-14	negative
PRJNA729271	stage_E18_vs_E10	ordinary_muscle_development	ENSGALG00010025381	MRPL37	0.69745892	0.122550806	5.691181821	1.26163E-08	3.86135E-08	positive
PRJNA729271	stage_E18_vs_E10	ordinary_muscle_development	ENSGALG00010025382	MAB21L3	0.094197613	0.301164601	0.312777838	0.754449454	0.793998754	positive
PRJNA729271	stage_E18_vs_E10	ordinary_muscle_development	ENSGALG00010025386	TPCN2	-0.758071182	0.100405119	-7.550124841	4.34841E-14	2.05243E-13	negative
PRJNA729271	stage_E18_vs_E10	ordinary_muscle_development	ENSGALG00010025387	PSMD9	0.043899793	0.069079693	0.635494895	0.525105695	0.581467307	positive
PRJNA729271	stage_E18_vs_E10	ordinary_muscle_development	ENSGALG00010025389	SNED1	-0.041073422	0.14782042	-0.277860271	0.781119622	0.816494478	negative
PRJNA729271	stage_E18_vs_E10	ordinary_muscle_development	ENSGALG00010025390	BCL7A	-0.181257371	0.086634349	-2.092211366	0.036419612	0.052377722	negative
PRJNA729271	stage_E18_vs_E10	ordinary_muscle_development	ENSGALG00010025392	SLC6A2	-0.266431106	0.242178732	-1.10014246	0.271270056	0.326420344	negative
PRJNA729271	stage_E18_vs_E10	ordinary_muscle_development	ENSGALG00010025398	TSPAN2	-0.565464462	0.147409934	-3.835999702	0.000125055	0.000257737	negative
PRJNA729271	stage_E18_vs_E10	ordinary_muscle_development	ENSGALG00010025400	SLC22A15	-0.138588631	0.154538371	-0.896791067	0.369830415	0.429473792	negative
PRJNA729271	stage_E18_vs_E10	ordinary_muscle_development	ENSGALG00010025403	ENSGALG00010025403	0.402918206	0.129650884	3.107716623	0.001885388	0.003322637	positive
PRJNA729271	stage_E18_vs_E10	ordinary_muscle_development	ENSGALG00010025404	CERS6	-0.854728261	0.280467722	-3.04751026	0.002307456	0.004010634	negative
PRJNA729271	stage_E18_vs_E10	ordinary_muscle_development	ENSGALG00010025405	TAMALIN	1.844007885	0.164380833	11.21790081	3.33083E-29	3.89987E-28	positive
PRJNA729271	stage_E18_vs_E10	ordinary_muscle_development	ENSGALG00010025406	ENSGALG00010025406	0.215189465	0.148453357	1.449542603	0.147186112	0.188695432	positive
PRJNA729271	stage_E18_vs_E10	ordinary_muscle_development	ENSGALG00010025407	MLXIP	2.361402914	0.127708949	18.49050464	2.46239E-76	1.54513E-74	positive
PRJNA729271	stage_E18_vs_E10	ordinary_muscle_development	ENSGALG00010025413	NECAB3	0.921805585	0.121630136	7.578759797	3.48874E-14	1.65855E-13	positive
PRJNA729271	stage_E18_vs_E10	ordinary_muscle_development	ENSGALG00010025414	MMP2	0.414636734	0.221383242	1.872936405	0.061077167	0.084379865	positive
PRJNA729271	stage_E18_vs_E10	ordinary_muscle_development	ENSGALG00010025415	ENSGALG00010025415	1.834007525	0.295657306	6.203153078	5.5343E-10	1.91775E-09	positive
PRJNA729271	stage_E18_vs_E10	ordinary_muscle_development	ENSGALG00010025416	ENSGALG00010025416	0.042208979	0.694048592	0.060815596	0.951506069	0.960116984	positive
PRJNA729271	stage_E18_vs_E10	ordinary_muscle_development	ENSGALG00010025417	B3GAT1	-1.134964032	0.190011725	-5.973126299	2.3275E-09	7.607E-09	negative
PRJNA729271	stage_E18_vs_E10	ordinary_muscle_development	ENSGALG00010025418	CASQ2	3.577862819	0.209430599	17.08376347	1.96055E-65	9.22669E-64	positive
PRJNA729271	stage_E18_vs_E10	ordinary_muscle_development	ENSGALG00010025419	CCND1	-0.276907183	0.134442294	-2.059673149	0.039429798	0.056392198	negative
PRJNA729271	stage_E18_vs_E10	ordinary_muscle_development	ENSGALG00010025420	STK39	-0.650890938	0.206788502	-3.147616682	0.001646074	0.002923642	negative
PRJNA729271	stage_E18_vs_E10	ordinary_muscle_development	ENSGALG00010025421	NR4A1	4.860457418	0.400930339	12.1229474	7.98354E-34	1.1607E-32	positive
PRJNA729271	stage_E18_vs_E10	ordinary_muscle_development	ENSGALG00010025422	SSBP3	-0.912486934	0.157734956	-5.784937952	7.25391E-09	2.26753E-08	negative
PRJNA729271	stage_E18_vs_E10	ordinary_muscle_development	ENSGALG00010025423	NGF	0.364486044	0.333490889	1.092941536	0.27441947	0.329812363	positive
PRJNA729271	stage_E18_vs_E10	ordinary_muscle_development	ENSGALG00010025424	IRX6	-3.354688845	0.40829886	-8.216258173	2.09951E-16	1.17773E-15	negative
PRJNA729271	stage_E18_vs_E10	ordinary_muscle_development	ENSGALG00010025425	ENSGALG00010025425	0.462425269	0.139218518	3.321578735	0.000895097	0.00164171	positive
PRJNA729271	stage_E18_vs_E10	ordinary_muscle_development	ENSGALG00010025426	ENSGALG00010025426	1.790068604	0.152816738	11.7138255	1.08281E-31	1.43599E-30	positive
PRJNA729271	stage_E18_vs_E10	ordinary_muscle_development	ENSGALG00010025427	SLC35G2	-0.434121386	0.13260091	-3.273894471	0.001060762	0.001926061	negative
PRJNA729271	stage_E18_vs_E10	ordinary_muscle_development	ENSGALG00010025428	VANGL1	-0.364605702	0.11258299	-3.238550528	0.001201387	0.002167012	negative
PRJNA729271	stage_E18_vs_E10	ordinary_muscle_development	ENSGALG00010025429	SCN3B	-2.37253959	0.226884731	-10.45702624	1.36059E-25	1.33616E-24	negative
PRJNA729271	stage_E18_vs_E10	ordinary_muscle_development	ENSGALG00010025430	IRX5	-4.125914708	0.870542193	-4.739477006	2.14271E-06	5.33377E-06	negative
PRJNA729271	stage_E18_vs_E10	ordinary_muscle_development	ENSGALG00010025432	IRX3	-3.714917176	0.685243093	-5.421312835	5.91629E-08	1.7067E-07	negative
PRJNA729271	stage_E18_vs_E10	ordinary_muscle_development	ENSGALG00010025433	CHPF2	0.348659208	0.122012603	2.857567156	0.004269023	0.007142138	positive
PRJNA729271	stage_E18_vs_E10	ordinary_muscle_development	ENSGALG00010025434	SYT6	0.949545249	0.234983029	4.040909912	5.32442E-05	0.000114337	positive
PRJNA729271	stage_E18_vs_E10	ordinary_muscle_development	ENSGALG00010025435	SCLY	0.039930544	0.099989686	0.399346628	0.689637814	0.735292396	positive
PRJNA729271	stage_E18_vs_E10	ordinary_muscle_development	ENSGALG00010025438	EXOSC6	0.011803781	0.12244305	0.096402212	0.923201136	0.936838453	positive
PRJNA729271	stage_E18_vs_E10	ordinary_muscle_development	ENSGALG00010025442	ABCF2	-1.02892975	0.148425246	-6.932309568	4.14025E-12	1.69098E-11	negative
PRJNA729271	stage_E18_vs_E10	ordinary_muscle_development	ENSGALG00010025445	B3GNT4	1.767628735	0.298283283	5.926006705	3.1039E-09	1.00337E-08	positive
PRJNA729271	stage_E18_vs_E10	ordinary_muscle_development	ENSGALG00010025448	CHD1L	-0.478949087	0.110204444	-4.346005205	1.38639E-05	3.18072E-05	negative
PRJNA729271	stage_E18_vs_E10	ordinary_muscle_development	ENSGALG00010025449	ENSGALG00010025449	0.451415113	0.116683019	3.868730149	0.000109404	0.000227258	positive
PRJNA729271	stage_E18_vs_E10	ordinary_muscle_development	ENSGALG00010025451	LRRC43	2.550720807	0.312195187	8.170275872	3.07685E-16	1.70616E-15	positive
PRJNA729271	stage_E18_vs_E10	ordinary_muscle_development	ENSGALG00010025452	TRIM33	-0.09486622	0.126635051	-0.749130823	0.453778359	0.513467393	negative
PRJNA729271	stage_E18_vs_E10	ordinary_muscle_development	ENSGALG00010025458	BCAS2	0.354906202	0.11569203	3.067680654	0.00215727	0.00377106	positive
PRJNA729271	stage_E18_vs_E10	ordinary_muscle_development	ENSGALG00010025459	DIABLO	-0.423344297	0.079706308	-5.311302324	1.08845E-07	3.07784E-07	negative
PRJNA729271	stage_E18_vs_E10	ordinary_muscle_development	ENSGALG00010025464	VPS33A	-0.9071422	0.094717528	-9.577342404	9.95741E-22	7.84843E-21	negative
PRJNA729271	stage_E18_vs_E10	ordinary_muscle_development	ENSGALG00010025465	ENSGALG00010025465	-0.351604784	0.084367229	-4.167551623	3.07889E-05	6.77935E-05	negative
PRJNA729271	stage_E18_vs_E10	ordinary_muscle_development	ENSGALG00010025470	ORAOV1	-0.314181542	0.125296639	-2.507501759	0.012158798	0.018997502	negative
PRJNA729271	stage_E18_vs_E10	ordinary_muscle_development	ENSGALG00010025472	ZCCHC8	0.250595924	0.097822949	2.56172939	0.010415244	0.016438257	positive
PRJNA729271	stage_E18_vs_E10	ordinary_muscle_development	ENSGALG00010025473	PARS2	0.280750559	0.079996539	3.509533796	0.000448893	0.000859311	positive
PRJNA729271	stage_E18_vs_E10	ordinary_muscle_development	ENSGALG00010025477	ENSGALG00010025477	-0.00483242	0.070945038	-0.068114981	0.945694105	0.955272409	negative
PRJNA729271	stage_E18_vs_E10	ordinary_muscle_development	ENSGALG00010025480	FGF19	-0.170089247	0.376981387	-0.451187388	0.651854498	0.699950437	negative
PRJNA729271	stage_E18_vs_E10	ordinary_muscle_development	ENSGALG00010025481	TMEM39A	0.168952452	0.089910399	1.879120252	0.060228075	0.083310044	positive
PRJNA729271	stage_E18_vs_E10	ordinary_muscle_development	ENSGALG00010025483	FGF4	-0.53091193	0.362009644	-1.466568471	0.142493482	0.183317922	negative
PRJNA729271	stage_E18_vs_E10	ordinary_muscle_development	ENSGALG00010025485	DENND2C	1.804575988	0.159450958	11.31743587	1.07572E-29	1.29614E-28	positive
PRJNA729271	stage_E18_vs_E10	ordinary_muscle_development	ENSGALG00010025486	B3GALT1	-1.051690645	0.18991828	-5.537595675	3.06652E-08	9.10845E-08	negative
PRJNA729271	stage_E18_vs_E10	ordinary_muscle_development	ENSGALG00010025487	FGF3	-4.199309656	0.78214998	-5.368931486	7.92045E-08	2.26354E-07	negative
PRJNA729271	stage_E18_vs_E10	ordinary_muscle_development	ENSGALG00010025488	SLC4A8	-1.017490281	0.180252493	-5.644805603	1.65368E-08	5.01257E-08	negative
PRJNA729271	stage_E18_vs_E10	ordinary_muscle_development	ENSGALG00010025491	ZMYND11	-0.023279058	0.064060786	-0.363390136	0.716313469	0.759599366	negative
PRJNA729271	stage_E18_vs_E10	ordinary_muscle_development	ENSGALG00010025496	ATG101	0.319612809	0.112264551	2.846961109	0.004413875	0.007369169	positive
PRJNA729271	stage_E18_vs_E10	ordinary_muscle_development	ENSGALG00010025499	STAC3	4.137241162	0.158929924	26.03185763	2.1593E-149	6.8042E-147	positive
PRJNA729271	stage_E18_vs_E10	ordinary_muscle_development	ENSGALG00010025504	AMPD1	3.552247923	0.197998027	17.94082483	5.66086E-72	3.13184E-70	positive
PRJNA729271	stage_E18_vs_E10	ordinary_muscle_development	ENSGALG00010025505	ENSGALG00010025505	-1.068207036	0.265185677	-4.028147555	5.62181E-05	0.000120491	negative
PRJNA729271	stage_E18_vs_E10	ordinary_muscle_development	ENSGALG00010025506	R3HDM2	0.417577572	0.179211171	2.330086741	0.019801567	0.029873409	positive
PRJNA729271	stage_E18_vs_E10	ordinary_muscle_development	ENSGALG00010025508	ENSGALG00010025508	0.443702211	0.13257099	3.346902755	0.000817199	0.001508955	positive

PRJNA729271	stage_E18_vs_E10	ordinary_muscle_development	ENSGALG00010025512	NRAS	-0.34423609	0.07839461	-4.39106836	1.12795E-05	2.61129E-05	negative
PRJNA729271	stage_E18_vs_E10	ordinary_muscle_development	ENSGALG00010025513	RSRC2	-0.247295381	0.088336334	-2.799475256	0.005118574	0.008474441	negative
PRJNA729271	stage_E18_vs_E10	ordinary_muscle_development	ENSGALG00010025520	ACOT11	0.347492901	0.297149431	1.169421391	0.242233895	0.29525622	positive
PRJNA729271	stage_E18_vs_E10	ordinary_muscle_development	ENSGALG00010025522	SUMO3	-0.075152698	0.105265273	-0.713936283	0.47526658	0.533920912	negative
PRJNA729271	stage_E18_vs_E10	ordinary_muscle_development	ENSGALG00010025523	B4GALT4	-0.813365892	0.132310764	-6.147390198	7.87682E-10	2.68646E-09	negative
PRJNA729271	stage_E18_vs_E10	ordinary_muscle_development	ENSGALG00010025527	KNTC1	-1.709836675	0.239333275	-7.144166109	9.05439E-13	3.90257E-12	negative
PRJNA729271	stage_E18_vs_E10	ordinary_muscle_development	ENSGALG00010025531	ENSGALG00010025531	-0.141020653	0.098097813	-1.437551451	0.150561364	0.192671226	negative
PRJNA729271	stage_E18_vs_E10	ordinary_muscle_development	ENSGALG00010025533	INHBC	-0.088777025	0.592360662	-0.149869886	0.880867271	0.901388711	negative
PRJNA729271	stage_E18_vs_E10	ordinary_muscle_development	ENSGALG00010025538	ANO1	4.144103361	0.208634623	19.86297054	8.51238E-88	7.43295E-86	positive
PRJNA729271	stage_E18_vs_E10	ordinary_muscle_development	ENSGALG00010025541	TTC21B	0.083208825	0.127696845	0.651612221	0.514651362	0.571418728	positive
PRJNA729271	stage_E18_vs_E10	ordinary_muscle_development	ENSGALG00010025544	CSDE1	-0.416198952	0.086112793	-4.833183753	1.34367E-06	3.42714E-06	negative
PRJNA729271	stage_E18_vs_E10	ordinary_muscle_development	ENSGALG00010025548	ENSGALG00010025548	-0.45998811	0.197056649	-2.33429378	0.019580344	0.029570441	negative
PRJNA729271	stage_E18_vs_E10	ordinary_muscle_development	ENSGALG00010025549	DHCR24	-3.06837399	0.254718658	-12.04612967	2.03272E-33	2.87177E-32	negative
PRJNA729271	stage_E18_vs_E10	ordinary_muscle_development	ENSGALG00010025553	UBE2G2	0.126933749	0.096446233	1.316108935	0.188137483	0.235374832	positive
PRJNA729271	stage_E18_vs_E10	ordinary_muscle_development	ENSGALG00010025554	DIP2C	0.469516592	0.070596569	6.65069987	2.91703E-11	1.10571E-10	positive
PRJNA729271	stage_E18_vs_E10	ordinary_muscle_development	ENSGALG00010025557	VPS29	-0.728183198	0.078581274	-9.266625037	1.92129E-20	1.39525E-19	negative
PRJNA729271	stage_E18_vs_E10	ordinary_muscle_development	ENSGALG00010025558	GALNT3	-2.321245854	0.233016195	-9.961736168	2.24114E-23	1.91428E-22	negative
PRJNA729271	stage_E18_vs_E10	ordinary_muscle_development	ENSGALG00010025559	SLC6A17	-5.028324005	1.199739244	-4.191180733	2.77506E-05	6.15055E-05	negative
PRJNA729271	stage_E18_vs_E10	ordinary_muscle_development	ENSGALG00010025562	GRAMD1B	1.268345979	0.182284765	6.958047089	3.45022E-12	1.41674E-11	positive
PRJNA729271	stage_E18_vs_E10	ordinary_muscle_development	ENSGALG00010025563	TSPEAR	-0.158424499	0.242691983	-0.652780106	0.51389805	0.570626034	negative
PRJNA729271	stage_E18_vs_E10	ordinary_muscle_development	ENSGALG00010025565	ENSGALG00010025565	0.842362723	0.247633916	3.401645199	0.000669815	0.001251318	positive
PRJNA729271	stage_E18_vs_E10	ordinary_muscle_development	ENSGALG00010025568	MYL3	-1.456413717	0.961099055	-1.51536276	0.129680563	0.168313015	negative
PRJNA729271	stage_E18_vs_E10	ordinary_muscle_development	ENSGALG00010025569	PPTC7	-0.003825677	0.193858686	-0.019734358	0.984255283	0.986637643	negative
PRJNA729271	stage_E18_vs_E10	ordinary_muscle_development	ENSGALG00010025571	SRSF3	-0.94381254	0.229162469	-4.118530159	3.81297E-05	8.31487E-05	negative
PRJNA729271	stage_E18_vs_E10	ordinary_muscle_development	ENSGALG00010025572	ENSGALG00010025572	-0.12338634	0.112314172	-1.098582117	0.271950382	0.32710321	negative
PRJNA729271	stage_E18_vs_E10	ordinary_muscle_development	ENSGALG00010025573	CLMP	-0.838822579	0.168212593	-4.986681208	6.14253E-07	1.61942E-06	negative
PRJNA729271	stage_E18_vs_E10	ordinary_muscle_development	ENSGALG00010025574	ARHGAP31	0.006223186	0.099258475	0.062696768	0.950007971	0.958738811	positive
PRJNA729271	stage_E18_vs_E10	ordinary_muscle_development	ENSGALG00010025575	TRPM2	-0.800741337	0.476537743	-1.680331409	0.092892853	0.123962613	negative
PRJNA729271	stage_E18_vs_E10	ordinary_muscle_development	ENSGALG00010025577	PTH2R	-1.849789067	0.190045186	-9.733417128	2.17172E-22	1.77148E-21	negative
PRJNA729271	stage_E18_vs_E10	ordinary_muscle_development	ENSGALG00010025578	ORMDL2	-0.182126266	0.150015614	-1.214048728	0.224729117	0.275914667	negative
PRJNA729271	stage_E18_vs_E10	ordinary_muscle_development	ENSGALG00010025579	CSRNP3	-1.212127331	0.228095503	-5.31412199	1.07173E-07	3.03175E-07	negative
PRJNA729271	stage_E18_vs_E10	ordinary_muscle_development	ENSGALG00010025580	RASSF7	2.094033694	0.138204591	15.15169415	7.38384E-52	2.29675E-50	positive
PRJNA729271	stage_E18_vs_E10	ordinary_muscle_development	ENSGALG00010025582	DMAP1	-0.433547374	0.11497909	-3.770662777	0.000162815	0.000330195	negative
PRJNA729271	stage_E18_vs_E10	ordinary_muscle_development	ENSGALG00010025585	SARNP	-0.876348168	0.096952881	-9.038907946	1.58245E-19	1.08555E-18	negative
PRJNA729271	stage_E18_vs_E10	ordinary_muscle_development	ENSGALG00010025586	TCTN1	0.655129814	0.125468094	5.221485328	1.77494E-07	4.91268E-07	positive
PRJNA729271	stage_E18_vs_E10	ordinary_muscle_development	ENSGALG00010025587	LRRC56	-0.386553675	0.213955366	-1.806702405	0.070808692	0.096800151	negative
PRJNA729271	stage_E18_vs_E10	ordinary_muscle_development	ENSGALG00010025588	ENSGALG00010025588	-0.32062507	0.311143933	-1.030471867	0.302788552	0.359895044	negative
PRJNA729271	stage_E18_vs_E10	ordinary_muscle_development	ENSGALG00010025590	HRAS	2.102079984	0.113355392	18.54415524	9.09179E-77	5.80553E-75	positive
PRJNA729271	stage_E18_vs_E10	ordinary_muscle_development	ENSGALG00010025591	OTOS	1.610741532	0.340633508	4.728664375	2.26002E-06	5.61806E-06	positive
PRJNA729271	stage_E18_vs_E10	ordinary_muscle_development	ENSGALG00010025592	HSPA8	-2.065663649	0.208384077	-9.91277108	3.66342E-23	3.09809E-22	negative
PRJNA729271	stage_E18_vs_E10	ordinary_muscle_development	ENSGALG00010025594	UPK1B	-2.34759265	0.426683129	-5.501957986	3.75596E-08	1.10746E-07	negative
PRJNA729271	stage_E18_vs_E10	ordinary_muscle_development	ENSGALG00010025595	ERI3	-0.05711732	0.093952607	-0.607937575	0.543228877	0.598199694	negative
PRJNA729271	stage_E18_vs_E10	ordinary_muscle_development	ENSGALG00010025597	SCN2A	-4.254550348	0.669411637	-6.355656388	2.07538E-10	7.38588E-10	negative
PRJNA729271	stage_E18_vs_E10	ordinary_muscle_development	ENSGALG00010025598	ENSGALG00010025598	1.542740846	0.429365242	3.593073434	0.0003268	0.000636433	positive
PRJNA729271	stage_E18_vs_E10	ordinary_muscle_development	ENSGALG00010025599	COPS9	0.724352474	0.143374621	5.052166604	4.36826E-07	1.16801E-06	positive
PRJNA729271	stage_E18_vs_E10	ordinary_muscle_development	ENSGALG00010025600	ENSGALG00010025600	-0.937368148	0.11200774	-8.368780157	5.82177E-17	3.39858E-16	negative
PRJNA729271	stage_E18_vs_E10	ordinary_muscle_development	ENSGALG00010025604	HVCN1	0.392754745	0.133111834	2.950562188	0.003171962	0.005412949	positive
PRJNA729271	stage_E18_vs_E10	ordinary_muscle_development	ENSGALG00010025606	CCDC12	-0.682905033	0.132456021	-5.155711524	2.5267E-07	6.90637E-07	negative
PRJNA729271	stage_E18_vs_E10	ordinary_muscle_development	ENSGALG00010025608	ENSGALG00010025608	-1.996252213	0.541583046	-3.685957728	0.000227844	0.000453382	negative
PRJNA729271	stage_E18_vs_E10	ordinary_muscle_development	ENSGALG00010025610	RNH1	-1.142867873	0.134376201	-8.50498723	1.81616E-17	1.0946E-16	negative
PRJNA729271	stage_E18_vs_E10	ordinary_muscle_development	ENSGALG00010025612	TMEM53	-0.422361543	0.156730726	-2.694822854	0.007042606	0.011383076	negative
PRJNA729271	stage_E18_vs_E10	ordinary_muscle_development	ENSGALG00010025613	CDKN1A	1.33429915	0.277352094	4.810849386	1.5029E-06	3.81316E-06	positive
PRJNA729271	stage_E18_vs_E10	ordinary_muscle_development	ENSGALG00010025618	ADIPOQ	6.87208573	0.221995605	30.95595397	2.1126E-210	1.3919E-207	positive
PRJNA729271	stage_E18_vs_E10	ordinary_muscle_development	ENSGALG00010025621	PPP1CC	-0.500856625	0.117787477	-4.25220609	2.11675E-05	4.75694E-05	negative
PRJNA729271	stage_E18_vs_E10	ordinary_muscle_development	ENSGALG00010025623	ENSGALG00010025623	0.138022351	0.435632393	0.316832158	0.751370962	0.791218459	positive
PRJNA729271	stage_E18_vs_E10	ordinary_muscle_development	ENSGALG00010025624	ARMH1	0.636219164	0.368110698	1.728336524	0.083927908	0.112966388	positive
PRJNA729271	stage_E18_vs_E10	ordinary_muscle_development	ENSGALG00010025628	ENSGALG00010025628	-0.867920168	0.267717023	-3.241931187	0.001187227	0.00214227	negative
PRJNA729271	stage_E18_vs_E10	ordinary_muscle_development	ENSGALG00010025629	ST6GAL1	3.105316871	0.143463198	21.64538996	6.7173E-104	8.8516E-102	positive
PRJNA729271	stage_E18_vs_E10	ordinary_muscle_development	ENSGALG00010025635	CHKA	-1.04104131	0.164493389	-6.328772933	2.47118E-10	8.75148E-10	negative
PRJNA729271	stage_E18_vs_E10	ordinary_muscle_development	ENSGALG00010025637	C6orf89	0.756370179	0.127262745	5.943374695	2.79213E-09	9.07036E-09	positive
PRJNA729271	stage_E18_vs_E10	ordinary_muscle_development	ENSGALG00010025639	MYL2	-3.071503481	0.436257556	-7.040573716	1.9145E-12	8.01347E-12	negative
PRJNA729271	stage_E18_vs_E10	ordinary_muscle_development	ENSGALG00010025640	SLC38A11	-3.320791738	0.655520013	-5.065889178	4.06498E-07	1.09135E-06	negative
PRJNA729271	stage_E18_vs_E10	ordinary_muscle_development	ENSGALG00010025642	RPS8	0.609142947	0.117537356	5.182547624	2.18875E-07	6.00985E-07	positive
PRJNA729271	stage_E18_vs_E10	ordinary_muscle_development	ENSGALG00010025644	ENSGALG00010025644	3.880811352	0.28239918	13.74228975	5.66552E-43	1.21662E-41	positive
PRJNA729271	stage_E18_vs_E10	ordinary_muscle_development	ENSGALG00010025645	NBEAL2	0.133011603	0.19102208	0.696315334	0.486231372	0.544241215	positive
PRJNA729271	stage_E18_vs_E10	ordinary_muscle_development	ENSGALG00010025647	JHY	-2.041194394	0.3566088	-5.723903601	1.04104E-08	3.20992E-08	negative
PRJNA729271	stage_E18_vs_E10	ordinary_muscle_development	ENSGALG00010025648	ENSGALG00010025648	1.644188076	0.524669483	3.13375969	0.001725821	0.003058912	positive
PRJNA729271	stage_E18_vs_E10	ordinary_muscle_development	ENSGALG00010025650	PPP1R1A	2.850731914	0.460608825	6.189051877	6.05272E-10	2.08941E-09	positive
PRJNA729271	stage_E18_vs_E10	ordinary_muscle_development	ENSGALG00010025652	KMT5B	-0.016251388	0.076836319	-0.211506595	0.832491986	0.86094259	negative
PRJNA729271	stage_E18_vs_E10	ordinary_muscle_development	ENSGALG00010025653	IGSF11	2.77809122	0.19236717	14.44160778	2.83159E-47	7.48976E-46	positive
PRJNA729271	stage_E18_vs_E10	ordinary_muscle_development	ENSGALG00010025654	CRTAM	0.679473905	0.864567034	0.785912345	0.431918842	0.491842534	positive
PRJNA729271	stage_E18_vs_E10	ordinary_muscle_development	ENSGALG00010025658	PDE1B	-3.532190061	0.504255046	-7.004768894	2.47394E-12	1.02662E-11	negative
PRJNA729271	stage_E18_vs_E10	ordinary_muscle_development	ENSGALG00010025659	COBLL1	0.872001325	0.131082313	6.652318735	2.88511E-11	1.09476E-10	positive
PRJNA729271	stage_E18_vs_E10	ordinary_muscle_development	ENSGALG00010025660	KIF2C	-2.155066719	0.201100884	-10.7163463	8.5307E-27	8.96682E-26	negative
PRJNA729271	stage_E18_vs_E10	ordinary_muscle_development	ENSGALG00010025661	SNRPE	-0.735988125	0.130151098	-5.654874504	1.5596E-08	4.74228E-08	negative
PRJNA729271	stage_E18_vs_E10	ordinary_muscle_development	ENSGALG00010025664	GPR55	1.378664268	0.695428891	1.982466196	0.047427087	0.066847105	positive
PRJNA729271	stage_E18_vs_E10	ordinary_muscle_development	ENSGALG00010025666	CUX2	-2.534250475	0.354984862	-7.139038166	9.39862E-13	4.04613E-12	negative
PRJNA729271	stage_E18_vs_E10	ordinary_muscle_development	ENSGALG00010025674	GRB14	3.011462797	0.428971006	7.02020126	2.21549E-12	9.23067E-12	positive
PRJNA729271	stage_E18_vs_E10	ordinary_muscle_development	ENSGALG00010025679	ENSGALG00010025679	3.117268293	1.157010645	2.6942434	0.007054862	0.011400347	positive
PRJNA729271	stage_E18_vs_E10	ordinary_muscle_development	ENSGALG00010025685	NCKAP1L	1.069613242	0.196366484	5.447025466	5.12191E-08	1.48663E-07	positive
PRJNA729271	stage_E18_vs_E10	ordinary_muscle_development	ENSGALG00010025688	BEST4	1.538665741	0.523105934	2.94140372	0.003267284	0.005555993	positive
PRJNA729271	stage_E18_vs_E10	ordinary_muscle_development	ENSGALG00010025691	ENSGALG00010025691	-0.644231899	0.164367115	-3.919469522	8.87441E-05	0.000186049	negative
PRJNA729271	stage_E18_vs_E10	ordinary_muscle_development	ENSGALG00010025692	ENSGALG00010025692	0.792104699	1.243858583	0.636812504	0.52424698	0.580782633	positive
PRJNA729271	stage_E18_vs_E10	ordinary_muscle_development	ENSGALG00010025696	MTCH1	0.221832856	0.105219534	2.108285866	0.03500627	0.050504218	positive
PRJNA729271	stage_E18_vs_E10	ordinary_muscle_development	ENSGALG00010025701	ENSGALG00010025701	5.954419553	3.158527272	1.885188583	0.059404382	0.082304197	positive
PRJNA729271	stage_E18_vs_E10	ordinary_muscle_development	ENSGALG00010025702	GTSF2	0.594691156	0.173176206	3.434023472	0.000594693	0.001118328	positive
PRJNA729271	stage_E18_vs_E10	ordinary_muscle_development	ENSGALG00010025703	UBASH3B	-0.98378409	0.142420863	-6.907584111	4.92977E-12	1.99992E-11	negative
PRJNA729271	stage_E18_vs_E10	ordinary_muscle_development	ENSGALG00010025705	ACSL3	-1.128408968	0.085246802	-13.23696537	5.36741E-40	1.03184E-38	negative
PRJNA729271	stage_E18_vs_E10	ordinary_muscle_development	ENSGALG00010025706	ENSGALG00010025706	4.193575396	2.982594477	1.406015946	0.15971939	0.203099619	positive
PRJNA729271	stage_E18_vs_E10	ordinary_muscle_development	ENSGALG00010025708	HOXC8	-3.194423004	0.264715042	-12.06740267	1.57012E-33	2.23785E-32	negative
PRJNA729271	stage_E18_vs_E10	ordinary_muscle_development	ENSGALG00010025710	ENSGALG00010025710	2.142760369	2.019060992	1.061265795	0.288569126	0.344689697	positive
PRJNA729271	stage_E18_vs_E10	ordinary_muscle_development	ENSGALG00010025719	HOXC11	-6.732362078	2.679982774	-2.512091549	0.012001795	0.018768585	negative
PRJNA729271	stage_E18_vs_E10	ordinary_muscle_development	ENSGALG00010025720	PHETA1	-1.389155983	0.261121174	-5.319966828	1.03786E-07	2.93939E-07	negative
PRJNA729271	stage_E18_vs_E10	ordinary_muscle_development	ENSGALG00010025723	SETD2	0.17434353	0.122242574	1.426209577	0.153807856	0.196323078	positive
PRJNA729271	stage_E18_vs_E10	ordinary_muscle_development	ENSGALG00010025727	GLI1	-1.687529954	0.214166347	-7.879529062	3.28617E-15	1.70361E-14	negative
PRJNA729271	stage_E18_vs_E10	ordinary_muscle_development	ENSGALG00010025734	PLK3	0.027775742	0.185524156	0.149714961	0.880989504	0.901450153	positive
PRJNA729271	stage_E18_vs_E10	ordinary_muscle_development	ENSGALG00010025736	KIF9	1.770064327	0.320550876	5.521945065	3.35267E-08	9.93118E-08	positive
PRJNA729271	stage_E18_vs_E10	ordinary_muscle_development	ENSGALG00010025737	ENSGALG00010025737	1.364318634	0.149156057	9.146920763	5.85795E-20	4.10198E-19	positive
PRJNA729271	stage_E18_vs_E10	ordinary_muscle_development	ENSGALG00010025738	SOX13	0.933307674	0.151530478	6.159207594	7.31098E-10	2.50349E-09	positive
PRJNA729271	stage_E18_vs_E10	ordinary_muscle_development	ENSGALG00010025743	SH2B3	0.086974661	0.110841959	0.784672715	0.432645484	0.492476542	positive
PRJNA729271	stage_E18_vs_E10	ordinary_muscle_development	ENSGALG00010025745	COX14	0.926145672	0.229040595	4.043587428	5.26395E-05	0.000113106	positive
PRJNA729271	stage_E18_vs_E10	ordinary_muscle_development	ENSGALG00010025748	ETNK2	3.376136393	0.191752861	17.60670678	2.18811E-69	1.14089E-67	positive
PRJNA729271	stage_E18_vs_E10	ordinary_muscle_development	ENSGALG00010025753	SORL1	-0.424061053	0.171935029	-2.4664029	0.013647773	0.021187155	negative
PRJNA729271	stage_E18_vs_E10	ordinary_muscle_development	ENSGALG00010025755	ENSGALG00010025755	-0.209193343	0.273871253	-0.763838267	0.444963629	0.504438277	negative
PRJNA729271	stage_E18_vs_E10	ordinary_muscle_development	ENSGALG00010025782	ENSGALG00010025782	3.387452724	4.535523341	0.746871412	0.455141184	0.514768372	positive
PRJNA729271	stage_E18_vs_E10	ordinary_muscle_development	ENSGALG00010025784	ENSGALG00010025784	1.71979906	2.459378008	0.699282117	0.484375741	0.54241551	positive
PRJNA729271	stage_E18_vs_E10	ordinary_muscle_development	ENSGALG00010025787	KCNE4	3.02104606	0.289767254	10.42576764	1.89126E-25	1.84356E-24	positive
PRJNA729271	stage_E18_vs_E10	ordinary_muscle_development	ENSGALG00010025790	LRP5	0.327448365	0.077990481	4.198568333	2.68608E-05	5.9597E-05	positive
PRJNA729271	stage_E18_vs_E10	ordinary_muscle_development	ENSGALG00010025791	FIGN	0.272952136	0.237657661	1.148509729	0.250758199	0.304381169	positive
PRJNA729271	stage_E18_vs_E10	ordinary_muscle_development	ENSGALG00010025794	ENSGALG00010025794	4.52995371	4.031235332	1.123713536	0.261134572	0.31545509	positive
PRJNA729271	stage_E18_vs_E10	ordinary_muscle_development	ENSGALG00010025799	SC5D	-0.501190621	0.288406225	-1.737794048	0.08224711	0.11096164	negative
PRJNA729271	stage_E18_vs_E10	ordinary_muscle_development	ENSGALG00010025800	ARL2BP	-0.14641146	0.136340163	-1.0738689	0.282881444	0.338481635	negative
PRJNA729271	stage_E18_vs_E10	ordinary_muscle_development	ENSGALG00010025801	PTPN7	-1.275600811	0.456858609	-2.792112891	0.005236508	0.008657829	negative
PRJNA729271	stage_E18_vs_E10	ordinary_muscle_development	ENSGALG00010025804	ENSGALG00010025804	4.6917021	2.128929619	2.203784502	0.027539503	0.040514066	positive
PRJNA729271	stage_E18_vs_E10	ordinary_muscle_development	ENSGALG00010025805	ATXN2	-0.200707734	0.094502518	-2.123834771	0.033683962	0.048761513	negative
PRJNA729271	stage_E18_vs_E10	ordinary_muscle_development	ENSGALG00010025809	ENSGALG00010025809	0.952715168	0.198974735	4.788121315	1.6835E-06	4.24684E-06	positive
PRJNA729271	stage_E18_vs_E10	ordinary_muscle_development	ENSGALG00010025822	KLF7	-0.154846249	0.099576783	-1.555043705	0.119935661	0.156788186	negative
PRJNA729271	stage_E18_vs_E10	ordinary_muscle_development	ENSGALG00010025829	ENSGALG00010025829	0.603181671	0.258228799	2.335841986	0.019499478	0.029460594	positive
PRJNA729271	stage_E18_vs_E10	ordinary_muscle_development	ENSGALG00010025830	TBCEL	0.653973993	0.191269466	3.419123852	0.000628231	0.001177426	positive
PRJNA729271	stage_E18_vs_E10	ordinary_muscle_development	ENSGALG00010025831	BRAP	-0.042508585	0.0708011	-0.600394414	0.54824341	0.602544409	negative
PRJNA729271	stage_E18_vs_E10	ordinary_muscle_development	ENSGALG00010025832	ENKD1	1.407444139	0.14537252	9.681638145	3.60886E-22	2.90675E-21	positive
PRJNA729271	stage_E18_vs_E10	ordinary_muscle_development	ENSGALG00010025835	PPP6R3	-0.060517012	0.119505919	-0.506393424	0.61258049	0.663281856	negative
PRJNA729271	stage_E18_vs_E10	ordinary_muscle_development	ENSGALG00010025836	ENSGALG00010025836	0.33875211	0.114262156	2.964692087	0.00302986	0.005185103	positive
PRJNA729271	stage_E18_vs_E10	ordinary_muscle_development	ENSGALG00010025837	ENSGALG00010025837	1.195867612	0.190010234	6.293701067	3.09985E-10	1.09032E-09	positive
PRJNA729271	stage_E18_vs_E10	ordinary_muscle_development	ENSGALG00010025846	GRIK4	-0.549696077	0.233131693	-2.357877945	0.018379735	0.027876348	negative
PRJNA729271	stage_E18_vs_E10	ordinary_muscle_development	ENSGALG00010025847	DEF6	0.227363472	0.126029772	1.804045735	0.071224138	0.097322198	positive
PRJNA729271	stage_E18_vs_E10	ordinary_muscle_development	ENSGALG00010025849	OBSCN	3.269876522	0.229095399	14.27299077	3.22448E-46	8.04455E-45	positive
PRJNA729271	stage_E18_vs_E10	ordinary_muscle_development	ENSGALG00010025852	BTBD19	-0.150297305	0.248450824	-0.604937842	0.545220298	0.599982404	negative
PRJNA729271	stage_E18_vs_E10	ordinary_muscle_development	ENSGALG00010025858	ENSGALG00010025858	5.806639142	2.872248496	2.021635367	0.043214036	0.06132636	positive
PRJNA729271	stage_E18_vs_E10	ordinary_muscle_development	ENSGALG00010025862	PTCH2	-2.68595263	0.285270058	-9.415473366	4.70955E-21	3.56103E-20	negative
PRJNA729271	stage_E18_vs_E10	ordinary_muscle_development	ENSGALG00010025865	LGR6	-1.723495509	0.51632224	-3.33802299	0.000843768	0.001554055	negative

PRJNA729271	stage_E18_vs_E10	ordinary_muscle_development	ENSGALG00010025869	FASTKD2	-0.557545412	0.09543992	-5.841847008	5.16252E-09	1.63779E-08	negative
PRJNA729271	stage_E18_vs_E10	ordinary_muscle_development	ENSGALG00010025870	ENSGALG00010025870	1.947766839	0.164189281	11.86293539	1.84395E-32	2.50497E-31	positive
PRJNA729271	stage_E18_vs_E10	ordinary_muscle_development	ENSGALG00010025871	ENSGALG00010025871	3.410926909	1.419007995	2.403740444	0.016228291	0.02490338	positive
PRJNA729271	stage_E18_vs_E10	ordinary_muscle_development	ENSGALG00010025874	MDH1B	-0.984385572	0.29403967	-3.347798514	0.000814562	0.001504278	negative
PRJNA729271	stage_E18_vs_E10	ordinary_muscle_development	ENSGALG00010025876	ENSGALG00010025876	7.062402637	1.588981435	4.444609913	8.80515E-06	2.06255E-05	positive
PRJNA729271	stage_E18_vs_E10	ordinary_muscle_development	ENSGALG00010025877	ENSGALG00010025877	3.082756248	3.479582904	0.885955683	0.375641409	0.435628628	positive
PRJNA729271	stage_E18_vs_E10	ordinary_muscle_development	ENSGALG00010025881	GAL	0.487538538	0.670452993	0.727177807	0.467117039	0.526096464	positive
PRJNA729271	stage_E18_vs_E10	ordinary_muscle_development	ENSGALG00010025882	LDHD	2.903547267	0.172535496	16.82869515	1.5038E-63	6.85457E-62	positive
PRJNA729271	stage_E18_vs_E10	ordinary_muscle_development	ENSGALG00010025886	ALDH2	-0.421586243	0.110351535	-3.820393083	0.000133239	0.000273788	negative
PRJNA729271	stage_E18_vs_E10	ordinary_muscle_development	ENSGALG00010025887	ZNF76	0.077880984	0.113808686	0.684314937	0.493776317	0.551154144	positive
PRJNA729271	stage_E18_vs_E10	ordinary_muscle_development	ENSGALG00010025889	TESMIN	1.493227133	0.580468098	2.572453399	0.010098055	0.015967198	positive
PRJNA729271	stage_E18_vs_E10	ordinary_muscle_development	ENSGALG00010025890	SCG2	-0.743449417	0.520564048	-1.428161279	0.153245443	0.195743099	negative
PRJNA729271	stage_E18_vs_E10	ordinary_muscle_development	ENSGALG00010025891	NUP93	-0.765239266	0.107800453	-7.09866468	1.25968E-12	5.35771E-12	negative
PRJNA729271	stage_E18_vs_E10	ordinary_muscle_development	ENSGALG00010025892	ZC3H11A	0.397725115	0.086201183	4.613917155	3.9515E-06	9.55679E-06	positive
PRJNA729271	stage_E18_vs_E10	ordinary_muscle_development	ENSGALG00010025894	DYTN	0.429147805	0.45085348	0.951856475	0.341169776	0.399584349	positive
PRJNA729271	stage_E18_vs_E10	ordinary_muscle_development	ENSGALG00010025897	MAPKAPK5	-0.340924293	0.122802834	-2.776192401	0.005499965	0.009057606	negative
PRJNA729271	stage_E18_vs_E10	ordinary_muscle_development	ENSGALG00010025898	ENSGALG00010025898	5.285940509	1.852350607	2.853639311	0.004322157	0.007223436	positive
PRJNA729271	stage_E18_vs_E10	ordinary_muscle_development	ENSGALG00010025899	ENSGALG00010025899	-0.19152035	0.17724273	-1.080554052	0.279895531	0.335462686	negative
PRJNA729271	stage_E18_vs_E10	ordinary_muscle_development	ENSGALG00010025900	FAM237A	-1.82536797	0.713037633	-2.559988262	0.01046757	0.016515449	negative
PRJNA729271	stage_E18_vs_E10	ordinary_muscle_development	ENSGALG00010025902	ENSGALG00010025902	3.667032395	1.779915389	2.060228491	0.039376702	0.056321818	positive
PRJNA729271	stage_E18_vs_E10	ordinary_muscle_development	ENSGALG00010025904	RPL10A	0.804129419	0.138805446	5.793212312	6.90527E-09	2.16415E-08	positive
PRJNA729271	stage_E18_vs_E10	ordinary_muscle_development	ENSGALG00010025905	CPT1A	-0.17677179	0.175230754	-1.008794322	0.313073285	0.370630289	negative
PRJNA729271	stage_E18_vs_E10	ordinary_muscle_development	ENSGALG00010025907	ARHGEF12	0.184382337	0.071720047	2.570861903	0.010144577	0.016035512	positive
PRJNA729271	stage_E18_vs_E10	ordinary_muscle_development	ENSGALG00010025910	HEATR3	-0.855854359	0.11462076	-7.466835472	8.21464E-14	3.80906E-13	negative
PRJNA729271	stage_E18_vs_E10	ordinary_muscle_development	ENSGALG00010025913	EIF2B3	-0.808968437	0.080044675	-10.1064616	5.17185E-24	4.59914E-23	negative
PRJNA729271	stage_E18_vs_E10	ordinary_muscle_development	ENSGALG00010025914	ENSGALG00010025914	-5.554457999	1.762371208	-3.151695837	0.001623253	0.002886291	negative
PRJNA729271	stage_E18_vs_E10	ordinary_muscle_development	ENSGALG00010025917	SCUBE3	-4.990256435	0.444587868	-11.2244548	3.09288E-29	3.63007E-28	negative
PRJNA729271	stage_E18_vs_E10	ordinary_muscle_development	ENSGALG00010025919	RGS9BP	-1.243787239	0.34347523	-3.621184673	0.000293257	0.000575282	negative
PRJNA729271	stage_E18_vs_E10	ordinary_muscle_development	ENSGALG00010025920	GTSF1	-0.037972796	0.262981272	-0.144393538	0.885189719	0.905349571	negative
PRJNA729271	stage_E18_vs_E10	ordinary_muscle_development	ENSGALG00010025924	TMEM116	2.692747761	0.168366275	15.9933916	1.42081E-57	5.22705E-56	positive
PRJNA729271	stage_E18_vs_E10	ordinary_muscle_development	ENSGALG00010025925	KLHL18	0.935709403	0.090815137	10.30345192	6.79773E-25	6.41492E-24	positive
PRJNA729271	stage_E18_vs_E10	ordinary_muscle_development	ENSGALG00010025926	ENSGALG00010025926	0.202749934	0.434573252	0.466549503	0.640822223	0.690046662	positive
PRJNA729271	stage_E18_vs_E10	ordinary_muscle_development	ENSGALG00010025927	ENSGALG00010025927	-2.423302828	1.163504819	-2.082761316	0.037272985	0.053508163	negative
PRJNA729271	stage_E18_vs_E10	ordinary_muscle_development	ENSGALG00010025929	NELFE	0.347147977	0.090260551	3.846065337	0.00012003	0.000247733	positive
PRJNA729271	stage_E18_vs_E10	ordinary_muscle_development	ENSGALG00010025931	TMEM136	-0.752855405	0.179243149	-4.200190688	2.6669E-05	5.91896E-05	negative
PRJNA729271	stage_E18_vs_E10	ordinary_muscle_development	ENSGALG00010025932	AP1S3	-2.374564255	0.343350233	-6.915866149	4.65013E-12	1.89018E-11	negative
PRJNA729271	stage_E18_vs_E10	ordinary_muscle_development	ENSGALG00010025933	SKIC2	0.12714976	0.103108191	1.233168373	0.217512952	0.268213547	positive
PRJNA729271	stage_E18_vs_E10	ordinary_muscle_development	ENSGALG00010025935	CATH3	2.221020048	0.372555713	5.961578278	2.49813E-09	8.14266E-09	positive
PRJNA729271	stage_E18_vs_E10	ordinary_muscle_development	ENSGALG00010025936	GOSR2	-0.130010742	0.067948248	-1.913378868	0.055699568	0.077571602	negative
PRJNA729271	stage_E18_vs_E10	ordinary_muscle_development	ENSGALG00010025938	ERP29	-0.504799653	0.089903941	-5.614877925	1.96701E-08	5.92392E-08	negative
PRJNA729271	stage_E18_vs_E10	ordinary_muscle_development	ENSGALG00010025939	TMEM136-1	-0.863317609	0.578681054	-1.49187122	0.135732909	0.175414454	negative
PRJNA729271	stage_E18_vs_E10	ordinary_muscle_development	ENSGALG00010025940	HECTD3	0.305931848	0.089067095	3.434847049	0.000592888	0.001115224	positive
PRJNA729271	stage_E18_vs_E10	ordinary_muscle_development	ENSGALG00010025941	ENSGALG00010025941	-3.144126729	0.769249193	-4.087266853	4.36485E-05	9.46E-05	negative
PRJNA729271	stage_E18_vs_E10	ordinary_muscle_development	ENSGALG00010025942	ENSGALG00010025942	-0.211855323	1.318310545	-0.160702138	0.872328005	0.894734959	negative
PRJNA729271	stage_E18_vs_E10	ordinary_muscle_development	ENSGALG00010025943	CATH1	3.935943555	0.71378498	5.514186579	3.50397E-08	1.03632E-07	positive
PRJNA729271	stage_E18_vs_E10	ordinary_muscle_development	ENSGALG00010025946	KRT5	-5.977733393	1.012319219	-5.904988547	3.52671E-09	1.13675E-08	negative
PRJNA729271	stage_E18_vs_E10	ordinary_muscle_development	ENSGALG00010025948	WNT9B	-6.4662149	0.762733529	-8.477685401	2.29718E-17	1.37423E-16	negative
PRJNA729271	stage_E18_vs_E10	ordinary_muscle_development	ENSGALG00010025949	GPATCH1	-0.106424175	0.122963112	-0.865496759	0.386766194	0.44693001	negative
PRJNA729271	stage_E18_vs_E10	ordinary_muscle_development	ENSGALG00010025951	TBRG4	-0.258072862	0.115578918	-2.232871413	0.025557425	0.037828539	negative
PRJNA729271	stage_E18_vs_E10	ordinary_muscle_development	ENSGALG00010025953	POU2F3	-0.585846502	0.851682102	-0.687869922	0.491534699	0.549117184	negative
PRJNA729271	stage_E18_vs_E10	ordinary_muscle_development	ENSGALG00010025954	TUBA1A	-1.64855036	0.158629638	-10.39244859	2.68373E-25	2.58476E-24	negative
PRJNA729271	stage_E18_vs_E10	ordinary_muscle_development	ENSGALG00010025956	ENSGALG00010025956	1.329133448	0.507391952	2.61953987	0.008804848	0.014052661	positive
PRJNA729271	stage_E18_vs_E10	ordinary_muscle_development	ENSGALG00010025957	ENSGALG00010025957	-1.1062381	0.117760083	-9.393999018	5.77644E-21	4.34282E-20	negative
PRJNA729271	stage_E18_vs_E10	ordinary_muscle_development	ENSGALG00010025958	HOXC9	-3.926621802	0.417293597	-9.409734123	4.97393E-21	3.75311E-20	negative
PRJNA729271	stage_E18_vs_E10	ordinary_muscle_development	ENSGALG00010025959	UROD	0.312530495	0.102932022	3.03628054	0.002395164	0.004155344	positive
PRJNA729271	stage_E18_vs_E10	ordinary_muscle_development	ENSGALG00010025960	CMKLR2	1.945646682	0.15829159	12.29153543	1.00577E-34	1.52977E-33	positive
PRJNA729271	stage_E18_vs_E10	ordinary_muscle_development	ENSGALG00010025962	ENSGALG00010025962	0.922622444	0.186208092	4.954792416	7.24075E-07	1.89826E-06	positive
PRJNA729271	stage_E18_vs_E10	ordinary_muscle_development	ENSGALG00010025963	OAF	0.821587999	0.245322263	3.349015239	0.000810993	0.001498069	positive
PRJNA729271	stage_E18_vs_E10	ordinary_muscle_development	ENSGALG00010025964	HOXC10	-7.16393601	0.987986917	-7.251043399	4.13573E-13	1.82322E-12	negative
PRJNA729271	stage_E18_vs_E10	ordinary_muscle_development	ENSGALG00010025968	FADS2	-1.912583099	0.161196519	-11.86491566	1.80084E-32	2.4487E-31	negative
PRJNA729271	stage_E18_vs_E10	ordinary_muscle_development	ENSGALG00010025970	KLHL12	-0.224672619	0.111729624	-2.010859883	0.044340259	0.062801647	negative
PRJNA729271	stage_E18_vs_E10	ordinary_muscle_development	ENSGALG00010025971	TRAFD1	-0.559069781	0.201002013	-2.781413841	0.005412269	0.008925011	negative
PRJNA729271	stage_E18_vs_E10	ordinary_muscle_development	ENSGALG00010025972	WNT3	-7.396998718	1.318245216	-5.611246394	2.00875E-08	6.04709E-08	negative
PRJNA729271	stage_E18_vs_E10	ordinary_muscle_development	ENSGALG00010025974	EEF1B2	0.675191098	0.095547738	7.066531443	1.58854E-12	6.69942E-12	positive
PRJNA729271	stage_E18_vs_E10	ordinary_muscle_development	ENSGALG00010025976	NAA25	-1.18946094	0.100591351	-11.82468407	2.90995E-32	3.93101E-31	negative
PRJNA729271	stage_E18_vs_E10	ordinary_muscle_development	ENSGALG00010025978	TRIM29	-4.397228059	0.504642779	-8.713545986	2.94514E-18	1.87483E-17	negative
PRJNA729271	stage_E18_vs_E10	ordinary_muscle_development	ENSGALG00010025980	HECTD4	-0.079885805	0.064460128	-1.2393057	0.215232309	0.265763039	negative
PRJNA729271	stage_E18_vs_E10	ordinary_muscle_development	ENSGALG00010025982	SERPINE2	-0.118803943	0.198846192	-0.597466526	0.550195949	0.60431085	negative
PRJNA729271	stage_E18_vs_E10	ordinary_muscle_development	ENSGALG00010025983	RNF26	-0.5804856	0.083364878	-6.963191404	3.32649E-12	1.36671E-11	negative
PRJNA729271	stage_E18_vs_E10	ordinary_muscle_development	ENSGALG00010025984	NDUFS1	0.734930509	0.117121373	6.274947862	3.49751E-10	1.22544E-09	positive
PRJNA729271	stage_E18_vs_E10	ordinary_muscle_development	ENSGALG00010025985	CHCHD10	1.055859036	0.320625873	3.293118636	0.000990827	0.001805863	positive
PRJNA729271	stage_E18_vs_E10	ordinary_muscle_development	ENSGALG00010025986	RAB3IL1	-0.099847665	0.108262046	-0.922277647	0.356383771	0.415723705	negative
PRJNA729271	stage_E18_vs_E10	ordinary_muscle_development	ENSGALG00010025988	NSF	-0.418679429	0.060312527	-6.941831959	3.87047E-12	1.58348E-11	negative
PRJNA729271	stage_E18_vs_E10	ordinary_muscle_development	ENSGALG00010025990	FAM124B	1.082273212	0.241286747	4.485423368	7.27693E-06	1.71762E-05	positive
PRJNA729271	stage_E18_vs_E10	ordinary_muscle_development	ENSGALG00010025992	MMP11	-0.829469832	0.302308172	-2.743788984	0.006073456	0.009936202	negative
PRJNA729271	stage_E18_vs_E10	ordinary_muscle_development	ENSGALG00010025994	INO80D	0.589595556	0.109039472	5.407175437	6.40264E-08	1.84213E-07	positive
PRJNA729271	stage_E18_vs_E10	ordinary_muscle_development	ENSGALG00010025996	CUL3	0.349258574	0.097146502	3.595173945	0.000324175	0.000631575	positive
PRJNA729271	stage_E18_vs_E10	ordinary_muscle_development	ENSGALG00010025997	CCDC43	-0.122469002	0.150285284	-0.814910139	0.415123749	0.475556685	negative
PRJNA729271	stage_E18_vs_E10	ordinary_muscle_development	ENSGALG00010025998	SMARCB1	-0.601738436	0.143710679	-4.18715186	2.82477E-05	6.25497E-05	negative
PRJNA729271	stage_E18_vs_E10	ordinary_muscle_development	ENSGALG00010025999	DBF4B	-1.147489991	0.161267261	-7.115455304	1.11544E-12	4.76239E-12	negative
PRJNA729271	stage_E18_vs_E10	ordinary_muscle_development	ENSGALG00010026001	ANKS1A	0.823644924	0.092291438	8.924391455	4.48156E-19	2.9977E-18	positive
PRJNA729271	stage_E18_vs_E10	ordinary_muscle_development	ENSGALG00010026002	FTH1	1.009144331	0.141872891	7.113017321	1.13533E-12	4.84018E-12	positive
PRJNA729271	stage_E18_vs_E10	ordinary_muscle_development	ENSGALG00010026003	NECTIN1	-0.087344521	0.155449633	-0.561883098	0.57419567	0.627343502	negative
PRJNA729271	stage_E18_vs_E10	ordinary_muscle_development	ENSGALG00010026005	DERL3	0.027884917	0.118203494	0.235906027	0.813505591	0.844924301	positive
PRJNA729271	stage_E18_vs_E10	ordinary_muscle_development	ENSGALG00010026006	ITGA2B	0.078190058	0.302020625	0.258889795	0.795720272	0.829304382	positive
PRJNA729271	stage_E18_vs_E10	ordinary_muscle_development	ENSGALG00010026008	NRP2	1.959817296	0.100143715	19.57004786	2.7842E-85	2.22967E-83	positive
PRJNA729271	stage_E18_vs_E10	ordinary_muscle_development	ENSGALG00010026009	ENSGALG00010026009	-0.10439683	0.303818996	-0.343615214	0.731135681	0.772773202	negative
PRJNA729271	stage_E18_vs_E10	ordinary_muscle_development	ENSGALG00010026010	ZSWIM5	0.866703243	0.125806883	6.889156022	5.61244E-12	2.26293E-11	positive
PRJNA729271	stage_E18_vs_E10	ordinary_muscle_development	ENSGALG00010026011	GJC2	-0.053836269	0.16407791	-0.328114062	0.742825422	0.783243944	negative
PRJNA729271	stage_E18_vs_E10	ordinary_muscle_development	ENSGALG00010026013	RABIF	-0.781362526	0.114906875	-6.799963269	1.04646E-11	4.10845E-11	negative
PRJNA729271	stage_E18_vs_E10	ordinary_muscle_development	ENSGALG00010026016	SLC2A11	-2.618857752	0.493595608	-5.305674748	1.12257E-07	3.17062E-07	negative
PRJNA729271	stage_E18_vs_E10	ordinary_muscle_development	ENSGALG00010026017	PLEKHM1	0.77398629	0.146294258	5.290612909	1.21907E-07	3.42783E-07	positive
PRJNA729271	stage_E18_vs_E10	ordinary_muscle_development	ENSGALG00010026023	THY1	-1.86063305	0.305994527	-6.080608922	1.19727E-09	4.00795E-09	negative
PRJNA729271	stage_E18_vs_E10	ordinary_muscle_development	ENSGALG00010026030	IBA57	0.047992254	0.085024972	0.564448928	0.572448648	0.625859342	positive
PRJNA729271	stage_E18_vs_E10	ordinary_muscle_development	ENSGALG00010026036	TUB5A	-1.744690722	0.207475185	-8.409153718	4.1298E-17	2.43636E-16	negative
PRJNA729271	stage_E18_vs_E10	ordinary_muscle_development	ENSGALG00010026040	GUK1	-0.258440307	0.115692762	-2.233850258	0.025492927	0.037748491	negative
PRJNA729271	stage_E18_vs_E10	ordinary_muscle_development	ENSGALG00010026051	TAF11	-0.475482553	0.13049058	-3.643807502	0.000268634	0.000529993	negative
PRJNA729271	stage_E18_vs_E10	ordinary_muscle_development	ENSGALG00010026061	MPEG1	1.303494007	0.344582399	3.782822365	0.00015506	0.000315629	positive
PRJNA729271	stage_E18_vs_E10	ordinary_muscle_development	ENSGALG00010026062	ITGA4	-1.792084091	0.161198188	-11.11727195	1.03385E-28	1.18557E-27	negative
PRJNA729271	stage_E18_vs_E10	ordinary_muscle_development	ENSGALG00010026064	ENSGALG00010026064	0.118007394	0.150415479	0.78454289	0.432721626	0.492524536	positive
PRJNA729271	stage_E18_vs_E10	ordinary_muscle_development	ENSGALG00010026066	SELENOP2	2.516292139	0.566155053	4.444528271	8.80849E-06	2.063E-05	positive
PRJNA729271	stage_E18_vs_E10	ordinary_muscle_development	ENSGALG00010026068	BLTP3A	0.651739222	0.111405025	5.850177975	4.91047E-09	1.56159E-08	positive
PRJNA729271	stage_E18_vs_E10	ordinary_muscle_development	ENSGALG00010026073	HPDL	-1.323394516	0.2657508	-4.979832672	6.36393E-07	1.67505E-06	negative
PRJNA729271	stage_E18_vs_E10	ordinary_muscle_development	ENSGALG00010026076	C1orf35	0.100441671	0.136792621	0.734262346	0.462788862	0.52203304	positive
PRJNA729271	stage_E18_vs_E10	ordinary_muscle_development	ENSGALG00010026078	UBE2E3	0.349709381	0.106870101	3.272284563	0.001066821	0.001936336	positive
PRJNA729271	stage_E18_vs_E10	ordinary_muscle_development	ENSGALG00010026081	KPNA1	-0.882682138	0.110345905	-7.999228786	1.25201E-15	6.6843E-15	negative
PRJNA729271	stage_E18_vs_E10	ordinary_muscle_development	ENSGALG00010026083	MUTYH	-1.75086078	0.133439916	-13.12096737	2.49727E-39	4.66468E-38	negative
PRJNA729271	stage_E18_vs_E10	ordinary_muscle_development	ENSGALG00010026088	SNRPC	-0.355810174	0.082378497	-4.319211789	1.56587E-05	3.57214E-05	negative
PRJNA729271	stage_E18_vs_E10	ordinary_muscle_development	ENSGALG00010026090	USP2	2.520939193	0.235004345	10.72720248	7.5858E-27	8.00263E-26	positive
PRJNA729271	stage_E18_vs_E10	ordinary_muscle_development	ENSGALG00010026091	DTX4	0.106683837	0.163156883	0.653872734	0.513193802	0.569975033	positive
PRJNA729271	stage_E18_vs_E10	ordinary_muscle_development	ENSGALG00010026093	TOE1	-1.100415822	0.132837596	-8.283918522	1.19187E-16	6.81775E-16	negative
PRJNA729271	stage_E18_vs_E10	ordinary_muscle_development	ENSGALG00010026094	C6orf106	0.522809004	0.078426464	6.666232024	2.62454E-11	9.99547E-11	positive
PRJNA729271	stage_E18_vs_E10	ordinary_muscle_development	ENSGALG00010026095	MIF	-2.084042229	0.203664113	-10.23274156	1.41457E-24	1.30683E-23	negative
PRJNA729271	stage_E18_vs_E10	ordinary_muscle_development	ENSGALG00010026097	ARF1	-0.518785173	0.056074857	-9.251653943	2.21045E-20	1.59564E-19	negative
PRJNA729271	stage_E18_vs_E10	ordinary_muscle_development	ENSGALG00010026098	STAT3	0.600774326	0.108296875	5.54747608	2.89823E-08	8.62802E-08	positive
PRJNA729271	stage_E18_vs_E10	ordinary_muscle_development	ENSGALG00010026102	IPO9	-1.041796811	0.059770373	-17.42998686	4.88572E-68	2.46754E-66	negative
PRJNA729271	stage_E18_vs_E10	ordinary_muscle_development	ENSGALG00010026104	DDX51	-0.899233569	0.107756263	-8.345070066	7.11717E-17	4.12324E-16	negative
PRJNA729271	stage_E18_vs_E10	ordinary_muscle_development	ENSGALG00010026105	CNTF	1.79433478	0.191615781	9.364232809	7.66048E-21	5.70893E-20	positive
PRJNA729271	stage_E18_vs_E10	ordinary_muscle_development	ENSGALG00010026106	TESK2	0.345125471	0.119620511	2.885169679	0.003912028	0.00657518	positive
PRJNA729271	stage_E18_vs_E10	ordinary_muscle_development	ENSGALG00010026111	ENSGALG00010026111	-1.869874499	0.160284584	-11.66596597	1.90232E-31	2.50902E-30	negative
PRJNA729271	stage_E18_vs_E10	ordinary_muscle_development	ENSGALG00010026112	ZFP91	0.41718158	0.121056305	3.446178041	0.000568576	0.001072554	positive
PRJNA729271	stage_E18_vs_E10	ordinary_muscle_development	ENSGALG00010026113	MFRP	1.177825594	0.522636605	2.253622466	0.024219929	0.036006961	positive
PRJNA729271	stage_E18_vs_E10	ordinary_muscle_development	ENSGALG00010026114	CWC22	-0.40762209	0.083774348	-4.865714851	1.14044E-06	2.93045E-06	negative
PRJNA729271	stage_E18_vs_E10	ordinary_muscle_development	ENSGALG00010026115	MMACHC	-0.333499124	0.195463147	-1.706199503	0.087970941	0.117871516	negative
PRJNA729271	stage_E18_vs_E10	ordinary_muscle_development	ENSGALG00010026116	DLX3	-2.708900596	0.60191644	-4.500459561	6.78067E-06	1.60467E-05	negative
PRJNA729271	stage_E18_vs_E10	ordinary_muscle_development	ENSGALG00010026117	LZTR1	0.193721928	0.07147192	2.710462056	0.006718954	0.010907294	positive
PRJNA729271	stage_E18_vs_E10	ordinary_muscle_development	ENSGALG00010026120	C1QTNF5	0.095555919	0.198854928	0.480530805	0.630850004	0.680775075	positive
PRJNA729271	stage_E18_vs_E10	ordinary_muscle_development	ENSGALG00010026121	ENSGALG00010026121	0.125277887	0.260133931	0.481589952	0.630097265	0.680215972	positive
PRJNA729271	stage_E18_vs_E10	ordinary_muscle_development	ENSGALG00010026124	PACSIN1	-1.686489252	0.352388487	-4.785880683	1.70239E-06	4.29151E-06	negative
PRJNA729271	stage_E18_vs_E10	ordinary_muscle_development	ENSGALG00010026127	ENSGALG00010026127	2.152983585	0.595634009	3.614608218	0.000300802	0.000588809	positive
PRJNA729271	stage_E18_vs_E10	ordinary_muscle_development	ENSGALG00010026128	PRDX1	-1.308902812	0.142162008	-9.207121035	3.34989E-20	2.37673E-19	negative

PRJNA729271	stage_E18_vs_E10	ordinary_muscle_development	ENSGALG00010026132	DDTL	0.246881294	0.185411994	1.331528176	0.18301528	0.229779687	positive
PRJNA729271	stage_E18_vs_E10	ordinary_muscle_development	ENSGALG00010026137	LPXN	0.15312308	0.394036486	0.388601274	0.697571124	0.742603808	positive
PRJNA729271	stage_E18_vs_E10	ordinary_muscle_development	ENSGALG00010026141	WNT3A	-7.965427564	0.966025017	-8.245570689	1.64374E-16	9.28888E-16	negative
PRJNA729271	stage_E18_vs_E10	ordinary_muscle_development	ENSGALG00010026143	RNPEP	0.63347105	0.139854481	4.529501276	5.91231E-06	1.40952E-05	positive
PRJNA729271	stage_E18_vs_E10	ordinary_muscle_development	ENSGALG00010026148	RPS10	1.003934655	0.134060077	7.488692239	6.95632E-14	3.24114E-13	positive
PRJNA729271	stage_E18_vs_E10	ordinary_muscle_development	ENSGALG00010026149	LASP1	-0.195939892	0.128940326	-1.519616851	0.1286073	0.167159507	negative
PRJNA729271	stage_E18_vs_E10	ordinary_muscle_development	ENSGALG00010026154	ZNF385B	0.110366211	0.172794816	0.638712515	0.52300997	0.579545104	positive
PRJNA729271	stage_E18_vs_E10	ordinary_muscle_development	ENSGALG00010026162	MED1	-0.365678635	0.152417892	-2.399184441	0.016431636	0.025169244	negative
PRJNA729271	stage_E18_vs_E10	ordinary_muscle_development	ENSGALG00010026163	CTNND1	-0.091971402	0.081796284	-1.124395851	0.260845131	0.315157971	negative
PRJNA729271	stage_E18_vs_E10	ordinary_muscle_development	ENSGALG00010026166	WNT9A	-0.463286303	0.242165034	-1.913101559	0.055735052	0.077606108	negative
PRJNA729271	stage_E18_vs_E10	ordinary_muscle_development	ENSGALG00010026167	CABIN1	-0.160583835	0.059515163	-2.69820036	0.006971547	0.01127693	negative
PRJNA729271	stage_E18_vs_E10	ordinary_muscle_development	ENSGALG00010026173	CDK12	-0.164858155	0.163059301	-1.011031904	0.31200116	0.369572347	negative
PRJNA729271	stage_E18_vs_E10	ordinary_muscle_development	ENSGALG00010026183	SESTD1	0.369295002	0.168382604	2.193189752	0.028293711	0.041539282	positive
PRJNA729271	stage_E18_vs_E10	ordinary_muscle_development	ENSGALG00010026184	NUDT3	0.113393866	0.104632862	1.083730905	0.278484129	0.334074935	positive
PRJNA729271	stage_E18_vs_E10	ordinary_muscle_development	ENSGALG00010026192	SMIM29	1.301445865	0.128436167	10.13301697	3.94292E-24	3.53668E-23	positive
PRJNA729271	stage_E18_vs_E10	ordinary_muscle_development	ENSGALG00010026199	TBX6	-1.054858437	0.424848055	-2.482907535	0.013031491	0.020278203	negative
PRJNA729271	stage_E18_vs_E10	ordinary_muscle_development	ENSGALG00010026203	TMX2	0.024207828	0.105579187	0.229285988	0.818646643	0.849148255	positive
PRJNA729271	stage_E18_vs_E10	ordinary_muscle_development	ENSGALG00010026205	ENSGALG00010026205	3.848125441	1.307010014	2.94422032	0.003237694	0.005511495	positive
PRJNA729271	stage_E18_vs_E10	ordinary_muscle_development	ENSGALG00010026209	MED19	0.988256951	0.171909639	5.748700047	8.99322E-09	2.79017E-08	positive
PRJNA729271	stage_E18_vs_E10	ordinary_muscle_development	ENSGALG00010026213	AKR1A1	-1.185366036	0.124338716	-9.533362368	1.5227E-21	1.186E-20	negative
PRJNA729271	stage_E18_vs_E10	ordinary_muscle_development	ENSGALG00010026218	FTSJ3	-0.969837955	0.16868715	-5.74932919	8.95982E-09	2.781E-08	negative
PRJNA729271	stage_E18_vs_E10	ordinary_muscle_development	ENSGALG00010026220	ENSGALG00010026220	0.14180193	0.096865518	1.463905134	0.14321987	0.184154353	positive
PRJNA729271	stage_E18_vs_E10	ordinary_muscle_development	ENSGALG00010026221	CRKL	-0.071175614	0.147254698	-0.483350376	0.628846977	0.679017947	negative
PRJNA729271	stage_E18_vs_E10	ordinary_muscle_development	ENSGALG00010026229	CRHR2	0.27751061	0.215959277	1.285013612	0.198787527	0.247481337	positive
PRJNA729271	stage_E18_vs_E10	ordinary_muscle_development	ENSGALG00010026232	PSMC5	-0.633611997	0.131734982	-4.809747479	1.51121E-06	3.83223E-06	negative
PRJNA729271	stage_E18_vs_E10	ordinary_muscle_development	ENSGALG00010026234	ZDHHC5	0.549257821	0.160458729	3.423047312	0.000619233	0.001162214	positive
PRJNA729271	stage_E18_vs_E10	ordinary_muscle_development	ENSGALG00010026237	KLHL22	-0.341776931	0.143575446	-2.38046923	0.017290605	0.026367945	negative
PRJNA729271	stage_E18_vs_E10	ordinary_muscle_development	ENSGALG00010026239	GIP	-2.656344441	1.014939679	-2.617243661	0.008864304	0.014136658	negative
PRJNA729271	stage_E18_vs_E10	ordinary_muscle_development	ENSGALG00010026242	CCDC141	1.839603463	0.253467977	7.257735222	3.93625E-13	1.73845E-12	positive
PRJNA729271	stage_E18_vs_E10	ordinary_muscle_development	ENSGALG00010026244	CLP1	-0.771122301	0.131815886	-5.849995192	4.91587E-09	1.56296E-08	negative
PRJNA729271	stage_E18_vs_E10	ordinary_muscle_development	ENSGALG00010026249	YPEL4	-0.150904958	0.58609427	-0.257475573	0.79681167	0.830203073	negative
PRJNA729271	stage_E18_vs_E10	ordinary_muscle_development	ENSGALG00010026252	ENO2	0.549954663	0.334088501	1.646134663	0.099736037	0.132266593	positive
PRJNA729271	stage_E18_vs_E10	ordinary_muscle_development	ENSGALG00010026254	ENSGALG00010026254	-0.56551534	0.594529448	-0.951198198	0.341503773	0.399910905	negative
PRJNA729271	stage_E18_vs_E10	ordinary_muscle_development	ENSGALG00010026259	MED15	-0.116312222	0.089745432	-1.296023879	0.194967258	0.243059294	negative
PRJNA729271	stage_E18_vs_E10	ordinary_muscle_development	ENSGALG00010026260	ENSGALG00010026260	-18.01640486	3.258908464	-5.528355602	3.23246E-08	9.58367E-08	negative
PRJNA729271	stage_E18_vs_E10	ordinary_muscle_development	ENSGALG00010026261	IFT70A	-0.475865831	0.108133135	-4.40074016	1.07882E-05	2.50521E-05	negative
PRJNA729271	stage_E18_vs_E10	ordinary_muscle_development	ENSGALG00010026262	NAV1	0.985831515	0.159461306	6.182261637	6.31897E-10	2.17717E-09	positive
PRJNA729271	stage_E18_vs_E10	ordinary_muscle_development	ENSGALG00010026264	FZD7	-0.247395918	0.138878757	-1.78138057	0.0748503	0.10175906	negative
PRJNA729271	stage_E18_vs_E10	ordinary_muscle_development	ENSGALG00010026266	ENSGALG00010026266	-1.158284406	0.332637794	-3.482119072	0.000497462	0.000945792	negative
PRJNA729271	stage_E18_vs_E10	ordinary_muscle_development	ENSGALG00010026268	GARS1	-0.870547955	0.151423993	-5.749075415	8.97328E-09	2.78458E-08	negative
PRJNA729271	stage_E18_vs_E10	ordinary_muscle_development	ENSGALG00010026272	LRRC58	1.633965544	0.094661918	17.26106525	9.23907E-67	4.5091E-65	positive
PRJNA729271	stage_E18_vs_E10	ordinary_muscle_development	ENSGALG00010026277	SMARCD2	0.324536893	0.124472827	2.607291106	0.009126174	0.014514361	positive
PRJNA729271	stage_E18_vs_E10	ordinary_muscle_development	ENSGALG00010026287	NASP	-1.289056206	0.090121353	-14.30356031	2.07896E-46	5.25907E-45	negative
PRJNA729271	stage_E18_vs_E10	ordinary_muscle_development	ENSGALG00010026291	SERPING1	1.165057407	0.171986291	6.77412949	1.25158E-11	4.88596E-11	positive
PRJNA729271	stage_E18_vs_E10	ordinary_muscle_development	ENSGALG00010026295	SMPD4	-0.830100692	0.136304502	-6.090046033	1.12878E-09	3.79182E-09	negative
PRJNA729271	stage_E18_vs_E10	ordinary_muscle_development	ENSGALG00010026298	SNF8	-0.388050359	0.117580307	-3.300300616	0.000965813	0.001762934	negative
PRJNA729271	stage_E18_vs_E10	ordinary_muscle_development	ENSGALG00010026300	HMGA1	-0.902492493	0.108974745	-8.281666486	1.21463E-16	6.937E-16	negative
PRJNA729271	stage_E18_vs_E10	ordinary_muscle_development	ENSGALG00010026305	ATP5MC1	1.36240687	0.167049207	8.155721857	3.471E-16	1.92031E-15	positive
PRJNA729271	stage_E18_vs_E10	ordinary_muscle_development	ENSGALG00010026315	SYT2	-0.57536499	0.274473173	-2.096252187	0.036059826	0.051915691	negative
PRJNA729271	stage_E18_vs_E10	ordinary_muscle_development	ENSGALG00010026316	ENSGALG00010026316	0.848593165	0.350380099	2.421921697	0.015438677	0.023773889	positive
PRJNA729271	stage_E18_vs_E10	ordinary_muscle_development	ENSGALG00010026320	ENSGALG00010026320	0.185320534	0.175634361	1.055149643	0.291356891	0.347647195	positive
PRJNA729271	stage_E18_vs_E10	ordinary_muscle_development	ENSGALG00010026321	GPBP1L1	0.19305284	0.047844996	4.034964032	5.46107E-05	0.000117185	positive
PRJNA729271	stage_E18_vs_E10	ordinary_muscle_development	ENSGALG00010026325	TMEM69	-0.485123989	0.140034878	-3.464308279	0.000531597	0.001006202	negative
PRJNA729271	stage_E18_vs_E10	ordinary_muscle_development	ENSGALG00010026329	ENSGALG00010026329	3.367422651	0.269200354	12.50898301	6.66724E-36	1.0738E-34	positive
PRJNA729271	stage_E18_vs_E10	ordinary_muscle_development	ENSGALG00010026334	ENSGALG00010026334	0.396640901	0.127872962	3.10183556	0.001923248	0.00338401	positive
PRJNA729271	stage_E18_vs_E10	ordinary_muscle_development	ENSGALG00010026335	IPP	-0.778964709	0.104355352	-7.464540062	8.35911E-14	3.87233E-13	negative
PRJNA729271	stage_E18_vs_E10	ordinary_muscle_development	ENSGALG00010026344	TIMM10	0.46356532	0.171943934	2.696026022	0.007017218	0.01134954	positive
PRJNA729271	stage_E18_vs_E10	ordinary_muscle_development	ENSGALG00010026346	FZD2	1.042240252	0.162940615	6.396442365	1.59039E-10	5.71176E-10	positive
PRJNA729271	stage_E18_vs_E10	ordinary_muscle_development	ENSGALG00010026354	ENSGALG00010026354	1.407174669	0.246688877	5.704248555	1.16857E-08	3.58638E-08	positive
PRJNA729271	stage_E18_vs_E10	ordinary_muscle_development	ENSGALG00010026355	PPP1R12B	2.012533993	0.137182137	14.67052514	9.95715E-49	2.74912E-47	positive
PRJNA729271	stage_E18_vs_E10	ordinary_muscle_development	ENSGALG00010026360	CTDSPL	0.74559272	0.126719426	5.883807585	4.00934E-09	1.28545E-08	positive
PRJNA729271	stage_E18_vs_E10	ordinary_muscle_development	ENSGALG00010026362	ENSGALG00010026362	-0.803518937	0.407686406	-1.970924037	0.048732565	0.068560471	negative
PRJNA729271	stage_E18_vs_E10	ordinary_muscle_development	ENSGALG00010026367	RBP	1.625468874	0.558609906	2.90984613	0.003616067	0.006103982	positive
PRJNA729271	stage_E18_vs_E10	ordinary_muscle_development	ENSGALG00010026377	ENSGALG00010026377	-2.323975907	0.183455756	-12.66777318	8.9211E-37	1.49839E-35	negative
PRJNA729271	stage_E18_vs_E10	ordinary_muscle_development	ENSGALG00010026388	SNRPD3	-0.436911008	0.080008363	-5.460816723	4.73949E-08	1.38172E-07	negative
PRJNA729271	stage_E18_vs_E10	ordinary_muscle_development	ENSGALG00010026393	SLC43A3	-0.102526584	0.124791611	-0.821582345	0.411314641	0.47152845	negative
PRJNA729271	stage_E18_vs_E10	ordinary_muscle_development	ENSGALG00010026402	TFEB	2.044861512	0.164844665	12.40477819	2.46194E-35	3.8496E-34	positive
PRJNA729271	stage_E18_vs_E10	ordinary_muscle_development	ENSGALG00010026403	GUCD1	0.717177192	0.117536734	6.101728082	1.04928E-09	3.53458E-09	positive
PRJNA729271	stage_E18_vs_E10	ordinary_muscle_development	ENSGALG00010026409	VILL	-2.186273775	0.266424845	-8.205968075	2.2874E-16	1.28064E-15	negative
PRJNA729271	stage_E18_vs_E10	ordinary_muscle_development	ENSGALG00010026410	ENSGALG00010026410	3.483838699	0.315157393	11.05428202	2.09002E-28	2.35758E-27	positive
PRJNA729271	stage_E18_vs_E10	ordinary_muscle_development	ENSGALG00010026416	ENSGALG00010026416	-1.080386738	1.484442726	-0.727806279	0.46673218	0.525826438	negative
PRJNA729271	stage_E18_vs_E10	ordinary_muscle_development	ENSGALG00010026422	P2RX3	-2.224366136	0.321994357	-6.908090436	4.91221E-12	1.99447E-11	negative
PRJNA729271	stage_E18_vs_E10	ordinary_muscle_development	ENSGALG00010026427	PGC	0.721650613	1.084840708	0.665213434	0.505913989	0.562936911	positive
PRJNA729271	stage_E18_vs_E10	ordinary_muscle_development	ENSGALG00010026433	VPS11	-0.442311174	0.07351366	-6.016720893	1.77986E-09	5.87543E-09	negative
PRJNA729271	stage_E18_vs_E10	ordinary_muscle_development	ENSGALG00010026434	ENSGALG00010026434	0.322337137	0.144450964	2.231464076	0.025650405	0.037942914	positive
PRJNA729271	stage_E18_vs_E10	ordinary_muscle_development	ENSGALG00010026436	PLCD1	-0.703161953	0.077124152	-9.11727302	7.70383E-20	5.36861E-19	negative
PRJNA729271	stage_E18_vs_E10	ordinary_muscle_development	ENSGALG00010026438	TOMM6	0.48113856	0.121430263	3.962262359	7.42429E-05	0.000156965	positive
PRJNA729271	stage_E18_vs_E10	ordinary_muscle_development	ENSGALG00010026443	UPB1	1.113930601	0.361576657	3.080759171	0.002064736	0.00361978	positive
PRJNA729271	stage_E18_vs_E10	ordinary_muscle_development	ENSGALG00010026448	ADORA2A	-0.028745557	0.112692611	-0.255079338	0.798661823	0.83177203	negative
PRJNA729271	stage_E18_vs_E10	ordinary_muscle_development	ENSGALG00010026457	SSRP1	-1.034913766	0.099166404	-10.43613287	1.69579E-25	1.65972E-24	negative
PRJNA729271	stage_E18_vs_E10	ordinary_muscle_development	ENSGALG00010026458	DLEC1	2.104550049	0.177890901	11.83056603	2.71306E-32	3.67875E-31	positive
PRJNA729271	stage_E18_vs_E10	ordinary_muscle_development	ENSGALG00010026480	USP49	-0.2357986	0.214861264	-1.09744584	0.272446544	0.327591261	negative
PRJNA729271	stage_E18_vs_E10	ordinary_muscle_development	ENSGALG00010026481	DIXDC1	-1.160433291	0.137294679	-8.452135943	2.86021E-17	1.70262E-16	negative
PRJNA729271	stage_E18_vs_E10	ordinary_muscle_development	ENSGALG00010026484	TNKS1BP1	0.154039464	0.111654314	1.379610495	0.167706604	0.212084037	positive
PRJNA729271	stage_E18_vs_E10	ordinary_muscle_development	ENSGALG00010026485	ACAA1	0.999190915	0.104804415	9.533862796	1.51537E-21	1.18093E-20	positive
PRJNA729271	stage_E18_vs_E10	ordinary_muscle_development	ENSGALG00010026492	SPECC1L	-0.428126206	0.07545219	-5.674138871	1.39388E-08	4.25175E-08	negative
PRJNA729271	stage_E18_vs_E10	ordinary_muscle_development	ENSGALG00010026498	ENSGALG00010026498	-0.006805138	0.087709893	-0.077586893	0.938156669	0.949422671	negative
PRJNA729271	stage_E18_vs_E10	ordinary_muscle_development	ENSGALG00010026507	APLNR	1.556265016	0.285436134	5.452235474	4.97405E-08	1.44603E-07	positive
PRJNA729271	stage_E18_vs_E10	ordinary_muscle_development	ENSGALG00010026508	CSRP1	0.362181303	0.156028145	2.321256225	0.020273019	0.030536986	positive
PRJNA729271	stage_E18_vs_E10	ordinary_muscle_development	ENSGALG00010026516	ENSGALG00010026516	0.805062304	0.167351233	4.810614709	1.50467E-06	3.81634E-06	positive
PRJNA729271	stage_E18_vs_E10	ordinary_muscle_development	ENSGALG00010026522	MED20	0.023525265	0.129614077	0.181502388	0.855973259	0.880827232	positive
PRJNA729271	stage_E18_vs_E10	ordinary_muscle_development	ENSGALG00010026536	BCR	-0.225362118	0.097371101	-2.314466161	0.020642165	0.031051078	negative
PRJNA729271	stage_E18_vs_E10	ordinary_muscle_development	ENSGALG00010026537	FJX1	-2.275246742	0.177364709	-12.82806909	1.14174E-37	1.98198E-36	negative
PRJNA729271	stage_E18_vs_E10	ordinary_muscle_development	ENSGALG00010026539	ENSGALG00010026539	-0.115002557	0.383414457	-0.299943194	0.764220486	0.802185238	negative
PRJNA729271	stage_E18_vs_E10	ordinary_muscle_development	ENSGALG00010026550	MYD88	-0.094016221	0.106483621	-0.882917206	0.377281007	0.437355102	negative
PRJNA729271	stage_E18_vs_E10	ordinary_muscle_development	ENSGALG00010026553	PLPP3	1.565139481	0.127461643	12.27929789	1.17009E-34	1.77597E-33	positive
PRJNA729271	stage_E18_vs_E10	ordinary_muscle_development	ENSGALG00010026559	RAB36	-0.975671337	0.455946803	-2.139879764	0.032364485	0.047006333	negative
PRJNA729271	stage_E18_vs_E10	ordinary_muscle_development	ENSGALG00010026560	TNNI1	0.070593494	0.588059354	0.120044845	0.904447623	0.921161348	positive
PRJNA729271	stage_E18_vs_E10	ordinary_muscle_development	ENSGALG00010026565	RSPH14	-0.790052358	0.405691563	-1.947421221	0.051484256	0.072137679	negative
PRJNA729271	stage_E18_vs_E10	ordinary_muscle_development	ENSGALG00010026566	ENSGALG00010026566	0.623816366	0.122559386	5.08991101	3.58232E-07	9.67139E-07	positive
PRJNA729271	stage_E18_vs_E10	ordinary_muscle_development	ENSGALG00010026568	BYSL	-0.119544217	0.111836078	-1.068923541	0.285104123	0.340859804	negative
PRJNA729271	stage_E18_vs_E10	ordinary_muscle_development	ENSGALG00010026569	GNAZ	-0.234966273	0.203123256	-1.156766965	0.247367582	0.300628247	negative
PRJNA729271	stage_E18_vs_E10	ordinary_muscle_development	ENSGALG00010026581	DNER	-1.964006101	0.181894818	-10.79748245	3.53771E-27	3.77607E-26	negative
PRJNA729271	stage_E18_vs_E10	ordinary_muscle_development	ENSGALG00010026584	ENSGALG00010026584	-0.941173339	0.111941963	-8.407690133	4.18164E-17	2.46594E-16	negative
PRJNA729271	stage_E18_vs_E10	ordinary_muscle_development	ENSGALG00010026588	CCND3	-0.48069037	0.147962501	-3.248731048	0.00115921	0.00209589	negative
PRJNA729271	stage_E18_vs_E10	ordinary_muscle_development	ENSGALG00010026595	PRKAA2	2.464904475	0.26132443	9.432353793	4.00988E-21	3.04151E-20	positive
PRJNA729271	stage_E18_vs_E10	ordinary_muscle_development	ENSGALG00010026601	ENSGALG00010026601	0.651637914	0.488373998	1.334301	0.18210523	0.228716343	positive
PRJNA729271	stage_E18_vs_E10	ordinary_muscle_development	ENSGALG00010026612	MFF	-0.562323305	0.085587469	-6.57015933	5.02615E-11	1.87816E-10	negative
PRJNA729271	stage_E18_vs_E10	ordinary_muscle_development	ENSGALG00010026625	ENSGALG00010026625	3.263709922	0.657863062	4.961077939	7.01031E-07	1.84051E-06	positive
PRJNA729271	stage_E18_vs_E10	ordinary_muscle_development	ENSGALG00010026628	FYB2	-4.483378745	1.093081549	-4.101595851	4.1031E-05	8.91672E-05	negative
PRJNA729271	stage_E18_vs_E10	ordinary_muscle_development	ENSGALG00010026630	XIRP1	1.890972899	0.243762591	7.757436824	8.6663E-15	4.34966E-14	positive
PRJNA729271	stage_E18_vs_E10	ordinary_muscle_development	ENSGALG00010026633	SLC5A1	3.086309375	0.498165731	6.19534661	5.81569E-10	2.01046E-09	positive
PRJNA729271	stage_E18_vs_E10	ordinary_muscle_development	ENSGALG00010026635	C8A	7.033095696	2.522462313	2.788186631	0.0053004	0.008754478	positive
PRJNA729271	stage_E18_vs_E10	ordinary_muscle_development	ENSGALG00010026638	LAD1	-2.800545127	0.455118881	-6.153436488	7.58217E-10	2.59329E-09	negative
PRJNA729271	stage_E18_vs_E10	ordinary_muscle_development	ENSGALG00010026643	YWHAH	-0.978391413	0.11233042	-8.709941703	3.04031E-18	1.93286E-17	negative
PRJNA729271	stage_E18_vs_E10	ordinary_muscle_development	ENSGALG00010026659	LIPH	0.238157131	0.248829095	0.957111269	0.338511096	0.396823157	positive
PRJNA729271	stage_E18_vs_E10	ordinary_muscle_development	ENSGALG00010026666	ENSGALG00010026666	3.207233143	0.418141993	7.670201019	1.71727E-14	8.40089E-14	positive
PRJNA729271	stage_E18_vs_E10	ordinary_muscle_development	ENSGALG00010026671	TAF8	0.93455564	0.154770853	6.038318085	1.55729E-09	5.16779E-09	positive
PRJNA729271	stage_E18_vs_E10	ordinary_muscle_development	ENSGALG00010026673	DEPDC5	0.276975188	0.122160168	2.267311775	0.023371187	0.034820161	positive
PRJNA729271	stage_E18_vs_E10	ordinary_muscle_development	ENSGALG00010026681	PLXDC1	2.599052902	0.257861205	10.07927077	6.82306E-24	6.0305E-23	positive
PRJNA729271	stage_E18_vs_E10	ordinary_muscle_development	ENSGALG00010026686	ENSGALG00010026686	0.377838623	0.42009424	0.899413958	0.368432207	0.428021547	positive
PRJNA729271	stage_E18_vs_E10	ordinary_muscle_development	ENSGALG00010026690	KRT14	-3.75173245	0.434579225	-8.633023004	5.97514E-18	3.72195E-17	negative
PRJNA729271	stage_E18_vs_E10	ordinary_muscle_development	ENSGALG00010026692	NMNAT3	2.459560469	0.525186443	4.683213936	2.82411E-06	6.94411E-06	positive
PRJNA729271	stage_E18_vs_E10	ordinary_muscle_development	ENSGALG00010026696	CSRNP1	-0.826185018	0.151276742	-5.461414663	4.72355E-08	1.37735E-07	negative
PRJNA729271	stage_E18_vs_E10	ordinary_muscle_development	ENSGALG00010026697	CHN1	-0.132446513	0.11543961	-1.147322941	0.251248176	0.304884245	negative
PRJNA729271	stage_E18_vs_E10	ordinary_muscle_development	ENSGALG00010026700	C8B	1.812021835	1.015825133	1.783793073	0.074457296	0.10126276	positive
PRJNA729271	stage_E18_vs_E10	ordinary_muscle_development	ENSGALG00010026704	WDFY1	-0.214924935	0.070234573	-3.060101702	0.002212618	0.003857114	negative
PRJNA729271	stage_E18_vs_E10	ordinary_muscle_development	ENSGALG00010026706	KRT24	-4.933830157	0.553609285	-8.912115976	5.00677E-19	3.34747E-18	negative
PRJNA729271	stage_E18_vs_E10	ordinary_muscle_development	ENSGALG00010026707	GORASP1	-0.60017494	0.08298393	-7.232423642	4.74449E-13	2.0802E-12	negative
PRJNA729271	stage_E18_vs_E10	ordinary_muscle_development	ENSGALG00010026715	WDR48	-0.117972709	0.067470854	-1.748498818	0.08037769	0.108672196	negative
PRJNA729271	stage_E18_vs_E10	ordinary_muscle_development	ENSGALG00010026719	ENSGALG00010026719	-2.735701161	0.855367686	-3.198275087	0.001382523	0.002477705	negative

PRJNA729271	stage_E18_vs_E10	ordinary_muscle_development	ENSGALG00010026724	OLA1	0.123860627	0.098414549	1.258560122	0.208189258	0.257945406	positive
PRJNA729271	stage_E18_vs_E10	ordinary_muscle_development	ENSGALG00010026731	ENSGALG00010026731	-0.247486653	0.460116583	-0.537878142	0.59066118	0.642717048	negative
PRJNA729271	stage_E18_vs_E10	ordinary_muscle_development	ENSGALG00010026737	SCN11A	-6.823963112	1.151887948	-5.924155315	3.13907E-09	1.01428E-08	negative
PRJNA729271	stage_E18_vs_E10	ordinary_muscle_development	ENSGALG00010026740	DAB1	-1.738448166	0.132058443	-13.16423344	1.40961E-39	2.657E-38	negative
PRJNA729271	stage_E18_vs_E10	ordinary_muscle_development	ENSGALG00010026743	TNNT2	-0.179911245	0.586459464	-0.306775244	0.759014453	0.797876169	negative
PRJNA729271	stage_E18_vs_E10	ordinary_muscle_development	ENSGALG00010026746	HOXD10	-2.954106871	0.448640208	-6.584578955	4.56176E-11	1.70948E-10	negative
PRJNA729271	stage_E18_vs_E10	ordinary_muscle_development	ENSGALG00010026755	ENSGALG00010026755	0.023956122	0.187281392	0.127915121	0.898216145	0.91558671	positive
PRJNA729271	stage_E18_vs_E10	ordinary_muscle_development	ENSGALG00010026759	OMA1	-0.26603083	0.110825159	-2.400455212	0.016374694	0.025095284	negative
PRJNA729271	stage_E18_vs_E10	ordinary_muscle_development	ENSGALG00010026767	ENSGALG00010026767	14.24252612	2.638380861	5.398207032	6.73102E-08	1.93353E-07	positive
PRJNA729271	stage_E18_vs_E10	ordinary_muscle_development	ENSGALG00010026770	ENSGALG00010026770	-0.68582214	0.587789647	-1.166781592	0.243298583	0.296378936	negative
PRJNA729271	stage_E18_vs_E10	ordinary_muscle_development	ENSGALG00010026775	BTG2	1.747406513	0.366159296	4.772257681	1.82172E-06	4.57561E-06	positive
PRJNA729271	stage_E18_vs_E10	ordinary_muscle_development	ENSGALG00010026780	MYSM1	0.560804376	0.078643313	7.13098615	9.96523E-13	4.27862E-12	positive
PRJNA729271	stage_E18_vs_E10	ordinary_muscle_development	ENSGALG00010026782	HOXB9	-4.233187394	0.603067586	-7.019424513	2.22784E-12	9.27413E-12	negative
PRJNA729271	stage_E18_vs_E10	ordinary_muscle_development	ENSGALG00010026786	ENSGALG00010026786	1.339133963	0.611653945	2.189365367	0.028570295	0.041898667	positive
PRJNA729271	stage_E18_vs_E10	ordinary_muscle_development	ENSGALG00010026796	DCAKD	0.345471731	0.217658782	1.587217058	0.112463493	0.147766346	positive
PRJNA729271	stage_E18_vs_E10	ordinary_muscle_development	ENSGALG00010026801	FMOD	3.60870797	0.489979114	7.365024069	1.77115E-13	7.98781E-13	positive
PRJNA729271	stage_E18_vs_E10	ordinary_muscle_development	ENSGALG00010026822	PRELP	2.043654478	0.19935782	10.25118792	1.16898E-24	1.08757E-23	positive
PRJNA729271	stage_E18_vs_E10	ordinary_muscle_development	ENSGALG00010026825	ADAM23	-0.353308479	0.135794476	-2.601788293	0.009273908	0.01473154	negative
PRJNA729271	stage_E18_vs_E10	ordinary_muscle_development	ENSGALG00010026827	FAM117A	1.367560654	0.128677104	10.62784766	2.21158E-26	2.27192E-25	positive
PRJNA729271	stage_E18_vs_E10	ordinary_muscle_development	ENSGALG00010026834	ENSGALG00010026834	5.650716965	1.938725806	2.914655052	0.003560819	0.006016813	positive
PRJNA729271	stage_E18_vs_E10	ordinary_muscle_development	ENSGALG00010026836	ENSGALG00010026836	3.279663181	0.582911197	5.626351313	1.84061E-08	5.55133E-08	positive
PRJNA729271	stage_E18_vs_E10	ordinary_muscle_development	ENSGALG00010026839	FGGY	0.68634165	0.116456125	5.893564192	3.77953E-09	1.215E-08	positive
PRJNA729271	stage_E18_vs_E10	ordinary_muscle_development	ENSGALG00010026846	MICAL1	-0.828144433	0.232913243	-3.555591863	0.000377129	0.000728282	negative
PRJNA729271	stage_E18_vs_E10	ordinary_muscle_development	ENSGALG00010026851	ENSGALG00010026851	6.813797585	1.116656027	6.101966425	1.04771E-09	3.53013E-09	positive
PRJNA729271	stage_E18_vs_E10	ordinary_muscle_development	ENSGALG00010026854	SMPD2	0.571603094	0.147196801	3.883257577	0.000103066	0.000214678	positive
PRJNA729271	stage_E18_vs_E10	ordinary_muscle_development	ENSGALG00010026856	UBE2Z	0.003665892	0.077884865	0.047068096	0.962458955	0.969303122	positive
PRJNA729271	stage_E18_vs_E10	ordinary_muscle_development	ENSGALG00010026874	TEAD3	0.923875339	0.160262142	5.764775919	8.17664E-09	2.54664E-08	positive
PRJNA729271	stage_E18_vs_E10	ordinary_muscle_development	ENSGALG00010026885	TULP1	3.81154743	0.631810335	6.032739924	1.61203E-09	5.34332E-09	positive
PRJNA729271	stage_E18_vs_E10	ordinary_muscle_development	ENSGALG00010026886	JUN	1.677645771	0.192813832	8.700857977	3.29384E-18	2.09129E-17	positive
PRJNA729271	stage_E18_vs_E10	ordinary_muscle_development	ENSGALG00010026889	ENSGALG00010026889	-0.047030697	0.27669932	-0.169970409	0.865033408	0.8885734	negative
PRJNA729271	stage_E18_vs_E10	ordinary_muscle_development	ENSGALG00010026893	FKBP5	2.210716443	0.38638213	5.721580461	1.05538E-08	3.24999E-08	positive
PRJNA729271	stage_E18_vs_E10	ordinary_muscle_development	ENSGALG00010026894	IP6K1	0.057787055	0.066226629	0.872565249	0.382900089	0.44330166	positive
PRJNA729271	stage_E18_vs_E10	ordinary_muscle_development	ENSGALG00010026897	ENSGALG00010026897	0.195558235	0.215832066	0.90606664	0.364900581	0.424448737	positive
PRJNA729271	stage_E18_vs_E10	ordinary_muscle_development	ENSGALG00010026898	PNPLA2	0.99410396	0.272159445	3.652652799	0.000259545	0.000513248	positive
PRJNA729271	stage_E18_vs_E10	ordinary_muscle_development	ENSGALG00010026899	HOOK1	-0.065244906	0.167373403	-0.389816452	0.696672278	0.741701408	negative
PRJNA729271	stage_E18_vs_E10	ordinary_muscle_development	ENSGALG00010026901	FAM212A	-0.235312179	0.173734917	-1.354432275	0.175598506	0.221215049	negative
PRJNA729271	stage_E18_vs_E10	ordinary_muscle_development	ENSGALG00010026907	ENSGALG00010026907	0.032171215	0.132263352	0.24323605	0.807822542	0.840045035	positive
PRJNA729271	stage_E18_vs_E10	ordinary_muscle_development	ENSGALG00010026909	ENSGALG00010026909	-0.221217831	0.15859831	-1.394830947	0.163066824	0.206974923	negative
PRJNA729271	stage_E18_vs_E10	ordinary_muscle_development	ENSGALG00010026916	ENSGALG00010026916	0.994012385	0.212774083	4.671679806	2.98746E-06	7.32588E-06	positive
PRJNA729271	stage_E18_vs_E10	ordinary_muscle_development	ENSGALG00010026917	AMT	-0.940274935	0.143002577	-6.575230689	4.85778E-11	1.81712E-10	negative
PRJNA729271	stage_E18_vs_E10	ordinary_muscle_development	ENSGALG00010026919	NICN1	0.521553456	0.162143211	3.216622227	0.001297093	0.002331229	positive
PRJNA729271	stage_E18_vs_E10	ordinary_muscle_development	ENSGALG00010026920	GATA2	1.785391369	0.202830465	8.802382658	1.33942E-18	8.7023E-18	positive
PRJNA729271	stage_E18_vs_E10	ordinary_muscle_development	ENSGALG00010026924	TPH1	-1.52769929	0.380378967	-4.016255948	5.913E-05	0.000126489	negative
PRJNA729271	stage_E18_vs_E10	ordinary_muscle_development	ENSGALG00010026942	CDK18	-0.428322344	0.178741938	-2.396317001	0.016560761	0.025351901	negative
PRJNA729271	stage_E18_vs_E10	ordinary_muscle_development	ENSGALG00010026943	EEFSEC	-0.335035225	0.072438578	-4.625093894	3.74429E-06	9.08342E-06	negative
PRJNA729271	stage_E18_vs_E10	ordinary_muscle_development	ENSGALG00010026950	LHFPL5	1.587214756	0.265552387	5.977030657	2.27241E-09	7.43201E-09	positive
PRJNA729271	stage_E18_vs_E10	ordinary_muscle_development	ENSGALG00010026951	RUVBL1	-0.971318185	0.106831735	-9.092037896	9.71998E-20	6.72832E-19	negative
PRJNA729271	stage_E18_vs_E10	ordinary_muscle_development	ENSGALG00010026954	MFSD4A	1.90241503	0.172948524	10.9998917	3.82591E-28	4.27576E-27	positive
PRJNA729271	stage_E18_vs_E10	ordinary_muscle_development	ENSGALG00010026958	CLTCL1	0.01176778	0.084307238	0.139582083	0.888990198	0.90841073	positive
PRJNA729271	stage_E18_vs_E10	ordinary_muscle_development	ENSGALG00010026960	SEC61A1	-0.871511632	0.102327377	-8.516896064	1.63886E-17	9.91458E-17	negative
PRJNA729271	stage_E18_vs_E10	ordinary_muscle_development	ENSGALG00010026964	SRPK1	-0.856038248	0.060878142	-14.06150413	6.54795E-45	1.53085E-43	negative
PRJNA729271	stage_E18_vs_E10	ordinary_muscle_development	ENSGALG00010026972	TNNT3	4.705338911	0.158362233	29.71250675	5.2936E-194	2.7404E-191	positive
PRJNA729271	stage_E18_vs_E10	ordinary_muscle_development	ENSGALG00010026973	MGLL	1.332705747	0.125433578	10.62479253	2.2852E-26	2.34423E-25	positive
PRJNA729271	stage_E18_vs_E10	ordinary_muscle_development	ENSGALG00010026977	ELK4	0.335548817	0.098118063	3.419847554	0.000626562	0.001174754	positive
PRJNA729271	stage_E18_vs_E10	ordinary_muscle_development	ENSGALG00010026980	LIMK2	-0.946662827	0.11902795	-7.953281809	1.81635E-15	9.57031E-15	negative
PRJNA729271	stage_E18_vs_E10	ordinary_muscle_development	ENSGALG00010026982	ABTB1	1.336012599	0.085287602	15.66479271	2.63287E-55	9.06494E-54	positive
PRJNA729271	stage_E18_vs_E10	ordinary_muscle_development	ENSGALG00010026984	PGR2/3	4.290970681	0.882083949	4.86458311	1.14698E-06	2.94622E-06	positive
PRJNA729271	stage_E18_vs_E10	ordinary_muscle_development	ENSGALG00010026999	MLEC	-0.537979108	0.082825004	-6.495370715	8.28292E-11	3.04106E-10	negative
PRJNA729271	stage_E18_vs_E10	ordinary_muscle_development	ENSGALG00010027001	SLC26A8	0.877632415	0.247544855	3.54534702	0.000392097	0.000755676	positive
PRJNA729271	stage_E18_vs_E10	ordinary_muscle_development	ENSGALG00010027003	SEPTIN2L	-0.253091064	0.082255955	-3.07687223	0.002091849	0.00366377	negative
PRJNA729271	stage_E18_vs_E10	ordinary_muscle_development	ENSGALG00010027012	RILPL1	0.738516435	0.192191634	3.84260448	0.000121736	0.000251111	positive
PRJNA729271	stage_E18_vs_E10	ordinary_muscle_development	ENSGALG00010027015	ENSGALG00010027015	3.261285208	0.507602804	6.424876272	1.31977E-10	4.76702E-10	positive
PRJNA729271	stage_E18_vs_E10	ordinary_muscle_development	ENSGALG00010027025	ORAI1	1.124456213	0.129692347	8.670181689	4.31431E-18	2.71777E-17	positive
PRJNA729271	stage_E18_vs_E10	ordinary_muscle_development	ENSGALG00010027026	SLC45A3	0.952212168	0.207050746	4.598931348	4.24664E-06	1.02387E-05	positive
PRJNA729271	stage_E18_vs_E10	ordinary_muscle_development	ENSGALG00010027027	ARHGEF3	0.246339307	0.102858165	2.394941677	0.016623009	0.02543383	positive
PRJNA729271	stage_E18_vs_E10	ordinary_muscle_development	ENSGALG00010027036	NUCKS1	-0.881962797	0.06579456	-13.40479823	5.66772E-41	1.12848E-39	negative
PRJNA729271	stage_E18_vs_E10	ordinary_muscle_development	ENSGALG00010027040	CLIP1	0.39108736	0.108855782	3.592710944	0.000327256	0.000637234	positive
PRJNA729271	stage_E18_vs_E10	ordinary_muscle_development	ENSGALG00010027041	MAPK14	1.303912184	0.073496762	17.74108335	2.02003E-70	1.08849E-68	positive
PRJNA729271	stage_E18_vs_E10	ordinary_muscle_development	ENSGALG00010027043	IL17RD	-1.451091663	0.175698345	-8.258994481	1.46905E-16	8.33092E-16	negative
PRJNA729271	stage_E18_vs_E10	ordinary_muscle_development	ENSGALG00010027045	MAPK13	1.193850989	0.185634842	6.431179484	1.26618E-10	4.57914E-10	positive
PRJNA729271	stage_E18_vs_E10	ordinary_muscle_development	ENSGALG00010027046	HESX1	0.023048386	0.305271356	0.075501305	0.939815859	0.950825996	positive
PRJNA729271	stage_E18_vs_E10	ordinary_muscle_development	ENSGALG00010027050	GSTT1	3.683432833	0.151238353	24.35515046	5.1128E-131	1.1402E-128	positive
PRJNA729271	stage_E18_vs_E10	ordinary_muscle_development	ENSGALG00010027052	APPL1	0.416136915	0.084681249	4.914156556	8.91655E-07	2.3208E-06	positive
PRJNA729271	stage_E18_vs_E10	ordinary_muscle_development	ENSGALG00010027059	ASB14	5.2608997	0.213437566	24.64842438	3.8261E-134	9.0917E-132	positive
PRJNA729271	stage_E18_vs_E10	ordinary_muscle_development	ENSGALG00010027063	RAB7L1	0.068571497	0.089803158	0.76357557	0.445120213	0.504576326	positive
PRJNA729271	stage_E18_vs_E10	ordinary_muscle_development	ENSGALG00010027073	DNAH12	-1.918272161	0.333100726	-5.75883513	8.46964E-09	2.63506E-08	negative
PRJNA729271	stage_E18_vs_E10	ordinary_muscle_development	ENSGALG00010027074	BRPF3	0.268016096	0.080279562	3.33853463	0.000842215	0.001551393	positive
PRJNA729271	stage_E18_vs_E10	ordinary_muscle_development	ENSGALG00010027075	SLC41A1	0.77519328	0.096204981	8.057725032	7.77274E-16	4.21181E-15	positive
PRJNA729271	stage_E18_vs_E10	ordinary_muscle_development	ENSGALG00010027076	VDHAP	1.388533341	0.172415538	8.053411878	8.05174E-16	4.3581E-15	positive
PRJNA729271	stage_E18_vs_E10	ordinary_muscle_development	ENSGALG00010027077	ARF4	-0.497674406	0.104630913	-4.756475821	1.97002E-06	4.92505E-06	negative
PRJNA729271	stage_E18_vs_E10	ordinary_muscle_development	ENSGALG00010027078	PDE12	-1.616446094	0.130378374	-12.39811516	2.67543E-35	4.17442E-34	negative
PRJNA729271	stage_E18_vs_E10	ordinary_muscle_development	ENSGALG00010027079	PM20D1	0.861614575	0.09451523	9.116145338	7.78438E-20	5.42214E-19	positive
PRJNA729271	stage_E18_vs_E10	ordinary_muscle_development	ENSGALG00010027080	TBC1D22B	-0.030989584	0.157662902	-0.196555964	0.844175034	0.871160268	negative
PRJNA729271	stage_E18_vs_E10	ordinary_muscle_development	ENSGALG00010027081	PDE4B	-0.222245321	0.2945272	-0.75458335	0.450499017	0.510075242	negative
PRJNA729271	stage_E18_vs_E10	ordinary_muscle_development	ENSGALG00010027082	SLC26A9	-1.899462525	0.639278373	-2.971260414	0.002965802	0.005082679	negative
PRJNA729271	stage_E18_vs_E10	ordinary_muscle_development	ENSGALG00010027083	DENND6A	-0.642340452	0.088377468	-7.268147264	3.64451E-13	1.61305E-12	negative
PRJNA729271	stage_E18_vs_E10	ordinary_muscle_development	ENSGALG00010027084	OSBPL9	0.044534741	0.094759903	0.469974524	0.638373219	0.687767192	positive
PRJNA729271	stage_E18_vs_E10	ordinary_muscle_development	ENSGALG00010027085	PIM1	-0.356118855	0.156493419	-2.275615526	0.022869031	0.034135167	negative
PRJNA729271	stage_E18_vs_E10	ordinary_muscle_development	ENSGALG00010027086	CACNA1S	4.069907325	0.201215435	20.22661598	5.7089E-91	5.48017E-89	positive
PRJNA729271	stage_E18_vs_E10	ordinary_muscle_development	ENSGALG00010027087	RAB7B	-0.237099688	0.348970384	-0.67942639	0.496867733	0.5541354	negative
PRJNA729271	stage_E18_vs_E10	ordinary_muscle_development	ENSGALG00010027088	AGBL4	0.366624646	0.260333799	1.408286773	0.159046171	0.202385589	positive
PRJNA729271	stage_E18_vs_E10	ordinary_muscle_development	ENSGALG00010027092	ENSGALG00010027092	-1.345034576	0.199813562	-6.73144787	1.67983E-11	6.48273E-11	negative
PRJNA729271	stage_E18_vs_E10	ordinary_muscle_development	ENSGALG00010027093	SLMAP	0.908335428	0.076092373	11.93727291	7.56635E-33	1.04352E-31	positive
PRJNA729271	stage_E18_vs_E10	ordinary_muscle_development	ENSGALG00010027095	SCN5A	-1.048817623	0.400638486	-2.617865378	0.00884817	0.014117142	negative
PRJNA729271	stage_E18_vs_E10	ordinary_muscle_development	ENSGALG00010027096	YIPF1	0.579783514	0.111327756	5.207897262	1.90993E-07	5.27221E-07	positive
PRJNA729271	stage_E18_vs_E10	ordinary_muscle_development	ENSGALG00010027097	ENSGALG00010027097	-0.315173076	0.148753656	-2.118758525	0.034110879	0.049310581	negative
PRJNA729271	stage_E18_vs_E10	ordinary_muscle_development	ENSGALG00010027098	EXOG	0.27424344	0.146523863	1.871664011	0.061253101	0.084598733	positive
PRJNA729271	stage_E18_vs_E10	ordinary_muscle_development	ENSGALG00010027099	GNA11	-0.255096048	0.085606116	-2.979881129	0.002883603	0.00495479	negative
PRJNA729271	stage_E18_vs_E10	ordinary_muscle_development	ENSGALG00010027100	MIER2	-0.205783507	0.090484036	-2.274252076	0.022950835	0.03424669	negative
PRJNA729271	stage_E18_vs_E10	ordinary_muscle_development	ENSGALG00010027101	RNF220	0.063277715	0.079413404	0.796814037	0.425559041	0.485745201	positive
PRJNA729271	stage_E18_vs_E10	ordinary_muscle_development	ENSGALG00010027102	DUS3L	-0.533830393	0.143732231	-3.714061836	0.000203959	0.00040851	negative
PRJNA729271	stage_E18_vs_E10	ordinary_muscle_development	ENSGALG00010027103	ACVR2B	-0.328099597	0.179624965	-1.826581271	0.067762751	0.092881426	negative
PRJNA729271	stage_E18_vs_E10	ordinary_muscle_development	ENSGALG00010027105	CHERP	-0.553175495	0.129416613	-4.274377786	1.91672E-05	4.32552E-05	negative
PRJNA729271	stage_E18_vs_E10	ordinary_muscle_development	ENSGALG00010027106	CYP2J23	0.226785288	0.150383578	1.508045571	0.131542864	0.170440137	positive
PRJNA729271	stage_E18_vs_E10	ordinary_muscle_development	ENSGALG00010027107	KIF21B	-1.155320643	0.204178671	-5.65838067	1.52808E-08	4.64935E-08	negative
PRJNA729271	stage_E18_vs_E10	ordinary_muscle_development	ENSGALG00010027108	ENSGALG00010027108	-0.142407333	0.145329054	-0.979895821	0.327137546	0.385203796	negative
PRJNA729271	stage_E18_vs_E10	ordinary_muscle_development	ENSGALG00010027109	C19orf44	-0.063283926	0.127746844	-0.495385435	0.620328074	0.671119229	negative
PRJNA729271	stage_E18_vs_E10	ordinary_muscle_development	ENSGALG00010027110	IKBKE	-0.606526257	0.225157856	-2.693782346	0.007064628	0.011413485	negative
PRJNA729271	stage_E18_vs_E10	ordinary_muscle_development	ENSGALG00010027111	CALR3	-2.341865558	0.827552323	-2.829870079	0.004656691	0.007750458	negative
PRJNA729271	stage_E18_vs_E10	ordinary_muscle_development	ENSGALG00010027112	XYLB	-0.5403317	0.182802156	-2.955827828	0.003118311	0.005327038	negative
PRJNA729271	stage_E18_vs_E10	ordinary_muscle_development	ENSGALG00010027113	PIAS4	-0.852780763	0.139861927	-6.097304559	1.07872E-09	3.63122E-09	negative
PRJNA729271	stage_E18_vs_E10	ordinary_muscle_development	ENSGALG00010027114	FLNB	-0.765999111	0.113740247	-6.73463558	1.64341E-11	6.35234E-11	negative
PRJNA729271	stage_E18_vs_E10	ordinary_muscle_development	ENSGALG00010027115	C9ORF152	-0.25677068	0.613092657	-0.418812193	0.675353393	0.721867667	negative
PRJNA729271	stage_E18_vs_E10	ordinary_muscle_development	ENSGALG00010027116	CCDC94	0.262913441	0.103015357	2.552177155	0.010705207	0.016875691	positive
PRJNA729271	stage_E18_vs_E10	ordinary_muscle_development	ENSGALG00010027117	RASSF5	0.6100165	0.251817509	2.422454664	0.015416049	0.02374409	positive
PRJNA729271	stage_E18_vs_E10	ordinary_muscle_development	ENSGALG00010027118	ABHD6	-0.048782914	0.168902575	-0.288822798	0.772716985	0.809295778	negative
PRJNA729271	stage_E18_vs_E10	ordinary_muscle_development	ENSGALG00010027119	NUB1	0.675578137	0.098975745	6.825693886	8.75013E-12	3.45218E-11	positive
PRJNA729271	stage_E18_vs_E10	ordinary_muscle_development	ENSGALG00010027121	ELF3	-1.617228751	0.977678899	-1.654151227	0.09809679	0.130295333	negative
PRJNA729271	stage_E18_vs_E10	ordinary_muscle_development	ENSGALG00010027122	DNASE1L3	2.534719878	0.251367868	10.0837068	6.52183E-24	5.76778E-23	positive
PRJNA729271	stage_E18_vs_E10	ordinary_muscle_development	ENSGALG00010027123	WDR86	-0.933666112	0.220273451	-4.238668386	2.2485E-05	5.03195E-05	negative
PRJNA729271	stage_E18_vs_E10	ordinary_muscle_development	ENSGALG00010027124	IP6K2	-0.078623835	0.174762093	-0.449890666	0.652789278	0.700902265	negative
PRJNA729271	stage_E18_vs_E10	ordinary_muscle_development	ENSGALG00010027125	CRYGN	0.330406014	0.445357167	0.741889968	0.458154005	0.517632523	positive
PRJNA729271	stage_E18_vs_E10	ordinary_muscle_development	ENSGALG00010027126	ENSGALG00010027126	6.732810014	0.537257152	12.53182018	5.00018E-36	8.09805E-35	positive
PRJNA729271	stage_E18_vs_E10	ordinary_muscle_development	ENSGALG00010027127	S1PR4	0.767611554	0.705763807	1.087632359	0.276757439	0.332251042	positive
PRJNA729271	stage_E18_vs_E10	ordinary_muscle_development	ENSGALG00010027128	EIF2D	0.246293997	0.090486329	2.721891816	0.006490939	0.010562231	positive
PRJNA729271	stage_E18_vs_E10	ordinary_muscle_development	ENSGALG00010027129	NCLN	0.091244971	0.129393127	0.705176329	0.480700511	0.538799405	positive
PRJNA729271	stage_E18_vs_E10	ordinary_muscle_development	ENSGALG00010027130	NCKIPSD	0.378655706	0.144223052	2.625486716	0.008652519	0.013824764	positive
PRJNA729271	stage_E18_vs_E10	ordinary_muscle_development	ENSGALG00010027131	UBA52	0.956252102	0.152191284	6.283225139	3.3162E-10	1.1636E-09	positive
PRJNA729271	stage_E18_vs_E10	ordinary_muscle_development	ENSGALG00010027133	CELF5	-1.735603072	0.254065246	-6.831328165	8.4132E-12	3.3274E-11	negative
PRJNA729271	stage_E18_vs_E10	ordinary_muscle_development	ENSGALG00010027134	DYRK3	1.865626885	0.148311271	12.57913088	2.75039E-36	4.53549E-35	positive
PRJNA729271	stage_E18_vs_E10	ordinary_muscle_development	ENSGALG00010027135	PRKAG2	-0.617297377	0.140928672	-4.380211414	1.18564E-05	2.73835E-05	negative

PRJNA729271	stage_E18_vs_E10	ordinary_muscle_development	ENSGALG00010027136	MAPKAPK2	1.749566316	0.143930941	12.15559563	5.35699E-34	7.86723E-33	positive
PRJNA729271	stage_E18_vs_E10	ordinary_muscle_development	ENSGALG00010027137	FKBP8	0.25499775	0.091252353	2.794423833	0.005199229	0.008599135	positive
PRJNA729271	stage_E18_vs_E10	ordinary_muscle_development	ENSGALG00010027139	HSD11B1L	0.533619168	0.138389593	3.855919775	0.000115295	0.00023871	positive
PRJNA729271	stage_E18_vs_E10	ordinary_muscle_development	ENSGALG00010027141	PIGR	4.010944416	1.479620282	2.710793077	0.00671225	0.010898853	positive
PRJNA729271	stage_E18_vs_E10	ordinary_muscle_development	ENSGALG00010027143	GALNT11	-1.151277914	0.122690595	-9.38358734	6.37655E-21	4.78158E-20	negative
PRJNA729271	stage_E18_vs_E10	ordinary_muscle_development	ENSGALG00010027144	ENSGALG00010027144	-2.959879952	0.782618842	-3.782019794	0.000155561	0.000316604	negative
PRJNA729271	stage_E18_vs_E10	ordinary_muscle_development	ENSGALG00010027145	CELSR3	-0.572016203	0.166235444	-3.441000233	0.000579568	0.001091869	negative
PRJNA729271	stage_E18_vs_E10	ordinary_muscle_development	ENSGALG00010027146	KXD1	0.192466032	0.129310421	1.488403105	0.136644618	0.176498284	positive
PRJNA729271	stage_E18_vs_E10	ordinary_muscle_development	ENSGALG00010027147	KMT2C	0.486787135	0.103694269	4.694445912	2.6733E-06	6.59006E-06	positive
PRJNA729271	stage_E18_vs_E10	ordinary_muscle_development	ENSGALG00010027148	ELL	1.25184701	0.165512118	7.563476493	3.92437E-14	1.85834E-13	positive
PRJNA729271	stage_E18_vs_E10	ordinary_muscle_development	ENSGALG00010027149	C1orf116	0.630697427	0.240591921	2.62144059	0.008755902	0.013979161	positive
PRJNA729271	stage_E18_vs_E10	ordinary_muscle_development	ENSGALG00010027150	YOD1	-0.797499352	0.09169903	-8.696922428	3.41006E-18	2.16319E-17	negative
PRJNA729271	stage_E18_vs_E10	ordinary_muscle_development	ENSGALG00010027151	MICOS13	1.126104065	0.143710065	7.835944283	4.65332E-15	2.38171E-14	positive
PRJNA729271	stage_E18_vs_E10	ordinary_muscle_development	ENSGALG00010027152	C4BPS	0.454099763	0.466393687	0.97364046	0.33023509	0.38836368	positive
PRJNA729271	stage_E18_vs_E10	ordinary_muscle_development	ENSGALG00010027153	ENSGALG00010027153	-0.510823441	0.231878084	-2.202982845	0.027595958	0.04058888	negative
PRJNA729271	stage_E18_vs_E10	ordinary_muscle_development	ENSGALG00010027154	PLPP2	0.537705219	0.136958543	3.926043643	8.63545E-05	0.000181302	positive
PRJNA729271	stage_E18_vs_E10	ordinary_muscle_development	ENSGALG00010027155	ENSGALG00010027155	0.514809063	0.296809744	1.734474941	0.082833848	0.111628545	positive
PRJNA729271	stage_E18_vs_E10	ordinary_muscle_development	ENSGALG00010027156	ISYNA1	-0.567805599	0.146953707	-3.86383992	0.000111618	0.000231626	negative
PRJNA729271	stage_E18_vs_E10	ordinary_muscle_development	ENSGALG00010027157	ENSGALG00010027157	1.30825093	0.251382249	5.204229555	1.94803E-07	5.37228E-07	positive
PRJNA729271	stage_E18_vs_E10	ordinary_muscle_development	ENSGALG00010027158	XRCC2	-0.648885673	0.138603594	-4.681593423	2.84654E-06	6.99568E-06	negative
PRJNA729271	stage_E18_vs_E10	ordinary_muscle_development	ENSGALG00010027159	TMUB1	0.484077502	0.165170245	2.930779092	0.003381131	0.005728085	positive
PRJNA729271	stage_E18_vs_E10	ordinary_muscle_development	ENSGALG00010027160	FASTK	0.145060324	0.167408101	0.866507191	0.386212085	0.446458582	positive
PRJNA729271	stage_E18_vs_E10	ordinary_muscle_development	ENSGALG00010027161	C4BPG	0.493732877	0.204338341	2.416251768	0.015681214	0.024126866	positive
PRJNA729271	stage_E18_vs_E10	ordinary_muscle_development	ENSGALG00010027162	SLC4A2	0.006779791	0.132506109	0.051165872	0.959193347	0.966393797	positive
PRJNA729271	stage_E18_vs_E10	ordinary_muscle_development	ENSGALG00010027163	ENSGALG00010027163	-0.514676657	0.079312481	-6.489226579	8.62781E-11	3.16288E-10	negative
PRJNA729271	stage_E18_vs_E10	ordinary_muscle_development	ENSGALG00010027164	ENSGALG00010027164	1.897482434	0.46263678	4.101451758	4.10566E-05	8.9196E-05	positive
PRJNA729271	stage_E18_vs_E10	ordinary_muscle_development	ENSGALG00010027165	ENSGALG00010027165	-2.810550321	0.223687042	-12.56465414	3.30316E-36	5.4285E-35	negative
PRJNA729271	stage_E18_vs_E10	ordinary_muscle_development	ENSGALG00010027166	CDK5	0.14069646	0.100249352	1.403465037	0.160478211	0.20402874	positive
PRJNA729271	stage_E18_vs_E10	ordinary_muscle_development	ENSGALG00010027167	ENSGALG00010027167	-0.002647229	0.349492563	-0.007574493	0.993956486	0.994848727	negative
PRJNA729271	stage_E18_vs_E10	ordinary_muscle_development	ENSGALG00010027168	SSBP4	-0.874018118	0.18294232	-4.777561128	1.77434E-06	4.46357E-06	negative
PRJNA729271	stage_E18_vs_E10	ordinary_muscle_development	ENSGALG00010027169	ABCB8	0.505691447	0.11165841	4.528914993	5.92873E-06	1.41299E-05	positive
PRJNA729271	stage_E18_vs_E10	ordinary_muscle_development	ENSGALG00010027170	TLE2	-0.475141222	0.175073303	-2.713955897	0.006648502	0.010803815	negative
PRJNA729271	stage_E18_vs_E10	ordinary_muscle_development	ENSGALG00010027171	ATG9B	2.955846527	0.274268377	10.77720503	4.41085E-27	4.69077E-26	positive
PRJNA729271	stage_E18_vs_E10	ordinary_muscle_development	ENSGALG00010027172	ENSGALG00010027172	1.573707554	0.439894127	3.57746889	0.000346937	0.000673117	positive
PRJNA729271	stage_E18_vs_E10	ordinary_muscle_development	ENSGALG00010027173	ENSGALG00010027173	0.476895332	0.096875996	4.9227399	8.53408E-07	2.22405E-06	positive
PRJNA729271	stage_E18_vs_E10	ordinary_muscle_development	ENSGALG00010027174	SHISA5	0.466725287	0.132422495	3.52451664	0.000424256	0.000814731	positive
PRJNA729271	stage_E18_vs_E10	ordinary_muscle_development	ENSGALG00010027175	NOS3	1.095782098	0.164252394	6.67133108	2.53494E-11	9.67199E-11	positive
PRJNA729271	stage_E18_vs_E10	ordinary_muscle_development	ENSGALG00010027176	ENSGALG00010027176	0.194479842	0.171000177	1.137307839	0.255409619	0.309286752	positive
PRJNA729271	stage_E18_vs_E10	ordinary_muscle_development	ENSGALG00010027178	C4BPM	0.366185752	0.082160165	4.456974385	8.31245E-06	1.95282E-05	positive
PRJNA729271	stage_E18_vs_E10	ordinary_muscle_development	ENSGALG00010027183	CD34	1.609431276	0.15608034	10.31155668	6.2481E-25	5.90777E-24	positive
PRJNA729271	stage_E18_vs_E10	ordinary_muscle_development	ENSGALG00010027184	MPND	-0.775744966	0.36674844	-2.115196365	0.034413215	0.049717886	negative
PRJNA729271	stage_E18_vs_E10	ordinary_muscle_development	ENSGALG00010027185	ATRIP	-0.621581443	0.081753223	-7.603142956	2.89024E-14	1.3831E-13	negative
PRJNA729271	stage_E18_vs_E10	ordinary_muscle_development	ENSGALG00010027186	PLXNA2	1.323816177	0.12278632	10.78146309	4.21133E-27	4.48518E-26	positive
PRJNA729271	stage_E18_vs_E10	ordinary_muscle_development	ENSGALG00010027187	ENSGALG00010027187	-0.050948187	0.135048707	-0.377257867	0.70598198	0.75056541	negative
PRJNA729271	stage_E18_vs_E10	ordinary_muscle_development	ENSGALG00010027188	KCNH2	2.295607227	0.253343275	9.06125187	1.28962E-19	8.88029E-19	positive
PRJNA729271	stage_E18_vs_E10	ordinary_muscle_development	ENSGALG00010027189	ENSGALG00010027189	-0.002482059	0.27163387	-0.009137517	0.992709418	0.993763408	negative
PRJNA729271	stage_E18_vs_E10	ordinary_muscle_development	ENSGALG00010027190	IPPK	-0.590724309	0.117722478	-5.017939822	5.22285E-07	1.38731E-06	negative
PRJNA729271	stage_E18_vs_E10	ordinary_muscle_development	ENSGALG00010027192	SH3GL1	0.128089782	0.115903703	1.10513969	0.269099065	0.324130875	positive
PRJNA729271	stage_E18_vs_E10	ordinary_muscle_development	ENSGALG00010027193	CAMK1G	-2.863854726	0.601240306	-4.76324474	1.90505E-06	4.77332E-06	negative
PRJNA729271	stage_E18_vs_E10	ordinary_muscle_development	ENSGALG00010027194	ENSGALG00010027194	-1.826237834	0.265154389	-6.887450899	5.6801E-12	2.2883E-11	negative
PRJNA729271	stage_E18_vs_E10	ordinary_muscle_development	ENSGALG00010027195	CENPP	-1.623939581	0.201369381	-8.064481169	7.35475E-16	3.99278E-15	negative
PRJNA729271	stage_E18_vs_E10	ordinary_muscle_development	ENSGALG00010027196	ENSGALG00010027196	0.587413383	0.331801966	1.770373427	0.076664954	0.104011466	positive
PRJNA729271	stage_E18_vs_E10	ordinary_muscle_development	ENSGALG00010027197	ENSGALG00010027197	-1.344184782	0.540890485	-2.485132976	0.012950305	0.020160527	negative
PRJNA729271	stage_E18_vs_E10	ordinary_muscle_development	ENSGALG00010027198	ECM2	1.135869613	0.203095999	5.592771987	2.23473E-08	6.69817E-08	positive
PRJNA729271	stage_E18_vs_E10	ordinary_muscle_development	ENSGALG00010027199	LAMB3	0.847708118	0.292431397	2.898827308	0.003745611	0.006310896	positive
PRJNA729271	stage_E18_vs_E10	ordinary_muscle_development	ENSGALG00010027200	G0S2	5.404694391	0.317225272	17.03740169	4.33571E-65	2.02077E-63	positive
PRJNA729271	stage_E18_vs_E10	ordinary_muscle_development	ENSGALG00010027201	ASPN	-0.328653868	0.316845002	-1.037270166	0.299609966	0.356614095	negative
PRJNA729271	stage_E18_vs_E10	ordinary_muscle_development	ENSGALG00010027202	CHAF1A	-1.512542292	0.086993443	-17.38685406	1.03776E-67	5.20497E-66	negative
PRJNA729271	stage_E18_vs_E10	ordinary_muscle_development	ENSGALG00010027203	ENSGALG00010027203	-0.321939819	0.299731292	-1.074094791	0.282780199	0.338416363	negative
PRJNA729271	stage_E18_vs_E10	ordinary_muscle_development	ENSGALG00010027204	OMD	-0.624884383	0.788516871	-0.792480676	0.428080455	0.48812352	negative
PRJNA729271	stage_E18_vs_E10	ordinary_muscle_development	ENSGALG00010027205	OGN	1.920019632	0.221831594	8.655302855	4.91605E-18	3.0821E-17	positive
PRJNA729271	stage_E18_vs_E10	ordinary_muscle_development	ENSGALG00010027206	ENSGALG00010027206	7.9508303	0.965719807	8.233061225	1.82489E-16	1.02845E-15	positive
PRJNA729271	stage_E18_vs_E10	ordinary_muscle_development	ENSGALG00010027207	NOL8	-0.68193449	0.125181561	-5.44756341	5.10645E-08	1.48273E-07	negative
PRJNA729271	stage_E18_vs_E10	ordinary_muscle_development	ENSGALG00010027208	UBXN6	0.355143944	0.096540239	3.678714152	0.000234413	0.000466028	positive
PRJNA729271	stage_E18_vs_E10	ordinary_muscle_development	ENSGALG00010027209	TRAF3IP3	0.549651487	0.265127419	2.073159724	0.038157417	0.05471822	positive
PRJNA729271	stage_E18_vs_E10	ordinary_muscle_development	ENSGALG00010027210	ENSGALG00010027210	-1.431975443	0.276281602	-5.183028584	2.18312E-07	5.9955E-07	negative
PRJNA729271	stage_E18_vs_E10	ordinary_muscle_development	ENSGALG00010027211	HDGFRP2	-0.446376254	0.101869401	-4.381848232	1.17677E-05	2.71829E-05	negative
PRJNA729271	stage_E18_vs_E10	ordinary_muscle_development	ENSGALG00010027212	IRF6	-3.127694401	0.55505339	-5.634943331	1.75116E-08	5.29365E-08	negative
PRJNA729271	stage_E18_vs_E10	ordinary_muscle_development	ENSGALG00010027213	KCNC4	0.208507361	0.349688043	0.596266773	0.550997024	0.605099013	positive
PRJNA729271	stage_E18_vs_E10	ordinary_muscle_development	ENSGALG00010027214	PLIN4	1.574211365	0.266146407	5.914832298	3.32214E-09	1.072E-08	positive
PRJNA729271	stage_E18_vs_E10	ordinary_muscle_development	ENSGALG00010027215	ENSGALG00010027215	-1.681561938	0.194861542	-8.629521889	6.1609E-18	3.83271E-17	negative
PRJNA729271	stage_E18_vs_E10	ordinary_muscle_development	ENSGALG00010027216	ENSGALG00010027216	1.031778124	0.609219018	1.69360787	0.090339821	0.120811487	positive
PRJNA729271	stage_E18_vs_E10	ordinary_muscle_development	ENSGALG00010027217	SEMA6B	1.903680036	0.219919272	8.656267444	4.87464E-18	3.05878E-17	positive
PRJNA729271	stage_E18_vs_E10	ordinary_muscle_development	ENSGALG00010027218	IARS1	-0.672429567	0.164709211	-4.082525592	4.45489E-05	9.63642E-05	negative
PRJNA729271	stage_E18_vs_E10	ordinary_muscle_development	ENSGALG00010027219	ENSGALG00010027219	0.12840052	0.13806804	0.929980028	0.352381425	0.411518592	positive
PRJNA729271	stage_E18_vs_E10	ordinary_muscle_development	ENSGALG00010027220	ANKRD24	-1.244953246	0.619625091	-2.009204057	0.044515498	0.063043687	negative
PRJNA729271	stage_E18_vs_E10	ordinary_muscle_development	ENSGALG00010027221	LRRC61	-1.139972152	0.280883007	-4.058530148	4.93826E-05	0.000106407	negative
PRJNA729271	stage_E18_vs_E10	ordinary_muscle_development	ENSGALG00010027222	GUCA1B	5.813749981	0.822721293	7.066487798	1.58904E-12	6.69958E-12	positive
PRJNA729271	stage_E18_vs_E10	ordinary_muscle_development	ENSGALG00010027223	SIRT6	-0.225799997	0.083602344	-2.700881191	0.006915604	0.011192684	negative
PRJNA729271	stage_E18_vs_E10	ordinary_muscle_development	ENSGALG00010027224	RARRES2	0.948728212	0.121138909	7.831738095	4.81171E-15	2.46104E-14	positive
PRJNA729271	stage_E18_vs_E10	ordinary_muscle_development	ENSGALG00010027225	CREB3L3	0.674504839	0.70833664	0.952237681	0.340976455	0.399390199	positive
PRJNA729271	stage_E18_vs_E10	ordinary_muscle_development	ENSGALG00010027227	ENSGALG00010027227	0.439437837	0.184716703	2.378982677	0.017360492	0.026463385	positive
PRJNA729271	stage_E18_vs_E10	ordinary_muscle_development	ENSGALG00010027229	MDM4	0.13582281	0.082004203	1.656290844	0.097662934	0.12979043	positive
PRJNA729271	stage_E18_vs_E10	ordinary_muscle_development	ENSGALG00010027230	MAP2K2	-1.213747135	0.08475333	-14.32093739	1.61924E-46	4.1322E-45	negative
PRJNA729271	stage_E18_vs_E10	ordinary_muscle_development	ENSGALG00010027231	ENSGALG00010027231	0.685875689	0.153791626	4.459772674	8.20466E-06	1.92844E-05	positive
PRJNA729271	stage_E18_vs_E10	ordinary_muscle_development	ENSGALG00010027232	ARRDC2	1.779820571	0.327728475	5.430777941	5.61089E-08	1.62303E-07	positive
PRJNA729271	stage_E18_vs_E10	ordinary_muscle_development	ENSGALG00010027233	ENSGALG00010027233	0.729429833	0.270743718	2.694170849	0.007056398	0.01140146	positive
PRJNA729271	stage_E18_vs_E10	ordinary_muscle_development	ENSGALG00010027234	SLC6A11	-2.87436367	0.431606339	-6.659688265	2.74409E-11	1.04316E-10	negative
PRJNA729271	stage_E18_vs_E10	ordinary_muscle_development	ENSGALG00010027236	PEX11G	0.180830662	0.281523076	0.642329802	0.520659068	0.577646628	positive
PRJNA729271	stage_E18_vs_E10	ordinary_muscle_development	ENSGALG00010027237	CTC-575C13.4	0.823264291	0.153903225	5.349233519	8.83275E-08	2.51287E-07	positive
PRJNA729271	stage_E18_vs_E10	ordinary_muscle_development	ENSGALG00010027239	ENSGALG00010027239	0.033574758	0.083387198	0.402636846	0.6872154	0.733033131	positive
PRJNA729271	stage_E18_vs_E10	ordinary_muscle_development	ENSGALG00010027240	SLC6A1	-0.781612179	0.351249627	-2.225232766	0.026065616	0.038513874	negative
PRJNA729271	stage_E18_vs_E10	ordinary_muscle_development	ENSGALG00010027241	LRRN2	-1.809391345	0.274762686	-6.585287725	4.54005E-11	1.70223E-10	negative
PRJNA729271	stage_E18_vs_E10	ordinary_muscle_development	ENSGALG00010027242	ENSGALG00010027242	0.395576474	0.190999052	2.071091294	0.038350265	0.054962141	positive
PRJNA729271	stage_E18_vs_E10	ordinary_muscle_development	ENSGALG00010027243	HRH1	-2.015230381	0.2244471	-8.97864297	2.74127E-19	1.85157E-18	negative
PRJNA729271	stage_E18_vs_E10	ordinary_muscle_development	ENSGALG00010027244	ARHGEF18	0.122171874	0.091015641	1.342317348	0.179493121	0.225650719	positive
PRJNA729271	stage_E18_vs_E10	ordinary_muscle_development	ENSGALG00010027245	LRIG2	0.317006778	0.06591891	4.809041543	1.51656E-06	3.84511E-06	positive
PRJNA729271	stage_E18_vs_E10	ordinary_muscle_development	ENSGALG00010027246	ZNF767	-0.807886079	0.101246947	-7.979362344	1.47091E-15	7.80412E-15	negative
PRJNA729271	stage_E18_vs_E10	ordinary_muscle_development	ENSGALG00010027247	ATG7	-0.378796073	0.082297503	-4.602765073	4.16918E-06	1.00603E-05	negative
PRJNA729271	stage_E18_vs_E10	ordinary_muscle_development	ENSGALG00010027248	ENSGALG00010027248	-0.374527484	0.604920579	-0.619134969	0.535827448	0.591352129	negative
PRJNA729271	stage_E18_vs_E10	ordinary_muscle_development	ENSGALG00010027249	C2CD4C	10.11306046	0.93199402	10.85099287	1.97271E-27	2.1387E-26	positive
PRJNA729271	stage_E18_vs_E10	ordinary_muscle_development	ENSGALG00010027250	ENSGALG00010027250	-0.151035883	0.173371873	-0.871167163	0.383662887	0.443972022	negative
PRJNA729271	stage_E18_vs_E10	ordinary_muscle_development	ENSGALG00010027251	ENSGALG00010027251	-0.065535323	0.204118453	-0.321065156	0.748161015	0.788296425	negative
PRJNA729271	stage_E18_vs_E10	ordinary_muscle_development	ENSGALG00010027252	MAGI3	0.641263099	0.071107947	9.018163562	1.91269E-19	1.30468E-18	positive
PRJNA729271	stage_E18_vs_E10	ordinary_muscle_development	ENSGALG00010027253	VGLL4	-0.513240888	0.087767392	-5.847740007	4.98297E-09	1.5836E-08	negative
PRJNA729271	stage_E18_vs_E10	ordinary_muscle_development	ENSGALG00010027254	INSR	0.99416892	0.133201546	7.463643989	8.41618E-14	3.89628E-13	positive
PRJNA729271	stage_E18_vs_E10	ordinary_muscle_development	ENSGALG00010027255	ENSGALG00010027255	-0.278223303	0.182731815	-1.522577242	0.127864507	0.166268595	negative
PRJNA729271	stage_E18_vs_E10	ordinary_muscle_development	ENSGALG00010027256	PHTF1	-1.487402504	0.091330795	-16.28588155	1.24325E-59	4.95079E-58	negative
PRJNA729271	stage_E18_vs_E10	ordinary_muscle_development	ENSGALG00010027257	TAFA3	1.970114469	0.267740685	7.35829324	1.86276E-13	8.39055E-13	positive
PRJNA729271	stage_E18_vs_E10	ordinary_muscle_development	ENSGALG00010027258	ENSGALG00010027258	-0.995202495	0.173059492	-5.750638009	8.89073E-09	2.76014E-08	negative
PRJNA729271	stage_E18_vs_E10	ordinary_muscle_development	ENSGALG00010027259	ENSGALG00010027259	0.061394267	0.083003816	0.739655953	0.459508781	0.518913415	positive
PRJNA729271	stage_E18_vs_E10	ordinary_muscle_development	ENSGALG00010027260	LEMD2	0.305123791	0.100793352	3.027221403	0.002468131	0.004275282	positive
PRJNA729271	stage_E18_vs_E10	ordinary_muscle_development	ENSGALG00010027261	ENSGALG00010027261	-0.07258929	0.111870479	-0.648869038	0.516423029	0.573210185	negative
PRJNA729271	stage_E18_vs_E10	ordinary_muscle_development	ENSGALG00010027262	USE1	-0.846205123	0.137093618	-6.172461811	6.72348E-10	2.31269E-09	negative
PRJNA729271	stage_E18_vs_E10	ordinary_muscle_development	ENSGALG00010027263	TAMM41	-1.177880974	0.128495193	-9.166731832	4.87577E-20	3.42912E-19	negative
PRJNA729271	stage_E18_vs_E10	ordinary_muscle_development	ENSGALG00010027264	ENSGALG00010027264	0.728798571	0.094855618	7.68324101	1.55113E-14	7.62676E-14	positive
PRJNA729271	stage_E18_vs_E10	ordinary_muscle_development	ENSGALG00010027265	IP6K3	3.704777878	0.208069471	17.8054852	6.40746E-71	3.50476E-69	positive
PRJNA729271	stage_E18_vs_E10	ordinary_muscle_development	ENSGALG00010027267	MYO9B	-0.586952005	0.096083074	-6.108797086	1.00385E-09	3.38627E-09	negative
PRJNA729271	stage_E18_vs_E10	ordinary_muscle_development	ENSGALG00010027268	UQCRC1	0.778961635	0.126732253	6.146514554	7.92041E-10	2.70069E-09	positive
PRJNA729271	stage_E18_vs_E10	ordinary_muscle_development	ENSGALG00010027269	CDC25A	-0.879136529	0.096246614	-9.134207332	6.58877E-20	4.60261E-19	negative
PRJNA729271	stage_E18_vs_E10	ordinary_muscle_development	ENSGALG00010027270	ENSGALG00010027270	-1.217711641	0.394593177	-3.085992646	0.002028738	0.003559686	negative
PRJNA729271	stage_E18_vs_E10	ordinary_muscle_development	ENSGALG00010027271	INKA2	1.360417024	0.140604019	9.675520174	3.83138E-22	3.07508E-21	positive
PRJNA729271	stage_E18_vs_E10	ordinary_muscle_development	ENSGALG00010027272	DDX20	-0.971022318	0.106030711	-9.157934613	5.29001E-20	3.71505E-19	negative
PRJNA729271	stage_E18_vs_E10	ordinary_muscle_development	ENSGALG00010027273	HAUS8	-1.268393122	0.133008917	-9.536151054	1.48232E-21	1.15579E-20	negative
PRJNA729271	stage_E18_vs_E10	ordinary_muscle_development	ENSGALG00010027274	COPG1	-0.382991407	0.060765298	-6.302798191	2.92319E-10	1.03019E-09	negative
PRJNA729271	stage_E18_vs_E10	ordinary_muscle_development	ENSGALG00010027275	CPAMD8	0.805274004	0.227121813	3.545559949	0.00039178	0.000755166	positive
PRJNA729271	stage_E18_vs_E10	ordinary_muscle_development	ENSGALG00010027276	ENSGALG00010027276	0.139972036	0.138576813	1.010068225	0.312462604	0.369967771	positive
PRJNA729271	stage_E18_vs_E10	ordinary_muscle_development	ENSGALG00010027277	KCND3	1.334364658	0.398245707	3.350606505	0.000806348	0.001490058	positive
PRJNA729271	stage_E18_vs_E10	ordinary_muscle_development	ENSGALG00010027278	H1-10	-0.271520692	0.129206138	-2.101453512	0.035601175	0.05131653	negative
PRJNA729271	stage_E18_vs_E10	ordinary_muscle_development	ENSGALG00010027279	F2RL3	1.414744074	0.343413728	4.11964915	3.7945E-05	8.27709E-05	positive
PRJNA729271	stage_E18_vs_E10	ordinary_muscle_development	ENSGALG00010027280	HMCES	-0.874286599	0.12773313	-6.844634607	7.66713E-12	3.04563E-11	negative

PRJNA729271	stage_E18_vs_E10	ordinary_muscle_development	ENSGALG00010027281	ENSGALG00010027281	-0.119758625	0.25065748	-0.477777983	0.632808233	0.682481796	negative
PRJNA729271	stage_E18_vs_E10	ordinary_muscle_development	ENSGALG00010027282	ENSGALG00010027282	-0.349288214	0.131972263	-2.646678985	0.008128648	0.013027946	negative
PRJNA729271	stage_E18_vs_E10	ordinary_muscle_development	ENSGALG00010027283	RAB7A	0.145911765	0.063198779	2.30877505	0.020956067	0.031490586	positive
PRJNA729271	stage_E18_vs_E10	ordinary_muscle_development	ENSGALG00010027284	CTTNBP2NL	0.794160135	0.09081058	8.745238012	2.22546E-18	1.42798E-17	positive
PRJNA729271	stage_E18_vs_E10	ordinary_muscle_development	ENSGALG00010027285	PTPRS	-0.026469274	0.111871188	-0.236604926	0.812963298	0.84442157	negative
PRJNA729271	stage_E18_vs_E10	ordinary_muscle_development	ENSGALG00010027286	RPN1	0.587087655	0.062720306	9.360407991	7.94294E-21	5.91031E-20	positive
PRJNA729271	stage_E18_vs_E10	ordinary_muscle_development	ENSGALG00010027287	DHX30	-0.65840672	0.090166132	-7.3021511	2.83203E-13	1.26192E-12	negative
PRJNA729271	stage_E18_vs_E10	ordinary_muscle_development	ENSGALG00010027288	DAG1	2.140022094	0.167668984	12.76337486	2.62574E-37	4.48293E-36	positive
PRJNA729271	stage_E18_vs_E10	ordinary_muscle_development	ENSGALG00010027289	SMARCC1	-0.629798228	0.139379645	-4.518581094	6.22554E-06	1.47957E-05	negative
PRJNA729271	stage_E18_vs_E10	ordinary_muscle_development	ENSGALG00010027290	CSPG5	-1.642308282	0.221753641	-7.406003699	1.30162E-13	5.93489E-13	negative
PRJNA729271	stage_E18_vs_E10	ordinary_muscle_development	ENSGALG00010027291	MST1	0.77094153	0.278068923	2.772483601	0.005563032	0.009155916	positive
PRJNA729271	stage_E18_vs_E10	ordinary_muscle_development	ENSGALG00010027292	KDM4B	0.296113931	0.060969534	4.856752388	1.19327E-06	3.06023E-06	positive
PRJNA729271	stage_E18_vs_E10	ordinary_muscle_development	ENSGALG00010027293	ELP6	-0.220331667	0.151939712	-1.450125612	0.147023494	0.188526676	negative
PRJNA729271	stage_E18_vs_E10	ordinary_muscle_development	ENSGALG00010027295	ENSGALG00010027295	1.483375014	0.248699049	5.964538355	2.45327E-09	8.00723E-09	positive
PRJNA729271	stage_E18_vs_E10	ordinary_muscle_development	ENSGALG00010027297	ENSGALG00010027297	1.178997628	2.096527412	0.562357364	0.573872562	0.62708502	positive
PRJNA729271	stage_E18_vs_E10	ordinary_muscle_development	ENSGALG00010027299	PGPEP1	1.716053073	0.161196141	10.64574536	1.82518E-26	1.88971E-25	positive
PRJNA729271	stage_E18_vs_E10	ordinary_muscle_development	ENSGALG00010027301	SCAP	-0.389067713	0.065286442	-5.959395287	2.53173E-09	8.24847E-09	negative
PRJNA729271	stage_E18_vs_E10	ordinary_muscle_development	ENSGALG00010027303	APEH	-0.253626161	0.141591433	-1.791253573	0.073252613	0.099774161	negative
PRJNA729271	stage_E18_vs_E10	ordinary_muscle_development	ENSGALG00010027304	PTPN23	0.872415215	0.138030201	6.32046617	2.60775E-10	9.21937E-10	positive
PRJNA729271	stage_E18_vs_E10	ordinary_muscle_development	ENSGALG00010027305	LSM4	-1.278243983	0.131736351	-9.703046815	2.92627E-22	2.37228E-21	negative
PRJNA729271	stage_E18_vs_E10	ordinary_muscle_development	ENSGALG00010027306	ENSGALG00010027306	1.005123463	0.339641767	2.959363547	0.003082752	0.005271884	positive
PRJNA729271	stage_E18_vs_E10	ordinary_muscle_development	ENSGALG00010027307	JUND	0.411885131	0.176358282	2.335502069	0.019517208	0.029484307	positive
PRJNA729271	stage_E18_vs_E10	ordinary_muscle_development	ENSGALG00010027309	ENSGALG00010027309	1.533723194	0.711748558	2.154866597	0.031172275	0.045420399	positive
PRJNA729271	stage_E18_vs_E10	ordinary_muscle_development	ENSGALG00010027310	ARHGEF2	0.124349982	0.132658601	0.93736841	0.348569085	0.407328997	positive
PRJNA729271	stage_E18_vs_E10	ordinary_muscle_development	ENSGALG00010027311	NFASC	0.65184798	0.244657163	2.664332295	0.007714132	0.012403366	positive
PRJNA729271	stage_E18_vs_E10	ordinary_muscle_development	ENSGALG00010027312	ENSGALG00010027312	0.582077237	0.766031564	0.759860643	0.447337889	0.506892019	positive
PRJNA729271	stage_E18_vs_E10	ordinary_muscle_development	ENSGALG00010027313	BSN	-0.490867947	0.238100173	-2.06160265	0.039245582	0.056143104	negative
PRJNA729271	stage_E18_vs_E10	ordinary_muscle_development	ENSGALG00010027314	ENSGALG00010027314	1.658655011	0.358319075	4.628988872	3.67456E-06	8.92023E-06	positive
PRJNA729271	stage_E18_vs_E10	ordinary_muscle_development	ENSGALG00010027315	RAB3A	-0.243862368	0.233263077	-1.045439212	0.295820022	0.35256629	negative
PRJNA729271	stage_E18_vs_E10	ordinary_muscle_development	ENSGALG00010027316	TRAIP	-2.114392115	0.211832041	-9.981455594	1.83751E-23	1.5807E-22	negative
PRJNA729271	stage_E18_vs_E10	ordinary_muscle_development	ENSGALG00010027317	MPV17L2	0.412625406	0.147562053	2.796283985	0.005169396	0.008551745	positive
PRJNA729271	stage_E18_vs_E10	ordinary_muscle_development	ENSGALG00010027319	MST1R	1.203429527	0.341357618	3.525421623	0.000422809	0.000812168	positive
PRJNA729271	stage_E18_vs_E10	ordinary_muscle_development	ENSGALG00010027320	MON1A	-0.032479015	0.16279968	-0.199502939	0.841869345	0.869214058	negative
PRJNA729271	stage_E18_vs_E10	ordinary_muscle_development	ENSGALG00010027321	IFI30	0.787226185	0.206171434	3.818308729	0.00013437	0.000275894	positive
PRJNA729271	stage_E18_vs_E10	ordinary_muscle_development	ENSGALG00010027322	CRABP2	-3.657025391	0.178716474	-20.4627213	4.62817E-93	4.69128E-91	negative
PRJNA729271	stage_E18_vs_E10	ordinary_muscle_development	ENSGALG00010027323	ISG20L2	-0.604295516	0.132759089	-4.551820284	5.31838E-06	1.27295E-05	negative
PRJNA729271	stage_E18_vs_E10	ordinary_muscle_development	ENSGALG00010027324	PIK3R2	0.122690245	0.097039378	1.26433462	0.206109948	0.255675114	positive
PRJNA729271	stage_E18_vs_E10	ordinary_muscle_development	ENSGALG00010027325	METTL25B	-0.779334972	0.307869088	-2.531384289	0.01136133	0.017845955	negative
PRJNA729271	stage_E18_vs_E10	ordinary_muscle_development	ENSGALG00010027326	MRPL24	0.082783277	0.118565486	0.69820721	0.485047618	0.543083981	positive
PRJNA729271	stage_E18_vs_E10	ordinary_muscle_development	ENSGALG00010027327	RBM6	-0.225889245	0.089123843	-2.534554594	0.011259038	0.017696786	negative
PRJNA729271	stage_E18_vs_E10	ordinary_muscle_development	ENSGALG00010027328	RBM15	-0.56293837	0.132468608	-4.249598275	2.14154E-05	4.80819E-05	negative
PRJNA729271	stage_E18_vs_E10	ordinary_muscle_development	ENSGALG00010027329	MINDY4	-0.863862851	0.12017308	-7.188488891	6.55123E-13	2.84738E-12	negative
PRJNA729271	stage_E18_vs_E10	ordinary_muscle_development	ENSGALG00010027330	ENSGALG00010027330	-0.920540964	0.214428696	-4.29299334	1.7628E-05	3.99747E-05	negative
PRJNA729271	stage_E18_vs_E10	ordinary_muscle_development	ENSGALG00010027331	SLC16A4	0.442303113	0.169526119	2.609055848	0.009079242	0.014455582	positive
PRJNA729271	stage_E18_vs_E10	ordinary_muscle_development	ENSGALG00010027332	HDGF	0.084505249	0.177178389	0.476950094	0.633397659	0.683015851	positive
PRJNA729271	stage_E18_vs_E10	ordinary_muscle_development	ENSGALG00010027333	PROK1	-0.436194357	0.309967591	-1.407225692	0.159360475	0.20272824	negative
PRJNA729271	stage_E18_vs_E10	ordinary_muscle_development	ENSGALG00010027334	PRCC	-0.320502943	0.115295951	-2.779828252	0.005438765	0.008964624	negative
PRJNA729271	stage_E18_vs_E10	ordinary_muscle_development	ENSGALG00010027335	MAST3	-0.218326848	0.179759199	-1.214551739	0.224537105	0.275702274	negative
PRJNA729271	stage_E18_vs_E10	ordinary_muscle_development	ENSGALG00010027336	RAD54L2	-0.414714653	0.139578245	-2.97119836	0.002966401	0.005083105	negative
PRJNA729271	stage_E18_vs_E10	ordinary_muscle_development	ENSGALG00010027337	AQP1	0.882975063	0.265298686	3.328230063	0.000873997	0.001605693	positive
PRJNA729271	stage_E18_vs_E10	ordinary_muscle_development	ENSGALG00010027339	LAMTOR5	0.219635647	0.109733889	2.001529785	0.045335327	0.064123299	positive
PRJNA729271	stage_E18_vs_E10	ordinary_muscle_development	ENSGALG00010027340	ENSGALG00010027340	0.660957074	0.893698584	0.739574937	0.459557954	0.518913495	positive
PRJNA729271	stage_E18_vs_E10	ordinary_muscle_development	ENSGALG00010027341	ENSGALG00010027341	0.390810623	0.755311764	0.517416306	0.604865579	0.655761149	positive
PRJNA729271	stage_E18_vs_E10	ordinary_muscle_development	ENSGALG00010027346	SIN3B	0.178788321	0.089114389	2.006278936	0.044826496	0.063459328	positive
PRJNA729271	stage_E18_vs_E10	ordinary_muscle_development	ENSGALG00010027348	ENSGALG00010027348	0.357827944	0.210054282	1.703502256	0.088474116	0.11849139	positive
PRJNA729271	stage_E18_vs_E10	ordinary_muscle_development	ENSGALG00010027350	INSRR	0.658879628	0.383000467	1.720310245	0.085376062	0.114691939	positive
PRJNA729271	stage_E18_vs_E10	ordinary_muscle_development	ENSGALG00010027351	TMEM38A	2.957473551	0.147974441	19.98638096	7.23573E-89	6.47419E-87	positive
PRJNA729271	stage_E18_vs_E10	ordinary_muscle_development	ENSGALG00010027354	ENSGALG00010027354	3.035095936	0.848742812	3.57599015	0.000348905	0.000676662	positive
PRJNA729271	stage_E18_vs_E10	ordinary_muscle_development	ENSGALG00010027355	ADCYAP1R1	0.539460126	0.235042622	2.295158731	0.021724029	0.032543386	positive
PRJNA729271	stage_E18_vs_E10	ordinary_muscle_development	ENSGALG00010027356	ENSGALG00010027356	1.834937978	0.214469994	8.555686253	1.1717E-17	7.17218E-17	positive
PRJNA729271	stage_E18_vs_E10	ordinary_muscle_development	ENSGALG00010027358	RNF126	-0.27673312	0.102109214	-2.710167952	0.006724915	0.010910862	negative
PRJNA729271	stage_E18_vs_E10	ordinary_muscle_development	ENSGALG00010027359	ENSGALG00010027359	1.105033667	0.116634026	9.47436787	2.68381E-21	2.0583E-20	positive
PRJNA729271	stage_E18_vs_E10	ordinary_muscle_development	ENSGALG00010027360	SMIM7	0.481714408	0.113831801	4.231808709	2.3182E-05	5.18233E-05	positive
PRJNA729271	stage_E18_vs_E10	ordinary_muscle_development	ENSGALG00010027361	FANCD2	-0.184524934	0.193165414	-0.955269012	0.339441674	0.397817518	negative
PRJNA729271	stage_E18_vs_E10	ordinary_muscle_development	ENSGALG00010027362	ENSGALG00010027362	0.83549853	0.144347405	5.788109125	7.11832E-09	2.22707E-08	positive
PRJNA729271	stage_E18_vs_E10	ordinary_muscle_development	ENSGALG00010027363	VIPR1	-1.323971897	0.267393453	-4.951399831	7.36815E-07	1.93036E-06	negative
PRJNA729271	stage_E18_vs_E10	ordinary_muscle_development	ENSGALG00010027364	FSTL3	3.102189095	0.230395032	13.46465271	2.52503E-41	5.09753E-40	positive
PRJNA729271	stage_E18_vs_E10	ordinary_muscle_development	ENSGALG00010027365	PLEKHA6	1.224907429	0.17162795	7.136992718	9.53949E-13	4.10433E-12	positive
PRJNA729271	stage_E18_vs_E10	ordinary_muscle_development	ENSGALG00010027366	USP4	0.034034151	0.083056286	0.409772129	0.681973113	0.728352511	positive
PRJNA729271	stage_E18_vs_E10	ordinary_muscle_development	ENSGALG00010027367	SEC22C	-0.210782343	0.094771041	-2.224121856	0.026140247	0.038618331	negative
PRJNA729271	stage_E18_vs_E10	ordinary_muscle_development	ENSGALG00010027368	EMC3	0.009500134	0.090707245	0.104734017	0.916586869	0.931104258	positive
PRJNA729271	stage_E18_vs_E10	ordinary_muscle_development	ENSGALG00010027369	ENSGALG00010027369	0.247875429	0.102741316	2.41261684	0.01583846	0.024345544	positive
PRJNA729271	stage_E18_vs_E10	ordinary_muscle_development	ENSGALG00010027370	NUP210L	-0.701643022	0.343925658	-2.040100837	0.041340283	0.058840083	negative
PRJNA729271	stage_E18_vs_E10	ordinary_muscle_development	ENSGALG00010027372	PIK3C2B	0.307137569	0.172584595	1.779634898	0.075135731	0.102127947	positive
PRJNA729271	stage_E18_vs_E10	ordinary_muscle_development	ENSGALG00010027373	MLN	3.479926817	1.156904371	3.007964101	0.002630042	0.004543797	positive
PRJNA729271	stage_E18_vs_E10	ordinary_muscle_development	ENSGALG00010027374	NKTR	0.003686064	0.118760151	0.031037888	0.975239324	0.97956441	positive
PRJNA729271	stage_E18_vs_E10	ordinary_muscle_development	ENSGALG00010027375	FUT7	0.706461678	0.454043159	1.555935077	0.119723531	0.156553233	positive
PRJNA729271	stage_E18_vs_E10	ordinary_muscle_development	ENSGALG00010027376	DSTYK	0.179842765	0.080551775	2.232635661	0.02557298	0.037847698	positive
PRJNA729271	stage_E18_vs_E10	ordinary_muscle_development	ENSGALG00010027378	ZBTB47	0.612698867	0.164389904	3.727107649	0.00019369	0.000389071	positive
PRJNA729271	stage_E18_vs_E10	ordinary_muscle_development	ENSGALG00010027379	OPTC	1.318906584	0.593454346	2.222422992	0.026254733	0.038773546	positive
PRJNA729271	stage_E18_vs_E10	ordinary_muscle_development	ENSGALG00010027380	GPX1	0.013346885	0.132757934	0.100535497	0.919919203	0.934031161	positive
PRJNA729271	stage_E18_vs_E10	ordinary_muscle_development	ENSGALG00010027381	KLHL40	1.772505792	0.196927764	9.000791728	2.24096E-19	1.52001E-18	positive
PRJNA729271	stage_E18_vs_E10	ordinary_muscle_development	ENSGALG00010027382	PALM	0.879309735	0.198900067	4.420861931	9.8308E-06	2.29059E-05	positive
PRJNA729271	stage_E18_vs_E10	ordinary_muscle_development	ENSGALG00010027383	ASS1	1.317152186	0.28587817	4.607389866	4.07755E-06	9.8474E-06	positive
PRJNA729271	stage_E18_vs_E10	ordinary_muscle_development	ENSGALG00010027384	HHATL	1.977393996	0.218550223	9.047778423	1.45906E-19	1.00233E-18	positive
PRJNA729271	stage_E18_vs_E10	ordinary_muscle_development	ENSGALG00010027385	CNTN2	-1.945636655	0.997522138	-1.95046965	0.051120167	0.071696839	negative
PRJNA729271	stage_E18_vs_E10	ordinary_muscle_development	ENSGALG00010027386	RHOA	-0.300091097	0.06609388	-4.540376431	5.61539E-06	1.34094E-05	negative
PRJNA729271	stage_E18_vs_E10	ordinary_muscle_development	ENSGALG00010027387	ENSGALG00010027387	-0.639598523	0.269626803	-2.372162248	0.017684323	0.026914559	negative
PRJNA729271	stage_E18_vs_E10	ordinary_muscle_development	ENSGALG00010027388	URM1	1.241432693	0.143760941	8.635396262	5.85238E-18	3.65018E-17	positive
PRJNA729271	stage_E18_vs_E10	ordinary_muscle_development	ENSGALG00010027391	TPM3	-0.513759967	0.114518469	-4.48626296	7.24833E-06	1.71115E-05	negative
PRJNA729271	stage_E18_vs_E10	ordinary_muscle_development	ENSGALG00010027392	RBBP5	-0.439199523	0.127706505	-3.439131968	0.000583583	0.001099146	negative
PRJNA729271	stage_E18_vs_E10	ordinary_muscle_development	ENSGALG00010027393	TEX264	0.627519822	0.190374308	3.296242173	0.000979875	0.001786577	positive
PRJNA729271	stage_E18_vs_E10	ordinary_muscle_development	ENSGALG00010027394	SLC27A4	-0.451866037	0.087412381	-5.169359652	2.34897E-07	6.43637E-07	negative
PRJNA729271	stage_E18_vs_E10	ordinary_muscle_development	ENSGALG00010027395	BCL2L15	-0.825716188	0.30461394	-2.710697311	0.006714189	0.01090078	negative
PRJNA729271	stage_E18_vs_E10	ordinary_muscle_development	ENSGALG00010027397	RFX5	0.664130798	0.126201736	5.262453729	1.42145E-07	3.96383E-07	positive
PRJNA729271	stage_E18_vs_E10	ordinary_muscle_development	ENSGALG00010027399	MAPKAP1	0.106027124	0.112020411	0.946498259	0.34389451	0.402320494	positive
PRJNA729271	stage_E18_vs_E10	ordinary_muscle_development	ENSGALG00010027401	ENSGALG00010027401	1.36247574	0.313492076	4.346124976	1.38564E-05	3.17948E-05	positive
PRJNA729271	stage_E18_vs_E10	ordinary_muscle_development	ENSGALG00010027402	AP4B1	0.747291909	0.085616758	8.728336926	2.58445E-18	1.65247E-17	positive
PRJNA729271	stage_E18_vs_E10	ordinary_muscle_development	ENSGALG00010027403	ACKR2	1.242540827	0.370185975	3.356531338	0.000789268	0.001460171	positive
PRJNA729271	stage_E18_vs_E10	ordinary_muscle_development	ENSGALG00010027405	DCLRE1B	-0.370611577	0.16846522	-2.199929327	0.02781191	0.040866754	negative
PRJNA729271	stage_E18_vs_E10	ordinary_muscle_development	ENSGALG00010027407	ENSGALG00010027407	0.191443016	1.140045511	0.167925767	0.866641679	0.890025076	positive
PRJNA729271	stage_E18_vs_E10	ordinary_muscle_development	ENSGALG00010027410	ALAS1	-0.025136454	0.088661223	-0.283511246	0.776784962	0.812955567	negative
PRJNA729271	stage_E18_vs_E10	ordinary_muscle_development	ENSGALG00010027412	ENSGALG00010027412	1.093978524	0.282270902	3.875633352	0.000106348	0.000221227	positive
PRJNA729271	stage_E18_vs_E10	ordinary_muscle_development	ENSGALG00010027414	PBX3	-1.220694668	0.073986717	-16.4988355	3.74036E-61	1.53588E-59	negative
PRJNA729271	stage_E18_vs_E10	ordinary_muscle_development	ENSGALG00010027417	WNT2B	-1.468569252	0.364744005	-4.026301276	5.66611E-05	0.000121405	negative
PRJNA729271	stage_E18_vs_E10	ordinary_muscle_development	ENSGALG00010027418	ENSGALG00010027418	0.744078318	1.036455285	0.71790682	0.472814742	0.531563615	positive
PRJNA729271	stage_E18_vs_E10	ordinary_muscle_development	ENSGALG00010027419	PTBP1	-1.209053201	0.084686788	-14.27676296	3.05464E-46	7.64715E-45	negative
PRJNA729271	stage_E18_vs_E10	ordinary_muscle_development	ENSGALG00010027421	PLPPR3	-0.750844436	0.233688012	-3.213020771	0.001313468	0.002359489	negative
PRJNA729271	stage_E18_vs_E10	ordinary_muscle_development	ENSGALG00010027423	PEX11B	1.156094181	0.21179187	5.458633429	4.79813E-08	1.39825E-07	positive
PRJNA729271	stage_E18_vs_E10	ordinary_muscle_development	ENSGALG00010027425	CFD	3.033148005	0.238945288	12.69390173	6.39203E-37	1.07987E-35	positive
PRJNA729271	stage_E18_vs_E10	ordinary_muscle_development	ENSGALG00010027427	UBAC1	0.888100089	0.11613458	7.647163204	2.05461E-14	9.97042E-14	positive
PRJNA729271	stage_E18_vs_E10	ordinary_muscle_development	ENSGALG00010027438	ST7L	0.084287884	0.08461194	0.996170092	0.319167508	0.376829277	positive
PRJNA729271	stage_E18_vs_E10	ordinary_muscle_development	ENSGALG00010027440	TWF2	-0.192918944	0.190867303	-1.010749048	0.312136555	0.369690724	negative
PRJNA729271	stage_E18_vs_E10	ordinary_muscle_development	ENSGALG00010027443	MED16	-0.235359383	0.156851365	-1.500524922	0.133478483	0.172747376	negative
PRJNA729271	stage_E18_vs_E10	ordinary_muscle_development	ENSGALG00010027444	SELENBP1	0.662469236	0.121812564	5.438431077	5.37518E-08	1.55733E-07	positive
PRJNA729271	stage_E18_vs_E10	ordinary_muscle_development	ENSGALG00010027445	PPM1M	1.542510087	0.16249875	9.492442767	2.25683E-21	1.7345E-20	positive
PRJNA729271	stage_E18_vs_E10	ordinary_muscle_development	ENSGALG00010027448	ADAMTSL5	5.149298949	0.311151667	16.54916074	1.6235E-61	6.76225E-60	positive
PRJNA729271	stage_E18_vs_E10	ordinary_muscle_development	ENSGALG00010027450	CAMSAP1	-0.096108158	0.144458072	-0.665301403	0.505857733	0.562936911	negative
PRJNA729271	stage_E18_vs_E10	ordinary_muscle_development	ENSGALG00010027455	R3HDM4	0.338638274	0.149813368	2.260400916	0.02379638	0.035408935	positive
PRJNA729271	stage_E18_vs_E10	ordinary_muscle_development	ENSGALG00010027457	CAPZA1	-0.631865061	0.128358011	-4.922677257	8.53682E-07	2.22436E-06	negative
PRJNA729271	stage_E18_vs_E10	ordinary_muscle_development	ENSGALG00010027460	WDR82	-0.540940112	0.073857892	-7.3240665	2.40567E-13	1.07525E-12	negative
PRJNA729271	stage_E18_vs_E10	ordinary_muscle_development	ENSGALG00010027467	GLYCTK	0.078111085	0.135881288	0.574847991	0.565394107	0.619220822	positive
PRJNA729271	stage_E18_vs_E10	ordinary_muscle_development	ENSGALG00010027468	BSG	0.652838718	0.115250729	5.664508359	1.47447E-08	4.49189E-08	positive
PRJNA729271	stage_E18_vs_E10	ordinary_muscle_development	ENSGALG00010027471	ENSGALG00010027471	0.558575643	0.130140098	4.292110202	1.76983E-05	4.0109E-05	positive
PRJNA729271	stage_E18_vs_E10	ordinary_muscle_development	ENSGALG00010027473	DNAH1	-1.389406248	0.357012157	-3.891761729	9.9519E-05	0.000207567	negative
PRJNA729271	stage_E18_vs_E10	ordinary_muscle_development	ENSGALG00010027475	ARID3A	-0.019359906	0.166157438	-0.116515435	0.907244054	0.923440726	negative
PRJNA729271	stage_E18_vs_E10	ordinary_muscle_development	ENSGALG00010027479	HCN2	-0.365226018	0.280139293	-1.303730063	0.192325636	0.240055118	negative
PRJNA729271	stage_E18_vs_E10	ordinary_muscle_development	ENSGALG00010027487	MPZ	4.861128408	0.53391646	9.104661073	8.65362E-20	6.01315E-19	positive
PRJNA729271	stage_E18_vs_E10	ordinary_muscle_development	ENSGALG00010027490	ATP2B4	-0.063621998	0.068096196	-0.934295918	0.350151269	0.408980068	negative
PRJNA729271	stage_E18_vs_E10	ordinary_muscle_development	ENSGALG00010027492	PCP4L1	1.355231162	0.506264201	2.676924734	0.007430132	0.011974624	positive
PRJNA729271	stage_E18_vs_E10	ordinary_muscle_development	ENSGALG00010027497	KCNT1	3.247423854	0.339686525	9.560060861	1.17687E-21	9.22097E-21	positive

PRJNA729271	stage_E18_vs_E10	ordinary_muscle_development	ENSGALG00010027499	MOV10	-0.155127829	0.409996084	-0.378364173	0.705160079	0.749911618	negative
PRJNA729271	stage_E18_vs_E10	ordinary_muscle_development	ENSGALG00010027501	BAP1	-0.229281108	0.066907507	-3.426836796	0.000610656	0.00114671	negative
PRJNA729271	stage_E18_vs_E10	ordinary_muscle_development	ENSGALG00010027502	THOP1	-0.594332206	0.139657822	-4.255631361	2.0846E-05	4.68833E-05	negative
PRJNA729271	stage_E18_vs_E10	ordinary_muscle_development	ENSGALG00010027510	RHOC	2.165386596	0.184677387	11.7252395	9.4633E-32	1.25729E-30	positive
PRJNA729271	stage_E18_vs_E10	ordinary_muscle_development	ENSGALG00010027511	POLRMT	-0.225279704	0.0839758	-2.6826741	0.007303613	0.011785135	negative
PRJNA729271	stage_E18_vs_E10	ordinary_muscle_development	ENSGALG00010027513	SEMA3G	1.138150734	0.182133247	6.249000388	4.13088E-10	1.44247E-09	positive
PRJNA729271	stage_E18_vs_E10	ordinary_muscle_development	ENSGALG00010027516	PPM1J	0.501234322	0.234961022	2.133265838	0.032902925	0.047707102	positive
PRJNA729271	stage_E18_vs_E10	ordinary_muscle_development	ENSGALG00010027520	FANCE	-0.52634945	0.165756771	-3.175432578	0.001496133	0.002669758	negative
PRJNA729271	stage_E18_vs_E10	ordinary_muscle_development	ENSGALG00010027522	PSMD5	-0.160743253	0.105435194	-1.524569237	0.127366575	0.165740058	negative
PRJNA729271	stage_E18_vs_E10	ordinary_muscle_development	ENSGALG00010027524	ENSGALG00010027524	1.310414496	0.111245854	11.77944568	4.98196E-32	6.68644E-31	positive
PRJNA729271	stage_E18_vs_E10	ordinary_muscle_development	ENSGALG00010027525	ENSGALG00010027525	3.780503811	0.265967227	14.2141716	7.48317E-46	1.83224E-44	positive
PRJNA729271	stage_E18_vs_E10	ordinary_muscle_development	ENSGALG00010027527	ENSGALG00010027527	-0.845995969	0.503012813	-1.681857692	0.092596438	0.123601195	negative
PRJNA729271	stage_E18_vs_E10	ordinary_muscle_development	ENSGALG00010027528	ENSGALG00010027528	8.012051743	2.329191476	3.439842462			positive
PRJNA729271	stage_E18_vs_E10	ordinary_muscle_development	ENSGALG00010027529	ACY1	-0.672679704	0.148604949	-4.526630581	5.99316E-06	1.42762E-05	negative
PRJNA729271	stage_E18_vs_E10	ordinary_muscle_development	ENSGALG00010027530	PHF19	-0.444492971	0.149082118	-2.981531098	0.002868109	0.004930998	negative
PRJNA729271	stage_E18_vs_E10	ordinary_muscle_development	ENSGALG00010027531	UFC1	-0.189347119	0.173642773	-1.09044054	0.275519132	0.331011175	negative
PRJNA729271	stage_E18_vs_E10	ordinary_muscle_development	ENSGALG00010027533	WDR18	-1.162671658	0.097458605	-11.92990246	8.26703E-33	1.13799E-31	negative
PRJNA729271	stage_E18_vs_E10	ordinary_muscle_development	ENSGALG00010027534	KCNN3	1.465525917	0.186343781	7.864635528	3.70174E-15	1.90949E-14	positive
PRJNA729271	stage_E18_vs_E10	ordinary_muscle_development	ENSGALG00010027536	USP21	-0.254258855	0.325706641	-0.780637612	0.435015664	0.494785942	negative
PRJNA729271	stage_E18_vs_E10	ordinary_muscle_development	ENSGALG00010027537	SGTA	-0.227712358	0.068092364	-3.344168795	0.000825295	0.00152216	negative
PRJNA729271	stage_E18_vs_E10	ordinary_muscle_development	ENSGALG00010027538	ABHD14A	0.55431967	0.140403562	3.948045642	7.87918E-05	0.000166049	positive
PRJNA729271	stage_E18_vs_E10	ordinary_muscle_development	ENSGALG00010027539	DDR2L	0.394960811	0.107778359	3.664565069	0.000247759	0.000490812	positive
PRJNA729271	stage_E18_vs_E10	ordinary_muscle_development	ENSGALG00010027540	PPOX	-0.454647357	0.117683805	-3.863295867	0.000111867	0.000232076	negative
PRJNA729271	stage_E18_vs_E10	ordinary_muscle_development	ENSGALG00010027543	TUBB4B	-0.487393626	0.152193213	-3.202466237	0.001362563	0.002444047	negative
PRJNA729271	stage_E18_vs_E10	ordinary_muscle_development	ENSGALG00010027545	PPARD	0.606627456	0.202099776	3.001623591	0.00268544	0.004630675	positive
PRJNA729271	stage_E18_vs_E10	ordinary_muscle_development	ENSGALG00010027547	B4GALT3	0.289434986	0.225848838	1.281542951	0.200003024	0.248844964	positive
PRJNA729271	stage_E18_vs_E10	ordinary_muscle_development	ENSGALG00010027548	PCBP4	0.401511053	0.146610511	2.738623923	0.00616969	0.010081125	positive
PRJNA729271	stage_E18_vs_E10	ordinary_muscle_development	ENSGALG00010027553	FGF22	0.068144995	0.303819161	0.224294593	0.822528083	0.852523744	positive
PRJNA729271	stage_E18_vs_E10	ordinary_muscle_development	ENSGALG00010027554	ENSGALG00010027554	0.401063826	0.148814419	2.69506026	0.00703759	0.01137868	positive
PRJNA729271	stage_E18_vs_E10	ordinary_muscle_development	ENSGALG00010027555	ENSGALG00010027555	-0.351724719	0.40585619	-0.866624011	0.386148053	0.446420165	negative
PRJNA729271	stage_E18_vs_E10	ordinary_muscle_development	ENSGALG00010027557	C6orf132	-1.702948965	0.824100633	-2.066433268	0.038787587	0.055544959	negative
PRJNA729271	stage_E18_vs_E10	ordinary_muscle_development	ENSGALG00010027559	ENSGALG00010027559	0.325921194	0.283784907	1.14847966	0.250770605	0.304381169	positive
PRJNA729271	stage_E18_vs_E10	ordinary_muscle_development	ENSGALG00010027561	ADAMTS4	0.238754607	0.362754626	0.658171087	0.510428201	0.567252507	positive
PRJNA729271	stage_E18_vs_E10	ordinary_muscle_development	ENSGALG00010027562	SLC39A3	0.417941672	0.147022732	2.842701032	0.004473301	0.007462348	positive
PRJNA729271	stage_E18_vs_E10	ordinary_muscle_development	ENSGALG00010027563	WDR77	-1.483512797	0.124259533	-11.93882486	7.4265E-33	1.02521E-31	negative
PRJNA729271	stage_E18_vs_E10	ordinary_muscle_development	ENSGALG00010027567	PARP3	1.049791711	0.132375702	7.930395769	2.18449E-15	1.14228E-14	positive
PRJNA729271	stage_E18_vs_E10	ordinary_muscle_development	ENSGALG00010027568	NELFB	-0.687938209	0.077496875	-8.876980015	6.87001E-19	4.55123E-18	negative
PRJNA729271	stage_E18_vs_E10	ordinary_muscle_development	ENSGALG00010027569	ATP5PB	0.374336934	0.125462845	2.983647744	0.002848345	0.004897599	positive
PRJNA729271	stage_E18_vs_E10	ordinary_muscle_development	ENSGALG00010027570	NDUFS2	0.658663027	0.137733905	4.782141512	1.73438E-06	4.36833E-06	positive
PRJNA729271	stage_E18_vs_E10	ordinary_muscle_development	ENSGALG00010027571	RBM10	0.452882635	0.101629438	4.456215103	8.34193E-06	1.95943E-05	positive
PRJNA729271	stage_E18_vs_E10	ordinary_muscle_development	ENSGALG00010027574	GNG7	-0.067248021	0.095468437	-0.704400564	0.481183355	0.539173835	negative
PRJNA729271	stage_E18_vs_E10	ordinary_muscle_development	ENSGALG00010027575	FCER1G	0.444729113	0.352365048	1.262126068	0.206903426	0.256505744	positive
PRJNA729271	stage_E18_vs_E10	ordinary_muscle_development	ENSGALG00010027576	GADD45B	2.084332471	0.417005648	4.998331513	5.78285E-07	1.52905E-06	positive
PRJNA729271	stage_E18_vs_E10	ordinary_muscle_development	ENSGALG00010027578	ENSGALG00010027578	0.610141875	0.247966125	2.460585598	0.013871048	0.021510735	positive
PRJNA729271	stage_E18_vs_E10	ordinary_muscle_development	ENSGALG00010027579	C26H6ORF125	-0.66232462	0.15987557	-4.142750649	3.43165E-05	7.5184E-05	negative
PRJNA729271	stage_E18_vs_E10	ordinary_muscle_development	ENSGALG00010027583	GLE1	-1.113070555	0.074689126	-14.90271228	3.16468E-50	9.32357E-49	negative
PRJNA729271	stage_E18_vs_E10	ordinary_muscle_development	ENSGALG00010027586	HJV	4.142285712	0.289268327	14.31987304	1.64423E-46	4.1886E-45	positive
PRJNA729271	stage_E18_vs_E10	ordinary_muscle_development	ENSGALG00010027590	SEMA3F	-0.333360541	0.235459745	-1.415785703	0.156838257	0.199821617	negative
PRJNA729271	stage_E18_vs_E10	ordinary_muscle_development	ENSGALG00010027592	MAP1S	-0.1925137	0.169415658	-1.136339471	0.255814517	0.309751184	negative
PRJNA729271	stage_E18_vs_E10	ordinary_muscle_development	ENSGALG00010027593	ENSGALG00010027593	3.098475983	0.44734107	6.926428606	4.31598E-12	1.75978E-11	positive
PRJNA729271	stage_E18_vs_E10	ordinary_muscle_development	ENSGALG00010027594	ITPR3	0.178685282	0.138919523	1.286250332	0.198355711	0.246986172	positive
PRJNA729271	stage_E18_vs_E10	ordinary_muscle_development	ENSGALG00010027595	GNAT1	0.170936824	0.44129168	0.387355646	0.698492935	0.743475921	positive
PRJNA729271	stage_E18_vs_E10	ordinary_muscle_development	ENSGALG00010027597	EXOSC2	-0.924619602	0.11720914	-7.888630553	3.05521E-15	1.58558E-14	negative
PRJNA729271	stage_E18_vs_E10	ordinary_muscle_development	ENSGALG00010027600	KIRREL1	0.183635165	0.141035989	1.302044724	0.192901097	0.240690489	positive
PRJNA729271	stage_E18_vs_E10	ordinary_muscle_development	ENSGALG00010027605	FCHO1	-1.442650682	0.387879593	-3.719326069	0.000199755	0.000400532	negative
PRJNA729271	stage_E18_vs_E10	ordinary_muscle_development	ENSGALG00010027607	ABL1	-0.285139139	0.079119477	-3.603905764	0.000313471	0.000611954	negative
PRJNA729271	stage_E18_vs_E10	ordinary_muscle_development	ENSGALG00010027608	GNAI2	0.001073313	0.144190773	0.007443699	0.994060842	0.994884479	positive
PRJNA729271	stage_E18_vs_E10	ordinary_muscle_development	ENSGALG00010027609	RPL36	0.987876259	0.186761373	5.289510571	1.22644E-07	3.44721E-07	positive
PRJNA729271	stage_E18_vs_E10	ordinary_muscle_development	ENSGALG00010027612	BAK1	-0.932558178	0.164589183	-5.665974873	1.46191E-08	4.4555E-08	negative
PRJNA729271	stage_E18_vs_E10	ordinary_muscle_development	ENSGALG00010027614	LONP1	-0.61570678	0.080706786	-7.628934472	2.36702E-14	1.14176E-13	negative
PRJNA729271	stage_E18_vs_E10	ordinary_muscle_development	ENSGALG00010027616	SLC38A3	5.06826321	0.266429868	19.02287927	1.10259E-80	7.87292E-79	positive
PRJNA729271	stage_E18_vs_E10	ordinary_muscle_development	ENSGALG00010027618	PPP1R26	0.077658768	0.079650905	0.974989146	0.329565644	0.38771642	positive
PRJNA729271	stage_E18_vs_E10	ordinary_muscle_development	ENSGALG00010027619	JAK3	0.040887369	0.397175985	0.102945221	0.918006447	0.932284975	positive
PRJNA729271	stage_E18_vs_E10	ordinary_muscle_development	ENSGALG00010027621	TSPO2	-1.096193135	0.394088405	-2.781591952	0.0054093	0.00892113	negative
PRJNA729271	stage_E18_vs_E10	ordinary_muscle_development	ENSGALG00010027622	FIBCD1	-4.442564502	1.222693808	-3.633423571	0.000279685	0.000550372	negative
PRJNA729271	stage_E18_vs_E10	ordinary_muscle_development	ENSGALG00010027623	APOBEC2	2.490058322	0.182700275	13.62919851	2.68513E-42	5.62442E-41	positive
PRJNA729271	stage_E18_vs_E10	ordinary_muscle_development	ENSGALG00010027624	ENSGALG00010027624	0.907153428	0.44923491	2.01932977	0.043452956	0.061629216	positive
PRJNA729271	stage_E18_vs_E10	ordinary_muscle_development	ENSGALG00010027626	SEMA3B	3.219819106	0.208220684	15.46349306	6.11885E-54	1.99309E-52	positive
PRJNA729271	stage_E18_vs_E10	ordinary_muscle_development	ENSGALG00010027627	OARD1	1.091596646	0.093658706	11.65504732	2.1626E-31	2.84197E-30	positive
PRJNA729271	stage_E18_vs_E10	ordinary_muscle_development	ENSGALG00010027628	PIERCE1	-0.295342292	0.246730224	-1.197025184	0.231296739	0.283090959	negative
PRJNA729271	stage_E18_vs_E10	ordinary_muscle_development	ENSGALG00010027632	LAMC3	-1.455336086	0.407271678	-3.573379061	0.000352404	0.000683234	negative
PRJNA729271	stage_E18_vs_E10	ordinary_muscle_development	ENSGALG00010027634	OASL	1.286703813	0.717945451	1.792202752	0.073100494	0.099604405	positive
PRJNA729271	stage_E18_vs_E10	ordinary_muscle_development	ENSGALG00010027635	CD48	0.447594856	0.235580148	1.899968482	0.057437256	0.079815265	positive
PRJNA729271	stage_E18_vs_E10	ordinary_muscle_development	ENSGALG00010027636	SLC1A6	0.095260998	0.604134876	0.157681672	0.874707643	0.896604716	positive
PRJNA729271	stage_E18_vs_E10	ordinary_muscle_development	ENSGALG00010027638	MRPS2	0.897167603	0.141001381	6.362828491	1.98072E-10	7.05938E-10	positive
PRJNA729271	stage_E18_vs_E10	ordinary_muscle_development	ENSGALG00010027641	HYAL3	0.787568923	0.165769893	4.750976838	2.02436E-06	5.05481E-06	positive
PRJNA729271	stage_E18_vs_E10	ordinary_muscle_development	ENSGALG00010027642	DIPK1B	-1.592281615	0.178740034	-8.908365859	5.17902E-19	3.45626E-18	negative
PRJNA729271	stage_E18_vs_E10	ordinary_muscle_development	ENSGALG00010027644	NAA80	1.850054661	0.143123188	12.92630976	3.19804E-38	5.70881E-37	positive
PRJNA729271	stage_E18_vs_E10	ordinary_muscle_development	ENSGALG00010027645	SLAMF1	-0.019041404	1.133366269	-0.016800751	0.986595571	0.988641742	negative
PRJNA729271	stage_E18_vs_E10	ordinary_muscle_development	ENSGALG00010027648	NFYA	-0.622728706	0.074019357	-8.41305204	3.99478E-17	2.35767E-16	negative
PRJNA729271	stage_E18_vs_E10	ordinary_muscle_development	ENSGALG00010027649	FBLN2	-0.615565259	0.191640424	-3.212084626	0.001317756	0.002366898	negative
PRJNA729271	stage_E18_vs_E10	ordinary_muscle_development	ENSGALG00010027650	CCDC124	-0.15741378	0.192619371	-0.817227149	0.41379863	0.4741136	negative
PRJNA729271	stage_E18_vs_E10	ordinary_muscle_development	ENSGALG00010027653	WNT7A	-6.789065012	0.678534424	-10.00548355	1.44186E-23	1.24774E-22	negative
PRJNA729271	stage_E18_vs_E10	ordinary_muscle_development	ENSGALG00010027654	AIF1L	0.611516213	0.091428311	6.688477603	2.25504E-11	8.63817E-11	positive
PRJNA729271	stage_E18_vs_E10	ordinary_muscle_development	ENSGALG00010027663	COPA	-0.425326345	0.070706813	-6.015351626	1.79497E-09	5.92396E-09	negative
PRJNA729271	stage_E18_vs_E10	ordinary_muscle_development	ENSGALG00010027664	TREMB1	0.630447424	0.349386446	1.804441562	0.071162112	0.097246613	positive
PRJNA729271	stage_E18_vs_E10	ordinary_muscle_development	ENSGALG00010027665	TRAF1	-0.914758966	0.149417197	-6.122179937	9.23037E-10	3.12896E-09	negative
PRJNA729271	stage_E18_vs_E10	ordinary_muscle_development	ENSGALG00010027667	PTPDC1	-0.212883543	0.153384843	-1.387904688	0.16516607	0.209236339	negative
PRJNA729271	stage_E18_vs_E10	ordinary_muscle_development	ENSGALG00010027668	RANBP3	-0.394736874	0.054274131	-7.273020614	3.51537E-13	1.55826E-12	negative
PRJNA729271	stage_E18_vs_E10	ordinary_muscle_development	ENSGALG00010027671	BARX1	-2.670215939	0.54142223	-4.931855017	8.14524E-07	2.12653E-06	negative
PRJNA729271	stage_E18_vs_E10	ordinary_muscle_development	ENSGALG00010027672	CNTRL	-1.20456779	0.175484979	-6.864221644	6.68548E-12	2.66738E-11	negative
PRJNA729271	stage_E18_vs_E10	ordinary_muscle_development	ENSGALG00010027674	TREM2	0.588354678	0.28529744	2.062250111	0.03918393	0.056073755	positive
PRJNA729271	stage_E18_vs_E10	ordinary_muscle_development	ENSGALG00010027685	ENSGALG00010027685	0.483647503	0.333985799	1.448107985	0.147586853	0.189148668	positive
PRJNA729271	stage_E18_vs_E10	ordinary_muscle_development	ENSGALG00010027692	PHF2	0.608506795	0.112760315	5.396462369	6.79677E-08	1.95126E-07	positive
PRJNA729271	stage_E18_vs_E10	ordinary_muscle_development	ENSGALG00010027693	RAB14	-0.383495759	0.060136738	-6.377062817	1.80516E-10	6.45592E-10	negative
PRJNA729271	stage_E18_vs_E10	ordinary_muscle_development	ENSGALG00010027695	TREMB2	0.57526285	0.310856656	1.85057273	0.064231046	0.088358072	positive
PRJNA729271	stage_E18_vs_E10	ordinary_muscle_development	ENSGALG00010027697	CATH2	4.867094235	0.872518883	5.578210775	2.43005E-08	7.27009E-08	positive
PRJNA729271	stage_E18_vs_E10	ordinary_muscle_development	ENSGALG00010027698	FAM120A	-0.204747417	0.066813649	-3.064454955	0.00218067	0.003807373	negative
PRJNA729271	stage_E18_vs_E10	ordinary_muscle_development	ENSGALG00010027699	VANGL2	-1.468729119	0.124153687	-11.82992754	2.73378E-32	3.70337E-31	negative
PRJNA729271	stage_E18_vs_E10	ordinary_muscle_development	ENSGALG00010027700	TCF3	-0.794983586	0.09605772	-8.276103011	1.27272E-16	7.26016E-16	negative
PRJNA729271	stage_E18_vs_E10	ordinary_muscle_development	ENSGALG00010027702	FOXP4	-1.851197542	0.246955258	-7.496084735	6.57523E-14	3.0695E-13	negative
PRJNA729271	stage_E18_vs_E10	ordinary_muscle_development	ENSGALG00010027703	NCSTN	-0.177583652	0.101965537	-1.741604645	0.081577648	0.110115976	negative
PRJNA729271	stage_E18_vs_E10	ordinary_muscle_development	ENSGALG00010027704	MDFI	0.015007743	0.170301662	0.088124467	0.929777751	0.942522449	positive
PRJNA729271	stage_E18_vs_E10	ordinary_muscle_development	ENSGALG00010027705	S100A11	0.152979484	0.22997634	0.665196619	0.505924743	0.562936911	positive
PRJNA729271	stage_E18_vs_E10	ordinary_muscle_development	ENSGALG00010027706	GSN	1.824763281	0.171966006	10.61118605	2.64372E-26	2.69484E-25	positive
PRJNA729271	stage_E18_vs_E10	ordinary_muscle_development	ENSGALG00010027707	HIGD1C	-0.685174679	0.214191939	-3.198881726	0.001379618	0.002472803	negative
PRJNA729271	stage_E18_vs_E10	ordinary_muscle_development	ENSGALG00010027709	PSMA5	1.424125943	0.106736465	13.34244991	1.3108E-40	2.57453E-39	positive
PRJNA729271	stage_E18_vs_E10	ordinary_muscle_development	ENSGALG00010027710	ENSGALG00010027710	4.676642376	0.281978058	16.58512869	8.92743E-62	3.78371E-60	positive
PRJNA729271	stage_E18_vs_E10	ordinary_muscle_development	ENSGALG00010027711	WNK2	1.363397518	0.186016226	7.32945478	2.31091E-13	1.03385E-12	positive
PRJNA729271	stage_E18_vs_E10	ordinary_muscle_development	ENSGALG00010027712	STOM	1.612967916	0.157290775	10.25468858	1.12739E-24	1.0509E-23	positive
PRJNA729271	stage_E18_vs_E10	ordinary_muscle_development	ENSGALG00010027713	ENSGALG00010027713	0.908395931	0.139609545	6.506689306	7.68251E-11	2.82706E-10	positive
PRJNA729271	stage_E18_vs_E10	ordinary_muscle_development	ENSGALG00010027714	SYPL2	7.440651511	0.162059443	45.91310055	0	0	positive
PRJNA729271	stage_E18_vs_E10	ordinary_muscle_development	ENSGALG00010027715	NINJ1	0.264740332	0.12270479	2.157538696	0.030963715	0.045131684	positive
PRJNA729271	stage_E18_vs_E10	ordinary_muscle_development	ENSGALG00010027717	ATXN7L2	1.63761164	0.226843552	7.219123593	5.23237E-13	2.28512E-12	positive
PRJNA729271	stage_E18_vs_E10	ordinary_muscle_development	ENSGALG00010027718	ENSGALG00010027718	1.942165923	0.478740527	4.056823713	4.97446E-05	0.000107171	positive
PRJNA729271	stage_E18_vs_E10	ordinary_muscle_development	ENSGALG00010027719	GPR61	-4.563009117	1.118336045	-4.080177096	4.50014E-05	9.72849E-05	negative
PRJNA729271	stage_E18_vs_E10	ordinary_muscle_development	ENSGALG00010027720	DAB2IP	1.25612391	0.086727587	14.48355659	1.53922E-47	4.08627E-46	positive
PRJNA729271	stage_E18_vs_E10	ordinary_muscle_development	ENSGALG00010027721	SUSD3	0.738712763	0.167930328	4.398924072	1.08789E-05	2.52344E-05	positive
PRJNA729271	stage_E18_vs_E10	ordinary_muscle_development	ENSGALG00010027722	GNAI3	-0.628390146	0.085290211	-7.367670197	1.73636E-13	7.8431E-13	negative
PRJNA729271	stage_E18_vs_E10	ordinary_muscle_development	ENSGALG00010027723	ENSGALG00010027723	6.531582137	0.950856967	6.869153157	6.45841E-12	2.58247E-11	positive
PRJNA729271	stage_E18_vs_E10	ordinary_muscle_development	ENSGALG00010027724	TTLL11	0.224807697	0.109915972	2.045268692	0.040828375	0.058208646	positive
PRJNA729271	stage_E18_vs_E10	ordinary_muscle_development	ENSGALG00010027725	BTN1A1	0.82779793	0.282305297	2.932279125	0.003364842	0.005704489	positive
PRJNA729271	stage_E18_vs_E10	ordinary_muscle_development	ENSGALG00010027727	GSTM2	0.72409968	0.171840642	4.213785942	2.51125E-05	5.59579E-05	positive
PRJNA729271	stage_E18_vs_E10	ordinary_muscle_development	ENSGALG00010027728	CRNN	5.011484708	1.48238952	3.380680071	0.000723067	0.001344337	positive
PRJNA729271	stage_E18_vs_E10	ordinary_muscle_development	ENSGALG00010027729	EPS8L3	0.421401838	0.255791434	1.647443119	0.099466999	0.131958095	positive
PRJNA729271	stage_E18_vs_E10	ordinary_muscle_development	ENSGALG00010027730	ENSGALG00010027730	-1.02310933	0.140486289	-7.282627608	3.2738E-13	1.4543E-12	negative
PRJNA729271	stage_E18_vs_E10	ordinary_muscle_development	ENSGALG00010027731	OLFML2A	3.681475189	0.101144033	36.39834289	4.5219E-290	7.2827E-287	positive
PRJNA729271	stage_E18_vs_E10	ordinary_muscle_development	ENSGALG00010027732	EEF2	0.978465171	0.117038873	8.360172508	6.26266E-17	3.63837E-16	positive
PRJNA729271	stage_E18_vs_E10	ordinary_muscle_development	ENSGALG00010027733	RPL35	1.114130279	0.154013665	7.233970318	4.69074E-13	2.05788E-12	positive
PRJNA729271	stage_E18_vs_E10	ordinary_muscle_development	ENSGALG00010027734	CSF1	0.81116319	0.14132842	5.739561736	9.49219E-09	2.94057E-08	positive
PRJNA729271	stage_E18_vs_E10	ordinary_muscle_development	ENSGALG00010027736	ENSGALG00010027736	6.548199547	1.451176851	4.512337378	6.41171E-06	1.52033E-05	positive

PRJNA729271	stage_E18_vs_E10	ordinary_muscle_development	ENSGALG00010027738	ENSGALG00010027738	1.535715304	0.242685408	6.328008413	2.48346E-10	8.79279E-10	positive
PRJNA729271	stage_E18_vs_E10	ordinary_muscle_development	ENSGALG00010027739	FGD3	-1.159832928	0.073279669	-15.82748588	2.01097E-56	7.14437E-55	negative
PRJNA729271	stage_E18_vs_E10	ordinary_muscle_development	ENSGALG00010027740	MARCHF2	0.001881065	0.145762156	0.012905029	0.989703563	0.991139501	positive
PRJNA729271	stage_E18_vs_E10	ordinary_muscle_development	ENSGALG00010027741	RAB11B	-0.779270359	0.062079234	-12.55283466	3.8353E-36	6.23936E-35	negative
PRJNA729271	stage_E18_vs_E10	ordinary_muscle_development	ENSGALG00010027743	C1orf74	0.608632776	0.195107798	3.119469251	0.001811772	0.003204983	positive
PRJNA729271	stage_E18_vs_E10	ordinary_muscle_development	ENSGALG00010027744	ENSGALG00010027744	7.438163305	1.055184797	7.049157004	1.80005E-12	7.55406E-12	positive
PRJNA729271	stage_E18_vs_E10	ordinary_muscle_development	ENSGALG00010027745	BICD2	0.563738567	0.138501169	4.070280208	4.69566E-05	0.000101406	positive
PRJNA729271	stage_E18_vs_E10	ordinary_muscle_development	ENSGALG00010027746	UQCR11	1.601035417	0.184711205	8.667776378	4.40641E-18	2.76977E-17	positive
PRJNA729271	stage_E18_vs_E10	ordinary_muscle_development	ENSGALG00010027748	ENSGALG00010027748	4.39439492	2.805393159	1.566409651	0.117252745	0.153557873	positive
PRJNA729271	stage_E18_vs_E10	ordinary_muscle_development	ENSGALG00010027751	ANGPTL4	-0.714912652	0.210693019	-3.393148271	0.000690942	0.001288627	negative
PRJNA729271	stage_E18_vs_E10	ordinary_muscle_development	ENSGALG00010027752	SCAI	-0.869144266	0.084902089	-10.23701859	1.35343E-24	1.25274E-23	negative
PRJNA729271	stage_E18_vs_E10	ordinary_muscle_development	ENSGALG00010027759	ENSGALG00010027759	2.048115268	0.723969134	2.829009099	0.004669237	0.00776893	positive
PRJNA729271	stage_E18_vs_E10	ordinary_muscle_development	ENSGALG00010027766	ENSGALG00010027766	0.009228377	0.599152779	0.015402377	0.987711167	0.989554421	positive
PRJNA729271	stage_E18_vs_E10	ordinary_muscle_development	ENSGALG00010027767	ENSGALG00010027767	0.83584125	0.980139272	0.852778043	0.393782403	0.453798372	positive
PRJNA729271	stage_E18_vs_E10	ordinary_muscle_development	ENSGALG00010027772	ENSGALG00010027772	1.867175015	0.864900002	2.158833404	0.030863094	0.045006091	positive
PRJNA729271	stage_E18_vs_E10	ordinary_muscle_development	ENSGALG00010027775	MVB12B	0.632297614	0.08826367	7.163735819	7.85076E-13	3.39591E-12	positive
PRJNA729271	stage_E18_vs_E10	ordinary_muscle_development	ENSGALG00010027776	TKT	-0.94058908	0.127991131	-7.348861362	1.99902E-13	8.98198E-13	negative
PRJNA729271	stage_E18_vs_E10	ordinary_muscle_development	ENSGALG00010027784	PEAR1	1.521540472	0.190793188	7.974815511	1.52609E-15	8.08798E-15	positive
PRJNA729271	stage_E18_vs_E10	ordinary_muscle_development	ENSGALG00010027787	DCP1A	-0.290143016	0.139568693	-2.078854579	0.037630719	0.053991413	negative
PRJNA729271	stage_E18_vs_E10	ordinary_muscle_development	ENSGALG00010027789	UAP1L1	-0.356536527	0.133938328	-2.66194549	0.007769046	0.012487505	negative
PRJNA729271	stage_E18_vs_E10	ordinary_muscle_development	ENSGALG00010027801	MBD3	-1.615961	0.127980586	-12.62661042	1.50628E-36	2.51249E-35	negative
PRJNA729271	stage_E18_vs_E10	ordinary_muscle_development	ENSGALG00010027803	SAPCD2	-2.396241436	0.175354372	-13.66513658	1.63996E-42	3.48041E-41	negative
PRJNA729271	stage_E18_vs_E10	ordinary_muscle_development	ENSGALG00010027811	MEX3D	-0.630766706	0.100208168	-6.294563797	3.08265E-10	1.08454E-09	negative
PRJNA729271	stage_E18_vs_E10	ordinary_muscle_development	ENSGALG00010027813	LRRC71	-0.542971684	0.266304981	-2.038909232	0.041459087	0.0589918	negative
PRJNA729271	stage_E18_vs_E10	ordinary_muscle_development	ENSGALG00010027814	ENSGALG00010027814	0.447863554	0.131579812	3.403740649	0.000664698	0.001242559	positive
PRJNA729271	stage_E18_vs_E10	ordinary_muscle_development	ENSGALG00010027816	ZMYND19	-1.046936429	0.123193471	-8.498311025	1.92369E-17	1.15653E-16	negative
PRJNA729271	stage_E18_vs_E10	ordinary_muscle_development	ENSGALG00010027821	NMRK2	1.824647692	0.22736638	8.02514289	1.01408E-15	5.44813E-15	positive
PRJNA729271	stage_E18_vs_E10	ordinary_muscle_development	ENSGALG00010027822	DPH7	-0.271430273	0.091188713	-2.976577506	0.002914854	0.005002464	negative
PRJNA729271	stage_E18_vs_E10	ordinary_muscle_development	ENSGALG00010027826	ARHGEF11	0.054581191	0.165178992	0.330436637	0.741070062	0.781848064	positive
PRJNA729271	stage_E18_vs_E10	ordinary_muscle_development	ENSGALG00010027830	CACNA1D	0.32893426	0.316188797	1.04030966	0.298196058	0.355164491	positive
PRJNA729271	stage_E18_vs_E10	ordinary_muscle_development	ENSGALG00010027831	ATCAY	-1.068811818	0.201539828	-5.303228794	1.13772E-07	3.21029E-07	negative
PRJNA729271	stage_E18_vs_E10	ordinary_muscle_development	ENSGALG00010027838	ENSGALG00010027838	-1.268338519	0.703512231	-1.802866338	0.071409209	0.097538304	negative
PRJNA729271	stage_E18_vs_E10	ordinary_muscle_development	ENSGALG00010027842	SLAMF8	1.789251419	0.185724767	9.633886994	5.75119E-22	4.58293E-21	positive
PRJNA729271	stage_E18_vs_E10	ordinary_muscle_development	ENSGALG00010027847	NRTN	-2.731910238	0.476751152	-5.730264579	1.00274E-08	3.09645E-08	negative
PRJNA729271	stage_E18_vs_E10	ordinary_muscle_development	ENSGALG00010027860	ENSGALG00010027860	-0.057308501	0.297030478	-0.192938118	0.847007432	0.873337084	negative
PRJNA729271	stage_E18_vs_E10	ordinary_muscle_development	ENSGALG00010027861	PNPLA7	-0.571315941	0.080566697	-7.09121713	1.32938E-12	5.63595E-12	negative
PRJNA729271	stage_E18_vs_E10	ordinary_muscle_development	ENSGALG00010027863	CHDH	-0.035252321	0.148846503	-0.236836742	0.812783446	0.84435577	negative
PRJNA729271	stage_E18_vs_E10	ordinary_muscle_development	ENSGALG00010027864	ACTR8	-0.958044887	0.085347366	-11.22524253	3.06544E-29	3.60078E-28	negative
PRJNA729271	stage_E18_vs_E10	ordinary_muscle_development	ENSGALG00010027865	SELENOK	0.063670735	0.122528979	0.519638175	0.603315786	0.654500265	positive
PRJNA729271	stage_E18_vs_E10	ordinary_muscle_development	ENSGALG00010027866	KCNA2	-0.230209429	0.137272088	-1.677030138	0.093536589	0.124764227	negative
PRJNA729271	stage_E18_vs_E10	ordinary_muscle_development	ENSGALG00010027867	UBAP2L	-0.400322982	0.169045748	-2.368133997	0.01787806	0.027186579	negative
PRJNA729271	stage_E18_vs_E10	ordinary_muscle_development	ENSGALG00010027868	CACNA2D3	-2.541733319	0.286946633	-8.857860755	8.15645E-19	5.36911E-18	negative
PRJNA729271	stage_E18_vs_E10	ordinary_muscle_development	ENSGALG00010027869	NSMF	-0.619580699	0.165512962	-3.743396842	0.000181549	0.000366053	negative
PRJNA729271	stage_E18_vs_E10	ordinary_muscle_development	ENSGALG00010027870	HAX1	0.648786553	0.123403398	5.25744478	1.46071E-07	4.06859E-07	positive
PRJNA729271	stage_E18_vs_E10	ordinary_muscle_development	ENSGALG00010027872	KDM5B	-0.057611162	0.091696526	-0.628280747	0.529820041	0.585882019	negative
PRJNA729271	stage_E18_vs_E10	ordinary_muscle_development	ENSGALG00010027873	RFX2	-0.788800988	0.204354283	-3.85996798	0.000113402	0.000234991	negative
PRJNA729271	stage_E18_vs_E10	ordinary_muscle_development	ENSGALG00010027874	FRS3	0.342739905	0.133042505	2.576168461	0.009990196	0.015803546	positive
PRJNA729271	stage_E18_vs_E10	ordinary_muscle_development	ENSGALG00010027875	WNT5A	-1.284152397	0.169330011	-7.583725946	3.3577E-14	1.59783E-13	negative
PRJNA729271	stage_E18_vs_E10	ordinary_muscle_development	ENSGALG00010027876	ANAPC2	0.289964742	0.109184681	2.655727336	0.007913757	0.012703202	positive
PRJNA729271	stage_E18_vs_E10	ordinary_muscle_development	ENSGALG00010027877	OAZ1	0.913259139	0.11434104	7.987150886	1.38093E-15	7.34823E-15	positive
PRJNA729271	stage_E18_vs_E10	ordinary_muscle_development	ENSGALG00010027879	ENSGALG00010027879	4.198816613	0.416949604	10.07032162	7.47333E-24	6.58516E-23	positive
PRJNA729271	stage_E18_vs_E10	ordinary_muscle_development	ENSGALG00010027880	AMH	2.037667465	0.339721796	5.998047491	1.99704E-09	6.56844E-09	positive
PRJNA729271	stage_E18_vs_E10	ordinary_muscle_development	ENSGALG00010027881	ENSGALG00010027881	-0.410617852	0.283588151	-1.447937266	0.147634596	0.189193128	negative
PRJNA729271	stage_E18_vs_E10	ordinary_muscle_development	ENSGALG00010027882	CHIA	0.874703239	1.51275168	0.578219976	0.563115614	0.617005127	positive
PRJNA729271	stage_E18_vs_E10	ordinary_muscle_development	ENSGALG00010027883	ATP8B2	0.350496136	0.117263169	2.988970344	0.002799193	0.004817656	positive
PRJNA729271	stage_E18_vs_E10	ordinary_muscle_development	ENSGALG00010027884	SF3A2	-0.280060407	0.189836254	-1.475273565	0.14013901	0.180602606	negative
PRJNA729271	stage_E18_vs_E10	ordinary_muscle_development	ENSGALG00010027885	PLEKHJ1	-0.699341027	0.074295033	-9.413025355	4.82058E-21	3.64118E-20	negative
PRJNA729271	stage_E18_vs_E10	ordinary_muscle_development	ENSGALG00010027886	IL6R	1.906941549	0.192964163	9.882361155	4.9649E-23	4.16954E-22	positive
PRJNA729271	stage_E18_vs_E10	ordinary_muscle_development	ENSGALG00010027887	ERC2	0.317049577	0.252452206	1.255879606	0.209159624	0.259014844	positive
PRJNA729271	stage_E18_vs_E10	ordinary_muscle_development	ENSGALG00010027888	PFKFB2	-0.708872314	0.14821818	-4.782627314	1.73019E-06	4.35854E-06	negative
PRJNA729271	stage_E18_vs_E10	ordinary_muscle_development	ENSGALG00010027889	RBM8A	-0.797816885	0.165256639	-4.827744828	1.38088E-06	3.51525E-06	negative
PRJNA729271	stage_E18_vs_E10	ordinary_muscle_development	ENSGALG00010027890	ENSGALG00010027890	4.825116903	0.832031289	5.799201266	6.66315E-09	2.09279E-08	positive
PRJNA729271	stage_E18_vs_E10	ordinary_muscle_development	ENSGALG00010027891	CCDC66	-1.430728515	0.146812053	-9.745306895	1.93194E-22	1.58033E-21	negative
PRJNA729271	stage_E18_vs_E10	ordinary_muscle_development	ENSGALG00010027892	SHE	1.035220516	0.166901493	6.202583914	5.55436E-10	1.92424E-09	positive
PRJNA729271	stage_E18_vs_E10	ordinary_muscle_development	ENSGALG00010027893	DOT1L	1.24208029	0.157487282	7.886860904	3.09882E-15	1.60764E-14	positive
PRJNA729271	stage_E18_vs_E10	ordinary_muscle_development	ENSGALG00010027894	RSBN1	-0.116213844	0.069392033	-1.674743311	0.093984606	0.125304163	negative
PRJNA729271	stage_E18_vs_E10	ordinary_muscle_development	ENSGALG00010027895	LIX1L	1.004346399	0.175858081	5.711118833	1.12236E-08	3.44893E-08	positive
PRJNA729271	stage_E18_vs_E10	ordinary_muscle_development	ENSGALG00010027896	TOR4A	1.138534473	0.158689329	7.174612668	7.25122E-13	3.14219E-12	positive
PRJNA729271	stage_E18_vs_E10	ordinary_muscle_development	ENSGALG00010027897	AP3D1	-0.342950468	0.098754773	-3.472748278	0.000515158	0.000977513	negative
PRJNA729271	stage_E18_vs_E10	ordinary_muscle_development	ENSGALG00010027898	TASOR	-0.112956779	0.092283866	-1.224014375	0.220946805	0.271869604	negative
PRJNA729271	stage_E18_vs_E10	ordinary_muscle_development	ENSGALG00010027899	PMVK	-0.20340216	0.177378227	-1.146714356	0.251499695	0.305111103	negative
PRJNA729271	stage_E18_vs_E10	ordinary_muscle_development	ENSGALG00010027901	MOB3A	-0.190761273	0.09746918	-1.957144543	0.050330477	0.070637128	negative
PRJNA729271	stage_E18_vs_E10	ordinary_muscle_development	ENSGALG00010027902	UBE2Q1	-0.093721409	0.070944822	-1.321046502	0.186485859	0.233570598	negative
PRJNA729271	stage_E18_vs_E10	ordinary_muscle_development	ENSGALG00010027903	NDUFA8	0.154646883	0.125988762	1.227465695	0.219647608	0.270385739	positive
PRJNA729271	stage_E18_vs_E10	ordinary_muscle_development	ENSGALG00010027904	MORN5	-1.101211425	0.511937267	-2.151067122	0.031470902	0.045818675	negative
PRJNA729271	stage_E18_vs_E10	ordinary_muscle_development	ENSGALG00010027905	SYN2	1.12011703	0.261010597	4.291461894	1.77501E-05	4.02011E-05	positive
PRJNA729271	stage_E18_vs_E10	ordinary_muscle_development	ENSGALG00010027906	LHX6	1.750572404	0.174211205	10.04856379	9.3216E-24	8.15922E-23	positive
PRJNA729271	stage_E18_vs_E10	ordinary_muscle_development	ENSGALG00010027907	MKNK2	1.917710265	0.188521678	10.17235942	2.63448E-24	2.38967E-23	positive
PRJNA729271	stage_E18_vs_E10	ordinary_muscle_development	ENSGALG00010027908	PBXIP1	1.848804176	0.106175337	17.41274594	6.6039E-68	3.32373E-66	positive
PRJNA729271	stage_E18_vs_E10	ordinary_muscle_development	ENSGALG00010027909	TIMP4	-2.029411816	0.25666343	-7.906899	2.6388E-15	1.37489E-14	negative
PRJNA729271	stage_E18_vs_E10	ordinary_muscle_development	ENSGALG00010027910	CHRNB2	0.304012978	0.184654494	1.646388196	0.099683862	0.132209496	positive
PRJNA729271	stage_E18_vs_E10	ordinary_muscle_development	ENSGALG00010027911	PTPN22	-1.058746304	0.448293461	-2.36172596	0.018190083	0.027617602	negative
PRJNA729271	stage_E18_vs_E10	ordinary_muscle_development	ENSGALG00010027912	RGS3	0.073762747	0.128760268	0.572868852	0.566733496	0.620453325	positive
PRJNA729271	stage_E18_vs_E10	ordinary_muscle_development	ENSGALG00010027913	PYGO2	-0.830819977	0.231254114	-3.592671113	0.000327306	0.000637246	negative
PRJNA729271	stage_E18_vs_E10	ordinary_muscle_development	ENSGALG00010027914	BTBD2	0.493062664	0.126610037	3.894341054	9.8466E-05	0.00020548	positive
PRJNA729271	stage_E18_vs_E10	ordinary_muscle_development	ENSGALG00010027915	GSTM3	0.298732534	0.083676279	3.570098231	0.000356847	0.000691604	positive
PRJNA729271	stage_E18_vs_E10	ordinary_muscle_development	ENSGALG00010027916	ADAR	-0.364693696	0.130082109	-2.803565368	0.005054099	0.008373432	negative
PRJNA729271	stage_E18_vs_E10	ordinary_muscle_development	ENSGALG00010027917	PPARG	-0.470123811	0.453651465	-1.036310576	0.300057274	0.357029239	negative
PRJNA729271	stage_E18_vs_E10	ordinary_muscle_development	ENSGALG00010027918	CSNK1G2	0.208614432	0.101380628	2.057734656	0.039615612	0.05661326	positive
PRJNA729271	stage_E18_vs_E10	ordinary_muscle_development	ENSGALG00010027919	TSEN2	-0.531166415	0.122109798	-4.349908238	1.36195E-05	3.12809E-05	negative
PRJNA729271	stage_E18_vs_E10	ordinary_muscle_development	ENSGALG00010027920	SCAMP4	-0.42306354	0.114087051	-3.708252048	0.000208695	0.000417419	negative
PRJNA729271	stage_E18_vs_E10	ordinary_muscle_development	ENSGALG00010027921	MKRN2OS	0.83170113	0.232519605	3.576907541	0.000347683	0.000674474	positive
PRJNA729271	stage_E18_vs_E10	ordinary_muscle_development	ENSGALG00010027922	SHC1	0.019967323	0.113686057	0.175635632	0.860580207	0.884800962	positive
PRJNA729271	stage_E18_vs_E10	ordinary_muscle_development	ENSGALG00010027923	AMPD2	-0.554024839	0.161566048	-3.429091977	0.000605604	0.001137814	negative
PRJNA729271	stage_E18_vs_E10	ordinary_muscle_development	ENSGALG00010027925	MKRN2	-0.594847432	0.071140984	-8.361529405	6.19104E-17	3.59964E-16	negative
PRJNA729271	stage_E18_vs_E10	ordinary_muscle_development	ENSGALG00010027926	CKS1B	-1.468812103	0.249453273	-5.888125206	3.90601E-09	1.25371E-08	negative
PRJNA729271	stage_E18_vs_E10	ordinary_muscle_development	ENSGALG00010027927	ENSGALG00010027927	0.659756745	0.196088256	3.364590821	0.000766573	0.001420904	positive
PRJNA729271	stage_E18_vs_E10	ordinary_muscle_development	ENSGALG00010027928	FLAD1	-0.443715924	0.167440522	-2.649991289	0.008049385	0.012909474	negative
PRJNA729271	stage_E18_vs_E10	ordinary_muscle_development	ENSGALG00010027929	CDK5RAP2	-1.785773951	0.139154191	-12.83305902	1.07052E-37	1.86505E-36	negative
PRJNA729271	stage_E18_vs_E10	ordinary_muscle_development	ENSGALG00010027930	RAF1	0.737556224	0.08958797	8.232759601	1.82949E-16	1.03064E-15	positive
PRJNA729271	stage_E18_vs_E10	ordinary_muscle_development	ENSGALG00010027931	ABHD17A	-0.353268933	0.173913594	-2.031289933	0.042225592	0.059970601	negative
PRJNA729271	stage_E18_vs_E10	ordinary_muscle_development	ENSGALG00010027932	POLE3	-1.108705826	0.075203528	-14.74273691	3.42613E-49	9.69957E-48	negative
PRJNA729271	stage_E18_vs_E10	ordinary_muscle_development	ENSGALG00010027933	ENSGALG00010027933	2.263309945	0.221562011	10.21524374	1.69453E-24	1.55753E-23	positive
PRJNA729271	stage_E18_vs_E10	ordinary_muscle_development	ENSGALG00010027934	CNBP	0.215796583	0.085133158	2.534812369	0.011250757	0.017685687	positive
PRJNA729271	stage_E18_vs_E10	ordinary_muscle_development	ENSGALG00010027935	ISY1	-0.269092768	0.087797787	-3.064915175	0.002177317	0.003801978	negative
PRJNA729271	stage_E18_vs_E10	ordinary_muscle_development	ENSGALG00010027936	ENSGALG00010027936	-0.502895578	0.08710633	-5.773352822	7.77095E-09	2.42445E-08	negative
PRJNA729271	stage_E18_vs_E10	ordinary_muscle_development	ENSGALG00010027937	HDHD3	0.50036295	0.093106423	5.374097038	7.69674E-08	2.20135E-07	positive
PRJNA729271	stage_E18_vs_E10	ordinary_muscle_development	ENSGALG00010027938	ENSGALG00010027938	-0.805728224	0.120864164	-6.666394709	2.62164E-11	9.98702E-11	negative
PRJNA729271	stage_E18_vs_E10	ordinary_muscle_development	ENSGALG00010027939	BSPRY	-1.519374487	0.429620678	-3.536548784	0.000405392	0.000780055	negative
PRJNA729271	stage_E18_vs_E10	ordinary_muscle_development	ENSGALG00010027940	ZBTB7B	0.899673106	0.418555076	2.149473647	0.031596873	0.045983602	positive
PRJNA729271	stage_E18_vs_E10	ordinary_muscle_development	ENSGALG00010027941	KLF2	0.721798069	0.151454847	4.76576407	1.88139E-06	4.71568E-06	positive
PRJNA729271	stage_E18_vs_E10	ordinary_muscle_development	ENSGALG00010027942	KHDC4	0.313164182	0.124230344	2.520834856	0.011707678	0.018350216	positive
PRJNA729271	stage_E18_vs_E10	ordinary_muscle_development	ENSGALG00010027943	RAB43	-0.339722558	0.094668224	-3.588559519	0.00033251	0.000647031	negative
PRJNA729271	stage_E18_vs_E10	ordinary_muscle_development	ENSGALG00010027944	WDR31	-1.287505321	0.271527955	-4.741704477	2.11928E-06	5.28179E-06	negative
PRJNA729271	stage_E18_vs_E10	ordinary_muscle_development	ENSGALG00010027946	AP1M1	-0.727895993	0.093314403	-7.800467783	6.16781E-15	3.13253E-14	negative
PRJNA729271	stage_E18_vs_E10	ordinary_muscle_development	ENSGALG00010027947	GP9	1.164384334	0.349117024	3.335226455	0.0008523	0.001568772	positive
PRJNA729271	stage_E18_vs_E10	ordinary_muscle_development	ENSGALG00010027948	PRPF4	-0.313223093	0.114390993	-2.73817969	0.006178031	0.010093616	negative
PRJNA729271	stage_E18_vs_E10	ordinary_muscle_development	ENSGALG00010027950	CDC26	0.45943459	0.127595287	3.600717566	0.00031734	0.000618841	positive
PRJNA729271	stage_E18_vs_E10	ordinary_muscle_development	ENSGALG00010027951	FAM32A	0.245214577	0.104778359	2.340317027	0.019267377	0.029131181	positive
PRJNA729271	stage_E18_vs_E10	ordinary_muscle_development	ENSGALG00010027952	CIB3	-0.513780333	0.55566871	-0.924616275	0.355165544	0.414569542	negative
PRJNA729271	stage_E18_vs_E10	ordinary_muscle_development	ENSGALG00010027953	SLC31A1	-1.273480359	0.12038285	-10.57858622	3.74567E-26	3.77826E-25	negative
PRJNA729271	stage_E18_vs_E10	ordinary_muscle_development	ENSGALG00010027954	CFAP92	-0.631032755	0.232837079	-2.710190133	0.006724465	0.010910862	negative
PRJNA729271	stage_E18_vs_E10	ordinary_muscle_development	ENSGALG00010027955	ENSGALG00010027955	0.233050852	0.1178442	1.977618345	0.047971778	0.067562274	positive
PRJNA729271	stage_E18_vs_E10	ordinary_muscle_development	ENSGALG00010027956	RAB8A	-0.708275409	0.066122158	-10.71161963	8.97772E-27	9.40941E-26	negative
PRJNA729271	stage_E18_vs_E10	ordinary_muscle_development	ENSGALG00010027957	ENTREP3	-1.096810881	0.507295482	-2.162075003	0.030612395	0.044670084	negative
PRJNA729271	stage_E18_vs_E10	ordinary_muscle_development	ENSGALG00010027958	ENSGALG00010027958	1.460224774	0.138468831	10.54551236	5.32816E-26	5.33368E-25	positive
PRJNA729271	stage_E18_vs_E10	ordinary_muscle_development	ENSGALG00010027959	TPM4	-1.865642932	0.171614859	-10.87110373	1.58274E-27	1.72625E-26	negative
PRJNA729271	stage_E18_vs_E10	ordinary_muscle_development	ENSGALG00010027960	PPP6C	0.026514119	0.121196257	0.218770115	0.826829128	0.856063444	positive
PRJNA729271	stage_E18_vs_E10	ordinary_muscle_development	ENSGALG00010027961	TINCR	-3.771262891	0.969043971	-3.89173557	9.95297E-05	0.000207567	negative
PRJNA729271	stage_E18_vs_E10	ordinary_muscle_development	ENSGALG00010027962	SCAMP3	-0.01814778	0.133448819	-0.135990561	0.891828743	0.910452173	negative
PRJNA729271	stage_E18_vs_E10	ordinary_muscle_development	ENSGALG00010027964	RABEPK	0.165643021	0.135906274	1.218803341	0.222918843	0.273924096	positive

PRJNA729271	stage_E18_vs_E10	ordinary_muscle_development	ENSGALG00010027965	ACAD9	1.221036727	0.109412189	11.1599698	6.40133E-29	7.41705E-28	positive
PRJNA729271	stage_E18_vs_E10	ordinary_muscle_development	ENSGALG00010027966	CLK2	0.270331915	0.100109436	2.700363982	0.006926365	0.011208849	positive
PRJNA729271	stage_E18_vs_E10	ordinary_muscle_development	ENSGALG00010027968	FKBP15	-0.201716982	0.097755687	-2.063480791	0.039066971	0.055917423	negative
PRJNA729271	stage_E18_vs_E10	ordinary_muscle_development	ENSGALG00010027969	HSPA5	-0.697168659	0.129508579	-5.383185147	7.31792E-08	2.09672E-07	negative
PRJNA729271	stage_E18_vs_E10	ordinary_muscle_development	ENSGALG00010027970	GRIN3B	0.634119806	0.174843328	3.626788702	0.000286968	0.000563861	positive
PRJNA729271	stage_E18_vs_E10	ordinary_muscle_development	ENSGALG00010027971	ENSGALG00010027971	1.457629002	0.386195192	3.774332333	0.000160437	0.000325796	positive
PRJNA729271	stage_E18_vs_E10	ordinary_muscle_development	ENSGALG00010027972	TMEM259	-0.09142375	0.107499342	-0.850458694	0.395070116	0.455065268	negative
PRJNA729271	stage_E18_vs_E10	ordinary_muscle_development	ENSGALG00010027973	GAPVD1	0.551401968	0.064836321	8.504522814	1.82344E-17	1.09853E-16	positive
PRJNA729271	stage_E18_vs_E10	ordinary_muscle_development	ENSGALG00010027974	ENSGALG00010027974	-0.802148218	0.362197964	-2.214667934	0.026782865	0.039501184	negative
PRJNA729271	stage_E18_vs_E10	ordinary_muscle_development	ENSGALG00010027975	IQSEC1	0.663429429	0.117367513	5.652581487	1.58056E-08	4.80298E-08	positive
PRJNA729271	stage_E18_vs_E10	ordinary_muscle_development	ENSGALG00010027976	LRSAM1	0.25707769	0.098203922	2.617794527	0.008850008	0.014118519	positive
PRJNA729271	stage_E18_vs_E10	ordinary_muscle_development	ENSGALG00010027977	BOLA1	0.305108475	0.158180534	1.928862338	0.053747954	0.075033863	positive
PRJNA729271	stage_E18_vs_E10	ordinary_muscle_development	ENSGALG00010027979	RPL12	0.585025701	0.119548931	4.893608805	9.90035E-07	2.55894E-06	positive
PRJNA729271	stage_E18_vs_E10	ordinary_muscle_development	ENSGALG00010027980	NUP210	0.345652488	0.144326936	2.394927081	0.016623671	0.02543383	positive
PRJNA729271	stage_E18_vs_E10	ordinary_muscle_development	ENSGALG00010027981	CNN2	-1.458182862	0.16210468	-8.995316244	2.35553E-19	1.59549E-18	negative
PRJNA729271	stage_E18_vs_E10	ordinary_muscle_development	ENSGALG00010027982	ENTPD2	3.031770892	0.248855774	12.18284329	3.83669E-34	5.6922E-33	positive
PRJNA729271	stage_E18_vs_E10	ordinary_muscle_development	ENSGALG00010027983	POGZ	0.088791928	0.146157475	0.607508636	0.543513413	0.598376647	positive
PRJNA729271	stage_E18_vs_E10	ordinary_muscle_development	ENSGALG00010027984	ARHGAP45	0.607356747	0.175417477	3.462350257	0.00053548	0.001013287	positive
PRJNA729271	stage_E18_vs_E10	ordinary_muscle_development	ENSGALG00010027985	PSMB4	-0.175888011	0.095166807	-1.848207549	0.064572324	0.088802261	negative
PRJNA729271	stage_E18_vs_E10	ordinary_muscle_development	ENSGALG00010027986	CGN	-0.028422569	0.254395176	-0.111726053	0.911040621	0.926444072	negative
PRJNA729271	stage_E18_vs_E10	ordinary_muscle_development	ENSGALG00010027987	TMEM141	0.54791768	0.138684995	3.950807217	7.7888E-05	0.000164196	positive
PRJNA729271	stage_E18_vs_E10	ordinary_muscle_development	ENSGALG00010027988	HDAC11	0.374281763	0.130312609	2.872183794	0.004076458	0.006837336	positive
PRJNA729271	stage_E18_vs_E10	ordinary_muscle_development	ENSGALG00010027989	ENSGALG00010027989	0.550610984	0.1406344	3.915194174	9.03315E-05	0.000189268	positive
PRJNA729271	stage_E18_vs_E10	ordinary_muscle_development	ENSGALG00010027990	ENSGALG00010027990	0.977941735	0.192333049	5.084626592	3.6835E-07	9.92792E-07	positive
PRJNA729271	stage_E18_vs_E10	ordinary_muscle_development	ENSGALG00010027991	ENSGALG00010027991	0.432462225	0.146288004	2.956238478	0.003114162	0.005321205	positive
PRJNA729271	stage_E18_vs_E10	ordinary_muscle_development	ENSGALG00010027993	CLIC3	1.643961705	0.161130756	10.20265617	1.92923E-24	1.76765E-23	positive
PRJNA729271	stage_E18_vs_E10	ordinary_muscle_development	ENSGALG00010027994	TUFT1	0.066962056	0.158321272	0.422950469	0.672331399	0.718902599	positive
PRJNA729271	stage_E18_vs_E10	ordinary_muscle_development	ENSGALG00010027995	POLR2E	-0.421926369	0.083243666	-5.068570249	4.00815E-07	1.07649E-06	negative
PRJNA729271	stage_E18_vs_E10	ordinary_muscle_development	ENSGALG00010027996	GPX4	0.545980478	0.122048607	4.473467508	7.69613E-06	1.81332E-05	positive
PRJNA729271	stage_E18_vs_E10	ordinary_muscle_development	ENSGALG00010027998	SNX27	-0.138818802	0.093808784	-1.47980601	0.138925024	0.179236157	negative
PRJNA729271	stage_E18_vs_E10	ordinary_muscle_development	ENSGALG00010027999	ENSGALG00010027999	-1.147791313	0.504176586	-2.276566077	0.022812151	0.034057279	negative
PRJNA729271	stage_E18_vs_E10	ordinary_muscle_development	ENSGALG00010028000	MRPL9	0.418873745	0.166864711	2.510259609	0.012064243	0.018860316	positive
PRJNA729271	stage_E18_vs_E10	ordinary_muscle_development	ENSGALG00010028001	SBNO2	0.242472046	0.118794229	2.041109635	0.041239931	0.058726083	positive
PRJNA729271	stage_E18_vs_E10	ordinary_muscle_development	ENSGALG00010028002	ABCA2	0.227318608	0.120605004	1.884819039	0.059454274	0.082365449	positive
PRJNA729271	stage_E18_vs_E10	ordinary_muscle_development	ENSGALG00010028004	ENSGALG00010028004	-2.146425656	0.348513683	-6.158798802	7.32988E-10	2.50937E-09	negative
PRJNA729271	stage_E18_vs_E10	ordinary_muscle_development	ENSGALG00010028005	IER5L	0.525588038	0.143130378	3.672092846	0.000240572	0.000477553	positive
PRJNA729271	stage_E18_vs_E10	ordinary_muscle_development	ENSGALG00010028006	ENSGALG00010028006	2.587634955	0.83462605	3.100352494	0.001932905	0.003400176	positive
PRJNA729271	stage_E18_vs_E10	ordinary_muscle_development	ENSGALG00010028007	STK11	1.03256069	0.119609731	8.632748171	5.98952E-18	3.7293E-17	positive
PRJNA729271	stage_E18_vs_E10	ordinary_muscle_development	ENSGALG00010028008	NTMT1	0.333361344	0.113611485	2.934222209	0.003343847	0.005673935	positive
PRJNA729271	stage_E18_vs_E10	ordinary_muscle_development	ENSGALG00010028009	RRP9	-0.965136783	0.180576118	-5.344764264	9.05348E-08	2.57466E-07	negative
PRJNA729271	stage_E18_vs_E10	ordinary_muscle_development	ENSGALG00010028010	ASB6	-0.320625498	0.073572788	-4.357935944	1.31295E-05	3.01938E-05	negative
PRJNA729271	stage_E18_vs_E10	ordinary_muscle_development	ENSGALG00010028011	ENSGALG00010028011	-0.21661128	0.091636703	-2.363804805	0.01808834	0.02748328	negative
PRJNA729271	stage_E18_vs_E10	ordinary_muscle_development	ENSGALG00010028012	PRRX2	-3.423815224	0.255208148	-13.41577551	4.88793E-41	9.78599E-40	negative
PRJNA729271	stage_E18_vs_E10	ordinary_muscle_development	ENSGALG00010028013	CBARP	-0.322304537	0.218602723	-1.474384822	0.140378009	0.180869266	negative
PRJNA729271	stage_E18_vs_E10	ordinary_muscle_development	ENSGALG00010028014	ENSGALG00010028014	0.816338612	0.293637128	2.7800933	0.005434328	0.008958329	positive
PRJNA729271	stage_E18_vs_E10	ordinary_muscle_development	ENSGALG00010028015	DUSP7	-0.673616256	0.101931906	-6.608492722	3.88253E-11	1.45947E-10	negative
PRJNA729271	stage_E18_vs_E10	ordinary_muscle_development	ENSGALG00010028016	PRF1	0.762334948	1.470453767	0.518435169	0.604154682	0.655186452	positive
PRJNA729271	stage_E18_vs_E10	ordinary_muscle_development	ENSGALG00010028017	ENSGALG00010028017	-0.639963621	1.331089302	-0.480781883	0.630671528	0.680683827	negative
PRJNA729271	stage_E18_vs_E10	ordinary_muscle_development	ENSGALG00010028019	RPL29	1.514660719	0.227099605	6.669587644	2.56523E-11	9.78243E-11	positive
PRJNA729271	stage_E18_vs_E10	ordinary_muscle_development	ENSGALG00010028020	ENSGALG00010028020	1.637987237	0.144531845	11.33305425	9.00103E-30	1.08906E-28	positive
PRJNA729271	stage_E18_vs_E10	ordinary_muscle_development	ENSGALG00010028021	ATP5F1D	1.271355587	0.145225266	8.754369195	2.05248E-18	1.31874E-17	positive
PRJNA729271	stage_E18_vs_E10	ordinary_muscle_development	ENSGALG00010028022	ENSGALG00010028022	-0.636401549	0.144088818	-4.416730988	1.00205E-05	2.33328E-05	negative
PRJNA729271	stage_E18_vs_E10	ordinary_muscle_development	ENSGALG00010028023	ENSGALG00010028023	-0.021662003	0.957711577	-0.022618504	0.981954584	0.985217117	negative
PRJNA729271	stage_E18_vs_E10	ordinary_muscle_development	ENSGALG00010028024	RBM3	-0.350086323	0.112799293	-3.103621612	0.001911677	0.003366102	negative
PRJNA729271	stage_E18_vs_E10	ordinary_muscle_development	ENSGALG00010028025	TNNC1	3.646509473	0.237098672	15.37971275	2.23939E-53	7.22939E-52	positive
PRJNA729271	stage_E18_vs_E10	ordinary_muscle_development	ENSGALG00010028026	ANKMY1	0.88846766	0.167134567	5.315882153	1.06142E-07	3.00493E-07	positive
PRJNA729271	stage_E18_vs_E10	ordinary_muscle_development	ENSGALG00010028027	ENSGALG00010028027	1.023559879	0.232489923	4.40259889	1.06962E-05	2.48583E-05	positive
PRJNA729271	stage_E18_vs_E10	ordinary_muscle_development	ENSGALG00010028028	FAM174C	-0.624568941	0.170178784	-3.670075231	0.000242479	0.000481009	negative
PRJNA729271	stage_E18_vs_E10	ordinary_muscle_development	ENSGALG00010028029	EFNA2	-2.931564265	0.13822519	-21.20861087	7.95271E-100	9.07673E-98	negative
PRJNA729271	stage_E18_vs_E10	ordinary_muscle_development	ENSGALG00010028030	PFDN2	0.179041629	0.135479603	1.321539375	0.186321583	0.23344553	positive
PRJNA729271	stage_E18_vs_E10	ordinary_muscle_development	ENSGALG00010028031	NISCH	0.747707825	0.081103939	9.21913089	2.99518E-20	2.13552E-19	positive
PRJNA729271	stage_E18_vs_E10	ordinary_muscle_development	ENSGALG00010028032	ENSGALG00010028032	-0.90848744	0.216532588	-4.195615304	2.72132E-05	6.03511E-05	negative
PRJNA729271	stage_E18_vs_E10	ordinary_muscle_development	ENSGALG00010028033	ENSGALG00010028033	3.374671972	0.29064437	11.61100064	3.6235E-31	4.70633E-30	positive
PRJNA729271	stage_E18_vs_E10	ordinary_muscle_development	ENSGALG00010028034	MUM1L1	-0.618576857	0.07888318	-7.841682568	4.44549E-15	2.27855E-14	negative
PRJNA729271	stage_E18_vs_E10	ordinary_muscle_development	ENSGALG00010028035	ENSGALG00010028035	0.286615482	1.132301307	0.253126514	0.800170451	0.833211966	positive
PRJNA729271	stage_E18_vs_E10	ordinary_muscle_development	ENSGALG00010028036	NDUFS7	1.070028868	0.12256114	8.730572091	2.53388E-18	1.62157E-17	positive
PRJNA729271	stage_E18_vs_E10	ordinary_muscle_development	ENSGALG00010028037	ENSGALG00010028037	-0.451367164	0.288579364	-1.564100627	0.117793939	0.154224834	negative
PRJNA729271	stage_E18_vs_E10	ordinary_muscle_development	ENSGALG00010028039	STAB1	1.7084444	0.162433319	10.5178199	7.1509E-26	7.10434E-25	positive
PRJNA729271	stage_E18_vs_E10	ordinary_muscle_development	ENSGALG00010028042	DPP7	-0.38657152	0.192687964	-2.00620481	0.044834401	0.06346432	negative
PRJNA729271	stage_E18_vs_E10	ordinary_muscle_development	ENSGALG00010028043	NT5DC2	1.640867913	0.115660751	14.18690355	1.10428E-45	2.66776E-44	positive
PRJNA729271	stage_E18_vs_E10	ordinary_muscle_development	ENSGALG00010028044	GAMT	3.751709072	0.153430465	24.45217816	4.771E-132	1.1154E-129	positive
PRJNA729271	stage_E18_vs_E10	ordinary_muscle_development	ENSGALG00010028045	ENSGALG00010028045	-1.401952581	0.662291435	-2.116821247	0.034275021	0.049533044	negative
PRJNA729271	stage_E18_vs_E10	ordinary_muscle_development	ENSGALG00010028046	SMIM4	2.018438948	0.20341123	9.92294745	3.30839E-23	2.8044E-22	positive
PRJNA729271	stage_E18_vs_E10	ordinary_muscle_development	ENSGALG00010028047	ENSGALG00010028047	1.058885553	0.28986437	3.653037984	0.000259156	0.000512548	positive
PRJNA729271	stage_E18_vs_E10	ordinary_muscle_development	ENSGALG00010028048	S100A10	1.443390079	0.218521988	6.605239542	3.96875E-11	1.4915E-10	positive
PRJNA729271	stage_E18_vs_E10	ordinary_muscle_development	ENSGALG00010028049	MAN1B1	-0.053709519	0.060874958	-0.882292497	0.377618653	0.437711505	negative
PRJNA729271	stage_E18_vs_E10	ordinary_muscle_development	ENSGALG00010028050	PBRM1	-0.651424431	0.087853735	-7.414874656	1.2174E-13	5.56487E-13	negative
PRJNA729271	stage_E18_vs_E10	ordinary_muscle_development	ENSGALG00010028051	GNL3	-0.955035231	0.093785971	-10.18313525	2.35838E-24	2.14593E-23	negative
PRJNA729271	stage_E18_vs_E10	ordinary_muscle_development	ENSGALG00010028052	ENSGALG00010028052	-1.74375581	0.213592292	-8.163945412	3.24255E-16	1.79598E-15	negative
PRJNA729271	stage_E18_vs_E10	ordinary_muscle_development	ENSGALG00010028053	ORM1	3.221803002	2.149251393	1.499034972	0.13386456	0.173216104	positive
PRJNA729271	stage_E18_vs_E10	ordinary_muscle_development	ENSGALG00010028054	RPS15	0.764128013	0.140747719	5.429061446	5.66512E-08	1.63773E-07	positive
PRJNA729271	stage_E18_vs_E10	ordinary_muscle_development	ENSGALG00010028055	ENSGALG00010028055	-0.200221518	0.118173016	-1.694308273	0.090206719	0.12064462	negative
PRJNA729271	stage_E18_vs_E10	ordinary_muscle_development	ENSGALG00010028056	SLC25A25	-0.773827131	0.240266063	-3.220709253	0.001278738	0.002300001	negative
PRJNA729271	stage_E18_vs_E10	ordinary_muscle_development	ENSGALG00010028057	APC2	-0.607316563	0.370920132	-1.637324349	0.101562716	0.134455344	negative
PRJNA729271	stage_E18_vs_E10	ordinary_muscle_development	ENSGALG00010028058	GLT8D1	0.092225769	0.113792533	0.810472942	0.41766841	0.477980705	positive
PRJNA729271	stage_E18_vs_E10	ordinary_muscle_development	ENSGALG00010028059	GZMM	0.207088033	0.689816946	0.300207228	0.764019092	0.802090008	positive
PRJNA729271	stage_E18_vs_E10	ordinary_muscle_development	ENSGALG00010028060	ENSGALG00010028060	0.483324941	0.142539121	3.390823077	0.000696831	0.001299275	positive
PRJNA729271	stage_E18_vs_E10	ordinary_muscle_development	ENSGALG00010028061	NAIF1	-0.151376733	0.094996795	-1.593493059	0.111049624	0.146094055	negative
PRJNA729271	stage_E18_vs_E10	ordinary_muscle_development	ENSGALG00010028062	ENSGALG00010028062	1.222381151	0.486443402	2.512894914	0.0119745	0.018733985	positive
PRJNA729271	stage_E18_vs_E10	ordinary_muscle_development	ENSGALG00010028064	SPCS1	-0.12746196	0.08789516	-1.450159037	0.147014175	0.188526676	negative
PRJNA729271	stage_E18_vs_E10	ordinary_muscle_development	ENSGALG00010028065	REEP6	2.508895176	0.772993015	3.245689322	0.001171666	0.002116299	positive
PRJNA729271	stage_E18_vs_E10	ordinary_muscle_development	ENSGALG00010028066	ENSGALG00010028066	1.76628889	0.184826805	9.556454159	1.2186E-21	9.53761E-21	positive
PRJNA729271	stage_E18_vs_E10	ordinary_muscle_development	ENSGALG00010028067	FAM102A	-0.01626666	0.112165586	-0.145023622	0.884692221	0.904919465	negative
PRJNA729271	stage_E18_vs_E10	ordinary_muscle_development	ENSGALG00010028068	POLE4	-0.212484312	0.110103314	-1.929862991	0.053623816	0.074874984	negative
PRJNA729271	stage_E18_vs_E10	ordinary_muscle_development	ENSGALG00010028069	NEK4	-0.746227644	0.115301684	-6.471957893	9.67411E-11	3.53215E-10	negative
PRJNA729271	stage_E18_vs_E10	ordinary_muscle_development	ENSGALG00010028070	MEX3A	-1.596058835	0.129822367	-12.29417448	9.73462E-35	1.48218E-33	negative
PRJNA729271	stage_E18_vs_E10	ordinary_muscle_development	ENSGALG00010028071	DPM2	0.263874714	0.113670826	2.321393469	0.020265618	0.030529009	positive
PRJNA729271	stage_E18_vs_E10	ordinary_muscle_development	ENSGALG00010028072	ENSGALG00010028072	-5.313797791	0.7226742	-7.352964579	1.93858E-13	8.71311E-13	negative
PRJNA729271	stage_E18_vs_E10	ordinary_muscle_development	ENSGALG00010028073	ST6GALNAC4	3.188860498	0.120635751	26.43379326	5.6043E-154	2.0829E-151	positive
PRJNA729271	stage_E18_vs_E10	ordinary_muscle_development	ENSGALG00010028074	ENSGALG00010028074	-0.006376602	0.48870923	-0.013047844	0.989589622	0.991093869	negative
PRJNA729271	stage_E18_vs_E10	ordinary_muscle_development	ENSGALG00010028075	RAB25	-1.465877555	0.57468538	-2.550747948	0.010749203	0.016937678	negative
PRJNA729271	stage_E18_vs_E10	ordinary_muscle_development	ENSGALG00010028076	ST6GALNAC6	2.687811371	0.15179564	17.70677579	3.71768E-70	1.98117E-68	positive
PRJNA729271	stage_E18_vs_E10	ordinary_muscle_development	ENSGALG00010028078	LAMTOR2	1.064469041	0.130584139	8.15159519	3.59155E-16	1.98397E-15	positive
PRJNA729271	stage_E18_vs_E10	ordinary_muscle_development	ENSGALG00010028079	ITIH3	5.989747416	1.53256123	3.90832503	9.29382E-05	0.000194477	positive
PRJNA729271	stage_E18_vs_E10	ordinary_muscle_development	ENSGALG00010028080	MUSTN1	1.60499385	0.40554061	3.95766493	7.5686E-05	0.000159853	positive
PRJNA729271	stage_E18_vs_E10	ordinary_muscle_development	ENSGALG00010028081	UBQLN4	-0.397575068	0.162887097	-2.440801483	0.014654708	0.022662967	negative
PRJNA729271	stage_E18_vs_E10	ordinary_muscle_development	ENSGALG00010028082	AK1	4.238094206	0.137255678	30.87736896	2.4048E-209	1.5155E-206	positive
PRJNA729271	stage_E18_vs_E10	ordinary_muscle_development	ENSGALG00010028083	ELAVL1	-0.524289035	0.049808944	-10.5260018	6.55603E-26	6.53125E-25	negative
PRJNA729271	stage_E18_vs_E10	ordinary_muscle_development	ENSGALG00010028084	ENG	0.572176667	0.194248265	2.945594736	0.003223344	0.005488356	positive
PRJNA729271	stage_E18_vs_E10	ordinary_muscle_development	ENSGALG00010028085	ENSGALG00010028085	-0.784944717	0.289896275	-2.70767438	0.006775646	0.010984565	negative
PRJNA729271	stage_E18_vs_E10	ordinary_muscle_development	ENSGALG00010028086	MATK	-0.008808589	0.286599747	-0.030734812	0.975481028	0.979671413	negative
PRJNA729271	stage_E18_vs_E10	ordinary_muscle_development	ENSGALG00010028087	STIMATE	-0.874885838	0.09046416	-9.671076763	4.00146E-22	3.20448E-21	negative
PRJNA729271	stage_E18_vs_E10	ordinary_muscle_development	ENSGALG00010028088	NAXE	1.028133167	0.114743216	8.960295889	3.23808E-19	2.18104E-18	positive
PRJNA729271	stage_E18_vs_E10	ordinary_muscle_development	ENSGALG00010028091	FPGS	-0.182648803	0.097896134	-1.865740714	0.062077651	0.08561518	negative
PRJNA729271	stage_E18_vs_E10	ordinary_muscle_development	ENSGALG00010028093	SFMBT1	-0.730389728	0.145792855	-5.009777246	5.44931E-07	1.44507E-06	negative
PRJNA729271	stage_E18_vs_E10	ordinary_muscle_development	ENSGALG00010028095	CDK9	-0.389433054	0.09512565	-4.093880605	4.24213E-05	9.20366E-05	negative
PRJNA729271	stage_E18_vs_E10	ordinary_muscle_development	ENSGALG00010028096	TOR2A	0.160615754	0.1475456	1.088583832	0.27633745	0.331829288	positive
PRJNA729271	stage_E18_vs_E10	ordinary_muscle_development	ENSGALG00010028097	TTC24	2.588001445	0.21658017	11.94939246	6.54016E-33	9.06306E-32	positive
PRJNA729271	stage_E18_vs_E10	ordinary_muscle_development	ENSGALG00010028098	RFT1	-0.50828166	0.121028474	-4.199686581	2.67285E-05	5.93125E-05	negative
PRJNA729271	stage_E18_vs_E10	ordinary_muscle_development	ENSGALG00010028099	PCASP2	1.117614403	0.272531985	4.100855919	4.11625E-05	8.94126E-05	positive
PRJNA729271	stage_E18_vs_E10	ordinary_muscle_development	ENSGALG00010028100	ENSGALG00010028100	1.285452691	0.471891029	2.724045623	0.00644876	0.010499273	positive
PRJNA729271	stage_E18_vs_E10	ordinary_muscle_development	ENSGALG00010028101	MRPL54	0.386296951	0.127736739	3.024164796	0.002493206	0.004317169	positive
PRJNA729271	stage_E18_vs_E10	ordinary_muscle_development	ENSGALG00010028102	CERCAM	1.045806016	0.179079886	5.839885442	5.22367E-09	1.65574E-08	positive
PRJNA729271	stage_E18_vs_E10	ordinary_muscle_development	ENSGALG00010028103	APBA3	0.382078414	0.133017659	2.87238865	0.004073816	0.006833696	positive
PRJNA729271	stage_E18_vs_E10	ordinary_muscle_development	ENSGALG00010028104	GOLGA1	0.266151589	0.115669196	2.300972079	0.021393209	0.032100887	positive
PRJNA729271	stage_E18_vs_E10	ordinary_muscle_development	ENSGALG00010028105	PRKCD	-0.855203931	0.143774774	-5.948219611	2.71075E-09	8.81586E-09	negative
PRJNA729271	stage_E18_vs_E10	ordinary_muscle_development	ENSGALG00010028106	ARPC5L	-0.588915045	0.112504801	-5.23457703	1.65363E-07	4.58393E-07	negative
PRJNA729271	stage_E18_vs_E10	ordinary_muscle_development	ENSGALG00010028107	NOTCH1	-0.067179173	0.124329522	-0.540331632	0.588968347	0.641067522	negative
PRJNA729271	stage_E18_vs_E10	ordinary_muscle_development	ENSGALG00010028108	IQGAP3	-1.133075968	0.210002309	-5.395540522	6.83176E-08	1.96092E-07	negative
PRJNA729271	stage_E18_vs_E10	ordinary_muscle_development	ENSGALG00010028109	TJP3	0.090648296	0.216771765	0.418173907	0.675819972	0.722313117	positive
PRJNA729271	stage_E18_vs_E10	ordinary_muscle_development	ENSGALG00010028110	COQ4	0.439951211	0.101922825	4.316513098	1.58513E-05	3.61323E-05	positive

PRJNA729271	stage_E18_vs_E10	ordinary_muscle_development	ENSGALG00010028111	TRUB2	-0.781640155	0.092294792	-8.468951876	2.47611E-17	1.47761E-16	negative
PRJNA729271	stage_E18_vs_E10	ordinary_muscle_development	ENSGALG00010028112	SWI5	-0.329312616	0.083422747	-3.947515844	7.89663E-05	0.000166393	negative
PRJNA729271	stage_E18_vs_E10	ordinary_muscle_development	ENSGALG00010028113	PIP5K1C	-0.394214127	0.096468916	-4.086436766	4.38049E-05	9.49106E-05	negative
PRJNA729271	stage_E18_vs_E10	ordinary_muscle_development	ENSGALG00010028114	PFKFB4	0.707084843	0.089870145	7.867850268	3.60787E-15	1.86372E-14	positive
PRJNA729271	stage_E18_vs_E10	ordinary_muscle_development	ENSGALG00010028115	CACTIN	-0.012391082	0.175747131	-0.070505175	0.943791582	0.954059487	negative
PRJNA729271	stage_E18_vs_E10	ordinary_muscle_development	ENSGALG00010028116	HYAL1	1.336373008	0.148277942	9.012621766	2.01188E-19	1.36912E-18	positive
PRJNA729271	stage_E18_vs_E10	ordinary_muscle_development	ENSGALG00010028117	ENSGALG00010028117	0.135319715	0.248004423	0.545634282	0.585317354	0.637842819	positive
PRJNA729271	stage_E18_vs_E10	ordinary_muscle_development	ENSGALG00010028118	ENSGALG00010028118	0.147546053	0.077605361	1.901235319	0.057271197	0.07959977	positive
PRJNA729271	stage_E18_vs_E10	ordinary_muscle_development	ENSGALG00010028119	HYAL2	0.508635191	0.127625337	3.985377855	6.73728E-05	0.000143297	positive
PRJNA729271	stage_E18_vs_E10	ordinary_muscle_development	ENSGALG00010028120	TBXA2R	0.39765596	0.237153989	1.676783767	0.093584773	0.124817012	positive
PRJNA729271	stage_E18_vs_E10	ordinary_muscle_development	ENSGALG00010028121	TUSC2	-0.021034453	0.09754869	-0.2156303	0.829275935	0.858167679	negative
PRJNA729271	stage_E18_vs_E10	ordinary_muscle_development	ENSGALG00010028122	SEC16A	-0.020727581	0.088872612	-0.233227989	0.815584373	0.846779993	negative
PRJNA729271	stage_E18_vs_E10	ordinary_muscle_development	ENSGALG00010028123	RASSF1	0.129464054	0.107431591	1.205083645	0.228171013	0.279619448	positive
PRJNA729271	stage_E18_vs_E10	ordinary_muscle_development	ENSGALG00010028124	SUGP2	-0.357211177	0.089353293	-3.99773936	6.39503E-05	0.000136318	negative
PRJNA729271	stage_E18_vs_E10	ordinary_muscle_development	ENSGALG00010028125	HMG20B	-0.742322964	0.121748283	-6.097194541	1.07946E-09	3.63287E-09	negative
PRJNA729271	stage_E18_vs_E10	ordinary_muscle_development	ENSGALG00010028126	ZMYND10	-0.272224441	0.088477466	-3.076765807	0.002092596	0.003664635	negative
PRJNA729271	stage_E18_vs_E10	ordinary_muscle_development	ENSGALG00010028127	MFSD12	-0.529252972	0.179454583	-2.949230738	0.00318566	0.005432973	negative
PRJNA729271	stage_E18_vs_E10	ordinary_muscle_development	ENSGALG00010028128	DNM1	-1.669917335	0.143474366	-11.63913378	2.60649E-31	3.40064E-30	negative
PRJNA729271	stage_E18_vs_E10	ordinary_muscle_development	ENSGALG00010028129	CYB561D2	0.493098247	0.106968861	4.609736353	4.0318E-06	9.74178E-06	positive
PRJNA729271	stage_E18_vs_E10	ordinary_muscle_development	ENSGALG00010028130	TMEM115	0.328513332	0.127288321	2.580859973	0.009855455	0.015605726	positive
PRJNA729271	stage_E18_vs_E10	ordinary_muscle_development	ENSGALG00010028131	TMEM161A	0.639865001	0.205069022	3.120242123	0.001807024	0.003196975	positive
PRJNA729271	stage_E18_vs_E10	ordinary_muscle_development	ENSGALG00010028132	CACNA2D2	0.572503063	0.129421698	4.423547759	9.70932E-06	2.2641E-05	positive
PRJNA729271	stage_E18_vs_E10	ordinary_muscle_development	ENSGALG00010028133	ENSGALG00010028133	0.21262554	0.325615792	0.65299517	0.513759392	0.570515773	positive
PRJNA729271	stage_E18_vs_E10	ordinary_muscle_development	ENSGALG00010028134	INPP5E	-0.74063092	0.072090985	-10.27355808	9.27205E-25	8.68768E-24	negative
PRJNA729271	stage_E18_vs_E10	ordinary_muscle_development	ENSGALG00010028136	C3orf18	-0.091397634	0.161804914	-0.564863154	0.572166847	0.625667356	negative
PRJNA729271	stage_E18_vs_E10	ordinary_muscle_development	ENSGALG00010028137	NUP214	-0.476520066	0.066977656	-7.114612462	1.12228E-12	4.78883E-12	negative
PRJNA729271	stage_E18_vs_E10	ordinary_muscle_development	ENSGALG00010028138	ENSGALG00010028138	-0.379030361	0.510920539	-0.74185775	0.458173527	0.517632523	negative
PRJNA729271	stage_E18_vs_E10	ordinary_muscle_development	ENSGALG00010028139	FZR1	-0.482272087	0.087632396	-5.503353904	3.72633E-08	1.09984E-07	negative
PRJNA729271	stage_E18_vs_E10	ordinary_muscle_development	ENSGALG00010028140	HEMK1	-0.403830934	0.11094888	-3.639792787	0.000272858	0.00053752	negative
PRJNA729271	stage_E18_vs_E10	ordinary_muscle_development	ENSGALG00010028141	CISH	0.812735776	0.187617578	4.331874365	1.47845E-05	3.38175E-05	positive
PRJNA729271	stage_E18_vs_E10	ordinary_muscle_development	ENSGALG00010028142	PMPCA	-0.139077884	0.122452021	-1.135774513	0.256050946	0.309963589	negative
PRJNA729271	stage_E18_vs_E10	ordinary_muscle_development	ENSGALG00010028144	FAM78A	2.008549916	0.184536779	10.88427971	1.36977E-27	1.49622E-26	positive
PRJNA729271	stage_E18_vs_E10	ordinary_muscle_development	ENSGALG00010028145	ENSGALG00010028145	0.145891786	0.140131261	1.04110807	0.297825395	0.354810479	positive
PRJNA729271	stage_E18_vs_E10	ordinary_muscle_development	ENSGALG00010028146	MAPKAPK3	1.985763764	0.104373611	19.02553483	1.04812E-80	7.52879E-79	positive
PRJNA729271	stage_E18_vs_E10	ordinary_muscle_development	ENSGALG00010028147	SLC25A42	3.525370299	0.197064465	17.88942667	1.42568E-71	7.85748E-70	positive
PRJNA729271	stage_E18_vs_E10	ordinary_muscle_development	ENSGALG00010028148	ENSGALG00010028148	5.643397646	0.256498461	22.00168229	2.7749E-107	3.9051E-105	positive
PRJNA729271	stage_E18_vs_E10	ordinary_muscle_development	ENSGALG00010028149	SNAPC4	-0.111898198	0.090876781	-1.231317806	0.218204025	0.268997052	negative
PRJNA729271	stage_E18_vs_E10	ordinary_muscle_development	ENSGALG00010028150	ARMC6	-0.346816602	0.082811127	-4.188043507	2.8137E-05	6.2314E-05	negative
PRJNA729271	stage_E18_vs_E10	ordinary_muscle_development	ENSGALG00010028151	DOCK3	1.20650401	0.221977479	5.43525414	5.47184E-08	1.5847E-07	positive
PRJNA729271	stage_E18_vs_E10	ordinary_muscle_development	ENSGALG00010028152	ENSGALG00010028152	-0.402180452	0.372854831	-1.078651577	0.280743079	0.336391848	negative
PRJNA729271	stage_E18_vs_E10	ordinary_muscle_development	ENSGALG00010028153	MANF	-0.680166185	0.148360905	-4.584537868	4.54992E-06	1.09499E-05	negative
PRJNA729271	stage_E18_vs_E10	ordinary_muscle_development	ENSGALG00010028154	ENSGALG00010028154	-0.20133749	0.126384517	-1.593055025	0.111147848	0.146210006	negative
PRJNA729271	stage_E18_vs_E10	ordinary_muscle_development	ENSGALG00010028155	CIZ1	-0.440954633	0.079101385	-5.574550057	2.4817E-08	7.4185E-08	negative
PRJNA729271	stage_E18_vs_E10	ordinary_muscle_development	ENSGALG00010028156	DOHH	-0.167189843	0.098593307	-1.695752456	0.08993277	0.120300433	negative
PRJNA729271	stage_E18_vs_E10	ordinary_muscle_development	ENSGALG00010028157	PRRC2B	-0.208077165	0.145135885	-1.433671379	0.151666063	0.193913698	negative
PRJNA729271	stage_E18_vs_E10	ordinary_muscle_development	ENSGALG00010028158	DDX49	-0.743373657	0.100713244	-7.381091346	1.56997E-13	7.11593E-13	negative
PRJNA729271	stage_E18_vs_E10	ordinary_muscle_development	ENSGALG00010028159	DCAF1	-0.050741034	0.09079929	-0.558826326	0.576280254	0.629336418	negative
PRJNA729271	stage_E18_vs_E10	ordinary_muscle_development	ENSGALG00010028160	ENSGALG00010028160	2.214884078	0.568252382	3.89771191	9.71058E-05	0.000202788	positive
PRJNA729271	stage_E18_vs_E10	ordinary_muscle_development	ENSGALG00010028161	ENSGALG00010028161	1.136916998	0.592819998	1.91781148	0.05513492	0.076832568	positive
PRJNA729271	stage_E18_vs_E10	ordinary_muscle_development	ENSGALG00010028162	CARD9	0.857655156	0.176125155	4.869577878	1.11837E-06	2.87731E-06	positive
PRJNA729271	stage_E18_vs_E10	ordinary_muscle_development	ENSGALG00010028163	GBP	1.06158026	0.363071666	2.923886276	0.00345691	0.005849628	positive
PRJNA729271	stage_E18_vs_E10	ordinary_muscle_development	ENSGALG00010028164	BBLN	-0.105431388	0.141952535	-0.742722827	0.457649507	0.517161425	negative
PRJNA729271	stage_E18_vs_E10	ordinary_muscle_development	ENSGALG00010028165	NFIC	1.385851852	0.157189351	8.816448705	1.18147E-18	7.70029E-18	positive
PRJNA729271	stage_E18_vs_E10	ordinary_muscle_development	ENSGALG00010028168	PTGES2	0.133163907	0.138372214	0.962360169	0.335868714	0.394267656	positive
PRJNA729271	stage_E18_vs_E10	ordinary_muscle_development	ENSGALG00010028169	DNLZ	0.398580983	0.147001059	2.711415723	0.006699657	0.010879624	positive
PRJNA729271	stage_E18_vs_E10	ordinary_muscle_development	ENSGALG00010028170	COPE	-0.852590808	0.094645284	-9.00827566	2.09322E-19	1.42313E-18	negative
PRJNA729271	stage_E18_vs_E10	ordinary_muscle_development	ENSGALG00010028171	RNF123	0.985059875	0.158905377	6.199034245	5.68107E-10	1.96579E-09	positive
PRJNA729271	stage_E18_vs_E10	ordinary_muscle_development	ENSGALG00010028172	CERS1	-0.454893525	0.212523664	-2.140437049	0.032319463	0.046950353	negative
PRJNA729271	stage_E18_vs_E10	ordinary_muscle_development	ENSGALG00010028173	GDF3	-0.46477888	0.230815802	-2.013635447	0.044047822	0.062430523	negative
PRJNA729271	stage_E18_vs_E10	ordinary_muscle_development	ENSGALG00010028174	GMPPB	-0.032542607	0.153968001	-0.211359549	0.832606719	0.860999814	negative
PRJNA729271	stage_E18_vs_E10	ordinary_muscle_development	ENSGALG00010028175	POMT1	0.433403908	0.093008176	4.659847408	3.16444E-06	7.74022E-06	positive
PRJNA729271	stage_E18_vs_E10	ordinary_muscle_development	ENSGALG00010028176	UPF1	-0.102378536	0.095079683	-1.076765641	0.281584979	0.337224564	negative
PRJNA729271	stage_E18_vs_E10	ordinary_muscle_development	ENSGALG00010028177	ENSGALG00010028177	3.014903584	0.388685219	7.756671561	8.71873E-15	4.37446E-14	positive
PRJNA729271	stage_E18_vs_E10	ordinary_muscle_development	ENSGALG00010028178	ENSGALG00010028178	-0.938641062	0.16371914	-5.733239628	9.85303E-09	3.04584E-08	negative
PRJNA729271	stage_E18_vs_E10	ordinary_muscle_development	ENSGALG00010028179	MCM2	-1.575741712	0.143698693	-10.96559527	5.59313E-28	6.21244E-27	negative
PRJNA729271	stage_E18_vs_E10	ordinary_muscle_development	ENSGALG00010028180	ODF2	-0.699948494	0.10514777	-6.656807802	2.79839E-11	1.06268E-10	negative
PRJNA729271	stage_E18_vs_E10	ordinary_muscle_development	ENSGALG00010028181	BGLAP	0.256709276	0.272273759	0.942835171	0.345765219	0.404443742	positive
PRJNA729271	stage_E18_vs_E10	ordinary_muscle_development	ENSGALG00010028182	SMG5	-0.029499621	0.147310372	-0.200254884	0.841281246	0.868792509	negative
PRJNA729271	stage_E18_vs_E10	ordinary_muscle_development	ENSGALG00010028183	CRTC1	0.455255905	0.097590186	4.664976291	3.08653E-06	7.55602E-06	positive
PRJNA729271	stage_E18_vs_E10	ordinary_muscle_development	ENSGALG00010028184	TMEM79	-0.154315904	0.255511763	-0.603948334	0.545877993	0.600569374	negative
PRJNA729271	stage_E18_vs_E10	ordinary_muscle_development	ENSGALG00010028185	GLMP	-0.168343888	0.091235094	-1.845165935	0.065013404	0.08934104	negative
PRJNA729271	stage_E18_vs_E10	ordinary_muscle_development	ENSGALG00010028186	CCT3	-0.403899858	0.132490099	-3.048528627	0.00229965	0.003997772	negative
PRJNA729271	stage_E18_vs_E10	ordinary_muscle_development	ENSGALG00010028187	TPRA1	0.108258272	0.071274532	1.518891383	0.128789838	0.167321745	positive
PRJNA729271	stage_E18_vs_E10	ordinary_muscle_development	ENSGALG00010028188	ENSGALG00010028188	0.310236708	0.110271213	2.81339706	0.004902108	0.0081365	positive
PRJNA729271	stage_E18_vs_E10	ordinary_muscle_development	ENSGALG00010028189	GPSM1	1.839260897	0.104325577	17.63000933	1.44943E-69	7.63982E-68	positive
PRJNA729271	stage_E18_vs_E10	ordinary_muscle_development	ENSGALG00010028190	ILF2	-0.830917558	0.081054025	-10.25140405	1.16637E-24	1.08584E-23	negative
PRJNA729271	stage_E18_vs_E10	ordinary_muscle_development	ENSGALG00010028191	KLHL26	0.561130393	0.100115646	5.60482217	2.08469E-08	6.2679E-08	positive
PRJNA729271	stage_E18_vs_E10	ordinary_muscle_development	ENSGALG00010028192	ENSGALG00010028192	1.213044935	0.349961896	3.466220033	0.000527831	0.000999466	positive
PRJNA729271	stage_E18_vs_E10	ordinary_muscle_development	ENSGALG00010028193	BRINP1	-3.938648758	0.215518212	-18.2752479	1.30297E-74	7.74036E-73	negative
PRJNA729271	stage_E18_vs_E10	ordinary_muscle_development	ENSGALG00010028194	ENSGALG00010028194	2.987937982	0.180832655	16.52322129	2.49698E-61	1.03411E-59	positive
PRJNA729271	stage_E18_vs_E10	ordinary_muscle_development	ENSGALG00010028195	ENSGALG00010028195	2.316392808	0.295107321	7.849323416	4.18288E-15	2.14927E-14	positive
PRJNA729271	stage_E18_vs_E10	ordinary_muscle_development	ENSGALG00010028196	ENSGALG00010028196	-0.824185855	0.095806271	-8.602629498	7.79102E-18	4.80148E-17	negative
PRJNA729271	stage_E18_vs_E10	ordinary_muscle_development	ENSGALG00010028197	INTS3	0.598537954	0.153308925	3.904129865	9.4565E-05	0.000197681	positive
PRJNA729271	stage_E18_vs_E10	ordinary_muscle_development	ENSGALG00010028198	UCK1	-0.323121538	0.093091274	-3.471018531	0.000518488	0.000983445	negative
PRJNA729271	stage_E18_vs_E10	ordinary_muscle_development	ENSGALG00010028199	DPP9	-0.58592886	0.108275567	-5.411459651	6.25131E-08	1.80001E-07	negative
PRJNA729271	stage_E18_vs_E10	ordinary_muscle_development	ENSGALG00010028201	ENSGALG00010028201	3.406805425	0.159939664	21.30056633	1.12157E-100	1.31106E-98	positive
PRJNA729271	stage_E18_vs_E10	ordinary_muscle_development	ENSGALG00010028202	QSOX2	-0.306865894	0.06739018	-4.553569894	5.27431E-06	1.26261E-05	negative
PRJNA729271	stage_E18_vs_E10	ordinary_muscle_development	ENSGALG00010028205	PRRT1B	-1.16183291	0.492819775	-2.357520881	0.018397421	0.027900252	negative
PRJNA729271	stage_E18_vs_E10	ordinary_muscle_development	ENSGALG00010028206	RBM15B	-0.462964469	0.096202358	-4.812402513	1.49127E-06	3.78629E-06	negative
PRJNA729271	stage_E18_vs_E10	ordinary_muscle_development	ENSGALG00010028207	ENSGALG00010028207	0.174395826	0.281946795	0.618541616	0.536218374	0.591693463	positive
PRJNA729271	stage_E18_vs_E10	ordinary_muscle_development	ENSGALG00010028211	GATAD2B	-0.168454414	0.1497075	-1.125223613	0.260494289	0.314760314	negative
PRJNA729271	stage_E18_vs_E10	ordinary_muscle_development	ENSGALG00010028216	NKX2-3	6.20319504	1.353821961	4.581987307	4.60578E-06	1.10795E-05	positive
PRJNA729271	stage_E18_vs_E10	ordinary_muscle_development	ENSGALG00010028221	TNFAIP8L1	-1.387806927	0.191005712	-7.265787567	3.70871E-13	1.64046E-12	negative
PRJNA729271	stage_E18_vs_E10	ordinary_muscle_development	ENSGALG00010028232	C9orf69	0.450036113	0.109891989	4.095258609	4.21697E-05	9.1532E-05	positive
PRJNA729271	stage_E18_vs_E10	ordinary_muscle_development	ENSGALG00010028239	EDMPN2	7.070724197	1.43176559	4.938464961	7.87399E-07	2.05831E-06	positive
PRJNA729271	stage_E18_vs_E10	ordinary_muscle_development	ENSGALG00010028242	ENSGALG00010028242	1.072759956	0.278743697	3.84855323	0.000118817	0.000245371	positive
PRJNA729271	stage_E18_vs_E10	ordinary_muscle_development	ENSGALG00010028245	JAC1	5.457429567	1.244937317	4.383698274	1.16681E-05	2.69658E-05	positive
PRJNA729271	stage_E18_vs_E10	ordinary_muscle_development	ENSGALG00010028247	MVP	0.463934645	0.093016625	4.987652938	6.11172E-07	1.61189E-06	positive
PRJNA729271	stage_E18_vs_E10	ordinary_muscle_development	ENSGALG00010028262	C1orf43	-0.013118887	0.145362327	-0.090249564	0.928088899	0.941073703	negative
PRJNA729271	stage_E18_vs_E10	ordinary_muscle_development	ENSGALG00010028270	RAPGEF1	0.299970728	0.182840065	1.640618145	0.100876706	0.133608174	positive
PRJNA729271	stage_E18_vs_E10	ordinary_muscle_development	ENSGALG00010028279	EDBETA	1.910591032	1.564249909	1.221410352	0.22193069	0.272894923	positive
PRJNA729271	stage_E18_vs_E10	ordinary_muscle_development	ENSGALG00010028283	ENSGALG00010028283	1.548687397	0.151068082	10.25158575	1.16418E-24	1.0845E-23	positive
PRJNA729271	stage_E18_vs_E10	ordinary_muscle_development	ENSGALG00010028286	KCTD12A	7.280829774	1.18877395	6.124654544	9.08806E-10	3.0836E-09	positive
PRJNA729271	stage_E18_vs_E10	ordinary_muscle_development	ENSGALG00010028292	ENSGALG00010028292	0.665439429	0.440931582	1.509167081	0.13125609	0.170098975	positive
PRJNA729271	stage_E18_vs_E10	ordinary_muscle_development	ENSGALG00010028301	FBN3	-1.996083722	0.184819863	-10.800158	3.43612E-27	3.67576E-26	negative
PRJNA729271	stage_E18_vs_E10	ordinary_muscle_development	ENSGALG00010028311	ENSGALG00010028311	-1.874462697	0.229165818	-8.179503884	2.85015E-16	1.58287E-15	negative
PRJNA729271	stage_E18_vs_E10	ordinary_muscle_development	ENSGALG00010028314	MEF2D	1.407292189	0.214465882	6.561846473	5.31455E-11	1.98185E-10	positive
PRJNA729271	stage_E18_vs_E10	ordinary_muscle_development	ENSGALG00010028316	TIMM44	-0.186941393	0.081034259	-2.306942707	0.021058014	0.031637222	negative
PRJNA729271	stage_E18_vs_E10	ordinary_muscle_development	ENSGALG00010028318	NACC2	0.456785397	0.20117111	2.270631191	0.023169314	0.034540697	positive
PRJNA729271	stage_E18_vs_E10	ordinary_muscle_development	ENSGALG00010028321	RHBG	0.723799379	0.190156465	3.806335899	0.000141041	0.000288918	positive
PRJNA729271	stage_E18_vs_E10	ordinary_muscle_development	ENSGALG00010028322	STRBP	-1.869806623	0.116990379	-15.98256747	1.69038E-57	6.15629E-56	negative
PRJNA729271	stage_E18_vs_E10	ordinary_muscle_development	ENSGALG00010028326	CRB2	-1.366322555	0.25571365	-5.343174123	9.1333E-08	2.59633E-07	negative
PRJNA729271	stage_E18_vs_E10	ordinary_muscle_development	ENSGALG00010028328	HNRNPM	-1.434327419	0.16617289	-8.631536804	6.05331E-18	3.7674E-17	negative
PRJNA729271	stage_E18_vs_E10	ordinary_muscle_development	ENSGALG00010028329	HAPLN2	2.039299616	0.564216768	3.614390304	0.000301055	0.000589224	positive
PRJNA729271	stage_E18_vs_E10	ordinary_muscle_development	ENSGALG00010028337	ENSGALG00010028337	2.446396165	0.204303816	11.97430482	4.84481E-33	6.74597E-32	positive
PRJNA729271	stage_E18_vs_E10	ordinary_muscle_development	ENSGALG00010028339	BCAN	1.598703659	0.38628723	4.138639682	3.49371E-05	7.64398E-05	positive
PRJNA729271	stage_E18_vs_E10	ordinary_muscle_development	ENSGALG00010028344	LSM7	-1.034257754	0.09423677	-10.97509769	5.03514E-28	5.59696E-27	negative
PRJNA729271	stage_E18_vs_E10	ordinary_muscle_development	ENSGALG00010028346	DENND1A	1.018371745	0.072907852	13.96792961	2.44635E-44	5.61074E-43	positive
PRJNA729271	stage_E18_vs_E10	ordinary_muscle_development	ENSGALG00010028348	NES	-0.218283658	0.276109599	-0.790568886	0.429195609	0.489279619	negative
PRJNA729271	stage_E18_vs_E10	ordinary_muscle_development	ENSGALG00010028353	ENSGALG00010028353	4.272889	1.470833574	2.905079865	0.003671594	0.006192664	positive
PRJNA729271	stage_E18_vs_E10	ordinary_muscle_development	ENSGALG00010028355	RBM18	-0.226699942	0.074794891	-3.030954897	0.002437817	0.004225801	negative
PRJNA729271	stage_E18_vs_E10	ordinary_muscle_development	ENSGALG00010028357	SPPL2C	-0.89679872	0.094108721	-9.529390137	1.5821E-21	1.23095E-20	negative
PRJNA729271	stage_E18_vs_E10	ordinary_muscle_development	ENSGALG00010028360	ENSGALG00010028360	-3.828207878	0.719266795	-5.322375377	1.02421E-07	2.90243E-07	negative
PRJNA729271	stage_E18_vs_E10	ordinary_muscle_development	ENSGALG00010028362	TMPRSS9	-2.845459164	0.445528843	-6.386700237	1.69503E-10	6.07405E-10	negative
PRJNA729271	stage_E18_vs_E10	ordinary_muscle_development	ENSGALG00010028364	MHM2	3.28725476	0.696925346	4.716796112	2.39588E-06	5.94153E-06	positive
PRJNA729271	stage_E18_vs_E10	ordinary_muscle_development	ENSGALG00010028366	MRRF	0.114703084	0.087809618	1.306270164	0.1914607	0.239099064	positive
PRJNA729271	stage_E18_vs_E10	ordinary_muscle_development	ENSGALG00010028368	TIMM13	0.584243278	0.120799998	4.836451054	1.32178E-06	3.37547E-06	positive
PRJNA729271	stage_E18_vs_E10	ordinary_muscle_development	ENSGALG00010028370	S100A12	3.949033681	0.657983539	6.001721091	1.95237E-09	6.42734E-09	positive
PRJNA729271	stage_E18_vs_E10	ordinary_muscle_development	ENSGALG00010028372	PTGS1	0.900336622	0.160974536	5.593037529	2.23131E-08	6.68932E-08	positive
PRJNA729271	stage_E18_vs_E10	ordinary_muscle_development	ENSGALG00010028374	FDPS	-0.945056092	0.136474885	-6.924761976	4.36709E-12	1.77962E-11	negative
PRJNA729271	stage_E18_vs_E10	ordinary_muscle_development	ENSGALG00010028375	LMNB2	-1.160729953	0.152646197	-7.604054201	2.86995E-14	1.37384E-13	negative

PRJNA729271	stage_E18_vs_E10	ordinary_muscle_development	ENSGALG00010028377	PDCL	0.011729286	0.059638198	0.196674045	0.844082622	0.871126921	positive
PRJNA729271	stage_E18_vs_E10	ordinary_muscle_development	ENSGALG00010028380	RUSC1	1.14079542	0.141354954	8.070431127	7.00504E-16	3.8072E-15	positive
PRJNA729271	stage_E18_vs_E10	ordinary_muscle_development	ENSGALG00010028384	ENSGALG00010028384	6.851332096	0.819118356	8.364276114	6.04851E-17	3.5196E-16	positive
PRJNA729271	stage_E18_vs_E10	ordinary_muscle_development	ENSGALG00010028386	NCAN	-4.326252476	0.512084061	-8.4483248	2.95512E-17	1.75695E-16	negative
PRJNA729271	stage_E18_vs_E10	ordinary_muscle_development	ENSGALG00010028391	MED26	0.144019074	0.115765844	1.244054968	0.213479333	0.263800761	positive
PRJNA729271	stage_E18_vs_E10	ordinary_muscle_development	ENSGALG00010028392	ASH1L	0.607179273	0.12096913	5.019291063	5.18625E-07	1.37809E-06	positive
PRJNA729271	stage_E18_vs_E10	ordinary_muscle_development	ENSGALG00010028394	ZBTB26	-0.346674711	0.104354071	-3.322100497	0.000893425	0.001638851	negative
PRJNA729271	stage_E18_vs_E10	ordinary_muscle_development	ENSGALG00010028399	SLC35E1	-0.224733308	0.080310087	-2.798319824	0.005136922	0.008503847	negative
PRJNA729271	stage_E18_vs_E10	ordinary_muscle_development	ENSGALG00010028405	S100A6	-0.168307647	0.280257127	-0.60054725	0.548141581	0.602510975	negative
PRJNA729271	stage_E18_vs_E10	ordinary_muscle_development	ENSGALG00010028411	LHX2	-3.283999886	0.727733741	-4.512639309	6.40259E-06	1.51841E-05	negative
PRJNA729271	stage_E18_vs_E10	ordinary_muscle_development	ENSGALG00010028412	ENSGALG00010028412	-1.378484568	0.339014971	-4.066146594	4.77969E-05	0.000103113	negative
PRJNA729271	stage_E18_vs_E10	ordinary_muscle_development	ENSGALG00010028414	KBTBD12	3.482937173	0.359012602	9.7014343	2.9729E-22	2.40873E-21	positive
PRJNA729271	stage_E18_vs_E10	ordinary_muscle_development	ENSGALG00010028415	FSD1	-0.544057534	0.171991251	-3.163286098	0.001559989	0.002775847	negative
PRJNA729271	stage_E18_vs_E10	ordinary_muscle_development	ENSGALG00010028418	ENSGALG00010028418	1.786075067	0.124156896	14.38562913	6.36962E-47	1.65166E-45	positive
PRJNA729271	stage_E18_vs_E10	ordinary_muscle_development	ENSGALG00010028419	ENSGALG00010028419	2.024814464	0.373492233	5.42130273	5.91663E-08	1.7067E-07	positive
PRJNA729271	stage_E18_vs_E10	ordinary_muscle_development	ENSGALG00010028420	ENSGALG00010028420	1.885833065	0.841987708	2.239739426	0.025107844	0.03723153	positive
PRJNA729271	stage_E18_vs_E10	ordinary_muscle_development	ENSGALG00010028421	ZBTB7A	0.868805534	0.191992563	4.525204095	6.03372E-06	1.43681E-05	positive
PRJNA729271	stage_E18_vs_E10	ordinary_muscle_development	ENSGALG00010028422	RXRA	0.858489544	0.210639291	4.075638212	4.58883E-05	9.91727E-05	positive
PRJNA729271	stage_E18_vs_E10	ordinary_muscle_development	ENSGALG00010028425	DAP3	0.021752864	0.130745671	0.166375409	0.867861525	0.891099583	positive
PRJNA729271	stage_E18_vs_E10	ordinary_muscle_development	ENSGALG00010028426	GPR21	-0.211500142	0.152938673	-1.382908186	0.16669302	0.210967897	negative
PRJNA729271	stage_E18_vs_E10	ordinary_muscle_development	ENSGALG00010028427	ENSGALG00010028427	0.004165967	0.210695076	0.019772493	0.984224861	0.986637643	positive
PRJNA729271	stage_E18_vs_E10	ordinary_muscle_development	ENSGALG00010028428	RABGAP1	0.235242586	0.067629791	3.478387026	0.000504441	0.000957804	positive
PRJNA729271	stage_E18_vs_E10	ordinary_muscle_development	ENSGALG00010028429	REXO1	-0.374236864	0.089527051	-4.180154021	2.91312E-05	6.43782E-05	negative
PRJNA729271	stage_E18_vs_E10	ordinary_muscle_development	ENSGALG00010028430	NR2C2AP	0.241179699	0.115961881	2.079818788	0.037542157	0.053883906	positive
PRJNA729271	stage_E18_vs_E10	ordinary_muscle_development	ENSGALG00010028431	ENSGALG00010028431	-0.663154518	0.275460859	-2.407436471	0.016064957	0.024672764	negative
PRJNA729271	stage_E18_vs_E10	ordinary_muscle_development	ENSGALG00010028432	RFXANK	0.565959338	0.134684453	4.202113341	2.64435E-05	5.8734E-05	positive
PRJNA729271	stage_E18_vs_E10	ordinary_muscle_development	ENSGALG00010028433	MSTO1	-0.478258549	0.095577988	-5.003856621	5.61946E-07	1.48829E-06	negative
PRJNA729271	stage_E18_vs_E10	ordinary_muscle_development	ENSGALG00010028434	COL5A1	1.940435131	0.195863153	9.907096373	3.87751E-23	3.27151E-22	positive
PRJNA729271	stage_E18_vs_E10	ordinary_muscle_development	ENSGALG00010028435	ATP8B3	0.523009996	0.379756198	1.377225701	0.168442478	0.21297747	positive
PRJNA729271	stage_E18_vs_E10	ordinary_muscle_development	ENSGALG00010028436	S100A1	3.851653401	0.184618558	20.86276394	1.16727E-96	1.27215E-94	positive
PRJNA729271	stage_E18_vs_E10	ordinary_muscle_development	ENSGALG00010028437	ENSGALG00010028437	0.217312767	0.067358532	3.226209938	0.001254413	0.002259284	positive
PRJNA729271	stage_E18_vs_E10	ordinary_muscle_development	ENSGALG00010028438	MED27	-0.153631254	0.072166815	-2.128835154	0.033267901	0.048207361	negative
PRJNA729271	stage_E18_vs_E10	ordinary_muscle_development	ENSGALG00010028439	BORCS8	0.260807887	0.11967895	2.179229398	0.029314631	0.042899099	positive
PRJNA729271	stage_E18_vs_E10	ordinary_muscle_development	ENSGALG00010028440	PSMB7	-0.058554798	0.078605913	-0.744915947	0.456322534	0.51586298	negative
PRJNA729271	stage_E18_vs_E10	ordinary_muscle_development	ENSGALG00010028442	CHTOP	-0.167536194	0.090464454	-1.851956056	0.064032133	0.088109529	negative
PRJNA729271	stage_E18_vs_E10	ordinary_muscle_development	ENSGALG00010028443	ENSGALG00010028443	0.385677682	0.349261994	1.104264674	0.269478342	0.324506818	positive
PRJNA729271	stage_E18_vs_E10	ordinary_muscle_development	ENSGALG00010028444	MEF2B	0.143112404	0.328984542	0.435012548	0.663553319	0.711142726	positive
PRJNA729271	stage_E18_vs_E10	ordinary_muscle_development	ENSGALG00010028445	SYT11	-1.685956573	0.203563137	-8.282229296	1.20891E-16	6.90973E-16	negative
PRJNA729271	stage_E18_vs_E10	ordinary_muscle_development	ENSGALG00010028446	ENSGALG00010028446	-0.197249598	0.22072376	-0.893649139	0.371509646	0.431216554	negative
PRJNA729271	stage_E18_vs_E10	ordinary_muscle_development	ENSGALG00010028447	OLFM1	-1.088494697	0.158245885	-6.878502361	6.0485E-12	2.42593E-11	negative
PRJNA729271	stage_E18_vs_E10	ordinary_muscle_development	ENSGALG00010028448	RIT1	-0.141510738	0.057731247	-2.451198381	0.014238146	0.022054063	negative
PRJNA729271	stage_E18_vs_E10	ordinary_muscle_development	ENSGALG00010028449	EFCAB12	-0.19737934	0.325246569	-0.606860638	0.543943402	0.598804558	negative
PRJNA729271	stage_E18_vs_E10	ordinary_muscle_development	ENSGALG00010028450	PTGDS	4.602732238	0.436618778	10.54176428	5.54488E-26	5.54297E-25	positive
PRJNA729271	stage_E18_vs_E10	ordinary_muscle_development	ENSGALG00010028451	TMEM59L	-0.534136588	0.306696896	-1.741578066	0.081582302	0.110115976	negative
PRJNA729271	stage_E18_vs_E10	ordinary_muscle_development	ENSGALG00010028452	GOLPH3L	-1.221969829	0.688525522	-1.774763302	0.075936978	0.103110679	negative
PRJNA729271	stage_E18_vs_E10	ordinary_muscle_development	ENSGALG00010028453	ENSGALG00010028453	-0.076832164	0.095396866	-0.805395058	0.420591743	0.481090891	negative
PRJNA729271	stage_E18_vs_E10	ordinary_muscle_development	ENSGALG00010028454	C8G	1.721074538	0.226048189	7.613750599	2.66254E-14	1.27878E-13	positive
PRJNA729271	stage_E18_vs_E10	ordinary_muscle_development	ENSGALG00010028455	ENSA	0.647491147	0.06998945	9.251267815	2.21845E-20	1.60062E-19	positive
PRJNA729271	stage_E18_vs_E10	ordinary_muscle_development	ENSGALG00010028456	FBXW5	-0.345428811	0.09446139	-3.656825384	0.000255358	0.000505378	negative
PRJNA729271	stage_E18_vs_E10	ordinary_muscle_development	ENSGALG00010028457	KANK3	1.502299375	0.10442773	14.38601968	6.33377E-47	1.6453E-45	positive
PRJNA729271	stage_E18_vs_E10	ordinary_muscle_development	ENSGALG00010028458	TRAF2	0.097936601	0.109519013	0.894242904	0.371191941	0.430916802	positive
PRJNA729271	stage_E18_vs_E10	ordinary_muscle_development	ENSGALG00010028459	MCL1	0.88560079	0.21030051	4.211120497	2.54107E-05	5.65789E-05	positive
PRJNA729271	stage_E18_vs_E10	ordinary_muscle_development	ENSGALG00010028460	CRLF1	-0.799328295	0.248066916	-3.22222853	0.001271976	0.002288926	negative
PRJNA729271	stage_E18_vs_E10	ordinary_muscle_development	ENSGALG00010028461	ENSGALG00010028461	1.539448038	0.323563727	4.757789303	1.95725E-06	4.89565E-06	positive
PRJNA729271	stage_E18_vs_E10	ordinary_muscle_development	ENSGALG00010028462	EDF1	0.294883684	0.105832153	2.786333601	0.005330798	0.008799672	positive
PRJNA729271	stage_E18_vs_E10	ordinary_muscle_development	ENSGALG00010028463	RPS28	1.725334665	0.169237755	10.19473853	2.09307E-24	1.91052E-23	positive
PRJNA729271	stage_E18_vs_E10	ordinary_muscle_development	ENSGALG00010028464	ENSGALG00010028464	0.746304085	0.110995321	6.723743647	1.77114E-11	6.82239E-11	positive
PRJNA729271	stage_E18_vs_E10	ordinary_muscle_development	ENSGALG00010028465	ENSGALG00010028465	-0.148200076	0.141230833	-1.049346473	0.294018684	0.350621212	negative
PRJNA729271	stage_E18_vs_E10	ordinary_muscle_development	ENSGALG00010028466	MAMDC4	0.462455602	0.672998928	0.687156521	0.4919841	0.549492181	positive
PRJNA729271	stage_E18_vs_E10	ordinary_muscle_development	ENSGALG00010028467	ENSGALG00010028467	0.227834311	0.2665422	0.854777631	0.392674264	0.452629301	positive
PRJNA729271	stage_E18_vs_E10	ordinary_muscle_development	ENSGALG00010028468	PHPT1	-0.56871578	0.104108386	-5.462727839	4.68873E-08	1.36774E-07	negative
PRJNA729271	stage_E18_vs_E10	ordinary_muscle_development	ENSGALG00010028470	AJM1	1.33092046	0.463707196	2.870174262	0.004102456	0.006876964	positive
PRJNA729271	stage_E18_vs_E10	ordinary_muscle_development	ENSGALG00010028471	TPGS1	0.254490215	0.167796905	1.516656192	0.12935351	0.167933643	positive
PRJNA729271	stage_E18_vs_E10	ordinary_muscle_development	ENSGALG00010028472	RPRD2	0.198672209	0.13965647	1.422577915	0.15485855	0.197525051	positive
PRJNA729271	stage_E18_vs_E10	ordinary_muscle_development	ENSGALG00010028473	CDC34	0.634034511	0.142746037	4.441696057	8.92526E-06	2.08799E-05	positive
PRJNA729271	stage_E18_vs_E10	ordinary_muscle_development	ENSGALG00010028474	PEX19	0.169624101	0.121700152	1.3937871	0.163381905	0.207320381	positive
PRJNA729271	stage_E18_vs_E10	ordinary_muscle_development	ENSGALG00010028475	AMBP	8.061859521	1.369319499	5.887493404			positive
PRJNA729271	stage_E18_vs_E10	ordinary_muscle_development	ENSGALG00010028476	ENSGALG00010028476	0.623543469	0.156515369	3.983912074	6.779E-05	0.000144057	positive
PRJNA729271	stage_E18_vs_E10	ordinary_muscle_development	ENSGALG00010028477	NRARP	0.707375941	0.210925267	3.353680438	0.000797444	0.001474732	positive
PRJNA729271	stage_E18_vs_E10	ordinary_muscle_development	ENSGALG00010028478	PEA15	0.294021595	0.161187652	1.824095028	0.068137707	0.093342412	positive
PRJNA729271	stage_E18_vs_E10	ordinary_muscle_development	ENSGALG00010028479	CASQ1	5.447548614	0.318032877	17.12888509	9.03699E-66	4.32314E-64	positive
PRJNA729271	stage_E18_vs_E10	ordinary_muscle_development	ENSGALG00010028480	F11R	0.536917311	0.215646049	2.489808242	0.012781203	0.019912247	positive
PRJNA729271	stage_E18_vs_E10	ordinary_muscle_development	ENSGALG00010028481	ENSGALG00010028481	0.852235424	0.144681462	5.890425857	3.85202E-09	1.23693E-08	positive
PRJNA729271	stage_E18_vs_E10	ordinary_muscle_development	ENSGALG00010028482	CD320	0.59223336	0.177068523	3.344656358	0.000823846	0.001519873	positive
PRJNA729271	stage_E18_vs_E10	ordinary_muscle_development	ENSGALG00010028483	EXD3	0.307609479	0.093275488	3.297859766	0.000974248	0.001776764	positive
PRJNA729271	stage_E18_vs_E10	ordinary_muscle_development	ENSGALG00010028484	NOXA1	-1.085495906	0.572662882	-1.895523423	0.058023092	0.080521275	negative
PRJNA729271	stage_E18_vs_E10	ordinary_muscle_development	ENSGALG00010028485	USF1	-0.057998715	0.185737064	-0.312262476	0.754841055	0.794353208	negative
PRJNA729271	stage_E18_vs_E10	ordinary_muscle_development	ENSGALG00010028486	ARHGAP30	0.818307374	0.234184892	3.494279102	0.000475344	0.000906235	positive
PRJNA729271	stage_E18_vs_E10	ordinary_muscle_development	ENSGALG00010028488	PTGES	1.833947722	0.129404131	14.17225024	1.36069E-45	3.26544E-44	positive
PRJNA729271	stage_E18_vs_E10	ordinary_muscle_development	ENSGALG00010028489	ENSGALG00010028489	-2.949365533	1.131777492	-2.605958816	0.009161748	0.014567742	negative
PRJNA729271	stage_E18_vs_E10	ordinary_muscle_development	ENSGALG00010028490	NECTIN4	-1.68295555	0.357017741	-4.713926945	2.42988E-06	6.02276E-06	negative
PRJNA729271	stage_E18_vs_E10	ordinary_muscle_development	ENSGALG00010028491	TOR1B	-0.298698389	0.099112979	-3.013716193	0.002580691	0.004461727	negative
PRJNA729271	stage_E18_vs_E10	ordinary_muscle_development	ENSGALG00010028492	ENSGALG00010028492	2.535591253	0.24547703	10.32924037	5.19705E-25	4.93006E-24	positive
PRJNA729271	stage_E18_vs_E10	ordinary_muscle_development	ENSGALG00010028493	TOR1A	-0.634985414	0.088257174	-7.194177236	6.25903E-13	2.72365E-12	negative
PRJNA729271	stage_E18_vs_E10	ordinary_muscle_development	ENSGALG00010028494	ENSGALG00010028494	-0.662218764	0.196853696	-3.364014894	0.000768174	0.00142369	negative
PRJNA729271	stage_E18_vs_E10	ordinary_muscle_development	ENSGALG00010028495	LINGO3	-0.827078618	0.257786189	-3.208389948	0.001334804	0.002396035	negative
PRJNA729271	stage_E18_vs_E10	ordinary_muscle_development	ENSGALG00010028496	ENSGALG00010028496	0.041417227	0.157474269	0.263009491	0.792543264	0.826618337	positive
PRJNA729271	stage_E18_vs_E10	ordinary_muscle_development	ENSGALG00010028498	C9orf78	-0.164397815	0.106188587	-1.548168395	0.121581758	0.158768251	negative
PRJNA729271	stage_E18_vs_E10	ordinary_muscle_development	ENSGALG00010028500	VPS45	0.539541218	0.070088145	7.698038191	1.38171E-14	6.81685E-14	positive
PRJNA729271	stage_E18_vs_E10	ordinary_muscle_development	ENSGALG00010028501	USP20	0.261607777	0.088616336	2.952139397	0.003155804	0.005389818	positive
PRJNA729271	stage_E18_vs_E10	ordinary_muscle_development	ENSGALG00010028502	PLEKHO1	0.463910162	0.150099061	3.090693305	0.001996898	0.003506789	positive
PRJNA729271	stage_E18_vs_E10	ordinary_muscle_development	ENSGALG00010028503	NDOR1	0.165973845	0.126884974	1.308065407	0.190851126	0.238433618	positive
PRJNA729271	stage_E18_vs_E10	ordinary_muscle_development	ENSGALG00010028506	SHD	-4.320970448	0.358621061	-12.04884741	1.96681E-33	2.78407E-32	negative
PRJNA729271	stage_E18_vs_E10	ordinary_muscle_development	ENSGALG00010028507	ANP32E	-0.648406506	0.056849648	-11.40563797	3.91894E-30	4.82215E-29	negative
PRJNA729271	stage_E18_vs_E10	ordinary_muscle_development	ENSGALG00010028510	CILP2	2.029569251	0.582832015	3.482254235	0.000497211	0.000945439	positive
PRJNA729271	stage_E18_vs_E10	ordinary_muscle_development	ENSGALG00010028511	YJEFN3	1.403882532	0.406275708	3.455492178	0.000549289	0.001037928	positive
PRJNA729271	stage_E18_vs_E10	ordinary_muscle_development	ENSGALG00010028512	NDUFA13	0.617050927	0.122865531	5.022164656	5.10924E-07	1.35862E-06	positive
PRJNA729271	stage_E18_vs_E10	ordinary_muscle_development	ENSGALG00010028513	ENSGALG00010028513	3.907283826	1.046957602	3.732036347	0.000189938	0.000381799	positive
PRJNA729271	stage_E18_vs_E10	ordinary_muscle_development	ENSGALG00010028514	FNBP1	0.632339662	0.0595606	10.61674432	2.49098E-26	2.54452E-25	positive
PRJNA729271	stage_E18_vs_E10	ordinary_muscle_development	ENSGALG00010028515	ENSGALG00010028515	2.630150108	3.384325242	0.777156426	0.437066487	0.496611956	positive
PRJNA729271	stage_E18_vs_E10	ordinary_muscle_development	ENSGALG00010028516	GATAD2A	-0.504550057	0.063695746	-7.921252026	2.35131E-15	1.2273E-14	negative
PRJNA729271	stage_E18_vs_E10	ordinary_muscle_development	ENSGALG00010028517	RNF208	-1.21886366	0.334265641	-3.646392306	0.000265948	0.000525264	negative
PRJNA729271	stage_E18_vs_E10	ordinary_muscle_development	ENSGALG00010028518	MAU2	0.462997161	0.115859837	3.996183442	6.43719E-05	0.000137115	positive
PRJNA729271	stage_E18_vs_E10	ordinary_muscle_development	ENSGALG00010028519	ENSGALG00010028519	5.343195262	1.957118857	2.730133248	0.006330873	0.01033052	positive
PRJNA729271	stage_E18_vs_E10	ordinary_muscle_development	ENSGALG00010028523	SUGP1	-0.273039399	0.115150485	-2.371152839	0.017732697	0.026982515	negative
PRJNA729271	stage_E18_vs_E10	ordinary_muscle_development	ENSGALG00010028528	UHRF1	-1.937982468	0.184361833	-10.51184202	7.61906E-26	7.55392E-25	negative
PRJNA729271	stage_E18_vs_E10	ordinary_muscle_development	ENSGALG00010028534	ENSGALG00010028534	0.763001422	4.345747042	0.175574283	0.860628407	0.884800962	positive
PRJNA729271	stage_E18_vs_E10	ordinary_muscle_development	ENSGALG00010028535	TICAM1	-0.286074902	0.109240586	-2.618760229	0.008824995	0.014083266	negative
PRJNA729271	stage_E18_vs_E10	ordinary_muscle_development	ENSGALG00010028536	GPR107	-0.134135049	0.06426467	-2.087228485	0.036867486	0.052973256	negative
PRJNA729271	stage_E18_vs_E10	ordinary_muscle_development	ENSGALG00010028537	ENSGALG00010028537	3.586391771	2.022615233	1.773145832	0.076204545	0.103444922	positive
PRJNA729271	stage_E18_vs_E10	ordinary_muscle_development	ENSGALG00010028538	FEM1A	0.645441093	0.117200998	5.507129668	3.64732E-08	1.07718E-07	positive
PRJNA729271	stage_E18_vs_E10	ordinary_muscle_development	ENSGALG00010028539	ENSGALG00010028539	2.203726648	3.347138525	0.65839123	0.51028677	0.567180947	positive
PRJNA729271	stage_E18_vs_E10	ordinary_muscle_development	ENSGALG00010028541	ENSGALG00010028541	-0.07358022	0.310364591	-0.237076721	0.812597271	0.844283381	negative
PRJNA729271	stage_E18_vs_E10	ordinary_muscle_development	ENSGALG00010028542	ENSGALG00010028542	5.841809835	1.839485684	3.175784343	0.00149432	0.002666851	positive
PRJNA729271	stage_E18_vs_E10	ordinary_muscle_development	ENSGALG00010028543	ANP32B	-1.130112628	0.093798611	-12.04828747	1.98021E-33	2.80031E-32	negative
PRJNA729271	stage_E18_vs_E10	ordinary_muscle_development	ENSGALG00010028544	ZNF414	0.884829174	0.168346267	5.25600708	1.47217E-07	4.09736E-07	positive
PRJNA729271	stage_E18_vs_E10	ordinary_muscle_development	ENSGALG00010028545	ENSGALG00010028545	8.34471341	1.309370479	6.37307282	1.85278E-10	6.61804E-10	positive
PRJNA729271	stage_E18_vs_E10	ordinary_muscle_development	ENSGALG00010028546	ENSGALG00010028546	6.367861617	1.53494209	4.148600562	3.34514E-05	7.33774E-05	positive
PRJNA729271	stage_E18_vs_E10	ordinary_muscle_development	ENSGALG00010028547	ZNF618	-0.12878322	0.105460853	-1.221147148	0.222030311	0.272940118	negative
PRJNA729271	stage_E18_vs_E10	ordinary_muscle_development	ENSGALG00010028548	MYO1F	0.220812436	0.253512938	0.871010519	0.383748409	0.444035539	positive
PRJNA729271	stage_E18_vs_E10	ordinary_muscle_development	ENSGALG00010028549	ADAMTS10	-0.69998953	0.177987321	-3.93280558	8.39601E-05	0.000176454	negative
PRJNA729271	stage_E18_vs_E10	ordinary_muscle_development	ENSGALG00010028550	ZAP70	-4.302010309	1.002304866	-4.292117555	1.76977E-05	4.0109E-05	negative
PRJNA729271	stage_E18_vs_E10	ordinary_muscle_development	ENSGALG00010028551	NCS1	-2.551510983	0.234324781	-10.88877999	1.30375E-27	1.42518E-26	negative
PRJNA729271	stage_E18_vs_E10	ordinary_muscle_development	ENSGALG00010028552	ENSGALG00010028552	-1.285002528	0.660076416	-1.946748128	0.051564938	0.072229782	negative
PRJNA729271	stage_E18_vs_E10	ordinary_muscle_development	ENSGALG00010028553	NFILZ	0.842443497	1.229979154	0.68492502	0.493391237	0.550766729	positive
PRJNA729271	stage_E18_vs_E10	ordinary_muscle_development	ENSGALG00010028554	ENSGALG00010028554	-0.009524201	0.767777562	-0.012404896	0.990102579	0.991402105	negative
PRJNA729271	stage_E18_vs_E10	ordinary_muscle_development	ENSGALG00010028555	ENSGALG00010028555	5.347780309	1.166011162	4.586388608	4.50979E-06	1.08569E-05	positive
PRJNA729271	stage_E18_vs_E10	ordinary_muscle_development	ENSGALG00010028556	SPTAN1	0.392526112	0.093340386	4.20531912	2.60714E-05	5.79787E-05	positive
PRJNA729271	stage_E18_vs_E10	ordinary_muscle_development	ENSGALG00010028558	ENSGALG00010028558	5.771867314	1.37189233	4.207230544	2.58519E-05	5.75171E-05	positive
PRJNA729271	stage_E18_vs_E10	ordinary_muscle_development	ENSGALG00010028559	DYNC2I2	-0.784194241	0.127961341	-6.128368434	8.87848E-10	3.01602E-09	negative
PRJNA729271	stage_E18_vs_E10	ordinary_muscle_development	ENSGALG00010028560	ENSGALG00010028560	5.218588279	3.423571245	1.524311284	0.12743097	0.165808968	positive

PRJNA729271	stage_E18_vs_E10	ordinary_muscle_development	ENSGALG00010028561	SET	-1.109133851	0.05174263	-21.43559079	6.2239E-102	7.7107E-100	negative
PRJNA729271	stage_E18_vs_E10	ordinary_muscle_development	ENSGALG00010028562	ACSBG2	0.760189197	0.200256262	3.796082022	0.000147001	0.00030011	positive
PRJNA729271	stage_E18_vs_E10	ordinary_muscle_development	ENSGALG00010028563	ENSGALG00010028563	4.686439333	2.187267535	2.14259996	0.032145234	0.046734721	positive
PRJNA729271	stage_E18_vs_E10	ordinary_muscle_development	ENSGALG00010028565	PKN1	-0.290780251	0.124347912	-2.338440954	0.019364385	0.029271745	negative
PRJNA729271	stage_E18_vs_E10	ordinary_muscle_development	ENSGALG00010028566	ZDHHC12	0.306621779	0.126903995	2.416171217	0.015684684	0.024129643	positive
PRJNA729271	stage_E18_vs_E10	ordinary_muscle_development	ENSGALG00010028567	ENSGALG00010028567	-2.648437854	1.200756272	-2.20564149	0.027409112	0.04033862	negative
PRJNA729271	stage_E18_vs_E10	ordinary_muscle_development	ENSGALG00010028568	MLLT1	-0.855791931	0.065830151	-12.99999952	1.22344E-38	2.22507E-37	negative
PRJNA729271	stage_E18_vs_E10	ordinary_muscle_development	ENSGALG00010028569	ZER1	0.194814289	0.069853417	2.788901352	0.005288717	0.008738169	positive
PRJNA729271	stage_E18_vs_E10	ordinary_muscle_development	ENSGALG00010028572	TBC1D13	0.002186329	0.068820178	0.031768721	0.974656491	0.979114688	positive
PRJNA729271	stage_E18_vs_E10	ordinary_muscle_development	ENSGALG00010028573	KCNN1	1.479629208	0.262564107	5.635306455	1.74747E-08	5.28361E-08	positive
PRJNA729271	stage_E18_vs_E10	ordinary_muscle_development	ENSGALG00010028574	ENDOG	0.265623046	0.11895811	2.232912467	0.025554717	0.037828539	positive
PRJNA729271	stage_E18_vs_E10	ordinary_muscle_development	ENSGALG00010028575	SETDB1	-0.004962908	0.119316058	-0.041594638	0.966821848	0.972457337	negative
PRJNA729271	stage_E18_vs_E10	ordinary_muscle_development	ENSGALG00010028577	CERS2	0.183171465	0.132669361	1.380661386	0.167383099	0.211730345	positive
PRJNA729271	stage_E18_vs_E10	ordinary_muscle_development	ENSGALG00010028578	SPOUT1	-0.271034791	0.1240259	-2.185307997	0.028866271	0.042302761	negative
PRJNA729271	stage_E18_vs_E10	ordinary_muscle_development	ENSGALG00010028579	OCLN	0.170474048	0.273894016	0.622408808	0.53367309	0.589423304	positive
PRJNA729271	stage_E18_vs_E10	ordinary_muscle_development	ENSGALG00010028580	PRUNE1	-0.195297771	0.156165156	-1.2505848	0.211085999	0.261132675	negative
PRJNA729271	stage_E18_vs_E10	ordinary_muscle_development	ENSGALG00010028581	NR2F6	0.285575524	0.124198087	2.29935526	0.021484774	0.032222115	positive
PRJNA729271	stage_E18_vs_E10	ordinary_muscle_development	ENSGALG00010028582	KYAT1	-0.488486362	0.218546461	-2.235160252	0.02540683	0.037640229	negative
PRJNA729271	stage_E18_vs_E10	ordinary_muscle_development	ENSGALG00010028583	USHBP1	1.619158202	0.196935251	8.221779472	2.00506E-16	1.12692E-15	positive
PRJNA729271	stage_E18_vs_E10	ordinary_muscle_development	ENSGALG00010028584	LRRC8A	-0.267638825	0.077135663	-3.469715751	0.000521009	0.000987839	negative
PRJNA729271	stage_E18_vs_E10	ordinary_muscle_development	ENSGALG00010028585	MINDY1	0.356434932	0.121888713	2.924265292	0.003452703	0.005843874	positive
PRJNA729271	stage_E18_vs_E10	ordinary_muscle_development	ENSGALG00010028586	BABAM1	-0.04460722	0.121626515	-0.366755723	0.713801237	0.757489489	negative
PRJNA729271	stage_E18_vs_E10	ordinary_muscle_development	ENSGALG00010028587	ANKLE1	-0.755956937	0.191858114	-3.94018748	8.1418E-05	0.00017126	negative
PRJNA729271	stage_E18_vs_E10	ordinary_muscle_development	ENSGALG00010028588	ABHD8	-1.714339079	0.679532158	-2.522822592	0.011641712	0.018264597	negative
PRJNA729271	stage_E18_vs_E10	ordinary_muscle_development	ENSGALG00010028589	PHYHD1	1.046019544	0.184355895	5.67391427	1.39571E-08	4.25643E-08	positive
PRJNA729271	stage_E18_vs_E10	ordinary_muscle_development	ENSGALG00010028590	ENSGALG00010028590	0.516451134	0.229244775	2.2528371	0.024269422	0.036073141	positive
PRJNA729271	stage_E18_vs_E10	ordinary_muscle_development	ENSGALG00010028591	GABPB2	-0.365291	0.121613947	-3.003693321	0.00266724	0.004599839	negative
PRJNA729271	stage_E18_vs_E10	ordinary_muscle_development	ENSGALG00010028592	SEMA6C	2.402786952	0.163703333	14.67769113	8.95894E-49	2.48298E-47	positive
PRJNA729271	stage_E18_vs_E10	ordinary_muscle_development	ENSGALG00010028593	DOLK	-0.376852673	0.12347712	-3.052004067	0.00227319	0.00395462	negative
PRJNA729271	stage_E18_vs_E10	ordinary_muscle_development	ENSGALG00010028594	ENSGALG00010028594	0.244142828	0.148009146	1.649511763	0.099042835	0.131455535	positive
PRJNA729271	stage_E18_vs_E10	ordinary_muscle_development	ENSGALG00010028595	SCNM1	0.724993056	0.11236691	6.452015602	1.10372E-10	4.00662E-10	positive
PRJNA729271	stage_E18_vs_E10	ordinary_muscle_development	ENSGALG00010028596	DDA1	-0.034885267	0.088981963	-0.392048748	0.695022195	0.740216511	negative
PRJNA729271	stage_E18_vs_E10	ordinary_muscle_development	ENSGALG00010028597	NUP188	-0.797751923	0.057225232	-13.94056258	3.59098E-44	8.17132E-43	negative
PRJNA729271	stage_E18_vs_E10	ordinary_muscle_development	ENSGALG00010028598	TMOD4	4.71753721	0.251439317	18.76213025	1.54098E-78	1.01993E-76	positive
PRJNA729271	stage_E18_vs_E10	ordinary_muscle_development	ENSGALG00010028599	VPS72	0.173342478	0.118883341	1.458088885	0.144816045	0.185975775	positive
PRJNA729271	stage_E18_vs_E10	ordinary_muscle_development	ENSGALG00010028600	PSMD4	0.159803763	0.133125343	1.20040076	0.229983734	0.281650408	positive
PRJNA729271	stage_E18_vs_E10	ordinary_muscle_development	ENSGALG00010028601	ANO8	2.682372302	0.22229788	12.06656716	1.58614E-33	2.25846E-32	positive
PRJNA729271	stage_E18_vs_E10	ordinary_muscle_development	ENSGALG00010028602	ZNF687	0.093739724	0.172134232	0.5445734	0.586046953	0.638463028	positive
PRJNA729271	stage_E18_vs_E10	ordinary_muscle_development	ENSGALG00010028603	SH3GLB2	0.015641634	0.338589053	0.046196516	0.96315362	0.969777141	positive
PRJNA729271	stage_E18_vs_E10	ordinary_muscle_development	ENSGALG00010028604	PI4KB	-0.063898627	0.140675813	-0.454226109	0.649666093	0.698169485	negative
PRJNA729271	stage_E18_vs_E10	ordinary_muscle_development	ENSGALG00010028605	GTPBP3	0.718040036	0.219333496	3.273736334	0.001061356	0.001926898	positive
PRJNA729271	stage_E18_vs_E10	ordinary_muscle_development	ENSGALG00010028606	PRPF3	-0.475248275	0.137333833	-3.460533102	0.000539107	0.001020018	negative
PRJNA729271	stage_E18_vs_E10	ordinary_muscle_development	ENSGALG00010028607	ENSGALG00010028607	0.995743818	0.23928006	4.161415782	3.16281E-05	6.95462E-05	positive
PRJNA729271	stage_E18_vs_E10	ordinary_muscle_development	ENSGALG00010028608	MIGA2	0.432388239	0.159258722	2.715005078	0.006627475	0.010770855	positive
PRJNA729271	stage_E18_vs_E10	ordinary_muscle_development	ENSGALG00010028609	MRPS21	0.097827761	0.146028684	0.669921544	0.502907805	0.560009882	positive
PRJNA729271	stage_E18_vs_E10	ordinary_muscle_development	ENSGALG00010028610	ENSGALG00010028610	1.353836021	0.531377819	2.547784217	0.01084095	0.017072966	positive
PRJNA729271	stage_E18_vs_E10	ordinary_muscle_development	ENSGALG00010028612	DIRAS1	-1.979209384	0.775430764	-2.552399873	0.010698365	0.01686674	negative
PRJNA729271	stage_E18_vs_E10	ordinary_muscle_development	ENSGALG00010028613	ENSGALG00010028613	0.363403333	0.370911421	0.979757733	0.32720572	0.385221485	positive
PRJNA729271	stage_E18_vs_E10	ordinary_muscle_development	ENSGALG00010028618	DOLPP1	-1.31197777	0.107471694	-12.20765879	2.82913E-34	4.21462E-33	negative
PRJNA729271	stage_E18_vs_E10	ordinary_muscle_development	ENSGALG00010028626	CRAT	2.064684026	0.173115878	11.92660113	8.60142E-33	1.18178E-31	positive
PRJNA729271	stage_E18_vs_E10	ordinary_muscle_development	ENSGALG00010028642	APH1A	0.092270365	0.140576268	0.656372273	0.511584626	0.568362011	positive
PRJNA729271	stage_E18_vs_E10	ordinary_muscle_development	ENSGALG00010028649	ENSGALG00010028649	0.572609972	0.172735372	3.314954927	0.000916579	0.001678043	positive
PRJNA729271	stage_E18_vs_E10	ordinary_muscle_development	ENSGALG00010028658	PTPA	-0.061418916	0.081925546	-0.749691875	0.453440302	0.513124926	negative
PRJNA729271	stage_E18_vs_E10	ordinary_muscle_development	ENSGALG00010028662	FCRL4	0.099031502	0.23016306	0.430266705	0.667001644	0.71415193	positive
PRJNA729271	stage_E18_vs_E10	ordinary_muscle_development	ENSGALG00010028669	ZBTB34	0.392153786	0.11088167	3.536687215	0.000405179	0.000779853	positive
PRJNA729271	stage_E18_vs_E10	ordinary_muscle_development	ENSGALG00010028671	CADM3	0.729431427	0.216777722	3.364881871	0.000765764	0.001419587	positive
PRJNA729271	stage_E18_vs_E10	ordinary_muscle_development	ENSGALG00010028675	ZBTB43	0.144127183	0.071092199	2.027327676	0.042628913	0.060537483	positive
PRJNA729271	stage_E18_vs_E10	ordinary_muscle_development	ENSGALG00010028678	ENSGALG00010028678	1.044561886	0.368120867	2.83755141	0.004546103	0.007577709	positive
PRJNA729271	stage_E18_vs_E10	ordinary_muscle_development	ENSGALG00010028686	TAGLN2	-0.178356518	0.120252075	-1.483188689	0.138024285	0.178185075	negative
PRJNA729271	stage_E18_vs_E10	ordinary_muscle_development	ENSGALG00010028689	CFAP45	0.904718617	0.622299573	1.453831333	0.145993074	0.187304798	positive
PRJNA729271	stage_E18_vs_E10	ordinary_muscle_development	ENSGALG00010028692	RALGPS1	0.753176548	0.110353575	6.82512139	8.7851E-12	3.46503E-11	positive
PRJNA729271	stage_E18_vs_E10	ordinary_muscle_development	ENSGALG00010028695	VSIG8	1.980968791	0.887644793	2.231713415	0.02563391	0.037926256	positive
PRJNA729271	stage_E18_vs_E10	ordinary_muscle_development	ENSGALG00010028700	ANGPTL2	1.968375481	0.197560555	9.963403264	2.20387E-23	1.88466E-22	positive
PRJNA729271	stage_E18_vs_E10	ordinary_muscle_development	ENSGALG00010028707	ENSGALG00010028707	-3.618952352	1.23336907	-2.934200671	0.003344079	0.005673935	negative
PRJNA729271	stage_E18_vs_E10	ordinary_muscle_development	ENSGALG00010028712	FBXW2	-0.071875622	0.085515809	-0.840495147	0.400630822	0.460445906	negative
PRJNA729271	stage_E18_vs_E10	ordinary_muscle_development	ENSGALG00010028726	SLC2A8	0.171983984	0.112349307	1.530797021	0.125819566	0.163932999	positive
PRJNA729271	stage_E18_vs_E10	ordinary_muscle_development	ENSGALG00010028727	ENSGALG00010028727	-0.419342785	0.178071876	-2.354907433	0.01852732	0.02808773	negative
PRJNA729271	stage_E18_vs_E10	ordinary_muscle_development	ENSGALG00010028734	ENSGALG00010028734	2.046688081	0.3573472	5.727449613	1.01952E-08	3.14557E-08	positive
PRJNA729271	stage_E18_vs_E10	ordinary_muscle_development	ENSGALG00010028735	ENSGALG00010028735	-0.40036665	0.342113356	-1.170274831	0.241890387	0.294936168	negative
PRJNA729271	stage_E18_vs_E10	ordinary_muscle_development	ENSGALG00010028738	MIDN	-0.709580214	0.186229892	-3.810238025	0.000138833	0.000284557	negative
PRJNA729271	stage_E18_vs_E10	ordinary_muscle_development	ENSGALG00010028739	GARNL3	-0.413749644	0.078181255	-5.292184714	1.20864E-07	3.39915E-07	negative
PRJNA729271	stage_E18_vs_E10	ordinary_muscle_development	ENSGALG00010028740	DAZAP1	-0.382330525	0.106391146	-3.593631035	0.000326102	0.000635243	negative
PRJNA729271	stage_E18_vs_E10	ordinary_muscle_development	ENSGALG00010028742	ENSGALG00010028742	0.306335624	0.142821707	2.14488141	0.03196233	0.046487455	positive
PRJNA729271	stage_E18_vs_E10	ordinary_muscle_development	ENSGALG00010028743	ARRDC1	1.287979581	0.186685909	6.899179413	5.23038E-12	2.11418E-11	positive
PRJNA729271	stage_E18_vs_E10	ordinary_muscle_development	ENSGALG00010028744	HOMER3	0.491130249	0.208426034	2.356376693	0.018454193	0.027983422	positive
PRJNA729271	stage_E18_vs_E10	ordinary_muscle_development	ENSGALG00010028745	ENSGALG00010028745	0.646055576	0.138489557	4.665012927	3.08598E-06	7.55595E-06	positive
PRJNA729271	stage_E18_vs_E10	ordinary_muscle_development	ENSGALG00010028746	MYDGF	0.066882943	0.12335631	0.542193124	0.587685472	0.639911427	positive
PRJNA729271	stage_E18_vs_E10	ordinary_muscle_development	ENSGALG00010028747	ENSGALG00010028747	-2.020428549	0.186306709	-10.84463659	2.11475E-27	2.28245E-26	negative
PRJNA729271	stage_E18_vs_E10	ordinary_muscle_development	ENSGALG00010028748	PIAS3	0.750205106	0.202427336	3.706046423	0.00021052	0.000420894	positive
PRJNA729271	stage_E18_vs_E10	ordinary_muscle_development	ENSGALG00010028750	OTUD7B	-0.061348411	0.122250791	-0.501824247	0.615791156	0.666459553	negative
PRJNA729271	stage_E18_vs_E10	ordinary_muscle_development	ENSGALG00010028751	EHMT1	-0.360698501	0.095546316	-3.77511679	0.000159933	0.000324909	negative
PRJNA729271	stage_E18_vs_E10	ordinary_muscle_development	ENSGALG00010028752	MTMR11	0.583895021	0.148101175	3.942541444	8.06227E-05	0.000169686	positive
PRJNA729271	stage_E18_vs_E10	ordinary_muscle_development	ENSGALG00010028753	SV2A	0.721024096	0.725331972	0.994060822	0.320193258	0.377855676	positive
PRJNA729271	stage_E18_vs_E10	ordinary_muscle_development	ENSGALG00010028756	ENSGALG00010028756	-1.470693391	0.619955448	-2.37225658	0.017679808	0.026912463	negative
PRJNA729271	stage_E18_vs_E10	ordinary_muscle_development	ENSGALG00010028757	MAT2A	-1.154711934	1.266221949	-0.911934859	0.36180301	0.421197866	negative
PRJNA729271	stage_E18_vs_E10	ordinary_muscle_development	ENSGALG00010028760	SSR2	0.338782587	0.097266561	3.48303243	0.000495768	0.000942818	positive
PRJNA729271	stage_E18_vs_E10	ordinary_muscle_development	ENSGALG00010028761	CTSS	0.530016557	0.239490955	2.213096345	0.026891004	0.039635132	positive
PRJNA729271	stage_E18_vs_E10	ordinary_muscle_development	ENSGALG00010028762	CACNA1B	-1.938464527	0.627304951	-3.090147025	0.002000574	0.003511969	negative
PRJNA729271	stage_E18_vs_E10	ordinary_muscle_development	ENSGALG00010028763	CTSK	1.779503067	0.217285725	8.189691538	2.61897E-16	1.45783E-15	positive
PRJNA729271	stage_E18_vs_E10	ordinary_muscle_development	ENSGALG00010028764	ARNT	0.366128383	0.12553936	2.916442962	0.003540475	0.005984744	positive
PRJNA729271	stage_E18_vs_E10	ordinary_muscle_development	ENSGALG00010028768	NTNG2	-0.89114161	0.35663446	-2.498753514	0.012463096	0.019447343	negative
PRJNA729271	stage_E18_vs_E10	ordinary_muscle_development	ENSGALG00010028771	SETX	-0.265784399	0.072974595	-3.642149725	0.000270371	0.000533128	negative
PRJNA729271	stage_E18_vs_E10	ordinary_muscle_development	ENSGALG00010028772	TTF1	-0.456659297	0.075635092	-6.03766431	1.56361E-09	5.18757E-09	negative
PRJNA729271	stage_E18_vs_E10	ordinary_muscle_development	ENSGALG00010028782	MLLT11	0.434419421	0.254745328	1.705308686	0.088136869	0.118061539	positive
PRJNA729271	stage_E18_vs_E10	ordinary_muscle_development	ENSGALG00010028783	DDX31	0.002490503	0.074149867	0.033587419	0.973206155	0.977996618	positive
PRJNA729271	stage_E18_vs_E10	ordinary_muscle_development	ENSGALG00010028786	GTF3C4	-0.315742234	0.067701503	-4.663740362	3.10513E-06	7.60028E-06	negative
PRJNA729271	stage_E18_vs_E10	ordinary_muscle_development	ENSGALG00010028800	ENSGALG00010028800	1.851400602	0.394883067	4.688478072	2.75244E-06	6.77478E-06	positive
PRJNA729271	stage_E18_vs_E10	ordinary_muscle_development	ENSGALG00010028806	SPACA9	1.101932236	0.402165589	2.739996327	0.006143987	0.010040776	positive
PRJNA729271	stage_E18_vs_E10	ordinary_muscle_development	ENSGALG00010028818	TSC1	1.11965648	0.116943686	9.574321768	1.02528E-21	8.07687E-21	positive
PRJNA729271	stage_E18_vs_E10	ordinary_muscle_development	ENSGALG00010028823	GFI1B	0.459564084	0.3219237	1.427555921	0.153419718	0.195896654	positive
PRJNA729271	stage_E18_vs_E10	ordinary_muscle_development	ENSGALG00010028826	CEL	-0.452691742	2.846051572	-0.159059571	0.87362194	0.895815602	negative
PRJNA729271	stage_E18_vs_E10	ordinary_muscle_development	ENSGALG00010028829	GTF3C5	-0.327247281	0.114820015	-2.850089153	0.004370698	0.007300445	negative
PRJNA729271	stage_E18_vs_E10	ordinary_muscle_development	ENSGALG00010028833	GBGT1	1.586711974	0.169092476	9.383693543	6.37012E-21	4.77924E-20	positive
PRJNA729271	stage_E18_vs_E10	ordinary_muscle_development	ENSGALG00010028849	ENSGALG00010028849	2.416463818	0.335394551	7.204839231	5.81124E-13	2.53335E-12	positive
PRJNA729271	stage_E18_vs_E10	ordinary_muscle_development	ENSGALG00010028853	ENSGALG00010028853	1.722611722	1.440009812	1.196249989	0.231599019	0.283413067	positive
PRJNA729271	stage_E18_vs_E10	ordinary_muscle_development	ENSGALG00010028854	RPL7A	1.058389563	0.140324266	7.542455703	4.61203E-14	2.17191E-13	positive
PRJNA729271	stage_E18_vs_E10	ordinary_muscle_development	ENSGALG00010028855	SURF6	-0.39754885	0.169783721	-2.341501577	0.019206345	0.029044963	negative
PRJNA729271	stage_E18_vs_E10	ordinary_muscle_development	ENSGALG00010028856	ETV3	0.096367887	0.109690768	0.878541458	0.379649949	0.439925335	positive
PRJNA729271	stage_E18_vs_E10	ordinary_muscle_development	ENSGALG00010028857	MED22	0.422212473	0.07879073	5.358656708	8.3843E-08	2.39092E-07	positive
PRJNA729271	stage_E18_vs_E10	ordinary_muscle_development	ENSGALG00010028858	ENSGALG00010028858	0.398407797	0.458536056	0.868869072	0.384918748	0.44531864	positive
PRJNA729271	stage_E18_vs_E10	ordinary_muscle_development	ENSGALG00010028859	SURF1	0.484697333	0.110962978	4.368099524	1.25332E-05	2.88914E-05	positive
PRJNA729271	stage_E18_vs_E10	ordinary_muscle_development	ENSGALG00010028860	SURF2	0.262036485	0.172960956	1.51500368	0.129771472	0.168415927	positive
PRJNA729271	stage_E18_vs_E10	ordinary_muscle_development	ENSGALG00010028861	SURF4	-0.152653601	0.074725483	-2.042858671	0.041066431	0.058513507	negative
PRJNA729271	stage_E18_vs_E10	ordinary_muscle_development	ENSGALG00010028862	ENSGALG00010028862	6.125462332	1.145018629	5.349661723	8.81188E-08	2.50742E-07	positive
PRJNA729271	stage_E18_vs_E10	ordinary_muscle_development	ENSGALG00010028865	STKLD1	0.919793575	0.168849887	5.447404134	5.11102E-08	1.48376E-07	positive
PRJNA729271	stage_E18_vs_E10	ordinary_muscle_development	ENSGALG00010028866	HORMAD1	-0.853670828	0.499229473	-1.709976825	0.087270159	0.116987048	negative
PRJNA729271	stage_E18_vs_E10	ordinary_muscle_development	ENSGALG00010028867	REXO4	-1.011482479	0.121743629	-8.308299082	9.70836E-17	5.57317E-16	negative
PRJNA729271	stage_E18_vs_E10	ordinary_muscle_development	ENSGALG00010028868	ENSGALG00010028868	0.754656726	4.241825273	0.177908489	0.858794844	0.883114194	positive
PRJNA729271	stage_E18_vs_E10	ordinary_muscle_development	ENSGALG00010028869	ADAMTS13	3.851595323	0.162792074	23.65959983	9.4016E-124	1.7698E-121	positive
PRJNA729271	stage_E18_vs_E10	ordinary_muscle_development	ENSGALG00010028870	CACFD1	-2.844770764	0.234020059	-12.15609798	5.32416E-34	7.82695E-33	negative
PRJNA729271	stage_E18_vs_E10	ordinary_muscle_development	ENSGALG00010028871	PIP5K1A	0.025074069	0.108599103	0.230886524	0.81740296	0.848338851	positive
PRJNA729271	stage_E18_vs_E10	ordinary_muscle_development	ENSGALG00010028872	SLC2A6	0.4009669	0.365489993	1.097066698	0.272612237	0.327763302	positive
PRJNA729271	stage_E18_vs_E10	ordinary_muscle_development	ENSGALG00010028873	TMEM8C	-1.04660735	0.28151072	-3.717824132	0.000200946	0.000402754	negative
PRJNA729271	stage_E18_vs_E10	ordinary_muscle_development	ENSGALG00010028874	CRPL1	2.128667309	0.496914014	4.28377395	1.8375E-05	4.15645E-05	positive
PRJNA729271	stage_E18_vs_E10	ordinary_muscle_development	ENSGALG00010028875	DUSP23	1.46893336	0.119667026	12.27517231	1.23129E-34	1.8669E-33	positive
PRJNA729271	stage_E18_vs_E10	ordinary_muscle_development	ENSGALG00010028876	ADAMTSL2	-0.695963526	0.212080057	-3.2816076	0.001032171	0.001876499	negative
PRJNA729271	stage_E18_vs_E10	ordinary_muscle_development	ENSGALG00010028877	FAM163B	-4.68182686	0.938679902	-4.987671358	6.11114E-07	1.61189E-06	negative
PRJNA729271	stage_E18_vs_E10	ordinary_muscle_development	ENSGALG00010028878	IGSF9	-0.107128337	0.162249692	-0.660268352	0.509081636	0.566058477	negative
PRJNA729271	stage_E18_vs_E10	ordinary_muscle_development	ENSGALG00010028881	SARDH	-0.13815252	0.230396086	-0.599630502	0.548752515	0.602999598	negative
PRJNA729271	stage_E18_vs_E10	ordinary_muscle_development	ENSGALG00010028884	VAV2	-0.91969402	0.129925029	-7.078651615	1.45564E-12	6.15504E-12	negative
PRJNA729271	stage_E18_vs_E10	ordinary_muscle_development	ENSGALG00010028885	BRD3	-0.452248785	0.141880741	-3.187527645	0.001434947	0.00256563	negative

PRJNA729271	stage_E18_vs_E10	ordinary_muscle_development	ENSGALG00010028886	ENSGALG00010028886	3.861993561	1.709782359	2.258763251	0.023898116	0.035542101	positive
PRJNA729271	stage_E18_vs_E10	ordinary_muscle_development	ENSGALG00010028887	SF3B4	0.020890665	0.175202905	0.119236977	0.905087613	0.921590428	positive
PRJNA729271	stage_E18_vs_E10	ordinary_muscle_development	ENSGALG00010028888	WDR5B	-1.049882186	0.08260703	-12.70935644	5.24644E-37	8.88402E-36	negative
PRJNA729271	stage_E18_vs_E10	ordinary_muscle_development	ENSGALG00010028889	FUBP3	-0.467986309	0.08265722	-5.661771737	1.49818E-08	4.56221E-08	negative
PRJNA729271	stage_E18_vs_E10	ordinary_muscle_development	ENSGALG00010028890	ENSGALG00010028890	-1.134380956	0.222179387	-5.105698468	3.29575E-07	8.93099E-07	negative
PRJNA729271	stage_E18_vs_E10	ordinary_muscle_development	ENSGALG00010028891	SLC31A2	0.572040959	0.250086433	2.287373022	0.02217406	0.033172979	positive
PRJNA729271	stage_E18_vs_E10	ordinary_muscle_development	ENSGALG00010028892	NIBAN2	0.287571018	0.140860076	2.041536721	0.041197508	0.058677201	positive
PRJNA729271	stage_E18_vs_E10	ordinary_muscle_development	ENSGALG00010028893	STXBP1	0.72004715	0.201740487	3.569175228	0.000358107	0.000693952	positive
PRJNA729271	stage_E18_vs_E10	ordinary_muscle_development	ENSGALG00010028894	AKNA	-1.28017944	0.160702496	-7.966145317	1.637E-15	8.64737E-15	negative
PRJNA729271	stage_E18_vs_E10	ordinary_muscle_development	ENSGALG00010028895	WHRN	-4.09495028	0.838868132	-4.88151847	1.05272E-06	2.71612E-06	negative
PRJNA729271	stage_E18_vs_E10	ordinary_muscle_development	ENSGALG00010028896	ATP6V1G1	0.477703039	0.099038267	4.8234188	1.41118E-06	3.58861E-06	positive
PRJNA729271	stage_E18_vs_E10	ordinary_muscle_development	ENSGALG00010028897	TMEM268	-0.475571402	0.287345607	-1.655050192	0.097914317	0.130088728	negative
PRJNA729271	stage_E18_vs_E10	ordinary_muscle_development	ENSGALG00010028898	TNFSF15	-1.476255355	0.254297988	-5.805218382	6.42822E-09	2.02339E-08	negative
PRJNA729271	stage_E18_vs_E10	ordinary_muscle_development	ENSGALG00010028899	TNFSF8	-2.334729823	0.972617568	-2.400460262	0.016374469	0.025095284	negative
PRJNA729271	stage_E18_vs_E10	ordinary_muscle_development	ENSGALG00010028900	TNC	-2.676678888	0.549669623	-4.86961399	1.11816E-06	2.87729E-06	negative
PRJNA729271	stage_E18_vs_E10	ordinary_muscle_development	ENSGALG00010028901	PAPPA	-0.307506935	0.112735353	-2.727688576	0.006377979	0.010401531	negative
PRJNA729271	stage_E18_vs_E10	ordinary_muscle_development	ENSGALG00010028902	ENSGALG00010028902	-0.545873201	0.430839335	-1.26699945	0.205155486	0.254534689	negative
PRJNA729271	stage_E18_vs_E10	ordinary_muscle_development	ENSGALG00010028903	ASTN2	-2.59848033	0.225333776	-11.53169476	9.13279E-31	1.16021E-29	negative
PRJNA729271	stage_E18_vs_E10	ordinary_muscle_development	ENSGALG00010028904	TRIM32	-0.246289076	0.082784898	-2.97504838	0.002929423	0.005024493	negative
PRJNA729271	stage_E18_vs_E10	ordinary_muscle_development	ENSGALG00010028905	COLGALT1	0.468654853	0.085936043	5.453530739	4.93794E-08	1.4361E-07	positive
PRJNA729271	stage_E18_vs_E10	ordinary_muscle_development	ENSGALG00010028909	ENSGALG00010028909	-1.060050733	0.13882695	-7.635770506	2.24474E-14	1.08567E-13	negative
PRJNA729271	stage_E18_vs_E10	ordinary_muscle_development	ENSGALG00010028911	LMX1B	-2.248342876	0.526993264	-4.266359803	1.98688E-05	4.47829E-05	negative
PRJNA729271	stage_E18_vs_E10	ordinary_muscle_development	ENSGALG00010028912	EXFABP	2.048723935	0.496980441	4.12234319	3.75038E-05	8.18676E-05	positive
PRJNA729271	stage_E18_vs_E10	ordinary_muscle_development	ENSGALG00010028914	TLR4	0.478535071	0.233333302	2.050864866	0.040280108	0.057494847	positive
PRJNA729271	stage_E18_vs_E10	ordinary_muscle_development	ENSGALG00010028915	ADGRD2	-0.055290606	0.511353471	-0.108126001	0.913895743	0.929021586	negative
PRJNA729271	stage_E18_vs_E10	ordinary_muscle_development	ENSGALG00010028917	NR6A1	0.24796522	0.127443375	1.945689371	0.051692064	0.072379875	positive
PRJNA729271	stage_E18_vs_E10	ordinary_muscle_development	ENSGALG00010028918	AGPAT2	1.84899918	0.18415862	10.04025321	1.01413E-23	8.86069E-23	positive
PRJNA729271	stage_E18_vs_E10	ordinary_muscle_development	ENSGALG00010028919	EGFL7	1.701654484	0.16858018	10.09403648	5.87047E-24	5.20493E-23	positive
PRJNA729271	stage_E18_vs_E10	ordinary_muscle_development	ENSGALG00010028921	MRPL41	-0.379998095	0.123475049	-3.077529413	0.002087242	0.003656142	negative
PRJNA729271	stage_E18_vs_E10	ordinary_muscle_development	ENSGALG00010028923	ENSGALG00010028923	0.70785032	0.19356324	3.656946015	0.000255238	0.000505213	positive
PRJNA729271	stage_E18_vs_E10	ordinary_muscle_development	ENSGALG00010028924	TMEM203	0.007831076	0.121514512	0.0644456	0.948615422	0.957647846	positive
PRJNA729271	stage_E18_vs_E10	ordinary_muscle_development	ENSGALG00010028925	ENSGALG00010028925	0.593170677	0.348402706	1.702543258	0.088653576	0.118720768	positive
PRJNA729271	stage_E18_vs_E10	ordinary_muscle_development	ENSGALG00010028926	ENSGALG00010028926	-1.104083804	0.640985282	-1.722479182	0.084982751	0.114195325	negative
PRJNA729271	stage_E18_vs_E10	ordinary_muscle_development	ENSGALG00010029140	NPDC1	0.498621017	0.192028622	2.596597388	0.009415221	0.014946187	positive
PRJNA729271	stage_E18_vs_E10	ordinary_muscle_development	ENSGALG00010029141	C5	0.326914255	0.567133419	0.576432713	0.564322738	0.618140867	positive
PRJNA729271	stage_E18_vs_E10	ordinary_muscle_development	ENSGALG00010029142	ENSGALG00010029142	1.151378577	0.181226785	6.353247269	2.10816E-10	7.49518E-10	positive
PRJNA729271	stage_E18_vs_E10	ordinary_muscle_development	ENSGALG00010029143	SH2D3C	0.34618671	0.108989629	3.176327076	0.001491527	0.002662522	positive
PRJNA729271	stage_E18_vs_E10	ordinary_muscle_development	ENSGALG00010029144	ENSGALG00010029144	4.693912031	0.219677266	21.36730906	2.6921E-101	3.25181E-99	positive
PRJNA729271	stage_E18_vs_E10	ordinary_muscle_development	ENSGALG00010029145	RC3H2	0.25281502	0.134568769	1.878705	0.060284785	0.08338053	positive
PRJNA729271	stage_E18_vs_E10	ordinary_muscle_development	ENSGALG00010029146	NEK6	-1.078765449	0.058905316	-18.31354993	6.45228E-75	3.8647E-73	negative
PRJNA729271	stage_E18_vs_E10	ordinary_muscle_development	ENSGALG00010029147	RABL6	-0.241825192	0.103631308	-2.333514811	0.019621142	0.02962588	negative
PRJNA729271	stage_E18_vs_E10	ordinary_muscle_development	ENSGALG00010029148	RALGDS	0.457275707	0.11868818	3.852748511	0.000116799	0.00024172	positive
PRJNA729271	stage_E18_vs_E10	ordinary_muscle_development	ENSGALG00010029149	ENSGALG00010029149	-1.011196621	0.226328475	-4.467827662	7.90179E-06	1.85966E-05	negative
PRJNA729271	stage_E18_vs_E10	ordinary_muscle_development	ENSGALG00010029150	NARF	-0.067312311	0.123374557	-0.545593129	0.585345648	0.637842819	negative
PRJNA729271	stage_E18_vs_E10	ordinary_muscle_development	ENSGALG00010029151	CYBC1	-0.702487717	0.114504152	-6.135041437	8.5137E-10	2.89618E-09	negative
PRJNA729271	stage_E18_vs_E10	ordinary_muscle_development	ENSGALG00010029152	HEXD	-0.173910972	0.131131183	-1.326236579	0.184761347	0.231660373	negative
PRJNA729271	stage_E18_vs_E10	ordinary_muscle_development	ENSGALG00010029153	OGFOD3	-0.413777871	0.078332527	-5.282325085	1.27555E-07	3.58037E-07	negative
PRJNA729271	stage_E18_vs_E10	ordinary_muscle_development	ENSGALG00010029154	UTS2R	0.030477721	0.141526452	0.215349997	0.829494452	0.85827126	positive
PRJNA729271	stage_E18_vs_E10	ordinary_muscle_development	ENSGALG00010029155	ENSGALG00010029155	0.175762362	0.580218905	0.302924226	0.761947609	0.800263067	positive
PRJNA729271	stage_E18_vs_E10	ordinary_muscle_development	ENSGALG00010029156	ENSGALG00010029156	-2.388696601	1.004671518	-2.377589649	0.017426206	0.026552388	negative
PRJNA729271	stage_E18_vs_E10	ordinary_muscle_development	ENSGALG00010029158	CD7	-1.59068744	0.492091615	-3.232502632	0.00122711	0.002212033	negative
PRJNA729271	stage_E18_vs_E10	ordinary_muscle_development	ENSGALG00010029159	SSC4D	-3.369277119	0.980306964	-3.436961322	0.00058828	0.001107102	negative
PRJNA729271	stage_E18_vs_E10	ordinary_muscle_development	ENSGALG00010029160	DUS1L	-0.088367303	0.093839559	-0.941684977	0.346353949	0.405034327	negative
PRJNA729271	stage_E18_vs_E10	ordinary_muscle_development	ENSGALG00010029162	ELN	1.487799303	0.31088725	4.785655575	1.7043E-06	4.29557E-06	positive
PRJNA729271	stage_E18_vs_E10	ordinary_muscle_development	ENSGALG00010029163	ANKFY1	-0.685453476	0.094651736	-7.241847925	4.42612E-13	1.9465E-12	negative
PRJNA729271	stage_E18_vs_E10	ordinary_muscle_development	ENSGALG00010029164	CYB5D2	0.338758771	0.083587328	4.052752735	5.06185E-05	0.000108973	positive
PRJNA729271	stage_E18_vs_E10	ordinary_muscle_development	ENSGALG00010029165	RAC3	0.065958281	0.120459611	0.547555155	0.583997389	0.636708699	positive
PRJNA729271	stage_E18_vs_E10	ordinary_muscle_development	ENSGALG00010029166	ZZEF1	-0.589369405	0.125079103	-4.711973382	2.45329E-06	6.0756E-06	negative
PRJNA729271	stage_E18_vs_E10	ordinary_muscle_development	ENSGALG00010029167	ENSGALG00010029167	0.198088804	0.127941554	1.548275734	0.121555924	0.158748817	positive
PRJNA729271	stage_E18_vs_E10	ordinary_muscle_development	ENSGALG00010029168	RFNG	0.425848459	0.080829141	5.268501569	1.37542E-07	3.84285E-07	positive
PRJNA729271	stage_E18_vs_E10	ordinary_muscle_development	ENSGALG00010029169	SBDS	0.285826553	0.07889544	3.622852662	0.000291372	0.000571894	positive
PRJNA729271	stage_E18_vs_E10	ordinary_muscle_development	ENSGALG00010029170	DCXR	0.586618466	0.13943805	4.207018561	2.58762E-05	5.75622E-05	positive
PRJNA729271	stage_E18_vs_E10	ordinary_muscle_development	ENSGALG00010029171	ENSGALG00010029171	0.022826412	0.147264253	0.155003078	0.876818891	0.898070225	positive
PRJNA729271	stage_E18_vs_E10	ordinary_muscle_development	ENSGALG00010029172	LRRC45	0.049465898	0.074302034	0.665740837	0.505576767	0.562808942	positive
PRJNA729271	stage_E18_vs_E10	ordinary_muscle_development	ENSGALG00010029173	ZNHIT3	0.107105216	0.13602368	0.787401257	0.431047011	0.491119826	positive
PRJNA729271	stage_E18_vs_E10	ordinary_muscle_development	ENSGALG00010029174	CALN1	-2.884817861	0.300390814	-9.603548865	7.72382E-22	6.1279E-21	negative
PRJNA729271	stage_E18_vs_E10	ordinary_muscle_development	ENSGALG00010029175	CENPX	0.010830257	0.130133208	0.083224392	0.933673118	0.94583334	positive
PRJNA729271	stage_E18_vs_E10	ordinary_muscle_development	ENSGALG00010029176	ENSGALG00010029176	0.355842017	0.515373041	0.690455241	0.489907948	0.547721998	positive
PRJNA729271	stage_E18_vs_E10	ordinary_muscle_development	ENSGALG00010029177	ASPSCR1	0.496475523	0.080760395	6.14751233	7.87076E-10	2.68502E-09	positive
PRJNA729271	stage_E18_vs_E10	ordinary_muscle_development	ENSGALG00010029178	NOTUM	-0.449292384	0.210611586	-2.133274775	0.032902192	0.047707102	negative
PRJNA729271	stage_E18_vs_E10	ordinary_muscle_development	ENSGALG00010029179	PIGW	0.130462353	0.153289494	0.851084767	0.394722267	0.454700728	positive
PRJNA729271	stage_E18_vs_E10	ordinary_muscle_development	ENSGALG00010029180	CA4	4.299001451	0.435315539	9.875598428	5.3115E-23	4.45545E-22	positive
PRJNA729271	stage_E18_vs_E10	ordinary_muscle_development	ENSGALG00010029181	MYO19	0.007144292	0.105606568	0.067650071	0.946064196	0.95549056	positive
PRJNA729271	stage_E18_vs_E10	ordinary_muscle_development	ENSGALG00010029182	HEATR6	-0.760223362	0.071557889	-10.6238931	2.30733E-26	2.36526E-25	negative
PRJNA729271	stage_E18_vs_E10	ordinary_muscle_development	ENSGALG00010029183	ENSGALG00010029183	0.54537881	0.139797405	3.901208394	9.57137E-05	0.000199938	positive
PRJNA729271	stage_E18_vs_E10	ordinary_muscle_development	ENSGALG00010029184	DYNLL2	-0.211441166	0.096444683	-2.192356902	0.028353746	0.041618992	negative
PRJNA729271	stage_E18_vs_E10	ordinary_muscle_development	ENSGALG00010029186	ZNF302	0.277375197	0.141134819	1.965320811	0.049377118	0.069393187	positive
PRJNA729271	stage_E18_vs_E10	ordinary_muscle_development	ENSGALG00010029187	SRSF1	-1.045359374	0.052539358	-19.8966911	4.34695E-88	3.86559E-86	negative
PRJNA729271	stage_E18_vs_E10	ordinary_muscle_development	ENSGALG00010029188	ENSGALG00010029188	0.439780621	0.265753023	1.654847108	0.097955516	0.130131303	positive
PRJNA729271	stage_E18_vs_E10	ordinary_muscle_development	ENSGALG00010029189	ENSGALG00010029189	0.435183589	0.355341518	1.224691085	0.220691634	0.271624798	positive
PRJNA729271	stage_E18_vs_E10	ordinary_muscle_development	ENSGALG00010029190	DHRS7C	5.469438763	0.163335947	33.4858239	7.7518E-246	7.4908E-243	positive
PRJNA729271	stage_E18_vs_E10	ordinary_muscle_development	ENSGALG00010029192	DTX2	0.298241265	0.101697463	2.932632293	0.003361017	0.005699338	positive
PRJNA729271	stage_E18_vs_E10	ordinary_muscle_development	ENSGALG00010029194	DHRS11	-0.40788043	0.125319084	-3.254735163	0.001134982	0.002054134	negative
PRJNA729271	stage_E18_vs_E10	ordinary_muscle_development	ENSGALG00010029196	MRM1	-0.428826276	0.101840911	-4.210746672	2.54528E-05	5.66639E-05	negative
PRJNA729271	stage_E18_vs_E10	ordinary_muscle_development	ENSGALG00010029197	LHX1	5.355034587	0.550081268	9.734988071	2.13843E-22	1.7453E-21	positive
PRJNA729271	stage_E18_vs_E10	ordinary_muscle_development	ENSGALG00010029198	PPM1D	0.049216117	0.175285561	0.280776789	0.778881608	0.814566299	positive
PRJNA729271	stage_E18_vs_E10	ordinary_muscle_development	ENSGALG00010029199	APPBP2	0.019003044	0.089606031	0.212073273	0.832049871	0.860546763	positive
PRJNA729271	stage_E18_vs_E10	ordinary_muscle_development	ENSGALG00010029200	GAS7	1.094452499	0.124732956	8.774365132	1.71871E-18	1.1092E-17	positive
PRJNA729271	stage_E18_vs_E10	ordinary_muscle_development	ENSGALG00010029201	PIGS	-0.767109651	0.113174268	-6.778127799	1.21743E-11	4.75395E-11	negative
PRJNA729271	stage_E18_vs_E10	ordinary_muscle_development	ENSGALG00010029202	PSMD12	-0.544504687	0.144244676	-3.774868537	0.000160092	0.000325187	negative
PRJNA729271	stage_E18_vs_E10	ordinary_muscle_development	ENSGALG00010029203	ALDOC	0.690267075	0.172441166	4.002913526	6.25672E-05	0.000133513	positive
PRJNA729271	stage_E18_vs_E10	ordinary_muscle_development	ENSGALG00010029204	HELZ	0.347604696	0.098160076	3.541202382	0.000398308	0.000766831	positive
PRJNA729271	stage_E18_vs_E10	ordinary_muscle_development	ENSGALG00010029205	CACNG1	4.084108323	0.114161778	35.77474339	2.7289E-280	3.9555E-277	positive
PRJNA729271	stage_E18_vs_E10	ordinary_muscle_development	ENSGALG00010029206	CACNG4	0.646662297	0.299598993	2.158426136	0.030894716	0.045047671	positive
PRJNA729271	stage_E18_vs_E10	ordinary_muscle_development	ENSGALG00010029207	SPAG5	-0.841810144	0.220232508	-3.822370058	0.000132175	0.000271717	negative
PRJNA729271	stage_E18_vs_E10	ordinary_muscle_development	ENSGALG00010029208	CACNG5	3.459264283	0.227960992	15.17480798	5.19295E-52	1.62926E-50	positive
PRJNA729271	stage_E18_vs_E10	ordinary_muscle_development	ENSGALG00010029209	PRKCA	0.662771919	0.096557472	6.864014813	6.69517E-12	2.66949E-11	positive
PRJNA729271	stage_E18_vs_E10	ordinary_muscle_development	ENSGALG00010029210	RSKR	5.392253683	0.252611444	21.34603875	4.2443E-101	5.04268E-99	positive
PRJNA729271	stage_E18_vs_E10	ordinary_muscle_development	ENSGALG00010029211	SDF2	-0.602596013	0.168304498	-3.580391609	0.00034308	0.000666078	negative
PRJNA729271	stage_E18_vs_E10	ordinary_muscle_development	ENSGALG00010029212	SUPT6H	-0.070254554	0.113808467	-0.617305159	0.537033465	0.592277263	negative
PRJNA729271	stage_E18_vs_E10	ordinary_muscle_development	ENSGALG00010029213	APOH	5.218210295	1.515370479	3.443521151	0.000574192	0.001082444	positive
PRJNA729271	stage_E18_vs_E10	ordinary_muscle_development	ENSGALG00010029214	ENSGALG00010029214	3.661471907	0.381217838	9.604670995	7.64015E-22	6.06484E-21	positive
PRJNA729271	stage_E18_vs_E10	ordinary_muscle_development	ENSGALG00010029215	ENSGALG00010029215	7.198422294	0.237245851	30.34161509	3.2414E-202	1.8793E-199	positive
PRJNA729271	stage_E18_vs_E10	ordinary_muscle_development	ENSGALG00010029216	RAB34	-1.095039715	0.135026643	-8.109804805	5.07011E-16	2.77664E-15	negative
PRJNA729271	stage_E18_vs_E10	ordinary_muscle_development	ENSGALG00010029217	CEP112	-0.532026061	0.091892506	-5.789656658	7.05304E-09	2.2076E-08	negative
PRJNA729271	stage_E18_vs_E10	ordinary_muscle_development	ENSGALG00010029218	RPL23A	1.302026971	0.150208458	8.668133555	4.39261E-18	2.7635E-17	positive
PRJNA729271	stage_E18_vs_E10	ordinary_muscle_development	ENSGALG00010029219	TLCD1	0.076304259	0.204088938	0.373877487	0.708495466	0.752851095	positive
PRJNA729271	stage_E18_vs_E10	ordinary_muscle_development	ENSGALG00010029220	NEK8	0.481694867	0.126031837	3.822009415	0.000132369	0.000272076	positive
PRJNA729271	stage_E18_vs_E10	ordinary_muscle_development	ENSGALG00010029221	AXIN2	-0.238459702	0.121874641	-1.956598188	0.050394728	0.070713609	negative
PRJNA729271	stage_E18_vs_E10	ordinary_muscle_development	ENSGALG00010029222	TRAF4	-0.14524964	0.233077989	-0.623180425	0.533165964	0.589082337	negative
PRJNA729271	stage_E18_vs_E10	ordinary_muscle_development	ENSGALG00010029223	NME2	0.451937368	0.1458138	3.099414229	0.001939037	0.003409309	positive
PRJNA729271	stage_E18_vs_E10	ordinary_muscle_development	ENSGALG00010029224	FAM222B	-0.226829914	0.162346596	-1.397195378	0.162354826	0.206125357	negative
PRJNA729271	stage_E18_vs_E10	ordinary_muscle_development	ENSGALG00010029226	MBTD1	-0.144710252	0.075499754	-1.916698324	0.055276268	0.077004278	negative
PRJNA729271	stage_E18_vs_E10	ordinary_muscle_development	ENSGALG00010029227	UTP18	-0.652172697	0.081322203	-8.019614228	1.06078E-15	5.68428E-15	negative
PRJNA729271	stage_E18_vs_E10	ordinary_muscle_development	ENSGALG00010029228	ERAL1	-0.062260718	0.119884368	-0.519339755	0.603523835	0.654636154	negative
PRJNA729271	stage_E18_vs_E10	ordinary_muscle_development	ENSGALG00010029229	CBX4	0.481062444	0.222204018	2.164958355	0.030390875	0.044375514	positive
PRJNA729271	stage_E18_vs_E10	ordinary_muscle_development	ENSGALG00010029230	PPM1E	-0.262720408	0.167049895	-1.572706215	0.11578686	0.15180269	negative
PRJNA729271	stage_E18_vs_E10	ordinary_muscle_development	ENSGALG00010029231	CBX8	-0.232266248	0.108773436	-2.135321416	0.032734765	0.047491784	negative
PRJNA729271	stage_E18_vs_E10	ordinary_muscle_development	ENSGALG00010029232	CA10	-1.542711276	0.382979666	-4.028180642	5.62101E-05	0.000120491	negative
PRJNA729271	stage_E18_vs_E10	ordinary_muscle_development	ENSGALG00010029233	CBX2	-1.934146636	0.160390073	-12.05901715	1.73845E-33	2.46806E-32	negative
PRJNA729271	stage_E18_vs_E10	ordinary_muscle_development	ENSGALG00010029234	TOM1L1	-1.624481001	0.23876344	-6.803725908	1.01947E-11	4.00794E-11	negative
PRJNA729271	stage_E18_vs_E10	ordinary_muscle_development	ENSGALG00010029235	TRIM37	-0.475411	0.076319555	-6.229216083	4.68775E-10	1.6326E-09	negative
PRJNA729271	stage_E18_vs_E10	ordinary_muscle_development	ENSGALG00010029237	COX11	-0.826911658	0.109838836	-7.528408817	5.13624E-14	2.41328E-13	negative
PRJNA729271	stage_E18_vs_E10	ordinary_muscle_development	ENSGALG00010029238	CAMKK1	-0.286325757	0.249713529	-1.146616917	0.251539981	0.305111103	negative
PRJNA729271	stage_E18_vs_E10	ordinary_muscle_development	ENSGALG00010029239	RBFOX3	0.105274987	0.381755475	0.275765494	0.782728189	0.81788099	positive
PRJNA729271	stage_E18_vs_E10	ordinary_muscle_development	ENSGALG00010029240	MIS12	-1.633582858	0.250327804	-6.52577473	6.76509E-11	2.50089E-10	negative
PRJNA729271	stage_E18_vs_E10	ordinary_muscle_development	ENSGALG00010029241	STXBP4	-1.032454834	0.093969937	-10.98707588	4.40982E-28	4.91317E-27	negative
PRJNA729271	stage_E18_vs_E10	ordinary_muscle_development	ENSGALG00010029242	DERL2	-0.227573838	0.099471663	-2.287825811	0.022147667	0.033136915	negative
PRJNA729271	stage_E18_vs_E10	ordinary_muscle_development	ENSGALG00010029243	NME1	-0.061401575	0.35017096	-0.175347422	0.860806652	0.884858692	negative
PRJNA729271	stage_E18_vs_E10	ordinary_muscle_development	ENSGALG00010029244	HLF	2.480592526	0.182538031	13.58945595	4.62506E-42	9.60461E-41	positive
PRJNA729271	stage_E18_vs_E10	ordinary_muscle_development	ENSGALG00010029245	SKA2	-0.808507358	0.114919784	-7.035406164	1.98681E-12	8.29699E-12	negative

PRJNA729271	stage_E18_vs_E10	ordinary_muscle_development	ENSGALG00010029246	DHX33	-1.171619615	0.136513808	-8.582425675	9.28932E-18	5.70787E-17	negative
PRJNA729271	stage_E18_vs_E10	ordinary_muscle_development	ENSGALG00010029247	CCDC92B	3.628603624	0.557012252	6.5144054	7.29782E-11	2.69028E-10	positive
PRJNA729271	stage_E18_vs_E10	ordinary_muscle_development	ENSGALG00010029248	C1QBP	-0.205843066	0.081594496	-2.522756763	0.011643891	0.018266039	negative
PRJNA729271	stage_E18_vs_E10	ordinary_muscle_development	ENSGALG00010029249	PRR11	-1.160088068	0.276743929	-4.191918766	2.76605E-05	6.1315E-05	negative
PRJNA729271	stage_E18_vs_E10	ordinary_muscle_development	ENSGALG00010029250	SPAG9	0.195701879	0.087699703	2.231499905	0.025648034	0.037942914	positive
PRJNA729271	stage_E18_vs_E10	ordinary_muscle_development	ENSGALG00010029251	ENGASE	-1.649277427	0.220710057	-7.47259754	7.8627E-14	3.65171E-13	negative
PRJNA729271	stage_E18_vs_E10	ordinary_muscle_development	ENSGALG00010029252	RPAIN	0.154626927	0.144849038	1.067504002	0.285744307	0.341540673	positive
PRJNA729271	stage_E18_vs_E10	ordinary_muscle_development	ENSGALG00010029253	NUP88	-0.657826973	0.086939996	-7.566448133	3.83567E-14	1.81871E-13	negative
PRJNA729271	stage_E18_vs_E10	ordinary_muscle_development	ENSGALG00010029254	MMD	-0.339575931	0.119291285	-2.846611386	0.004418727	0.007376419	negative
PRJNA729271	stage_E18_vs_E10	ordinary_muscle_development	ENSGALG00010029255	RAP1GAP2	1.406806675	0.185916192	7.566886233	3.82276E-14	1.81319E-13	positive
PRJNA729271	stage_E18_vs_E10	ordinary_muscle_development	ENSGALG00010029256	RABEP1	-0.30917353	0.097209944	-3.180472267	0.001470352	0.00262537	negative
PRJNA729271	stage_E18_vs_E10	ordinary_muscle_development	ENSGALG00010029257	YPEL2	2.627890578	0.160194242	16.40440099	1.77872E-60	7.24231E-59	positive
PRJNA729271	stage_E18_vs_E10	ordinary_muscle_development	ENSGALG00010029258	TMEM100	0.381616264	0.235219202	1.622385676	0.104720794	0.138345599	positive
PRJNA729271	stage_E18_vs_E10	ordinary_muscle_development	ENSGALG00010029259	PTRH2	-0.267580753	0.108909727	-2.45690408	0.01401401	0.021725462	negative
PRJNA729271	stage_E18_vs_E10	ordinary_muscle_development	ENSGALG00010029260	DHX40	-0.102959247	0.104110059	-0.988946202	0.322689464	0.380553558	negative
PRJNA729271	stage_E18_vs_E10	ordinary_muscle_development	ENSGALG00010029261	IFT22	-0.753057214	0.093401568	-8.062575681	7.47035E-16	4.05401E-15	negative
PRJNA729271	stage_E18_vs_E10	ordinary_muscle_development	ENSGALG00010029262	CLTC	-0.598750245	0.065329927	-9.165022384	4.95367E-20	3.48222E-19	negative
PRJNA729271	stage_E18_vs_E10	ordinary_muscle_development	ENSGALG00010029263	TOB1	1.855547639	0.188691745	9.833751027	8.0561E-23	6.69955E-22	positive
PRJNA729271	stage_E18_vs_E10	ordinary_muscle_development	ENSGALG00010029264	COL26A1	-3.715995528	0.178318194	-20.83912718	1.91295E-96	2.06927E-94	negative
PRJNA729271	stage_E18_vs_E10	ordinary_muscle_development	ENSGALG00010029266	CANT1	-0.01840417	0.113428625	-0.162253308	0.871106382	0.893861462	negative
PRJNA729271	stage_E18_vs_E10	ordinary_muscle_development	ENSGALG00010029267	ENSGALG00010029267	2.363312121	0.285415241	8.280258996	1.22908E-16	7.01673E-16	positive
PRJNA729271	stage_E18_vs_E10	ordinary_muscle_development	ENSGALG00010029268	PCTP	0.288178648	0.182266093	1.581087534	0.113858025	0.149490223	positive
PRJNA729271	stage_E18_vs_E10	ordinary_muscle_development	ENSGALG00010029269	WSCD1	1.149875748	0.153617697	7.485307831	7.13796E-14	3.32257E-13	positive
PRJNA729271	stage_E18_vs_E10	ordinary_muscle_development	ENSGALG00010029270	TIMP2	0.740596642	0.149571618	4.951451694	7.36619E-07	1.93036E-06	positive
PRJNA729271	stage_E18_vs_E10	ordinary_muscle_development	ENSGALG00010029271	MYL10	-2.604240495	0.799754295	-3.256300729	0.001128741	0.00204335	negative
PRJNA729271	stage_E18_vs_E10	ordinary_muscle_development	ENSGALG00010029273	SLC13A5	-1.774612335	0.448946877	-3.952833682	7.72311E-05	0.000162997	negative
PRJNA729271	stage_E18_vs_E10	ordinary_muscle_development	ENSGALG00010029274	VMP1	-0.484525978	0.148370074	-3.265658386	0.001092099	0.00198048	negative
PRJNA729271	stage_E18_vs_E10	ordinary_muscle_development	ENSGALG00010029275	PCYT2	0.079515318	0.072090769	1.102988895	0.270031988	0.325092497	positive
PRJNA729271	stage_E18_vs_E10	ordinary_muscle_development	ENSGALG00010029276	TUBD1	0.894198313	0.171619471	5.210354687	1.8848E-07	5.20781E-07	positive
PRJNA729271	stage_E18_vs_E10	ordinary_muscle_development	ENSGALG00010029277	WFIKKN2	2.918434289	0.739604511	3.945939008	7.94878E-05	0.000167443	positive
PRJNA729271	stage_E18_vs_E10	ordinary_muscle_development	ENSGALG00010029278	MED31	-0.322383235	0.070568852	-4.568350261	4.91578E-06	1.17932E-05	negative
PRJNA729271	stage_E18_vs_E10	ordinary_muscle_development	ENSGALG00010029279	ENSGALG00010029279	0.85381108	0.15860261	5.383335635	7.3118E-08	2.09538E-07	positive
PRJNA729271	stage_E18_vs_E10	ordinary_muscle_development	ENSGALG00010029280	USP36	0.002555349	0.184408727	0.013856986	0.988944078	0.990721157	positive
PRJNA729271	stage_E18_vs_E10	ordinary_muscle_development	ENSGALG00010029281	KIAA0753	-0.55034616	0.133765151	-4.114271589	3.88404E-05	8.46349E-05	negative
PRJNA729271	stage_E18_vs_E10	ordinary_muscle_development	ENSGALG00010029282	LUC7L3	0.020034904	0.140890255	0.142202197	0.886920296	0.90670072	positive
PRJNA729271	stage_E18_vs_E10	ordinary_muscle_development	ENSGALG00010029283	RPS6KB1	-0.462628237	0.078247198	-5.912393642	3.37172E-09	1.08728E-08	negative
PRJNA729271	stage_E18_vs_E10	ordinary_muscle_development	ENSGALG00010029284	CYTH1	0.613901591	0.09385972	6.54062884	6.12607E-11	2.27045E-10	positive
PRJNA729271	stage_E18_vs_E10	ordinary_muscle_development	ENSGALG00010029285	CUX1	0.477029603	0.069558727	6.85794034	6.98604E-12	2.78118E-11	positive
PRJNA729271	stage_E18_vs_E10	ordinary_muscle_development	ENSGALG00010029286	MAFG	0.587435647	0.184184122	3.189393523	0.001425716	0.002550384	positive
PRJNA729271	stage_E18_vs_E10	ordinary_muscle_development	ENSGALG00010029287	ANKRD40	0.374329746	0.127239131	2.94193888	0.003261643	0.005547702	positive
PRJNA729271	stage_E18_vs_E10	ordinary_muscle_development	ENSGALG00010029288	PYCR1	-0.097331734	0.293174819	-0.331992133	0.739895199	0.780945536	negative
PRJNA729271	stage_E18_vs_E10	ordinary_muscle_development	ENSGALG00010029289	PITPNM3	-1.441656967	0.22877521	-6.301631052	2.9453E-10	1.03773E-09	negative
PRJNA729271	stage_E18_vs_E10	ordinary_muscle_development	ENSGALG00010029290	ENSGALG00010029290	-1.525165657	0.198442673	-7.685673821	1.52194E-14	7.48575E-14	negative
PRJNA729271	stage_E18_vs_E10	ordinary_muscle_development	ENSGALG00010029291	RNFT1	-0.773102137	0.116777209	-6.620316975	3.58429E-11	1.35122E-10	negative
PRJNA729271	stage_E18_vs_E10	ordinary_muscle_development	ENSGALG00010029292	ENSGALG00010029292	-0.338961074	0.252754011	-1.341070996	0.179897403	0.226080532	negative
PRJNA729271	stage_E18_vs_E10	ordinary_muscle_development	ENSGALG00010029293	DNAH17	-1.102827079	0.141865671	-7.773741698	7.6201E-15	3.8417E-14	negative
PRJNA729271	stage_E18_vs_E10	ordinary_muscle_development	ENSGALG00010029294	PGS1	-0.397948533	0.076317785	-5.214361646	1.84451E-07	5.10038E-07	negative
PRJNA729271	stage_E18_vs_E10	ordinary_muscle_development	ENSGALG00010029295	SOCS3	1.70935404	0.251757553	6.789683258	1.1238E-11	4.40375E-11	positive
PRJNA729271	stage_E18_vs_E10	ordinary_muscle_development	ENSGALG00010029296	FBXO39	-0.931417607	0.1597516	-5.830411744	5.52908E-09	1.7491E-08	negative
PRJNA729271	stage_E18_vs_E10	ordinary_muscle_development	ENSGALG00010029297	SH2B2	1.765357383	0.253766194	6.956629479	3.4851E-12	1.43028E-11	positive
PRJNA729271	stage_E18_vs_E10	ordinary_muscle_development	ENSGALG00010029298	ENDOV	0.237914837	0.147778665	1.609940355	0.107410878	0.14162837	positive
PRJNA729271	stage_E18_vs_E10	ordinary_muscle_development	ENSGALG00010029299	PRKRIP1	-0.044413304	0.091618248	-0.484764821	0.627843179	0.678085604	negative
PRJNA729271	stage_E18_vs_E10	ordinary_muscle_development	ENSGALG00010029300	TEKT1	1.495010328	0.345730272	4.324210088	1.53079E-05	3.49651E-05	positive
PRJNA729271	stage_E18_vs_E10	ordinary_muscle_development	ENSGALG00010029301	RNF213	1.044003621	0.465903775	2.240813827	0.025038137	0.037139561	positive
PRJNA729271	stage_E18_vs_E10	ordinary_muscle_development	ENSGALG00010029303	SLC26A11	-0.168999547	0.15575075	-1.085064098	0.277893267	0.333448916	negative
PRJNA729271	stage_E18_vs_E10	ordinary_muscle_development	ENSGALG00010029304	ORAI3	-0.364273894	0.119202815	-3.05591688	0.002243734	0.003907597	negative
PRJNA729271	stage_E18_vs_E10	ordinary_muscle_development	ENSGALG00010029305	SMTNL2	2.218571788	0.189961596	11.67905425	1.631E-31	2.15706E-30	positive
PRJNA729271	stage_E18_vs_E10	ordinary_muscle_development	ENSGALG00010029306	CEP95	-0.567198918	0.15900936	-3.567078804	0.000360983	0.000699151	negative
PRJNA729271	stage_E18_vs_E10	ordinary_muscle_development	ENSGALG00010029307	SGSH	-0.274388045	0.175114717	-1.566904538	0.117137007	0.153447891	negative
PRJNA729271	stage_E18_vs_E10	ordinary_muscle_development	ENSGALG00010029308	GAA	0.586105495	0.136976341	4.278881229	1.87835E-05	4.24888E-05	positive
PRJNA729271	stage_E18_vs_E10	ordinary_muscle_development	ENSGALG00010029309	ALKBH4	0.447363525	0.186346799	2.400704101	0.016363562	0.025086181	positive
PRJNA729271	stage_E18_vs_E10	ordinary_muscle_development	ENSGALG00010029310	LRWD1	-0.608779255	0.091644106	-6.642863147	3.07647E-11	1.16432E-10	negative
PRJNA729271	stage_E18_vs_E10	ordinary_muscle_development	ENSGALG00010029311	ENSGALG00010029311	0.511684907	0.167178588	3.060708388	0.00220814	0.003849771	positive
PRJNA729271	stage_E18_vs_E10	ordinary_muscle_development	ENSGALG00010029312	SMURF2	0.461336108	0.094196813	4.897576625	9.70258E-07	2.51186E-06	positive
PRJNA729271	stage_E18_vs_E10	ordinary_muscle_development	ENSGALG00010029313	CCDC40	-0.93768779	0.238632589	-3.929420516	8.51508E-05	0.000178827	negative
PRJNA729271	stage_E18_vs_E10	ordinary_muscle_development	ENSGALG00010029314	MED13	0.240642304	0.12618286	1.907091846	0.056508695	0.078577661	positive
PRJNA729271	stage_E18_vs_E10	ordinary_muscle_development	ENSGALG00010029315	TBC1D16	-0.67677079	0.097985129	-6.906872493	4.95455E-12	2.00941E-11	negative
PRJNA729271	stage_E18_vs_E10	ordinary_muscle_development	ENSGALG00010029316	INTS2	-0.538379233	0.095290278	-5.649886262	1.60554E-08	4.8748E-08	negative
PRJNA729271	stage_E18_vs_E10	ordinary_muscle_development	ENSGALG00010029317	KPNA2	-2.118161171	0.204827173	-10.34121174	4.587E-25	4.35705E-24	negative
PRJNA729271	stage_E18_vs_E10	ordinary_muscle_development	ENSGALG00010029318	C17orf58	4.347466992	0.201912238	21.53146855	7.8991E-103	1.01325E-100	positive
PRJNA729271	stage_E18_vs_E10	ordinary_muscle_development	ENSGALG00010029319	BRIP1	-0.843662615	0.124908787	-6.754229513	1.43596E-11	5.58773E-11	negative
PRJNA729271	stage_E18_vs_E10	ordinary_muscle_development	ENSGALG00010029320	TBX4	-1.845471042	0.604363139	-3.05357975	0.002261286	0.003936588	negative
PRJNA729271	stage_E18_vs_E10	ordinary_muscle_development	ENSGALG00010029321	TBX2	-0.56038639	0.171907578	-3.259812019	0.001114861	0.002019231	negative
PRJNA729271	stage_E18_vs_E10	ordinary_muscle_development	ENSGALG00010029322	BPTF	-0.507685619	0.097397302	-5.212522406	1.8629E-07	5.14926E-07	negative
PRJNA729271	stage_E18_vs_E10	ordinary_muscle_development	ENSGALG00010029323	NOL11	-0.255525505	0.091910243	-2.780163516	0.005433153	0.008957411	negative
PRJNA729271	stage_E18_vs_E10	ordinary_muscle_development	ENSGALG00010029324	BCAS3	0.899024396	0.126884443	7.085379244	1.38664E-12	5.865E-12	positive
PRJNA729271	stage_E18_vs_E10	ordinary_muscle_development	ENSGALG00010029325	LRRC75A	-0.458958176	0.167419025	-2.741374088	0.006118281	0.010001633	negative
PRJNA729271	stage_E18_vs_E10	ordinary_muscle_development	ENSGALG00010029326	NCBP3	0.312908495	0.079770835	3.922592685	8.76012E-05	0.000183786	positive
PRJNA729271	stage_E18_vs_E10	ordinary_muscle_development	ENSGALG00010029327	CRK	-0.417673598	0.054909582	-7.606570319	2.81465E-14	1.34782E-13	negative
PRJNA729271	stage_E18_vs_E10	ordinary_muscle_development	ENSGALG00010029328	ENSGALG00010029328	0.214362412	0.081922394	2.616652192	0.008879677	0.014156502	positive
PRJNA729271	stage_E18_vs_E10	ordinary_muscle_development	ENSGALG00010029329	ENSGALG00010029329	1.625527538	0.177021204	9.182671332	4.20527E-20	2.96765E-19	positive
PRJNA729271	stage_E18_vs_E10	ordinary_muscle_development	ENSGALG00010029330	AFMID	0.544836401	0.130768507	4.166419051	3.09422E-05	6.81207E-05	positive
PRJNA729271	stage_E18_vs_E10	ordinary_muscle_development	ENSGALG00010029331	TK1	-1.360310999	0.27728995	-4.905734946	9.30781E-07	2.41527E-06	negative
PRJNA729271	stage_E18_vs_E10	ordinary_muscle_development	ENSGALG00010029332	YWHAE	-0.577569246	0.148753757	-3.882720388	0.000103294	0.000215122	negative
PRJNA729271	stage_E18_vs_E10	ordinary_muscle_development	ENSGALG00010029333	ENSGALG00010029333	-0.752418268	0.175290932	-4.292396991	1.76755E-05	4.00697E-05	negative
PRJNA729271	stage_E18_vs_E10	ordinary_muscle_development	ENSGALG00010029335	RNF222	0.690585166	1.496064616	0.461601163	0.64436736	0.693400511	positive
PRJNA729271	stage_E18_vs_E10	ordinary_muscle_development	ENSGALG00010029336	SYNGR2	1.03604923	0.111204284	9.316630564	1.20094E-20	8.81395E-20	positive
PRJNA729271	stage_E18_vs_E10	ordinary_muscle_development	ENSGALG00010029337	MYO1C	1.181809004	0.133836685	8.830232218	1.04459E-18	6.83271E-18	positive
PRJNA729271	stage_E18_vs_E10	ordinary_muscle_development	ENSGALG00010029338	INPP5K	-0.035789421	0.120732982	-0.296434495	0.766898285	0.804355329	negative
PRJNA729271	stage_E18_vs_E10	ordinary_muscle_development	ENSGALG00010029339	PITPNA	0.634899299	0.127088516	4.995725157	5.86151E-07	1.54843E-06	positive
PRJNA729271	stage_E18_vs_E10	ordinary_muscle_development	ENSGALG00010029340	NDEL1	0.37551718	0.104675193	3.587451514	0.000333926	0.000649437	positive
PRJNA729271	stage_E18_vs_E10	ordinary_muscle_development	ENSGALG00010029341	SLC43A2	1.775696778	0.326495994	5.438647974	5.36864E-08	1.55636E-07	positive
PRJNA729271	stage_E18_vs_E10	ordinary_muscle_development	ENSGALG00010029342	TMC6	-0.483246882	0.131782049	-3.667016008	0.000245397	0.000486332	negative
PRJNA729271	stage_E18_vs_E10	ordinary_muscle_development	ENSGALG00010029343	SCARF1	1.504658493	0.130891116	11.4954975	1.38978E-30	1.74414E-29	positive
PRJNA729271	stage_E18_vs_E10	ordinary_muscle_development	ENSGALG00010029344	ENSGALG00010029344	1.100900861	0.16578383	6.640580454	3.1245E-11	1.18126E-10	positive
PRJNA729271	stage_E18_vs_E10	ordinary_muscle_development	ENSGALG00010029345	RILP	1.468805024	0.246136709	5.96743586	2.41011E-09	7.86991E-09	positive
PRJNA729271	stage_E18_vs_E10	ordinary_muscle_development	ENSGALG00010029346	ARL16	-0.469988247	0.125366246	-3.748921759	0.000177596	0.000358582	negative
PRJNA729271	stage_E18_vs_E10	ordinary_muscle_development	ENSGALG00010029347	PRPF8	-0.622535439	0.070157503	-8.873397901	7.09473E-19	4.68938E-18	negative
PRJNA729271	stage_E18_vs_E10	ordinary_muscle_development	ENSGALG00010029348	HGS	0.205730382	0.124171604	1.656823106	0.097555244	0.129659202	positive
PRJNA729271	stage_E18_vs_E10	ordinary_muscle_development	ENSGALG00010029349	MYH10	-1.669975707	0.107141577	-15.58662613	8.97501E-55	3.02541E-53	negative
PRJNA729271	stage_E18_vs_E10	ordinary_muscle_development	ENSGALG00010029350	MRPL12	0.372988279	0.121846424	3.061134382	0.002205001	0.003845223	positive
PRJNA729271	stage_E18_vs_E10	ordinary_muscle_development	ENSGALG00010029351	ENSGALG00010029351	-0.951908491	0.202844532	-4.692798379	2.69493E-06	6.63998E-06	negative
PRJNA729271	stage_E18_vs_E10	ordinary_muscle_development	ENSGALG00010029352	AATF	-0.52118605	0.11074388	-4.706228907	2.52341E-06	6.23859E-06	negative
PRJNA729271	stage_E18_vs_E10	ordinary_muscle_development	ENSGALG00010029353	GCGR	4.866495984	0.885525775	5.495600606	3.89383E-08	1.14624E-07	positive
PRJNA729271	stage_E18_vs_E10	ordinary_muscle_development	ENSGALG00010029354	TLCD2	-1.085903243	0.29442549	-3.688210697	0.000225837	0.000449595	negative
PRJNA729271	stage_E18_vs_E10	ordinary_muscle_development	ENSGALG00010029355	MCRIP1	0.752432289	0.172510258	4.36166695	1.29075E-05	2.97211E-05	positive
PRJNA729271	stage_E18_vs_E10	ordinary_muscle_development	ENSGALG00010029357	ARHGDIA	-0.513170389	0.097427535	-5.267200799	1.3852E-07	3.86868E-07	negative
PRJNA729271	stage_E18_vs_E10	ordinary_muscle_development	ENSGALG00010029359	ENSGALG00010029359	-1.053214352	0.513185611	-2.052306864	0.040139849	0.057305931	negative
PRJNA729271	stage_E18_vs_E10	ordinary_muscle_development	ENSGALG00010029360	ACACA	-0.708551871	0.086274887	-8.212724401	2.16225E-16	1.21198E-15	negative
PRJNA729271	stage_E18_vs_E10	ordinary_muscle_development	ENSGALG00010029361	ALYREF	-0.905489013	0.134433001	-6.73561555	1.63237E-11	6.31135E-11	negative
PRJNA729271	stage_E18_vs_E10	ordinary_muscle_development	ENSGALG00010029362	TADA2A	0.049842575	0.11165016	0.446417407	0.655295761	0.703385076	positive
PRJNA729271	stage_E18_vs_E10	ordinary_muscle_development	ENSGALG00010029363	PIK3R6	1.779446788	0.192612018	9.238503415	2.49969E-20	1.79638E-19	positive
PRJNA729271	stage_E18_vs_E10	ordinary_muscle_development	ENSGALG00010029364	ANAPC11	-0.658696934	0.133927352	-4.918315235	8.72923E-07	2.27409E-06	negative
PRJNA729271	stage_E18_vs_E10	ordinary_muscle_development	ENSGALG00010029365	PIK3R5	1.396902381	0.188271595	7.419613029	1.17463E-13	5.37276E-13	positive
PRJNA729271	stage_E18_vs_E10	ordinary_muscle_development	ENSGALG00010029366	MGAT5B	1.125350699	0.348044209	3.233355617	0.001223452	0.002205987	positive
PRJNA729271	stage_E18_vs_E10	ordinary_muscle_development	ENSGALG00010029367	DUSP14	-0.229665047	0.117479708	-1.954933758	0.050590889	0.07097512	negative
PRJNA729271	stage_E18_vs_E10	ordinary_muscle_development	ENSGALG00010029368	DDX52	-0.476409139	0.094181888	-5.05839446	4.22801E-07	1.13239E-06	negative
PRJNA729271	stage_E18_vs_E10	ordinary_muscle_development	ENSGALG00010029369	MFSD11	0.184408998	0.085014217	2.169154808	0.030070933	0.043930475	positive
PRJNA729271	stage_E18_vs_E10	ordinary_muscle_development	ENSGALG00010029370	SRSF2	-0.953181093	0.126289594	-7.547582208	4.43413E-14	2.09085E-13	negative
PRJNA729271	stage_E18_vs_E10	ordinary_muscle_development	ENSGALG00010029371	AUTS2	-0.671817656	0.169330855	-3.967485177	7.2635E-05	0.000153835	negative
PRJNA729271	stage_E18_vs_E10	ordinary_muscle_development	ENSGALG00010029372	METTL23	-1.180174874	0.167413327	-7.049467882	1.79603E-12	7.54157E-12	negative
PRJNA729271	stage_E18_vs_E10	ordinary_muscle_development	ENSGALG00010029373	GATSL2	1.500328593	0.168818354	8.887236246	6.26477E-19	4.15597E-18	positive
PRJNA729271	stage_E18_vs_E10	ordinary_muscle_development	ENSGALG00010029374	RCC1L	-0.156649187	0.118143853	-1.325919069	0.184866506	0.231742628	negative
PRJNA729271	stage_E18_vs_E10	ordinary_muscle_development	ENSGALG00010029375	JMJD6	-0.729075712	0.163121364	-4.469529283	7.83919E-06	1.84643E-05	negative
PRJNA729271	stage_E18_vs_E10	ordinary_muscle_development	ENSGALG00010029376	ENSGALG00010029376	0.255036664	0.206419502	1.235526011	0.216634802	0.267267125	positive
PRJNA729271	stage_E18_vs_E10	ordinary_muscle_development	ENSGALG00010029377	NCF1C	0.852865915	0.303554611	2.809596315	0.004960367	0.008227546	positive
PRJNA729271	stage_E18_vs_E10	ordinary_muscle_development	ENSGALG00010029378	ST6GALNAC1	-2.571390418	0.369041275	-6.967758331	3.22031E-12	1.32496E-11	negative
PRJNA729271	stage_E18_vs_E10	ordinary_muscle_development	ENSGALG00010029379	GTF2I	0.091635507	0.112911125	0.81571996	0.417037265	0.477333793	positive
PRJNA729271	stage_E18_vs_E10	ordinary_muscle_development	ENSGALG00010029380	RGS9	0.742009709	0.147044579	5.0461548	4.5079E-07	1.20402E-06	positive
PRJNA729271	stage_E18_vs_E10	ordinary_muscle_development	ENSGALG00010029382	GTF2IRD1	-0.138719959	0.206873471	-0.67054608	0.502504307	0.55968956	negative
PRJNA729271	stage_E18_vs_E10	ordinary_muscle_development	ENSGALG00010029383	GNA13	-0.282410186	0.079588608	-3.5483745	0.000387617	0.000747639	negative

PRJNA729271	stage_E18_vs_E10	ordinary_muscle_development	ENSGALG00010029384	WDR81	-0.352699829	0.076288321	-4.623248036	3.77778E-06	9.15853E-06	negative
PRJNA729271	stage_E18_vs_E10	ordinary_muscle_development	ENSGALG00010029385	CLIP2	-0.424138696	0.122566617	-3.46047485	0.000539224	0.001020105	negative
PRJNA729271	stage_E18_vs_E10	ordinary_muscle_development	ENSGALG00010029386	SLC16A6	0.163063529	0.237933812	0.685331467	0.49313478	0.550565249	positive
PRJNA729271	stage_E18_vs_E10	ordinary_muscle_development	ENSGALG00010029387	SERPINF1	1.671971272	0.175971056	9.501399325	2.07089E-21	1.59667E-20	positive
PRJNA729271	stage_E18_vs_E10	ordinary_muscle_development	ENSGALG00010029388	ST6GALNAC2	0.892371403	0.339842504	2.625838123	0.008643591	0.013812023	positive
PRJNA729271	stage_E18_vs_E10	ordinary_muscle_development	ENSGALG00010029389	SMYD4	-0.182726348	0.098100461	-1.862645148	0.062512208	0.086165315	negative
PRJNA729271	stage_E18_vs_E10	ordinary_muscle_development	ENSGALG00010029390	RPA1	-1.161716329	0.080923232	-14.35578263	9.80128E-47	2.52793E-45	negative
PRJNA729271	stage_E18_vs_E10	ordinary_muscle_development	ENSGALG00010029391	ARSG	0.14923108	0.14140315	1.055358954	0.291261188	0.347579672	positive
PRJNA729271	stage_E18_vs_E10	ordinary_muscle_development	ENSGALG00010029392	RTN4RL1	1.48935904	0.552461312	2.695861246	0.00702069	0.011353889	positive
PRJNA729271	stage_E18_vs_E10	ordinary_muscle_development	ENSGALG00010029394	CYGB	-0.115779049	0.142985667	-0.809724863	0.418098325	0.478434927	negative
PRJNA729271	stage_E18_vs_E10	ordinary_muscle_development	ENSGALG00010029395	DPH1	-0.485694515	0.122354832	-3.969557282	7.20063E-05	0.000152548	negative
PRJNA729271	stage_E18_vs_E10	ordinary_muscle_development	ENSGALG00010029396	WIPI1	0.74113721	0.154759049	4.788974941	1.67635E-06	4.22955E-06	positive
PRJNA729271	stage_E18_vs_E10	ordinary_muscle_development	ENSGALG00010029397	OVCA2	-0.671781032	0.157530044	-4.264462916	2.00384E-05	4.51439E-05	negative
PRJNA729271	stage_E18_vs_E10	ordinary_muscle_development	ENSGALG00010029398	RHBDF2	0.182856598	0.126796121	1.442130852	0.149265463	0.191164772	positive
PRJNA729271	stage_E18_vs_E10	ordinary_muscle_development	ENSGALG00010029399	PRKAR1A	-0.401268396	0.053555192	-7.492614219	6.75151E-14	3.14875E-13	negative
PRJNA729271	stage_E18_vs_E10	ordinary_muscle_development	ENSGALG00010029400	AANAT	-0.107749714	0.711965976	-0.151341099	0.87970665	0.900391745	negative
PRJNA729271	stage_E18_vs_E10	ordinary_muscle_development	ENSGALG00010029401	HIC1	-0.605607106	0.202324641	-2.993244436	0.002760286	0.004753516	negative
PRJNA729271	stage_E18_vs_E10	ordinary_muscle_development	ENSGALG00010029402	SMG6	0.522154522	0.088442281	5.903901572	3.55004E-09	1.14376E-08	positive
PRJNA729271	stage_E18_vs_E10	ordinary_muscle_development	ENSGALG00010029403	FAM20A	2.462083587	0.156560975	15.72603635	1.00296E-55	3.51158E-54	positive
PRJNA729271	stage_E18_vs_E10	ordinary_muscle_development	ENSGALG00010029404	SRR	1.717702155	0.175198227	9.804335279	1.07855E-22	8.8928E-22	positive
PRJNA729271	stage_E18_vs_E10	ordinary_muscle_development	ENSGALG00010029405	POLDIP2	0.814735237	0.078896639	10.32661523	5.34122E-25	5.06019E-24	positive
PRJNA729271	stage_E18_vs_E10	ordinary_muscle_development	ENSGALG00010029406	TMEM199	0.145045157	0.103774798	1.397691541	0.162205715	0.205954086	positive
PRJNA729271	stage_E18_vs_E10	ordinary_muscle_development	ENSGALG00010029407	UBE2O	-0.429684354	0.190241924	-2.258620736	0.023906987	0.035549012	negative
PRJNA729271	stage_E18_vs_E10	ordinary_muscle_development	ENSGALG00010029408	KIF12	2.581558327	0.459579255	5.617221188	1.94053E-08	5.84903E-08	positive
PRJNA729271	stage_E18_vs_E10	ordinary_muscle_development	ENSGALG00010029409	ENSGALG00010029409	2.72780667	0.189216716	14.41630915	4.08617E-47	1.07105E-45	positive
PRJNA729271	stage_E18_vs_E10	ordinary_muscle_development	ENSGALG00010029410	VTN	4.65560924	0.879617998	5.292762597	1.20482E-07	3.38973E-07	positive
PRJNA729271	stage_E18_vs_E10	ordinary_muscle_development	ENSGALG00010029411	SARM1	-0.51366266	0.167252098	-3.071188143	0.002132088	0.003729288	negative
PRJNA729271	stage_E18_vs_E10	ordinary_muscle_development	ENSGALG00010029412	SLC46A1	1.009600716	0.105536517	9.566363815	1.10732E-21	8.71307E-21	positive
PRJNA729271	stage_E18_vs_E10	ordinary_muscle_development	ENSGALG00010029413	B3GNTL1	0.316700077	0.218330946	1.450550563	0.14690505	0.188424803	positive
PRJNA729271	stage_E18_vs_E10	ordinary_muscle_development	ENSGALG00010029416	UNC119	-2.106611668	0.451875954	-4.661924688	3.13266E-06	7.66378E-06	negative
PRJNA729271	stage_E18_vs_E10	ordinary_muscle_development	ENSGALG00010029417	ENSGALG00010029417	1.93745279	0.211776672	9.148565665	5.76945E-20	4.04196E-19	positive
PRJNA729271	stage_E18_vs_E10	ordinary_muscle_development	ENSGALG00010029418	ABCA5	0.106977877	0.133095901	0.803765381	0.421532487	0.481998943	positive
PRJNA729271	stage_E18_vs_E10	ordinary_muscle_development	ENSGALG00010029419	RFC2	-0.459380918	0.133028785	-3.453244479	0.000553887	0.001045798	negative
PRJNA729271	stage_E18_vs_E10	ordinary_muscle_development	ENSGALG00010029420	LAT2	1.434635193	0.297609491	4.820529031	1.43178E-06	3.63844E-06	positive
PRJNA729271	stage_E18_vs_E10	ordinary_muscle_development	ENSGALG00010029421	TBCD	-1.076150823	0.094865922	-11.34391365	7.95075E-30	9.6521E-29	negative
PRJNA729271	stage_E18_vs_E10	ordinary_muscle_development	ENSGALG00010029422	EIF4H	-0.249710517	0.087313133	-2.859942238	0.004237182	0.007091323	negative
PRJNA729271	stage_E18_vs_E10	ordinary_muscle_development	ENSGALG00010029423	MYBBP1A	-1.200784186	0.122150433	-9.830371884	8.33106E-23	6.9163E-22	negative
PRJNA729271	stage_E18_vs_E10	ordinary_muscle_development	ENSGALG00010029424	MAP2K6	-0.591904505	0.218987447	-2.702915228	0.006873427	0.011134369	negative
PRJNA729271	stage_E18_vs_E10	ordinary_muscle_development	ENSGALG00010029425	SPNS2	0.57007169	0.171070905	3.332370809	0.000861094	0.001583553	positive
PRJNA729271	stage_E18_vs_E10	ordinary_muscle_development	ENSGALG00010029426	ZNF750	-6.943845257	1.11331863	-6.23706913	4.45845E-10	1.55461E-09	negative
PRJNA729271	stage_E18_vs_E10	ordinary_muscle_development	ENSGALG00010029427	SPNS3	-0.943795121	0.321463046	-2.935936592	0.003325423	0.005644924	negative
PRJNA729271	stage_E18_vs_E10	ordinary_muscle_development	ENSGALG00010029428	FN3K	0.375934571	0.26214343	1.4340797	0.15154952	0.193781783	positive
PRJNA729271	stage_E18_vs_E10	ordinary_muscle_development	ENSGALG00010029429	UBE2G1	0.775119642	0.089919333	8.620166705	6.68567E-18	4.14849E-17	positive
PRJNA729271	stage_E18_vs_E10	ordinary_muscle_development	ENSGALG00010029430	FN3KRP	-0.305168887	0.09690503	-3.149154245	0.001637437	0.002909729	negative
PRJNA729271	stage_E18_vs_E10	ordinary_muscle_development	ENSGALG00010029432	ENSGALG00010029432	-0.708121651	5.461157295	-0.129665126			negative
PRJNA729271	stage_E18_vs_E10	ordinary_muscle_development	ENSGALG00010029436	WDR45B	-0.316320575	0.080491841	-3.92984647	8.50001E-05	0.000178536	negative
PRJNA729271	stage_E18_vs_E10	ordinary_muscle_development	ENSGALG00010029437	ENSGALG00010029437	-6.751173328	2.92589985	-2.307383599	0.021033445	0.031603585	negative
PRJNA729271	stage_E18_vs_E10	ordinary_muscle_development	ENSGALG00010029438	ENSGALG00010029438	-0.277366747	0.269124205	-1.030627278	0.302715639	0.359837887	negative
PRJNA729271	stage_E18_vs_E10	ordinary_muscle_development	ENSGALG00010029439	ENSGALG00010029439	-0.538142379	0.208593339	-2.579863674	0.009883933	0.015647401	negative
PRJNA729271	stage_E18_vs_E10	ordinary_muscle_development	ENSGALG00010029440	FOXK2	0.561865996	0.181674999	3.092698499	0.001983455	0.003484026	positive
PRJNA729271	stage_E18_vs_E10	ordinary_muscle_development	ENSGALG00010029442	ENSGALG00010029442	-0.137880666	0.281077849	-0.490542626	0.623749974	0.674267721	negative
PRJNA729271	stage_E18_vs_E10	ordinary_muscle_development	ENSGALG00010029443	ENSGALG00010029443	-0.850729505	0.318369679	-2.672143613	0.007536839	0.0121358	negative
PRJNA729271	stage_E18_vs_E10	ordinary_muscle_development	ENSGALG00010029445	SPHK1	0.026021683	0.139238473	0.186885724	0.851750232	0.877603044	positive
PRJNA729271	stage_E18_vs_E10	ordinary_muscle_development	ENSGALG00010029446	PRPSAP1	0.070769101	0.081312577	0.870334009	0.384117898	0.444427597	positive
PRJNA729271	stage_E18_vs_E10	ordinary_muscle_development	ENSGALG00010029449	UBALD2	1.250521856	0.182024059	6.87009104	6.41609E-12	2.56626E-11	positive
PRJNA729271	stage_E18_vs_E10	ordinary_muscle_development	ENSGALG00010029451	ENSGALG00010029451	-1.46114357	0.222847805	-6.556688197	5.50159E-11	2.04949E-10	negative
PRJNA729271	stage_E18_vs_E10	ordinary_muscle_development	ENSGALG00010029452	ENSGALG00010029452	-1.926191561	1.028194325	-1.873373073	0.061016886	0.084304619	negative
PRJNA729271	stage_E18_vs_E10	ordinary_muscle_development	ENSGALG00010029453	ENSGALG00010029453	-2.161109631	1.561707364	-1.383812154	0.166415981	0.21066693	negative
PRJNA729271	stage_E18_vs_E10	ordinary_muscle_development	ENSGALG00010029454	ENSGALG00010029454	-0.269118762	0.289985884	-0.928040903	0.353386353	0.41265892	negative
PRJNA729271	stage_E18_vs_E10	ordinary_muscle_development	ENSGALG00010029455	ENSGALG00010029455	-0.319023183	0.253863369	-1.25667277	0.208872152	0.258680951	negative
PRJNA729271	stage_E18_vs_E10	ordinary_muscle_development	ENSGALG00010029456	RNF157	-2.3446977	0.50431143	-4.649305096	3.33055E-06	8.12323E-06	negative
PRJNA729271	stage_E18_vs_E10	ordinary_muscle_development	ENSGALG00010029457	ENSGALG00010029457	-0.282466445	0.190674632	-1.481405483	0.138498555	0.178749581	negative
PRJNA729271	stage_E18_vs_E10	ordinary_muscle_development	ENSGALG00010029460	ENSGALG00010029460	-0.747550893	0.28154914	-2.655134704	0.007927674	0.012722724	negative
PRJNA729271	stage_E18_vs_E10	ordinary_muscle_development	ENSGALG00010029461	ENSGALG00010029461	0.096751939	0.200855597	0.481698992	0.630019792	0.680182995	positive
PRJNA729271	stage_E18_vs_E10	ordinary_muscle_development	ENSGALG00010029462	ENSGALG00010029462	2.829745256	1.804596575	1.568076375	0.116863309	0.153117027	positive
PRJNA729271	stage_E18_vs_E10	ordinary_muscle_development	ENSGALG00010029463	ENSGALG00010029463	0.158329076	0.419620123	0.377315261	0.705939332	0.75056541	positive
PRJNA729271	stage_E18_vs_E10	ordinary_muscle_development	ENSGALG00010029464	ENSGALG00010029464	-0.035025352	0.556481861	-0.06294069	0.949813733	0.958609529	negative
PRJNA729271	stage_E18_vs_E10	ordinary_muscle_development	ENSGALG00010029465	ENSGALG00010029465	-2.782321604	2.431993258	-1.144049884	0.25260295	0.306246216	negative
PRJNA729271	stage_E18_vs_E10	ordinary_muscle_development	ENSGALG00010029466	SLC39A11	-0.408240417	0.118178479	-3.45443961	0.000551438	0.001041581	negative
PRJNA729271	stage_E18_vs_E10	ordinary_muscle_development	ENSGALG00010029468	ENSGALG00010029468	-1.315626478	0.241272192	-5.452872414	4.95626E-08	1.44114E-07	negative
PRJNA729271	stage_E18_vs_E10	ordinary_muscle_development	ENSGALG00010029469	ENSGALG00010029469	-1.923074257	0.405752357	-4.739527016	2.14218E-06	5.33337E-06	negative
PRJNA729271	stage_E18_vs_E10	ordinary_muscle_development	ENSGALG00010029470	SSTR2	1.949995608	0.919296028	2.121183545	0.033906359	0.04904917	positive
PRJNA729271	stage_E18_vs_E10	ordinary_muscle_development	ENSGALG00010029472	ENSGALG00010029472	-0.200956162	0.79687427	-0.252180512	0.80090154	0.833805058	negative
PRJNA729271	stage_E18_vs_E10	ordinary_muscle_development	ENSGALG00010029473	COG1	0.72878787	0.100765408	7.232520445	4.74111E-13	2.07935E-12	positive
PRJNA729271	stage_E18_vs_E10	ordinary_muscle_development	ENSGALG00010029474	ENSGALG00010029474	-0.347922205	1.058810575	-0.328597214	0.742460154	0.783029681	negative
PRJNA729271	stage_E18_vs_E10	ordinary_muscle_development	ENSGALG00010029477	FAM104A	-0.229010556	0.059483529	-3.849982666	0.000118126	0.000244187	negative
PRJNA729271	stage_E18_vs_E10	ordinary_muscle_development	ENSGALG00010029479	ENSGALG00010029479	0.527669626	0.545856599	0.966681774	0.333703141	0.392011268	positive
PRJNA729271	stage_E18_vs_E10	ordinary_muscle_development	ENSGALG00010029480	ENSGALG00010029480	0.577301858	0.116224313	4.967135024	6.79493E-07	1.78558E-06	positive
PRJNA729271	stage_E18_vs_E10	ordinary_muscle_development	ENSGALG00010029481	ENSGALG00010029481	0.032090942	0.274968343	0.116707771	0.907091632	0.923400042	positive
PRJNA729271	stage_E18_vs_E10	ordinary_muscle_development	ENSGALG00010029482	DDX25	-0.146818219	0.18611442	-0.788859991	0.430193842	0.490186285	negative
PRJNA729271	stage_E18_vs_E10	ordinary_muscle_development	ENSGALG00010029484	ENSGALG00010029484	-1.126921869	0.538590124	-2.092355241	0.03640675	0.052368347	negative
PRJNA729271	stage_E18_vs_E10	ordinary_muscle_development	ENSGALG00010029487	CDC42EP4	-1.534516826	0.145055591	-10.57881891	3.73638E-26	3.77151E-25	negative
PRJNA729271	stage_E18_vs_E10	ordinary_muscle_development	ENSGALG00010029489	ENSGALG00010029489	-2.023855004	1.012947742	-1.997985603	0.045718221	0.064620733	negative
PRJNA729271	stage_E18_vs_E10	ordinary_muscle_development	ENSGALG00010029490	ENSGALG00010029490	-1.220094787	0.642182894	-1.899917918	0.057443892	0.079816835	negative
PRJNA729271	stage_E18_vs_E10	ordinary_muscle_development	ENSGALG00010029491	ENSGALG00010029491	-0.045440227	0.199268099	-0.228035633	0.819618539	0.850055146	negative
PRJNA729271	stage_E18_vs_E10	ordinary_muscle_development	ENSGALG00010029492	SDK2	0.986457515	0.224320172	4.397542613	1.09483E-05	2.53833E-05	positive
PRJNA729271	stage_E18_vs_E10	ordinary_muscle_development	ENSGALG00010029494	MYH7	-1.088676279	0.503176465	-2.163607312	0.0304945	0.044517854	negative
PRJNA729271	stage_E18_vs_E10	ordinary_muscle_development	ENSGALG00010029495	RPL38	0.897696793	0.188581741	4.760252971	1.9335E-06	4.8396E-06	positive
PRJNA729271	stage_E18_vs_E10	ordinary_muscle_development	ENSGALG00010029496	TTYH2	0.053122765	0.145141661	0.366006319	0.71436036	0.757916368	positive
PRJNA729271	stage_E18_vs_E10	ordinary_muscle_development	ENSGALG00010029497	ENSGALG00010029497	-0.361769209	0.585086277	-0.618317713	0.536365928	0.591766184	negative
PRJNA729271	stage_E18_vs_E10	ordinary_muscle_development	ENSGALG00010029498	ENSGALG00010029498	0.471219391	0.148528494	3.172585812	0.001510879	0.002695076	positive
PRJNA729271	stage_E18_vs_E10	ordinary_muscle_development	ENSGALG00010029501	ENSGALG00010029501	-0.564722875	0.391870061	-1.44109727	0.149557205	0.191521485	negative
PRJNA729271	stage_E18_vs_E10	ordinary_muscle_development	ENSGALG00010029502	BTBD17	1.226761542	0.418362651	2.932292213	0.0033647	0.005704489	positive
PRJNA729271	stage_E18_vs_E10	ordinary_muscle_development	ENSGALG00010029504	GPR142	-1.198164001	0.42720268	-2.804673419	0.005036758	0.008346612	negative
PRJNA729271	stage_E18_vs_E10	ordinary_muscle_development	ENSGALG00010029505	VEZF1	0.262219848	0.110048921	2.382757095	0.017183529	0.026218447	positive
PRJNA729271	stage_E18_vs_E10	ordinary_muscle_development	ENSGALG00010029506	OXLD1	-0.271466988	0.152978136	-1.774547621	0.075972612	0.103149402	negative
PRJNA729271	stage_E18_vs_E10	ordinary_muscle_development	ENSGALG00010029507	VPS37D	-0.063793087	0.172922251	-0.368911961	0.712193347	0.756115328	negative
PRJNA729271	stage_E18_vs_E10	ordinary_muscle_development	ENSGALG00010029508	ENSGALG00010029508	-0.897751322	0.145773256	-6.158546129	7.34158E-10	2.51278E-09	negative
PRJNA729271	stage_E18_vs_E10	ordinary_muscle_development	ENSGALG00010029509	DNAJC30	0.791414859	0.170438081	4.643415693	3.42696E-06	8.34292E-06	positive
PRJNA729271	stage_E18_vs_E10	ordinary_muscle_development	ENSGALG00010029510	PDE6G	-0.32032567	0.287021231	-1.11603476	0.264407256	0.319142574	negative
PRJNA729271	stage_E18_vs_E10	ordinary_muscle_development	ENSGALG00010029511	CUEDC1	0.632931466	0.072273057	8.757502303	1.99624E-18	1.28488E-17	positive
PRJNA729271	stage_E18_vs_E10	ordinary_muscle_development	ENSGALG00010029512	TSPAN10	-2.199187841	0.787885569	-2.791252851	0.005250444	0.00867889	negative
PRJNA729271	stage_E18_vs_E10	ordinary_muscle_development	ENSGALG00010029513	WBSCR22	-0.098077142	0.085748854	-1.143772046	0.252718186	0.3063603	negative
PRJNA729271	stage_E18_vs_E10	ordinary_muscle_development	ENSGALG00010029514	MRPS23	0.033326574	0.09895826	0.336774048	0.736287238	0.777621583	positive
PRJNA729271	stage_E18_vs_E10	ordinary_muscle_development	ENSGALG00010029515	NPLOC4	0.462694076	0.152671559	3.030650101	0.002440279	0.004229563	positive
PRJNA729271	stage_E18_vs_E10	ordinary_muscle_development	ENSGALG00010029516	ENSGALG00010029516	3.243417231	0.227941128	14.22918832	6.03785E-46	1.49095E-44	positive
PRJNA729271	stage_E18_vs_E10	ordinary_muscle_development	ENSGALG00010029517	MSI2	-0.490272498	0.115873033	-4.231118181	2.32532E-05	5.19667E-05	negative
PRJNA729271	stage_E18_vs_E10	ordinary_muscle_development	ENSGALG00010029518	FAAP100	0.088485183	0.186867303	0.47351881	0.635843084	0.68529597	positive
PRJNA729271	stage_E18_vs_E10	ordinary_muscle_development	ENSGALG00010029519	STX1A	1.391430805	0.105780404	13.15395623	1.61498E-39	3.03621E-38	positive
PRJNA729271	stage_E18_vs_E10	ordinary_muscle_development	ENSGALG00010029520	MKS1	-0.335981074	0.111426884	-3.015260442	0.002567587	0.00444013	negative
PRJNA729271	stage_E18_vs_E10	ordinary_muscle_development	ENSGALG00010029521	ACTG1	-0.2013743	0.098387589	-2.046744931	0.040683135	0.05801299	negative
PRJNA729271	stage_E18_vs_E10	ordinary_muscle_development	ENSGALG00010029522	AKAP1	0.530683074	0.112303056	4.725455336	2.296E-06	5.70361E-06	positive
PRJNA729271	stage_E18_vs_E10	ordinary_muscle_development	ENSGALG00010029523	FSCN2	1.636698894	0.695063199	2.35474831	0.018535255	0.028091648	positive
PRJNA729271	stage_E18_vs_E10	ordinary_muscle_development	ENSGALG00010029524	ENSGALG00010029524	1.370707765	0.147840175	9.271551304	1.83458E-20	1.33428E-19	positive
PRJNA729271	stage_E18_vs_E10	ordinary_muscle_development	ENSGALG00010029525	SLC38A10	-0.659151281	0.138436116	-4.761411251	1.92244E-06	4.8144E-06	negative
PRJNA729271	stage_E18_vs_E10	ordinary_muscle_development	ENSGALG00010029526	BCL7B	0.306743551	0.113684371	2.698203349	0.006971484	0.01127693	positive
PRJNA729271	stage_E18_vs_E10	ordinary_muscle_development	ENSGALG00010029527	NDUFAF8	1.658235659	0.152182872	10.89633568	1.19993E-27	1.31367E-26	positive
PRJNA729271	stage_E18_vs_E10	ordinary_muscle_development	ENSGALG00010029528	TBL2	-0.114457599	0.096880459	-1.181431222	0.237431461	0.290036156	negative
PRJNA729271	stage_E18_vs_E10	ordinary_muscle_development	ENSGALG00010029529	TEPSIN	-0.065631705	0.13661552	-0.480411779	0.63093462	0.680815702	negative
PRJNA729271	stage_E18_vs_E10	ordinary_muscle_development	ENSGALG00010029530	ENSGALG00010029530	0.782682762	0.376586084	2.078363477	0.037675894	0.054049098	positive
PRJNA729271	stage_E18_vs_E10	ordinary_muscle_development	ENSGALG00010029531	CEP131	-0.239671875	0.171410179	-1.398235954	0.162042223	0.20578255	negative
PRJNA729271	stage_E18_vs_E10	ordinary_muscle_development	ENSGALG00010029532	EVI2A	0.509430176	0.485772727	1.048700653	0.294315914	0.350917921	positive
PRJNA729271	stage_E18_vs_E10	ordinary_muscle_development	ENSGALG00010029533	MLXIPL	-0.275060255	0.750262122	-0.366618875	0.713903327	0.757542366	negative
PRJNA729271	stage_E18_vs_E10	ordinary_muscle_development	ENSGALG00010029534	NF1	-0.138835157	0.090974316	-1.526091788	0.126987007	0.165295824	negative
PRJNA729271	stage_E18_vs_E10	ordinary_muscle_development	ENSGALG00010029536	BAZ1B	-1.096597882	0.078298048	-14.00543073	1.44402E-44	3.33828E-43	negative
PRJNA729271	stage_E18_vs_E10	ordinary_muscle_development	ENSGALG00010029537	MYH1B	2.073950671	0.206084322	10.06360238	8.00157E-24	7.02926E-23	positive
PRJNA729271	stage_E18_vs_E10	ordinary_muscle_development	ENSGALG00010029538	FZD9	-0.556505814	0.174481334	-3.189486249	0.001425259	0.002549881	negative
PRJNA729271	stage_E18_vs_E10	ordinary_muscle_development	ENSGALG00010029539	FKBP6	-1.605364448	0.391407903	-4.101512604	4.10458E-05	8.91859E-05	negative
PRJNA729271	stage_E18_vs_E10	ordinary_muscle_development	ENSGALG00010029541	ENSGALG00010029541	0.749459851	0.23457027	3.195033408	0.001398147	0.002503537	positive
PRJNA729271	stage_E18_vs_E10	ordinary_muscle_development	ENSGALG00010029542	AATK	1.90688083	0.141316355	13.49370232	1.70328E-41	3.45784E-40	positive
PRJNA729271	stage_E18_vs_E10	ordinary_muscle_development	ENSGALG00010029543	NSUN5	0.212791801	0.134806293	1.578500505	0.114450672	0.150200316	positive

PRJNA729271	stage_E18_vs_E10	ordinary_muscle_development	ENSGALG00010029544	ENSGALG00010029544	-0.995272222	0.179606205	-5.541413344	3.0004E-08	8.91936E-08	negative
PRJNA729271	stage_E18_vs_E10	ordinary_muscle_development	ENSGALG00010029545	OMG	0.907295201	0.329879729	2.750381797	0.005952586	0.009749462	positive
PRJNA729271	stage_E18_vs_E10	ordinary_muscle_development	ENSGALG00010029546	WSB1	0.243456704	0.165370497	1.472189463	0.140969722	0.181550966	positive
PRJNA729271	stage_E18_vs_E10	ordinary_muscle_development	ENSGALG00010029547	HIP1	0.398899191	0.100139597	3.983431163	6.79274E-05	0.000144328	positive
PRJNA729271	stage_E18_vs_E10	ordinary_muscle_development	ENSGALG00010029548	BAIAP2	0.440825906	0.224794538	1.961016976	0.049877043	0.070061802	positive
PRJNA729271	stage_E18_vs_E10	ordinary_muscle_development	ENSGALG00010029549	KSR1	1.710914879	0.124302439	13.76412959	4.18899E-43	9.07614E-42	positive
PRJNA729271	stage_E18_vs_E10	ordinary_muscle_development	ENSGALG00010029550	CHMP6	-0.013413447	0.239921787	-0.055907583	0.95541543	0.963056096	negative
PRJNA729271	stage_E18_vs_E10	ordinary_muscle_development	ENSGALG00010029552	NOS2	0.759643993	0.124242237	6.114216966	9.70322E-10	3.27928E-09	positive
PRJNA729271	stage_E18_vs_E10	ordinary_muscle_development	ENSGALG00010029553	MYH1C	5.821676943	0.194788222	29.88721235	2.8852E-196	1.5489E-193	positive
PRJNA729271	stage_E18_vs_E10	ordinary_muscle_development	ENSGALG00010029554	ENSGALG00010029554	3.541531751	0.379859724	9.323262065	1.12817E-20	8.30095E-20	positive
PRJNA729271	stage_E18_vs_E10	ordinary_muscle_development	ENSGALG00010029555	LYRM9	0.893313098	0.156008795	5.726043199	1.028E-08	3.17107E-08	positive
PRJNA729271	stage_E18_vs_E10	ordinary_muscle_development	ENSGALG00010029556	NPTX1	2.047500149	0.563340063	3.634572229	0.000278442	0.000548	positive
PRJNA729271	stage_E18_vs_E10	ordinary_muscle_development	ENSGALG00010029557	ENSGALG00010029557	6.687268327	0.542248966	12.33246857	6.05625E-35	9.29929E-34	positive
PRJNA729271	stage_E18_vs_E10	ordinary_muscle_development	ENSGALG00010029558	ENSGALG00010029558	2.652357211	0.6577836	4.032264121	5.52421E-05	0.000118504	positive
PRJNA729271	stage_E18_vs_E10	ordinary_muscle_development	ENSGALG00010029560	ENSGALG00010029560	2.450031038	0.176233572	13.90218115	6.14401E-44	1.38288E-42	positive
PRJNA729271	stage_E18_vs_E10	ordinary_muscle_development	ENSGALG00010029561	SCO1	-0.167783764	0.104776544	-1.601348513	0.10929975	0.143974906	negative
PRJNA729271	stage_E18_vs_E10	ordinary_muscle_development	ENSGALG00010029562	ENSGALG00010029562	2.158214756	0.73143194	2.950670646	0.003170848	0.005411686	positive
PRJNA729271	stage_E18_vs_E10	ordinary_muscle_development	ENSGALG00010029563	ADPRM	0.802443121	0.125872055	6.3750697	1.8288E-10	6.53722E-10	positive
PRJNA729271	stage_E18_vs_E10	ordinary_muscle_development	ENSGALG00010029564	NLK	-0.15172416	0.091649634	-1.655480259	0.097827118	0.12998479	negative
PRJNA729271	stage_E18_vs_E10	ordinary_muscle_development	ENSGALG00010029566	TMEM97	-0.286015618	0.145759729	-1.962240329	0.049734511	0.069875132	negative
PRJNA729271	stage_E18_vs_E10	ordinary_muscle_development	ENSGALG00010029567	IFT20	-0.318898569	0.119876137	-2.660233941	0.007808639	0.012548362	negative
PRJNA729271	stage_E18_vs_E10	ordinary_muscle_development	ENSGALG00010029568	ENSGALG00010029568	-2.47590024	1.148581047	-2.155616487	0.031113624	0.045339497	negative
PRJNA729271	stage_E18_vs_E10	ordinary_muscle_development	ENSGALG00010029569	TMEM220	1.523134704	0.200117861	7.611188206	2.71587E-14	1.30353E-13	positive
PRJNA729271	stage_E18_vs_E10	ordinary_muscle_development	ENSGALG00010029570	TNFAIP1	-0.577545221	0.079713224	-7.245287458	4.31523E-13	1.89888E-12	negative
PRJNA729271	stage_E18_vs_E10	ordinary_muscle_development	ENSGALG00010029571	EPX	2.376555404	0.7014593	3.388016102	0.000704001	0.00131079	positive
PRJNA729271	stage_E18_vs_E10	ordinary_muscle_development	ENSGALG00010029572	TSPOAP1	0.235723418	0.118402933	1.990857928	0.046496509	0.06561843	positive
PRJNA729271	stage_E18_vs_E10	ordinary_muscle_development	ENSGALG00010029573	MAP2K4	-0.496853257	0.062234572	-7.983557067	1.42176E-15	7.55992E-15	negative
PRJNA729271	stage_E18_vs_E10	ordinary_muscle_development	ENSGALG00010029574	SGSM2	-0.175846053	0.133097558	-1.321181662	0.186440799	0.233554523	negative
PRJNA729271	stage_E18_vs_E10	ordinary_muscle_development	ENSGALG00010029575	ANKFN1	-4.219409048	0.843538228	-5.002036551	5.67278E-07	1.50159E-06	negative
PRJNA729271	stage_E18_vs_E10	ordinary_muscle_development	ENSGALG00010029576	ATP2A3	1.458771586	0.13085648	11.14787424	7.33375E-29	8.47709E-28	positive
PRJNA729271	stage_E18_vs_E10	ordinary_muscle_development	ENSGALG00010029577	NOG	-4.739781337	0.426880671	-11.10329339	1.20905E-28	1.38429E-27	negative
PRJNA729271	stage_E18_vs_E10	ordinary_muscle_development	ENSGALG00010029578	C17orf67	-0.572503854	0.513321376	-1.115293226	0.264724787	0.319472633	negative
PRJNA729271	stage_E18_vs_E10	ordinary_muscle_development	ENSGALG00010029579	GALNT17	1.24668568	0.183398208	6.797698267	1.06304E-11	4.17129E-11	positive
PRJNA729271	stage_E18_vs_E10	ordinary_muscle_development	ENSGALG00010029580	P2RX1	2.282128696	0.326425177	6.99127658	2.72396E-12	1.1265E-11	positive
PRJNA729271	stage_E18_vs_E10	ordinary_muscle_development	ENSGALG00010029581	DGKE	-0.269904832	0.106607131	-2.531770898	0.011348812	0.017830156	negative
PRJNA729271	stage_E18_vs_E10	ordinary_muscle_development	ENSGALG00010029582	SCPEP1	0.41299981	0.150037921	2.752636185	0.005911755	0.009684775	positive
PRJNA729271	stage_E18_vs_E10	ordinary_muscle_development	ENSGALG00010029583	LIMK1	-0.802975878	0.204010294	-3.935957654	8.28656E-05	0.000174255	negative
PRJNA729271	stage_E18_vs_E10	ordinary_muscle_development	ENSGALG00010029584	TRIM25	0.150037637	0.233737925	0.641905404	0.520934605	0.57777543	positive
PRJNA729271	stage_E18_vs_E10	ordinary_muscle_development	ENSGALG00010029585	ENSGALG00010029585	0.33850803	0.369645641	0.915763617	0.35979089	0.419360643	positive
PRJNA729271	stage_E18_vs_E10	ordinary_muscle_development	ENSGALG00010029586	COIL	-0.735823042	0.191157958	-3.849293271	0.000118459	0.000244736	negative
PRJNA729271	stage_E18_vs_E10	ordinary_muscle_development	ENSGALG00010029588	RAB11FIP4	-1.376111191	0.139944798	-9.83324288	8.09686E-23	6.72959E-22	negative
PRJNA729271	stage_E18_vs_E10	ordinary_muscle_development	ENSGALG00010029590	UTP6	-0.448781551	0.099205159	-4.523772309	6.07471E-06	1.44586E-05	negative
PRJNA729271	stage_E18_vs_E10	ordinary_muscle_development	ENSGALG00010029591	SUZ12	-0.872671816	0.080621985	-10.82424132	2.64259E-27	2.83735E-26	negative
PRJNA729271	stage_E18_vs_E10	ordinary_muscle_development	ENSGALG00010029592	MNT	0.558529232	0.167011976	3.344246597	0.000825064	0.001521926	positive
PRJNA729271	stage_E18_vs_E10	ordinary_muscle_development	ENSGALG00010029593	PAFAH1B1	0.178442187	0.078989499	2.259062136	0.02387952	0.035519101	positive
PRJNA729271	stage_E18_vs_E10	ordinary_muscle_development	ENSGALG00010029594	CRLF3	-0.332304326	0.067946235	-4.890695201	1.0048E-06	2.59573E-06	negative
PRJNA729271	stage_E18_vs_E10	ordinary_muscle_development	ENSGALG00010029595	CLUH	0.942910611	0.135090662	6.979835596	2.95526E-12	1.21902E-11	positive
PRJNA729271	stage_E18_vs_E10	ordinary_muscle_development	ENSGALG00010029596	ATAD5	-1.318173732	0.130645877	-10.08966956	6.13758E-24	5.43459E-23	negative
PRJNA729271	stage_E18_vs_E10	ordinary_muscle_development	ENSGALG00010029597	ADAP2	1.048490553	0.21007827	4.990951965	6.00824E-07	1.58546E-06	positive
PRJNA729271	stage_E18_vs_E10	ordinary_muscle_development	ENSGALG00010029598	SUPT4H1	0.324722825	0.149269044	2.175419744	0.029598676	0.043279816	positive
PRJNA729271	stage_E18_vs_E10	ordinary_muscle_development	ENSGALG00010029599	RNF43	-2.110119487	0.365283429	-5.77666359	7.61964E-09	2.37827E-08	negative
PRJNA729271	stage_E18_vs_E10	ordinary_muscle_development	ENSGALG00010029600	RHOT1	0.60565188	0.109347698	5.538771197	3.04601E-08	9.04939E-08	positive
PRJNA729271	stage_E18_vs_E10	ordinary_muscle_development	ENSGALG00010029601	HSF5	0.4138249	0.492546011	0.840175111	0.400810211	0.460615556	positive
PRJNA729271	stage_E18_vs_E10	ordinary_muscle_development	ENSGALG00010029602	MTMR4	-0.894567762	0.088527627	-10.10495586	5.25192E-24	4.66462E-23	negative
PRJNA729271	stage_E18_vs_E10	ordinary_muscle_development	ENSGALG00010029603	RHBDL3	0.305986125	0.420618808	0.727466579	0.466940182	0.525959051	positive
PRJNA729271	stage_E18_vs_E10	ordinary_muscle_development	ENSGALG00010029604	ENSGALG00010029604	0.241535328	0.119950027	2.013632968	0.044048082	0.062430523	positive
PRJNA729271	stage_E18_vs_E10	ordinary_muscle_development	ENSGALG00010029605	C17orf75	-0.974129338	0.095228578	-10.22938033	1.46453E-24	1.35127E-23	negative
PRJNA729271	stage_E18_vs_E10	ordinary_muscle_development	ENSGALG00010029606	ABHD11	0.776673393	0.213328519	3.640738688	0.000271857	0.00053584	positive
PRJNA729271	stage_E18_vs_E10	ordinary_muscle_development	ENSGALG00010029607	ZNF207	-0.63617658	0.065500866	-9.712491164	2.66737E-22	2.16481E-21	negative
PRJNA729271	stage_E18_vs_E10	ordinary_muscle_development	ENSGALG00010029608	ENSGALG00010029608	-1.492010825	0.315497827	-4.729068473	2.25552E-06	5.60786E-06	negative
PRJNA729271	stage_E18_vs_E10	ordinary_muscle_development	ENSGALG00010029609	METTL27	0.162223732	0.141763506	1.144326463	0.252488273	0.3061584	positive
PRJNA729271	stage_E18_vs_E10	ordinary_muscle_development	ENSGALG00010029610	ERN1	-0.182348252	0.080987136	-2.251570566	0.024349423	0.036177212	negative
PRJNA729271	stage_E18_vs_E10	ordinary_muscle_development	ENSGALG00010029611	YWHAG	0.138839909	0.131450726	1.056212571	0.290871109	0.347230735	positive
PRJNA729271	stage_E18_vs_E10	ordinary_muscle_development	ENSGALG00010029612	HSPB1	2.738584104	0.195431221	14.01303276	1.29744E-44	3.00421E-43	positive
PRJNA729271	stage_E18_vs_E10	ordinary_muscle_development	ENSGALG00010029614	MDH2	0.420613628	0.128779654	3.266149695	0.001090206	0.001977542	positive
PRJNA729271	stage_E18_vs_E10	ordinary_muscle_development	ENSGALG00010029615	ENSGALG00010029615	-1.031383921	0.310348684	-3.323306894	0.00088957	0.001633019	negative
PRJNA729271	stage_E18_vs_E10	ordinary_muscle_development	ENSGALG00010029616	TMEM120A	1.134273199	0.135297763	8.383532514	5.13625E-17	3.00565E-16	positive
PRJNA729271	stage_E18_vs_E10	ordinary_muscle_development	ENSGALG00010029617	POR	-0.005348385	0.09505166	-0.056268195	0.955128155	0.96283348	negative
PRJNA729271	stage_E18_vs_E10	ordinary_muscle_development	ENSGALG00010029618	TEX2	1.124905251	0.112720097	9.979633415	1.87157E-23	1.60904E-22	positive
PRJNA729271	stage_E18_vs_E10	ordinary_muscle_development	ENSGALG00010029619	ENSGALG00010029619	0.368132297	0.105392241	3.492973436	0.000477674	0.000910558	positive
PRJNA729271	stage_E18_vs_E10	ordinary_muscle_development	ENSGALG00010029620	ENSGALG00010029620	0.798385532	0.390444825	2.044810127	0.040873581	0.058267364	positive
PRJNA729271	stage_E18_vs_E10	ordinary_muscle_development	ENSGALG00010029621	TAF15	-1.449161081	0.122001468	-11.87822661	1.53591E-32	2.09239E-31	negative
PRJNA729271	stage_E18_vs_E10	ordinary_muscle_development	ENSGALG00010029622	MMP28	0.63838786	0.156932438	4.067915267	4.74356E-05	0.000102379	positive
PRJNA729271	stage_E18_vs_E10	ordinary_muscle_development	ENSGALG00010029623	ENSGALG00010029623	0.926571127	0.386925432	2.394702057	0.016633876	0.025446757	positive
PRJNA729271	stage_E18_vs_E10	ordinary_muscle_development	ENSGALG00010029624	RASL10B	0.669420898	0.276320905	2.42262126	0.015408982	0.023735728	positive
PRJNA729271	stage_E18_vs_E10	ordinary_muscle_development	ENSGALG00010029625	PECAM1	1.756900902	0.174410647	10.07335806	7.24609E-24	6.38881E-23	positive
PRJNA729271	stage_E18_vs_E10	ordinary_muscle_development	ENSGALG00010029626	AP2B1	-0.378441979	0.109176584	-3.466329197	0.000527617	0.000999191	negative
PRJNA729271	stage_E18_vs_E10	ordinary_muscle_development	ENSGALG00010029627	PEX12	-0.316792608	0.148586412	-2.132042922	0.033003318	0.047843094	negative
PRJNA729271	stage_E18_vs_E10	ordinary_muscle_development	ENSGALG00010029628	UNC45B	1.14995434	0.160679857	7.156804602	8.25793E-13	3.56883E-12	positive
PRJNA729271	stage_E18_vs_E10	ordinary_muscle_development	ENSGALG00010029629	ENSGALG00010029629	2.219460901	0.257412288	8.622202607	6.56784E-18	4.07887E-17	positive
PRJNA729271	stage_E18_vs_E10	ordinary_muscle_development	ENSGALG00010029630	RAD51D	0.413206872	0.119409141	3.460429146	0.000539315	0.001020145	positive
PRJNA729271	stage_E18_vs_E10	ordinary_muscle_development	ENSGALG00010029631	LIG3	-0.444637103	0.08781986	-5.063058645	4.12583E-07	1.10645E-06	negative
PRJNA729271	stage_E18_vs_E10	ordinary_muscle_development	ENSGALG00010029632	TMEM132E	0.359720592	0.229391199	1.568153413	0.116845334	0.153107314	positive
PRJNA729271	stage_E18_vs_E10	ordinary_muscle_development	ENSGALG00010029633	POLG2	0.832499357	0.174442843	4.772333111	1.82104E-06	4.57469E-06	positive
PRJNA729271	stage_E18_vs_E10	ordinary_muscle_development	ENSGALG00010029634	DDX5	-1.430543018	0.091467916	-15.63983388	3.89743E-55	1.33239E-53	negative
PRJNA729271	stage_E18_vs_E10	ordinary_muscle_development	ENSGALG00010029635	ENSGALG00010029635	-0.803338099	0.594334142	-1.351660695	0.176483884	0.222177887	negative
PRJNA729271	stage_E18_vs_E10	ordinary_muscle_development	ENSGALG00010029636	SPATA20	0.324293723	0.077736216	4.171719964	3.02309E-05	6.66155E-05	positive
PRJNA729271	stage_E18_vs_E10	ordinary_muscle_development	ENSGALG00010029638	EPN3	2.061945886	0.195452093	10.54962295	5.10014E-26	5.11248E-25	positive
PRJNA729271	stage_E18_vs_E10	ordinary_muscle_development	ENSGALG00010029639	ENSGALG00010029639	-1.744888308	0.787956577	-2.214447292	0.026798025	0.039519521	negative
PRJNA729271	stage_E18_vs_E10	ordinary_muscle_development	ENSGALG00010029641	ENSGALG00010029641	-0.507097158	0.724108946	-0.700305059	0.483736813	0.541783736	negative
PRJNA729271	stage_E18_vs_E10	ordinary_muscle_development	ENSGALG00010029642	NAT9	0.101599876	0.146708272	0.692529978	0.488604567	0.546517725	positive
PRJNA729271	stage_E18_vs_E10	ordinary_muscle_development	ENSGALG00010029644	MYCBPAP	1.640293304	0.15962744	10.27576023	9.06271E-25	8.49702E-24	positive
PRJNA729271	stage_E18_vs_E10	ordinary_muscle_development	ENSGALG00010029645	ENSGALG00010029645	-1.656506609	0.388715681	-4.261486449	2.03072E-05	4.57282E-05	negative
PRJNA729271	stage_E18_vs_E10	ordinary_muscle_development	ENSGALG00010029646	MRPS17	-0.29617982	0.109206635	-2.712104612	0.006685749	0.010859472	negative
PRJNA729271	stage_E18_vs_E10	ordinary_muscle_development	ENSGALG00010029647	RSAD1	1.000938064	0.152158955	6.578239593	4.76051E-11	1.78166E-10	positive
PRJNA729271	stage_E18_vs_E10	ordinary_muscle_development	ENSGALG00010029648	NIPSNAP2	0.975256339	0.121941303	7.997752325	1.26711E-15	6.75994E-15	positive
PRJNA729271	stage_E18_vs_E10	ordinary_muscle_development	ENSGALG00010029649	PSPH	2.047034781	0.259874441	7.877014647	3.35296E-15	1.73699E-14	positive
PRJNA729271	stage_E18_vs_E10	ordinary_muscle_development	ENSGALG00010029650	ACSF2	-0.476512657	0.136307938	-3.495854056	0.000472547	0.000901659	negative
PRJNA729271	stage_E18_vs_E10	ordinary_muscle_development	ENSGALG00010029651	ENSGALG00010029651	2.23300755	0.276407835	8.078669522	6.54772E-16	3.56802E-15	positive
PRJNA729271	stage_E18_vs_E10	ordinary_muscle_development	ENSGALG00010029652	LRRC59	-0.400574775	0.101370639	-3.951585777	7.7635E-05	0.000163778	negative
PRJNA729271	stage_E18_vs_E10	ordinary_muscle_development	ENSGALG00010029653	CCT6A	-0.586602857	0.090416826	-6.487762129	8.71207E-11	3.19241E-10	negative
PRJNA729271	stage_E18_vs_E10	ordinary_muscle_development	ENSGALG00010029654	EME1	-1.851194529	0.129853246	-14.25605127	4.11065E-46	1.02378E-44	negative
PRJNA729271	stage_E18_vs_E10	ordinary_muscle_development	ENSGALG00010029655	PHKG1	1.149229215	0.129493225	8.874821162	7.00459E-19	4.63403E-18	positive
PRJNA729271	stage_E18_vs_E10	ordinary_muscle_development	ENSGALG00010029656	MRPL27	-0.340970771	0.115807847	-2.944280369	0.003237066	0.005511073	negative
PRJNA729271	stage_E18_vs_E10	ordinary_muscle_development	ENSGALG00010029657	CHCHD2	0.914485096	0.146858253	6.226991519	4.75477E-10	1.65435E-09	positive
PRJNA729271	stage_E18_vs_E10	ordinary_muscle_development	ENSGALG00010029658	XYLT2	0.359611878	0.125908759	2.856130758	0.004288385	0.00717122	positive
PRJNA729271	stage_E18_vs_E10	ordinary_muscle_development	ENSGALG00010029659	VKORC1L1	-0.997040432	0.146946257	-6.7850686	1.16031E-11	4.53825E-11	negative
PRJNA729271	stage_E18_vs_E10	ordinary_muscle_development	ENSGALG00010029660	CD300LG	-0.153417648	0.320120117	-0.479250257	0.631760605	0.681605506	negative
PRJNA729271	stage_E18_vs_E10	ordinary_muscle_development	ENSGALG00010029661	KRABZFP	1.590220193	0.658460745	2.415056943	0.01573275	0.024198452	positive
PRJNA729271	stage_E18_vs_E10	ordinary_muscle_development	ENSGALG00010029663	GUSB	-0.211718752	0.154596733	-1.36949047	0.170846012	0.215847027	negative
PRJNA729271	stage_E18_vs_E10	ordinary_muscle_development	ENSGALG00010029664	RAB37	-0.160800729	0.482514764	-0.333255563	0.73894138	0.780113277	negative
PRJNA729271	stage_E18_vs_E10	ordinary_muscle_development	ENSGALG00010029665	ASL1	0.947265715	0.191313087	4.951390039	7.36853E-07	1.93036E-06	positive
PRJNA729271	stage_E18_vs_E10	ordinary_muscle_development	ENSGALG00010029666	SLC9A3R1	-0.345181934	0.201341305	-1.714411921	0.086453101	0.115999046	negative
PRJNA729271	stage_E18_vs_E10	ordinary_muscle_development	ENSGALG00010029667	TMEM104	-0.186709212	0.095051264	-1.964300139	0.049495296	0.069545784	negative
PRJNA729271	stage_E18_vs_E10	ordinary_muscle_development	ENSGALG00010029668	CRCP	0.359272054	0.181567684	1.978722465	0.047847262	0.067406557	positive
PRJNA729271	stage_E18_vs_E10	ordinary_muscle_development	ENSGALG00010029669	HID1	0.458891498	0.074923992	6.124760359	9.08202E-10	3.08227E-09	positive
PRJNA729271	stage_E18_vs_E10	ordinary_muscle_development	ENSGALG00010029670	TPST1	0.36544146	0.169823949	2.151884121	0.031406482	0.045734073	positive
PRJNA729271	stage_E18_vs_E10	ordinary_muscle_development	ENSGALG00010029671	GRIN2C	3.045692241	0.200462279	15.19334339	3.91435E-52	1.23077E-50	positive
PRJNA729271	stage_E18_vs_E10	ordinary_muscle_development	ENSGALG00010029672	KCTD7	-1.346847193	0.233621873	-5.765073163	8.16224E-09	2.5427E-08	negative
PRJNA729271	stage_E18_vs_E10	ordinary_muscle_development	ENSGALG00010029673	TMEM248	-0.230692417	0.09041082	-2.551601873	0.010722897	0.016899901	negative
PRJNA729271	stage_E18_vs_E10	ordinary_muscle_development	ENSGALG00010029674	FDXR	-0.482182773	0.113950014	-4.23152887	2.32108E-05	5.18799E-05	negative
PRJNA729271	stage_E18_vs_E10	ordinary_muscle_development	ENSGALG00010029675	DHRS13	0.128447448	0.227988391	0.563394685	0.573166155	0.62650203	positive
PRJNA729271	stage_E18_vs_E10	ordinary_muscle_development	ENSGALG00010029676	FADS6	0.738055569	1.024185246	0.720627027	0.471139021	0.530012372	positive
PRJNA729271	stage_E18_vs_E10	ordinary_muscle_development	ENSGALG00010029677	PHF12	0.439700509	0.100227985	4.387003399	1.14923E-05	2.65722E-05	positive
PRJNA729271	stage_E18_vs_E10	ordinary_muscle_development	ENSGALG00010029682	CDR2L	-0.958696193	0.127702466	-7.507264551	6.03756E-14	2.82396E-13	negative
PRJNA729271	stage_E18_vs_E10	ordinary_muscle_development	ENSGALG00010029683	MRPL58	0.184925552	0.111528312	1.658104109	0.097296455	0.129338968	positive
PRJNA729271	stage_E18_vs_E10	ordinary_muscle_development	ENSGALG00010029684	ENSGALG00010029684	0.170088167	0.565821013	0.300604189	0.763716337	0.801830241	positive
PRJNA729271	stage_E18_vs_E10	ordinary_muscle_development	ENSGALG00010029685	ATP5PD	0.912511403	0.115059164	7.930801575	2.17736E-15	1.13897E-14	positive
PRJNA729271	stage_E18_vs_E10	ordinary_muscle_development	ENSGALG00010029686	KCTD2	-0.248562046	0.060193436	-4.129387904	3.6373E-05	7.94855E-05	negative
PRJNA729271	stage_E18_vs_E10	ordinary_muscle_development	ENSGALG00010029687	SLC16A5	0.120602114	0.427551379	0.282076306	0.777885001	0.813700158	positive
PRJNA729271	stage_E18_vs_E10	ordinary_muscle_development	ENSGALG00010029689	ENSGALG00010029689	1.440783385	0.175925822	8.18972094	2.61833E-16	1.45783E-15	positive

PRJNA729271	stage_E18_vs_E10	ordinary_muscle_development	ENSGALG00010029690	ENSGALG00010029690	1.29389913	0.161239819	8.024687332	1.01785E-15	5.46517E-15	positive
PRJNA729271	stage_E18_vs_E10	ordinary_muscle_development	ENSGALG00010029691	NUFIP2	0.621792568	0.141693473	4.38829366	1.14243E-05	2.64319E-05	positive
PRJNA729271	stage_E18_vs_E10	ordinary_muscle_development	ENSGALG00010029692	CRYBA1	-0.360732523	0.523077497	-0.689634949	0.490423783	0.548087335	negative
PRJNA729271	stage_E18_vs_E10	ordinary_muscle_development	ENSGALG00010029693	HN1	-1.179879838	0.127072009	-9.285127759	1.61513E-20	1.17763E-19	negative
PRJNA729271	stage_E18_vs_E10	ordinary_muscle_development	ENSGALG00010029694	ARMC7	0.33102603	0.091537887	3.616273445	0.000298875	0.000585273	positive
PRJNA729271	stage_E18_vs_E10	ordinary_muscle_development	ENSGALG00010029695	TAOK1	0.108973937	0.086677816	1.257229844	0.208670419	0.258453194	positive
PRJNA729271	stage_E18_vs_E10	ordinary_muscle_development	ENSGALG00010029696	SUMO2	-0.793100719	0.108990746	-7.276771195	3.41904E-13	1.51649E-12	negative
PRJNA729271	stage_E18_vs_E10	ordinary_muscle_development	ENSGALG00010029697	GIT1	0.177586128	0.170037985	1.044390924	0.296304561	0.353056688	positive
PRJNA729271	stage_E18_vs_E10	ordinary_muscle_development	ENSGALG00010029698	NUP85	-0.623135336	0.086304905	-7.22016134	5.19259E-13	2.26843E-12	negative
PRJNA729271	stage_E18_vs_E10	ordinary_muscle_development	ENSGALG00010029699	ANKRD13B	-0.700551294	0.17582984	-3.984257125	6.76915E-05	0.000143912	negative
PRJNA729271	stage_E18_vs_E10	ordinary_muscle_development	ENSGALG00010029700	GGA3	0.070311608	0.080625334	0.872078347	0.383165638	0.443538246	positive
PRJNA729271	stage_E18_vs_E10	ordinary_muscle_development	ENSGALG00010029701	MRPS7	0.097125648	0.066817499	1.453595971	0.146058355	0.187371967	positive
PRJNA729271	stage_E18_vs_E10	ordinary_muscle_development	ENSGALG00010029702	CORO6	3.229021499	0.170611317	18.92618583	6.94097E-80	4.81385E-78	positive
PRJNA729271	stage_E18_vs_E10	ordinary_muscle_development	ENSGALG00010029703	MIF4GD	0.279898743	0.115174292	2.430218915	0.015089706	0.023288467	positive
PRJNA729271	stage_E18_vs_E10	ordinary_muscle_development	ENSGALG00010029704	SLC25A19	0.800914774	0.101224239	7.912282483	2.52712E-15	1.31812E-14	positive
PRJNA729271	stage_E18_vs_E10	ordinary_muscle_development	ENSGALG00010029705	GRB2	-0.669368955	0.068214188	-9.812752666	9.92242E-23	8.20453E-22	negative
PRJNA729271	stage_E18_vs_E10	ordinary_muscle_development	ENSGALG00010029706	SSH2	1.333826151	0.217937903	6.120211918	9.3451E-10	3.16489E-09	positive
PRJNA729271	stage_E18_vs_E10	ordinary_muscle_development	ENSGALG00010029707	NSRP1	0.081001734	0.224494857	0.360817771	0.718235677	0.761236372	positive
PRJNA729271	stage_E18_vs_E10	ordinary_muscle_development	ENSGALG00010029708	TMEM94	-0.108489655	0.085253737	-1.272550147	0.203177719	0.252448228	negative
PRJNA729271	stage_E18_vs_E10	ordinary_muscle_development	ENSGALG00010029709	SLC6A4	0.203870769	0.205231156	0.993371442	0.320528973	0.378221058	positive
PRJNA729271	stage_E18_vs_E10	ordinary_muscle_development	ENSGALG00010029710	BLMH	-1.104712238	0.083797298	-13.18314868	1.09714E-39	2.07612E-38	negative
PRJNA729271	stage_E18_vs_E10	ordinary_muscle_development	ENSGALG00010029711	CASKIN2	1.586445559	0.110621104	14.34125584	1.20849E-46	3.11137E-45	positive
PRJNA729271	stage_E18_vs_E10	ordinary_muscle_development	ENSGALG00010029713	CPD	0.211932914	0.116745649	1.815338867	0.06947185	0.095098164	positive
PRJNA729271	stage_E18_vs_E10	ordinary_muscle_development	ENSGALG00010029716	GOSR1	0.244835637	0.063208491	3.873461191	0.000107301	0.000223177	positive
PRJNA729271	stage_E18_vs_E10	ordinary_muscle_development	ENSGALG00010029717	TRARG1	0.347745363	0.411632852	0.844794969	0.398225324	0.458141888	positive
PRJNA729271	stage_E18_vs_E10	ordinary_muscle_development	ENSGALG00010029718	ABR	-0.024321864	0.112708251	-0.215794884	0.829147636	0.858096172	negative
PRJNA729271	stage_E18_vs_E10	ordinary_muscle_development	ENSGALG00010029720	TIMM22	-0.568839979	0.112322334	-5.064353275	4.09789E-07	1.09978E-06	negative
PRJNA729271	stage_E18_vs_E10	ordinary_muscle_development	ENSGALG00010029721	EXOC7	-0.207177141	0.10595019	-1.955420192	0.050533495	0.070901462	negative
PRJNA729271	stage_E18_vs_E10	ordinary_muscle_development	ENSGALG00010029722	SPECC1	1.720733886	0.141682601	12.14499078	6.09895E-34	8.92071E-33	positive
PRJNA729271	stage_E18_vs_E10	ordinary_muscle_development	ENSGALG00010029723	ADORA2B	0.949453683	0.181025113	5.244872761	1.5639E-07	4.34351E-07	positive
PRJNA729271	stage_E18_vs_E10	ordinary_muscle_development	ENSGALG00010029724	CACNA1G	-1.914964318	0.217144931	-8.8188304	1.15661E-18	7.54506E-18	negative
PRJNA729271	stage_E18_vs_E10	ordinary_muscle_development	ENSGALG00010029725	TSEN54	-0.09946112	0.068004635	-1.462563836	0.143586765	0.184527898	negative
PRJNA729271	stage_E18_vs_E10	ordinary_muscle_development	ENSGALG00010029726	SEPTIN9	-0.888010305	0.125495229	-7.076048344	1.48323E-12	6.26989E-12	negative
PRJNA729271	stage_E18_vs_E10	ordinary_muscle_development	ENSGALG00010029727	ZSWIM7	0.332733767	0.138396743	2.404202299	0.016207801	0.024876332	positive
PRJNA729271	stage_E18_vs_E10	ordinary_muscle_development	ENSGALG00010029728	TNRC6C	0.671517537	0.103320449	6.499367218	8.06586E-11	2.96512E-10	positive
PRJNA729271	stage_E18_vs_E10	ordinary_muscle_development	ENSGALG00010029729	RASL10A	5.874015765	0.756395805	7.765796329	8.11338E-15	4.08513E-14	positive
PRJNA729271	stage_E18_vs_E10	ordinary_muscle_development	ENSGALG00010029730	TTC19	0.897427612	0.133600548	6.717244999	1.85192E-11	7.12789E-11	positive
PRJNA729271	stage_E18_vs_E10	ordinary_muscle_development	ENSGALG00010029731	NCOR1	0.083009933	0.184858904	0.449044816	0.653399328	0.701505315	positive
PRJNA729271	stage_E18_vs_E10	ordinary_muscle_development	ENSGALG00010029732	PIGL	0.71745905	0.179234671	4.002903263	6.25699E-05	0.000133513	positive
PRJNA729271	stage_E18_vs_E10	ordinary_muscle_development	ENSGALG00010029733	ENSGALG00010029733	2.666698016	0.164319625	16.22872507	3.15954E-59	1.23443E-57	positive
PRJNA729271	stage_E18_vs_E10	ordinary_muscle_development	ENSGALG00010029734	SEC14L1	-0.181032456	0.084142691	-2.151493545	0.031437265	0.0457743	negative
PRJNA729271	stage_E18_vs_E10	ordinary_muscle_development	ENSGALG00010029735	SRP68	-0.299428817	0.114632646	-2.612072809	0.008999509	0.014334932	negative
PRJNA729271	stage_E18_vs_E10	ordinary_muscle_development	ENSGALG00010029736	ENSGALG00010029736	1.438586616	0.133077447	10.81014588	3.0818E-27	3.29916E-26	positive
PRJNA729271	stage_E18_vs_E10	ordinary_muscle_development	ENSGALG00010029737	ENSGALG00010029737	-5.382584757	0.551085636	-9.76723834	1.55637E-22	1.27599E-21	negative
PRJNA729271	stage_E18_vs_E10	ordinary_muscle_development	ENSGALG00010029738	TRPV2	0.730399152	0.167010867	4.373363035	1.22347E-05	2.82302E-05	positive
PRJNA729271	stage_E18_vs_E10	ordinary_muscle_development	ENSGALG00010029739	P2RX5	3.173244192	0.202658761	15.65806569	2.92664E-55	1.00525E-53	positive
PRJNA729271	stage_E18_vs_E10	ordinary_muscle_development	ENSGALG00010029740	HASPIN	-1.561812754	0.21188756	-7.370950695	1.69415E-13	7.66202E-13	negative
PRJNA729271	stage_E18_vs_E10	ordinary_muscle_development	ENSGALG00010029741	EMC6	-0.336749916	0.167005508	-2.016400064	0.043758159	0.062049943	negative
PRJNA729271	stage_E18_vs_E10	ordinary_muscle_development	ENSGALG00010029742	TAX1BP3	-0.630306303	0.11887943	-5.302063642	1.14501E-07	3.22834E-07	negative
PRJNA729271	stage_E18_vs_E10	ordinary_muscle_development	ENSGALG00010029743	MYOCD	4.085844813	0.253676056	16.10654499	2.29493E-58	8.70814E-57	positive
PRJNA729271	stage_E18_vs_E10	ordinary_muscle_development	ENSGALG00010029744	CTNS	0.186615957	0.091846109	2.03183301	0.042170564	0.059910059	positive
PRJNA729271	stage_E18_vs_E10	ordinary_muscle_development	ENSGALG00010029745	SHPK	1.008845576	0.168192001	5.998178097	1.99544E-09	6.56465E-09	positive
PRJNA729271	stage_E18_vs_E10	ordinary_muscle_development	ENSGALG00010029746	TRPV1	0.710805615	0.312164615	2.277021741	0.022784927	0.03402715	positive
PRJNA729271	stage_E18_vs_E10	ordinary_muscle_development	ENSGALG00010029747	TRPV3	1.392891972	0.333010469	4.182727274	2.88033E-05	6.37021E-05	positive
PRJNA729271	stage_E18_vs_E10	ordinary_muscle_development	ENSGALG00010029749	ASPA	0.724724424	0.30135044	2.404922407	0.0161759	0.024832628	positive
PRJNA729271	stage_E18_vs_E10	ordinary_muscle_development	ENSGALG00010029750	TEN1	-1.13109519	0.227721795	-4.967004543	6.7995E-07	1.78646E-06	negative
PRJNA729271	stage_E18_vs_E10	ordinary_muscle_development	ENSGALG00010029752	ENSGALG00010029752	1.182881368	0.169159008	6.99271875	2.6961E-12	1.11529E-11	positive
PRJNA729271	stage_E18_vs_E10	ordinary_muscle_development	ENSGALG00010029753	AKAP10	-0.404847326	0.076780888	-5.272761688	1.34386E-07	3.75902E-07	negative
PRJNA729271	stage_E18_vs_E10	ordinary_muscle_development	ENSGALG00010029754	NLE1	-1.114559301	0.182159503	-6.11858994	9.4407E-10	3.19577E-09	negative
PRJNA729271	stage_E18_vs_E10	ordinary_muscle_development	ENSGALG00010029755	ACOX1	-0.27607317	0.120516471	-2.290750531	0.021977846	0.032910008	negative
PRJNA729271	stage_E18_vs_E10	ordinary_muscle_development	ENSGALG00010029756	ELAC2	-0.533256013	0.085912842	-6.206941825	5.40256E-10	1.87255E-09	negative
PRJNA729271	stage_E18_vs_E10	ordinary_muscle_development	ENSGALG00010029757	ULK2	-0.031231474	0.209571013	-0.149025732	0.881533329	0.901942938	negative
PRJNA729271	stage_E18_vs_E10	ordinary_muscle_development	ENSGALG00010029758	DNAH9	1.923629477	0.298874588	6.436243002	1.22467E-10	4.43346E-10	positive
PRJNA729271	stage_E18_vs_E10	ordinary_muscle_development	ENSGALG00010029759	ALDH3A2	0.370207101	0.107933934	3.429941697	0.000603711	0.001134404	positive
PRJNA729271	stage_E18_vs_E10	ordinary_muscle_development	ENSGALG00010029760	SHISA6	-3.807084484	0.511097361	-7.448843953	9.41617E-14	4.33293E-13	negative
PRJNA729271	stage_E18_vs_E10	ordinary_muscle_development	ENSGALG00010029761	MRPL38	0.244186826	0.118967839	2.052544862	0.04011674	0.057284223	positive
PRJNA729271	stage_E18_vs_E10	ordinary_muscle_development	ENSGALG00010029762	SLC47A2	2.896304305	0.48311144	5.995106029	2.03353E-09	6.68238E-09	positive
PRJNA729271	stage_E18_vs_E10	ordinary_muscle_development	ENSGALG00010029763	GEMIN4	-0.532139557	0.099312332	-5.35824248	8.40354E-08	2.39593E-07	negative
PRJNA729271	stage_E18_vs_E10	ordinary_muscle_development	ENSGALG00010029764	NXN	-0.918206145	0.125878959	-7.294357607	3.00087E-13	1.33592E-12	negative
PRJNA729271	stage_E18_vs_E10	ordinary_muscle_development	ENSGALG00010029765	KCNJ16	-1.082719275	0.304140512	-3.559931122	0.000370952	0.000717405	negative
PRJNA729271	stage_E18_vs_E10	ordinary_muscle_development	ENSGALG00010029766	ENSGALG00010029766	-0.071651281	0.149017176	-0.480825656	0.630640414	0.680683827	negative
PRJNA729271	stage_E18_vs_E10	ordinary_muscle_development	ENSGALG00010029767	GLOD4	-0.083725855	0.087061023	-0.961691599	0.336204544	0.394629918	negative
PRJNA729271	stage_E18_vs_E10	ordinary_muscle_development	ENSGALG00010029768	KCNJ2	0.989327694	0.175446158	5.638924813	1.71115E-08	5.18027E-08	positive
PRJNA729271	stage_E18_vs_E10	ordinary_muscle_development	ENSGALG00010029770	TLCD3A	-0.716124926	0.231738895	-3.090223275	0.002000061	0.003511492	negative
PRJNA729271	stage_E18_vs_E10	ordinary_muscle_development	ENSGALG00010029771	SOX9	-3.899018081	0.264559517	-14.73777293	3.68746E-49	1.03988E-47	negative
PRJNA729271	stage_E18_vs_E10	ordinary_muscle_development	ENSGALG00010029772	VPS53	0.219002555	0.069000314	3.173935644	0.00150387	0.002683235	positive
PRJNA729271	stage_E18_vs_E10	ordinary_muscle_development	ENSGALG00010029773	TRIM65	0.896887279	0.13457586	6.664547995	2.65481E-11	1.00975E-10	positive
PRJNA729271	stage_E18_vs_E10	ordinary_muscle_development	ENSGALG00010029774	RFLNB	0.432082848	0.124549325	3.469170541	0.000522068	0.000989632	positive
PRJNA729271	stage_E18_vs_E10	ordinary_muscle_development	ENSGALG00010029775	ENSGALG00010029775	-0.126224342	0.142629495	-0.884980647	0.376167069	0.436148041	negative
PRJNA729271	stage_E18_vs_E10	ordinary_muscle_development	ENSGALG00010029776	WBP2	0.832352999	0.137537672	6.051818287	1.4322E-09	4.76687E-09	positive
PRJNA729271	stage_E18_vs_E10	ordinary_muscle_development	ENSGALG00010029777	ENSGALG00010029777	0.34640949	0.137201187	2.524828665	0.011575471	0.018164604	positive
PRJNA729271	stage_E18_vs_E10	ordinary_muscle_development	ENSGALG00010029778	DOC2B	1.862496501	0.342268217	5.441628548	5.27957E-08	1.53116E-07	positive
PRJNA729271	stage_E18_vs_E10	ordinary_muscle_development	ENSGALG00010029780	TEX14	0.585888055	0.252704215	2.318473617	0.020423595	0.030751014	positive
PRJNA729271	stage_E18_vs_E10	ordinary_muscle_development	ENSGALG00010029781	UNC13D	0.489427983	0.259286203	1.88759748	0.059080009	0.08188609	positive
PRJNA729271	stage_E18_vs_E10	ordinary_muscle_development	ENSGALG00010029782	UNK	0.16758459	0.121838609	1.375463748	0.168987717	0.213610967	positive
PRJNA729271	stage_E18_vs_E10	ordinary_muscle_development	ENSGALG00010029783	RAD51C	0.055482505	0.121031082	0.458415342	0.646654071	0.695489309	positive
PRJNA729271	stage_E18_vs_E10	ordinary_muscle_development	ENSGALG00010029784	CLDN3	-2.881017398	0.681270991	-4.228886063	2.34851E-05	5.24606E-05	negative
PRJNA729271	stage_E18_vs_E10	ordinary_muscle_development	ENSGALG00010029785	ENSGALG00010029785	2.617799043	0.244105484	10.72404846	7.84908E-27	8.26834E-26	positive
PRJNA729271	stage_E18_vs_E10	ordinary_muscle_development	ENSGALG00010029787	H3-3B	-0.000205752	0.063907312	-0.003219542	0.997431182	0.997706506	negative
PRJNA729271	stage_E18_vs_E10	ordinary_muscle_development	ENSGALG00010029791	GALK1	0.40888299	0.10924704	3.74273747	0.000182026	0.000366964	positive
PRJNA729271	stage_E18_vs_E10	ordinary_muscle_development	ENSGALG00010029804	ITGB4	-0.143529226	0.231498092	-0.620001768	0.535256623	0.590947121	negative
PRJNA729271	stage_E18_vs_E10	ordinary_muscle_development	ENSGALG00010029812	SAP30BP	0.012430491	0.115745638	0.1073949	0.9144757	0.929480771	positive
PRJNA729271	stage_E18_vs_E10	ordinary_muscle_development	ENSGALG00010029835	RECQL5	-0.600130708	0.11150077	-5.382301029	7.35396E-08	2.10622E-07	negative
PRJNA729271	stage_E18_vs_E10	ordinary_muscle_development	ENSGALG00010029841	SMIM5	2.380179201	0.128420392	18.53427762	1.09247E-76	6.915E-75	positive
PRJNA729271	stage_E18_vs_E10	ordinary_muscle_development	ENSGALG00010029859	CSNK1D	-0.203385041	0.05281204	-3.851111266	0.000117583	0.000243203	negative
PRJNA729271	stage_E18_vs_E10	ordinary_muscle_development	ENSGALG00010029864	SLC16A3	0.702324537	0.339678341	2.067616486	0.038676101	0.055400418	positive
PRJNA729271	stage_E18_vs_E10	ordinary_muscle_development	ENSGALG00010029879	ENSGALG00010029879	0.049645149	0.316542632	0.156835584	0.875374428	0.897161304	positive
PRJNA729271	stage_E18_vs_E10	ordinary_muscle_development	ENSGALG00010029895	FASN	-1.933139825	0.168683709	-11.46014539	2.09163E-30	2.60466E-29	negative
PRJNA729271	stage_E18_vs_E10	ordinary_muscle_development	ENSGALG00010029907	NTN1	-0.96520349	0.281205497	-3.432377744	0.000598314	0.001124845	negative
PRJNA729271	stage_E18_vs_E10	ordinary_muscle_development	ENSGALG00010029921	STX8	0.056528845	0.105221011	0.537239139	0.591102439	0.643100642	positive
PRJNA729271	stage_E18_vs_E10	ordinary_muscle_development	ENSGALG00010029925	GGNBP2	0.114589146	0.126679622	0.904558637	0.365699257	0.425289692	positive
PRJNA729271	stage_E18_vs_E10	ordinary_muscle_development	ENSGALG00010029926	BLTP2	0.206648706	0.132742669	1.556761727	0.119527067	0.156324537	positive
PRJNA729271	stage_E18_vs_E10	ordinary_muscle_development	ENSGALG00010029927	ENSGALG00010029927	0.435014387	0.693498336	0.627275314	0.530478782	0.586476237	positive
PRJNA729271	stage_E18_vs_E10	ordinary_muscle_development	ENSGALG00010029928	USP43	1.407216424	0.13105845	10.73731931	6.79899E-27	7.19353E-26	positive
PRJNA729271	stage_E18_vs_E10	ordinary_muscle_development	ENSGALG00010029929	GDPD1	-0.333035424	0.095288183	-3.495033848	0.000474002	0.000904271	negative
PRJNA729271	stage_E18_vs_E10	ordinary_muscle_development	ENSGALG00010029930	ARHGAP44	2.638606122	0.203309945	12.97824425	1.62564E-38	2.93812E-37	positive
PRJNA729271	stage_E18_vs_E10	ordinary_muscle_development	ENSGALG00010029932	RASA4B	1.89695377	0.267245628	7.098165771	1.26423E-12	5.37393E-12	positive
PRJNA729271	stage_E18_vs_E10	ordinary_muscle_development	ENSGALG00010029933	TVP23B	-0.032842491	0.094668302	-0.346921725	0.728650125	0.770820578	negative
PRJNA729271	stage_E18_vs_E10	ordinary_muscle_development	ENSGALG00010029934	TEKT3	3.117332073	0.445939361	6.990484243	2.73939E-12	1.13223E-11	positive
PRJNA729271	stage_E18_vs_E10	ordinary_muscle_development	ENSGALG00010029935	SYNRG	-0.15194449	0.074090889	-2.050785072	0.040287882	0.057500281	negative
PRJNA729271	stage_E18_vs_E10	ordinary_muscle_development	ENSGALG00010029936	SERPINF2	2.674284056	0.501181213	5.3359623			positive
PRJNA729271	stage_E18_vs_E10	ordinary_muscle_development	ENSGALG00010029937	PMP22	3.194306428	0.123648714	25.83372138	3.7079E-147	1.1197E-144	positive
PRJNA729271	stage_E18_vs_E10	ordinary_muscle_development	ENSGALG00010029938	ENSGALG00010029938	-1.340987392	0.315747089	-4.2470301	2.16623E-05	4.85835E-05	negative
PRJNA729271	stage_E18_vs_E10	ordinary_muscle_development	ENSGALG00010029939	ENSGALG00010029939	3.468985764	1.485676256	2.334954031	0.019545822	0.029524457	positive
PRJNA729271	stage_E18_vs_E10	ordinary_muscle_development	ENSGALG00010029941	COX10	0.514543317	0.174355451	2.9511169	0.003166271	0.005406419	positive
PRJNA729271	stage_E18_vs_E10	ordinary_muscle_development	ENSGALG00010029942	ENSGALG00010029942	-4.683582537	1.668981805	-2.806251405	0.005012157	0.008309644	negative
PRJNA729271	stage_E18_vs_E10	ordinary_muscle_development	ENSGALG00010029943	HS3ST3A1	-2.627193629	0.228253747	-11.50996933	1.1752E-30	1.47869E-29	negative
PRJNA729271	stage_E18_vs_E10	ordinary_muscle_development	ENSGALG00010029945	CCL4	-0.152693013	0.870508426	-0.175406703	0.860760074	0.884858692	negative
PRJNA729271	stage_E18_vs_E10	ordinary_muscle_development	ENSGALG00010029946	METRNL	-0.356614053	0.137038649	-2.602288152	0.009260401	0.014711696	negative
PRJNA729271	stage_E18_vs_E10	ordinary_muscle_development	ENSGALG00010029947	CCL5	1.242381134	1.27993687	0.970658134	0.331718546	0.389901097	positive
PRJNA729271	stage_E18_vs_E10	ordinary_muscle_development	ENSGALG00010029948	MYADML2	2.995172069	0.217783435	13.75298389	4.88715E-43	1.05259E-41	positive
PRJNA729271	stage_E18_vs_E10	ordinary_muscle_development	ENSGALG00010029950	RFFL	-0.529892688	0.107899009	-4.911006085	9.06103E-07	2.3546E-06	negative
PRJNA729271	stage_E18_vs_E10	ordinary_muscle_development	ENSGALG00010029956	CCL15	4.420215186	0.182421213	24.23081788	1.0535E-129	2.2791E-127	positive
PRJNA729271	stage_E18_vs_E10	ordinary_muscle_development	ENSGALG00010029962	ENSGALG00010029962	0.381599958	0.756200907	0.504627745	0.613820319	0.664475394	positive
PRJNA729271	stage_E18_vs_E10	ordinary_muscle_development	ENSGALG00010029966	RABGEF1	-0.168622254	0.081883923	-2.05928401	0.039467039	0.056434323	negative
PRJNA729271	stage_E18_vs_E10	ordinary_muscle_development	ENSGALG00010030004	FLOT2	1.386303914	0.069824037	19.85425028	1.01262E-87	8.78922E-86	positive
PRJNA729271	stage_E18_vs_E10	ordinary_muscle_development	ENSGALG00010030014	ENSGALG00010030014	-0.530763581	0.308112381	-1.722629837	0.084955486	0.114179858	negative
PRJNA729271	stage_E18_vs_E10	ordinary_muscle_development	ENSGALG00010030102	GPS1	-0.210350919	0.084198386	-2.498277325	0.012479852	0.019469966	negative
PRJNA729271	stage_E18_vs_E10	ordinary_muscle_development	ENSGALG00010030103	C1QTNF1	1.257834532	0.21451488	5.863623673	4.52873E-09	1.44431E-08	positive
PRJNA729271	stage_E18_vs_E10	ordinary_muscle_development	ENSGALG00010030104	P4HB	-0.605500746	0.11003873	-5.502614808	3.74199E-08	1.10356E-07	negative
PRJNA729271	stage_E18_vs_E10	ordinary_muscle_development	ENSGALG00010030105	RAB40B	-0.764627826	0.206457298	-3.703564048	0.000212591	0.000424639	negative

PRJNA729271	stage_E18_vs_E10	ordinary_muscle_development	ENSGALG00010030106	GPRC5C	0.750293127	0.16415388	4.570669455	4.86169E-06	1.1673E-05	positive
PRJNA729271	stage_E18_vs_E10	ordinary_muscle_development	ENSGALG00010030107	RPTOR	0.411834384	0.139216118	2.958237806	0.003094033	0.005288057	positive
PRJNA729271	stage_E18_vs_E10	ordinary_muscle_development	ENSGALG00010030108	LLGL2	0.239923115	0.120043196	1.998639849	0.045647336	0.064526831	positive
PRJNA729271	stage_E18_vs_E10	ordinary_muscle_development	ENSGALG00010030109	FBF1	0.189034818	0.089775752	2.105633369	0.035236212	0.050820785	positive
PRJNA729271	stage_1D_vs_E10	ordinary_muscle_development	ENSGALG00010000007	ND1	1.234489947	0.242793234	5.084531926	3.68534E-07	8.49944E-07	positive
PRJNA729271	stage_1D_vs_E10	ordinary_muscle_development	ENSGALG00010000011	ND2	1.04719932	0.252128075	4.153441932	3.27511E-05	6.36023E-05	positive
PRJNA729271	stage_1D_vs_E10	ordinary_muscle_development	ENSGALG00010000017	COX1	2.333510082	0.272389999	8.566797927	1.06402E-17	5.01399E-17	positive
PRJNA729271	stage_1D_vs_E10	ordinary_muscle_development	ENSGALG00010000020	COX2	1.956299804	0.238988628	8.185744304	2.70626E-16	1.17941E-15	positive
PRJNA729271	stage_1D_vs_E10	ordinary_muscle_development	ENSGALG00010000022	ATP8	1.128341656	0.26951486	4.186565647	2.83207E-05	5.53619E-05	positive
PRJNA729271	stage_1D_vs_E10	ordinary_muscle_development	ENSGALG00010000023	ATP6	1.068529358	0.198661247	5.378650202	7.50464E-08	1.831E-07	positive
PRJNA729271	stage_1D_vs_E10	ordinary_muscle_development	ENSGALG00010000024	COX3	1.724268942	0.221494775	7.784693531	6.98824E-15	2.77671E-14	positive
PRJNA729271	stage_1D_vs_E10	ordinary_muscle_development	ENSGALG00010000026	ND3	1.587596789	0.317787836	4.995775822	5.85997E-07	1.33281E-06	positive
PRJNA729271	stage_1D_vs_E10	ordinary_muscle_development	ENSGALG00010000028	ND4L	2.445911529	0.269913753	9.061826255	1.28284E-19	6.72021E-19	positive
PRJNA729271	stage_1D_vs_E10	ordinary_muscle_development	ENSGALG00010000029	ND4	2.225141447	0.253845488	8.765731708	1.85567E-18	9.11797E-18	positive
PRJNA729271	stage_1D_vs_E10	ordinary_muscle_development	ENSGALG00010000033	ND5	1.989126529	0.23187883	8.578301558	9.62843E-18	4.54754E-17	positive
PRJNA729271	stage_1D_vs_E10	ordinary_muscle_development	ENSGALG00010000034	CYTB	1.844039228	0.218807897	8.427663044	3.52637E-17	1.61245E-16	positive
PRJNA729271	stage_1D_vs_E10	ordinary_muscle_development	ENSGALG00010000037	ND6	0.776718149	0.190763556	4.071627546	4.66858E-05	8.95001E-05	positive
PRJNA729271	stage_1D_vs_E10	ordinary_muscle_development	ENSGALG00010000039	PDIA3	-0.733389773	0.065745566	-11.15496929	6.77162E-29	5.56432E-28	negative
PRJNA729271	stage_1D_vs_E10	ordinary_muscle_development	ENSGALG00010000042	CKMT1B	3.909585677	0.446258033	8.760818603	1.93838E-18	9.51142E-18	positive
PRJNA729271	stage_1D_vs_E10	ordinary_muscle_development	ENSGALG00010000043	PPIP5K1	0.729740276	0.121481298	6.007017438	1.88967E-09	5.22825E-09	positive
PRJNA729271	stage_1D_vs_E10	ordinary_muscle_development	ENSGALG00010000044	MAP1A	0.571495104	0.132420223	4.315769098	1.59048E-05	3.18118E-05	positive
PRJNA729271	stage_1D_vs_E10	ordinary_muscle_development	ENSGALG00010000045	TP53BP1	-1.038538993	0.072803308	-14.26499739	3.61605E-46	5.92927E-45	negative
PRJNA729271	stage_1D_vs_E10	ordinary_muscle_development	ENSGALG00010000046	TUBGCP4	-0.838210554	0.062593374	-13.39136228	6.79234E-41	9.24459E-40	negative
PRJNA729271	stage_1D_vs_E10	ordinary_muscle_development	ENSGALG00010000047	IQGAP1	-0.782524025	0.097211114	-8.049738282	8.29713E-16	3.5053E-15	negative
PRJNA729271	stage_1D_vs_E10	ordinary_muscle_development	ENSGALG00010000048	MAN2A2	0.782195817	0.14828396	5.274986043	1.32766E-07	3.17723E-07	positive
PRJNA729271	stage_1D_vs_E10	ordinary_muscle_development	ENSGALG00010000049	FES	0.595707427	0.194797822	3.05808053	0.002227597	0.003563516	positive
PRJNA729271	stage_1D_vs_E10	ordinary_muscle_development	ENSGALG00010000050	CIB1	0.223385498	0.130702951	1.709108293	0.087430892	0.112260676	positive
PRJNA729271	stage_1D_vs_E10	ordinary_muscle_development	ENSGALG00010000051	GDPGP1	-0.064160557	0.189330225	-0.338881744	0.734698818	0.768470152	negative
PRJNA729271	stage_1D_vs_E10	ordinary_muscle_development	ENSGALG00010000052	TTLL13	1.056794318	0.131068659	8.062906307	7.45016E-16	3.15761E-15	positive
PRJNA729271	stage_1D_vs_E10	ordinary_muscle_development	ENSGALG00010000053	HDDC3	1.016766519	0.112969928	9.000329003	2.25042E-19	1.1625E-18	positive
PRJNA729271	stage_1D_vs_E10	ordinary_muscle_development	ENSGALG00010000054	VPS33B	0.266064688	0.099318797	2.678895597	0.007386542	0.011113549	positive
PRJNA729271	stage_1D_vs_E10	ordinary_muscle_development	ENSGALG00010000055	PRC1	-0.920178075	0.211629066	-4.348070395	1.37341E-05	2.76685E-05	negative
PRJNA729271	stage_1D_vs_E10	ordinary_muscle_development	ENSGALG00010000056	UNC45A	0.022003149	0.081576461	0.269724236	0.787372414	0.815328128	positive
PRJNA729271	stage_1D_vs_E10	ordinary_muscle_development	ENSGALG00010000058	FURIN	0.587417438	0.172747637	3.40043689	0.000672783	0.001141919	positive
PRJNA729271	stage_1D_vs_E10	ordinary_muscle_development	ENSGALG00010000059	MFAP1	-0.372535394	0.110700295	-3.365261085	0.000764713	0.001292202	negative
PRJNA729271	stage_1D_vs_E10	ordinary_muscle_development	ENSGALG00010000060	HYPK	0.413055876	0.245102746	1.68523561	0.091943123	0.117668689	positive
PRJNA729271	stage_1D_vs_E10	ordinary_muscle_development	ENSGALG00010000061	SERINC4	2.712693988	0.33473538	8.103995425	5.31831E-16	2.28006E-15	positive
PRJNA729271	stage_1D_vs_E10	ordinary_muscle_development	ENSGALG00010000063	SERF2	-0.43066892	0.127668467	-3.373338232	0.000742627	0.001256493	negative
PRJNA729271	stage_1D_vs_E10	ordinary_muscle_development	ENSGALG00010000069	ENSGALG00010000069	1.935856919	0.233340162	8.296286867	1.07416E-16	4.78192E-16	positive
PRJNA729271	stage_1D_vs_E10	ordinary_muscle_development	ENSGALG00010000076	ENSGALG00010000076	1.678107519	0.185873102	9.028242923	1.7445E-19	9.06328E-19	positive
PRJNA729271	stage_1D_vs_E10	ordinary_muscle_development	ENSGALG00010000078	ENSGALG00010000078	0.488538081	0.469972898	1.039502667	0.298571018	0.345449147	positive
PRJNA729271	stage_1D_vs_E10	ordinary_muscle_development	ENSGALG00010000081	ENSGALG00010000081	5.922969409	0.299293839	19.78981401	3.64384E-87	2.04719E-85	positive
PRJNA729271	stage_1D_vs_E10	ordinary_muscle_development	ENSGALG00010000084	ENSGALG00010000084	1.211833796	0.26265379	4.613806631	3.9536E-06	8.37706E-06	positive
PRJNA729271	stage_1D_vs_E10	ordinary_muscle_development	ENSGALG00010000085	ENSGALG00010000085	-0.052312617	0.146443268	-0.357221042	0.720926335	0.755901679	negative
PRJNA729271	stage_1D_vs_E10	ordinary_muscle_development	ENSGALG00010000089	ENSGALG00010000089	2.168835102	0.281443592	7.706109366	1.29711E-14	5.06784E-14	positive
PRJNA729271	stage_1D_vs_E10	ordinary_muscle_development	ENSGALG00010000091	ENSGALG00010000091	3.533054517	0.881391772	4.008495008	6.11069E-05	0.00011592	positive
PRJNA729271	stage_1D_vs_E10	ordinary_muscle_development	ENSGALG00010000092	INPPL1	1.438681927	0.170197403	8.453019265	2.83865E-17	1.30789E-16	positive
PRJNA729271	stage_1D_vs_E10	ordinary_muscle_development	ENSGALG00010000093	ENSGALG00010000093	2.252158649	0.327675963	6.873127424	6.28094E-12	2.05652E-11	positive
PRJNA729271	stage_1D_vs_E10	ordinary_muscle_development	ENSGALG00010000098	ENSGALG00010000098	1.16818519	0.190937198	6.118164523	9.46593E-10	2.68248E-09	positive
PRJNA729271	stage_1D_vs_E10	ordinary_muscle_development	ENSGALG00010000105	CACNA1F	0.176770259	0.292484519	0.604374753	0.545594517	0.591547051	positive
PRJNA729271	stage_1D_vs_E10	ordinary_muscle_development	ENSGALG00010000107	ENSGALG00010000107	-0.636379352	0.539869616	-1.178764897	0.238491806	0.282222118	negative
PRJNA729271	stage_1D_vs_E10	ordinary_muscle_development	ENSGALG00010000121	ENSGALG00010000121	0.463475144	0.133057082	3.483280542	0.000495309	0.000853281	positive
PRJNA729271	stage_1D_vs_E10	ordinary_muscle_development	ENSGALG00010000123	ENSGALG00010000123	1.059572436	0.232354593	4.560152746	5.11164E-06	1.07195E-05	positive
PRJNA729271	stage_1D_vs_E10	ordinary_muscle_development	ENSGALG00010000124	ENSGALG00010000124	1.252316977	0.182077004	6.877952465	6.07189E-12	1.98897E-11	positive
PRJNA729271	stage_1D_vs_E10	ordinary_muscle_development	ENSGALG00010000126	ENSGALG00010000126	0.202651194	0.203921413	0.993771037	0.32033435	0.368394668	positive
PRJNA729271	stage_1D_vs_E10	ordinary_muscle_development	ENSGALG00010000127	ENSGALG00010000127	1.476549413	0.203221144	7.265727312	3.71036E-13	1.32435E-12	positive
PRJNA729271	stage_1D_vs_E10	ordinary_muscle_development	ENSGALG00010000133	USP39	-0.221577707	0.141132202	-1.570001064	0.116414864	0.14641505	negative
PRJNA729271	stage_1D_vs_E10	ordinary_muscle_development	ENSGALG00010000134	ENSGALG00010000134	0.467494316	0.127198934	3.675300587	0.00023757	0.000423355	positive
PRJNA729271	stage_1D_vs_E10	ordinary_muscle_development	ENSGALG00010000137	ENSGALG00010000137	0.820688175	0.539782085	1.520406471	0.128408849	0.160220906	positive
PRJNA729271	stage_1D_vs_E10	ordinary_muscle_development	ENSGALG00010000140	SEPHS3	-0.505083182	0.12741159	-3.964185543	7.3647E-05	0.000138458	negative
PRJNA729271	stage_1D_vs_E10	ordinary_muscle_development	ENSGALG00010000141	ENSGALG00010000141	-0.411580443	0.139744359	-2.94523834	0.00322706	0.005067298	negative
PRJNA729271	stage_1D_vs_E10	ordinary_muscle_development	ENSGALG00010000143	SCARF2	-1.324610344	0.159440359	-8.307873584	9.74322E-17	4.34949E-16	negative
PRJNA729271	stage_1D_vs_E10	ordinary_muscle_development	ENSGALG00010000145	ENSGALG00010000145	0.626569744	0.27973154	2.239896668	0.025097632	0.035014932	positive
PRJNA729271	stage_1D_vs_E10	ordinary_muscle_development	ENSGALG00010000146	ACADM	3.838119711	0.157639175	24.34749938	6.1619E-131	7.8348E-129	positive
PRJNA729271	stage_1D_vs_E10	ordinary_muscle_development	ENSGALG00010000147	ENSGALG00010000147	0.242130265	0.108337372	2.234965277	0.025419628	0.035435022	positive
PRJNA729271	stage_1D_vs_E10	ordinary_muscle_development	ENSGALG00010000148	ENSGALG00010000148	1.327961505	0.275924308	4.812774624	1.48849E-06	3.27848E-06	positive
PRJNA729271	stage_1D_vs_E10	ordinary_muscle_development	ENSGALG00010000149	ENSGALG00010000149	-0.319779038	0.137595925	-2.324044396	0.020123115	0.028457029	negative
PRJNA729271	stage_1D_vs_E10	ordinary_muscle_development	ENSGALG00010000150	B3GAT3	0.083599623	0.126239165	0.662232071	0.507822504	0.555832303	positive
PRJNA729271	stage_1D_vs_E10	ordinary_muscle_development	ENSGALG00010000151	TBX5	-3.750671136	0.241972267	-15.50041742	3.44646E-54	7.66203E-53	negative
PRJNA729271	stage_1D_vs_E10	ordinary_muscle_development	ENSGALG00010000153	NF2	-0.075886394	0.093549112	-0.811193102	0.417254786	0.466711021	negative
PRJNA729271	stage_1D_vs_E10	ordinary_muscle_development	ENSGALG00010000154	ENSGALG00010000154	-21.09418731	4.021492466	-5.245362882	1.55975E-07	3.7118E-07	negative
PRJNA729271	stage_1D_vs_E10	ordinary_muscle_development	ENSGALG00010000155	MAZ	-0.61122922	0.166337198	-3.674639401	0.000238186	0.000424349	negative
PRJNA729271	stage_1D_vs_E10	ordinary_muscle_development	ENSGALG00010000156	ENSGALG00010000156	0.250648006	0.52469215	0.47770489	0.632860263	0.674309726	positive
PRJNA729271	stage_1D_vs_E10	ordinary_muscle_development	ENSGALG00010000157	ENSGALG00010000157	-18.99968692	5.370802047	-3.537588382			negative
PRJNA729271	stage_1D_vs_E10	ordinary_muscle_development	ENSGALG00010000161	CDC42EP2	1.413730651	0.311489677	4.538611571	5.66258E-06	1.1827E-05	positive
PRJNA729271	stage_1D_vs_E10	ordinary_muscle_development	ENSGALG00010000166	PFDN6	0.018553838	0.12965287	0.143103948	0.886208087	0.90315589	positive
PRJNA729271	stage_1D_vs_E10	ordinary_muscle_development	ENSGALG00010000171	DPF2	0.518953792	0.199756112	2.597936982	0.00937857	0.01391854	positive
PRJNA729271	stage_1D_vs_E10	ordinary_muscle_development	ENSGALG00010000172	ENSGALG00010000172	0.709514743	1.11940309	0.633833111	0.526189742	0.573122957	positive
PRJNA729271	stage_1D_vs_E10	ordinary_muscle_development	ENSGALG00010000173	NIPSNAP1	-1.103515465	0.128084165	-8.615549492	6.96069E-18	3.31565E-17	negative
PRJNA729271	stage_1D_vs_E10	ordinary_muscle_development	ENSGALG00010000174	ENSGALG00010000174	0.75816684	0.096296424	7.873260553	3.45516E-15	1.40445E-14	positive
PRJNA729271	stage_1D_vs_E10	ordinary_muscle_development	ENSGALG00010000175	ENSGALG00010000175	-0.810339997	0.877351353	-0.923620843	0.355683758	0.404553992	negative
PRJNA729271	stage_1D_vs_E10	ordinary_muscle_development	ENSGALG00010000176	ENSGALG00010000176	0.580818661	0.493955289	1.175852702	0.239653742	0.283481393	positive
PRJNA729271	stage_1D_vs_E10	ordinary_muscle_development	ENSGALG00010000177	ENSGALG00010000177	0.593219475	0.208938225	2.83920989	0.00452254	0.006994688	positive
PRJNA729271	stage_1D_vs_E10	ordinary_muscle_development	ENSGALG00010000184	ENSGALG00010000184	5.48629749	3.404834479	1.611325756	0.10710874	0.135427528	positive
PRJNA729271	stage_1D_vs_E10	ordinary_muscle_development	ENSGALG00010000186	THOC5	-0.49205266	0.079814488	-6.164954123	7.05036E-10	2.0125E-09	negative
PRJNA729271	stage_1D_vs_E10	ordinary_muscle_development	ENSGALG00010000188	ENSGALG00010000188	-0.411956566	1.341241724	-0.307145654	0.75873251	0.789790142	negative
PRJNA729271	stage_1D_vs_E10	ordinary_muscle_development	ENSGALG00010000189	ENSGALG00010000189	1.002164727	0.58478172	1.713741544	0.086576203	0.111271685	positive
PRJNA729271	stage_1D_vs_E10	ordinary_muscle_development	ENSGALG00010000191	NEFH	3.619598119	0.3868209	9.357297177	8.18024E-21	4.57986E-20	positive
PRJNA729271	stage_1D_vs_E10	ordinary_muscle_development	ENSGALG00010000192	ENSGALG00010000192	1.440324385	0.679480143	2.119744632	0.034027587	0.046579457	positive
PRJNA729271	stage_1D_vs_E10	ordinary_muscle_development	ENSGALG00010000196	KSR2	-4.516358902	1.016376219	-4.443589704	8.84702E-06	1.81768E-05	negative
PRJNA729271	stage_1D_vs_E10	ordinary_muscle_development	ENSGALG00010000197	ENSGALG00010000197	-0.399246773	0.51194351	-0.779864898	0.435470402	0.484840884	negative
PRJNA729271	stage_1D_vs_E10	ordinary_muscle_development	ENSGALG00010000200	GAL3ST1	-0.94347998	0.22856431	-4.127853472	3.66165E-05	7.08148E-05	negative
PRJNA729271	stage_1D_vs_E10	ordinary_muscle_development	ENSGALG00010000202	ENSGALG00010000202	-0.750711616	0.302500806	-2.481684679	0.013076293	0.019022568	negative
PRJNA729271	stage_1D_vs_E10	ordinary_muscle_development	ENSGALG00010000205	RNF215	0.565104212	0.110932474	5.094127923	3.5035E-07	8.09644E-07	positive
PRJNA729271	stage_1D_vs_E10	ordinary_muscle_development	ENSGALG00010000210	CCDC157	-0.558006755	0.523884038	-1.065134104	0.286815248	0.333150655	negative
PRJNA729271	stage_1D_vs_E10	ordinary_muscle_development	ENSGALG00010000215	SF3A1	-0.007229583	0.089652044	-0.080640469	0.935727881	0.945248843	negative
PRJNA729271	stage_1D_vs_E10	ordinary_muscle_development	ENSGALG00010000221	TBC1D10A	-0.324276313	0.08825997	-3.67410405	0.000238685	0.000425082	negative
PRJNA729271	stage_1D_vs_E10	ordinary_muscle_development	ENSGALG00010000223	CASTOR1	0.921277437	0.153195133	6.013751363	1.81278E-09	5.02415E-09	positive
PRJNA729271	stage_1D_vs_E10	ordinary_muscle_development	ENSGALG00010000224	MYBPC2	9.564494927	0.251807906	37.98329881	1.0889E-315	1.7538E-312	positive
PRJNA729271	stage_1D_vs_E10	ordinary_muscle_development	ENSGALG00010000225	LIF	3.449072507	0.455874847	7.565831997	3.8539E-14	1.46505E-13	positive
PRJNA729271	stage_1D_vs_E10	ordinary_muscle_development	ENSGALG00010000226	ENSGALG00010000226	1.377928338	0.407264001	3.383378682	0.000715998	0.001212996	positive
PRJNA729271	stage_1D_vs_E10	ordinary_muscle_development	ENSGALG00010000227	ENSGALG00010000227	-0.51572349	0.293420805	-1.757624137	0.078811484	0.102024869	negative
PRJNA729271	stage_1D_vs_E10	ordinary_muscle_development	ENSGALG00010000231	ENSGALG00010000231	3.431235227	0.440312605	7.792725423	6.5579E-15	2.61073E-14	positive
PRJNA729271	stage_1D_vs_E10	ordinary_muscle_development	ENSGALG00010000232	ENSGALG00010000232	0.106421072	0.162546457	0.654711729	0.512653372	0.560343129	positive
PRJNA729271	stage_1D_vs_E10	ordinary_muscle_development	ENSGALG00010000233	HORMAD2	-1.308879375	0.503371065	-2.600227678	0.009316193	0.013831631	negative
PRJNA729271	stage_1D_vs_E10	ordinary_muscle_development	ENSGALG00010000234	ENSGALG00010000234	-3.546621709	4.6114594	-0.769088785			negative
PRJNA729271	stage_1D_vs_E10	ordinary_muscle_development	ENSGALG00010000235	ENSGALG00010000235	-1.252112574	0.225128345	-5.561772208	2.67049E-08	6.72727E-08	negative
PRJNA729271	stage_1D_vs_E10	ordinary_muscle_development	ENSGALG00010000239	ENSGALG00010000239	-8.750700671	4.691732336	-1.865132118	0.062162888	0.081631732	negative
PRJNA729271	stage_1D_vs_E10	ordinary_muscle_development	ENSGALG00010000242	ENSGALG00010000242	2.277152445	0.192676558	11.81852361	3.13139E-32	3.00992E-31	positive
PRJNA729271	stage_1D_vs_E10	ordinary_muscle_development	ENSGALG00010000246	ENSGALG00010000246	-19.27784589	4.315478855	-4.467139461	7.92725E-06	1.63543E-05	negative
PRJNA729271	stage_1D_vs_E10	ordinary_muscle_development	ENSGALG00010000247	ENSGALG00010000247	5.444414033	0.223344234	24.3767835	3.0155E-131	3.9378E-129	positive
PRJNA729271	stage_1D_vs_E10	ordinary_muscle_development	ENSGALG00010000249	ENSGALG00010000249	-22.32713205	4.458072001	-5.008248418			negative
PRJNA729271	stage_1D_vs_E10	ordinary_muscle_development	ENSGALG00010000254	ENSGALG00010000254	-1.88276822	0.61853227	-3.04392885	0.002335104	0.003725905	negative
PRJNA729271	stage_1D_vs_E10	ordinary_muscle_development	ENSGALG00010000255	ENSGALG00010000255	0.128801227	0.145144253	0.887401492	0.374862781	0.424138319	positive
PRJNA729271	stage_1D_vs_E10	ordinary_muscle_development	ENSGALG00010000256	ENSGALG00010000256	-0.701925749	0.399194962	-1.758353226	0.078687426	0.10188247	negative
PRJNA729271	stage_1D_vs_E10	ordinary_muscle_development	ENSGALG00010000262	HNRNPKL	-6.039312435	3.528838283	-1.711466605	0.087004234	0.111742546	negative
PRJNA729271	stage_1D_vs_E10	ordinary_muscle_development	ENSGALG00010000263	MTMR3	1.108561191	0.091289145	12.14340644	6.21823E-34	6.44269E-33	positive
PRJNA729271	stage_1D_vs_E10	ordinary_muscle_development	ENSGALG00010000267	ENSGALG00010000267	0.040006774	0.122564799	0.326413248	0.744111715	0.776676759	positive
PRJNA729271	stage_1D_vs_E10	ordinary_muscle_development	ENSGALG00010000269	ENSGALG00010000269	-0.881767937	0.476603384	-1.850108424	0.064297924	0.084313227	negative
PRJNA729271	stage_1D_vs_E10	ordinary_muscle_development	ENSGALG00010000271	RTN4R	-2.752352799	0.391648046	-7.027617853	2.10089E-12	7.11506E-12	negative
PRJNA729271	stage_1D_vs_E10	ordinary_muscle_development	ENSGALG00010000272	ENSGALG00010000272	-0.884774671	0.416846875	-2.122541212	0.033792316	0.04629675	negative
PRJNA729271	stage_1D_vs_E10	ordinary_muscle_development	ENSGALG00010000277	ENSGALG00010000277	-0.015856169	0.687789561	-0.023053808	0.981607351	0.984119419	negative
PRJNA729271	stage_1D_vs_E10	ordinary_muscle_development	ENSGALG00010000279	ENSGALG00010000279	-1.117804323	0.135526872	-8.247842701	1.61279E-16	7.11643E-16	negative
PRJNA729271	stage_1D_vs_E10	ordinary_muscle_development	ENSGALG00010000281	OSBP2	2.70742791	0.202893087	13.34411118	1.2819E-40	1.72527E-39	positive
PRJNA729271	stage_1D_vs_E10	ordinary_muscle_development	ENSGALG00010000282	ENSGALG00010000282	-21.77393171	4.991011543	-4.362629003	1.28509E-05	2.59904E-05	negative
PRJNA729271	stage_1D_vs_E10	ordinary_muscle_development	ENSGALG00010000284	ENSGALG00010000284	-0.95463689	0.154052616	-6.196823623	5.7614E-10	1.65504E-09	negative
PRJNA729271	stage_1D_vs_E10	ordinary_muscle_development	ENSGALG00010000285	MYLPF	4.576795416	0.145696747	31.41316141	1.3378E-216	8.4308E-214	positive
PRJNA729271	stage_1D_vs_E10	ordinary_muscle_development	ENSGALG00010000286	TCN2	2.333509584	0.135970274	17.16190982	5.12019E-66	1.64927E-64	positive
PRJNA729271	stage_1D_vs_E10	ordinary_muscle_development	ENSGALG00010000287	IQCD	-2.763704344	0.243725192	-11.33942831	8.36894E-30	7.14415E-29	negative
PRJNA729271	stage_1D_vs_E10	ordinary_muscle_development	ENSGALG00010000288	VARS2	0.543932867	0.152711512	3.561832766	0.000368275	0.000643383	positive
PRJNA729271	stage_1D_vs_E10	ordinary_muscle_development	ENSGALG00010000289	QPRT	-2.060140605	0.46151898	-4.463826394	8.05088E-06	1.65905E-05	negative

PRJNA729271	stage_1D_vs_E10	ordinary_muscle_development	ENSGALG00010000292	ABHD16A	0.869756489	0.128100599	6.789636379	1.12417E-11	3.6267E-11	positive
PRJNA729271	stage_1D_vs_E10	ordinary_muscle_development	ENSGALG00010000293	ENSGALG00010000293	0.564596845	0.372996537	1.513678514	0.130107397	0.162214581	positive
PRJNA729271	stage_1D_vs_E10	ordinary_muscle_development	ENSGALG00010000294	DUSP21	-1.549505504	0.302675652	-5.119359605	3.06575E-07	7.11692E-07	negative
PRJNA729271	stage_1D_vs_E10	ordinary_muscle_development	ENSGALG00010000298	SLC35E4	1.140665289	0.214305631	5.322609987	1.02289E-07	2.46907E-07	positive
PRJNA729271	stage_1D_vs_E10	ordinary_muscle_development	ENSGALG00010000301	UQCR10	1.461412763	0.154712682	9.445979091	3.52097E-21	2.0101E-20	positive
PRJNA729271	stage_1D_vs_E10	ordinary_muscle_development	ENSGALG00010000302	ENSGALG00010000302	-0.181466568	0.235365715	-0.770998307	0.440707935	0.489957165	negative
PRJNA729271	stage_1D_vs_E10	ordinary_muscle_development	ENSGALG00010000303	ENSGALG00010000303	-0.308289403	0.112995124	-2.728342524	0.006365347	0.009652235	negative
PRJNA729271	stage_1D_vs_E10	ordinary_muscle_development	ENSGALG00010000306	ENSGALG00010000306	1.733046095	0.205631452	8.427923255	3.51854E-17	1.60938E-16	positive
PRJNA729271	stage_1D_vs_E10	ordinary_muscle_development	ENSGALG00010000307	ZMAT5	0.366794592	0.112838617	3.25061226	0.001151568	0.001907655	positive
PRJNA729271	stage_1D_vs_E10	ordinary_muscle_development	ENSGALG00010000308	ENSGALG00010000308	-0.153154145	0.345126306	-0.443762596	0.657214234	0.69652119	negative
PRJNA729271	stage_1D_vs_E10	ordinary_muscle_development	ENSGALG00010000309	ENSGALG00010000309	-22.56039938	4.357230699	-5.177692195	2.24647E-07	5.2733E-07	negative
PRJNA729271	stage_1D_vs_E10	ordinary_muscle_development	ENSGALG00010000312	CABP7	-2.118217287	0.523505888	-4.046214825	5.20525E-05	9.92633E-05	negative
PRJNA729271	stage_1D_vs_E10	ordinary_muscle_development	ENSGALG00010000313	ENSGALG00010000313	-6.073149793	3.863480329	-1.571937547	0.115965036	0.145975961	negative
PRJNA729271	stage_1D_vs_E10	ordinary_muscle_development	ENSGALG00010000318	ENSGALG00010000318	-22.39108995	4.79588298	-4.668814908	3.02942E-06	6.49674E-06	negative
PRJNA729271	stage_1D_vs_E10	ordinary_muscle_development	ENSGALG00010000323	AP1B1	0.593439769	0.144612104	4.10366596	4.06654E-05	7.82796E-05	positive
PRJNA729271	stage_1D_vs_E10	ordinary_muscle_development	ENSGALG00010000324	ENSGALG00010000324	0.380611	0.166531819	2.285515172	0.022282638	0.031348814	positive
PRJNA729271	stage_1D_vs_E10	ordinary_muscle_development	ENSGALG00010000326	ENSGALG00010000326	-3.814462192	1.847502838	-2.064658366	0.038955337	0.052801347	negative
PRJNA729271	stage_1D_vs_E10	ordinary_muscle_development	ENSGALG00010000331	ENSGALG00010000331	-0.312342802	0.169065293	-1.847468495	0.06467927	0.08479027	negative
PRJNA729271	stage_1D_vs_E10	ordinary_muscle_development	ENSGALG00010000338	ENSGALG00010000338	-0.462350178	0.191445227	-2.415051998	0.015732963	0.022605998	negative
PRJNA729271	stage_1D_vs_E10	ordinary_muscle_development	ENSGALG00010000340	NAA10	-0.387210791	0.133471589	-2.901072769	0.003718875	0.005802486	negative
PRJNA729271	stage_1D_vs_E10	ordinary_muscle_development	ENSGALG00010000341	EWSR1	-0.849884976	0.147609424	-5.75766066	8.52876E-09	2.23998E-08	negative
PRJNA729271	stage_1D_vs_E10	ordinary_muscle_development	ENSGALG00010000346	ENSGALG00010000346	0.024401819	0.112596494	0.216719176	0.828427203	0.852784057	positive
PRJNA729271	stage_1D_vs_E10	ordinary_muscle_development	ENSGALG00010000349	CHIR-IG1-5	1.276690949	0.45402428	2.811944216	0.004924304	0.00757726	positive
PRJNA729271	stage_1D_vs_E10	ordinary_muscle_development	ENSGALG00010000350	ENSGALG00010000350	0.304629843	0.165540024	1.840218673	0.06573614	0.086066782	positive
PRJNA729271	stage_1D_vs_E10	ordinary_muscle_development	ENSGALG00010000353	ENSGALG00010000353	0.695224159	0.110471699	6.293233181	3.10921E-10	9.10648E-10	positive
PRJNA729271	stage_1D_vs_E10	ordinary_muscle_development	ENSGALG00010000356	ENSGALG00010000356	1.347859686	0.118788533	11.34671543	7.70012E-30	6.59442E-29	positive
PRJNA729271	stage_1D_vs_E10	ordinary_muscle_development	ENSGALG00010000366	EMID1	-0.937202843	0.21954509	-4.268839917	1.96492E-05	3.89518E-05	negative
PRJNA729271	stage_1D_vs_E10	ordinary_muscle_development	ENSGALG00010000367	HSD17B10	0.646828584	0.138763764	4.661365235	3.14119E-06	6.72647E-06	positive
PRJNA729271	stage_1D_vs_E10	ordinary_muscle_development	ENSGALG00010000373	ENSGALG00010000373	1.168015446	0.50357487	2.319447446	0.020370788	0.028787615	positive
PRJNA729271	stage_1D_vs_E10	ordinary_muscle_development	ENSGALG00010000376	ASCC2	-0.716443074	0.085053459	-8.423444346	3.65575E-17	1.67003E-16	negative
PRJNA729271	stage_1D_vs_E10	ordinary_muscle_development	ENSGALG00010000377	ENSGALG00010000377	1.211232438	0.227875998	5.31531381	1.06474E-07	2.56785E-07	positive
PRJNA729271	stage_1D_vs_E10	ordinary_muscle_development	ENSGALG00010000381	ENSGALG00010000381	-1.154374145	0.309846324	-3.725634469	0.000194825	0.000350283	negative
PRJNA729271	stage_1D_vs_E10	ordinary_muscle_development	ENSGALG00010000382	ENSGALG00010000382	-0.169913492	0.50500103	-0.336461674	0.736522748	0.770100067	negative
PRJNA729271	stage_1D_vs_E10	ordinary_muscle_development	ENSGALG00010000384	ENSGALG00010000384	0.589633496	0.142765922	4.130071712	3.6265E-05	7.01537E-05	positive
PRJNA729271	stage_1D_vs_E10	ordinary_muscle_development	ENSGALG00010000386	ENSGALG00010000386	0.730823347	0.352304035	2.074410947	0.038041161	0.051654017	positive
PRJNA729271	stage_1D_vs_E10	ordinary_muscle_development	ENSGALG00010000387	DTX1	-1.860883063	0.480519733	-3.872646501	0.00010766	0.000199149	negative
PRJNA729271	stage_1D_vs_E10	ordinary_muscle_development	ENSGALG00010000388	ENSGALG00010000388	-0.446122591	0.184811036	-2.413939128	0.015781099	0.022662006	negative
PRJNA729271	stage_1D_vs_E10	ordinary_muscle_development	ENSGALG00010000391	ENSGALG00010000391	1.074625371	0.698339938	1.538828459	0.12384618	0.155061794	positive
PRJNA729271	stage_1D_vs_E10	ordinary_muscle_development	ENSGALG00010000402	PFKFB1	7.765478116	0.318671405	24.36829285	3.7101E-131	4.8015E-129	positive
PRJNA729271	stage_1D_vs_E10	ordinary_muscle_development	ENSGALG00010000405	ENSGALG00010000405	2.721854631	0.384290268	7.082809159	1.41261E-12	4.83148E-12	positive
PRJNA729271	stage_1D_vs_E10	ordinary_muscle_development	ENSGALG00010000406	NSMCE3	-0.160731336	0.097535591	-1.647924976	0.099368068	0.126401065	negative
PRJNA729271	stage_1D_vs_E10	ordinary_muscle_development	ENSGALG00010000407	WSB2	0.19028612	0.085373286	2.228871922	0.025822428	0.035934725	positive
PRJNA729271	stage_1D_vs_E10	ordinary_muscle_development	ENSGALG00010000408	ENSGALG00010000408	9.058423123	0.349974071	25.88312643	1.0315E-147	1.78E-145	positive
PRJNA729271	stage_1D_vs_E10	ordinary_muscle_development	ENSGALG00010000411	ENSGALG00010000411	4.000236194	0.884413193	4.523039936	6.09577E-06	1.27043E-05	positive
PRJNA729271	stage_1D_vs_E10	ordinary_muscle_development	ENSGALG00010000412	RFC5	-0.46043204	0.105213709	-4.376160166	1.20788E-05	2.44768E-05	negative
PRJNA729271	stage_1D_vs_E10	ordinary_muscle_development	ENSGALG00010000422	ITIH6	-1.965688202	0.566483873	-3.469980871	0.000520495	0.000894438	negative
PRJNA729271	stage_1D_vs_E10	ordinary_muscle_development	ENSGALG00010000423	ENSGALG00010000423	1.557689795	0.205203951	7.590934727	3.17606E-14	1.21342E-13	positive
PRJNA729271	stage_1D_vs_E10	ordinary_muscle_development	ENSGALG00010000428	GNL3L	-0.707481785	0.147772216	-4.787650909	1.68745E-06	3.69815E-06	negative
PRJNA729271	stage_1D_vs_E10	ordinary_muscle_development	ENSGALG00010000431	AIFM3	-4.395844561	1.177696049	-3.732579867	0.000189529	0.000341439	negative
PRJNA729271	stage_1D_vs_E10	ordinary_muscle_development	ENSGALG00010000432	ENSGALG00010000432	1.218440369	0.181653754	6.707487971	1.98003E-11	6.28296E-11	positive
PRJNA729271	stage_1D_vs_E10	ordinary_muscle_development	ENSGALG00010000434	TP53	0.346830307	0.216805663	1.599729005	0.109658714	0.138386128	positive
PRJNA729271	stage_1D_vs_E10	ordinary_muscle_development	ENSGALG00010000435	P2RX6	-0.032048191	0.297105152	-0.107868177	0.914100261	0.92701905	negative
PRJNA729271	stage_1D_vs_E10	ordinary_muscle_development	ENSGALG00010000436	ENSGALG00010000436	1.413214522	0.378604008	3.73269826	0.000189439	0.000341321	positive
PRJNA729271	stage_1D_vs_E10	ordinary_muscle_development	ENSGALG00010000440	ENSGALG00010000440	-1.555440209	0.441959253	-3.519419944	0.000432492	0.00074907	negative
PRJNA729271	stage_1D_vs_E10	ordinary_muscle_development	ENSGALG00010000441	SLC7A4	-0.936822662	0.202851464	-4.618269162	3.86954E-06	8.20655E-06	negative
PRJNA729271	stage_1D_vs_E10	ordinary_muscle_development	ENSGALG00010000442	DGCR6	-0.848231993	0.130445749	-6.502565266	7.89617E-11	2.40806E-10	negative
PRJNA729271	stage_1D_vs_E10	ordinary_muscle_development	ENSGALG00010000443	TBX3	-0.378349254	0.156735546	-2.413933933	0.015781324	0.022662006	negative
PRJNA729271	stage_1D_vs_E10	ordinary_muscle_development	ENSGALG00010000444	RBM19	-0.451278405	0.118701944	-3.801777698	0.000143662	0.000262528	negative
PRJNA729271	stage_1D_vs_E10	ordinary_muscle_development	ENSGALG00010000449	PLBD2	1.571332944	0.107156051	14.66396843	1.09671E-48	2.01226E-47	positive
PRJNA729271	stage_1D_vs_E10	ordinary_muscle_development	ENSGALG00010000450	ENSGALG00010000450	0.341355152	0.189175461	1.80443674	0.071162868	0.092661316	positive
PRJNA729271	stage_1D_vs_E10	ordinary_muscle_development	ENSGALG00010000452	SLC8B1	1.59381387	0.225363396	7.072194949	1.52502E-12	5.20734E-12	positive
PRJNA729271	stage_1D_vs_E10	ordinary_muscle_development	ENSGALG00010000453	ACHE	1.183040077	0.241996552	4.888665019	1.01522E-06	2.26848E-06	positive
PRJNA729271	stage_1D_vs_E10	ordinary_muscle_development	ENSGALG00010000457	TPCN1	-0.105576348	0.080523491	-1.311124827	0.189815594	0.229338754	negative
PRJNA729271	stage_1D_vs_E10	ordinary_muscle_development	ENSGALG00010000458	ENSGALG00010000458	0.564736684	0.199426347	2.831805785	0.004628595	0.007147846	positive
PRJNA729271	stage_1D_vs_E10	ordinary_muscle_development	ENSGALG00010000462	ENSGALG00010000462	-0.473332838	0.223367807	-2.11907367	0.034084242	0.046639392	negative
PRJNA729271	stage_1D_vs_E10	ordinary_muscle_development	ENSGALG00010000465	ENSGALG00010000465	0.482954533	0.192549095	2.508215029	0.01213428	0.017744793	positive
PRJNA729271	stage_1D_vs_E10	ordinary_muscle_development	ENSGALG00010000466	FBXO21	-0.186584339	0.079893504	-2.33541314	0.019521849	0.027663427	negative
PRJNA729271	stage_1D_vs_E10	ordinary_muscle_development	ENSGALG00010000467	RNASEK	0.178711123	0.199281405	0.896777716	0.369837541	0.419008532	positive
PRJNA729271	stage_1D_vs_E10	ordinary_muscle_development	ENSGALG00010000468	TESC	-3.223725078	0.31866026	-10.11649549	4.66832E-24	3.04534E-23	negative
PRJNA729271	stage_1D_vs_E10	ordinary_muscle_development	ENSGALG00010000469	ENSGALG00010000469	7.695539078	0.384754272	20.00117901	5.37859E-89	3.14365E-87	positive
PRJNA729271	stage_1D_vs_E10	ordinary_muscle_development	ENSGALG00010000470	FBXW8	0.158528892	0.10785039	1.469896328	0.141589834	0.175324163	positive
PRJNA729271	stage_1D_vs_E10	ordinary_muscle_development	ENSGALG00010000471	ENSGALG00010000471	-0.026688981	0.199034303	-0.13409237	0.893329533	0.909135125	negative
PRJNA729271	stage_1D_vs_E10	ordinary_muscle_development	ENSGALG00010000475	RNFT2	-0.877639138	0.110752621	-7.924319365	2.29399E-15	9.43036E-15	negative
PRJNA729271	stage_1D_vs_E10	ordinary_muscle_development	ENSGALG00010000476	ENSGALG00010000476	5.870555334	0.214137968	27.41482693	1.8257E-165	4.3383E-163	positive
PRJNA729271	stage_1D_vs_E10	ordinary_muscle_development	ENSGALG00010000479	C12orf49	-0.482227185	0.114887882	-4.197372038	2.7003E-05	5.28359E-05	negative
PRJNA729271	stage_1D_vs_E10	ordinary_muscle_development	ENSGALG00010000483	ENSGALG00010000483	0.971519027	0.243019482	3.99770018	6.39609E-05	0.000121112	positive
PRJNA729271	stage_1D_vs_E10	ordinary_muscle_development	ENSGALG00010000485	MED13L	1.674429267	0.125584981	13.33303749	1.48717E-40	1.99782E-39	positive
PRJNA729271	stage_1D_vs_E10	ordinary_muscle_development	ENSGALG00010000491	ENSGALG00010000491	1.30798563	0.154329767	8.475264702	2.34546E-17	1.08583E-16	positive
PRJNA729271	stage_1D_vs_E10	ordinary_muscle_development	ENSGALG00010000495	ENSGALG00010000495	1.204521659	0.536142963	2.246642671	0.024662873	0.034463351	positive
PRJNA729271	stage_1D_vs_E10	ordinary_muscle_development	ENSGALG00010000501	ENSGALG00010000501	0.05516356	0.163490804	0.33741078	0.735807259	0.769518486	positive
PRJNA729271	stage_1D_vs_E10	ordinary_muscle_development	ENSGALG00010000507	TRPT1	1.513482096	0.287883436	5.257273978	1.46206E-07	3.4871E-07	positive
PRJNA729271	stage_1D_vs_E10	ordinary_muscle_development	ENSGALG00010000509	ENSGALG00010000509	-4.910366333	1.158226018	-4.239557959	2.2396E-05	4.41794E-05	negative
PRJNA729271	stage_1D_vs_E10	ordinary_muscle_development	ENSGALG00010000511	FERMT3	-0.38044096	0.164682028	-2.310154701	0.020879591	0.029469296	negative
PRJNA729271	stage_1D_vs_E10	ordinary_muscle_development	ENSGALG00010000525	ENSGALG00010000525	-1.924678763	0.533751788	-3.605943452	0.000311021	0.000547317	negative
PRJNA729271	stage_1D_vs_E10	ordinary_muscle_development	ENSGALG00010000526	POLD1	-1.154926771	0.158004379	-7.309460537	2.68217E-13	9.63998E-13	negative
PRJNA729271	stage_1D_vs_E10	ordinary_muscle_development	ENSGALG00010000529	STIP1	-0.239321725	0.192821308	-1.241158078	0.214547358	0.256441325	negative
PRJNA729271	stage_1D_vs_E10	ordinary_muscle_development	ENSGALG00010000532	ENSGALG00010000532	1.982737053	0.471675706	4.203602237	2.627E-05	5.14573E-05	positive
PRJNA729271	stage_1D_vs_E10	ordinary_muscle_development	ENSGALG00010000546	FLRT1	-3.969140838	0.650118198	-6.105260326	1.02633E-09	2.89824E-09	negative
PRJNA729271	stage_1D_vs_E10	ordinary_muscle_development	ENSGALG00010000555	OTUB1	-1.118246661	0.102999733	-10.85679182	1.8514E-27	1.41093E-26	negative
PRJNA729271	stage_1D_vs_E10	ordinary_muscle_development	ENSGALG00010000561	ENSGALG00010000561	-0.728601147	0.277281621	-2.627657555	0.008597502	0.012815795	negative
PRJNA729271	stage_1D_vs_E10	ordinary_muscle_development	ENSGALG00010000564	NAA40	-1.006248615	0.08997908	-11.18313963	4.93139E-29	4.0846E-28	negative
PRJNA729271	stage_1D_vs_E10	ordinary_muscle_development	ENSGALG00010000569	SIPA1	0.366658092	0.23360188	1.569585369	0.116511605	0.146511296	positive
PRJNA729271	stage_1D_vs_E10	ordinary_muscle_development	ENSGALG00010000574	ENSGALG00010000574	3.911329834	0.171204138	22.84600055	1.6015E-115	1.6701E-113	positive
PRJNA729271	stage_1D_vs_E10	ordinary_muscle_development	ENSGALG00010000575	ENSGALG00010000575	0.975614459	0.159309058	6.124036321	9.12341E-10	2.58845E-09	positive
PRJNA729271	stage_1D_vs_E10	ordinary_muscle_development	ENSGALG00010000576	NOS1	2.844584854	0.430586764	6.606298873	3.94047E-11	1.22674E-10	positive
PRJNA729271	stage_1D_vs_E10	ordinary_muscle_development	ENSGALG00010000577	ENSGALG00010000577	-0.38689716	0.273577259	-1.414215351	0.157298682	0.193109545	negative
PRJNA729271	stage_1D_vs_E10	ordinary_muscle_development	ENSGALG00010000578	ENSGALG00010000578	0.456149837	0.39311436	1.160348955	0.24590676	0.289978725	positive
PRJNA729271	stage_1D_vs_E10	ordinary_muscle_development	ENSGALG00010000579	ENSGALG00010000579	-0.979797939	0.928024636	-1.055788716	0.291064755	0.337599713	negative
PRJNA729271	stage_1D_vs_E10	ordinary_muscle_development	ENSGALG00010000580	ENSGALG00010000580	0.78360062	0.4727426	1.657562952	0.097405713	0.124100888	positive
PRJNA729271	stage_1D_vs_E10	ordinary_muscle_development	ENSGALG00010000581	ENSGALG00010000581	0.15526461	0.129972018	1.194600293	0.232243235	0.27557021	positive
PRJNA729271	stage_1D_vs_E10	ordinary_muscle_development	ENSGALG00010000583	CCS	-0.137525748	0.140388621	-0.979607518	0.327279892	0.375547977	negative
PRJNA729271	stage_1D_vs_E10	ordinary_muscle_development	ENSGALG00010000584	RBM14	0.876216832	0.209442162	4.183574227	2.86961E-05	5.60429E-05	positive
PRJNA729271	stage_1D_vs_E10	ordinary_muscle_development	ENSGALG00010000585	SPTBN2	-2.546441049	0.538421284	-4.729458371	2.2512E-06	4.88928E-06	negative
PRJNA729271	stage_1D_vs_E10	ordinary_muscle_development	ENSGALG00010000586	ENSGALG00010000586	3.570805866	0.254441463	14.0338993	9.66859E-45	1.49889E-43	positive
PRJNA729271	stage_1D_vs_E10	ordinary_muscle_development	ENSGALG00010000589	ENSGALG00010000589	0.800917229	0.328595465	2.437395866	0.014793474	0.021340706	positive
PRJNA729271	stage_1D_vs_E10	ordinary_muscle_development	ENSGALG00010000590	DNAH5	-0.687640841	0.456242064	-1.507184225	0.131763442	0.164053522	negative
PRJNA729271	stage_1D_vs_E10	ordinary_muscle_development	ENSGALG00010000591	SBK2	3.554837404	0.305054817	11.65311021	2.21234E-31	2.03994E-30	positive
PRJNA729271	stage_1D_vs_E10	ordinary_muscle_development	ENSGALG00010000592	CCT5	-1.091134409	0.119183476	-9.155081292	5.43169E-20	2.9074E-19	negative
PRJNA729271	stage_1D_vs_E10	ordinary_muscle_development	ENSGALG00010000593	ATPSCKMT	-0.881196936	0.145287848	-6.065179892	1.31806E-09	3.68614E-09	negative
PRJNA729271	stage_1D_vs_E10	ordinary_muscle_development	ENSGALG00010000595	MYO1O	-0.967032822	0.138475242	-6.983434787	2.88049E-12	9.66722E-12	negative
PRJNA729271	stage_1D_vs_E10	ordinary_muscle_development	ENSGALG00010000596	ENSGALG00010000596	-0.904840805	0.679257496	-1.332102789	0.182826414	0.221710992	negative
PRJNA729271	stage_1D_vs_E10	ordinary_muscle_development	ENSGALG00010000597	CDH9	-1.03510166	0.588447395	-1.759038563	0.078570959	0.101758939	negative
PRJNA729271	stage_1D_vs_E10	ordinary_muscle_development	ENSGALG00010000598	ENSGALG00010000598	-2.069326733	0.532967468	-3.882651116	0.000103324	0.000191348	negative
PRJNA729271	stage_1D_vs_E10	ordinary_muscle_development	ENSGALG00010000599	RETREG1	5.27926188	0.374598519	14.09312001	4.18649E-45	6.58883E-44	positive
PRJNA729271	stage_1D_vs_E10	ordinary_muscle_development	ENSGALG00010000601	ENSGALG00010000601	-1.428212653	0.338262106	-4.222207067	2.41922E-05	4.75543E-05	negative
PRJNA729271	stage_1D_vs_E10	ordinary_muscle_development	ENSGALG00010000603	ZNF622	-0.724845088	0.109761921	-6.603793781	4.00767E-11	1.24686E-10	negative
PRJNA729271	stage_1D_vs_E10	ordinary_muscle_development	ENSGALG00010000604	ENSGALG00010000604	-0.585215445	0.132464018	-4.417920098	9.96552E-06	2.03451E-05	negative
PRJNA729271	stage_1D_vs_E10	ordinary_muscle_development	ENSGALG00010000605	MARCHF11	-3.895880857	0.962912804	-4.045933175	5.21151E-05	9.93566E-05	negative
PRJNA729271	stage_1D_vs_E10	ordinary_muscle_development	ENSGALG00010000606	UBE2QL1	-0.693620831	0.158088087	-4.387559135	1.1463E-05	2.32777E-05	negative
PRJNA729271	stage_1D_vs_E10	ordinary_muscle_development	ENSGALG00010000607	ENSGALG00010000607	-0.809521338	0.259283094	-3.122152421	0.00179534	0.002908948	negative
PRJNA729271	stage_1D_vs_E10	ordinary_muscle_development	ENSGALG00010000608	ENSGALG00010000608	-1.693413538	1.004547049	-1.685748358	0.091844278	0.117552566	negative
PRJNA729271	stage_1D_vs_E10	ordinary_muscle_development	ENSGALG00010000610	ENSGALG00010000610	-0.505156388	0.079786743	-6.331332338	2.43053E-10	7.17819E-10	negative
PRJNA729271	stage_1D_vs_E10	ordinary_muscle_development	ENSGALG00010000611	C7orf57	1.526258231	0.31669978	4.819258894	1.44092E-06	3.17661E-06	positive
PRJNA729271	stage_1D_vs_E10	ordinary_muscle_development	ENSGALG00010000612	ENSGALG00010000612	-0.348733873	0.394014482	-0.88507882	0.376114122	0.42532175	negative
PRJNA729271	stage_1D_vs_E10	ordinary_muscle_development	ENSGALG00010000613	ENSGALG00010000613	-0.075030256	0.176621672	-0.424807754	0.670976825	0.709447011	negative
PRJNA729271	stage_1D_vs_E10	ordinary_muscle_development	ENSGALG00010000614	KAT8	1.910128485	0.181753181	10.50946387	7.81365E-26	5.54647E-25	positive
PRJNA729271	stage_1D_vs_E10	ordinary_muscle_development	ENSGALG00010000615	ENSGALG00010000615	-1.223386759	0.605777818	-2.019530465	0.043432114	0.058475617	negative
PRJNA729271	stage_1D_vs_E10	ordinary_muscle_development	ENSGALG00010000616	PSENEN	0.734912611	0.500099279	1.469533434	0.14168816	0.175430929	positive
PRJNA729271	stage_1D_vs_E10	ordinary_muscle_development	ENSGALG00010000619	ENSGALG00010000619	-0.199281304	0.310133278	-0.642566659	0.520505323	0.567784818	negative
PRJNA729271	stage_1D_vs_E10	ordinary_muscle_development	ENSGALG00010000621	UPP1	-0.97388673	0.240408306	-4.050969558	5.10058E-05	9.73827E-05	negative
PRJNA729271	stage_1D_vs_E10	ordinary_muscle_development	ENSGALG00010000622	ENSGALG00010000622	-0.157415566	0.433663682	-0.362989967	0.716612379	0.751885374	negative

PRJNA729271	stage_1D_vs_E10	ordinary_muscle_development	ENSGALG00010000623	ENSGALG00010000623	0.674822282	0.235394817	2.866767799	0.004146871	0.006435643	positive
PRJNA729271	stage_1D_vs_E10	ordinary_muscle_development	ENSGALG00010000625	ENSGALG00010000625	3.171682153	0.354298047	8.952017025	3.49045E-19	1.78148E-18	positive
PRJNA729271	stage_1D_vs_E10	ordinary_muscle_development	ENSGALG00010000626	ABCA13	0.824749377	0.598981544	1.376919515	0.168537133	0.205756401	positive
PRJNA729271	stage_1D_vs_E10	ordinary_muscle_development	ENSGALG00010000627	ENSGALG00010000627	0.988789559	0.224820542	4.398128179	1.09188E-05	2.22194E-05	positive
PRJNA729271	stage_1D_vs_E10	ordinary_muscle_development	ENSGALG00010000630	ENSGALG00010000630	0.424008892	0.367873672	1.152593743	0.249077173	0.293407039	positive
PRJNA729271	stage_1D_vs_E10	ordinary_muscle_development	ENSGALG00010000632	ENSGALG00010000632	0.517068886	0.359249237	1.439304063	0.150064392	0.185074735	positive
PRJNA729271	stage_1D_vs_E10	ordinary_muscle_development	ENSGALG00010000633	VWC2	-2.438015744	0.453936416	-5.370830934	7.83747E-08	1.90899E-07	negative
PRJNA729271	stage_1D_vs_E10	ordinary_muscle_development	ENSGALG00010000634	ENSGALG00010000634	1.253998771	0.182879711	6.8569595	7.03416E-12	2.29692E-11	positive
PRJNA729271	stage_1D_vs_E10	ordinary_muscle_development	ENSGALG00010000635	ENSGALG00010000635	-0.153211872	0.126667296	-1.209561396	0.226447239	0.269288106	negative
PRJNA729271	stage_1D_vs_E10	ordinary_muscle_development	ENSGALG00010000636	ENSGALG00010000636	0.688836671	0.139779208	4.928033851	8.30612E-07	1.86691E-06	positive
PRJNA729271	stage_1D_vs_E10	ordinary_muscle_development	ENSGALG00010000639	SCN1B	3.829489127	0.267113873	14.33654152	1.29342E-46	2.15744E-45	positive
PRJNA729271	stage_1D_vs_E10	ordinary_muscle_development	ENSGALG00010000641	ENSGALG00010000641	0.569189102	0.411016358	1.384833209	0.166103474	0.203076067	positive
PRJNA729271	stage_1D_vs_E10	ordinary_muscle_development	ENSGALG00010000643	VKORC1	1.33834422	0.199854585	6.696590034	2.13339E-11	6.75185E-11	positive
PRJNA729271	stage_1D_vs_E10	ordinary_muscle_development	ENSGALG00010000645	ENSGALG00010000645	-0.680359461	0.528393862	-1.2875991	0.197885553	0.237867861	negative
PRJNA729271	stage_1D_vs_E10	ordinary_muscle_development	ENSGALG00010000646	ENSGALG00010000646	0.655951208	0.501604111	1.307707003	0.190972707	0.230602249	positive
PRJNA729271	stage_1D_vs_E10	ordinary_muscle_development	ENSGALG00010000649	PAPD7	0.783507489	0.08203621	9.550751902	1.28758E-21	7.51652E-21	positive
PRJNA729271	stage_1D_vs_E10	ordinary_muscle_development	ENSGALG00010000650	BCKDK	1.700176103	0.167656447	10.14083343	3.63981E-24	2.38621E-23	positive
PRJNA729271	stage_1D_vs_E10	ordinary_muscle_development	ENSGALG00010000651	ENSGALG00010000651	-0.624930386	0.648696194	-0.963363731	0.335365021	0.383853125	negative
PRJNA729271	stage_1D_vs_E10	ordinary_muscle_development	ENSGALG00010000652	SRD5A1	0.662278669	0.223736724	2.960080293	0.003075589	0.00484204	positive
PRJNA729271	stage_1D_vs_E10	ordinary_muscle_development	ENSGALG00010000653	NSUN2	-0.877795903	0.09255033	-9.484524795	2.4349E-21	1.40222E-20	negative
PRJNA729271	stage_1D_vs_E10	ordinary_muscle_development	ENSGALG00010000654	ENSGALG00010000654	-0.956540526	0.168463247	-5.678036866	1.36249E-08	3.51537E-08	negative
PRJNA729271	stage_1D_vs_E10	ordinary_muscle_development	ENSGALG00010000655	ENSGALG00010000655	-1.869295685	0.884097127	-2.114355571	0.034484909	0.047156487	negative
PRJNA729271	stage_1D_vs_E10	ordinary_muscle_development	ENSGALG00010000656	ENSGALG00010000656	0.854097967	0.413240122	2.066832141	0.038749974	0.052532816	positive
PRJNA729271	stage_1D_vs_E10	ordinary_muscle_development	ENSGALG00010000659	ENSGALG00010000659	2.464706468	0.545691426	4.516667021	6.28206E-06	1.30775E-05	positive
PRJNA729271	stage_1D_vs_E10	ordinary_muscle_development	ENSGALG00010000660	ENSGALG00010000660	1.877206366	0.172036789	10.91165659	1.01392E-27	7.8299E-27	positive
PRJNA729271	stage_1D_vs_E10	ordinary_muscle_development	ENSGALG00010000661	ENSGALG00010000661	-1.040473622	0.613965366	-1.694678037	0.090136514	0.115499361	negative
PRJNA729271	stage_1D_vs_E10	ordinary_muscle_development	ENSGALG00010000670	ENSGALG00010000670	-1.554800044	0.54768873	-2.838838851	0.004527802	0.007001332	negative
PRJNA729271	stage_1D_vs_E10	ordinary_muscle_development	ENSGALG00010000674	GRB10	1.780067861	0.168913927	10.53831315	5.75214E-26	4.10725E-25	positive
PRJNA729271	stage_1D_vs_E10	ordinary_muscle_development	ENSGALG00010000684	COBL	2.970540301	0.134475233	22.08986914	3.9554E-108	3.3333E-106	positive
PRJNA729271	stage_1D_vs_E10	ordinary_muscle_development	ENSGALG00010000686	ENSGALG00010000686	-0.065778097	0.132162141	-0.497707559	0.618690179	0.660863239	negative
PRJNA729271	stage_1D_vs_E10	ordinary_muscle_development	ENSGALG00010000687	IKZF1	-0.744428077	0.300935321	-2.473714531	0.01337165	0.019421259	negative
PRJNA729271	stage_1D_vs_E10	ordinary_muscle_development	ENSGALG00010000688	ENSGALG00010000688	1.571138411	0.27794786	5.652637201	1.58005E-08	4.06077E-08	positive
PRJNA729271	stage_1D_vs_E10	ordinary_muscle_development	ENSGALG00010000695	FIGNL1	-1.344216571	0.143022933	-9.398608596	5.52894E-21	3.12444E-20	negative
PRJNA729271	stage_1D_vs_E10	ordinary_muscle_development	ENSGALG00010000697	ENSGALG00010000697	1.343939807	0.242299208	5.546612457	2.91257E-08	7.31297E-08	positive
PRJNA729271	stage_1D_vs_E10	ordinary_muscle_development	ENSGALG00010000702	DDC	-0.610099293	0.372063644	-1.639771321	0.101052723	0.128420062	negative
PRJNA729271	stage_1D_vs_E10	ordinary_muscle_development	ENSGALG00010000704	ENSGALG00010000704	-0.145829176	0.091943071	-1.586081195	0.112720892	0.14206498	negative
PRJNA729271	stage_1D_vs_E10	ordinary_muscle_development	ENSGALG00010000705	ENSGALG00010000705	4.199196296	1.016102084	4.132651984	3.58601E-05	6.93982E-05	positive
PRJNA729271	stage_1D_vs_E10	ordinary_muscle_development	ENSGALG00010000710	ENSGALG00010000710	1.65974124	0.483699227	3.431349787	0.000600586	0.001024055	positive
PRJNA729271	stage_1D_vs_E10	ordinary_muscle_development	ENSGALG00010000711	SPRY2	0.581427716	0.110508278	5.261395111	1.42966E-07	3.41569E-07	positive
PRJNA729271	stage_1D_vs_E10	ordinary_muscle_development	ENSGALG00010000714	FBXL7	-1.392595535	0.09615784	-14.48239203	1.56553E-47	2.71764E-46	negative
PRJNA729271	stage_1D_vs_E10	ordinary_muscle_development	ENSGALG00010000715	PCDH20	-0.940865918	0.223987238	-4.200533595	2.66287E-05	5.21387E-05	negative
PRJNA729271	stage_1D_vs_E10	ordinary_muscle_development	ENSGALG00010000717	NDFIP2	0.834089221	0.06658072	12.52748872	5.28085E-36	5.96616E-35	positive
PRJNA729271	stage_1D_vs_E10	ordinary_muscle_development	ENSGALG00010000718	EEF1G	0.455150982	0.150860854	3.017025103	0.002552687	0.004054037	positive
PRJNA729271	stage_1D_vs_E10	ordinary_muscle_development	ENSGALG00010000720	RBM26	0.012907311	0.050872205	0.253720307	0.799711645	0.826688082	positive
PRJNA729271	stage_1D_vs_E10	ordinary_muscle_development	ENSGALG00010000721	ENSGALG00010000721	-1.10676139	0.151335307	-7.313335867	2.60649E-13	9.3796E-13	negative
PRJNA729271	stage_1D_vs_E10	ordinary_muscle_development	ENSGALG00010000722	ANKH	0.873079288	0.169416364	5.153453103	2.55733E-07	5.97397E-07	positive
PRJNA729271	stage_1D_vs_E10	ordinary_muscle_development	ENSGALG00010000724	OBI1	-1.561685962	0.146983213	-10.62492734	2.2819E-26	1.66128E-25	negative
PRJNA729271	stage_1D_vs_E10	ordinary_muscle_development	ENSGALG00010000725	ENSGALG00010000725	-1.621249592	0.614205202	-2.639589482	0.008300651	0.01240391	negative
PRJNA729271	stage_1D_vs_E10	ordinary_muscle_development	ENSGALG00010000727	ENSGALG00010000727	-0.192124203	0.147730979	-1.300500443	0.193429505	0.233296777	negative
PRJNA729271	stage_1D_vs_E10	ordinary_muscle_development	ENSGALG00010000728	OTULIN	-0.419710511	0.274312755	-1.53004373	0.126005905	0.157466643	negative
PRJNA729271	stage_1D_vs_E10	ordinary_muscle_development	ENSGALG00010000729	EDNRB	-0.821083132	0.15731529	-5.2193473	1.79555E-07	4.2513E-07	negative
PRJNA729271	stage_1D_vs_E10	ordinary_muscle_development	ENSGALG00010000730	INTS5	0.851884347	0.222986832	3.820334762	0.000133271	0.000244341	positive
PRJNA729271	stage_1D_vs_E10	ordinary_muscle_development	ENSGALG00010000731	ENSGALG00010000731	-1.220349539	0.448820551	-2.719014398	0.006547676	0.009915228	negative
PRJNA729271	stage_1D_vs_E10	ordinary_muscle_development	ENSGALG00010000732	ENSGALG00010000732	-0.559616938	0.146682204	-3.815165854	0.000136091	0.000249197	negative
PRJNA729271	stage_1D_vs_E10	ordinary_muscle_development	ENSGALG00010000733	ENSGALG00010000733	-1.04983253	0.435660733	-2.409747883	0.015963548	0.022905526	negative
PRJNA729271	stage_1D_vs_E10	ordinary_muscle_development	ENSGALG00010000734	SLAIN1	-0.245763269	0.298841155	-0.82238763	0.410856317	0.460549247	negative
PRJNA729271	stage_1D_vs_E10	ordinary_muscle_development	ENSGALG00010000735	ENSGALG00010000735	0.42503383	0.54946671	0.773538819	0.439203564	0.48843453	positive
PRJNA729271	stage_1D_vs_E10	ordinary_muscle_development	ENSGALG00010000737	ENSGALG00010000737	-0.581511239	0.755665898	-0.769534844	0.441575868	0.490846795	negative
PRJNA729271	stage_1D_vs_E10	ordinary_muscle_development	ENSGALG00010000741	FAM105A	-1.247435568	0.197540238	-6.314842886	2.70436E-10	7.96095E-10	negative
PRJNA729271	stage_1D_vs_E10	ordinary_muscle_development	ENSGALG00010000753	TRIO	0.012110732	0.068233904	0.177488477	0.859124716	0.879574287	positive
PRJNA729271	stage_1D_vs_E10	ordinary_muscle_development	ENSGALG00010000757	SCEL	-3.307455845	0.48257597	-6.853751636	7.19379E-12	2.34746E-11	negative
PRJNA729271	stage_1D_vs_E10	ordinary_muscle_development	ENSGALG00010000760	CDH10	0.492326453	0.269413618	1.827400031	0.067639645	0.088375397	positive
PRJNA729271	stage_1D_vs_E10	ordinary_muscle_development	ENSGALG00010000762	ENSGALG00010000762	0.702678387	0.293828462	2.391457863	0.01678161	0.02401752	positive
PRJNA729271	stage_1D_vs_E10	ordinary_muscle_development	ENSGALG00010000768	ENSGALG00010000768	-0.465199075	0.343271163	-1.355194159	0.175355705	0.213346061	negative
PRJNA729271	stage_1D_vs_E10	ordinary_muscle_development	ENSGALG00010000770	ENSGALG00010000770	-2.400432684	0.639155271	-3.755633088	0.000172904	0.000313319	negative
PRJNA729271	stage_1D_vs_E10	ordinary_muscle_development	ENSGALG00010000777	MYCBP2	-0.018610077	0.082525653	-0.225506566	0.821585218	0.846574784	negative
PRJNA729271	stage_1D_vs_E10	ordinary_muscle_development	ENSGALG00010000783	DAP	-0.965099393	0.162930892	-5.923366535	3.15417E-09	8.57941E-09	negative
PRJNA729271	stage_1D_vs_E10	ordinary_muscle_development	ENSGALG00010000784	FBXL3	0.529424607	0.095254552	5.557997983	2.72887E-08	6.86836E-08	positive
PRJNA729271	stage_1D_vs_E10	ordinary_muscle_development	ENSGALG00010000785	CLN5	0.226510722	0.092457772	2.449882982	0.014290265	0.020666207	positive
PRJNA729271	stage_1D_vs_E10	ordinary_muscle_development	ENSGALG00010000786	ANKRD33B	2.797190853	0.303943023	9.203010581	3.48059E-20	1.8797E-19	positive
PRJNA729271	stage_1D_vs_E10	ordinary_muscle_development	ENSGALG00010000787	SNRNP48	0.107455264	0.10429731	1.030278375	0.302879348	0.349931145	positive
PRJNA729271	stage_1D_vs_E10	ordinary_muscle_development	ENSGALG00010000788	GATD3AL1	0.392390127	0.104598763	3.751384026	0.000175861	0.000318519	positive
PRJNA729271	stage_1D_vs_E10	ordinary_muscle_development	ENSGALG00010000789	ROPN1L	0.867041549	0.225693234	3.841681614	0.000122194	0.000224858	positive
PRJNA729271	stage_1D_vs_E10	ordinary_muscle_development	ENSGALG00010000790	MARCHF6	0.856842006	0.09906312	8.649455108	5.17459E-18	2.48117E-17	positive
PRJNA729271	stage_1D_vs_E10	ordinary_muscle_development	ENSGALG00010000791	ACOD1	-0.785440149	0.537913004	-1.460162038	0.144245546	0.178429697	negative
PRJNA729271	stage_1D_vs_E10	ordinary_muscle_development	ENSGALG00010000792	ENSGALG00010000792	-2.512429644	1.832728758	-1.370868239	0.170416034	0.207752768	negative
PRJNA729271	stage_1D_vs_E10	ordinary_muscle_development	ENSGALG00010000793	ENSGALG00010000793	0.750794373	1.375477879	0.545842564	0.585174162	0.629562791	positive
PRJNA729271	stage_1D_vs_E10	ordinary_muscle_development	ENSGALG00010000795	ENSGALG00010000795	-1.55494859	0.243121766	-6.395760516	1.5975E-10	4.76744E-10	negative
PRJNA729271	stage_1D_vs_E10	ordinary_muscle_development	ENSGALG00010000796	ENSGALG00010000796	-1.589120743	0.207111433	-7.672781375	1.68306E-14	6.53873E-14	negative
PRJNA729271	stage_1D_vs_E10	ordinary_muscle_development	ENSGALG00010000797	ENSGALG00010000797	2.404950347	0.414637966	5.800120931	6.62671E-09	1.75762E-08	positive
PRJNA729271	stage_1D_vs_E10	ordinary_muscle_development	ENSGALG00010000798	ENSGALG00010000798	-4.160445972	0.496517324	-8.379256414	5.32631E-17	2.41416E-16	negative
PRJNA729271	stage_1D_vs_E10	ordinary_muscle_development	ENSGALG00010000799	ENSGALG00010000799	-1.189618723	0.293762923	-4.049587703	5.13079E-05	9.79208E-05	negative
PRJNA729271	stage_1D_vs_E10	ordinary_muscle_development	ENSGALG00010000800	LMO7	1.604089888	0.151391736	10.59562384	3.12252E-26	2.25403E-25	positive
PRJNA729271	stage_1D_vs_E10	ordinary_muscle_development	ENSGALG00010000802	SEMA5A	-1.173634611	0.126296501	-9.292692973	1.50433E-20	8.29413E-20	negative
PRJNA729271	stage_1D_vs_E10	ordinary_muscle_development	ENSGALG00010000803	MTRR	-0.452104603	0.099119147	-4.56122369	5.08564E-06	1.06696E-05	negative
PRJNA729271	stage_1D_vs_E10	ordinary_muscle_development	ENSGALG00010000804	ENSGALG00010000804	0.164483495	0.431578506	0.381120682	0.703113701	0.739862845	positive
PRJNA729271	stage_1D_vs_E10	ordinary_muscle_development	ENSGALG00010000805	FASTKD3	0.5173143	0.128985502	4.010639107	6.05546E-05	0.000114902	positive
PRJNA729271	stage_1D_vs_E10	ordinary_muscle_development	ENSGALG00010000806	CFAP90	0.203654138	0.447583834	0.455007805	0.649103624	0.689033836	positive
PRJNA729271	stage_1D_vs_E10	ordinary_muscle_development	ENSGALG00010000807	ENSGALG00010000807	-0.144474018	0.874925132	-0.165127292	0.868843792	0.888269908	negative
PRJNA729271	stage_1D_vs_E10	ordinary_muscle_development	ENSGALG00010000808	ADCY2	0.394792675	0.171772339	2.298348367	0.02154197	0.03035983	positive
PRJNA729271	stage_1D_vs_E10	ordinary_muscle_development	ENSGALG00010000810	UCHL3	-1.413625496	0.138475399	-10.20849554	1.8166E-24	1.2112E-23	negative
PRJNA729271	stage_1D_vs_E10	ordinary_muscle_development	ENSGALG00010000811	ENSGALG00010000811	7.819099595	1.044546358	7.485641529	7.11985E-14	2.65506E-13	positive
PRJNA729271	stage_1D_vs_E10	ordinary_muscle_development	ENSGALG00010000812	ENSGALG00010000812	-0.949675028	0.096288584	-9.862799832	6.03428E-23	3.75879E-22	negative
PRJNA729271	stage_1D_vs_E10	ordinary_muscle_development	ENSGALG00010000813	ENSGALG00010000813	-1.406148806	0.452144752	-3.109952729	0.001871173	0.003024044	negative
PRJNA729271	stage_1D_vs_E10	ordinary_muscle_development	ENSGALG00010000814	CDH18	-1.970478701	0.292228961	-6.742927505	1.55227E-11	4.96691E-11	negative
PRJNA729271	stage_1D_vs_E10	ordinary_muscle_development	ENSGALG00010000815	ENSGALG00010000815	-1.408531552	0.168748009	-8.346952111	7.00471E-17	3.15713E-16	negative
PRJNA729271	stage_1D_vs_E10	ordinary_muscle_development	ENSGALG00010000816	TBC1D4	1.666555002	0.103484414	16.10440586	2.37569E-58	6.10561E-57	positive
PRJNA729271	stage_1D_vs_E10	ordinary_muscle_development	ENSGALG00010000817	ENSGALG00010000817	0.928439982	0.115359587	8.048225594	8.4003E-16	3.54682E-15	positive
PRJNA729271	stage_1D_vs_E10	ordinary_muscle_development	ENSGALG00010000818	KLF12	-1.190144984	0.077662764	-15.32452528	5.24362E-53	1.12602E-51	negative
PRJNA729271	stage_1D_vs_E10	ordinary_muscle_development	ENSGALG00010000820	KLF5	2.624148447	0.191452091	13.70655415	9.27596E-43	1.33786E-41	positive
PRJNA729271	stage_1D_vs_E10	ordinary_muscle_development	ENSGALG00010000821	ENSGALG00010000821	3.341834161	0.148392762	22.52019659	2.632E-112	2.5265E-110	positive
PRJNA729271	stage_1D_vs_E10	ordinary_muscle_development	ENSGALG00010000822	BASP1	-3.592737218	0.166037183	-21.63814851	7.8596E-104	6.125E-102	negative
PRJNA729271	stage_1D_vs_E10	ordinary_muscle_development	ENSGALG00010000823	PIBF1	1.130088436	0.158774578	7.117565363	1.0985E-12	3.78034E-12	positive
PRJNA729271	stage_1D_vs_E10	ordinary_muscle_development	ENSGALG00010000843	DIS3	-0.522614116	0.082797575	-6.311949549	2.75542E-10	8.10467E-10	negative
PRJNA729271	stage_1D_vs_E10	ordinary_muscle_development	ENSGALG00010000853	ENSGALG00010000853	1.046854541	0.303484291	3.449452153	0.000561725	0.000961413	positive
PRJNA729271	stage_1D_vs_E10	ordinary_muscle_development	ENSGALG00010000857	ENSGALG00010000857	-1.582634584	0.785140855	-2.015733322			negative
PRJNA729271	stage_1D_vs_E10	ordinary_muscle_development	ENSGALG00010000859	BORA	-2.324609045	0.192421634	-12.08080921	1.334E-33	1.3675E-32	negative
PRJNA729271	stage_1D_vs_E10	ordinary_muscle_development	ENSGALG00010000864	MZT1	-0.581607354	0.129446519	-4.493032004	7.02162E-06	1.45585E-05	negative
PRJNA729271	stage_1D_vs_E10	ordinary_muscle_development	ENSGALG00010000870	DACH1	0.58838619	0.139136856	4.228830565	2.34909E-05	4.6226E-05	positive
PRJNA729271	stage_1D_vs_E10	ordinary_muscle_development	ENSGALG00010000901	PCDH9	-1.005022218	0.240638796	-4.176476259	2.9606E-05	5.77653E-05	negative
PRJNA729271	stage_1D_vs_E10	ordinary_muscle_development	ENSGALG00010001066	SLC33A1	-0.3570019	0.085296304	-4.185432212	2.84624E-05	5.56164E-05	negative
PRJNA729271	stage_1D_vs_E10	ordinary_muscle_development	ENSGALG00010001070	C3orf33	-0.063366293	0.179768043	-0.352489198	0.724471418	0.758977537	negative
PRJNA729271	stage_1D_vs_E10	ordinary_muscle_development	ENSGALG00010001082	P2RY1	2.658592018	0.180205779	14.75308971	2.93896E-49	5.4968E-48	positive
PRJNA729271	stage_1D_vs_E10	ordinary_muscle_development	ENSGALG00010001104	MBNL1	1.589757624	0.122686547	12.95788055	2.12025E-38	2.62004E-37	positive
PRJNA729271	stage_1D_vs_E10	ordinary_muscle_development	ENSGALG00010001111	SUCNR1	3.881913912	0.421731628	9.204701885	3.42621E-20	1.85171E-19	positive
PRJNA729271	stage_1D_vs_E10	ordinary_muscle_development	ENSGALG00010001112	TSC22D2	0.161315446	0.089475586	1.802899019	0.071404075	0.092967041	positive
PRJNA729271	stage_1D_vs_E10	ordinary_muscle_development	ENSGALG00010001113	IGSF10	1.344676314	0.297241127	4.523856872	6.07228E-06	1.26589E-05	positive
PRJNA729271	stage_1D_vs_E10	ordinary_muscle_development	ENSGALG00010001114	MED12L	0.000146921	0.102953433	0.001427062	0.99886137	0.99899921	positive
PRJNA729271	stage_1D_vs_E10	ordinary_muscle_development	ENSGALG00010001115	PFN2	-1.039859493	0.095877287	-10.84573338	2.08953E-27	1.58908E-26	negative
PRJNA729271	stage_1D_vs_E10	ordinary_muscle_development	ENSGALG00010001116	RNF13	0.412572632	0.069972846	5.896181914	3.72009E-09	1.00658E-08	positive
PRJNA729271	stage_1D_vs_E10	ordinary_muscle_development	ENSGALG00010001117	P2RY13	-0.745591734	0.325915663	-2.28768304	0.022155986	0.03117971	negative
PRJNA729271	stage_1D_vs_E10	ordinary_muscle_development	ENSGALG00010001118	P2RY12	-1.049882922	0.346384225	-3.030977871	0.002437631	0.003881519	negative
PRJNA729271	stage_1D_vs_E10	ordinary_muscle_development	ENSGALG00010001119	COMMD2	-0.80532008	0.099279638	-8.111633957	4.99436E-16	2.14498E-15	negative
PRJNA729271	stage_1D_vs_E10	ordinary_muscle_development	ENSGALG00010001120	GPR87	-3.596749184	1.147030109	-3.135705991	0.00171441	0.002783108	negative
PRJNA729271	stage_1D_vs_E10	ordinary_muscle_development	ENSGALG00010001121	WWTR1	0.742154233	0.091462543	8.114296929	4.88605E-16	2.09971E-15	positive
PRJNA729271	stage_1D_vs_E10	ordinary_muscle_development	ENSGALG00010001122	P2RY14	2.823807984	0.424015356	6.659683295	2.74418E-11	8.62466E-11	positive
PRJNA729271	stage_1D_vs_E10	ordinary_muscle_development	ENSGALG00010001123	CP	-0.50684322	0.153087513	-3.310807065	0.000930273	0.00155672	negative
PRJNA729271	stage_1D_vs_E10	ordinary_muscle_development	ENSGALG00010001124	GPR171	0.938275522	0.66257285	1.416109221	0.156743529	0.192509528	positive
PRJNA729271	stage_1D_vs_E10	ordinary_muscle_development	ENSGALG00010001125	CLRN1	-0.750296273	0.300883434	-2.493644344	0.012643916	0.01843984	negative
PRJNA729271	stage_1D_vs_E10	ordinary_muscle_development	ENSGALG00010001126	MINDY4B	1.87608059	1.222110277	1.535115631	0.124755427	0.156065411	positive
PRJNA729271	stage_1D_vs_E10	ordinary_muscle_development	ENSGALG00010001127	SIAH2	0.294867483	0.101210551	2.91340656	0.003575088	0.00558957	positive

PRJNA729271	stage_1D_vs_E10	ordinary_muscle_development	ENSGALG00010001129	TM4SF4	-2.353869076	1.091299711	-2.156940987	0.031010263	0.042682913	negative
PRJNA729271	stage_1D_vs_E10	ordinary_muscle_development	ENSGALG00010001131	HPS3	1.084332369	0.098455358	11.01344198	3.29182E-28	2.60169E-27	positive
PRJNA729271	stage_1D_vs_E10	ordinary_muscle_development	ENSGALG00010001132	SELENOT	0.717963163	0.094044111	7.634323493	2.27009E-14	8.75366E-14	positive
PRJNA729271	stage_1D_vs_E10	ordinary_muscle_development	ENSGALG00010001133	EIF2A	-0.408368084	0.07394922	-5.522277065	3.34634E-08	8.36586E-08	negative
PRJNA729271	stage_1D_vs_E10	ordinary_muscle_development	ENSGALG00010001134	ENSGALG00010001134	-1.22835017	0.715953793	-1.715683586	0.086219971	0.110882661	negative
PRJNA729271	stage_1D_vs_E10	ordinary_muscle_development	ENSGALG00010001135	GYG1	1.477353426	0.15933673	9.271894976	1.82868E-20	1.00328E-19	positive
PRJNA729271	stage_1D_vs_E10	ordinary_muscle_development	ENSGALG00010001136	SERP1	-0.826080767	0.126295388	-6.540862481	6.11651E-11	1.87876E-10	negative
PRJNA729271	stage_1D_vs_E10	ordinary_muscle_development	ENSGALG00010001138	SPTSSB	-7.217341426	0.994755276	-7.255393967	4.00494E-13	1.42738E-12	negative
PRJNA729271	stage_1D_vs_E10	ordinary_muscle_development	ENSGALG00010001140	NMD3	0.398893924	0.12076706	3.303002685	0.000956555	0.001598854	positive
PRJNA729271	stage_1D_vs_E10	ordinary_muscle_development	ENSGALG00010001141	MTAP	-1.12912507	0.112476688	-10.03874749	1.02973E-23	6.6132E-23	negative
PRJNA729271	stage_1D_vs_E10	ordinary_muscle_development	ENSGALG00010001142	PPM1L	2.668418406	0.145576664	18.32998732	4.77016E-75	1.99837E-73	positive
PRJNA729271	stage_1D_vs_E10	ordinary_muscle_development	ENSGALG00010001143	CDKN2A	0.523788954	0.487211065	1.075076065	0.282340675	0.328426938	positive
PRJNA729271	stage_1D_vs_E10	ordinary_muscle_development	ENSGALG00010001144	CDKN2B	3.039577737	1.459433084	2.082711274	0.037277549	0.050707402	positive
PRJNA729271	stage_1D_vs_E10	ordinary_muscle_development	ENSGALG00010001145	TRIM36	0.301638627	0.207870261	1.45109082	0.146754572	0.181332156	positive
PRJNA729271	stage_1D_vs_E10	ordinary_muscle_development	ENSGALG00010001146	DIPK1C	1.929982773	0.240580581	8.022188497	1.03878E-15	4.35805E-15	positive
PRJNA729271	stage_1D_vs_E10	ordinary_muscle_development	ENSGALG00010001147	PGGT1B	-0.26846158	0.112616398	-2.383858701	0.017132179	0.024485401	negative
PRJNA729271	stage_1D_vs_E10	ordinary_muscle_development	ENSGALG00010001149	CIDEA	2.549550252	0.620314223	4.110094781	3.95497E-05	7.6233E-05	positive
PRJNA729271	stage_1D_vs_E10	ordinary_muscle_development	ENSGALG00010001150	ENSGALG00010001150	0.699411269	0.616767244	1.133995482	0.256796443	0.301764446	positive
PRJNA729271	stage_1D_vs_E10	ordinary_muscle_development	ENSGALG00010001151	CCDC112	1.525319077	0.215225863	7.08706219	1.36989E-12	4.68757E-12	positive
PRJNA729271	stage_1D_vs_E10	ordinary_muscle_development	ENSGALG00010001152	FEM1C	3.178277565	0.197267218	16.11153439	2.11705E-58	5.46025E-57	positive
PRJNA729271	stage_1D_vs_E10	ordinary_muscle_development	ENSGALG00010001153	TSHZ1	-1.434948099	0.112665896	-12.73631289	3.71543E-37	4.3856E-36	negative
PRJNA729271	stage_1D_vs_E10	ordinary_muscle_development	ENSGALG00010001154	PTGR3	-0.130737209	0.084368634	-1.549594947	0.121238767	0.15208619	negative
PRJNA729271	stage_1D_vs_E10	ordinary_muscle_development	ENSGALG00010001155	CYB5A	-0.250132225	0.093805867	-2.666488051	0.007664833	0.011505981	negative
PRJNA729271	stage_1D_vs_E10	ordinary_muscle_development	ENSGALG00010001156	ALDH7A1	0.320582297	0.18108191	1.770371749	0.076665233	0.099379588	positive
PRJNA729271	stage_1D_vs_E10	ordinary_muscle_development	ENSGALG00010001157	TIMM21	1.036620071	0.092004132	11.26710345	1.90742E-29	1.60279E-28	positive
PRJNA729271	stage_1D_vs_E10	ordinary_muscle_development	ENSGALG00010001158	FRMPD1	-0.803354955	0.378362803	-2.123239783	0.033733764	0.046220901	negative
PRJNA729271	stage_1D_vs_E10	ordinary_muscle_development	ENSGALG00010001159	RTTN	-0.510465291	0.111372136	-4.583420126	4.57432E-06	9.62752E-06	negative
PRJNA729271	stage_1D_vs_E10	ordinary_muscle_development	ENSGALG00010001160	TRMT10B	-0.186112467	0.127431431	-1.460491076	0.144155158	0.178333107	negative
PRJNA729271	stage_1D_vs_E10	ordinary_muscle_development	ENSGALG00010001161	B3GALNT1	-0.100493253	0.098865667	-1.016462603	0.309409147	0.356707674	negative
PRJNA729271	stage_1D_vs_E10	ordinary_muscle_development	ENSGALG00010001162	ENSGALG00010001162	0.13729759	0.416349622	0.329765138	0.741577431	0.774435509	positive
PRJNA729271	stage_1D_vs_E10	ordinary_muscle_development	ENSGALG00010001163	ARL14	-0.404545778	0.473342471	-0.854657678	0.392740687	0.441882811	negative
PRJNA729271	stage_1D_vs_E10	ordinary_muscle_development	ENSGALG00010001164	KPNA4	0.639870197	0.074589597	8.578544822	9.60809E-18	4.53941E-17	positive
PRJNA729271	stage_1D_vs_E10	ordinary_muscle_development	ENSGALG00010001165	ENSGALG00010001165	1.0642558	0.159681192	6.664878839	2.64884E-11	8.33404E-11	positive
PRJNA729271	stage_1D_vs_E10	ordinary_muscle_development	ENSGALG00010001166	TRIM59	-0.371165399	0.096378456	-3.851124145	0.000117577	0.000216802	negative
PRJNA729271	stage_1D_vs_E10	ordinary_muscle_development	ENSGALG00010001167	SMC4	-1.265298668	0.1536057	-8.237315865	1.76117E-16	7.7499E-16	negative
PRJNA729271	stage_1D_vs_E10	ordinary_muscle_development	ENSGALG00010001168	DOK6	-1.170255932	0.146042656	-8.013110465	1.11843E-15	4.6841E-15	negative
PRJNA729271	stage_1D_vs_E10	ordinary_muscle_development	ENSGALG00010001169	ENSGALG00010001169	0.113495807	0.103140205	1.100403158	0.271156503	0.316713418	positive
PRJNA729271	stage_1D_vs_E10	ordinary_muscle_development	ENSGALG00010001170	DCAF10	-0.014052166	0.06909166	-0.203384405	0.838834588	0.86208929	negative
PRJNA729271	stage_1D_vs_E10	ordinary_muscle_development	ENSGALG00010001171	POLR1E	-0.575064306	0.127645884	-4.505153549	6.6325E-06	1.37773E-05	negative
PRJNA729271	stage_1D_vs_E10	ordinary_muscle_development	ENSGALG00010001172	TMEM200C	-2.267744165	0.283718329	-7.992942073	1.31756E-15	5.49742E-15	negative
PRJNA729271	stage_1D_vs_E10	ordinary_muscle_development	ENSGALG00010001173	IFT80	-0.187779744	0.110784103	-1.695006214	0.090074242	0.115439977	negative
PRJNA729271	stage_1D_vs_E10	ordinary_muscle_development	ENSGALG00010001174	ENSGALG00010001174	-1.669086872	0.141901652	-11.76227938	6.10632E-32	5.81545E-31	negative
PRJNA729271	stage_1D_vs_E10	ordinary_muscle_development	ENSGALG00010001177	SEH1L	-0.521200454	0.071201301	-7.320097313	2.47791E-13	8.93244E-13	negative
PRJNA729271	stage_1D_vs_E10	ordinary_muscle_development	ENSGALG00010001178	ZBTB5	0.043665968	0.064153216	0.680651277	0.496092173	0.544225842	positive
PRJNA729271	stage_1D_vs_E10	ordinary_muscle_development	ENSGALG00010001179	GRHPR	-0.372809771	0.190323687	-1.958819618	0.050133915	0.066883672	negative
PRJNA729271	stage_1D_vs_E10	ordinary_muscle_development	ENSGALG00010001180	SPIRE1	-0.525063783	0.164273665	-3.196274854	0.001392144	0.002285292	negative
PRJNA729271	stage_1D_vs_E10	ordinary_muscle_development	ENSGALG00010001181	MFSD1	0.529070644	0.104517026	5.062052232	4.14767E-07	9.52782E-07	positive
PRJNA729271	stage_1D_vs_E10	ordinary_muscle_development	ENSGALG00010001182	ZCCHC7	-0.328726104	0.084052031	-3.910983453	9.19211E-05	0.000171237	negative
PRJNA729271	stage_1D_vs_E10	ordinary_muscle_development	ENSGALG00010001183	RARRES1	0.672502265	0.188800267	3.561977299	0.000368072	0.000643106	positive
PRJNA729271	stage_1D_vs_E10	ordinary_muscle_development	ENSGALG00010001185	PTPN2	-0.663717997	0.067311039	-9.860462795	6.1764E-23	3.84401E-22	negative
PRJNA729271	stage_1D_vs_E10	ordinary_muscle_development	ENSGALG00010001186	ENSGALG00010001186	0.108466019	0.353333069	0.306979528	0.758858955	0.789808325	positive
PRJNA729271	stage_1D_vs_E10	ordinary_muscle_development	ENSGALG00010001187	GFM1	0.577352934	0.181758394	3.17648568	0.001490711	0.002436336	positive
PRJNA729271	stage_1D_vs_E10	ordinary_muscle_development	ENSGALG00010001188	PSMG2	-0.427031909	0.113333579	-3.767920433	0.000164613	0.000299006	negative
PRJNA729271	stage_1D_vs_E10	ordinary_muscle_development	ENSGALG00010001190	LXN	-0.308664526	0.430732718	-0.716603389	0.473618853	0.522259815	negative
PRJNA729271	stage_1D_vs_E10	ordinary_muscle_development	ENSGALG00010001191	TOMM5	0.336810202	0.151077129	2.229392394	0.025787808	0.035898942	positive
PRJNA729271	stage_1D_vs_E10	ordinary_muscle_development	ENSGALG00010001192	ENSGALG00010001192	2.484347966	0.220613539	11.26108568	2.04227E-29	1.71411E-28	positive
PRJNA729271	stage_1D_vs_E10	ordinary_muscle_development	ENSGALG00010001193	PRELID3A	-0.237185189	0.230603134	-1.028542785	0.303694576	0.350789137	negative
PRJNA729271	stage_1D_vs_E10	ordinary_muscle_development	ENSGALG00010001194	POT1	-1.027543335	0.110285518	-9.317119401	1.19542E-20	6.63129E-20	negative
PRJNA729271	stage_1D_vs_E10	ordinary_muscle_development	ENSGALG00010001195	ZNF608	-1.066873804	0.142621341	-7.480463962	7.40607E-14	2.75825E-13	negative
PRJNA729271	stage_1D_vs_E10	ordinary_muscle_development	ENSGALG00010001196	LSMEM1	4.176885748	0.205981132	20.27800168	2.01138E-91	1.23538E-89	positive
PRJNA729271	stage_1D_vs_E10	ordinary_muscle_development	ENSGALG00010001197	CEP76	-0.804786703	0.143871008	-5.593807359	2.22143E-08	5.63918E-08	negative
PRJNA729271	stage_1D_vs_E10	ordinary_muscle_development	ENSGALG00010001198	IFRD1	0.550350554	0.176220771	3.123074267	0.001789726	0.0029005	positive
PRJNA729271	stage_1D_vs_E10	ordinary_muscle_development	ENSGALG00010001199	AFG3L2	0.200314192	0.090183162	2.221192828	0.026337904	0.036595851	positive
PRJNA729271	stage_1D_vs_E10	ordinary_muscle_development	ENSGALG00010001200	CTTNBP2	0.475230303	0.128201185	3.706910375	0.000209803	0.000376001	positive
PRJNA729271	stage_1D_vs_E10	ordinary_muscle_development	ENSGALG00010001201	TUBB6	-0.995847129	0.165361248	-6.022252137	1.72007E-09	4.77357E-09	negative
PRJNA729271	stage_1D_vs_E10	ordinary_muscle_development	ENSGALG00010001202	CFTR	2.510280803	0.248168533	10.115226	4.72924E-24	3.08282E-23	positive
PRJNA729271	stage_1D_vs_E10	ordinary_muscle_development	ENSGALG00010001203	TMX3	-0.907902413	0.100482069	-9.03546695	1.63304E-19	8.49332E-19	negative
PRJNA729271	stage_1D_vs_E10	ordinary_muscle_development	ENSGALG00010001204	SNX2	-0.17763557	0.081652424	-2.175508826	0.029592007	0.040819961	negative
PRJNA729271	stage_1D_vs_E10	ordinary_muscle_development	ENSGALG00010001205	CEP192	-0.051898646	0.104130083	-0.498402046	0.618200695	0.660389054	negative
PRJNA729271	stage_1D_vs_E10	ordinary_muscle_development	ENSGALG00010001206	RSRC1	-0.427661465	0.077592451	-5.511637552	3.5551E-08	8.87093E-08	negative
PRJNA729271	stage_1D_vs_E10	ordinary_muscle_development	ENSGALG00010001208	MOV10L1	-1.495049901	0.672018502	-2.224715385	0.026100351	0.036297091	negative
PRJNA729271	stage_1D_vs_E10	ordinary_muscle_development	ENSGALG00010001209	CDH7	-0.349811574	0.241780481	-1.44681478	0.147948806	0.182656568	negative
PRJNA729271	stage_1D_vs_E10	ordinary_muscle_development	ENSGALG00010001210	PANX2	-2.258722431	0.204510834	-11.04451233	2.33029E-28	1.85898E-27	negative
PRJNA729271	stage_1D_vs_E10	ordinary_muscle_development	ENSGALG00010001213	PPIC	-0.811791303	0.218770718	-3.710694507	0.000206691	0.000370653	negative
PRJNA729271	stage_1D_vs_E10	ordinary_muscle_development	ENSGALG00010001214	TES	-2.567635777	0.17348105	-14.80067004	1.45031E-49	2.76244E-48	negative
PRJNA729271	stage_1D_vs_E10	ordinary_muscle_development	ENSGALG00010001215	PRDM6	-1.330643751	0.586588349	-2.268445587	0.023302062	0.032694162	negative
PRJNA729271	stage_1D_vs_E10	ordinary_muscle_development	ENSGALG00010001216	SHOX2	-4.281553793	0.462347873	-9.260459579	2.03554E-20	1.11089E-19	negative
PRJNA729271	stage_1D_vs_E10	ordinary_muscle_development	ENSGALG00010001218	ENSGALG00010001218	-1.001799166	0.112414281	-8.911671708	5.02688E-19	2.54061E-18	negative
PRJNA729271	stage_1D_vs_E10	ordinary_muscle_development	ENSGALG00010001219	TFEC	-0.526138459	0.422470847	-1.24538406	0.21299061	0.254664595	negative
PRJNA729271	stage_1D_vs_E10	ordinary_muscle_development	ENSGALG00010001220	PLXNB2	-0.427788298	0.082564064	-5.181289273	2.20357E-07	5.17595E-07	negative
PRJNA729271	stage_1D_vs_E10	ordinary_muscle_development	ENSGALG00010001221	GRAMD2B	-0.466127831	0.154980892	-3.007647105	0.002632787	0.004177585	negative
PRJNA729271	stage_1D_vs_E10	ordinary_muscle_development	ENSGALG00010001222	ZNF800	0.228454496	0.096175942	2.375380912	0.017530848	0.02500587	positive
PRJNA729271	stage_1D_vs_E10	ordinary_muscle_development	ENSGALG00010001223	GRM8	-1.575083327	0.506797424	-3.107915021	0.001884122	0.003043615	negative
PRJNA729271	stage_1D_vs_E10	ordinary_muscle_development	ENSGALG00010001224	DNAJB9	1.503328803	0.188531552	7.973884415	1.53763E-15	6.38259E-15	positive
PRJNA729271	stage_1D_vs_E10	ordinary_muscle_development	ENSGALG00010001225	SOCS6	0.002622391	0.097228621	0.026971393	0.978482551	0.981733548	positive
PRJNA729271	stage_1D_vs_E10	ordinary_muscle_development	ENSGALG00010001226	VEPH1	-0.822541572	0.523833391	-1.570235091	0.116360429	0.146396241	negative
PRJNA729271	stage_1D_vs_E10	ordinary_muscle_development	ENSGALG00010001227	ENSGALG00010001227	-4.611055409	0.995401478	-4.632357406	3.61525E-06	7.6965E-06	negative
PRJNA729271	stage_1D_vs_E10	ordinary_muscle_development	ENSGALG00010001228	THAP5	-0.550963775	0.111613928	-4.936335323	7.96042E-07	1.79199E-06	negative
PRJNA729271	stage_1D_vs_E10	ordinary_muscle_development	ENSGALG00010001229	ZNF407	0.111052731	0.103216191	1.075923548	0.28196145	0.328117471	positive
PRJNA729271	stage_1D_vs_E10	ordinary_muscle_development	ENSGALG00010001230	PTX3	-2.206568262	0.582633728	-3.787230563	0.000152336	0.000277749	negative
PRJNA729271	stage_1D_vs_E10	ordinary_muscle_development	ENSGALG00010001231	CNDP1	1.475908271	0.52002502	2.838148578	0.004537606	0.007014995	positive
PRJNA729271	stage_1D_vs_E10	ordinary_muscle_development	ENSGALG00010001232	TSPAN12	0.323663231	0.101995272	3.173316011	0.001507084	0.002461984	positive
PRJNA729271	stage_1D_vs_E10	ordinary_muscle_development	ENSGALG00010001233	CNDP2	-1.127654726	0.097071454	-11.61674906	3.38784E-31	3.09431E-30	negative
PRJNA729271	stage_1D_vs_E10	ordinary_muscle_development	ENSGALG00010001234	AASS	1.755818707	0.098311751	17.8597033	2.42927E-71	9.31542E-70	positive
PRJNA729271	stage_1D_vs_E10	ordinary_muscle_development	ENSGALG00010001235	CCDC102B	-0.70420837	0.296378011	-2.376047965	0.017499188	0.024963166	negative
PRJNA729271	stage_1D_vs_E10	ordinary_muscle_development	ENSGALG00010001237	MELK	-2.061456118	0.269933527	-7.636902849	2.22509E-14	8.58927E-14	negative
PRJNA729271	stage_1D_vs_E10	ordinary_muscle_development	ENSGALG00010001238	GOLIM4	-0.173795655	0.121990256	-1.424668338	0.154253096	0.189659735	negative
PRJNA729271	stage_1D_vs_E10	ordinary_muscle_development	ENSGALG00010001239	ENSGALG00010001239	0.580494994	0.243558695	2.383388499	0.01715408	0.024514285	positive
PRJNA729271	stage_1D_vs_E10	ordinary_muscle_development	ENSGALG00010001242	ENSGALG00010001242	-2.337926226	0.662084929	-3.531157595	0.000413745	0.000719007	negative
PRJNA729271	stage_1D_vs_E10	ordinary_muscle_development	ENSGALG00010001244	ENSGALG00010001244	0.660217983	0.150673917	4.38176691	1.17721E-05	2.38786E-05	positive
PRJNA729271	stage_1D_vs_E10	ordinary_muscle_development	ENSGALG00010001245	SERPINI1	-1.300577148	0.225541187	-5.766472926	8.09477E-09	2.13256E-08	negative
PRJNA729271	stage_1D_vs_E10	ordinary_muscle_development	ENSGALG00010001248	PTPRZ1	1.338092036	0.237326505	5.638190461	1.71846E-08	4.4048E-08	positive
PRJNA729271	stage_1D_vs_E10	ordinary_muscle_development	ENSGALG00010001249	PDCD10	0.404390994	0.13882365	2.912983434	0.003579936	0.005595339	positive
PRJNA729271	stage_1D_vs_E10	ordinary_muscle_development	ENSGALG00010001250	ENSGALG00010001250	-0.557587478	0.656537675	-0.849284816	0.395722828	0.445030832	negative
PRJNA729271	stage_1D_vs_E10	ordinary_muscle_development	ENSGALG00010001251	ZNF277	-0.357013974	0.122956873	-2.903570709	0.003689337	0.005759497	negative
PRJNA729271	stage_1D_vs_E10	ordinary_muscle_development	ENSGALG00010001252	SNCAIP	-1.87601171	0.123333984	-15.21082552	2.99739E-52	6.26041E-51	negative
PRJNA729271	stage_1D_vs_E10	ordinary_muscle_development	ENSGALG00010001253	ENSGALG00010001253	-3.854472798	1.096282307	-3.515949106	0.000438185	0.000758387	negative
PRJNA729271	stage_1D_vs_E10	ordinary_muscle_development	ENSGALG00010001254	NETO1	-0.831627002	0.395966475	-2.100245991	0.035707208	0.048717618	negative
PRJNA729271	stage_1D_vs_E10	ordinary_muscle_development	ENSGALG00010001258	ENSGALG00010001258	-0.628478207	0.126566198	-4.965608651	6.84859E-07	1.5494E-06	negative
PRJNA729271	stage_1D_vs_E10	ordinary_muscle_development	ENSGALG00010001259	ENSGALG00010001259	0.80725687	0.164494967	4.90748674	9.22509E-07	2.06801E-06	positive
PRJNA729271	stage_1D_vs_E10	ordinary_muscle_development	ENSGALG00010001260	ENSGALG00010001260	-3.656530965	0.920927783	-3.970486104	7.17261E-05	0.000135022	negative
PRJNA729271	stage_1D_vs_E10	ordinary_muscle_development	ENSGALG00010001262	ENSGALG00010001262	0.110442796	0.145301574	0.760093596	0.44719864	0.496335704	positive
PRJNA729271	stage_1D_vs_E10	ordinary_muscle_development	ENSGALG00010001264	FAM3C	0.309627775	0.113134646	2.736807728	0.006203854	0.009428063	positive
PRJNA729271	stage_1D_vs_E10	ordinary_muscle_development	ENSGALG00010001265	OPN1LW	0.371924285	0.649287764	0.572818873	0.566767339	0.612031035	positive
PRJNA729271	stage_1D_vs_E10	ordinary_muscle_development	ENSGALG00010001267	BCHE	0.690702741	0.249253578	2.771084554	0.005586992	0.008531758	positive
PRJNA729271	stage_1D_vs_E10	ordinary_muscle_development	ENSGALG00010001268	CBLN2	-3.462400345	0.428853326	-8.073623619	6.8242E-16	2.89909E-15	negative
PRJNA729271	stage_1D_vs_E10	ordinary_muscle_development	ENSGALG00010001270	DRAP1	-0.615186258	0.110922007	-5.546115469	2.92086E-08	7.33123E-08	negative
PRJNA729271	stage_1D_vs_E10	ordinary_muscle_development	ENSGALG00010001271	ENSGALG00010001271	0.60795278	0.184440588	3.296198455	0.000980028	0.00163526	positive
PRJNA729271	stage_1D_vs_E10	ordinary_muscle_development	ENSGALG00010001273	ENSGALG00010001273	0.251780934	0.558054995	0.451175846	0.651862817	0.691557603	positive
PRJNA729271	stage_1D_vs_E10	ordinary_muscle_development	ENSGALG00010001274	DLGAP1	-0.785073183	0.388698845	-2.01974663	0.043409676	0.058450836	negative
PRJNA729271	stage_1D_vs_E10	ordinary_muscle_development	ENSGALG00010001276	ENSGALG00010001276	3.00514629	1.191082238	2.523038456			positive
PRJNA729271	stage_1D_vs_E10	ordinary_muscle_development	ENSGALG00010001277	ENSGALG00010001277	0.268539442	0.162667693	1.650846814	0.098769858	0.125706304	positive
PRJNA729271	stage_1D_vs_E10	ordinary_muscle_development	ENSGALG00010001279	LPIN2	-0.846188855	0.137144394	-6.17005792	6.8265E-10	1.95052E-09	negative
PRJNA729271	stage_1D_vs_E10	ordinary_muscle_development	ENSGALG00010001281	LRRN3	0.019808392	0.145390225	0.136242944	0.891629227	0.907723392	positive
PRJNA729271	stage_1D_vs_E10	ordinary_muscle_development	ENSGALG00010001283	ENSGALG00010001283	0.735199856	0.17915295	4.103755242	4.06498E-05	7.82598E-05	positive
PRJNA729271	stage_1D_vs_E10	ordinary_muscle_development	ENSGALG00010001285	ENSGALG00010001285	0.845473523	0.221104495	3.823864026	0.000131376	0.000240959	positive
PRJNA729271	stage_1D_vs_E10	ordinary_muscle_development	ENSGALG00010001286	SRFBP1	-0.589851541	0.111548104	-5.287867004	1.23751E-07	2.97031E-07	negative
PRJNA729271	stage_1D_vs_E10	ordinary_muscle_development	ENSGALG00010001287	PLPP4	-4.661767312	0.428540546	-10.87824094	1.4636E-27	1.12011E-26	negative
PRJNA729271	stage_1D_vs_E10	ordinary_muscle_development	ENSGALG00010001288	TGIF1	0.020342857	0.153658011	0.132390476	0.894675447	0.910249218	positive
PRJNA729271	stage_1D_vs_E10	ordinary_muscle_development	ENSGALG00010001289	ENSGALG00010001289	1.537576965	0.342951123	4.483370555	7.34732E-06	1.52033E-05	positive
PRJNA729271	stage_1D_vs_E10	ordinary_muscle_development	ENSGALG00010001291	ENSGALG00010001291	0.701732067	0.143590039	4.887052553	1.02357E-06	2.28607E-06	positive

PRJNA729271	stage_1D_vs_E10	ordinary_muscle_development	ENSGALG00010001292	LOX	2.091069987	0.141872023	14.73912856	3.61418E-49	6.73362E-48	positive
PRJNA729271	stage_1D_vs_E10	ordinary_muscle_development	ENSGALG00010001295	ENSGALG00010001295	0.977979478	0.126949963	7.703661021	1.32222E-14	5.16454E-14	positive
PRJNA729271	stage_1D_vs_E10	ordinary_muscle_development	ENSGALG00010001297	MYL12A	-0.300881327	0.22285671	-1.350111137	0.176980337	0.215122011	negative
PRJNA729271	stage_1D_vs_E10	ordinary_muscle_development	ENSGALG00010001298	DOCK4	0.155476372	0.132308502	1.175104926	0.23995274	0.283696164	positive
PRJNA729271	stage_1D_vs_E10	ordinary_muscle_development	ENSGALG00010001300	MRPL49	0.456509605	0.180273545	2.532316121	0.011331178	0.016640874	positive
PRJNA729271	stage_1D_vs_E10	ordinary_muscle_development	ENSGALG00010001301	GMPS	-0.736207422	0.075919549	-9.697204913	3.09871E-22	1.8676E-21	negative
PRJNA729271	stage_1D_vs_E10	ordinary_muscle_development	ENSGALG00010001302	ENSGALG00010001302	-1.09898011	0.388850313	-2.826229201	0.004709955	0.007261306	negative
PRJNA729271	stage_1D_vs_E10	ordinary_muscle_development	ENSGALG00010001304	SYVN1	0.261438202	0.176698722	1.479570419	0.138987926	0.172278945	positive
PRJNA729271	stage_1D_vs_E10	ordinary_muscle_development	ENSGALG00010001305	WNT16	-0.151161897	0.391358971	-0.386248709	0.699312483	0.736292179	negative
PRJNA729271	stage_1D_vs_E10	ordinary_muscle_development	ENSGALG00010001306	CEP120	-0.242502563	0.085179591	-2.846956172	0.004413944	0.006836943	negative
PRJNA729271	stage_1D_vs_E10	ordinary_muscle_development	ENSGALG00010001307	CALU	-0.368751606	0.155315434	-2.37421096	0.017586499	0.025077846	negative
PRJNA729271	stage_1D_vs_E10	ordinary_muscle_development	ENSGALG00010001308	MGMT	0.025450611	0.138310793	0.184010301	0.854005374	0.875136649	positive
PRJNA729271	stage_1D_vs_E10	ordinary_muscle_development	ENSGALG00010001313	EMILIN2	1.184483395	0.176344673	6.716865187	1.85676E-11	5.90212E-11	positive
PRJNA729271	stage_1D_vs_E10	ordinary_muscle_development	ENSGALG00010001320	CPED1	0.20140972	0.196400448	1.025505401	0.305124779	0.352216586	positive
PRJNA729271	stage_1D_vs_E10	ordinary_muscle_development	ENSGALG00010001330	ENSGALG00010001330	1.038029988	0.141408257	7.340660394	2.12542E-13	7.69239E-13	positive
PRJNA729271	stage_1D_vs_E10	ordinary_muscle_development	ENSGALG00010001333	PLCH1	-1.056124944	0.287891129	-3.668487283	0.00024399	0.000433943	negative
PRJNA729271	stage_1D_vs_E10	ordinary_muscle_development	ENSGALG00010001334	EBF3	-0.318943568	0.15866589	-2.010158375	0.044414429	0.059681761	negative
PRJNA729271	stage_1D_vs_E10	ordinary_muscle_development	ENSGALG00010001343	ENSGALG00010001343	-0.140450549	0.287378878	-0.488729549	0.625033181	0.666950527	negative
PRJNA729271	stage_1D_vs_E10	ordinary_muscle_development	ENSGALG00010001347	MYOM1	1.930962306	0.164811145	11.71621195	1.05275E-31	9.88316E-31	positive
PRJNA729271	stage_1D_vs_E10	ordinary_muscle_development	ENSGALG00010001349	ING3	0.690448365	0.10437259	6.61522689	3.70982E-11	1.15693E-10	positive
PRJNA729271	stage_1D_vs_E10	ordinary_muscle_development	ENSGALG00010001351	CAPN1	1.350684254	0.154564948	8.738619422	2.35976E-18	1.15245E-17	positive
PRJNA729271	stage_1D_vs_E10	ordinary_muscle_development	ENSGALG00010001353	GLRX3	-0.561289585	0.079198043	-7.087164823	1.36887E-12	4.6852E-12	negative
PRJNA729271	stage_1D_vs_E10	ordinary_muscle_development	ENSGALG00010001358	TCERG1L	-3.194102751	0.548601246	-5.822266668	5.80548E-09	1.54859E-08	negative
PRJNA729271	stage_1D_vs_E10	ordinary_muscle_development	ENSGALG00010001363	HIBADH	1.632215768	0.210202436	7.764970763	8.16641E-15	3.23156E-14	positive
PRJNA729271	stage_1D_vs_E10	ordinary_muscle_development	ENSGALG00010001364	SMCHD1	0.077421839	0.18408383	0.420579247	0.67406235	0.71239765	positive
PRJNA729271	stage_1D_vs_E10	ordinary_muscle_development	ENSGALG00010001365	ENSGALG00010001365	3.479045802	0.252464757	13.7803226	3.34773E-43	4.91645E-42	positive
PRJNA729271	stage_1D_vs_E10	ordinary_muscle_development	ENSGALG00010001367	ENSGALG00010001367	0.196597487	0.431074769	0.456063544	0.648344285	0.688379022	positive
PRJNA729271	stage_1D_vs_E10	ordinary_muscle_development	ENSGALG00010001368	WIPF3	2.191695116	0.139471859	15.71424615	1.2081E-55	2.85201E-54	positive
PRJNA729271	stage_1D_vs_E10	ordinary_muscle_development	ENSGALG00010001369	PPP2R2D	0.280736952	0.123839888	2.2669348	0.023394209	0.032810746	positive
PRJNA729271	stage_1D_vs_E10	ordinary_muscle_development	ENSGALG00010001372	ENSGALG00010001372	1.589156077	0.278205689	5.712162403	1.1155E-08	2.89873E-08	positive
PRJNA729271	stage_1D_vs_E10	ordinary_muscle_development	ENSGALG00010001373	IMPA2	0.485955616	0.139971839	3.471809891	0.000516962	0.000888682	positive
PRJNA729271	stage_1D_vs_E10	ordinary_muscle_development	ENSGALG00010001378	POLA2	-1.207970061	0.129138787	-9.354045283	8.43581E-21	4.72112E-20	negative
PRJNA729271	stage_1D_vs_E10	ordinary_muscle_development	ENSGALG00010001380	SCRN1	-3.336149207	0.16895661	-19.74559749	8.75235E-87	4.87944E-85	negative
PRJNA729271	stage_1D_vs_E10	ordinary_muscle_development	ENSGALG00010001381	MDFIC	0.324280466	0.130317483	2.488388043	0.012832363	0.01869586	positive
PRJNA729271	stage_1D_vs_E10	ordinary_muscle_development	ENSGALG00010001382	BNIP3	2.018629101	0.192929605	10.46303443	1.27699E-25	9.01169E-25	positive
PRJNA729271	stage_1D_vs_E10	ordinary_muscle_development	ENSGALG00010001385	MME	2.51606727	0.252678005	9.957603026	2.33627E-23	1.47686E-22	positive
PRJNA729271	stage_1D_vs_E10	ordinary_muscle_development	ENSGALG00010001386	FKBP14	-1.431308829	0.18500198	-7.736721687	1.02013E-14	4.01379E-14	negative
PRJNA729271	stage_1D_vs_E10	ordinary_muscle_development	ENSGALG00010001388	MPPE1	0.719553062	0.164205591	4.382025348	1.17581E-05	2.38569E-05	positive
PRJNA729271	stage_1D_vs_E10	ordinary_muscle_development	ENSGALG00010001395	GNAL	0.335108924	0.104954622	3.192893428	0.001408549	0.002310129	positive
PRJNA729271	stage_1D_vs_E10	ordinary_muscle_development	ENSGALG00010001397	KLHL7	-0.193405519	0.063473764	-3.047015123	0.002311261	0.003690837	negative
PRJNA729271	stage_1D_vs_E10	ordinary_muscle_development	ENSGALG00010001398	WDR11	-0.383854393	0.096591296	-3.974006021	7.06738E-05	0.000133093	negative
PRJNA729271	stage_1D_vs_E10	ordinary_muscle_development	ENSGALG00010001400	SNX24	0.592049748	0.159666859	3.708031526	0.000208877	0.000374433	positive
PRJNA729271	stage_1D_vs_E10	ordinary_muscle_development	ENSGALG00010001402	NUP42	-0.961050937	0.138248614	-6.95161353	3.61132E-12	1.20474E-11	negative
PRJNA729271	stage_1D_vs_E10	ordinary_muscle_development	ENSGALG00010001406	EML3	0.995519253	0.158830159	6.267822546	3.66132E-10	1.06718E-09	positive
PRJNA729271	stage_1D_vs_E10	ordinary_muscle_development	ENSGALG00010001407	FOXP2	-0.799209049	0.149259341	-5.354499374	8.57936E-08	2.08235E-07	negative
PRJNA729271	stage_1D_vs_E10	ordinary_muscle_development	ENSGALG00010001409	GPNMB	-2.986045637	0.964881484	-3.094727888	0.001969935	0.003174101	negative
PRJNA729271	stage_1D_vs_E10	ordinary_muscle_development	ENSGALG00010001411	FGFR2	-5.085014571	0.369300229	-13.76932416	3.89843E-43	5.71362E-42	negative
PRJNA729271	stage_1D_vs_E10	ordinary_muscle_development	ENSGALG00010001412	ENSGALG00010001412	-1.53722664	0.218279179	-7.04247949	1.88849E-12	6.41068E-12	negative
PRJNA729271	stage_1D_vs_E10	ordinary_muscle_development	ENSGALG00010001414	CCDC126	0.815859806	0.171953557	4.744652095	2.08865E-06	4.54852E-06	positive
PRJNA729271	stage_1D_vs_E10	ordinary_muscle_development	ENSGALG00010001415	MC5R	-0.300575226	0.266588801	-1.127486317	0.259536922	0.304639055	negative
PRJNA729271	stage_1D_vs_E10	ordinary_muscle_development	ENSGALG00010001416	AP4M1	0.219758895	0.092317221	2.380475638	0.017290304	0.024689485	positive
PRJNA729271	stage_1D_vs_E10	ordinary_muscle_development	ENSGALG00010001418	RNMT	-0.959361553	0.066455934	-14.43605559	3.06911E-47	5.24607E-46	negative
PRJNA729271	stage_1D_vs_E10	ordinary_muscle_development	ENSGALG00010001419	KCND2	-1.589535426	0.228732683	-6.949314827	3.67065E-12	1.22341E-11	negative
PRJNA729271	stage_1D_vs_E10	ordinary_muscle_development	ENSGALG00010001420	FAM221A	-4.830840273	0.687280687	-7.028918991	2.0814E-12	7.05068E-12	negative
PRJNA729271	stage_1D_vs_E10	ordinary_muscle_development	ENSGALG00010001422	AP1S1	-1.455751224	0.14094599	-10.32843309	5.24097E-25	3.59867E-24	negative
PRJNA729271	stage_1D_vs_E10	ordinary_muscle_development	ENSGALG00010001423	FAM210A	1.084739221	0.136613053	7.940231158	2.01805E-15	8.3172E-15	positive
PRJNA729271	stage_1D_vs_E10	ordinary_muscle_development	ENSGALG00010001426	ATE1	0.257066615	0.09089354	2.828216545	0.004680813	0.007220986	positive
PRJNA729271	stage_1D_vs_E10	ordinary_muscle_development	ENSGALG00010001427	ASB11	8.65353334	0.46273151	18.70098137	4.8602E-78	2.19466E-76	positive
PRJNA729271	stage_1D_vs_E10	ordinary_muscle_development	ENSGALG00010001432	NSMCE4A	-0.089628722	0.114035094	-0.7859749	0.431882192	0.481400521	negative
PRJNA729271	stage_1D_vs_E10	ordinary_muscle_development	ENSGALG00010001436	ENSGALG00010001436	-1.177801896	1.319051009	-0.89291611	0.371902099	0.421103112	negative
PRJNA729271	stage_1D_vs_E10	ordinary_muscle_development	ENSGALG00010001440	CDCA7L	-1.122016864	0.149249598	-7.517721155	5.57392E-14	2.09691E-13	negative
PRJNA729271	stage_1D_vs_E10	ordinary_muscle_development	ENSGALG00010001441	HMGCLL1	-2.840250996	0.330806436	-8.585839583	9.01754E-18	4.27015E-17	negative
PRJNA729271	stage_1D_vs_E10	ordinary_muscle_development	ENSGALG00010001442	LDLRAD4	0.791446508	0.11360603	6.966588906	3.24718E-12	1.08677E-11	positive
PRJNA729271	stage_1D_vs_E10	ordinary_muscle_development	ENSGALG00010001443	ANKRD26	0.054793026	0.144399041	0.379455606	0.704349565	0.740896004	positive
PRJNA729271	stage_1D_vs_E10	ordinary_muscle_development	ENSGALG00010001448	GPR37	-2.000253651	0.322879256	-6.195051602	5.82659E-10	1.67207E-09	negative
PRJNA729271	stage_1D_vs_E10	ordinary_muscle_development	ENSGALG00010001450	GPR149	-0.417703741	0.449393289	-0.929483711	0.352638463	0.401499845	negative
PRJNA729271	stage_1D_vs_E10	ordinary_muscle_development	ENSGALG00010001453	TAX1BP1	0.551069455	0.073559833	7.491445123	6.81194E-14	2.54351E-13	positive
PRJNA729271	stage_1D_vs_E10	ordinary_muscle_development	ENSGALG00010001454	VEGFD	2.211313958	0.164526807	13.44044782	3.5031E-41	4.82217E-40	positive
PRJNA729271	stage_1D_vs_E10	ordinary_muscle_development	ENSGALG00010001456	LSM8	-1.152766672	0.115436529	-9.986151521	1.75254E-23	1.11368E-22	negative
PRJNA729271	stage_1D_vs_E10	ordinary_muscle_development	ENSGALG00010001457	NDC80	-1.830711566	0.225532751	-8.117275905	4.76764E-16	2.05065E-15	negative
PRJNA729271	stage_1D_vs_E10	ordinary_muscle_development	ENSGALG00010001458	JAZF1	-0.621857345	0.15709668	-3.95843722	7.54418E-05	0.000141649	negative
PRJNA729271	stage_1D_vs_E10	ordinary_muscle_development	ENSGALG00010001460	MALSU1	-0.079822686	0.106360702	-0.750490402	0.452959402	0.501922371	negative
PRJNA729271	stage_1D_vs_E10	ordinary_muscle_development	ENSGALG00010001461	ASB9	-2.669351955	0.228501463	-11.68199065	1.57562E-31	1.46683E-30	negative
PRJNA729271	stage_1D_vs_E10	ordinary_muscle_development	ENSGALG00010001462	DHX36	-0.400527904	0.108847917	-3.679702053	0.000233507	0.00041632	negative
PRJNA729271	stage_1D_vs_E10	ordinary_muscle_development	ENSGALG00010001463	DSEL	0.08821756	0.15818977	0.557669184	0.577070304	0.621813415	positive
PRJNA729271	stage_1D_vs_E10	ordinary_muscle_development	ENSGALG00010001465	TACC2	1.925113195	0.157214391	12.24514612	1.78356E-34	1.89535E-33	positive
PRJNA729271	stage_1D_vs_E10	ordinary_muscle_development	ENSGALG00010001466	PRKX	0.099846074	0.129863182	0.768855897	0.441978861	0.491257081	positive
PRJNA729271	stage_1D_vs_E10	ordinary_muscle_development	ENSGALG00010001468	ANOS1	-0.210052112	0.152077088	-1.381221287	0.167210932	0.204309403	negative
PRJNA729271	stage_1D_vs_E10	ordinary_muscle_development	ENSGALG00010001469	ARHGEF26	0.698340757	0.119702188	5.833984903	5.4119E-09	1.446E-08	positive
PRJNA729271	stage_1D_vs_E10	ordinary_muscle_development	ENSGALG00010001470	GPR85	-1.211566927	0.315796666	-3.836541219	0.000124779	0.000229265	negative
PRJNA729271	stage_1D_vs_E10	ordinary_muscle_development	ENSGALG00010001471	BMP5	-3.211511318	0.372135601	-8.62994916	6.13793E-18	2.93434E-17	negative
PRJNA729271	stage_1D_vs_E10	ordinary_muscle_development	ENSGALG00010001472	ENSGALG00010001472	1.386868188	0.175656938	7.895322573	2.89565E-15	1.18133E-14	positive
PRJNA729271	stage_1D_vs_E10	ordinary_muscle_development	ENSGALG00010001475	BMT2	-1.217319697	0.138263675	-8.804334891	1.31631E-18	6.52304E-18	negative
PRJNA729271	stage_1D_vs_E10	ordinary_muscle_development	ENSGALG00010001476	IGF2BP3	-0.596334277	0.193163231	-3.087203886	0.00202049	0.003250499	negative
PRJNA729271	stage_1D_vs_E10	ordinary_muscle_development	ENSGALG00010001477	NLGN4	-0.450267632	0.211757434	-2.126336833	0.033475223	0.045884006	negative
PRJNA729271	stage_1D_vs_E10	ordinary_muscle_development	ENSGALG00010001479	TMEM168	0.037413554	0.154719565	0.241815276	0.808923302	0.834722236	positive
PRJNA729271	stage_1D_vs_E10	ordinary_muscle_development	ENSGALG00010001480	JAKMIP3	0.013039406	0.276195334	0.047210813	0.96234521	0.967820288	positive
PRJNA729271	stage_1D_vs_E10	ordinary_muscle_development	ENSGALG00010001482	TRA2A	-0.69534026	0.065547026	-10.60826558	2.72765E-26	1.97588E-25	negative
PRJNA729271	stage_1D_vs_E10	ordinary_muscle_development	ENSGALG00010001483	ENSGALG00010001483	-2.667951316	0.341331458	-7.816306558	5.43958E-15	2.17869E-14	negative
PRJNA729271	stage_1D_vs_E10	ordinary_muscle_development	ENSGALG00010001486	WNT2	1.759718987	0.261407058	6.731719478	1.6767E-11	5.34971E-11	positive
PRJNA729271	stage_1D_vs_E10	ordinary_muscle_development	ENSGALG00010001487	CDH19	2.15698864	0.224109565	9.624705856	6.28866E-22	3.74811E-21	positive
PRJNA729271	stage_1D_vs_E10	ordinary_muscle_development	ENSGALG00010001488	GRPR	-4.614259057	0.5160151	-8.942100843	3.81841E-19	1.94339E-18	negative
PRJNA729271	stage_1D_vs_E10	ordinary_muscle_development	ENSGALG00010001491	PHF3	0.56381576	0.088070407	6.401875249	1.5348E-10	4.59078E-10	positive
PRJNA729271	stage_1D_vs_E10	ordinary_muscle_development	ENSGALG00010001492	ENSGALG00010001492	2.087248281	0.274687038	7.59864133	2.99256E-14	1.14542E-13	positive
PRJNA729271	stage_1D_vs_E10	ordinary_muscle_development	ENSGALG00010001495	CCNL1	0.978380649	0.107778012	9.077738876	1.10854E-19	5.83028E-19	positive
PRJNA729271	stage_1D_vs_E10	ordinary_muscle_development	ENSGALG00010001496	PTP4A1	1.197460565	0.190119296	6.298469387	3.00599E-10	8.81842E-10	positive
PRJNA729271	stage_1D_vs_E10	ordinary_muscle_development	ENSGALG00010001497	LEKR1	-0.677468866	0.275445142	-2.459541893	0.013911446	0.020146509	negative
PRJNA729271	stage_1D_vs_E10	ordinary_muscle_development	ENSGALG00010001499	TIPARP	1.483585654	0.18634144	7.961651749	1.69758E-15	7.02638E-15	positive
PRJNA729271	stage_1D_vs_E10	ordinary_muscle_development	ENSGALG00010001501	CAPZA2	0.146314738	0.10648196	1.374080063	0.169416828	0.206632586	positive
PRJNA729271	stage_1D_vs_E10	ordinary_muscle_development	ENSGALG00010001502	LGSN	1.011900217	0.947983328	1.067424065	0.285780386	0.332028431	positive
PRJNA729271	stage_1D_vs_E10	ordinary_muscle_development	ENSGALG00010001504	RBMS3	0.32341097	0.098675353	3.277525346	0.001047213	0.001742951	positive
PRJNA729271	stage_1D_vs_E10	ordinary_muscle_development	ENSGALG00010001505	SSR3	-0.429812706	0.096975747	-4.432166979	9.32907E-06	1.91239E-05	negative
PRJNA729271	stage_1D_vs_E10	ordinary_muscle_development	ENSGALG00010001506	DPYSL4	-0.948163433	0.259183065	-3.658276951	0.000253917	0.000450713	negative
PRJNA729271	stage_1D_vs_E10	ordinary_muscle_development	ENSGALG00010001507	METTL4	-0.284091138	0.096708628	-2.937598665	0.003307649	0.00519047	negative
PRJNA729271	stage_1D_vs_E10	ordinary_muscle_development	ENSGALG00010001508	CD109	-1.25871386	0.290583528	-4.331676573	1.47978E-05	2.97043E-05	negative
PRJNA729271	stage_1D_vs_E10	ordinary_muscle_development	ENSGALG00010001510	AP1S2	-0.009704762	0.098155399	-0.098871399	0.921240378	0.93301359	negative
PRJNA729271	stage_1D_vs_E10	ordinary_muscle_development	ENSGALG00010001512	PLCL2	3.99535337	0.172404266	23.17433012	8.266E-119	9.288E-117	positive
PRJNA729271	stage_1D_vs_E10	ordinary_muscle_development	ENSGALG00010001513	ZRSR2	0.143794641	0.092513943	1.55430238	0.120112306	0.150751396	positive
PRJNA729271	stage_1D_vs_E10	ordinary_muscle_development	ENSGALG00010001514	MET	1.720941904	0.149805005	11.48787991	1.51791E-30	1.35066E-29	positive
PRJNA729271	stage_1D_vs_E10	ordinary_muscle_development	ENSGALG00010001516	CLTRN	-2.42796332	0.643774913	-3.771447552	0.000162303	0.000295032	negative
PRJNA729271	stage_1D_vs_E10	ordinary_muscle_development	ENSGALG00010001517	CAV2	3.150036335	0.163674905	19.24568914	1.5341E-82	7.72109E-81	positive
PRJNA729271	stage_1D_vs_E10	ordinary_muscle_development	ENSGALG00010001518	SLC17A5	-1.247969556	0.166799957	-7.48183379	7.32926E-14	2.73175E-13	negative
PRJNA729271	stage_1D_vs_E10	ordinary_muscle_development	ENSGALG00010001520	KCNAB1	-1.914747251	0.202677482	-9.447261873	3.4781E-21	1.98876E-20	negative
PRJNA729271	stage_1D_vs_E10	ordinary_muscle_development	ENSGALG00010001521	TBC1D5	-0.363194967	0.07448545	-4.876052503	1.0823E-06	2.41241E-06	negative
PRJNA729271	stage_1D_vs_E10	ordinary_muscle_development	ENSGALG00010001524	ACE2	1.873634593	1.523900304	1.229499455	0.218884602	0.261173223	positive
PRJNA729271	stage_1D_vs_E10	ordinary_muscle_development	ENSGALG00010001525	EEF1A1	-0.872059177	0.176700897	-4.935227792	8.00573E-07	1.80163E-06	negative
PRJNA729271	stage_1D_vs_E10	ordinary_muscle_development	ENSGALG00010001526	PPP1R3A	5.240917108	0.149286519	35.10643243	5.3773E-270	6.4954E-267	positive
PRJNA729271	stage_1D_vs_E10	ordinary_muscle_development	ENSGALG00010001527	STK32C	1.241038362	0.21855061	5.678494164	1.35886E-08	3.50661E-08	positive
PRJNA729271	stage_1D_vs_E10	ordinary_muscle_development	ENSGALG00010001528	NR1D2	2.939876631	0.180306365	16.30489654	9.10931E-60	2.50075E-58	positive
PRJNA729271	stage_1D_vs_E10	ordinary_muscle_development	ENSGALG00010001531	ENSGALG00010001531	-10.41216927	5.524679758	-1.884664764			negative
PRJNA729271	stage_1D_vs_E10	ordinary_muscle_development	ENSGALG00010001532	ENSGALG00010001532	-0.859430131	0.276832514	-3.104512968	0.001905926	0.003074724	negative
PRJNA729271	stage_1D_vs_E10	ordinary_muscle_development	ENSGALG00010001533	ENSGALG00010001533	1.047059105	0.146446268	7.149783473	8.69149E-13	3.02118E-12	positive
PRJNA729271	stage_1D_vs_E10	ordinary_muscle_development	ENSGALG00010001534	SLITRK3	-0.952916574	0.25577795	-3.725561857	0.000194881	0.00035034	negative
PRJNA729271	stage_1D_vs_E10	ordinary_muscle_development	ENSGALG00010001535	RAP2B	-0.438472063	0.104925295	-4.178897594	2.92926E-05	5.71692E-05	negative
PRJNA729271	stage_1D_vs_E10	ordinary_muscle_development	ENSGALG00010001540	TRABD	-0.759080242	0.130131925	-5.83315923	5.43876E-09	1.45264E-08	negative
PRJNA729271	stage_1D_vs_E10	ordinary_muscle_development	ENSGALG00010001542	MTO1	0.023583925	0.128500938	0.183531153	0.854381277	0.875398078	positive
PRJNA729271	stage_1D_vs_E10	ordinary_muscle_development	ENSGALG00010001545	SELENOO	1.102290856	0.132631826	8.310907629	9.49728E-17	4.24624E-16	positive
PRJNA729271	stage_1D_vs_E10	ordinary_muscle_development	ENSGALG00010001546	CGAS	-0.499350654	0.162749284	-3.068220274	0.002153378	0.003449737	negative
PRJNA729271	stage_1D_vs_E10	ordinary_muscle_development	ENSGALG00010001547	PWWP2B	0.810523581	0.10964345	7.392357524	1.44248E-13	5.27732E-13	positive
PRJNA729271	stage_1D_vs_E10	ordinary_muscle_development	ENSGALG00010001548	AKAP17A	0.236602403	0.09502701	2.489843724	0.012779928	0.01862508	positive
PRJNA729271	stage_1D_vs_E10	ordinary_muscle_development	ENSGALG00010001549	ENSGALG00010001549	-1.247014614	0.492812409	-2.53040425	0.011393118	0.01671998	negative

PRJNA729271	stage_1D_vs_E10	ordinary_muscle_development	ENSGALG00010001551	FHIP1B	1.31554884	0.160267266	8.208468729	2.24027E-16	9.80161E-16	positive
PRJNA729271	stage_1D_vs_E10	ordinary_muscle_development	ENSGALG00010001552	TUBGCP6	0.047360544	0.108088626	0.438163991	0.66126741	0.700407096	positive
PRJNA729271	stage_1D_vs_E10	ordinary_muscle_development	ENSGALG00010001553	CNGA4	0.687411159	0.393078782	1.748787239	0.080327803	0.103802399	positive
PRJNA729271	stage_1D_vs_E10	ordinary_muscle_development	ENSGALG00010001554	ENSGALG00010001554	0.43993816	0.182699199	2.407991736	0.016040544	0.023006896	positive
PRJNA729271	stage_1D_vs_E10	ordinary_muscle_development	ENSGALG00010001556	EPB41L3	-0.379907261	0.117102697	-3.244222992	0.001177715	0.00194941	negative
PRJNA729271	stage_1D_vs_E10	ordinary_muscle_development	ENSGALG00010001557	THRB	-0.987555667	0.314847181	-3.136619056	0.00170908	0.002775389	negative
PRJNA729271	stage_1D_vs_E10	ordinary_muscle_development	ENSGALG00010001558	CCKBR	-1.803535031	0.626895138	-2.876932553	0.004015614	0.006239289	negative
PRJNA729271	stage_1D_vs_E10	ordinary_muscle_development	ENSGALG00010001559	HDAC10	-1.154144261	0.125446277	-9.200307033	3.56928E-20	1.92402E-19	negative
PRJNA729271	stage_1D_vs_E10	ordinary_muscle_development	ENSGALG00010001567	INPP5A	-0.066154808	0.082181186	-0.804987265	0.420827029	0.470343726	negative
PRJNA729271	stage_1D_vs_E10	ordinary_muscle_development	ENSGALG00010001570	SMPD1	1.946408115	0.162129024	12.00530338	3.3324E-33	3.35438E-32	positive
PRJNA729271	stage_1D_vs_E10	ordinary_muscle_development	ENSGALG00010001585	ENSGALG00010001585	-0.448381969	0.137930235	-3.250788129	0.001150856	0.001906693	negative
PRJNA729271	stage_1D_vs_E10	ordinary_muscle_development	ENSGALG00010001586	ZBTB14	0.289167079	0.093930649	3.078516783	0.002080338	0.003340479	positive
PRJNA729271	stage_1D_vs_E10	ordinary_muscle_development	ENSGALG00010001588	ENSGALG00010001588	0.957635266	0.585926506	1.634394854	0.102175968	0.129779237	positive
PRJNA729271	stage_1D_vs_E10	ordinary_muscle_development	ENSGALG00010001595	AKAIN1	1.605417383	0.253120631	6.342499141	2.26067E-10	6.69018E-10	positive
PRJNA729271	stage_1D_vs_E10	ordinary_muscle_development	ENSGALG00010001599	TBLX1	-0.390820393	0.075006517	-5.210485832	1.88347E-07	4.45292E-07	negative
PRJNA729271	stage_1D_vs_E10	ordinary_muscle_development	ENSGALG00010001604	ENSGALG00010001604	5.745095251	0.420759572	13.65410471	1.9082E-42	2.73044E-41	positive
PRJNA729271	stage_1D_vs_E10	ordinary_muscle_development	ENSGALG00010001605	APBB1	0.23331341	0.112453032	2.074763175	0.038008489	0.051614488	positive
PRJNA729271	stage_1D_vs_E10	ordinary_muscle_development	ENSGALG00010001606	PIGA	-0.570410079	0.110114957	-5.180132592	2.21728E-07	5.20646E-07	negative
PRJNA729271	stage_1D_vs_E10	ordinary_muscle_development	ENSGALG00010001608	ENSGALG00010001608	0.064543158	0.477580691	0.13514608	0.892496378	0.908542383	positive
PRJNA729271	stage_1D_vs_E10	ordinary_muscle_development	ENSGALG00010001609	ENSGALG00010001609	0.433468221	0.470251535	0.921779492	0.356643606	0.405486632	positive
PRJNA729271	stage_1D_vs_E10	ordinary_muscle_development	ENSGALG00010001611	MAPK12	-1.430931277	0.162788274	-8.790137272	1.49377E-18	7.38229E-18	negative
PRJNA729271	stage_1D_vs_E10	ordinary_muscle_development	ENSGALG00010001612	HPX	2.747828195	0.932931718	2.945369037	0.003225697	0.005065707	positive
PRJNA729271	stage_1D_vs_E10	ordinary_muscle_development	ENSGALG00010001616	OPNVA	-1.314603892	0.586459374	-2.241594134	0.024987615	0.034873434	negative
PRJNA729271	stage_1D_vs_E10	ordinary_muscle_development	ENSGALG00010001617	TRIM3	-0.537534929	0.093450328	-5.752092471	8.81456E-09	2.31043E-08	negative
PRJNA729271	stage_1D_vs_E10	ordinary_muscle_development	ENSGALG00010001620	MOSPD2	0.405577143	0.143306519	2.830137432	0.004652801	0.007180065	positive
PRJNA729271	stage_1D_vs_E10	ordinary_muscle_development	ENSGALG00010001621	DLGAP2	0.454104478	0.236635531	1.919003778	0.054983856	0.072831124	positive
PRJNA729271	stage_1D_vs_E10	ordinary_muscle_development	ENSGALG00010001627	ARFIP2	-0.079663532	0.118781228	-0.670674427	0.502427958	0.550551349	negative
PRJNA729271	stage_1D_vs_E10	ordinary_muscle_development	ENSGALG00010001628	MAPK11	-1.587631297	0.2085547	-7.612541453	2.68758E-14	1.03087E-13	negative
PRJNA729271	stage_1D_vs_E10	ordinary_muscle_development	ENSGALG00010001632	FANCB	0.247076668	0.271389648	0.910413018	0.362604735	0.411586189	positive
PRJNA729271	stage_1D_vs_E10	ordinary_muscle_development	ENSGALG00010001633	B3GAT2	-2.817888492	0.407206658	-6.920045234	4.51499E-12	1.49315E-11	negative
PRJNA729271	stage_1D_vs_E10	ordinary_muscle_development	ENSGALG00010001635	TIMM10B	0.047155659	0.149079607	0.316311932	0.751765755	0.783607408	positive
PRJNA729271	stage_1D_vs_E10	ordinary_muscle_development	ENSGALG00010001639	ENSGALG00010001639	-0.918727169	0.160373731	-5.728663684	1.01225E-08	2.63752E-08	negative
PRJNA729271	stage_1D_vs_E10	ordinary_muscle_development	ENSGALG00010001642	ENSGALG00010001642	1.182084508	0.120221862	9.832525345	8.15478E-23	5.04927E-22	positive
PRJNA729271	stage_1D_vs_E10	ordinary_muscle_development	ENSGALG00010001647	ARL8BL	-0.150236434	0.152315825	-0.98634816	0.323962295	0.372153547	negative
PRJNA729271	stage_1D_vs_E10	ordinary_muscle_development	ENSGALG00010001649	UBE2E2	-0.515424274	0.22188516	-2.322932612	0.020182773	0.028530261	negative
PRJNA729271	stage_1D_vs_E10	ordinary_muscle_development	ENSGALG00010001654	RPS11	0.264941031	0.169400249	1.563994344	0.117818897	0.148103799	positive
PRJNA729271	stage_1D_vs_E10	ordinary_muscle_development	ENSGALG00010001657	SMAP1	-1.094117213	0.098137017	-11.14887371	7.25184E-29	5.94209E-28	negative
PRJNA729271	stage_1D_vs_E10	ordinary_muscle_development	ENSGALG00010001661	TCTN3	-0.490475777	0.104688926	-4.685077934	2.79853E-06	6.02208E-06	negative
PRJNA729271	stage_1D_vs_E10	ordinary_muscle_development	ENSGALG00010001664	SPAM1	0.757383321	2.0670095	0.366415017	0.714055416	0.749473805	positive
PRJNA729271	stage_1D_vs_E10	ordinary_muscle_development	ENSGALG00010001666	PIEZO2	-3.024134775	0.183411276	-16.48827077	4.45518E-61	1.26376E-59	negative
PRJNA729271	stage_1D_vs_E10	ordinary_muscle_development	ENSGALG00010001668	UBE2E1	-0.231338753	0.085603593	-2.702442094	0.006883217	0.010387531	negative
PRJNA729271	stage_1D_vs_E10	ordinary_muscle_development	ENSGALG00010001670	SHOX	-0.81613055	0.160261311	-5.092498902	3.53375E-07	8.16152E-07	negative
PRJNA729271	stage_1D_vs_E10	ordinary_muscle_development	ENSGALG00010001672	ENSGALG00010001672	1.828628018	0.552653357	3.308815541	0.000936915	0.001567292	positive
PRJNA729271	stage_1D_vs_E10	ordinary_muscle_development	ENSGALG00010001674	ENSGALG00010001674	0.289730233	0.104257413	2.778989268	0.005452833	0.008343588	positive
PRJNA729271	stage_1D_vs_E10	ordinary_muscle_development	ENSGALG00010001677	SDHAF4	-0.06397169	0.107600047	-0.594532173	0.55215623	0.598079851	negative
PRJNA729271	stage_1D_vs_E10	ordinary_muscle_development	ENSGALG00010001678	SYAP1	0.171949613	0.058989192	2.914934185	0.003557636	0.005563483	positive
PRJNA729271	stage_1D_vs_E10	ordinary_muscle_development	ENSGALG00010001681	NAPG	-0.050109363	0.091717219	-0.546346296	0.584827919	0.629283697	negative
PRJNA729271	stage_1D_vs_E10	ordinary_muscle_development	ENSGALG00010001686	WASL	0.769320093	0.084827266	9.069254895	1.19834E-19	6.29344E-19	positive
PRJNA729271	stage_1D_vs_E10	ordinary_muscle_development	ENSGALG00010001688	ENSGALG00010001688	-0.556666596	0.165799745	-3.357463526	0.000786611	0.001326731	negative
PRJNA729271	stage_1D_vs_E10	ordinary_muscle_development	ENSGALG00010001689	ILK	0.27712262	0.067024442	4.134650142	3.55496E-05	6.8834E-05	positive
PRJNA729271	stage_1D_vs_E10	ordinary_muscle_development	ENSGALG00010001691	CTPS2	0.988795161	0.133531236	7.404972757	1.31177E-13	4.81371E-13	positive
PRJNA729271	stage_1D_vs_E10	ordinary_muscle_development	ENSGALG00010001692	ALDH18A1	-1.245043483	0.255112958	-4.880361594	1.05892E-06	2.36211E-06	negative
PRJNA729271	stage_1D_vs_E10	ordinary_muscle_development	ENSGALG00010001693	NKIRAS1	1.030293432	0.19533025	5.274623015	1.33029E-07	3.183E-07	positive
PRJNA729271	stage_1D_vs_E10	ordinary_muscle_development	ENSGALG00010001694	ENSGALG00010001694	-0.859746883	0.218948261	-3.926712542	8.61148E-05	0.000160855	negative
PRJNA729271	stage_1D_vs_E10	ordinary_muscle_development	ENSGALG00010001695	LMOD2	4.883142855	0.468651397	10.41956322	2.01878E-25	1.41227E-24	positive
PRJNA729271	stage_1D_vs_E10	ordinary_muscle_development	ENSGALG00010001696	FAM135A	0.181700569	0.090599907	2.005527108	0.044906726	0.060315325	positive
PRJNA729271	stage_1D_vs_E10	ordinary_muscle_development	ENSGALG00010001697	TAF10	-0.736094162	0.11986193	-6.141183945	8.19087E-10	2.33071E-09	negative
PRJNA729271	stage_1D_vs_E10	ordinary_muscle_development	ENSGALG00010001698	RPL15	0.204850556	0.176437239	1.161039233	0.245625945	0.289765449	positive
PRJNA729271	stage_1D_vs_E10	ordinary_muscle_development	ENSGALG00010001699	ASB15	6.663837678	0.249944987	26.66121752	1.3266E-156	2.6341E-154	positive
PRJNA729271	stage_1D_vs_E10	ordinary_muscle_development	ENSGALG00010001700	ENSGALG00010001700	1.256183797	0.200928901	6.251882103	4.05535E-10	1.17682E-09	positive
PRJNA729271	stage_1D_vs_E10	ordinary_muscle_development	ENSGALG00010001701	NDUFA5	2.065295326	0.168844739	12.2319199	2.09918E-34	2.21614E-33	positive
PRJNA729271	stage_1D_vs_E10	ordinary_muscle_development	ENSGALG00010001702	PLEKHA1	0.345106802	0.083018888	4.156967285	3.225E-05	6.26879E-05	positive
PRJNA729271	stage_1D_vs_E10	ordinary_muscle_development	ENSGALG00010001704	ENSGALG00010001704	-0.433150266	0.145661249	-2.97368222	0.002942496	0.004643904	negative
PRJNA729271	stage_1D_vs_E10	ordinary_muscle_development	ENSGALG00010001705	HTRA1	2.4730248	0.154517263	16.00484474	1.18208E-57	2.96953E-56	positive
PRJNA729271	stage_1D_vs_E10	ordinary_muscle_development	ENSGALG00010001706	IQUB	4.056090392	0.337291071	12.02548997	2.61035E-33	2.6441E-32	positive
PRJNA729271	stage_1D_vs_E10	ordinary_muscle_development	ENSGALG00010001707	ENSGALG00010001707	3.246494504	1.06354137	3.052532413	0.002269192	0.003626854	positive
PRJNA729271	stage_1D_vs_E10	ordinary_muscle_development	ENSGALG00010001708	AADAC	3.56064327	0.249862435	14.25041451	4.45628E-46	7.29874E-45	positive
PRJNA729271	stage_1D_vs_E10	ordinary_muscle_development	ENSGALG00010001710	LCMT2	-0.740571951	0.080297819	-9.222815275	2.894E-20	1.567E-19	negative
PRJNA729271	stage_1D_vs_E10	ordinary_muscle_development	ENSGALG00010001711	CREB5	0.31972289	0.173916065	1.838374675	0.066007214	0.086406084	positive
PRJNA729271	stage_1D_vs_E10	ordinary_muscle_development	ENSGALG00010001712	ATP6AP1	0.999369586	0.159055308	6.283157735	3.31764E-10	9.70127E-10	positive
PRJNA729271	stage_1D_vs_E10	ordinary_muscle_development	ENSGALG00010001713	PNPLA4	-0.520940295	0.234016716	-2.226081553	0.026008719	0.036180076	negative
PRJNA729271	stage_1D_vs_E10	ordinary_muscle_development	ENSGALG00010001716	TRIL	-1.364578406	0.151696494	-8.995451171	2.35264E-19	1.21271E-18	negative
PRJNA729271	stage_1D_vs_E10	ordinary_muscle_development	ENSGALG00010001717	STARD10	0.563335763	0.485142756	1.161175253	0.245570636	0.289723781	positive
PRJNA729271	stage_1D_vs_E10	ordinary_muscle_development	ENSGALG00010001721	TM4SF1	-0.166193048	0.161620315	-1.028293058	0.303811995	0.350868845	negative
PRJNA729271	stage_1D_vs_E10	ordinary_muscle_development	ENSGALG00010001725	MTA2	-0.2473905	0.186029071	-1.329848607	0.183568157	0.222513835	negative
PRJNA729271	stage_1D_vs_E10	ordinary_muscle_development	ENSGALG00010001727	COL9A1	-7.951540507	0.776887521	-10.23512451	1.38018E-24	9.25758E-24	negative
PRJNA729271	stage_1D_vs_E10	ordinary_muscle_development	ENSGALG00010001728	STS	0.961285622	0.323868647	2.968134246	0.002996134	0.004724135	positive
PRJNA729271	stage_1D_vs_E10	ordinary_muscle_development	ENSGALG00010001729	ENSGALG00010001729	2.238279731	0.241656675	9.262230099	2.00206E-20	1.09385E-19	positive
PRJNA729271	stage_1D_vs_E10	ordinary_muscle_development	ENSGALG00010001731	ENSGALG00010001731	0.367000426	0.199782157	1.83700302	0.06620945	0.086616062	positive
PRJNA729271	stage_1D_vs_E10	ordinary_muscle_development	ENSGALG00010001738	PUDP	0.062044422	0.122878781	0.50492381	0.61361235	0.656116186	positive
PRJNA729271	stage_1D_vs_E10	ordinary_muscle_development	ENSGALG00010001741	SATB1	-0.612041323	0.198577549	-3.082127492	0.002055268	0.003302051	negative
PRJNA729271	stage_1D_vs_E10	ordinary_muscle_development	ENSGALG00010001745	CADPS2	-0.561428785	0.104106812	-5.392815069	6.93623E-08	1.69775E-07	negative
PRJNA729271	stage_1D_vs_E10	ordinary_muscle_development	ENSGALG00010001749	VAPA	1.027337979	0.105985178	9.69322312	3.22197E-22	1.94108E-21	positive
PRJNA729271	stage_1D_vs_E10	ordinary_muscle_development	ENSGALG00010001751	AVPR2C	0.805839799	0.415308918	1.940338297	0.05233859	0.06958157	positive
PRJNA729271	stage_1D_vs_E10	ordinary_muscle_development	ENSGALG00010001752	ARRB1	1.922476563	0.104804701	18.3434191	3.72608E-75	1.57127E-73	positive
PRJNA729271	stage_1D_vs_E10	ordinary_muscle_development	ENSGALG00010001753	PNPLA8	0.444305683	0.204518767	2.17244456	0.029822147	0.04112568	positive
PRJNA729271	stage_1D_vs_E10	ordinary_muscle_development	ENSGALG00010001754	APCDD1	-3.468193195	0.194638663	-17.81862426	5.06673E-71	1.92762E-69	negative
PRJNA729271	stage_1D_vs_E10	ordinary_muscle_development	ENSGALG00010001755	CUZD1	0.850599787	1.443833443	0.589125976	0.555776769	0.601462167	positive
PRJNA729271	stage_1D_vs_E10	ordinary_muscle_development	ENSGALG00010001756	ENSGALG00010001756	0.041594217	0.219207367	0.189748263	0.849506399	0.871378011	positive
PRJNA729271	stage_1D_vs_E10	ordinary_muscle_development	ENSGALG00010001758	COL19A1	2.305826756	0.276720987	8.332677556	7.90342E-17	3.55225E-16	positive
PRJNA729271	stage_1D_vs_E10	ordinary_muscle_development	ENSGALG00010001759	C10orf88	-0.138827184	0.089077015	-1.558507357	0.119113025	0.149552759	negative
PRJNA729271	stage_1D_vs_E10	ordinary_muscle_development	ENSGALG00010001760	RAB5A	0.34254129	0.095684564	3.579901249	0.000343724	0.000602452	positive
PRJNA729271	stage_1D_vs_E10	ordinary_muscle_development	ENSGALG00010001761	ELOB	-0.160724791	0.150089293	-1.070861134	0.284231874	0.330467715	negative
PRJNA729271	stage_1D_vs_E10	ordinary_muscle_development	ENSGALG00010001763	ENSGALG00010001763	0.486067096	0.262986834	1.84825639	0.064565261	0.084656125	positive
PRJNA729271	stage_1D_vs_E10	ordinary_muscle_development	ENSGALG00010001764	ENSGALG00010001764	-1.058533315	0.494765855	-2.139463151	0.032398177	0.044479217	negative
PRJNA729271	stage_1D_vs_E10	ordinary_muscle_development	ENSGALG00010001767	ENSGALG00010001767	1.333988897	0.168629998	7.910744913	2.55853E-15	1.04615E-14	positive
PRJNA729271	stage_1D_vs_E10	ordinary_muscle_development	ENSGALG00010001771	ENSGALG00010001771	-0.520162366	0.137964613	-3.770259308	0.000163078	0.000296292	negative
PRJNA729271	stage_1D_vs_E10	ordinary_muscle_development	ENSGALG00010001773	NRCAM	-0.450732816	0.233017935	-1.934326715	0.053072971	0.070467409	negative
PRJNA729271	stage_1D_vs_E10	ordinary_muscle_development	ENSGALG00010001776	LMBRD1	1.031779392	0.13120304	7.86399001	3.72087E-15	1.50864E-14	positive
PRJNA729271	stage_1D_vs_E10	ordinary_muscle_development	ENSGALG00010001778	KAT2B	2.605917186	0.147245556	17.69776453	4.36285E-70	1.60506E-68	positive
PRJNA729271	stage_1D_vs_E10	ordinary_muscle_development	ENSGALG00010001779	ENSGALG00010001779	0.682469865	0.604464051	1.129049551	0.258876931	0.303938208	positive
PRJNA729271	stage_1D_vs_E10	ordinary_muscle_development	ENSGALG00010001780	ENSGALG00010001780	2.06491549	0.235714858	8.760226275	1.94859E-18	9.5583E-18	positive
PRJNA729271	stage_1D_vs_E10	ordinary_muscle_development	ENSGALG00010001783	SGO1	-1.950514829	0.246215311	-7.921988372	2.33742E-15	9.598E-15	negative
PRJNA729271	stage_1D_vs_E10	ordinary_muscle_development	ENSGALG00010001785	CNTN1	1.464169416	0.212332895	6.895631587	5.3626E-12	1.76438E-11	positive
PRJNA729271	stage_1D_vs_E10	ordinary_muscle_development	ENSGALG00010001786	P2RY8	-1.064834258	0.212420345	-5.012863808	5.36259E-07	1.22314E-06	negative
PRJNA729271	stage_1D_vs_E10	ordinary_muscle_development	ENSGALG00010001788	HOXA1	-1.34291865	0.390746195	-3.436805441	0.000588618	0.001004595	negative
PRJNA729271	stage_1D_vs_E10	ordinary_muscle_development	ENSGALG00010001790	HOXA2	-1.026591674	0.233530878	-4.395956901	1.10286E-05	2.24238E-05	negative
PRJNA729271	stage_1D_vs_E10	ordinary_muscle_development	ENSGALG00010001793	IKZF5	0.709727913	0.103013983	6.889626941	5.59389E-12	1.83821E-11	positive
PRJNA729271	stage_1D_vs_E10	ordinary_muscle_development	ENSGALG00010001794	ENSGALG00010001794	-1.1030315	0.544623117	-2.025311571	0.042835381	0.057747289	negative
PRJNA729271	stage_1D_vs_E10	ordinary_muscle_development	ENSGALG00010001795	ENSGALG00010001795	1.642166754	0.472216685	3.477570372	0.00050598	0.000870836	positive
PRJNA729271	stage_1D_vs_E10	ordinary_muscle_development	ENSGALG00010001796	SLCO2B1	1.965847151	0.155692928	12.62643832	1.50958E-36	1.73799E-35	positive
PRJNA729271	stage_1D_vs_E10	ordinary_muscle_development	ENSGALG00010001798	PSTK	-0.041199807	0.13018506	-0.31647108	0.751644973	0.783537856	negative
PRJNA729271	stage_1D_vs_E10	ordinary_muscle_development	ENSGALG00010001800	ENSGALG00010001800	1.010575193	0.179240336	5.638101424	1.71935E-08	4.4063E-08	positive
PRJNA729271	stage_1D_vs_E10	ordinary_muscle_development	ENSGALG00010001801	HOXA3	-0.391695151	0.138650095	-2.825062264	0.004727143	0.007286254	negative
PRJNA729271	stage_1D_vs_E10	ordinary_muscle_development	ENSGALG00010001803	PPP4R1	-0.903474969	0.097044151	-9.309937394	1.27908E-20	7.06833E-20	negative
PRJNA729271	stage_1D_vs_E10	ordinary_muscle_development	ENSGALG00010001805	ENSGALG00010001805	1.507281354	0.257035881	5.864089281	4.51604E-09	1.21335E-08	positive
PRJNA729271	stage_1D_vs_E10	ordinary_muscle_development	ENSGALG00010001806	ENSGALG00010001806	2.99434805	0.912541262	3.281328939	0.001033192	0.001720799	positive
PRJNA729271	stage_1D_vs_E10	ordinary_muscle_development	ENSGALG00010001807	ASMTL	-0.35663145	0.105044002	-3.395067236	0.000686117	0.00116387	negative
PRJNA729271	stage_1D_vs_E10	ordinary_muscle_development	ENSGALG00010001808	HOXA4	-0.521207806	0.143738694	-3.626078627	0.000287758	0.000508169	negative
PRJNA729271	stage_1D_vs_E10	ordinary_muscle_development	ENSGALG00010001811	ACADSB	0.636063848	0.148738745	4.27638304	1.89954E-05	3.76918E-05	positive
PRJNA729271	stage_1D_vs_E10	ordinary_muscle_development	ENSGALG00010001813	HOXA5	-0.585839006	0.103414851	-5.664940757	1.47075E-08	3.78862E-08	negative
PRJNA729271	stage_1D_vs_E10	ordinary_muscle_development	ENSGALG00010001817	SLC25A6	-0.596382315	0.112898936	-5.282444077	1.27472E-07	3.05557E-07	negative
PRJNA729271	stage_1D_vs_E10	ordinary_muscle_development	ENSGALG00010001818	ADGRB3	-0.270692408	0.223051917	-1.213584763	0.224906328	0.267609361	negative
PRJNA729271	stage_1D_vs_E10	ordinary_muscle_development	ENSGALG00010001819	HOXA6	0.286091003	0.143333252	1.995984875	0.045935569	0.061577368	positive
PRJNA729271	stage_1D_vs_E10	ordinary_muscle_development	ENSGALG00010001820	PGM2L1	0.568347957	0.158024077	3.596590888	0.000322415	0.000566199	positive
PRJNA729271	stage_1D_vs_E10	ordinary_muscle_development	ENSGALG00010001822	KCNE3	2.029416513	0.305995754	6.632172128	3.30783E-11	1.03468E-10	positive
PRJNA729271	stage_1D_vs_E10	ordinary_muscle_development	ENSGALG00010001823	RALBP1	-0.250803641	0.076081479	-3.296513732	0.000978928	0.001633613	negative
PRJNA729271	stage_1D_vs_E10	ordinary_muscle_development	ENSGALG00010001825	LIPT2	1.466016902	0.144177813	10.16811718	2.75176E-24	1.81634E-23	positive
PRJNA729271	stage_1D_vs_E10	ordinary_muscle_development	ENSGALG00010001826	HOXA7	1.245236988	0.132997339	9.362871415	7.75986E-21	4.34954E-20	positive
PRJNA729271	stage_1D_vs_E10	ordinary_muscle_development	ENSGALG00010001827	PDZRN4	-2.042064957	0.182261531	-11.20403709	3.89567E-29	3.23969E-28	negative
PRJNA729271	stage_1D_vs_E10	ordinary_muscle_development	ENSGALG00010001828	ENSGALG00010001828	-2.985444061	1.102729578	-2.707322013	0.006782843	0.01024032	negative

PRJNA729271	stage_1D_vs_E10	ordinary_muscle_development	ENSGALG00010001829	POLD3	-0.245613885	0.121357628	-2.023885012	0.042981984	0.057917994	negative
PRJNA729271	stage_1D_vs_E10	ordinary_muscle_development	ENSGALG00010001831	CSF2RA	-0.169508965	0.257529352	-0.658212215	0.510401777	0.558276015	negative
PRJNA729271	stage_1D_vs_E10	ordinary_muscle_development	ENSGALG00010001832	BUB3	-1.109382109	0.086655021	-12.80228302	1.59201E-37	1.90712E-36	negative
PRJNA729271	stage_1D_vs_E10	ordinary_muscle_development	ENSGALG00010001834	ZCRB1	-0.25194549	0.085080544	-2.961258564	0.003063846	0.004824077	negative
PRJNA729271	stage_1D_vs_E10	ordinary_muscle_development	ENSGALG00010001835	MECP2	0.218827347	0.190792686	1.146937822	0.25140732	0.29598352	positive
PRJNA729271	stage_1D_vs_E10	ordinary_muscle_development	ENSGALG00010001836	TWSG1	-1.226003566	0.140282199	-8.739551937	2.34037E-18	1.14414E-17	negative
PRJNA729271	stage_1D_vs_E10	ordinary_muscle_development	ENSGALG00010001837	TAB3	0.588159571	0.138564523	4.244662052	2.18923E-05	4.32211E-05	positive
PRJNA729271	stage_1D_vs_E10	ordinary_muscle_development	ENSGALG00010001838	PPHLN1	-0.725308638	0.064141743	-11.30790344	1.19921E-29	1.01771E-28	negative
PRJNA729271	stage_1D_vs_E10	ordinary_muscle_development	ENSGALG00010001839	ENSGALG00010001839	0.645470215	0.719928705	0.896575189	0.369945642	0.419098248	positive
PRJNA729271	stage_1D_vs_E10	ordinary_muscle_development	ENSGALG00010001840	HOXA9	0.222639645	0.144424731	1.541561776	0.12318012	0.154334501	positive
PRJNA729271	stage_1D_vs_E10	ordinary_muscle_development	ENSGALG00010001842	GPR26	0.256523844	0.483799318	0.530227793	0.595954004	0.639262435	positive
PRJNA729271	stage_1D_vs_E10	ordinary_muscle_development	ENSGALG00010001843	PRICKLE1	-1.729093573	0.121721823	-14.20528822	8.49524E-46	1.36821E-44	negative
PRJNA729271	stage_1D_vs_E10	ordinary_muscle_development	ENSGALG00010001844	HOXA10	-1.10267739	0.165910417	-6.646221559	3.00713E-11	9.43061E-11	negative
PRJNA729271	stage_1D_vs_E10	ordinary_muscle_development	ENSGALG00010001845	ANKRD12	1.451427517	0.133273711	10.89057626	1.27829E-27	9.80878E-27	positive
PRJNA729271	stage_1D_vs_E10	ordinary_muscle_development	ENSGALG00010001848	HOXA11	-1.680589437	0.372174427	-4.515596225	6.31389E-06	1.31387E-05	negative
PRJNA729271	stage_1D_vs_E10	ordinary_muscle_development	ENSGALG00010001849	NDUFV2	0.857785445	0.094439853	9.082875716	1.05743E-19	5.57363E-19	positive
PRJNA729271	stage_1D_vs_E10	ordinary_muscle_development	ENSGALG00010001850	MYOM2	3.971800589	0.187795057	21.14965463	2.77969E-99	2.03493E-97	positive
PRJNA729271	stage_1D_vs_E10	ordinary_muscle_development	ENSGALG00010001852	CPXM2	-0.800163777	0.380804814	-2.101243857	0.035619565	0.048611769	negative
PRJNA729271	stage_1D_vs_E10	ordinary_muscle_development	ENSGALG00010001853	ENSGALG00010001853	0.643902914	0.188461645	3.416625774	0.000634024	0.001078406	positive
PRJNA729271	stage_1D_vs_E10	ordinary_muscle_development	ENSGALG00010001854	CHRDL2	2.002500241	0.283530957	7.062721699	1.63273E-12	5.56201E-12	positive
PRJNA729271	stage_1D_vs_E10	ordinary_muscle_development	ENSGALG00010001856	CHST15	-0.387940767	0.116433419	-3.331867862	0.000862652	0.001448244	negative
PRJNA729271	stage_1D_vs_E10	ordinary_muscle_development	ENSGALG00010001857	HNRNPA2B1	-0.513523373	0.187663021	-2.736412165	0.006211317	0.009438416	negative
PRJNA729271	stage_1D_vs_E10	ordinary_muscle_development	ENSGALG00010001858	ADAMTS20	2.2414225	0.160163035	13.99463051	1.68101E-44	2.5894E-43	positive
PRJNA729271	stage_1D_vs_E10	ordinary_muscle_development	ENSGALG00010001860	RNF169	0.646476031	0.126897989	5.094454501	3.49747E-07	8.08417E-07	positive
PRJNA729271	stage_1D_vs_E10	ordinary_muscle_development	ENSGALG00010001861	CBX3	-0.986017221	0.074362083	-13.25967719	3.96599E-40	5.21188E-39	negative
PRJNA729271	stage_1D_vs_E10	ordinary_muscle_development	ENSGALG00010001864	ENSGALG00010001864	-0.120687193	0.196833791	-0.613142654	0.539782019	0.586209662	negative
PRJNA729271	stage_1D_vs_E10	ordinary_muscle_development	ENSGALG00010001865	CYBB	-0.727160506	0.326906524	-2.224368292	0.026123675	0.036326043	negative
PRJNA729271	stage_1D_vs_E10	ordinary_muscle_development	ENSGALG00010001866	PUS7L	-0.125275442	0.118517357	-1.057021907	0.290501589	0.337000443	negative
PRJNA729271	stage_1D_vs_E10	ordinary_muscle_development	ENSGALG00010001868	ENSGALG00010001868	-0.276868166	0.123577138	-2.240448107	0.025061846	0.034970298	negative
PRJNA729271	stage_1D_vs_E10	ordinary_muscle_development	ENSGALG00010001869	CRLF2	7.199994687	0.922602849	7.804002229	5.99741E-15	2.39352E-14	positive
PRJNA729271	stage_1D_vs_E10	ordinary_muscle_development	ENSGALG00010001870	PLP2	1.958746129	0.163687014	11.96641124	5.32839E-33	5.31555E-32	positive
PRJNA729271	stage_1D_vs_E10	ordinary_muscle_development	ENSGALG00010001871	ENSGALG00010001871	-0.573417393	0.123134087	-4.6568534	3.21079E-06	6.87044E-06	negative
PRJNA729271	stage_1D_vs_E10	ordinary_muscle_development	ENSGALG00010001873	ENSGALG00010001873	0.160699858	0.197301383	0.814489262	0.415364721	0.464812139	positive
PRJNA729271	stage_1D_vs_E10	ordinary_muscle_development	ENSGALG00010001877	OAT	0.328011285	0.13674053	2.398786118	0.01644952	0.023560849	positive
PRJNA729271	stage_1D_vs_E10	ordinary_muscle_development	ENSGALG00010001878	XK	0.30348548	0.181011067	1.676612842	0.093618214	0.119632902	positive
PRJNA729271	stage_1D_vs_E10	ordinary_muscle_development	ENSGALG00010001879	IRAK4	0.492194033	0.164616107	2.989950624	0.002790225	0.004414837	positive
PRJNA729271	stage_1D_vs_E10	ordinary_muscle_development	ENSGALG00010001880	MTCL1	1.348816292	0.144370801	9.342722235	9.38885E-21	5.24437E-20	positive
PRJNA729271	stage_1D_vs_E10	ordinary_muscle_development	ENSGALG00010001883	KBTBD11	-0.476156561	0.08777392	-5.424806855	5.80172E-08	1.42656E-07	negative
PRJNA729271	stage_1D_vs_E10	ordinary_muscle_development	ENSGALG00010001885	SMPX	5.350623512	0.371045392	14.42040147	3.85096E-47	6.5516E-46	positive
PRJNA729271	stage_1D_vs_E10	ordinary_muscle_development	ENSGALG00010001886	NEU3	-0.453050897	0.084542676	-5.358842641	8.37568E-08	2.03496E-07	negative
PRJNA729271	stage_1D_vs_E10	ordinary_muscle_development	ENSGALG00010001888	SNX10	1.094011669	0.200392699	5.45933895	4.77911E-08	1.18395E-07	positive
PRJNA729271	stage_1D_vs_E10	ordinary_muscle_development	ENSGALG00010001890	KLHL34	6.665791131	0.250759929	26.58236165	1.0857E-155	2.0984E-153	positive
PRJNA729271	stage_1D_vs_E10	ordinary_muscle_development	ENSGALG00010001892	RAB12	0.89989448	0.070710813	12.7264055	4.21816E-37	4.96284E-36	positive
PRJNA729271	stage_1D_vs_E10	ordinary_muscle_development	ENSGALG00010001895	P4HA3	-1.622892721	0.240378521	-6.751404874	1.4642E-11	4.69653E-11	negative
PRJNA729271	stage_1D_vs_E10	ordinary_muscle_development	ENSGALG00010001896	ARHGEF10	0.657733228	0.133454765	4.928510639	8.28588E-07	1.86265E-06	positive
PRJNA729271	stage_1D_vs_E10	ordinary_muscle_development	ENSGALG00010001897	TLR7	0.629492304	0.182282334	3.453391732	0.000553584	0.000948712	positive
PRJNA729271	stage_1D_vs_E10	ordinary_muscle_development	ENSGALG00010001898	LHPP	0.135995011	0.132265311	1.028198629	0.303856403	0.350892174	positive
PRJNA729271	stage_1D_vs_E10	ordinary_muscle_development	ENSGALG00010001899	TWF1	-0.425809046	0.112496521	-3.785086345	0.000153655	0.000279998	negative
PRJNA729271	stage_1D_vs_E10	ordinary_muscle_development	ENSGALG00010001901	PRPS2	2.496493532	0.181310754	13.76914206	3.90827E-43	5.72226E-42	positive
PRJNA729271	stage_1D_vs_E10	ordinary_muscle_development	ENSGALG00010001902	WDR45	0.16470411	0.14204324	1.159535014	0.246238173	0.290298684	positive
PRJNA729271	stage_1D_vs_E10	ordinary_muscle_development	ENSGALG00010001903	NARS2	-0.092412804	0.11599492	-0.796696987	0.425627034	0.475085774	negative
PRJNA729271	stage_1D_vs_E10	ordinary_muscle_development	ENSGALG00010001906	SKAP2	0.060992839	0.127398875	0.478754924	0.632112987	0.673612539	positive
PRJNA729271	stage_1D_vs_E10	ordinary_muscle_development	ENSGALG00010001908	FAM83B	-2.211791977	0.373772845	-5.917476374	3.26919E-09	8.87894E-09	negative
PRJNA729271	stage_1D_vs_E10	ordinary_muscle_development	ENSGALG00010001910	FAM53B	-0.148614644	0.096253306	-1.543995211	0.122589493	0.153740673	negative
PRJNA729271	stage_1D_vs_E10	ordinary_muscle_development	ENSGALG00010001911	USP35	-3.609718637	0.515563784	-7.001497676	2.53241E-12	8.52863E-12	negative
PRJNA729271	stage_1D_vs_E10	ordinary_muscle_development	ENSGALG00010001912	TMEM117	3.835770756	0.205946907	18.62504671	2.01317E-77	8.92381E-76	positive
PRJNA729271	stage_1D_vs_E10	ordinary_muscle_development	ENSGALG00010001914	ENSGALG00010001914	-0.416667645	0.15236913	-2.734593587	0.006245734	0.009485741	negative
PRJNA729271	stage_1D_vs_E10	ordinary_muscle_development	ENSGALG00010001915	EEF1AKMT2	-0.918365131	0.170826935	-5.375997231	7.616E-08	1.85661E-07	negative
PRJNA729271	stage_1D_vs_E10	ordinary_muscle_development	ENSGALG00010001918	CNKSR2	1.196278681	0.142054393	8.421272016	3.72418E-17	1.70022E-16	positive
PRJNA729271	stage_1D_vs_E10	ordinary_muscle_development	ENSGALG00010001921	ENSGALG00010001921	0.031462494	0.280196666	0.112287181	0.910595706	0.923788127	positive
PRJNA729271	stage_1D_vs_E10	ordinary_muscle_development	ENSGALG00010001923	RAPGEF5	2.275063967	0.10060911	22.61290215	3.2355E-113	3.2344E-111	positive
PRJNA729271	stage_1D_vs_E10	ordinary_muscle_development	ENSGALG00010001925	FRMPD4	-1.327438872	0.180979169	-7.334760558	2.22118E-13	8.02493E-13	negative
PRJNA729271	stage_1D_vs_E10	ordinary_muscle_development	ENSGALG00010001928	KCTD21	-0.641699968	0.172697249	-3.715750965	0.000202601	0.000363498	negative
PRJNA729271	stage_1D_vs_E10	ordinary_muscle_development	ENSGALG00010001929	POLR2I	-0.148634945	0.122982362	-1.208587499	0.226821361	0.269688757	negative
PRJNA729271	stage_1D_vs_E10	ordinary_muscle_development	ENSGALG00010001935	ABRAXAS2	0.265488392	0.129279517	2.053599814	0.04001444	0.054160922	positive
PRJNA729271	stage_1D_vs_E10	ordinary_muscle_development	ENSGALG00010001938	ALG8	-0.585540325	0.10107409	-5.793179263	6.90663E-09	1.83004E-08	negative
PRJNA729271	stage_1D_vs_E10	ordinary_muscle_development	ENSGALG00010001940	PTPRM	1.520564824	0.110170418	13.80193393	2.48106E-43	3.66222E-42	positive
PRJNA729271	stage_1D_vs_E10	ordinary_muscle_development	ENSGALG00010001947	TINAG	6.609023526	0.3010346	21.95436512	7.8673E-107	6.4427E-105	positive
PRJNA729271	stage_1D_vs_E10	ordinary_muscle_development	ENSGALG00010001948	EMX2	-1.768411547	0.253751817	-6.96905964	3.19066E-12	1.06859E-11	negative
PRJNA729271	stage_1D_vs_E10	ordinary_muscle_development	ENSGALG00010001952	ENSGALG00010001952	-0.19664833	0.073039272	-2.692364309	0.00709474	0.010685628	negative
PRJNA729271	stage_1D_vs_E10	ordinary_muscle_development	ENSGALG00010001957	NDUFC2	1.057833558	0.111557748	9.48238538	2.48535E-21	1.43014E-20	positive
PRJNA729271	stage_1D_vs_E10	ordinary_muscle_development	ENSGALG00010001958	RAB11FIP2	0.180678193	0.111622325	1.618656422	0.105521208	0.133641757	positive
PRJNA729271	stage_1D_vs_E10	ordinary_muscle_development	ENSGALG00010001966	ARHGAP6	0.930433816	0.1411727	6.590748896	4.37613E-11	1.35858E-10	positive
PRJNA729271	stage_1D_vs_E10	ordinary_muscle_development	ENSGALG00010001967	LAMA1	-3.775368301	0.233626035	-16.15987831	9.67525E-59	2.54063E-57	negative
PRJNA729271	stage_1D_vs_E10	ordinary_muscle_development	ENSGALG00010001969	PLEKHA8	-1.787219244	0.12039799	-14.84426142	7.57849E-50	1.45497E-48	negative
PRJNA729271	stage_1D_vs_E10	ordinary_muscle_development	ENSGALG00010001971	MLIP	6.032331786	0.203623262	29.62496394	7.1287E-193	3.0391E-190	positive
PRJNA729271	stage_1D_vs_E10	ordinary_muscle_development	ENSGALG00010001972	FAM204A	-0.088310161	0.158410165	-0.557477871	0.577200974	0.62184232	negative
PRJNA729271	stage_1D_vs_E10	ordinary_muscle_development	ENSGALG00010001974	ENSGALG00010001974	1.126487857	0.392714091	2.868468137	0.004124647	0.006401838	positive
PRJNA729271	stage_1D_vs_E10	ordinary_muscle_development	ENSGALG00010001976	THRSP	10.67160302	1.422639639	7.501269283	6.32028E-14	2.36602E-13	positive
PRJNA729271	stage_1D_vs_E10	ordinary_muscle_development	ENSGALG00010001982	DMD	3.079741185	0.121769722	25.29151854	3.9599E-141	6.239E-139	positive
PRJNA729271	stage_1D_vs_E10	ordinary_muscle_development	ENSGALG00010001987	KCTD14	-3.787629666	0.528193186	-7.170917315	7.44969E-13	2.60263E-12	negative
PRJNA729271	stage_1D_vs_E10	ordinary_muscle_development	ENSGALG00010001991	TOMM7	0.564846182	0.130259508	4.336314406	1.44892E-05	2.91129E-05	positive
PRJNA729271	stage_1D_vs_E10	ordinary_muscle_development	ENSGALG00010001995	LRRC1	-1.295949417	0.138214699	-9.376350207	6.8296E-21	3.83702E-20	negative
PRJNA729271	stage_1D_vs_E10	ordinary_muscle_development	ENSGALG00010001997	INTS4	-0.083808642	0.076772054	-1.09165559	0.274984512	0.32066778	negative
PRJNA729271	stage_1D_vs_E10	ordinary_muscle_development	ENSGALG00010002005	COLQ	2.454954621	0.20898434	11.74707453	7.31062E-32	6.9305E-31	positive
PRJNA729271	stage_1D_vs_E10	ordinary_muscle_development	ENSGALG00010002006	ENSGALG00010002006	3.216530661	0.401306642	8.015144345	1.10008E-15	4.60989E-15	positive
PRJNA729271	stage_1D_vs_E10	ordinary_muscle_development	ENSGALG00010002012	MID1	-2.47448448	0.142028206	-17.42248632	5.57031E-68	1.91331E-66	negative
PRJNA729271	stage_1D_vs_E10	ordinary_muscle_development	ENSGALG00010002017	ARHGAP28	-0.889970064	0.118004382	-7.541839094	4.63389E-14	1.75237E-13	negative
PRJNA729271	stage_1D_vs_E10	ordinary_muscle_development	ENSGALG00010002022	ERNI	-0.365116149	0.654027067	-0.558258469	0.5766679	0.621472209	negative
PRJNA729271	stage_1D_vs_E10	ordinary_muscle_development	ENSGALG00010002023	CLCN4	-0.024710666	0.242840457	-0.101756791	0.918949723	0.931025109	negative
PRJNA729271	stage_1D_vs_E10	ordinary_muscle_development	ENSGALG00010002024	KLHL31	3.755523396	0.165784133	22.65309307	1.3005E-113	1.3276E-111	positive
PRJNA729271	stage_1D_vs_E10	ordinary_muscle_development	ENSGALG00010002027	ENSGALG00010002027	6.76259199	0.536174914	12.61266019	1.79819E-36	2.06372E-35	positive
PRJNA729271	stage_1D_vs_E10	ordinary_muscle_development	ENSGALG00010002034	WWC3	0.220112229	0.098619226	2.231940338	0.025618906	0.035682334	positive
PRJNA729271	stage_1D_vs_E10	ordinary_muscle_development	ENSGALG00010002039	AAMDC	0.496433594	0.129044005	3.847010138	0.000119568	0.000220301	positive
PRJNA729271	stage_1D_vs_E10	ordinary_muscle_development	ENSGALG00010002041	GCLC	0.929642579	0.138067067	6.733268086	1.65894E-11	5.29538E-11	positive
PRJNA729271	stage_1D_vs_E10	ordinary_muscle_development	ENSGALG00010002046	FAM126A	-0.274413908	0.087805041	-3.125263709	0.001776457	0.002880285	negative
PRJNA729271	stage_1D_vs_E10	ordinary_muscle_development	ENSGALG00010002050	ENSGALG00010002050	-0.047575871	1.101139799	-0.043206023	0.965537304	0.970599877	negative
PRJNA729271	stage_1D_vs_E10	ordinary_muscle_development	ENSGALG00010002054	RSF1	-0.015954541	0.099481228	-0.160377401	0.87258379	0.891464762	negative
PRJNA729271	stage_1D_vs_E10	ordinary_muscle_development	ENSGALG00010002056	PRLHR	-2.304928283	0.474130407	-4.861380428	1.1657E-06	2.58876E-06	negative
PRJNA729271	stage_1D_vs_E10	ordinary_muscle_development	ENSGALG00010002062	GSTA4	-1.73845007	0.372899882	-4.661975377	3.13189E-06	6.70854E-06	negative
PRJNA729271	stage_1D_vs_E10	ordinary_muscle_development	ENSGALG00010002065	CLNS1A	-0.586958326	0.06285135	-9.338834023	9.74008E-21	5.42801E-20	negative
PRJNA729271	stage_1D_vs_E10	ordinary_muscle_development	ENSGALG00010002072	NEURL4	0.270440928	0.160287669	1.687222291	0.091560617	0.117220556	positive
PRJNA729271	stage_1D_vs_E10	ordinary_muscle_development	ENSGALG00010002079	ARX	3.2659798	0.188411629	17.33427925	2.59297E-67	8.68017E-66	positive
PRJNA729271	stage_1D_vs_E10	ordinary_muscle_development	ENSGALG00010002084	HACL1	-0.815900395	0.091510092	-8.915960847	4.83604E-19	2.44757E-18	negative
PRJNA729271	stage_1D_vs_E10	ordinary_muscle_development	ENSGALG00010002087	POLA1	-0.811600066	0.072809397	-11.14691369	7.41332E-29	6.06754E-28	negative
PRJNA729271	stage_1D_vs_E10	ordinary_muscle_development	ENSGALG00010002088	OZFL1	-0.227692793	0.136047934	-1.673621843	0.094204943	0.120308427	negative
PRJNA729271	stage_1D_vs_E10	ordinary_muscle_development	ENSGALG00010002090	AQP11	0.572071239	0.285565643	2.003291548	0.045146004	0.060619855	positive
PRJNA729271	stage_1D_vs_E10	ordinary_muscle_development	ENSGALG00010002097	PCYT1B	-3.34185239	0.517528987	-6.457324079	1.06571E-10	3.21955E-10	negative
PRJNA729271	stage_1D_vs_E10	ordinary_muscle_development	ENSGALG00010002098	ENSGALG00010002098	0.676764624	0.421765993	1.60459742	0.10858243	0.137159243	positive
PRJNA729271	stage_1D_vs_E10	ordinary_muscle_development	ENSGALG00010002100	BTD	0.522666218	0.115614076	4.520783613	6.16111E-06	1.28294E-05	positive
PRJNA729271	stage_1D_vs_E10	ordinary_muscle_development	ENSGALG00010002102	PAK1	-1.594938325	0.274141995	-5.817927759	5.95816E-09	1.58727E-08	negative
PRJNA729271	stage_1D_vs_E10	ordinary_muscle_development	ENSGALG00010002106	ENSGALG00010002106	2.718501452	0.871429588	3.119588189	0.00181104	0.002931766	positive
PRJNA729271	stage_1D_vs_E10	ordinary_muscle_development	ENSGALG00010002113	GDPD4	-6.893282284	0.333969839	-20.64043357	1.1899E-94	7.91177E-93	negative
PRJNA729271	stage_1D_vs_E10	ordinary_muscle_development	ENSGALG00010002118	ENSGALG00010002118	0.873016459	0.269274304	3.242108314	0.001186489	0.00196326	positive
PRJNA729271	stage_1D_vs_E10	ordinary_muscle_development	ENSGALG00010002122	ENSGALG00010002122	0.505848169	0.525651056	0.962326933	0.335885404	0.384418392	positive
PRJNA729271	stage_1D_vs_E10	ordinary_muscle_development	ENSGALG00010002126	ENSGALG00010002126	2.468805073	0.297121792	8.309067652	9.64569E-17	4.30861E-16	positive
PRJNA729271	stage_1D_vs_E10	ordinary_muscle_development	ENSGALG00010002134	ANKRD28	0.414147124	0.120819912	3.42780522	0.000608482	0.001036909	positive
PRJNA729271	stage_1D_vs_E10	ordinary_muscle_development	ENSGALG00010002135	ELOVL5	-0.626937242	0.168534129	-3.719942348	0.000199268	0.000357829	negative
PRJNA729271	stage_1D_vs_E10	ordinary_muscle_development	ENSGALG00010002136	ACAP1	-1.661967004	0.270693359	-6.139666701	8.26948E-10	2.35124E-09	negative
PRJNA729271	stage_1D_vs_E10	ordinary_muscle_development	ENSGALG00010002137	PDK3	1.965806621	0.131340799	14.96721996	1.20244E-50	2.38431E-49	positive
PRJNA729271	stage_1D_vs_E10	ordinary_muscle_development	ENSGALG00010002138	MYO7A	-1.150121349	0.172594453	-6.663721389	2.66979E-11	8.39815E-11	negative
PRJNA729271	stage_1D_vs_E10	ordinary_muscle_development	ENSGALG00010002140	ENSGALG00010002140	0.238350568	0.427459606	0.557597875	0.577119008	0.62181967	positive
PRJNA729271	stage_1D_vs_E10	ordinary_muscle_development	ENSGALG00010002142	CLDN34	0.763618598	0.242021383	3.155169962	0.001604046	0.00261302	positive
PRJNA729271	stage_1D_vs_E10	ordinary_muscle_development	ENSGALG00010002145	ENSGALG00010002145	0.412656124	0.462015622	0.893164871	0.371768888	0.420999221	positive
PRJNA729271	stage_1D_vs_E10	ordinary_muscle_development	ENSGALG00010002146	CACUL1	0.032962611	0.088798656	0.371206193	0.710483961	0.746211507	positive
PRJNA729271	stage_1D_vs_E10	ordinary_muscle_development	ENSGALG00010002148	POLR2A	1.749385088	0.17619935	9.928442331	3.13108E-23	1.96898E-22	positive
PRJNA729271	stage_1D_vs_E10	ordinary_muscle_development	ENSGALG00010002149	ENSGALG00010002149	-3.331051067	0.394729478	-8.438820131	3.20557E-17	1.4718E-16	negative
PRJNA729271	stage_1D_vs_E10	ordinary_muscle_development	ENSGALG00010002150	EIF3A	0.276662737	0.13240507	2.089517697	0.036661147	0.049925152	positive
PRJNA729271	stage_1D_vs_E10	ordinary_muscle_development	ENSGALG00010002159	GALNT15	2.552575923	0.188497283	13.54171205	8.86973E-42	1.23741E-40	positive
PRJNA729271	stage_1D_vs_E10	ordinary_muscle_development	ENSGALG00010002160	CAPN5	0.645275011	0.165287063	3.903965606	9.46292E-05	0.000175988	positive
PRJNA729271	stage_1D_vs_E10	ordinary_muscle_development	ENSGALG00010002161	CDKL5	-0.466024328	0.174666484	-2.668081007	0.007628586	0.011453942	negative

PRJNA729271	stage_1D_vs_E10	ordinary_muscle_development	ENSGALG00010002173	GCM1	-1.055066581	0.897659672	-1.175352547	0.2398537	0.283659628	negative
PRJNA729271	stage_1D_vs_E10	ordinary_muscle_development	ENSGALG00010002174	ENSGALG00010002174	0.122936923	0.455665826	0.269796233	0.787317021	0.815328128	positive
PRJNA729271	stage_1D_vs_E10	ordinary_muscle_development	ENSGALG00010002177	FBXO9	0.049666665	0.155556028	0.319284736	0.749510607	0.781593974	positive
PRJNA729271	stage_1D_vs_E10	ordinary_muscle_development	ENSGALG00010002181	FIS1	0.217857924	0.257950031	0.844574136	0.398348655	0.447740676	positive
PRJNA729271	stage_1D_vs_E10	ordinary_muscle_development	ENSGALG00010002185	ENSGALG00010002185	0.224133268	0.327478912	0.684420461	0.493709699	0.542008729	positive
PRJNA729271	stage_1D_vs_E10	ordinary_muscle_development	ENSGALG00010002186	CILK1	0.20739062	0.07302026	2.840179137	0.004508821	0.006974958	positive
PRJNA729271	stage_1D_vs_E10	ordinary_muscle_development	ENSGALG00010002192	ENSGALG00010002192	1.207513044	0.194277053	6.215417742	5.11883E-10	1.47598E-09	positive
PRJNA729271	stage_1D_vs_E10	ordinary_muscle_development	ENSGALG00010002194	SHROOM2	-1.884886378	0.131466226	-14.33741904	1.27718E-46	2.13279E-45	negative
PRJNA729271	stage_1D_vs_E10	ordinary_muscle_development	ENSGALG00010002196	ENSGALG00010002196	-1.36257587	0.102040231	-13.35332009	1.13285E-40	1.53035E-39	negative
PRJNA729271	stage_1D_vs_E10	ordinary_muscle_development	ENSGALG00010002197	GSTA2	-1.112787888	0.189366007	-5.876386728	4.19318E-09	1.12765E-08	negative
PRJNA729271	stage_1D_vs_E10	ordinary_muscle_development	ENSGALG00010002202	SFXN4	0.141856161	0.101249151	1.401060249	0.161196057	0.19757626	positive
PRJNA729271	stage_1D_vs_E10	ordinary_muscle_development	ENSGALG00010002206	DPH3P1	0.823061199	0.108986888	7.551928634	4.28859E-14	1.62433E-13	positive
PRJNA729271	stage_1D_vs_E10	ordinary_muscle_development	ENSGALG00010002210	GPR143	-4.518376251	1.211712295	-3.728918383	0.000192303	0.000346051	negative
PRJNA729271	stage_1D_vs_E10	ordinary_muscle_development	ENSGALG00010002211	ACER3	-0.570827024	0.17426432	-3.275639123	0.001054232	0.001754028	negative
PRJNA729271	stage_1D_vs_E10	ordinary_muscle_development	ENSGALG00010002215	YLEC18	0.416039283	0.353162952	1.17803773	0.238781565	0.282541942	positive
PRJNA729271	stage_1D_vs_E10	ordinary_muscle_development	ENSGALG00010002225	PRDX3	0.266652118	0.105574845	2.525716395	0.011546265	0.016929305	positive
PRJNA729271	stage_1D_vs_E10	ordinary_muscle_development	ENSGALG00010002226	OXNAD1	-0.147689927	0.133502021	-1.10627484	0.268607578	0.313938627	negative
PRJNA729271	stage_1D_vs_E10	ordinary_muscle_development	ENSGALG00010002227	ENSGALG00010002227	2.592630542	1.113571511	2.328211988	0.01990085	0.028164696	positive
PRJNA729271	stage_1D_vs_E10	ordinary_muscle_development	ENSGALG00010002231	TMEM14A	-0.795945217	0.135315189	-5.882157248	4.04953E-09	1.09064E-08	negative
PRJNA729271	stage_1D_vs_E10	ordinary_muscle_development	ENSGALG00010002235	MXRA5	-0.768744852	0.201272716	-3.819419083	0.000133766	0.000245188	negative
PRJNA729271	stage_1D_vs_E10	ordinary_muscle_development	ENSGALG00010002238	ENSGALG00010002238	-0.118950626	0.409843647	-0.290234157	0.771637106	0.801009182	negative
PRJNA729271	stage_1D_vs_E10	ordinary_muscle_development	ENSGALG00010002239	AGPAT5	0.197283256	0.128038308	1.540814295	0.123361989	0.154522298	positive
PRJNA729271	stage_1D_vs_E10	ordinary_muscle_development	ENSGALG00010002243	ARSH	-0.639420823	0.178167917	-3.588866249	0.000332119	0.000582958	negative
PRJNA729271	stage_1D_vs_E10	ordinary_muscle_development	ENSGALG00010002247	RFTN1	-0.552393152	0.18529447	-2.98116372	0.002871552	0.004538067	negative
PRJNA729271	stage_1D_vs_E10	ordinary_muscle_development	ENSGALG00010002251	GRK5	-0.100205691	0.148821581	-0.673327687	0.500738842	0.548824916	negative
PRJNA729271	stage_1D_vs_E10	ordinary_muscle_development	ENSGALG00010002253	ENSGALG00010002253	0.777228444	0.320147873	2.427717032	0.015194193	0.021888275	positive
PRJNA729271	stage_1D_vs_E10	ordinary_muscle_development	ENSGALG00010002254	ENSGALG00010002254	4.672633371	0.451326624	10.35310821	4.05116E-25	2.79893E-24	positive
PRJNA729271	stage_1D_vs_E10	ordinary_muscle_development	ENSGALG00010002259	TASL	-1.061538836	0.638439251	-1.662709232	0.096370653	0.122900987	negative
PRJNA729271	stage_1D_vs_E10	ordinary_muscle_development	ENSGALG00010002261	NROB1	-1.94944987	0.374448249	-5.206193039	1.92754E-07	4.55267E-07	negative
PRJNA729271	stage_1D_vs_E10	ordinary_muscle_development	ENSGALG00010002269	MCPH1	-1.523197037	0.139904179	-10.88743056	1.32321E-27	1.01481E-26	negative
PRJNA729271	stage_1D_vs_E10	ordinary_muscle_development	ENSGALG00010002270	IMMP2L	-0.448238251	0.251492757	-1.782310776	0.074698566	0.096995048	negative
PRJNA729271	stage_1D_vs_E10	ordinary_muscle_development	ENSGALG00010002271	ENSGALG00010002271	3.220603969	0.677854256	4.751174665	2.02238E-06	4.40752E-06	positive
PRJNA729271	stage_1D_vs_E10	ordinary_muscle_development	ENSGALG00010002274	ENSGALG00010002274	4.101969265	0.777765907	5.274040978	1.33452E-07	3.19153E-07	positive
PRJNA729271	stage_1D_vs_E10	ordinary_muscle_development	ENSGALG00010002275	IL1RAPL1	-2.570984655	0.269749044	-9.531024171	1.55739E-21	9.06422E-21	negative
PRJNA729271	stage_1D_vs_E10	ordinary_muscle_development	ENSGALG00010002277	RGS10	-1.62576298	0.178819115	-9.091662164	9.75364E-20	5.15229E-19	negative
PRJNA729271	stage_1D_vs_E10	ordinary_muscle_development	ENSGALG00010002279	ENSGALG00010002279	6.205184979	0.693541082	8.947105138	3.64927E-19	1.85927E-18	positive
PRJNA729271	stage_1D_vs_E10	ordinary_muscle_development	ENSGALG00010002280	ST7	1.626929854	0.141054499	11.53405152	8.88612E-31	7.9953E-30	positive
PRJNA729271	stage_1D_vs_E10	ordinary_muscle_development	ENSGALG00010002281	TGFBR2	2.65528053	0.154628455	17.1720045	4.303E-66	1.39223E-64	positive
PRJNA729271	stage_1D_vs_E10	ordinary_muscle_development	ENSGALG00010002282	TSKU	-1.280447139	0.165541739	-7.73488998	1.03493E-14	4.07044E-14	negative
PRJNA729271	stage_1D_vs_E10	ordinary_muscle_development	ENSGALG00010002283	ENSGALG00010002283	4.335192658	0.65364995	6.632284849	3.3053E-11	1.03411E-10	positive
PRJNA729271	stage_1D_vs_E10	ordinary_muscle_development	ENSGALG00010002285	CAV1	3.006039804	0.15735073	19.10407277	2.33539E-81	1.13596E-79	positive
PRJNA729271	stage_1D_vs_E10	ordinary_muscle_development	ENSGALG00010002288	ANGPT2	-0.720433818	0.178053546	-4.046163832	5.20638E-05	9.92719E-05	negative
PRJNA729271	stage_1D_vs_E10	ordinary_muscle_development	ENSGALG00010002289	ENSGALG00010002289	1.904637241	0.937413507	2.031800508	0.042173856	0.056897807	positive
PRJNA729271	stage_1D_vs_E10	ordinary_muscle_development	ENSGALG00010002291	RNASEH1	0.161254875	0.116316419	1.386346624	0.165641087	0.202579105	positive
PRJNA729271	stage_1D_vs_E10	ordinary_muscle_development	ENSGALG00010002293	GYG2	-3.503009151	0.386513186	-9.063103878	1.2679E-19	6.64434E-19	negative
PRJNA729271	stage_1D_vs_E10	ordinary_muscle_development	ENSGALG00010002294	DAZL	2.292145123	0.808362035	2.83554277	0.004574789	0.007069463	positive
PRJNA729271	stage_1D_vs_E10	ordinary_muscle_development	ENSGALG00010002295	YAF2	0.299179572	0.113563672	2.634465479	0.008426987	0.012575845	positive
PRJNA729271	stage_1D_vs_E10	ordinary_muscle_development	ENSGALG00010002298	ENSGALG00010002298	-1.322920841	0.428998614	-3.083741526	0.002044151	0.003285648	negative
PRJNA729271	stage_1D_vs_E10	ordinary_muscle_development	ENSGALG00010002303	ENSGALG00010002303	0.125890438	0.897038328	0.140340088	0.888391292	0.904808304	positive
PRJNA729271	stage_1D_vs_E10	ordinary_muscle_development	ENSGALG00010002308	GXYLT1	-1.363071898	0.104199257	-13.08139746	4.20635E-39	5.34834E-38	negative
PRJNA729271	stage_1D_vs_E10	ordinary_muscle_development	ENSGALG00010002309	GADL1	3.145074238	0.326905103	9.620756029	6.53491E-22	3.88689E-21	positive
PRJNA729271	stage_1D_vs_E10	ordinary_muscle_development	ENSGALG00010002310	PRRG1	0.202167862	0.104198423	1.94021998	0.052352961	0.069587912	positive
PRJNA729271	stage_1D_vs_E10	ordinary_muscle_development	ENSGALG00010002312	ENSGALG00010002312	-0.095408387	0.190890544	-0.499806774	0.617211141	0.65952639	negative
PRJNA729271	stage_1D_vs_E10	ordinary_muscle_development	ENSGALG00010002315	ENSGALG00010002315	0.157924182	0.158035849	0.999293405	0.317652579	0.365513546	positive
PRJNA729271	stage_1D_vs_E10	ordinary_muscle_development	ENSGALG00010002324	SNTG2	-1.866663866	0.334946046	-5.573028509	2.50349E-08	6.33299E-08	negative
PRJNA729271	stage_1D_vs_E10	ordinary_muscle_development	ENSGALG00010002328	ENSGALG00010002328	0.553341391	0.247924103	2.23189833	0.025621683	0.035682773	positive
PRJNA729271	stage_1D_vs_E10	ordinary_muscle_development	ENSGALG00010002329	STT3B	-0.067880648	0.072456171	-0.93685116	0.348835125	0.397669299	negative
PRJNA729271	stage_1D_vs_E10	ordinary_muscle_development	ENSGALG00010002330	KHDRBS2	-0.588891869	0.280660584	-2.098235027	0.035884389	0.048945536	negative
PRJNA729271	stage_1D_vs_E10	ordinary_muscle_development	ENSGALG00010002331	ENSGALG00010002331	-2.438016195	0.307929538	-7.917448302	2.42434E-15	9.93522E-15	negative
PRJNA729271	stage_1D_vs_E10	ordinary_muscle_development	ENSGALG00010002332	ENSGALG00010002332	-1.898106547	0.611339605	-3.104831636	0.001903874	0.003072097	negative
PRJNA729271	stage_1D_vs_E10	ordinary_muscle_development	ENSGALG00010002333	ENSGALG00010002333	-0.037384508	0.702641219	-0.053205686	0.957568025	0.964220113	negative
PRJNA729271	stage_1D_vs_E10	ordinary_muscle_development	ENSGALG00010002334	OSBPL10	-0.765846408	0.168176745	-4.553818717	5.26808E-06	1.10412E-05	negative
PRJNA729271	stage_1D_vs_E10	ordinary_muscle_development	ENSGALG00010002335	TMEM47	1.117104766	0.133808372	8.348541666	6.9111E-17	3.1159E-16	positive
PRJNA729271	stage_1D_vs_E10	ordinary_muscle_development	ENSGALG00010002336	PRIM2	-1.357785406	0.118374857	-11.47021795	1.8619E-30	1.65268E-29	negative
PRJNA729271	stage_1D_vs_E10	ordinary_muscle_development	ENSGALG00010002338	CD99	0.099901747	0.148290093	0.673691312	0.500507587	0.548654426	positive
PRJNA729271	stage_1D_vs_E10	ordinary_muscle_development	ENSGALG00010002339	ZNFY4	0.058852986	0.310266977	0.189684984	0.849555989	0.871378011	positive
PRJNA729271	stage_1D_vs_E10	ordinary_muscle_development	ENSGALG00010002340	RAB23	-0.427491173	0.134653725	-3.174744497	0.001499685	0.002450725	negative
PRJNA729271	stage_1D_vs_E10	ordinary_muscle_development	ENSGALG00010002341	FAM49A	-0.714306708	0.111028071	-6.433568574	1.24642E-10	3.74988E-10	negative
PRJNA729271	stage_1D_vs_E10	ordinary_muscle_development	ENSGALG00010002342	BAG2	1.840895025	0.123905401	14.85726218	6.24246E-50	1.20165E-48	positive
PRJNA729271	stage_1D_vs_E10	ordinary_muscle_development	ENSGALG00010002343	GPD1L	0.3978	0.081398258	4.887082478	1.02341E-06	2.28607E-06	positive
PRJNA729271	stage_1D_vs_E10	ordinary_muscle_development	ENSGALG00010002345	SCML2	-1.674558294	0.104596959	-16.009627	1.09464E-57	2.76908E-56	negative
PRJNA729271	stage_1D_vs_E10	ordinary_muscle_development	ENSGALG00010002346	FBXO15	-0.730469527	0.362077792	-2.017438085	0.043649816	0.05873599	negative
PRJNA729271	stage_1D_vs_E10	ordinary_muscle_development	ENSGALG00010002347	ZNF451	-0.260697621	0.061434066	-4.243535177	2.20026E-05	4.34151E-05	negative
PRJNA729271	stage_1D_vs_E10	ordinary_muscle_development	ENSGALG00010002348	THAP12	-0.357029299	0.153462509	-2.326491996	0.019992319	0.028283102	negative
PRJNA729271	stage_1D_vs_E10	ordinary_muscle_development	ENSGALG00010002349	ZBED1	0.73349355	0.129083651	5.682311792	1.32886E-08	3.43287E-08	positive
PRJNA729271	stage_1D_vs_E10	ordinary_muscle_development	ENSGALG00010002350	BEND6	-0.26904438	0.249527935	-1.078213465	0.280938504	0.327032093	negative
PRJNA729271	stage_1D_vs_E10	ordinary_muscle_development	ENSGALG00010002352	ENSGALG00010002352	-0.020739185	0.106926531	-0.193957331	0.846209292	0.868559956	negative
PRJNA729271	stage_1D_vs_E10	ordinary_muscle_development	ENSGALG00010002353	CMTM8	0.630310201	0.136458741	4.619053323	3.85495E-06	8.17879E-06	positive
PRJNA729271	stage_1D_vs_E10	ordinary_muscle_development	ENSGALG00010002355	UFM1	-0.839252957	0.100807521	-8.325300991	8.41147E-17	3.77241E-16	negative
PRJNA729271	stage_1D_vs_E10	ordinary_muscle_development	ENSGALG00010002356	WNT11	-3.85973458	0.328328844	-11.75569754	6.60133E-32	6.26629E-31	negative
PRJNA729271	stage_1D_vs_E10	ordinary_muscle_development	ENSGALG00010002357	RAB31	-0.792117946	0.142143621	-5.572659117	2.5088E-08	6.34422E-08	negative
PRJNA729271	stage_1D_vs_E10	ordinary_muscle_development	ENSGALG00010002358	FREM2	-0.330512097	0.233800804	-1.413648251	0.157465207	0.193297609	negative
PRJNA729271	stage_1D_vs_E10	ordinary_muscle_development	ENSGALG00010002359	CMTM6	-0.041442681	0.115778062	-0.357949339	0.720381226	0.755442315	negative
PRJNA729271	stage_1D_vs_E10	ordinary_muscle_development	ENSGALG00010002360	DHRSX	0.946646218	0.163140767	5.802634362	6.5281E-09	1.73239E-08	positive
PRJNA729271	stage_1D_vs_E10	ordinary_muscle_development	ENSGALG00010002361	ENSGALG00010002361	0.102391135	0.099038913	1.033847519	0.301207453	0.348166031	positive
PRJNA729271	stage_1D_vs_E10	ordinary_muscle_development	ENSGALG00010002362	TRPC4	0.079493356	0.314969288	0.252384466	0.800743906	0.827578104	positive
PRJNA729271	stage_1D_vs_E10	ordinary_muscle_development	ENSGALG00010002364	VSNL1	-0.98679363	0.396847663	-2.48658042	0.012897741	0.018777899	negative
PRJNA729271	stage_1D_vs_E10	ordinary_muscle_development	ENSGALG00010002365	RAI2	1.249449608	0.137604093	9.080032286	1.08543E-19	5.71703E-19	positive
PRJNA729271	stage_1D_vs_E10	ordinary_muscle_development	ENSGALG00010002366	ENSGALG00010002366	1.899892685	0.756704319	2.510746453	0.012047619	0.017625176	positive
PRJNA729271	stage_1D_vs_E10	ordinary_muscle_development	ENSGALG00010002367	STK31	-4.314680988	0.722993243	-5.967802645	2.4047E-09	6.59904E-09	negative
PRJNA729271	stage_1D_vs_E10	ordinary_muscle_development	ENSGALG00010002368	UVRAG	0.503917693	0.095539827	5.274425432	1.33173E-07	3.18538E-07	positive
PRJNA729271	stage_1D_vs_E10	ordinary_muscle_development	ENSGALG00010002369	GEMIN8	-1.027174626	0.121429903	-8.458992418	2.69698E-17	1.24341E-16	negative
PRJNA729271	stage_1D_vs_E10	ordinary_muscle_development	ENSGALG00010002370	ENSGALG00010002370	0.851683962	0.149236152	5.706954704	1.15015E-08	2.98611E-08	positive
PRJNA729271	stage_1D_vs_E10	ordinary_muscle_development	ENSGALG00010002371	BAG3	2.029526522	0.154852608	13.10618238	3.03495E-39	3.89651E-38	positive
PRJNA729271	stage_1D_vs_E10	ordinary_muscle_development	ENSGALG00010002372	SMC6	-0.204870135	0.126443774	-1.62024692	0.105179246	0.133255238	negative
PRJNA729271	stage_1D_vs_E10	ordinary_muscle_development	ENSGALG00010002373	POSTN	0.311253894	0.25560421	1.217718183	0.223331085	0.265997048	positive
PRJNA729271	stage_1D_vs_E10	ordinary_muscle_development	ENSGALG00010002374	DST	1.408742929	0.11418917	12.33692242	5.73047E-35	6.22196E-34	positive
PRJNA729271	stage_1D_vs_E10	ordinary_muscle_development	ENSGALG00010002375	NPY	-4.235928464	0.53905946	-7.857998568	3.9032E-15	1.58168E-14	negative
PRJNA729271	stage_1D_vs_E10	ordinary_muscle_development	ENSGALG00010002376	GEN1	-1.230547482	0.192096334	-6.405887378	1.49497E-10	4.4735E-10	negative
PRJNA729271	stage_1D_vs_E10	ordinary_muscle_development	ENSGALG00010002377	ART1A	3.589388229	0.247257865	14.51678083	9.48617E-48	1.66467E-46	positive
PRJNA729271	stage_1D_vs_E10	ordinary_muscle_development	ENSGALG00010002378	ENSGALG00010002378	1.226896044	0.447524903	2.741514576	0.006115665	0.009301843	positive
PRJNA729271	stage_1D_vs_E10	ordinary_muscle_development	ENSGALG00010002379	NHS	-0.987277625	0.130895309	-7.542498153	4.61053E-14	1.74444E-13	negative
PRJNA729271	stage_1D_vs_E10	ordinary_muscle_development	ENSGALG00010002380	CMPK2	2.209952191	0.743175248	2.973662263	0.002942688	0.004643904	positive
PRJNA729271	stage_1D_vs_E10	ordinary_muscle_development	ENSGALG00010002381	TDRD3	0.320623841	0.096348274	3.327759043	0.000875476	0.001468582	positive
PRJNA729271	stage_1D_vs_E10	ordinary_muscle_development	ENSGALG00010002382	ART1D	-0.626574586	0.667130978	-0.939207751	0.34762409	0.396475819	negative
PRJNA729271	stage_1D_vs_E10	ordinary_muscle_development	ENSGALG00010002383	INPP5F	0.329411915	0.123848361	2.659800355	0.007818698	0.011722386	positive
PRJNA729271	stage_1D_vs_E10	ordinary_muscle_development	ENSGALG00010002384	ENSGALG00010002384	-0.5143371	0.306116027	-1.680203111	0.092917804	0.118832149	negative
PRJNA729271	stage_1D_vs_E10	ordinary_muscle_development	ENSGALG00010002385	PALS2	-0.736901781	0.123946221	-5.945334787	2.75892E-09	7.54397E-09	negative
PRJNA729271	stage_1D_vs_E10	ordinary_muscle_development	ENSGALG00010002386	MHCY2B2	-1.096192293	0.389056954	-2.817562526	0.004838969	0.007449114	negative
PRJNA729271	stage_1D_vs_E10	ordinary_muscle_development	ENSGALG00010002387	MCMBP	-0.194331463	0.108793979	-1.786233618	0.07406144	0.096210842	negative
PRJNA729271	stage_1D_vs_E10	ordinary_muscle_development	ENSGALG00010002388	ENSGALG00010002388	1.928921403	0.804096809	2.398867128	0.016445881	0.023557965	positive
PRJNA729271	stage_1D_vs_E10	ordinary_muscle_development	ENSGALG00010002389	COL21A1	-1.855556987	0.323532807	-5.735297768	9.73412E-09	2.54136E-08	negative
PRJNA729271	stage_1D_vs_E10	ordinary_muscle_development	ENSGALG00010002391	REPS2	0.688450059	0.158967839	4.330750574	1.48602E-05	2.98129E-05	positive
PRJNA729271	stage_1D_vs_E10	ordinary_muscle_development	ENSGALG00010002392	DFNA5	-1.795804745	0.20780639	-8.641720509	5.53728E-18	2.65069E-17	negative
PRJNA729271	stage_1D_vs_E10	ordinary_muscle_development	ENSGALG00010002393	GPM6B	-0.141958498	0.106864493	-1.328397249	0.184046908	0.223019556	negative
PRJNA729271	stage_1D_vs_E10	ordinary_muscle_development	ENSGALG00010002395	CLN8	0.24037011	0.090351663	2.6603839	0.007805163	0.011704515	positive
PRJNA729271	stage_1D_vs_E10	ordinary_muscle_development	ENSGALG00010002396	SEC23IP	0.216064826	0.087437362	2.471081249	0.013470521	0.019554332	positive
PRJNA729271	stage_1D_vs_E10	ordinary_muscle_development	ENSGALG00010002398	ENSGALG00010002398	-3.42600692	0.462233177	-7.411858532	1.24542E-13	4.57716E-13	negative
PRJNA729271	stage_1D_vs_E10	ordinary_muscle_development	ENSGALG00010002399	CSMD1	-0.802529949	0.39142538	-2.05027571	0.040337533	0.054567667	negative
PRJNA729271	stage_1D_vs_E10	ordinary_muscle_development	ENSGALG00010002400	RBBP7	-1.419239288	0.155669411	-9.117008154	7.72268E-20	4.09438E-19	negative
PRJNA729271	stage_1D_vs_E10	ordinary_muscle_development	ENSGALG00010002401	ZRANB1	0.662249187	0.161250803	4.10695126	4.00916E-05	7.72262E-05	positive
PRJNA729271	stage_1D_vs_E10	ordinary_muscle_development	ENSGALG00010002402	TXLNG	0.720409871	0.125389259	5.745387427	9.17108E-09	2.39954E-08	positive
PRJNA729271	stage_1D_vs_E10	ordinary_muscle_development	ENSGALG00010002403	ZNFY3	-0.154897809	0.263623208	-0.587572731	0.556819119	0.60245526	negative
PRJNA729271	stage_1D_vs_E10	ordinary_muscle_development	ENSGALG00010002404	OSBPL3	-1.295829346	0.255205448	-5.077592808	3.82247E-07	8.80449E-07	negative
PRJNA729271	stage_1D_vs_E10	ordinary_muscle_development	ENSGALG00010002405	KCNQ5	1.913690935	0.196294562	9.749077671	1.86152E-22	1.13468E-21	positive
PRJNA729271	stage_1D_vs_E10	ordinary_muscle_development	ENSGALG00010002406	DIAPH3	-1.071616506	0.246117703	-4.354081365	1.33626E-05	2.69877E-05	negative
PRJNA729271	stage_1D_vs_E10	ordinary_muscle_development	ENSGALG00010002408	OFD1	-0.17749343	0.115040629	-1.542876033	0.122860857	0.154014367	negative
PRJNA729271	stage_1D_vs_E10	ordinary_muscle_development	ENSGALG00010002409	CYCS	1.43909896	0.166706338	8.632538969	6.00049E-18	2.86958E-17	positive
PRJNA729271	stage_1D_vs_E10	ordinary_muscle_development	ENSGALG00010002410	RNF121	0.152026943	0.104540508	1.454239563	0.1458799	0.180297506	positive
PRJNA729271	stage_1D_vs_E10	ordinary_muscle_development	ENSGALG00010002411	ENSGALG00010002411	-0.982236316	0.178588277	-5.500004437	3.79782E-08	9.4619E-08	negative
PRJNA729271	stage_1D_vs_E10	ordinary_muscle_development	ENSGALG00010002413	TSSC1	-0.487376521	0.083416113	-5.842714329	5.1357E-09	1.37398E-08	negative

PRJNA729271	stage_1D_vs_E10	ordinary_muscle_development	ENSGALG00010002414	ENSGALG00010002414	-0.154221877	0.370452299	-0.416306978	0.677185387	0.715212966	negative
PRJNA729271	stage_1D_vs_E10	ordinary_muscle_development	ENSGALG00010002415	PCDH17	-4.129516778	0.334742327	-12.33640458	5.76743E-35	6.24339E-34	negative
PRJNA729271	stage_1D_vs_E10	ordinary_muscle_development	ENSGALG00010002416	SH3BGRL2	-1.310396156	0.273287584	-4.794934834	1.62728E-06	3.57222E-06	negative
PRJNA729271	stage_1D_vs_E10	ordinary_muscle_development	ENSGALG00010002418	TRAPPC2	-0.652304727	0.083744304	-7.789242899	6.74119E-15	2.68076E-14	negative
PRJNA729271	stage_1D_vs_E10	ordinary_muscle_development	ENSGALG00010002419	CTBP2	-1.465407613	0.114963551	-12.74671495	3.25161E-37	3.85066E-36	negative
PRJNA729271	stage_1D_vs_E10	ordinary_muscle_development	ENSGALG00010002420	TMEM272	-3.042759875	0.959637105	-3.170740125	0.001520511	0.002482799	negative
PRJNA729271	stage_1D_vs_E10	ordinary_muscle_development	ENSGALG00010002421	ENSGALG00010002421	0.124863809	0.18094739	0.690055872	0.490159052	0.538528596	positive
PRJNA729271	stage_1D_vs_E10	ordinary_muscle_development	ENSGALG00010002422	TRAPPC12	0.183143178	0.102791149	1.781701828	0.074797869	0.097063125	positive
PRJNA729271	stage_1D_vs_E10	ordinary_muscle_development	ENSGALG00010002423	ENSGALG00010002423	0.557199506	0.577278769	0.965217387	0.33443594	0.383001418	positive
PRJNA729271	stage_1D_vs_E10	ordinary_muscle_development	ENSGALG00010002424	CASK	-0.49291411	0.056888692	-8.664535777	4.53356E-18	2.18102E-17	negative
PRJNA729271	stage_1D_vs_E10	ordinary_muscle_development	ENSGALG00010002425	FDX2	0.144109656	0.13416165	1.074149404	0.282755725	0.328883344	positive
PRJNA729271	stage_1D_vs_E10	ordinary_muscle_development	ENSGALG00010002427	CYB5R4	-0.103833228	0.112474923	-0.923167807	0.355919763	0.404727148	negative
PRJNA729271	stage_1D_vs_E10	ordinary_muscle_development	ENSGALG00010002428	IBSP	-5.381649956	0.798344656	-6.74101081	1.57289E-11	5.02734E-11	negative
PRJNA729271	stage_1D_vs_E10	ordinary_muscle_development	ENSGALG00010002429	ENSGALG00010002429	0.685866001	0.542452911	1.264378875	0.206094071	0.246934792	positive
PRJNA729271	stage_1D_vs_E10	ordinary_muscle_development	ENSGALG00010002430	NUMA1	0.933041551	0.174300575	5.353060663	8.64788E-08	2.09828E-07	positive
PRJNA729271	stage_1D_vs_E10	ordinary_muscle_development	ENSGALG00010002431	BCKDHB	2.017055357	0.147057134	13.71613398	8.12855E-43	1.17588E-41	positive
PRJNA729271	stage_1D_vs_E10	ordinary_muscle_development	ENSGALG00010002432	RAVER1	0.382781792	0.162904876	2.349725807	0.018787245	0.026666776	positive
PRJNA729271	stage_1D_vs_E10	ordinary_muscle_development	ENSGALG00010002433	GRHL1	-2.426259059	0.334160506	-7.26075948	3.84923E-13	1.3729E-12	negative
PRJNA729271	stage_1D_vs_E10	ordinary_muscle_development	ENSGALG00010002434	GPR34	1.414785039	0.247927006	5.706457963	1.15351E-08	2.9943E-08	positive
PRJNA729271	stage_1D_vs_E10	ordinary_muscle_development	ENSGALG00010002435	ATP7B	-3.076645821	0.74040862	-4.155334954	3.24811E-05	6.31118E-05	negative
PRJNA729271	stage_1D_vs_E10	ordinary_muscle_development	ENSGALG00010002436	ENSGALG00010002436	0.868292633	0.879432275	0.987333145	0.323479347	0.371687129	positive
PRJNA729271	stage_1D_vs_E10	ordinary_muscle_development	ENSGALG00010002437	TTK	-2.08829268	0.19587778	-10.6612025	1.54586E-26	1.13512E-25	negative
PRJNA729271	stage_1D_vs_E10	ordinary_muscle_development	ENSGALG00010002438	RARB	-1.136113181	0.091108944	-12.46983149	1.09056E-35	1.21504E-34	negative
PRJNA729271	stage_1D_vs_E10	ordinary_muscle_development	ENSGALG00010002439	GPR82	0.496266249	1.095228042	0.453116822	0.650464623	0.690377441	positive
PRJNA729271	stage_1D_vs_E10	ordinary_muscle_development	ENSGALG00010002440	LRRC51	0.119010599	0.272442847	0.436827763	0.662236263	0.701330798	positive
PRJNA729271	stage_1D_vs_E10	ordinary_muscle_development	ENSGALG00010002441	USO1	-0.331737398	0.067899352	-4.885722589	1.0305E-06	2.30085E-06	negative
PRJNA729271	stage_1D_vs_E10	ordinary_muscle_development	ENSGALG00010002442	ENSGALG00010002442	0.526752536	0.443088952	1.188818933	0.234510926	0.277919702	positive
PRJNA729271	stage_1D_vs_E10	ordinary_muscle_development	ENSGALG00010002443	TYK2	0.798659496	0.135276184	5.903918004	3.54969E-09	9.62632E-09	positive
PRJNA729271	stage_1D_vs_E10	ordinary_muscle_development	ENSGALG00010002444	KLF11	1.830073059	0.180191947	10.15624224	3.10826E-24	2.04513E-23	positive
PRJNA729271	stage_1D_vs_E10	ordinary_muscle_development	ENSGALG00010002445	EDRF1	0.338615813	0.111358431	3.040773914	0.002359709	0.003762815	positive
PRJNA729271	stage_1D_vs_E10	ordinary_muscle_development	ENSGALG00010002446	RIMS1	-3.451163231	0.270109242	-12.7769165	2.20645E-37	2.62367E-36	negative
PRJNA729271	stage_1D_vs_E10	ordinary_muscle_development	ENSGALG00010002447	YLEC12	-0.097845659	0.562982844	-0.173798651	0.862023704	0.882106148	negative
PRJNA729271	stage_1D_vs_E10	ordinary_muscle_development	ENSGALG00010002448	RAB9A	0.018706187	0.135809319	0.137738612	0.890447002	0.9065835	positive
PRJNA729271	stage_1D_vs_E10	ordinary_muscle_development	ENSGALG00010002449	ALG11	-0.741024997	0.140008452	-5.292716166	1.20513E-07	2.89595E-07	negative
PRJNA729271	stage_1D_vs_E10	ordinary_muscle_development	ENSGALG00010002453	TOP2B	-0.480540868	0.062827169	-7.64861568	2.03154E-14	7.85366E-14	negative
PRJNA729271	stage_1D_vs_E10	ordinary_muscle_development	ENSGALG00010002454	NEK5	-3.835425656	0.738623026	-5.192670039	2.07299E-07	4.8811E-07	negative
PRJNA729271	stage_1D_vs_E10	ordinary_muscle_development	ENSGALG00010002455	NYX	1.463965617	0.357332216	4.096931515	4.18663E-05	8.05056E-05	positive
PRJNA729271	stage_1D_vs_E10	ordinary_muscle_development	ENSGALG00010002456	ELOVL4	-2.465854005	0.531428145	-4.640051582	3.48322E-06	7.42381E-06	negative
PRJNA729271	stage_1D_vs_E10	ordinary_muscle_development	ENSGALG00010002457	ENSGALG00010002457	0.307083363	0.446063303	0.688430007	0.491182033	0.539451702	positive
PRJNA729271	stage_1D_vs_E10	ordinary_muscle_development	ENSGALG00010002458	ENSGALG00010002458	0.025579727	0.197882442	0.12926729	0.897146153	0.912314683	positive
PRJNA729271	stage_1D_vs_E10	ordinary_muscle_development	ENSGALG00010002459	G3BP2	-0.937732163	0.074412012	-12.60189227	2.06139E-36	2.36018E-35	negative
PRJNA729271	stage_1D_vs_E10	ordinary_muscle_development	ENSGALG00010002460	LAMTOR1	0.33044062	0.18230576	1.8125627	0.069899295	0.091155221	positive
PRJNA729271	stage_1D_vs_E10	ordinary_muscle_development	ENSGALG00010002461	TOMT	0.09961626	0.328148285	0.30357087	0.761454847	0.792055113	positive
PRJNA729271	stage_1D_vs_E10	ordinary_muscle_development	ENSGALG00010002462	RRM2	-2.44324121	0.27519569	-8.878195758	6.79536E-19	3.41298E-18	negative
PRJNA729271	stage_1D_vs_E10	ordinary_muscle_development	ENSGALG00010002463	ASRGL1	-0.462917912	0.104746985	-4.419391265	9.89793E-06	2.02157E-05	negative
PRJNA729271	stage_1D_vs_E10	ordinary_muscle_development	ENSGALG00010002464	NGLY1	0.529870452	0.082954081	6.387515221	1.68603E-10	5.02446E-10	positive
PRJNA729271	stage_1D_vs_E10	ordinary_muscle_development	ENSGALG00010002466	UROS	-1.079226624	0.185092693	-5.830735985	5.51834E-09	1.47335E-08	negative
PRJNA729271	stage_1D_vs_E10	ordinary_muscle_development	ENSGALG00010002467	NEK3	-1.904296312	0.227127618	-8.384256976	5.10472E-17	2.31517E-16	negative
PRJNA729271	stage_1D_vs_E10	ordinary_muscle_development	ENSGALG00010002468	ENSGALG00010002468	0.787640694	0.545149508	1.444815931	0.148509595	0.183266353	positive
PRJNA729271	stage_1D_vs_E10	ordinary_muscle_development	ENSGALG00010002469	MEPE	-4.845610234	1.384328532	-3.500332559	0.000464678	0.000802898	negative
PRJNA729271	stage_1D_vs_E10	ordinary_muscle_development	ENSGALG00010002470	OXSM	0.006466667	0.162632654	0.039762412	0.968282543	0.97276515	positive
PRJNA729271	stage_1D_vs_E10	ordinary_muscle_development	ENSGALG00010002471	OGFRL1	-0.111292831	0.152231077	-0.731078263	0.464731351	0.513553475	negative
PRJNA729271	stage_1D_vs_E10	ordinary_muscle_development	ENSGALG00010002472	CDC37	-0.484567237	0.214813508	-2.255757763	0.024085806	0.033712221	negative
PRJNA729271	stage_1D_vs_E10	ordinary_muscle_development	ENSGALG00010002473	ENSGALG00010002473	0.064018526	0.645757177	0.09913715	0.921029376	0.932871274	positive
PRJNA729271	stage_1D_vs_E10	ordinary_muscle_development	ENSGALG00010002474	HPCAL1	-0.345321372	0.141558596	-2.439423547	0.014710715	0.021236113	negative
PRJNA729271	stage_1D_vs_E10	ordinary_muscle_development	ENSGALG00010002475	ENSGALG00010002475	0.149199516	0.182866112	0.815894833	0.414560288	0.464055245	positive
PRJNA729271	stage_1D_vs_E10	ordinary_muscle_development	ENSGALG00010002476	DDX3X	0.440534649	0.144958145	3.039047227	0.002373276	0.003783617	positive
PRJNA729271	stage_1D_vs_E10	ordinary_muscle_development	ENSGALG00010002478	PPME1	-0.919682868	0.056405308	-16.30489932	9.10889E-60	2.50075E-58	negative
PRJNA729271	stage_1D_vs_E10	ordinary_muscle_development	ENSGALG00010002481	CKAP2	-1.835347904	0.245393692	-7.479197578	7.47778E-14	2.78352E-13	negative
PRJNA729271	stage_1D_vs_E10	ordinary_muscle_development	ENSGALG00010002483	COQ2	0.883654077	0.138713202	6.370367516	1.88576E-10	5.60469E-10	positive
PRJNA729271	stage_1D_vs_E10	ordinary_muscle_development	ENSGALG00010002487	BCCIP	-1.209649551	0.11937423	-10.13325528	3.93332E-24	2.57397E-23	negative
PRJNA729271	stage_1D_vs_E10	ordinary_muscle_development	ENSGALG00010002492	RPL8	0.353243214	0.129566218	2.726352748	0.006403852	0.009707575	positive
PRJNA729271	stage_1D_vs_E10	ordinary_muscle_development	ENSGALG00010002500	OSR1	-1.181027019	0.197288133	-5.986305422	2.14661E-09	5.91655E-09	negative
PRJNA729271	stage_1D_vs_E10	ordinary_muscle_development	ENSGALG00010002501	TCEANC	-0.442618101	0.096879539	-4.568746953	4.90649E-06	1.03087E-05	negative
PRJNA729271	stage_1D_vs_E10	ordinary_muscle_development	ENSGALG00010002504	ENSGALG00010002504	0.258126263	0.065673233	3.930463786	8.47822E-05	0.000158427	positive
PRJNA729271	stage_1D_vs_E10	ordinary_muscle_development	ENSGALG00010002507	C2CD3	-0.146336722	0.108799076	-1.345018063	0.178619401	0.216914228	negative
PRJNA729271	stage_1D_vs_E10	ordinary_muscle_development	ENSGALG00010002511	VPS36	-0.348000234	0.077293192	-4.502340074	6.72093E-06	1.3957E-05	negative
PRJNA729271	stage_1D_vs_E10	ordinary_muscle_development	ENSGALG00010002512	DAXX	0.857632363	0.228823256	3.748012236	0.000178242	0.000322548	positive
PRJNA729271	stage_1D_vs_E10	ordinary_muscle_development	ENSGALG00010002518	MAST1	-0.16284785	0.37020793	-0.439882122	0.660022485	0.699241772	negative
PRJNA729271	stage_1D_vs_E10	ordinary_muscle_development	ENSGALG00010002520	TTC32	0.839316398	0.163321085	5.139057189	2.7612E-07	6.43157E-07	positive
PRJNA729271	stage_1D_vs_E10	ordinary_muscle_development	ENSGALG00010002522	HPSE	0.038310656	0.307771598	0.124477556	0.90093717	0.915643323	positive
PRJNA729271	stage_1D_vs_E10	ordinary_muscle_development	ENSGALG00010002523	DNAJB13	2.332400042	0.418616312	5.571689338	2.52281E-08	6.37631E-08	positive
PRJNA729271	stage_1D_vs_E10	ordinary_muscle_development	ENSGALG00010002526	ZBTB22	-0.152364574	0.106493445	-1.430741341	0.15250436	0.187716602	negative
PRJNA729271	stage_1D_vs_E10	ordinary_muscle_development	ENSGALG00010002527	LRRC3B	1.47825398	0.156606833	9.439268735	3.7539E-21	2.13887E-20	positive
PRJNA729271	stage_1D_vs_E10	ordinary_muscle_development	ENSGALG00010002528	MED14	0.289337204	0.115229383	2.510967226	0.012040087	0.017615935	positive
PRJNA729271	stage_1D_vs_E10	ordinary_muscle_development	ENSGALG00010002530	STAG3	0.317297515	0.12131201	2.615549078	0.008908412	0.013254715	positive
PRJNA729271	stage_1D_vs_E10	ordinary_muscle_development	ENSGALG00010002533	LPAR2	-0.942355313	0.144253745	-6.532622841	6.46277E-11	1.98051E-10	negative
PRJNA729271	stage_1D_vs_E10	ordinary_muscle_development	ENSGALG00010002536	RGL2	1.382657716	0.14202583	9.735255344	2.13281E-22	1.29678E-21	positive
PRJNA729271	stage_1D_vs_E10	ordinary_muscle_development	ENSGALG00010002539	SNAP91	-1.409962426	0.210784727	-6.68911094	2.2453E-11	7.09985E-11	negative
PRJNA729271	stage_1D_vs_E10	ordinary_muscle_development	ENSGALG00010002543	UCP3	8.318202043	0.503236537	16.52940802	2.25346E-61	6.50675E-60	positive
PRJNA729271	stage_1D_vs_E10	ordinary_muscle_development	ENSGALG00010002544	THSD1	0.542048905	0.125217531	4.328857966	1.49885E-05	3.00453E-05	positive
PRJNA729271	stage_1D_vs_E10	ordinary_muscle_development	ENSGALG00010002546	ATP6AP2	0.750317804	0.0621725	12.06832287	1.55266E-33	1.58604E-32	positive
PRJNA729271	stage_1D_vs_E10	ordinary_muscle_development	ENSGALG00010002547	PBX4	-0.932377057	0.103019368	-9.050502607	1.42312E-19	7.42285E-19	negative
PRJNA729271	stage_1D_vs_E10	ordinary_muscle_development	ENSGALG00010002551	WDR35	-0.867003701	0.110524272	-7.844464271	4.34806E-15	1.75313E-14	negative
PRJNA729271	stage_1D_vs_E10	ordinary_muscle_development	ENSGALG00010002553	ENSGALG00010002553	0.76127264	0.216262164	3.520137904	0.000431322	0.000747224	positive
PRJNA729271	stage_1D_vs_E10	ordinary_muscle_development	ENSGALG00010002554	EGFL6	-3.133673201	0.293183025	-10.68845374	1.15277E-26	8.5035E-26	negative
PRJNA729271	stage_1D_vs_E10	ordinary_muscle_development	ENSGALG00010002556	ENSGALG00010002556	4.675755327	0.229455493	20.37761339	2.64228E-92	1.66521E-90	positive
PRJNA729271	stage_1D_vs_E10	ordinary_muscle_development	ENSGALG00010002557	ENSGALG00010002557	0.362352703	0.408913643	0.886135028	0.375544769	0.424777326	positive
PRJNA729271	stage_1D_vs_E10	ordinary_muscle_development	ENSGALG00010002559	NEK10	-0.509035734	0.539442198	-0.943633508	0.345356963	0.394169227	negative
PRJNA729271	stage_1D_vs_E10	ordinary_muscle_development	ENSGALG00010002561	PAAF1	-0.269030711	0.081662641	-3.294415975	0.000986264	0.001645287	negative
PRJNA729271	stage_1D_vs_E10	ordinary_muscle_development	ENSGALG00010002565	MRPS18C	0.067465885	0.116281194	0.580196012	0.561782443	0.607281416	positive
PRJNA729271	stage_1D_vs_E10	ordinary_muscle_development	ENSGALG00010002566	TMSB4X	-1.682930991	0.115780013	-14.53559166	7.2088E-48	1.2774E-46	negative
PRJNA729271	stage_1D_vs_E10	ordinary_muscle_development	ENSGALG00010002567	COA4	-0.232012479	0.231413459	-1.002588528	0.316059433	0.363766991	negative
PRJNA729271	stage_1D_vs_E10	ordinary_muscle_development	ENSGALG00010002569	GTPBP6	0.043031485	0.116452165	0.369520694	0.711739651	0.747422027	positive
PRJNA729271	stage_1D_vs_E10	ordinary_muscle_development	ENSGALG00010002570	ADAM12	-1.663859705	0.26775342	-6.214149219	5.16035E-10	1.48736E-09	negative
PRJNA729271	stage_1D_vs_E10	ordinary_muscle_development	ENSGALG00010002571	BCOR	1.326976128	0.139610609	9.504837313	2.00361E-21	1.15799E-20	positive
PRJNA729271	stage_1D_vs_E10	ordinary_muscle_development	ENSGALG00010002573	MVB12A	0.868327527	0.094800305	9.159543576	5.21173E-20	2.79172E-19	positive
PRJNA729271	stage_1D_vs_E10	ordinary_muscle_development	ENSGALG00010002575	BGV	-0.02575329	0.40165516	-0.064117912	0.948876339	0.956730839	negative
PRJNA729271	stage_1D_vs_E10	ordinary_muscle_development	ENSGALG00010002576	HELQ	-0.464003088	0.106627862	-4.35161202	1.3514E-05	2.72632E-05	negative
PRJNA729271	stage_1D_vs_E10	ordinary_muscle_development	ENSGALG00010002577	MRPL48	-0.016149133	0.102988165	-0.156805717	0.875397967	0.893646985	negative
PRJNA729271	stage_1D_vs_E10	ordinary_muscle_development	ENSGALG00010002579	MATN3	-0.995896289	0.571664913	-1.74209798	0.081491301	0.105193375	negative
PRJNA729271	stage_1D_vs_E10	ordinary_muscle_development	ENSGALG00010002581	ATP13A1	0.214521055	0.076586517	2.801029012	0.005093994	0.007825079	positive
PRJNA729271	stage_1D_vs_E10	ordinary_muscle_development	ENSGALG00010002582	ENSGALG00010002582	-2.492014508	0.435705784	-5.71948916	1.06845E-08	2.77996E-08	negative
PRJNA729271	stage_1D_vs_E10	ordinary_muscle_development	ENSGALG00010002584	ENSGALG00010002584	0.33390325	0.444435602	0.751297261	0.452473777	0.501499266	positive
PRJNA729271	stage_1D_vs_E10	ordinary_muscle_development	ENSGALG00010002586	GPR63	-0.479876386	0.147469547	-3.254071079	0.001137638	0.001885872	negative
PRJNA729271	stage_1D_vs_E10	ordinary_muscle_development	ENSGALG00010002587	DOCK1	-0.080540478	0.066352463	-1.213828006	0.22481341	0.26752076	negative
PRJNA729271	stage_1D_vs_E10	ordinary_muscle_development	ENSGALG00010002588	FAM175A	-0.517003834	0.084626908	-6.109213308	1.00123E-09	2.83123E-09	negative
PRJNA729271	stage_1D_vs_E10	ordinary_muscle_development	ENSGALG00010002589	SLC4A7	1.334342789	0.143759802	9.281751721	1.66715E-20	9.16394E-20	positive
PRJNA729271	stage_1D_vs_E10	ordinary_muscle_development	ENSGALG00010002590	ATP6V1C2	-1.777865211	0.260425919	-6.826759852	8.68539E-12	2.82085E-11	negative
PRJNA729271	stage_1D_vs_E10	ordinary_muscle_development	ENSGALG00010002591	ENSGALG00010002591	0.46657313	0.212972332	2.19076875	0.028468531	0.039348847	positive
PRJNA729271	stage_1D_vs_E10	ordinary_muscle_development	ENSGALG00010002592	RPS6KA3	1.088827433	0.101028393	10.77743992	4.3996E-27	3.3094E-26	positive
PRJNA729271	stage_1D_vs_E10	ordinary_muscle_development	ENSGALG00010002597	ENSGALG00010002597	-0.608345897	0.117043097	-5.197623039	2.01853E-07	4.76059E-07	negative
PRJNA729271	stage_1D_vs_E10	ordinary_muscle_development	ENSGALG00010002598	NMT2	0.277972999	0.10197385	2.725924334	0.00641217	0.009719167	positive
PRJNA729271	stage_1D_vs_E10	ordinary_muscle_development	ENSGALG00010002599	KIFC1	-1.023468484	0.219221103	-4.668658573	3.03173E-06	6.50072E-06	negative
PRJNA729271	stage_1D_vs_E10	ordinary_muscle_development	ENSGALG00010002600	TRPA1	-1.599119462	0.561909539	-2.845866375	0.004429078	0.006858186	negative
PRJNA729271	stage_1D_vs_E10	ordinary_muscle_development	ENSGALG00010002601	PLCXD1	0.864055229	0.189374753	4.562673831	5.05062E-06	1.06008E-05	positive
PRJNA729271	stage_1D_vs_E10	ordinary_muscle_development	ENSGALG00010002603	INSYN2A	0.055985565	0.256416206	0.218338639	0.827165271	0.851666473	positive
PRJNA729271	stage_1D_vs_E10	ordinary_muscle_development	ENSGALG00010002604	BLEC3	0.898565556	0.377335513	2.381343727	0.017249608	0.0246338	positive
PRJNA729271	stage_1D_vs_E10	ordinary_muscle_development	ENSGALG00010002605	EOMES	-2.847470691	1.067171369	-2.668241272	0.007624948	0.011449666	negative
PRJNA729271	stage_1D_vs_E10	ordinary_muscle_development	ENSGALG00010002608	RPP38	-0.335793156	0.1272637	-2.638561951	0.008325849	0.012433873	negative
PRJNA729271	stage_1D_vs_E10	ordinary_muscle_development	ENSGALG00010002609	PDIA6	-1.014851613	0.125297916	-8.099509102	5.51814E-16	2.36085E-15	negative
PRJNA729271	stage_1D_vs_E10	ordinary_muscle_development	ENSGALG00010002611	NPS	-0.586489631	0.419224151	-1.398988177	0.161816528	0.198269702	negative
PRJNA729271	stage_1D_vs_E10	ordinary_muscle_development	ENSGALG00010002612	JPH1	3.726196522	0.214369144	17.38214956	1.12651E-67	3.82406E-66	positive
PRJNA729271	stage_1D_vs_E10	ordinary_muscle_development	ENSGALG00010002613	ENSGALG00010002613	-0.262161965	0.104726724	-2.50329576	0.012304267	0.017978867	negative
PRJNA729271	stage_1D_vs_E10	ordinary_muscle_development	ENSGALG00010002614	GPAT3	0.646442049	0.144256617	4.481195135	7.42262E-06	1.5346E-05	positive
PRJNA729271	stage_1D_vs_E10	ordinary_muscle_development	ENSGALG00010002616	ZNFB3	-0.926367144	0.170681974	-5.4274457	5.71663E-08	1.40683E-07	negative
PRJNA729271	stage_1D_vs_E10	ordinary_muscle_development	ENSGALG00010002619	RAB6A	-0.466657545	0.067799382	-6.882917366	5.8639E-12	1.92345E-11	negative
PRJNA729271	stage_1D_vs_E10	ordinary_muscle_development	ENSGALG00010002621	SLC25A15	-0.413337655	0.161813428	-2.554408871	0.010636827	0.015664005	negative
PRJNA729271	stage_1D_vs_E10	ordinary_muscle_development	ENSGALG00010002622	AZI2	-0.054127689	0.080343006	-0.673707541	0.500497267	0.548654426	negative
PRJNA729271	stage_1D_vs_E10	ordinary_muscle_development	ENSGALG00010002623	MID1IP1	0.542381419	0.136676202	3.96836765	7.23666E-05	0.000136157	positive
PRJNA729271	stage_1D_vs_E10	ordinary_muscle_development	ENSGALG00010002625	FHL5	0.018060064	0.21983977	0.08215103	0.934526614	0.944364422	positive

PRJNA729271	stage_1D_vs_E10	ordinary_muscle_development	ENSGALG00010002626	CHD7	0.86877582	0.135483424	6.412414114	1.43233E-10	4.29314E-10	positive
PRJNA729271	stage_1D_vs_E10	ordinary_muscle_development	ENSGALG00010002627	MRPS31	0.829774499	0.146192549	5.675901439	1.3796E-08	3.55888E-08	positive
PRJNA729271	stage_1D_vs_E10	ordinary_muscle_development	ENSGALG00010002628	TRIM7.2	-0.188491172	0.192010114	-0.981673145	0.3262609	0.374526946	negative
PRJNA729271	stage_1D_vs_E10	ordinary_muscle_development	ENSGALG00010002632	HPGD	3.216223363	0.272021118	11.82343265	2.95364E-32	2.84094E-31	positive
PRJNA729271	stage_1D_vs_E10	ordinary_muscle_development	ENSGALG00010002633	ENSGALG00010002633	-0.799895158	0.126458294	-6.325367321	2.52631E-10	7.45346E-10	negative
PRJNA729271	stage_1D_vs_E10	ordinary_muscle_development	ENSGALG00010002634	RGN	-0.068436275	0.185759502	-0.368413319	0.712565068	0.74807204	negative
PRJNA729271	stage_1D_vs_E10	ordinary_muscle_development	ENSGALG00010002638	FABP9	7.744308332	0.49523899	15.63751743	4.04181E-55	9.34386E-54	positive
PRJNA729271	stage_1D_vs_E10	ordinary_muscle_development	ENSGALG00010002644	KCNF1	0.53772738	0.482859873	1.113630288	0.265437827	0.310609615	positive
PRJNA729271	stage_1D_vs_E10	ordinary_muscle_development	ENSGALG00010002646	FOXO1	1.082989212	0.169330003	6.395731385	1.59781E-10	4.76744E-10	positive
PRJNA729271	stage_1D_vs_E10	ordinary_muscle_development	ENSGALG00010002647	FAM168A	-0.706627856	0.098559621	-7.169547189	7.52462E-13	2.62755E-12	negative
PRJNA729271	stage_1D_vs_E10	ordinary_muscle_development	ENSGALG00010002649	UFL1	0.170891926	0.079157636	2.158881135	0.03085939	0.042491389	positive
PRJNA729271	stage_1D_vs_E10	ordinary_muscle_development	ENSGALG00010002653	EIF1AX	-0.590475425	0.063704379	-9.26899268	1.87912E-20	1.02901E-19	negative
PRJNA729271	stage_1D_vs_E10	ordinary_muscle_development	ENSGALG00010002655	FABP4	8.784851267	0.946209323	9.284257779	1.62838E-20	8.95424E-20	positive
PRJNA729271	stage_1D_vs_E10	ordinary_muscle_development	ENSGALG00010002656	GLRA3	2.201263912	0.549506511	4.005892316	6.17838E-05	0.000117158	positive
PRJNA729271	stage_1D_vs_E10	ordinary_muscle_development	ENSGALG00010002658	FBXL12	-0.393449729	0.14497641	-2.713887934	0.006649866	0.010057367	negative
PRJNA729271	stage_1D_vs_E10	ordinary_muscle_development	ENSGALG00010002661	ACBD7	0.12930036	0.191480776	0.675265487	0.499507107	0.547640535	positive
PRJNA729271	stage_1D_vs_E10	ordinary_muscle_development	ENSGALG00010002664	ZNFB1	-0.119134569	0.139326647	-0.855073825	0.392510279	0.441692143	negative
PRJNA729271	stage_1D_vs_E10	ordinary_muscle_development	ENSGALG00010002667	ENSGALG00010002667	-1.882096882	1.757251322	-1.071045933			negative
PRJNA729271	stage_1D_vs_E10	ordinary_muscle_development	ENSGALG00010002669	IL4I1	1.962226943	1.192332447	1.645704558	0.0998246	0.126959514	positive
PRJNA729271	stage_1D_vs_E10	ordinary_muscle_development	ENSGALG00010002673	SLC66A3	-0.237269987	0.148625127	-1.59643253	0.110392258	0.139287586	negative
PRJNA729271	stage_1D_vs_E10	ordinary_muscle_development	ENSGALG00010002678	CCNI	1.504779409	0.172707302	8.712888184	2.96229E-18	1.4404E-17	positive
PRJNA729271	stage_1D_vs_E10	ordinary_muscle_development	ENSGALG00010002681	PLEKHB1	-0.128218571	0.158113338	-0.810928243	0.41740688	0.466809099	negative
PRJNA729271	stage_1D_vs_E10	ordinary_muscle_development	ENSGALG00010002682	UBL5	-0.430100098	0.166472108	-2.583616584	0.009777042	0.014469902	negative
PRJNA729271	stage_1D_vs_E10	ordinary_muscle_development	ENSGALG00010002685	CA8	1.393900301	0.177573097	7.849726795	4.16945E-15	1.68487E-14	positive
PRJNA729271	stage_1D_vs_E10	ordinary_muscle_development	ENSGALG00010002689	COG6	-0.454505857	0.060813877	-7.47371953	7.79592E-14	2.89823E-13	negative
PRJNA729271	stage_1D_vs_E10	ordinary_muscle_development	ENSGALG00010002693	MSC	-0.400692574	0.168778706	-2.374070667	0.017593183	0.025084909	negative
PRJNA729271	stage_1D_vs_E10	ordinary_muscle_development	ENSGALG00010002700	LHFPL6	-0.594755858	0.170466273	-3.488994322	0.000484841	0.000836142	negative
PRJNA729271	stage_1D_vs_E10	ordinary_muscle_development	ENSGALG00010002701	TMEM196	-1.663549946	0.508033692	-3.274487447	0.001058538	0.001760789	negative
PRJNA729271	stage_1D_vs_E10	ordinary_muscle_development	ENSGALG00010002703	PIN1	-0.229474342	0.096788132	-2.370893394	0.017745149	0.025279207	negative
PRJNA729271	stage_1D_vs_E10	ordinary_muscle_development	ENSGALG00010002704	KCNB2	1.530473041	0.251377981	6.088333727	1.14092E-09	3.20683E-09	positive
PRJNA729271	stage_1D_vs_E10	ordinary_muscle_development	ENSGALG00010002705	JADE3	-0.809940273	0.053893644	-15.02849335	4.77749E-51	9.56487E-50	negative
PRJNA729271	stage_1D_vs_E10	ordinary_muscle_development	ENSGALG00010002706	ROCK2	1.207090632	0.144102435	8.376615095	5.44716E-17	2.46662E-16	positive
PRJNA729271	stage_1D_vs_E10	ordinary_muscle_development	ENSGALG00010002707	RELT	-3.560700985	0.254858459	-13.97128824	2.33367E-44	3.58332E-43	negative
PRJNA729271	stage_1D_vs_E10	ordinary_muscle_development	ENSGALG00010002712	PTPRE	1.377639104	0.122768743	11.22141576	3.20104E-29	2.66968E-28	positive
PRJNA729271	stage_1D_vs_E10	ordinary_muscle_development	ENSGALG00010002713	OLFM2	0.109875007	0.177212925	0.620016893	0.535246665	0.581938225	positive
PRJNA729271	stage_1D_vs_E10	ordinary_muscle_development	ENSGALG00010002716	NHLRC3	-1.057987478	0.111467621	-9.491433176	2.27879E-21	1.31493E-20	negative
PRJNA729271	stage_1D_vs_E10	ordinary_muscle_development	ENSGALG00010002722	TRIM7.1	2.423831034	0.149113963	16.25488975	2.06239E-59	5.5773E-58	positive
PRJNA729271	stage_1D_vs_E10	ordinary_muscle_development	ENSGALG00010002725	TSPAN7	-0.413714924	0.179235763	-2.308216374	0.020987105	0.029618157	negative
PRJNA729271	stage_1D_vs_E10	ordinary_muscle_development	ENSGALG00010002728	RP2	-1.010312872	0.11250031	-8.980534117	2.69455E-19	1.38551E-18	negative
PRJNA729271	stage_1D_vs_E10	ordinary_muscle_development	ENSGALG00010002730	E2F6	0.18884338	0.101159338	1.866791374	0.061930728	0.081356344	positive
PRJNA729271	stage_1D_vs_E10	ordinary_muscle_development	ENSGALG00010002733	RDH8	0.724421623	0.36367294	1.991959104	0.046375545	0.062132686	positive
PRJNA729271	stage_1D_vs_E10	ordinary_muscle_development	ENSGALG00010002739	HEP21	-2.247198121	1.015077512	-2.213819234	0.026841216	0.037248773	negative
PRJNA729271	stage_1D_vs_E10	ordinary_muscle_development	ENSGALG00010002741	PDK4	9.147131433	0.574088164	15.93332176	3.72056E-57	9.29819E-56	positive
PRJNA729271	stage_1D_vs_E10	ordinary_muscle_development	ENSGALG00010002742	PROSER1	0.681638903	0.117236641	5.81421386	6.09195E-09	1.62202E-08	positive
PRJNA729271	stage_1D_vs_E10	ordinary_muscle_development	ENSGALG00010002747	FUT9	0.539568838	0.496466434	1.086818365	0.27711709	0.322946793	positive
PRJNA729271	stage_1D_vs_E10	ordinary_muscle_development	ENSGALG00010002750	ENSGALG00010002750	-0.964821323	0.234542818	-4.113625528	3.89493E-05	7.51058E-05	negative
PRJNA729271	stage_1D_vs_E10	ordinary_muscle_development	ENSGALG00010002752	NDUFA4	1.475946635	0.154154309	9.574475363	1.02376E-21	6.02247E-21	positive
PRJNA729271	stage_1D_vs_E10	ordinary_muscle_development	ENSGALG00010002753	SLC9A7	-0.036264713	0.153261034	-0.236620569	0.812951162	0.838639747	negative
PRJNA729271	stage_1D_vs_E10	ordinary_muscle_development	ENSGALG00010002758	GREB1	-4.003710761	0.314238313	-12.74100131	3.49875E-37	4.1332E-36	negative
PRJNA729271	stage_1D_vs_E10	ordinary_muscle_development	ENSGALG00010002760	RAB2A	0.174801193	0.092571012	1.888292987	0.058986628	0.077728289	positive
PRJNA729271	stage_1D_vs_E10	ordinary_muscle_development	ENSGALG00010002762	PPAN	-0.517993552	0.145349029	-3.563790944	0.000365537	0.000638985	negative
PRJNA729271	stage_1D_vs_E10	ordinary_muscle_development	ENSGALG00010002763	SEPTIN11	-1.806641326	0.11580377	-15.6008852	7.17917E-55	1.63877E-53	negative
PRJNA729271	stage_1D_vs_E10	ordinary_muscle_development	ENSGALG00010002765	MAP7D2	-3.90624665	0.430002776	-9.084235887	1.0443E-19	5.5084E-19	negative
PRJNA729271	stage_1D_vs_E10	ordinary_muscle_development	ENSGALG00010002766	NANOS1	-2.103107592	0.341566074	-6.157249653	7.40191E-10	2.11036E-09	negative
PRJNA729271	stage_1D_vs_E10	ordinary_muscle_development	ENSGALG00010002768	TRIM39.2	3.382856042	0.947622418	3.569835387	0.000357206	0.000624879	positive
PRJNA729271	stage_1D_vs_E10	ordinary_muscle_development	ENSGALG00010002774	TBX18	-0.515573922	0.117257099	-4.39695272	1.09781E-05	2.23369E-05	negative
PRJNA729271	stage_1D_vs_E10	ordinary_muscle_development	ENSGALG00010002779	FBXO8	0.840994994	0.083589768	10.06098011	8.21761E-24	5.30104E-23	positive
PRJNA729271	stage_1D_vs_E10	ordinary_muscle_development	ENSGALG00010002781	EIF3G	0.67733321	0.137620059	4.921762252	8.57684E-07	1.92537E-06	positive
PRJNA729271	stage_1D_vs_E10	ordinary_muscle_development	ENSGALG00010002782	GSTA3	-0.850610144	0.148214872	-5.739033674	9.52183E-09	2.48772E-08	negative
PRJNA729271	stage_1D_vs_E10	ordinary_muscle_development	ENSGALG00010002784	TRIM27.2	-0.459208043	0.575695902	-0.797657307	0.425069385	0.47457296	negative
PRJNA729271	stage_1D_vs_E10	ordinary_muscle_development	ENSGALG00010002787	CEP44	-0.361553529	0.129239357	-2.797549739	0.005149184	0.007901484	negative
PRJNA729271	stage_1D_vs_E10	ordinary_muscle_development	ENSGALG00010002788	ENSGALG00010002788	5.406204319	0.629979605	8.581554508	9.35995E-18	4.42795E-17	positive
PRJNA729271	stage_1D_vs_E10	ordinary_muscle_development	ENSGALG00010002790	BCLAF3	0.340899383	0.101853223	3.346967078	0.000817009	0.00137624	positive
PRJNA729271	stage_1D_vs_E10	ordinary_muscle_development	ENSGALG00010002792	ENSGALG00010002792	-1.212194698	0.2320928	-5.222887995	1.76154E-07	4.17419E-07	negative
PRJNA729271	stage_1D_vs_E10	ordinary_muscle_development	ENSGALG00010002796	PRDM8	3.693404864	0.696036858	5.306335176	1.11851E-07	2.69495E-07	positive
PRJNA729271	stage_1D_vs_E10	ordinary_muscle_development	ENSGALG00010002800	TUBGCP5	0.15092231	0.074695108	2.0205113	0.043330379	0.058354905	positive
PRJNA729271	stage_1D_vs_E10	ordinary_muscle_development	ENSGALG00010002801	FGF5	-0.627898496	0.361222567	-1.738259328	0.08216513	0.105978248	negative
PRJNA729271	stage_1D_vs_E10	ordinary_muscle_development	ENSGALG00010002805	FAM171A1	-0.44147699	0.098686347	-4.473536675	7.69364E-06	1.58954E-05	negative
PRJNA729271	stage_1D_vs_E10	ordinary_muscle_development	ENSGALG00010002807	P2RY6	0.961008679	0.2259522	4.253150344	2.10784E-05	4.16994E-05	positive
PRJNA729271	stage_1D_vs_E10	ordinary_muscle_development	ENSGALG00010002809	SH3KBP1	-0.688416738	0.110310163	-6.240737199	4.35513E-10	1.2618E-09	negative
PRJNA729271	stage_1D_vs_E10	ordinary_muscle_development	ENSGALG00010002810	TRIM27.1	-0.096988335	0.315232158	-0.30767272	0.75833138	0.789542691	negative
PRJNA729271	stage_1D_vs_E10	ordinary_muscle_development	ENSGALG00010002812	TRIM13	-0.82955671	0.102609729	-8.084581395	6.23781E-16	2.65776E-15	negative
PRJNA729271	stage_1D_vs_E10	ordinary_muscle_development	ENSGALG00010002814	TERF1	-1.761569399	0.151847552	-11.60090745	4.07732E-31	3.71236E-30	negative
PRJNA729271	stage_1D_vs_E10	ordinary_muscle_development	ENSGALG00010002817	SHFM1	-0.148399902	0.091893702	-1.614908307	0.106330553	0.134549225	negative
PRJNA729271	stage_1D_vs_E10	ordinary_muscle_development	ENSGALG00010002819	BMP3	-2.590057202	0.351223829	-7.374377793	1.65114E-13	6.01491E-13	negative
PRJNA729271	stage_1D_vs_E10	ordinary_muscle_development	ENSGALG00010002824	NXPH1	-3.857481678	0.626966449	-6.152612608	7.62168E-10	2.17216E-09	negative
PRJNA729271	stage_1D_vs_E10	ordinary_muscle_development	ENSGALG00010002825	TRIM41	0.456788409	0.120892083	3.778480747	0.000157788	0.000287185	positive
PRJNA729271	stage_1D_vs_E10	ordinary_muscle_development	ENSGALG00010002827	SBSPON	0.306017413	0.333765454	0.916863651	0.359214093	0.408140784	positive
PRJNA729271	stage_1D_vs_E10	ordinary_muscle_development	ENSGALG00010002832	LPIN1	2.580325257	0.20113895	12.82857077	1.13437E-37	1.36568E-36	positive
PRJNA729271	stage_1D_vs_E10	ordinary_muscle_development	ENSGALG00010002834	MRPL53	-0.116605424	0.112901417	-1.032807442	0.30169402	0.348672845	negative
PRJNA729271	stage_1D_vs_E10	ordinary_muscle_development	ENSGALG00010002835	CYFIP1	-0.781329224	0.084492449	-9.24732604	2.30179E-20	1.25113E-19	negative
PRJNA729271	stage_1D_vs_E10	ordinary_muscle_development	ENSGALG00010002837	DNMT1	-0.253097501	0.129807528	-1.949790633	0.051201078	0.068200665	negative
PRJNA729271	stage_1D_vs_E10	ordinary_muscle_development	ENSGALG00010002838	ENSGALG00010002838	-0.443830826	0.285850902	-1.552665471	0.120503075	0.151202569	negative
PRJNA729271	stage_1D_vs_E10	ordinary_muscle_development	ENSGALG00010002840	SRPX	1.235210427	0.169095083	7.304827589	2.77622E-13	9.97555E-13	positive
PRJNA729271	stage_1D_vs_E10	ordinary_muscle_development	ENSGALG00010002848	TRIB2	-0.964427645	0.107069269	-9.007511268	2.10785E-19	1.09002E-18	negative
PRJNA729271	stage_1D_vs_E10	ordinary_muscle_development	ENSGALG00010002853	CEP162	0.122513579	0.167618689	0.730906439	0.464836303	0.513630295	positive
PRJNA729271	stage_1D_vs_E10	ordinary_muscle_development	ENSGALG00010002855	RACK1	0.229478601	0.125288475	1.831601838	0.067010763	0.087601101	positive
PRJNA729271	stage_1D_vs_E10	ordinary_muscle_development	ENSGALG00010002857	DCAF15	-0.449403145	0.144616838	-3.107543719	0.001886491	0.003046761	negative
PRJNA729271	stage_1D_vs_E10	ordinary_muscle_development	ENSGALG00010002860	NIPA2	-0.48565983	0.109170048	-4.448654562	8.64099E-06	1.77737E-05	negative
PRJNA729271	stage_1D_vs_E10	ordinary_muscle_development	ENSGALG00010002864	RNASEH2B	-0.537351585	0.09308566	-5.772656969	7.80312E-09	2.05947E-08	negative
PRJNA729271	stage_1D_vs_E10	ordinary_muscle_development	ENSGALG00010002866	ENSGALG00010002866	1.878349568	0.252858173	7.428470844	1.0986E-13	4.0561E-13	positive
PRJNA729271	stage_1D_vs_E10	ordinary_muscle_development	ENSGALG00010002868	PPP1R9A	1.097866939	0.066516765	16.50511617	3.37084E-61	9.61817E-60	positive
PRJNA729271	stage_1D_vs_E10	ordinary_muscle_development	ENSGALG00010002869	ENSGALG00010002869	0.720605957	0.295666527	2.437225357	0.014800452	0.021348647	positive
PRJNA729271	stage_1D_vs_E10	ordinary_muscle_development	ENSGALG00010002870	FABP5	4.353769146	0.216091955	20.14776142	2.81561E-90	1.67952E-88	positive
PRJNA729271	stage_1D_vs_E10	ordinary_muscle_development	ENSGALG00010002878	P2RY2	5.198664649	0.250266614	20.77250563	7.67484E-96	5.27236E-94	positive
PRJNA729271	stage_1D_vs_E10	ordinary_muscle_development	ENSGALG00010002880	RASGEF1B	-1.137842927	0.209181813	-5.43949262	5.34325E-08	1.31718E-07	negative
PRJNA729271	stage_1D_vs_E10	ordinary_muscle_development	ENSGALG00010002881	ENSGALG00010002881	3.872194901	0.315738268	12.26393914	1.41453E-34	1.51542E-33	positive
PRJNA729271	stage_1D_vs_E10	ordinary_muscle_development	ENSGALG00010002883	MAP3K15	6.356094314	0.412025289	15.4264665	1.08659E-53	2.37916E-52	positive
PRJNA729271	stage_1D_vs_E10	ordinary_muscle_development	ENSGALG00010002884	ADHFE1	3.51983746	0.252519131	13.93889423	3.67591E-44	5.58515E-43	positive
PRJNA729271	stage_1D_vs_E10	ordinary_muscle_development	ENSGALG00010002886	HERC2	0.952289463	0.105685849	9.010567395	2.04993E-19	1.06083E-18	positive
PRJNA729271	stage_1D_vs_E10	ordinary_muscle_development	ENSGALG00010002888	ENSGALG00010002888	-3.090382811	0.344572436	-8.96874645	2.99908E-19	1.53773E-18	negative
PRJNA729271	stage_1D_vs_E10	ordinary_muscle_development	ENSGALG00010002890	VXN	0.874383966	0.162901863	5.367550445	7.98132E-08	1.9424E-07	positive
PRJNA729271	stage_1D_vs_E10	ordinary_muscle_development	ENSGALG00010002894	FCHSD2	-0.330355275	0.070952398	-4.656012853	3.22392E-06	6.89447E-06	negative
PRJNA729271	stage_1D_vs_E10	ordinary_muscle_development	ENSGALG00010002896	OCA2	-1.453856493	0.247324746	-5.878330074	4.14426E-09	1.1149E-08	negative
PRJNA729271	stage_1D_vs_E10	ordinary_muscle_development	ENSGALG00010002904	PON1	2.350393099	0.179020879	13.12915627	2.2414E-39	2.88535E-38	positive
PRJNA729271	stage_1D_vs_E10	ordinary_muscle_development	ENSGALG00010002907	GABRG3	-1.420325299	0.355810013	-3.991808125	6.55714E-05	0.000124	negative
PRJNA729271	stage_1D_vs_E10	ordinary_muscle_development	ENSGALG00010002908	S1PR2	-0.073055238	0.180262373	-0.405271696	0.685277809	0.723091057	negative
PRJNA729271	stage_1D_vs_E10	ordinary_muscle_development	ENSGALG00010002911	MYBL1	0.20779624	0.220652163	0.941736705	0.346327458	0.395214652	positive
PRJNA729271	stage_1D_vs_E10	ordinary_muscle_development	ENSGALG00010002912	RPS3	0.094739676	0.192281708	0.492712892	0.622215467	0.664237236	positive
PRJNA729271	stage_1D_vs_E10	ordinary_muscle_development	ENSGALG00010002913	NXNL1	4.999799908	0.560912875	8.91368362	4.93646E-19	2.49578E-18	positive
PRJNA729271	stage_1D_vs_E10	ordinary_muscle_development	ENSGALG00010002914	SLC27A1	3.241261457	0.30414216	10.65706069	1.61625E-26	1.185E-25	positive
PRJNA729271	stage_1D_vs_E10	ordinary_muscle_development	ENSGALG00010002916	SDCBP	-1.269141499	0.122932067	-10.32392551	5.49304E-25	3.76639E-24	negative
PRJNA729271	stage_1D_vs_E10	ordinary_muscle_development	ENSGALG00010002917	PGLS	0.04817083	0.096004568	0.501755606	0.615839444	0.658254756	positive
PRJNA729271	stage_1D_vs_E10	ordinary_muscle_development	ENSGALG00010002918	LRATD1	-3.894374859	0.400201753	-9.731028999	2.22332E-22	1.35124E-21	negative
PRJNA729271	stage_1D_vs_E10	ordinary_muscle_development	ENSGALG00010002919	ENSGALG00010002919	1.627989584	0.202501815	8.03938267	9.02922E-16	3.80793E-15	positive
PRJNA729271	stage_1D_vs_E10	ordinary_muscle_development	ENSGALG00010002920	VCPIP1	0.76502247	0.102026943	7.498239666	6.46806E-14	2.41885E-13	positive
PRJNA729271	stage_1D_vs_E10	ordinary_muscle_development	ENSGALG00010002922	TMEM106B	0.26997021	0.090741266	2.975164678	0.002928313	0.00462424	positive
PRJNA729271	stage_1D_vs_E10	ordinary_muscle_development	ENSGALG00010002923	SERPINH1	0.000779032	0.158463753	0.004916155	0.996077492	0.996971637	positive
PRJNA729271	stage_1D_vs_E10	ordinary_muscle_development	ENSGALG00010002925	INTS6	0.238917536	0.12140356	1.967961544	0.049072463	0.065553523	positive
PRJNA729271	stage_1D_vs_E10	ordinary_muscle_development	ENSGALG00010002926	GALNTL6	-0.958317019	0.327738733	-2.924027352	0.003455344	0.005415788	negative
PRJNA729271	stage_1D_vs_E10	ordinary_muscle_development	ENSGALG00010002927	GABRA5	-7.412694799	0.971973834	-7.626434516	2.41335E-14	9.28879E-14	negative
PRJNA729271	stage_1D_vs_E10	ordinary_muscle_development	ENSGALG00010002928	ASB4	9.52588432	0.545933586	17.44879701	3.51559E-68	1.22496E-66	positive
PRJNA729271	stage_1D_vs_E10	ordinary_muscle_development	ENSGALG00010002929	PDHA2	1.317128204	0.112413419	11.71682362	1.04518E-31	9.81844E-31	positive
PRJNA729271	stage_1D_vs_E10	ordinary_muscle_development	ENSGALG00010002930	SERAF	-1.792365659	0.278551582	-6.434591565	1.23806E-10	3.72626E-10	negative
PRJNA729271	stage_1D_vs_E10	ordinary_muscle_development	ENSGALG00010002934	VWDE	-5.577053545	0.741059976	-7.525778923	5.24069E-14	1.97551E-13	negative
PRJNA729271	stage_1D_vs_E10	ordinary_muscle_development	ENSGALG00010002935	SYTL5	-5.168761551	0.896231232	-5.767218733	8.05904E-09	2.12392E-08	negative
PRJNA729271	stage_1D_vs_E10	ordinary_muscle_development	ENSGALG00010002937	GALNT7	-0.37466413	0.108446918	-3.454815847	0.000550669	0.000943939	negative
PRJNA729271	stage_1D_vs_E10	ordinary_muscle_development	ENSGALG00010002939	HMGB2	-1.629291156	0.171132732	-9.520628451	1.72135E-21	9.98837E-21	negative
PRJNA729271	stage_1D_vs_E10	ordinary_muscle_development	ENSGALG00010002940	BG1	-0.212014285	0.464756286	-0.456183791	0.64825782	0.688379022	negative
PRJNA729271	stage_1D_vs_E10	ordinary_muscle_development	ENSGALG00010002942	GABRB3	-3.499804179	0.519649597	-6.734930996	1.64008E-11	5.23748E-11	negative
PRJNA729271	stage_1D_vs_E10	ordinary_muscle_development	ENSGALG00010002943	SAP30	-0.613482405	0.14950254	-4.103491522	4.06961E-05	7.83192E-05	negative

PRJNA729271	stage_1D_vs_E10	ordinary_muscle_development	ENSGALG00010002946	GDPD5	0.095891168	0.11613267	0.825703638	0.408972228	0.458650092	positive
PRJNA729271	stage_1D_vs_E10	ordinary_muscle_development	ENSGALG00010002947	ENSGALG00010002947	-0.804196498	0.672591436	-1.195668656	0.231825888	0.275120046	negative
PRJNA729271	stage_1D_vs_E10	ordinary_muscle_development	ENSGALG00010002948	DLX6	-4.087233671	1.566216174	-2.609622949	0.009064207	0.013475454	negative
PRJNA729271	stage_1D_vs_E10	ordinary_muscle_development	ENSGALG00010002949	ENSGALG00010002949	2.272774064	1.182970393	1.921243404	0.054701029	0.072489615	positive
PRJNA729271	stage_1D_vs_E10	ordinary_muscle_development	ENSGALG00010002951	ENSGALG00010002951	-2.054084587	4.282463454	-0.479650232	0.631476122	0.673032823	negative
PRJNA729271	stage_1D_vs_E10	ordinary_muscle_development	ENSGALG00010002952	NBAS	0.09561458	0.064286324	1.487323812	0.136929308	0.169901586	positive
PRJNA729271	stage_1D_vs_E10	ordinary_muscle_development	ENSGALG00010002953	WDFY2	-0.705394627	0.133462032	-5.285358058	1.25459E-07	3.00832E-07	negative
PRJNA729271	stage_1D_vs_E10	ordinary_muscle_development	ENSGALG00010002954	CHST7	0.993009708	0.12141492	8.178646471	2.8705E-16	1.24911E-15	positive
PRJNA729271	stage_1D_vs_E10	ordinary_muscle_development	ENSGALG00010002955	BLEC1	-0.349777596	0.480218867	-0.728371206	0.466386384	0.515146738	negative
PRJNA729271	stage_1D_vs_E10	ordinary_muscle_development	ENSGALG00010002956	SCRG1	2.666946573	0.329993533	8.081814674	6.381E-16	2.71717E-15	positive
PRJNA729271	stage_1D_vs_E10	ordinary_muscle_development	ENSGALG00010002957	ADGRG2	-1.91413001	0.294478712	-6.500062413	8.02867E-11	2.44589E-10	negative
PRJNA729271	stage_1D_vs_E10	ordinary_muscle_development	ENSGALG00010002958	ENSGALG00010002958	0.833455597	0.172753208	4.824544823	1.40323E-06	3.09776E-06	positive
PRJNA729271	stage_1D_vs_E10	ordinary_muscle_development	ENSGALG00010002959	DLX5	-5.385903637	0.686155208	-7.849395552	4.18047E-15	1.68791E-14	negative
PRJNA729271	stage_1D_vs_E10	ordinary_muscle_development	ENSGALG00010002960	BLEC2	-0.420325348	0.841085858	-0.499741309	0.617257241	0.659527032	negative
PRJNA729271	stage_1D_vs_E10	ordinary_muscle_development	ENSGALG00010002965	PRSS35	-0.378493546	0.211599835	-1.788723257	0.073659395	0.095740041	negative
PRJNA729271	stage_1D_vs_E10	ordinary_muscle_development	ENSGALG00010002967	OLFM4	-3.282829562	0.926540185	-3.543105433	0.000395445	0.000688856	negative
PRJNA729271	stage_1D_vs_E10	ordinary_muscle_development	ENSGALG00010002972	ME1	-0.732017333	0.155444835	-4.709177584	2.48718E-06	5.37764E-06	negative
PRJNA729271	stage_1D_vs_E10	ordinary_muscle_development	ENSGALG00010002978	DYNLT3	0.648961917	0.101908917	6.368058192	1.91436E-10	5.68504E-10	positive
PRJNA729271	stage_1D_vs_E10	ordinary_muscle_development	ENSGALG00010002981	KLHL35	-5.053998864	0.330196073	-15.3060538	6.96644E-53	1.48936E-51	negative
PRJNA729271	stage_1D_vs_E10	ordinary_muscle_development	ENSGALG00010002982	HAND2	-2.139585727	0.291141383	-7.348957753	1.99758E-13	7.24055E-13	negative
PRJNA729271	stage_1D_vs_E10	ordinary_muscle_development	ENSGALG00010002983	NSMAF	0.735812955	0.111370869	6.606870919	3.92528E-11	1.22228E-10	positive
PRJNA729271	stage_1D_vs_E10	ordinary_muscle_development	ENSGALG00010002985	ENSGALG00010002985	-0.399870923	0.218664857	-1.828693136	0.067445591	0.088129798	negative
PRJNA729271	stage_1D_vs_E10	ordinary_muscle_development	ENSGALG00010002987	PCDH8	-1.860550938	0.364467265	-5.104850606	3.31056E-07	7.66436E-07	negative
PRJNA729271	stage_1D_vs_E10	ordinary_muscle_development	ENSGALG00010002991	BLB1	0.394625525	0.314837212	1.2534272	0.210050278	0.251356293	positive
PRJNA729271	stage_1D_vs_E10	ordinary_muscle_development	ENSGALG00010002992	ENSGALG00010002992	-0.827167713	0.250424832	-3.30305787	0.000956366	0.001598724	negative
PRJNA729271	stage_1D_vs_E10	ordinary_muscle_development	ENSGALG00010002994	DUSP4	-3.312807249	0.15947488	-20.77322298	7.56107E-96	5.21894E-94	negative
PRJNA729271	stage_1D_vs_E10	ordinary_muscle_development	ENSGALG00010002995	RWDD2A	-1.140736024	0.129066809	-8.838337576	9.71533E-19	4.8493E-18	negative
PRJNA729271	stage_1D_vs_E10	ordinary_muscle_development	ENSGALG00010002998	LECT1	-4.991108034	0.282890622	-17.64324316	1.14686E-69	4.17681E-68	negative
PRJNA729271	stage_1D_vs_E10	ordinary_muscle_development	ENSGALG00010002999	CMTM7	-0.707434413	0.212890577	-3.322995421	0.000890564	0.00149251	negative
PRJNA729271	stage_1D_vs_E10	ordinary_muscle_development	ENSGALG00010003001	MAP6	-2.568238091	0.309335817	-8.302427175	1.02006E-16	4.54666E-16	negative
PRJNA729271	stage_1D_vs_E10	ordinary_muscle_development	ENSGALG00010003002	PHKA2	-1.322417107	0.188779538	-7.005087117	2.46832E-12	8.32248E-12	negative
PRJNA729271	stage_1D_vs_E10	ordinary_muscle_development	ENSGALG00010003006	CYP7B1	0.741673501	0.200198274	3.70469478	0.000211645	0.000379068	positive
PRJNA729271	stage_1D_vs_E10	ordinary_muscle_development	ENSGALG00010003009	TNKS	0.021351679	0.05780611	0.369367157	0.711854075	0.747488033	positive
PRJNA729271	stage_1D_vs_E10	ordinary_muscle_development	ENSGALG00010003017	PGM3	-1.60232048	0.155932607	-10.27572427	9.06609E-25	6.12362E-24	negative
PRJNA729271	stage_1D_vs_E10	ordinary_muscle_development	ENSGALG00010003018	MOGAT2	-2.644395531	1.104776132	-2.393603062	0.016683793	0.023882242	negative
PRJNA729271	stage_1D_vs_E10	ordinary_muscle_development	ENSGALG00010003019	TAPBP	1.52975371	0.253379183	6.03740879	1.56609E-09	4.36043E-09	positive
PRJNA729271	stage_1D_vs_E10	ordinary_muscle_development	ENSGALG00010003022	BLB2	1.622876767	0.269871134	6.013524836	1.81532E-09	5.03022E-09	positive
PRJNA729271	stage_1D_vs_E10	ordinary_muscle_development	ENSGALG00010003024	SUGT1	-0.354555732	0.072934162	-4.861312181	1.1661E-06	2.58925E-06	negative
PRJNA729271	stage_1D_vs_E10	ordinary_muscle_development	ENSGALG00010003025	MFHAS1	0.877144946	0.122885742	7.137890274	9.47742E-13	3.28413E-12	positive
PRJNA729271	stage_1D_vs_E10	ordinary_muscle_development	ENSGALG00010003027	SCIN	-3.017093568	0.257181202	-11.73139229	8.79992E-32	8.30435E-31	negative
PRJNA729271	stage_1D_vs_E10	ordinary_muscle_development	ENSGALG00010003030	PPEF1	-2.298788228	0.825311128	-2.785359543	0.00534684	0.00818832	negative
PRJNA729271	stage_1D_vs_E10	ordinary_muscle_development	ENSGALG00010003034	ERI1	-0.689754713	0.062228691	-11.08419118	1.49697E-28	1.20951E-27	negative
PRJNA729271	stage_1D_vs_E10	ordinary_muscle_development	ENSGALG00010003035	DGAT2	2.358574599	0.456869772	5.162465863	2.43718E-07	5.70525E-07	positive
PRJNA729271	stage_1D_vs_E10	ordinary_muscle_development	ENSGALG00010003042	TOX	-3.576406308	0.311901344	-11.46646648	1.94438E-30	1.72378E-29	negative
PRJNA729271	stage_1D_vs_E10	ordinary_muscle_development	ENSGALG00010003052	ENSGALG00010003052	0.527460421	0.093086532	5.666345153	1.45876E-08	3.75972E-08	positive
PRJNA729271	stage_1D_vs_E10	ordinary_muscle_development	ENSGALG00010003057	DDX1	-0.714996292	0.089902705	-7.953000836	1.82047E-15	7.5286E-15	negative
PRJNA729271	stage_1D_vs_E10	ordinary_muscle_development	ENSGALG00010003061	ENSGALG00010003061	0.547602976	0.279863881	1.956676134	0.050385558	0.067176109	positive
PRJNA729271	stage_1D_vs_E10	ordinary_muscle_development	ENSGALG00010003063	GGH	0.189825223	0.088151873	2.153388414	0.031288165	0.043028649	positive
PRJNA729271	stage_1D_vs_E10	ordinary_muscle_development	ENSGALG00010003064	VAV1	1.330919106	0.254857117	5.222216761	1.76794E-07	4.18867E-07	positive
PRJNA729271	stage_1D_vs_E10	ordinary_muscle_development	ENSGALG00010003068	BRD2	0.46192859	0.126041793	3.664884314	0.00024745	0.00043972	positive
PRJNA729271	stage_1D_vs_E10	ordinary_muscle_development	ENSGALG00010003072	ELF1	0.93009904	0.073535472	12.6483044	1.14315E-36	1.3256E-35	positive
PRJNA729271	stage_1D_vs_E10	ordinary_muscle_development	ENSGALG00010003074	TTPA	5.397754152	0.344640212	15.66199756	2.75119E-55	6.41132E-54	positive
PRJNA729271	stage_1D_vs_E10	ordinary_muscle_development	ENSGALG00010003077	EIF2S3	0.238716782	0.081735023	2.920618033	0.003493378	0.005471852	positive
PRJNA729271	stage_1D_vs_E10	ordinary_muscle_development	ENSGALG00010003081	DOP1A	0.769615087	0.102793451	7.487005084	7.0463E-14	2.62966E-13	positive
PRJNA729271	stage_1D_vs_E10	ordinary_muscle_development	ENSGALG00010003087	TWISTNB	-0.444100935	0.180343824	-2.462523666	0.013796307	0.019993748	negative
PRJNA729271	stage_1D_vs_E10	ordinary_muscle_development	ENSGALG00010003090	DMA	-0.581885553	0.205514488	-2.831360247	0.004635048	0.007155717	negative
PRJNA729271	stage_1D_vs_E10	ordinary_muscle_development	ENSGALG00010003092	ART1F	1.288912433	1.54906624	0.832057661	0.405376398	0.455075193	positive
PRJNA729271	stage_1D_vs_E10	ordinary_muscle_development	ENSGALG00010003097	TFPI2	1.027497201	0.13577093	7.567873346	3.79384E-14	1.44297E-13	positive
PRJNA729271	stage_1D_vs_E10	ordinary_muscle_development	ENSGALG00010003101	KLHL15	0.185942392	0.07077011	2.627414194	0.008603654	0.012823646	positive
PRJNA729271	stage_1D_vs_E10	ordinary_muscle_development	ENSGALG00010003103	YTHDF3	1.125451876	0.085922397	13.09846933	3.35965E-39	4.29816E-38	positive
PRJNA729271	stage_1D_vs_E10	ordinary_muscle_development	ENSGALG00010003107	GNG11	0.031683077	0.181265341	0.17478839	0.861245911	0.881434688	positive
PRJNA729271	stage_1D_vs_E10	ordinary_muscle_development	ENSGALG00010003108	PPP1R3B	0.100335842	0.181516185	0.552765265	0.580424156	0.624869886	positive
PRJNA729271	stage_1D_vs_E10	ordinary_muscle_development	ENSGALG00010003109	ART5	1.678383368	0.630159367	2.663426834	0.007734923	0.011605187	positive
PRJNA729271	stage_1D_vs_E10	ordinary_muscle_development	ENSGALG00010003110	BF1	2.813875302	0.257611916	10.92292368	8.95654E-28	6.94252E-27	positive
PRJNA729271	stage_1D_vs_E10	ordinary_muscle_development	ENSGALG00010003113	WBP4	0.001266069	0.116334855	0.010882969	0.991316819	0.992686514	positive
PRJNA729271	stage_1D_vs_E10	ordinary_muscle_development	ENSGALG00010003115	UBE3D	-0.817289896	0.156270099	-5.229982576	1.69526E-07	4.02305E-07	negative
PRJNA729271	stage_1D_vs_E10	ordinary_muscle_development	ENSGALG00010003117	ID2	-2.081461099	0.126681921	-16.43060886	1.15493E-60	3.22558E-59	negative
PRJNA729271	stage_1D_vs_E10	ordinary_muscle_development	ENSGALG00010003121	ARMC1	0.106512111	0.08205724	1.298022093	0.194279743	0.234205329	positive
PRJNA729271	stage_1D_vs_E10	ordinary_muscle_development	ENSGALG00010003125	APOO	0.596233626	0.106227641	5.612791727	1.99088E-08	5.07703E-08	positive
PRJNA729271	stage_1D_vs_E10	ordinary_muscle_development	ENSGALG00010003126	ENSGALG00010003126	-2.720027008	0.512667489	-5.305635852	1.12281E-07	2.70441E-07	negative
PRJNA729271	stage_1D_vs_E10	ordinary_muscle_development	ENSGALG00010003131	TAP1	1.529913733	0.356211816	4.294955035	1.74729E-05	3.47993E-05	positive
PRJNA729271	stage_1D_vs_E10	ordinary_muscle_development	ENSGALG00010003132	TPBG	-1.73568004	0.136336207	-12.73088108	3.98319E-37	4.6902E-36	negative
PRJNA729271	stage_1D_vs_E10	ordinary_muscle_development	ENSGALG00010003133	SAT1	1.641757313	0.167304911	9.812965473	9.90151E-23	6.10734E-22	positive
PRJNA729271	stage_1D_vs_E10	ordinary_muscle_development	ENSGALG00010003136	NUP98	0.169794988	0.104532065	1.624333999	0.104304545	0.132239515	positive
PRJNA729271	stage_1D_vs_E10	ordinary_muscle_development	ENSGALG00010003152	ENSGALG00010003152	1.622464888	0.126532017	12.822564	1.22579E-37	1.47206E-36	positive
PRJNA729271	stage_1D_vs_E10	ordinary_muscle_development	ENSGALG00010003154	MTFR1	-0.736498435	0.081393223	-9.048645638	1.44753E-19	7.54744E-19	negative
PRJNA729271	stage_1D_vs_E10	ordinary_muscle_development	ENSGALG00010003159	TAP2	2.091947597	0.268616214	7.787867926	6.81494E-15	2.70934E-14	positive
PRJNA729271	stage_1D_vs_E10	ordinary_muscle_development	ENSGALG00010003160	KIDINS220	0.299178381	0.151368352	1.976492294	0.048099048	0.064311013	positive
PRJNA729271	stage_1D_vs_E10	ordinary_muscle_development	ENSGALG00010003164	C3	2.946437003	0.459345	6.414431428	1.41349E-10	4.23931E-10	positive
PRJNA729271	stage_1D_vs_E10	ordinary_muscle_development	ENSGALG00010003165	BMP2K	0.847088086	0.109254991	7.75331248	8.9526E-15	3.53495E-14	positive
PRJNA729271	stage_1D_vs_E10	ordinary_muscle_development	ENSGALG00010003166	PGAP2	0.216256307	0.111007879	1.948116742	0.051400996	0.068442342	positive
PRJNA729271	stage_1D_vs_E10	ordinary_muscle_development	ENSGALG00010003169	MTRF1	-0.086080586	0.109141362	-0.788707275	0.430283115	0.479818392	negative
PRJNA729271	stage_1D_vs_E10	ordinary_muscle_development	ENSGALG00010003170	C4	0.078746284	0.408430176	0.192802315	0.84711379	0.869242134	positive
PRJNA729271	stage_1D_vs_E10	ordinary_muscle_development	ENSGALG00010003171	PAQR3	-0.29249603	0.101773403	-2.873992833	0.004053182	0.006294286	negative
PRJNA729271	stage_1D_vs_E10	ordinary_muscle_development	ENSGALG00010003176	PRAG1	-1.250017851	0.258268306	-4.839997091	1.29841E-06	2.8716E-06	negative
PRJNA729271	stage_1D_vs_E10	ordinary_muscle_development	ENSGALG00010003178	CENPA	-1.131416794	0.21124531	-5.355938042	8.51137E-08	2.06723E-07	negative
PRJNA729271	stage_1D_vs_E10	ordinary_muscle_development	ENSGALG00010003180	IBTK	0.316189271	0.126705112	2.495473682	0.012578909	0.018348725	positive
PRJNA729271	stage_1D_vs_E10	ordinary_muscle_development	ENSGALG00010003183	NAA16	0.892097418	0.087588951	10.18504513	2.31253E-24	1.5341E-23	positive
PRJNA729271	stage_1D_vs_E10	ordinary_muscle_development	ENSGALG00010003184	BET1	-0.17386421	0.08051371	-2.159436064	0.030816353	0.042440193	negative
PRJNA729271	stage_1D_vs_E10	ordinary_muscle_development	ENSGALG00010003186	CYP21A1	-1.152213275	0.239625127	-4.808399233	1.52144E-06	3.34748E-06	negative
PRJNA729271	stage_1D_vs_E10	ordinary_muscle_development	ENSGALG00010003187	RHOG	0.668375558	0.245424169	2.723348567	0.006462383	0.009792207	positive
PRJNA729271	stage_1D_vs_E10	ordinary_muscle_development	ENSGALG00010003189	ANTXR2	1.236268416	0.126515065	9.771709134	1.48918E-22	9.11944E-22	positive
PRJNA729271	stage_1D_vs_E10	ordinary_muscle_development	ENSGALG00010003191	CC2D1A	1.331738369	0.159964344	8.325220072	8.41722E-17	3.77382E-16	positive
PRJNA729271	stage_1D_vs_E10	ordinary_muscle_development	ENSGALG00010003192	RGCC	1.142511125	0.308426166	3.70432619	0.000211953	0.000379526	positive
PRJNA729271	stage_1D_vs_E10	ordinary_muscle_development	ENSGALG00010003193	ACOT9	-0.132159179	0.137032724	-0.96443517	0.334827797	0.383359314	negative
PRJNA729271	stage_1D_vs_E10	ordinary_muscle_development	ENSGALG00010003198	PRKG2	-2.463944936	0.351880429	-7.002222155	2.51934E-12	8.48858E-12	negative
PRJNA729271	stage_1D_vs_E10	ordinary_muscle_development	ENSGALG00010003201	MBOAT2	-1.076749643	0.129266475	-8.329689859	8.10544E-17	3.63854E-16	negative
PRJNA729271	stage_1D_vs_E10	ordinary_muscle_development	ENSGALG00010003202	FAM46A	1.269284164	0.102373363	12.39857833	2.66001E-35	2.92985E-34	positive
PRJNA729271	stage_1D_vs_E10	ordinary_muscle_development	ENSGALG00010003204	PKD2	-0.058597374	0.143253591	-0.409046458	0.682505568	0.720323155	negative
PRJNA729271	stage_1D_vs_E10	ordinary_muscle_development	ENSGALG00010003205	ENSGALG00010003205	7.293176744	0.263896812	27.63647153	4.0584E-168	1.032E-165	positive
PRJNA729271	stage_1D_vs_E10	ordinary_muscle_development	ENSGALG00010003206	ENSGALG00010003206	-2.062820936	0.620726687	-3.323235461	0.000889798	0.001491399	negative
PRJNA729271	stage_1D_vs_E10	ordinary_muscle_development	ENSGALG00010003210	EYA1	2.114649169	0.199699955	10.58913191	3.34681E-26	2.41473E-25	positive
PRJNA729271	stage_1D_vs_E10	ordinary_muscle_development	ENSGALG00010003212	LTB4R	1.361727639	0.776877448	1.752821687	0.079632619	0.103023458	positive
PRJNA729271	stage_1D_vs_E10	ordinary_muscle_development	ENSGALG00010003214	PRDX4	-0.83090142	0.094880797	-8.757319157	1.99949E-18	9.80135E-18	negative
PRJNA729271	stage_1D_vs_E10	ordinary_muscle_development	ENSGALG00010003218	MAP3K7	-0.261713418	0.060778704	-4.306005205	1.66229E-05	3.3184E-05	negative
PRJNA729271	stage_1D_vs_E10	ordinary_muscle_development	ENSGALG00010003220	SPP1	-6.17346867	0.999992937	-6.173512273	6.67894E-10	1.90987E-09	negative
PRJNA729271	stage_1D_vs_E10	ordinary_muscle_development	ENSGALG00010003222	ITGA8	-2.51430975	0.310176131	-8.106071043	5.22829E-16	2.24346E-15	negative
PRJNA729271	stage_1D_vs_E10	ordinary_muscle_development	ENSGALG00010003223	AREG	-1.339463182	0.814833739	-1.643848453	0.10020751	0.127435327	negative
PRJNA729271	stage_1D_vs_E10	ordinary_muscle_development	ENSGALG00010003229	STIM1	1.025717012	0.105144917	9.755269576	1.75135E-22	1.06848E-21	positive
PRJNA729271	stage_1D_vs_E10	ordinary_muscle_development	ENSGALG00010003239	RRM1	-1.381408012	0.118496185	-11.65782686	2.09317E-31	1.93621E-30	negative
PRJNA729271	stage_1D_vs_E10	ordinary_muscle_development	ENSGALG00010003240	PLACL2	2.306297139	0.889022525	2.594194268	0.00948129	0.014063784	positive
PRJNA729271	stage_1D_vs_E10	ordinary_muscle_development	ENSGALG00010003242	ASAP2	-1.263872301	0.124561542	-10.14656915	3.43218E-24	2.25212E-23	negative
PRJNA729271	stage_1D_vs_E10	ordinary_muscle_development	ENSGALG00010003243	VPS50	-0.076067754	0.091719798	-0.829349342	0.406906758	0.456545666	negative
PRJNA729271	stage_1D_vs_E10	ordinary_muscle_development	ENSGALG00010003245	ENSGALG00010003245	-0.076188378	0.41402399	-0.184019234	0.853998366	0.875136649	negative
PRJNA729271	stage_1D_vs_E10	ordinary_muscle_development	ENSGALG00010003246	VWA8	1.827828205	0.108347274	16.87008949	7.46831E-64	2.30326E-62	positive
PRJNA729271	stage_1D_vs_E10	ordinary_muscle_development	ENSGALG00010003247	ENSGALG00010003247	0.272155049	0.210828735	1.290882141	0.196744549	0.236684558	positive
PRJNA729271	stage_1D_vs_E10	ordinary_muscle_development	ENSGALG00010003248	MRI1	0.395635207	0.095557836	4.140269614	3.46898E-05	6.72141E-05	positive
PRJNA729271	stage_1D_vs_E10	ordinary_muscle_development	ENSGALG00010003249	YJU2B	2.66699251	0.306792923	8.693135687	3.52571E-18	1.70806E-17	positive
PRJNA729271	stage_1D_vs_E10	ordinary_muscle_development	ENSGALG00010003250	CD1G	0.056272291	0.786109621	0.071583262	0.942933561	0.951466896	positive
PRJNA729271	stage_1D_vs_E10	ordinary_muscle_development	ENSGALG00010003251	ELOF1	-0.668554687	0.155750885	-4.292461558	1.76703E-05	3.51635E-05	negative
PRJNA729271	stage_1D_vs_E10	ordinary_muscle_development	ENSGALG00010003252	PHEX	-1.742213298	0.195831713	-8.896481949	5.76448E-19	2.9063E-18	negative
PRJNA729271	stage_1D_vs_E10	ordinary_muscle_development	ENSGALG00010003253	ITGB1BP1	-0.281122473	0.114022177	-2.465506966	0.013681951	0.019847867	negative
PRJNA729271	stage_1D_vs_E10	ordinary_muscle_development	ENSGALG00010003254	DGKH	1.34964894	0.166915981	8.085798206	6.17584E-16	2.63368E-15	positive
PRJNA729271	stage_1D_vs_E10	ordinary_muscle_development	ENSGALG00010003255	CALCR	0.924255193	0.319588354	2.892017753	0.003827763	0.005964677	positive
PRJNA729271	stage_1D_vs_E10	ordinary_muscle_development	ENSGALG00010003256	ENSGALG00010003256	0.586418679	1.338193016	0.438216813	0.661229122	0.700407096	positive
PRJNA729271	stage_1D_vs_E10	ordinary_muscle_development	ENSGALG00010003257	ENSGALG00010003257	0.940000591	0.794414301	1.183262424	0.23670516	0.280290932	positive
PRJNA729271	stage_1D_vs_E10	ordinary_muscle_development	ENSGALG00010003259	VEGFC	0.982782753	0.14016559	7.011583586	2.35636E-12	7.96163E-12	positive
PRJNA729271	stage_1D_vs_E10	ordinary_muscle_development	ENSGALG00010003260	ACP5	-0.522128092	0.276030666	-1.891558278	0.058549859	0.077173794	negative
PRJNA729271	stage_1D_vs_E10	ordinary_muscle_development	ENSGALG00010003261	PEX2	-0.649821936	0.173397148	-3.747592991	0.00017854	0.000323047	negative
PRJNA729271	stage_1D_vs_E10	ordinary_muscle_development	ENSGALG00010003262	ENSGALG00010003262	-1.16233153	0.583752566	-1.991137338	0.046465791	0.062247841	negative
PRJNA729271	stage_1D_vs_E10	ordinary_muscle_development	ENSGALG00010003263	ENSGALG00010003263	-0.293212686	0.834646431	-0.351301671	0.725362041	0.75970232	negative

PRJNA729271	stage_1D_vs_E10	ordinary_muscle_development	ENSGALG00010003264	ENSGALG00010003264	-0.37964267	0.388788451	-0.976476201	0.328828536	0.377205573	negative
PRJNA729271	stage_1D_vs_E10	ordinary_muscle_development	ENSGALG00010003268	SMS	-0.489029569	0.063558697	-7.694140927	1.42448E-14	5.55049E-14	negative
PRJNA729271	stage_1D_vs_E10	ordinary_muscle_development	ENSGALG00010003269	ENSGALG00010003269	2.377300055	0.40377323	5.887710918	3.91581E-09	1.05717E-08	positive
PRJNA729271	stage_1D_vs_E10	ordinary_muscle_development	ENSGALG00010003271	AKAP11	0.656766839	0.083300646	7.88429465	3.16317E-15	1.28865E-14	positive
PRJNA729271	stage_1D_vs_E10	ordinary_muscle_development	ENSGALG00010003272	CPSF3	-0.682374052	0.06720608	-10.15345711	3.19829E-24	2.1015E-23	negative
PRJNA729271	stage_1D_vs_E10	ordinary_muscle_development	ENSGALG00010003273	NFIX	2.157220988	0.196309544	10.98887474	4.32282E-28	3.39433E-27	positive
PRJNA729271	stage_1D_vs_E10	ordinary_muscle_development	ENSGALG00010003274	BACH2	0.614301215	0.1471081	4.175849025	2.96877E-05	5.79169E-05	positive
PRJNA729271	stage_1D_vs_E10	ordinary_muscle_development	ENSGALG00010003275	MBTPS2	0.620422669	0.126501527	4.904467816	9.3681E-07	2.09877E-06	positive
PRJNA729271	stage_1D_vs_E10	ordinary_muscle_development	ENSGALG00010003276	TNFSF11	-2.601568912	0.496909596	-5.235497425	1.64541E-07	3.90859E-07	negative
PRJNA729271	stage_1D_vs_E10	ordinary_muscle_development	ENSGALG00010003278	ENSGALG00010003278	0.583609261	0.502196011	1.162114489	0.245188963	0.289344135	positive
PRJNA729271	stage_1D_vs_E10	ordinary_muscle_development	ENSGALG00010003279	ENSGALG00010003279	2.631067228	0.354220949	7.427757269	1.10454E-13	4.07492E-13	positive
PRJNA729271	stage_1D_vs_E10	ordinary_muscle_development	ENSGALG00010003280	ENSGALG00010003280	0.244283533	0.165751141	1.47379699	0.140536259	0.174138577	positive
PRJNA729271	stage_1D_vs_E10	ordinary_muscle_development	ENSGALG00010003287	ENSGALG00010003287	-4.774235794	1.938069576	-2.463397524	0.013762723	0.019949067	negative
PRJNA729271	stage_1D_vs_E10	ordinary_muscle_development	ENSGALG00010003292	GADD45GIP1	0.518259889	0.124222825	4.172018221	3.01913E-05	5.88362E-05	positive
PRJNA729271	stage_1D_vs_E10	ordinary_muscle_development	ENSGALG00010003294	ENSGALG00010003294	-0.432169779	4.422254251	-0.097726127	0.922149769	0.933679862	negative
PRJNA729271	stage_1D_vs_E10	ordinary_muscle_development	ENSGALG00010003295	IAH1	-0.815822892	0.135511153	-6.020337619	1.74054E-09	4.82946E-09	negative
PRJNA729271	stage_1D_vs_E10	ordinary_muscle_development	ENSGALG00010003296	ENSGALG00010003296	3.154178679	0.534826447	5.89757424	3.68884E-09	9.99062E-09	positive
PRJNA729271	stage_1D_vs_E10	ordinary_muscle_development	ENSGALG00010003298	IL7	4.721200841	1.019188797	4.632312339	3.61604E-06	7.6967E-06	positive
PRJNA729271	stage_1D_vs_E10	ordinary_muscle_development	ENSGALG00010003299	RAD23A	1.133676977	0.120449147	9.412079757	4.86415E-21	2.75521E-20	positive
PRJNA729271	stage_1D_vs_E10	ordinary_muscle_development	ENSGALG00010003300	CASP8AP2	0.113362781	0.110632888	1.024675243	0.305516448	0.352640621	positive
PRJNA729271	stage_1D_vs_E10	ordinary_muscle_development	ENSGALG00010003303	MTHFD2L	-0.07318953	0.192409399	-0.380384381	0.703660107	0.740278216	negative
PRJNA729271	stage_1D_vs_E10	ordinary_muscle_development	ENSGALG00010003304	ENSGALG00010003304	-0.464276466	0.65238218	-0.711663316	0.476673289	0.525308243	negative
PRJNA729271	stage_1D_vs_E10	ordinary_muscle_development	ENSGALG00010003305	CALR	-0.597776077	0.078154985	-7.648598224	2.03182E-14	7.85366E-14	negative
PRJNA729271	stage_1D_vs_E10	ordinary_muscle_development	ENSGALG00010003307	COL1A2	1.263523922	0.220380095	5.733384972	9.84458E-09	2.56742E-08	positive
PRJNA729271	stage_1D_vs_E10	ordinary_muscle_development	ENSGALG00010003308	MDN1	-0.57144633	0.099169105	-5.762342304	8.29545E-09	2.18187E-08	negative
PRJNA729271	stage_1D_vs_E10	ordinary_muscle_development	ENSGALG00010003311	ENSGALG00010003311	2.449110591	1.160694554	2.110038841	0.03485501	0.047640111	positive
PRJNA729271	stage_1D_vs_E10	ordinary_muscle_development	ENSGALG00010003315	EPGN	-0.75232701	0.351696972	-2.139134172	0.032424804	0.044511557	negative
PRJNA729271	stage_1D_vs_E10	ordinary_muscle_development	ENSGALG00010003317	LYRM2	0.248299081	0.17784611	1.396145699	0.162670625	0.199248834	positive
PRJNA729271	stage_1D_vs_E10	ordinary_muscle_development	ENSGALG00010003320	OR51E2	1.905739477	0.452239388	4.214005962	2.50881E-05	4.92686E-05	positive
PRJNA729271	stage_1D_vs_E10	ordinary_muscle_development	ENSGALG00010003323	STMN2	-1.981673266	0.477206664	-4.152652126	3.28644E-05	6.38138E-05	negative
PRJNA729271	stage_1D_vs_E10	ordinary_muscle_development	ENSGALG00010003324	ENSGALG00010003324	0.843976231	0.587058543	1.43763555	0.150537489	0.185595041	positive
PRJNA729271	stage_1D_vs_E10	ordinary_muscle_development	ENSGALG00010003330	HEY1	0.671690524	0.120018873	5.596540847	2.18671E-08	5.55394E-08	positive
PRJNA729271	stage_1D_vs_E10	ordinary_muscle_development	ENSGALG00010003331	EPSTI1	1.156402315	0.622075301	1.858942662	0.063035266	0.082739852	positive
PRJNA729271	stage_1D_vs_E10	ordinary_muscle_development	ENSGALG00010003333	ENSGALG00010003333	18.18878049	2.694812279	6.749553813	1.483E-11	4.75368E-11	positive
PRJNA729271	stage_1D_vs_E10	ordinary_muscle_development	ENSGALG00010003334	TAC1	-4.129874367	0.955386429	-4.322726639	1.54113E-05	3.0863E-05	negative
PRJNA729271	stage_1D_vs_E10	ordinary_muscle_development	ENSGALG00010003339	ENSGALG00010003339	-0.449798639	0.467995368	-0.961117716	0.336492983	0.38502256	negative
PRJNA729271	stage_1D_vs_E10	ordinary_muscle_development	ENSGALG00010003344	ZFAND1	-0.068884753	0.190264837	-0.362046682	0.717317146	0.752570356	negative
PRJNA729271	stage_1D_vs_E10	ordinary_muscle_development	ENSGALG00010003346	ENSGALG00010003346	4.064437297	5.41235147	0.750955905	0.452679193	0.501688578	positive
PRJNA729271	stage_1D_vs_E10	ordinary_muscle_development	ENSGALG00010003347	GPM6A	-1.381711332	0.362570363	-3.810877755	0.000138474	0.000253272	negative
PRJNA729271	stage_1D_vs_E10	ordinary_muscle_development	ENSGALG00010003348	ADAM17	-0.130691428	0.083233854	-1.57017153	0.116375211	0.146396241	negative
PRJNA729271	stage_1D_vs_E10	ordinary_muscle_development	ENSGALG00010003351	ENSGALG00010003351	-0.519995457	1.578083962	-0.329510641	0.741769753	0.774524749	negative
PRJNA729271	stage_1D_vs_E10	ordinary_muscle_development	ENSGALG00010003352	FARSA	0.12220479	0.101357948	1.205675457	0.227942652	0.270910851	positive
PRJNA729271	stage_1D_vs_E10	ordinary_muscle_development	ENSGALG00010003356	ENSGALG00010003356	2.290627798	1.109614584	2.064345432	0.038984976	0.052826702	positive
PRJNA729271	stage_1D_vs_E10	ordinary_muscle_development	ENSGALG00010003358	DNAJC15	-0.187845816	0.134872758	-1.392763213	0.163691406	0.200397545	negative
PRJNA729271	stage_1D_vs_E10	ordinary_muscle_development	ENSGALG00010003363	HBE1	-4.811174587	0.505829241	-9.511459994	1.88005E-21	1.08875E-20	negative
PRJNA729271	stage_1D_vs_E10	ordinary_muscle_development	ENSGALG00010003364	ANKRD6	-3.065885278	0.169425591	-18.09576262	3.44147E-73	1.38183E-71	negative
PRJNA729271	stage_1D_vs_E10	ordinary_muscle_development	ENSGALG00010003366	CASD1	0.642587888	0.107595677	5.972246335	2.34009E-09	6.43269E-09	positive
PRJNA729271	stage_1D_vs_E10	ordinary_muscle_development	ENSGALG00010003371	ENSGALG00010003371	-2.864739588	0.378660051	-7.565465598	3.86478E-14	1.4688E-13	negative
PRJNA729271	stage_1D_vs_E10	ordinary_muscle_development	ENSGALG00010003372	ENOX1	-1.092123534	0.141608897	-7.712252257	1.23616E-14	4.83622E-14	negative
PRJNA729271	stage_1D_vs_E10	ordinary_muscle_development	ENSGALG00010003374	SYCE2	0.654866379	0.403328443	1.623655338	0.104449388	0.132391628	positive
PRJNA729271	stage_1D_vs_E10	ordinary_muscle_development	ENSGALG00010003375	YWHAQ	-0.664117419	0.08601534	-7.720918366	1.15495E-14	4.52702E-14	negative
PRJNA729271	stage_1D_vs_E10	ordinary_muscle_development	ENSGALG00010003379	ENSGALG00010003379	-2.60227425	0.483819308	-5.378607688	7.50641E-08	1.83112E-07	negative
PRJNA729271	stage_1D_vs_E10	ordinary_muscle_development	ENSGALG00010003382	ASNS	-1.57699762	0.600138542	-2.627722615	0.008595858	0.012814662	negative
PRJNA729271	stage_1D_vs_E10	ordinary_muscle_development	ENSGALG00010003383	UBE2J1	0.495450719	0.085971272	5.762979997	8.26416E-09	2.17442E-08	positive
PRJNA729271	stage_1D_vs_E10	ordinary_muscle_development	ENSGALG00010003388	PXDN	-0.532838778	0.186042122	-2.864076003	0.004182276	0.006488505	negative
PRJNA729271	stage_1D_vs_E10	ordinary_muscle_development	ENSGALG00010003389	ENSGALG00010003389	-3.023389195	0.397619402	-7.603726519	2.87723E-14	1.10215E-13	negative
PRJNA729271	stage_1D_vs_E10	ordinary_muscle_development	ENSGALG00010003395	ENSGALG00010003395	1.076887923	1.000164969	1.076710299	0.28160971	0.327760779	positive
PRJNA729271	stage_1D_vs_E10	ordinary_muscle_development	ENSGALG00010003397	SPATA4	2.197329704	0.390573595	5.625904386	1.84539E-08	4.71512E-08	positive
PRJNA729271	stage_1D_vs_E10	ordinary_muscle_development	ENSGALG00010003400	PKIA	2.031404665	0.114311537	17.77077547	1.19034E-70	4.48155E-69	positive
PRJNA729271	stage_1D_vs_E10	ordinary_muscle_development	ENSGALG00010003401	ENSGALG00010003401	-0.54304853	1.724647941	-0.314875006	0.75285656	0.784462356	negative
PRJNA729271	stage_1D_vs_E10	ordinary_muscle_development	ENSGALG00010003403	GCDH	0.791134818	0.147311516	5.370488615	7.85236E-08	1.9123E-07	positive
PRJNA729271	stage_1D_vs_E10	ordinary_muscle_development	ENSGALG00010003405	GABRR2	-1.796032237	0.261617295	-6.865112785	6.64387E-12	2.1729E-11	negative
PRJNA729271	stage_1D_vs_E10	ordinary_muscle_development	ENSGALG00010003406	C1GALT1	-0.667006998	0.105664962	-6.312470919	2.74615E-10	8.07905E-10	negative
PRJNA729271	stage_1D_vs_E10	ordinary_muscle_development	ENSGALG00010003407	CHMP4C	0.552924774	0.209516603	2.639049915	0.008313874	0.012419829	positive
PRJNA729271	stage_1D_vs_E10	ordinary_muscle_development	ENSGALG00010003408	ENSGALG00010003408	9.157843168	3.663435181	2.499796698	0.01242646	0.018139127	positive
PRJNA729271	stage_1D_vs_E10	ordinary_muscle_development	ENSGALG00010003412	ASB5	1.232710911	0.146817148	8.39623249	4.61032E-17	2.09554E-16	positive
PRJNA729271	stage_1D_vs_E10	ordinary_muscle_development	ENSGALG00010003419	ENSGALG00010003419	-1.827531797	0.920976111	-1.984342238	0.047217701	0.063202684	negative
PRJNA729271	stage_1D_vs_E10	ordinary_muscle_development	ENSGALG00010003420	ENSGALG00010003420	1.417367343	0.63156711	2.244207021	0.024819084	0.034660969	positive
PRJNA729271	stage_1D_vs_E10	ordinary_muscle_development	ENSGALG00010003428	SNX16	0.45195412	0.154835718	2.918926752	0.003512387	0.00549925	positive
PRJNA729271	stage_1D_vs_E10	ordinary_muscle_development	ENSGALG00010003431	PM20D2	5.171525105	0.285087109	18.14015768	1.5359E-73	6.21867E-72	positive
PRJNA729271	stage_1D_vs_E10	ordinary_muscle_development	ENSGALG00010003432	LACC1	1.171086306	0.314124647	3.728094293	0.000192933	0.000347098	positive
PRJNA729271	stage_1D_vs_E10	ordinary_muscle_development	ENSGALG00010003437	PNRC1	1.455463437	0.178947649	8.133459386	4.1721E-16	1.80252E-15	positive
PRJNA729271	stage_1D_vs_E10	ordinary_muscle_development	ENSGALG00010003438	KCNS3	-0.438913479	0.166060782	-2.643089319	0.008215336	0.012282754	negative
PRJNA729271	stage_1D_vs_E10	ordinary_muscle_development	ENSGALG00010003439	GMIP	-1.39387168	0.242963438	-5.736960635	9.63906E-09	2.51745E-08	negative
PRJNA729271	stage_1D_vs_E10	ordinary_muscle_development	ENSGALG00010003442	SPCS3	0.031228875	0.089577282	0.348624942	0.7273709	0.761519769	positive
PRJNA729271	stage_1D_vs_E10	ordinary_muscle_development	ENSGALG00010003447	ZC2HC1A	0.229920909	0.124932944	1.840354535	0.065716204	0.086048453	positive
PRJNA729271	stage_1D_vs_E10	ordinary_muscle_development	ENSGALG00010003449	BORCS6	0.818928289	0.109998721	7.444889198	9.70258E-14	3.59323E-13	positive
PRJNA729271	stage_1D_vs_E10	ordinary_muscle_development	ENSGALG00010003454	BPNT2	-1.34403588	0.120464467	-11.15711477	6.61021E-29	5.43477E-28	negative
PRJNA729271	stage_1D_vs_E10	ordinary_muscle_development	ENSGALG00010003456	CDS1	-4.082224825	0.414030147	-9.859728469	6.22174E-23	3.87056E-22	negative
PRJNA729271	stage_1D_vs_E10	ordinary_muscle_development	ENSGALG00010003466	TSC22D1	-0.801812838	0.101095165	-7.931267884	2.1692E-15	8.92746E-15	negative
PRJNA729271	stage_1D_vs_E10	ordinary_muscle_development	ENSGALG00010003468	ILVBL	0.676709071	0.135531631	4.992997329	5.94494E-07	1.35087E-06	positive
PRJNA729271	stage_1D_vs_E10	ordinary_muscle_development	ENSGALG00010003470	METTL3	0.643173614	0.186620061	3.44643342	0.000568039	0.000971072	positive
PRJNA729271	stage_1D_vs_E10	ordinary_muscle_development	ENSGALG00010003471	ADI1	0.393851902	0.133612371	2.947720339	0.003201265	0.005028429	positive
PRJNA729271	stage_1D_vs_E10	ordinary_muscle_development	ENSGALG00010003472	FAM110B	-2.068202524	0.130163305	-15.88929014	7.5174E-57	1.8563E-55	negative
PRJNA729271	stage_1D_vs_E10	ordinary_muscle_development	ENSGALG00010003473	ENSGALG00010003473	0.228108279	0.239689429	0.951682685	0.341257933	0.390166725	positive
PRJNA729271	stage_1D_vs_E10	ordinary_muscle_development	ENSGALG00010003474	RPS7	0.135877913	0.16610518	0.818023329	0.413343866	0.462908085	positive
PRJNA729271	stage_1D_vs_E10	ordinary_muscle_development	ENSGALG00010003475	ENSGALG00010003475	0.859482968	2.232576104	0.384973648	0.700256939	0.737233029	positive
PRJNA729271	stage_1D_vs_E10	ordinary_muscle_development	ENSGALG00010003478	COLEC11	1.086005424	0.178008646	6.100857731	1.05501E-09	2.97632E-09	positive
PRJNA729271	stage_1D_vs_E10	ordinary_muscle_development	ENSGALG00010003479	SYDE1	0.675795526	0.199833589	3.381791461	0.000720148	0.001219741	positive
PRJNA729271	stage_1D_vs_E10	ordinary_muscle_development	ENSGALG00010003485	RNGTT	-0.916311991	0.102924691	-8.902742224	5.44836E-19	2.75267E-18	negative
PRJNA729271	stage_1D_vs_E10	ordinary_muscle_development	ENSGALG00010003489	SGCE	-0.007178765	0.118546703	-0.06055643	0.951712474	0.95919012	negative
PRJNA729271	stage_1D_vs_E10	ordinary_muscle_development	ENSGALG00010003503	NUFIP1	-0.57970568	0.086569698	-6.696404063	2.13611E-11	6.75897E-11	negative
PRJNA729271	stage_1D_vs_E10	ordinary_muscle_development	ENSGALG00010003506	DYNC1I1	-1.542983114	0.165449841	-9.325987292	1.09955E-20	6.11119E-20	negative
PRJNA729271	stage_1D_vs_E10	ordinary_muscle_development	ENSGALG00010003507	WDFY3	0.27023875	0.097478696	2.772285246	0.005566424	0.00850214	positive
PRJNA729271	stage_1D_vs_E10	ordinary_muscle_development	ENSGALG00010003509	GPALPP1	-0.531923853	0.072540353	-7.332799336	2.25394E-13	8.13721E-13	negative
PRJNA729271	stage_1D_vs_E10	ordinary_muscle_development	ENSGALG00010003511	UBXN2B	-0.77511612	0.142127584	-5.453664222	4.93423E-08	1.22051E-07	negative
PRJNA729271	stage_1D_vs_E10	ordinary_muscle_development	ENSGALG00010003512	ENSGALG00010003512	-1.401767313	0.519458439	-2.69851678	0.006964923	0.010504272	negative
PRJNA729271	stage_1D_vs_E10	ordinary_muscle_development	ENSGALG00010003517	GTF2F2	-1.162965002	0.089130047	-13.04795681	6.52746E-39	8.24895E-38	negative
PRJNA729271	stage_1D_vs_E10	ordinary_muscle_development	ENSGALG00010003519	KCTD4	-2.632869917	0.699427359	-3.764322176	0.000167001	0.000303116	negative
PRJNA729271	stage_1D_vs_E10	ordinary_muscle_development	ENSGALG00010003520	ENSGALG00010003520	0.859508019	1.808471231	0.475267731	0.634596156	0.67581157	positive
PRJNA729271	stage_1D_vs_E10	ordinary_muscle_development	ENSGALG00010003522	ENSGALG00010003522	2.131240491	0.640908574	3.325342455	0.0008831	0.001480686	positive
PRJNA729271	stage_1D_vs_E10	ordinary_muscle_development	ENSGALG00010003525	NOL10	-0.652850853	0.066420922	-9.828994137	8.44581E-23	5.22724E-22	negative
PRJNA729271	stage_1D_vs_E10	ordinary_muscle_development	ENSGALG00010003528	GDAP1	-0.938915135	0.198252574	-4.735954311	2.18027E-06	4.74092E-06	negative
PRJNA729271	stage_1D_vs_E10	ordinary_muscle_development	ENSGALG00010003531	PPM1K	-0.454699971	0.15030295	-3.025223201	0.002484497	0.003951026	negative
PRJNA729271	stage_1D_vs_E10	ordinary_muscle_development	ENSGALG00010003539	ENSGALG00010003539	0.951538039	0.20508829	4.639650753	3.48998E-06	7.43713E-06	positive
PRJNA729271	stage_1D_vs_E10	ordinary_muscle_development	ENSGALG00010003541	CNR1	0.138867744	0.218447012	0.63570448	0.524969056	0.572051306	positive
PRJNA729271	stage_1D_vs_E10	ordinary_muscle_development	ENSGALG00010003543	SOX11	-2.323208595	0.080408918	-28.89242466	1.4865E-183	5.2552E-181	negative
PRJNA729271	stage_1D_vs_E10	ordinary_muscle_development	ENSGALG00010003552	PI15	-2.090188401	0.389035302	-5.37274738	7.75459E-08	1.88944E-07	negative
PRJNA729271	stage_1D_vs_E10	ordinary_muscle_development	ENSGALG00010003556	ENSGALG00010003556	1.322150514	2.007218613	0.658697815	0.510089836	0.557976921	positive
PRJNA729271	stage_1D_vs_E10	ordinary_muscle_development	ENSGALG00010003558	TPT1	1.613731117	0.184651703	8.739324321	2.34509E-18	1.14567E-17	positive
PRJNA729271	stage_1D_vs_E10	ordinary_muscle_development	ENSGALG00010003560	ADGRL3	-1.148526959	0.137878689	-8.329981716	8.08548E-17	3.6307E-16	negative
PRJNA729271	stage_1D_vs_E10	ordinary_muscle_development	ENSGALG00010003571	RPL7	0.159895254	0.157445449	1.015559706	0.309839101	0.357174946	positive
PRJNA729271	stage_1D_vs_E10	ordinary_muscle_development	ENSGALG00010003577	ENSGALG00010003577	0.859506016	1.755449981	0.489621479	0.624401774	0.666325828	positive
PRJNA729271	stage_1D_vs_E10	ordinary_muscle_development	ENSGALG00010003582	RDH10	-1.418195783	0.142134508	-9.977842842	1.90565E-23	1.20991E-22	negative
PRJNA729271	stage_1D_vs_E10	ordinary_muscle_development	ENSGALG00010003583	RSAD2	1.845449776	0.805067452	2.29229212	0.021888792	0.030824642	positive
PRJNA729271	stage_1D_vs_E10	ordinary_muscle_development	ENSGALG00010003585	PNPLA6	1.344731174	0.141409016	9.509515122	1.91553E-21	1.10797E-20	positive
PRJNA729271	stage_1D_vs_E10	ordinary_muscle_development	ENSGALG00010003589	SLC25A30	0.996672373	0.388125862	2.567910232	0.010231365	0.015102203	positive
PRJNA729271	stage_1D_vs_E10	ordinary_muscle_development	ENSGALG00010003595	SNX13	0.687568744	0.09398773	7.315516013	2.56394E-13	9.23567E-13	positive
PRJNA729271	stage_1D_vs_E10	ordinary_muscle_development	ENSGALG00010003596	CRISPLD1	-1.737008829	0.151690821	-11.45098176	2.32497E-30	2.0524E-29	negative
PRJNA729271	stage_1D_vs_E10	ordinary_muscle_development	ENSGALG00010003597	RNF144A	0.340628071	0.141423543	2.40856695	0.016015289	0.022972945	positive
PRJNA729271	stage_1D_vs_E10	ordinary_muscle_development	ENSGALG00010003604	ODC	-1.436893927	0.21630797	-6.642815451	3.07747E-11	9.64496E-11	negative
PRJNA729271	stage_1D_vs_E10	ordinary_muscle_development	ENSGALG00010003617	COG3	0.097630079	0.081944983	1.191410088	0.233492635	0.276894031	positive
PRJNA729271	stage_1D_vs_E10	ordinary_muscle_development	ENSGALG00010003619	HNF4G	-1.204825531	0.532844478	-2.26112042	0.023751801	0.033273641	negative
PRJNA729271	stage_1D_vs_E10	ordinary_muscle_development	ENSGALG00010003627	MCOLN1	0.376538967	0.119623438	3.147702263	0.001645592	0.002677389	positive
PRJNA729271	stage_1D_vs_E10	ordinary_muscle_development	ENSGALG00010003632	TECRL	2.853610854	0.305408273	9.343593831	9.31185E-21	5.20337E-20	positive
PRJNA729271	stage_1D_vs_E10	ordinary_muscle_development	ENSGALG00010003634	CYP4F11	1.468382263	0.298587887	4.917755625	8.75421E-07	1.96428E-06	positive
PRJNA729271	stage_1D_vs_E10	ordinary_muscle_development	ENSGALG00010003639	ENSGALG00010003639	-0.585934267	0.79133155	-0.740440927	0.459032493	0.507914197	negative
PRJNA729271	stage_1D_vs_E10	ordinary_muscle_development	ENSGALG00010003641	ENSGALG00010003641	0.792239621	2.578076982	0.307298667	0.75861605	0.789725628	positive
PRJNA729271	stage_1D_vs_E10	ordinary_muscle_development	ENSGALG00010003650	SPACA1	-1.365403258	0.846635964	-1.612739495	0.106801116	0.135073918	negative
PRJNA729271	stage_1D_vs_E10	ordinary_muscle_development	ENSGALG00010003652	MYCN	0.186038713	0.138929542	1.339086784	0.180542423	0.21910268	positive
PRJNA729271	stage_1D_vs_E10	ordinary_muscle_development	ENSGALG00010003660	IGFBP7	3.06031613	0.16478789	18.57124408	5.49157E-77	2.3976E-75	positive

PRJNA729271	stage_1D_vs_E10	ordinary_muscle_development	ENSGALG00010003665	RDH14	0.583979671	0.09395233	6.215701852	5.10957E-10	1.4736E-09	positive
PRJNA729271	stage_1D_vs_E10	ordinary_muscle_development	ENSGALG00010003666	SLC25A13	-0.195135465	0.075962533	-2.568838312	0.010204006	0.015063353	negative
PRJNA729271	stage_1D_vs_E10	ordinary_muscle_development	ENSGALG00010003669	ENSGALG00010003669	1.339927169	1.676252672	0.799358708	0.424082446	0.473580513	positive
PRJNA729271	stage_1D_vs_E10	ordinary_muscle_development	ENSGALG00010003671	ENSGALG00010003671	0.60728938	0.372327813	1.631061011	0.102877447	0.130601558	positive
PRJNA729271	stage_1D_vs_E10	ordinary_muscle_development	ENSGALG00010003674	AKIRIN2	0.171035432	0.132221269	1.29355461	0.195819309	0.235728003	positive
PRJNA729271	stage_1D_vs_E10	ordinary_muscle_development	ENSGALG00010003676	POLR2B	0.17640272	0.077943552	2.26321122	0.023622672	0.033102342	positive
PRJNA729271	stage_1D_vs_E10	ordinary_muscle_development	ENSGALG00010003678	ZNFY1	-0.252775932	0.443915837	-0.5694231	0.569069044	0.614196262	negative
PRJNA729271	stage_1D_vs_E10	ordinary_muscle_development	ENSGALG00010003680	TRAPPC5	0.322921656	0.106538873	3.031021876	0.002437276	0.003881379	positive
PRJNA729271	stage_1D_vs_E10	ordinary_muscle_development	ENSGALG00010003681	NT5C1B	-0.400563324	0.12990821	-3.083433485	0.002046268	0.003288322	negative
PRJNA729271	stage_1D_vs_E10	ordinary_muscle_development	ENSGALG00010003683	ENSGALG00010003683	0.380895185	1.755426205	0.216981599	0.828222685	0.852634078	positive
PRJNA729271	stage_1D_vs_E10	ordinary_muscle_development	ENSGALG00010003691	ERICH1	0.413486206	0.142544072	2.900760444	0.003722583	0.005807647	positive
PRJNA729271	stage_1D_vs_E10	ordinary_muscle_development	ENSGALG00010003692	ZNFY2	-0.527469876	0.259123046	-2.035596153	0.041790924	0.056407436	negative
PRJNA729271	stage_1D_vs_E10	ordinary_muscle_development	ENSGALG00010003695	NOA1	0.082122829	0.101114016	0.812180469	0.416688085	0.466149092	positive
PRJNA729271	stage_1D_vs_E10	ordinary_muscle_development	ENSGALG00010003698	MACC1	-6.410640174	1.330622444	-4.817775473	1.45168E-06	3.19982E-06	negative
PRJNA729271	stage_1D_vs_E10	ordinary_muscle_development	ENSGALG00010003700	REST	-0.303848968	0.066858395	-4.544664423	5.50228E-06	1.15004E-05	negative
PRJNA729271	stage_1D_vs_E10	ordinary_muscle_development	ENSGALG00010003701	SARAF	1.300425975	0.081496615	15.95680969	2.5546E-57	6.39533E-56	positive
PRJNA729271	stage_1D_vs_E10	ordinary_muscle_development	ENSGALG00010003703	TDRP	-0.802046049	0.227168634	-3.530619674	0.000414587	0.000720298	negative
PRJNA729271	stage_1D_vs_E10	ordinary_muscle_development	ENSGALG00010003713	SPERT	0.696590506	0.255812672	2.72304926	0.006468241	0.009800058	positive
PRJNA729271	stage_1D_vs_E10	ordinary_muscle_development	ENSGALG00010003714	ORC3	-1.203791633	0.112468253	-10.70339053	9.81209E-27	7.27128E-26	negative
PRJNA729271	stage_1D_vs_E10	ordinary_muscle_development	ENSGALG00010003716	YLEC8	0.806496069	0.681854152	1.1827985	0.236889015	0.280485727	positive
PRJNA729271	stage_1D_vs_E10	ordinary_muscle_development	ENSGALG00010003717	LEPROTL1	-0.013527865	0.09717166	-0.139216157	0.889279341	0.905521886	negative
PRJNA729271	stage_1D_vs_E10	ordinary_muscle_development	ENSGALG00010003719	RPGR	-0.370243717	0.112417437	-3.293472328	0.000989581	0.00165044	negative
PRJNA729271	stage_1D_vs_E10	ordinary_muscle_development	ENSGALG00010003720	ENSGALG00010003720	0.859462659	2.731725995	0.314622572	0.753048239	0.78460568	positive
PRJNA729271	stage_1D_vs_E10	ordinary_muscle_development	ENSGALG00010003722	MHCY11	1.689049502	0.654997653	2.578710768	0.009916978	0.01466802	positive
PRJNA729271	stage_1D_vs_E10	ordinary_muscle_development	ENSGALG00010003724	ENSGALG00010003724	0.052817371	0.21510701	0.245539982	0.806038354	0.832575069	positive
PRJNA729271	stage_1D_vs_E10	ordinary_muscle_development	ENSGALG00010003726	ENPEP	-1.494838495	0.438987375	-3.405197007	0.000661163	0.001122986	negative
PRJNA729271	stage_1D_vs_E10	ordinary_muscle_development	ENSGALG00010003727	FBXO25	0.155796656	0.153593979	1.014340902	0.310420113	0.357787813	positive
PRJNA729271	stage_1D_vs_E10	ordinary_muscle_development	ENSGALG00010003729	RARS2	-0.617267625	0.10310727	-5.986654713	2.14201E-09	5.90499E-09	negative
PRJNA729271	stage_1D_vs_E10	ordinary_muscle_development	ENSGALG00010003731	ENSGALG00010003731	0.862758819	0.586018569	1.472238022	0.140956613	0.174614658	positive
PRJNA729271	stage_1D_vs_E10	ordinary_muscle_development	ENSGALG00010003733	STAU2	0.057801881	0.070596984	0.818758511	0.412924206	0.462509572	positive
PRJNA729271	stage_1D_vs_E10	ordinary_muscle_development	ENSGALG00010003734	ZC3H13	-0.658888202	0.141984828	-4.640553597	3.47477E-06	7.40688E-06	negative
PRJNA729271	stage_1D_vs_E10	ordinary_muscle_development	ENSGALG00010003736	ENSGALG00010003736	0.859441271	3.115575725	0.275853116	0.782660885	0.810912762	positive
PRJNA729271	stage_1D_vs_E10	ordinary_muscle_development	ENSGALG00010003737	SRP72	-0.433839026	0.056341005	-7.700235823	1.35815E-14	5.29917E-14	negative
PRJNA729271	stage_1D_vs_E10	ordinary_muscle_development	ENSGALG00010003740	PITX2	-1.40115622	0.354156951	-3.956314325	7.6115E-05	0.000142857	negative
PRJNA729271	stage_1D_vs_E10	ordinary_muscle_development	ENSGALG00010003742	ZFHX4	-0.683752068	0.165956373	-4.12007116	3.78755E-05	7.31618E-05	negative
PRJNA729271	stage_1D_vs_E10	ordinary_muscle_development	ENSGALG00010003743	ARL9	-0.008863115	0.175803	-0.050415037	0.959791654	0.965922379	negative
PRJNA729271	stage_1D_vs_E10	ordinary_muscle_development	ENSGALG00010003746	HOPX	3.482294756	0.406616885	8.564068253	1.08953E-17	5.1242E-17	positive
PRJNA729271	stage_1D_vs_E10	ordinary_muscle_development	ENSGALG00010003749	MHCY15	3.325441954	0.945018609	3.518916899	0.000433312	0.000750402	positive
PRJNA729271	stage_1D_vs_E10	ordinary_muscle_development	ENSGALG00010003753	SLCO5A1	-0.907788513	0.163276691	-5.559816958	2.70058E-08	6.80071E-08	negative
PRJNA729271	stage_1D_vs_E10	ordinary_muscle_development	ENSGALG00010003754	FAM110C	0.653690224	0.246030603	2.656946804	0.007885188	0.011819628	positive
PRJNA729271	stage_1D_vs_E10	ordinary_muscle_development	ENSGALG00010003755	SLC35A1	-0.629242984	0.089334851	-7.043645109	1.87275E-12	6.35874E-12	negative
PRJNA729271	stage_1D_vs_E10	ordinary_muscle_development	ENSGALG00010003756	MHCY7	-0.244444301	0.380887757	-0.64177516	0.52101918	0.568259821	negative
PRJNA729271	stage_1D_vs_E10	ordinary_muscle_development	ENSGALG00010003761	UGT8	5.013040036	0.324017679	15.4715016	5.40318E-54	1.19207E-52	positive
PRJNA729271	stage_1D_vs_E10	ordinary_muscle_development	ENSGALG00010003762	EVI5L	-0.155071184	0.099321855	-1.561299711	0.118453052	0.148823523	negative
PRJNA729271	stage_1D_vs_E10	ordinary_muscle_development	ENSGALG00010003764	HDAC9	1.242085992	0.192800621	6.442333981	1.1765E-10	3.54687E-10	positive
PRJNA729271	stage_1D_vs_E10	ordinary_muscle_development	ENSGALG00010003765	ENSGALG00010003765	1.721276289	0.867438067	1.984321826	0.047219975	0.063202684	positive
PRJNA729271	stage_1D_vs_E10	ordinary_muscle_development	ENSGALG00010003767	ENSGALG00010003767	-0.306898011	0.413558179	-0.742091505	0.458031896	0.50700056	negative
PRJNA729271	stage_1D_vs_E10	ordinary_muscle_development	ENSGALG00010003768	ARSJ	-0.830990189	0.128387763	-6.472503061	9.63926E-11	2.91937E-10	negative
PRJNA729271	stage_1D_vs_E10	ordinary_muscle_development	ENSGALG00010003769	C6orf163	0.427012616	0.483051358	0.883990096	0.376701559	0.425853151	positive
PRJNA729271	stage_1D_vs_E10	ordinary_muscle_development	ENSGALG00010003772	ENSGALG00010003772	0.500694597	0.445153397	1.124768677	0.260687071	0.305766232	positive
PRJNA729271	stage_1D_vs_E10	ordinary_muscle_development	ENSGALG00010003775	NCOA2	0.592906945	0.114055396	5.198412045	2.00998E-07	4.74275E-07	positive
PRJNA729271	stage_1D_vs_E10	ordinary_muscle_development	ENSGALG00010003777	ENSGALG00010003777	3.054321213	0.816324329	3.741553578	0.000182886	0.000330418	positive
PRJNA729271	stage_1D_vs_E10	ordinary_muscle_development	ENSGALG00010003778	LCA5	-0.277830591	0.125930714	-2.20621787	0.027368749	0.037937268	negative
PRJNA729271	stage_1D_vs_E10	ordinary_muscle_development	ENSGALG00010003785	ENSGALG00010003785	0.764384085	1.293538305	0.590924971	0.554570694	0.600246581	positive
PRJNA729271	stage_1D_vs_E10	ordinary_muscle_development	ENSGALG00010003786	ENSGALG00010003786	-0.285386736	0.601702606	-0.474298655	0.635286951	0.676249126	negative
PRJNA729271	stage_1D_vs_E10	ordinary_muscle_development	ENSGALG00010003787	SMIM8	0.233143525	0.190512128	1.223772613	0.221038019	0.263525751	positive
PRJNA729271	stage_1D_vs_E10	ordinary_muscle_development	ENSGALG00010003788	NUDT9	-0.165174727	0.126445974	-1.306286964	0.191454989	0.2311076	negative
PRJNA729271	stage_1D_vs_E10	ordinary_muscle_development	ENSGALG00010003790	ELOVL6	-0.565810703	0.181213343	-3.122345714	0.001794161	0.002907363	negative
PRJNA729271	stage_1D_vs_E10	ordinary_muscle_development	ENSGALG00010003791	ITGB8	-0.438748692	0.168499478	-2.603857879	0.009218097	0.013694406	negative
PRJNA729271	stage_1D_vs_E10	ordinary_muscle_development	ENSGALG00010003793	TRAM1L1	-0.585486095	0.084934362	-6.893394894	5.44764E-12	1.79096E-11	negative
PRJNA729271	stage_1D_vs_E10	ordinary_muscle_development	ENSGALG00010003795	LCP1	-0.31207693	0.202231878	-1.543163887	0.122791017	0.153966764	negative
PRJNA729271	stage_1D_vs_E10	ordinary_muscle_development	ENSGALG00010003796	ENSGALG00010003796	0.919286449	0.310385226	2.961759686	0.003058864	0.004817803	positive
PRJNA729271	stage_1D_vs_E10	ordinary_muscle_development	ENSGALG00010003797	ENSGALG00010003797	1.437323815	0.169764996	8.466549937	2.52768E-17	1.16758E-16	positive
PRJNA729271	stage_1D_vs_E10	ordinary_muscle_development	ENSGALG00010003803	LACTB2	1.768986124	0.131689962	13.43296102	3.87598E-41	5.31526E-40	positive
PRJNA729271	stage_1D_vs_E10	ordinary_muscle_development	ENSGALG00010003804	PSME1	2.320513467	0.256194015	9.057641211	1.33302E-19	6.97046E-19	positive
PRJNA729271	stage_1D_vs_E10	ordinary_muscle_development	ENSGALG00010003806	EGF	5.185498785	0.314866351	16.46888836	6.13881E-61	1.72446E-59	positive
PRJNA729271	stage_1D_vs_E10	ordinary_muscle_development	ENSGALG00010003808	TWIST1	-2.52287034	0.164145994	-15.36967356	2.61483E-53	5.65702E-52	negative
PRJNA729271	stage_1D_vs_E10	ordinary_muscle_development	ENSGALG00010003810	HMGN2P46	0.510799407	0.090902423	5.619205619	1.91837E-08	4.89557E-08	positive
PRJNA729271	stage_1D_vs_E10	ordinary_muscle_development	ENSGALG00010003812	BG8	-0.195231715	0.405099006	-0.481935803	0.629851551	0.67149884	negative
PRJNA729271	stage_1D_vs_E10	ordinary_muscle_development	ENSGALG00010003813	LRIT3	-2.515969509	0.597458845	-4.211117688	2.5411E-05	4.98758E-05	negative
PRJNA729271	stage_1D_vs_E10	ordinary_muscle_development	ENSGALG00010003814	TPP1	1.21489917	0.132224538	9.188152137	3.99647E-20	2.15029E-19	positive
PRJNA729271	stage_1D_vs_E10	ordinary_muscle_development	ENSGALG00010003815	RASAL3	1.678972664	0.624680165	2.687731672	0.007193918	0.010831603	positive
PRJNA729271	stage_1D_vs_E10	ordinary_muscle_development	ENSGALG00010003816	KLHL8	-0.419886952	0.17823856	-2.355758215	0.018484945	0.026258259	negative
PRJNA729271	stage_1D_vs_E10	ordinary_muscle_development	ENSGALG00010003817	BF2	3.171046082	0.282713731	11.21645586	3.38569E-29	2.82205E-28	positive
PRJNA729271	stage_1D_vs_E10	ordinary_muscle_development	ENSGALG00010003818	GAB2	0.812152473	0.15645089	5.191101643	2.09053E-07	4.92E-07	positive
PRJNA729271	stage_1D_vs_E10	ordinary_muscle_development	ENSGALG00010003819	PHIP	-0.085743625	0.094051143	-0.911670206	0.361942353	0.410963058	negative
PRJNA729271	stage_1D_vs_E10	ordinary_muscle_development	ENSGALG00010003820	SH3YL1	-1.11940627	0.302252216	-3.703550251	0.000212603	0.000380501	negative
PRJNA729271	stage_1D_vs_E10	ordinary_muscle_development	ENSGALG00010003821	RUBCNL	0.018393706	0.157502369	0.116783676	0.90703148	0.920752245	positive
PRJNA729271	stage_1D_vs_E10	ordinary_muscle_development	ENSGALG00010003822	ENSGALG00010003822	-0.268965467	0.156256871	-1.721303293	0.085195802	0.109645825	negative
PRJNA729271	stage_1D_vs_E10	ordinary_muscle_development	ENSGALG00010003823	PTPN13	0.599424012	0.148621212	4.033233225	5.50146E-05	0.000104719	positive
PRJNA729271	stage_1D_vs_E10	ordinary_muscle_development	ENSGALG00010003824	SP4	0.438145693	0.158033993	2.772477517	0.005563136	0.008499092	positive
PRJNA729271	stage_1D_vs_E10	ordinary_muscle_development	ENSGALG00010003826	ENSGALG00010003826	0.191146218	0.106270155	1.798682022	0.072068994	0.093790633	positive
PRJNA729271	stage_1D_vs_E10	ordinary_muscle_development	ENSGALG00010003827	RRH	-0.195017754	0.692171284	-0.281747826	0.77813688	0.807033062	negative
PRJNA729271	stage_1D_vs_E10	ordinary_muscle_development	ENSGALG00010003829	ENSGALG00010003829	0.222465833	0.125342504	1.774863477	0.075920432	0.098440528	positive
PRJNA729271	stage_1D_vs_E10	ordinary_muscle_development	ENSGALG00010003830	DMB2	-1.772014515	0.256280544	-6.914354426	4.69999E-12	1.55256E-11	negative
PRJNA729271	stage_1D_vs_E10	ordinary_muscle_development	ENSGALG00010003831	IRAK1BP1	-0.503495163	0.169424182	-2.971802244	0.002960573	0.004669587	negative
PRJNA729271	stage_1D_vs_E10	ordinary_muscle_development	ENSGALG00010003832	ART1B	4.570499406	0.949631638	4.812918216	1.48742E-06	3.27662E-06	positive
PRJNA729271	stage_1D_vs_E10	ordinary_muscle_development	ENSGALG00010003833	ACP1	0.561996673	0.157321198	3.572288283	0.000353876	0.000619571	positive
PRJNA729271	stage_1D_vs_E10	ordinary_muscle_development	ENSGALG00010003834	MAPK10	-1.02029725	0.428645014	-2.380284889	0.017299258	0.024699837	negative
PRJNA729271	stage_1D_vs_E10	ordinary_muscle_development	ENSGALG00010003835	ENSGALG00010003835	1.335003768	0.16581779	8.051028598	8.21011E-16	3.47157E-15	positive
PRJNA729271	stage_1D_vs_E10	ordinary_muscle_development	ENSGALG00010003836	CD1F	1.606379145	0.368989306	4.35345718	1.34007E-05	2.70534E-05	positive
PRJNA729271	stage_1D_vs_E10	ordinary_muscle_development	ENSGALG00010003837	ALKAL2	3.080824007	0.232655868	13.24197854	5.02085E-40	6.57427E-39	positive
PRJNA729271	stage_1D_vs_E10	ordinary_muscle_development	ENSGALG00010003838	GAR1	-1.069186022	0.181013334	-5.906669956	3.49092E-09	9.4705E-09	negative
PRJNA729271	stage_1D_vs_E10	ordinary_muscle_development	ENSGALG00010003839	ENSGALG00010003839	2.016429019	0.755623943	2.668561575	0.007617681	0.011439939	positive
PRJNA729271	stage_1D_vs_E10	ordinary_muscle_development	ENSGALG00010003841	UBE2W	0.549645515	0.142071677	3.868790236	0.000109377	0.000202221	positive
PRJNA729271	stage_1D_vs_E10	ordinary_muscle_development	ENSGALG00010003842	ENSGALG00010003842	0.318443761	0.144189979	2.208501335	0.027209345	0.037723525	positive
PRJNA729271	stage_1D_vs_E10	ordinary_muscle_development	ENSGALG00010003843	LRCH1	1.106143885	0.106934	10.34417383	4.44736E-25	3.06536E-24	positive
PRJNA729271	stage_1D_vs_E10	ordinary_muscle_development	ENSGALG00010003844	TMEM18	-1.009161491	0.112476098	-8.972230664	2.90569E-19	1.49249E-18	negative
PRJNA729271	stage_1D_vs_E10	ordinary_muscle_development	ENSGALG00010003845	CFI	-0.972457425	0.923035589	-1.053542719			negative
PRJNA729271	stage_1D_vs_E10	ordinary_muscle_development	ENSGALG00010003848	ESD	0.201931261	0.123786613	1.631285131	0.102830169	0.130552974	positive
PRJNA729271	stage_1D_vs_E10	ordinary_muscle_development	ENSGALG00010003850	PLA2G12A	-0.530621493	0.099694441	-5.322478221	1.02363E-07	2.47045E-07	negative
PRJNA729271	stage_1D_vs_E10	ordinary_muscle_development	ENSGALG00010003851	PHF14	-0.970608223	0.06294656	-15.41955936	1.20927E-53	2.64379E-52	negative
PRJNA729271	stage_1D_vs_E10	ordinary_muscle_development	ENSGALG00010003852	TCEB1	-0.490633758	0.107547945	-4.562000318	5.06686E-06	1.06333E-05	negative
PRJNA729271	stage_1D_vs_E10	ordinary_muscle_development	ENSGALG00010003853	ENSGALG00010003853	0.861682508	0.22429742	3.841696033	0.000122187	0.000224858	positive
PRJNA729271	stage_1D_vs_E10	ordinary_muscle_development	ENSGALG00010003858	SUCLA2	1.782502103	0.124195748	14.35236017	1.02972E-46	1.72553E-45	positive
PRJNA729271	stage_1D_vs_E10	ordinary_muscle_development	ENSGALG00010003860	TMEM70	0.532537645	0.113578524	4.688717782	2.74922E-06	5.91949E-06	positive
PRJNA729271	stage_1D_vs_E10	ordinary_muscle_development	ENSGALG00010003861	NUDT15	-0.558735773	0.102349017	-5.459122032	4.78495E-08	1.1852E-07	negative
PRJNA729271	stage_1D_vs_E10	ordinary_muscle_development	ENSGALG00010003862	PCK2	-0.299964709	0.145004602	-2.068656474	0.038578334	0.0523197	negative
PRJNA729271	stage_1D_vs_E10	ordinary_muscle_development	ENSGALG00010003863	HTR1B	-2.767792641	0.306586626	-9.027767064	1.7521E-19	9.09951E-19	negative
PRJNA729271	stage_1D_vs_E10	ordinary_muscle_development	ENSGALG00010003865	AKAP8L	-0.495491792	0.189695051	-2.612043853	0.009000271	0.013384521	negative
PRJNA729271	stage_1D_vs_E10	ordinary_muscle_development	ENSGALG00010003868	HBBA	-2.535314816	0.398916799	-6.355497751	2.07753E-10	6.16075E-10	negative
PRJNA729271	stage_1D_vs_E10	ordinary_muscle_development	ENSGALG00010003869	LY96	-0.186139124	0.293333591	-0.634564637	0.525712398	0.572732146	negative
PRJNA729271	stage_1D_vs_E10	ordinary_muscle_development	ENSGALG00010003870	MED4	-0.996159087	0.095793977	-10.39897414	2.50617E-25	1.74649E-24	negative
PRJNA729271	stage_1D_vs_E10	ordinary_muscle_development	ENSGALG00010003871	ENSGALG00010003871	-0.155302464	0.193907869	-0.80090852	0.423184607	0.472759991	negative
PRJNA729271	stage_1D_vs_E10	ordinary_muscle_development	ENSGALG00010003874	YES1	-0.069770064	0.097209744	-0.717727058	0.472925596	0.521654099	negative
PRJNA729271	stage_1D_vs_E10	ordinary_muscle_development	ENSGALG00010003875	AKAP8	-0.787898057	0.111137878	-7.089374676	1.34719E-12	4.61426E-12	negative
PRJNA729271	stage_1D_vs_E10	ordinary_muscle_development	ENSGALG00010003878	ARHGAP24	0.88525917	0.088231602	10.03335713	1.08755E-23	6.9814E-23	positive
PRJNA729271	stage_1D_vs_E10	ordinary_muscle_development	ENSGALG00010003879	CASP6	-1.460606155	0.119687654	-12.20348217	2.9781E-34	3.12582E-33	negative
PRJNA729271	stage_1D_vs_E10	ordinary_muscle_development	ENSGALG00010003882	ENSGALG00010003882	-0.274317159	0.121725789	-2.25356648	0.024223454	0.033891792	negative
PRJNA729271	stage_1D_vs_E10	ordinary_muscle_development	ENSGALG00010003884	TYMS	-2.148823548	0.19180828	-11.20297595	3.94263E-29	3.27686E-28	negative
PRJNA729271	stage_1D_vs_E10	ordinary_muscle_development	ENSGALG00010003887	GIPC1	-0.688164356	0.143189302	-4.805976051	1.53998E-06	3.38645E-06	negative
PRJNA729271	stage_1D_vs_E10	ordinary_muscle_development	ENSGALG00010003889	ITM2B	0.625535751	0.087458514	7.15237114	8.52916E-13	2.96689E-12	positive
PRJNA729271	stage_1D_vs_E10	ordinary_muscle_development	ENSGALG00010003892	OPRK1	-5.065881662	2.563370122	-1.976258371	0.048125522	0.064340476	negative
PRJNA729271	stage_1D_vs_E10	ordinary_muscle_development	ENSGALG00010003894	COPS4	-0.218178828	0.124632736	-1.750574003	0.080019316	0.103468331	negative
PRJNA729271	stage_1D_vs_E10	ordinary_muscle_development	ENSGALG00010003895	THSD7A	1.118706452	0.156658687	7.141043201	9.26252E-13	3.21197E-12	positive
PRJNA729271	stage_1D_vs_E10	ordinary_muscle_development	ENSGALG00010003896	MCUB	-1.436388829	0.13549209	-10.60127447	2.93947E-26	2.12613E-25	negative
PRJNA729271	stage_1D_vs_E10	ordinary_muscle_development	ENSGALG00010003898	NDST4	-5.567475186	0.681089408	-8.174367592	2.97422E-16	1.29269E-15	negative
PRJNA729271	stage_1D_vs_E10	ordinary_muscle_development	ENSGALG00010003899	LIN54	0.765796237	0.163633279	4.679954097	2.86939E-06	6.16815E-06	positive
PRJNA729271	stage_1D_vs_E10	ordinary_muscle_development	ENSGALG00010003900	RB1	-0.597440928	0.087519821	-6.826349962	8.71023E-12	2.82829E-11	negative
PRJNA729271	stage_1D_vs_E10	ordinary_muscle_development	ENSGALG00010003902	ENSGALG00010003902	0.763694352	0.124740821	6.122248898	9.22637E-10	2.61612E-09	positive
PRJNA729271	stage_1D_vs_E10	ordinary_muscle_development	ENSGALG00010003903	COL28A1	5.356872187	0.277886688	19.27718172	8.35039E-83	4.24698E-81	positive

PRJNA729271	stage_1D_vs_E10	ordinary_muscle_development	ENSGALG00010003909	DNAJB1	-0.673748672	0.15770631	-4.272173196	1.93577E-05	3.83897E-05	negative
PRJNA729271	stage_1D_vs_E10	ordinary_muscle_development	ENSGALG00010003910	THAP9	0.918060903	0.147639771	6.218249321	5.02732E-10	1.45075E-09	positive
PRJNA729271	stage_1D_vs_E10	ordinary_muscle_development	ENSGALG00010003913	PSMB5	-0.119293622	0.117166894	-1.018151267	0.308606075	0.35586675	negative
PRJNA729271	stage_1D_vs_E10	ordinary_muscle_development	ENSGALG00010003914	CAMK2D	0.335355742	0.105921602	3.166075051	0.001545109	0.00252151	positive
PRJNA729271	stage_1D_vs_E10	ordinary_muscle_development	ENSGALG00010003915	MIOS	0.120735189	0.092038158	1.311794929	0.189589336	0.229103579	positive
PRJNA729271	stage_1D_vs_E10	ordinary_muscle_development	ENSGALG00010003918	HTR1E	-0.607600817	0.649117775	-0.936040947	0.349252106	0.398082038	negative
PRJNA729271	stage_1D_vs_E10	ordinary_muscle_development	ENSGALG00010003920	LPAR6	-0.439061834	0.158220542	-2.774998921	0.005520189	0.008439525	negative
PRJNA729271	stage_1D_vs_E10	ordinary_muscle_development	ENSGALG00010003921	COLEC12	-0.186397699	0.168317765	-1.107415478	0.268114337	0.313412687	negative
PRJNA729271	stage_1D_vs_E10	ordinary_muscle_development	ENSGALG00010003923	ENSGALG00010003923	-1.168843828	0.121266056	-9.638672747	5.48931E-22	3.2852E-21	negative
PRJNA729271	stage_1D_vs_E10	ordinary_muscle_development	ENSGALG00010003925	SEC31A	-0.6692017	0.085799971	-7.799556261	6.21252E-15	2.47664E-14	negative
PRJNA729271	stage_1D_vs_E10	ordinary_muscle_development	ENSGALG00010003928	ENSGALG00010003928	0.164140689	0.156241269	1.050559113	0.293461128	0.340079873	positive
PRJNA729271	stage_1D_vs_E10	ordinary_muscle_development	ENSGALG00010003929	RCBTB2	-0.131207724	0.098309781	-1.334635508	0.181995671	0.220755418	negative
PRJNA729271	stage_1D_vs_E10	ordinary_muscle_development	ENSGALG00010003933	TECR	0.862384726	0.207714488	4.151779369	3.299E-05	6.40491E-05	positive
PRJNA729271	stage_1D_vs_E10	ordinary_muscle_development	ENSGALG00010003937	ENSGALG00010003937	1.440018746	0.205660133	7.001934332	2.52452E-12	8.50406E-12	positive
PRJNA729271	stage_1D_vs_E10	ordinary_muscle_development	ENSGALG00010003948	SCD5	-2.729023694	0.141833855	-19.24098934	1.67972E-82	8.42474E-81	negative
PRJNA729271	stage_1D_vs_E10	ordinary_muscle_development	ENSGALG00010003958	CYSLTR2	2.177908587	0.372298937	5.84989203	4.91892E-09	1.31768E-08	positive
PRJNA729271	stage_1D_vs_E10	ordinary_muscle_development	ENSGALG00010003960	ASPH	1.466202074	0.195955539	7.482320145	7.30218E-14	2.72235E-13	positive
PRJNA729271	stage_1D_vs_E10	ordinary_muscle_development	ENSGALG00010003962	UMAD1	-0.087397065	0.205258872	-0.425789463	0.670261265	0.708793831	negative
PRJNA729271	stage_1D_vs_E10	ordinary_muscle_development	ENSGALG00010003965	SNTG1	-0.546250581	0.491133864	-1.112223412	0.266042104	0.311266782	negative
PRJNA729271	stage_1D_vs_E10	ordinary_muscle_development	ENSGALG00010003968	DSN1	-0.076956451	0.173001847	-0.444830225	0.65644245	0.695804995	negative
PRJNA729271	stage_1D_vs_E10	ordinary_muscle_development	ENSGALG00010003972	PENK	-1.028885778	0.240569779	-4.276870441	1.89539E-05	3.76146E-05	negative
PRJNA729271	stage_1D_vs_E10	ordinary_muscle_development	ENSGALG00010003977	MYO6	-1.249208213	0.118708616	-10.52331543	6.74573E-26	4.80016E-25	negative
PRJNA729271	stage_1D_vs_E10	ordinary_muscle_development	ENSGALG00010003978	ANK2	0.434717273	0.11976419	3.629776756	0.000283666	0.000501188	positive
PRJNA729271	stage_1D_vs_E10	ordinary_muscle_development	ENSGALG00010003980	CHCHD7	-0.585465138	0.122430044	-4.782038123	1.73527E-06	3.79893E-06	negative
PRJNA729271	stage_1D_vs_E10	ordinary_muscle_development	ENSGALG00010003981	TMEM150C	-0.950327461	0.16988606	-5.59391078	2.22011E-08	5.6368E-08	negative
PRJNA729271	stage_1D_vs_E10	ordinary_muscle_development	ENSGALG00010003982	GLCCI1	-0.846011417	0.121744828	-6.949054296	3.67743E-12	1.22539E-11	negative
PRJNA729271	stage_1D_vs_E10	ordinary_muscle_development	ENSGALG00010003985	PRMT5	0.076045604	0.133938502	0.567765078	0.570194496	0.615273521	positive
PRJNA729271	stage_1D_vs_E10	ordinary_muscle_development	ENSGALG00010003986	FNDC3A	-0.055821185	0.064032944	-0.871757274	0.383340808	0.432380749	negative
PRJNA729271	stage_1D_vs_E10	ordinary_muscle_development	ENSGALG00010003988	ENOPH1	-0.886875962	0.089381051	-9.922415878	3.32606E-23	2.09069E-22	negative
PRJNA729271	stage_1D_vs_E10	ordinary_muscle_development	ENSGALG00010003990	ICA1	-1.09902152	0.17436218	-6.303095774	2.91758E-10	8.56254E-10	negative
PRJNA729271	stage_1D_vs_E10	ordinary_muscle_development	ENSGALG00010003993	ENSGALG00010003993	0.470516329	0.13416796	3.506920203	0.000453325	0.000783842	positive
PRJNA729271	stage_1D_vs_E10	ordinary_muscle_development	ENSGALG00010003994	SDR16C5	1.417277167	0.08402821	16.86668289	7.91163E-64	2.42964E-62	positive
PRJNA729271	stage_1D_vs_E10	ordinary_muscle_development	ENSGALG00010003995	MLNR	-2.804117959	0.251093981	-11.16760327	5.87446E-29	4.84359E-28	negative
PRJNA729271	stage_1D_vs_E10	ordinary_muscle_development	ENSGALG00010004000	ENSGALG00010004000	1.160444221	0.175709195	6.60434543	3.99278E-11	1.24276E-10	positive
PRJNA729271	stage_1D_vs_E10	ordinary_muscle_development	ENSGALG00010004001	LARP7	-0.921427689	0.11402624	-8.080839006	6.43227E-16	2.7374E-15	negative
PRJNA729271	stage_1D_vs_E10	ordinary_muscle_development	ENSGALG00010004002	CDADC1	1.240260736	0.120604803	10.28367612	8.34814E-25	5.65186E-24	positive
PRJNA729271	stage_1D_vs_E10	ordinary_muscle_development	ENSGALG00010004003	HNRNPDL	-0.345574568	0.068128779	-5.072372833	3.92886E-07	9.04092E-07	negative
PRJNA729271	stage_1D_vs_E10	ordinary_muscle_development	ENSGALG00010004006	NKAIN3	-1.749837469	0.331599295	-5.276963779	1.31342E-07	3.14522E-07	negative
PRJNA729271	stage_1D_vs_E10	ordinary_muscle_development	ENSGALG00010004007	ENSGALG00010004007	-1.017643151	0.158761114	-6.409901802	1.45613E-10	4.35998E-10	negative
PRJNA729271	stage_1D_vs_E10	ordinary_muscle_development	ENSGALG00010004016	ENSGALG00010004016	-1.937646263	0.504209643	-3.842937736	0.00012157	0.000223795	negative
PRJNA729271	stage_1D_vs_E10	ordinary_muscle_development	ENSGALG00010004018	HNRNPD	-0.427139694	0.161031451	-2.652523416	0.007989259	0.0119682	negative
PRJNA729271	stage_1D_vs_E10	ordinary_muscle_development	ENSGALG00010004029	ARL4A	-0.38651428	0.194743131	-1.984738959	0.047173522	0.063155094	negative
PRJNA729271	stage_1D_vs_E10	ordinary_muscle_development	ENSGALG00010004034	NEDD8	-0.294084023	0.11353365	-2.590280712	0.00958977	0.014215966	negative
PRJNA729271	stage_1D_vs_E10	ordinary_muscle_development	ENSGALG00010004036	ADCYAP1	-1.003322028	0.546754084	-1.83505173	0.066498025	0.086962186	negative
PRJNA729271	stage_1D_vs_E10	ordinary_muscle_development	ENSGALG00010004038	SENP6	0.007642052	0.087643251	0.087194989	0.930516522	0.940932079	positive
PRJNA729271	stage_1D_vs_E10	ordinary_muscle_development	ENSGALG00010004039	ALPK1	2.692454788	0.229829534	11.71500782	1.06781E-31	1.00116E-30	positive
PRJNA729271	stage_1D_vs_E10	ordinary_muscle_development	ENSGALG00010004040	LGALS1B	-3.352914182	0.226559123	-14.79929007	1.48037E-49	2.816E-48	negative
PRJNA729271	stage_1D_vs_E10	ordinary_muscle_development	ENSGALG00010004042	PLAG1	0.124726482	0.119773048	1.041356831	0.29770997	0.344590435	positive
PRJNA729271	stage_1D_vs_E10	ordinary_muscle_development	ENSGALG00010004043	CAB39L	0.930339901	0.200638639	4.636893002	3.53685E-06	7.53368E-06	positive
PRJNA729271	stage_1D_vs_E10	ordinary_muscle_development	ENSGALG00010004044	RNF138	-0.925660179	0.135322132	-6.840419715	7.89615E-12	2.56971E-11	negative
PRJNA729271	stage_1D_vs_E10	ordinary_muscle_development	ENSGALG00010004046	ENSGALG00010004046	0.415447819	0.120835377	3.43813071	0.000585745	0.000999926	positive
PRJNA729271	stage_1D_vs_E10	ordinary_muscle_development	ENSGALG00010004047	PDE7A	-1.061158544	0.118032548	-8.990389177	2.46357E-19	1.26899E-18	negative
PRJNA729271	stage_1D_vs_E10	ordinary_muscle_development	ENSGALG00010004049	SETDB2	-0.174083809	0.138233456	-1.259346426	0.207905231	0.248933283	negative
PRJNA729271	stage_1D_vs_E10	ordinary_muscle_development	ENSGALG00010004055	FILIP1	2.587705218	0.175131561	14.7757789	2.09916E-49	3.96706E-48	positive
PRJNA729271	stage_1D_vs_E10	ordinary_muscle_development	ENSGALG00010004056	SHROOM3	0.54315215	0.180958122	3.001535066	0.002686221	0.004257246	positive
PRJNA729271	stage_1D_vs_E10	ordinary_muscle_development	ENSGALG00010004057	ENSGALG00010004057	-0.723761032	0.413320637	-1.751088545	0.079930659	0.103372136	negative
PRJNA729271	stage_1D_vs_E10	ordinary_muscle_development	ENSGALG00010004058	TMEM30A	0.214287753	0.061565971	3.480620019	0.000500255	0.000861596	positive
PRJNA729271	stage_1D_vs_E10	ordinary_muscle_development	ENSGALG00010004060	ETV1	-1.095611802	0.092018377	-11.90644558	1.09548E-32	1.07874E-31	negative
PRJNA729271	stage_1D_vs_E10	ordinary_muscle_development	ENSGALG00010004063	ENSGALG00010004063	2.249206714	0.405444459	5.54750883	2.89769E-08	7.27684E-08	positive
PRJNA729271	stage_1D_vs_E10	ordinary_muscle_development	ENSGALG00010004065	TIFA	0.00244449	0.282127981	0.008664472	0.993086838	0.994321594	positive
PRJNA729271	stage_1D_vs_E10	ordinary_muscle_development	ENSGALG00010004070	ARL11	-1.488756495	0.411323242	-3.61943198	0.00029525	0.000520703	negative
PRJNA729271	stage_1D_vs_E10	ordinary_muscle_development	ENSGALG00010004072	SGK3	-0.364478301	0.135343602	-2.692985082	0.007081544	0.010669055	negative
PRJNA729271	stage_1D_vs_E10	ordinary_muscle_development	ENSGALG00010004076	COX7A2	-0.832188006	0.136850673	-6.08099317	1.1944E-09	3.35261E-09	negative
PRJNA729271	stage_1D_vs_E10	ordinary_muscle_development	ENSGALG00010004079	AP1AR	0.253010657	0.148439816	1.704466255	0.088294015	0.113298668	positive
PRJNA729271	stage_1D_vs_E10	ordinary_muscle_development	ENSGALG00010004081	RPS20	0.105700967	0.167956453	0.629335554	0.5291294	0.575935319	positive
PRJNA729271	stage_1D_vs_E10	ordinary_muscle_development	ENSGALG00010004082	FAM241A	1.373558596	0.121100632	11.34229088	8.0996E-30	6.92239E-29	positive
PRJNA729271	stage_1D_vs_E10	ordinary_muscle_development	ENSGALG00010004083	PHF11	-0.477491493	0.184443622	-2.58882084	0.009630519	0.014269076	negative
PRJNA729271	stage_1D_vs_E10	ordinary_muscle_development	ENSGALG00010004085	RB1CC1	1.187479863	0.140574642	8.447326274	2.9805E-17	1.36933E-16	positive
PRJNA729271	stage_1D_vs_E10	ordinary_muscle_development	ENSGALG00010004086	UTP3	-0.040435475	0.257283672	-0.157163005	0.875116384	0.893422453	negative
PRJNA729271	stage_1D_vs_E10	ordinary_muscle_development	ENSGALG00010004087	NCOA7	-0.304663897	0.109125645	-2.791863433	0.005240547	0.008034027	negative
PRJNA729271	stage_1D_vs_E10	ordinary_muscle_development	ENSGALG00010004094	DGKB	-0.296757164	0.169464479	-1.751146712	0.079920642	0.103368404	negative
PRJNA729271	stage_1D_vs_E10	ordinary_muscle_development	ENSGALG00010004095	ANKRD50	-0.706924004	0.11348997	-6.228955783	4.69554E-10	1.35771E-09	negative
PRJNA729271	stage_1D_vs_E10	ordinary_muscle_development	ENSGALG00010004096	COL12A1	-1.584607044	0.414069513	-3.826910689	0.000129762	0.000238089	negative
PRJNA729271	stage_1D_vs_E10	ordinary_muscle_development	ENSGALG00010004097	ENSGALG00010004097	-0.76802518	0.116837838	-6.573428544	4.91697E-11	1.52094E-10	negative
PRJNA729271	stage_1D_vs_E10	ordinary_muscle_development	ENSGALG00010004101	ARID1B	0.687332794	0.166133388	4.137234565	3.51517E-05	6.80726E-05	positive
PRJNA729271	stage_1D_vs_E10	ordinary_muscle_development	ENSGALG00010004102	PCMTD1	2.350557585	0.187651351	12.52619589	5.36761E-36	6.05947E-35	positive
PRJNA729271	stage_1D_vs_E10	ordinary_muscle_development	ENSGALG00010004104	SCARB2	0.617870705	0.094613073	6.530500327	6.55503E-11	2.00751E-10	positive
PRJNA729271	stage_1D_vs_E10	ordinary_muscle_development	ENSGALG00010004108	PERPB	1.327912055	0.240843539	5.513588039	3.51591E-08	8.77866E-08	positive
PRJNA729271	stage_1D_vs_E10	ordinary_muscle_development	ENSGALG00010004109	AGMO	-0.070257748	0.492426775	-0.14267654	0.886545646	0.903270125	negative
PRJNA729271	stage_1D_vs_E10	ordinary_muscle_development	ENSGALG00010004113	ECHDC1	0.815445276	0.181604938	4.49021532	7.11512E-06	1.47418E-05	positive
PRJNA729271	stage_1D_vs_E10	ordinary_muscle_development	ENSGALG00010004116	SPRY1	0.648141111	0.094984021	6.823685755	8.87339E-12	2.88062E-11	positive
PRJNA729271	stage_1D_vs_E10	ordinary_muscle_development	ENSGALG00010004117	STBD1	2.540290992	0.190570746	13.32991049	1.55084E-40	2.0795E-39	positive
PRJNA729271	stage_1D_vs_E10	ordinary_muscle_development	ENSGALG00010004122	TRIM55	1.533761499	0.194817267	7.872821139	3.46732E-15	1.40899E-14	positive
PRJNA729271	stage_1D_vs_E10	ordinary_muscle_development	ENSGALG00010004123	NDUFAF4	0.060594349	0.109862328	0.551548017	0.58125806	0.625674705	positive
PRJNA729271	stage_1D_vs_E10	ordinary_muscle_development	ENSGALG00010004125	MANEA	0.219477061	0.092505749	2.372577542	0.017664455	0.025176625	positive
PRJNA729271	stage_1D_vs_E10	ordinary_muscle_development	ENSGALG00010004127	EPHA7	-3.445775836	0.183070762	-18.82209804	4.97741E-79	2.31986E-77	negative
PRJNA729271	stage_1D_vs_E10	ordinary_muscle_development	ENSGALG00010004131	NUDT6	1.045225332	0.156049098	6.698054305	2.11213E-11	6.6904E-11	positive
PRJNA729271	stage_1D_vs_E10	ordinary_muscle_development	ENSGALG00010004133	CRH	2.03830791	0.996388508	2.045695923	0.040786297	0.055123299	positive
PRJNA729271	stage_1D_vs_E10	ordinary_muscle_development	ENSGALG00010004135	NUP54	-0.796806666	0.065462602	-12.17193691	4.38551E-34	4.56666E-33	negative
PRJNA729271	stage_1D_vs_E10	ordinary_muscle_development	ENSGALG00010004137	CROT	-0.087297795	0.0838555	-1.041050319	0.297852195	0.344727529	negative
PRJNA729271	stage_1D_vs_E10	ordinary_muscle_development	ENSGALG00010004139	PPEF2	-0.239802723	0.682268836	-0.351478348	0.725229513	0.759661931	negative
PRJNA729271	stage_1D_vs_E10	ordinary_muscle_development	ENSGALG00010004141	KPNA3	0.360854165	0.140997021	2.559303467	0.010488214	0.015453005	positive
PRJNA729271	stage_1D_vs_E10	ordinary_muscle_development	ENSGALG00010004142	RBBP8	-1.363315888	0.11393098	-11.96615609	5.34479E-33	5.32825E-32	negative
PRJNA729271	stage_1D_vs_E10	ordinary_muscle_development	ENSGALG00010004143	TCF24	-1.550644639	0.440975605	-3.516395511	0.000437449	0.000757294	negative
PRJNA729271	stage_1D_vs_E10	ordinary_muscle_development	ENSGALG00010004146	NAAA	1.358245923	0.175682271	7.73126345	1.06484E-14	4.18517E-14	positive
PRJNA729271	stage_1D_vs_E10	ordinary_muscle_development	ENSGALG00010004147	SPRYD7	0.211371398	0.091779725	2.303029315	0.021277194	0.030007095	positive
PRJNA729271	stage_1D_vs_E10	ordinary_muscle_development	ENSGALG00010004148	ENSGALG00010004148	0.688569794	0.152487343	4.515586539	6.31418E-06	1.31387E-05	positive
PRJNA729271	stage_1D_vs_E10	ordinary_muscle_development	ENSGALG00010004149	SDAD1	-0.711723223	0.121461282	-5.859671582	4.63783E-09	1.24538E-08	negative
PRJNA729271	stage_1D_vs_E10	ordinary_muscle_development	ENSGALG00010004154	COPS5	-0.060168316	0.130088273	-0.462519139	0.643709081	0.683858335	negative
PRJNA729271	stage_1D_vs_E10	ordinary_muscle_development	ENSGALG00010004158	JCHAIN	0.637778749	0.494024114	1.29098708	0.196708158	0.23666042	positive
PRJNA729271	stage_1D_vs_E10	ordinary_muscle_development	ENSGALG00010004172	ST8SIA6	-0.580693442	0.125911219	-4.611927745	3.98952E-06	8.44945E-06	negative
PRJNA729271	stage_1D_vs_E10	ordinary_muscle_development	ENSGALG00010004177	CSPP1	0.273922098	0.126695556	2.162049772	0.03061434	0.042182021	positive
PRJNA729271	stage_1D_vs_E10	ordinary_muscle_development	ENSGALG00010004181	BBS12	-0.611448859	0.131142771	-4.662467134	3.12441E-06	6.69351E-06	negative
PRJNA729271	stage_1D_vs_E10	ordinary_muscle_development	ENSGALG00010004187	ARFGEF1	0.88112099	0.093935668	9.3800471	6.59433E-21	3.70771E-20	positive
PRJNA729271	stage_1D_vs_E10	ordinary_muscle_development	ENSGALG00010004188	BMI1	0.365770592	0.114532252	3.193603437	0.00140509	0.00230576	positive
PRJNA729271	stage_1D_vs_E10	ordinary_muscle_development	ENSGALG00010004191	CABLES1	0.551330237	0.12542161	4.395815333	1.10358E-05	2.24353E-05	positive
PRJNA729271	stage_1D_vs_E10	ordinary_muscle_development	ENSGALG00010004200	ABCB1	2.804861708	0.181724833	15.4346638	9.56978E-54	2.10173E-52	positive
PRJNA729271	stage_1D_vs_E10	ordinary_muscle_development	ENSGALG00010004201	CPA6	-6.138085301	0.512236716	-11.98290771	4.36732E-33	4.36281E-32	negative
PRJNA729271	stage_1D_vs_E10	ordinary_muscle_development	ENSGALG00010004210	SYNCRIP	-0.634510619	0.070490679	-9.001340742	2.22978E-19	1.15225E-18	negative
PRJNA729271	stage_1D_vs_E10	ordinary_muscle_development	ENSGALG00010004211	RNF146	-0.050015415	0.075336543	-0.663893151	0.5067587	0.554788481	negative
PRJNA729271	stage_1D_vs_E10	ordinary_muscle_development	ENSGALG00010004212	RSPO3	-0.113491912	0.378341048	-0.299972504	0.764198129	0.794395574	negative
PRJNA729271	stage_1D_vs_E10	ordinary_muscle_development	ENSGALG00010004214	REPS1	0.14774547	0.125201932	1.180057427	0.237977376	0.281677758	positive
PRJNA729271	stage_1D_vs_E10	ordinary_muscle_development	ENSGALG00010004216	SNX14	0.349400227	0.073282954	4.767823981	1.86226E-06	4.06651E-06	positive
PRJNA729271	stage_1D_vs_E10	ordinary_muscle_development	ENSGALG00010004217	CCDC28A	-0.775298067	0.113928614	-6.805121584	1.00964E-11	3.26595E-11	negative
PRJNA729271	stage_1D_vs_E10	ordinary_muscle_development	ENSGALG00010004219	FGF2	0.844098051	0.181327233	4.655109097	3.23809E-06	6.92376E-06	positive
PRJNA729271	stage_1D_vs_E10	ordinary_muscle_development	ENSGALG00010004222	SPAG6	-2.917419501	0.261150918	-11.17139285	5.62912E-29	4.64923E-28	negative
PRJNA729271	stage_1D_vs_E10	ordinary_muscle_development	ENSGALG00010004224	NT5E	1.51665218	0.25750732	5.889743949	3.86794E-09	1.04503E-08	positive
PRJNA729271	stage_1D_vs_E10	ordinary_muscle_development	ENSGALG00010004225	CETN4	-1.838684223	0.128158995	-14.34689947	1.11408E-46	1.86473E-45	negative
PRJNA729271	stage_1D_vs_E10	ordinary_muscle_development	ENSGALG00010004226	RUNDC3B	-0.863822164	0.199447093	-4.331084246	1.48377E-05	2.97719E-05	negative
PRJNA729271	stage_1D_vs_E10	ordinary_muscle_development	ENSGALG00010004230	TAF1B	0.110524113	0.115304855	0.958538241	0.337791418	0.386416747	positive
PRJNA729271	stage_1D_vs_E10	ordinary_muscle_development	ENSGALG00010004231	ARMC3	-1.115635615	0.416643605	-2.677673681	0.007413541	0.011151855	negative
PRJNA729271	stage_1D_vs_E10	ordinary_muscle_development	ENSGALG00010004233	NHSL1	-2.20175782	0.12170552	-18.09086245	3.76154E-73	1.50618E-71	negative
PRJNA729271	stage_1D_vs_E10	ordinary_muscle_development	ENSGALG00010004235	ADAD1	-1.42089046	0.425890634	-3.336280127	0.000849076	0.001426443	negative
PRJNA729271	stage_1D_vs_E10	ordinary_muscle_development	ENSGALG00010004236	DBF4	-1.627068184	0.125811226	-12.93261527	2.94624E-38	3.61913E-37	negative
PRJNA729271	stage_1D_vs_E10	ordinary_muscle_development	ENSGALG00010004237	RUFY3	-0.264268429	0.091779523	-2.879383331	0.003984537	0.006194323	negative
PRJNA729271	stage_1D_vs_E10	ordinary_muscle_development	ENSGALG00010004238	CCNC	-0.719574577	0.124256997	-5.791018548	6.99608E-09	1.85322E-08	negative
PRJNA729271	stage_1D_vs_E10	ordinary_muscle_development	ENSGALG00010004240	ENSGALG00010004240	0.005349781	0.179428087	0.029815739	0.976214006	0.979728728	positive
PRJNA729271	stage_1D_vs_E10	ordinary_muscle_development	ENSGALG00010004241	PREX2	0.642046833	0.114171345	5.623537437	1.87086E-08	4.77937E-08	positive
PRJNA729271	stage_1D_vs_E10	ordinary_muscle_development	ENSGALG00010004242	ADAM22	-1.584379749	0.174255176	-9.092296615	9.69687E-20	5.12418E-19	negative
PRJNA729271	stage_1D_vs_E10	ordinary_muscle_development	ENSGALG00010004243	BLTP1	0.397695411	0.112717324	3.528254547	0.00041831	0.000726156	positive
PRJNA729271	stage_1D_vs_E10	ordinary_muscle_development	ENSGALG00010004244	TMEM241	-0.187163815	0.081422192	-2.298683069	0.021522943	0.030335964	negative

PRJNA729271	stage_1D_vs_E10	ordinary_muscle_development	ENSGALG00010004245	ARFGEF3	-0.906882472	0.223477758	-4.058043542	4.94855E-05	9.45798E-05	negative
PRJNA729271	stage_1D_vs_E10	ordinary_muscle_development	ENSGALG00010004246	C8orf34	-3.644342622	0.349590684	-10.42459879	1.91466E-25	1.34202E-24	negative
PRJNA729271	stage_1D_vs_E10	ordinary_muscle_development	ENSGALG00010004247	MEOX2	-0.916615188	0.104430555	-8.777270093	1.6749E-18	8.25493E-18	negative
PRJNA729271	stage_1D_vs_E10	ordinary_muscle_development	ENSGALG00010004248	GRSF1	0.088116262	0.159181648	0.553557922	0.57988143	0.624471124	positive
PRJNA729271	stage_1D_vs_E10	ordinary_muscle_development	ENSGALG00010004249	USP45	0.986935119	0.101291256	9.743537186	1.9659E-22	1.1973E-21	positive
PRJNA729271	stage_1D_vs_E10	ordinary_muscle_development	ENSGALG00010004250	MOB1B	-0.295745154	0.087542811	-3.378291716	0.000729377	0.001234939	negative
PRJNA729271	stage_1D_vs_E10	ordinary_muscle_development	ENSGALG00010004251	CCN2	0.661801659	0.265597384	2.491747651	0.012711632	0.018531137	positive
PRJNA729271	stage_1D_vs_E10	ordinary_muscle_development	ENSGALG00010004255	RPS12	0.095740609	0.151080431	0.63370622	0.526272565	0.573170098	positive
PRJNA729271	stage_1D_vs_E10	ordinary_muscle_development	ENSGALG00010004256	ENSGALG00010004256	-5.137280336	0.57270587	-8.970189772	2.96004E-19	1.51825E-18	negative
PRJNA729271	stage_1D_vs_E10	ordinary_muscle_development	ENSGALG00010004257	ENSGALG00010004257	-0.470377464	0.633319058	-0.742717999	0.45765243	0.506619213	negative
PRJNA729271	stage_1D_vs_E10	ordinary_muscle_development	ENSGALG00010004265	PIP4K2A	0.711082417	0.092050734	7.724896742	1.11944E-14	4.39261E-14	positive
PRJNA729271	stage_1D_vs_E10	ordinary_muscle_development	ENSGALG00010004275	ADARB2	-2.464030205	0.675150835	-3.649599579	0.000262649	0.000465587	negative
PRJNA729271	stage_1D_vs_E10	ordinary_muscle_development	ENSGALG00010004281	VIM	-1.421485707	0.171648795	-8.281361425	1.21775E-16	5.4029E-16	negative
PRJNA729271	stage_1D_vs_E10	ordinary_muscle_development	ENSGALG00010004283	DCK	-1.033829309	0.127308662	-8.120651775	4.63687E-16	1.99677E-15	negative
PRJNA729271	stage_1D_vs_E10	ordinary_muscle_development	ENSGALG00010004285	SLC18B1	-0.241579772	0.118600303	-2.036923739	0.041657685	0.056243307	negative
PRJNA729271	stage_1D_vs_E10	ordinary_muscle_development	ENSGALG00010004296	ENSGALG00010004296	-6.675784992	0.852489406	-7.830930154	4.84274E-15	1.94825E-14	negative
PRJNA729271	stage_1D_vs_E10	ordinary_muscle_development	ENSGALG00010004297	SULF1	-2.301810163	0.184054904	-12.50610615	6.91308E-36	7.76783E-35	negative
PRJNA729271	stage_1D_vs_E10	ordinary_muscle_development	ENSGALG00010004300	VNN2	3.595026128	0.244406333	14.70921841	5.62534E-49	1.0427E-47	positive
PRJNA729271	stage_1D_vs_E10	ordinary_muscle_development	ENSGALG00010004302	CRPPA	0.239716407	0.127460211	1.880715596	0.060010617	0.078987144	positive
PRJNA729271	stage_1D_vs_E10	ordinary_muscle_development	ENSGALG00010004306	PNISR	0.615243224	0.143485765	4.287834578	1.80423E-05	3.58645E-05	positive
PRJNA729271	stage_1D_vs_E10	ordinary_muscle_development	ENSGALG00010004307	NPC2	0.959853977	0.14072888	6.820589888	9.06674E-12	2.94076E-11	positive
PRJNA729271	stage_1D_vs_E10	ordinary_muscle_development	ENSGALG00010004308	DSG2	-5.640497205	0.536011549	-10.52308895	6.76196E-26	4.80936E-25	negative
PRJNA729271	stage_1D_vs_E10	ordinary_muscle_development	ENSGALG00010004309	MRPS28	-0.507655838	0.136975778	-3.706172337	0.000210415	0.000377051	negative
PRJNA729271	stage_1D_vs_E10	ordinary_muscle_development	ENSGALG00010004312	PDLIM5	3.513544831	0.181614491	19.34617013	2.19564E-83	1.12857E-81	positive
PRJNA729271	stage_1D_vs_E10	ordinary_muscle_development	ENSGALG00010004319	TAAR1	5.042520278	0.826772942	6.099038832	1.06708E-09	3.00921E-09	positive
PRJNA729271	stage_1D_vs_E10	ordinary_muscle_development	ENSGALG00010004323	TRDMT1	-0.262587043	0.158733327	-1.654265351	0.09807361	0.124864029	negative
PRJNA729271	stage_1D_vs_E10	ordinary_muscle_development	ENSGALG00010004325	LTBP2	1.154254056	0.112912695	10.22253566	1.57175E-24	1.05231E-23	positive
PRJNA729271	stage_1D_vs_E10	ordinary_muscle_development	ENSGALG00010004326	ENSGALG00010004326	-0.150181246	0.946277523	-0.158707401	0.873899407	0.892305713	negative
PRJNA729271	stage_1D_vs_E10	ordinary_muscle_development	ENSGALG00010004328	ENSGALG00010004328	-0.792947603	0.693416137	-1.143537856	0.252815347	0.297460547	negative
PRJNA729271	stage_1D_vs_E10	ordinary_muscle_development	ENSGALG00010004329	COQ3	0.781366391	0.120457246	6.486669909	8.77543E-11	2.66722E-10	positive
PRJNA729271	stage_1D_vs_E10	ordinary_muscle_development	ENSGALG00010004330	STX7	-0.153784551	0.103417671	-1.487023924	0.137008493	0.169977794	negative
PRJNA729271	stage_1D_vs_E10	ordinary_muscle_development	ENSGALG00010004332	ENSGALG00010004332	-0.494315495	0.891287226	-0.554608527	0.579162455	0.623743205	negative
PRJNA729271	stage_1D_vs_E10	ordinary_muscle_development	ENSGALG00010004333	DIO2	-1.070740808	0.320053879	-3.345501733	0.000821338	0.00138289	negative
PRJNA729271	stage_1D_vs_E10	ordinary_muscle_development	ENSGALG00010004334	ENSGALG00010004334	0.130907734	0.231473157	0.565541749	0.571705336	0.616766065	positive
PRJNA729271	stage_1D_vs_E10	ordinary_muscle_development	ENSGALG00010004336	ENSGALG00010004336	-3.821499553	0.545130048	-7.010253003	2.37888E-12	8.03397E-12	negative
PRJNA729271	stage_1D_vs_E10	ordinary_muscle_development	ENSGALG00010004337	BMPR1B	-1.377762668	0.2352763	-5.855934773	4.74334E-09	1.27277E-08	negative
PRJNA729271	stage_1D_vs_E10	ordinary_muscle_development	ENSGALG00010004338	ENSGALG00010004338	0.910250204	0.166292997	5.473773512	4.40552E-08	1.09215E-07	positive
PRJNA729271	stage_1D_vs_E10	ordinary_muscle_development	ENSGALG00010004339	B4GALT6	-2.813678061	0.122382652	-22.99082445	5.7585E-117	6.183E-115	negative
PRJNA729271	stage_1D_vs_E10	ordinary_muscle_development	ENSGALG00010004340	SLC4A4	1.20936113	0.16933981	7.141623293	9.22351E-13	3.1992E-12	positive
PRJNA729271	stage_1D_vs_E10	ordinary_muscle_development	ENSGALG00010004341	ENSGALG00010004341	-1.187019802	0.168795873	-7.03227977	2.03186E-12	6.8877E-12	negative
PRJNA729271	stage_1D_vs_E10	ordinary_muscle_development	ENSGALG00010004342	ZNF438	0.331911636	0.129297388	2.56704053	0.010257062	0.01513551	positive
PRJNA729271	stage_1D_vs_E10	ordinary_muscle_development	ENSGALG00010004344	SOSTDC1	-6.007962872	0.407907236	-14.72874795	4.21442E-49	7.83179E-48	negative
PRJNA729271	stage_1D_vs_E10	ordinary_muscle_development	ENSGALG00010004345	FAXC	-2.13301194	0.187608329	-11.36949493	5.93299E-30	5.10984E-29	negative
PRJNA729271	stage_1D_vs_E10	ordinary_muscle_development	ENSGALG00010004346	CEP128	0.147022128	0.098460597	1.493207774	0.135382808	0.168112207	positive
PRJNA729271	stage_1D_vs_E10	ordinary_muscle_development	ENSGALG00010004350	MOXD1	-0.641883527	0.240802579	-2.665600709	0.007685091	0.011534002	negative
PRJNA729271	stage_1D_vs_E10	ordinary_muscle_development	ENSGALG00010004352	CHMP2A	0.210083866	0.056446486	3.721823646	0.000197789	0.000355393	positive
PRJNA729271	stage_1D_vs_E10	ordinary_muscle_development	ENSGALG00010004355	ANKMY2	0.373234888	0.12287114	3.037612306	0.002384605	0.003799588	positive
PRJNA729271	stage_1D_vs_E10	ordinary_muscle_development	ENSGALG00010004356	TRAPPC8	0.319512434	0.077147588	4.141573864	3.44931E-05	6.68687E-05	positive
PRJNA729271	stage_1D_vs_E10	ordinary_muscle_development	ENSGALG00010004358	GC	-16.35242069	2.261167356	-7.231848917	4.76462E-13	1.69065E-12	negative
PRJNA729271	stage_1D_vs_E10	ordinary_muscle_development	ENSGALG00010004362	FBXL4	2.107087204	0.120336423	17.5099704	1.20257E-68	4.26191E-67	positive
PRJNA729271	stage_1D_vs_E10	ordinary_muscle_development	ENSGALG00010004364	TSHR	1.284112767	0.449987261	2.853664709	0.004321812	0.006698531	positive
PRJNA729271	stage_1D_vs_E10	ordinary_muscle_development	ENSGALG00010004365	TPD52	-1.804392972	0.156781137	-11.50899278	1.18858E-30	1.0648E-29	negative
PRJNA729271	stage_1D_vs_E10	ordinary_muscle_development	ENSGALG00010004366	UNC5C	-3.532422289	0.347068654	-10.17787764	2.48931E-24	1.64911E-23	negative
PRJNA729271	stage_1D_vs_E10	ordinary_muscle_development	ENSGALG00010004367	TSPAN13	-0.381318246	0.178984718	-2.130451424	0.033134362	0.045446875	negative
PRJNA729271	stage_1D_vs_E10	ordinary_muscle_development	ENSGALG00010004368	GJD4	-7.080230727	0.421022473	-16.81675249	1.83971E-63	5.55553E-62	negative
PRJNA729271	stage_1D_vs_E10	ordinary_muscle_development	ENSGALG00010004369	UBE2M	0.545319689	0.234076634	2.329663062	0.019823966	0.028061366	positive
PRJNA729271	stage_1D_vs_E10	ordinary_muscle_development	ENSGALG00010004370	ATP6V1H	0.042540264	0.074872503	0.568169379	0.569919962	0.615068859	positive
PRJNA729271	stage_1D_vs_E10	ordinary_muscle_development	ENSGALG00010004372	SYNJ2	1.575561208	0.183937871	8.565724946	1.07398E-17	5.05761E-17	positive
PRJNA729271	stage_1D_vs_E10	ordinary_muscle_development	ENSGALG00010004373	MMS22L	-0.903829494	0.121347488	-7.44827526	9.45684E-14	3.5058E-13	negative
PRJNA729271	stage_1D_vs_E10	ordinary_muscle_development	ENSGALG00010004374	ILDR1	-6.318522027	0.986305001	-6.406255696	1.49137E-10	4.46363E-10	negative
PRJNA729271	stage_1D_vs_E10	ordinary_muscle_development	ENSGALG00010004375	ENSGALG00010004375	-0.262831337	0.216916534	-1.211670369	0.225638586	0.268414551	negative
PRJNA729271	stage_1D_vs_E10	ordinary_muscle_development	ENSGALG00010004377	AGR2	-5.035096697	0.8431654	-5.971659528	2.34852E-09	6.45343E-09	negative
PRJNA729271	stage_1D_vs_E10	ordinary_muscle_development	ENSGALG00010004378	TRIM28	0.16565313	0.154025263	1.075493247	0.282153954	0.328315129	positive
PRJNA729271	stage_1D_vs_E10	ordinary_muscle_development	ENSGALG00010004379	ZBTB10	0.15170515	0.059100737	2.566890978	0.010261487	0.015140498	positive
PRJNA729271	stage_1D_vs_E10	ordinary_muscle_development	ENSGALG00010004380	ISCA2	0.325302135	0.084196957	3.863585415	0.000111735	0.000206423	positive
PRJNA729271	stage_1D_vs_E10	ordinary_muscle_development	ENSGALG00010004381	ENSGALG00010004381	0.049863818	0.438051905	0.113830843	0.909371888	0.922748848	positive
PRJNA729271	stage_1D_vs_E10	ordinary_muscle_development	ENSGALG00010004383	ENSGALG00010004383	-1.180367207	0.519316463	-2.272924684	0.023030719	0.032341627	negative
PRJNA729271	stage_1D_vs_E10	ordinary_muscle_development	ENSGALG00010004384	ENSGALG00010004384	1.073228031	0.558946261	1.920091618	0.054846328	0.072655595	positive
PRJNA729271	stage_1D_vs_E10	ordinary_muscle_development	ENSGALG00010004385	ADAMTS3	-2.777945969	0.206137812	-13.47615921	2.16065E-41	2.997E-40	negative
PRJNA729271	stage_1D_vs_E10	ordinary_muscle_development	ENSGALG00010004386	CFAP44	-1.750184299	0.442878644	-3.951837197	7.75535E-05	0.000145444	negative
PRJNA729271	stage_1D_vs_E10	ordinary_muscle_development	ENSGALG00010004389	ENSGALG00010004389	0.046704567	0.131770505	0.3544387	0.723010136	0.757830062	positive
PRJNA729271	stage_1D_vs_E10	ordinary_muscle_development	ENSGALG00010004390	PNLDC1	-0.066747562	0.50903481	-0.131125732	0.895675846	0.911075185	negative
PRJNA729271	stage_1D_vs_E10	ordinary_muscle_development	ENSGALG00010004393	ZBTB45	0.284436002	0.212950756	1.335689091	0.181650917	0.22039257	positive
PRJNA729271	stage_1D_vs_E10	ordinary_muscle_development	ENSGALG00010004394	CCNY	-0.908079715	0.087590735	-10.36730333	3.49241E-25	2.42097E-24	negative
PRJNA729271	stage_1D_vs_E10	ordinary_muscle_development	ENSGALG00010004395	BOC	-0.73361255	0.190302524	-3.85498067	0.000115739	0.000213548	negative
PRJNA729271	stage_1D_vs_E10	ordinary_muscle_development	ENSGALG00010004397	ENSGALG00010004397	0.629809655	0.165420626	3.807322395	0.00014048	0.000256852	positive
PRJNA729271	stage_1D_vs_E10	ordinary_muscle_development	ENSGALG00010004398	COX18	-0.255806366	0.122412324	-2.089710877	0.036643779	0.04990619	negative
PRJNA729271	stage_1D_vs_E10	ordinary_muscle_development	ENSGALG00010004399	POU3F2	0.424069642	0.484547886	0.87518624	0.381472579	0.430508022	positive
PRJNA729271	stage_1D_vs_E10	ordinary_muscle_development	ENSGALG00010004404	RGS20	-2.135543429	0.483919836	-4.413010729	1.01943E-05	2.07946E-05	negative
PRJNA729271	stage_1D_vs_E10	ordinary_muscle_development	ENSGALG00010004406	ENSGALG00010004406	-0.743455341	0.141807133	-5.242721746	1.58225E-07	3.76287E-07	negative
PRJNA729271	stage_1D_vs_E10	ordinary_muscle_development	ENSGALG00010004408	SERAC1	-0.221750683	0.110029262	-2.015379166	0.043864936	0.05901998	negative
PRJNA729271	stage_1D_vs_E10	ordinary_muscle_development	ENSGALG00010004416	STXBP6	-0.398926588	0.212799534	-1.874659122	0.060839634	0.080017285	negative
PRJNA729271	stage_1D_vs_E10	ordinary_muscle_development	ENSGALG00010004420	ENSGALG00010004420	1.737752464	0.281248359	6.178711475	6.46269E-10	1.85059E-09	positive
PRJNA729271	stage_1D_vs_E10	ordinary_muscle_development	ENSGALG00010004421	RAP1GDS1	-1.181636689	0.075387044	-15.67426746	2.26825E-55	5.32011E-54	negative
PRJNA729271	stage_1D_vs_E10	ordinary_muscle_development	ENSGALG00010004424	MRPL18	0.124743812	0.145630892	0.856575206	0.391679693	0.440894397	positive
PRJNA729271	stage_1D_vs_E10	ordinary_muscle_development	ENSGALG00010004425	CREM	2.183677719	0.241083467	9.057766384	1.33149E-19	6.96498E-19	positive
PRJNA729271	stage_1D_vs_E10	ordinary_muscle_development	ENSGALG00010004429	ENSGALG00010004429	0.824778957	0.346238199	2.382114278	0.017213555	0.024589581	positive
PRJNA729271	stage_1D_vs_E10	ordinary_muscle_development	ENSGALG00010004430	TCEA1	-0.793074692	0.094083582	-8.429469538	3.47236E-17	1.58876E-16	negative
PRJNA729271	stage_1D_vs_E10	ordinary_muscle_development	ENSGALG00010004433	AHR1A	-1.611600017	0.159720089	-10.09015227	6.10747E-24	3.96097E-23	negative
PRJNA729271	stage_1D_vs_E10	ordinary_muscle_development	ENSGALG00010004434	TCP1	-0.790380213	0.086622688	-9.124401877	7.21335E-20	3.82854E-19	negative
PRJNA729271	stage_1D_vs_E10	ordinary_muscle_development	ENSGALG00010004436	NOVA1	0.782973398	0.149757161	5.228286869	1.71088E-07	4.05728E-07	positive
PRJNA729271	stage_1D_vs_E10	ordinary_muscle_development	ENSGALG00010004440	GTF2H5	-0.674953394	0.10482524	-6.438844236	1.20387E-10	3.62636E-10	negative
PRJNA729271	stage_1D_vs_E10	ordinary_muscle_development	ENSGALG00010004443	ANKRD17	-0.06893654	0.093581649	-0.736645918	0.461337703	0.510153342	negative
PRJNA729271	stage_1D_vs_E10	ordinary_muscle_development	ENSGALG00010004445	EPHA6	-3.559451617	0.550446454	-6.466481149	1.00311E-10	3.03615E-10	negative
PRJNA729271	stage_1D_vs_E10	ordinary_muscle_development	ENSGALG00010004446	ACAT2	-2.262415753	0.129440058	-17.47848219	2.08988E-68	7.33483E-67	negative
PRJNA729271	stage_1D_vs_E10	ordinary_muscle_development	ENSGALG00010004451	CLDND1	-0.211117756	0.084674032	-2.493299901	0.01265619	0.018455882	negative
PRJNA729271	stage_1D_vs_E10	ordinary_muscle_development	ENSGALG00010004454	SRI	-0.808365359	0.164321284	-4.919419684	8.68012E-07	1.94795E-06	negative
PRJNA729271	stage_1D_vs_E10	ordinary_muscle_development	ENSGALG00010004457	AGA	-0.019106005	0.126604153	-0.150911363	0.880045637	0.897749902	negative
PRJNA729271	stage_1D_vs_E10	ordinary_muscle_development	ENSGALG00010004460	ENSGALG00010004460	-0.098620963	0.585785328	-0.168356833	0.866302565	0.886046831	negative
PRJNA729271	stage_1D_vs_E10	ordinary_muscle_development	ENSGALG00010004468	LYPLA1	0.484457281	0.122262329	3.962441115	7.41873E-05	0.000139438	positive
PRJNA729271	stage_1D_vs_E10	ordinary_muscle_development	ENSGALG00010004471	C1H3ORF52	0.968414799	0.251412139	3.851901516	0.000117204	0.000216142	positive
PRJNA729271	stage_1D_vs_E10	ordinary_muscle_development	ENSGALG00010004472	NEIL3	-0.915206061	0.194776399	-4.698752339	2.61756E-06	5.64934E-06	negative
PRJNA729271	stage_1D_vs_E10	ordinary_muscle_development	ENSGALG00010004473	JDP2	0.502301796	0.132474185	3.791695693	0.000149622	0.000273076	positive
PRJNA729271	stage_1D_vs_E10	ordinary_muscle_development	ENSGALG00010004477	TULP4	-0.016497377	0.125636813	-0.131310056	0.895530037	0.910990799	negative
PRJNA729271	stage_1D_vs_E10	ordinary_muscle_development	ENSGALG00010004481	ZNF704	0.332122474	0.078284331	4.242515317	2.21028E-05	4.3607E-05	positive
PRJNA729271	stage_1D_vs_E10	ordinary_muscle_development	ENSGALG00010004483	STEAP4	-0.208447212	0.64936156	-0.321003312	0.748207881	0.780516246	negative
PRJNA729271	stage_1D_vs_E10	ordinary_muscle_development	ENSGALG00010004487	ABHD10	0.045153171	0.099070372	0.455768662	0.648556341	0.688553737	positive
PRJNA729271	stage_1D_vs_E10	ordinary_muscle_development	ENSGALG00010004489	LIG1	-1.588427332	0.256927921	-6.182385038	6.31403E-10	1.80945E-09	negative
PRJNA729271	stage_1D_vs_E10	ordinary_muscle_development	ENSGALG00010004494	WTAP	-0.098855477	0.05690727	-1.737132671	0.082363756	0.106215537	negative
PRJNA729271	stage_1D_vs_E10	ordinary_muscle_development	ENSGALG00010004495	MRPL15	0.764522257	0.112997886	6.76581026	1.32565E-11	4.26344E-11	positive
PRJNA729271	stage_1D_vs_E10	ordinary_muscle_development	ENSGALG00010004501	TAGLN3	-2.231270212	0.333953361	-6.681382704	2.36698E-11	7.47319E-11	negative
PRJNA729271	stage_1D_vs_E10	ordinary_muscle_development	ENSGALG00010004503	ENSGALG00010004503	0.376180135	0.181375891	2.074036036	0.038075964	0.051696431	positive
PRJNA729271	stage_1D_vs_E10	ordinary_muscle_development	ENSGALG00010004504	ZNF804B	-2.433017744	0.681572048	-3.569714679	0.00035737	0.000625085	negative
PRJNA729271	stage_1D_vs_E10	ordinary_muscle_development	ENSGALG00010004505	ENSGALG00010004505	1.171574715	0.130778853	8.958441572	3.29299E-19	1.68545E-18	positive
PRJNA729271	stage_1D_vs_E10	ordinary_muscle_development	ENSGALG00010004507	CUL2	0.17311268	0.090134346	1.920607262	0.054781239	0.072582638	positive
PRJNA729271	stage_1D_vs_E10	ordinary_muscle_development	ENSGALG00010004515	ENSGALG00010004515	-0.433000516	0.254000527	-1.704722901	0.088246117	0.11324723	negative
PRJNA729271	stage_1D_vs_E10	ordinary_muscle_development	ENSGALG00010004516	ENSGALG00010004516	0.410087425	0.284413023	1.441872878	0.149338239	0.184241874	positive
PRJNA729271	stage_1D_vs_E10	ordinary_muscle_development	ENSGALG00010004522	TSPAN5	-0.194060446	0.062850994	-3.087627342	0.002017613	0.003246232	negative
PRJNA729271	stage_1D_vs_E10	ordinary_muscle_development	ENSGALG00010004533	PAG1	0.86722405	0.112014756	7.742051831	9.78251E-15	3.85529E-14	positive
PRJNA729271	stage_1D_vs_E10	ordinary_muscle_development	ENSGALG00010004534	CUBN	2.820395697	0.212007313	13.30329438	2.2149E-40	2.93196E-39	positive
PRJNA729271	stage_1D_vs_E10	ordinary_muscle_development	ENSGALG00010004542	ERMARD	-0.340064942	0.129895143	-2.617995829	0.008844789	0.013165456	negative
PRJNA729271	stage_1D_vs_E10	ordinary_muscle_development	ENSGALG00010004545	TMPRSS7	1.170705253	0.552485848	2.118977811	0.034092342	0.046646073	positive
PRJNA729271	stage_1D_vs_E10	ordinary_muscle_development	ENSGALG00010004553	BATF	-0.630041705	0.374580841	-1.681991272	0.092570532	0.118429821	negative
PRJNA729271	stage_1D_vs_E10	ordinary_muscle_development	ENSGALG00010004565	ENSGALG00010004565	3.821582521	0.376283249	10.15613247	3.11176E-24	2.04651E-23	positive
PRJNA729271	stage_1D_vs_E10	ordinary_muscle_development	ENSGALG00010004568	RSU1	-0.177819047	0.067946717	-2.617036646	0.008869682	0.013201154	negative
PRJNA729271	stage_1D_vs_E10	ordinary_muscle_development	ENSGALG00010004570	ENSGALG00010004570	-0.322398026	0.595753305	-0.541160282	0.588397111	0.632513804	negative
PRJNA729271	stage_1D_vs_E10	ordinary_muscle_development	ENSGALG00010004576	STEAP2	0.876075802	0.209810852	4.175550481	2.97266E-05	5.79851E-05	positive
PRJNA729271	stage_1D_vs_E10	ordinary_muscle_development	ENSGALG00010004577	ZPLD1	-5.580997312	0.490946995	-11.3678205	6.04789E-30	5.20571E-29	negative
PRJNA729271	stage_1D_vs_E10	ordinary_muscle_development	ENSGALG00010004583	XKR4	1.123697862	0.107121805	10.48990782	9.61221E-26	6.79985E-25	positive
PRJNA729271	stage_1D_vs_E10	ordinary_muscle_development	ENSGALG00010004584	EIF4E	-0.28945645	0.066538979	-4.350178686	1.36027E-05	2.74229E-05	negative
PRJNA729271	stage_1D_vs_E10	ordinary_muscle_development	ENSGALG00010004586	ENSGALG00010004586	-5.604405641	0.474639543	-11.80770909	3.56133E-32	3.41412E-31	negative

PRJNA729271	stage_1D_vs_E10	ordinary_muscle_development	ENSGALG00010004588	MINDY3	-0.99224365	0.15388802	-6.447829101	1.13464E-10	3.42351E-10	negative
PRJNA729271	stage_1D_vs_E10	ordinary_muscle_development	ENSGALG00010004591	RRS1	-0.091239498	0.161987986	-0.563248548	0.573265649	0.618173306	negative
PRJNA729271	stage_1D_vs_E10	ordinary_muscle_development	ENSGALG00010004602	DYNLT2	-1.206386133	0.354413282	-3.403896505	0.000664319	0.001127818	negative
PRJNA729271	stage_1D_vs_E10	ordinary_muscle_development	ENSGALG00010004609	CFAP69	-2.582546788	0.618576323	-4.174984865	2.98006E-05	5.81216E-05	negative
PRJNA729271	stage_1D_vs_E10	ordinary_muscle_development	ENSGALG00010004610	TGS1	-0.463395043	0.122743631	-3.775308253	0.00015981	0.000290828	negative
PRJNA729271	stage_1D_vs_E10	ordinary_muscle_development	ENSGALG00010004622	ENSGALG00010004622	1.894064647	0.223248641	8.484103828	2.17388E-17	1.00736E-16	positive
PRJNA729271	stage_1D_vs_E10	ordinary_muscle_development	ENSGALG00010004623	MBIP	-0.477239972	0.125449605	-3.804236535	0.000142242	0.000259967	negative
PRJNA729271	stage_1D_vs_E10	ordinary_muscle_development	ENSGALG00010004624	PHF10	0.108004214	0.075883229	1.423294923	0.154650678	0.190084082	positive
PRJNA729271	stage_1D_vs_E10	ordinary_muscle_development	ENSGALG00010004630	ENSGALG00010004630	1.831789069	0.308704809	5.933788583	2.96023E-09	8.07006E-09	positive
PRJNA729271	stage_1D_vs_E10	ordinary_muscle_development	ENSGALG00010004639	HBS1L	0.717187319	0.079223589	9.052699197	1.39477E-19	7.27761E-19	positive
PRJNA729271	stage_1D_vs_E10	ordinary_muscle_development	ENSGALG00010004641	METAP1	-0.379268904	0.080491455	-4.711915115	2.454E-06	5.30906E-06	negative
PRJNA729271	stage_1D_vs_E10	ordinary_muscle_development	ENSGALG00010004650	GTPBP10	-1.021845925	0.129386738	-7.897609423	2.84303E-15	1.16051E-14	negative
PRJNA729271	stage_1D_vs_E10	ordinary_muscle_development	ENSGALG00010004666	C6orf120	-0.601138161	0.118150837	-5.087887443	3.62074E-07	8.35578E-07	negative
PRJNA729271	stage_1D_vs_E10	ordinary_muscle_development	ENSGALG00010004669	ELMOD1	-3.643091485	0.397297832	-9.169673715	4.74453E-20	2.54617E-19	negative
PRJNA729271	stage_1D_vs_E10	ordinary_muscle_development	ENSGALG00010004673	RAB18	-0.049811832	0.084993145	-0.586068823	0.557829267	0.603471752	negative
PRJNA729271	stage_1D_vs_E10	ordinary_muscle_development	ENSGALG00010004689	BBX	0.281419742	0.08364266	3.364547978	0.000766692	0.00129515	positive
PRJNA729271	stage_1D_vs_E10	ordinary_muscle_development	ENSGALG00010004705	ENSGALG00010004705	3.694120508	0.226689776	16.29592906	1.05488E-59	2.87416E-58	positive
PRJNA729271	stage_1D_vs_E10	ordinary_muscle_development	ENSGALG00010004707	OTUD1	4.632242042	0.29625192	15.63615872	4.12895E-55	9.51497E-54	positive
PRJNA729271	stage_1D_vs_E10	ordinary_muscle_development	ENSGALG00010004715	WDR27	-0.94248782	0.345305606	-2.729430981	0.006344373	0.00962345	negative
PRJNA729271	stage_1D_vs_E10	ordinary_muscle_development	ENSGALG00010004716	ADH6	-2.165519068	1.112323083	-1.946843593	0.051553489	0.068619635	negative
PRJNA729271	stage_1D_vs_E10	ordinary_muscle_development	ENSGALG00010004717	ALKBH8	-0.231152127	0.175501096	-1.317097906	0.187805809	0.227156642	negative
PRJNA729271	stage_1D_vs_E10	ordinary_muscle_development	ENSGALG00010004721	LAMA2	3.453452947	0.15881276	21.74543755	7.6302E-105	5.9783E-103	positive
PRJNA729271	stage_1D_vs_E10	ordinary_muscle_development	ENSGALG00010004728	ENSGALG00010004728	-0.190756188	0.216413229	-0.881444213	0.378077436	0.427241946	negative
PRJNA729271	stage_1D_vs_E10	ordinary_muscle_development	ENSGALG00010004729	ENSGALG00010004729	5.739221016	1.112753121	5.157676852	2.50033E-07	5.84741E-07	positive
PRJNA729271	stage_1D_vs_E10	ordinary_muscle_development	ENSGALG00010004730	CLDN12	0.257261203	0.061240363	4.200843884	2.65922E-05	5.20743E-05	positive
PRJNA729271	stage_1D_vs_E10	ordinary_muscle_development	ENSGALG00010004735	ENSGALG00010004735	2.757511243	0.16445879	16.76718678	4.24119E-63	1.26494E-61	positive
PRJNA729271	stage_1D_vs_E10	ordinary_muscle_development	ENSGALG00010004736	CD47	0.152529349	0.101107785	1.508581649	0.131405727	0.163692507	positive
PRJNA729271	stage_1D_vs_E10	ordinary_muscle_development	ENSGALG00010004739	ENSGALG00010004739	-0.723720996	0.734400012	-0.985458857	0.324398731	0.372625375	negative
PRJNA729271	stage_1D_vs_E10	ordinary_muscle_development	ENSGALG00010004746	LYN	-0.620188252	0.142600545	-4.349129616	1.36679E-05	2.75429E-05	negative
PRJNA729271	stage_1D_vs_E10	ordinary_muscle_development	ENSGALG00010004751	PTER	0.373720954	0.125560973	2.976410148	0.002916445	0.004606503	positive
PRJNA729271	stage_1D_vs_E10	ordinary_muscle_development	ENSGALG00010004755	PAX9	-3.585998245	0.686861052	-5.220849595	1.78104E-07	4.21902E-07	negative
PRJNA729271	stage_1D_vs_E10	ordinary_muscle_development	ENSGALG00010004757	TRMT10A	-0.081805578	0.076837161	-1.064661651	0.28702907	0.333345595	negative
PRJNA729271	stage_1D_vs_E10	ordinary_muscle_development	ENSGALG00010004758	FLT3	2.055410279	0.850407858	2.416969996	0.015650307	0.022493922	positive
PRJNA729271	stage_1D_vs_E10	ordinary_muscle_development	ENSGALG00010004760	THBS2	-1.190866452	0.240405092	-4.953582484	7.28595E-07	1.64373E-06	negative
PRJNA729271	stage_1D_vs_E10	ordinary_muscle_development	ENSGALG00010004762	IFT57	-1.575333605	0.114073547	-13.80980642	2.22428E-43	3.28655E-42	negative
PRJNA729271	stage_1D_vs_E10	ordinary_muscle_development	ENSGALG00010004765	ENSGALG00010004765	2.126503118	0.2004394	10.60920715	2.70031E-26	1.95705E-25	positive
PRJNA729271	stage_1D_vs_E10	ordinary_muscle_development	ENSGALG00010004769	HTR2A	-0.442407725	0.36766402	-1.203293498	0.228862764	0.27195981	negative
PRJNA729271	stage_1D_vs_E10	ordinary_muscle_development	ENSGALG00010004777	CDK14	-0.877469948	0.1236647	-7.095557192	1.28831E-12	4.41573E-12	negative
PRJNA729271	stage_1D_vs_E10	ordinary_muscle_development	ENSGALG00010004778	ENSGALG00010004778	-2.201379451	0.466802308	-4.715870967	2.40679E-06	5.21315E-06	negative
PRJNA729271	stage_1D_vs_E10	ordinary_muscle_development	ENSGALG00010004780	ENSGALG00010004780	0.209169187	0.258446453	0.809332785	0.418323753	0.46765408	positive
PRJNA729271	stage_1D_vs_E10	ordinary_muscle_development	ENSGALG00010004781	ENSGALG00010004781	-0.833571689	0.081813744	-10.188651	2.22835E-24	1.47961E-23	negative
PRJNA729271	stage_1D_vs_E10	ordinary_muscle_development	ENSGALG00010004787	RIOK3	0.076844354	0.114881251	0.668902481	0.503557687	0.551664173	positive
PRJNA729271	stage_1D_vs_E10	ordinary_muscle_development	ENSGALG00010004791	FZD1	-0.20432004	0.118013569	-1.731326679	0.083393516	0.107419267	negative
PRJNA729271	stage_1D_vs_E10	ordinary_muscle_development	ENSGALG00010004798	PTPRK	-2.376818111	0.112199798	-21.18380027	1.34714E-99	9.91207E-98	negative
PRJNA729271	stage_1D_vs_E10	ordinary_muscle_development	ENSGALG00010004800	ZC3H12C	0.243306276	0.276406069	0.880249398	0.378724219	0.427706081	positive
PRJNA729271	stage_1D_vs_E10	ordinary_muscle_development	ENSGALG00010004803	RMC1	0.340602419	0.140906358	2.417225335	0.015639332	0.022482606	positive
PRJNA729271	stage_1D_vs_E10	ordinary_muscle_development	ENSGALG00010004808	THEMIS	-1.091106119	1.028500116	-1.060871168	0.288748454	0.335181296	negative
PRJNA729271	stage_1D_vs_E10	ordinary_muscle_development	ENSGALG00010004824	ATM	-0.011820128	0.111126843	-0.10636609	0.915291897	0.928162589	negative
PRJNA729271	stage_1D_vs_E10	ordinary_muscle_development	ENSGALG00010004832	C6orf58	4.830849297	1.849924977	2.61137579	0.009017874	0.013407948	positive
PRJNA729271	stage_1D_vs_E10	ordinary_muscle_development	ENSGALG00010004835	HHLA2	-0.971186021	0.410871871	-2.363719911	0.018092485	0.025738598	negative
PRJNA729271	stage_1D_vs_E10	ordinary_muscle_development	ENSGALG00010004837	CENPC	-1.537913542	0.135646314	-11.33767294	8.53848E-30	7.28459E-29	negative
PRJNA729271	stage_1D_vs_E10	ordinary_muscle_development	ENSGALG00010004841	AKAP9	0.398579065	0.194521854	2.049019462	0.040460212	0.054718303	positive
PRJNA729271	stage_1D_vs_E10	ordinary_muscle_development	ENSGALG00010004846	TTLL2	-0.714742757	0.374138706	-1.910368388	0.056085798	0.074189053	negative
PRJNA729271	stage_1D_vs_E10	ordinary_muscle_development	ENSGALG00010004851	MTCL3	-2.699104562	0.451121481	-5.983099176	2.18931E-09	6.03195E-09	negative
PRJNA729271	stage_1D_vs_E10	ordinary_muscle_development	ENSGALG00010004852	ENSGALG00010004852	3.859405003	0.22206952	17.37926487	1.18462E-67	4.01194E-66	positive
PRJNA729271	stage_1D_vs_E10	ordinary_muscle_development	ENSGALG00010004855	ZEB1	0.556320281	0.091689764	6.067419711	1.29982E-09	3.63793E-09	positive
PRJNA729271	stage_1D_vs_E10	ordinary_muscle_development	ENSGALG00010004859	NPC1	1.614482843	0.191187217	8.444512486	3.05317E-17	1.40227E-16	positive
PRJNA729271	stage_1D_vs_E10	ordinary_muscle_development	ENSGALG00010004866	SLC25A21	-0.710764426	0.303910924	-2.338726152	0.019349611	0.027427402	negative
PRJNA729271	stage_1D_vs_E10	ordinary_muscle_development	ENSGALG00010004868	NPAT	0.024736288	0.112954644	0.218993103	0.82665542	0.851262455	positive
PRJNA729271	stage_1D_vs_E10	ordinary_muscle_development	ENSGALG00010004871	C11orf87	0.334128556	0.31124683	1.073516334	0.283039514	0.329187014	positive
PRJNA729271	stage_1D_vs_E10	ordinary_muscle_development	ENSGALG00010004872	KIAA0408	-0.293903454	0.252954066	-1.161884681	0.24528231	0.28943073	negative
PRJNA729271	stage_1D_vs_E10	ordinary_muscle_development	ENSGALG00010004876	ENSGALG00010004876	-0.804790351	0.705108553	-1.141370853	0.253715629	0.298434475	negative
PRJNA729271	stage_1D_vs_E10	ordinary_muscle_development	ENSGALG00010004879	ENSGALG00010004879	-2.319675151	0.656685387	-3.532399529	0.000411807	0.00071581	negative
PRJNA729271	stage_1D_vs_E10	ordinary_muscle_development	ENSGALG00010004880	STAP1	-0.236117692	0.751202027	-0.314319828	0.75327814	0.784788805	negative
PRJNA729271	stage_1D_vs_E10	ordinary_muscle_development	ENSGALG00010004881	MIPOL1	-0.783096665	0.151399452	-5.172387699	2.31121E-07	5.42088E-07	negative
PRJNA729271	stage_1D_vs_E10	ordinary_muscle_development	ENSGALG00010004883	KRIT1	-0.117052594	0.073791653	-1.586257916	0.112680815	0.142026818	negative
PRJNA729271	stage_1D_vs_E10	ordinary_muscle_development	ENSGALG00010004887	MYH15	-8.217408469	0.747355473	-10.9953145	4.02508E-28	3.16912E-27	negative
PRJNA729271	stage_1D_vs_E10	ordinary_muscle_development	ENSGALG00010004890	ACAT1	0.175049537	0.098228777	1.782059628	0.074739508	0.097030826	positive
PRJNA729271	stage_1D_vs_E10	ordinary_muscle_development	ENSGALG00010004891	ANKIB1	-0.512705771	0.077415948	-6.622740957	3.52599E-11	1.10102E-10	negative
PRJNA729271	stage_1D_vs_E10	ordinary_muscle_development	ENSGALG00010004892	HEY2	-1.480487364	0.269193751	-5.499709255	3.80418E-08	9.47613E-08	negative
PRJNA729271	stage_1D_vs_E10	ordinary_muscle_development	ENSGALG00010004896	UBA6	-0.373827314	0.082528375	-4.529682208	5.90725E-06	1.23255E-05	negative
PRJNA729271	stage_1D_vs_E10	ordinary_muscle_development	ENSGALG00010004899	GATAD1	-0.16007068	0.106158122	-1.507851463	0.131592547	0.163868898	negative
PRJNA729271	stage_1D_vs_E10	ordinary_muscle_development	ENSGALG00010004901	CUL5	0.435948631	0.092544087	4.710712958	2.46852E-06	5.33888E-06	positive
PRJNA729271	stage_1D_vs_E10	ordinary_muscle_development	ENSGALG00010004902	ANKRD29	1.363032681	0.306457663	4.447703043	8.67934E-06	1.785E-05	positive
PRJNA729271	stage_1D_vs_E10	ordinary_muscle_development	ENSGALG00010004903	ENSGALG00010004903	2.134050133	0.483628628	4.412580253	1.02146E-05	2.08331E-05	positive
PRJNA729271	stage_1D_vs_E10	ordinary_muscle_development	ENSGALG00010004907	ENSGALG00010004907	-0.561336807	0.116248457	-4.828767823	1.3738E-06	3.03371E-06	negative
PRJNA729271	stage_1D_vs_E10	ordinary_muscle_development	ENSGALG00010004917	ENSGALG00010004917	-0.14081647	0.090427056	-1.557238239	0.119413933	0.149900836	negative
PRJNA729271	stage_1D_vs_E10	ordinary_muscle_development	ENSGALG00010004921	RRAGD	1.372883729	0.133275293	10.30111204	6.96514E-25	4.75105E-24	positive
PRJNA729271	stage_1D_vs_E10	ordinary_muscle_development	ENSGALG00010004924	ENSGALG00010004924	0.732587501	0.349566466	2.095703029	0.036108543	0.049228116	positive
PRJNA729271	stage_1D_vs_E10	ordinary_muscle_development	ENSGALG00010004927	KIF25	-2.402036568	0.244288325	-9.832793135	8.13312E-23	5.04007E-22	negative
PRJNA729271	stage_1D_vs_E10	ordinary_muscle_development	ENSGALG00010004928	HDDC2	-0.88245002	0.123210697	-7.162121817	7.94378E-13	2.76858E-12	negative
PRJNA729271	stage_1D_vs_E10	ordinary_muscle_development	ENSGALG00010004929	ENSGALG00010004929	1.258864521	0.159218973	7.906498203	2.6473E-15	1.08122E-14	positive
PRJNA729271	stage_1D_vs_E10	ordinary_muscle_development	ENSGALG00010004933	SPTLC2	-0.397321557	0.090585307	-4.386158969	1.1537E-05	2.34214E-05	negative
PRJNA729271	stage_1D_vs_E10	ordinary_muscle_development	ENSGALG00010004934	CIP2A	-2.075017708	0.162857385	-12.74131783	3.48459E-37	4.11983E-36	negative
PRJNA729271	stage_1D_vs_E10	ordinary_muscle_development	ENSGALG00010004935	SVIL	0.559527485	0.069042016	8.104159181	5.31115E-16	2.27767E-15	positive
PRJNA729271	stage_1D_vs_E10	ordinary_muscle_development	ENSGALG00010004939	DDX10	0.023039551	0.108272717	0.212791842	0.831489327	0.855450195	positive
PRJNA729271	stage_1D_vs_E10	ordinary_muscle_development	ENSGALG00010004942	GABRR1	-0.228453969	0.459574933	-0.497098411	0.619119655	0.661273259	negative
PRJNA729271	stage_1D_vs_E10	ordinary_muscle_development	ENSGALG00010004944	ALKBH1	0.225235133	0.096050606	2.344963156	0.01902896	0.026999293	positive
PRJNA729271	stage_1D_vs_E10	ordinary_muscle_development	ENSGALG00010004946	SH2D1B	0.999687621	0.469711018	2.128303538	0.033311925	0.045673132	positive
PRJNA729271	stage_1D_vs_E10	ordinary_muscle_development	ENSGALG00010004949	RAB39A	-0.423447436	0.325918795	-1.299242148	0.193860841	0.233758663	negative
PRJNA729271	stage_1D_vs_E10	ordinary_muscle_development	ENSGALG00010004950	SLIRP	-0.194061905	0.110827198	-1.751031415	0.079940499	0.103375638	negative
PRJNA729271	stage_1D_vs_E10	ordinary_muscle_development	ENSGALG00010004951	AFDN	-0.512925908	0.061958155	-8.278585881	1.24647E-16	5.52862E-16	negative
PRJNA729271	stage_1D_vs_E10	ordinary_muscle_development	ENSGALG00010004952	PEX1	0.587827698	0.121716268	4.829491646	1.36882E-06	3.02363E-06	positive
PRJNA729271	stage_1D_vs_E10	ordinary_muscle_development	ENSGALG00010004957	ENSGALG00010004957	0.33625234	0.138454626	2.428610362	0.015156811	0.021840937	positive
PRJNA729271	stage_1D_vs_E10	ordinary_muscle_development	ENSGALG00010004958	TPD52L1	-2.805599278	0.426894445	-6.572114742	4.96056E-11	1.53377E-10	negative
PRJNA729271	stage_1D_vs_E10	ordinary_muscle_development	ENSGALG00010004962	ENSGALG00010004962	-0.416299368	0.095004255	-4.381902367	1.17648E-05	2.38671E-05	negative
PRJNA729271	stage_1D_vs_E10	ordinary_muscle_development	ENSGALG00010004963	ENSGALG00010004963	0.957154057	0.231603535	4.132726449	3.58485E-05	6.9385E-05	positive
PRJNA729271	stage_1D_vs_E10	ordinary_muscle_development	ENSGALG00010004965	JCAD	0.407337116	0.100665141	4.046456523	5.19988E-05	9.9174E-05	positive
PRJNA729271	stage_1D_vs_E10	ordinary_muscle_development	ENSGALG00010004966	LAMA3	1.643996814	0.210842074	7.797290071	6.32507E-15	2.52012E-14	positive
PRJNA729271	stage_1D_vs_E10	ordinary_muscle_development	ENSGALG00010004967	SLC35F2	-2.186802085	0.29068491	-7.522929512	5.35623E-14	2.01711E-13	negative
PRJNA729271	stage_1D_vs_E10	ordinary_muscle_development	ENSGALG00010004968	TRAT1	1.339533287	1.239544825	1.080665467	0.27984595	0.325995905	positive
PRJNA729271	stage_1D_vs_E10	ordinary_muscle_development	ENSGALG00010004969	YTHDC1	0.446483575	0.077069885	5.793230092	6.90454E-09	1.82997E-08	positive
PRJNA729271	stage_1D_vs_E10	ordinary_muscle_development	ENSGALG00010004970	RBM48	0.438375723	0.16608247	2.639506285	0.008302688	0.012405677	positive
PRJNA729271	stage_1D_vs_E10	ordinary_muscle_development	ENSGALG00010004973	SLN	9.199913298	0.194945506	47.19223081	0	0	positive
PRJNA729271	stage_1D_vs_E10	ordinary_muscle_development	ENSGALG00010004974	MTPAP	0.078522284	0.060986633	1.287532682	0.197908686	0.237867861	positive
PRJNA729271	stage_1D_vs_E10	ordinary_muscle_development	ENSGALG00010004975	SNW1	0.1182781	0.068946179	1.715513483	0.086251125	0.110891933	positive
PRJNA729271	stage_1D_vs_E10	ordinary_muscle_development	ENSGALG00010004976	ENSGALG00010004976	2.614292921	0.225081261	11.61488484	3.46255E-31	3.16055E-30	positive
PRJNA729271	stage_1D_vs_E10	ordinary_muscle_development	ENSGALG00010004977	RNF217	0.444684061	0.132977272	3.34406065	0.000825617	0.001389609	positive
PRJNA729271	stage_1D_vs_E10	ordinary_muscle_development	ENSGALG00010004980	FNDC1	0.772839038	0.37744857	2.047534685	0.040605615	0.054894459	positive
PRJNA729271	stage_1D_vs_E10	ordinary_muscle_development	ENSGALG00010004981	ENSGALG00010004981	0.397265858	0.198731951	1.999003461	0.045607979	0.061166628	positive
PRJNA729271	stage_1D_vs_E10	ordinary_muscle_development	ENSGALG00010004983	CLXN	-1.687227396	0.276727265	-6.097076833	1.08026E-09	3.044E-09	negative
PRJNA729271	stage_1D_vs_E10	ordinary_muscle_development	ENSGALG00010004987	MAP3K8	4.014143371	0.256492542	15.6501368	3.31506E-55	7.68829E-54	positive
PRJNA729271	stage_1D_vs_E10	ordinary_muscle_development	ENSGALG00010004988	HJURP	-1.24350697	0.216595561	-5.741147098	9.40374E-09	2.45909E-08	negative
PRJNA729271	stage_1D_vs_E10	ordinary_muscle_development	ENSGALG00010004990	ENSGALG00010004990	-1.586016856	2.51362712	-0.630967435	0.528061817	0.574989186	negative
PRJNA729271	stage_1D_vs_E10	ordinary_muscle_development	ENSGALG00010004991	FAM133B	-0.508651254	0.063284718	-8.037505217	9.1686E-16	3.86222E-15	negative
PRJNA729271	stage_1D_vs_E10	ordinary_muscle_development	ENSGALG00010004993	SULT1B	3.136533596	0.239672134	13.08676795	3.91933E-39	4.98777E-38	positive
PRJNA729271	stage_1D_vs_E10	ordinary_muscle_development	ENSGALG00010004995	PIT54	2.990348552	2.303484535	1.298184774	0.194223849	0.234157422	positive
PRJNA729271	stage_1D_vs_E10	ordinary_muscle_development	ENSGALG00010004996	GPR158	0.15179509	0.214235295	0.708543802	0.478607621	0.52723951	positive
PRJNA729271	stage_1D_vs_E10	ordinary_muscle_development	ENSGALG00010004999	ADCK1	0.016113187	0.184872966	0.087158156	0.930545799	0.940932079	positive
PRJNA729271	stage_1D_vs_E10	ordinary_muscle_development	ENSGALG00010005003	EXPH5	-1.074803761	0.450279841	-2.38696842	0.01698795	0.024291244	negative
PRJNA729271	stage_1D_vs_E10	ordinary_muscle_development	ENSGALG00010005004	CDK6	0.398832384	0.095277729	4.185997994	2.83916E-05	5.5493E-05	positive
PRJNA729271	stage_1D_vs_E10	ordinary_muscle_development	ENSGALG00010005010	NKAIN2	-3.206818584	0.332359198	-9.648653024	4.98056E-22	2.98566E-21	negative
PRJNA729271	stage_1D_vs_E10	ordinary_muscle_development	ENSGALG00010005012	ENSGALG00010005012	5.866603999	1.064064122	5.513393297	3.5198E-08	8.78587E-08	positive
PRJNA729271	stage_1D_vs_E10	ordinary_muscle_development	ENSGALG00010005014	SULT1B1	0.837925584	0.151823108	5.519091248	3.40757E-08	8.51599E-08	positive
PRJNA729271	stage_1D_vs_E10	ordinary_muscle_development	ENSGALG00010005015	NRXN3	-0.880060083	0.391340097	-2.248836981	0.02452287	0.034281261	negative
PRJNA729271	stage_1D_vs_E10	ordinary_muscle_development	ENSGALG00010005016	STX11	-3.68935E-05	0.172900227	-0.00021338	0.999829747	0.999829747	negative
PRJNA729271	stage_1D_vs_E10	ordinary_muscle_development	ENSGALG00010005017	ENSGALG00010005017	1.79282989	0.169594566	10.57126967	4.04974E-26	2.91176E-25	positive
PRJNA729271	stage_1D_vs_E10	ordinary_muscle_development	ENSGALG00010005023	POGLUT3	-0.211798177	0.180665288	-1.172323578	0.241067169	0.284874336	negative
PRJNA729271	stage_1D_vs_E10	ordinary_muscle_development	ENSGALG00010005025	THOC1	-0.94299206	0.058179192	-16.20840775	4.39816E-59	1.16761E-57	negative
PRJNA729271	stage_1D_vs_E10	ordinary_muscle_development	ENSGALG00010005029	FAT4	-0.694616034	0.197359043	-3.519555143	0.000432271	0.000748778	negative
PRJNA729271	stage_1D_vs_E10	ordinary_muscle_development	ENSGALG00010005033	USP14	-0.664440113	0.135534041	-4.902385454	9.46798E-07	2.11984E-06	negative

PRJNA729271	stage_1D_vs_E10	ordinary_muscle_development	ENSGALG00010005035	NECTIN3	0.332063435	0.097173737	3.417213785	0.000632656	0.001076332	positive
PRJNA729271	stage_1D_vs_E10	ordinary_muscle_development	ENSGALG00010005037	THNSL1	0.297019258	0.141609612	2.097451247	0.035953649	0.049030778	positive
PRJNA729271	stage_1D_vs_E10	ordinary_muscle_development	ENSGALG00010005042	SLC46A3	1.741864964	0.200515633	8.686928499	3.7237E-18	1.80037E-17	positive
PRJNA729271	stage_1D_vs_E10	ordinary_muscle_development	ENSGALG00010005045	UTRN	0.334962194	0.099293307	3.373461972	0.000742293	0.001256075	positive
PRJNA729271	stage_1D_vs_E10	ordinary_muscle_development	ENSGALG00010005048	ROCK1	0.154006154	0.114302432	1.347356761	0.177865359	0.216125273	positive
PRJNA729271	stage_1D_vs_E10	ordinary_muscle_development	ENSGALG00010005057	FLVCR2	-1.045665113	0.288704217	-3.62192532	0.000292419	0.000516022	negative
PRJNA729271	stage_1D_vs_E10	ordinary_muscle_development	ENSGALG00010005060	POMP	-0.425538571	0.138535092	-3.07170236	0.002128418	0.003412391	negative
PRJNA729271	stage_1D_vs_E10	ordinary_muscle_development	ENSGALG00010005065	ENKUR	-1.362853856	0.436217042	-3.124256331	0.001782551	0.002889196	negative
PRJNA729271	stage_1D_vs_E10	ordinary_muscle_development	ENSGALG00010005068	PLCXD2	2.109528639	0.258711584	8.153978289	3.52144E-16	1.52596E-15	positive
PRJNA729271	stage_1D_vs_E10	ordinary_muscle_development	ENSGALG00010005070	ALB	0.266580275	1.415293672	0.188356862	0.850596914	0.872148916	positive
PRJNA729271	stage_1D_vs_E10	ordinary_muscle_development	ENSGALG00010005075	TRDN	6.016492648	0.16704818	36.01651131	4.614E-284	6.0799E-281	positive
PRJNA729271	stage_1D_vs_E10	ordinary_muscle_development	ENSGALG00010005077	ERG28	-0.661017255	0.103793001	-6.368611087	1.90748E-10	5.66575E-10	negative
PRJNA729271	stage_1D_vs_E10	ordinary_muscle_development	ENSGALG00010005081	GREB1L	0.564298088	0.179233613	3.148394315	0.001641701	0.002671357	positive
PRJNA729271	stage_1D_vs_E10	ordinary_muscle_development	ENSGALG00010005082	ENSGALG00010005082	-0.647836974	0.086359545	-7.501625589	6.30312E-14	2.36021E-13	negative
PRJNA729271	stage_1D_vs_E10	ordinary_muscle_development	ENSGALG00010005087	ENPP1	1.727292117	0.214083676	8.068303722	7.12815E-16	3.02467E-15	positive
PRJNA729271	stage_1D_vs_E10	ordinary_muscle_development	ENSGALG00010005092	ESCO1	1.186422071	0.14027536	8.457808048	2.7245E-17	1.2557E-16	positive
PRJNA729271	stage_1D_vs_E10	ordinary_muscle_development	ENSGALG00010005104	ENPP3	-0.744139785	0.200715382	-3.707437751	0.000209367	0.000375266	negative
PRJNA729271	stage_1D_vs_E10	ordinary_muscle_development	ENSGALG00010005107	TTLL5	-0.887746549	0.074324001	-11.94427819	6.95515E-33	6.89569E-32	negative
PRJNA729271	stage_1D_vs_E10	ordinary_muscle_development	ENSGALG00010005109	ABHD3	1.430140163	0.359714803	3.975761218	7.01545E-05	0.000132149	positive
PRJNA729271	stage_1D_vs_E10	ordinary_muscle_development	ENSGALG00010005111	RASSF6	-0.505894112	0.403311271	-1.254351535	0.20971426	0.251016366	negative
PRJNA729271	stage_1D_vs_E10	ordinary_muscle_development	ENSGALG00010005114	PHLDB2	-0.606543738	0.099890893	-6.072062447	1.26278E-09	3.53837E-09	negative
PRJNA729271	stage_1D_vs_E10	ordinary_muscle_development	ENSGALG00010005118	PRTFDC1	-1.131973318	0.138012641	-8.201953913	2.36511E-16	1.03384E-15	negative
PRJNA729271	stage_1D_vs_E10	ordinary_muscle_development	ENSGALG00010005119	MIB1	-1.217869859	0.056584829	-21.52290447	9.502E-103	7.2111E-101	negative
PRJNA729271	stage_1D_vs_E10	ordinary_muscle_development	ENSGALG00010005122	IL8L1	-1.689331432	1.206984276	-1.399630025	0.161624138	0.198050713	negative
PRJNA729271	stage_1D_vs_E10	ordinary_muscle_development	ENSGALG00010005123	MTIF3	0.602316434	0.105976842	5.683472168	1.31987E-08	3.41087E-08	positive
PRJNA729271	stage_1D_vs_E10	ordinary_muscle_development	ENSGALG00010005126	MED23	-0.715507321	0.058153259	-12.30382149	8.63881E-35	9.28929E-34	negative
PRJNA729271	stage_1D_vs_E10	ordinary_muscle_development	ENSGALG00010005127	GATA6	-1.007386147	0.313437028	-3.213998523	0.001309003	0.002155647	negative
PRJNA729271	stage_1D_vs_E10	ordinary_muscle_development	ENSGALG00010005131	IL8L2	1.527536394	0.690577183	2.211970554	0.0269687	0.03741136	positive
PRJNA729271	stage_1D_vs_E10	ordinary_muscle_development	ENSGALG00010005132	LNX2	-0.289908824	0.094919842	-3.05424892	0.002256248	0.003606961	negative
PRJNA729271	stage_1D_vs_E10	ordinary_muscle_development	ENSGALG00010005138	ENSGALG00010005138	1.418739012	0.552516217	2.567778047	0.010235267	0.015106424	positive
PRJNA729271	stage_1D_vs_E10	ordinary_muscle_development	ENSGALG00010005140	DCT	-0.316607881	0.896915857	-0.352996191	0.724091296	0.758688979	negative
PRJNA729271	stage_1D_vs_E10	ordinary_muscle_development	ENSGALG00010005141	CCDC170	-1.872475435	0.324651903	-5.767640404	8.03891E-09	2.11977E-08	negative
PRJNA729271	stage_1D_vs_E10	ordinary_muscle_development	ENSGALG00010005143	GTF3A	-0.70701835	0.136569415	-5.176988945	2.25496E-07	5.2915E-07	negative
PRJNA729271	stage_1D_vs_E10	ordinary_muscle_development	ENSGALG00010005145	AKAP7	2.421469815	0.174415744	13.88332128	7.99517E-44	1.19969E-42	positive
PRJNA729271	stage_1D_vs_E10	ordinary_muscle_development	ENSGALG00010005146	TGFB3	-0.829770425	0.204819599	-4.051225702	5.095E-05	9.7289E-05	negative
PRJNA729271	stage_1D_vs_E10	ordinary_muscle_development	ENSGALG00010005148	TTC39C	-0.182999578	0.082246893	-2.225002928	0.026081042	0.036273719	negative
PRJNA729271	stage_1D_vs_E10	ordinary_muscle_development	ENSGALG00010005150	ALCAM	-1.63171759	0.186971517	-8.727091797	2.61305E-18	1.27272E-17	negative
PRJNA729271	stage_1D_vs_E10	ordinary_muscle_development	ENSGALG00010005154	CWF19L2	-0.231366908	0.148355457	-1.559544303	0.118867607	0.149266739	negative
PRJNA729271	stage_1D_vs_E10	ordinary_muscle_development	ENSGALG00010005157	IMPA1	-0.619254956	0.109754876	-5.642163491	1.67927E-08	4.31042E-08	negative
PRJNA729271	stage_1D_vs_E10	ordinary_muscle_development	ENSGALG00010005158	IFT43	-1.355334912	0.200596642	-6.756518445	1.41347E-11	4.53681E-11	negative
PRJNA729271	stage_1D_vs_E10	ordinary_muscle_development	ENSGALG00010005159	ARHGAP21	0.572800485	0.098057334	5.841485405	5.17374E-09	1.38364E-08	positive
PRJNA729271	stage_1D_vs_E10	ordinary_muscle_development	ENSGALG00010005163	GPC6	-2.297812976	0.375698009	-6.116116992	9.58829E-10	2.71503E-09	negative
PRJNA729271	stage_1D_vs_E10	ordinary_muscle_development	ENSGALG00010005165	METTL21C	4.040816284	0.560999024	7.202893614	5.8948E-13	2.07441E-12	positive
PRJNA729271	stage_1D_vs_E10	ordinary_muscle_development	ENSGALG00010005166	CBLB	0.145021506	0.123388013	1.175328967	0.23986313	0.283659628	positive
PRJNA729271	stage_1D_vs_E10	ordinary_muscle_development	ENSGALG00010005167	GUCY1A2	0.535465253	0.177511233	3.016514756	0.002556988	0.004060423	positive
PRJNA729271	stage_1D_vs_E10	ordinary_muscle_development	ENSGALG00010005170	GPATCH2L	0.338865897	0.058851262	5.758005594	8.51135E-09	2.23581E-08	positive
PRJNA729271	stage_1D_vs_E10	ordinary_muscle_development	ENSGALG00010005172	OSBPL1A	1.754671194	0.079755027	22.00075987	2.8319E-107	2.3323E-105	positive
PRJNA729271	stage_1D_vs_E10	ordinary_muscle_development	ENSGALG00010005174	TPP2	0.437851964	0.185114542	2.365302903	0.01801533	0.025636384	positive
PRJNA729271	stage_1D_vs_E10	ordinary_muscle_development	ENSGALG00010005176	BAMBI	-3.707091703	0.315187412	-11.76154746	6.15949E-32	5.86223E-31	negative
PRJNA729271	stage_1D_vs_E10	ordinary_muscle_development	ENSGALG00010005177	AASDHPPT	0.421280062	0.127143422	3.313423978	0.000921612	0.001543473	positive
PRJNA729271	stage_1D_vs_E10	ordinary_muscle_development	ENSGALG00010005178	EPHA5	-0.29948731	0.198970026	-1.505188072	0.132275731	0.164646383	negative
PRJNA729271	stage_1D_vs_E10	ordinary_muscle_development	ENSGALG00010005180	WAC	0.537581789	0.115500922	4.654350618	3.25003E-06	6.94827E-06	positive
PRJNA729271	stage_1D_vs_E10	ordinary_muscle_development	ENSGALG00010005181	IMPACT	0.548579083	0.132381159	4.14393626	3.41395E-05	6.62186E-05	positive
PRJNA729271	stage_1D_vs_E10	ordinary_muscle_development	ENSGALG00010005182	NEPRO	-0.427632876	0.085318378	-5.012201177	5.38109E-07	1.22717E-06	negative
PRJNA729271	stage_1D_vs_E10	ordinary_muscle_development	ENSGALG00010005184	FGF14	-3.284714588	0.330674671	-9.933372208	2.98003E-23	1.87644E-22	negative
PRJNA729271	stage_1D_vs_E10	ordinary_muscle_development	ENSGALG00010005186	ESR1	0.426264015	0.191203785	2.229370174	0.025789285	0.035898942	positive
PRJNA729271	stage_1D_vs_E10	ordinary_muscle_development	ENSGALG00010005187	KBTBD3	1.57145595	0.248567073	6.322060001	2.58099E-10	7.6055E-10	positive
PRJNA729271	stage_1D_vs_E10	ordinary_muscle_development	ENSGALG00010005190	ENSGALG00010005190	1.635407218	0.283107287	5.776634137	7.62098E-09	2.0125E-08	positive
PRJNA729271	stage_1D_vs_E10	ordinary_muscle_development	ENSGALG00010005191	MSANTD4	-0.645602257	0.105494421	-6.119776275	9.37068E-10	2.65652E-09	negative
PRJNA729271	stage_1D_vs_E10	ordinary_muscle_development	ENSGALG00010005193	EPB41L2	0.821962973	0.098245785	8.36639425	5.94082E-17	2.68429E-16	positive
PRJNA729271	stage_1D_vs_E10	ordinary_muscle_development	ENSGALG00010005194	ENSGALG00010005194	1.89541044	1.113224553	1.702630826	0.088637178	0.113708814	positive
PRJNA729271	stage_1D_vs_E10	ordinary_muscle_development	ENSGALG00010005198	TRPC3	-1.231830033	0.507930385	-2.425194612	0.015300182	0.022032202	negative
PRJNA729271	stage_1D_vs_E10	ordinary_muscle_development	ENSGALG00010005203	ENSGALG00010005203	0.621806443	0.105438024	5.897364346	3.69354E-09	1.00015E-08	positive
PRJNA729271	stage_1D_vs_E10	ordinary_muscle_development	ENSGALG00010005204	ITGBL1	1.090457466	0.310309121	3.514100596	0.000441246	0.000763593	positive
PRJNA729271	stage_1D_vs_E10	ordinary_muscle_development	ENSGALG00010005208	ENSGALG00010005208	-0.358843656	0.110515634	-3.246994495	0.001166306	0.001930967	negative
PRJNA729271	stage_1D_vs_E10	ordinary_muscle_development	ENSGALG00010005211	ESRRB	3.388080702	0.331516227	10.21995435	1.61417E-24	1.07971E-23	positive
PRJNA729271	stage_1D_vs_E10	ordinary_muscle_development	ENSGALG00010005212	TAF13	-0.292526119	0.102277997	-2.860108026	0.004234967	0.006567439	negative
PRJNA729271	stage_1D_vs_E10	ordinary_muscle_development	ENSGALG00010005213	ENSGALG00010005213	-0.071026591	0.160480528	-0.44258697	0.658064511	0.697269378	negative
PRJNA729271	stage_1D_vs_E10	ordinary_muscle_development	ENSGALG00010005214	BBS7	-0.519863104	0.118802935	-4.375843958	1.20964E-05	2.45089E-05	negative
PRJNA729271	stage_1D_vs_E10	ordinary_muscle_development	ENSGALG00010005215	VASH1	-1.325431493	0.142326158	-9.312634508	1.247E-20	6.9016E-20	negative
PRJNA729271	stage_1D_vs_E10	ordinary_muscle_development	ENSGALG00010005216	ENSGALG00010005216	-0.246249382	0.164859484	-1.493692547	0.135255998	0.16796913	negative
PRJNA729271	stage_1D_vs_E10	ordinary_muscle_development	ENSGALG00010005217	CCNA2	-1.610265191	0.1440217	-11.18071231	5.06815E-29	4.19309E-28	negative
PRJNA729271	stage_1D_vs_E10	ordinary_muscle_development	ENSGALG00010005218	GTPBP8	0.278917128	0.117290591	2.378000866	0.017406785	0.024841129	positive
PRJNA729271	stage_1D_vs_E10	ordinary_muscle_development	ENSGALG00010005219	NALCN	0.397550075	0.181246041	2.193427636	0.028276583	0.039105913	positive
PRJNA729271	stage_1D_vs_E10	ordinary_muscle_development	ENSGALG00010005220	ANGEL1	0.196371674	0.08553078	2.295918187	0.02168056	0.030540302	positive
PRJNA729271	stage_1D_vs_E10	ordinary_muscle_development	ENSGALG00010005221	ENSGALG00010005221	1.207453479	0.300680621	4.01573428	5.9261E-05	0.000112507	positive
PRJNA729271	stage_1D_vs_E10	ordinary_muscle_development	ENSGALG00010005222	ABCB1	2.954811873	1.011405217	2.921491626	0.003483596	0.00545712	positive
PRJNA729271	stage_1D_vs_E10	ordinary_muscle_development	ENSGALG00010005223	GRIA4	-1.989659	0.429514142	-4.632348055	3.61542E-06	7.6965E-06	negative
PRJNA729271	stage_1D_vs_E10	ordinary_muscle_development	ENSGALG00010005224	ENSGALG00010005224	1.53524156	0.392996565	3.906501218	9.36422E-05	0.000174264	positive
PRJNA729271	stage_1D_vs_E10	ordinary_muscle_development	ENSGALG00010005226	MYCT1	1.611954426	0.165414419	9.744945066	1.93884E-22	1.18131E-21	positive
PRJNA729271	stage_1D_vs_E10	ordinary_muscle_development	ENSGALG00010005227	EXOSC9	-0.10122892	0.103351525	-0.979462273	0.327351621	0.375600549	negative
PRJNA729271	stage_1D_vs_E10	ordinary_muscle_development	ENSGALG00010005228	ZNF521	-1.406612668	0.131962405	-10.65919239	1.57963E-26	1.15933E-25	negative
PRJNA729271	stage_1D_vs_E10	ordinary_muscle_development	ENSGALG00010005229	CIPC	-0.363940415	0.073422353	-4.956806722	7.16612E-07	1.61821E-06	negative
PRJNA729271	stage_1D_vs_E10	ordinary_muscle_development	ENSGALG00010005231	ENSGALG00010005231	-0.04836591	0.441794187	-0.109476113	0.912824867	0.925919975	negative
PRJNA729271	stage_1D_vs_E10	ordinary_muscle_development	ENSGALG00010005232	GATA3	-3.977796949	0.525214767	-7.573657862	3.62859E-14	1.3823E-13	negative
PRJNA729271	stage_1D_vs_E10	ordinary_muscle_development	ENSGALG00010005236	TMTC4	-1.245370721	0.090918892	-13.69760113	1.04934E-42	1.51045E-41	negative
PRJNA729271	stage_1D_vs_E10	ordinary_muscle_development	ENSGALG00010005237	RAP2A	-2.087549516	0.111878176	-18.65913084	1.06449E-77	4.76226E-76	negative
PRJNA729271	stage_1D_vs_E10	ordinary_muscle_development	ENSGALG00010005238	TMEM63C	-0.774144266	0.167860277	-4.611837182	3.99126E-06	8.4519E-06	negative
PRJNA729271	stage_1D_vs_E10	ordinary_muscle_development	ENSGALG00010005239	ENSGALG00010005239	-1.681195522	0.185371662	-9.069323233	1.19759E-19	6.29177E-19	negative
PRJNA729271	stage_1D_vs_E10	ordinary_muscle_development	ENSGALG00010005240	VIP	-4.205013608	0.406309662	-10.34928282	4.21632E-25	2.90781E-24	negative
PRJNA729271	stage_1D_vs_E10	ordinary_muscle_development	ENSGALG00010005241	C4orf54	3.695902361	0.187416347	19.72027747	1.44434E-86	7.99073E-85	positive
PRJNA729271	stage_1D_vs_E10	ordinary_muscle_development	ENSGALG00010005242	CCDC80	0.45134882	0.233890758	1.929742006	0.053638812	0.071147016	positive
PRJNA729271	stage_1D_vs_E10	ordinary_muscle_development	ENSGALG00010005243	NGB	-2.531937641	0.517305525	-4.894472451	9.85698E-07	2.20319E-06	negative
PRJNA729271	stage_1D_vs_E10	ordinary_muscle_development	ENSGALG00010005244	FBXO5	-0.509867781	0.090445413	-5.637298422	1.72739E-08	4.4261E-08	negative
PRJNA729271	stage_1D_vs_E10	ordinary_muscle_development	ENSGALG00010005245	KIAA1217	1.572467749	0.13490781	11.65586892	2.14184E-31	1.97871E-30	positive
PRJNA729271	stage_1D_vs_E10	ordinary_muscle_development	ENSGALG00010005246	ENSGALG00010005246	-1.581785816	0.198511737	-7.96822313	1.60972E-15	6.67417E-15	negative
PRJNA729271	stage_1D_vs_E10	ordinary_muscle_development	ENSGALG00010005247	THAP7	0.120761072	0.118091875	1.022602712	0.306495727	0.353686455	positive
PRJNA729271	stage_1D_vs_E10	ordinary_muscle_development	ENSGALG00010005248	TMEM200A	-1.744515436	0.240884739	-7.242116876	4.41735E-13	1.57128E-12	negative
PRJNA729271	stage_1D_vs_E10	ordinary_muscle_development	ENSGALG00010005249	PDGFD	-0.171324741	0.228819793	-0.748732173	0.454018648	0.502942323	negative
PRJNA729271	stage_1D_vs_E10	ordinary_muscle_development	ENSGALG00010005250	SLC35A5	-0.640422594	0.090715528	-7.059679958	1.66887E-12	5.68245E-12	negative
PRJNA729271	stage_1D_vs_E10	ordinary_muscle_development	ENSGALG00010005251	MTRF1L	0.246481874	0.101545717	2.427299558	0.01521169	0.021911303	positive
PRJNA729271	stage_1D_vs_E10	ordinary_muscle_development	ENSGALG00010005252	KLHL14	-2.598535268	0.223538484	-11.62455437	3.09203E-31	2.83126E-30	negative
PRJNA729271	stage_1D_vs_E10	ordinary_muscle_development	ENSGALG00010005253	POMT2	-0.634567048	0.096261121	-6.592142699	4.33524E-11	1.34646E-10	negative
PRJNA729271	stage_1D_vs_E10	ordinary_muscle_development	ENSGALG00010005254	ENSGALG00010005254	-1.265388286	0.755043535	-1.675914338	0.093754973	0.119786543	negative
PRJNA729271	stage_1D_vs_E10	ordinary_muscle_development	ENSGALG00010005255	BZW2	-0.268050902	0.093125108	-2.878395619	0.003997035	0.00621242	negative
PRJNA729271	stage_1D_vs_E10	ordinary_muscle_development	ENSGALG00010005256	OPRM1	-6.017654364	0.686210852	-8.769395509	1.79628E-18	8.83511E-18	negative
PRJNA729271	stage_1D_vs_E10	ordinary_muscle_development	ENSGALG00010005258	ITIH5	3.01707537	0.278808607	10.8213136	2.72839E-27	2.06733E-26	positive
PRJNA729271	stage_1D_vs_E10	ordinary_muscle_development	ENSGALG00010005259	SLC27A5	-0.283380256	0.718968343	-0.394148448	0.693471444	0.730832382	negative
PRJNA729271	stage_1D_vs_E10	ordinary_muscle_development	ENSGALG00010005260	ANXA5	-0.183913698	0.149963957	-1.226385999	0.220053452	0.262438275	negative
PRJNA729271	stage_1D_vs_E10	ordinary_muscle_development	ENSGALG00010005261	ATG3	-0.615266303	0.110585041	-5.563738965	2.64055E-08	6.65764E-08	negative
PRJNA729271	stage_1D_vs_E10	ordinary_muscle_development	ENSGALG00010005262	ENSGALG00010005262	-0.387355819	0.124879243	-3.101843114	0.001923199	0.003101553	negative
PRJNA729271	stage_1D_vs_E10	ordinary_muscle_development	ENSGALG00010005263	ENSGALG00010005263	0.497186414	0.342070077	1.453463622	0.146095073	0.18054805	positive
PRJNA729271	stage_1D_vs_E10	ordinary_muscle_development	ENSGALG00010005264	ITIH2	1.199125934	1.056609669	1.134880713			positive
PRJNA729271	stage_1D_vs_E10	ordinary_muscle_development	ENSGALG00010005265	DYNC2H1	-0.705405099	0.094727679	-7.446662942	9.57308E-14	3.54617E-13	negative
PRJNA729271	stage_1D_vs_E10	ordinary_muscle_development	ENSGALG00010005266	SLC19A2	1.253642666	0.262533524	4.775171752	1.79554E-06	3.92318E-06	positive
PRJNA729271	stage_1D_vs_E10	ordinary_muscle_development	ENSGALG00010005267	CYP51A1	-1.906711882	0.18386287	-10.37029328	3.38484E-25	2.34978E-24	negative
PRJNA729271	stage_1D_vs_E10	ordinary_muscle_development	ENSGALG00010005268	MTTP	2.087140476	0.911939873	2.288682112	0.022097829	0.031103907	positive
PRJNA729271	stage_1D_vs_E10	ordinary_muscle_development	ENSGALG00010005269	KIN	0.494322254	0.104453173	4.732477124	2.21796E-06	4.81855E-06	positive
PRJNA729271	stage_1D_vs_E10	ordinary_muscle_development	ENSGALG00010005270	GSTZ1	-1.008580479	0.218602002	-4.613775128	3.9542E-06	8.37711E-06	negative
PRJNA729271	stage_1D_vs_E10	ordinary_muscle_development	ENSGALG00010005272	MBNL2	1.238162797	0.093049605	13.30648095	2.12245E-40	2.81731E-39	positive
PRJNA729271	stage_1D_vs_E10	ordinary_muscle_development	ENSGALG00010005273	ENSGALG00010005273	2.115517889	0.648879747	3.260261856	0.001113094	0.001847086	positive
PRJNA729271	stage_1D_vs_E10	ordinary_muscle_development	ENSGALG00010005275	L3MBTL3	1.038708287	0.171010063	6.073960015	1.24794E-09	3.49814E-09	positive
PRJNA729271	stage_1D_vs_E10	ordinary_muscle_development	ENSGALG00010005277	ENSGALG00010005277	0.650350168	0.206522189	3.149057111	0.001637982	0.002665605	positive
PRJNA729271	stage_1D_vs_E10	ordinary_muscle_development	ENSGALG00010005278	DAPP1	-0.464341577	0.131708258	-3.525531229	0.000422634	0.000733136	negative
PRJNA729271	stage_1D_vs_E10	ordinary_muscle_development	ENSGALG00010005279	CCDC181	-0.210842297	0.327729923	-0.643341613	0.52000246	0.567321666	negative
PRJNA729271	stage_1D_vs_E10	ordinary_muscle_development	ENSGALG00010005280	TMEM244	-4.787433904	0.940001968	-5.093004127	3.52434E-07	8.14109E-07	negative
PRJNA729271	stage_1D_vs_E10	ordinary_muscle_development	ENSGALG00010005281	TMED8	-1.108548052	0.068686271	-16.13929592	1.35073E-58	3.51506E-57	negative
PRJNA729271	stage_1D_vs_E10	ordinary_muscle_development	ENSGALG00010005282	FLT1	1.843894735	0.1249201	14.76059281	2.62959E-49	4.9373E-48	positive
PRJNA729271	stage_1D_vs_E10	ordinary_muscle_development	ENSGALG00010005283	ATP5C1	1.22501387	0.132047824	9.277046986	1.74241E-20	9.57399E-20	positive
PRJNA729271	stage_1D_vs_E10	ordinary_muscle_development	ENSGALG00010005284	ENSGALG00010005284	0.968527837	0.208604136	4.642898531	3.43555E-06	7.32759E-06	positive
PRJNA729271	stage_1D_vs_E10	ordinary_muscle_development	ENSGALG00010005285	MPP7	1.135387177	0.126964911	8.942527221	3.8037E-19	1.93659E-18	positive

PRJNA729271	stage_1D_vs_E10	ordinary_muscle_development	ENSGALG00010005286	ENSGALG00010005286	-0.208193832	0.66796998	-0.311681419	0.75528265	0.786651003	negative
PRJNA729271	stage_1D_vs_E10	ordinary_muscle_development	ENSGALG00010005287	ENSGALG00010005287	-0.466761548	0.391025924	-1.193684407	0.232601442	0.275950066	negative
PRJNA729271	stage_1D_vs_E10	ordinary_muscle_development	ENSGALG00010005288	ENSGALG00010005288	-0.507738727	0.094936593	-5.348187792	8.88393E-08	2.15339E-07	negative
PRJNA729271	stage_1D_vs_E10	ordinary_muscle_development	ENSGALG00010005291	IFNGR1	0.322794626	0.095963608	3.363719157	0.000768998	0.001298837	positive
PRJNA729271	stage_1D_vs_E10	ordinary_muscle_development	ENSGALG00010005292	TAF3	0.013912987	0.098566449	0.14115337	0.887748783	0.904217455	positive
PRJNA729271	stage_1D_vs_E10	ordinary_muscle_development	ENSGALG00010005293	BLZF1	-0.423421447	0.097252474	-4.353837281	1.33775E-05	2.70141E-05	negative
PRJNA729271	stage_1D_vs_E10	ordinary_muscle_development	ENSGALG00010005295	ENSGALG00010005295	-1.136231088	0.566831887	-2.004529232	0.045013399	0.060452999	negative
PRJNA729271	stage_1D_vs_E10	ordinary_muscle_development	ENSGALG00010005296	PAN3	1.071366773	0.100636938	10.64586018	1.82293E-26	1.33317E-25	positive
PRJNA729271	stage_1D_vs_E10	ordinary_muscle_development	ENSGALG00010005297	IL22RA2	-2.926471453	0.826493988	-3.540826061	0.000398876	0.000694333	negative
PRJNA729271	stage_1D_vs_E10	ordinary_muscle_development	ENSGALG00010005299	ODAD2	-3.784387405	0.627412255	-6.031739702	1.62204E-09	4.51016E-09	negative
PRJNA729271	stage_1D_vs_E10	ordinary_muscle_development	ENSGALG00010005300	QRFPR	-0.675838485	0.827614847	-0.81660991	0.41415139	0.463669143	negative
PRJNA729271	stage_1D_vs_E10	ordinary_muscle_development	ENSGALG00010005301	ENSGALG00010005301	0.517887211	0.375773751	1.378188896	0.168144975	0.20534682	positive
PRJNA729271	stage_1D_vs_E10	ordinary_muscle_development	ENSGALG00010005302	PSPC1	-0.869772485	0.079770656	-10.90341398	1.11012E-27	8.54553E-27	negative
PRJNA729271	stage_1D_vs_E10	ordinary_muscle_development	ENSGALG00010005303	ENSGALG00010005303	0.766735465	0.300939164	2.54780885	0.010840185	0.01595699	positive
PRJNA729271	stage_1D_vs_E10	ordinary_muscle_development	ENSGALG00010005304	ENSGALG00010005304	0.539253776	0.20918089	2.577930405	0.009939401	0.014698186	positive
PRJNA729271	stage_1D_vs_E10	ordinary_muscle_development	ENSGALG00010005305	SCAF8	0.10885629	0.086095771	1.264362809	0.206099835	0.246934792	positive
PRJNA729271	stage_1D_vs_E10	ordinary_muscle_development	ENSGALG00010005307	TIAM2	-2.193472491	0.157603534	-13.91766058	4.94837E-44	7.47932E-43	negative
PRJNA729271	stage_1D_vs_E10	ordinary_muscle_development	ENSGALG00010005308	ENSGALG00010005308	1.253909079	0.575894054	2.177325967	0.029456255	0.040652044	positive
PRJNA729271	stage_1D_vs_E10	ordinary_muscle_development	ENSGALG00010005309	ASXL3	-1.562672519	0.139489681	-11.20278222	3.95126E-29	3.28215E-28	negative
PRJNA729271	stage_1D_vs_E10	ordinary_muscle_development	ENSGALG00010005310	LAMTOR3	0.512089102	0.076073739	6.731483267	1.67942E-11	5.35604E-11	positive
PRJNA729271	stage_1D_vs_E10	ordinary_muscle_development	ENSGALG00010005311	YME1L1	0.057961282	0.078235234	0.740859054	0.458778905	0.507711118	positive
PRJNA729271	stage_1D_vs_E10	ordinary_muscle_development	ENSGALG00010005313	MPHOSPH8	0.408252412	0.13880873	2.941114806	0.003270333	0.005134136	positive
PRJNA729271	stage_1D_vs_E10	ordinary_muscle_development	ENSGALG00010005314	IL20RA	-2.768633536	0.660603548	-4.191066705	2.77646E-05	5.42968E-05	negative
PRJNA729271	stage_1D_vs_E10	ordinary_muscle_development	ENSGALG00010005315	ENSGALG00010005315	0.9477402	0.539653096	1.756202656	0.079053811	0.10231116	positive
PRJNA729271	stage_1D_vs_E10	ordinary_muscle_development	ENSGALG00010005316	PARP4	0.31915027	0.10776904	2.961428164	0.003062159	0.004822468	positive
PRJNA729271	stage_1D_vs_E10	ordinary_muscle_development	ENSGALG00010005317	CHCHD3	0.866319153	0.109414243	7.917791428	2.41766E-15	9.91065E-15	positive
PRJNA729271	stage_1D_vs_E10	ordinary_muscle_development	ENSGALG00010005318	SLC35D3	-3.976863794	0.621303866	-6.400835421	1.54529E-10	4.62025E-10	negative
PRJNA729271	stage_1D_vs_E10	ordinary_muscle_development	ENSGALG00010005319	NOL4	-1.760285481	0.761129617	-2.312727611	0.02073762	0.029283176	negative
PRJNA729271	stage_1D_vs_E10	ordinary_muscle_development	ENSGALG00010005321	HS6ST3	-3.492361949	0.295512813	-11.81797132	3.15204E-32	3.02776E-31	negative
PRJNA729271	stage_1D_vs_E10	ordinary_muscle_development	ENSGALG00010005322	TFB1M	0.305223209	0.12217911	2.498161991	0.012483913	0.018217489	positive
PRJNA729271	stage_1D_vs_E10	ordinary_muscle_development	ENSGALG00010005323	DNAJB14	0.255587691	0.063581362	4.019852384	5.82346E-05	0.00011066	positive
PRJNA729271	stage_1D_vs_E10	ordinary_muscle_development	ENSGALG00010005324	CENPJ	-1.295661189	0.182993147	-7.080380949	1.43759E-12	4.91574E-12	negative
PRJNA729271	stage_1D_vs_E10	ordinary_muscle_development	ENSGALG00010005326	MASTL	-1.716232887	0.182950405	-9.380864127	6.54342E-21	3.68052E-20	negative
PRJNA729271	stage_1D_vs_E10	ordinary_muscle_development	ENSGALG00010005329	VIPAS39	0.077938822	0.117291985	0.66448549	0.506379632	0.554462364	positive
PRJNA729271	stage_1D_vs_E10	ordinary_muscle_development	ENSGALG00010005331	ENSGALG00010005331	2.312921987	0.777567685	2.974560326	0.002934088	0.004631845	positive
PRJNA729271	stage_1D_vs_E10	ordinary_muscle_development	ENSGALG00010005332	ENSGALG00010005332	1.491885095	0.705411358	2.114915047	0.034437188	0.047100118	positive
PRJNA729271	stage_1D_vs_E10	ordinary_muscle_development	ENSGALG00010005333	PLXNA4	1.451909792	0.151727192	9.569212854	1.07723E-21	6.32418E-21	positive
PRJNA729271	stage_1D_vs_E10	ordinary_muscle_development	ENSGALG00010005334	NME7	-0.692069324	0.134341549	-5.15156576	2.58321E-07	6.0315E-07	negative
PRJNA729271	stage_1D_vs_E10	ordinary_muscle_development	ENSGALG00010005335	AHSA1	-0.229995658	0.134065837	-1.715542624	0.086245788	0.110891933	negative
PRJNA729271	stage_1D_vs_E10	ordinary_muscle_development	ENSGALG00010005336	PEX7	-0.845632055	0.128689128	-6.571122742	4.99373E-11	1.54337E-10	negative
PRJNA729271	stage_1D_vs_E10	ordinary_muscle_development	ENSGALG00010005337	DCTN1	0.081812975	0.153876056	0.531680997	0.594946956	0.638401921	positive
PRJNA729271	stage_1D_vs_E10	ordinary_muscle_development	ENSGALG00010005338	PCGF1	0.875926634	0.113856949	7.69322064	1.43477E-14	5.58908E-14	positive
PRJNA729271	stage_1D_vs_E10	ordinary_muscle_development	ENSGALG00010005341	UGGT2	-0.17713504	0.118745617	-1.491718553	0.135772943	0.168538906	negative
PRJNA729271	stage_1D_vs_E10	ordinary_muscle_development	ENSGALG00010005342	MAP3K5	0.601195873	0.144996277	4.146284891	3.37913E-05	6.55609E-05	positive
PRJNA729271	stage_1D_vs_E10	ordinary_muscle_development	ENSGALG00010005343	RNF17	-4.757310716	1.784097237	-2.666508651	0.007664363	0.011505981	negative
PRJNA729271	stage_1D_vs_E10	ordinary_muscle_development	ENSGALG00010005344	ENSGALG00010005344	-0.714195855	0.813971092	-0.877421646	0.38025766	0.429270622	negative
PRJNA729271	stage_1D_vs_E10	ordinary_muscle_development	ENSGALG00010005345	NOX3	-0.643225937	0.570850066	-1.12678613	0.259832916	0.304912413	negative
PRJNA729271	stage_1D_vs_E10	ordinary_muscle_development	ENSGALG00010005346	FBXO30	1.904828028	0.220025942	8.657288384	4.83119E-18	2.32189E-17	positive
PRJNA729271	stage_1D_vs_E10	ordinary_muscle_development	ENSGALG00010005347	DOK1	-0.828288956	0.155562378	-5.324481187	1.01242E-07	2.4442E-07	negative
PRJNA729271	stage_1D_vs_E10	ordinary_muscle_development	ENSGALG00010005349	ACBD5	0.719333902	0.103728166	6.934798239	4.06801E-12	1.34933E-11	positive
PRJNA729271	stage_1D_vs_E10	ordinary_muscle_development	ENSGALG00010005350	ARHGAP20	-0.964830413	0.276755365	-3.486221168	0.000489896	0.000844256	negative
PRJNA729271	stage_1D_vs_E10	ordinary_muscle_development	ENSGALG00010005351	DTNA	3.709601666	0.16275948	22.79192385	5.5139E-115	5.7089E-113	positive
PRJNA729271	stage_1D_vs_E10	ordinary_muscle_development	ENSGALG00010005352	ENSGALG00010005352	0.116576979	0.710163154	0.164155207	0.869608963	0.888989485	positive
PRJNA729271	stage_1D_vs_E10	ordinary_muscle_development	ENSGALG00010005353	ENSGALG00010005353	-0.072479063	0.10396473	-0.697150498	0.485708615	0.53424999	negative
PRJNA729271	stage_1D_vs_E10	ordinary_muscle_development	ENSGALG00010005354	M1AP	-0.831843917	0.355007408	-2.343173405	0.019120494	0.027121202	negative
PRJNA729271	stage_1D_vs_E10	ordinary_muscle_development	ENSGALG00010005355	FDX1	0.756895039	0.102080221	7.414708107	1.21893E-13	4.48209E-13	positive
PRJNA729271	stage_1D_vs_E10	ordinary_muscle_development	ENSGALG00010005356	DNAJC3	0.45029915	0.08405452	5.357227095	8.45089E-08	2.05288E-07	positive
PRJNA729271	stage_1D_vs_E10	ordinary_muscle_development	ENSGALG00010005357	TNIP3	-4.632563766	0.904581007	-5.121225994	3.03556E-07	7.04908E-07	negative
PRJNA729271	stage_1D_vs_E10	ordinary_muscle_development	ENSGALG00010005358	SHPRH	0.564409009	0.088508589	6.376884042	1.80727E-10	5.37803E-10	positive
PRJNA729271	stage_1D_vs_E10	ordinary_muscle_development	ENSGALG00010005359	MEIS2	-1.901020026	0.130257709	-14.59429953	3.05333E-48	5.48426E-47	negative
PRJNA729271	stage_1D_vs_E10	ordinary_muscle_development	ENSGALG00010005360	CAMK1D	-0.869026731	0.151977449	-5.718129472	1.07703E-08	2.80179E-08	negative
PRJNA729271	stage_1D_vs_E10	ordinary_muscle_development	ENSGALG00010005361	ENSGALG00010005361	0.102055339	0.484236592	0.210755115	0.833078365	0.856902561	positive
PRJNA729271	stage_1D_vs_E10	ordinary_muscle_development	ENSGALG00010005362	ATP1B1	1.725989028	0.174114715	9.91294178	3.65716E-23	2.29582E-22	positive
PRJNA729271	stage_1D_vs_E10	ordinary_muscle_development	ENSGALG00010005363	RDX	0.210067826	0.085448665	2.45840968	0.013955387	0.020206107	positive
PRJNA729271	stage_1D_vs_E10	ordinary_muscle_development	ENSGALG00010005364	MAP7	-1.164258649	0.335150875	-3.473834429	0.000513078	0.000882213	negative
PRJNA729271	stage_1D_vs_E10	ordinary_muscle_development	ENSGALG00010005366	AUP1	0.638456988	0.113779704	5.611343354	2.00762E-08	5.11705E-08	positive
PRJNA729271	stage_1D_vs_E10	ordinary_muscle_development	ENSGALG00010005367	H2AZ1	-1.434083819	0.095675554	-14.98903078	8.66079E-51	1.72206E-49	negative
PRJNA729271	stage_1D_vs_E10	ordinary_muscle_development	ENSGALG00010005368	CCDC3	-1.044203577	0.226477592	-4.610626464	4.01457E-06	8.49634E-06	negative
PRJNA729271	stage_1D_vs_E10	ordinary_muscle_development	ENSGALG00010005369	DPT	3.148682204	0.312885298	10.06337539	8.02005E-24	5.17589E-23	positive
PRJNA729271	stage_1D_vs_E10	ordinary_muscle_development	ENSGALG00010005371	GRM1	-4.201527145	1.325544304	-3.169661801	0.001526165	0.00249175	negative
PRJNA729271	stage_1D_vs_E10	ordinary_muscle_development	ENSGALG00010005372	CDIN1	-0.851483687	0.10888078	-7.820330484	5.26848E-15	2.11249E-14	negative
PRJNA729271	stage_1D_vs_E10	ordinary_muscle_development	ENSGALG00010005376	HTRA2	0.047726471	0.095168641	0.501493667	0.616023734	0.65838865	positive
PRJNA729271	stage_1D_vs_E10	ordinary_muscle_development	ENSGALG00010005377	EMCN	2.386476242	0.140452039	16.99139624	9.50982E-65	2.98366E-63	positive
PRJNA729271	stage_1D_vs_E10	ordinary_muscle_development	ENSGALG00010005378	ENSGALG00010005378	-7.936186353	1.050399425	-7.555398605	4.17579E-14	1.58285E-13	negative
PRJNA729271	stage_1D_vs_E10	ordinary_muscle_development	ENSGALG00010005379	DZIP1	-0.339076416	0.166770708	-2.03318928	0.042033404	0.056713598	negative
PRJNA729271	stage_1D_vs_E10	ordinary_muscle_development	ENSGALG00010005380	XCL1	1.684571786	1.201274981	1.40231988	0.160819747	0.197148361	positive
PRJNA729271	stage_1D_vs_E10	ordinary_muscle_development	ENSGALG00010005381	FGF9	-2.011931938	0.184974682	-10.87679629	1.48698E-27	1.1374E-26	negative
PRJNA729271	stage_1D_vs_E10	ordinary_muscle_development	ENSGALG00010005382	RAB32	-0.654214041	0.128053487	-5.108912343	3.24019E-07	7.50863E-07	negative
PRJNA729271	stage_1D_vs_E10	ordinary_muscle_development	ENSGALG00010005383	OPTN	1.06605937	0.139026908	7.668007493	1.74689E-14	6.77942E-14	positive
PRJNA729271	stage_1D_vs_E10	ordinary_muscle_development	ENSGALG00010005384	MAPRE2	-0.693365725	0.173381508	-3.999075413	6.35904E-05	0.000120442	negative
PRJNA729271	stage_1D_vs_E10	ordinary_muscle_development	ENSGALG00010005385	PDSS1	0.364951243	0.098534003	3.703810169	0.000212385	0.000380159	positive
PRJNA729271	stage_1D_vs_E10	ordinary_muscle_development	ENSGALG00010005386	CPOX	-1.155486113	0.142136587	-8.129406637	4.31397E-16	1.86104E-15	negative
PRJNA729271	stage_1D_vs_E10	ordinary_muscle_development	ENSGALG00010005387	CLDN10	-3.345003329	0.579237387	-5.774840167	7.70262E-09	2.03332E-08	negative
PRJNA729271	stage_1D_vs_E10	ordinary_muscle_development	ENSGALG00010005388	ENSGALG00010005388	0.287565575	0.504627561	0.569857054	0.568774655	0.61396996	positive
PRJNA729271	stage_1D_vs_E10	ordinary_muscle_development	ENSGALG00010005389	MCM10	-1.991678289	0.185610386	-10.73042481	7.32586E-27	5.45956E-26	negative
PRJNA729271	stage_1D_vs_E10	ordinary_muscle_development	ENSGALG00010005391	ADGB	1.023796466	0.378148139	2.707395229	0.006781347	0.010240195	positive
PRJNA729271	stage_1D_vs_E10	ordinary_muscle_development	ENSGALG00010005392	ENSGALG00010005392	0.620995641	0.104935968	5.917853097	3.26171E-09	8.86029E-09	positive
PRJNA729271	stage_1D_vs_E10	ordinary_muscle_development	ENSGALG00010005394	PHYH	2.279014162	0.135395997	16.83221224	1.41707E-63	4.30617E-62	positive
PRJNA729271	stage_1D_vs_E10	ordinary_muscle_development	ENSGALG00010005395	ENSGALG00010005395	-3.10952542	0.397010723	-7.832346177	4.78849E-15	1.92696E-14	negative
PRJNA729271	stage_1D_vs_E10	ordinary_muscle_development	ENSGALG00010005396	MICU2	0.784239275	0.122319364	6.411407393	1.44182E-10	4.31891E-10	positive
PRJNA729271	stage_1D_vs_E10	ordinary_muscle_development	ENSGALG00010005398	SEPHS1	-0.695909549	0.085888884	-8.102440261	5.38676E-16	2.30736E-15	negative
PRJNA729271	stage_1D_vs_E10	ordinary_muscle_development	ENSGALG00010005399	NDNF	-4.659657261	0.242531745	-19.21256646	2.90541E-82	1.44226E-80	negative
PRJNA729271	stage_1D_vs_E10	ordinary_muscle_development	ENSGALG00010005401	BCLAF1	-0.866901264	0.086351564	-10.03920746	1.02494E-23	6.58827E-23	negative
PRJNA729271	stage_1D_vs_E10	ordinary_muscle_development	ENSGALG00010005402	ABCC4	1.125347532	0.169006867	6.658590578	2.76466E-11	8.68713E-11	positive
PRJNA729271	stage_1D_vs_E10	ordinary_muscle_development	ENSGALG00010005403	ENSGALG00010005403	-0.543804711	0.139436822	-3.900007914	9.61895E-05	0.000178798	negative
PRJNA729271	stage_1D_vs_E10	ordinary_muscle_development	ENSGALG00010005404	BEND7	0.913610096	0.186985116	4.88600438	1.02903E-06	2.29792E-06	positive
PRJNA729271	stage_1D_vs_E10	ordinary_muscle_development	ENSGALG00010005405	CEBPD	5.527453609	0.47398014	11.66178314	1.99813E-31	1.84949E-30	positive
PRJNA729271	stage_1D_vs_E10	ordinary_muscle_development	ENSGALG00010005407	DPH6	-0.372550638	0.119875353	-3.107816822	0.001884748	0.003044287	negative
PRJNA729271	stage_1D_vs_E10	ordinary_muscle_development	ENSGALG00010005408	APBB1IP	-0.108835124	0.207369948	-0.524835564	0.599697506	0.64266615	negative
PRJNA729271	stage_1D_vs_E10	ordinary_muscle_development	ENSGALG00010005409	PRKCQ	0.772425894	0.218319933	3.538045663	0.0004031	0.000701518	positive
PRJNA729271	stage_1D_vs_E10	ordinary_muscle_development	ENSGALG00010005411	STXBP5	0.571623851	0.096254692	5.938659585	2.87362E-09	7.84427E-09	positive
PRJNA729271	stage_1D_vs_E10	ordinary_muscle_development	ENSGALG00010005412	MTFR2	-1.487968843	0.161108336	-9.235827752	2.56297E-20	1.38931E-19	negative
PRJNA729271	stage_1D_vs_E10	ordinary_muscle_development	ENSGALG00010005413	GAD2	-0.593560812	0.251096391	-2.363876317	0.018084849	0.02573026	negative
PRJNA729271	stage_1D_vs_E10	ordinary_muscle_development	ENSGALG00010005414	ENSGALG00010005414	-0.903029773	0.21641298	-4.172715387	3.00991E-05	5.86879E-05	negative
PRJNA729271	stage_1D_vs_E10	ordinary_muscle_development	ENSGALG00010005415	PRKDC	-0.220790567	0.087247413	-2.53062596	0.01138592	0.0167128	negative
PRJNA729271	stage_1D_vs_E10	ordinary_muscle_development	ENSGALG00010005416	PPP3CA	1.157607846	0.162091566	7.141690786	9.21898E-13	3.1984E-12	positive
PRJNA729271	stage_1D_vs_E10	ordinary_muscle_development	ENSGALG00010005417	LOXL3	1.560214796	0.163272238	9.555909901	1.22503E-21	7.16576E-21	positive
PRJNA729271	stage_1D_vs_E10	ordinary_muscle_development	ENSGALG00010005420	DHRS4	0.42981771	0.117506915	3.657807805	0.000254382	0.000451483	positive
PRJNA729271	stage_1D_vs_E10	ordinary_muscle_development	ENSGALG00010005421	ENSGALG00010005421	0.097470826	0.34067159	0.286113749	0.774791001	0.804023165	positive
PRJNA729271	stage_1D_vs_E10	ordinary_muscle_development	ENSGALG00010005423	ST3GAL6	1.322251263	0.149612347	8.837848517	9.75795E-19	4.8689E-18	positive
PRJNA729271	stage_1D_vs_E10	ordinary_muscle_development	ENSGALG00010005424	ENSGALG00010005424	0.137653459	0.138633329	0.992931928	0.320743129	0.368806255	positive
PRJNA729271	stage_1D_vs_E10	ordinary_muscle_development	ENSGALG00010005425	ELAPOR2	-0.603380833	0.24986273	-2.414849275	0.015741722	0.022616341	negative
PRJNA729271	stage_1D_vs_E10	ordinary_muscle_development	ENSGALG00010005426	ENSGALG00010005426	1.349816146	0.981393608	1.375407516	0.16900514	0.206240908	positive
PRJNA729271	stage_1D_vs_E10	ordinary_muscle_development	ENSGALG00010005427	ENSGALG00010005427	0.099245306	0.142318915	0.697344454	0.485587254	0.53423839	positive
PRJNA729271	stage_1D_vs_E10	ordinary_muscle_development	ENSGALG00010005428	ZNF770	0.273696234	0.10185011	2.687245357	0.007204401	0.01084626	positive
PRJNA729271	stage_1D_vs_E10	ordinary_muscle_development	ENSGALG00010005429	ZDHHC20	-1.747617692	0.118270543	-14.77644097	2.07864E-49	3.9334E-48	negative
PRJNA729271	stage_1D_vs_E10	ordinary_muscle_development	ENSGALG00010005430	MCM4	-1.094043014	0.134088734	-8.159097224	3.37538E-16	1.46354E-15	negative
PRJNA729271	stage_1D_vs_E10	ordinary_muscle_development	ENSGALG00010005431	GRM3	-5.545940761	0.954320895	-5.81140033	6.19524E-09	1.64892E-08	negative
PRJNA729271	stage_1D_vs_E10	ordinary_muscle_development	ENSGALG00010005433	GPC5	-0.161941148	0.132004269	-1.226787204	0.219902581	0.262301507	negative
PRJNA729271	stage_1D_vs_E10	ordinary_muscle_development	ENSGALG00010005434	PRDM5	-1.337773094	0.221160247	-6.048885882	1.45851E-09	4.06794E-09	negative
PRJNA729271	stage_1D_vs_E10	ordinary_muscle_development	ENSGALG00010005435	MAD2L1	-2.208489059	0.170610342	-12.94463767	2.51949E-38	3.10281E-37	negative
PRJNA729271	stage_1D_vs_E10	ordinary_muscle_development	ENSGALG00010005436	AQR	-0.995042062	0.055143412	-18.04462257	8.69565E-73	3.45324E-71	negative
PRJNA729271	stage_1D_vs_E10	ordinary_muscle_development	ENSGALG00010005437	PDE7B	2.793819788	0.157797955	17.70504433	3.83379E-70	1.41762E-68	positive
PRJNA729271	stage_1D_vs_E10	ordinary_muscle_development	ENSGALG00010005438	GPR180	-0.238784763	0.137326477	-1.738810818	0.082068045	0.105862447	negative
PRJNA729271	stage_1D_vs_E10	ordinary_muscle_development	ENSGALG00010005439	TBX19	0.827116888	0.507398872	1.6301118	0.10307787	0.130810167	positive
PRJNA729271	stage_1D_vs_E10	ordinary_muscle_development	ENSGALG00010005440	ENSGALG00010005440	1.188776412	0.54373279	2.186324667	0.028791861	0.039776784	positive
PRJNA729271	stage_1D_vs_E10	ordinary_muscle_development	ENSGALG00010005441	UBE2V2	-0.440480693	0.095756604	-4.600003311	4.22484E-06	8.91788E-06	negative
PRJNA729271	stage_1D_vs_E10	ordinary_muscle_development	ENSGALG00010005442	SASH1	0.67429	0.097219758	6.935730103	4.04129E-12	1.342E-11	positive
PRJNA729271	stage_1D_vs_E10	ordinary_muscle_development	ENSGALG00010005443	BANK1	-0.040731553	0.433104338	-0.09404559	0.925072942	0.936220762	negative
PRJNA729271	stage_1D_vs_E10	ordinary_muscle_development	ENSGALG00010005444	ACTC1	-1.399674676	0.426589838	-3.28107834	0.00103411	0.001722131	negative
PRJNA729271	stage_1D_vs_E10	ordinary_muscle_development	ENSGALG00010005445	SFT2D2	-1.256547776	0.104535409	-12.0203077	2.77935E-33	2.81135E-32	negative

PRJNA729271	stage_1D_vs_E10	ordinary_muscle_development	ENSGALG00010005446	ENSGALG00010005446	-0.282231179	0.437689398	-0.644820688	0.519043394	0.566445866	negative
PRJNA729271	stage_1D_vs_E10	ordinary_muscle_development	ENSGALG00010005447	MYO3A	-2.941838343	0.390465581	-7.534180961	4.91409E-14	1.85543E-13	negative
PRJNA729271	stage_1D_vs_E10	ordinary_muscle_development	ENSGALG00010005448	ENSGALG00010005448	0.011139553	0.147665527	0.075437736	0.939866436	0.948857631	positive
PRJNA729271	stage_1D_vs_E10	ordinary_muscle_development	ENSGALG00010005449	TIPRL	-0.120014566	0.085999344	-1.395528858	0.162856419	0.199459551	negative
PRJNA729271	stage_1D_vs_E10	ordinary_muscle_development	ENSGALG00010005450	ENSGALG00010005450	1.787380943	0.439015851	4.071335783	4.67443E-05	8.96005E-05	positive
PRJNA729271	stage_1D_vs_E10	ordinary_muscle_development	ENSGALG00010005453	UST	0.349396531	0.113874357	3.068263486	0.002153067	0.003449619	positive
PRJNA729271	stage_1D_vs_E10	ordinary_muscle_development	ENSGALG00010005454	GPR161	-1.089928566	0.096474761	-11.29755129	1.34928E-29	1.1424E-28	negative
PRJNA729271	stage_1D_vs_E10	ordinary_muscle_development	ENSGALG00010005455	ENSGALG00010005455	1.112198898	0.708338278	1.570152189	0.116379709	0.146396241	positive
PRJNA729271	stage_1D_vs_E10	ordinary_muscle_development	ENSGALG00010005456	RPL7L1	-0.768724368	0.070404068	-10.91874931	9.37782E-28	7.2613E-27	negative
PRJNA729271	stage_1D_vs_E10	ordinary_muscle_development	ENSGALG00010005457	SEMA3D	0.184939604	0.15951134	1.159413518	0.246287669	0.290333423	positive
PRJNA729271	stage_1D_vs_E10	ordinary_muscle_development	ENSGALG00010005458	PRKD1	0.508650341	0.174235378	2.919328703	0.003507861	0.005492757	positive
PRJNA729271	stage_1D_vs_E10	ordinary_muscle_development	ENSGALG00010005459	ZC3H12D	2.969630817	0.52105903	5.699221476	1.20356E-08	3.1203E-08	positive
PRJNA729271	stage_1D_vs_E10	ordinary_muscle_development	ENSGALG00010005460	TGDS	-0.997950508	0.100788688	-9.901413804	4.10431E-23	2.56763E-22	negative
PRJNA729271	stage_1D_vs_E10	ordinary_muscle_development	ENSGALG00010005462	AHI1	0.781164978	0.149733079	5.217050134	1.81795E-07	4.30153E-07	positive
PRJNA729271	stage_1D_vs_E10	ordinary_muscle_development	ENSGALG00010005463	SKA3	-2.028152238	0.247967502	-8.179105011	2.8596E-16	1.24474E-15	negative
PRJNA729271	stage_1D_vs_E10	ordinary_muscle_development	ENSGALG00010005464	SLC39A8	-4.294598368	0.328389945	-13.07774014	4.41368E-39	5.60703E-38	negative
PRJNA729271	stage_1D_vs_E10	ordinary_muscle_development	ENSGALG00010005465	ENSGALG00010005465	-1.04557366	0.090418112	-11.56376348	6.28907E-31	5.67976E-30	negative
PRJNA729271	stage_1D_vs_E10	ordinary_muscle_development	ENSGALG00010005466	LATS2	0.872530675	0.088467409	9.862735774	6.03813E-23	3.75957E-22	positive
PRJNA729271	stage_1D_vs_E10	ordinary_muscle_development	ENSGALG00010005467	ENSGALG00010005467	1.541195915	0.260279741	5.921305696	3.19396E-09	8.68274E-09	positive
PRJNA729271	stage_1D_vs_E10	ordinary_muscle_development	ENSGALG00010005468	PPIL4	-0.312557135	0.103963192	-3.006421117	0.002643426	0.00419355	negative
PRJNA729271	stage_1D_vs_E10	ordinary_muscle_development	ENSGALG00010005470	SEMA3A	-1.258632974	0.141002326	-8.926327736	4.40384E-19	2.23351E-18	negative
PRJNA729271	stage_1D_vs_E10	ordinary_muscle_development	ENSGALG00010005472	PFKP	-1.416941718	0.11514517	-12.30569823	8.44033E-35	9.0826E-34	negative
PRJNA729271	stage_1D_vs_E10	ordinary_muscle_development	ENSGALG00010005473	ENSGALG00010005473	-1.215787323	1.01648697	-1.196067789	0.231670106	0.274980199	negative
PRJNA729271	stage_1D_vs_E10	ordinary_muscle_development	ENSGALG00010005474	COL8A1	-0.328354065	0.34535965	-0.950759782	0.341726333	0.390640631	negative
PRJNA729271	stage_1D_vs_E10	ordinary_muscle_development	ENSGALG00010005475	TMEM181	-0.642646394	0.092284361	-6.963762706	3.31302E-12	1.10829E-11	negative
PRJNA729271	stage_1D_vs_E10	ordinary_muscle_development	ENSGALG00010005476	NFKB1	0.967437638	0.114713313	8.433525359	3.35405E-17	1.53657E-16	positive
PRJNA729271	stage_1D_vs_E10	ordinary_muscle_development	ENSGALG00010005477	TMEM161B	1.134133758	0.160789498	7.053531307	1.74433E-12	5.93245E-12	positive
PRJNA729271	stage_1D_vs_E10	ordinary_muscle_development	ENSGALG00010005479	ENSGALG00010005479	0.33326971	0.617096354	0.540061059	0.589154925	0.633187561	positive
PRJNA729271	stage_1D_vs_E10	ordinary_muscle_development	ENSGALG00010005481	ENSGALG00010005481	-1.558733681	0.555908661	-2.803938471	0.005048254	0.00775975	negative
PRJNA729271	stage_1D_vs_E10	ordinary_muscle_development	ENSGALG00010005482	XPO4	-1.109717862	0.074777873	-14.84019027	8.05272E-50	1.54397E-48	negative
PRJNA729271	stage_1D_vs_E10	ordinary_muscle_development	ENSGALG00010005484	GINM1	1.324585912	0.122324391	10.82846932	2.52338E-27	1.91299E-26	positive
PRJNA729271	stage_1D_vs_E10	ordinary_muscle_development	ENSGALG00010005485	PITRM1	-0.565316166	0.096450842	-5.861184346	4.59577E-09	1.23454E-08	negative
PRJNA729271	stage_1D_vs_E10	ordinary_muscle_development	ENSGALG00010005486	IRF5	0.650878896	0.328831288	1.979370329	0.047774327	0.06388863	positive
PRJNA729271	stage_1D_vs_E10	ordinary_muscle_development	ENSGALG00010005487	ENSGALG00010005487	-1.847318595	0.212360841	-8.698960615	3.34938E-18	1.62481E-17	negative
PRJNA729271	stage_1D_vs_E10	ordinary_muscle_development	ENSGALG00010005489	G2E3	-0.994693112	0.08544437	-11.64141195	2.53779E-31	2.33557E-30	negative
PRJNA729271	stage_1D_vs_E10	ordinary_muscle_development	ENSGALG00010005490	DCBLD2	-0.0047158	0.114813555	-0.041073544	0.967237266	0.971930965	negative
PRJNA729271	stage_1D_vs_E10	ordinary_muscle_development	ENSGALG00010005491	GGACT	0.676273026	0.168388278	4.016152627	5.9156E-05	0.000112322	positive
PRJNA729271	stage_1D_vs_E10	ordinary_muscle_development	ENSGALG00010005492	TNPO3	-0.135229451	0.096852173	-1.396245915	0.162640455	0.199228715	negative
PRJNA729271	stage_1D_vs_E10	ordinary_muscle_development	ENSGALG00010005493	KLF6	0.526348679	0.181099042	2.90641338	0.003655981	0.00570927	positive
PRJNA729271	stage_1D_vs_E10	ordinary_muscle_development	ENSGALG00010005494	PPDPFL	7.548498536	0.43284631	17.43921194	4.15767E-68	1.44522E-66	positive
PRJNA729271	stage_1D_vs_E10	ordinary_muscle_development	ENSGALG00010005495	TXN	-1.230721294	0.146585764	-8.395912807	4.62289E-17	2.10059E-16	negative
PRJNA729271	stage_1D_vs_E10	ordinary_muscle_development	ENSGALG00010005497	MKX	-2.094265186	0.202358685	-10.34927256	4.21677E-25	2.90781E-24	negative
PRJNA729271	stage_1D_vs_E10	ordinary_muscle_development	ENSGALG00010005498	SMO	0.397266834	0.136695379	2.906219918	0.003658242	0.005712186	positive
PRJNA729271	stage_1D_vs_E10	ordinary_muscle_development	ENSGALG00010005499	PDE5A	-0.178904685	0.112472629	-1.590650873	0.111688169	0.140861394	negative
PRJNA729271	stage_1D_vs_E10	ordinary_muscle_development	ENSGALG00010005500	KATNA1	0.167728392	0.129550198	1.294695075	0.19542544	0.235393418	positive
PRJNA729271	stage_1D_vs_E10	ordinary_muscle_development	ENSGALG00010005502	SCFD1	-0.52524818	0.071619271	-7.333894529	2.23559E-13	8.07498E-13	negative
PRJNA729271	stage_1D_vs_E10	ordinary_muscle_development	ENSGALG00010005503	DYNLT1	-1.984296058	0.152184563	-13.03874727	7.36573E-39	9.28403E-38	negative
PRJNA729271	stage_1D_vs_E10	ordinary_muscle_development	ENSGALG00010005504	LATS1	0.241917353	0.077908785	3.105135746	0.001901917	0.003069965	positive
PRJNA729271	stage_1D_vs_E10	ordinary_muscle_development	ENSGALG00010005505	FABP2	-0.728632457	1.014203714	-0.71842811	0.47249336	0.521296335	negative
PRJNA729271	stage_1D_vs_E10	ordinary_muscle_development	ENSGALG00010005507	USP53	1.355110098	0.119279004	11.36084349	6.5509E-30	5.63385E-29	positive
PRJNA729271	stage_1D_vs_E10	ordinary_muscle_development	ENSGALG00010005508	SS18	-0.681913206	0.063982602	-10.65779112	1.60361E-26	1.17633E-25	negative
PRJNA729271	stage_1D_vs_E10	ordinary_muscle_development	ENSGALG00010005509	COCH	3.292826748	0.443479853	7.424974831	1.12801E-13	4.15622E-13	positive
PRJNA729271	stage_1D_vs_E10	ordinary_muscle_development	ENSGALG00010005510	MYOZ2	0.700251714	0.317529719	2.205310785	0.027432294	0.038014445	positive
PRJNA729271	stage_1D_vs_E10	ordinary_muscle_development	ENSGALG00010005511	AHCYL2	0.3166515	0.098689712	3.208556313	0.001334032	0.002194868	positive
PRJNA729271	stage_1D_vs_E10	ordinary_muscle_development	ENSGALG00010005512	SMARCAD1	-1.764099248	0.110746518	-15.9291622	3.97649E-57	9.9207E-56	negative
PRJNA729271	stage_1D_vs_E10	ordinary_muscle_development	ENSGALG00010005513	TMEM30CP	0.976255458	0.25447061	3.836417322	0.000124842	0.000229352	positive
PRJNA729271	stage_1D_vs_E10	ordinary_muscle_development	ENSGALG00010005514	EEF1AKMT1	-0.157423535	0.144044297	-1.0928828	0.274445261	0.320141965	negative
PRJNA729271	stage_1D_vs_E10	ordinary_muscle_development	ENSGALG00010005515	PCCA	0.81904648	0.095258295	8.598164405	8.10012E-18	3.84451E-17	positive
PRJNA729271	stage_1D_vs_E10	ordinary_muscle_development	ENSGALG00010005516	LARP4B	0.790314884	0.131691879	6.00124238	1.95813E-09	5.41457E-09	positive
PRJNA729271	stage_1D_vs_E10	ordinary_muscle_development	ENSGALG00010005518	HPGDS	-1.774042853	0.654842246	-2.709114851	0.006746298	0.010191516	negative
PRJNA729271	stage_1D_vs_E10	ordinary_muscle_development	ENSGALG00010005519	PTBP3	-1.46606486	0.255503664	-5.737940659	9.58347E-09	2.50338E-08	negative
PRJNA729271	stage_1D_vs_E10	ordinary_muscle_development	ENSGALG00010005521	SYTL3	1.323142785	0.40224725	3.289376828	0.001004095	0.001673876	positive
PRJNA729271	stage_1D_vs_E10	ordinary_muscle_development	ENSGALG00010005522	SYNPO2	0.566654032	0.178497043	3.174584979	0.001500509	0.002451796	positive
PRJNA729271	stage_1D_vs_E10	ordinary_muscle_development	ENSGALG00010005523	ENSGALG00010005523	0.206888834	0.747161306	0.276899824	0.781857027	0.810253636	positive
PRJNA729271	stage_1D_vs_E10	ordinary_muscle_development	ENSGALG00010005524	STRIP2	4.411535066	0.272679621	16.1784553	7.15675E-59	1.88613E-57	positive
PRJNA729271	stage_1D_vs_E10	ordinary_muscle_development	ENSGALG00010005526	STRN3	0.197621828	0.069808833	2.83090004	0.004641722	0.007164494	positive
PRJNA729271	stage_1D_vs_E10	ordinary_muscle_development	ENSGALG00010005527	NUP43	-1.194415461	0.096521599	-12.37459251	3.5871E-35	3.93306E-34	negative
PRJNA729271	stage_1D_vs_E10	ordinary_muscle_development	ENSGALG00010005529	ENSGALG00010005529	-0.14746404	0.399780257	-0.368862738	0.712230039	0.747774476	negative
PRJNA729271	stage_1D_vs_E10	ordinary_muscle_development	ENSGALG00010005530	IL17D	0.222040414	0.211080213	1.051924344	0.292834262	0.339516288	positive
PRJNA729271	stage_1D_vs_E10	ordinary_muscle_development	ENSGALG00010005531	SUSD1	-0.237898293	0.25525868	-0.931989041	0.351342189	0.400338419	negative
PRJNA729271	stage_1D_vs_E10	ordinary_muscle_development	ENSGALG00010005532	GTPBP4	0.184972054	0.09723267	1.902365268	0.057123418	0.075417064	positive
PRJNA729271	stage_1D_vs_E10	ordinary_muscle_development	ENSGALG00010005533	OPN4-1	-2.114254403	0.746616668	-2.831780342	0.004628964	0.007147846	negative
PRJNA729271	stage_1D_vs_E10	ordinary_muscle_development	ENSGALG00010005534	ENSGALG00010005534	-1.951729599	0.165838967	-11.76882393	5.65068E-32	5.38505E-31	negative
PRJNA729271	stage_1D_vs_E10	ordinary_muscle_development	ENSGALG00010005535	AP4S1	-0.019802676	0.157193231	-0.125976646	0.899750411	0.914577995	negative
PRJNA729271	stage_1D_vs_E10	ordinary_muscle_development	ENSGALG00010005536	TAF4B	-1.506960616	0.198769467	-7.581449189	3.41716E-14	1.30278E-13	negative
PRJNA729271	stage_1D_vs_E10	ordinary_muscle_development	ENSGALG00010005537	GRIN3A	-2.258408016	0.296599792	-7.614327704	2.65068E-14	1.01779E-13	negative
PRJNA729271	stage_1D_vs_E10	ordinary_muscle_development	ENSGALG00010005538	ENSGALG00010005538	0.211100347	0.360208731	0.586050057	0.557841877	0.603471752	positive
PRJNA729271	stage_1D_vs_E10	ordinary_muscle_development	ENSGALG00010005539	IFT88	-1.344288149	0.134265548	-10.01215997	1.34777E-23	8.62515E-23	negative
PRJNA729271	stage_1D_vs_E10	ordinary_muscle_development	ENSGALG00010005540	PCMT1	0.715463456	0.13726121	5.212422769	1.8639E-07	4.40738E-07	positive
PRJNA729271	stage_1D_vs_E10	ordinary_muscle_development	ENSGALG00010005542	KCTD1	-0.417566069	0.134439862	-3.105969187	0.001896565	0.00306269	negative
PRJNA729271	stage_1D_vs_E10	ordinary_muscle_development	ENSGALG00010005543	SEC24D	-0.733861699	0.202107238	-3.631051049	0.000282269	0.000498963	negative
PRJNA729271	stage_1D_vs_E10	ordinary_muscle_development	ENSGALG00010005544	AQP4	8.245774597	0.308645894	26.71597048	3.0706E-157	6.1817E-155	positive
PRJNA729271	stage_1D_vs_E10	ordinary_muscle_development	ENSGALG00010005545	RPL34	0.455877038	0.166911085	2.73125681	0.006309329	0.0095743	positive
PRJNA729271	stage_1D_vs_E10	ordinary_muscle_development	ENSGALG00010005546	NRF1	1.124998313	0.15199128	7.401729334	1.34422E-13	4.9278E-13	positive
PRJNA729271	stage_1D_vs_E10	ordinary_muscle_development	ENSGALG00010005547	LRP11	1.514185319	0.214234092	7.067900839	1.57295E-12	5.36594E-12	positive
PRJNA729271	stage_1D_vs_E10	ordinary_muscle_development	ENSGALG00010005548	EZR	-0.792995545	0.178743426	-4.436501886	9.14325E-06	1.87562E-05	negative
PRJNA729271	stage_1D_vs_E10	ordinary_muscle_development	ENSGALG00010005549	RNF20	-0.466181838	0.113678084	-4.100894563	4.11556E-05	7.91601E-05	negative
PRJNA729271	stage_1D_vs_E10	ordinary_muscle_development	ENSGALG00010005550	ENSGALG00010005550	0.587197339	0.608992288	0.964211454	0.334939923	0.383444186	positive
PRJNA729271	stage_1D_vs_E10	ordinary_muscle_development	ENSGALG00010005551	METTL14	-1.402994258	0.083006341	-16.90225397	4.33033E-64	1.3412E-62	negative
PRJNA729271	stage_1D_vs_E10	ordinary_muscle_development	ENSGALG00010005552	WDR37	0.041304892	0.086192201	0.479218435	0.631783241	0.673310644	positive
PRJNA729271	stage_1D_vs_E10	ordinary_muscle_development	ENSGALG00010005553	PPP1R14C	1.422853993	0.162992781	8.729552216	2.55683E-18	1.24617E-17	positive
PRJNA729271	stage_1D_vs_E10	ordinary_muscle_development	ENSGALG00010005554	CRYL1	0.532136344	0.162775746	3.2691378	0.001078758	0.001792365	positive
PRJNA729271	stage_1D_vs_E10	ordinary_muscle_development	ENSGALG00010005555	PRSS12	-3.981793463	0.171079031	-23.27458504	8.021E-120	9.0832E-118	negative
PRJNA729271	stage_1D_vs_E10	ordinary_muscle_development	ENSGALG00010005556	IYD	-0.883569106	0.752828008	-1.173666624	0.240528584	0.284330601	negative
PRJNA729271	stage_1D_vs_E10	ordinary_muscle_development	ENSGALG00010005557	CMSS1	0.046637392	0.165662115	0.281521168	0.778310694	0.807097833	positive
PRJNA729271	stage_1D_vs_E10	ordinary_muscle_development	ENSGALG00010005558	TAGAP	-0.313350108	0.228414441	-1.371848934	0.17011047	0.207415147	negative
PRJNA729271	stage_1D_vs_E10	ordinary_muscle_development	ENSGALG00010005559	FILIP1L	1.286903446	0.130235829	9.881331858	5.01617E-23	3.12863E-22	positive
PRJNA729271	stage_1D_vs_E10	ordinary_muscle_development	ENSGALG00010005560	RSPH3	-2.648617797	0.429911822	-6.160839638	7.23602E-10	2.06469E-09	negative
PRJNA729271	stage_1D_vs_E10	ordinary_muscle_development	ENSGALG00010005561	CLYBL	1.502647386	0.190762413	7.877062162	3.35168E-15	1.36353E-14	positive
PRJNA729271	stage_1D_vs_E10	ordinary_muscle_development	ENSGALG00010005562	HECTD1	0.354193782	0.085418192	4.146584839	3.37471E-05	6.54839E-05	positive
PRJNA729271	stage_1D_vs_E10	ordinary_muscle_development	ENSGALG00010005564	TM9SF2	0.065252771	0.057896082	1.127067136	0.259714097	0.304822335	positive
PRJNA729271	stage_1D_vs_E10	ordinary_muscle_development	ENSGALG00010005565	PGAP4	-0.835418245	0.17622903	-4.740525706	2.13164E-06	4.63728E-06	negative
PRJNA729271	stage_1D_vs_E10	ordinary_muscle_development	ENSGALG00010005567	NDST3	-0.851029289	0.238685283	-3.565487068	0.000363181	0.000634943	negative
PRJNA729271	stage_1D_vs_E10	ordinary_muscle_development	ENSGALG00010005568	PLEKHG1	0.398435947	0.131972937	3.019073119	0.002535493	0.004028055	positive
PRJNA729271	stage_1D_vs_E10	ordinary_muscle_development	ENSGALG00010005572	ENSGALG00010005572	0.33294574	0.157408103	2.115175353	0.034415005	0.047074219	positive
PRJNA729271	stage_1D_vs_E10	ordinary_muscle_development	ENSGALG00010005573	CHST9	0.890067353	0.281867948	3.157745882	0.001589941	0.002590915	positive
PRJNA729271	stage_1D_vs_E10	ordinary_muscle_development	ENSGALG00010005575	ENSGALG00010005575	1.495691462	0.465422448	3.213621235	0.001310725	0.002158236	positive
PRJNA729271	stage_1D_vs_E10	ordinary_muscle_development	ENSGALG00010005578	HEATR5A	0.461834161	0.120141579	3.844082659	0.000121004	0.000222781	positive
PRJNA729271	stage_1D_vs_E10	ordinary_muscle_development	ENSGALG00010005581	GR42L5	-0.316277265	1.002747413	-0.315410702	0.752449842	0.784218207	negative
PRJNA729271	stage_1D_vs_E10	ordinary_muscle_development	ENSGALG00010005583	ALDOB	-3.640725504	1.017113024	-3.579469948			negative
PRJNA729271	stage_1D_vs_E10	ordinary_muscle_development	ENSGALG00010005587	LEF1	-5.495742686	0.220558068	-24.91744116	4.8151E-137	6.9104E-135	negative
PRJNA729271	stage_1D_vs_E10	ordinary_muscle_development	ENSGALG00010005588	PLAGL1	0.001496969	0.126912882	0.011795252	0.990588968	0.992050002	positive
PRJNA729271	stage_1D_vs_E10	ordinary_muscle_development	ENSGALG00010005590	TBC1D23	-0.255209021	0.056815371	-4.491900976	7.05902E-06	1.46319E-05	negative
PRJNA729271	stage_1D_vs_E10	ordinary_muscle_development	ENSGALG00010005593	ENSGALG00010005593	-1.808494144	1.326211404	-1.363654496	0.172676332	0.210313707	negative
PRJNA729271	stage_1D_vs_E10	ordinary_muscle_development	ENSGALG00010005594	MRPL50	0.065678584	0.150682826	0.435873058	0.662928834	0.701961681	positive
PRJNA729271	stage_1D_vs_E10	ordinary_muscle_development	ENSGALG00010005596	PARD3	-0.137729209	0.053723955	-2.563646139	0.010357909	0.01527033	negative
PRJNA729271	stage_1D_vs_E10	ordinary_muscle_development	ENSGALG00010005597	ENSGALG00010005597	0.368991607	0.551314338	0.669294414	0.503307689	0.551431969	positive
PRJNA729271	stage_1D_vs_E10	ordinary_muscle_development	ENSGALG00010005601	TMEM215	-1.696562461	0.394343124	-4.302249381	1.69073E-05	3.37192E-05	negative
PRJNA729271	stage_1D_vs_E10	ordinary_muscle_development	ENSGALG00010005609	GJB6	-3.813314992	0.567173777	-6.723362653	1.77578E-11	5.65215E-11	negative
PRJNA729271	stage_1D_vs_E10	ordinary_muscle_development	ENSGALG00010005610	MTHFD1L	-0.726827915	0.19065507	-3.812266386	0.000137698	0.000251954	negative
PRJNA729271	stage_1D_vs_E10	ordinary_muscle_development	ENSGALG00010005617	DTD2	1.272181902	0.208070669	6.114181827	9.70536E-10	2.74764E-09	positive
PRJNA729271	stage_1D_vs_E10	ordinary_muscle_development	ENSGALG00010005618	PHACTR2	-0.822697553	0.068583907	-11.99548977	3.75191E-33	3.77143E-32	negative
PRJNA729271	stage_1D_vs_E10	ordinary_muscle_development	ENSGALG00010005619	CELF2	0.417533545	0.076709749	5.443031071	5.23815E-08	1.29237E-07	positive
PRJNA729271	stage_1D_vs_E10	ordinary_muscle_development	ENSGALG00010005621	CDH2	-2.524139	0.142604914	-17.70022456	4.17639E-70	1.54037E-68	negative
PRJNA729271	stage_1D_vs_E10	ordinary_muscle_development	ENSGALG00010005629	NRP1	1.020711279	0.173134987	5.895465118	3.73627E-09	1.01059E-08	positive
PRJNA729271	stage_1D_vs_E10	ordinary_muscle_development	ENSGALG00010005630	NIT2	-0.249819963	0.164648135	-1.517296038	0.129191958	0.161170276	negative
PRJNA729271	stage_1D_vs_E10	ordinary_muscle_development	ENSGALG00010005632	ITGB1	0.221263236	0.093430882	2.368202378	0.017874755	0.025456335	positive
PRJNA729271	stage_1D_vs_E10	ordinary_muscle_development	ENSGALG00010005634	HADH	0.635998305	0.086740478	7.332197382	2.26409E-13	8.17182E-13	positive
PRJNA729271	stage_1D_vs_E10	ordinary_muscle_development	ENSGALG00010005636	ENSGALG00010005636	0.86020105	0.666080929	1.29143624	0.196552451	0.236506792	positive
PRJNA729271	stage_1D_vs_E10	ordinary_muscle_development	ENSGALG00010005637	TOPORS	-0.166581657	0.070986104	-2.346679824	0.018941524	0.026877864	negative
PRJNA729271	stage_1D_vs_E10	ordinary_muscle_development	ENSGALG00010005643	ENSGALG00010005643	-2.791931473	1.222340705	-2.284086149	0.022366468	0.0314637	negative

PRJNA729271	stage_1D_vs_E10	ordinary_muscle_development	ENSGALG00010005645	FUCA2	0.10243147	0.157195252	0.651619364	0.514646753	0.562325093	positive
PRJNA729271	stage_1D_vs_E10	ordinary_muscle_development	ENSGALG00010005649	TOMM70	-0.073228335	0.078636882	-0.931221242	0.351739132	0.400727715	negative
PRJNA729271	stage_1D_vs_E10	ordinary_muscle_development	ENSGALG00010005651	USP6NL	0.390150224	0.120457131	3.238913463	0.00119986	0.001984478	positive
PRJNA729271	stage_1D_vs_E10	ordinary_muscle_development	ENSGALG00010005656	CYP2U1	0.33566725	0.13379457	2.50882566	0.012113325	0.017717725	positive
PRJNA729271	stage_1D_vs_E10	ordinary_muscle_development	ENSGALG00010005660	UBAC2	0.653553016	0.100993728	6.471223789	9.72123E-11	2.94358E-10	positive
PRJNA729271	stage_1D_vs_E10	ordinary_muscle_development	ENSGALG00010005668	LNP1	-2.499126243	0.43862044	-5.697696725	1.21437E-08	3.14776E-08	negative
PRJNA729271	stage_1D_vs_E10	ordinary_muscle_development	ENSGALG00010005670	SGMS2	-1.330479299	0.208541078	-6.379938735	1.77159E-10	5.27618E-10	negative
PRJNA729271	stage_1D_vs_E10	ordinary_muscle_development	ENSGALG00010005675	GJA3	0.669347939	0.280401585	2.387104687	0.016981655	0.024284638	positive
PRJNA729271	stage_1D_vs_E10	ordinary_muscle_development	ENSGALG00010005679	TMEM45A	-2.29405525	0.207143228	-11.07472968	1.66382E-28	1.33983E-27	negative
PRJNA729271	stage_1D_vs_E10	ordinary_muscle_development	ENSGALG00010005680	ENSGALG00010005680	0.826080034	0.495288277	1.66787722	0.095340103	0.121640242	positive
PRJNA729271	stage_1D_vs_E10	ordinary_muscle_development	ENSGALG00010005681	EPC1	0.477876208	0.065530444	7.292430534	3.04413E-13	1.09246E-12	positive
PRJNA729271	stage_1D_vs_E10	ordinary_muscle_development	ENSGALG00010005688	ENSGALG00010005688	0.422276386	0.581337486	0.726387677	0.467601143	0.516252462	positive
PRJNA729271	stage_1D_vs_E10	ordinary_muscle_development	ENSGALG00010005692	SEC24B	0.675396303	0.143740345	4.698724662	2.61791E-06	5.64934E-06	positive
PRJNA729271	stage_1D_vs_E10	ordinary_muscle_development	ENSGALG00010005694	ENSGALG00010005694	-0.173581217	0.073124802	-2.373766681	0.017607673	0.025100631	negative
PRJNA729271	stage_1D_vs_E10	ordinary_muscle_development	ENSGALG00010005695	KIF5B	-0.212215242	0.083621752	-2.53779953	0.011155186	0.016394041	negative
PRJNA729271	stage_1D_vs_E10	ordinary_muscle_development	ENSGALG00010005696	NUBPL	-1.099004692	0.140883445	-7.800807933	6.15121E-15	2.45287E-14	negative
PRJNA729271	stage_1D_vs_E10	ordinary_muscle_development	ENSGALG00010005699	PRPF18	0.441154508	0.092577881	4.765225816	1.88642E-06	4.11555E-06	positive
PRJNA729271	stage_1D_vs_E10	ordinary_muscle_development	ENSGALG00010005704	AKAP12	-0.720064001	0.093636444	-7.689997265	1.47138E-14	5.72863E-14	negative
PRJNA729271	stage_1D_vs_E10	ordinary_muscle_development	ENSGALG00010005705	ECHDC3	0.286610362	0.125234168	2.288595568	0.022102862	0.03110797	positive
PRJNA729271	stage_1D_vs_E10	ordinary_muscle_development	ENSGALG00010005712	ENSGALG00010005712	0.042588848	0.109845098	0.38771733	0.69822523	0.735254247	positive
PRJNA729271	stage_1D_vs_E10	ordinary_muscle_development	ENSGALG00010005714	ARHGAP5	-0.392283385	0.067968196	-5.771572725	7.85351E-09	2.07239E-08	negative
PRJNA729271	stage_1D_vs_E10	ordinary_muscle_development	ENSGALG00010005716	ZBTB2	-0.315553955	0.11185653	-2.821059753	0.004786529	0.007372302	negative
PRJNA729271	stage_1D_vs_E10	ordinary_muscle_development	ENSGALG00010005720	COL25A1	-2.173785555	0.185460642	-11.72100737	9.94819E-32	9.36358E-31	negative
PRJNA729271	stage_1D_vs_E10	ordinary_muscle_development	ENSGALG00010005724	PROSER2	0.5064123	0.118046011	4.289956891	1.78708E-05	3.55478E-05	positive
PRJNA729271	stage_1D_vs_E10	ordinary_muscle_development	ENSGALG00010005738	ELAVL2	-2.534445383	0.364652484	-6.950303355	3.64502E-12	1.21542E-11	negative
PRJNA729271	stage_1D_vs_E10	ordinary_muscle_development	ENSGALG00010005740	ENSGALG00010005740	0.186185372	0.356977403	0.521560667	0.601976255	0.644910999	positive
PRJNA729271	stage_1D_vs_E10	ordinary_muscle_development	ENSGALG00010005742	GPR183	-1.421878864	0.211391377	-6.726286031	1.74048E-11	5.5459E-11	negative
PRJNA729271	stage_1D_vs_E10	ordinary_muscle_development	ENSGALG00010005743	ETNPPL	5.792818905	0.223243992	25.94837533	1.8967E-148	3.3124E-146	positive
PRJNA729271	stage_1D_vs_E10	ordinary_muscle_development	ENSGALG00010005748	AIG1	0.660075572	0.178680974	3.694157007	0.000220617	0.000394067	positive
PRJNA729271	stage_1D_vs_E10	ordinary_muscle_development	ENSGALG00010005752	RMND1	-0.240593444	0.099359516	-2.421443388	0.015459009	0.022249859	negative
PRJNA729271	stage_1D_vs_E10	ordinary_muscle_development	ENSGALG00010005755	ZMYM2	-0.057046423	0.097449637	-0.585393892	0.558282895	0.603903773	negative
PRJNA729271	stage_1D_vs_E10	ordinary_muscle_development	ENSGALG00010005756	TET2	0.961900931	0.163427836	5.885783945	3.96172E-09	1.06877E-08	positive
PRJNA729271	stage_1D_vs_E10	ordinary_muscle_development	ENSGALG00010005757	ARHGAP12	0.522325194	0.133232553	3.920402189	8.84013E-05	0.000164935	positive
PRJNA729271	stage_1D_vs_E10	ordinary_muscle_development	ENSGALG00010005758	HIVEP2	1.440720694	0.10499259	13.72211783	7.48468E-43	1.08599E-41	positive
PRJNA729271	stage_1D_vs_E10	ordinary_muscle_development	ENSGALG00010005760	GPR18	-2.822870326	0.387749862	-7.280132375	3.33493E-13	1.19357E-12	negative
PRJNA729271	stage_1D_vs_E10	ordinary_muscle_development	ENSGALG00010005763	AKAP6	1.612123532	0.142608007	11.30457933	1.2455E-29	1.05638E-28	positive
PRJNA729271	stage_1D_vs_E10	ordinary_muscle_development	ENSGALG00010005768	CXXC4	-3.578666902	0.644706138	-5.550849742	2.84284E-08	7.14407E-08	negative
PRJNA729271	stage_1D_vs_E10	ordinary_muscle_development	ENSGALG00010005769	TFG	-0.110144967	0.105141585	-1.047587092	0.294828887	0.341555684	negative
PRJNA729271	stage_1D_vs_E10	ordinary_muscle_development	ENSGALG00010005772	SVEP1	2.430072775	0.140687958	17.27278451	7.54143E-67	2.47314E-65	positive
PRJNA729271	stage_1D_vs_E10	ordinary_muscle_development	ENSGALG00010005777	ENSGALG00010005777	-0.410600297	1.023018523	-0.401361547	0.688153956	0.725803955	negative
PRJNA729271	stage_1D_vs_E10	ordinary_muscle_development	ENSGALG00010005779	ARMT1	-0.081293092	0.111544998	-0.72879191	0.466128961	0.51494088	negative
PRJNA729271	stage_1D_vs_E10	ordinary_muscle_development	ENSGALG00010005784	RASL11A	-0.004607686	0.440437501	-0.010461612	0.991652994	0.992954556	negative
PRJNA729271	stage_1D_vs_E10	ordinary_muscle_development	ENSGALG00010005785	RPL21	0.187777334	0.139810123	1.343088249	0.1792434	0.217617312	positive
PRJNA729271	stage_1D_vs_E10	ordinary_muscle_development	ENSGALG00010005789	RNASET2	-0.650860911	0.156585932	-4.156573355	3.23056E-05	6.27877E-05	negative
PRJNA729271	stage_1D_vs_E10	ordinary_muscle_development	ENSGALG00010005790	MUSK	1.145094856	0.146964169	7.791660123	6.61344E-15	2.63068E-14	positive
PRJNA729271	stage_1D_vs_E10	ordinary_muscle_development	ENSGALG00010005792	ADGRG6	-1.704317175	0.160689826	-10.6062544	2.78699E-26	2.01785E-25	negative
PRJNA729271	stage_1D_vs_E10	ordinary_muscle_development	ENSGALG00010005799	UFSP2	-0.802309778	0.098665097	-8.131647409	4.23495E-16	1.8275E-15	negative
PRJNA729271	stage_1D_vs_E10	ordinary_muscle_development	ENSGALG00010005800	FRMD4A	-0.372849281	0.108396293	-3.439686624	0.000582388	0.00099443	negative
PRJNA729271	stage_1D_vs_E10	ordinary_muscle_development	ENSGALG00010005814	UPF2	-0.104275129	0.123981666	-0.841052812	0.400318349	0.449815075	negative
PRJNA729271	stage_1D_vs_E10	ordinary_muscle_development	ENSGALG00010005817	VTA1	-0.319320121	0.070129832	-4.553270844	5.28182E-06	1.10668E-05	negative
PRJNA729271	stage_1D_vs_E10	ordinary_muscle_development	ENSGALG00010005821	DOCK9	1.112705487	0.153911374	7.229520867	4.84701E-13	1.71821E-12	positive
PRJNA729271	stage_1D_vs_E10	ordinary_muscle_development	ENSGALG00010005824	NMBR	-3.937162539	1.230267694	-3.20024866	0.001373091	0.002255547	negative
PRJNA729271	stage_1D_vs_E10	ordinary_muscle_development	ENSGALG00010005826	LRP2BP	-0.642818275	0.209788615	-3.064123743	0.002183086	0.003495011	negative
PRJNA729271	stage_1D_vs_E10	ordinary_muscle_development	ENSGALG00010005828	LPAR1	-1.812394422	0.154834178	-11.70538986	1.19608E-31	1.1207E-30	negative
PRJNA729271	stage_1D_vs_E10	ordinary_muscle_development	ENSGALG00010005829	NPAS3	0.302685816	0.131775312	2.296984242	0.021619669	0.03046341	positive
PRJNA729271	stage_1D_vs_E10	ordinary_muscle_development	ENSGALG00010005830	USP12	0.146793374	0.183085192	0.801776337	0.422682349	0.472308098	positive
PRJNA729271	stage_1D_vs_E10	ordinary_muscle_development	ENSGALG00010005831	SEC61A2	0.120431703	0.10524252	1.144325533	0.252488658	0.297135918	positive
PRJNA729271	stage_1D_vs_E10	ordinary_muscle_development	ENSGALG00010005836	NEBL	0.744912191	0.244617013	3.045218244	0.002325115	0.003710917	positive
PRJNA729271	stage_1D_vs_E10	ordinary_muscle_development	ENSGALG00010005839	RPS6KA2	-0.362825018	0.183698484	-1.975111658	0.048255478	0.064508268	negative
PRJNA729271	stage_1D_vs_E10	ordinary_muscle_development	ENSGALG00010005841	ENSGALG00010005841	-0.074438057	0.330653139	-0.225124303	0.821882575	0.846768635	negative
PRJNA729271	stage_1D_vs_E10	ordinary_muscle_development	ENSGALG00010005843	GPR12	0.871679193	0.433601812	2.010321842	0.044397137	0.059664055	positive
PRJNA729271	stage_1D_vs_E10	ordinary_muscle_development	ENSGALG00010005848	WASF3	1.257511653	0.186535392	6.741410502	1.56856E-11	5.01684E-11	positive
PRJNA729271	stage_1D_vs_E10	ordinary_muscle_development	ENSGALG00010005849	SNX25	-0.050594746	0.097661135	-0.518064279	0.604413422	0.647187158	negative
PRJNA729271	stage_1D_vs_E10	ordinary_muscle_development	ENSGALG00010005851	SLC15A1	-1.770107424	1.013777752	-1.74605077	0.08080213	0.104378119	negative
PRJNA729271	stage_1D_vs_E10	ordinary_muscle_development	ENSGALG00010005853	COMMD3	-0.626569724	0.140212098	-4.468727959	7.86861E-06	1.62403E-05	negative
PRJNA729271	stage_1D_vs_E10	ordinary_muscle_development	ENSGALG00010005858	CFAP97	-0.978944827	0.11268544	-8.687411889	3.7079E-18	1.79333E-17	negative
PRJNA729271	stage_1D_vs_E10	ordinary_muscle_development	ENSGALG00010005859	ENSGALG00010005859	-0.051063505	0.586681594	-0.087037851	0.930641426	0.940963132	negative
PRJNA729271	stage_1D_vs_E10	ordinary_muscle_development	ENSGALG00010005860	ABI3BP	1.322166238	0.21220286	6.230671165	4.64441E-10	1.34346E-09	positive
PRJNA729271	stage_1D_vs_E10	ordinary_muscle_development	ENSGALG00010005861	MPC1L	1.416091944	0.131722397	10.75057831	5.88912E-27	4.40469E-26	positive
PRJNA729271	stage_1D_vs_E10	ordinary_muscle_development	ENSGALG00010005866	DNAJC1	0.62304361	0.148106232	4.206734604	2.59087E-05	5.07839E-05	positive
PRJNA729271	stage_1D_vs_E10	ordinary_muscle_development	ENSGALG00010005871	EGLN3	0.839320204	0.196445624	4.272531956	1.93266E-05	3.83332E-05	positive
PRJNA729271	stage_1D_vs_E10	ordinary_muscle_development	ENSGALG00010005872	STK24	0.188622699	0.13518765	1.395265751	0.162935716	0.199539811	positive
PRJNA729271	stage_1D_vs_E10	ordinary_muscle_development	ENSGALG00010005875	CDK8	-0.869831671	0.151306765	-5.748795648	8.98814E-09	2.35465E-08	negative
PRJNA729271	stage_1D_vs_E10	ordinary_muscle_development	ENSGALG00010005879	FAM107B	-2.390665135	0.124422922	-19.21402506	2.82491E-82	1.40711E-80	negative
PRJNA729271	stage_1D_vs_E10	ordinary_muscle_development	ENSGALG00010005880	SLC25A4	5.242561423	0.179720404	29.17065231	4.5713E-187	1.699E-184	positive
PRJNA729271	stage_1D_vs_E10	ordinary_muscle_development	ENSGALG00010005881	CORO2A	-0.043438265	0.270394099	-0.160647975	0.872370667	0.891309848	negative
PRJNA729271	stage_1D_vs_E10	ordinary_muscle_development	ENSGALG00010005884	MYB	-1.560218818	0.275029654	-5.672911244	1.40391E-08	3.62094E-08	negative
PRJNA729271	stage_1D_vs_E10	ordinary_muscle_development	ENSGALG00010005885	SLITRK5	2.283007922	0.328149312	6.957222942	3.47046E-12	1.15935E-11	positive
PRJNA729271	stage_1D_vs_E10	ordinary_muscle_development	ENSGALG00010005887	CCNH	-0.257920364	0.084700162	-3.045098845	0.002326039	0.003711982	negative
PRJNA729271	stage_1D_vs_E10	ordinary_muscle_development	ENSGALG00010005888	IMPG2	0.546880962	0.225705646	2.422983084	0.015393643	0.022160181	positive
PRJNA729271	stage_1D_vs_E10	ordinary_muscle_development	ENSGALG00010005890	SNX32	-0.786774719	0.08940208	-8.800407311	1.3632E-18	6.75082E-18	negative
PRJNA729271	stage_1D_vs_E10	ordinary_muscle_development	ENSGALG00010005891	RNF6	0.167314784	0.116925106	1.430956866	0.152442578	0.187656489	positive
PRJNA729271	stage_1D_vs_E10	ordinary_muscle_development	ENSGALG00010005892	ENSGALG00010005892	-1.320484237	0.983600435	-1.342500664	0.179433715	0.217830126	negative
PRJNA729271	stage_1D_vs_E10	ordinary_muscle_development	ENSGALG00010005893	SFT2D1	-0.081344896	0.066892387	-1.216056112	0.223963548	0.266662693	negative
PRJNA729271	stage_1D_vs_E10	ordinary_muscle_development	ENSGALG00010005895	MLLT10	0.446637296	0.099063116	4.508613427	6.52527E-06	1.35662E-05	positive
PRJNA729271	stage_1D_vs_E10	ordinary_muscle_development	ENSGALG00010005899	ENSGALG00010005899	-0.623077214	0.088336914	-7.053418362	1.74575E-12	5.93588E-12	negative
PRJNA729271	stage_1D_vs_E10	ordinary_muscle_development	ENSGALG00010005900	SPTSSA	-0.091091524	0.08680125	-1.049426412	0.293981907	0.340631006	negative
PRJNA729271	stage_1D_vs_E10	ordinary_muscle_development	ENSGALG00010005901	ENSGALG00010005901	0.457017537	0.244339486	1.870420308	0.061425474	0.08075848	positive
PRJNA729271	stage_1D_vs_E10	ordinary_muscle_development	ENSGALG00010005902	EAPP	-0.585795088	0.101336098	-5.780714868	7.43839E-09	1.96643E-08	negative
PRJNA729271	stage_1D_vs_E10	ordinary_muscle_development	ENSGALG00010005903	PRR18	-0.985886292	0.248414346	-3.968717208	7.22606E-05	0.000135975	negative
PRJNA729271	stage_1D_vs_E10	ordinary_muscle_development	ENSGALG00010005905	CITED2	0.985760358	0.199216954	4.948175042	7.49125E-07	1.68926E-06	positive
PRJNA729271	stage_1D_vs_E10	ordinary_muscle_development	ENSGALG00010005909	BRMS1L	-0.822021607	0.102255443	-8.038903211	9.06461E-16	3.82063E-15	negative
PRJNA729271	stage_1D_vs_E10	ordinary_muscle_development	ENSGALG00010005910	ENSGALG00010005910	1.13685098	0.46141967	2.463811262	0.013746848	0.019932035	positive
PRJNA729271	stage_1D_vs_E10	ordinary_muscle_development	ENSGALG00010005911	TBXT	-1.94727081	0.447600521	-4.350465916	1.35849E-05	2.73946E-05	negative
PRJNA729271	stage_1D_vs_E10	ordinary_muscle_development	ENSGALG00010005913	NFKBIA	2.501743481	0.142333179	17.57667111	3.71773E-69	1.33387E-67	positive
PRJNA729271	stage_1D_vs_E10	ordinary_muscle_development	ENSGALG00010005915	PAPSS1	-2.04282294	0.100111487	-20.40547988	1.49487E-92	9.46209E-91	negative
PRJNA729271	stage_1D_vs_E10	ordinary_muscle_development	ENSGALG00010005926	NUDT5	0.015761316	0.230385278	0.06841286	0.945456985	0.953614849	positive
PRJNA729271	stage_1D_vs_E10	ordinary_muscle_development	ENSGALG00010005934	SLITRK6	-4.615157413	0.284029669	-16.24885678	2.27568E-59	6.11986E-58	negative
PRJNA729271	stage_1D_vs_E10	ordinary_muscle_development	ENSGALG00010005935	ATP8A2	-2.689265953	0.209575283	-12.83198052	1.08553E-37	1.31014E-36	negative
PRJNA729271	stage_1D_vs_E10	ordinary_muscle_development	ENSGALG00010005941	TXLNB	4.476800328	0.181405558	24.67840776	1.8242E-134	2.5183E-132	positive
PRJNA729271	stage_1D_vs_E10	ordinary_muscle_development	ENSGALG00010005943	PROX2	-2.980592335	0.971851359	-3.06692202	0.002162753	0.003464372	negative
PRJNA729271	stage_1D_vs_E10	ordinary_muscle_development	ENSGALG00010005949	SLITRK1	0.978447802	0.343554703	2.848011667	0.004399331	0.006815037	positive
PRJNA729271	stage_1D_vs_E10	ordinary_muscle_development	ENSGALG00010005956	TXNL4B	0.160210001	0.11068404	1.447453502	0.147769951	0.182462342	positive
PRJNA729271	stage_1D_vs_E10	ordinary_muscle_development	ENSGALG00010005963	UGCG	1.576384076	0.102393488	15.39535484	1.75857E-53	3.82166E-52	positive
PRJNA729271	stage_1D_vs_E10	ordinary_muscle_development	ENSGALG00010005964	HSPA14	-0.693560857	0.114394728	-6.062874282	1.3371E-09	3.73867E-09	negative
PRJNA729271	stage_1D_vs_E10	ordinary_muscle_development	ENSGALG00010005966	PLXDC2	2.045904638	0.152826575	13.38710001	7.1935E-41	9.78141E-40	positive
PRJNA729271	stage_1D_vs_E10	ordinary_muscle_development	ENSGALG00010005967	GJD2	-3.657411396	0.291590183	-12.54298537	4.3432E-36	4.91449E-35	negative
PRJNA729271	stage_1D_vs_E10	ordinary_muscle_development	ENSGALG00010005969	HECA	0.430804816	0.065954188	6.531879603	6.49494E-11	1.98994E-10	positive
PRJNA729271	stage_1D_vs_E10	ordinary_muscle_development	ENSGALG00010005970	SHISA2	-2.366907269	0.120212311	-19.68939158	2.65864E-86	1.44876E-84	negative
PRJNA729271	stage_1D_vs_E10	ordinary_muscle_development	ENSGALG00010005975	ABRACL	-1.125956896	0.108764648	-10.35223229	4.0884E-25	2.82332E-24	negative
PRJNA729271	stage_1D_vs_E10	ordinary_muscle_development	ENSGALG00010005978	VPS16	0.301410864	0.114264444	2.637836003	0.008343692	0.012457954	positive
PRJNA729271	stage_1D_vs_E10	ordinary_muscle_development	ENSGALG00010005979	SUV39H2	0.206686284	0.091842569	2.250441	0.024420965	0.034151653	positive
PRJNA729271	stage_1D_vs_E10	ordinary_muscle_development	ENSGALG00010005981	AREL1	0.033105124	0.099166055	0.333835247	0.738503885	0.771948785	positive
PRJNA729271	stage_1D_vs_E10	ordinary_muscle_development	ENSGALG00010005984	IDH3B	1.976655407	0.117410158	16.83547183	1.34115E-63	4.08403E-62	positive
PRJNA729271	stage_1D_vs_E10	ordinary_muscle_development	ENSGALG00010005985	ENSGALG00010005985	-3.675342797	0.32259121	-11.39318952	4.52131E-30	3.91964E-29	negative
PRJNA729271	stage_1D_vs_E10	ordinary_muscle_development	ENSGALG00010005988	NUP58	-0.231378233	0.089040886	-2.598561651	0.009361523	0.013897509	negative
PRJNA729271	stage_1D_vs_E10	ordinary_muscle_development	ENSGALG00010005989	FCF1	-0.562847043	0.100809428	-5.58327782	2.36027E-08	5.98324E-08	negative
PRJNA729271	stage_1D_vs_E10	ordinary_muscle_development	ENSGALG00010005990	SLC2A12	1.23012093	0.151841068	8.101371692	5.43429E-16	2.32703E-15	positive
PRJNA729271	stage_1D_vs_E10	ordinary_muscle_development	ENSGALG00010005991	FARP1	-0.460797678	0.088129143	-5.228664011	1.70739E-07	4.04986E-07	negative
PRJNA729271	stage_1D_vs_E10	ordinary_muscle_development	ENSGALG00010005997	ENSGALG00010005997	1.658450554	0.229897839	7.213858815	5.43881E-13	1.92001E-12	positive
PRJNA729271	stage_1D_vs_E10	ordinary_muscle_development	ENSGALG00010005998	YLPM1	0.573599748	0.132505843	4.328863809	1.49881E-05	3.00453E-05	positive
PRJNA729271	stage_1D_vs_E10	ordinary_muscle_development	ENSGALG00010005999	PDE10A	0.255460595	0.293301286	0.870983548	0.383763136	0.432823425	positive
PRJNA729271	stage_1D_vs_E10	ordinary_muscle_development	ENSGALG00010006001	NOP56	-0.589871593	0.140022643	-4.212687176	2.5235E-05	4.95438E-05	negative
PRJNA729271	stage_1D_vs_E10	ordinary_muscle_development	ENSGALG00010006004	DCLRE1C	-1.224956112	0.149588569	-8.188835033	2.63767E-16	1.14986E-15	negative
PRJNA729271	stage_1D_vs_E10	ordinary_muscle_development	ENSGALG00010006009	MALRD1	0.434460576	0.629651162	0.690002024	0.490192915	0.538528596	positive
PRJNA729271	stage_1D_vs_E10	ordinary_muscle_development	ENSGALG00010006010	IPO5	-0.298463682	0.080777725	-3.694876048	0.000219994	0.000393002	negative
PRJNA729271	stage_1D_vs_E10	ordinary_muscle_development	ENSGALG00010006013	TRMT10C	-0.408315872	0.106270366	-3.842236425	0.000121918	0.000224407	negative
PRJNA729271	stage_1D_vs_E10	ordinary_muscle_development	ENSGALG00010006015	CDC123	-0.828771647	0.112290231	-7.380621122	1.57553E-13	5.74957E-13	negative
PRJNA729271	stage_1D_vs_E10	ordinary_muscle_development	ENSGALG00010006016	MTMR6	0.071928094	0.076607729	0.938914326	0.347774733	0.396616425	positive
PRJNA729271	stage_1D_vs_E10	ordinary_muscle_development	ENSGALG00010006019	SLC10A2	-2.480064808	1.023336049	-2.423509666	0.015371344	0.022130277	negative
PRJNA729271	stage_1D_vs_E10	ordinary_muscle_development	ENSGALG00010006021	ENSGALG00010006021	0.425906465	0.12536548	3.397318492	0.000680497	0.001154607	positive

PRJNA729271	stage_1D_vs_E10	ordinary_muscle_development	ENSGALG00010006022	AFG2A	-0.552348148	0.090363997	-6.112480294	9.80944E-10	2.77587E-09	negative
PRJNA729271	stage_1D_vs_E10	ordinary_muscle_development	ENSGALG00010006024	AMER2	-1.37780243	0.356498031	-3.864824801	0.000111169	0.000205404	negative
PRJNA729271	stage_1D_vs_E10	ordinary_muscle_development	ENSGALG00010006025	TNFAIP3	1.146192841	0.148016683	7.743673361	9.65849E-15	3.80745E-14	positive
PRJNA729271	stage_1D_vs_E10	ordinary_muscle_development	ENSGALG00010006028	ARL5B	0.201000175	0.12774665	1.573428151	0.115619713	0.145566554	positive
PRJNA729271	stage_1D_vs_E10	ordinary_muscle_development	ENSGALG00010006029	HSDL2	0.68892535	0.134336815	5.12834364	2.92303E-07	6.79866E-07	positive
PRJNA729271	stage_1D_vs_E10	ordinary_muscle_development	ENSGALG00010006031	ERCC5	0.289982372	0.096904849	2.992444395	0.002767531	0.004380362	positive
PRJNA729271	stage_1D_vs_E10	ordinary_muscle_development	ENSGALG00010006033	NSUN6	-0.965245463	0.180422138	-5.349928073	8.79892E-08	2.1335E-07	negative
PRJNA729271	stage_1D_vs_E10	ordinary_muscle_development	ENSGALG00010006035	ADH1C	-0.817482495	0.723076301	-1.130561871	0.258239544	0.303263565	negative
PRJNA729271	stage_1D_vs_E10	ordinary_muscle_development	ENSGALG00010006037	METTL21EP	4.695753834	0.683062538	6.874559174	6.21818E-12	2.03643E-11	positive
PRJNA729271	stage_1D_vs_E10	ordinary_muscle_development	ENSGALG00010006038	ENSGALG00010006038	0.01709931	0.197104139	0.086752669	0.93086811	0.941061045	positive
PRJNA729271	stage_1D_vs_E10	ordinary_muscle_development	ENSGALG00010006039	PCNP	-0.575297083	0.125498384	-4.584099535	4.55947E-06	9.60045E-06	negative
PRJNA729271	stage_1D_vs_E10	ordinary_muscle_development	ENSGALG00010006041	QKI	0.588205885	0.085734898	6.86075216	6.84989E-12	2.23876E-11	positive
PRJNA729271	stage_1D_vs_E10	ordinary_muscle_development	ENSGALG00010006047	ZBTB11	0.404140629	0.13444175	3.006064928	0.002646525	0.004197546	positive
PRJNA729271	stage_1D_vs_E10	ordinary_muscle_development	ENSGALG00010006049	AIMP1	-0.251128146	0.124215627	-2.021711383	0.043206177	0.05820927	negative
PRJNA729271	stage_1D_vs_E10	ordinary_muscle_development	ENSGALG00010006052	RALGAPA1	-0.10686222	0.08136464	-1.313374216	0.189056881	0.22853636	negative
PRJNA729271	stage_1D_vs_E10	ordinary_muscle_development	ENSGALG00010006053	TMEM243	-1.315130383	0.244919737	-5.369638223	7.88947E-08	1.92069E-07	negative
PRJNA729271	stage_1D_vs_E10	ordinary_muscle_development	ENSGALG00010006054	ENSGALG00010006054	0.443340398	2.294838746	0.193190218	0.846809999	0.869053451	positive
PRJNA729271	stage_1D_vs_E10	ordinary_muscle_development	ENSGALG00010006055	ENSGALG00010006055	0.506390002	0.645047875	0.78504251	0.432428645	0.481898455	positive
PRJNA729271	stage_1D_vs_E10	ordinary_muscle_development	ENSGALG00010006059	BIVM	-0.568213548	0.055940483	-10.15746585	3.06951E-24	2.02055E-23	negative
PRJNA729271	stage_1D_vs_E10	ordinary_muscle_development	ENSGALG00010006060	ENSGALG00010006060	-0.316532355	0.400927234	-0.789500757	0.429819387	0.479417903	negative
PRJNA729271	stage_1D_vs_E10	ordinary_muscle_development	ENSGALG00010006061	RPL24	0.08793323	0.159033845	0.552921483	0.580317176	0.624801119	positive
PRJNA729271	stage_1D_vs_E10	ordinary_muscle_development	ENSGALG00010006062	ENSGALG00010006062	0.002554309	0.680987274	0.003750892	0.997007229	0.997695532	positive
PRJNA729271	stage_1D_vs_E10	ordinary_muscle_development	ENSGALG00010006063	POGLUT2	-0.866975919	0.101420126	-8.548361671	1.24848E-17	5.85465E-17	negative
PRJNA729271	stage_1D_vs_E10	ordinary_muscle_development	ENSGALG00010006064	DMTF1	0.161965674	0.080736983	2.006090247	0.04484662	0.060251345	positive
PRJNA729271	stage_1D_vs_E10	ordinary_muscle_development	ENSGALG00010006065	WDR54	-0.531790617	0.166436721	-3.195151979	0.001397572	0.002293943	negative
PRJNA729271	stage_1D_vs_E10	ordinary_muscle_development	ENSGALG00010006066	ARHGAP18	-0.272490799	0.120807918	-2.255570681	0.024097531	0.033725375	negative
PRJNA729271	stage_1D_vs_E10	ordinary_muscle_development	ENSGALG00010006068	PACRG	2.136221426	0.25691162	8.315005077	9.17483E-17	4.10461E-16	positive
PRJNA729271	stage_1D_vs_E10	ordinary_muscle_development	ENSGALG00010006069	TTR	-0.415698619	1.498967223	-0.277323355	0.781531828	0.810032453	negative
PRJNA729271	stage_1D_vs_E10	ordinary_muscle_development	ENSGALG00010006070	TMEM242	0.432552596	0.114060511	3.792308058	0.000149254	0.000272438	positive
PRJNA729271	stage_1D_vs_E10	ordinary_muscle_development	ENSGALG00010006071	TEX30	0.50819403	0.236365393	2.15003569	0.031552392	0.043379676	positive
PRJNA729271	stage_1D_vs_E10	ordinary_muscle_development	ENSGALG00010006072	ENSGALG00010006072	0.214097404	0.233236232	0.917942301	0.358649074	0.407542986	positive
PRJNA729271	stage_1D_vs_E10	ordinary_muscle_development	ENSGALG00010006073	CEP97	0.298520958	0.102971837	2.899054419	0.003742899	0.005838085	positive
PRJNA729271	stage_1D_vs_E10	ordinary_muscle_development	ENSGALG00010006075	SOX21	-3.788025247	0.390257025	-9.706488289	2.82917E-22	1.70657E-21	negative
PRJNA729271	stage_1D_vs_E10	ordinary_muscle_development	ENSGALG00010006076	PODXL	3.008571963	0.162986078	18.45907329	4.4084E-76	1.87941E-74	positive
PRJNA729271	stage_1D_vs_E10	ordinary_muscle_development	ENSGALG00010006077	TBCK	-0.742079853	0.100797714	-7.362070266	1.8108E-13	6.57832E-13	negative
PRJNA729271	stage_1D_vs_E10	ordinary_muscle_development	ENSGALG00010006078	SPATA13	-1.1852059	0.162072098	-7.312831223	2.61571E-13	9.41047E-13	negative
PRJNA729271	stage_1D_vs_E10	ordinary_muscle_development	ENSGALG00010006079	ENSGALG00010006079	-0.798399561	0.819551729	-0.974190563	0.329961929	0.378395821	negative
PRJNA729271	stage_1D_vs_E10	ordinary_muscle_development	ENSGALG00010006080	ENSGALG00010006080	-0.820268659	0.207457263	-3.953916326	7.68823E-05	0.000144241	negative
PRJNA729271	stage_1D_vs_E10	ordinary_muscle_development	ENSGALG00010006081	ZDHHC14	0.611069816	0.077851916	7.849130046	4.18933E-15	1.69007E-14	positive
PRJNA729271	stage_1D_vs_E10	ordinary_muscle_development	ENSGALG00010006083	CACNB2	-0.554586771	0.169560682	-3.270727415	0.001072713	0.001783343	negative
PRJNA729271	stage_1D_vs_E10	ordinary_muscle_development	ENSGALG00010006084	TMEM68	-0.904265221	0.150130878	-6.023179452	1.71024E-09	4.7472E-09	negative
PRJNA729271	stage_1D_vs_E10	ordinary_muscle_development	ENSGALG00010006085	DLST	0.789133378	0.089831639	8.784581793	1.56947E-18	7.75111E-18	positive
PRJNA729271	stage_1D_vs_E10	ordinary_muscle_development	ENSGALG00010006086	ENSGALG00010006086	0.555252266	0.284168328	1.953955498	0.05070648	0.0675729	positive
PRJNA729271	stage_1D_vs_E10	ordinary_muscle_development	ENSGALG00010006087	ENSGALG00010006087	-0.286822541	0.583640117	-0.49143733	0.623117167	0.665101865	negative
PRJNA729271	stage_1D_vs_E10	ordinary_muscle_development	ENSGALG00010006091	RTKN	-0.561861991	0.10161445	-5.529351325	3.21417E-08	8.04514E-08	negative
PRJNA729271	stage_1D_vs_E10	ordinary_muscle_development	ENSGALG00010006096	EDIL3	1.148355707	0.235641644	4.873313932	1.09742E-06	2.44385E-06	positive
PRJNA729271	stage_1D_vs_E10	ordinary_muscle_development	ENSGALG00010006100	RPS6KL1	-1.417249633	0.20462845	-6.925965731	4.33011E-12	1.43332E-11	negative
PRJNA729271	stage_1D_vs_E10	ordinary_muscle_development	ENSGALG00010006101	ENSGALG00010006101	-0.070076438	0.116919464	-0.599356475	0.548935195	0.594902105	negative
PRJNA729271	stage_1D_vs_E10	ordinary_muscle_development	ENSGALG00010006103	NXPE3	-0.448548683	0.118531807	-3.784205231	0.000154201	0.000280937	negative
PRJNA729271	stage_1D_vs_E10	ordinary_muscle_development	ENSGALG00010006106	SHOC1	2.22556291	0.368797933	6.034640416	1.59317E-09	4.43244E-09	positive
PRJNA729271	stage_1D_vs_E10	ordinary_muscle_development	ENSGALG00010006107	PGF	1.634776882	0.195228231	8.373670502	5.58508E-17	2.52829E-16	positive
PRJNA729271	stage_1D_vs_E10	ordinary_muscle_development	ENSGALG00010006108	MKLN1	0.294637368	0.061015038	4.828930349	1.37268E-06	3.0317E-06	positive
PRJNA729271	stage_1D_vs_E10	ordinary_muscle_development	ENSGALG00010006111	WBP1	-0.550850577	0.085357768	-6.453432302	1.09345E-10	3.30199E-10	negative
PRJNA729271	stage_1D_vs_E10	ordinary_muscle_development	ENSGALG00010006113	EIF2B2	-0.484877985	0.168921184	-2.870439189	0.00409902	0.006363425	negative
PRJNA729271	stage_1D_vs_E10	ordinary_muscle_development	ENSGALG00010006118	NPNT	-2.905748319	0.209171515	-13.89170182	7.11251E-44	1.07057E-42	negative
PRJNA729271	stage_1D_vs_E10	ordinary_muscle_development	ENSGALG00010006120	MOGS	0.504734902	0.134240284	3.759936181	0.000169957	0.00030821	positive
PRJNA729271	stage_1D_vs_E10	ordinary_muscle_development	ENSGALG00010006121	ENSGALG00010006121	-0.690323614	0.325302104	-2.12210006	0.033829336	0.046334332	negative
PRJNA729271	stage_1D_vs_E10	ordinary_muscle_development	ENSGALG00010006123	MLH3	-0.118052092	0.103354088	-1.142210191	0.253366661	0.298072376	negative
PRJNA729271	stage_1D_vs_E10	ordinary_muscle_development	ENSGALG00010006126	ENSGALG00010006126	4.865615487	0.28159906	17.27852177	6.82755E-67	2.24921E-65	positive
PRJNA729271	stage_1D_vs_E10	ordinary_muscle_development	ENSGALG00010006128	ACYP1	0.403307854	0.125331471	3.217929638	0.001291195	0.002128254	positive
PRJNA729271	stage_1D_vs_E10	ordinary_muscle_development	ENSGALG00010006131	ENSGALG00010006131	0.846818051	0.125807319	6.731071433	1.68418E-11	5.37005E-11	positive
PRJNA729271	stage_1D_vs_E10	ordinary_muscle_development	ENSGALG00010006132	MIPEP	-0.078234591	0.132914376	-0.588608945	0.556123632	0.601792612	negative
PRJNA729271	stage_1D_vs_E10	ordinary_muscle_development	ENSGALG00010006134	K123	2.277730301	0.725254634	3.140593928	0.001686056	0.002740149	positive
PRJNA729271	stage_1D_vs_E10	ordinary_muscle_development	ENSGALG00010006135	ZC2HC1C	-0.483617808	0.147865124	-3.270668528	0.001072936	0.00178351	negative
PRJNA729271	stage_1D_vs_E10	ordinary_muscle_development	ENSGALG00010006139	GAREM1	0.02702908	0.129869696	0.208124609	0.835131673	0.85870991	positive
PRJNA729271	stage_1D_vs_E10	ordinary_muscle_development	ENSGALG00010006146	CCDC142	0.369552013	0.183159491	2.017651447	0.043627575	0.058711512	positive
PRJNA729271	stage_1D_vs_E10	ordinary_muscle_development	ENSGALG00010006147	ENSGALG00010006147	2.877887122	0.215642831	13.34561926	1.25622E-40	1.69228E-39	positive
PRJNA729271	stage_1D_vs_E10	ordinary_muscle_development	ENSGALG00010006151	ENSGALG00010006151	-0.096608319	0.157180401	-0.614633366	0.538796874	0.585227478	negative
PRJNA729271	stage_1D_vs_E10	ordinary_muscle_development	ENSGALG00010006155	TNFRSF19	-1.642637894	0.194370681	-8.451057955	2.88675E-17	1.32878E-16	negative
PRJNA729271	stage_1D_vs_E10	ordinary_muscle_development	ENSGALG00010006156	NFKBIZ	1.100060854	0.19763797	5.566040049	2.60593E-08	6.57379E-08	positive
PRJNA729271	stage_1D_vs_E10	ordinary_muscle_development	ENSGALG00010006157	SNX9	0.357692474	0.073551664	4.863145904	1.15535E-06	2.56694E-06	positive
PRJNA729271	stage_1D_vs_E10	ordinary_muscle_development	ENSGALG00010006163	ENSGALG00010006163	0.598866654	0.528558029	1.133019689	0.257205985	0.302221203	positive
PRJNA729271	stage_1D_vs_E10	ordinary_muscle_development	ENSGALG00010006172	ENSGALG00010006172	0.532373751	0.192136816	2.770805516	0.005591781	0.008538173	positive
PRJNA729271	stage_1D_vs_E10	ordinary_muscle_development	ENSGALG00010006173	TBPL1	-0.324061514	0.082568996	-3.924736045	8.68249E-05	0.000162139	negative
PRJNA729271	stage_1D_vs_E10	ordinary_muscle_development	ENSGALG00010006175	IL2RA	4.325538725	0.500104828	8.649264084	5.18326E-18	2.4845E-17	positive
PRJNA729271	stage_1D_vs_E10	ordinary_muscle_development	ENSGALG00010006176	MMRIL3	1.570220942	0.217228241	7.228438313	4.8858E-13	1.73153E-12	positive
PRJNA729271	stage_1D_vs_E10	ordinary_muscle_development	ENSGALG00010006177	NEK9	-0.481401897	0.113852793	-4.228283601	2.35481E-05	4.63322E-05	negative
PRJNA729271	stage_1D_vs_E10	ordinary_muscle_development	ENSGALG00010006178	RPS23	-0.075277799	0.140878695	-0.534344808	0.593102997	0.636674896	negative
PRJNA729271	stage_1D_vs_E10	ordinary_muscle_development	ENSGALG00010006180	CD200L	4.74900424	1.07554536	4.415438361			positive
PRJNA729271	stage_1D_vs_E10	ordinary_muscle_development	ENSGALG00010006183	PRKN	1.640185659	0.137936611	11.89086532	1.32032E-32	1.29486E-31	positive
PRJNA729271	stage_1D_vs_E10	ordinary_muscle_development	ENSGALG00010006184	LBX2	-0.151306293	0.281404175	-0.537683185	0.59079579	0.634621682	negative
PRJNA729271	stage_1D_vs_E10	ordinary_muscle_development	ENSGALG00010006187	TCF21	-0.561881272	0.600314599	-0.935978024	0.349284503	0.398087661	negative
PRJNA729271	stage_1D_vs_E10	ordinary_muscle_development	ENSGALG00010006188	TMED10	0.142266986	0.074719869	1.904004774	0.056909562	0.075162105	positive
PRJNA729271	stage_1D_vs_E10	ordinary_muscle_development	ENSGALG00010006193	RBM17	-0.617568304	0.099170849	-6.227316879	4.74491E-10	1.37171E-09	negative
PRJNA729271	stage_1D_vs_E10	ordinary_muscle_development	ENSGALG00010006197	ENSGALG00010006197	-0.669591302	0.429345808	-1.559561754	0.118863481	0.149266739	negative
PRJNA729271	stage_1D_vs_E10	ordinary_muscle_development	ENSGALG00010006198	ENSGALG00010006198	0.624918022	0.706223737	0.884872583	0.376225357	0.42541435	positive
PRJNA729271	stage_1D_vs_E10	ordinary_muscle_development	ENSGALG00010006200	GSTCD	-1.330389141	0.100940064	-13.1799911	1.14404E-39	1.48459E-38	negative
PRJNA729271	stage_1D_vs_E10	ordinary_muscle_development	ENSGALG00010006201	SSTR1	-2.451183788	0.363798806	-6.737745552	1.60863E-11	5.13932E-11	negative
PRJNA729271	stage_1D_vs_E10	ordinary_muscle_development	ENSGALG00010006202	SACS	-0.461903031	0.200266067	-2.306446804	0.021085679	0.029751477	negative
PRJNA729271	stage_1D_vs_E10	ordinary_muscle_development	ENSGALG00010006203	MMRIL4	1.806824188	0.174878343	10.33189222	5.05534E-25	3.47285E-24	positive
PRJNA729271	stage_1D_vs_E10	ordinary_muscle_development	ENSGALG00010006205	CGGBP1	-0.346027486	0.073667801	-4.697133357	2.63838E-06	5.69097E-06	negative
PRJNA729271	stage_1D_vs_E10	ordinary_muscle_development	ENSGALG00010006207	ENSGALG00010006207	-1.176252154	0.215739602	-5.452184678	4.97547E-08	1.22966E-07	negative
PRJNA729271	stage_1D_vs_E10	ordinary_muscle_development	ENSGALG00010006212	FOS	4.718872026	0.365773566	12.9010745	4.43865E-38	5.43293E-37	positive
PRJNA729271	stage_1D_vs_E10	ordinary_muscle_development	ENSGALG00010006219	CLEC14A	1.934620308	0.182215107	10.61723331	2.47797E-26	1.79951E-25	positive
PRJNA729271	stage_1D_vs_E10	ordinary_muscle_development	ENSGALG00010006220	SGCG	1.953578241	0.170838478	11.4352356	2.78779E-30	2.45349E-29	positive
PRJNA729271	stage_1D_vs_E10	ordinary_muscle_development	ENSGALG00010006223	ATG10	0.081480225	0.11192499	0.727989566	0.466619973	0.515319642	positive
PRJNA729271	stage_1D_vs_E10	ordinary_muscle_development	ENSGALG00010006227	AGPAT4	-1.263182204	0.146090579	-8.646568538	5.30712E-18	2.54219E-17	negative
PRJNA729271	stage_1D_vs_E10	ordinary_muscle_development	ENSGALG00010006229	INTS12	-0.570115884	0.134565498	-4.236716631	2.26812E-05	4.47237E-05	negative
PRJNA729271	stage_1D_vs_E10	ordinary_muscle_development	ENSGALG00010006232	ZNF654	0.034050533	0.103521244	0.328923143	0.742213781	0.774932562	positive
PRJNA729271	stage_1D_vs_E10	ordinary_muscle_development	ENSGALG00010006236	C3orf38	0.088251662	0.084652034	1.042522643	0.297169433	0.344019721	positive
PRJNA729271	stage_1D_vs_E10	ordinary_muscle_development	ENSGALG00010006237	ENSGALG00010006237	0.492508747	0.322104749	1.529032868	0.126256296	0.15775235	positive
PRJNA729271	stage_1D_vs_E10	ordinary_muscle_development	ENSGALG00010006239	EYA4	0.779401258	0.162662335	4.791528777	1.65515E-06	3.63122E-06	positive
PRJNA729271	stage_1D_vs_E10	ordinary_muscle_development	ENSGALG00010006241	MRC1	1.800865283	0.244476019	7.366224675	1.75528E-13	6.38303E-13	positive
PRJNA729271	stage_1D_vs_E10	ordinary_muscle_development	ENSGALG00010006242	ENSGALG00010006242	-0.368557198	0.598958695	-0.615329906	0.538336871	0.58481548	negative
PRJNA729271	stage_1D_vs_E10	ordinary_muscle_development	ENSGALG00010006244	EPHA3	-1.284410174	0.162098506	-7.92363982	2.30657E-15	9.47669E-15	negative
PRJNA729271	stage_1D_vs_E10	ordinary_muscle_development	ENSGALG00010006245	PFKFB3	0.520629383	0.509352729	1.022139183	0.30671503	0.353911349	positive
PRJNA729271	stage_1D_vs_E10	ordinary_muscle_development	ENSGALG00010006246	SUPT20H	-0.370036315	0.099859645	-3.705564096	0.000210921	0.000377911	negative
PRJNA729271	stage_1D_vs_E10	ordinary_muscle_development	ENSGALG00010006249	C9orf72	0.334664923	0.104612272	3.199098125	0.001378582	0.002264055	positive
PRJNA729271	stage_1D_vs_E10	ordinary_muscle_development	ENSGALG00010006251	SEMA3E	-3.212108599	0.374113778	-8.585913658	9.01173E-18	4.26879E-17	negative
PRJNA729271	stage_1D_vs_E10	ordinary_muscle_development	ENSGALG00010006252	COX7C	1.051746277	0.136573747	7.700940362	1.35069E-14	5.27145E-14	positive
PRJNA729271	stage_1D_vs_E10	ordinary_muscle_development	ENSGALG00010006254	PROS1	2.060357281	0.136976818	15.04164951	3.91668E-51	7.86319E-50	positive
PRJNA729271	stage_1D_vs_E10	ordinary_muscle_development	ENSGALG00010006259	PPA2	-0.158281374	0.087687652	-1.805058865	0.071065471	0.09255944	negative
PRJNA729271	stage_1D_vs_E10	ordinary_muscle_development	ENSGALG00010006260	MAP3K4	0.655017782	0.159245383	4.11326073	3.90109E-05	7.52146E-05	positive
PRJNA729271	stage_1D_vs_E10	ordinary_muscle_development	ENSGALG00010006261	PCLO	-1.608536046	0.490714818	-3.27794472	0.001045659	0.001740763	negative
PRJNA729271	stage_1D_vs_E10	ordinary_muscle_development	ENSGALG00010006263	EXOSC8	-1.133102943	0.15046389	-7.530730101	5.04574E-14	1.90414E-13	negative
PRJNA729271	stage_1D_vs_E10	ordinary_muscle_development	ENSGALG00010006266	CENPW	-1.883787713	0.21914409	-8.596114603	8.24605E-18	3.91249E-17	negative
PRJNA729271	stage_1D_vs_E10	ordinary_muscle_development	ENSGALG00010006269	ENSGALG00010006269	1.315850513	1.279599437	1.028330019	0.303794615	0.350868845	positive
PRJNA729271	stage_1D_vs_E10	ordinary_muscle_development	ENSGALG00010006270	STAM	0.662853356	0.091823867	7.218747995	5.24684E-13	1.85359E-12	positive
PRJNA729271	stage_1D_vs_E10	ordinary_muscle_development	ENSGALG00010006273	ARL13B	-0.192685202	0.098119469	-1.963781541	0.049555432	0.066154539	negative
PRJNA729271	stage_1D_vs_E10	ordinary_muscle_development	ENSGALG00010006277	GNG10	-1.799052349	0.163857735	-10.97935564	4.80338E-28	3.76555E-27	negative
PRJNA729271	stage_1D_vs_E10	ordinary_muscle_development	ENSGALG00010006279	HACD1	3.243772517	0.141363695	22.94629125	1.6046E-116	1.7102E-114	positive
PRJNA729271	stage_1D_vs_E10	ordinary_muscle_development	ENSGALG00010006281	TRMT11	-0.013201527	0.080600677	-0.16378928	0.869897031	0.889165383	negative
PRJNA729271	stage_1D_vs_E10	ordinary_muscle_development	ENSGALG00010006283	DNAJC25	-0.028482829	0.107415266	-0.265165556	0.790881928	0.818611364	negative
PRJNA729271	stage_1D_vs_E10	ordinary_muscle_development	ENSGALG00010006285	FZD8	-0.621957789	0.153774404	-4.04461192	5.24099E-05	9.99054E-05	negative
PRJNA729271	stage_1D_vs_E10	ordinary_muscle_development	ENSGALG00010006287	ECPAS	0.917830137	0.122482361	7.493569937	6.70251E-14	2.50394E-13	positive
PRJNA729271	stage_1D_vs_E10	ordinary_muscle_development	ENSGALG00010006288	ARF5	-3.870160177	0.196383674	-19.70713804	1.87263E-86	1.02429E-84	negative
PRJNA729271	stage_1D_vs_E10	ordinary_muscle_development	ENSGALG00010006289	SKIDA1	-2.092185866	0.151662412	-13.79501911	2.73081E-43	4.02676E-42	negative
PRJNA729271	stage_1D_vs_E10	ordinary_muscle_development	ENSGALG00010006291	ENSGALG00010006291	1.306248992	0.197126698	6.626443844	3.4387E-11	1.07469E-10	positive
PRJNA729271	stage_1D_vs_E10	ordinary_muscle_development	ENSGALG00010006293	SLC22A3	1.614009273	0.337090466	4.788059688	1.68402E-06	3.69119E-06	positive
PRJNA729271	stage_1D_vs_E10	ordinary_muscle_development	ENSGALG00010006300	GCC1	0.065188761	0.119124815	0.547230744	0.584220217	0.628677573	positive
PRJNA729271	stage_1D_vs_E10	ordinary_muscle_development	ENSGALG00010006301	ENSGALG00010006301	0.263747857	0.147248218	1.7911786	0.073264639	0.095261118	positive

PRJNA729271	stage_1D_vs_E10	ordinary_muscle_development	ENSGALG00010006310	ALG5	-0.281605992	0.110179471	-2.555884416	0.01059183	0.015599326	negative
PRJNA729271	stage_1D_vs_E10	ordinary_muscle_development	ENSGALG00010006311	ABCD4	-0.299293314	0.11448473	-2.614264041	0.00894199	0.01330331	negative
PRJNA729271	stage_1D_vs_E10	ordinary_muscle_development	ENSGALG00010006323	ANAPC10	-0.135859662	0.105166896	-1.291848177	0.196409727	0.236399485	negative
PRJNA729271	stage_1D_vs_E10	ordinary_muscle_development	ENSGALG00010006327	DENND6B	0.059201709	0.110735223	0.534624008	0.592909879	0.63656238	positive
PRJNA729271	stage_1D_vs_E10	ordinary_muscle_development	ENSGALG00010006328	SYNDIG1L	-1.712665543	0.439161116	-3.899856982	9.62495E-05	0.000178887	negative
PRJNA729271	stage_1D_vs_E10	ordinary_muscle_development	ENSGALG00010006330	STX19	0.804529011	0.412507216	1.950339241	0.051135699	0.068119837	positive
PRJNA729271	stage_1D_vs_E10	ordinary_muscle_development	ENSGALG00010006331	SMAD9	-0.917820163	0.154146153	-5.95422037	2.61315E-09	7.16021E-09	negative
PRJNA729271	stage_1D_vs_E10	ordinary_muscle_development	ENSGALG00010006336	ALDH8A1	1.487534872	0.879636762	1.691078564	0.090821799	0.116326056	positive
PRJNA729271	stage_1D_vs_E10	ordinary_muscle_development	ENSGALG00010006339	ENSGALG00010006339	1.459089628	0.756446988	1.928872281	0.05374672	0.071283622	positive
PRJNA729271	stage_1D_vs_E10	ordinary_muscle_development	ENSGALG00010006341	HHIP	-1.396956511	0.221290258	-6.312779074	2.74069E-10	8.06461E-10	negative
PRJNA729271	stage_1D_vs_E10	ordinary_muscle_development	ENSGALG00010006342	RFXAP	-0.514380369	0.085382649	-6.024413345	1.69724E-09	4.71293E-09	negative
PRJNA729271	stage_1D_vs_E10	ordinary_muscle_development	ENSGALG00010006343	SEC23A	-0.291519441	0.094843067	-3.07370324	0.002114196	0.003392215	negative
PRJNA729271	stage_1D_vs_E10	ordinary_muscle_development	ENSGALG00010006344	HINT3	0.204611838	0.105421264	1.940897209	0.052270747	0.069510502	positive
PRJNA729271	stage_1D_vs_E10	ordinary_muscle_development	ENSGALG00010006345	SMC2	-1.641243209	0.249715023	-6.572464843	4.94891E-11	1.5305E-10	negative
PRJNA729271	stage_1D_vs_E10	ordinary_muscle_development	ENSGALG00010006346	NSUN3	0.587500646	0.128593437	4.568667411	4.90835E-06	1.03111E-05	positive
PRJNA729271	stage_1D_vs_E10	ordinary_muscle_development	ENSGALG00010006347	PPP6R2	1.45172595	0.135516185	10.71256509	8.88649E-27	6.60562E-26	positive
PRJNA729271	stage_1D_vs_E10	ordinary_muscle_development	ENSGALG00010006351	IGF2R	1.166883854	0.17394317	6.708420077	1.96743E-11	6.24434E-11	positive
PRJNA729271	stage_1D_vs_E10	ordinary_muscle_development	ENSGALG00010006352	GEMIN2	-0.140610836	0.098105412	-1.433262779	0.151782755	0.186907742	negative
PRJNA729271	stage_1D_vs_E10	ordinary_muscle_development	ENSGALG00010006353	ABCE1	-0.673298825	0.080804429	-8.332449541	7.91866E-17	3.558E-16	negative
PRJNA729271	stage_1D_vs_E10	ordinary_muscle_development	ENSGALG00010006355	PTGR1	-0.847898866	0.152200711	-5.570925787	2.53389E-08	6.4032E-08	negative
PRJNA729271	stage_1D_vs_E10	ordinary_muscle_development	ENSGALG00010006359	SGK1	1.840988909	0.163689211	11.24685551	2.39996E-29	2.01084E-28	positive
PRJNA729271	stage_1D_vs_E10	ordinary_muscle_development	ENSGALG00010006361	SERTM1	-1.606833865	0.26006199	-6.178657108	6.46491E-10	1.85086E-09	negative
PRJNA729271	stage_1D_vs_E10	ordinary_muscle_development	ENSGALG00010006363	TRAPPC6A	-0.632465258	0.103504904	-6.110485927	9.93282E-10	2.80929E-09	negative
PRJNA729271	stage_1D_vs_E10	ordinary_muscle_development	ENSGALG00010006372	SBF1	0.202672436	0.113309448	1.788663172	0.073669077	0.09574404	positive
PRJNA729271	stage_1D_vs_E10	ordinary_muscle_development	ENSGALG00010006378	CAAP1	-1.075659911	0.103189522	-10.42411957	1.92433E-25	1.3475E-24	negative
PRJNA729271	stage_1D_vs_E10	ordinary_muscle_development	ENSGALG00010006381	SPART	1.305881033	0.156454792	8.346698875	7.01974E-17	3.16292E-16	positive
PRJNA729271	stage_1D_vs_E10	ordinary_muscle_development	ENSGALG00010006382	PNN	-0.424433072	0.196587313	-2.159005404	0.030849748	0.042482148	negative
PRJNA729271	stage_1D_vs_E10	ordinary_muscle_development	ENSGALG00010006387	OTUD4	-0.376835128	0.135849933	-2.773907349	0.005538745	0.008465216	negative
PRJNA729271	stage_1D_vs_E10	ordinary_muscle_development	ENSGALG00010006404	SMAD1	0.204832815	0.109048866	1.878358044	0.060332201	0.079393123	positive
PRJNA729271	stage_1D_vs_E10	ordinary_muscle_development	ENSGALG00010006408	ENSGALG00010006408	-0.730627579	0.669812779	-1.09079373	0.275363656	0.321011031	negative
PRJNA729271	stage_1D_vs_E10	ordinary_muscle_development	ENSGALG00010006411	RGS17	-1.942066011	0.403756309	-4.809995456	1.50934E-06	3.32288E-06	negative
PRJNA729271	stage_1D_vs_E10	ordinary_muscle_development	ENSGALG00010006421	LMF2	-0.921501576	0.113080399	-8.149083171	3.66694E-16	1.58806E-15	negative
PRJNA729271	stage_1D_vs_E10	ordinary_muscle_development	ENSGALG00010006434	MMAA	0.2570492	0.115468963	2.226132412	0.026005314	0.03617881	positive
PRJNA729271	stage_1D_vs_E10	ordinary_muscle_development	ENSGALG00010006446	LSAMP	-0.257625636	0.183842344	-1.401340035	0.161112415	0.19749044	negative
PRJNA729271	stage_1D_vs_E10	ordinary_muscle_development	ENSGALG00010006454	SOD2	0.751158126	0.105541344	7.117193104	1.10147E-12	3.78966E-12	positive
PRJNA729271	stage_1D_vs_E10	ordinary_muscle_development	ENSGALG00010006457	NCAPH2	-1.520994641	0.200543544	-7.58436103	3.3413E-14	1.27486E-13	negative
PRJNA729271	stage_1D_vs_E10	ordinary_muscle_development	ENSGALG00010006463	SCO2	-0.006411157	0.135139821	-0.047440914	0.962161821	0.967702997	negative
PRJNA729271	stage_1D_vs_E10	ordinary_muscle_development	ENSGALG00010006465	PLAA	-0.32198762	0.11640687	-2.766053402	0.005673924	0.008660861	negative
PRJNA729271	stage_1D_vs_E10	ordinary_muscle_development	ENSGALG00010006472	DCLK1	-3.285384587	0.327257553	-10.03914059	1.02564E-23	6.58982E-23	negative
PRJNA729271	stage_1D_vs_E10	ordinary_muscle_development	ENSGALG00010006478	ENSGALG00010006478	0.429682668	0.13185278	3.258806286	0.00111882	0.001856164	positive
PRJNA729271	stage_1D_vs_E10	ordinary_muscle_development	ENSGALG00010006480	IFT74	-0.903670253	0.119815492	-7.542182059	4.62172E-14	1.74822E-13	negative
PRJNA729271	stage_1D_vs_E10	ordinary_muscle_development	ENSGALG00010006486	CEP43	-0.262226893	0.11267639	-2.327256777	0.019951603	0.028231012	negative
PRJNA729271	stage_1D_vs_E10	ordinary_muscle_development	ENSGALG00010006492	GAP43	-4.600351947	0.496602302	-9.263654085	1.97553E-20	1.08017E-19	negative
PRJNA729271	stage_1D_vs_E10	ordinary_muscle_development	ENSGALG00010006496	UBE2H	0.753527554	0.113696898	6.627511967	3.41392E-11	1.06717E-10	positive
PRJNA729271	stage_1D_vs_E10	ordinary_muscle_development	ENSGALG00010006499	CCR6	-0.970981428	0.816036196	-1.18987544	0.234095353	0.277540662	negative
PRJNA729271	stage_1D_vs_E10	ordinary_muscle_development	ENSGALG00010006503	LRRC19	1.339997287	1.27540687	1.05064299	0.293422589	0.340062399	positive
PRJNA729271	stage_1D_vs_E10	ordinary_muscle_development	ENSGALG00010006506	ZNF827	0.293020468	0.103687191	2.826004504	0.00471326	0.007265629	positive
PRJNA729271	stage_1D_vs_E10	ordinary_muscle_development	ENSGALG00010006515	FBXO33	-0.157932146	0.103341409	-1.528256176	0.126448946	0.157965826	negative
PRJNA729271	stage_1D_vs_E10	ordinary_muscle_development	ENSGALG00010006516	NBEA	-0.275288093	0.125840381	-2.187597419	0.02869894	0.039655971	negative
PRJNA729271	stage_1D_vs_E10	ordinary_muscle_development	ENSGALG00010006517	ZBTB20	3.900795	0.144657377	26.96575224	3.7284E-160	8.0662E-158	positive
PRJNA729271	stage_1D_vs_E10	ordinary_muscle_development	ENSGALG00010006521	ZNF410	-0.907097104	0.104376699	-8.690609213	3.60501E-18	1.74589E-17	negative
PRJNA729271	stage_1D_vs_E10	ordinary_muscle_development	ENSGALG00010006524	TAB2	-0.069071978	0.091527246	-0.754660286	0.450452841	0.499756137	negative
PRJNA729271	stage_1D_vs_E10	ordinary_muscle_development	ENSGALG00010006526	LSM6	-1.129296456	0.16099963	-7.014279809	2.31136E-12	7.81507E-12	negative
PRJNA729271	stage_1D_vs_E10	ordinary_muscle_development	ENSGALG00010006528	ZC3HC1	-0.422188957	0.081284808	-5.193946638	2.05882E-07	4.85009E-07	negative
PRJNA729271	stage_1D_vs_E10	ordinary_muscle_development	ENSGALG00010006531	FAM161B	-1.299798485	0.145609454	-8.926607812	4.39271E-19	2.22865E-18	negative
PRJNA729271	stage_1D_vs_E10	ordinary_muscle_development	ENSGALG00010006532	KLHDC10	0.190334909	0.103138556	1.845429249	0.064975122	0.085154473	positive
PRJNA729271	stage_1D_vs_E10	ordinary_muscle_development	ENSGALG00010006537	DRD3	-2.860672387	0.355369796	-8.049846724	8.28978E-16	3.50322E-15	negative
PRJNA729271	stage_1D_vs_E10	ordinary_muscle_development	ENSGALG00010006538	TEK	1.236534357	0.104993105	11.77729102	5.11094E-32	4.8803E-31	positive
PRJNA729271	stage_1D_vs_E10	ordinary_muscle_development	ENSGALG00010006542	RFC3	-1.555164356	0.140513	-11.06776139	1.79837E-28	1.44498E-27	negative
PRJNA729271	stage_1D_vs_E10	ordinary_muscle_development	ENSGALG00010006544	MOB3B	-2.44944609	0.148751934	-16.46665038	6.37013E-61	1.78598E-59	negative
PRJNA729271	stage_1D_vs_E10	ordinary_muscle_development	ENSGALG00010006546	SMOC2	-0.43007307	0.208352748	-2.064158374	0.039002703	0.052845782	negative
PRJNA729271	stage_1D_vs_E10	ordinary_muscle_development	ENSGALG00010006548	TMEM209	-0.723010035	0.079548891	-9.088876379	1.00068E-19	5.28024E-19	negative
PRJNA729271	stage_1D_vs_E10	ordinary_muscle_development	ENSGALG00010006549	ZDHHC23	-0.587634354	0.183553379	-3.201435778	0.001367446	0.002246783	negative
PRJNA729271	stage_1D_vs_E10	ordinary_muscle_development	ENSGALG00010006552	CPA2	3.903655474	1.712149812	2.279973076	0.022609285	0.031789853	positive
PRJNA729271	stage_1D_vs_E10	ordinary_muscle_development	ENSGALG00010006557	COQ6	0.367380098	0.108386598	3.389534373	0.000700114	0.00118678	positive
PRJNA729271	stage_1D_vs_E10	ordinary_muscle_development	ENSGALG00010006558	USP10	-0.424228141	0.088529704	-4.791929943	1.65185E-06	3.62451E-06	negative
PRJNA729271	stage_1D_vs_E10	ordinary_muscle_development	ENSGALG00010006564	SLC10A7	-1.10750229	0.144098228	-7.68574537	1.52109E-14	5.91579E-14	negative
PRJNA729271	stage_1D_vs_E10	ordinary_muscle_development	ENSGALG00010006566	CPA5	5.407345386	2.814949776	1.920938495	0.054739462	0.072533915	positive
PRJNA729271	stage_1D_vs_E10	ordinary_muscle_development	ENSGALG00010006568	ENTPD5	-0.707604125	0.199474512	-3.547341053	0.00038914	0.000678608	negative
PRJNA729271	stage_1D_vs_E10	ordinary_muscle_development	ENSGALG00010006570	ENSGALG00010006570	1.051771352	0.48728674	2.158423912	0.030894888	0.042536228	positive
PRJNA729271	stage_1D_vs_E10	ordinary_muscle_development	ENSGALG00010006574	CPA1	6.563247017	2.557898315	2.565874874	0.010291595	0.015180285	positive
PRJNA729271	stage_1D_vs_E10	ordinary_muscle_development	ENSGALG00010006575	SSBP2	-0.427217405	0.116417124	-3.669712753	0.000242823	0.000431921	negative
PRJNA729271	stage_1D_vs_E10	ordinary_muscle_development	ENSGALG00010006576	CCDC191	0.078469769	0.109883327	0.714118974	0.475153614	0.523832646	positive
PRJNA729271	stage_1D_vs_E10	ordinary_muscle_development	ENSGALG00010006580	ALDH6A1	1.679295229	0.138150912	12.1555132	5.3624E-34	5.56791E-33	positive
PRJNA729271	stage_1D_vs_E10	ordinary_muscle_development	ENSGALG00010006581	CEP41	-1.470569279	0.105399051	-13.9523958	3.04212E-44	4.63675E-43	negative
PRJNA729271	stage_1D_vs_E10	ordinary_muscle_development	ENSGALG00010006585	TTC29	-0.142293989	0.181285368	-0.784917119	0.432502164	0.481943333	negative
PRJNA729271	stage_1D_vs_E10	ordinary_muscle_development	ENSGALG00010006588	ACOT12	-2.223195471	0.76058128	-2.92302155	0.003466525	0.005432726	negative
PRJNA729271	stage_1D_vs_E10	ordinary_muscle_development	ENSGALG00010006592	STARD13	0.392593448	0.114266162	3.435780474	0.00059085	0.001008165	positive
PRJNA729271	stage_1D_vs_E10	ordinary_muscle_development	ENSGALG00010006598	ENSGALG00010006598	0.954639164	0.150626054	6.337809033	2.33055E-10	6.88994E-10	positive
PRJNA729271	stage_1D_vs_E10	ordinary_muscle_development	ENSGALG00010006602	EDNRA	-0.730621696	0.118328778	-6.174505573	6.63708E-10	1.89827E-09	negative
PRJNA729271	stage_1D_vs_E10	ordinary_muscle_development	ENSGALG00010006603	MEST	-1.795971453	0.240128317	-7.4792156	7.47675E-14	2.78352E-13	negative
PRJNA729271	stage_1D_vs_E10	ordinary_muscle_development	ENSGALG00010006606	SULT	-3.494947329	1.260291334	-2.773126527			negative
PRJNA729271	stage_1D_vs_E10	ordinary_muscle_development	ENSGALG00010006608	KL	2.731236929	0.410212595	6.658101095	2.77388E-11	8.71421E-11	positive
PRJNA729271	stage_1D_vs_E10	ordinary_muscle_development	ENSGALG00010006614	TMEM184C	-0.504011785	0.055204613	-9.129885276	6.85719E-20	3.64351E-19	negative
PRJNA729271	stage_1D_vs_E10	ordinary_muscle_development	ENSGALG00010006615	GRAMD1C	-0.817257305	0.104100491	-7.850657559	4.13862E-15	1.67334E-14	negative
PRJNA729271	stage_1D_vs_E10	ordinary_muscle_development	ENSGALG00010006616	COPG2	-0.744937158	0.068624861	-10.85520824	1.88377E-27	1.4341E-26	negative
PRJNA729271	stage_1D_vs_E10	ordinary_muscle_development	ENSGALG00010006619	LIN52	-0.030746691	0.101502349	-0.302916055	0.761953835	0.792403562	negative
PRJNA729271	stage_1D_vs_E10	ordinary_muscle_development	ENSGALG00010006621	DACT2	-2.340116253	0.210588444	-11.11227287	1.0934E-28	8.87894E-28	negative
PRJNA729271	stage_1D_vs_E10	ordinary_muscle_development	ENSGALG00010006623	ENSGALG00010006623	0.800023364	0.18332704	4.363913597	1.27756E-05	2.58454E-05	positive
PRJNA729271	stage_1D_vs_E10	ordinary_muscle_development	ENSGALG00010006627	SF3B5	-1.013556714	0.111607779	-9.081416352	1.07171E-19	5.64683E-19	negative
PRJNA729271	stage_1D_vs_E10	ordinary_muscle_development	ENSGALG00010006633	FBXO18	0.074660162	0.093443596	0.798986397	0.424298299	0.473785059	positive
PRJNA729271	stage_1D_vs_E10	ordinary_muscle_development	ENSGALG00010006634	PRMT9	0.154115962	0.095716694	1.610126247	0.107370298	0.135722724	positive
PRJNA729271	stage_1D_vs_E10	ordinary_muscle_development	ENSGALG00010006635	ENSGALG00010006635	-1.810951785	0.474193887	-3.819011246	0.000133988	0.000245531	negative
PRJNA729271	stage_1D_vs_E10	ordinary_muscle_development	ENSGALG00010006641	ENSGALG00010006641	-5.366561824	0.725179843	-7.400318521	1.35858E-13	4.97918E-13	negative
PRJNA729271	stage_1D_vs_E10	ordinary_muscle_development	ENSGALG00010006651	ENSGALG00010006651	0.39856538	0.111873237	3.562651731	0.000367128	0.000641533	positive
PRJNA729271	stage_1D_vs_E10	ordinary_muscle_development	ENSGALG00010006653	CFL2	0.288502333	0.104670508	2.75629056	0.005846104	0.008911481	positive
PRJNA729271	stage_1D_vs_E10	ordinary_muscle_development	ENSGALG00010006657	PDS5B	-0.715462841	0.088174807	-8.114141262	4.89232E-16	2.10178E-15	negative
PRJNA729271	stage_1D_vs_E10	ordinary_muscle_development	ENSGALG00010006664	ENSGALG00010006664	3.280486332	1.337800604	2.45214894	0.014200587	0.020546767	positive
PRJNA729271	stage_1D_vs_E10	ordinary_muscle_development	ENSGALG00010006670	ANKRD16	-0.356001547	0.191239485	-1.861548345	0.06266678	0.082278531	negative
PRJNA729271	stage_1D_vs_E10	ordinary_muscle_development	ENSGALG00010006673	BAZ1A	-0.570722602	0.081591032	-6.994918303	2.65414E-12	8.93029E-12	negative
PRJNA729271	stage_1D_vs_E10	ordinary_muscle_development	ENSGALG00010006678	ENSGALG00010006678	-4.069322699	1.290444571	-3.15342696	0.001613656	0.002628084	negative
PRJNA729271	stage_1D_vs_E10	ordinary_muscle_development	ENSGALG00010006681	CRISPLD2	-0.267527074	0.202202449	-1.323065445	0.185813615	0.224953508	negative
PRJNA729271	stage_1D_vs_E10	ordinary_muscle_development	ENSGALG00010006682	ARHGAP10	-0.936916532	0.121277714	-7.725380848	1.11519E-14	4.37713E-14	negative
PRJNA729271	stage_1D_vs_E10	ordinary_muscle_development	ENSGALG00010006685	ENSGALG00010006685	-0.417676407	0.117082943	-3.567354865	0.000360603	0.000630664	negative
PRJNA729271	stage_1D_vs_E10	ordinary_muscle_development	ENSGALG00010006694	ENSGALG00010006694	-6.188058927	0.975042403	-6.346451095	2.20339E-10	6.5233E-10	negative
PRJNA729271	stage_1D_vs_E10	ordinary_muscle_development	ENSGALG00010006701	LINGO2	-3.536476617	0.362513359	-9.755438053	1.74844E-22	1.068E-21	negative
PRJNA729271	stage_1D_vs_E10	ordinary_muscle_development	ENSGALG00010006715	IRF8	0.544501782	0.161924354	3.362692323	0.000771863	0.00130307	positive
PRJNA729271	stage_1D_vs_E10	ordinary_muscle_development	ENSGALG00010006716	ENSGALG00010006716	0.761196418	0.097437226	7.812172508	5.62106E-15	2.24827E-14	positive
PRJNA729271	stage_1D_vs_E10	ordinary_muscle_development	ENSGALG00010006720	SRP54	-0.32595295	0.091307629	-3.569832609	0.000357209	0.000624879	negative
PRJNA729271	stage_1D_vs_E10	ordinary_muscle_development	ENSGALG00010006728	ENSGALG00010006728	0.195273745	0.136299497	1.432681333	0.151948926	0.187096474	positive
PRJNA729271	stage_1D_vs_E10	ordinary_muscle_development	ENSGALG00010006730	HAPLN1	-6.411003116	0.491097447	-13.05444195	5.99477E-39	7.58901E-38	negative
PRJNA729271	stage_1D_vs_E10	ordinary_muscle_development	ENSGALG00010006735	NR3C2	-0.326276375	0.112719525	-2.894586152	0.003796587	0.005918005	negative
PRJNA729271	stage_1D_vs_E10	ordinary_muscle_development	ENSGALG00010006739	VCAN	-3.377815135	0.253797015	-13.30912083	2.04878E-40	2.72701E-39	negative
PRJNA729271	stage_1D_vs_E10	ordinary_muscle_development	ENSGALG00010006740	BRCA2	-0.509315093	0.152091065	-3.348750917	0.000811767	0.001368047	negative
PRJNA729271	stage_1D_vs_E10	ordinary_muscle_development	ENSGALG00010006748	FAM177A1	0.648072465	0.077146708	8.400520064	4.44506E-17	2.02295E-16	positive
PRJNA729271	stage_1D_vs_E10	ordinary_muscle_development	ENSGALG00010006752	ZAR1L	-1.858123916	0.498460546	-3.727725158	0.000193216	0.00034752	negative
PRJNA729271	stage_1D_vs_E10	ordinary_muscle_development	ENSGALG00010006760	TASOR2	0.4421869	0.102089984	4.331344567	1.48202E-05	2.9745E-05	positive
PRJNA729271	stage_1D_vs_E10	ordinary_muscle_development	ENSGALG00010006761	PPP2R3C	-0.19575571	0.093992539	-2.082672869	0.037281052	0.050707408	negative
PRJNA729271	stage_1D_vs_E10	ordinary_muscle_development	ENSGALG00010006767	ENSGALG00010006767	7.440295598	0.330118089	22.5382851	1.7497E-112	1.6907E-110	positive
PRJNA729271	stage_1D_vs_E10	ordinary_muscle_development	ENSGALG00010006794	PERPA	-6.194271435	0.606609486	-10.21129999	1.76484E-24	1.17887E-23	negative
PRJNA729271	stage_1D_vs_E10	ordinary_muscle_development	ENSGALG00010006803	COX4I1	1.755233803	0.103792276	16.91102523	3.73159E-64	1.16072E-62	positive
PRJNA729271	stage_1D_vs_E10	ordinary_muscle_development	ENSGALG00010006804	PSMA6	-0.295654679	0.10243022	-2.886400892	0.003896755	0.006066966	negative
PRJNA729271	stage_1D_vs_E10	ordinary_muscle_development	ENSGALG00010006814	ENSGALG00010006814	-0.712696727	0.393022095	-1.813375726	0.069773891	0.090999869	negative
PRJNA729271	stage_1D_vs_E10	ordinary_muscle_development	ENSGALG00010006816	ENSGALG00010006816	4.393923442	0.507991465	8.649600913	5.16798E-18	2.47893E-17	positive
PRJNA729271	stage_1D_vs_E10	ordinary_muscle_development	ENSGALG00010006819	EMC8	0.263942071	0.131871299	2.001512632	0.045337174	0.060848364	positive
PRJNA729271	stage_1D_vs_E10	ordinary_muscle_development	ENSGALG00010006821	XRCC4	-1.002127708	0.127684005	-7.848498375	4.21048E-15	1.69813E-14	negative
PRJNA729271	stage_1D_vs_E10	ordinary_muscle_development	ENSGALG00010006828	GTF2A1	0.08195822	0.105893746	0.773966581	0.438950554	0.488190615	positive
PRJNA729271	stage_1D_vs_E10	ordinary_muscle_development	ENSGALG00010006833	LTV1	-0.539129241	0.086731135	-6.216098105	5.09669E-10	1.47018E-09	negative
PRJNA729271	stage_1D_vs_E10	ordinary_muscle_development	ENSGALG00010006835	FRY	2.562683061	0.151983493	16.8615881	8.62403E-64	2.63724E-62	positive
PRJNA729271	stage_1D_vs_E10	ordinary_muscle_development	ENSGALG00010006838	ASB13	0.807036528	0.123673027	6.525566227	6.77451E-11	2.07209E-10	positive
PRJNA729271	stage_1D_vs_E10	ordinary_muscle_development	ENSGALG00010006840	ENSGALG00010006840	-1.924266502	0.277237095	-6.940869511	3.89694E-12	1.29615E-11	negative

PRJNA729271	stage_1D_vs_E10	ordinary_muscle_development	ENSGALG00010006843	STON2	1.262222942	0.192301713	6.563763369	5.24664E-11	1.62016E-10	positive
PRJNA729271	stage_1D_vs_E10	ordinary_muscle_development	ENSGALG00010006845	CALML3	-8.091461312	0.748440567	-10.81109399	3.05011E-27	2.30507E-26	negative
PRJNA729271	stage_1D_vs_E10	ordinary_muscle_development	ENSGALG00010006847	SEL1L	0.097468535	0.086575645	1.125819334	0.260241999	0.305293604	positive
PRJNA729271	stage_1D_vs_E10	ordinary_muscle_development	ENSGALG00010006850	IRF2BPL	-0.403172927	0.159839896	-2.522354783	0.011657207	0.017081603	negative
PRJNA729271	stage_1D_vs_E10	ordinary_muscle_development	ENSGALG00010006851	TMEM167A	-0.509288687	0.104663443	-4.865965373	1.13899E-06	2.53217E-06	negative
PRJNA729271	stage_1D_vs_E10	ordinary_muscle_development	ENSGALG00010006853	NET1	-0.312131725	0.083669951	-3.730511642	0.000191091	0.00034404	negative
PRJNA729271	stage_1D_vs_E10	ordinary_muscle_development	ENSGALG00010006855	DCLK2	-0.284078796	0.123971302	-2.291488365	0.021935184	0.030884502	negative
PRJNA729271	stage_1D_vs_E10	ordinary_muscle_development	ENSGALG00010006865	EFCAB2	-1.549754137	0.367907453	-4.212347766	2.5273E-05	4.96116E-05	negative
PRJNA729271	stage_1D_vs_E10	ordinary_muscle_development	ENSGALG00010006877	GSE1	1.440019387	0.240123426	5.996996684	2.01E-09	5.55163E-09	positive
PRJNA729271	stage_1D_vs_E10	ordinary_muscle_development	ENSGALG00010006883	LRBA	-0.615960611	0.070806416	-8.699220242	3.34172E-18	1.62164E-17	negative
PRJNA729271	stage_1D_vs_E10	ordinary_muscle_development	ENSGALG00010006885	RHOU	-0.624554045	0.153207949	-4.076512026	4.57163E-05	8.77111E-05	negative
PRJNA729271	stage_1D_vs_E10	ordinary_muscle_development	ENSGALG00010006901	TUBAL3	-2.608020027	0.261923849	-9.957169002	2.34649E-23	1.48267E-22	negative
PRJNA729271	stage_1D_vs_E10	ordinary_muscle_development	ENSGALG00010006903	B3GLCT	-0.742568917	0.145097697	-5.117716753	3.09257E-07	7.17802E-07	negative
PRJNA729271	stage_1D_vs_E10	ordinary_muscle_development	ENSGALG00010006912	ENSGALG00010006912	-0.893144104	0.181347892	-4.925031638	8.43467E-07	1.89492E-06	negative
PRJNA729271	stage_1D_vs_E10	ordinary_muscle_development	ENSGALG00010006913	HSPH1	-1.339873308	0.185995862	-7.203780198	5.85657E-13	2.06246E-12	negative
PRJNA729271	stage_1D_vs_E10	ordinary_muscle_development	ENSGALG00010006922	SMYD3	-0.519567499	0.158799424	-3.271847515	0.001068472	0.001776905	negative
PRJNA729271	stage_1D_vs_E10	ordinary_muscle_development	ENSGALG00010006923	ENSGALG00010006923	0.541148166	0.218685709	2.474547464	0.013340509	0.019381646	positive
PRJNA729271	stage_1D_vs_E10	ordinary_muscle_development	ENSGALG00010006926	ID4	-2.479932174	0.227293623	-10.91069845	1.02466E-27	7.90865E-27	negative
PRJNA729271	stage_1D_vs_E10	ordinary_muscle_development	ENSGALG00010006927	UCN3	-2.809328846	0.846117421	-3.320258841	0.00089934	0.001507045	negative
PRJNA729271	stage_1D_vs_E10	ordinary_muscle_development	ENSGALG00010006939	FBXO31	0.782661978	0.12736422	6.145069435	7.99285E-10	2.27571E-09	positive
PRJNA729271	stage_1D_vs_E10	ordinary_muscle_development	ENSGALG00010006942	ALOX5AP	-0.527608367	0.188128271	-2.804513983	0.00503925	0.007746732	negative
PRJNA729271	stage_1D_vs_E10	ordinary_muscle_development	ENSGALG00010006944	MAB21L2	-6.059679481	0.554984153	-10.91865318	9.38775E-28	7.26511E-27	negative
PRJNA729271	stage_1D_vs_E10	ordinary_muscle_development	ENSGALG00010006946	PLPPR1	-5.323452434	0.352610348	-15.0972666	1.68787E-51	3.42176E-50	negative
PRJNA729271	stage_1D_vs_E10	ordinary_muscle_development	ENSGALG00010006948	ENSGALG00010006948	-7.361329578	0.301596558	-24.40786996	1.4109E-131	1.8936E-129	negative
PRJNA729271	stage_1D_vs_E10	ordinary_muscle_development	ENSGALG00010006953	MAP1LC3B	0.331673398	0.12822417	2.586668312	0.009690883	0.014354113	positive
PRJNA729271	stage_1D_vs_E10	ordinary_muscle_development	ENSGALG00010006957	PALM2AKAP2	0.159282021	0.091845129	1.734245707	0.082874496	0.106836164	positive
PRJNA729271	stage_1D_vs_E10	ordinary_muscle_development	ENSGALG00010006958	KIF26B	-3.705881422	0.233670698	-15.85941864	1.21009E-56	2.96288E-55	negative
PRJNA729271	stage_1D_vs_E10	ordinary_muscle_development	ENSGALG00010006961	ZCCHC14	1.100108418	0.115982353	9.485136244	2.42067E-21	1.39458E-20	positive
PRJNA729271	stage_1D_vs_E10	ordinary_muscle_development	ENSGALG00010006963	ENSGALG00010006963	-0.438165522	0.446303946	-0.981764839	0.326215714	0.374526946	negative
PRJNA729271	stage_1D_vs_E10	ordinary_muscle_development	ENSGALG00010006966	PGBD5	-2.612689534	0.243344024	-10.73660857	6.85152E-27	5.11394E-26	negative
PRJNA729271	stage_1D_vs_E10	ordinary_muscle_development	ENSGALG00010006983	GTPBP2	0.641992844	0.12410499	5.172981708	2.30388E-07	5.40454E-07	positive
PRJNA729271	stage_1D_vs_E10	ordinary_muscle_development	ENSGALG00010006988	USPL1	0.124777933	0.103953509	1.200324394	0.230013379	0.273215106	positive
PRJNA729271	stage_1D_vs_E10	ordinary_muscle_development	ENSGALG00010006992	SLC35B4	-1.768852756	0.104868266	-16.86737871	7.819E-64	2.40629E-62	negative
PRJNA729271	stage_1D_vs_E10	ordinary_muscle_development	ENSGALG00010006998	POLH	0.324192134	0.121681476	2.664268596	0.007715593	0.011577383	positive
PRJNA729271	stage_1D_vs_E10	ordinary_muscle_development	ENSGALG00010007000	LRGUK	-3.330895208	0.682026211	-4.883822871	1.04049E-06	2.32243E-06	negative
PRJNA729271	stage_1D_vs_E10	ordinary_muscle_development	ENSGALG00010007001	SNX30	-1.4359845	0.094037496	-15.27033953	1.20544E-52	2.55825E-51	negative
PRJNA729271	stage_1D_vs_E10	ordinary_muscle_development	ENSGALG00010007002	EXOC4	-0.022687884	0.119493899	-0.189866463	0.849413773	0.871378011	negative
PRJNA729271	stage_1D_vs_E10	ordinary_muscle_development	ENSGALG00010007004	ENSGALG00010007004	0.773295794	0.561875932	1.376274993	0.168736512	0.205947772	positive
PRJNA729271	stage_1D_vs_E10	ordinary_muscle_development	ENSGALG00010007007	ACSL1	2.618125306	0.216753355	12.07882251	1.36663E-33	1.39995E-32	positive
PRJNA729271	stage_1D_vs_E10	ordinary_muscle_development	ENSGALG00010007010	ENSGALG00010007010	1.086733356	0.995799913	1.091316982	0.275133428	0.32078982	positive
PRJNA729271	stage_1D_vs_E10	ordinary_muscle_development	ENSGALG00010007011	HMGB1	-1.207881715	0.101734111	-11.87292736	1.6364E-32	1.59728E-31	negative
PRJNA729271	stage_1D_vs_E10	ordinary_muscle_development	ENSGALG00010007014	ENSGALG00010007014	0.278574543	0.571345232	0.48757656	0.625849802	0.667719927	positive
PRJNA729271	stage_1D_vs_E10	ordinary_muscle_development	ENSGALG00010007017	SRD5A2	-0.41167735	0.354724757	-1.160554322	0.24582319	0.289945427	negative
PRJNA729271	stage_1D_vs_E10	ordinary_muscle_development	ENSGALG00010007021	SULT6B1	-0.181229448	0.246001065	-0.736701884	0.461303661	0.510153342	negative
PRJNA729271	stage_1D_vs_E10	ordinary_muscle_development	ENSGALG00010007025	ENSGALG00010007025	2.471736545	0.71221274	3.470503132	0.000519484	0.000892912	positive
PRJNA729271	stage_1D_vs_E10	ordinary_muscle_development	ENSGALG00010007028	KATNAL1	-0.525115394	0.139417228	-3.766502911	0.00016555	0.00030067	negative
PRJNA729271	stage_1D_vs_E10	ordinary_muscle_development	ENSGALG00010007033	CEBPZOS	0.694558845	0.186086317	3.73245522	0.000189622	0.000341565	positive
PRJNA729271	stage_1D_vs_E10	ordinary_muscle_development	ENSGALG00010007038	CENPU	-1.443669042	0.201813343	-7.153486576	8.46011E-13	2.94357E-12	negative
PRJNA729271	stage_1D_vs_E10	ordinary_muscle_development	ENSGALG00010007041	NAE1	-0.791982846	0.080453564	-9.843974611	7.27775E-23	4.51782E-22	negative
PRJNA729271	stage_1D_vs_E10	ordinary_muscle_development	ENSGALG00010007049	ENSGALG00010007049	-0.620386789	0.520062844	-1.19290735	0.23290566	0.276220549	negative
PRJNA729271	stage_1D_vs_E10	ordinary_muscle_development	ENSGALG00010007051	E2F3	-2.213799719	0.155568665	-14.2303704	5.93665E-46	9.67961E-45	negative
PRJNA729271	stage_1D_vs_E10	ordinary_muscle_development	ENSGALG00010007066	INIP	-0.706227146	0.153449267	-4.602349421	4.17752E-06	8.82312E-06	negative
PRJNA729271	stage_1D_vs_E10	ordinary_muscle_development	ENSGALG00010007067	CASP3	-1.167646091	0.107510522	-10.86076111	1.77266E-27	1.35235E-26	negative
PRJNA729271	stage_1D_vs_E10	ordinary_muscle_development	ENSGALG00010007068	NCAM2	-2.002981928	0.222173502	-9.015395207	1.96162E-19	1.01621E-18	negative
PRJNA729271	stage_1D_vs_E10	ordinary_muscle_development	ENSGALG00010007070	UBL3	0.755499806	0.052344327	14.43327015	3.19563E-47	5.44949E-46	positive
PRJNA729271	stage_1D_vs_E10	ordinary_muscle_development	ENSGALG00010007085	PRIMPOL	-0.666147867	0.168851908	-3.945160442	7.97466E-05	0.000149402	negative
PRJNA729271	stage_1D_vs_E10	ordinary_muscle_development	ENSGALG00010007090	CEBPZ	-0.303999903	0.093163756	-3.263070492	0.001102121	0.001829506	negative
PRJNA729271	stage_1D_vs_E10	ordinary_muscle_development	ENSGALG00010007092	DYNC1LI2	0.290116744	0.061066754	4.750813229	2.026E-06	4.41475E-06	positive
PRJNA729271	stage_1D_vs_E10	ordinary_muscle_development	ENSGALG00010007097	ZCCHC9	0.276582954	0.167012754	1.656058883	0.097709895	0.124455618	positive
PRJNA729271	stage_1D_vs_E10	ordinary_muscle_development	ENSGALG00010007098	NDUFAF7	0.105770471	0.128827984	0.821020929	0.411634347	0.461243032	positive
PRJNA729271	stage_1D_vs_E10	ordinary_muscle_development	ENSGALG00010007102	CMTM3	-1.413369523	0.165631722	-8.533205531	1.42347E-17	6.64303E-17	negative
PRJNA729271	stage_1D_vs_E10	ordinary_muscle_development	ENSGALG00010007106	SLC7A1	-1.500027382	0.2406799	-6.232458054	4.59173E-10	1.32848E-09	negative
PRJNA729271	stage_1D_vs_E10	ordinary_muscle_development	ENSGALG00010007108	MBOAT1	-0.576084274	0.191555003	-3.007409183	0.002634848	0.004180399	negative
PRJNA729271	stage_1D_vs_E10	ordinary_muscle_development	ENSGALG00010007110	ARL6	-0.100701853	0.102590569	-0.981589772	0.326301988	0.374544451	negative
PRJNA729271	stage_1D_vs_E10	ordinary_muscle_development	ENSGALG00010007114	CKLF	-0.650302108	0.152615097	-4.261060154	2.03459E-05	4.02834E-05	negative
PRJNA729271	stage_1D_vs_E10	ordinary_muscle_development	ENSGALG00010007115	ENSGALG00010007115	1.063293707	0.277846614	3.826908995	0.000129762	0.000238089	positive
PRJNA729271	stage_1D_vs_E10	ordinary_muscle_development	ENSGALG00010007118	ENSGALG00010007118	-0.654220003	0.124680404	-5.247175819	1.54448E-07	3.6791E-07	negative
PRJNA729271	stage_1D_vs_E10	ordinary_muscle_development	ENSGALG00010007123	ENSGALG00010007123	2.69898263	0.242676777	11.12171781	9.83597E-29	7.99621E-28	positive
PRJNA729271	stage_1D_vs_E10	ordinary_muscle_development	ENSGALG00010007126	ENSGALG00010007126	3.244166439	1.070501976	3.03050953	0.002441415	0.003886689	positive
PRJNA729271	stage_1D_vs_E10	ordinary_muscle_development	ENSGALG00010007130	IRF2	1.59561366	0.121772287	13.10325769	3.15423E-39	4.04249E-38	positive
PRJNA729271	stage_1D_vs_E10	ordinary_muscle_development	ENSGALG00010007133	BLVRA	1.394667782	0.1867934	7.466365426	8.24402E-14	3.06324E-13	positive
PRJNA729271	stage_1D_vs_E10	ordinary_muscle_development	ENSGALG00010007135	TK2	-0.394870633	0.116713706	-3.383241337	0.000716357	0.001213461	negative
PRJNA729271	stage_1D_vs_E10	ordinary_muscle_development	ENSGALG00010007137	ENSGALG00010007137	1.457243275	0.359242031	4.056438688	4.98266E-05	9.52006E-05	positive
PRJNA729271	stage_1D_vs_E10	ordinary_muscle_development	ENSGALG00010007141	PRKD3	0.068075932	0.081986284	0.830333179	0.406350434	0.455956769	positive
PRJNA729271	stage_1D_vs_E10	ordinary_muscle_development	ENSGALG00010007143	CKMT2	9.299088297	0.31684501	29.34901295	2.4598E-189	9.6366E-187	positive
PRJNA729271	stage_1D_vs_E10	ordinary_muscle_development	ENSGALG00010007146	ENSGALG00010007146	0.628527422	0.14268317	4.40505648	1.05756E-05	2.15482E-05	positive
PRJNA729271	stage_1D_vs_E10	ordinary_muscle_development	ENSGALG00010007155	FOXL1	-1.048225999	0.604418871	-1.7342708	0.082870046	0.106836164	negative
PRJNA729271	stage_1D_vs_E10	ordinary_muscle_development	ENSGALG00010007160	ENSGALG00010007160	1.258367078	0.293880744	4.281897005	1.85307E-05	3.67999E-05	positive
PRJNA729271	stage_1D_vs_E10	ordinary_muscle_development	ENSGALG00010007162	ENSGALG00010007162	-1.307549928	0.434023935	-3.012621705	0.002590016	0.004111519	negative
PRJNA729271	stage_1D_vs_E10	ordinary_muscle_development	ENSGALG00010007165	ENSGALG00010007165	0.643586877	0.324520036	1.983196124	0.047345527	0.063361962	positive
PRJNA729271	stage_1D_vs_E10	ordinary_muscle_development	ENSGALG00010007166	RASGRF2	2.697818065	0.180771225	14.9239353	2.30282E-50	4.49857E-49	positive
PRJNA729271	stage_1D_vs_E10	ordinary_muscle_development	ENSGALG00010007168	MINA	-0.393593488	0.107502169	-3.661260907	0.000250977	0.000445713	negative
PRJNA729271	stage_1D_vs_E10	ordinary_muscle_development	ENSGALG00010007169	QPCT	-0.399335586	0.218742213	-1.825599098	0.067910672	0.088713517	negative
PRJNA729271	stage_1D_vs_E10	ordinary_muscle_development	ENSGALG00010007175	FOXC2	-3.196201902	0.412514023	-7.748104851	9.32741E-15	3.67994E-14	negative
PRJNA729271	stage_1D_vs_E10	ordinary_muscle_development	ENSGALG00010007181	GJB2	-0.61479171	0.630765967	-0.974674828	0.329721583	0.378170149	negative
PRJNA729271	stage_1D_vs_E10	ordinary_muscle_development	ENSGALG00010007182	ENSGALG00010007182	1.515215863	0.292013517	5.188855226	2.11591E-07	4.97649E-07	positive
PRJNA729271	stage_1D_vs_E10	ordinary_muscle_development	ENSGALG00010007183	VOPP1	1.3329867	0.314046625	4.244550316	2.19032E-05	4.32367E-05	positive
PRJNA729271	stage_1D_vs_E10	ordinary_muscle_development	ENSGALG00010007184	VGLL3	0.574140798	0.09258627	6.201144077	5.60542E-10	1.61275E-09	positive
PRJNA729271	stage_1D_vs_E10	ordinary_muscle_development	ENSGALG00010007187	ENPP6	-0.037434873	0.277844649	-0.134733109	0.892822895	0.908747129	negative
PRJNA729271	stage_1D_vs_E10	ordinary_muscle_development	ENSGALG00010007188	MAB21L1	-0.819504384	0.208318119	-3.933908335	8.35757E-05	0.000156293	negative
PRJNA729271	stage_1D_vs_E10	ordinary_muscle_development	ENSGALG00010007190	ENSGALG00010007190	-0.271937581	0.149291539	-1.82152038	0.068527792	0.089463239	negative
PRJNA729271	stage_1D_vs_E10	ordinary_muscle_development	ENSGALG00010007201	ENSGALG00010007201	2.125822599	0.255609714	8.316673765	9.04663E-17	4.0485E-16	positive
PRJNA729271	stage_1D_vs_E10	ordinary_muscle_development	ENSGALG00010007203	SLC30A6	0.270316263	0.103920352	2.601186933	0.009290182	0.013795839	positive
PRJNA729271	stage_1D_vs_E10	ordinary_muscle_development	ENSGALG00010007204	LANCL2	-0.822101252	0.086969631	-9.45273939	3.30077E-21	1.88885E-20	negative
PRJNA729271	stage_1D_vs_E10	ordinary_muscle_development	ENSGALG00010007207	EPM2A	1.177545484	0.103457761	11.38189611	5.14684E-30	4.44597E-29	positive
PRJNA729271	stage_1D_vs_E10	ordinary_muscle_development	ENSGALG00010007214	MTHFSD	0.179901941	0.140950103	1.276351963	0.201831129	0.242240806	positive
PRJNA729271	stage_1D_vs_E10	ordinary_muscle_development	ENSGALG00010007217	MSH3	0.029374405	0.14309666	0.205276662	0.837355977	0.860691737	positive
PRJNA729271	stage_1D_vs_E10	ordinary_muscle_development	ENSGALG00010007223	FOXF1	-0.271205838	0.445316323	-0.609018407	0.542512238	0.588689541	negative
PRJNA729271	stage_1D_vs_E10	ordinary_muscle_development	ENSGALG00010007224	DHFR	-2.060524235	0.215198404	-9.574997746	1.0186E-21	5.99453E-21	negative
PRJNA729271	stage_1D_vs_E10	ordinary_muscle_development	ENSGALG00010007226	ENSGALG00010007226	-3.0007598	1.661995145	-1.805516586	0.070993882	0.092474508	negative
PRJNA729271	stage_1D_vs_E10	ordinary_muscle_development	ENSGALG00010007230	ENSGALG00010007230	-0.477035977	0.34691229	-1.375091026	0.169103226	0.206336346	negative
PRJNA729271	stage_1D_vs_E10	ordinary_muscle_development	ENSGALG00010007243	BEAN1	-1.181392197	0.188700645	-6.260668567	3.8333E-10	1.1144E-09	negative
PRJNA729271	stage_1D_vs_E10	ordinary_muscle_development	ENSGALG00010007251	SPAST	-0.441886104	0.063422488	-6.967341096	3.22987E-12	1.08122E-11	negative
PRJNA729271	stage_1D_vs_E10	ordinary_muscle_development	ENSGALG00010007252	NFATC1	0.56161537	0.153184496	3.666267703	0.000246116	0.000437617	positive
PRJNA729271	stage_1D_vs_E10	ordinary_muscle_development	ENSGALG00010007259	ANKRD34B	-0.882187678	0.096181474	-9.172116452	4.63822E-20	2.49003E-19	negative
PRJNA729271	stage_1D_vs_E10	ordinary_muscle_development	ENSGALG00010007265	CD200R1A	-1.591965437	0.308151589	-5.166176297	2.38931E-07	5.59772E-07	negative
PRJNA729271	stage_1D_vs_E10	ordinary_muscle_development	ENSGALG00010007270	CDH5	0.436527985	0.108367186	4.028230315	5.61983E-05	0.000106944	positive
PRJNA729271	stage_1D_vs_E10	ordinary_muscle_development	ENSGALG00010007271	CADM2	-2.785287854	0.301066073	-9.251417242	2.21535E-20	1.2072E-19	negative
PRJNA729271	stage_1D_vs_E10	ordinary_muscle_development	ENSGALG00010007275	DPY30	-0.36872962	0.100258564	-3.677786764	0.000235267	0.000419354	negative
PRJNA729271	stage_1D_vs_E10	ordinary_muscle_development	ENSGALG00010007277	FAM151B	-0.493733877	0.187317355	-2.635814904	0.00839355	0.012531106	negative
PRJNA729271	stage_1D_vs_E10	ordinary_muscle_development	ENSGALG00010007283	STOX2	-0.73105718	0.121366201	-6.023564816	1.70617E-09	4.73681E-09	negative
PRJNA729271	stage_1D_vs_E10	ordinary_muscle_development	ENSGALG00010007284	ENSGALG00010007284	0.089931025	0.081749232	1.100084035	0.271295509	0.316822489	positive
PRJNA729271	stage_1D_vs_E10	ordinary_muscle_development	ENSGALG00010007287	ENSGALG00010007287	1.304984359	0.620325509	2.103709004	0.035403839	0.048344667	positive
PRJNA729271	stage_1D_vs_E10	ordinary_muscle_development	ENSGALG00010007294	TRAPPC11	0.27510637	0.064422017	4.270378113	1.95142E-05	3.86947E-05	positive
PRJNA729271	stage_1D_vs_E10	ordinary_muscle_development	ENSGALG00010007300	CCDC125	-0.871860988	0.282065394	-3.090988853	0.001994911	0.003212558	negative
PRJNA729271	stage_1D_vs_E10	ordinary_muscle_development	ENSGALG00010007302	CDH11	-2.726446669	0.17510551	-15.57030765	1.15849E-54	2.62379E-53	negative
PRJNA729271	stage_1D_vs_E10	ordinary_muscle_development	ENSGALG00010007304	F5	0.838793006	0.356059675	2.355765244	0.018484595	0.026258259	positive
PRJNA729271	stage_1D_vs_E10	ordinary_muscle_development	ENSGALG00010007318	RMDN2	1.172978833	0.123197339	9.521137726	1.71293E-21	9.94352E-21	positive
PRJNA729271	stage_1D_vs_E10	ordinary_muscle_development	ENSGALG00010007319	ATP9B	0.382064749	0.100791611	3.790640352	0.000150259	0.000274205	positive
PRJNA729271	stage_1D_vs_E10	ordinary_muscle_development	ENSGALG00010007320	ENSGALG00010007320	1.57840666	0.302745553	5.213641113	1.8517E-07	4.37923E-07	positive
PRJNA729271	stage_1D_vs_E10	ordinary_muscle_development	ENSGALG00010007321	CD200	1.973619603	0.148289518	13.30923207	2.04574E-40	2.72545E-39	positive
PRJNA729271	stage_1D_vs_E10	ordinary_muscle_development	ENSGALG00010007322	AK6	-0.405215324	0.10841169	-3.737745663	0.000185678	0.000335043	negative
PRJNA729271	stage_1D_vs_E10	ordinary_muscle_development	ENSGALG00010007324	ENSGALG00010007324	0.295229483	0.13864893	2.129331132	0.033226874	0.045569452	positive
PRJNA729271	stage_1D_vs_E10	ordinary_muscle_development	ENSGALG00010007326	RWDD4	0.127624991	0.116159427	1.098705415	0.27189658	0.317449934	positive
PRJNA729271	stage_1D_vs_E10	ordinary_muscle_development	ENSGALG00010007328	NOVA1	0.620256693	0.132384435	4.685269011	2.79592E-06	6.01736E-06	positive
PRJNA729271	stage_1D_vs_E10	ordinary_muscle_development	ENSGALG00010007330	MMP13	-0.604007818	0.707788236	-0.853373631	0.393452139	0.442648926	negative
PRJNA729271	stage_1D_vs_E10	ordinary_muscle_development	ENSGALG00010007333	QTRT2	-1.332281826	0.071890384	-18.53212843	1.13699E-76	4.93434E-75	negative
PRJNA729271	stage_1D_vs_E10	ordinary_muscle_development	ENSGALG00010007337	PRORP	-0.798816055	0.100189101	-7.973083345	1.54764E-15	6.42044E-15	negative
PRJNA729271	stage_1D_vs_E10	ordinary_muscle_development	ENSGALG00010007339	ENSGALG00010007339	0.791970808	0.223564298	3.542474432	0.000396392	0.00069034	positive
PRJNA729271	stage_1D_vs_E10	ordinary_muscle_development	ENSGALG00010007344	RAD17	0.463689056	0.106578136	4.350695875	1.35706E-05	2.73697E-05	positive
PRJNA729271	stage_1D_vs_E10	ordinary_muscle_development	ENSGALG00010007353	ING2	-0.844318431	0.123977787	-6.8102396	9.74363E-12	3.15677E-11	negative

PRJNA729271	stage_1D_vs_E10	ordinary_muscle_development	ENSGALG00010007357	NOX4	-0.155208175	0.119395602	-1.299948842	0.193618504	0.233505301	negative
PRJNA729271	stage_1D_vs_E10	ordinary_muscle_development	ENSGALG00010007360	ROBO1	-2.766923925	0.186157301	-14.86336508	5.69892E-50	1.09994E-48	negative
PRJNA729271	stage_1D_vs_E10	ordinary_muscle_development	ENSGALG00010007365	MEMO1	0.583630565	0.087300093	6.685337262	2.30393E-11	7.2773E-11	positive
PRJNA729271	stage_1D_vs_E10	ordinary_muscle_development	ENSGALG00010007369	ENSGALG00010007369	-0.137235065	0.21191655	-0.647590123	0.517250087	0.564786442	negative
PRJNA729271	stage_1D_vs_E10	ordinary_muscle_development	ENSGALG00010007374	CMIP	0.02513327	0.133499141	0.188265407	0.850668602	0.872148916	positive
PRJNA729271	stage_1D_vs_E10	ordinary_muscle_development	ENSGALG00010007375	CHORDC1	-0.174593242	0.074314632	-2.349379087	0.018804751	0.02668901	negative
PRJNA729271	stage_1D_vs_E10	ordinary_muscle_development	ENSGALG00010007376	CDKN2AIP	-0.390376509	0.059745627	-6.53397623	6.40461E-11	1.96351E-10	negative
PRJNA729271	stage_1D_vs_E10	ordinary_muscle_development	ENSGALG00010007381	EGFR	-0.004088523	0.097948002	-0.04174177	0.966704555	0.97153037	negative
PRJNA729271	stage_1D_vs_E10	ordinary_muscle_development	ENSGALG00010007382	EIF2AK2	0.230140919	0.257749772	0.892885052	0.371918733	0.421103112	positive
PRJNA729271	stage_1D_vs_E10	ordinary_muscle_development	ENSGALG00010007387	ENSGALG00010007387	-1.639935399	0.50055303	-3.276247074	0.001051965	0.001750457	negative
PRJNA729271	stage_1D_vs_E10	ordinary_muscle_development	ENSGALG00010007388	CYP1B1	-2.706758254	0.199673973	-13.55588918	7.31207E-42	1.02503E-40	negative
PRJNA729271	stage_1D_vs_E10	ordinary_muscle_development	ENSGALG00010007391	NANP	-0.02544023	0.139020635	-0.182996072	0.8548011	0.875580662	negative
PRJNA729271	stage_1D_vs_E10	ordinary_muscle_development	ENSGALG00010007392	NAALAD2	2.470986629	0.199383875	12.3931117	2.84775E-35	3.1295E-34	positive
PRJNA729271	stage_1D_vs_E10	ordinary_muscle_development	ENSGALG00010007393	PLCG2	0.462646474	0.128494564	3.600513967	0.000317589	0.0005584	positive
PRJNA729271	stage_1D_vs_E10	ordinary_muscle_development	ENSGALG00010007399	WWC2	-0.361996082	0.107827056	-3.357191546	0.000787385	0.001327883	negative
PRJNA729271	stage_1D_vs_E10	ordinary_muscle_development	ENSGALG00010007401	SEC61G	-1.054521256	0.220521601	-4.781940867	1.73611E-06	3.79962E-06	negative
PRJNA729271	stage_1D_vs_E10	ordinary_muscle_development	ENSGALG00010007403	SDR42E1	0.779868838	0.216568883	3.60101982	0.000316971	0.000557382	positive
PRJNA729271	stage_1D_vs_E10	ordinary_muscle_development	ENSGALG00010007404	FOLH1	2.012805554	0.169503431	11.87471863	1.60172E-32	1.56486E-31	positive
PRJNA729271	stage_1D_vs_E10	ordinary_muscle_development	ENSGALG00010007408	HCLS1	-0.059829232	0.257056834	-0.232747096	0.815957796	0.841322255	negative
PRJNA729271	stage_1D_vs_E10	ordinary_muscle_development	ENSGALG00010007411	ENSGALG00010007411	1.469082721	0.573034886	2.563688105	0.010356656	0.015270037	positive
PRJNA729271	stage_1D_vs_E10	ordinary_muscle_development	ENSGALG00010007413	GPATCH11	-1.029115396	0.100275905	-10.26283831	1.03616E-24	6.98562E-24	negative
PRJNA729271	stage_1D_vs_E10	ordinary_muscle_development	ENSGALG00010007414	ROBO2	-4.078841857	0.232432136	-17.54852803	6.10416E-69	2.18469E-67	negative
PRJNA729271	stage_1D_vs_E10	ordinary_muscle_development	ENSGALG00010007416	DCTD	-1.445030219	0.250594778	-5.766401971	8.09818E-09	2.13307E-08	negative
PRJNA729271	stage_1D_vs_E10	ordinary_muscle_development	ENSGALG00010007418	NME6	0.764538561	0.14494972	5.274508713	1.33112E-07	3.18445E-07	positive
PRJNA729271	stage_1D_vs_E10	ordinary_muscle_development	ENSGALG00010007419	RABL2A	-0.9470299	0.155854287	-6.076380193	1.22926E-09	3.44777E-09	negative
PRJNA729271	stage_1D_vs_E10	ordinary_muscle_development	ENSGALG00010007423	HEATR5B	-0.247686925	0.065065698	-3.806720473	0.000140822	0.000257436	negative
PRJNA729271	stage_1D_vs_E10	ordinary_muscle_development	ENSGALG00010007425	ARSA	0.011134977	0.128309203	0.086782369	0.930844502	0.941061045	positive
PRJNA729271	stage_1D_vs_E10	ordinary_muscle_development	ENSGALG00010007429	C21orf91	1.25982508	0.208009306	6.05658038	1.39046E-09	3.88561E-09	positive
PRJNA729271	stage_1D_vs_E10	ordinary_muscle_development	ENSGALG00010007430	MEF2C	0.966723871	0.146880314	6.581711608	4.65063E-11	1.44256E-10	positive
PRJNA729271	stage_1D_vs_E10	ordinary_muscle_development	ENSGALG00010007432	NAT	1.263117639	0.189207494	6.675833049	2.45832E-11	7.74974E-11	positive
PRJNA729271	stage_1D_vs_E10	ordinary_muscle_development	ENSGALG00010007433	RNF144B	3.236192992	0.267153081	12.1136278	8.94486E-34	9.21505E-33	positive
PRJNA729271	stage_1D_vs_E10	ordinary_muscle_development	ENSGALG00010007434	ENSGALG00010007434	0.422322358	0.250642427	1.684959577	0.091996371	0.117716048	positive
PRJNA729271	stage_1D_vs_E10	ordinary_muscle_development	ENSGALG00010007436	PNAT3	1.100157972	0.264483225	4.159651227	3.18734E-05	6.20057E-05	positive
PRJNA729271	stage_1D_vs_E10	ordinary_muscle_development	ENSGALG00010007437	TUSC1	0.435968199	0.137701147	3.166046247	0.001545262	0.00252151	positive
PRJNA729271	stage_1D_vs_E10	ordinary_muscle_development	ENSGALG00010007439	HSD17B2	-1.80965056	0.332078283	-5.449469751	5.05202E-08	1.24773E-07	negative
PRJNA729271	stage_1D_vs_E10	ordinary_muscle_development	ENSGALG00010007441	STRN	1.180111587	0.135347055	8.719152335	2.80292E-18	1.36383E-17	positive
PRJNA729271	stage_1D_vs_E10	ordinary_muscle_development	ENSGALG00010007455	TRPC6	1.214235935	0.206379117	5.883521327	4.01628E-09	1.08261E-08	positive
PRJNA729271	stage_1D_vs_E10	ordinary_muscle_development	ENSGALG00010007457	SHANK3	0.846205793	0.176024332	4.807322863	1.52965E-06	3.36504E-06	positive
PRJNA729271	stage_1D_vs_E10	ordinary_muscle_development	ENSGALG00010007465	PNAT10	1.217740166	0.19676443	6.188822668	6.06152E-10	1.73812E-09	positive
PRJNA729271	stage_1D_vs_E10	ordinary_muscle_development	ENSGALG00010007467	CHODL	-1.592582992	0.33115178	-4.809223711	1.51518E-06	3.33421E-06	negative
PRJNA729271	stage_1D_vs_E10	ordinary_muscle_development	ENSGALG00010007479	NFX1	-0.124613531	0.09636081	-1.29319721	0.19594286	0.235857147	negative
PRJNA729271	stage_1D_vs_E10	ordinary_muscle_development	ENSGALG00010007480	TENM3	0.030111065	0.105482794	0.285459494	0.775292133	0.804485609	positive
PRJNA729271	stage_1D_vs_E10	ordinary_muscle_development	ENSGALG00010007485	CTSC	-0.792776367	0.156488195	-5.066045839	4.06164E-07	9.33907E-07	negative
PRJNA729271	stage_1D_vs_E10	ordinary_muscle_development	ENSGALG00010007500	MPHOSPH6	0.452849514	0.116298504	3.893855033	9.86636E-05	0.000183068	positive
PRJNA729271	stage_1D_vs_E10	ordinary_muscle_development	ENSGALG00010007506	RAB38	-2.315208401	0.591602213	-3.913454598	9.0985E-05	0.000169559	negative
PRJNA729271	stage_1D_vs_E10	ordinary_muscle_development	ENSGALG00010007513	GALNT12	-0.65484293	0.129905398	-5.040921629	4.63295E-07	1.0619E-06	negative
PRJNA729271	stage_1D_vs_E10	ordinary_muscle_development	ENSGALG00010007514	VIT	-3.790255786	0.36822069	-10.29343513	7.54361E-25	5.13114E-24	negative
PRJNA729271	stage_1D_vs_E10	ordinary_muscle_development	ENSGALG00010007521	SLC12A7	-0.738776128	0.137034281	-5.391177476	6.99975E-08	1.71272E-07	negative
PRJNA729271	stage_1D_vs_E10	ordinary_muscle_development	ENSGALG00010007529	CDH13	2.104014909	0.134237425	15.67383249	2.28383E-55	5.348E-54	positive
PRJNA729271	stage_1D_vs_E10	ordinary_muscle_development	ENSGALG00010007532	DCUN1D5	-0.572520967	0.26685963	-2.145401187	0.031920784	0.043865355	negative
PRJNA729271	stage_1D_vs_E10	ordinary_muscle_development	ENSGALG00010007536	GABBR2	0.253174654	0.195548693	1.294688549	0.195427692	0.235393418	positive
PRJNA729271	stage_1D_vs_E10	ordinary_muscle_development	ENSGALG00010007539	RPS3A	0.075143895	0.175347687	0.428542264	0.66825637	0.706931549	positive
PRJNA729271	stage_1D_vs_E10	ordinary_muscle_development	ENSGALG00010007542	MRPL36	0.571398471	0.109985221	5.195229541	2.04467E-07	4.81911E-07	positive
PRJNA729271	stage_1D_vs_E10	ordinary_muscle_development	ENSGALG00010007544	MLYCD	0.114863447	0.104745091	1.09659981	0.27281637	0.318472521	positive
PRJNA729271	stage_1D_vs_E10	ordinary_muscle_development	ENSGALG00010007547	ENSGALG00010007547	-0.008271482	0.60210999	-0.013737493	0.989039411	0.990748187	negative
PRJNA729271	stage_1D_vs_E10	ordinary_muscle_development	ENSGALG00010007548	HSBP1	-0.612339334	0.087243411	-7.018745897	2.23868E-12	7.5711E-12	negative
PRJNA729271	stage_1D_vs_E10	ordinary_muscle_development	ENSGALG00010007549	SH3D19	0.665302192	0.157694718	4.218925029	2.4547E-05	4.82452E-05	positive
PRJNA729271	stage_1D_vs_E10	ordinary_muscle_development	ENSGALG00010007551	FEZ2	0.072807306	0.111211684	0.654673168	0.512678204	0.560343129	positive
PRJNA729271	stage_1D_vs_E10	ordinary_muscle_development	ENSGALG00010007555	NDUFS6	1.427774248	0.129583689	11.01816328	3.12368E-28	2.47014E-27	positive
PRJNA729271	stage_1D_vs_E10	ordinary_muscle_development	ENSGALG00010007564	IRX4	-8.702201104	0.921505784	-9.443457932	3.60676E-21	2.05584E-20	negative
PRJNA729271	stage_1D_vs_E10	ordinary_muscle_development	ENSGALG00010007566	FHIP1A	0.723918273	0.172043751	4.207756859	2.57918E-05	5.05821E-05	positive
PRJNA729271	stage_1D_vs_E10	ordinary_muscle_development	ENSGALG00010007567	TRIP13	-2.439526164	0.135874088	-17.95431494	4.44026E-72	1.73015E-70	negative
PRJNA729271	stage_1D_vs_E10	ordinary_muscle_development	ENSGALG00010007569	GPR89A	-0.04275308	0.081014046	-0.527724292	0.59769071	0.640983046	negative
PRJNA729271	stage_1D_vs_E10	ordinary_muscle_development	ENSGALG00010007574	ENSGALG00010007574	0.885045795	0.182612392	4.846581237	1.25607E-06	2.78263E-06	positive
PRJNA729271	stage_1D_vs_E10	ordinary_muscle_development	ENSGALG00010007575	MMP27	-4.073535986	0.358074806	-11.37621503	5.49325E-30	4.73674E-29	negative
PRJNA729271	stage_1D_vs_E10	ordinary_muscle_development	ENSGALG00010007577	WFDC1	0.214082553	0.119274375	1.794874659	0.072673672	0.094543608	positive
PRJNA729271	stage_1D_vs_E10	ordinary_muscle_development	ENSGALG00010007579	GATB	1.854904185	0.177704357	10.4381469	1.6602E-25	1.16648E-24	positive
PRJNA729271	stage_1D_vs_E10	ordinary_muscle_development	ENSGALG00010007580	BRD9	-0.563312875	0.101022538	-5.576110917	2.45955E-08	6.22728E-08	negative
PRJNA729271	stage_1D_vs_E10	ordinary_muscle_development	ENSGALG00010007581	LPCAT1	-0.772635234	0.115781689	-6.673207501	2.50272E-11	7.88801E-11	negative
PRJNA729271	stage_1D_vs_E10	ordinary_muscle_development	ENSGALG00010007584	ELP2	-0.407272649	0.114786927	-3.548075215	0.000388057	0.000676801	negative
PRJNA729271	stage_1D_vs_E10	ordinary_muscle_development	ENSGALG00010007585	KCNG4	-4.029127056	0.923410895	-4.363308988	1.2811E-05	2.59134E-05	negative
PRJNA729271	stage_1D_vs_E10	ordinary_muscle_development	ENSGALG00010007586	MMP7	-1.764402589	1.697970734	-1.039124264	0.298746948	0.34562511	negative
PRJNA729271	stage_1D_vs_E10	ordinary_muscle_development	ENSGALG00010007588	TTC27	-0.963469221	0.103418704	-9.316198988	1.20583E-20	6.68138E-20	negative
PRJNA729271	stage_1D_vs_E10	ordinary_muscle_development	ENSGALG00010007590	FBXW7	0.147644563	0.138220598	1.068180615	0.285439047	0.331685024	positive
PRJNA729271	stage_1D_vs_E10	ordinary_muscle_development	ENSGALG00010007593	SEC61B	-1.626138221	0.119437145	-13.61501245	3.2609E-42	4.6159E-41	negative
PRJNA729271	stage_1D_vs_E10	ordinary_muscle_development	ENSGALG00010007595	ATP2C2	0.8108179	0.15777503	5.139076197	2.76092E-07	6.43157E-07	positive
PRJNA729271	stage_1D_vs_E10	ordinary_muscle_development	ENSGALG00010007596	ENSGALG00010007596	-2.632165972	0.504657509	-5.215747162	1.83078E-07	4.33047E-07	negative
PRJNA729271	stage_1D_vs_E10	ordinary_muscle_development	ENSGALG00010007597	SMKR1	-3.752249677	1.001706256	-3.745858285	0.000179778	0.000325166	negative
PRJNA729271	stage_1D_vs_E10	ordinary_muscle_development	ENSGALG00010007601	TMEM154	-0.461362282	0.320576048	-1.439166415	0.150103378	0.185107067	negative
PRJNA729271	stage_1D_vs_E10	ordinary_muscle_development	ENSGALG00010007603	ALG2	-0.439012864	0.105398678	-4.16525968	3.10998E-05	6.05334E-05	negative
PRJNA729271	stage_1D_vs_E10	ordinary_muscle_development	ENSGALG00010007604	ENSGALG00010007604	-2.715814278	0.529692212	-5.127155385	2.94153E-07	6.8406E-07	negative
PRJNA729271	stage_1D_vs_E10	ordinary_muscle_development	ENSGALG00010007610	TLDC1	-0.634828049	0.159251491	-3.986324047	6.71048E-05	0.000126718	negative
PRJNA729271	stage_1D_vs_E10	ordinary_muscle_development	ENSGALG00010007614	BIRC6	0.528812977	0.087883942	6.01717409	1.77488E-09	4.92287E-09	positive
PRJNA729271	stage_1D_vs_E10	ordinary_muscle_development	ENSGALG00010007624	TPPP	2.541622387	0.246820644	10.29744653	7.23565E-25	4.92629E-24	positive
PRJNA729271	stage_1D_vs_E10	ordinary_muscle_development	ENSGALG00010007626	DLK2	-3.942560244	0.394143103	-10.00286499	1.48051E-23	9.46205E-23	negative
PRJNA729271	stage_1D_vs_E10	ordinary_muscle_development	ENSGALG00010007633	ENSGALG00010007633	0.216236365	0.102980616	2.099777353	0.035748431	0.048769272	positive
PRJNA729271	stage_1D_vs_E10	ordinary_muscle_development	ENSGALG00010007640	TMEM123	-1.847525183	0.219146965	-8.430530539	3.44102E-17	1.57541E-16	negative
PRJNA729271	stage_1D_vs_E10	ordinary_muscle_development	ENSGALG00010007642	ZNF318	0.882505214	0.106245232	8.30630417	9.8729E-17	4.40331E-16	positive
PRJNA729271	stage_1D_vs_E10	ordinary_muscle_development	ENSGALG00010007643	COTL1	-0.715315003	0.164045852	-4.36045772	1.29791E-05	2.62387E-05	negative
PRJNA729271	stage_1D_vs_E10	ordinary_muscle_development	ENSGALG00010007650	KLHL36	-0.194569363	0.087869882	-2.214289566	0.026808866	0.037214567	negative
PRJNA729271	stage_1D_vs_E10	ordinary_muscle_development	ENSGALG00010007651	CTNNAL1	-0.252985024	0.135343264	-1.869210302	0.061593562	0.080958766	negative
PRJNA729271	stage_1D_vs_E10	ordinary_muscle_development	ENSGALG00010007658	BIRC2	0.074027829	0.119603376	0.618944308	0.535953048	0.582487773	positive
PRJNA729271	stage_1D_vs_E10	ordinary_muscle_development	ENSGALG00010007660	FAM98A	-0.325399458	0.097612516	-3.33358336	0.00085735	0.00144001	negative
PRJNA729271	stage_1D_vs_E10	ordinary_muscle_development	ENSGALG00010007663	ARFIP1	-0.165573557	0.110090724	-1.50397373	0.132588131	0.164910328	negative
PRJNA729271	stage_1D_vs_E10	ordinary_muscle_development	ENSGALG00010007671	FAM206A	-0.146577352	0.1026034	-1.428581817	0.153124463	0.188399889	negative
PRJNA729271	stage_1D_vs_E10	ordinary_muscle_development	ENSGALG00010007673	FHDC1	-1.465716602	0.330108616	-4.440104041	8.99154E-06	1.84581E-05	negative
PRJNA729271	stage_1D_vs_E10	ordinary_muscle_development	ENSGALG00010007678	CDH8	0.642475199	0.283230921	2.268379444	0.02330609	0.032695166	positive
PRJNA729271	stage_1D_vs_E10	ordinary_muscle_development	ENSGALG00010007679	TMEM135	1.025986232	0.141525438	7.249482848	4.18366E-13	1.48961E-12	positive
PRJNA729271	stage_1D_vs_E10	ordinary_muscle_development	ENSGALG00010007680	CHMP2B	-0.409838116	0.126009219	-3.25244549	0.001144165	0.001896042	negative
PRJNA729271	stage_1D_vs_E10	ordinary_muscle_development	ENSGALG00010007695	RASGRP3	0.640073483	0.143336202	4.465539578	7.98672E-06	1.647E-05	positive
PRJNA729271	stage_1D_vs_E10	ordinary_muscle_development	ENSGALG00010007698	ADAMTS18	-2.454568924	0.403092989	-6.089336687	1.13379E-09	3.18804E-09	negative
PRJNA729271	stage_1D_vs_E10	ordinary_muscle_development	ENSGALG00010007699	SLC39A6	-1.199416753	0.105706296	-11.34669169	7.70221E-30	6.59442E-29	negative
PRJNA729271	stage_1D_vs_E10	ordinary_muscle_development	ENSGALG00010007707	ENSGALG00010007707	3.035178451	0.741914909	4.091006141	4.29506E-05	8.25141E-05	positive
PRJNA729271	stage_1D_vs_E10	ordinary_muscle_development	ENSGALG00010007713	SCLT1	-1.052445477	0.214503271	-4.9064309	9.27486E-07	2.07853E-06	negative
PRJNA729271	stage_1D_vs_E10	ordinary_muscle_development	ENSGALG00010007719	LTBP1	-1.562142598	0.190879302	-8.183928693	2.74737E-16	1.19697E-15	negative
PRJNA729271	stage_1D_vs_E10	ordinary_muscle_development	ENSGALG00010007724	RBM11	1.17562796	0.338416213	3.473911463	0.00051293	0.000882065	positive
PRJNA729271	stage_1D_vs_E10	ordinary_muscle_development	ENSGALG00010007730	COG2	-0.353397618	0.063716385	-5.546416674	2.91583E-08	7.31989E-08	negative
PRJNA729271	stage_1D_vs_E10	ordinary_muscle_development	ENSGALG00010007731	ENSGALG00010007731	-1.210078767	0.274197597	-4.413163278	1.01871E-05	2.07829E-05	negative
PRJNA729271	stage_1D_vs_E10	ordinary_muscle_development	ENSGALG00010007741	ENSGALG00010007741	-0.818526633	0.208188226	-3.93166631	8.43591E-05	0.000157718	negative
PRJNA729271	stage_1D_vs_E10	ordinary_muscle_development	ENSGALG00010007748	CSGALNACT1	-3.560345143	0.273945555	-12.99654287	1.28E-38	1.59395E-37	negative
PRJNA729271	stage_1D_vs_E10	ordinary_muscle_development	ENSGALG00010007751	HSPA13	-0.282156857	0.095635909	-2.95032336	0.003174415	0.004989498	negative
PRJNA729271	stage_1D_vs_E10	ordinary_muscle_development	ENSGALG00010007753	PRSS23	1.267478359	0.227642498	5.567845936	2.57908E-08	6.50926E-08	positive
PRJNA729271	stage_1D_vs_E10	ordinary_muscle_development	ENSGALG00010007758	DEK	-0.619529911	0.076783393	-8.068540388	7.11435E-16	3.01969E-15	negative
PRJNA729271	stage_1D_vs_E10	ordinary_muscle_development	ENSGALG00010007759	AGT	0.876873525	1.271373109	0.689705893			positive
PRJNA729271	stage_1D_vs_E10	ordinary_muscle_development	ENSGALG00010007761	ENSGALG00010007761	1.247753102	0.115763016	10.77851241	4.34861E-27	3.27275E-26	positive
PRJNA729271	stage_1D_vs_E10	ordinary_muscle_development	ENSGALG00010007762	SH2D4A	-1.373093671	0.345681057	-3.972140338	7.12297E-05	0.000134105	negative
PRJNA729271	stage_1D_vs_E10	ordinary_muscle_development	ENSGALG00010007763	FZD4	1.082773712	0.099401289	10.89295439	1.24534E-27	9.56099E-27	positive
PRJNA729271	stage_1D_vs_E10	ordinary_muscle_development	ENSGALG00010007765	NUDT7	0.207543504	0.096083806	2.160025835	0.03077067	0.042381305	positive
PRJNA729271	stage_1D_vs_E10	ordinary_muscle_development	ENSGALG00010007766	ENSGALG00010007766	1.245464231	0.128998469	9.654876089	4.68726E-22	2.81449E-21	positive
PRJNA729271	stage_1D_vs_E10	ordinary_muscle_development	ENSGALG00010007767	VAT1L	-1.458351365	0.32168064	-4.533537876	5.80039E-06	1.21078E-05	negative
PRJNA729271	stage_1D_vs_E10	ordinary_muscle_development	ENSGALG00010007770	ENSGALG00010007770	-0.048002383	0.390371733	-0.122965827	0.902134159	0.916551106	negative
PRJNA729271	stage_1D_vs_E10	ordinary_muscle_development	ENSGALG00010007771	CAPN9	-1.864914201	0.471526735	-3.955055064	7.6517E-05	0.000143593	negative
PRJNA729271	stage_1D_vs_E10	ordinary_muscle_development	ENSGALG00010007772	CLEC3A	0.299657125	0.536854026	0.558172446	0.576726635	0.6214893	positive
PRJNA729271	stage_1D_vs_E10	ordinary_muscle_development	ENSGALG00010007774	ENSGALG00010007774	1.182463673	0.265350016	4.456241194	8.34092E-06	1.71711E-05	positive
PRJNA729271	stage_1D_vs_E10	ordinary_muscle_development	ENSGALG00010007777	KIF13A	0.962292465	0.124241405	7.745344353	9.53232E-15	3.75975E-14	positive
PRJNA729271	stage_1D_vs_E10	ordinary_muscle_development	ENSGALG00010007780	SIRT5	-0.911120183	0.145693583	-6.253674088	4.00907E-10	1.16433E-09	negative
PRJNA729271	stage_1D_vs_E10	ordinary_muscle_development	ENSGALG00010007781	MBP	6.27202769	0.234763547	26.71636111	3.0387E-157	6.1817E-155	positive
PRJNA729271	stage_1D_vs_E10	ordinary_muscle_development	ENSGALG00010007783	NOL7	-0.737980979	0.110601103	-6.67245586	2.51558E-11	7.9268E-11	negative
PRJNA729271	stage_1D_vs_E10	ordinary_muscle_development	ENSGALG00010007784	INTS10	-0.823057533	0.094821757	-8.6800494	3.95597E-18	1.90885E-17	negative
PRJNA729271	stage_1D_vs_E10	ordinary_muscle_development	ENSGALG00010007786	SAMSN1	0.057642923	0.236122536	0.244122919	0.807135623	0.833352151	positive
PRJNA729271	stage_1D_vs_E10	ordinary_muscle_development	ENSGALG00010007787	C1orf198	-2.144109886	0.120003928	-17.8669975	2.13163E-71	8.19575E-70	negative

PRJNA729271	stage_1D_vs_E10	ordinary_muscle_development	ENSGALG00010007788	PALM2AKAP2	0.962982101	0.165804709	5.807929743	6.325E-09	1.6816E-08	positive
PRJNA729271	stage_1D_vs_E10	ordinary_muscle_development	ENSGALG00010007789	WWOX	-0.692163844	0.112711204	-6.141038526	8.19837E-10	2.33239E-09	negative
PRJNA729271	stage_1D_vs_E10	ordinary_muscle_development	ENSGALG00010007792	ENSGALG00010007792	1.06989834	0.223400367	4.789152115	1.67487E-06	3.67226E-06	positive
PRJNA729271	stage_1D_vs_E10	ordinary_muscle_development	ENSGALG00010007795	ZNF236	0.274985665	0.115576978	2.379242551	0.017348257	0.024767357	positive
PRJNA729271	stage_1D_vs_E10	ordinary_muscle_development	ENSGALG00010007796	MCUR1	-0.505829298	0.101648878	-4.976240839	6.4831E-07	1.46947E-06	negative
PRJNA729271	stage_1D_vs_E10	ordinary_muscle_development	ENSGALG00010007798	HGSNAT	0.337257993	0.097538516	3.457690415	0.000544827	0.00093492	positive
PRJNA729271	stage_1D_vs_E10	ordinary_muscle_development	ENSGALG00010007800	MAF	0.998037401	0.113762258	8.773009777	1.73953E-18	8.5647E-18	positive
PRJNA729271	stage_1D_vs_E10	ordinary_muscle_development	ENSGALG00010007801	TTC13	0.096350318	0.160242023	0.601279969	0.547653529	0.593690667	positive
PRJNA729271	stage_1D_vs_E10	ordinary_muscle_development	ENSGALG00010007802	ENSGALG00010007802	6.154911074	0.715485041	8.60243153	7.80447E-18	3.70783E-17	positive
PRJNA729271	stage_1D_vs_E10	ordinary_muscle_development	ENSGALG00010007803	ME3	1.507733231	0.176363396	8.549014497	1.24144E-17	5.82352E-17	positive
PRJNA729271	stage_1D_vs_E10	ordinary_muscle_development	ENSGALG00010007805	ENSGALG00010007805	8.978748421	1.517800454	5.915631662	3.30604E-09	8.97452E-09	positive
PRJNA729271	stage_1D_vs_E10	ordinary_muscle_development	ENSGALG00010007806	DYNLRB2	-1.271875624	0.397832363	-3.197013972	0.001388582	0.002280219	negative
PRJNA729271	stage_1D_vs_E10	ordinary_muscle_development	ENSGALG00010007809	ARV1	-0.082078478	0.117755162	-0.697026581	0.485786159	0.53429474	negative
PRJNA729271	stage_1D_vs_E10	ordinary_muscle_development	ENSGALG00010007811	PRCP	0.286443801	0.182700176	1.567835388	0.116919554	0.14699878	positive
PRJNA729271	stage_1D_vs_E10	ordinary_muscle_development	ENSGALG00010007812	POMK	0.124422668	0.120895503	1.029175313	0.303397301	0.350501624	positive
PRJNA729271	stage_1D_vs_E10	ordinary_muscle_development	ENSGALG00010007817	CDYL2	-1.149824727	0.13665338	-8.414169686	3.95688E-17	1.80418E-16	negative
PRJNA729271	stage_1D_vs_E10	ordinary_muscle_development	ENSGALG00010007818	ENSGALG00010007818	1.076268423	0.216756163	4.965341741	6.85802E-07	1.55105E-06	positive
PRJNA729271	stage_1D_vs_E10	ordinary_muscle_development	ENSGALG00010007819	NRIP1	1.509768689	0.166446363	9.070601873	1.18361E-19	6.22062E-19	positive
PRJNA729271	stage_1D_vs_E10	ordinary_muscle_development	ENSGALG00010007821	FAM89A	-1.820105411	0.125726586	-14.47669475	1.70081E-47	2.94543E-46	negative
PRJNA729271	stage_1D_vs_E10	ordinary_muscle_development	ENSGALG00010007826	ENSGALG00010007826	8.936225999	1.37431866	6.502295473	7.91035E-11	2.41188E-10	positive
PRJNA729271	stage_1D_vs_E10	ordinary_muscle_development	ENSGALG00010007830	SMIM19	0.885558043	0.130187438	6.802177364	1.0305E-11	3.33119E-11	positive
PRJNA729271	stage_1D_vs_E10	ordinary_muscle_development	ENSGALG00010007835	GUCY1A1	1.531193209	0.185972117	8.233455838	1.81888E-16	7.99416E-16	positive
PRJNA729271	stage_1D_vs_E10	ordinary_muscle_development	ENSGALG00010007845	TFAP2B	-8.058541692	1.094265546	-7.364338317	1.78028E-13	6.47069E-13	negative
PRJNA729271	stage_1D_vs_E10	ordinary_muscle_development	ENSGALG00010007846	ENSGALG00010007846	0.168081304	0.148549695	1.131481988	0.25785228	0.302857856	positive
PRJNA729271	stage_1D_vs_E10	ordinary_muscle_development	ENSGALG00010007847	CMC2	-0.132896706	0.11671887	-1.138605149	0.25486788	0.299643922	negative
PRJNA729271	stage_1D_vs_E10	ordinary_muscle_development	ENSGALG00010007849	CXADR	-4.147076273	0.318583398	-13.01723914	9.76377E-39	1.22427E-37	negative
PRJNA729271	stage_1D_vs_E10	ordinary_muscle_development	ENSGALG00010007850	ENSGALG00010007850	-1.742581069	0.277390077	-6.282059859	3.34116E-10	9.76808E-10	negative
PRJNA729271	stage_1D_vs_E10	ordinary_muscle_development	ENSGALG00010007851	AvBD7	5.532518394	0.893408544	6.192596246	5.91812E-10	1.698E-09	positive
PRJNA729271	stage_1D_vs_E10	ordinary_muscle_development	ENSGALG00010007852	SLC20A2	2.471685167	0.207096461	11.93494643	7.7809E-33	7.69332E-32	positive
PRJNA729271	stage_1D_vs_E10	ordinary_muscle_development	ENSGALG00010007853	C1orf131	-0.427949435	0.113371386	-3.774757026	0.000160164	0.000291399	negative
PRJNA729271	stage_1D_vs_E10	ordinary_muscle_development	ENSGALG00010007854	FAM181B	0.747849067	0.117263408	6.377514345	1.79985E-10	5.35705E-10	positive
PRJNA729271	stage_1D_vs_E10	ordinary_muscle_development	ENSGALG00010007855	RNF182	-1.47623793	0.219481007	-6.726039536	1.74343E-11	5.55408E-11	negative
PRJNA729271	stage_1D_vs_E10	ordinary_muscle_development	ENSGALG00010007856	PDCD2	0.53203063	0.129688792	4.10236398	4.0895E-05	7.86902E-05	positive
PRJNA729271	stage_1D_vs_E10	ordinary_muscle_development	ENSGALG00010007857	ENSGALG00010007857	0.084698969	0.331445365	0.255544286	0.798302743	0.825467132	positive
PRJNA729271	stage_1D_vs_E10	ordinary_muscle_development	ENSGALG00010007859	CENPN	-1.548070292	0.159087289	-9.730948948	2.22507E-22	1.35174E-21	negative
PRJNA729271	stage_1D_vs_E10	ordinary_muscle_development	ENSGALG00010007860	DEFB4A	5.556980515	0.885298382	6.276957719	3.45262E-10	1.00797E-09	positive
PRJNA729271	stage_1D_vs_E10	ordinary_muscle_development	ENSGALG00010007861	INTU	-2.275529517	0.17903915	-12.70967558	5.22508E-37	6.14254E-36	negative
PRJNA729271	stage_1D_vs_E10	ordinary_muscle_development	ENSGALG00010007862	ATMIN	0.706884468	0.085261427	8.290788631	1.125E-16	5.00211E-16	positive
PRJNA729271	stage_1D_vs_E10	ordinary_muscle_development	ENSGALG00010007863	AvBD1	5.421555168	0.819928185	6.612231735	3.78569E-11	1.18033E-10	positive
PRJNA729271	stage_1D_vs_E10	ordinary_muscle_development	ENSGALG00010007864	TYR	-1.93829594	0.992227605	-1.953479152	0.050762845	0.067641795	negative
PRJNA729271	stage_1D_vs_E10	ordinary_muscle_development	ENSGALG00010007865	GABRR3	-0.042699254	0.435001763	-0.098158807	0.921806191	0.933462397	negative
PRJNA729271	stage_1D_vs_E10	ordinary_muscle_development	ENSGALG00010007866	TBP	-0.180879293	0.084767947	-2.133817063	0.032857759	0.045080285	negative
PRJNA729271	stage_1D_vs_E10	ordinary_muscle_development	ENSGALG00010007867	ZNF516	-1.0473985	0.105920795	-9.888506759	4.66941E-23	2.91361E-22	negative
PRJNA729271	stage_1D_vs_E10	ordinary_muscle_development	ENSGALG00010007868	INHBA	-2.063245582	0.299369294	-6.891974652	5.50232E-12	1.80853E-11	negative
PRJNA729271	stage_1D_vs_E10	ordinary_muscle_development	ENSGALG00010007869	GCSH	-0.820829603	0.123692232	-6.63606427	3.22169E-11	1.00882E-10	negative
PRJNA729271	stage_1D_vs_E10	ordinary_muscle_development	ENSGALG00010007870	AvBD3	0.371110598	0.499123401	0.743524742	0.457164048	0.506117228	positive
PRJNA729271	stage_1D_vs_E10	ordinary_muscle_development	ENSGALG00010007871	HSPA4L	-0.248342943	0.08806158	-2.820105463	0.004800787	0.007392692	negative
PRJNA729271	stage_1D_vs_E10	ordinary_muscle_development	ENSGALG00010007872	KLHL2	-0.566722735	0.076951968	-7.364629478	1.7764E-13	6.4582E-13	negative
PRJNA729271	stage_1D_vs_E10	ordinary_muscle_development	ENSGALG00010007873	ENSGALG00010007873	2.987498051	0.766906481	3.895518065	9.79891E-05	0.000181886	positive
PRJNA729271	stage_1D_vs_E10	ordinary_muscle_development	ENSGALG00010007874	ENSGALG00010007874	-1.414879509	0.583588752	-2.42444616	0.015331757	0.022075475	negative
PRJNA729271	stage_1D_vs_E10	ordinary_muscle_development	ENSGALG00010007875	AvBD4	5.666119901	1.379388073	4.107705446	3.99609E-05	7.6995E-05	positive
PRJNA729271	stage_1D_vs_E10	ordinary_muscle_development	ENSGALG00010007877	MSMO1	-1.026307409	0.188082176	-5.456696792	4.85074E-08	1.20067E-07	negative
PRJNA729271	stage_1D_vs_E10	ordinary_muscle_development	ENSGALG00010007878	PSMB1	-0.460834353	0.122424815	-3.764223395	0.000167067	0.000303198	negative
PRJNA729271	stage_1D_vs_E10	ordinary_muscle_development	ENSGALG00010007879	PLK4	-1.391241641	0.189543534	-7.339958326	2.1366E-13	7.73092E-13	negative
PRJNA729271	stage_1D_vs_E10	ordinary_muscle_development	ENSGALG00010007880	BCO1	1.188700352	0.283128425	4.198449345	2.68749E-05	5.25995E-05	positive
PRJNA729271	stage_1D_vs_E10	ordinary_muscle_development	ENSGALG00010007881	AvBD14	-3.505079117	0.947122137	-3.700767811	0.000214948	0.000384461	negative
PRJNA729271	stage_1D_vs_E10	ordinary_muscle_development	ENSGALG00010007882	CPE	-0.551551322	0.227157795	-2.428053691	0.015180096	0.021872315	negative
PRJNA729271	stage_1D_vs_E10	ordinary_muscle_development	ENSGALG00010007883	DHTKD1	0.801684943	0.120072272	6.676686692	2.44405E-11	7.70811E-11	positive
PRJNA729271	stage_1D_vs_E10	ordinary_muscle_development	ENSGALG00010007884	GLI3	-0.9272297	0.110383592	-8.400068194	4.46219E-17	2.02948E-16	negative
PRJNA729271	stage_1D_vs_E10	ordinary_muscle_development	ENSGALG00010007885	USP38	0.708742894	0.10367919	6.835922351	8.14791E-12	2.64926E-11	positive
PRJNA729271	stage_1D_vs_E10	ordinary_muscle_development	ENSGALG00010007886	GRM5	-1.94118357	0.785428776	-2.471495353	0.01345493	0.019534176	negative
PRJNA729271	stage_1D_vs_E10	ordinary_muscle_development	ENSGALG00010007887	CDT1	-0.369259363	0.122890382	-3.004786531	0.002657673	0.004213845	negative
PRJNA729271	stage_1D_vs_E10	ordinary_muscle_development	ENSGALG00010007889	DCAF6	1.639687408	0.097088973	16.88850288	5.46728E-64	1.68973E-62	positive
PRJNA729271	stage_1D_vs_E10	ordinary_muscle_development	ENSGALG00010007890	ENSGALG00010007890	-0.999992375	0.125754212	-7.951959317	1.83585E-15	7.59001E-15	negative
PRJNA729271	stage_1D_vs_E10	ordinary_muscle_development	ENSGALG00010007891	GDI1	-0.978043934	0.055943396	-17.48274142	1.93946E-68	6.84003E-67	negative
PRJNA729271	stage_1D_vs_E10	ordinary_muscle_development	ENSGALG00010007892	ENSGALG00010007892	0.241143965	0.44725409	0.539165477	0.589772679	0.633663552	positive
PRJNA729271	stage_1D_vs_E10	ordinary_muscle_development	ENSGALG00010007893	MFSD8	-0.038730932	0.150391042	-0.257534832	0.79676593	0.824171994	negative
PRJNA729271	stage_1D_vs_E10	ordinary_muscle_development	ENSGALG00010007894	CD88	0.381955222	0.265454308	1.438873698	0.150186311	0.185193583	positive
PRJNA729271	stage_1D_vs_E10	ordinary_muscle_development	ENSGALG00010007895	GOLGB1	-0.484042118	0.111849819	-4.327607519	1.50738E-05	3.02122E-05	negative
PRJNA729271	stage_1D_vs_E10	ordinary_muscle_development	ENSGALG00010007896	TIMMDC1	-0.259102501	0.105925677	-2.446078309	0.014441963	0.020871012	negative
PRJNA729271	stage_1D_vs_E10	ordinary_muscle_development	ENSGALG00010007897	EFHC1	-0.759359789	0.367807113	-2.064559826	0.038964668	0.052806064	negative
PRJNA729271	stage_1D_vs_E10	ordinary_muscle_development	ENSGALG00010007898	GAB1	0.493250788	0.088787153	5.555429739	2.7693E-08	6.96529E-08	positive
PRJNA729271	stage_1D_vs_E10	ordinary_muscle_development	ENSGALG00010007899	APRT	-0.592135385	0.152028292	-3.894902558	9.82382E-05	0.000182326	negative
PRJNA729271	stage_1D_vs_E10	ordinary_muscle_development	ENSGALG00010007900	FHOD3	0.52936953	0.199933992	2.647721504	0.008103625	0.012126992	positive
PRJNA729271	stage_1D_vs_E10	ordinary_muscle_development	ENSGALG00010007901	FSTL5	1.324928475	0.287439504	4.609416784	4.038E-06	8.5434E-06	positive
PRJNA729271	stage_1D_vs_E10	ordinary_muscle_development	ENSGALG00010007902	C7orf25	0.840363374	0.143470847	5.857380707	4.70224E-09	1.2622E-08	positive
PRJNA729271	stage_1D_vs_E10	ordinary_muscle_development	ENSGALG00010007903	PSMA2	-0.464182746	0.103831032	-4.470558927	7.80154E-06	1.6111E-05	negative
PRJNA729271	stage_1D_vs_E10	ordinary_muscle_development	ENSGALG00010007904	ENSGALG00010007904	-0.228926181	0.123733726	-1.850151843	0.064291668	0.08431265	negative
PRJNA729271	stage_1D_vs_E10	ordinary_muscle_development	ENSGALG00010007905	MRPL32	0.546299646	0.153719222	3.553879859	0.000379593	0.000662516	positive
PRJNA729271	stage_1D_vs_E10	ordinary_muscle_development	ENSGALG00010007906	NAF1	-0.766809491	0.110938553	-6.912019972	4.77801E-12	1.57654E-11	negative
PRJNA729271	stage_1D_vs_E10	ordinary_muscle_development	ENSGALG00010007907	IRX2	-7.83269465	0.987577504	-7.931220197	2.17003E-15	8.92835E-15	negative
PRJNA729271	stage_1D_vs_E10	ordinary_muscle_development	ENSGALG00010007908	IRX1	-6.245144852	0.832094604	-7.505330309	6.12739E-14	2.29619E-13	negative
PRJNA729271	stage_1D_vs_E10	ordinary_muscle_development	ENSGALG00010007909	GALNS	-0.396762551	0.107660316	-3.68531848	0.000228417	0.000407696	negative
PRJNA729271	stage_1D_vs_E10	ordinary_muscle_development	ENSGALG00010007910	ANKS6	0.25656092	0.146561843	1.75053012	0.080026881	0.103468883	positive
PRJNA729271	stage_1D_vs_E10	ordinary_muscle_development	ENSGALG00010007911	IFI6	3.812977233	0.802152269	4.753433209	1.99991E-06	4.35986E-06	positive
PRJNA729271	stage_1D_vs_E10	ordinary_muscle_development	ENSGALG00010007912	TRAPPC2L	-0.488785613	0.115038533	-4.248885996	2.14836E-05	4.24315E-05	negative
PRJNA729271	stage_1D_vs_E10	ordinary_muscle_development	ENSGALG00010007914	PAQR8	0.497102082	0.122416355	4.060748921	4.89156E-05	9.3552E-05	positive
PRJNA729271	stage_1D_vs_E10	ordinary_muscle_development	ENSGALG00010007915	HECW1	0.745726452	0.131901761	5.653650481	1.57075E-08	4.03761E-08	positive
PRJNA729271	stage_1D_vs_E10	ordinary_muscle_development	ENSGALG00010007916	MCM3	-1.625573472	0.202264994	-8.036850253	9.21772E-16	3.88065E-15	negative
PRJNA729271	stage_1D_vs_E10	ordinary_muscle_development	ENSGALG00010007917	POGLUT1	-2.212312863	0.080801111	-27.37973329	4.7813E-165	1.1178E-162	negative
PRJNA729271	stage_1D_vs_E10	ordinary_muscle_development	ENSGALG00010007918	NPY1R	-1.425557808	0.675303403	-2.110988634	0.034773289	0.047541862	negative
PRJNA729271	stage_1D_vs_E10	ordinary_muscle_development	ENSGALG00010007920	FAT3	-1.953438887	0.17899237	-10.91353161	9.93213E-28	7.6782E-27	negative
PRJNA729271	stage_1D_vs_E10	ordinary_muscle_development	ENSGALG00010007921	FAM120B	-0.354551154	0.100834686	-3.516162626	0.000437833	0.000757868	negative
PRJNA729271	stage_1D_vs_E10	ordinary_muscle_development	ENSGALG00010007922	IL17A	-0.038775971	0.444294523	-0.087275375	0.930452627	0.940932079	negative
PRJNA729271	stage_1D_vs_E10	ordinary_muscle_development	ENSGALG00010007923	YAP1	-1.37457563	0.091461516	-15.02900553	4.74069E-51	9.50434E-50	negative
PRJNA729271	stage_1D_vs_E10	ordinary_muscle_development	ENSGALG00010007924	ABHD18	0.818314922	0.114886517	7.122810759	1.05748E-12	3.64783E-12	positive
PRJNA729271	stage_1D_vs_E10	ordinary_muscle_development	ENSGALG00010007925	STK17A	3.656885893	0.234503984	15.59413118	7.98017E-55	1.8159E-53	positive
PRJNA729271	stage_1D_vs_E10	ordinary_muscle_development	ENSGALG00010007926	EIF3E	0.2699077	0.131376136	2.054465209	0.039930687	0.054062704	positive
PRJNA729271	stage_1D_vs_E10	ordinary_muscle_development	ENSGALG00010007927	DLL1	0.326012802	0.161469031	2.019042285	0.043482825	0.058538455	positive
PRJNA729271	stage_1D_vs_E10	ordinary_muscle_development	ENSGALG00010007928	C11orf70	-3.589033526	0.812398288	-4.417825072	9.96991E-06	2.03512E-05	negative
PRJNA729271	stage_1D_vs_E10	ordinary_muscle_development	ENSGALG00010007929	LRRC14B	7.867346328	0.329337146	23.88842687	4.0403E-126	5.0055E-124	positive
PRJNA729271	stage_1D_vs_E10	ordinary_muscle_development	ENSGALG00010007930	CBFA2T3	0.62029386	0.196421923	3.157966529	0.001588738	0.002589246	positive
PRJNA729271	stage_1D_vs_E10	ordinary_muscle_development	ENSGALG00010007931	GBE1	1.748800931	0.188905778	9.257530144	2.09216E-20	1.14093E-19	positive
PRJNA729271	stage_1D_vs_E10	ordinary_muscle_development	ENSGALG00010007932	NPY5R	1.152436105	0.175737746	6.557703906	5.46426E-11	1.6852E-10	positive
PRJNA729271	stage_1D_vs_E10	ordinary_muscle_development	ENSGALG00010007933	CEP126	0.139294811	0.451575337	0.30846417	0.75772916	0.788972357	positive
PRJNA729271	stage_1D_vs_E10	ordinary_muscle_development	ENSGALG00010007934	PKHD1	-0.824324048	0.34813904	-2.36780123	0.017894147	0.025481448	negative
PRJNA729271	stage_1D_vs_E10	ordinary_muscle_development	ENSGALG00010007935	LARP1B	1.150269729	0.122759227	9.370128482	7.24443E-21	4.06692E-20	positive
PRJNA729271	stage_1D_vs_E10	ordinary_muscle_development	ENSGALG00010007936	ENSGALG00010007936	3.5421677	0.138940321	25.49416668	2.2879E-143	3.6848E-141	positive
PRJNA729271	stage_1D_vs_E10	ordinary_muscle_development	ENSGALG00010007937	ANGPTL5	2.974932319	0.40204517	7.39949773	1.36701E-13	5.00878E-13	positive
PRJNA729271	stage_1D_vs_E10	ordinary_muscle_development	ENSGALG00010007938	BCL9	-0.226390056	0.101854818	-2.222674015	0.026237789	0.036463735	negative
PRJNA729271	stage_1D_vs_E10	ordinary_muscle_development	ENSGALG00010007940	GLIPR2	-3.348392348	0.273559256	-12.24009888	1.89801E-34	2.01256E-33	negative
PRJNA729271	stage_1D_vs_E10	ordinary_muscle_development	ENSGALG00010007941	MRPL19	-0.932821886	0.074594757	-12.5051937	6.99292E-36	7.85146E-35	negative
PRJNA729271	stage_1D_vs_E10	ordinary_muscle_development	ENSGALG00010007942	PGRMC2	-0.529803601	0.085841656	-6.171870668	6.74867E-10	1.92905E-09	negative
PRJNA729271	stage_1D_vs_E10	ordinary_muscle_development	ENSGALG00010007943	BLOC1S4	-0.515588486	0.101366471	-5.086380925	3.64961E-07	8.42105E-07	negative
PRJNA729271	stage_1D_vs_E10	ordinary_muscle_development	ENSGALG00010007944	ACSF3	0.050446011	0.135925718	0.371129259	0.710541259	0.746217617	positive
PRJNA729271	stage_1D_vs_E10	ordinary_muscle_development	ENSGALG00010007945	ENSGALG00010007945	-0.844957281	0.117310988	-7.202712193	5.90265E-13	2.07667E-12	negative
PRJNA729271	stage_1D_vs_E10	ordinary_muscle_development	ENSGALG00010007946	ENSGALG00010007946	-0.173563606	0.151781185	-1.143512003	0.252826074	0.297460547	negative
PRJNA729271	stage_1D_vs_E10	ordinary_muscle_development	ENSGALG00010007948	ENSGALG00010007948	-1.80923702	0.463095988	-3.906829399	9.35151E-05	0.00017405	negative
PRJNA729271	stage_1D_vs_E10	ordinary_muscle_development	ENSGALG00010007950	ARC	-0.768515632	0.558395761	-1.376292026	0.168731241	0.205947772	negative
PRJNA729271	stage_1D_vs_E10	ordinary_muscle_development	ENSGALG00010007951	TGFBR1	-0.192802126	0.113755716	-1.694878576	0.090098458	0.115460803	negative
PRJNA729271	stage_1D_vs_E10	ordinary_muscle_development	ENSGALG00010007952	EMC2	-0.279801793	0.109262222	-2.560828314	0.010442295	0.015388477	negative
PRJNA729271	stage_1D_vs_E10	ordinary_muscle_development	ENSGALG00010007953	SCGN	-1.990693907	0.490387341	-4.059431676	4.91923E-05	9.40441E-05	negative
PRJNA729271	stage_1D_vs_E10	ordinary_muscle_development	ENSGALG00010007954	PSCA	1.705906633	0.580770052	2.937318524	0.003310639	0.005194599	positive
PRJNA729271	stage_1D_vs_E10	ordinary_muscle_development	ENSGALG00010007955	TMA16	-0.796230561	0.122948592	-6.476125874	9.41076E-11	2.85494E-10	negative
PRJNA729271	stage_1D_vs_E10	ordinary_muscle_development	ENSGALG00010007956	HS1BP3	0.049315559	0.13186236	0.37399269	0.708409755	0.744572503	positive
PRJNA729271	stage_1D_vs_E10	ordinary_muscle_development	ENSGALG00010007957	CDH15	1.107339355	0.16286274	6.799218502	1.05188E-11	3.39956E-11	positive
PRJNA729271	stage_1D_vs_E10	ordinary_muscle_development	ENSGALG00010007958	ENSGALG00010007958	-4.601495894	0.95517705	-4.81742719	1.45421E-06	3.20493E-06	negative
PRJNA729271	stage_1D_vs_E10	ordinary_muscle_development	ENSGALG00010007960	TSNAX	-0.896267055	0.063028172	-14.22010238	6.87523E-46	1.11473E-44	negative
PRJNA729271	stage_1D_vs_E10	ordinary_muscle_development	ENSGALG00010007962	ADCY8	-2.348197315	0.485628835	-4.835374561	1.32895E-06	2.93646E-06	negative
PRJNA729271	stage_1D_vs_E10	ordinary_muscle_development	ENSGALG00010007963	LDAH	0.120036723	0.083594136	1.435946687	0.151017512	0.186091885	positive
PRJNA729271	stage_1D_vs_E10	ordinary_muscle_development	ENSGALG00010007964	RECQL4	-1.820459713	0.158353152	-11.4962013	1.3785E-30	1.22886E-29	negative

PRJNA729271	stage_1D_vs_E10	ordinary_muscle_development	ENSGALG00010007965	EGLN1	1.15722574	0.157491704	7.347852037	2.01417E-13	7.29703E-13	positive
PRJNA729271	stage_1D_vs_E10	ordinary_muscle_development	ENSGALG00010007966	TMEM74	-1.155399119	0.214535749	-5.385578518	7.2212E-08	1.76511E-07	negative
PRJNA729271	stage_1D_vs_E10	ordinary_muscle_development	ENSGALG00010007968	SLC22A31	-0.003041195	0.207578155	-0.014650841	0.988310738	0.990086678	negative
PRJNA729271	stage_1D_vs_E10	ordinary_muscle_development	ENSGALG00010007970	SPRTN	0.238728834	0.106950664	2.232139805	0.025605724	0.035667401	positive
PRJNA729271	stage_1D_vs_E10	ordinary_muscle_development	ENSGALG00010007972	EXOC8	-0.461803208	0.112585927	-4.101784465	4.09976E-05	7.88667E-05	negative
PRJNA729271	stage_1D_vs_E10	ordinary_muscle_development	ENSGALG00010007973	CARMIL1	-0.877750707	0.135194432	-6.492506356	8.442E-11	2.56965E-10	negative
PRJNA729271	stage_1D_vs_E10	ordinary_muscle_development	ENSGALG00010007975	ANKRD11	0.958316439	0.175741654	5.452984053	4.95315E-08	1.22477E-07	positive
PRJNA729271	stage_1D_vs_E10	ordinary_muscle_development	ENSGALG00010007976	ENSGALG00010007976	0.068517067	0.362469908	0.18902829	0.850070644	0.871844194	positive
PRJNA729271	stage_1D_vs_E10	ordinary_muscle_development	ENSGALG00010007977	APOB	-0.006476596	1.382423316	-0.004684959	0.996261957	0.997087418	negative
PRJNA729271	stage_1D_vs_E10	ordinary_muscle_development	ENSGALG00010007978	JADE1	0.862969391	0.116283804	7.421234633	1.16034E-13	4.27097E-13	positive
PRJNA729271	stage_1D_vs_E10	ordinary_muscle_development	ENSGALG00010007979	RAB30	0.009402825	0.088728142	0.105973429	0.915603434	0.928391454	positive
PRJNA729271	stage_1D_vs_E10	ordinary_muscle_development	ENSGALG00010007980	MARCHF1	-1.332373349	0.231222135	-5.762308814	8.2971E-09	2.1819E-08	negative
PRJNA729271	stage_1D_vs_E10	ordinary_muscle_development	ENSGALG00010007981	TDRD15	-2.550213407	0.675878135	-3.773185244	0.000161177	0.000293057	negative
PRJNA729271	stage_1D_vs_E10	ordinary_muscle_development	ENSGALG00010007982	RECK	-0.180068736	0.146131339	-1.232239002	0.217859818	0.260100326	negative
PRJNA729271	stage_1D_vs_E10	ordinary_muscle_development	ENSGALG00010007983	RCHY1	-0.307288182	0.08769638	-3.504000773	0.000458324	0.000792297	negative
PRJNA729271	stage_1D_vs_E10	ordinary_muscle_development	ENSGALG00010007984	EIF3H	0.329674532	0.122029952	2.701587001	0.006900942	0.010413196	positive
PRJNA729271	stage_1D_vs_E10	ordinary_muscle_development	ENSGALG00010007985	APELA	-4.535147593	0.377493133	-12.01385456	3.00505E-33	3.03541E-32	negative
PRJNA729271	stage_1D_vs_E10	ordinary_muscle_development	ENSGALG00010007986	MYC	0.535984259	0.187083673	2.864944066	0.004170829	0.006471437	positive
PRJNA729271	stage_1D_vs_E10	ordinary_muscle_development	ENSGALG00010007987	ENSGALG00010007987	0.669681839	0.293386198	2.2825949	0.022454242	0.031581043	positive
PRJNA729271	stage_1D_vs_E10	ordinary_muscle_development	ENSGALG00010007988	TARBP1	-0.196060233	0.123484971	-1.587725467	0.112348432	0.141632503	negative
PRJNA729271	stage_1D_vs_E10	ordinary_muscle_development	ENSGALG00010007989	SPG7	0.481943268	0.097190007	4.958773899	7.09395E-07	1.60291E-06	positive
PRJNA729271	stage_1D_vs_E10	ordinary_muscle_development	ENSGALG00010007990	TRHR	-4.387453563	1.154799176	-3.799321695	0.000145093	0.000265077	negative
PRJNA729271	stage_1D_vs_E10	ordinary_muscle_development	ENSGALG00010007991	PCF11	0.839121407	0.1094959	7.663496139	1.80939E-14	7.01071E-14	positive
PRJNA729271	stage_1D_vs_E10	ordinary_muscle_development	ENSGALG00010007992	LAPTM4A	-0.127994306	0.099097844	-1.291595263	0.196497345	0.236465672	negative
PRJNA729271	stage_1D_vs_E10	ordinary_muscle_development	ENSGALG00010007993	LRRC14	0.543787109	0.171790943	3.165400331	0.001548697	0.002526831	positive
PRJNA729271	stage_1D_vs_E10	ordinary_muscle_development	ENSGALG00010007994	FAM65B	1.621534731	0.165873085	9.775755557	1.43086E-22	8.77338E-22	positive
PRJNA729271	stage_1D_vs_E10	ordinary_muscle_development	ENSGALG00010007995	KIAA0319	-4.523513441	0.813506395	-5.560513682	2.68982E-08	6.77479E-08	negative
PRJNA729271	stage_1D_vs_E10	ordinary_muscle_development	ENSGALG00010007996	NUDCD1	-0.69879786	0.07489342	-9.330564098	1.05309E-20	5.8597E-20	negative
PRJNA729271	stage_1D_vs_E10	ordinary_muscle_development	ENSGALG00010007997	TMEM192	-0.206071121	0.136480986	-1.509888866	0.131071784	0.163346704	negative
PRJNA729271	stage_1D_vs_E10	ordinary_muscle_development	ENSGALG00010007998	RPL13	0.110912265	0.144810756	0.765911786	0.443728793	0.492975309	positive
PRJNA729271	stage_1D_vs_E10	ordinary_muscle_development	ENSGALG00010007999	SDC1	-0.484226648	0.207219525	-2.336780999	0.019450574	0.027565123	negative
PRJNA729271	stage_1D_vs_E10	ordinary_muscle_development	ENSGALG00010008000	C8orf82	0.087040127	0.169523517	0.513439835	0.607643686	0.650213734	positive
PRJNA729271	stage_1D_vs_E10	ordinary_muscle_development	ENSGALG00010008001	ALDH5A1	-0.250784231	0.136697843	-1.83458806	0.066566749	0.087044206	negative
PRJNA729271	stage_1D_vs_E10	ordinary_muscle_development	ENSGALG00010008002	GJA8	-1.227964577	0.167876293	-7.314699153	2.57959E-13	9.28741E-13	negative
PRJNA729271	stage_1D_vs_E10	ordinary_muscle_development	ENSGALG00010008004	ENY2	-1.28634249	0.171498142	-7.500620581	6.35164E-14	2.37715E-13	negative
PRJNA729271	stage_1D_vs_E10	ordinary_muscle_development	ENSGALG00010008005	ANKRD42	-0.068993447	0.329866917	-0.209155399	0.834326929	0.858004174	negative
PRJNA729271	stage_1D_vs_E10	ordinary_muscle_development	ENSGALG00010008006	CPNE7	0.453931006	0.499507405	0.908757311	0.363478245	0.412416216	positive
PRJNA729271	stage_1D_vs_E10	ordinary_muscle_development	ENSGALG00010008007	CBR4	0.381170921	0.121823323	3.128882978	0.001754722	0.002846317	positive
PRJNA729271	stage_1D_vs_E10	ordinary_muscle_development	ENSGALG00010008008	BTC	3.246215426	0.199876354	16.24111789	2.58176E-59	6.91731E-58	positive
PRJNA729271	stage_1D_vs_E10	ordinary_muscle_development	ENSGALG00010008009	GPLD1	-3.678971263	1.480981486	-2.484143994	0.012986328	0.018895486	negative
PRJNA729271	stage_1D_vs_E10	ordinary_muscle_development	ENSGALG00010008010	CERK	-0.125416322	0.091633572	-1.368672193	0.171101766	0.208536122	negative
PRJNA729271	stage_1D_vs_E10	ordinary_muscle_development	ENSGALG00010008011	ADNP2	-0.149624912	0.099937791	-1.497180505	0.134346296	0.166896603	negative
PRJNA729271	stage_1D_vs_E10	ordinary_muscle_development	ENSGALG00010008012	CCDC90B	-0.344922328	0.079664926	-4.32966356	1.49337E-05	2.99439E-05	negative
PRJNA729271	stage_1D_vs_E10	ordinary_muscle_development	ENSGALG00010008013	ENSGALG00010008013	1.071461546	0.226939934	4.721344221	2.34291E-06	5.08086E-06	positive
PRJNA729271	stage_1D_vs_E10	ordinary_muscle_development	ENSGALG00010008015	ARHGAP39	0.248288869	0.149422775	1.661653446	0.096582281	0.123138372	positive
PRJNA729271	stage_1D_vs_E10	ordinary_muscle_development	ENSGALG00010008016	GSR	-0.809740381	0.14354814	-5.640897751	1.69166E-08	4.33839E-08	negative
PRJNA729271	stage_1D_vs_E10	ordinary_muscle_development	ENSGALG00010008017	GMNN	-1.487696144	0.133910787	-11.10960645	1.12655E-28	9.13784E-28	negative
PRJNA729271	stage_1D_vs_E10	ordinary_muscle_development	ENSGALG00010008018	ENSGALG00010008018	0.720036127	0.205184095	3.509219994	0.000449423	0.000777188	positive
PRJNA729271	stage_1D_vs_E10	ordinary_muscle_development	ENSGALG00010008019	PPP2CB	-0.626927197	0.099890445	-6.276147823	3.47064E-10	1.01282E-09	negative
PRJNA729271	stage_1D_vs_E10	ordinary_muscle_development	ENSGALG00010008020	COA6	0.305106996	0.131902653	2.313122507	0.020715905	0.029258213	positive
PRJNA729271	stage_1D_vs_E10	ordinary_muscle_development	ENSGALG00010008022	SLC35F3	-0.994124364	0.392736556	-2.531275351	0.011364859	0.016685266	negative
PRJNA729271	stage_1D_vs_E10	ordinary_muscle_development	ENSGALG00010008023	ATAD2	-0.192692574	0.076173591	-2.529650655	0.011417615	0.016754234	negative
PRJNA729271	stage_1D_vs_E10	ordinary_muscle_development	ENSGALG00010008024	MRS2	0.645092061	0.094257903	6.843904196	7.70635E-12	2.51132E-11	positive
PRJNA729271	stage_1D_vs_E10	ordinary_muscle_development	ENSGALG00010008025	TBC1D22A	-0.037695148	0.096860752	-0.389168439	0.697151549	0.734283658	negative
PRJNA729271	stage_1D_vs_E10	ordinary_muscle_development	ENSGALG00010008026	PURG	0.79877096	0.121897107	6.552829527	5.6457E-11	1.74005E-10	positive
PRJNA729271	stage_1D_vs_E10	ordinary_muscle_development	ENSGALG00010008027	C6orf62	1.288259439	0.106885972	12.05265211	1.87808E-33	1.91171E-32	positive
PRJNA729271	stage_1D_vs_E10	ordinary_muscle_development	ENSGALG00010008028	KCNK1	-3.457449489	0.170967537	-20.22284207	6.16284E-91	3.73767E-89	negative
PRJNA729271	stage_1D_vs_E10	ordinary_muscle_development	ENSGALG00010008029	LY6E	-0.457317476	0.314455551	-1.454315165	0.145858948	0.180286983	negative
PRJNA729271	stage_1D_vs_E10	ordinary_muscle_development	ENSGALG00010008030	GJA5	-2.004260366	0.147847046	-13.55630987	7.27026E-42	1.02016E-40	negative
PRJNA729271	stage_1D_vs_E10	ordinary_muscle_development	ENSGALG00010008031	MAP3K21	-2.55444598	0.186222322	-13.71718466	8.01164E-43	1.16013E-41	negative
PRJNA729271	stage_1D_vs_E10	ordinary_muscle_development	ENSGALG00010008032	PUM2	-0.173056225	0.053840679	-3.214228116	0.001307957	0.002154169	negative
PRJNA729271	stage_1D_vs_E10	ordinary_muscle_development	ENSGALG00010008033	ACOT13	1.386733722	0.118495088	11.70287937	1.23201E-31	1.15287E-30	positive
PRJNA729271	stage_1D_vs_E10	ordinary_muscle_development	ENSGALG00010008034	DPEP1	2.06356846	0.307994782	6.700011098	2.08404E-11	6.60431E-11	positive
PRJNA729271	stage_1D_vs_E10	ordinary_muscle_development	ENSGALG00010008035	TDP2	-0.513593028	0.103947801	-4.940874368	7.7773E-07	1.75186E-06	negative
PRJNA729271	stage_1D_vs_E10	ordinary_muscle_development	ENSGALG00010008036	SPOCK3	-2.846558102	0.168780725	-16.86542173	8.08232E-64	2.47681E-62	negative
PRJNA729271	stage_1D_vs_E10	ordinary_muscle_development	ENSGALG00010008037	ENSGALG00010008037	1.58456745	0.914938742	1.731883653	0.08329428	0.107310513	positive
PRJNA729271	stage_1D_vs_E10	ordinary_muscle_development	ENSGALG00010008038	WRN	0.192904073	0.073989768	2.607172279	0.009129341	0.013566722	positive
PRJNA729271	stage_1D_vs_E10	ordinary_muscle_development	ENSGALG00010008039	CHMP1A	-0.223165857	0.089733068	-2.486996845	0.012882654	0.018761587	negative
PRJNA729271	stage_1D_vs_E10	ordinary_muscle_development	ENSGALG00010008040	ENSGALG00010008040	1.112291668	0.258460212	4.303531521	1.68097E-05	3.35476E-05	positive
PRJNA729271	stage_1D_vs_E10	ordinary_muscle_development	ENSGALG00010008041	ENSGALG00010008041	-0.410469588	0.191041824	-2.148584953	0.031667316	0.04352942	negative
PRJNA729271	stage_1D_vs_E10	ordinary_muscle_development	ENSGALG00010008042	ENSGALG00010008042	2.07868763	0.777061651	2.675061404	0.007471556	0.011233296	positive
PRJNA729271	stage_1D_vs_E10	ordinary_muscle_development	ENSGALG00010008043	IL17REL	-2.445503263	0.361520877	-6.764486978	1.33782E-11	4.30163E-11	negative
PRJNA729271	stage_1D_vs_E10	ordinary_muscle_development	ENSGALG00010008045	DCDC2	-1.552468685	0.839100229	-1.850158815	0.064290663	0.08431265	negative
PRJNA729271	stage_1D_vs_E10	ordinary_muscle_development	ENSGALG00010008046	RHOB	0.299953093	0.167860623	1.786917547	0.073950816	0.096075744	positive
PRJNA729271	stage_1D_vs_E10	ordinary_muscle_development	ENSGALG00010008047	ASAP1	-1.343341758	0.12905425	-10.40912449	2.25284E-25	1.57373E-24	negative
PRJNA729271	stage_1D_vs_E10	ordinary_muscle_development	ENSGALG00010008048	ENSGALG00010008048	1.958956734	0.756902133	2.588124209	0.009650018	0.014296505	positive
PRJNA729271	stage_1D_vs_E10	ordinary_muscle_development	ENSGALG00010008050	CDK10	-0.435932506	0.154116014	-2.828599665	0.004675214	0.007213116	negative
PRJNA729271	stage_1D_vs_E10	ordinary_muscle_development	ENSGALG00010008051	ENSGALG00010008051	0.072849997	0.076642515	0.950516779	0.341849732	0.390750877	positive
PRJNA729271	stage_1D_vs_E10	ordinary_muscle_development	ENSGALG00010008052	RBFA	0.391657298	0.121832911	3.214708514	0.00130577	0.002150812	positive
PRJNA729271	stage_1D_vs_E10	ordinary_muscle_development	ENSGALG00010008054	CRISP3	-5.916906185	0.969254158	-6.104597165	1.0306E-09	2.90974E-09	negative
PRJNA729271	stage_1D_vs_E10	ordinary_muscle_development	ENSGALG00010008056	GTF2E2	-0.797425505	0.071428957	-11.16389673	6.12469E-29	5.04371E-28	negative
PRJNA729271	stage_1D_vs_E10	ordinary_muscle_development	ENSGALG00010008057	BAG1	-0.477456883	0.132524132	-3.602792005	0.000314817	0.000553729	negative
PRJNA729271	stage_1D_vs_E10	ordinary_muscle_development	ENSGALG00010008058	USP25	1.789438027	0.138315998	12.93731788	2.77137E-38	3.40721E-37	positive
PRJNA729271	stage_1D_vs_E10	ordinary_muscle_development	ENSGALG00010008059	ENSGALG00010008059	0.210541856	0.093198685	2.259064648	0.023879364	0.033436184	positive
PRJNA729271	stage_1D_vs_E10	ordinary_muscle_development	ENSGALG00010008062	VPS9D1	-0.359767554	0.142593442	-2.523030158	0.011634842	0.017050555	negative
PRJNA729271	stage_1D_vs_E10	ordinary_muscle_development	ENSGALG00010008063	KCNV1	0.03642719	0.270241215	0.134795093	0.892773886	0.908747129	positive
PRJNA729271	stage_1D_vs_E10	ordinary_muscle_development	ENSGALG00010008064	DDX60	-1.596339549	0.664640161	-2.401810248	0.016314168	0.023380999	negative
PRJNA729271	stage_1D_vs_E10	ordinary_muscle_development	ENSGALG00010008065	CHMP5	-0.192154806	0.086395637	-2.224126279	0.026139949	0.036345186	negative
PRJNA729271	stage_1D_vs_E10	ordinary_muscle_development	ENSGALG00010008066	PCNX2	-3.901378114	0.186016647	-20.97327405	1.15067E-97	8.17593E-96	negative
PRJNA729271	stage_1D_vs_E10	ordinary_muscle_development	ENSGALG00010008067	MPC2	0.865275544	0.145671098	5.939926007	2.85151E-09	7.78538E-09	positive
PRJNA729271	stage_1D_vs_E10	ordinary_muscle_development	ENSGALG00010008069	DLG2	-2.676593775	0.223290989	-11.987021	4.15584E-33	4.1544E-32	negative
PRJNA729271	stage_1D_vs_E10	ordinary_muscle_development	ENSGALG00010008071	SMIM18	-2.392979921	0.385635045	-6.20529682	5.45938E-10	1.57198E-09	negative
PRJNA729271	stage_1D_vs_E10	ordinary_muscle_development	ENSGALG00010008072	NRSN1	0.114654263	0.200523864	0.57177366	0.567475324	0.612749912	positive
PRJNA729271	stage_1D_vs_E10	ordinary_muscle_development	ENSGALG00010008073	ZNF276	0.090882981	0.082409259	1.10282488	0.270103221	0.315559458	positive
PRJNA729271	stage_1D_vs_E10	ordinary_muscle_development	ENSGALG00010008074	RHAG	-0.972148276	0.432206197	-2.249269638	0.024495346	0.034252368	negative
PRJNA729271	stage_1D_vs_E10	ordinary_muscle_development	ENSGALG00010008075	SDHA	1.551942617	0.136395708	11.37823641	5.36742E-30	4.63099E-29	positive
PRJNA729271	stage_1D_vs_E10	ordinary_muscle_development	ENSGALG00010008076	ENSGALG00010008076	1.960759187	1.764915016	1.110965213	0.266583323	0.311723562	positive
PRJNA729271	stage_1D_vs_E10	ordinary_muscle_development	ENSGALG00010008077	ENSGALG00010008077	1.188504704	1.496703776	0.79408145	0.427148016	0.476526629	positive
PRJNA729271	stage_1D_vs_E10	ordinary_muscle_development	ENSGALG00010008078	GUCY1B1	0.156753214	0.141411658	1.108488623	0.26765085	0.312921364	positive
PRJNA729271	stage_1D_vs_E10	ordinary_muscle_development	ENSGALG00010008079	CCDC127	0.079092411	0.10858073	0.728420324	0.466356326	0.515146738	positive
PRJNA729271	stage_1D_vs_E10	ordinary_muscle_development	ENSGALG00010008081	TOP1MT	-0.903859054	0.141816265	-6.373451285	1.84821E-10	5.4976E-10	negative
PRJNA729271	stage_1D_vs_E10	ordinary_muscle_development	ENSGALG00010008083	FANCA	-1.894879236	0.249635289	-7.590590444	3.18451E-14	1.21632E-13	negative
PRJNA729271	stage_1D_vs_E10	ordinary_muscle_development	ENSGALG00010008084	CYP2AC1	3.427888886	1.334170612	2.569303247			positive
PRJNA729271	stage_1D_vs_E10	ordinary_muscle_development	ENSGALG00010008086	RHPN1	-0.46415773	0.210867914	-2.201177604	0.027723453	0.038381228	negative
PRJNA729271	stage_1D_vs_E10	ordinary_muscle_development	ENSGALG00010008087	CPNE8	-0.245334345	0.182263004	-1.346045769	0.178287757	0.216584062	negative
PRJNA729271	stage_1D_vs_E10	ordinary_muscle_development	ENSGALG00010008088	SLC35A4	0.544896962	0.069697153	7.818066253	5.3641E-15	2.14964E-14	positive
PRJNA729271	stage_1D_vs_E10	ordinary_muscle_development	ENSGALG00010008091	ENSGALG00010008091	1.125580983	0.271996052	4.138225444	3.50002E-05	6.77975E-05	positive
PRJNA729271	stage_1D_vs_E10	ordinary_muscle_development	ENSGALG00010008092	SPIRE2	-1.850746152	0.753781408	-2.455282303	0.014077399	0.02037056	negative
PRJNA729271	stage_1D_vs_E10	ordinary_muscle_development	ENSGALG00010008093	EFR3A	0.050134527	0.092318159	0.543062464	0.587086796	0.631433042	positive
PRJNA729271	stage_1D_vs_E10	ordinary_muscle_development	ENSGALG00010008094	PIM3	0.821213637	0.141467617	5.80495845	6.4382E-09	1.71013E-08	positive
PRJNA729271	stage_1D_vs_E10	ordinary_muscle_development	ENSGALG00010008096	CPSF1	-0.594293331	0.147761624	-4.021973463	5.77126E-05	0.000109696	negative
PRJNA729271	stage_1D_vs_E10	ordinary_muscle_development	ENSGALG00010008098	ENSGALG00010008098	-0.093296247	0.167465879	-0.557106007	0.577455004	0.622042976	negative
PRJNA729271	stage_1D_vs_E10	ordinary_muscle_development	ENSGALG00010008099	PANK3	-0.173923287	0.076673591	-2.268359747	0.023307289	0.032695166	negative
PRJNA729271	stage_1D_vs_E10	ordinary_muscle_development	ENSGALG00010008100	ENSGALG00010008100	1.909378701	0.233314333	8.183717966	2.75218E-16	1.1987E-15	positive
PRJNA729271	stage_1D_vs_E10	ordinary_muscle_development	ENSGALG00010008101	CENPQ	-2.13798782	0.205599762	-10.39878548	2.51113E-25	1.74911E-24	negative
PRJNA729271	stage_1D_vs_E10	ordinary_muscle_development	ENSGALG00010008103	RBPMS	0.04500274	0.108007355	0.416663664	0.676924436	0.71505755	positive
PRJNA729271	stage_1D_vs_E10	ordinary_muscle_development	ENSGALG00010008104	TCF25	-0.100707088	0.094836482	-1.061902398	0.288279997	0.334717923	negative
PRJNA729271	stage_1D_vs_E10	ordinary_muscle_development	ENSGALG00010008105	MMUT	0.790034849	0.094970434	8.31874531	8.88993E-17	3.9796E-16	positive
PRJNA729271	stage_1D_vs_E10	ordinary_muscle_development	ENSGALG00010008106	ENSGALG00010008106	0.3811025	0.371264788	1.026497831	0.304656985	0.351704604	positive
PRJNA729271	stage_1D_vs_E10	ordinary_muscle_development	ENSGALG00010008107	TUBB3	-1.737806905	0.208584648	-8.33142286	7.98765E-17	3.58788E-16	negative
PRJNA729271	stage_1D_vs_E10	ordinary_muscle_development	ENSGALG00010008108	ENSGALG00010008108	2.173960684	0.307206156	7.076553133	1.47784E-12	5.051E-12	positive
PRJNA729271	stage_1D_vs_E10	ordinary_muscle_development	ENSGALG00010008110	DEF8	0.003583712	0.153703648	0.023315727	0.981398427	0.984046085	positive
PRJNA729271	stage_1D_vs_E10	ordinary_muscle_development	ENSGALG00010008111	SYTL2	-0.586533726	0.258975645	-2.264821955	0.023523608	0.032976276	negative
PRJNA729271	stage_1D_vs_E10	ordinary_muscle_development	ENSGALG00010008113	DBNDD1	-1.890099126	0.186066375	-10.15819826	3.04654E-24	2.00726E-23	negative
PRJNA729271	stage_1D_vs_E10	ordinary_muscle_development	ENSGALG00010008115	ENSGALG00010008115	-0.332805681	0.201465364	-1.651925049	0.098549831	0.125448301	negative
PRJNA729271	stage_1D_vs_E10	ordinary_muscle_development	ENSGALG00010008116	URAH	1.950164237	0.372433783	5.236271056	1.63853E-07	3.89289E-07	positive
PRJNA729271	stage_1D_vs_E10	ordinary_muscle_development	ENSGALG00010008117	ENSGALG00010008117	2.804265258	0.172926827	16.21648475	3.85644E-59	1.02567E-57	positive
PRJNA729271	stage_1D_vs_E10	ordinary_muscle_development	ENSGALG00010008118	ENSGALG00010008118	0.269510739	0.605294624	0.445255464	0.656135148	0.695530128	positive
PRJNA729271	stage_1D_vs_E10	ordinary_muscle_development	ENSGALG00010008119	CCDC83	-2.364436496	0.59249726	-3.990628573	6.58984E-05	0.000124569	negative
PRJNA729271	stage_1D_vs_E10	ordinary_muscle_development	ENSGALG00010008120	CDC5L	-0.383092636	0.064592842	-5.93088374	3.01309E-09	8.20644E-09	negative
PRJNA729271	stage_1D_vs_E10	ordinary_muscle_development	ENSGALG00010008121	MPZL1	-0.529320926	0.135413253	-3.908930004	9.27058E-05	0.000172588	negative

PRJNA729271	stage_1D_vs_E10	ordinary_muscle_development	ENSGALG00010008123	ENSGALG00010008123	-0.405718068	0.161380352	-2.514048715	0.011935395	0.017471576	negative
PRJNA729271	stage_1D_vs_E10	ordinary_muscle_development	ENSGALG00010008124	ENSGALG00010008124	0.423164953	0.204482327	2.069445113	0.038504337	0.052229119	positive
PRJNA729271	stage_1D_vs_E10	ordinary_muscle_development	ENSGALG00010008126	CDH3	-3.197206278	0.306004692	-10.44822632	1.49291E-25	1.05098E-24	negative
PRJNA729271	stage_1D_vs_E10	ordinary_muscle_development	ENSGALG00010008127	TBC1D2	-0.75540552	0.135600714	-5.570807836	2.53561E-08	6.40531E-08	negative
PRJNA729271	stage_1D_vs_E10	ordinary_muscle_development	ENSGALG00010008128	RARS	-0.482779929	0.070030988	-6.893804338	5.43197E-12	1.78662E-11	negative
PRJNA729271	stage_1D_vs_E10	ordinary_muscle_development	ENSGALG00010008129	TANGO6	0.564069843	0.116687958	4.834002168	1.33815E-06	2.95633E-06	positive
PRJNA729271	stage_1D_vs_E10	ordinary_muscle_development	ENSGALG00010008130	RCSD1	2.401657291	0.119141606	20.15800666	2.2892E-90	1.37116E-88	positive
PRJNA729271	stage_1D_vs_E10	ordinary_muscle_development	ENSGALG00010008131	OC90	-1.461398192	0.607835485	-2.404266003	0.016204977	0.023233545	negative
PRJNA729271	stage_1D_vs_E10	ordinary_muscle_development	ENSGALG00010008132	TDO2	0.6389508	0.650225951	0.982659641	0.32577498	0.374087645	positive
PRJNA729271	stage_1D_vs_E10	ordinary_muscle_development	ENSGALG00010008133	CREG1	2.634872801	0.167209584	15.7579054	6.06087E-56	1.44257E-54	positive
PRJNA729271	stage_1D_vs_E10	ordinary_muscle_development	ENSGALG00010008134	SLC2A13	-0.402906801	0.076025063	-5.299657587	1.1602E-07	2.79151E-07	negative
PRJNA729271	stage_1D_vs_E10	ordinary_muscle_development	ENSGALG00010008135	WWC1	-0.061221769	0.130359712	-0.469637189	0.63861425	0.679292108	negative
PRJNA729271	stage_1D_vs_E10	ordinary_muscle_development	ENSGALG00010008136	ELP1	0.049992614	0.113182119	0.441700637	0.658705849	0.697897908	positive
PRJNA729271	stage_1D_vs_E10	ordinary_muscle_development	ENSGALG00010008137	HHLA1	0.99671287	0.498386387	1.999879806	0.045513244	0.061062058	positive
PRJNA729271	stage_1D_vs_E10	ordinary_muscle_development	ENSGALG00010008139	ENSGALG00010008139	1.264398609	0.3931128	3.216376083	0.001298206	0.002139567	positive
PRJNA729271	stage_1D_vs_E10	ordinary_muscle_development	ENSGALG00010008141	APTX	-0.256675836	0.154879196	-1.657264776	0.097465955	0.124166727	negative
PRJNA729271	stage_1D_vs_E10	ordinary_muscle_development	ENSGALG00010008142	CD247	1.664853538	0.330669509	5.034796046	4.78358E-07	1.09556E-06	positive
PRJNA729271	stage_1D_vs_E10	ordinary_muscle_development	ENSGALG00010008143	DCTN6	0.40131103	0.115189304	3.483926149	0.000494116	0.000851326	positive
PRJNA729271	stage_1D_vs_E10	ordinary_muscle_development	ENSGALG00010008144	NTPCR	-0.335820627	0.133669529	-2.512319975	0.011994028	0.017555634	negative
PRJNA729271	stage_1D_vs_E10	ordinary_muscle_development	ENSGALG00010008145	ENSGALG00010008145	1.522837079	0.206957683	7.358205085	1.864E-13	6.76988E-13	positive
PRJNA729271	stage_1D_vs_E10	ordinary_muscle_development	ENSGALG00010008146	SOX4	-1.581229721	0.118855256	-13.30382661	2.19919E-40	2.91382E-39	negative
PRJNA729271	stage_1D_vs_E10	ordinary_muscle_development	ENSGALG00010008147	CDKAL1	0.298204588	0.112360612	2.653995768	0.007954482	0.011917336	positive
PRJNA729271	stage_1D_vs_E10	ordinary_muscle_development	ENSGALG00010008148	ABCD2	1.316444986	0.138324902	9.517049856	1.78165E-21	1.033E-20	positive
PRJNA729271	stage_1D_vs_E10	ordinary_muscle_development	ENSGALG00010008149	DNAJA1	1.018812741	0.281501907	3.619203691	0.000295511	0.000521099	positive
PRJNA729271	stage_1D_vs_E10	ordinary_muscle_development	ENSGALG00010008150	TERT	-3.810346772	0.731408762	-5.209599572	1.89249E-07	4.47278E-07	negative
PRJNA729271	stage_1D_vs_E10	ordinary_muscle_development	ENSGALG00010008151	SIPA1L2	0.114072953	0.120749316	0.9447089	0.344807512	0.393716726	positive
PRJNA729271	stage_1D_vs_E10	ordinary_muscle_development	ENSGALG00010008152	ENSGALG00010008152	-0.997389204	0.166083628	-6.005343302	1.90927E-09	5.28047E-09	negative
PRJNA729271	stage_1D_vs_E10	ordinary_muscle_development	ENSGALG00010008153	SMU1	-0.473514432	0.061706459	-7.673660725	1.67156E-14	6.49752E-14	negative
PRJNA729271	stage_1D_vs_E10	ordinary_muscle_development	ENSGALG00010008154	KCNQ3	0.004462443	0.224224601	0.019901664	0.984121818	0.986299229	positive
PRJNA729271	stage_1D_vs_E10	ordinary_muscle_development	ENSGALG00010008155	MAP10	-0.270777074	0.227405308	-1.190724509	0.233761753	0.277167821	negative
PRJNA729271	stage_1D_vs_E10	ordinary_muscle_development	ENSGALG00010008156	HAS3	-4.306012246	1.154238576	-3.730608504	0.000191018	0.000343951	negative
PRJNA729271	stage_1D_vs_E10	ordinary_muscle_development	ENSGALG00010008157	CTSO	-0.826209265	0.155964849	-5.297406869	1.17459E-07	2.82537E-07	negative
PRJNA729271	stage_1D_vs_E10	ordinary_muscle_development	ENSGALG00010008158	CLPTM1L	-0.409014341	0.075011194	-5.452710731	4.96077E-08	1.22623E-07	negative
PRJNA729271	stage_1D_vs_E10	ordinary_muscle_development	ENSGALG00010008159	SUPT3H	-2.395970724	0.140938142	-17.00015833	8.18984E-65	2.57509E-63	negative
PRJNA729271	stage_1D_vs_E10	ordinary_muscle_development	ENSGALG00010008160	TXNL4A	-0.463129088	0.092452355	-5.009381202	5.46053E-07	1.2447E-06	negative
PRJNA729271	stage_1D_vs_E10	ordinary_muscle_development	ENSGALG00010008161	TMEFF1	-1.781643284	0.354376613	-5.027541945	4.96807E-07	1.13666E-06	negative
PRJNA729271	stage_1D_vs_E10	ordinary_muscle_development	ENSGALG00010008162	HSBP1L1	0.33117032	0.207746586	1.594107156	0.110912036	0.139931236	positive
PRJNA729271	stage_1D_vs_E10	ordinary_muscle_development	ENSGALG00010008163	CHTF8	-1.662912493	0.219176557	-7.587091048	3.27167E-14	1.24929E-13	negative
PRJNA729271	stage_1D_vs_E10	ordinary_muscle_development	ENSGALG00010008164	TG	0.41847973	0.291317325	1.436508216	0.15085778	0.185926666	positive
PRJNA729271	stage_1D_vs_E10	ordinary_muscle_development	ENSGALG00010008165	MRPL1	0.22435538	0.084694945	2.648981936	0.008073465	0.012086849	positive
PRJNA729271	stage_1D_vs_E10	ordinary_muscle_development	ENSGALG00010008166	RUNX2	-4.394129974	0.420916842	-10.4394254	1.63799E-25	1.15144E-24	negative
PRJNA729271	stage_1D_vs_E10	ordinary_muscle_development	ENSGALG00010008167	UTP4	0.229300594	0.17384468	1.318996896	0.18717015	0.226463383	positive
PRJNA729271	stage_1D_vs_E10	ordinary_muscle_development	ENSGALG00010008168	CAVIN4	5.280778829	0.124849608	42.29711991	0	0	positive
PRJNA729271	stage_1D_vs_E10	ordinary_muscle_development	ENSGALG00010008169	HTR4	-1.252855442	0.355088435	-3.528291314	0.000418252	0.000726142	negative
PRJNA729271	stage_1D_vs_E10	ordinary_muscle_development	ENSGALG00010008170	PICALM	-0.394164383	0.07073517	-5.572396078	2.51259E-08	6.3527E-08	negative
PRJNA729271	stage_1D_vs_E10	ordinary_muscle_development	ENSGALG00010008171	KIF21A	-1.346959759	0.181226995	-7.432445468	1.06608E-13	3.94004E-13	negative
PRJNA729271	stage_1D_vs_E10	ordinary_muscle_development	ENSGALG00010008172	SNTB2	-0.312906082	0.13088551	-2.390685433	0.016816954	0.024063352	negative
PRJNA729271	stage_1D_vs_E10	ordinary_muscle_development	ENSGALG00010008174	SLC26A2	-0.591872089	0.122904337	-4.815713633	1.46675E-06	3.23157E-06	negative
PRJNA729271	stage_1D_vs_E10	ordinary_muscle_development	ENSGALG00010008175	ENSGALG00010008175	0.683549366	0.440166425	1.552933906	0.120438926	0.151135159	positive
PRJNA729271	stage_1D_vs_E10	ordinary_muscle_development	ENSGALG00010008176	SLA	0.466561259	0.31021215	1.504007046	0.132579552	0.164910328	positive
PRJNA729271	stage_1D_vs_E10	ordinary_muscle_development	ENSGALG00010008177	NREP	-1.996703293	0.184672069	-10.8121564	3.01498E-27	2.27972E-26	negative
PRJNA729271	stage_1D_vs_E10	ordinary_muscle_development	ENSGALG00010008178	PGR	0.615698357	0.280804398	2.192623624	0.028334508	0.039182283	positive
PRJNA729271	stage_1D_vs_E10	ordinary_muscle_development	ENSGALG00010008179	SEMA6A	-0.320641735	0.095695805	-3.350635219	0.000806264	0.001359247	negative
PRJNA729271	stage_1D_vs_E10	ordinary_muscle_development	ENSGALG00010008180	POU2F1	1.064745391	0.15548528	6.84788548	7.49495E-12	2.44408E-11	positive
PRJNA729271	stage_1D_vs_E10	ordinary_muscle_development	ENSGALG00010008181	DISC1	0.145731489	0.129645375	1.124077809	0.260980019	0.306085069	positive
PRJNA729271	stage_1D_vs_E10	ordinary_muscle_development	ENSGALG00010008182	CLIC5	2.618908728	0.291933252	8.970916147	2.94058E-19	1.50881E-18	positive
PRJNA729271	stage_1D_vs_E10	ordinary_muscle_development	ENSGALG00010008183	ALG10	-0.62447433	0.084532459	-7.387391047	1.49738E-13	5.4685E-13	negative
PRJNA729271	stage_1D_vs_E10	ordinary_muscle_development	ENSGALG00010008184	SLC66A2	-0.282949615	0.085764281	-3.299154526	0.000969765	0.001619627	negative
PRJNA729271	stage_1D_vs_E10	ordinary_muscle_development	ENSGALG00010008185	PDGFC	-1.254548136	0.08881475	-14.12544809	2.64711E-45	4.20722E-44	negative
PRJNA729271	stage_1D_vs_E10	ordinary_muscle_development	ENSGALG00010008186	NOP53	0.376780314	0.121340627	3.105145604	0.001901854	0.003069965	positive
PRJNA729271	stage_1D_vs_E10	ordinary_muscle_development	ENSGALG00010008187	ENSGALG00010008187	2.082850361	0.377605838	5.515937917	3.46925E-08	8.66415E-08	positive
PRJNA729271	stage_1D_vs_E10	ordinary_muscle_development	ENSGALG00010008188	VPS4A	0.32297594	0.113034251	2.857328086	0.00427224	0.006623114	positive
PRJNA729271	stage_1D_vs_E10	ordinary_muscle_development	ENSGALG00010008189	EXOC3	0.011551853	0.114345447	0.10102591	0.919529892	0.931547791	positive
PRJNA729271	stage_1D_vs_E10	ordinary_muscle_development	ENSGALG00010008190	TSNARE1	-0.428776488	0.169448339	-2.530426027	0.01139241	0.01671998	negative
PRJNA729271	stage_1D_vs_E10	ordinary_muscle_development	ENSGALG00010008192	HNRNPU	-0.317703176	0.082784769	-3.837700809	0.000124192	0.000228215	negative
PRJNA729271	stage_1D_vs_E10	ordinary_muscle_development	ENSGALG00010008193	CNOT6L	1.328249114	0.129685391	10.24208743	1.28435E-24	8.63481E-24	positive
PRJNA729271	stage_1D_vs_E10	ordinary_muscle_development	ENSGALG00010008194	PDF	0.895132287	0.162737713	5.500460031	3.78802E-08	9.44073E-08	positive
PRJNA729271	stage_1D_vs_E10	ordinary_muscle_development	ENSGALG00010008195	ENSGALG00010008195	4.204512119	0.40708629	10.32830685	5.24787E-25	3.6017E-24	positive
PRJNA729271	stage_1D_vs_E10	ordinary_muscle_development	ENSGALG00010008196	PDCD6	-0.212132865	0.147160616	-1.44150569	0.149441871	0.184354036	negative
PRJNA729271	stage_1D_vs_E10	ordinary_muscle_development	ENSGALG00010008197	STARD4	-0.801514033	0.158600963	-5.053651757	4.33442E-07	9.94261E-07	negative
PRJNA729271	stage_1D_vs_E10	ordinary_muscle_development	ENSGALG00010008198	GLRB	-3.790921884	0.406121378	-9.334455375	1.01512E-20	5.65276E-20	negative
PRJNA729271	stage_1D_vs_E10	ordinary_muscle_development	ENSGALG00010008199	TPRKB	-0.363191606	0.114185871	-3.18070532	0.00146917	0.002402484	negative
PRJNA729271	stage_1D_vs_E10	ordinary_muscle_development	ENSGALG00010008200	COG8	-0.335152785	0.128207592	-2.614141483	0.008945199	0.013306717	negative
PRJNA729271	stage_1D_vs_E10	ordinary_muscle_development	ENSGALG00010008201	ENPP4	0.92575691	0.241923747	3.826647526	0.0001299	0.000238312	positive
PRJNA729271	stage_1D_vs_E10	ordinary_muscle_development	ENSGALG00010008202	KCNG2	1.818564803	0.191960781	9.473626845	2.70292E-21	1.55348E-20	positive
PRJNA729271	stage_1D_vs_E10	ordinary_muscle_development	ENSGALG00010008203	CXCL13	0.116715145	1.48034204	0.078843363	0.937157213	0.946496566	positive
PRJNA729271	stage_1D_vs_E10	ordinary_muscle_development	ENSGALG00010008204	ENSGALG00010008204	0.854083538	0.151765428	5.62765546	1.82676E-08	4.67141E-08	positive
PRJNA729271	stage_1D_vs_E10	ordinary_muscle_development	ENSGALG00010008205	ENSGALG00010008205	-0.366821984	0.173401256	-2.11545171	0.034391467	0.047046462	negative
PRJNA729271	stage_1D_vs_E10	ordinary_muscle_development	ENSGALG00010008206	CXCL13L2	-3.774295939	1.034863663	-3.647143168	0.000265172	0.000469772	negative
PRJNA729271	stage_1D_vs_E10	ordinary_muscle_development	ENSGALG00010008207	CAMK4	-0.687275012	0.157466704	-4.364573542	1.27371E-05	2.57747E-05	negative
PRJNA729271	stage_1D_vs_E10	ordinary_muscle_development	ENSGALG00010008208	ILDR2	2.115534337	0.150847283	14.02434497	1.10629E-44	1.71138E-43	positive
PRJNA729271	stage_1D_vs_E10	ordinary_muscle_development	ENSGALG00010008209	PPARGC1B	3.422769402	0.231714523	14.7714928	2.23703E-49	4.21661E-48	positive
PRJNA729271	stage_1D_vs_E10	ordinary_muscle_development	ENSGALG00010008210	CTDP1	0.244540605	0.08483114	2.882674979	0.003943141	0.006134574	positive
PRJNA729271	stage_1D_vs_E10	ordinary_muscle_development	ENSGALG00010008211	ADGRB1	-0.567164648	0.236325627	-2.399928675	0.016398267	0.023496726	negative
PRJNA729271	stage_1D_vs_E10	ordinary_muscle_development	ENSGALG00010008212	GTSE1	-2.094877908	0.225952666	-9.271313075	1.83868E-20	1.00839E-19	negative
PRJNA729271	stage_1D_vs_E10	ordinary_muscle_development	ENSGALG00010008213	ISCA1	0.488277988	0.117092313	4.170025982	3.04565E-05	5.9329E-05	positive
PRJNA729271	stage_1D_vs_E10	ordinary_muscle_development	ENSGALG00010008214	CCNG2	-0.663372603	0.135994109	-4.877951006	1.07194E-06	2.39042E-06	negative
PRJNA729271	stage_1D_vs_E10	ordinary_muscle_development	ENSGALG00010008215	SELENOW	1.888621461	0.127868318	14.77005009	2.28543E-49	4.30225E-48	positive
PRJNA729271	stage_1D_vs_E10	ordinary_muscle_development	ENSGALG00010008217	ARHGEF37	3.426318038	0.162331906	21.10686753	6.87832E-99	4.98506E-97	positive
PRJNA729271	stage_1D_vs_E10	ordinary_muscle_development	ENSGALG00010008218	ARHGAP42	-0.142170415	0.101518065	-1.400444487	0.161380256	0.197768584	negative
PRJNA729271	stage_1D_vs_E10	ordinary_muscle_development	ENSGALG00010008219	NIP7	-0.163045829	0.118660693	-1.374050869	0.169425891	0.206632586	negative
PRJNA729271	stage_1D_vs_E10	ordinary_muscle_development	ENSGALG00010008221	ENSGALG00010008221	0.552777996	0.520477524	1.062059302	0.288208764	0.334662024	positive
PRJNA729271	stage_1D_vs_E10	ordinary_muscle_development	ENSGALG00010008222	STYXL2	2.098995553	0.274949168	7.634122218	2.27364E-14	8.76501E-14	positive
PRJNA729271	stage_1D_vs_E10	ordinary_muscle_development	ENSGALG00010008223	ENSGALG00010008223	-1.613108599	0.153366125	-10.51802411	7.13542E-26	5.07248E-25	negative
PRJNA729271	stage_1D_vs_E10	ordinary_muscle_development	ENSGALG00010008224	ENSGALG00010008224	0.285261814	0.211673003	1.347653267	0.177769929	0.216027425	positive
PRJNA729271	stage_1D_vs_E10	ordinary_muscle_development	ENSGALG00010008226	COX20	-0.569195204	0.165002446	-3.449616773	0.000561383	0.000961081	negative
PRJNA729271	stage_1D_vs_E10	ordinary_muscle_development	ENSGALG00010008227	DUSP1	3.913843577	0.176500523	22.17468549	6.0295E-109	5.1715E-107	positive
PRJNA729271	stage_1D_vs_E10	ordinary_muscle_development	ENSGALG00010008228	TMED6	2.399210983	0.679556528	3.530553949	0.00041469	0.000720346	positive
PRJNA729271	stage_1D_vs_E10	ordinary_muscle_development	ENSGALG00010008229	PIGY	0.528027255	0.122573902	4.307827738	1.64866E-05	3.29209E-05	positive
PRJNA729271	stage_1D_vs_E10	ordinary_muscle_development	ENSGALG00010008230	TRMU	0.489321055	0.112764369	4.3393233	1.42922E-05	2.87331E-05	positive
PRJNA729271	stage_1D_vs_E10	ordinary_muscle_development	ENSGALG00010008231	ZCCHC6	0.360791085	0.109710748	3.288566459	0.00100699	0.00167851	positive
PRJNA729271	stage_1D_vs_E10	ordinary_muscle_development	ENSGALG00010008232	PYURF	0.291129443	0.127791954	2.278151592	0.022717547	0.031938977	positive
PRJNA729271	stage_1D_vs_E10	ordinary_muscle_development	ENSGALG00010008233	CYRIB	-0.490116256	0.070758435	-6.926612437	4.31037E-12	1.42744E-11	negative
PRJNA729271	stage_1D_vs_E10	ordinary_muscle_development	ENSGALG00010008234	COMMD10	-1.478900166	0.120679454	-12.25478004	1.58378E-34	1.6905E-33	negative
PRJNA729271	stage_1D_vs_E10	ordinary_muscle_development	ENSGALG00010008236	ENSGALG00010008236	2.7039141	0.620915453	4.354721869	1.33236E-05	2.69127E-05	positive
PRJNA729271	stage_1D_vs_E10	ordinary_muscle_development	ENSGALG00010008237	GRIA2	-3.251012999	0.403026662	-8.066496098	7.23444E-16	3.06707E-15	negative
PRJNA729271	stage_1D_vs_E10	ordinary_muscle_development	ENSGALG00010008238	NTAQ1	-0.325105325	0.161681671	-2.010774156	0.044349317	0.05960905	negative
PRJNA729271	stage_1D_vs_E10	ordinary_muscle_development	ENSGALG00010008240	RCAN2	-0.28684532	0.137666694	-2.083621762	0.037194586	0.050608798	negative
PRJNA729271	stage_1D_vs_E10	ordinary_muscle_development	ENSGALG00010008241	HERC3	0.008678088	0.083519963	0.103904361	0.917245247	0.929688123	positive
PRJNA729271	stage_1D_vs_E10	ordinary_muscle_development	ENSGALG00010008242	CNTN5	-2.105884694	1.162981624	-1.810763515			negative
PRJNA729271	stage_1D_vs_E10	ordinary_muscle_development	ENSGALG00010008243	YTHDC2	-0.060134196	0.103402289	-0.581555756	0.560865952	0.606426374	negative
PRJNA729271	stage_1D_vs_E10	ordinary_muscle_development	ENSGALG00010008245	FBXO32	4.095012727	0.491846158	8.325799969	8.37611E-17	3.75772E-16	positive
PRJNA729271	stage_1D_vs_E10	ordinary_muscle_development	ENSGALG00010008246	TERF2	-0.443980443	0.057433764	-7.730303795	1.0729E-14	4.2157E-14	negative
PRJNA729271	stage_1D_vs_E10	ordinary_muscle_development	ENSGALG00010008247	GASK1B	0.260798428	0.16115339	1.618324181	0.105592752	0.133709002	positive
PRJNA729271	stage_1D_vs_E10	ordinary_muscle_development	ENSGALG00010008248	TPK1	-0.391621227	0.162230837	-2.413975259	0.015779534	0.022662006	negative
PRJNA729271	stage_1D_vs_E10	ordinary_muscle_development	ENSGALG00010008250	KLHL38	6.458806062	0.592448103	10.9018934	1.12883E-27	8.68496E-27	positive
PRJNA729271	stage_1D_vs_E10	ordinary_muscle_development	ENSGALG00010008251	AHRR	-2.662660488	0.282339013	-9.430721095	4.07279E-21	2.31601E-20	negative
PRJNA729271	stage_1D_vs_E10	ordinary_muscle_development	ENSGALG00010008252	ZFP92	-1.983414984	0.126819136	-15.63971371	3.9048E-55	9.04154E-54	negative
PRJNA729271	stage_1D_vs_E10	ordinary_muscle_development	ENSGALG00010008253	ENSGALG00010008253	-0.524809427	0.719468758	-0.729440189	0.465732442	0.514542054	negative
PRJNA729271	stage_1D_vs_E10	ordinary_muscle_development	ENSGALG00010008255	MSANTD3	-2.330070279	0.132169432	-17.62941891	1.46465E-69	5.32081E-68	negative
PRJNA729271	stage_1D_vs_E10	ordinary_muscle_development	ENSGALG00010008257	DESI2	-0.550048518	0.05940511	-9.25927958	2.05816E-20	1.12281E-19	negative
PRJNA729271	stage_1D_vs_E10	ordinary_muscle_development	ENSGALG00010008258	CCDC82	0.755654664	0.13102473	5.767267499	8.05671E-09	2.1237E-08	positive
PRJNA729271	stage_1D_vs_E10	ordinary_muscle_development	ENSGALG00010008259	MCC	-0.32198423	0.108680931	-2.962656164	0.003049971	0.004805361	negative
PRJNA729271	stage_1D_vs_E10	ordinary_muscle_development	ENSGALG00010008261	CRX	-1.080963311	0.40613948	-2.66155684	0.007778021	0.011667434	negative
PRJNA729271	stage_1D_vs_E10	ordinary_muscle_development	ENSGALG00010008262	CYB5B	-0.857565294	0.071784142	-11.94644487	6.77624E-33	6.7229E-32	negative
PRJNA729271	stage_1D_vs_E10	ordinary_muscle_development	ENSGALG00010008263	C1orf101	-2.070889131	0.571644975	-3.622684048	0.000291562	0.000514573	negative
PRJNA729271	stage_1D_vs_E10	ordinary_muscle_development	ENSGALG00010008264	CNTNAP2	-2.750799934	0.33315115	-8.256912622	1.49489E-16	6.60622E-16	negative
PRJNA729271	stage_1D_vs_E10	ordinary_muscle_development	ENSGALG00010008265	ENSGALG00010008265	-5.829624051	1.047020867	-5.56782031	2.57946E-08	6.50926E-08	negative
PRJNA729271	stage_1D_vs_E10	ordinary_muscle_development	ENSGALG00010008266	ECSCR	0.540405862	0.1875123	2.881975548	0.003951904	0.006146888	positive
PRJNA729271	stage_1D_vs_E10	ordinary_muscle_development	ENSGALG00010008267	MAML2	-0.151191244	0.117389322	-1.287947161	0.197764357	0.237773255	negative
PRJNA729271	stage_1D_vs_E10	ordinary_muscle_development	ENSGALG00010008268	ENSGALG00010008268	-1.003628279	0.304173649	-3.299524082	0.000968489	0.001617869	negative

PRJNA729271	stage_1D_vs_E10	ordinary_muscle_development	ENSGALG00010008269	ENSGALG00010008269	1.096976762	0.296164352	3.703945978	0.000212271	0.000380002	positive
PRJNA729271	stage_1D_vs_E10	ordinary_muscle_development	ENSGALG00010008270	ENSGALG00010008270	1.130798993	0.15988953	7.072376726	1.52302E-12	5.20175E-12	positive
PRJNA729271	stage_1D_vs_E10	ordinary_muscle_development	ENSGALG00010008272	DCP2	-0.921600923	0.08420329	-10.94495151	7.02553E-28	5.46912E-27	negative
PRJNA729271	stage_1D_vs_E10	ordinary_muscle_development	ENSGALG00010008273	TMEM144	0.081953046	0.120107429	0.682331198	0.49502955	0.543183446	positive
PRJNA729271	stage_1D_vs_E10	ordinary_muscle_development	ENSGALG00010008274	NFAT5	0.068610636	0.108975109	0.629599151	0.528956879	0.575790775	positive
PRJNA729271	stage_1D_vs_E10	ordinary_muscle_development	ENSGALG00010008275	CELSR1	-0.67432565	0.1707316	-3.949624147	7.8274E-05	0.000146719	negative
PRJNA729271	stage_1D_vs_E10	ordinary_muscle_development	ENSGALG00010008276	DNAJC18	0.759292374	0.092958098	8.168114306	3.13247E-16	1.35984E-15	positive
PRJNA729271	stage_1D_vs_E10	ordinary_muscle_development	ENSGALG00010008277	MZB1	4.026822535	1.06962426	3.764707559	0.000166744	0.000302687	positive
PRJNA729271	stage_1D_vs_E10	ordinary_muscle_development	ENSGALG00010008278	CYP39A1	0.843014804	0.159429423	5.287699035	1.23865E-07	2.97254E-07	positive
PRJNA729271	stage_1D_vs_E10	ordinary_muscle_development	ENSGALG00010008279	MAPK15	-0.773029492	0.172140304	-4.490694348	7.09914E-06	1.47108E-05	negative
PRJNA729271	stage_1D_vs_E10	ordinary_muscle_development	ENSGALG00010008282	FAM83H	-3.043359718	0.295828498	-10.28758129	8.01648E-25	5.43747E-24	negative
PRJNA729271	stage_1D_vs_E10	ordinary_muscle_development	ENSGALG00010008283	NQO1	-1.275278473	0.200138141	-6.371991185	1.8659E-10	5.54793E-10	negative
PRJNA729271	stage_1D_vs_E10	ordinary_muscle_development	ENSGALG00010008284	BCKDHA	1.841198467	0.128169709	14.36531676	8.54153E-47	1.4363E-45	positive
PRJNA729271	stage_1D_vs_E10	ordinary_muscle_development	ENSGALG00010008285	ADSS2	-1.845219064	0.090297979	-20.43477709	8.20666E-93	5.28691E-91	negative
PRJNA729271	stage_1D_vs_E10	ordinary_muscle_development	ENSGALG00010008289	MTMR2	-0.135773116	0.068542442	-1.980861956	0.047606757	0.063682165	negative
PRJNA729271	stage_1D_vs_E10	ordinary_muscle_development	ENSGALG00010008290	FAM13A	0.974455347	0.163230973	5.969794396	2.37553E-09	6.52269E-09	positive
PRJNA729271	stage_1D_vs_E10	ordinary_muscle_development	ENSGALG00010008291	PTGFRN	0.247121224	0.160843718	1.536405817	0.124438881	0.155709736	positive
PRJNA729271	stage_1D_vs_E10	ordinary_muscle_development	ENSGALG00010008292	REEP5	0.229204051	0.113572113	2.018136718	0.043577025	0.058654376	positive
PRJNA729271	stage_1D_vs_E10	ordinary_muscle_development	ENSGALG00010008293	PLA2G7	0.366194576	0.114566171	3.196358697	0.00139174	0.002284887	positive
PRJNA729271	stage_1D_vs_E10	ordinary_muscle_development	ENSGALG00010008294	NOB1	0.3025281	0.154142763	1.96264874	0.049687003	0.06631186	positive
PRJNA729271	stage_1D_vs_E10	ordinary_muscle_development	ENSGALG00010008295	DMAC2	0.300445972	0.131429548	2.285984964	0.022255138	0.031313165	positive
PRJNA729271	stage_1D_vs_E10	ordinary_muscle_development	ENSGALG00010008296	PALLD	-0.394766105	0.153086002	-2.578721101	0.009916682	0.01466802	negative
PRJNA729271	stage_1D_vs_E10	ordinary_muscle_development	ENSGALG00010008297	ENSGALG00010008297	-0.446924429	0.295349619	-1.513204693	0.130227673	0.162350573	negative
PRJNA729271	stage_1D_vs_E10	ordinary_muscle_development	ENSGALG00010008298	ENSGALG00010008298	-1.510060293	0.132048963	-11.43560892	2.77582E-30	2.44445E-29	negative
PRJNA729271	stage_1D_vs_E10	ordinary_muscle_development	ENSGALG00010008299	ANKRD66	-1.112189335	0.353134099	-3.149481563	0.001635604	0.002662334	negative
PRJNA729271	stage_1D_vs_E10	ordinary_muscle_development	ENSGALG00010008300	CUL1	-0.076929666	0.085036145	-0.904670198	0.365640134	0.414674418	negative
PRJNA729271	stage_1D_vs_E10	ordinary_muscle_development	ENSGALG00010008301	FAM91A1	-0.641943291	0.105507833	-6.084318794	1.16988E-09	3.28504E-09	negative
PRJNA729271	stage_1D_vs_E10	ordinary_muscle_development	ENSGALG00010008302	ENSGALG00010008302	0.728221169	0.143963029	5.058390155	4.22811E-07	9.70489E-07	positive
PRJNA729271	stage_1D_vs_E10	ordinary_muscle_development	ENSGALG00010008303	TEX10	-1.028862425	0.071201683	-14.44997331	2.50783E-47	4.30189E-46	negative
PRJNA729271	stage_1D_vs_E10	ordinary_muscle_development	ENSGALG00010008306	ENSGALG00010008306	0.514974001	0.286441166	1.797835162	0.072203133	0.093956766	positive
PRJNA729271	stage_1D_vs_E10	ordinary_muscle_development	ENSGALG00010008307	ENSGALG00010008307	-0.642092687	0.986994675	-0.650553345	0.515334856	0.562992066	negative
PRJNA729271	stage_1D_vs_E10	ordinary_muscle_development	ENSGALG00010008308	WWP2	-1.987804549	0.125393075	-15.85258634	1.34912E-56	3.29218E-55	negative
PRJNA729271	stage_1D_vs_E10	ordinary_muscle_development	ENSGALG00010008309	ENSGALG00010008309	0.711118277	0.276324347	2.573491209	0.01006782	0.014871401	positive
PRJNA729271	stage_1D_vs_E10	ordinary_muscle_development	ENSGALG00010008310	EXOSC5	0.27456566	0.179835365	1.526761218	0.126820399	0.158416208	positive
PRJNA729271	stage_1D_vs_E10	ordinary_muscle_development	ENSGALG00010008311	SRP19	-0.926884966	0.115513649	-8.024029847	1.02331E-15	4.29691E-15	negative
PRJNA729271	stage_1D_vs_E10	ordinary_muscle_development	ENSGALG00010008312	ZBTB18	0.734727168	0.217491285	3.37819131	0.000729643	0.001235246	positive
PRJNA729271	stage_1D_vs_E10	ordinary_muscle_development	ENSGALG00010008313	PSMD7	0.12193258	0.178373049	0.683581858	0.494239244	0.542439452	positive
PRJNA729271	stage_1D_vs_E10	ordinary_muscle_development	ENSGALG00010008314	INVS	0.056034943	0.105452554	0.531375879	0.595158333	0.638503445	positive
PRJNA729271	stage_1D_vs_E10	ordinary_muscle_development	ENSGALG00010008316	ADGRF5	2.729786704	0.134000436	20.37147635	2.99511E-92	1.8794E-90	positive
PRJNA729271	stage_1D_vs_E10	ordinary_muscle_development	ENSGALG00010008317	CEP57	-0.023260685	0.109674484	-0.212088395	0.832038073	0.855954001	negative
PRJNA729271	stage_1D_vs_E10	ordinary_muscle_development	ENSGALG00010008318	ADPRH	1.944326132	0.133044323	14.61412324	2.28274E-48	4.13088E-47	positive
PRJNA729271	stage_1D_vs_E10	ordinary_muscle_development	ENSGALG00010008319	ERP44	-0.063482596	0.064667609	-0.981675324	0.326259826	0.374526946	negative
PRJNA729271	stage_1D_vs_E10	ordinary_muscle_development	ENSGALG00010008320	GPRIN3	0.000198075	0.115060512	0.001721487	0.998626453	0.998971045	positive
PRJNA729271	stage_1D_vs_E10	ordinary_muscle_development	ENSGALG00010008321	ZFHX3	0.543326712	0.100993778	5.379803822	7.45671E-08	1.82022E-07	positive
PRJNA729271	stage_1D_vs_E10	ordinary_muscle_development	ENSGALG00010008323	AKT3	0.785444881	0.10313727	7.6155291	2.62613E-14	1.00863E-13	positive
PRJNA729271	stage_1D_vs_E10	ordinary_muscle_development	ENSGALG00010008324	SCRIB	-0.007478511	0.103890285	-0.071984703	0.942614081	0.951276969	negative
PRJNA729271	stage_1D_vs_E10	ordinary_muscle_development	ENSGALG00010008325	AADAT	0.315578294	0.130793928	2.412790089	0.015830934	0.022728743	positive
PRJNA729271	stage_1D_vs_E10	ordinary_muscle_development	ENSGALG00010008327	TGFB1	0.364113859	0.127914191	2.846547806	0.004419609	0.006844256	positive
PRJNA729271	stage_1D_vs_E10	ordinary_muscle_development	ENSGALG00010008328	ENSGALG00010008328	0.099295691	0.441222153	0.22504693	0.821942765	0.846770461	positive
PRJNA729271	stage_1D_vs_E10	ordinary_muscle_development	ENSGALG00010008330	EZH2	-1.922357528	0.078896807	-24.36546663	3.975E-131	5.0989E-129	negative
PRJNA729271	stage_1D_vs_E10	ordinary_muscle_development	ENSGALG00010008331	CCDC97	0.285620419	0.26434059	1.080501558	0.279918893	0.326054674	positive
PRJNA729271	stage_1D_vs_E10	ordinary_muscle_development	ENSGALG00010008332	DHX38	-0.200455659	0.093314239	-2.148178671	0.031699564	0.043569618	negative
PRJNA729271	stage_1D_vs_E10	ordinary_muscle_development	ENSGALG00010008333	APC	-0.285226625	0.092012679	-3.099862198	0.001936107	0.003120982	negative
PRJNA729271	stage_1D_vs_E10	ordinary_muscle_development	ENSGALG00010008334	STX17	0.129517778	0.134173951	0.965297485	0.334395831	0.382985744	positive
PRJNA729271	stage_1D_vs_E10	ordinary_muscle_development	ENSGALG00010008335	HNRNPUL1	-0.213465696	0.18773663	-1.137048726	0.255517917	0.30033508	negative
PRJNA729271	stage_1D_vs_E10	ordinary_muscle_development	ENSGALG00010008336	PAIP2B	0.682368197	0.108986871	6.26101281	3.82485E-10	1.11216E-09	positive
PRJNA729271	stage_1D_vs_E10	ordinary_muscle_development	ENSGALG00010008339	FAM76B	-1.187118809	0.113052744	-10.50057496	8.58556E-26	6.07951E-25	negative
PRJNA729271	stage_1D_vs_E10	ordinary_muscle_development	ENSGALG00010008340	PDIA4	-0.657690226	0.105633217	-6.226168653	4.7798E-10	1.38124E-09	negative
PRJNA729271	stage_1D_vs_E10	ordinary_muscle_development	ENSGALG00010008341	DHODH	-1.632177749	0.109621511	-14.88921048	3.87316E-50	7.52567E-49	negative
PRJNA729271	stage_1D_vs_E10	ordinary_muscle_development	ENSGALG00010008342	PLD3	0.79983909	0.147717047	5.414670188	6.14018E-08	1.508E-07	positive
PRJNA729271	stage_1D_vs_E10	ordinary_muscle_development	ENSGALG00010008343	TNFRSF21	1.088679538	0.223740077	4.865822662	1.13982E-06	2.53361E-06	positive
PRJNA729271	stage_1D_vs_E10	ordinary_muscle_development	ENSGALG00010008344	MFAP3L	1.657844365	0.27905596	5.940902915	2.83456E-09	7.74058E-09	positive
PRJNA729271	stage_1D_vs_E10	ordinary_muscle_development	ENSGALG00010008346	NR4A3	4.083075264	0.522529749	7.814053218	5.53777E-15	2.21618E-14	positive
PRJNA729271	stage_1D_vs_E10	ordinary_muscle_development	ENSGALG00010008347	PUF60	-0.44047649	0.054468976	-8.086740739	6.12825E-16	2.61429E-15	negative
PRJNA729271	stage_1D_vs_E10	ordinary_muscle_development	ENSGALG00010008348	ENSGALG00010008348	-1.252550623	0.101720567	-12.31364178	7.64921E-35	8.24965E-34	negative
PRJNA729271	stage_1D_vs_E10	ordinary_muscle_development	ENSGALG00010008350	LRRC6	-0.302768739	0.175755934	-1.722665815	0.084948976	0.109354832	negative
PRJNA729271	stage_1D_vs_E10	ordinary_muscle_development	ENSGALG00010008351	IST1	-0.252726812	0.067982689	-3.717517144	0.00020119	0.000361101	negative
PRJNA729271	stage_1D_vs_E10	ordinary_muscle_development	ENSGALG00010008352	ENSGALG00010008352	0.303952932	0.152734153	1.990078355	0.046582305	0.062386631	positive
PRJNA729271	stage_1D_vs_E10	ordinary_muscle_development	ENSGALG00010008354	TMEM71	1.784673435	0.223604154	7.98139659	1.44687E-15	6.02135E-15	positive
PRJNA729271	stage_1D_vs_E10	ordinary_muscle_development	ENSGALG00010008355	MAFA	4.399808607	0.177378847	24.80458456	7.9999E-136	1.1368E-133	positive
PRJNA729271	stage_1D_vs_E10	ordinary_muscle_development	ENSGALG00010008356	ENSGALG00010008356	-0.674967967	0.125708515	-5.36930983	7.90385E-08	1.92387E-07	negative
PRJNA729271	stage_1D_vs_E10	ordinary_muscle_development	ENSGALG00010008358	HPF1	-1.463483258	0.114508555	-12.78055829	2.10553E-37	2.50984E-36	negative
PRJNA729271	stage_1D_vs_E10	ordinary_muscle_development	ENSGALG00010008360	ZC3H3	0.091552389	0.06114816	1.497222301	0.134335425	0.166896603	positive
PRJNA729271	stage_1D_vs_E10	ordinary_muscle_development	ENSGALG00010008361	MATR3	-0.800791206	0.056760553	-14.10823477	3.37931E-45	5.35921E-44	negative
PRJNA729271	stage_1D_vs_E10	ordinary_muscle_development	ENSGALG00010008362	RAMP3	0.397932826	0.261704145	1.520544606	0.128374157	0.160191409	positive
PRJNA729271	stage_1D_vs_E10	ordinary_muscle_development	ENSGALG00010008364	ENSGALG00010008364	-1.27973333	0.596587334	-2.145089676	0.031945678	0.043895402	negative
PRJNA729271	stage_1D_vs_E10	ordinary_muscle_development	ENSGALG00010008366	RPRD1A	0.227403284	0.105777203	2.14983264	0.031568455	0.043397644	positive
PRJNA729271	stage_1D_vs_E10	ordinary_muscle_development	ENSGALG00010008367	ENSGALG00010008367	-1.61948877	0.628902805	-2.575101841	0.010021058	0.014812979	negative
PRJNA729271	stage_1D_vs_E10	ordinary_muscle_development	ENSGALG00010008368	SNCA	3.44731128	0.312536284	11.0301154	2.73514E-28	2.16762E-27	positive
PRJNA729271	stage_1D_vs_E10	ordinary_muscle_development	ENSGALG00010008372	CLCN3	-0.049073464	0.068918632	-0.712049298	0.476434249	0.525084736	negative
PRJNA729271	stage_1D_vs_E10	ordinary_muscle_development	ENSGALG00010008373	MACIR	0.012338222	0.163617835	0.075408785	0.93988947	0.948857631	positive
PRJNA729271	stage_1D_vs_E10	ordinary_muscle_development	ENSGALG00010008374	ZNF821	-0.360445271	0.086184689	-4.18224251	2.88648E-05	5.63571E-05	negative
PRJNA729271	stage_1D_vs_E10	ordinary_muscle_development	ENSGALG00010008375	ENSGALG00010008375	-0.559153242	0.09822575	-5.692532145	1.25169E-08	3.24218E-08	negative
PRJNA729271	stage_1D_vs_E10	ordinary_muscle_development	ENSGALG00010008376	MMRN1	1.291215763	0.258085095	5.003062122	5.64267E-07	1.2848E-06	positive
PRJNA729271	stage_1D_vs_E10	ordinary_muscle_development	ENSGALG00010008377	AKT2	-0.081919927	0.116831226	-0.701181781	0.483189578	0.531761668	negative
PRJNA729271	stage_1D_vs_E10	ordinary_muscle_development	ENSGALG00010008380	SIL1	0.943865808	0.170068706	5.549908786	2.85819E-08	7.18139E-08	positive
PRJNA729271	stage_1D_vs_E10	ordinary_muscle_development	ENSGALG00010008381	C18orf21	0.386885097	0.112485658	3.439417102	0.000582968	0.000995303	positive
PRJNA729271	stage_1D_vs_E10	ordinary_muscle_development	ENSGALG00010008382	ENSGALG00010008382	1.144895349	0.128152688	8.933837981	4.1148E-19	2.09057E-18	positive
PRJNA729271	stage_1D_vs_E10	ordinary_muscle_development	ENSGALG00010008383	PLD5	-1.792930856	0.418740134	-4.281726807	1.85449E-05	3.68179E-05	negative
PRJNA729271	stage_1D_vs_E10	ordinary_muscle_development	ENSGALG00010008385	CTNNA1	-0.47387769	0.091249811	-5.193190896	2.0672E-07	4.86904E-07	negative
PRJNA729271	stage_1D_vs_E10	ordinary_muscle_development	ENSGALG00010008386	SESN3	-0.610127932	0.228005691	-2.675932904	0.007452156	0.011207616	negative
PRJNA729271	stage_1D_vs_E10	ordinary_muscle_development	ENSGALG00010008387	PHF20L1	0.348503268	0.112077239	3.10949191	0.001874094	0.00302809	positive
PRJNA729271	stage_1D_vs_E10	ordinary_muscle_development	ENSGALG00010008388	ADCY1	-1.416559002	0.429677825	-3.296793367	0.000977954	0.001632552	negative
PRJNA729271	stage_1D_vs_E10	ordinary_muscle_development	ENSGALG00010008389	GALNT1	-0.914690758	0.088666617	-10.3160669	5.96155E-25	4.07427E-24	negative
PRJNA729271	stage_1D_vs_E10	ordinary_muscle_development	ENSGALG00010008391	ENSGALG00010008391	-0.445716847	0.072154573	-6.177250137	6.52277E-10	1.86668E-09	negative
PRJNA729271	stage_1D_vs_E10	ordinary_muscle_development	ENSGALG00010008392	CD36	5.03585111	0.369036213	13.64595378	2.13403E-42	3.04456E-41	positive
PRJNA729271	stage_1D_vs_E10	ordinary_muscle_development	ENSGALG00010008394	CD2AP	0.86441821	0.110523286	7.821141042	5.23467E-15	2.09952E-14	positive
PRJNA729271	stage_1D_vs_E10	ordinary_muscle_development	ENSGALG00010008395	ENDOD1	1.363746721	0.144069447	9.465898234	2.91049E-21	1.66881E-20	positive
PRJNA729271	stage_1D_vs_E10	ordinary_muscle_development	ENSGALG00010008396	LRRTM2	-2.611686944	0.408233082	-6.397538714	1.57901E-10	4.71622E-10	negative
PRJNA729271	stage_1D_vs_E10	ordinary_muscle_development	ENSGALG00010008397	ATXN1L	-0.094300109	0.071603463	-1.316976922	0.18784636	0.227186733	negative
PRJNA729271	stage_1D_vs_E10	ordinary_muscle_development	ENSGALG00010008399	INO80C	-0.843795551	0.113343389	-7.444594352	9.72428E-14	3.60034E-13	negative
PRJNA729271	stage_1D_vs_E10	ordinary_muscle_development	ENSGALG00010008400	MAP1LC3C	-2.132502772	0.264218208	-8.070990962	6.97299E-16	2.96056E-15	negative
PRJNA729271	stage_1D_vs_E10	ordinary_muscle_development	ENSGALG00010008401	PSMD8	-0.206842233	0.156677811	-1.320175659	0.186776377	0.226005809	negative
PRJNA729271	stage_1D_vs_E10	ordinary_muscle_development	ENSGALG00010008403	GNAT3	0.428098479	0.396616825	1.079375489	0.280420373	0.326533845	positive
PRJNA729271	stage_1D_vs_E10	ordinary_muscle_development	ENSGALG00010008405	LRATD2	-0.048314081	0.144822463	-0.33360903	0.738674604	0.772071559	negative
PRJNA729271	stage_1D_vs_E10	ordinary_muscle_development	ENSGALG00010008407	GNAI1	-0.328685338	0.194266764	-1.691927798	0.090659741	0.116139014	negative
PRJNA729271	stage_1D_vs_E10	ordinary_muscle_development	ENSGALG00010008408	SMARCA4	-0.583186328	0.153701181	-3.794286576	0.000148069	0.000270343	negative
PRJNA729271	stage_1D_vs_E10	ordinary_muscle_development	ENSGALG00010008409	CWC15	-0.122722862	0.084968327	-1.444336566	0.148644325	0.183416999	negative
PRJNA729271	stage_1D_vs_E10	ordinary_muscle_development	ENSGALG00010008410	NAPRT	-0.631009021	0.132596386	-4.758870424	1.94679E-06	4.24598E-06	negative
PRJNA729271	stage_1D_vs_E10	ordinary_muscle_development	ENSGALG00010008411	ENSGALG00010008411	0.622075045	0.378594801	1.643115658	0.100359006	0.127583212	positive
PRJNA729271	stage_1D_vs_E10	ordinary_muscle_development	ENSGALG00010008412	NEK1	-1.088602214	0.10494051	-10.37351744	3.27252E-25	2.27289E-24	negative
PRJNA729271	stage_1D_vs_E10	ordinary_muscle_development	ENSGALG00010008415	ENSGALG00010008415	0.591217115	0.357063938	1.655773804	0.097767635	0.12451822	positive
PRJNA729271	stage_1D_vs_E10	ordinary_muscle_development	ENSGALG00010008416	MSX2	-5.924890784	0.530472202	-11.16908815	5.77709E-29	4.76602E-28	negative
PRJNA729271	stage_1D_vs_E10	ordinary_muscle_development	ENSGALG00010008417	IGFBP1	-0.822071529	0.914401263	-0.899027114	0.368638219	0.417813041	negative
PRJNA729271	stage_1D_vs_E10	ordinary_muscle_development	ENSGALG00010008418	TMEM245	0.897583354	0.089367458	10.04373821	9.78924E-24	6.29805E-23	positive
PRJNA729271	stage_1D_vs_E10	ordinary_muscle_development	ENSGALG00010008422	EXO1	-2.158184339	0.227479127	-9.487395016	2.3688E-21	1.36578E-20	negative
PRJNA729271	stage_1D_vs_E10	ordinary_muscle_development	ENSGALG00010008424	PPIP5K2	-0.625213335	0.120859954	-5.173039652	2.30316E-07	5.40374E-07	negative
PRJNA729271	stage_1D_vs_E10	ordinary_muscle_development	ENSGALG00010008425	MAGI2	0.072217101	0.10416773	0.693277088	0.488135679	0.536512486	positive
PRJNA729271	stage_1D_vs_E10	ordinary_muscle_development	ENSGALG00010008426	AP1G1	-0.340766561	0.055532731	-6.136319216	8.44554E-10	2.40082E-09	negative
PRJNA729271	stage_1D_vs_E10	ordinary_muscle_development	ENSGALG00010008429	IGFBP3	1.422299637	0.118470279	12.00553966	3.32289E-33	3.34714E-32	positive
PRJNA729271	stage_1D_vs_E10	ordinary_muscle_development	ENSGALG00010008430	ENSGALG00010008430	0.069900334	0.392018648	0.178308695	0.85848055	0.878976872	positive
PRJNA729271	stage_1D_vs_E10	ordinary_muscle_development	ENSGALG00010008431	SH3RF1	-0.169153991	0.125404844	-1.34886329	0.177380881	0.215590799	negative
PRJNA729271	stage_1D_vs_E10	ordinary_muscle_development	ENSGALG00010008433	EBAG9	0.17052709	0.095941726	1.77402773	0.075501995	0.097906729	positive
PRJNA729271	stage_1D_vs_E10	ordinary_muscle_development	ENSGALG00010008434	PTCHD4	-3.503889505	0.646491525	-5.419853734	5.96478E-08	1.46566E-07	negative
PRJNA729271	stage_1D_vs_E10	ordinary_muscle_development	ENSGALG00010008437	SHKBP1	-0.169083507	0.16284617	-1.038302019	0.299129469	0.346012422	negative
PRJNA729271	stage_1D_vs_E10	ordinary_muscle_development	ENSGALG00010008438	SYBU	0.407193423	0.261856226	1.555026703	0.11993971	0.150547809	positive
PRJNA729271	stage_1D_vs_E10	ordinary_muscle_development	ENSGALG00010008440	ENSGALG00010008440	0.624058025	0.30770729	2.02808983	0.042551081	0.057390706	positive
PRJNA729271	stage_1D_vs_E10	ordinary_muscle_development	ENSGALG00010008441	PHLPP2	0.061126913	0.086090458	0.710031217	0.477684778	0.526262891	positive
PRJNA729271	stage_1D_vs_E10	ordinary_muscle_development	ENSGALG00010008443	RSFR	2.770062339	0.440552085	6.287706799	3.2219E-10	9.42701E-10	positive
PRJNA729271	stage_1D_vs_E10	ordinary_muscle_development	ENSGALG00010008444	SLC7A11	-3.567876761	0.474928586	-7.512448961	5.80314E-14	2.18031E-13	negative
PRJNA729271	stage_1D_vs_E10	ordinary_muscle_development	ENSGALG00010008447	FRRS1L	-0.246973298	0.155342977	-1.589858155	0.111866782	0.141074387	negative

PRJNA729271	stage_1D_vs_E10	ordinary_muscle_development	ENSGALG00010008448	TNS3	-0.640600384	0.080760634	-7.932087156	2.15493E-15	8.87127E-15	negative
PRJNA729271	stage_1D_vs_E10	ordinary_muscle_development	ENSGALG00010008449	SRA1	0.245479757	0.108280392	2.267074882	0.023385652	0.032801918	positive
PRJNA729271	stage_1D_vs_E10	ordinary_muscle_development	ENSGALG00010008450	GIN1	-0.842156458	0.179204844	-4.699406778	2.60918E-06	5.63218E-06	negative
PRJNA729271	stage_1D_vs_E10	ordinary_muscle_development	ENSGALG00010008451	OPN3	-0.007692115	0.315111918	-0.02441074	0.980524981	0.983374359	negative
PRJNA729271	stage_1D_vs_E10	ordinary_muscle_development	ENSGALG00010008452	ENSGALG00010008452	1.054064755	0.392464982	2.685754918	0.007236615	0.010893627	positive
PRJNA729271	stage_1D_vs_E10	ordinary_muscle_development	ENSGALG00010008453	AMOTL1	2.363043096	0.119468698	19.77960036	4.46213E-87	2.49724E-85	positive
PRJNA729271	stage_1D_vs_E10	ordinary_muscle_development	ENSGALG00010008455	FKBP1B	0.837736096	0.151309696	5.536565848	3.0846E-08	7.72904E-08	positive
PRJNA729271	stage_1D_vs_E10	ordinary_muscle_development	ENSGALG00010008456	NHLRC1	-0.069404596	0.129195097	-0.537207658	0.591124182	0.634927382	negative
PRJNA729271	stage_1D_vs_E10	ordinary_muscle_development	ENSGALG00010008457	MARVELD3	0.375974333	0.119339055	3.150471853	0.00163007	0.002653623	positive
PRJNA729271	stage_1D_vs_E10	ordinary_muscle_development	ENSGALG00010008459	EPB41L4B	-2.014845805	0.21644936	-9.30862443	1.29499E-20	7.15351E-20	negative
PRJNA729271	stage_1D_vs_E10	ordinary_muscle_development	ENSGALG00010008464	SF3B6	-0.940220679	0.111823614	-8.408069179	4.16815E-17	1.89813E-16	negative
PRJNA729271	stage_1D_vs_E10	ordinary_muscle_development	ENSGALG00010008466	C11orf97	-2.438474355	0.457708579	-5.327569693	9.95356E-08	2.40421E-07	negative
PRJNA729271	stage_1D_vs_E10	ordinary_muscle_development	ENSGALG00010008468	EPPK1	-1.77146914	0.293111159	-6.043676887	1.50641E-09	4.19831E-09	negative
PRJNA729271	stage_1D_vs_E10	ordinary_muscle_development	ENSGALG00010008469	NOCT	1.225117374	0.277069966	4.421689549	9.79321E-06	2.00131E-05	positive
PRJNA729271	stage_1D_vs_E10	ordinary_muscle_development	ENSGALG00010008473	CSMD3	-3.389257336	0.380917908	-8.897605653	5.70644E-19	2.87804E-18	negative
PRJNA729271	stage_1D_vs_E10	ordinary_muscle_development	ENSGALG00010008474	ENSGALG00010008474	3.027522059	0.137252979	22.05796975	8.0098E-108	6.6725E-106	positive
PRJNA729271	stage_1D_vs_E10	ordinary_muscle_development	ENSGALG00010008476	RNF166	0.614597208	0.133720309	4.596139592	4.3039E-06	9.07552E-06	positive
PRJNA729271	stage_1D_vs_E10	ordinary_muscle_development	ENSGALG00010008486	CTU2	-0.423284498	0.137202877	-3.085099286	0.002034842	0.003272499	negative
PRJNA729271	stage_1D_vs_E10	ordinary_muscle_development	ENSGALG00010008488	CCSER1	-0.537299909	0.152027086	-3.534238029	0.000408953	0.000711019	negative
PRJNA729271	stage_1D_vs_E10	ordinary_muscle_development	ENSGALG00010008492	ANKRD49	-0.744125329	0.127166399	-5.851587666	4.86903E-09	1.30456E-08	negative
PRJNA729271	stage_1D_vs_E10	ordinary_muscle_development	ENSGALG00010008497	PAM	-0.241696655	0.128907269	-1.874965293	0.060797498	0.079969123	negative
PRJNA729271	stage_1D_vs_E10	ordinary_muscle_development	ENSGALG00010008498	ENSGALG00010008498	2.61706917	0.147604128	17.73032505	2.44616E-70	9.11493E-69	positive
PRJNA729271	stage_1D_vs_E10	ordinary_muscle_development	ENSGALG00010008500	ENSGALG00010008500	1.021561557	0.149756382	6.821489286	9.01015E-12	2.92306E-11	positive
PRJNA729271	stage_1D_vs_E10	ordinary_muscle_development	ENSGALG00010008501	ENSGALG00010008501	-2.066447396	0.440026793	-4.696185387	2.65065E-06	5.71488E-06	negative
PRJNA729271	stage_1D_vs_E10	ordinary_muscle_development	ENSGALG00010008504	RNF139	0.481384908	0.08764796	5.492254549	3.96835E-08	9.86811E-08	positive
PRJNA729271	stage_1D_vs_E10	ordinary_muscle_development	ENSGALG00010008505	PTPN3	-1.795224191	0.275653837	-6.512603662	7.38592E-11	2.25482E-10	negative
PRJNA729271	stage_1D_vs_E10	ordinary_muscle_development	ENSGALG00010008509	MGARP	2.662511637	0.350637816	7.593338519	3.11767E-14	1.19205E-13	positive
PRJNA729271	stage_1D_vs_E10	ordinary_muscle_development	ENSGALG00010008514	PIEZO1	2.096059666	0.165929747	12.63221159	1.40279E-36	1.61891E-35	positive
PRJNA729271	stage_1D_vs_E10	ordinary_muscle_development	ENSGALG00010008515	TAFA5	-0.659040738	0.210434404	-3.131810789	0.001737318	0.002819033	negative
PRJNA729271	stage_1D_vs_E10	ordinary_muscle_development	ENSGALG00010008516	MRE11	-1.154476112	0.078230663	-14.75733521	2.75971E-49	5.17491E-48	negative
PRJNA729271	stage_1D_vs_E10	ordinary_muscle_development	ENSGALG00010008522	NUDT12	0.051393114	0.151578758	0.339052218	0.734570393	0.768391271	positive
PRJNA729271	stage_1D_vs_E10	ordinary_muscle_development	ENSGALG00010008527	CHUK	0.063139974	0.05506621	1.146619198	0.251539039	0.296114543	positive
PRJNA729271	stage_1D_vs_E10	ordinary_muscle_development	ENSGALG00010008529	GPR83	-0.515633643	0.512663808	-1.005792948	0.31451518	0.362075891	negative
PRJNA729271	stage_1D_vs_E10	ordinary_muscle_development	ENSGALG00010008533	ENSGALG00010008533	-0.001490056	0.550035963	-0.002709016	0.99783852	0.998320635	negative
PRJNA729271	stage_1D_vs_E10	ordinary_muscle_development	ENSGALG00010008534	RAB33B	-0.073564432	0.072801257	-1.010482989	0.312263945	0.359712778	negative
PRJNA729271	stage_1D_vs_E10	ordinary_muscle_development	ENSGALG00010008542	MOCOS	1.839276555	0.15608984	11.78344829	4.75089E-32	4.5395E-31	positive
PRJNA729271	stage_1D_vs_E10	ordinary_muscle_development	ENSGALG00010008561	EEF1D	0.922241178	0.114711748	8.039640187	9.01027E-16	3.80104E-15	positive
PRJNA729271	stage_1D_vs_E10	ordinary_muscle_development	ENSGALG00010008580	PANX1	-0.433080426	0.243057232	-1.781804318	0.074781148	0.097050115	negative
PRJNA729271	stage_1D_vs_E10	ordinary_muscle_development	ENSGALG00010008588	PYCR3	-1.125382659	0.179962319	-6.253434985	4.01522E-10	1.16588E-09	negative
PRJNA729271	stage_1D_vs_E10	ordinary_muscle_development	ENSGALG00010008592	KIAA0513	1.363273087	0.138720022	9.8275149	8.57076E-23	5.30005E-22	positive
PRJNA729271	stage_1D_vs_E10	ordinary_muscle_development	ENSGALG00010008596	ENSGALG00010008596	-1.2082596	0.159820319	-7.560112533	4.02721E-14	1.52813E-13	negative
PRJNA729271	stage_1D_vs_E10	ordinary_muscle_development	ENSGALG00010008618	EIF4EBP3	0.316214445	0.116554016	2.713029156	0.006667124	0.010080313	positive
PRJNA729271	stage_1D_vs_E10	ordinary_muscle_development	ENSGALG00010008623	TRPS1	-1.821419144	0.132294891	-13.76787211	3.97757E-43	5.81785E-42	negative
PRJNA729271	stage_1D_vs_E10	ordinary_muscle_development	ENSGALG00010008636	AGTPBP1	3.600796329	0.23887585	15.07392367	2.40402E-51	4.85413E-50	positive
PRJNA729271	stage_1D_vs_E10	ordinary_muscle_development	ENSGALG00010008637	RGS7	-4.249486823	0.792161181	-5.364421947	8.12089E-08	1.9757E-07	negative
PRJNA729271	stage_1D_vs_E10	ordinary_muscle_development	ENSGALG00010008653	ENSGALG00010008653	-0.888682833	0.155770209	-5.70508855	1.16283E-08	3.01739E-08	negative
PRJNA729271	stage_1D_vs_E10	ordinary_muscle_development	ENSGALG00010008656	CCDC6	-0.202761804	0.071794403	-2.824200704	0.004739869	0.007305094	negative
PRJNA729271	stage_1D_vs_E10	ordinary_muscle_development	ENSGALG00010008658	TSTA3	-1.248890821	0.117000569	-10.67422859	1.34369E-26	9.89673E-26	negative
PRJNA729271	stage_1D_vs_E10	ordinary_muscle_development	ENSGALG00010008666	SLC16A9	0.459796979	0.189097168	2.431538154	0.015034865	0.021675986	positive
PRJNA729271	stage_1D_vs_E10	ordinary_muscle_development	ENSGALG00010008668	ENSGALG00010008668	0.248585741	0.302683813	0.821272002	0.411491352	0.461127936	positive
PRJNA729271	stage_1D_vs_E10	ordinary_muscle_development	ENSGALG00010008674	RXFP1	0.614679955	0.275594308	2.230379719	0.025722245	0.03581594	positive
PRJNA729271	stage_1D_vs_E10	ordinary_muscle_development	ENSGALG00010008686	ENSGALG00010008686	-3.672106778	0.470363423	-7.806956484	5.85855E-15	2.34132E-14	negative
PRJNA729271	stage_1D_vs_E10	ordinary_muscle_development	ENSGALG00010008688	HBEGF	1.337257861	0.232787946	5.744532249	9.21754E-09	2.41127E-08	positive
PRJNA729271	stage_1D_vs_E10	ordinary_muscle_development	ENSGALG00010008690	YIF1B	-0.571460318	0.124246995	-4.599389436	4.23731E-06	8.94159E-06	negative
PRJNA729271	stage_1D_vs_E10	ordinary_muscle_development	ENSGALG00010008693	C4orf46	-0.557732217	0.104558254	-5.334176856	9.59789E-08	2.32063E-07	negative
PRJNA729271	stage_1D_vs_E10	ordinary_muscle_development	ENSGALG00010008696	PFN4	0.192559156	0.204792544	0.940264485	0.347081912	0.395919754	positive
PRJNA729271	stage_1D_vs_E10	ordinary_muscle_development	ENSGALG00010008699	NAA35	0.174178768	0.100086061	1.740289972	0.081808114	0.10556473	positive
PRJNA729271	stage_1D_vs_E10	ordinary_muscle_development	ENSGALG00010008700	ENSGALG00010008700	0.293056101	0.255387941	1.147493884	0.251177559	0.295761066	positive
PRJNA729271	stage_1D_vs_E10	ordinary_muscle_development	ENSGALG00010008708	ZC3H4	1.360915634	0.243443381	5.590275775	2.26709E-08	5.75105E-08	positive
PRJNA729271	stage_1D_vs_E10	ordinary_muscle_development	ENSGALG00010008716	TATDN1	-0.894607342	0.113412722	-7.88806868	3.06899E-15	1.25098E-14	negative
PRJNA729271	stage_1D_vs_E10	ordinary_muscle_development	ENSGALG00010008729	TMEM254	-0.61125439	0.119030069	-5.135293926	2.81703E-07	6.55634E-07	negative
PRJNA729271	stage_1D_vs_E10	ordinary_muscle_development	ENSGALG00010008731	NDUFB9	0.833543414	0.087425606	9.53431669	1.50876E-21	8.78644E-21	positive
PRJNA729271	stage_1D_vs_E10	ordinary_muscle_development	ENSGALG00010008739	VSTM5	-0.677019487	0.246308437	-2.748665432	0.005983843	0.00910899	negative
PRJNA729271	stage_1D_vs_E10	ordinary_muscle_development	ENSGALG00010008745	GRAMD4	-0.109186979	0.153686495	-0.710452661	0.477423477	0.526014997	negative
PRJNA729271	stage_1D_vs_E10	ordinary_muscle_development	ENSGALG00010008750	PFDN1	0.129859321	0.166162433	0.781520344	0.434496516	0.484016831	positive
PRJNA729271	stage_1D_vs_E10	ordinary_muscle_development	ENSGALG00010008752	GOLM1	0.265644142	0.172577855	1.539271321	0.123738072	0.154953206	positive
PRJNA729271	stage_1D_vs_E10	ordinary_muscle_development	ENSGALG00010008756	ITSN2	2.24486099	0.071859675	31.23950938	3.0995E-214	1.7971E-211	positive
PRJNA729271	stage_1D_vs_E10	ordinary_muscle_development	ENSGALG00010008762	SAE1	-0.89786554	0.10268305	-8.744048186	2.24903E-18	1.10023E-17	negative
PRJNA729271	stage_1D_vs_E10	ordinary_muscle_development	ENSGALG00010008764	ETFDH	1.700977993	0.123427513	13.78118985	3.30775E-43	4.8676E-42	positive
PRJNA729271	stage_1D_vs_E10	ordinary_muscle_development	ENSGALG00010008768	PRXL2C	1.590663712	0.148650405	10.70070215	1.0101E-26	7.47772E-26	positive
PRJNA729271	stage_1D_vs_E10	ordinary_muscle_development	ENSGALG00010008775	MED17	-0.130161892	0.089833691	-1.448920667	0.147359739	0.182017845	negative
PRJNA729271	stage_1D_vs_E10	ordinary_muscle_development	ENSGALG00010008780	KARS1	0.064311331	0.057284347	1.12266849	0.261578315	0.306748694	positive
PRJNA729271	stage_1D_vs_E10	ordinary_muscle_development	ENSGALG00010008782	CCDC9	0.015875423	0.283804663	0.055937851	0.955391317	0.962429435	positive
PRJNA729271	stage_1D_vs_E10	ordinary_muscle_development	ENSGALG00010008790	GPN1	0.140230946	0.095308961	1.471330136	0.141201857	0.174903514	positive
PRJNA729271	stage_1D_vs_E10	ordinary_muscle_development	ENSGALG00010008793	C11orf54	0.796212625	0.11312598	7.038282697	1.94624E-12	6.60053E-12	positive
PRJNA729271	stage_1D_vs_E10	ordinary_muscle_development	ENSGALG00010008799	ENSGALG00010008799	0.681340665	0.170578189	3.994301196	6.48853E-05	0.000122782	positive
PRJNA729271	stage_1D_vs_E10	ordinary_muscle_development	ENSGALG00010008807	BRD1	0.32937935	0.061452028	5.359942736	8.32483E-08	2.02362E-07	positive
PRJNA729271	stage_1D_vs_E10	ordinary_muscle_development	ENSGALG00010008811	TERF2IP	0.607154869	0.094291331	6.439137761	1.20154E-10	3.62011E-10	positive
PRJNA729271	stage_1D_vs_E10	ordinary_muscle_development	ENSGALG00010008814	ENSGALG00010008814	0.034487451	0.241749537	0.142657774	0.886560467	0.903270125	positive
PRJNA729271	stage_1D_vs_E10	ordinary_muscle_development	ENSGALG00010008821	ENSGALG00010008821	0.324780997	0.136811161	2.373936426	0.01759958	0.025091563	positive
PRJNA729271	stage_1D_vs_E10	ordinary_muscle_development	ENSGALG00010008824	GREM2	0.100597925	0.303809561	0.331121658	0.740552593	0.773699714	positive
PRJNA729271	stage_1D_vs_E10	ordinary_muscle_development	ENSGALG00010008825	PPID	-0.490974594	0.109219921	-4.495284279	6.94771E-06	1.44135E-05	negative
PRJNA729271	stage_1D_vs_E10	ordinary_muscle_development	ENSGALG00010008830	DHX34	0.632066224	0.192481091	3.283783468	0.001024236	0.001706471	positive
PRJNA729271	stage_1D_vs_E10	ordinary_muscle_development	ENSGALG00010008832	MTSS1	0.555077339	0.123853059	4.481741045	7.40365E-06	1.53112E-05	positive
PRJNA729271	stage_1D_vs_E10	ordinary_muscle_development	ENSGALG00010008837	ENSGALG00010008837	-0.682867044	0.098449408	-6.936222924	4.02722E-12	1.33764E-11	negative
PRJNA729271	stage_1D_vs_E10	ordinary_muscle_development	ENSGALG00010008839	ZNF512	-0.382994911	0.074265898	-5.157076442	2.50835E-07	5.86429E-07	negative
PRJNA729271	stage_1D_vs_E10	ordinary_muscle_development	ENSGALG00010008846	SLC35D2	-0.296373926	0.290447943	-1.020402909	0.307537415	0.354803791	negative
PRJNA729271	stage_1D_vs_E10	ordinary_muscle_development	ENSGALG00010008850	CYSTM1	2.352992828	0.123352403	19.07537078	4.04543E-81	1.95462E-79	positive
PRJNA729271	stage_1D_vs_E10	ordinary_muscle_development	ENSGALG00010008852	KHDRBS3	0.235679532	0.083464732	2.823702009	0.00474725	0.007314913	positive
PRJNA729271	stage_1D_vs_E10	ordinary_muscle_development	ENSGALG00010008859	ENSGALG00010008859	-1.018856508	0.213072044	-4.781746531	1.73779E-06	3.80272E-06	negative
PRJNA729271	stage_1D_vs_E10	ordinary_muscle_development	ENSGALG00010008860	SQLE	-3.03432329	0.250730352	-12.10193846	1.03146E-33	1.06036E-32	negative
PRJNA729271	stage_1D_vs_E10	ordinary_muscle_development	ENSGALG00010008862	ZNF367	-0.157440062	0.140566281	-1.120041454	0.262696097	0.307848648	negative
PRJNA729271	stage_1D_vs_E10	ordinary_muscle_development	ENSGALG00010008864	ADAT1	0.851784688	0.136934	6.22040318	4.95879E-10	1.4324E-09	positive
PRJNA729271	stage_1D_vs_E10	ordinary_muscle_development	ENSGALG00010008866	WASHC5	-0.592693807	0.073737303	-8.037910009	9.13837E-16	3.8506E-15	negative
PRJNA729271	stage_1D_vs_E10	ordinary_muscle_development	ENSGALG00010008871	PRR7	-1.045705518	0.189532485	-5.517289122	3.44269E-08	8.5993E-08	negative
PRJNA729271	stage_1D_vs_E10	ordinary_muscle_development	ENSGALG00010008877	FNDC4	1.584450503	0.242188294	6.542225782	6.06099E-11	1.86289E-10	positive
PRJNA729271	stage_1D_vs_E10	ordinary_muscle_development	ENSGALG00010008879	HABP4	-1.932707952	0.179184656	-10.78612421	4.00318E-27	3.01434E-26	negative
PRJNA729271	stage_1D_vs_E10	ordinary_muscle_development	ENSGALG00010008880	FMN2	0.184965432	0.279236107	0.662397976	0.5077162	0.555757916	positive
PRJNA729271	stage_1D_vs_E10	ordinary_muscle_development	ENSGALG00010008884	FAM135B	3.542695415	0.443781316	7.982975597	1.42847E-15	5.94991E-15	positive
PRJNA729271	stage_1D_vs_E10	ordinary_muscle_development	ENSGALG00010008888	FBXO46	0.214411552	0.142524018	1.504388911	0.132481258	0.164805684	positive
PRJNA729271	stage_1D_vs_E10	ordinary_muscle_development	ENSGALG00010008892	ENSGALG00010008892	-0.059685104	0.120614746	-0.494840851	0.620712466	0.66292567	negative
PRJNA729271	stage_1D_vs_E10	ordinary_muscle_development	ENSGALG00010008893	ENSGALG00010008893	4.259327199	0.138282116	30.80172141	2.485E-208	1.3341E-205	positive
PRJNA729271	stage_1D_vs_E10	ordinary_muscle_development	ENSGALG00010008897	NSMCE2	-0.274159356	0.164077289	-1.670915931	0.094738284	0.120936278	negative
PRJNA729271	stage_1D_vs_E10	ordinary_muscle_development	ENSGALG00010008903	ENSGALG00010008903	3.709450409	0.396984801	9.344061533	9.2708E-21	5.18242E-20	positive
PRJNA729271	stage_1D_vs_E10	ordinary_muscle_development	ENSGALG00010008905	GABARAPL2	0.335010917	0.094032664	3.562707905	0.000367049	0.000641473	positive
PRJNA729271	stage_1D_vs_E10	ordinary_muscle_development	ENSGALG00010008906	DBN1	-3.465045146	0.21318933	-16.25337044	2.11415E-59	5.70663E-58	negative
PRJNA729271	stage_1D_vs_E10	ordinary_muscle_development	ENSGALG00010008910	TRIB1	2.071046092	0.260912074	7.937716577	2.05937E-15	8.48511E-15	positive
PRJNA729271	stage_1D_vs_E10	ordinary_muscle_development	ENSGALG00010008918	PLAC9	0.069479637	0.245790995	0.282677715	0.777423901	0.806409005	positive
PRJNA729271	stage_1D_vs_E10	ordinary_muscle_development	ENSGALG00010008921	OPA3	0.871377223	0.125512487	6.942554045	3.85074E-12	1.28206E-11	positive
PRJNA729271	stage_1D_vs_E10	ordinary_muscle_development	ENSGALG00010008922	ENSGALG00010008922	6.517123489	1.138943874	5.722076072	1.0523E-08	2.73844E-08	positive
PRJNA729271	stage_1D_vs_E10	ordinary_muscle_development	ENSGALG00010008928	CFC1	-3.128416616	0.530148111	-5.901023786	3.61253E-09	9.78942E-09	negative
PRJNA729271	stage_1D_vs_E10	ordinary_muscle_development	ENSGALG00010008931	FNIP2	0.114338254	0.131774499	0.867681192	0.385568885	0.434589501	positive
PRJNA729271	stage_1D_vs_E10	ordinary_muscle_development	ENSGALG00010008932	VASP	1.029364497	0.120383127	8.550737336	1.22305E-17	5.74467E-17	positive
PRJNA729271	stage_1D_vs_E10	ordinary_muscle_development	ENSGALG00010008936	IFT172	-0.547774056	0.094513912	-5.795697645	6.80377E-09	1.80393E-08	negative
PRJNA729271	stage_1D_vs_E10	ordinary_muscle_development	ENSGALG00010008938	ZBED4	-0.716308729	0.090959287	-7.8750478	3.40613E-15	1.3849E-14	negative
PRJNA729271	stage_1D_vs_E10	ordinary_muscle_development	ENSGALG00010008939	RANBP9	1.046943672	0.102607087	10.20342459	1.91402E-24	1.27265E-23	positive
PRJNA729271	stage_1D_vs_E10	ordinary_muscle_development	ENSGALG00010008943	ENSGALG00010008943	3.855222425	0.446380098	8.636635997	5.78925E-18	2.77039E-17	positive
PRJNA729271	stage_1D_vs_E10	ordinary_muscle_development	ENSGALG00010008945	FER1L6	1.462784553	0.531664786	2.75132864	0.005935406	0.009040954	positive
PRJNA729271	stage_1D_vs_E10	ordinary_muscle_development	ENSGALG00010008946	COL22A1	-3.295950902	0.622949554	-5.290879304	1.2173E-07	2.92325E-07	negative
PRJNA729271	stage_1D_vs_E10	ordinary_muscle_development	ENSGALG00010008955	KRTCAP3	-2.277590213	0.400027041	-5.693590627	1.24395E-08	3.22329E-08	negative
PRJNA729271	stage_1D_vs_E10	ordinary_muscle_development	ENSGALG00010008956	LVRN	0.839628292	0.482845871	1.738915755	0.082049582	0.105848051	positive
PRJNA729271	stage_1D_vs_E10	ordinary_muscle_development	ENSGALG00010008960	LRRC2	10.46217216	0.397288129	26.33396623	7.8345E-153	1.4559E-150	positive
PRJNA729271	stage_1D_vs_E10	ordinary_muscle_development	ENSGALG00010008966	NRBP1	0.176477252	0.115716265	1.525085962	0.127237658	0.158841603	positive
PRJNA729271	stage_1D_vs_E10	ordinary_muscle_development	ENSGALG00010008969	PPP1R37	0.402706487	0.186996686	2.153548784	0.031275574	0.043019496	positive
PRJNA729271	stage_1D_vs_E10	ordinary_muscle_development	ENSGALG00010008971	ANXA11	0.466067609	0.103697839	4.494477547	6.9741E-06	1.44641E-05	positive
PRJNA729271	stage_1D_vs_E10	ordinary_muscle_development	ENSGALG00010008973	KCNK9	-2.090587094	0.661517815	-3.160288427	0.00157613	0.002569855	negative
PRJNA729271	stage_1D_vs_E10	ordinary_muscle_development	ENSGALG00010008974	SMCO4	-0.55371946	0.10196045	-5.430727903	5.61247E-08	1.38244E-07	negative
PRJNA729271	stage_1D_vs_E10	ordinary_muscle_development	ENSGALG00010008980	ENSGALG00010008980	-0.299461126	0.25274345	-1.184842283	0.236079811	0.279641813	negative
PRJNA729271	stage_1D_vs_E10	ordinary_muscle_development	ENSGALG00010008983	ENSGALG00010008983	-0.332931723	0.335757671	-0.99158337	0.321400805	0.369474555	negative
PRJNA729271	stage_1D_vs_E10	ordinary_muscle_development	ENSGALG00010008988	PPM1G	-0.830124821	0.080302637	-10.33745403	4.77043E-25	3.28491E-24	negative
PRJNA729271	stage_1D_vs_E10	ordinary_muscle_development	ENSGALG00010008991	ENSGALG00010008991	0.121101151	0.129258827	0.936888828	0.348815747	0.397669299	positive

PRJNA729271	stage_1D_vs_E10	ordinary_muscle_development	ENSGALG00010008995	ECD	-0.618238344	0.099422861	-6.218271521	5.02661E-10	1.45075E-09	negative
PRJNA729271	stage_1D_vs_E10	ordinary_muscle_development	ENSGALG00010008996	CHRM3	-0.980972472	0.23817933	-4.118629735	3.81132E-05	7.3611E-05	negative
PRJNA729271	stage_1D_vs_E10	ordinary_muscle_development	ENSGALG00010008997	CHST6	-1.330211781	0.131257534	-10.13436514	3.88891E-24	2.54606E-23	negative
PRJNA729271	stage_1D_vs_E10	ordinary_muscle_development	ENSGALG00010008999	TRAPPC9	-0.551290187	0.064667331	-8.525018389	1.52786E-17	7.12557E-17	negative
PRJNA729271	stage_1D_vs_E10	ordinary_muscle_development	ENSGALG00010009000	FAM149B1	-0.276721204	0.102143296	-2.709147002	0.006745644	0.010191516	negative
PRJNA729271	stage_1D_vs_E10	ordinary_muscle_development	ENSGALG00010009002	ENSGALG00010009002	0.846517517	0.096989217	8.72795495	2.59319E-18	1.26347E-17	positive
PRJNA729271	stage_1D_vs_E10	ordinary_muscle_development	ENSGALG00010009003	ALG12	-1.161906747	0.136021577	-8.542076701	1.31831E-17	6.17013E-17	negative
PRJNA729271	stage_1D_vs_E10	ordinary_muscle_development	ENSGALG00010009007	TMEM65	0.704276977	0.098996812	7.114137959	1.12614E-12	3.86995E-12	positive
PRJNA729271	stage_1D_vs_E10	ordinary_muscle_development	ENSGALG00010009011	ACP6	-0.569237163	0.120445371	-4.726102437	2.2887E-06	4.96702E-06	negative
PRJNA729271	stage_1D_vs_E10	ordinary_muscle_development	ENSGALG00010009012	DEUP1	-2.222507888	0.814224549	-2.729600688	0.006341108	0.009619504	negative
PRJNA729271	stage_1D_vs_E10	ordinary_muscle_development	ENSGALG00010009013	LIPG	0.231477589	0.222104223	1.042202556	0.297317778	0.344163967	positive
PRJNA729271	stage_1D_vs_E10	ordinary_muscle_development	ENSGALG00010009015	TMEM231	0.033780612	0.090604182	0.372837226	0.709269595	0.745255276	positive
PRJNA729271	stage_1D_vs_E10	ordinary_muscle_development	ENSGALG00010009016	DNAJC9	-1.220502413	0.110233944	-11.07192908	1.71665E-28	1.38161E-27	negative
PRJNA729271	stage_1D_vs_E10	ordinary_muscle_development	ENSGALG00010009019	ZNF513	-0.024021991	0.135721027	-0.176995355	0.859512038	0.879908679	negative
PRJNA729271	stage_1D_vs_E10	ordinary_muscle_development	ENSGALG00010009020	ARHGAP35	-0.330073698	0.182922011	-1.804450412	0.071160726	0.092661316	negative
PRJNA729271	stage_1D_vs_E10	ordinary_muscle_development	ENSGALG00010009021	HNF4beta	0.223518183	0.414314561	0.539489085	0.589549426	0.633517602	positive
PRJNA729271	stage_1D_vs_E10	ordinary_muscle_development	ENSGALG00010009024	PMAIP1	-0.454636671	0.26164969	-1.737577719	0.082285249	0.106123737	negative
PRJNA729271	stage_1D_vs_E10	ordinary_muscle_development	ENSGALG00010009025	UTP23	-0.010106236	0.096114355	-0.105148036	0.916258344	0.928882689	negative
PRJNA729271	stage_1D_vs_E10	ordinary_muscle_development	ENSGALG00010009026	RAPGEF2	-0.014178695	0.136811412	-0.103636789	0.917457591	0.929838329	negative
PRJNA729271	stage_1D_vs_E10	ordinary_muscle_development	ENSGALG00010009028	PDLIM7	-1.080454381	0.112715875	-9.585645193	9.18816E-22	5.41612E-21	negative
PRJNA729271	stage_1D_vs_E10	ordinary_muscle_development	ENSGALG00010009029	IGSF3	-0.737909397	0.163151886	-4.522837073	6.10162E-06	1.27146E-05	negative
PRJNA729271	stage_1D_vs_E10	ordinary_muscle_development	ENSGALG00010009030	ZDHHC7	-0.026477661	0.088348009	-0.299697319	0.764408044	0.794556802	negative
PRJNA729271	stage_1D_vs_E10	ordinary_muscle_development	ENSGALG00010009031	SPIN1	-0.465687642	0.079404052	-5.864784377	4.49717E-09	1.2085E-08	negative
PRJNA729271	stage_1D_vs_E10	ordinary_muscle_development	ENSGALG00010009034	SNX17	0.47636705	0.119442423	3.988256759	6.65606E-05	0.000125772	positive
PRJNA729271	stage_1D_vs_E10	ordinary_muscle_development	ENSGALG00010009035	ENSGALG00010009035	-1.168350644	0.080386865	-14.53409884	7.36767E-48	1.30396E-46	negative
PRJNA729271	stage_1D_vs_E10	ordinary_muscle_development	ENSGALG00010009037	ENSGALG00010009037	0.892757438	0.187701403	4.756264055	1.97208E-06	4.29985E-06	positive
PRJNA729271	stage_1D_vs_E10	ordinary_muscle_development	ENSGALG00010009038	RAD21	-0.151792925	0.123880568	-1.225316674	0.220455928	0.262896642	negative
PRJNA729271	stage_1D_vs_E10	ordinary_muscle_development	ENSGALG00010009039	SLC36A4	-0.329735169	0.168939404	-1.9517955	0.050962489	0.067895338	negative
PRJNA729271	stage_1D_vs_E10	ordinary_muscle_development	ENSGALG00010009040	CRELD2	-1.173206248	0.142487759	-8.233733606	1.81467E-16	7.97805E-16	negative
PRJNA729271	stage_1D_vs_E10	ordinary_muscle_development	ENSGALG00010009043	TFAM	0.06190104	0.082168631	0.753341496	0.451244729	0.500443179	positive
PRJNA729271	stage_1D_vs_E10	ordinary_muscle_development	ENSGALG00010009047	SUPT5H	0.006971594	0.110110783	0.063314366	0.949516177	0.9572428	positive
PRJNA729271	stage_1D_vs_E10	ordinary_muscle_development	ENSGALG00010009050	PIK3CG	1.338984196	0.091999917	14.55418913	5.49334E-48	9.75809E-47	positive
PRJNA729271	stage_1D_vs_E10	ordinary_muscle_development	ENSGALG00010009051	TROJANZ	1.612963271	0.24711849	6.527084521	6.70622E-11	2.05207E-10	positive
PRJNA729271	stage_1D_vs_E10	ordinary_muscle_development	ENSGALG00010009054	RYR2	-1.713955047	0.498902473	-3.435451092	0.000591568	0.001009273	negative
PRJNA729271	stage_1D_vs_E10	ordinary_muscle_development	ENSGALG00010009057	NXNL2	1.086746981	0.361495413	3.006253857	0.002644881	0.004195398	positive
PRJNA729271	stage_1D_vs_E10	ordinary_muscle_development	ENSGALG00010009059	MED29	0.785373269	0.125864977	6.239807855	4.38109E-10	1.26906E-09	positive
PRJNA729271	stage_1D_vs_E10	ordinary_muscle_development	ENSGALG00010009065	EIF2B4	-0.100980219	0.095530381	-1.057048219	0.290489582	0.337000443	negative
PRJNA729271	stage_1D_vs_E10	ordinary_muscle_development	ENSGALG00010009067	ENSGALG00010009067	-0.963383392	1.621080296	-0.594284808	0.552321638	0.598214313	negative
PRJNA729271	stage_1D_vs_E10	ordinary_muscle_development	ENSGALG00010009068	AGO2	0.875375744	0.078786971	11.11066624	1.11326E-28	9.03509E-28	positive
PRJNA729271	stage_1D_vs_E10	ordinary_muscle_development	ENSGALG00010009069	GRID2	-3.844029493	0.349879002	-10.98673963	4.42628E-28	3.47368E-27	negative
PRJNA729271	stage_1D_vs_E10	ordinary_muscle_development	ENSGALG00010009070	ENSGALG00010009070	-4.762464084	1.260065585	-3.77953667	0.00015712	0.000286041	negative
PRJNA729271	stage_1D_vs_E10	ordinary_muscle_development	ENSGALG00010009073	PRKAR2B	1.659706429	0.402421288	4.124300774	3.71863E-05	7.18687E-05	positive
PRJNA729271	stage_1D_vs_E10	ordinary_muscle_development	ENSGALG00010009076	TMEM214	-0.337443535	0.116852246	-2.887779612	0.003879716	0.006041737	negative
PRJNA729271	stage_1D_vs_E10	ordinary_muscle_development	ENSGALG00010009078	PTTG2	-1.916213369	0.16985141	-11.28170421	1.61585E-29	1.36252E-28	negative
PRJNA729271	stage_1D_vs_E10	ordinary_muscle_development	ENSGALG00010009079	KCTD15	-0.117390626	0.095515302	-1.229024281	0.219062702	0.261364217	negative
PRJNA729271	stage_1D_vs_E10	ordinary_muscle_development	ENSGALG00010009080	TLL1	-0.780715021	0.169861743	-4.59617925	4.30309E-06	9.07511E-06	negative
PRJNA729271	stage_1D_vs_E10	ordinary_muscle_development	ENSGALG00010009081	HBP1	1.112133738	0.121852502	9.126884689	7.04987E-20	3.74315E-19	positive
PRJNA729271	stage_1D_vs_E10	ordinary_muscle_development	ENSGALG00010009083	MED30	-0.455040835	0.129138931	-3.523653412	0.000425641	0.000738086	negative
PRJNA729271	stage_1D_vs_E10	ordinary_muscle_development	ENSGALG00010009085	PAF1	-0.191606285	0.088599379	-2.162614309	0.030570857	0.042126112	negative
PRJNA729271	stage_1D_vs_E10	ordinary_muscle_development	ENSGALG00010009086	UBE2D1	1.795082647	0.170467879	10.53032783	6.2616E-26	4.46443E-25	positive
PRJNA729271	stage_1D_vs_E10	ordinary_muscle_development	ENSGALG00010009088	ENSGALG00010009088	-0.513369021	0.130222289	-3.942251541	8.07203E-05	0.000151109	negative
PRJNA729271	stage_1D_vs_E10	ordinary_muscle_development	ENSGALG00010009089	AGBL5	-0.017437926	0.152071988	-0.114668892	0.908707572	0.922259925	negative
PRJNA729271	stage_1D_vs_E10	ordinary_muscle_development	ENSGALG00010009091	EXT1	-0.105498589	0.111343014	-0.947509732	0.343379099	0.39234432	negative
PRJNA729271	stage_1D_vs_E10	ordinary_muscle_development	ENSGALG00010009092	EED	-0.78186685	0.112086835	-6.975545782	3.04686E-12	1.02137E-11	negative
PRJNA729271	stage_1D_vs_E10	ordinary_muscle_development	ENSGALG00010009093	SLU7	0.63048962	0.131302765	4.801800005	1.57246E-06	3.4545E-06	positive
PRJNA729271	stage_1D_vs_E10	ordinary_muscle_development	ENSGALG00010009094	DARS	-0.615366606	0.095598172	-6.437012239	1.21848E-10	3.66886E-10	negative
PRJNA729271	stage_1D_vs_E10	ordinary_muscle_development	ENSGALG00010009096	ENSGALG00010009096	1.77798313	1.305958684	1.361439034	0.173374988	0.211058238	positive
PRJNA729271	stage_1D_vs_E10	ordinary_muscle_development	ENSGALG00010009097	C1QTNF2	0.34866668	0.181474133	1.921302357	0.0546936	0.072486398	positive
PRJNA729271	stage_1D_vs_E10	ordinary_muscle_development	ENSGALG00010009098	CAD	-0.852382703	0.197733843	-4.31075779	1.62696E-05	3.25011E-05	negative
PRJNA729271	stage_1D_vs_E10	ordinary_muscle_development	ENSGALG00010009099	CXCR4	-0.740576637	0.193292576	-3.831376513	0.000127428	0.000233984	negative
PRJNA729271	stage_1D_vs_E10	ordinary_muscle_development	ENSGALG00010009100	ENSGALG00010009100	-0.054524604	0.15300824	-0.356350766	0.721577895	0.756492955	negative
PRJNA729271	stage_1D_vs_E10	ordinary_muscle_development	ENSGALG00010009101	HIKESHI	-0.160309514	0.098341266	-1.630134732	0.103073024	0.130810167	negative
PRJNA729271	stage_1D_vs_E10	ordinary_muscle_development	ENSGALG00010009102	HTRA3	2.166294484	0.216154595	10.02196824	1.22048E-23	7.82434E-23	positive
PRJNA729271	stage_1D_vs_E10	ordinary_muscle_development	ENSGALG00010009103	AP3S1	-1.511296651	0.124032316	-12.18470076	3.75027E-34	3.91927E-33	negative
PRJNA729271	stage_1D_vs_E10	ordinary_muscle_development	ENSGALG00010009104	MTR	0.360317303	0.122634733	2.938134195	0.003301941	0.005182073	positive
PRJNA729271	stage_1D_vs_E10	ordinary_muscle_development	ENSGALG00010009106	THSD7B	-1.260345345	0.238120604	-5.29288656	1.20401E-07	2.89373E-07	negative
PRJNA729271	stage_1D_vs_E10	ordinary_muscle_development	ENSGALG00010009109	ENSGALG00010009109	-0.918802256	0.223649577	-4.108222637	3.98716E-05	7.68433E-05	negative
PRJNA729271	stage_1D_vs_E10	ordinary_muscle_development	ENSGALG00010009111	LRFN1	0.507578636	0.243668377	2.083071432	0.037244713	0.050667575	positive
PRJNA729271	stage_1D_vs_E10	ordinary_muscle_development	ENSGALG00010009112	ENSGALG00010009112	4.150523964	0.266396918	15.58022518	9.92047E-55	2.25387E-53	positive
PRJNA729271	stage_1D_vs_E10	ordinary_muscle_development	ENSGALG00010009113	RPLP1	0.040965516	0.176372224	0.232267391	0.816330338	0.841646508	positive
PRJNA729271	stage_1D_vs_E10	ordinary_muscle_development	ENSGALG00010009114	SETD7	0.561291663	0.150016943	3.741521804	0.000182909	0.000330418	positive
PRJNA729271	stage_1D_vs_E10	ordinary_muscle_development	ENSGALG00010009115	COG5	-0.302541882	0.114900133	-2.633085557	0.008461303	0.012625755	negative
PRJNA729271	stage_1D_vs_E10	ordinary_muscle_development	ENSGALG00010009116	S1PR3	-0.542784645	0.184068105	-2.948825086	0.003189845	0.005012662	negative
PRJNA729271	stage_1D_vs_E10	ordinary_muscle_development	ENSGALG00010009117	ERGIC1	-0.530938851	0.067137053	-7.908283577	2.60962E-15	1.06673E-14	negative
PRJNA729271	stage_1D_vs_E10	ordinary_muscle_development	ENSGALG00010009118	ACTN2	4.128045519	0.143905134	28.68588078	5.7237E-181	1.9294E-178	positive
PRJNA729271	stage_1D_vs_E10	ordinary_muscle_development	ENSGALG00010009119	CISD1	0.922740831	0.153018446	6.030258791	1.63697E-09	4.55081E-09	positive
PRJNA729271	stage_1D_vs_E10	ordinary_muscle_development	ENSGALG00010009120	ATG12	0.22497072	0.088359164	2.546093793	0.010893591	0.016033977	positive
PRJNA729271	stage_1D_vs_E10	ordinary_muscle_development	ENSGALG00010009121	SYCN	-0.24859402	0.78856527	-0.315248501	0.752572983	0.784223248	negative
PRJNA729271	stage_1D_vs_E10	ordinary_muscle_development	ENSGALG00010009123	ENSGALG00010009123	-1.067707947	0.133442652	-8.00124948	1.23163E-15	5.14036E-15	negative
PRJNA729271	stage_1D_vs_E10	ordinary_muscle_development	ENSGALG00010009125	CD72L1	4.732898289	0.958741921	4.936571756	7.95078E-07	1.7901E-06	positive
PRJNA729271	stage_1D_vs_E10	ordinary_muscle_development	ENSGALG00010009126	JPH3	-1.605191308	0.143914109	-11.15381472	6.86009E-29	5.63383E-28	negative
PRJNA729271	stage_1D_vs_E10	ordinary_muscle_development	ENSGALG00010009127	SCOC	-0.817940807	0.075752412	-10.79755469	3.53493E-27	2.6673E-26	negative
PRJNA729271	stage_1D_vs_E10	ordinary_muscle_development	ENSGALG00010009129	ENSGALG00010009129	0.920550507	0.377461204	2.438795027	0.014736324	0.021264611	positive
PRJNA729271	stage_1D_vs_E10	ordinary_muscle_development	ENSGALG00010009130	HEATR1	-0.79810581	0.072997925	-10.93326709	7.99182E-28	6.21134E-27	negative
PRJNA729271	stage_1D_vs_E10	ordinary_muscle_development	ENSGALG00010009131	CDKL2	-2.992646146	0.318330119	-9.401077597	5.40071E-21	3.05436E-20	negative
PRJNA729271	stage_1D_vs_E10	ordinary_muscle_development	ENSGALG00010009133	BOD1L1	0.595140764	0.128964819	4.614752822	3.93563E-06	8.34265E-06	positive
PRJNA729271	stage_1D_vs_E10	ordinary_muscle_development	ENSGALG00010009135	TENM4	-1.70529117	0.185466014	-9.194628869	3.76291E-20	2.02764E-19	negative
PRJNA729271	stage_1D_vs_E10	ordinary_muscle_development	ENSGALG00010009136	KHK	-0.557335022	0.390265082	-1.428093489	0.153264951	0.188556737	negative
PRJNA729271	stage_1D_vs_E10	ordinary_muscle_development	ENSGALG00010009137	IPMK	-0.072630026	0.092030317	-0.789196743	0.429997026	0.479557351	negative
PRJNA729271	stage_1D_vs_E10	ordinary_muscle_development	ENSGALG00010009138	ENSGALG00010009138	0.569324742	0.102975387	5.528745809	3.22528E-08	8.06877E-08	positive
PRJNA729271	stage_1D_vs_E10	ordinary_muscle_development	ENSGALG00010009139	CKS2	-2.168941407	0.289090521	-7.502637586	6.25463E-14	2.34266E-13	negative
PRJNA729271	stage_1D_vs_E10	ordinary_muscle_development	ENSGALG00010009141	MAML3	0.636441892	0.114794047	5.544206405	2.9529E-08	7.41038E-08	positive
PRJNA729271	stage_1D_vs_E10	ordinary_muscle_development	ENSGALG00010009142	LGALS8	-0.755082866	0.135602587	-5.568351468	2.57161E-08	6.49172E-08	negative
PRJNA729271	stage_1D_vs_E10	ordinary_muscle_development	ENSGALG00010009143	MCCC2	1.21638596	0.134544719	9.040755866	1.55592E-19	8.10388E-19	positive
PRJNA729271	stage_1D_vs_E10	ordinary_muscle_development	ENSGALG00010009144	CREBZF	0.59066489	0.152023092	3.885362968	0.000102177	0.000189345	positive
PRJNA729271	stage_1D_vs_E10	ordinary_muscle_development	ENSGALG00010009147	HNMT	4.696695363	0.226288425	20.75534958	1.09682E-95	7.46402E-94	positive
PRJNA729271	stage_1D_vs_E10	ordinary_muscle_development	ENSGALG00010009149	FUT4	-1.567861196	0.211928622	-7.398062519	1.38186E-13	5.06192E-13	negative
PRJNA729271	stage_1D_vs_E10	ordinary_muscle_development	ENSGALG00010009150	ENSGALG00010009150	0.188983224	0.395761161	0.47751837	0.632993043	0.674401629	positive
PRJNA729271	stage_1D_vs_E10	ordinary_muscle_development	ENSGALG00010009151	CCNJL	0.739707171	0.357793848	2.067411652	0.038695381	0.052473529	positive
PRJNA729271	stage_1D_vs_E10	ordinary_muscle_development	ENSGALG00010009152	RPP25L	0.186104476	0.123103938	1.511767041	0.130593137	0.162792184	positive
PRJNA729271	stage_1D_vs_E10	ordinary_muscle_development	ENSGALG00010009153	SECISBP2	0.210917989	0.124110661	1.699434898	0.089237271	0.114387535	positive
PRJNA729271	stage_1D_vs_E10	ordinary_muscle_development	ENSGALG00010009155	PAK4	-0.144348078	0.219453515	-0.657761523	0.510691383	0.558550637	negative
PRJNA729271	stage_1D_vs_E10	ordinary_muscle_development	ENSGALG00010009157	ABHD1	0.821051228	0.137518358	5.970484519	2.3655E-09	6.49762E-09	positive
PRJNA729271	stage_1D_vs_E10	ordinary_muscle_development	ENSGALG00010009159	PTK2	-0.051136989	0.098935232	-0.516873398	0.605244539	0.648029221	negative
PRJNA729271	stage_1D_vs_E10	ordinary_muscle_development	ENSGALG00010009160	ENSGALG00010009160	-1.38630456	0.391458911	-3.541379495	0.000398041	0.000693045	negative
PRJNA729271	stage_1D_vs_E10	ordinary_muscle_development	ENSGALG00010009163	SAMD12	-0.811242079	0.178431303	-4.546523311	5.45393E-06	1.14109E-05	negative
PRJNA729271	stage_1D_vs_E10	ordinary_muscle_development	ENSGALG00010009164	TMEM128	0.128099516	0.100937846	1.269093025	0.204407888	0.245029138	positive
PRJNA729271	stage_1D_vs_E10	ordinary_muscle_development	ENSGALG00010009166	CLGN	-1.014921707	0.23348383	-4.346860791	1.381E-05	2.7806E-05	negative
PRJNA729271	stage_1D_vs_E10	ordinary_muscle_development	ENSGALG00010009167	DCTN3	-0.565576989	0.111080022	-5.091617525	3.55022E-07	8.19825E-07	negative
PRJNA729271	stage_1D_vs_E10	ordinary_muscle_development	ENSGALG00010009168	MRPS12	0.258942443	0.175236625	1.477673079	0.139495303	0.172893067	positive
PRJNA729271	stage_1D_vs_E10	ordinary_muscle_development	ENSGALG00010009170	RPL26L1	0.340907899	0.19128168	1.78222974	0.074711775	0.097003509	positive
PRJNA729271	stage_1D_vs_E10	ordinary_muscle_development	ENSGALG00010009172	SPOPL	0.710033639	0.131542666	5.397744018	6.74841E-08	1.65289E-07	positive
PRJNA729271	stage_1D_vs_E10	ordinary_muscle_development	ENSGALG00010009173	LYAR	-0.334211107	0.142084762	-2.352195281	0.018662974	0.026500765	negative
PRJNA729271	stage_1D_vs_E10	ordinary_muscle_development	ENSGALG00010009178	ENSGALG00010009178	0.510873315	0.25286938	2.020305166	0.043351743	0.058378253	positive
PRJNA729271	stage_1D_vs_E10	ordinary_muscle_development	ENSGALG00010009180	NFKBIB	0.586907149	0.146507725	4.0059809	6.17606E-05	0.000117133	positive
PRJNA729271	stage_1D_vs_E10	ordinary_muscle_development	ENSGALG00010009181	ARID3C	-4.857324915	0.770336932	-6.305455074	2.87348E-10	8.43824E-10	negative
PRJNA729271	stage_1D_vs_E10	ordinary_muscle_development	ENSGALG00010009186	PREB	-0.204707378	0.097412081	-2.10145781	0.035600798	0.048590731	negative
PRJNA729271	stage_1D_vs_E10	ordinary_muscle_development	ENSGALG00010009188	ENSGALG00010009188	-0.240595027	0.42203223	-0.570086856	0.568618789	0.613847423	negative
PRJNA729271	stage_1D_vs_E10	ordinary_muscle_development	ENSGALG00010009189	ENSGALG00010009189	-0.819723023	0.219515801	-3.734232432	0.000188289	0.000339416	negative
PRJNA729271	stage_1D_vs_E10	ordinary_muscle_development	ENSGALG00010009190	ZBTB49	0.58554801	0.136733639	4.282399094	1.84889E-05	3.6727E-05	positive
PRJNA729271	stage_1D_vs_E10	ordinary_muscle_development	ENSGALG00010009192	LSM14A	-0.042728987	0.059126573	-0.722669776	0.469882789	0.518574008	negative
PRJNA729271	stage_1D_vs_E10	ordinary_muscle_development	ENSGALG00010009193	EDARADD	-2.147959013	0.228637238	-9.394615835	5.7427E-21	3.24145E-20	negative
PRJNA729271	stage_1D_vs_E10	ordinary_muscle_development	ENSGALG00010009195	ENSGALG00010009195	-0.487875003	0.929592164	-0.524826931	0.599703508	0.64266615	negative
PRJNA729271	stage_1D_vs_E10	ordinary_muscle_development	ENSGALG00010009197	FH	1.175021964	0.102240361	11.49274074	1.43486E-30	1.27754E-29	positive
PRJNA729271	stage_1D_vs_E10	ordinary_muscle_development	ENSGALG00010009198	KCNN2	0.362863131	0.170984287	2.122201624	0.03382081	0.046327031	positive
PRJNA729271	stage_1D_vs_E10	ordinary_muscle_development	ENSGALG00010009199	SEMA4D	-2.290984905	0.151453735	-15.12663198	1.08087E-51	2.20977E-50	negative
PRJNA729271	stage_1D_vs_E10	ordinary_muscle_development	ENSGALG00010009200	ADGRA3	-1.386372164	0.091084631	-15.2207036	2.57744E-52	5.41449E-51	negative
PRJNA729271	stage_1D_vs_E10	ordinary_muscle_development	ENSGALG00010009202	FABP6	0.210494571	0.298042385	0.706257169	0.480028224	0.528563438	positive
PRJNA729271	stage_1D_vs_E10	ordinary_muscle_development	ENSGALG00010009205	SIRT2	-0.179454754	0.09848658	-1.822123919	0.068436185	0.089351694	negative
PRJNA729271	stage_1D_vs_E10	ordinary_muscle_development	ENSGALG00010009208	SLC5A6	0.51767935	0.189207468	2.736040789	0.006218332	0.009447093	positive
PRJNA729271	stage_1D_vs_E10	ordinary_muscle_development	ENSGALG00010009209	SLC6A19	4.140528717	1.734718767	2.386858778	0.016993017	0.024296093	positive

PRJNA729271	stage_1D_vs_E10	ordinary_muscle_development	ENSGALG00010009210	PCDH15	-1.93719202	0.293512351	-6.600035795	4.11059E-11	1.2786E-10	negative
PRJNA729271	stage_1D_vs_E10	ordinary_muscle_development	ENSGALG00010009212	NXPH2	0.283650005	0.253984496	1.116800472	0.264079647	0.309220008	positive
PRJNA729271	stage_1D_vs_E10	ordinary_muscle_development	ENSGALG00010009213	DUSAL	-0.826856366	0.092514568	-8.937580167	3.97785E-19	2.02383E-18	negative
PRJNA729271	stage_1D_vs_E10	ordinary_muscle_development	ENSGALG00010009214	PCDH10	-4.438822184	0.263791876	-16.82698593	1.54783E-63	4.6839E-62	negative
PRJNA729271	stage_1D_vs_E10	ordinary_muscle_development	ENSGALG00010009216	NSG1	-2.530450056	0.213214365	-11.86810305	1.73354E-32	1.68869E-31	negative
PRJNA729271	stage_1D_vs_E10	ordinary_muscle_development	ENSGALG00010009219	TNFRSF11B	-0.441755414	0.183400425	-2.408693512	0.016009736	0.022967253	negative
PRJNA729271	stage_1D_vs_E10	ordinary_muscle_development	ENSGALG00010009220	GADD45G	0.758573979	0.189851612	3.995615167	6.45265E-05	0.000122135	positive
PRJNA729271	stage_1D_vs_E10	ordinary_muscle_development	ENSGALG00010009223	ENSGALG00010009223	0.068032654	0.108640775	0.626216575	0.531172897	0.578072764	positive
PRJNA729271	stage_1D_vs_E10	ordinary_muscle_development	ENSGALG00010009224	ENSGALG00010009224	4.960053293	0.446821237	11.10075546	1.24388E-28	1.00614E-27	positive
PRJNA729271	stage_1D_vs_E10	ordinary_muscle_development	ENSGALG00010009225	HNRNPL	-0.585539144	0.124991137	-4.684645297	2.80445E-06	6.03392E-06	negative
PRJNA729271	stage_1D_vs_E10	ordinary_muscle_development	ENSGALG00010009227	ENSGALG00010009227	0.247507899	0.173174675	1.429238425	0.152935717	0.188199611	positive
PRJNA729271	stage_1D_vs_E10	ordinary_muscle_development	ENSGALG00010009228	ATP6V0E1	0.401076141	0.09984126	4.017138199	5.89092E-05	0.000111897	positive
PRJNA729271	stage_1D_vs_E10	ordinary_muscle_development	ENSGALG00010009232	ECH1	1.374705245	0.163282933	8.419160654	3.79191E-17	1.7306E-16	positive
PRJNA729271	stage_1D_vs_E10	ordinary_muscle_development	ENSGALG00010009234	PCDH18	-0.422665436	0.127483571	-3.315450248	0.000914956	0.001532504	negative
PRJNA729271	stage_1D_vs_E10	ordinary_muscle_development	ENSGALG00010009235	KLHDC4	-0.517630195	0.131263762	-3.943435628	8.03226E-05	0.000150423	negative
PRJNA729271	stage_1D_vs_E10	ordinary_muscle_development	ENSGALG00010009236	DTWD2	-0.258169848	0.115774418	-2.22993864	0.025751517	0.035853255	negative
PRJNA729271	stage_1D_vs_E10	ordinary_muscle_development	ENSGALG00010009237	DENND3	-0.794077346	0.177716608	-4.468222496	7.88722E-06	1.62764E-05	negative
PRJNA729271	stage_1D_vs_E10	ordinary_muscle_development	ENSGALG00010009239	STX18	0.047607801	0.082021636	0.580429794	0.561624818	0.607156305	positive
PRJNA729271	stage_1D_vs_E10	ordinary_muscle_development	ENSGALG00010009243	BCAP29	-0.707893162	0.100336718	-7.055175584	1.72383E-12	5.86685E-12	negative
PRJNA729271	stage_1D_vs_E10	ordinary_muscle_development	ENSGALG00010009246	RUSC2	3.046727491	0.141144782	21.58583155	2.4406E-103	1.8718E-101	positive
PRJNA729271	stage_1D_vs_E10	ordinary_muscle_development	ENSGALG00010009250	ERO1B	-0.303508966	0.108451026	-2.798580851	0.005132772	0.007880509	negative
PRJNA729271	stage_1D_vs_E10	ordinary_muscle_development	ENSGALG00010009252	SIRT1	-0.182503387	0.096419481	-1.89280615	0.058383653	0.076961719	negative
PRJNA729271	stage_1D_vs_E10	ordinary_muscle_development	ENSGALG00010009253	SLC45A4	-0.481771854	0.141252429	-3.410715524	0.000647927	0.001101019	negative
PRJNA729271	stage_1D_vs_E10	ordinary_muscle_development	ENSGALG00010009254	SLC26A4	1.921830373	0.649270594	2.959983698	0.003076553	0.004843032	positive
PRJNA729271	stage_1D_vs_E10	ordinary_muscle_development	ENSGALG00010009256	CREBRF	1.589716048	0.17276219	9.201759057	3.52137E-20	1.90102E-19	positive
PRJNA729271	stage_1D_vs_E10	ordinary_muscle_development	ENSGALG00010009257	ENSGALG00010009257	3.594683934	0.185841547	19.34273577	2.34688E-83	1.20205E-81	positive
PRJNA729271	stage_1D_vs_E10	ordinary_muscle_development	ENSGALG00010009258	DNAJC12	-0.300032226	0.167783454	-1.788211054	0.073741963	0.095830174	negative
PRJNA729271	stage_1D_vs_E10	ordinary_muscle_development	ENSGALG00010009259	MGAT4D	1.268584562	0.808090833	1.569853921	0.1164491	0.146445402	positive
PRJNA729271	stage_1D_vs_E10	ordinary_muscle_development	ENSGALG00010009263	COLEC10	-0.708259669	0.222605659	-3.181678638	0.001464242	0.002396318	negative
PRJNA729271	stage_1D_vs_E10	ordinary_muscle_development	ENSGALG00010009266	PCDH7	-2.211875087	0.242805363	-9.109663218	8.26381E-20	4.37647E-19	negative
PRJNA729271	stage_1D_vs_E10	ordinary_muscle_development	ENSGALG00010009268	PWWP2A	0.436153073	0.130916994	3.331523737	0.000863719	0.001449868	positive
PRJNA729271	stage_1D_vs_E10	ordinary_muscle_development	ENSGALG00010009269	ENSGALG00010009269	-3.227083318	0.456331176	-7.071801119	1.52936E-12	5.22091E-12	negative
PRJNA729271	stage_1D_vs_E10	ordinary_muscle_development	ENSGALG00010009273	MAL2	-0.963581494	0.49313421	-1.95399442	0.050701877	0.0675729	negative
PRJNA729271	stage_1D_vs_E10	ordinary_muscle_development	ENSGALG00010009275	CBLL1	0.697465813	0.137764024	5.062757247	4.13236E-07	9.49715E-07	positive
PRJNA729271	stage_1D_vs_E10	ordinary_muscle_development	ENSGALG00010009276	ENSGALG00010009276	0.043993078	0.309465296	0.142158358	0.886954923	0.903599354	positive
PRJNA729271	stage_1D_vs_E10	ordinary_muscle_development	ENSGALG00010009277	ACTN4	-0.098032391	0.128145069	-0.765011028	0.444264978	0.493495352	negative
PRJNA729271	stage_1D_vs_E10	ordinary_muscle_development	ENSGALG00010009279	ELMOD2	-1.287614325	0.112880603	-11.40686962	3.86386E-30	3.3678E-29	negative
PRJNA729271	stage_1D_vs_E10	ordinary_muscle_development	ENSGALG00010009280	HSD17B4	0.135854783	0.083069309	1.635438946	0.101957064	0.129523895	positive
PRJNA729271	stage_1D_vs_E10	ordinary_muscle_development	ENSGALG00010009284	CCN3	-0.96992289	0.674786677	-1.437377059	0.150610884	0.185669736	negative
PRJNA729271	stage_1D_vs_E10	ordinary_muscle_development	ENSGALG00010009286	ENSGALG00010009286	0.467077491	0.182032086	2.565907488	0.010290627	0.015180285	positive
PRJNA729271	stage_1D_vs_E10	ordinary_muscle_development	ENSGALG00010009289	GPR137B	-0.60791577	0.149727865	-4.060137853	4.90438E-05	9.37725E-05	negative
PRJNA729271	stage_1D_vs_E10	ordinary_muscle_development	ENSGALG00010009294	ACOX3	0.274907733	0.14047793	1.956946063	0.05035381	0.067152312	positive
PRJNA729271	stage_1D_vs_E10	ordinary_muscle_development	ENSGALG00010009297	CTNNA3	5.087470838	0.203794816	24.96369113	1.5165E-137	2.2204E-135	positive
PRJNA729271	stage_1D_vs_E10	ordinary_muscle_development	ENSGALG00010009299	MAP4K1	2.054290672	0.300553536	6.835024144	8.19912E-12	2.66531E-11	positive
PRJNA729271	stage_1D_vs_E10	ordinary_muscle_development	ENSGALG00010009303	TTC1	-0.252964028	0.096952566	-2.609152485	0.009076679	0.013492612	negative
PRJNA729271	stage_1D_vs_E10	ordinary_muscle_development	ENSGALG00010009306	TBC1D9	-1.280629624	0.107868381	-11.87215022	1.65167E-32	1.6111E-31	negative
PRJNA729271	stage_1D_vs_E10	ordinary_muscle_development	ENSGALG00010009307	DIRAS2	3.275508057	0.310784522	10.53948258	5.68106E-26	4.0585E-25	positive
PRJNA729271	stage_1D_vs_E10	ordinary_muscle_development	ENSGALG00010009309	TRIM54	2.129596064	0.149099083	14.28309293	2.78941E-46	4.59461E-45	positive
PRJNA729271	stage_1D_vs_E10	ordinary_muscle_development	ENSGALG00010009311	BNIP1	-0.08218689	0.110977941	-0.740569609	0.45895444	0.507866602	negative
PRJNA729271	stage_1D_vs_E10	ordinary_muscle_development	ENSGALG00010009313	UNC13B	0.248358246	0.175921412	1.411756782	0.158021588	0.193931328	positive
PRJNA729271	stage_1D_vs_E10	ordinary_muscle_development	ENSGALG00010009317	LRRTM3	-2.237024827	0.478549764	-4.674591849	2.94539E-06	6.32401E-06	negative
PRJNA729271	stage_1D_vs_E10	ordinary_muscle_development	ENSGALG00010009320	GPR20	0.808096604	0.206131597	3.920294682	8.84408E-05	0.000164987	positive
PRJNA729271	stage_1D_vs_E10	ordinary_muscle_development	ENSGALG00010009322	ENSGALG00010009322	1.526908259	0.168405272	9.066867337	1.22488E-19	6.42585E-19	positive
PRJNA729271	stage_1D_vs_E10	ordinary_muscle_development	ENSGALG00010009323	RNF150	-0.245924801	0.108387392	-2.268942875	0.0232718	0.032658025	negative
PRJNA729271	stage_1D_vs_E10	ordinary_muscle_development	ENSGALG00010009326	DLD	0.692957133	0.131725566	5.260612298	1.43576E-07	3.42857E-07	positive
PRJNA729271	stage_1D_vs_E10	ordinary_muscle_development	ENSGALG00010009327	NID1	2.703392142	0.16304468	16.5806829	9.61302E-62	2.79801E-60	positive
PRJNA729271	stage_1D_vs_E10	ordinary_muscle_development	ENSGALG00010009328	ENSGALG00010009328	-1.151236974	0.132841273	-8.666259733	4.46548E-18	2.1504E-17	negative
PRJNA729271	stage_1D_vs_E10	ordinary_muscle_development	ENSGALG00010009330	EIF3K	0.226979185	0.141496455	1.604133364	0.108684659	0.137276415	positive
PRJNA729271	stage_1D_vs_E10	ordinary_muscle_development	ENSGALG00010009332	ENSGALG00010009332	0.37944408	0.163019468	2.327599798	0.019933364	0.028207958	positive
PRJNA729271	stage_1D_vs_E10	ordinary_muscle_development	ENSGALG00010009333	ADRA1B	1.884011177	0.315256135	5.976128503	2.28503E-09	6.2873E-09	positive
PRJNA729271	stage_1D_vs_E10	ordinary_muscle_development	ENSGALG00010009334	SS18L2	-0.031028149	0.166414007	-0.186451547	0.852090673	0.873483331	negative
PRJNA729271	stage_1D_vs_E10	ordinary_muscle_development	ENSGALG00010009335	PTP4A3	1.392230647	0.195240441	7.130851782	9.97497E-13	3.45159E-12	positive
PRJNA729271	stage_1D_vs_E10	ordinary_muscle_development	ENSGALG00010009336	LAMB1	1.647099156	0.109858383	14.99293104	8.16681E-51	1.62607E-49	positive
PRJNA729271	stage_1D_vs_E10	ordinary_muscle_development	ENSGALG00010009337	ZNF330	-0.061229107	0.100274488	-0.610615012	0.541454482	0.587629733	negative
PRJNA729271	stage_1D_vs_E10	ordinary_muscle_development	ENSGALG00010009338	KYNU	-0.090850815	0.566862528	-0.160269573	0.872668727	0.891488702	negative
PRJNA729271	stage_1D_vs_E10	ordinary_muscle_development	ENSGALG00010009340	SYK	0.682112017	0.140923303	4.840306772	1.29639E-06	2.86756E-06	positive
PRJNA729271	stage_1D_vs_E10	ordinary_muscle_development	ENSGALG00010009344	ENPP2	-0.2434305	0.257378109	-0.945808879	0.344246076	0.393148982	negative
PRJNA729271	stage_1D_vs_E10	ordinary_muscle_development	ENSGALG00010009345	ZFAT	0.48379317	0.124109508	3.898115286	9.69443E-05	0.000180062	positive
PRJNA729271	stage_1D_vs_E10	ordinary_muscle_development	ENSGALG00010009346	STC2	1.913090785	0.306632116	6.239042447	4.40258E-10	1.27503E-09	positive
PRJNA729271	stage_1D_vs_E10	ordinary_muscle_development	ENSGALG00010009347	TESK1	2.411902706	0.141467508	17.04916378	3.5457E-65	1.13205E-63	positive
PRJNA729271	stage_1D_vs_E10	ordinary_muscle_development	ENSGALG00010009348	ERGIC2	-0.426953292	0.082584742	-5.169881022	2.34243E-07	5.49232E-07	negative
PRJNA729271	stage_1D_vs_E10	ordinary_muscle_development	ENSGALG00010009351	GTF3C2	0.232033647	0.121815743	1.904791949	0.056807121	0.075054162	positive
PRJNA729271	stage_1D_vs_E10	ordinary_muscle_development	ENSGALG00010009352	HSF1	1.114656889	0.178695912	6.237730199	4.43966E-10	1.28551E-09	positive
PRJNA729271	stage_1D_vs_E10	ordinary_muscle_development	ENSGALG00010009355	GARRE1	0.574755467	0.071629761	8.023975792	1.02376E-15	4.29756E-15	positive
PRJNA729271	stage_1D_vs_E10	ordinary_muscle_development	ENSGALG00010009357	LYST	0.408145913	0.11618698	3.512836926	0.000443349	0.000767142	positive
PRJNA729271	stage_1D_vs_E10	ordinary_muscle_development	ENSGALG00010009359	REEP3	-0.879915825	0.110720947	-7.94714868	1.90853E-15	7.87705E-15	negative
PRJNA729271	stage_1D_vs_E10	ordinary_muscle_development	ENSGALG00010009360	TNFAIP8	-0.242722459	0.128771765	-1.884904346	0.059442753	0.078286635	negative
PRJNA729271	stage_1D_vs_E10	ordinary_muscle_development	ENSGALG00010009361	LRRK2	1.2287154	0.086869861	14.14432331	2.02455E-45	3.22836E-44	positive
PRJNA729271	stage_1D_vs_E10	ordinary_muscle_development	ENSGALG00010009362	IL15	3.443879162	0.310846329	11.07904079	1.58562E-28	1.27829E-27	positive
PRJNA729271	stage_1D_vs_E10	ordinary_muscle_development	ENSGALG00010009363	TAF2	-0.19392638	0.077057807	-2.516635067	0.011848148	0.017350869	negative
PRJNA729271	stage_1D_vs_E10	ordinary_muscle_development	ENSGALG00010009365	CA5B	0.395658515	0.160807658	2.460445721	0.013876456	0.020103871	positive
PRJNA729271	stage_1D_vs_E10	ordinary_muscle_development	ENSGALG00010009367	DMXL1	0.884275816	0.119022751	7.429468816	1.09035E-13	4.02767E-13	positive
PRJNA729271	stage_1D_vs_E10	ordinary_muscle_development	ENSGALG00010009368	DSCC1	-1.776962486	0.196276832	-9.053348127	1.3865E-19	7.23969E-19	negative
PRJNA729271	stage_1D_vs_E10	ordinary_muscle_development	ENSGALG00010009370	ENSGALG00010009370	1.930776357	0.450148342	4.289200201	1.79318E-05	3.56642E-05	positive
PRJNA729271	stage_1D_vs_E10	ordinary_muscle_development	ENSGALG00010009371	PHTF2	2.666951583	0.154283802	17.2860116	5.99601E-67	1.9843E-65	positive
PRJNA729271	stage_1D_vs_E10	ordinary_muscle_development	ENSGALG00010009372	RAB28	-0.220161534	0.097127139	-2.266735495	0.023406389	0.032824652	negative
PRJNA729271	stage_1D_vs_E10	ordinary_muscle_development	ENSGALG00010009373	ARHGAP15	-1.342086768	0.226588766	-5.923006658	3.16108E-09	8.5966E-09	negative
PRJNA729271	stage_1D_vs_E10	ordinary_muscle_development	ENSGALG00010009376	CDO1	0.173657764	0.249590275	0.695771355	0.486572031	0.535077884	positive
PRJNA729271	stage_1D_vs_E10	ordinary_muscle_development	ENSGALG00010009378	ENSGALG00010009378	-0.997553644	0.083706589	-11.91726552	9.62148E-33	9.48731E-32	negative
PRJNA729271	stage_1D_vs_E10	ordinary_muscle_development	ENSGALG00010009379	RYR1	4.306931677	0.219471907	19.62406826	9.63269E-86	5.19055E-84	positive
PRJNA729271	stage_1D_vs_E10	ordinary_muscle_development	ENSGALG00010009381	INPP4B	-1.082630758	0.115406758	-9.380999688	6.53501E-21	3.67721E-20	negative
PRJNA729271	stage_1D_vs_E10	ordinary_muscle_development	ENSGALG00010009383	ASXL2	0.944080883	0.110887137	8.513889978	1.68194E-17	7.83408E-17	positive
PRJNA729271	stage_1D_vs_E10	ordinary_muscle_development	ENSGALG00010009385	GPI	0.663271984	0.178024273	3.725739033	0.000194744	0.000350181	positive
PRJNA729271	stage_1D_vs_E10	ordinary_muscle_development	ENSGALG00010009386	TMEM60	-0.057543234	0.111651817	-0.515381075	0.606286755	0.649028776	negative
PRJNA729271	stage_1D_vs_E10	ordinary_muscle_development	ENSGALG00010009387	DEPTOR	3.044330093	0.138142929	22.03753834	1.2579E-107	1.0419E-105	positive
PRJNA729271	stage_1D_vs_E10	ordinary_muscle_development	ENSGALG00010009388	TMED7	-0.188475482	0.073467556	-2.56542469	0.010304959	0.015198451	negative
PRJNA729271	stage_1D_vs_E10	ordinary_muscle_development	ENSGALG00010009389	CPEB4	2.335927196	0.124711368	18.73066777	2.78402E-78	1.27704E-76	positive
PRJNA729271	stage_1D_vs_E10	ordinary_muscle_development	ENSGALG00010009391	RSBN1L	0.839349786	0.105415952	7.962265356	1.68918E-15	6.99761E-15	positive
PRJNA729271	stage_1D_vs_E10	ordinary_muscle_development	ENSGALG00010009392	MAPRE3	0.400133644	0.107048161	3.737884327	0.000185575	0.0003349	positive
PRJNA729271	stage_1D_vs_E10	ordinary_muscle_development	ENSGALG00010009393	MARVELD2	-0.403768972	0.131107423	-3.079680487	0.002072228	0.003327824	negative
PRJNA729271	stage_1D_vs_E10	ordinary_muscle_development	ENSGALG00010009394	BANP	0.355145424	0.091247219	3.892123245	9.93707E-05	0.000184286	positive
PRJNA729271	stage_1D_vs_E10	ordinary_muscle_development	ENSGALG00010009396	AUH	1.150638235	0.110970605	10.36885607	3.43613E-25	2.38424E-24	positive
PRJNA729271	stage_1D_vs_E10	ordinary_muscle_development	ENSGALG00010009398	WT1P	0.238888249	0.137790433	1.733707072	0.082970071	0.10694986	positive
PRJNA729271	stage_1D_vs_E10	ordinary_muscle_development	ENSGALG00010009400	CNTFR	0.805459566	0.281083622	2.865551396	0.004162837	0.006459728	positive
PRJNA729271	stage_1D_vs_E10	ordinary_muscle_development	ENSGALG00010009404	GNG4	-1.178428409	0.405069023	-2.909203967	0.003623504	0.005660382	negative
PRJNA729271	stage_1D_vs_E10	ordinary_muscle_development	ENSGALG00010009407	PTPN12	0.21197388	0.086489775	2.450854809	0.014251743	0.020616668	positive
PRJNA729271	stage_1D_vs_E10	ordinary_muscle_development	ENSGALG00010009408	ENSGALG00010009408	-0.187404241	0.090495988	-2.070856901	0.038372171	0.052064459	negative
PRJNA729271	stage_1D_vs_E10	ordinary_muscle_development	ENSGALG00010009410	JMJD1C	0.535334671	0.107839722	4.964169613	6.89956E-07	1.56021E-06	positive
PRJNA729271	stage_1D_vs_E10	ordinary_muscle_development	ENSGALG00010009411	SMARCA5	-1.462236387	0.097083681	-15.06160838	2.89654E-51	5.8232E-50	negative
PRJNA729271	stage_1D_vs_E10	ordinary_muscle_development	ENSGALG00010009415	HMP19	-3.964080843	0.432631091	-9.16272762	5.06018E-20	2.71355E-19	negative
PRJNA729271	stage_1D_vs_E10	ordinary_muscle_development	ENSGALG00010009416	COL14A1	-2.220094914	0.47356454	-4.688051425	2.75819E-06	5.93702E-06	negative
PRJNA729271	stage_1D_vs_E10	ordinary_muscle_development	ENSGALG00010009424	ENSGALG00010009424	5.099933217	0.986619232	5.169099744	2.35224E-07	5.51355E-07	positive
PRJNA729271	stage_1D_vs_E10	ordinary_muscle_development	ENSGALG00010009431	SMN	-0.112583412	0.149614314	-0.752490912	0.451755893	0.50085679	negative
PRJNA729271	stage_1D_vs_E10	ordinary_muscle_development	ENSGALG00010009432	BOP1	0.253617686	0.177390169	1.429716693	0.152798347	0.188046531	positive
PRJNA729271	stage_1D_vs_E10	ordinary_muscle_development	ENSGALG00010009435	GTDC1	-0.45726515	0.089595199	-5.103679186	3.33113E-07	7.70952E-07	negative
PRJNA729271	stage_1D_vs_E10	ordinary_muscle_development	ENSGALG00010009437	DPYSL5	-4.567694695	0.22935101	-19.91573829	2.97239E-88	1.71652E-86	negative
PRJNA729271	stage_1D_vs_E10	ordinary_muscle_development	ENSGALG00010009439	B3GALNT2	0.369387362	0.098788362	3.739178928	0.000184622	0.000333322	positive
PRJNA729271	stage_1D_vs_E10	ordinary_muscle_development	ENSGALG00010009440	DOK3	-0.184081342	0.212995864	-0.864248433	0.387451431	0.436507733	negative
PRJNA729271	stage_1D_vs_E10	ordinary_muscle_development	ENSGALG00010009441	ZNF518B	-0.729261599	0.098495499	-7.404009421	1.32133E-13	4.84633E-13	negative
PRJNA729271	stage_1D_vs_E10	ordinary_muscle_development	ENSGALG00010009444	NFIL3	2.494583484	0.221182725	11.27838299	1.67802E-29	1.41412E-28	positive
PRJNA729271	stage_1D_vs_E10	ordinary_muscle_development	ENSGALG00010009446	ENSGALG00010009446	0.007766533	0.249774873	0.031094131	0.97519447	0.978908854	positive
PRJNA729271	stage_1D_vs_E10	ordinary_muscle_development	ENSGALG00010009447	DDX41	-0.294982426	0.105495826	-2.79615258	0.005171498	0.007932366	negative
PRJNA729271	stage_1D_vs_E10	ordinary_muscle_development	ENSGALG00010009451	ROR2	-0.866395262	0.137781652	-6.288175867	3.21218E-10	9.40047E-10	negative
PRJNA729271	stage_1D_vs_E10	ordinary_muscle_development	ENSGALG00010009452	TBCE	-0.771689562	0.088341904	-8.73526068	2.43095E-18	1.18522E-17	negative
PRJNA729271	stage_1D_vs_E10	ordinary_muscle_development	ENSGALG00010009453	GSAP	0.317211048	0.113544239	2.793722076	0.005210524	0.007990536	positive
PRJNA729271	stage_1D_vs_E10	ordinary_muscle_development	ENSGALG00010009457	BDP1	0.122506727	0.12949131	0.94606138	0.34411728	0.393094805	positive
PRJNA729271	stage_1D_vs_E10	ordinary_muscle_development	ENSGALG00010009458	SCX	-2.992177025	0.448206151	-6.6758946	2.45729E-11	7.74817E-11	negative
PRJNA729271	stage_1D_vs_E10	ordinary_muscle_development	ENSGALG00010009461	ZEB2	0.371956963	0.125441395	2.965185158	0.003025008	0.004767586	positive
PRJNA729271	stage_1D_vs_E10	ordinary_muscle_development	ENSGALG00010009462	NRBF2	0.305920396	0.120944384	2.529430355	0.011424785	0.016761362	positive
PRJNA729271	stage_1D_vs_E10	ordinary_muscle_development	ENSGALG00010009463	MRPL13	-0.113177374	0.109105197	-1.037323406	0.299585161	0.346484234	negative
PRJNA729271	stage_1D_vs_E10	ordinary_muscle_development	ENSGALG00010009464	SPTLC1	0.493212495	0.147421275	3.345599177	0.00082105	0.001382565	positive

PRJNA729271	stage_1D_vs_E10	ordinary_muscle_development	ENSGALG00010009465	GGPS1	-0.371656568	0.101678314	-3.655219619	0.000256962	0.000455895	negative
PRJNA729271	stage_1D_vs_E10	ordinary_muscle_development	ENSGALG00010009466	FAM193B	-0.012399327	0.108276368	-0.114515538	0.90882913	0.922318717	negative
PRJNA729271	stage_1D_vs_E10	ordinary_muscle_development	ENSGALG00010009467	EGR2	1.406285225	0.301989813	4.65673067	3.2127E-06	6.87352E-06	positive
PRJNA729271	stage_1D_vs_E10	ordinary_muscle_development	ENSGALG00010009469	DNMT3A	0.421654303	0.210697016	2.001235289	0.04536704	0.060877175	positive
PRJNA729271	stage_1D_vs_E10	ordinary_muscle_development	ENSGALG00010009470	ZNF469	-0.116847194	0.21058684	-0.554864655	0.578987239	0.623600835	negative
PRJNA729271	stage_1D_vs_E10	ordinary_muscle_development	ENSGALG00010009472	ADO	-0.167273189	0.084783871	-1.972936453	0.048502803	0.064826937	negative
PRJNA729271	stage_1D_vs_E10	ordinary_muscle_development	ENSGALG00010009474	ACVR2A	-0.262711874	0.095531495	-2.750002762	0.005959476	0.009074757	negative
PRJNA729271	stage_1D_vs_E10	ordinary_muscle_development	ENSGALG00010009477	ZNF365	0.640924622	0.24355985	2.631487173	0.008501208	0.012680079	positive
PRJNA729271	stage_1D_vs_E10	ordinary_muscle_development	ENSGALG00010009479	MTBP	-1.916496086	0.128316766	-14.93566387	1.93141E-50	3.79347E-49	negative
PRJNA729271	stage_1D_vs_E10	ordinary_muscle_development	ENSGALG00010009482	ARID4B	0.449765767	0.107518582	4.183144505	2.87505E-05	5.61414E-05	positive
PRJNA729271	stage_1D_vs_E10	ordinary_muscle_development	ENSGALG00010009484	RTKN2	-1.031031037	0.131061189	-7.86679141	3.63853E-15	1.4765E-14	negative
PRJNA729271	stage_1D_vs_E10	ordinary_muscle_development	ENSGALG00010009486	B4GALT1	0.152161414	0.127911211	1.189586221	0.234209064	0.277630061	positive
PRJNA729271	stage_1D_vs_E10	ordinary_muscle_development	ENSGALG00010009489	MROH1	-0.127960877	0.138114154	-0.926486344	0.354193291	0.403080141	negative
PRJNA729271	stage_1D_vs_E10	ordinary_muscle_development	ENSGALG00010009490	SNTB1	1.150802765	0.159405677	7.219333648	5.22429E-13	1.84653E-12	positive
PRJNA729271	stage_1D_vs_E10	ordinary_muscle_development	ENSGALG00010009491	RBM34	0.002351273	0.101829832	0.02309022	0.981578307	0.984119419	positive
PRJNA729271	stage_1D_vs_E10	ordinary_muscle_development	ENSGALG00010009492	CDC42SE2	-1.683742794	0.127135854	-13.2436503	4.9103E-40	6.44116E-39	negative
PRJNA729271	stage_1D_vs_E10	ordinary_muscle_development	ENSGALG00010009493	ENSGALG00010009493	-0.662911058	0.444301921	-1.492028341	0.135691716	0.168466931	negative
PRJNA729271	stage_1D_vs_E10	ordinary_muscle_development	ENSGALG00010009494	UBA2	-0.45934184	0.074488279	-6.166632468	6.97596E-10	1.99166E-09	negative
PRJNA729271	stage_1D_vs_E10	ordinary_muscle_development	ENSGALG00010009497	HS3ST1	2.823290375	0.186218577	15.16116397	6.39263E-52	1.32373E-50	positive
PRJNA729271	stage_1D_vs_E10	ordinary_muscle_development	ENSGALG00010009498	ORC4	-2.188143085	0.117697836	-18.59119217	3.78667E-77	1.66832E-75	negative
PRJNA729271	stage_1D_vs_E10	ordinary_muscle_development	ENSGALG00010009499	MXD3	-0.355344985	0.241055172	-1.474123048	0.140448464	0.174044668	negative
PRJNA729271	stage_1D_vs_E10	ordinary_muscle_development	ENSGALG00010009500	CCDC146	0.322218358	0.613729024	0.525017304	0.599571162	0.642619343	positive
PRJNA729271	stage_1D_vs_E10	ordinary_muscle_development	ENSGALG00010009501	HGH1	-0.064625099	0.110613621	-0.584241784	0.559057653	0.604696716	negative
PRJNA729271	stage_1D_vs_E10	ordinary_muscle_development	ENSGALG00010009502	ARID5B	-0.167210727	0.094023553	-1.778391918	0.075339509	0.09771351	negative
PRJNA729271	stage_1D_vs_E10	ordinary_muscle_development	ENSGALG00010009503	IL11RA	-1.872129411	0.134937783	-13.87401934	9.10295E-44	1.36169E-42	negative
PRJNA729271	stage_1D_vs_E10	ordinary_muscle_development	ENSGALG00010009506	HAS2	-2.171525341	0.259445321	-8.369876668	5.76786E-17	2.6094E-16	negative
PRJNA729271	stage_1D_vs_E10	ordinary_muscle_development	ENSGALG00010009508	TOMM20	0.948479725	0.089917275	10.54835926	5.16919E-26	3.70377E-25	positive
PRJNA729271	stage_1D_vs_E10	ordinary_muscle_development	ENSGALG00010009511	ZFPM1	-1.370079724	0.261985918	-5.229592998	1.69884E-07	4.03088E-07	negative
PRJNA729271	stage_1D_vs_E10	ordinary_muscle_development	ENSGALG00010009514	PRELID1	-1.179890349	0.091502394	-12.89463908	4.82517E-38	5.89223E-37	negative
PRJNA729271	stage_1D_vs_E10	ordinary_muscle_development	ENSGALG00010009515	MBD5	0.947561641	0.095237522	9.949457129	2.53563E-23	1.59939E-22	positive
PRJNA729271	stage_1D_vs_E10	ordinary_muscle_development	ENSGALG00010009518	FGL2	6.237617825	0.288384205	21.62954046	9.4722E-104	7.3032E-102	positive
PRJNA729271	stage_1D_vs_E10	ordinary_muscle_development	ENSGALG00010009520	SPINK4	-0.749577533	0.643326857	-1.165158154	0.243954981	0.288028626	negative
PRJNA729271	stage_1D_vs_E10	ordinary_muscle_development	ENSGALG00010009522	TAF5L	0.581568863	0.080395135	7.233881302	4.69382E-13	1.66635E-12	positive
PRJNA729271	stage_1D_vs_E10	ordinary_muscle_development	ENSGALG00010009524	FAM185A	1.700486645	0.1598501	10.63800799	1.98324E-26	1.44821E-25	positive
PRJNA729271	stage_1D_vs_E10	ordinary_muscle_development	ENSGALG00010009525	ENSGALG00010009525	1.272075663	0.154933031	8.210487146	2.20293E-16	9.64114E-16	positive
PRJNA729271	stage_1D_vs_E10	ordinary_muscle_development	ENSGALG00010009526	ENSGALG00010009526	-0.879221684	0.241745826	-3.636967381	0.000275867	0.000488002	negative
PRJNA729271	stage_1D_vs_E10	ordinary_muscle_development	ENSGALG00010009527	ENSGALG00010009527	-2.879205707	0.594652476	-4.841829172	1.28649E-06	2.84655E-06	negative
PRJNA729271	stage_1D_vs_E10	ordinary_muscle_development	ENSGALG00010009530	KIF3C	-1.58725368	0.157551752	-10.07449085	7.16308E-24	4.63315E-23	negative
PRJNA729271	stage_1D_vs_E10	ordinary_muscle_development	ENSGALG00010009531	FBXL13	-0.85892538	0.44599435	-1.925866056	0.054121101	0.071760461	negative
PRJNA729271	stage_1D_vs_E10	ordinary_muscle_development	ENSGALG00010009534	XPA	-0.008763658	0.13117728	-0.066807743	0.946734759	0.954770774	negative
PRJNA729271	stage_1D_vs_E10	ordinary_muscle_development	ENSGALG00010009538	CRIM1	1.526177227	0.073778069	20.68605538	4.62541E-95	3.08965E-93	positive
PRJNA729271	stage_1D_vs_E10	ordinary_muscle_development	ENSGALG00010009540	RAB10	0.048131734	0.090086761	0.53428199	0.59314645	0.636674896	positive
PRJNA729271	stage_1D_vs_E10	ordinary_muscle_development	ENSGALG00010009541	PMPCB	-0.245316593	0.092545202	-2.650775919	0.00803071	0.012025325	negative
PRJNA729271	stage_1D_vs_E10	ordinary_muscle_development	ENSGALG00010009542	WDR1	-0.363008836	0.143159621	-2.535692908	0.01122251	0.016490152	negative
PRJNA729271	stage_1D_vs_E10	ordinary_muscle_development	ENSGALG00010009543	MAF1	1.387332528	0.129670662	10.69889293	1.03001E-26	7.62126E-26	positive
PRJNA729271	stage_1D_vs_E10	ordinary_muscle_development	ENSGALG00010009546	GAREM2	-1.758263057	0.550568546	-3.193540696	0.001405395	0.002306	negative
PRJNA729271	stage_1D_vs_E10	ordinary_muscle_development	ENSGALG00010009548	PIK3C3	0.053242657	0.081466312	0.653554278	0.513399009	0.561088641	positive
PRJNA729271	stage_1D_vs_E10	ordinary_muscle_development	ENSGALG00010009551	HADHA	1.919567506	0.130018265	14.76382952	2.50636E-49	4.71202E-48	positive
PRJNA729271	stage_1D_vs_E10	ordinary_muscle_development	ENSGALG00010009554	ABCB10	0.526213427	0.101440989	5.187384648	2.13268E-07	5.01349E-07	positive
PRJNA729271	stage_1D_vs_E10	ordinary_muscle_development	ENSGALG00010009555	EPC2	0.407818436	0.075479048	5.403068067	6.55106E-08	1.60673E-07	positive
PRJNA729271	stage_1D_vs_E10	ordinary_muscle_development	ENSGALG00010009557	CIDEC	4.877831506	1.052253527	4.635604807	3.55895E-06	7.57964E-06	positive
PRJNA729271	stage_1D_vs_E10	ordinary_muscle_development	ENSGALG00010009558	TMEM26	-3.515001107	0.201719499	-17.42519253	5.31293E-68	1.83359E-66	negative
PRJNA729271	stage_1D_vs_E10	ordinary_muscle_development	ENSGALG00010009560	RAB24	-0.085326939	0.146590561	-0.582076626	0.560515068	0.606137415	negative
PRJNA729271	stage_1D_vs_E10	ordinary_muscle_development	ENSGALG00010009562	HADHB	2.62976292	0.189205799	13.89895516	6.42728E-44	9.69442E-43	positive
PRJNA729271	stage_1D_vs_E10	ordinary_muscle_development	ENSGALG00010009565	TMEM8B	0.381197212	0.222135635	1.716056104	0.086151774	0.110804789	positive
PRJNA729271	stage_1D_vs_E10	ordinary_muscle_development	ENSGALG00010009567	NSD1	0.391155571	0.106207747	3.682928817	0.00023057	0.000411336	positive
PRJNA729271	stage_1D_vs_E10	ordinary_muscle_development	ENSGALG00010009569	ZC3H18	-0.187595538	0.143272487	-1.309361911	0.190411787	0.229987505	negative
PRJNA729271	stage_1D_vs_E10	ordinary_muscle_development	ENSGALG00010009570	SHARPIN	1.39573335	0.135816972	10.27657539	8.98641E-25	6.07263E-24	positive
PRJNA729271	stage_1D_vs_E10	ordinary_muscle_development	ENSGALG00010009571	DNAJC2	-0.583568519	0.185849102	-3.140012575	0.001689406	0.002745284	negative
PRJNA729271	stage_1D_vs_E10	ordinary_muscle_development	ENSGALG00010009574	TAF1C	0.05776772	0.137617317	0.419770721	0.674652956	0.712865913	positive
PRJNA729271	stage_1D_vs_E10	ordinary_muscle_development	ENSGALG00010009575	RHOBTB1	-2.338844901	0.245257412	-9.536286304	1.48039E-21	8.62816E-21	negative
PRJNA729271	stage_1D_vs_E10	ordinary_muscle_development	ENSGALG00010009578	KIF5C	-2.319892578	0.159692958	-14.52720648	8.14759E-48	1.43848E-46	negative
PRJNA729271	stage_1D_vs_E10	ordinary_muscle_development	ENSGALG00010009582	TIGD5	-1.060190382	0.138845932	-7.635732403	2.2454E-14	8.66306E-14	negative
PRJNA729271	stage_1D_vs_E10	ordinary_muscle_development	ENSGALG00010009583	LYPD6B	-2.746956605	0.304826735	-9.01153439	2.03193E-19	1.05189E-18	negative
PRJNA729271	stage_1D_vs_E10	ordinary_muscle_development	ENSGALG00010009591	LYPD6	-1.719929453	0.453856152	-3.78959158	0.000150895	0.000275296	negative
PRJNA729271	stage_1D_vs_E10	ordinary_muscle_development	ENSGALG00010009593	NCBP1	-1.018342996	0.062982616	-16.16863599	8.39362E-59	2.20809E-57	negative
PRJNA729271	stage_1D_vs_E10	ordinary_muscle_development	ENSGALG00010009594	HINT2	1.982853177	0.15875827	12.48976309	8.49061E-36	9.51093E-35	positive
PRJNA729271	stage_1D_vs_E10	ordinary_muscle_development	ENSGALG00010009595	ENSGALG00010009595	0.728013351	0.186015909	3.913715532	9.08867E-05	0.000169419	positive
PRJNA729271	stage_1D_vs_E10	ordinary_muscle_development	ENSGALG00010009596	URB2	-0.609307099	0.096859877	-6.29060372	3.16234E-10	9.25835E-10	negative
PRJNA729271	stage_1D_vs_E10	ordinary_muscle_development	ENSGALG00010009597	CDK1	-2.243298025	0.259352935	-8.649595673	5.16822E-18	2.47893E-17	negative
PRJNA729271	stage_1D_vs_E10	ordinary_muscle_development	ENSGALG00010009600	LRRC17	-1.355335578	0.217319755	-6.236596294	4.47194E-10	1.2946E-09	negative
PRJNA729271	stage_1D_vs_E10	ordinary_muscle_development	ENSGALG00010009601	ZHX2	-0.030395494	0.136352248	-0.222918904	0.823598623	0.848295462	negative
PRJNA729271	stage_1D_vs_E10	ordinary_muscle_development	ENSGALG00010009602	TSTD2	0.007173637	0.080559302	0.089047908	0.929043836	0.939806714	positive
PRJNA729271	stage_1D_vs_E10	ordinary_muscle_development	ENSGALG00010009603	HINT1	0.406186246	0.177259203	2.291481855	0.02193556	0.030884502	positive
PRJNA729271	stage_1D_vs_E10	ordinary_muscle_development	ENSGALG00010009605	ENSGALG00010009605	0.97588205	0.156893168	6.220041719	4.97023E-10	1.43513E-09	positive
PRJNA729271	stage_1D_vs_E10	ordinary_muscle_development	ENSGALG00010009606	CHSY3	-0.126607665	0.21188432	-0.597532017	0.550152237	0.596087358	negative
PRJNA729271	stage_1D_vs_E10	ordinary_muscle_development	ENSGALG00010009608	AFAP1	-0.914514399	0.106776652	-8.56474126	1.08319E-17	5.09767E-17	negative
PRJNA729271	stage_1D_vs_E10	ordinary_muscle_development	ENSGALG00010009609	MMADHC	-0.139712268	0.071753002	-1.947127834	0.051519411	0.068580573	negative
PRJNA729271	stage_1D_vs_E10	ordinary_muscle_development	ENSGALG00010009612	DERL1	-0.3519661	0.063509352	-5.541957065	2.9911E-08	7.50103E-08	negative
PRJNA729271	stage_1D_vs_E10	ordinary_muscle_development	ENSGALG00010009615	ENSGALG00010009615	1.591805022	0.395558178	4.024199502	5.71694E-05	0.000108707	positive
PRJNA729271	stage_1D_vs_E10	ordinary_muscle_development	ENSGALG00010009616	ARMC10	-0.407676961	0.177316142	-2.299153121	0.021496246	0.030304229	negative
PRJNA729271	stage_1D_vs_E10	ordinary_muscle_development	ENSGALG00010009617	SLC25A46	0.852033027	0.097103637	8.774470782	1.71709E-18	8.45711E-18	positive
PRJNA729271	stage_1D_vs_E10	ordinary_muscle_development	ENSGALG00010009619	ENSGALG00010009619	0.302496083	0.188125719	1.607946457	0.107846906	0.136253892	positive
PRJNA729271	stage_1D_vs_E10	ordinary_muscle_development	ENSGALG00010009620	TMOD1	1.346590471	0.231895793	5.806877532	6.36487E-09	1.69127E-08	positive
PRJNA729271	stage_1D_vs_E10	ordinary_muscle_development	ENSGALG00010009623	XPO5	-0.222719458	0.110722465	-2.01151101	0.044271509	0.059522819	negative
PRJNA729271	stage_1D_vs_E10	ordinary_muscle_development	ENSGALG00010009629	ENSGALG00010009629	0.987019277	0.536452263	1.839901413	0.065782713	0.08611998	positive
PRJNA729271	stage_1D_vs_E10	ordinary_muscle_development	ENSGALG00010009634	TBC1D31	-0.51724372	0.103219714	-5.011094289	5.41214E-07	1.23406E-06	negative
PRJNA729271	stage_1D_vs_E10	ordinary_muscle_development	ENSGALG00010009635	TDRD7	0.404681021	0.139606163	2.898733213	0.003746736	0.005842183	positive
PRJNA729271	stage_1D_vs_E10	ordinary_muscle_development	ENSGALG00010009637	CYBA	0.316930404	0.135981622	2.330685575	0.019769945	0.027990365	positive
PRJNA729271	stage_1D_vs_E10	ordinary_muscle_development	ENSGALG00010009638	RND3	-1.126069129	0.150231963	-7.495536279	6.60278E-14	2.46796E-13	negative
PRJNA729271	stage_1D_vs_E10	ordinary_muscle_development	ENSGALG00010009640	NPR2	1.330988288	0.186891125	7.12173085	1.0658E-12	3.67565E-12	positive
PRJNA729271	stage_1D_vs_E10	ordinary_muscle_development	ENSGALG00010009643	PSMC2	-0.207254992	0.108801617	-1.904888903	0.056794514	0.075051188	negative
PRJNA729271	stage_1D_vs_E10	ordinary_muscle_development	ENSGALG00010009644	SLC27A6	-0.30198458	0.219665699	-1.374746174	0.169210151	0.206404202	negative
PRJNA729271	stage_1D_vs_E10	ordinary_muscle_development	ENSGALG00010009646	MVD	0.093720859	0.168126811	0.557441481	0.577225831	0.62184232	positive
PRJNA729271	stage_1D_vs_E10	ordinary_muscle_development	ENSGALG00010009647	GALNT2	-1.270891953	0.077787435	-16.33801073	5.29525E-60	1.45921E-58	negative
PRJNA729271	stage_1D_vs_E10	ordinary_muscle_development	ENSGALG00010009649	TENM2	-1.194914637	0.312736639	-3.820833537	0.000133001	0.000243878	negative
PRJNA729271	stage_1D_vs_E10	ordinary_muscle_development	ENSGALG00010009650	KLHL29	-0.405180329	0.192350825	-2.106465259	0.035163958	0.04804879	negative
PRJNA729271	stage_1D_vs_E10	ordinary_muscle_development	ENSGALG00010009652	CIAO2B	-0.26505598	0.082336605	-3.219175475	0.001285598	0.002119511	negative
PRJNA729271	stage_1D_vs_E10	ordinary_muscle_development	ENSGALG00010009653	ABLIM2	4.210363981	0.182758164	23.03789821	1.945E-117	2.1198E-115	positive
PRJNA729271	stage_1D_vs_E10	ordinary_muscle_development	ENSGALG00010009654	NDUFA12	1.269825694	0.139629582	9.094245485	9.52455E-20	5.03679E-19	positive
PRJNA729271	stage_1D_vs_E10	ordinary_muscle_development	ENSGALG00010009657	MSMP	2.19476303	0.670830153	3.271711954	0.001068984	0.001777553	positive
PRJNA729271	stage_1D_vs_E10	ordinary_muscle_development	ENSGALG00010009658	NAPEPLD	0.340845382	0.124190112	2.74454526	0.00605948	0.009220256	positive
PRJNA729271	stage_1D_vs_E10	ordinary_muscle_development	ENSGALG00010009659	APOLD1	1.514789701	0.251982117	6.011496837	1.83818E-09	5.09162E-09	positive
PRJNA729271	stage_1D_vs_E10	ordinary_muscle_development	ENSGALG00010009663	FER	-0.145359875	0.122299891	-1.188552775	0.234615701	0.27802114	negative
PRJNA729271	stage_1D_vs_E10	ordinary_muscle_development	ENSGALG00010009665	DGKQ	0.216793906	0.169986797	1.275357319	0.202182798	0.242642794	positive
PRJNA729271	stage_1D_vs_E10	ordinary_muscle_development	ENSGALG00010009666	RRAD	1.301891512	0.364274768	3.573927227	0.000351667	0.000615852	positive
PRJNA729271	stage_1D_vs_E10	ordinary_muscle_development	ENSGALG00010009668	TFAP2A	-6.194840457	0.862492151	-7.182489078	6.84535E-13	2.39786E-12	negative
PRJNA729271	stage_1D_vs_E10	ordinary_muscle_development	ENSGALG00010009671	MINAR2	3.086959373	0.808220706	3.819450989	0.000133749	0.000245187	positive
PRJNA729271	stage_1D_vs_E10	ordinary_muscle_development	ENSGALG00010009672	DDX47	-0.164640057	0.119121536	-1.382118318	0.166935375	0.204041509	negative
PRJNA729271	stage_1D_vs_E10	ordinary_muscle_development	ENSGALG00010009676	ACO1	-0.631593034	0.095935478	-6.583518937	4.59442E-11	1.42574E-10	negative
PRJNA729271	stage_1D_vs_E10	ordinary_muscle_development	ENSGALG00010009677	SLC26A5	0.944926448	0.161950439	5.834664322	5.3899E-09	1.44065E-08	positive
PRJNA729271	stage_1D_vs_E10	ordinary_muscle_development	ENSGALG00010009678	JARID2	-0.163968986	0.099768713	-1.643491047	0.100281376	0.127518076	negative
PRJNA729271	stage_1D_vs_E10	ordinary_muscle_development	ENSGALG00010009679	ANK3	2.369466229	0.102700735	23.07156069	8.9381E-118	9.815E-116	positive
PRJNA729271	stage_1D_vs_E10	ordinary_muscle_development	ENSGALG00010009683	ADAMTS19	2.409120949	0.238221994	10.11292412	4.84172E-24	3.15136E-23	positive
PRJNA729271	stage_1D_vs_E10	ordinary_muscle_development	ENSGALG00010009686	MAT2B	0.789251043	0.086883647	9.083999968	1.04656E-19	5.51835E-19	positive
PRJNA729271	stage_1D_vs_E10	ordinary_muscle_development	ENSGALG00010009689	ENSGALG00010009689	-2.497184655	0.537406763	-4.646730984	3.37236E-06	7.2013E-06	negative
PRJNA729271	stage_1D_vs_E10	ordinary_muscle_development	ENSGALG00010009690	RBM43	-0.427134383	0.187218935	-2.281469997	0.02252065	0.031671371	negative
PRJNA729271	stage_1D_vs_E10	ordinary_muscle_development	ENSGALG00010009695	ATAD2B	0.589433866	0.07530308	7.827486868	4.97719E-15	1.99957E-14	positive
PRJNA729271	stage_1D_vs_E10	ordinary_muscle_development	ENSGALG00010009697	ENSGALG00010009697	-0.399633607	0.266289609	-1.500748031	0.133420745	0.165838194	negative
PRJNA729271	stage_1D_vs_E10	ordinary_muscle_development	ENSGALG00010009699	POLR1C	-0.771339795	0.109340991	-7.054443054	1.73293E-12	5.89645E-12	negative
PRJNA729271	stage_1D_vs_E10	ordinary_muscle_development	ENSGALG00010009700	SH3TC1	0.635923925	0.206608551	3.077916777	0.002084531	0.00334647	positive
PRJNA729271	stage_1D_vs_E10	ordinary_muscle_development	ENSGALG00010009702	ENSGALG00010009702	-0.565938404	0.122225525	-4.630280013	3.65172E-06	7.76921E-06	negative
PRJNA729271	stage_1D_vs_E10	ordinary_muscle_development	ENSGALG00010009704	RGP1	0.07337889	0.130404636	0.562701545	0.573638132	0.618482946	positive
PRJNA729271	stage_1D_vs_E10	ordinary_muscle_development	ENSGALG00010009705	DTNBP1	-0.348058548	0.070935281	-4.906705715	9.26188E-07	2.07594E-06	negative
PRJNA729271	stage_1D_vs_E10	ordinary_muscle_development	ENSGALG00010009710	ENSGALG00010009710	-0.672020937	0.111127984	-6.047270131	1.47321E-09	4.10814E-09	negative
PRJNA729271	stage_1D_vs_E10	ordinary_muscle_development	ENSGALG00010009712	UBXN2A	1.78043291	0.178458182	9.976751339	1.92672E-23	1.22222E-22	positive
PRJNA729271	stage_1D_vs_E10	ordinary_muscle_development	ENSGALG00010009715	NUDCD2	-0.308201157	0.092610754	-3.32791974	0.000874971	0.001467905	negative
PRJNA729271	stage_1D_vs_E10	ordinary_muscle_development	ENSGALG00010009717	EDN1	2.129436619	0.252984835	8.417250053	3.85424E-17	1.75794E-16	positive
PRJNA729271	stage_1D_vs_E10	ordinary_muscle_development	ENSGALG00010009718	ENSGALG00010009718	-0.705718792	0.180972324	-3.899595122	9.63537E-05	0.000179057	negative
PRJNA729271	stage_1D_vs_E10	ordinary_muscle_development	ENSGALG00010009719	YIPF3	-0.164029193	0.082253022	-1.994202633	0.046129915	0.061815024	negative
PRJNA729271	stage_1D_vs_E10	ordinary_muscle_development	ENSGALG00010009720	RELN	0.131781516	0.167956245	0.784618137	0.432677493	0.482101642	positive

PRJNA729271	stage_1D_vs_E10	ordinary_muscle_development	ENSGALG00010009721	ZHX1	1.184256167	0.155857061	7.598347845	2.99935E-14	1.14772E-13	positive
PRJNA729271	stage_1D_vs_E10	ordinary_muscle_development	ENSGALG00010009722	GBA2	-0.624750011	0.099386225	-6.286082539	3.25577E-10	9.52419E-10	negative
PRJNA729271	stage_1D_vs_E10	ordinary_muscle_development	ENSGALG00010009724	ISOC1	0.410728773	0.14284235	2.875399164	0.00403517	0.006266987	positive
PRJNA729271	stage_1D_vs_E10	ordinary_muscle_development	ENSGALG00010009726	PRR16	0.583047714	0.139895315	4.16774296	3.07631E-05	5.9902E-05	positive
PRJNA729271	stage_1D_vs_E10	ordinary_muscle_development	ENSGALG00010009727	CDH20	3.888596503	0.211989131	18.34337674	3.72899E-75	1.57127E-73	positive
PRJNA729271	stage_1D_vs_E10	ordinary_muscle_development	ENSGALG00010009728	CCDC149	-1.368398683	0.103922095	-13.16754326	1.34917E-39	1.74453E-38	negative
PRJNA729271	stage_1D_vs_E10	ordinary_muscle_development	ENSGALG00010009730	C8orf76	-0.146179338	0.114334579	-1.278522552	0.201065238	0.241361543	negative
PRJNA729271	stage_1D_vs_E10	ordinary_muscle_development	ENSGALG00010009731	NMI	-0.533407337	0.243060698	-2.194543755	0.028196342	0.039006106	negative
PRJNA729271	stage_1D_vs_E10	ordinary_muscle_development	ENSGALG00010009732	HMMR	-1.808161847	0.24225404	-7.46390793	8.39933E-14	3.11935E-13	negative
PRJNA729271	stage_1D_vs_E10	ordinary_muscle_development	ENSGALG00010009736	WISP1	-1.546177251	0.330738305	-4.674926449	2.94059E-06	6.31464E-06	negative
PRJNA729271	stage_1D_vs_E10	ordinary_muscle_development	ENSGALG00010009738	ENSGALG00010009738	-0.571423662	0.258148152	-2.213549301	0.026859798	0.037263857	negative
PRJNA729271	stage_1D_vs_E10	ordinary_muscle_development	ENSGALG00010009740	LRRC73	-2.802754578	0.372421744	-7.52575439	5.24168E-14	1.97551E-13	negative
PRJNA729271	stage_1D_vs_E10	ordinary_muscle_development	ENSGALG00010009741	C2H6orf52	0.013857192	0.069848432	0.198389452	0.84274037	0.865367077	positive
PRJNA729271	stage_1D_vs_E10	ordinary_muscle_development	ENSGALG00010009743	CCNG1	0.595130025	0.172896402	3.442119192	0.000577176	0.000985878	positive
PRJNA729271	stage_1D_vs_E10	ordinary_muscle_development	ENSGALG00010009744	DAPK1	-0.517264793	0.103712391	-4.987492684	6.11679E-07	1.38883E-06	negative
PRJNA729271	stage_1D_vs_E10	ordinary_muscle_development	ENSGALG00010009749	NDRG1	1.685144472	0.166812259	10.10204213	5.41036E-24	3.51516E-23	positive
PRJNA729271	stage_1D_vs_E10	ordinary_muscle_development	ENSGALG00010009751	ENSGALG00010009751	0.538365328	0.198997767	2.705383768	0.006822552	0.010299197	positive
PRJNA729271	stage_1D_vs_E10	ordinary_muscle_development	ENSGALG00010009761	PDP2	0.846733471	0.11572759	7.316608537	2.54316E-13	9.1631E-13	positive
PRJNA729271	stage_1D_vs_E10	ordinary_muscle_development	ENSGALG00010009762	MAN2A1	0.313910959	0.102452285	3.063972264	0.002184192	0.003496395	positive
PRJNA729271	stage_1D_vs_E10	ordinary_muscle_development	ENSGALG00010009765	STX10	-0.655037893	0.098259865	-6.666382995	2.62185E-11	8.2509E-11	negative
PRJNA729271	stage_1D_vs_E10	ordinary_muscle_development	ENSGALG00010009769	TMCC3	1.725905392	0.225411186	7.656698079	1.90774E-14	7.38194E-14	positive
PRJNA729271	stage_1D_vs_E10	ordinary_muscle_development	ENSGALG00010009772	ENSGALG00010009772	-0.145766702	0.566629277	-0.25725233	0.796983989	0.824279909	negative
PRJNA729271	stage_1D_vs_E10	ordinary_muscle_development	ENSGALG00010009774	PIAS2	-0.3779617	0.059978141	-6.301657516	2.94479E-10	8.64064E-10	negative
PRJNA729271	stage_1D_vs_E10	ordinary_muscle_development	ENSGALG00010009775	KIAA1614	-0.412227679	0.138506354	-2.976236591	0.002918096	0.004608608	negative
PRJNA729271	stage_1D_vs_E10	ordinary_muscle_development	ENSGALG00010009777	ST3GAL1	-2.374423808	0.191946726	-12.37022299	3.78768E-35	4.14046E-34	negative
PRJNA729271	stage_1D_vs_E10	ordinary_muscle_development	ENSGALG00010009778	ENSGALG00010009778	0.339110865	0.110398313	3.071703323	0.002128412	0.003412391	positive
PRJNA729271	stage_1D_vs_E10	ordinary_muscle_development	ENSGALG00010009779	PPP1R3E	2.642320153	0.343650563	7.688973743	1.4832E-14	5.77154E-14	positive
PRJNA729271	stage_1D_vs_E10	ordinary_muscle_development	ENSGALG00010009780	MFSD2B	-0.704003281	0.253534988	-2.776750015	0.005490539	0.008397738	negative
PRJNA729271	stage_1D_vs_E10	ordinary_muscle_development	ENSGALG00010009784	ENSGALG00010009784	-0.686560268	0.178441302	-3.84754123	0.000119309	0.000219884	negative
PRJNA729271	stage_1D_vs_E10	ordinary_muscle_development	ENSGALG00010009786	DIPK2B	0.339983996	0.16024308	2.121676625	0.033864903	0.046369901	positive
PRJNA729271	stage_1D_vs_E10	ordinary_muscle_development	ENSGALG00010009788	PAK1IP1	-0.239091057	0.09251017	-2.584484023	0.009752482	0.014435028	negative
PRJNA729271	stage_1D_vs_E10	ordinary_muscle_development	ENSGALG00010009791	GABRG2	1.41936811	0.273667527	5.186468876	2.14319E-07	5.03738E-07	positive
PRJNA729271	stage_1D_vs_E10	ordinary_muscle_development	ENSGALG00010009793	TNFAIP6	-2.345137392	0.295305678	-7.941389448	1.99929E-15	8.24458E-15	negative
PRJNA729271	stage_1D_vs_E10	ordinary_muscle_development	ENSGALG00010009795	NDUFA6	1.033146748	0.114485764	9.024237719	1.80951E-19	9.38083E-19	positive
PRJNA729271	stage_1D_vs_E10	ordinary_muscle_development	ENSGALG00010009796	ENSGALG00010009796	-0.60962943	0.195418043	-3.119616905	0.001810864	0.002931766	negative
PRJNA729271	stage_1D_vs_E10	ordinary_muscle_development	ENSGALG00010009798	YIPF4	0.617394879	0.077253603	7.991793944	1.32989E-15	5.54728E-15	positive
PRJNA729271	stage_1D_vs_E10	ordinary_muscle_development	ENSGALG00010009801	ENSGALG00010009801	-0.592613635	0.60346858	-0.982012411	0.326093734	0.374424008	negative
PRJNA729271	stage_1D_vs_E10	ordinary_muscle_development	ENSGALG00010009804	XPR1	-0.286727311	0.117815369	-2.433700406	0.014945359	0.021553376	negative
PRJNA729271	stage_1D_vs_E10	ordinary_muscle_development	ENSGALG00010009806	WDCP	-1.269098743	0.175773764	-7.220069221	5.19611E-13	1.83746E-12	negative
PRJNA729271	stage_1D_vs_E10	ordinary_muscle_development	ENSGALG00010009809	SMDT1	0.775999942	0.090007595	8.621493999	6.60861E-18	3.15209E-17	positive
PRJNA729271	stage_1D_vs_E10	ordinary_muscle_development	ENSGALG00010009811	ENSGALG00010009811	0.542038414	0.08635823	6.276627146	3.45996E-10	1.00991E-09	positive
PRJNA729271	stage_1D_vs_E10	ordinary_muscle_development	ENSGALG00010009822	ENSGALG00010009822	-0.208430948	0.200338118	-1.040395857	0.298156026	0.345051624	negative
PRJNA729271	stage_1D_vs_E10	ordinary_muscle_development	ENSGALG00010009830	RNASEL	-1.317035246	0.198714471	-6.627777242	3.40779E-11	1.06559E-10	negative
PRJNA729271	stage_1D_vs_E10	ordinary_muscle_development	ENSGALG00010009831	N6AMT1	-0.893074122	0.121780975	-7.333445342	2.2431E-13	8.10008E-13	negative
PRJNA729271	stage_1D_vs_E10	ordinary_muscle_development	ENSGALG00010009833	SOD3	3.339129661	0.311430853	10.72189743	8.03381E-27	5.98407E-26	positive
PRJNA729271	stage_1D_vs_E10	ordinary_muscle_development	ENSGALG00010009840	ENSGALG00010009840	0.015119411	0.17679369	0.085520084	0.93184793	0.941985894	positive
PRJNA729271	stage_1D_vs_E10	ordinary_muscle_development	ENSGALG00010009841	GABRA1	3.231752478	0.227989052	14.17503363	1.3078E-45	2.09697E-44	positive
PRJNA729271	stage_1D_vs_E10	ordinary_muscle_development	ENSGALG00010009842	PHETA2	0.259586761	0.089972009	2.885194682	0.003911717	0.0060883	positive
PRJNA729271	stage_1D_vs_E10	ordinary_muscle_development	ENSGALG00010009847	ENSGALG00010009847	-1.232132335	0.119651559	-10.29767052	7.21882E-25	4.91714E-24	negative
PRJNA729271	stage_1D_vs_E10	ordinary_muscle_development	ENSGALG00010009850	RIF1	-1.154579054	0.104837152	-11.01307157	3.30539E-28	2.61099E-27	negative
PRJNA729271	stage_1D_vs_E10	ordinary_muscle_development	ENSGALG00010009852	ENSGALG00010009852	-1.986126814	0.27777571	-7.150109752	8.67085E-13	3.01473E-12	negative
PRJNA729271	stage_1D_vs_E10	ordinary_muscle_development	ENSGALG00010009857	ORC5	-0.914076823	0.138017609	-6.622899993	3.52219E-11	1.10007E-10	negative
PRJNA729271	stage_1D_vs_E10	ordinary_muscle_development	ENSGALG00010009858	ST8SIA5	-0.480459797	0.24855278	-1.933029262	0.053232593	0.07065346	negative
PRJNA729271	stage_1D_vs_E10	ordinary_muscle_development	ENSGALG00010009866	NAGA	0.579574627	0.114547812	5.059674378	4.19973E-07	9.64434E-07	positive
PRJNA729271	stage_1D_vs_E10	ordinary_muscle_development	ENSGALG00010009868	LTN1	0.880635201	0.094517466	9.317168969	1.19486E-20	6.63073E-20	positive
PRJNA729271	stage_1D_vs_E10	ordinary_muscle_development	ENSGALG00010009876	SELENOI	-0.025473852	0.107740129	-0.236437919	0.813092875	0.838726247	negative
PRJNA729271	stage_1D_vs_E10	ordinary_muscle_development	ENSGALG00010009883	ENSGALG00010009883	-0.014684734	0.200466615	-0.073252764	0.941604979	0.950390932	negative
PRJNA729271	stage_1D_vs_E10	ordinary_muscle_development	ENSGALG00010009884	GFOD1	1.300157426	0.111231849	11.68871536	1.45572E-31	1.35958E-30	positive
PRJNA729271	stage_1D_vs_E10	ordinary_muscle_development	ENSGALG00010009910	ENSGALG00010009910	0.651553472	0.173893056	3.746863081	0.00017906	0.000323948	positive
PRJNA729271	stage_1D_vs_E10	ordinary_muscle_development	ENSGALG00010009914	RWDD2B	-0.601900327	0.124788269	-4.823372693	1.41151E-06	3.1146E-06	negative
PRJNA729271	stage_1D_vs_E10	ordinary_muscle_development	ENSGALG00010009941	CACNA1E	-1.636486461	0.384857325	-4.252189976	2.1169E-05	4.18615E-05	negative
PRJNA729271	stage_1D_vs_E10	ordinary_muscle_development	ENSGALG00010009943	DHX15	-0.447078986	0.068672005	-6.510352879	7.49745E-11	2.28791E-10	negative
PRJNA729271	stage_1D_vs_E10	ordinary_muscle_development	ENSGALG00010009948	WBP2NL	0.668864558	0.064736893	10.33204608	5.04723E-25	3.46892E-24	positive
PRJNA729271	stage_1D_vs_E10	ordinary_muscle_development	ENSGALG00010009972	PTK2B	-0.165365729	0.285991209	-0.578219622	0.563115853	0.60863204	negative
PRJNA729271	stage_1D_vs_E10	ordinary_muscle_development	ENSGALG00010009985	USP16	-0.618965427	0.115411145	-5.363133918	8.17903E-08	1.98918E-07	negative
PRJNA729271	stage_1D_vs_E10	ordinary_muscle_development	ENSGALG00010009989	TBC1D7	-1.338866131	0.137245503	-9.755264137	1.75144E-22	1.06848E-21	negative
PRJNA729271	stage_1D_vs_E10	ordinary_muscle_development	ENSGALG00010009991	LHFPL3	-3.303646189	0.544388069	-6.06854995	1.2907E-09	3.61312E-09	negative
PRJNA729271	stage_1D_vs_E10	ordinary_muscle_development	ENSGALG00010009993	ENSGALG00010009993	-0.474764821	0.361150098	-1.314591421	0.188647255	0.228079236	negative
PRJNA729271	stage_1D_vs_E10	ordinary_muscle_development	ENSGALG00010009995	SEPTIN3	2.830732662	0.32595297	8.684481882	3.80473E-18	1.83648E-17	positive
PRJNA729271	stage_1D_vs_E10	ordinary_muscle_development	ENSGALG00010009999	GABRB2	0.25461617	0.225804271	1.127596787	0.259490244	0.304608932	positive
PRJNA729271	stage_1D_vs_E10	ordinary_muscle_development	ENSGALG00010010006	PPARGC1A	3.662027824	0.264331874	13.85390178	1.20486E-43	1.7949E-42	positive
PRJNA729271	stage_1D_vs_E10	ordinary_muscle_development	ENSGALG00010010007	CTIF	0.072505222	0.095387977	0.760108606	0.447189669	0.496335704	positive
PRJNA729271	stage_1D_vs_E10	ordinary_muscle_development	ENSGALG00010010009	ELF2	0.180144759	0.087799319	2.051778545	0.040191189	0.054384922	positive
PRJNA729271	stage_1D_vs_E10	ordinary_muscle_development	ENSGALG00010010011	EPB41L4A	0.342458028	0.12356971	2.771375185	0.005582007	0.008525044	positive
PRJNA729271	stage_1D_vs_E10	ordinary_muscle_development	ENSGALG00010010014	GLUL	2.616390183	0.392404123	6.667590959	2.60036E-11	8.18507E-11	positive
PRJNA729271	stage_1D_vs_E10	ordinary_muscle_development	ENSGALG00010010017	CCDC59	-0.684865465	0.106132174	-6.452948612	1.09695E-10	3.31186E-10	negative
PRJNA729271	stage_1D_vs_E10	ordinary_muscle_development	ENSGALG00010010022	KMT2E	0.779403749	0.100881357	7.725944314	1.11027E-14	4.36017E-14	positive
PRJNA729271	stage_1D_vs_E10	ordinary_muscle_development	ENSGALG00010010035	ATP10B	0.100589872	0.285589562	0.352218308	0.724674548	0.759135475	positive
PRJNA729271	stage_1D_vs_E10	ordinary_muscle_development	ENSGALG00010010037	NAA15	0.02553205	0.138247536	0.184683579	0.853477227	0.874842826	positive
PRJNA729271	stage_1D_vs_E10	ordinary_muscle_development	ENSGALG00010010042	METTL25	-1.223412304	0.118553246	-10.31951759	5.75114E-25	3.93406E-24	negative
PRJNA729271	stage_1D_vs_E10	ordinary_muscle_development	ENSGALG00010010049	ZBTB7C	-0.028797915	0.272683498	-0.105609306	0.915892341	0.928576588	negative
PRJNA729271	stage_1D_vs_E10	ordinary_muscle_development	ENSGALG00010010052	ZNF346	-0.755721283	0.114836063	-6.580870686	4.67701E-11	1.45043E-10	negative
PRJNA729271	stage_1D_vs_E10	ordinary_muscle_development	ENSGALG00010010053	CHRNA2	-6.829265012	0.942351996	-7.247042552	4.2597E-13	1.51594E-12	negative
PRJNA729271	stage_1D_vs_E10	ordinary_muscle_development	ENSGALG00010010059	ENSGALG00010010059	-1.154770497	0.12895246	-8.955009445	3.39707E-19	1.73626E-18	negative
PRJNA729271	stage_1D_vs_E10	ordinary_muscle_development	ENSGALG00010010061	CYP2D6	2.521966601	0.202302395	12.46632103	1.13967E-35	1.26878E-34	positive
PRJNA729271	stage_1D_vs_E10	ordinary_muscle_development	ENSGALG00010010062	RGS7BP	-0.34729103	0.171492789	-2.025105727	0.042856509	0.057770399	negative
PRJNA729271	stage_1D_vs_E10	ordinary_muscle_development	ENSGALG00010010064	ENSGALG00010010064	-0.085955881	0.088234702	-0.97417319	0.329970554	0.378395821	negative
PRJNA729271	stage_1D_vs_E10	ordinary_muscle_development	ENSGALG00010010071	GLT8D2	-1.651324145	0.269413452	-6.129330706	8.82495E-10	2.50622E-09	negative
PRJNA729271	stage_1D_vs_E10	ordinary_muscle_development	ENSGALG00010010077	CCT8	-1.059472146	0.115430437	-9.178446965	4.37353E-20	2.34968E-19	negative
PRJNA729271	stage_1D_vs_E10	ordinary_muscle_development	ENSGALG00010010080	CDC42EP3	1.009763948	0.118283827	8.536787904	1.38005E-17	6.45283E-17	positive
PRJNA729271	stage_1D_vs_E10	ordinary_muscle_development	ENSGALG00010010084	HK3	1.214189437	0.21048051	5.768654965	7.99067E-09	2.10744E-08	positive
PRJNA729271	stage_1D_vs_E10	ordinary_muscle_development	ENSGALG00010010085	TDG	-1.134755989	0.103517264	-10.96199748	5.82004E-28	4.54779E-27	negative
PRJNA729271	stage_1D_vs_E10	ordinary_muscle_development	ENSGALG00010010094	SLC2A9	1.394380154	0.245296303	5.68447277	1.31217E-08	3.39217E-08	positive
PRJNA729271	stage_1D_vs_E10	ordinary_muscle_development	ENSGALG00010010095	C12orf73	0.29620908	0.098699256	3.00112779	0.002689817	0.004262479	positive
PRJNA729271	stage_1D_vs_E10	ordinary_muscle_development	ENSGALG00010010100	IRF2BP2	1.188111081	0.162147981	7.32732577	2.34791E-13	8.47011E-13	positive
PRJNA729271	stage_1D_vs_E10	ordinary_muscle_development	ENSGALG00010010106	C7orf73	-0.761280942	0.206721726	-3.682636352	0.000230834	0.000411758	negative
PRJNA729271	stage_1D_vs_E10	ordinary_muscle_development	ENSGALG00010010107	MGST2	-1.462725308	0.355415921	-4.115531192	3.86289E-05	7.45375E-05	negative
PRJNA729271	stage_1D_vs_E10	ordinary_muscle_development	ENSGALG00010010111	CNOT4	0.909769993	0.133166122	6.831842659	8.38308E-12	2.7245E-11	positive
PRJNA729271	stage_1D_vs_E10	ordinary_muscle_development	ENSGALG00010010115	NEB	4.188524185	0.17169199	24.39557134	1.9057E-131	2.5342E-129	positive
PRJNA729271	stage_1D_vs_E10	ordinary_muscle_development	ENSGALG00010010116	DRD5	-4.794037854	0.714984154	-6.705096649	2.01273E-11	6.38253E-11	negative
PRJNA729271	stage_1D_vs_E10	ordinary_muscle_development	ENSGALG00010010119	SRPK2	-0.865360746	0.124783531	-6.934895505	4.06522E-12	1.34871E-11	negative
PRJNA729271	stage_1D_vs_E10	ordinary_muscle_development	ENSGALG00010010122	THBS4	-2.846836864	0.246738196	-11.53788472	8.49895E-31	7.65645E-30	negative
PRJNA729271	stage_1D_vs_E10	ordinary_muscle_development	ENSGALG00010010124	ENSGALG00010010124	0.644746291	0.145911361	4.418753176	9.92719E-06	2.02726E-05	positive
PRJNA729271	stage_1D_vs_E10	ordinary_muscle_development	ENSGALG00010010125	AHCTF1	0.000297595	0.066491796	0.004475668	0.996428945	0.997185692	positive
PRJNA729271	stage_1D_vs_E10	ordinary_muscle_development	ENSGALG00010010127	FAM13C	2.387484439	0.326513483	7.312054668	2.63088E-13	9.46033E-13	positive
PRJNA729271	stage_1D_vs_E10	ordinary_muscle_development	ENSGALG00010010136	EPHX2	0.451016063	0.114920035	3.924607776	8.68712E-05	0.000162205	positive
PRJNA729271	stage_1D_vs_E10	ordinary_muscle_development	ENSGALG00010010142	ENSGALG00010010142	0.766203828	0.291319117	2.630118598	0.008535509	0.012727312	positive
PRJNA729271	stage_1D_vs_E10	ordinary_muscle_development	ENSGALG00010010146	PHACTR1	-4.833041447	0.368359957	-13.12043114	2.515E-39	3.23469E-38	negative
PRJNA729271	stage_1D_vs_E10	ordinary_muscle_development	ENSGALG00010010150	NTRK2	0.970490193	0.296958173	3.268103997	0.001082706	0.001798719	positive
PRJNA729271	stage_1D_vs_E10	ordinary_muscle_development	ENSGALG00010010153	ENSGALG00010010153	0.082439409	0.455716172	0.180900775	0.856445461	0.877079055	positive
PRJNA729271	stage_1D_vs_E10	ordinary_muscle_development	ENSGALG00010010154	IQSEC3	-2.952973253	0.327266424	-9.023147623	1.82761E-19	9.4713E-19	negative
PRJNA729271	stage_1D_vs_E10	ordinary_muscle_development	ENSGALG00010010155	BRINP3	-1.700427302	0.356001129	-4.776466039	1.78403E-06	3.89861E-06	negative
PRJNA729271	stage_1D_vs_E10	ordinary_muscle_development	ENSGALG00010010156	ENSGALG00010010156	-0.342571115	0.097836698	-3.501458275	0.000462719	0.000799704	negative
PRJNA729271	stage_1D_vs_E10	ordinary_muscle_development	ENSGALG00010010161	SEPSECS	0.155105323	0.063121789	2.45723901	0.01400095	0.020266005	positive
PRJNA729271	stage_1D_vs_E10	ordinary_muscle_development	ENSGALG00010010164	ENSGALG00010010164	1.130473253	0.39648974	2.851204302	0.004355398	0.006749143	positive
PRJNA729271	stage_1D_vs_E10	ordinary_muscle_development	ENSGALG00010010167	PUS7	-0.7971464	0.06980829	-11.41907933	3.35772E-30	2.94079E-29	negative
PRJNA729271	stage_1D_vs_E10	ordinary_muscle_development	ENSGALG00010010168	SNCB	-3.546986098	0.329159087	-10.77590209	4.47375E-27	3.36343E-26	negative
PRJNA729271	stage_1D_vs_E10	ordinary_muscle_development	ENSGALG00010010171	ENSGALG00010010171	0.188575086	0.401429183	0.469759286	0.638527006	0.679249152	positive
PRJNA729271	stage_1D_vs_E10	ordinary_muscle_development	ENSGALG00010010178	LGI2	-2.659218369	0.184849154	-14.38588332	6.34626E-47	1.07213E-45	negative
PRJNA729271	stage_1D_vs_E10	ordinary_muscle_development	ENSGALG00010010179	CDHR2	-0.047965643	0.869684198	-0.055152943	0.956016618	0.9629255	negative
PRJNA729271	stage_1D_vs_E10	ordinary_muscle_development	ENSGALG00010010183	NKX3-2	-4.071148149	0.407055171	-10.00146524	1.50159E-23	9.58832E-23	negative
PRJNA729271	stage_1D_vs_E10	ordinary_muscle_development	ENSGALG00010010185	PHYHIPL	-0.792014207	0.32575515	-2.43131753	0.015044024	0.021687034	negative
PRJNA729271	stage_1D_vs_E10	ordinary_muscle_development	ENSGALG00010010186	TNPO1	0.600828276	0.072019734	8.342550591	7.2705E-17	3.27387E-16	positive
PRJNA729271	stage_1D_vs_E10	ordinary_muscle_development	ENSGALG00010010188	DSP	-5.022108072	0.353041236	-14.22527332	6.38544E-46	1.03763E-44	negative
PRJNA729271	stage_1D_vs_E10	ordinary_muscle_development	ENSGALG00010010190	RNF44	0.490017783	0.176715274	2.772922628	0.005555533	0.008489084	positive
PRJNA729271	stage_1D_vs_E10	ordinary_muscle_development	ENSGALG00010010191	RINT1	-0.588307628	0.093845729	-6.268880168	3.63654E-10	1.06017E-09	negative
PRJNA729271	stage_1D_vs_E10	ordinary_muscle_development	ENSGALG00010010195	ENSGALG00010010195	5.174142673	0.276235768	18.73089324	2.77225E-78	1.27568E-76	positive
PRJNA729271	stage_1D_vs_E10	ordinary_muscle_development	ENSGALG00010010196	EFCAB10	-0.243768893	0.342820976	-0.711067614	0.477042338	0.52567498	negative
PRJNA729271	stage_1D_vs_E10	ordinary_muscle_development	ENSGALG00010010197	SCCPDH	-0.590782832	0.121523339	-4.861476277	1.16514E-06	2.5879E-06	negative
PRJNA729271	stage_1D_vs_E10	ordinary_muscle_development	ENSGALG00010010201	RIOK1	-0.632642209	0.098913231	-6.395931105	1.59572E-10	4.76317E-10	negative
PRJNA729271	stage_1D_vs_E10	ordinary_muscle_development	ENSGALG00010010205	ARL5A	-0.114603164	0.082992862	-1.380879756	0.167315935	0.204411015	negative

PRJNA729271	stage_1D_vs_E10	ordinary_muscle_development	ENSGALG00010010206	SCARA5	2.066501475	0.157822614	13.09382362	3.57165E-39	4.56133E-38	positive
PRJNA729271	stage_1D_vs_E10	ordinary_muscle_development	ENSGALG00010010214	FAF2	-0.767188843	0.076706717	-10.00158619	1.49975E-23	9.58084E-23	negative
PRJNA729271	stage_1D_vs_E10	ordinary_muscle_development	ENSGALG00010010215	ENSGALG00010010215	0.616467579	0.123155235	5.005614073	5.56842E-07	1.26869E-06	positive
PRJNA729271	stage_1D_vs_E10	ordinary_muscle_development	ENSGALG00010010216	ATXN7L1	1.04318203	0.127226306	8.199420875	2.41548E-16	1.05491E-15	positive
PRJNA729271	stage_1D_vs_E10	ordinary_muscle_development	ENSGALG00010010218	PBK	-1.940095493	0.219213419	-8.85025882	8.73166E-19	4.36583E-18	negative
PRJNA729271	stage_1D_vs_E10	ordinary_muscle_development	ENSGALG00010010220	LHX9	-9.381923261	0.902676048	-10.39345542	2.65554E-25	1.84703E-24	negative
PRJNA729271	stage_1D_vs_E10	ordinary_muscle_development	ENSGALG00010010222	PAH	-1.880816442	1.093294199	-1.720320518	0.085374196	0.10986319	negative
PRJNA729271	stage_1D_vs_E10	ordinary_muscle_development	ENSGALG00010010223	ENSGALG00010010223	0.487612076	0.212410837	2.295608279	0.021698289	0.030562307	positive
PRJNA729271	stage_1D_vs_E10	ordinary_muscle_development	ENSGALG00010010226	BICC1	0.013250859	0.163965004	0.080815165	0.935588948	0.945174365	positive
PRJNA729271	stage_1D_vs_E10	ordinary_muscle_development	ENSGALG00010010231	C1orf53	-0.79734155	0.242273914	-3.291074707	0.000998054	0.001664189	negative
PRJNA729271	stage_1D_vs_E10	ordinary_muscle_development	ENSGALG00010010234	ENSGALG00010010234	1.336457878	0.268438877	4.978630127	6.40359E-07	1.45213E-06	positive
PRJNA729271	stage_1D_vs_E10	ordinary_muscle_development	ENSGALG00010010243	ESCO2	-0.379512782	0.107641141	-3.525722397	0.000422329	0.000732695	negative
PRJNA729271	stage_1D_vs_E10	ordinary_muscle_development	ENSGALG00010010246	CLTB	0.41791145	0.066532963	6.281269147	3.3582E-10	9.81394E-10	positive
PRJNA729271	stage_1D_vs_E10	ordinary_muscle_development	ENSGALG00010010252	WDR7	1.053632938	0.09512372	11.07644803	1.6322E-28	1.3151E-27	positive
PRJNA729271	stage_1D_vs_E10	ordinary_muscle_development	ENSGALG00010010253	IL10RB	0.60177573	0.123053455	4.890360278	1.00652E-06	2.24938E-06	positive
PRJNA729271	stage_1D_vs_E10	ordinary_muscle_development	ENSGALG00010010254	WDR41	0.466978566	0.11343702	4.116632886	3.84448E-05	7.42119E-05	positive
PRJNA729271	stage_1D_vs_E10	ordinary_muscle_development	ENSGALG00010010255	CACNB4	0.133002399	0.175700366	0.756984187	0.449059336	0.498286388	positive
PRJNA729271	stage_1D_vs_E10	ordinary_muscle_development	ENSGALG00010010257	ENSGALG00010010257	1.25758933	0.133419781	9.425808704	4.26804E-21	2.42324E-20	positive
PRJNA729271	stage_1D_vs_E10	ordinary_muscle_development	ENSGALG00010010259	IGF1	0.377644685	0.395971216	0.953717516	0.340226651	0.389018325	positive
PRJNA729271	stage_1D_vs_E10	ordinary_muscle_development	ENSGALG00010010262	HSPA9	0.567574624	0.145599516	3.898190322	9.69142E-05	0.00018003	positive
PRJNA729271	stage_1D_vs_E10	ordinary_muscle_development	ENSGALG00010010263	RAB4A	-0.905139056	0.137487054	-6.583449333	4.59657E-11	1.4261E-10	negative
PRJNA729271	stage_1D_vs_E10	ordinary_muscle_development	ENSGALG00010010266	EFNA5	-1.332200976	0.20731725	-6.425905107	1.31087E-10	3.93886E-10	negative
PRJNA729271	stage_1D_vs_E10	ordinary_muscle_development	ENSGALG00010010267	CCSER2	0.290982643	0.107356626	2.710430223	0.006719599	0.010154356	positive
PRJNA729271	stage_1D_vs_E10	ordinary_muscle_development	ENSGALG00010010270	SSR1	-0.905701041	0.077772957	-11.64544953	2.42042E-31	2.22897E-30	negative
PRJNA729271	stage_1D_vs_E10	ordinary_muscle_development	ENSGALG00010010271	HUNK	-1.789927757	0.110280743	-16.23064645	3.06217E-59	8.17426E-58	negative
PRJNA729271	stage_1D_vs_E10	ordinary_muscle_development	ENSGALG00010010274	MIS18A	-1.575241373	0.09484103	-16.60928165	5.97039E-62	1.75184E-60	negative
PRJNA729271	stage_1D_vs_E10	ordinary_muscle_development	ENSGALG00010010275	PDE8B	0.152568656	0.848146791	0.17988473	0.857243064	0.877833854	positive
PRJNA729271	stage_1D_vs_E10	ordinary_muscle_development	ENSGALG00010010279	CCSAP	0.971958147	0.240029396	4.049329638	5.13646E-05	9.8016E-05	positive
PRJNA729271	stage_1D_vs_E10	ordinary_muscle_development	ENSGALG00010010280	HSP90B1	-0.696111759	0.135456653	-5.139000153	2.76204E-07	6.43249E-07	negative
PRJNA729271	stage_1D_vs_E10	ordinary_muscle_development	ENSGALG00010010284	CCDC25	0.34603999	0.094398715	3.665727773	0.000246636	0.000438434	positive
PRJNA729271	stage_1D_vs_E10	ordinary_muscle_development	ENSGALG00010010285	ENSGALG00010010285	1.770965053	0.585887317	3.022705904	0.002505256	0.003981327	positive
PRJNA729271	stage_1D_vs_E10	ordinary_muscle_development	ENSGALG00010010287	STAM2	0.030934035	0.096980649	0.318971211	0.749748344	0.781785645	positive
PRJNA729271	stage_1D_vs_E10	ordinary_muscle_development	ENSGALG00010010288	ENSGALG00010010288	-0.339360599	0.163800249	-2.071795375	0.038284528	0.051965	negative
PRJNA729271	stage_1D_vs_E10	ordinary_muscle_development	ENSGALG00010010290	ENSGALG00010010290	1.208194507	0.162653369	7.428032478	1.10225E-13	4.06749E-13	positive
PRJNA729271	stage_1D_vs_E10	ordinary_muscle_development	ENSGALG00010010291	TCF20	-0.323684213	0.117359318	-2.758061466	0.005814526	0.008864278	negative
PRJNA729271	stage_1D_vs_E10	ordinary_muscle_development	ENSGALG00010010293	ENSGALG00010010293	-2.378028064	0.757147787	-3.140771333	0.001685035	0.002738797	negative
PRJNA729271	stage_1D_vs_E10	ordinary_muscle_development	ENSGALG00010010295	ENSGALG00010010295	6.171015105	0.181418338	34.01538765	1.3195E-253	1.2751E-250	positive
PRJNA729271	stage_1D_vs_E10	ordinary_muscle_development	ENSGALG00010010298	KCNIP4	-1.656189967	0.413430104	-4.005973325	6.17626E-05	0.000117133	negative
PRJNA729271	stage_1D_vs_E10	ordinary_muscle_development	ENSGALG00010010300	DENND1B	1.147672831	0.092145104	12.45506029	1.31252E-35	1.45562E-34	positive
PRJNA729271	stage_1D_vs_E10	ordinary_muscle_development	ENSGALG00010010301	ENSGALG00010010301	-0.887850018	0.531233404	-1.671299302	0.094662574	0.120860919	negative
PRJNA729271	stage_1D_vs_E10	ordinary_muscle_development	ENSGALG00010010302	WDR36	-0.20598645	0.103882059	-1.98288764	0.047379982	0.063396366	negative
PRJNA729271	stage_1D_vs_E10	ordinary_muscle_development	ENSGALG00010010303	ETF1	0.475221256	0.18454479	2.575099829	0.010021116	0.014812979	positive
PRJNA729271	stage_1D_vs_E10	ordinary_muscle_development	ENSGALG00010010305	URB1	-0.55873203	0.101824659	-5.487197645	4.0836E-08	1.01373E-07	negative
PRJNA729271	stage_1D_vs_E10	ordinary_muscle_development	ENSGALG00010010308	PACRGL	-0.452989471	0.212895956	-2.127750478	0.033357777	0.045731673	negative
PRJNA729271	stage_1D_vs_E10	ordinary_muscle_development	ENSGALG00010010310	NT5DC3	1.897898458	0.117501055	16.15218222	1.09614E-58	2.86797E-57	positive
PRJNA729271	stage_1D_vs_E10	ordinary_muscle_development	ENSGALG00010010311	CTSV	1.05340872	0.189382579	5.562331695	2.66194E-08	6.7069E-08	positive
PRJNA729271	stage_1D_vs_E10	ordinary_muscle_development	ENSGALG00010010313	GRID1	-2.366476095	0.198533073	-11.91980789	9.33235E-33	9.21474E-32	negative
PRJNA729271	stage_1D_vs_E10	ordinary_muscle_development	ENSGALG00010010314	CRB1	0.854871226	0.277490742	3.080719815	0.002065009	0.003316966	positive
PRJNA729271	stage_1D_vs_E10	ordinary_muscle_development	ENSGALG00010010317	ENSGALG00010010317	0.009466302	0.106964886	0.088499159	0.929479953	0.940182269	positive
PRJNA729271	stage_1D_vs_E10	ordinary_muscle_development	ENSGALG00010010320	SYPL1	-0.44748922	0.607747173	-0.736308189	0.461543164	0.510341609	negative
PRJNA729271	stage_1D_vs_E10	ordinary_muscle_development	ENSGALG00010010321	NUP133	-0.64963833	0.071562604	-9.077902336	1.10687E-19	5.82364E-19	negative
PRJNA729271	stage_1D_vs_E10	ordinary_muscle_development	ENSGALG00010010322	ENSGALG00010010322	0.08191477	0.067126758	1.220299812	0.222351241	0.264960641	positive
PRJNA729271	stage_1D_vs_E10	ordinary_muscle_development	ENSGALG00010010323	FBP2	6.770617399	0.241414043	28.04566505	4.5117E-173	1.211E-170	positive
PRJNA729271	stage_1D_vs_E10	ordinary_muscle_development	ENSGALG00010010327	FBP1	-2.105771966	0.584429597	-3.603123415	0.000314416	0.00055309	negative
PRJNA729271	stage_1D_vs_E10	ordinary_muscle_development	ENSGALG00010010330	ALPK2	1.930721245	0.153207754	12.60198125	2.05906E-36	2.35938E-35	positive
PRJNA729271	stage_1D_vs_E10	ordinary_muscle_development	ENSGALG00010010333	WAPL	0.140604227	0.063126649	2.22733552	0.025924858	0.036073804	positive
PRJNA729271	stage_1D_vs_E10	ordinary_muscle_development	ENSGALG00010010334	ZBTB41	0.798635681	0.086232092	9.261467102	2.01642E-20	1.10128E-19	positive
PRJNA729271	stage_1D_vs_E10	ordinary_muscle_development	ENSGALG00010010336	FMNL2	-0.383183686	0.087328939	-4.387820219	1.14492E-05	2.32563E-05	negative
PRJNA729271	stage_1D_vs_E10	ordinary_muscle_development	ENSGALG00010010337	SCARA3	-0.163698097	0.154191861	-1.061651995	0.2883937	0.334823122	negative
PRJNA729271	stage_1D_vs_E10	ordinary_muscle_development	ENSGALG00010010338	CNST	1.080724239	0.155681819	6.941878288	3.86921E-12	1.28751E-11	positive
PRJNA729271	stage_1D_vs_E10	ordinary_muscle_development	ENSGALG00010010345	ENSGALG00010010345	0.209472541	0.134117142	1.561862545	0.118320374	0.148695493	positive
PRJNA729271	stage_1D_vs_E10	ordinary_muscle_development	ENSGALG00010010346	TFB2M	0.180833812	0.139761336	1.293875812	0.195708321	0.235613962	positive
PRJNA729271	stage_1D_vs_E10	ordinary_muscle_development	ENSGALG00010010347	FBXW11	-0.093575035	0.083888523	-1.115468852	0.264649559	0.309787237	negative
PRJNA729271	stage_1D_vs_E10	ordinary_muscle_development	ENSGALG00010010349	RREB1	1.122018256	0.081733744	13.72772365	6.9276E-43	1.00718E-41	positive
PRJNA729271	stage_1D_vs_E10	ordinary_muscle_development	ENSGALG00010010351	NAMPTP1	2.620256577	0.212589954	12.3254017	6.61132E-35	7.14092E-34	positive
PRJNA729271	stage_1D_vs_E10	ordinary_muscle_development	ENSGALG00010010352	MALT1	0.051228858	0.115016203	0.44540557	0.656026687	0.695466016	positive
PRJNA729271	stage_1D_vs_E10	ordinary_muscle_development	ENSGALG00010010356	STAB2	1.664639654	0.277408105	6.00068861	1.96482E-09	5.43203E-09	positive
PRJNA729271	stage_1D_vs_E10	ordinary_muscle_development	ENSGALG00010010358	ASPM	-1.36756362	0.181300841	-7.543062749	4.5906E-14	1.73736E-13	negative
PRJNA729271	stage_1D_vs_E10	ordinary_muscle_development	ENSGALG00010010361	PIGM	-0.462557183	0.159572112	-2.898734486	0.00374672	0.005842183	negative
PRJNA729271	stage_1D_vs_E10	ordinary_muscle_development	ENSGALG00010010366	CLU	1.167975719	0.235759914	4.954089528	7.26697E-07	1.63996E-06	positive
PRJNA729271	stage_1D_vs_E10	ordinary_muscle_development	ENSGALG00010010368	ENSGALG00010010368	0.089591894	0.079540535	1.126367754	0.26000989	0.305070694	positive
PRJNA729271	stage_1D_vs_E10	ordinary_muscle_development	ENSGALG00010010369	ENSGALG00010010369	-0.01280609	0.23888971	-0.053606704	0.957248515	0.964032323	negative
PRJNA729271	stage_1D_vs_E10	ordinary_muscle_development	ENSGALG00010010371	SOCS1L	-2.335820213	0.212177672	-11.00879365	3.46614E-28	2.73498E-27	negative
PRJNA729271	stage_1D_vs_E10	ordinary_muscle_development	ENSGALG00010010374	CMTM4	-0.127507094	0.111741786	-1.141086955	0.253833739	0.298500734	negative
PRJNA729271	stage_1D_vs_E10	ordinary_muscle_development	ENSGALG00010010379	SLC35F6	-0.272170063	0.189557363	-1.435819	0.151053852	0.186120842	negative
PRJNA729271	stage_1D_vs_E10	ordinary_muscle_development	ENSGALG00010010381	MAST4	0.837420372	0.131693916	6.358838737	2.03285E-10	6.02949E-10	positive
PRJNA729271	stage_1D_vs_E10	ordinary_muscle_development	ENSGALG00010010382	SEC11C	1.122273384	0.149540377	7.504818497	6.15138E-14	2.30458E-13	positive
PRJNA729271	stage_1D_vs_E10	ordinary_muscle_development	ENSGALG00010010383	SLIT2	-0.496799629	0.159713881	-3.11056011	0.001867329	0.003018505	negative
PRJNA729271	stage_1D_vs_E10	ordinary_muscle_development	ENSGALG00010010386	PRPF40A	0.380418806	0.146284687	2.600537437	0.009307786	0.013820566	positive
PRJNA729271	stage_1D_vs_E10	ordinary_muscle_development	ENSGALG00010010390	KCNK3	2.36302833	0.411715079	5.739474816	9.49706E-09	2.48215E-08	positive
PRJNA729271	stage_1D_vs_E10	ordinary_muscle_development	ENSGALG00010010393	FANCC	-1.740729777	0.143110269	-12.16355604	4.85963E-34	5.05673E-33	negative
PRJNA729271	stage_1D_vs_E10	ordinary_muscle_development	ENSGALG00010010394	MRPL42	-0.131301242	0.124244058	-1.05680097	0.290602432	0.337090449	negative
PRJNA729271	stage_1D_vs_E10	ordinary_muscle_development	ENSGALG00010010395	GRP	0.12590676	0.834679184	0.150844495	0.880098386	0.897749902	positive
PRJNA729271	stage_1D_vs_E10	ordinary_muscle_development	ENSGALG00010010397	PICK1	-0.647631676	0.09248821	-7.002316074	2.51765E-12	8.48486E-12	negative
PRJNA729271	stage_1D_vs_E10	ordinary_muscle_development	ENSGALG00010010399	FGF18	2.157039902	0.174390308	12.36903542	3.8441E-35	4.19896E-34	positive
PRJNA729271	stage_1D_vs_E10	ordinary_muscle_development	ENSGALG00010010403	ENSGALG00010010403	-1.159689447	0.450429664	-2.574629382	0.010034755	0.014831627	negative
PRJNA729271	stage_1D_vs_E10	ordinary_muscle_development	ENSGALG00010010404	COP1	0.127079659	0.116171057	1.093901211	0.273998308	0.319718909	positive
PRJNA729271	stage_1D_vs_E10	ordinary_muscle_development	ENSGALG00010010405	EVA1C	2.158256666	0.23060559	9.359082156	8.04323E-21	4.5049E-20	positive
PRJNA729271	stage_1D_vs_E10	ordinary_muscle_development	ENSGALG00010010406	PTCH1	-1.074155917	0.094101601	-11.41485277	3.525E-30	3.08172E-29	negative
PRJNA729271	stage_1D_vs_E10	ordinary_muscle_development	ENSGALG00010010407	SOX10	0.886187303	0.286769793	3.090239365	0.001999952	0.003219961	positive
PRJNA729271	stage_1D_vs_E10	ordinary_muscle_development	ENSGALG00010010408	GAS8	-0.864759594	0.119775517	-7.219836035	5.20503E-13	1.84017E-12	negative
PRJNA729271	stage_1D_vs_E10	ordinary_muscle_development	ENSGALG00010010410	CACNA2D1	2.898532331	0.158022603	18.34251727	3.78842E-75	1.59168E-73	positive
PRJNA729271	stage_1D_vs_E10	ordinary_muscle_development	ENSGALG00010010411	CD180	-0.085092724	0.464082078	-0.183357057	0.854517868	0.875476145	negative
PRJNA729271	stage_1D_vs_E10	ordinary_muscle_development	ENSGALG00010010416	SLC35B3	-0.378703639	0.126667271	-2.989751307	0.002792047	0.004417236	negative
PRJNA729271	stage_1D_vs_E10	ordinary_muscle_development	ENSGALG00010010417	OTOF	1.152968165	0.632339892	1.823336121	0.068252499	0.089127925	positive
PRJNA729271	stage_1D_vs_E10	ordinary_muscle_development	ENSGALG00010010418	BRINP2	-5.076123958	0.522616333	-9.712907235	2.6565E-22	1.60508E-21	negative
PRJNA729271	stage_1D_vs_E10	ordinary_muscle_development	ENSGALG00010010420	NPM1	-0.833732157	0.112297714	-7.424302134	1.13376E-13	4.17527E-13	negative
PRJNA729271	stage_1D_vs_E10	ordinary_muscle_development	ENSGALG00010010421	POLR2F	0.33337474	0.090595346	3.67982193	0.000233397	0.000416175	positive
PRJNA729271	stage_1D_vs_E10	ordinary_muscle_development	ENSGALG00010010422	CDH1	-5.304479345	0.939242909	-5.647611813	1.62692E-08	4.17902E-08	negative
PRJNA729271	stage_1D_vs_E10	ordinary_muscle_development	ENSGALG00010010423	C21orf59	-0.159880076	0.09872627	-1.619427904	0.105355227	0.133443203	negative
PRJNA729271	stage_1D_vs_E10	ordinary_muscle_development	ENSGALG00010010425	BLOC1S5	0.582848657	0.078475206	7.427169499	1.10946E-13	4.09098E-13	positive
PRJNA729271	stage_1D_vs_E10	ordinary_muscle_development	ENSGALG00010010430	ASTN1	-4.953121819	0.531661289	-9.316310819	1.20456E-20	6.67689E-20	negative
PRJNA729271	stage_1D_vs_E10	ordinary_muscle_development	ENSGALG00010010431	SOCS2	-1.705327684	0.253812319	-6.718853089	1.8316E-11	5.82601E-11	negative
PRJNA729271	stage_1D_vs_E10	ordinary_muscle_development	ENSGALG00010010433	ENSGALG00010010433	0.556846483	1.563621192	0.356126206	0.721746053	0.756614525	positive
PRJNA729271	stage_1D_vs_E10	ordinary_muscle_development	ENSGALG00010010434	PROM1	-1.754989705	0.148079508	-11.85167163	2.1094E-32	2.04384E-31	negative
PRJNA729271	stage_1D_vs_E10	ordinary_muscle_development	ENSGALG00010010435	GAS1	-0.112493855	0.165225084	-0.680852158	0.495965043	0.544168745	negative
PRJNA729271	stage_1D_vs_E10	ordinary_muscle_development	ENSGALG00010010438	LDB3	3.432211394	0.18521736	18.53072196	1.1671E-76	5.03488E-75	positive
PRJNA729271	stage_1D_vs_E10	ordinary_muscle_development	ENSGALG00010010439	HGF	2.006158922	0.237812465	8.43588632	3.28702E-17	1.50777E-16	positive
PRJNA729271	stage_1D_vs_E10	ordinary_muscle_development	ENSGALG00010010444	TXNDC5	-1.658701375	0.139873704	-11.85856476	1.94277E-32	1.88743E-31	negative
PRJNA729271	stage_1D_vs_E10	ordinary_muscle_development	ENSGALG00010010445	LMAN1L	-0.563552021	0.08297951	-6.791459977	1.11004E-11	3.58274E-11	negative
PRJNA729271	stage_1D_vs_E10	ordinary_muscle_development	ENSGALG00010010446	C22orf23	-1.735548767	0.623798304	-2.782227454	0.005398719	0.008263403	negative
PRJNA729271	stage_1D_vs_E10	ordinary_muscle_development	ENSGALG00010010447	BTF3	0.194994399	0.09623711	2.026187185	0.042745606	0.057642345	positive
PRJNA729271	stage_1D_vs_E10	ordinary_muscle_development	ENSGALG00010010448	GALNT13	-1.165278251	0.373806733	-3.117328149	0.001824983	0.002953017	negative
PRJNA729271	stage_1D_vs_E10	ordinary_muscle_development	ENSGALG00010010449	PAPPA2	-3.33572508	0.760951364	-4.383624551	1.16721E-05	2.36857E-05	negative
PRJNA729271	stage_1D_vs_E10	ordinary_muscle_development	ENSGALG00010010451	RANBP17	-0.671900514	0.473166277	-1.420009302	0.155604973	0.191192175	negative
PRJNA729271	stage_1D_vs_E10	ordinary_muscle_development	ENSGALG00010010453	DRC1	-0.737757772	0.40655373	-1.814662411	0.069575805	0.090757856	negative
PRJNA729271	stage_1D_vs_E10	ordinary_muscle_development	ENSGALG00010010456	BMP6	-0.659141102	0.17700534	-3.723848689	0.000196209	0.00035264	negative
PRJNA729271	stage_1D_vs_E10	ordinary_muscle_development	ENSGALG00010010457	AGGF1	-0.271695927	0.090132951	-3.014390693	0.00257496	0.004088514	negative
PRJNA729271	stage_1D_vs_E10	ordinary_muscle_development	ENSGALG00010010460	SEMA3C	1.33319875	0.141940114	9.392684803	5.849E-21	3.29888E-20	positive
PRJNA729271	stage_1D_vs_E10	ordinary_muscle_development	ENSGALG00010010461	SLC7A5	-1.792985642	0.263570555	-6.802678089	1.02692E-11	3.32037E-11	negative
PRJNA729271	stage_1D_vs_E10	ordinary_muscle_development	ENSGALG00010010463	ACAA2	2.030095139	0.127860132	15.87746787	9.07694E-57	2.23E-55	positive
PRJNA729271	stage_1D_vs_E10	ordinary_muscle_development	ENSGALG00010010465	BMPR1A	-0.429667598	0.097613034	-4.401744101	1.07384E-05	2.18737E-05	negative
PRJNA729271	stage_1D_vs_E10	ordinary_muscle_development	ENSGALG00010010466	CRADD	0.442857879	0.124439516	3.55882033	0.000372524	0.000650493	positive
PRJNA729271	stage_1D_vs_E10	ordinary_muscle_development	ENSGALG00010010467	FGFBP2	-8.220317704	0.545400363	-15.07207964	2.47207E-51	4.98369E-50	negative
PRJNA729271	stage_1D_vs_E10	ordinary_muscle_development	ENSGALG00010010468	ENSGALG00010010468	0.958200839	1.461371829	0.655685856	0.512026271	0.559841653	positive
PRJNA729271	stage_1D_vs_E10	ordinary_muscle_development	ENSGALG00010010471	KCNJ3	-0.218542316	0.152069731	-1.437119103	0.150684154	0.185739208	negative
PRJNA729271	stage_1D_vs_E10	ordinary_muscle_development	ENSGALG00010010472	MMRN2	1.3367403	0.136490085	9.793680617	1.19852E-22	7.38E-22	positive
PRJNA729271	stage_1D_vs_E10	ordinary_muscle_development	ENSGALG00010010473	TRIM35	0.427061661	0.178484824	2.392705738	0.016724648	0.023938361	positive
PRJNA729271	stage_1D_vs_E10	ordinary_muscle_development	ENSGALG00010010475	CCDC71L	0.358301457	0.108231966	3.3104957	0.000931309	0.001558273	positive
PRJNA729271	stage_1D_vs_E10	ordinary_muscle_development	ENSGALG00010010478	ENSGALG00010010478	0.899322336	0.20496249	4.387741071	1.14534E-05	2.32615E-05	positive
PRJNA729271	stage_1D_vs_E10	ordinary_muscle_development	ENSGALG00010010483	FGFBP1	-3.904266001	0.961197411	-4.06187736	4.86797E-05	9.31254E-05	negative

PRJNA729271	stage_1D_vs_E10	ordinary_muscle_development	ENSGALG00010010486	ENSGALG00010010486	-3.639635351	0.481930641	-7.552197436	4.27975E-14	1.62141E-13	negative
PRJNA729271	stage_1D_vs_E10	ordinary_muscle_development	ENSGALG00010010494	ENSGALG00010010494	1.311256463	0.262057518	5.003697173	5.62411E-07	1.28078E-06	positive
PRJNA729271	stage_1D_vs_E10	ordinary_muscle_development	ENSGALG00010010496	MRPL17	0.33030253	0.142349679	2.3203602	0.0203214	0.028723422	positive
PRJNA729271	stage_1D_vs_E10	ordinary_muscle_development	ENSGALG00010010499	NR4A2	3.738365298	0.450026665	8.306986206	9.81634E-17	4.37943E-16	positive
PRJNA729271	stage_1D_vs_E10	ordinary_muscle_development	ENSGALG00010010505	CD38	1.243087608	0.198954587	6.248097258	4.15483E-10	1.20473E-09	positive
PRJNA729271	stage_1D_vs_E10	ordinary_muscle_development	ENSGALG00010010511	GABRP	-2.913859776	0.495341838	-5.882523041	4.04059E-09	1.08863E-08	negative
PRJNA729271	stage_1D_vs_E10	ordinary_muscle_development	ENSGALG00010010512	PLXNC1	-1.218650548	0.20046999	-6.078967477	1.20959E-09	3.39326E-09	negative
PRJNA729271	stage_1D_vs_E10	ordinary_muscle_development	ENSGALG00010010515	RXRG	-1.142563258	0.380742333	-3.000883163	0.002691978	0.004265438	negative
PRJNA729271	stage_1D_vs_E10	ordinary_muscle_development	ENSGALG00010010517	STMN4	-5.404451227	0.311241753	-17.36415883	1.54142E-67	5.17197E-66	negative
PRJNA729271	stage_1D_vs_E10	ordinary_muscle_development	ENSGALG00010010521	SNCG	-0.180026681	0.387253431	-0.464880791	0.642016827	0.682310573	negative
PRJNA729271	stage_1D_vs_E10	ordinary_muscle_development	ENSGALG00010010526	ENSGALG00010010526	0.996512266	0.242171003	4.114911593	3.87328E-05	7.47181E-05	positive
PRJNA729271	stage_1D_vs_E10	ordinary_muscle_development	ENSGALG00010010528	GPD2	1.099738273	0.124839603	8.809209927	1.26031E-18	6.25195E-18	positive
PRJNA729271	stage_1D_vs_E10	ordinary_muscle_development	ENSGALG00010010534	SYNJ1	0.98845146	0.119353586	8.28170728	1.21422E-16	5.38888E-16	positive
PRJNA729271	stage_1D_vs_E10	ordinary_muscle_development	ENSGALG00010010535	LMX1A	0.189392341	0.408380162	0.463764792	0.642816271	0.683110106	positive
PRJNA729271	stage_1D_vs_E10	ordinary_muscle_development	ENSGALG00010010537	ENSGALG00010010537	0.559740509	0.204916544	2.731553523	0.00630365	0.009567687	positive
PRJNA729271	stage_1D_vs_E10	ordinary_muscle_development	ENSGALG00010010544	ENSGALG00010010544	-0.382948586	0.162216743	-2.360721701	0.018239412	0.025932348	negative
PRJNA729271	stage_1D_vs_E10	ordinary_muscle_development	ENSGALG00010010546	MICALL1	0.030550734	0.167612383	0.182270146	0.855370726	0.876040322	positive
PRJNA729271	stage_1D_vs_E10	ordinary_muscle_development	ENSGALG00010010547	BST1	0.46132925	0.165235719	2.791946269	0.005239206	0.00803282	positive
PRJNA729271	stage_1D_vs_E10	ordinary_muscle_development	ENSGALG00010010567	ERMN	-0.367342834	0.491021712	-0.74811933	0.454388183	0.503274755	negative
PRJNA729271	stage_1D_vs_E10	ordinary_muscle_development	ENSGALG00010010572	AQP3	3.440529327	0.146096808	23.54965433	1.2656E-122	1.4915E-120	positive
PRJNA729271	stage_1D_vs_E10	ordinary_muscle_development	ENSGALG00010010573	CEP83	-1.07783885	0.158355297	-6.806459104	1.0003E-11	3.23719E-11	negative
PRJNA729271	stage_1D_vs_E10	ordinary_muscle_development	ENSGALG00010010575	PAXBP1	0.286282284	0.122805558	2.33118345	0.019743688	0.027961384	positive
PRJNA729271	stage_1D_vs_E10	ordinary_muscle_development	ENSGALG00010010577	PBX1	-0.177502053	0.145626335	-1.21888705	0.222887066	0.265533658	negative
PRJNA729271	stage_1D_vs_E10	ordinary_muscle_development	ENSGALG00010010578	GLUD2	0.761517203	0.111229405	6.846365868	7.57496E-12	2.46961E-11	positive
PRJNA729271	stage_1D_vs_E10	ordinary_muscle_development	ENSGALG00010010585	FBXL5	1.005961939	0.163256652	6.161843496	7.19029E-10	2.05204E-09	positive
PRJNA729271	stage_1D_vs_E10	ordinary_muscle_development	ENSGALG00010010586	CYTIP	-1.10864734	0.280873707	-3.947138201	7.90909E-05	0.000148212	negative
PRJNA729271	stage_1D_vs_E10	ordinary_muscle_development	ENSGALG00010010588	SLC25A3	0.692448282	0.100990912	6.856540523	7.05481E-12	2.30314E-11	positive
PRJNA729271	stage_1D_vs_E10	ordinary_muscle_development	ENSGALG00010010593	EIF3L	0.529207095	0.103704141	5.103046915	3.34228E-07	7.73409E-07	positive
PRJNA729271	stage_1D_vs_E10	ordinary_muscle_development	ENSGALG00010010596	SHLD2	0.251134365	0.133526358	1.880784951	0.060001178	0.078986203	positive
PRJNA729271	stage_1D_vs_E10	ordinary_muscle_development	ENSGALG00010010599	F2RL1	-1.952818416	0.20295416	-9.621967913	6.45836E-22	3.84451E-21	negative
PRJNA729271	stage_1D_vs_E10	ordinary_muscle_development	ENSGALG00010010604	HIVEP1	0.223980065	0.099820199	2.243835091	0.024843014	0.034688323	positive
PRJNA729271	stage_1D_vs_E10	ordinary_muscle_development	ENSGALG00010010605	NUF2	-1.706951749	0.266860428	-6.396421383	1.59061E-10	4.74889E-10	negative
PRJNA729271	stage_1D_vs_E10	ordinary_muscle_development	ENSGALG00010010614	IKBIP	-0.186376783	0.141631852	-1.315924215	0.18819948	0.227556846	negative
PRJNA729271	stage_1D_vs_E10	ordinary_muscle_development	ENSGALG00010010618	APAF1	-0.731705526	0.137037707	-5.339446645	9.32307E-08	2.25568E-07	negative
PRJNA729271	stage_1D_vs_E10	ordinary_muscle_development	ENSGALG00010010619	LRRC52	-1.165154687	0.274121989	-4.250496986	2.13297E-05	4.21562E-05	negative
PRJNA729271	stage_1D_vs_E10	ordinary_muscle_development	ENSGALG00010010620	ANKRD54	0.299394283	0.110586537	2.707330308	0.006782674	0.01024032	positive
PRJNA729271	stage_1D_vs_E10	ordinary_muscle_development	ENSGALG00010010623	SMAD2	-0.3470878	0.076922136	-4.512196599	6.41597E-06	1.33447E-05	negative
PRJNA729271	stage_1D_vs_E10	ordinary_muscle_development	ENSGALG00010010625	FAM169A	-4.009306935	0.269527717	-14.8753048	4.76809E-50	9.23976E-49	negative
PRJNA729271	stage_1D_vs_E10	ordinary_muscle_development	ENSGALG00010010626	ADTRP	1.760725618	0.576856745	3.052275343	0.002271137	0.003629562	positive
PRJNA729271	stage_1D_vs_E10	ordinary_muscle_development	ENSGALG00010010629	ENSGALG00010010629	-0.551576451	0.324133874	-1.701693326	0.088812872	0.113903962	negative
PRJNA729271	stage_1D_vs_E10	ordinary_muscle_development	ENSGALG00010010633	EPC1P	1.391891252	0.320349304	4.344917364	1.39328E-05	2.80495E-05	positive
PRJNA729271	stage_1D_vs_E10	ordinary_muscle_development	ENSGALG00010010635	ALDH9A1	-0.323449412	0.076208734	-4.244256451	2.19319E-05	4.32875E-05	negative
PRJNA729271	stage_1D_vs_E10	ordinary_muscle_development	ENSGALG00010010638	ACVR1C	2.697635075	0.431509531	6.251623389	4.06208E-10	1.17854E-09	positive
PRJNA729271	stage_1D_vs_E10	ordinary_muscle_development	ENSGALG00010010642	CC2D2A	-0.485256209	0.119413035	-4.063678731	4.83053E-05	9.24703E-05	negative
PRJNA729271	stage_1D_vs_E10	ordinary_muscle_development	ENSGALG00010010645	FAM219A	0.078626414	0.08548752	0.919741429	0.357707897	0.406601001	positive
PRJNA729271	stage_1D_vs_E10	ordinary_muscle_development	ENSGALG00010010646	MCM3AP	-0.328192251	0.057716118	-5.686318873	1.29807E-08	3.35811E-08	negative
PRJNA729271	stage_1D_vs_E10	ordinary_muscle_development	ENSGALG00010010648	CENPM	-1.105864189	0.241832966	-4.572843007	4.81151E-06	1.01135E-05	negative
PRJNA729271	stage_1D_vs_E10	ordinary_muscle_development	ENSGALG00010010651	NUAK1	0.895941234	0.133536339	6.709343998	1.95501E-11	6.20629E-11	positive
PRJNA729271	stage_1D_vs_E10	ordinary_muscle_development	ENSGALG00010010655	C1QTNF7	1.324318161	0.247547227	5.349759631	8.80711E-08	2.13512E-07	positive
PRJNA729271	stage_1D_vs_E10	ordinary_muscle_development	ENSGALG00010010657	TMEM170B	-1.537984034	0.17986747	-8.550651397	1.22396E-17	5.74709E-17	negative
PRJNA729271	stage_1D_vs_E10	ordinary_muscle_development	ENSGALG00010010659	ADAMTS1	1.719055437	0.151324706	11.36004479	6.61107E-30	5.68035E-29	positive
PRJNA729271	stage_1D_vs_E10	ordinary_muscle_development	ENSGALG00010010661	CPEB2	0.1629912	0.180362152	0.903688486	0.366160606	0.4152322	positive
PRJNA729271	stage_1D_vs_E10	ordinary_muscle_development	ENSGALG00010010662	MGST3	-1.503331654	0.153879239	-9.769554751	1.52119E-22	9.31151E-22	negative
PRJNA729271	stage_1D_vs_E10	ordinary_muscle_development	ENSGALG00010010663	EFR3B	-1.479735258	0.173082834	-8.549289506	1.23849E-17	5.81154E-17	negative
PRJNA729271	stage_1D_vs_E10	ordinary_muscle_development	ENSGALG00010010666	C12orf75	0.550040197	0.377635775	1.456536256	0.145244435	0.179634648	positive
PRJNA729271	stage_1D_vs_E10	ordinary_muscle_development	ENSGALG00010010671	SIGMAR1	-0.822351584	0.141283483	-5.820578366	5.86443E-09	1.56374E-08	negative
PRJNA729271	stage_1D_vs_E10	ordinary_muscle_development	ENSGALG00010010678	KCNIP1	-1.617271979	0.367534339	-4.400328909	1.08087E-05	2.20045E-05	negative
PRJNA729271	stage_1D_vs_E10	ordinary_muscle_development	ENSGALG00010010680	ENSGALG00010010680	-0.098421772	0.574686628	-0.171261637	0.864018044	0.883959737	negative
PRJNA729271	stage_1D_vs_E10	ordinary_muscle_development	ENSGALG00010010682	SMIM13	-0.583358551	0.105364782	-5.536561076	3.08468E-08	7.72904E-08	negative
PRJNA729271	stage_1D_vs_E10	ordinary_muscle_development	ENSGALG00010010685	STIM2	-0.11169276	0.108315418	-1.031180627	0.302456124	0.349497889	negative
PRJNA729271	stage_1D_vs_E10	ordinary_muscle_development	ENSGALG00010010688	IQGAP2	-1.794141473	0.170188288	-10.54209721	5.52528E-26	3.95306E-25	negative
PRJNA729271	stage_1D_vs_E10	ordinary_muscle_development	ENSGALG00010010689	POFUT2	-0.481953833	0.149857653	-3.216077547	0.001299557	0.002141307	negative
PRJNA729271	stage_1D_vs_E10	ordinary_muscle_development	ENSGALG00010010690	MTPN	-0.506297499	0.135221101	-3.744219631	0.000180955	0.000327173	negative
PRJNA729271	stage_1D_vs_E10	ordinary_muscle_development	ENSGALG00010010693	KCNMB1	-0.185430894	0.34311753	-0.540429673	0.588900749	0.633008258	negative
PRJNA729271	stage_1D_vs_E10	ordinary_muscle_development	ENSGALG00010010697	NEDD9	-0.289169396	0.138280216	-2.091184156	0.036511559	0.049735462	negative
PRJNA729271	stage_1D_vs_E10	ordinary_muscle_development	ENSGALG00010010698	GPRIN2	-1.750614265	0.181367475	-9.652305433	4.80628E-22	2.88477E-21	negative
PRJNA729271	stage_1D_vs_E10	ordinary_muscle_development	ENSGALG00010010699	DNAJC27	-0.342090212	0.151118814	-2.263716886	0.023591533	0.033065101	negative
PRJNA729271	stage_1D_vs_E10	ordinary_muscle_development	ENSGALG00010010706	SREBF2	0.215171962	0.138636123	1.552062745	0.120647211	0.15135722	positive
PRJNA729271	stage_1D_vs_E10	ordinary_muscle_development	ENSGALG00010010710	UCK2	-0.706768742	0.101084352	-6.991870922	2.71244E-12	9.12013E-12	negative
PRJNA729271	stage_1D_vs_E10	ordinary_muscle_development	ENSGALG00010010711	ENSGALG00010010711	-2.338254861	0.442792961	-5.280695639	1.28694E-07	3.08437E-07	negative
PRJNA729271	stage_1D_vs_E10	ordinary_muscle_development	ENSGALG00010010712	ADAMTS5	1.32572423	0.144884839	9.150192947	5.6832E-20	3.03306E-19	positive
PRJNA729271	stage_1D_vs_E10	ordinary_muscle_development	ENSGALG00010010713	NSA2	0.389816082	0.090590012	4.303080145	1.6844E-05	3.36022E-05	positive
PRJNA729271	stage_1D_vs_E10	ordinary_muscle_development	ENSGALG00010010719	SH3BP4	0.628892015	0.164718831	3.817972786	0.000134553	0.000246504	positive
PRJNA729271	stage_1D_vs_E10	ordinary_muscle_development	ENSGALG00010010722	TBC1D19	-1.174130313	0.167862619	-6.994590682	2.66034E-12	8.94911E-12	negative
PRJNA729271	stage_1D_vs_E10	ordinary_muscle_development	ENSGALG00010010724	ADCY3	0.431960882	0.14278028	3.025353937	0.002483423	0.003950095	positive
PRJNA729271	stage_1D_vs_E10	ordinary_muscle_development	ENSGALG00010010728	ELOVL2	1.61446971	0.858441027	1.880699617	0.060012792	0.078987144	positive
PRJNA729271	stage_1D_vs_E10	ordinary_muscle_development	ENSGALG00010010732	CCKAR	-5.22873531	1.104151025	-4.735525479	2.18488E-06	4.74953E-06	negative
PRJNA729271	stage_1D_vs_E10	ordinary_muscle_development	ENSGALG00010010733	GFM2	-0.171979775	0.126174944	-1.363026365	0.172874202	0.210509448	negative
PRJNA729271	stage_1D_vs_E10	ordinary_muscle_development	ENSGALG00010010734	AMN1	0.180064468	0.145672843	1.236088102	0.216425817	0.258473446	positive
PRJNA729271	stage_1D_vs_E10	ordinary_muscle_development	ENSGALG00010010740	CCDC134	0.954417007	0.095216232	10.02367969	1.19952E-23	7.69338E-23	positive
PRJNA729271	stage_1D_vs_E10	ordinary_muscle_development	ENSGALG00010010742	LCP2	0.541173975	0.2668516	2.027995988	0.042560657	0.057398282	positive
PRJNA729271	stage_1D_vs_E10	ordinary_muscle_development	ENSGALG00010010743	KLHL20	-0.13059578	0.083697575	-1.560329316	0.118682079	0.149059591	negative
PRJNA729271	stage_1D_vs_E10	ordinary_muscle_development	ENSGALG00010010745	ATVR1	-0.124437694	0.063264021	-1.966958352	0.049188012	0.065700354	negative
PRJNA729271	stage_1D_vs_E10	ordinary_muscle_development	ENSGALG00010010748	SF3B1	0.007406123	0.071705653	0.103285066	0.917736728	0.930056203	positive
PRJNA729271	stage_1D_vs_E10	ordinary_muscle_development	ENSGALG00010010749	NCOA4	-0.505599352	0.098409821	-5.137692009	2.78133E-07	6.47637E-07	negative
PRJNA729271	stage_1D_vs_E10	ordinary_muscle_development	ENSGALG00010010751	SLC30A5	-0.312959985	0.078546305	-3.984401131	6.76505E-05	0.000127682	negative
PRJNA729271	stage_1D_vs_E10	ordinary_muscle_development	ENSGALG00010010752	ENSGALG00010010752	-1.917545179	0.272803662	-7.029030209	2.07974E-12	7.04671E-12	negative
PRJNA729271	stage_1D_vs_E10	ordinary_muscle_development	ENSGALG00010010753	ENSGALG00010010753	2.541086683	1.160913846	2.188867582			positive
PRJNA729271	stage_1D_vs_E10	ordinary_muscle_development	ENSGALG00010010754	DNAI1	-2.429899425	0.284653382	-8.536344823	1.38535E-17	6.47343E-17	negative
PRJNA729271	stage_1D_vs_E10	ordinary_muscle_development	ENSGALG00010010756	CENPL	-1.890893002	0.19550055	-9.672059738	3.9632E-22	2.38368E-21	negative
PRJNA729271	stage_1D_vs_E10	ordinary_muscle_development	ENSGALG00010010759	ENSGALG00010010759	-0.757731475	0.289360734	-2.618639595	0.008828116	0.013143339	negative
PRJNA729271	stage_1D_vs_E10	ordinary_muscle_development	ENSGALG00010010760	FOXI1	0.61719995	0.753488267	0.819123504	0.412715953	0.462347766	positive
PRJNA729271	stage_1D_vs_E10	ordinary_muscle_development	ENSGALG00010010761	MEI1	0.34836181	0.265863217	1.310304654	0.190092794	0.229654529	positive
PRJNA729271	stage_1D_vs_E10	ordinary_muscle_development	ENSGALG00010010762	MRPL39	0.032167467	0.110595963	0.290855707	0.771161678	0.800658156	positive
PRJNA729271	stage_1D_vs_E10	ordinary_muscle_development	ENSGALG00010010765	ENSGALG00010010765	3.400912876	0.785226394	4.331123995	1.4835E-05	2.97707E-05	positive
PRJNA729271	stage_1D_vs_E10	ordinary_muscle_development	ENSGALG00010010766	CENPO	-1.858302634	0.23610204	-7.87076059	3.52492E-15	1.43119E-14	negative
PRJNA729271	stage_1D_vs_E10	ordinary_muscle_development	ENSGALG00010010768	ENSGALG00010010768	0.199476525	0.091746058	2.174224489	0.02968828	0.040948865	positive
PRJNA729271	stage_1D_vs_E10	ordinary_muscle_development	ENSGALG00010010769	HEXB	-0.572232632	0.122001651	-4.69036792	2.72714E-06	5.87365E-06	negative
PRJNA729271	stage_1D_vs_E10	ordinary_muscle_development	ENSGALG00010010771	ENSGALG00010010771	-0.385247129	0.07171091	-5.372224814	7.77711E-08	1.89461E-07	negative
PRJNA729271	stage_1D_vs_E10	ordinary_muscle_development	ENSGALG00010010774	KPTN	-0.780120989	0.137977174	-5.653985861	1.56769E-08	4.03117E-08	negative
PRJNA729271	stage_1D_vs_E10	ordinary_muscle_development	ENSGALG00010010776	GCAT	0.46372695	0.194076161	2.389407068	0.016875593	0.024142491	positive
PRJNA729271	stage_1D_vs_E10	ordinary_muscle_development	ENSGALG00010010778	UPP2	2.869443752	0.365448617	7.851839133	4.0998E-15	1.65903E-14	positive
PRJNA729271	stage_1D_vs_E10	ordinary_muscle_development	ENSGALG00010010779	DOCK2	-0.53787742	0.190069422	-2.829899805	0.004656258	0.007184635	negative
PRJNA729271	stage_1D_vs_E10	ordinary_muscle_development	ENSGALG00010010780	PARG	-0.724051707	0.049285671	-14.69091705	7.37095E-49	1.35931E-47	negative
PRJNA729271	stage_1D_vs_E10	ordinary_muscle_development	ENSGALG00010010781	NAPA	0.305440318	0.097508677	3.132442457	0.001733584	0.002813289	positive
PRJNA729271	stage_1D_vs_E10	ordinary_muscle_development	ENSGALG00010010783	F2RL2	-0.217593347	0.142559902	-1.526329243	0.126927889	0.158509499	negative
PRJNA729271	stage_1D_vs_E10	ordinary_muscle_development	ENSGALG00010010784	CCDC148	2.035425222	0.284172376	7.162642809	7.91364E-13	2.7594E-12	positive
PRJNA729271	stage_1D_vs_E10	ordinary_muscle_development	ENSGALG00010010786	NABP1	0.151114455	0.154228825	0.979806818	0.327181486	0.37547027	positive
PRJNA729271	stage_1D_vs_E10	ordinary_muscle_development	ENSGALG00010010789	ENSGALG00010010789	0.387077037	0.257128931	1.505381115	0.132226122	0.164601308	positive
PRJNA729271	stage_1D_vs_E10	ordinary_muscle_development	ENSGALG00010010791	H-10	-0.377080766	0.294645963	-1.279775776	0.200624006	0.24089173	negative
PRJNA729271	stage_1D_vs_E10	ordinary_muscle_development	ENSGALG00010010792	XRCC6	-1.225839919	0.14874462	-8.2412387	1.70437E-16	7.50906E-16	negative
PRJNA729271	stage_1D_vs_E10	ordinary_muscle_development	ENSGALG00010010796	CAVIN2	2.40670407	0.145948081	16.49013853	4.31958E-61	1.22769E-59	positive
PRJNA729271	stage_1D_vs_E10	ordinary_muscle_development	ENSGALG00010010799	DARS2	-0.159668699	0.105848872	-1.508459142	0.131437056	0.163717464	negative
PRJNA729271	stage_1D_vs_E10	ordinary_muscle_development	ENSGALG00010010800	BICRA	1.185301059	0.186649793	6.350401148	2.14754E-10	6.36317E-10	positive
PRJNA729271	stage_1D_vs_E10	ordinary_muscle_development	ENSGALG00010010805	PTRHD1	-1.156420662	0.17196506	-6.724742014	1.75904E-11	5.60133E-11	negative
PRJNA729271	stage_1D_vs_E10	ordinary_muscle_development	ENSGALG00010010806	INSYN2B	-0.678384772	0.826032118	-0.821257137	0.411499817	0.461127936	negative
PRJNA729271	stage_1D_vs_E10	ordinary_muscle_development	ENSGALG00010010809	CENPH	-1.875700682	0.15401608	-12.17860289	4.04146E-34	4.21749E-33	negative
PRJNA729271	stage_1D_vs_E10	ordinary_muscle_development	ENSGALG00010010810	GALT	0.138283579	0.113174057	1.221866415	0.221758148	0.264297349	positive
PRJNA729271	stage_1D_vs_E10	ordinary_muscle_development	ENSGALG00010010813	EHD2	2.578164921	0.147669125	17.45906549	2.93703E-68	1.02831E-66	positive
PRJNA729271	stage_1D_vs_E10	ordinary_muscle_development	ENSGALG00010010816	AP2S1	-1.150702861	0.112770754	-10.20391208	1.90443E-24	1.26686E-23	negative
PRJNA729271	stage_1D_vs_E10	ordinary_muscle_development	ENSGALG00010010821	RGS8	-1.607625754	0.432241717	-3.719274869	0.000199796	0.000358702	negative
PRJNA729271	stage_1D_vs_E10	ordinary_muscle_development	ENSGALG00010010822	OGDHL	-1.847551842	0.38642896	-4.781090534	1.74347E-06	3.814E-06	negative
PRJNA729271	stage_1D_vs_E10	ordinary_muscle_development	ENSGALG00010010823	TMEFF2	-1.333682964	0.167513268	-7.961655676	1.69752E-15	7.02638E-15	negative
PRJNA729271	stage_1D_vs_E10	ordinary_muscle_development	ENSGALG00010010824	PKP4	-0.262931929	0.109658658	-2.397730686	0.016496989	0.023621838	negative
PRJNA729271	stage_1D_vs_E10	ordinary_muscle_development	ENSGALG00010010825	MAK	1.869722898	0.381556816	4.900247669	9.57159E-07	2.14271E-06	positive
PRJNA729271	stage_1D_vs_E10	ordinary_muscle_development	ENSGALG00010010826	NCOA1	0.546058588	0.161512998	3.380895631	0.0007225	0.001223582	positive
PRJNA729271	stage_1D_vs_E10	ordinary_muscle_development	ENSGALG00010010828	SPDL1	-1.07401208	0.124727834	-8.610845274	7.25238E-18	3.45119E-17	negative
PRJNA729271	stage_1D_vs_E10	ordinary_muscle_development	ENSGALG00010010829	LAMB4	-2.356004537	0.250761103	-9.395414635	5.69929E-21	3.2182E-20	negative
PRJNA729271	stage_1D_vs_E10	ordinary_muscle_development	ENSGALG00010010831	DESI1	-0.086064941	0.092508543	-0.93034588	0.35219203	0.401086068	negative
PRJNA729271	stage_1D_vs_E10	ordinary_muscle_development	ENSGALG00010010834	KGD4	0.592014381	0.125441854	4.719432643	2.36503E-06	5.12654E-06	positive
PRJNA729271	stage_1D_vs_E10	ordinary_muscle_development	ENSGALG00010010836	DIP2A	0.261476694	0.105232381	2.484755083	0.012964059	0.018864977	positive

PRJNA729271	stage_1D_vs_E10	ordinary_muscle_development	ENSGALG00010010837	RBPJ	0.197623909	0.122544048	1.612676517	0.106814805	0.135079445	positive
PRJNA729271	stage_1D_vs_E10	ordinary_muscle_development	ENSGALG00010010838	JAM2	1.114184428	0.173913908	6.406528606	1.4887E-10	4.45657E-10	positive
PRJNA729271	stage_1D_vs_E10	ordinary_muscle_development	ENSGALG00010010840	RGS16	-0.696208648	0.615931113	-1.130335248	0.258334987	0.303351073	negative
PRJNA729271	stage_1D_vs_E10	ordinary_muscle_development	ENSGALG00010010841	PMM1	0.849149543	0.13015532	6.524124715	6.83998E-11	2.09124E-10	positive
PRJNA729271	stage_1D_vs_E10	ordinary_muscle_development	ENSGALG00010010843	ZNF395	1.093901101	0.178656752	6.122920568	9.18755E-10	2.60563E-09	positive
PRJNA729271	stage_1D_vs_E10	ordinary_muscle_development	ENSGALG00010010844	CDC14B	-0.021859115	0.174919622	-0.124966623	0.900549974	0.915326546	negative
PRJNA729271	stage_1D_vs_E10	ordinary_muscle_development	ENSGALG00010010845	FKRP	1.333414028	0.248979591	5.355515385	8.53129E-08	2.07137E-07	positive
PRJNA729271	stage_1D_vs_E10	ordinary_muscle_development	ENSGALG00010010848	RGR	0.945988123	0.5699233	1.659851639	0.096944302	0.123578196	positive
PRJNA729271	stage_1D_vs_E10	ordinary_muscle_development	ENSGALG00010010849	ACBD6	-0.616104206	0.109725788	-5.614944446	1.96625E-08	5.01599E-08	negative
PRJNA729271	stage_1D_vs_E10	ordinary_muscle_development	ENSGALG00010010850	ENSGALG00010010850	2.064093869	0.106021688	19.46860038	2.02735E-84	1.0686E-82	positive
PRJNA729271	stage_1D_vs_E10	ordinary_muscle_development	ENSGALG00010010851	PNOC	6.835270718	0.744385894	9.182429125	4.21474E-20	2.26605E-19	positive
PRJNA729271	stage_1D_vs_E10	ordinary_muscle_development	ENSGALG00010010853	SMIM20	0.126685874	0.146167775	0.866715486	0.386097919	0.435118125	positive
PRJNA729271	stage_1D_vs_E10	ordinary_muscle_development	ENSGALG00010010854	LRIT1	1.981874833	0.550582686	3.599595274	0.000318713	0.00056024	positive
PRJNA729271	stage_1D_vs_E10	ordinary_muscle_development	ENSGALG00010010855	ATP5PF	0.839466873	0.148478521	5.653793345	1.56945E-08	4.03497E-08	positive
PRJNA729271	stage_1D_vs_E10	ordinary_muscle_development	ENSGALG00010010856	CSDC2	-4.797108434	0.748007918	-6.413178685	1.42516E-10	4.27254E-10	negative
PRJNA729271	stage_1D_vs_E10	ordinary_muscle_development	ENSGALG00010010857	SEL1L3	0.091222729	0.060767277	1.501181785	0.13330855	0.165721049	positive
PRJNA729271	stage_1D_vs_E10	ordinary_muscle_development	ENSGALG00010010859	ENSGALG00010010859	-0.815314081	0.267175607	-3.051603733	0.002276224	0.00363689	negative
PRJNA729271	stage_1D_vs_E10	ordinary_muscle_development	ENSGALG00010010861	ENSGALG00010010861	0.936883612	0.236092398	3.968292161	7.23896E-05	0.000136183	positive
PRJNA729271	stage_1D_vs_E10	ordinary_muscle_development	ENSGALG00010010862	STRN4	0.300092736	0.15043712	1.994805116	0.046064139	0.061738298	positive
PRJNA729271	stage_1D_vs_E10	ordinary_muscle_development	ENSGALG00010010865	ENSGALG00010010865	0.665282658	0.156759821	4.243961577	2.19608E-05	4.33386E-05	positive
PRJNA729271	stage_1D_vs_E10	ordinary_muscle_development	ENSGALG00010010867	QSOX1	0.046484499	0.16149414	0.28784016	0.773469094	0.802708851	positive
PRJNA729271	stage_1D_vs_E10	ordinary_muscle_development	ENSGALG00010010868	SLC39A10	-1.870599361	0.109140216	-17.13941409	7.54067E-66	2.42355E-64	negative
PRJNA729271	stage_1D_vs_E10	ordinary_muscle_development	ENSGALG00010010869	ENSGALG00010010869	4.927810449	0.173497719	28.40273914	1.8706E-177	5.4228E-175	positive
PRJNA729271	stage_1D_vs_E10	ordinary_muscle_development	ENSGALG00010010870	NOL6	-0.442519632	0.121568116	-3.640096156	0.000272536	0.000482228	negative
PRJNA729271	stage_1D_vs_E10	ordinary_muscle_development	ENSGALG00010010871	GCNT2	-3.697344671	1.118006622	-3.307086559	0.000942718	0.001576816	negative
PRJNA729271	stage_1D_vs_E10	ordinary_muscle_development	ENSGALG00010010874	ENSGALG00010010874	0.107837025	0.275580035	0.391309278	0.695568642	0.732935989	positive
PRJNA729271	stage_1D_vs_E10	ordinary_muscle_development	ENSGALG00010010876	ENSGALG00010010876	0.227408252	0.158955647	1.430639656	0.152533516	0.187736547	positive
PRJNA729271	stage_1D_vs_E10	ordinary_muscle_development	ENSGALG00010010880	ELP3	-0.034890809	0.106234057	-0.328433372	0.742584014	0.775263273	negative
PRJNA729271	stage_1D_vs_E10	ordinary_muscle_development	ENSGALG00010010882	CEP350	1.518180375	0.098117996	15.4730063	5.27833E-54	1.1663E-52	positive
PRJNA729271	stage_1D_vs_E10	ordinary_muscle_development	ENSGALG00010010884	DNAH7	-3.032045416	0.31388896	-9.659611514	4.47558E-22	2.6885E-21	negative
PRJNA729271	stage_1D_vs_E10	ordinary_muscle_development	ENSGALG00010010885	SNRPD2	-0.678494622	0.273687671	-2.479083618	0.013172042	0.019152247	negative
PRJNA729271	stage_1D_vs_E10	ordinary_muscle_development	ENSGALG00010010887	GABPA	-0.242912789	0.066269874	-3.66550856	0.000246848	0.000438756	negative
PRJNA729271	stage_1D_vs_E10	ordinary_muscle_development	ENSGALG00010010888	GNPAT	0.422070796	0.118921153	3.549165016	0.000386455	0.000674249	positive
PRJNA729271	stage_1D_vs_E10	ordinary_muscle_development	ENSGALG00010010889	CCL19	-1.189644898	0.681081804	-1.746698988	0.080689566	0.104242002	negative
PRJNA729271	stage_1D_vs_E10	ordinary_muscle_development	ENSGALG00010010890	NOL12	-0.450428489	0.134469606	-3.349667646	0.000809086	0.001363844	negative
PRJNA729271	stage_1D_vs_E10	ordinary_muscle_development	ENSGALG00010010891	POLR3H	-0.233593728	0.10451937	-2.234932405	0.025421787	0.035435022	negative
PRJNA729271	stage_1D_vs_E10	ordinary_muscle_development	ENSGALG00010010893	CCL21	-3.164563483	0.526515563	-6.010389255	1.85078E-09	5.12457E-09	negative
PRJNA729271	stage_1D_vs_E10	ordinary_muscle_development	ENSGALG00010010894	SPAG1	-0.2572215	0.144056259	-1.785562825	0.074170071	0.096334693	negative
PRJNA729271	stage_1D_vs_E10	ordinary_muscle_development	ENSGALG00010010896	LY86	3.225042497	0.292472562	11.02682068	2.83718E-28	2.24604E-27	positive
PRJNA729271	stage_1D_vs_E10	ordinary_muscle_development	ENSGALG00010010897	CEP170	-0.535142819	0.090822041	-5.892213106	3.81057E-09	1.02991E-08	negative
PRJNA729271	stage_1D_vs_E10	ordinary_muscle_development	ENSGALG00010010898	PHF24	-4.91687868	3.569493153	-1.377472506	0.168366209	0.20559968	negative
PRJNA729271	stage_1D_vs_E10	ordinary_muscle_development	ENSGALG00010010900	SLC34A2	-1.242894338	1.036060362	-1.199635063	0.230281103	0.273465884	negative
PRJNA729271	stage_1D_vs_E10	ordinary_muscle_development	ENSGALG00010010904	TOR1AIP1	-0.483516288	0.081354565	-5.943320958	2.79305E-09	7.6344E-09	negative
PRJNA729271	stage_1D_vs_E10	ordinary_muscle_development	ENSGALG00010010905	ENC1	-0.111420417	0.147872491	-0.753489823	0.451155625	0.500382644	negative
PRJNA729271	stage_1D_vs_E10	ordinary_muscle_development	ENSGALG00010010906	ATP6V1C1	0.024344351	0.139769294	0.174175245	0.861727739	0.881865545	positive
PRJNA729271	stage_1D_vs_E10	ordinary_muscle_development	ENSGALG00010010908	STK17B	0.614420804	0.122928177	4.998209676	5.7865E-07	1.31652E-06	positive
PRJNA729271	stage_1D_vs_E10	ordinary_muscle_development	ENSGALG00010010909	SLIT3	-2.126663776	0.329145324	-6.461169641	1.03897E-10	3.14139E-10	negative
PRJNA729271	stage_1D_vs_E10	ordinary_muscle_development	ENSGALG00010010911	ENSGALG00010010911	-2.515458257	0.391585755	-6.42377366	1.32937E-10	3.99196E-10	negative
PRJNA729271	stage_1D_vs_E10	ordinary_muscle_development	ENSGALG00010010912	BAALC	-0.854162894	0.344951286	-2.476184112	0.013279508	0.019298824	negative
PRJNA729271	stage_1D_vs_E10	ordinary_muscle_development	ENSGALG00010010913	IFT56	-1.609898471	0.145910072	-11.03349792	2.63416E-28	2.08988E-27	negative
PRJNA729271	stage_1D_vs_E10	ordinary_muscle_development	ENSGALG00010010915	APP	-1.087448933	0.110204882	-9.867520509	5.75701E-23	3.58761E-22	negative
PRJNA729271	stage_1D_vs_E10	ordinary_muscle_development	ENSGALG00010010916	ENSGALG00010010916	-2.976340945	0.709822581	-4.19307729	2.75196E-05	5.38394E-05	negative
PRJNA729271	stage_1D_vs_E10	ordinary_muscle_development	ENSGALG00010010917	TANC1	0.119688272	0.068589807	1.744986273	0.080987258	0.10459862	positive
PRJNA729271	stage_1D_vs_E10	ordinary_muscle_development	ENSGALG00010010918	TOR1AIP2	0.696239445	0.11927868	5.837082059	5.31229E-09	1.42017E-08	positive
PRJNA729271	stage_1D_vs_E10	ordinary_muscle_development	ENSGALG00010010919	CDHR1	-2.211721828	0.554176536	-3.991005906	6.57936E-05	0.000124387	negative
PRJNA729271	stage_1D_vs_E10	ordinary_muscle_development	ENSGALG00010010920	UBN2	0.743591469	0.113227901	6.567210602	5.12665E-11	1.58378E-10	positive
PRJNA729271	stage_1D_vs_E10	ordinary_muscle_development	ENSGALG00010010921	ENSGALG00010010921	-0.194157625	0.128615127	-1.509601782	0.131145066	0.16342398	negative
PRJNA729271	stage_1D_vs_E10	ordinary_muscle_development	ENSGALG00010010922	DYM	0.25532094	0.10211611	2.500300301	0.012408807	0.018117008	positive
PRJNA729271	stage_1D_vs_E10	ordinary_muscle_development	ENSGALG00010010923	F13A1	2.245879244	0.2308818	9.72739838	2.3041E-22	1.39858E-21	positive
PRJNA729271	stage_1D_vs_E10	ordinary_muscle_development	ENSGALG00010010924	ENSGALG00010010924	-0.954403483	0.199379472	-4.786869356	1.69403E-06	3.71201E-06	negative
PRJNA729271	stage_1D_vs_E10	ordinary_muscle_development	ENSGALG00010010928	HECW2	-0.455096565	0.116570175	-3.904056631	9.45936E-05	0.000175944	negative
PRJNA729271	stage_1D_vs_E10	ordinary_muscle_development	ENSGALG00010010929	FMC1	0.483006544	0.147796556	3.268050072	0.001082912	0.001798855	positive
PRJNA729271	stage_1D_vs_E10	ordinary_muscle_development	ENSGALG00010010930	FAM163A	-3.475371886	0.502795672	-6.912095867	4.77545E-12	1.57605E-11	negative
PRJNA729271	stage_1D_vs_E10	ordinary_muscle_development	ENSGALG00010010931	CYYR1	-2.785442937	0.197312027	-14.11694454	2.98663E-45	4.74164E-44	negative
PRJNA729271	stage_1D_vs_E10	ordinary_muscle_development	ENSGALG00010010932	GHITM	0.609500032	0.088205599	6.909992519	4.84679E-12	1.59887E-11	positive
PRJNA729271	stage_1D_vs_E10	ordinary_muscle_development	ENSGALG00010010933	ANAPC4	-0.735554833	0.111080867	-6.621795943	3.54861E-11	1.1076E-10	negative
PRJNA729271	stage_1D_vs_E10	ordinary_muscle_development	ENSGALG00010010934	ENSGALG00010010934	0.580628601	0.110357509	5.261342054	1.43008E-07	3.41611E-07	positive
PRJNA729271	stage_1D_vs_E10	ordinary_muscle_development	ENSGALG00010010935	LGALS1A	0.524205299	0.227747938	2.301690651	0.021352624	0.030110544	positive
PRJNA729271	stage_1D_vs_E10	ordinary_muscle_development	ENSGALG00010010937	CDK7	-0.213004847	0.116045225	-1.835533051	0.066426747	0.086892491	negative
PRJNA729271	stage_1D_vs_E10	ordinary_muscle_development	ENSGALG00010010938	FZD6	-0.275949597	0.120976734	-2.281013779	0.022547632	0.03170624	negative
PRJNA729271	stage_1D_vs_E10	ordinary_muscle_development	ENSGALG00010010939	ZCCHC4	0.035921163	0.136572133	0.263019712	0.792535386	0.820205656	positive
PRJNA729271	stage_1D_vs_E10	ordinary_muscle_development	ENSGALG00010010940	NRG3	1.733065456	0.3160489	5.483535796	4.16908E-08	1.0346E-07	positive
PRJNA729271	stage_1D_vs_E10	ordinary_muscle_development	ENSGALG00010010941	RIPK4	-6.441592733	0.852215089	-7.558646656	4.07285E-14	1.54504E-13	negative
PRJNA729271	stage_1D_vs_E10	ordinary_muscle_development	ENSGALG00010010942	NRN1	3.314891104	0.336898054	9.839448645	7.61269E-23	4.72169E-22	positive
PRJNA729271	stage_1D_vs_E10	ordinary_muscle_development	ENSGALG00010010943	WDSUB1	0.228314372	0.10288813	2.219054529	0.026483015	0.036790426	positive
PRJNA729271	stage_1D_vs_E10	ordinary_muscle_development	ENSGALG00010010945	PI4K2B	-0.457540988	0.097936893	-4.671793985	2.9858E-06	6.40889E-06	negative
PRJNA729271	stage_1D_vs_E10	ordinary_muscle_development	ENSGALG00010010946	SH2D4B	1.595367116	0.29808296	5.352090964	8.69437E-08	2.10885E-07	positive
PRJNA729271	stage_1D_vs_E10	ordinary_muscle_development	ENSGALG00010010947	LUC7L2	-0.314237807	0.070121137	-4.481356386	7.41701E-06	1.53366E-05	negative
PRJNA729271	stage_1D_vs_E10	ordinary_muscle_development	ENSGALG00010010948	PRDM15	-0.418747376	0.084298982	-4.967407295	6.7854E-07	1.53607E-06	negative
PRJNA729271	stage_1D_vs_E10	ordinary_muscle_development	ENSGALG00010010949	FZD3	-1.481745146	0.224595533	-6.597393666	4.18449E-11	1.30075E-10	negative
PRJNA729271	stage_1D_vs_E10	ordinary_muscle_development	ENSGALG00010010951	ENSGALG00010010951	-1.211114414	0.134780832	-8.985806039	2.56845E-19	1.32208E-18	negative
PRJNA729271	stage_1D_vs_E10	ordinary_muscle_development	ENSGALG00010010952	ENSGALG00010010952	-0.461630692	0.515742612	-0.895079602	0.370744537	0.419904834	negative
PRJNA729271	stage_1D_vs_E10	ordinary_muscle_development	ENSGALG00010010953	EXTL3	0.213286196	0.109927452	1.94024506	0.052349915	0.069587912	positive
PRJNA729271	stage_1D_vs_E10	ordinary_muscle_development	ENSGALG00010010954	MTERF2	0.919196267	0.224523666	4.093983875	4.24024E-05	8.15149E-05	positive
PRJNA729271	stage_1D_vs_E10	ordinary_muscle_development	ENSGALG00010010955	GTF3C3	0.056784625	0.081846665	0.693792773	0.487812176	0.536238244	positive
PRJNA729271	stage_1D_vs_E10	ordinary_muscle_development	ENSGALG00010010956	AXDND1	4.648198409	0.544522102	8.536289701	1.38601E-17	6.47443E-17	positive
PRJNA729271	stage_1D_vs_E10	ordinary_muscle_development	ENSGALG00010010957	ZMAT2	-0.134094219	0.075377585	-1.778966771	0.07524521	0.097599939	negative
PRJNA729271	stage_1D_vs_E10	ordinary_muscle_development	ENSGALG00010010958	LCORL	0.578331259	0.159916884	3.616449017	0.000298672	0.000526353	positive
PRJNA729271	stage_1D_vs_E10	ordinary_muscle_development	ENSGALG00010010959	TMEM263	-1.11067007	0.089961595	-12.34604684	5.11644E-35	5.55943E-34	negative
PRJNA729271	stage_1D_vs_E10	ordinary_muscle_development	ENSGALG00010010960	POLDIP3	-0.320333734	0.069723421	-4.594349068	4.34102E-06	9.14846E-06	negative
PRJNA729271	stage_1D_vs_E10	ordinary_muscle_development	ENSGALG00010010961	C2orf66	0.4376474	0.372186679	1.175881419	0.239642265	0.283481393	positive
PRJNA729271	stage_1D_vs_E10	ordinary_muscle_development	ENSGALG00010010962	ANKDD1B	0.808216538	0.263678351	3.065160772	0.0021753	0.003483684	positive
PRJNA729271	stage_1D_vs_E10	ordinary_muscle_development	ENSGALG00010010963	TSPAN14	-0.184490566	0.073490972	-2.510384095	0.012059991	0.017641494	negative
PRJNA729271	stage_1D_vs_E10	ordinary_muscle_development	ENSGALG00010010964	INTS9	-0.440738395	0.090749313	-4.856658203	1.19383E-06	2.6484E-06	negative
PRJNA729271	stage_1D_vs_E10	ordinary_muscle_development	ENSGALG00010010965	BAZ2B	0.538021195	0.131683334	4.085719722	4.39404E-05	8.43597E-05	positive
PRJNA729271	stage_1D_vs_E10	ordinary_muscle_development	ENSGALG00010010966	ENSGALG00010010966	2.871133143	0.511673824	5.611256642	2.00863E-08	5.11868E-08	positive
PRJNA729271	stage_1D_vs_E10	ordinary_muscle_development	ENSGALG00010010967	PRXL2A	1.092574567	0.389398278	2.805802254	0.005019148	0.007717466	positive
PRJNA729271	stage_1D_vs_E10	ordinary_muscle_development	ENSGALG00010010968	RRP7A	0.531136188	0.245892748	2.160031938	0.030770197	0.042381305	positive
PRJNA729271	stage_1D_vs_E10	ordinary_muscle_development	ENSGALG00010010970	TPM2	1.373335214	0.352424484	3.896821243	9.74635E-05	0.000180957	positive
PRJNA729271	stage_1D_vs_E10	ordinary_muscle_development	ENSGALG00010010971	LCORL	0.331379471	0.123867529	2.67527312	0.007466839	0.011227369	positive
PRJNA729271	stage_1D_vs_E10	ordinary_muscle_development	ENSGALG00010010972	EXOSC3	-0.144494343	0.161968492	-0.8921139	0.372331885	0.421537974	negative
PRJNA729271	stage_1D_vs_E10	ordinary_muscle_development	ENSGALG00010010973	PGAP1	-0.188833766	0.112464667	-1.679049701	0.093142358	0.119098313	negative
PRJNA729271	stage_1D_vs_E10	ordinary_muscle_development	ENSGALG00010010974	NCAPG	-1.623323848	0.191144779	-8.492640252	2.01995E-17	9.37533E-17	negative
PRJNA729271	stage_1D_vs_E10	ordinary_muscle_development	ENSGALG00010010975	ATP8B1	-6.582092744	1.184112735	-5.558670681	2.71837E-08	6.84314E-08	negative
PRJNA729271	stage_1D_vs_E10	ordinary_muscle_development	ENSGALG00010010976	DYDC2	-1.602819874	1.010606368	-1.585998194	0.112739719	0.142076355	negative
PRJNA729271	stage_1D_vs_E10	ordinary_muscle_development	ENSGALG00010010977	FARS2	-0.198956554	0.08188498	-2.429707548	0.01511101	0.021781434	negative
PRJNA729271	stage_1D_vs_E10	ordinary_muscle_development	ENSGALG00010010978	SERHL	-0.207519286	0.134968542	-1.537538183	0.124161572	0.155416407	negative
PRJNA729271	stage_1D_vs_E10	ordinary_muscle_development	ENSGALG00010010979	ENSGALG00010010979	-2.458403322	0.41451561	-5.93078587	3.01488E-09	8.20979E-09	negative
PRJNA729271	stage_1D_vs_E10	ordinary_muscle_development	ENSGALG00010010980	POLK	1.14762453	0.124265072	9.235294424	2.57577E-20	1.39521E-19	positive
PRJNA729271	stage_1D_vs_E10	ordinary_muscle_development	ENSGALG00010010981	RIC8B	-1.028613558	0.064907177	-15.84745493	1.46391E-56	3.56627E-55	negative
PRJNA729271	stage_1D_vs_E10	ordinary_muscle_development	ENSGALG00010010982	ZBTB21	1.159301808	0.118837444	9.755357968	1.74982E-22	1.06839E-21	positive
PRJNA729271	stage_1D_vs_E10	ordinary_muscle_development	ENSGALG00010010983	MED28	-0.136610542	0.089121852	-1.532851242	0.125312512	0.156667661	negative
PRJNA729271	stage_1D_vs_E10	ordinary_muscle_development	ENSGALG00010010984	MAT1A	-0.717196096	0.390137504	-1.838316208	0.066015824	0.086409551	negative
PRJNA729271	stage_1D_vs_E10	ordinary_muscle_development	ENSGALG00010010985	CERT1	0.924989089	0.107917043	8.571297566	1.02325E-17	4.82501E-17	positive
PRJNA729271	stage_1D_vs_E10	ordinary_muscle_development	ENSGALG00010010986	NARS	0.04865105	0.129754985	0.374945515	0.707700988	0.743881495	positive
PRJNA729271	stage_1D_vs_E10	ordinary_muscle_development	ENSGALG00010010987	MARCHF7	-0.080973148	0.080716241	-1.003182849	0.315772647	0.363465776	negative
PRJNA729271	stage_1D_vs_E10	ordinary_muscle_development	ENSGALG00010010988	ANKRD44	-1.115575592	0.086699641	-12.86713041	6.89152E-38	8.37323E-37	negative
PRJNA729271	stage_1D_vs_E10	ordinary_muscle_development	ENSGALG00010010989	TRIQK	-1.343691572	0.17934296	-7.492301745	6.76761E-14	2.52761E-13	negative
PRJNA729271	stage_1D_vs_E10	ordinary_muscle_development	ENSGALG00010010990	MED18	0.150671273	0.119478811	1.26107107	0.207283243	0.248270584	positive
PRJNA729271	stage_1D_vs_E10	ordinary_muscle_development	ENSGALG00010010991	C2CD2	-0.004395144	0.161866918	-0.027152825	0.978337842	0.981656308	negative
PRJNA729271	stage_1D_vs_E10	ordinary_muscle_development	ENSGALG00010010992	PCDHA6	1.764274185	0.289736156	6.089244143	1.13445E-09	3.18927E-09	positive
PRJNA729271	stage_1D_vs_E10	ordinary_muscle_development	ENSGALG00010010993	ENSGALG00010010993	4.974487573	0.328698366	15.13389807	9.67883E-52	1.98437E-50	positive
PRJNA729271	stage_1D_vs_E10	ordinary_muscle_development	ENSGALG00010010994	NAA20	0.064433237	0.149550351	0.430846442	0.666580028	0.705467108	positive
PRJNA729271	stage_1D_vs_E10	ordinary_muscle_development	ENSGALG00010010995	HEBP1	-1.09064879	0.183975665	-5.92822313	3.0623E-09	8.33266E-09	negative
PRJNA729271	stage_1D_vs_E10	ordinary_muscle_development	ENSGALG00010010996	RFX4	1.558006273	0.542164917	2.873675929	0.00405725	0.00629993	positive
PRJNA729271	stage_1D_vs_E10	ordinary_muscle_development	ENSGALG00010010997	LAP3	-0.803276395	0.149413121	-5.376210526	7.60698E-08	1.85472E-07	negative
PRJNA729271	stage_1D_vs_E10	ordinary_muscle_development	ENSGALG00010010998	SOAT1	1.053615461	0.165862071	6.352359241	2.12037E-10	6.28524E-10	positive
PRJNA729271	stage_1D_vs_E10	ordinary_muscle_development	ENSGALG00010011001	ANGPT1	-2.836452588	0.258930103	-10.95451072	6.32191E-28	4.93463E-27	negative
PRJNA729271	stage_1D_vs_E10	ordinary_muscle_development	ENSGALG00010011002	RPRM	-2.2258512	0.703290635	-3.164909483	0.001551312	0.002530812	negative
PRJNA729271	stage_1D_vs_E10	ordinary_muscle_development	ENSGALG00010011003	FECH	-0.709643456	0.112291343	-6.319663079	2.62134E-10	7.72284E-10	negative
PRJNA729271	stage_1D_vs_E10	ordinary_muscle_development	ENSGALG00010011004	UMODL1	0.073178983	0.385576932	0.189790873	0.849473008	0.871378011	positive

PRJNA729271	stage_1D_vs_E10	ordinary_muscle_development	ENSGALG00010011006	TMEM64	-0.197254155	0.100521553	-1.962307075	0.049726744	0.066352679	negative
PRJNA729271	stage_1D_vs_E10	ordinary_muscle_development	ENSGALG00010011007	ENSGALG00010011007	-0.534613264	0.172983435	-3.090546012	0.001997888	0.003216995	negative
PRJNA729271	stage_1D_vs_E10	ordinary_muscle_development	ENSGALG00010011009	SFTPA1	0.520546835	1.229042946	0.423538361	0.671902516	0.710270345	positive
PRJNA729271	stage_1D_vs_E10	ordinary_muscle_development	ENSGALG00010011013	ENSGALG00010011013	3.750219583	0.334442637	11.21334176	3.50698E-29	2.9198E-28	positive
PRJNA729271	stage_1D_vs_E10	ordinary_muscle_development	ENSGALG00010011015	LYRM4	0.177786267	0.18284773	0.972318699	0.330892024	0.379362484	positive
PRJNA729271	stage_1D_vs_E10	ordinary_muscle_development	ENSGALG00010011018	QDPR	-0.333256406	0.10375089	-3.212082376	0.001317766	0.002169584	negative
PRJNA729271	stage_1D_vs_E10	ordinary_muscle_development	ENSGALG00010011019	NECAB1	-2.629063603	0.225524268	-11.65756409	2.09963E-31	1.94096E-30	negative
PRJNA729271	stage_1D_vs_E10	ordinary_muscle_development	ENSGALG00010011020	SFTPA2	0.461501892	0.876739374	0.526384357	0.598621182	0.641790979	positive
PRJNA729271	stage_1D_vs_E10	ordinary_muscle_development	ENSGALG00010011021	PPP1R3G	1.379794156	0.18909199	7.296946615	2.94372E-13	1.05695E-12	positive
PRJNA729271	stage_1D_vs_E10	ordinary_muscle_development	ENSGALG00010011022	C5orf22	-0.205909155	0.194455174	-1.058902937	0.289643986	0.336140079	negative
PRJNA729271	stage_1D_vs_E10	ordinary_muscle_development	ENSGALG00010011025	POLR3B	-0.615003719	0.091582309	-6.715311337	1.87665E-11	5.96275E-11	negative
PRJNA729271	stage_1D_vs_E10	ordinary_muscle_development	ENSGALG00010011027	FAM234B	0.27288526	0.09296403	2.935385433	0.003331336	0.005225943	positive
PRJNA729271	stage_1D_vs_E10	ordinary_muscle_development	ENSGALG00010011028	HMBOX1	-0.379108295	0.083848218	-4.521363773	6.14425E-06	1.27961E-05	negative
PRJNA729271	stage_1D_vs_E10	ordinary_muscle_development	ENSGALG00010011029	HMGCR	-0.626805189	0.125457172	-4.996168642	5.84805E-07	1.33031E-06	negative
PRJNA729271	stage_1D_vs_E10	ordinary_muscle_development	ENSGALG00010011033	DROSHA	-0.604795003	0.099248953	-6.093716703	1.10319E-09	3.10499E-09	negative
PRJNA729271	stage_1D_vs_E10	ordinary_muscle_development	ENSGALG00010011034	FAM180A	1.823295564	0.388734283	4.69033899	2.72753E-06	5.87365E-06	positive
PRJNA729271	stage_1D_vs_E10	ordinary_muscle_development	ENSGALG00010011038	GSG1	-2.714323968	0.487424833	-5.568702667	2.56643E-08	6.47978E-08	negative
PRJNA729271	stage_1D_vs_E10	ordinary_muscle_development	ENSGALG00010011040	CDH6	-2.010669225	0.217589133	-9.240669321	2.4496E-20	1.32985E-19	negative
PRJNA729271	stage_1D_vs_E10	ordinary_muscle_development	ENSGALG00010011042	SLC13A4	0.365401443	0.502879002	0.726619012	0.467459379	0.51613526	positive
PRJNA729271	stage_1D_vs_E10	ordinary_muscle_development	ENSGALG00010011044	MBL2	0.85161548	0.614850602	1.385077084	0.166028898	0.203002014	positive
PRJNA729271	stage_1D_vs_E10	ordinary_muscle_development	ENSGALG00010011045	ENSGALG00010011045	-1.40374957	0.407053218	-3.448565219	0.000563573	0.000964348	negative
PRJNA729271	stage_1D_vs_E10	ordinary_muscle_development	ENSGALG00010011049	ABL2	-0.162629633	0.12882213	-1.262435518	0.206792115	0.247723282	negative
PRJNA729271	stage_1D_vs_E10	ordinary_muscle_development	ENSGALG00010011050	SYT10	2.775696547	0.4016971	6.909924279	4.84912E-12	1.59927E-11	positive
PRJNA729271	stage_1D_vs_E10	ordinary_muscle_development	ENSGALG00010011055	TOR3A	0.121898026	0.131764079	0.925123351	0.354901748	0.403759582	positive
PRJNA729271	stage_1D_vs_E10	ordinary_muscle_development	ENSGALG00010011056	ENSGALG00010011056	-1.428453057	0.536900764	-2.660553223	0.00780124	0.011699842	negative
PRJNA729271	stage_1D_vs_E10	ordinary_muscle_development	ENSGALG00010011059	ENSGALG00010011059	-1.232130253	0.207535226	-5.936969244	2.90339E-09	7.92256E-09	negative
PRJNA729271	stage_1D_vs_E10	ordinary_muscle_development	ENSGALG00010011061	KIF13B	0.904365799	0.154402404	5.857200263	4.70735E-09	1.26334E-08	positive
PRJNA729271	stage_1D_vs_E10	ordinary_muscle_development	ENSGALG00010011062	TMEM55A	-0.568897267	0.122034659	-4.661767993	3.13504E-06	6.71431E-06	negative
PRJNA729271	stage_1D_vs_E10	ordinary_muscle_development	ENSGALG00010011064	MYLIP	1.12579393	0.096222073	11.69995505	1.27521E-31	1.19253E-30	positive
PRJNA729271	stage_1D_vs_E10	ordinary_muscle_development	ENSGALG00010011067	TMTC2	-0.251966669	0.102204346	-2.465322465	0.013688999	0.019852131	negative
PRJNA729271	stage_1D_vs_E10	ordinary_muscle_development	ENSGALG00010011069	ENSGALG00010011069	-1.760674396	0.545343821	-3.228558441	0.001244158	0.002053995	negative
PRJNA729271	stage_1D_vs_E10	ordinary_muscle_development	ENSGALG00010011076	PKP2	-1.964693906	0.296537237	-6.625454273	3.46182E-11	1.08168E-10	negative
PRJNA729271	stage_1D_vs_E10	ordinary_muscle_development	ENSGALG00010011077	RASGEF1A	0.802852343	0.144494235	5.556293264	2.75564E-08	6.93454E-08	positive
PRJNA729271	stage_1D_vs_E10	ordinary_muscle_development	ENSGALG00010011080	EMP1	-0.580614716	0.256459608	-2.263961649	0.023576474	0.033047189	negative
PRJNA729271	stage_1D_vs_E10	ordinary_muscle_development	ENSGALG00010011085	UGT1A1	0.697093334	1.383744373	0.503773202	0.614420761	0.65688368	positive
PRJNA729271	stage_1D_vs_E10	ordinary_muscle_development	ENSGALG00010011086	OTUD6A	-0.127550775	0.113359201	-1.125191196	0.260508023	0.30558095	negative
PRJNA729271	stage_1D_vs_E10	ordinary_muscle_development	ENSGALG00010011090	GRIN2B	-2.252829704	0.379570912	-5.935201132	2.93485E-09	8.0039E-09	negative
PRJNA729271	stage_1D_vs_E10	ordinary_muscle_development	ENSGALG00010011091	YARS2	0.535602713	0.098793783	5.421421227	5.91271E-08	1.45336E-07	positive
PRJNA729271	stage_1D_vs_E10	ordinary_muscle_development	ENSGALG00010011094	CSGALNACT2	-0.153706447	0.089867476	-1.710367903	0.087197863	0.111971387	negative
PRJNA729271	stage_1D_vs_E10	ordinary_muscle_development	ENSGALG00010011095	TNN	-2.902316363	0.478184278	-6.069451665	1.28348E-09	3.59428E-09	negative
PRJNA729271	stage_1D_vs_E10	ordinary_muscle_development	ENSGALG00010011096	LDB2	-0.267560872	0.114287976	-2.341111303	0.019226434	0.027260801	negative
PRJNA729271	stage_1D_vs_E10	ordinary_muscle_development	ENSGALG00010011099	CCBE1	0.19492869	0.196721482	0.990886648	0.321740933	0.369777578	positive
PRJNA729271	stage_1D_vs_E10	ordinary_muscle_development	ENSGALG00010011101	ENSGALG00010011101	-1.740865156	0.843219902	-2.064544672	0.038966103	0.052806064	negative
PRJNA729271	stage_1D_vs_E10	ordinary_muscle_development	ENSGALG00010011104	ABCG1	1.49703886	0.245603689	6.095343532	1.09203E-09	3.07657E-09	positive
PRJNA729271	stage_1D_vs_E10	ordinary_muscle_development	ENSGALG00010011106	USP40	0.680549713	0.114718649	5.93233723	2.98652E-09	8.13716E-09	positive
PRJNA729271	stage_1D_vs_E10	ordinary_muscle_development	ENSGALG00010011107	ENSGALG00010011107	1.079877321	0.185884673	5.809394079	6.26993E-09	1.66757E-08	positive
PRJNA729271	stage_1D_vs_E10	ordinary_muscle_development	ENSGALG00010011109	ENSGALG00010011109	-1.99143062	1.259184451	-1.581524151	0.113758241	0.143272718	negative
PRJNA729271	stage_1D_vs_E10	ordinary_muscle_development	ENSGALG00010011111	MRPS14	0.508446037	0.116872874	4.350419559	1.35877E-05	2.73966E-05	positive
PRJNA729271	stage_1D_vs_E10	ordinary_muscle_development	ENSGALG00010011116	TAPT1	1.267798298	0.126878059	9.992257996	1.64785E-23	1.04899E-22	positive
PRJNA729271	stage_1D_vs_E10	ordinary_muscle_development	ENSGALG00010011117	SLC6A15	-5.831923452	0.501984294	-11.61774087	3.34874E-31	3.06053E-30	negative
PRJNA729271	stage_1D_vs_E10	ordinary_muscle_development	ENSGALG00010011118	GMPR2	3.117523373	0.135488896	23.00943822	3.75E-117	4.0564E-115	positive
PRJNA729271	stage_1D_vs_E10	ordinary_muscle_development	ENSGALG00010011119	ENSGALG00010011119	-1.349901351	0.295799814	-4.563563887	5.02925E-06	1.05574E-05	negative
PRJNA729271	stage_1D_vs_E10	ordinary_muscle_development	ENSGALG00010011120	ENSGALG00010011120	1.527578714	3.005218453	0.508308709	0.611236863	0.653817307	positive
PRJNA729271	stage_1D_vs_E10	ordinary_muscle_development	ENSGALG00010011121	NUP35	-0.250506669	0.102165283	-2.451974501	0.014207473	0.020554678	negative
PRJNA729271	stage_1D_vs_E10	ordinary_muscle_development	ENSGALG00010011124	CACYBP	0.077691323	0.067790911	1.14604334	0.25177722	0.296346796	positive
PRJNA729271	stage_1D_vs_E10	ordinary_muscle_development	ENSGALG00010011126	ATXN1	1.021507892	0.137921433	7.406447793	1.29727E-13	4.7617E-13	positive
PRJNA729271	stage_1D_vs_E10	ordinary_muscle_development	ENSGALG00010011128	GCNT4	-0.985212874	0.250492643	-3.93310103	8.3857E-05	0.000156799	negative
PRJNA729271	stage_1D_vs_E10	ordinary_muscle_development	ENSGALG00010011129	ENSGALG00010011129	0.533324625	0.115311258	4.625087211	3.74441E-06	7.95591E-06	positive
PRJNA729271	stage_1D_vs_E10	ordinary_muscle_development	ENSGALG00010011130	DNM1L	-0.20854478	0.115238414	-1.809681099	0.070345255	0.091703793	negative
PRJNA729271	stage_1D_vs_E10	ordinary_muscle_development	ENSGALG00010011131	RET	0.64339218	0.495012378	1.299749679	0.193686777	0.233568206	positive
PRJNA729271	stage_1D_vs_E10	ordinary_muscle_development	ENSGALG00010011133	CDC37L1	-0.020739013	0.120314904	-0.172372767	0.863144481	0.883190685	negative
PRJNA729271	stage_1D_vs_E10	ordinary_muscle_development	ENSGALG00010011136	ENSGALG00010011136	-1.487819307	0.406860777	-3.65682659	0.000255357	0.000453159	negative
PRJNA729271	stage_1D_vs_E10	ordinary_muscle_development	ENSGALG00010011139	UBASH3A	-0.344796508	0.908240447	-0.379631307	0.704219118	0.74081255	negative
PRJNA729271	stage_1D_vs_E10	ordinary_muscle_development	ENSGALG00010011141	MSX1	-6.292497703	0.971931487	-6.474219416	9.53033E-11	2.88759E-10	negative
PRJNA729271	stage_1D_vs_E10	ordinary_muscle_development	ENSGALG00010011142	DUSP19	0.051154087	0.150132341	0.340726632	0.733309388	0.767182936	positive
PRJNA729271	stage_1D_vs_E10	ordinary_muscle_development	ENSGALG00010011143	AK3	1.280985053	0.165075639	7.759988459	8.49371E-15	3.35833E-14	positive
PRJNA729271	stage_1D_vs_E10	ordinary_muscle_development	ENSGALG00010011144	ATF7IP	0.295968692	0.060928575	4.857633614	1.18797E-06	2.63579E-06	positive
PRJNA729271	stage_1D_vs_E10	ordinary_muscle_development	ENSGALG00010011146	BMS1	-0.727386962	0.128934256	-5.64153379	1.68542E-08	4.32469E-08	negative
PRJNA729271	stage_1D_vs_E10	ordinary_muscle_development	ENSGALG00010011148	ENSGALG00010011148	-0.499787874	0.184125709	-2.714383971	0.006639916	0.010045463	negative
PRJNA729271	stage_1D_vs_E10	ordinary_muscle_development	ENSGALG00010011150	FGD4	0.781865502	0.167128836	4.678220225	2.89376E-06	6.21684E-06	positive
PRJNA729271	stage_1D_vs_E10	ordinary_muscle_development	ENSGALG00010011152	SLC18A3	1.782698001	0.460988123	3.867123494	0.000110127	0.000203556	positive
PRJNA729271	stage_1D_vs_E10	ordinary_muscle_development	ENSGALG00010011154	SLC26A7	-3.250513751	0.501246795	-6.484856925	8.8816E-11	2.69723E-10	negative
PRJNA729271	stage_1D_vs_E10	ordinary_muscle_development	ENSGALG00010011157	ENSGALG00010011157	-5.232278717	1.574356484	-3.323439621	0.000889147	0.00149048	negative
PRJNA729271	stage_1D_vs_E10	ordinary_muscle_development	ENSGALG00010011161	ENSGALG00010011161	0.031539203	0.094243398	0.334656895	0.737883921	0.771412004	positive
PRJNA729271	stage_1D_vs_E10	ordinary_muscle_development	ENSGALG00010011166	SOX7	1.143247097	0.140285822	8.149412944	3.65695E-16	1.58421E-15	positive
PRJNA729271	stage_1D_vs_E10	ordinary_muscle_development	ENSGALG00010011170	RBM24	1.143074574	0.142755981	8.007192202	1.17357E-15	4.90793E-15	positive
PRJNA729271	stage_1D_vs_E10	ordinary_muscle_development	ENSGALG00010011175	GPR52	0.190994894	0.183986432	1.038092277	0.299227097	0.346097731	positive
PRJNA729271	stage_1D_vs_E10	ordinary_muscle_development	ENSGALG00010011178	RSPO2	-2.27910856	0.319577162	-7.131637776	9.91816E-13	3.43357E-12	negative
PRJNA729271	stage_1D_vs_E10	ordinary_muscle_development	ENSGALG00010011181	ENSGALG00010011181	-0.306455019	0.277386422	-1.104794591	0.269248605	0.314637095	negative
PRJNA729271	stage_1D_vs_E10	ordinary_muscle_development	ENSGALG00010011184	FAM8A1	0.924689311	0.135231001	6.837850059	8.03905E-12	2.61504E-11	positive
PRJNA729271	stage_1D_vs_E10	ordinary_muscle_development	ENSGALG00010011187	STK32B	-8.475527411	0.885049854	-9.576327672	1.00557E-21	5.92028E-21	negative
PRJNA729271	stage_1D_vs_E10	ordinary_muscle_development	ENSGALG00010011190	ENSGALG00010011190	-0.886089488	0.215321999	-4.115183262	3.86872E-05	7.46401E-05	negative
PRJNA729271	stage_1D_vs_E10	ordinary_muscle_development	ENSGALG00010011192	RPL17	0.328329579	0.123490464	2.658744394	0.007843244	0.011757971	positive
PRJNA729271	stage_1D_vs_E10	ordinary_muscle_development	ENSGALG00010011193	FAM20B	-0.587873958	0.075055731	-7.83249923	4.78266E-15	1.92515E-14	negative
PRJNA729271	stage_1D_vs_E10	ordinary_muscle_development	ENSGALG00010011198	PINX1	-0.747150326	0.066032795	-11.31483718	1.10808E-29	9.42029E-29	negative
PRJNA729271	stage_1D_vs_E10	ordinary_muscle_development	ENSGALG00010011199	ENSGALG00010011199	-5.544047396	1.718897299	-3.225351159	0.001258182	0.002076201	negative
PRJNA729271	stage_1D_vs_E10	ordinary_muscle_development	ENSGALG00010011200	PCDHB4	-1.038846234	0.59671752	-1.740934696	0.081695026	0.105437575	negative
PRJNA729271	stage_1D_vs_E10	ordinary_muscle_development	ENSGALG00010011203	ENSGALG00010011203	-0.16708524	0.10383288	-1.609174669	0.107578154	0.135973608	negative
PRJNA729271	stage_1D_vs_E10	ordinary_muscle_development	ENSGALG00010011205	CYTL1	-7.997749468	0.873385913	-9.15717708	5.32726E-20	2.85255E-19	negative
PRJNA729271	stage_1D_vs_E10	ordinary_muscle_development	ENSGALG00010011210	NUP153	0.129862937	0.097275429	1.335002462	0.181875541	0.220628167	positive
PRJNA729271	stage_1D_vs_E10	ordinary_muscle_development	ENSGALG00010011211	SLC37A1	1.522758895	0.148230183	10.27293402	9.33224E-25	6.30046E-24	positive
PRJNA729271	stage_1D_vs_E10	ordinary_muscle_development	ENSGALG00010011212	NCKAP1	-0.293306153	0.062894944	-4.663429788	3.10982E-06	6.66424E-06	negative
PRJNA729271	stage_1D_vs_E10	ordinary_muscle_development	ENSGALG00010011213	XKR6	1.255236205	0.169876064	7.389129308	1.47793E-13	5.40021E-13	positive
PRJNA729271	stage_1D_vs_E10	ordinary_muscle_development	ENSGALG00010011218	ENSGALG00010011218	-1.439780953	0.603691346	-2.384962055	0.017080883	0.024414849	negative
PRJNA729271	stage_1D_vs_E10	ordinary_muscle_development	ENSGALG00010011222	RCL1	-0.021695748	0.088552013	-0.245005696	0.806452021	0.832824312	negative
PRJNA729271	stage_1D_vs_E10	ordinary_muscle_development	ENSGALG00010011231	PLBD1	-0.863335413	0.20857779	-4.139153128	3.4859E-05	6.7533E-05	negative
PRJNA729271	stage_1D_vs_E10	ordinary_muscle_development	ENSGALG00010011235	ENSGALG00010011235	-0.679145114	0.092424689	-7.348091987	2.01056E-13	7.28577E-13	negative
PRJNA729271	stage_1D_vs_E10	ordinary_muscle_development	ENSGALG00010011237	ENSGALG00010011237	-0.806976774	0.262894969	-3.069578614	0.00214361	0.003435606	negative
PRJNA729271	stage_1D_vs_E10	ordinary_muscle_development	ENSGALG00010011239	RALGPS2	-1.074477922	0.074753703	-14.37357445	7.58151E-47	1.27635E-45	negative
PRJNA729271	stage_1D_vs_E10	ordinary_muscle_development	ENSGALG00010011243	ALX1	-6.022911054	0.595450567	-10.11488005	4.74598E-24	3.09095E-23	negative
PRJNA729271	stage_1D_vs_E10	ordinary_muscle_development	ENSGALG00010011244	PDE9A	-0.927353001	0.170065699	-5.452910308	4.95521E-08	1.22507E-07	negative
PRJNA729271	stage_1D_vs_E10	ordinary_muscle_development	ENSGALG00010011246	DNAJB5	3.087476746	0.232380184	13.2863168	2.77926E-40	3.66897E-39	positive
PRJNA729271	stage_1D_vs_E10	ordinary_muscle_development	ENSGALG00010011247	ENSGALG00010011247	-0.023016154	0.094479343	-0.243610436	0.807532545	0.833643205	negative
PRJNA729271	stage_1D_vs_E10	ordinary_muscle_development	ENSGALG00010011250	ENSGALG00010011250	0.033811817	0.099108631	0.341159161	0.732983766	0.766897624	positive
PRJNA729271	stage_1D_vs_E10	ordinary_muscle_development	ENSGALG00010011251	FRZB	0.297956472	0.1752634	1.700049598	0.089121597	0.114258088	positive
PRJNA729271	stage_1D_vs_E10	ordinary_muscle_development	ENSGALG00010011253	KDM4C	0.999498568	0.107588134	9.29004468	1.54224E-20	8.49989E-20	positive
PRJNA729271	stage_1D_vs_E10	ordinary_muscle_development	ENSGALG00010011262	ENSGALG00010011262	-0.713058857	0.201597598	-3.537040432	0.000404638	0.000704024	negative
PRJNA729271	stage_1D_vs_E10	ordinary_muscle_development	ENSGALG00010011267	EVC2	-1.007701683	0.165099585	-6.103599132	1.03706E-09	2.92626E-09	negative
PRJNA729271	stage_1D_vs_E10	ordinary_muscle_development	ENSGALG00010011271	ANGPTL1	-2.915590266	0.162162672	-17.97941673	2.82448E-72	1.10951E-70	negative
PRJNA729271	stage_1D_vs_E10	ordinary_muscle_development	ENSGALG00010011273	RUNX1T1	-0.526736152	0.098215293	-5.363076695	8.18162E-08	1.98948E-07	negative
PRJNA729271	stage_1D_vs_E10	ordinary_muscle_development	ENSGALG00010011274	ENSGALG00010011274	-1.297945307	0.544835056	-2.382272017	0.017206182	0.024581472	negative
PRJNA729271	stage_1D_vs_E10	ordinary_muscle_development	ENSGALG00010011275	WDR4	-0.536832263	0.103304689	-5.196591441	2.02976E-07	4.78629E-07	negative
PRJNA729271	stage_1D_vs_E10	ordinary_muscle_development	ENSGALG00010011278	ENSGALG00010011278	-1.096654547	0.524404108	-2.091239429	0.036506606	0.049733389	negative
PRJNA729271	stage_1D_vs_E10	ordinary_muscle_development	ENSGALG00010011280	FAM199X	0.072493033	0.080674392	0.898587907	0.368872203	0.417998539	positive
PRJNA729271	stage_1D_vs_E10	ordinary_muscle_development	ENSGALG00010011281	ENSGALG00010011281	-0.926601767	0.61567705	-1.50501268	0.132320817	0.164648488	negative
PRJNA729271	stage_1D_vs_E10	ordinary_muscle_development	ENSGALG00010011282	CRY1	1.066631638	0.126814147	8.410983005	4.06589E-17	1.85272E-16	positive
PRJNA729271	stage_1D_vs_E10	ordinary_muscle_development	ENSGALG00010011283	LONRF3	1.145628128	0.141457364	8.098752132	5.55258E-16	2.37418E-15	positive
PRJNA729271	stage_1D_vs_E10	ordinary_muscle_development	ENSGALG00010011285	ENSGALG00010011285	1.580927128	0.262368248	6.025603851	1.68479E-09	4.68016E-09	positive
PRJNA729271	stage_1D_vs_E10	ordinary_muscle_development	ENSGALG00010011287	ENSGALG00010011287	-0.438453532	0.118133944	-3.711494903	0.000206039	0.000369575	negative
PRJNA729271	stage_1D_vs_E10	ordinary_muscle_development	ENSGALG00010011288	ENSGALG00010011288	-0.382810958	0.294787985	-1.29859756	0.194082076	0.234005963	negative
PRJNA729271	stage_1D_vs_E10	ordinary_muscle_development	ENSGALG00010011293	NDUFV3	1.361782998	0.167333329	8.138145607	4.01378E-16	1.73567E-15	positive
PRJNA729271	stage_1D_vs_E10	ordinary_muscle_development	ENSGALG00010011295	FAM167A	-0.889554653	0.375307088	-2.370204778	0.017778236	0.025323853	negative
PRJNA729271	stage_1D_vs_E10	ordinary_muscle_development	ENSGALG00010011297	OSR2	0.643270586	0.183241832	3.510500737	0.000447264	0.000773638	positive
PRJNA729271	stage_1D_vs_E10	ordinary_muscle_development	ENSGALG00010011300	EVC	-0.760275618	0.16170105	-4.701735801	2.57959E-06	5.57246E-06	negative
PRJNA729271	stage_1D_vs_E10	ordinary_muscle_development	ENSGALG00010011304	COMMD5	-0.990153617	0.093197439	-10.62425783	2.29833E-26	1.6724E-25	negative
PRJNA729271	stage_1D_vs_E10	ordinary_muscle_development	ENSGALG00010011306	ECI2	0.776650121	0.137787314	5.636586571	1.73454E-08	4.44207E-08	positive
PRJNA729271	stage_1D_vs_E10	ordinary_muscle_development	ENSGALG00010011307	ENSGALG00010011307	-1.762359373	0.46640472	-3.778605352	0.000157709	0.000287077	negative
PRJNA729271	stage_1D_vs_E10	ordinary_muscle_development	ENSGALG00010011308	SPIC	0.291936386	1.593907683	0.183157651	0.854674322	0.875574549	positive
PRJNA729271	stage_1D_vs_E10	ordinary_muscle_development	ENSGALG00010011311	DOCK8	-0.600176863	0.192993954	-3.109822099	0.001872001	0.003025044	negative
PRJNA729271	stage_1D_vs_E10	ordinary_muscle_development	ENSGALG00010011312	VPS13B	0.2107223	0.05233846	4.02614638	5.66984E-05	0.000107882	positive
PRJNA729271	stage_1D_vs_E10	ordinary_muscle_development	ENSGALG00010011313	RASSF9	-0.742083142	0.251106851	-2.955248484	0.003124173	0.004915325	negative

PRJNA729271	stage_1D_vs_E10	ordinary_muscle_development	ENSGALG00010011316	PARPBP	-2.054174437	0.202160677	-10.16109793	2.95727E-24	1.95021E-23	negative
PRJNA729271	stage_1D_vs_E10	ordinary_muscle_development	ENSGALG00010011317	BLK	-1.320255112	0.420902647	-3.13672323	0.001708473	0.002774983	negative
PRJNA729271	stage_1D_vs_E10	ordinary_muscle_development	ENSGALG00010011318	PRP4K	-0.02167592	0.097448716	-0.222434129	0.823975946	0.848563505	negative
PRJNA729271	stage_1D_vs_E10	ordinary_muscle_development	ENSGALG00010011321	MAP2	-2.138385828	0.151172742	-14.14531353	1.99625E-45	3.18674E-44	negative
PRJNA729271	stage_1D_vs_E10	ordinary_muscle_development	ENSGALG00010011322	GATA4	-5.005663881	1.415744785	-3.535710627	0.00040668	0.000707153	negative
PRJNA729271	stage_1D_vs_E10	ordinary_muscle_development	ENSGALG00010011323	GUCY2C	-1.887855806	0.913222282	-2.067246762	0.038710908	0.052489674	negative
PRJNA729271	stage_1D_vs_E10	ordinary_muscle_development	ENSGALG00010011326	COX6C	0.783903216	0.135528266	5.784057009	7.29202E-09	1.9295E-08	positive
PRJNA729271	stage_1D_vs_E10	ordinary_muscle_development	ENSGALG00010011329	PCDHGC5	0.140705435	0.11676912	1.204988405	0.228207778	0.271203718	positive
PRJNA729271	stage_1D_vs_E10	ordinary_muscle_development	ENSGALG00010011333	ENSGALG00010011333	-0.321356782	0.130543716	-2.461679446	0.01382882	0.020038863	negative
PRJNA729271	stage_1D_vs_E10	ordinary_muscle_development	ENSGALG00010011335	VCP	-0.399175205	0.173798099	-2.296775434	0.021631584	0.030477236	negative
PRJNA729271	stage_1D_vs_E10	ordinary_muscle_development	ENSGALG00010011339	ENSGALG00010011339	-0.609266692	0.302593408	-2.013483028	0.044063839	0.059265597	negative
PRJNA729271	stage_1D_vs_E10	ordinary_muscle_development	ENSGALG00010011340	PKNOX1	0.057404642	0.107867585	0.532176946	0.594603449	0.63809706	positive
PRJNA729271	stage_1D_vs_E10	ordinary_muscle_development	ENSGALG00010011342	RASAL2	0.428007562	0.065991577	6.485790756	8.82676E-11	2.68226E-10	positive
PRJNA729271	stage_1D_vs_E10	ordinary_muscle_development	ENSGALG00010011343	PXDC1	-0.624148666	0.140999358	-4.426606442	9.57272E-06	1.95901E-05	negative
PRJNA729271	stage_1D_vs_E10	ordinary_muscle_development	ENSGALG00010011345	CRMP1	-2.173197911	0.228265876	-9.520467732	1.72401E-21	9.99983E-21	negative
PRJNA729271	stage_1D_vs_E10	ordinary_muscle_development	ENSGALG00010011347	RGS22	-1.07067959	0.399278959	-2.68153271	0.007328575	0.011028623	negative
PRJNA729271	stage_1D_vs_E10	ordinary_muscle_development	ENSGALG00010011348	SLC22A23	0.74674626	0.161373221	4.627448446	3.70198E-06	7.86807E-06	positive
PRJNA729271	stage_1D_vs_E10	ordinary_muscle_development	ENSGALG00010011349	ENSGALG00010011349	-0.248204611	0.089078335	-2.78636337	0.005330308	0.008164728	negative
PRJNA729271	stage_1D_vs_E10	ordinary_muscle_development	ENSGALG00010011351	KANK1	-1.527083314	0.11003334	-13.87836921	8.5671E-44	1.28285E-42	negative
PRJNA729271	stage_1D_vs_E10	ordinary_muscle_development	ENSGALG00010011352	NTS	-1.773582081	0.553037909	-3.206981027	0.001341358	0.002205921	negative
PRJNA729271	stage_1D_vs_E10	ordinary_muscle_development	ENSGALG00010011353	ENSGALG00010011353	-2.527723996	0.211675894	-11.94148258	7.19295E-33	7.12171E-32	negative
PRJNA729271	stage_1D_vs_E10	ordinary_muscle_development	ENSGALG00010011357	UNC80	-1.425710849	0.435390986	-3.274552981	0.001058293	0.001760583	negative
PRJNA729271	stage_1D_vs_E10	ordinary_muscle_development	ENSGALG00010011359	RNF19A	-0.048863832	0.086542773	-0.564620594	0.572331854	0.617218676	negative
PRJNA729271	stage_1D_vs_E10	ordinary_muscle_development	ENSGALG00010011360	TCF4	0.422422714	0.171340396	2.465400587	0.013686015	0.019849788	positive
PRJNA729271	stage_1D_vs_E10	ordinary_muscle_development	ENSGALG00010011361	NUP37	-1.493199183	0.091599018	-16.30147591	9.63374E-60	2.63474E-58	negative
PRJNA729271	stage_1D_vs_E10	ordinary_muscle_development	ENSGALG00010011363	FDFT1	-0.199823791	0.147326568	-1.356332351	0.174993447	0.212974812	negative
PRJNA729271	stage_1D_vs_E10	ordinary_muscle_development	ENSGALG00010011365	PSMG4	-0.086923737	0.178820653	-0.486094509	0.62690016	0.668745792	negative
PRJNA729271	stage_1D_vs_E10	ordinary_muscle_development	ENSGALG00010011367	IER3IP1	0.496849773	0.112248519	4.426336983	9.58468E-06	1.96062E-05	positive
PRJNA729271	stage_1D_vs_E10	ordinary_muscle_development	ENSGALG00010011368	ENSGALG00010011368	-1.906566069	0.28001924	-6.808696676	9.84869E-12	3.18938E-11	negative
PRJNA729271	stage_1D_vs_E10	ordinary_muscle_development	ENSGALG00010011370	MGAT4C	-5.200013258	1.574826278	-3.301959925	0.000960118	0.00160444	negative
PRJNA729271	stage_1D_vs_E10	ordinary_muscle_development	ENSGALG00010011371	ENSGALG00010011371	0.143036166	0.202302535	0.707040896	0.479541063	0.528187516	positive
PRJNA729271	stage_1D_vs_E10	ordinary_muscle_development	ENSGALG00010011372	VMA21	-0.271267414	0.09239109	-2.936077639	0.003323911	0.00521486	negative
PRJNA729271	stage_1D_vs_E10	ordinary_muscle_development	ENSGALG00010011374	ENSGALG00010011374	-0.138319207	0.092022728	-1.503098309	0.132813695	0.165176706	negative
PRJNA729271	stage_1D_vs_E10	ordinary_muscle_development	ENSGALG00010011375	C12orf50	0.49855137	0.136649014	3.648408115	0.00026387	0.000467652	positive
PRJNA729271	stage_1D_vs_E10	ordinary_muscle_development	ENSGALG00010011377	SEC16B	0.948930501	0.309626325	3.064760403	0.002178444	0.003487965	positive
PRJNA729271	stage_1D_vs_E10	ordinary_muscle_development	ENSGALG00010011378	TUBB2B	-3.159123534	0.193802349	-16.30074943	9.74894E-60	2.66122E-58	negative
PRJNA729271	stage_1D_vs_E10	ordinary_muscle_development	ENSGALG00010011379	CELF4	-1.149148225	0.469362809	-2.44831547	0.014352593	0.020752203	negative
PRJNA729271	stage_1D_vs_E10	ordinary_muscle_development	ENSGALG00010011381	RLIG1	-0.568721362	0.120763702	-4.709373348	2.4848E-06	5.37328E-06	negative
PRJNA729271	stage_1D_vs_E10	ordinary_muscle_development	ENSGALG00010011382	ENSGALG00010011382	0.236283582	0.19380965	1.219152827	0.222786192	0.2654353	positive
PRJNA729271	stage_1D_vs_E10	ordinary_muscle_development	ENSGALG00010011384	PUM3	-1.133225855	0.081588696	-13.88949586	7.33498E-44	1.10291E-42	negative
PRJNA729271	stage_1D_vs_E10	ordinary_muscle_development	ENSGALG00010011386	CTSB	0.979426102	0.126170663	7.76270868	8.31344E-15	3.28795E-14	positive
PRJNA729271	stage_1D_vs_E10	ordinary_muscle_development	ENSGALG00010011388	GPR50	0.447238599	0.161846098	2.763357316	0.00572101	0.008726863	positive
PRJNA729271	stage_1D_vs_E10	ordinary_muscle_development	ENSGALG00010011389	RC3H1	0.823777962	0.085378365	9.648556323	4.98526E-22	2.98724E-21	positive
PRJNA729271	stage_1D_vs_E10	ordinary_muscle_development	ENSGALG00010011394	SERPINC1	1.756978526	0.918149972	1.913607341	0.055670346	0.073673119	positive
PRJNA729271	stage_1D_vs_E10	ordinary_muscle_development	ENSGALG00010011398	ENSGALG00010011398	0.914958098	0.180366669	5.072767073	3.92072E-07	9.02482E-07	positive
PRJNA729271	stage_1D_vs_E10	ordinary_muscle_development	ENSGALG00010011399	JAKMIP1	-2.452051379	0.257691558	-9.515450956	1.80926E-21	1.04817E-20	negative
PRJNA729271	stage_1D_vs_E10	ordinary_muscle_development	ENSGALG00010011400	BPHL	0.99908769	0.198348076	5.037042504	4.7278E-07	1.08313E-06	positive
PRJNA729271	stage_1D_vs_E10	ordinary_muscle_development	ENSGALG00010011401	ANKRD46	-0.229259619	0.067569233	-3.392958722	0.000691421	0.001172317	negative
PRJNA729271	stage_1D_vs_E10	ordinary_muscle_development	ENSGALG00010011403	SV2C	-4.016087579	0.53722243	-7.475651338	7.68224E-14	2.85743E-13	negative
PRJNA729271	stage_1D_vs_E10	ordinary_muscle_development	ENSGALG00010011406	UBE2R2	-0.498381807	0.115899641	-4.300115183	1.70709E-05	3.40315E-05	negative
PRJNA729271	stage_1D_vs_E10	ordinary_muscle_development	ENSGALG00010011407	CEP290	-0.766844009	0.180363211	-4.251665327	2.12187E-05	4.1954E-05	negative
PRJNA729271	stage_1D_vs_E10	ordinary_muscle_development	ENSGALG00010011411	ENSGALG00010011411	0.267514319	0.205353774	1.302699794	0.192677273	0.232466877	positive
PRJNA729271	stage_1D_vs_E10	ordinary_muscle_development	ENSGALG00010011412	KANSL1L	0.831835015	0.119899958	6.937742328	3.98416E-12	1.32425E-11	positive
PRJNA729271	stage_1D_vs_E10	ordinary_muscle_development	ENSGALG00010011413	ZBTB37	0.43277574	0.103919438	4.164531177	3.11993E-05	6.07188E-05	positive
PRJNA729271	stage_1D_vs_E10	ordinary_muscle_development	ENSGALG00010011414	PABPC1	0.656324178	0.114037768	5.755322881	8.64763E-09	2.26955E-08	positive
PRJNA729271	stage_1D_vs_E10	ordinary_muscle_development	ENSGALG00010011416	ENSGALG00010011416	0.962157324	0.427931402	2.248391491	0.024551237	0.034317279	positive
PRJNA729271	stage_1D_vs_E10	ordinary_muscle_development	ENSGALG00010011417	WFS1	0.626664214	0.144393621	4.339971585	1.42501E-05	2.86564E-05	positive
PRJNA729271	stage_1D_vs_E10	ordinary_muscle_development	ENSGALG00010011418	RIPK1	-0.871636624	0.087085547	-10.00896996	1.39194E-23	8.90388E-23	negative
PRJNA729271	stage_1D_vs_E10	ordinary_muscle_development	ENSGALG00010011419	RNF180	0.628033246	0.146888944	4.275565115	1.90653E-05	3.78253E-05	positive
PRJNA729271	stage_1D_vs_E10	ordinary_muscle_development	ENSGALG00010011420	HMGB3	-1.447991222	0.099534345	-14.54765414	6.04404E-48	1.07232E-46	negative
PRJNA729271	stage_1D_vs_E10	ordinary_muscle_development	ENSGALG00010011421	ENSGALG00010011421	1.10536783	0.116677464	9.473704649	2.70091E-21	1.55294E-20	positive
PRJNA729271	stage_1D_vs_E10	ordinary_muscle_development	ENSGALG00010011422	KATNAL2	-1.880558719	0.238035644	-7.900324021	2.78179E-15	1.13583E-14	negative
PRJNA729271	stage_1D_vs_E10	ordinary_muscle_development	ENSGALG00010011423	GDF6	-3.454159164	0.504149012	-6.851464706	7.30976E-12	2.38423E-11	negative
PRJNA729271	stage_1D_vs_E10	ordinary_muscle_development	ENSGALG00010011424	ACADL	2.470651734	0.096850196	25.51003329	1.5256E-143	2.4846E-141	positive
PRJNA729271	stage_1D_vs_E10	ordinary_muscle_development	ENSGALG00010011425	WASHC3	-0.179675159	0.079641227	-2.256057136	0.024067053	0.033689226	negative
PRJNA729271	stage_1D_vs_E10	ordinary_muscle_development	ENSGALG00010011426	NQO2	0.100747816	0.127865482	0.78792036	0.430743291	0.480241828	positive
PRJNA729271	stage_1D_vs_E10	ordinary_muscle_development	ENSGALG00010011427	TMTC3	-0.38452034	0.087362028	-4.401458506	1.07526E-05	2.18964E-05	negative
PRJNA729271	stage_1D_vs_E10	ordinary_muscle_development	ENSGALG00010011428	UQCRB	1.314112813	0.162887085	8.067630475	7.16756E-16	3.0405E-15	positive
PRJNA729271	stage_1D_vs_E10	ordinary_muscle_development	ENSGALG00010011429	UBAP2	0.349903292	0.1070037	3.270011131	0.001075433	0.00178725	positive
PRJNA729271	stage_1D_vs_E10	ordinary_muscle_development	ENSGALG00010011430	ENSGALG00010011430	0.184555769	0.327780779	0.563046345	0.573403326	0.618275772	positive
PRJNA729271	stage_1D_vs_E10	ordinary_muscle_development	ENSGALG00010011431	DRAM1	0.529271156	0.425051916	1.24519179	0.21306126	0.254728057	positive
PRJNA729271	stage_1D_vs_E10	ordinary_muscle_development	ENSGALG00010011433	RNASE6	3.248470365	0.334223352	9.7194596	2.491E-22	1.51075E-21	positive
PRJNA729271	stage_1D_vs_E10	ordinary_muscle_development	ENSGALG00010011434	NPL	0.727188917	0.179822972	4.043915567	5.25659E-05	0.00010019	positive
PRJNA729271	stage_1D_vs_E10	ordinary_muscle_development	ENSGALG00010011436	UBAP2L2	-4.623863584	2.344903802	-1.971877729			negative
PRJNA729271	stage_1D_vs_E10	ordinary_muscle_development	ENSGALG00010011437	DMRT1	-1.694160339	0.533244479	-3.177079941	0.00148766	0.002431623	negative
PRJNA729271	stage_1D_vs_E10	ordinary_muscle_development	ENSGALG00010011438	ENSGALG00010011438	6.354775251	0.439388184	14.46278139	2.08211E-47	3.58434E-46	positive
PRJNA729271	stage_1D_vs_E10	ordinary_muscle_development	ENSGALG00010011439	ENSGALG00010011439	0.173696446	0.313946369	0.553267891	0.580079985	0.624592139	positive
PRJNA729271	stage_1D_vs_E10	ordinary_muscle_development	ENSGALG00010011440	FUNDC1	-0.333291237	0.111886196	-2.978841436	0.002893405	0.004571605	negative
PRJNA729271	stage_1D_vs_E10	ordinary_muscle_development	ENSGALG00010011441	CD99L2	0.965711305	0.07963398	12.12687478	7.60985E-34	7.85647E-33	positive
PRJNA729271	stage_1D_vs_E10	ordinary_muscle_development	ENSGALG00010011442	ENSGALG00010011442	-3.991143992	2.095255091	-1.904848727	0.056799738	0.075051249	negative
PRJNA729271	stage_1D_vs_E10	ordinary_muscle_development	ENSGALG00010011443	ENSGALG00010011443	-0.67750045	0.166510352	-4.068818791	4.72521E-05	9.05379E-05	negative
PRJNA729271	stage_1D_vs_E10	ordinary_muscle_development	ENSGALG00010011445	MTERF3	0.063049469	0.079970959	0.788404569	0.430460099	0.479963011	positive
PRJNA729271	stage_1D_vs_E10	ordinary_muscle_development	ENSGALG00010011446	ENSGALG00010011446	0.461983022	0.220731295	2.092965663	0.03635222	0.049546349	positive
PRJNA729271	stage_1D_vs_E10	ordinary_muscle_development	ENSGALG00010011447	LAMC1	2.379847023	0.130277374	18.26753908	1.50067E-74	6.23274E-73	positive
PRJNA729271	stage_1D_vs_E10	ordinary_muscle_development	ENSGALG00010011448	AvBD10	-0.226709258	2.732083914	-0.082980342	0.93386717	0.943961272	negative
PRJNA729271	stage_1D_vs_E10	ordinary_muscle_development	ENSGALG00010011449	DUSP22	0.300408734	0.144639521	2.076947788	0.037806378	0.051363707	positive
PRJNA729271	stage_1D_vs_E10	ordinary_muscle_development	ENSGALG00010011450	PTDSS1	-0.361827149	0.067729736	-5.34221995	9.18152E-08	2.22292E-07	negative
PRJNA729271	stage_1D_vs_E10	ordinary_muscle_development	ENSGALG00010011451	CRHBP	-10.60132491	0.9606156	-11.03596997	2.56271E-28	2.03431E-27	negative
PRJNA729271	stage_1D_vs_E10	ordinary_muscle_development	ENSGALG00010011452	KITLG	1.066357469	0.188374675	5.660832421	1.5064E-08	3.87564E-08	positive
PRJNA729271	stage_1D_vs_E10	ordinary_muscle_development	ENSGALG00010011453	GNPTAB	0.164664747	0.124348634	1.324218388	0.185430525	0.22452723	positive
PRJNA729271	stage_1D_vs_E10	ordinary_muscle_development	ENSGALG00010011456	KIAA1328	-0.527761039	0.094598683	-5.578947	2.41979E-08	6.12768E-08	negative
PRJNA729271	stage_1D_vs_E10	ordinary_muscle_development	ENSGALG00010011457	ENSGALG00010011457	1.023133516	0.261438655	3.91347453	9.09775E-05	0.000169559	positive
PRJNA729271	stage_1D_vs_E10	ordinary_muscle_development	ENSGALG00010011458	TPGS2	-1.400805894	0.133050271	-10.52839566	6.39145E-26	4.55253E-25	negative
PRJNA729271	stage_1D_vs_E10	ordinary_muscle_development	ENSGALG00010011459	ENSGALG00010011459	0.392151256	0.27335314	1.43459576	0.151402324	0.18650265	positive
PRJNA729271	stage_1D_vs_E10	ordinary_muscle_development	ENSGALG00010011460	LAMC2	-2.37394913	0.671378489	-3.535932667	0.000406338	0.000706728	negative
PRJNA729271	stage_1D_vs_E10	ordinary_muscle_development	ENSGALG00010011461	IRF4	-0.887918936	0.393037275	-2.259121442	0.023875832	0.033434468	negative
PRJNA729271	stage_1D_vs_E10	ordinary_muscle_development	ENSGALG00010011463	IMP3	-0.802796071	0.130754075	-6.13974036	8.26565E-10	2.35061E-09	negative
PRJNA729271	stage_1D_vs_E10	ordinary_muscle_development	ENSGALG00010011464	FANCG	-1.348501491	0.157405782	-8.567039122	1.0618E-17	5.00513E-17	negative
PRJNA729271	stage_1D_vs_E10	ordinary_muscle_development	ENSGALG00010011465	PPP2R2C	-1.173533896	0.339521858	-3.456431063	0.000547379	0.000938744	negative
PRJNA729271	stage_1D_vs_E10	ordinary_muscle_development	ENSGALG00010011466	MTMR1	-0.015777202	0.066012686	-0.239002571	0.811103589	0.836793347	negative
PRJNA729271	stage_1D_vs_E10	ordinary_muscle_development	ENSGALG00010011467	ENSGALG00010011467	-21.35968284	4.05365818	-5.269236303			negative
PRJNA729271	stage_1D_vs_E10	ordinary_muscle_development	ENSGALG00010011468	EFHC2	-2.141897489	0.321056849	-6.671396339	2.53381E-11	7.98078E-11	negative
PRJNA729271	stage_1D_vs_E10	ordinary_muscle_development	ENSGALG00010011469	CPS1	0.496180357	0.334324064	1.484129952	0.137774448	0.170862477	positive
PRJNA729271	stage_1D_vs_E10	ordinary_muscle_development	ENSGALG00010011471	NMNAT2	-2.759248212	0.411427107	-6.706529943	1.99307E-11	6.32295E-11	negative
PRJNA729271	stage_1D_vs_E10	ordinary_muscle_development	ENSGALG00010011475	NDP	-2.26432134	0.308259999	-7.34549195	2.05004E-13	7.42511E-13	negative
PRJNA729271	stage_1D_vs_E10	ordinary_muscle_development	ENSGALG00010011477	SDC2	0.023229356	0.167271358	0.138872286	0.889551072	0.905734952	positive
PRJNA729271	stage_1D_vs_E10	ordinary_muscle_development	ENSGALG00010011478	HDAC3	-0.568784066	0.07665498	-7.42005372	1.17073E-13	4.30813E-13	negative
PRJNA729271	stage_1D_vs_E10	ordinary_muscle_development	ENSGALG00010011480	BLTP3B	-0.20866123	0.064405901	-3.239784348	0.001196201	0.001978879	negative
PRJNA729271	stage_1D_vs_E10	ordinary_muscle_development	ENSGALG00010011482	DMRT2	0.575616301	0.148847407	3.867157062	0.000110112	0.000203554	positive
PRJNA729271	stage_1D_vs_E10	ordinary_muscle_development	ENSGALG00010011484	CHPT1	1.393162467	0.077568738	17.96036016	3.98216E-72	1.55583E-70	positive
PRJNA729271	stage_1D_vs_E10	ordinary_muscle_development	ENSGALG00010011485	MTM1	0.659521516	0.076566127	8.613750503	7.07085E-18	3.36591E-17	positive
PRJNA729271	stage_1D_vs_E10	ordinary_muscle_development	ENSGALG00010011487	MAN2B2	-1.209413535	0.159271241	-7.593420697	3.11569E-14	1.19161E-13	negative
PRJNA729271	stage_1D_vs_E10	ordinary_muscle_development	ENSGALG00010011493	ENSGALG00010011493	0.956414652	0.262146368	3.648399395	0.000263879	0.000467652	positive
PRJNA729271	stage_1D_vs_E10	ordinary_muscle_development	ENSGALG00010011496	ACTR6	-1.418630899	0.087843607	-16.14950638	1.14474E-58	2.98436E-57	negative
PRJNA729271	stage_1D_vs_E10	ordinary_muscle_development	ENSGALG00010011497	CPQ	0.358904015	0.249159546	1.440458618	0.14973769	0.184703243	positive
PRJNA729271	stage_1D_vs_E10	ordinary_muscle_development	ENSGALG00010011499	SMG7	0.421144129	0.14618771	2.880844962	0.003966107	0.006166332	positive
PRJNA729271	stage_1D_vs_E10	ordinary_muscle_development	ENSGALG00010011503	NCF2	-0.893314236	0.435119726	-2.053030885	0.040069582	0.054230494	negative
PRJNA729271	stage_1D_vs_E10	ordinary_muscle_development	ENSGALG00010011505	MYL1	4.324537057	0.152239882	28.40607203	1.7014E-177	5.0331E-175	positive
PRJNA729271	stage_1D_vs_E10	ordinary_muscle_development	ENSGALG00010011509	EXOC2	0.173740565	0.067097198	2.589386295	0.009614717	0.01425149	positive
PRJNA729271	stage_1D_vs_E10	ordinary_muscle_development	ENSGALG00010011510	ENSGALG00010011510	0.24993907	0.268961636	0.929274055	0.352747077	0.401591964	positive
PRJNA729271	stage_1D_vs_E10	ordinary_muscle_development	ENSGALG00010011515	SERINC5	-0.24741689	0.0857829	-2.884221549	0.003923826	0.006106491	negative
PRJNA729271	stage_1D_vs_E10	ordinary_muscle_development	ENSGALG00010011523	PIGO	0.160580952	0.119379794	1.345126732	0.178584312	0.216889786	positive
PRJNA729271	stage_1D_vs_E10	ordinary_muscle_development	ENSGALG00010011525	ENSGALG00010011525	-2.108617371	0.242746725	-8.686491538	3.73805E-18	1.8067E-17	negative
PRJNA729271	stage_1D_vs_E10	ordinary_muscle_development	ENSGALG00010011529	SCYL2	0.198418227	0.081996978	2.419823663	0.015528035	0.02234477	positive
PRJNA729271	stage_1D_vs_E10	ordinary_muscle_development	ENSGALG00010011530	ARPC5	-1.369425259	0.120045443	-11.4075572	3.83344E-30	3.3433E-29	negative
PRJNA729271	stage_1D_vs_E10	ordinary_muscle_development	ENSGALG00010011531	MTDH	0.190446819	0.071805174	2.652271523	0.007995222	0.011975895	positive
PRJNA729271	stage_1D_vs_E10	ordinary_muscle_development	ENSGALG00010011533	ENSGALG00010011533	-0.762199918	0.273916058	-2.782604001	0.005392458	0.008254692	negative
PRJNA729271	stage_1D_vs_E10	ordinary_muscle_development	ENSGALG00010011536	FCHSD1	-1.352981375	0.184044077	-7.351398639	1.96143E-13	7.11486E-13	negative
PRJNA729271	stage_1D_vs_E10	ordinary_muscle_development	ENSGALG00010011538	ENSGALG00010011538	0.938189537	0.258510686	3.629209893	0.00028429	0.000502228	positive
PRJNA729271	stage_1D_vs_E10	ordinary_muscle_development	ENSGALG00010011539	LANCL1	-0.298125739	0.106528832	-2.798545095	0.00513334	0.007880509	negative
PRJNA729271	stage_1D_vs_E10	ordinary_muscle_development	ENSGALG00010011544	STOML2	-0.595551857	0.092434593	-6.442954317	1.1717E-10	3.53387E-10	negative

PRJNA729271	stage_1D_vs_E10	ordinary_muscle_development	ENSGALG00010011545	USP12-like	-0.057155675	0.07558997	-0.756127757	0.449572601	0.498817732	negative
PRJNA729271	stage_1D_vs_E10	ordinary_muscle_development	ENSGALG00010011546	MAOA	2.20436785	0.125696094	17.53728205	7.44018E-69	2.65629E-67	positive
PRJNA729271	stage_1D_vs_E10	ordinary_muscle_development	ENSGALG00010011549	KIAA0232	1.047024714	0.143652501	7.288593678	3.13207E-13	1.12291E-12	positive
PRJNA729271	stage_1D_vs_E10	ordinary_muscle_development	ENSGALG00010011551	GMDS	-1.892342393	0.157028324	-12.05096214	1.91699E-33	1.94995E-32	negative
PRJNA729271	stage_1D_vs_E10	ordinary_muscle_development	ENSGALG00010011555	ENSGALG00010011555	-4.793529315	4.289209579	-1.117578712	0.263746965	0.308880364	negative
PRJNA729271	stage_1D_vs_E10	ordinary_muscle_development	ENSGALG00010011556	LAPTM4B	0.492100491	0.143179159	3.436956155	0.000588291	0.001004154	positive
PRJNA729271	stage_1D_vs_E10	ordinary_muscle_development	ENSGALG00010011558	MYBPC1	3.006721608	0.559845689	5.370625633			positive
PRJNA729271	stage_1D_vs_E10	ordinary_muscle_development	ENSGALG00010011559	MTX3	0.237104936	0.126038815	1.881205698	0.059943943	0.078918025	positive
PRJNA729271	stage_1D_vs_E10	ordinary_muscle_development	ENSGALG00010011562	ENSGALG00010011562	-9.170355174	0.892338086	-10.27677213	8.96809E-25	6.06591E-24	negative
PRJNA729271	stage_1D_vs_E10	ordinary_muscle_development	ENSGALG00010011564	ARAP3	1.34079547	0.121339056	11.04999098	2.19237E-28	1.75184E-27	positive
PRJNA729271	stage_1D_vs_E10	ordinary_muscle_development	ENSGALG00010011570	RGL1	-0.538806773	0.136606093	-3.944236742	8.00545E-05	0.000149941	negative
PRJNA729271	stage_1D_vs_E10	ordinary_muscle_development	ENSGALG00010011571	ATOSB	0.756761035	0.114792592	6.592420505	4.32713E-11	1.34423E-10	positive
PRJNA729271	stage_1D_vs_E10	ordinary_muscle_development	ENSGALG00010011572	PTCD2	0.51025121	0.102175227	4.99388381	5.9177E-07	1.34489E-06	positive
PRJNA729271	stage_1D_vs_E10	ordinary_muscle_development	ENSGALG00010011573	CMAS	-0.910028416	0.179978884	-5.05630657	4.27454E-07	9.80837E-07	negative
PRJNA729271	stage_1D_vs_E10	ordinary_muscle_development	ENSGALG00010011574	GAS2L3	0.657620587	0.137597839	4.779294455	1.75911E-06	3.84707E-06	positive
PRJNA729271	stage_1D_vs_E10	ordinary_muscle_development	ENSGALG00010011575	ENSGALG00010011575	-4.483419597	0.624379394	-7.180601471	6.94054E-13	2.43003E-12	negative
PRJNA729271	stage_1D_vs_E10	ordinary_muscle_development	ENSGALG00010011577	ENSGALG00010011577	0.752520767	0.159199454	4.726905459	2.27967E-06	4.94891E-06	positive
PRJNA729271	stage_1D_vs_E10	ordinary_muscle_development	ENSGALG00010011578	MYLK4	-0.300283772	0.329956104	-0.91007188	0.362784603	0.41175811	negative
PRJNA729271	stage_1D_vs_E10	ordinary_muscle_development	ENSGALG00010011584	ENSGALG00010011584	-2.055895987	0.97614479	-2.106138359	0.035192336	0.048078502	negative
PRJNA729271	stage_1D_vs_E10	ordinary_muscle_development	ENSGALG00010011586	ENSGALG00010011586	0.327952428	0.252769888	1.297434713	0.194481655	0.234429238	positive
PRJNA729271	stage_1D_vs_E10	ordinary_muscle_development	ENSGALG00010011588	COLGALT2	-0.450596432	0.198922471	-2.265186177	0.023501257	0.032951318	negative
PRJNA729271	stage_1D_vs_E10	ordinary_muscle_development	ENSGALG00010011589	RERG	-1.166377777	0.224549047	-5.194311859	2.05478E-07	4.84216E-07	negative
PRJNA729271	stage_1D_vs_E10	ordinary_muscle_development	ENSGALG00010011593	WRNIP1	-0.968395102	0.102238469	-9.471924901	2.74734E-21	1.57776E-20	negative
PRJNA729271	stage_1D_vs_E10	ordinary_muscle_development	ENSGALG00010011595	ENSGALG00010011595	0.785109053	0.1613083	4.867133644	1.13228E-06	2.51802E-06	positive
PRJNA729271	stage_1D_vs_E10	ordinary_muscle_development	ENSGALG00010011597	TBC1D14	-0.309518425	0.130342064	-2.37466261	0.017564997	0.025052114	negative
PRJNA729271	stage_1D_vs_E10	ordinary_muscle_development	ENSGALG00010011601	ANO4	-5.548626982	0.923097906	-6.01087593	1.84524E-09	5.11018E-09	negative
PRJNA729271	stage_1D_vs_E10	ordinary_muscle_development	ENSGALG00010011603	ENSGALG00010011603	0.780392585	0.271299542	2.876497979	0.004021148	0.006246548	positive
PRJNA729271	stage_1D_vs_E10	ordinary_muscle_development	ENSGALG00010011605	APPL2	-0.363860102	0.14132931	-2.574555143	0.010036909	0.014831786	negative
PRJNA729271	stage_1D_vs_E10	ordinary_muscle_development	ENSGALG00010011607	TSEN15	-0.422922572	0.164991995	-2.563291454	0.010368497	0.015284386	negative
PRJNA729271	stage_1D_vs_E10	ordinary_muscle_development	ENSGALG00010011609	ENSGALG00010011609	1.243857569	0.808224206	1.539000639	0.12380414	0.155022548	positive
PRJNA729271	stage_1D_vs_E10	ordinary_muscle_development	ENSGALG00010011612	ENSGALG00010011612	-0.47134284	0.459074525	-1.026724015	0.304550438	0.351609605	negative
PRJNA729271	stage_1D_vs_E10	ordinary_muscle_development	ENSGALG00010011617	MAMLD1	1.437407471	0.095324031	15.07917206	2.22036E-51	4.49498E-50	positive
PRJNA729271	stage_1D_vs_E10	ordinary_muscle_development	ENSGALG00010011619	PCDH1	0.365998858	0.15907908	2.300735319	0.021406596	0.030180782	positive
PRJNA729271	stage_1D_vs_E10	ordinary_muscle_development	ENSGALG00010011621	WASHC4	0.160800245	0.088060423	1.826021731	0.067846989	0.088638314	positive
PRJNA729271	stage_1D_vs_E10	ordinary_muscle_development	ENSGALG00010011622	BACH1	0.736006226	0.159866973	4.603866642	4.14718E-06	8.768E-06	positive
PRJNA729271	stage_1D_vs_E10	ordinary_muscle_development	ENSGALG00010011625	ERBB4	2.240458226	0.21597497	10.37369389	3.26648E-25	2.26978E-24	positive
PRJNA729271	stage_1D_vs_E10	ordinary_muscle_development	ENSGALG00010011626	SERPINB1	0.323018726	0.599521844	0.538793922	0.590029058	0.633845045	positive
PRJNA729271	stage_1D_vs_E10	ordinary_muscle_development	ENSGALG00010011628	MATN2	3.492211694	0.191521982	18.23399938	2.77282E-74	1.14507E-72	positive
PRJNA729271	stage_1D_vs_E10	ordinary_muscle_development	ENSGALG00010011630	DELE1	0.816131547	0.100220076	8.14339383	3.8435E-16	1.66303E-15	positive
PRJNA729271	stage_1D_vs_E10	ordinary_muscle_development	ENSGALG00010011633	TADA2B	-0.175277433	0.084966294	-2.062905488	0.039121609	0.053001936	negative
PRJNA729271	stage_1D_vs_E10	ordinary_muscle_development	ENSGALG00010011634	AVD	3.267122168	0.889094969	3.674660505	0.000238166	0.000424349	positive
PRJNA729271	stage_1D_vs_E10	ordinary_muscle_development	ENSGALG00010011635	PCDH12	-0.667610168	0.197224945	-3.385018914	0.000711734	0.001206053	negative
PRJNA729271	stage_1D_vs_E10	ordinary_muscle_development	ENSGALG00010011636	GRIK1	-0.706356469	0.400389735	-1.76417227	0.07770298	0.100670781	negative
PRJNA729271	stage_1D_vs_E10	ordinary_muscle_development	ENSGALG00010011637	CECR2	-0.242517602	0.155126436	-1.56335444	0.11796925	0.148279941	negative
PRJNA729271	stage_1D_vs_E10	ordinary_muscle_development	ENSGALG00010011638	GRPEL1	-0.103155749	0.081892103	-1.259654414	0.207794057	0.24884128	negative
PRJNA729271	stage_1D_vs_E10	ordinary_muscle_development	ENSGALG00010011639	ENSGALG00010011639	0.198261366	0.143509623	1.381519662	0.167119237	0.204249017	positive
PRJNA729271	stage_1D_vs_E10	ordinary_muscle_development	ENSGALG00010011641	ENSGALG00010011641	0.197658542	0.456231955	0.433241336	0.664839452	0.703830547	positive
PRJNA729271	stage_1D_vs_E10	ordinary_muscle_development	ENSGALG00010011643	C1orf21	0.354433865	0.080654464	4.39447301	1.11042E-05	2.25649E-05	positive
PRJNA729271	stage_1D_vs_E10	ordinary_muscle_development	ENSGALG00010011647	FBXO38	0.298355007	0.082547745	3.614332607	0.000301122	0.000530477	positive
PRJNA729271	stage_1D_vs_E10	ordinary_muscle_development	ENSGALG00010011648	ENSGALG00010011648	-0.548362756	0.224474094	-2.442877689	0.014570674	0.021044433	negative
PRJNA729271	stage_1D_vs_E10	ordinary_muscle_development	ENSGALG00010011649	MRPS27	0.359486596	0.136663409	2.63045242	0.008527131	0.012716127	positive
PRJNA729271	stage_1D_vs_E10	ordinary_muscle_development	ENSGALG00010011650	RPL30	0.173524858	0.155340765	1.117059378	0.263968938	0.309115346	positive
PRJNA729271	stage_1D_vs_E10	ordinary_muscle_development	ENSGALG00010011651	SERPINB6	-0.352341883	0.128516189	-2.74161479	0.006113799	0.009299981	negative
PRJNA729271	stage_1D_vs_E10	ordinary_muscle_development	ENSGALG00010011653	NR2C1	-0.046894789	0.087765378	-0.534319913	0.593120217	0.636674896	negative
PRJNA729271	stage_1D_vs_E10	ordinary_muscle_development	ENSGALG00010011654	CLDN8	-3.88865136	1.364434345	-2.850009878	0.004371787	0.006773817	negative
PRJNA729271	stage_1D_vs_E10	ordinary_muscle_development	ENSGALG00010011655	SORCS2	0.026291938	0.184315456	0.14264641	0.886569442	0.903270125	positive
PRJNA729271	stage_1D_vs_E10	ordinary_muscle_development	ENSGALG00010011657	ALDH1L2	0.184425495	0.142343978	1.295632577	0.1951021	0.235086203	positive
PRJNA729271	stage_1D_vs_E10	ordinary_muscle_development	ENSGALG00010011658	TENM1	-1.580797561	0.30014028	-5.266862425	1.38775E-07	3.31665E-07	negative
PRJNA729271	stage_1D_vs_E10	ordinary_muscle_development	ENSGALG00010011659	CD14	1.011057087	0.152923504	6.611521837	3.80389E-11	1.18575E-10	positive
PRJNA729271	stage_1D_vs_E10	ordinary_muscle_development	ENSGALG00010011660	ENSGALG00010011660	-0.730613987	0.086634046	-8.433335667	3.35949E-17	1.53857E-16	negative
PRJNA729271	stage_1D_vs_E10	ordinary_muscle_development	ENSGALG00010011662	RIDA	-1.490280129	0.172213571	-8.653674178	4.98674E-18	2.39506E-17	negative
PRJNA729271	stage_1D_vs_E10	ordinary_muscle_development	ENSGALG00010011663	UTP20	-0.11389432	0.136788304	-0.832632002	0.405052304	0.454746585	negative
PRJNA729271	stage_1D_vs_E10	ordinary_muscle_development	ENSGALG00010011664	SERPINB10B	4.737875933	0.536210149	8.835856514	9.93342E-19	4.95134E-18	positive
PRJNA729271	stage_1D_vs_E10	ordinary_muscle_development	ENSGALG00010011667	ENSGALG00010011667	-1.092883564	0.41419297	-2.638585499	0.00832527	0.012433873	negative
PRJNA729271	stage_1D_vs_E10	ordinary_muscle_development	ENSGALG00010011669	ENSGALG00010011669	-3.617579117	0.753361649	-4.80191568	1.57155E-06	3.45302E-06	negative
PRJNA729271	stage_1D_vs_E10	ordinary_muscle_development	ENSGALG00010011670	TMCO6	0.036535481	0.170782331	0.213930099	0.830601568	0.85471885	positive
PRJNA729271	stage_1D_vs_E10	ordinary_muscle_development	ENSGALG00010011671	SLC41A2	-2.388692713	0.197394001	-12.10114141	1.04153E-33	1.06995E-32	negative
PRJNA729271	stage_1D_vs_E10	ordinary_muscle_development	ENSGALG00010011673	POP1	-0.973839415	0.121924426	-7.987238075	1.37995E-15	5.75113E-15	negative
PRJNA729271	stage_1D_vs_E10	ordinary_muscle_development	ENSGALG00010011674	ENSGALG00010011674	-1.90389157	0.510439067	-3.729909585	0.000191548	0.000344778	negative
PRJNA729271	stage_1D_vs_E10	ordinary_muscle_development	ENSGALG00010011677	EDEM3	-0.287144208	0.079692453	-3.603154336	0.000314379	0.00055309	negative
PRJNA729271	stage_1D_vs_E10	ordinary_muscle_development	ENSGALG00010011679	OVAL	-7.689394505	1.214507677	-6.331285223	2.43127E-10	7.17892E-10	negative
PRJNA729271	stage_1D_vs_E10	ordinary_muscle_development	ENSGALG00010011681	MAP1B	-1.824979452	0.358813455	-5.086151113	3.65403E-07	8.42992E-07	negative
PRJNA729271	stage_1D_vs_E10	ordinary_muscle_development	ENSGALG00010011682	USP15	0.78047402	0.122583236	6.366890307	1.92899E-10	5.72613E-10	positive
PRJNA729271	stage_1D_vs_E10	ordinary_muscle_development	ENSGALG00010011683	NIPAL2	-0.191934954	0.270973815	-0.708315504	0.478749351	0.527355563	negative
PRJNA729271	stage_1D_vs_E10	ordinary_muscle_development	ENSGALG00010011685	NDUFA2	1.121321894	0.111305454	10.07427625	7.17874E-24	4.6412E-23	positive
PRJNA729271	stage_1D_vs_E10	ordinary_muscle_development	ENSGALG00010011688	PDE3A	0.486312627	0.146806014	3.312620617	0.000924263	0.001547734	positive
PRJNA729271	stage_1D_vs_E10	ordinary_muscle_development	ENSGALG00010011692	ARL1	-0.804022495	0.089563227	-8.977149685	2.77872E-19	1.42828E-18	negative
PRJNA729271	stage_1D_vs_E10	ordinary_muscle_development	ENSGALG00010011696	ZFC3H1	0.768511473	0.108695409	7.070321395	1.54575E-12	5.27565E-12	positive
PRJNA729271	stage_1D_vs_E10	ordinary_muscle_development	ENSGALG00010011699	TIAM1	-1.615657324	0.145193996	-11.12757667	9.21055E-29	7.50039E-28	negative
PRJNA729271	stage_1D_vs_E10	ordinary_muscle_development	ENSGALG00010011702	NUP205	-0.407341396	0.083660728	-4.868967836	1.12183E-06	2.49591E-06	negative
PRJNA729271	stage_1D_vs_E10	ordinary_muscle_development	ENSGALG00010011703	YWHAZ	-1.204643028	0.09434616	-12.76833134	2.4638E-37	2.92248E-36	negative
PRJNA729271	stage_1D_vs_E10	ordinary_muscle_development	ENSGALG00010011708	IK	0.196619312	0.091440127	2.150251958	0.03153529	0.043360276	positive
PRJNA729271	stage_1D_vs_E10	ordinary_muscle_development	ENSGALG00010011712	DCAF12L2	-0.072802082	0.075826411	-0.960115101	0.336997289	0.38553873	negative
PRJNA729271	stage_1D_vs_E10	ordinary_muscle_development	ENSGALG00010011713	AVPR1A	-2.317691966	0.664000224	-3.49049877	0.00048212	0.000831944	negative
PRJNA729271	stage_1D_vs_E10	ordinary_muscle_development	ENSGALG00010011715	SLCO1B3	-3.09515299	1.905139523	-1.624633237	0.104240731	0.132193298	negative
PRJNA729271	stage_1D_vs_E10	ordinary_muscle_development	ENSGALG00010011720	IKZF2	0.637510836	0.172368482	3.698534839	0.000216848	0.000387667	positive
PRJNA729271	stage_1D_vs_E10	ordinary_muscle_development	ENSGALG00010011723	SPRY3	2.442283541	0.155298877	15.7263439	9.98104E-56	2.36397E-54	positive
PRJNA729271	stage_1D_vs_E10	ordinary_muscle_development	ENSGALG00010011724	ARHGEF28	-0.208686402	0.149915074	-1.392030805	0.16391307	0.200651968	negative
PRJNA729271	stage_1D_vs_E10	ordinary_muscle_development	ENSGALG00010011726	WDR55	-0.480344718	0.085725904	-5.603262218	2.10355E-08	5.3521E-08	negative
PRJNA729271	stage_1D_vs_E10	ordinary_muscle_development	ENSGALG00010011729	FAM129A	-1.022590684	0.232580513	-4.396716962	1.09901E-05	2.2358E-05	negative
PRJNA729271	stage_1D_vs_E10	ordinary_muscle_development	ENSGALG00010011734	ENSGALG00010011734	-2.305970635	0.303642181	-7.594368574	3.09297E-14	1.18323E-13	negative
PRJNA729271	stage_1D_vs_E10	ordinary_muscle_development	ENSGALG00010011738	FGD6	1.06654385	0.116512248	9.15392043	5.4904E-20	2.93774E-19	positive
PRJNA729271	stage_1D_vs_E10	ordinary_muscle_development	ENSGALG00010011745	AFAP1L1	0.582222924	0.190896245	3.049944348	0.002288838	0.003655835	positive
PRJNA729271	stage_1D_vs_E10	ordinary_muscle_development	ENSGALG00010011746	SOD1	-0.907665322	0.143031365	-6.345918082	2.21103E-10	6.54459E-10	negative
PRJNA729271	stage_1D_vs_E10	ordinary_muscle_development	ENSGALG00010011747	UTP15	-0.541823253	0.116362614	-4.656334505	3.21889E-06	6.88472E-06	negative
PRJNA729271	stage_1D_vs_E10	ordinary_muscle_development	ENSGALG00010011751	ZNF706	1.750842838	0.109270889	16.0229578	8.8346E-58	2.24269E-56	positive
PRJNA729271	stage_1D_vs_E10	ordinary_muscle_development	ENSGALG00010011752	RNF2	-2.175147992	0.125739256	-17.29887754	4.79643E-67	1.59826E-65	negative
PRJNA729271	stage_1D_vs_E10	ordinary_muscle_development	ENSGALG00010011758	SERPINB11	0.56646448	0.471090319	1.202454089	0.229187644	0.272278903	positive
PRJNA729271	stage_1D_vs_E10	ordinary_muscle_development	ENSGALG00010011759	CREB3	-0.100692035	0.130777909	-0.769946822	0.441331438	0.490612715	negative
PRJNA729271	stage_1D_vs_E10	ordinary_muscle_development	ENSGALG00010011764	PCYOX1L	-0.12137528	0.127792046	-0.949787438	0.34222027	0.391143575	negative
PRJNA729271	stage_1D_vs_E10	ordinary_muscle_development	ENSGALG00010011770	CHST11	-4.215611223	0.310856881	-13.56126076	6.79579E-42	9.54505E-41	negative
PRJNA729271	stage_1D_vs_E10	ordinary_muscle_development	ENSGALG00010011772	GRHL2	-4.301549253	0.497574519	-8.64503524	5.37887E-18	2.57571E-17	negative
PRJNA729271	stage_1D_vs_E10	ordinary_muscle_development	ENSGALG00010011774	ANKRA2	-0.633849288	0.093587228	-6.772818303	1.26298E-11	4.06367E-11	negative
PRJNA729271	stage_1D_vs_E10	ordinary_muscle_development	ENSGALG00010011775	VEZT	0.080223262	0.080097411	1.001571229	0.316550723	0.364303511	positive
PRJNA729271	stage_1D_vs_E10	ordinary_muscle_development	ENSGALG00010011776	IL17RA	-1.389767541	0.139055401	-9.994344188	1.61352E-23	1.02849E-22	negative
PRJNA729271	stage_1D_vs_E10	ordinary_muscle_development	ENSGALG00010011780	SERPINB5	-6.373470546	0.938364494	-6.792105397	1.10509E-11	3.56833E-11	negative
PRJNA729271	stage_1D_vs_E10	ordinary_muscle_development	ENSGALG00010011784	GRPEL2	0.575164881	0.133698253	4.301962584	1.69292E-05	3.37536E-05	positive
PRJNA729271	stage_1D_vs_E10	ordinary_muscle_development	ENSGALG00010011789	TXNRD1	-0.97036691	0.135488365	-7.161994371	7.95117E-13	2.77049E-12	negative
PRJNA729271	stage_1D_vs_E10	ordinary_muscle_development	ENSGALG00010011790	AMY2A	1.663918972	1.109798961	1.499297648	0.133796432	0.166270515	positive
PRJNA729271	stage_1D_vs_E10	ordinary_muscle_development	ENSGALG00010011792	HDHD5	1.146158898	0.141039669	8.126358413	4.4238E-16	1.90559E-15	positive
PRJNA729271	stage_1D_vs_E10	ordinary_muscle_development	ENSGALG00010011793	TLN1	0.670643038	0.113923109	5.886804217	3.93735E-09	1.06279E-08	positive
PRJNA729271	stage_1D_vs_E10	ordinary_muscle_development	ENSGALG00010011796	METAP2	-0.207027294	0.089526195	-2.312477303	0.020751395	0.029299773	negative
PRJNA729271	stage_1D_vs_E10	ordinary_muscle_development	ENSGALG00010011797	SCAF4	0.465067501	0.155316121	2.994328581	0.002750496	0.004355778	positive
PRJNA729271	stage_1D_vs_E10	ordinary_muscle_development	ENSGALG00010011802	VPS4B	-0.412032726	0.093069553	-4.427148427	9.5487E-06	1.95459E-05	negative
PRJNA729271	stage_1D_vs_E10	ordinary_muscle_development	ENSGALG00010011808	SMARCA2	0.615107554	0.122584908	5.017808166	5.22643E-07	1.1934E-06	positive
PRJNA729271	stage_1D_vs_E10	ordinary_muscle_development	ENSGALG00010011810	FGFR4	0.874059608	0.125311076	6.975118517	3.05613E-12	1.02425E-11	positive
PRJNA729271	stage_1D_vs_E10	ordinary_muscle_development	ENSGALG00010011814	ENSGALG00010011814	3.964702819	0.524117249	7.564534137	3.89257E-14	1.47859E-13	positive
PRJNA729271	stage_1D_vs_E10	ordinary_muscle_development	ENSGALG00010011817	KDSR	0.835720895	0.088874752	9.403355575	5.28501E-21	2.99126E-20	positive
PRJNA729271	stage_1D_vs_E10	ordinary_muscle_development	ENSGALG00010011819	USP44	0.995347028	0.13764047	7.231499793	4.77689E-13	1.69459E-12	positive
PRJNA729271	stage_1D_vs_E10	ordinary_muscle_development	ENSGALG00010011823	ENSGALG00010011823	-3.439843561	0.655197253	-5.250088494	1.52026E-07	3.62199E-07	negative
PRJNA729271	stage_1D_vs_E10	ordinary_muscle_development	ENSGALG00010011825	TAFA2	-4.075001942	0.3722493	-10.94697006	6.87076E-28	5.35151E-27	negative
PRJNA729271	stage_1D_vs_E10	ordinary_muscle_development	ENSGALG00010011837	VLDLR	1.488182654	0.134484581	11.06582362	1.83767E-28	1.47573E-27	positive
PRJNA729271	stage_1D_vs_E10	ordinary_muscle_development	ENSGALG00010011839	CBS	1.957616387	0.459686458	4.258590513	2.0572E-05	4.07142E-05	positive
PRJNA729271	stage_1D_vs_E10	ordinary_muscle_development	ENSGALG00010011841	KCNMB4	-3.055198761	0.256893896	-11.89284297	1.28941E-32	1.26626E-31	negative
PRJNA729271	stage_1D_vs_E10	ordinary_muscle_development	ENSGALG00010011843	BCL2	1.967422464	0.110634527	17.7830784	9.55846E-71	3.61749E-69	positive
PRJNA729271	stage_1D_vs_E10	ordinary_muscle_development	ENSGALG00010011846	UBAP1	0.463328138	0.104572035	4.430707845	9.39243E-06	1.92483E-05	positive
PRJNA729271	stage_1D_vs_E10	ordinary_muscle_development	ENSGALG00010011850	SPAG16	-1.452641585	0.358338444	-4.053825678	5.03868E-05	9.62388E-05	negative
PRJNA729271	stage_1D_vs_E10	ordinary_muscle_development	ENSGALG00010011856	NFYB	-0.255269197	0.128780242	-1.982207774	0.047455991	0.063492209	negative
PRJNA729271	stage_1D_vs_E10	ordinary_muscle_development	ENSGALG00010011857	ENSGALG00010011857	1.033598772	0.2431864	4.250232629	2.13549E-05	4.22002E-05	positive
PRJNA729271	stage_1D_vs_E10	ordinary_muscle_development	ENSGALG00010011859	NTN4	2.556819442	0.198547501	12.87762088	6.01616E-38	7.32809E-37	positive
PRJNA729271	stage_1D_vs_E10	ordinary_muscle_development	ENSGALG00010011860	CECR1	0.852162687	0.154430185	5.518109596	3.42666E-08	8.56074E-08	positive

PRJNA729271	stage_1D_vs_E10	ordinary_muscle_development	ENSGALG00010011861	ENSGALG00010011861	-0.100883643	0.38479737	-0.26217342	0.793187744	0.820705001	negative
PRJNA729271	stage_1D_vs_E10	ordinary_muscle_development	ENSGALG00010011866	KLF8	0.492026173	0.186870499	2.63297939	0.008463948	0.012628403	positive
PRJNA729271	stage_1D_vs_E10	ordinary_muscle_development	ENSGALG00010011867	ENSGALG00010011867	0.442535325	0.272140886	1.626125833	0.10392289	0.13181329	positive
PRJNA729271	stage_1D_vs_E10	ordinary_muscle_development	ENSGALG00010011868	SNRPF	-1.397588831	0.124838166	-11.19520479	4.30407E-29	3.57316E-28	negative
PRJNA729271	stage_1D_vs_E10	ordinary_muscle_development	ENSGALG00010011870	AvBD6	5.156564225	1.338681776	3.851971633	0.000117171	0.000216109	positive
PRJNA729271	stage_1D_vs_E10	ordinary_muscle_development	ENSGALG00010011871	CCT2	-1.169647882	0.097963765	-11.93959707	7.35788E-33	7.28003E-32	negative
PRJNA729271	stage_1D_vs_E10	ordinary_muscle_development	ENSGALG00010011873	RFX3	0.333684071	0.189869131	1.757442454	0.078842422	0.102055806	positive
PRJNA729271	stage_1D_vs_E10	ordinary_muscle_development	ENSGALG00010011874	KCNS2	-6.612605156	0.804035709	-8.224268002	1.96387E-16	8.61047E-16	negative
PRJNA729271	stage_1D_vs_E10	ordinary_muscle_development	ENSGALG00010011875	PSD2	-1.657264059	0.291784249	-5.679758473	1.34885E-08	3.48327E-08	negative
PRJNA729271	stage_1D_vs_E10	ordinary_muscle_development	ENSGALG00010011876	AMDHD1	-0.927789271	0.461226288	-2.011570666	0.044265214	0.059519878	negative
PRJNA729271	stage_1D_vs_E10	ordinary_muscle_development	ENSGALG00010011880	U2AF1	-0.457940292	0.156294577	-2.929981972	0.003389817	0.005315382	negative
PRJNA729271	stage_1D_vs_E10	ordinary_muscle_development	ENSGALG00010011881	RIOK2	-0.801362884	0.096522628	-8.30233184	1.02088E-16	4.54891E-16	negative
PRJNA729271	stage_1D_vs_E10	ordinary_muscle_development	ENSGALG00010011882	ENSGALG00010011882	0.241557965	0.282197224	0.855989868	0.392003384	0.441190236	positive
PRJNA729271	stage_1D_vs_E10	ordinary_muscle_development	ENSGALG00010011883	HCFC2	1.107988235	0.106399727	10.41344995	2.15276E-25	1.50527E-24	positive
PRJNA729271	stage_1D_vs_E10	ordinary_muscle_development	ENSGALG00010011885	NCALD	-0.923141398	0.212554032	-4.343090492	1.40492E-05	2.82642E-05	negative
PRJNA729271	stage_1D_vs_E10	ordinary_muscle_development	ENSGALG00010011886	NTNG1	-3.118499693	0.176068915	-17.71181301	3.39944E-70	1.26023E-68	negative
PRJNA729271	stage_1D_vs_E10	ordinary_muscle_development	ENSGALG00010011887	PGM5	2.184307086	0.132218413	16.52044553	2.6146E-61	7.51956E-60	positive
PRJNA729271	stage_1D_vs_E10	ordinary_muscle_development	ENSGALG00010011888	CAND1	-0.7856858	0.069353393	-11.32872916	9.45668E-30	8.05847E-29	negative
PRJNA729271	stage_1D_vs_E10	ordinary_muscle_development	ENSGALG00010011891	HAL	-0.567052072	0.57486271	-0.986413037	0.32393047	0.372146482	negative
PRJNA729271	stage_1D_vs_E10	ordinary_muscle_development	ENSGALG00010011893	LIX1	-3.366395804	0.489392993	-6.878716797	6.03941E-12	1.97923E-11	negative
PRJNA729271	stage_1D_vs_E10	ordinary_muscle_development	ENSGALG00010011894	VWC2L	-1.026004274	0.330916785	-3.100490276	0.001932006	0.003114717	negative
PRJNA729271	stage_1D_vs_E10	ordinary_muscle_development	ENSGALG00010011895	PHF21B	-2.709085296	0.780247411	-3.472084953	0.000516433	0.000887877	negative
PRJNA729271	stage_1D_vs_E10	ordinary_muscle_development	ENSGALG00010011896	SIK1	3.575289636	0.298179212	11.99040542	3.98948E-33	3.99637E-32	positive
PRJNA729271	stage_1D_vs_E10	ordinary_muscle_development	ENSGALG00010011898	ENSGALG00010011898	-0.260504368	0.110999745	-2.346891592	0.018930762	0.026865224	negative
PRJNA729271	stage_1D_vs_E10	ordinary_muscle_development	ENSGALG00010011899	PHLPP1	0.271749544	0.083899676	3.238982054	0.001199571	0.001984227	positive
PRJNA729271	stage_1D_vs_E10	ordinary_muscle_development	ENSGALG00010011902	TP53I3	-1.411939059	0.254709035	-5.543341086	2.96754E-08	7.44453E-08	negative
PRJNA729271	stage_1D_vs_E10	ordinary_muscle_development	ENSGALG00010011903	LTA4H	-0.888121942	0.116252985	-7.639562476	2.17961E-14	8.41819E-14	negative
PRJNA729271	stage_1D_vs_E10	ordinary_muscle_development	ENSGALG00010011904	KIF24	-2.562891618	0.335650551	-7.635594839	2.2478E-14	8.67001E-14	negative
PRJNA729271	stage_1D_vs_E10	ordinary_muscle_development	ENSGALG00010011906	ENSGALG00010011906	-3.083580096	0.948521316	-3.250933896	0.001150266	0.001905933	negative
PRJNA729271	stage_1D_vs_E10	ordinary_muscle_development	ENSGALG00010011907	BIK	-0.400479324	0.220643023	-1.81505546	0.069515387	0.090687205	negative
PRJNA729271	stage_1D_vs_E10	ordinary_muscle_development	ENSGALG00010011908	ENSGALG00010011908	-0.891294551	0.194990326	-4.570968058	4.85476E-06	1.0203E-05	negative
PRJNA729271	stage_1D_vs_E10	ordinary_muscle_development	ENSGALG00010011909	C8orf48	-0.65748765	0.203172041	-3.236112842	0.001211695	0.002002681	negative
PRJNA729271	stage_1D_vs_E10	ordinary_muscle_development	ENSGALG00010011911	NUDT2	0.054965106	0.138067592	0.398102882	0.69055435	0.727916901	positive
PRJNA729271	stage_1D_vs_E10	ordinary_muscle_development	ENSGALG00010011912	TCP11X2	1.957348023	0.125179875	15.63628359	4.12087E-55	9.51146E-54	positive
PRJNA729271	stage_1D_vs_E10	ordinary_muscle_development	ENSGALG00010011913	CXXC5	0.788591696	0.110770461	7.119151527	1.08593E-12	3.74242E-12	positive
PRJNA729271	stage_1D_vs_E10	ordinary_muscle_development	ENSGALG00010011915	RRM2B	0.060262496	0.122122627	0.493458888	0.621688382	0.663754386	positive
PRJNA729271	stage_1D_vs_E10	ordinary_muscle_development	ENSGALG00010011916	MCAT	-0.109840144	0.097870935	-1.122295849	0.26173667	0.306897997	negative
PRJNA729271	stage_1D_vs_E10	ordinary_muscle_development	ENSGALG00010011917	HSF2BP	-0.544015229	0.178033758	-3.055685819	0.002245464	0.003590909	negative
PRJNA729271	stage_1D_vs_E10	ordinary_muscle_development	ENSGALG00010011921	MEGF10	0.600369115	0.167217307	3.590352738	0.000330231	0.000579854	positive
PRJNA729271	stage_1D_vs_E10	ordinary_muscle_development	ENSGALG00010011922	PIK3R1	1.913992388	0.172882429	11.07106372	1.73331E-28	1.39425E-27	positive
PRJNA729271	stage_1D_vs_E10	ordinary_muscle_development	ENSGALG00010011923	BARD1	-1.565720967	0.152473223	-10.26882579	9.73827E-25	6.56846E-24	negative
PRJNA729271	stage_1D_vs_E10	ordinary_muscle_development	ENSGALG00010011925	RRP1B	-0.181371474	0.446734441	-0.405993936	0.684747056	0.722583618	negative
PRJNA729271	stage_1D_vs_E10	ordinary_muscle_development	ENSGALG00010011926	DYRK2	1.261630758	0.194780451	6.477193938	9.34441E-11	2.8354E-10	positive
PRJNA729271	stage_1D_vs_E10	ordinary_muscle_development	ENSGALG00010011927	ENSGALG00010011927	-0.216891199	0.460186838	-0.471311175	0.637418537	0.678119757	negative
PRJNA729271	stage_1D_vs_E10	ordinary_muscle_development	ENSGALG00010011929	ELK3	-0.757850362	0.134444593	-5.636897307	1.73141E-08	4.43485E-08	negative
PRJNA729271	stage_1D_vs_E10	ordinary_muscle_development	ENSGALG00010011930	TSPO	0.078527283	0.150590042	0.52146398	0.602043592	0.644935471	positive
PRJNA729271	stage_1D_vs_E10	ordinary_muscle_development	ENSGALG00010011931	ENSGALG00010011931	-2.774128835	1.303417128	-2.128350759			negative
PRJNA729271	stage_1D_vs_E10	ordinary_muscle_development	ENSGALG00010011932	VAV3	-0.577597582	0.167511637	-3.448104209	0.000564536	0.000965882	negative
PRJNA729271	stage_1D_vs_E10	ordinary_muscle_development	ENSGALG00010011935	ST8SIA4	-0.726901778	0.126778964	-5.733615047	9.83123E-09	2.56474E-08	negative
PRJNA729271	stage_1D_vs_E10	ordinary_muscle_development	ENSGALG00010011936	CKAP4	-1.676702995	0.175575869	-9.549734864	1.30029E-21	7.58762E-21	negative
PRJNA729271	stage_1D_vs_E10	ordinary_muscle_development	ENSGALG00010011937	SLC1A1	-1.897847498	0.334202471	-5.678735688	1.35694E-08	3.50229E-08	negative
PRJNA729271	stage_1D_vs_E10	ordinary_muscle_development	ENSGALG00010011941	ENSGALG00010011941	-3.040081776	0.243500595	-12.4849049	9.02511E-36	1.00941E-34	negative
PRJNA729271	stage_1D_vs_E10	ordinary_muscle_development	ENSGALG00010011943	TTLL12	-0.586281489	0.054758939	-10.70658968	9.47896E-27	7.03881E-26	negative
PRJNA729271	stage_1D_vs_E10	ordinary_muscle_development	ENSGALG00010011945	ENSGALG00010011945	0.575452135	0.458278601	1.25568188	0.209231331	0.250479695	positive
PRJNA729271	stage_1D_vs_E10	ordinary_muscle_development	ENSGALG00010011947	THAP1	-0.186678301	0.118840212	-1.570834468	0.116221104	0.146272892	negative
PRJNA729271	stage_1D_vs_E10	ordinary_muscle_development	ENSGALG00010011949	ABCA12	-0.039235771	0.418228321	-0.093814237	0.925256722	0.936237081	negative
PRJNA729271	stage_1D_vs_E10	ordinary_muscle_development	ENSGALG00010011950	PDXK	1.358357392	0.164904426	8.237240345	1.76228E-16	7.75244E-16	positive
PRJNA729271	stage_1D_vs_E10	ordinary_muscle_development	ENSGALG00010011952	UBE2D2	0.64977496	0.173573663	3.743511255	0.000181466	0.000328056	positive
PRJNA729271	stage_1D_vs_E10	ordinary_muscle_development	ENSGALG00010011953	CDK17	0.127722292	0.058287822	2.19123458	0.028434821	0.039306002	positive
PRJNA729271	stage_1D_vs_E10	ordinary_muscle_development	ENSGALG00010011954	ENSGALG00010011954	1.72441779	0.436457581	3.95094017	7.78448E-05	0.000145953	positive
PRJNA729271	stage_1D_vs_E10	ordinary_muscle_development	ENSGALG00010011957	ENSGALG00010011957	3.792022769	0.209824665	18.07234036	5.26329E-73	2.10169E-71	positive
PRJNA729271	stage_1D_vs_E10	ordinary_muscle_development	ENSGALG00010011960	CFAP54	-0.795672749	0.293008119	-2.715531404	0.00661695	0.010013854	negative
PRJNA729271	stage_1D_vs_E10	ordinary_muscle_development	ENSGALG00010011961	AGPAT3	0.882586068	0.102902789	8.576891628	9.74714E-18	4.60061E-17	positive
PRJNA729271	stage_1D_vs_E10	ordinary_muscle_development	ENSGALG00010011962	CPSF6	-0.596480406	0.058415122	-10.21106148	1.76919E-24	1.18122E-23	negative
PRJNA729271	stage_1D_vs_E10	ordinary_muscle_development	ENSGALG00010011963	ZCCHC2	0.383453516	0.099863388	3.839780747	0.000123144	0.000226462	positive
PRJNA729271	stage_1D_vs_E10	ordinary_muscle_development	ENSGALG00010011964	ENSGALG00010011964	-4.783956721	4.386377894	-1.090639438	0.275431569	0.321059959	negative
PRJNA729271	stage_1D_vs_E10	ordinary_muscle_development	ENSGALG00010011965	ATIC	0.774668003	0.146700774	5.280599291	1.28762E-07	3.08541E-07	positive
PRJNA729271	stage_1D_vs_E10	ordinary_muscle_development	ENSGALG00010011966	SLC25A24	-0.532401279	0.138257839	-3.850785467	0.00011774	0.000217074	negative
PRJNA729271	stage_1D_vs_E10	ordinary_muscle_development	ENSGALG00010011967	MTMR9	-0.021097015	0.137286994	-0.153670896	0.877869234	0.895854304	negative
PRJNA729271	stage_1D_vs_E10	ordinary_muscle_development	ENSGALG00010011968	ENSGALG00010011968	5.981157106	0.223378469	26.77588911	6.1697E-158	1.2961E-155	positive
PRJNA729271	stage_1D_vs_E10	ordinary_muscle_development	ENSGALG00010011969	TMEM173	1.855092514	0.291958628	6.353956815	2.09846E-10	6.22155E-10	positive
PRJNA729271	stage_1D_vs_E10	ordinary_muscle_development	ENSGALG00010011971	RNF170	-0.655283866	0.090730347	-7.22232294	5.11069E-13	1.80902E-12	negative
PRJNA729271	stage_1D_vs_E10	ordinary_muscle_development	ENSGALG00010011972	IPO11	-0.582843788	0.081901096	-7.116434528	1.10755E-12	3.80966E-12	negative
PRJNA729271	stage_1D_vs_E10	ordinary_muscle_development	ENSGALG00010011973	TRAPPC10	-0.034258445	0.049960274	-0.685713723	0.492893652	0.541208505	negative
PRJNA729271	stage_1D_vs_E10	ordinary_muscle_development	ENSGALG00010011976	TNFRSF11A	-0.178776717	0.090251037	-1.980882683	0.047604431	0.063682165	negative
PRJNA729271	stage_1D_vs_E10	ordinary_muscle_development	ENSGALG00010011977	GATD3A	-0.060775315	0.118946254	-0.510947701	0.609387678	0.651935523	negative
PRJNA729271	stage_1D_vs_E10	ordinary_muscle_development	ENSGALG00010011978	ENSGALG00010011978	-2.654413989	5.478910529	-0.484478433	0.628046367	0.669919206	negative
PRJNA729271	stage_1D_vs_E10	ordinary_muscle_development	ENSGALG00010011979	NEDD1	-0.319514757	0.077432865	-4.126345547	3.68573E-05	7.12614E-05	negative
PRJNA729271	stage_1D_vs_E10	ordinary_muscle_development	ENSGALG00010011980	AvBD9	13.06889903	2.419053955	5.402483481	6.57245E-08	1.6117E-07	positive
PRJNA729271	stage_1D_vs_E10	ordinary_muscle_development	ENSGALG00010011981	SCUBE1	-3.940185809	0.347942075	-11.32425795	9.9518E-30	8.4754E-29	negative
PRJNA729271	stage_1D_vs_E10	ordinary_muscle_development	ENSGALG00010011982	STK3	0.108367947	0.104788423	1.034159538	0.301061586	0.348025177	positive
PRJNA729271	stage_1D_vs_E10	ordinary_muscle_development	ENSGALG00010011983	LYZ	1.791546847	0.530006803	3.380233681	0.000724242	0.001226389	positive
PRJNA729271	stage_1D_vs_E10	ordinary_muscle_development	ENSGALG00010011984	FN1	0.503269237	0.24021838	2.09504883	0.036166652	0.049298065	positive
PRJNA729271	stage_1D_vs_E10	ordinary_muscle_development	ENSGALG00010011987	H2BC32	-1.820716047	0.375207554	-4.852557009	1.2188E-06	2.70253E-06	negative
PRJNA729271	stage_1D_vs_E10	ordinary_muscle_development	ENSGALG00010011988	HINTW	-11.06706335	3.243015517	-3.412584149	0.000643501	0.001094011	negative
PRJNA729271	stage_1D_vs_E10	ordinary_muscle_development	ENSGALG00010011989	H1-010	-0.458856237	0.136600713	-3.359105726	0.000781951	0.001319333	negative
PRJNA729271	stage_1D_vs_E10	ordinary_muscle_development	ENSGALG00010011991	CSNK1A1	0.151730316	0.056225909	2.698583623	0.006963524	0.010503255	positive
PRJNA729271	stage_1D_vs_E10	ordinary_muscle_development	ENSGALG00010011992	YEATS4	-1.413484487	0.085582749	-16.51599766	2.81468E-61	8.07896E-60	negative
PRJNA729271	stage_1D_vs_E10	ordinary_muscle_development	ENSGALG00010011993	RELCH	-0.040635295	0.050752029	-0.800663463	0.4233265	0.472882061	negative
PRJNA729271	stage_1D_vs_E10	ordinary_muscle_development	ENSGALG00010011996	LRRC70	1.953675953	0.217087153	8.999500539	2.26747E-19	1.1705E-18	positive
PRJNA729271	stage_1D_vs_E10	ordinary_muscle_development	ENSGALG00010011997	DENND5B	-0.327974787	0.132176011	-2.481348801	0.013088623	0.019038565	negative
PRJNA729271	stage_1D_vs_E10	ordinary_muscle_development	ENSGALG00010011998	MPPED1	-0.919408058	0.075810091	-12.12777936	7.52627E-34	7.77572E-33	negative
PRJNA729271	stage_1D_vs_E10	ordinary_muscle_development	ENSGALG00010012001	EEIG2	-0.587121499	0.254121357	-2.310398092	0.020866125	0.029456026	negative
PRJNA729271	stage_1D_vs_E10	ordinary_muscle_development	ENSGALG00010012004	LGR5	-2.87523046	0.197300633	-14.57283948	4.18143E-48	7.45509E-47	negative
PRJNA729271	stage_1D_vs_E10	ordinary_muscle_development	ENSGALG00010012007	MREG	-0.490471358	0.198221462	-2.474360514	0.013347493	0.019389849	negative
PRJNA729271	stage_1D_vs_E10	ordinary_muscle_development	ENSGALG00010012008	ZNF366	3.975471217	0.176093872	22.57586351	7.4834E-113	7.379E-111	positive
PRJNA729271	stage_1D_vs_E10	ordinary_muscle_development	ENSGALG00010012010	HENMT1	-1.787013782	0.18348737	-9.739165033	2.05234E-22	1.24889E-21	negative
PRJNA729271	stage_1D_vs_E10	ordinary_muscle_development	ENSGALG00010012011	HOOK3	0.370879652	0.068345463	5.426543844	5.74557E-08	1.41372E-07	positive
PRJNA729271	stage_1D_vs_E10	ordinary_muscle_development	ENSGALG00010012013	FOXD1	-3.835296821	0.754750294	-5.081543989	3.74379E-07	8.62739E-07	negative
PRJNA729271	stage_1D_vs_E10	ordinary_muscle_development	ENSGALG00010012015	ENSGALG00010012015	1.055734829	0.215238642	4.904950231	9.3451E-07	2.09394E-06	positive
PRJNA729271	stage_1D_vs_E10	ordinary_muscle_development	ENSGALG00010012016	WDR91	-0.616658426	0.064144472	-9.613586485	7.0065E-22	4.16056E-21	negative
PRJNA729271	stage_1D_vs_E10	ordinary_muscle_development	ENSGALG00010012017	N4BP3	-0.387022303	0.129037546	-2.999299936	0.002706008	0.00428673	negative
PRJNA729271	stage_1D_vs_E10	ordinary_muscle_development	ENSGALG00010012019	ENSGALG00010012019	6.806465081	0.300776554	22.62963979	2.214E-113	2.2442E-111	positive
PRJNA729271	stage_1D_vs_E10	ordinary_muscle_development	ENSGALG00010012021	ENSGALG00010012021	0.073903004	0.130105039	0.568025687	0.570017526	0.615128353	positive
PRJNA729271	stage_1D_vs_E10	ordinary_muscle_development	ENSGALG00010012024	UBR5	0.415629676	0.075636576	5.495088444	3.90515E-08	9.71761E-08	positive
PRJNA729271	stage_1D_vs_E10	ordinary_muscle_development	ENSGALG00010012026	SYT1	-2.254046122	0.247266389	-9.115861373	7.8048E-20	4.1364E-19	negative
PRJNA729271	stage_1D_vs_E10	ordinary_muscle_development	ENSGALG00010012028	TMEM140	3.501747341	0.172239237	20.33071792	6.87887E-92	4.27936E-90	positive
PRJNA729271	stage_1D_vs_E10	ordinary_muscle_development	ENSGALG00010012031	GRK6	-0.321663566	0.108173023	-2.973602445	0.002943261	0.004644304	negative
PRJNA729271	stage_1D_vs_E10	ordinary_muscle_development	ENSGALG00010012034	ARHGEF9	1.326044365	0.150366332	8.818758487	1.15736E-18	5.74911E-18	positive
PRJNA729271	stage_1D_vs_E10	ordinary_muscle_development	ENSGALG00010012035	ENSGALG00010012035	0.033281754	0.476922563	0.069784399	0.944365265	0.952646288	positive
PRJNA729271	stage_1D_vs_E10	ordinary_muscle_development	ENSGALG00010012039	ODF1	-1.956124981	0.75554035	-2.589041051	0.009624362	0.014261412	negative
PRJNA729271	stage_1D_vs_E10	ordinary_muscle_development	ENSGALG00010012040	B4GALT7	-0.181712888	0.15118673	-1.201910299	0.229398284	0.272506812	negative
PRJNA729271	stage_1D_vs_E10	ordinary_muscle_development	ENSGALG00010012041	ICOSLG	-0.830500515	0.154092996	-5.389605849	7.06124E-08	1.72631E-07	negative
PRJNA729271	stage_1D_vs_E10	ordinary_muscle_development	ENSGALG00010012042	FNDC7	-4.019244216	0.413834215	-9.712208577	2.67477E-22	1.61545E-21	negative
PRJNA729271	stage_1D_vs_E10	ordinary_muscle_development	ENSGALG00010012043	NUP50	-0.752107697	0.09575962	-7.854121588	4.02584E-15	1.63092E-14	negative
PRJNA729271	stage_1D_vs_E10	ordinary_muscle_development	ENSGALG00010012044	ENSGALG00010012044	2.63367211	0.610397705	4.314682193	1.59833E-05	3.19643E-05	positive
PRJNA729271	stage_1D_vs_E10	ordinary_muscle_development	ENSGALG00010012045	ENSGALG00010012045	0.626034782	0.300605273	2.082580843	0.037289447	0.050714068	positive
PRJNA729271	stage_1D_vs_E10	ordinary_muscle_development	ENSGALG00010012046	LOXHD1	-0.845531328	0.278799842	-3.032753976	0.002423331	0.003859596	negative
PRJNA729271	stage_1D_vs_E10	ordinary_muscle_development	ENSGALG00010012047	LMAN2	0.079893807	0.090399175	0.883789124	0.376810058	0.425942587	positive
PRJNA729271	stage_1D_vs_E10	ordinary_muscle_development	ENSGALG00010012048	PDE6H	0.756890683	0.458671025	1.650182028	0.098905711	0.125857105	positive
PRJNA729271	stage_1D_vs_E10	ordinary_muscle_development	ENSGALG00010012049	IFNAR1	0.75332	0.144621384	5.208911551	1.89952E-07	4.48867E-07	positive
PRJNA729271	stage_1D_vs_E10	ordinary_muscle_development	ENSGALG00010012051	ARK2C	-1.757700297	0.198350549	-8.861585236	7.88846E-19	3.95104E-18	negative
PRJNA729271	stage_1D_vs_E10	ordinary_muscle_development	ENSGALG00010012052	KLF10	2.145097921	0.122705767	17.48163899	1.97733E-68	6.95665E-67	positive
PRJNA729271	stage_1D_vs_E10	ordinary_muscle_development	ENSGALG00010012054	FAM60A	-1.078034945	0.087562111	-12.31166	7.8394E-35	8.44848E-34	negative
PRJNA729271	stage_1D_vs_E10	ordinary_muscle_development	ENSGALG00010012055	ENSGALG00010012055	1.683920777	0.156276035	10.77529758	4.50324E-27	3.38385E-26	positive
PRJNA729271	stage_1D_vs_E10	ordinary_muscle_development	ENSGALG00010012058	ENSGALG00010012058	-1.867683657	0.301362003	-6.197475584	5.73759E-10	1.64882E-09	negative
PRJNA729271	stage_1D_vs_E10	ordinary_muscle_development	ENSGALG00010012059	ARHGDIB	-0.679410487	0.145454004	-4.670964474	2.99789E-06	6.43292E-06	negative
PRJNA729271	stage_1D_vs_E10	ordinary_muscle_development	ENSGALG00010012060	ENSGALG00010012060	0.694322402	0.124417254	5.58059577	2.39696E-08	6.07306E-08	positive
PRJNA729271	stage_1D_vs_E10	ordinary_muscle_development	ENSGALG00010012061	AZIN1	0.905445391	0.12035104	7.523369886	5.33821E-14	2.01085E-13	positive
PRJNA729271	stage_1D_vs_E10	ordinary_muscle_development	ENSGALG00010012062	HMGA2	-3.959994299	0.154660307	-25.60446419	1.3605E-144	2.2931E-142	negative
PRJNA729271	stage_1D_vs_E10	ordinary_muscle_development	ENSGALG00010012063	PTPRD	-0.91025039	0.196662642	-4.628486536	3.68348E-06	7.83104E-06	negative

PRJNA729271	stage_1D_vs_E10	ordinary_muscle_development	ENSGALG00010012064	RGS14	-1.810353423	0.182809663	-9.902941658	4.04207E-23	2.53197E-22	negative
PRJNA729271	stage_1D_vs_E10	ordinary_muscle_development	ENSGALG00010012066	STXBP3	-0.553339341	0.12176021	-4.544500544	5.50657E-06	1.15077E-05	negative
PRJNA729271	stage_1D_vs_E10	ordinary_muscle_development	ENSGALG00010012067	ENSGALG00010012067	0.105489091	0.083242115	1.267256263	0.205063674	0.245794919	positive
PRJNA729271	stage_1D_vs_E10	ordinary_muscle_development	ENSGALG00010012069	ERP27	1.556465612	0.415007975	3.750447476	0.000176519	0.000319631	positive
PRJNA729271	stage_1D_vs_E10	ordinary_muscle_development	ENSGALG00010012070	DNAJC28	0.952199658	0.158426608	6.01035188	1.85121E-09	5.12477E-09	positive
PRJNA729271	stage_1D_vs_E10	ordinary_muscle_development	ENSGALG00010012071	ENSGALG00010012071	-0.947176315	0.152270755	-6.220342919	4.96069E-10	1.43266E-09	negative
PRJNA729271	stage_1D_vs_E10	ordinary_muscle_development	ENSGALG00010012072	CAPRIN2	-1.027515892	0.153877709	-6.677483676	2.4308E-11	7.66799E-11	negative
PRJNA729271	stage_1D_vs_E10	ordinary_muscle_development	ENSGALG00010012074	KIAA0930	-0.666227257	0.088575723	-7.52155599	5.41281E-14	2.03736E-13	negative
PRJNA729271	stage_1D_vs_E10	ordinary_muscle_development	ENSGALG00010012075	PIGN	-0.168338743	0.094428646	-1.782708431	0.074633778	0.096919604	negative
PRJNA729271	stage_1D_vs_E10	ordinary_muscle_development	ENSGALG00010012077	IFNGR2	0.502596543	0.068431799	7.344488232	2.06548E-13	7.47917E-13	positive
PRJNA729271	stage_1D_vs_E10	ordinary_muscle_development	ENSGALG00010012078	TMBIM4	-0.46698661	0.083178277	-5.614285685	1.97376E-08	5.03425E-08	negative
PRJNA729271	stage_1D_vs_E10	ordinary_muscle_development	ENSGALG00010012079	ENSGALG00010012079	-1.658835809	0.380280238	-4.36214045	1.28796E-05	2.60449E-05	negative
PRJNA729271	stage_1D_vs_E10	ordinary_muscle_development	ENSGALG00010012081	CHIC1	0.068201598	0.124310542	0.548638892	0.58325329	0.627729168	positive
PRJNA729271	stage_1D_vs_E10	ordinary_muscle_development	ENSGALG00010012082	HAUS1	-2.120920472	0.206147746	-10.28835152	7.95262E-25	5.39669E-24	negative
PRJNA729271	stage_1D_vs_E10	ordinary_muscle_development	ENSGALG00010012083	MGP	5.243611387	0.277640412	18.88634061	1.47739E-79	6.95285E-78	positive
PRJNA729271	stage_1D_vs_E10	ordinary_muscle_development	ENSGALG00010012084	TMEM50B	0.209548018	0.105872593	1.979247051	0.047788198	0.063901285	positive
PRJNA729271	stage_1D_vs_E10	ordinary_muscle_development	ENSGALG00010012085	IPO8	-0.330438874	0.096653183	-3.418810036	0.000628956	0.001070289	negative
PRJNA729271	stage_1D_vs_E10	ordinary_muscle_development	ENSGALG00010012086	CIBAR1	-1.816711984	0.133877952	-13.56991165	6.03948E-42	8.50751E-41	negative
PRJNA729271	stage_1D_vs_E10	ordinary_muscle_development	ENSGALG00010012087	TMEM171	-2.020145326	0.204148953	-9.895447871	4.35656E-23	2.72191E-22	negative
PRJNA729271	stage_1D_vs_E10	ordinary_muscle_development	ENSGALG00010012088	ATP5F1AZ	1.227628941	0.169838541	7.228211791	4.89395E-13	1.734E-12	positive
PRJNA729271	stage_1D_vs_E10	ordinary_muscle_development	ENSGALG00010012089	ENSGALG00010012089	-1.009262933	0.248628933	-4.059314104	4.92171E-05	9.40791E-05	negative
PRJNA729271	stage_1D_vs_E10	ordinary_muscle_development	ENSGALG00010012090	HELB	0.128899148	0.223501177	0.576726933	0.564123935	0.609584427	positive
PRJNA729271	stage_1D_vs_E10	ordinary_muscle_development	ENSGALG00010012091	ENSGALG00010012091	-0.744799425	0.444609949	-1.675174894	0.093899922	0.119946688	negative
PRJNA729271	stage_1D_vs_E10	ordinary_muscle_development	ENSGALG00010012092	OC3	8.061616881	0.69818705	11.54650016	7.68881E-31	6.93524E-30	positive
PRJNA729271	stage_1D_vs_E10	ordinary_muscle_development	ENSGALG00010012093	ENSGALG00010012093	-1.277941241	0.068395038	-18.68470708	6.59398E-78	2.96831E-76	negative
PRJNA729271	stage_1D_vs_E10	ordinary_muscle_development	ENSGALG00010012094	ENSGALG00010012094	-1.180325857	0.366321335	-3.222105141	0.001272524	0.002098673	negative
PRJNA729271	stage_1D_vs_E10	ordinary_muscle_development	ENSGALG00010012095	RNF38	0.445618445	0.084992233	5.243049052	1.57945E-07	3.75682E-07	positive
PRJNA729271	stage_1D_vs_E10	ordinary_muscle_development	ENSGALG00010012096	ENSGALG00010012096	-1.744507915	0.164833363	-10.58346369	3.55566E-26	2.56287E-25	negative
PRJNA729271	stage_1D_vs_E10	ordinary_muscle_development	ENSGALG00010012097	GART	-0.853612331	0.192628593	-4.431389541	9.36278E-06	1.91903E-05	negative
PRJNA729271	stage_1D_vs_E10	ordinary_muscle_development	ENSGALG00010012098	ART4	2.708547969	0.513206474	5.277696412	1.30818E-07	3.13319E-07	positive
PRJNA729271	stage_1D_vs_E10	ordinary_muscle_development	ENSGALG00010012099	UPK3A	-6.278580679	0.634905773	-9.888996047	4.64664E-23	2.90065E-22	negative
PRJNA729271	stage_1D_vs_E10	ordinary_muscle_development	ENSGALG00010012100	UBE2F	0.551170838	0.111341159	4.950288304	7.41036E-07	1.67154E-06	positive
PRJNA729271	stage_1D_vs_E10	ordinary_muscle_development	ENSGALG00010012101	ENSGALG00010012101	-1.519840311	0.1358963	-11.18382408	4.89349E-29	4.05553E-28	negative
PRJNA729271	stage_1D_vs_E10	ordinary_muscle_development	ENSGALG00010012102	SON	0.396631918	0.104824149	3.783783803	0.000154462	0.000281378	positive
PRJNA729271	stage_1D_vs_E10	ordinary_muscle_development	ENSGALG00010012103	ENSGALG00010012103	0.981320868	0.207697753	4.724754388	2.30394E-06	4.99859E-06	positive
PRJNA729271	stage_1D_vs_E10	ordinary_muscle_development	ENSGALG00010012105	DONSON	-1.511106056	0.204110982	-7.403354973	1.32786E-13	4.86905E-13	negative
PRJNA729271	stage_1D_vs_E10	ordinary_muscle_development	ENSGALG00010012106	TMTC1	0.026907315	0.1383925	0.194427549	0.845841121	0.868243541	positive
PRJNA729271	stage_1D_vs_E10	ordinary_muscle_development	ENSGALG00010012107	CLCC1	0.072050754	0.084803831	0.849616736	0.395538204	0.444857718	positive
PRJNA729271	stage_1D_vs_E10	ordinary_muscle_development	ENSGALG00010012108	PSTPIP2	-1.530320499	0.363912951	-4.205182846	2.60871E-05	5.11128E-05	negative
PRJNA729271	stage_1D_vs_E10	ordinary_muscle_development	ENSGALG00010012110	ENSGALG00010012110	0.766511594	0.112659431	6.803794288	1.01899E-11	3.29546E-11	positive
PRJNA729271	stage_1D_vs_E10	ordinary_muscle_development	ENSGALG00010012111	DRD1	-0.469989154	0.330601289	-1.42161924	0.155136816	0.190665436	negative
PRJNA729271	stage_1D_vs_E10	ordinary_muscle_development	ENSGALG00010012112	MBD2	1.131153621	0.190388488	5.941292105	2.82784E-09	7.72368E-09	positive
PRJNA729271	stage_1D_vs_E10	ordinary_muscle_development	ENSGALG00010012113	WDR47	-0.533189014	0.095204649	-5.600451439	2.13794E-08	5.43484E-08	negative
PRJNA729271	stage_1D_vs_E10	ordinary_muscle_development	ENSGALG00010012114	TAL2	-0.372013833	0.313694081	-1.185912822	0.235656729	0.279202357	negative
PRJNA729271	stage_1D_vs_E10	ordinary_muscle_development	ENSGALG00010012116	RBM12B	0.006379357	0.077226908	0.082605365	0.934165335	0.944196815	positive
PRJNA729271	stage_1D_vs_E10	ordinary_muscle_development	ENSGALG00010012118	ENSGALG00010012118	-0.969148048	0.762435041	-1.271122123	0.203685206	0.244263842	negative
PRJNA729271	stage_1D_vs_E10	ordinary_muscle_development	ENSGALG00010012119	EPG5	0.852283333	0.121922443	6.99037285	2.74157E-12	9.21591E-12	positive
PRJNA729271	stage_1D_vs_E10	ordinary_muscle_development	ENSGALG00010012120	ENSGALG00010012120	-0.838932142	0.190428532	-4.405496022	1.05542E-05	2.15076E-05	negative
PRJNA729271	stage_1D_vs_E10	ordinary_muscle_development	ENSGALG00010012121	SREK1	-0.081502173	0.100100619	-0.814202485	0.415528962	0.464905573	negative
PRJNA729271	stage_1D_vs_E10	ordinary_muscle_development	ENSGALG00010012122	GRIP1	-3.215115031	0.254795482	-12.61841461	1.67153E-36	1.91987E-35	negative
PRJNA729271	stage_1D_vs_E10	ordinary_muscle_development	ENSGALG00010012123	ENSGALG00010012123	-1.747772568	0.21002323	-8.321805973	8.6633E-17	3.88055E-16	negative
PRJNA729271	stage_1D_vs_E10	ordinary_muscle_development	ENSGALG00010012124	ERAP1	1.964956031	0.146827536	13.38274881	7.62735E-41	1.03616E-39	positive
PRJNA729271	stage_1D_vs_E10	ordinary_muscle_development	ENSGALG00010012125	RNF152	0.945088511	0.146894789	6.433778354	1.2447E-10	3.74548E-10	positive
PRJNA729271	stage_1D_vs_E10	ordinary_muscle_development	ENSGALG00010012127	ENSGALG00010012127	-0.139930693	0.115959255	-1.206722945	0.22753886	0.270475297	negative
PRJNA729271	stage_1D_vs_E10	ordinary_muscle_development	ENSGALG00010012128	ENSGALG00010012128	4.069769695	0.736986667	5.522175471	3.34828E-08	8.36925E-08	positive
PRJNA729271	stage_1D_vs_E10	ordinary_muscle_development	ENSGALG00010012129	CRYZL1	-1.283721023	0.100136089	-12.81976388	1.27087E-37	1.52494E-36	negative
PRJNA729271	stage_1D_vs_E10	ordinary_muscle_development	ENSGALG00010012130	TMEM67	-1.423312631	0.168186757	-8.462691447	2.61276E-17	1.20611E-16	negative
PRJNA729271	stage_1D_vs_E10	ordinary_muscle_development	ENSGALG00010012131	SLC14A1	0.614511594	0.24310675	2.527743853	0.011479807	0.016836973	positive
PRJNA729271	stage_1D_vs_E10	ordinary_muscle_development	ENSGALG00010012132	MC4R	-5.29496869	1.155122634	-4.583901771	4.56379E-06	9.60742E-06	negative
PRJNA729271	stage_1D_vs_E10	ordinary_muscle_development	ENSGALG00010012133	RAB3IP	-3.149899687	0.244551495	-12.88031253	5.80998E-38	7.0829E-37	negative
PRJNA729271	stage_1D_vs_E10	ordinary_muscle_development	ENSGALG00010012135	CAMSAP2	0.11843348	0.091411867	1.295602893	0.195112332	0.235086203	positive
PRJNA729271	stage_1D_vs_E10	ordinary_muscle_development	ENSGALG00010012136	ENSGALG00010012136	0.852193038	0.171905717	4.957328069	7.14692E-07	1.61413E-06	positive
PRJNA729271	stage_1D_vs_E10	ordinary_muscle_development	ENSGALG00010012137	ENSGALG00010012137	-0.289996767	0.965963922	-0.300214905	0.764013237	0.794309281	negative
PRJNA729271	stage_1D_vs_E10	ordinary_muscle_development	ENSGALG00010012138	DDX11	-2.157968186	0.171633029	-12.57315214	2.96656E-36	3.37787E-35	negative
PRJNA729271	stage_1D_vs_E10	ordinary_muscle_development	ENSGALG00010012140	GLOD5	1.706531547	0.571477696	2.986173493	0.002824923	0.004466324	positive
PRJNA729271	stage_1D_vs_E10	ordinary_muscle_development	ENSGALG00010012141	ENSGALG00010012141	0.253900006	0.093699666	2.709721585	0.006733971	0.010175014	positive
PRJNA729271	stage_1D_vs_E10	ordinary_muscle_development	ENSGALG00010012144	H1-01	-0.671548588	0.157611248	-4.260791007	2.03705E-05	4.03264E-05	negative
PRJNA729271	stage_1D_vs_E10	ordinary_muscle_development	ENSGALG00010012145	ENSGALG00010012145	2.535146391	1.018988039	2.487905936	0.012849771	0.018717459	positive
PRJNA729271	stage_1D_vs_E10	ordinary_muscle_development	ENSGALG00010012147	ARFGAP3	-0.784909853	0.123068297	-6.377839571	1.79604E-10	5.34679E-10	negative
PRJNA729271	stage_1D_vs_E10	ordinary_muscle_development	ENSGALG00010012148	SETBP1	0.459613065	0.108817334	4.223711867	2.40311E-05	4.72506E-05	positive
PRJNA729271	stage_1D_vs_E10	ordinary_muscle_development	ENSGALG00010012149	SFXN1	-0.642527105	0.173626519	-3.700627706	0.000215067	0.000384626	negative
PRJNA729271	stage_1D_vs_E10	ordinary_muscle_development	ENSGALG00010012151	MYRFL	-1.930703487	0.593572857	-3.25268156	0.001143215	0.001894684	negative
PRJNA729271	stage_1D_vs_E10	ordinary_muscle_development	ENSGALG00010012154	CWC27	-0.18966805	0.113496026	-1.671142657	0.094693503	0.12088976	negative
PRJNA729271	stage_1D_vs_E10	ordinary_muscle_development	ENSGALG00010012156	ENSGALG00010012156	-2.098845578	0.564207835	-3.71998659	0.000199233	0.00035781	negative
PRJNA729271	stage_1D_vs_E10	ordinary_muscle_development	ENSGALG00010012157	H2BC32	-1.633737837	0.198511476	-8.229941508	1.87305E-16	8.22224E-16	negative
PRJNA729271	stage_1D_vs_E10	ordinary_muscle_development	ENSGALG00010012159	WASHC1	-0.044218328	0.101308129	-0.436473642	0.662493119	0.70155156	negative
PRJNA729271	stage_1D_vs_E10	ordinary_muscle_development	ENSGALG00010012160	SYT4	-3.483175664	0.362746435	-9.602232658	7.82312E-22	4.63219E-21	negative
PRJNA729271	stage_1D_vs_E10	ordinary_muscle_development	ENSGALG00010012163	PDP1	0.580063289	0.220996244	2.624765377	0.008670869	0.012918517	positive
PRJNA729271	stage_1D_vs_E10	ordinary_muscle_development	ENSGALG00010012168	ENSGALG00010012168	-2.150506659	0.57521412	-3.738619387	0.000185034	0.000334005	negative
PRJNA729271	stage_1D_vs_E10	ordinary_muscle_development	ENSGALG00010012169	H3C33	-1.608518886	0.228039505	-7.053685212	1.7424E-12	5.92728E-12	negative
PRJNA729271	stage_1D_vs_E10	ordinary_muscle_development	ENSGALG00010012171	CDH17	-0.111589017	0.391491983	-0.285035254	0.775617133	0.804765236	negative
PRJNA729271	stage_1D_vs_E10	ordinary_muscle_development	ENSGALG00010012172	CNOT2	-0.337599274	0.088902111	-3.797426986	0.000146206	0.000267009	negative
PRJNA729271	stage_1D_vs_E10	ordinary_muscle_development	ENSGALG00010012175	PACSIN2	0.355016003	0.154495326	2.297907723	0.021567042	0.03039221	positive
PRJNA729271	stage_1D_vs_E10	ordinary_muscle_development	ENSGALG00010012179	ENSGALG00010012179	-1.000125915	0.180259307	-5.548262294	2.88523E-08	7.24682E-08	negative
PRJNA729271	stage_1D_vs_E10	ordinary_muscle_development	ENSGALG00010012180	DDX59	-0.485059794	0.10694499	-4.535600915	5.74397E-06	1.19918E-05	negative
PRJNA729271	stage_1D_vs_E10	ordinary_muscle_development	ENSGALG00010012186	KIF20A	-1.76619787	0.24753629	-7.135106806	9.6712E-13	3.34888E-12	negative
PRJNA729271	stage_1D_vs_E10	ordinary_muscle_development	ENSGALG00010012187	ENSGALG00010012187	-1.608378585	0.843103601	-1.907687955	0.056431559	0.074625988	negative
PRJNA729271	stage_1D_vs_E10	ordinary_muscle_development	ENSGALG00010012188	ADAMTS6	-0.587237312	0.182659683	-3.214925717	0.001304783	0.002149429	negative
PRJNA729271	stage_1D_vs_E10	ordinary_muscle_development	ENSGALG00010012190	CAST	0.845609726	0.132302646	6.391480091	1.64288E-10	4.90091E-10	positive
PRJNA729271	stage_1D_vs_E10	ordinary_muscle_development	ENSGALG00010012191	BEST3	4.391749592	0.374734263	11.71963768	1.01104E-31	9.51008E-31	positive
PRJNA729271	stage_1D_vs_E10	ordinary_muscle_development	ENSGALG00010012193	TTLL1	-1.657321044	0.231362465	-7.16330995	7.8752E-13	2.74666E-12	negative
PRJNA729271	stage_1D_vs_E10	ordinary_muscle_development	ENSGALG00010012194	LRRFIP1	2.049930006	0.159618107	12.84271595	9.45E-38	1.14339E-36	positive
PRJNA729271	stage_1D_vs_E10	ordinary_muscle_development	ENSGALG00010012195	ITSN1	0.281851058	0.071886287	3.920790338	8.8259E-05	0.00016469	positive
PRJNA729271	stage_1D_vs_E10	ordinary_muscle_development	ENSGALG00010012196	CENPK	-2.247077168	0.26160619	-8.589541265	8.73172E-18	4.13886E-17	negative
PRJNA729271	stage_1D_vs_E10	ordinary_muscle_development	ENSGALG00010012198	TMEM5	0.221270321	0.101699772	2.17572092	0.029576135	0.040805833	positive
PRJNA729271	stage_1D_vs_E10	ordinary_muscle_development	ENSGALG00010012199	ENSGALG00010012199	0.694575909	0.288637163	2.406398059	0.0161107	0.023105233	positive
PRJNA729271	stage_1D_vs_E10	ordinary_muscle_development	ENSGALG00010012200	GEM	1.236341194	0.238725873	5.178915803	2.23179E-07	5.23969E-07	positive
PRJNA729271	stage_1D_vs_E10	ordinary_muscle_development	ENSGALG00010012205	SLC6A13	-5.767399691	0.538740742	-10.70533419	9.60833E-27	7.12393E-26	negative
PRJNA729271	stage_1D_vs_E10	ordinary_muscle_development	ENSGALG00010012206	RIBC2	-4.686473011	0.901028012	-5.201251178	1.97951E-07	4.67237E-07	negative
PRJNA729271	stage_1D_vs_E10	ordinary_muscle_development	ENSGALG00010012207	GRIA3	-2.019290143	0.262152653	-7.702726329	1.33193E-14	5.20107E-14	negative
PRJNA729271	stage_1D_vs_E10	ordinary_muscle_development	ENSGALG00010012209	KIF14	-0.898318613	0.226933834	-3.95850455	7.54205E-05	0.000141646	negative
PRJNA729271	stage_1D_vs_E10	ordinary_muscle_development	ENSGALG00010012210	PPWD1	-1.031019639	0.075664667	-13.6261703	2.79887E-42	3.96963E-41	negative
PRJNA729271	stage_1D_vs_E10	ordinary_muscle_development	ENSGALG00010012211	ENSGALG00010012211	-6.220358547	4.076154142	-1.526036143	0.127000864	0.158573307	negative
PRJNA729271	stage_1D_vs_E10	ordinary_muscle_development	ENSGALG00010012212	RAD54B	-2.105447552	0.187397977	-11.23516691	2.73976E-29	2.29024E-28	negative
PRJNA729271	stage_1D_vs_E10	ordinary_muscle_development	ENSGALG00010012215	RAB17	4.153673887	0.771487652	5.383979741	7.28567E-08	1.77997E-07	positive
PRJNA729271	stage_1D_vs_E10	ordinary_muscle_development	ENSGALG00010012216	BRD8	0.01307401	0.057041442	0.229201954	0.818711954	0.843981919	positive
PRJNA729271	stage_1D_vs_E10	ordinary_muscle_development	ENSGALG00010012219	KDM5A	-0.409765916	0.077119013	-5.313422715	1.07585E-07	2.59303E-07	negative
PRJNA729271	stage_1D_vs_E10	ordinary_muscle_development	ENSGALG00010012226	SLCO4C1	-0.55298129	0.53756412	-1.028679685	0.303630219	0.35074275	negative
PRJNA729271	stage_1D_vs_E10	ordinary_muscle_development	ENSGALG00010012228	NHP2	-0.90667747	0.113622463	-7.97973784	1.46644E-15	6.09931E-15	negative
PRJNA729271	stage_1D_vs_E10	ordinary_muscle_development	ENSGALG00010012229	FSBP	-0.282789032	0.203310192	-1.390924032	0.164248464	0.200989573	negative
PRJNA729271	stage_1D_vs_E10	ordinary_muscle_development	ENSGALG00010012230	PTPRQ	-0.598710614	0.401352841	-1.491731349	0.135769587	0.168538906	negative
PRJNA729271	stage_1D_vs_E10	ordinary_muscle_development	ENSGALG00010012232	PCSK1	0.27863062	0.430700492	0.646924314	0.517680929	0.565214302	positive
PRJNA729271	stage_1D_vs_E10	ordinary_muscle_development	ENSGALG00010012234	C1GALT1C1	0.039715695	0.06060318	0.655340117	0.512248797	0.560042715	positive
PRJNA729271	stage_1D_vs_E10	ordinary_muscle_development	ENSGALG00010012236	PRLH	-3.159566205	0.770521574	-4.100555147	4.1216E-05	7.92658E-05	negative
PRJNA729271	stage_1D_vs_E10	ordinary_muscle_development	ENSGALG00010012240	TRIM14	-1.418386561	0.263749368	-5.377781831	7.54091E-08	1.83892E-07	negative
PRJNA729271	stage_1D_vs_E10	ordinary_muscle_development	ENSGALG00010012242	CCDC77	-2.072972731	0.13870306	-14.94540016	1.66886E-50	3.2867E-49	negative
PRJNA729271	stage_1D_vs_E10	ordinary_muscle_development	ENSGALG00010012243	ENSGALG00010012243	-4.439042636	1.680890327	-2.640887728	0.008268912	0.01235903	negative
PRJNA729271	stage_1D_vs_E10	ordinary_muscle_development	ENSGALG00010012244	ENSGALG00010012244	-0.45102185	0.080904367	-5.574752819	2.47882E-08	6.27387E-08	negative
PRJNA729271	stage_1D_vs_E10	ordinary_muscle_development	ENSGALG00010012245	ENSGALG00010012245	-0.482279023	0.07862959	-6.133556347	8.5936E-10	2.44148E-09	negative
PRJNA729271	stage_1D_vs_E10	ordinary_muscle_development	ENSGALG00010012247	TRIM23	0.988483682	0.125474991	7.877933906	3.32839E-15	1.35443E-14	positive
PRJNA729271	stage_1D_vs_E10	ordinary_muscle_development	ENSGALG00010012248	H2AC39	-1.18385406	0.20982623	-5.642068966	1.68019E-08	4.31203E-08	negative
PRJNA729271	stage_1D_vs_E10	ordinary_muscle_development	ENSGALG00010012250	NANS	-1.38581266	0.112076608	-12.3648698	4.04867E-35	4.41576E-34	negative
PRJNA729271	stage_1D_vs_E10	ordinary_muscle_development	ENSGALG00010012253	MYF6	1.951606389	0.289350727	6.744777897	1.53261E-11	4.91053E-11	positive
PRJNA729271	stage_1D_vs_E10	ordinary_muscle_development	ENSGALG00010012259	IL1RAPL2	-0.370690282	0.219212492	-1.691008934	0.090835096	0.116332808	negative
PRJNA729271	stage_1D_vs_E10	ordinary_muscle_development	ENSGALG00010012260	ENSGALG00010012260	1.704617585	0.302772288	5.630031721	1.80176E-08	4.61016E-08	positive
PRJNA729271	stage_1D_vs_E10	ordinary_muscle_development	ENSGALG00010012263	SHLD3	0.437858732	0.126681637	3.456370959	0.000547501	0.000938842	positive
PRJNA729271	stage_1D_vs_E10	ordinary_muscle_development	ENSGALG00010012264	MYF5	2.285099314	0.153932727	14.8449196	7.51877E-50	1.44542E-48	positive
PRJNA729271	stage_1D_vs_E10	ordinary_muscle_development	ENSGALG00010012266	H3C33	-1.235455967	0.202847848	-6.090554973	1.1252E-09	3.16449E-09	negative
PRJNA729271	stage_1D_vs_E10	ordinary_muscle_development	ENSGALG00010012268	ENSGALG00010012268	-0.502894958	0.166234377	-3.025216369	0.002484553	0.003951026	negative
PRJNA729271	stage_1D_vs_E10	ordinary_muscle_development	ENSGALG00010012271	ENSGALG00010012271	1.013897889	0.26044277	3.892977673	9.90213E-05	0.000183685	positive
PRJNA729271	stage_1D_vs_E10	ordinary_muscle_development	ENSGALG00010012274	B4GALNT3	1.42580139	0.276333883	5.159705257	2.47339E-07	5.78908E-07	positive

PRJNA729271	stage_1D_vs_E10	ordinary_muscle_development	ENSGALG00010012275	VIRMA	0.058951449	0.073302114	0.804225773	0.421266597	0.470798714	positive
PRJNA729271	stage_1D_vs_E10	ordinary_muscle_development	ENSGALG00010012277	H3C33	-1.076233745	0.209939393	-5.12640211	2.95331E-07	6.86691E-07	negative
PRJNA729271	stage_1D_vs_E10	ordinary_muscle_development	ENSGALG00010012282	ENSGALG00010012282	-3.137336464	1.552861332	-2.020358417			negative
PRJNA729271	stage_1D_vs_E10	ordinary_muscle_development	ENSGALG00010012283	CUPIN1A	0.557424761	0.755469703	0.737851907	0.460604447	0.509420224	positive
PRJNA729271	stage_1D_vs_E10	ordinary_muscle_development	ENSGALG00010012285	FBLN1	-2.156107989	0.199109863	-10.82873525	2.51606E-27	1.90844E-26	negative
PRJNA729271	stage_1D_vs_E10	ordinary_muscle_development	ENSGALG00010012286	ENSGALG00010012286	-7.406616901	2.755012633	-2.68841486	0.007179214	0.01081171	negative
PRJNA729271	stage_1D_vs_E10	ordinary_muscle_development	ENSGALG00010012288	MCTS1	-0.088958018	0.114098185	-0.779661987	0.43558986	0.484936637	negative
PRJNA729271	stage_1D_vs_E10	ordinary_muscle_development	ENSGALG00010012290	ENSGALG00010012290	-0.165060611	0.096899179	-1.703426318	0.088488316	0.113537943	negative
PRJNA729271	stage_1D_vs_E10	ordinary_muscle_development	ENSGALG00010012291	NR5A2	0.111443698	0.189599049	0.587786167	0.55667583	0.60234519	positive
PRJNA729271	stage_1D_vs_E10	ordinary_muscle_development	ENSGALG00010012293	SRGAP1	-1.747141573	0.106819245	-16.35605613	3.93821E-60	1.09357E-58	negative
PRJNA729271	stage_1D_vs_E10	ordinary_muscle_development	ENSGALG00010012294	ENSGALG00010012294	0.643005681	0.482920147	1.331494834	0.183026244	0.221922343	positive
PRJNA729271	stage_1D_vs_E10	ordinary_muscle_development	ENSGALG00010012295	CUL4B	-0.219988006	0.065375329	-3.365000378	0.000765436	0.001293273	negative
PRJNA729271	stage_1D_vs_E10	ordinary_muscle_development	ENSGALG00010012297	ABLIM3	0.230201787	0.190125513	1.210788515	0.225976468	0.268794428	positive
PRJNA729271	stage_1D_vs_E10	ordinary_muscle_development	ENSGALG00010012300	NINJ2	-1.009999011	0.206222987	-4.897606355	9.70111E-07	2.17036E-06	negative
PRJNA729271	stage_1D_vs_E10	ordinary_muscle_development	ENSGALG00010012303	CA9	0.853115587	0.211930299	4.025453613	5.68656E-05	0.000108171	positive
PRJNA729271	stage_1D_vs_E10	ordinary_muscle_development	ENSGALG00010012304	ESRP1	-6.993984402	0.97013099	-7.209319641	5.62321E-13	1.98221E-12	negative
PRJNA729271	stage_1D_vs_E10	ordinary_muscle_development	ENSGALG00010012307	ENSGALG00010012307	-1.168090968	0.17288257	-6.756557181	1.41309E-11	4.53661E-11	negative
PRJNA729271	stage_1D_vs_E10	ordinary_muscle_development	ENSGALG00010012308	TRABD2A	0.436819478	0.361097442	1.209699727	0.226394135	0.269247045	positive
PRJNA729271	stage_1D_vs_E10	ordinary_muscle_development	ENSGALG00010012309	TRAPPC13	0.110983763	0.124142331	0.894004184	0.371319653	0.420523351	positive
PRJNA729271	stage_1D_vs_E10	ordinary_muscle_development	ENSGALG00010012311	ENSGALG00010012311	-2.826284784	0.519527361	-5.440107677	5.32484E-08	1.31287E-07	negative
PRJNA729271	stage_1D_vs_E10	ordinary_muscle_development	ENSGALG00010012312	SH3TC2	1.144812552	0.177303698	6.456788933	1.06948E-10	3.23028E-10	positive
PRJNA729271	stage_1D_vs_E10	ordinary_muscle_development	ENSGALG00010012314	ADRB2	3.889083961	0.262800929	14.79859292	1.49579E-49	2.84161E-48	positive
PRJNA729271	stage_1D_vs_E10	ordinary_muscle_development	ENSGALG00010012315	ATP6V1E1	0.045989594	0.101146777	0.454681751	0.649338212	0.68923238	positive
PRJNA729271	stage_1D_vs_E10	ordinary_muscle_development	ENSGALG00010012316	ENSGALG00010012316	-2.873042754	0.960391195	-2.991533833	0.002775798	0.004392967	negative
PRJNA729271	stage_1D_vs_E10	ordinary_muscle_development	ENSGALG00010012317	ENSGALG00010012317	-1.84120805	1.148470205	-1.603183122	0.108894231	0.137517152	negative
PRJNA729271	stage_1D_vs_E10	ordinary_muscle_development	ENSGALG00010012318	POLR1D	-0.462152445	0.138280972	-3.342126095	0.000831393	0.001398519	negative
PRJNA729271	stage_1D_vs_E10	ordinary_muscle_development	ENSGALG00010012320	DPY19L4	-0.238545188	0.060905605	-3.916637681	8.97925E-05	0.000167401	negative
PRJNA729271	stage_1D_vs_E10	ordinary_muscle_development	ENSGALG00010012322	ENSGALG00010012322	-1.109541552	0.267363817	-4.149931593	3.32575E-05	6.45424E-05	negative
PRJNA729271	stage_1D_vs_E10	ordinary_muscle_development	ENSGALG00010012323	SMAD7B	-2.934269059	3.603976252	-0.81417547	0.415544436	0.464905573	negative
PRJNA729271	stage_1D_vs_E10	ordinary_muscle_development	ENSGALG00010012324	ENSGALG00010012324	-1.408865458	0.163513058	-8.616225973	6.91971E-18	3.29721E-17	negative
PRJNA729271	stage_1D_vs_E10	ordinary_muscle_development	ENSGALG00010012325	MLPH	-3.103091384	0.590899876	-5.251467311	1.50892E-07	3.59616E-07	negative
PRJNA729271	stage_1D_vs_E10	ordinary_muscle_development	ENSGALG00010012326	SGTB	-0.275358834	0.11665006	-2.360554589	0.018247632	0.02594149	negative
PRJNA729271	stage_1D_vs_E10	ordinary_muscle_development	ENSGALG00010012327	UBTD2	-0.493885436	0.08638483	-5.717270485	1.08249E-08	2.81548E-08	negative
PRJNA729271	stage_1D_vs_E10	ordinary_muscle_development	ENSGALG00010012329	ATXN10	-0.592467165	0.107765315	-5.497753742	3.8466E-08	9.57849E-08	negative
PRJNA729271	stage_1D_vs_E10	ordinary_muscle_development	ENSGALG00010012331	ENSGALG00010012331	-0.703755384	0.260774826	-2.698709058	0.0069609	0.010501483	negative
PRJNA729271	stage_1D_vs_E10	ordinary_muscle_development	ENSGALG00010012334	ARHGEF39	-1.208781389	0.223320614	-5.412762261	6.20599E-08	1.52364E-07	negative
PRJNA729271	stage_1D_vs_E10	ordinary_muscle_development	ENSGALG00010012336	ENSGALG00010012336	-21.11578489	4.176925169	-5.055341916	4.2962E-07	9.85652E-07	negative
PRJNA729271	stage_1D_vs_E10	ordinary_muscle_development	ENSGALG00010012337	ACSS3	0.833173089	0.151946705	5.483324497	4.17406E-08	1.03566E-07	positive
PRJNA729271	stage_1D_vs_E10	ordinary_muscle_development	ENSGALG00010012338	H3C33	-1.323838165	0.225135117	-5.880194008	4.09786E-09	1.10263E-08	negative
PRJNA729271	stage_1D_vs_E10	ordinary_muscle_development	ENSGALG00010012339	WNK1	0.74432798	0.154770693	4.809230786	1.51512E-06	3.33421E-06	positive
PRJNA729271	stage_1D_vs_E10	ordinary_muscle_development	ENSGALG00010012340	ENHO	-1.295537897	0.163832734	-7.907686466	2.62216E-15	1.07126E-14	negative
PRJNA729271	stage_1D_vs_E10	ordinary_muscle_development	ENSGALG00010012341	GOPC	-0.074679507	0.080254781	-0.930530321	0.352096573	0.401008865	negative
PRJNA729271	stage_1D_vs_E10	ordinary_muscle_development	ENSGALG00010012342	ENSGALG00010012342	-0.993829826	1.653377769	-0.601090594	0.547779648	0.593782979	negative
PRJNA729271	stage_1D_vs_E10	ordinary_muscle_development	ENSGALG00010012343	BCL2L13	1.211674579	0.17026994	7.116197822	1.10945E-12	3.81439E-12	positive
PRJNA729271	stage_1D_vs_E10	ordinary_muscle_development	ENSGALG00010012345	H2BC32	-1.39344122	0.165149927	-8.437431641	3.24387E-17	1.48891E-16	negative
PRJNA729271	stage_1D_vs_E10	ordinary_muscle_development	ENSGALG00010012346	NLN	-0.661077522	0.139673153	-4.733032136	2.2119E-06	4.80611E-06	negative
PRJNA729271	stage_1D_vs_E10	ordinary_muscle_development	ENSGALG00010012347	ENSGALG00010012347	-2.022370443	0.491531436	-4.114427471	3.88142E-05	7.48651E-05	negative
PRJNA729271	stage_1D_vs_E10	ordinary_muscle_development	ENSGALG00010012350	H2AC32	-1.392330451	0.192176429	-7.24506359	4.32236E-13	1.53787E-12	negative
PRJNA729271	stage_1D_vs_E10	ordinary_muscle_development	ENSGALG00010012352	PTPRC	0.032050923	0.197035903	0.162665395	0.870781895	0.889980969	positive
PRJNA729271	stage_1D_vs_E10	ordinary_muscle_development	ENSGALG00010012353	PIGG	0.292183386	0.151997658	1.922288731	0.054569437	0.07233507	positive
PRJNA729271	stage_1D_vs_E10	ordinary_muscle_development	ENSGALG00010012354	DCBLD1	1.350309207	0.148732934	9.078750583	1.09828E-19	5.78264E-19	positive
PRJNA729271	stage_1D_vs_E10	ordinary_muscle_development	ENSGALG00010012358	STK10	0.486268029	0.148848413	3.266867419	0.001087446	0.001805973	positive
PRJNA729271	stage_1D_vs_E10	ordinary_muscle_development	ENSGALG00010012360	ROS1	3.863618436	0.36523113	10.57855729	3.74683E-26	2.69798E-25	positive
PRJNA729271	stage_1D_vs_E10	ordinary_muscle_development	ENSGALG00010012361	WIF1	-6.02526557	0.387197725	-15.5612112	1.33548E-54	3.01522E-53	negative
PRJNA729271	stage_1D_vs_E10	ordinary_muscle_development	ENSGALG00010012362	COL6A3	2.692326341	0.292461443	9.205747993	3.393E-20	1.83445E-19	positive
PRJNA729271	stage_1D_vs_E10	ordinary_muscle_development	ENSGALG00010012366	INTS8	-0.406140413	0.067589703	-6.008909574	1.86775E-09	5.16958E-09	negative
PRJNA729271	stage_1D_vs_E10	ordinary_muscle_development	ENSGALG00010012367	RAD52	-1.780158258	0.139350392	-12.77469145	2.27046E-37	2.69757E-36	negative
PRJNA729271	stage_1D_vs_E10	ordinary_muscle_development	ENSGALG00010012371	ATP6V1G3	-2.590187188	0.557732411	-4.644139619	3.41497E-06	7.28798E-06	negative
PRJNA729271	stage_1D_vs_E10	ordinary_muscle_development	ENSGALG00010012372	LEMD3	0.228247993	0.072537561	3.146617971	0.001651706	0.002686733	positive
PRJNA729271	stage_1D_vs_E10	ordinary_muscle_development	ENSGALG00010012373	WNT7B	-7.216988539	0.812901846	-8.878056522	6.80387E-19	3.41607E-18	negative
PRJNA729271	stage_1D_vs_E10	ordinary_muscle_development	ENSGALG00010012376	VGLL2	2.097356127	0.185672201	11.29601588	1.37307E-29	1.16186E-28	positive
PRJNA729271	stage_1D_vs_E10	ordinary_muscle_development	ENSGALG00010012378	RPS19BP1	-0.187908858	0.091254881	-2.059165002	0.039478435	0.053470371	negative
PRJNA729271	stage_1D_vs_E10	ordinary_muscle_development	ENSGALG00010012382	SH3PXD2B	-1.015895311	0.133142169	-7.630154453	2.34473E-14	9.02944E-14	negative
PRJNA729271	stage_1D_vs_E10	ordinary_muscle_development	ENSGALG00010012384	NRK	-0.393962234	0.122101612	-3.226511316	0.001253093	0.002068038	negative
PRJNA729271	stage_1D_vs_E10	ordinary_muscle_development	ENSGALG00010012387	ERBIN	-0.098865409	0.088907425	-1.112003971	0.266136443	0.311301464	negative
PRJNA729271	stage_1D_vs_E10	ordinary_muscle_development	ENSGALG00010012390	MMP16	-0.797628312	0.174360358	-4.574596663	4.77138E-06	1.00306E-05	negative
PRJNA729271	stage_1D_vs_E10	ordinary_muscle_development	ENSGALG00010012391	NEURL1B	-0.298143718	0.15695682	-1.899527	0.05749522	0.075866474	negative
PRJNA729271	stage_1D_vs_E10	ordinary_muscle_development	ENSGALG00010012393	ENSGALG00010012393	-3.60919464	1.63214074	-2.211325624	0.027013296	0.037462469	negative
PRJNA729271	stage_1D_vs_E10	ordinary_muscle_development	ENSGALG00010012398	ENSGALG00010012398	-4.338659917	0.820871757	-5.285429642	1.2541E-07	3.00798E-07	negative
PRJNA729271	stage_1D_vs_E10	ordinary_muscle_development	ENSGALG00010012399	ERC1	-0.380427902	0.084406804	-4.507076261	6.5727E-06	1.36609E-05	negative
PRJNA729271	stage_1D_vs_E10	ordinary_muscle_development	ENSGALG00010012401	RIPK2	0.297266426	0.131338127	2.263367325	0.023613055	0.033092066	positive
PRJNA729271	stage_1D_vs_E10	ordinary_muscle_development	ENSGALG00010012404	VAMP7	-0.077786913	0.052552944	-1.480162817	0.138829801	0.172127103	negative
PRJNA729271	stage_1D_vs_E10	ordinary_muscle_development	ENSGALG00010012405	MSRB3	2.260897205	0.125610942	17.99920583	1.97633E-72	7.80568E-71	positive
PRJNA729271	stage_1D_vs_E10	ordinary_muscle_development	ENSGALG00010012406	ENSGALG00010012406	4.280328871	0.238597796	17.93951552	5.79582E-72	2.25229E-70	positive
PRJNA729271	stage_1D_vs_E10	ordinary_muscle_development	ENSGALG00010012407	ATF4	0.651083669	0.128844698	5.053243767	4.34369E-07	9.96231E-07	positive
PRJNA729271	stage_1D_vs_E10	ordinary_muscle_development	ENSGALG00010012410	CACNA1I	-2.185222015	0.402474962	-5.429460769	5.65246E-08	1.39151E-07	negative
PRJNA729271	stage_1D_vs_E10	ordinary_muscle_development	ENSGALG00010012412	PURA	1.012990507	0.131632613	7.695589142	1.40844E-14	5.49241E-14	positive
PRJNA729271	stage_1D_vs_E10	ordinary_muscle_development	ENSGALG00010012413	NEK7	0.585688778	0.072049904	8.12893216	4.33089E-16	1.86778E-15	positive
PRJNA729271	stage_1D_vs_E10	ordinary_muscle_development	ENSGALG00010012414	ENSGALG00010012414	6.961551112	0.20332182	34.23907535	6.3422E-257	7.0715E-254	positive
PRJNA729271	stage_1D_vs_E10	ordinary_muscle_development	ENSGALG00010012415	BID	-1.730485083	0.114874	-15.06420149	2.7851E-51	5.60695E-50	negative
PRJNA729271	stage_1D_vs_E10	ordinary_muscle_development	ENSGALG00010012418	ENSGALG00010012418	1.013178678	0.766327867	1.322121669	0.186127639	0.225277232	positive
PRJNA729271	stage_1D_vs_E10	ordinary_muscle_development	ENSGALG00010012419	HACE1	0.262335401	0.096483928	2.718954388	0.006548864	0.009915991	positive
PRJNA729271	stage_1D_vs_E10	ordinary_muscle_development	ENSGALG00010012426	MIEF1	-0.051814104	0.080216198	-0.645930698	0.518324238	0.565831438	negative
PRJNA729271	stage_1D_vs_E10	ordinary_muscle_development	ENSGALG00010012427	C7	3.408937204	0.447703629	7.614272	2.65182E-14	1.01796E-13	positive
PRJNA729271	stage_1D_vs_E10	ordinary_muscle_development	ENSGALG00010012428	CCNE2	-2.063844938	0.215812198	-9.5631524	1.14223E-21	6.69498E-21	negative
PRJNA729271	stage_1D_vs_E10	ordinary_muscle_development	ENSGALG00010012429	CXCR7	2.194222614	0.100543422	21.82363182	1.3842E-105	1.1024E-103	positive
PRJNA729271	stage_1D_vs_E10	ordinary_muscle_development	ENSGALG00010012431	LAMP2	0.492199894	0.079413116	6.197967315	5.7197E-10	1.644E-09	positive
PRJNA729271	stage_1D_vs_E10	ordinary_muscle_development	ENSGALG00010012433	ENSGALG00010012433	0.908950372	0.70749165	1.28475067	0.198879426	0.238915736	positive
PRJNA729271	stage_1D_vs_E10	ordinary_muscle_development	ENSGALG00010012436	ENSGALG00010012436	3.790237562	0.199754647	18.97446503	2.7732E-80	1.31795E-78	positive
PRJNA729271	stage_1D_vs_E10	ordinary_muscle_development	ENSGALG00010012437	ENSGALG00010012437	-21.55372565	3.874110008	-5.563529586	2.64372E-08	6.66447E-08	negative
PRJNA729271	stage_1D_vs_E10	ordinary_muscle_development	ENSGALG00010012439	TENT2	0.364785744	0.101334779	3.599807971	0.000318452	0.00055985	positive
PRJNA729271	stage_1D_vs_E10	ordinary_muscle_development	ENSGALG00010012441	FST	2.281006005	0.23842179	9.56710376	1.09942E-21	6.45188E-21	positive
PRJNA729271	stage_1D_vs_E10	ordinary_muscle_development	ENSGALG00010012442	CPLX1	-4.401254562	0.34566605	-12.73267813	3.89255E-37	4.58719E-36	negative
PRJNA729271	stage_1D_vs_E10	ordinary_muscle_development	ENSGALG00010012445	SOBP	-0.133849366	0.162520505	-0.823584482	0.410175696	0.459893001	negative
PRJNA729271	stage_1D_vs_E10	ordinary_muscle_development	ENSGALG00010012447	COPS8	-0.436880062	0.11718467	-3.728133212	0.000192903	0.000347087	negative
PRJNA729271	stage_1D_vs_E10	ordinary_muscle_development	ENSGALG00010012449	SUB1	-1.253744476	0.109550973	-11.44439385	2.50852E-30	2.21039E-29	negative
PRJNA729271	stage_1D_vs_E10	ordinary_muscle_development	ENSGALG00010012450	PPFIA2	-1.160358842	0.251997097	-4.604651624	4.13157E-06	8.73754E-06	negative
PRJNA729271	stage_1D_vs_E10	ordinary_muscle_development	ENSGALG00010012454	IQCA1	2.954712208	0.400533678	7.376938237	1.61971E-13	5.90337E-13	positive
PRJNA729271	stage_1D_vs_E10	ordinary_muscle_development	ENSGALG00010012456	ENSGALG00010012456	-0.927251735	0.088035586	-10.53269221	6.10626E-26	4.35797E-25	negative
PRJNA729271	stage_1D_vs_E10	ordinary_muscle_development	ENSGALG00010012458	MRPS30	0.49035149	0.107514215	4.560806113	5.09576E-06	1.06893E-05	positive
PRJNA729271	stage_1D_vs_E10	ordinary_muscle_development	ENSGALG00010012464	ENSGALG00010012464	1.154730598	0.234179246	4.930968985	8.18227E-07	1.84022E-06	positive
PRJNA729271	stage_1D_vs_E10	ordinary_muscle_development	ENSGALG00010012466	PDSS2	0.318947986	0.121026283	2.635361328	0.008404776	0.01254399	positive
PRJNA729271	stage_1D_vs_E10	ordinary_muscle_development	ENSGALG00010012469	CETN2	-1.184096259	0.131604659	-8.997373378	2.31182E-19	1.19295E-18	negative
PRJNA729271	stage_1D_vs_E10	ordinary_muscle_development	ENSGALG00010012470	CHD1	0.294619123	0.10535179	2.79652699	0.00516551	0.00792402	positive
PRJNA729271	stage_1D_vs_E10	ordinary_muscle_development	ENSGALG00010012474	ASB18	2.803208568	0.930418026	3.012848516	0.002588081	0.004108897	positive
PRJNA729271	stage_1D_vs_E10	ordinary_muscle_development	ENSGALG00010012477	ENSGALG00010012477	-0.893705683	0.260904662	-3.425410935	0.00061387	0.001045476	negative
PRJNA729271	stage_1D_vs_E10	ordinary_muscle_development	ENSGALG00010012483	ENSGALG00010012483	1.025751647	0.252607864	4.060648113	4.89367E-05	9.35801E-05	positive
PRJNA729271	stage_1D_vs_E10	ordinary_muscle_development	ENSGALG00010012485	C6	1.775613602	1.337275101	1.327784837	0.184249198	0.223227359	positive
PRJNA729271	stage_1D_vs_E10	ordinary_muscle_development	ENSGALG00010012486	RADX	0.756240601	0.10730886	7.047326744	1.82388E-12	6.19571E-12	positive
PRJNA729271	stage_1D_vs_E10	ordinary_muscle_development	ENSGALG00010012489	GBX2	-4.58778123	1.068441194	-4.293901485	1.7556E-05	3.49553E-05	negative
PRJNA729271	stage_1D_vs_E10	ordinary_muscle_development	ENSGALG00010012497	PLCXD3	-0.306243862	0.41044771	-0.746121502	0.455594021	0.504494678	negative
PRJNA729271	stage_1D_vs_E10	ordinary_muscle_development	ENSGALG00010012498	KCNT2	0.026260552	0.21717946	0.12091637	0.903757276	0.917814174	positive
PRJNA729271	stage_1D_vs_E10	ordinary_muscle_development	ENSGALG00010012506	TP53INP1	-1.492314497	0.128437944	-11.61895348	3.30156E-31	3.01931E-30	negative
PRJNA729271	stage_1D_vs_E10	ordinary_muscle_development	ENSGALG00010012508	MDM1	-0.599987369	0.06783666	-8.844588899	9.18659E-19	4.59013E-18	negative
PRJNA729271	stage_1D_vs_E10	ordinary_muscle_development	ENSGALG00010012509	MICAL3	0.965315867	0.093145884	10.36348388	3.63477E-25	2.51845E-24	positive
PRJNA729271	stage_1D_vs_E10	ordinary_muscle_development	ENSGALG00010012511	OXCT1	-0.935927973	0.122231032	-7.65704058	1.90267E-14	7.36622E-14	negative
PRJNA729271	stage_1D_vs_E10	ordinary_muscle_development	ENSGALG00010012517	MGAT3	0.97278137	0.175198652	5.552447791	2.81697E-08	7.08151E-08	positive
PRJNA729271	stage_1D_vs_E10	ordinary_muscle_development	ENSGALG00010012519	ENSGALG00010012519	-4.991471432	1.836559503	-2.717838123	0.006570998	0.009948466	negative
PRJNA729271	stage_1D_vs_E10	ordinary_muscle_development	ENSGALG00010012521	HOMER1	1.148893519	0.13057439	8.798766128	1.38329E-18	6.84559E-18	positive
PRJNA729271	stage_1D_vs_E10	ordinary_muscle_development	ENSGALG00010012529	BEND3	-0.810898674	0.12521768	-6.475911978	9.4241E-11	2.85779E-10	negative
PRJNA729271	stage_1D_vs_E10	ordinary_muscle_development	ENSGALG00010012532	FAM174A	0.093589522	0.152981944	0.611768419	0.540690987	0.586976922	positive
PRJNA729271	stage_1D_vs_E10	ordinary_muscle_development	ENSGALG00010012535	JMY	0.079258279	0.102574469	0.772690123	0.439705794	0.488880531	positive
PRJNA729271	stage_1D_vs_E10	ordinary_muscle_development	ENSGALG00010012536	FBXO4	-0.223402267	0.134625194	-1.65943877	0.097027409	0.123662384	negative
PRJNA729271	stage_1D_vs_E10	ordinary_muscle_development	ENSGALG00010012538	RAP1B	-0.65463865	0.088045606	-7.43522227	1.04392E-13	3.86012E-13	negative
PRJNA729271	stage_1D_vs_E10	ordinary_muscle_development	ENSGALG00010012547	ENSGALG00010012547	-1.793997381	0.426431	-4.207005075	2.58777E-05	5.073E-05	negative
PRJNA729271	stage_1D_vs_E10	ordinary_muscle_development	ENSGALG00010012550	TAB1	0.41897848	0.093752261	4.468996026	7.85876E-06	1.62246E-05	positive
PRJNA729271	stage_1D_vs_E10	ordinary_muscle_development	ENSGALG00010012551	PCGF3	0.146643668	0.155933103	0.940426793	0.346998685	0.395855968	positive
PRJNA729271	stage_1D_vs_E10	ordinary_muscle_development	ENSGALG00010012553	ENSGALG00010012553	0.29772828	0.22394678	1.329459972	0.183696263	0.2226505	positive
PRJNA729271	stage_1D_vs_E10	ordinary_muscle_development	ENSGALG00010012555	ENSGALG00010012555	-0.666300531	0.212994567	-3.128251297	0.001758498	0.002851804	negative
PRJNA729271	stage_1D_vs_E10	ordinary_muscle_development	ENSGALG00010012561	OSGIN2	-0.567400033	0.123889486	-4.57988851	4.65224E-06	9.78584E-06	negative
PRJNA729271	stage_1D_vs_E10	ordinary_muscle_development	ENSGALG00010012565	SYNGR1	1.015029925	0.121964876	8.322313406	8.62629E-17	3.86516E-16	positive

PRJNA729271	stage_1D_vs_E10	ordinary_muscle_development	ENSGALG00010012569	C6orf203	0.413521714	0.121744574	3.396633628	0.000682202	0.001157365	positive
PRJNA729271	stage_1D_vs_E10	ordinary_muscle_development	ENSGALG00010012571	AGAP1	0.173730213	0.081861354	2.122249441	0.033816797	0.046325911	positive
PRJNA729271	stage_1D_vs_E10	ordinary_muscle_development	ENSGALG00010012574	PHAX	-0.520916044	0.105152683	-4.953901601	7.274E-07	1.64129E-06	negative
PRJNA729271	stage_1D_vs_E10	ordinary_muscle_development	ENSGALG00010012578	NDUFS4	0.706263946	0.152771514	4.6230081	3.78215E-06	8.03257E-06	positive
PRJNA729271	stage_1D_vs_E10	ordinary_muscle_development	ENSGALG00010012582	CD24	-3.246355758	0.226613554	-14.3255145	1.51601E-46	2.51714E-45	negative
PRJNA729271	stage_1D_vs_E10	ordinary_muscle_development	ENSGALG00010012583	NDUFAF6	-0.172514712	0.103528444	-1.666350863	0.095643552	0.122016659	negative
PRJNA729271	stage_1D_vs_E10	ordinary_muscle_development	ENSGALG00010012586	CDC73	0.372618217	0.133638287	2.788259447	0.005299209	0.008120523	positive
PRJNA729271	stage_1D_vs_E10	ordinary_muscle_development	ENSGALG00010012587	MBLAC2	1.093509969	0.144741927	7.554894363	4.192E-14	1.58858E-13	positive
PRJNA729271	stage_1D_vs_E10	ordinary_muscle_development	ENSGALG00010012588	PEX26	0.277996838	0.104987244	2.647910615	0.008099094	0.012121462	positive
PRJNA729271	stage_1D_vs_E10	ordinary_muscle_development	ENSGALG00010012593	TUBA8B	3.431584612	0.958699839	3.57941503	0.000344364	0.000603355	positive
PRJNA729271	stage_1D_vs_E10	ordinary_muscle_development	ENSGALG00010012599	DMGDH	-1.504013022	0.19743623	-7.617715454	2.58205E-14	9.92753E-14	negative
PRJNA729271	stage_1D_vs_E10	ordinary_muscle_development	ENSGALG00010012600	NUP107	-1.250672686	0.100327098	-12.46595094	1.14498E-35	1.27371E-34	negative
PRJNA729271	stage_1D_vs_E10	ordinary_muscle_development	ENSGALG00010012601	TUBA8A	2.431510828	0.239347403	10.15891878	3.02411E-24	1.99338E-23	positive
PRJNA729271	stage_1D_vs_E10	ordinary_muscle_development	ENSGALG00010012604	ENSGALG00010012604	-5.95454012	2.227587256	-2.673089507			negative
PRJNA729271	stage_1D_vs_E10	ordinary_muscle_development	ENSGALG00010012606	ATP5PO	0.824677227	0.114063158	7.230005217	4.82975E-13	1.71251E-12	positive
PRJNA729271	stage_1D_vs_E10	ordinary_muscle_development	ENSGALG00010012607	LMNB1	-2.540160485	0.164140269	-15.47554727	5.07399E-54	1.12286E-52	negative
PRJNA729271	stage_1D_vs_E10	ordinary_muscle_development	ENSGALG00010012610	RPL3	0.226683823	0.154957966	1.462872987	0.143502137	0.177540413	positive
PRJNA729271	stage_1D_vs_E10	ordinary_muscle_development	ENSGALG00010012614	LHFPL2	-0.444410204	0.144601466	-3.07334508	0.002116736	0.003395914	negative
PRJNA729271	stage_1D_vs_E10	ordinary_muscle_development	ENSGALG00010012618	B3GALT2	1.289753118	0.32552473	3.962074145	7.43015E-05	0.000139634	positive
PRJNA729271	stage_1D_vs_E10	ordinary_muscle_development	ENSGALG00010012624	PDGFB	0.687251145	0.18282606	3.759043677	0.000170564	0.000309273	positive
PRJNA729271	stage_1D_vs_E10	ordinary_muscle_development	ENSGALG00010012629	NUS1	-0.245266769	0.110548069	-2.21864362	0.02651098	0.036825745	negative
PRJNA729271	stage_1D_vs_E10	ordinary_muscle_development	ENSGALG00010012631	GLRX2	-0.233560286	0.155667297	-1.500381199	0.133515687	0.165935855	negative
PRJNA729271	stage_1D_vs_E10	ordinary_muscle_development	ENSGALG00010012633	USP18	0.06412634	0.542788259	0.11814246	0.905954781	0.919852518	positive
PRJNA729271	stage_1D_vs_E10	ordinary_muscle_development	ENSGALG00010012640	PRRC1	-0.415837842	0.073425155	-5.66342481	1.48381E-08	3.8209E-08	negative
PRJNA729271	stage_1D_vs_E10	ordinary_muscle_development	ENSGALG00010012648	MRPS6	0.089758671	0.130571446	0.687429559	0.491812076	0.540102738	positive
PRJNA729271	stage_1D_vs_E10	ordinary_muscle_development	ENSGALG00010012650	DSE	-0.401050802	0.104490934	-3.838139717	0.00012397	0.000227894	negative
PRJNA729271	stage_1D_vs_E10	ordinary_muscle_development	ENSGALG00010012653	CBX7	2.38812025	0.249551988	9.569630255	1.07289E-21	6.30125E-21	positive
PRJNA729271	stage_1D_vs_E10	ordinary_muscle_development	ENSGALG00010012654	TENT5B	2.773020916	0.308613277	8.985423248	2.5774E-19	1.32621E-18	positive
PRJNA729271	stage_1D_vs_E10	ordinary_muscle_development	ENSGALG00010012662	SLC35E3	-1.775879459	0.108920536	-16.30435839	9.18989E-60	2.5181E-58	negative
PRJNA729271	stage_1D_vs_E10	ordinary_muscle_development	ENSGALG00010012667	SCAMP1	-0.408928153	0.088899822	-4.599875946	4.22743E-06	8.92204E-06	negative
PRJNA729271	stage_1D_vs_E10	ordinary_muscle_development	ENSGALG00010012669	ERCC8	0.199539195	0.147661904	1.351324816	0.176591406	0.214739298	positive
PRJNA729271	stage_1D_vs_E10	ordinary_muscle_development	ENSGALG00010012670	ENSGALG00010012670	0.254081352	0.203438713	1.248933147	0.211689527	0.253213376	positive
PRJNA729271	stage_1D_vs_E10	ordinary_muscle_development	ENSGALG00010012671	NBN	-0.458174464	0.184325994	-2.485674729	0.012930609	0.01882008	negative
PRJNA729271	stage_1D_vs_E10	ordinary_muscle_development	ENSGALG00010012672	RO60	-0.852616896	0.090423833	-9.42911697	4.13556E-21	2.35078E-20	negative
PRJNA729271	stage_1D_vs_E10	ordinary_muscle_development	ENSGALG00010012673	ENSGALG00010012673	-1.067888891	0.323683483	-3.29917635	0.00096969	0.001619627	negative
PRJNA729271	stage_1D_vs_E10	ordinary_muscle_development	ENSGALG00010012674	ENSGALG00010012674	0.879465021	0.126849671	6.933128115	4.11635E-12	1.36474E-11	positive
PRJNA729271	stage_1D_vs_E10	ordinary_muscle_development	ENSGALG00010012676	ENSGALG00010012676	0.901772108	0.207439919	4.347148383	1.37919E-05	2.77735E-05	positive
PRJNA729271	stage_1D_vs_E10	ordinary_muscle_development	ENSGALG00010012680	TRPM8	0.37773319	0.27664324	1.365416302	0.172122243	0.209691705	positive
PRJNA729271	stage_1D_vs_E10	ordinary_muscle_development	ENSGALG00010012682	ENSGALG00010012682	-0.056924725	0.110529903	-0.515016514	0.60654148	0.649178081	negative
PRJNA729271	stage_1D_vs_E10	ordinary_muscle_development	ENSGALG00010012683	MDM2	0.584223767	0.086036573	6.79041191	1.11814E-11	3.60806E-11	positive
PRJNA729271	stage_1D_vs_E10	ordinary_muscle_development	ENSGALG00010012685	NPTXR	1.900471043	0.237128507	8.014519502	1.10568E-15	4.63205E-15	positive
PRJNA729271	stage_1D_vs_E10	ordinary_muscle_development	ENSGALG00010012688	NDUFAF2	0.03116328	0.113470017	0.274638893	0.783593683	0.811705169	positive
PRJNA729271	stage_1D_vs_E10	ordinary_muscle_development	ENSGALG00010012690	NT5DC1	-1.333432208	0.338551946	-3.938634006	8.19468E-05	0.000153307	negative
PRJNA729271	stage_1D_vs_E10	ordinary_muscle_development	ENSGALG00010012693	UCHL5	-0.675950309	0.077892919	-8.677942977	4.02991E-18	1.94323E-17	negative
PRJNA729271	stage_1D_vs_E10	ordinary_muscle_development	ENSGALG00010012696	DNAL4	-1.213934377	0.093932952	-12.9234135	3.32076E-38	4.07573E-37	negative
PRJNA729271	stage_1D_vs_E10	ordinary_muscle_development	ENSGALG00010012699	RNF128	0.241591285	0.143026887	1.689131955	0.091194145	0.116761694	positive
PRJNA729271	stage_1D_vs_E10	ordinary_muscle_development	ENSGALG00010012702	PTPRO	-2.930192282	0.352662187	-8.308779297	9.66916E-17	4.31776E-16	negative
PRJNA729271	stage_1D_vs_E10	ordinary_muscle_development	ENSGALG00010012704	SMIM15	-0.470230497	0.068272754	-6.887527861	5.67703E-12	1.86468E-11	negative
PRJNA729271	stage_1D_vs_E10	ordinary_muscle_development	ENSGALG00010012705	CPM	3.171074532	0.183557961	17.27560334	7.18184E-67	2.36056E-65	positive
PRJNA729271	stage_1D_vs_E10	ordinary_muscle_development	ENSGALG00010012706	TBC1D8B	0.577031226	0.104367627	5.528833437	3.22367E-08	8.06614E-08	positive
PRJNA729271	stage_1D_vs_E10	ordinary_muscle_development	ENSGALG00010012708	SPP2	-0.950824545	1.134764941	-0.837904407	0.402084394	0.451589438	negative
PRJNA729271	stage_1D_vs_E10	ordinary_muscle_development	ENSGALG00010012709	PPP1R8	-0.942369953	0.057174005	-16.48248976	4.90237E-61	1.38789E-59	negative
PRJNA729271	stage_1D_vs_E10	ordinary_muscle_development	ENSGALG00010012711	ELL2	-0.238358695	0.125333899	-1.901789509	0.057198678	0.075507367	negative
PRJNA729271	stage_1D_vs_E10	ordinary_muscle_development	ENSGALG00010012712	EEF1E1	0.185597871	0.133108284	1.394337491	0.163215715	0.199865826	positive
PRJNA729271	stage_1D_vs_E10	ordinary_muscle_development	ENSGALG00010012714	RGS2	1.328139212	0.171909791	7.725791564	1.1116E-14	4.36422E-14	positive
PRJNA729271	stage_1D_vs_E10	ordinary_muscle_development	ENSGALG00010012719	COL10A1	-0.776275418	0.768916661	-1.009570292	0.312701209	0.360130623	negative
PRJNA729271	stage_1D_vs_E10	ordinary_muscle_development	ENSGALG00010012720	ACO2	1.996041078	0.119801387	16.66125189	2.50721E-62	7.41674E-61	positive
PRJNA729271	stage_1D_vs_E10	ordinary_muscle_development	ENSGALG00010012722	ENSGALG00010012722	-0.064106909	0.098815089	-0.648756274	0.516495924	0.564005456	negative
PRJNA729271	stage_1D_vs_E10	ordinary_muscle_development	ENSGALG00010012725	ENSGALG00010012725	1.703660543	0.255040796	6.679953055	2.39019E-11	7.54481E-11	positive
PRJNA729271	stage_1D_vs_E10	ordinary_muscle_development	ENSGALG00010012726	IL7R	5.027083023	0.467729498	10.74784259	6.06639E-27	4.53493E-26	positive
PRJNA729271	stage_1D_vs_E10	ordinary_muscle_development	ENSGALG00010012730	CAP2	1.098334825	0.168008374	6.53738144	6.26052E-11	1.92137E-10	positive
PRJNA729271	stage_1D_vs_E10	ordinary_muscle_development	ENSGALG00010012732	MGST1	-0.666236187	0.174581985	-3.81617947	0.000135534	0.000248208	negative
PRJNA729271	stage_1D_vs_E10	ordinary_muscle_development	ENSGALG00010012733	ENSGALG00010012733	-1.20892425	1.020861202	-1.184219997	0.236325988	0.27988767	negative
PRJNA729271	stage_1D_vs_E10	ordinary_muscle_development	ENSGALG00010012734	TMEM19	-1.02324445	0.14596313	-7.010293974	2.37818E-12	8.03349E-12	negative
PRJNA729271	stage_1D_vs_E10	ordinary_muscle_development	ENSGALG00010012738	RAB21	0.337764281	0.068706106	4.916073685	8.82972E-07	1.9803E-06	positive
PRJNA729271	stage_1D_vs_E10	ordinary_muscle_development	ENSGALG00010012741	ENSGALG00010012741	0.550284713	0.149755443	3.674555687	0.000238264	0.000424436	positive
PRJNA729271	stage_1D_vs_E10	ordinary_muscle_development	ENSGALG00010012742	ENSGALG00010012742	-1.346937581	0.174066896	-7.738045626	1.00957E-14	3.9733E-14	negative
PRJNA729271	stage_1D_vs_E10	ordinary_muscle_development	ENSGALG00010012743	ENSGALG00010012743	-0.102032621	0.516397879	-0.197585283	0.84336955	0.865951805	negative
PRJNA729271	stage_1D_vs_E10	ordinary_muscle_development	ENSGALG00010012744	ARL4C	-2.87054402	0.234596885	-12.23607046	1.99458E-34	2.11186E-33	negative
PRJNA729271	stage_1D_vs_E10	ordinary_muscle_development	ENSGALG00010012745	MARCHF3	-0.935104507	0.325809523	-2.870095684	0.004103476	0.00636966	negative
PRJNA729271	stage_1D_vs_E10	ordinary_muscle_development	ENSGALG00010012747	TOB2	1.273624896	0.145205802	8.771170857	1.76818E-18	8.7028E-18	positive
PRJNA729271	stage_1D_vs_E10	ordinary_muscle_development	ENSGALG00010012748	ENSGALG00010012748	1.015227941	0.204417888	4.966433961	6.81953E-07	1.54307E-06	positive
PRJNA729271	stage_1D_vs_E10	ordinary_muscle_development	ENSGALG00010012749	RGS4	2.094169718	0.176965632	11.83376508	2.61159E-32	2.51863E-31	positive
PRJNA729271	stage_1D_vs_E10	ordinary_muscle_development	ENSGALG00010012751	AP3B1	-0.398275696	0.074927042	-5.315513385	1.06357E-07	2.56556E-07	negative
PRJNA729271	stage_1D_vs_E10	ordinary_muscle_development	ENSGALG00010012753	GTPBP1	-0.437948276	0.052699543	-8.310286058	9.54716E-17	4.26723E-16	negative
PRJNA729271	stage_1D_vs_E10	ordinary_muscle_development	ENSGALG00010012755	CLVS2	-2.216954104	0.384786773	-5.761513279	8.33631E-09	2.19182E-08	negative
PRJNA729271	stage_1D_vs_E10	ordinary_muscle_development	ENSGALG00010012756	RPA2	-1.268584892	0.103513297	-12.25528436	1.57396E-34	1.68125E-33	negative
PRJNA729271	stage_1D_vs_E10	ordinary_muscle_development	ENSGALG00010012758	NAB1	-0.3039629	0.128620353	-2.363256613	0.018115121	0.025765743	negative
PRJNA729271	stage_1D_vs_E10	ordinary_muscle_development	ENSGALG00010012759	ATP1B4	4.697494263	0.197933439	23.73269664	1.6581E-124	2.0197E-122	positive
PRJNA729271	stage_1D_vs_E10	ordinary_muscle_development	ENSGALG00010012760	PLEKHF2	1.017890253	0.104657672	9.725901966	2.33824E-22	1.4187E-21	positive
PRJNA729271	stage_1D_vs_E10	ordinary_muscle_development	ENSGALG00010012761	SMPDL3A	0.831880602	0.226604225	3.671072769	0.000241535	0.00042984	positive
PRJNA729271	stage_1D_vs_E10	ordinary_muscle_development	ENSGALG00010012763	SMPDL3B	-0.820019068	0.138136758	-5.936284301	2.91554E-09	7.95422E-09	negative
PRJNA729271	stage_1D_vs_E10	ordinary_muscle_development	ENSGALG00010012765	PHF5A	0.115730229	0.106688137	1.084752555	0.278031264	0.323978054	positive
PRJNA729271	stage_1D_vs_E10	ordinary_muscle_development	ENSGALG00010012767	JOSD1	-0.596696537	0.070597881	-8.452046031	2.86242E-17	1.31842E-16	negative
PRJNA729271	stage_1D_vs_E10	ordinary_muscle_development	ENSGALG00010012768	EPS8	-0.798518441	0.116414091	-6.859293714	6.92018E-12	2.26123E-11	negative
PRJNA729271	stage_1D_vs_E10	ordinary_muscle_development	ENSGALG00010012769	NEMP2	-0.715311164	0.08123075	-8.805916003	1.29788E-18	6.43393E-18	negative
PRJNA729271	stage_1D_vs_E10	ordinary_muscle_development	ENSGALG00010012771	ITGA2	-0.852020742	0.18435917	-4.621526242	3.80927E-06	8.08543E-06	negative
PRJNA729271	stage_1D_vs_E10	ordinary_muscle_development	ENSGALG00010012772	TOMM22	-0.476969931	0.074186812	-6.429308934	1.28185E-10	3.85247E-10	negative
PRJNA729271	stage_1D_vs_E10	ordinary_muscle_development	ENSGALG00010012773	XKR8	-0.800595083	0.128970843	-6.207566509	5.38113E-10	1.54976E-09	negative
PRJNA729271	stage_1D_vs_E10	ordinary_muscle_development	ENSGALG00010012775	KCNE2	1.845689891	0.145758942	12.66261865	9.52676E-37	1.10916E-35	positive
PRJNA729271	stage_1D_vs_E10	ordinary_muscle_development	ENSGALG00010012778	TBC1D15	0.111184218	0.15152753	0.733755893	0.463097527	0.511876679	positive
PRJNA729271	stage_1D_vs_E10	ordinary_muscle_development	ENSGALG00010012780	FABP7	-2.685615539	0.251032523	-10.6982773	1.03688E-26	7.66815E-26	negative
PRJNA729271	stage_1D_vs_E10	ordinary_muscle_development	ENSGALG00010012781	SPIN1L	-6.736984209	3.275261939	-2.056929899	0.039692969	0.053750896	negative
PRJNA729271	stage_1D_vs_E10	ordinary_muscle_development	ENSGALG00010012786	TEF	1.000314333	0.087704861	11.40546058	3.92693E-30	3.41867E-29	positive
PRJNA729271	stage_1D_vs_E10	ordinary_muscle_development	ENSGALG00010012788	RGS1	1.001595354	0.665480755	1.505070351	0.132305991	0.164646383	positive
PRJNA729271	stage_1D_vs_E10	ordinary_muscle_development	ENSGALG00010012790	PITX1	-4.412602971	0.464700265	-9.495589538	2.18969E-21	1.26402E-20	negative
PRJNA729271	stage_1D_vs_E10	ordinary_muscle_development	ENSGALG00010012791	CFAP418	-0.038680093	0.191246933	-0.202252097	0.839719647	0.862693053	negative
PRJNA729271	stage_1D_vs_E10	ordinary_muscle_development	ENSGALG00010012792	CBY1	-1.455839228	0.14991873	-9.710856213	2.71051E-22	1.63635E-21	negative
PRJNA729271	stage_1D_vs_E10	ordinary_muscle_development	ENSGALG00010012795	TBCA	-0.814308817	0.136432075	-5.968602456	2.39294E-09	6.56802E-09	negative
PRJNA729271	stage_1D_vs_E10	ordinary_muscle_development	ENSGALG00010012796	MOCS2	-0.480729434	0.123894758	-3.880143447	0.000104395	0.000193257	negative
PRJNA729271	stage_1D_vs_E10	ordinary_muscle_development	ENSGALG00010012797	ZC3H7B	0.148745933	0.070167535	2.119868287	0.034017155	0.046569574	positive
PRJNA729271	stage_1D_vs_E10	ordinary_muscle_development	ENSGALG00010012798	MFSD6	0.164029739	0.096313479	1.703081861	0.08855275	0.11361056	positive
PRJNA729271	stage_1D_vs_E10	ordinary_muscle_development	ENSGALG00010012801	DMC1	1.794315895	0.438582838	4.0911676	4.29207E-05	8.24785E-05	positive
PRJNA729271	stage_1D_vs_E10	ordinary_muscle_development	ENSGALG00010012803	NUP155	-1.008395901	0.090273551	-11.17044684	5.68939E-29	4.69634E-28	negative
PRJNA729271	stage_1D_vs_E10	ordinary_muscle_development	ENSGALG00010012804	ENSGALG00010012804	0.973844778	0.158071109	6.160801841	7.23775E-10	2.06477E-09	positive
PRJNA729271	stage_1D_vs_E10	ordinary_muscle_development	ENSGALG00010012808	EYA3	-0.52867603	0.092753159	-5.699816975	1.19936E-08	3.10997E-08	negative
PRJNA729271	stage_1D_vs_E10	ordinary_muscle_development	ENSGALG00010012809	RHOBTB3	2.261252604	0.243524049	9.285541239	1.60887E-20	8.85369E-20	positive
PRJNA729271	stage_1D_vs_E10	ordinary_muscle_development	ENSGALG00010012812	DECR1	1.933162891	0.239488838	8.072037552	6.91346E-16	2.93614E-15	positive
PRJNA729271	stage_1D_vs_E10	ordinary_muscle_development	ENSGALG00010012818	RGS18	-0.20123825	0.199789717	-1.00725029	0.313814512	0.361326662	negative
PRJNA729271	stage_1D_vs_E10	ordinary_muscle_development	ENSGALG00010012819	RANGAP1	-1.031459219	0.068990786	-14.95068083	1.54165E-50	3.04444E-49	negative
PRJNA729271	stage_1D_vs_E10	ordinary_muscle_development	ENSGALG00010012821	ZBTB33	-0.073670281	0.051742332	-1.423791275	0.154506902	0.189939576	negative
PRJNA729271	stage_1D_vs_E10	ordinary_muscle_development	ENSGALG00010012824	HIBCH	0.841093907	0.11725433	7.173243885	7.32412E-13	2.56123E-12	positive
PRJNA729271	stage_1D_vs_E10	ordinary_muscle_development	ENSGALG00010012827	ENSGALG00010012827	0.476856232	0.185374246	2.572397415	0.010099688	0.014913914	positive
PRJNA729271	stage_1D_vs_E10	ordinary_muscle_development	ENSGALG00010012829	RGS5	4.032776168	0.180645593	22.32424328	2.149E-110	1.9347E-108	positive
PRJNA729271	stage_1D_vs_E10	ordinary_muscle_development	ENSGALG00010012830	ARRDC3	0.72696226	0.149184603	4.872904086	1.0997E-06	2.44818E-06	positive
PRJNA729271	stage_1D_vs_E10	ordinary_muscle_development	ENSGALG00010012831	ENSGALG00010012831	-0.097784481	0.380381862	-0.257069253	0.797125312	0.824367252	negative
PRJNA729271	stage_1D_vs_E10	ordinary_muscle_development	ENSGALG00010012832	CHADL	-2.467233965	0.348565791	-7.078244709	1.45992E-12	4.99092E-12	negative
PRJNA729271	stage_1D_vs_E10	ordinary_muscle_development	ENSGALG00010012833	JAK2	0.776429239	0.105202921	7.380301157	1.57932E-13	5.76195E-13	positive
PRJNA729271	stage_1D_vs_E10	ordinary_muscle_development	ENSGALG00010012834	TPH2	-6.434833247	0.968330476	-6.645286299	3.02628E-11	9.48864E-11	negative
PRJNA729271	stage_1D_vs_E10	ordinary_muscle_development	ENSGALG00010012836	ENSGALG00010012836	3.337251593	1.15195027	2.897044845			positive
PRJNA729271	stage_1D_vs_E10	ordinary_muscle_development	ENSGALG00010012837	PKIB	0.616413686	0.270736536	2.276802736	0.022798008	0.032039667	positive
PRJNA729271	stage_1D_vs_E10	ordinary_muscle_development	ENSGALG00010012838	C2orf88	-0.35260802	0.234754236	-1.502030487	0.133089236	0.165462601	negative
PRJNA729271	stage_1D_vs_E10	ordinary_muscle_development	ENSGALG00010012839	TGFBI	-1.569256254	0.180113448	-8.712599018	2.96986E-18	1.4436E-17	negative
PRJNA729271	stage_1D_vs_E10	ordinary_muscle_development	ENSGALG00010012841	L3MBTL2	-0.762050678	0.093314635	-8.166464765	3.17558E-16	1.37815E-15	negative
PRJNA729271	stage_1D_vs_E10	ordinary_muscle_development	ENSGALG00010012842	DDX17	0.228501449	0.048853082	4.67731897	2.9065E-06	6.24329E-06	positive
PRJNA729271	stage_1D_vs_E10	ordinary_muscle_development	ENSGALG00010012843	ENSGALG00010012843	0.504447877	0.155262777	3.248994295	0.001158138	0.001918319	positive
PRJNA729271	stage_1D_vs_E10	ordinary_muscle_development	ENSGALG00010012845	HDHD2	0.108907195	0.101812258	1.069686464	0.284760461	0.330976095	positive
PRJNA729271	stage_1D_vs_E10	ordinary_muscle_development	ENSGALG00010012847	RLN3	-2.504041108	0.968261185	-2.586121542	0.00970627	0.014373966	negative
PRJNA729271	stage_1D_vs_E10	ordinary_muscle_development	ENSGALG00010012848	SERINC1	0.420678106	0.09251902	4.546936465	5.44324E-06	1.13902E-05	positive
PRJNA729271	stage_1D_vs_E10	ordinary_muscle_development	ENSGALG00010012849	TRHDE	-3.003055381	0.336832048	-8.915586866	4.85239E-19	2.45499E-18	negative
PRJNA729271	stage_1D_vs_E10	ordinary_muscle_development	ENSGALG00010012850	CALB1	1.250856034	1.115098809	1.12174457	0.26197106	0.307123141	positive

PRJNA729271	stage_1D_vs_E10	ordinary_muscle_development	ENSGALG00010012851	ENSGALG00010012851	-1.160563809	0.259827375	-4.466672578	7.94456E-06	1.63877E-05	negative
PRJNA729271	stage_1D_vs_E10	ordinary_muscle_development	ENSGALG00010012852	MSTN	-1.023381244	0.420189452	-2.435523405	0.014870263	0.02144721	negative
PRJNA729271	stage_1D_vs_E10	ordinary_muscle_development	ENSGALG00010012854	LECT2	5.515915134	0.744610004	7.407790798	1.28421E-13	4.71733E-13	positive
PRJNA729271	stage_1D_vs_E10	ordinary_muscle_development	ENSGALG00010012856	AVR2	-1.419731097	1.220029437	-1.163685936	0.244551311	0.288685663	negative
PRJNA729271	stage_1D_vs_E10	ordinary_muscle_development	ENSGALG00010012857	NSDHL	-2.433826982	0.226676121	-10.73702412	6.82076E-27	5.09361E-26	negative
PRJNA729271	stage_1D_vs_E10	ordinary_muscle_development	ENSGALG00010012859	FBXL21	0.801335366	0.111100379	7.212714933	5.48472E-13	1.93527E-12	positive
PRJNA729271	stage_1D_vs_E10	ordinary_muscle_development	ENSGALG00010012860	ENSGALG00010012860	1.58915089	0.253056402	6.279828843	3.38946E-10	9.90129E-10	positive
PRJNA729271	stage_1D_vs_E10	ordinary_muscle_development	ENSGALG00010012861	WDR70	-0.429169629	0.082588416	-5.196486999	2.0309E-07	4.7882E-07	negative
PRJNA729271	stage_1D_vs_E10	ordinary_muscle_development	ENSGALG00010012863	KCNE1	0.114594555	0.466194829	0.245808293	0.805830637	0.832419832	positive
PRJNA729271	stage_1D_vs_E10	ordinary_muscle_development	ENSGALG00010012865	EP300	0.861300377	0.122660881	7.021801671	2.19025E-12	7.41076E-12	positive
PRJNA729271	stage_1D_vs_E10	ordinary_muscle_development	ENSGALG00010012867	A4GALT	0.239116879	0.102170053	2.340381268	0.019264063	0.027311482	positive
PRJNA729271	stage_1D_vs_E10	ordinary_muscle_development	ENSGALG00010012868	CTHRC1	-6.093321299	0.608331938	-10.01644155	1.29066E-23	8.26333E-23	negative
PRJNA729271	stage_1D_vs_E10	ordinary_muscle_development	ENSGALG00010012869	ENSGALG00010012869	-0.577403829	0.222644527	-2.593388829	0.009503526	0.014093882	negative
PRJNA729271	stage_1D_vs_E10	ordinary_muscle_development	ENSGALG00010012870	KCNC2	-0.935786753	0.188648278	-4.960483928	7.03178E-07	1.58936E-06	negative
PRJNA729271	stage_1D_vs_E10	ordinary_muscle_development	ENSGALG00010012871	ENSGALG00010012871	-0.498640081	0.361292883	-1.380154731	0.167539009	0.204658514	negative
PRJNA729271	stage_1D_vs_E10	ordinary_muscle_development	ENSGALG00010012873	PTAFR	0.814647094	0.224720984	3.625149203	0.000288795	0.000509876	positive
PRJNA729271	stage_1D_vs_E10	ordinary_muscle_development	ENSGALG00010012875	HSF2	-0.527519008	0.133229366	-3.959479986	7.51131E-05	0.000141123	negative
PRJNA729271	stage_1D_vs_E10	ordinary_muscle_development	ENSGALG00010012876	KDELR3	-0.718959293	0.16123245	-4.459147598	8.22862E-06	1.69544E-05	negative
PRJNA729271	stage_1D_vs_E10	ordinary_muscle_development	ENSGALG00010012877	SLC25A32	-0.180202706	0.094500481	-1.906897241	0.056533896	0.074734046	negative
PRJNA729271	stage_1D_vs_E10	ordinary_muscle_development	ENSGALG00010012878	GDNF	0.531863325	0.31386351	1.694568841	0.090157242	0.11551571	positive
PRJNA729271	stage_1D_vs_E10	ordinary_muscle_development	ENSGALG00010012879	RCAN1	1.092707107	0.173206029	6.308712901	2.81365E-10	8.26925E-10	positive
PRJNA729271	stage_1D_vs_E10	ordinary_muscle_development	ENSGALG00010012880	CLK4	1.178853378	0.139124348	8.473379371	2.38375E-17	1.10285E-16	positive
PRJNA729271	stage_1D_vs_E10	ordinary_muscle_development	ENSGALG00010012882	ENSGALG00010012882	2.040439669	0.537143226	3.798688262	0.000145464	0.000265721	positive
PRJNA729271	stage_1D_vs_E10	ordinary_muscle_development	ENSGALG00010012883	DCAF13	-0.610392019	0.066701968	-9.151034632	5.63908E-20	3.01174E-19	negative
PRJNA729271	stage_1D_vs_E10	ordinary_muscle_development	ENSGALG00010012884	ENSGALG00010012884	-0.365815665	0.213508562	-1.713353604	0.086647506	0.111343582	negative
PRJNA729271	stage_1D_vs_E10	ordinary_muscle_development	ENSGALG00010012885	PMS1	0.041872327	0.129470375	0.32341242	0.746382925	0.778836692	positive
PRJNA729271	stage_1D_vs_E10	ordinary_muscle_development	ENSGALG00010012887	KCNJ4	0.958348399	0.383322675	2.500108818	0.012415516	0.018124978	positive
PRJNA729271	stage_1D_vs_E10	ordinary_muscle_development	ENSGALG00010012888	ENSGALG00010012888	-4.359032383	3.675783325	-1.185878491	0.235670289	0.279202357	negative
PRJNA729271	stage_1D_vs_E10	ordinary_muscle_development	ENSGALG00010012889	RIC1	0.658870921	0.104685119	6.293835522	3.09716E-10	9.0767E-10	positive
PRJNA729271	stage_1D_vs_E10	ordinary_muscle_development	ENSGALG00010012890	MFAP3	-0.487140961	0.070015961	-6.957570207	3.46191E-12	1.15703E-11	negative
PRJNA729271	stage_1D_vs_E10	ordinary_muscle_development	ENSGALG00010012891	CAPS2	-2.700989466	0.314436137	-8.589946102	8.70101E-18	4.12565E-17	negative
PRJNA729271	stage_1D_vs_E10	ordinary_muscle_development	ENSGALG00010012892	ORMDL1	0.210889148	0.160545768	1.3135765	0.188988761	0.228473069	positive
PRJNA729271	stage_1D_vs_E10	ordinary_muscle_development	ENSGALG00010012893	ENSGALG00010012893	-0.428736186	0.190964497	-2.245109397	0.02476111	0.034587289	negative
PRJNA729271	stage_1D_vs_E10	ordinary_muscle_development	ENSGALG00010012894	CLIC6	-0.867470279	0.677824143	-1.279786635	0.200620185	0.24089173	negative
PRJNA729271	stage_1D_vs_E10	ordinary_muscle_development	ENSGALG00010012896	STK38L	0.344158176	0.09896726	3.477495238	0.000506122	0.000870977	positive
PRJNA729271	stage_1D_vs_E10	ordinary_muscle_development	ENSGALG00010012897	ENSGALG00010012897	1.080057673	0.286420295	3.770883877	0.00016267	0.000295625	positive
PRJNA729271	stage_1D_vs_E10	ordinary_muscle_development	ENSGALG00010012898	FAM114A2	-0.117446144	0.115816051	-1.014074846	0.310547039	0.357905648	negative
PRJNA729271	stage_1D_vs_E10	ordinary_muscle_development	ENSGALG00010012899	ENSGALG00010012899	-0.0126031	0.134192709	-0.093917923	0.925174356	0.936220762	negative
PRJNA729271	stage_1D_vs_E10	ordinary_muscle_development	ENSGALG00010012900	RBX1	-0.061852626	0.172371449	-0.358833358	0.719719756	0.75492712	negative
PRJNA729271	stage_1D_vs_E10	ordinary_muscle_development	ENSGALG00010012903	RGMB	0.797304784	0.091566486	8.707386507	3.10961E-18	1.51001E-17	positive
PRJNA729271	stage_1D_vs_E10	ordinary_muscle_development	ENSGALG00010012904	ERMP1	-0.666433663	0.136686132	-4.875649458	1.08451E-06	2.4166E-06	negative
PRJNA729271	stage_1D_vs_E10	ordinary_muscle_development	ENSGALG00010012905	NKAIN1	-2.261488741	0.228484401	-9.897781783	4.2561E-23	2.66029E-22	negative
PRJNA729271	stage_1D_vs_E10	ordinary_muscle_development	ENSGALG00010012908	STRAP	-0.637907349	0.083136004	-7.673057633	1.67944E-14	6.5264E-14	negative
PRJNA729271	stage_1D_vs_E10	ordinary_muscle_development	ENSGALG00010012910	XPNPEP3	-0.037655619	0.099423563	-0.378739388	0.704881401	0.741347838	negative
PRJNA729271	stage_1D_vs_E10	ordinary_muscle_development	ENSGALG00010012911	CSNK1E	-0.275687906	0.083588435	-3.298158494	0.000973212	0.001625196	negative
PRJNA729271	stage_1D_vs_E10	ordinary_muscle_development	ENSGALG00010012912	NKAPL	0.313333678	0.098275761	3.188310895	0.001431066	0.00234467	positive
PRJNA729271	stage_1D_vs_E10	ordinary_muscle_development	ENSGALG00010012913	EGFLAM	-0.706993521	0.153588318	-4.603172491	4.16103E-06	8.79344E-06	negative
PRJNA729271	stage_1D_vs_E10	ordinary_muscle_development	ENSGALG00010012915	ENSGALG00010012915	3.005299842	0.284040841	10.58052017	3.66916E-26	2.64336E-25	positive
PRJNA729271	stage_1D_vs_E10	ordinary_muscle_development	ENSGALG00010012917	RUNX1	-1.648935925	0.265311948	-6.215083563	5.12973E-10	1.47883E-09	negative
PRJNA729271	stage_1D_vs_E10	ordinary_muscle_development	ENSGALG00010012918	GLRX	-0.600124743	0.287411938	-2.088029977	0.036795131	0.050093493	negative
PRJNA729271	stage_1D_vs_E10	ordinary_muscle_development	ENSGALG00010012921	EBF1	0.007179188	0.139981229	0.051286787	0.959096996	0.965557354	positive
PRJNA729271	stage_1D_vs_E10	ordinary_muscle_development	ENSGALG00010012922	SNRNP40	-1.321131095	0.129832209	-10.17568066	2.54614E-24	1.68445E-23	negative
PRJNA729271	stage_1D_vs_E10	ordinary_muscle_development	ENSGALG00010012923	MLANA	-3.655168743	0.870086658	-4.200924942	2.65827E-05	5.20627E-05	negative
PRJNA729271	stage_1D_vs_E10	ordinary_muscle_development	ENSGALG00010012924	GLIPR1	0.246520941	0.200445409	1.229865741	0.218747385	0.261030983	positive
PRJNA729271	stage_1D_vs_E10	ordinary_muscle_development	ENSGALG00010012925	SETD4	-1.550681538	0.193789075	-8.001903824	1.2251E-15	5.11458E-15	negative
PRJNA729271	stage_1D_vs_E10	ordinary_muscle_development	ENSGALG00010012926	OSGEPL1	0.453755329	0.164222848	2.763046287	0.005726465	0.008733702	positive
PRJNA729271	stage_1D_vs_E10	ordinary_muscle_development	ENSGALG00010012927	ENSGALG00010012927	-8.058703245	3.77582095	-2.134291682			negative
PRJNA729271	stage_1D_vs_E10	ordinary_muscle_development	ENSGALG00010012930	IL6ST	0.735362859	0.090795084	8.099148383	5.53452E-16	2.36716E-15	positive
PRJNA729271	stage_1D_vs_E10	ordinary_muscle_development	ENSGALG00010012932	ATF6	0.677350055	0.11753195	5.763114261	8.25759E-09	2.17309E-08	positive
PRJNA729271	stage_1D_vs_E10	ordinary_muscle_development	ENSGALG00010012933	ZCCHC17	-0.47953982	0.145902323	-3.286718202	0.001013622	0.001689176	negative
PRJNA729271	stage_1D_vs_E10	ordinary_muscle_development	ENSGALG00010012934	NDUFA1	0.9021931	0.151679548	5.948020745	2.71404E-09	7.42685E-09	positive
PRJNA729271	stage_1D_vs_E10	ordinary_muscle_development	ENSGALG00010012938	UPF3B	0.165566005	0.150282546	1.101698164	0.270592915	0.316106086	positive
PRJNA729271	stage_1D_vs_E10	ordinary_muscle_development	ENSGALG00010012939	GJA1	-4.020764016	0.207751241	-19.35374251	1.89564E-83	9.81334E-82	negative
PRJNA729271	stage_1D_vs_E10	ordinary_muscle_development	ENSGALG00010012940	FBN2	-1.270976158	0.223455734	-5.687820728	1.28671E-08	3.32991E-08	negative
PRJNA729271	stage_1D_vs_E10	ordinary_muscle_development	ENSGALG00010012943	ASNSD1	-0.47710561	0.122850904	-3.88361496	0.000102915	0.000190639	negative
PRJNA729271	stage_1D_vs_E10	ordinary_muscle_development	ENSGALG00010012944	NCK1	-0.386206514	0.052217253	-7.396147713	1.40192E-13	5.13152E-13	negative
PRJNA729271	stage_1D_vs_E10	ordinary_muscle_development	ENSGALG00010012945	CLINT1	-0.088684772	0.062802996	-1.412110535	0.157917417	0.193819895	negative
PRJNA729271	stage_1D_vs_E10	ordinary_muscle_development	ENSGALG00010012948	ST13P5	-0.763914354	0.095273827	-8.018092461	1.074E-15	4.50192E-15	negative
PRJNA729271	stage_1D_vs_E10	ordinary_muscle_development	ENSGALG00010012949	ENSGALG00010012949	-0.656390564	0.173405899	-3.785283932	0.000153533	0.000279827	negative
PRJNA729271	stage_1D_vs_E10	ordinary_muscle_development	ENSGALG00010012952	BRD10	0.725237439	0.186508862	3.888487823	0.000100871	0.000186948	positive
PRJNA729271	stage_1D_vs_E10	ordinary_muscle_development	ENSGALG00010012954	RIMS2	-1.239982502	0.341979025	-3.625902209	0.000287954	0.000508454	negative
PRJNA729271	stage_1D_vs_E10	ordinary_muscle_development	ENSGALG00010012955	RPL39L	-0.1656721	0.155202151	-1.067460076	0.285764133	0.332028431	negative
PRJNA729271	stage_1D_vs_E10	ordinary_muscle_development	ENSGALG00010012956	FABP3	4.367416758	0.168119958	25.97797917	8.7843E-149	1.5528E-146	positive
PRJNA729271	stage_1D_vs_E10	ordinary_muscle_development	ENSGALG00010012957	DERA	-0.658087282	0.228739912	-2.877011168	0.004014614	0.006238404	negative
PRJNA729271	stage_1D_vs_E10	ordinary_muscle_development	ENSGALG00010012958	OLFML2B	1.896512917	0.204559731	9.271193815	1.84074E-20	1.00913E-19	positive
PRJNA729271	stage_1D_vs_E10	ordinary_muscle_development	ENSGALG00010012959	TBC1D32	0.028224913	0.146067402	0.193232117	0.846777187	0.869053451	positive
PRJNA729271	stage_1D_vs_E10	ordinary_muscle_development	ENSGALG00010012962	SLC25A17	-2.149298944	0.213033634	-10.08901225	6.17881E-24	4.00366E-23	negative
PRJNA729271	stage_1D_vs_E10	ordinary_muscle_development	ENSGALG00010012963	UIMC1	-0.174280961	0.089840304	-1.939897277	0.052392175	0.069633649	negative
PRJNA729271	stage_1D_vs_E10	ordinary_muscle_development	ENSGALG00010012964	CBR3	-0.207378635	0.071933906	-2.882905245	0.003940259	0.006130749	negative
PRJNA729271	stage_1D_vs_E10	ordinary_muscle_development	ENSGALG00010012965	ENSGALG00010012965	-0.179409755	0.084016994	-2.135398399	0.032728481	0.044907169	negative
PRJNA729271	stage_1D_vs_E10	ordinary_muscle_development	ENSGALG00010012966	GLIPR1L	-0.256980647	0.272061304	-0.944568899	0.344879011	0.393716726	negative
PRJNA729271	stage_1D_vs_E10	ordinary_muscle_development	ENSGALG00010012967	ECRG4	-0.567754117	0.209062808	-2.715710766	0.006613367	0.010009476	negative
PRJNA729271	stage_1D_vs_E10	ordinary_muscle_development	ENSGALG00010012968	LSM11	-0.45402221	0.076170874	-5.960575072	2.51352E-09	6.89505E-09	negative
PRJNA729271	stage_1D_vs_E10	ordinary_muscle_development	ENSGALG00010012969	SLC12A2	-0.166264691	0.142369551	-1.167838843	0.242871777	0.286866558	negative
PRJNA729271	stage_1D_vs_E10	ordinary_muscle_development	ENSGALG00010012970	UNC5A	-0.773981339	0.177532278	-4.359665463	1.30261E-05	2.63302E-05	negative
PRJNA729271	stage_1D_vs_E10	ordinary_muscle_development	ENSGALG00010012971	ENSGALG00010012971	2.231671352	0.399716129	5.583140611	2.36214E-08	5.98691E-08	positive
PRJNA729271	stage_1D_vs_E10	ordinary_muscle_development	ENSGALG00010012973	GLDC	-3.539684792	0.320652439	-11.03900787	2.47754E-28	1.96993E-27	negative
PRJNA729271	stage_1D_vs_E10	ordinary_muscle_development	ENSGALG00010012974	SLC49A3	-1.624220976	0.222595147	-7.296749276	2.94803E-13	1.05824E-12	negative
PRJNA729271	stage_1D_vs_E10	ordinary_muscle_development	ENSGALG00010012975	KRR1	-0.29150262	0.092495691	-3.151526497	0.001624194	0.00264495	negative
PRJNA729271	stage_1D_vs_E10	ordinary_muscle_development	ENSGALG00010012976	SLC9A2	3.61493985	0.225436212	16.03531135	7.24197E-58	1.84811E-56	positive
PRJNA729271	stage_1D_vs_E10	ordinary_muscle_development	ENSGALG00010012977	NKRF	-0.255250507	0.098685213	-2.586512188	0.009695274	0.01435915	negative
PRJNA729271	stage_1D_vs_E10	ordinary_muscle_development	ENSGALG00010012978	HSD17B7	-0.967574148	0.213146405	-4.53948144	5.63927E-06	1.178E-05	negative
PRJNA729271	stage_1D_vs_E10	ordinary_muscle_development	ENSGALG00010012979	HTR1A	2.294504011	0.51856386	4.424727964	9.65639E-06	1.97502E-05	positive
PRJNA729271	stage_1D_vs_E10	ordinary_muscle_development	ENSGALG00010012980	KICS2	-0.070947873	0.075356415	-0.94149745	0.346449996	0.395323364	negative
PRJNA729271	stage_1D_vs_E10	ordinary_muscle_development	ENSGALG00010012982	DDR2	-0.418416043	0.243249237	-1.720112463	0.085412001	0.109902082	negative
PRJNA729271	stage_1D_vs_E10	ordinary_muscle_development	ENSGALG00010012985	ARNTL2	-0.097658988	0.170264531	-0.57357212	0.566257383	0.61152591	negative
PRJNA729271	stage_1D_vs_E10	ordinary_muscle_development	ENSGALG00010012986	TMEM184B	0.887755294	0.13987234	6.346896721	2.19702E-10	6.50606E-10	positive
PRJNA729271	stage_1D_vs_E10	ordinary_muscle_development	ENSGALG00010012988	RPL18A	0.241661012	0.134884643	1.7916125	0.073195059	0.095179186	positive
PRJNA729271	stage_1D_vs_E10	ordinary_muscle_development	ENSGALG00010012991	MFSD9	0.222198824	0.10072119	2.206078228	0.027378523	0.037947188	positive
PRJNA729271	stage_1D_vs_E10	ordinary_muscle_development	ENSGALG00010012992	HARS1	-0.24939287	0.126877369	-1.965621381	0.049342362	0.065888304	negative
PRJNA729271	stage_1D_vs_E10	ordinary_muscle_development	ENSGALG00010012993	DOP1B	-0.150413761	0.098820866	-1.522085033	0.127987776	0.159723014	negative
PRJNA729271	stage_1D_vs_E10	ordinary_muscle_development	ENSGALG00010012995	SERINC2	1.250224668	0.147606479	8.46998504	2.45425E-17	1.13475E-16	positive
PRJNA729271	stage_1D_vs_E10	ordinary_muscle_development	ENSGALG00010012997	UBE2A	0.103789586	0.057954818	1.790870723	0.073314043	0.095308256	positive
PRJNA729271	stage_1D_vs_E10	ordinary_muscle_development	ENSGALG00010012998	MAFF	0.093252158	0.199832905	0.466650663	0.640749834	0.681363718	positive
PRJNA729271	stage_1D_vs_E10	ordinary_muscle_development	ENSGALG00010013001	ADGRV1	-1.500939241	0.325885633	-4.605723875	4.11034E-06	8.6939E-06	negative
PRJNA729271	stage_1D_vs_E10	ordinary_muscle_development	ENSGALG00010013004	UAP1	-0.098089427	0.15295433	-0.641298788	0.521328576	0.568511715	negative
PRJNA729271	stage_1D_vs_E10	ordinary_muscle_development	ENSGALG00010013005	SLC40A1	0.596752726	0.090707363	6.578878591	4.7401E-11	1.46905E-10	positive
PRJNA729271	stage_1D_vs_E10	ordinary_muscle_development	ENSGALG00010013006	GRIK2	-4.360106966	1.018246037	-4.281977841	1.85239E-05	3.67915E-05	negative
PRJNA729271	stage_1D_vs_E10	ordinary_muscle_development	ENSGALG00010013008	STEEP1	-0.487832062	0.109772953	-4.444009651	8.82976E-06	1.8144E-05	negative
PRJNA729271	stage_1D_vs_E10	ordinary_muscle_development	ENSGALG00010013012	TMEM182	2.821538492	0.151397756	18.6365939	1.62249E-77	7.23628E-76	positive
PRJNA729271	stage_1D_vs_E10	ordinary_muscle_development	ENSGALG00010013013	DCSTAMP	1.450739331	1.244194453	1.166006911	0.243611651	0.2876467	positive
PRJNA729271	stage_1D_vs_E10	ordinary_muscle_development	ENSGALG00010013015	PLA2G6	0.9165753	0.148099687	6.188907766	6.05825E-10	1.73752E-09	positive
PRJNA729271	stage_1D_vs_E10	ordinary_muscle_development	ENSGALG00010013016	QRSL1	0.717484604	0.121886535	5.886496008	3.94469E-09	1.06457E-08	positive
PRJNA729271	stage_1D_vs_E10	ordinary_muscle_development	ENSGALG00010013017	SCMH1	0.835518268	0.157655695	5.299638998	1.16032E-07	2.79151E-07	positive
PRJNA729271	stage_1D_vs_E10	ordinary_muscle_development	ENSGALG00010013018	ID1	-0.068091999	0.192278763	-0.354131665	0.723240212	0.757906801	negative
PRJNA729271	stage_1D_vs_E10	ordinary_muscle_development	ENSGALG00010013019	DPYS	-2.633453602	1.035785611	-2.542469768	0.011007212	0.016189705	negative
PRJNA729271	stage_1D_vs_E10	ordinary_muscle_development	ENSGALG00010013021	SLC25A5	-0.875558252	0.162199745	-5.39802482	6.73785E-08	1.65059E-07	negative
PRJNA729271	stage_1D_vs_E10	ordinary_muscle_development	ENSGALG00010013027	UHMK1	0.685653939	0.125481732	5.464173363	4.65069E-08	1.15253E-07	positive
PRJNA729271	stage_1D_vs_E10	ordinary_muscle_development	ENSGALG00010013029	MYLK2	7.524968108	0.253869275	29.64111395	4.415E-193	1.9393E-190	positive
PRJNA729271	stage_1D_vs_E10	ordinary_muscle_development	ENSGALG00010013032	BAIAP2L2	1.535581903	0.385755018	3.980717892	6.87075E-05	0.000129575	positive
PRJNA729271	stage_1D_vs_E10	ordinary_muscle_development	ENSGALG00010013033	MAP3K1	-0.196936026	0.125363219	-1.570923496	0.11620042	0.14625956	negative
PRJNA729271	stage_1D_vs_E10	ordinary_muscle_development	ENSGALG00010013036	ENSGALG00010013036	0.834985075	0.420586177	1.985288916	0.047112336	0.063079006	positive
PRJNA729271	stage_1D_vs_E10	ordinary_muscle_development	ENSGALG00010013037	MORC4	0.755849224	0.089448129	8.450140111	2.90953E-17	1.33885E-16	positive
PRJNA729271	stage_1D_vs_E10	ordinary_muscle_development	ENSGALG00010013040	SLC25A43	1.296236444	0.204465217	6.339642829	2.30298E-10	6.8126E-10	positive
PRJNA729271	stage_1D_vs_E10	ordinary_muscle_development	ENSGALG00010013041	LRP12	0.114948531	0.07493082	1.534062102	0.125014376	0.156362359	positive
PRJNA729271	stage_1D_vs_E10	ordinary_muscle_development	ENSGALG00010013042	SLC25A48	-1.771910086	0.318504382	-5.563220426	2.64841E-08	6.67514E-08	negative
PRJNA729271	stage_1D_vs_E10	ordinary_muscle_development	ENSGALG00010013044	ENSGALG00010013044	1.191451221	0.124675552	9.556414274	1.21907E-21	7.13382E-21	positive
PRJNA729271	stage_1D_vs_E10	ordinary_muscle_development	ENSGALG00010013045	FSD1L	1.086484183	0.144507104	7.518552038	5.53862E-14	2.08417E-13	positive
PRJNA729271	stage_1D_vs_E10	ordinary_muscle_development	ENSGALG00010013046	SLC12A5	0.826964446	0.35337502	2.340189313	0.019273967	0.027322851	positive
PRJNA729271	stage_1D_vs_E10	ordinary_muscle_development	ENSGALG00010013047	CTPS1	-0.528143333	0.10095989	-5.231219395	1.68396E-07	3.99849E-07	negative
PRJNA729271	stage_1D_vs_E10	ordinary_muscle_development	ENSGALG00010013048	WDR75	-1.023736787	0.087626284	-11.68298761	1.55724E-31	1.45066E-30	negative

PRJNA729271	stage_1D_vs_E10	ordinary_muscle_development	ENSGALG00010013051	XPOT	-0.051943305	0.078701587	-0.660003269	0.509251731	0.557144226	negative
PRJNA729271	stage_1D_vs_E10	ordinary_muscle_development	ENSGALG00010013053	MRTFA	0.956572747	0.173980556	5.498158931	3.83777E-08	9.55816E-08	positive
PRJNA729271	stage_1D_vs_E10	ordinary_muscle_development	ENSGALG00010013056	ENSGALG00010013056	2.754189014	0.546751995	5.037364364	4.71986E-07	1.08148E-06	positive
PRJNA729271	stage_1D_vs_E10	ordinary_muscle_development	ENSGALG00010013058	ENSGALG00010013058	0.642329428	0.274259402	2.342050713	0.019178109	0.027197602	positive
PRJNA729271	stage_1D_vs_E10	ordinary_muscle_development	ENSGALG00010013062	ENSGALG00010013062	-0.335220871	0.108215875	-3.097705128	0.001950254	0.003143087	negative
PRJNA729271	stage_1D_vs_E10	ordinary_muscle_development	ENSGALG00010013063	PARP12	0.289699275	0.218340546	1.326823077	0.184567215	0.223593964	positive
PRJNA729271	stage_1D_vs_E10	ordinary_muscle_development	ENSGALG00010013065	FKTN	-0.065515359	0.102652933	-0.63822199	0.523329184	0.570538046	negative
PRJNA729271	stage_1D_vs_E10	ordinary_muscle_development	ENSGALG00010013071	ENSGALG00010013071	-0.363946731	0.225809957	-1.611739075	0.107018731	0.135325526	negative
PRJNA729271	stage_1D_vs_E10	ordinary_muscle_development	ENSGALG00010013076	RTN4IP1	-0.09119861	0.080532301	-1.132447588	0.257446307	0.302454549	negative
PRJNA729271	stage_1D_vs_E10	ordinary_muscle_development	ENSGALG00010013078	COL5A2	-0.355528793	0.241989239	-1.469192577	0.141780563	0.175530344	negative
PRJNA729271	stage_1D_vs_E10	ordinary_muscle_development	ENSGALG00010013079	ENSGALG00010013079	-0.756307761	0.205685619	-3.677008465	0.000235985	0.000420584	negative
PRJNA729271	stage_1D_vs_E10	ordinary_muscle_development	ENSGALG00010013080	ARHGEF7	0.340415454	0.09879199	3.445779895	0.000569414	0.000973194	positive
PRJNA729271	stage_1D_vs_E10	ordinary_muscle_development	ENSGALG00010013081	NCOA5	-0.511480191	0.099962709	-5.116709957	3.10911E-07	7.21181E-07	negative
PRJNA729271	stage_1D_vs_E10	ordinary_muscle_development	ENSGALG00010013082	CHAF1B	-1.642171872	0.113712445	-14.44144369	2.83834E-47	4.86309E-46	negative
PRJNA729271	stage_1D_vs_E10	ordinary_muscle_development	ENSGALG00010013084	SGSM3	0.570519548	0.115491996	4.939905508	7.81604E-07	1.76031E-06	positive
PRJNA729271	stage_1D_vs_E10	ordinary_muscle_development	ENSGALG00010013085	LYSMD3	0.214406136	0.0878996	2.439216272	0.014719156	0.021244067	positive
PRJNA729271	stage_1D_vs_E10	ordinary_muscle_development	ENSGALG00010013086	CXCL14	-3.169522513	0.517590192	-6.123613933	9.14764E-10	2.59481E-09	negative
PRJNA729271	stage_1D_vs_E10	ordinary_muscle_development	ENSGALG00010013087	GPN2	-0.199217664	0.091487377	-2.17754264	0.029440104	0.040633623	negative
PRJNA729271	stage_1D_vs_E10	ordinary_muscle_development	ENSGALG00010013089	TBK1	0.191961904	0.07641465	2.512108651	0.012001213	0.017564377	positive
PRJNA729271	stage_1D_vs_E10	ordinary_muscle_development	ENSGALG00010013093	GPATCH3	0.191659348	0.157886671	1.213904548	0.224784176	0.267520048	positive
PRJNA729271	stage_1D_vs_E10	ordinary_muscle_development	ENSGALG00010013095	ADSL	2.253483848	0.127637118	17.65539584	9.2483E-70	3.37668E-68	positive
PRJNA729271	stage_1D_vs_E10	ordinary_muscle_development	ENSGALG00010013096	CLDN14	-0.908173568	0.410632121	-2.211647658	0.02699102	0.037435158	negative
PRJNA729271	stage_1D_vs_E10	ordinary_muscle_development	ENSGALG00010013097	ENSGALG00010013097	-0.233780402	0.41276151	-0.566381304	0.571134602	0.616206399	negative
PRJNA729271	stage_1D_vs_E10	ordinary_muscle_development	ENSGALG00010013099	SIM2	1.188063962	0.201529135	5.895246663	3.74122E-09	1.01174E-08	positive
PRJNA729271	stage_1D_vs_E10	ordinary_muscle_development	ENSGALG00010013100	ZFPM2	-0.478524308	0.153120569	-3.125147135	0.001777161	0.002881104	negative
PRJNA729271	stage_1D_vs_E10	ordinary_muscle_development	ENSGALG00010013103	AIRIM	0.089667214	0.14401398	0.62262854	0.533528652	0.580331518	positive
PRJNA729271	stage_1D_vs_E10	ordinary_muscle_development	ENSGALG00010013104	HLCS	-0.091253171	0.109083832	-0.836541669	0.402850245	0.452344434	negative
PRJNA729271	stage_1D_vs_E10	ordinary_muscle_development	ENSGALG00010013106	SETD9	-0.168030632	0.180505812	-0.930887656	0.351911681	0.400861282	negative
PRJNA729271	stage_1D_vs_E10	ordinary_muscle_development	ENSGALG00010013109	ENSGALG00010013109	2.22162887	1.467028366	1.514373492	0.129931138	0.162036639	positive
PRJNA729271	stage_1D_vs_E10	ordinary_muscle_development	ENSGALG00010013110	ANKRD10	1.424705358	0.154238799	9.237010196	2.53481E-20	1.37456E-19	positive
PRJNA729271	stage_1D_vs_E10	ordinary_muscle_development	ENSGALG00010013111	RFESD	2.501513815	0.272369477	9.184266318	4.14342E-20	2.22853E-19	positive
PRJNA729271	stage_1D_vs_E10	ordinary_muscle_development	ENSGALG00010013112	CD40	0.198122196	0.162502629	1.219193791	0.222770648	0.2654353	positive
PRJNA729271	stage_1D_vs_E10	ordinary_muscle_development	ENSGALG00010013114	PIGP	0.001430192	0.121558596	0.011765451	0.990612744	0.992050002	positive
PRJNA729271	stage_1D_vs_E10	ordinary_muscle_development	ENSGALG00010013115	ENSGALG00010013115	-2.442354687	0.38286971	-6.379075246	1.7816E-10	5.30492E-10	negative
PRJNA729271	stage_1D_vs_E10	ordinary_muscle_development	ENSGALG00010013116	TARS	-0.857012481	0.168236075	-5.094106499	3.5039E-07	8.09644E-07	negative
PRJNA729271	stage_1D_vs_E10	ordinary_muscle_development	ENSGALG00010013117	ARSK	-0.089153538	0.131652764	-0.677186982	0.498287327	0.546385869	negative
PRJNA729271	stage_1D_vs_E10	ordinary_muscle_development	ENSGALG00010013119	RNF114	0.37411223	0.078812366	4.746872219	2.06586E-06	4.50025E-06	positive
PRJNA729271	stage_1D_vs_E10	ordinary_muscle_development	ENSGALG00010013121	PCBD2	-0.761049946	0.167152977	-4.553014611	5.28826E-06	1.10787E-05	negative
PRJNA729271	stage_1D_vs_E10	ordinary_muscle_development	ENSGALG00010013123	PPFIBP1	0.139397423	0.108168289	1.288708768	0.197499353	0.237494037	positive
PRJNA729271	stage_1D_vs_E10	ordinary_muscle_development	ENSGALG00010013125	POU3F3	-4.430540721	0.427570618	-10.36212624	3.68674E-25	2.55202E-24	negative
PRJNA729271	stage_1D_vs_E10	ordinary_muscle_development	ENSGALG00010013129	RASSF3	-0.078043389	0.111035256	-0.702870348	0.482136552	0.530643076	negative
PRJNA729271	stage_1D_vs_E10	ordinary_muscle_development	ENSGALG00010013131	SPATA2	0.718565191	0.115338121	6.230075382	4.66211E-10	1.34831E-09	positive
PRJNA729271	stage_1D_vs_E10	ordinary_muscle_development	ENSGALG00010013132	SKIC3	-1.050949479	0.090063384	-11.66899837	1.83572E-31	1.70569E-30	negative
PRJNA729271	stage_1D_vs_E10	ordinary_muscle_development	ENSGALG00010013133	TXNDC15	-0.234647919	0.119536875	-1.962975182	0.049649058	0.06626732	negative
PRJNA729271	stage_1D_vs_E10	ordinary_muscle_development	ENSGALG00010013134	ENSGALG00010013134	-4.645837164	5.435844367	-0.854667067	0.392735487	0.441882811	negative
PRJNA729271	stage_1D_vs_E10	ordinary_muscle_development	ENSGALG00010013135	TTC3	-0.205504686	0.084609608	-2.428857554	0.015146482	0.021828222	negative
PRJNA729271	stage_1D_vs_E10	ordinary_muscle_development	ENSGALG00010013137	TMEM121	-4.080793986	0.196652028	-20.7513445	1.19211E-95	8.07458E-94	negative
PRJNA729271	stage_1D_vs_E10	ordinary_muscle_development	ENSGALG00010013139	TMEM185A	0.583151949	0.082219181	7.092650933	1.31567E-12	4.50736E-12	positive
PRJNA729271	stage_1D_vs_E10	ordinary_muscle_development	ENSGALG00010013143	ENSGALG00010013143	0.887805981	0.406024257	2.186583599	0.028772936	0.039754428	positive
PRJNA729271	stage_1D_vs_E10	ordinary_muscle_development	ENSGALG00010013144	TEDC1	-1.600355202	0.171319654	-9.341340372	9.51222E-21	5.30714E-20	negative
PRJNA729271	stage_1D_vs_E10	ordinary_muscle_development	ENSGALG00010013145	ENSGALG00010013145	-0.37864962	0.285763088	-1.325047339	0.185155451	0.224244031	negative
PRJNA729271	stage_1D_vs_E10	ordinary_muscle_development	ENSGALG00010013146	TNRC6B	0.949246147	0.119160476	7.966115745	1.6374E-15	6.78504E-15	positive
PRJNA729271	stage_1D_vs_E10	ordinary_muscle_development	ENSGALG00010013149	MCTP1	0.364099758	0.198094947	1.838006289	0.066061479	0.086453696	positive
PRJNA729271	stage_1D_vs_E10	ordinary_muscle_development	ENSGALG00010013150	GNS	0.466165538	0.103132748	4.520053485	6.1824E-06	1.28719E-05	positive
PRJNA729271	stage_1D_vs_E10	ordinary_muscle_development	ENSGALG00010013152	TBXAS1	-1.052855687	0.232605219	-4.526363132	6.00074E-06	1.25134E-05	negative
PRJNA729271	stage_1D_vs_E10	ordinary_muscle_development	ENSGALG00010013154	ENSGALG00010013154	-0.561387006	0.370807276	-1.513958981	0.130036243	0.162141513	negative
PRJNA729271	stage_1D_vs_E10	ordinary_muscle_development	ENSGALG00010013156	ABCC9	1.391339143	0.129810237	10.71825436	8.35656E-27	6.21809E-26	positive
PRJNA729271	stage_1D_vs_E10	ordinary_muscle_development	ENSGALG00010013158	CRIP1	2.614336913	0.184893974	14.13965454	2.16344E-45	3.44605E-44	positive
PRJNA729271	stage_1D_vs_E10	ordinary_muscle_development	ENSGALG00010013159	ENSGALG00010013159	-1.142071973	1.430437294	-0.798407577	0.424634008	0.474123397	negative
PRJNA729271	stage_1D_vs_E10	ordinary_muscle_development	ENSGALG00010013160	CRYBG1	-5.01470741	0.606174446	-8.272713309	1.30944E-16	5.8044E-16	negative
PRJNA729271	stage_1D_vs_E10	ordinary_muscle_development	ENSGALG00010013162	IDS	0.265285417	0.109738277	2.41743742	0.015630221	0.022476196	positive
PRJNA729271	stage_1D_vs_E10	ordinary_muscle_development	ENSGALG00010013163	ENSGALG00010013163	-0.217940847	0.456924224	-0.47697372	0.633380835	0.674744601	negative
PRJNA729271	stage_1D_vs_E10	ordinary_muscle_development	ENSGALG00010013164	FAM83F	-3.120756032	0.520582766	-5.994735585	2.03817E-09	5.62728E-09	negative
PRJNA729271	stage_1D_vs_E10	ordinary_muscle_development	ENSGALG00010013165	NUDC	-0.585010227	0.081239183	-7.201084523	5.97355E-13	2.1006E-12	negative
PRJNA729271	stage_1D_vs_E10	ordinary_muscle_development	ENSGALG00010013168	VPS26C	0.065610688	0.137805678	0.476110195	0.633995874	0.675271526	positive
PRJNA729271	stage_1D_vs_E10	ordinary_muscle_development	ENSGALG00010013170	COL3A1	3.047753706	0.234904674	12.9744277	1.70868E-38	2.11867E-37	positive
PRJNA729271	stage_1D_vs_E10	ordinary_muscle_development	ENSGALG00010013171	SLC9A8	0.610288187	0.135646101	4.499120751	6.82351E-06	1.41619E-05	positive
PRJNA729271	stage_1D_vs_E10	ordinary_muscle_development	ENSGALG00010013172	TNFSF13B	0.495093284	0.458442981	1.079945173	0.280166596	0.326316979	positive
PRJNA729271	stage_1D_vs_E10	ordinary_muscle_development	ENSGALG00010013173	KCNJ8	0.623999212	0.155001284	4.025768009	5.67897E-05	0.000108041	positive
PRJNA729271	stage_1D_vs_E10	ordinary_muscle_development	ENSGALG00010013174	ADAMTS12	0.601733765	0.165194015	3.64258816	0.00026991	0.000477699	positive
PRJNA729271	stage_1D_vs_E10	ordinary_muscle_development	ENSGALG00010013175	HIPK2	1.249524532	0.163345102	7.649599035	2.01607E-14	7.79694E-14	positive
PRJNA729271	stage_1D_vs_E10	ordinary_muscle_development	ENSGALG00010013177	OXR1	0.613935152	0.069322402	8.856230215	8.27659E-19	4.14258E-18	positive
PRJNA729271	stage_1D_vs_E10	ordinary_muscle_development	ENSGALG00010013179	TBC1D30	-0.309021278	0.187188465	-1.650856419	0.098767897	0.125706304	negative
PRJNA729271	stage_1D_vs_E10	ordinary_muscle_development	ENSGALG00010013180	C5orf24	0.230484516	0.058826674	3.918027316	8.92766E-05	0.000166482	positive
PRJNA729271	stage_1D_vs_E10	ordinary_muscle_development	ENSGALG00010013181	DYRK1A	1.357019619	0.078645354	17.25492412	1.02757E-66	3.35463E-65	positive
PRJNA729271	stage_1D_vs_E10	ordinary_muscle_development	ENSGALG00010013183	CLTA	-1.168539056	0.137611268	-8.491594294	2.03821E-17	9.45708E-17	negative
PRJNA729271	stage_1D_vs_E10	ordinary_muscle_development	ENSGALG00010013184	LDHB	-0.597243186	0.114183337	-5.230563425	1.68994E-07	4.01174E-07	negative
PRJNA729271	stage_1D_vs_E10	ordinary_muscle_development	ENSGALG00010013185	ENSGALG00010013185	-0.57032497	0.313526809	-1.819062848	0.068901842	0.089927265	negative
PRJNA729271	stage_1D_vs_E10	ordinary_muscle_development	ENSGALG00010013186	DDX46	-0.461135236	0.080119799	-5.755571552	8.63491E-09	2.26663E-08	negative
PRJNA729271	stage_1D_vs_E10	ordinary_muscle_development	ENSGALG00010013187	KDF1	-3.557454426	0.267312994	-13.30819864	2.07423E-40	2.75581E-39	negative
PRJNA729271	stage_1D_vs_E10	ordinary_muscle_development	ENSGALG00010013188	ENSGALG00010013188	0.103088226	0.36425281	0.283012851	0.777166986	0.806200205	positive
PRJNA729271	stage_1D_vs_E10	ordinary_muscle_development	ENSGALG00010013190	SLC45A2	-2.407066068	0.445572222	-5.4021906	6.58319E-08	1.61406E-07	negative
PRJNA729271	stage_1D_vs_E10	ordinary_muscle_development	ENSGALG00010013191	ABRA	5.892852241	0.418845327	14.06928014	5.86635E-45	9.1729E-44	positive
PRJNA729271	stage_1D_vs_E10	ordinary_muscle_development	ENSGALG00010013192	B4GALT5	0.913785339	0.080993357	11.28222573	1.6063E-29	1.35525E-28	positive
PRJNA729271	stage_1D_vs_E10	ordinary_muscle_development	ENSGALG00010013194	GRAP2	-2.704942129	0.391999751	-6.90036696	5.18684E-12	1.70871E-11	negative
PRJNA729271	stage_1D_vs_E10	ordinary_muscle_development	ENSGALG00010013196	MRPS9	-0.327180141	0.114331496	-2.861679865	0.004214023	0.006535659	negative
PRJNA729271	stage_1D_vs_E10	ordinary_muscle_development	ENSGALG00010013198	CAMLG	-0.455284324	0.086281777	-5.276714764	1.3152E-07	3.14897E-07	negative
PRJNA729271	stage_1D_vs_E10	ordinary_muscle_development	ENSGALG00010013199	RALYL	-2.996666134	0.557730167	-5.372967628	7.74512E-08	1.88745E-07	negative
PRJNA729271	stage_1D_vs_E10	ordinary_muscle_development	ENSGALG00010013202	ENSGALG00010013202	-0.157669262	0.649145132	-0.242887536	0.808092521	0.834043086	negative
PRJNA729271	stage_1D_vs_E10	ordinary_muscle_development	ENSGALG00010013203	AMACR	0.424431355	0.119862838	3.54097536	0.000398651	0.000694024	positive
PRJNA729271	stage_1D_vs_E10	ordinary_muscle_development	ENSGALG00010013204	ENSGALG00010013204	1.272200777	0.328578028	3.871837639	0.000108018	0.000199786	positive
PRJNA729271	stage_1D_vs_E10	ordinary_muscle_development	ENSGALG00010013205	KCNJ6	-1.407254896	0.383009545	-3.674203196	0.000238593	0.00042497	negative
PRJNA729271	stage_1D_vs_E10	ordinary_muscle_development	ENSGALG00010013206	AFF2	-0.348808372	0.111426911	-3.130378186	0.001745814	0.002832469	negative
PRJNA729271	stage_1D_vs_E10	ordinary_muscle_development	ENSGALG00010013207	C1QTNF3	-1.888122136	0.199030693	-9.486587782	2.38721E-21	1.37585E-20	negative
PRJNA729271	stage_1D_vs_E10	ordinary_muscle_development	ENSGALG00010013208	ENSGALG00010013208	0.368028635	0.300913336	1.223038634	0.221315105	0.263812701	positive
PRJNA729271	stage_1D_vs_E10	ordinary_muscle_development	ENSGALG00010013209	KIAA1549	-0.155884083	0.101595278	-1.534363458	0.124940262	0.156283146	negative
PRJNA729271	stage_1D_vs_E10	ordinary_muscle_development	ENSGALG00010013212	ENSGALG00010013212	0.956681928	0.151771884	6.303420014	2.91148E-10	8.54637E-10	positive
PRJNA729271	stage_1D_vs_E10	ordinary_muscle_development	ENSGALG00010013213	SH2D1A	1.312377945	0.371010825	3.537303649	0.000404235	0.000703407	positive
PRJNA729271	stage_1D_vs_E10	ordinary_muscle_development	ENSGALG00010013214	RAI14	-0.753807282	0.169391398	-4.450091871	8.58336E-06	1.76576E-05	negative
PRJNA729271	stage_1D_vs_E10	ordinary_muscle_development	ENSGALG00010013215	SF3A3	-1.00258773	0.082315206	-12.17986048	3.97962E-34	4.15595E-33	negative
PRJNA729271	stage_1D_vs_E10	ordinary_muscle_development	ENSGALG00010013217	TGFBRAP1	0.286243334	0.082644486	3.463550275	0.000533097	0.000915658	positive
PRJNA729271	stage_1D_vs_E10	ordinary_muscle_development	ENSGALG00010013220	CSTF1	0.129045435	0.086276947	1.495711655	0.134728812	0.167343113	positive
PRJNA729271	stage_1D_vs_E10	ordinary_muscle_development	ENSGALG00010013221	C2orf49	0.044914338	0.118031833	0.380527323	0.703554019	0.740220331	positive
PRJNA729271	stage_1D_vs_E10	ordinary_muscle_development	ENSGALG00010013224	ATP6V0A4	1.504734441	0.692210106	2.173811722	0.029719278	0.04098772	positive
PRJNA729271	stage_1D_vs_E10	ordinary_muscle_development	ENSGALG00010013225	GNE	-1.080307253	0.096757762	-11.16507069	6.04431E-29	4.9808E-28	negative
PRJNA729271	stage_1D_vs_E10	ordinary_muscle_development	ENSGALG00010013226	DUSP6	-0.504375331	0.113120713	-4.458735413	8.24446E-06	1.69846E-05	negative
PRJNA729271	stage_1D_vs_E10	ordinary_muscle_development	ENSGALG00010013227	CITED4	1.497116884	0.16165594	9.261131305	2.02277E-20	1.10434E-19	positive
PRJNA729271	stage_1D_vs_E10	ordinary_muscle_development	ENSGALG00010013228	RTFDC1	-0.070718038	0.085034087	-0.831643412	0.40561025	0.455267197	negative
PRJNA729271	stage_1D_vs_E10	ordinary_muscle_development	ENSGALG00010013230	ENSGALG00010013230	3.404360848	0.341194957	9.977758401	1.90727E-23	1.21041E-22	positive
PRJNA729271	stage_1D_vs_E10	ordinary_muscle_development	ENSGALG00010013232	FHL2	-1.38579305	0.422748611	-3.278054649	0.001045252	0.001740285	negative
PRJNA729271	stage_1D_vs_E10	ordinary_muscle_development	ENSGALG00010013233	LRRCC1	-0.506009165	0.126991079	-3.984604039	6.75927E-05	0.000127589	negative
PRJNA729271	stage_1D_vs_E10	ordinary_muscle_development	ENSGALG00010013235	GCNT7	0.208723813	0.252851426	0.825480069	0.409099093	0.458756875	positive
PRJNA729271	stage_1D_vs_E10	ordinary_muscle_development	ENSGALG00010013236	MON2	0.089892047	0.082618763	1.088034287	0.276579971	0.322346763	positive
PRJNA729271	stage_1D_vs_E10	ordinary_muscle_development	ENSGALG00010013238	RAD1	-0.054290641	0.131327566	-0.413398667	0.679314551	0.717246747	negative
PRJNA729271	stage_1D_vs_E10	ordinary_muscle_development	ENSGALG00010013241	SEC24A	0.295379699	0.135541001	2.179264553	0.029312021	0.040472256	positive
PRJNA729271	stage_1D_vs_E10	ordinary_muscle_development	ENSGALG00010013242	SULT4A1	-1.291055418	0.162842273	-7.928257173	2.22243E-15	9.13875E-15	negative
PRJNA729271	stage_1D_vs_E10	ordinary_muscle_development	ENSGALG00010013244	TRIM24	0.029107744	0.147704287	0.197067703	0.843774557	0.86630629	positive
PRJNA729271	stage_1D_vs_E10	ordinary_muscle_development	ENSGALG00010013246	LNPEP	2.080545525	0.174783022	11.90359054	1.13363E-32	1.11478E-31	positive
PRJNA729271	stage_1D_vs_E10	ordinary_muscle_development	ENSGALG00010013247	ATG5	-0.26259915	0.092893388	-2.826887416	0.004700285	0.007247866	negative
PRJNA729271	stage_1D_vs_E10	ordinary_muscle_development	ENSGALG00010013248	ENSGALG00010013248	-0.699151667	0.686241215	-1.018813287	0.308291617	0.355617299	negative
PRJNA729271	stage_1D_vs_E10	ordinary_muscle_development	ENSGALG00010013249	AURKA	-3.072360413	0.228772448	-13.42976586	4.04689E-41	5.53393E-40	negative
PRJNA729271	stage_1D_vs_E10	ordinary_muscle_development	ENSGALG00010013250	E2F5	-0.14848434	0.094565427	-1.570175737	0.116374232	0.146396241	negative
PRJNA729271	stage_1D_vs_E10	ordinary_muscle_development	ENSGALG00010013251	MTA1	-0.235774977	0.081597656	-2.889482236	0.003858768	0.006009761	negative
PRJNA729271	stage_1D_vs_E10	ordinary_muscle_development	ENSGALG00010013252	PPM1H	-2.959401016	0.202013703	-14.6495063	1.35698E-48	2.47726E-47	negative
PRJNA729271	stage_1D_vs_E10	ordinary_muscle_development	ENSGALG00010013253	SHB	0.570268247	0.098708193	5.77731422	7.59025E-09	2.00511E-08	positive
PRJNA729271	stage_1D_vs_E10	ordinary_muscle_development	ENSGALG00010013254	SAR1B	0.547895585	0.141749257	3.865244837	0.000110978	0.000205077	positive
PRJNA729271	stage_1D_vs_E10	ordinary_muscle_development	ENSGALG00010013256	BRIX1	-0.643085583	0.117759873	-5.460990788	4.73485E-08	1.17319E-07	negative
PRJNA729271	stage_1D_vs_E10	ordinary_muscle_development	ENSGALG00010013257	STX12	-0.719964327	0.090190626	-7.982695727	1.43172E-15	5.96171E-15	negative
PRJNA729271	stage_1D_vs_E10	ordinary_muscle_development	ENSGALG00010013258	RBIS	-0.094922527	0.146258807	-0.649003838	0.516335896	0.563915673	negative
PRJNA729271	stage_1D_vs_E10	ordinary_muscle_development	ENSGALG00010013259	PTPRB	2.457635505	0.1391147	17.66625316	7.62994E-70	2.79283E-68	positive
PRJNA729271	stage_1D_vs_E10	ordinary_muscle_development	ENSGALG00010013260	FAM210B	0.123026273	0.080662807	1.525192058	0.127211201	0.158822253	positive

PRJNA729271	stage_1D_vs_E10	ordinary_muscle_development	ENSGALG00010013261	JADE2	1.398061338	0.197632406	7.074049071	1.50477E-12	5.14183E-12	positive
PRJNA729271	stage_1D_vs_E10	ordinary_muscle_development	ENSGALG00010013262	CA13	-1.190482205	0.263272346	-4.52186576	6.12969E-06	1.2769E-05	negative
PRJNA729271	stage_1D_vs_E10	ordinary_muscle_development	ENSGALG00010013263	ATP11A	-0.431276045	0.112949306	-3.818315132	0.000134366	0.000246194	negative
PRJNA729271	stage_1D_vs_E10	ordinary_muscle_development	ENSGALG00010013264	DNAJC21	-0.09062439	0.122133268	-0.742012318	0.458079873	0.507014947	negative
PRJNA729271	stage_1D_vs_E10	ordinary_muscle_development	ENSGALG00010013265	PACS2	1.320634643	0.121777187	10.84468011	2.11374E-27	1.60664E-26	positive
PRJNA729271	stage_1D_vs_E10	ordinary_muscle_development	ENSGALG00010013266	KIAA1755	1.329595319	0.18927573	7.024647685	2.14607E-12	7.26297E-12	positive
PRJNA729271	stage_1D_vs_E10	ordinary_muscle_development	ENSGALG00010013267	PNPLA3	-0.456052678	0.179534633	-2.540193331	0.011079121	0.016287206	negative
PRJNA729271	stage_1D_vs_E10	ordinary_muscle_development	ENSGALG00010013268	PTPRR	1.825505393	0.257558856	7.087721317	1.36338E-12	4.66751E-12	positive
PRJNA729271	stage_1D_vs_E10	ordinary_muscle_development	ENSGALG00010013269	INPP5B	0.370238396	0.101618049	3.643431448	0.000269027	0.000476252	positive
PRJNA729271	stage_1D_vs_E10	ordinary_muscle_development	ENSGALG00010013271	GULP1	0.963554456	0.13871952	6.946062495	3.75622E-12	1.25107E-11	positive
PRJNA729271	stage_1D_vs_E10	ordinary_muscle_development	ENSGALG00010013273	STAG2	0.903678191	0.116194445	7.777292519	7.40931E-15	2.94E-14	positive
PRJNA729271	stage_1D_vs_E10	ordinary_muscle_development	ENSGALG00010013274	MTF1	0.969088975	0.119359204	8.1190972	4.69664E-16	2.02131E-15	positive
PRJNA729271	stage_1D_vs_E10	ordinary_muscle_development	ENSGALG00010013275	MAPRE1	-1.552090056	0.076432331	-20.30672148	1.12145E-91	6.94675E-90	negative
PRJNA729271	stage_1D_vs_E10	ordinary_muscle_development	ENSGALG00010013276	POC1B	-1.666674115	0.109758119	-15.18497333	4.44744E-52	9.23577E-51	negative
PRJNA729271	stage_1D_vs_E10	ordinary_muscle_development	ENSGALG00010013277	YRDC	-0.601801269	0.090359485	-6.660078551	2.73681E-11	8.60336E-11	negative
PRJNA729271	stage_1D_vs_E10	ordinary_muscle_development	ENSGALG00010013278	SAMM50	0.672085223	0.102764097	6.540078142	6.14867E-11	1.88824E-10	positive
PRJNA729271	stage_1D_vs_E10	ordinary_muscle_development	ENSGALG00010013280	FRS2	0.164012204	0.110211569	1.488157782	0.136709288	0.169643107	positive
PRJNA729271	stage_1D_vs_E10	ordinary_muscle_development	ENSGALG00010013281	BIRC8	2.133896672	0.138412706	15.41691318	1.25984E-53	2.74607E-52	positive
PRJNA729271	stage_1D_vs_E10	ordinary_muscle_development	ENSGALG00010013282	SEC22B	-1.204427664	0.090165323	-13.35799202	1.06396E-40	1.43997E-39	negative
PRJNA729271	stage_1D_vs_E10	ordinary_muscle_development	ENSGALG00010013283	PAWR	-1.500563027	0.282310652	-5.315290138	1.06488E-07	2.56785E-07	negative
PRJNA729271	stage_1D_vs_E10	ordinary_muscle_development	ENSGALG00010013284	MANEAL	-1.93275966	0.338302015	-5.713118978	1.10924E-08	2.88403E-08	negative
PRJNA729271	stage_1D_vs_E10	ordinary_muscle_development	ENSGALG00010013285	ATP2B1	-0.12827869	0.107533186	-1.192921869	0.232899974	0.276220549	negative
PRJNA729271	stage_1D_vs_E10	ordinary_muscle_development	ENSGALG00010013286	RNF14	0.334565737	0.119908229	2.790181625	0.005267848	0.008075027	positive
PRJNA729271	stage_1D_vs_E10	ordinary_muscle_development	ENSGALG00010013288	CA3A	2.917360393	0.778366377	3.748055516	0.000178211	0.000322533	positive
PRJNA729271	stage_1D_vs_E10	ordinary_muscle_development	ENSGALG00010013289	TM9SF4	-0.591367949	0.073402555	-8.0565036	7.85077E-16	3.32254E-15	negative
PRJNA729271	stage_1D_vs_E10	ordinary_muscle_development	ENSGALG00010013290	AKR1D1	-2.012482172	0.338013377	-5.953853635	2.61901E-09	7.17385E-09	negative
PRJNA729271	stage_1D_vs_E10	ordinary_muscle_development	ENSGALG00010013292	PRDM1	-2.891029053	0.301752527	-9.580794825	9.63009E-22	5.67201E-21	negative
PRJNA729271	stage_1D_vs_E10	ordinary_muscle_development	ENSGALG00010013293	TUBGCP3	-0.445670247	0.055860095	-7.978329558	1.48327E-15	6.16752E-15	negative
PRJNA729271	stage_1D_vs_E10	ordinary_muscle_development	ENSGALG00010013295	CA3B	-1.790356449	0.414699198	-4.317241164	1.57992E-05	3.16136E-05	negative
PRJNA729271	stage_1D_vs_E10	ordinary_muscle_development	ENSGALG00010013296	AGXT2	-2.219771289	0.484409736	-4.582425007	4.59615E-06	9.67206E-06	negative
PRJNA729271	stage_1D_vs_E10	ordinary_muscle_development	ENSGALG00010013297	FCHO2	0.541037306	0.124432552	4.348036725	1.37362E-05	2.76689E-05	positive
PRJNA729271	stage_1D_vs_E10	ordinary_muscle_development	ENSGALG00010013298	EPYC	-7.87210942	0.424793719	-18.53160503	1.14811E-76	4.96771E-75	negative
PRJNA729271	stage_1D_vs_E10	ordinary_muscle_development	ENSGALG00010013299	PLAGL2	-0.666909101	0.109133173	-6.110965901	9.90299E-10	2.8014E-09	negative
PRJNA729271	stage_1D_vs_E10	ordinary_muscle_development	ENSGALG00010013300	GNPDA1	-1.409918341	0.099295644	-14.19919624	9.26692E-46	1.49083E-44	negative
PRJNA729271	stage_1D_vs_E10	ordinary_muscle_development	ENSGALG00010013301	PARVB	-1.983758523	0.141338813	-14.0354831	9.45499E-45	1.46735E-43	negative
PRJNA729271	stage_1D_vs_E10	ordinary_muscle_development	ENSGALG00010013302	ENSGALG00010013302	0.495584538	0.650389331	0.761981347	0.446071137	0.495311869	positive
PRJNA729271	stage_1D_vs_E10	ordinary_muscle_development	ENSGALG00010013303	TFPI	-0.417821396	0.233726866	-1.787648137	0.073832794	0.09593101	negative
PRJNA729271	stage_1D_vs_E10	ordinary_muscle_development	ENSGALG00010013304	KCNJ15	-0.086548923	0.236439676	-0.366050758	0.714327201	0.749704784	negative
PRJNA729271	stage_1D_vs_E10	ordinary_muscle_development	ENSGALG00010013305	THOC2	0.154285187	0.090728399	1.700517035	0.089033715	0.114177094	positive
PRJNA729271	stage_1D_vs_E10	ordinary_muscle_development	ENSGALG00010013306	ARSB	-0.332723178	0.150185487	-2.215414991	0.026731593	0.037110856	negative
PRJNA729271	stage_1D_vs_E10	ordinary_muscle_development	ENSGALG00010013307	POFUT1	-0.567218467	0.086800933	-6.534704748	6.37351E-11	1.95546E-10	negative
PRJNA729271	stage_1D_vs_E10	ordinary_muscle_development	ENSGALG00010013309	ENSGALG00010013309	4.51134847	0.285143612	15.8213205	2.21792E-56	5.35812E-55	positive
PRJNA729271	stage_1D_vs_E10	ordinary_muscle_development	ENSGALG00010013310	MYO16	-1.009808067	0.295773474	-3.414126542	0.000639868	0.001088219	negative
PRJNA729271	stage_1D_vs_E10	ordinary_muscle_development	ENSGALG00010013311	RSPO1	-3.784005483	0.262119849	-14.43616536	3.06422E-47	5.24391E-46	negative
PRJNA729271	stage_1D_vs_E10	ordinary_muscle_development	ENSGALG00010013312	ENSGALG00010013312	-3.850294907	0.902689577	-4.265358774	1.99581E-05	3.95425E-05	negative
PRJNA729271	stage_1D_vs_E10	ordinary_muscle_development	ENSGALG00010013313	GAK	0.058434486	0.093514717	0.624869405	0.532056774	0.578947747	positive
PRJNA729271	stage_1D_vs_E10	ordinary_muscle_development	ENSGALG00010013314	KIT	-0.257739662	0.206586248	-1.247612868	0.212172864	0.25377058	negative
PRJNA729271	stage_1D_vs_E10	ordinary_muscle_development	ENSGALG00010013316	KDM7A	1.707698068	0.129510221	13.18581695	1.05901E-39	1.37547E-38	positive
PRJNA729271	stage_1D_vs_E10	ordinary_muscle_development	ENSGALG00010013317	PREP	-0.707776277	0.109595979	-6.458049677	1.06061E-10	3.20482E-10	negative
PRJNA729271	stage_1D_vs_E10	ordinary_muscle_development	ENSGALG00010013318	KERA	-3.860972761	0.486639653	-7.933946069	2.1229E-15	8.7419E-15	negative
PRJNA729271	stage_1D_vs_E10	ordinary_muscle_development	ENSGALG00010013319	ENSGALG00010013319	-3.565136251	1.579546771	-2.257062796			negative
PRJNA729271	stage_1D_vs_E10	ordinary_muscle_development	ENSGALG00010013320	CALCRL	0.388674113	0.123528768	3.146425879	0.001652791	0.002688197	positive
PRJNA729271	stage_1D_vs_E10	ordinary_muscle_development	ENSGALG00010013322	IRS2	2.225450898	0.510904188	4.35590655	1.32517E-05	2.67713E-05	positive
PRJNA729271	stage_1D_vs_E10	ordinary_muscle_development	ENSGALG00010013323	NOTCH2	-0.573461024	0.108685276	-5.276345113	1.31786E-07	3.15481E-07	negative
PRJNA729271	stage_1D_vs_E10	ordinary_muscle_development	ENSGALG00010013324	PARVG	0.017128136	0.151235785	0.113254518	0.909828775	0.923139303	positive
PRJNA729271	stage_1D_vs_E10	ordinary_muscle_development	ENSGALG00010013325	CA2	1.322115075	0.317276679	4.16707298	3.08536E-05	6.00702E-05	positive
PRJNA729271	stage_1D_vs_E10	ordinary_muscle_development	ENSGALG00010013326	FGF20	-7.774293193	1.03478446	-7.512958975	5.78056E-14	2.1724E-13	negative
PRJNA729271	stage_1D_vs_E10	ordinary_muscle_development	ENSGALG00010013327	SLF1	-0.916290619	0.096766268	-9.469111878	2.82233E-21	1.62019E-20	negative
PRJNA729271	stage_1D_vs_E10	ordinary_muscle_development	ENSGALG00010013328	SLC37A3	-0.320977966	0.077862755	-4.12235561	3.75018E-05	7.24591E-05	negative
PRJNA729271	stage_1D_vs_E10	ordinary_muscle_development	ENSGALG00010013329	LIFR	0.433755281	0.1223936	3.543937611	0.000394199	0.000686933	positive
PRJNA729271	stage_1D_vs_E10	ordinary_muscle_development	ENSGALG00010013330	ERG	-0.225742307	0.128432652	-1.757670679	0.07880356	0.102023722	negative
PRJNA729271	stage_1D_vs_E10	ordinary_muscle_development	ENSGALG00010013331	KIF3B	-0.86015879	0.150629788	-5.710416248	1.127E-08	2.92758E-08	negative
PRJNA729271	stage_1D_vs_E10	ordinary_muscle_development	ENSGALG00010013332	LUM	-0.591826311	0.320704992	-1.845391639	0.064980588	0.085154473	negative
PRJNA729271	stage_1D_vs_E10	ordinary_muscle_development	ENSGALG00010013333	PPP1R12A	0.496150088	0.097734328	5.07651812	3.84414E-07	8.8516E-07	positive
PRJNA729271	stage_1D_vs_E10	ordinary_muscle_development	ENSGALG00010013334	SH3BGRL3	-0.056884293	0.12138072	-0.468643562	0.639324435	0.679997628	negative
PRJNA729271	stage_1D_vs_E10	ordinary_muscle_development	ENSGALG00010013335	POPDC3	2.367924394	0.176095199	13.44684243	3.21302E-41	4.43128E-40	positive
PRJNA729271	stage_1D_vs_E10	ordinary_muscle_development	ENSGALG00010013336	MTNR1A	1.769950936	0.671579924	2.635503048	0.008401267	0.012540044	positive
PRJNA729271	stage_1D_vs_E10	ordinary_muscle_development	ENSGALG00010013337	RAB19	-0.02989645	0.255941843	-0.116809545	0.90701098	0.920752245	negative
PRJNA729271	stage_1D_vs_E10	ordinary_muscle_development	ENSGALG00010013339	ATP6V0D2	-0.746024253	0.593010866	-1.258027965	0.208381643	0.249483102	negative
PRJNA729271	stage_1D_vs_E10	ordinary_muscle_development	ENSGALG00010013340	LIN28B	-4.155513085	0.38247131	-10.86490144	1.69406E-27	1.29307E-26	negative
PRJNA729271	stage_1D_vs_E10	ordinary_muscle_development	ENSGALG00010013343	SHISAL1	-4.820482571	0.388713185	-12.40112957	2.57665E-35	2.8402E-34	negative
PRJNA729271	stage_1D_vs_E10	ordinary_muscle_development	ENSGALG00010013344	OSMR	1.324560595	0.123047012	10.76467094	5.05418E-27	3.78998E-26	positive
PRJNA729271	stage_1D_vs_E10	ordinary_muscle_development	ENSGALG00010013345	DCN	0.522240943	0.213771938	2.442981748	0.014566474	0.021040462	positive
PRJNA729271	stage_1D_vs_E10	ordinary_muscle_development	ENSGALG00010013346	HMGCS2	-0.800669596	0.713199093	-1.122645281	0.261588176	0.306748694	negative
PRJNA729271	stage_1D_vs_E10	ordinary_muscle_development	ENSGALG00010013347	ENSGALG00010013347	0.290681958	0.31356476	0.92702368	0.35391424	0.402825827	positive
PRJNA729271	stage_1D_vs_E10	ordinary_muscle_development	ENSGALG00010013348	MKRN4P	0.097344679	0.070123909	1.388181016	0.165081932	0.201929333	positive
PRJNA729271	stage_1D_vs_E10	ordinary_muscle_development	ENSGALG00010013349	ETS2	1.127422004	0.099830882	11.29331913	1.41587E-29	1.19668E-28	positive
PRJNA729271	stage_1D_vs_E10	ordinary_muscle_development	ENSGALG00010013351	EDAR	-4.91703882	0.443794067	-11.07955059	1.57662E-28	1.27174E-27	negative
PRJNA729271	stage_1D_vs_E10	ordinary_muscle_development	ENSGALG00010013352	ASXL1	-0.102494357	0.086542395	-1.184325409	0.236284274	0.279861134	negative
PRJNA729271	stage_1D_vs_E10	ordinary_muscle_development	ENSGALG00010013353	NDFIP1	-0.250087406	0.084712885	-2.952176707	0.003155423	0.004962337	negative
PRJNA729271	stage_1D_vs_E10	ordinary_muscle_development	ENSGALG00010013354	WWP1	2.424636637	0.145535636	16.66008896	2.55644E-62	7.53162E-61	positive
PRJNA729271	stage_1D_vs_E10	ordinary_muscle_development	ENSGALG00010013355	FAM171B	-2.185718039	0.150551708	-14.51805539	9.31145E-48	1.63798E-46	negative
PRJNA729271	stage_1D_vs_E10	ordinary_muscle_development	ENSGALG00010013356	AMER1	-0.332779172	0.064628709	-5.149092045	2.6175E-07	6.10962E-07	negative
PRJNA729271	stage_1D_vs_E10	ordinary_muscle_development	ENSGALG00010013357	RICTOR	0.846564471	0.109310245	7.744603204	9.58808E-15	3.78072E-14	positive
PRJNA729271	stage_1D_vs_E10	ordinary_muscle_development	ENSGALG00010013358	RPS16	0.267132195	0.174149508	1.533924486	0.125048231	0.156391209	positive
PRJNA729271	stage_1D_vs_E10	ordinary_muscle_development	ENSGALG00010013359	BTG1	0.743185042	0.132457803	5.610730554	2.01474E-08	5.13336E-08	positive
PRJNA729271	stage_1D_vs_E10	ordinary_muscle_development	ENSGALG00010013360	PRR5	-0.395155072	0.11322451	-3.490013528	0.000482996	0.000833257	negative
PRJNA729271	stage_1D_vs_E10	ordinary_muscle_development	ENSGALG00010013361	ENSGALG00010013361	-0.348031223	0.232408044	-1.497500766	0.134263007	0.166821728	negative
PRJNA729271	stage_1D_vs_E10	ordinary_muscle_development	ENSGALG00010013362	ENSGALG00010013362	3.771934848	0.358190104	10.53053897	6.24757E-26	4.45662E-25	positive
PRJNA729271	stage_1D_vs_E10	ordinary_muscle_development	ENSGALG00010013363	HS3ST5	2.332836128	0.2277134	10.24461506	1.25122E-24	8.42379E-24	positive
PRJNA729271	stage_1D_vs_E10	ordinary_muscle_development	ENSGALG00010013364	OTOGL	3.672466075	0.349361058	10.51195028	7.61032E-26	5.40478E-25	positive
PRJNA729271	stage_1D_vs_E10	ordinary_muscle_development	ENSGALG00010013365	CEP85	2.289138403	0.22704758	10.08219686	6.62285E-24	4.28564E-23	positive
PRJNA729271	stage_1D_vs_E10	ordinary_muscle_development	ENSGALG00010013367	NOL4L	0.631892948	0.165795095	3.811288554	0.000138244	0.000252884	positive
PRJNA729271	stage_1D_vs_E10	ordinary_muscle_development	ENSGALG00010013369	MTMR8	-0.346348779	0.0621568	-5.572178449	2.51574E-08	6.35954E-08	negative
PRJNA729271	stage_1D_vs_E10	ordinary_muscle_development	ENSGALG00010013370	CNKSR1	3.588816612	0.233112966	15.39518235	1.76327E-53	3.82613E-52	positive
PRJNA729271	stage_1D_vs_E10	ordinary_muscle_development	ENSGALG00010013371	FAT1	-0.498441403	0.156485882	-3.185216435	0.001446458	0.002368283	negative
PRJNA729271	stage_1D_vs_E10	ordinary_muscle_development	ENSGALG00010013372	SPRY4	-1.398516424	0.297816291	-4.695903027	2.65431E-06	5.72193E-06	negative
PRJNA729271	stage_1D_vs_E10	ordinary_muscle_development	ENSGALG00010013373	ENSGALG00010013373	0.273361374	0.565296014	0.483572088	0.628689588	0.670470549	positive
PRJNA729271	stage_1D_vs_E10	ordinary_muscle_development	ENSGALG00010013374	RMDN1	0.409783528	0.165833434	2.47105495	0.013471512	0.019554332	positive
PRJNA729271	stage_1D_vs_E10	ordinary_muscle_development	ENSGALG00010013375	PSMG1	-0.444556569	0.088295544	-5.034869802	4.78174E-07	1.09531E-06	negative
PRJNA729271	stage_1D_vs_E10	ordinary_muscle_development	ENSGALG00010013376	ARHGAP8	1.024347506	0.40669045	2.518740002	0.011777559	0.017250982	positive
PRJNA729271	stage_1D_vs_E10	ordinary_muscle_development	ENSGALG00010013378	SH3RF3	-0.287299349	0.143435772	-2.002982559	0.045179161	0.060653138	negative
PRJNA729271	stage_1D_vs_E10	ordinary_muscle_development	ENSGALG00010013379	ZNF593	0.566754553	0.131704777	4.303219412	1.68334E-05	3.35857E-05	positive
PRJNA729271	stage_1D_vs_E10	ordinary_muscle_development	ENSGALG00010013380	PHGDH	-1.385197056	0.610553852	-2.268754923	0.023283234	0.032670907	negative
PRJNA729271	stage_1D_vs_E10	ordinary_muscle_development	ENSGALG00010013381	DENND2A	-0.400720277	0.088498898	-4.527969118	5.95533E-06	1.24223E-05	negative
PRJNA729271	stage_1D_vs_E10	ordinary_muscle_development	ENSGALG00010013382	ASB12	1.511130597	0.220863101	6.84193326	7.81315E-12	2.54441E-11	positive
PRJNA729271	stage_1D_vs_E10	ordinary_muscle_development	ENSGALG00010013383	SEPTIN10	0.822774974	0.138174704	5.954599138	2.6071E-09	7.145E-09	positive
PRJNA729271	stage_1D_vs_E10	ordinary_muscle_development	ENSGALG00010013385	PGRMC1	-0.323058698	0.155609863	-2.076081117	0.037886449	0.051453582	negative
PRJNA729271	stage_1D_vs_E10	ordinary_muscle_development	ENSGALG00010013386	ITGAV	0.151664841	0.121814164	1.245050957	0.21311302	0.254768926	positive
PRJNA729271	stage_1D_vs_E10	ordinary_muscle_development	ENSGALG00010013387	NDST1	0.013025346	0.091940003	0.141672243	0.887338901	0.903927006	positive
PRJNA729271	stage_1D_vs_E10	ordinary_muscle_development	ENSGALG00010013388	SOWAHC	0.323595583	0.209912881	1.541570872	0.123177908	0.154334501	positive
PRJNA729271	stage_1D_vs_E10	ordinary_muscle_development	ENSGALG00010013389	NAP1L1	-1.407891964	0.062247739	-22.61755979	2.9114E-113	2.9306E-111	negative
PRJNA729271	stage_1D_vs_E10	ordinary_muscle_development	ENSGALG00010013390	SLC16A2	-2.123462754	0.134865261	-15.74506833	7.42509E-56	1.76148E-54	negative
PRJNA729271	stage_1D_vs_E10	ordinary_muscle_development	ENSGALG00010013391	HMGN1	-0.519127195	0.087936797	-5.903412609	3.56058E-09	9.65407E-09	negative
PRJNA729271	stage_1D_vs_E10	ordinary_muscle_development	ENSGALG00010013392	HDAC2	-0.893631618	0.069015208	-12.94832895	2.40124E-38	2.95969E-37	negative
PRJNA729271	stage_1D_vs_E10	ordinary_muscle_development	ENSGALG00010013393	ADCK2	-0.804233726	0.163259028	-4.926120999	8.38781E-07	1.88498E-06	negative
PRJNA729271	stage_1D_vs_E10	ordinary_muscle_development	ENSGALG00010013394	DNMT3B	-5.733266852	0.201160182	-28.50100246	1.1384E-178	3.4377E-176	negative
PRJNA729271	stage_1D_vs_E10	ordinary_muscle_development	ENSGALG00010013396	FYB1	-0.376373978	0.190126413	-1.979598582	0.047748652	0.063860188	negative
PRJNA729271	stage_1D_vs_E10	ordinary_muscle_development	ENSGALG00010013397	MATN1	-9.173618973	0.494519908	-18.55055543	8.07136E-77	3.51335E-75	negative
PRJNA729271	stage_1D_vs_E10	ordinary_muscle_development	ENSGALG00010013398	CHAMP1	1.234829547	0.154856911	7.974003504	1.53615E-15	6.37826E-15	positive
PRJNA729271	stage_1D_vs_E10	ordinary_muscle_development	ENSGALG00010013399	FGF1	3.823274652	0.321968145	11.8746985	1.60211E-32	1.56486E-31	positive
PRJNA729271	stage_1D_vs_E10	ordinary_muscle_development	ENSGALG00010013400	BBS10	0.639531938	0.160979088	3.972764073	7.10434E-05	0.000133772	positive
PRJNA729271	stage_1D_vs_E10	ordinary_muscle_development	ENSGALG00010013402	MARCKS	-0.857069511	0.155011398	-5.529074132	3.21925E-08	8.05647E-08	negative
PRJNA729271	stage_1D_vs_E10	ordinary_muscle_development	ENSGALG00010013403	CPNE3	1.186830862	0.102696465	11.55668656	6.82934E-31	6.16384E-30	positive
PRJNA729271	stage_1D_vs_E10	ordinary_muscle_development	ENSGALG00010013404	ENSGALG00010013404	0.259883275	0.220018134	1.181190247	0.237527155	0.281218338	positive
PRJNA729271	stage_1D_vs_E10	ordinary_muscle_development	ENSGALG00010013405	RPS14	0.1697749	0.175347881	0.968217574	0.33293572	0.381494329	positive
PRJNA729271	stage_1D_vs_E10	ordinary_muscle_development	ENSGALG00010013406	ENSGALG00010013406	0.796093654	0.270195303	2.946363779	0.00321534	0.00504999	positive
PRJNA729271	stage_1D_vs_E10	ordinary_muscle_development	ENSGALG00010013407	ZC3H15	0.445328057	0.112738983	3.950080493	7.81249E-05	0.000146459	positive
PRJNA729271	stage_1D_vs_E10	ordinary_muscle_development	ENSGALG00010013408	RLIM	-0.227597048	0.065128059	-3.494608192	0.000474758	0.000819631	negative
PRJNA729271	stage_1D_vs_E10	ordinary_muscle_development	ENSGALG00010013409	DAB2	0.062468798	0.147240387	0.42426402	0.671373276	0.709814416	positive
PRJNA729271	stage_1D_vs_E10	ordinary_muscle_development	ENSGALG00010013410	KIAA0825	-0.566412011	0.102193576	-5.542540289	2.98115E-08	7.47737E-08	negative

PRJNA729271	stage_1D_vs_E10	ordinary_muscle_development	ENSGALG00010013411	ENSGALG00010013411	1.484303714	0.441853815	3.359264224	0.000781503	0.00131873	positive
PRJNA729271	stage_1D_vs_E10	ordinary_muscle_development	ENSGALG00010013412	COMMD7	-0.797360893	0.100775937	-7.912215158	2.52849E-15	1.03474E-14	negative
PRJNA729271	stage_1D_vs_E10	ordinary_muscle_development	ENSGALG00010013413	WRB	-0.340737794	0.083694926	-4.071188186	4.67739E-05	8.96454E-05	negative
PRJNA729271	stage_1D_vs_E10	ordinary_muscle_development	ENSGALG00010013414	LAPTM5	0.113622292	0.175130971	0.648784685	0.516477558	0.564005456	positive
PRJNA729271	stage_1D_vs_E10	ordinary_muscle_development	ENSGALG00010013416	NDUFB2	0.771054	0.110348176	6.98746486	2.79898E-12	9.398E-12	positive
PRJNA729271	stage_1D_vs_E10	ordinary_muscle_development	ENSGALG00010013417	AKR1B1	-0.272366757	0.08828658	-3.085030117	0.002035315	0.003272897	negative
PRJNA729271	stage_1D_vs_E10	ordinary_muscle_development	ENSGALG00010013418	HCN1	0.926826839	0.654520066	1.416040375	0.156763684	0.19251797	positive
PRJNA729271	stage_1D_vs_E10	ordinary_muscle_development	ENSGALG00010013419	ENSGALG00010013419	1.942671521	0.335968974	5.78229442	7.36886E-09	1.94841E-08	positive
PRJNA729271	stage_1D_vs_E10	ordinary_muscle_development	ENSGALG00010013420	ENSGALG00010013420	-22.21021301	4.276721409	-5.193280293			negative
PRJNA729271	stage_1D_vs_E10	ordinary_muscle_development	ENSGALG00010013423	ZNF804A	-0.113331472	0.383720746	-0.295348826	0.767727421	0.797549557	negative
PRJNA729271	stage_1D_vs_E10	ordinary_muscle_development	ENSGALG00010013425	ENSGALG00010013425	0.30645387	0.222345536	1.378277593	0.168117599	0.205330687	positive
PRJNA729271	stage_1D_vs_E10	ordinary_muscle_development	ENSGALG00010013427	NEXMIF	-1.666260421	0.174885162	-9.527740402	1.60744E-21	9.34609E-21	negative
PRJNA729271	stage_1D_vs_E10	ordinary_muscle_development	ENSGALG00010013428	BPGM	0.155661609	0.153318119	1.015285148	0.309969922	0.357297337	positive
PRJNA729271	stage_1D_vs_E10	ordinary_muscle_development	ENSGALG00010013429	PDE4DIP	2.65631561	0.187500267	14.16699638	1.4664E-45	2.34866E-44	positive
PRJNA729271	stage_1D_vs_E10	ordinary_muscle_development	ENSGALG00010013431	TMEM175	-0.705099944	0.087785804	-8.032049724	9.58575E-16	4.03208E-15	negative
PRJNA729271	stage_1D_vs_E10	ordinary_muscle_development	ENSGALG00010013434	CORIN	-3.231459918	0.412549689	-7.832898691	4.76748E-15	1.91957E-14	negative
PRJNA729271	stage_1D_vs_E10	ordinary_muscle_development	ENSGALG00010013435	SDC3	-1.756371362	0.179936652	-9.761053897	1.65427E-22	1.01176E-21	negative
PRJNA729271	stage_1D_vs_E10	ordinary_muscle_development	ENSGALG00010013436	NPAS2	0.55015953	0.205631762	2.675459883	0.00746268	0.01122228	positive
PRJNA729271	stage_1D_vs_E10	ordinary_muscle_development	ENSGALG00010013437	ENSGALG00010013437	0.745642659	0.123172258	6.053657459	1.41594E-09	3.95224E-09	positive
PRJNA729271	stage_1D_vs_E10	ordinary_muscle_development	ENSGALG00010013438	RPL37	0.458381888	0.187491994	2.444807792	0.014492936	0.020942588	positive
PRJNA729271	stage_1D_vs_E10	ordinary_muscle_development	ENSGALG00010013440	EEA1	0.068122424	0.098202896	0.693690578	0.487876276	0.536268038	positive
PRJNA729271	stage_1D_vs_E10	ordinary_muscle_development	ENSGALG00010013442	ENSGALG00010013442	0.860275664	0.104293053	8.248638221	1.6021E-16	7.07137E-16	positive
PRJNA729271	stage_1D_vs_E10	ordinary_muscle_development	ENSGALG00010013444	OSBPL8	-1.062227077	0.103156932	-10.29719533	7.25456E-25	4.93685E-24	negative
PRJNA729271	stage_1D_vs_E10	ordinary_muscle_development	ENSGALG00010013446	ATP5I	1.269014364	0.145267997	8.735677447	2.422E-18	1.18126E-17	positive
PRJNA729271	stage_1D_vs_E10	ordinary_muscle_development	ENSGALG00010013449	LCA5L	-1.115106766	0.236584565	-4.713353833	2.43673E-06	5.27327E-06	negative
PRJNA729271	stage_1D_vs_E10	ordinary_muscle_development	ENSGALG00010013450	CD74	1.291219639	0.191043059	6.758788531	1.3915E-11	4.46928E-11	positive
PRJNA729271	stage_1D_vs_E10	ordinary_muscle_development	ENSGALG00010013451	PRKAB2	2.488379402	0.167866311	14.82357829	1.03141E-49	1.97232E-48	positive
PRJNA729271	stage_1D_vs_E10	ordinary_muscle_development	ENSGALG00010013452	LSS	-1.807669698	0.228176273	-7.922250967	2.33249E-15	9.58046E-15	negative
PRJNA729271	stage_1D_vs_E10	ordinary_muscle_development	ENSGALG00010013453	PUM1	0.077596246	0.089117967	0.870713831	0.383910425	0.432922173	positive
PRJNA729271	stage_1D_vs_E10	ordinary_muscle_development	ENSGALG00010013457	SH3BGR	1.805811186	0.147865424	12.21253177	2.66466E-34	2.79886E-33	positive
PRJNA729271	stage_1D_vs_E10	ordinary_muscle_development	ENSGALG00010013458	LAMA4	1.032200343	0.143648498	7.185597889	6.69137E-13	2.34619E-12	positive
PRJNA729271	stage_1D_vs_E10	ordinary_muscle_development	ENSGALG00010013459	ATP10D	-1.399718315	0.146224007	-9.572424828	1.04427E-21	6.13817E-21	negative
PRJNA729271	stage_1D_vs_E10	ordinary_muscle_development	ENSGALG00010013461	S100B	4.333292437	0.565064704	7.668665925	1.73794E-14	6.74833E-14	positive
PRJNA729271	stage_1D_vs_E10	ordinary_muscle_development	ENSGALG00010013463	ENSGALG00010013463	1.804196999	0.208951453	8.63452718	5.89704E-18	2.82104E-17	positive
PRJNA729271	stage_1D_vs_E10	ordinary_muscle_development	ENSGALG00010013464	ABCB7	0.002261029	0.07404894	0.03053425	0.975640978	0.979221436	positive
PRJNA729271	stage_1D_vs_E10	ordinary_muscle_development	ENSGALG00010013465	CALD1	-0.707896112	0.157155952	-4.504418095	6.65551E-06	1.38231E-05	negative
PRJNA729271	stage_1D_vs_E10	ordinary_muscle_development	ENSGALG00010013466	BRAF	0.653989682	0.060184624	10.86639139	1.66663E-27	1.27347E-26	positive
PRJNA729271	stage_1D_vs_E10	ordinary_muscle_development	ENSGALG00010013468	DEPDC1B	-1.88304921	0.270651738	-6.957462108	3.46457E-12	1.15765E-11	negative
PRJNA729271	stage_1D_vs_E10	ordinary_muscle_development	ENSGALG00010013470	RPL31	-0.106529382	0.146354043	-0.727888208	0.466682022	0.515319642	negative
PRJNA729271	stage_1D_vs_E10	ordinary_muscle_development	ENSGALG00010013472	ENSGALG00010013472	0.497658941	0.2368704	2.100975641	0.035643105	0.048638219	positive
PRJNA729271	stage_1D_vs_E10	ordinary_muscle_development	ENSGALG00010013476	ZDHHC17	-0.57390314	0.129631519	-4.427188277	9.54694E-06	1.95456E-05	negative
PRJNA729271	stage_1D_vs_E10	ordinary_muscle_development	ENSGALG00010013477	RFC1	-0.417675022	0.089668483	-4.657991409	3.1931E-06	6.83359E-06	negative
PRJNA729271	stage_1D_vs_E10	ordinary_muscle_development	ENSGALG00010013480	TBC1D8	1.799664388	0.151408409	11.88615874	1.39684E-32	1.36806E-31	positive
PRJNA729271	stage_1D_vs_E10	ordinary_muscle_development	ENSGALG00010013482	ENSGALG00010013482	-1.552362235	0.238315015	-6.513908635	7.322E-11	2.23578E-10	negative
PRJNA729271	stage_1D_vs_E10	ordinary_muscle_development	ENSGALG00010013483	ENSGALG00010013483	-0.541480562	0.20578846	-2.631248424	0.008507183	0.012687685	negative
PRJNA729271	stage_1D_vs_E10	ordinary_muscle_development	ENSGALG00010013484	TCOF1	-0.302633413	0.101638899	-2.977535321	0.002905762	0.004590128	negative
PRJNA729271	stage_1D_vs_E10	ordinary_muscle_development	ENSGALG00010013486	PLPP6	0.886366486	0.162187813	5.465062213	4.62744E-08	1.14697E-07	positive
PRJNA729271	stage_1D_vs_E10	ordinary_muscle_development	ENSGALG00010013487	ENSGALG00010013487	0.083799304	0.092296795	0.907932974	0.363913635	0.412877907	positive
PRJNA729271	stage_1D_vs_E10	ordinary_muscle_development	ENSGALG00010013488	UPRT	-0.608789878	0.119414391	-5.098128242	3.43028E-07	7.93393E-07	negative
PRJNA729271	stage_1D_vs_E10	ordinary_muscle_development	ENSGALG00010013491	CNOT11	-0.228871338	0.111789426	-2.047343343	0.040624385	0.054914712	negative
PRJNA729271	stage_1D_vs_E10	ordinary_muscle_development	ENSGALG00010013492	B3GALT5	-2.326465137	0.614330777	-3.786991024	0.000152483	0.000277982	negative
PRJNA729271	stage_1D_vs_E10	ordinary_muscle_development	ENSGALG00010013493	ENSGALG00010013493	0.851482199	0.127103034	6.699149303	2.09636E-11	6.64192E-11	positive
PRJNA729271	stage_1D_vs_E10	ordinary_muscle_development	ENSGALG00010013494	ASB1	-1.631219271	0.145926517	-11.17836089	5.20423E-29	4.30076E-28	negative
PRJNA729271	stage_1D_vs_E10	ordinary_muscle_development	ENSGALG00010013497	FAM172A	-0.069128377	0.107300349	-0.644251185	0.519412564	0.566763408	negative
PRJNA729271	stage_1D_vs_E10	ordinary_muscle_development	ENSGALG00010013498	KDR	1.116845562	0.124671683	8.958293757	3.29741E-19	1.68711E-18	positive
PRJNA729271	stage_1D_vs_E10	ordinary_muscle_development	ENSGALG00010013501	AKR1B10	-0.277052236	0.133001837	-2.08307075	0.037244775	0.050667575	negative
PRJNA729271	stage_1D_vs_E10	ordinary_muscle_development	ENSGALG00010013502	PAFAH2	0.867251632	0.208052074	4.168435403	3.06698E-05	5.97284E-05	positive
PRJNA729271	stage_1D_vs_E10	ordinary_muscle_development	ENSGALG00010013504	IGSF5	-1.678502092	0.807716291	-2.078083741	0.037701647	0.051231403	negative
PRJNA729271	stage_1D_vs_E10	ordinary_muscle_development	ENSGALG00010013506	RNF149	-0.444032165	0.104447153	-4.251261551	2.1257E-05	4.20183E-05	negative
PRJNA729271	stage_1D_vs_E10	ordinary_muscle_development	ENSGALG00010013508	TUBE1	0.071490287	0.191772847	0.372786285	0.709307511	0.745255276	positive
PRJNA729271	stage_1D_vs_E10	ordinary_muscle_development	ENSGALG00010013509	ENSGALG00010013509	-0.684755778	0.195807583	-3.49708509	0.000470372	0.000812542	negative
PRJNA729271	stage_1D_vs_E10	ordinary_muscle_development	ENSGALG00010013510	MRPS33	-0.34935511	0.088164538	-3.962535494	7.4158E-05	0.000139401	negative
PRJNA729271	stage_1D_vs_E10	ordinary_muscle_development	ENSGALG00010013511	FAM78B	1.22642303	0.226055218	5.425325014	5.78492E-08	1.42267E-07	positive
PRJNA729271	stage_1D_vs_E10	ordinary_muscle_development	ENSGALG00010013512	ENSGALG00010013512	-8.0713388	3.526157266	-2.288990023			negative
PRJNA729271	stage_1D_vs_E10	ordinary_muscle_development	ENSGALG00010013514	LMBRD2	0.663620057	0.135320395	4.904065322	9.38732E-07	2.10275E-06	positive
PRJNA729271	stage_1D_vs_E10	ordinary_muscle_development	ENSGALG00010013516	CSRP2	-0.376538914	0.13332756	-2.824164134	0.00474041	0.007305151	negative
PRJNA729271	stage_1D_vs_E10	ordinary_muscle_development	ENSGALG00010013517	STMN1	-2.612087946	0.217774701	-11.99445085	3.79929E-33	3.81112E-32	negative
PRJNA729271	stage_1D_vs_E10	ordinary_muscle_development	ENSGALG00010013520	CREG2	-0.49109858	0.186972008	-2.626588796	0.008624549	0.012852147	negative
PRJNA729271	stage_1D_vs_E10	ordinary_muscle_development	ENSGALG00010013523	TMEM178B	-1.763186118	0.187054908	-9.426035036	4.25884E-21	2.41896E-20	negative
PRJNA729271	stage_1D_vs_E10	ordinary_muscle_development	ENSGALG00010013524	ZDHHC15	0.111591927	0.122371027	0.911914608	0.361813671	0.410849132	positive
PRJNA729271	stage_1D_vs_E10	ordinary_muscle_development	ENSGALG00010013526	SIRPA	-0.824963795	0.259324578	-3.181201721	0.001466655	0.002399183	negative
PRJNA729271	stage_1D_vs_E10	ordinary_muscle_development	ENSGALG00010013527	ADARB1	0.073717409	0.099386231	0.741726579	0.458253013	0.507167857	positive
PRJNA729271	stage_1D_vs_E10	ordinary_muscle_development	ENSGALG00010013528	PRDX6	0.257127944	0.131631254	1.953395837	0.050772709	0.06764872	positive
PRJNA729271	stage_1D_vs_E10	ordinary_muscle_development	ENSGALG00010013531	ARSI	-2.882864809	0.238500956	-12.08743504	1.23069E-33	1.26248E-32	negative
PRJNA729271	stage_1D_vs_E10	ordinary_muscle_development	ENSGALG00010013532	WDR19	-0.232857302	0.073979136	-3.147607758	0.001646124	0.002677954	negative
PRJNA729271	stage_1D_vs_E10	ordinary_muscle_development	ENSGALG00010013533	SKP2	-0.834906466	0.08118453	-10.28405866	8.31506E-25	5.63209E-24	negative
PRJNA729271	stage_1D_vs_E10	ordinary_muscle_development	ENSGALG00010013537	LMO3	-2.446794172	0.409865232	-5.969752933	2.37613E-09	6.52311E-09	negative
PRJNA729271	stage_1D_vs_E10	ordinary_muscle_development	ENSGALG00010013538	E2F7	-1.776659333	0.265297049	-6.696868059	2.12934E-11	6.7405E-11	negative
PRJNA729271	stage_1D_vs_E10	ordinary_muscle_development	ENSGALG00010013539	NSFL1C	-0.468107291	0.092470911	-5.062211302	4.14421E-07	9.52138E-07	negative
PRJNA729271	stage_1D_vs_E10	ordinary_muscle_development	ENSGALG00010013544	ENSGALG00010013544	-0.438164044	0.287567144	-1.523693002	0.127585418	0.159248311	negative
PRJNA729271	stage_1D_vs_E10	ordinary_muscle_development	ENSGALG00010013546	IDUA	0.225983646	0.131119073	1.723499423	0.084798249	0.109170497	positive
PRJNA729271	stage_1D_vs_E10	ordinary_muscle_development	ENSGALG00010013549	PCP4	-3.131028066	0.303338954	-10.32187928	5.61139E-25	3.84573E-24	negative
PRJNA729271	stage_1D_vs_E10	ordinary_muscle_development	ENSGALG00010013550	KLHL5	-0.454670328	0.124858885	-3.641473546	0.000271082	0.000479714	negative
PRJNA729271	stage_1D_vs_E10	ordinary_muscle_development	ENSGALG00010013551	UBE2N	-0.68749209	0.111782788	-6.150249997	7.73609E-10	2.20347E-09	negative
PRJNA729271	stage_1D_vs_E10	ordinary_muscle_development	ENSGALG00010013552	FGF16	-0.842167167	0.40655119	-2.071491087	0.038312927	0.051998677	negative
PRJNA729271	stage_1D_vs_E10	ordinary_muscle_development	ENSGALG00010013553	TADA1	-0.191381613	0.112427763	-1.702262925	0.088706092	0.113777081	negative
PRJNA729271	stage_1D_vs_E10	ordinary_muscle_development	ENSGALG00010013554	ENSGALG00010013554	-4.708307854	5.466207082	-0.861348241	0.389046274	0.438202327	negative
PRJNA729271	stage_1D_vs_E10	ordinary_muscle_development	ENSGALG00010013556	RASL11B	-2.440832416	0.167519671	-14.57042266	4.33202E-48	7.71409E-47	negative
PRJNA729271	stage_1D_vs_E10	ordinary_muscle_development	ENSGALG00010013558	SNAPC3	-0.31909767	0.084225257	-3.788622078	0.000151485	0.000276233	negative
PRJNA729271	stage_1D_vs_E10	ordinary_muscle_development	ENSGALG00010013561	AGK	-0.211202564	0.10855718	-1.945542109	0.051709767	0.068802375	negative
PRJNA729271	stage_1D_vs_E10	ordinary_muscle_development	ENSGALG00010013562	RERGL	0.332280606	0.158586662	2.095261997	0.036147709	0.049276877	positive
PRJNA729271	stage_1D_vs_E10	ordinary_muscle_development	ENSGALG00010013563	FKBP1A	0.04705038	0.099185231	0.474368806	0.635236934	0.676245546	positive
PRJNA729271	stage_1D_vs_E10	ordinary_muscle_development	ENSGALG00010013565	ENSGALG00010013565	-0.830257743	5.324803546	-0.155922699	0.876093953	0.894231522	negative
PRJNA729271	stage_1D_vs_E10	ordinary_muscle_development	ENSGALG00010013569	ENSGALG00010013569	-1.018152009	0.536547722	-1.897598232	0.057749027	0.076173642	negative
PRJNA729271	stage_1D_vs_E10	ordinary_muscle_development	ENSGALG00010013572	DENND11	0.497153977	0.102407292	4.854673598	1.20585E-06	2.67465E-06	positive
PRJNA729271	stage_1D_vs_E10	ordinary_muscle_development	ENSGALG00010013573	ENSGALG00010013573	-3.920942078	0.581279285	-6.745366952	1.52641E-11	4.89173E-11	negative
PRJNA729271	stage_1D_vs_E10	ordinary_muscle_development	ENSGALG00010013574	NR2F1	-1.181903661	0.151100208	-7.821985669	5.19965E-15	2.08605E-14	negative
PRJNA729271	stage_1D_vs_E10	ordinary_muscle_development	ENSGALG00010013576	CAMK2A	0.301478997	0.186085954	1.620106137	0.105209479	0.133281892	positive
PRJNA729271	stage_1D_vs_E10	ordinary_muscle_development	ENSGALG00010013579	H2AC32	-1.23727037	0.176763931	-6.999563573	2.56761E-12	8.64318E-12	negative
PRJNA729271	stage_1D_vs_E10	ordinary_muscle_development	ENSGALG00010013580	RANBP3L	1.117839335	0.30195497	3.702006747	0.000213901	0.00038273	positive
PRJNA729271	stage_1D_vs_E10	ordinary_muscle_development	ENSGALG00010013582	MAP4K4	-0.626182541	0.106695974	-5.86884885	4.38831E-09	1.17968E-08	negative
PRJNA729271	stage_1D_vs_E10	ordinary_muscle_development	ENSGALG00010013583	FASLG	-0.316519089	0.375894479	-0.8420424	0.399764221	0.449227256	negative
PRJNA729271	stage_1D_vs_E10	ordinary_muscle_development	ENSGALG00010013587	SLC26A1	-1.066965914	0.523546178	-2.037959515	0.041553982	0.05610852	negative
PRJNA729271	stage_1D_vs_E10	ordinary_muscle_development	ENSGALG00010013589	DSCAM	-3.926621785	0.625823272	-6.274330083	3.51143E-10	1.02452E-09	negative
PRJNA729271	stage_1D_vs_E10	ordinary_muscle_development	ENSGALG00010013592	H3C33	-1.363503647	0.18384161	-7.416730344	1.20047E-13	4.41645E-13	negative
PRJNA729271	stage_1D_vs_E10	ordinary_muscle_development	ENSGALG00010013596	SDCBP2	-0.126842458	0.121640821	-1.042762262	0.297058414	0.343946138	negative
PRJNA729271	stage_1D_vs_E10	ordinary_muscle_development	ENSGALG00010013598	PAQR7	1.322251906	0.227549787	5.810824635	6.21659E-09	1.65399E-08	positive
PRJNA729271	stage_1D_vs_E10	ordinary_muscle_development	ENSGALG00010013604	FYN	-0.506366107	0.111419744	-4.544671233	5.50211E-06	1.15004E-05	negative
PRJNA729271	stage_1D_vs_E10	ordinary_muscle_development	ENSGALG00010013609	PIGC	0.528950741	0.163681699	3.23158144	0.001231072	0.002033317	positive
PRJNA729271	stage_1D_vs_E10	ordinary_muscle_development	ENSGALG00010013612	TWIST2	-4.071944722	0.290377992	-14.02291093	1.12888E-44	1.7426E-43	negative
PRJNA729271	stage_1D_vs_E10	ordinary_muscle_development	ENSGALG00010013614	ATRX	-0.442844537	0.086559748	-5.116056243	3.1199E-07	7.23567E-07	negative
PRJNA729271	stage_1D_vs_E10	ordinary_muscle_development	ENSGALG00010013617	PIK3C2G	0.153698209	0.161849341	0.949637534	0.34229646	0.391199809	positive
PRJNA729271	stage_1D_vs_E10	ordinary_muscle_development	ENSGALG00010013619	IL1R2	1.108666375	0.19472077	5.69362157	1.24373E-08	3.22328E-08	positive
PRJNA729271	stage_1D_vs_E10	ordinary_muscle_development	ENSGALG00010013621	PDGFRA	0.579474764	0.164595572	3.520597528	0.000430576	0.000746019	positive
PRJNA729271	stage_1D_vs_E10	ordinary_muscle_development	ENSGALG00010013624	SST1	-8.67699827	1.740043611	-4.98665563	6.14334E-07	1.39442E-06	negative
PRJNA729271	stage_1D_vs_E10	ordinary_muscle_development	ENSGALG00010013625	SSBP1	-0.821562751	0.113040643	-7.267852757	3.65247E-13	1.304E-12	negative
PRJNA729271	stage_1D_vs_E10	ordinary_muscle_development	ENSGALG00010013627	MYCBP	-0.198998339	0.147885157	-1.345627531	0.178422668	0.216711629	negative
PRJNA729271	stage_1D_vs_E10	ordinary_muscle_development	ENSGALG00010013632	SLC1A3	-4.79907857	0.524892003	-9.142982832	6.0753E-20	3.23994E-19	negative
PRJNA729271	stage_1D_vs_E10	ordinary_muscle_development	ENSGALG00010013635	BCL6	0.702413096	0.159234629	4.411183034	1.02807E-05	2.09591E-05	positive
PRJNA729271	stage_1D_vs_E10	ordinary_muscle_development	ENSGALG00010013638	COMMD8	-0.862054413	0.118296696	-7.287223055	3.16409E-13	1.13411E-12	negative
PRJNA729271	stage_1D_vs_E10	ordinary_muscle_development	ENSGALG00010013639	ENSGALG00010013639	1.177596852	0.445216885	2.644995936	0.00816919	0.01221628	positive
PRJNA729271	stage_1D_vs_E10	ordinary_muscle_development	ENSGALG00010013640	SNPH	-3.996656009	0.862948054	-4.631398138	3.63205E-06	7.72963E-06	negative
PRJNA729271	stage_1D_vs_E10	ordinary_muscle_development	ENSGALG00010013645	RRAGC	-0.199903009	0.120845509	-1.654203045	0.098086264	0.124869173	negative
PRJNA729271	stage_1D_vs_E10	ordinary_muscle_development	ENSGALG00010013646	CLDN1	0.546052414	0.335768107	1.626278384	0.103890448	0.131783674	positive
PRJNA729271	stage_1D_vs_E10	ordinary_muscle_development	ENSGALG00010013648	NAV3	-0.927592334	0.118686396	-7.815489958	5.47496E-15	2.19225E-14	negative
PRJNA729271	stage_1D_vs_E10	ordinary_muscle_development	ENSGALG00010013650	BACE2	-0.821467883	0.160518278	-5.11759715	3.09453E-07	7.18142E-07	negative
PRJNA729271	stage_1D_vs_E10	ordinary_muscle_development	ENSGALG00010013651	ENSGALG00010013651	0.297853501	0.156623011	1.901722486	0.057207445	0.075507367	positive
PRJNA729271	stage_1D_vs_E10	ordinary_muscle_development	ENSGALG00010013654	SLC6A7	-3.270695638	0.505199989	-6.474061177	9.54032E-11	2.89001E-10	negative
PRJNA729271	stage_1D_vs_E10	ordinary_muscle_development	ENSGALG00010013657	MAGT1	-0.397617275	0.104299595	-3.812260985	0.000137701	0.000251954	negative
PRJNA729271	stage_1D_vs_E10	ordinary_muscle_development	ENSGALG00010013661	GABRA4	-1.758017152	0.37895005	-4.639179095	3.49796E-06	7.45302E-06	negative
PRJNA729271	stage_1D_vs_E10	ordinary_muscle_development	ENSGALG00010013663	OPA1	0.369000764	0.106168692	3.475608101	0.000509697	0.000876816	positive

PRJNA729271	stage_1D_vs_E10	ordinary_muscle_development	ENSGALG00010013664	REV3L	0.722325	0.095797088	7.540156131	4.69409E-14	1.77467E-13	positive
PRJNA729271	stage_1D_vs_E10	ordinary_muscle_development	ENSGALG00010013666	COX7B	0.962899175	0.143373449	6.716021581	1.86753E-11	5.93507E-11	positive
PRJNA729271	stage_1D_vs_E10	ordinary_muscle_development	ENSGALG00010013667	SOWAHA	-5.201676618	0.922732446	-5.637253398	1.72784E-08	4.42648E-08	negative
PRJNA729271	stage_1D_vs_E10	ordinary_muscle_development	ENSGALG00010013669	NPR3	-0.380213086	0.255690972	-1.487002387	0.13701418	0.169977794	negative
PRJNA729271	stage_1D_vs_E10	ordinary_muscle_development	ENSGALG00010013672	ENSGALG00010013672	-5.624459566	0.54275205	-10.36285274	3.65884E-25	2.53392E-24	negative
PRJNA729271	stage_1D_vs_E10	ordinary_muscle_development	ENSGALG00010013675	CHRNA6	1.364713093	0.364823203	3.740751908	0.000183471	0.00033139	positive
PRJNA729271	stage_1D_vs_E10	ordinary_muscle_development	ENSGALG00010013677	GABRG1	-3.203739139	0.440397578	-7.274652039	3.47315E-13	1.24151E-12	negative
PRJNA729271	stage_1D_vs_E10	ordinary_muscle_development	ENSGALG00010013678	ATP7A	0.162908923	0.101731537	1.601361067	0.109296971	0.137953639	positive
PRJNA729271	stage_1D_vs_E10	ordinary_muscle_development	ENSGALG00010013679	MFSD4B	0.600975816	0.214635293	2.799986003	0.005110482	0.007847912	positive
PRJNA729271	stage_1D_vs_E10	ordinary_muscle_development	ENSGALG00010013686	UQCRQ	2.377735925	0.137905081	17.24182969	1.28892E-66	4.1984E-65	positive
PRJNA729271	stage_1D_vs_E10	ordinary_muscle_development	ENSGALG00010013690	CREB3L2	-0.597277163	0.101544711	-5.881913069	4.05551E-09	1.09204E-08	negative
PRJNA729271	stage_1D_vs_E10	ordinary_muscle_development	ENSGALG00010013696	CETN3	-0.377929767	0.109321933	-3.457035175	0.000546154	0.000936974	negative
PRJNA729271	stage_1D_vs_E10	ordinary_muscle_development	ENSGALG00010013697	HDAC4	1.002450762	0.066022827	15.18339643	4.55568E-52	9.44702E-51	positive
PRJNA729271	stage_1D_vs_E10	ordinary_muscle_development	ENSGALG00010013698	TFAP2E	-6.28672153	0.955685501	-6.578232616	4.76073E-11	1.47482E-10	negative
PRJNA729271	stage_1D_vs_E10	ordinary_muscle_development	ENSGALG00010013709	PSMF1	0.235767547	0.100651337	2.342418433	0.019159221	0.027173475	positive
PRJNA729271	stage_1D_vs_E10	ordinary_muscle_development	ENSGALG00010013715	IL1R1	0.721590763	0.233385157	3.091845137	0.001989166	0.003203662	positive
PRJNA729271	stage_1D_vs_E10	ordinary_muscle_development	ENSGALG00010013719	NCDN	-0.271898878	0.139746762	-1.945654235	0.051696287	0.068790754	negative
PRJNA729271	stage_1D_vs_E10	ordinary_muscle_development	ENSGALG00010013720	SLC16A10	0.286171338	0.189977655	1.506342088	0.131979379	0.164308262	positive
PRJNA729271	stage_1D_vs_E10	ordinary_muscle_development	ENSGALG00010013732	FNTA	0.052630203	0.075738812	0.694890795	0.487123742	0.535603325	positive
PRJNA729271	stage_1D_vs_E10	ordinary_muscle_development	ENSGALG00010013737	AFF4	0.865703231	0.084172185	10.2849086	8.24202E-25	5.58523E-24	positive
PRJNA729271	stage_1D_vs_E10	ordinary_muscle_development	ENSGALG00010013740	GNPDA2	-1.474110611	0.120690704	-12.21395316	2.6185E-34	2.75237E-33	negative
PRJNA729271	stage_1D_vs_E10	ordinary_muscle_development	ENSGALG00010013742	RSPO4	0.156066679	1.11465276	0.140013719	0.888649151	0.905007338	positive
PRJNA729271	stage_1D_vs_E10	ordinary_muscle_development	ENSGALG00010013744	ENSGALG00010013744	-0.518636999	0.31286047	-1.657726206	0.097372742	0.124069787	negative
PRJNA729271	stage_1D_vs_E10	ordinary_muscle_development	ENSGALG00010013746	ENSGALG00010013746	0.386030835	0.086592869	4.457998002	8.27287E-06	1.70407E-05	positive
PRJNA729271	stage_1D_vs_E10	ordinary_muscle_development	ENSGALG00010013749	NELL2	-3.748019194	0.465740894	-8.047434192	8.45478E-16	3.56878E-15	negative
PRJNA729271	stage_1D_vs_E10	ordinary_muscle_development	ENSGALG00010013751	FUT10	0.18821109	0.169361159	1.111300203	0.266439152	0.311630397	positive
PRJNA729271	stage_1D_vs_E10	ordinary_muscle_development	ENSGALG00010013753	PLEKHA5	1.013294295	0.123406399	8.211035284	2.19289E-16	9.60012E-16	positive
PRJNA729271	stage_1D_vs_E10	ordinary_muscle_development	ENSGALG00010013755	ANGPT4	2.777801029	0.152395938	18.22752669	3.12121E-74	1.28528E-72	positive
PRJNA729271	stage_1D_vs_E10	ordinary_muscle_development	ENSGALG00010013757	DGKI	-0.269614357	0.225921122	-1.193400398	0.232712599	0.276043563	negative
PRJNA729271	stage_1D_vs_E10	ordinary_muscle_development	ENSGALG00010013758	FAM3B	-5.126513535	0.885586434	-5.788834766	7.08764E-09	1.87713E-08	negative
PRJNA729271	stage_1D_vs_E10	ordinary_muscle_development	ENSGALG00010013760	RPF2	-0.75947428	0.115217038	-6.591683796	4.34866E-11	1.35034E-10	negative
PRJNA729271	stage_1D_vs_E10	ordinary_muscle_development	ENSGALG00010013766	DNM3	-0.069988723	0.106703797	-0.655915957	0.511878201	0.559764204	negative
PRJNA729271	stage_1D_vs_E10	ordinary_muscle_development	ENSGALG00010013767	PGK2	1.038708566	0.172021731	6.038240398	1.55804E-09	4.33969E-09	positive
PRJNA729271	stage_1D_vs_E10	ordinary_muscle_development	ENSGALG00010013768	FAM110A	0.186012617	0.220361895	0.844123332	0.398600491	0.447988999	positive
PRJNA729271	stage_1D_vs_E10	ordinary_muscle_development	ENSGALG00010013771	PELO	-0.396044704	0.10381967	-3.814736697	0.000136328	0.000249599	negative
PRJNA729271	stage_1D_vs_E10	ordinary_muscle_development	ENSGALG00010013772	KIAA0319L	0.651026962	0.143347263	4.541607209	5.5827E-06	1.1665E-05	positive
PRJNA729271	stage_1D_vs_E10	ordinary_muscle_development	ENSGALG00010013775	DBX2	-3.660222963	0.451499953	-8.10680696	5.19673E-16	2.23124E-15	negative
PRJNA729271	stage_1D_vs_E10	ordinary_muscle_development	ENSGALG00010013777	CPLANE1	-0.68120249	0.094266897	-7.226317104	4.96268E-13	1.75749E-12	negative
PRJNA729271	stage_1D_vs_E10	ordinary_muscle_development	ENSGALG00010013778	ENSGALG00010013778	-1.924622677	0.667936145	-2.881447115	0.003958537	0.006155883	negative
PRJNA729271	stage_1D_vs_E10	ordinary_muscle_development	ENSGALG00010013779	SLC52A3	-0.602264777	0.243466138	-2.473710643	0.013371795	0.019421259	negative
PRJNA729271	stage_1D_vs_E10	ordinary_muscle_development	ENSGALG00010013783	NDUFA10	0.827363195	0.095604592	8.654011085	4.97204E-18	2.38879E-17	positive
PRJNA729271	stage_1D_vs_E10	ordinary_muscle_development	ENSGALG00010013784	ANO6	1.446469972	0.094395588	15.32349138	5.32772E-53	1.14239E-51	positive
PRJNA729271	stage_1D_vs_E10	ordinary_muscle_development	ENSGALG00010013787	ZCCHC10	-0.004924443	0.102312843	-0.048131232	0.961611655	0.967216775	negative
PRJNA729271	stage_1D_vs_E10	ordinary_muscle_development	ENSGALG00010013791	AEBP2	0.407222196	0.062727652	6.491908754	8.47556E-11	2.57932E-10	positive
PRJNA729271	stage_1D_vs_E10	ordinary_muscle_development	ENSGALG00010013792	FGF10	0.941678273	0.225784416	4.170696503	3.0367E-05	5.91626E-05	positive
PRJNA729271	stage_1D_vs_E10	ordinary_muscle_development	ENSGALG00010013793	METTL13	-0.784688948	0.129200266	-6.073431342	1.25206E-09	3.509E-09	negative
PRJNA729271	stage_1D_vs_E10	ordinary_muscle_development	ENSGALG00010013794	GTF3C6	-1.474121681	0.181529099	-8.120580624	4.63959E-16	1.99735E-15	negative
PRJNA729271	stage_1D_vs_E10	ordinary_muscle_development	ENSGALG00010013797	IL1RL1	1.263318316	0.315570531	4.003283555	6.24694E-05	0.000118365	positive
PRJNA729271	stage_1D_vs_E10	ordinary_muscle_development	ENSGALG00010013802	PYROXD1	-0.409339681	0.14386441	-2.845315816	0.004436741	0.006869319	negative
PRJNA729271	stage_1D_vs_E10	ordinary_muscle_development	ENSGALG00010013805	HSPA4	0.158278082	0.145268213	1.089557576	0.27590808	0.321589548	positive
PRJNA729271	stage_1D_vs_E10	ordinary_muscle_development	ENSGALG00010013808	PLAAT1	2.054464604	0.13500556	15.21762955	2.70143E-52	5.65856E-51	positive
PRJNA729271	stage_1D_vs_E10	ordinary_muscle_development	ENSGALG00010013809	MX1	4.323547605	1.2132868	3.563500077	0.000365943	0.000639616	positive
PRJNA729271	stage_1D_vs_E10	ordinary_muscle_development	ENSGALG00010013814	AMD1	0.554304654	0.109609896	5.057067604	4.25752E-07	9.77087E-07	positive
PRJNA729271	stage_1D_vs_E10	ordinary_muscle_development	ENSGALG00010013815	SRXN1	-0.46426589	0.252559575	-1.838243071	0.066026596	0.086415847	negative
PRJNA729271	stage_1D_vs_E10	ordinary_muscle_development	ENSGALG00010013820	MASP1	-3.512366762	0.494043314	-7.109430815	1.16522E-12	4.00141E-12	negative
PRJNA729271	stage_1D_vs_E10	ordinary_muscle_development	ENSGALG00010013823	GUF1	0.254208405	0.111023555	2.289679931	0.022039878	0.031028363	positive
PRJNA729271	stage_1D_vs_E10	ordinary_muscle_development	ENSGALG00010013828	PTN	-5.403295307	0.27404084	-19.71711699	1.53746E-86	8.47357E-85	negative
PRJNA729271	stage_1D_vs_E10	ordinary_muscle_development	ENSGALG00010013831	TCERG1	0.068970047	0.112803647	0.611416819	0.540923671	0.587185547	positive
PRJNA729271	stage_1D_vs_E10	ordinary_muscle_development	ENSGALG00010013833	TMPRSS2	-3.722424909	1.330114275	-2.798575265	0.005132861	0.007880509	negative
PRJNA729271	stage_1D_vs_E10	ordinary_muscle_development	ENSGALG00010013836	IL18R1	-0.896908205	0.269853863	-3.323681175	0.000888377	0.001489362	negative
PRJNA729271	stage_1D_vs_E10	ordinary_muscle_development	ENSGALG00010013837	IFI27L2	0.636398046	0.33761789	1.884965414	0.059434507	0.078282888	positive
PRJNA729271	stage_1D_vs_E10	ordinary_muscle_development	ENSGALG00010013842	CDK19	0.758915729	0.1104883	6.868742936	6.47701E-12	2.11881E-11	positive
PRJNA729271	stage_1D_vs_E10	ordinary_muscle_development	ENSGALG00010013846	YIPF7	7.913270067	0.319092287	24.79931477	9.1188E-136	1.2833E-133	positive
PRJNA729271	stage_1D_vs_E10	ordinary_muscle_development	ENSGALG00010013848	ITPA	-1.13691467	0.100853488	-11.27293362	1.78521E-29	1.50183E-28	negative
PRJNA729271	stage_1D_vs_E10	ordinary_muscle_development	ENSGALG00010013857	IL18RAP	-1.986282884	0.906234353	-2.191798267	0.028394076	0.039257167	negative
PRJNA729271	stage_1D_vs_E10	ordinary_muscle_development	ENSGALG00010013859	MYOC	2.593393616	0.242978264	10.67335643	1.35637E-26	9.97996E-26	positive
PRJNA729271	stage_1D_vs_E10	ordinary_muscle_development	ENSGALG00010013861	TCF15	1.811045092	0.155969789	11.61151213	3.60189E-31	3.28568E-30	positive
PRJNA729271	stage_1D_vs_E10	ordinary_muscle_development	ENSGALG00010013866	KCTD8	-3.219046769	0.420412654	-7.656874125	1.90513E-14	7.3738E-14	negative
PRJNA729271	stage_1D_vs_E10	ordinary_muscle_development	ENSGALG00010013867	RECQL	-0.652991926	0.124120046	-5.26097072	1.43297E-07	3.42245E-07	negative
PRJNA729271	stage_1D_vs_E10	ordinary_muscle_development	ENSGALG00010013871	ENSGALG00010013871	0.856781203	0.222814456	3.84526758	0.000120421	0.000221735	positive
PRJNA729271	stage_1D_vs_E10	ordinary_muscle_development	ENSGALG00010013872	GLS2	0.130489698	0.197363176	0.661165373	0.508506265	0.556454651	positive
PRJNA729271	stage_1D_vs_E10	ordinary_muscle_development	ENSGALG00010013878	ARID2	0.193965476	0.081401028	2.382838174	0.017179745	0.024548541	positive
PRJNA729271	stage_1D_vs_E10	ordinary_muscle_development	ENSGALG00010013880	ZMYM4	-0.401747593	0.089265968	-4.50056837	6.7772E-06	1.40718E-05	negative
PRJNA729271	stage_1D_vs_E10	ordinary_muscle_development	ENSGALG00010013889	ENSGALG00010013889	-0.227131336	0.421457257	-0.538919029	0.589942726	0.633799275	negative
PRJNA729271	stage_1D_vs_E10	ordinary_muscle_development	ENSGALG00010013890	SFPQ	-0.942933475	0.072029189	-13.09099117	3.70738E-39	4.72634E-38	negative
PRJNA729271	stage_1D_vs_E10	ordinary_muscle_development	ENSGALG00010013893	CSNK2A1	-0.473951167	0.078990056	-6.000137098	1.97151E-09	5.44843E-09	negative
PRJNA729271	stage_1D_vs_E10	ordinary_muscle_development	ENSGALG00010013897	AMOT	-0.783869158	0.074624838	-10.50413205	8.26798E-26	5.86036E-25	negative
PRJNA729271	stage_1D_vs_E10	ordinary_muscle_development	ENSGALG00010013898	EMB	-2.185437467	0.297029981	-7.357632585	1.872E-13	6.79727E-13	negative
PRJNA729271	stage_1D_vs_E10	ordinary_muscle_development	ENSGALG00010013899	WNT5B	-0.453945762	0.142180835	-3.192735241	0.001409321	0.002311133	negative
PRJNA729271	stage_1D_vs_E10	ordinary_muscle_development	ENSGALG00010013901	NFXL1	-0.278325544	0.121253532	-2.295401537	0.021710123	0.030576004	negative
PRJNA729271	stage_1D_vs_E10	ordinary_muscle_development	ENSGALG00010013908	TBC1D20	0.663186444	0.115675294	5.733172729	9.85692E-09	2.57017E-08	positive
PRJNA729271	stage_1D_vs_E10	ordinary_muscle_development	ENSGALG00010013910	ADIPOR2	1.110155217	0.207978906	5.337826033	9.40677E-08	2.27517E-07	positive
PRJNA729271	stage_1D_vs_E10	ordinary_muscle_development	ENSGALG00010013911	TNIK	0.055568311	0.14800724	0.375443196	0.707330885	0.743600317	positive
PRJNA729271	stage_1D_vs_E10	ordinary_muscle_development	ENSGALG00010013914	NRG1	2.183693481	0.22635828	9.647066949	5.05818E-22	3.02968E-21	positive
PRJNA729271	stage_1D_vs_E10	ordinary_muscle_development	ENSGALG00010013915	ENSGALG00010013915	1.680700408	0.188850336	8.899642144	5.6027E-19	2.82867E-18	positive
PRJNA729271	stage_1D_vs_E10	ordinary_muscle_development	ENSGALG00010013917	TRPC5	-0.865308388	0.492513213	-1.756924211	0.078930728	0.102160988	negative
PRJNA729271	stage_1D_vs_E10	ordinary_muscle_development	ENSGALG00010013921	PPP2R2B	0.988414802	0.105082399	9.406092844	5.14923E-21	2.91555E-20	positive
PRJNA729271	stage_1D_vs_E10	ordinary_muscle_development	ENSGALG00010013922	ENSGALG00010013922	-2.890193985	0.895224287	-3.22845797	0.001244595	0.002054483	negative
PRJNA729271	stage_1D_vs_E10	ordinary_muscle_development	ENSGALG00010013923	FMR1NB	1.928141842	0.495185623	3.893775896	9.86958E-05	0.000183105	positive
PRJNA729271	stage_1D_vs_E10	ordinary_muscle_development	ENSGALG00010013928	CCNO	3.871346562	0.455303538	8.502781637	1.85101E-17	8.59948E-17	positive
PRJNA729271	stage_1D_vs_E10	ordinary_muscle_development	ENSGALG00010013930	SLC22A16	2.590770053	0.30329357	8.542119951	1.31782E-17	6.16981E-17	positive
PRJNA729271	stage_1D_vs_E10	ordinary_muscle_development	ENSGALG00010013934	STAT1	0.328354945	0.282915042	1.160613242	0.245799218	0.289945427	positive
PRJNA729271	stage_1D_vs_E10	ordinary_muscle_development	ENSGALG00010013936	DLGAP3	-2.092476191	0.384377482	-5.443805349	5.21542E-08	1.28699E-07	negative
PRJNA729271	stage_1D_vs_E10	ordinary_muscle_development	ENSGALG00010013938	ENSGALG00010013938	0.627438657	0.407757161	1.538755702	0.123863948	0.155070645	positive
PRJNA729271	stage_1D_vs_E10	ordinary_muscle_development	ENSGALG00010013940	UPF3A	-0.26024991	0.232607237	-1.118838406	0.263209084	0.308350091	negative
PRJNA729271	stage_1D_vs_E10	ordinary_muscle_development	ENSGALG00010013942	CACNA2D4	2.854932529	0.446905689	6.388221492	1.67826E-10	5.00337E-10	positive
PRJNA729271	stage_1D_vs_E10	ordinary_muscle_development	ENSGALG00010013944	METTL24	-1.508734283	0.215860359	-6.989399486	2.76065E-12	9.27792E-12	negative
PRJNA729271	stage_1D_vs_E10	ordinary_muscle_development	ENSGALG00010013945	SCAF11	-0.126078841	0.076723279	-1.643293193	0.100322286	0.127547718	negative
PRJNA729271	stage_1D_vs_E10	ordinary_muscle_development	ENSGALG00010013947	ENSGALG00010013947	-0.101906308	0.176716405	-0.576665807	0.564165235	0.609584427	negative
PRJNA729271	stage_1D_vs_E10	ordinary_muscle_development	ENSGALG00010013949	PRRC2C	0.027538575	0.12608122	0.218419328	0.827102407	0.851662243	positive
PRJNA729271	stage_1D_vs_E10	ordinary_muscle_development	ENSGALG00010013953	DHX29	-0.11710141	0.08084374	-1.448490759	0.147479849	0.182150682	negative
PRJNA729271	stage_1D_vs_E10	ordinary_muscle_development	ENSGALG00010013954	LPL	1.719978296	0.333987368	5.149830382	2.60722E-07	6.0866E-07	positive
PRJNA729271	stage_1D_vs_E10	ordinary_muscle_development	ENSGALG00010013958	TUSC3	-0.092675369	0.178093294	-0.5203754	0.602801953	0.645700141	negative
PRJNA729271	stage_1D_vs_E10	ordinary_muscle_development	ENSGALG00010013959	STK32A	-2.512854528	0.508468343	-4.942007821	7.73221E-07	1.74251E-06	negative
PRJNA729271	stage_1D_vs_E10	ordinary_muscle_development	ENSGALG00010013964	SLC38A1	-1.166176923	0.160545912	-7.263821969	3.76303E-13	1.34248E-12	negative
PRJNA729271	stage_1D_vs_E10	ordinary_muscle_development	ENSGALG00010013965	SLC2A2	0.368968867	1.059735309	0.348170778			positive
PRJNA729271	stage_1D_vs_E10	ordinary_muscle_development	ENSGALG00010013967	MLLT3	-0.636953237	0.154654317	-4.118561003	3.81246E-05	7.36174E-05	negative
PRJNA729271	stage_1D_vs_E10	ordinary_muscle_development	ENSGALG00010013971	ENSGALG00010013971	-1.222939653	0.384167261	-3.183352087	0.001455805	0.002383048	negative
PRJNA729271	stage_1D_vs_E10	ordinary_muscle_development	ENSGALG00010013973	ENSGALG00010013973	0.762042075	0.238611602	3.193650556	0.001404861	0.002305645	positive
PRJNA729271	stage_1D_vs_E10	ordinary_muscle_development	ENSGALG00010013976	CDC40	-0.193745966	0.074957679	-2.584738047	0.009745301	0.014427345	negative
PRJNA729271	stage_1D_vs_E10	ordinary_muscle_development	ENSGALG00010013977	SLC38A2	1.64920444	0.192562752	8.56450389	1.08542E-17	5.10652E-17	positive
PRJNA729271	stage_1D_vs_E10	ordinary_muscle_development	ENSGALG00010013980	FMO4	0.565576221	0.385198073	1.468273755	0.142029877	0.175808972	positive
PRJNA729271	stage_1D_vs_E10	ordinary_muscle_development	ENSGALG00010013981	RLF	0.222324034	0.107079069	2.076260426	0.037869871	0.051435887	positive
PRJNA729271	stage_1D_vs_E10	ordinary_muscle_development	ENSGALG00010013989	ENSGALG00010013989	1.665857433	0.200536226	8.307014984	9.81396E-17	4.37943E-16	positive
PRJNA729271	stage_1D_vs_E10	ordinary_muscle_development	ENSGALG00010013994	CHRM2	-3.153383134	0.425689144	-7.407713301	1.28496E-13	4.71889E-13	negative
PRJNA729271	stage_1D_vs_E10	ordinary_muscle_development	ENSGALG00010013995	REM1	0.399926673	0.210026019	1.904176798	0.056887162	0.075143568	positive
PRJNA729271	stage_1D_vs_E10	ordinary_muscle_development	ENSGALG00010014000	STAT4	3.680286894	1.019402656	3.610238675	0.000305915	0.000538594	positive
PRJNA729271	stage_1D_vs_E10	ordinary_muscle_development	ENSGALG00010014001	CDC16	-0.822586926	0.085017607	-9.675489088	3.83255E-22	2.30605E-21	negative
PRJNA729271	stage_1D_vs_E10	ordinary_muscle_development	ENSGALG00010014002	ZDHHC18	-0.219652372	0.269679037	-0.814495539	0.415361127	0.464812139	negative
PRJNA729271	stage_1D_vs_E10	ordinary_muscle_development	ENSGALG00010014010	EIF5A2	-0.623088418	0.102471634	-6.080594146	1.19738E-09	3.36031E-09	negative
PRJNA729271	stage_1D_vs_E10	ordinary_muscle_development	ENSGALG00010014011	SFN	-6.034182347	0.651459204	-9.262563658	1.99582E-20	1.09085E-19	negative
PRJNA729271	stage_1D_vs_E10	ordinary_muscle_development	ENSGALG00010014014	PARP8	-0.070726366	0.201431202	-0.351119216	0.725498912	0.75970232	negative
PRJNA729271	stage_1D_vs_E10	ordinary_muscle_development	ENSGALG00010014018	DPYSL3	-2.342505665	0.239419373	-9.784110754	1.31748E-22	8.09191E-22	negative
PRJNA729271	stage_1D_vs_E10	ordinary_muscle_development	ENSGALG00010014019	IRAG2	-0.301349473	0.244606552	-1.231976295	0.217957939	0.260196041	negative
PRJNA729271	stage_1D_vs_E10	ordinary_muscle_development	ENSGALG00010014020	PDXP	-0.220422077	0.100318801	-2.19721603	0.028005021	0.038748834	negative
PRJNA729271	stage_1D_vs_E10	ordinary_muscle_development	ENSGALG00010014026	SH3BP1	0.141119581	0.391689327	0.360284469	0.718634413	0.753897793	positive
PRJNA729271	stage_1D_vs_E10	ordinary_muscle_development	ENSGALG00010014030	WASF1	-1.734275354	0.333808963	-5.195412784	2.04266E-07	4.81515E-07	negative
PRJNA729271	stage_1D_vs_E10	ordinary_muscle_development	ENSGALG00010014031	RPL22L1	-0.580808749	0.188583727	-3.079845527	0.00207108	0.003326349	negative
PRJNA729271	stage_1D_vs_E10	ordinary_muscle_development	ENSGALG00010014032	PSD3	-0.81249026	0.077944992	-10.42389301	1.92892E-25	1.35006E-24	negative
PRJNA729271	stage_1D_vs_E10	ordinary_muscle_development	ENSGALG00010014033	SLC38A4	4.177764056	0.473264566	8.827544579	1.06999E-18	5.32422E-18	positive
PRJNA729271	stage_1D_vs_E10	ordinary_muscle_development	ENSGALG00010014035	PSIP1	-0.849903229	0.117530505	-7.231341593	4.78246E-13	1.69615E-12	negative

PRJNA729271	stage_1D_vs_E10	ordinary_muscle_development	ENSGALG00010014041	SKIV2L2	0.129188501	0.098390757	1.313014606	0.189178027	0.228644667	positive
PRJNA729271	stage_1D_vs_E10	ordinary_muscle_development	ENSGALG00010014042	SGCZ	-0.535746183	0.321723486	-1.665238027	0.095865277	0.122288761	negative
PRJNA729271	stage_1D_vs_E10	ordinary_muscle_development	ENSGALG00010014045	GOLT1B	-1.114880473	0.110368081	-10.10147554	5.44172E-24	3.53395E-23	negative
PRJNA729271	stage_1D_vs_E10	ordinary_muscle_development	ENSGALG00010014047	HM13	-0.148258345	0.167241827	-0.886490825	0.375353096	0.424626796	negative
PRJNA729271	stage_1D_vs_E10	ordinary_muscle_development	ENSGALG00010014056	FMO3	5.66078858	0.253447504	22.33515222	1.6835E-110	1.5348E-108	positive
PRJNA729271	stage_1D_vs_E10	ordinary_muscle_development	ENSGALG00010014062	GGA1	0.417576907	0.103842468	4.0212537	5.78892E-05	0.000110018	positive
PRJNA729271	stage_1D_vs_E10	ordinary_muscle_development	ENSGALG00010014065	CHUNK-1	-1.498460417	0.676861464	-2.213836209	0.026840048	0.037248773	negative
PRJNA729271	stage_1D_vs_E10	ordinary_muscle_development	ENSGALG00010014066	BCL2L1	-0.500820673	0.117525089	-4.26139371	2.03156E-05	4.02288E-05	negative
PRJNA729271	stage_1D_vs_E10	ordinary_muscle_development	ENSGALG00010014076	PRRX1	-2.028759138	0.245801687	-8.253642035	1.53639E-16	6.78756E-16	negative
PRJNA729271	stage_1D_vs_E10	ordinary_muscle_development	ENSGALG00010014077	SLITRK2	0.773345666	0.098475651	7.853166336	4.05663E-15	1.64202E-14	positive
PRJNA729271	stage_1D_vs_E10	ordinary_muscle_development	ENSGALG00010014080	MYO1B	-0.20919598	0.160712368	-1.301679406	0.193026002	0.232858688	negative
PRJNA729271	stage_1D_vs_E10	ordinary_muscle_development	ENSGALG00010014082	HNRNPR	-0.639195593	0.042723039	-14.96137929	1.31277E-50	2.59953E-49	negative
PRJNA729271	stage_1D_vs_E10	ordinary_muscle_development	ENSGALG00010014083	ENSGALG00010014083	-1.144767425	0.146636324	-7.806847544	5.86361E-15	2.3427E-14	negative
PRJNA729271	stage_1D_vs_E10	ordinary_muscle_development	ENSGALG00010014089	FIG4	-0.471223157	0.115877389	-4.066566914	4.77108E-05	9.13685E-05	negative
PRJNA729271	stage_1D_vs_E10	ordinary_muscle_development	ENSGALG00010014091	CHIC2	0.948997844	0.097272677	9.756057641	1.7378E-22	1.06195E-21	positive
PRJNA729271	stage_1D_vs_E10	ordinary_muscle_development	ENSGALG00010014092	TYRP1	0.195762291	0.917903981	0.21327099	0.831115599	0.855187095	positive
PRJNA729271	stage_1D_vs_E10	ordinary_muscle_development	ENSGALG00010014093	PLD1	0.557830578	0.092881079	6.005858081	1.90323E-09	5.26474E-09	positive
PRJNA729271	stage_1D_vs_E10	ordinary_muscle_development	ENSGALG00010014096	JAKMIP2	-3.164863597	0.161159758	-19.63805128	7.31518E-86	3.95648E-84	negative
PRJNA729271	stage_1D_vs_E10	ordinary_muscle_development	ENSGALG00010014099	TPX2	-1.871807168	0.143565961	-13.03795942	7.44223E-39	9.3723E-38	negative
PRJNA729271	stage_1D_vs_E10	ordinary_muscle_development	ENSGALG00010014102	TRIM2	-0.464716642	0.13816695	-3.363442856	0.000769768	0.001299835	negative
PRJNA729271	stage_1D_vs_E10	ordinary_muscle_development	ENSGALG00010014104	ALDH1A1	0.365767122	0.535644524	0.682854217	0.494698966	0.542896549	positive
PRJNA729271	stage_1D_vs_E10	ordinary_muscle_development	ENSGALG00010014105	AHR1B	-2.502922482	0.576162558	-4.344125534	1.39832E-05	2.81469E-05	negative
PRJNA729271	stage_1D_vs_E10	ordinary_muscle_development	ENSGALG00010014109	AMIGO2	-0.753500249	0.162944616	-4.624272158	3.75916E-06	7.98608E-06	negative
PRJNA729271	stage_1D_vs_E10	ordinary_muscle_development	ENSGALG00010014111	GORAB	-0.069575703	0.08088262	-0.860205844	0.389675584	0.438877047	negative
PRJNA729271	stage_1D_vs_E10	ordinary_muscle_development	ENSGALG00010014114	ANXA1	2.260308757	0.332117447	6.805751326	1.00523E-11	3.25242E-11	positive
PRJNA729271	stage_1D_vs_E10	ordinary_muscle_development	ENSGALG00010014120	SOX5	-1.719779679	0.132172761	-13.01160444	1.05112E-38	1.31571E-37	negative
PRJNA729271	stage_1D_vs_E10	ordinary_muscle_development	ENSGALG00010014122	ABCA1	0.957425893	0.140624148	6.808403158	9.8688E-12	3.19447E-11	positive
PRJNA729271	stage_1D_vs_E10	ordinary_muscle_development	ENSGALG00010014125	PLPP1	3.096427942	0.138675304	22.32861831	1.9486E-110	1.7653E-108	positive
PRJNA729271	stage_1D_vs_E10	ordinary_muscle_development	ENSGALG00010014128	COL4A1	3.089042749	0.144538347	21.37178686	2.4459E-101	1.8275E-99	positive
PRJNA729271	stage_1D_vs_E10	ordinary_muscle_development	ENSGALG00010014131	CDC23	-0.684826222	0.116531779	-5.87673362	4.18441E-09	1.1255E-08	negative
PRJNA729271	stage_1D_vs_E10	ordinary_muscle_development	ENSGALG00010014134	RPAP3	-0.422155029	0.113994768	-3.70328424	0.000212826	0.000380854	negative
PRJNA729271	stage_1D_vs_E10	ordinary_muscle_development	ENSGALG00010014135	ZBTB24	0.023363124	0.154541407	0.151177116	0.879836002	0.89760859	positive
PRJNA729271	stage_1D_vs_E10	ordinary_muscle_development	ENSGALG00010014142	PPIL6	1.852026089	0.476158088	3.88951933	0.000100443	0.000186179	positive
PRJNA729271	stage_1D_vs_E10	ordinary_muscle_development	ENSGALG00010014143	NFATC2	-0.308337785	0.153344827	-2.010747879	0.044352094	0.05960905	negative
PRJNA729271	stage_1D_vs_E10	ordinary_muscle_development	ENSGALG00010014145	NTMT2	4.650718461	0.251895105	18.46291714	4.10557E-76	1.75546E-74	positive
PRJNA729271	stage_1D_vs_E10	ordinary_muscle_development	ENSGALG00010014147	ENSGALG00010014147	-1.839093363	0.365511594	-5.031559584	4.86506E-07	1.11404E-06	negative
PRJNA729271	stage_1D_vs_E10	ordinary_muscle_development	ENSGALG00010014148	KCNG1	0.61688293	0.350004468	1.762500159	0.07798483	0.101017882	positive
PRJNA729271	stage_1D_vs_E10	ordinary_muscle_development	ENSGALG00010014149	LGALS2	2.061538538	0.539979356	3.817809911	0.000134642	0.000246636	positive
PRJNA729271	stage_1D_vs_E10	ordinary_muscle_development	ENSGALG00010014151	ATP23	-0.252834049	0.090891212	-2.781721614	0.00540714	0.008275419	negative
PRJNA729271	stage_1D_vs_E10	ordinary_muscle_development	ENSGALG00010014153	FOCAD	-0.084281744	0.132663923	-0.635302662	0.525231038	0.57229376	negative
PRJNA729271	stage_1D_vs_E10	ordinary_muscle_development	ENSGALG00010014154	DNAI7	-1.527402728	0.440535597	-3.467149389	0.000526009	0.000903699	negative
PRJNA729271	stage_1D_vs_E10	ordinary_muscle_development	ENSGALG00010014155	ENSGALG00010014155	-2.280361071	1.276706487	-1.786127896	0.074078552	0.096224448	negative
PRJNA729271	stage_1D_vs_E10	ordinary_muscle_development	ENSGALG00010014156	CD164	-0.142007425	0.092295275	-1.538620746	0.123896911	0.155098517	negative
PRJNA729271	stage_1D_vs_E10	ordinary_muscle_development	ENSGALG00010014159	CDC42EP1	0.079932613	0.149162878	0.535874706	0.592045147	0.635728158	positive
PRJNA729271	stage_1D_vs_E10	ordinary_muscle_development	ENSGALG00010014161	GFRA3	1.067734467	0.247831195	4.308313441	1.64504E-05	3.28532E-05	positive
PRJNA729271	stage_1D_vs_E10	ordinary_muscle_development	ENSGALG00010014162	COL18A1	1.387369623	0.18823568	7.370386007	1.70135E-13	6.19624E-13	positive
PRJNA729271	stage_1D_vs_E10	ordinary_muscle_development	ENSGALG00010014165	LUZP1	-1.030711594	0.118220172	-8.718576372	2.81721E-18	1.37032E-17	negative
PRJNA729271	stage_1D_vs_E10	ordinary_muscle_development	ENSGALG00010014166	FGF12	-1.351120521	0.200375009	-6.742959261	1.55193E-11	4.96691E-11	negative
PRJNA729271	stage_1D_vs_E10	ordinary_muscle_development	ENSGALG00010014167	SLC38A9	0.077542915	0.10807042	0.7175221	0.473052005	0.521753828	positive
PRJNA729271	stage_1D_vs_E10	ordinary_muscle_development	ENSGALG00010014170	CEMIP2	-1.132418828	0.107149277	-10.56860914	4.16628E-26	2.99406E-25	negative
PRJNA729271	stage_1D_vs_E10	ordinary_muscle_development	ENSGALG00010014171	DLC1	0.902685636	0.132581251	6.808546664	9.85896E-12	3.192E-11	positive
PRJNA729271	stage_1D_vs_E10	ordinary_muscle_development	ENSGALG00010014173	MND1	-2.762992986	0.205292428	-13.45881589	2.7326E-41	3.77949E-40	negative
PRJNA729271	stage_1D_vs_E10	ordinary_muscle_development	ENSGALG00010014176	ABHD17B	-0.460323632	0.090135102	-5.107040653	3.27243E-07	7.57852E-07	negative
PRJNA729271	stage_1D_vs_E10	ordinary_muscle_development	ENSGALG00010014178	LRTM2	1.04947433	0.341292723	3.074997672	0.002105042	0.003378649	positive
PRJNA729271	stage_1D_vs_E10	ordinary_muscle_development	ENSGALG00010014179	CTBPL	-0.856675657	0.042608377	-20.10580345	6.56488E-90	3.884E-88	negative
PRJNA729271	stage_1D_vs_E10	ordinary_muscle_development	ENSGALG00010014180	SLC19A1	0.186905414	0.142746777	1.309349443	0.190416009	0.229987505	positive
PRJNA729271	stage_1D_vs_E10	ordinary_muscle_development	ENSGALG00010014184	SLC44A1	1.633708256	0.132041106	12.37272468	3.67152E-35	4.01954E-34	positive
PRJNA729271	stage_1D_vs_E10	ordinary_muscle_development	ENSGALG00010014186	MOCS3	-0.436818055	0.107883431	-4.048981874	5.14409E-05	9.81359E-05	negative
PRJNA729271	stage_1D_vs_E10	ordinary_muscle_development	ENSGALG00010014189	DDX4	-4.041805272	0.789398739	-5.120106064	3.05364E-07	7.08994E-07	negative
PRJNA729271	stage_1D_vs_E10	ordinary_muscle_development	ENSGALG00010014190	HACD4	1.715483773	0.220789738	7.769762244	7.86336E-15	3.11419E-14	positive
PRJNA729271	stage_1D_vs_E10	ordinary_muscle_development	ENSGALG00010014192	KDM1A	-1.293484498	0.061298084	-21.10154876	7.69733E-99	5.55088E-97	negative
PRJNA729271	stage_1D_vs_E10	ordinary_muscle_development	ENSGALG00010014193	CARD10	-2.728925129	0.393943001	-6.927208049	4.29227E-12	1.42242E-11	negative
PRJNA729271	stage_1D_vs_E10	ordinary_muscle_development	ENSGALG00010014194	LRIG3	-0.893477804	0.091225668	-9.79414922	1.19297E-22	7.34899E-22	negative
PRJNA729271	stage_1D_vs_E10	ordinary_muscle_development	ENSGALG00010014197	SH3GL2	-2.920449559	0.359087359	-8.132977915	4.18871E-16	1.80916E-15	negative
PRJNA729271	stage_1D_vs_E10	ordinary_muscle_development	ENSGALG00010014198	KDM3B	-0.093866293	0.044524576	-2.108190617	0.035014505	0.047849085	negative
PRJNA729271	stage_1D_vs_E10	ordinary_muscle_development	ENSGALG00010014199	LNX1	1.127779098	0.21858172	5.159530706	2.4757E-07	5.79261E-07	positive
PRJNA729271	stage_1D_vs_E10	ordinary_muscle_development	ENSGALG00010014200	DCP1B	0.269839044	0.088648928	3.043906455	0.002335277	0.003725905	positive
PRJNA729271	stage_1D_vs_E10	ordinary_muscle_development	ENSGALG00010014203	TMEM38B	1.033971297	0.16915802	6.112458026	9.81081E-10	2.77587E-09	positive
PRJNA729271	stage_1D_vs_E10	ordinary_muscle_development	ENSGALG00010014204	KIFAP3	-0.961445269	0.084628197	-11.36081472	6.55306E-30	5.63385E-29	negative
PRJNA729271	stage_1D_vs_E10	ordinary_muscle_development	ENSGALG00010014205	DPM1	-0.193263844	0.084998701	-2.273727033	0.022982404	0.032276906	negative
PRJNA729271	stage_1D_vs_E10	ordinary_muscle_development	ENSGALG00010014209	CCDC50	0.209034237	0.107386946	1.946551648	0.05158851	0.068653641	positive
PRJNA729271	stage_1D_vs_E10	ordinary_muscle_development	ENSGALG00010014210	ENSGALG00010014210	0.271472966	0.248427621	1.092764822	0.274497071	0.320176635	positive
PRJNA729271	stage_1D_vs_E10	ordinary_muscle_development	ENSGALG00010014213	ETFRF1	0.854966327	0.150890108	5.666152255	1.4604E-08	3.76328E-08	positive
PRJNA729271	stage_1D_vs_E10	ordinary_muscle_development	ENSGALG00010014214	REEP2	-0.063435534	0.133637656	-0.474683082	0.635012878	0.676056678	negative
PRJNA729271	stage_1D_vs_E10	ordinary_muscle_development	ENSGALG00010014217	RASA3	0.341762717	0.109546011	3.119809777	0.001809679	0.002930216	positive
PRJNA729271	stage_1D_vs_E10	ordinary_muscle_development	ENSGALG00010014218	IL31RA	1.255851216	0.166561281	7.539874878	4.70423E-14	1.77804E-13	positive
PRJNA729271	stage_1D_vs_E10	ordinary_muscle_development	ENSGALG00010014220	C9orf85	-1.076639346	0.134010725	-8.03397894	9.43614E-16	3.9703E-15	negative
PRJNA729271	stage_1D_vs_E10	ordinary_muscle_development	ENSGALG00010014221	SCYL3	0.520640767	0.131531259	3.958304459	7.54837E-05	0.000141709	positive
PRJNA729271	stage_1D_vs_E10	ordinary_muscle_development	ENSGALG00010014222	TBX22	-1.40163638	0.403437536	-3.474233937	0.000512314	0.00088111	negative
PRJNA729271	stage_1D_vs_E10	ordinary_muscle_development	ENSGALG00010014224	NMUR2	-5.706152075	0.961059557	-5.937355323	2.89656E-09	7.90542E-09	negative
PRJNA729271	stage_1D_vs_E10	ordinary_muscle_development	ENSGALG00010014225	ZMPSTE24	-0.403041074	0.075962685	-5.305777112	1.12194E-07	2.70276E-07	negative
PRJNA729271	stage_1D_vs_E10	ordinary_muscle_development	ENSGALG00010014226	SLC16A7	1.750348184	0.257916063	6.786503185	1.14884E-11	3.70383E-11	positive
PRJNA729271	stage_1D_vs_E10	ordinary_muscle_development	ENSGALG00010014234	GLRA1	-0.632297304	0.702344093	-0.900267134	0.367978107	0.417130105	negative
PRJNA729271	stage_1D_vs_E10	ordinary_muscle_development	ENSGALG00010014236	WDR44	-0.099175426	0.081804311	-1.212349619	0.225378578	0.268127256	negative
PRJNA729271	stage_1D_vs_E10	ordinary_muscle_development	ENSGALG00010014238	PHLDA1	-2.808787821	0.122498916	-22.92908303	2.383E-116	2.5213E-114	negative
PRJNA729271	stage_1D_vs_E10	ordinary_muscle_development	ENSGALG00010014240	PIGV	0.80804198	0.12627324	6.399154554	1.5624E-10	4.66851E-10	positive
PRJNA729271	stage_1D_vs_E10	ordinary_muscle_development	ENSGALG00010014241	ENSGALG00010014241	-0.750506252	0.864077613	-0.868563473	0.385085941	0.434146435	negative
PRJNA729271	stage_1D_vs_E10	ordinary_muscle_development	ENSGALG00010014243	ENSGALG00010014243	0.024502512	0.144465851	0.169607642	0.865318712	0.885165471	positive
PRJNA729271	stage_1D_vs_E10	ordinary_muscle_development	ENSGALG00010014244	SESN1	4.09127926	0.369808686	11.06323192	1.89156E-28	1.51649E-27	positive
PRJNA729271	stage_1D_vs_E10	ordinary_muscle_development	ENSGALG00010014246	KRAS	-1.055200748	0.114690196	-9.200444215	3.56473E-20	1.92228E-19	negative
PRJNA729271	stage_1D_vs_E10	ordinary_muscle_development	ENSGALG00010014249	ENSGALG00010014249	0.058488993	0.101208	0.577908791	0.5633257	0.608768062	positive
PRJNA729271	stage_1D_vs_E10	ordinary_muscle_development	ENSGALG00010014250	ENSGALG00010014250	5.975920378	0.252899218	23.6296515	1.9111E-123	2.2894E-121	positive
PRJNA729271	stage_1D_vs_E10	ordinary_muscle_development	ENSGALG00010014257	NOP16	-0.536831932	0.124771398	-4.302523992	1.68863E-05	3.36821E-05	negative
PRJNA729271	stage_1D_vs_E10	ordinary_muscle_development	ENSGALG00010014258	ENSGALG00010014258	-1.375189107	0.513504204	-2.678048389	0.007405252	0.011140542	negative
PRJNA729271	stage_1D_vs_E10	ordinary_muscle_development	ENSGALG00010014260	ADNP	-0.2531066	0.07536532	-3.358396153	0.000783962	0.00132257	negative
PRJNA729271	stage_1D_vs_E10	ordinary_muscle_development	ENSGALG00010014263	ZNF131	-0.173917865	0.118869887	-1.463094393	0.143441552	0.177480606	negative
PRJNA729271	stage_1D_vs_E10	ordinary_muscle_development	ENSGALG00010014265	ZNF462	-0.699250849	0.139715444	-5.004821427	5.59138E-07	1.27352E-06	negative
PRJNA729271	stage_1D_vs_E10	ordinary_muscle_development	ENSGALG00010014267	SCFD2	-0.41406575	0.073967948	-5.597907767	2.16954E-08	5.51227E-08	negative
PRJNA729271	stage_1D_vs_E10	ordinary_muscle_development	ENSGALG00010014272	PARD6B	-1.258867667	0.254531251	-4.945827528	7.58211E-07	1.70895E-06	negative
PRJNA729271	stage_1D_vs_E10	ordinary_muscle_development	ENSGALG00010014274	FIRRM	-0.769292661	0.149212912	-5.155670852	2.52724E-07	5.9056E-07	negative
PRJNA729271	stage_1D_vs_E10	ordinary_muscle_development	ENSGALG00010014278	G3BP1	-0.71110227	0.139482968	-5.098129746	3.43026E-07	7.93393E-07	negative
PRJNA729271	stage_1D_vs_E10	ordinary_muscle_development	ENSGALG00010014284	TMEM131L	-0.608645115	0.08431433	-7.218762402	5.24628E-13	1.85359E-12	negative
PRJNA729271	stage_1D_vs_E10	ordinary_muscle_development	ENSGALG00010014288	ARMC2	-2.153649135	0.253129503	-8.508092148	1.76819E-17	8.22524E-17	negative
PRJNA729271	stage_1D_vs_E10	ordinary_muscle_development	ENSGALG00010014291	OSTN	-4.695334572	0.541678056	-8.668127725	4.39284E-18	2.11612E-17	negative
PRJNA729271	stage_1D_vs_E10	ordinary_muscle_development	ENSGALG00010014292	NIM1K	2.028935166	0.449534987	4.513408795	6.37939E-06	1.32706E-05	positive
PRJNA729271	stage_1D_vs_E10	ordinary_muscle_development	ENSGALG00010014294	TMPO	-0.231973455	0.101982432	-2.274641342	0.022927454	0.032209095	negative
PRJNA729271	stage_1D_vs_E10	ordinary_muscle_development	ENSGALG00010014295	CACNA1C	1.249299599	0.173511422	7.200100069	6.01684E-13	2.11531E-12	positive
PRJNA729271	stage_1D_vs_E10	ordinary_muscle_development	ENSGALG00010014298	METTL18	-0.945888705	0.226305457	-4.179699061	2.91895E-05	5.69834E-05	negative
PRJNA729271	stage_1D_vs_E10	ordinary_muscle_development	ENSGALG00010014300	ELFN2	-1.05807011	0.475869744	-2.223444803	0.026185821	0.036401984	negative
PRJNA729271	stage_1D_vs_E10	ordinary_muscle_development	ENSGALG00010014301	ARID1A	0.397275582	0.118832866	3.343145685	0.000828344	0.001394038	positive
PRJNA729271	stage_1D_vs_E10	ordinary_muscle_development	ENSGALG00010014302	FOXO3	2.140451171	0.233156764	9.180309151	4.29856E-20	2.31025E-19	positive
PRJNA729271	stage_1D_vs_E10	ordinary_muscle_development	ENSGALG00010014303	GABRA2	1.081744493	0.355043922	3.046790627	0.002312988	0.003692781	positive
PRJNA729271	stage_1D_vs_E10	ordinary_muscle_development	ENSGALG00010014305	ARL10	-1.601191073	0.302285973	-5.296941362	1.17758E-07	2.8321E-07	negative
PRJNA729271	stage_1D_vs_E10	ordinary_muscle_development	ENSGALG00010014306	ENSGALG00010014306	-0.703750116	0.322167268	-2.18442463	0.02893106	0.039961475	negative
PRJNA729271	stage_1D_vs_E10	ordinary_muscle_development	ENSGALG00010014308	ENSGALG00010014308	0.412260636	0.202979616	2.031044516	0.042250479	0.056995877	positive
PRJNA729271	stage_1D_vs_E10	ordinary_muscle_development	ENSGALG00010014310	COL6A1	1.933700801	0.237723272	8.134251162	4.14492E-16	1.79131E-15	positive
PRJNA729271	stage_1D_vs_E10	ordinary_muscle_development	ENSGALG00010014312	KIAA1191	-0.047957585	0.082165367	-0.583671524	0.559441329	0.605066562	negative
PRJNA729271	stage_1D_vs_E10	ordinary_muscle_development	ENSGALG00010014314	ENSGALG00010014314	0.943982062	0.157899042	5.97839004	2.25354E-09	6.20654E-09	positive
PRJNA729271	stage_1D_vs_E10	ordinary_muscle_development	ENSGALG00010014317	RAD23B	0.640528028	0.146258422	4.37942663	1.18992E-05	2.41263E-05	positive
PRJNA729271	stage_1D_vs_E10	ordinary_muscle_development	ENSGALG00010014318	ENSGALG00010014318	0.351917419	0.469712621	0.749218573	0.453725476	0.502732822	positive
PRJNA729271	stage_1D_vs_E10	ordinary_muscle_development	ENSGALG00010014319	ENSGALG00010014319	0.20449375	0.115285909	1.773796569	0.076096806	0.09865157	positive
PRJNA729271	stage_1D_vs_E10	ordinary_muscle_development	ENSGALG00010014320	UTS2B	-3.588120691	1.076611172	-3.332791618	0.000859793	0.001443779	negative
PRJNA729271	stage_1D_vs_E10	ordinary_muscle_development	ENSGALG00010014323	KLF4	1.109945938	0.176238483	6.297977149	3.01555E-10	8.84468E-10	positive
PRJNA729271	stage_1D_vs_E10	ordinary_muscle_development	ENSGALG00010014324	COL4A2	3.063658399	0.142207214	21.54362148	6.0765E-103	4.6358E-101	positive
PRJNA729271	stage_1D_vs_E10	ordinary_muscle_development	ENSGALG00010014328	RASSF8	-0.426474359	0.090824654	-4.69557921	2.65852E-06	5.73016E-06	negative
PRJNA729271	stage_1D_vs_E10	ordinary_muscle_development	ENSGALG00010014329	IER5	1.265067507	0.153320762	8.251116767	1.56921E-16	6.92833E-16	positive
PRJNA729271	stage_1D_vs_E10	ordinary_muscle_development	ENSGALG00010014334	HMGCS1	0.273713431	0.157944742	1.73296957	0.083101078	0.10709016	positive
PRJNA729271	stage_1D_vs_E10	ordinary_muscle_development	ENSGALG00010014338	BHLHE41	1.115989246	0.222745614	5.010151381	5.43872E-07	1.23992E-06	positive
PRJNA729271	stage_1D_vs_E10	ordinary_muscle_development	ENSGALG00010014340	EFNB1	-1.545708552	0.13740156	-11.2495706	2.32722E-29	1.95102E-28	negative
PRJNA729271	stage_1D_vs_E10	ordinary_muscle_development	ENSGALG00010014344	LSG1	-0.400311891	0.086504561	-4.627639148	3.69858E-06	7.86199E-06	negative
PRJNA729271	stage_1D_vs_E10	ordinary_muscle_development	ENSGALG00010014346	AFG1L	1.046563709	0.157712578	6.635892481	3.22545E-11	1.00978E-10	positive

PRJNA729271	stage_1D_vs_E10	ordinary_muscle_development	ENSGALG00010014347	KIAA0040	2.195454406	0.331562575	6.621538648	3.55479E-11	1.10929E-10	positive
PRJNA729271	stage_1D_vs_E10	ordinary_muscle_development	ENSGALG00010014350	PTGER4	-0.804381468	0.174802792	-4.60165114	4.19155E-06	8.84889E-06	negative
PRJNA729271	stage_1D_vs_E10	ordinary_muscle_development	ENSGALG00010014355	RAB20	0.98584087	0.165085376	5.971703222	2.34789E-09	6.45292E-09	positive
PRJNA729271	stage_1D_vs_E10	ordinary_muscle_development	ENSGALG00010014359	TMEM271	-0.879067276	0.449255293	-1.956721021	0.050380277	0.067175248	negative
PRJNA729271	stage_1D_vs_E10	ordinary_muscle_development	ENSGALG00010014361	SSPN	2.536586254	0.148996836	17.02443036	5.41165E-65	1.72022E-63	positive
PRJNA729271	stage_1D_vs_E10	ordinary_muscle_development	ENSGALG00010014362	NAXD	1.362035789	0.106679362	12.76756589	2.48814E-37	2.94895E-36	positive
PRJNA729271	stage_1D_vs_E10	ordinary_muscle_development	ENSGALG00010014367	OPHN1	0.175780145	0.108903271	1.614094258	0.106506983	0.134737189	positive
PRJNA729271	stage_1D_vs_E10	ordinary_muscle_development	ENSGALG00010014371	TTC33	-0.444696641	0.101277442	-4.39087551	1.12895E-05	2.29383E-05	negative
PRJNA729271	stage_1D_vs_E10	ordinary_muscle_development	ENSGALG00010014372	CCDC171	-0.528119079	0.125521367	-4.207403825	2.58321E-05	5.06543E-05	negative
PRJNA729271	stage_1D_vs_E10	ordinary_muscle_development	ENSGALG00010014375	H2BC35	-1.602789463	0.246926571	-6.490955809	8.52935E-11	2.59515E-10	negative
PRJNA729271	stage_1D_vs_E10	ordinary_muscle_development	ENSGALG00010014379	CARS2	-0.414036086	0.089100078	-4.646865593	3.37017E-06	7.19767E-06	negative
PRJNA729271	stage_1D_vs_E10	ordinary_muscle_development	ENSGALG00010014380	SNX3	-0.377899967	0.117669297	-3.211542651	0.001320244	0.002172923	negative
PRJNA729271	stage_1D_vs_E10	ordinary_muscle_development	ENSGALG00010014388	MFNG	1.03425299	0.236446239	4.374157079	1.21903E-05	2.46957E-05	positive
PRJNA729271	stage_1D_vs_E10	ordinary_muscle_development	ENSGALG00010014389	COL6A2	1.628241975	0.264472724	6.156559176	7.43424E-10	2.11916E-09	positive
PRJNA729271	stage_1D_vs_E10	ordinary_muscle_development	ENSGALG00010014390	SPARC	1.310894044	0.159038098	8.242641587	1.6845E-16	7.42377E-16	positive
PRJNA729271	stage_1D_vs_E10	ordinary_muscle_development	ENSGALG00010014393	ATP8A1	0.908940726	0.144428359	6.293367379	3.10652E-10	9.10044E-10	positive
PRJNA729271	stage_1D_vs_E10	ordinary_muscle_development	ENSGALG00010014396	ING1	0.455577954	0.116878846	3.897864938	9.70445E-05	0.000180226	positive
PRJNA729271	stage_1D_vs_E10	ordinary_muscle_development	ENSGALG00010014397	PRKAA1	-0.236979063	0.076182542	-3.110674124	0.001866608	0.003018299	negative
PRJNA729271	stage_1D_vs_E10	ordinary_muscle_development	ENSGALG00010014399	GAS6	2.11679095	0.190176354	11.13067375	8.89605E-29	7.25652E-28	positive
PRJNA729271	stage_1D_vs_E10	ordinary_muscle_development	ENSGALG00010014400	RIPOR3	-0.600520541	0.100921856	-5.950351703	2.67567E-09	7.32461E-09	negative
PRJNA729271	stage_1D_vs_E10	ordinary_muscle_development	ENSGALG00010014403	H2BC37	-0.092157584	0.18789479	-0.490474401	0.62379824	0.665730782	negative
PRJNA729271	stage_1D_vs_E10	ordinary_muscle_development	ENSGALG00010014407	YIPF6	-0.337118171	0.090137709	-3.740034836	0.000183995	0.000332254	negative
PRJNA729271	stage_1D_vs_E10	ordinary_muscle_development	ENSGALG00010014408	ENSGALG00010014408	-0.471104693	0.316898619	-1.486610117	0.137117814	0.170091803	negative
PRJNA729271	stage_1D_vs_E10	ordinary_muscle_development	ENSGALG00010014411	ATP13A3	-0.223139384	0.080483952	-2.772470536	0.005563256	0.008499092	negative
PRJNA729271	stage_1D_vs_E10	ordinary_muscle_development	ENSGALG00010014414	TMEM255B	0.952976099	0.500831565	1.902787615	0.057068264	0.07535111	positive
PRJNA729271	stage_1D_vs_E10	ordinary_muscle_development	ENSGALG00010014416	H2BC32	-1.159962427	0.269797311	-4.299384684	1.71273E-05	3.41345E-05	negative
PRJNA729271	stage_1D_vs_E10	ordinary_muscle_development	ENSGALG00010014418	RAC2	0.508019652	0.309427083	1.641807323	0.100629941	0.127905208	positive
PRJNA729271	stage_1D_vs_E10	ordinary_muscle_development	ENSGALG00010014427	TMEM267	-0.888123431	0.136119807	-6.524571632	6.81962E-11	2.08545E-10	negative
PRJNA729271	stage_1D_vs_E10	ordinary_muscle_development	ENSGALG00010014431	ENSGALG00010014431	-0.580574979	0.157280154	-3.691342897	0.000223073	0.000398355	negative
PRJNA729271	stage_1D_vs_E10	ordinary_muscle_development	ENSGALG00010014439	ENSGALG00010014439	2.038111533	0.839320962	2.428286227			positive
PRJNA729271	stage_1D_vs_E10	ordinary_muscle_development	ENSGALG00010014442	SHISA3	-3.742918517	0.850633225	-4.400155565	1.08173E-05	2.2019E-05	negative
PRJNA729271	stage_1D_vs_E10	ordinary_muscle_development	ENSGALG00010014449	ENSGALG00010014449	-0.761217164	0.154600077	-4.923782563	8.48872E-07	1.90648E-06	negative
PRJNA729271	stage_1D_vs_E10	ordinary_muscle_development	ENSGALG00010014453	NR2E1	-0.088935815	0.358116505	-0.248343244	0.803868843	0.83057088	negative
PRJNA729271	stage_1D_vs_E10	ordinary_muscle_development	ENSGALG00010014455	C4orf19	-1.81746085	0.242013985	-7.50973482	5.92473E-14	2.22427E-13	negative
PRJNA729271	stage_1D_vs_E10	ordinary_muscle_development	ENSGALG00010014465	SIMC1	0.787381983	0.152286513	5.170398675	2.33595E-07	5.47801E-07	positive
PRJNA729271	stage_1D_vs_E10	ordinary_muscle_development	ENSGALG00010014469	TMCO3	-0.472919548	0.12634732	-3.743012115	0.000181827	0.000328668	negative
PRJNA729271	stage_1D_vs_E10	ordinary_muscle_development	ENSGALG00010014470	RPS6KA1	-1.321816527	0.130046947	-10.16414882	2.86614E-24	1.89098E-23	negative
PRJNA729271	stage_1D_vs_E10	ordinary_muscle_development	ENSGALG00010014474	PTPN1	-0.218386949	0.077378068	-2.822336524	0.004767512	0.007344573	negative
PRJNA729271	stage_1D_vs_E10	ordinary_muscle_development	ENSGALG00010014476	C5orf34	-0.13440378	0.178515889	-0.752895335	0.451512811	0.500663877	negative
PRJNA729271	stage_1D_vs_E10	ordinary_muscle_development	ENSGALG00010014477	ITPR2	-0.117015324	0.09839886	-1.189193893	0.234363377	0.277787803	negative
PRJNA729271	stage_1D_vs_E10	ordinary_muscle_development	ENSGALG00010014482	THOC3	-1.200354983	0.083709643	-14.33950658	1.23933E-46	2.07199E-45	negative
PRJNA729271	stage_1D_vs_E10	ordinary_muscle_development	ENSGALG00010014487	OSTM1	-0.070598888	0.084093524	-0.839528236	0.401172951	0.450600692	negative
PRJNA729271	stage_1D_vs_E10	ordinary_muscle_development	ENSGALG00010014488	ENSGALG00010014488	1.868162055	0.409357477	4.563644637	5.02731E-06	1.05549E-05	positive
PRJNA729271	stage_1D_vs_E10	ordinary_muscle_development	ENSGALG00010014492	CPLX2	-2.109699881	0.336747497	-6.264931144	3.7299E-10	1.0863E-09	negative
PRJNA729271	stage_1D_vs_E10	ordinary_muscle_development	ENSGALG00010014494	KLHL9	-0.715019978	0.06668962	-10.7216082	8.05897E-27	5.99973E-26	negative
PRJNA729271	stage_1D_vs_E10	ordinary_muscle_development	ENSGALG00010014504	SEC63	-0.773056863	0.04603453	-16.79297836	2.74706E-63	8.26114E-62	negative
PRJNA729271	stage_1D_vs_E10	ordinary_muscle_development	ENSGALG00010014505	CYTH4	0.810683125	0.219609299	3.691479044	0.000222954	0.000398191	positive
PRJNA729271	stage_1D_vs_E10	ordinary_muscle_development	ENSGALG00010014512	HMGN4	-0.778522012	0.110689554	-7.033382861	2.01585E-12	6.83504E-12	negative
PRJNA729271	stage_1D_vs_E10	ordinary_muscle_development	ENSGALG00010014515	ETNK1	-1.690603839	0.113288137	-14.92304391	2.3338E-50	4.55294E-49	negative
PRJNA729271	stage_1D_vs_E10	ordinary_muscle_development	ENSGALG00010014517	DCUN1D2	1.702542992	0.126703641	13.43720658	3.65994E-41	5.02851E-40	positive
PRJNA729271	stage_1D_vs_E10	ordinary_muscle_development	ENSGALG00010014522	FAT2	-5.237976929	0.469211234	-11.16336641	6.16134E-29	5.0686E-28	negative
PRJNA729271	stage_1D_vs_E10	ordinary_muscle_development	ENSGALG00010014537	GKAP1	1.085832776	0.122083259	8.894198786	5.88423E-19	2.96564E-18	positive
PRJNA729271	stage_1D_vs_E10	ordinary_muscle_development	ENSGALG00010014539	PAIP1	-0.255178591	0.084957059	-3.003618452	0.002667897	0.00422913	negative
PRJNA729271	stage_1D_vs_E10	ordinary_muscle_development	ENSGALG00010014547	DHDDS	0.262325176	0.1060786	2.472932109	0.01340096	0.019461668	positive
PRJNA729271	stage_1D_vs_E10	ordinary_muscle_development	ENSGALG00010014550	SCML4	-6.414068388	0.984979851	-6.511877765	7.42171E-11	2.26527E-10	negative
PRJNA729271	stage_1D_vs_E10	ordinary_muscle_development	ENSGALG00010014555	VGLL1	-7.936133482	1.013545443	-7.830071695	4.87592E-15	1.96106E-14	negative
PRJNA729271	stage_1D_vs_E10	ordinary_muscle_development	ENSGALG00010014567	UTP11	-0.586008427	0.116861784	-5.014542873	5.31597E-07	1.21308E-06	negative
PRJNA729271	stage_1D_vs_E10	ordinary_muscle_development	ENSGALG00010014570	KIF27	-1.693021983	0.168373121	-10.05517968	8.71623E-24	5.61519E-23	negative
PRJNA729271	stage_1D_vs_E10	ordinary_muscle_development	ENSGALG00010014572	IL1RAP	0.06281833	0.124474113	0.504669831	0.613790754	0.656258537	positive
PRJNA729271	stage_1D_vs_E10	ordinary_muscle_development	ENSGALG00010014573	CEBPB	4.295348987	0.347351148	12.36601349	3.99145E-35	4.35663E-34	positive
PRJNA729271	stage_1D_vs_E10	ordinary_muscle_development	ENSGALG00010014576	HRH2	0.02212854	0.271350504	0.081549655	0.935004837	0.944715956	positive
PRJNA729271	stage_1D_vs_E10	ordinary_muscle_development	ENSGALG00010014578	ENSGALG00010014578	6.594332667	0.300498575	21.94463876	9.7439E-107	7.9347E-105	positive
PRJNA729271	stage_1D_vs_E10	ordinary_muscle_development	ENSGALG00010014579	NNT	1.281514567	0.144531736	8.866665555	7.53689E-19	3.77756E-18	positive
PRJNA729271	stage_1D_vs_E10	ordinary_muscle_development	ENSGALG00010014592	QNG1	-0.541308266	0.093794631	-5.771207386	7.87055E-09	2.07651E-08	negative
PRJNA729271	stage_1D_vs_E10	ordinary_muscle_development	ENSGALG00010014599	SSTR3	-1.413952273	0.395974841	-3.57081341	0.000355874	0.000622694	negative
PRJNA729271	stage_1D_vs_E10	ordinary_muscle_development	ENSGALG00010014602	FRK	-4.965310443	1.364705842	-3.638374138	0.000274365	0.000485404	negative
PRJNA729271	stage_1D_vs_E10	ordinary_muscle_development	ENSGALG00010014603	ENSGALG00010014603	4.723262244	0.221466779	21.32718172	6.3522E-101	4.72177E-99	positive
PRJNA729271	stage_1D_vs_E10	ordinary_muscle_development	ENSGALG00010014611	TMEM189	0.292701378	0.10377249	2.820606679	0.004793293	0.007381937	positive
PRJNA729271	stage_1D_vs_E10	ordinary_muscle_development	ENSGALG00010014614	GHR	1.17188009	0.090161573	12.99755591	1.26316E-38	1.57569E-37	positive
PRJNA729271	stage_1D_vs_E10	ordinary_muscle_development	ENSGALG00010014617	GRTP1	1.157910861	0.203119047	5.700651295	1.19351E-08	3.09534E-08	positive
PRJNA729271	stage_1D_vs_E10	ordinary_muscle_development	ENSGALG00010014624	GPRC6A	0.326337322	0.418146837	0.78043714	0.435133614	0.484577576	positive
PRJNA729271	stage_1D_vs_E10	ordinary_muscle_development	ENSGALG00010014626	C2CD5	-0.054439548	0.090713912	-0.600123475	0.548423949	0.594392489	negative
PRJNA729271	stage_1D_vs_E10	ordinary_muscle_development	ENSGALG00010014632	NWD2	-0.912375452	0.166082463	-5.493508679	3.94026E-08	9.80162E-08	negative
PRJNA729271	stage_1D_vs_E10	ordinary_muscle_development	ENSGALG00010014634	CCDC152	2.212771723	0.453009152	4.884607101	1.03635E-06	2.31356E-06	positive
PRJNA729271	stage_1D_vs_E10	ordinary_muscle_development	ENSGALG00010014641	DOCK11	0.17522874	0.09621426	1.821234609	0.068571202	0.089511849	positive
PRJNA729271	stage_1D_vs_E10	ordinary_muscle_development	ENSGALG00010014645	SLC36A1	-0.337028526	0.120902808	-2.78759883	0.005310025	0.008135379	negative
PRJNA729271	stage_1D_vs_E10	ordinary_muscle_development	ENSGALG00010014648	C1QTNF6	0.340752008	0.274578347	1.241001018	0.214605373	0.256486755	positive
PRJNA729271	stage_1D_vs_E10	ordinary_muscle_development	ENSGALG00010014653	FAM162B	0.415789558	0.510528813	0.81442917	0.415399133	0.464814763	positive
PRJNA729271	stage_1D_vs_E10	ordinary_muscle_development	ENSGALG00010014654	LAMP1	0.683766533	0.104517247	6.542140696	6.06444E-11	1.86356E-10	positive
PRJNA729271	stage_1D_vs_E10	ordinary_muscle_development	ENSGALG00010014655	FHL3	-0.315708473	0.134111654	-2.354071875	0.01856902	0.026369937	negative
PRJNA729271	stage_1D_vs_E10	ordinary_muscle_development	ENSGALG00010014658	SELENOP1	1.995750068	0.173508189	11.50233937	1.28388E-30	1.14663E-29	positive
PRJNA729271	stage_1D_vs_E10	ordinary_muscle_development	ENSGALG00010014668	GP5	-0.111252469	0.148650058	-0.748418605	0.454207704	0.503113302	negative
PRJNA729271	stage_1D_vs_E10	ordinary_muscle_development	ENSGALG00010014672	ISL1	-3.821776958	1.213613025	-3.149090262	0.001637796	0.002665602	negative
PRJNA729271	stage_1D_vs_E10	ordinary_muscle_development	ENSGALG00010014676	UBE2V1	-0.825401941	0.058678343	-14.06655167	6.09707E-45	9.52339E-44	negative
PRJNA729271	stage_1D_vs_E10	ordinary_muscle_development	ENSGALG00010014677	ENSGALG00010014677	-1.09963429	0.089975506	-12.22148488	2.38685E-34	2.51069E-33	negative
PRJNA729271	stage_1D_vs_E10	ordinary_muscle_development	ENSGALG00010014682	IL13RA1	-0.230251199	0.127459138	-1.806470703	0.070844846	0.09232186	negative
PRJNA729271	stage_1D_vs_E10	ordinary_muscle_development	ENSGALG00010014687	CUL4A	1.23812783	0.115023432	10.76413571	5.08363E-27	3.81009E-26	positive
PRJNA729271	stage_1D_vs_E10	ordinary_muscle_development	ENSGALG00010014690	CSF3R	2.230476211	0.370833937	6.014757515	1.80156E-09	4.99496E-09	positive
PRJNA729271	stage_1D_vs_E10	ordinary_muscle_development	ENSGALG00010014691	PABIR2	0.138472215	0.053784149	2.574591548	0.010035852	0.014831737	positive
PRJNA729271	stage_1D_vs_E10	ordinary_muscle_development	ENSGALG00010014696	SPINK5	-0.471621117	0.633681989	-0.744255203	0.456722097	0.505666575	negative
PRJNA729271	stage_1D_vs_E10	ordinary_muscle_development	ENSGALG00010014697	ENSGALG00010014697	-0.582358204	0.757916899	-0.768366828	0.442269282	0.491504504	negative
PRJNA729271	stage_1D_vs_E10	ordinary_muscle_development	ENSGALG00010014702	ENSGALG00010014702	0.670571554	0.262168278	2.557790587	0.010533951	0.015518814	positive
PRJNA729271	stage_1D_vs_E10	ordinary_muscle_development	ENSGALG00010014703	PCID2	-0.806330919	0.080189047	-10.05537478	8.69898E-24	5.60657E-23	negative
PRJNA729271	stage_1D_vs_E10	ordinary_muscle_development	ENSGALG00010014705	ARAP2	-0.92922789	0.219947983	-4.224762052	2.39193E-05	4.70458E-05	negative
PRJNA729271	stage_1D_vs_E10	ordinary_muscle_development	ENSGALG00010014707	KPNA5	1.172002145	0.129370714	9.059253907	1.31346E-19	6.87562E-19	positive
PRJNA729271	stage_1D_vs_E10	ordinary_muscle_development	ENSGALG00010014710	NPEPL1	0.31096097	0.099973266	3.110441236	0.001868081	0.003019383	positive
PRJNA729271	stage_1D_vs_E10	ordinary_muscle_development	ENSGALG00010014713	ST8SIA1	-2.806206617	0.137020695	-20.48016632	3.23552E-93	2.11256E-91	negative
PRJNA729271	stage_1D_vs_E10	ordinary_muscle_development	ENSGALG00010014716	TMEM35A	-1.84974574	0.124819078	-14.81941524	1.09736E-49	2.09567E-48	negative
PRJNA729271	stage_1D_vs_E10	ordinary_muscle_development	ENSGALG00010014717	ELOVL7	2.148886326	0.181106538	11.86531612	1.79224E-32	1.7447E-31	positive
PRJNA729271	stage_1D_vs_E10	ordinary_muscle_development	ENSGALG00010014719	ENSGALG00010014719	5.486413122	0.850644193	6.449715601	1.1206E-10	3.38258E-10	positive
PRJNA729271	stage_1D_vs_E10	ordinary_muscle_development	ENSGALG00010014722	KCTD17	-3.346974519	0.442296559	-7.567263302	3.81169E-14	1.44938E-13	negative
PRJNA729271	stage_1D_vs_E10	ordinary_muscle_development	ENSGALG00010014726	PWP2	-1.255402765	0.060157519	-20.86859286	1.03331E-96	7.30624E-95	negative
PRJNA729271	stage_1D_vs_E10	ordinary_muscle_development	ENSGALG00010014728	INTS13	-0.173071985	0.112116944	-1.543673764	0.122667385	0.15382505	negative
PRJNA729271	stage_1D_vs_E10	ordinary_muscle_development	ENSGALG00010014729	MRPS15	0.019016594	0.095662066	0.198789288	0.842427576	0.86510717	positive
PRJNA729271	stage_1D_vs_E10	ordinary_muscle_development	ENSGALG00010014734	ZUP1	-0.552261079	0.096429418	-5.727101675	1.02161E-08	2.66096E-08	negative
PRJNA729271	stage_1D_vs_E10	ordinary_muscle_development	ENSGALG00010014735	STX16	-0.006065946	0.105128601	-0.057700247	0.953987397	0.9612824	negative
PRJNA729271	stage_1D_vs_E10	ordinary_muscle_development	ENSGALG00010014736	KIF2A	-0.984158456	0.088942306	-11.06513316	1.85188E-28	1.4855E-27	negative
PRJNA729271	stage_1D_vs_E10	ordinary_muscle_development	ENSGALG00010014737	RMI1	-0.726338374	0.115500911	-6.288594318	3.20353E-10	9.37707E-10	negative
PRJNA729271	stage_1D_vs_E10	ordinary_muscle_development	ENSGALG00010014738	BRWD1	0.422472237	0.08642097	4.888538468	1.01587E-06	2.26959E-06	positive
PRJNA729271	stage_1D_vs_E10	ordinary_muscle_development	ENSGALG00010014739	TRMT2B	0.086962569	0.178378754	0.487516403	0.625892421	0.667719927	positive
PRJNA729271	stage_1D_vs_E10	ordinary_muscle_development	ENSGALG00010014740	TRMT9B	-3.02992582	0.493285246	-6.142340248	8.13144E-10	2.31471E-09	negative
PRJNA729271	stage_1D_vs_E10	ordinary_muscle_development	ENSGALG00010014742	PROZ	13.48013509	2.946630084	4.574763274	4.76759E-06	1.00241E-05	positive
PRJNA729271	stage_1D_vs_E10	ordinary_muscle_development	ENSGALG00010014744	IL2RB	1.458407509	0.470873574	3.097237964	0.00195333	0.003147695	positive
PRJNA729271	stage_1D_vs_E10	ordinary_muscle_development	ENSGALG00010014745	LSM10	0.590385811	0.186563682	3.164527009	0.001553352	0.002533856	positive
PRJNA729271	stage_1D_vs_E10	ordinary_muscle_development	ENSGALG00010014748	FGFR1OP2	0.220797666	0.107425121	2.05536344	0.039843914	0.05395026	positive
PRJNA729271	stage_1D_vs_E10	ordinary_muscle_development	ENSGALG00010014750	LONRF1	-0.042543238	0.182596022	-0.232991046	0.815768359	0.841186766	negative
PRJNA729271	stage_1D_vs_E10	ordinary_muscle_development	ENSGALG00010014753	ENSGALG00010014753	-3.478673538	0.513179529	-6.778667785	1.21289E-11	3.90512E-11	negative
PRJNA729271	stage_1D_vs_E10	ordinary_muscle_development	ENSGALG00010014754	DIMT1	-0.620323796	0.143192127	-4.332108272	1.47688E-05	2.96502E-05	negative
PRJNA729271	stage_1D_vs_E10	ordinary_muscle_development	ENSGALG00010014758	FXN	-0.757165607	0.101214733	-7.480784509	7.38802E-14	2.75223E-13	negative
PRJNA729271	stage_1D_vs_E10	ordinary_muscle_development	ENSGALG00010014762	STK40	-0.289341892	0.104759394	-2.761966082	0.005745445	0.008759884	negative
PRJNA729271	stage_1D_vs_E10	ordinary_muscle_development	ENSGALG00010014764	ENSGALG00010014764	4.431060294	2.337596634	1.895562404	0.058017933	0.076507454	positive
PRJNA729271	stage_1D_vs_E10	ordinary_muscle_development	ENSGALG00010014766	F10	-0.19976292	0.217884244	-0.916830499	0.359231468	0.408140784	negative
PRJNA729271	stage_1D_vs_E10	ordinary_muscle_development	ENSGALG00010014767	LRRC15	0.914356327	0.247134678	3.69983012	0.000215744	0.000385789	positive
PRJNA729271	stage_1D_vs_E10	ordinary_muscle_development	ENSGALG00010014769	CCDC69	2.499766041	0.321138571	7.784072876	7.02263E-15	2.78961E-14	positive
PRJNA729271	stage_1D_vs_E10	ordinary_muscle_development	ENSGALG00010014777	APCDD1L	-0.879280901	0.220071848	-3.995426532	6.45779E-05	0.000122217	negative
PRJNA729271	stage_1D_vs_E10	ordinary_muscle_development	ENSGALG00010014780	EVA1B	0.305265402	0.125130036	2.439585349	0.014704129	0.02122872	positive
PRJNA729271	stage_1D_vs_E10	ordinary_muscle_development	ENSGALG00010014783	ENSGALG00010014783	-0.155585213	0.119277975	-1.304391808	0.192100027	0.231809015	negative
PRJNA729271	stage_1D_vs_E10	ordinary_muscle_development	ENSGALG00010014789	F7	-1.414958449	0.972866842	-1.454421498	0.145829483	0.180265936	negative
PRJNA729271	stage_1D_vs_E10	ordinary_muscle_development	ENSGALG00010014792	ENSGALG00010014792	0.081016575	0.290708823	0.278686331	0.780485553	0.809121591	positive
PRJNA729271	stage_1D_vs_E10	ordinary_muscle_development	ENSGALG00010014793	TLR2A	-0.240749163	0.176356036	-1.365131409	0.172211752	0.209765491	negative
PRJNA729271	stage_1D_vs_E10	ordinary_muscle_development	ENSGALG00010014795	THRAP3	-0.464027161	0.095357851	-4.866166306	1.13784E-06	2.52998E-06	negative

PRJNA729271	stage_1D_vs_E10	ordinary_muscle_development	ENSGALG00010014796	PRLR	-0.683603106	0.248066669	-2.755723325	0.005856251	0.00892526	negative
PRJNA729271	stage_1D_vs_E10	ordinary_muscle_development	ENSGALG00010014797	ZDHHC9	-0.334815042	0.092478717	-3.620455115	0.000294085	0.000518837	negative
PRJNA729271	stage_1D_vs_E10	ordinary_muscle_development	ENSGALG00010014801	MPST	-0.607455895	0.087294309	-6.958711304	3.43399E-12	1.14796E-11	negative
PRJNA729271	stage_1D_vs_E10	ordinary_muscle_development	ENSGALG00010014802	ENSGALG00010014802	-0.71623653	0.405929025	-1.764437838	0.077658292	0.100621878	negative
PRJNA729271	stage_1D_vs_E10	ordinary_muscle_development	ENSGALG00010014805	SPEF2	-0.778867801	0.354986765	-2.194075603	0.028229975	0.039048906	negative
PRJNA729271	stage_1D_vs_E10	ordinary_muscle_development	ENSGALG00010014806	MAP7D1	1.647916706	0.226140217	7.28714569	3.16591E-13	1.13448E-12	positive
PRJNA729271	stage_1D_vs_E10	ordinary_muscle_development	ENSGALG00010014807	HES1	-0.630414859	0.226028236	-2.789097818	0.00528551	0.008100388	negative
PRJNA729271	stage_1D_vs_E10	ordinary_muscle_development	ENSGALG00010014810	ENTREP1	2.054411295	0.219837006	9.345156833	9.17534E-21	5.13104E-20	positive
PRJNA729271	stage_1D_vs_E10	ordinary_muscle_development	ENSGALG00010014812	RWDD1	-0.984679585	0.084680123	-11.62822568	2.9619E-31	2.71383E-30	negative
PRJNA729271	stage_1D_vs_E10	ordinary_muscle_development	ENSGALG00010014813	SASH3	0.698129884	0.26870468	2.598130721	0.00937328	0.013912113	positive
PRJNA729271	stage_1D_vs_E10	ordinary_muscle_development	ENSGALG00010014816	TST	0.448863752	0.108330735	4.143457092	3.42109E-05	6.63483E-05	positive
PRJNA729271	stage_1D_vs_E10	ordinary_muscle_development	ENSGALG00010014818	ENSGALG00010014818	2.195151013	0.385671606	5.691762061	1.25735E-08	3.25568E-08	positive
PRJNA729271	stage_1D_vs_E10	ordinary_muscle_development	ENSGALG00010014821	C3AR1L	0.878071203	0.232158975	3.782197966	0.00015545	0.000283142	positive
PRJNA729271	stage_1D_vs_E10	ordinary_muscle_development	ENSGALG00010014824	ANXA6	0.398359495	0.090606359	4.39659534	1.09962E-05	2.23674E-05	positive
PRJNA729271	stage_1D_vs_E10	ordinary_muscle_development	ENSGALG00010014829	ENSGALG00010014829	1.585512418	0.405535468	3.909676325	9.24199E-05	0.000172078	positive
PRJNA729271	stage_1D_vs_E10	ordinary_muscle_development	ENSGALG00010014830	TRAPPC3L	-1.615879958	0.431876662	-3.74153109	0.000182903	0.000330418	negative
PRJNA729271	stage_1D_vs_E10	ordinary_muscle_development	ENSGALG00010014831	VAPB	0.81100269	0.05985888	13.54857784	8.078E-42	1.13131E-40	positive
PRJNA729271	stage_1D_vs_E10	ordinary_muscle_development	ENSGALG00010014837	APBA1	-0.465701645	0.112468026	-4.140747028	3.46177E-05	6.71012E-05	negative
PRJNA729271	stage_1D_vs_E10	ordinary_muscle_development	ENSGALG00010014839	TM7SF3	0.465592282	0.106799001	4.359519085	1.30349E-05	2.63442E-05	positive
PRJNA729271	stage_1D_vs_E10	ordinary_muscle_development	ENSGALG00010014840	NCF4	0.078203418	0.221130322	0.353653072	0.723598895	0.75822786	positive
PRJNA729271	stage_1D_vs_E10	ordinary_muscle_development	ENSGALG00010014847	PAICS	-1.060565827	0.091481162	-11.59327016	4.45781E-31	4.0537E-30	negative
PRJNA729271	stage_1D_vs_E10	ordinary_muscle_development	ENSGALG00010014851	CALHM4	0.659971725	0.439325772	1.502237671	0.133035739	0.16541028	positive
PRJNA729271	stage_1D_vs_E10	ordinary_muscle_development	ENSGALG00010014856	MMGT1	0.146852419	0.071709461	2.047880677	0.040571693	0.054853716	positive
PRJNA729271	stage_1D_vs_E10	ordinary_muscle_development	ENSGALG00010014859	TRAPPC3	-0.306524075	0.128650266	-2.382615174	0.017190154	0.024560993	negative
PRJNA729271	stage_1D_vs_E10	ordinary_muscle_development	ENSGALG00010014861	MED21	-0.369975714	0.096090574	-3.850281003	0.000117982	0.000217494	negative
PRJNA729271	stage_1D_vs_E10	ordinary_muscle_development	ENSGALG00010014862	ENSGALG00010014862	-0.032958218	0.315884411	-0.104336324	0.916902453	0.929405668	negative
PRJNA729271	stage_1D_vs_E10	ordinary_muscle_development	ENSGALG00010014865	TLR2	1.162135291	0.37822779	3.072580392	0.002122167	0.003403497	positive
PRJNA729271	stage_1D_vs_E10	ordinary_muscle_development	ENSGALG00010014866	FAM26E	0.539521149	0.149051148	3.619704763	0.000294939	0.000520217	positive
PRJNA729271	stage_1D_vs_E10	ordinary_muscle_development	ENSGALG00010014867	TP63	-0.724064216	0.272768796	-2.654497972	0.007942652	0.011900841	negative
PRJNA729271	stage_1D_vs_E10	ordinary_muscle_development	ENSGALG00010014870	ENSGALG00010014870	1.029999804	2.033987753	0.5063943	0.612579875	0.655157183	positive
PRJNA729271	stage_1D_vs_E10	ordinary_muscle_development	ENSGALG00010014873	CALHM6	-0.198703619	0.723191044	-0.274759514	0.783501005	0.811667172	negative
PRJNA729271	stage_1D_vs_E10	ordinary_muscle_development	ENSGALG00010014876	PCNT	-0.002618783	0.10314307	-0.025389812	0.979744037	0.982785096	negative
PRJNA729271	stage_1D_vs_E10	ordinary_muscle_development	ENSGALG00010014877	ENSGALG00010014877	-0.296342021	0.090986229	-3.256998595	0.00112597	0.001867597	negative
PRJNA729271	stage_1D_vs_E10	ordinary_muscle_development	ENSGALG00010014878	TMCO1	0.256102253	0.130281012	1.965768065	0.049325408	0.065871733	positive
PRJNA729271	stage_1D_vs_E10	ordinary_muscle_development	ENSGALG00010014880	SFRP2	-1.330314789	0.29269227	-4.545097104	5.49099E-06	1.14835E-05	negative
PRJNA729271	stage_1D_vs_E10	ordinary_muscle_development	ENSGALG00010014881	ENSGALG00010014881	1.123909304	0.399167588	2.815632672	0.004868129	0.007493207	positive
PRJNA729271	stage_1D_vs_E10	ordinary_muscle_development	ENSGALG00010014882	XPNPEP2	-1.210391945	0.670132586	-1.806197714	0.070887462	0.092360788	negative
PRJNA729271	stage_1D_vs_E10	ordinary_muscle_development	ENSGALG00010014883	PPAT	-0.984250046	0.121762699	-8.083346185	6.30134E-16	2.68404E-15	negative
PRJNA729271	stage_1D_vs_E10	ordinary_muscle_development	ENSGALG00010014886	RABGAP1L	0.41879826	0.069489242	6.026807118	1.6723E-09	4.64636E-09	positive
PRJNA729271	stage_1D_vs_E10	ordinary_muscle_development	ENSGALG00010014887	MTMR12	-0.234804224	0.131776351	-1.781838872	0.074775511	0.097050115	negative
PRJNA729271	stage_1D_vs_E10	ordinary_muscle_development	ENSGALG00010014889	PVALB	1.041367029	0.418279631	2.489643174	0.01278714	0.018633719	positive
PRJNA729271	stage_1D_vs_E10	ordinary_muscle_development	ENSGALG00010014893	SLC9A6	-0.449112217	0.089368676	-5.025387411	5.02417E-07	1.14848E-06	negative
PRJNA729271	stage_1D_vs_E10	ordinary_muscle_development	ENSGALG00010014894	RAB22A	-0.147934859	0.077557986	-1.907409759	0.056467547	0.074666766	negative
PRJNA729271	stage_1D_vs_E10	ordinary_muscle_development	ENSGALG00010014897	COL8A2	-3.31696304	0.251362271	-13.19594634	9.25848E-40	1.20468E-38	negative
PRJNA729271	stage_1D_vs_E10	ordinary_muscle_development	ENSGALG00010014898	ENSGALG00010014898	2.269637417	0.658829008	3.44495672	0.000571151	0.000975932	positive
PRJNA729271	stage_1D_vs_E10	ordinary_muscle_development	ENSGALG00010014900	UBLCP1	-0.465474339	0.083653328	-5.564325427	2.63168E-08	6.63644E-08	negative
PRJNA729271	stage_1D_vs_E10	ordinary_muscle_development	ENSGALG00010014902	AASDH	0.254806582	0.125806472	2.025385318	0.042827814	0.057742458	positive
PRJNA729271	stage_1D_vs_E10	ordinary_muscle_development	ENSGALG00010014903	MCF2L	1.755854726	0.153550719	11.43501465	2.7949E-30	2.45825E-29	positive
PRJNA729271	stage_1D_vs_E10	ordinary_muscle_development	ENSGALG00010014906	IFT27	-0.433702204	0.109118466	-3.974599546	7.04978E-05	0.000132779	negative
PRJNA729271	stage_1D_vs_E10	ordinary_muscle_development	ENSGALG00010014907	ENSGALG00010014907	-0.309663234	0.070085925	-4.418337005	9.94632E-06	2.03088E-05	negative
PRJNA729271	stage_1D_vs_E10	ordinary_muscle_development	ENSGALG00010014908	ENSGALG00010014908	-0.25838426	0.101689658	-2.540909902	0.011056441	0.016255514	negative
PRJNA729271	stage_1D_vs_E10	ordinary_muscle_development	ENSGALG00010014909	FBXL14	0.124253359	0.096021425	1.294017028	0.195659539	0.2355748	positive
PRJNA729271	stage_1D_vs_E10	ordinary_muscle_development	ENSGALG00010014912	ENSGALG00010014912	1.200101899	0.33284153	3.605625471	0.000311402	0.000547921	positive
PRJNA729271	stage_1D_vs_E10	ordinary_muscle_development	ENSGALG00010014914	PTAR1	0.811613441	0.178321557	4.551403959	5.32891E-06	1.1159E-05	positive
PRJNA729271	stage_1D_vs_E10	ordinary_muscle_development	ENSGALG00010014916	DYNLT2B	-0.718751933	0.157596261	-4.560716929	5.09793E-06	1.06923E-05	negative
PRJNA729271	stage_1D_vs_E10	ordinary_muscle_development	ENSGALG00010014917	EIF3D	0.403184825	0.10497967	3.8405991	0.000122734	0.000225766	positive
PRJNA729271	stage_1D_vs_E10	ordinary_muscle_development	ENSGALG00010014918	PLRG1	-0.549439155	0.084225474	-6.52343205	6.87166E-11	2.10048E-10	negative
PRJNA729271	stage_1D_vs_E10	ordinary_muscle_development	ENSGALG00010014922	MAN1A1	0.033309115	0.164816565	0.202098099	0.839840034	0.862695307	positive
PRJNA729271	stage_1D_vs_E10	ordinary_muscle_development	ENSGALG00010014923	CIMIP1	-2.523471635	0.406753738	-6.203929802	5.50704E-10	1.58539E-09	negative
PRJNA729271	stage_1D_vs_E10	ordinary_muscle_development	ENSGALG00010014928	SUCO	-0.453594881	0.080672193	-5.62269183	1.88005E-08	4.80199E-08	negative
PRJNA729271	stage_1D_vs_E10	ordinary_muscle_development	ENSGALG00010014929	FHL1	2.910329896	0.284786394	10.21934321	1.62438E-24	1.08604E-23	positive
PRJNA729271	stage_1D_vs_E10	ordinary_muscle_development	ENSGALG00010014931	RNF145	0.016285706	0.108049178	0.150724937	0.880192701	0.897782929	positive
PRJNA729271	stage_1D_vs_E10	ordinary_muscle_development	ENSGALG00010014934	MAMDC2	0.281234469	0.204810159	1.373147066	0.169706632	0.206940155	positive
PRJNA729271	stage_1D_vs_E10	ordinary_muscle_development	ENSGALG00010014935	PCYT1A	0.140793941	0.131651377	1.069445255	0.284869085	0.3310758	positive
PRJNA729271	stage_1D_vs_E10	ordinary_muscle_development	ENSGALG00010014936	FOXRED2	-0.411376756	0.17005425	-2.419091293	0.015559335	0.022387587	negative
PRJNA729271	stage_1D_vs_E10	ordinary_muscle_development	ENSGALG00010014940	VAMP4	-0.160522455	0.084542444	-1.898720294	0.057601262	0.075992563	negative
PRJNA729271	stage_1D_vs_E10	ordinary_muscle_development	ENSGALG00010014942	PMEPA1	-1.844994212	0.146866836	-12.56236095	3.40033E-36	3.86267E-35	negative
PRJNA729271	stage_1D_vs_E10	ordinary_muscle_development	ENSGALG00010014943	APLN	-0.091155458	0.303696463	-0.300153178	0.764060318	0.794309281	negative
PRJNA729271	stage_1D_vs_E10	ordinary_muscle_development	ENSGALG00010014944	FGB	-1.661793924	0.931533997	-1.783932663			negative
PRJNA729271	stage_1D_vs_E10	ordinary_muscle_development	ENSGALG00010014945	TXN2	0.568688489	0.104371004	5.448721079	5.07333E-08	1.25278E-07	positive
PRJNA729271	stage_1D_vs_E10	ordinary_muscle_development	ENSGALG00010014946	PCK1	1.821804896	1.067306508	1.706918193	0.087837259	0.112752486	positive
PRJNA729271	stage_1D_vs_E10	ordinary_muscle_development	ENSGALG00010014948	RBM38	2.081539425	0.259230836	8.029675241	9.7731E-16	4.10731E-15	positive
PRJNA729271	stage_1D_vs_E10	ordinary_muscle_development	ENSGALG00010014949	AGO3	0.148122108	0.108082327	1.370456325	0.1705445	0.207874414	positive
PRJNA729271	stage_1D_vs_E10	ordinary_muscle_development	ENSGALG00010014952	C21orf58	-1.151860971	0.609040988	-1.891270036	0.058588306	0.077217449	negative
PRJNA729271	stage_1D_vs_E10	ordinary_muscle_development	ENSGALG00010014953	ENSGALG00010014953	-0.139238228	0.388839454	-0.358086677	0.720278449	0.755403815	negative
PRJNA729271	stage_1D_vs_E10	ordinary_muscle_development	ENSGALG00010014956	FGG	-0.774761944	1.607380533	-0.482002816	0.629803946	0.671497476	negative
PRJNA729271	stage_1D_vs_E10	ordinary_muscle_development	ENSGALG00010014965	ABHD13	-0.086997706	0.098965369	-0.879072219	0.37936212	0.428393107	negative
PRJNA729271	stage_1D_vs_E10	ordinary_muscle_development	ENSGALG00010014967	HNRNPH1	-0.765413511	0.107696905	-7.107107766	1.185E-12	4.06738E-12	negative
PRJNA729271	stage_1D_vs_E10	ordinary_muscle_development	ENSGALG00010014970	SLC51A	0.265161919	0.518865359	0.511041862	0.609321744	0.6519131	positive
PRJNA729271	stage_1D_vs_E10	ordinary_muscle_development	ENSGALG00010014971	SMC5	0.065231391	0.124003822	0.526043396	0.598858055	0.6419025	positive
PRJNA729271	stage_1D_vs_E10	ordinary_muscle_development	ENSGALG00010014972	FGA	-3.782090742	1.808274149	-2.091547205	0.036479039	0.049705177	negative
PRJNA729271	stage_1D_vs_E10	ordinary_muscle_development	ENSGALG00010014974	LIG4	0.105748299	0.112024556	0.943974275	0.345182794	0.394001465	positive
PRJNA729271	stage_1D_vs_E10	ordinary_muscle_development	ENSGALG00010014976	ITGB2	0.331046711	0.215899857	1.533334556	0.125193445	0.156532302	positive
PRJNA729271	stage_1D_vs_E10	ordinary_muscle_development	ENSGALG00010014980	ENSGALG00010014980	0.046510293	0.727504907	0.063931243	0.949024974	0.956814148	positive
PRJNA729271	stage_1D_vs_E10	ordinary_muscle_development	ENSGALG00010014982	ENSGALG00010014982	1.109876823	0.322051205	3.446274397	0.000568373	0.000971529	positive
PRJNA729271	stage_1D_vs_E10	ordinary_muscle_development	ENSGALG00010014984	LPP	1.778193881	0.09067599	19.61041603	1.25997E-85	6.76418E-84	positive
PRJNA729271	stage_1D_vs_E10	ordinary_muscle_development	ENSGALG00010014985	ENSGALG00010014985	0.190963354	0.107392842	1.778175806	0.075374985	0.097750775	positive
PRJNA729271	stage_1D_vs_E10	ordinary_muscle_development	ENSGALG00010014987	KLF9	7.480342799	0.289950993	25.79864525	9.183E-147	1.566E-144	positive
PRJNA729271	stage_1D_vs_E10	ordinary_muscle_development	ENSGALG00010014990	AGO1	-0.847339032	0.047319545	-17.90674495	1.04469E-71	4.04887E-70	negative
PRJNA729271	stage_1D_vs_E10	ordinary_muscle_development	ENSGALG00010014992	ARL15	-0.43732451	0.096671336	-4.523828129	6.0731E-06	1.26589E-05	negative
PRJNA729271	stage_1D_vs_E10	ordinary_muscle_development	ENSGALG00010014994	ENSGALG00010014994	0.239265178	0.118942305	2.011607033	0.044261377	0.059519878	positive
PRJNA729271	stage_1D_vs_E10	ordinary_muscle_development	ENSGALG00010014995	LRAT	-0.649530711	0.234952696	-2.764516957	0.005700714	0.008699006	negative
PRJNA729271	stage_1D_vs_E10	ordinary_muscle_development	ENSGALG00010014997	RAE1	-0.381957583	0.089274439	-4.278465225	1.88186E-05	3.73512E-05	negative
PRJNA729271	stage_1D_vs_E10	ordinary_muscle_development	ENSGALG00010014998	NALF1	0.426935908	0.461320772	0.925464307	0.354724442	0.403589543	positive
PRJNA729271	stage_1D_vs_E10	ordinary_muscle_development	ENSGALG00010015002	ADAM19	-0.436795549	0.200263388	-2.181105364	0.029175624	0.040291604	negative
PRJNA729271	stage_1D_vs_E10	ordinary_muscle_development	ENSGALG00010015006	HSPB3	3.630060832	0.329947662	11.00192925	3.74043E-28	2.94868E-27	positive
PRJNA729271	stage_1D_vs_E10	ordinary_muscle_development	ENSGALG00010015009	AGO4	0.718054179	0.10388286	6.912152613	4.77354E-12	1.57578E-11	positive
PRJNA729271	stage_1D_vs_E10	ordinary_muscle_development	ENSGALG00010015013	SNX18	-0.439579174	0.107636777	-4.083912453	4.42837E-05	8.50076E-05	negative
PRJNA729271	stage_1D_vs_E10	ordinary_muscle_development	ENSGALG00010015016	ARGLU1	0.117366211	0.079796488	1.470819242	0.141340007	0.175059677	positive
PRJNA729271	stage_1D_vs_E10	ordinary_muscle_development	ENSGALG00010015017	FAM184A	0.165465549	0.188830435	0.87626525	0.380885851	0.429879344	positive
PRJNA729271	stage_1D_vs_E10	ordinary_muscle_development	ENSGALG00010015018	MAP7D3	-0.082745604	0.068367507	-1.210306003	0.226161495	0.268992441	negative
PRJNA729271	stage_1D_vs_E10	ordinary_muscle_development	ENSGALG00010015020	NIPAL4	-2.142660947	0.874526198	-2.450082057	0.014282366	0.020658906	negative
PRJNA729271	stage_1D_vs_E10	ordinary_muscle_development	ENSGALG00010015021	RBM46	-3.146265857	1.051248081	-2.992886182	0.002763528	0.004374504	negative
PRJNA729271	stage_1D_vs_E10	ordinary_muscle_development	ENSGALG00010015024	CRACD	-0.484096756	0.372040473	-1.301193796	0.193192128	0.233029866	negative
PRJNA729271	stage_1D_vs_E10	ordinary_muscle_development	ENSGALG00010015025	TRAF3IP1	-0.680438896	0.186740282	-3.643771383	0.000268672	0.000475681	negative
PRJNA729271	stage_1D_vs_E10	ordinary_muscle_development	ENSGALG00010015027	CLSPN	-1.566198061	0.17743894	-8.826687446	1.07822E-18	5.35965E-18	negative
PRJNA729271	stage_1D_vs_E10	ordinary_muscle_development	ENSGALG00010015028	MCM9	-2.374634793	0.271779907	-8.737344936	2.38653E-18	1.16435E-17	negative
PRJNA729271	stage_1D_vs_E10	ordinary_muscle_development	ENSGALG00010015029	TFRC	-0.348240612	0.182243633	-1.910852012	0.056023601	0.074113544	negative
PRJNA729271	stage_1D_vs_E10	ordinary_muscle_development	ENSGALG00010015031	MYH9	0.394017231	0.109523365	3.597563245	0.000321212	0.00056436	positive
PRJNA729271	stage_1D_vs_E10	ordinary_muscle_development	ENSGALG00010015032	C1orf216	-1.83547416	0.25709576	-7.139262669	9.38329E-13	3.25229E-12	negative
PRJNA729271	stage_1D_vs_E10	ordinary_muscle_development	ENSGALG00010015033	BMP7	-1.467420333	0.324518238	-4.521842419	6.13037E-06	1.2769E-05	negative
PRJNA729271	stage_1D_vs_E10	ordinary_muscle_development	ENSGALG00010015035	SLC35F1	-1.590529345	0.11504254	-13.82557573	1.78677E-43	2.64278E-42	negative
PRJNA729271	stage_1D_vs_E10	ordinary_muscle_development	ENSGALG00010015037	CYFIP2	-0.409333958	0.192240712	-2.129278209	0.03323125	0.045571141	negative
PRJNA729271	stage_1D_vs_E10	ordinary_muscle_development	ENSGALG00010015038	PCSK5	-0.092714474	0.206007114	-0.45005472	0.652670985	0.692262983	negative
PRJNA729271	stage_1D_vs_E10	ordinary_muscle_development	ENSGALG00010015039	FAM114A1	-0.418829251	0.127034084	-3.296983286	0.000977293	0.001631636	negative
PRJNA729271	stage_1D_vs_E10	ordinary_muscle_development	ENSGALG00010015040	ZC4H2	-0.635278892	0.086036966	-7.38379	1.53846E-13	5.61571E-13	negative
PRJNA729271	stage_1D_vs_E10	ordinary_muscle_development	ENSGALG00010015041	TFAP2C	-5.628456012	0.562795005	-10.00089902	1.5102E-23	9.63906E-23	negative
PRJNA729271	stage_1D_vs_E10	ordinary_muscle_development	ENSGALG00010015042	EFNB2	0.023303363	0.132472682	0.175910707	0.860364093	0.880656559	positive
PRJNA729271	stage_1D_vs_E10	ordinary_muscle_development	ENSGALG00010015043	COQ10B	2.251107309	0.221856267	10.14669243	3.42784E-24	2.2503E-23	positive
PRJNA729271	stage_1D_vs_E10	ordinary_muscle_development	ENSGALG00010015044	ZC3H12B	0.282639242	0.125661573	2.249209799	0.024499151	0.034254384	positive
PRJNA729271	stage_1D_vs_E10	ordinary_muscle_development	ENSGALG00010015046	PSMB2	-0.607647089	0.094242932	-6.447667483	1.13585E-10	3.42645E-10	negative
PRJNA729271	stage_1D_vs_E10	ordinary_muscle_development	ENSGALG00010015047	NPY2R	-2.564527421	0.207464907	-12.36125887	4.23473E-35	4.61521E-34	negative
PRJNA729271	stage_1D_vs_E10	ordinary_muscle_development	ENSGALG00010015049	DCK2	-1.448081556	0.249019731	-5.815127777	6.05876E-09	1.61348E-08	negative
PRJNA729271	stage_1D_vs_E10	ordinary_muscle_development	ENSGALG00010015050	HIVEP3	-0.660021691	0.178167533	-3.704500375	0.000211808	0.000379312	negative
PRJNA729271	stage_1D_vs_E10	ordinary_muscle_development	ENSGALG00010015052	ENSGALG00010015052	-0.722964214	0.108737658	-6.648701346	2.9569E-11	9.27512E-11	negative
PRJNA729271	stage_1D_vs_E10	ordinary_muscle_development	ENSGALG00010015054	ENSGALG00010015054	-0.670666629	0.174335742	-3.846982963	0.000119581	0.000220301	negative
PRJNA729271	stage_1D_vs_E10	ordinary_muscle_development	ENSGALG00010015055	ZNF335	0.30545553	0.182176703	1.676699188	0.09360132	0.119621859	positive
PRJNA729271	stage_1D_vs_E10	ordinary_muscle_development	ENSGALG00010015056	ENSGALG00010015056	-1.121184115	0.47072074	-2.381845585	0.017226119	0.024605104	negative
PRJNA729271	stage_1D_vs_E10	ordinary_muscle_development	ENSGALG00010015057	LAS1L	-0.759977894	0.11321657	-6.712603056	1.91183E-11	6.07318E-11	negative
PRJNA729271	stage_1D_vs_E10	ordinary_muscle_development	ENSGALG00010015059	ENSGALG00010015059	0.513221491	0.162416503	3.159909747	0.00157818	0.002572908	positive
PRJNA729271	stage_1D_vs_E10	ordinary_muscle_development	ENSGALG00010015061	MMP9	-0.600520695	0.471271152	-1.274257278	0.202572252	0.243009583	negative

PRJNA729271	stage_1D_vs_E10	ordinary_muscle_development	ENSGALG00010015062	ENSGALG00010015062	0.444430859	0.227316114	1.955122547	0.050568608	0.067407759	positive
PRJNA729271	stage_1D_vs_E10	ordinary_muscle_development	ENSGALG00010015063	EEF1AKMT4	-0.932101819	0.215690351	-4.321481307	1.54985E-05	3.10249E-05	negative
PRJNA729271	stage_1D_vs_E10	ordinary_muscle_development	ENSGALG00010015064	RFK	-0.210973996	0.146993128	-1.435264344	0.151211784	0.186283768	negative
PRJNA729271	stage_1D_vs_E10	ordinary_muscle_development	ENSGALG00010015065	F11	4.807393664	1.218370093	3.945758101	7.95479E-05	0.000149049	positive
PRJNA729271	stage_1D_vs_E10	ordinary_muscle_development	ENSGALG00010015066	RUFY1	-0.28469399	0.070183515	-4.056422516	4.98301E-05	9.52006E-05	negative
PRJNA729271	stage_1D_vs_E10	ordinary_muscle_development	ENSGALG00010015068	ESM1	-2.280234063	0.331419541	-6.880204042	5.97669E-12	1.96E-11	negative
PRJNA729271	stage_1D_vs_E10	ordinary_muscle_development	ENSGALG00010015069	HSPD1	-0.670462352	0.10245148	-6.544193923	5.98171E-11	1.83931E-10	negative
PRJNA729271	stage_1D_vs_E10	ordinary_muscle_development	ENSGALG00010015070	EDN2	0.23166225	0.440033142	0.52646546	0.598564845	0.641778047	positive
PRJNA729271	stage_1D_vs_E10	ordinary_muscle_development	ENSGALG00010015071	SNAI1	-1.980969808	0.127229668	-15.57003052	1.16352E-54	2.63107E-53	negative
PRJNA729271	stage_1D_vs_E10	ordinary_muscle_development	ENSGALG00010015072	MAP9	-0.266353934	0.137352598	-1.939198366	0.052477189	0.069733851	negative
PRJNA729271	stage_1D_vs_E10	ordinary_muscle_development	ENSGALG00010015075	ENSGALG00010015075	1.802400782	0.167795548	10.74164842	6.48755E-27	4.84727E-26	positive
PRJNA729271	stage_1D_vs_E10	ordinary_muscle_development	ENSGALG00010015076	ITK	-2.403174901	0.999190538	-2.405121757	0.016167078	0.023183795	negative
PRJNA729271	stage_1D_vs_E10	ordinary_muscle_development	ENSGALG00010015080	ASF1A	-0.980181287	0.078315436	-12.51581219	6.11789E-36	6.89035E-35	negative
PRJNA729271	stage_1D_vs_E10	ordinary_muscle_development	ENSGALG00010015083	CEP135	-1.724795348	0.189447161	-9.104361022	8.67757E-20	4.59224E-19	negative
PRJNA729271	stage_1D_vs_E10	ordinary_muscle_development	ENSGALG00010015084	FOXO6	1.808801512	0.201611022	8.971739234	2.91869E-19	1.4981E-18	positive
PRJNA729271	stage_1D_vs_E10	ordinary_muscle_development	ENSGALG00010015085	PSMD2	-0.312860031	0.163688843	-1.91130944	0.055964827	0.074042549	negative
PRJNA729271	stage_1D_vs_E10	ordinary_muscle_development	ENSGALG00010015086	FOXN3	0.681559403	0.095648004	7.125704374	1.0355E-12	3.5754E-12	positive
PRJNA729271	stage_1D_vs_E10	ordinary_muscle_development	ENSGALG00010015087	GABRA3	-4.027798758	0.558538668	-7.211315862	5.54137E-13	1.95479E-12	negative
PRJNA729271	stage_1D_vs_E10	ordinary_muscle_development	ENSGALG00010015088	ENSGALG00010015088	2.138693951	1.079435873	1.981307093			positive
PRJNA729271	stage_1D_vs_E10	ordinary_muscle_development	ENSGALG00010015090	GZMA	-2.141667504	0.693112335	-3.089928421	0.002002047	0.003222618	negative
PRJNA729271	stage_1D_vs_E10	ordinary_muscle_development	ENSGALG00010015091	MSN	-0.840386656	0.121319337	-6.927062705	4.29668E-12	1.42355E-11	negative
PRJNA729271	stage_1D_vs_E10	ordinary_muscle_development	ENSGALG00010015092	HSPE1	-0.133646833	0.12245678	-1.091379611	0.27510588	0.320783504	negative
PRJNA729271	stage_1D_vs_E10	ordinary_muscle_development	ENSGALG00010015094	CDK5RAP1	0.418259746	0.213621196	1.957950589	0.050235811	0.067007277	positive
PRJNA729271	stage_1D_vs_E10	ordinary_muscle_development	ENSGALG00010015095	RBFOX2	0.020677026	0.133080615	0.155372185	0.876527911	0.894548481	positive
PRJNA729271	stage_1D_vs_E10	ordinary_muscle_development	ENSGALG00010015096	GABRE	-3.256689949	1.087911234	-2.993525436	0.002757745	0.004366304	negative
PRJNA729271	stage_1D_vs_E10	ordinary_muscle_development	ENSGALG00010015097	MFSD2A	-0.410806634	0.160666267	-2.556894126	0.010561136	0.015557283	negative
PRJNA729271	stage_1D_vs_E10	ordinary_muscle_development	ENSGALG00010015098	HAVCR1	0.073706131	0.222315882	0.331537857	0.740238251	0.773482803	positive
PRJNA729271	stage_1D_vs_E10	ordinary_muscle_development	ENSGALG00010015100	MOB4	0.392253011	0.088836034	4.415471906	1.0079E-05	2.05681E-05	positive
PRJNA729271	stage_1D_vs_E10	ordinary_muscle_development	ENSGALG00010015101	CAP1	-1.137566466	0.099548925	-11.42720996	3.05776E-30	2.68295E-29	negative
PRJNA729271	stage_1D_vs_E10	ordinary_muscle_development	ENSGALG00010015103	ITPK1	-0.835063755	0.09218733	-9.058335413	1.32456E-19	6.93125E-19	negative
PRJNA729271	stage_1D_vs_E10	ordinary_muscle_development	ENSGALG00010015104	CNGA2	2.069386238	0.265035288	7.807964953	5.81187E-15	2.32395E-14	positive
PRJNA729271	stage_1D_vs_E10	ordinary_muscle_development	ENSGALG00010015105	ENSGALG00010015105	4.230545853	0.984166584	4.298607492	1.71875E-05	3.4245E-05	positive
PRJNA729271	stage_1D_vs_E10	ordinary_muscle_development	ENSGALG00010015107	MB	0.216383051	0.564126258	0.383572026	0.701295677	0.738219378	positive
PRJNA729271	stage_1D_vs_E10	ordinary_muscle_development	ENSGALG00010015108	ENSGALG00010015108	0.312622201	0.088331856	3.53917844	0.000401374	0.000698598	positive
PRJNA729271	stage_1D_vs_E10	ordinary_muscle_development	ENSGALG00010015109	PPT1	0.425187214	0.108286804	3.926491485	8.61939E-05	0.000160982	positive
PRJNA729271	stage_1D_vs_E10	ordinary_muscle_development	ENSGALG00010015110	ENSGALG00010015110	2.517761653	0.721926751	3.487558331	0.000487453	0.000840445	positive
PRJNA729271	stage_1D_vs_E10	ordinary_muscle_development	ENSGALG00010015111	ADAMTS2	2.466621862	0.141519226	17.42958837	4.91989E-68	1.702E-66	positive
PRJNA729271	stage_1D_vs_E10	ordinary_muscle_development	ENSGALG00010015112	CBLN4	-2.517353426	0.376898677	-6.679125132	2.40373E-11	7.5859E-11	negative
PRJNA729271	stage_1D_vs_E10	ordinary_muscle_development	ENSGALG00010015115	CLDN2	-0.482584605	0.421037869	-1.146178621	0.251721252	0.296304982	negative
PRJNA729271	stage_1D_vs_E10	ordinary_muscle_development	ENSGALG00010015117	RASD2	-0.217990308	0.205194868	-1.062357503	0.288073419	0.334531662	negative
PRJNA729271	stage_1D_vs_E10	ordinary_muscle_development	ENSGALG00010015118	GPX8	-0.722803993	0.185707434	-3.892165095	9.93536E-05	0.000184278	negative
PRJNA729271	stage_1D_vs_E10	ordinary_muscle_development	ENSGALG00010015119	TLR1A	-0.131102975	0.230110564	-0.569739051	0.568854699	0.614010639	negative
PRJNA729271	stage_1D_vs_E10	ordinary_muscle_development	ENSGALG00010015121	UNC79	-4.439689821	0.291282922	-15.24184734	1.86521E-52	3.94114E-51	negative
PRJNA729271	stage_1D_vs_E10	ordinary_muscle_development	ENSGALG00010015122	ENSGALG00010015122	-2.297999861	0.205437518	-11.18588213	4.78127E-29	3.96479E-28	negative
PRJNA729271	stage_1D_vs_E10	ordinary_muscle_development	ENSGALG00010015123	CEP85L	1.349349678	0.250719786	5.381903428	7.37023E-08	1.79972E-07	positive
PRJNA729271	stage_1D_vs_E10	ordinary_muscle_development	ENSGALG00010015124	UXS1	0.195622629	0.095445302	2.049578388	0.040405591	0.054654633	positive
PRJNA729271	stage_1D_vs_E10	ordinary_muscle_development	ENSGALG00010015126	MCM5	-1.698380477	0.179266897	-9.474032873	2.69243E-21	1.54868E-20	negative
PRJNA729271	stage_1D_vs_E10	ordinary_muscle_development	ENSGALG00010015127	RPL11	0.219809187	0.151571264	1.450203563	0.147001762	0.181591149	positive
PRJNA729271	stage_1D_vs_E10	ordinary_muscle_development	ENSGALG00010015128	OCRL	0.172879699	0.106739713	1.619638045	0.105310052	0.133397641	positive
PRJNA729271	stage_1D_vs_E10	ordinary_muscle_development	ENSGALG00010015129	PRRG3	-0.63963922	0.129914686	-4.9235328	8.49956E-07	1.90862E-06	negative
PRJNA729271	stage_1D_vs_E10	ordinary_muscle_development	ENSGALG00010015131	DOK5	3.648265171	0.239727993	15.2183528	2.67174E-52	5.60446E-51	positive
PRJNA729271	stage_1D_vs_E10	ordinary_muscle_development	ENSGALG00010015132	GCNT1	-1.556983184	0.292607237	-5.321068607	1.03159E-07	2.48926E-07	negative
PRJNA729271	stage_1D_vs_E10	ordinary_muscle_development	ENSGALG00010015133	RFTN2	-0.268240595	0.127676068	-2.100946563	0.035645657	0.048638219	negative
PRJNA729271	stage_1D_vs_E10	ordinary_muscle_development	ENSGALG00010015134	PLN	5.899671252	0.519987822	11.34578735	7.78226E-30	6.65902E-29	positive
PRJNA729271	stage_1D_vs_E10	ordinary_muscle_development	ENSGALG00010015136	TCEB3	0.484168299	0.090439946	5.35347844	8.62793E-08	2.09379E-07	positive
PRJNA729271	stage_1D_vs_E10	ordinary_muscle_development	ENSGALG00010015137	AMN	2.165262951	0.406940111	5.320839336	1.0329E-07	2.49198E-07	positive
PRJNA729271	stage_1D_vs_E10	ordinary_muscle_development	ENSGALG00010015138	ENSGALG00010015138	-0.087150321	0.458135589	-0.190228227	0.849130293	0.871249635	negative
PRJNA729271	stage_1D_vs_E10	ordinary_muscle_development	ENSGALG00010015140	ASCC3	-0.015257059	0.073093569	-0.208733265	0.83465647	0.858282175	negative
PRJNA729271	stage_1D_vs_E10	ordinary_muscle_development	ENSGALG00010015141	PFDN4	0.414860326	0.135734306	3.056414684	0.002240012	0.003582586	positive
PRJNA729271	stage_1D_vs_E10	ordinary_muscle_development	ENSGALG00010015142	HNRNPAB	-0.842631762	0.128625865	-6.55102894	5.7142E-11	1.75891E-10	negative
PRJNA729271	stage_1D_vs_E10	ordinary_muscle_development	ENSGALG00010015143	EXOC1	-0.289577723	0.080165118	-3.61226591	0.000303533	0.000534465	negative
PRJNA729271	stage_1D_vs_E10	ordinary_muscle_development	ENSGALG00010015145	CLIC2	1.586293271	0.12448535	12.74281085	3.41853E-37	4.04502E-36	positive
PRJNA729271	stage_1D_vs_E10	ordinary_muscle_development	ENSGALG00010015146	ENSGALG00010015146	0.948382251	0.127567273	7.434369556	1.05068E-13	3.88411E-13	positive
PRJNA729271	stage_1D_vs_E10	ordinary_muscle_development	ENSGALG00010015148	ENSGALG00010015148	-0.113502231	0.075412255	-1.50509001	0.132300937	0.164646383	negative
PRJNA729271	stage_1D_vs_E10	ordinary_muscle_development	ENSGALG00010015149	ENSGALG00010015149	1.653844144	0.138277127	11.96035944	5.73133E-33	5.70183E-32	positive
PRJNA729271	stage_1D_vs_E10	ordinary_muscle_development	ENSGALG00010015151	ENSGALG00010015151	-2.945954405	0.390411709	-7.545763442	4.49646E-14	1.70262E-13	negative
PRJNA729271	stage_1D_vs_E10	ordinary_muscle_development	ENSGALG00010015152	BOLL	2.81701964	0.276097001	10.20300703	1.92227E-24	1.27755E-23	positive
PRJNA729271	stage_1D_vs_E10	ordinary_muscle_development	ENSGALG00010015153	ERCC6L	-1.70291707	0.241083867	-7.063587822	1.62258E-12	5.53003E-12	negative
PRJNA729271	stage_1D_vs_E10	ordinary_muscle_development	ENSGALG00010015154	CYP24A1	-4.00925646	0.892704159	-4.491136755	7.0844E-06	1.46824E-05	negative
PRJNA729271	stage_1D_vs_E10	ordinary_muscle_development	ENSGALG00010015155	HMOX1	-0.996202726	0.179464347	-5.550978455	2.84075E-08	7.14005E-08	negative
PRJNA729271	stage_1D_vs_E10	ordinary_muscle_development	ENSGALG00010015156	CNOT6	-0.265452077	0.073801702	-3.596828664	0.000322121	0.000565819	negative
PRJNA729271	stage_1D_vs_E10	ordinary_muscle_development	ENSGALG00010015157	CDC42BPB	-0.258619684	0.069817972	-3.704199302	0.000212059	0.000379669	negative
PRJNA729271	stage_1D_vs_E10	ordinary_muscle_development	ENSGALG00010015158	PIN4	-0.283910648	0.165485512	-1.715622384	0.086231179	0.110887237	negative
PRJNA729271	stage_1D_vs_E10	ordinary_muscle_development	ENSGALG00010015160	SIM1	-6.536395418	0.563929771	-11.59079686	4.58844E-31	4.16465E-30	negative
PRJNA729271	stage_1D_vs_E10	ordinary_muscle_development	ENSGALG00010015161	GFPT2	-0.176810289	0.200441182	-0.882105601	0.377719704	0.426870973	negative
PRJNA729271	stage_1D_vs_E10	ordinary_muscle_development	ENSGALG00010015163	PLK2	-0.917879487	0.209555386	-4.380128355	1.18609E-05	2.40521E-05	negative
PRJNA729271	stage_1D_vs_E10	ordinary_muscle_development	ENSGALG00010015165	KLF3	0.922428755	0.115632917	7.977216018	1.49671E-15	6.21983E-15	positive
PRJNA729271	stage_1D_vs_E10	ordinary_muscle_development	ENSGALG00010015166	YTHDF2	-0.229631994	0.181250182	-1.266933864	0.205178939	0.245912744	negative
PRJNA729271	stage_1D_vs_E10	ordinary_muscle_development	ENSGALG00010015167	RAB3C	-1.9267893	0.391865953	-4.916960208	8.78984E-07	1.97197E-06	negative
PRJNA729271	stage_1D_vs_E10	ordinary_muscle_development	ENSGALG00010015169	ENSGALG00010015169	1.232047769	0.532722281	2.312739326	0.020736975	0.029283176	positive
PRJNA729271	stage_1D_vs_E10	ordinary_muscle_development	ENSGALG00010015170	EXOC1L	-1.877997258	0.84169222	-2.231216131	0.025666816	0.035742194	negative
PRJNA729271	stage_1D_vs_E10	ordinary_muscle_development	ENSGALG00010015171	NHSL2	2.190494154	0.198123787	11.05618962	2.04606E-28	1.63764E-27	positive
PRJNA729271	stage_1D_vs_E10	ordinary_muscle_development	ENSGALG00010015172	PPP4R3A	0.348942462	0.076163082	4.581517099	4.61615E-06	9.71274E-06	positive
PRJNA729271	stage_1D_vs_E10	ordinary_muscle_development	ENSGALG00010015173	ENS-1	-1.049178988	0.697099509	-1.505063444	0.132307766	0.164646383	negative
PRJNA729271	stage_1D_vs_E10	ordinary_muscle_development	ENSGALG00010015174	ST6GAL2	-4.039870479	0.216819048	-18.63245192	1.75306E-77	7.79467E-76	negative
PRJNA729271	stage_1D_vs_E10	ordinary_muscle_development	ENSGALG00010015175	MAPK9	0.265754485	0.068481066	3.880700166	0.000104156	0.000192865	positive
PRJNA729271	stage_1D_vs_E10	ordinary_muscle_development	ENSGALG00010015177	TC2N	-0.018317847	0.159041968	-0.115176184	0.908305476	0.921960695	negative
PRJNA729271	stage_1D_vs_E10	ordinary_muscle_development	ENSGALG00010015180	PITHD1	0.555283503	0.125597158	4.421147029	9.81783E-06	2.00605E-05	positive
PRJNA729271	stage_1D_vs_E10	ordinary_muscle_development	ENSGALG00010015181	VPS13A	1.3709741	0.119941372	11.43036866	2.94854E-30	2.58878E-29	positive
PRJNA729271	stage_1D_vs_E10	ordinary_muscle_development	ENSGALG00010015182	PHYKPL	-1.110133654	0.32653931	-3.399693755	0.000674614	0.001144892	negative
PRJNA729271	stage_1D_vs_E10	ordinary_muscle_development	ENSGALG00010015183	SLC5A7	-4.25591539	0.698581942	-6.092220731	1.11355E-09	3.13294E-09	negative
PRJNA729271	stage_1D_vs_E10	ordinary_muscle_development	ENSGALG00010015186	ENSGALG00010015186	0.45474608	0.364052625	1.249121826	0.211620519	0.253151722	positive
PRJNA729271	stage_1D_vs_E10	ordinary_muscle_development	ENSGALG00010015188	SULT1C3	-3.581689268	1.312121433	-2.72969344	0.006339325	0.009617805	negative
PRJNA729271	stage_1D_vs_E10	ordinary_muscle_development	ENSGALG00010015190	FBLN5	1.288419242	0.191999315	6.710540818	1.93904E-11	6.15694E-11	positive
PRJNA729271	stage_1D_vs_E10	ordinary_muscle_development	ENSGALG00010015191	TOM1	2.87135543	0.187287557	15.33126641	4.7268E-53	1.01654E-51	positive
PRJNA729271	stage_1D_vs_E10	ordinary_muscle_development	ENSGALG00010015192	ENSGALG00010015192	2.159986902	0.216289785	9.986541438	1.74566E-23	1.1098E-22	positive
PRJNA729271	stage_1D_vs_E10	ordinary_muscle_development	ENSGALG00010015193	LYPLA2	-0.693377704	0.106640395	-6.502017392	7.92499E-11	2.41532E-10	negative
PRJNA729271	stage_1D_vs_E10	ordinary_muscle_development	ENSGALG00010015194	TMLHE	0.325741703	0.113028954	2.881931507	0.003952456	0.006147087	positive
PRJNA729271	stage_1D_vs_E10	ordinary_muscle_development	ENSGALG00010015196	PDE4D	0.838609648	0.218006371	3.846720834	0.000119709	0.000220509	positive
PRJNA729271	stage_1D_vs_E10	ordinary_muscle_development	ENSGALG00010015198	NME5	-0.843561578	0.231133759	-3.649668408	0.000262579	0.000465519	negative
PRJNA729271	stage_1D_vs_E10	ordinary_muscle_development	ENSGALG00010015199	ENSGALG00010015199	-0.947457658	0.172631368	-5.488328502	4.05755E-08	1.00796E-07	negative
PRJNA729271	stage_1D_vs_E10	ordinary_muscle_development	ENSGALG00010015200	TBC1D1	-0.21449924	0.085590039	-2.506123871	0.012206285	0.01784469	negative
PRJNA729271	stage_1D_vs_E10	ordinary_muscle_development	ENSGALG00010015201	GCC2	0.265266944	0.108411583	2.446850568	0.014411057	0.020830502	positive
PRJNA729271	stage_1D_vs_E10	ordinary_muscle_development	ENSGALG00010015203	HMGXB4	-0.156003126	0.064387619	-2.422874604	0.015398241	0.022164598	negative
PRJNA729271	stage_1D_vs_E10	ordinary_muscle_development	ENSGALG00010015204	ENSGALG00010015204	0.49327741	0.137545455	3.586286501	0.00033542	0.000588325	positive
PRJNA729271	stage_1D_vs_E10	ordinary_muscle_development	ENSGALG00010015207	AKR1E2	-1.003808852	0.179590426	-5.589434095	2.27811E-08	5.77798E-08	negative
PRJNA729271	stage_1D_vs_E10	ordinary_muscle_development	ENSGALG00010015208	GNA14	0.088172723	0.658077751	0.13398526	0.893414224	0.909157483	positive
PRJNA729271	stage_1D_vs_E10	ordinary_muscle_development	ENSGALG00010015212	GNAQ	0.289478957	0.097182863	2.978703752	0.002894705	0.004573161	positive
PRJNA729271	stage_1D_vs_E10	ordinary_muscle_development	ENSGALG00010015213	RUBCN	0.305350316	0.118999962	2.565969857	0.010288777	0.015179218	positive
PRJNA729271	stage_1D_vs_E10	ordinary_muscle_development	ENSGALG00010015218	PLCL1	-1.423783195	0.157490262	-9.040452253	1.56025E-19	8.1235E-19	negative
PRJNA729271	stage_1D_vs_E10	ordinary_muscle_development	ENSGALG00010015227	FYTTD1	-1.036769389	0.075895636	-13.66046117	1.74873E-42	2.5072E-41	negative
PRJNA729271	stage_1D_vs_E10	ordinary_muscle_development	ENSGALG00010015233	ENSGALG00010015233	0.535821159	0.686186528	0.780868084	0.434880085	0.484406888	positive
PRJNA729271	stage_1D_vs_E10	ordinary_muscle_development	ENSGALG00010015234	TRIP11	0.520410874	0.143412737	3.628763296	0.000284782	0.000502975	positive
PRJNA729271	stage_1D_vs_E10	ordinary_muscle_development	ENSGALG00010015241	ENSGALG00010015241	0.634883943	0.154152354	4.11854849	3.81266E-05	7.36174E-05	positive
PRJNA729271	stage_1D_vs_E10	ordinary_muscle_development	ENSGALG00010015248	ENSGALG00010015248	0.42817992	0.134591061	3.181339945	0.001465955	0.002398309	positive
PRJNA729271	stage_1D_vs_E10	ordinary_muscle_development	ENSGALG00010015250	RORB	-1.91407023	0.473329387	-4.04384406	5.25819E-05	0.000100207	negative
PRJNA729271	stage_1D_vs_E10	ordinary_muscle_development	ENSGALG00010015253	ENSGALG00010015253	-0.127622705	0.072224733	-1.767022176	0.077224515	0.100086672	negative
PRJNA729271	stage_1D_vs_E10	ordinary_muscle_development	ENSGALG00010015260	RASGEF1C	1.999070086	0.138167877	14.46841428	1.91844E-47	3.31045E-46	positive
PRJNA729271	stage_1D_vs_E10	ordinary_muscle_development	ENSGALG00010015261	IQCG	-0.331234898	0.41264558	-0.802710397	0.422142142	0.471740833	negative
PRJNA729271	stage_1D_vs_E10	ordinary_muscle_development	ENSGALG00010015283	ENSGALG00010015283	-1.000268211	0.624327179	-1.602153877	0.109121586	0.137756262	negative
PRJNA729271	stage_1D_vs_E10	ordinary_muscle_development	ENSGALG00010015285	ALG13	0.2404798	0.405921291	0.592429629	0.55356293	0.599424374	positive
PRJNA729271	stage_1D_vs_E10	ordinary_muscle_development	ENSGALG00010015291	ZFP64	0.294416519	0.091667206	3.211797677	0.001319072	0.002171488	positive
PRJNA729271	stage_1D_vs_E10	ordinary_muscle_development	ENSGALG00010015296	RPL35A	0.111670488	0.195379983	0.57155542	0.567623205	0.612863937	positive
PRJNA729271	stage_1D_vs_E10	ordinary_muscle_development	ENSGALG00010015298	ENSGALG00010015298	2.556937257	0.234561989	10.90090199	1.1412E-27	8.77081E-27	positive
PRJNA729271	stage_1D_vs_E10	ordinary_muscle_development	ENSGALG00010015299	GALE	-0.730708764	0.095286101	-7.668576557	1.73916E-14	6.75122E-14	negative
PRJNA729271	stage_1D_vs_E10	ordinary_muscle_development	ENSGALG00010015300	ATXN3	1.063205436	0.126781974	8.386093108	5.02565E-17	2.28145E-16	positive
PRJNA729271	stage_1D_vs_E10	ordinary_muscle_development	ENSGALG00010015306	BCAT1	0.738101996	0.090762842	8.132204531	4.21553E-16	1.81966E-15	positive
PRJNA729271	stage_1D_vs_E10	ordinary_muscle_development	ENSGALG00010015310	PGM2	-1.585663015	0.094228311	-16.82788329	1.52456E-63	4.62311E-62	negative
PRJNA729271	stage_1D_vs_E10	ordinary_muscle_development	ENSGALG00010015314	GMEB1	-0.044128778	0.066848927	-0.660126937	0.509172374	0.557139186	negative
PRJNA729271	stage_1D_vs_E10	ordinary_muscle_development	ENSGALG00010015318	SALL4	0.26634939	0.167994527	1.585464687	0.112860794	0.14221657	positive
PRJNA729271	stage_1D_vs_E10	ordinary_muscle_development	ENSGALG00010015320	TRPM6	-3.38660655	0.439299454	-7.709107125	1.26701E-14	4.95381E-14	negative

PRJNA729271	stage_1D_vs_E10	ordinary_muscle_development	ENSGALG00010015327	LARGE1	-0.199374311	0.126260045	-1.579076829	0.114318436	0.143953238	negative
PRJNA729271	stage_1D_vs_E10	ordinary_muscle_development	ENSGALG00010015328	ENSGALG00010015328	0.536054803	0.400196149	1.339480165	0.180414407	0.218965657	positive
PRJNA729271	stage_1D_vs_E10	ordinary_muscle_development	ENSGALG00010015333	MED7	-0.317923273	0.068830329	-4.61894162	3.85702E-06	8.18199E-06	negative
PRJNA729271	stage_1D_vs_E10	ordinary_muscle_development	ENSGALG00010015339	ENSGALG00010015339	0.518221529	0.77983181	0.664529868	0.506351239	0.554462364	positive
PRJNA729271	stage_1D_vs_E10	ordinary_muscle_development	ENSGALG00010015348	CPSF2	-0.517297085	0.06052258	-8.547175042	1.26138E-17	5.91131E-17	negative
PRJNA729271	stage_1D_vs_E10	ordinary_muscle_development	ENSGALG00010015353	RELL1	0.391497208	0.091244959	4.290617383	1.78177E-05	3.54471E-05	positive
PRJNA729271	stage_1D_vs_E10	ordinary_muscle_development	ENSGALG00010015356	ENSGALG00010015356	-0.156065342	0.084058848	-1.856620037	0.063365231	0.083165431	negative
PRJNA729271	stage_1D_vs_E10	ordinary_muscle_development	ENSGALG00010015362	ATP9A	0.331551445	0.127600878	2.598347675	0.00936736	0.013904749	positive
PRJNA729271	stage_1D_vs_E10	ordinary_muscle_development	ENSGALG00010015364	CLOCK	2.757274167	0.20217104	13.6383241	2.36943E-42	3.37377E-41	positive
PRJNA729271	stage_1D_vs_E10	ordinary_muscle_development	ENSGALG00010015370	ENSGALG00010015370	1.589954258	0.14259031	11.15050704	7.11995E-29	5.84061E-28	positive
PRJNA729271	stage_1D_vs_E10	ordinary_muscle_development	ENSGALG00010015374	LIMS1	-1.43126802	0.151156551	-9.468779292	2.83133E-21	1.62471E-20	negative
PRJNA729271	stage_1D_vs_E10	ordinary_muscle_development	ENSGALG00010015384	TAF12	0.102317725	0.116165673	0.880791398	0.378430737	0.427474558	positive
PRJNA729271	stage_1D_vs_E10	ordinary_muscle_development	ENSGALG00010015386	SLC24A4	-0.444174855	0.262613607	-1.691362684	0.090767555	0.116266853	negative
PRJNA729271	stage_1D_vs_E10	ordinary_muscle_development	ENSGALG00010015388	PSMD1	-0.588371021	0.1225222	-4.802158477	1.56964E-06	3.44988E-06	negative
PRJNA729271	stage_1D_vs_E10	ordinary_muscle_development	ENSGALG00010015390	CCNB3	-1.96494047	0.204984036	-9.58582194	9.17244E-22	5.40905E-21	negative
PRJNA729271	stage_1D_vs_E10	ordinary_muscle_development	ENSGALG00010015391	SYN3	-1.490093283	0.415409477	-3.587046914	0.000334444	0.000586826	negative
PRJNA729271	stage_1D_vs_E10	ordinary_muscle_development	ENSGALG00010015392	TMEM165	-0.726955101	0.081962971	-8.869311207	7.35997E-19	3.69272E-18	negative
PRJNA729271	stage_1D_vs_E10	ordinary_muscle_development	ENSGALG00010015393	DCX	-2.31667528	0.37797707	-6.129142389	8.8354E-10	2.5087E-09	negative
PRJNA729271	stage_1D_vs_E10	ordinary_muscle_development	ENSGALG00010015397	SRD5A3	-0.566569333	0.084948294	-6.669578694	2.56539E-11	8.07849E-11	negative
PRJNA729271	stage_1D_vs_E10	ordinary_muscle_development	ENSGALG00010015404	BEND4	-2.629287646	0.283621399	-9.270413493	1.85426E-20	1.01616E-19	negative
PRJNA729271	stage_1D_vs_E10	ordinary_muscle_development	ENSGALG00010015407	TIMP3	0.55789841	0.17549146	3.179063009	0.00147752	0.002415321	positive
PRJNA729271	stage_1D_vs_E10	ordinary_muscle_development	ENSGALG00010015409	FAM13B	1.342274847	0.131540444	10.20427485	1.89733E-24	1.26271E-23	positive
PRJNA729271	stage_1D_vs_E10	ordinary_muscle_development	ENSGALG00010015410	HTR2B	-2.590359438	0.234212875	-11.05985075	1.96424E-28	1.57389E-27	negative
PRJNA729271	stage_1D_vs_E10	ordinary_muscle_development	ENSGALG00010015413	CARNMT1	0.061148643	0.106556683	0.573860234	0.566062385	0.611360872	positive
PRJNA729271	stage_1D_vs_E10	ordinary_muscle_development	ENSGALG00010015416	CAPN6	2.391820677	0.1622477	14.74178482	3.47479E-49	6.48224E-48	positive
PRJNA729271	stage_1D_vs_E10	ordinary_muscle_development	ENSGALG00010015422	ENSGALG00010015422	-0.989505055	0.503889583	-1.963733898	0.049560959	0.066155825	negative
PRJNA729271	stage_1D_vs_E10	ordinary_muscle_development	ENSGALG00010015430	HMGCL	0.903805336	0.129814421	6.962287639	3.34791E-12	1.1197E-11	positive
PRJNA729271	stage_1D_vs_E10	ordinary_muscle_development	ENSGALG00010015431	RIN3	-0.738417748	0.158856856	-4.648321545	3.34647E-06	7.14811E-06	negative
PRJNA729271	stage_1D_vs_E10	ordinary_muscle_development	ENSGALG00010015436	SLC30A9	-0.093043619	0.098358649	-0.945962757	0.344167582	0.393121285	negative
PRJNA729271	stage_1D_vs_E10	ordinary_muscle_development	ENSGALG00010015440	ENSGALG00010015440	0.462821507	0.377374364	1.226425405	0.22003863	0.262438275	positive
PRJNA729271	stage_1D_vs_E10	ordinary_muscle_development	ENSGALG00010015443	PAK3	-0.223889415	0.101639702	-2.202775199	0.027610597	0.038239595	negative
PRJNA729271	stage_1D_vs_E10	ordinary_muscle_development	ENSGALG00010015449	HDX	-0.581287704	0.092387665	-6.291832406	3.1374E-10	9.1872E-10	negative
PRJNA729271	stage_1D_vs_E10	ordinary_muscle_development	ENSGALG00010015466	NMRK1	-0.093839909	0.126036336	-0.744546466	0.456545941	0.505510153	negative
PRJNA729271	stage_1D_vs_E10	ordinary_muscle_development	ENSGALG00010015469	TMEM33	-0.169698174	0.072881252	-2.328420131	0.019889806	0.028151815	negative
PRJNA729271	stage_1D_vs_E10	ordinary_muscle_development	ENSGALG00010015472	LGMN	-0.133734314	0.134383633	-0.995168167	0.319654481	0.36767114	negative
PRJNA729271	stage_1D_vs_E10	ordinary_muscle_development	ENSGALG00010015477	ARMC9	-1.122850101	0.103811477	-10.8162424	2.88359E-27	2.1815E-26	negative
PRJNA729271	stage_1D_vs_E10	ordinary_muscle_development	ENSGALG00010015478	FBXO7	0.193045025	0.061844658	3.121450279	0.001799626	0.002915568	positive
PRJNA729271	stage_1D_vs_E10	ordinary_muscle_development	ENSGALG00010015481	GOLGA5	0.128391827	0.070629434	1.817823251	0.069091152	0.090166223	positive
PRJNA729271	stage_1D_vs_E10	ordinary_muscle_development	ENSGALG00010015483	SGCD	1.667041423	0.162122728	10.28258927	8.44284E-25	5.7133E-24	positive
PRJNA729271	stage_1D_vs_E10	ordinary_muscle_development	ENSGALG00010015485	BCAS1	3.053990898	0.18203408	16.77702818	3.59376E-63	1.07405E-61	positive
PRJNA729271	stage_1D_vs_E10	ordinary_muscle_development	ENSGALG00010015486	MTFR1L	0.342114791	0.120577584	2.837300107	0.004549683	0.007032351	positive
PRJNA729271	stage_1D_vs_E10	ordinary_muscle_development	ENSGALG00010015487	RPS6KA6	-0.074756105	0.117435456	-0.636571848	0.524403767	0.571521249	negative
PRJNA729271	stage_1D_vs_E10	ordinary_muscle_development	ENSGALG00010015490	RANBP2	0.105514788	0.082876474	1.273157304	0.202962228	0.243416969	positive
PRJNA729271	stage_1D_vs_E10	ordinary_muscle_development	ENSGALG00010015493	OSTF1	-0.757092408	0.141988257	-5.332077611	9.70954E-08	2.34645E-07	negative
PRJNA729271	stage_1D_vs_E10	ordinary_muscle_development	ENSGALG00010015494	CHRDL1	-0.760328748	0.2399586	-3.168583029	0.00153184	0.002500453	negative
PRJNA729271	stage_1D_vs_E10	ordinary_muscle_development	ENSGALG00010015498	MID2	1.57636158	0.132081485	11.93476571	7.79781E-33	7.70479E-32	positive
PRJNA729271	stage_1D_vs_E10	ordinary_muscle_development	ENSGALG00010015502	BPIFB6	-0.288060414	1.462328405	-0.196987498	0.843837321	0.866309368	negative
PRJNA729271	stage_1D_vs_E10	ordinary_muscle_development	ENSGALG00010015505	CHGA	-0.848640874	0.468683744	-1.810689798	0.070188883	0.091516404	negative
PRJNA729271	stage_1D_vs_E10	ordinary_muscle_development	ENSGALG00010015507	AMMECR1	-0.737895123	0.069401447	-10.63227289	2.10909E-26	1.53779E-25	negative
PRJNA729271	stage_1D_vs_E10	ordinary_muscle_development	ENSGALG00010015508	FUCA1	0.820926649	0.103294942	7.947404149	1.9046E-15	7.86306E-15	positive
PRJNA729271	stage_1D_vs_E10	ordinary_muscle_development	ENSGALG00010015511	BPIFB4	-2.992255005	0.508726967	-5.881848617	4.05709E-09	1.09227E-08	negative
PRJNA729271	stage_1D_vs_E10	ordinary_muscle_development	ENSGALG00010015516	SELENON	-0.574074354	0.112813649	-5.088695897	3.60534E-07	8.32157E-07	negative
PRJNA729271	stage_1D_vs_E10	ordinary_muscle_development	ENSGALG00010015520	UBE3A	0.21776588	0.078550143	2.77231677	0.005565884	0.00850214	positive
PRJNA729271	stage_1D_vs_E10	ordinary_muscle_development	ENSGALG00010015521	DKC1	-0.481078401	0.081628424	-5.89351574	3.78064E-09	1.0222E-08	negative
PRJNA729271	stage_1D_vs_E10	ordinary_muscle_development	ENSGALG00010015523	MRPL22	-0.10074183	0.144622991	-0.696582396	0.486064176	0.534559957	negative
PRJNA729271	stage_1D_vs_E10	ordinary_muscle_development	ENSGALG00010015532	NXT2	-0.436664903	0.110752716	-3.94270155	8.05689E-05	0.000150846	negative
PRJNA729271	stage_1D_vs_E10	ordinary_muscle_development	ENSGALG00010015533	PSAT1	-1.259695258	0.277726362	-4.535742475	5.74012E-06	1.19855E-05	negative
PRJNA729271	stage_1D_vs_E10	ordinary_muscle_development	ENSGALG00010015540	ACSL4	0.465843496	0.152058274	3.063585323	0.002187018	0.003500147	positive
PRJNA729271	stage_1D_vs_E10	ordinary_muscle_development	ENSGALG00010015541	RASEF	-0.511882811	0.306636653	-1.669346462	0.095048737	0.121289854	negative
PRJNA729271	stage_1D_vs_E10	ordinary_muscle_development	ENSGALG00010015542	CNGA3	-1.115614837	0.287727201	-3.877335313	0.000105607	0.000195426	negative
PRJNA729271	stage_1D_vs_E10	ordinary_muscle_development	ENSGALG00010015544	RTCB	-0.523537508	0.07361026	-7.112289904	1.14133E-12	3.92029E-12	negative
PRJNA729271	stage_1D_vs_E10	ordinary_muscle_development	ENSGALG00010015551	VWA3B	-1.309506708	0.420631393	-3.113193	0.001850749	0.002993374	negative
PRJNA729271	stage_1D_vs_E10	ordinary_muscle_development	ENSGALG00010015553	ASCL4	-5.517877195	0.910647551	-6.059289556	1.36724E-09	3.82147E-09	negative
PRJNA729271	stage_1D_vs_E10	ordinary_muscle_development	ENSGALG00010015555	PNRC2	0.142066232	0.102069472	1.391858207	0.16396534	0.200699004	positive
PRJNA729271	stage_1D_vs_E10	ordinary_muscle_development	ENSGALG00010015557	IRS4	0.592281966	0.192074031	3.083612928	0.002045035	0.003286704	positive
PRJNA729271	stage_1D_vs_E10	ordinary_muscle_development	ENSGALG00010015558	MPP1	0.601417825	0.259496799	2.317631001	0.020469384	0.02892131	positive
PRJNA729271	stage_1D_vs_E10	ordinary_muscle_development	ENSGALG00010015562	IRF9	2.621368082	0.266324157	9.842772475	7.36527E-23	4.57018E-22	positive
PRJNA729271	stage_1D_vs_E10	ordinary_muscle_development	ENSGALG00010015563	B3GNT7	-2.605610244	0.321463129	-8.105471544	5.25413E-16	2.25388E-15	negative
PRJNA729271	stage_1D_vs_E10	ordinary_muscle_development	ENSGALG00010015564	PKD2L2	-0.976030849	0.304728935	-3.202947725	0.001360287	0.002235528	negative
PRJNA729271	stage_1D_vs_E10	ordinary_muscle_development	ENSGALG00010015565	ENSGALG00010015565	-0.280135222	0.451535473	-0.62040579	0.534990662	0.581703521	negative
PRJNA729271	stage_1D_vs_E10	ordinary_muscle_development	ENSGALG00010015567	ENSGALG00010015567	4.781818779	0.425890762	11.22780583	2.97782E-29	2.48637E-28	positive
PRJNA729271	stage_1D_vs_E10	ordinary_muscle_development	ENSGALG00010015570	LIMCH1	0.760541555	0.12787923	5.947342292	2.72531E-09	7.45488E-09	positive
PRJNA729271	stage_1D_vs_E10	ordinary_muscle_development	ENSGALG00010015571	DUSP15	1.359761885	0.294889973	4.611082132	4.00578E-06	8.48018E-06	positive
PRJNA729271	stage_1D_vs_E10	ordinary_muscle_development	ENSGALG00010015573	MAN1C1	1.044835577	0.122718859	8.514058778	1.67949E-17	7.82771E-17	positive
PRJNA729271	stage_1D_vs_E10	ordinary_muscle_development	ENSGALG00010015574	C14orf132	0.416782062	0.253663751	1.643049356	0.100372722	0.127589459	positive
PRJNA729271	stage_1D_vs_E10	ordinary_muscle_development	ENSGALG00010015580	GEMIN5	-0.628498933	0.071736805	-8.761178162	1.9322E-18	9.48434E-18	negative
PRJNA729271	stage_1D_vs_E10	ordinary_muscle_development	ENSGALG00010015583	PRDM4	-0.168046391	0.136507816	-1.231038602	0.218308427	0.260592988	negative
PRJNA729271	stage_1D_vs_E10	ordinary_muscle_development	ENSGALG00010015584	TTLL9	2.182603604	0.229568899	9.507401118	1.95485E-21	1.13026E-20	positive
PRJNA729271	stage_1D_vs_E10	ordinary_muscle_development	ENSGALG00010015585	BDKRB1	1.042730749	0.301646815	3.456793499	0.000546644	0.000937593	positive
PRJNA729271	stage_1D_vs_E10	ordinary_muscle_development	ENSGALG00010015588	BDKRB2	-0.926424176	0.454519221	-2.038250821	0.041524855	0.056074416	negative
PRJNA729271	stage_1D_vs_E10	ordinary_muscle_development	ENSGALG00010015589	FRMD3	2.830050104	0.178629199	15.84315506	1.56754E-56	3.81234E-55	positive
PRJNA729271	stage_1D_vs_E10	ordinary_muscle_development	ENSGALG00010015596	UCHL1	-5.925258557	0.513984513	-11.52808773	9.52356E-31	8.55291E-30	negative
PRJNA729271	stage_1D_vs_E10	ordinary_muscle_development	ENSGALG00010015605	LDLRAP1	-0.404686244	0.058333417	-6.937468553	3.99188E-12	1.3262E-11	negative
PRJNA729271	stage_1D_vs_E10	ordinary_muscle_development	ENSGALG00010015606	TMEM131	0.410681319	0.108377124	3.789372746	0.000151028	0.000275504	positive
PRJNA729271	stage_1D_vs_E10	ordinary_muscle_development	ENSGALG00010015610	ENSGALG00010015610	2.235409692	0.286434307	7.804266595	5.98486E-15	2.38916E-14	positive
PRJNA729271	stage_1D_vs_E10	ordinary_muscle_development	ENSGALG00010015613	PWP1	-0.357095032	0.080129074	-4.456497678	8.33095E-06	1.7153E-05	negative
PRJNA729271	stage_1D_vs_E10	ordinary_muscle_development	ENSGALG00010015614	ATG2B	0.641692703	0.106982616	5.998102539	1.99636E-09	5.51502E-09	positive
PRJNA729271	stage_1D_vs_E10	ordinary_muscle_development	ENSGALG00010015616	NADK2	-0.452188122	0.11814493	-3.827401826	0.000129503	0.000237674	negative
PRJNA729271	stage_1D_vs_E10	ordinary_muscle_development	ENSGALG00010015617	SRSF10	0.04481598	0.055467338	0.807970617	0.419107497	0.468494114	positive
PRJNA729271	stage_1D_vs_E10	ordinary_muscle_development	ENSGALG00010015618	NCL	-0.981154525	0.100960693	-9.718183385	2.52241E-22	1.52917E-21	negative
PRJNA729271	stage_1D_vs_E10	ordinary_muscle_development	ENSGALG00010015620	PDRG1	1.267734293	0.14319173	8.853404404	8.48894E-19	4.24593E-18	positive
PRJNA729271	stage_1D_vs_E10	ordinary_muscle_development	ENSGALG00010015621	IDNK	-0.680700758	0.167376047	-4.066894704	4.76438E-05	9.12521E-05	negative
PRJNA729271	stage_1D_vs_E10	ordinary_muscle_development	ENSGALG00010015624	ITGA1	1.526098185	0.128942087	11.83553194	2.55717E-32	2.46779E-31	positive
PRJNA729271	stage_1D_vs_E10	ordinary_muscle_development	ENSGALG00010015626	XKR7	0.6917016	0.488662702	1.415499068	0.156922221	0.192680017	positive
PRJNA729271	stage_1D_vs_E10	ordinary_muscle_development	ENSGALG00010015628	UBQLN1	0.263759004	0.118479174	2.226205625	0.026000411	0.036175462	positive
PRJNA729271	stage_1D_vs_E10	ordinary_muscle_development	ENSGALG00010015631	GSKIP	-0.420774519	0.105782462	-3.977734221	6.95751E-05	0.000131143	negative
PRJNA729271	stage_1D_vs_E10	ordinary_muscle_development	ENSGALG00010015632	COL4A5	2.86926583	0.161384609	17.77905492	1.02697E-70	3.87653E-69	positive
PRJNA729271	stage_1D_vs_E10	ordinary_muscle_development	ENSGALG00010015633	CCM2L	0.860526349	0.184199422	4.671710357	2.98702E-06	6.41055E-06	positive
PRJNA729271	stage_1D_vs_E10	ordinary_muscle_development	ENSGALG00010015634	ENSGALG00010015634	3.035741085	1.057431968	2.870861839	0.004093544	0.006355604	positive
PRJNA729271	stage_1D_vs_E10	ordinary_muscle_development	ENSGALG00010015635	ABTB3	-1.92177959	0.128059643	-15.00691039	6.61583E-51	1.32271E-49	negative
PRJNA729271	stage_1D_vs_E10	ordinary_muscle_development	ENSGALG00010015636	APBB2	0.136162413	0.124247532	1.095896318	0.273124151	0.318806133	positive
PRJNA729271	stage_1D_vs_E10	ordinary_muscle_development	ENSGALG00010015642	ENSGALG00010015642	-1.531222591	0.859353624	-1.78183061	0.074776859	0.097050115	negative
PRJNA729271	stage_1D_vs_E10	ordinary_muscle_development	ENSGALG00010015643	ENSGALG00010015643	-2.427541546	0.121564914	-19.96909682	1.02286E-88	5.93056E-87	negative
PRJNA729271	stage_1D_vs_E10	ordinary_muscle_development	ENSGALG00010015645	MACO1	0.195761959	0.094124105	2.079828094	0.037541303	0.051032654	positive
PRJNA729271	stage_1D_vs_E10	ordinary_muscle_development	ENSGALG00010015647	PDZD2	0.020429959	0.13654437	0.149621394	0.881063329	0.898544498	positive
PRJNA729271	stage_1D_vs_E10	ordinary_muscle_development	ENSGALG00010015650	ENSGALG00010015650	3.279508354	1.208955039	2.712680165	0.006674149	0.010089882	positive
PRJNA729271	stage_1D_vs_E10	ordinary_muscle_development	ENSGALG00010015653	HCK	1.46985251	0.524472186	2.802536627	0.005070246	0.007790935	positive
PRJNA729271	stage_1D_vs_E10	ordinary_muscle_development	ENSGALG00010015655	ENSGALG00010015655	5.608963542	0.361533506	15.51436715	2.77356E-54	6.19456E-53	positive
PRJNA729271	stage_1D_vs_E10	ordinary_muscle_development	ENSGALG00010015657	NMUR1	-3.994692679	1.196859156	-3.337646422	0.000844912	0.001420271	negative
PRJNA729271	stage_1D_vs_E10	ordinary_muscle_development	ENSGALG00010015660	KLHL3	0.370535999	0.122040122	3.036181811	0.002395948	0.003817243	positive
PRJNA729271	stage_1D_vs_E10	ordinary_muscle_development	ENSGALG00010015668	ZNF217	0.459023264	0.134803556	3.405127271	0.000661332	0.001123141	positive
PRJNA729271	stage_1D_vs_E10	ordinary_muscle_development	ENSGALG00010015669	INPP4A	0.103553495	0.13037517	0.794273136	0.42703644	0.476444959	positive
PRJNA729271	stage_1D_vs_E10	ordinary_muscle_development	ENSGALG00010015670	ENSGALG00010015670	-1.153865388	0.130712703	-8.827492356	1.07049E-18	5.32488E-18	negative
PRJNA729271	stage_1D_vs_E10	ordinary_muscle_development	ENSGALG00010015674	RFKL	0.392869964	0.193911236	2.026029914	0.042761719	0.057658709	positive
PRJNA729271	stage_1D_vs_E10	ordinary_muscle_development	ENSGALG00010015676	TGM2	2.753078698	0.192103851	14.33119997	1.39686E-46	2.32462E-45	positive
PRJNA729271	stage_1D_vs_E10	ordinary_muscle_development	ENSGALG00010015677	GRHL3	-2.678293517	0.257415858	-10.40453972	2.36396E-25	1.64976E-24	negative
PRJNA729271	stage_1D_vs_E10	ordinary_muscle_development	ENSGALG00010015680	ENSGALG00010015680	-0.445821397	0.103941268	-4.289166425	1.79345E-05	3.56648E-05	negative
PRJNA729271	stage_1D_vs_E10	ordinary_muscle_development	ENSGALG00010015684	PDE6D	-0.438950056	0.098121604	-4.473531191	7.69383E-06	1.58954E-05	negative
PRJNA729271	stage_1D_vs_E10	ordinary_muscle_development	ENSGALG00010015686	CNOT8	-0.666667261	0.052663996	-12.65888098	9.99134E-37	1.16231E-35	negative
PRJNA729271	stage_1D_vs_E10	ordinary_muscle_development	ENSGALG00010015691	EXOC3L4	-3.837185411	1.037363167	-3.698979809	0.000216468	0.000387036	negative
PRJNA729271	stage_1D_vs_E10	ordinary_muscle_development	ENSGALG00010015693	COA5	-0.271027269	0.108059139	-2.508138336	0.012136914	0.017746855	negative
PRJNA729271	stage_1D_vs_E10	ordinary_muscle_development	ENSGALG00010015695	ASAH1	0.409548897	0.094133709	4.350714524	1.35695E-05	2.73697E-05	positive
PRJNA729271	stage_1D_vs_E10	ordinary_muscle_development	ENSGALG00010015697	FAXDC2	1.129612753	0.326890456	3.455630876	0.000549007	0.000941312	positive
PRJNA729271	stage_1D_vs_E10	ordinary_muscle_development	ENSGALG00010015698	TNFAIP2	-0.837696849	0.201488245	-4.157547007	3.21683E-05	6.25375E-05	negative
PRJNA729271	stage_1D_vs_E10	ordinary_muscle_development	ENSGALG00010015701	UNC50	-0.136814864	0.085399091	-1.602064629	0.109141319	0.137769173	negative
PRJNA729271	stage_1D_vs_E10	ordinary_muscle_development	ENSGALG00010015703	GK5	0.638344737	0.185190787	3.44695731	0.000566938	0.000969534	positive
PRJNA729271	stage_1D_vs_E10	ordinary_muscle_development	ENSGALG00010015705	NPY6R	-4.187366877	1.045950184	-4.003409475	6.24361E-05	0.000118318	negative
PRJNA729271	stage_1D_vs_E10	ordinary_muscle_development	ENSGALG00010015706	ENSGALG00010015706	1.099678286	0.162250748	6.77764694	1.22149E-11	3.93193E-11	positive
PRJNA729271	stage_1D_vs_E10	ordinary_muscle_development	ENSGALG00010015709	HNRNPA0	-1.087141142	0.112643987	-9.651124529	4.86196E-22	2.91577E-21	negative
PRJNA729271	stage_1D_vs_E10	ordinary_muscle_development	ENSGALG00010015711	LBFABP	-0.517190458	0.70447374	-0.734151508	0.462856404	0.5116766	negative
PRJNA729271	stage_1D_vs_E10	ordinary_muscle_development	ENSGALG00010015716	MGAT4A	-0.129723191	0.13200516	-0.982713032	0.325748695	0.374087097	negative

PRJNA729271	stage_1D_vs_E10	ordinary_muscle_development	ENSGALG00010015719	XRN1	0.514281768	0.099127287	5.188094856	2.12456E-07	4.99603E-07	positive
PRJNA729271	stage_1D_vs_E10	ordinary_muscle_development	ENSGALG00010015729	RHCE	-0.516249873	0.199391318	-2.589129149	0.0096219	0.014259221	negative
PRJNA729271	stage_1D_vs_E10	ordinary_muscle_development	ENSGALG00010015731	PHF6	-1.30814021	0.069353892	-18.86181403	2.35017E-79	1.10245E-77	negative
PRJNA729271	stage_1D_vs_E10	ordinary_muscle_development	ENSGALG00010015733	COL4A6	2.763445054	0.152364632	18.13705065	1.62522E-73	6.54377E-72	positive
PRJNA729271	stage_1D_vs_E10	ordinary_muscle_development	ENSGALG00010015734	TSHZ2	-1.316361425	0.166383756	-7.911598217	2.54105E-15	1.03959E-14	negative
PRJNA729271	stage_1D_vs_E10	ordinary_muscle_development	ENSGALG00010015738	CRACDL	-3.278407587	0.441396263	-7.427356925	1.10789E-13	4.08623E-13	negative
PRJNA729271	stage_1D_vs_E10	ordinary_muscle_development	ENSGALG00010015742	HPRT1	0.777302621	0.086035966	9.034624173	1.64567E-19	8.55595E-19	positive
PRJNA729271	stage_1D_vs_E10	ordinary_muscle_development	ENSGALG00010015746	PAPOLB	-0.187193087	0.072079669	-2.597030349	0.009403362	0.013952475	negative
PRJNA729271	stage_1D_vs_E10	ordinary_muscle_development	ENSGALG00010015749	ATR	-0.849920139	0.133898036	-6.347517599	2.18817E-10	6.48222E-10	negative
PRJNA729271	stage_1D_vs_E10	ordinary_muscle_development	ENSGALG00010015751	MYOM3	-0.70929312	0.538450324	-1.317286086	0.187742747	0.227099318	negative
PRJNA729271	stage_1D_vs_E10	ordinary_muscle_development	ENSGALG00010015753	RPRD1B	-0.207843811	0.078228969	-2.656865005	0.007887102	0.011821274	negative
PRJNA729271	stage_1D_vs_E10	ordinary_muscle_development	ENSGALG00010015756	PCM1	-0.218616119	0.103149132	-2.119417924	0.034055163	0.046608403	negative
PRJNA729271	stage_1D_vs_E10	ordinary_muscle_development	ENSGALG00010015758	PCDH19	0.707870865	0.169196122	4.183729845	2.86765E-05	5.60121E-05	positive
PRJNA729271	stage_1D_vs_E10	ordinary_muscle_development	ENSGALG00010015759	E1F5	-0.221947497	0.091770541	-2.418504843	0.015584438	0.022417032	negative
PRJNA729271	stage_1D_vs_E10	ordinary_muscle_development	ENSGALG00010015764	TTI1	0.460988365	0.094844037	4.860488668	1.17096E-06	2.59885E-06	positive
PRJNA729271	stage_1D_vs_E10	ordinary_muscle_development	ENSGALG00010015765	ENSGALG00010015765	0.88272348	0.362729038	2.433561658	0.014951089	0.021559494	positive
PRJNA729271	stage_1D_vs_E10	ordinary_muscle_development	ENSGALG00010015766	TLE1	-0.349044111	0.098539587	-3.54217146	0.000396847	0.00069105	negative
PRJNA729271	stage_1D_vs_E10	ordinary_muscle_development	ENSGALG00010015769	TMEM50A	0.140183591	0.099628138	1.407068263	0.159407148	0.195511555	positive
PRJNA729271	stage_1D_vs_E10	ordinary_muscle_development	ENSGALG00010015774	PLS1	-1.594222859	0.358822959	-4.442923225	8.87448E-06	1.82255E-05	negative
PRJNA729271	stage_1D_vs_E10	ordinary_muscle_development	ENSGALG00010015776	SPOCK1	-3.754323395	0.195747615	-19.17940807	5.5003E-82	2.7118E-80	negative
PRJNA729271	stage_1D_vs_E10	ordinary_muscle_development	ENSGALG00010015778	PABIR3	-1.230708199	0.087245358	-14.10628867	3.47385E-45	5.4971E-44	negative
PRJNA729271	stage_1D_vs_E10	ordinary_muscle_development	ENSGALG00010015782	ATG4A	0.429968328	0.081241514	5.292470635	1.20675E-07	2.89936E-07	positive
PRJNA729271	stage_1D_vs_E10	ordinary_muscle_development	ENSGALG00010015785	FGL1	2.485318566	0.190045235	13.07751054	4.42703E-39	5.61907E-38	positive
PRJNA729271	stage_1D_vs_E10	ordinary_muscle_development	ENSGALG00010015788	TRPC1	-0.079299384	0.128043609	-0.619315445	0.535708572	0.582374469	negative
PRJNA729271	stage_1D_vs_E10	ordinary_muscle_development	ENSGALG00010015792	MARK3	0.156114828	0.081404843	1.917758489	0.055141642	0.073020107	positive
PRJNA729271	stage_1D_vs_E10	ordinary_muscle_development	ENSGALG00010015793	RSRP1	1.924433508	0.232788806	8.266864476	1.37528E-16	6.09065E-16	positive
PRJNA729271	stage_1D_vs_E10	ordinary_muscle_development	ENSGALG00010015795	PSMD10	-0.328033907	0.134290467	-2.442719246	0.014577072	0.021051576	negative
PRJNA729271	stage_1D_vs_E10	ordinary_muscle_development	ENSGALG00010015796	LARP1	0.438510848	0.140846412	3.113397366	0.001849468	0.002991635	positive
PRJNA729271	stage_1D_vs_E10	ordinary_muscle_development	ENSGALG00010015797	PIP5K1B	-0.436629327	0.163396136	-2.67221329	0.007535274	0.011318528	negative
PRJNA729271	stage_1D_vs_E10	ordinary_muscle_development	ENSGALG00010015798	IL22RA1	-2.640606515	0.304057549	-8.684561607	3.80206E-18	1.8358E-17	negative
PRJNA729271	stage_1D_vs_E10	ordinary_muscle_development	ENSGALG00010015800	BVES	2.500105461	0.181476324	13.77648284	3.53059E-43	5.17974E-42	positive
PRJNA729271	stage_1D_vs_E10	ordinary_muscle_development	ENSGALG00010015801	VSTM2L	-2.176747796	0.25804179	-8.435640572	3.29394E-17	1.51046E-16	negative
PRJNA729271	stage_1D_vs_E10	ordinary_muscle_development	ENSGALG00010015802	TRPC7	-3.219004191	0.910278851	-3.536283622	0.000405799	0.000705875	negative
PRJNA729271	stage_1D_vs_E10	ordinary_muscle_development	ENSGALG00010015803	SYF2	0.332632248	0.159615151	2.083964118	0.037163431	0.050571154	positive
PRJNA729271	stage_1D_vs_E10	ordinary_muscle_development	ENSGALG00010015804	CKB	-3.015624711	0.161023046	-18.72790753	2.93216E-78	1.34075E-76	negative
PRJNA729271	stage_1D_vs_E10	ordinary_muscle_development	ENSGALG00010015807	ENSGALG00010015807	0.454596954	0.940055719	0.483585116	0.62868034	0.670470549	positive
PRJNA729271	stage_1D_vs_E10	ordinary_muscle_development	ENSGALG00010015809	PCOLCE2	2.945555147	0.232277729	12.68117765	7.51936E-37	8.79686E-36	positive
PRJNA729271	stage_1D_vs_E10	ordinary_muscle_development	ENSGALG00010015810	LIPT1	-0.04831234	0.103570086	-0.46647002	0.640879102	0.681451187	negative
PRJNA729271	stage_1D_vs_E10	ordinary_muscle_development	ENSGALG00010015811	CTNNBL1	-0.635379068	0.055273174	-11.49525207	1.39374E-30	1.24168E-29	negative
PRJNA729271	stage_1D_vs_E10	ordinary_muscle_development	ENSGALG00010015813	ENSGALG00010015813	0.164169056	0.139034541	1.18077893	0.237690556	0.281388812	positive
PRJNA729271	stage_1D_vs_E10	ordinary_muscle_development	ENSGALG00010015814	PAQR9	3.112881281	0.401755792	7.748192676	9.32096E-15	3.67839E-14	positive
PRJNA729271	stage_1D_vs_E10	ordinary_muscle_development	ENSGALG00010015816	MITD1	-0.752176781	0.21611688	-3.480416621	0.000500635	0.000862148	negative
PRJNA729271	stage_1D_vs_E10	ordinary_muscle_development	ENSGALG00010015820	ENSGALG00010015820	0.166035042	0.524376578	0.316633215	0.75152193	0.783522288	positive
PRJNA729271	stage_1D_vs_E10	ordinary_muscle_development	ENSGALG00010015822	WFDC2	-0.050350413	0.491593667	-0.102422827	0.918421066	0.930619598	negative
PRJNA729271	stage_1D_vs_E10	ordinary_muscle_development	ENSGALG00010015825	RUNX3	-6.184551863	0.909663706	-6.798723334	1.0555E-11	3.4105E-11	negative
PRJNA729271	stage_1D_vs_E10	ordinary_muscle_development	ENSGALG00010015827	ENSGALG00010015827	2.016852118	0.146512783	13.76570748	4.09852E-43	5.98872E-42	positive
PRJNA729271	stage_1D_vs_E10	ordinary_muscle_development	ENSGALG00010015829	SMAD5	-0.056596475	0.04767498	-1.187131589	0.23517572	0.278661985	negative
PRJNA729271	stage_1D_vs_E10	ordinary_muscle_development	ENSGALG00010015831	LYG2	4.656069045	0.620797161	7.500145514	6.3747E-14	2.38517E-13	positive
PRJNA729271	stage_1D_vs_E10	ordinary_muscle_development	ENSGALG00010015832	RAB39B	-1.08017865	0.085514422	-12.63153773	1.41486E-36	1.63153E-35	negative
PRJNA729271	stage_1D_vs_E10	ordinary_muscle_development	ENSGALG00010015834	STARD8	1.726334924	0.132725537	13.00680303	1.11927E-38	1.39861E-37	positive
PRJNA729271	stage_1D_vs_E10	ordinary_muscle_development	ENSGALG00010015837	GDA	2.292711645	0.160318073	14.30101802	2.15632E-46	3.56395E-45	positive
PRJNA729271	stage_1D_vs_E10	ordinary_muscle_development	ENSGALG00010015838	IFNLR1	-0.913902122	1.26967875	-0.719790043	0.471654276	0.520410195	negative
PRJNA729271	stage_1D_vs_E10	ordinary_muscle_development	ENSGALG00010015839	U2SURP	-0.186322133	0.070695485	-2.635559153	0.008399878	0.012539262	negative
PRJNA729271	stage_1D_vs_E10	ordinary_muscle_development	ENSGALG00010015840	MTUS1	2.597845659	0.145376928	17.86972456	2.02994E-71	7.82553E-70	positive
PRJNA729271	stage_1D_vs_E10	ordinary_muscle_development	ENSGALG00010015843	ENSGALG00010015843	-1.160750737	0.406029851	-2.858781772	0.004252712	0.006593546	negative
PRJNA729271	stage_1D_vs_E10	ordinary_muscle_development	ENSGALG00010015845	SAP30L	1.074285256	0.125249487	8.577162908	9.72418E-18	4.59127E-17	positive
PRJNA729271	stage_1D_vs_E10	ordinary_muscle_development	ENSGALG00010015848	UBE2B	1.715843273	0.161357362	10.63380843	2.07464E-26	1.51343E-25	positive
PRJNA729271	stage_1D_vs_E10	ordinary_muscle_development	ENSGALG00010015852	ZFAND5	1.67119997	0.28575514	5.848363635	4.96432E-09	1.32911E-08	positive
PRJNA729271	stage_1D_vs_E10	ordinary_muscle_development	ENSGALG00010015855	NIPAL3	-0.61163184	0.080333822	-7.613628062	2.66507E-14	1.02277E-13	negative
PRJNA729271	stage_1D_vs_E10	ordinary_muscle_development	ENSGALG00010015856	TXNDC9	-0.390340928	0.133608665	-2.921524059	0.003483234	0.00545712	negative
PRJNA729271	stage_1D_vs_E10	ordinary_muscle_development	ENSGALG00010015858	CHST2	-0.954322382	0.091688958	-10.40825857	2.27342E-25	1.58734E-24	negative
PRJNA729271	stage_1D_vs_E10	ordinary_muscle_development	ENSGALG00010015861	PPP2CA	-0.642554133	0.067272337	-9.551535786	1.27788E-21	7.46587E-21	negative
PRJNA729271	stage_1D_vs_E10	ordinary_muscle_development	ENSGALG00010015862	PDGFRL	0.685543733	0.208863993	3.282249478	0.001029824	0.001715585	positive
PRJNA729271	stage_1D_vs_E10	ordinary_muscle_development	ENSGALG00010015863	ENSGALG00010015863	-3.191708257	0.578882532	-5.513568093	3.51631E-08	8.77866E-08	negative
PRJNA729271	stage_1D_vs_E10	ordinary_muscle_development	ENSGALG00010015865	VRK1	-1.690270272	0.140755573	-12.00854954	3.20416E-33	3.23203E-32	negative
PRJNA729271	stage_1D_vs_E10	ordinary_muscle_development	ENSGALG00010015866	ARHGEF6	0.174954612	0.070954106	2.465743295	0.013672928	0.019836763	positive
PRJNA729271	stage_1D_vs_E10	ordinary_muscle_development	ENSGALG00010015867	PICALML	-0.082068203	0.056796302	-1.444956806	0.148470019	0.183233114	negative
PRJNA729271	stage_1D_vs_E10	ordinary_muscle_development	ENSGALG00010015869	SLC7A2	3.200078905	0.193445318	16.54255029	1.81187E-61	5.24211E-60	positive
PRJNA729271	stage_1D_vs_E10	ordinary_muscle_development	ENSGALG00010015870	EIF5B	-0.273289213	0.156266722	-1.748863804	0.080314565	0.103794545	negative
PRJNA729271	stage_1D_vs_E10	ordinary_muscle_development	ENSGALG00010015872	TMC1	-1.302653733	0.363260978	-3.585999635	0.000335789	0.000588901	negative
PRJNA729271	stage_1D_vs_E10	ordinary_muscle_development	ENSGALG00010015874	CLIC4	-0.644617435	0.114546262	-5.627572875	1.82763E-08	4.67222E-08	negative
PRJNA729271	stage_1D_vs_E10	ordinary_muscle_development	ENSGALG00010015875	DNTTIP1	-0.133145989	0.140496768	-0.947680086	0.343292342	0.392276113	negative
PRJNA729271	stage_1D_vs_E10	ordinary_muscle_development	ENSGALG00010015876	SKP1	-0.61145493	0.077462547	-7.893555742	2.93696E-15	1.19784E-14	negative
PRJNA729271	stage_1D_vs_E10	ordinary_muscle_development	ENSGALG00010015877	REV1	0.597636329	0.102668292	5.821040944	5.84822E-09	1.55971E-08	positive
PRJNA729271	stage_1D_vs_E10	ordinary_muscle_development	ENSGALG00010015879	TRMT61A	-1.491644996	0.123122345	-12.11514445	8.78093E-34	9.0526E-33	negative
PRJNA729271	stage_1D_vs_E10	ordinary_muscle_development	ENSGALG00010015881	GAB3	1.689139864	0.17583293	9.60650468	7.50536E-22	4.45132E-21	positive
PRJNA729271	stage_1D_vs_E10	ordinary_muscle_development	ENSGALG00010015886	AFF3	-0.562546725	0.208518587	-2.697825327	0.006979405	0.010522829	negative
PRJNA729271	stage_1D_vs_E10	ordinary_muscle_development	ENSGALG00010015887	SLC9A9	-0.050969918	0.181042372	-0.281535851	0.778299434	0.807097833	negative
PRJNA729271	stage_1D_vs_E10	ordinary_muscle_development	ENSGALG00010015889	SH3BP5	1.229458836	0.165004962	7.451041595	9.26062E-14	3.43482E-13	positive
PRJNA729271	stage_1D_vs_E10	ordinary_muscle_development	ENSGALG00010015892	UBE2C	-1.584748758	0.221660026	-7.149456701	8.7122E-13	3.02765E-12	negative
PRJNA729271	stage_1D_vs_E10	ordinary_muscle_development	ENSGALG00010015893	TCF7	-2.067611364	0.444571607	-4.650794908	3.30658E-06	7.06603E-06	negative
PRJNA729271	stage_1D_vs_E10	ordinary_muscle_development	ENSGALG00010015894	CEP78	-1.935902242	0.116904311	-16.55971647	1.36234E-61	3.95735E-60	negative
PRJNA729271	stage_1D_vs_E10	ordinary_muscle_development	ENSGALG00010015895	RBMXL1	-0.673491234	0.084729908	-7.948683653	1.88504E-15	7.78672E-15	negative
PRJNA729271	stage_1D_vs_E10	ordinary_muscle_development	ENSGALG00010015896	LONRF2	0.106838136	0.078131246	1.367418823	0.171494066	0.208996677	positive
PRJNA729271	stage_1D_vs_E10	ordinary_muscle_development	ENSGALG00010015898	ENSGALG00010015898	0.819791973	0.219595234	3.733195648	0.000189066	0.000340732	positive
PRJNA729271	stage_1D_vs_E10	ordinary_muscle_development	ENSGALG00010015899	SMARCA1	-0.032739316	0.115153826	-0.284309408	0.776173277	0.805284636	negative
PRJNA729271	stage_1D_vs_E10	ordinary_muscle_development	ENSGALG00010015900	ENSGALG00010015900	0.961924805	0.160531965	5.992107582	2.07139E-09	5.71683E-09	positive
PRJNA729271	stage_1D_vs_E10	ordinary_muscle_development	ENSGALG00010015902	ENSGALG00010015902	0.62247391	0.502373871	1.239065059	0.215321406	0.257239246	positive
PRJNA729271	stage_1D_vs_E10	ordinary_muscle_development	ENSGALG00010015904	MTMR7	1.196491149	0.259990625	4.602054983	4.18343E-06	8.83303E-06	positive
PRJNA729271	stage_1D_vs_E10	ordinary_muscle_development	ENSGALG00010015905	ANPEP	1.960877126	0.216518646	9.056389211	1.3484E-19	7.04582E-19	positive
PRJNA729271	stage_1D_vs_E10	ordinary_muscle_development	ENSGALG00010015906	BAG5	0.966483524	0.100411205	9.625255724	6.25511E-22	3.72965E-21	positive
PRJNA729271	stage_1D_vs_E10	ordinary_muscle_development	ENSGALG00010015908	CHRD	1.336060395	0.135597223	9.85315452	6.64257E-23	4.13059E-22	positive
PRJNA729271	stage_1D_vs_E10	ordinary_muscle_development	ENSGALG00010015909	TNNC2	3.425586723	0.106587867	32.13861771	1.2738E-226	8.7926E-224	positive
PRJNA729271	stage_1D_vs_E10	ordinary_muscle_development	ENSGALG00010015910	C5orf15	-0.134837469	0.093154094	-1.447466911	0.147766198	0.182462342	negative
PRJNA729271	stage_1D_vs_E10	ordinary_muscle_development	ENSGALG00010015911	TM9SF2L	0.130248756	0.078245617	1.664614087	0.095989773	0.122436797	positive
PRJNA729271	stage_1D_vs_E10	ordinary_muscle_development	ENSGALG00010015912	ENSGALG00010015912	0.783021191	0.256354902	3.054442039	0.002254796	0.003605037	positive
PRJNA729271	stage_1D_vs_E10	ordinary_muscle_development	ENSGALG00010015913	CHST10	-0.777044359	0.104655853	-7.424757786	1.12986E-13	4.16198E-13	negative
PRJNA729271	stage_1D_vs_E10	ordinary_muscle_development	ENSGALG00010015914	SRRM1	-0.126090416	0.117284174	-1.075084657	0.282336828	0.328426938	negative
PRJNA729271	stage_1D_vs_E10	ordinary_muscle_development	ENSGALG00010015916	COA8	0.809518291	0.145078554	5.579861853	2.4071E-08	6.09767E-08	positive
PRJNA729271	stage_1D_vs_E10	ordinary_muscle_development	ENSGALG00010015917	TMEM63A	0.555826027	0.10776989	5.157526156	2.50234E-07	5.85117E-07	positive
PRJNA729271	stage_1D_vs_E10	ordinary_muscle_development	ENSGALG00010015918	BICD1	-0.651423953	0.186683184	-3.489462411	0.000483993	0.000834878	negative
PRJNA729271	stage_1D_vs_E10	ordinary_muscle_development	ENSGALG00010015919	ENSGALG00010015919	1.043802337	0.226135604	4.615824838	3.91537E-06	8.30212E-06	positive
PRJNA729271	stage_1D_vs_E10	ordinary_muscle_development	ENSGALG00010015921	SNX21	1.344413506	0.110193695	12.20045766	3.09082E-34	3.24178E-33	positive
PRJNA729271	stage_1D_vs_E10	ordinary_muscle_development	ENSGALG00010015923	RCAN3	-0.452859892	0.120428784	-3.760395793	0.000169645	0.000307683	negative
PRJNA729271	stage_1D_vs_E10	ordinary_muscle_development	ENSGALG00010015927	GLA	1.566295608	0.168119169	9.316579541	1.20151E-20	6.66256E-20	positive
PRJNA729271	stage_1D_vs_E10	ordinary_muscle_development	ENSGALG00010015928	CAPN7	0.55773407	0.120767173	4.618258873	3.86973E-06	8.20655E-06	positive
PRJNA729271	stage_1D_vs_E10	ordinary_muscle_development	ENSGALG00010015929	SLC6A12	-4.114864476	0.951267926	-4.325663004	1.52074E-05	3.04589E-05	negative
PRJNA729271	stage_1D_vs_E10	ordinary_muscle_development	ENSGALG00010015931	RBM47	0.26546437	0.280660522	0.945855752	0.344222165	0.393148982	positive
PRJNA729271	stage_1D_vs_E10	ordinary_muscle_development	ENSGALG00010015932	ACOT8	0.64067718	0.145199911	4.412379985	1.0224E-05	2.08494E-05	positive
PRJNA729271	stage_1D_vs_E10	ordinary_muscle_development	ENSGALG00010015933	ENSGALG00010015933	5.59172368	0.65518579	8.534561904	1.40687E-17	6.56768E-17	positive
PRJNA729271	stage_1D_vs_E10	ordinary_muscle_development	ENSGALG00010015934	TMC2	0.441731892	1.131912839	0.390252568	0.696349794	0.733620602	positive
PRJNA729271	stage_1D_vs_E10	ordinary_muscle_development	ENSGALG00010015935	EPHX1	0.367247356	0.110039773	3.337405626	0.000845644	0.001421338	positive
PRJNA729271	stage_1D_vs_E10	ordinary_muscle_development	ENSGALG00010015936	RPL36AL	-0.045990437	0.13954266	-0.329579764	0.741717515	0.774524749	negative
PRJNA729271	stage_1D_vs_E10	ordinary_muscle_development	ENSGALG00010015937	ENSGALG00010015937	1.490444553	0.190073611	7.84140704	4.45526E-15	1.79536E-14	positive
PRJNA729271	stage_1D_vs_E10	ordinary_muscle_development	ENSGALG00010015939	SUN2	0.905367286	0.07917817	11.43455691	2.80967E-30	2.46975E-29	positive
PRJNA729271	stage_1D_vs_E10	ordinary_muscle_development	ENSGALG00010015942	AP3S2	0.260923804	0.130353635	2.001661131	0.04532119	0.060832544	positive
PRJNA729271	stage_1D_vs_E10	ordinary_muscle_development	ENSGALG00010015946	TLE4	0.609546342	0.097895886	6.226475569	4.77045E-10	1.37882E-09	positive
PRJNA729271	stage_1D_vs_E10	ordinary_muscle_development	ENSGALG00010015954	CTSA	0.424358659	0.096797602	4.383979046	1.16531E-05	2.36505E-05	positive
PRJNA729271	stage_1D_vs_E10	ordinary_muscle_development	ENSGALG00010015957	ENSGALG00010015957	1.628245087	0.216862788	7.508181113	5.99545E-14	2.24907E-13	positive
PRJNA729271	stage_1D_vs_E10	ordinary_muscle_development	ENSGALG00010015959	TRNAU1AP	0.742880442	0.129734747	5.726148627	1.02736E-08	2.67546E-08	positive
PRJNA729271	stage_1D_vs_E10	ordinary_muscle_development	ENSGALG00010015960	CHRNA9	-2.614820621	0.962298886	-2.717264519	0.006582398	0.009964685	negative
PRJNA729271	stage_1D_vs_E10	ordinary_muscle_development	ENSGALG00010015963	DIPK2A	0.302581887	0.108234729	2.795608107	0.005180217	0.0079449	positive
PRJNA729271	stage_1D_vs_E10	ordinary_muscle_development	ENSGALG00010015964	KLC1	-0.218577974	0.087471479	-2.498848505	0.012459756	0.018184068	negative
PRJNA729271	stage_1D_vs_E10	ordinary_muscle_development	ENSGALG00010015967	ZSWIM1	-0.818317882	0.217966656	-3.754325989	0.000173809	0.000314919	negative
PRJNA729271	stage_1D_vs_E10	ordinary_muscle_development	ENSGALG00010015970	THPO	0.886741644	0.374326332	2.368899991	0.017841077	0.025410869	positive
PRJNA729271	stage_1D_vs_E10	ordinary_muscle_development	ENSGALG00010015971	ENSGALG00010015971	-1.556072235	0.160206472	-9.712917439	2.65623E-22	1.60508E-21	negative
PRJNA729271	stage_1D_vs_E10	ordinary_muscle_development	ENSGALG00010015972	FSTL4	-1.456686992	0.300590908	-4.84607802	1.25926E-06	2.78927E-06	negative
PRJNA729271	stage_1D_vs_E10	ordinary_muscle_development	ENSGALG00010015974	VPS37A	0.878823855	0.164795043	5.332829416	9.66941E-08	2.33714E-07	positive
PRJNA729271	stage_1D_vs_E10	ordinary_muscle_development	ENSGALG00010015975	BTK	0.490860465	0.180820431	2.714629438	0.006634997	0.010039068	positive
PRJNA729271	stage_1D_vs_E10	ordinary_muscle_development	ENSGALG00010015979	ENSGALG00010015979	2.655733437	0.203568358	13.04590488	6.70561E-39	8.45934E-38	positive
PRJNA729271	stage_1D_vs_E10	ordinary_muscle_development	ENSGALG00010015980	EXOSC7	-1.098830251	0.091407568	-12.02121742	2.74891E-33	2.78251E-32	negative

PRJNA729271	stage_1D_vs_E10	ordinary_muscle_development	ENSGALG00010015983	CNOT7	-0.626477602	0.065051797	-9.6304427	5.94727E-22	3.55194E-21	negative
PRJNA729271	stage_1D_vs_E10	ordinary_muscle_development	ENSGALG00010015984	PDCL3	-0.275068298	0.108180197	-2.542686235	0.011000396	0.016181321	negative
PRJNA729271	stage_1D_vs_E10	ordinary_muscle_development	ENSGALG00010015985	TIMM8A	0.165194498	0.110485041	1.495175243	0.134868713	0.167502527	positive
PRJNA729271	stage_1D_vs_E10	ordinary_muscle_development	ENSGALG00010015986	NEURL2	1.998812174	0.236920632	8.436631935	3.26613E-17	1.49866E-16	positive
PRJNA729271	stage_1D_vs_E10	ordinary_muscle_development	ENSGALG00010015987	POLR2H	-0.529924061	0.096509683	-5.490890081	3.99913E-08	9.94295E-08	negative
PRJNA729271	stage_1D_vs_E10	ordinary_muscle_development	ENSGALG00010015989	ENAH	-0.13533343	0.16296728	-0.830433139	0.406293936	0.455928668	negative
PRJNA729271	stage_1D_vs_E10	ordinary_muscle_development	ENSGALG00010015992	ATP10A	-0.88867172	0.157536421	-5.641055681	1.69011E-08	4.33595E-08	negative
PRJNA729271	stage_1D_vs_E10	ordinary_muscle_development	ENSGALG00010015994	ENSGALG00010015994	-0.060268725	0.106412771	-0.566367405	0.571144048	0.616206399	negative
PRJNA729271	stage_1D_vs_E10	ordinary_muscle_development	ENSGALG00010016004	XRCC3	-0.642922252	0.169857454	-3.7850694	0.000153666	0.000279998	negative
PRJNA729271	stage_1D_vs_E10	ordinary_muscle_development	ENSGALG00010016006	PLTP	-1.611623359	0.32240427	-4.998765552	5.76985E-07	1.31314E-06	negative
PRJNA729271	stage_1D_vs_E10	ordinary_muscle_development	ENSGALG00010016008	TAF7	-0.492664691	0.09382777	-5.25073432	1.51494E-07	3.60991E-07	negative
PRJNA729271	stage_1D_vs_E10	ordinary_muscle_development	ENSGALG00010016011	ADD1	-0.542603563	0.08076157	-6.718586091	1.83496E-11	5.83541E-11	negative
PRJNA729271	stage_1D_vs_E10	ordinary_muscle_development	ENSGALG00010016015	ZDHHC3	-0.189848225	0.083155923	-2.283039119	0.022428064	0.031547287	negative
PRJNA729271	stage_1D_vs_E10	ordinary_muscle_development	ENSGALG00010016016	ZDHHC2	-0.426389652	0.27833124	-1.531950391	0.125534677	0.15693188	negative
PRJNA729271	stage_1D_vs_E10	ordinary_muscle_development	ENSGALG00010016017	RAPGEF6	0.846582382	0.070774652	11.96166075	5.64221E-33	5.61702E-32	positive
PRJNA729271	stage_1D_vs_E10	ordinary_muscle_development	ENSGALG00010016022	EPHB3	-0.844629592	0.165362382	-5.107749322	3.26019E-07	7.55257E-07	negative
PRJNA729271	stage_1D_vs_E10	ordinary_muscle_development	ENSGALG00010016025	PCIF1	0.453807937	0.10762168	4.216696282	2.47908E-05	4.87045E-05	positive
PRJNA729271	stage_1D_vs_E10	ordinary_muscle_development	ENSGALG00010016027	COPS2	-0.088735264	0.150146189	-0.59099245	0.55452548	0.600246581	negative
PRJNA729271	stage_1D_vs_E10	ordinary_muscle_development	ENSGALG00010016030	TGM4	-1.123760805	0.758005204	-1.482523864	0.138200959	0.171376756	negative
PRJNA729271	stage_1D_vs_E10	ordinary_muscle_development	ENSGALG00010016040	RPSA2	0.041497592	0.105915067	0.391800653	0.695205512	0.732606608	positive
PRJNA729271	stage_1D_vs_E10	ordinary_muscle_development	ENSGALG00010016041	ENSGALG00010016041	0.743189788	0.089153899	8.336032404	7.68249E-17	3.45509E-16	positive
PRJNA729271	stage_1D_vs_E10	ordinary_muscle_development	ENSGALG00010016043	ADAMTSL1	0.311191333	0.192048562	1.620378354	0.105151027	0.133231131	positive
PRJNA729271	stage_1D_vs_E10	ordinary_muscle_development	ENSGALG00010016047	SRP9	-0.221851502	0.089076917	-2.49056108	0.012754157	0.018589392	negative
PRJNA729271	stage_1D_vs_E10	ordinary_muscle_development	ENSGALG00010016049	ZFYVE21	0.677385448	0.121548802	5.572950409	2.50461E-08	6.33472E-08	positive
PRJNA729271	stage_1D_vs_E10	ordinary_muscle_development	ENSGALG00010016053	DRP2	4.241889218	0.456282444	9.296630346	1.44967E-20	7.99886E-20	positive
PRJNA729271	stage_1D_vs_E10	ordinary_muscle_development	ENSGALG00010016057	SOHO1	-3.644236993	0.699788415	-5.207626928	1.91271E-07	4.51837E-07	negative
PRJNA729271	stage_1D_vs_E10	ordinary_muscle_development	ENSGALG00010016059	EPDR1	-0.870045445	0.202180757	-4.303304919	1.68269E-05	3.35774E-05	negative
PRJNA729271	stage_1D_vs_E10	ordinary_muscle_development	ENSGALG00010016060	ENSGALG00010016060	1.508884035	1.364349334	1.105936726	0.268753907	0.314084325	positive
PRJNA729271	stage_1D_vs_E10	ordinary_muscle_development	ENSGALG00010016066	C14orf2	1.087916054	0.149464916	7.278738583	3.36956E-13	1.20508E-12	positive
PRJNA729271	stage_1D_vs_E10	ordinary_muscle_development	ENSGALG00010016067	AHSG	6.249396322	0.845861555	7.388202346	1.48827E-13	5.43662E-13	positive
PRJNA729271	stage_1D_vs_E10	ordinary_muscle_development	ENSGALG00010016069	GALK2	0.587960544	0.14472453	4.062618434	4.85253E-05	9.28547E-05	positive
PRJNA729271	stage_1D_vs_E10	ordinary_muscle_development	ENSGALG00010016073	SAXO1	2.489876063	1.047513935	2.376938368	0.017457005	0.024910345	positive
PRJNA729271	stage_1D_vs_E10	ordinary_muscle_development	ENSGALG00010016076	RAB3GAP2	0.16449587	0.090402975	1.819584705	0.068822273	0.089831503	positive
PRJNA729271	stage_1D_vs_E10	ordinary_muscle_development	ENSGALG00010016083	CDH22	-3.311157763	0.618357297	-5.3547646	8.56679E-08	2.07964E-07	negative
PRJNA729271	stage_1D_vs_E10	ordinary_muscle_development	ENSGALG00010016086	TDRD9	-1.38257661	0.341624147	-4.04706934	5.18629E-05	9.89278E-05	negative
PRJNA729271	stage_1D_vs_E10	ordinary_muscle_development	ENSGALG00010016087	IARS2	0.339377917	0.089911357	3.774583426	0.000160275	0.000291565	positive
PRJNA729271	stage_1D_vs_E10	ordinary_muscle_development	ENSGALG00010016091	HMX1	-4.159033915	0.992226786	-4.191616244	2.76974E-05	5.41727E-05	negative
PRJNA729271	stage_1D_vs_E10	ordinary_muscle_development	ENSGALG00010016094	FGF7	1.177108826	0.184436549	6.382188516	1.74575E-10	5.20029E-10	positive
PRJNA729271	stage_1D_vs_E10	ordinary_muscle_development	ENSGALG00010016097	FNIP1	1.25179785	0.109921658	11.38809106	4.79377E-30	4.15088E-29	positive
PRJNA729271	stage_1D_vs_E10	ordinary_muscle_development	ENSGALG00010016100	SFRP4	-3.941384054	0.457086783	-8.622835314	6.53164E-18	3.11639E-17	negative
PRJNA729271	stage_1D_vs_E10	ordinary_muscle_development	ENSGALG00010016103	BPNT1	-1.124429654	0.090163256	-12.47104091	1.07414E-35	1.19766E-34	negative
PRJNA729271	stage_1D_vs_E10	ordinary_muscle_development	ENSGALG00010016107	UVSSA	-0.099990407	0.091410614	-1.093859918	0.27401642	0.319718909	negative
PRJNA729271	stage_1D_vs_E10	ordinary_muscle_development	ENSGALG00010016109	MLH1	-0.034930807	0.09949597	-0.351077605	0.725530128	0.75970232	negative
PRJNA729271	stage_1D_vs_E10	ordinary_muscle_development	ENSGALG00010016111	EPB41	0.093802294	0.10201579	0.919487992	0.357840384	0.406719702	positive
PRJNA729271	stage_1D_vs_E10	ordinary_muscle_development	ENSGALG00010016113	DTWD1	0.021363293	0.148220873	0.144131475	0.885396651	0.902525718	positive
PRJNA729271	stage_1D_vs_E10	ordinary_muscle_development	ENSGALG00010016114	HAUS6	-1.222434327	0.151544404	-8.066509203	7.23366E-16	3.06707E-15	negative
PRJNA729271	stage_1D_vs_E10	ordinary_muscle_development	ENSGALG00010016115	RD3L	0.348567764	0.458556886	0.760140726	0.447170471	0.496335704	positive
PRJNA729271	stage_1D_vs_E10	ordinary_muscle_development	ENSGALG00010016117	ENSGALG00010016117	0.283153373	0.236330374	1.198125184	0.230868286	0.274095815	positive
PRJNA729271	stage_1D_vs_E10	ordinary_muscle_development	ENSGALG00010016118	EPRS	-0.0935398	0.087546339	-1.068460445	0.285312864	0.331564977	negative
PRJNA729271	stage_1D_vs_E10	ordinary_muscle_development	ENSGALG00010016119	FETUB	-1.804964677	1.604193291	-1.125154112			negative
PRJNA729271	stage_1D_vs_E10	ordinary_muscle_development	ENSGALG00010016121	SLC35C2	-0.092682833	0.097059724	-0.954905171	0.339625655	0.388392415	negative
PRJNA729271	stage_1D_vs_E10	ordinary_muscle_development	ENSGALG00010016122	AFG2B	0.010247362	0.131757853	0.077774207	0.938007664	0.947287751	positive
PRJNA729271	stage_1D_vs_E10	ordinary_muscle_development	ENSGALG00010016127	ENSGALG00010016127	0.059433313	0.169284718	0.351084928	0.725524635	0.75970232	positive
PRJNA729271	stage_1D_vs_E10	ordinary_muscle_development	ENSGALG00010016129	CENPI	-1.679880125	0.233512454	-7.193963743	6.29369E-13	2.21103E-12	negative
PRJNA729271	stage_1D_vs_E10	ordinary_muscle_development	ENSGALG00010016131	SMYD1	-0.312068991	0.217688388	-1.433558281	0.151698356	0.186835557	negative
PRJNA729271	stage_1D_vs_E10	ordinary_muscle_development	ENSGALG00010016137	STARD3NL	-0.348881365	0.140825754	-2.47739745	0.013234443	0.019239119	negative
PRJNA729271	stage_1D_vs_E10	ordinary_muscle_development	ENSGALG00010016139	LURAP1L	-0.184448322	0.190931547	-0.96604424	0.334022044	0.382587873	negative
PRJNA729271	stage_1D_vs_E10	ordinary_muscle_development	ENSGALG00010016141	RRP15	-0.735652407	0.176936109	-4.157729092	3.21427E-05	6.25045E-05	negative
PRJNA729271	stage_1D_vs_E10	ordinary_muscle_development	ENSGALG00010016147	FABP1	0.848092095	0.83653359	1.013817143	0.310670013	0.35801891	positive
PRJNA729271	stage_1D_vs_E10	ordinary_muscle_development	ENSGALG00010016150	POLG	0.419264503	0.093663398	4.476289694	7.59514E-06	1.5696E-05	positive
PRJNA729271	stage_1D_vs_E10	ordinary_muscle_development	ENSGALG00010016159	ASPG	-2.606264898	0.196679672	-13.2513181	4.43348E-40	5.82094E-39	negative
PRJNA729271	stage_1D_vs_E10	ordinary_muscle_development	ENSGALG00010016165	KK34	2.51483155	0.9528788	2.639193516	0.008310353	0.012415849	positive
PRJNA729271	stage_1D_vs_E10	ordinary_muscle_development	ENSGALG00010016168	PLIN2	1.900738681	0.229252879	8.291013351	1.12288E-16	4.9942E-16	positive
PRJNA729271	stage_1D_vs_E10	ordinary_muscle_development	ENSGALG00010016169	RHOH	-0.44981987	0.123216066	-3.650659231	0.000261568	0.00046384	negative
PRJNA729271	stage_1D_vs_E10	ordinary_muscle_development	ENSGALG00010016173	ENSGALG00010016173	-1.534396788	0.331862261	-4.623595299	3.77145E-06	8.01102E-06	negative
PRJNA729271	stage_1D_vs_E10	ordinary_muscle_development	ENSGALG00010016177	ENSGALG00010016177	0.207210511	1.69094903	0.122540956	0.902470612	0.916764421	positive
PRJNA729271	stage_1D_vs_E10	ordinary_muscle_development	ENSGALG00010016181	FANCI	-1.548298104	0.173499952	-8.92391085	4.50106E-19	2.28122E-18	negative
PRJNA729271	stage_1D_vs_E10	ordinary_muscle_development	ENSGALG00010016182	ELMO2	-0.639794231	0.062574191	-10.22457049	1.53909E-24	1.03092E-23	negative
PRJNA729271	stage_1D_vs_E10	ordinary_muscle_development	ENSGALG00010016190	THNSL2	-0.307814962	0.154208201	-1.99609982	0.045923059	0.061566291	negative
PRJNA729271	stage_1D_vs_E10	ordinary_muscle_development	ENSGALG00010016193	TCAIM	0.505429734	0.13406529	3.770026783	0.00016323	0.000296531	positive
PRJNA729271	stage_1D_vs_E10	ordinary_muscle_development	ENSGALG00010016197	SNRPN	-1.440287935	0.116073268	-12.40843787	2.35197E-35	2.5945E-34	negative
PRJNA729271	stage_1D_vs_E10	ordinary_muscle_development	ENSGALG00010016199	RLBP1	-4.598562075	0.446930607	-10.28920822	7.88218E-25	5.3539E-24	negative
PRJNA729271	stage_1D_vs_E10	ordinary_muscle_development	ENSGALG00010016200	MICU3	0.997994738	0.116731644	8.549479015	1.23645E-17	5.80388E-17	positive
PRJNA729271	stage_1D_vs_E10	ordinary_muscle_development	ENSGALG00010016205	CD40LG	2.161193625	1.262773638	1.711465587	0.086995199	0.111740842	positive
PRJNA729271	stage_1D_vs_E10	ordinary_muscle_development	ENSGALG00010016206	KNG1	0.020355384	1.197415042	0.016999439	0.986437063	0.988414574	positive
PRJNA729271	stage_1D_vs_E10	ordinary_muscle_development	ENSGALG00010016209	ADAMTS7	-0.607934841	0.165527627	-3.672709221	0.000239993	0.000427166	negative
PRJNA729271	stage_1D_vs_E10	ordinary_muscle_development	ENSGALG00010016211	TACC3	-1.672616383	0.161611614	-10.34960509	4.20216E-25	2.90049E-24	negative
PRJNA729271	stage_1D_vs_E10	ordinary_muscle_development	ENSGALG00010016215	SPATA17	-3.004874194	0.365950836	-8.211141766	2.19095E-16	9.59451E-16	negative
PRJNA729271	stage_1D_vs_E10	ordinary_muscle_development	ENSGALG00010016218	N4BP2	0.104852611	0.075504786	1.38868827	0.164927563	0.201757534	positive
PRJNA729271	stage_1D_vs_E10	ordinary_muscle_development	ENSGALG00010016221	TMEM200B	-0.353448802	0.201444756	-1.754569389	0.079332991	0.102654143	negative
PRJNA729271	stage_1D_vs_E10	ordinary_muscle_development	ENSGALG00010016224	MOSPD1	1.033792981	0.115521354	8.94893407	3.58931E-19	1.83065E-18	positive
PRJNA729271	stage_1D_vs_E10	ordinary_muscle_development	ENSGALG00010016225	P4HA2	1.611477478	0.155956662	10.33285439	5.00487E-25	3.44144E-24	positive
PRJNA729271	stage_1D_vs_E10	ordinary_muscle_development	ENSGALG00010016229	ENSGALG00010016229	0.963850805	0.218044117	4.420439402	9.85004E-06	2.01207E-05	positive
PRJNA729271	stage_1D_vs_E10	ordinary_muscle_development	ENSGALG00010016235	DDX27	-0.46709608	0.123564004	-3.780195404	0.000156705	0.000285321	negative
PRJNA729271	stage_1D_vs_E10	ordinary_muscle_development	ENSGALG00010016236	GPATCH2	-0.403163907	0.065024456	-6.200188892	5.63954E-10	1.62193E-09	negative
PRJNA729271	stage_1D_vs_E10	ordinary_muscle_development	ENSGALG00010016240	EIF4A2	0.725865212	0.100320696	7.235448304	4.63994E-13	1.64803E-12	positive
PRJNA729271	stage_1D_vs_E10	ordinary_muscle_development	ENSGALG00010016241	TOPAZ1	-0.505164658	0.824016521	-0.613051614	0.539842212	0.586231111	negative
PRJNA729271	stage_1D_vs_E10	ordinary_muscle_development	ENSGALG00010016242	PDS5A	0.044370573	0.092275906	0.480846784	0.630625397	0.672175537	positive
PRJNA729271	stage_1D_vs_E10	ordinary_muscle_development	ENSGALG00010016245	FAM96A	-0.453196266	0.105118007	-4.311309534	1.62291E-05	3.24299E-05	negative
PRJNA729271	stage_1D_vs_E10	ordinary_muscle_development	ENSGALG00010016247	KIF26A	-2.803893881	0.167655907	-16.72409836	8.74902E-63	2.59871E-61	negative
PRJNA729271	stage_1D_vs_E10	ordinary_muscle_development	ENSGALG00010016251	MPDZ	-0.136416137	0.072700294	-1.87641796	0.06059791	0.079713832	negative
PRJNA729271	stage_1D_vs_E10	ordinary_muscle_development	ENSGALG00010016253	EDN3	-0.369845808	0.204518035	-1.808377476	0.070547771	0.091951258	negative
PRJNA729271	stage_1D_vs_E10	ordinary_muscle_development	ENSGALG00010016257	ENSGALG00010016257	-0.902000958	0.111668905	-8.077458603	6.61305E-16	2.81185E-15	negative
PRJNA729271	stage_1D_vs_E10	ordinary_muscle_development	ENSGALG00010016258	CTNNB1	-1.564921538	0.077125974	-20.29046054	1.56126E-91	9.62998E-90	negative
PRJNA729271	stage_1D_vs_E10	ordinary_muscle_development	ENSGALG00010016260	INTS6L	1.099304868	0.143957629	7.636308538	2.23538E-14	8.6267E-14	positive
PRJNA729271	stage_1D_vs_E10	ordinary_muscle_development	ENSGALG00010016262	SPG11	0.666520425	0.060213444	11.0692958	1.76784E-28	1.42124E-27	positive
PRJNA729271	stage_1D_vs_E10	ordinary_muscle_development	ENSGALG00010016263	HGFAC	-0.716746158	1.405632097	-0.50991021			negative
PRJNA729271	stage_1D_vs_E10	ordinary_muscle_development	ENSGALG00010016266	PDLIM4	-0.387168331	0.120736202	-3.20672941	0.001342532	0.002207601	negative
PRJNA729271	stage_1D_vs_E10	ordinary_muscle_development	ENSGALG00010016270	RFC4	-1.240914812	0.110482207	-11.23180684	2.84598E-29	2.37767E-28	negative
PRJNA729271	stage_1D_vs_E10	ordinary_muscle_development	ENSGALG00010016274	ZNF831	-0.249727805	0.553219595	-0.451408097	0.651695449	0.691430649	negative
PRJNA729271	stage_1D_vs_E10	ordinary_muscle_development	ENSGALG00010016276	ACTR2	-1.11232868	0.107774321	-10.32090645	5.66854E-25	3.88123E-24	negative
PRJNA729271	stage_1D_vs_E10	ordinary_muscle_development	ENSGALG00010016279	PRELID3B	-0.086785785	0.105787876	-0.820375534	0.412002058	0.461619373	negative
PRJNA729271	stage_1D_vs_E10	ordinary_muscle_development	ENSGALG00010016281	SLC22A4	-1.664086962	0.220541202	-7.54546973	4.5066E-14	1.70601E-13	negative
PRJNA729271	stage_1D_vs_E10	ordinary_muscle_development	ENSGALG00010016286	ULK4	-1.293303295	0.138709847	-9.323803078	1.12243E-20	6.2312E-20	negative
PRJNA729271	stage_1D_vs_E10	ordinary_muscle_development	ENSGALG00010016287	BCL11B	-7.536585339	0.407649995	-18.48788284	2.58506E-76	1.10859E-74	negative
PRJNA729271	stage_1D_vs_E10	ordinary_muscle_development	ENSGALG00010016289	IRF1	2.203066829	0.260349145	8.461970664	2.62897E-17	1.21282E-16	positive
PRJNA729271	stage_1D_vs_E10	ordinary_muscle_development	ENSGALG00010016292	PHKA1	2.445550701	0.14845738	16.47308267	5.72758E-61	1.61835E-59	positive
PRJNA729271	stage_1D_vs_E10	ordinary_muscle_development	ENSGALG00010016295	UBE2K	-0.600803924	0.093617323	-6.417657576	1.38387E-10	4.15305E-10	negative
PRJNA729271	stage_1D_vs_E10	ordinary_muscle_development	ENSGALG00010016299	SRSF4	-1.050398044	0.109491975	-9.59337928	8.52463E-22	5.03523E-21	negative
PRJNA729271	stage_1D_vs_E10	ordinary_muscle_development	ENSGALG00010016303	ENSGALG00010016303	0.978562133	0.203334687	4.812568613	1.49003E-06	3.28136E-06	positive
PRJNA729271	stage_1D_vs_E10	ordinary_muscle_development	ENSGALG00010016304	EIF3J	0.317944591	0.099928243	3.181729009	0.001463987	0.002396172	positive
PRJNA729271	stage_1D_vs_E10	ordinary_muscle_development	ENSGALG00010016305	DENND4C	1.24047006	0.095620945	12.97278604	1.74568E-38	2.16085E-37	positive
PRJNA729271	stage_1D_vs_E10	ordinary_muscle_development	ENSGALG00010016307	RAD50	-0.094119972	0.127315578	-0.739265167	0.459745996	0.50858721	negative
PRJNA729271	stage_1D_vs_E10	ordinary_muscle_development	ENSGALG00010016308	RPS6	-0.204239366	0.140715452	-1.451435239	0.146658703	0.181229148	negative
PRJNA729271	stage_1D_vs_E10	ordinary_muscle_development	ENSGALG00010016310	HDAC8	-0.59729915	0.092755788	-6.439481169	1.19883E-10	3.61343E-10	negative
PRJNA729271	stage_1D_vs_E10	ordinary_muscle_development	ENSGALG00010016312	MECR	-0.748536966	0.150289633	-4.980629391	6.33778E-07	1.43743E-06	negative
PRJNA729271	stage_1D_vs_E10	ordinary_muscle_development	ENSGALG00010016314	ACER2	0.487467688	0.15510006	3.142923918	0.001672693	0.002719651	positive
PRJNA729271	stage_1D_vs_E10	ordinary_muscle_development	ENSGALG00010016315	RPS4Y1	0.259088383	0.136423392	1.899149253	0.057544855	0.075925056	positive
PRJNA729271	stage_1D_vs_E10	ordinary_muscle_development	ENSGALG00010016317	CTDSPL2	-0.150196877	0.056348522	-2.665498098	0.007687437	0.011536328	negative
PRJNA729271	stage_1D_vs_E10	ordinary_muscle_development	ENSGALG00010016319	RBM41	-0.052331133	0.18931297	-0.276426559	0.78222046	0.810514374	negative
PRJNA729271	stage_1D_vs_E10	ordinary_muscle_development	ENSGALG00010016322	EBF4	-0.442125918	0.132270079	-3.342599634	0.000829976	0.001396621	negative
PRJNA729271	stage_1D_vs_E10	ordinary_muscle_development	ENSGALG00010016324	TUBB1	-1.801289811	0.214383613	-8.402180474	4.38264E-17	1.99517E-16	negative
PRJNA729271	stage_1D_vs_E10	ordinary_muscle_development	ENSGALG00010016328	USH2A	-0.620724359	0.655785135	-0.946536184	0.343875176	0.392849202	negative
PRJNA729271	stage_1D_vs_E10	ordinary_muscle_development	ENSGALG00010016332	ENSGALG00010016332	-0.382096508	0.232956388	-1.64020618	0.100962305	0.128316406	negative
PRJNA729271	stage_1D_vs_E10	ordinary_muscle_development	ENSGALG00010016335	ABHD4	2.556004898	0.183110817	13.95878705	2.78127E-44	4.2481E-43	positive
PRJNA729271	stage_1D_vs_E10	ordinary_muscle_development	ENSGALG00010016336	SLC24A2	-0.586091917	0.364316812	-1.608742438	0.107672672	0.136069344	negative
PRJNA729271	stage_1D_vs_E10	ordinary_muscle_development	ENSGALG00010016338	PTPRU	-0.681866361	0.149833541	-4.550825899	5.34358E-06	1.11881E-05	negative
PRJNA729271	stage_1D_vs_E10	ordinary_muscle_development	ENSGALG00010016339	SETD3	0.766191784	0.121741868	6.293576702	3.10233E-10	9.09001E-10	positive
PRJNA729271	stage_1D_vs_E10	ordinary_muscle_development	ENSGALG00010016340	RGS12	-1.542520522	0.09882722	-15.60825565	6.39619E-55	1.46235E-53	negative
PRJNA729271	stage_1D_vs_E10	ordinary_muscle_development	ENSGALG00010016341	SLX4	-0.511554886	0.06346194	-8.060813847	7.57882E-16	3.21026E-15	negative
PRJNA729271	stage_1D_vs_E10	ordinary_muscle_development	ENSGALG00010016344	SMIM14	0.108857104	0.111902384	0.972786287	0.330659529	0.37912592	positive

PRJNA729271	stage_1D_vs_E10	ordinary_muscle_development	ENSGALG00010016345	CTSZ	0.548960884	0.110527747	4.966724639	6.80932E-07	1.54124E-06	positive
PRJNA729271	stage_1D_vs_E10	ordinary_muscle_development	ENSGALG00010016346	TRAP1	-0.395629871	0.104165933	-3.798073516	0.000145825	0.000266348	negative
PRJNA729271	stage_1D_vs_E10	ordinary_muscle_development	ENSGALG00010016347	ANO10	0.198257311	0.089551038	2.213902984	0.026835453	0.037247907	positive
PRJNA729271	stage_1D_vs_E10	ordinary_muscle_development	ENSGALG00010016350	CASC4	0.201912901	0.062732847	3.21861531	0.001288112	0.002123414	positive
PRJNA729271	stage_1D_vs_E10	ordinary_muscle_development	ENSGALG00010016353	C1orf232	0.347875948	0.140880385	2.469300093	0.013537764	0.019644597	positive
PRJNA729271	stage_1D_vs_E10	ordinary_muscle_development	ENSGALG00010016355	MCF2L2	0.350430471	0.229241914	1.528649212	0.126351429	0.157857608	positive
PRJNA729271	stage_1D_vs_E10	ordinary_muscle_development	ENSGALG00010016361	NELFCD	-1.073331816	0.083089859	-12.91772334	3.57565E-38	4.38116E-37	negative
PRJNA729271	stage_1D_vs_E10	ordinary_muscle_development	ENSGALG00010016362	NUP62	0.073503036	0.168253662	0.436858462	0.662213998	0.701330798	positive
PRJNA729271	stage_1D_vs_E10	ordinary_muscle_development	ENSGALG00010016366	KIF3A	-0.364872275	0.098152937	-3.717385199	0.000201295	0.000361245	negative
PRJNA729271	stage_1D_vs_E10	ordinary_muscle_development	ENSGALG00010016368	CCNK	0.500244691	0.145013867	3.449633486	0.000561348	0.000961081	positive
PRJNA729271	stage_1D_vs_E10	ordinary_muscle_development	ENSGALG00010016369	MSANTD1	-3.308524401	0.300792531	-10.99935691	3.84867E-28	3.03187E-27	negative
PRJNA729271	stage_1D_vs_E10	ordinary_muscle_development	ENSGALG00010016370	TMEM255A	2.470475441	0.185836772	13.29379225	2.51501E-40	3.3262E-39	positive
PRJNA729271	stage_1D_vs_E10	ordinary_muscle_development	ENSGALG00010016372	DNAAF6	-1.497578465	0.207496729	-7.217359389	5.30068E-13	1.87216E-12	negative
PRJNA729271	stage_1D_vs_E10	ordinary_muscle_development	ENSGALG00010016373	SRL	3.577711546	0.131620021	27.18212254	1.0567E-162	2.3934E-160	positive
PRJNA729271	stage_1D_vs_E10	ordinary_muscle_development	ENSGALG00010016374	FRMD5	4.54079006	0.337842387	13.44055759	3.49791E-41	4.8196E-40	positive
PRJNA729271	stage_1D_vs_E10	ordinary_muscle_development	ENSGALG00010016375	CCNI2	-1.876414097	0.140369815	-13.3676467	9.34509E-41	1.26714E-39	negative
PRJNA729271	stage_1D_vs_E10	ordinary_muscle_development	ENSGALG00010016377	FRMPD3	3.700315901	0.277310277	13.34359455	1.29082E-40	1.73566E-39	positive
PRJNA729271	stage_1D_vs_E10	ordinary_muscle_development	ENSGALG00010016378	WDR76	-0.345108268	0.17207004	-2.005626714	0.04489609	0.06030867	negative
PRJNA729271	stage_1D_vs_E10	ordinary_muscle_development	ENSGALG00010016379	TFAP4	-0.859515769	0.15335229	-5.604844705	2.08442E-08	5.30436E-08	negative
PRJNA729271	stage_1D_vs_E10	ordinary_muscle_development	ENSGALG00010016382	UGDH	-0.945825808	0.186109055	-5.082105261	3.73274E-07	8.6033E-07	negative
PRJNA729271	stage_1D_vs_E10	ordinary_muscle_development	ENSGALG00010016383	FAM110D	1.262676748	0.174194438	7.248662816	4.20906E-13	1.49829E-12	positive
PRJNA729271	stage_1D_vs_E10	ordinary_muscle_development	ENSGALG00010016384	GNAS	0.367626357	0.073808804	4.980792773	6.33243E-07	1.43644E-06	positive
PRJNA729271	stage_1D_vs_E10	ordinary_muscle_development	ENSGALG00010016385	HTT	0.716946621	0.106205879	6.750536086	1.473E-11	4.7237E-11	positive
PRJNA729271	stage_1D_vs_E10	ordinary_muscle_development	ENSGALG00010016387	CCDC85C	-0.915846215	0.159324975	-5.748290332	9.01503E-09	2.36127E-08	negative
PRJNA729271	stage_1D_vs_E10	ordinary_muscle_development	ENSGALG00010016390	ENSGALG00010016390	3.226924487	0.308080302	10.4742967	1.13377E-25	8.01269E-25	positive
PRJNA729271	stage_1D_vs_E10	ordinary_muscle_development	ENSGALG00010016392	STMN3	0.353355781	0.315384781	1.12039579	0.262545138	0.307696618	positive
PRJNA729271	stage_1D_vs_E10	ordinary_muscle_development	ENSGALG00010016393	BLM	-1.472863762	0.122563249	-12.01717294	2.8868E-33	2.918E-32	negative
PRJNA729271	stage_1D_vs_E10	ordinary_muscle_development	ENSGALG00010016394	PDIK1L	0.499548533	0.095320256	5.240738497	1.59935E-07	3.80292E-07	positive
PRJNA729271	stage_1D_vs_E10	ordinary_muscle_development	ENSGALG00010016395	ENSGALG00010016395	-1.058022938	0.162697953	-6.502988628	7.87398E-11	2.40179E-10	negative
PRJNA729271	stage_1D_vs_E10	ordinary_muscle_development	ENSGALG00010016397	LIAS	0.585662159	0.10292165	5.690368931	1.26765E-08	3.28177E-08	positive
PRJNA729271	stage_1D_vs_E10	ordinary_muscle_development	ENSGALG00010016398	SEPTIN6	-0.721434051	0.068670573	-10.50572352	8.1297E-26	5.76516E-25	negative
PRJNA729271	stage_1D_vs_E10	ordinary_muscle_development	ENSGALG00010016399	ACSM5	7.166770563	0.326931468	21.92132375	1.6266E-106	1.3172E-104	positive
PRJNA729271	stage_1D_vs_E10	ordinary_muscle_development	ENSGALG00010016400	ENSGALG00010016400	-0.285593657	0.086803577	-3.290113933	0.001001468	0.00166969	negative
PRJNA729271	stage_1D_vs_E10	ordinary_muscle_development	ENSGALG00010016402	SEPTIN8	-0.844130677	0.077430566	-10.90177586	1.13029E-27	8.69157E-27	negative
PRJNA729271	stage_1D_vs_E10	ordinary_muscle_development	ENSGALG00010016403	GRK4	-0.847309425	0.134297114	-6.309215435	2.80454E-10	8.24412E-10	negative
PRJNA729271	stage_1D_vs_E10	ordinary_muscle_development	ENSGALG00010016404	ESRRG	1.800193365	0.148670019	12.10865094	9.50445E-34	9.78459E-33	positive
PRJNA729271	stage_1D_vs_E10	ordinary_muscle_development	ENSGALG00010016407	RPL9	0.124567699	0.180722066	0.689277749	0.490648498	0.538947406	positive
PRJNA729271	stage_1D_vs_E10	ordinary_muscle_development	ENSGALG00010016409	GMEB2	0.800598477	0.207109132	3.865587528	0.000110822	0.000204815	positive
PRJNA729271	stage_1D_vs_E10	ordinary_muscle_development	ENSGALG00010016410	TRAK1	1.145023381	0.096597467	11.8535549	2.06252E-32	2.00108E-31	positive
PRJNA729271	stage_1D_vs_E10	ordinary_muscle_development	ENSGALG00010016411	CTSH	0.208457081	0.123974771	1.681447606	0.092676005	0.118554293	positive
PRJNA729271	stage_1D_vs_E10	ordinary_muscle_development	ENSGALG00010016412	TMSB15B	-3.894475147	0.216252027	-18.00896479	1.65698E-72	6.56229E-71	negative
PRJNA729271	stage_1D_vs_E10	ordinary_muscle_development	ENSGALG00010016415	RASGRF1	3.2806925	0.511620971	6.412349539	1.43294E-10	4.29407E-10	positive
PRJNA729271	stage_1D_vs_E10	ordinary_muscle_development	ENSGALG00010016416	CYP46A1	0.546734779	0.112471736	4.861085992	1.16744E-06	2.59182E-06	positive
PRJNA729271	stage_1D_vs_E10	ordinary_muscle_development	ENSGALG00010016417	NOP14	-1.292910136	0.108262025	-11.942416	7.11267E-33	7.04704E-32	negative
PRJNA729271	stage_1D_vs_E10	ordinary_muscle_development	ENSGALG00010016418	NFIB	0.459866175	0.097780583	4.703041857	2.56314E-06	5.53856E-06	positive
PRJNA729271	stage_1D_vs_E10	ordinary_muscle_development	ENSGALG00010016421	ENSGALG00010016421	-3.64161803	0.633966543	-5.744180148	9.23674E-09	2.41585E-08	negative
PRJNA729271	stage_1D_vs_E10	ordinary_muscle_development	ENSGALG00010016422	KCTD3	-0.84991908	0.091145135	-9.324897935	1.1109E-20	6.17193E-20	negative
PRJNA729271	stage_1D_vs_E10	ordinary_muscle_development	ENSGALG00010016423	TRMT44	-0.787414716	0.109917786	-7.163669717	7.85455E-13	2.74011E-12	negative
PRJNA729271	stage_1D_vs_E10	ordinary_muscle_development	ENSGALG00010016425	TRIM63	4.199271145	0.413912338	10.14531521	3.47654E-24	2.2802E-23	positive
PRJNA729271	stage_1D_vs_E10	ordinary_muscle_development	ENSGALG00010016426	HELZ2	1.114903433	0.368729715	3.023633266	0.00249759	0.003970015	positive
PRJNA729271	stage_1D_vs_E10	ordinary_muscle_development	ENSGALG00010016428	ADRA1D	1.087544181	0.197960844	5.493733809	3.93524E-08	9.79081E-08	positive
PRJNA729271	stage_1D_vs_E10	ordinary_muscle_development	ENSGALG00010016429	ENSGALG00010016429	-2.308640549	0.504029141	-4.580371175	4.64151E-06	9.7647E-06	negative
PRJNA729271	stage_1D_vs_E10	ordinary_muscle_development	ENSGALG00010016431	B3GNT5	-3.71231026	0.160436572	-23.1388031	1.8847E-118	2.1015E-116	negative
PRJNA729271	stage_1D_vs_E10	ordinary_muscle_development	ENSGALG00010016433	TMED3	-0.099835986	0.143190227	-0.697226253	0.485661212	0.53423839	negative
PRJNA729271	stage_1D_vs_E10	ordinary_muscle_development	ENSGALG00010016434	GPR139	-1.797580719	0.525536763	-3.420466169	0.000625139	0.001063918	negative
PRJNA729271	stage_1D_vs_E10	ordinary_muscle_development	ENSGALG00010016436	SLC30A2	2.030189243	0.541508392	3.749137178	0.000177444	0.000321225	positive
PRJNA729271	stage_1D_vs_E10	ordinary_muscle_development	ENSGALG00010016441	ENSGALG00010016441	-1.827879056	0.661742376	-2.762221557	0.005740951	0.008753953	negative
PRJNA729271	stage_1D_vs_E10	ordinary_muscle_development	ENSGALG00010016445	ZDHHC21	-0.541479027	0.140816015	-3.845294343	0.000120408	0.000221735	negative
PRJNA729271	stage_1D_vs_E10	ordinary_muscle_development	ENSGALG00010016447	ID3	-1.526313637	0.113271801	-13.47478911	2.20113E-41	3.05023E-40	negative
PRJNA729271	stage_1D_vs_E10	ordinary_muscle_development	ENSGALG00010016449	FMR1	-0.013145538	0.106775769	-0.12311349	0.902017229	0.916496547	negative
PRJNA729271	stage_1D_vs_E10	ordinary_muscle_development	ENSGALG00010016450	ENSGALG00010016450	-0.312888571	0.262997612	-1.189701186	0.234163859	0.277599177	negative
PRJNA729271	stage_1D_vs_E10	ordinary_muscle_development	ENSGALG00010016451	USP46	-0.372360921	0.147966528	-2.516521306	0.011851973	0.017354718	negative
PRJNA729271	stage_1D_vs_E10	ordinary_muscle_development	ENSGALG00010016452	GLCE	-0.315515382	0.114213759	-2.762498896	0.005736076	0.00874744	negative
PRJNA729271	stage_1D_vs_E10	ordinary_muscle_development	ENSGALG00010016453	MYCL	-1.149436054	0.177791118	-6.465092663	1.01237E-10	3.06352E-10	negative
PRJNA729271	stage_1D_vs_E10	ordinary_muscle_development	ENSGALG00010016454	ANKS3	0.255611219	0.09317697	2.743287517	0.00608274	0.009254678	positive
PRJNA729271	stage_1D_vs_E10	ordinary_muscle_development	ENSGALG00010016455	RBM27	-0.405706185	0.091114462	-4.452708998	8.47936E-06	1.74536E-05	negative
PRJNA729271	stage_1D_vs_E10	ordinary_muscle_development	ENSGALG00010016456	LAMP3	-1.43309954	0.37160289	-3.85653497	0.000115006	0.000212249	negative
PRJNA729271	stage_1D_vs_E10	ordinary_muscle_development	ENSGALG00010016457	SMOX	1.37727224	0.176421076	7.806733018	5.86894E-15	2.34418E-14	positive
PRJNA729271	stage_1D_vs_E10	ordinary_muscle_development	ENSGALG00010016458	ZC3H12A	0.862611771	0.089125739	9.678593222	3.71797E-22	2.23804E-21	positive
PRJNA729271	stage_1D_vs_E10	ordinary_muscle_development	ENSGALG00010016459	PAQR5	2.326096082	0.276112272	8.42445744	3.62426E-17	1.65669E-16	positive
PRJNA729271	stage_1D_vs_E10	ordinary_muscle_development	ENSGALG00010016460	LBR	-1.150943215	0.069183929	-16.63599093	3.82377E-62	1.12425E-60	negative
PRJNA729271	stage_1D_vs_E10	ordinary_muscle_development	ENSGALG00010016461	HPCAL4	-4.103234202	0.416792887	-9.844779827	7.21971E-23	4.48509E-22	negative
PRJNA729271	stage_1D_vs_E10	ordinary_muscle_development	ENSGALG00010016462	RNF24	-1.074395635	0.157061266	-6.840614892	7.8854E-12	2.56678E-11	negative
PRJNA729271	stage_1D_vs_E10	ordinary_muscle_development	ENSGALG00010016463	FREM1	-0.308348287	0.142533005	-2.163346567	0.030514534	0.042052498	negative
PRJNA729271	stage_1D_vs_E10	ordinary_muscle_development	ENSGALG00010016464	PANK2	-0.336333464	0.087062183	-3.863140692	0.000111938	0.000206773	negative
PRJNA729271	stage_1D_vs_E10	ordinary_muscle_development	ENSGALG00010016465	STK35	-1.427673876	0.198179731	-7.203934877	5.84993E-13	2.06063E-12	negative
PRJNA729271	stage_1D_vs_E10	ordinary_muscle_development	ENSGALG00010016466	TMEM164	-1.103598487	0.236662441	-4.663175464	3.11367E-06	6.67149E-06	negative
PRJNA729271	stage_1D_vs_E10	ordinary_muscle_development	ENSGALG00010016468	TTC39B	-0.024759166	0.161657865	-0.15315782	0.87827382	0.896204085	negative
PRJNA729271	stage_1D_vs_E10	ordinary_muscle_development	ENSGALG00010016469	MAVS	1.026000234	0.101215462	10.13679347	3.79347E-24	2.4847E-23	positive
PRJNA729271	stage_1D_vs_E10	ordinary_muscle_development	ENSGALG00010016470	TNIP1	1.2571982	0.158146561	7.949576586	1.8715E-15	7.73301E-15	positive
PRJNA729271	stage_1D_vs_E10	ordinary_muscle_development	ENSGALG00010016471	POMGNT2	-0.102983128	0.137846633	-0.747084827	0.455012358	0.503889077	negative
PRJNA729271	stage_1D_vs_E10	ordinary_muscle_development	ENSGALG00010016472	TFDP1	-1.495411298	0.122191994	-12.23821017	1.94269E-34	2.05843E-33	negative
PRJNA729271	stage_1D_vs_E10	ordinary_muscle_development	ENSGALG00010016473	RAB9B	-0.977375611	0.160888188	-6.074874876	1.24084E-09	3.47892E-09	negative
PRJNA729271	stage_1D_vs_E10	ordinary_muscle_development	ENSGALG00010016474	MAPK8IP3	0.086590055	0.10992816	0.787696753	0.430874105	0.480350727	positive
PRJNA729271	stage_1D_vs_E10	ordinary_muscle_development	ENSGALG00010016475	MCCC1	1.274786846	0.082958201	15.36661627	2.74116E-53	5.91267E-52	positive
PRJNA729271	stage_1D_vs_E10	ordinary_muscle_development	ENSGALG00010016477	CNIH3	0.413283255	0.181517768	2.276819836	0.022796987	0.032039667	positive
PRJNA729271	stage_1D_vs_E10	ordinary_muscle_development	ENSGALG00010016479	ENSGALG00010016479	-3.427810615	0.461705723	-7.424232459	1.13436E-13	4.17641E-13	negative
PRJNA729271	stage_1D_vs_E10	ordinary_muscle_development	ENSGALG00010016480	GPX3	4.140061204	0.133226006	31.07547345	5.1669E-212	2.8805E-209	positive
PRJNA729271	stage_1D_vs_E10	ordinary_muscle_development	ENSGALG00010016482	EML1	1.578058789	0.139985511	11.2730152	1.78356E-29	1.50131E-28	positive
PRJNA729271	stage_1D_vs_E10	ordinary_muscle_development	ENSGALG00010016483	WDR26	0.270196104	0.102653924	2.632106927	0.008485715	0.012659576	positive
PRJNA729271	stage_1D_vs_E10	ordinary_muscle_development	ENSGALG00010016489	CCK	-5.161110173	0.574417345	-8.984948338	2.58856E-19	1.33148E-18	negative
PRJNA729271	stage_1D_vs_E10	ordinary_muscle_development	ENSGALG00010016491	DNAJC8	-0.534361906	0.054079138	-9.881109973	5.02729E-23	3.13422E-22	negative
PRJNA729271	stage_1D_vs_E10	ordinary_muscle_development	ENSGALG00010016496	PLP1	5.189391889	0.32399143	16.01706531	9.71274E-58	2.4613E-56	positive
PRJNA729271	stage_1D_vs_E10	ordinary_muscle_development	ENSGALG00010016498	KIF23	-1.889107616	0.206759549	-9.136736977	6.43651E-20	3.42626E-19	negative
PRJNA729271	stage_1D_vs_E10	ordinary_muscle_development	ENSGALG00010016500	ENSGALG00010016500	0.932600914	0.10663954	8.745357642	2.2231E-18	1.08791E-17	positive
PRJNA729271	stage_1D_vs_E10	ordinary_muscle_development	ENSGALG00010016502	YIPF5	-1.364636477	0.132789579	-10.27668352	8.97634E-25	6.06866E-24	negative
PRJNA729271	stage_1D_vs_E10	ordinary_muscle_development	ENSGALG00010016504	SNRK	1.217602998	0.084088041	14.48009718	1.61868E-47	2.80655E-46	positive
PRJNA729271	stage_1D_vs_E10	ordinary_muscle_development	ENSGALG00010016505	KCTD16	-2.694321528	0.348920893	-7.721869296	1.14636E-14	4.49458E-14	negative
PRJNA729271	stage_1D_vs_E10	ordinary_muscle_development	ENSGALG00010016507	SPATA18	-0.410539105	0.142373709	-2.88353172	0.00393243	0.006119225	negative
PRJNA729271	stage_1D_vs_E10	ordinary_muscle_development	ENSGALG00010016508	CREBBP	0.807658901	0.119444244	6.761806817	1.36281E-11	4.38004E-11	positive
PRJNA729271	stage_1D_vs_E10	ordinary_muscle_development	ENSGALG00010016510	THG1L	-0.757042562	0.112269651	-6.743073985	1.5507E-11	4.9641E-11	negative
PRJNA729271	stage_1D_vs_E10	ordinary_muscle_development	ENSGALG00010016514	GASK1A	-0.564690784	0.238058562	-2.372066685	0.017688898	0.025206506	negative
PRJNA729271	stage_1D_vs_E10	ordinary_muscle_development	ENSGALG00010016518	TMEM42	-0.313001743	0.143732677	-2.177665852	0.029430923	0.040624819	negative
PRJNA729271	stage_1D_vs_E10	ordinary_muscle_development	ENSGALG00010016519	MEX3B	-3.11154878	0.179884735	-17.29745871	4.91601E-67	1.63435E-65	negative
PRJNA729271	stage_1D_vs_E10	ordinary_muscle_development	ENSGALG00010016520	CHMP1B	-0.15239449	0.08291579	-1.837942937	0.066070814	0.086458107	negative
PRJNA729271	stage_1D_vs_E10	ordinary_muscle_development	ENSGALG00010016526	EIF1B	0.618624114	0.115846639	5.340026435	9.2933E-08	2.24923E-07	positive
PRJNA729271	stage_1D_vs_E10	ordinary_muscle_development	ENSGALG00010016527	MRPS34	-0.336004548	0.097862426	-3.433437767	0.000595979	0.001016439	negative
PRJNA729271	stage_1D_vs_E10	ordinary_muscle_development	ENSGALG00010016529	DCUN1D1	-0.017916683	0.097422614	-0.183906814	0.854086559	0.875157972	negative
PRJNA729271	stage_1D_vs_E10	ordinary_muscle_development	ENSGALG00010016533	CNIH4	0.153623024	0.109416958	1.404014767	0.160314452	0.196578799	positive
PRJNA729271	stage_1D_vs_E10	ordinary_muscle_development	ENSGALG00010016534	TRIT1	-0.33887865	0.144575105	-2.343962672	0.019080081	0.027069176	negative
PRJNA729271	stage_1D_vs_E10	ordinary_muscle_development	ENSGALG00010016535	BNC2	-0.72945215	0.130334456	-5.596771349	2.1838E-08	5.54754E-08	negative
PRJNA729271	stage_1D_vs_E10	ordinary_muscle_development	ENSGALG00010016541	UBE2J2	-0.436961578	0.079373959	-5.505099976	3.68959E-08	9.20017E-08	negative
PRJNA729271	stage_1D_vs_E10	ordinary_muscle_development	ENSGALG00010016542	ENOX2	-0.207568635	0.076795313	-2.702881571	0.006874123	0.010374887	negative
PRJNA729271	stage_1D_vs_E10	ordinary_muscle_development	ENSGALG00010016543	TCEA3	5.060363317	0.173945251	29.09170152	4.5716E-186	1.6566E-183	positive
PRJNA729271	stage_1D_vs_E10	ordinary_muscle_development	ENSGALG00010016544	ADCY9	1.045433583	0.135566704	7.711580735	1.24269E-14	4.86043E-14	positive
PRJNA729271	stage_1D_vs_E10	ordinary_muscle_development	ENSGALG00010016546	C1QTNF12	-1.813098168	0.427875806	-4.237440264	2.26083E-05	4.45859E-05	negative
PRJNA729271	stage_1D_vs_E10	ordinary_muscle_development	ENSGALG00010016547	SGCB	2.31241343	0.143169767	16.15154847	1.10746E-58	2.89237E-57	positive
PRJNA729271	stage_1D_vs_E10	ordinary_muscle_development	ENSGALG00010016548	EVL	0.142943254	0.108548945	1.316855304	0.187887131	0.227217083	positive
PRJNA729271	stage_1D_vs_E10	ordinary_muscle_development	ENSGALG00010016549	PTPRA	-0.76716492	0.10311065	-7.440210295	1.00525E-13	3.71997E-13	negative
PRJNA729271	stage_1D_vs_E10	ordinary_muscle_development	ENSGALG00010016550	FBLIM1	-2.184247219	0.146097838	-14.95057869	1.54402E-50	3.04497E-49	negative
PRJNA729271	stage_1D_vs_E10	ordinary_muscle_development	ENSGALG00010016552	ENSGALG00010016552	2.819561299	0.44516836	6.333696539	2.39356E-10	7.07333E-10	positive
PRJNA729271	stage_1D_vs_E10	ordinary_muscle_development	ENSGALG00010016553	SLC25A14	-0.590885373	0.096596906	-6.117021703	9.53404E-10	2.70069E-09	negative
PRJNA729271	stage_1D_vs_E10	ordinary_muscle_development	ENSGALG00010016556	ASAP3	-0.018756029	0.195825962	-0.095779074	0.923696037	0.935114825	negative
PRJNA729271	stage_1D_vs_E10	ordinary_muscle_development	ENSGALG00010016558	NR2F2	-0.650818025	0.109425345	-5.947598548	2.72105E-09	7.44462E-09	negative
PRJNA729271	stage_1D_vs_E10	ordinary_muscle_development	ENSGALG00010016559	ROGDI	-0.782762492	0.116234796	-6.734321567	1.64697E-11	5.25832E-11	negative
PRJNA729271	stage_1D_vs_E10	ordinary_muscle_development	ENSGALG00010016560	DCUN1D4	1.303359101	0.169067975	7.709083298	1.26725E-14	4.95381E-14	positive
PRJNA729271	stage_1D_vs_E10	ordinary_muscle_development	ENSGALG00010016561	DEGS2	0.93167907	0.596178777	1.562751151	0.118111138	0.148445413	positive
PRJNA729271	stage_1D_vs_E10	ordinary_muscle_development	ENSGALG00010016562	GLIS2	1.595097291	0.218809284	7.289897689	3.1019E-13	1.11265E-12	positive
PRJNA729271	stage_1D_vs_E10	ordinary_muscle_development	ENSGALG00010016564	SPEN	1.493223082	0.165699609	9.011627052	2.03022E-19	1.05138E-18	positive
PRJNA729271	stage_1D_vs_E10	ordinary_muscle_development	ENSGALG00010016565	NVL	-0.459976159	0.097111463	-4.736579439	2.17356E-06	4.72704E-06	negative
PRJNA729271	stage_1D_vs_E10	ordinary_muscle_development	ENSGALG00010016566	CNTLN	-1.293751339	0.206926684	-6.252220898	4.04656E-10	1.17451E-09	negative

PRJNA729271	stage_1D_vs_E10	ordinary_muscle_development	ENSGALG00010016567	E2F2	-0.8382301	0.267374157	-3.135045318	0.001718276	0.002789071	negative
PRJNA729271	stage_1D_vs_E10	ordinary_muscle_development	ENSGALG00010016568	PAM16	1.340335939	0.134288663	9.981005924	1.84586E-23	1.17247E-22	positive
PRJNA729271	stage_1D_vs_E10	ordinary_muscle_development	ENSGALG00010016569	YY1	0.231751096	0.086723437	2.672300624	0.007533313	0.011316755	positive
PRJNA729271	stage_1D_vs_E10	ordinary_muscle_development	ENSGALG00010016572	RAB33A	-0.132343963	0.114604788	-1.15478564	0.248178219	0.292419386	negative
PRJNA729271	stage_1D_vs_E10	ordinary_muscle_development	ENSGALG00010016573	DEGS1	-0.661339553	0.128649098	-5.140646635	2.73795E-07	6.38252E-07	negative
PRJNA729271	stage_1D_vs_E10	ordinary_muscle_development	ENSGALG00010016575	CWH43	-0.269993634	0.390390454	-0.691598965	0.489189212	0.537588903	negative
PRJNA729271	stage_1D_vs_E10	ordinary_muscle_development	ENSGALG00010016576	MRPS26	0.240469448	0.106362513	2.260847754	0.023768686	0.033294077	positive
PRJNA729271	stage_1D_vs_E10	ordinary_muscle_development	ENSGALG00010016577	ZBTB17	0.798877437	0.185631569	4.303564526	1.68072E-05	3.35473E-05	positive
PRJNA729271	stage_1D_vs_E10	ordinary_muscle_development	ENSGALG00010016580	MARS2	0.200098032	0.104543442	1.91401801	0.055617855	0.073610373	positive
PRJNA729271	stage_1D_vs_E10	ordinary_muscle_development	ENSGALG00010016581	H2AFYMACROH2A1	-0.041221894	0.076420166	-0.539411212	0.589603146	0.633528362	negative
PRJNA729271	stage_1D_vs_E10	ordinary_muscle_development	ENSGALG00010016583	VPS41	-0.361727701	0.083402048	-4.337156127	1.44338E-05	2.90097E-05	negative
PRJNA729271	stage_1D_vs_E10	ordinary_muscle_development	ENSGALG00010016584	EHBP1	0.019944003	0.160539969	0.124230761	0.901132567	0.91572481	positive
PRJNA729271	stage_1D_vs_E10	ordinary_muscle_development	ENSGALG00010016588	ATP11B	-0.2885356	0.100324256	-2.876030312	0.00402711	0.00625514	negative
PRJNA729271	stage_1D_vs_E10	ordinary_muscle_development	ENSGALG00010016589	FBXO28	-0.060389279	0.077567719	-0.778536225	0.43625296	0.485600265	negative
PRJNA729271	stage_1D_vs_E10	ordinary_muscle_development	ENSGALG00010016591	HOMER2	-0.99929196	0.204768812	-4.880098446	1.06033E-06	2.3649E-06	negative
PRJNA729271	stage_1D_vs_E10	ordinary_muscle_development	ENSGALG00010016592	TMEM129	0.267427939	0.103045499	2.595241348	0.009452452	0.014022443	positive
PRJNA729271	stage_1D_vs_E10	ordinary_muscle_development	ENSGALG00010016597	FRYL	0.663368133	0.125278286	5.295156523	1.18915E-07	2.85896E-07	positive
PRJNA729271	stage_1D_vs_E10	ordinary_muscle_development	ENSGALG00010016598	AIFM1	0.25262549	0.088251296	2.862569762	0.004202207	0.006518728	positive
PRJNA729271	stage_1D_vs_E10	ordinary_muscle_development	ENSGALG00010016599	ENSGALG00010016599	-0.237379844	0.280650856	-0.845819063	0.39765369	0.447063541	negative
PRJNA729271	stage_1D_vs_E10	ordinary_muscle_development	ENSGALG00010016600	GNL2	-0.524826973	0.109761032	-4.781541886	1.73956E-06	3.80602E-06	negative
PRJNA729271	stage_1D_vs_E10	ordinary_muscle_development	ENSGALG00010016602	HSPB7	6.011981139	0.513003931	11.71917167	1.01661E-31	9.55628E-31	positive
PRJNA729271	stage_1D_vs_E10	ordinary_muscle_development	ENSGALG00010016603	ENSGALG00010016603	0.483611703	0.124633554	3.880268887	0.000104341	0.000193182	positive
PRJNA729271	stage_1D_vs_E10	ordinary_muscle_development	ENSGALG00010016604	TP53BP2	-0.580451704	0.062311279	-9.315355345	1.21545E-20	6.73212E-20	negative
PRJNA729271	stage_1D_vs_E10	ordinary_muscle_development	ENSGALG00010016605	WDPCP	-0.333507608	0.163888722	-2.034963741	0.041854522	0.056488016	negative
PRJNA729271	stage_1D_vs_E10	ordinary_muscle_development	ENSGALG00010016606	VASN	-0.321707103	0.178839442	-1.798859915	0.072040843	0.093762415	negative
PRJNA729271	stage_1D_vs_E10	ordinary_muscle_development	ENSGALG00010016607	FAM103A1	-0.630588136	0.114889845	-5.488632511	4.05057E-08	1.00639E-07	negative
PRJNA729271	stage_1D_vs_E10	ordinary_muscle_development	ENSGALG00010016609	SOX2	-0.542112682	0.467458607	-1.159702	0.246170155	0.290242102	negative
PRJNA729271	stage_1D_vs_E10	ordinary_muscle_development	ENSGALG00010016611	DNALI1	-0.449153725	0.209218325	-2.146818282	0.031807751	0.04371417	negative
PRJNA729271	stage_1D_vs_E10	ordinary_muscle_development	ENSGALG00010016612	GRIA1	-1.233939341	0.335134643	-3.681921186	0.000231483	0.000412813	negative
PRJNA729271	stage_1D_vs_E10	ordinary_muscle_development	ENSGALG00010016613	SNIP1	-0.213809532	0.075601477	-2.828113146	0.004682325	0.007222551	negative
PRJNA729271	stage_1D_vs_E10	ordinary_muscle_development	ENSGALG00010016614	DNAJC19	0.319204596	0.139432004	2.289320873	0.022060716	0.031054683	positive
PRJNA729271	stage_1D_vs_E10	ordinary_muscle_development	ENSGALG00010016615	BCORL1	1.543912221	0.122118975	12.64268903	1.22782E-36	1.42067E-35	positive
PRJNA729271	stage_1D_vs_E10	ordinary_muscle_development	ENSGALG00010016616	SLBP	-1.846304612	0.235429055	-7.842297185	4.42378E-15	1.78317E-14	negative
PRJNA729271	stage_1D_vs_E10	ordinary_muscle_development	ENSGALG00010016618	TRPM3	-2.503883943	0.530888779	-4.716400196	2.40054E-06	5.20117E-06	negative
PRJNA729271	stage_1D_vs_E10	ordinary_muscle_development	ENSGALG00010016619	SLC25A47	1.323418535	0.325343449	4.067758364	4.74676E-05	9.09267E-05	positive
PRJNA729271	stage_1D_vs_E10	ordinary_muscle_development	ENSGALG00010016620	ENSGALG00010016620	-0.015122403	0.121549646	-0.124413384	0.900987976	0.915643323	negative
PRJNA729271	stage_1D_vs_E10	ordinary_muscle_development	ENSGALG00010016622	KIAA1143	-0.867113747	0.134594501	-6.442415838	1.17587E-10	3.5457E-10	negative
PRJNA729271	stage_1D_vs_E10	ordinary_muscle_development	ENSGALG00010016623	DNAJA3	1.079762388	0.141143651	7.650095339	2.0083E-14	7.76898E-14	positive
PRJNA729271	stage_1D_vs_E10	ordinary_muscle_development	ENSGALG00010016624	MDH1	1.331153724	0.154636469	8.608278053	7.41662E-18	3.52819E-17	positive
PRJNA729271	stage_1D_vs_E10	ordinary_muscle_development	ENSGALG00010016626	CAPN2	-0.47200568	0.230442722	-2.048255965	0.040534925	0.054809118	negative
PRJNA729271	stage_1D_vs_E10	ordinary_muscle_development	ENSGALG00010016629	LARS1	-0.576666885	0.071365053	-8.08052203	6.44901E-16	2.74372E-15	negative
PRJNA729271	stage_1D_vs_E10	ordinary_muscle_development	ENSGALG00010016630	ENSGALG00010016630	0.848340502	0.357500536	2.372976865	0.017645369	0.025151896	positive
PRJNA729271	stage_1D_vs_E10	ordinary_muscle_development	ENSGALG00010016631	FAM53A	1.71255325	0.091098567	18.79890434	7.70924E-79	3.55877E-77	positive
PRJNA729271	stage_1D_vs_E10	ordinary_muscle_development	ENSGALG00010016637	POU6F2	-0.670248625	0.550623936	-1.217252977	0.223507979	0.266163996	negative
PRJNA729271	stage_1D_vs_E10	ordinary_muscle_development	ENSGALG00010016640	OCIAD1	0.651684165	0.103712191	6.283583066	3.30857E-10	9.67671E-10	positive
PRJNA729271	stage_1D_vs_E10	ordinary_muscle_development	ENSGALG00010016643	BTBD1	1.147508129	0.173216625	6.624699746	3.47955E-11	1.08698E-10	positive
PRJNA729271	stage_1D_vs_E10	ordinary_muscle_development	ENSGALG00010016644	NMRAL1	-2.877934827	0.212086142	-13.56965053	6.06103E-42	8.52958E-41	negative
PRJNA729271	stage_1D_vs_E10	ordinary_muscle_development	ENSGALG00010016647	EGR1	2.992855626	0.203698158	14.69260033	7.19008E-49	1.32765E-47	positive
PRJNA729271	stage_1D_vs_E10	ordinary_muscle_development	ENSGALG00010016648	ADRA2C	-0.481417602	0.246205898	-1.955345526	0.050542301	0.067378889	negative
PRJNA729271	stage_1D_vs_E10	ordinary_muscle_development	ENSGALG00010016651	ENSGALG00010016651	3.027150103	0.178722097	16.93774942	2.3702E-64	7.38841E-63	positive
PRJNA729271	stage_1D_vs_E10	ordinary_muscle_development	ENSGALG00010016652	FXR1	1.732089978	0.098889579	17.51539445	1.09326E-68	3.884E-67	positive
PRJNA729271	stage_1D_vs_E10	ordinary_muscle_development	ENSGALG00010016653	EPHA2	0.230172423	0.205582823	1.119609215	0.262880327	0.308039639	positive
PRJNA729271	stage_1D_vs_E10	ordinary_muscle_development	ENSGALG00010016654	SMIM3	1.1872317	0.182055662	6.521256659	6.97208E-11	2.13072E-10	positive
PRJNA729271	stage_1D_vs_E10	ordinary_muscle_development	ENSGALG00010016655	TRANK1	0.043185221	0.323808496	0.133366548	0.8939035	0.909527673	positive
PRJNA729271	stage_1D_vs_E10	ordinary_muscle_development	ENSGALG00010016656	CAPN8	-5.099978395	1.188356961	-4.291621595	1.77373E-05	3.5292E-05	negative
PRJNA729271	stage_1D_vs_E10	ordinary_muscle_development	ENSGALG00010016657	SLC25A29	1.311760409	0.279381449	4.695230896	2.66306E-06	5.73907E-06	positive
PRJNA729271	stage_1D_vs_E10	ordinary_muscle_development	ENSGALG00010016658	ZAR1	-3.222856432	0.69021451	-4.669354797	3.02147E-06	6.48065E-06	negative
PRJNA729271	stage_1D_vs_E10	ordinary_muscle_development	ENSGALG00010016661	UBE4B	-0.741726131	0.128680397	-5.764095778	8.20968E-09	2.16166E-08	negative
PRJNA729271	stage_1D_vs_E10	ordinary_muscle_development	ENSGALG00010016664	TM6SF1	3.933517696	0.248086989	15.85539701	1.2901E-56	3.15345E-55	positive
PRJNA729271	stage_1D_vs_E10	ordinary_muscle_development	ENSGALG00010016665	ENSGALG00010016665	4.933750013	1.011985695	4.875315964	1.08634E-06	2.42031E-06	positive
PRJNA729271	stage_1D_vs_E10	ordinary_muscle_development	ENSGALG00010016667	WARS1	-0.480069502	0.143511287	-3.345168945	0.000822325	0.00138439	negative
PRJNA729271	stage_1D_vs_E10	ordinary_muscle_development	ENSGALG00010016671	CASS4	-1.080878358	0.249246759	-4.336579397	1.44717E-05	2.90819E-05	negative
PRJNA729271	stage_1D_vs_E10	ordinary_muscle_development	ENSGALG00010016672	DCTN4	-0.036847138	0.080602924	-0.457143934	0.647567594	0.687755881	negative
PRJNA729271	stage_1D_vs_E10	ordinary_muscle_development	ENSGALG00010016673	LRPAP1	0.172651109	0.079168672	2.180800866	0.029198148	0.040318868	positive
PRJNA729271	stage_1D_vs_E10	ordinary_muscle_development	ENSGALG00010016674	SLC10A4	-0.928519159	0.364416078	-2.547964302	0.010835356	0.015951501	negative
PRJNA729271	stage_1D_vs_E10	ordinary_muscle_development	ENSGALG00010016675	SKIL	-0.068540103	0.13918431	-0.492441305	0.622407406	0.664393206	negative
PRJNA729271	stage_1D_vs_E10	ordinary_muscle_development	ENSGALG00010016676	NLRC3	-0.59869276	0.535647888	-1.117698349	0.263695848	0.308845452	negative
PRJNA729271	stage_1D_vs_E10	ordinary_muscle_development	ENSGALG00010016677	MEAF6	0.410786071	0.087802401	4.678528901	2.8894E-06	6.20841E-06	positive
PRJNA729271	stage_1D_vs_E10	ordinary_muscle_development	ENSGALG00010016678	NOX1	2.581269843	0.316101204	8.165990167	3.18888E-16	1.3835E-15	positive
PRJNA729271	stage_1D_vs_E10	ordinary_muscle_development	ENSGALG00010016680	BPIFB3	-0.788722908	0.963147259	-0.818901679	0.412842512	0.462453803	negative
PRJNA729271	stage_1D_vs_E10	ordinary_muscle_development	ENSGALG00010016681	SUSD4	2.332744043	0.278281575	8.382675144	5.17381E-17	2.34577E-16	positive
PRJNA729271	stage_1D_vs_E10	ordinary_muscle_development	ENSGALG00010016682	GPR141	-0.327432356	0.45269527	-0.723295288	0.469498487	0.518228797	negative
PRJNA729271	stage_1D_vs_E10	ordinary_muscle_development	ENSGALG00010016683	RBM22	-0.373980027	0.073605028	-5.080903294	3.75644E-07	8.65517E-07	negative
PRJNA729271	stage_1D_vs_E10	ordinary_muscle_development	ENSGALG00010016691	ENSGALG00010016691	0.348632564	0.316758137	1.100627019	0.271059021	0.316625072	positive
PRJNA729271	stage_1D_vs_E10	ordinary_muscle_development	ENSGALG00010016692	PRKCI	-1.249405136	0.074132876	-16.85359045	9.87341E-64	3.01295E-62	negative
PRJNA729271	stage_1D_vs_E10	ordinary_muscle_development	ENSGALG00010016696	DOK7	1.629376453	0.12885912	12.64463435	1.1978E-36	1.38786E-35	positive
PRJNA729271	stage_1D_vs_E10	ordinary_muscle_development	ENSGALG00010016699	SYNPO	-0.004501796	0.145467968	-0.030946992	0.975311813	0.978958849	negative
PRJNA729271	stage_1D_vs_E10	ordinary_muscle_development	ENSGALG00010016700	CLUAP1	-0.929261063	0.125933189	-7.379000499	1.59482E-13	5.81705E-13	negative
PRJNA729271	stage_1D_vs_E10	ordinary_muscle_development	ENSGALG00010016702	WDR25	0.46169361	0.09423722	4.899270269	9.61932E-07	2.15306E-06	positive
PRJNA729271	stage_1D_vs_E10	ordinary_muscle_development	ENSGALG00010016704	CSTF2	-0.424353263	0.071293557	-5.952196521	2.64567E-09	7.24524E-09	negative
PRJNA729271	stage_1D_vs_E10	ordinary_muscle_development	ENSGALG00010016706	RTN4	1.901221429	0.12544778	15.1554809	6.97033E-52	1.4413E-50	positive
PRJNA729271	stage_1D_vs_E10	ordinary_muscle_development	ENSGALG00010016707	KIF1B	1.649827598	0.13040631	12.65143997	1.09843E-36	1.27578E-35	positive
PRJNA729271	stage_1D_vs_E10	ordinary_muscle_development	ENSGALG00010016712	ENSGALG00010016712	0.482304634	0.155971001	3.0922712	0.001986313	0.003199778	positive
PRJNA729271	stage_1D_vs_E10	ordinary_muscle_development	ENSGALG00010016720	INTS15	-0.183403378	0.098025679	-1.870972782	0.061348854	0.080665061	negative
PRJNA729271	stage_1D_vs_E10	ordinary_muscle_development	ENSGALG00010016724	PGD	-1.693350917	0.110748735	-15.29002487	8.91161E-53	1.89404E-51	negative
PRJNA729271	stage_1D_vs_E10	ordinary_muscle_development	ENSGALG00010016729	ELMO1	0.679874823	0.094140242	7.221936209	5.12525E-13	1.81373E-12	positive
PRJNA729271	stage_1D_vs_E10	ordinary_muscle_development	ENSGALG00010016731	C4orf48	-1.231130398	0.272916658	-4.511012284	6.4519E-06	1.34175E-05	negative
PRJNA729271	stage_1D_vs_E10	ordinary_muscle_development	ENSGALG00010016732	ARHGAP26	0.084932935	0.075923324	1.118667229	0.263282131	0.308410739	positive
PRJNA729271	stage_1D_vs_E10	ordinary_muscle_development	ENSGALG00010016733	PHC3	0.890775466	0.152188664	5.853099983	4.82494E-09	1.2937E-08	positive
PRJNA729271	stage_1D_vs_E10	ordinary_muscle_development	ENSGALG00010016735	ENSGALG00010016735	-1.490293993	0.34567131	-4.31130368	1.62295E-05	3.24299E-05	negative
PRJNA729271	stage_1D_vs_E10	ordinary_muscle_development	ENSGALG00010016736	CENPS	-2.256520515	0.268191136	-8.413851966	3.96761E-17	1.80851E-16	negative
PRJNA729271	stage_1D_vs_E10	ordinary_muscle_development	ENSGALG00010016738	NAA60	0.919198648	0.14966726	6.141614742	8.16868E-10	2.32486E-09	positive
PRJNA729271	stage_1D_vs_E10	ordinary_muscle_development	ENSGALG00010016741	NELFA	-0.057437258	0.089339965	-0.642906648	0.520284675	0.567586842	negative
PRJNA729271	stage_1D_vs_E10	ordinary_muscle_development	ENSGALG00010016742	TRMT12	-0.162772969	0.136130479	-1.195712897	0.231808617	0.275120046	negative
PRJNA729271	stage_1D_vs_E10	ordinary_muscle_development	ENSGALG00010016744	CRYBG2	-7.965418212	1.035850956	-7.689733903	1.47441E-14	5.73889E-14	negative
PRJNA729271	stage_1D_vs_E10	ordinary_muscle_development	ENSGALG00010016745	BEGAIN	-1.253681269	0.394993104	-3.173932044	0.001503889	0.002457041	negative
PRJNA729271	stage_1D_vs_E10	ordinary_muscle_development	ENSGALG00010016746	HDGFL3	-1.56630413	0.123589475	-12.67344269	8.29905E-37	9.67E-36	negative
PRJNA729271	stage_1D_vs_E10	ordinary_muscle_development	ENSGALG00010016750	SYTL4	0.294304964	0.126883239	2.319494419	0.020368243	0.028786826	positive
PRJNA729271	stage_1D_vs_E10	ordinary_muscle_development	ENSGALG00010016751	DFFA	-0.746446534	0.087153329	-8.564750692	1.0831E-17	5.09767E-17	negative
PRJNA729271	stage_1D_vs_E10	ordinary_muscle_development	ENSGALG00010016752	ENSGALG00010016752	2.133326285	0.170676872	12.49921127	7.53956E-36	8.45212E-35	positive
PRJNA729271	stage_1D_vs_E10	ordinary_muscle_development	ENSGALG00010016754	ENSGALG00010016754	0.501462123	0.183371964	2.734671718	0.006244252	0.009484484	positive
PRJNA729271	stage_1D_vs_E10	ordinary_muscle_development	ENSGALG00010016756	NUDT16L1	-0.663228119	0.107147649	-6.189852272	6.02206E-10	1.72748E-09	negative
PRJNA729271	stage_1D_vs_E10	ordinary_muscle_development	ENSGALG00010016757	SRPX2	-0.838427916	0.279064389	-3.004424601	0.002660837	0.0042184	negative
PRJNA729271	stage_1D_vs_E10	ordinary_muscle_development	ENSGALG00010016758	NR3C1	0.650311455	0.067991269	9.56463187	1.12601E-21	6.60526E-21	positive
PRJNA729271	stage_1D_vs_E10	ordinary_muscle_development	ENSGALG00010016760	DLK1	1.780409288	0.259169854	6.869661957	6.43542E-12	2.10615E-11	positive
PRJNA729271	stage_1D_vs_E10	ordinary_muscle_development	ENSGALG00010016761	GPR160	-0.777206975	0.367582896	-2.114371978	0.034483509	0.047156487	negative
PRJNA729271	stage_1D_vs_E10	ordinary_muscle_development	ENSGALG00010016762	EML6	0.168923165	0.121634855	1.388772692	0.164901882	0.201743144	positive
PRJNA729271	stage_1D_vs_E10	ordinary_muscle_development	ENSGALG00010016763	UBALD1	0.766639816	0.172538081	4.443307876	8.85863E-06	1.81955E-05	positive
PRJNA729271	stage_1D_vs_E10	ordinary_muscle_development	ENSGALG00010016766	AOAH	-0.910159105	0.369799306	-2.461224481	0.01384637	0.020062289	negative
PRJNA729271	stage_1D_vs_E10	ordinary_muscle_development	ENSGALG00010016767	SLAIN2	0.531812722	0.092534564	5.747179185	9.07446E-09	2.37598E-08	positive
PRJNA729271	stage_1D_vs_E10	ordinary_muscle_development	ENSGALG00010016768	GALNT10	-0.147863859	0.100388725	-1.472912999	0.140774497	0.17440396	negative
PRJNA729271	stage_1D_vs_E10	ordinary_muscle_development	ENSGALG00010016769	BNC1	-7.175047662	0.328811488	-21.8211587	1.4611E-105	1.1573E-103	negative
PRJNA729271	stage_1D_vs_E10	ordinary_muscle_development	ENSGALG00010016772	TSPAN6	-1.389377797	0.126879571	-10.95036647	6.61793E-28	5.16014E-27	negative
PRJNA729271	stage_1D_vs_E10	ordinary_muscle_development	ENSGALG00010016773	RPS27A	0.260708633	0.161269282	1.616604417	0.105963698	0.134120136	positive
PRJNA729271	stage_1D_vs_E10	ordinary_muscle_development	ENSGALG00010016774	PEX14	0.298256612	0.115916227	2.573035886	0.010081075	0.014887946	positive
PRJNA729271	stage_1D_vs_E10	ordinary_muscle_development	ENSGALG00010016777	TNMD	-0.035709207	0.487305557	-0.073278882	0.941584196	0.950390932	negative
PRJNA729271	stage_1D_vs_E10	ordinary_muscle_development	ENSGALG00010016778	MTIF2	-0.110253602	0.108224101	-1.018752765	0.308320356	0.35562215	negative
PRJNA729271	stage_1D_vs_E10	ordinary_muscle_development	ENSGALG00010016779	CTNNA2	-2.463740072	0.153068081	-16.0957141	2.73399E-58	7.01401E-57	negative
PRJNA729271	stage_1D_vs_E10	ordinary_muscle_development	ENSGALG00010016780	PRORSD1P	-1.611052739	0.141165498	-11.41251058	3.62124E-30	3.16225E-29	negative
PRJNA729271	stage_1D_vs_E10	ordinary_muscle_development	ENSGALG00010016781	CDIP1	1.429107878	0.143471199	9.960939128	2.25918E-23	1.42937E-22	positive
PRJNA729271	stage_1D_vs_E10	ordinary_muscle_development	ENSGALG00010016783	CLBA1	-1.040383541	0.31805465	-3.271084207	0.00107136	0.001781299	negative
PRJNA729271	stage_1D_vs_E10	ordinary_muscle_development	ENSGALG00010016785	PDGFRB	0.735754896	0.19419195	3.788802243	0.000151375	0.000276068	positive
PRJNA729271	stage_1D_vs_E10	ordinary_muscle_development	ENSGALG00010016786	DISP1	0.058825692	0.110649563	0.53163962	0.594975616	0.638401921	positive
PRJNA729271	stage_1D_vs_E10	ordinary_muscle_development	ENSGALG00010016787	PPP2R5C	-0.566304813	0.075452873	-7.505410848	6.12362E-14	2.29537E-13	negative
PRJNA729271	stage_1D_vs_E10	ordinary_muscle_development	ENSGALG00010016788	ANLN	-1.997798604	0.288477934	-6.925308206	4.35027E-12	1.43966E-11	negative
PRJNA729271	stage_1D_vs_E10	ordinary_muscle_development	ENSGALG00010016789	DISP3	1.379436645	0.111143432	12.41131949	2.26883E-35	2.50469E-34	positive
PRJNA729271	stage_1D_vs_E10	ordinary_muscle_development	ENSGALG00010016791	LRRTM1	-2.306339067	0.443419903	-5.201252919	1.9795E-07	4.67237E-07	negative
PRJNA729271	stage_1D_vs_E10	ordinary_muscle_development	ENSGALG00010016792	HMOX2	-0.662024094	0.121913742	-5.43026638	5.627E-08	1.38548E-07	negative
PRJNA729271	stage_1D_vs_E10	ordinary_muscle_development	ENSGALG00010016793	SLC9A1	0.549134117	0.154948773	3.543972017	0.000394147	0.000686926	positive
PRJNA729271	stage_1D_vs_E10	ordinary_muscle_development	ENSGALG00010016795	GUCA2B	0.30750049	1.264657252	0.243149272	0.807889763	0.833893193	positive
PRJNA729271	stage_1D_vs_E10	ordinary_muscle_development	ENSGALG00010016796	BROX	0.187196736	0.084953811	2.203511925	0.027558687	0.03817135	positive

PRJNA729271	stage_1D_vs_E10	ordinary_muscle_development	ENSGALG00010016797	SEC62	0.091982892	0.108767063	0.845687012	0.39772737	0.447111698	positive
PRJNA729271	stage_1D_vs_E10	ordinary_muscle_development	ENSGALG00010016799	WDTC1	1.170707427	0.132017684	8.867807651	7.46E-19	3.74162E-18	positive
PRJNA729271	stage_1D_vs_E10	ordinary_muscle_development	ENSGALG00010016800	RPL14	-0.077419201	0.169049371	-0.457967992	0.646975437	0.687177325	negative
PRJNA729271	stage_1D_vs_E10	ordinary_muscle_development	ENSGALG00010016802	TMEM222	-0.24067319	0.084088187	-2.862152215	0.004207748	0.006526624	negative
PRJNA729271	stage_1D_vs_E10	ordinary_muscle_development	ENSGALG00010016804	CSF1R	1.348904579	0.136170479	9.905998668	3.92034E-23	2.45784E-22	positive
PRJNA729271	stage_1D_vs_E10	ordinary_muscle_development	ENSGALG00010016805	ENSGALG00010016805	-6.455566654	1.063593272	-6.069582071	1.28244E-09	3.59206E-09	negative
PRJNA729271	stage_1D_vs_E10	ordinary_muscle_development	ENSGALG00010016807	VSIG4	0.932030401	0.259142596	3.596592827	0.000322413	0.000566199	positive
PRJNA729271	stage_1D_vs_E10	ordinary_muscle_development	ENSGALG00010016808	ENTPD3	-3.67994694	0.686586657	-5.359770544	8.33277E-08	2.02521E-07	negative
PRJNA729271	stage_1D_vs_E10	ordinary_muscle_development	ENSGALG00010016809	SYTL1	-3.422472833	0.611254721	-5.599094313	2.15475E-08	5.47564E-08	negative
PRJNA729271	stage_1D_vs_E10	ordinary_muscle_development	ENSGALG00010016810	SH3GL3	-1.531935845	0.198433764	-7.720137023	1.16205E-14	4.55363E-14	negative
PRJNA729271	stage_1D_vs_E10	ordinary_muscle_development	ENSGALG00010016812	LRRC31	-1.040277027	0.131344176	-7.920237181	2.37058E-15	9.72588E-15	negative
PRJNA729271	stage_1D_vs_E10	ordinary_muscle_development	ENSGALG00010016813	ENSGALG00010016813	-2.170427314	0.514728834	-4.216642183	2.47967E-05	4.87096E-05	negative
PRJNA729271	stage_1D_vs_E10	ordinary_muscle_development	ENSGALG00010016814	HMGXB3	0.42112976	0.099502357	4.232359629	2.31252E-05	4.55621E-05	positive
PRJNA729271	stage_1D_vs_E10	ordinary_muscle_development	ENSGALG00010016816	MAP3K6	1.484139818	0.277580724	5.346696265	8.95741E-08	2.17084E-07	positive
PRJNA729271	stage_1D_vs_E10	ordinary_muscle_development	ENSGALG00010016817	GGCT	-1.27775796	0.105159154	-12.15070594	5.68724E-34	5.89761E-33	negative
PRJNA729271	stage_1D_vs_E10	ordinary_muscle_development	ENSGALG00010016820	EIF2AK3	-0.439540166	0.108549124	-4.049228122	5.13868E-05	9.80456E-05	negative
PRJNA729271	stage_1D_vs_E10	ordinary_muscle_development	ENSGALG00010016823	HSF3	1.193635437	0.078501776	15.20520287	3.26617E-52	6.81197E-51	positive
PRJNA729271	stage_1D_vs_E10	ordinary_muscle_development	ENSGALG00010016825	ENSGALG00010016825	3.661060917	0.290614679	12.59764625	2.1754E-36	2.48678E-35	positive
PRJNA729271	stage_1D_vs_E10	ordinary_muscle_development	ENSGALG00010016828	LACTBL1	5.691814118	0.373945712	15.22096374	2.56721E-52	5.40083E-51	positive
PRJNA729271	stage_1D_vs_E10	ordinary_muscle_development	ENSGALG00010016831	AIDA	-0.809134509	0.067394211	-12.00599423	3.30468E-33	3.33111E-32	negative
PRJNA729271	stage_1D_vs_E10	ordinary_muscle_development	ENSGALG00010016832	DYNC1H1	-0.150072716	0.094175199	-1.593548177	0.11103727	0.140074849	negative
PRJNA729271	stage_1D_vs_E10	ordinary_muscle_development	ENSGALG00010016834	ENSGALG00010016834	1.425420101	0.074046313	19.25038583	1.40116E-82	7.07657E-81	positive
PRJNA729271	stage_1D_vs_E10	ordinary_muscle_development	ENSGALG00010016835	CCDC88A	-1.505112713	0.151896538	-9.90880195	3.8119E-23	2.39089E-22	negative
PRJNA729271	stage_1D_vs_E10	ordinary_muscle_development	ENSGALG00010016836	HEPH	1.465638601	0.149264137	9.819094031	9.31771E-23	5.75214E-22	positive
PRJNA729271	stage_1D_vs_E10	ordinary_muscle_development	ENSGALG00010016840	LRRC34	-0.913589721	0.215013125	-4.248995132	2.14732E-05	4.24167E-05	negative
PRJNA729271	stage_1D_vs_E10	ordinary_muscle_development	ENSGALG00010016842	NPY7R	0.019288151	0.430324098	0.044822383	0.964248884	0.96953299	positive
PRJNA729271	stage_1D_vs_E10	ordinary_muscle_development	ENSGALG00010016845	MIA3	1.102357984	0.137458686	8.019558554	1.06126E-15	4.45108E-15	positive
PRJNA729271	stage_1D_vs_E10	ordinary_muscle_development	ENSGALG00010016847	EPHB2	-0.550262919	0.106003215	-5.19100216	2.09165E-07	4.92183E-07	negative
PRJNA729271	stage_1D_vs_E10	ordinary_muscle_development	ENSGALG00010016848	ENSGALG00010016848	0.260139868	0.169267256	1.536858776	0.124327896	0.15559656	positive
PRJNA729271	stage_1D_vs_E10	ordinary_muscle_development	ENSGALG00010016849	WASF2	0.70544925	0.152696441	4.619945595	3.83841E-06	8.14488E-06	positive
PRJNA729271	stage_1D_vs_E10	ordinary_muscle_development	ENSGALG00010016851	TEC	1.293117608	0.139060357	9.298966567	1.41817E-20	7.83102E-20	positive
PRJNA729271	stage_1D_vs_E10	ordinary_muscle_development	ENSGALG00010016852	AHDC1	0.616433004	0.168600345	3.656178772	0.000256003	0.000454249	positive
PRJNA729271	stage_1D_vs_E10	ordinary_muscle_development	ENSGALG00010016854	TAF1A	-0.056377839	0.103927393	-0.542473335	0.587492472	0.631728737	negative
PRJNA729271	stage_1D_vs_E10	ordinary_muscle_development	ENSGALG00010016856	NOD1	-0.780630958	0.140277388	-5.564909414	2.62289E-08	6.61541E-08	negative
PRJNA729271	stage_1D_vs_E10	ordinary_muscle_development	ENSGALG00010016858	ENSGALG00010016858	-2.254004211	0.18059577	-12.48093582	9.48651E-36	1.06019E-34	negative
PRJNA729271	stage_1D_vs_E10	ordinary_muscle_development	ENSGALG00010016859	RPIA	0.419419915	0.130409896	3.216166324	0.001299155	0.002140888	positive
PRJNA729271	stage_1D_vs_E10	ordinary_muscle_development	ENSGALG00010016860	VSX1	-1.497101031	0.490516085	-3.052093657	0.002272512	0.003631359	negative
PRJNA729271	stage_1D_vs_E10	ordinary_muscle_development	ENSGALG00010016861	FGR	0.86812651	0.121536272	7.142941725	9.13544E-13	3.17017E-12	positive
PRJNA729271	stage_1D_vs_E10	ordinary_muscle_development	ENSGALG00010016862	TXK	3.324765969	0.530117105	6.271757581	3.56995E-10	1.04118E-09	positive
PRJNA729271	stage_1D_vs_E10	ordinary_muscle_development	ENSGALG00010016863	MYNN	-0.042732436	0.081222972	-0.526112682	0.598809917	0.64189837	negative
PRJNA729271	stage_1D_vs_E10	ordinary_muscle_development	ENSGALG00010016864	ENSGALG00010016864	1.09709454	0.262876771	4.173417589	3.00064E-05	5.85151E-05	positive
PRJNA729271	stage_1D_vs_E10	ordinary_muscle_development	ENSGALG00010016865	CFAP36	-1.77725567	0.139888303	-12.70481975	5.55974E-37	6.52538E-36	negative
PRJNA729271	stage_1D_vs_E10	ordinary_muscle_development	ENSGALG00010016866	ZNRF2	0.761763295	0.100141407	7.606876261	2.808E-14	1.07649E-13	positive
PRJNA729271	stage_1D_vs_E10	ordinary_muscle_development	ENSGALG00010016867	FLT4	0.621673879	0.118704463	5.237156727	1.63069E-07	3.87681E-07	positive
PRJNA729271	stage_1D_vs_E10	ordinary_muscle_development	ENSGALG00010016868	NIPAL1	-0.793817053	0.695666864	-1.141087918	0.253833338	0.298500734	negative
PRJNA729271	stage_1D_vs_E10	ordinary_muscle_development	ENSGALG00010016869	HSP90AA1	0.168714739	0.156347918	1.07909809	0.280544003	0.326625327	positive
PRJNA729271	stage_1D_vs_E10	ordinary_muscle_development	ENSGALG00010016872	EDA2R	2.276939805	0.28011833	8.128492708	4.34661E-16	1.87401E-15	positive
PRJNA729271	stage_1D_vs_E10	ordinary_muscle_development	ENSGALG00010016873	ENTPD6	-0.829970118	0.14173718	-5.855697965	4.75011E-09	1.27411E-08	negative
PRJNA729271	stage_1D_vs_E10	ordinary_muscle_development	ENSGALG00010016874	ADAMTSL3	0.162498121	0.145027415	1.120464851	0.262515723	0.307687022	positive
PRJNA729271	stage_1D_vs_E10	ordinary_muscle_development	ENSGALG00010016875	SESN2	3.317946856	0.217384481	15.26303464	1.34829E-52	2.85724E-51	positive
PRJNA729271	stage_1D_vs_E10	ordinary_muscle_development	ENSGALG00010016876	MTURN	-1.264359307	0.192953392	-6.552666915	5.65185E-11	1.7412E-10	negative
PRJNA729271	stage_1D_vs_E10	ordinary_muscle_development	ENSGALG00010016877	UBE2D3	0.569923209	0.135514439	4.205627178	2.60359E-05	5.10194E-05	positive
PRJNA729271	stage_1D_vs_E10	ordinary_muscle_development	ENSGALG00010016878	SUCLG1	1.5482704	0.125283763	12.35810896	4.40395E-35	4.79244E-34	positive
PRJNA729271	stage_1D_vs_E10	ordinary_muscle_development	ENSGALG00010016879	RNF130	-0.380325844	0.100859545	-3.770846338	0.000162695	0.000295633	negative
PRJNA729271	stage_1D_vs_E10	ordinary_muscle_development	ENSGALG00010016880	AR	2.799339635	0.200798374	13.94104734	3.56668E-44	5.42486E-43	positive
PRJNA729271	stage_1D_vs_E10	ordinary_muscle_development	ENSGALG00010016881	C1QB	1.09133536	0.292561352	3.730278635	0.000191268	0.000344316	positive
PRJNA729271	stage_1D_vs_E10	ordinary_muscle_development	ENSGALG00010016882	ENSGALG00010016882	-0.381250204	0.048715555	-7.826046646	5.03451E-15	2.02203E-14	negative
PRJNA729271	stage_1D_vs_E10	ordinary_muscle_development	ENSGALG00010016883	TMEM251	-0.242107358	0.077832679	-3.110613214	0.001866993	0.003018299	negative
PRJNA729271	stage_1D_vs_E10	ordinary_muscle_development	ENSGALG00010016884	SBK1	0.004113978	0.25298219	0.016261926	0.987025432	0.988867407	positive
PRJNA729271	stage_1D_vs_E10	ordinary_muscle_development	ENSGALG00010016885	CDCA4	-0.603288365	0.15346458	-3.931124466	8.45495E-05	0.000158053	negative
PRJNA729271	stage_1D_vs_E10	ordinary_muscle_development	ENSGALG00010016887	TJP2	0.412574119	0.138692581	2.974738209	0.002932387	0.004630169	positive
PRJNA729271	stage_1D_vs_E10	ordinary_muscle_development	ENSGALG00010016888	ENSGALG00010016888	0.036438108	0.081481649	0.447194037	0.654734966	0.694198181	positive
PRJNA729271	stage_1D_vs_E10	ordinary_muscle_development	ENSGALG00010016889	FUNDC2	0.888793764	0.087062027	10.20874196	1.81199E-24	1.20869E-23	positive
PRJNA729271	stage_1D_vs_E10	ordinary_muscle_development	ENSGALG00010016890	UBR7	-0.883912724	0.075656406	-11.6832503	1.55244E-31	1.44711E-30	negative
PRJNA729271	stage_1D_vs_E10	ordinary_muscle_development	ENSGALG00010016891	ENSGALG00010016891	2.656201549	1.164670159	2.280647038			positive
PRJNA729271	stage_1D_vs_E10	ordinary_muscle_development	ENSGALG00010016892	MAL	0.460445662	0.441400312	1.043147568	0.296879953	0.343766969	positive
PRJNA729271	stage_1D_vs_E10	ordinary_muscle_development	ENSGALG00010016893	TBC1D9B	-0.004860628	0.05070021	-0.095869981	0.923623836	0.935107041	negative
PRJNA729271	stage_1D_vs_E10	ordinary_muscle_development	ENSGALG00010016894	BTBD7	0.744899446	0.091139873	8.173145516	3.00451E-16	1.30547E-15	positive
PRJNA729271	stage_1D_vs_E10	ordinary_muscle_development	ENSGALG00010016895	ENSGALG00010016895	0.204948955	0.161968176	1.265365673	0.205740308	0.246565173	positive
PRJNA729271	stage_1D_vs_E10	ordinary_muscle_development	ENSGALG00010016896	SPTBN1	0.385359541	0.0987069	3.904079056	9.45849E-05	0.000175944	positive
PRJNA729271	stage_1D_vs_E10	ordinary_muscle_development	ENSGALG00010016898	ENSGALG00010016898	-0.46592377	0.660312711	-0.705610784	0.480430216	0.528925716	negative
PRJNA729271	stage_1D_vs_E10	ordinary_muscle_development	ENSGALG00010016899	SAXO2	-1.731024588	0.591288733	-2.92754536	0.003416492	0.005356631	negative
PRJNA729271	stage_1D_vs_E10	ordinary_muscle_development	ENSGALG00010016900	CISD2	-0.374664779	0.089803746	-4.172039543	3.01885E-05	5.88362E-05	negative
PRJNA729271	stage_1D_vs_E10	ordinary_muscle_development	ENSGALG00010016901	MECOM	-0.471343524	0.191931063	-2.455795936	0.014057296	0.0203435	negative
PRJNA729271	stage_1D_vs_E10	ordinary_muscle_development	ENSGALG00010016902	MRNIP	-1.236580421	0.143308926	-8.628774601	6.20128E-18	2.96194E-17	negative
PRJNA729271	stage_1D_vs_E10	ordinary_muscle_development	ENSGALG00010016903	PPM1A	1.603809324	0.147008026	10.90967183	1.0363E-27	7.99422E-27	positive
PRJNA729271	stage_1D_vs_E10	ordinary_muscle_development	ENSGALG00010016904	EAF1	0.200200333	0.070493221	2.839994138	0.004511436	0.006978259	positive
PRJNA729271	stage_1D_vs_E10	ordinary_muscle_development	ENSGALG00010016905	C1QC	1.919134901	0.373306071	5.140915328	2.73403E-07	6.37443E-07	positive
PRJNA729271	stage_1D_vs_E10	ordinary_muscle_development	ENSGALG00010016907	SQSTM1	2.741306286	0.159636947	17.1721292	4.29376E-66	1.39223E-64	positive
PRJNA729271	stage_1D_vs_E10	ordinary_muscle_development	ENSGALG00010016908	CMC4	0.813740425	0.160650693	5.065278042	4.07805E-07	9.3753E-07	positive
PRJNA729271	stage_1D_vs_E10	ordinary_muscle_development	ENSGALG00010016909	MRPS5	-0.104602001	0.133778647	-0.781903566	0.434271249	0.483803071	negative
PRJNA729271	stage_1D_vs_E10	ordinary_muscle_development	ENSGALG00010016910	NSD2	-0.774071868	0.110454486	-7.008061806	2.41642E-12	8.15697E-12	negative
PRJNA729271	stage_1D_vs_E10	ordinary_muscle_development	ENSGALG00010016911	AXIN1	-0.189045825	0.098641797	-1.916488055	0.055303002	0.073227096	negative
PRJNA729271	stage_1D_vs_E10	ordinary_muscle_development	ENSGALG00010016912	CCDC88C	0.938901659	0.159656236	5.880770363	4.08361E-09	1.099E-08	positive
PRJNA729271	stage_1D_vs_E10	ordinary_muscle_development	ENSGALG00010016913	BRCC3	-0.566915438	0.111769173	-5.072198547	3.93246E-07	9.04777E-07	negative
PRJNA729271	stage_1D_vs_E10	ordinary_muscle_development	ENSGALG00010016915	PLOD2	-1.265926105	0.167913035	-7.53917709	4.72946E-14	1.78665E-13	negative
PRJNA729271	stage_1D_vs_E10	ordinary_muscle_development	ENSGALG00010016916	ACYP2	2.597409134	0.157764292	16.46385944	6.67081E-61	1.86667E-59	positive
PRJNA729271	stage_1D_vs_E10	ordinary_muscle_development	ENSGALG00010016917	METTL6	-0.284538068	0.17197068	-1.654573136	0.098011116	0.124803755	negative
PRJNA729271	stage_1D_vs_E10	ordinary_muscle_development	ENSGALG00010016918	RCOR1	-0.513598276	0.074502377	-6.893716638	5.43532E-12	1.78732E-11	negative
PRJNA729271	stage_1D_vs_E10	ordinary_muscle_development	ENSGALG00010016919	ARHGDIG	0.343433186	0.197387989	1.739888975	0.081878515	0.10564617	positive
PRJNA729271	stage_1D_vs_E10	ordinary_muscle_development	ENSGALG00010016920	C1QA	1.886117286	0.319722794	5.899226843	3.65209E-09	9.89292E-09	positive
PRJNA729271	stage_1D_vs_E10	ordinary_muscle_development	ENSGALG00010016921	EFL1	-0.429936255	0.074985488	-5.733592834	9.83252E-09	2.56474E-08	negative
PRJNA729271	stage_1D_vs_E10	ordinary_muscle_development	ENSGALG00010016922	ENSGALG00010016922	1.079896197	0.189976034	5.6843812	1.31287E-08	3.39338E-08	positive
PRJNA729271	stage_1D_vs_E10	ordinary_muscle_development	ENSGALG00010016923	ENSGALG00010016923	-1.936575804	0.165469115	-11.70354845	1.22233E-31	1.14455E-30	negative
PRJNA729271	stage_1D_vs_E10	ordinary_muscle_development	ENSGALG00010016924	LETM1	0.265529793	0.11701198	2.269253054	0.023252942	0.03263788	positive
PRJNA729271	stage_1D_vs_E10	ordinary_muscle_development	ENSGALG00010016925	PHACTR4	0.127746777	0.133329106	0.958131203	0.337996603	0.386620956	positive
PRJNA729271	stage_1D_vs_E10	ordinary_muscle_development	ENSGALG00010016926	ENSGALG00010016926	-0.017359687	0.182793975	-0.094968596	0.924339771	0.93569154	negative
PRJNA729271	stage_1D_vs_E10	ordinary_muscle_development	ENSGALG00010016927	MGAT4B	0.819875721	0.115495906	7.09874272	1.25897E-12	4.31719E-12	positive
PRJNA729271	stage_1D_vs_E10	ordinary_muscle_development	ENSGALG00010016928	RGS11	3.737543367	0.374702557	9.974694046	1.96707E-23	1.24727E-22	positive
PRJNA729271	stage_1D_vs_E10	ordinary_muscle_development	ENSGALG00010016930	C10H15ORF48	3.582299159	0.392177896	9.134372938	6.57869E-20	3.49938E-19	positive
PRJNA729271	stage_1D_vs_E10	ordinary_muscle_development	ENSGALG00010016931	SLC30A4	0.207485014	0.082124704	2.526462868	0.011521757	0.01689508	positive
PRJNA729271	stage_1D_vs_E10	ordinary_muscle_development	ENSGALG00010016932	OGT	1.283778127	0.091922192	13.9659216	2.51629E-44	3.85557E-43	positive
PRJNA729271	stage_1D_vs_E10	ordinary_muscle_development	ENSGALG00010016934	SLC25A38	-0.233764301	0.13961346	-1.674367944	0.094058309	0.120131746	negative
PRJNA729271	stage_1D_vs_E10	ordinary_muscle_development	ENSGALG00010016935	PRIMA1	-0.000239315	0.248795673	-0.000961892	0.999232522	0.999301463	negative
PRJNA729271	stage_1D_vs_E10	ordinary_muscle_development	ENSGALG00010016936	RCC1	-0.40312649	0.162669617	-2.478191677	0.013205018	0.019198269	negative
PRJNA729271	stage_1D_vs_E10	ordinary_muscle_development	ENSGALG00010016937	DHRS7	0.584944896	0.170679806	3.427147657	0.000609957	0.001039056	positive
PRJNA729271	stage_1D_vs_E10	ordinary_muscle_development	ENSGALG00010016938	PSME4	0.808621596	0.166383391	4.859989878	1.17392E-06	2.60501E-06	positive
PRJNA729271	stage_1D_vs_E10	ordinary_muscle_development	ENSGALG00010016939	FAM234A	0.329177587	0.079249598	4.153681498	3.27168E-05	6.35442E-05	positive
PRJNA729271	stage_1D_vs_E10	ordinary_muscle_development	ENSGALG00010016940	FGFR3	-3.191252712	0.214165662	-14.90086075	3.25361E-50	6.33034E-49	negative
PRJNA729271	stage_1D_vs_E10	ordinary_muscle_development	ENSGALG00010016941	SLC8A1	0.690333938	0.172935849	3.991849821	6.55599E-05	0.000123994	positive
PRJNA729271	stage_1D_vs_E10	ordinary_muscle_development	ENSGALG00010016942	ZBTB40	1.092026473	0.14881166	7.338312549	2.16304E-13	7.82461E-13	positive
PRJNA729271	stage_1D_vs_E10	ordinary_muscle_development	ENSGALG00010016943	PCNX4	0.419962246	0.107581862	3.903652878	9.47517E-05	0.000176193	positive
PRJNA729271	stage_1D_vs_E10	ordinary_muscle_development	ENSGALG00010016944	OPRD1	-4.359518876	0.622182628	-7.006815488	2.43803E-12	8.2261E-12	negative
PRJNA729271	stage_1D_vs_E10	ordinary_muscle_development	ENSGALG00010016945	BDH2	-0.106909987	0.145841862	-0.733054182	0.463525383	0.512298927	negative
PRJNA729271	stage_1D_vs_E10	ordinary_muscle_development	ENSGALG00010016946	TAF1	0.027128555	0.106503395	0.254720098	0.798939292	0.82594858	positive
PRJNA729271	stage_1D_vs_E10	ordinary_muscle_development	ENSGALG00010016947	CANX	-0.015854724	0.07092928	-0.223528615	0.823124115	0.847866973	negative
PRJNA729271	stage_1D_vs_E10	ordinary_muscle_development	ENSGALG00010016948	CCR8	-3.710518763	0.950816104	-3.902456793	9.52212E-05	0.000177021	negative
PRJNA729271	stage_1D_vs_E10	ordinary_muscle_development	ENSGALG00010016949	LUC7L	-0.189778509	0.094946056	-1.998803487	0.04562962	0.061189874	negative
PRJNA729271	stage_1D_vs_E10	ordinary_muscle_development	ENSGALG00010016950	ENSGALG00010016950	-1.631748345	1.267757455	-1.287113981	0.198054563	0.238023455	negative
PRJNA729271	stage_1D_vs_E10	ordinary_muscle_development	ENSGALG00010016952	GINS1	-1.180055919	0.203516108	-5.79834161	6.69739E-09	1.77605E-08	negative
PRJNA729271	stage_1D_vs_E10	ordinary_muscle_development	ENSGALG00010016953	GJA4	1.78454585	0.159850334	11.16383436	6.12761E-29	5.04371E-28	positive
PRJNA729271	stage_1D_vs_E10	ordinary_muscle_development	ENSGALG00010016955	ENSGALG00010016955	-5.93553318	0.253120953	-23.44939488	1.3408E-121	1.5674E-119	negative
PRJNA729271	stage_1D_vs_E10	ordinary_muscle_development	ENSGALG00010016956	CENPE	-1.70656629	0.255935447	-6.667955965	2.59391E-11	8.16652E-11	negative
PRJNA729271	stage_1D_vs_E10	ordinary_muscle_development	ENSGALG00010016957	WNT4	0.876967133	0.200742193	4.368623843	1.25032E-05	2.5312E-05	positive
PRJNA729271	stage_1D_vs_E10	ordinary_muscle_development	ENSGALG00010016958	TLR15	-1.82102249	0.699929946	-2.601721073	0.009275726	0.013775784	negative
PRJNA729271	stage_1D_vs_E10	ordinary_muscle_development	ENSGALG00010016959	PERCC1	3.64582571	1.081811708	3.370111158	0.000751379	0.001270411	positive
PRJNA729271	stage_1D_vs_E10	ordinary_muscle_development	ENSGALG00010016960	GJB3	-5.730683683	1.067158386	-5.370040435	7.8719E-08	1.91673E-07	negative
PRJNA729271	stage_1D_vs_E10	ordinary_muscle_development	ENSGALG00010016961	EIF4A3	-0.666423994	0.074644042	-8.928026663	4.33675E-19	2.20179E-18	negative
PRJNA729271	stage_1D_vs_E10	ordinary_muscle_development	ENSGALG00010016962	SQOR	1.56280697	0.136995459	11.40772826	3.82591E-30	3.33875E-29	positive
PRJNA729271	stage_1D_vs_E10	ordinary_muscle_development	ENSGALG00010016963	CD8A	-0.117160521	0.90125249	-0.129997445	0.896568448	0.911855154	negative
PRJNA729271	stage_1D_vs_E10	ordinary_muscle_development	ENSGALG00010016964	MAML1	0.55112875	0.084114487	6.552126381	5.67235E-11	1.74715E-10	positive

PRJNA729271	stage_1D_vs_E10	ordinary_muscle_development	ENSGALG00010016965	SLC25A33	0.005638082	0.338582704	0.016652007	0.986714235	0.98862396	positive
PRJNA729271	stage_1D_vs_E10	ordinary_muscle_development	ENSGALG00010016966	KDM3A	-0.095896552	0.15838075	-0.605481106	0.544859375	0.590926797	negative
PRJNA729271	stage_1D_vs_E10	ordinary_muscle_development	ENSGALG00010016967	ERLEC1	-0.11858412	0.075088363	-1.579260956	0.114276214	0.143912573	negative
PRJNA729271	stage_1D_vs_E10	ordinary_muscle_development	ENSGALG00010016968	C15orf61	0.291365906	0.147995041	1.968754529	0.048981286	0.065442321	positive
PRJNA729271	stage_1D_vs_E10	ordinary_muscle_development	ENSGALG00010016969	CHMP3	0.60737638	0.08373735	7.253350855	4.06585E-13	1.44802E-12	positive
PRJNA729271	stage_1D_vs_E10	ordinary_muscle_development	ENSGALG00010016970	SYNE3	1.504429356	0.098385207	15.29121498	8.75023E-53	1.86248E-51	positive
PRJNA729271	stage_1D_vs_E10	ordinary_muscle_development	ENSGALG00010016972	ASB3	-1.215637651	0.108989394	-11.15372434	6.86706E-29	5.63636E-28	negative
PRJNA729271	stage_1D_vs_E10	ordinary_muscle_development	ENSGALG00010016973	PLSCR1	1.953254619	0.121815606	16.03451879	7.33496E-58	1.86854E-56	positive
PRJNA729271	stage_1D_vs_E10	ordinary_muscle_development	ENSGALG00010016974	CFAP96	-2.991434661	0.801364847	-3.732924737	0.000189269	0.000341056	negative
PRJNA729271	stage_1D_vs_E10	ordinary_muscle_development	ENSGALG00010016975	MALL	0.881104876	0.220988582	3.987105888	6.68842E-05	0.000126317	positive
PRJNA729271	stage_1D_vs_E10	ordinary_muscle_development	ENSGALG00010016976	ENSGALG00010016976	4.352198304	0.937684606	4.641430897	3.46005E-06	7.37658E-06	positive
PRJNA729271	stage_1D_vs_E10	ordinary_muscle_development	ENSGALG00010016977	CLCN7	0.386712086	0.118406443	3.265971662	0.001090892	0.001811073	positive
PRJNA729271	stage_1D_vs_E10	ordinary_muscle_development	ENSGALG00010016978	FAM181A	3.400333136	0.352715439	9.640443149	5.39545E-22	3.23036E-21	positive
PRJNA729271	stage_1D_vs_E10	ordinary_muscle_development	ENSGALG00010016979	PLSCR5	1.392437507	0.440325362	3.16229231	0.001565324	0.002552809	positive
PRJNA729271	stage_1D_vs_E10	ordinary_muscle_development	ENSGALG00010016980	NPHP1	-0.509310706	0.101576003	-5.014084915	5.32865E-07	1.21559E-06	negative
PRJNA729271	stage_1D_vs_E10	ordinary_muscle_development	ENSGALG00010016981	ENSGALG00010016981	-0.456791416	0.520184604	-0.878133285	0.379871392	0.428867966	negative
PRJNA729271	stage_1D_vs_E10	ordinary_muscle_development	ENSGALG00010016982	GLRX5	0.089747675	0.140782235	0.6374929	0.523803833	0.570996207	positive
PRJNA729271	stage_1D_vs_E10	ordinary_muscle_development	ENSGALG00010016983	ZFAND2A	-0.905633821	0.288722327	-3.136694796	0.001708639	0.002774983	negative
PRJNA729271	stage_1D_vs_E10	ordinary_muscle_development	ENSGALG00010016984	RNF103	1.087023987	0.114619885	9.483729509	2.45354E-21	1.41239E-20	positive
PRJNA729271	stage_1D_vs_E10	ordinary_muscle_development	ENSGALG00010016985	ENSGALG00010016985	1.567130922	0.184621886	8.488326917	2.09633E-17	9.71741E-17	positive
PRJNA729271	stage_1D_vs_E10	ordinary_muscle_development	ENSGALG00010016986	GFER	0.243200267	0.085715637	2.837291705	0.004549802	0.007032351	positive
PRJNA729271	stage_1D_vs_E10	ordinary_muscle_development	ENSGALG00010016987	ASB2	4.434280527	0.317930881	13.9473099	3.26697E-44	4.97424E-43	positive
PRJNA729271	stage_1D_vs_E10	ordinary_muscle_development	ENSGALG00010016988	ECI1	1.113433408	0.109413668	10.17636489	2.5283E-24	1.67418E-23	positive
PRJNA729271	stage_1D_vs_E10	ordinary_muscle_development	ENSGALG00010016989	RHOG2	0.06308818	0.092768102	0.680063282	0.496464392	0.54459296	positive
PRJNA729271	stage_1D_vs_E10	ordinary_muscle_development	ENSGALG00010016991	PDLIM3	4.103991334	0.199159995	20.6065045	2.39946E-94	1.58092E-92	positive
PRJNA729271	stage_1D_vs_E10	ordinary_muscle_development	ENSGALG00010016993	ENSGALG00010016993	1.211640519	0.179789899	6.739202389	1.59258E-11	5.08918E-11	positive
PRJNA729271	stage_1D_vs_E10	ordinary_muscle_development	ENSGALG00010016994	ATL2	0.790315284	0.11554105	6.84012554	7.91238E-12	2.57441E-11	positive
PRJNA729271	stage_1D_vs_E10	ordinary_muscle_development	ENSGALG00010016998	DNASE1L2	0.612372238	0.346270304	1.768480376	0.07698063	0.099779508	positive
PRJNA729271	stage_1D_vs_E10	ordinary_muscle_development	ENSGALG00010016999	ITGB1BP2	2.441460861	0.172701177	14.13690918	2.24949E-45	3.57919E-44	positive
PRJNA729271	stage_1D_vs_E10	ordinary_muscle_development	ENSGALG00010017001	TPCN3	-1.119238326	0.119253064	-9.385405186	6.2675E-21	3.52943E-20	negative
PRJNA729271	stage_1D_vs_E10	ordinary_muscle_development	ENSGALG00010017003	CPZ	1.56933938	0.39711114	3.951889589	7.75365E-05	0.000145431	positive
PRJNA729271	stage_1D_vs_E10	ordinary_muscle_development	ENSGALG00010017004	CX3CR1	-5.926213631	0.760521492	-7.792302645	6.57989E-15	2.61805E-14	negative
PRJNA729271	stage_1D_vs_E10	ordinary_muscle_development	ENSGALG00010017006	DNAH3	0.586374602	0.244365136	2.399583723	0.016413726	0.023516552	positive
PRJNA729271	stage_1D_vs_E10	ordinary_muscle_development	ENSGALG00010017008	RTN1	-1.023512804	0.169078489	-6.05347734	1.41752E-09	3.9559E-09	negative
PRJNA729271	stage_1D_vs_E10	ordinary_muscle_development	ENSGALG00010017010	NONO	-1.222237837	0.055049317	-22.20259768	3.2417E-109	2.8136E-107	negative
PRJNA729271	stage_1D_vs_E10	ordinary_muscle_development	ENSGALG00010017012	SYNGR3	0.179389888	0.18422032	0.973779048	0.330166258	0.378590295	positive
PRJNA729271	stage_1D_vs_E10	ordinary_muscle_development	ENSGALG00010017016	ZIC1	-6.614756252	0.928185678	-7.126544191	1.0292E-12	3.55705E-12	negative
PRJNA729271	stage_1D_vs_E10	ordinary_muscle_development	ENSGALG00010017017	ENSGALG00010017017	0.66817979	0.267619702	2.496751117	0.012533689	0.018286444	positive
PRJNA729271	stage_1D_vs_E10	ordinary_muscle_development	ENSGALG00010017018	HP1BP3	-0.850441874	0.08359874	-10.17290303	2.61982E-24	1.73004E-23	negative
PRJNA729271	stage_1D_vs_E10	ordinary_muscle_development	ENSGALG00010017020	GJB5	-3.048988214	0.451066946	-6.759502636	1.38466E-11	4.44829E-11	negative
PRJNA729271	stage_1D_vs_E10	ordinary_muscle_development	ENSGALG00010017022	CCR4	-1.337131514	0.727712628	-1.83744443	0.066144313	0.08653866	negative
PRJNA729271	stage_1D_vs_E10	ordinary_muscle_development	ENSGALG00010017024	BUB1	-2.397481187	0.217233698	-11.03641474	2.55006E-28	2.02538E-27	negative
PRJNA729271	stage_1D_vs_E10	ordinary_muscle_development	ENSGALG00010017033	LDAF1	0.286104908	0.111138915	2.574299986	0.010044314	0.014838192	positive
PRJNA729271	stage_1D_vs_E10	ordinary_muscle_development	ENSGALG00010017034	ZMYM3	0.055644912	0.089936554	0.618712968	0.536105466	0.582566064	positive
PRJNA729271	stage_1D_vs_E10	ordinary_muscle_development	ENSGALG00010017035	PIK3CD	-0.01593085	0.206286673	-0.077226752	0.93844316	0.947652287	negative
PRJNA729271	stage_1D_vs_E10	ordinary_muscle_development	ENSGALG00010017038	ZNF598	-0.303072754	0.131508185	-2.304592315	0.021189417	0.029892029	negative
PRJNA729271	stage_1D_vs_E10	ordinary_muscle_development	ENSGALG00010017039	MAP2K5	-0.485652832	0.077497576	-6.266684117	3.68817E-10	1.07479E-09	negative
PRJNA729271	stage_1D_vs_E10	ordinary_muscle_development	ENSGALG00010017041	JKAMP	-0.053639329	0.090035154	-0.595759836	0.551335679	0.597301311	negative
PRJNA729271	stage_1D_vs_E10	ordinary_muscle_development	ENSGALG00010017042	HNRNPLL	-1.089028319	0.070236882	-15.50507778	3.20528E-54	7.1368E-53	negative
PRJNA729271	stage_1D_vs_E10	ordinary_muscle_development	ENSGALG00010017045	GLB1	-0.081936507	0.098983158	-0.827782313	0.407793793	0.45747009	negative
PRJNA729271	stage_1D_vs_E10	ordinary_muscle_development	ENSGALG00010017049	TRAF3	0.21643188	0.087858297	2.463419942	0.013761862	0.019949067	positive
PRJNA729271	stage_1D_vs_E10	ordinary_muscle_development	ENSGALG00010017050	L3HYPDH	-1.041481809	0.15578558	-6.685354365	2.30367E-11	7.2773E-11	negative
PRJNA729271	stage_1D_vs_E10	ordinary_muscle_development	ENSGALG00010017054	GALM	-1.127059989	0.152973606	-7.367676159	1.73628E-13	6.32028E-13	negative
PRJNA729271	stage_1D_vs_E10	ordinary_muscle_development	ENSGALG00010017055	GPR135	-2.045162642	0.354709488	-5.765739881	8.13004E-09	2.14108E-08	negative
PRJNA729271	stage_1D_vs_E10	ordinary_muscle_development	ENSGALG00010017056	AGTR1	4.888478787	0.202195372	24.17700632	3.8836E-129	4.895E-127	positive
PRJNA729271	stage_1D_vs_E10	ordinary_muscle_development	ENSGALG00010017057	NDUFAB1	1.19135196	0.121013951	9.844748875	7.22194E-23	4.48509E-22	positive
PRJNA729271	stage_1D_vs_E10	ordinary_muscle_development	ENSGALG00010017060	CLSTN1	-1.200193546	0.102265332	-11.73607436	8.32623E-32	7.87271E-31	negative
PRJNA729271	stage_1D_vs_E10	ordinary_muscle_development	ENSGALG00010017063	TMPPE	0.633355692	0.104597226	6.055186299	1.40255E-09	3.91866E-09	positive
PRJNA729271	stage_1D_vs_E10	ordinary_muscle_development	ENSGALG00010017065	VRK2	-0.616269484	0.134953978	-4.566515876	4.95897E-06	1.04144E-05	negative
PRJNA729271	stage_1D_vs_E10	ordinary_muscle_development	ENSGALG00010017068	ZP2	-5.554404044	1.197543657	-4.638164137	3.51518E-06	7.48861E-06	negative
PRJNA729271	stage_1D_vs_E10	ordinary_muscle_development	ENSGALG00010017070	BLOC1S6	-0.031147936	0.105366018	-0.295616522	0.767522954	0.797394296	negative
PRJNA729271	stage_1D_vs_E10	ordinary_muscle_development	ENSGALG00010017077	CTNNBIP1	-1.222666815	0.106082136	-11.5256617	9.79567E-31	8.7864E-30	negative
PRJNA729271	stage_1D_vs_E10	ordinary_muscle_development	ENSGALG00010017080	PALB2	-0.75356257	0.098947577	-7.615775883	2.62112E-14	1.00697E-13	negative
PRJNA729271	stage_1D_vs_E10	ordinary_muscle_development	ENSGALG00010017083	ENSGALG00010017083	0.781746429	0.39283033	1.990035824	0.046586989	0.062387141	positive
PRJNA729271	stage_1D_vs_E10	ordinary_muscle_development	ENSGALG00010017085	ENSGALG00010017085	-0.672528381	0.119630168	-5.62172895	1.89056E-08	4.82713E-08	negative
PRJNA729271	stage_1D_vs_E10	ordinary_muscle_development	ENSGALG00010017089	CRTAP	0.24085971	0.156848125	1.535623781	0.124630678	0.155936269	positive
PRJNA729271	stage_1D_vs_E10	ordinary_muscle_development	ENSGALG00010017094	SORBS2	0.734108554	0.147189405	4.987509498	6.11626E-07	1.38883E-06	positive
PRJNA729271	stage_1D_vs_E10	ordinary_muscle_development	ENSGALG00010017096	DCTN5	-0.635758236	0.091132636	-6.976186172	3.03301E-12	1.01697E-11	negative
PRJNA729271	stage_1D_vs_E10	ordinary_muscle_development	ENSGALG00010017098	DAAM1	-0.665471102	0.123197508	-5.401660409	6.60269E-08	1.61857E-07	negative
PRJNA729271	stage_1D_vs_E10	ordinary_muscle_development	ENSGALG00010017100	SUSD5	-7.034750357	0.593787842	-11.84724552	2.22381E-32	2.15325E-31	negative
PRJNA729271	stage_1D_vs_E10	ordinary_muscle_development	ENSGALG00010017101	CSMD2	-0.04280561	0.232379183	-0.184205873	0.853851952	0.875103164	negative
PRJNA729271	stage_1D_vs_E10	ordinary_muscle_development	ENSGALG00010017104	FANCL	-2.526265936	0.179421207	-14.0800855	5.03488E-45	7.91547E-44	negative
PRJNA729271	stage_1D_vs_E10	ordinary_muscle_development	ENSGALG00010017105	PLK1	-2.319598292	0.234013554	-9.912239054	3.68298E-23	2.31103E-22	negative
PRJNA729271	stage_1D_vs_E10	ordinary_muscle_development	ENSGALG00010017106	CRYM	-0.22718829	0.113894023	-1.994734088	0.046071889	0.061742977	negative
PRJNA729271	stage_1D_vs_E10	ordinary_muscle_development	ENSGALG00010017109	ITGB1BP3	0.437366917	0.127505398	3.430183547	0.000603173	0.001028225	positive
PRJNA729271	stage_1D_vs_E10	ordinary_muscle_development	ENSGALG00010017110	BRF1	-0.300798751	0.071888439	-4.184243734	2.86117E-05	5.58931E-05	negative
PRJNA729271	stage_1D_vs_E10	ordinary_muscle_development	ENSGALG00010017111	GJB1	0.491750006	0.444138699	1.107199187	0.268207819	0.313496681	positive
PRJNA729271	stage_1D_vs_E10	ordinary_muscle_development	ENSGALG00010017112	LZIC	-0.63861082	0.063294477	-10.08951894	6.147E-24	3.98483E-23	negative
PRJNA729271	stage_1D_vs_E10	ordinary_muscle_development	ENSGALG00010017115	LGALSL	2.059557564	0.191901301	10.7323794	7.17251E-27	5.35078E-26	positive
PRJNA729271	stage_1D_vs_E10	ordinary_muscle_development	ENSGALG00010017116	ENSGALG00010017116	-1.755990983	0.277926003	-6.31819609	2.64634E-10	7.7949E-10	negative
PRJNA729271	stage_1D_vs_E10	ordinary_muscle_development	ENSGALG00010017119	BTBD6	-0.769626272	0.15016145	-5.125325238	2.97025E-07	6.90406E-07	negative
PRJNA729271	stage_1D_vs_E10	ordinary_muscle_development	ENSGALG00010017120	ERN2	1.101554908	0.141497112	7.784999259	6.97136E-15	2.77077E-14	positive
PRJNA729271	stage_1D_vs_E10	ordinary_muscle_development	ENSGALG00010017122	NCBP2	-1.127685422	0.192876414	-5.846673516	5.015E-09	1.34243E-08	negative
PRJNA729271	stage_1D_vs_E10	ordinary_muscle_development	ENSGALG00010017127	SOX8	0.125321009	0.13266291	0.944657474	0.344833774	0.393716726	positive
PRJNA729271	stage_1D_vs_E10	ordinary_muscle_development	ENSGALG00010017131	TLR3	1.308913782	0.385202893	3.397985338	0.000678841	0.001151931	positive
PRJNA729271	stage_1D_vs_E10	ordinary_muscle_development	ENSGALG00010017132	TMEM108	-1.61342195	0.277206317	-5.820292866	5.87446E-09	1.56613E-08	negative
PRJNA729271	stage_1D_vs_E10	ordinary_muscle_development	ENSGALG00010017133	WDR20	0.628050525	0.111996562	5.607766108	2.04955E-08	5.21929E-08	positive
PRJNA729271	stage_1D_vs_E10	ordinary_muscle_development	ENSGALG00010017135	ENSGALG00010017135	0.708362043	0.132174403	5.359298225	8.35458E-08	2.03017E-07	positive
PRJNA729271	stage_1D_vs_E10	ordinary_muscle_development	ENSGALG00010017136	ENSGALG00010017136	0.084788149	0.39809332	0.212985611	0.831338186	0.855355409	positive
PRJNA729271	stage_1D_vs_E10	ordinary_muscle_development	ENSGALG00010017139	COL9A2	-7.194013653	0.567609664	-12.67422686	8.21648E-37	9.58149E-36	negative
PRJNA729271	stage_1D_vs_E10	ordinary_muscle_development	ENSGALG00010017140	UQCRC2	1.456545664	0.114912696	12.67523704	8.11131E-37	9.47409E-36	positive
PRJNA729271	stage_1D_vs_E10	ordinary_muscle_development	ENSGALG00010017141	AFTPH	0.473862238	0.0754845	6.277609798	3.43817E-10	1.00396E-09	positive
PRJNA729271	stage_1D_vs_E10	ordinary_muscle_development	ENSGALG00010017142	RBP7	2.785734268	0.694381087	4.011823364	6.02516E-05	0.000114342	positive
PRJNA729271	stage_1D_vs_E10	ordinary_muscle_development	ENSGALG00010017143	FAM149A	-0.420691709	0.274320627	-1.533576655	0.125133835	0.156471268	negative
PRJNA729271	stage_1D_vs_E10	ordinary_muscle_development	ENSGALG00010017144	CPB1	2.161416092	0.726938145	2.973315003	0.002946019	0.004647644	positive
PRJNA729271	stage_1D_vs_E10	ordinary_muscle_development	ENSGALG00010017147	SRSF7L	-0.818494003	0.07061771	-11.59049197	4.6048E-31	4.17688E-30	negative
PRJNA729271	stage_1D_vs_E10	ordinary_muscle_development	ENSGALG00010017148	MOK	-0.521437592	0.467639327	-1.115042217	0.264832331	0.30997615	negative
PRJNA729271	stage_1D_vs_E10	ordinary_muscle_development	ENSGALG00010017152	CYP4V2	3.253129091	0.196186803	16.58179372	9.43695E-62	2.75229E-60	positive
PRJNA729271	stage_1D_vs_E10	ordinary_muscle_development	ENSGALG00010017153	SLC35F4	1.936325938	0.548128507	3.532613089	0.000411474	0.000715318	positive
PRJNA729271	stage_1D_vs_E10	ordinary_muscle_development	ENSGALG00010017154	PIGZ	0.216859007	0.189019771	1.147282134	0.251265035	0.295840036	positive
PRJNA729271	stage_1D_vs_E10	ordinary_muscle_development	ENSGALG00010017162	BCL11A	-2.182454057	0.204015864	-10.69747232	1.04592E-26	7.73109E-26	negative
PRJNA729271	stage_1D_vs_E10	ordinary_muscle_development	ENSGALG00010017163	CDV3	-0.734435104	0.124799244	-5.884932317	3.98217E-09	1.07409E-08	negative
PRJNA729271	stage_1D_vs_E10	ordinary_muscle_development	ENSGALG00010017167	SMAP2	-0.285569598	0.096653777	-2.954562228	0.00313113	0.004924667	negative
PRJNA729271	stage_1D_vs_E10	ordinary_muscle_development	ENSGALG00010017169	CCDC198	2.619090098	0.213599374	12.26169367	1.45428E-34	1.55456E-33	positive
PRJNA729271	stage_1D_vs_E10	ordinary_muscle_development	ENSGALG00010017174	DHX57	-0.950647603	0.098321345	-9.668781554	4.09221E-22	2.46025E-21	negative
PRJNA729271	stage_1D_vs_E10	ordinary_muscle_development	ENSGALG00010017180	ENSGALG00010017180	-3.522705519	0.639422867	-5.509195403	3.60478E-08	8.99333E-08	negative
PRJNA729271	stage_1D_vs_E10	ordinary_muscle_development	ENSGALG00010017183	NFYC	0.210749861	0.093382502	2.256845312	0.024017742	0.033626695	positive
PRJNA729271	stage_1D_vs_E10	ordinary_muscle_development	ENSGALG00010017184	CARD11	0.416858625	0.21480226	1.940662193	0.052299265	0.069542046	positive
PRJNA729271	stage_1D_vs_E10	ordinary_muscle_development	ENSGALG00010017185	NAA30	0.033293321	0.064246456	0.518212565	0.604309969	0.647124188	positive
PRJNA729271	stage_1D_vs_E10	ordinary_muscle_development	ENSGALG00010017187	ZNF839	1.129938344	0.137425012	8.222217538	1.99774E-16	8.75372E-16	positive
PRJNA729271	stage_1D_vs_E10	ordinary_muscle_development	ENSGALG00010017188	ENSGALG00010017188	0.608242691	0.158641729	3.834064932	0.000126043	0.000231499	positive
PRJNA729271	stage_1D_vs_E10	ordinary_muscle_development	ENSGALG00010017190	STK26	-0.44352083	0.067885507	-6.533365494	6.43079E-11	1.97112E-10	negative
PRJNA729271	stage_1D_vs_E10	ordinary_muscle_development	ENSGALG00010017198	TOPBP1	-0.588928299	0.067213375	-8.762070058	1.91697E-18	9.41277E-18	negative
PRJNA729271	stage_1D_vs_E10	ordinary_muscle_development	ENSGALG00010017200	KCNQ4	0.59385596	0.309657407	1.917783807	0.05513843	0.073020107	positive
PRJNA729271	stage_1D_vs_E10	ordinary_muscle_development	ENSGALG00010017201	AP5M1	-2.144597949	0.158340385	-13.54422596	8.57126E-42	1.19808E-40	negative
PRJNA729271	stage_1D_vs_E10	ordinary_muscle_development	ENSGALG00010017207	ENSGALG00010017207	7.030095506	0.280813069	25.03478748	2.5569E-138	3.8209E-136	positive
PRJNA729271	stage_1D_vs_E10	ordinary_muscle_development	ENSGALG00010017208	CINP	-1.451989105	0.1764019	-8.231142095	1.85437E-16	8.14517E-16	negative
PRJNA729271	stage_1D_vs_E10	ordinary_muscle_development	ENSGALG00010017211	UBFD1	0.344005907	0.129021502	2.666268031	0.007669852	0.011512323	positive
PRJNA729271	stage_1D_vs_E10	ordinary_muscle_development	ENSGALG00010017212	TINAGL1	3.330144685	0.134642052	24.73331797	4.6875E-135	6.5333E-133	positive
PRJNA729271	stage_1D_vs_E10	ordinary_muscle_development	ENSGALG00010017215	FRMD7	1.424620696	0.634725601	2.244467049	0.024802367	0.034641579	positive
PRJNA729271	stage_1D_vs_E10	ordinary_muscle_development	ENSGALG00010017219	EARS2	-0.947479388	0.139103927	-6.811305814	9.67168E-12	3.13416E-11	negative
PRJNA729271	stage_1D_vs_E10	ordinary_muscle_development	ENSGALG00010017221	SPPL2A	0.328179466	0.075571656	4.342626361	1.40789E-05	2.83201E-05	positive
PRJNA729271	stage_1D_vs_E10	ordinary_muscle_development	ENSGALG00010017223	NOC2L	-1.211493842	0.076533635	-15.82956107	1.94574E-56	4.70843E-55	negative
PRJNA729271	stage_1D_vs_E10	ordinary_muscle_development	ENSGALG00010017226	PEF1	-0.354236836	0.075428399	-4.696332399	2.64874E-06	5.71162E-06	negative
PRJNA729271	stage_1D_vs_E10	ordinary_muscle_development	ENSGALG00010017228	RAP2C	-1.001689444	0.072307463	-13.85319583	1.21676E-43	1.81077E-42	negative
PRJNA729271	stage_1D_vs_E10	ordinary_muscle_development	ENSGALG00010017240	MELTF	0.011973558	0.237250353	0.050468031	0.959749424	0.965922379	positive
PRJNA729271	stage_1D_vs_E10	ordinary_muscle_development	ENSGALG00010017246	GGA2	-0.036079068	0.133522274	-0.270210108	0.786998617	0.815057513	negative
PRJNA729271	stage_1D_vs_E10	ordinary_muscle_development	ENSGALG00010017248	TECPR2	0.322936373	0.084101564	3.839837883	0.000123116	0.000226438	positive
PRJNA729271	stage_1D_vs_E10	ordinary_muscle_development	ENSGALG00010017250	COL16A1	0.859903565	0.16951454	5.072742213	3.92124E-07	9.02482E-07	positive
PRJNA729271	stage_1D_vs_E10	ordinary_muscle_development	ENSGALG00010017252	EXOC5	-0.72639428	0.088211858	-8.234655732	1.80075E-16	7.91924E-16	negative
PRJNA729271	stage_1D_vs_E10	ordinary_muscle_development	ENSGALG00010017256	CARHSP1	0.104466572	0.128696049	0.811730992	0.416946007	0.466401634	positive

PRJNA729271	stage_1D_vs_E10	ordinary_muscle_development	ENSGALG00010017263	COG7	-0.352541744	0.071434455	-4.935177928	8.00777E-07	1.80181E-06	negative
PRJNA729271	stage_1D_vs_E10	ordinary_muscle_development	ENSGALG00010017264	PIAS1	0.703578753	0.085568738	8.222380877	1.99502E-16	8.74444E-16	positive
PRJNA729271	stage_1D_vs_E10	ordinary_muscle_development	ENSGALG00010017266	PMM2	-1.174718702	0.109119851	-10.76539873	5.0144E-27	3.7621E-26	negative
PRJNA729271	stage_1D_vs_E10	ordinary_muscle_development	ENSGALG00010017272	NRXN1	1.847464879	0.134060254	13.78085471	3.32314E-43	4.88529E-42	positive
PRJNA729271	stage_1D_vs_E10	ordinary_muscle_development	ENSGALG00010017278	CCR5	-0.366114644	0.236506659	-1.54800988	0.121619918	0.152537917	negative
PRJNA729271	stage_1D_vs_E10	ordinary_muscle_development	ENSGALG00010017280	SOS1	0.886766847	0.154930402	5.723646451	1.04262E-08	2.71421E-08	positive
PRJNA729271	stage_1D_vs_E10	ordinary_muscle_development	ENSGALG00010017283	INF2	1.082315695	0.13628889	7.941334741	2.00017E-15	8.24587E-15	positive
PRJNA729271	stage_1D_vs_E10	ordinary_muscle_development	ENSGALG00010017284	ENSGALG00010017284	0.24468587	0.198226002	1.234378274	0.217061981	0.259211848	positive
PRJNA729271	stage_1D_vs_E10	ordinary_muscle_development	ENSGALG00010017285	IL21R	-0.281388984	1.130581596	-0.2488887	0.803446876	0.830194074	negative
PRJNA729271	stage_1D_vs_E10	ordinary_muscle_development	ENSGALG00010017289	ADGRB2	-1.391624995	0.301743194	-4.611951565	3.98906E-06	8.44945E-06	negative
PRJNA729271	stage_1D_vs_E10	ordinary_muscle_development	ENSGALG00010017290	DACT1	-0.661990459	0.156392006	-4.232891922	2.30705E-05	4.54667E-05	negative
PRJNA729271	stage_1D_vs_E10	ordinary_muscle_development	ENSGALG00010017296	SCNN1G	2.209162481	0.223273689	9.894414751	4.40177E-23	2.74898E-22	positive
PRJNA729271	stage_1D_vs_E10	ordinary_muscle_development	ENSGALG00010017299	USP31	1.601811193	0.10930694	14.65424972	1.26547E-48	2.31603E-47	positive
PRJNA729271	stage_1D_vs_E10	ordinary_muscle_development	ENSGALG00010017300	TRPM7	-0.376914294	0.104152281	-3.618877009	0.000295884	0.00052163	negative
PRJNA729271	stage_1D_vs_E10	ordinary_muscle_development	ENSGALG00010017301	LHCGR	0.380143069	0.431818834	0.880329988	0.378680572	0.427690111	positive
PRJNA729271	stage_1D_vs_E10	ordinary_muscle_development	ENSGALG00010017304	ANKRD9	1.878129259	0.300740818	6.245009476	4.23774E-10	1.22844E-09	positive
PRJNA729271	stage_1D_vs_E10	ordinary_muscle_development	ENSGALG00010017305	HS3ST2	4.069302002	0.461755741	8.812672245	1.22197E-18	6.06593E-18	positive
PRJNA729271	stage_1D_vs_E10	ordinary_muscle_development	ENSGALG00010017307	TMEM186	0.022491699	0.157488362	0.142814992	0.886436296	0.903270125	positive
PRJNA729271	stage_1D_vs_E10	ordinary_muscle_development	ENSGALG00010017310	SAMD11	-1.440931521	0.178229413	-8.084701023	6.23169E-16	2.65593E-15	negative
PRJNA729271	stage_1D_vs_E10	ordinary_muscle_development	ENSGALG00010017312	PTP4A2	0.689176657	0.154778539	4.452662878	8.48119E-06	1.74549E-05	positive
PRJNA729271	stage_1D_vs_E10	ordinary_muscle_development	ENSGALG00010017313	MAP4K3	0.452328143	0.082421046	5.488017514	4.0647E-08	1.00921E-07	positive
PRJNA729271	stage_1D_vs_E10	ordinary_muscle_development	ENSGALG00010017314	CALML4	-1.362653255	0.225437149	-6.044492945	1.49881E-09	4.17873E-09	negative
PRJNA729271	stage_1D_vs_E10	ordinary_muscle_development	ENSGALG00010017315	HS3ST4	-1.257661578	0.838891464	-1.499194631	0.133823147	0.166289458	negative
PRJNA729271	stage_1D_vs_E10	ordinary_muscle_development	ENSGALG00010017318	ADSS1	3.485414252	0.123710403	28.17397859	1.2187E-174	3.3332E-172	positive
PRJNA729271	stage_1D_vs_E10	ordinary_muscle_development	ENSGALG00010017322	KHDRBS1	-1.057402722	0.069966777	-15.11292595	1.33096E-51	2.70959E-50	negative
PRJNA729271	stage_1D_vs_E10	ordinary_muscle_development	ENSGALG00010017323	ENSGALG00010017323	1.442039189	0.104070361	13.85638693	1.16387E-43	1.73563E-42	positive
PRJNA729271	stage_1D_vs_E10	ordinary_muscle_development	ENSGALG00010017326	STON1	0.506351317	0.119704009	4.230028056	2.33662E-05	4.60056E-05	positive
PRJNA729271	stage_1D_vs_E10	ordinary_muscle_development	ENSGALG00010017327	CLN6	-0.417686631	0.134509796	-3.105250653	0.001901178	0.003069457	negative
PRJNA729271	stage_1D_vs_E10	ordinary_muscle_development	ENSGALG00010017332	FGFR1	0.324924437	0.158605417	2.04863392	0.040497925	0.054764197	positive
PRJNA729271	stage_1D_vs_E10	ordinary_muscle_development	ENSGALG00010017334	KDM8	-0.53905279	0.154552674	-3.48782571	0.000486965	0.000839705	negative
PRJNA729271	stage_1D_vs_E10	ordinary_muscle_development	ENSGALG00010017335	THUMPD2	-0.256725102	0.13755342	-1.866366555	0.061990099	0.081426958	negative
PRJNA729271	stage_1D_vs_E10	ordinary_muscle_development	ENSGALG00010017337	ABAT	-1.26253695	0.205001584	-6.158669243	7.33588E-10	2.09236E-09	negative
PRJNA729271	stage_1D_vs_E10	ordinary_muscle_development	ENSGALG00010017338	MBNL3	0.134942519	0.074638438	1.807949396	0.070614377	0.092029796	positive
PRJNA729271	stage_1D_vs_E10	ordinary_muscle_development	ENSGALG00010017340	SIVA1	-0.454953726	0.103621145	-4.39054912	1.13065E-05	2.29695E-05	negative
PRJNA729271	stage_1D_vs_E10	ordinary_muscle_development	ENSGALG00010017341	TA3	-0.326675965	0.079609343	-4.103487771	4.06968E-05	7.83192E-05	negative
PRJNA729271	stage_1D_vs_E10	ordinary_muscle_development	ENSGALG00010017343	DLG1	0.333102482	0.099086095	3.361748023	0.000774508	0.00130723	positive
PRJNA729271	stage_1D_vs_E10	ordinary_muscle_development	ENSGALG00010017344	RERE	2.018794714	0.152543197	13.23424941	5.56499E-40	7.27363E-39	positive
PRJNA729271	stage_1D_vs_E10	ordinary_muscle_development	ENSGALG00010017346	GM2A	0.210355211	0.149862253	1.403657071	0.160420992	0.196692799	positive
PRJNA729271	stage_1D_vs_E10	ordinary_muscle_development	ENSGALG00010017347	TMEM39B	-0.368804015	0.115591419	-3.190583001	0.001419861	0.00232789	negative
PRJNA729271	stage_1D_vs_E10	ordinary_muscle_development	ENSGALG00010017349	ENSGALG00010017349	1.213407882	0.112250182	10.80985224	3.09168E-27	2.33527E-26	positive
PRJNA729271	stage_1D_vs_E10	ordinary_muscle_development	ENSGALG00010017351	CCR2	1.511670991	0.207369376	7.289750404	3.1053E-13	1.11359E-12	positive
PRJNA729271	stage_1D_vs_E10	ordinary_muscle_development	ENSGALG00010017352	GPCPD1	0.93213752	0.238409655	3.909814472	9.2367E-05	0.00017202	positive
PRJNA729271	stage_1D_vs_E10	ordinary_muscle_development	ENSGALG00010017354	FEM1B	-0.11393553	0.105037034	-1.084717703	0.278046705	0.323978054	negative
PRJNA729271	stage_1D_vs_E10	ordinary_muscle_development	ENSGALG00010017356	NSMCE1	-0.544208426	0.117751232	-4.621679236	3.80646E-06	8.08065E-06	negative
PRJNA729271	stage_1D_vs_E10	ordinary_muscle_development	ENSGALG00010017359	EHD3	0.177067087	0.095474683	1.854597266	0.063653759	0.083528991	positive
PRJNA729271	stage_1D_vs_E10	ordinary_muscle_development	ENSGALG00010017361	METTL22	-0.082404137	0.151367306	-0.544398519	0.586167264	0.630537625	negative
PRJNA729271	stage_1D_vs_E10	ordinary_muscle_development	ENSGALG00010017362	ENO1	-0.449122401	0.130189004	-3.449772154	0.00056106	0.000960842	negative
PRJNA729271	stage_1D_vs_E10	ordinary_muscle_development	ENSGALG00010017363	TIMM9	0.326986498	0.140663551	2.32460005	0.020093356	0.028417719	positive
PRJNA729271	stage_1D_vs_E10	ordinary_muscle_development	ENSGALG00010017364	AKT1	-0.251317923	0.073135038	-3.436354573	0.000589599	0.00100615	negative
PRJNA729271	stage_1D_vs_E10	ordinary_muscle_development	ENSGALG00010017367	TIMD4	-1.100467767	0.324964857	-3.386420848	0.000708107	0.001200188	negative
PRJNA729271	stage_1D_vs_E10	ordinary_muscle_development	ENSGALG00010017368	ERLIN2	-0.155925594	0.050431237	-3.091845536	0.001989163	0.003203662	negative
PRJNA729271	stage_1D_vs_E10	ordinary_muscle_development	ENSGALG00010017369	CAPN14	0.397998507	0.488622935	0.814530957	0.415340845	0.464812139	positive
PRJNA729271	stage_1D_vs_E10	ordinary_muscle_development	ENSGALG00010017371	TMEM114	-2.710216912	1.332980689	-2.033200432	0.042032277	0.056713598	negative
PRJNA729271	stage_1D_vs_E10	ordinary_muscle_development	ENSGALG00010017372	ENSGALG00010017372	0.526253537	0.110425348	4.765695055	1.88204E-06	4.10668E-06	positive
PRJNA729271	stage_1D_vs_E10	ordinary_muscle_development	ENSGALG00010017373	RP1-270S.3	-0.140566386	0.105693701	-1.329941002	0.183537711	0.222495535	negative
PRJNA729271	stage_1D_vs_E10	ordinary_muscle_development	ENSGALG00010017375	BDH1A	-1.255413907	0.161820301	-7.758074217	8.62287E-15	3.40847E-14	negative
PRJNA729271	stage_1D_vs_E10	ordinary_muscle_development	ENSGALG00010017376	GALNT14	-1.514752869	0.301613846	-5.022159593	5.10937E-07	1.16704E-06	negative
PRJNA729271	stage_1D_vs_E10	ordinary_muscle_development	ENSGALG00010017377	USP8	0.478829495	0.075118332	6.374336067	1.83757E-10	5.46708E-10	positive
PRJNA729271	stage_1D_vs_E10	ordinary_muscle_development	ENSGALG00010017378	ENSGALG00010017378	-3.341127304	0.657912689	-5.078374926	3.80677E-07	8.76973E-07	negative
PRJNA729271	stage_1D_vs_E10	ordinary_muscle_development	ENSGALG00010017380	MARCKSL1	-1.407186505	0.134839783	-10.43598909	1.69836E-25	1.19272E-24	negative
PRJNA729271	stage_1D_vs_E10	ordinary_muscle_development	ENSGALG00010017381	CA6	-0.855560853	0.708546123	-1.207487875	0.227244312	0.270147322	negative
PRJNA729271	stage_1D_vs_E10	ordinary_muscle_development	ENSGALG00010017383	ENSGALG00010017383	-0.922059857	0.154365645	-5.973219364	2.32617E-09	6.39685E-09	negative
PRJNA729271	stage_1D_vs_E10	ordinary_muscle_development	ENSGALG00010017384	ARID4A	0.091879741	0.092309987	0.995339111	0.319571362	0.367604705	positive
PRJNA729271	stage_1D_vs_E10	ordinary_muscle_development	ENSGALG00010017386	ENSGALG00010017386	-3.422749157	0.95781103	-3.573511944	0.000352225	0.000616756	negative
PRJNA729271	stage_1D_vs_E10	ordinary_muscle_development	ENSGALG00010017387	HS6ST2	-0.251200666	0.11331777	-2.216780882	0.026638069	0.036991646	negative
PRJNA729271	stage_1D_vs_E10	ordinary_muscle_development	ENSGALG00010017388	PLPBP	0.566168656	0.082105578	6.895617475	5.36313E-12	1.76438E-11	positive
PRJNA729271	stage_1D_vs_E10	ordinary_muscle_development	ENSGALG00010017390	DDX24	-0.643190111	0.102188518	-6.29415242	3.09084E-10	9.06184E-10	negative
PRJNA729271	stage_1D_vs_E10	ordinary_muscle_development	ENSGALG00010017391	CRAMP1	0.212639032	0.116495898	1.825292015	0.067956974	0.088758006	positive
PRJNA729271	stage_1D_vs_E10	ordinary_muscle_development	ENSGALG00010017393	VDAC1	3.293041757	0.163579304	20.13116379	3.9364E-90	2.33845E-88	positive
PRJNA729271	stage_1D_vs_E10	ordinary_muscle_development	ENSGALG00010017395	BRF2	-0.329411076	0.124426367	-2.647437876	0.008110426	0.012135917	negative
PRJNA729271	stage_1D_vs_E10	ordinary_muscle_development	ENSGALG00010017396	CCR8L	4.933337109	0.269620714	18.29732235	8.6917E-75	3.63073E-73	positive
PRJNA729271	stage_1D_vs_E10	ordinary_muscle_development	ENSGALG00010017398	SLC22A5	-0.227071901	0.183206398	-1.239432157	0.215185499	0.257098072	negative
PRJNA729271	stage_1D_vs_E10	ordinary_muscle_development	ENSGALG00010017399	CPXM1	1.003121287	0.289979451	3.459284038	0.000541613	0.000929846	positive
PRJNA729271	stage_1D_vs_E10	ordinary_muscle_development	ENSGALG00010017400	GPC4	-1.258847205	0.119029159	-10.57595645	3.85227E-26	2.77252E-25	negative
PRJNA729271	stage_1D_vs_E10	ordinary_muscle_development	ENSGALG00010017402	SLC2A5	1.27216383	0.229973815	5.531776869	3.17003E-08	7.9374E-08	positive
PRJNA729271	stage_1D_vs_E10	ordinary_muscle_development	ENSGALG00010017403	ITGA11	-0.710631284	0.292843431	-2.426659466	0.015238552	0.021947815	negative
PRJNA729271	stage_1D_vs_E10	ordinary_muscle_development	ENSGALG00010017405	XCR1	-0.04880136	1.219872021	-0.040005311	0.968088892	0.972719291	negative
PRJNA729271	stage_1D_vs_E10	ordinary_muscle_development	ENSGALG00010017406	IFT140	-1.223225725	0.124967572	-9.788345159	1.26347E-22	7.77003E-22	negative
PRJNA729271	stage_1D_vs_E10	ordinary_muscle_development	ENSGALG00010017407	EPN2	0.159526907	0.085003554	1.876708672	0.060558033	0.079675837	positive
PRJNA729271	stage_1D_vs_E10	ordinary_muscle_development	ENSGALG00010017409	GPC3	-1.582082679	0.331018175	-4.779443542	1.75781E-06	3.8448E-06	negative
PRJNA729271	stage_1D_vs_E10	ordinary_muscle_development	ENSGALG00010017411	RAB11FIP1	0.414173132	0.136984952	3.023493633	0.002498743	0.003971412	positive
PRJNA729271	stage_1D_vs_E10	ordinary_muscle_development	ENSGALG00010017413	ZIC3	-4.321555644	0.800049802	-5.401608292	6.6046E-08	1.61876E-07	negative
PRJNA729271	stage_1D_vs_E10	ordinary_muscle_development	ENSGALG00010017414	ENSGALG00010017414	0.837590442	0.196719163	4.257797922	2.0645E-05	4.08532E-05	positive
PRJNA729271	stage_1D_vs_E10	ordinary_muscle_development	ENSGALG00010017415	HDAC1	-1.451139588	0.063734511	-22.76850602	9.4099E-115	9.6735E-113	negative
PRJNA729271	stage_1D_vs_E10	ordinary_muscle_development	ENSGALG00010017418	CHGB	-2.035175243	0.290164247	-7.013873231	2.31809E-12	7.836E-12	negative
PRJNA729271	stage_1D_vs_E10	ordinary_muscle_development	ENSGALG00010017419	B9D1	-1.363905694	0.103155416	-13.22185252	6.56274E-40	8.56228E-39	negative
PRJNA729271	stage_1D_vs_E10	ordinary_muscle_development	ENSGALG00010017421	GPR157	2.348207468	0.261708978	8.972590409	2.89621E-19	1.48815E-18	positive
PRJNA729271	stage_1D_vs_E10	ordinary_muscle_development	ENSGALG00010017422	ENSGALG00010017422	0.198031576	0.224720347	0.88123563	0.378190298	0.427328494	positive
PRJNA729271	stage_1D_vs_E10	ordinary_muscle_development	ENSGALG00010017423	TMEM204	0.508395575	0.132360551	3.840990163	0.000122539	0.000225435	positive
PRJNA729271	stage_1D_vs_E10	ordinary_muscle_development	ENSGALG00010017424	APOD	1.04947538	0.518468126	2.024184955	0.042951125	0.057881792	positive
PRJNA729271	stage_1D_vs_E10	ordinary_muscle_development	ENSGALG00010017425	H6PD	1.135184881	0.124065872	9.149856117	5.70094E-20	3.04141E-19	positive
PRJNA729271	stage_1D_vs_E10	ordinary_muscle_development	ENSGALG00010017428	PSMA3	-0.531167905	0.108326143	-4.903413814	9.41853E-07	2.10942E-06	negative
PRJNA729271	stage_1D_vs_E10	ordinary_muscle_development	ENSGALG00010017429	TELO2	-0.204673004	0.102820738	-1.990580958	0.046526975	0.062324047	negative
PRJNA729271	stage_1D_vs_E10	ordinary_muscle_development	ENSGALG00010017430	CACNA1H	-1.536292738	0.204152686	-7.525214411	5.26339E-14	1.98318E-13	negative
PRJNA729271	stage_1D_vs_E10	ordinary_muscle_development	ENSGALG00010017431	FYCO1	3.351905019	0.117231788	28.59211713	8.4199E-180	2.6532E-177	positive
PRJNA729271	stage_1D_vs_E10	ordinary_muscle_development	ENSGALG00010017433	RNPS1	-0.656042119	0.079011752	-8.303095414	1.01433E-16	4.52254E-16	negative
PRJNA729271	stage_1D_vs_E10	ordinary_muscle_development	ENSGALG00010017434	FGF13	2.631101577	0.148237833	17.74919077	1.74855E-70	6.54917E-69	positive
PRJNA729271	stage_1D_vs_E10	ordinary_muscle_development	ENSGALG00010017435	ACTR10	-0.715390003	0.090358332	-7.917255564	2.4281E-15	9.94781E-15	negative
PRJNA729271	stage_1D_vs_E10	ordinary_muscle_development	ENSGALG00010017437	DRG2	0.120417541	0.139541213	0.862953233	0.388163181	0.437275612	positive
PRJNA729271	stage_1D_vs_E10	ordinary_muscle_development	ENSGALG00010017438	ADRB3	4.593477462	0.769073519	5.97274158	2.33299E-09	6.4144E-09	positive
PRJNA729271	stage_1D_vs_E10	ordinary_muscle_development	ENSGALG00010017439	PPP4R4	-2.164008877	0.274920402	-7.871401551	3.5069E-15	1.42428E-14	negative
PRJNA729271	stage_1D_vs_E10	ordinary_muscle_development	ENSGALG00010017441	SPSB1	0.259381358	0.256778271	1.010137488	0.312429424	0.359874801	positive
PRJNA729271	stage_1D_vs_E10	ordinary_muscle_development	ENSGALG00010017442	LCK	0.676149377	0.261880035	2.581905015	0.009825662	0.014538891	positive
PRJNA729271	stage_1D_vs_E10	ordinary_muscle_development	ENSGALG00010017445	F9	3.893223033	1.175721065	3.311349221	0.000928473	0.001554066	positive
PRJNA729271	stage_1D_vs_E10	ordinary_muscle_development	ENSGALG00010017447	CAPN13	-1.04881195	0.561699203	-1.867212817	0.061871874	0.081301135	negative
PRJNA729271	stage_1D_vs_E10	ordinary_muscle_development	ENSGALG00010017449	TRMT6	-0.753094085	0.084627828	-8.898894219	5.64058E-19	2.8468E-18	negative
PRJNA729271	stage_1D_vs_E10	ordinary_muscle_development	ENSGALG00010017450	ENSGALG00010017450	1.145900803	0.210858257	5.434460187	5.49626E-08	1.35467E-07	positive
PRJNA729271	stage_1D_vs_E10	ordinary_muscle_development	ENSGALG00010017451	GABPB1	-0.44613417	0.076862597	-5.804307769	6.46325E-09	1.71584E-08	negative
PRJNA729271	stage_1D_vs_E10	ordinary_muscle_development	ENSGALG00010017452	ARMH4	-5.708262752	0.502763297	-11.35377779	7.10262E-30	6.08826E-29	negative
PRJNA729271	stage_1D_vs_E10	ordinary_muscle_development	ENSGALG00010017453	PPP1R2	-0.262923345	0.138816832	-1.894030719	0.058220932	0.076754199	negative
PRJNA729271	stage_1D_vs_E10	ordinary_muscle_development	ENSGALG00010017454	ENSGALG00010017454	0.809128117	0.158869578	5.093033713	3.52379E-07	8.14109E-07	positive
PRJNA729271	stage_1D_vs_E10	ordinary_muscle_development	ENSGALG00010017455	CCR9	2.779932851	0.695273379	3.998330632	6.37908E-05	0.000120806	positive
PRJNA729271	stage_1D_vs_E10	ordinary_muscle_development	ENSGALG00010017457	ZMAT4	-0.834201611	0.249760409	-3.340007388	0.000837762	0.001408905	negative
PRJNA729271	stage_1D_vs_E10	ordinary_muscle_development	ENSGALG00010017458	ENSGALG00010017458	0.24610483	0.172490125	1.426776344	0.153644372	0.188991444	positive
PRJNA729271	stage_1D_vs_E10	ordinary_muscle_development	ENSGALG00010017459	FAM167B	1.954211726	0.281769641	6.935494241	4.04803E-12	1.34341E-11	positive
PRJNA729271	stage_1D_vs_E10	ordinary_muscle_development	ENSGALG00010017462	MCM8	-0.137665458	0.11748733	-1.171747275	0.241298537	0.285116493	negative
PRJNA729271	stage_1D_vs_E10	ordinary_muscle_development	ENSGALG00010017463	PPP1R36	0.642237916	0.333093922	1.928098572	0.053842867	0.071404607	positive
PRJNA729271	stage_1D_vs_E10	ordinary_muscle_development	ENSGALG00010017464	SH3BP2	0.043055163	0.130265719	0.330517985	0.741008605	0.774120341	positive
PRJNA729271	stage_1D_vs_E10	ordinary_muscle_development	ENSGALG00010017465	TMEM234	-0.215682329	0.235898534	-0.914301268	0.360558564	0.409520169	negative
PRJNA729271	stage_1D_vs_E10	ordinary_muscle_development	ENSGALG00010017466	SPIA4	-14.20742336	2.838419055	-5.005400218			negative
PRJNA729271	stage_1D_vs_E10	ordinary_muscle_development	ENSGALG00010017467	SFRP1	-3.582426408	0.109990424	-32.57034829	1.0788E-232	8.687E-230	negative
PRJNA729271	stage_1D_vs_E10	ordinary_muscle_development	ENSGALG00010017468	AMDHD2	-0.443056011	0.129110716	-3.431597512	0.000600037	0.00102324	negative
PRJNA729271	stage_1D_vs_E10	ordinary_muscle_development	ENSGALG00010017470	HSPA2	2.127954025	0.169960276	12.52030223	5.78146E-36	6.5165E-35	positive
PRJNA729271	stage_1D_vs_E10	ordinary_muscle_development	ENSGALG00010017471	CRLS1	0.343204042	0.089512494	3.834146782	0.000126001	0.000231451	positive
PRJNA729271	stage_1D_vs_E10	ordinary_muscle_development	ENSGALG00010017472	LZTFL1	-1.632961223	0.171926951	-9.497994432	2.13971E-21	1.23566E-20	negative
PRJNA729271	stage_1D_vs_E10	ordinary_muscle_development	ENSGALG00010017473	EIF3I	0.06190246	0.104528164	0.592208433	0.553711023	0.599477102	positive
PRJNA729271	stage_1D_vs_E10	ordinary_muscle_development	ENSGALG00010017475	STUM	4.314917271	0.246114527	17.53215192	8.14285E-69	2.90002E-67	positive
PRJNA729271	stage_1D_vs_E10	ordinary_muscle_development	ENSGALG00010017476	ATP6V0C	1.255882419	0.111989979	11.21423925	3.47159E-29	2.89199E-28	positive
PRJNA729271	stage_1D_vs_E10	ordinary_muscle_development	ENSGALG00010017477	MCF2	2.166104372	0.181854886	11.91116947	1.03515E-32	1.02002E-31	positive
PRJNA729271	stage_1D_vs_E10	ordinary_muscle_development	ENSGALG00010017479	SSTR5	-1.92353122	0.654171992	-2.940405955	0.003277825	0.00514534	negative
PRJNA729271	stage_1D_vs_E10	ordinary_muscle_development	ENSGALG00010017480	DCDC2B	0.398398861	0.198782712	2.004192699	0.045049422	0.060495773	positive

PRJNA729271	stage_1D_vs_E10	ordinary_muscle_development	ENSGALG00010017481	SPIA1	-14.72104816	2.61245505	-5.634947922			negative
PRJNA729271	stage_1D_vs_E10	ordinary_muscle_development	ENSGALG00010017482	CORO2B	-0.658032956	0.143189921	-4.595525669	4.3166E-06	9.09963E-06	negative
PRJNA729271	stage_1D_vs_E10	ordinary_muscle_development	ENSGALG00010017484	IQCC	0.008211237	0.132390498	0.062022854	0.950544632	0.958146345	positive
PRJNA729271	stage_1D_vs_E10	ordinary_muscle_development	ENSGALG00010017485	LBH	-0.901908567	0.10796693	-8.353563141	6.6234E-17	2.98898E-16	negative
PRJNA729271	stage_1D_vs_E10	ordinary_muscle_development	ENSGALG00010017487	C1QTNF8	-2.2586945	0.519454651	-4.348203436	1.37257E-05	2.76556E-05	negative
PRJNA729271	stage_1D_vs_E10	ordinary_muscle_development	ENSGALG00010017489	TNFRSF1B	0.771411734	0.177963172	4.334670631	1.45979E-05	2.93232E-05	positive
PRJNA729271	stage_1D_vs_E10	ordinary_muscle_development	ENSGALG00010017491	CCDC28B	0.765905782	0.199505093	3.839028731	0.000123522	0.000227128	positive
PRJNA729271	stage_1D_vs_E10	ordinary_muscle_development	ENSGALG00010017493	YPEL5	-0.114531694	0.134537493	-0.851299448	0.394603031	0.443848703	negative
PRJNA729271	stage_1D_vs_E10	ordinary_muscle_development	ENSGALG00010017494	TBC1D24	-1.208287332	0.094567396	-12.77699697	2.20417E-37	2.62311E-36	negative
PRJNA729271	stage_1D_vs_E10	ordinary_muscle_development	ENSGALG00010017495	NT5M	-0.21955826	0.134800264	-1.628767288	0.103362292	0.131136667	negative
PRJNA729271	stage_1D_vs_E10	ordinary_muscle_development	ENSGALG00010017496	PARP1	-1.51315766	0.103687375	-14.59346098	3.0911E-48	5.54523E-47	negative
PRJNA729271	stage_1D_vs_E10	ordinary_muscle_development	ENSGALG00010017499	SNRPD1	-1.291818849	0.098580826	-13.10415935	3.11697E-39	3.99827E-38	negative
PRJNA729271	stage_1D_vs_E10	ordinary_muscle_development	ENSGALG00010017500	ACAP2	0.172220836	0.100475016	1.714066271	0.086516555	0.111204884	positive
PRJNA729271	stage_1D_vs_E10	ordinary_muscle_development	ENSGALG00010017502	NAT8B	1.053973161	0.116767595	9.026247053	1.7766E-19	9.22014E-19	positive
PRJNA729271	stage_1D_vs_E10	ordinary_muscle_development	ENSGALG00010017503	MED9	0.027452978	0.126042655	0.217807047	0.827579453	0.852032401	positive
PRJNA729271	stage_1D_vs_E10	ordinary_muscle_development	ENSGALG00010017505	TNFRSF8	0.827634805	0.302957552	2.731850718	0.006297967	0.009562065	positive
PRJNA729271	stage_1D_vs_E10	ordinary_muscle_development	ENSGALG00010017509	RASD1	1.41025288	0.294219887	4.793193595	1.64147E-06	3.60283E-06	positive
PRJNA729271	stage_1D_vs_E10	ordinary_muscle_development	ENSGALG00010017510	HDC	5.133493472	0.376029591	13.65183377	1.96862E-42	2.81134E-41	positive
PRJNA729271	stage_1D_vs_E10	ordinary_muscle_development	ENSGALG00010017513	TNIP2	0.514773591	0.143567358	3.585589346	0.000336318	0.000589756	positive
PRJNA729271	stage_1D_vs_E10	ordinary_muscle_development	ENSGALG00010017514	TXLNA	-0.606275896	0.097017226	-6.249157189	4.12673E-10	1.19682E-09	negative
PRJNA729271	stage_1D_vs_E10	ordinary_muscle_development	ENSGALG00010017516	AAK1	0.198757547	0.079760333	2.49193477	0.012704937	0.018523241	positive
PRJNA729271	stage_1D_vs_E10	ordinary_muscle_development	ENSGALG00010017517	LIN9	-0.112426525	0.097855442	-1.148904169	0.250595498	0.295147619	negative
PRJNA729271	stage_1D_vs_E10	ordinary_muscle_development	ENSGALG00010017518	PEMT	0.748615731	0.147289889	5.082600938	3.72301E-07	8.58223E-07	positive
PRJNA729271	stage_1D_vs_E10	ordinary_muscle_development	ENSGALG00010017519	NTN3	-1.597307639	0.196084903	-8.146000088	3.7616E-16	1.62857E-15	negative
PRJNA729271	stage_1D_vs_E10	ordinary_muscle_development	ENSGALG00010017520	ZBTB1	-0.598946903	0.080859495	-7.407255009	1.2894E-13	4.73402E-13	negative
PRJNA729271	stage_1D_vs_E10	ordinary_muscle_development	ENSGALG00010017521	MIIP	0.452434599	0.114860333	3.938997811	8.18227E-05	0.000153114	positive
PRJNA729271	stage_1D_vs_E10	ordinary_muscle_development	ENSGALG00010017522	XXYLT1	-0.051230602	0.106516078	-0.480965911	0.630540727	0.672134714	negative
PRJNA729271	stage_1D_vs_E10	ordinary_muscle_development	ENSGALG00010017524	ACBD3	-0.413671154	0.05984347	-6.912552941	4.76008E-12	1.57169E-11	negative
PRJNA729271	stage_1D_vs_E10	ordinary_muscle_development	ENSGALG00010017525	ENSGALG00010017525	-4.29109625	0.691111315	-6.208979881	5.33296E-10	1.53619E-09	negative
PRJNA729271	stage_1D_vs_E10	ordinary_muscle_development	ENSGALG00010017526	PLEKHA2	0.834365909	0.172449431	4.838322203	1.3094E-06	2.89501E-06	positive
PRJNA729271	stage_1D_vs_E10	ordinary_muscle_development	ENSGALG00010017528	RAI1	0.065970595	0.177510366	0.371643617	0.71015821	0.74597748	positive
PRJNA729271	stage_1D_vs_E10	ordinary_muscle_development	ENSGALG00010017529	MFN2	-0.205802215	0.118880921	-1.731162695	0.083422751	0.107447376	negative
PRJNA729271	stage_1D_vs_E10	ordinary_muscle_development	ENSGALG00010017530	TLR1B	-0.340823742	0.217019989	-1.570471661	0.116305422	0.146353598	negative
PRJNA729271	stage_1D_vs_E10	ordinary_muscle_development	ENSGALG00010017531	SACM1L	-0.179483227	0.111617358	-1.608022533	0.107830244	0.136244716	negative
PRJNA729271	stage_1D_vs_E10	ordinary_muscle_development	ENSGALG00010017532	MIXL1	-0.591223571	0.35196701	-1.679769851	0.093002103	0.118929465	negative
PRJNA729271	stage_1D_vs_E10	ordinary_muscle_development	ENSGALG00010017533	FAM193A	0.475293414	0.076345559	6.225554211	4.79857E-10	1.38639E-09	positive
PRJNA729271	stage_1D_vs_E10	ordinary_muscle_development	ENSGALG00010017534	ATP11C	-0.823847601	0.084212382	-9.78297468	1.33236E-22	8.17982E-22	negative
PRJNA729271	stage_1D_vs_E10	ordinary_muscle_development	ENSGALG00010017535	LIMD1	-0.539265365	0.090538063	-5.956228212	2.58126E-09	7.07819E-09	negative
PRJNA729271	stage_1D_vs_E10	ordinary_muscle_development	ENSGALG00010017536	GSC	-4.726291751	0.518936148	-9.107655664	8.41813E-20	4.45656E-19	negative
PRJNA729271	stage_1D_vs_E10	ordinary_muscle_development	ENSGALG00010017537	MANBA	1.141529214	0.186913853	6.107247778	1.01364E-09	2.86358E-09	positive
PRJNA729271	stage_1D_vs_E10	ordinary_muscle_development	ENSGALG00010017538	H3-3A	-0.202962442	0.075897973	-2.674148385	0.007491929	0.011259255	negative
PRJNA729271	stage_1D_vs_E10	ordinary_muscle_development	ENSGALG00010017539	KPNA6	0.749959116	0.138430044	5.417603693	6.04031E-08	1.48397E-07	positive
PRJNA729271	stage_1D_vs_E10	ordinary_muscle_development	ENSGALG00010017540	EIF4G1	0.696074825	0.164512188	4.231144417	2.32505E-05	4.57841E-05	positive
PRJNA729271	stage_1D_vs_E10	ordinary_muscle_development	ENSGALG00010017541	SREBF1	0.837340514	0.228571129	3.663369559	0.000248919	0.000442275	positive
PRJNA729271	stage_1D_vs_E10	ordinary_muscle_development	ENSGALG00010017542	PLOD1	-0.580304047	0.125306865	-4.631063502	3.63792E-06	7.741E-06	negative
PRJNA729271	stage_1D_vs_E10	ordinary_muscle_development	ENSGALG00010017543	ZBTB25	-0.138558679	0.178284021	-0.77719459	0.4370529	0.48637864	negative
PRJNA729271	stage_1D_vs_E10	ordinary_muscle_development	ENSGALG00010017544	SDE2	0.11878918	0.068537215	1.733206993	0.083058884	0.107054822	positive
PRJNA729271	stage_1D_vs_E10	ordinary_muscle_development	ENSGALG00010017545	DICER1	0.224120947	0.120157277	1.865229911	0.062149185	0.081621132	positive
PRJNA729271	stage_1D_vs_E10	ordinary_muscle_development	ENSGALG00010017546	NFU1	1.016521219	0.107973755	9.414521305	4.75243E-21	2.69403E-20	positive
PRJNA729271	stage_1D_vs_E10	ordinary_muscle_development	ENSGALG00010017547	RNF4	-0.287419445	0.065830549	-4.366049637	1.26514E-05	2.56048E-05	negative
PRJNA729271	stage_1D_vs_E10	ordinary_muscle_development	ENSGALG00010017550	TM2D2	0.151483526	0.076783511	1.972865305	0.04851091	0.064831795	positive
PRJNA729271	stage_1D_vs_E10	ordinary_muscle_development	ENSGALG00010017551	NALF2	-0.170938571	0.2770948	-0.616895629	0.53730357	0.58378047	negative
PRJNA729271	stage_1D_vs_E10	ordinary_muscle_development	ENSGALG00010017552	LARS2	-0.108126394	0.10281174	-1.051693059	0.292940396	0.339612177	negative
PRJNA729271	stage_1D_vs_E10	ordinary_muscle_development	ENSGALG00010017553	EDA	0.703884903	0.108563162	6.483644101	8.95333E-11	2.71844E-10	positive
PRJNA729271	stage_1D_vs_E10	ordinary_muscle_development	ENSGALG00010017554	FAM131A	-2.660811515	0.342446727	-7.770001313	7.84853E-15	3.10917E-14	negative
PRJNA729271	stage_1D_vs_E10	ordinary_muscle_development	ENSGALG00010017555	CELA2A	1.236766383	1.22054047	1.013294039	0.310919734	0.358249725	positive
PRJNA729271	stage_1D_vs_E10	ordinary_muscle_development	ENSGALG00010017556	TOM1L2	0.272183635	0.11452369	2.376657926	0.017470281	0.024926836	positive
PRJNA729271	stage_1D_vs_E10	ordinary_muscle_development	ENSGALG00010017558	ENSGALG00010017558	-1.007473934	0.074387166	-13.54365263	8.63844E-42	1.2063E-40	negative
PRJNA729271	stage_1D_vs_E10	ordinary_muscle_development	ENSGALG00010017559	DUSP10	2.039606489	0.149671616	13.62720962	2.7593E-42	3.91735E-41	positive
PRJNA729271	stage_1D_vs_E10	ordinary_muscle_development	ENSGALG00010017561	CFAP99	-1.824819867	0.68900953	-2.648468254	0.008085744	0.012102733	negative
PRJNA729271	stage_1D_vs_E10	ordinary_muscle_development	ENSGALG00010017562	SLC30A10	-3.958482548	0.619716546	-6.387569561	1.68543E-10	5.02371E-10	negative
PRJNA729271	stage_1D_vs_E10	ordinary_muscle_development	ENSGALG00010017563	ALK	3.381874951	0.150680975	22.44394124	1.4665E-111	1.3627E-109	positive
PRJNA729271	stage_1D_vs_E10	ordinary_muscle_development	ENSGALG00010017565	ENSGALG00010017565	-0.555766245	0.187642822	-2.96183056	0.00305816	0.004817217	negative
PRJNA729271	stage_1D_vs_E10	ordinary_muscle_development	ENSGALG00010017568	CDCP1	-2.778345035	0.249389526	-11.14058428	7.95954E-29	6.50725E-28	negative
PRJNA729271	stage_1D_vs_E10	ordinary_muscle_development	ENSGALG00010017569	CLMN	-0.723254861	0.245120918	-2.950604406	0.003171528	0.004985501	negative
PRJNA729271	stage_1D_vs_E10	ordinary_muscle_development	ENSGALG00010017570	DRC3	-1.692210502	0.447029021	-3.78546005	0.000153424	0.000279664	negative
PRJNA729271	stage_1D_vs_E10	ordinary_muscle_development	ENSGALG00010017571	ADAM9	1.188931914	0.149612144	7.946760756	1.91452E-15	7.89949E-15	positive
PRJNA729271	stage_1D_vs_E10	ordinary_muscle_development	ENSGALG00010017573	ENSGALG00010017573	0.873891894	0.219247125	3.985876185	6.72316E-05	0.00012694	positive
PRJNA729271	stage_1D_vs_E10	ordinary_muscle_development	ENSGALG00010017574	DYNC1LI1	-0.566607778	0.056394183	-10.04727347	9.44443E-24	6.07891E-23	negative
PRJNA729271	stage_1D_vs_E10	ordinary_muscle_development	ENSGALG00010017575	CLCN2	3.027122873	0.697549381	4.339653872	1.42707E-05	2.86939E-05	positive
PRJNA729271	stage_1D_vs_E10	ordinary_muscle_development	ENSGALG00010017576	ENSGALG00010017576	-3.581712937	0.307991513	-11.62925855	2.92628E-31	2.68289E-30	negative
PRJNA729271	stage_1D_vs_E10	ordinary_muscle_development	ENSGALG00010017577	CLPX	2.075921158	0.137384165	15.11033791	1.38428E-51	2.81419E-50	positive
PRJNA729271	stage_1D_vs_E10	ordinary_muscle_development	ENSGALG00010017578	BSDC1	0.252899637	0.100078118	2.52702231	0.011503419	0.016869897	positive
PRJNA729271	stage_1D_vs_E10	ordinary_muscle_development	ENSGALG00010017579	P3H2	0.703425753	0.141422799	4.973920447	6.56122E-07	1.48694E-06	positive
PRJNA729271	stage_1D_vs_E10	ordinary_muscle_development	ENSGALG00010017580	LYPLAL1	-0.80807863	0.156362151	-5.167993834	2.3662E-07	5.54537E-07	negative
PRJNA729271	stage_1D_vs_E10	ordinary_muscle_development	ENSGALG00010017581	EFCAB11	-2.303607308	0.427333484	-5.390654822	7.02014E-08	1.71713E-07	negative
PRJNA729271	stage_1D_vs_E10	ordinary_muscle_development	ENSGALG00010017582	CLCN6	0.282242844	0.101570194	2.778795944	0.005456079	0.008347674	positive
PRJNA729271	stage_1D_vs_E10	ordinary_muscle_development	ENSGALG00010017583	MTHFD1	-0.806822831	0.105574771	-7.642193535	2.13552E-14	8.25009E-14	negative
PRJNA729271	stage_1D_vs_E10	ordinary_muscle_development	ENSGALG00010017584	WNT11B	-3.449154862	0.935696298	-3.686190562	0.000227636	0.000406403	negative
PRJNA729271	stage_1D_vs_E10	ordinary_muscle_development	ENSGALG00010017585	ENSGALG00010017585	-0.379802228	0.117859013	-3.222513229	0.001270713	0.002095925	negative
PRJNA729271	stage_1D_vs_E10	ordinary_muscle_development	ENSGALG00010017586	ZFYVE28	-0.041270108	0.161794311	-0.25507762	0.798663149	0.825721994	negative
PRJNA729271	stage_1D_vs_E10	ordinary_muscle_development	ENSGALG00010017587	MB21D2	0.585299049	0.143618321	4.075378725	4.59395E-05	8.81278E-05	positive
PRJNA729271	stage_1D_vs_E10	ordinary_muscle_development	ENSGALG00010017588	NOX5	-2.114642879	0.539433054	-3.920121068	8.85045E-05	0.000165085	negative
PRJNA729271	stage_1D_vs_E10	ordinary_muscle_development	ENSGALG00010017589	ATPAF2	0.03338528	0.146844247	0.227351634	0.820150326	0.845224243	positive
PRJNA729271	stage_1D_vs_E10	ordinary_muscle_development	ENSGALG00010017590	ENSGALG00010017590	-0.685475051	0.191901589	-3.57201342	0.000354247	0.000620072	negative
PRJNA729271	stage_1D_vs_E10	ordinary_muscle_development	ENSGALG00010017591	ESR2	-3.093173455	0.295473647	-10.46852566	1.20505E-25	8.50815E-25	negative
PRJNA729271	stage_1D_vs_E10	ordinary_muscle_development	ENSGALG00010017592	GID4	0.609762349	0.111065339	5.490122808	4.01654E-08	9.98453E-08	positive
PRJNA729271	stage_1D_vs_E10	ordinary_muscle_development	ENSGALG00010017593	MTHFR	0.350386457	0.1326348	2.641738491	0.008248171	0.012329302	positive
PRJNA729271	stage_1D_vs_E10	ordinary_muscle_development	ENSGALG00010017594	CEP68	0.646935851	0.099900843	6.4757797	9.43236E-11	2.8591E-10	positive
PRJNA729271	stage_1D_vs_E10	ordinary_muscle_development	ENSGALG00010017595	CCNF	-2.206946642	0.127036928	-17.37248113	1.33334E-67	4.50507E-66	negative
PRJNA729271	stage_1D_vs_E10	ordinary_muscle_development	ENSGALG00010017596	TDP1	-1.652044309	0.0969247	-17.04461617	3.83252E-65	1.22093E-63	negative
PRJNA729271	stage_1D_vs_E10	ordinary_muscle_development	ENSGALG00010017597	CNOT10	-0.094995853	0.134995913	-0.703694287	0.481623179	0.530118307	negative
PRJNA729271	stage_1D_vs_E10	ordinary_muscle_development	ENSGALG00010017598	ENSGALG00010017598	0.298761679	0.117893851	2.534158275	0.011271781	0.016556999	positive
PRJNA729271	stage_1D_vs_E10	ordinary_muscle_development	ENSGALG00010017599	KCNK13	-0.714959668	0.238143004	-3.002228311	0.002680111	0.004248027	negative
PRJNA729271	stage_1D_vs_E10	ordinary_muscle_development	ENSGALG00010017600	MXD4	0.354030968	0.103331524	3.426166126	0.000612166	0.001042696	positive
PRJNA729271	stage_1D_vs_E10	ordinary_muscle_development	ENSGALG00010017601	ENSGALG00010017601	1.214944571	0.114037348	10.65391813	1.67177E-26	1.22509E-25	positive
PRJNA729271	stage_1D_vs_E10	ordinary_muscle_development	ENSGALG00010017603	CYP3A5	-0.247352344	1.379917728	-0.179251515	0.857740215	0.8782809	negative
PRJNA729271	stage_1D_vs_E10	ordinary_muscle_development	ENSGALG00010017604	GATM	4.576845099	0.241146391	18.97952971	2.5184E-80	1.2008E-78	positive
PRJNA729271	stage_1D_vs_E10	ordinary_muscle_development	ENSGALG00010017605	GFPT1	-1.42478246	0.071874434	-19.82321639	1.87719E-87	1.06288E-85	negative
PRJNA729271	stage_1D_vs_E10	ordinary_muscle_development	ENSGALG00010017606	ENSGALG00010017606	0.967288708	0.244715714	3.952703707	7.72731E-05	0.000144956	positive
PRJNA729271	stage_1D_vs_E10	ordinary_muscle_development	ENSGALG00010017608	RBBP4	-0.932553842	0.080842974	-11.53537275	8.75073E-31	7.87837E-30	negative
PRJNA729271	stage_1D_vs_E10	ordinary_muscle_development	ENSGALG00010017609	TRIM71	2.081113396	0.192007063	10.83873354	2.25572E-27	1.71276E-26	positive
PRJNA729271	stage_1D_vs_E10	ordinary_muscle_development	ENSGALG00010017610	PSMC1	-0.502387415	0.119501046	-4.204042002	2.6219E-05	5.13643E-05	negative
PRJNA729271	stage_1D_vs_E10	ordinary_muscle_development	ENSGALG00010017612	CLDN11	0.64948214	0.399014295	1.627716473	0.103585019	0.131407741	positive
PRJNA729271	stage_1D_vs_E10	ordinary_muscle_development	ENSGALG00010017613	HAUS3	-1.218529816	0.119647908	-10.18429692	2.33038E-24	1.54453E-23	negative
PRJNA729271	stage_1D_vs_E10	ordinary_muscle_development	ENSGALG00010017615	AGTRAP	0.356923448	0.134642841	2.650890657	0.008027983	0.012022483	positive
PRJNA729271	stage_1D_vs_E10	ordinary_muscle_development	ENSGALG00010017616	PDCD7	0.737723355	0.155585928	4.741581485	2.12056E-06	4.61594E-06	positive
PRJNA729271	stage_1D_vs_E10	ordinary_muscle_development	ENSGALG00010017619	POLN	0.261428037	0.328017873	0.796993267	0.425454942	0.474930256	positive
PRJNA729271	stage_1D_vs_E10	ordinary_muscle_development	ENSGALG00010017620	UBAP1L	0.784197335	0.316293562	2.479333848	0.013162804	0.019140735	positive
PRJNA729271	stage_1D_vs_E10	ordinary_muscle_development	ENSGALG00010017621	SYNE2	2.052377608	0.155879992	13.16639537	1.36983E-39	1.76967E-38	positive
PRJNA729271	stage_1D_vs_E10	ordinary_muscle_development	ENSGALG00010017622	RAB1B	-0.328188726	0.112583468	-2.915070331	0.003556084	0.005561657	negative
PRJNA729271	stage_1D_vs_E10	ordinary_muscle_development	ENSGALG00010017625	YBX1	-0.76600314	0.091288723	-8.390994116	4.82048E-17	2.18969E-16	negative
PRJNA729271	stage_1D_vs_E10	ordinary_muscle_development	ENSGALG00010017626	COPS7B	-0.816764324	0.140557709	-5.810882451	6.21444E-09	1.65372E-08	negative
PRJNA729271	stage_1D_vs_E10	ordinary_muscle_development	ENSGALG00010017628	NRDE2	-0.091368522	0.103687216	-0.881193703	0.378212987	0.427328494	negative
PRJNA729271	stage_1D_vs_E10	ordinary_muscle_development	ENSGALG00010017629	ENSGALG00010017629	-0.127904357	0.141125616	-0.906315668	0.364768795	0.413783353	negative
PRJNA729271	stage_1D_vs_E10	ordinary_muscle_development	ENSGALG00010017631	NAT8L	-2.986538043	0.260492707	-11.46495836	1.97855E-30	1.75193E-29	negative
PRJNA729271	stage_1D_vs_E10	ordinary_muscle_development	ENSGALG00010017632	AWAT1	1.024030798	0.591332651	1.7317339	0.083320952	0.107335336	positive
PRJNA729271	stage_1D_vs_E10	ordinary_muscle_development	ENSGALG00010017633	HLX	0.988800522	0.14745464	6.705794539	2.00313E-11	6.35349E-11	positive
PRJNA729271	stage_1D_vs_E10	ordinary_muscle_development	ENSGALG00010017634	PPIH	-0.693152085	0.097610913	-7.101174083	1.23701E-12	4.24291E-12	negative
PRJNA729271	stage_1D_vs_E10	ordinary_muscle_development	ENSGALG00010017636	ZBTB8OS	-0.430741631	0.158668394	-2.714728626	0.00663301	0.01003711	negative
PRJNA729271	stage_1D_vs_E10	ordinary_muscle_development	ENSGALG00010017638	GFRA2	0.034682859	0.258271815	0.134288208	0.893174678	0.909041353	positive
PRJNA729271	stage_1D_vs_E10	ordinary_muscle_development	ENSGALG00010017639	ENSGALG00010017639	-0.671273323	0.127968596	-5.245609826	1.55766E-07	3.70805E-07	negative
PRJNA729271	stage_1D_vs_E10	ordinary_muscle_development	ENSGALG00010017641	GPRC5B	1.495342222	0.159134204	9.396736764	5.62815E-21	3.17927E-20	positive
PRJNA729271	stage_1D_vs_E10	ordinary_muscle_development	ENSGALG00010017642	MTARC1	1.625252959	0.121935021	13.32884473	1.57315E-40	2.10747E-39	positive
PRJNA729271	stage_1D_vs_E10	ordinary_muscle_development	ENSGALG00010017643	CALM1	-0.838235481	0.061164971	-13.70450232	9.54193E-43	1.37485E-41	negative
PRJNA729271	stage_1D_vs_E10	ordinary_muscle_development	ENSGALG00010017644	RBBP6	0.754402847	0.096406437	7.825233141	5.06717E-15	2.03402E-14	positive
PRJNA729271	stage_1D_vs_E10	ordinary_muscle_development	ENSGALG00010017645	SYNC	2.316252633	0.268433845	8.628765259	6.20178E-18	2.96194E-17	positive
PRJNA729271	stage_1D_vs_E10	ordinary_muscle_development	ENSGALG00010017646	MAEA	-0.716398549	0.087038231	-8.230849133	1.85891E-16	8.16265E-16	negative
PRJNA729271	stage_1D_vs_E10	ordinary_muscle_development	ENSGALG00010017647	ITGA9	0.192869931	0.166787984	1.156377852	0.247526637	0.291699073	positive
PRJNA729271	stage_1D_vs_E10	ordinary_muscle_development	ENSGALG00010017648	ENSGALG00010017648	-0.586799575	0.148116788	-3.961735759	7.44069E-05	0.000139814	negative
PRJNA729271	stage_1D_vs_E10	ordinary_muscle_development	ENSGALG00010017649	ENSGALG00010017649	-0.836582233	0.114100357	-7.331986086	2.26767E-13	8.18268E-13	negative

PRJNA729271	stage_1D_vs_E10	ordinary_muscle_development	ENSGALG00010017651	PPCS	-0.678684591	0.124695489	-5.442735723	5.24685E-08	1.2943E-07	negative
PRJNA729271	stage_1D_vs_E10	ordinary_muscle_development	ENSGALG00010017652	C1orf115	2.103806553	0.170016778	12.37411141	3.60866E-35	3.9537E-34	positive
PRJNA729271	stage_1D_vs_E10	ordinary_muscle_development	ENSGALG00010017653	ENSGALG00010017653	1.382016968	0.172110824	8.029808576	9.76249E-16	4.10404E-15	positive
PRJNA729271	stage_1D_vs_E10	ordinary_muscle_development	ENSGALG00010017654	KBTBD13	6.897693548	0.310541704	22.21181069	2.6408E-109	2.3059E-107	positive
PRJNA729271	stage_1D_vs_E10	ordinary_muscle_development	ENSGALG00010017655	ENSGALG00010017655	-1.220264984	0.110507706	-11.04235198	2.38702E-28	1.90213E-27	negative
PRJNA729271	stage_1D_vs_E10	ordinary_muscle_development	ENSGALG00010017656	FSD2	6.700191962	0.208171879	32.18586487	2.7832E-227	2.0171E-224	positive
PRJNA729271	stage_1D_vs_E10	ordinary_muscle_development	ENSGALG00010017657	DIS3L2	0.183677475	0.093991745	1.954187298	0.050679071	0.067548794	positive
PRJNA729271	stage_1D_vs_E10	ordinary_muscle_development	ENSGALG00010017658	WHAMM	2.339103448	0.161607817	14.47394987	1.77008E-47	3.06174E-46	positive
PRJNA729271	stage_1D_vs_E10	ordinary_muscle_development	ENSGALG00010017659	TNRC6A	0.00215817	0.078428296	0.027517751	0.978046782	0.981432198	positive
PRJNA729271	stage_1D_vs_E10	ordinary_muscle_development	ENSGALG00010017660	CLIP4	2.729682196	0.2471391	11.04512478	2.31446E-28	1.84838E-27	positive
PRJNA729271	stage_1D_vs_E10	ordinary_muscle_development	ENSGALG00010017661	NHSL3	1.203884749	0.165087891	7.292386738	3.04512E-13	1.09255E-12	positive
PRJNA729271	stage_1D_vs_E10	ordinary_muscle_development	ENSGALG00010017662	GOLGA4	1.002603108	0.165201228	6.068980949	1.28724E-09	3.60413E-09	positive
PRJNA729271	stage_1D_vs_E10	ordinary_muscle_development	ENSGALG00010017663	ZMYND12	-0.538396447	0.221326513	-2.432589027	0.014991306	0.021615336	negative
PRJNA729271	stage_1D_vs_E10	ordinary_muscle_development	ENSGALG00010017664	ENSGALG00010017664	0.287899794	0.074388929	3.870196795	0.000108748	0.000201109	positive
PRJNA729271	stage_1D_vs_E10	ordinary_muscle_development	ENSGALG00010017665	CTBP1	-0.298258415	0.062978791	-4.735854913	2.18134E-06	4.74253E-06	negative
PRJNA729271	stage_1D_vs_E10	ordinary_muscle_development	ENSGALG00010017667	SLC2A1	-0.774957336	0.111398186	-6.956642336	3.48478E-12	1.16387E-11	negative
PRJNA729271	stage_1D_vs_E10	ordinary_muscle_development	ENSGALG00010017668	ENSGALG00010017668	0.388420479	0.774597041	0.501448442	0.616055555	0.65838865	positive
PRJNA729271	stage_1D_vs_E10	ordinary_muscle_development	ENSGALG00010017669	ENSGALG00010017669	0.869423839	0.174043583	4.995437491	5.87025E-07	1.33494E-06	positive
PRJNA729271	stage_1D_vs_E10	ordinary_muscle_development	ENSGALG00010017670	FAM179A	2.228436331	0.331751744	6.717180453	1.85274E-11	5.89066E-11	positive
PRJNA729271	stage_1D_vs_E10	ordinary_muscle_development	ENSGALG00010017671	ANTXR1	-0.375831768	0.184588287	-2.036054253	0.041744908	0.05635582	negative
PRJNA729271	stage_1D_vs_E10	ordinary_muscle_development	ENSGALG00010017672	SPRED2	-0.407737937	0.077512366	-5.260295358	1.43824E-07	3.43392E-07	negative
PRJNA729271	stage_1D_vs_E10	ordinary_muscle_development	ENSGALG00010017673	WDR89	-0.248729316	0.090962999	-2.734401002	0.006249388	0.009490297	negative
PRJNA729271	stage_1D_vs_E10	ordinary_muscle_development	ENSGALG00010017675	ENSGALG00010017675	0.716593323	0.472735062	1.515845513	0.12955842	0.161585726	positive
PRJNA729271	stage_1D_vs_E10	ordinary_muscle_development	ENSGALG00010017676	SPON2	3.665330452	0.262566551	13.95962446	2.74878E-44	4.20291E-43	positive
PRJNA729271	stage_1D_vs_E10	ordinary_muscle_development	ENSGALG00010017677	WDR43	-0.431895629	0.110073208	-3.923712553	8.71948E-05	0.000162767	negative
PRJNA729271	stage_1D_vs_E10	ordinary_muscle_development	ENSGALG00010017678	YARS	-0.462956654	0.137686014	-3.362408727	0.000772657	0.001304257	negative
PRJNA729271	stage_1D_vs_E10	ordinary_muscle_development	ENSGALG00010017679	RASL12	4.16392807	0.174973465	23.79748305	3.5463E-125	4.3563E-123	positive
PRJNA729271	stage_1D_vs_E10	ordinary_muscle_development	ENSGALG00010017681	ENSGALG00010017681	4.264186655	1.244951722	3.425182341	0.000614387	0.001045988	positive
PRJNA729271	stage_1D_vs_E10	ordinary_muscle_development	ENSGALG00010017684	TRMT61B	0.304615898	0.094884677	3.21038031	0.001325595	0.002181234	positive
PRJNA729271	stage_1D_vs_E10	ordinary_muscle_development	ENSGALG00010017685	PHC2	0.18728902	0.183903633	1.018408484	0.308483872	0.355754135	positive
PRJNA729271	stage_1D_vs_E10	ordinary_muscle_development	ENSGALG00010017687	TTC7B	0.81650558	0.095902323	8.513929135	1.68137E-17	7.83395E-17	positive
PRJNA729271	stage_1D_vs_E10	ordinary_muscle_development	ENSGALG00010017688	SPDYA	-1.10544077	0.568634975	-1.944025288	0.051892402	0.069026036	negative
PRJNA729271	stage_1D_vs_E10	ordinary_muscle_development	ENSGALG00010017691	LZTS1	-1.919524417	0.250331628	-7.667926058	1.748E-14	6.78191E-14	negative
PRJNA729271	stage_1D_vs_E10	ordinary_muscle_development	ENSGALG00010017692	EPCAM	-6.848265551	1.041017527	-6.578434441	4.75427E-11	1.47313E-10	negative
PRJNA729271	stage_1D_vs_E10	ordinary_muscle_development	ENSGALG00010017693	MTFMT	0.119890266	0.11668479	1.027471242	0.30419862	0.35125938	positive
PRJNA729271	stage_1D_vs_E10	ordinary_muscle_development	ENSGALG00010017694	ATP6V1B2	-0.218584787	0.070792175	-3.087697018	0.00201714	0.003245831	negative
PRJNA729271	stage_1D_vs_E10	ordinary_muscle_development	ENSGALG00010017695	FNDC5	-0.372551747	0.205431751	-1.813506162	0.069753789	0.090981839	negative
PRJNA729271	stage_1D_vs_E10	ordinary_muscle_development	ENSGALG00010017698	ENSGALG00010017698	2.900664948	0.700219351	4.142508977	3.43527E-05	6.66128E-05	positive
PRJNA729271	stage_1D_vs_E10	ordinary_muscle_development	ENSGALG00010017699	RNF19B	-0.071435532	0.246519473	-0.289776426	0.771987284	0.801285763	negative
PRJNA729271	stage_1D_vs_E10	ordinary_muscle_development	ENSGALG00010017700	VPS35L	-0.166945254	0.078703121	-2.121202466	0.033904768	0.046420101	negative
PRJNA729271	stage_1D_vs_E10	ordinary_muscle_development	ENSGALG00010017701	PPP2R5E	0.438847491	0.098456781	4.457260187	8.30138E-06	1.7097E-05	positive
PRJNA729271	stage_1D_vs_E10	ordinary_muscle_development	ENSGALG00010017703	RPS6KA5	0.834202498	0.139816093	5.96642688	2.42505E-09	6.65363E-09	positive
PRJNA729271	stage_1D_vs_E10	ordinary_muscle_development	ENSGALG00010017704	ZNF362	0.155675665	0.195251421	0.797308742	0.425271745	0.474762318	positive
PRJNA729271	stage_1D_vs_E10	ordinary_muscle_development	ENSGALG00010017705	MAK16	0.600724022	0.103226206	5.819491462	5.90269E-09	1.57307E-08	positive
PRJNA729271	stage_1D_vs_E10	ordinary_muscle_development	ENSGALG00010017706	FGFRL1	-0.007036231	0.142811241	-0.049269447	0.960704567	0.966438525	negative
PRJNA729271	stage_1D_vs_E10	ordinary_muscle_development	ENSGALG00010017707	AK2	-0.712192456	0.095705878	-7.441470374	9.95707E-14	3.68559E-13	negative
PRJNA729271	stage_1D_vs_E10	ordinary_muscle_development	ENSGALG00010017708	MEIS1	-1.749668441	0.104438537	-16.75309222	5.37579E-63	1.60004E-61	negative
PRJNA729271	stage_1D_vs_E10	ordinary_muscle_development	ENSGALG00010017710	RAB41	-0.120968183	0.115294039	-1.049214556	0.294079381	0.340714621	negative
PRJNA729271	stage_1D_vs_E10	ordinary_muscle_development	ENSGALG00010017711	LRRFIP2	0.755737868	0.134243316	5.629612629	1.80615E-08	4.62056E-08	positive
PRJNA729271	stage_1D_vs_E10	ordinary_muscle_development	ENSGALG00010017712	MSH2	-1.235835288	0.072639715	-17.01321762	6.55375E-65	2.07416E-63	negative
PRJNA729271	stage_1D_vs_E10	ordinary_muscle_development	ENSGALG00010017713	ALKBH5	1.154044237	0.14943749	7.722588461	1.13991E-14	4.4705E-14	positive
PRJNA729271	stage_1D_vs_E10	ordinary_muscle_development	ENSGALG00010017714	ATAD3A	-0.703520616	0.153422408	-4.585514097	4.52871E-06	9.53845E-06	negative
PRJNA729271	stage_1D_vs_E10	ordinary_muscle_development	ENSGALG00010017715	CILP	3.059743986	0.16854972	18.1533614	1.20778E-73	4.91765E-72	positive
PRJNA729271	stage_1D_vs_E10	ordinary_muscle_development	ENSGALG00010017717	LLGL1	-0.623348521	0.150164451	-4.151105778	3.30873E-05	6.42207E-05	negative
PRJNA729271	stage_1D_vs_E10	ordinary_muscle_development	ENSGALG00010017718	ATOH8	-0.265272437	0.299653436	-0.885264125	0.376014194	0.425241924	negative
PRJNA729271	stage_1D_vs_E10	ordinary_muscle_development	ENSGALG00010017719	ENSGALG00010017719	3.680565926	1.770184424	2.079199137	0.037599051	0.051106362	positive
PRJNA729271	stage_1D_vs_E10	ordinary_muscle_development	ENSGALG00010017720	ENSGALG00010017720	-1.779128195	0.348984394	-5.098016491	3.43231E-07	7.93735E-07	negative
PRJNA729271	stage_1D_vs_E10	ordinary_muscle_development	ENSGALG00010017721	VWA1	1.862911446	0.119744028	15.55744768	1.41635E-54	3.18789E-53	positive
PRJNA729271	stage_1D_vs_E10	ordinary_muscle_development	ENSGALG00010017723	KCNK12	-1.973318284	0.366102139	-5.390075811	7.0428E-08	1.72209E-07	negative
PRJNA729271	stage_1D_vs_E10	ordinary_muscle_development	ENSGALG00010017724	FLII	0.998082404	0.127932801	7.801614581	6.11201E-15	2.43858E-14	positive
PRJNA729271	stage_1D_vs_E10	ordinary_muscle_development	ENSGALG00010017725	ENSGALG00010017725	1.079296279	0.394286016	2.737343541	0.006193757	0.009414693	positive
PRJNA729271	stage_1D_vs_E10	ordinary_muscle_development	ENSGALG00010017726	TTI2	0.40928399	0.119543515	3.423723916	0.000617693	0.001051493	positive
PRJNA729271	stage_1D_vs_E10	ordinary_muscle_development	ENSGALG00010017727	AZIN2	0.607325155	0.150227717	4.04269708	5.28399E-05	0.000100685	positive
PRJNA729271	stage_1D_vs_E10	ordinary_muscle_development	ENSGALG00010017729	MIEF2	1.758770263	0.139608366	12.59788584	2.1688E-36	2.4812E-35	positive
PRJNA729271	stage_1D_vs_E10	ordinary_muscle_development	ENSGALG00010017730	TARP	3.427784516	0.555064258	6.175473324	6.59655E-10	1.88705E-09	positive
PRJNA729271	stage_1D_vs_E10	ordinary_muscle_development	ENSGALG00010017732	RHOJ	-0.035961693	0.077296279	-0.465244813	0.641756151	0.682133564	negative
PRJNA729271	stage_1D_vs_E10	ordinary_muscle_development	ENSGALG00010017733	TRIM62	-0.452604826	0.132135519	-3.425307814	0.000614103	0.001045645	negative
PRJNA729271	stage_1D_vs_E10	ordinary_muscle_development	ENSGALG00010017734	TOP3A	-0.316068775	0.116462283	-2.713915321	0.006649316	0.010057367	negative
PRJNA729271	stage_1D_vs_E10	ordinary_muscle_development	ENSGALG00010017736	ENSGALG00010017736	5.768955702	4.323770137	1.334241997	0.18212456	0.220893272	positive
PRJNA729271	stage_1D_vs_E10	ordinary_muscle_development	ENSGALG00010017737	ENSGALG00010017737	-0.715366899	0.201370723	-3.552487112	0.000381608	0.000665873	negative
PRJNA729271	stage_1D_vs_E10	ordinary_muscle_development	ENSGALG00010017738	NDUFS5	0.496460846	0.111853761	4.438481472	9.05958E-06	1.85925E-05	positive
PRJNA729271	stage_1D_vs_E10	ordinary_muscle_development	ENSGALG00010017739	MRPL20	-0.444434404	0.099383053	-4.471933521	7.75155E-06	1.60101E-05	negative
PRJNA729271	stage_1D_vs_E10	ordinary_muscle_development	ENSGALG00010017741	SMCR8	0.837872445	0.138859881	6.033941843	1.60008E-09	4.4508E-09	positive
PRJNA729271	stage_1D_vs_E10	ordinary_muscle_development	ENSGALG00010017742	DGLUCY	0.94802088	0.238360261	3.97726062	6.97137E-05	0.000131336	positive
PRJNA729271	stage_1D_vs_E10	ordinary_muscle_development	ENSGALG00010017743	KCNH5	-2.092801703	0.329508041	-6.351291749	2.13514E-10	6.32772E-10	negative
PRJNA729271	stage_1D_vs_E10	ordinary_muscle_development	ENSGALG00010017744	ETAA1	-0.568146025	0.098592573	-5.762564116	8.28456E-09	2.17939E-08	negative
PRJNA729271	stage_1D_vs_E10	ordinary_muscle_development	ENSGALG00010017745	SHMT1	0.004590781	0.184192778	0.024923786	0.980115755	0.983031959	positive
PRJNA729271	stage_1D_vs_E10	ordinary_muscle_development	ENSGALG00010017746	MSH6	-1.105782277	0.08950035	-12.35506098	4.57409E-35	4.97385E-34	negative
PRJNA729271	stage_1D_vs_E10	ordinary_muscle_development	ENSGALG00010017747	DUSP26	2.94343759	0.117674718	25.01333873	4.3771E-138	6.4741E-136	positive
PRJNA729271	stage_1D_vs_E10	ordinary_muscle_development	ENSGALG00010017748	ENSGALG00010017748	-1.681938874	0.486309286	-3.458578564	0.000543034	0.000932174	negative
PRJNA729271	stage_1D_vs_E10	ordinary_muscle_development	ENSGALG00010017751	ST3GAL5	-0.148976915	0.126233437	-1.180169997	0.237932609	0.281652362	negative
PRJNA729271	stage_1D_vs_E10	ordinary_muscle_development	ENSGALG00010017753	GNRHR	1.581712115	0.320472437	4.935563669	7.99196E-07	1.79881E-06	positive
PRJNA729271	stage_1D_vs_E10	ordinary_muscle_development	ENSGALG00010017754	PDZD11	-0.735616241	0.159516166	-4.611546646	3.99684E-06	8.46249E-06	negative
PRJNA729271	stage_1D_vs_E10	ordinary_muscle_development	ENSGALG00010017755	C1D	-0.75995331	0.139910159	-5.431723584	5.58123E-08	1.37515E-07	negative
PRJNA729271	stage_1D_vs_E10	ordinary_muscle_development	ENSGALG00010017756	EIF2B5	-0.054905359	0.118428576	-0.463615797	0.642923034	0.683173476	negative
PRJNA729271	stage_1D_vs_E10	ordinary_muscle_development	ENSGALG00010017757	ENSGALG00010017757	0.998860935	0.138538537	7.209986185	5.59576E-13	1.97349E-12	positive
PRJNA729271	stage_1D_vs_E10	ordinary_muscle_development	ENSGALG00010017759	ENSGALG00010017759	9.930869629	0.835515548	11.8859184	1.40087E-32	1.37107E-31	positive
PRJNA729271	stage_1D_vs_E10	ordinary_muscle_development	ENSGALG00010017761	PARP16	1.350855365	0.105553695	12.79780271	1.68656E-37	2.01705E-36	positive
PRJNA729271	stage_1D_vs_E10	ordinary_muscle_development	ENSGALG00010017762	FBXO11	-0.114125515	0.055247491	-2.065713999	0.038855493	0.052670941	negative
PRJNA729271	stage_1D_vs_E10	ordinary_muscle_development	ENSGALG00010017763	POLR1A	-0.105828074	0.086035513	-1.230051069	0.218677982	0.260969648	negative
PRJNA729271	stage_1D_vs_E10	ordinary_muscle_development	ENSGALG00010017764	IGDCC3	-1.727469791	0.237927895	-7.260476096	3.8573E-13	1.37544E-12	negative
PRJNA729271	stage_1D_vs_E10	ordinary_muscle_development	ENSGALG00010017765	SYT16	-3.704181384	0.308823068	-11.99451001	3.79657E-33	3.81103E-32	negative
PRJNA729271	stage_1D_vs_E10	ordinary_muscle_development	ENSGALG00010017766	DNAAF10	0.404186939	0.15389099	2.626449671	0.008628075	0.01285608	positive
PRJNA729271	stage_1D_vs_E10	ordinary_muscle_development	ENSGALG00010017767	KIF4B	-2.197251818	0.220819318	-9.950451047	2.51043E-23	1.58418E-22	negative
PRJNA729271	stage_1D_vs_E10	ordinary_muscle_development	ENSGALG00010017768	FOXN2	-0.191430554	0.08327548	-2.298762544	0.021518427	0.030332549	negative
PRJNA729271	stage_1D_vs_E10	ordinary_muscle_development	ENSGALG00010017770	PTCD3	0.021278272	0.069610514	0.305676124	0.759851254	0.790663452	positive
PRJNA729271	stage_1D_vs_E10	ordinary_muscle_development	ENSGALG00010017771	PNO1	-0.226003456	0.158350658	-1.427234079	0.153512435	0.188845179	negative
PRJNA729271	stage_1D_vs_E10	ordinary_muscle_development	ENSGALG00010017772	ENSGALG00010017772	1.516367554	0.360167033	4.210178651	2.55169E-05	5.007E-05	positive
PRJNA729271	stage_1D_vs_E10	ordinary_muscle_development	ENSGALG00010017773	DVL3	-0.972242789	0.080945235	-12.0111182	3.10616E-33	3.13536E-32	negative
PRJNA729271	stage_1D_vs_E10	ordinary_muscle_development	ENSGALG00010017774	ASH2L	0.390921665	0.163148748	2.396105818	0.016570306	0.023722132	positive
PRJNA729271	stage_1D_vs_E10	ordinary_muscle_development	ENSGALG00010017775	PRPSAP2	-1.144714301	0.086124628	-13.2913701	2.59777E-40	3.43252E-39	negative
PRJNA729271	stage_1D_vs_E10	ordinary_muscle_development	ENSGALG00010017776	GPR68	-0.439349478	0.367993896	-1.193904254	0.232515423	0.275870595	negative
PRJNA729271	stage_1D_vs_E10	ordinary_muscle_development	ENSGALG00010017778	PPP3R1	0.479715508	0.078703838	6.095198432	1.09302E-09	3.07876E-09	positive
PRJNA729271	stage_1D_vs_E10	ordinary_muscle_development	ENSGALG00010017779	SNAPC1	-0.305542711	0.124884355	-2.44660518	0.014420871	0.02084261	negative
PRJNA729271	stage_1D_vs_E10	ordinary_muscle_development	ENSGALG00010017780	TAF9	-0.02420772	0.13738535	-0.176203068	0.860134411	0.880483636	negative
PRJNA729271	stage_1D_vs_E10	ordinary_muscle_development	ENSGALG00010017781	CYP26C1	1.314800921	0.297291384	4.422600162	9.75201E-06	1.99373E-05	positive
PRJNA729271	stage_1D_vs_E10	ordinary_muscle_development	ENSGALG00010017782	IGDCC4	0.319612525	0.114099606	2.801171152	0.005091751	0.007822462	positive
PRJNA729271	stage_1D_vs_E10	ordinary_muscle_development	ENSGALG00010017783	STAR	0.125988934	0.394252399	0.319564154	0.749298752	0.781429269	positive
PRJNA729271	stage_1D_vs_E10	ordinary_muscle_development	ENSGALG00010017786	KAZALD1	-1.082669487	0.16608441	-6.51879058	7.08765E-11	2.16559E-10	negative
PRJNA729271	stage_1D_vs_E10	ordinary_muscle_development	ENSGALG00010017787	CNRIP1	0.540934795	0.241327338	2.241498205	0.024993822	0.034878737	positive
PRJNA729271	stage_1D_vs_E10	ordinary_muscle_development	ENSGALG00010017789	IMMT	0.930717599	0.130154344	7.150876174	8.62257E-13	2.99866E-12	positive
PRJNA729271	stage_1D_vs_E10	ordinary_muscle_development	ENSGALG00010017790	R3HCC1L	-0.137646281	0.177705381	-0.774575759	0.438590386	0.487845894	negative
PRJNA729271	stage_1D_vs_E10	ordinary_muscle_development	ENSGALG00010017791	CCNL2	0.490662278	0.111217736	4.41172692	1.02549E-05	2.09095E-05	positive
PRJNA729271	stage_1D_vs_E10	ordinary_muscle_development	ENSGALG00010017792	LSM1	-0.477526371	0.125201481	-3.814063291	0.0001367	0.000250249	negative
PRJNA729271	stage_1D_vs_E10	ordinary_muscle_development	ENSGALG00010017793	FAM83G	-4.883745608	0.518302165	-9.422583844	4.40122E-21	2.49592E-20	negative
PRJNA729271	stage_1D_vs_E10	ordinary_muscle_development	ENSGALG00010017795	MACF1	-0.080635241	0.114162903	-0.706317367	0.479990795	0.528562378	negative
PRJNA729271	stage_1D_vs_E10	ordinary_muscle_development	ENSGALG00010017796	MXI1	1.895661542	0.131760008	14.38722997	6.22392E-47	1.05269E-45	positive
PRJNA729271	stage_1D_vs_E10	ordinary_muscle_development	ENSGALG00010017797	PPP1R21	-0.462090497	0.092258994	-5.008622777	5.48209E-07	1.24942E-06	negative
PRJNA729271	stage_1D_vs_E10	ordinary_muscle_development	ENSGALG00010017798	BAG4	0.175885504	0.15788193	1.114031888	0.265265507	0.310458096	positive
PRJNA729271	stage_1D_vs_E10	ordinary_muscle_development	ENSGALG00010017799	GRAPL	-0.051136217	0.35528889	-0.143928557	0.885556885	0.902555692	negative
PRJNA729271	stage_1D_vs_E10	ordinary_muscle_development	ENSGALG00010017801	SMNDC1	-0.227864179	0.071358432	-3.193234099	0.001406888	0.002308055	negative
PRJNA729271	stage_1D_vs_E10	ordinary_muscle_development	ENSGALG00010017803	ENSGALG00010017803	1.167008355	0.117852227	9.902302071	4.06801E-23	2.54712E-22	positive
PRJNA729271	stage_1D_vs_E10	ordinary_muscle_development	ENSGALG00010017804	DUSP5	0.308173577	0.240613068	1.280784873	0.200269239	0.240525529	positive
PRJNA729271	stage_1D_vs_E10	ordinary_muscle_development	ENSGALG00010017805	DPP8	-0.317304434	0.10750704	-2.951475852	0.003162593	0.004972973	negative
PRJNA729271	stage_1D_vs_E10	ordinary_muscle_development	ENSGALG00010017806	ENSGALG00010017806	2.578389625	0.752747842	3.425303243	0.000614114	0.001045645	positive
PRJNA729271	stage_1D_vs_E10	ordinary_muscle_development	ENSGALG00010017807	MRPL35	-0.108306542	0.147153499	-0.736010649	0.461724217	0.510502862	negative
PRJNA729271	stage_1D_vs_E10	ordinary_muscle_development	ENSGALG00010017808	SFRP5	3.713850561	0.313074283	11.86252198	1.85308E-32	1.8015E-31	positive
PRJNA729271	stage_1D_vs_E10	ordinary_muscle_development	ENSGALG00010017809	MOSMO	0.161198043	0.099246091	1.624225624	0.104327664	0.132257258	positive
PRJNA729271	stage_1D_vs_E10	ordinary_muscle_development	ENSGALG00010017810	HIF1A	-0.613978876	0.078462843	-7.825090903	5.0729E-15	2.03576E-14	negative
PRJNA729271	stage_1D_vs_E10	ordinary_muscle_development	ENSGALG00010017811	HACD3	-0.773879823	0.065920572	-11.7395799	7.98823E-32	7.55805E-31	negative
PRJNA729271	stage_1D_vs_E10	ordinary_muscle_development	ENSGALG00010017812	ENSGALG00010017812	4.038491828	1.736204199	2.326046574	0.020016066	0.028313934	positive

PRJNA729271	stage_1D_vs_E10	ordinary_muscle_development	ENSGALG00010017813	DDHD2	0.049813622	0.160457454	0.310447538	0.756220649	0.787514786	positive
PRJNA729271	stage_1D_vs_E10	ordinary_muscle_development	ENSGALG00010017814	ENSGALG00010017814	-2.380370616	0.252555894	-9.42512398	4.29598E-21	2.43814E-20	negative
PRJNA729271	stage_1D_vs_E10	ordinary_muscle_development	ENSGALG00010017815	VWA9	-0.768968671	0.08546952	-8.996992982	2.31984E-19	1.19666E-18	negative
PRJNA729271	stage_1D_vs_E10	ordinary_muscle_development	ENSGALG00010017816	PRKCH	-0.825090864	0.293894194	-2.807441868	0.004993669	0.007680715	negative
PRJNA729271	stage_1D_vs_E10	ordinary_muscle_development	ENSGALG00010017817	REEP1	2.501038293	0.116483496	21.47118165	2.8956E-102	2.18604E-100	positive
PRJNA729271	stage_1D_vs_E10	ordinary_muscle_development	ENSGALG00010017818	SLC38A6	-0.095201517	0.136973534	-0.695035854	0.487032833	0.535543992	negative
PRJNA729271	stage_1D_vs_E10	ordinary_muscle_development	ENSGALG00010017820	FLRT2	-0.040960664	0.134763441	-0.303944929	0.761169847	0.791815483	negative
PRJNA729271	stage_1D_vs_E10	ordinary_muscle_development	ENSGALG00010017821	ENSGALG00010017821	-5.230254935	0.48212156	-10.84841535	2.02913E-27	1.54395E-26	negative
PRJNA729271	stage_1D_vs_E10	ordinary_muscle_development	ENSGALG00010017822	VWA3A	-0.591054378	0.262895158	-2.248251289	0.024560171	0.034326456	negative
PRJNA729271	stage_1D_vs_E10	ordinary_muscle_development	ENSGALG00010017823	PPIE	-0.326675662	0.089947707	-3.63183979	0.000281408	0.0004975	negative
PRJNA729271	stage_1D_vs_E10	ordinary_muscle_development	ENSGALG00010017824	YAE1	-0.08711597	0.123537492	-0.70517839	0.480699228	0.529181689	negative
PRJNA729271	stage_1D_vs_E10	ordinary_muscle_development	ENSGALG00010017825	PLPP5	-1.183699594	0.146135591	-8.100008965	5.49551E-16	2.35186E-15	negative
PRJNA729271	stage_1D_vs_E10	ordinary_muscle_development	ENSGALG00010017826	PABPC4	3.83785133	0.139832474	27.44606609	7.7409E-166	1.9018E-163	positive
PRJNA729271	stage_1D_vs_E10	ordinary_muscle_development	ENSGALG00010017828	ENSGALG00010017828	0.347363091	0.236594042	1.468181908	0.142054817	0.175824829	positive
PRJNA729271	stage_1D_vs_E10	ordinary_muscle_development	ENSGALG00010017830	DENND4A	0.761016727	0.127776118	5.955860467	2.58707E-09	7.09279E-09	positive
PRJNA729271	stage_1D_vs_E10	ordinary_muscle_development	ENSGALG00010017831	PLEK	0.207934471	0.186953974	1.112222793	0.26604237	0.311266782	positive
PRJNA729271	stage_1D_vs_E10	ordinary_muscle_development	ENSGALG00010017832	POU3F1	-0.092086685	0.401093818	-0.229588892	0.818411239	0.843731928	negative
PRJNA729271	stage_1D_vs_E10	ordinary_muscle_development	ENSGALG00010017834	ENSGALG00010017834	-0.218775078	0.332994544	-0.65699298	0.51118543	0.559048801	negative
PRJNA729271	stage_1D_vs_E10	ordinary_muscle_development	ENSGALG00010017838	B3GNT2	-0.810713161	0.125430492	-6.463445577	1.02345E-10	3.09578E-10	negative
PRJNA729271	stage_1D_vs_E10	ordinary_muscle_development	ENSGALG00010017841	GALC	-1.257573525	0.267601549	-4.69942543	2.60894E-06	5.63218E-06	negative
PRJNA729271	stage_1D_vs_E10	ordinary_muscle_development	ENSGALG00010017843	NSD3	0.172145693	0.070852775	2.429625283	0.01511444	0.021784211	positive
PRJNA729271	stage_1D_vs_E10	ordinary_muscle_development	ENSGALG00010017848	CYP3A4	0.939851397	0.196540059	4.781983907	1.73574E-06	3.79938E-06	positive
PRJNA729271	stage_1D_vs_E10	ordinary_muscle_development	ENSGALG00010017849	FBXO48	1.792123551	0.178034772	10.06614343	7.79759E-24	5.03681E-23	positive
PRJNA729271	stage_1D_vs_E10	ordinary_muscle_development	ENSGALG00010017850	TRMT5	-0.633795458	0.091196715	-6.949761923	3.65903E-12	1.21982E-11	negative
PRJNA729271	stage_1D_vs_E10	ordinary_muscle_development	ENSGALG00010017852	LETM2	0.445322456	0.113382078	3.927626507	8.57883E-05	0.000160266	positive
PRJNA729271	stage_1D_vs_E10	ordinary_muscle_development	ENSGALG00010017855	SDR42E2	-0.349215426	0.28153902	-1.2403802	0.214834801	0.256700226	negative
PRJNA729271	stage_1D_vs_E10	ordinary_muscle_development	ENSGALG00010017856	FASTKD5	0.36884723	0.114846751	3.211647052	0.001319764	0.00217238	positive
PRJNA729271	stage_1D_vs_E10	ordinary_muscle_development	ENSGALG00010017858	GPR65	1.158732867	0.288453529	4.017052151	5.89307E-05	0.000111924	positive
PRJNA729271	stage_1D_vs_E10	ordinary_muscle_development	ENSGALG00010017859	EXOC6	1.780307241	0.133799608	13.30577321	2.14265E-40	2.84151E-39	positive
PRJNA729271	stage_1D_vs_E10	ordinary_muscle_development	ENSGALG00010017860	CYSLTR1	-0.394766079	0.207761484	-1.900092703	0.057420955	0.075782277	negative
PRJNA729271	stage_1D_vs_E10	ordinary_muscle_development	ENSGALG00010017861	PRSS56	0.222769532	0.51540811	0.432219687	0.665581752	0.704513473	positive
PRJNA729271	stage_1D_vs_E10	ordinary_muscle_development	ENSGALG00010017865	UBOX5	-0.015925026	0.135548458	-0.117485852	0.906475055	0.920316307	negative
PRJNA729271	stage_1D_vs_E10	ordinary_muscle_development	ENSGALG00010017866	RALA	-0.161323062	0.094883758	-1.70021788	0.08908995	0.114228999	negative
PRJNA729271	stage_1D_vs_E10	ordinary_muscle_development	ENSGALG00010017867	APLF	0.701485763	0.109347164	6.415216817	1.40623E-10	4.21926E-10	positive
PRJNA729271	stage_1D_vs_E10	ordinary_muscle_development	ENSGALG00010017868	CHRND	1.282735627	0.240904458	5.324665372	1.01139E-07	2.44213E-07	positive
PRJNA729271	stage_1D_vs_E10	ordinary_muscle_development	ENSGALG00010017870	RAB11A	0.134906536	0.073732183	1.829683207	0.067297323	0.087951916	positive
PRJNA729271	stage_1D_vs_E10	ordinary_muscle_development	ENSGALG00010017873	HSPG2	2.281378904	0.109589281	20.81753696	3.00226E-96	2.08219E-94	positive
PRJNA729271	stage_1D_vs_E10	ordinary_muscle_development	ENSGALG00010017876	COMMD1	-0.329476183	0.13726458	-2.400300081	0.016381636	0.023475217	negative
PRJNA729271	stage_1D_vs_E10	ordinary_muscle_development	ENSGALG00010017878	CHRNG	0.39459971	0.248495578	1.587954656	0.112296593	0.141579466	positive
PRJNA729271	stage_1D_vs_E10	ordinary_muscle_development	ENSGALG00010017880	CDK13	0.372202739	0.084009684	4.430474197	9.40261E-06	1.92664E-05	positive
PRJNA729271	stage_1D_vs_E10	ordinary_muscle_development	ENSGALG00010017881	KCNK10	-1.721626874	0.488486268	-3.524412019	0.000424424	0.000736064	negative
PRJNA729271	stage_1D_vs_E10	ordinary_muscle_development	ENSGALG00010017882	LZTS3	2.102941531	0.199277598	10.55282456	4.92926E-26	3.53886E-25	positive
PRJNA729271	stage_1D_vs_E10	ordinary_muscle_development	ENSGALG00010017886	DDRGK1	-0.08875134	0.102235113	-0.868110151	0.385334035	0.434358569	negative
PRJNA729271	stage_1D_vs_E10	ordinary_muscle_development	ENSGALG00010017887	PROKR1	6.403797307	0.387466698	16.52734892	2.33176E-61	6.71946E-60	positive
PRJNA729271	stage_1D_vs_E10	ordinary_muscle_development	ENSGALG00010017888	MPLKIP	-0.26206871	0.109093773	-2.402233431	0.016295306	0.023358432	negative
PRJNA729271	stage_1D_vs_E10	ordinary_muscle_development	ENSGALG00010017890	ANAPC1	-0.478397211	0.065150821	-7.342919156	2.08985E-13	7.56552E-13	negative
PRJNA729271	stage_1D_vs_E10	ordinary_muscle_development	ENSGALG00010017891	GOLGA7B	3.968157938	0.32466489	12.22231926	2.36247E-34	2.48686E-33	positive
PRJNA729271	stage_1D_vs_E10	ordinary_muscle_development	ENSGALG00010017894	KAT6A	0.580437802	0.080760593	7.187141439	6.61619E-13	2.32103E-12	positive
PRJNA729271	stage_1D_vs_E10	ordinary_muscle_development	ENSGALG00010017895	MNAT1	0.082638351	0.08122446	1.017407208	0.308959755	0.356217916	positive
PRJNA729271	stage_1D_vs_E10	ordinary_muscle_development	ENSGALG00010017898	NHERF2	0.924442503	0.11445184	8.077130966	6.63083E-16	2.81859E-15	positive
PRJNA729271	stage_1D_vs_E10	ordinary_muscle_development	ENSGALG00010017901	HTR7L	-3.100764553	0.781574813	-3.967329171	7.26826E-05	0.000136681	negative
PRJNA729271	stage_1D_vs_E10	ordinary_muscle_development	ENSGALG00010017903	ENSGALG00010017903	-3.248161933	0.404789975	-8.024314164	1.02095E-15	4.28821E-15	negative
PRJNA729271	stage_1D_vs_E10	ordinary_muscle_development	ENSGALG00010017904	MEGF11	-2.580057749	0.286545907	-9.003994433	2.17651E-19	1.12513E-18	negative
PRJNA729271	stage_1D_vs_E10	ordinary_muscle_development	ENSGALG00010017905	EIF4E2	-0.530331705	0.087837661	-6.037634627	1.5639E-09	4.35517E-09	negative
PRJNA729271	stage_1D_vs_E10	ordinary_muscle_development	ENSGALG00010017909	NTHL1	-0.500186198	0.137540149	-3.636655942	0.0002762	0.000488533	negative
PRJNA729271	stage_1D_vs_E10	ordinary_muscle_development	ENSGALG00010017915	BFSP1	2.917980122	0.433471932	6.731647214	1.67753E-11	5.35119E-11	positive
PRJNA729271	stage_1D_vs_E10	ordinary_muscle_development	ENSGALG00010017918	SIX4	0.952520172	0.200453312	4.751830543	2.01583E-06	4.39391E-06	positive
PRJNA729271	stage_1D_vs_E10	ordinary_muscle_development	ENSGALG00010017920	AP3M2	-1.075360827	0.08185425	-13.13750758	2.0073E-39	2.5863E-38	negative
PRJNA729271	stage_1D_vs_E10	ordinary_muscle_development	ENSGALG00010017921	SPATA7	2.255947482	0.196196746	11.49839394	1.34393E-30	1.19878E-29	positive
PRJNA729271	stage_1D_vs_E10	ordinary_muscle_development	ENSGALG00010017922	MERTK	0.359134843	0.131044363	2.740559267	0.006133472	0.009325991	positive
PRJNA729271	stage_1D_vs_E10	ordinary_muscle_development	ENSGALG00010017927	PCSK2	-0.185426077	0.127333541	-1.456223359	0.145330884	0.1797109	negative
PRJNA729271	stage_1D_vs_E10	ordinary_muscle_development	ENSGALG00010017930	TSC2	0.009456242	0.077283166	0.122358365	0.902615211	0.916847055	positive
PRJNA729271	stage_1D_vs_E10	ordinary_muscle_development	ENSGALG00010017933	SIX1	0.110720805	0.196088446	0.564647267	0.572313709	0.617218676	positive
PRJNA729271	stage_1D_vs_E10	ordinary_muscle_development	ENSGALG00010017937	PTPN21	3.288669095	0.118745316	27.69514805	7.9878E-169	2.0676E-166	positive
PRJNA729271	stage_1D_vs_E10	ordinary_muscle_development	ENSGALG00010017941	DIS3L	0.556397483	0.136004845	4.091012233	4.29494E-05	8.25141E-05	positive
PRJNA729271	stage_1D_vs_E10	ordinary_muscle_development	ENSGALG00010017946	PTGDR	-0.126041632	0.260656474	-0.483554581	0.628702015	0.670470549	negative
PRJNA729271	stage_1D_vs_E10	ordinary_muscle_development	ENSGALG00010017948	PLAT	1.16955804	0.157263129	7.436950099	1.03036E-13	3.81096E-13	positive
PRJNA729271	stage_1D_vs_E10	ordinary_muscle_development	ENSGALG00010017949	SFXN3	0.666846689	0.13875401	4.805963375	1.54008E-06	3.38645E-06	positive
PRJNA729271	stage_1D_vs_E10	ordinary_muscle_development	ENSGALG00010017950	FBLN7	-2.303562694	0.321503278	-7.164974216	7.78012E-13	2.7148E-12	negative
PRJNA729271	stage_1D_vs_E10	ordinary_muscle_development	ENSGALG00010017954	PTGER2	1.013308221	0.258201535	3.92448566	8.69152E-05	0.000162266	positive
PRJNA729271	stage_1D_vs_E10	ordinary_muscle_development	ENSGALG00010017955	IKBKB	0.202405414	0.063793506	3.172821625	0.001509652	0.002465902	positive
PRJNA729271	stage_1D_vs_E10	ordinary_muscle_development	ENSGALG00010017956	TIPIN	-0.566031918	0.080099444	-7.066614853	1.58759E-12	5.41333E-12	negative
PRJNA729271	stage_1D_vs_E10	ordinary_muscle_development	ENSGALG00010017957	THUMPD1	-0.733845153	0.099586351	-7.368933064	1.71999E-13	6.26256E-13	negative
PRJNA729271	stage_1D_vs_E10	ordinary_muscle_development	ENSGALG00010017958	CAPN10	-1.610545886	0.095035879	-16.94671434	2.03514E-64	6.35762E-63	negative
PRJNA729271	stage_1D_vs_E10	ordinary_muscle_development	ENSGALG00010017959	PDZD7	0.406146584	0.290367136	1.398734683	0.16189256	0.198329332	positive
PRJNA729271	stage_1D_vs_E10	ordinary_muscle_development	ENSGALG00010017962	TXNDC16	-0.693222473	0.150754037	-4.598367551	4.25814E-06	8.98294E-06	negative
PRJNA729271	stage_1D_vs_E10	ordinary_muscle_development	ENSGALG00010017963	LPAR4	-1.660338364	0.16974551	-9.78133895	1.35407E-22	8.30608E-22	negative
PRJNA729271	stage_1D_vs_E10	ordinary_muscle_development	ENSGALG00010017970	AMPH	0.534626158	0.713744663	0.749044001	0.453830685	0.50277253	positive
PRJNA729271	stage_1D_vs_E10	ordinary_muscle_development	ENSGALG00010017972	RNPEPL1	1.486116082	0.171108339	8.685234691	3.77961E-18	1.82557E-17	positive
PRJNA729271	stage_1D_vs_E10	ordinary_muscle_development	ENSGALG00010017973	PKD1	1.618216217	0.128994419	12.54485452	4.24193E-36	4.80365E-35	positive
PRJNA729271	stage_1D_vs_E10	ordinary_muscle_development	ENSGALG00010017974	MXRA8	-0.163971213	0.184078154	-0.890769544	0.373052817	0.422189303	negative
PRJNA729271	stage_1D_vs_E10	ordinary_muscle_development	ENSGALG00010017976	GPR137C	-0.837117073	0.106337695	-7.872251414	3.48315E-15	1.41503E-14	negative
PRJNA729271	stage_1D_vs_E10	ordinary_muscle_development	ENSGALG00010017979	ENSGALG00010017979	-0.671693986	0.167763859	-4.003806238	6.23314E-05	0.000118135	negative
PRJNA729271	stage_1D_vs_E10	ordinary_muscle_development	ENSGALG00010017983	SLC4A11	1.805949357	0.202507127	8.91795454	4.74979E-19	2.40476E-18	positive
PRJNA729271	stage_1D_vs_E10	ordinary_muscle_development	ENSGALG00010017985	ABCC5	2.229668689	0.267357187	8.339662427	7.45029E-17	3.35275E-16	positive
PRJNA729271	stage_1D_vs_E10	ordinary_muscle_development	ENSGALG00010017987	ERI2	-1.182919763	0.218024404	-5.425630065	5.77505E-08	1.42049E-07	negative
PRJNA729271	stage_1D_vs_E10	ordinary_muscle_development	ENSGALG00010017991	RAB26	-1.055440727	0.245499772	-4.299151561	1.71453E-05	3.41657E-05	negative
PRJNA729271	stage_1D_vs_E10	ordinary_muscle_development	ENSGALG00010017992	ENSGALG00010017992	1.292262832	0.659228863	1.960264349	0.049964902	0.066664327	positive
PRJNA729271	stage_1D_vs_E10	ordinary_muscle_development	ENSGALG00010017993	REXO5	-0.667642255	0.092287423	-7.234379616	4.67662E-13	1.66065E-12	negative
PRJNA729271	stage_1D_vs_E10	ordinary_muscle_development	ENSGALG00010017996	ZC3H14	-0.371970451	0.092335268	-4.028476455	5.61395E-05	0.000106846	negative
PRJNA729271	stage_1D_vs_E10	ordinary_muscle_development	ENSGALG00010017997	ENSGALG00010017997	-0.360351707	0.346556572	-1.039806302	0.298429901	0.345313436	negative
PRJNA729271	stage_1D_vs_E10	ordinary_muscle_development	ENSGALG00010017999	GPR174	-0.042138055	0.719148749	-0.058594352	0.95327521	0.960631547	negative
PRJNA729271	stage_1D_vs_E10	ordinary_muscle_development	ENSGALG00010018000	MAP2K1	-0.099149526	0.073302481	-1.352608055	0.176180878	0.214276038	negative
PRJNA729271	stage_1D_vs_E10	ordinary_muscle_development	ENSGALG00010018001	TRAF7	0.152999245	0.084728339	1.805762351	0.070955469	0.09243663	positive
PRJNA729271	stage_1D_vs_E10	ordinary_muscle_development	ENSGALG00010018002	ZC3H8	0.56219644	0.078841548	7.130712838	9.98504E-13	3.45425E-12	positive
PRJNA729271	stage_1D_vs_E10	ordinary_muscle_development	ENSGALG00010018004	CDC42BPA	0.700259581	0.061664059	11.35604094	6.92106E-30	5.94317E-29	positive
PRJNA729271	stage_1D_vs_E10	ordinary_muscle_development	ENSGALG00010018006	DCUN1D3	0.00774013	0.102786717	0.075302823	0.939973776	0.948876654	positive
PRJNA729271	stage_1D_vs_E10	ordinary_muscle_development	ENSGALG00010018007	DNAAF9	0.139140458	0.179478076	0.775250445	0.438191685	0.487458824	positive
PRJNA729271	stage_1D_vs_E10	ordinary_muscle_development	ENSGALG00010018008	ABCF3	0.808914281	0.132200603	6.118839593	9.42592E-10	2.67166E-09	positive
PRJNA729271	stage_1D_vs_E10	ordinary_muscle_development	ENSGALG00010018010	ENSGALG00010018010	-1.120784082	0.119979076	-9.341496202	9.49823E-21	5.30138E-20	negative
PRJNA729271	stage_1D_vs_E10	ordinary_muscle_development	ENSGALG00010018011	LIPA	-0.775387247	0.186388674	-4.160055589	3.1817E-05	6.19044E-05	negative
PRJNA729271	stage_1D_vs_E10	ordinary_muscle_development	ENSGALG00010018012	DVL1	1.26368472	0.137959102	9.159850299	5.19694E-20	2.78483E-19	positive
PRJNA729271	stage_1D_vs_E10	ordinary_muscle_development	ENSGALG00010018014	LYRM1	0.251097246	0.09514811	2.639014552	0.008314741	0.012419845	positive
PRJNA729271	stage_1D_vs_E10	ordinary_muscle_development	ENSGALG00010018016	IFIT5	4.947687025	0.857561482	5.769483741	7.95147E-09	2.09748E-08	positive
PRJNA729271	stage_1D_vs_E10	ordinary_muscle_development	ENSGALG00010018019	NAPB	0.497206874	0.353104512	1.408101163	0.159101117	0.195188763	positive
PRJNA729271	stage_1D_vs_E10	ordinary_muscle_development	ENSGALG00010018020	SNAPC5	-0.976721159	0.104276942	-9.366607206	7.49015E-21	4.20162E-20	negative
PRJNA729271	stage_1D_vs_E10	ordinary_muscle_development	ENSGALG00010018021	ATRN	1.463888027	0.070601966	20.73438059	1.69625E-95	1.14359E-93	positive
PRJNA729271	stage_1D_vs_E10	ordinary_muscle_development	ENSGALG00010018022	XPO6	-0.427985454	0.116200671	-3.6831582	0.000230362	0.000411017	negative
PRJNA729271	stage_1D_vs_E10	ordinary_muscle_development	ENSGALG00010018025	CPTP	0.171988294	0.131635941	1.306545103	0.191367251	0.231040172	positive
PRJNA729271	stage_1D_vs_E10	ordinary_muscle_development	ENSGALG00010018026	CCDC39	-2.090838086	0.734986375	-2.844730401	0.004444903	0.00688122	negative
PRJNA729271	stage_1D_vs_E10	ordinary_muscle_development	ENSGALG00010018027	EML5	-0.160515686	0.276866161	-0.579759134	0.56207706	0.607554585	negative
PRJNA729271	stage_1D_vs_E10	ordinary_muscle_development	ENSGALG00010018030	POLB	-0.084100002	0.101772536	-0.826352624	0.40860409	0.458272693	negative
PRJNA729271	stage_1D_vs_E10	ordinary_muscle_development	ENSGALG00010018032	ERO1A	-0.138489521	0.1237653	-1.118968892	0.26315341	0.308334709	negative
PRJNA729271	stage_1D_vs_E10	ordinary_muscle_development	ENSGALG00010018033	COQ8A	3.059547534	0.213105134	14.35698649	9.63254E-47	1.61601E-45	positive
PRJNA729271	stage_1D_vs_E10	ordinary_muscle_development	ENSGALG00010018035	ITM2A	1.03181552	0.086347447	11.94957765	6.5256E-33	6.48311E-32	positive
PRJNA729271	stage_1D_vs_E10	ordinary_muscle_development	ENSGALG00010018036	RPL4	0.137592945	0.107040279	1.285431492	0.198641542	0.238649743	positive
PRJNA729271	stage_1D_vs_E10	ordinary_muscle_development	ENSGALG00010018037	GZF1	0.194795472	0.081676732	2.384956732	0.01708113	0.024414849	positive
PRJNA729271	stage_1D_vs_E10	ordinary_muscle_development	ENSGALG00010018039	TTC14	0.428454877	0.131034969	3.269775092	0.00107633	0.001788537	positive
PRJNA729271	stage_1D_vs_E10	ordinary_muscle_development	ENSGALG00010018042	ZWILCH	-1.331846748	0.138587219	-9.610170102	7.24292E-22	4.29743E-21	negative
PRJNA729271	stage_1D_vs_E10	ordinary_muscle_development	ENSGALG00010018043	PSMC6	-0.171877177	0.09109877	-1.886712387	0.059199021	0.077979808	negative
PRJNA729271	stage_1D_vs_E10	ordinary_muscle_development	ENSGALG00010018044	INTS11	-0.65167198	0.059186114	-11.01055528	3.39902E-28	2.68349E-27	negative
PRJNA729271	stage_1D_vs_E10	ordinary_muscle_development	ENSGALG00010018045	PSEN2	0.698287255	0.073266353	9.53080407	1.5607E-21	9.07797E-21	positive
PRJNA729271	stage_1D_vs_E10	ordinary_muscle_development	ENSGALG00010018046	CAPN15	-0.216116551	0.1027926	-2.102452433	0.035513662	0.048485498	negative
PRJNA729271	stage_1D_vs_E10	ordinary_muscle_development	ENSGALG00010018048	GFRA4	-2.509860279	0.160080295	-15.67875843	2.11345E-55	4.96507E-54	negative
PRJNA729271	stage_1D_vs_E10	ordinary_muscle_development	ENSGALG00010018049	SUGCT	-0.987128815	0.167131379	-5.90630448	3.49867E-09	9.48975E-09	negative
PRJNA729271	stage_1D_vs_E10	ordinary_muscle_development	ENSGALG00010018051	GNPNAT1	-0.577814124	0.133999342	-4.312066882	1.61735E-05	3.23359E-05	negative
PRJNA729271	stage_1D_vs_E10	ordinary_muscle_development	ENSGALG00010018052	PPP1RL	-0.419112209	0.131480207	-3.18764488	0.001434366	0.002349812	negative
PRJNA729271	stage_1D_vs_E10	ordinary_muscle_development	ENSGALG00010018053	DIAPH2	1.751720666	0.091258465	19.19515806	4.06245E-82	2.00973E-80	positive
PRJNA729271	stage_1D_vs_E10	ordinary_muscle_development	ENSGALG00010018054	SMAD6	-0.603496221	0.157876263	-3.822589979	0.000132057	0.000242177	negative
PRJNA729271	stage_1D_vs_E10	ordinary_muscle_development	ENSGALG00010018055	PEX5L	-0.398705234	0.494360519	-0.80650703	0.419950556	0.469400317	negative
PRJNA729271	stage_1D_vs_E10	ordinary_muscle_development	ENSGALG00010018057	SSTR4	-0.343041258	0.177567816	-1.931888698	0.053373245	0.070820687	negative

PRJNA729271	stage_1D_vs_E10	ordinary_muscle_development	ENSGALG00010018058	ITPKB	0.116834934	0.09054948	1.290288302	0.196950577	0.236912748	positive
PRJNA729271	stage_1D_vs_E10	ordinary_muscle_development	ENSGALG00010018060	CASKIN1	-2.485415784	0.346325695	-7.176527242	7.15044E-13	2.50171E-12	negative
PRJNA729271	stage_1D_vs_E10	ordinary_muscle_development	ENSGALG00010018061	NDUFAF5	0.246315986	0.136090311	1.809945066	0.070304307	0.091658655	positive
PRJNA729271	stage_1D_vs_E10	ordinary_muscle_development	ENSGALG00010018062	GOLGA7	-0.692707589	0.093735306	-7.390039241	1.46785E-13	5.36474E-13	negative
PRJNA729271	stage_1D_vs_E10	ordinary_muscle_development	ENSGALG00010018063	DECR2	0.053282276	0.098351004	0.541756298	0.587986401	0.632166064	positive
PRJNA729271	stage_1D_vs_E10	ordinary_muscle_development	ENSGALG00010018064	TTC8	-1.909027438	0.162594262	-11.74105046	7.85052E-32	7.43262E-31	negative
PRJNA729271	stage_1D_vs_E10	ordinary_muscle_development	ENSGALG00010018067	ENSGALG00010018067	0.217919115	0.17816715	1.223116131	0.221285837	0.263799507	positive
PRJNA729271	stage_1D_vs_E10	ordinary_muscle_development	ENSGALG00010018068	ADAM33	-0.053759918	0.138303736	-0.388709082	0.697491363	0.734588194	negative
PRJNA729271	stage_1D_vs_E10	ordinary_muscle_development	ENSGALG00010018069	ENSGALG00010018069	-0.917364251	0.374575697	-2.449075735	0.014322333	0.020710516	negative
PRJNA729271	stage_1D_vs_E10	ordinary_muscle_development	ENSGALG00010018071	GPAT4	0.129238096	0.078640322	1.643407517	0.100298646	0.127528848	positive
PRJNA729271	stage_1D_vs_E10	ordinary_muscle_development	ENSGALG00010018075	MLST8	0.786288652	0.102882641	7.642578389	2.12914E-14	8.22765E-14	positive
PRJNA729271	stage_1D_vs_E10	ordinary_muscle_development	ENSGALG00010018076	FOXA2	-7.047778292	1.564284352	-4.50543297	6.62377E-06	1.37612E-05	negative
PRJNA729271	stage_1D_vs_E10	ordinary_muscle_development	ENSGALG00010018078	NKX6-3	5.022610308	0.69886631	7.186797015	6.63289E-13	2.32625E-12	positive
PRJNA729271	stage_1D_vs_E10	ordinary_muscle_development	ENSGALG00010018080	BRICD5	0.906710135	0.330821123	2.740786703	0.006129228	0.009320516	positive
PRJNA729271	stage_1D_vs_E10	ordinary_muscle_development	ENSGALG00010018081	DIO3	-3.018321832	0.244659439	-12.33682971	5.73707E-35	6.22232E-34	negative
PRJNA729271	stage_1D_vs_E10	ordinary_muscle_development	ENSGALG00010018082	RAB11FIP3	-0.531517634	0.177176939	-2.999925602	0.002700456	0.004278402	negative
PRJNA729271	stage_1D_vs_E10	ordinary_muscle_development	ENSGALG00010018083	PAX1	-5.777295103	1.00531095	-5.746774273	9.0962E-09	2.38038E-08	negative
PRJNA729271	stage_1D_vs_E10	ordinary_muscle_development	ENSGALG00010018085	PGP	1.115217379	0.159993608	6.970387102	3.1607E-12	1.05905E-11	positive
PRJNA729271	stage_1D_vs_E10	ordinary_muscle_development	ENSGALG00010018087	ENSGALG00010018087	-0.533987282	0.054826454	-9.739591722	2.04374E-22	1.24418E-21	negative
PRJNA729271	stage_1D_vs_E10	ordinary_muscle_development	ENSGALG00010018089	TASP1	0.381444136	0.091272794	4.179165769	2.9258E-05	5.71095E-05	positive
PRJNA729271	stage_1D_vs_E10	ordinary_muscle_development	ENSGALG00010018092	ENSGALG00010018092	-3.233817221	0.213569513	-15.14175492	8.58908E-52	1.76845E-50	negative
PRJNA729271	stage_1D_vs_E10	ordinary_muscle_development	ENSGALG00010018095	E4F1	-0.174428956	0.069111261	-2.523886154	0.011606551	0.017012535	negative
PRJNA729271	stage_1D_vs_E10	ordinary_muscle_development	ENSGALG00010018099	SMAD3	-1.253119449	0.130784555	-9.581555352	9.55943E-22	5.63268E-21	negative
PRJNA729271	stage_1D_vs_E10	ordinary_muscle_development	ENSGALG00010018102	ZDHHC4	0.539993544	0.166940911	3.23463877	0.001217967	0.00201236	positive
PRJNA729271	stage_1D_vs_E10	ordinary_muscle_development	ENSGALG00010018103	BTRC	-0.053641149	0.107157279	-0.500583344	0.616664387	0.658990732	negative
PRJNA729271	stage_1D_vs_E10	ordinary_muscle_development	ENSGALG00010018111	NKX2-2	1.40876284	0.599502348	2.34988711	0.018779106	0.026657833	positive
PRJNA729271	stage_1D_vs_E10	ordinary_muscle_development	ENSGALG00010018115	KLHL4	-1.183324784	0.190973257	-6.196285303	5.78113E-10	1.66001E-09	negative
PRJNA729271	stage_1D_vs_E10	ordinary_muscle_development	ENSGALG00010018119	GRID2IP	-1.737245624	0.597898421	-2.905586574	0.003665654	0.005723143	negative
PRJNA729271	stage_1D_vs_E10	ordinary_muscle_development	ENSGALG00010018121	FERMT2	1.239360607	0.15960815	7.76502081	8.16318E-15	3.23117E-14	positive
PRJNA729271	stage_1D_vs_E10	ordinary_muscle_development	ENSGALG00010018123	USP13	3.582399697	0.140734009	25.45511008	6.1973E-143	9.8714E-141	positive
PRJNA729271	stage_1D_vs_E10	ordinary_muscle_development	ENSGALG00010018128	SEPTIN12	-0.476971474	0.192597357	-2.476521384	0.013266968	0.019284466	negative
PRJNA729271	stage_1D_vs_E10	ordinary_muscle_development	ENSGALG00010018137	NDUFB5	1.443970918	0.127437757	11.33079358	9.23641E-30	7.87539E-29	positive
PRJNA729271	stage_1D_vs_E10	ordinary_muscle_development	ENSGALG00010018140	CHPF	-0.846946928	0.103635902	-8.172331332	3.02486E-16	1.31352E-15	negative
PRJNA729271	stage_1D_vs_E10	ordinary_muscle_development	ENSGALG00010018142	DDHD1	-1.106636683	0.143071145	-7.73486982	1.03509E-14	4.07044E-14	negative
PRJNA729271	stage_1D_vs_E10	ordinary_muscle_development	ENSGALG00010018143	MRPL47	0.145240739	0.123581933	1.175258673	0.239891243	0.283660947	positive
PRJNA729271	stage_1D_vs_E10	ordinary_muscle_development	ENSGALG00010018145	ENSGALG00010018145	-1.776345149	0.36535127	-4.862019917	1.16194E-06	2.5812E-06	negative
PRJNA729271	stage_1D_vs_E10	ordinary_muscle_development	ENSGALG00010018147	ISM1	0.542728824	0.284560881	1.907250295	0.056488183	0.074680428	positive
PRJNA729271	stage_1D_vs_E10	ordinary_muscle_development	ENSGALG00010018148	XRN2	-0.222543806	0.054649213	-4.072223446	4.65665E-05	8.92832E-05	negative
PRJNA729271	stage_1D_vs_E10	ordinary_muscle_development	ENSGALG00010018149	ENSGALG00010018149	-0.353418077	0.295732511	-1.195059938	0.232063613	0.275379621	negative
PRJNA729271	stage_1D_vs_E10	ordinary_muscle_development	ENSGALG00010018150	AAGAB	-1.667547194	0.080865687	-20.62119609	1.77125E-94	1.17234E-92	negative
PRJNA729271	stage_1D_vs_E10	ordinary_muscle_development	ENSGALG00010018154	SLC16A12	0.134860499	0.217856137	0.61903466	0.535893526	0.582466756	positive
PRJNA729271	stage_1D_vs_E10	ordinary_muscle_development	ENSGALG00010018157	FBXL2	-0.502936827	0.15253211	-3.297252137	0.000976358	0.001630263	negative
PRJNA729271	stage_1D_vs_E10	ordinary_muscle_development	ENSGALG00010018160	ENSGALG00010018160	0.751181174	0.163695533	4.588892323	4.45604E-06	9.38813E-06	positive
PRJNA729271	stage_1D_vs_E10	ordinary_muscle_development	ENSGALG00010018162	ENSGALG00010018162	0.868097	0.323794804	2.681009666	0.00734004	0.011044729	positive
PRJNA729271	stage_1D_vs_E10	ordinary_muscle_development	ENSGALG00010018163	CCT7	-0.888576453	0.081103142	-10.9561286	6.20994E-28	4.84984E-27	negative
PRJNA729271	stage_1D_vs_E10	ordinary_muscle_development	ENSGALG00010018165	DACH2	1.18932958	0.17931563	6.632604099	3.29816E-11	1.0321E-10	positive
PRJNA729271	stage_1D_vs_E10	ordinary_muscle_development	ENSGALG00010018166	MSS51	4.178835511	0.238791503	17.49993384	1.43436E-68	5.07098E-67	positive
PRJNA729271	stage_1D_vs_E10	ordinary_muscle_development	ENSGALG00010018168	ENSGALG00010018168	-1.646731159	0.21689603	-7.5922605	3.14372E-14	1.20138E-13	negative
PRJNA729271	stage_1D_vs_E10	ordinary_muscle_development	ENSGALG00010018172	ACTL6A	-1.380410405	0.093110036	-14.82558127	1.0011E-49	1.91689E-48	negative
PRJNA729271	stage_1D_vs_E10	ordinary_muscle_development	ENSGALG00010018174	BMP4	1.086366765	0.203056918	5.350060351	8.79249E-08	2.13229E-07	positive
PRJNA729271	stage_1D_vs_E10	ordinary_muscle_development	ENSGALG00010018177	KIF20B	-2.261664374	0.18737108	-12.07050937	1.51196E-33	1.54554E-32	negative
PRJNA729271	stage_1D_vs_E10	ordinary_muscle_development	ENSGALG00010018179	PRADC1	0.270660563	0.117636622	2.300818891	0.02140187	0.030177053	positive
PRJNA729271	stage_1D_vs_E10	ordinary_muscle_development	ENSGALG00010018183	CYP17A1	1.951053975	0.289326465	6.743434189	1.54686E-11	4.95399E-11	positive
PRJNA729271	stage_1D_vs_E10	ordinary_muscle_development	ENSGALG00010018185	GNB4	-0.564003401	0.069488365	-8.116515659	4.79759E-16	2.06292E-15	negative
PRJNA729271	stage_1D_vs_E10	ordinary_muscle_development	ENSGALG00010018186	SPTLC3	-4.392020633	0.663619931	-6.618277165	3.63409E-11	1.1338E-10	negative
PRJNA729271	stage_1D_vs_E10	ordinary_muscle_development	ENSGALG00010018188	UBP1	0.047237024	0.051702319	0.913634534	0.360908914	0.409853863	positive
PRJNA729271	stage_1D_vs_E10	ordinary_muscle_development	ENSGALG00010018189	MIS18BP1	-0.820644553	0.118485841	-6.92609803	4.32607E-12	1.43231E-11	negative
PRJNA729271	stage_1D_vs_E10	ordinary_muscle_development	ENSGALG00010018192	IQCH	-1.824872376	0.222468971	-8.202817524	2.34817E-16	1.02675E-15	negative
PRJNA729271	stage_1D_vs_E10	ordinary_muscle_development	ENSGALG00010018193	ANK1	4.334799829	0.246465793	17.5878355	3.05319E-69	1.09816E-67	positive
PRJNA729271	stage_1D_vs_E10	ordinary_muscle_development	ENSGALG00010018198	BTBD3	-0.550212054	0.102307347	-5.378030709	7.5305E-08	1.83669E-07	negative
PRJNA729271	stage_1D_vs_E10	ordinary_muscle_development	ENSGALG00010018199	BORCS7	-0.719200577	0.120315496	-5.977622183	2.26418E-09	6.23349E-09	negative
PRJNA729271	stage_1D_vs_E10	ordinary_muscle_development	ENSGALG00010018206	KIZ	-0.079498725	0.12480189	-0.636999373	0.524125252	0.571303619	negative
PRJNA729271	stage_1D_vs_E10	ordinary_muscle_development	ENSGALG00010018209	GMPPA	0.061134448	0.131218542	0.465897936	0.641288559	0.681786538	positive
PRJNA729271	stage_1D_vs_E10	ordinary_muscle_development	ENSGALG00010018215	CFAP70	1.903831369	0.273920605	6.950303595	3.64501E-12	1.21542E-11	positive
PRJNA729271	stage_1D_vs_E10	ordinary_muscle_development	ENSGALG00010018216	FANCM	0.389313926	0.083848342	4.64307246	3.43266E-06	7.32358E-06	positive
PRJNA729271	stage_1D_vs_E10	ordinary_muscle_development	ENSGALG00010018217	ENSGALG00010018217	-1.066783377	0.431848414	-2.470272768	0.013501006	0.019595182	negative
PRJNA729271	stage_1D_vs_E10	ordinary_muscle_development	ENSGALG00010018219	SLC24A5	-3.752245143	0.646732966	-5.801846113	6.55887E-09	1.73995E-08	negative
PRJNA729271	stage_1D_vs_E10	ordinary_muscle_development	ENSGALG00010018226	JAG1	0.137396226	0.101523346	1.353346112	0.175945084	0.214007216	positive
PRJNA729271	stage_1D_vs_E10	ordinary_muscle_development	ENSGALG00010018229	AS3MT	0.473232688	0.189128396	2.502176821	0.012343226	0.01803034	positive
PRJNA729271	stage_1D_vs_E10	ordinary_muscle_development	ENSGALG00010018231	SMYD5	-0.607320936	0.078307955	-7.755545906	8.79643E-15	3.47518E-14	negative
PRJNA729271	stage_1D_vs_E10	ordinary_muscle_development	ENSGALG00010018233	RASSF2	-0.95189982	0.189345983	-5.027304008	4.97423E-07	1.13779E-06	negative
PRJNA729271	stage_1D_vs_E10	ordinary_muscle_development	ENSGALG00010018240	SOUL	-0.245058405	0.659145806	-0.371781787	0.710055325	0.745923463	negative
PRJNA729271	stage_1D_vs_E10	ordinary_muscle_development	ENSGALG00010018241	RALGAPA2	0.079345066	0.091770719	0.864601117	0.387257759	0.436323453	positive
PRJNA729271	stage_1D_vs_E10	ordinary_muscle_development	ENSGALG00010018244	CHM	0.071530502	0.081787363	0.874591133	0.381796414	0.430772867	positive
PRJNA729271	stage_1D_vs_E10	ordinary_muscle_development	ENSGALG00010018251	USP48	0.590427925	0.165862522	3.55974283	0.000371218	0.00064829	positive
PRJNA729271	stage_1D_vs_E10	ordinary_muscle_development	ENSGALG00010018255	MFN1	0.688977818	0.11278389	6.108831812	1.00363E-09	2.83745E-09	positive
PRJNA729271	stage_1D_vs_E10	ordinary_muscle_development	ENSGALG00010018258	OSCP1	-1.456567727	0.289930812	-5.023845916	5.06469E-07	1.15702E-06	negative
PRJNA729271	stage_1D_vs_E10	ordinary_muscle_development	ENSGALG00010018259	WDR90	-0.764409891	0.130626416	-5.851878303	4.86052E-09	1.30252E-08	negative
PRJNA729271	stage_1D_vs_E10	ordinary_muscle_development	ENSGALG00010018261	PRNP	0.760122605	0.194415265	3.909788688	9.23769E-05	0.00017202	positive
PRJNA729271	stage_1D_vs_E10	ordinary_muscle_development	ENSGALG00010018263	SLX4IP	-1.222216025	0.110546979	-11.056078	2.04861E-28	1.63877E-27	negative
PRJNA729271	stage_1D_vs_E10	ordinary_muscle_development	ENSGALG00010018264	CFAP61	-2.05732636	0.38978934	-5.278046748	1.30568E-07	3.12772E-07	negative
PRJNA729271	stage_1D_vs_E10	ordinary_muscle_development	ENSGALG00010018265	PDCD4	-0.308425817	0.065512094	-4.707921802	2.50255E-06	5.41006E-06	negative
PRJNA729271	stage_1D_vs_E10	ordinary_muscle_development	ENSGALG00010018267	ADPRHL2	-0.373622644	0.076551962	-4.880641013	1.05742E-06	2.35913E-06	negative
PRJNA729271	stage_1D_vs_E10	ordinary_muscle_development	ENSGALG00010018273	PROM2	-6.200539356	1.077725316	-5.753357804	8.7488E-09	2.29444E-08	negative
PRJNA729271	stage_1D_vs_E10	ordinary_muscle_development	ENSGALG00010018274	ZNF711	0.007446078	0.097217687	0.076591803	0.93894828	0.948039518	positive
PRJNA729271	stage_1D_vs_E10	ordinary_muscle_development	ENSGALG00010018275	ZNF639	-0.175609481	0.083443457	-2.104532672	0.035332008	0.04826022	negative
PRJNA729271	stage_1D_vs_E10	ordinary_muscle_development	ENSGALG00010018276	ARHGAP17	0.280580918	0.071385816	3.93048553	8.47745E-05	0.000158427	positive
PRJNA729271	stage_1D_vs_E10	ordinary_muscle_development	ENSGALG00010018278	PUSL1	0.484968054	0.150158092	3.229716041	0.001239131	0.002046161	positive
PRJNA729271	stage_1D_vs_E10	ordinary_muscle_development	ENSGALG00010018283	POF1B	0.918028332	0.174466799	5.261908507	1.42568E-07	3.40672E-07	positive
PRJNA729271	stage_1D_vs_E10	ordinary_muscle_development	ENSGALG00010018284	ENSGALG00010018284	1.155110151	0.167073444	6.913786687	4.71885E-12	1.55843E-11	positive
PRJNA729271	stage_1D_vs_E10	ordinary_muscle_development	ENSGALG00010018285	BBIP1	-0.139645997	0.191197494	-0.730375665	0.465160589	0.513949443	negative
PRJNA729271	stage_1D_vs_E10	ordinary_muscle_development	ENSGALG00010018287	MKKS	0.229949097	0.102381531	2.246001745	0.024703896	0.034514022	positive
PRJNA729271	stage_1D_vs_E10	ordinary_muscle_development	ENSGALG00010018288	CRNKL1	-0.333098419	0.061643894	-5.403591486	6.53196E-08	1.60231E-07	negative
PRJNA729271	stage_1D_vs_E10	ordinary_muscle_development	ENSGALG00010018289	HEYL	-1.224360693	0.210632362	-5.812785268	6.14419E-09	1.63563E-08	negative
PRJNA729271	stage_1D_vs_E10	ordinary_muscle_development	ENSGALG00010018290	RHOT2	-0.191943783	0.133805994	-1.434493153	0.151431582	0.18652284	negative
PRJNA729271	stage_1D_vs_E10	ordinary_muscle_development	ENSGALG00010018294	FKBP3	0.826181315	0.177683696	4.649730574	3.32369E-06	7.10154E-06	positive
PRJNA729271	stage_1D_vs_E10	ordinary_muscle_development	ENSGALG00010018296	PIK3CA	0.85640568	0.09630906	8.892264958	5.98757E-19	3.01668E-18	positive
PRJNA729271	stage_1D_vs_E10	ordinary_muscle_development	ENSGALG00010018298	ENSGALG00010018298	0.635679992	0.195737359	3.247617083	0.001163758	0.001926967	positive
PRJNA729271	stage_1D_vs_E10	ordinary_muscle_development	ENSGALG00010018299	SHOC2	0.143663372	0.082792877	1.735214154	0.08270288	0.106633895	positive
PRJNA729271	stage_1D_vs_E10	ordinary_muscle_development	ENSGALG00010018301	ENSGALG00010018301	-0.200057619	0.308188308	-0.649140848	0.516247341	0.563903934	negative
PRJNA729271	stage_1D_vs_E10	ordinary_muscle_development	ENSGALG00010018302	RAB11FIP5	0.502540172	0.101204784	4.965577224	6.8497E-07	1.54941E-06	positive
PRJNA729271	stage_1D_vs_E10	ordinary_muscle_development	ENSGALG00010018306	DES	2.396660131	0.150975137	15.87453527	9.51128E-57	2.33276E-55	positive
PRJNA729271	stage_1D_vs_E10	ordinary_muscle_development	ENSGALG00010018308	ACAP3	-0.606590217	0.176259806	-3.441455158	0.000578594	0.000988068	negative
PRJNA729271	stage_1D_vs_E10	ordinary_muscle_development	ENSGALG00010018309	DPYSL2	-1.328198027	0.124208142	-10.693325	1.09378E-26	8.07659E-26	negative
PRJNA729271	stage_1D_vs_E10	ordinary_muscle_development	ENSGALG00010018310	APOOL	0.457872884	0.111825278	4.094538286	4.23011E-05	8.13309E-05	positive
PRJNA729271	stage_1D_vs_E10	ordinary_muscle_development	ENSGALG00010018311	NKX2-3	0.786313589	0.816193786	0.963390806	0.335351439	0.383853125	positive
PRJNA729271	stage_1D_vs_E10	ordinary_muscle_development	ENSGALG00010018312	PRPF39	-0.21590215	0.078803464	-2.73975456	0.006148508	0.009347873	negative
PRJNA729271	stage_1D_vs_E10	ordinary_muscle_development	ENSGALG00010018313	MYEF2	-1.085175746	0.081392471	-13.33263053	1.49531E-40	2.00689E-39	negative
PRJNA729271	stage_1D_vs_E10	ordinary_muscle_development	ENSGALG00010018314	SLC24A3	-1.035971162	0.127476921	-8.126735045	4.41008E-16	1.90024E-15	negative
PRJNA729271	stage_1D_vs_E10	ordinary_muscle_development	ENSGALG00010018315	CLASP2	0.942200735	0.117180715	8.04057848	8.94153E-16	3.77315E-15	positive
PRJNA729271	stage_1D_vs_E10	ordinary_muscle_development	ENSGALG00010018317	ZMAT3	-0.218014919	0.131767621	-1.654540914	0.098017657	0.124803755	negative
PRJNA729271	stage_1D_vs_E10	ordinary_muscle_development	ENSGALG00010018319	STUB1	0.238231933	0.201891243	1.180001321	0.23799969	0.281677758	positive
PRJNA729271	stage_1D_vs_E10	ordinary_muscle_development	ENSGALG00010018322	NT5C1A	3.428114066	0.241806683	14.1770857	1.27013E-45	2.03881E-44	positive
PRJNA729271	stage_1D_vs_E10	ordinary_muscle_development	ENSGALG00010018323	GOT1	2.286170624	0.142298974	16.06596707	4.41918E-58	1.13173E-56	positive
PRJNA729271	stage_1D_vs_E10	ordinary_muscle_development	ENSGALG00010018325	ENSGALG00010018325	0.770873302	0.193186196	3.990312549	6.59863E-05	0.000124719	positive
PRJNA729271	stage_1D_vs_E10	ordinary_muscle_development	ENSGALG00010018326	LCMT1	-0.920490165	0.110601134	-8.32261057	8.60468E-17	3.85667E-16	negative
PRJNA729271	stage_1D_vs_E10	ordinary_muscle_development	ENSGALG00010018327	SNAP25	0.211844923	0.455043782	0.46554844	0.641538757	0.68199344	positive
PRJNA729271	stage_1D_vs_E10	ordinary_muscle_development	ENSGALG00010018328	ENSGALG00010018328	2.812104228	0.545158627	5.158322898	2.49172E-07	5.82821E-07	positive
PRJNA729271	stage_1D_vs_E10	ordinary_muscle_development	ENSGALG00010018329	ENSGALG00010018329	0.3278367	0.238427007	1.374998179	0.169132009	0.206343614	positive
PRJNA729271	stage_1D_vs_E10	ordinary_muscle_development	ENSGALG00010018333	ENSGALG00010018333	0.726806482	0.216898582	3.350904713	0.00080548	0.00135824	positive
PRJNA729271	stage_1D_vs_E10	ordinary_muscle_development	ENSGALG00010018336	JMJD8	-0.268392724	0.146660153	-1.830031661	0.067245205	0.087891726	negative
PRJNA729271	stage_1D_vs_E10	ordinary_muscle_development	ENSGALG00010018337	TMEM151B	-1.419396698	0.32285657	-4.396369256	1.10077E-05	2.23844E-05	negative
PRJNA729271	stage_1D_vs_E10	ordinary_muscle_development	ENSGALG00010018338	PDCD6IP	0.375564497	0.084623819	4.438047133	9.07787E-06	1.86274E-05	positive
PRJNA729271	stage_1D_vs_E10	ordinary_muscle_development	ENSGALG00010018339	FAM76A	-0.048301337	0.115859091	-0.416897253	0.676753563	0.714929152	negative
PRJNA729271	stage_1D_vs_E10	ordinary_muscle_development	ENSGALG00010018340	DNPEP	0.088312368	0.097817999	0.902823287	0.36661969	0.415687766	positive
PRJNA729271	stage_1D_vs_E10	ordinary_muscle_development	ENSGALG00010018342	BNIP3L	-0.37054647	0.106170009	-3.490123739	0.000482797	0.000833013	negative
PRJNA729271	stage_1D_vs_E10	ordinary_muscle_development	ENSGALG00010018344	SFXN5	-1.128470406	0.193208956	-5.840673384	5.19902E-09	1.39015E-08	negative
PRJNA729271	stage_1D_vs_E10	ordinary_muscle_development	ENSGALG00010018345	WDR24	-0.052252663	0.171910535	-0.30395265	0.761163965	0.791815483	negative
PRJNA729271	stage_1D_vs_E10	ordinary_muscle_development	ENSGALG00010018347	CTXN2	-4.75619304	0.911376834	-5.218689856	1.80193E-07	4.26502E-07	negative
PRJNA729271	stage_1D_vs_E10	ordinary_muscle_development	ENSGALG00010018348	TOGARAM1	0.395353744	0.107284449	3.685098336	0.000228614	0.000407999	positive
PRJNA729271	stage_1D_vs_E10	ordinary_muscle_development	ENSGALG00010018349	KCNMB2	0.147568178	0.196150538	0.752321044	0.451858016	0.500931697	positive

PRJNA729271	stage_1D_vs_E10	ordinary_muscle_development	ENSGALG00010018350	CNNM1	-2.380729431	0.296272671	-8.035602553	9.31201E-16	3.91921E-15	negative
PRJNA729271	stage_1D_vs_E10	ordinary_muscle_development	ENSGALG00010018351	RIN2	0.962995675	0.086448829	11.13948774	8.05815E-29	6.58415E-28	positive
PRJNA729271	stage_1D_vs_E10	ordinary_muscle_development	ENSGALG00010018354	AARS2	0.376442128	0.116092984	3.242591538	0.001184479	0.001960382	positive
PRJNA729271	stage_1D_vs_E10	ordinary_muscle_development	ENSGALG00010018355	PRKCB	-1.880671414	0.162234798	-11.59228131	4.50959E-31	4.09821E-30	negative
PRJNA729271	stage_1D_vs_E10	ordinary_muscle_development	ENSGALG00010018356	ADRA1A	-0.809251113	0.35389251	-2.28671444	0.022212496	0.031256202	negative
PRJNA729271	stage_1D_vs_E10	ordinary_muscle_development	ENSGALG00010018359	CACNG3	-3.826063057	0.685534445	-5.581139046	2.38949E-08	6.05517E-08	negative
PRJNA729271	stage_1D_vs_E10	ordinary_muscle_development	ENSGALG00010018361	ASIC4	0.177489038	0.210768498	0.842104201	0.39972963	0.449223212	positive
PRJNA729271	stage_1D_vs_E10	ordinary_muscle_development	ENSGALG00010018363	TCTE1	-0.858882792	0.41281145	-2.080569207	0.037473355	0.050945065	negative
PRJNA729271	stage_1D_vs_E10	ordinary_muscle_development	ENSGALG00010018365	HPSE2	-2.993646301	0.275713575	-10.85781251	1.83082E-27	1.39599E-26	negative
PRJNA729271	stage_1D_vs_E10	ordinary_muscle_development	ENSGALG00010018367	PAK5	-0.374520913	0.208393857	-1.797178279	0.072307322	0.094083898	negative
PRJNA729271	stage_1D_vs_E10	ordinary_muscle_development	ENSGALG00010018370	ZBTB8A	0.06827151	0.110001533	0.620641437	0.534835569	0.581664462	positive
PRJNA729271	stage_1D_vs_E10	ordinary_muscle_development	ENSGALG00010018371	ENSGALG00010018371	0.205267678	0.136571649	1.503003576	0.132838122	0.165192911	positive
PRJNA729271	stage_1D_vs_E10	ordinary_muscle_development	ENSGALG00010018372	NLGN3	0.389618076	0.460812867	0.84550173	0.397830767	0.447193251	positive
PRJNA729271	stage_1D_vs_E10	ordinary_muscle_development	ENSGALG00010018374	HPS1	-0.151382996	0.132188883	-1.145202173	0.252125418	0.296732538	negative
PRJNA729271	stage_1D_vs_E10	ordinary_muscle_development	ENSGALG00010018376	PYGB	-0.273851217	0.141774692	-1.931594508	0.053409574	0.07085592	negative
PRJNA729271	stage_1D_vs_E10	ordinary_muscle_development	ENSGALG00010018377	RAP1GAP	2.309320232	0.259849545	8.887143658	6.26999E-19	3.15677E-18	positive
PRJNA729271	stage_1D_vs_E10	ordinary_muscle_development	ENSGALG00010018378	ARPP21	0.440596505	0.145013081	3.038322492	0.002378992	0.003791061	positive
PRJNA729271	stage_1D_vs_E10	ordinary_muscle_development	ENSGALG00010018379	SRBD1	0.032998229	0.12627822	0.261313698	0.793850604	0.821332227	positive
PRJNA729271	stage_1D_vs_E10	ordinary_muscle_development	ENSGALG00010018380	SLC20A1	-1.202375246	0.138202011	-8.70012842	3.31509E-18	1.60925E-17	negative
PRJNA729271	stage_1D_vs_E10	ordinary_muscle_development	ENSGALG00010018381	HAPSTR1	0.223426266	0.067455016	3.312226129	0.000925567	0.00154956	positive
PRJNA729271	stage_1D_vs_E10	ordinary_muscle_development	ENSGALG00010018383	METRN	-1.301681078	0.161958319	-8.037136238	9.19624E-16	3.87273E-15	negative
PRJNA729271	stage_1D_vs_E10	ordinary_muscle_development	ENSGALG00010018384	LAMP5	-0.11111941	0.238712543	-0.465494645	0.641577271	0.68199344	negative
PRJNA729271	stage_1D_vs_E10	ordinary_muscle_development	ENSGALG00010018385	SPR	-1.318598417	0.142212162	-9.272050963	1.826E-20	1.00219E-19	negative
PRJNA729271	stage_1D_vs_E10	ordinary_muscle_development	ENSGALG00010018387	NT5DC4	-0.145972421	0.311663353	-0.468365688	0.639523101	0.680159025	negative
PRJNA729271	stage_1D_vs_E10	ordinary_muscle_development	ENSGALG00010018388	SMC3	-0.517541843	0.065360125	-7.918311701	2.40757E-15	9.87486E-15	negative
PRJNA729271	stage_1D_vs_E10	ordinary_muscle_development	ENSGALG00010018391	STAC	-2.288717167	0.229642178	-9.966449467	2.13733E-23	1.35346E-22	negative
PRJNA729271	stage_1D_vs_E10	ordinary_muscle_development	ENSGALG00010018393	PRKCE	0.171830512	0.075430521	2.277997149	0.022726747	0.031948812	positive
PRJNA729271	stage_1D_vs_E10	ordinary_muscle_development	ENSGALG00010018394	KLHL28	0.026772911	0.096600193	0.277151736	0.781663597	0.8101111	positive
PRJNA729271	stage_1D_vs_E10	ordinary_muscle_development	ENSGALG00010018395	MED12	0.308017808	0.07820352	3.938669342	8.19348E-05	0.000153304	positive
PRJNA729271	stage_1D_vs_E10	ordinary_muscle_development	ENSGALG00010018396	TBL1XR1	0.216530631	0.074235987	2.916787945	0.003536561	0.005534109	positive
PRJNA729271	stage_1D_vs_E10	ordinary_muscle_development	ENSGALG00010018397	FAM173A	1.462444038	0.160035235	9.138262841	6.34635E-20	3.37951E-19	positive
PRJNA729271	stage_1D_vs_E10	ordinary_muscle_development	ENSGALG00010018398	LRFN5	0.038056548	0.184746761	0.205993045	0.836796344	0.860238511	positive
PRJNA729271	stage_1D_vs_E10	ordinary_muscle_development	ENSGALG00010018399	ABHD12	0.142021653	0.162764982	0.872556556	0.382904829	0.431956222	positive
PRJNA729271	stage_1D_vs_E10	ordinary_muscle_development	ENSGALG00010018401	ENSGALG00010018401	-0.631492499	0.102206712	-6.178581506	6.46801E-10	1.85138E-09	negative
PRJNA729271	stage_1D_vs_E10	ordinary_muscle_development	ENSGALG00010018404	IL2RG	0.754361361	0.261244221	2.887571482	0.003882284	0.006045086	positive
PRJNA729271	stage_1D_vs_E10	ordinary_muscle_development	ENSGALG00010018405	DCLK3	-3.031126364	0.200281594	-15.13432315	9.6165E-52	1.97438E-50	negative
PRJNA729271	stage_1D_vs_E10	ordinary_muscle_development	ENSGALG00010018406	TMEM260	-0.440920052	0.125367892	-3.517009375	0.000436438	0.000755635	negative
PRJNA729271	stage_1D_vs_E10	ordinary_muscle_development	ENSGALG00010018410	EXOC6B	0.649755395	0.097101706	6.691493114	2.20905E-11	6.98673E-11	positive
PRJNA729271	stage_1D_vs_E10	ordinary_muscle_development	ENSGALG00010018413	SLC15A2	-1.532542618	0.358884678	-4.270292693	1.95217E-05	3.87042E-05	negative
PRJNA729271	stage_1D_vs_E10	ordinary_muscle_development	ENSGALG00010018414	EPAS1	1.686514224	0.137663008	12.2510342	1.65867E-34	1.76913E-33	positive
PRJNA729271	stage_1D_vs_E10	ordinary_muscle_development	ENSGALG00010018415	PELI2	-1.08066545	0.127917134	-8.448168092	2.95909E-17	1.36036E-16	negative
PRJNA729271	stage_1D_vs_E10	ordinary_muscle_development	ENSGALG00010018416	DUT	-1.329631407	0.133583755	-9.953541166	2.43366E-23	1.53641E-22	negative
PRJNA729271	stage_1D_vs_E10	ordinary_muscle_development	ENSGALG00010018418	PLCB4	0.162124665	0.117408553	1.38085907	0.167322297	0.204411015	positive
PRJNA729271	stage_1D_vs_E10	ordinary_muscle_development	ENSGALG00010018419	USP7	0.683727982	0.133476109	5.122474622	3.01552E-07	7.00479E-07	positive
PRJNA729271	stage_1D_vs_E10	ordinary_muscle_development	ENSGALG00010018420	RHOQ	0.622666699	0.132153377	4.711697209	2.45662E-06	5.31394E-06	positive
PRJNA729271	stage_1D_vs_E10	ordinary_muscle_development	ENSGALG00010018422	ENSGALG00010018422	0.953775893	0.212559826	4.487093872	7.22013E-06	1.4953E-05	positive
PRJNA729271	stage_1D_vs_E10	ordinary_muscle_development	ENSGALG00010018425	CYP26B1	-2.734210232	0.233973071	-11.68600393	1.50293E-31	1.40187E-30	negative
PRJNA729271	stage_1D_vs_E10	ordinary_muscle_development	ENSGALG00010018426	ENSGALG00010018426	4.779192942	0.34434608	13.87903978	8.48733E-44	1.27222E-42	positive
PRJNA729271	stage_1D_vs_E10	ordinary_muscle_development	ENSGALG00010018427	PIGF	-0.028856551	0.1417649	-0.203552156	0.838703484	0.862015671	negative
PRJNA729271	stage_1D_vs_E10	ordinary_muscle_development	ENSGALG00010018428	PPP2R5D	-0.050754602	0.062612648	-0.810612614	0.417588171	0.466975817	negative
PRJNA729271	stage_1D_vs_E10	ordinary_muscle_development	ENSGALG00010018430	LOXL4	-1.366469671	0.25373317	-5.385459337	7.22599E-08	1.76599E-07	negative
PRJNA729271	stage_1D_vs_E10	ordinary_muscle_development	ENSGALG00010018434	HSPBAP1	0.910596827	0.160604243	5.669818001	1.42949E-08	3.68627E-08	positive
PRJNA729271	stage_1D_vs_E10	ordinary_muscle_development	ENSGALG00010018436	FOXO4	1.251490315	0.144265233	8.674926649	4.13817E-18	1.99477E-17	positive
PRJNA729271	stage_1D_vs_E10	ordinary_muscle_development	ENSGALG00010018438	PPP2R2A	-0.054365798	0.098480269	-0.552047617	0.58091573	0.625352655	negative
PRJNA729271	stage_1D_vs_E10	ordinary_muscle_development	ENSGALG00010018440	MEA1	0.170789434	0.101646954	1.680221861	0.092914158	0.118832149	positive
PRJNA729271	stage_1D_vs_E10	ordinary_muscle_development	ENSGALG00010018441	CRIPT	0.226468479	0.093687677	2.417270734	0.015637381	0.022482032	positive
PRJNA729271	stage_1D_vs_E10	ordinary_muscle_development	ENSGALG00010018443	PLCB1	2.697076264	0.146165963	18.45214997	5.01112E-76	2.13009E-74	positive
PRJNA729271	stage_1D_vs_E10	ordinary_muscle_development	ENSGALG00010018444	NUBP2	-0.798746183	0.0997918	-8.004126382	1.20318E-15	5.0274E-15	negative
PRJNA729271	stage_1D_vs_E10	ordinary_muscle_development	ENSGALG00010018446	FBN1	2.063240391	0.232067243	8.890700651	6.07248E-19	3.05839E-18	positive
PRJNA729271	stage_1D_vs_E10	ordinary_muscle_development	ENSGALG00010018448	KLHDC3	2.600993974	0.164259446	15.83466908	1.794E-56	4.34851E-55	positive
PRJNA729271	stage_1D_vs_E10	ordinary_muscle_development	ENSGALG00010018451	PIK3R4	-0.48082734	0.111982684	-4.293765094	1.75668E-05	3.49672E-05	negative
PRJNA729271	stage_1D_vs_E10	ordinary_muscle_development	ENSGALG00010018452	SPSB3	0.47517714	0.118901566	3.996390934	6.43155E-05	0.000121752	positive
PRJNA729271	stage_1D_vs_E10	ordinary_muscle_development	ENSGALG00010018453	TMX4	0.002381706	0.092207805	0.025829769	0.979393117	0.982511124	positive
PRJNA729271	stage_1D_vs_E10	ordinary_muscle_development	ENSGALG00010018455	RBM20	3.517467034	0.246456838	14.27214221	3.26396E-46	5.37016E-45	positive
PRJNA729271	stage_1D_vs_E10	ordinary_muscle_development	ENSGALG00010018458	SEMA5B	-0.673302974	0.106193067	-6.340366605	2.29219E-10	6.78206E-10	negative
PRJNA729271	stage_1D_vs_E10	ordinary_muscle_development	ENSGALG00010018459	SNX12	0.135135979	0.102472792	1.31874984	0.187252759	0.226525516	positive
PRJNA729271	stage_1D_vs_E10	ordinary_muscle_development	ENSGALG00010018462	KTN1	0.34790623	0.116401125	2.988856262	0.002800238	0.004429229	positive
PRJNA729271	stage_1D_vs_E10	ordinary_muscle_development	ENSGALG00010018464	ENSGALG00010018464	-4.101739199	1.179339516	-3.477996917	0.000505176	0.000869648	negative
PRJNA729271	stage_1D_vs_E10	ordinary_muscle_development	ENSGALG00010018465	NAALADL2	-2.311336284	0.202526645	-11.41250471	3.62149E-30	3.16225E-29	negative
PRJNA729271	stage_1D_vs_E10	ordinary_muscle_development	ENSGALG00010018466	DYSF	2.825558513	0.182356477	15.4946979	3.76727E-54	8.36243E-53	positive
PRJNA729271	stage_1D_vs_E10	ordinary_muscle_development	ENSGALG00010018467	TLX1	-5.100567425	1.148744715	-4.440122647	8.99076E-06	1.84581E-05	negative
PRJNA729271	stage_1D_vs_E10	ordinary_muscle_development	ENSGALG00010018469	KLHDC7A	0.331040003	0.198136479	1.67076757	0.094767596	0.120963042	positive
PRJNA729271	stage_1D_vs_E10	ordinary_muscle_development	ENSGALG00010018470	IQCB1	0.774510526	0.158094882	4.899023392	9.63142E-07	2.15543E-06	positive
PRJNA729271	stage_1D_vs_E10	ordinary_muscle_development	ENSGALG00010018471	HHEX	1.044235489	0.180752641	5.77715204	7.59756E-09	2.00668E-08	positive
PRJNA729271	stage_1D_vs_E10	ordinary_muscle_development	ENSGALG00010018472	LMF1	0.522793608	0.139887457	3.737244338	0.000186048	0.000335669	positive
PRJNA729271	stage_1D_vs_E10	ordinary_muscle_development	ENSGALG00010018476	ENSGALG00010018476	-0.321025352	0.376260533	-0.853199643	0.393548602	0.442723087	negative
PRJNA729271	stage_1D_vs_E10	ordinary_muscle_development	ENSGALG00010018480	EME2	-0.420925057	0.245867952	-1.711996437	0.086897325	0.111644808	negative
PRJNA729271	stage_1D_vs_E10	ordinary_muscle_development	ENSGALG00010018482	TWNK	-0.244981317	0.127845261	-1.916233074	0.055335435	0.073263348	negative
PRJNA729271	stage_1D_vs_E10	ordinary_muscle_development	ENSGALG00010018484	NLGN1	-0.842418991	0.238657495	-3.52982416	0.000415836	0.000722207	negative
PRJNA729271	stage_1D_vs_E10	ordinary_muscle_development	ENSGALG00010018485	ATP2C1	-0.047766142	0.066500353	-0.718284037	0.472582171	0.521354636	negative
PRJNA729271	stage_1D_vs_E10	ordinary_muscle_development	ENSGALG00010018486	ENSGALG00010018486	0.602364237	0.100123244	6.016227739	1.78528E-09	4.95077E-09	positive
PRJNA729271	stage_1D_vs_E10	ordinary_muscle_development	ENSGALG00010018487	ENSGALG00010018487	-1.151880515	0.123303871	-9.341803324	9.47071E-21	5.28806E-20	negative
PRJNA729271	stage_1D_vs_E10	ordinary_muscle_development	ENSGALG00010018488	ENSGALG00010018488	0.933698575	0.153205734	6.094410114	1.09842E-09	3.09277E-09	positive
PRJNA729271	stage_1D_vs_E10	ordinary_muscle_development	ENSGALG00010018489	SLC7A3	-0.813138696	0.206310751	-3.941329723	8.10311E-05	0.000151672	negative
PRJNA729271	stage_1D_vs_E10	ordinary_muscle_development	ENSGALG00010018490	NME3	-0.659669025	0.121231888	-5.441382084	5.28688E-08	1.30395E-07	negative
PRJNA729271	stage_1D_vs_E10	ordinary_muscle_development	ENSGALG00010018491	TIA1	0.646074454	0.119282305	5.416347848	6.08287E-08	1.49417E-07	positive
PRJNA729271	stage_1D_vs_E10	ordinary_muscle_development	ENSGALG00010018492	ENSGALG00010018492	1.066777016	0.170258644	6.265626156	3.7133E-10	1.0819E-09	positive
PRJNA729271	stage_1D_vs_E10	ordinary_muscle_development	ENSGALG00010018494	HAO1	0.601108321	0.28482657	2.110436258	0.034820796	0.047597835	positive
PRJNA729271	stage_1D_vs_E10	ordinary_muscle_development	ENSGALG00010018495	ENSGALG00010018495	1.149035845	0.635073267	1.80929651	0.070404951	0.09177336	positive
PRJNA729271	stage_1D_vs_E10	ordinary_muscle_development	ENSGALG00010018496	GNG13	-2.215297494	0.548847617	-4.036270592	5.43076E-05	0.000103387	negative
PRJNA729271	stage_1D_vs_E10	ordinary_muscle_development	ENSGALG00010018497	ASTE1	-0.964865503	0.108625222	-8.882518093	6.53636E-19	3.28632E-18	negative
PRJNA729271	stage_1D_vs_E10	ordinary_muscle_development	ENSGALG00010018498	ENSGALG00010018498	4.966961416	0.378761495	13.11369156	2.74886E-39	3.53233E-38	positive
PRJNA729271	stage_1D_vs_E10	ordinary_muscle_development	ENSGALG00010018501	ATG14	0.130519535	0.135325089	0.964488823	0.334800909	0.38335881	positive
PRJNA729271	stage_1D_vs_E10	ordinary_muscle_development	ENSGALG00010018502	ARHGEF10L	0.739404529	0.122137751	6.053857409	1.41418E-09	3.9481E-09	positive
PRJNA729271	stage_1D_vs_E10	ordinary_muscle_development	ENSGALG00010018503	BMP2	-3.324462417	0.193664116	-17.16612493	4.76167E-66	1.5372E-64	negative
PRJNA729271	stage_1D_vs_E10	ordinary_muscle_development	ENSGALG00010018504	SLCO3A1	-0.599977888	0.182071744	-3.295282817	0.000983227	0.001640409	negative
PRJNA729271	stage_1D_vs_E10	ordinary_muscle_development	ENSGALG00010018505	CHTF18	-1.654153739	0.145314101	-11.38329815	5.06474E-30	4.38027E-29	negative
PRJNA729271	stage_1D_vs_E10	ordinary_muscle_development	ENSGALG00010018506	ENSGALG00010018506	-0.079913054	0.148080797	-0.539658456	0.589432595	0.633439017	negative
PRJNA729271	stage_1D_vs_E10	ordinary_muscle_development	ENSGALG00010018508	ENSGALG00010018508	-0.377514509	0.101265316	-3.727974413	0.000193025	0.00034722	negative
PRJNA729271	stage_1D_vs_E10	ordinary_muscle_development	ENSGALG00010018509	ENSGALG00010018509	-0.831207458	0.110382442	-7.530250661	5.0643E-14	1.91065E-13	negative
PRJNA729271	stage_1D_vs_E10	ordinary_muscle_development	ENSGALG00010018510	ENSGALG00010018510	0.2633797	0.126240288	2.086336337	0.036948167	0.050291584	positive
PRJNA729271	stage_1D_vs_E10	ordinary_muscle_development	ENSGALG00010018511	SOCS5	0.117770162	0.100372398	1.173332157	0.240662632	0.284419474	positive
PRJNA729271	stage_1D_vs_E10	ordinary_muscle_development	ENSGALG00010018512	FERMT1	-0.577877292	0.174508413	-3.311458073	0.000928111	0.001553641	negative
PRJNA729271	stage_1D_vs_E10	ordinary_muscle_development	ENSGALG00010018513	RPUSD1	-0.508570228	0.158642026	-3.205772392	0.001347005	0.002214453	negative
PRJNA729271	stage_1D_vs_E10	ordinary_muscle_development	ENSGALG00010018514	EAF2	-1.916693576	0.250033841	-7.665736628	1.77808E-14	6.89492E-14	negative
PRJNA729271	stage_1D_vs_E10	ordinary_muscle_development	ENSGALG00010018515	SEMA4G	0.210058332	0.172990601	1.214275984	0.224642353	0.267405018	positive
PRJNA729271	stage_1D_vs_E10	ordinary_muscle_development	ENSGALG00010018516	LRRN4	1.097179852	0.152705082	7.184959642	6.7227E-13	2.35603E-12	positive
PRJNA729271	stage_1D_vs_E10	ordinary_muscle_development	ENSGALG00010018517	ECT2	-1.612311273	0.197189616	-8.176451211	2.92326E-16	1.27131E-15	negative
PRJNA729271	stage_1D_vs_E10	ordinary_muscle_development	ENSGALG00010018518	ENSGALG00010018518	-1.52536476	0.934859504	-1.631651338	0.102752957	0.130466372	negative
PRJNA729271	stage_1D_vs_E10	ordinary_muscle_development	ENSGALG00010018519	OGDH	2.118446039	0.136145459	15.56016666	1.35745E-54	3.06007E-53	positive
PRJNA729271	stage_1D_vs_E10	ordinary_muscle_development	ENSGALG00010018520	ENSGALG00010018520	0.254929579	0.12412997	2.053731084	0.040001726	0.054153793	positive
PRJNA729271	stage_1D_vs_E10	ordinary_muscle_development	ENSGALG00010018521	ENSGALG00010018521	0.581027141	0.158775724	3.659420506	0.000252786	0.000448871	positive
PRJNA729271	stage_1D_vs_E10	ordinary_muscle_development	ENSGALG00010018522	NEK11	-0.630459917	0.260713875	-2.418206232	0.015597234	0.022433212	negative
PRJNA729271	stage_1D_vs_E10	ordinary_muscle_development	ENSGALG00010018523	TEX11	-0.01821902	0.167515404	-0.108760268	0.913392638	0.926366239	negative
PRJNA729271	stage_1D_vs_E10	ordinary_muscle_development	ENSGALG00010018524	SEC22A	-0.49686427	0.116972488	-4.247701999	2.15974E-05	4.26505E-05	negative
PRJNA729271	stage_1D_vs_E10	ordinary_muscle_development	ENSGALG00010018525	ST8SIA2	-3.125611084	0.292789188	-10.67529546	1.32834E-26	9.79366E-26	negative
PRJNA729271	stage_1D_vs_E10	ordinary_muscle_development	ENSGALG00010018526	ENSGALG00010018526	0.62403189	0.10034123	6.219097494	5.00023E-10	1.4435E-09	positive
PRJNA729271	stage_1D_vs_E10	ordinary_muscle_development	ENSGALG00010018527	CCT4	-0.936903891	0.081442423	-11.50388035	1.26115E-30	1.12703E-29	negative
PRJNA729271	stage_1D_vs_E10	ordinary_muscle_development	ENSGALG00010018528	ENSGALG00010018528	0.895630864	0.629012367	1.423868449	0.154484557	0.189928214	positive
PRJNA729271	stage_1D_vs_E10	ordinary_muscle_development	ENSGALG00010018529	UBR2	0.499531698	0.092673816	5.390213987	7.03738E-08	1.72105E-07	positive
PRJNA729271	stage_1D_vs_E10	ordinary_muscle_development	ENSGALG00010018530	FAM161A	-1.061170495	0.230487328	-4.60402966	4.14393E-06	8.76241E-06	negative
PRJNA729271	stage_1D_vs_E10	ordinary_muscle_development	ENSGALG00010018531	ADCY5	1.049654367	0.134700204	7.792522474	6.56845E-15	2.61421E-14	positive
PRJNA729271	stage_1D_vs_E10	ordinary_muscle_development	ENSGALG00010018532	MFSD13A	-0.716720759	0.12971199	-5.525478099	3.2859E-08	8.21757E-08	negative
PRJNA729271	stage_1D_vs_E10	ordinary_muscle_development	ENSGALG00010018533	PCYOX1	1.263485916	0.172791378	7.312204649	2.62794E-13	9.45212E-13	positive
PRJNA729271	stage_1D_vs_E10	ordinary_muscle_development	ENSGALG00010018534	NCEH1	1.554711637	0.195022084	7.971977362	1.56155E-15	6.47632E-15	positive
PRJNA729271	stage_1D_vs_E10	ordinary_muscle_development	ENSGALG00010018535	FAM174B	0.254271216	0.102266675	2.486354583	0.01290593	0.018787934	positive
PRJNA729271	stage_1D_vs_E10	ordinary_muscle_development	ENSGALG00010018536	ENSGALG00010018536	0.655005336	0.242747267	2.69830159	0.006969427	0.010509971	positive
PRJNA729271	stage_1D_vs_E10	ordinary_muscle_development	ENSGALG00010018537	ENSGALG00010018537	-1.606114413	0.717601422	-2.23817061	0.025209931	0.035160007	negative
PRJNA729271	stage_1D_vs_E10	ordinary_muscle_development	ENSGALG00010018539	CHD2	0.235434116	0.088047965	2.673930252	0.007496804	0.011264246	positive
PRJNA729271	stage_1D_vs_E10	ordinary_muscle_development	ENSGALG00010018540	TNFSF10	1.130752065	0.108718921	10.4006925	2.46138E-25	1.7161E-24	positive
PRJNA729271	stage_1D_vs_E10	ordinary_muscle_development	ENSGALG00010018542	ZMIZ2	2.209266174	0.262270474	8.423617569	3.65034E-17	1.66809E-16	positive

PRJNA729271	stage_1D_vs_E10	ordinary_muscle_development	ENSGALG00010018543	MRPL3	-0.603562374	0.077442515	-7.793682493	6.5084E-15	2.59174E-14	negative
PRJNA729271	stage_1D_vs_E10	ordinary_muscle_development	ENSGALG00010018544	HACD2	-0.528151946	0.100668541	-5.246444809	1.55062E-07	3.6925E-07	negative
PRJNA729271	stage_1D_vs_E10	ordinary_muscle_development	ENSGALG00010018546	SNRPGP15	-1.24821233	0.136369497	-9.153163691	5.52901E-20	2.95731E-19	negative
PRJNA729271	stage_1D_vs_E10	ordinary_muscle_development	ENSGALG00010018548	RGMA	-0.234897134	0.123516535	-1.901746471	0.057204307	0.075507367	negative
PRJNA729271	stage_1D_vs_E10	ordinary_muscle_development	ENSGALG00010018549	GHSR	-2.514268187	0.519704134	-4.837883756	1.31229E-06	2.90096E-06	negative
PRJNA729271	stage_1D_vs_E10	ordinary_muscle_development	ENSGALG00010018551	FBXO34	1.610860056	0.110998203	14.51248774	1.0099E-47	1.76794E-46	positive
PRJNA729271	stage_1D_vs_E10	ordinary_muscle_development	ENSGALG00010018552	DTD1	0.210775774	0.103142554	2.043538431	0.040999167	0.0553955	positive
PRJNA729271	stage_1D_vs_E10	ordinary_muscle_development	ENSGALG00010018553	ENSGALG00010018553	-0.269727531	0.425508808	-0.63389412	0.526149923	0.573122653	negative
PRJNA729271	stage_1D_vs_E10	ordinary_muscle_development	ENSGALG00010018554	PPIA	-1.044406659	0.123612976	-8.449005048	2.93796E-17	1.35107E-16	negative
PRJNA729271	stage_1D_vs_E10	ordinary_muscle_development	ENSGALG00010018555	UBN1	-0.23039596	0.07274597	-3.167130237	0.001539514	0.002512696	negative
PRJNA729271	stage_1D_vs_E10	ordinary_muscle_development	ENSGALG00010018556	MRPL2	1.635297995	0.125817796	12.99735055	1.26656E-38	1.57857E-37	positive
PRJNA729271	stage_1D_vs_E10	ordinary_muscle_development	ENSGALG00010018557	TBCC	0.191612581	0.10232501	1.872587951	0.061125307	0.080378419	positive
PRJNA729271	stage_1D_vs_E10	ordinary_muscle_development	ENSGALG00010018558	ENSGALG00010018558	0.240270015	0.237670402	1.010937889	0.312046158	0.359490468	positive
PRJNA729271	stage_1D_vs_E10	ordinary_muscle_development	ENSGALG00010018559	DLG3	-1.586065239	0.130049289	-12.19587781	3.26962E-34	3.42683E-33	negative
PRJNA729271	stage_1D_vs_E10	ordinary_muscle_development	ENSGALG00010018560	ENSGALG00010018560	0.571527346	0.080294255	7.117910836	1.09575E-12	3.77446E-12	positive
PRJNA729271	stage_1D_vs_E10	ordinary_muscle_development	ENSGALG00010018561	ACTR1A	0.036103409	0.059118421	0.610696436	0.541400566	0.587615215	positive
PRJNA729271	stage_1D_vs_E10	ordinary_muscle_development	ENSGALG00010018562	ENSGALG00010018562	-2.465067493	0.764290497	-3.225301769	0.001258399	0.002076323	negative
PRJNA729271	stage_1D_vs_E10	ordinary_muscle_development	ENSGALG00010018564	DLGAP5	-2.005206679	0.153542463	-13.05962302	5.60044E-39	7.09601E-38	negative
PRJNA729271	stage_1D_vs_E10	ordinary_muscle_development	ENSGALG00010018566	ENSGALG00010018566	1.532686345	0.141795642	10.80912167	3.1164E-27	2.35272E-26	positive
PRJNA729271	stage_1D_vs_E10	ordinary_muscle_development	ENSGALG00010018568	FAM136A	-0.325700356	0.193292556	-1.685012417	0.091986176	0.117713394	negative
PRJNA729271	stage_1D_vs_E10	ordinary_muscle_development	ENSGALG00010018573	SEC23B	-0.857269736	0.079677236	-10.75928054	5.35869E-27	4.01003E-26	negative
PRJNA729271	stage_1D_vs_E10	ordinary_muscle_development	ENSGALG00010018574	ENSGALG00010018574	-1.110986899	0.228994419	-4.851589406	1.22476E-06	2.71534E-06	negative
PRJNA729271	stage_1D_vs_E10	ordinary_muscle_development	ENSGALG00010018576	PTK7	-1.510678569	0.113412447	-13.32021848	1.7659E-40	2.36132E-39	negative
PRJNA729271	stage_1D_vs_E10	ordinary_muscle_development	ENSGALG00010018577	NARFL	0.633303952	0.080769094	7.840919334	4.4726E-15	1.80184E-14	positive
PRJNA729271	stage_1D_vs_E10	ordinary_muscle_development	ENSGALG00010018579	FNDC3B	0.90556228	0.10102129	8.964073597	3.129E-19	1.60321E-18	positive
PRJNA729271	stage_1D_vs_E10	ordinary_muscle_development	ENSGALG00010018581	SUFU	-0.561783184	0.091688453	-6.127087619	8.95022E-10	2.54031E-09	negative
PRJNA729271	stage_1D_vs_E10	ordinary_muscle_development	ENSGALG00010018582	RCC2	-1.765363076	0.111435764	-15.84197944	1.59713E-56	3.87779E-55	negative
PRJNA729271	stage_1D_vs_E10	ordinary_muscle_development	ENSGALG00010018583	PPL	2.777978292	0.21292161	13.04695326	6.61399E-39	8.35103E-38	positive
PRJNA729271	stage_1D_vs_E10	ordinary_muscle_development	ENSGALG00010018585	GDPD2	-2.639291865	0.638992044	-4.130398631	3.62135E-05	7.00727E-05	negative
PRJNA729271	stage_1D_vs_E10	ordinary_muscle_development	ENSGALG00010018587	SRF	0.605953754	0.173383454	3.494876463	0.000474281	0.000818905	positive
PRJNA729271	stage_1D_vs_E10	ordinary_muscle_development	ENSGALG00010018588	POLR3F	0.465833988	0.102122459	4.561523403	5.07838E-06	1.06559E-05	positive
PRJNA729271	stage_1D_vs_E10	ordinary_muscle_development	ENSGALG00010018590	TGFA	-0.752957635	0.520023341	-1.447930461	0.1476365	0.182328624	negative
PRJNA729271	stage_1D_vs_E10	ordinary_muscle_development	ENSGALG00010018593	LGALS3	-2.005843043	0.358753763	-5.591141469	2.25582E-08	5.72345E-08	negative
PRJNA729271	stage_1D_vs_E10	ordinary_muscle_development	ENSGALG00010018594	BICRAL	0.514942298	0.103868195	4.957651353	7.13505E-07	1.61195E-06	positive
PRJNA729271	stage_1D_vs_E10	ordinary_muscle_development	ENSGALG00010018598	ENSGALG00010018598	0.390483485	0.077661651	5.028009034	4.95599E-07	1.13415E-06	positive
PRJNA729271	stage_1D_vs_E10	ordinary_muscle_development	ENSGALG00010018601	CPNE4	-2.552870122	0.295833044	-8.629428582	6.16592E-18	2.94675E-17	negative
PRJNA729271	stage_1D_vs_E10	ordinary_muscle_development	ENSGALG00010018602	MCTP2	-1.214747342	0.224098623	-5.420592626	5.94018E-08	1.45987E-07	negative
PRJNA729271	stage_1D_vs_E10	ordinary_muscle_development	ENSGALG00010018604	FAM43A	1.465573239	0.123608218	11.85660035	1.98888E-32	1.93093E-31	positive
PRJNA729271	stage_1D_vs_E10	ordinary_muscle_development	ENSGALG00010018606	DZANK1	-0.808509751	0.247495709	-3.266762702	0.001087848	0.001806434	negative
PRJNA729271	stage_1D_vs_E10	ordinary_muscle_development	ENSGALG00010018607	HAGHL	-0.389463173	0.118466539	-3.287537354	0.001010678	0.001684463	negative
PRJNA729271	stage_1D_vs_E10	ordinary_muscle_development	ENSGALG00010018608	TRIM8	0.664606792	0.12550644	5.295399898	1.18756E-07	2.85563E-07	positive
PRJNA729271	stage_1D_vs_E10	ordinary_muscle_development	ENSGALG00010018609	ENSGALG00010018609	0.018510414	0.125092241	0.147974119	0.882363194	0.899743545	positive
PRJNA729271	stage_1D_vs_E10	ordinary_muscle_development	ENSGALG00010018611	NUDCD3	0.144033551	0.113008671	1.274535396	0.202473737	0.242931613	positive
PRJNA729271	stage_1D_vs_E10	ordinary_muscle_development	ENSGALG00010018613	PADI3	4.009076375	0.350148156	11.44965726	2.36077E-30	2.08147E-29	positive
PRJNA729271	stage_1D_vs_E10	ordinary_muscle_development	ENSGALG00010018615	HTR2C	0.006085157	0.253202468	0.024032771	0.980826468	0.983608666	positive
PRJNA729271	stage_1D_vs_E10	ordinary_muscle_development	ENSGALG00010018616	ENSGALG00010018616	2.770748998	0.849311822	3.262345968	0.001104942	0.001833979	positive
PRJNA729271	stage_1D_vs_E10	ordinary_muscle_development	ENSGALG00010018618	FAHD1	0.558098928	0.170054352	3.281885586	0.001031154	0.001717603	positive
PRJNA729271	stage_1D_vs_E10	ordinary_muscle_development	ENSGALG00010018619	MAPK1IP1L	-0.054798391	0.075648227	-0.724384344	0.468829808	0.517569541	negative
PRJNA729271	stage_1D_vs_E10	ordinary_muscle_development	ENSGALG00010018623	ARRDC4	1.859406816	0.130856223	14.20954065	7.99482E-46	1.29192E-44	positive
PRJNA729271	stage_1D_vs_E10	ordinary_muscle_development	ENSGALG00010018624	ENSGALG00010018624	0.037254029	0.402061816	0.092657466	0.926175688	0.937101536	positive
PRJNA729271	stage_1D_vs_E10	ordinary_muscle_development	ENSGALG00010018627	MYLK	-0.71586437	0.179983128	-3.977397092	6.96738E-05	0.000131295	negative
PRJNA729271	stage_1D_vs_E10	ordinary_muscle_development	ENSGALG00010018628	XDH	0.349000811	0.590557077	0.590968807	0.554541321	0.600246581	positive
PRJNA729271	stage_1D_vs_E10	ordinary_muscle_development	ENSGALG00010018631	KAT14	-0.415919446	0.052248062	-7.96047607	1.71379E-15	7.09144E-15	negative
PRJNA729271	stage_1D_vs_E10	ordinary_muscle_development	ENSGALG00010018633	ENSGALG00010018633	0.851309628	0.182069272	4.67574578	2.92887E-06	6.29041E-06	positive
PRJNA729271	stage_1D_vs_E10	ordinary_muscle_development	ENSGALG00010018638	SEC14L5	0.727772195	0.162811088	4.470040735	7.82047E-06	1.61478E-05	positive
PRJNA729271	stage_1D_vs_E10	ordinary_muscle_development	ENSGALG00010018641	ENSGALG00010018641	-7.494075238	1.05106112	-7.130008996	1.00362E-12	3.47031E-12	negative
PRJNA729271	stage_1D_vs_E10	ordinary_muscle_development	ENSGALG00010018644	ESF1	-0.43373132	0.096704156	-4.485136298	7.28673E-06	1.50801E-05	negative
PRJNA729271	stage_1D_vs_E10	ordinary_muscle_development	ENSGALG00010018649	HAGH	1.24945361	0.106852441	11.69326224	1.37983E-31	1.28953E-30	positive
PRJNA729271	stage_1D_vs_E10	ordinary_muscle_development	ENSGALG00010018654	ENSGALG00010018654	0.066186869	0.227709021	0.290664239	0.771308125	0.800752848	positive
PRJNA729271	stage_1D_vs_E10	ordinary_muscle_development	ENSGALG00010018655	BIRC5	-1.810869451	0.208997604	-8.664546472	4.53314E-18	2.18102E-17	negative
PRJNA729271	stage_1D_vs_E10	ordinary_muscle_development	ENSGALG00010018662	PADI1	0.784026798	0.880076211	0.890862391	0.373002999	0.422165883	positive
PRJNA729271	stage_1D_vs_E10	ordinary_muscle_development	ENSGALG00010018665	IL13RA2	-1.373802217	0.333796327	-4.115690036	3.86023E-05	7.44961E-05	negative
PRJNA729271	stage_1D_vs_E10	ordinary_muscle_development	ENSGALG00010018668	OVOL2	-6.274099399	1.085633824	-5.779204054	7.50549E-09	1.98345E-08	negative
PRJNA729271	stage_1D_vs_E10	ordinary_muscle_development	ENSGALG00010018670	MRAS	1.880559343	0.21291313	8.832519343	1.02344E-18	5.09961E-18	positive
PRJNA729271	stage_1D_vs_E10	ordinary_muscle_development	ENSGALG00010018672	PKDCC	-1.493434633	0.098612398	-15.14449166	8.23892E-52	1.69876E-50	negative
PRJNA729271	stage_1D_vs_E10	ordinary_muscle_development	ENSGALG00010018673	NAGPA	-0.046970874	0.090276925	-0.520297668	0.602856121	0.645710447	negative
PRJNA729271	stage_1D_vs_E10	ordinary_muscle_development	ENSGALG00010018684	PADI2	8.451820983	0.962227806	8.783596705	1.58329E-18	7.81667E-18	positive
PRJNA729271	stage_1D_vs_E10	ordinary_muscle_development	ENSGALG00010018685	ARL3	-2.137965407	0.104751524	-20.40987393	1.36637E-92	8.68662E-91	negative
PRJNA729271	stage_1D_vs_E10	ordinary_muscle_development	ENSGALG00010018693	ENSGALG00010018693	0.441757824	0.163984293	2.69390328	0.007062065	0.010641921	positive
PRJNA729271	stage_1D_vs_E10	ordinary_muscle_development	ENSGALG00010018694	MGME1	-1.06812245	0.101636464	-10.5092445	7.83184E-26	5.55666E-25	negative
PRJNA729271	stage_1D_vs_E10	ordinary_muscle_development	ENSGALG00010018698	MRPS16	0.664503891	0.114473386	5.80487672	6.44134E-09	1.71065E-08	positive
PRJNA729271	stage_1D_vs_E10	ordinary_muscle_development	ENSGALG00010018701	SFXN2	-1.14947864	0.120793617	-9.516054498	1.79879E-21	1.04252E-20	negative
PRJNA729271	stage_1D_vs_E10	ordinary_muscle_development	ENSGALG00010018704	PGPEP1L	1.065128635	0.436954532	2.437618921	0.01478435	0.021329667	positive
PRJNA729271	stage_1D_vs_E10	ordinary_muscle_development	ENSGALG00010018707	SLC45A1	0.49871981	0.098890062	5.043174225	4.57872E-07	1.0498E-06	positive
PRJNA729271	stage_1D_vs_E10	ordinary_muscle_development	ENSGALG00010018709	SNX5	-0.375136616	0.113671788	-3.300173453	0.000966251	0.001614316	negative
PRJNA729271	stage_1D_vs_E10	ordinary_muscle_development	ENSGALG00010018713	ENSGALG00010018713	-0.395155211	0.13589906	-2.907711141	0.003640845	0.005686858	negative
PRJNA729271	stage_1D_vs_E10	ordinary_muscle_development	ENSGALG00010018715	DNAJC13	-0.080028717	0.10310207	-0.776208631	0.437625811	0.486941439	negative
PRJNA729271	stage_1D_vs_E10	ordinary_muscle_development	ENSGALG00010018717	MEIOB	-5.316728906	0.986228426	-5.390971063	7.0078E-08	1.7144E-07	negative
PRJNA729271	stage_1D_vs_E10	ordinary_muscle_development	ENSGALG00010018719	ERRFI1	-0.445140595	0.08597719	-5.177426629	2.24967E-07	5.27996E-07	negative
PRJNA729271	stage_1D_vs_E10	ordinary_muscle_development	ENSGALG00010018721	LRCH2	0.385688865	0.188503798	2.046053549	0.040751102	0.055080868	positive
PRJNA729271	stage_1D_vs_E10	ordinary_muscle_development	ENSGALG00010018724	RRBP1	-0.471700369	0.141290142	-3.338522866	0.000842251	0.001416127	negative
PRJNA729271	stage_1D_vs_E10	ordinary_muscle_development	ENSGALG00010018726	EBF2	-0.203789172	0.155577595	-1.309887663	0.190233841	0.229805778	negative
PRJNA729271	stage_1D_vs_E10	ordinary_muscle_development	ENSGALG00010018727	PARK7	-0.623954766	0.133210598	-4.683972431	2.81368E-06	6.05288E-06	negative
PRJNA729271	stage_1D_vs_E10	ordinary_muscle_development	ENSGALG00010018729	HS3ST6	-6.061830278	0.535350029	-11.32311562	1.00824E-29	8.58156E-29	negative
PRJNA729271	stage_1D_vs_E10	ordinary_muscle_development	ENSGALG00010018730	IGF1R	0.387783273	0.086440771	4.486115382	7.25335E-06	1.50153E-05	positive
PRJNA729271	stage_1D_vs_E10	ordinary_muscle_development	ENSGALG00010018732	ENSGALG00010018732	3.187831582	0.218694237	14.57666022	3.95392E-48	7.05814E-47	positive
PRJNA729271	stage_1D_vs_E10	ordinary_muscle_development	ENSGALG00010018736	EEF2KMT	0.201244161	0.130536084	1.541674569	0.123152695	0.154334501	positive
PRJNA729271	stage_1D_vs_E10	ordinary_muscle_development	ENSGALG00010018747	FLRT3	-1.783362772	0.122218296	-14.59161874	3.17572E-48	5.68297E-47	negative
PRJNA729271	stage_1D_vs_E10	ordinary_muscle_development	ENSGALG00010018750	SOCS4	-0.020780549	0.125615132	-0.165430299	0.868605307	0.888095996	negative
PRJNA729271	stage_1D_vs_E10	ordinary_muscle_development	ENSGALG00010018752	PLS3	-0.586843658	0.140870928	-4.165825177	3.10228E-05	6.03916E-05	negative
PRJNA729271	stage_1D_vs_E10	ordinary_muscle_development	ENSGALG00010018753	SYNM	1.792642651	0.153708393	11.66262051	1.97858E-31	1.83255E-30	positive
PRJNA729271	stage_1D_vs_E10	ordinary_muscle_development	ENSGALG00010018756	EML4	-1.749520024	0.06249019	-27.99671484	1.7815E-172	4.6951E-170	negative
PRJNA729271	stage_1D_vs_E10	ordinary_muscle_development	ENSGALG00010018758	ENSGALG00010018758	-1.813690818	0.140879074	-12.87409669	6.29715E-38	7.66391E-37	negative
PRJNA729271	stage_1D_vs_E10	ordinary_muscle_development	ENSGALG00010018764	TMEM177	-0.065802466	0.091234461	-0.721245737	0.470758336	0.519461182	negative
PRJNA729271	stage_1D_vs_E10	ordinary_muscle_development	ENSGALG00010018767	NDUFB6	1.672300632	0.168912983	9.900367663	4.14747E-23	2.59351E-22	positive
PRJNA729271	stage_1D_vs_E10	ordinary_muscle_development	ENSGALG00010018774	MSRB1	0.056010981	0.124306318	0.450588367	0.652286252	0.691905542	positive
PRJNA729271	stage_1D_vs_E10	ordinary_muscle_development	ENSGALG00010018780	WDHD1	-1.92180566	0.204390489	-9.402617848	5.32221E-21	3.01114E-20	negative
PRJNA729271	stage_1D_vs_E10	ordinary_muscle_development	ENSGALG00010018783	ENSGALG00010018783	-0.820865312	0.179062808	-4.584231207	4.5566E-06	9.5958E-06	negative
PRJNA729271	stage_1D_vs_E10	ordinary_muscle_development	ENSGALG00010018786	DSTN	-1.477307228	0.114919495	-12.85514902	8.04706E-38	9.75269E-37	negative
PRJNA729271	stage_1D_vs_E10	ordinary_muscle_development	ENSGALG00010018796	PER3	0.923461339	0.309416348	2.984526654	0.002840175	0.004489458	positive
PRJNA729271	stage_1D_vs_E10	ordinary_muscle_development	ENSGALG00010018799	ENSGALG00010018799	-3.278680081	0.431049883	-7.606266035	2.82128E-14	1.08129E-13	negative
PRJNA729271	stage_1D_vs_E10	ordinary_muscle_development	ENSGALG00010018806	GCH1	-0.679925095	0.317774739	-2.139644886	0.032383476	0.044463246	negative
PRJNA729271	stage_1D_vs_E10	ordinary_muscle_development	ENSGALG00010018807	RD3	-3.395801176	0.267852542	-12.67787549	7.84289E-37	9.16796E-36	negative
PRJNA729271	stage_1D_vs_E10	ordinary_muscle_development	ENSGALG00010018808	MATCAP2	-1.238930107	0.090710611	-13.65805047	1.80758E-42	2.58903E-41	negative
PRJNA729271	stage_1D_vs_E10	ordinary_muscle_development	ENSGALG00010018811	VAMP3	0.565196529	0.123724944	4.568169613	4.92002E-06	1.03341E-05	positive
PRJNA729271	stage_1D_vs_E10	ordinary_muscle_development	ENSGALG00010018815	ENSGALG00010018815	0.84544458	0.170333025	4.963480104	6.92411E-07	1.56527E-06	positive
PRJNA729271	stage_1D_vs_E10	ordinary_muscle_development	ENSGALG00010018819	LRRC28	-0.124786078	0.078605798	-1.587492034	0.11240125	0.141686765	negative
PRJNA729271	stage_1D_vs_E10	ordinary_muscle_development	ENSGALG00010018822	TRAF5	-0.894598967	0.220094895	-4.064605716	4.81137E-05	9.21158E-05	negative
PRJNA729271	stage_1D_vs_E10	ordinary_muscle_development	ENSGALG00010018823	RAB40C	-0.392967752	0.075340119	-5.215916252	1.82911E-07	4.32723E-07	negative
PRJNA729271	stage_1D_vs_E10	ordinary_muscle_development	ENSGALG00010018829	PTPN4	1.989494836	0.138040746	14.41237382	4.32583E-47	7.34226E-46	positive
PRJNA729271	stage_1D_vs_E10	ordinary_muscle_development	ENSGALG00010018831	WBP1L	1.039352579	0.240228961	4.32650824	1.51492E-05	3.03549E-05	positive
PRJNA729271	stage_1D_vs_E10	ordinary_muscle_development	ENSGALG00010018832	EEPD1	1.734585304	0.183921871	9.431098595	4.05816E-21	2.3086E-20	positive
PRJNA729271	stage_1D_vs_E10	ordinary_muscle_development	ENSGALG00010018834	SAMD4A	1.650249382	0.171220232	9.638168114	5.51636E-22	3.30003E-21	positive
PRJNA729271	stage_1D_vs_E10	ordinary_muscle_development	ENSGALG00010018835	CAMK2B	1.602169214	0.170863466	9.376897515	6.79425E-21	3.81864E-20	positive
PRJNA729271	stage_1D_vs_E10	ordinary_muscle_development	ENSGALG00010018837	RCOR3	-1.235136291	0.095192322	-12.97516715	1.69227E-38	2.10012E-37	negative
PRJNA729271	stage_1D_vs_E10	ordinary_muscle_development	ENSGALG00010018838	WFIKKN1	-3.137996638	0.232593836	-13.49131468	1.75935E-41	2.44504E-40	negative
PRJNA729271	stage_1D_vs_E10	ordinary_muscle_development	ENSGALG00010018839	RPL3L	3.897696195	0.253609805	15.36887025	2.64745E-53	5.71905E-52	positive
PRJNA729271	stage_1D_vs_E10	ordinary_muscle_development	ENSGALG00010018843	MCRIP2	2.597052202	0.235698439	11.01853799	3.1107E-28	2.46123E-27	positive
PRJNA729271	stage_1D_vs_E10	ordinary_muscle_development	ENSGALG00010018847	CGRRF1	-0.303956581	0.157040404	-1.93553107	0.05292516	0.07028403	negative
PRJNA729271	stage_1D_vs_E10	ordinary_muscle_development	ENSGALG00010018848	MEF2A	1.74683546	0.11446336	15.26108838	1.38912E-52	2.93946E-51	positive
PRJNA729271	stage_1D_vs_E10	ordinary_muscle_development	ENSGALG00010018849	KCNH1	-1.476956335	0.325207623	-4.54157968	5.58343E-06	1.1665E-05	negative
PRJNA729271	stage_1D_vs_E10	ordinary_muscle_development	ENSGALG00010018852	NDUFB10	1.701370526	0.11577324	14.69571486	6.867E-49	1.26961E-47	positive
PRJNA729271	stage_1D_vs_E10	ordinary_muscle_development	ENSGALG00010018854	LYSMD4	0.514223458	0.17109452	3.005493448	0.002651503	0.004204522	positive
PRJNA729271	stage_1D_vs_E10	ordinary_muscle_development	ENSGALG00010018855	SYNDIG1	-1.65761589	0.614809446	-2.696145775	0.007014696	0.010573837	negative
PRJNA729271	stage_1D_vs_E10	ordinary_muscle_development	ENSGALG00010018857	ENSGALG00010018857	0.172944072	0.082267417	2.102218341	0.035534154	0.048508906	positive
PRJNA729271	stage_1D_vs_E10	ordinary_muscle_development	ENSGALG00010018858	EPB41L5	-0.315635734	0.113799065	-2.773623271	0.005543583	0.008471717	negative
PRJNA729271	stage_1D_vs_E10	ordinary_muscle_development	ENSGALG00010018860	AGTR2	2.331490288	0.214583716	10.86517807	1.68893E-27	1.28984E-26	positive
PRJNA729271	stage_1D_vs_E10	ordinary_muscle_development	ENSGALG00010018864	ABCA3	1.077726906	0.11883695	9.0689546	1.20164E-19	6.30852E-19	positive
PRJNA729271	stage_1D_vs_E10	ordinary_muscle_development	ENSGALG00010018865	ENSGALG00010018865	-1.601747293	0.231234633	-6.9269351	4.30056E-12	1.42451E-11	negative
PRJNA729271	stage_1D_vs_E10	ordinary_muscle_development	ENSGALG00010018867	RPS2	-0.150759307	0.155936004	-0.966802428	0.33364281	0.382213905	negative
PRJNA729271	stage_1D_vs_E10	ordinary_muscle_development	ENSGALG00010018872	KCTD9	0.043443211	0.10075724	0.431167141	0.666346844	0.705271815	positive

PRJNA729271	stage_1D_vs_E10	ordinary_muscle_development	ENSGALG00010018874	ENSGALG00010018874	-0.70301087	0.116363219	-6.041521337	1.52668E-09	4.25398E-09	negative
PRJNA729271	stage_1D_vs_E10	ordinary_muscle_development	ENSGALG00010018876	GMFB	-1.367942329	0.098280335	-13.91877963	4.87151E-44	7.37083E-43	negative
PRJNA729271	stage_1D_vs_E10	ordinary_muscle_development	ENSGALG00010018877	CST7	1.931638145	0.896258142	2.155225212	0.031144215	0.042846943	positive
PRJNA729271	stage_1D_vs_E10	ordinary_muscle_development	ENSGALG00010018880	TMEM17	-0.205564849	0.253766125	-0.810056303	0.417907817	0.46726117	negative
PRJNA729271	stage_1D_vs_E10	ordinary_muscle_development	ENSGALG00010018881	ENSGALG00010018881	1.031662326	0.632024154	1.632314714	0.102613205	0.130300342	positive
PRJNA729271	stage_1D_vs_E10	ordinary_muscle_development	ENSGALG00010018882	HHAT	-0.144318937	0.155885042	-0.925803624	0.354548043	0.403420511	negative
PRJNA729271	stage_1D_vs_E10	ordinary_muscle_development	ENSGALG00010018883	TBL3	-0.845325748	0.07467254	-11.32043656	1.03953E-29	8.84271E-29	negative
PRJNA729271	stage_1D_vs_E10	ordinary_muscle_development	ENSGALG00010018885	EFEMP1	1.326621306	0.2072368	6.401475537	1.53882E-10	4.60187E-10	positive
PRJNA729271	stage_1D_vs_E10	ordinary_muscle_development	ENSGALG00010018887	ADAMTS17	-2.334495661	0.201739128	-11.57185363	5.72326E-31	5.17522E-30	negative
PRJNA729271	stage_1D_vs_E10	ordinary_muscle_development	ENSGALG00010018891	GNRH1	-2.032016021	0.343413426	-5.917112922	3.27642E-09	8.8969E-09	negative
PRJNA729271	stage_1D_vs_E10	ordinary_muscle_development	ENSGALG00010018892	APMAP	-0.121546705	0.123113463	-0.987273872	0.323508396	0.371691043	negative
PRJNA729271	stage_1D_vs_E10	ordinary_muscle_development	ENSGALG00010018894	SERTAD4	-0.787596935	0.092065688	-8.554728165	1.18147E-17	5.55298E-17	negative
PRJNA729271	stage_1D_vs_E10	ordinary_muscle_development	ENSGALG00010018895	SEPTIN7	-1.013505215	0.089824117	-11.2832193	1.58825E-29	1.34081E-28	negative
PRJNA729271	stage_1D_vs_E10	ordinary_muscle_development	ENSGALG00010018897	CCDC85A	-0.449791632	0.166881729	-2.695271881	0.007033122	0.010600509	negative
PRJNA729271	stage_1D_vs_E10	ordinary_muscle_development	ENSGALG00010018899	ACSS1	0.29080196	0.098034379	2.966326315	0.003013805	0.004751176	positive
PRJNA729271	stage_1D_vs_E10	ordinary_muscle_development	ENSGALG00010018901	TCF7L2	0.40920339	0.146781869	2.787833362	0.005306183	0.008130351	positive
PRJNA729271	stage_1D_vs_E10	ordinary_muscle_development	ENSGALG00010018902	NOXO1	-2.909875631	0.767659126	-3.790582997	0.000150294	0.000274234	negative
PRJNA729271	stage_1D_vs_E10	ordinary_muscle_development	ENSGALG00010018903	CAMTA1	-0.278845132	0.096424114	-2.891860983	0.003829674	0.005967013	negative
PRJNA729271	stage_1D_vs_E10	ordinary_muscle_development	ENSGALG00010018904	UGP2	2.836758574	0.271167903	10.46126234	1.30111E-25	9.17738E-25	positive
PRJNA729271	stage_1D_vs_E10	ordinary_muscle_development	ENSGALG00010018908	ENSGALG00010018908	0.330624001	0.231854061	1.426000473	0.153868205	0.1892186	positive
PRJNA729271	stage_1D_vs_E10	ordinary_muscle_development	ENSGALG00010018909	TTBK1	-0.009340791	0.131107099	-0.071245501	0.943202369	0.951671887	negative
PRJNA729271	stage_1D_vs_E10	ordinary_muscle_development	ENSGALG00010018911	CNIH1	-0.106424565	0.093947869	-1.132804467	0.257296375	0.302302907	negative
PRJNA729271	stage_1D_vs_E10	ordinary_muscle_development	ENSGALG00010018913	ENSGALG00010018913	-0.107677106	0.227422131	-0.473468019	0.635879312	0.676680907	negative
PRJNA729271	stage_1D_vs_E10	ordinary_muscle_development	ENSGALG00010018915	DOCK5	-0.29485756	0.187865125	-1.56951728	0.116527456	0.146518518	negative
PRJNA729271	stage_1D_vs_E10	ordinary_muscle_development	ENSGALG00010018916	CERS3	-1.314879579	0.330316867	-3.980661329	6.87238E-05	0.000129589	negative
PRJNA729271	stage_1D_vs_E10	ordinary_muscle_development	ENSGALG00010018917	DNAJC11	-0.154504671	0.105412564	-1.465713997	0.142726219	0.176610598	negative
PRJNA729271	stage_1D_vs_E10	ordinary_muscle_development	ENSGALG00010018919	RALB	-0.289370447	0.107643408	-2.688231923	0.007183149	0.010816512	negative
PRJNA729271	stage_1D_vs_E10	ordinary_muscle_development	ENSGALG00010018920	ACAD11	-0.333430862	0.115383322	-2.889766516	0.003855281	0.006004974	negative
PRJNA729271	stage_1D_vs_E10	ordinary_muscle_development	ENSGALG00010018922	EEF2K	1.37257769	0.114117635	12.02774386	2.54007E-33	2.57471E-32	positive
PRJNA729271	stage_1D_vs_E10	ordinary_muscle_development	ENSGALG00010018923	VPS54	-0.09820056	0.091505974	-1.073160106	0.283199287	0.329346411	negative
PRJNA729271	stage_1D_vs_E10	ordinary_muscle_development	ENSGALG00010018925	LINS1	-0.475442429	0.130080979	-3.654972721	0.000257209	0.000456278	negative
PRJNA729271	stage_1D_vs_E10	ordinary_muscle_development	ENSGALG00010018926	SLC22A7	-0.30323499	1.089843615	-0.278237157	0.780830313	0.80942111	negative
PRJNA729271	stage_1D_vs_E10	ordinary_muscle_development	ENSGALG00010018932	CDKN3	-1.895617546	0.233994755	-8.101111266	5.44594E-16	2.33133E-15	negative
PRJNA729271	stage_1D_vs_E10	ordinary_muscle_development	ENSGALG00010018933	PHF13	0.007214967	0.08758932	0.08237268	0.93435036	0.94426964	positive
PRJNA729271	stage_1D_vs_E10	ordinary_muscle_development	ENSGALG00010018936	SYT14	2.782896545	0.214244142	12.98937054	1.40578E-38	1.74909E-37	positive
PRJNA729271	stage_1D_vs_E10	ordinary_muscle_development	ENSGALG00010018937	INHBB	-3.344426815	0.363460842	-9.20161521	3.52609E-20	1.90285E-19	negative
PRJNA729271	stage_1D_vs_E10	ordinary_muscle_development	ENSGALG00010018941	KLHL21	2.491329627	0.143447396	17.36754865	1.45302E-67	4.89803E-66	positive
PRJNA729271	stage_1D_vs_E10	ordinary_muscle_development	ENSGALG00010018943	ACKR4	2.036186133	0.34962551	5.823906087	5.74879E-09	1.53375E-08	positive
PRJNA729271	stage_1D_vs_E10	ordinary_muscle_development	ENSGALG00010018944	NEFL	-2.219334279	0.150882293	-14.70904391	5.63986E-49	1.04406E-47	negative
PRJNA729271	stage_1D_vs_E10	ordinary_muscle_development	ENSGALG00010018945	HABP2	-1.143298708	0.690224414	-1.65641592	0.097637619	0.124374487	negative
PRJNA729271	stage_1D_vs_E10	ordinary_muscle_development	ENSGALG00010018947	ENSGALG00010018947	-0.556811326	0.232988805	-2.389863007	0.016854658	0.024114922	negative
PRJNA729271	stage_1D_vs_E10	ordinary_muscle_development	ENSGALG00010018948	POLR3E	0.105126909	0.108655591	0.967524155	0.333282072	0.381830828	positive
PRJNA729271	stage_1D_vs_E10	ordinary_muscle_development	ENSGALG00010018950	TTL	-0.995787427	0.097349212	-10.22902397	1.46993E-24	9.85052E-24	negative
PRJNA729271	stage_1D_vs_E10	ordinary_muscle_development	ENSGALG00010018951	YKT6	-0.786324	0.089702117	-8.765946997	1.85213E-18	9.10364E-18	negative
PRJNA729271	stage_1D_vs_E10	ordinary_muscle_development	ENSGALG00010018955	ZBTB48	0.555012929	0.142115832	3.905356089	9.40867E-05	0.000175069	positive
PRJNA729271	stage_1D_vs_E10	ordinary_muscle_development	ENSGALG00010018958	PELI3	-0.142886655	0.117653161	-1.214473579	0.224566933	0.267337196	negative
PRJNA729271	stage_1D_vs_E10	ordinary_muscle_development	ENSGALG00010018959	UBA5	-0.135233907	0.115415341	-1.171715178	0.241311427	0.285116493	negative
PRJNA729271	stage_1D_vs_E10	ordinary_muscle_development	ENSGALG00010018960	RBFOX1	-0.283644076	0.218928485	-1.295601512	0.195112808	0.235086203	negative
PRJNA729271	stage_1D_vs_E10	ordinary_muscle_development	ENSGALG00010018961	ASB7	0.052371009	0.097906867	0.534906388	0.592714591	0.636399851	positive
PRJNA729271	stage_1D_vs_E10	ordinary_muscle_development	ENSGALG00010018968	GLI2	-1.117222983	0.195831004	-5.705036273	1.16318E-08	3.01778E-08	negative
PRJNA729271	stage_1D_vs_E10	ordinary_muscle_development	ENSGALG00010018969	DIEXF	-0.596192459	0.089621098	-6.652367287	2.88416E-11	9.05281E-11	negative
PRJNA729271	stage_1D_vs_E10	ordinary_muscle_development	ENSGALG00010018971	POLR1B	-0.447538323	0.099519461	-4.496993045	6.89212E-06	1.43023E-05	negative
PRJNA729271	stage_1D_vs_E10	ordinary_muscle_development	ENSGALG00010018973	NEFM	-5.175643758	0.513045418	-10.08808104	6.2377E-24	4.03821E-23	negative
PRJNA729271	stage_1D_vs_E10	ordinary_muscle_development	ENSGALG00010018974	TAS1R1	0.599122219	0.290926343	2.059360498	0.039459717	0.053450014	positive
PRJNA729271	stage_1D_vs_E10	ordinary_muscle_development	ENSGALG00010018977	NPHP3	0.051346004	0.110175572	0.466038006	0.641188296	0.681729946	positive
PRJNA729271	stage_1D_vs_E10	ordinary_muscle_development	ENSGALG00010018983	MDGA2	-0.828298236	0.166159436	-4.984960571	6.19744E-07	1.40648E-06	negative
PRJNA729271	stage_1D_vs_E10	ordinary_muscle_development	ENSGALG00010018985	NOL9	-0.319359547	0.089027319	-3.587208393	0.000334237	0.000586534	negative
PRJNA729271	stage_1D_vs_E10	ordinary_muscle_development	ENSGALG00010018986	CTDSP1	1.777100693	0.191191783	9.294859169	1.47401E-20	8.13006E-20	positive
PRJNA729271	stage_1D_vs_E10	ordinary_muscle_development	ENSGALG00010018987	MRPS10	-0.352223785	0.108692513	-3.240552407	0.001192983	0.001973781	negative
PRJNA729271	stage_1D_vs_E10	ordinary_muscle_development	ENSGALG00010018990	GCK	-2.19992566	0.428045441	-5.139467561	2.75518E-07	6.42167E-07	negative
PRJNA729271	stage_1D_vs_E10	ordinary_muscle_development	ENSGALG00010018991	RYBP	-1.099893352	0.130480554	-8.429557641	3.46974E-17	1.58806E-16	negative
PRJNA729271	stage_1D_vs_E10	ordinary_muscle_development	ENSGALG00010018993	ENSGALG00010018993	-0.74351733	0.528044053	-1.408059281	0.159113517	0.195188763	negative
PRJNA729271	stage_1D_vs_E10	ordinary_muscle_development	ENSGALG00010018998	RPS29	1.875554742	0.439936005	4.263244471	2.0148E-05	3.99078E-05	positive
PRJNA729271	stage_1D_vs_E10	ordinary_muscle_development	ENSGALG00010018999	THUMPD3	-0.825969933	0.072105247	-11.45505996	2.2181E-30	1.96045E-29	negative
PRJNA729271	stage_1D_vs_E10	ordinary_muscle_development	ENSGALG00010019000	TRERF1	0.333104354	0.118170222	2.818851897	0.004819575	0.007420047	positive
PRJNA729271	stage_1D_vs_E10	ordinary_muscle_development	ENSGALG00010019001	ALDH1A3	0.653150371	0.159799727	4.087305914	4.36412E-05	8.37963E-05	positive
PRJNA729271	stage_1D_vs_E10	ordinary_muscle_development	ENSGALG00010019004	ECE2	-0.266360183	0.203930077	-1.306134859	0.1915067	0.231150772	negative
PRJNA729271	stage_1D_vs_E10	ordinary_muscle_development	ENSGALG00010019005	ENSGALG00010019005	-1.240716724	0.219003314	-5.665287428	1.46778E-08	3.78164E-08	negative
PRJNA729271	stage_1D_vs_E10	ordinary_muscle_development	ENSGALG00010019006	ENSGALG00010019006	-0.405168419	0.077652427	-5.217717393	1.81142E-07	4.28677E-07	negative
PRJNA729271	stage_1D_vs_E10	ordinary_muscle_development	ENSGALG00010019008	HERPUD2	0.100873214	0.044745902	2.254356494	0.02417375	0.033825515	positive
PRJNA729271	stage_1D_vs_E10	ordinary_muscle_development	ENSGALG00010019009	NRAP	4.819745507	0.218328144	22.07569496	5.4126E-108	4.535E-106	positive
PRJNA729271	stage_1D_vs_E10	ordinary_muscle_development	ENSGALG00010019011	VIL1	2.181327493	0.390091314	5.591838156	2.24678E-08	5.70152E-08	positive
PRJNA729271	stage_1D_vs_E10	ordinary_muscle_development	ENSGALG00010019012	ADAM28	-0.884589467	0.367839548	-2.404824257	0.016180245	0.023200381	negative
PRJNA729271	stage_1D_vs_E10	ordinary_muscle_development	ENSGALG00010019013	LRRK1	-0.49411887	0.102467892	-4.822182468	1.41996E-06	3.1323E-06	negative
PRJNA729271	stage_1D_vs_E10	ordinary_muscle_development	ENSGALG00010019015	GSG1L	-3.142818622	0.869747954	-3.613482054	0.000302112	0.000532092	negative
PRJNA729271	stage_1D_vs_E10	ordinary_muscle_development	ENSGALG00010019016	PLEKHG5	0.564361741	0.143681959	3.927853879	8.57073E-05	0.000160135	positive
PRJNA729271	stage_1D_vs_E10	ordinary_muscle_development	ENSGALG00010019017	IRAK2	-0.330521734	0.083101578	-3.977321987	6.96958E-05	0.000131319	negative
PRJNA729271	stage_1D_vs_E10	ordinary_muscle_development	ENSGALG00010019018	SH3BGRL	-1.253865011	0.11007636	-11.39086553	4.64354E-30	4.0232E-29	negative
PRJNA729271	stage_1D_vs_E10	ordinary_muscle_development	ENSGALG00010019019	TBX20	-0.646297703	0.320152452	-2.01871858	0.043516478	0.05857832	negative
PRJNA729271	stage_1D_vs_E10	ordinary_muscle_development	ENSGALG00010019020	KCNK2	0.68472794	0.443794472	1.542894252	0.122856436	0.154014367	positive
PRJNA729271	stage_1D_vs_E10	ordinary_muscle_development	ENSGALG00010019021	LRR1	-1.181101407	0.149264111	-7.912829153	2.51605E-15	1.03023E-14	negative
PRJNA729271	stage_1D_vs_E10	ordinary_muscle_development	ENSGALG00010019023	AP2M1	-0.160053885	0.071519887	-2.237893423	0.025228006	0.035181831	negative
PRJNA729271	stage_1D_vs_E10	ordinary_muscle_development	ENSGALG00010019024	CNPY3	-0.048242826	0.101557601	-0.475029198	0.634766162	0.675843655	negative
PRJNA729271	stage_1D_vs_E10	ordinary_muscle_development	ENSGALG00010019025	DNAAF2	-0.66273173	0.242603196	-2.731751851	0.006299857	0.009563933	negative
PRJNA729271	stage_1D_vs_E10	ordinary_muscle_development	ENSGALG00010019026	CHSY1	-1.452000344	0.12475922	-11.63842116	2.62836E-31	2.41586E-30	negative
PRJNA729271	stage_1D_vs_E10	ordinary_muscle_development	ENSGALG00010019027	GNLY	-0.41945942	0.330238143	-1.270172539	0.204023177	0.244628667	negative
PRJNA729271	stage_1D_vs_E10	ordinary_muscle_development	ENSGALG00010019028	TNFRSF25	5.750436699	0.707080318	8.13264993	4.20006E-16	1.81352E-15	positive
PRJNA729271	stage_1D_vs_E10	ordinary_muscle_development	ENSGALG00010019029	CENPF	-1.304377736	0.259550412	-5.025527508	5.02051E-07	1.14783E-06	negative
PRJNA729271	stage_1D_vs_E10	ordinary_muscle_development	ENSGALG00010019030	CDR2	-1.98900094	0.152284914	-13.06105042	5.49641E-39	6.97029E-38	negative
PRJNA729271	stage_1D_vs_E10	ordinary_muscle_development	ENSGALG00010019032	GNMT	-0.666873374	0.188817306	-3.531844563	0.000412672	0.000717228	negative
PRJNA729271	stage_1D_vs_E10	ordinary_muscle_development	ENSGALG00010019033	CYP2AB4	-3.916448877	1.168804554	-3.350815895	0.000805739	0.001358518	negative
PRJNA729271	stage_1D_vs_E10	ordinary_muscle_development	ENSGALG00010019034	CASP7	0.064450846	0.170669887	0.377634551	0.705702094	0.742103297	positive
PRJNA729271	stage_1D_vs_E10	ordinary_muscle_development	ENSGALG00010019035	ENSGALG00010019035	-1.405091475	0.480824152	-2.922256441	0.003475053	0.005445502	negative
PRJNA729271	stage_1D_vs_E10	ordinary_muscle_development	ENSGALG00010019036	REL	0.274398143	0.109948166	2.495704587	0.012570724	0.018338632	positive
PRJNA729271	stage_1D_vs_E10	ordinary_muscle_development	ENSGALG00010019038	PLEKHS1	-4.110853349	0.543177588	-7.568157159	3.78556E-14	1.4402E-13	negative
PRJNA729271	stage_1D_vs_E10	ordinary_muscle_development	ENSGALG00010019040	HMGN5	-1.917464086	0.082244846	-23.31409418	3.1901E-120	3.6698E-118	negative
PRJNA729271	stage_1D_vs_E10	ordinary_muscle_development	ENSGALG00010019041	KATNIP	-0.522951292	0.089467726	-5.845138971	5.06146E-09	1.35461E-08	negative
PRJNA729271	stage_1D_vs_E10	ordinary_muscle_development	ENSGALG00010019042	PEX6	0.397449715	0.105750693	3.758365129	0.000171027	0.000310035	positive
PRJNA729271	stage_1D_vs_E10	ordinary_muscle_development	ENSGALG00010019043	METTL9	-1.455065212	0.104730262	-13.89345524	6.94048E-44	1.04576E-42	negative
PRJNA729271	stage_1D_vs_E10	ordinary_muscle_development	ENSGALG00010019045	LBX1	2.132672622	0.26363129	8.089603567	5.98592E-16	2.55645E-15	positive
PRJNA729271	stage_1D_vs_E10	ordinary_muscle_development	ENSGALG00010019046	SELENOS	0.252041672	0.08117399	3.104956043	0.001903073	0.003071489	positive
PRJNA729271	stage_1D_vs_E10	ordinary_muscle_development	ENSGALG00010019047	GHRL	-0.744721213	0.445087725	-1.673200969	0.09428774	0.120403558	negative
PRJNA729271	stage_1D_vs_E10	ordinary_muscle_development	ENSGALG00010019049	POLD2	-0.696975606	0.162632285	-4.285591929	1.82253E-05	3.62183E-05	negative
PRJNA729271	stage_1D_vs_E10	ordinary_muscle_development	ENSGALG00010019050	TNS1	1.755324576	0.133230953	13.17505084	1.22145E-39	1.58363E-38	positive
PRJNA729271	stage_1D_vs_E10	ordinary_muscle_development	ENSGALG00010019051	IGSF6	0.738112003	0.264604125	2.789495445	0.005279024	0.008091303	positive
PRJNA729271	stage_1D_vs_E10	ordinary_muscle_development	ENSGALG00010019059	BRWD3	0.853366108	0.076816914	11.10909128	1.13307E-28	9.18557E-28	positive
PRJNA729271	stage_1D_vs_E10	ordinary_muscle_development	ENSGALG00010019062	PUS10	-1.181229066	0.099996526	-11.81270099	3.35604E-32	3.21944E-31	negative
PRJNA729271	stage_1D_vs_E10	ordinary_muscle_development	ENSGALG00010019064	ENSGALG00010019064	-1.086659385	0.172198615	-6.310500137	2.78135E-10	8.17763E-10	negative
PRJNA729271	stage_1D_vs_E10	ordinary_muscle_development	ENSGALG00010019065	CAND2	3.259754101	0.120888354	26.96499701	3.8052E-160	8.1113E-158	positive
PRJNA729271	stage_1D_vs_E10	ordinary_muscle_development	ENSGALG00010019067	GTF3C1	-0.226064445	0.057002583	-3.965863208	7.31308E-05	0.000137506	negative
PRJNA729271	stage_1D_vs_E10	ordinary_muscle_development	ENSGALG00010019068	SNRPA1	-0.188427636	0.163468848	-1.152682227	0.249040839	0.293388082	negative
PRJNA729271	stage_1D_vs_E10	ordinary_muscle_development	ENSGALG00010019072	AEBP1	1.699388388	0.21110134	8.050107053	8.27216E-16	3.49679E-15	positive
PRJNA729271	stage_1D_vs_E10	ordinary_muscle_development	ENSGALG00010019076	ESPN	-0.566624847	0.393574397	-1.439689297	0.149955323	0.184955957	negative
PRJNA729271	stage_1D_vs_E10	ordinary_muscle_development	ENSGALG00010019079	CAPG	1.18004016	0.242413797	4.867875414	1.12804E-06	2.50936E-06	positive
PRJNA729271	stage_1D_vs_E10	ordinary_muscle_development	ENSGALG00010019081	PCSK6	0.976728976	0.135852957	7.189604106	6.49795E-13	2.28113E-12	positive
PRJNA729271	stage_1D_vs_E10	ordinary_muscle_development	ENSGALG00010019082	POLR3K	-0.098913505	0.182322736	-0.542518764	0.587461185	0.631728737	negative
PRJNA729271	stage_1D_vs_E10	ordinary_muscle_development	ENSGALG00010019083	OTOA	-0.173898977	0.228403303	-0.761368049	0.446437266	0.495642476	negative
PRJNA729271	stage_1D_vs_E10	ordinary_muscle_development	ENSGALG00010019087	PEX13	0.302402513	0.086901899	3.479814772	0.000501761	0.000863984	positive
PRJNA729271	stage_1D_vs_E10	ordinary_muscle_development	ENSGALG00010019093	RPL32	0.140517054	0.151768863	0.925862201	0.354517597	0.403417536	positive
PRJNA729271	stage_1D_vs_E10	ordinary_muscle_development	ENSGALG00010019094	SNRNP25	-0.100745492	0.168532465	-0.597780923	0.549986121	0.59595192	negative
PRJNA729271	stage_1D_vs_E10	ordinary_muscle_development	ENSGALG00010019097	STC1	-2.529834651	0.263766269	-9.59119852	8.70677E-22	5.14072E-21	negative
PRJNA729271	stage_1D_vs_E10	ordinary_muscle_development	ENSGALG00010019101	POLE2	-1.628159255	0.121465295	-13.40431646	5.70464E-41	7.77882E-40	negative
PRJNA729271	stage_1D_vs_E10	ordinary_muscle_development	ENSGALG00010019104	POLL	-0.530139788	0.122545828	-4.326053337	1.51805E-05	3.04092E-05	negative
PRJNA729271	stage_1D_vs_E10	ordinary_muscle_development	ENSGALG00010019108	KDELR2	-0.498397336	0.099575913	-5.005199782	5.58041E-07	1.27123E-06	negative
PRJNA729271	stage_1D_vs_E10	ordinary_muscle_development	ENSGALG00010019110	MBD4	0.034627798	0.111194417	0.311416694	0.755483865	0.786804039	positive
PRJNA729271	stage_1D_vs_E10	ordinary_muscle_development	ENSGALG00010019113	KLHDC1	1.350417636	0.132981326	10.15494186	3.14998E-24	2.0707E-23	positive
PRJNA729271	stage_1D_vs_E10	ordinary_muscle_development	ENSGALG00010019114	TENT5D	-1.53360783	0.363648538	-4.217280345	2.47266E-05	4.85918E-05	negative
PRJNA729271	stage_1D_vs_E10	ordinary_muscle_development	ENSGALG00010019115	DBNL	-1.227150099	0.103196348	-11.89141014	1.31173E-32	1.28731E-31	negative
PRJNA729271	stage_1D_vs_E10	ordinary_muscle_development	ENSGALG00010019120	DAGLB	0.609950933	0.080535053	7.573732295	3.62651E-14	1.38187E-13	positive
PRJNA729271	stage_1D_vs_E10	ordinary_muscle_development	ENSGALG00010019123	PLEKHO2	1.481121179	0.117663516	12.58776917	2.46543E-36	2.81611E-35	positive
PRJNA729271	stage_1D_vs_E10	ordinary_muscle_development	ENSGALG00010019124	BMPER	-0.341275053	0.248980091	-1.370692133	0.170470948	0.207802236	negative

PRJNA729271	stage_1D_vs_E10	ordinary_muscle_development	ENSGALG00010019125	ENSGALG00010019125	2.142294876	0.196903769	10.8799079	1.43708E-27	1.1004E-26	positive
PRJNA729271	stage_1D_vs_E10	ordinary_muscle_development	ENSGALG00010019127	TMBIM1	1.262203431	0.121854762	10.35826103	3.83878E-25	2.65599E-24	positive
PRJNA729271	stage_1D_vs_E10	ordinary_muscle_development	ENSGALG00010019130	RPS6KC1	-0.308792616	0.08237402	-3.748665118	0.000177778	0.00032179	negative
PRJNA729271	stage_1D_vs_E10	ordinary_muscle_development	ENSGALG00010019132	ENSGALG00010019132	-0.343389606	0.15318798	-2.241622388	0.024985788	0.034873434	negative
PRJNA729271	stage_1D_vs_E10	ordinary_muscle_development	ENSGALG00010019135	KIAA1841	-2.374774038	0.21085222	-11.26274146	2.00425E-29	1.68318E-28	negative
PRJNA729271	stage_1D_vs_E10	ordinary_muscle_development	ENSGALG00010019139	DPCD	-1.818839429	0.137114494	-13.26511422	3.68858E-40	4.85613E-39	negative
PRJNA729271	stage_1D_vs_E10	ordinary_muscle_development	ENSGALG00010019141	ACOT7	-0.87348475	0.154401089	-5.657244754	1.53822E-08	3.95609E-08	negative
PRJNA729271	stage_1D_vs_E10	ordinary_muscle_development	ENSGALG00010019144	IFT122	-1.063829566	0.073438914	-14.48591096	1.48738E-47	2.58507E-46	negative
PRJNA729271	stage_1D_vs_E10	ordinary_muscle_development	ENSGALG00010019145	RAC1	-0.205001781	0.076641192	-2.674825045	0.007476826	0.011240053	negative
PRJNA729271	stage_1D_vs_E10	ordinary_muscle_development	ENSGALG00010019146	KLHDC2	0.438682738	0.085636702	5.122601981	3.01348E-07	7.00119E-07	positive
PRJNA729271	stage_1D_vs_E10	ordinary_muscle_development	ENSGALG00010019148	ENSGALG00010019148	1.057751172	0.211522009	5.000667192	5.71323E-07	1.30046E-06	positive
PRJNA729271	stage_1D_vs_E10	ordinary_muscle_development	ENSGALG00010019149	COX5B	1.491043005	0.179965832	8.285144958	1.17965E-16	5.23708E-16	positive
PRJNA729271	stage_1D_vs_E10	ordinary_muscle_development	ENSGALG00010019158	ENSGALG00010019158	2.788374581	0.249355021	11.18234783	4.9756E-29	4.11886E-28	positive
PRJNA729271	stage_1D_vs_E10	ordinary_muscle_development	ENSGALG00010019166	PNPT1	-0.064496826	0.108637528	-0.593688266	0.552720633	0.598601731	negative
PRJNA729271	stage_1D_vs_E10	ordinary_muscle_development	ENSGALG00010019169	GPR153	-2.809470579	0.23355203	-12.02931347	2.49224E-33	2.528E-32	negative
PRJNA729271	stage_1D_vs_E10	ordinary_muscle_development	ENSGALG00010019172	NEMF	0.221378892	0.086488811	2.559624636	0.010478527	0.015440302	positive
PRJNA729271	stage_1D_vs_E10	ordinary_muscle_development	ENSGALG00010019173	RHO	-1.778925103	0.768445298	-2.314966475	0.020614767	0.029118207	negative
PRJNA729271	stage_1D_vs_E10	ordinary_muscle_development	ENSGALG00010019174	ENSGALG00010019174	0.111501201	0.433117141	0.2574389	0.796839977	0.824189772	positive
PRJNA729271	stage_1D_vs_E10	ordinary_muscle_development	ENSGALG00010019183	ANGEL2	0.160891024	0.078775776	2.042392116	0.041112651	0.055533303	positive
PRJNA729271	stage_1D_vs_E10	ordinary_muscle_development	ENSGALG00010019184	PNKD	-0.589957536	0.185200241	-3.185511708	0.001444983	0.002366402	negative
PRJNA729271	stage_1D_vs_E10	ordinary_muscle_development	ENSGALG00010019185	ANKDD1A	0.263709526	0.208872211	1.262540021	0.206754535	0.247698734	positive
PRJNA729271	stage_1D_vs_E10	ordinary_muscle_development	ENSGALG00010019188	ENSGALG00010019188	1.889116483	0.372517126	5.071220494	3.95272E-07	9.09296E-07	positive
PRJNA729271	stage_1D_vs_E10	ordinary_muscle_development	ENSGALG00010019189	ICMT	-0.150300038	0.093661623	-1.604713158	0.108556945	0.137139003	negative
PRJNA729271	stage_1D_vs_E10	ordinary_muscle_development	ENSGALG00010019193	ENSGALG00010019193	-0.065005937	1.288149547	-0.050464588	0.959752168	0.965922379	negative
PRJNA729271	stage_1D_vs_E10	ordinary_muscle_development	ENSGALG00010019194	ENSGALG00010019194	0.1196745	0.188210199	0.635855551	0.524870576	0.571986993	positive
PRJNA729271	stage_1D_vs_E10	ordinary_muscle_development	ENSGALG00010019204	ARF6	-0.639005597	0.230175005	-2.776172839	0.005500296	0.008411774	negative
PRJNA729271	stage_1D_vs_E10	ordinary_muscle_development	ENSGALG00010019205	ENSGALG00010019205	-0.367865733	0.259102948	-1.419766682	0.155675618	0.191262763	negative
PRJNA729271	stage_1D_vs_E10	ordinary_muscle_development	ENSGALG00010019206	RNF207	2.20817821	0.281320804	7.849324255	4.18285E-15	1.6884E-14	positive
PRJNA729271	stage_1D_vs_E10	ordinary_muscle_development	ENSGALG00010019208	PLXND1	0.686068536	0.157965596	4.343151637	1.40453E-05	2.82602E-05	positive
PRJNA729271	stage_1D_vs_E10	ordinary_muscle_development	ENSGALG00010019209	VCPKMT	0.296490623	0.084163753	3.522782804	0.000427041	0.000740426	positive
PRJNA729271	stage_1D_vs_E10	ordinary_muscle_development	ENSGALG00010019212	RPL22	-0.027809464	0.113868665	-0.24422403	0.807057317	0.83333066	negative
PRJNA729271	stage_1D_vs_E10	ordinary_muscle_development	ENSGALG00010019213	RHBDF1	0.282105719	0.109634308	2.573151813	0.010077699	0.014884476	positive
PRJNA729271	stage_1D_vs_E10	ordinary_muscle_development	ENSGALG00010019215	ENSGALG00010019215	-0.0463465	0.10097279	-0.458999896	0.64623424	0.686490679	negative
PRJNA729271	stage_1D_vs_E10	ordinary_muscle_development	ENSGALG00010019218	RMND5A	0.855041359	0.137540384	6.216656767	5.07859E-10	1.46525E-09	positive
PRJNA729271	stage_1D_vs_E10	ordinary_muscle_development	ENSGALG00010019221	VASH2	-0.850637254	0.231765999	-3.670241783	0.000242321	0.000431081	negative
PRJNA729271	stage_1D_vs_E10	ordinary_muscle_development	ENSGALG00010019223	AAMP	-0.2832276	0.125751677	-2.252276914	0.024304778	0.033999012	negative
PRJNA729271	stage_1D_vs_E10	ordinary_muscle_development	ENSGALG00010019225	PROK2	-4.148712799	0.582868699	-7.117748487	1.09704E-12	3.77711E-12	negative
PRJNA729271	stage_1D_vs_E10	ordinary_muscle_development	ENSGALG00010019227	CLCN5	-0.076860668	0.072986982	-1.053073657	0.292307235	0.338932355	negative
PRJNA729271	stage_1D_vs_E10	ordinary_muscle_development	ENSGALG00010019230	SPG21	-0.627786774	0.098130034	-6.397498759	1.57943E-10	4.71648E-10	negative
PRJNA729271	stage_1D_vs_E10	ordinary_muscle_development	ENSGALG00010019231	ENSGALG00010019231	3.348838096	0.311162619	10.76234063	5.18366E-27	3.88105E-26	positive
PRJNA729271	stage_1D_vs_E10	ordinary_muscle_development	ENSGALG00010019232	SOS2	0.778038355	0.093166026	8.351095198	6.76329E-17	3.05116E-16	positive
PRJNA729271	stage_1D_vs_E10	ordinary_muscle_development	ENSGALG00010019233	ARPC2	-0.91598599	0.053872122	-17.00296851	7.80647E-65	2.45989E-63	negative
PRJNA729271	stage_1D_vs_E10	ordinary_muscle_development	ENSGALG00010019236	MPG	-0.789582446	0.211744002	-3.728948339	0.000192281	0.000346051	negative
PRJNA729271	stage_1D_vs_E10	ordinary_muscle_development	ENSGALG00010019237	SORCS3	-2.7583128	0.345770345	-7.977297194	1.49572E-15	6.21753E-15	negative
PRJNA729271	stage_1D_vs_E10	ordinary_muscle_development	ENSGALG00010019239	CYTH3	0.035330662	0.08109519	0.43566902	0.663076886	0.702067163	positive
PRJNA729271	stage_1D_vs_E10	ordinary_muscle_development	ENSGALG00010019240	ENSGALG00010019240	0.386448387	0.194752779	1.9843023	0.04722215	0.063202684	positive
PRJNA729271	stage_1D_vs_E10	ordinary_muscle_development	ENSGALG00010019243	SERTAD2	1.467772668	0.152081566	9.651220141	4.85743E-22	2.91426E-21	positive
PRJNA729271	stage_1D_vs_E10	ordinary_muscle_development	ENSGALG00010019246	IL17RC	0.906735181	0.134787426	6.727149615	1.73019E-11	5.51431E-11	positive
PRJNA729271	stage_1D_vs_E10	ordinary_muscle_development	ENSGALG00010019247	HBZ	-5.266491745	0.493811082	-10.66499302	1.4841E-26	1.09032E-25	negative
PRJNA729271	stage_1D_vs_E10	ordinary_muscle_development	ENSGALG00010019248	RMI2	-0.12554785	0.165101228	-0.760429533	0.446997876	0.496226868	negative
PRJNA729271	stage_1D_vs_E10	ordinary_muscle_development	ENSGALG00010019250	CXCR1	0.151209833	0.249134226	0.606941229	0.543889915	0.590002787	positive
PRJNA729271	stage_1D_vs_E10	ordinary_muscle_development	ENSGALG00010019252	TATDN3	-0.005022679	0.11638635	-0.043155221	0.965577801	0.970599877	negative
PRJNA729271	stage_1D_vs_E10	ordinary_muscle_development	ENSGALG00010019253	HBAD	-3.029670828	0.426596586	-7.101957505	1.23002E-12	4.21992E-12	negative
PRJNA729271	stage_1D_vs_E10	ordinary_muscle_development	ENSGALG00010019255	ENSGALG00010019255	3.789465284	0.197766189	19.16134052	7.78424E-82	3.82483E-80	positive
PRJNA729271	stage_1D_vs_E10	ordinary_muscle_development	ENSGALG00010019256	BBS9	-1.113657793	0.104943135	-10.61201188	2.62045E-26	1.90107E-25	negative
PRJNA729271	stage_1D_vs_E10	ordinary_muscle_development	ENSGALG00010019257	SLC1A4	-1.234870095	0.158818569	-7.775350849	7.52386E-15	2.98463E-14	negative
PRJNA729271	stage_1D_vs_E10	ordinary_muscle_development	ENSGALG00010019259	L2HGDH	0.426049242	0.107905158	3.948367721	7.86859E-05	0.000147472	positive
PRJNA729271	stage_1D_vs_E10	ordinary_muscle_development	ENSGALG00010019260	TRAIL-LIKE	-0.053869287	0.175808374	-0.306409106	0.759293176	0.790146786	negative
PRJNA729271	stage_1D_vs_E10	ordinary_muscle_development	ENSGALG00010019262	TMC3	-4.073679837	0.514914218	-7.911375715	2.5456E-15	1.04115E-14	negative
PRJNA729271	stage_1D_vs_E10	ordinary_muscle_development	ENSGALG00010019263	SNRPB2	-0.962939239	0.10251478	-9.393174699	5.82185E-21	3.28485E-20	negative
PRJNA729271	stage_1D_vs_E10	ordinary_muscle_development	ENSGALG00010019264	HK2	0.957355805	0.214428426	4.464686989	8.01859E-06	1.65263E-05	positive
PRJNA729271	stage_1D_vs_E10	ordinary_muscle_development	ENSGALG00010019265	RUFY4	-0.980812569	1.046677491	-0.937072382	0.348721327	0.397602111	negative
PRJNA729271	stage_1D_vs_E10	ordinary_muscle_development	ENSGALG00010019266	ATP5S	-0.953486489	0.163602802	-5.828057203	5.60764E-09	1.49664E-08	negative
PRJNA729271	stage_1D_vs_E10	ordinary_muscle_development	ENSGALG00010019267	STARD5	-1.062124489	0.19169498	-5.540700596	3.01264E-08	7.55375E-08	negative
PRJNA729271	stage_1D_vs_E10	ordinary_muscle_development	ENSGALG00010019268	NSL1	-0.686383996	0.093174816	-7.366625716	1.75001E-13	6.36547E-13	negative
PRJNA729271	stage_1D_vs_E10	ordinary_muscle_development	ENSGALG00010019269	SOCS1	-0.005822038	0.255215249	-0.022812266	0.981800024	0.984176442	negative
PRJNA729271	stage_1D_vs_E10	ordinary_muscle_development	ENSGALG00010019270	ENSGALG00010019270	-2.198602481	0.219477405	-10.0174434	1.27765E-23	8.18363E-23	negative
PRJNA729271	stage_1D_vs_E10	ordinary_muscle_development	ENSGALG00010019271	CDKL1	-0.967407692	0.199738502	-4.843371111	1.27655E-06	2.8254E-06	negative
PRJNA729271	stage_1D_vs_E10	ordinary_muscle_development	ENSGALG00010019274	ENSGALG00010019274	-3.847948491	0.985613764	-3.904113995	9.45712E-05	0.000175944	negative
PRJNA729271	stage_1D_vs_E10	ordinary_muscle_development	ENSGALG00010019275	CTAGE1	0.904932735	0.591163527	1.530765504	0.125827358	0.157270635	positive
PRJNA729271	stage_1D_vs_E10	ordinary_muscle_development	ENSGALG00010019277	IGFBP5	1.164286505	0.132600533	8.780405909	1.62886E-18	8.03618E-18	positive
PRJNA729271	stage_1D_vs_E10	ordinary_muscle_development	ENSGALG00010019278	JPT2	-1.83197789	0.073427115	-24.94961005	2.1563E-137	3.1255E-135	negative
PRJNA729271	stage_1D_vs_E10	ordinary_muscle_development	ENSGALG00010019280	TLNRD1	0.119633934	0.115992803	1.031391001	0.302357499	0.34941178	positive
PRJNA729271	stage_1D_vs_E10	ordinary_muscle_development	ENSGALG00010019281	ENSGALG00010019281	-1.938034184	1.24143143	-1.561128659	0.118493397	0.14886131	negative
PRJNA729271	stage_1D_vs_E10	ordinary_muscle_development	ENSGALG00010019282	IGFBP2	-1.661265374	0.154358239	-10.76240173	5.18022E-27	3.88048E-26	negative
PRJNA729271	stage_1D_vs_E10	ordinary_muscle_development	ENSGALG00010019283	NT5C3A	0.611207717	0.255851086	2.388919769	0.016897992	0.024169765	positive
PRJNA729271	stage_1D_vs_E10	ordinary_muscle_development	ENSGALG00010019284	CHD5	1.036465361	0.131605316	7.875558493	3.39224E-15	1.37964E-14	positive
PRJNA729271	stage_1D_vs_E10	ordinary_muscle_development	ENSGALG00010019285	BATF3	-0.811102336	0.278704147	-2.910262889	0.003611249	0.005643063	negative
PRJNA729271	stage_1D_vs_E10	ordinary_muscle_development	ENSGALG00010019287	AHSA2	-0.539794183	0.096104597	-5.616736341	1.94598E-08	4.96514E-08	negative
PRJNA729271	stage_1D_vs_E10	ordinary_muscle_development	ENSGALG00010019288	RPL37A	0.031386679	0.156724598	0.200266448	0.841272202	0.863981902	positive
PRJNA729271	stage_1D_vs_E10	ordinary_muscle_development	ENSGALG00010019289	FKBP9	-1.705926835	0.166562809	-10.24194323	1.28627E-24	8.64368E-24	negative
PRJNA729271	stage_1D_vs_E10	ordinary_muscle_development	ENSGALG00010019291	ADD2	-0.517244872	0.270027795	-1.915524558	0.05542564	0.073369375	negative
PRJNA729271	stage_1D_vs_E10	ordinary_muscle_development	ENSGALG00010019292	ENSGALG00010019292	-0.029556301	0.092415348	-0.319820259	0.749104589	0.781339211	negative
PRJNA729271	stage_1D_vs_E10	ordinary_muscle_development	ENSGALG00010019294	SMARCAL1	-0.619471951	0.097832751	-6.331948584	2.42084E-10	7.15103E-10	negative
PRJNA729271	stage_1D_vs_E10	ordinary_muscle_development	ENSGALG00010019295	MAP4K5	-0.098068491	0.130139279	-0.753565655	0.451110074	0.50037041	negative
PRJNA729271	stage_1D_vs_E10	ordinary_muscle_development	ENSGALG00010019296	ENSGALG00010019296	0.03023863	0.10203453	0.296356827	0.766957592	0.796921169	positive
PRJNA729271	stage_1D_vs_E10	ordinary_muscle_development	ENSGALG00010019297	SLC25A26	0.414551752	0.148917739	2.783763401	0.005373221	0.008226983	positive
PRJNA729271	stage_1D_vs_E10	ordinary_muscle_development	ENSGALG00010019298	PGAP6	0.313589982	0.092924609	3.374670984	0.00073904	0.001250716	positive
PRJNA729271	stage_1D_vs_E10	ordinary_muscle_development	ENSGALG00010019300	MESDC2	-0.058991303	0.065672596	-0.898263608	0.369045029	0.418143336	negative
PRJNA729271	stage_1D_vs_E10	ordinary_muscle_development	ENSGALG00010019302	USP34	0.17431177	0.094341682	1.847664422	0.064650904	0.08476075	positive
PRJNA729271	stage_1D_vs_E10	ordinary_muscle_development	ENSGALG00010019305	SNRNP27	-0.074021264	0.11908111	-0.621603746	0.534202457	0.581020831	negative
PRJNA729271	stage_1D_vs_E10	ordinary_muscle_development	ENSGALG00010019307	ATF3	3.803216973	0.486016326	7.825286453	5.06502E-15	2.03373E-14	positive
PRJNA729271	stage_1D_vs_E10	ordinary_muscle_development	ENSGALG00010019310	CRELD1	1.363399968	0.096357233	14.14943042	1.88276E-45	3.00889E-44	positive
PRJNA729271	stage_1D_vs_E10	ordinary_muscle_development	ENSGALG00010019312	KBTBD2	0.261710869	0.071823885	3.643786026	0.000268657	0.000475681	positive
PRJNA729271	stage_1D_vs_E10	ordinary_muscle_development	ENSGALG00010019313	MRPL28	-0.277174575	0.099603995	-2.782765651	0.005389772	0.008251452	negative
PRJNA729271	stage_1D_vs_E10	ordinary_muscle_development	ENSGALG00010019314	GKN2	-2.475403406	1.226640485	-2.01803498	0.043587618	0.058663187	negative
PRJNA729271	stage_1D_vs_E10	ordinary_muscle_development	ENSGALG00010019316	CLEC16A	0.559484257	0.087217687	6.41480276	1.41005E-10	4.22987E-10	positive
PRJNA729271	stage_1D_vs_E10	ordinary_muscle_development	ENSGALG00010019318	NOG2	0.131752407	0.277977821	0.473967334	0.635523202	0.676450933	positive
PRJNA729271	stage_1D_vs_E10	ordinary_muscle_development	ENSGALG00010019319	ACTR3	-1.366521088	0.067186721	-20.33915448	5.79204E-92	3.61877E-90	negative
PRJNA729271	stage_1D_vs_E10	ordinary_muscle_development	ENSGALG00010019322	EGR3	1.211437999	0.311378084	3.890569255	0.000100009	0.000185423	positive
PRJNA729271	stage_1D_vs_E10	ordinary_muscle_development	ENSGALG00010019323	LRIG1	0.838705308	0.118925157	7.052379229	1.75884E-12	5.97899E-12	positive
PRJNA729271	stage_1D_vs_E10	ordinary_muscle_development	ENSGALG00010019325	SLITRK4	-3.288689445	0.21614462	-15.21522692	2.80246E-52	5.86171E-51	negative
PRJNA729271	stage_1D_vs_E10	ordinary_muscle_development	ENSGALG00010019331	CEMIP	0.074463263	0.306032336	0.243318285	0.807758841	0.833817433	positive
PRJNA729271	stage_1D_vs_E10	ordinary_muscle_development	ENSGALG00010019334	ATL1	-1.19458036	0.130534071	-9.151483193	5.61572E-20	3.00147E-19	negative
PRJNA729271	stage_1D_vs_E10	ordinary_muscle_development	ENSGALG00010019335	HIPK3	1.688352779	0.115052051	14.67468643	9.36475E-49	1.72261E-47	positive
PRJNA729271	stage_1D_vs_E10	ordinary_muscle_development	ENSGALG00010019336	DEXI	2.355653693	0.223476162	10.54096181	5.5924E-26	3.9991E-25	positive
PRJNA729271	stage_1D_vs_E10	ordinary_muscle_development	ENSGALG00010019337	XPO1	-0.168645704	0.08394405	-2.009025113	0.044534471	0.059837519	negative
PRJNA729271	stage_1D_vs_E10	ordinary_muscle_development	ENSGALG00010019344	DNAJC24	-0.28282236	0.08461296	-3.342541866	0.000830148	0.00139675	negative
PRJNA729271	stage_1D_vs_E10	ordinary_muscle_development	ENSGALG00010019345	ENSGALG00010019345	-0.145410051	0.18669665	-0.77885731	0.436063774	0.485426956	negative
PRJNA729271	stage_1D_vs_E10	ordinary_muscle_development	ENSGALG00010019346	POU3F4	0.830663931	0.462972476	1.794197223	0.072781695	0.094667146	positive
PRJNA729271	stage_1D_vs_E10	ordinary_muscle_development	ENSGALG00010019350	SAV1	-0.418384998	0.088744692	-4.714479134	2.4233E-06	5.24578E-06	negative
PRJNA729271	stage_1D_vs_E10	ordinary_muscle_development	ENSGALG00010019355	ENTPD4	0.165089738	0.098377391	1.678126822	0.093322344	0.119307406	positive
PRJNA729271	stage_1D_vs_E10	ordinary_muscle_development	ENSGALG00010019356	AVL9	-0.025987099	0.09455676	-0.274830679	0.783446327	0.811667172	negative
PRJNA729271	stage_1D_vs_E10	ordinary_muscle_development	ENSGALG00010019365	ENSGALG00010019365	0.478108546	0.174071765	2.746617447	0.006021332	0.009163172	positive
PRJNA729271	stage_1D_vs_E10	ordinary_muscle_development	ENSGALG00010019371	TMEM87A	0.931781612	0.076520668	12.17686198	4.12863E-34	4.30536E-33	positive
PRJNA729271	stage_1D_vs_E10	ordinary_muscle_development	ENSGALG00010019373	DENND5A	0.432680726	0.060818079	7.11434387	1.12446E-12	3.8651E-12	positive
PRJNA729271	stage_1D_vs_E10	ordinary_muscle_development	ENSGALG00010019376	ENSGALG00010019376	-0.039224235	0.370318828	-0.105920175	0.915645687	0.928391454	negative
PRJNA729271	stage_1D_vs_E10	ordinary_muscle_development	ENSGALG00010019388	GPR75	-0.690262763	0.137316682	-5.026794664	4.98746E-07	1.14063E-06	negative
PRJNA729271	stage_1D_vs_E10	ordinary_muscle_development	ENSGALG00010019390	TVP23A	-2.279242357	0.271351164	-8.399604122	4.47986E-17	2.03688E-16	negative
PRJNA729271	stage_1D_vs_E10	ordinary_muscle_development	ENSGALG00010019391	GANC	1.47247341	0.132315797	11.12847781	9.11792E-29	7.42913E-28	positive
PRJNA729271	stage_1D_vs_E10	ordinary_muscle_development	ENSGALG00010019392	NIN	-1.027949845	0.103253438	-9.955599168	2.38383E-23	1.5056E-22	negative
PRJNA729271	stage_1D_vs_E10	ordinary_muscle_development	ENSGALG00010019398	SLC35F5	1.004296906	0.120020394	8.367718805	5.87444E-17	2.65596E-16	positive
PRJNA729271	stage_1D_vs_E10	ordinary_muscle_development	ENSGALG00010019399	CD93	0.808418916	0.151266348	5.344340811	9.07467E-08	2.19852E-07	positive
PRJNA729271	stage_1D_vs_E10	ordinary_muscle_development	ENSGALG00010019403	PACC1	-0.071467508	0.103280299	-0.691976193	0.48895228	0.537369269	negative
PRJNA729271	stage_1D_vs_E10	ordinary_muscle_development	ENSGALG00010019411	LSM5	-1.257170256	0.158713887	-7.920984615	2.35637E-15	9.67307E-15	negative
PRJNA729271	stage_1D_vs_E10	ordinary_muscle_development	ENSGALG00010019416	GPR39	3.89246901	0.412120857	9.444969703	3.55507E-21	2.02717E-20	positive
PRJNA729271	stage_1D_vs_E10	ordinary_muscle_development	ENSGALG00010019418	ZNF703	0.955016535	0.228892017	4.172345316	3.0148E-05	5.87676E-05	positive
PRJNA729271	stage_1D_vs_E10	ordinary_muscle_development	ENSGALG00010019422	IMMP1L	0.425479981	0.139208534	3.056421666	0.00223996	0.003582586	positive
PRJNA729271	stage_1D_vs_E10	ordinary_muscle_development	ENSGALG00010019423	LYPD1	-1.400827772	0.181897303	-7.70120143	1.34793E-14	5.26211E-14	negative
PRJNA729271	stage_1D_vs_E10	ordinary_muscle_development	ENSGALG00010019426	GRM7	-2.254953144	0.258615645	-8.719322253	2.79872E-18	1.36224E-17	negative
PRJNA729271	stage_1D_vs_E10	ordinary_muscle_development	ENSGALG00010019430	ENSGALG00010019430	1.132890076	0.40001393	2.83212656	0.004623954	0.007141632	positive

PRJNA729271	stage_1D_vs_E10	ordinary_muscle_development	ENSGALG00010019441	LMCD1	3.180636458	0.136589754	23.28605445	6.1384E-120	7.006E-118	positive
PRJNA729271	stage_1D_vs_E10	ordinary_muscle_development	ENSGALG00010019443	NCKAP5	0.54592352	0.131259636	4.159111964	3.19487E-05	6.2144E-05	positive
PRJNA729271	stage_1D_vs_E10	ordinary_muscle_development	ENSGALG00010019451	CAV3	4.032140361	0.142784151	28.23941121	1.9203E-175	5.3527E-173	positive
PRJNA729271	stage_1D_vs_E10	ordinary_muscle_development	ENSGALG00010019458	ELP4	-0.193234107	0.101000484	-1.913199811	0.055722478	0.073735376	negative
PRJNA729271	stage_1D_vs_E10	ordinary_muscle_development	ENSGALG00010019461	MGAT5	0.588074098	0.069601359	8.449175462	2.93367E-17	1.34953E-16	positive
PRJNA729271	stage_1D_vs_E10	ordinary_muscle_development	ENSGALG00010019470	TGFB2	-0.99115522	0.200266377	-4.949184362	7.45251E-07	1.68079E-06	negative
PRJNA729271	stage_1D_vs_E10	ordinary_muscle_development	ENSGALG00010019471	NUBP1	0.03123641	0.093185036	0.335208431	0.737467863	0.771032651	positive
PRJNA729271	stage_1D_vs_E10	ordinary_muscle_development	ENSGALG00010019472	CAPN3	7.533253897	0.255626857	29.46972786	7.0359E-191	2.9139E-188	positive
PRJNA729271	stage_1D_vs_E10	ordinary_muscle_development	ENSGALG00010019473	OXTR	0.489620018	0.441910651	1.107961568	0.267878414	0.313162159	positive
PRJNA729271	stage_1D_vs_E10	ordinary_muscle_development	ENSGALG00010019474	TMEM163	-0.887350883	0.542142039	-1.636749816	0.101682755	0.129186742	negative
PRJNA729271	stage_1D_vs_E10	ordinary_muscle_development	ENSGALG00010019477	NCAPH	-1.648806489	0.175687017	-9.384907966	6.29714E-21	3.54474E-20	negative
PRJNA729271	stage_1D_vs_E10	ordinary_muscle_development	ENSGALG00010019481	PYGL	-0.150104185	0.098736473	-1.520250635	0.128447996	0.160255956	negative
PRJNA729271	stage_1D_vs_E10	ordinary_muscle_development	ENSGALG00010019483	SCUBE2	-3.678379173	0.267750538	-13.7380832	6.00445E-43	8.7384E-42	negative
PRJNA729271	stage_1D_vs_E10	ordinary_muscle_development	ENSGALG00010019488	TEKT5	-3.368929841	1.292933232	-2.605648736	0.009170045	0.013624416	negative
PRJNA729271	stage_1D_vs_E10	ordinary_muscle_development	ENSGALG00010019495	EMP2	0.435045147	0.196777034	2.210853263	0.027046	0.037504235	positive
PRJNA729271	stage_1D_vs_E10	ordinary_muscle_development	ENSGALG00010019498	ENSGALG00010019498	-0.395047908	0.276426826	-1.429122902	0.152968912	0.188224481	negative
PRJNA729271	stage_1D_vs_E10	ordinary_muscle_development	ENSGALG00010019509	ZNF106	0.503290247	0.222196049	2.265072894	0.023508207	0.032957874	positive
PRJNA729271	stage_1D_vs_E10	ordinary_muscle_development	ENSGALG00010019515	ENSGALG00010019515	-0.509045312	0.109319357	-4.656497511	3.21634E-06	6.88029E-06	negative
PRJNA729271	stage_1D_vs_E10	ordinary_muscle_development	ENSGALG00010019520	CD6	0.771592255	1.23597098	0.624280236	0.532443561	0.57928165	positive
PRJNA729271	stage_1D_vs_E10	ordinary_muscle_development	ENSGALG00010019524	HHIPL2	0.261985098	0.380479636	0.688565361	0.491096826	0.539398991	positive
PRJNA729271	stage_1D_vs_E10	ordinary_muscle_development	ENSGALG00010019529	SNAP23	-0.15565371	0.067352311	-2.31103741	0.020830789	0.029409008	negative
PRJNA729271	stage_1D_vs_E10	ordinary_muscle_development	ENSGALG00010019534	PPP2R5A	0.94505559	0.104348429	9.056730427	1.34419E-19	7.02635E-19	positive
PRJNA729271	stage_1D_vs_E10	ordinary_muscle_development	ENSGALG00010019540	UNC5D	-0.624094798	0.362316496	-1.722512787	0.084976668	0.109380766	negative
PRJNA729271	stage_1D_vs_E10	ordinary_muscle_development	ENSGALG00010019546	HSD17B12	-1.012271454	0.077779583	-13.01461659	1.01048E-38	1.26594E-37	negative
PRJNA729271	stage_1D_vs_E10	ordinary_muscle_development	ENSGALG00010019557	TRIM9	-0.891016278	0.335596205	-2.655024892	0.007930256	0.011883496	negative
PRJNA729271	stage_1D_vs_E10	ordinary_muscle_development	ENSGALG00010019572	NFKBIE	-1.072055155	0.196709275	-5.449947151	5.03848E-08	1.24459E-07	negative
PRJNA729271	stage_1D_vs_E10	ordinary_muscle_development	ENSGALG00010019577	MXD1	0.006595164	0.187675356	0.03514134	0.971967037	0.976142327	positive
PRJNA729271	stage_1D_vs_E10	ordinary_muscle_development	ENSGALG00010019580	CD5	-0.505749896	0.608372669	-0.831315939	0.405795173	0.455439491	negative
PRJNA729271	stage_1D_vs_E10	ordinary_muscle_development	ENSGALG00010019581	SLC35B2	-0.032110771	0.132842286	-0.241721003	0.808996354	0.834738194	negative
PRJNA729271	stage_1D_vs_E10	ordinary_muscle_development	ENSGALG00010019584	RAD18	-1.192702616	0.116788146	-10.21253149	1.74258E-24	1.16453E-23	negative
PRJNA729271	stage_1D_vs_E10	ordinary_muscle_development	ENSGALG00010019585	PDE1C	-0.01667839	0.207209539	-0.080490455	0.935847188	0.945303483	negative
PRJNA729271	stage_1D_vs_E10	ordinary_muscle_development	ENSGALG00010019586	GLYR1	-0.382367614	0.090325525	-4.233217748	2.30371E-05	4.5407E-05	negative
PRJNA729271	stage_1D_vs_E10	ordinary_muscle_development	ENSGALG00010019587	CCNT2	0.354884921	0.126868515	2.797265512	0.005153716	0.007907602	positive
PRJNA729271	stage_1D_vs_E10	ordinary_muscle_development	ENSGALG00010019588	CHMP7	0.331052505	0.09284282	3.56573083	0.000362844	0.000634429	positive
PRJNA729271	stage_1D_vs_E10	ordinary_muscle_development	ENSGALG00010019591	ENSGALG00010019591	-2.27204189	0.227344624	-9.993822817	1.62203E-23	1.03346E-22	negative
PRJNA729271	stage_1D_vs_E10	ordinary_muscle_development	ENSGALG00010019592	ALKBH3	0.582053149	0.129763847	4.485480068	7.27499E-06	1.5058E-05	positive
PRJNA729271	stage_1D_vs_E10	ordinary_muscle_development	ENSGALG00010019594	TMX1	-0.349706022	0.32766948	-1.067252348	0.2858579	0.33209187	negative
PRJNA729271	stage_1D_vs_E10	ordinary_muscle_development	ENSGALG00010019595	GRIN2A	-1.141876414	0.221931736	-5.145169562	2.67279E-07	6.23666E-07	negative
PRJNA729271	stage_1D_vs_E10	ordinary_muscle_development	ENSGALG00010019598	KCNAB2	-1.659704616	0.139251622	-11.91874527	9.45213E-33	9.32666E-32	negative
PRJNA729271	stage_1D_vs_E10	ordinary_muscle_development	ENSGALG00010019600	MAP3K19	-1.101133849	0.227321017	-4.843959714	1.27277E-06	2.81833E-06	negative
PRJNA729271	stage_1D_vs_E10	ordinary_muscle_development	ENSGALG00010019602	SLC29A1	1.840614524	0.193526337	9.510925237	1.88974E-21	1.09392E-20	positive
PRJNA729271	stage_1D_vs_E10	ordinary_muscle_development	ENSGALG00010019610	CORO7	-0.139990345	0.101648314	-1.377202823	0.168449549	0.205666797	negative
PRJNA729271	stage_1D_vs_E10	ordinary_muscle_development	ENSGALG00010019613	FRMD6	-0.421289538	0.123768115	-3.403861642	0.000664404	0.001127829	negative
PRJNA729271	stage_1D_vs_E10	ordinary_muscle_development	ENSGALG00010019614	MICALL2	1.078307832	0.154926072	6.960144399	3.39924E-12	1.13661E-11	positive
PRJNA729271	stage_1D_vs_E10	ordinary_muscle_development	ENSGALG00010019617	ANO3	-1.025461296	0.143057528	-7.168174277	7.60045E-13	2.65275E-12	negative
PRJNA729271	stage_1D_vs_E10	ordinary_muscle_development	ENSGALG00010019618	NPHP4	-0.045909157	0.101702	-0.451408597	0.651695088	0.691430649	negative
PRJNA729271	stage_1D_vs_E10	ordinary_muscle_development	ENSGALG00010019621	ENSGALG00010019621	0.022713213	0.151213146	0.150206602	0.880601617	0.898136817	positive
PRJNA729271	stage_1D_vs_E10	ordinary_muscle_development	ENSGALG00010019622	C2orf42	0.587269314	0.142343849	4.12570912	3.69594E-05	7.14493E-05	positive
PRJNA729271	stage_1D_vs_E10	ordinary_muscle_development	ENSGALG00010019624	SRGAP3	0.195895051	0.129649149	1.510962882	0.130797908	0.163033423	positive
PRJNA729271	stage_1D_vs_E10	ordinary_muscle_development	ENSGALG00010019625	INTS1	-0.03489493	0.076098094	-0.458551956	0.646555944	0.686782091	negative
PRJNA729271	stage_1D_vs_E10	ordinary_muscle_development	ENSGALG00010019630	AJAP1	-0.771221095	0.371930527	-2.073562238	0.038119985	0.051751352	negative
PRJNA729271	stage_1D_vs_E10	ordinary_muscle_development	ENSGALG00010019634	GNG2	-3.10652409	0.30898739	-10.05388631	8.83143E-24	5.68688E-23	negative
PRJNA729271	stage_1D_vs_E10	ordinary_muscle_development	ENSGALG00010019635	LRRC57	0.554741168	0.077172727	7.188305865	6.56002E-13	2.30236E-12	positive
PRJNA729271	stage_1D_vs_E10	ordinary_muscle_development	ENSGALG00010019640	MGRN1	0.044540361	0.116205918	0.383288229	0.701506066	0.738387221	positive
PRJNA729271	stage_1D_vs_E10	ordinary_muscle_development	ENSGALG00010019641	CAPN11	0.611546546	0.081778716	7.478064903	7.54249E-14	2.80689E-13	positive
PRJNA729271	stage_1D_vs_E10	ordinary_muscle_development	ENSGALG00010019643	RAB3GAP1	-0.021245001	0.070222455	-0.302538572	0.762241534	0.792645888	negative
PRJNA729271	stage_1D_vs_E10	ordinary_muscle_development	ENSGALG00010019645	ENSGALG00010019645	2.319879416	0.162193527	14.30315657	2.09106E-46	3.46398E-45	positive
PRJNA729271	stage_1D_vs_E10	ordinary_muscle_development	ENSGALG00010019646	VPS37C	0.109239727	0.127246346	0.85849009	0.3906219	0.439806155	positive
PRJNA729271	stage_1D_vs_E10	ordinary_muscle_development	ENSGALG00010019648	PRDM16	-1.009027366	0.187412054	-5.384004626	7.28466E-08	1.77997E-07	negative
PRJNA729271	stage_1D_vs_E10	ordinary_muscle_development	ENSGALG00010019649	TMEM63B	-0.340309096	0.076685115	-4.437746434	9.09056E-06	1.86508E-05	negative
PRJNA729271	stage_1D_vs_E10	ordinary_muscle_development	ENSGALG00010019650	KBTBD8	0.529654825	0.086839463	6.09924113	1.06573E-09	3.00599E-09	positive
PRJNA729271	stage_1D_vs_E10	ordinary_muscle_development	ENSGALG00010019652	TNFRSF10B	0.709368093	0.320446927	2.213683556	0.026850555	0.037254599	positive
PRJNA729271	stage_1D_vs_E10	ordinary_muscle_development	ENSGALG00010019655	RTRAF	-0.100012796	0.098663002	-1.01368086	0.31073506	0.358065402	negative
PRJNA729271	stage_1D_vs_E10	ordinary_muscle_development	ENSGALG00010019657	PPP1R17	-2.805799463	0.27718455	-10.12249588	4.39074E-24	2.86942E-23	negative
PRJNA729271	stage_1D_vs_E10	ordinary_muscle_development	ENSGALG00010019659	MRPL14	0.453089909	0.125319807	3.615469248	0.000299804	0.000528283	positive
PRJNA729271	stage_1D_vs_E10	ordinary_muscle_development	ENSGALG00010019660	ENSGALG00010019660	1.056283712	0.480168079	2.199820766	0.027819614	0.038499647	positive
PRJNA729271	stage_1D_vs_E10	ordinary_muscle_development	ENSGALG00010019667	ITPRID1	0.652536983	0.60690326	1.075191099	0.28228918	0.328419749	positive
PRJNA729271	stage_1D_vs_E10	ordinary_muscle_development	ENSGALG00010019670	ENSGALG00010019670	-0.826782926	0.119838002	-6.899171477	5.23067E-12	1.72276E-11	negative
PRJNA729271	stage_1D_vs_E10	ordinary_muscle_development	ENSGALG00010019673	NID2	0.642856141	0.132537498	4.850371794	1.2323E-06	2.73165E-06	positive
PRJNA729271	stage_1D_vs_E10	ordinary_muscle_development	ENSGALG00010019675	MAFK	1.691984885	0.136880444	12.36104173	4.24618E-35	4.62422E-34	positive
PRJNA729271	stage_1D_vs_E10	ordinary_muscle_development	ENSGALG00010019679	RHOBTB2	-1.604651709	0.170292576	-9.422910535	4.38754E-21	2.48913E-20	negative
PRJNA729271	stage_1D_vs_E10	ordinary_muscle_development	ENSGALG00010019680	CCDC34	-0.653240578	0.201735106	-3.23811057	0.001203242	0.001989164	negative
PRJNA729271	stage_1D_vs_E10	ordinary_muscle_development	ENSGALG00010019682	TMEM158	-0.993560997	0.357204353	-2.781491847	0.005410969	0.008280405	negative
PRJNA729271	stage_1D_vs_E10	ordinary_muscle_development	ENSGALG00010019683	TMEM184A	0.55281357	0.285035232	1.939456981	0.052445718	0.069698421	positive
PRJNA729271	stage_1D_vs_E10	ordinary_muscle_development	ENSGALG00010019684	PIGQ	-0.018454074	0.126359636	-0.146044059	0.883886607	0.901106792	negative
PRJNA729271	stage_1D_vs_E10	ordinary_muscle_development	ENSGALG00010019690	ENSGALG00010019690	-0.25658513	0.090543701	-2.833826409	0.004599431	0.00710527	negative
PRJNA729271	stage_1D_vs_E10	ordinary_muscle_development	ENSGALG00010019692	MMEL1	-0.783074273	0.302427241	-2.589298077	0.009617181	0.014253684	negative
PRJNA729271	stage_1D_vs_E10	ordinary_muscle_development	ENSGALG00010019693	HAUS2	-0.518502018	0.118605661	-4.371646473	1.23313E-05	2.49745E-05	negative
PRJNA729271	stage_1D_vs_E10	ordinary_muscle_development	ENSGALG00010019695	PGA5	2.15932066	2.487218221	0.868166951	0.385302944	0.4343573	positive
PRJNA729271	stage_1D_vs_E10	ordinary_muscle_development	ENSGALG00010019697	EIF4EBP1	2.715939546	0.145164046	18.70945061	4.14627E-78	1.87813E-76	positive
PRJNA729271	stage_1D_vs_E10	ordinary_muscle_development	ENSGALG00010019700	HES5B	-2.970723019	0.2671135	-11.12157572	9.85165E-29	8.00446E-28	negative
PRJNA729271	stage_1D_vs_E10	ordinary_muscle_development	ENSGALG00010019706	NPRL3	0.378856571	0.098273552	3.855122377	0.000115672	0.000213452	positive
PRJNA729271	stage_1D_vs_E10	ordinary_muscle_development	ENSGALG00010019713	NUDT14	0.184522083	0.114098375	1.617219194	0.105830975	0.133975544	positive
PRJNA729271	stage_1D_vs_E10	ordinary_muscle_development	ENSGALG00010019716	VEGFA	1.810208032	0.128968339	14.03606537	9.37765E-45	1.4569E-43	positive
PRJNA729271	stage_1D_vs_E10	ordinary_muscle_development	ENSGALG00010019717	ZRANB3	-0.575054659	0.139785013	-4.113850599	3.89113E-05	7.50425E-05	negative
PRJNA729271	stage_1D_vs_E10	ordinary_muscle_development	ENSGALG00010019724	HES5A	-7.436060187	0.631583309	-11.77368065	5.33452E-32	5.09044E-31	negative
PRJNA729271	stage_1D_vs_E10	ordinary_muscle_development	ENSGALG00010019726	PEBP4	3.523921316	0.226914569	15.52972706	2.18307E-54	4.89082E-53	positive
PRJNA729271	stage_1D_vs_E10	ordinary_muscle_development	ENSGALG00010019731	PANK4	0.57453105	0.134181331	4.281751005	1.85428E-05	3.68179E-05	positive
PRJNA729271	stage_1D_vs_E10	ordinary_muscle_development	ENSGALG00010019732	HBA1	-2.074127368	0.403571969	-5.139423771	2.75582E-07	6.42213E-07	negative
PRJNA729271	stage_1D_vs_E10	ordinary_muscle_development	ENSGALG00010019735	ENSGALG00010019735	1.122989561	0.17113933	6.561843861	5.31465E-11	1.64046E-10	positive
PRJNA729271	stage_1D_vs_E10	ordinary_muscle_development	ENSGALG00010019738	ADRA2A	-1.314760057	0.354300278	-3.710863747	0.000206553	0.000370452	negative
PRJNA729271	stage_1D_vs_E10	ordinary_muscle_development	ENSGALG00010019743	KCNU1	-1.691980181	0.823872106	-2.053692761	0.040005438	0.054153793	negative
PRJNA729271	stage_1D_vs_E10	ordinary_muscle_development	ENSGALG00010019744	KBP	0.435607534	0.415092622	1.049422492	0.293983711	0.340631006	positive
PRJNA729271	stage_1D_vs_E10	ordinary_muscle_development	ENSGALG00010019752	GPAM	0.862467744	0.166317278	5.185677353	2.15231E-07	5.058E-07	positive
PRJNA729271	stage_1D_vs_E10	ordinary_muscle_development	ENSGALG00010019761	MRPS18A	0.053588374	0.088023528	0.608796023	0.54265965	0.588805422	positive
PRJNA729271	stage_1D_vs_E10	ordinary_muscle_development	ENSGALG00010019767	SUCLG2	-0.397354064	0.07727807	-5.14187353	2.72012E-07	6.34403E-07	negative
PRJNA729271	stage_1D_vs_E10	ordinary_muscle_development	ENSGALG00010019771	VWCE	0.152844073	0.339927294	0.449637542	0.652971814	0.692531385	positive
PRJNA729271	stage_1D_vs_E10	ordinary_muscle_development	ENSGALG00010019772	ENSGALG00010019772	0.085601779	0.164887416	0.519152894	0.603654125	0.646517404	positive
PRJNA729271	stage_1D_vs_E10	ordinary_muscle_development	ENSGALG00010019778	ABHD17C	0.033174603	0.13159446	0.252097264	0.800965885	0.827748503	positive
PRJNA729271	stage_1D_vs_E10	ordinary_muscle_development	ENSGALG00010019785	ACSL5	0.074663527	0.227754458	0.327824657	0.743044243	0.775632025	positive
PRJNA729271	stage_1D_vs_E10	ordinary_muscle_development	ENSGALG00010019786	R3HDM1	0.359636919	0.087331769	4.118053749	3.82086E-05	7.37657E-05	positive
PRJNA729271	stage_1D_vs_E10	ordinary_muscle_development	ENSGALG00010019789	ARNT2	-1.970761652	0.173775307	-11.34086126	8.23302E-30	7.03227E-29	negative
PRJNA729271	stage_1D_vs_E10	ordinary_muscle_development	ENSGALG00010019794	RSPH9	-1.943317209	0.529400463	-3.670788646	0.000241803	0.000430265	negative
PRJNA729271	stage_1D_vs_E10	ordinary_muscle_development	ENSGALG00010019799	DOK2	-0.351991994	0.2854293	-1.233202035	0.217500396	0.259692607	negative
PRJNA729271	stage_1D_vs_E10	ordinary_muscle_development	ENSGALG00010019800	EXT2	-0.079276276	0.059782398	-1.326080573	0.18481301	0.223854315	negative
PRJNA729271	stage_1D_vs_E10	ordinary_muscle_development	ENSGALG00010019801	FIBIN	-4.102340602	0.187426249	-21.88775919	3.3981E-106	2.7364E-104	negative
PRJNA729271	stage_1D_vs_E10	ordinary_muscle_development	ENSGALG00010019803	FAH	-2.24116684	0.507610924	-4.415127287	1.00951E-05	2.0598E-05	negative
PRJNA729271	stage_1D_vs_E10	ordinary_muscle_development	ENSGALG00010019804	ELFN1	-1.815270116	0.283677925	-6.399053141	1.56343E-10	4.67065E-10	negative
PRJNA729271	stage_1D_vs_E10	ordinary_muscle_development	ENSGALG00010019807	ZDHHC6	-0.301982963	0.079619495	-3.792826921	0.000148942	0.000271904	negative
PRJNA729271	stage_1D_vs_E10	ordinary_muscle_development	ENSGALG00010019810	ZFAND6	0.087357095	0.124982166	0.698956483	0.484579227	0.533210043	positive
PRJNA729271	stage_1D_vs_E10	ordinary_muscle_development	ENSGALG00010019811	UBXN4	0.447648483	0.110527775	4.050099451	5.11959E-05	9.77327E-05	positive
PRJNA729271	stage_1D_vs_E10	ordinary_muscle_development	ENSGALG00010019813	ENSGALG00010019813	2.856360343	0.219235518	13.02872987	8.39942E-39	1.05594E-37	positive
PRJNA729271	stage_1D_vs_E10	ordinary_muscle_development	ENSGALG00010019816	ENSGALG00010019816	2.6765055	0.716191341	3.737137473	0.000186127	0.000335759	positive
PRJNA729271	stage_1D_vs_E10	ordinary_muscle_development	ENSGALG00010019820	BBOX1	-0.248427567	0.154411697	-1.608864947	0.107645876	0.136047342	negative
PRJNA729271	stage_1D_vs_E10	ordinary_muscle_development	ENSGALG00010019823	LCT	-0.658569056	0.203347727	-3.238634954	0.001201032	0.00198619	negative
PRJNA729271	stage_1D_vs_E10	ordinary_muscle_development	ENSGALG00010019825	BCL2A1	0.557876927	0.294232997	1.896037943	0.057955027	0.07643841	positive
PRJNA729271	stage_1D_vs_E10	ordinary_muscle_development	ENSGALG00010019830	JAG2	0.704386465	0.097797875	7.202472079	5.91306E-13	2.07983E-12	positive
PRJNA729271	stage_1D_vs_E10	ordinary_muscle_development	ENSGALG00010019832	MAD2L1BP	-2.291722206	0.141424845	-16.20452337	4.68505E-59	1.23923E-57	negative
PRJNA729271	stage_1D_vs_E10	ordinary_muscle_development	ENSGALG00010019833	MTHFS	-0.616407456	0.129137797	-4.7732536	1.81273E-06	3.95955E-06	negative
PRJNA729271	stage_1D_vs_E10	ordinary_muscle_development	ENSGALG00010019834	PLCH2	-0.317716233	0.225460206	-1.409189842	0.15877904	0.194827918	negative
PRJNA729271	stage_1D_vs_E10	ordinary_muscle_development	ENSGALG00010019836	MINAR1	1.934384101	0.481597872	4.016596031	5.90448E-05	0.000112126	positive
PRJNA729271	stage_1D_vs_E10	ordinary_muscle_development	ENSGALG00010019838	ALX4	-1.296849625	0.171344465	-7.568669482	3.77066E-14	1.43491E-13	negative
PRJNA729271	stage_1D_vs_E10	ordinary_muscle_development	ENSGALG00010019843	MCM6	-1.278297298	0.109570439	-11.66644311	1.89168E-31	1.75544E-30	negative
PRJNA729271	stage_1D_vs_E10	ordinary_muscle_development	ENSGALG00010019846	ENSGALG00010019846	0.407170737	0.071028196	5.732522605	9.89478E-09	2.57912E-08	positive
PRJNA729271	stage_1D_vs_E10	ordinary_muscle_development	ENSGALG00010019848	MAD1L1	-0.595657994	0.125714615	-4.73817619	2.1565E-06	4.69065E-06	negative
PRJNA729271	stage_1D_vs_E10	ordinary_muscle_development	ENSGALG00010019854	CCDC14	-1.030530107	0.149811174	-6.878860097	6.03334E-12	1.97768E-11	negative
PRJNA729271	stage_1D_vs_E10	ordinary_muscle_development	ENSGALG00010019855	TTC7A	-0.026188456	0.126406713	-0.207176148	0.835872294	0.859410477	negative
PRJNA729271	stage_1D_vs_E10	ordinary_muscle_development	ENSGALG00010019859	BIN3	0.006696636	0.135250802	0.049512723	0.960510698	0.966438525	positive
PRJNA729271	stage_1D_vs_E10	ordinary_muscle_development	ENSGALG00010019865	ENSGALG00010019865	-0.306756727	0.136992252	-2.239226843	0.02514116	0.035067467	negative
PRJNA729271	stage_1D_vs_E10	ordinary_muscle_development	ENSGALG00010019867	PEX11A	-0.63346958	0.375708934	-1.686064725	0.091783333	0.117484936	negative
PRJNA729271	stage_1D_vs_E10	ordinary_muscle_development	ENSGALG00010019871	TAFA1	-2.937639156	0.590187063	-4.977471279	6.44203E-07	1.46062E-06	negative

PRJNA729271	stage_1D_vs_E10	ordinary_muscle_development	ENSGALG00010019872	VTI1A	-0.109662623	0.109037982	-1.005728655	0.314546115	0.362082746	negative
PRJNA729271	stage_1D_vs_E10	ordinary_muscle_development	ENSGALG00010019873	LGR4	-1.343255539	0.09808658	-13.69459045	1.09375E-42	1.57281E-41	negative
PRJNA729271	stage_1D_vs_E10	ordinary_muscle_development	ENSGALG00010019875	PLEKHH1	-2.061517261	0.271832405	-7.583780398	3.35629E-14	1.28025E-13	negative
PRJNA729271	stage_1D_vs_E10	ordinary_muscle_development	ENSGALG00010019876	MRM2	0.20773668	0.224144944	0.926796188	0.354032364	0.402928637	positive
PRJNA729271	stage_1D_vs_E10	ordinary_muscle_development	ENSGALG00010019878	MCFD2	0.098242976	0.149988457	0.655003582	0.512465448	0.560195073	positive
PRJNA729271	stage_1D_vs_E10	ordinary_muscle_development	ENSGALG00010019891	PIGH	-0.934077719	0.104779559	-8.914694096	4.89165E-19	2.47399E-18	negative
PRJNA729271	stage_1D_vs_E10	ordinary_muscle_development	ENSGALG00010019896	DDB1	0.018448433	0.09082703	0.203116108	0.839044282	0.862243663	positive
PRJNA729271	stage_1D_vs_E10	ordinary_muscle_development	ENSGALG00010019898	NUDT1	-1.257611183	0.186999363	-6.725216399	1.75332E-11	5.58434E-11	negative
PRJNA729271	stage_1D_vs_E10	ordinary_muscle_development	ENSGALG00010019899	ENSGALG00010019899	0.388677542	0.446578623	0.870345158	0.384111808	0.43308188	positive
PRJNA729271	stage_1D_vs_E10	ordinary_muscle_development	ENSGALG00010019905	ENSGALG00010019905	-0.213023295	0.12444687	-1.711760973	0.086940727	0.111688398	negative
PRJNA729271	stage_1D_vs_E10	ordinary_muscle_development	ENSGALG00010019906	ENSGALG00010019906	2.091330428	0.231723263	9.025120732	1.79497E-19	9.30882E-19	positive
PRJNA729271	stage_1D_vs_E10	ordinary_muscle_development	ENSGALG00010019910	ENSGALG00010019910	0.000674096	0.244805192	0.002753602	0.997802946	0.998320635	positive
PRJNA729271	stage_1D_vs_E10	ordinary_muscle_development	ENSGALG00010019912	ARG2	-4.989609793	0.230668374	-21.63109624	9.1581E-104	7.0987E-102	negative
PRJNA729271	stage_1D_vs_E10	ordinary_muscle_development	ENSGALG00010019916	NOC3L	0.021222891	0.113211136	0.18746293	0.851297686	0.872732156	positive
PRJNA729271	stage_1D_vs_E10	ordinary_muscle_development	ENSGALG00010019919	LIN7C	0.133969531	0.106892731	1.253308153	0.210093582	0.25138736	positive
PRJNA729271	stage_1D_vs_E10	ordinary_muscle_development	ENSGALG00010019921	CALM2	-0.352126935	0.112702899	-3.124382236	0.001781788	0.002888282	negative
PRJNA729271	stage_1D_vs_E10	ordinary_muscle_development	ENSGALG00010019925	RDH11	-2.197777971	0.147838033	-14.86612026	5.46923E-50	1.05702E-48	negative
PRJNA729271	stage_1D_vs_E10	ordinary_muscle_development	ENSGALG00010019926	KIF7	-0.874449814	0.100549108	-8.696743609	3.41544E-18	1.6563E-17	negative
PRJNA729271	stage_1D_vs_E10	ordinary_muscle_development	ENSGALG00010019931	ZFYVE26	1.095930894	0.133791685	8.191322901	2.5837E-16	1.12668E-15	positive
PRJNA729271	stage_1D_vs_E10	ordinary_muscle_development	ENSGALG00010019932	KALRN	-0.075558932	0.115698226	-0.65306906	0.513711758	0.561345792	negative
PRJNA729271	stage_1D_vs_E10	ordinary_muscle_development	ENSGALG00010019934	TKFC	-0.57628098	0.153823436	-3.746379576	0.000179405	0.000324532	negative
PRJNA729271	stage_1D_vs_E10	ordinary_muscle_development	ENSGALG00010019935	ENSGALG00010019935	6.142677851	1.195621677	5.137643428	2.78205E-07	6.477E-07	positive
PRJNA729271	stage_1D_vs_E10	ordinary_muscle_development	ENSGALG00010019938	TAFA4	-4.056125227	0.423039657	-9.58804964	8.97658E-22	5.29571E-21	negative
PRJNA729271	stage_1D_vs_E10	ordinary_muscle_development	ENSGALG00010019947	ENSGALG00010019947	-1.806070921	0.308812877	-5.848431382	4.9623E-09	1.32881E-08	negative
PRJNA729271	stage_1D_vs_E10	ordinary_muscle_development	ENSGALG00010019948	TICRR	-1.148054435	0.127958726	-8.97206832	2.90998E-19	1.49416E-18	negative
PRJNA729271	stage_1D_vs_E10	ordinary_muscle_development	ENSGALG00010019954	CYB561A3	0.563591771	0.130150297	4.33031492	1.48896E-05	2.98637E-05	positive
PRJNA729271	stage_1D_vs_E10	ordinary_muscle_development	ENSGALG00010019956	SLC29A3	-1.041252379	0.177050713	-5.881096789	4.07557E-09	1.09704E-08	negative
PRJNA729271	stage_1D_vs_E10	ordinary_muscle_development	ENSGALG00010019961	HTATSF1	-0.212480236	0.069104775	-3.074754735	0.002106757	0.003381028	negative
PRJNA729271	stage_1D_vs_E10	ordinary_muscle_development	ENSGALG00010019962	SNX8	0.85266214	0.141533588	6.024450801	1.69685E-09	4.71274E-09	positive
PRJNA729271	stage_1D_vs_E10	ordinary_muscle_development	ENSGALG00010019967	CEP170B	-1.285001356	0.109502929	-11.73485835	8.44676E-32	7.98147E-31	negative
PRJNA729271	stage_1D_vs_E10	ordinary_muscle_development	ENSGALG00010019968	RHCG	4.012800436	0.793112985	5.059557105	4.20231E-07	9.64875E-07	positive
PRJNA729271	stage_1D_vs_E10	ordinary_muscle_development	ENSGALG00010019972	PPP1CB	0.360033312	0.091395236	3.939300634	8.17195E-05	0.00015294	positive
PRJNA729271	stage_1D_vs_E10	ordinary_muscle_development	ENSGALG00010019975	ENSGALG00010019975	0.279346846	0.19426502	1.437967814	0.150443187	0.185494555	positive
PRJNA729271	stage_1D_vs_E10	ordinary_muscle_development	ENSGALG00010019977	ZMIZ1	0.790656728	0.0864063	9.150452295	5.66957E-20	3.0269E-19	positive
PRJNA729271	stage_1D_vs_E10	ordinary_muscle_development	ENSGALG00010019979	ENSGALG00010019979	-0.279414377	0.284086067	-0.98355537	0.325334178	0.373640671	negative
PRJNA729271	stage_1D_vs_E10	ordinary_muscle_development	ENSGALG00010019982	PLCE1	0.153440827	0.112391807	1.365231425	0.172180324	0.209744836	positive
PRJNA729271	stage_1D_vs_E10	ordinary_muscle_development	ENSGALG00010019985	UMPS	-1.415710702	0.085759389	-16.5079383	3.21687E-61	9.19694E-60	negative
PRJNA729271	stage_1D_vs_E10	ordinary_muscle_development	ENSGALG00010019989	ENSGALG00010019989	2.029527055	0.271461475	7.476298633	7.64451E-14	2.84413E-13	positive
PRJNA729271	stage_1D_vs_E10	ordinary_muscle_development	ENSGALG00010019990	MARCHF8	0.399327452	0.10679588	3.739165349	0.000184632	0.000333322	positive
PRJNA729271	stage_1D_vs_E10	ordinary_muscle_development	ENSGALG00010019991	EIF3B	0.13933388	0.075168421	1.853622546	0.06379318	0.083704367	positive
PRJNA729271	stage_1D_vs_E10	ordinary_muscle_development	ENSGALG00010019992	CHAC2	-0.136718891	0.136575353	-1.001050974	0.316802165	0.36456394	negative
PRJNA729271	stage_1D_vs_E10	ordinary_muscle_development	ENSGALG00010019994	PLD4	-2.186477625	0.997658564	-2.191609138	0.028407742	0.039272314	negative
PRJNA729271	stage_1D_vs_E10	ordinary_muscle_development	ENSGALG00010019996	ITGB5	-0.089337376	0.124618417	-0.716887425	0.473443562	0.52210624	negative
PRJNA729271	stage_1D_vs_E10	ordinary_muscle_development	ENSGALG00010019997	FSHR	2.533151232	0.401598047	6.307678167	2.83252E-10	8.31964E-10	positive
PRJNA729271	stage_1D_vs_E10	ordinary_muscle_development	ENSGALG00010019998	PLEK2	-5.220725243	0.723059305	-7.220327855	5.18624E-13	1.83442E-12	negative
PRJNA729271	stage_1D_vs_E10	ordinary_muscle_development	ENSGALG00010019999	ERLIN1	-0.684501934	0.063551104	-10.77088982	4.72413E-27	3.54799E-26	negative
PRJNA729271	stage_1D_vs_E10	ordinary_muscle_development	ENSGALG00010020003	ENSGALG00010020003	1.901850934	0.186896847	10.17593909	2.53939E-24	1.68075E-23	positive
PRJNA729271	stage_1D_vs_E10	ordinary_muscle_development	ENSGALG00010020005	EOGT	0.188843287	0.163664579	1.153843352	0.248564397	0.2928506	positive
PRJNA729271	stage_1D_vs_E10	ordinary_muscle_development	ENSGALG00010020006	PLB1	-0.016786534	0.366569314	-0.045793615	0.963474748	0.968821816	negative
PRJNA729271	stage_1D_vs_E10	ordinary_muscle_development	ENSGALG00010020008	EIF2S1	-0.58140143	0.079088264	-7.351298402	1.96291E-13	7.11842E-13	negative
PRJNA729271	stage_1D_vs_E10	ordinary_muscle_development	ENSGALG00010020009	MACROD2	-1.041033617	0.118297424	-8.800137665	1.36648E-18	6.76475E-18	negative
PRJNA729271	stage_1D_vs_E10	ordinary_muscle_development	ENSGALG00010020010	SORBS3	2.714794759	0.203869576	13.31633101	1.86027E-40	2.48522E-39	positive
PRJNA729271	stage_1D_vs_E10	ordinary_muscle_development	ENSGALG00010020011	TLE3	0.451955278	0.202289971	2.234195182	0.025470233	0.035499138	positive
PRJNA729271	stage_1D_vs_E10	ordinary_muscle_development	ENSGALG00010020013	ADAM8	-3.306148441	0.74673318	-4.427482973	9.53391E-06	1.95244E-05	negative
PRJNA729271	stage_1D_vs_E10	ordinary_muscle_development	ENSGALG00010020015	ATP6V1D	0.491780003	0.078391411	6.273391412	3.53267E-10	1.03051E-09	positive
PRJNA729271	stage_1D_vs_E10	ordinary_muscle_development	ENSGALG00010020017	ENSGALG00010020017	2.84159951	0.291125133	9.760749533	1.65924E-22	1.01437E-21	positive
PRJNA729271	stage_1D_vs_E10	ordinary_muscle_development	ENSGALG00010020018	ENSGALG00010020018	1.960325659	0.203648743	9.626014049	6.20914E-22	3.70377E-21	positive
PRJNA729271	stage_1D_vs_E10	ordinary_muscle_development	ENSGALG00010020019	PEX10	0.368673834	0.098652005	3.737114454	0.000186144	0.000335759	positive
PRJNA729271	stage_1D_vs_E10	ordinary_muscle_development	ENSGALG00010020023	FOSL2	0.399815074	0.208695928	1.915778033	0.055393355	0.073333335	positive
PRJNA729271	stage_1D_vs_E10	ordinary_muscle_development	ENSGALG00010020025	LOXL2	-0.81087196	0.140102999	-5.787684539	7.13633E-09	1.88933E-08	negative
PRJNA729271	stage_1D_vs_E10	ordinary_muscle_development	ENSGALG00010020027	RER1	-0.191552485	0.070372292	-2.721987302	0.006489064	0.009830579	negative
PRJNA729271	stage_1D_vs_E10	ordinary_muscle_development	ENSGALG00010020030	MRPL33	0.009535437	0.135726142	0.070254979	0.943990715	0.952334731	positive
PRJNA729271	stage_1D_vs_E10	ordinary_muscle_development	ENSGALG00010020032	CHST12	-0.159490317	0.123130194	-1.295298188	0.195217384	0.235179187	negative
PRJNA729271	stage_1D_vs_E10	ordinary_muscle_development	ENSGALG00010020036	TMF1	0.065747866	0.083718171	0.785347621	0.432249782	0.481736167	positive
PRJNA729271	stage_1D_vs_E10	ordinary_muscle_development	ENSGALG00010020037	MPP5	0.029206106	0.088425471	0.330290653	0.741180355	0.774188171	positive
PRJNA729271	stage_1D_vs_E10	ordinary_muscle_development	ENSGALG00010020038	SKI	0.023801606	0.090549718	0.262856772	0.792660976	0.820277065	positive
PRJNA729271	stage_1D_vs_E10	ordinary_muscle_development	ENSGALG00010020041	CDH23	-0.01435165	0.152813932	-0.093915848	0.925176005	0.936220762	negative
PRJNA729271	stage_1D_vs_E10	ordinary_muscle_development	ENSGALG00010020046	BDNF	-0.253992488	0.227242274	-1.117716713	0.263688002	0.308845452	negative
PRJNA729271	stage_1D_vs_E10	ordinary_muscle_development	ENSGALG00010020048	ENSGALG00010020048	-0.306605175	0.264197135	-1.160516656	0.245838516	0.289945427	negative
PRJNA729271	stage_1D_vs_E10	ordinary_muscle_development	ENSGALG00010020053	LFNG	-0.021616255	0.135136683	-0.159958456	0.8729138	0.891667302	negative
PRJNA729271	stage_1D_vs_E10	ordinary_muscle_development	ENSGALG00010020055	R3HCC1	-0.774375278	0.084413139	-9.173634376	4.57334E-20	2.45612E-19	negative
PRJNA729271	stage_1D_vs_E10	ordinary_muscle_development	ENSGALG00010020056	UACA	-2.105714984	0.1661361	-12.67463837	8.17348E-37	9.53901E-36	negative
PRJNA729271	stage_1D_vs_E10	ordinary_muscle_development	ENSGALG00010020057	RBKS	-0.23160817	0.093562667	-2.475433594	0.013307451	0.019337493	negative
PRJNA729271	stage_1D_vs_E10	ordinary_muscle_development	ENSGALG00010020059	PRKCZ	-0.430574622	0.140554906	-3.063390911	0.00218844	0.003502035	negative
PRJNA729271	stage_1D_vs_E10	ordinary_muscle_development	ENSGALG00010020061	UBA3	-0.055933811	0.072387849	-0.772696131	0.439702237	0.488880531	negative
PRJNA729271	stage_1D_vs_E10	ordinary_muscle_development	ENSGALG00010020063	ENSGALG00010020063	1.55136856	0.128443516	12.07821625	1.37674E-33	1.40931E-32	positive
PRJNA729271	stage_1D_vs_E10	ordinary_muscle_development	ENSGALG00010020064	TMEM138	-0.548901516	0.134713031	-4.074598527	4.60938E-05	8.84003E-05	negative
PRJNA729271	stage_1D_vs_E10	ordinary_muscle_development	ENSGALG00010020065	TTYH3	-2.088770866	0.112448974	-18.57527723	5.09413E-77	2.2308E-75	negative
PRJNA729271	stage_1D_vs_E10	ordinary_muscle_development	ENSGALG00010020066	ENSGALG00010020066	1.999479572	0.383107649	5.219106373	1.79788E-07	4.25614E-07	positive
PRJNA729271	stage_1D_vs_E10	ordinary_muscle_development	ENSGALG00010020069	TMEM216	-1.051094193	0.203717886	-5.159557724	2.47534E-07	5.79261E-07	negative
PRJNA729271	stage_1D_vs_E10	ordinary_muscle_development	ENSGALG00010020070	ARL6IP5	0.353217508	0.11448759	3.085203461	0.00203413	0.003271716	positive
PRJNA729271	stage_1D_vs_E10	ordinary_muscle_development	ENSGALG00010020071	ENSGALG00010020071	0.166970457	0.143647261	1.162364359	0.245087494	0.289247942	positive
PRJNA729271	stage_1D_vs_E10	ordinary_muscle_development	ENSGALG00010020074	ENSGALG00010020074	-0.944484065	0.208418523	-4.531670462	5.85191E-06	1.22118E-05	negative
PRJNA729271	stage_1D_vs_E10	ordinary_muscle_development	ENSGALG00010020075	CPSF7	-0.05901214	0.139545204	-0.422889061	0.672376204	0.71071925	negative
PRJNA729271	stage_1D_vs_E10	ordinary_muscle_development	ENSGALG00010020080	SLC4A1AP	-0.216388345	0.102936773	-2.102148134	0.035540302	0.048512729	negative
PRJNA729271	stage_1D_vs_E10	ordinary_muscle_development	ENSGALG00010020083	CFAP74	-2.051247261	0.457993184	-4.478772468	7.50735E-06	1.55168E-05	negative
PRJNA729271	stage_1D_vs_E10	ordinary_muscle_development	ENSGALG00010020084	ENSGALG00010020084	-0.454222566	0.087294285	-5.203348276	1.9573E-07	4.62219E-07	negative
PRJNA729271	stage_1D_vs_E10	ordinary_muscle_development	ENSGALG00010020087	ENSGALG00010020087	1.2001896	0.495959834	2.419933064	0.015523364	0.022340267	positive
PRJNA729271	stage_1D_vs_E10	ordinary_muscle_development	ENSGALG00010020088	LMOD3	2.654090837	0.194245444	13.66359378	1.67508E-42	2.404E-41	positive
PRJNA729271	stage_1D_vs_E10	ordinary_muscle_development	ENSGALG00010020090	MORF4L2	-0.688672454	0.05353143	-12.86482447	7.1003E-38	8.61967E-37	negative
PRJNA729271	stage_1D_vs_E10	ordinary_muscle_development	ENSGALG00010020091	SUPT7L	0.074440997	0.082554268	0.901721965	0.367204582	0.416285814	positive
PRJNA729271	stage_1D_vs_E10	ordinary_muscle_development	ENSGALG00010020092	SDHAF2	0.987406852	0.136989654	7.207893606	5.6824E-13	2.00259E-12	positive
PRJNA729271	stage_1D_vs_E10	ordinary_muscle_development	ENSGALG00010020094	CLEC3B	3.248790016	0.227704871	14.26754727	3.48626E-46	5.72291E-45	positive
PRJNA729271	stage_1D_vs_E10	ordinary_muscle_development	ENSGALG00010020095	LRFN2	-0.816359829	0.322731193	-2.529534942	0.01142138	0.016758063	negative
PRJNA729271	stage_1D_vs_E10	ordinary_muscle_development	ENSGALG00010020096	GPHN	0.789749203	0.116278379	6.7918835	1.10679E-11	3.57303E-11	positive
PRJNA729271	stage_1D_vs_E10	ordinary_muscle_development	ENSGALG00010020097	RETSAT	2.170216952	0.227839876	9.525184925	1.64748E-21	9.57509E-21	positive
PRJNA729271	stage_1D_vs_E10	ordinary_muscle_development	ENSGALG00010020098	GABRD	-0.586022283	0.350577847	-1.671589597	0.094605278	0.120798405	negative
PRJNA729271	stage_1D_vs_E10	ordinary_muscle_development	ENSGALG00010020100	MOCS1	1.365547508	0.157533807	8.668282298	4.38688E-18	2.11396E-17	positive
PRJNA729271	stage_1D_vs_E10	ordinary_muscle_development	ENSGALG00010020101	KIF15	-1.929711506	0.205540527	-9.388472136	6.08769E-21	3.43217E-20	negative
PRJNA729271	stage_1D_vs_E10	ordinary_muscle_development	ENSGALG00010020102	TMEM127	0.273433493	0.202742868	1.348671328	0.177442559	0.215647681	positive
PRJNA729271	stage_1D_vs_E10	ordinary_muscle_development	ENSGALG00010020103	PSAP	0.121091215	0.105068123	1.152501937	0.249114874	0.293427605	positive
PRJNA729271	stage_1D_vs_E10	ordinary_muscle_development	ENSGALG00010020104	BRAT1	0.31043035	0.112231138	2.765991293	0.005675005	0.008661598	positive
PRJNA729271	stage_1D_vs_E10	ordinary_muscle_development	ENSGALG00010020106	ENSGALG00010020106	2.330039259	0.161749763	14.40520971	4.79859E-47	8.13516E-46	positive
PRJNA729271	stage_1D_vs_E10	ordinary_muscle_development	ENSGALG00010020108	RPAP1	-0.308907919	0.116028776	-2.662338864	0.007759971	0.011641563	negative
PRJNA729271	stage_1D_vs_E10	ordinary_muscle_development	ENSGALG00010020109	WDFY4	-0.891884096	0.364396127	-2.447567443	0.014382421	0.020791182	negative
PRJNA729271	stage_1D_vs_E10	ordinary_muscle_development	ENSGALG00010020111	ADRA2B	1.419580108	0.120463463	11.78432091	4.70195E-32	4.49569E-31	positive
PRJNA729271	stage_1D_vs_E10	ordinary_muscle_development	ENSGALG00010020113	CIAO1	-0.622188663	0.105741798	-5.884037107	4.00378E-09	1.07952E-08	negative
PRJNA729271	stage_1D_vs_E10	ordinary_muscle_development	ENSGALG00010020116	GNA12	-0.390898366	0.072318034	-5.405268145	6.47115E-08	1.58794E-07	negative
PRJNA729271	stage_1D_vs_E10	ordinary_muscle_development	ENSGALG00010020117	AKAP13	-0.29029784	0.102257801	-2.838882104	0.004527188	0.00700113	negative
PRJNA729271	stage_1D_vs_E10	ordinary_muscle_development	ENSGALG00010020118	GNB1	-0.794051878	0.09336522	-8.504793112	1.8192E-17	8.45711E-17	negative
PRJNA729271	stage_1D_vs_E10	ordinary_muscle_development	ENSGALG00010020120	DAAM2	-0.608417931	0.101571559	-5.990042253	2.09787E-09	5.78439E-09	negative
PRJNA729271	stage_1D_vs_E10	ordinary_muscle_development	ENSGALG00010020121	ENSGALG00010020121	-2.415416323	0.262836029	-9.18982201	3.93492E-20	2.11875E-19	negative
PRJNA729271	stage_1D_vs_E10	ordinary_muscle_development	ENSGALG00010020123	ENSGALG00010020123	0.213624046	0.145569574	1.467504788	0.142238788	0.176037503	positive
PRJNA729271	stage_1D_vs_E10	ordinary_muscle_development	ENSGALG00010020125	NAV2	0.969892461	0.12951678	7.488546714	6.96404E-14	2.59963E-13	positive
PRJNA729271	stage_1D_vs_E10	ordinary_muscle_development	ENSGALG00010020126	NADK	0.029462	0.104442844	0.282087303	0.777876569	0.806820813	positive
PRJNA729271	stage_1D_vs_E10	ordinary_muscle_development	ENSGALG00010020127	FRMD4B	-1.100329072	0.140537327	-7.829443567	4.90034E-15	1.97033E-14	negative
PRJNA729271	stage_1D_vs_E10	ordinary_muscle_development	ENSGALG00010020128	KIF6	1.038142604	0.486685244	2.133088308	0.032917483	0.045157951	positive
PRJNA729271	stage_1D_vs_E10	ordinary_muscle_development	ENSGALG00010020129	MGAT2	-0.323368592	0.116138862	-2.784327185	0.00536389	0.008213562	negative
PRJNA729271	stage_1D_vs_E10	ordinary_muscle_development	ENSGALG00010020130	PDXDC1	-0.386661858	0.073895004	-5.232584587	1.67156E-07	3.97006E-07	negative
PRJNA729271	stage_1D_vs_E10	ordinary_muscle_development	ENSGALG00010020131	SNRNP200	-0.301014851	0.124537851	-2.417055115	0.015646648	0.022490892	negative
PRJNA729271	stage_1D_vs_E10	ordinary_muscle_development	ENSGALG00010020132	SHC4	0.579215776	0.227828421	2.542333274	0.011011512	0.016194386	positive
PRJNA729271	stage_1D_vs_E10	ordinary_muscle_development	ENSGALG00010020133	ENSGALG00010020133	2.107692743	0.36721897	5.739607464	9.48963E-09	2.48065E-08	positive
PRJNA729271	stage_1D_vs_E10	ordinary_muscle_development	ENSGALG00010020136	KCNK17	-0.486980548	0.307764478	-1.582315642	0.113577532	0.143069985	negative
PRJNA729271	stage_1D_vs_E10	ordinary_muscle_development	ENSGALG00010020137	NTAN1	-0.429365529	0.082813808	-5.184709398	2.16352E-07	5.08352E-07	negative
PRJNA729271	stage_1D_vs_E10	ordinary_muscle_development	ENSGALG00010020138	ENSGALG00010020138	0.211726878	0.085140092	2.486805847	0.012889572	0.018769776	positive
PRJNA729271	stage_1D_vs_E10	ordinary_muscle_development	ENSGALG00010020140	SECISBP2L	0.968207571	0.094100155	10.28911773	7.88959E-25	5.35642E-24	positive
PRJNA729271	stage_1D_vs_E10	ordinary_muscle_development	ENSGALG00010020141	THOC7	-0.569030359	0.127969697	-4.446602386	8.72391E-06	1.79392E-05	negative
PRJNA729271	stage_1D_vs_E10	ordinary_muscle_development	ENSGALG00010020142	SLC35E2B	-1.396270008	0.070246517	-19.87671507	6.47359E-88	3.70888E-86	negative
PRJNA729271	stage_1D_vs_E10	ordinary_muscle_development	ENSGALG00010020143	KCNK5	-0.752967578	0.179013417	-4.206207493	2.59692E-05	5.08955E-05	negative
PRJNA729271	stage_1D_vs_E10	ordinary_muscle_development	ENSGALG00010020147	ABHD2	1.660089603	0.145675666	11.39579208	4.38821E-30	3.80653E-29	positive
PRJNA729271	stage_1D_vs_E10	ordinary_muscle_development	ENSGALG00010020149	FAHD2A	0.849018713	0.172421514	4.924088021	8.47547E-07	1.9038E-06	positive

PRJNA729271	stage_1D_vs_E10	ordinary_muscle_development	ENSGALG00010020152	ATXN7	0.700477784	0.107070064	6.542237473	6.06052E-11	1.86289E-10	positive
PRJNA729271	stage_1D_vs_E10	ordinary_muscle_development	ENSGALG00010020153	AXL	-0.462901833	0.1212061	-3.819129834	0.000133923	0.000245444	negative
PRJNA729271	stage_1D_vs_E10	ordinary_muscle_development	ENSGALG00010020155	MFGE8	1.546679705	0.134336466	11.51347622	1.12836E-30	1.01148E-29	positive
PRJNA729271	stage_1D_vs_E10	ordinary_muscle_development	ENSGALG00010020160	SYT7	-3.987337999	0.347567938	-11.4721111	1.8216E-30	1.61889E-29	negative
PRJNA729271	stage_1D_vs_E10	ordinary_muscle_development	ENSGALG00010020163	CDC2L1	-0.246776142	0.095459575	-2.585137647	0.009734013	0.014413578	negative
PRJNA729271	stage_1D_vs_E10	ordinary_muscle_development	ENSGALG00010020164	C10orf71	4.230221718	0.147830772	28.61529878	4.3349E-180	1.3963E-177	positive
PRJNA729271	stage_1D_vs_E10	ordinary_muscle_development	ENSGALG00010020166	HAPLN3	-0.788875753	0.177605519	-4.441729964	8.92385E-06	1.83243E-05	negative
PRJNA729271	stage_1D_vs_E10	ordinary_muscle_development	ENSGALG00010020168	COX7A2L	1.026214083	0.114682293	8.948321966	3.60927E-19	1.84018E-18	positive
PRJNA729271	stage_1D_vs_E10	ordinary_muscle_development	ENSGALG00010020170	SORCS1	-1.465302295	0.307449514	-4.765993211	1.87926E-06	4.10176E-06	negative
PRJNA729271	stage_1D_vs_E10	ordinary_muscle_development	ENSGALG00010020171	RRN3	-0.049136799	0.07523935	-0.653073143	0.513709126	0.561345792	negative
PRJNA729271	stage_1D_vs_E10	ordinary_muscle_development	ENSGALG00010020175	ACAN	-8.382519748	0.515680269	-16.25526563	2.04978E-59	5.55356E-58	negative
PRJNA729271	stage_1D_vs_E10	ordinary_muscle_development	ENSGALG00010020176	ENSGALG00010020176	0.163561436	0.247065475	0.662016562	0.507960608	0.555941483	positive
PRJNA729271	stage_1D_vs_E10	ordinary_muscle_development	ENSGALG00010020177	KCNG3	-1.828527085	0.243803017	-7.50001827	6.38089E-14	2.38687E-13	negative
PRJNA729271	stage_1D_vs_E10	ordinary_muscle_development	ENSGALG00010020179	PSMD6	-0.250844892	0.135390427	-1.85275205	0.063917906	0.083860431	negative
PRJNA729271	stage_1D_vs_E10	ordinary_muscle_development	ENSGALG00010020180	TCF7L1	0.045957874	0.202071301	0.22743395	0.820086323	0.845218377	positive
PRJNA729271	stage_1D_vs_E10	ordinary_muscle_development	ENSGALG00010020181	LEUTX	3.966951366	0.208951113	18.98506932	2.26637E-80	1.08419E-78	positive
PRJNA729271	stage_1D_vs_E10	ordinary_muscle_development	ENSGALG00010020183	XPNPEP1	-0.871905681	0.069083659	-12.6210118	1.6173E-36	1.86054E-35	negative
PRJNA729271	stage_1D_vs_E10	ordinary_muscle_development	ENSGALG00010020184	RSL1D1	-0.84453795	0.069749283	-12.10819538	9.55738E-34	9.83209E-33	negative
PRJNA729271	stage_1D_vs_E10	ordinary_muscle_development	ENSGALG00010020186	MGA	0.531448045	0.09453412	5.621759052	1.89023E-08	4.82713E-08	positive
PRJNA729271	stage_1D_vs_E10	ordinary_muscle_development	ENSGALG00010020187	MMP23B	-0.917574841	0.208348078	-4.404047535	1.0625E-05	2.16457E-05	negative
PRJNA729271	stage_1D_vs_E10	ordinary_muscle_development	ENSGALG00010020193	VSTM4	-0.924302607	0.178012981	-5.192332601	2.07676E-07	4.88916E-07	negative
PRJNA729271	stage_1D_vs_E10	ordinary_muscle_development	ENSGALG00010020194	PRICKLE2	-0.499531126	0.153751433	-3.248952653	0.001158308	0.001918381	negative
PRJNA729271	stage_1D_vs_E10	ordinary_muscle_development	ENSGALG00010020199	DET1	-0.20952617	0.083268253	-2.516279149	0.01186012	0.017364894	negative
PRJNA729271	stage_1D_vs_E10	ordinary_muscle_development	ENSGALG00010020202	MAPKBP1	1.279990923	0.090015073	14.21973984	6.91094E-46	1.11926E-44	positive
PRJNA729271	stage_1D_vs_E10	ordinary_muscle_development	ENSGALG00010020204	GSPT2	-0.170129187	0.086575364	-1.965099302	0.049402745	0.065962859	negative
PRJNA729271	stage_1D_vs_E10	ordinary_muscle_development	ENSGALG00010020206	MRPS11	-0.092650434	0.081929191	-1.130859863	0.258114079	0.303140786	negative
PRJNA729271	stage_1D_vs_E10	ordinary_muscle_development	ENSGALG00010020211	ADD3	0.167541883	0.083143169	2.015100994	0.043894068	0.059053696	positive
PRJNA729271	stage_1D_vs_E10	ordinary_muscle_development	ENSGALG00010020212	JMJD7	-0.110875686	0.139436617	-0.795169078	0.426515157	0.475967141	negative
PRJNA729271	stage_1D_vs_E10	ordinary_muscle_development	ENSGALG00010020215	PRMT3	-0.755460714	0.081119518	-9.312933963	1.24349E-20	6.88479E-20	negative
PRJNA729271	stage_1D_vs_E10	ordinary_muscle_development	ENSGALG00010020224	MRPL46	-0.136154751	0.087229638	-1.56087717	0.118552735	0.148910043	negative
PRJNA729271	stage_1D_vs_E10	ordinary_muscle_development	ENSGALG00010020226	TCIM	2.680956356	0.197203808	13.59485085	4.29636E-42	6.07568E-41	positive
PRJNA729271	stage_1D_vs_E10	ordinary_muscle_development	ENSGALG00010020228	TM9SF3	-0.507985171	0.083044923	-6.116992472	9.53579E-10	2.70069E-09	negative
PRJNA729271	stage_1D_vs_E10	ordinary_muscle_development	ENSGALG00010020230	ENSGALG00010020230	0.66223821	0.215416453	3.074222973	0.002110516	0.003386686	positive
PRJNA729271	stage_1D_vs_E10	ordinary_muscle_development	ENSGALG00010020236	SLC35G1	1.994787281	0.286789439	6.955581383	3.51111E-12	1.17212E-11	positive
PRJNA729271	stage_1D_vs_E10	ordinary_muscle_development	ENSGALG00010020237	MIB2	0.091660619	0.075289285	1.217445737	0.22343467	0.266098558	positive
PRJNA729271	stage_1D_vs_E10	ordinary_muscle_development	ENSGALG00010020240	SV2B	-3.308959291	0.479696827	-6.898022051	5.27316E-12	1.73596E-11	negative
PRJNA729271	stage_1D_vs_E10	ordinary_muscle_development	ENSGALG00010020244	SLC25A37	-1.672126705	0.215760101	-7.74993474	9.19398E-15	3.62927E-14	negative
PRJNA729271	stage_1D_vs_E10	ordinary_muscle_development	ENSGALG00010020246	ADAMTS9	-0.611660334	0.129339465	-4.729108261	2.25508E-06	4.89699E-06	negative
PRJNA729271	stage_1D_vs_E10	ordinary_muscle_development	ENSGALG00010020248	NOLC1	-0.832615658	0.080836447	-10.30000308	7.0459E-25	4.80387E-24	negative
PRJNA729271	stage_1D_vs_E10	ordinary_muscle_development	ENSGALG00010020250	ENSGALG00010020250	0.115753281	0.24460694	0.473221575	0.636055107	0.67676861	positive
PRJNA729271	stage_1D_vs_E10	ordinary_muscle_development	ENSGALG00010020252	DMTN	1.079478863	0.187593006	5.754366254	8.69674E-09	2.28162E-08	positive
PRJNA729271	stage_1D_vs_E10	ordinary_muscle_development	ENSGALG00010020254	ENSGALG00010020254	-1.718632076	0.135519739	-12.6817841	7.4614E-37	8.7361E-36	negative
PRJNA729271	stage_1D_vs_E10	ordinary_muscle_development	ENSGALG00010020257	ZNF710	0.234319632	0.161962005	1.446756795	0.147965051	0.182656568	positive
PRJNA729271	stage_1D_vs_E10	ordinary_muscle_development	ENSGALG00010020259	ENSGALG00010020259	-2.137566251	0.231453383	-9.235407237	2.57305E-20	1.39426E-19	negative
PRJNA729271	stage_1D_vs_E10	ordinary_muscle_development	ENSGALG00010020261	SSU72	0.442929872	0.090901034	4.872660444	1.10105E-06	2.45083E-06	positive
PRJNA729271	stage_1D_vs_E10	ordinary_muscle_development	ENSGALG00010020265	ENSGALG00010020265	-0.594792138	0.227323069	-2.616505841	0.008883484	0.01322034	negative
PRJNA729271	stage_1D_vs_E10	ordinary_muscle_development	ENSGALG00010020268	CPLANE2	-0.396110896	0.099829642	-3.967868533	7.25183E-05	0.000136407	negative
PRJNA729271	stage_1D_vs_E10	ordinary_muscle_development	ENSGALG00010020269	ENSGALG00010020269	-1.690154309	0.664451926	-2.543681855	0.010969094	0.016138553	negative
PRJNA729271	stage_1D_vs_E10	ordinary_muscle_development	ENSGALG00010020272	ENSGALG00010020272	-0.967568221	0.090822367	-10.65341344	1.68086E-26	1.23113E-25	negative
PRJNA729271	stage_1D_vs_E10	ordinary_muscle_development	ENSGALG00010020275	ELOVL3	-1.172199616	0.457120502	-2.564312061	0.010338055	0.015244161	negative
PRJNA729271	stage_1D_vs_E10	ordinary_muscle_development	ENSGALG00010020277	IDH2	1.282827781	0.124640511	10.29222181	7.6393E-25	5.19379E-24	positive
PRJNA729271	stage_1D_vs_E10	ordinary_muscle_development	ENSGALG00010020282	MAGI1	-0.266222448	0.136546743	-1.949679957	0.051214277	0.068211977	negative
PRJNA729271	stage_1D_vs_E10	ordinary_muscle_development	ENSGALG00010020283	FBXO42	0.101237441	0.221964796	0.456096836	0.648320345	0.688379022	positive
PRJNA729271	stage_1D_vs_E10	ordinary_muscle_development	ENSGALG00010020284	HEG1	1.949131267	0.116033902	16.79794637	2.52641E-63	7.61337E-62	positive
PRJNA729271	stage_1D_vs_E10	ordinary_muscle_development	ENSGALG00010020285	ADAMTS14	-2.660620658	0.264324849	-10.06572278	7.831E-24	5.05614E-23	negative
PRJNA729271	stage_1D_vs_E10	ordinary_muscle_development	ENSGALG00010020287	GALNT16	0.084113004	0.160998069	0.522447287	0.601358939	0.644344901	positive
PRJNA729271	stage_1D_vs_E10	ordinary_muscle_development	ENSGALG00010020290	NELL1	-5.058691271	0.610014233	-8.292743016	1.10666E-16	4.9236E-16	negative
PRJNA729271	stage_1D_vs_E10	ordinary_muscle_development	ENSGALG00010020291	SNX29	0.129407113	0.14508222	0.891957074	0.372415941	0.421600209	positive
PRJNA729271	stage_1D_vs_E10	ordinary_muscle_development	ENSGALG00010020292	NPM2	-1.497134775	0.255830685	-5.852053186	4.85541E-09	1.30139E-08	negative
PRJNA729271	stage_1D_vs_E10	ordinary_muscle_development	ENSGALG00010020293	SEMA4B	0.128642764	0.110537286	1.16379521	0.244507014	0.288656879	positive
PRJNA729271	stage_1D_vs_E10	ordinary_muscle_development	ENSGALG00010020295	LGI1	0.693285501	0.139786041	4.959618963	7.06316E-07	1.5962E-06	positive
PRJNA729271	stage_1D_vs_E10	ordinary_muscle_development	ENSGALG00010020298	ENSGALG00010020298	-0.412782827	0.174576962	-2.364474803	0.018055656	0.025691246	negative
PRJNA729271	stage_1D_vs_E10	ordinary_muscle_development	ENSGALG00010020300	EHD4	1.370388202	0.128440729	10.66942092	1.41506E-26	1.04065E-25	positive
PRJNA729271	stage_1D_vs_E10	ordinary_muscle_development	ENSGALG00010020302	HAAO	-0.537915889	1.58006252	-0.340439624	0.733525483	0.767353629	negative
PRJNA729271	stage_1D_vs_E10	ordinary_muscle_development	ENSGALG00010020303	EXD2	-0.321815851	0.072203767	-4.457050683	8.3095E-06	1.71113E-05	negative
PRJNA729271	stage_1D_vs_E10	ordinary_muscle_development	ENSGALG00010020304	ZFP36L2	1.751230869	0.154979878	11.29973058	1.31621E-29	1.11505E-28	positive
PRJNA729271	stage_1D_vs_E10	ordinary_muscle_development	ENSGALG00010020305	XPO7	0.037375945	0.137981884	0.270875741	0.786486604	0.814585447	positive
PRJNA729271	stage_1D_vs_E10	ordinary_muscle_development	ENSGALG00010020307	SHQ1	-0.800585927	0.226958383	-3.527456948	0.000419572	0.000728172	negative
PRJNA729271	stage_1D_vs_E10	ordinary_muscle_development	ENSGALG00010020309	GXYLT2	-0.203315018	0.159546265	-1.274332673	0.202545542	0.242997652	negative
PRJNA729271	stage_1D_vs_E10	ordinary_muscle_development	ENSGALG00010020311	SGPL1	-0.265759221	0.134021042	-1.982966392	0.047371184	0.063390446	negative
PRJNA729271	stage_1D_vs_E10	ordinary_muscle_development	ENSGALG00010020313	FRA10AC1	0.228280332	0.113963581	2.00309897	0.045166667	0.060641981	positive
PRJNA729271	stage_1D_vs_E10	ordinary_muscle_development	ENSGALG00010020314	THADA	-0.525130495	0.079627443	-6.594843137	4.25706E-11	1.32274E-10	negative
PRJNA729271	stage_1D_vs_E10	ordinary_muscle_development	ENSGALG00010020315	ANO5	0.298052214	0.108377707	2.750124744	0.005957258	0.009072332	positive
PRJNA729271	stage_1D_vs_E10	ordinary_muscle_development	ENSGALG00010020316	PDPK1	0.24835143	0.112283102	2.211832645	0.026978231	0.037421	positive
PRJNA729271	stage_1D_vs_E10	ordinary_muscle_development	ENSGALG00010020319	NTRK3	0.749401877	0.158009509	4.742764425	2.10821E-06	4.58975E-06	positive
PRJNA729271	stage_1D_vs_E10	ordinary_muscle_development	ENSGALG00010020321	SZRD1	0.819750907	0.169460719	4.837409586	1.31542E-06	2.907E-06	positive
PRJNA729271	stage_1D_vs_E10	ordinary_muscle_development	ENSGALG00010020323	ARHGAP25	1.298212167	0.152079978	8.536377932	1.38495E-17	6.47343E-17	positive
PRJNA729271	stage_1D_vs_E10	ordinary_muscle_development	ENSGALG00010020324	PLEKHH2	0.462583747	0.184684094	2.504729766	0.012254498	0.017909755	positive
PRJNA729271	stage_1D_vs_E10	ordinary_muscle_development	ENSGALG00010020329	PPP4R2	0.386566755	0.105246592	3.6729622	0.000239755	0.00042683	positive
PRJNA729271	stage_1D_vs_E10	ordinary_muscle_development	ENSGALG00010020334	CABP4	-0.388697379	0.517712975	-0.75079706	0.452774799	0.501756171	negative
PRJNA729271	stage_1D_vs_E10	ordinary_muscle_development	ENSGALG00010020336	OSBP	-0.046585162	0.134622792	-0.346042161	0.72931103	0.763385571	negative
PRJNA729271	stage_1D_vs_E10	ordinary_muscle_development	ENSGALG00010020338	NECAP2	-0.224818252	0.166406718	-1.351016685	0.176690088	0.214841274	negative
PRJNA729271	stage_1D_vs_E10	ordinary_muscle_development	ENSGALG00010020340	DCAF5	0.529146234	0.096612303	5.477006737	4.32581E-08	1.07311E-07	positive
PRJNA729271	stage_1D_vs_E10	ordinary_muscle_development	ENSGALG00010020341	DYNC2LI1	-1.374800487	0.130371278	-10.5452712	5.34185E-26	3.8256E-25	negative
PRJNA729271	stage_1D_vs_E10	ordinary_muscle_development	ENSGALG00010020342	CPPED1	-0.767412322	0.106775806	-7.18713678	6.61641E-13	2.32103E-12	negative
PRJNA729271	stage_1D_vs_E10	ordinary_muscle_development	ENSGALG00010020343	PATL1	0.731886368	0.174871394	4.185283555	2.8481E-05	5.56453E-05	positive
PRJNA729271	stage_1D_vs_E10	ordinary_muscle_development	ENSGALG00010020345	AGBL1	0.23937406	0.453370545	0.527987676	0.59750789	0.640834396	positive
PRJNA729271	stage_1D_vs_E10	ordinary_muscle_development	ENSGALG00010020346	PCBD1	-1.359600262	0.261898347	-5.191328142	2.08799E-07	4.91482E-07	negative
PRJNA729271	stage_1D_vs_E10	ordinary_muscle_development	ENSGALG00010020350	PDZRN3	0.130503706	0.108030098	1.208030991	0.227035342	0.269921037	positive
PRJNA729271	stage_1D_vs_E10	ordinary_muscle_development	ENSGALG00010020351	CROCC	-0.053764501	0.146553847	-0.366858341	0.713724687	0.749180921	negative
PRJNA729271	stage_1D_vs_E10	ordinary_muscle_development	ENSGALG00010020352	ABCG5	0.608145828	0.582830866	1.043434492	0.296747106	0.343640593	positive
PRJNA729271	stage_1D_vs_E10	ordinary_muscle_development	ENSGALG00010020356	STX3	-0.372342122	0.386174222	-0.964181712	0.334954832	0.383444186	negative
PRJNA729271	stage_1D_vs_E10	ordinary_muscle_development	ENSGALG00010020357	RBP4	0.453392754	0.732790724	0.618720652	0.536100403	0.582566064	positive
PRJNA729271	stage_1D_vs_E10	ordinary_muscle_development	ENSGALG00010020362	CDS2	0.102566737	0.126590353	0.810225536	0.417810563	0.467188468	positive
PRJNA729271	stage_1D_vs_E10	ordinary_muscle_development	ENSGALG00010020364	MRPL16	0.700082002	0.11067975	6.32529438	2.5275E-10	7.45395E-10	positive
PRJNA729271	stage_1D_vs_E10	ordinary_muscle_development	ENSGALG00010020365	UNC5B	-0.543690496	0.139109125	-3.908374076	9.29194E-05	0.000172963	negative
PRJNA729271	stage_1D_vs_E10	ordinary_muscle_development	ENSGALG00010020369	CNTN3	-3.519303666	0.305280605	-11.52809453	9.52281E-31	8.55291E-30	negative
PRJNA729271	stage_1D_vs_E10	ordinary_muscle_development	ENSGALG00010020370	CEP55	-1.159693872	0.221158719	-5.243717621	1.57373E-07	3.74445E-07	negative
PRJNA729271	stage_1D_vs_E10	ordinary_muscle_development	ENSGALG00010020373	MS4A4A	-1.44677215	0.217781329	-6.643233185	3.06876E-11	9.61973E-11	negative
PRJNA729271	stage_1D_vs_E10	ordinary_muscle_development	ENSGALG00010020374	PCNA	-1.391200132	0.095840438	-14.51579474	9.62358E-48	1.68674E-46	negative
PRJNA729271	stage_1D_vs_E10	ordinary_muscle_development	ENSGALG00010020375	LRPPRC	0.531001565	0.101584583	5.227186541	1.72109E-07	4.07967E-07	positive
PRJNA729271	stage_1D_vs_E10	ordinary_muscle_development	ENSGALG00010020377	TMEM230	-0.744264035	0.100876117	-7.378000422	1.60684E-13	5.85795E-13	negative
PRJNA729271	stage_1D_vs_E10	ordinary_muscle_development	ENSGALG00010020379	COL13A1	-0.809746955	0.194761948	-4.157624029	3.21575E-05	6.25248E-05	negative
PRJNA729271	stage_1D_vs_E10	ordinary_muscle_development	ENSGALG00010020382	MFAP2	-2.980740052	0.149840553	-19.89274588	4.70281E-88	2.70505E-86	negative
PRJNA729271	stage_1D_vs_E10	ordinary_muscle_development	ENSGALG00010020383	BAAT	-0.660263642	0.105794426	-6.241005924	4.34766E-10	1.25988E-09	negative
PRJNA729271	stage_1D_vs_E10	ordinary_muscle_development	ENSGALG00010020385	SLC12A8	-2.765324115	0.423176239	-6.534686634	6.37429E-11	1.95546E-10	negative
PRJNA729271	stage_1D_vs_E10	ordinary_muscle_development	ENSGALG00010020386	PPM1B	1.126314033	0.071735621	15.70090303	1.49104E-55	3.51426E-54	positive
PRJNA729271	stage_1D_vs_E10	ordinary_muscle_development	ENSGALG00010020388	MYOF	0.944842175	0.166368037	5.679228955	1.35303E-08	3.49282E-08	positive
PRJNA729271	stage_1D_vs_E10	ordinary_muscle_development	ENSGALG00010020389	ACTN1	-1.356734865	0.157450599	-8.616892394	6.87957E-18	3.27917E-17	negative
PRJNA729271	stage_1D_vs_E10	ordinary_muscle_development	ENSGALG00010020390	KLHL25	0.854799836	0.221912403	3.851959621	0.000117172	0.000216109	positive
PRJNA729271	stage_1D_vs_E10	ordinary_muscle_development	ENSGALG00010020392	SLC23A2	-0.126761187	0.079747051	-1.589540744	0.111938363	0.141152377	negative
PRJNA729271	stage_1D_vs_E10	ordinary_muscle_development	ENSGALG00010020393	SHISA9	-1.559229808	0.308691287	-5.05109757	4.39279E-07	1.00733E-06	negative
PRJNA729271	stage_1D_vs_E10	ordinary_muscle_development	ENSGALG00010020396	CHL1	1.139241469	0.188974065	6.028559898	1.65427E-09	4.59714E-09	positive
PRJNA729271	stage_1D_vs_E10	ordinary_muscle_development	ENSGALG00010020399	ATP13A2	0.479274337	0.130257826	3.679428346	0.000233757	0.000416715	positive
PRJNA729271	stage_1D_vs_E10	ordinary_muscle_development	ENSGALG00010020402	SLC3A1	3.265200509	0.307722827	10.61084919	2.65327E-26	1.92392E-25	positive
PRJNA729271	stage_1D_vs_E10	ordinary_muscle_development	ENSGALG00010020405	UBE2I	-0.650544689	0.081498367	-7.982303328	1.43628E-15	5.97898E-15	negative
PRJNA729271	stage_1D_vs_E10	ordinary_muscle_development	ENSGALG00010020413	CYP26A1	0.962260788	0.30165799	3.189906514	0.001423188	0.002332555	positive
PRJNA729271	stage_1D_vs_E10	ordinary_muscle_development	ENSGALG00010020415	TSR3	-0.127830838	0.092666269	-1.379475394	0.167748228	0.20489682	negative
PRJNA729271	stage_1D_vs_E10	ordinary_muscle_development	ENSGALG00010020416	ENSGALG00010020416	-5.431192772	1.103478365	-4.921884238	8.57149E-07	1.92447E-06	negative
PRJNA729271	stage_1D_vs_E10	ordinary_muscle_development	ENSGALG00010020417	ENSGALG00010020417	2.541073391	0.763971178	3.326137771	0.000880584	0.001476639	positive
PRJNA729271	stage_1D_vs_E10	ordinary_muscle_development	ENSGALG00010020422	ERCC4	-0.008599993	0.087918544	-0.097817738	0.922077022	0.933671424	negative
PRJNA729271	stage_1D_vs_E10	ordinary_muscle_development	ENSGALG00010020428	SEMA4C	0.230194331	0.148682641	1.548226002	0.121567893	0.152485861	positive
PRJNA729271	stage_1D_vs_E10	ordinary_muscle_development	ENSGALG00010020429	ZNF148	0.358116428	0.100653393	3.557917085	0.000373807	0.000652576	positive
PRJNA729271	stage_1D_vs_E10	ordinary_muscle_development	ENSGALG00010020433	GNPTG	-0.153079229	0.070831032	-2.16118875	0.030680762	0.042269522	negative
PRJNA729271	stage_1D_vs_E10	ordinary_muscle_development	ENSGALG00010020436	CNTN4	-2.973482526	0.267672387	-11.10866371	1.1385E-28	9.21934E-28	negative
PRJNA729271	stage_1D_vs_E10	ordinary_muscle_development	ENSGALG00010020437	SNX4	-1.353575676	0.085176795	-15.89136654	7.27247E-57	1.79888E-55	negative
PRJNA729271	stage_1D_vs_E10	ordinary_muscle_development	ENSGALG00010020438	HTR7	0.315805652	0.278882396	1.132397225	0.257467471	0.302454898	positive
PRJNA729271	stage_1D_vs_E10	ordinary_muscle_development	ENSGALG00010020440	PAX7	0.242689628	0.186029879	1.30457338	0.192038157	0.23175365	positive
PRJNA729271	stage_1D_vs_E10	ordinary_muscle_development	ENSGALG00010020443	PREPL	0.293160804	0.134745577	2.1756618	0.029580559	0.040808051	positive
PRJNA729271	stage_1D_vs_E10	ordinary_muscle_development	ENSGALG00010020444	ENSGALG00010020444	-3.243892528	1.199661159	-2.704007297	0.006850878	0.010340881	negative
PRJNA729271	stage_1D_vs_E10	ordinary_muscle_development	ENSGALG00010020449	PRPF19	-0.324747277	0.097333495	-3.336439072	0.00084859	0.001425793	negative

PRJNA729271	stage_1D_vs_E10	ordinary_muscle_development	ENSGALG00010020450	ANKRD39	0.808729926	0.179927694	4.494749584	6.96519E-06	1.44477E-05	positive
PRJNA729271	stage_1D_vs_E10	ordinary_muscle_development	ENSGALG00010020453	ENSGALG00010020453	-0.431242205	0.140622802	-3.066659168	0.002164655	0.003466853	negative
PRJNA729271	stage_1D_vs_E10	ordinary_muscle_development	ENSGALG00010020455	ENSGALG00010020455	-2.905747087	0.91963952	-3.159658785	0.00157954	0.002574835	negative
PRJNA729271	stage_1D_vs_E10	ordinary_muscle_development	ENSGALG00010020457	IL5RA	-0.51187709	1.061224912	-0.482345528	0.62956051	0.671287303	negative
PRJNA729271	stage_1D_vs_E10	ordinary_muscle_development	ENSGALG00010020460	MAPK8	0.71328727	0.140313307	5.083532588	3.70479E-07	8.54294E-07	positive
PRJNA729271	stage_1D_vs_E10	ordinary_muscle_development	ENSGALG00010020462	CNNM4	0.558163263	0.180719996	3.088552885	0.002011339	0.003236856	positive
PRJNA729271	stage_1D_vs_E10	ordinary_muscle_development	ENSGALG00010020467	ALDH4A1	0.26928251	0.131407673	2.049214507	0.040441144	0.054697619	positive
PRJNA729271	stage_1D_vs_E10	ordinary_muscle_development	ENSGALG00010020468	ENSGALG00010020468	-0.134483179	0.451714567	-0.297717162	0.765919043	0.796013231	negative
PRJNA729271	stage_1D_vs_E10	ordinary_muscle_development	ENSGALG00010020473	TMEM109	2.899133223	0.158937033	18.24076599	2.45001E-74	1.01465E-72	positive
PRJNA729271	stage_1D_vs_E10	ordinary_muscle_development	ENSGALG00010020476	TMEM132A	-0.693637781	0.110901408	-6.254544399	3.98678E-10	1.15808E-09	negative
PRJNA729271	stage_1D_vs_E10	ordinary_muscle_development	ENSGALG00010020478	ENSGALG00010020478	0.246897281	0.786642307	0.313862195	0.753625704	0.785094479	positive
PRJNA729271	stage_1D_vs_E10	ordinary_muscle_development	ENSGALG00010020481	RPP30	-0.966130899	0.12433645	-7.770295042	7.83035E-15	3.10281E-14	negative
PRJNA729271	stage_1D_vs_E10	ordinary_muscle_development	ENSGALG00010020484	TRNT1	-0.317479912	0.125037746	-2.539072572	0.011114677	0.016336163	negative
PRJNA729271	stage_1D_vs_E10	ordinary_muscle_development	ENSGALG00010020485	LMAN2L	-0.350758322	0.131160029	-2.674277559	0.007489044	0.011256086	negative
PRJNA729271	stage_1D_vs_E10	ordinary_muscle_development	ENSGALG00010020486	MRTFB	1.553080933	0.111518972	13.92660728	4.36608E-44	6.61991E-43	positive
PRJNA729271	stage_1D_vs_E10	ordinary_muscle_development	ENSGALG00010020489	IFFO2	-1.633182815	0.188768446	-8.651778651	5.07029E-18	2.43438E-17	negative
PRJNA729271	stage_1D_vs_E10	ordinary_muscle_development	ENSGALG00010020493	DEPDC7	0.801967746	0.143226197	5.599309082	2.15208E-08	5.46982E-08	positive
PRJNA729271	stage_1D_vs_E10	ordinary_muscle_development	ENSGALG00010020497	OSBPL11	-0.639772769	0.070034989	-9.135044942	6.53796E-20	3.47899E-19	negative
PRJNA729271	stage_1D_vs_E10	ordinary_muscle_development	ENSGALG00010020498	FRMPD2	-1.67617838	0.597057309	-2.807399481	0.004994326	0.007680715	negative
PRJNA729271	stage_1D_vs_E10	ordinary_muscle_development	ENSGALG00010020507	ANKRD1	4.517407369	0.585363885	7.717263541	1.18854E-14	4.65492E-14	positive
PRJNA729271	stage_1D_vs_E10	ordinary_muscle_development	ENSGALG00010020509	CRBN	0.429406753	0.10290928	4.172672788	3.01047E-05	5.8691E-05	positive
PRJNA729271	stage_1D_vs_E10	ordinary_muscle_development	ENSGALG00010020514	CAMKMT	-1.064494461	0.138319688	-7.695899808	1.40502E-14	5.48056E-14	negative
PRJNA729271	stage_1D_vs_E10	ordinary_muscle_development	ENSGALG00010020517	UBR4	0.444359592	0.094099921	4.722210031	2.33296E-06	5.06003E-06	positive
PRJNA729271	stage_1D_vs_E10	ordinary_muscle_development	ENSGALG00010020520	ARHGAP22	-0.9154154	0.202428977	-4.522155934	6.12129E-06	1.27538E-05	negative
PRJNA729271	stage_1D_vs_E10	ordinary_muscle_development	ENSGALG00010020521	ENSGALG00010020521	2.174589614	0.629065075	3.456859552	0.00054651	0.000937474	positive
PRJNA729271	stage_1D_vs_E10	ordinary_muscle_development	ENSGALG00010020524	ENSGALG00010020524	-0.624712026	0.412636174	-1.51395361	0.130037606	0.162141513	negative
PRJNA729271	stage_1D_vs_E10	ordinary_muscle_development	ENSGALG00010020525	ENSGALG00010020525	1.233589791	0.722545032	1.707284302	0.087769222	0.112678329	positive
PRJNA729271	stage_1D_vs_E10	ordinary_muscle_development	ENSGALG00010020530	SRSF5A	-0.427999699	0.069244434	-6.180997904	6.36977E-10	1.8247E-09	negative
PRJNA729271	stage_1D_vs_E10	ordinary_muscle_development	ENSGALG00010020533	ENSGALG00010020533	0.429179628	0.095454883	4.49615163	6.91944E-06	1.43569E-05	positive
PRJNA729271	stage_1D_vs_E10	ordinary_muscle_development	ENSGALG00010020535	LMLN	-0.563107647	0.14956553	-3.764956037	0.000166578	0.000302424	negative
PRJNA729271	stage_1D_vs_E10	ordinary_muscle_development	ENSGALG00010020537	CCDC167	-0.993875719	0.099336998	-10.00509116	1.44758E-23	9.25572E-23	negative
PRJNA729271	stage_1D_vs_E10	ordinary_muscle_development	ENSGALG00010020540	PCGF5	1.373832904	0.161649981	8.498812658	1.9154E-17	8.89577E-17	positive
PRJNA729271	stage_1D_vs_E10	ordinary_muscle_development	ENSGALG00010020544	BMERB1	-1.764046284	0.164954529	-10.69413671	1.08425E-26	8.01027E-26	negative
PRJNA729271	stage_1D_vs_E10	ordinary_muscle_development	ENSGALG00010020545	STYX	2.078034653	0.140627733	14.77684814	2.06611E-49	3.91481E-48	positive
PRJNA729271	stage_1D_vs_E10	ordinary_muscle_development	ENSGALG00010020546	LRRC20	2.237903781	0.237373406	9.427778027	4.18868E-21	2.38004E-20	positive
PRJNA729271	stage_1D_vs_E10	ordinary_muscle_development	ENSGALG00010020548	CMTR1	-0.399479307	0.185239498	-2.156555768	0.031040294	0.042712081	negative
PRJNA729271	stage_1D_vs_E10	ordinary_muscle_development	ENSGALG00010020550	HECTD2	1.061125698	0.212726965	4.988204946	6.09429E-07	1.38415E-06	positive
PRJNA729271	stage_1D_vs_E10	ordinary_muscle_development	ENSGALG00010020554	ZFP36L1	0.707196828	0.210634031	3.357467087	0.000786601	0.001326731	positive
PRJNA729271	stage_1D_vs_E10	ordinary_muscle_development	ENSGALG00010020556	EMC1	0.091575714	0.085569633	1.070189404	0.284534063	0.330765999	positive
PRJNA729271	stage_1D_vs_E10	ordinary_muscle_development	ENSGALG00010020557	ENSGALG00010020557	0.92389994	0.261665918	3.530837903	0.000414246	0.00071979	positive
PRJNA729271	stage_1D_vs_E10	ordinary_muscle_development	ENSGALG00010020558	EIF4EBP2	0.371033803	0.100030626	3.709202062	0.000207913	0.000372799	positive
PRJNA729271	stage_1D_vs_E10	ordinary_muscle_development	ENSGALG00010020562	LRRN1	-5.356089278	0.239245198	-22.38744739	5.2162E-111	4.7854E-109	negative
PRJNA729271	stage_1D_vs_E10	ordinary_muscle_development	ENSGALG00010020568	PLA2G4F	-1.440136537	0.431608498	-3.33667327	0.000847876	0.001424923	negative
PRJNA729271	stage_1D_vs_E10	ordinary_muscle_development	ENSGALG00010020569	DTX3L	1.710240001	0.253155413	6.755692021	1.42155E-11	4.56174E-11	positive
PRJNA729271	stage_1D_vs_E10	ordinary_muscle_development	ENSGALG00010020571	MRTO4	-0.302494404	0.080846618	-3.741583906	0.000182864	0.000330418	negative
PRJNA729271	stage_1D_vs_E10	ordinary_muscle_development	ENSGALG00010020572	PPP1R3C	3.391220138	0.271764059	12.47854535	9.77563E-36	1.09166E-34	positive
PRJNA729271	stage_1D_vs_E10	ordinary_muscle_development	ENSGALG00010020574	DTL	-1.270630898	0.178302915	-7.126248616	1.03141E-12	3.56385E-12	negative
PRJNA729271	stage_1D_vs_E10	ordinary_muscle_development	ENSGALG00010020576	AKR7L	0.232541463	0.104610842	2.222919335	0.02622124	0.036447724	positive
PRJNA729271	stage_1D_vs_E10	ordinary_muscle_development	ENSGALG00010020578	TNKS2	0.372658021	0.062652087	5.948054419	2.71348E-09	7.42673E-09	positive
PRJNA729271	stage_1D_vs_E10	ordinary_muscle_development	ENSGALG00010020580	ENSGALG00010020580	-0.144594978	0.114748827	-1.260099841	0.207633348	0.248669369	negative
PRJNA729271	stage_1D_vs_E10	ordinary_muscle_development	ENSGALG00010020582	KANSL3	0.326428944	0.148363251	2.200200806	0.027792651	0.038469679	positive
PRJNA729271	stage_1D_vs_E10	ordinary_muscle_development	ENSGALG00010020584	PARP9	1.516082797	0.289951849	5.228739893	1.70669E-07	4.04886E-07	positive
PRJNA729271	stage_1D_vs_E10	ordinary_muscle_development	ENSGALG00010020586	C11orf16	0.545604876	0.097319716	5.606313914	2.06681E-08	5.2614E-08	positive
PRJNA729271	stage_1D_vs_E10	ordinary_muscle_development	ENSGALG00010020588	PALD1	-1.328432075	0.110131648	-12.06221913	1.67216E-33	1.7045E-32	negative
PRJNA729271	stage_1D_vs_E10	ordinary_muscle_development	ENSGALG00010020590	INTS7	-0.459862132	0.076081512	-6.0443348	1.50028E-09	4.18202E-09	negative
PRJNA729271	stage_1D_vs_E10	ordinary_muscle_development	ENSGALG00010020594	PQLC2	-0.424251479	0.151504133	-2.800263396	0.005106092	0.007842001	negative
PRJNA729271	stage_1D_vs_E10	ordinary_muscle_development	ENSGALG00010020595	ZFYVE27	0.928000588	0.129177044	7.18394352	6.77289E-13	2.37305E-12	positive
PRJNA729271	stage_1D_vs_E10	ordinary_muscle_development	ENSGALG00010020596	SUMF1	0.416799288	0.120176604	3.468223202	0.000523912	0.000900202	positive
PRJNA729271	stage_1D_vs_E10	ordinary_muscle_development	ENSGALG00010020597	ARID5A	2.327352762	0.236999387	9.820079256	9.2271E-23	5.69863E-22	positive
PRJNA729271	stage_1D_vs_E10	ordinary_muscle_development	ENSGALG00010020599	PARN	-1.250243939	0.066493015	-18.80263581	7.18552E-79	3.32761E-77	negative
PRJNA729271	stage_1D_vs_E10	ordinary_muscle_development	ENSGALG00010020601	AKIP1	-0.72369115	0.166262851	-4.352693002	1.34475E-05	2.71328E-05	negative
PRJNA729271	stage_1D_vs_E10	ordinary_muscle_development	ENSGALG00010020602	DENND2B	-0.73622837	0.096451179	-7.63317127	2.29048E-14	8.82758E-14	negative
PRJNA729271	stage_1D_vs_E10	ordinary_muscle_development	ENSGALG00010020603	VPS39	0.034304706	0.093205644	0.368053952	0.712833006	0.748299132	positive
PRJNA729271	stage_1D_vs_E10	ordinary_muscle_development	ENSGALG00010020605	STAMBP	-0.169660238	0.087766675	-1.933082654	0.053226017	0.070651201	negative
PRJNA729271	stage_1D_vs_E10	ordinary_muscle_development	ENSGALG00010020607	ENSGALG00010020607	3.733352073	0.238477249	15.6549612	3.07301E-55	7.13834E-54	positive
PRJNA729271	stage_1D_vs_E10	ordinary_muscle_development	ENSGALG00010020608	CAPZB	0.239163133	0.099576298	2.401807851	0.016314275	0.023380999	positive
PRJNA729271	stage_1D_vs_E10	ordinary_muscle_development	ENSGALG00010020609	AVPI1	-0.732766006	0.23605985	-3.104153491	0.001908243	0.003077779	negative
PRJNA729271	stage_1D_vs_E10	ordinary_muscle_development	ENSGALG00010020611	LPGAT1	-0.166759266	0.099434832	-1.677070931	0.093528612	0.119550021	negative
PRJNA729271	stage_1D_vs_E10	ordinary_muscle_development	ENSGALG00010020612	RPL27A	0.262767321	0.19278515	1.363006025	0.172880612	0.210509448	positive
PRJNA729271	stage_1D_vs_E10	ordinary_muscle_development	ENSGALG00010020613	ACTG2	1.165324809	0.238998341	4.875869875	1.0833E-06	2.41427E-06	positive
PRJNA729271	stage_1D_vs_E10	ordinary_muscle_development	ENSGALG00010020614	FAIM	-0.734827671	0.110970343	-6.621838338	3.54759E-11	1.10752E-10	negative
PRJNA729271	stage_1D_vs_E10	ordinary_muscle_development	ENSGALG00010020616	PI4K2A	0.599148998	0.130894096	4.577356948	4.70888E-06	9.90353E-06	positive
PRJNA729271	stage_1D_vs_E10	ordinary_muscle_development	ENSGALG00010020617	DGUOK	0.247371202	0.110733194	2.233939018	0.025487086	0.035515796	positive
PRJNA729271	stage_1D_vs_E10	ordinary_muscle_development	ENSGALG00010020618	ENSGALG00010020618	-0.616675012	0.328433771	-1.87762364	0.060432669	0.079518114	negative
PRJNA729271	stage_1D_vs_E10	ordinary_muscle_development	ENSGALG00010020619	QARS	-0.424268597	0.099848915	-4.249105715	2.14626E-05	4.24015E-05	negative
PRJNA729271	stage_1D_vs_E10	ordinary_muscle_development	ENSGALG00010020620	MARF1	0.554279067	0.102974062	5.38270565	7.33745E-08	1.79202E-07	positive
PRJNA729271	stage_1D_vs_E10	ordinary_muscle_development	ENSGALG00010020622	ABCG2	1.561719238	0.116397952	13.41706798	4.80344E-41	6.56229E-40	positive
PRJNA729271	stage_1D_vs_E10	ordinary_muscle_development	ENSGALG00010020623	NEK2	-0.722745296	0.21611714	-3.344229408	0.000825115	0.001388926	negative
PRJNA729271	stage_1D_vs_E10	ordinary_muscle_development	ENSGALG00010020624	MINOS1	0.404406052	0.135948579	2.97469864	0.002932765	0.004630261	positive
PRJNA729271	stage_1D_vs_E10	ordinary_muscle_development	ENSGALG00010020625	SLC30A1	-0.378034737	0.076269508	-4.9565645	7.17506E-07	1.61998E-06	negative
PRJNA729271	stage_1D_vs_E10	ordinary_muscle_development	ENSGALG00010020626	MORN4	-1.108830259	0.120484105	-9.20312481	3.47689E-20	1.8784E-19	negative
PRJNA729271	stage_1D_vs_E10	ordinary_muscle_development	ENSGALG00010020629	TRIM66	-4.10223741	0.265624215	-15.44376297	8.31068E-54	1.82797E-52	negative
PRJNA729271	stage_1D_vs_E10	ordinary_muscle_development	ENSGALG00010020630	QRICH1	-0.076472155	0.126007266	-0.606886872	0.543925991	0.590002787	negative
PRJNA729271	stage_1D_vs_E10	ordinary_muscle_development	ENSGALG00010020632	BLOC1S2	-0.68706338	0.114524888	-5.999249515	1.98232E-09	5.47725E-09	negative
PRJNA729271	stage_1D_vs_E10	ordinary_muscle_development	ENSGALG00010020633	HOGA1	-0.774552967	0.367909727	-2.105279937	0.035266948	0.048175893	negative
PRJNA729271	stage_1D_vs_E10	ordinary_muscle_development	ENSGALG00010020635	NDE1	-1.268907934	0.135004932	-9.398974626	5.50974E-21	3.11481E-20	negative
PRJNA729271	stage_1D_vs_E10	ordinary_muscle_development	ENSGALG00010020636	NBL1	-0.754309881	0.17860866	-4.22325479	2.40799E-05	4.73401E-05	negative
PRJNA729271	stage_1D_vs_E10	ordinary_muscle_development	ENSGALG00010020638	IMPDH2	0.522078962	0.111533228	4.680927573	2.8558E-06	6.14075E-06	positive
PRJNA729271	stage_1D_vs_E10	ordinary_muscle_development	ENSGALG00010020640	PTPN14	0.31395651	0.091057723	3.447884466	0.000564996	0.000966554	positive
PRJNA729271	stage_1D_vs_E10	ordinary_muscle_development	ENSGALG00010020642	ANKRD2	3.745157239	0.62923009	5.95196781	2.64937E-09	7.254E-09	positive
PRJNA729271	stage_1D_vs_E10	ordinary_muscle_development	ENSGALG00010020646	CEP70	-0.772254646	0.116518153	-6.6277625	3.40813E-11	1.06559E-10	negative
PRJNA729271	stage_1D_vs_E10	ordinary_muscle_development	ENSGALG00010020648	TET3	-0.005236324	0.146281258	-0.035796276	0.971444803	0.97568545	negative
PRJNA729271	stage_1D_vs_E10	ordinary_muscle_development	ENSGALG00010020649	MYH11	4.001960765	0.211843495	18.8911194	1.34954E-79	6.37183E-78	positive
PRJNA729271	stage_1D_vs_E10	ordinary_muscle_development	ENSGALG00010020651	LMO1	-0.635987295	0.179392708	-3.545223781	0.00039228	0.000683918	negative
PRJNA729271	stage_1D_vs_E10	ordinary_muscle_development	ENSGALG00010020653	NDUFAF3	1.030000355	0.117340024	8.777911542	1.66538E-18	8.21079E-18	positive
PRJNA729271	stage_1D_vs_E10	ordinary_muscle_development	ENSGALG00010020654	SAYSD1	-0.503151148	0.151098751	-3.329949099	0.000868619	0.001457923	negative
PRJNA729271	stage_1D_vs_E10	ordinary_muscle_development	ENSGALG00010020655	RIC3	-1.464104997	0.136939816	-10.69159463	1.11438E-26	8.22453E-26	negative
PRJNA729271	stage_1D_vs_E10	ordinary_muscle_development	ENSGALG00010020657	DALRD3	0.184475478	0.094002424	1.962454484	0.049709595	0.066335903	positive
PRJNA729271	stage_1D_vs_E10	ordinary_muscle_development	ENSGALG00010020659	RAD51B	-1.552056878	0.141428101	-10.97417603	5.08675E-28	3.98338E-27	negative
PRJNA729271	stage_1D_vs_E10	ordinary_muscle_development	ENSGALG00010020662	BFAR	-0.207381251	0.086738146	-2.390888686	0.016807648	0.02405241	negative
PRJNA729271	stage_1D_vs_E10	ordinary_muscle_development	ENSGALG00010020664	ESYT3	0.095023196	0.459214585	0.206925474	0.836068062	0.859550788	positive
PRJNA729271	stage_1D_vs_E10	ordinary_muscle_development	ENSGALG00010020666	TMCO4	0.878770776	0.204984342	4.287014153	1.81091E-05	3.59922E-05	positive
PRJNA729271	stage_1D_vs_E10	ordinary_muscle_development	ENSGALG00010020667	UBTD1	-0.335991759	0.158148541	-2.12453278	0.033625618	0.046077078	negative
PRJNA729271	stage_1D_vs_E10	ordinary_muscle_development	ENSGALG00010020670	ENSGALG00010020670	0.111514748	0.129822831	0.858976402	0.390353535	0.439572288	positive
PRJNA729271	stage_1D_vs_E10	ordinary_muscle_development	ENSGALG00010020673	MMS19	-0.068412271	0.127485846	-0.536626404	0.591525701	0.635269994	negative
PRJNA729271	stage_1D_vs_E10	ordinary_muscle_development	ENSGALG00010020674	TRPV4	-8.457778654	0.94523584	-8.947797254	3.62646E-19	1.8483E-18	negative
PRJNA729271	stage_1D_vs_E10	ordinary_muscle_development	ENSGALG00010020675	SMYD2	-2.449453455	0.185716782	-13.1891875	1.01271E-39	1.31652E-38	negative
PRJNA729271	stage_1D_vs_E10	ordinary_muscle_development	ENSGALG00010020676	CYP2C23a	4.949142568	1.396538285	3.543864583	0.000394308	0.000687041	positive
PRJNA729271	stage_1D_vs_E10	ordinary_muscle_development	ENSGALG00010020677	CYR61	2.388638562	0.231956315	10.29779494	7.20949E-25	4.9131E-24	positive
PRJNA729271	stage_1D_vs_E10	ordinary_muscle_development	ENSGALG00010020679	OTUD3	-0.005819897	0.108802207	-0.053490618	0.957341005	0.964058488	negative
PRJNA729271	stage_1D_vs_E10	ordinary_muscle_development	ENSGALG00010020680	CFAP221	-1.393951795	0.626270505	-2.22579825	0.026027698	0.036203002	negative
PRJNA729271	stage_1D_vs_E10	ordinary_muscle_development	ENSGALG00010020681	BCL10	-0.271554066	0.087446983	-3.105356617	0.001900497	0.003068699	negative
PRJNA729271	stage_1D_vs_E10	ordinary_muscle_development	ENSGALG00010020682	ZDHHC16	0.415194051	0.167955394	2.472049516	0.013434091	0.019505875	positive
PRJNA729271	stage_1D_vs_E10	ordinary_muscle_development	ENSGALG00010020684	P4HTM	0.806302951	0.122689697	6.571888047	4.96812E-11	1.53578E-10	positive
PRJNA729271	stage_1D_vs_E10	ordinary_muscle_development	ENSGALG00010020685	RPL6	0.097208423	0.116033087	0.837764685	0.402162876	0.451642589	positive
PRJNA729271	stage_1D_vs_E10	ordinary_muscle_development	ENSGALG00010020686	C1orf52	-0.457197401	0.124716919	-3.665881146	0.000246488	0.000438225	negative
PRJNA729271	stage_1D_vs_E10	ordinary_muscle_development	ENSGALG00010020691	ENSGALG00010020691	0.532443179	0.163906607	3.24845465	0.001160337	0.001921522	positive
PRJNA729271	stage_1D_vs_E10	ordinary_muscle_development	ENSGALG00010020692	TUB	-0.525659664	0.153840599	-3.416911181	0.000633359	0.001077402	negative
PRJNA729271	stage_1D_vs_E10	ordinary_muscle_development	ENSGALG00010020693	PTGFR	-0.854854499	0.190682946	-4.483119837	7.35596E-06	1.5219E-05	negative
PRJNA729271	stage_1D_vs_E10	ordinary_muscle_development	ENSGALG00010020696	MOB1A	-0.828438781	0.101060522	-8.197452032	2.45536E-16	1.072E-15	negative
PRJNA729271	stage_1D_vs_E10	ordinary_muscle_development	ENSGALG00010020699	PLA2G10	-0.191996295	0.414914078	-0.462737479	0.64355255	0.683742154	negative
PRJNA729271	stage_1D_vs_E10	ordinary_muscle_development	ENSGALG00010020700	MTHFD2	1.861098489	0.131012813	14.20546929	8.47331E-46	1.36619E-44	positive
PRJNA729271	stage_1D_vs_E10	ordinary_muscle_development	ENSGALG00010020701	PTPN11	-0.54924263	0.084958589	-6.4648276	1.01414E-10	3.06826E-10	negative
PRJNA729271	stage_1D_vs_E10	ordinary_muscle_development	ENSGALG00010020704	PROX1	0.799385757	0.23092849	3.461616001	0.000536943	0.000922045	positive
PRJNA729271	stage_1D_vs_E10	ordinary_muscle_development	ENSGALG00010020705	EXOSC1	0.602319737	0.174392545	3.453815856	0.000552715	0.000947333	positive
PRJNA729271	stage_1D_vs_E10	ordinary_muscle_development	ENSGALG00010020707	SLC4A5	-2.284994748	0.492239605	-4.642037586	3.4499E-06	7.35603E-06	negative
PRJNA729271	stage_1D_vs_E10	ordinary_muscle_development	ENSGALG00010020708	FAM214A	0.190241329	0.133841076	1.421397186	0.155201324	0.190728545	positive
PRJNA729271	stage_1D_vs_E10	ordinary_muscle_development	ENSGALG00010020712	ZC3H7A	-0.785186123	0.0708554	-11.08152837	1.54217E-28	1.24534E-27	negative
PRJNA729271	stage_1D_vs_E10	ordinary_muscle_development	ENSGALG00010020713	SCTR	-0.188415014	1.07789628	-0.174798835	0.861237704	0.881434688	negative
PRJNA729271	stage_1D_vs_E10	ordinary_muscle_development	ENSGALG00010020714	PGAM1	1.579868148	0.144527461	10.93126621	8.17003E-28	6.34644E-27	positive
PRJNA729271	stage_1D_vs_E10	ordinary_muscle_development	ENSGALG00010020715	ENSGALG00010020715	-2.409404378	1.059010354	-2.27514714	0.022897105	0.032169578	negative

PRJNA729271	stage_1D_vs_E10	ordinary_muscle_development	ENSGALG00010020716	RRAS	0.048401699	0.179540322	0.269586788	0.787478166	0.815379385	positive
PRJNA729271	stage_1D_vs_E10	ordinary_muscle_development	ENSGALG00010020719	RPH3A	1.309984792	0.633767646	2.066979594	0.038736077	0.052518888	positive
PRJNA729271	stage_1D_vs_E10	ordinary_muscle_development	ENSGALG00010020720	ENSGALG00010020720	-4.866601734	0.406770575	-11.9639965	5.48568E-33	5.46494E-32	negative
PRJNA729271	stage_1D_vs_E10	ordinary_muscle_development	ENSGALG00010020721	PTDSS2	-0.073255979	0.170103504	-0.430655318	0.666719012	0.705562684	negative
PRJNA729271	stage_1D_vs_E10	ordinary_muscle_development	ENSGALG00010020723	RPS24	-0.101370763	0.190119444	-0.533195137	0.593898507	0.637387743	negative
PRJNA729271	stage_1D_vs_E10	ordinary_muscle_development	ENSGALG00010020724	TACR1	-2.097355399	0.264968458	-7.915490814	2.4628E-15	1.00871E-14	negative
PRJNA729271	stage_1D_vs_E10	ordinary_muscle_development	ENSGALG00010020725	FOPNL	-0.08305745	0.10723348	-0.774547742	0.438606947	0.487845894	negative
PRJNA729271	stage_1D_vs_E10	ordinary_muscle_development	ENSGALG00010020728	TMEM37	1.535023415	0.20756155	7.395509516	1.40867E-13	5.15493E-13	positive
PRJNA729271	stage_1D_vs_E10	ordinary_muscle_development	ENSGALG00010020730	RRP12	-0.050505872	0.461894528	-0.109345033	0.91292883	0.925960632	negative
PRJNA729271	stage_1D_vs_E10	ordinary_muscle_development	ENSGALG00010020732	ARIH2	0.098329995	0.143782363	0.683880787	0.494050447	0.542314368	positive
PRJNA729271	stage_1D_vs_E10	ordinary_muscle_development	ENSGALG00010020733	TMEM229B	-1.692170576	0.110561272	-15.30527406	7.05044E-53	1.5051E-51	negative
PRJNA729271	stage_1D_vs_E10	ordinary_muscle_development	ENSGALG00010020736	LRRTM4	0.444964347	0.219802395	2.024383527	0.042930705	0.057859654	positive
PRJNA729271	stage_1D_vs_E10	ordinary_muscle_development	ENSGALG00010020737	POLR3A	-0.343411564	0.115965905	-2.961314915	0.003063285	0.004823718	negative
PRJNA729271	stage_1D_vs_E10	ordinary_muscle_development	ENSGALG00010020739	PKP3	-4.913298699	0.771347816	-6.369757715	1.89327E-10	5.62586E-10	negative
PRJNA729271	stage_1D_vs_E10	ordinary_muscle_development	ENSGALG00010020742	THAP10	-1.937341698	0.214645768	-9.025762393	1.78448E-19	9.25773E-19	negative
PRJNA729271	stage_1D_vs_E10	ordinary_muscle_development	ENSGALG00010020743	PURB	1.418653636	0.161990921	8.757612016	1.9943E-18	9.77923E-18	positive
PRJNA729271	stage_1D_vs_E10	ordinary_muscle_development	ENSGALG00010020744	CHST3	-1.176267938	0.115214584	-10.20936674	1.80036E-24	1.20148E-23	negative
PRJNA729271	stage_1D_vs_E10	ordinary_muscle_development	ENSGALG00010020745	ABCC1	0.270044752	0.166564209	1.621265181	0.104960779	0.133001704	positive
PRJNA729271	stage_1D_vs_E10	ordinary_muscle_development	ENSGALG00010020747	DBI	0.182195424	0.120607233	1.510650897	0.130877419	0.163118503	positive
PRJNA729271	stage_1D_vs_E10	ordinary_muscle_development	ENSGALG00010020750	AIFM2	-1.098740009	0.296560788	-3.704940281	0.000211441	0.000378748	negative
PRJNA729271	stage_1D_vs_E10	ordinary_muscle_development	ENSGALG00010020753	SYDE2	1.237230294	0.095972272	12.89153907	5.02312E-38	6.1288E-37	positive
PRJNA729271	stage_1D_vs_E10	ordinary_muscle_development	ENSGALG00010020754	SLC25A20	-0.319958069	0.114820074	-2.786603915	0.005326354	0.008159533	negative
PRJNA729271	stage_1D_vs_E10	ordinary_muscle_development	ENSGALG00010020756	MACROH2A2	-3.610542433	0.157739387	-22.88928907	5.9401E-116	6.2393E-114	negative
PRJNA729271	stage_1D_vs_E10	ordinary_muscle_development	ENSGALG00010020759	ARHGAP19	-1.944557474	0.16902577	-11.50450295	1.25209E-30	1.11962E-29	negative
PRJNA729271	stage_1D_vs_E10	ordinary_muscle_development	ENSGALG00010020760	TXNDC11	-0.386920611	0.095101956	-4.068482163	4.73204E-05	9.06567E-05	negative
PRJNA729271	stage_1D_vs_E10	ordinary_muscle_development	ENSGALG00010020762	TTBK2	-0.185409361	0.141623356	-1.3091722	0.190476027	0.230040827	negative
PRJNA729271	stage_1D_vs_E10	ordinary_muscle_development	ENSGALG00010020764	PRKAR2A	0.039567179	0.098655892	0.401062504	0.688374107	0.725882633	positive
PRJNA729271	stage_1D_vs_E10	ordinary_muscle_development	ENSGALG00010020765	GLT1D1	-4.175831237	0.266288427	-15.68160991	2.02067E-55	4.75481E-54	negative
PRJNA729271	stage_1D_vs_E10	ordinary_muscle_development	ENSGALG00010020766	C2orf76	-0.333759742	0.122697675	-2.720179834	0.006524642	0.00988138	negative
PRJNA729271	stage_1D_vs_E10	ordinary_muscle_development	ENSGALG00010020767	UBXN10	-0.376343561	0.174629344	-2.155099214	0.031154071	0.042856436	negative
PRJNA729271	stage_1D_vs_E10	ordinary_muscle_development	ENSGALG00010020768	DNAH8	-0.106496249	0.627298252	-0.169769721	0.86519124	0.885097539	negative
PRJNA729271	stage_1D_vs_E10	ordinary_muscle_development	ENSGALG00010020770	B4GALNT4	-3.515678321	0.199491986	-17.62315569	1.63618E-69	5.91432E-68	negative
PRJNA729271	stage_1D_vs_E10	ordinary_muscle_development	ENSGALG00010020771	LINGO1	1.437629967	0.200891919	7.156235931	8.29224E-13	2.88724E-12	positive
PRJNA729271	stage_1D_vs_E10	ordinary_muscle_development	ENSGALG00010020773	ZCCHC24	1.329819866	0.089396566	14.87551396	4.75321E-50	9.22326E-49	positive
PRJNA729271	stage_1D_vs_E10	ordinary_muscle_development	ENSGALG00010020775	UBR1	0.42820616	0.081090851	5.280573045	1.2878E-07	3.08541E-07	positive
PRJNA729271	stage_1D_vs_E10	ordinary_muscle_development	ENSGALG00010020776	MCOLN3	-1.379256517	0.473465106	-2.913111231	0.003578471	0.005593652	negative
PRJNA729271	stage_1D_vs_E10	ordinary_muscle_development	ENSGALG00010020778	SLIT1	0.819826644	0.201171071	4.075271069	4.59608E-05	8.81569E-05	positive
PRJNA729271	stage_1D_vs_E10	ordinary_muscle_development	ENSGALG00010020779	SLC15A4	-0.565517742	0.06678834	-8.467312398	2.5112E-17	1.16034E-16	negative
PRJNA729271	stage_1D_vs_E10	ordinary_muscle_development	ENSGALG00010020781	SNN	0.027219153	0.135867211	0.200336438	0.841217467	0.863981902	positive
PRJNA729271	stage_1D_vs_E10	ordinary_muscle_development	ENSGALG00010020782	PPIF	1.030609113	0.15619659	6.598153729	4.16309E-11	1.29438E-10	positive
PRJNA729271	stage_1D_vs_E10	ordinary_muscle_development	ENSGALG00010020786	DDX19	-0.251906738	0.13119114	-1.920150535	0.054838888	0.072652379	negative
PRJNA729271	stage_1D_vs_E10	ordinary_muscle_development	ENSGALG00010020787	TMEM132C	-4.264688226	0.306282384	-13.92404022	4.5258E-44	6.85491E-43	negative
PRJNA729271	stage_1D_vs_E10	ordinary_muscle_development	ENSGALG00010020790	PRPH2L	0.717079918	0.332387516	2.15736116	0.030977535	0.042645965	positive
PRJNA729271	stage_1D_vs_E10	ordinary_muscle_development	ENSGALG00010020794	STEAP3	0.77702005	0.134956004	5.757580432	8.53281E-09	2.24064E-08	positive
PRJNA729271	stage_1D_vs_E10	ordinary_muscle_development	ENSGALG00010020797	DGCR8	-0.766277193	0.066135908	-11.58640162	4.83001E-31	4.37568E-30	negative
PRJNA729271	stage_1D_vs_E10	ordinary_muscle_development	ENSGALG00010020798	C1QL2	-3.449378835	0.67162886	-5.135840697	2.80886E-07	6.53836E-07	negative
PRJNA729271	stage_1D_vs_E10	ordinary_muscle_development	ENSGALG00010020803	ABCC6	1.756238778	0.157584256	11.14476042	7.59484E-29	6.2126E-28	positive
PRJNA729271	stage_1D_vs_E10	ordinary_muscle_development	ENSGALG00010020806	HTD2	0.087530056	0.173122071	0.505597327	0.613139358	0.655707171	positive
PRJNA729271	stage_1D_vs_E10	ordinary_muscle_development	ENSGALG00010020809	ENSGALG00010020809	0.137204486	0.221581319	0.619206016	0.535780649	0.58238774	positive
PRJNA729271	stage_1D_vs_E10	ordinary_muscle_development	ENSGALG00010020810	IFITM5	1.462706856	0.241605937	6.054101456	1.41204E-09	3.94287E-09	positive
PRJNA729271	stage_1D_vs_E10	ordinary_muscle_development	ENSGALG00010020811	MCOLN2	-1.547027772	0.70140646	-2.205608103	0.027411451	0.037989195	negative
PRJNA729271	stage_1D_vs_E10	ordinary_muscle_development	ENSGALG00010020813	NPY4R	0.18090129	0.545573347	0.331580147	0.740206313	0.773482803	positive
PRJNA729271	stage_1D_vs_E10	ordinary_muscle_development	ENSGALG00010020816	TRMT2A	0.022300215	0.090073036	0.247579255	0.804459963	0.831122392	positive
PRJNA729271	stage_1D_vs_E10	ordinary_muscle_development	ENSGALG00010020818	TMEM62	-0.633370383	0.198348609	-3.193218178	0.001406966	0.002308055	negative
PRJNA729271	stage_1D_vs_E10	ordinary_muscle_development	ENSGALG00010020820	VWA5B1	-0.460837322	0.452411898	-1.018623347	0.308381816	0.355664738	negative
PRJNA729271	stage_1D_vs_E10	ordinary_muscle_development	ENSGALG00010020827	ENSGALG00010020827	-0.588774118	0.091946657	-6.403431504	1.51923E-10	4.54515E-10	negative
PRJNA729271	stage_1D_vs_E10	ordinary_muscle_development	ENSGALG00010020828	CAMK2N1	-0.693011171	0.226226864	-3.063346057	0.002188768	0.003502174	negative
PRJNA729271	stage_1D_vs_E10	ordinary_muscle_development	ENSGALG00010020831	MUL1	0.335827431	0.14492698	2.317218162	0.020491851	0.028950232	positive
PRJNA729271	stage_1D_vs_E10	ordinary_muscle_development	ENSGALG00010020833	RANBP1	-1.715099137	0.082610497	-20.76127378	9.69623E-96	6.62957E-94	negative
PRJNA729271	stage_1D_vs_E10	ordinary_muscle_development	ENSGALG00010020835	PTGR2	0.0869519	0.114562048	0.758993938	0.447856183	0.497027438	positive
PRJNA729271	stage_1D_vs_E10	ordinary_muscle_development	ENSGALG00010020839	PGGHG	-0.508079527	0.14055533	-3.614800864	0.000300579	0.000529584	negative
PRJNA729271	stage_1D_vs_E10	ordinary_muscle_development	ENSGALG00010020840	LPAR3	-1.392731798	0.256799247	-5.423426339	5.84673E-08	1.43739E-07	negative
PRJNA729271	stage_1D_vs_E10	ordinary_muscle_development	ENSGALG00010020843	HMG20A	-1.069021445	0.083455766	-12.80943779	1.45183E-37	1.74063E-36	negative
PRJNA729271	stage_1D_vs_E10	ordinary_muscle_development	ENSGALG00010020845	RAB35	0.206720818	0.091923585	2.2488333	0.024523104	0.034281261	positive
PRJNA729271	stage_1D_vs_E10	ordinary_muscle_development	ENSGALG00010020847	MIDEAS	0.426594886	0.089308954	4.776619428	1.78267E-06	3.89622E-06	positive
PRJNA729271	stage_1D_vs_E10	ordinary_muscle_development	ENSGALG00010020854	ENSGALG00010020854	10.87284365	0.381039547	28.53468551	4.3512E-179	1.3419E-176	positive
PRJNA729271	stage_1D_vs_E10	ordinary_muscle_development	ENSGALG00010020855	DNAL1	-0.182777982	0.100224661	-1.823682705	0.068200055	0.089067465	negative
PRJNA729271	stage_1D_vs_E10	ordinary_muscle_development	ENSGALG00010020856	VTG2	-2.779408998	0.762076112	-3.647154077	0.000265161	0.000469772	negative
PRJNA729271	stage_1D_vs_E10	ordinary_muscle_development	ENSGALG00010020861	CDA	0.576581152	0.181027744	3.185043023	0.001447325	0.002369435	positive
PRJNA729271	stage_1D_vs_E10	ordinary_muscle_development	ENSGALG00010020865	BICDL1	-2.41681217	0.850028462	-2.843213232	0.004466118	0.006912587	negative
PRJNA729271	stage_1D_vs_E10	ordinary_muscle_development	ENSGALG00010020866	LCOR	-0.498042346	0.097377313	-5.114562448	3.14469E-07	7.29083E-07	negative
PRJNA729271	stage_1D_vs_E10	ordinary_muscle_development	ENSGALG00010020870	RIOX1	-0.334702608	0.147978054	-2.261839497	0.023707322	0.03321775	negative
PRJNA729271	stage_1D_vs_E10	ordinary_muscle_development	ENSGALG00010020873	PINK1	2.064367673	0.13652061	15.12128951	1.17224E-51	2.39319E-50	positive
PRJNA729271	stage_1D_vs_E10	ordinary_muscle_development	ENSGALG00010020875	ENSGALG00010020875	1.244033389	0.171854976	7.238855775	4.52486E-13	1.60754E-12	positive
PRJNA729271	stage_1D_vs_E10	ordinary_muscle_development	ENSGALG00010020878	XYLT1	-0.942815907	0.125338572	-7.522152929	5.38815E-14	2.0286E-13	negative
PRJNA729271	stage_1D_vs_E10	ordinary_muscle_development	ENSGALG00010020880	PXK	1.320578093	0.165354377	7.986350983	1.38992E-15	5.79098E-15	positive
PRJNA729271	stage_1D_vs_E10	ordinary_muscle_development	ENSGALG00010020886	GBF1	-0.206772172	0.086186575	-2.399122734	0.016434405	0.023543853	negative
PRJNA729271	stage_1D_vs_E10	ordinary_muscle_development	ENSGALG00010020887	ARPP19	-1.063725391	0.07124457	-14.93061701	2.08329E-50	4.08072E-49	negative
PRJNA729271	stage_1D_vs_E10	ordinary_muscle_development	ENSGALG00010020889	PPRC1	0.328433187	0.28105695	1.168564548	0.242579119	0.286544236	positive
PRJNA729271	stage_1D_vs_E10	ordinary_muscle_development	ENSGALG00010020890	RPS15A	0.067760485	0.188359428	0.35974034	0.719041322	0.754270079	positive
PRJNA729271	stage_1D_vs_E10	ordinary_muscle_development	ENSGALG00010020894	PSMD13	-0.165164112	0.120944395	-1.365620224	0.172058195	0.209631297	negative
PRJNA729271	stage_1D_vs_E10	ordinary_muscle_development	ENSGALG00010020896	MARCO	1.709969282	0.393707077	4.34325259	1.40389E-05	2.82512E-05	positive
PRJNA729271	stage_1D_vs_E10	ordinary_muscle_development	ENSGALG00010020898	ENSGALG00010020898	0.729878787	0.122589435	5.953847388	2.61911E-09	7.17385E-09	positive
PRJNA729271	stage_1D_vs_E10	ordinary_muscle_development	ENSGALG00010020900	PDHB	0.779904124	0.1159075	6.728676949	1.71213E-11	5.45795E-11	positive
PRJNA729271	stage_1D_vs_E10	ordinary_muscle_development	ENSGALG00010020903	AGMAT	0.315138721	0.394017188	0.799809579	0.423821132	0.473361636	positive
PRJNA729271	stage_1D_vs_E10	ordinary_muscle_development	ENSGALG00010020905	KCTD6	-0.320110968	0.086130284	-3.716590184	0.00020193	0.000362338	negative
PRJNA729271	stage_1D_vs_E10	ordinary_muscle_development	ENSGALG00010020906	SIRT3	0.515970748	0.111370541	4.632919453	3.60545E-06	7.67753E-06	positive
PRJNA729271	stage_1D_vs_E10	ordinary_muscle_development	ENSGALG00010020912	PDE8A	1.368941793	0.1431822	9.560837846	1.16807E-21	6.84089E-21	positive
PRJNA729271	stage_1D_vs_E10	ordinary_muscle_development	ENSGALG00010020913	TMEM132B	-1.952050897	0.248554139	-7.8536246	4.04183E-15	1.63649E-14	negative
PRJNA729271	stage_1D_vs_E10	ordinary_muscle_development	ENSGALG00010020915	EN1	-3.955110081	0.369433735	-10.70587148	9.55275E-27	7.08997E-26	negative
PRJNA729271	stage_1D_vs_E10	ordinary_muscle_development	ENSGALG00010020916	ARL6IP1	-0.517294153	0.097912941	-5.283205141	1.26943E-07	3.0434E-07	negative
PRJNA729271	stage_1D_vs_E10	ordinary_muscle_development	ENSGALG00010020918	ENSGALG00010020918	-2.723517939	0.360109184	-7.563033819	3.93775E-14	1.49496E-13	negative
PRJNA729271	stage_1D_vs_E10	ordinary_muscle_development	ENSGALG00010020920	ACOX2	1.2366318	0.206774964	5.980568317	2.2236E-09	6.12527E-09	positive
PRJNA729271	stage_1D_vs_E10	ordinary_muscle_development	ENSGALG00010020921	LDB1	-0.058238991	0.149244649	-0.390224987	0.696370187	0.733620602	negative
PRJNA729271	stage_1D_vs_E10	ordinary_muscle_development	ENSGALG00010020922	INSIG2	0.409010262	0.079929367	5.117146282	3.10193E-07	7.19745E-07	positive
PRJNA729271	stage_1D_vs_E10	ordinary_muscle_development	ENSGALG00010020924	DNAJC16	0.150541619	0.120356962	1.250792777	0.211010091	0.25244233	positive
PRJNA729271	stage_1D_vs_E10	ordinary_muscle_development	ENSGALG00010020925	RIC8A	0.040441022	0.103724307	0.389889534	0.696618233	0.733828582	positive
PRJNA729271	stage_1D_vs_E10	ordinary_muscle_development	ENSGALG00010020927	ENSGALG00010020927	-0.326694513	0.116245859	-2.810375482	0.004948373	0.00761268	negative
PRJNA729271	stage_1D_vs_E10	ordinary_muscle_development	ENSGALG00010020928	AACS	-1.390383456	0.151667243	-9.167328626	4.84886E-20	2.60119E-19	negative
PRJNA729271	stage_1D_vs_E10	ordinary_muscle_development	ENSGALG00010020932	CASP9	-0.514025241	0.125025714	-4.111356173	3.93342E-05	7.58278E-05	negative
PRJNA729271	stage_1D_vs_E10	ordinary_muscle_development	ENSGALG00010020934	ABCD3	0.139840662	0.153593181	0.910461399	0.36257923	0.411586189	positive
PRJNA729271	stage_1D_vs_E10	ordinary_muscle_development	ENSGALG00010020935	BRI3BP	0.233276759	0.064456088	3.61915787	0.000295563	0.000521128	positive
PRJNA729271	stage_1D_vs_E10	ordinary_muscle_development	ENSGALG00010020936	BET1L	-0.363974334	0.125866625	-2.891746186	0.003831073	0.005968552	negative
PRJNA729271	stage_1D_vs_E10	ordinary_muscle_development	ENSGALG00010020938	SMG1	0.406411373	0.079766636	5.095004533	3.48733E-07	8.06202E-07	positive
PRJNA729271	stage_1D_vs_E10	ordinary_muscle_development	ENSGALG00010020939	CTRC	6.835629626	2.11659822	3.229535753	0.001239914	0.002047221	positive
PRJNA729271	stage_1D_vs_E10	ordinary_muscle_development	ENSGALG00010020940	ENSGALG00010020940	-0.911247263	0.132337998	-6.885756751	5.74811E-12	1.88718E-11	negative
PRJNA729271	stage_1D_vs_E10	ordinary_muscle_development	ENSGALG00010020941	ENSGALG00010020941	2.674827406	0.18278703	14.63357334	1.71536E-48	3.11972E-47	positive
PRJNA729271	stage_1D_vs_E10	ordinary_muscle_development	ENSGALG00010020943	FAM107A	1.208792101	0.356131657	3.394228167	0.000688223	0.001167033	positive
PRJNA729271	stage_1D_vs_E10	ordinary_muscle_development	ENSGALG00010020944	MYO5A	-1.505471742	0.156823115	-9.599807655	8.00937E-22	4.74054E-21	negative
PRJNA729271	stage_1D_vs_E10	ordinary_muscle_development	ENSGALG00010020945	NUMB	0.650539086	0.085351616	7.621871888	2.50023E-14	9.61806E-14	positive
PRJNA729271	stage_1D_vs_E10	ordinary_muscle_development	ENSGALG00010020946	CTBS	0.484656447	0.215638365	2.247542764	0.024605361	0.0343863	positive
PRJNA729271	stage_1D_vs_E10	ordinary_muscle_development	ENSGALG00010020949	HPS6	-0.09523349	0.110913129	-0.858631357	0.390543932	0.439752529	negative
PRJNA729271	stage_1D_vs_E10	ordinary_muscle_development	ENSGALG00010020950	EFHD2	-4.21981452	0.384613663	-10.97156687	5.23571E-28	4.09561E-27	negative
PRJNA729271	stage_1D_vs_E10	ordinary_muscle_development	ENSGALG00010020951	ENSGALG00010020951	-2.325659424	0.290287648	-8.011568674	1.13254E-15	4.74184E-15	negative
PRJNA729271	stage_1D_vs_E10	ordinary_muscle_development	ENSGALG00010020952	GNG5	-1.015127076	0.099165204	-10.23672656	1.35752E-24	9.10983E-24	negative
PRJNA729271	stage_1D_vs_E10	ordinary_muscle_development	ENSGALG00010020954	PLIN1	11.96120795	1.405575286	8.509830867	1.74187E-17	8.10804E-17	positive
PRJNA729271	stage_1D_vs_E10	ordinary_muscle_development	ENSGALG00010020955	FHAD1	0.090654103	0.888418011	0.10203992	0.918724989	0.930862485	positive
PRJNA729271	stage_1D_vs_E10	ordinary_muscle_development	ENSGALG00010020957	RPF1	-0.210832195	0.101443406	-2.078323315	0.03767959	0.051206231	negative
PRJNA729271	stage_1D_vs_E10	ordinary_muscle_development	ENSGALG00010020959	ENSGALG00010020959	-0.47517527	0.353494274	-1.344223388	0.178876161	0.217207837	negative
PRJNA729271	stage_1D_vs_E10	ordinary_muscle_development	ENSGALG00010020960	CLEC19A	5.005990952	0.365748607	13.68697204	1.21466E-42	1.74495E-41	positive
PRJNA729271	stage_1D_vs_E10	ordinary_muscle_development	ENSGALG00010020962	MYO5C	-3.485774534	0.364480265	-9.563685247	1.13637E-21	6.66328E-21	negative
PRJNA729271	stage_1D_vs_E10	ordinary_muscle_development	ENSGALG00010020963	CCDC93	0.798766549	0.099361855	8.038965776	9.05999E-16	3.81979E-15	positive
PRJNA729271	stage_1D_vs_E10	ordinary_muscle_development	ENSGALG00010020965	DHX37	-0.375297089	0.092375453	-4.062736128	4.85008E-05	9.28201E-05	negative
PRJNA729271	stage_1D_vs_E10	ordinary_muscle_development	ENSGALG00010020966	PAPLN	3.057867165	0.245390191	12.46124449	1.2146E-35	1.34909E-34	positive
PRJNA729271	stage_1D_vs_E10	ordinary_muscle_development	ENSGALG00010020967	VPS51	-0.14460194	0.075880678	-1.905649027	0.056695757	0.074934348	negative
PRJNA729271	stage_1D_vs_E10	ordinary_muscle_development	ENSGALG00010020969	RBP3	-1.626469695	0.344864238	-4.716260819	2.40218E-06	5.20396E-06	negative
PRJNA729271	stage_1D_vs_E10	ordinary_muscle_development	ENSGALG00010020970	ARMH3	0.079423301	0.078900257	1.006629171	0.314113011	0.361641628	positive
PRJNA729271	stage_1D_vs_E10	ordinary_muscle_development	ENSGALG00010020971	TMEM51	-1.213743462	0.289057082	-4.198975003	2.68126E-05	5.24846E-05	negative
PRJNA729271	stage_1D_vs_E10	ordinary_muscle_development	ENSGALG00010020973	GNB5	-1.130870608	0.163980804	-6.896359678	5.3352E-12	1.75599E-11	negative
PRJNA729271	stage_1D_vs_E10	ordinary_muscle_development	ENSGALG00010020975	HCCS	0.572482148	0.13021199	4.396539431	1.0999E-05	2.237E-05	positive
PRJNA729271	stage_1D_vs_E10	ordinary_muscle_development	ENSGALG00010020976	GDF10	-1.392564053	0.309987809	-4.492318775	7.04519E-06	1.46053E-05	negative

PRJNA729271	stage_1D_vs_E10	ordinary_muscle_development	ENSGALG00010020978	BCL2L10	3.046895673	0.115068611	26.47894712	1.6942E-154	3.1893E-152	positive
PRJNA729271	stage_1D_vs_E10	ordinary_muscle_development	ENSGALG00010020979	ENSGALG00010020979	0.824304387	0.242102699	3.404771578	0.000662194	0.001124473	positive
PRJNA729271	stage_1D_vs_E10	ordinary_muscle_development	ENSGALG00010020981	CIT	-1.621863113	0.235107896	-6.898377907	5.25997E-12	1.73201E-11	negative
PRJNA729271	stage_1D_vs_E10	ordinary_muscle_development	ENSGALG00010020982	TLCD4	0.561339032	0.150659535	3.725877893	0.000194637	0.000350032	positive
PRJNA729271	stage_1D_vs_E10	ordinary_muscle_development	ENSGALG00010020984	PSEN1	-0.66122591	0.0585542	-11.29254453	1.42841E-29	1.20657E-28	negative
PRJNA729271	stage_1D_vs_E10	ordinary_muscle_development	ENSGALG00010020986	CFAP20DC	-1.943845599	0.319696699	-6.080280486	1.19972E-09	3.36624E-09	negative
PRJNA729271	stage_1D_vs_E10	ordinary_muscle_development	ENSGALG00010020987	NADSYN1	-0.298034095	0.093013986	-3.204185812	0.00135445	0.002226189	negative
PRJNA729271	stage_1D_vs_E10	ordinary_muscle_development	ENSGALG00010020989	MAPK4	0.331705453	0.152884872	2.169642081	0.030033971	0.041405974	positive
PRJNA729271	stage_1D_vs_E10	ordinary_muscle_development	ENSGALG00010020990	UBB	-1.257668042	0.106694681	-11.78754208	4.52556E-32	4.3299E-31	negative
PRJNA729271	stage_1D_vs_E10	ordinary_muscle_development	ENSGALG00010020991	ALG14	0.362783347	0.139343525	2.603517801	0.009227248	0.013706595	positive
PRJNA729271	stage_1D_vs_E10	ordinary_muscle_development	ENSGALG00010020992	KCNH7	3.358841565	0.429074074	7.828115872	4.95235E-15	1.99069E-14	positive
PRJNA729271	stage_1D_vs_E10	ordinary_muscle_development	ENSGALG00010020994	DHCR7	-1.500069959	0.144844652	-10.35640556	3.91395E-25	2.70671E-24	negative
PRJNA729271	stage_1D_vs_E10	ordinary_muscle_development	ENSGALG00010020998	SYT17	0.056623416	0.280179368	0.202097024	0.839840875	0.862695307	positive
PRJNA729271	stage_1D_vs_E10	ordinary_muscle_development	ENSGALG00010020999	IFIH1	0.849460972	0.337430182	2.517442173	0.011821037	0.017312917	positive
PRJNA729271	stage_1D_vs_E10	ordinary_muscle_development	ENSGALG00010021000	NFKB2	0.842289109	0.11448526	7.357183877	1.87831E-13	6.81844E-13	positive
PRJNA729271	stage_1D_vs_E10	ordinary_muscle_development	ENSGALG00010021002	SLC44A3	-1.00152845	0.163269688	-6.134197111	8.55903E-10	2.43213E-09	negative
PRJNA729271	stage_1D_vs_E10	ordinary_muscle_development	ENSGALG00010021003	KAZN	-0.497082159	0.118749662	-4.185966941	2.83955E-05	5.54931E-05	negative
PRJNA729271	stage_1D_vs_E10	ordinary_muscle_development	ENSGALG00010021004	PSD	0.316802745	0.169065943	1.873841298	0.060952302	0.080158195	positive
PRJNA729271	stage_1D_vs_E10	ordinary_muscle_development	ENSGALG00010021010	LEO1	-0.638503958	0.112286754	-5.686369339	1.29769E-08	3.35772E-08	negative
PRJNA729271	stage_1D_vs_E10	ordinary_muscle_development	ENSGALG00010021011	SCARB1	-0.00861643	0.109288422	-0.078841202	0.937158932	0.946496566	negative
PRJNA729271	stage_1D_vs_E10	ordinary_muscle_development	ENSGALG00010021012	KCNIP2	-3.437482047	0.989077925	-3.475441074	0.000510014	0.000877258	negative
PRJNA729271	stage_1D_vs_E10	ordinary_muscle_development	ENSGALG00010021013	FAP	-0.557516977	0.296758601	-1.87868852	0.060287036	0.079340893	negative
PRJNA729271	stage_1D_vs_E10	ordinary_muscle_development	ENSGALG00010021014	FBXL15	0.203764773	0.091144287	2.235628574	0.025376111	0.03537816	positive
PRJNA729271	stage_1D_vs_E10	ordinary_muscle_development	ENSGALG00010021015	RBM25	-0.41215085	0.135306223	-3.04605983	0.002318617	0.00370136	negative
PRJNA729271	stage_1D_vs_E10	ordinary_muscle_development	ENSGALG00010021016	FHIT	-0.934065125	0.221502929	-4.216942542	2.47637E-05	4.8658E-05	negative
PRJNA729271	stage_1D_vs_E10	ordinary_muscle_development	ENSGALG00010021017	COQ7	0.445543131	0.156673298	2.843771948	0.004458295	0.006901215	positive
PRJNA729271	stage_1D_vs_E10	ordinary_muscle_development	ENSGALG00010021018	F3	0.177049289	0.221207329	0.800377138	0.423492323	0.47303084	positive
PRJNA729271	stage_1D_vs_E10	ordinary_muscle_development	ENSGALG00010021019	VSIG10	-0.586126147	0.093585603	-6.262994844	3.77653E-10	1.09877E-09	negative
PRJNA729271	stage_1D_vs_E10	ordinary_muscle_development	ENSGALG00010021020	ZDHHC13	-0.278906037	0.119559383	-2.332782506	0.019659563	0.027847686	negative
PRJNA729271	stage_1D_vs_E10	ordinary_muscle_development	ENSGALG00010021021	GCG	0.949879375	0.351616078	2.70146741	0.006903425	0.010415857	positive
PRJNA729271	stage_1D_vs_E10	ordinary_muscle_development	ENSGALG00010021022	ZFYVE1	0.656139333	0.078656518	8.341830388	7.31493E-17	3.29286E-16	positive
PRJNA729271	stage_1D_vs_E10	ordinary_muscle_development	ENSGALG00010021023	MGEA5	0.216267977	0.097294287	2.222822984	0.026227739	0.036453262	positive
PRJNA729271	stage_1D_vs_E10	ordinary_muscle_development	ENSGALG00010021024	ENSGALG00010021024	0.253067766	0.186618606	1.356069319	0.175077114	0.213058749	positive
PRJNA729271	stage_1D_vs_E10	ordinary_muscle_development	ENSGALG00010021025	CUEDC2	-0.029487446	0.116373407	-0.25338646	0.79996959	0.826836794	negative
PRJNA729271	stage_1D_vs_E10	ordinary_muscle_development	ENSGALG00010021026	ADGRA2	0.615920393	0.132641084	4.643511465	3.42537E-06	7.3091E-06	positive
PRJNA729271	stage_1D_vs_E10	ordinary_muscle_development	ENSGALG00010021027	PLA2G4A	1.13603093	0.166028783	6.84237342	7.78917E-12	2.53717E-11	positive
PRJNA729271	stage_1D_vs_E10	ordinary_muscle_development	ENSGALG00010021028	ENSGALG00010021028	1.45843624	0.309947883	4.705424109	2.53339E-06	5.47509E-06	positive
PRJNA729271	stage_1D_vs_E10	ordinary_muscle_development	ENSGALG00010021029	PRDM2	-0.176318201	0.074621983	-2.362818507	0.018136549	0.025793689	negative
PRJNA729271	stage_1D_vs_E10	ordinary_muscle_development	ENSGALG00010021030	CSRP3	3.309381016	0.547055821	6.049439361	1.45351E-09	4.05477E-09	positive
PRJNA729271	stage_1D_vs_E10	ordinary_muscle_development	ENSGALG00010021031	DDX54	-0.190994081	0.109791192	-1.739612065	0.081927159	0.105699526	negative
PRJNA729271	stage_1D_vs_E10	ordinary_muscle_development	ENSGALG00010021032	TMC7	-0.326525822	0.119847436	-2.724512366	0.006439652	0.009758782	negative
PRJNA729271	stage_1D_vs_E10	ordinary_muscle_development	ENSGALG00010021033	RNF122	-1.001573847	0.231508533	-4.326293435	1.51639E-05	3.03803E-05	negative
PRJNA729271	stage_1D_vs_E10	ordinary_muscle_development	ENSGALG00010021034	NPM3	-1.815042178	0.280055246	-6.48101475	9.11078E-11	2.76567E-10	negative
PRJNA729271	stage_1D_vs_E10	ordinary_muscle_development	ENSGALG00010021036	E2F8	-1.150490925	0.195576826	-5.882552379	4.03987E-09	1.08863E-08	negative
PRJNA729271	stage_1D_vs_E10	ordinary_muscle_development	ENSGALG00010021038	PTGS2	-0.293952292	0.300812565	-0.977194196	0.328473021	0.376827577	negative
PRJNA729271	stage_1D_vs_E10	ordinary_muscle_development	ENSGALG00010021040	FGF8	-2.530495261	0.524835644	-4.821500391	1.42482E-06	3.14255E-06	negative
PRJNA729271	stage_1D_vs_E10	ordinary_muscle_development	ENSGALG00010021041	GLTP	-1.023044429	0.191488063	-5.342601578	9.1622E-08	2.21861E-07	negative
PRJNA729271	stage_1D_vs_E10	ordinary_muscle_development	ENSGALG00010021042	LRRC38	1.006890541	0.094777766	10.62369987	2.31211E-26	1.68159E-25	positive
PRJNA729271	stage_1D_vs_E10	ordinary_muscle_development	ENSGALG00010021043	PTPRG	0.302705445	0.089785993	3.37141054	0.000747843	0.001264876	positive
PRJNA729271	stage_1D_vs_E10	ordinary_muscle_development	ENSGALG00010021044	EIF4G2	-0.264321369	0.06804215	-3.884671033	0.000102469	0.000189861	negative
PRJNA729271	stage_1D_vs_E10	ordinary_muscle_development	ENSGALG00010021045	DPP4	1.581854337	0.260374232	6.075310627	1.23748E-09	3.47016E-09	positive
PRJNA729271	stage_1D_vs_E10	ordinary_muscle_development	ENSGALG00010021046	ENSGALG00010021046	-0.938921622	0.251028151	-3.740304098	0.000183798	0.00033194	negative
PRJNA729271	stage_1D_vs_E10	ordinary_muscle_development	ENSGALG00010021047	TMC5	-0.133944573	0.476472662	-0.281117017	0.77862065	0.807303742	negative
PRJNA729271	stage_1D_vs_E10	ordinary_muscle_development	ENSGALG00010021048	API5	-0.331659297	0.075636344	-4.384919736	1.16029E-05	2.35518E-05	negative
PRJNA729271	stage_1D_vs_E10	ordinary_muscle_development	ENSGALG00010021050	CTR9	0.091897246	0.094235325	0.975188931	0.329466552	0.377907547	positive
PRJNA729271	stage_1D_vs_E10	ordinary_muscle_development	ENSGALG00010021051	GDE1	-0.076915387	0.136064401	-0.565286628	0.571878822	0.616907311	negative
PRJNA729271	stage_1D_vs_E10	ordinary_muscle_development	ENSGALG00010021052	CEP15	-1.767057038	0.151191084	-11.68757437	1.4754E-31	1.37708E-30	negative
PRJNA729271	stage_1D_vs_E10	ordinary_muscle_development	ENSGALG00010021053	ENSGALG00010021053	-3.486875063	0.44698309	-7.800910466	6.14621E-15	2.45155E-14	negative
PRJNA729271	stage_1D_vs_E10	ordinary_muscle_development	ENSGALG00010021055	IRAG1	-0.28689824	0.222160204	-1.291402489	0.196564148	0.236506792	negative
PRJNA729271	stage_1D_vs_E10	ordinary_muscle_development	ENSGALG00010021057	ZDHHC8	0.525881584	0.077026359	6.827293766	8.65314E-12	2.81101E-11	positive
PRJNA729271	stage_1D_vs_E10	ordinary_muscle_development	ENSGALG00010021058	CCP110	0.063989879	0.093608179	0.683592824	0.494232317	0.542439452	positive
PRJNA729271	stage_1D_vs_E10	ordinary_muscle_development	ENSGALG00010021059	ENSGALG00010021059	-2.302612016	1.385081825	-1.662437536	0.096425078	0.122959577	negative
PRJNA729271	stage_1D_vs_E10	ordinary_muscle_development	ENSGALG00010021062	MYO9A	-0.532879437	0.100800335	-5.2864848	1.24689E-07	2.99184E-07	negative
PRJNA729271	stage_1D_vs_E10	ordinary_muscle_development	ENSGALG00010021064	LYVE1	2.522056113	0.285072742	8.847061617	8.98538E-19	4.49114E-18	positive
PRJNA729271	stage_1D_vs_E10	ordinary_muscle_development	ENSGALG00010021065	CNNM2	-0.263028583	0.110769328	-2.374561511	0.017569809	0.025056511	negative
PRJNA729271	stage_1D_vs_E10	ordinary_muscle_development	ENSGALG00010021067	SLC4A10	-0.297248548	0.214320157	-1.386936968	0.165460984	0.202375915	negative
PRJNA729271	stage_1D_vs_E10	ordinary_muscle_development	ENSGALG00010021069	PGAM5	0.29337167	0.070851623	4.140648544	3.46325E-05	6.71173E-05	positive
PRJNA729271	stage_1D_vs_E10	ordinary_muscle_development	ENSGALG00010021070	RNF141	-0.139172624	0.086009098	-1.618115154	0.105637783	0.133754339	negative
PRJNA729271	stage_1D_vs_E10	ordinary_muscle_development	ENSGALG00010021071	HIF1AN	0.451099652	0.095696496	4.713857562	2.43071E-06	5.26103E-06	positive
PRJNA729271	stage_1D_vs_E10	ordinary_muscle_development	ENSGALG00010021072	ENSGALG00010021072	1.043006826	0.089625724	11.63736012	2.66126E-31	2.44454E-30	positive
PRJNA729271	stage_1D_vs_E10	ordinary_muscle_development	ENSGALG00010021073	NDUFB8	0.401643707	0.107561206	3.734094508	0.000188392	0.00033956	positive
PRJNA729271	stage_1D_vs_E10	ordinary_muscle_development	ENSGALG00010021074	TTC17	0.521852932	0.068917404	7.57215019	3.67097E-14	1.39734E-13	positive
PRJNA729271	stage_1D_vs_E10	ordinary_muscle_development	ENSGALG00010021075	DHRS3	-1.681563784	0.133697015	-12.57742204	2.81053E-36	3.20272E-35	negative
PRJNA729271	stage_1D_vs_E10	ordinary_muscle_development	ENSGALG00010021077	SEC31B	0.44648393	0.078520866	5.686181935	1.29911E-08	3.36021E-08	positive
PRJNA729271	stage_1D_vs_E10	ordinary_muscle_development	ENSGALG00010021078	ENSGALG00010021078	-0.912959449	0.210642583	-4.33416376	1.46315E-05	2.93867E-05	negative
PRJNA729271	stage_1D_vs_E10	ordinary_muscle_development	ENSGALG00010021080	TTLL7	2.439914077	0.173319542	14.07754748	5.21898E-45	8.196E-44	positive
PRJNA729271	stage_1D_vs_E10	ordinary_muscle_development	ENSGALG00010021081	KCNH6	1.103452255	0.198122659	5.569540911	2.55411E-08	6.44981E-08	positive
PRJNA729271	stage_1D_vs_E10	ordinary_muscle_development	ENSGALG00010021082	SENP8	-1.079880838	0.164651389	-6.558589302	5.43192E-11	1.67558E-10	negative
PRJNA729271	stage_1D_vs_E10	ordinary_muscle_development	ENSGALG00010021083	SCD	-2.738199282	0.567799866	-4.822472577			negative
PRJNA729271	stage_1D_vs_E10	ordinary_muscle_development	ENSGALG00010021084	ENSGALG00010021084	-3.242579832	0.812108252	-3.992792615	6.52997E-05	0.000123534	negative
PRJNA729271	stage_1D_vs_E10	ordinary_muscle_development	ENSGALG00010021085	AMPD3	1.70148932	0.184036451	9.245392999	2.34378E-20	1.27335E-19	positive
PRJNA729271	stage_1D_vs_E10	ordinary_muscle_development	ENSGALG00010021086	PSMD14	-0.712287726	0.1097605	-6.489472344	8.61375E-11	2.62018E-10	negative
PRJNA729271	stage_1D_vs_E10	ordinary_muscle_development	ENSGALG00010021087	THBS1	-1.900509818	0.356328633	-5.33358715	9.62913E-08	2.32779E-07	negative
PRJNA729271	stage_1D_vs_E10	ordinary_muscle_development	ENSGALG00010021088	ANKLE2	0.240634549	0.086611709	2.77831428	0.005464174	0.008359177	positive
PRJNA729271	stage_1D_vs_E10	ordinary_muscle_development	ENSGALG00010021090	ADM	2.993247977	0.255495095	11.71548116	1.06187E-31	9.9623E-31	positive
PRJNA729271	stage_1D_vs_E10	ordinary_muscle_development	ENSGALG00010021091	TANK	-0.147776793	0.082401882	-1.793366732	0.072914303	0.09483112	negative
PRJNA729271	stage_1D_vs_E10	ordinary_muscle_development	ENSGALG00010021092	VPS13D	1.197434214	0.094601737	12.65763454	1.01512E-36	1.17997E-35	positive
PRJNA729271	stage_1D_vs_E10	ordinary_muscle_development	ENSGALG00010021093	GRAMD2	1.454179424	0.221112325	6.576654766	4.8115E-11	1.49005E-10	positive
PRJNA729271	stage_1D_vs_E10	ordinary_muscle_development	ENSGALG00010021094	ENSGALG00010021094	3.049748134	0.313931922	9.714679899	2.61068E-22	1.58003E-21	positive
PRJNA729271	stage_1D_vs_E10	ordinary_muscle_development	ENSGALG00010021095	NT5C2	1.120928369	0.105019412	10.67353499	1.35376E-26	9.96585E-26	positive
PRJNA729271	stage_1D_vs_E10	ordinary_muscle_development	ENSGALG00010021096	ZFAND4	-0.464436295	0.101053273	-4.595954994	4.30772E-06	9.08223E-06	negative
PRJNA729271	stage_1D_vs_E10	ordinary_muscle_development	ENSGALG00010021097	KATNBL1	-0.122012645	0.129237143	-0.944098897	0.345119113	0.3939598	negative
PRJNA729271	stage_1D_vs_E10	ordinary_muscle_development	ENSGALG00010021098	SDK1	-0.4443609	0.142036396	-3.128500254	0.001757009	0.002849708	negative
PRJNA729271	stage_1D_vs_E10	ordinary_muscle_development	ENSGALG00010021099	HMCN1	-1.568356117	0.264338309	-5.933139707	2.97196E-09	8.10051E-09	negative
PRJNA729271	stage_1D_vs_E10	ordinary_muscle_development	ENSGALG00010021100	EMC7	0.962524097	0.085146808	11.30428864	1.24963E-29	1.05926E-28	positive
PRJNA729271	stage_1D_vs_E10	ordinary_muscle_development	ENSGALG00010021101	TMEM240	-2.460302405	0.379995558	-6.4745557	9.50913E-11	2.88177E-10	negative
PRJNA729271	stage_1D_vs_E10	ordinary_muscle_development	ENSGALG00010021102	GOLGA3	0.612526507	0.112248601	5.456874303	4.84589E-08	1.19988E-07	positive
PRJNA729271	stage_1D_vs_E10	ordinary_muscle_development	ENSGALG00010021103	RBMS1	0.07745417	0.074009803	1.046539331	0.295312096	0.342060798	positive
PRJNA729271	stage_1D_vs_E10	ordinary_muscle_development	ENSGALG00010021104	INA	0.579402328	0.273698255	2.116938335	0.034265082	0.046877995	positive
PRJNA729271	stage_1D_vs_E10	ordinary_muscle_development	ENSGALG00010021105	ENSGALG00010021105	2.030954111	0.421140401	4.822510749	1.41762E-06	3.12762E-06	positive
PRJNA729271	stage_1D_vs_E10	ordinary_muscle_development	ENSGALG00010021106	ITGB6	-0.599412968	0.19435926	-3.084046356	0.002042058	0.003283011	negative
PRJNA729271	stage_1D_vs_E10	ordinary_muscle_development	ENSGALG00010021108	PCGF6	0.334696097	0.105069517	3.185472873	0.001445177	0.002366452	positive
PRJNA729271	stage_1D_vs_E10	ordinary_muscle_development	ENSGALG00010021110	PKLR	1.942277467	0.168809668	11.50572409	1.23449E-30	1.10456E-29	positive
PRJNA729271	stage_1D_vs_E10	ordinary_muscle_development	ENSGALG00010021111	AVEN	-0.024245039	0.079891502	-0.303474566	0.761528227	0.792074602	negative
PRJNA729271	stage_1D_vs_E10	ordinary_muscle_development	ENSGALG00010021112	TAF5	-0.575284124	0.070532614	-8.15628535	3.45486E-16	1.49755E-15	negative
PRJNA729271	stage_1D_vs_E10	ordinary_muscle_development	ENSGALG00010021113	SYP	1.305297118	0.296543476	4.401705725	1.07403E-05	2.18745E-05	positive
PRJNA729271	stage_1D_vs_E10	ordinary_muscle_development	ENSGALG00010021114	SBF2	-0.109574804	0.098195754	-1.115881286	0.264472953	0.309630519	negative
PRJNA729271	stage_1D_vs_E10	ordinary_muscle_development	ENSGALG00010021115	ALPL	-1.41551211	0.247797529	-5.712373778	1.11411E-08	2.89617E-08	negative
PRJNA729271	stage_1D_vs_E10	ordinary_muscle_development	ENSGALG00010021116	FAM21C	0.63495736	0.063572499	9.987925155	1.72147E-23	1.09538E-22	positive
PRJNA729271	stage_1D_vs_E10	ordinary_muscle_development	ENSGALG00010021118	USMG5	0.986115483	0.179755676	5.485865623	4.1145E-08	1.02123E-07	positive
PRJNA729271	stage_1D_vs_E10	ordinary_muscle_development	ENSGALG00010021120	PDCD11	-1.062448254	0.087143051	-12.19200202	3.42893E-34	3.59121E-33	negative
PRJNA729271	stage_1D_vs_E10	ordinary_muscle_development	ENSGALG00010021121	PLA2R1	1.914992237	0.09121401	20.99449679	7.36386E-98	5.25808E-96	positive
PRJNA729271	stage_1D_vs_E10	ordinary_muscle_development	ENSGALG00010021122	CYP1A1	0.319961302	1.014470317	0.315397402	0.752459939	0.784218207	positive
PRJNA729271	stage_1D_vs_E10	ordinary_muscle_development	ENSGALG00010021123	RYR3	5.926890713	0.216145596	27.42082574	1.5485E-165	3.7409E-163	positive
PRJNA729271	stage_1D_vs_E10	ordinary_muscle_development	ENSGALG00010021124	CALHM2	0.447529143	0.130314034	3.434235965	0.000594227	0.00101357	positive
PRJNA729271	stage_1D_vs_E10	ordinary_muscle_development	ENSGALG00010021125	LY75	1.280376234	0.124271301	10.30307261	6.8246E-25	4.65737E-24	positive
PRJNA729271	stage_1D_vs_E10	ordinary_muscle_development	ENSGALG00010021126	ECE1	0.90175674	0.10371222	8.694797349	3.47449E-18	1.68437E-17	positive
PRJNA729271	stage_1D_vs_E10	ordinary_muscle_development	ENSGALG00010021127	SWAP70	-0.392729543	0.0776317	-5.058881123	4.21724E-07	9.68148E-07	negative
PRJNA729271	stage_1D_vs_E10	ordinary_muscle_development	ENSGALG00010021129	TXNRD3	-0.113540808	0.085344668	-1.330379625	0.183393224	0.222338975	negative
PRJNA729271	stage_1D_vs_E10	ordinary_muscle_development	ENSGALG00010021130	ENSGALG00010021130	4.079593763	1.102165622	3.701434414	0.000214384	0.0003835	positive
PRJNA729271	stage_1D_vs_E10	ordinary_muscle_development	ENSGALG00010021131	EDC3	-0.164938234	0.115653768	-1.426138012	0.153828508	0.189201886	negative
PRJNA729271	stage_1D_vs_E10	ordinary_muscle_development	ENSGALG00010021133	ENSGALG00010021133	-0.303803369	0.101669692	-2.988140943	0.002806801	0.004439125	negative
PRJNA729271	stage_1D_vs_E10	ordinary_muscle_development	ENSGALG00010021134	CHFR	-0.853853521	0.093616003	-9.120807247	7.45668E-20	3.95624E-19	negative
PRJNA729271	stage_1D_vs_E10	ordinary_muscle_development	ENSGALG00010021135	CLK3	0.852569229	0.105049862	8.11585293	4.82385E-16	2.0736E-15	positive
PRJNA729271	stage_1D_vs_E10	ordinary_muscle_development	ENSGALG00010021136	WEE1	-1.714144991	0.149068512	-11.49904137	1.33389E-30	1.19056E-29	negative
PRJNA729271	stage_1D_vs_E10	ordinary_muscle_development	ENSGALG00010021137	PLAU	-0.527159999	0.195596389	-2.695141773	0.007035869	0.010603547	negative
PRJNA729271	stage_1D_vs_E10	ordinary_muscle_development	ENSGALG00010021138	DDX18	-0.522987611	0.099523629	-5.254908992	1.48098E-07	3.53014E-07	negative
PRJNA729271	stage_1D_vs_E10	ordinary_muscle_development	ENSGALG00010021139	ARHGAP29	0.674570676	0.095668485	7.051127387	1.77474E-12	6.03162E-12	positive
PRJNA729271	stage_1D_vs_E10	ordinary_muscle_development	ENSGALG00010021140	SLC11A1	-0.180214678	0.442520357	-0.407246074	0.683827265	0.721665541	negative
PRJNA729271	stage_1D_vs_E10	ordinary_muscle_development	ENSGALG00010021141	SGSM1	-0.490187123	0.152046159	-3.223936244	0.001264415	0.002086012	negative
PRJNA729271	stage_1D_vs_E10	ordinary_muscle_development	ENSGALG00010021142	WNT6	-5.81477353	0.605487674	-9.603454834	7.73087E-22	4.57944E-21	negative

PRJNA729271	stage_1D_vs_E10	ordinary_muscle_development	ENSGALG00010021143	ABCA4	-0.812484514	0.348056527	-2.334346436	0.019577589	0.027739702	negative
PRJNA729271	stage_1D_vs_E10	ordinary_muscle_development	ENSGALG00010021144	FOXK1	0.244574325	0.108443535	2.25531495	0.024113567	0.03374456	positive
PRJNA729271	stage_1D_vs_E10	ordinary_muscle_development	ENSGALG00010021145	EIF4G3	0.213051266	0.157283147	1.354571487	0.175554123	0.213567521	positive
PRJNA729271	stage_1D_vs_E10	ordinary_muscle_development	ENSGALG00010021146	AP5Z1	0.055317613	0.085000541	0.650791312	0.515181209	0.562866634	positive
PRJNA729271	stage_1D_vs_E10	ordinary_muscle_development	ENSGALG00010021147	ARID3B	0.126819243	0.082322852	1.540510799	0.123435893	0.154601509	positive
PRJNA729271	stage_1D_vs_E10	ordinary_muscle_development	ENSGALG00010021148	GPATCH8	0.51852265	0.104361648	4.968517266	6.74668E-07	1.52754E-06	positive
PRJNA729271	stage_1D_vs_E10	ordinary_muscle_development	ENSGALG00010021149	ENSGALG00010021149	-3.9432211	1.345538149	-2.930590338	0.003383186	0.005305559	negative
PRJNA729271	stage_1D_vs_E10	ordinary_muscle_development	ENSGALG00010021150	ENSGALG00010021150	1.134529684	0.24941412	4.548778889	5.39581E-06	1.12942E-05	positive
PRJNA729271	stage_1D_vs_E10	ordinary_muscle_development	ENSGALG00010021151	WNT10A	-5.946360794	0.942578278	-6.308612167	2.81549E-10	8.27295E-10	negative
PRJNA729271	stage_1D_vs_E10	ordinary_muscle_development	ENSGALG00010021152	STK11IP	0.728792691	0.126813437	5.746967447	9.08582E-09	2.37853E-08	positive
PRJNA729271	stage_1D_vs_E10	ordinary_muscle_development	ENSGALG00010021153	PTPRF	-1.668961489	0.157653973	-10.58623171	3.4521E-26	2.48946E-25	negative
PRJNA729271	stage_1D_vs_E10	ordinary_muscle_development	ENSGALG00010021154	CHST13	-2.194078571	0.211016116	-10.3976825	2.54036E-25	1.76862E-24	negative
PRJNA729271	stage_1D_vs_E10	ordinary_muscle_development	ENSGALG00010021155	RADIL	-1.811081336	0.217671941	-8.320233334	8.77903E-17	3.93117E-16	negative
PRJNA729271	stage_1D_vs_E10	ordinary_muscle_development	ENSGALG00010021156	DDOST	-0.86069463	0.080695282	-10.66598451	1.46836E-26	1.0793E-25	negative
PRJNA729271	stage_1D_vs_E10	ordinary_muscle_development	ENSGALG00010021157	ENSGALG00010021157	3.005547834	0.871287304	3.449548524	0.000561525	0.000961184	positive
PRJNA729271	stage_1D_vs_E10	ordinary_muscle_development	ENSGALG00010021158	ACTG1L	-2.552572447	0.129298346	-19.74172551	9.44953E-87	5.24793E-85	negative
PRJNA729271	stage_1D_vs_E10	ordinary_muscle_development	ENSGALG00010021159	MMD2	2.030034729	0.23773767	8.538969578	1.35424E-17	6.33421E-17	positive
PRJNA729271	stage_1D_vs_E10	ordinary_muscle_development	ENSGALG00010021160	ZNF143	0.386872011	0.163966559	2.35945679	0.018301712	0.026010718	positive
PRJNA729271	stage_1D_vs_E10	ordinary_muscle_development	ENSGALG00010021161	ENSGALG00010021161	-1.199896016	0.106070327	-11.31226845	1.14101E-29	9.68888E-29	negative
PRJNA729271	stage_1D_vs_E10	ordinary_muscle_development	ENSGALG00010021162	WIPI2	-0.196151227	0.094741726	-2.070378438	0.038416919	0.052120296	negative
PRJNA729271	stage_1D_vs_E10	ordinary_muscle_development	ENSGALG00010021163	INHA	-3.305154326	0.488822168	-6.761465714	1.36603E-11	4.38939E-11	negative
PRJNA729271	stage_1D_vs_E10	ordinary_muscle_development	ENSGALG00010021164	SLC41A3	0.532586348	0.212584545	2.505291943	0.012235036	0.017884918	positive
PRJNA729271	stage_1D_vs_E10	ordinary_muscle_development	ENSGALG00010021165	PTRF	4.091945257	0.10552533	38.77690086	0	0	positive
PRJNA729271	stage_1D_vs_E10	ordinary_muscle_development	ENSGALG00010021166	NEURL1	-0.888526601	0.313389553	-2.835214485	0.004579493	0.007075978	negative
PRJNA729271	stage_1D_vs_E10	ordinary_muscle_development	ENSGALG00010021167	KIAA1671	0.044999481	0.12598766	0.357173717	0.72096176	0.755901679	positive
PRJNA729271	stage_1D_vs_E10	ordinary_muscle_development	ENSGALG00010021168	IVNS1ABP	0.369304504	0.105771472	3.49153225	0.000480258	0.000828929	positive
PRJNA729271	stage_1D_vs_E10	ordinary_muscle_development	ENSGALG00010021169	CAMK2G	1.761528998	0.140119018	12.57166242	3.023E-36	3.43673E-35	positive
PRJNA729271	stage_1D_vs_E10	ordinary_muscle_development	ENSGALG00010021170	UBL7	-0.444970544	0.138366014	-3.215894783	0.001300385	0.002142428	negative
PRJNA729271	stage_1D_vs_E10	ordinary_muscle_development	ENSGALG00010021171	ENSGALG00010021171	-0.827956753	0.213700262	-3.874383424	0.000106895	0.000197784	negative
PRJNA729271	stage_1D_vs_E10	ordinary_muscle_development	ENSGALG00010021172	SLC29A4	0.269006518	0.260041182	1.0344766	0.300913411	0.347881631	positive
PRJNA729271	stage_1D_vs_E10	ordinary_muscle_development	ENSGALG00010021173	KLF15	3.551603582	0.287075242	12.37168194	3.7195E-35	4.06899E-34	positive
PRJNA729271	stage_1D_vs_E10	ordinary_muscle_development	ENSGALG00010021175	IPO7	0.4982776	0.097563142	5.107231973	3.26912E-07	7.57206E-07	positive
PRJNA729271	stage_1D_vs_E10	ordinary_muscle_development	ENSGALG00010021176	ENSGALG00010021176	0.167099735	0.117814908	1.418324191	0.156096137	0.191763159	positive
PRJNA729271	stage_1D_vs_E10	ordinary_muscle_development	ENSGALG00010021177	OBSL1	-0.733551471	0.19018794	-3.856982057	0.000114796	0.000211889	negative
PRJNA729271	stage_1D_vs_E10	ordinary_muscle_development	ENSGALG00010021178	NDST2	-0.216345514	0.096770606	-2.235653191	0.025374497	0.03537816	negative
PRJNA729271	stage_1D_vs_E10	ordinary_muscle_development	ENSGALG00010021180	TMEM198	-0.352662544	0.141818271	-2.4867215	0.012892628	0.01877234	negative
PRJNA729271	stage_1D_vs_E10	ordinary_muscle_development	ENSGALG00010021181	DAGLA	0.170822939	0.127660448	1.338103874	0.180862578	0.219454468	positive
PRJNA729271	stage_1D_vs_E10	ordinary_muscle_development	ENSGALG00010021182	ZSWIM8	0.51144593	0.068130789	7.506825332	6.05785E-14	2.27189E-13	positive
PRJNA729271	stage_1D_vs_E10	ordinary_muscle_development	ENSGALG00010021183	CFAP100	4.643568406	0.449325003	10.33454264	4.91752E-25	3.38298E-24	positive
PRJNA729271	stage_1D_vs_E10	ordinary_muscle_development	ENSGALG00010021184	DRAXIN	-1.839944975	0.468084511	-3.930796536	8.46649E-05	0.000158249	negative
PRJNA729271	stage_1D_vs_E10	ordinary_muscle_development	ENSGALG00010021186	SWT1	0.44645513	0.089762055	4.973762374	6.56658E-07	1.48793E-06	positive
PRJNA729271	stage_1D_vs_E10	ordinary_muscle_development	ENSGALG00010021187	SEMA7A	2.092999605	0.277950439	7.530117999	5.06945E-14	1.91209E-13	positive
PRJNA729271	stage_1D_vs_E10	ordinary_muscle_development	ENSGALG00010021188	TNRC18	0.229977338	0.133415947	1.723761982	0.08475082	0.109119128	positive
PRJNA729271	stage_1D_vs_E10	ordinary_muscle_development	ENSGALG00010021189	ENSGALG00010021189	4.088419033	0.358105424	11.41680288	3.44682E-30	3.01701E-29	positive
PRJNA729271	stage_1D_vs_E10	ordinary_muscle_development	ENSGALG00010021190	ENSGALG00010021190	0.60903096	0.101644737	5.991760872	2.07581E-09	5.72794E-09	positive
PRJNA729271	stage_1D_vs_E10	ordinary_muscle_development	ENSGALG00010021191	MYRF	-0.532060228	0.201080584	-2.646004984	0.008144862	0.012183671	negative
PRJNA729271	stage_1D_vs_E10	ordinary_muscle_development	ENSGALG00010021192	ENSGALG00010021192	-1.482879299	0.172986386	-8.572231221	1.01499E-17	4.7876E-17	negative
PRJNA729271	stage_1D_vs_E10	ordinary_muscle_development	ENSGALG00010021193	USP37	-0.033837135	0.156599052	-0.216074964	0.828929314	0.853179749	negative
PRJNA729271	stage_1D_vs_E10	ordinary_muscle_development	ENSGALG00010021194	CYP11A1	-0.527995513	0.370855629	-1.423722527	0.15452681	0.18994794	negative
PRJNA729271	stage_1D_vs_E10	ordinary_muscle_development	ENSGALG00010021195	GCLM	-0.752278247	0.090775118	-8.287273692	1.15874E-16	5.14896E-16	negative
PRJNA729271	stage_1D_vs_E10	ordinary_muscle_development	ENSGALG00010021196	ATP6V0A1	1.911620395	0.120037312	15.9252183	4.23533E-57	1.05483E-55	positive
PRJNA729271	stage_1D_vs_E10	ordinary_muscle_development	ENSGALG00010021197	CHCHD1	0.172305892	0.105222916	1.637531997	0.101519359	0.128990455	positive
PRJNA729271	stage_1D_vs_E10	ordinary_muscle_development	ENSGALG00010021199	TMEM258	-0.938789993	0.137218303	-6.841579985	7.83245E-12	2.55012E-11	negative
PRJNA729271	stage_1D_vs_E10	ordinary_muscle_development	ENSGALG00010021200	FTO	-0.172630543	0.1617656	-1.067164728	0.285897457	0.332111207	negative
PRJNA729271	stage_1D_vs_E10	ordinary_muscle_development	ENSGALG00010021201	TRMT1L	0.10087061	0.088381269	1.141312084	0.253740075	0.298439013	positive
PRJNA729271	stage_1D_vs_E10	ordinary_muscle_development	ENSGALG00010021202	UROC1	-1.569401196	0.55999878	-2.802508244	0.005070692	0.007790935	negative
PRJNA729271	stage_1D_vs_E10	ordinary_muscle_development	ENSGALG00010021203	FUT11	-0.062604407	0.08147331	-0.768403874	0.44224728	0.491504504	negative
PRJNA729271	stage_1D_vs_E10	ordinary_muscle_development	ENSGALG00010021204	NAGLU	0.5370101	0.09356924	5.739173498	9.51397E-09	2.48612E-08	positive
PRJNA729271	stage_1D_vs_E10	ordinary_muscle_development	ENSGALG00010021206	HSD17B1	-0.607308168	0.459536656	-1.32156632	0.186312605	0.225482275	negative
PRJNA729271	stage_1D_vs_E10	ordinary_muscle_development	ENSGALG00010021207	MAD2L2	-0.527095574	0.122915364	-4.288280631	1.80062E-05	3.57974E-05	negative
PRJNA729271	stage_1D_vs_E10	ordinary_muscle_development	ENSGALG00010021210	ENSGALG00010021210	-0.33576591	0.063452829	-5.291582982	1.21262E-07	2.9125E-07	negative
PRJNA729271	stage_1D_vs_E10	ordinary_muscle_development	ENSGALG00010021211	FEN1	-1.72469828	0.154906525	-11.13380006	8.5894E-29	7.01032E-28	negative
PRJNA729271	stage_1D_vs_E10	ordinary_muscle_development	ENSGALG00010021212	MYO18B	3.155466984	0.161082286	19.58916194	1.91313E-85	1.02328E-83	positive
PRJNA729271	stage_1D_vs_E10	ordinary_muscle_development	ENSGALG00010021213	FBXO44	-0.251767795	0.209860611	-1.19969056	0.23025954	0.273462682	negative
PRJNA729271	stage_1D_vs_E10	ordinary_muscle_development	ENSGALG00010021214	FBXL18	0.156326582	0.113506476	1.377248137	0.168435544	0.205666797	positive
PRJNA729271	stage_1D_vs_E10	ordinary_muscle_development	ENSGALG00010021215	SH3PXD2A	0.278098055	0.111467952	2.494870059	0.012600326	0.018378117	positive
PRJNA729271	stage_1D_vs_E10	ordinary_muscle_development	ENSGALG00010021217	FBXO2	-1.882663789	0.217616681	-8.651284376	5.0923E-18	2.44414E-17	negative
PRJNA729271	stage_1D_vs_E10	ordinary_muscle_development	ENSGALG00010021218	DPF3	0.220897556	0.173873946	1.270446552	0.20392561	0.244531909	positive
PRJNA729271	stage_1D_vs_E10	ordinary_muscle_development	ENSGALG00010021219	STRA6	-8.329020289	0.751187026	-11.08781169	1.43762E-28	1.1622E-27	negative
PRJNA729271	stage_1D_vs_E10	ordinary_muscle_development	ENSGALG00010021220	SYNPO2L	3.961775578	0.205620985	19.26736987	1.00937E-82	5.11567E-81	positive
PRJNA729271	stage_1D_vs_E10	ordinary_muscle_development	ENSGALG00010021222	ENSGALG00010021222	3.893701017	0.304542367	12.78541654	1.978E-37	2.35976E-36	positive
PRJNA729271	stage_1D_vs_E10	ordinary_muscle_development	ENSGALG00010021223	DFFB	0.312368054	0.109400746	2.855264377	0.004300102	0.006665595	positive
PRJNA729271	stage_1D_vs_E10	ordinary_muscle_development	ENSGALG00010021224	MYOZ1	6.301183949	0.141050454	44.67326253	0	0	positive
PRJNA729271	stage_1D_vs_E10	ordinary_muscle_development	ENSGALG00010021227	CHCHD4	-0.21626045	0.092944695	-2.326764855	0.019977784	0.028265298	negative
PRJNA729271	stage_1D_vs_E10	ordinary_muscle_development	ENSGALG00010021228	ESRP2	-8.970939261	1.177830093	-7.616496906	2.60653E-14	1.0019E-13	negative
PRJNA729271	stage_1D_vs_E10	ordinary_muscle_development	ENSGALG00010021229	GLO1	-0.084445098	0.178445641	-0.473225893	0.636052027	0.67676861	negative
PRJNA729271	stage_1D_vs_E10	ordinary_muscle_development	ENSGALG00010021230	C1orf174	-0.229673249	0.059227081	-3.877841808	0.000105387	0.000195044	negative
PRJNA729271	stage_1D_vs_E10	ordinary_muscle_development	ENSGALG00010021231	STN1	-1.179466592	0.116886227	-10.09072346	6.07203E-24	3.93975E-23	negative
PRJNA729271	stage_1D_vs_E10	ordinary_muscle_development	ENSGALG00010021232	ACTB	-1.318298045	0.109367599	-12.05382633	1.85151E-33	1.88599E-32	negative
PRJNA729271	stage_1D_vs_E10	ordinary_muscle_development	ENSGALG00010021233	COASY	0.208403502	0.061819768	3.371146631	0.00074856	0.001265941	positive
PRJNA729271	stage_1D_vs_E10	ordinary_muscle_development	ENSGALG00010021234	ISLR2	0.07931111	0.327442597	0.242213782	0.808614516	0.834463008	positive
PRJNA729271	stage_1D_vs_E10	ordinary_muscle_development	ENSGALG00010021235	XRCC5	-1.458279414	0.139771517	-10.43330889	1.74697E-25	1.22567E-24	negative
PRJNA729271	stage_1D_vs_E10	ordinary_muscle_development	ENSGALG00010021236	BTBD9	0.029472004	0.073906062	0.398776542	0.690057863	0.727446453	positive
PRJNA729271	stage_1D_vs_E10	ordinary_muscle_development	ENSGALG00010021237	FSCN1	-3.285159258	0.223186851	-14.71932257	4.84489E-49	8.9919E-48	negative
PRJNA729271	stage_1D_vs_E10	ordinary_muscle_development	ENSGALG00010021238	NDUFS8	1.388446381	0.146974593	9.446846211	3.49193E-21	1.99512E-20	positive
PRJNA729271	stage_1D_vs_E10	ordinary_muscle_development	ENSGALG00010021239	USP54	0.358458094	0.069338161	5.169708709	2.34459E-07	5.4965E-07	positive
PRJNA729271	stage_1D_vs_E10	ordinary_muscle_development	ENSGALG00010021240	SLK	-0.061326975	0.078588195	-0.780358615	0.43517982	0.484591803	negative
PRJNA729271	stage_1D_vs_E10	ordinary_muscle_development	ENSGALG00010021241	TMEM43	-0.09846629	0.171488375	-0.574186388	0.56584168	0.611168044	negative
PRJNA729271	stage_1D_vs_E10	ordinary_muscle_development	ENSGALG00010021242	BCAR3	3.190696369	0.224372567	14.220528	6.83354E-46	1.10921E-44	positive
PRJNA729271	stage_1D_vs_E10	ordinary_muscle_development	ENSGALG00010021243	DYL1	-1.296054031	0.095070592	-13.63254397	2.56481E-42	3.6448E-41	negative
PRJNA729271	stage_1D_vs_E10	ordinary_muscle_development	ENSGALG00010021244	PPP3CB	0.999754129	0.086679588	11.53390491	8.90127E-31	8.00396E-30	positive
PRJNA729271	stage_1D_vs_E10	ordinary_muscle_development	ENSGALG00010021245	ZFAND3	0.666407335	0.07038214	9.468415292	2.84121E-21	1.62973E-20	positive
PRJNA729271	stage_1D_vs_E10	ordinary_muscle_development	ENSGALG00010021246	PECR	0.526039642	0.113639741	4.62901127	3.67416E-06	7.81351E-06	positive
PRJNA729271	stage_1D_vs_E10	ordinary_muscle_development	ENSGALG00010021247	GNAO1	-1.585873605	0.222931983	-7.113710586	1.12964E-12	3.88104E-12	negative
PRJNA729271	stage_1D_vs_E10	ordinary_muscle_development	ENSGALG00010021248	ANXA7	0.612404617	0.167902229	3.647388257	0.000264919	0.000469439	positive
PRJNA729271	stage_1D_vs_E10	ordinary_muscle_development	ENSGALG00010021249	ENSGALG00010021249	-1.473988019	0.424769061	-3.470092704	0.000520279	0.000894171	negative
PRJNA729271	stage_1D_vs_E10	ordinary_muscle_development	ENSGALG00010021250	ISLR	0.578127699	0.145347171	3.977564153	6.96248E-05	0.00013122	positive
PRJNA729271	stage_1D_vs_E10	ordinary_muscle_development	ENSGALG00010021251	RNF216	-0.361183647	0.085251994	-4.236659246	2.2687E-05	4.47291E-05	negative
PRJNA729271	stage_1D_vs_E10	ordinary_muscle_development	ENSGALG00010021252	XPC	0.622375952	0.131152361	4.745442223	2.08051E-06	4.53149E-06	positive
PRJNA729271	stage_1D_vs_E10	ordinary_muscle_development	ENSGALG00010021253	TMEM169	-1.565939929	0.114207805	-13.71132152	8.68612E-43	1.25404E-41	negative
PRJNA729271	stage_1D_vs_E10	ordinary_muscle_development	ENSGALG00010021254	FNBP1L	-0.360969209	0.108184745	-3.336599892	0.000848099	0.001425133	negative
PRJNA729271	stage_1D_vs_E10	ordinary_muscle_development	ENSGALG00010021255	EIF3F	0.706077489	0.091759888	7.69483816	1.41673E-14	5.52235E-14	positive
PRJNA729271	stage_1D_vs_E10	ordinary_muscle_development	ENSGALG00010021256	ENSGALG00010021256	-0.129770367	0.292707821	-0.443344376	0.657516662	0.696739822	negative
PRJNA729271	stage_1D_vs_E10	ordinary_muscle_development	ENSGALG00010021257	RGS6	-1.065587769	0.316476441	-3.367036626	0.000759806	0.00128451	negative
PRJNA729271	stage_1D_vs_E10	ordinary_muscle_development	ENSGALG00010021258	LSM3	-1.085464654	0.11271362	-9.630288254	5.95622E-22	3.55582E-21	negative
PRJNA729271	stage_1D_vs_E10	ordinary_muscle_development	ENSGALG00010021259	CEP104	-1.32466713	0.135652369	-9.765160348	1.58861E-22	9.72006E-22	negative
PRJNA729271	stage_1D_vs_E10	ordinary_muscle_development	ENSGALG00010021260	KDM4A	-0.201263232	0.067849897	-2.966301195	0.003014052	0.004751176	negative
PRJNA729271	stage_1D_vs_E10	ordinary_muscle_development	ENSGALG00010021261	JUP	0.481481394	0.128144579	3.75732941	0.000171736	0.000311282	positive
PRJNA729271	stage_1D_vs_E10	ordinary_muscle_development	ENSGALG00010021262	MDGA1	-1.760610618	0.335784816	-5.243270492	1.57755E-07	3.75293E-07	negative
PRJNA729271	stage_1D_vs_E10	ordinary_muscle_development	ENSGALG00010021263	STOML1	-0.621057129	0.246440303	-2.520111852	0.011731755	0.01718715	negative
PRJNA729271	stage_1D_vs_E10	ordinary_muscle_development	ENSGALG00010021264	CBFB	0.011368965	0.169482625	0.067080414	0.946517686	0.954618276	positive
PRJNA729271	stage_1D_vs_E10	ordinary_muscle_development	ENSGALG00010021265	SIPA1L1	-0.245685702	0.089189383	-2.754651876	0.005875461	0.008951525	negative
PRJNA729271	stage_1D_vs_E10	ordinary_muscle_development	ENSGALG00010021266	COQ5	0.406064791	0.098529667	4.121243939	3.76832E-05	7.28E-05	positive
PRJNA729271	stage_1D_vs_E10	ordinary_muscle_development	ENSGALG00010021267	LRRC47	0.228554203	0.078267277	2.920175744	0.00349834	0.005479032	positive
PRJNA729271	stage_1D_vs_E10	ordinary_muscle_development	ENSGALG00010021269	NUDT13	0.464110112	0.165893864	2.797632778	0.00514786	0.00790029	positive
PRJNA729271	stage_1D_vs_E10	ordinary_muscle_development	ENSGALG00010021271	ENSGALG00010021271	-1.098856877	0.46759993	-2.349993674	0.01877373	0.026652813	negative
PRJNA729271	stage_1D_vs_E10	ordinary_muscle_development	ENSGALG00010021272	ENSGALG00010021272	-0.183909925	0.361941495	-0.508120589	0.611368777	0.653910155	negative
PRJNA729271	stage_1D_vs_E10	ordinary_muscle_development	ENSGALG00010021275	MAP3K2	0.370829092	0.09058783	4.093586209	4.24752E-05	8.16333E-05	positive
PRJNA729271	stage_1D_vs_E10	ordinary_muscle_development	ENSGALG00010021277	ENSGALG00010021277	-0.496397913	0.243852917	-2.03564476	0.04178604	0.056406095	negative
PRJNA729271	stage_1D_vs_E10	ordinary_muscle_development	ENSGALG00010021287	COL17A1	-6.628905888	0.459266568	-14.43367827	3.17677E-47	5.42371E-46	negative
PRJNA729271	stage_1D_vs_E10	ordinary_muscle_development	ENSGALG00010021290	ERCC3	-0.040762096	0.067783179	-0.601360048	0.547600203	0.593677258	negative
PRJNA729271	stage_1D_vs_E10	ordinary_muscle_development	ENSGALG00010021293	PCNX1	0.443054702	0.068569752	6.46137239	1.03758E-10	3.13784E-10	positive
PRJNA729271	stage_1D_vs_E10	ordinary_muscle_development	ENSGALG00010021296	GATC	-0.366851971	0.122977589	-2.983079868	0.002853635	0.004510243	negative
PRJNA729271	stage_1D_vs_E10	ordinary_muscle_development	ENSGALG00010021298	RPGRIP1L	-1.313517384	0.146137603	-8.988223145	2.51259E-19	1.29379E-18	negative
PRJNA729271	stage_1D_vs_E10	ordinary_muscle_development	ENSGALG00010021299	CYP27C1	-2.429321598	0.157056382	-15.46783119	5.7202E-54	1.2601E-52	negative
PRJNA729271	stage_1D_vs_E10	ordinary_muscle_development	ENSGALG00010021301	SMURF1	0.234119519	0.079773301	2.934810488	0.003337515	0.005235069	positive
PRJNA729271	stage_1D_vs_E10	ordinary_muscle_development	ENSGALG00010021302	P4HA1	-0.642808049	0.060066764	-10.70155952	1.00079E-26	7.41263E-26	negative
PRJNA729271	stage_1D_vs_E10	ordinary_muscle_development	ENSGALG00010021307	MAP3K9	-0.601802259	0.135935243	-4.427124613	9.54976E-06	1.95459E-05	negative
PRJNA729271	stage_1D_vs_E10	ordinary_muscle_development	ENSGALG00010021309	ST3GAL3	0.076612392	0.12670486	0.604652351	0.545410015	0.591391245	positive
PRJNA729271	stage_1D_vs_E10	ordinary_muscle_development	ENSGALG00010021311	TRIAP1	0.304269914	0.120173545	2.531920923	0.011343957	0.016656266	positive
PRJNA729271	stage_1D_vs_E10	ordinary_muscle_development	ENSGALG00010021313	KPNA7	-0.163632399	1.437638784	-0.113820246	0.909380289	0.922748848	negative

PRJNA729271	stage_1D_vs_E10	ordinary_muscle_development	ENSGALG00010021315	COX6A1	1.615056856	0.13466308	11.99331589	3.85172E-33	3.86104E-32	positive
PRJNA729271	stage_1D_vs_E10	ordinary_muscle_development	ENSGALG00010021318	TP73	-2.586919521	0.273643786	-9.453602301	3.27366E-21	1.87408E-20	negative
PRJNA729271	stage_1D_vs_E10	ordinary_muscle_development	ENSGALG00010021320	SLC6A6	1.373452648	0.195029316	7.042288198	1.89108E-12	6.41799E-12	positive
PRJNA729271	stage_1D_vs_E10	ordinary_muscle_development	ENSGALG00010021325	ARPC1A	-0.443392937	0.064621333	-6.861401858	6.8188E-12	2.22911E-11	negative
PRJNA729271	stage_1D_vs_E10	ordinary_muscle_development	ENSGALG00010021327	IGSF1	0.5085589	0.491033288	1.035691292	0.300346186	0.347308972	positive
PRJNA729271	stage_1D_vs_E10	ordinary_muscle_development	ENSGALG00010021329	PSMC3IP	-1.273376925	0.158105394	-8.053975213	8.01475E-16	3.39094E-15	negative
PRJNA729271	stage_1D_vs_E10	ordinary_muscle_development	ENSGALG00010021330	COMMD4	-0.946848266	0.13202973	-7.171477705	7.41925E-13	2.59325E-12	negative
PRJNA729271	stage_1D_vs_E10	ordinary_muscle_development	ENSGALG00010021332	OIT3	1.446938012	0.124733318	11.60025275	4.10863E-31	3.73852E-30	positive
PRJNA729271	stage_1D_vs_E10	ordinary_muscle_development	ENSGALG00010021335	NFATC3	0.450641582	0.101639734	4.43371471	9.26232E-06	1.89951E-05	positive
PRJNA729271	stage_1D_vs_E10	ordinary_muscle_development	ENSGALG00010021338	PDIA5	-0.68625491	0.158045817	-4.342126385	1.4111E-05	2.83807E-05	negative
PRJNA729271	stage_1D_vs_E10	ordinary_muscle_development	ENSGALG00010021341	L3MBTL1	-0.14617896	0.152980599	-0.955539204	0.339305089	0.388056436	negative
PRJNA729271	stage_1D_vs_E10	ordinary_muscle_development	ENSGALG00010021342	SFR1	-1.874381986	0.176885368	-10.59659147	3.09039E-26	2.23195E-25	negative
PRJNA729271	stage_1D_vs_E10	ordinary_muscle_development	ENSGALG00010021345	NEIL1	0.384016305	0.152909391	2.511397779	0.012025411	0.017598014	positive
PRJNA729271	stage_1D_vs_E10	ordinary_muscle_development	ENSGALG00010021346	RPS21	0.459245748	0.278464973	1.64920472	0.099105701	0.12609902	positive
PRJNA729271	stage_1D_vs_E10	ordinary_muscle_development	ENSGALG00010021348	SLC4A3	0.209969678	0.115517971	1.817636485	0.069119711	0.090187255	positive
PRJNA729271	stage_1D_vs_E10	ordinary_muscle_development	ENSGALG00010021350	CABLES2	-0.057238709	0.133628845	-0.428340967	0.668402897	0.707034955	negative
PRJNA729271	stage_1D_vs_E10	ordinary_muscle_development	ENSGALG00010021352	CFAP43	-1.43305961	0.236202879	-6.067070892	1.30264E-09	3.64513E-09	negative
PRJNA729271	stage_1D_vs_E10	ordinary_muscle_development	ENSGALG00010021353	IPO13	1.47502613	0.177988206	8.287212752	1.15933E-16	5.15002E-16	positive
PRJNA729271	stage_1D_vs_E10	ordinary_muscle_development	ENSGALG00010021354	ENSGALG00010021354	0.557749472	0.111579071	4.99869257	5.77203E-07	1.31343E-06	positive
PRJNA729271	stage_1D_vs_E10	ordinary_muscle_development	ENSGALG00010021358	MMP15	0.150015699	0.129283374	1.160363427	0.24590087	0.289978725	positive
PRJNA729271	stage_1D_vs_E10	ordinary_muscle_development	ENSGALG00010021360	PTPRN	-1.41938393	0.297021655	-4.778722051	1.76413E-06	3.85745E-06	negative
PRJNA729271	stage_1D_vs_E10	ordinary_muscle_development	ENSGALG00010021361	WRAP73	-0.377011735	0.089078032	-4.232376131	2.31235E-05	4.55621E-05	negative
PRJNA729271	stage_1D_vs_E10	ordinary_muscle_development	ENSGALG00010021362	ARPC1B	0.112593283	0.121074843	0.929947791	0.352398117	0.401257714	positive
PRJNA729271	stage_1D_vs_E10	ordinary_muscle_development	ENSGALG00010021363	ENSGALG00010021363	-0.253202274	0.074194199	-3.412696368	0.000643236	0.001093689	negative
PRJNA729271	stage_1D_vs_E10	ordinary_muscle_development	ENSGALG00010021367	GRIP2	1.256181408	0.176317469	7.124543102	1.04427E-12	3.60482E-12	positive
PRJNA729271	stage_1D_vs_E10	ordinary_muscle_development	ENSGALG00010021372	CD86	1.053024282	0.646042543	1.629961206	0.103109696	0.130839101	positive
PRJNA729271	stage_1D_vs_E10	ordinary_muscle_development	ENSGALG00010021373	CEP250	-0.197471841	0.152583467	-1.294188976	0.195600154	0.235540215	negative
PRJNA729271	stage_1D_vs_E10	ordinary_muscle_development	ENSGALG00010021375	TUBG1	-1.146476119	0.118067979	-9.71030526	2.7252E-22	1.64454E-21	negative
PRJNA729271	stage_1D_vs_E10	ordinary_muscle_development	ENSGALG00010021378	KIF11	-1.680597954	0.185643245	-9.052836532	1.39302E-19	7.27108E-19	negative
PRJNA729271	stage_1D_vs_E10	ordinary_muscle_development	ENSGALG00010021381	DNAJB2	-0.0236804	0.14810298	-0.159891445	0.872966587	0.891667302	negative
PRJNA729271	stage_1D_vs_E10	ordinary_muscle_development	ENSGALG00010021382	TPRG1L	-0.059666649	0.086931805	-0.686361565	0.492485135	0.540800912	negative
PRJNA729271	stage_1D_vs_E10	ordinary_muscle_development	ENSGALG00010021383	MSI1	-1.811519442	0.121257598	-14.93943033	1.82529E-50	3.5899E-49	negative
PRJNA729271	stage_1D_vs_E10	ordinary_muscle_development	ENSGALG00010021384	ARTN	2.981497151	0.346415808	8.606700634	7.51935E-18	3.57471E-17	positive
PRJNA729271	stage_1D_vs_E10	ordinary_muscle_development	ENSGALG00010021385	GDF5	-5.486998533	0.416767432	-13.16561256	1.3841E-39	1.78652E-38	negative
PRJNA729271	stage_1D_vs_E10	ordinary_muscle_development	ENSGALG00010021387	SLC2A14	0.43142277	0.094398351	4.570236301	4.87175E-06	1.02372E-05	positive
PRJNA729271	stage_1D_vs_E10	ordinary_muscle_development	ENSGALG00010021388	DPH2	-0.788764866	0.148734393	-5.303177363	1.13804E-07	2.74019E-07	negative
PRJNA729271	stage_1D_vs_E10	ordinary_muscle_development	ENSGALG00010021390	GSTO2	-1.494292555	0.216065439	-6.915925856	4.64817E-12	1.53579E-11	negative
PRJNA729271	stage_1D_vs_E10	ordinary_muscle_development	ENSGALG00010021391	ENSGALG00010021391	-0.23100968	0.385940507	-0.59856293	0.549464382	0.595431087	negative
PRJNA729271	stage_1D_vs_E10	ordinary_muscle_development	ENSGALG00010021393	CCDC174	-0.075789889	0.11340818	-0.668292965	0.503946603	0.552048519	negative
PRJNA729271	stage_1D_vs_E10	ordinary_muscle_development	ENSGALG00010021394	MAN2C1	-0.122968616	0.096462564	-1.274780705	0.202386872	0.242847492	negative
PRJNA729271	stage_1D_vs_E10	ordinary_muscle_development	ENSGALG00010021396	MED6	-0.37144872	0.079312051	-4.683383105	2.82178E-06	6.06941E-06	negative
PRJNA729271	stage_1D_vs_E10	ordinary_muscle_development	ENSGALG00010021397	ENSGALG00010021397	2.106540433	1.820777323	1.156945666	0.24729456	0.291449276	positive
PRJNA729271	stage_1D_vs_E10	ordinary_muscle_development	ENSGALG00010021400	B4GALT2	0.530142952	0.091679074	5.782594971	7.3557E-09	1.94564E-08	positive
PRJNA729271	stage_1D_vs_E10	ordinary_muscle_development	ENSGALG00010021401	PDAP1	-0.424164535	0.094548919	-4.486191274	7.25077E-06	1.50121E-05	negative
PRJNA729271	stage_1D_vs_E10	ordinary_muscle_development	ENSGALG00010021402	ENSGALG00010021402	-0.052614481	0.088319217	-0.595730834	0.551355056	0.597301311	negative
PRJNA729271	stage_1D_vs_E10	ordinary_muscle_development	ENSGALG00010021404	UQCC1	0.108649574	0.093778387	1.158577975	0.246628251	0.290711271	positive
PRJNA729271	stage_1D_vs_E10	ordinary_muscle_development	ENSGALG00010021405	IDE	0.745722916	0.114025901	6.539943218	6.15422E-11	1.88954E-10	positive
PRJNA729271	stage_1D_vs_E10	ordinary_muscle_development	ENSGALG00010021407	SIN3A	0.276810269	0.110609421	2.502592156	0.012328752	0.018011012	positive
PRJNA729271	stage_1D_vs_E10	ordinary_muscle_development	ENSGALG00010021408	TTC9	0.60940103	0.206216985	2.955144695	0.003125224	0.004916445	positive
PRJNA729271	stage_1D_vs_E10	ordinary_muscle_development	ENSGALG00010021409	MEGF6	-1.362381532	0.248047158	-5.492429523	3.96442E-08	9.86003E-08	negative
PRJNA729271	stage_1D_vs_E10	ordinary_muscle_development	ENSGALG00010021411	MCU	1.328392263	0.128858736	10.30890345	6.423E-25	4.38743E-24	positive
PRJNA729271	stage_1D_vs_E10	ordinary_muscle_development	ENSGALG00010021412	ZNF319	-0.078702012	0.124486822	-0.632211593	0.527248623	0.574146855	negative
PRJNA729271	stage_1D_vs_E10	ordinary_muscle_development	ENSGALG00010021413	SYNJ2BP	0.857308076	0.151678736	5.652130948	1.58471E-08	4.07203E-08	positive
PRJNA729271	stage_1D_vs_E10	ordinary_muscle_development	ENSGALG00010021414	BUD31	-0.101443047	0.11155138	-0.909384064	0.36314743	0.412105379	negative
PRJNA729271	stage_1D_vs_E10	ordinary_muscle_development	ENSGALG00010021415	SIRT4	0.544367052	0.149154894	3.649676113	0.000262571	0.000465519	positive
PRJNA729271	stage_1D_vs_E10	ordinary_muscle_development	ENSGALG00010021416	PLEKHH3	0.637253536	0.123360863	5.165767491	2.39454E-07	5.60816E-07	positive
PRJNA729271	stage_1D_vs_E10	ordinary_muscle_development	ENSGALG00010021417	STK16	-1.658083637	0.166012095	-9.987727909	1.7249E-23	1.09708E-22	negative
PRJNA729271	stage_1D_vs_E10	ordinary_muscle_development	ENSGALG00010021419	MANSC1	-1.595577286	0.20144341	-7.920722197	2.36135E-15	9.69076E-15	negative
PRJNA729271	stage_1D_vs_E10	ordinary_muscle_development	ENSGALG00010021420	PLXNB3	-1.284178084	0.189873583	-6.763332016	1.34854E-11	4.33512E-11	negative
PRJNA729271	stage_1D_vs_E10	ordinary_muscle_development	ENSGALG00010021421	GLB1L	0.008358051	0.165649336	0.050456291	0.959758779	0.965922379	positive
PRJNA729271	stage_1D_vs_E10	ordinary_muscle_development	ENSGALG00010021424	PTCD1	-0.126684726	0.107956699	-1.173477207	0.240604492	0.284373949	negative
PRJNA729271	stage_1D_vs_E10	ordinary_muscle_development	ENSGALG00010021425	PTPN9	-1.241725649	0.124892735	-9.942336923	2.72361E-23	1.71722E-22	negative
PRJNA729271	stage_1D_vs_E10	ordinary_muscle_development	ENSGALG00010021426	SLC38A7	-0.500757609	0.145801366	-3.434519327	0.000593606	0.00101263	negative
PRJNA729271	stage_1D_vs_E10	ordinary_muscle_development	ENSGALG00010021427	RPLP0	-0.165764954	0.135415861	-1.22411771	0.220907826	0.263392197	negative
PRJNA729271	stage_1D_vs_E10	ordinary_muscle_development	ENSGALG00010021429	MED8	0.427379559	0.114763769	3.723993766	0.000196096	0.000352481	positive
PRJNA729271	stage_1D_vs_E10	ordinary_muscle_development	ENSGALG00010021431	CPSF4	-0.742773889	0.107097617	-6.93548477	4.04831E-12	1.34341E-11	negative
PRJNA729271	stage_1D_vs_E10	ordinary_muscle_development	ENSGALG00010021432	SYT9	-0.108927997	0.29244952	-0.37246769	0.709544664	0.745441031	negative
PRJNA729271	stage_1D_vs_E10	ordinary_muscle_development	ENSGALG00010021433	ENSGALG00010021433	4.407439827	0.270937145	16.26738864	1.68179E-59	4.56507E-58	positive
PRJNA729271	stage_1D_vs_E10	ordinary_muscle_development	ENSGALG00010021434	ANKZF1	0.238559652	0.177269098	1.345748666	0.178383586	0.216682316	positive
PRJNA729271	stage_1D_vs_E10	ordinary_muscle_development	ENSGALG00010021435	GCN1	0.270422015	0.088996114	3.038582272	0.002376942	0.00378821	positive
PRJNA729271	stage_1D_vs_E10	ordinary_muscle_development	ENSGALG00010021438	GOT2	0.959140095	0.110099789	8.711552496	2.99741E-18	1.4565E-17	positive
PRJNA729271	stage_1D_vs_E10	ordinary_muscle_development	ENSGALG00010021439	ITPRIP	0.057241735	0.186269149	0.307306577	0.75861003	0.789725628	positive
PRJNA729271	stage_1D_vs_E10	ordinary_muscle_development	ENSGALG00010021441	ENSGALG00010021441	-0.707177369	0.327978615	-2.156169143	0.03107046	0.042749532	negative
PRJNA729271	stage_1D_vs_E10	ordinary_muscle_development	ENSGALG00010021443	SNUPN	-0.511613148	0.136955453	-3.735617218	0.000187255	0.000337595	negative
PRJNA729271	stage_1D_vs_E10	ordinary_muscle_development	ENSGALG00010021444	ATG9A	1.362559167	0.246184279	5.534712338	3.1174E-08	7.80965E-08	positive
PRJNA729271	stage_1D_vs_E10	ordinary_muscle_development	ENSGALG00010021446	HOXB8	-1.819289508	0.630542533	-2.885276429	0.003910701	0.006087373	negative
PRJNA729271	stage_1D_vs_E10	ordinary_muscle_development	ENSGALG00010021447	OCSTAMP	-0.109909437	0.705350943	-0.155822343	0.876173058	0.894249295	negative
PRJNA729271	stage_1D_vs_E10	ordinary_muscle_development	ENSGALG00010021448	SZT2	0.400608913	0.100038385	4.004551966	6.21351E-05	0.000117778	positive
PRJNA729271	stage_1D_vs_E10	ordinary_muscle_development	ENSGALG00010021450	OLFML1	2.784851479	0.232114112	11.99776893	3.65003E-33	3.67156E-32	positive
PRJNA729271	stage_1D_vs_E10	ordinary_muscle_development	ENSGALG00010021452	SLC25A1	-0.691002706	0.116546532	-5.928985582	3.04812E-09	8.29718E-09	negative
PRJNA729271	stage_1D_vs_E10	ordinary_muscle_development	ENSGALG00010021454	ATP5J2	1.345781592	0.174127958	7.728693366	1.08656E-14	4.26821E-14	positive
PRJNA729271	stage_1D_vs_E10	ordinary_muscle_development	ENSGALG00010021455	CFAP58	-0.891567312	0.528764932	-1.686131697	0.091770436	0.117478802	negative
PRJNA729271	stage_1D_vs_E10	ordinary_muscle_development	ENSGALG00010021457	USB1	0.712781361	0.112621727	6.328986273	2.46777E-10	7.28372E-10	positive
PRJNA729271	stage_1D_vs_E10	ordinary_muscle_development	ENSGALG00010021458	USP30	-0.010587106	0.080404067	-0.131673767	0.895242336	0.910762048	negative
PRJNA729271	stage_1D_vs_E10	ordinary_muscle_development	ENSGALG00010021459	ARHGEF16	0.225697477	0.123379668	1.829292302	0.067355831	0.088020445	positive
PRJNA729271	stage_1D_vs_E10	ordinary_muscle_development	ENSGALG00010021461	SNX33	0.411154431	0.109635358	3.750199193	0.000176694	0.000319908	positive
PRJNA729271	stage_1D_vs_E10	ordinary_muscle_development	ENSGALG00010021462	SETD5	-0.062235545	0.094286231	-0.660070345	0.509208688	0.557139186	negative
PRJNA729271	stage_1D_vs_E10	ordinary_muscle_development	ENSGALG00010021464	ENSGALG00010021464	1.235535067	0.413046553	2.991273161	0.002778169	0.004396239	positive
PRJNA729271	stage_1D_vs_E10	ordinary_muscle_development	ENSGALG00010021465	ABCB6	1.541047052	0.211661545	7.280713422	3.32059E-13	1.18874E-12	positive
PRJNA729271	stage_1D_vs_E10	ordinary_muscle_development	ENSGALG00010021466	HES4	-0.961809783	0.181399788	-5.30215494	1.14444E-07	2.75512E-07	negative
PRJNA729271	stage_1D_vs_E10	ordinary_muscle_development	ENSGALG00010021467	LHFPL4	-0.144332282	0.264506648	-0.54566599	0.585295555	0.629646658	negative
PRJNA729271	stage_1D_vs_E10	ordinary_muscle_development	ENSGALG00010021469	CSPG4	0.118344511	0.161620223	0.732238259	0.464023156	0.512809976	positive
PRJNA729271	stage_1D_vs_E10	ordinary_muscle_development	ENSGALG00010021470	ENSGALG00010021470	-0.722303076	0.566580506	-1.274846326	0.20236364	0.242839719	negative
PRJNA729271	stage_1D_vs_E10	ordinary_muscle_development	ENSGALG00010021473	SLC8A3	1.450067324	0.131326955	11.04165798	2.40553E-28	1.91583E-27	positive
PRJNA729271	stage_1D_vs_E10	ordinary_muscle_development	ENSGALG00010021474	PPFIBP2	1.294354523	0.180492271	7.171246256	7.43181E-13	2.59701E-12	positive
PRJNA729271	stage_1D_vs_E10	ordinary_muscle_development	ENSGALG00010021476	ALKBH2	-0.18884802	0.121674126	-1.552080357	0.120642998	0.15135722	negative
PRJNA729271	stage_1D_vs_E10	ordinary_muscle_development	ENSGALG00010021477	ZFAND2B	0.258203309	0.118610166	2.176907071	0.029487502	0.040687419	positive
PRJNA729271	stage_1D_vs_E10	ordinary_muscle_development	ENSGALG00010021478	SLC13A3	-0.816006958	0.196984958	-4.142483598	3.43565E-05	6.66128E-05	negative
PRJNA729271	stage_1D_vs_E10	ordinary_muscle_development	ENSGALG00010021479	SKAP1	-2.154212442	0.646257821	-3.333363826	0.000858027	0.00144098	negative
PRJNA729271	stage_1D_vs_E10	ordinary_muscle_development	ENSGALG00010021480	ENSGALG00010021480	-0.230945389	0.075260787	-3.068601845	0.00215063	0.003446096	negative
PRJNA729271	stage_1D_vs_E10	ordinary_muscle_development	ENSGALG00010021481	MICU1	0.848826573	0.174190101	4.872989723	1.09922E-06	2.44749E-06	positive
PRJNA729271	stage_1D_vs_E10	ordinary_muscle_development	ENSGALG00010021483	UNG	-1.093213508	0.154678091	-7.067668756	1.57558E-12	5.37366E-12	negative
PRJNA729271	stage_1D_vs_E10	ordinary_muscle_development	ENSGALG00010021484	MARCHF5	0.343045169	0.0711078	4.824297314	1.40498E-06	3.10113E-06	positive
PRJNA729271	stage_1D_vs_E10	ordinary_muscle_development	ENSGALG00010021485	SMOC1	-3.87139658	0.234456164	-16.51224053	2.99552E-61	8.58105E-60	negative
PRJNA729271	stage_1D_vs_E10	ordinary_muscle_development	ENSGALG00010021487	KLHL17	-1.710786115	0.149289804	-11.45949739	2.10734E-30	1.86369E-29	negative
PRJNA729271	stage_1D_vs_E10	ordinary_muscle_development	ENSGALG00010021488	HYI	-0.930485803	0.137076762	-6.788063771	1.13648E-11	3.66481E-11	negative
PRJNA729271	stage_1D_vs_E10	ordinary_muscle_development	ENSGALG00010021489	CNPPD1	0.360907041	0.08596885	4.198114118	2.69147E-05	5.26703E-05	positive
PRJNA729271	stage_1D_vs_E10	ordinary_muscle_development	ENSGALG00010021490	ACACB	7.393561362	0.328535982	22.50457108	3.7442E-112	3.5472E-110	positive
PRJNA729271	stage_1D_vs_E10	ordinary_muscle_development	ENSGALG00010021492	PARP6	0.376853329	0.156212061	2.412447061	0.015845838	0.022747888	positive
PRJNA729271	stage_1D_vs_E10	ordinary_muscle_development	ENSGALG00010021493	UNC93B1	0.203061224	0.132713441	1.53007278	0.125998715	0.157466643	positive
PRJNA729271	stage_1D_vs_E10	ordinary_muscle_development	ENSGALG00010021494	SLC2A10	-1.15998659	0.240688023	-4.819461212	1.43946E-06	3.17387E-06	negative
PRJNA729271	stage_1D_vs_E10	ordinary_muscle_development	ENSGALG00010021496	MTMR14	0.337355276	0.115805092	2.913129889	0.003578257	0.005593652	positive
PRJNA729271	stage_1D_vs_E10	ordinary_muscle_development	ENSGALG00010021498	B3GALT6	-1.036640825	0.107745771	-9.621174171	6.50839E-22	3.87271E-21	negative
PRJNA729271	stage_1D_vs_E10	ordinary_muscle_development	ENSGALG00010021501	SLC23A3	1.921038627	0.598923893	3.207483703	0.001339016	0.00220257	positive
PRJNA729271	stage_1D_vs_E10	ordinary_muscle_development	ENSGALG00010021502	DNAJB12	0.524479442	0.146094466	3.590002105	0.000330675	0.000580564	positive
PRJNA729271	stage_1D_vs_E10	ordinary_muscle_development	ENSGALG00010021503	KCNS1	-2.330524779	0.584463789	-3.987457946	6.6785E-05	0.000126163	negative
PRJNA729271	stage_1D_vs_E10	ordinary_muscle_development	ENSGALG00010021504	ODR4	-0.504555293	0.076999742	-6.552688071	5.65105E-11	1.7412E-10	negative
PRJNA729271	stage_1D_vs_E10	ordinary_muscle_development	ENSGALG00010021505	IHH	-4.451137766	0.550384545	-8.087323321	6.09902E-16	2.60322E-15	negative
PRJNA729271	stage_1D_vs_E10	ordinary_muscle_development	ENSGALG00010021506	MAX	0.631175561	0.249062519	2.53420532	0.011270267	0.016556453	positive
PRJNA729271	stage_1D_vs_E10	ordinary_muscle_development	ENSGALG00010021509	DDIT4	2.157868077	0.2777847	7.768131497	7.96523E-15	3.15368E-14	positive
PRJNA729271	stage_1D_vs_E10	ordinary_muscle_development	ENSGALG00010021510	NTSR1	0.86510283	0.391875036	2.207598724	0.027272258	0.037807133	positive
PRJNA729271	stage_1D_vs_E10	ordinary_muscle_development	ENSGALG00010021512	CPNE9	2.206950991	0.400813109	5.506184655	3.66694E-08	9.14684E-08	positive
PRJNA729271	stage_1D_vs_E10	ordinary_muscle_development	ENSGALG00010021513	SDF4	0.146230671	0.096978797	1.507862294	0.131589775	0.163868898	positive
PRJNA729271	stage_1D_vs_E10	ordinary_muscle_development	ENSGALG00010021514	TPR	-0.252555748	0.079115348	-3.192247218	0.001411704	0.00231478	negative
PRJNA729271	stage_1D_vs_E10	ordinary_muscle_development	ENSGALG00010021516	SRSF5	-0.51589358	0.151960057	-3.394928831	0.000686464	0.001164323	negative
PRJNA729271	stage_1D_vs_E10	ordinary_muscle_development	ENSGALG00010021518	CPEB3	2.742647247	0.145609023	18.83569568	3.85034E-79	1.80035E-77	positive
PRJNA729271	stage_1D_vs_E10	ordinary_muscle_development	ENSGALG00010021519	FNTB	0.260533836	0.099684402	2.613586801	0.008959732	0.013325602	positive
PRJNA729271	stage_1D_vs_E10	ordinary_muscle_development	ENSGALG00010021521	ENSGALG00010021521	0.858276873	0.263807645	3.253419262	0.001140251	0.001889988	positive
PRJNA729271	stage_1D_vs_E10	ordinary_muscle_development	ENSGALG00010021523	CELF6	-0.987712468	0.153392027	-6.439138247	1.20154E-10	3.62011E-10	negative
PRJNA729271	stage_1D_vs_E10	ordinary_muscle_development	ENSGALG00010021525	ENSGALG00010021525	0.479711181	0.766803018	0.625598973	0.531578012	0.578470217	positive
PRJNA729271	stage_1D_vs_E10	ordinary_muscle_development	ENSGALG00010021526	KCNJ12	1.312932253	0.216303434	6.069863204	1.28019E-09	3.58647E-09	positive

PRJNA729271	stage_1D_vs_E10	ordinary_muscle_development	ENSGALG00010021528	BRPF1	0.351176024	0.166384491	2.110629553	0.034804165	0.047579589	positive
PRJNA729271	stage_1D_vs_E10	ordinary_muscle_development	ENSGALG00010021529	BTAF1	0.620727365	0.075069233	8.268731896	1.35391E-16	5.99785E-16	positive
PRJNA729271	stage_1D_vs_E10	ordinary_muscle_development	ENSGALG00010021530	MLF2	-0.614959649	0.060795542	-10.11520964	4.73003E-24	3.08282E-23	negative
PRJNA729271	stage_1D_vs_E10	ordinary_muscle_development	ENSGALG00010021531	RAB15	-2.01651155	0.299889789	-6.724175426	1.7659E-11	5.62193E-11	negative
PRJNA729271	stage_1D_vs_E10	ordinary_muscle_development	ENSGALG00010021532	DPP10	-0.267453996	0.181501514	-1.473563446	0.140599169	0.174201638	negative
PRJNA729271	stage_1D_vs_E10	ordinary_muscle_development	ENSGALG00010021533	SSX2IP	-3.488331505	0.317896091	-10.9731815	5.14303E-28	4.02528E-27	negative
PRJNA729271	stage_1D_vs_E10	ordinary_muscle_development	ENSGALG00010021534	KIFC3	1.566516977	0.169041469	9.267057268	1.91352E-20	1.04706E-19	positive
PRJNA729271	stage_1D_vs_E10	ordinary_muscle_development	ENSGALG00010021535	SUSD6	0.24678108	0.085744504	2.878097937	0.004000809	0.006217619	positive
PRJNA729271	stage_1D_vs_E10	ordinary_muscle_development	ENSGALG00010021538	TNFRSF18	0.727093135	0.433772387	1.676208899	0.093697282	0.119723387	positive
PRJNA729271	stage_1D_vs_E10	ordinary_muscle_development	ENSGALG00010021539	HEXA	-0.708852792	0.120298139	-5.892466817	3.80473E-09	1.02852E-08	negative
PRJNA729271	stage_1D_vs_E10	ordinary_muscle_development	ENSGALG00010021540	STK4	-1.003259106	0.077959602	-12.86896141	6.7301E-38	8.18396E-37	negative
PRJNA729271	stage_1D_vs_E10	ordinary_muscle_development	ENSGALG00010021541	CYP27A1	2.416914707	0.27631448	8.746970879	2.19156E-18	1.07284E-17	positive
PRJNA729271	stage_1D_vs_E10	ordinary_muscle_development	ENSGALG00010021543	PLEKHD1	0.893125789	0.258690652	3.452485745	0.000555447	0.000951454	positive
PRJNA729271	stage_1D_vs_E10	ordinary_muscle_development	ENSGALG00010021545	CYB5R2	-1.29738372	0.138999583	-9.333723818	1.02215E-20	5.68974E-20	negative
PRJNA729271	stage_1D_vs_E10	ordinary_muscle_development	ENSGALG00010021546	OGG1	-0.659517258	0.172802372	-3.816598416	0.000135304	0.000247818	negative
PRJNA729271	stage_1D_vs_E10	ordinary_muscle_development	ENSGALG00010021549	ANAPC16	-0.324454624	0.091761853	-3.535833388	0.000406491	0.000706909	negative
PRJNA729271	stage_1D_vs_E10	ordinary_muscle_development	ENSGALG00010021550	MAP2K3	0.728806057	0.17224222	4.231285788	2.32359E-05	4.57615E-05	positive
PRJNA729271	stage_1D_vs_E10	ordinary_muscle_development	ENSGALG00010021552	ENSGALG00010021552	1.958677053	0.1708699	11.46297302	2.02444E-30	1.79147E-29	positive
PRJNA729271	stage_1D_vs_E10	ordinary_muscle_development	ENSGALG00010021553	SLC39A9	-0.392285138	0.086613133	-4.52916461	5.92174E-06	1.2354E-05	negative
PRJNA729271	stage_1D_vs_E10	ordinary_muscle_development	ENSGALG00010021554	SNX11	0.272369721	0.147738783	1.843589854	0.065242936	0.085467361	positive
PRJNA729271	stage_1D_vs_E10	ordinary_muscle_development	ENSGALG00010021556	CAMK1	-0.09917174	0.142232142	-0.697252668	0.485644684	0.53423839	negative
PRJNA729271	stage_1D_vs_E10	ordinary_muscle_development	ENSGALG00010021558	CHURC1	0.690086728	0.179801254	3.838052912	0.000124014	0.000227946	positive
PRJNA729271	stage_1D_vs_E10	ordinary_muscle_development	ENSGALG00010021559	MYO1H	-1.053932971	0.6390695	-1.649168004	0.099113221	0.12609902	negative
PRJNA729271	stage_1D_vs_E10	ordinary_muscle_development	ENSGALG00010021561	TGIF2	-1.290212873	0.097957824	-13.17110594	1.28699E-39	1.6671E-38	negative
PRJNA729271	stage_1D_vs_E10	ordinary_muscle_development	ENSGALG00010021562	LRP6	0.529939556	0.081663572	6.489301703	8.62351E-11	2.62215E-10	positive
PRJNA729271	stage_1D_vs_E10	ordinary_muscle_development	ENSGALG00010021564	PRKAG3	7.227518563	0.632910773	11.4194905	3.34187E-30	2.92869E-29	positive
PRJNA729271	stage_1D_vs_E10	ordinary_muscle_development	ENSGALG00010021565	CBX1	-1.381483251	0.104908861	-13.16841345	1.33371E-39	1.72608E-38	negative
PRJNA729271	stage_1D_vs_E10	ordinary_muscle_development	ENSGALG00010021566	ENSGALG00010021566	0.306840951	0.094754291	3.238280278	0.001202526	0.001988434	positive
PRJNA729271	stage_1D_vs_E10	ordinary_muscle_development	ENSGALG00010021568	TADA3	0.204399618	0.14223176	1.43708844	0.150692865	0.185739208	positive
PRJNA729271	stage_1D_vs_E10	ordinary_muscle_development	ENSGALG00010021570	KATNB1	-0.574914993	0.100712377	-5.708484024	1.13987E-08	2.95994E-08	negative
PRJNA729271	stage_1D_vs_E10	ordinary_muscle_development	ENSGALG00010021572	MYL9	-0.45148694	0.113190714	-3.988727739	6.64286E-05	0.000125539	negative
PRJNA729271	stage_1D_vs_E10	ordinary_muscle_development	ENSGALG00010021573	NATD1	1.012326574	0.195413966	5.180420805	2.21386E-07	5.19927E-07	positive
PRJNA729271	stage_1D_vs_E10	ordinary_muscle_development	ENSGALG00010021574	TTLL4	0.972156981	0.226462669	4.292791329	1.76441E-05	3.51162E-05	positive
PRJNA729271	stage_1D_vs_E10	ordinary_muscle_development	ENSGALG00010021575	ADGRL2	-0.040838093	0.082615814	-0.494313269	0.621084955	0.663225757	negative
PRJNA729271	stage_1D_vs_E10	ordinary_muscle_development	ENSGALG00010021576	ERH	-1.163298149	0.122153353	-9.523260101	1.6783E-21	9.75026E-21	negative
PRJNA729271	stage_1D_vs_E10	ordinary_muscle_development	ENSGALG00010021577	ARIH1	0.009738521	0.076875407	0.126679277	0.899194248	0.914204996	positive
PRJNA729271	stage_1D_vs_E10	ordinary_muscle_development	ENSGALG00010021578	ENSGALG00010021578	2.489389604	1.045199958	2.381735271	0.01723128	0.02461005	positive
PRJNA729271	stage_1D_vs_E10	ordinary_muscle_development	ENSGALG00010021579	C20H20ORF24	-0.336248582	0.112257534	-2.995331975	0.002741463	0.004341948	negative
PRJNA729271	stage_1D_vs_E10	ordinary_muscle_development	ENSGALG00010021580	KCTD10	-1.123996187	0.109786716	-10.23799808	1.3398E-24	8.99925E-24	negative
PRJNA729271	stage_1D_vs_E10	ordinary_muscle_development	ENSGALG00010021582	STK36	0.090179693	0.141303417	0.638198954	0.523344178	0.570538046	positive
PRJNA729271	stage_1D_vs_E10	ordinary_muscle_development	ENSGALG00010021583	NFE2L1	1.353267334	0.220907182	6.125954455	9.01416E-10	2.55795E-09	positive
PRJNA729271	stage_1D_vs_E10	ordinary_muscle_development	ENSGALG00010021584	OVCH2	-0.19126792	0.38363692	-0.498564945	0.618085906	0.660315095	negative
PRJNA729271	stage_1D_vs_E10	ordinary_muscle_development	ENSGALG00010021586	BBS4	-0.429517565	0.123190156	-3.486622463	0.000489161	0.000843191	negative
PRJNA729271	stage_1D_vs_E10	ordinary_muscle_development	ENSGALG00010021588	RNF25	0.254319265	0.138583907	1.835128411	0.066486665	0.086955176	positive
PRJNA729271	stage_1D_vs_E10	ordinary_muscle_development	ENSGALG00010021593	DCAF7	-0.565211334	0.055117654	-10.25463336	1.12803E-24	7.59797E-24	negative
PRJNA729271	stage_1D_vs_E10	ordinary_muscle_development	ENSGALG00010021594	ADPGK	-1.24321038	0.090615618	-13.71960379	7.74877E-43	1.12318E-41	negative
PRJNA729271	stage_1D_vs_E10	ordinary_muscle_development	ENSGALG00010021595	UBE3B	0.002921863	0.084377173	0.034628596	0.972375899	0.976485289	positive
PRJNA729271	stage_1D_vs_E10	ordinary_muscle_development	ENSGALG00010021597	GGT7	-0.710526197	0.187142451	-3.796713103	0.000146627	0.000267745	negative
PRJNA729271	stage_1D_vs_E10	ordinary_muscle_development	ENSGALG00010021599	ARPC4	-0.640328429	0.103754946	-6.171546048	6.76254E-10	1.93263E-09	negative
PRJNA729271	stage_1D_vs_E10	ordinary_muscle_development	ENSGALG00010021600	FMN1	-0.253251368	0.130883114	-1.93494302	0.052997288	0.070373369	negative
PRJNA729271	stage_1D_vs_E10	ordinary_muscle_development	ENSGALG00010021601	LPAR5	-0.673548121	0.403233363	-1.670368035	0.09484657	0.121042528	negative
PRJNA729271	stage_1D_vs_E10	ordinary_muscle_development	ENSGALG00010021602	PLEKHG4	0.815469351	0.097328858	8.378494997	5.36088E-17	2.42907E-16	positive
PRJNA729271	stage_1D_vs_E10	ordinary_muscle_development	ENSGALG00010021604	SPTB	2.452013577	0.189275477	12.95473467	2.20899E-38	2.72736E-37	positive
PRJNA729271	stage_1D_vs_E10	ordinary_muscle_development	ENSGALG00010021605	MAP3K3	0.285208732	0.111564051	2.5564573	0.010574405	0.015575246	positive
PRJNA729271	stage_1D_vs_E10	ordinary_muscle_development	ENSGALG00010021606	RNF8	-0.194796387	0.094133052	-2.069372912	0.038511106	0.052233413	negative
PRJNA729271	stage_1D_vs_E10	ordinary_muscle_development	ENSGALG00010021607	SLC9A5	-0.015138584	0.196207288	-0.077156075	0.938499385	0.947652287	negative
PRJNA729271	stage_1D_vs_E10	ordinary_muscle_development	ENSGALG00010021609	ENSGALG00010021609	1.924191192	0.203283056	9.465575902	2.91948E-21	1.6733E-20	positive
PRJNA729271	stage_1D_vs_E10	ordinary_muscle_development	ENSGALG00010021610	TMEM11	0.109421138	0.089539584	1.222042069	0.221691719	0.264239903	positive
PRJNA729271	stage_1D_vs_E10	ordinary_muscle_development	ENSGALG00010021611	GREM1	-1.048167057	0.600842448	-1.744495683	0.081072694	0.104690307	negative
PRJNA729271	stage_1D_vs_E10	ordinary_muscle_development	ENSGALG00010021612	BCS1L	0.005622265	0.131937113	0.042613221	0.966009857	0.970966845	positive
PRJNA729271	stage_1D_vs_E10	ordinary_muscle_development	ENSGALG00010021613	ADGRL4	2.671945889	0.149400481	17.88445311	1.55875E-71	6.02509E-70	positive
PRJNA729271	stage_1D_vs_E10	ordinary_muscle_development	ENSGALG00010021615	FLVCR1	-1.544460579	0.177805438	-8.686239294	3.74635E-18	1.81011E-17	negative
PRJNA729271	stage_1D_vs_E10	ordinary_muscle_development	ENSGALG00010021617	SORBS1	1.313172339	0.11088659	11.84248098	2.35385E-32	2.27612E-31	positive
PRJNA729271	stage_1D_vs_E10	ordinary_muscle_development	ENSGALG00010021618	NEO1	-0.845173983	0.106293987	-7.951286898	1.84584E-15	7.62915E-15	negative
PRJNA729271	stage_1D_vs_E10	ordinary_muscle_development	ENSGALG00010021619	FHOD1	3.615463499	0.178789311	20.22192201	6.27886E-91	3.79217E-89	positive
PRJNA729271	stage_1D_vs_E10	ordinary_muscle_development	ENSGALG00010021621	ZNF518A	0.177512398	0.136842513	1.297202118	0.194561652	0.234505416	positive
PRJNA729271	stage_1D_vs_E10	ordinary_muscle_development	ENSGALG00010021622	PTHLH	-2.79534043	0.268534819	-10.4096014	2.24158E-25	1.56662E-24	negative
PRJNA729271	stage_1D_vs_E10	ordinary_muscle_development	ENSGALG00010021623	FAR1	0.064105298	0.129920914	0.493417851	0.621717372	0.663754386	positive
PRJNA729271	stage_1D_vs_E10	ordinary_muscle_development	ENSGALG00010021624	HSD11B2	2.048366175	0.35257816	5.809679697	6.25925E-09	1.66504E-08	positive
PRJNA729271	stage_1D_vs_E10	ordinary_muscle_development	ENSGALG00010021625	HCN4	-1.006444798	0.74229752	-1.355850951	0.175146596	0.213125413	negative
PRJNA729271	stage_1D_vs_E10	ordinary_muscle_development	ENSGALG00010021626	ZNF142	0.495296539	0.213138822	2.323821318	0.020135073	0.028471162	positive
PRJNA729271	stage_1D_vs_E10	ordinary_muscle_development	ENSGALG00010021627	NCOA6	0.798800511	0.10302	7.75383917	8.91552E-15	3.52127E-14	positive
PRJNA729271	stage_1D_vs_E10	ordinary_muscle_development	ENSGALG00010021628	REC114	-0.503120079	0.552249934	-0.91103692	0.362275921	0.411309585	negative
PRJNA729271	stage_1D_vs_E10	ordinary_muscle_development	ENSGALG00010021629	DHRS7B	1.50593038	0.100834518	14.9346713	1.96038E-50	3.84516E-49	positive
PRJNA729271	stage_1D_vs_E10	ordinary_muscle_development	ENSGALG00010021630	VTG1	-1.447863891	0.280371562	-5.164089689	2.41612E-07	5.65686E-07	negative
PRJNA729271	stage_1D_vs_E10	ordinary_muscle_development	ENSGALG00010021632	LIMD2	-4.272513944	0.170617588	-25.04146252	2.1628E-138	3.3E-136	negative
PRJNA729271	stage_1D_vs_E10	ordinary_muscle_development	ENSGALG00010021633	CCNJ	-0.819738688	0.10427097	-7.861619439	3.79199E-15	1.53705E-14	negative
PRJNA729271	stage_1D_vs_E10	ordinary_muscle_development	ENSGALG00010021634	PLCD4	3.801756769	0.243415089	15.61841046	5.45486E-55	1.25505E-53	positive
PRJNA729271	stage_1D_vs_E10	ordinary_muscle_development	ENSGALG00010021635	FOXJ2	0.571321417	0.113705481	5.024572374	5.04556E-07	1.15301E-06	positive
PRJNA729271	stage_1D_vs_E10	ordinary_muscle_development	ENSGALG00010021636	C1orf159	0.412529709	0.192703825	2.140744784	0.032294625	0.044353855	positive
PRJNA729271	stage_1D_vs_E10	ordinary_muscle_development	ENSGALG00010021637	ATP6V0D1	-0.017075047	0.103221595	-0.16542127	0.868612413	0.888095996	negative
PRJNA729271	stage_1D_vs_E10	ordinary_muscle_development	ENSGALG00010021638	SRC	-1.338524359	0.176753674	-7.572823389	3.65199E-14	1.39048E-13	negative
PRJNA729271	stage_1D_vs_E10	ordinary_muscle_development	ENSGALG00010021639	SCG5	-2.928773325	0.37688087	-7.771085128	7.78166E-15	3.08521E-14	negative
PRJNA729271	stage_1D_vs_E10	ordinary_muscle_development	ENSGALG00010021640	PLEKHG3	0.140584024	0.105585538	1.331470453	0.183034261	0.221922343	positive
PRJNA729271	stage_1D_vs_E10	ordinary_muscle_development	ENSGALG00010021642	NPTN	-0.273639804	0.090312187	-3.029932217	0.002446086	0.003893271	negative
PRJNA729271	stage_1D_vs_E10	ordinary_muscle_development	ENSGALG00010021643	ENSGALG00010021643	-0.63498984	0.131517481	-4.828178247	1.37788E-06	3.04224E-06	negative
PRJNA729271	stage_1D_vs_E10	ordinary_muscle_development	ENSGALG00010021644	TARDBP	-0.274897941	0.153678533	-1.788785565	0.073649357	0.095735577	negative
PRJNA729271	stage_1D_vs_E10	ordinary_muscle_development	ENSGALG00010021645	ASCC1	-0.475603327	0.139148605	-3.417952531	0.000630941	0.00107354	negative
PRJNA729271	stage_1D_vs_E10	ordinary_muscle_development	ENSGALG00010021646	RPUSD3	0.125013919	0.166079491	0.752735444	0.451608907	0.500732127	positive
PRJNA729271	stage_1D_vs_E10	ordinary_muscle_development	ENSGALG00010021647	ARHGAP11B	-1.69838933	0.136648027	-12.42893418	1.82045E-35	2.01124E-34	negative
PRJNA729271	stage_1D_vs_E10	ordinary_muscle_development	ENSGALG00010021648	BRE	-0.296186703	0.067315922	-4.399950159	1.08276E-05	2.20367E-05	negative
PRJNA729271	stage_1D_vs_E10	ordinary_muscle_development	ENSGALG00010021649	PXN	0.294138777	0.096324455	3.053625142	0.002260944	0.00361407	positive
PRJNA729271	stage_1D_vs_E10	ordinary_muscle_development	ENSGALG00010021650	CD276	-1.906575149	0.112117581	-17.00513993	7.52254E-65	2.37558E-63	negative
PRJNA729271	stage_1D_vs_E10	ordinary_muscle_development	ENSGALG00010021651	RNF113B	-0.109449695	0.154881215	-0.706668624	0.479772432	0.528370047	negative
PRJNA729271	stage_1D_vs_E10	ordinary_muscle_development	ENSGALG00010021652	BORCS5	-0.35631793	0.096412131	-3.695778993	0.000219214	0.000391752	negative
PRJNA729271	stage_1D_vs_E10	ordinary_muscle_development	ENSGALG00010021655	USP22	-0.99815767	0.094113627	-10.60587824	2.79823E-26	2.02498E-25	negative
PRJNA729271	stage_1D_vs_E10	ordinary_muscle_development	ENSGALG00010021656	SETD6	-0.445045967	0.079530353	-5.595925975	2.19447E-08	5.57269E-08	negative
PRJNA729271	stage_1D_vs_E10	ordinary_muscle_development	ENSGALG00010021657	JAGN1	0.038075845	0.143051693	0.266168436	0.790109491	0.817987077	positive
PRJNA729271	stage_1D_vs_E10	ordinary_muscle_development	ENSGALG00010021658	STRADA	-0.842090591	0.091062592	-9.247382228	2.30058E-20	1.25113E-19	negative
PRJNA729271	stage_1D_vs_E10	ordinary_muscle_development	ENSGALG00010021659	SPOCK2	-1.131304507	0.148292537	-7.628870155	2.3682E-14	9.11741E-14	negative
PRJNA729271	stage_1D_vs_E10	ordinary_muscle_development	ENSGALG00010021660	RNF223	2.702865292	0.445568855	6.066100139	1.31053E-09	3.6658E-09	positive
PRJNA729271	stage_1D_vs_E10	ordinary_muscle_development	ENSGALG00010021661	DUSP16	0.159820758	0.120623558	1.324954765	0.185186155	0.224250068	positive
PRJNA729271	stage_1D_vs_E10	ordinary_muscle_development	ENSGALG00010021662	RASGRP1	0.636463545	0.162443895	3.918051495	8.92676E-05	0.000166482	positive
PRJNA729271	stage_1D_vs_E10	ordinary_muscle_development	ENSGALG00010021663	CNOT9	-0.31052382	0.140020555	-2.217701677	0.02657518	0.03690785	negative
PRJNA729271	stage_1D_vs_E10	ordinary_muscle_development	ENSGALG00010021664	TYSND1	-0.640895104	0.166516967	-3.848827631	0.000118684	0.00021876	negative
PRJNA729271	stage_1D_vs_E10	ordinary_muscle_development	ENSGALG00010021665	CNOT1	0.139817231	0.091385914	1.529964792	0.126025444	0.157477484	positive
PRJNA729271	stage_1D_vs_E10	ordinary_muscle_development	ENSGALG00010021666	CCDC47	0.8541875	0.091981295	9.286534786	1.59393E-20	8.7748E-20	positive
PRJNA729271	stage_1D_vs_E10	ordinary_muscle_development	ENSGALG00010021667	TBX15	0.066261229	0.191623683	0.345788309	0.729501812	0.763530129	positive
PRJNA729271	stage_1D_vs_E10	ordinary_muscle_development	ENSGALG00010021668	PYGO1	-0.644913384	0.129217974	-4.990895346	6.01001E-07	1.36523E-06	negative
PRJNA729271	stage_1D_vs_E10	ordinary_muscle_development	ENSGALG00010021669	ENSGALG00010021669	-0.825060759	0.074178541	-11.12263401	9.73546E-29	7.91894E-28	negative
PRJNA729271	stage_1D_vs_E10	ordinary_muscle_development	ENSGALG00010021670	BLCAP	0.636682615	0.10885821	5.848733109	4.95331E-09	1.32665E-08	positive
PRJNA729271	stage_1D_vs_E10	ordinary_muscle_development	ENSGALG00010021671	TNFRSF13B	2.278061222	0.743528954	3.06385005	0.002185084	0.003497437	positive
PRJNA729271	stage_1D_vs_E10	ordinary_muscle_development	ENSGALG00010021674	ING4	-1.094450372	0.123434889	-8.86662092	7.53991E-19	3.77777E-18	negative
PRJNA729271	stage_1D_vs_E10	ordinary_muscle_development	ENSGALG00010021675	DDX42	-0.124785795	0.058606205	-2.12922495	0.033235654	0.045572869	negative
PRJNA729271	stage_1D_vs_E10	ordinary_muscle_development	ENSGALG00010021676	TP53INP2	1.733142784	0.277525344	6.244989216	4.23829E-10	1.22844E-09	positive
PRJNA729271	stage_1D_vs_E10	ordinary_muscle_development	ENSGALG00010021677	ENSGALG00010021677	-0.112871942	0.152620813	-0.739557993	0.459568239	0.508429371	negative
PRJNA729271	stage_1D_vs_E10	ordinary_muscle_development	ENSGALG00010021678	DNAAF4	0.627002673	0.156640153	4.002822153	6.25914E-05	0.000118581	positive
PRJNA729271	stage_1D_vs_E10	ordinary_muscle_development	ENSGALG00010021679	MASP2	-2.759380024	1.120105751	-2.463499559	0.013758806	0.019947379	negative
PRJNA729271	stage_1D_vs_E10	ordinary_muscle_development	ENSGALG00010021680	PIGT	0.285849004	0.118319358	2.415910707	0.01569591	0.022554993	positive
PRJNA729271	stage_1D_vs_E10	ordinary_muscle_development	ENSGALG00010021681	BTBD10	-0.718390783	0.081296826	-8.836639956	9.86404E-19	4.92014E-18	negative
PRJNA729271	stage_1D_vs_E10	ordinary_muscle_development	ENSGALG00010021682	MYL4	-3.899004015	0.203570634	-19.15307695	9.12335E-82	4.46767E-80	negative
PRJNA729271	stage_1D_vs_E10	ordinary_muscle_development	ENSGALG00010021683	HFM1	-0.091068998	0.421228758	-0.216198435	0.828833074	0.853141273	negative
PRJNA729271	stage_1D_vs_E10	ordinary_muscle_development	ENSGALG00010021684	BIN1	2.687779453	0.121175663	22.18085214	5.2574E-109	4.536E-107	positive
PRJNA729271	stage_1D_vs_E10	ordinary_muscle_development	ENSGALG00010021685	DBNDD2	1.651033827	0.138669668	11.90623622	1.09824E-32	1.08071E-31	positive
PRJNA729271	stage_1D_vs_E10	ordinary_muscle_development	ENSGALG00010021686	CCPG1	2.344647581	0.164894462	14.21908018	6.97639E-46	1.1286E-44	positive
PRJNA729271	stage_1D_vs_E10	ordinary_muscle_development	ENSGALG00010021687	ZNF384	0.139637541	0.105535912	1.323128195	0.18579275	0.224947036	positive
PRJNA729271	stage_1D_vs_E10	ordinary_muscle_development	ENSGALG00010021688	ITPR1	0.063534009	0.116740568	0.544232484	0.586281501	0.63061371	positive
PRJNA729271	stage_1D_vs_E10	ordinary_muscle_development	ENSGALG00010021689	MPRIP	0.303010394	0.109223885	2.774213648	0.005533532	0.008458141	positive
PRJNA729271	stage_1D_vs_E10	ordinary_muscle_development	ENSGALG00010021691	PIGB	-0.595933533	0.146427584	-4.069817434	4.705E-05	9.01626E-05	negative
PRJNA729271	stage_1D_vs_E10	ordinary_muscle_development	ENSGALG00010021693	PPA1	-0.810161486	0.092371179	-8.770717167	1.77532E-18	8.73498E-18	negative
PRJNA729271	stage_1D_vs_E10	ordinary_muscle_development	ENSGALG00010021694	ENSGALG00010021694	3.254723405	1.104143907	2.947734787	0.003201116	0.005028429	positive

PRJNA729271	stage_1D_vs_E10	ordinary_muscle_development	ENSGALG00010021695	FNBP4	0.179488451	0.070376545	2.55040157	0.01075989	0.015842012	positive
PRJNA729271	stage_1D_vs_E10	ordinary_muscle_development	ENSGALG00010021697	PIANP	-1.290059195	0.224347163	-5.750280853	8.90953E-09	2.33491E-08	negative
PRJNA729271	stage_1D_vs_E10	ordinary_muscle_development	ENSGALG00010021698	GYPC	0.580018124	0.134546939	4.310897946	1.62593E-05	3.2485E-05	positive
PRJNA729271	stage_1D_vs_E10	ordinary_muscle_development	ENSGALG00010021699	SYS1	-0.289557404	0.083939191	-3.449609191	0.000561399	0.000961081	negative
PRJNA729271	stage_1D_vs_E10	ordinary_muscle_development	ENSGALG00010021700	FLCN	0.283678085	0.078267431	3.624471651	0.000289553	0.000511121	positive
PRJNA729271	stage_1D_vs_E10	ordinary_muscle_development	ENSGALG00010021701	DR1	-0.500553766	0.076648515	-6.530508369	6.55468E-11	2.00751E-10	negative
PRJNA729271	stage_1D_vs_E10	ordinary_muscle_development	ENSGALG00010021702	PLD6	0.169468218	0.294751941	0.574952	0.56532376	0.61065414	positive
PRJNA729271	stage_1D_vs_E10	ordinary_muscle_development	ENSGALG00010021703	CD82	-0.123990889	0.173530052	-0.714521131	0.474904995	0.52359838	negative
PRJNA729271	stage_1D_vs_E10	ordinary_muscle_development	ENSGALG00010021704	COPS7A	0.893693113	0.102281112	8.737616289	2.38081E-18	1.16195E-17	positive
PRJNA729271	stage_1D_vs_E10	ordinary_muscle_development	ENSGALG00010021705	CCDC18	-0.211198754	0.18933427	-1.11548086	0.264644416	0.309787237	negative
PRJNA729271	stage_1D_vs_E10	ordinary_muscle_development	ENSGALG00010021706	ENSGALG00010021706	3.087876459	0.158246062	19.51313298	8.4915E-85	4.50858E-83	positive
PRJNA729271	stage_1D_vs_E10	ordinary_muscle_development	ENSGALG00010021707	DLX2	-2.948405161	0.968008954	-3.045844925	0.002320275	0.003703599	negative
PRJNA729271	stage_1D_vs_E10	ordinary_muscle_development	ENSGALG00010021708	CNTNAP5	-4.833454646	0.359221134	-13.45537383	2.86288E-41	3.95213E-40	negative
PRJNA729271	stage_1D_vs_E10	ordinary_muscle_development	ENSGALG00010021710	ENSGALG00010021710	-0.799325018	0.321241105	-2.488240159	0.012837701	0.018701756	negative
PRJNA729271	stage_1D_vs_E10	ordinary_muscle_development	ENSGALG00010021711	ENSGALG00010021711	1.377766658	0.216328656	6.368858771	1.9044E-10	5.65777E-10	positive
PRJNA729271	stage_1D_vs_E10	ordinary_muscle_development	ENSGALG00010021713	RAB27A	0.339537335	0.079839352	4.252756631	2.11155E-05	4.17671E-05	positive
PRJNA729271	stage_1D_vs_E10	ordinary_muscle_development	ENSGALG00010021714	TSPAN18	0.636175141	0.115588108	5.503811373	3.71667E-08	9.26452E-08	positive
PRJNA729271	stage_1D_vs_E10	ordinary_muscle_development	ENSGALG00010021715	MMAB	0.186393756	0.128036198	1.455789533	0.14545081	0.179843853	positive
PRJNA729271	stage_1D_vs_E10	ordinary_muscle_development	ENSGALG00010021716	TFCP2L1	-1.513486716	0.395432425	-3.827421885	0.000129492	0.000237674	negative
PRJNA729271	stage_1D_vs_E10	ordinary_muscle_development	ENSGALG00010021717	SDC4	1.87294132	0.196712635	9.521204996	1.71182E-21	9.94106E-21	positive
PRJNA729271	stage_1D_vs_E10	ordinary_muscle_development	ENSGALG00010021718	SST7	-3.053313557	0.442162858	-6.905404887	5.00604E-12	1.6499E-11	negative
PRJNA729271	stage_1D_vs_E10	ordinary_muscle_development	ENSGALG00010021719	BHLHE40	3.342522661	0.214026623	15.61732191	5.54878E-55	1.27464E-53	positive
PRJNA729271	stage_1D_vs_E10	ordinary_muscle_development	ENSGALG00010021720	CACNB1	1.974987515	0.260379812	7.585025505	3.32422E-14	1.26868E-13	positive
PRJNA729271	stage_1D_vs_E10	ordinary_muscle_development	ENSGALG00010021721	TP53I11	-0.149503626	0.142787982	-1.047032278	0.295084692	0.341824711	negative
PRJNA729271	stage_1D_vs_E10	ordinary_muscle_development	ENSGALG00010021722	AARS1	-0.201910554	0.159030059	-1.26963767	0.204213725	0.244821134	negative
PRJNA729271	stage_1D_vs_E10	ordinary_muscle_development	ENSGALG00010021723	COPS3	-0.425166923	0.107158157	-3.967658018	7.25824E-05	0.00013651	negative
PRJNA729271	stage_1D_vs_E10	ordinary_muscle_development	ENSGALG00010021725	SH2D6	0.306893409	0.216990667	1.414316171	0.157269091	0.193089571	positive
PRJNA729271	stage_1D_vs_E10	ordinary_muscle_development	ENSGALG00010021726	ARNTL	0.127716406	0.146484252	0.871878062	0.383274903	0.432340056	positive
PRJNA729271	stage_1D_vs_E10	ordinary_muscle_development	ENSGALG00010021727	MVK	-0.216119418	0.101400502	-2.131344648	0.033060759	0.045350214	negative
PRJNA729271	stage_1D_vs_E10	ordinary_muscle_development	ENSGALG00010021728	TMED5	-0.323769042	0.06491898	-4.987278609	6.12357E-07	1.39015E-06	negative
PRJNA729271	stage_1D_vs_E10	ordinary_muscle_development	ENSGALG00010021729	RBPJL	-3.774205247	0.658137245	-5.734678099	9.76977E-09	2.5502E-08	negative
PRJNA729271	stage_1D_vs_E10	ordinary_muscle_development	ENSGALG00010021730	ARL8B	0.171399364	0.072049507	2.378910985	0.017363868	0.024785809	positive
PRJNA729271	stage_1D_vs_E10	ordinary_muscle_development	ENSGALG00010021731	SRM	-0.56852044	0.118790209	-4.785920016	1.70206E-06	3.72904E-06	negative
PRJNA729271	stage_1D_vs_E10	ordinary_muscle_development	ENSGALG00010021732	CASR	1.606290993	0.252069814	6.372405211	1.86086E-10	5.53411E-10	positive
PRJNA729271	stage_1D_vs_E10	ordinary_muscle_development	ENSGALG00010021733	ENSGALG00010021733	1.808459914	0.588554242	3.072715791	0.002121204	0.00340233	positive
PRJNA729271	stage_1D_vs_E10	ordinary_muscle_development	ENSGALG00010021734	PARD3B	0.357618855	0.063427472	5.638232797	1.71804E-08	4.40449E-08	positive
PRJNA729271	stage_1D_vs_E10	ordinary_muscle_development	ENSGALG00010021735	PIGBOS1	0.78453718	0.144992094	5.410896255	6.27101E-08	1.53934E-07	positive
PRJNA729271	stage_1D_vs_E10	ordinary_muscle_development	ENSGALG00010021736	EDEM1	0.231129693	0.082753718	2.79298258	0.00522245	0.008007978	positive
PRJNA729271	stage_1D_vs_E10	ordinary_muscle_development	ENSGALG00010021738	PRDM11	0.576770371	0.071174054	8.103660507	5.33298E-16	2.28568E-15	positive
PRJNA729271	stage_1D_vs_E10	ordinary_muscle_development	ENSGALG00010021739	EXOSC10	0.275190924	0.110115855	2.499103546	0.012450793	0.018172816	positive
PRJNA729271	stage_1D_vs_E10	ordinary_muscle_development	ENSGALG00010021740	PDLIM1	0.528353959	0.158746306	3.328291367	0.000873804	0.001466118	positive
PRJNA729271	stage_1D_vs_E10	ordinary_muscle_development	ENSGALG00010021741	RPL19	0.127121415	0.146024249	0.870550035	0.383999888	0.432989372	positive
PRJNA729271	stage_1D_vs_E10	ordinary_muscle_development	ENSGALG00010021742	MATN4	-5.297743368	0.483630236	-10.95411945	6.34929E-28	4.95333E-27	negative
PRJNA729271	stage_1D_vs_E10	ordinary_muscle_development	ENSGALG00010021743	SYT13	-4.583178425	0.419154786	-10.93433398	7.89838E-28	6.14201E-27	negative
PRJNA729271	stage_1D_vs_E10	ordinary_muscle_development	ENSGALG00010021744	LITAF	0.23950757	0.383569418	0.624417794	0.532353242	0.579226861	positive
PRJNA729271	stage_1D_vs_E10	ordinary_muscle_development	ENSGALG00010021745	RSL24D1	-0.052292052	0.125623465	-0.416260231	0.67721959	0.715212966	negative
PRJNA729271	stage_1D_vs_E10	ordinary_muscle_development	ENSGALG00010021746	MTOR	1.24894344	0.110001174	11.35391011	7.09187E-30	6.08265E-29	positive
PRJNA729271	stage_1D_vs_E10	ordinary_muscle_development	ENSGALG00010021747	AKTIP	1.207504443	0.114382053	10.55676486	4.72672E-26	3.39514E-25	positive
PRJNA729271	stage_1D_vs_E10	ordinary_muscle_development	ENSGALG00010021748	CDCA7	-1.307646715	0.176910032	-7.391591651	1.45082E-13	5.3038E-13	negative
PRJNA729271	stage_1D_vs_E10	ordinary_muscle_development	ENSGALG00010021749	CLASP1	0.268725345	0.09086695	2.957349686	0.00310296	0.00488301	positive
PRJNA729271	stage_1D_vs_E10	ordinary_muscle_development	ENSGALG00010021750	HELLS	-1.782293558	0.147745446	-12.06327234	1.65091E-33	1.68402E-32	negative
PRJNA729271	stage_1D_vs_E10	ordinary_muscle_development	ENSGALG00010021751	MTCH2	-0.515477846	0.092591441	-5.567229983	2.58821E-08	6.53021E-08	negative
PRJNA729271	stage_1D_vs_E10	ordinary_muscle_development	ENSGALG00010021752	CSTB	-0.72583137	0.111078777	-6.534383886	6.38719E-11	1.959E-10	negative
PRJNA729271	stage_1D_vs_E10	ordinary_muscle_development	ENSGALG00010021753	MTF2	-1.297132201	0.097328528	-13.32735871	1.60479E-40	2.14788E-39	negative
PRJNA729271	stage_1D_vs_E10	ordinary_muscle_development	ENSGALG00010021754	SLC17A9	-2.641040588	0.524262949	-5.037625857	4.71342E-07	1.08017E-06	negative
PRJNA729271	stage_1D_vs_E10	ordinary_muscle_development	ENSGALG00010021755	ENSGALG00010021755	2.149158558	0.153667003	13.98581685	1.9028E-44	2.92792E-43	positive
PRJNA729271	stage_1D_vs_E10	ordinary_muscle_development	ENSGALG00010021756	USP42	-0.895088084	0.123077934	-7.272530955	3.52814E-13	1.26055E-12	negative
PRJNA729271	stage_1D_vs_E10	ordinary_muscle_development	ENSGALG00010021757	ZP4	-0.156321508	0.361485671	-0.432441782	0.665420357	0.704394075	negative
PRJNA729271	stage_1D_vs_E10	ordinary_muscle_development	ENSGALG00010021758	RASSF10	-4.880757743	0.447210718	-10.91377631	9.90542E-28	7.66164E-27	negative
PRJNA729271	stage_1D_vs_E10	ordinary_muscle_development	ENSGALG00010021759	NIFK	-0.66158129	0.093682128	-7.061979762	1.64147E-12	5.59048E-12	negative
PRJNA729271	stage_1D_vs_E10	ordinary_muscle_development	ENSGALG00010021760	CSTA	0.462191638	0.397220223	1.163565223	0.244600252	0.288719922	positive
PRJNA729271	stage_1D_vs_E10	ordinary_muscle_development	ENSGALG00010021761	MAFB	-0.177458365	0.169841953	-1.044844116	0.296095022	0.342940259	negative
PRJNA729271	stage_1D_vs_E10	ordinary_muscle_development	ENSGALG00010021762	LAMB2	3.712770447	0.198325573	18.72058348	3.36446E-78	1.53358E-76	positive
PRJNA729271	stage_1D_vs_E10	ordinary_muscle_development	ENSGALG00010021764	FAM222A	-1.12669231	0.761345584	-1.479869763	0.138908006	0.172209336	negative
PRJNA729271	stage_1D_vs_E10	ordinary_muscle_development	ENSGALG00010021765	EIF2AK1	-0.032729683	0.104718175	-0.312550172	0.75462244	0.786019852	negative
PRJNA729271	stage_1D_vs_E10	ordinary_muscle_development	ENSGALG00010021766	AGRN	0.244408435	0.148466184	1.646222917	0.099717873	0.126834904	positive
PRJNA729271	stage_1D_vs_E10	ordinary_muscle_development	ENSGALG00010021767	CCDC58	0.062608299	0.212839809	0.294156903	0.768638011	0.798248775	positive
PRJNA729271	stage_1D_vs_E10	ordinary_muscle_development	ENSGALG00010021768	DIPK1A	0.633060239	0.150473841	4.207111574	2.58655E-05	5.0713E-05	positive
PRJNA729271	stage_1D_vs_E10	ordinary_muscle_development	ENSGALG00010021769	ENSGALG00010021769	-0.575929148	0.174155412	-3.306983921	0.000943063	0.001577212	negative
PRJNA729271	stage_1D_vs_E10	ordinary_muscle_development	ENSGALG00010021770	RALGAPB	0.232138283	0.097594295	2.378605042	0.017378285	0.024802899	positive
PRJNA729271	stage_1D_vs_E10	ordinary_muscle_development	ENSGALG00010021771	ANKRD61	0.017924429	0.144374261	0.124152527	0.90119451	0.91572481	positive
PRJNA729271	stage_1D_vs_E10	ordinary_muscle_development	ENSGALG00010021772	TSN	-0.841544	0.083413408	-10.08883362	6.19006E-24	4.00916E-23	negative
PRJNA729271	stage_1D_vs_E10	ordinary_muscle_development	ENSGALG00010021773	ENSGALG00010021773	-1.025818226	0.910888101	-1.126173703	0.260092002	0.305142337	negative
PRJNA729271	stage_1D_vs_E10	ordinary_muscle_development	ENSGALG00010021774	FBXL20	0.750606705	0.071668467	10.4733188	1.14555E-25	8.09198E-25	positive
PRJNA729271	stage_1D_vs_E10	ordinary_muscle_development	ENSGALG00010021775	CCDC36	2.471423053	0.31020651	7.967025114	1.6254E-15	6.73724E-15	positive
PRJNA729271	stage_1D_vs_E10	ordinary_muscle_development	ENSGALG00010021776	PMS2	-0.906573599	0.09489937	-9.552999182	1.25995E-21	7.3641E-21	negative
PRJNA729271	stage_1D_vs_E10	ordinary_muscle_development	ENSGALG00010021778	RBL2	0.451007405	0.103683819	4.349834049	1.36241E-05	2.74584E-05	positive
PRJNA729271	stage_1D_vs_E10	ordinary_muscle_development	ENSGALG00010021779	UNC13C	-0.18606668	0.247102313	-0.752994491	0.451453224	0.500636102	negative
PRJNA729271	stage_1D_vs_E10	ordinary_muscle_development	ENSGALG00010021781	RAPGEF4	-0.856575718	0.2692337	-3.181532326	0.001464982	0.002397258	negative
PRJNA729271	stage_1D_vs_E10	ordinary_muscle_development	ENSGALG00010021782	ADAM11	-0.810218722	0.306806319	-2.640814973	0.008270687	0.01236041	negative
PRJNA729271	stage_1D_vs_E10	ordinary_muscle_development	ENSGALG00010021783	GIT2	-0.795033473	0.048268964	-16.47090389	5.93765E-61	1.67444E-59	negative
PRJNA729271	stage_1D_vs_E10	ordinary_muscle_development	ENSGALG00010021784	ANGPTL7	5.275581793	0.842464387	6.262082853	3.79869E-10	1.105E-09	positive
PRJNA729271	stage_1D_vs_E10	ordinary_muscle_development	ENSGALG00010021785	CCDC71	-0.165694318	0.089833996	-1.844450043	0.06511758	0.085318568	negative
PRJNA729271	stage_1D_vs_E10	ordinary_muscle_development	ENSGALG00010021786	FAM162A	0.069632452	0.173065665	0.402347008	0.687428664	0.725149442	positive
PRJNA729271	stage_1D_vs_E10	ordinary_muscle_development	ENSGALG00010021788	RPL5	0.271287147	0.12390499	2.189477164	0.028562177	0.039470755	positive
PRJNA729271	stage_1D_vs_E10	ordinary_muscle_development	ENSGALG00010021789	CHD9	0.275020722	0.090665709	3.033348829	0.002418559	0.003852418	positive
PRJNA729271	stage_1D_vs_E10	ordinary_muscle_development	ENSGALG00010021790	PHF21A	0.123897334	0.128162226	0.966722702	0.333682675	0.382229364	positive
PRJNA729271	stage_1D_vs_E10	ordinary_muscle_development	ENSGALG00010021791	PLEKHM2	1.773202062	0.184015456	9.636158268	5.6254E-22	3.36387E-21	positive
PRJNA729271	stage_1D_vs_E10	ordinary_muscle_development	ENSGALG00010021792	KLHDC8B	4.056650239	0.256996829	15.78482606	3.95739E-56	9.46573E-55	positive
PRJNA729271	stage_1D_vs_E10	ordinary_muscle_development	ENSGALG00010021793	ENSGALG00010021793	0.927601626	0.157510303	5.889148878	3.8819E-09	1.04841E-08	positive
PRJNA729271	stage_1D_vs_E10	ordinary_muscle_development	ENSGALG00010021794	ENSGALG00010021794	-0.917012596	0.191130471	-4.79783569	1.60389E-06	3.52195E-06	negative
PRJNA729271	stage_1D_vs_E10	ordinary_muscle_development	ENSGALG00010021795	NDRG3	-0.605244137	0.106886819	-5.662476856	1.49204E-08	3.84139E-08	negative
PRJNA729271	stage_1D_vs_E10	ordinary_muscle_development	ENSGALG00010021797	SLC25A34	2.471628818	0.159603994	15.48600857	4.31246E-54	9.55797E-53	positive
PRJNA729271	stage_1D_vs_E10	ordinary_muscle_development	ENSGALG00010021798	RANBP10	0.536542876	0.18105858	2.963366194	0.003042943	0.004794811	positive
PRJNA729271	stage_1D_vs_E10	ordinary_muscle_development	ENSGALG00010021799	ENSGALG00010021799	-0.699126711	0.219752394	-3.18142933	0.001465503	0.00239784	negative
PRJNA729271	stage_1D_vs_E10	ordinary_muscle_development	ENSGALG00010021800	TMEM82	2.216627195	0.326736321	6.784146879	1.16774E-11	3.76144E-11	positive
PRJNA729271	stage_1D_vs_E10	ordinary_muscle_development	ENSGALG00010021801	VAMP1	1.262024826	0.246762526	5.114329345	3.14858E-07	7.29867E-07	positive
PRJNA729271	stage_1D_vs_E10	ordinary_muscle_development	ENSGALG00010021803	TLL2	-2.623812801	0.165234157	-15.87936088	8.80712E-57	2.16739E-55	negative
PRJNA729271	stage_1D_vs_E10	ordinary_muscle_development	ENSGALG00010021805	FUT8	-0.354889555	0.108050177	-3.284488407	0.001021677	0.001702404	negative
PRJNA729271	stage_1D_vs_E10	ordinary_muscle_development	ENSGALG00010021807	CDC42	-0.250826136	0.082949834	-3.02382926	0.002495973	0.003967879	negative
PRJNA729271	stage_1D_vs_E10	ordinary_muscle_development	ENSGALG00010021810	ANKRD13A	-1.216968653	0.097096784	-12.5335629	4.89148E-36	5.53058E-35	negative
PRJNA729271	stage_1D_vs_E10	ordinary_muscle_development	ENSGALG00010021812	TEAD1	0.439207768	0.083048962	5.288540113	1.23296E-07	2.95989E-07	positive
PRJNA729271	stage_1D_vs_E10	ordinary_muscle_development	ENSGALG00010021817	EVI5	-0.032177387	0.100652522	-0.319687836	0.749204982	0.781387697	negative
PRJNA729271	stage_1D_vs_E10	ordinary_muscle_development	ENSGALG00010021821	PPP1R19L	-0.888242641	0.119626684	-7.425121303	1.12677E-13	4.15268E-13	negative
PRJNA729271	stage_1D_vs_E10	ordinary_muscle_development	ENSGALG00010021823	ENSGALG00010021823	-0.300707377	0.25960464	-1.158328207	0.246730125	0.290807705	negative
PRJNA729271	stage_1D_vs_E10	ordinary_muscle_development	ENSGALG00010021826	FBXO40	4.906209176	0.208132335	23.57254659	7.373E-123	8.76E-121	positive
PRJNA729271	stage_1D_vs_E10	ordinary_muscle_development	ENSGALG00010021827	CCZ1	-0.573967474	0.096053869	-5.975474774	2.29421E-09	6.31136E-09	negative
PRJNA729271	stage_1D_vs_E10	ordinary_muscle_development	ENSGALG00010021829	ERCC6	0.065339186	0.096258407	0.678789398	0.497271313	0.545395557	positive
PRJNA729271	stage_1D_vs_E10	ordinary_muscle_development	ENSGALG00010021831	ENSGALG00010021831	0.986264792	0.363693931	2.711798869	0.006691918	0.010114636	positive
PRJNA729271	stage_1D_vs_E10	ordinary_muscle_development	ENSGALG00010021835	NUDT8	0.04692519	0.172622643	0.271836819	0.785747494	0.813878085	positive
PRJNA729271	stage_1D_vs_E10	ordinary_muscle_development	ENSGALG00010021837	ENSGALG00010021837	0.231541827	0.324967064	0.712508599	0.476149889	0.524811242	positive
PRJNA729271	stage_1D_vs_E10	ordinary_muscle_development	ENSGALG00010021839	C1orf50	-0.158383513	0.121326684	-1.305430165	0.191746412	0.231420837	negative
PRJNA729271	stage_1D_vs_E10	ordinary_muscle_development	ENSGALG00010021840	ENSGALG00010021840	0.301807363	0.218122899	1.383657401	0.166463383	0.203481762	positive
PRJNA729271	stage_1D_vs_E10	ordinary_muscle_development	ENSGALG00010021841	CENPT	-1.281351122	0.090343381	-14.18312115	1.16546E-45	1.87287E-44	negative
PRJNA729271	stage_1D_vs_E10	ordinary_muscle_development	ENSGALG00010021842	ENSGALG00010021842	-0.395889392	0.178650237	-2.216002608	0.026691324	0.037062049	negative
PRJNA729271	stage_1D_vs_E10	ordinary_muscle_development	ENSGALG00010021843	ENSGALG00010021843	-0.006858914	0.398185995	-0.017225403	0.986256796	0.988302265	negative
PRJNA729271	stage_1D_vs_E10	ordinary_muscle_development	ENSGALG00010021847	P3H1	-1.035648948	0.151287922	-6.845549448	7.61829E-12	2.48318E-11	negative
PRJNA729271	stage_1D_vs_E10	ordinary_muscle_development	ENSGALG00010021848	LARGE2	1.483816294	0.136323636	10.88451233	1.36628E-27	1.04673E-26	positive
PRJNA729271	stage_1D_vs_E10	ordinary_muscle_development	ENSGALG00010021851	ENSGALG00010021851	1.367266917	0.212787125	6.425515243	1.31424E-10	3.94816E-10	positive
PRJNA729271	stage_1D_vs_E10	ordinary_muscle_development	ENSGALG00010021853	ENSGALG00010021853	0.72265437	0.56218553	1.285437514	0.198639439	0.238649743	positive
PRJNA729271	stage_1D_vs_E10	ordinary_muscle_development	ENSGALG00010021854	TAPBPL	1.120947684	0.154078224	7.275185631	3.45944E-13	1.23692E-12	positive
PRJNA729271	stage_1D_vs_E10	ordinary_muscle_development	ENSGALG00010021860	ENSGALG00010021860	-0.045553659	0.280868878	-0.162188347	0.871157536	0.890258635	negative
PRJNA729271	stage_1D_vs_E10	ordinary_muscle_development	ENSGALG00010021862	PDK1	0.452267633	0.106031899	4.265392169	1.99551E-05	3.9542E-05	positive
PRJNA729271	stage_1D_vs_E10	ordinary_muscle_development	ENSGALG00010021863	CLDN19	4.039242879	0.282404021	14.30306434	2.09383E-46	3.46462E-45	positive
PRJNA729271	stage_1D_vs_E10	ordinary_muscle_development	ENSGALG00010021864	GFI1	2.272661068	0.469787646	4.837634811	1.31393E-06	2.90416E-06	positive
PRJNA729271	stage_1D_vs_E10	ordinary_muscle_development	ENSGALG00010021866	SCNN1A	0.870910796	0.374781701	2.323781531	0.020137206	0.028471401	positive
PRJNA729271	stage_1D_vs_E10	ordinary_muscle_development	ENSGALG00010021867	CXCL12	1.180791906	0.182019834	6.487160645	8.74691E-11	2.65911E-10	positive
PRJNA729271	stage_1D_vs_E10	ordinary_muscle_development	ENSGALG00010021869	GJC3	0.018502826	0.121255807	0.152593321	0.878718993	0.896532118	positive
PRJNA729271	stage_1D_vs_E10	ordinary_muscle_development	ENSGALG00010021870	ENSGALG00010021870	-0.388025441	0.168955104	-2.296618636	0.021640535	0.030486884	negative
PRJNA729271	stage_1D_vs_E10	ordinary_muscle_development	ENSGALG00010021871	ENSGALG00010021871	4.994270414	0.24964249	20.00569059	4.91334E-89	2.88336E-87	positive
PRJNA729271	stage_1D_vs_E10	ordinary_muscle_development	ENSGALG00010021874	TMCC1	0.687517111	0.078285809	8.782142215	1.6039E-18	7.91575E-18	positive

PRJNA729271	stage_1D_vs_E10	ordinary_muscle_development	ENSGALG00010021877	PLEKHN1	0.690256386	0.147469402	4.680675297	2.85932E-06	6.1474E-06	positive
PRJNA729271	stage_1D_vs_E10	ordinary_muscle_development	ENSGALG00010021879	CREBL2	0.862443678	0.154720827	5.574192531	2.48681E-08	6.29299E-08	positive
PRJNA729271	stage_1D_vs_E10	ordinary_muscle_development	ENSGALG00010021881	PHF20	-0.319565097	0.048175054	-6.633414389	3.28009E-11	1.02667E-10	negative
PRJNA729271	stage_1D_vs_E10	ordinary_muscle_development	ENSGALG00010021887	NUTF2	-1.361615053	0.111183549	-12.24655146	1.75293E-34	1.86417E-33	negative
PRJNA729271	stage_1D_vs_E10	ordinary_muscle_development	ENSGALG00010021892	NDUFV1	1.683825637	0.121607952	13.84634483	1.33852E-43	1.98789E-42	positive
PRJNA729271	stage_1D_vs_E10	ordinary_muscle_development	ENSGALG00010021897	ENSGALG00010021897	-0.522986652	0.220453701	-2.372319677	0.017676789	0.025191727	negative
PRJNA729271	stage_1D_vs_E10	ordinary_muscle_development	ENSGALG00010021902	LMTK2	0.669258368	0.119070667	5.620682089	1.90205E-08	4.85477E-08	positive
PRJNA729271	stage_1D_vs_E10	ordinary_muscle_development	ENSGALG00010021903	MORC2	-0.516721604	0.078484724	-6.583722024	4.58815E-11	1.42409E-10	negative
PRJNA729271	stage_1D_vs_E10	ordinary_muscle_development	ENSGALG00010021905	RBSN	-0.229291785	0.073116765	-3.135967305	0.001712883	0.002780941	negative
PRJNA729271	stage_1D_vs_E10	ordinary_muscle_development	ENSGALG00010021908	EDC4	-0.287911486	0.108649719	-2.649905494	0.008051429	0.012055104	negative
PRJNA729271	stage_1D_vs_E10	ordinary_muscle_development	ENSGALG00010021913	ENSGALG00010021913	2.040933612	0.174972363	11.6643199	1.93947E-31	1.79776E-30	positive
PRJNA729271	stage_1D_vs_E10	ordinary_muscle_development	ENSGALG00010021916	MRPS25	0.051848084	0.148757173	0.34854174	0.727433373	0.76153017	positive
PRJNA729271	stage_1D_vs_E10	ordinary_muscle_development	ENSGALG00010021919	PARVA	0.903898308	0.124146251	7.280915062	3.31563E-13	1.18726E-12	positive
PRJNA729271	stage_1D_vs_E10	ordinary_muscle_development	ENSGALG00010021922	MRPL51	-0.218650669	0.138172641	-1.582445462	0.113547913	0.143047378	negative
PRJNA729271	stage_1D_vs_E10	ordinary_muscle_development	ENSGALG00010021924	ENSGALG00010021924	-2.575227304	0.557183491	-4.621865768	3.80304E-06	8.07457E-06	negative
PRJNA729271	stage_1D_vs_E10	ordinary_muscle_development	ENSGALG00010021928	ENSGALG00010021928	-1.101898912	1.073086609	-1.026849933	0.304491133	0.351569139	negative
PRJNA729271	stage_1D_vs_E10	ordinary_muscle_development	ENSGALG00010021931	ITGA6	1.042720287	0.121580342	8.576388816	9.78982E-18	4.61925E-17	positive
PRJNA729271	stage_1D_vs_E10	ordinary_muscle_development	ENSGALG00010021932	RPAP2	0.699476325	0.161879265	4.320975421	1.55341E-05	3.10918E-05	positive
PRJNA729271	stage_1D_vs_E10	ordinary_muscle_development	ENSGALG00010021933	PRR14L	-0.049150067	0.089254899	-0.550670808	0.581859359	0.626275445	negative
PRJNA729271	stage_1D_vs_E10	ordinary_muscle_development	ENSGALG00010021934	EFTUD2	-0.739979852	0.066654415	-11.10173798	1.23028E-28	9.95695E-28	negative
PRJNA729271	stage_1D_vs_E10	ordinary_muscle_development	ENSGALG00010021935	CETP	4.578084736	0.297815165	15.37223513	2.51346E-53	5.44584E-52	positive
PRJNA729271	stage_1D_vs_E10	ordinary_muscle_development	ENSGALG00010021938	CABP2	-0.067485282	0.163073431	-0.413833706	0.678995897	0.716984449	negative
PRJNA729271	stage_1D_vs_E10	ordinary_muscle_development	ENSGALG00010021943	OCM3	-5.000189982	0.570189431	-8.769348768			negative
PRJNA729271	stage_1D_vs_E10	ordinary_muscle_development	ENSGALG00010021945	C1QTNF4	-2.012102343	0.216115423	-9.310313523	1.27456E-20	7.04872E-20	negative
PRJNA729271	stage_1D_vs_E10	ordinary_muscle_development	ENSGALG00010021947	CDK2AP2	0.789067908	0.247573963	3.187200702	0.001436571	0.002353157	positive
PRJNA729271	stage_1D_vs_E10	ordinary_muscle_development	ENSGALG00010021949	ENSGALG00010021949	-1.309913323	0.247148942	-5.300096821	1.15741E-07	2.78544E-07	negative
PRJNA729271	stage_1D_vs_E10	ordinary_muscle_development	ENSGALG00010021951	PSKH1	0.275673576	0.142350177	1.936587519	0.052795785	0.070125071	positive
PRJNA729271	stage_1D_vs_E10	ordinary_muscle_development	ENSGALG00010021953	PITPNM1	0.138650997	0.177594962	0.780714696	0.434970315	0.484470165	positive
PRJNA729271	stage_1D_vs_E10	ordinary_muscle_development	ENSGALG00010021958	BHLHA15	-0.708926628	0.408484017	-1.735506404	0.082651148	0.106576674	negative
PRJNA729271	stage_1D_vs_E10	ordinary_muscle_development	ENSGALG00010021960	CDKN1B	0.3582758	0.144775933	2.474691708	0.013335123	0.019375763	positive
PRJNA729271	stage_1D_vs_E10	ordinary_muscle_development	ENSGALG00010021965	PEX16	0.34715073	0.1012882	3.427356103	0.000609489	0.001038381	positive
PRJNA729271	stage_1D_vs_E10	ordinary_muscle_development	ENSGALG00010021966	ADGRA1	0.722695537	0.158306474	4.565167279	4.99096E-06	1.04801E-05	positive
PRJNA729271	stage_1D_vs_E10	ordinary_muscle_development	ENSGALG00010021969	CCDC103	-1.749803261	0.286218833	-6.113515465	9.74599E-10	2.7586E-09	negative
PRJNA729271	stage_1D_vs_E10	ordinary_muscle_development	ENSGALG00010021970	ERGIC3	-0.291767996	0.090246241	-3.233021056	0.001224885	0.00202356	negative
PRJNA729271	stage_1D_vs_E10	ordinary_muscle_development	ENSGALG00010021971	ICOS	-3.382430541	0.488931849	-6.918000016	4.58064E-12	1.51417E-11	negative
PRJNA729271	stage_1D_vs_E10	ordinary_muscle_development	ENSGALG00010021972	PISD	0.067067885	0.183548687	0.365395613	0.714816115	0.750163596	positive
PRJNA729271	stage_1D_vs_E10	ordinary_muscle_development	ENSGALG00010021974	GFAP	4.796999732	0.787114689	6.09441013	1.09842E-09	3.09277E-09	positive
PRJNA729271	stage_1D_vs_E10	ordinary_muscle_development	ENSGALG00010021978	NR2C2	0.570517983	0.073352159	7.77779403	7.38001E-15	2.92917E-14	positive
PRJNA729271	stage_1D_vs_E10	ordinary_muscle_development	ENSGALG00010021980	GLMN	0.102282929	0.13181781	0.775941653	0.437783437	0.487046069	positive
PRJNA729271	stage_1D_vs_E10	ordinary_muscle_development	ENSGALG00010021983	CTLA4	-6.02995033	0.962771854	-6.263114467	3.77363E-10	1.09815E-09	negative
PRJNA729271	stage_1D_vs_E10	ordinary_muscle_development	ENSGALG00010021988	FAM180B	3.149359206	0.263606124	11.94721563	6.7137E-33	6.66542E-32	positive
PRJNA729271	stage_1D_vs_E10	ordinary_muscle_development	ENSGALG00010021989	NCAPD2	-1.426907236	0.151047155	-9.446766741	3.49458E-21	1.99582E-20	negative
PRJNA729271	stage_1D_vs_E10	ordinary_muscle_development	ENSGALG00010021994	ENSGALG00010021994	-2.334307915	0.336058987	-6.946125556	3.75455E-12	1.2508E-11	negative
PRJNA729271	stage_1D_vs_E10	ordinary_muscle_development	ENSGALG00010021995	CD28	1.72504114	0.50532788	3.413706639	0.000640855	0.00108977	positive
PRJNA729271	stage_1D_vs_E10	ordinary_muscle_development	ENSGALG00010021999	ANKRD22	-0.898181881	0.448787893	-2.001350515	0.045354629	0.060866156	negative
PRJNA729271	stage_1D_vs_E10	ordinary_muscle_development	ENSGALG00010022002	KIF18B	-1.368699505	0.208814932	-6.554605517	5.57892E-11	1.7202E-10	negative
PRJNA729271	stage_1D_vs_E10	ordinary_muscle_development	ENSGALG00010022005	NLRC5	3.15471066	0.182529776	17.28326591	6.28842E-67	2.07633E-65	positive
PRJNA729271	stage_1D_vs_E10	ordinary_muscle_development	ENSGALG00010022006	SFI1	0.198692399	0.106479317	1.866018727	0.062038746	0.081483474	positive
PRJNA729271	stage_1D_vs_E10	ordinary_muscle_development	ENSGALG00010022012	ENSGALG00010022012	-0.96835656	2.998349023	-0.322963255	0.74672307	0.779135539	negative
PRJNA729271	stage_1D_vs_E10	ordinary_muscle_development	ENSGALG00010022017	CNP1	0.288252249	0.721667749	0.399425151	0.689579965	0.727048414	positive
PRJNA729271	stage_1D_vs_E10	ordinary_muscle_development	ENSGALG00010022019	BTBD8	0.414323545	0.152968928	2.70854709	0.006757852	0.010205779	positive
PRJNA729271	stage_1D_vs_E10	ordinary_muscle_development	ENSGALG00010022021	TECPR1	1.388334931	0.077266948	17.96803127	3.46805E-72	1.35863E-70	positive
PRJNA729271	stage_1D_vs_E10	ordinary_muscle_development	ENSGALG00010022022	STAMBPL1	-1.178803613	0.250028497	-4.714677036	2.42094E-06	5.24146E-06	negative
PRJNA729271	stage_1D_vs_E10	ordinary_muscle_development	ENSGALG00010022024	MICAL2	1.820382635	0.183282996	9.932086841	3.01871E-23	1.89996E-22	positive
PRJNA729271	stage_1D_vs_E10	ordinary_muscle_development	ENSGALG00010022027	EIF4ENIF1	-0.317415401	0.066585888	-4.767007104	1.86983E-06	4.08241E-06	negative
PRJNA729271	stage_1D_vs_E10	ordinary_muscle_development	ENSGALG00010022028	FGD5	0.30288069	0.115189571	2.629410704	0.008553299	0.012752527	positive
PRJNA729271	stage_1D_vs_E10	ordinary_muscle_development	ENSGALG00010022029	ENSGALG00010022029	0.061488036	0.647929312	0.094899296	0.924394815	0.93569154	positive
PRJNA729271	stage_1D_vs_E10	ordinary_muscle_development	ENSGALG00010022030	ZNF341	0.527497602	0.151768777	3.475666146	0.000509586	0.00087673	positive
PRJNA729271	stage_1D_vs_E10	ordinary_muscle_development	ENSGALG00010022032	KBTBD4	-0.240911175	0.083002793	-2.902446623	0.003702603	0.005777718	negative
PRJNA729271	stage_1D_vs_E10	ordinary_muscle_development	ENSGALG00010022033	RAPH1	0.316784951	0.11460332	2.764186511	0.005706491	0.008705988	positive
PRJNA729271	stage_1D_vs_E10	ordinary_muscle_development	ENSGALG00010022034	ENSGALG00010022034	1.280216759	0.105446613	12.14089979	6.4117E-34	6.6384E-33	positive
PRJNA729271	stage_1D_vs_E10	ordinary_muscle_development	ENSGALG00010022037	DRG1	-0.127745534	0.094614345	-1.350170885	0.176961175	0.215116759	negative
PRJNA729271	stage_1D_vs_E10	ordinary_muscle_development	ENSGALG00010022038	GAPDH	1.973468017	0.137187041	14.38523644	6.40588E-47	1.08095E-45	positive
PRJNA729271	stage_1D_vs_E10	ordinary_muscle_development	ENSGALG00010022041	MAPK8IP1	-2.090259955	0.161354267	-12.95447588	2.21645E-38	2.73425E-37	negative
PRJNA729271	stage_1D_vs_E10	ordinary_muscle_development	ENSGALG00010022042	AIP	0.281008312	0.084394781	3.329688253	0.000869433	0.001459121	positive
PRJNA729271	stage_1D_vs_E10	ordinary_muscle_development	ENSGALG00010022044	PXMP4	0.10704096	0.059656304	1.794294203	0.072766223	0.094655515	positive
PRJNA729271	stage_1D_vs_E10	ordinary_muscle_development	ENSGALG00010022046	CRY2	0.912674133	0.103336557	8.832054806	1.0277E-18	5.11908E-18	positive
PRJNA729271	stage_1D_vs_E10	ordinary_muscle_development	ENSGALG00010022049	EPHX4	-2.55640232	0.635173111	-4.024733219	5.70399E-05	0.000108489	negative
PRJNA729271	stage_1D_vs_E10	ordinary_muscle_development	ENSGALG00010022050	ENSGALG00010022050	0.095824832	0.20051431	0.477895226	0.632724779	0.674214929	positive
PRJNA729271	stage_1D_vs_E10	ordinary_muscle_development	ENSGALG00010022051	BAIAP2L1	0.228933455	0.110260586	2.076294571	0.037866715	0.051435887	positive
PRJNA729271	stage_1D_vs_E10	ordinary_muscle_development	ENSGALG00010022052	IFFO1	0.443102777	0.1040172	4.259899102	2.04519E-05	4.04821E-05	positive
PRJNA729271	stage_1D_vs_E10	ordinary_muscle_development	ENSGALG00010022054	ENSGALG00010022054	2.522116287	0.408546272	6.173391993	6.68402E-10	1.91094E-09	positive
PRJNA729271	stage_1D_vs_E10	ordinary_muscle_development	ENSGALG00010022055	E2F1	-0.519089818	0.160807273	-3.22802451	0.001246483	0.002057364	negative
PRJNA729271	stage_1D_vs_E10	ordinary_muscle_development	ENSGALG00010022058	NLRP3	-0.519995623	0.127148404	-4.089674783	4.31978E-05	8.29671E-05	negative
PRJNA729271	stage_1D_vs_E10	ordinary_muscle_development	ENSGALG00010022059	NPTX2	-1.467572446	0.250113521	-5.867625388	4.42081E-09	1.1882E-08	negative
PRJNA729271	stage_1D_vs_E10	ordinary_muscle_development	ENSGALG00010022062	MITF	1.949092248	0.17484438	11.14758309	7.35778E-29	6.02548E-28	positive
PRJNA729271	stage_1D_vs_E10	ordinary_muscle_development	ENSGALG00010022065	PABPC1L	0.286572831	0.243730451	1.175777705	0.239683718	0.283493716	positive
PRJNA729271	stage_1D_vs_E10	ordinary_muscle_development	ENSGALG00010022066	DAD1	-0.470888903	0.138825412	-3.391950351	0.00069397	0.001176503	negative
PRJNA729271	stage_1D_vs_E10	ordinary_muscle_development	ENSGALG00010022067	TMEM130	-0.60949047	0.231053419	-2.637876872	0.008342687	0.012457736	negative
PRJNA729271	stage_1D_vs_E10	ordinary_muscle_development	ENSGALG00010022069	NOP2	-0.531794998	0.134866323	-3.943126707	8.04261E-05	0.000150598	negative
PRJNA729271	stage_1D_vs_E10	ordinary_muscle_development	ENSGALG00010022071	ACTA2	-0.702744796	0.198186161	-3.545882281	0.000391301	0.000682294	negative
PRJNA729271	stage_1D_vs_E10	ordinary_muscle_development	ENSGALG00010022072	SMTN	1.759074751	0.312421544	5.630452776	1.79737E-08	4.59974E-08	positive
PRJNA729271	stage_1D_vs_E10	ordinary_muscle_development	ENSGALG00010022073	PTPMT1	-0.628179807	0.140015272	-4.486509216	7.23996E-06	1.49919E-05	negative
PRJNA729271	stage_1D_vs_E10	ordinary_muscle_development	ENSGALG00010022076	YWHAB	-1.355504964	0.069938079	-19.38150121	1.10574E-83	5.74469E-82	negative
PRJNA729271	stage_1D_vs_E10	ordinary_muscle_development	ENSGALG00010022079	ABI2	-0.525255822	0.076089758	-6.903108061	5.08769E-12	1.67643E-11	negative
PRJNA729271	stage_1D_vs_E10	ordinary_muscle_development	ENSGALG00010022081	FAS	0.784281673	0.177618334	4.415544578	1.00756E-05	2.0564E-05	positive
PRJNA729271	stage_1D_vs_E10	ordinary_muscle_development	ENSGALG00010022082	TGFBR3	1.916709047	0.144327367	13.28028831	3.01232E-40	3.97303E-39	positive
PRJNA729271	stage_1D_vs_E10	ordinary_muscle_development	ENSGALG00010022084	SELENOM	-1.066936453	0.201859337	-5.28554422	1.25332E-07	3.00676E-07	negative
PRJNA729271	stage_1D_vs_E10	ordinary_muscle_development	ENSGALG00010022085	MLLT6	0.661619542	0.177072545	3.736432112	0.00018665	0.000336587	positive
PRJNA729271	stage_1D_vs_E10	ordinary_muscle_development	ENSGALG00010022087	SLC35C1	-0.549130834	0.07522301	-7.30003805	2.87686E-13	1.0332E-12	negative
PRJNA729271	stage_1D_vs_E10	ordinary_muscle_development	ENSGALG00010022088	DKK3	-1.126299828	0.270131574	-4.169449024	3.05337E-05	5.94714E-05	negative
PRJNA729271	stage_1D_vs_E10	ordinary_muscle_development	ENSGALG00010022090	TRRAP	-0.415917821	0.090451934	-4.598219209	4.26118E-06	8.98803E-06	negative
PRJNA729271	stage_1D_vs_E10	ordinary_muscle_development	ENSGALG00010022092	CTRL	2.619516171	1.905432925	1.374761681	0.169205342	0.206404202	positive
PRJNA729271	stage_1D_vs_E10	ordinary_muscle_development	ENSGALG00010022093	CHD4	-0.764165963	0.136391116	-5.602754661	2.10972E-08	5.36592E-08	negative
PRJNA729271	stage_1D_vs_E10	ordinary_muscle_development	ENSGALG00010022097	INPP5J	-0.011582475	0.275633195	-0.042021336	0.96648169	0.971373741	negative
PRJNA729271	stage_1D_vs_E10	ordinary_muscle_development	ENSGALG00010022098	CDC7	-1.425157116	0.156777061	-9.090342088	9.87279E-20	5.21334E-19	negative
PRJNA729271	stage_1D_vs_E10	ordinary_muscle_development	ENSGALG00010022100	ENTPD1	-1.094878544	0.166771316	-6.565149024	5.19809E-11	1.6055E-10	negative
PRJNA729271	stage_1D_vs_E10	ordinary_muscle_development	ENSGALG00010022101	ENSGALG00010022101	1.867830745	0.178653895	10.4550239	1.38964E-25	9.78756E-25	positive
PRJNA729271	stage_1D_vs_E10	ordinary_muscle_development	ENSGALG00010022104	EXOC3L1	0.411377717	0.141031514	2.916920527	0.003535058	0.005532355	positive
PRJNA729271	stage_1D_vs_E10	ordinary_muscle_development	ENSGALG00010022105	DLG5	-0.928005137	0.08402092	-11.04492955	2.3195E-28	1.85138E-27	negative
PRJNA729271	stage_1D_vs_E10	ordinary_muscle_development	ENSGALG00010022108	USP47	0.375394805	0.088074782	4.262228021	2.02399E-05	4.00843E-05	positive
PRJNA729271	stage_1D_vs_E10	ordinary_muscle_development	ENSGALG00010022111	PCGF2	-1.203093782	0.171719956	-7.006138417	2.44986E-12	8.26214E-12	negative
PRJNA729271	stage_1D_vs_E10	ordinary_muscle_development	ENSGALG00010022115	E2F4	-0.00909827	0.184498562	-0.049313502	0.960669459	0.966438525	negative
PRJNA729271	stage_1D_vs_E10	ordinary_muscle_development	ENSGALG00010022116	CYP20A1	-0.52862451	0.101632148	-5.201351364	1.97845E-07	4.67138E-07	negative
PRJNA729271	stage_1D_vs_E10	ordinary_muscle_development	ENSGALG00010022117	FAM20C	0.256111187	0.120696944	2.121935964	0.033843116	0.046348825	positive
PRJNA729271	stage_1D_vs_E10	ordinary_muscle_development	ENSGALG00010022120	FOXL3	-3.767313437	0.624742978	-6.030181322	1.63776E-09	4.55212E-09	negative
PRJNA729271	stage_1D_vs_E10	ordinary_muscle_development	ENSGALG00010022122	PSMB3	-0.689033346	0.158273952	-4.353422259	1.34029E-05	2.7054E-05	negative
PRJNA729271	stage_1D_vs_E10	ordinary_muscle_development	ENSGALG00010022124	ENSGALG00010022124	-0.394571807	0.157753793	-2.501187443	0.012377765	0.018077149	negative
PRJNA729271	stage_1D_vs_E10	ordinary_muscle_development	ENSGALG00010022125	CBFA2T2	0.626222963	0.186035122	3.366154501	0.00076224	0.001288324	positive
PRJNA729271	stage_1D_vs_E10	ordinary_muscle_development	ENSGALG00010022129	ELMO3	0.71618448	0.236557439	3.027528892	0.002465621	0.003923072	positive
PRJNA729271	stage_1D_vs_E10	ordinary_muscle_development	ENSGALG00010022134	CH25H	-0.090959919	0.234764669	-0.387451482	0.698421997	0.735408023	negative
PRJNA729271	stage_1D_vs_E10	ordinary_muscle_development	ENSGALG00010022135	CHST1	-2.987427045	0.256310397	-11.65550472	2.15102E-31	1.98593E-30	negative
PRJNA729271	stage_1D_vs_E10	ordinary_muscle_development	ENSGALG00010022137	PDDC1	-0.867353085	0.128628124	-6.743106105	1.55036E-11	4.96409E-11	negative
PRJNA729271	stage_1D_vs_E10	ordinary_muscle_development	ENSGALG00010022138	SPAG17	1.367812968	0.205453345	6.657535659	2.78457E-11	8.7459E-11	positive
PRJNA729271	stage_1D_vs_E10	ordinary_muscle_development	ENSGALG00010022142	TMEM208	0.862185037	0.104817653	8.22557092	1.94263E-16	8.51996E-16	positive
PRJNA729271	stage_1D_vs_E10	ordinary_muscle_development	ENSGALG00010022143	MATCAP1	-0.007711711	0.193978995	-0.039755392	0.96828814	0.97276515	negative
PRJNA729271	stage_1D_vs_E10	ordinary_muscle_development	ENSGALG00010022144	SNTA1	3.672124312	0.186550683	19.6843252	2.93824E-86	1.59512E-84	positive
PRJNA729271	stage_1D_vs_E10	ordinary_muscle_development	ENSGALG00010022148	TALDO1	-0.983124109	0.086895103	-11.31391849	1.11975E-29	9.5139E-29	negative
PRJNA729271	stage_1D_vs_E10	ordinary_muscle_development	ENSGALG00010022149	NBEAL1	0.272347439	0.106084057	2.567279636	0.010249992	0.015126617	positive
PRJNA729271	stage_1D_vs_E10	ordinary_muscle_development	ENSGALG00010022150	CPNE2	-1.130857129	0.131794619	-8.580449909	9.45028E-18	4.46923E-17	negative
PRJNA729271	stage_1D_vs_E10	ordinary_muscle_development	ENSGALG00010022151	PIP4K2B	0.395166399	0.153801732	2.569323471	0.010189729	0.015045342	positive
PRJNA729271	stage_1D_vs_E10	ordinary_muscle_development	ENSGALG00010022152	ZNF644	0.108525401	0.100170555	1.083406198	0.278628166	0.324629472	positive
PRJNA729271	stage_1D_vs_E10	ordinary_muscle_development	ENSGALG00010022154	CPN1	-0.576943135	0.198924458	-2.900312712	0.003727905	0.005815324	negative
PRJNA729271	stage_1D_vs_E10	ordinary_muscle_development	ENSGALG00010022157	EPS8L2	0.031498561	0.137807529	0.228569231	0.819203742	0.844428832	positive
PRJNA729271	stage_1D_vs_E10	ordinary_muscle_development	ENSGALG00010022158	CWC25	-0.303358855	0.078578862	-3.860565671	0.000113125	0.000208911	negative
PRJNA729271	stage_1D_vs_E10	ordinary_muscle_development	ENSGALG00010022159	KIAA2013	-0.165648419	0.089816714	-1.844293917	0.065140318	0.085340646	negative
PRJNA729271	stage_1D_vs_E10	ordinary_muscle_development	ENSGALG00010022161	CELF1	0.274313063	0.094128663	2.914235211	0.003565611	0.005575355	positive
PRJNA729271	stage_1D_vs_E10	ordinary_muscle_development	ENSGALG00010022162	POLE	-1.243758025	0.153824679	-8.085555776	6.18813E-16	2.63815E-15	negative
PRJNA729271	stage_1D_vs_E10	ordinary_muscle_development	ENSGALG00010022164	PDGFA	-0.142582697	0.107802127	-1.322633424	0.185957313	0.225089875	negative
PRJNA729271	stage_1D_vs_E10	ordinary_muscle_development	ENSGALG00010022165	FAM192A	-0.629913397	0.08972737	-7.02030381	2.21386E-12	7.4889E-12	negative
PRJNA729271	stage_1D_vs_E10	ordinary_muscle_development	ENSGALG00010022166	SDHB	1.439879465	0.107681847	13.37160819	8.86042E-41	1.20254E-39	positive

PRJNA729271	stage_1D_vs_E10	ordinary_muscle_development	ENSGALG00010022167	WDR3	-0.846460082	0.072070184	-11.74494123	7.49746E-32	7.10299E-31	negative
PRJNA729271	stage_1D_vs_E10	ordinary_muscle_development	ENSGALG00010022169	TOX2	-0.613702468	0.238390144	-2.574361743	0.010042521	0.014837055	negative
PRJNA729271	stage_1D_vs_E10	ordinary_muscle_development	ENSGALG00010022170	RSPRY1	-0.558300055	0.140094663	-3.98516291	6.74338E-05	0.000127306	negative
PRJNA729271	stage_1D_vs_E10	ordinary_muscle_development	ENSGALG00010022171	FOXP1	-0.197455775	0.094182228	-2.096529016	0.036035289	0.049137489	negative
PRJNA729271	stage_1D_vs_E10	ordinary_muscle_development	ENSGALG00010022172	GIPC2	1.414569892	0.180537281	7.835333981	4.67598E-15	1.88325E-14	positive
PRJNA729271	stage_1D_vs_E10	ordinary_muscle_development	ENSGALG00010022173	RPL23	0.198121628	0.104038932	1.904302784	0.056870762	0.075131398	positive
PRJNA729271	stage_1D_vs_E10	ordinary_muscle_development	ENSGALG00010022174	KCNMA1	3.082244705	0.123108728	25.03676831	2.433E-138	3.6736E-136	positive
PRJNA729271	stage_1D_vs_E10	ordinary_muscle_development	ENSGALG00010022176	ARL2BPL	-1.165822103	0.139851043	-8.336170234	7.67354E-17	3.45214E-16	negative
PRJNA729271	stage_1D_vs_E10	ordinary_muscle_development	ENSGALG00010022178	ENSGALG00010022178	0.73430279	0.323482448	2.269992678	0.023208027	0.032577993	positive
PRJNA729271	stage_1D_vs_E10	ordinary_muscle_development	ENSGALG00010022180	PLLP	2.958910875	0.367241363	8.057128552	7.81075E-16	3.30657E-15	positive
PRJNA729271	stage_1D_vs_E10	ordinary_muscle_development	ENSGALG00010022181	ENSGALG00010022181	-1.625993906	0.233887753	-6.952026711	3.60075E-12	1.20149E-11	negative
PRJNA729271	stage_1D_vs_E10	ordinary_muscle_development	ENSGALG00010022182	CNTNAP1	-0.925087904	0.3034957	-3.048108765	0.002302866	0.003677836	negative
PRJNA729271	stage_1D_vs_E10	ordinary_muscle_development	ENSGALG00010022184	PRKAR1B	-0.517913816	0.092030485	-5.627633224	1.82699E-08	4.67141E-08	negative
PRJNA729271	stage_1D_vs_E10	ordinary_muscle_development	ENSGALG00010022186	DNAJB4	0.929749132	0.112564545	8.259697881	1.46042E-16	6.45782E-16	positive
PRJNA729271	stage_1D_vs_E10	ordinary_muscle_development	ENSGALG00010022190	ACVR1B	0.881878501	0.163537394	5.39251899	6.94768E-08	1.70026E-07	positive
PRJNA729271	stage_1D_vs_E10	ordinary_muscle_development	ENSGALG00010022193	GDAP2	-0.702006788	0.082428775	-8.516525837	1.64411E-17	7.66529E-17	negative
PRJNA729271	stage_1D_vs_E10	ordinary_muscle_development	ENSGALG00010022194	ENSGALG00010022194	-0.793147731	0.089969372	-8.815752702	1.18884E-18	5.90345E-18	negative
PRJNA729271	stage_1D_vs_E10	ordinary_muscle_development	ENSGALG00010022196	PLXNA1	0.274704184	0.108162055	2.539746348	0.011093289	0.016306381	positive
PRJNA729271	stage_1D_vs_E10	ordinary_muscle_development	ENSGALG00010022199	RBM39	-0.055981849	0.061256734	-0.913888898	0.360775228	0.409734148	negative
PRJNA729271	stage_1D_vs_E10	ordinary_muscle_development	ENSGALG00010022200	DNMBP	0.44238455	0.108812874	4.065553418	4.79186E-05	9.17543E-05	positive
PRJNA729271	stage_1D_vs_E10	ordinary_muscle_development	ENSGALG00010022202	CX3CL1	1.960903761	0.159834027	12.26837488	1.33914E-34	1.43571E-33	positive
PRJNA729271	stage_1D_vs_E10	ordinary_muscle_development	ENSGALG00010022203	DNAAF5	-0.000329209	0.13136332	-0.002506092	0.99800043	0.998413709	negative
PRJNA729271	stage_1D_vs_E10	ordinary_muscle_development	ENSGALG00010022204	ENSGALG00010022204	0.417786491	0.332801899	1.255360898	0.209347777	0.250598401	positive
PRJNA729271	stage_1D_vs_E10	ordinary_muscle_development	ENSGALG00010022205	GALNT18	-0.710732047	0.108514593	-6.549644893	5.76741E-11	1.77454E-10	negative
PRJNA729271	stage_1D_vs_E10	ordinary_muscle_development	ENSGALG00010022207	ENSGALG00010022207	2.040587891	0.306242863	6.663299419	2.67747E-11	8.42048E-11	positive
PRJNA729271	stage_1D_vs_E10	ordinary_muscle_development	ENSGALG00010022208	CCL17	-2.794726271	1.064769749	-2.624723584	0.008671934	0.012918775	negative
PRJNA729271	stage_1D_vs_E10	ordinary_muscle_development	ENSGALG00010022209	ZFYVE19	-0.792292458	0.121253243	-6.534196037	6.39521E-11	1.96105E-10	negative
PRJNA729271	stage_1D_vs_E10	ordinary_muscle_development	ENSGALG00010022210	EZH1	0.868501484	0.082692199	10.50282243	8.38353E-26	5.93936E-25	positive
PRJNA729271	stage_1D_vs_E10	ordinary_muscle_development	ENSGALG00010022211	ENSGALG00010022211	0.028558057	0.498079459	0.057336348	0.954277266	0.961507644	positive
PRJNA729271	stage_1D_vs_E10	ordinary_muscle_development	ENSGALG00010022213	IDH1	-0.885249393	0.080518428	-10.99436998	4.06744E-28	3.19726E-27	negative
PRJNA729271	stage_1D_vs_E10	ordinary_muscle_development	ENSGALG00010022214	ENSGALG00010022214	-2.441060018	0.277369844	-8.800740481	1.35916E-18	6.73311E-18	negative
PRJNA729271	stage_1D_vs_E10	ordinary_muscle_development	ENSGALG00010022215	ENSGALG00010022215	1.66553626	0.127875149	13.02470632	8.85411E-39	1.11213E-37	positive
PRJNA729271	stage_1D_vs_E10	ordinary_muscle_development	ENSGALG00010022216	ROMO1	-0.315045232	0.145143074	-2.170583988	0.029962634	0.041311555	negative
PRJNA729271	stage_1D_vs_E10	ordinary_muscle_development	ENSGALG00010022218	ENSGALG00010022218	2.927508565	0.247483784	11.82909249	2.76111E-32	2.65929E-31	positive
PRJNA729271	stage_1D_vs_E10	ordinary_muscle_development	ENSGALG00010022219	EIF4E3	0.340264852	0.13714579	2.481044816	0.01309979	0.019051014	positive
PRJNA729271	stage_1D_vs_E10	ordinary_muscle_development	ENSGALG00010022221	CUTC	0.11925963	0.083270888	1.432188766	0.152089806	0.187238129	positive
PRJNA729271	stage_1D_vs_E10	ordinary_muscle_development	ENSGALG00010022222	ENSGALG00010022222	-0.16394018	0.106675025	-1.536818754	0.1243377	0.15559656	negative
PRJNA729271	stage_1D_vs_E10	ordinary_muscle_development	ENSGALG00010022223	DOK4	-2.151086295	0.181326082	-11.86308266	1.84071E-32	1.79068E-31	negative
PRJNA729271	stage_1D_vs_E10	ordinary_muscle_development	ENSGALG00010022224	NHEJ1	-0.018943845	0.155529583	-0.121802198	0.903055673	0.917230186	negative
PRJNA729271	stage_1D_vs_E10	ordinary_muscle_development	ENSGALG00010022225	ENSGALG00010022225	-1.318652248	0.621506593	-2.12170275	0.033862708	0.046369901	negative
PRJNA729271	stage_1D_vs_E10	ordinary_muscle_development	ENSGALG00010022226	FBRSL1	0.491614239	0.130794662	3.758672026	0.000170818	0.000309694	positive
PRJNA729271	stage_1D_vs_E10	ordinary_muscle_development	ENSGALG00010022227	TMEM80	0.678189233	0.156136869	4.343555986	1.40195E-05	2.82161E-05	positive
PRJNA729271	stage_1D_vs_E10	ordinary_muscle_development	ENSGALG00010022228	FUBP1	-1.467347716	0.05525156	-26.55757973	2.0994E-155	4.004E-153	negative
PRJNA729271	stage_1D_vs_E10	ordinary_muscle_development	ENSGALG00010022229	RAPSN	0.977091712	0.178006508	5.489078602	4.04036E-08	1.00403E-07	positive
PRJNA729271	stage_1D_vs_E10	ordinary_muscle_development	ENSGALG00010022230	NFS1	0.282919183	0.086583497	3.267587855	0.001084682	0.001801589	positive
PRJNA729271	stage_1D_vs_E10	ordinary_muscle_development	ENSGALG00010022231	ZNF503	-2.039806606	0.206000677	-9.901941262	4.08272E-23	2.55523E-22	negative
PRJNA729271	stage_1D_vs_E10	ordinary_muscle_development	ENSGALG00010022232	GPR27	-2.483324652	0.202526038	-12.26175499	1.45318E-34	1.55453E-33	negative
PRJNA729271	stage_1D_vs_E10	ordinary_muscle_development	ENSGALG00010022233	FAM46C	3.189037804	0.163963728	19.44965415	2.93403E-84	1.5409E-82	positive
PRJNA729271	stage_1D_vs_E10	ordinary_muscle_development	ENSGALG00010022234	COX15	-0.295454592	0.096151661	-3.072797574	0.002120623	0.003401774	negative
PRJNA729271	stage_1D_vs_E10	ordinary_muscle_development	ENSGALG00010022235	RAMP2	0.965230063	0.112998737	8.541954424	1.3197E-17	6.17467E-17	positive
PRJNA729271	stage_1D_vs_E10	ordinary_muscle_development	ENSGALG00010022236	SHF	1.891928014	0.181352126	10.43234537	1.76478E-25	1.23756E-24	positive
PRJNA729271	stage_1D_vs_E10	ordinary_muscle_development	ENSGALG00010022237	SUN1	1.10515728	0.090668679	12.18896412	3.55917E-34	3.72223E-33	positive
PRJNA729271	stage_1D_vs_E10	ordinary_muscle_development	ENSGALG00010022238	COMTD1	-1.051969131	0.170987911	-6.152301211	7.63667E-10	2.17601E-09	negative
PRJNA729271	stage_1D_vs_E10	ordinary_muscle_development	ENSGALG00010022239	DEAF1	0.731648163	0.111179917	6.580758332	4.68055E-11	1.45122E-10	positive
PRJNA729271	stage_1D_vs_E10	ordinary_muscle_development	ENSGALG00010022240	ENSGALG00010022240	-0.409544851	0.130830422	-3.130348771	0.001745989	0.002832469	negative
PRJNA729271	stage_1D_vs_E10	ordinary_muscle_development	ENSGALG00010022241	POLR2C	-0.268965591	0.095146528	-2.826856614	0.004700737	0.007247866	negative
PRJNA729271	stage_1D_vs_E10	ordinary_muscle_development	ENSGALG00010022242	USP19	0.594692498	0.125857765	4.725115689	2.29984E-06	4.99045E-06	positive
PRJNA729271	stage_1D_vs_E10	ordinary_muscle_development	ENSGALG00010022243	ENSGALG00010022243	1.843653509	0.569346638	3.238191617	0.0012029	0.001988826	positive
PRJNA729271	stage_1D_vs_E10	ordinary_muscle_development	ENSGALG00010022244	ENTPD7	-1.211045401	0.173802184	-6.967952717	3.21586E-12	1.07678E-11	negative
PRJNA729271	stage_1D_vs_E10	ordinary_muscle_development	ENSGALG00010022245	PIKFYVE	1.176022401	0.09954029	11.81453661	3.28354E-32	3.15198E-31	positive
PRJNA729271	stage_1D_vs_E10	ordinary_muscle_development	ENSGALG00010022246	RHOV	-2.711570593	0.437129773	-6.203124929	5.53529E-10	1.59321E-09	negative
PRJNA729271	stage_1D_vs_E10	ordinary_muscle_development	ENSGALG00010022247	GALNT9	-2.687097996	0.229007696	-11.73365804	8.56744E-32	8.09023E-31	negative
PRJNA729271	stage_1D_vs_E10	ordinary_muscle_development	ENSGALG00010022248	FIGNL2	-0.287886829	0.21681627	-1.327791635	0.184246951	0.223227359	negative
PRJNA729271	stage_1D_vs_E10	ordinary_muscle_development	ENSGALG00010022250	SLC25A28	1.070729459	0.127980533	8.366346316	5.94323E-17	2.68455E-16	positive
PRJNA729271	stage_1D_vs_E10	ordinary_muscle_development	ENSGALG00010022251	WNK4	-0.102094205	0.149521954	-0.682804109	0.494730633	0.542896549	negative
PRJNA729271	stage_1D_vs_E10	ordinary_muscle_development	ENSGALG00010022252	COQ9	2.927634785	0.131724964	22.22536035	1.9531E-109	1.7158E-107	positive
PRJNA729271	stage_1D_vs_E10	ordinary_muscle_development	ENSGALG00010022253	ALPK3	2.988273826	0.21728626	13.75270495	4.90604E-43	7.15422E-42	positive
PRJNA729271	stage_1D_vs_E10	ordinary_muscle_development	ENSGALG00010022255	NOC4L	-1.635309746	0.092265099	-17.72403385	2.73573E-70	1.01678E-68	negative
PRJNA729271	stage_1D_vs_E10	ordinary_muscle_development	ENSGALG00010022256	PSMC3	-0.531799999	0.092347606	-5.758676588	8.4776E-09	2.22815E-08	negative
PRJNA729271	stage_1D_vs_E10	ordinary_muscle_development	ENSGALG00010022257	KCNK18	-0.338004603	1.148592618	-0.294277186	0.768546105	0.798248775	negative
PRJNA729271	stage_1D_vs_E10	ordinary_muscle_development	ENSGALG00010022258	ENSGALG00010022258	4.683867831	0.676392895	6.924773851	4.36673E-12	1.44478E-11	positive
PRJNA729271	stage_1D_vs_E10	ordinary_muscle_development	ENSGALG00010022259	GET4	0.069138704	0.085258343	0.810931832	0.417404819	0.466809099	positive
PRJNA729271	stage_1D_vs_E10	ordinary_muscle_development	ENSGALG00010022264	SCN8A	-1.011117168	0.254179755	-3.977961063	6.95088E-05	0.000131035	negative
PRJNA729271	stage_1D_vs_E10	ordinary_muscle_development	ENSGALG00010022266	COA3	0.358938603	0.144944058	2.47639405	0.013271701	0.019289412	positive
PRJNA729271	stage_1D_vs_E10	ordinary_muscle_development	ENSGALG00010022269	CNTD1	-0.156019697	0.373244322	-0.418009565	0.675940124	0.714121873	negative
PRJNA729271	stage_1D_vs_E10	ordinary_muscle_development	ENSGALG00010022271	SLC18A2	-1.074849307	0.229150731	-4.690577684	2.72435E-06	5.86854E-06	negative
PRJNA729271	stage_1D_vs_E10	ordinary_muscle_development	ENSGALG00010022274	CCDC91	2.115683875	0.173547496	12.19080608	3.47962E-34	3.64167E-33	positive
PRJNA729271	stage_1D_vs_E10	ordinary_muscle_development	ENSGALG00010022275	VDAC2	1.92492454	0.124007336	15.52266666	2.43709E-54	5.45148E-53	positive
PRJNA729271	stage_1D_vs_E10	ordinary_muscle_development	ENSGALG00010022276	BECN1	0.208885638	0.088627375	2.356897482	0.018428334	0.02618554	positive
PRJNA729271	stage_1D_vs_E10	ordinary_muscle_development	ENSGALG00010022279	EP400	0.639570137	0.148737633	4.299988684	1.70807E-05	3.40463E-05	positive
PRJNA729271	stage_1D_vs_E10	ordinary_muscle_development	ENSGALG00010022280	PDZD8	0.196915862	0.066931694	2.942042099	0.003260556	0.005119341	positive
PRJNA729271	stage_1D_vs_E10	ordinary_muscle_development	ENSGALG00010022282	TMT1A	2.150566254	0.223293915	9.631101047	5.90929E-22	3.53071E-21	positive
PRJNA729271	stage_1D_vs_E10	ordinary_muscle_development	ENSGALG00010022290	NEXN	1.761776804	0.220049384	8.006279207	1.18231E-15	4.94306E-15	positive
PRJNA729271	stage_1D_vs_E10	ordinary_muscle_development	ENSGALG00010022293	DNAJC10	-0.789648957	0.063792005	-12.37849415	3.41694E-35	3.75216E-34	negative
PRJNA729271	stage_1D_vs_E10	ordinary_muscle_development	ENSGALG00010022294	ENSGALG00010022294	1.041058751	0.653310558	1.593512821	0.111045195	0.140074849	positive
PRJNA729271	stage_1D_vs_E10	ordinary_muscle_development	ENSGALG00010022296	CIAPIN1	0.049114875	0.097206241	0.505264628	0.613372983	0.655908623	positive
PRJNA729271	stage_1D_vs_E10	ordinary_muscle_development	ENSGALG00010022297	VPS18	-0.428251653	0.057441688	-7.455415507	8.95851E-14	3.32446E-13	negative
PRJNA729271	stage_1D_vs_E10	ordinary_muscle_development	ENSGALG00010022299	MRGBP	-0.010312394	0.079578945	-0.129586965	0.896893217	0.91212146	negative
PRJNA729271	stage_1D_vs_E10	ordinary_muscle_development	ENSGALG00010022302	ENSGALG00010022302	-1.439177686	0.157446721	-9.140728212	6.20332E-20	3.30456E-19	negative
PRJNA729271	stage_1D_vs_E10	ordinary_muscle_development	ENSGALG00010022303	DUSP13A	3.577171455	0.206858766	17.29282027	5.32805E-67	1.76728E-65	positive
PRJNA729271	stage_1D_vs_E10	ordinary_muscle_development	ENSGALG00010022304	ADAP1	-1.971744466	0.193207277	-10.20533233	1.87678E-24	1.2496E-23	negative
PRJNA729271	stage_1D_vs_E10	ordinary_muscle_development	ENSGALG00010022307	ENSGALG00010022307	7.83416998	0.635024625	12.33679715	5.73939E-35	6.22232E-34	positive
PRJNA729271	stage_1D_vs_E10	ordinary_muscle_development	ENSGALG00010022308	OGFR	-0.163956648	0.181663657	-0.90252861	0.36677613	0.415832617	negative
PRJNA729271	stage_1D_vs_E10	ordinary_muscle_development	ENSGALG00010022309	WSCD2	-0.221552029	0.321138983	-0.689894534	0.490260513	0.538562042	negative
PRJNA729271	stage_1D_vs_E10	ordinary_muscle_development	ENSGALG00010022310	DCLRE1A	-1.609051891	0.099802868	-16.12230115	1.77861E-58	4.59554E-57	negative
PRJNA729271	stage_1D_vs_E10	ordinary_muscle_development	ENSGALG00010022311	DLL4	0.853170775	0.141334527	6.036534685	1.57459E-09	4.38243E-09	positive
PRJNA729271	stage_1D_vs_E10	ordinary_muscle_development	ENSGALG00010022312	ZNF592	0.45164762	0.053329308	8.469031997	2.47441E-17	1.1437E-16	positive
PRJNA729271	stage_1D_vs_E10	ordinary_muscle_development	ENSGALG00010022313	ENSGALG00010022313	-1.223124346	0.413399159	-2.958700614	0.003089391	0.004862713	negative
PRJNA729271	stage_1D_vs_E10	ordinary_muscle_development	ENSGALG00010022314	DUSP13B	7.20733999	0.550693573	13.08775032	3.86898E-39	4.92802E-38	positive
PRJNA729271	stage_1D_vs_E10	ordinary_muscle_development	ENSGALG00010022316	INO80	0.242704689	0.068907968	3.522157095	0.00042805	0.00074191	positive
PRJNA729271	stage_1D_vs_E10	ordinary_muscle_development	ENSGALG00010022317	PSME3	-1.3894354	0.073884924	-18.8053981	6.82082E-79	3.16884E-77	negative
PRJNA729271	stage_1D_vs_E10	ordinary_muscle_development	ENSGALG00010022320	COL9A3	-7.097094004	0.547522735	-12.96219051	2.00441E-38	2.479E-37	negative
PRJNA729271	stage_1D_vs_E10	ordinary_muscle_development	ENSGALG00010022322	ENSGALG00010022322	1.386611913	0.381414356	3.635447619	0.000277498	0.000490769	positive
PRJNA729271	stage_1D_vs_E10	ordinary_muscle_development	ENSGALG00010022323	DUSP29	4.4603681	0.382748192	11.65353148	2.20143E-31	2.03117E-30	positive
PRJNA729271	stage_1D_vs_E10	ordinary_muscle_development	ENSGALG00010022325	MIGA1	-0.036152965	0.106807192	-0.338488113	0.734995383	0.768724878	negative
PRJNA729271	stage_1D_vs_E10	ordinary_muscle_development	ENSGALG00010022326	COX19	1.08528859	0.093715311	11.58069672	5.16248E-31	4.67104E-30	positive
PRJNA729271	stage_1D_vs_E10	ordinary_muscle_development	ENSGALG00010022328	ENSGALG00010022328	-1.546576845	0.264165627	-5.854572614	4.78238E-09	1.28253E-08	negative
PRJNA729271	stage_1D_vs_E10	ordinary_muscle_development	ENSGALG00010022332	KAT6B	-0.355590304	0.06058826	-5.868963782	4.38527E-09	1.17909E-08	negative
PRJNA729271	stage_1D_vs_E10	ordinary_muscle_development	ENSGALG00010022333	ENSGALG00010022333	1.965266411	0.128950623	15.24045688	1.90533E-52	4.02006E-51	positive
PRJNA729271	stage_1D_vs_E10	ordinary_muscle_development	ENSGALG00010022334	PDE1A	-1.141154991	0.120797471	-9.446845017	3.49197E-21	1.99512E-20	negative
PRJNA729271	stage_1D_vs_E10	ordinary_muscle_development	ENSGALG00010022340	GFOD2	-0.65939147	0.094441093	-6.98203983	2.90925E-12	9.7592E-12	negative
PRJNA729271	stage_1D_vs_E10	ordinary_muscle_development	ENSGALG00010022343	GATA5	-4.539785707	0.678893149	-6.687040094	2.2773E-11	7.19786E-11	negative
PRJNA729271	stage_1D_vs_E10	ordinary_muscle_development	ENSGALG00010022345	USP33	0.06563593	0.122346711	0.536474822	0.591630433	0.63532991	positive
PRJNA729271	stage_1D_vs_E10	ordinary_muscle_development	ENSGALG00010022347	SEC11A	-1.189970841	0.104324753	-11.40640943	3.88434E-30	3.38363E-29	negative
PRJNA729271	stage_1D_vs_E10	ordinary_muscle_development	ENSGALG00010022348	G6PC1	2.863349217	0.615502852	4.652048654	3.28654E-06	7.02527E-06	positive
PRJNA729271	stage_1D_vs_E10	ordinary_muscle_development	ENSGALG00010022353	SLC39A13	-0.281360282	0.095431227	-2.948304136	0.003195225	0.00502003	negative
PRJNA729271	stage_1D_vs_E10	ordinary_muscle_development	ENSGALG00010022354	FAR2	-0.33390377	0.136805884	-2.440712061	0.014658337	0.021164717	negative
PRJNA729271	stage_1D_vs_E10	ordinary_muscle_development	ENSGALG00010022356	TCFL5	-0.865681155	0.216769466	-3.993556708	6.50895E-05	0.000123153	negative
PRJNA729271	stage_1D_vs_E10	ordinary_muscle_development	ENSGALG00010022357	ENSGALG00010022357	-5.292182717	0.78953324	-6.702925794	2.04287E-11	6.47669E-11	negative
PRJNA729271	stage_1D_vs_E10	ordinary_muscle_development	ENSGALG00010022359	ZZZ3	-0.134957193	0.078707171	-1.714674676	0.086404889	0.111071201	negative
PRJNA729271	stage_1D_vs_E10	ordinary_muscle_development	ENSGALG00010022363	KIF18A	-1.648854827	0.201670565	-8.175981592	2.93467E-16	1.27589E-15	negative
PRJNA729271	stage_1D_vs_E10	ordinary_muscle_development	ENSGALG00010022365	PLCG1	-1.191309208	0.109232533	-10.90617578	1.07692E-27	8.30316E-27	negative
PRJNA729271	stage_1D_vs_E10	ordinary_muscle_development	ENSGALG00010022366	AARSD1	0.159178229	0.113635924	1.400773837	0.161281715	0.197664535	positive
PRJNA729271	stage_1D_vs_E10	ordinary_muscle_development	ENSGALG00010022373	AK5	-3.330648364	0.204991467	-16.24774149	2.31746E-59	6.22066E-58	negative
PRJNA729271	stage_1D_vs_E10	ordinary_muscle_development	ENSGALG00010022375	NMB	-2.233715956	0.438204627	-5.097426679	3.44302E-07	7.96084E-07	negative
PRJNA729271	stage_1D_vs_E10	ordinary_muscle_development	ENSGALG00010022377	ZHX3	1.350963603	0.089306191	15.12732299	1.06959E-51	2.18978E-50	positive
PRJNA729271	stage_1D_vs_E10	ordinary_muscle_development	ENSGALG00010022379	METTL15	-0.420790497	0.114164112	-3.685838658	0.000227951	0.000406915	negative
PRJNA729271	stage_1D_vs_E10	ordinary_muscle_development	ENSGALG00010022382	CALB2	-5.202241644	0.99649173	-5.220556767	1.78386E-07	4.22501E-07	negative
PRJNA729271	stage_1D_vs_E10	ordinary_muscle_development	ENSGALG00010022385	ENSGALG00010022385	2.273858923	0.633545461	3.589101436	0.00033182	0.000582503	positive
PRJNA729271	stage_1D_vs_E10	ordinary_muscle_development	ENSGALG00010022386	SPI1	0.145635691	0.247177808	0.589194037	0.555731117	0.601457667	positive
PRJNA729271	stage_1D_vs_E10	ordinary_muscle_development	ENSGALG00010022388	CYP2W1	-0.539807086	0.439788956	-1.227423016	0.21966364	0.26203806	negative

PRJNA729271	stage_1D_vs_E10	ordinary_muscle_development	ENSGALG00010022391	ENSGALG00010022391	1.003544547	0.177251408	5.661701423	1.49879E-08	3.85743E-08	positive
PRJNA729271	stage_1D_vs_E10	ordinary_muscle_development	ENSGALG00010022392	EMILIN3	-3.212303326	0.188372528	-17.0529289	3.32449E-65	1.06376E-63	negative
PRJNA729271	stage_1D_vs_E10	ordinary_muscle_development	ENSGALG00010022395	PTGES3L	1.262470313	0.133612131	9.448770137	3.42835E-21	1.96109E-20	positive
PRJNA729271	stage_1D_vs_E10	ordinary_muscle_development	ENSGALG00010022396	PPP1R1C	0.852554879	0.322657543	2.64229025	0.008234745	0.012310503	positive
PRJNA729271	stage_1D_vs_E10	ordinary_muscle_development	ENSGALG00010022397	ATF1	-0.332549378	0.047445854	-7.009029308	2.39977E-12	8.10265E-12	negative
PRJNA729271	stage_1D_vs_E10	ordinary_muscle_development	ENSGALG00010022400	NHLRC2	0.390375535	0.071129721	5.488219676	4.06005E-08	1.00826E-07	positive
PRJNA729271	stage_1D_vs_E10	ordinary_muscle_development	ENSGALG00010022401	LCAT	4.22453889	0.435875861	9.692068936	3.2586E-22	1.96233E-21	positive
PRJNA729271	stage_1D_vs_E10	ordinary_muscle_development	ENSGALG00010022406	ITPRID2	1.014300498	0.103758909	9.775550922	1.43375E-22	8.78741E-22	positive
PRJNA729271	stage_1D_vs_E10	ordinary_muscle_development	ENSGALG00010022407	RUNDC1	0.345212304	0.09535556	3.620264041	0.000294303	0.000519157	positive
PRJNA729271	stage_1D_vs_E10	ordinary_muscle_development	ENSGALG00010022408	RAD51AP1	-0.41578738	0.137204613	-3.03041836	0.002442152	0.003887436	negative
PRJNA729271	stage_1D_vs_E10	ordinary_muscle_development	ENSGALG00010022409	CARNS1	6.567046147	0.339353024	19.35166531	1.97361E-83	1.01806E-81	positive
PRJNA729271	stage_1D_vs_E10	ordinary_muscle_development	ENSGALG00010022411	ADRB1	0.583569823	0.290968386	2.005612471	0.04489761	0.06030867	positive
PRJNA729271	stage_1D_vs_E10	ordinary_muscle_development	ENSGALG00010022412	CHAC1	3.678680434	0.650893104	5.651742833	1.58829E-08	4.08052E-08	positive
PRJNA729271	stage_1D_vs_E10	ordinary_muscle_development	ENSGALG00010022415	DIP2B	0.090079554	0.108923163	0.827000906	0.408236548	0.457908799	positive
PRJNA729271	stage_1D_vs_E10	ordinary_muscle_development	ENSGALG00010022416	CCDC186	0.235096394	0.085849127	2.738483238	0.006172331	0.009383108	positive
PRJNA729271	stage_1D_vs_E10	ordinary_muscle_development	ENSGALG00010022419	DUOX2	2.379431955	0.693543055	3.43083524	0.000601726	0.001025878	positive
PRJNA729271	stage_1D_vs_E10	ordinary_muscle_development	ENSGALG00010022421	CMKLR1	0.616442054	0.17829146	3.457496238	0.00054522	0.000935483	positive
PRJNA729271	stage_1D_vs_E10	ordinary_muscle_development	ENSGALG00010022422	RPS6KB2	0.407227311	0.157173626	2.590939212	0.00957144	0.014190244	positive
PRJNA729271	stage_1D_vs_E10	ordinary_muscle_development	ENSGALG00010022425	VWA2	-3.858779146	0.462157482	-8.349489723	6.85585E-17	3.09196E-16	negative
PRJNA729271	stage_1D_vs_E10	ordinary_muscle_development	ENSGALG00010022426	EXD1	-1.597050787	0.242951706	-6.57353189	4.91355E-11	1.52021E-10	negative
PRJNA729271	stage_1D_vs_E10	ordinary_muscle_development	ENSGALG00010022427	RPL27	0.438722408	0.138641791	3.164431192	0.001553864	0.002534405	positive
PRJNA729271	stage_1D_vs_E10	ordinary_muscle_development	ENSGALG00010022429	ADK	0.571479751	0.130021623	4.395267015	1.10637E-05	2.24857E-05	positive
PRJNA729271	stage_1D_vs_E10	ordinary_muscle_development	ENSGALG00010022431	LARP4	0.236583108	0.132570052	1.784589393	0.074327943	0.096531094	positive
PRJNA729271	stage_1D_vs_E10	ordinary_muscle_development	ENSGALG00010022433	LNPK	1.322611915	0.106858055	12.37727853	3.46908E-35	3.80653E-34	positive
PRJNA729271	stage_1D_vs_E10	ordinary_muscle_development	ENSGALG00010022435	PIGK	0.267334155	0.16373921	1.632682577	0.102535774	0.130213426	positive
PRJNA729271	stage_1D_vs_E10	ordinary_muscle_development	ENSGALG00010022436	AFAP1L2	0.021643091	0.155263329	0.139396026	0.889137212	0.905440768	positive
PRJNA729271	stage_1D_vs_E10	ordinary_muscle_development	ENSGALG00010022438	RPN2	-0.518370396	0.1010732	-5.128663157	2.91807E-07	6.78822E-07	negative
PRJNA729271	stage_1D_vs_E10	ordinary_muscle_development	ENSGALG00010022439	IFI35	0.859141385	0.205108947	4.188707496	2.80548E-05	5.48495E-05	positive
PRJNA729271	stage_1D_vs_E10	ordinary_muscle_development	ENSGALG00010022440	SORD	0.136707372	0.124887552	1.0946437	0.273672763	0.31936935	positive
PRJNA729271	stage_1D_vs_E10	ordinary_muscle_development	ENSGALG00010022441	FICD	-0.00039616	0.118426974	-0.003345181	0.997330937	0.997950568	negative
PRJNA729271	stage_1D_vs_E10	ordinary_muscle_development	ENSGALG00010022448	FERRY3	-0.714785617	0.121133222	-5.900822312	3.61694E-09	9.79955E-09	negative
PRJNA729271	stage_1D_vs_E10	ordinary_muscle_development	ENSGALG00010022449	ENSGALG00010022449	-0.69388392	0.102479727	-6.770938395	1.2795E-11	4.11592E-11	negative
PRJNA729271	stage_1D_vs_E10	ordinary_muscle_development	ENSGALG00010022451	CHP1	-0.094114539	0.121074675	-0.77732638	0.436966235	0.486319532	negative
PRJNA729271	stage_1D_vs_E10	ordinary_muscle_development	ENSGALG00010022452	AP3M1	0.191964605	0.081139361	2.365862911	0.017988104	0.025600236	positive
PRJNA729271	stage_1D_vs_E10	ordinary_muscle_development	ENSGALG00010022453	ENSGALG00010022453	1.802282985	0.23535348	7.65777072	1.89188E-14	7.32643E-14	positive
PRJNA729271	stage_1D_vs_E10	ordinary_muscle_development	ENSGALG00010022454	ABLIM1	2.308937437	0.140192177	16.46980226	6.04677E-61	1.7019E-59	positive
PRJNA729271	stage_1D_vs_E10	ordinary_muscle_development	ENSGALG00010022455	C7orf50	-1.324925879	0.15406178	-8.599964787	7.97406E-18	3.78591E-17	negative
PRJNA729271	stage_1D_vs_E10	ordinary_muscle_development	ENSGALG00010022456	ATP5G3	0.176210797	0.158207344	1.113796572	0.265366468	0.310551183	positive
PRJNA729271	stage_1D_vs_E10	ordinary_muscle_development	ENSGALG00010022459	FGF6	1.277635926	0.793315767	1.610501114	0.107288502	0.135631157	positive
PRJNA729271	stage_1D_vs_E10	ordinary_muscle_development	ENSGALG00010022460	VAT1	-0.314459397	0.1159357	-2.712360368	0.006680592	0.010098569	negative
PRJNA729271	stage_1D_vs_E10	ordinary_muscle_development	ENSGALG00010022462	ITGA5	0.404124915	0.128735787	3.139180844	0.001694209	0.00275278	positive
PRJNA729271	stage_1D_vs_E10	ordinary_muscle_development	ENSGALG00010022463	DUSP12	0.143036521	0.115314129	1.24040759	0.214824675	0.256700226	positive
PRJNA729271	stage_1D_vs_E10	ordinary_muscle_development	ENSGALG00010022466	NEDD4	-0.310964923	0.137825058	-2.256229228	0.024056279	0.033677397	negative
PRJNA729271	stage_1D_vs_E10	ordinary_muscle_development	ENSGALG00010022467	SART3	-0.359820654	0.115887509	-3.104913176	0.001903349	0.003071592	negative
PRJNA729271	stage_1D_vs_E10	ordinary_muscle_development	ENSGALG00010022468	MYBPC3	0.008280459	0.429231308	0.019291368	0.98460867	0.986718935	positive
PRJNA729271	stage_1D_vs_E10	ordinary_muscle_development	ENSGALG00010022470	ENSGALG00010022470	-1.043737844	0.179698187	-5.808282533	6.31169E-09	1.67837E-08	negative
PRJNA729271	stage_1D_vs_E10	ordinary_muscle_development	ENSGALG00010022471	ST6GALNAC5	-1.002966108	0.140182704	-7.154706527	8.38521E-13	2.91892E-12	negative
PRJNA729271	stage_1D_vs_E10	ordinary_muscle_development	ENSGALG00010022472	QSER1	0.594093027	0.087088343	6.821728475	8.99516E-12	2.91884E-11	positive
PRJNA729271	stage_1D_vs_E10	ordinary_muscle_development	ENSGALG00010022473	ZNF385A	2.519730239	0.390770366	6.448109835	1.13254E-10	3.41789E-10	positive
PRJNA729271	stage_1D_vs_E10	ordinary_muscle_development	ENSGALG00010022474	VCL	-0.087119492	0.119688845	-0.727883134	0.466685128	0.515319642	negative
PRJNA729271	stage_1D_vs_E10	ordinary_muscle_development	ENSGALG00010022476	OIP5	-0.623203802	0.111108719	-5.608954987	2.03552E-08	5.18448E-08	negative
PRJNA729271	stage_1D_vs_E10	ordinary_muscle_development	ENSGALG00010022478	ATP6V1A	0.200438788	0.096172546	2.084158074	0.037145791	0.050551896	positive
PRJNA729271	stage_1D_vs_E10	ordinary_muscle_development	ENSGALG00010022479	ENSGALG00010022479	0.263111003	0.650261109	0.404623619	0.685754194	0.723488394	positive
PRJNA729271	stage_1D_vs_E10	ordinary_muscle_development	ENSGALG00010022480	PIF1	-2.256669529	0.24682868	-9.142655241	6.09374E-20	3.24858E-19	negative
PRJNA729271	stage_1D_vs_E10	ordinary_muscle_development	ENSGALG00010022481	CORO1B	-0.600258277	0.148521522	-4.041557535	5.30973E-05	0.000101163	negative
PRJNA729271	stage_1D_vs_E10	ordinary_muscle_development	ENSGALG00010022482	PRTG	-1.743971795	0.33075165	-5.272753122	1.34392E-07	3.21349E-07	negative
PRJNA729271	stage_1D_vs_E10	ordinary_muscle_development	ENSGALG00010022483	TOP2A	-1.820226851	0.238495273	-7.63212969	2.30907E-14	8.89685E-14	negative
PRJNA729271	stage_1D_vs_E10	ordinary_muscle_development	ENSGALG00010022485	ATF2	-0.185388498	0.057117225	-3.245754617	0.001171398	0.001939174	negative
PRJNA729271	stage_1D_vs_E10	ordinary_muscle_development	ENSGALG00010022486	FAM183A	-2.581141549	0.746970473	-3.455480026	0.000549314	0.000941727	negative
PRJNA729271	stage_1D_vs_E10	ordinary_muscle_development	ENSGALG00010022489	NUSAP1	-1.167500152	0.238388969	-4.897458792	9.7084E-07	2.17132E-06	negative
PRJNA729271	stage_1D_vs_E10	ordinary_muscle_development	ENSGALG00010022493	ENSGALG00010022493	-0.460634895	0.164080327	-2.80737432	0.004994717	0.007680715	negative
PRJNA729271	stage_1D_vs_E10	ordinary_muscle_development	ENSGALG00010022494	FHIP2A	0.200046848	0.065158996	3.070134008	0.002139627	0.003429982	positive
PRJNA729271	stage_1D_vs_E10	ordinary_muscle_development	ENSGALG00010022496	TUBGCP2	-0.190244441	0.072283954	-2.631904182	0.008490781	0.012665829	negative
PRJNA729271	stage_1D_vs_E10	ordinary_muscle_development	ENSGALG00010022497	CORO1C	-0.654729992	0.149532983	-4.378498843	1.195E-05	2.42224E-05	negative
PRJNA729271	stage_1D_vs_E10	ordinary_muscle_development	ENSGALG00010022498	NAA50	0.332020602	0.113823904	2.916967271	0.003534529	0.005532123	positive
PRJNA729271	stage_1D_vs_E10	ordinary_muscle_development	ENSGALG00010022500	ENSGALG00010022500	0.642437817	0.411584745	1.560888309	0.118550106	0.148910043	positive
PRJNA729271	stage_1D_vs_E10	ordinary_muscle_development	ENSGALG00010022502	ENSGALG00010022502	-0.628515428	0.437872675	-1.435383991	0.151177705	0.186257615	negative
PRJNA729271	stage_1D_vs_E10	ordinary_muscle_development	ENSGALG00010022504	RAD9A	0.009044214	0.148145969	0.061049345	0.951319911	0.958861144	positive
PRJNA729271	stage_1D_vs_E10	ordinary_muscle_development	ENSGALG00010022505	ZNF511	0.958478012	0.119398192	8.027575611	9.94177E-16	4.17698E-15	positive
PRJNA729271	stage_1D_vs_E10	ordinary_muscle_development	ENSGALG00010022506	EBNA1BP2	-1.156019409	0.08619334	-13.41193428	5.14784E-41	7.02618E-40	negative
PRJNA729271	stage_1D_vs_E10	ordinary_muscle_development	ENSGALG00010022507	ENSGALG00010022507	-0.588314987	0.216630279	-2.715756035	0.006612462	0.010009152	negative
PRJNA729271	stage_1D_vs_E10	ordinary_muscle_development	ENSGALG00010022508	USF3	0.939289526	0.119994266	7.827786735	4.96533E-15	1.99536E-14	positive
PRJNA729271	stage_1D_vs_E10	ordinary_muscle_development	ENSGALG00010022511	IGFBP4	0.45278291	0.271284375	1.669034236	0.095110595	0.121358105	positive
PRJNA729271	stage_1D_vs_E10	ordinary_muscle_development	ENSGALG00010022512	PRAP1	0.808211574	0.882767573	0.915542888	0.359906698	0.408875898	positive
PRJNA729271	stage_1D_vs_E10	ordinary_muscle_development	ENSGALG00010022513	NDUFAF1	-0.41141543	0.085412119	-4.816827353	1.45859E-06	3.21408E-06	negative
PRJNA729271	stage_1D_vs_E10	ordinary_muscle_development	ENSGALG00010022515	CHRNA1	-1.675725647	0.1707444	-9.814234886	9.77769E-23	6.03353E-22	negative
PRJNA729271	stage_1D_vs_E10	ordinary_muscle_development	ENSGALG00010022516	TRUB1	-0.90732171	0.151790497	-5.977460549	2.26643E-09	6.23849E-09	negative
PRJNA729271	stage_1D_vs_E10	ordinary_muscle_development	ENSGALG00010022517	HNRNPA1	-0.514166841	0.181399296	-2.83444783	0.004590495	0.007092222	negative
PRJNA729271	stage_1D_vs_E10	ordinary_muscle_development	ENSGALG00010022518	ECHS1	1.175493047	0.11149471	10.54303876	5.47022E-26	3.9156E-25	positive
PRJNA729271	stage_1D_vs_E10	ordinary_muscle_development	ENSGALG00010022520	TMOD2	-1.95877026	0.203686944	-9.616572497	6.80613E-22	4.04323E-21	negative
PRJNA729271	stage_1D_vs_E10	ordinary_muscle_development	ENSGALG00010022521	RTF1	-0.360441437	0.091213274	-3.951633588	7.76195E-05	0.000145549	negative
PRJNA729271	stage_1D_vs_E10	ordinary_muscle_development	ENSGALG00010022522	TNS4	-1.269002805	0.35441956	-3.580510078	0.000342924	0.000601268	negative
PRJNA729271	stage_1D_vs_E10	ordinary_muscle_development	ENSGALG00010022524	SOX18	1.070429604	0.227683083	4.70140157	2.58382E-06	5.58075E-06	positive
PRJNA729271	stage_1D_vs_E10	ordinary_muscle_development	ENSGALG00010022525	ISCU	0.441500188	0.194266421	2.272653123	0.023047092	0.032361483	positive
PRJNA729271	stage_1D_vs_E10	ordinary_muscle_development	ENSGALG00010022526	PPP1CA	0.398946019	0.137620647	2.898882025	0.003744958	0.005840667	positive
PRJNA729271	stage_1D_vs_E10	ordinary_muscle_development	ENSGALG00010022528	SIDT1	-4.354122484	0.544949482	-7.989956188	1.34987E-15	5.62736E-15	negative
PRJNA729271	stage_1D_vs_E10	ordinary_muscle_development	ENSGALG00010022529	CCR7	-2.336087275	0.406034658	-5.753418411	8.74567E-09	2.29404E-08	negative
PRJNA729271	stage_1D_vs_E10	ordinary_muscle_development	ENSGALG00010022531	ENSGALG00010022531	-1.379726782	0.427038397	-3.230919731	0.001233926	0.002037798	negative
PRJNA729271	stage_1D_vs_E10	ordinary_muscle_development	ENSGALG00010022532	CBX5	-2.332155734	0.174677365	-13.3512189	1.16526E-40	1.57121E-39	negative
PRJNA729271	stage_1D_vs_E10	ordinary_muscle_development	ENSGALG00010022533	TMEM119	-0.649404148	0.132599752	-4.89747632	9.70753E-07	2.17132E-06	negative
PRJNA729271	stage_1D_vs_E10	ordinary_muscle_development	ENSGALG00010022534	CFAP57	-1.119013913	0.533785347	-2.096374355	0.036048996	0.049151556	negative
PRJNA729271	stage_1D_vs_E10	ordinary_muscle_development	ENSGALG00010022536	SMUG1	0.13008254	0.099539478	1.306843702	0.1912658	0.230936923	positive
PRJNA729271	stage_1D_vs_E10	ordinary_muscle_development	ENSGALG00010022538	WIPF1	0.931996069	0.156818246	5.943160926	2.79578E-09	7.64042E-09	positive
PRJNA729271	stage_1D_vs_E10	ordinary_muscle_development	ENSGALG00010022539	LYSMD2	0.5733051	0.101632317	5.640972449	1.69092E-08	4.33728E-08	positive
PRJNA729271	stage_1D_vs_E10	ordinary_muscle_development	ENSGALG00010022540	GPR146	2.120242285	0.237452448	8.929123717	4.29396E-19	2.18083E-18	positive
PRJNA729271	stage_1D_vs_E10	ordinary_muscle_development	ENSGALG00010022541	ITPKA	-2.047087175	0.127892107	-16.00636056	1.15363E-57	2.90816E-56	negative
PRJNA729271	stage_1D_vs_E10	ordinary_muscle_development	ENSGALG00010022542	ENSGALG00010022542	0.368626132	0.731936362	0.503631396	0.614520425	0.656941778	positive
PRJNA729271	stage_1D_vs_E10	ordinary_muscle_development	ENSGALG00010022544	SPICE1	-0.028688626	0.068893112	-0.416422277	0.67710103	0.715191972	negative
PRJNA729271	stage_1D_vs_E10	ordinary_muscle_development	ENSGALG00010022545	CSTF3	-0.654484713	0.066587554	-9.828934641	8.4508E-23	5.2281E-22	negative
PRJNA729271	stage_1D_vs_E10	ordinary_muscle_development	ENSGALG00010022546	MTG1	0.78506984	0.1575231	4.983839436	6.23348E-07	1.41422E-06	positive
PRJNA729271	stage_1D_vs_E10	ordinary_muscle_development	ENSGALG00010022547	SMARCE1	-0.808680238	0.061739242	-13.09831811	3.36635E-39	4.30293E-38	negative
PRJNA729271	stage_1D_vs_E10	ordinary_muscle_development	ENSGALG00010022548	ENSGALG00010022548	0.173249649	0.114197426	1.517106429	0.129239815	0.161216104	positive
PRJNA729271	stage_1D_vs_E10	ordinary_muscle_development	ENSGALG00010022550	TRIM45	-0.614655208	0.163446231	-3.760595777	0.000169509	0.000307475	negative
PRJNA729271	stage_1D_vs_E10	ordinary_muscle_development	ENSGALG00010022551	GPER1	-1.169166815	0.601427008	-1.943987882	0.051896912	0.069026036	negative
PRJNA729271	stage_1D_vs_E10	ordinary_muscle_development	ENSGALG00010022552	GPR155	0.099550815	0.105950917	0.939593711	0.347426003	0.396281076	positive
PRJNA729271	stage_1D_vs_E10	ordinary_muscle_development	ENSGALG00010022553	PRPF6	0.159274114	0.169330987	0.940608195	0.346905681	0.395781019	positive
PRJNA729271	stage_1D_vs_E10	ordinary_muscle_development	ENSGALG00010022554	SCG3	-3.074731455	0.353826113	-8.689950622	3.62597E-18	1.75546E-17	negative
PRJNA729271	stage_1D_vs_E10	ordinary_muscle_development	ENSGALG00010022557	WNT1	0.315443702	0.37528328	0.840548244	0.400601064	0.450097854	positive
PRJNA729271	stage_1D_vs_E10	ordinary_muscle_development	ENSGALG00010022558	NYAP2	-0.343154389	0.505938348	-0.678253369	0.497611059	0.545685603	negative
PRJNA729271	stage_1D_vs_E10	ordinary_muscle_development	ENSGALG00010022559	SSH1	0.60753407	0.084992678	7.148075411	8.8003E-13	3.05753E-12	positive
PRJNA729271	stage_1D_vs_E10	ordinary_muscle_development	ENSGALG00010022560	SAMD10	-3.742964894	1.448819092	-2.583459119	0.009781506	0.014475031	negative
PRJNA729271	stage_1D_vs_E10	ordinary_muscle_development	ENSGALG00010022562	GHRH	-3.893172192	1.098767534	-3.54321735	0.000395277	0.000688646	negative
PRJNA729271	stage_1D_vs_E10	ordinary_muscle_development	ENSGALG00010022565	ARF3	-0.872768902	0.166045529	-5.256202378	1.4706E-07	3.50657E-07	negative
PRJNA729271	stage_1D_vs_E10	ordinary_muscle_development	ENSGALG00010022567	PAOX	0.316474315	0.124925044	2.533313607	0.011298981	0.016595272	positive
PRJNA729271	stage_1D_vs_E10	ordinary_muscle_development	ENSGALG00010022568	SCRN3	1.212139888	0.16737612	7.242012111	4.42076E-13	1.5721E-12	positive
PRJNA729271	stage_1D_vs_E10	ordinary_muscle_development	ENSGALG00010022569	KIAA1549L	-2.576417917	0.236276577	-10.90424597	1.10001E-27	8.47561E-27	negative
PRJNA729271	stage_1D_vs_E10	ordinary_muscle_development	ENSGALG00010022571	RIPOR1	1.220967587	0.106627696	11.45075465	2.33107E-30	2.05654E-29	positive
PRJNA729271	stage_1D_vs_E10	ordinary_muscle_development	ENSGALG00010022572	ENSGALG00010022572	-3.951186937	0.36819661	-10.73118771	7.26563E-27	5.41745E-26	negative
PRJNA729271	stage_1D_vs_E10	ordinary_muscle_development	ENSGALG00010022573	AP4E1	-0.212075129	0.081773342	-2.593450668	0.009501817	0.01409279	negative
PRJNA729271	stage_1D_vs_E10	ordinary_muscle_development	ENSGALG00010022574	ITPRIPL2	-0.031583187	0.107884012	-0.29275132	0.76971224	0.799210524	negative
PRJNA729271	stage_1D_vs_E10	ordinary_muscle_development	ENSGALG00010022575	TMEM41B	-0.073971628	0.109857916	-0.673339077	0.500731598	0.548824916	negative
PRJNA729271	stage_1D_vs_E10	ordinary_muscle_development	ENSGALG00010022578	ZNF512B	-0.012411038	0.134622712	-0.092191262	0.92654608	0.937410863	negative
PRJNA729271	stage_1D_vs_E10	ordinary_muscle_development	ENSGALG00010022579	TNFAIP8L3	-1.187269434	0.148371666	-8.001995707	1.22419E-15	5.11224E-15	negative
PRJNA729271	stage_1D_vs_E10	ordinary_muscle_development	ENSGALG00010022581	TMEM125	-1.884845302	0.637083295	-2.958553953	0.003090861	0.004864499	negative
PRJNA729271	stage_1D_vs_E10	ordinary_muscle_development	ENSGALG00010022582	ENSGALG00010022582	-0.077156218	0.217767249	-0.354305884	0.723109659	0.75785525	negative
PRJNA729271	stage_1D_vs_E10	ordinary_muscle_development	ENSGALG00010022583	FKBP11	-0.042505416	0.118763938	-0.357898338	0.720419394	0.755442315	negative
PRJNA729271	stage_1D_vs_E10	ordinary_muscle_development	ENSGALG00010022585	CIR1	-0.334636869	0.094395611	-3.545046929	0.000392543	0.000684295	negative
PRJNA729271	stage_1D_vs_E10	ordinary_muscle_development	ENSGALG00010022587	ENSGALG00010022587	-5.415711078	1.147425245	-4.719881403	2.35982E-06	5.11677E-06	negative
PRJNA729271	stage_1D_vs_E10	ordinary_muscle_development	ENSGALG00010022588	TCP11L1	-0.73823123	0.079293179	-9.310148002	1.27654E-20	7.05702E-20	negative
PRJNA729271	stage_1D_vs_E10	ordinary_muscle_development	ENSGALG00010022590	ENSGALG00010022590	-0.329953328	0.157232906	-2.098500475	0.035860958	0.04891818	negative
PRJNA729271	stage_1D_vs_E10	ordinary_muscle_development	ENSGALG00010022592	CCDC65	-0.885541157	0.45479255	-1.947132066	0.051518904	0.068580573	negative

PRJNA729271	stage_1D_vs_E10	ordinary_muscle_development	ENSGALG00010022593	MANBAL	-1.639597116	0.139781236	-11.72973682	8.97373E-32	8.46286E-31	negative
PRJNA729271	stage_1D_vs_E10	ordinary_muscle_development	ENSGALG00010022597	SLCO4A1	2.412240175	0.216629449	11.13532895	8.44327E-29	6.89494E-28	positive
PRJNA729271	stage_1D_vs_E10	ordinary_muscle_development	ENSGALG00010022599	RNLS	-2.14893923	0.29296332	-7.335181854	2.21421E-13	8.00372E-13	negative
PRJNA729271	stage_1D_vs_E10	ordinary_muscle_development	ENSGALG00010022603	ATRNL1	0.196266866	0.168984041	1.161452081	0.2454581	0.289614583	positive
PRJNA729271	stage_1D_vs_E10	ordinary_muscle_development	ENSGALG00010022608	P3H4	-1.001548565	0.205913242	-4.8639347	1.15075E-06	2.55751E-06	negative
PRJNA729271	stage_1D_vs_E10	ordinary_muscle_development	ENSGALG00010022609	RBBP8NL	-7.229277544	1.061065745	-6.813223004	9.5436E-12	3.09335E-11	negative
PRJNA729271	stage_1D_vs_E10	ordinary_muscle_development	ENSGALG00010022614	DAO	-0.752871765	0.203641148	-3.69705127	0.000218118	0.000389843	negative
PRJNA729271	stage_1D_vs_E10	ordinary_muscle_development	ENSGALG00010022616	GLDN	4.620773815	0.329512344	14.02306743	1.12639E-44	1.74062E-43	positive
PRJNA729271	stage_1D_vs_E10	ordinary_muscle_development	ENSGALG00010022619	MADD	-0.936066358	0.133599821	-7.006494079	2.44364E-12	8.24309E-12	negative
PRJNA729271	stage_1D_vs_E10	ordinary_muscle_development	ENSGALG00010022622	MPPED2	-0.798381061	0.134961475	-5.915621925	3.30624E-09	8.97452E-09	negative
PRJNA729271	stage_1D_vs_E10	ordinary_muscle_development	ENSGALG00010022623	TOP1	-0.872398045	0.126259523	-6.909562316	4.86151E-12	1.60299E-11	negative
PRJNA729271	stage_1D_vs_E10	ordinary_muscle_development	ENSGALG00010022625	ENSGALG00010022625	0.299349009	0.12279365	2.437821578	0.014776065	0.021319835	positive
PRJNA729271	stage_1D_vs_E10	ordinary_muscle_development	ENSGALG00010022627	CTCF	-0.32567483	0.098205047	-3.316273844	0.000912264	0.001528171	negative
PRJNA729271	stage_1D_vs_E10	ordinary_muscle_development	ENSGALG00010022628	SVOP	0.46972873	0.563376202	0.833774534	0.404408048	0.454058456	positive
PRJNA729271	stage_1D_vs_E10	ordinary_muscle_development	ENSGALG00010022630	ENSGALG00010022630	-3.0873224	0.309915917	-9.961806497	2.23956E-23	1.41757E-22	negative
PRJNA729271	stage_1D_vs_E10	ordinary_muscle_development	ENSGALG00010022637	DMXL2	-1.06742899	0.110788487	-9.634836776	5.69825E-22	3.40603E-21	negative
PRJNA729271	stage_1D_vs_E10	ordinary_muscle_development	ENSGALG00010022642	SEC13	-0.634924838	0.068652587	-9.248374588	2.27932E-20	1.24019E-19	negative
PRJNA729271	stage_1D_vs_E10	ordinary_muscle_development	ENSGALG00010022643	SP3	-0.03632578	0.076717747	-0.473499049	0.635857179	0.676680907	negative
PRJNA729271	stage_1D_vs_E10	ordinary_muscle_development	ENSGALG00010022644	ARMC8	1.224310101	0.084335589	14.51712285	9.43897E-48	1.6584E-46	positive
PRJNA729271	stage_1D_vs_E10	ordinary_muscle_development	ENSGALG00010022645	ENSGALG00010022645	-0.800968588	0.662644404	-1.208745721	0.22676055	0.269638571	negative
PRJNA729271	stage_1D_vs_E10	ordinary_muscle_development	ENSGALG00010022647	SVIP	-1.025647966	0.134724432	-7.612932204	2.67946E-14	1.02803E-13	negative
PRJNA729271	stage_1D_vs_E10	ordinary_muscle_development	ENSGALG00010022649	PATZ1	-1.871844319	0.084200188	-22.23088062	1.7271E-109	1.5265E-107	negative
PRJNA729271	stage_1D_vs_E10	ordinary_muscle_development	ENSGALG00010022650	NR1H3	0.697575135	0.09886761	7.055648808	1.71797E-12	5.84829E-12	positive
PRJNA729271	stage_1D_vs_E10	ordinary_muscle_development	ENSGALG00010022652	CARMIL2	-1.423480522	0.285603346	-4.984117095	6.22454E-07	1.41241E-06	negative
PRJNA729271	stage_1D_vs_E10	ordinary_muscle_development	ENSGALG00010022655	NRIP3	-8.922312793	0.63387176	-14.07589572	5.34237E-45	8.38069E-44	negative
PRJNA729271	stage_1D_vs_E10	ordinary_muscle_development	ENSGALG00010022656	FKBP10	-1.355251387	0.211127724	-6.419106692	1.37076E-10	4.11456E-10	negative
PRJNA729271	stage_1D_vs_E10	ordinary_muscle_development	ENSGALG00010022657	MRPS35	0.223077533	0.111492768	2.000825134	0.045411238	0.060930843	positive
PRJNA729271	stage_1D_vs_E10	ordinary_muscle_development	ENSGALG00010022659	TIE1	1.195158467	0.15082935	7.923911801	2.30153E-15	9.45865E-15	positive
PRJNA729271	stage_1D_vs_E10	ordinary_muscle_development	ENSGALG00010022660	METAP1D	0.633509852	0.181186166	3.496458183	0.000471478	0.000814351	positive
PRJNA729271	stage_1D_vs_E10	ordinary_muscle_development	ENSGALG00010022661	ENSGALG00010022661	-1.708854194	0.413380164	-4.133856302	3.56727E-05	6.90538E-05	negative
PRJNA729271	stage_1D_vs_E10	ordinary_muscle_development	ENSGALG00010022662	GFRA1	-2.649593783	0.150533986	-17.60129959	2.40736E-69	8.68028E-68	negative
PRJNA729271	stage_1D_vs_E10	ordinary_muscle_development	ENSGALG00010022664	TRPC4AP	0.632823141	0.097351714	6.50038006	8.01173E-11	2.44125E-10	positive
PRJNA729271	stage_1D_vs_E10	ordinary_muscle_development	ENSGALG00010022666	ENSGALG00010022666	-0.609209946	0.102533063	-5.941595101	2.82262E-09	7.71087E-09	negative
PRJNA729271	stage_1D_vs_E10	ordinary_muscle_development	ENSGALG00010022667	PTEN	0.009646036	0.042329393	0.227880328	0.819739276	0.844920771	positive
PRJNA729271	stage_1D_vs_E10	ordinary_muscle_development	ENSGALG00010022669	MANSC4	-6.762522966	1.139271136	-5.935832789	2.92358E-09	7.97464E-09	negative
PRJNA729271	stage_1D_vs_E10	ordinary_muscle_development	ENSGALG00010022671	DBR1	-0.474207975	0.056135974	-8.447488217	2.97637E-17	1.36787E-16	negative
PRJNA729271	stage_1D_vs_E10	ordinary_muscle_development	ENSGALG00010022673	KLHL42	0.490283885	0.147271637	3.329112749	0.000871231	0.00146197	positive
PRJNA729271	stage_1D_vs_E10	ordinary_muscle_development	ENSGALG00010022674	ENSGALG00010022674	2.542728243	0.327826618	7.756320269	8.74291E-15	3.45497E-14	positive
PRJNA729271	stage_1D_vs_E10	ordinary_muscle_development	ENSGALG00010022681	PPP2R3A	1.20580143	0.092885882	12.98153607	1.55726E-38	1.93423E-37	positive
PRJNA729271	stage_1D_vs_E10	ordinary_muscle_development	ENSGALG00010022682	EIF1	0.744683077	0.155706065	4.782620882	1.73024E-06	3.7885E-06	positive
PRJNA729271	stage_1D_vs_E10	ordinary_muscle_development	ENSGALG00010022684	ENSGALG00010022684	-1.514897074	0.53684742	-2.821839161	0.004774912	0.007355191	negative
PRJNA729271	stage_1D_vs_E10	ordinary_muscle_development	ENSGALG00010022685	ATAD1	0.182721036	0.108167369	1.68924361	0.091172755	0.116744618	positive
PRJNA729271	stage_1D_vs_E10	ordinary_muscle_development	ENSGALG00010022689	MPL	0.462730362	0.416468073	1.111082438	0.266532866	0.311689705	positive
PRJNA729271	stage_1D_vs_E10	ordinary_muscle_development	ENSGALG00010022691	ASIC2	-4.675701923	0.655068559	-7.137729116	9.48854E-13	3.2872E-12	negative
PRJNA729271	stage_1D_vs_E10	ordinary_muscle_development	ENSGALG00010022695	PIK3IP1	2.275787412	0.202528594	11.23686968	2.68744E-29	2.2491E-28	positive
PRJNA729271	stage_1D_vs_E10	ordinary_muscle_development	ENSGALG00010022698	SEMA6D	1.363349492	0.116733243	11.67918802	1.62844E-31	1.51503E-30	positive
PRJNA729271	stage_1D_vs_E10	ordinary_muscle_development	ENSGALG00010022702	CDC20	-2.277675275	0.233017347	-9.774702643	1.44581E-22	8.85759E-22	negative
PRJNA729271	stage_1D_vs_E10	ordinary_muscle_development	ENSGALG00010022707	SALL1	-4.518304676	0.5470034	-8.260103454	1.45546E-16	6.43788E-16	negative
PRJNA729271	stage_1D_vs_E10	ordinary_muscle_development	ENSGALG00010022708	SPRED1	-0.62807202	0.130771837	-4.802807966	1.56456E-06	3.43923E-06	negative
PRJNA729271	stage_1D_vs_E10	ordinary_muscle_development	ENSGALG00010022709	RNF5	-0.450692575	0.084549297	-5.330530115	9.79265E-08	2.36614E-07	negative
PRJNA729271	stage_1D_vs_E10	ordinary_muscle_development	ENSGALG00010022711	LRRC49	-0.264732981	0.191303764	-1.383835717	0.166408764	0.203432153	negative
PRJNA729271	stage_1D_vs_E10	ordinary_muscle_development	ENSGALG00010022712	DDX23	0.04341056	0.201521433	0.215414111	0.82944447	0.853588753	positive
PRJNA729271	stage_1D_vs_E10	ordinary_muscle_development	ENSGALG00010022713	PNLIPRP1	4.420359256	2.822064099	1.566356788	0.117265113	0.147420453	positive
PRJNA729271	stage_1D_vs_E10	ordinary_muscle_development	ENSGALG00010022717	ELOVL1	1.294672339	0.170667733	7.585923339	3.30128E-14	1.26026E-13	positive
PRJNA729271	stage_1D_vs_E10	ordinary_muscle_development	ENSGALG00010022718	C12orf43	-0.443097783	0.134245469	-3.300653549	0.000964599	0.001611742	negative
PRJNA729271	stage_1D_vs_E10	ordinary_muscle_development	ENSGALG00010022719	MSL2	0.342043522	0.096378418	3.548963871	0.00038675	0.000674683	positive
PRJNA729271	stage_1D_vs_E10	ordinary_muscle_development	ENSGALG00010022724	LUZP2	-2.219456915	0.411510854	-5.393434686	6.91235E-08	1.69247E-07	negative
PRJNA729271	stage_1D_vs_E10	ordinary_muscle_development	ENSGALG00010022725	KRT222	-3.853207849	0.560131643	-6.879111185	6.02271E-12	1.97465E-11	negative
PRJNA729271	stage_1D_vs_E10	ordinary_muscle_development	ENSGALG00010022727	ACP2	-0.233643743	0.084206261	-2.774659993	0.005525945	0.008447434	negative
PRJNA729271	stage_1D_vs_E10	ordinary_muscle_development	ENSGALG00010022729	ENSGALG00010022729	-0.424183204	0.13225584	-3.207292801	0.001339905	0.002203782	negative
PRJNA729271	stage_1D_vs_E10	ordinary_muscle_development	ENSGALG00010022731	LRRC8B	-1.471923649	0.179475013	-8.201273386	2.37854E-16	1.0394E-15	negative
PRJNA729271	stage_1D_vs_E10	ordinary_muscle_development	ENSGALG00010022733	PCCB	0.827225488	0.11468951	7.212738891	5.48375E-13	1.93527E-12	positive
PRJNA729271	stage_1D_vs_E10	ordinary_muscle_development	ENSGALG00010022734	ENSGALG00010022734	0.870853431	0.118892191	7.324731987	2.39377E-13	8.6334E-13	positive
PRJNA729271	stage_1D_vs_E10	ordinary_muscle_development	ENSGALG00010022737	CYLD	0.526463905	0.078713719	6.688337314	2.2572E-11	7.13591E-11	positive
PRJNA729271	stage_1D_vs_E10	ordinary_muscle_development	ENSGALG00010022742	IFT46	-0.793593182	0.124672151	-6.365440685	1.9473E-10	5.7793E-10	negative
PRJNA729271	stage_1D_vs_E10	ordinary_muscle_development	ENSGALG00010022743	ZNF326	-0.967371507	0.151825887	-6.371584758	1.87085E-10	5.56152E-10	negative
PRJNA729271	stage_1D_vs_E10	ordinary_muscle_development	ENSGALG00010022745	PAPSS2	0.288792955	0.237074407	1.218153229	0.223165749	0.265821969	positive
PRJNA729271	stage_1D_vs_E10	ordinary_muscle_development	ENSGALG00010022747	NT5C3B	-1.014754522	0.139875884	-7.254678191	4.02618E-13	1.4346E-12	negative
PRJNA729271	stage_1D_vs_E10	ordinary_muscle_development	ENSGALG00010022748	ENSGALG00010022748	-0.637133744	0.324405466	-1.964004343	0.049529588	0.066126129	negative
PRJNA729271	stage_1D_vs_E10	ordinary_muscle_development	ENSGALG00010022749	HSPA12A	-0.717574003	0.129431737	-5.544034419	2.95581E-08	7.41638E-08	negative
PRJNA729271	stage_1D_vs_E10	ordinary_muscle_development	ENSGALG00010022751	SLC25A12	3.050597886	0.190975111	15.97379817	1.94568E-57	4.87936E-56	positive
PRJNA729271	stage_1D_vs_E10	ordinary_muscle_development	ENSGALG00010022754	SNX20	-0.351791404	0.18608806	-1.890456614	0.058696918	0.077353562	negative
PRJNA729271	stage_1D_vs_E10	ordinary_muscle_development	ENSGALG00010022755	MINPP1	-0.224684621	0.114796627	-1.957240623	0.050319185	0.06711231	negative
PRJNA729271	stage_1D_vs_E10	ordinary_muscle_development	ENSGALG00010022759	THSD4	0.517717836	0.119934954	4.316655128	1.58411E-05	3.16932E-05	positive
PRJNA729271	stage_1D_vs_E10	ordinary_muscle_development	ENSGALG00010022760	ELAVL4	-2.419638987	0.126728102	-19.09315257	2.87861E-81	1.3955E-79	negative
PRJNA729271	stage_1D_vs_E10	ordinary_muscle_development	ENSGALG00010022764	MFAP5	2.898969431	0.222831345	13.00970216	1.07761E-38	1.34771E-37	positive
PRJNA729271	stage_1D_vs_E10	ordinary_muscle_development	ENSGALG00010022767	CACNB3	-0.931877886	0.475883823	-1.958204588	0.050206011	0.066973691	negative
PRJNA729271	stage_1D_vs_E10	ordinary_muscle_development	ENSGALG00010022768	ENSGALG00010022768	-1.823245788	0.717413388	-2.541415896	0.01104045	0.016233651	negative
PRJNA729271	stage_1D_vs_E10	ordinary_muscle_development	ENSGALG00010022769	FXYD6	1.560240562	0.097227189	16.04736882	5.96393E-58	1.52464E-56	positive
PRJNA729271	stage_1D_vs_E10	ordinary_muscle_development	ENSGALG00010022770	HAT1	-1.460503687	0.092562247	-15.7786109	4.36693E-56	1.0411E-54	negative
PRJNA729271	stage_1D_vs_E10	ordinary_muscle_development	ENSGALG00010022771	SGMS1	1.556844319	0.219134619	7.104511041	1.20749E-12	4.14361E-12	positive
PRJNA729271	stage_1D_vs_E10	ordinary_muscle_development	ENSGALG00010022776	CDH4	1.333405413	0.175002434	7.619353534	2.54949E-14	9.80496E-14	positive
PRJNA729271	stage_1D_vs_E10	ordinary_muscle_development	ENSGALG00010022780	DYNC1I2	-0.53466847	0.057565322	-9.288030534	1.5717E-20	8.65567E-20	negative
PRJNA729271	stage_1D_vs_E10	ordinary_muscle_development	ENSGALG00010022782	TMPRSS13	-0.310547529	0.242767965	-1.279194843	0.200828452	0.241097267	negative
PRJNA729271	stage_1D_vs_E10	ordinary_muscle_development	ENSGALG00010022783	STAG1	0.187925776	0.054560493	3.444356287	0.000572421	0.000977872	positive
PRJNA729271	stage_1D_vs_E10	ordinary_muscle_development	ENSGALG00010022784	DMRTA2	-0.482620255	0.612854849	-0.787495205	0.430992034	0.480445246	negative
PRJNA729271	stage_1D_vs_E10	ordinary_muscle_development	ENSGALG00010022786	KRT40	0.606327659	0.545644947	1.111212818	0.266476755	0.311649231	positive
PRJNA729271	stage_1D_vs_E10	ordinary_muscle_development	ENSGALG00010022787	ENO4	-1.885574537	0.33189421	-5.681251677	1.33713E-08	3.45361E-08	negative
PRJNA729271	stage_1D_vs_E10	ordinary_muscle_development	ENSGALG00010022788	CYBRD1	2.527286481	0.173113223	14.59903774	2.84837E-48	5.12884E-47	positive
PRJNA729271	stage_1D_vs_E10	ordinary_muscle_development	ENSGALG00010022789	ENSGALG00010022789	-3.233576455	0.369251362	-8.757114484	2.00312E-18	9.81584E-18	negative
PRJNA729271	stage_1D_vs_E10	ordinary_muscle_development	ENSGALG00010022792	DDB2	-1.088895533	0.126758503	-8.590315486	8.67308E-18	4.11376E-17	negative
PRJNA729271	stage_1D_vs_E10	ordinary_muscle_development	ENSGALG00010022795	TAF4	0.601923651	0.109526065	5.495711468	3.89138E-08	9.68503E-08	positive
PRJNA729271	stage_1D_vs_E10	ordinary_muscle_development	ENSGALG00010022796	RIMKLB	-1.394476292	0.136634294	-10.20590258	1.86578E-24	1.24285E-23	negative
PRJNA729271	stage_1D_vs_E10	ordinary_muscle_development	ENSGALG00010022797	EFHD1	1.420094668	0.131550279	10.79507151	3.6318E-27	2.73754E-26	positive
PRJNA729271	stage_1D_vs_E10	ordinary_muscle_development	ENSGALG00010022799	DCAF17	-0.260116328	0.153611996	-1.693333436	0.090392017	0.115806283	negative
PRJNA729271	stage_1D_vs_E10	ordinary_muscle_development	ENSGALG00010022800	KLHL10	-0.835702682	0.452719173	-1.845962645	0.06489763	0.085061139	negative
PRJNA729271	stage_1D_vs_E10	ordinary_muscle_development	ENSGALG00010022801	LSM14B	-0.265507085	0.102727886	-2.584566813	0.009750141	0.014433037	negative
PRJNA729271	stage_1D_vs_E10	ordinary_muscle_development	ENSGALG00010022803	ENSGALG00010022803	-0.796630176	0.170724849	-4.666164184	3.06875E-06	6.57912E-06	negative
PRJNA729271	stage_1D_vs_E10	ordinary_muscle_development	ENSGALG00010022804	SNX19	0.457562086	0.110805245	4.129426244	3.6367E-05	7.03415E-05	positive
PRJNA729271	stage_1D_vs_E10	ordinary_muscle_development	ENSGALG00010022805	ENSGALG00010022805	-0.164157982	0.27146096	-0.604720407	0.545364786	0.591386443	negative
PRJNA729271	stage_1D_vs_E10	ordinary_muscle_development	ENSGALG00010022806	SPPL3	-0.208339702	0.06222559	-3.348135423	0.000813572	0.001370929	negative
PRJNA729271	stage_1D_vs_E10	ordinary_muscle_development	ENSGALG00010022807	ADCY6	-0.349071376	0.14816628	-2.355943437	0.018475731	0.026250315	negative
PRJNA729271	stage_1D_vs_E10	ordinary_muscle_development	ENSGALG00010022808	PSMA7	-0.648149178	0.137062315	-4.728864967	2.25778E-06	4.90213E-06	negative
PRJNA729271	stage_1D_vs_E10	ordinary_muscle_development	ENSGALG00010022809	MYO1E	-0.182711076	0.155227645	-1.177052422	0.239174584	0.282960789	negative
PRJNA729271	stage_1D_vs_E10	ordinary_muscle_development	ENSGALG00010022811	GIGYF2	0.413688082	0.097586556	4.239191345	2.24326E-05	4.42456E-05	positive
PRJNA729271	stage_1D_vs_E10	ordinary_muscle_development	ENSGALG00010022813	USP24	1.653988332	0.121849646	13.5740101	5.711E-42	8.06047E-41	positive
PRJNA729271	stage_1D_vs_E10	ordinary_muscle_development	ENSGALG00010022815	LRRC8D	-0.825197457	0.239142056	-3.450658042	0.000559222	0.000957807	negative
PRJNA729271	stage_1D_vs_E10	ordinary_muscle_development	ENSGALG00010022816	METTL8	-0.625892103	0.147376654	-4.24688772	2.16761E-05	4.27999E-05	negative
PRJNA729271	stage_1D_vs_E10	ordinary_muscle_development	ENSGALG00010022817	KRT23	-4.146264234	0.967756808	-4.284407198	1.83227E-05	3.64068E-05	negative
PRJNA729271	stage_1D_vs_E10	ordinary_muscle_development	ENSGALG00010022819	PACSIN3	2.495995462	0.108148203	23.07939844	7.4568E-118	8.2508E-116	positive
PRJNA729271	stage_1D_vs_E10	ordinary_muscle_development	ENSGALG00010022820	SHTN1	-1.217743532	0.170829499	-7.12841483	1.01531E-12	3.50989E-12	negative
PRJNA729271	stage_1D_vs_E10	ordinary_muscle_development	ENSGALG00010022821	ENSGALG00010022821	2.263106926	1.383659232	1.635595582			positive
PRJNA729271	stage_1D_vs_E10	ordinary_muscle_development	ENSGALG00010022823	IGHMBP2	-0.158064621	0.115269297	-1.37126385	0.17029272	0.207619899	negative
PRJNA729271	stage_1D_vs_E10	ordinary_muscle_development	ENSGALG00010022824	PUS1	-0.737305252	0.083231407	-8.858497949	8.10997E-19	4.06059E-18	negative
PRJNA729271	stage_1D_vs_E10	ordinary_muscle_development	ENSGALG00010022825	SS18L1	0.55584783	0.272143312	2.042482055	0.041103738	0.055526438	positive
PRJNA729271	stage_1D_vs_E10	ordinary_muscle_development	ENSGALG00010022826	SNORC	-5.980439082	0.598037164	-10.00011277	1.52224E-23	9.71163E-23	negative
PRJNA729271	stage_1D_vs_E10	ordinary_muscle_development	ENSGALG00010022827	CMTR2	-0.02846314	0.093436072	-0.304626884	0.760650342	0.791388652	negative
PRJNA729271	stage_1D_vs_E10	ordinary_muscle_development	ENSGALG00010022828	PRKG1	0.677660943	0.168910938	4.011942345	6.02212E-05	0.0001143	positive
PRJNA729271	stage_1D_vs_E10	ordinary_muscle_development	ENSGALG00010022830	MTG2	-0.568824399	0.129667801	-4.386782197	1.1504E-05	2.33577E-05	negative
PRJNA729271	stage_1D_vs_E10	ordinary_muscle_development	ENSGALG00010022831	LRRC8C	0.730035815	0.088455979	8.253097448	1.54341E-16	6.8165E-16	positive
PRJNA729271	stage_1D_vs_E10	ordinary_muscle_development	ENSGALG00010022833	HRH3	-0.553925733	0.521996362	-1.061167803	0.288613649	0.335051645	negative
PRJNA729271	stage_1D_vs_E10	ordinary_muscle_development	ENSGALG00010022834	CHRNA7	-1.682318268	0.233556818	-7.203036439	5.88862E-13	2.07274E-12	negative
PRJNA729271	stage_1D_vs_E10	ordinary_muscle_development	ENSGALG00010022837	ITGB3	-1.375990164	0.151571877	-9.078136336	1.1045E-19	5.81325E-19	negative
PRJNA729271	stage_1D_vs_E10	ordinary_muscle_development	ENSGALG00010022838	ARFGAP2	0.237171916	0.079347199	2.989039563	0.002798559	0.004427056	positive
PRJNA729271	stage_1D_vs_E10	ordinary_muscle_development	ENSGALG00010022839	SYT12	-3.018692676	0.351842139	-8.579679175	9.51381E-18	4.49634E-17	negative
PRJNA729271	stage_1D_vs_E10	ordinary_muscle_development	ENSGALG00010022840	MARVELD1	0.49033069	0.183454604	2.672763071	0.007522936	0.011302338	positive
PRJNA729271	stage_1D_vs_E10	ordinary_muscle_development	ENSGALG00010022841	ENSGALG00010022841	-4.582462288	0.341201285	-13.43037816	4.01357E-41	5.49355E-40	negative
PRJNA729271	stage_1D_vs_E10	ordinary_muscle_development	ENSGALG00010022842	PHC1	-0.748386838	0.140810931	-5.314834803	1.06754E-07	2.57343E-07	negative
PRJNA729271	stage_1D_vs_E10	ordinary_muscle_development	ENSGALG00010022845	HYDIN	2.527956562	0.317759848	7.955556926	1.78328E-15	7.37688E-15	positive
PRJNA729271	stage_1D_vs_E10	ordinary_muscle_development	ENSGALG00010022848	SGIP1	-1.547300587	0.747090683	-2.071101436	0.038349318	0.052043194	negative
PRJNA729271	stage_1D_vs_E10	ordinary_muscle_development	ENSGALG00010022850	ULK1	2.494326114	0.249602364	9.993199067	1.63227E-23	1.03953E-22	positive

PRJNA729271	stage_1D_vs_E10	ordinary_muscle_development	ENSGALG00010022858	HERC4	0.167849007	0.12727443	1.318795984	0.187237327	0.226525516	positive
PRJNA729271	stage_1D_vs_E10	ordinary_muscle_development	ENSGALG00010022860	MOB3C	-0.937058701	0.129692967	-7.225208276	5.00334E-13	1.77146E-12	negative
PRJNA729271	stage_1D_vs_E10	ordinary_muscle_development	ENSGALG00010022862	ACADS	1.216380133	0.122405401	9.937307689	2.86465E-23	1.80535E-22	positive
PRJNA729271	stage_1D_vs_E10	ordinary_muscle_development	ENSGALG00010022863	FAM81A	-1.837347881	0.37697553	-4.873918161	1.09406E-06	2.43714E-06	negative
PRJNA729271	stage_1D_vs_E10	ordinary_muscle_development	ENSGALG00010022864	CSTPP1	-0.779079524	0.19917877	-3.911458652	9.17404E-05	0.000170922	negative
PRJNA729271	stage_1D_vs_E10	ordinary_muscle_development	ENSGALG00010022866	KLHL11	-0.012638856	0.098042806	-0.128911614	0.897427586	0.912472844	negative
PRJNA729271	stage_1D_vs_E10	ordinary_muscle_development	ENSGALG00010022867	ATPAF1	-0.76121592	0.134506318	-5.659332059	1.51963E-08	3.90898E-08	negative
PRJNA729271	stage_1D_vs_E10	ordinary_muscle_development	ENSGALG00010022868	VAC14	-0.546600411	0.101348578	-5.393271655	6.91862E-08	1.69372E-07	negative
PRJNA729271	stage_1D_vs_E10	ordinary_muscle_development	ENSGALG00010022872	KRT13	-1.517696154	0.46913364	-3.235104081	0.001215984	0.002009312	negative
PRJNA729271	stage_1D_vs_E10	ordinary_muscle_development	ENSGALG00010022873	NGEF	-1.260965173	0.385558197	-3.270492454	0.001073604	0.001784416	negative
PRJNA729271	stage_1D_vs_E10	ordinary_muscle_development	ENSGALG00010022874	EFCAB14	0.78502434	0.076155456	10.30818252	6.47135E-25	4.41838E-24	positive
PRJNA729271	stage_1D_vs_E10	ordinary_muscle_development	ENSGALG00010022875	SPON1	-0.56927252	0.244250153	-2.33069463	0.019769468	0.027990365	negative
PRJNA729271	stage_1D_vs_E10	ordinary_muscle_development	ENSGALG00010022877	KYAT3	-0.246077719	0.10173894	-2.418717159	0.015575346	0.022408401	negative
PRJNA729271	stage_1D_vs_E10	ordinary_muscle_development	ENSGALG00010022879	ENSGALG00010022879	-0.647288506	0.287573211	-2.250865104	0.024394083	0.034117351	negative
PRJNA729271	stage_1D_vs_E10	ordinary_muscle_development	ENSGALG00010022881	CCNB1	-1.933620288	0.211291803	-9.151421227	5.61894E-20	3.00208E-19	negative
PRJNA729271	stage_1D_vs_E10	ordinary_muscle_development	ENSGALG00010022885	M6PR	0.001304879	0.099315718	0.013138697	0.989517138	0.991158242	positive
PRJNA729271	stage_1D_vs_E10	ordinary_muscle_development	ENSGALG00010022886	PLA2G15	1.977093465	0.28118701	7.031240396	2.04705E-12	6.93759E-12	positive
PRJNA729271	stage_1D_vs_E10	ordinary_muscle_development	ENSGALG00010022888	OSBPL2	0.567889946	0.090851684	6.250736622	4.08521E-10	1.18501E-09	positive
PRJNA729271	stage_1D_vs_E10	ordinary_muscle_development	ENSGALG00010022890	MYPN	5.073370826	0.166100668	30.54395189	6.8034E-205	3.4005E-202	positive
PRJNA729271	stage_1D_vs_E10	ordinary_muscle_development	ENSGALG00010022891	NTM	0.944359816	0.203960245	4.630117097	3.65459E-06	7.77304E-06	positive
PRJNA729271	stage_1D_vs_E10	ordinary_muscle_development	ENSGALG00010022900	MMP17	1.160258503	0.19814043	5.855738283	4.74896E-09	1.27404E-08	positive
PRJNA729271	stage_1D_vs_E10	ordinary_muscle_development	ENSGALG00010022911	SMPD3	-4.739410053	0.358441321	-13.22227594	6.5259E-40	8.52188E-39	negative
PRJNA729271	stage_1D_vs_E10	ordinary_muscle_development	ENSGALG00010022917	CYP4B7	-2.148939917	0.500474638	-4.293803833	1.75638E-05	3.49659E-05	negative
PRJNA729271	stage_1D_vs_E10	ordinary_muscle_development	ENSGALG00010022919	ACLY	-1.713527973	0.124971684	-13.71132981	8.68513E-43	1.25404E-41	negative
PRJNA729271	stage_1D_vs_E10	ordinary_muscle_development	ENSGALG00010022921	ENSGALG00010022921	0.943190873	0.194504912	4.849187947	1.23968E-06	2.74716E-06	positive
PRJNA729271	stage_1D_vs_E10	ordinary_muscle_development	ENSGALG00010022922	ATOH7	1.227901094	0.397358361	3.090160455	0.002000484	0.003220459	positive
PRJNA729271	stage_1D_vs_E10	ordinary_muscle_development	ENSGALG00010022923	CD63	1.474561585	0.126416564	11.66430675	1.93977E-31	1.79776E-30	positive
PRJNA729271	stage_1D_vs_E10	ordinary_muscle_development	ENSGALG00010022928	RNF111	0.433336932	0.082426434	5.257256794	1.4622E-07	3.4871E-07	positive
PRJNA729271	stage_1D_vs_E10	ordinary_muscle_development	ENSGALG00010022929	LRP4	0.846945998	0.113435548	7.46631908	8.24692E-14	3.06354E-13	positive
PRJNA729271	stage_1D_vs_E10	ordinary_muscle_development	ENSGALG00010022930	GTF2B	-0.223466351	0.117380863	-1.903771576	0.05693994	0.075195374	negative
PRJNA729271	stage_1D_vs_E10	ordinary_muscle_development	ENSGALG00010022931	RDH5	0.54347597	0.123279617	4.40848197	1.04098E-05	2.12192E-05	positive
PRJNA729271	stage_1D_vs_E10	ordinary_muscle_development	ENSGALG00010022932	OPCML	0.351208344	0.181170812	1.938548162	0.052556381	0.069832683	positive
PRJNA729271	stage_1D_vs_E10	ordinary_muscle_development	ENSGALG00010022933	UNC119B	-0.923243158	0.076503646	-12.0679628	1.55947E-33	1.59187E-32	negative
PRJNA729271	stage_1D_vs_E10	ordinary_muscle_development	ENSGALG00010022937	BLOC1S1	0.385801519	0.161566514	2.387880451	0.016945853	0.02423583	positive
PRJNA729271	stage_1D_vs_E10	ordinary_muscle_development	ENSGALG00010022942	NEU2	-1.338227692	0.496330304	-2.696244176	0.007012624	0.010571813	negative
PRJNA729271	stage_1D_vs_E10	ordinary_muscle_development	ENSGALG00010022945	KCNA4	-4.159536634	0.40169343	-10.35500291	3.97175E-25	2.74537E-24	negative
PRJNA729271	stage_1D_vs_E10	ordinary_muscle_development	ENSGALG00010022946	KRT17	-5.811478508	0.519885687	-11.17837758	5.20325E-29	4.30076E-28	negative
PRJNA729271	stage_1D_vs_E10	ordinary_muscle_development	ENSGALG00010022949	INSC	-1.970711257	0.26240717	-7.510127311	5.90699E-14	2.21818E-13	negative
PRJNA729271	stage_1D_vs_E10	ordinary_muscle_development	ENSGALG00010022950	ITGA7	1.623051428	0.155698434	10.42432726	1.92013E-25	1.34521E-24	positive
PRJNA729271	stage_1D_vs_E10	ordinary_muscle_development	ENSGALG00010022953	ENSGALG00010022953	-1.009759465	1.166613399	-0.865547632	0.386738284	0.435772033	negative
PRJNA729271	stage_1D_vs_E10	ordinary_muscle_development	ENSGALG00010022954	ADRM1	-0.345000891	0.14391931	-2.397182779	0.01652168	0.023654855	negative
PRJNA729271	stage_1D_vs_E10	ordinary_muscle_development	ENSGALG00010022956	PBLD	0.417733663	0.122569689	3.408131884	0.000654093	0.001111237	positive
PRJNA729271	stage_1D_vs_E10	ordinary_muscle_development	ENSGALG00010022957	SLTM	-0.080634124	0.142813369	-0.564611875	0.572337787	0.617218676	negative
PRJNA729271	stage_1D_vs_E10	ordinary_muscle_development	ENSGALG00010022958	SFSWAP	0.213131345	0.097163619	2.193530338	0.028269191	0.039099421	positive
PRJNA729271	stage_1D_vs_E10	ordinary_muscle_development	ENSGALG00010022959	ADAMTS8	0.534173839	0.264034634	2.023120343	0.043060742	0.058018726	positive
PRJNA729271	stage_1D_vs_E10	ordinary_muscle_development	ENSGALG00010022967	ADAMTS15	-0.006424847	0.161853165	-0.039695532	0.968335864	0.97276515	negative
PRJNA729271	stage_1D_vs_E10	ordinary_muscle_development	ENSGALG00010022969	TTN	4.868130479	0.575497386	8.458996683	2.69688E-17	1.24341E-16	positive
PRJNA729271	stage_1D_vs_E10	ordinary_muscle_development	ENSGALG00010022971	CKAP5	-1.101411644	0.058296504	-18.89327083	1.29564E-79	6.13733E-78	negative
PRJNA729271	stage_1D_vs_E10	ordinary_muscle_development	ENSGALG00010022972	ENSGALG00010022972	-3.481061794	1.112630869	-3.128676267			negative
PRJNA729271	stage_1D_vs_E10	ordinary_muscle_development	ENSGALG00010022974	INPP5D	0.631512036	0.156414809	4.037418455	5.40426E-05	0.000102896	positive
PRJNA729271	stage_1D_vs_E10	ordinary_muscle_development	ENSGALG00010022976	MINDY2	0.255200409	0.06514607	3.917356941	8.95251E-05	0.000166924	positive
PRJNA729271	stage_1D_vs_E10	ordinary_muscle_development	ENSGALG00010022978	ARL14EP	-0.44416964	0.086824059	-5.115743796	3.12507E-07	7.2465E-07	negative
PRJNA729271	stage_1D_vs_E10	ordinary_muscle_development	ENSGALG00010022979	LAMA5	0.675584534	0.148645681	4.544932161	5.49529E-06	1.14908E-05	positive
PRJNA729271	stage_1D_vs_E10	ordinary_muscle_development	ENSGALG00010022981	HNRNPH3	-1.312993534	0.129657684	-10.12661566	4.20968E-24	2.75358E-23	negative
PRJNA729271	stage_1D_vs_E10	ordinary_muscle_development	ENSGALG00010022983	PKN2	0.0763554	0.061039507	1.250917705	0.210964504	0.252408624	positive
PRJNA729271	stage_1D_vs_E10	ordinary_muscle_development	ENSGALG00010022986	ALG9	0.193771709	0.101596402	1.907269402	0.05648571	0.074680428	positive
PRJNA729271	stage_1D_vs_E10	ordinary_muscle_development	ENSGALG00010022987	F2	-0.7742599	1.48711621	-0.520645189			negative
PRJNA729271	stage_1D_vs_E10	ordinary_muscle_development	ENSGALG00010022990	SLC25A22	-0.900797054	0.099346329	-9.067240474	1.22069E-19	6.40621E-19	negative
PRJNA729271	stage_1D_vs_E10	ordinary_muscle_development	ENSGALG00010022991	TAL1	1.050349683	0.175330576	5.990681765	2.08963E-09	5.76388E-09	positive
PRJNA729271	stage_1D_vs_E10	ordinary_muscle_development	ENSGALG00010022992	SLC7A6	-0.197102267	0.106732963	-1.846685978	0.064792665	0.084931243	negative
PRJNA729271	stage_1D_vs_E10	ordinary_muscle_development	ENSGALG00010022994	LIPC	-3.149196503	0.89230185	-3.529295052	0.000416668	0.000723566	negative
PRJNA729271	stage_1D_vs_E10	ordinary_muscle_development	ENSGALG00010022997	ARHGAP1	-1.049406848	0.095120321	-11.0324149	2.66608E-28	2.11405E-27	negative
PRJNA729271	stage_1D_vs_E10	ordinary_muscle_development	ENSGALG00010022999	ENSGALG00010022999	1.976638841	0.478904676	4.127416041	3.66862E-05	7.09401E-05	positive
PRJNA729271	stage_1D_vs_E10	ordinary_muscle_development	ENSGALG00010023002	ATG16L1	0.274458886	0.134409893	2.041954493	0.041156046	0.055586739	positive
PRJNA729271	stage_1D_vs_E10	ordinary_muscle_development	ENSGALG00010023003	ENSGALG00010023003	-4.655414922	0.614742596	-7.572949969	3.64843E-14	1.38949E-13	negative
PRJNA729271	stage_1D_vs_E10	ordinary_muscle_development	ENSGALG00010023005	IRF7	0.596283299	0.355253222	1.67847401	0.0932546	0.119231316	positive
PRJNA729271	stage_1D_vs_E10	ordinary_muscle_development	ENSGALG00010023007	LMO4	-0.71574213	0.142689024	-5.016097997	5.27314E-07	1.20388E-06	negative
PRJNA729271	stage_1D_vs_E10	ordinary_muscle_development	ENSGALG00010023008	CDHR5	-1.644120401	0.997579128	-1.648110265	0.099330046	0.12636379	negative
PRJNA729271	stage_1D_vs_E10	ordinary_muscle_development	ENSGALG00010023012	ENSGALG00010023012	0.326997747	0.526920831	0.62058231	0.534874482	0.581664462	positive
PRJNA729271	stage_1D_vs_E10	ordinary_muscle_development	ENSGALG00010023013	ENSGALG00010023013	0.464883543	0.4210457	1.104116591	0.269542565	0.314955218	positive
PRJNA729271	stage_1D_vs_E10	ordinary_muscle_development	ENSGALG00010023015	METTL2B	-0.605968028	0.102994447	-5.883501933	4.01675E-09	1.08261E-08	negative
PRJNA729271	stage_1D_vs_E10	ordinary_muscle_development	ENSGALG00010023017	BUB1B	-1.994761196	0.200001447	-9.973733839	1.98618E-23	1.25884E-22	negative
PRJNA729271	stage_1D_vs_E10	ordinary_muscle_development	ENSGALG00010023019	CABP1	0.141384563	0.31547819	0.44815955	0.654038054	0.693611471	positive
PRJNA729271	stage_1D_vs_E10	ordinary_muscle_development	ENSGALG00010023024	PRMT7	-0.425001355	0.113312183	-3.750711909	0.000176333	0.000319334	negative
PRJNA729271	stage_1D_vs_E10	ordinary_muscle_development	ENSGALG00010023026	PHRF1	-0.034257748	0.091319726	-0.375140718	0.707555816	0.743782838	negative
PRJNA729271	stage_1D_vs_E10	ordinary_muscle_development	ENSGALG00010023028	TMEM61	-2.493802015	0.333617645	-7.47503033	7.71861E-14	2.87022E-13	negative
PRJNA729271	stage_1D_vs_E10	ordinary_muscle_development	ENSGALG00010023030	HS2ST1	-0.931335503	0.085469761	-10.89666672	1.19557E-27	9.18379E-27	negative
PRJNA729271	stage_1D_vs_E10	ordinary_muscle_development	ENSGALG00010023041	CD151	1.90571015	0.119059135	16.00641684	1.15259E-57	2.90816E-56	positive
PRJNA729271	stage_1D_vs_E10	ordinary_muscle_development	ENSGALG00010023042	ENSGALG00010023042	-5.592332169	1.039558525	-5.379526052	7.46822E-08	1.82273E-07	negative
PRJNA729271	stage_1D_vs_E10	ordinary_muscle_development	ENSGALG00010023043	CNP	1.871015778	0.144224257	12.97296179	1.74168E-38	2.15775E-37	positive
PRJNA729271	stage_1D_vs_E10	ordinary_muscle_development	ENSGALG00010023046	SLC7A6OS	-0.625106227	0.074114071	-8.434379841	3.32964E-17	1.52586E-16	negative
PRJNA729271	stage_1D_vs_E10	ordinary_muscle_development	ENSGALG00010023048	ARFGAP1	-0.766139746	0.083713063	-9.151973678	5.59027E-20	2.98897E-19	negative
PRJNA729271	stage_1D_vs_E10	ordinary_muscle_development	ENSGALG00010023050	RUFY2	-0.263410855	0.093036099	-2.831275788	0.004636273	0.007156845	negative
PRJNA729271	stage_1D_vs_E10	ordinary_muscle_development	ENSGALG00010023052	RAD54L	-1.271672373	0.129955612	-9.785436366	1.30033E-22	7.99334E-22	negative
PRJNA729271	stage_1D_vs_E10	ordinary_muscle_development	ENSGALG00010023053	MAN1A2	-0.806543834	0.047527853	-16.9699193	1.3712E-64	4.29279E-63	negative
PRJNA729271	stage_1D_vs_E10	ordinary_muscle_development	ENSGALG00010023054	AQP9	2.514599419	0.229813265	10.94192459	7.26413E-28	5.65182E-27	positive
PRJNA729271	stage_1D_vs_E10	ordinary_muscle_development	ENSGALG00010023056	SAG	0.928820871	0.442693652	2.098112019	0.035895251	0.048955746	positive
PRJNA729271	stage_1D_vs_E10	ordinary_muscle_development	ENSGALG00010023057	PAFAH1B2	-1.135904931	0.074965722	-15.15232437	7.31337E-52	1.51007E-50	negative
PRJNA729271	stage_1D_vs_E10	ordinary_muscle_development	ENSGALG00010023058	PPARA	2.370995175	0.201665087	11.75709298	6.49316E-32	6.16765E-31	positive
PRJNA729271	stage_1D_vs_E10	ordinary_muscle_development	ENSGALG00010023059	SELENOF	-0.231254756	0.093046047	-2.485379687	0.012941332	0.018833796	negative
PRJNA729271	stage_1D_vs_E10	ordinary_muscle_development	ENSGALG00010023060	ALDH1A2	-1.878731149	0.167949697	-11.1862729	4.76025E-29	3.94962E-28	negative
PRJNA729271	stage_1D_vs_E10	ordinary_muscle_development	ENSGALG00010023062	STX2	-0.232672653	0.103579764	-2.246313799	0.024683915	0.034489431	negative
PRJNA729271	stage_1D_vs_E10	ordinary_muscle_development	ENSGALG00010023063	ENSGALG00010023063	-0.557635436	0.104177119	-5.352763053	8.66212E-08	2.10138E-07	negative
PRJNA729271	stage_1D_vs_E10	ordinary_muscle_development	ENSGALG00010023064	ATG13	0.528919638	0.068522796	7.718885801	1.17351E-14	4.5973E-14	positive
PRJNA729271	stage_1D_vs_E10	ordinary_muscle_development	ENSGALG00010023065	DNA2	-1.502873102	0.224389692	-6.697603118	2.11866E-11	6.70962E-11	negative
PRJNA729271	stage_1D_vs_E10	ordinary_muscle_development	ENSGALG00010023066	TLR21	1.148705146	0.185400179	6.195814662	5.79843E-10	1.66465E-09	positive
PRJNA729271	stage_1D_vs_E10	ordinary_muscle_development	ENSGALG00010023068	CDPF1	0.049504183	0.123375379	0.401248475	0.688237194	0.725803955	positive
PRJNA729271	stage_1D_vs_E10	ordinary_muscle_development	ENSGALG00010023069	ENSGALG00010023069	-0.549754036	0.290190792	-1.894457201	0.05816435	0.07668658	negative
PRJNA729271	stage_1D_vs_E10	ordinary_muscle_development	ENSGALG00010023070	SIDT2	1.178241704	0.201026946	5.8611133	4.59774E-09	1.23484E-08	positive
PRJNA729271	stage_1D_vs_E10	ordinary_muscle_development	ENSGALG00010023071	POLR2M	-0.052380004	0.08274558	-0.63302479	0.526717454	0.573611533	negative
PRJNA729271	stage_1D_vs_E10	ordinary_muscle_development	ENSGALG00010023073	POMGNT1	0.007743311	0.085803661	0.090244535	0.928092895	0.938910281	positive
PRJNA729271	stage_1D_vs_E10	ordinary_muscle_development	ENSGALG00010023074	CCNT1	0.413043487	0.165174667	2.500646702	0.012396678	0.018101123	positive
PRJNA729271	stage_1D_vs_E10	ordinary_muscle_development	ENSGALG00010023075	PKDREJ	-1.433376592	0.275768459	-5.197753926	2.01711E-07	4.75801E-07	negative
PRJNA729271	stage_1D_vs_E10	ordinary_muscle_development	ENSGALG00010023076	DNAJC7	0.575246084	0.08005776	7.185388179	6.70165E-13	2.34922E-12	positive
PRJNA729271	stage_1D_vs_E10	ordinary_muscle_development	ENSGALG00010023077	KANSL2	0.289589596	0.151188222	1.915424316	0.055438413	0.073379581	positive
PRJNA729271	stage_1D_vs_E10	ordinary_muscle_development	ENSGALG00010023078	TAGLN	1.760287459	0.196620955	8.952695071	3.46907E-19	1.77244E-18	positive
PRJNA729271	stage_1D_vs_E10	ordinary_muscle_development	ENSGALG00010023079	SLC25A16	0.580847554	0.095155171	6.104214272	1.03308E-09	2.91615E-09	positive
PRJNA729271	stage_1D_vs_E10	ordinary_muscle_development	ENSGALG00010023080	MCRS1	-0.068567378	0.111931923	-0.612581076	0.540153374	0.58652507	negative
PRJNA729271	stage_1D_vs_E10	ordinary_muscle_development	ENSGALG00010023081	PRLHR2	-7.484363269	0.739670504	-10.11850983	4.57325E-24	2.986E-23	negative
PRJNA729271	stage_1D_vs_E10	ordinary_muscle_development	ENSGALG00010023083	HARBI1	-0.281870417	0.161584099	-1.744419268	0.081086008	0.104698172	negative
PRJNA729271	stage_1D_vs_E10	ordinary_muscle_development	ENSGALG00010023084	ENSGALG00010023084	-0.24798834	0.242151926	-1.024102281	0.305786967	0.352924762	negative
PRJNA729271	stage_1D_vs_E10	ordinary_muscle_development	ENSGALG00010023086	PCSK7	-0.157207743	0.092474413	-1.700013409	0.089128403	0.114258088	negative
PRJNA729271	stage_1D_vs_E10	ordinary_muscle_development	ENSGALG00010023087	SH3GLB1	0.137753976	0.118069803	1.166716402	0.243324917	0.287354951	positive
PRJNA729271	stage_1D_vs_E10	ordinary_muscle_development	ENSGALG00010023089	ENSGALG00010023089	2.9236263	0.685030703	4.267876296	1.97343E-05	3.9115E-05	positive
PRJNA729271	stage_1D_vs_E10	ordinary_muscle_development	ENSGALG00010023090	LRRC41	0.018438201	0.090826407	0.203004853	0.839131239	0.862271892	positive
PRJNA729271	stage_1D_vs_E10	ordinary_muscle_development	ENSGALG00010023091	ENSGALG00010023091	0.581381444	0.131251741	4.429514152	9.44456E-06	1.93469E-05	positive
PRJNA729271	stage_1D_vs_E10	ordinary_muscle_development	ENSGALG00010023093	POP5	-0.078523832	0.128326321	-0.611907451	0.540598992	0.586921008	negative
PRJNA729271	stage_1D_vs_E10	ordinary_muscle_development	ENSGALG00010023094	CCDC32	-0.33340741	0.102081285	-3.266097305	0.001090408	0.001810477	negative
PRJNA729271	stage_1D_vs_E10	ordinary_muscle_development	ENSGALG00010023096	SPATS2	-0.178041079	0.171360148	-1.038987656	0.298810477	0.345671019	negative
PRJNA729271	stage_1D_vs_E10	ordinary_muscle_development	ENSGALG00010023097	LURAP1	0.958607011	0.21025865	4.55917991	5.13538E-06	1.07677E-05	positive
PRJNA729271	stage_1D_vs_E10	ordinary_muscle_development	ENSGALG00010023101	CLCA2	-0.865061304	1.274734746	-0.678620636	0.497378264	0.545471585	negative
PRJNA729271	stage_1D_vs_E10	ordinary_muscle_development	ENSGALG00010023102	RNF10	0.12379447	0.101571833	1.218787401	0.222924895	0.265556899	positive
PRJNA729271	stage_1D_vs_E10	ordinary_muscle_development	ENSGALG00010023103	DGKD	0.841820199	0.124831115	6.743672812	1.54432E-11	4.94695E-11	positive
PRJNA729271	stage_1D_vs_E10	ordinary_muscle_development	ENSGALG00010023104	ENSGALG00010023104	0.793389988	0.157712012	5.030624979	4.88884E-07	1.11914E-06	positive
PRJNA729271	stage_1D_vs_E10	ordinary_muscle_development	ENSGALG00010023107	ZC3HAV1	-0.221592668	0.292664768	-0.757155257	0.448956852	0.498210808	negative
PRJNA729271	stage_1D_vs_E10	ordinary_muscle_development	ENSGALG00010023108	TET1	0.213221483	0.061188539	3.484663754	0.000492756	0.000849084	positive
PRJNA729271	stage_1D_vs_E10	ordinary_muscle_development	ENSGALG00010023110	LIMA1	-1.818759887	0.155123624	-11.72458354	9.53689E-32	8.98228E-31	negative
PRJNA729271	stage_1D_vs_E10	ordinary_muscle_development	ENSGALG00010023112	PCMT1L	1.676789752	0.13098618	12.80127221	1.61287E-37	1.93052E-36	positive
PRJNA729271	stage_1D_vs_E10	ordinary_muscle_development	ENSGALG00010023113	MYZAP	0.101658004	0.131468539	0.77324967	0.439374635	0.488587291	positive
PRJNA729271	stage_1D_vs_E10	ordinary_muscle_development	ENSGALG00010023114	UQCRHL	1.541412856	0.167367684	9.20973999	3.26916E-20	1.76815E-19	positive
PRJNA729271	stage_1D_vs_E10	ordinary_muscle_development	ENSGALG00010023115	RPLP2	0.071753753	0.136366321	0.526183828	0.598760489	0.641892855	positive

PRJNA729271	stage_1D_vs_E10	ordinary_muscle_development	ENSGALG00010023116	PROC	2.628538967	1.040829411	2.525427259	0.01155577	0.016941528	positive
PRJNA729271	stage_1D_vs_E10	ordinary_muscle_development	ENSGALG00010023117	RPUSD2	-0.360039308	0.103257774	-3.486800996	0.000488835	0.000842729	negative
PRJNA729271	stage_1D_vs_E10	ordinary_muscle_development	ENSGALG00010023118	PIDD1	0.777463574	0.134596768	5.776242533	7.63872E-09	2.01682E-08	positive
PRJNA729271	stage_1D_vs_E10	ordinary_muscle_development	ENSGALG00010023119	NDRG4	0.229194197	0.170126852	1.347195895	0.17791715	0.216151964	positive
PRJNA729271	stage_1D_vs_E10	ordinary_muscle_development	ENSGALG00010023120	CHRM4	-0.380757969	0.243807803	-1.561713629	0.118355467	0.1487267	negative
PRJNA729271	stage_1D_vs_E10	ordinary_muscle_development	ENSGALG00010023122	ENSGALG00010023122	1.64627956	0.669146767	2.460266777	0.013883377	0.020107869	positive
PRJNA729271	stage_1D_vs_E10	ordinary_muscle_development	ENSGALG00010023123	RNF214	0.509553557	0.142323169	3.580257245	0.000343256	0.000601705	positive
PRJNA729271	stage_1D_vs_E10	ordinary_muscle_development	ENSGALG00010023124	NSUN4	1.18626886	0.133586127	8.880180068	6.67522E-19	3.35497E-18	positive
PRJNA729271	stage_1D_vs_E10	ordinary_muscle_development	ENSGALG00010023125	COL20A1	1.552996821	0.282357546	5.500107372	3.7956E-08	9.45801E-08	positive
PRJNA729271	stage_1D_vs_E10	ordinary_muscle_development	ENSGALG00010023126	CGNL1	0.530934249	0.137062359	3.873669265	0.000107209	0.00019834	positive
PRJNA729271	stage_1D_vs_E10	ordinary_muscle_development	ENSGALG00010023127	CERS5	-0.857068365	0.097313011	-8.807335787	1.28155E-18	6.35516E-18	negative
PRJNA729271	stage_1D_vs_E10	ordinary_muscle_development	ENSGALG00010023128	IWS1	-0.083130547	0.137283207	-0.605540536	0.544819899	0.590926797	negative
PRJNA729271	stage_1D_vs_E10	ordinary_muscle_development	ENSGALG00010023130	DYNLT5	-0.299755368	0.505750941	-0.592693644	0.553386196	0.599277767	negative
PRJNA729271	stage_1D_vs_E10	ordinary_muscle_development	ENSGALG00010023131	SOX6	0.973986605	0.136867883	7.116253902	1.109E-12	3.81375E-12	positive
PRJNA729271	stage_1D_vs_E10	ordinary_muscle_development	ENSGALG00010023134	KNL1	-1.743188536	0.215440284	-8.091284071	5.9039E-16	2.52216E-15	negative
PRJNA729271	stage_1D_vs_E10	ordinary_muscle_development	ENSGALG00010023135	GINS3	-1.127381565	0.068355406	-16.4929393	4.12391E-61	1.17438E-59	negative
PRJNA729271	stage_1D_vs_E10	ordinary_muscle_development	ENSGALG00010023136	MDK	-5.117049783	0.410733189	-12.45833043	1.2598E-35	1.39823E-34	negative
PRJNA729271	stage_1D_vs_E10	ordinary_muscle_development	ENSGALG00010023137	ETV6	-0.287888681	0.11353501	-2.535682004	0.011222859	0.016490152	negative
PRJNA729271	stage_1D_vs_E10	ordinary_muscle_development	ENSGALG00010023140	CCAR1	-0.260165441	0.083453344	-3.117495692	0.001823946	0.002951669	negative
PRJNA729271	stage_1D_vs_E10	ordinary_muscle_development	ENSGALG00010023142	TLK2	0.410615667	0.168342962	2.439161471	0.014721389	0.021245174	positive
PRJNA729271	stage_1D_vs_E10	ordinary_muscle_development	ENSGALG00010023144	C11orf58	0.165527647	0.106209949	1.558494745	0.119116013	0.149552759	positive
PRJNA729271	stage_1D_vs_E10	ordinary_muscle_development	ENSGALG00010023147	RAD51	-2.370703825	0.190699406	-12.43162669	1.76016E-35	1.94611E-34	negative
PRJNA729271	stage_1D_vs_E10	ordinary_muscle_development	ENSGALG00010023149	ADGRD1	0.002201861	0.184363834	0.011943022	0.990471074	0.992045206	positive
PRJNA729271	stage_1D_vs_E10	ordinary_muscle_development	ENSGALG00010023151	ENSGALG00010023151	-2.642024792	0.551617134	-4.789598853	1.67115E-06	3.6652E-06	negative
PRJNA729271	stage_1D_vs_E10	ordinary_muscle_development	ENSGALG00010023155	CFAP263	-0.280837475	0.328435808	-0.855075689	0.392509247	0.441692143	negative
PRJNA729271	stage_1D_vs_E10	ordinary_muscle_development	ENSGALG00010023157	STOX1	-1.783405729	0.350686762	-5.085466348	3.66724E-07	8.45904E-07	negative
PRJNA729271	stage_1D_vs_E10	ordinary_muscle_development	ENSGALG00010023158	ENSGALG00010023158	-2.769373832	0.938477491	-2.950921956	0.00316827	0.004980918	negative
PRJNA729271	stage_1D_vs_E10	ordinary_muscle_development	ENSGALG00010023159	DAW1	-0.177073087	0.499829355	-0.354267082	0.723138735	0.75785525	negative
PRJNA729271	stage_1D_vs_E10	ordinary_muscle_development	ENSGALG00010023160	ENSGALG00010023160	-0.609640078	0.109599584	-5.562430591	2.66043E-08	6.70426E-08	negative
PRJNA729271	stage_1D_vs_E10	ordinary_muscle_development	ENSGALG00010023161	ODF2L	-1.190094506	0.417840512	-2.848202776	0.00439669	0.006811674	negative
PRJNA729271	stage_1D_vs_E10	ordinary_muscle_development	ENSGALG00010023162	CHRNA4	-1.956232409	0.273274708	-7.158483223	8.15746E-13	2.841E-12	negative
PRJNA729271	stage_1D_vs_E10	ordinary_muscle_development	ENSGALG00010023163	CRTAC1	-0.90121755	0.281900745	-3.196932133	0.001388976	0.002280608	negative
PRJNA729271	stage_1D_vs_E10	ordinary_muscle_development	ENSGALG00010023164	NKIRAS2	0.509923023	0.132870169	3.837754009	0.000124165	0.000228194	positive
PRJNA729271	stage_1D_vs_E10	ordinary_muscle_development	ENSGALG00010023165	CSNK2A2	0.147278636	0.129606311	1.136353891	0.255808485	0.300652233	positive
PRJNA729271	stage_1D_vs_E10	ordinary_muscle_development	ENSGALG00010023166	KDM2A	0.161338149	0.112553397	1.433436507	0.151733132	0.18686251	positive
PRJNA729271	stage_1D_vs_E10	ordinary_muscle_development	ENSGALG00010023167	GPD1	7.897828225	0.210072261	37.5957691	2.5206E-309	3.6535E-306	positive
PRJNA729271	stage_1D_vs_E10	ordinary_muscle_development	ENSGALG00010023168	SPHKAP	-2.775623229	0.332147146	-8.356607204	6.45478E-17	2.9138E-16	negative
PRJNA729271	stage_1D_vs_E10	ordinary_muscle_development	ENSGALG00010023169	RAN	-1.209411507	0.09701812	-12.46583119	1.1467E-35	1.27465E-34	negative
PRJNA729271	stage_1D_vs_E10	ordinary_muscle_development	ENSGALG00010023170	TCF12	-0.902697971	0.064801275	-13.93025023	4.14899E-44	6.29735E-43	negative
PRJNA729271	stage_1D_vs_E10	ordinary_muscle_development	ENSGALG00010023171	ENSGALG00010023171	-0.419379635	0.114330576	-3.66813193	0.000244329	0.000434493	negative
PRJNA729271	stage_1D_vs_E10	ordinary_muscle_development	ENSGALG00010023172	DHX58	1.90343793	0.327759144	5.807428918	6.34395E-09	1.68602E-08	positive
PRJNA729271	stage_1D_vs_E10	ordinary_muscle_development	ENSGALG00010023174	KCNQ2	-0.868704259	0.318340495	-2.728852506	0.006355512	0.009639338	negative
PRJNA729271	stage_1D_vs_E10	ordinary_muscle_development	ENSGALG00010023175	GRK2	0.444632828	0.136557764	3.256005477	0.001129916	0.001873499	positive
PRJNA729271	stage_1D_vs_E10	ordinary_muscle_development	ENSGALG00010023177	LZTS2	0.240504394	0.171103486	1.405607799	0.159840619	0.196014363	positive
PRJNA729271	stage_1D_vs_E10	ordinary_muscle_development	ENSGALG00010023178	CFAP20	-0.453898192	0.098446112	-4.610625864	4.01458E-06	8.49634E-06	negative
PRJNA729271	stage_1D_vs_E10	ordinary_muscle_development	ENSGALG00010023179	KAT2A	-0.510083305	0.128473742	-3.970331191	7.17728E-05	0.000135092	negative
PRJNA729271	stage_1D_vs_E10	ordinary_muscle_development	ENSGALG00010023180	EEF1A2	6.34721631	0.121648901	52.17651975	0	0	positive
PRJNA729271	stage_1D_vs_E10	ordinary_muscle_development	ENSGALG00010023181	PLEKHA7	0.647053447	0.163643634	3.954039829	7.68426E-05	0.000144186	positive
PRJNA729271	stage_1D_vs_E10	ordinary_muscle_development	ENSGALG00010023182	EPHB6	-2.176984301	0.143981482	-15.11989092	1.19741E-51	2.44113E-50	negative
PRJNA729271	stage_1D_vs_E10	ordinary_muscle_development	ENSGALG00010023183	ENSGALG00010023183	-1.721361014	0.561317894	-3.06664197	0.00216478	0.003466853	negative
PRJNA729271	stage_1D_vs_E10	ordinary_muscle_development	ENSGALG00010023184	SMARCD1	-0.777152819	0.053527606	-14.51872918	9.22038E-48	1.62393E-46	negative
PRJNA729271	stage_1D_vs_E10	ordinary_muscle_development	ENSGALG00010023185	FBXW4	1.621336442	0.117052071	13.85141181	1.24736E-43	1.85441E-42	positive
PRJNA729271	stage_1D_vs_E10	ordinary_muscle_development	ENSGALG00010023186	MIER1	-0.122996987	0.098611273	-1.247291345	0.21229069	0.253869611	negative
PRJNA729271	stage_1D_vs_E10	ordinary_muscle_development	ENSGALG00010023187	ENSGALG00010023187	3.051233022	0.230406695	13.24281406	4.96529E-40	6.50741E-39	positive
PRJNA729271	stage_1D_vs_E10	ordinary_muscle_development	ENSGALG00010023188	KIF1BP	0.055450183	0.058278121	0.951475135	0.341363235	0.390256337	positive
PRJNA729271	stage_1D_vs_E10	ordinary_muscle_development	ENSGALG00010023189	RMDN3	-0.389529571	0.084634134	-4.602511437	4.17427E-06	8.81754E-06	negative
PRJNA729271	stage_1D_vs_E10	ordinary_muscle_development	ENSGALG00010023190	SF3B3	-0.684380676	0.055895846	-12.2438557	1.81215E-34	1.92433E-33	negative
PRJNA729271	stage_1D_vs_E10	ordinary_muscle_development	ENSGALG00010023193	PPDPF	4.567985911	0.312607904	14.61250931	2.33747E-48	4.22465E-47	positive
PRJNA729271	stage_1D_vs_E10	ordinary_muscle_development	ENSGALG00010023194	MRC2	0.191696265	0.19323246	0.992050015	0.321173128	0.369242107	positive
PRJNA729271	stage_1D_vs_E10	ordinary_muscle_development	ENSGALG00010023195	ENSGALG00010023195	-3.417508413	0.189137708	-18.06888982	5.60298E-73	2.23119E-71	negative
PRJNA729271	stage_1D_vs_E10	ordinary_muscle_development	ENSGALG00010023196	DGKZ	0.261483373	0.128510776	2.034719435	0.041879112	0.056515941	positive
PRJNA729271	stage_1D_vs_E10	ordinary_muscle_development	ENSGALG00010023198	PTK6	-1.666711756	0.529728839	-3.146348911	0.001653227	0.002688603	negative
PRJNA729271	stage_1D_vs_E10	ordinary_muscle_development	ENSGALG00010023199	RPS13	0.396071993	0.196701352	2.013570265	0.044054671	0.059258765	positive
PRJNA729271	stage_1D_vs_E10	ordinary_muscle_development	ENSGALG00010023200	VPS26A	-0.182812579	0.082943665	-2.20405717	0.027520324	0.038123133	negative
PRJNA729271	stage_1D_vs_E10	ordinary_muscle_development	ENSGALG00010023201	ZNF280D	0.07504185	0.117820462	0.636916953	0.524178939	0.571319176	positive
PRJNA729271	stage_1D_vs_E10	ordinary_muscle_development	ENSGALG00010023202	PID1	0.161997688	0.156346027	1.036148414	0.300132909	0.347090036	positive
PRJNA729271	stage_1D_vs_E10	ordinary_muscle_development	ENSGALG00010023203	COG4	-0.083694306	0.063342247	-1.321303071	0.18640033	0.22556961	negative
PRJNA729271	stage_1D_vs_E10	ordinary_muscle_development	ENSGALG00010023204	SRGN	4.179691323	0.309021466	13.52556955	1.10485E-41	1.5384E-40	positive
PRJNA729271	stage_1D_vs_E10	ordinary_muscle_development	ENSGALG00010023205	DNAJC17	-0.78442351	0.113533508	-6.909180598	4.87461E-12	1.60695E-11	negative
PRJNA729271	stage_1D_vs_E10	ordinary_muscle_development	ENSGALG00010023206	HSPB9	1.345952546	0.315023834	4.272541957	1.93257E-05	3.83332E-05	positive
PRJNA729271	stage_1D_vs_E10	ordinary_muscle_development	ENSGALG00010023207	MNS1	-0.428250027	0.150858506	-2.838752943	0.004529021	0.00700247	negative
PRJNA729271	stage_1D_vs_E10	ordinary_muscle_development	ENSGALG00010023208	BACE1	-0.166672511	0.078030853	-2.135982187	0.032680866	0.044854573	negative
PRJNA729271	stage_1D_vs_E10	ordinary_muscle_development	ENSGALG00010023209	PIK3C2A	-0.095782427	0.106055783	-0.903132525	0.366455564	0.415534178	negative
PRJNA729271	stage_1D_vs_E10	ordinary_muscle_development	ENSGALG00010023210	ZNHIT6	-0.22302634	0.137256638	-1.624885637	0.104186929	0.13213663	negative
PRJNA729271	stage_1D_vs_E10	ordinary_muscle_development	ENSGALG00010023211	TLK1	0.126723749	0.075857087	1.67055912	0.094808793	0.121004971	positive
PRJNA729271	stage_1D_vs_E10	ordinary_muscle_development	ENSGALG00010023212	RIMBP2	2.697543646	0.213622442	12.62762294	1.48703E-36	1.71339E-35	positive
PRJNA729271	stage_1D_vs_E10	ordinary_muscle_development	ENSGALG00010023213	FCSK	0.737926463	0.113008911	6.529807723	6.58542E-11	2.01638E-10	positive
PRJNA729271	stage_1D_vs_E10	ordinary_muscle_development	ENSGALG00010023214	SUPV3L1	-0.132094819	0.088865783	-1.486453097	0.137159314	0.170128723	negative
PRJNA729271	stage_1D_vs_E10	ordinary_muscle_development	ENSGALG00010023215	TRIP12	0.077395272	0.056232832	1.376336015	0.168717628	0.205947772	positive
PRJNA729271	stage_1D_vs_E10	ordinary_muscle_development	ENSGALG00010023216	SLC35D1	-0.713396498	0.123888138	-5.758392283	8.49188E-09	2.23151E-08	negative
PRJNA729271	stage_1D_vs_E10	ordinary_muscle_development	ENSGALG00010023217	CREB3L1	0.071422807	0.205955923	0.346786858	0.728751451	0.762854935	positive
PRJNA729271	stage_1D_vs_E10	ordinary_muscle_development	ENSGALG00010023218	SSH3	0.124766506	0.130480176	0.956210433	0.33896593	0.387699137	positive
PRJNA729271	stage_1D_vs_E10	ordinary_muscle_development	ENSGALG00010023219	TEX9	-0.140582487	0.149990975	-0.937272972	0.348618162	0.397515754	negative
PRJNA729271	stage_1D_vs_E10	ordinary_muscle_development	ENSGALG00010023220	ST3GAL2	-1.286833688	0.08823327	-14.58444976	3.52755E-48	6.30478E-47	negative
PRJNA729271	stage_1D_vs_E10	ordinary_muscle_development	ENSGALG00010023221	AMY1A	2.676817125	0.135767523	19.71618156	1.56615E-86	8.59902E-85	positive
PRJNA729271	stage_1D_vs_E10	ordinary_muscle_development	ENSGALG00010023222	ENSGALG00010023222	-1.052539249	0.574171659	-1.833143857	0.06678118	0.087316725	negative
PRJNA729271	stage_1D_vs_E10	ordinary_muscle_development	ENSGALG00010023223	CEP164	-1.227112559	0.098172357	-12.49957321	7.50531E-36	8.42024E-35	negative
PRJNA729271	stage_1D_vs_E10	ordinary_muscle_development	ENSGALG00010023225	PDPR	0.653086524	0.119280918	5.475197016	4.37025E-08	1.08359E-07	positive
PRJNA729271	stage_1D_vs_E10	ordinary_muscle_development	ENSGALG00010023226	ENSGALG00010023226	0.378245242	0.198031768	1.910023055	0.056130245	0.074241071	positive
PRJNA729271	stage_1D_vs_E10	ordinary_muscle_development	ENSGALG00010023227	RNPC3	0.503535175	0.121918637	4.130091901	3.62618E-05	7.01537E-05	positive
PRJNA729271	stage_1D_vs_E10	ordinary_muscle_development	ENSGALG00010023228	GORASP2	0.513111386	0.1017452	5.043101647	4.58046E-07	1.05004E-06	positive
PRJNA729271	stage_1D_vs_E10	ordinary_muscle_development	ENSGALG00010023229	HKDC1	-1.791275381	0.474982283	-3.771246728	0.000162434	0.000295233	negative
PRJNA729271	stage_1D_vs_E10	ordinary_muscle_development	ENSGALG00010023230	ENSGALG00010023230	0.352435399	0.083397592	4.225966102	2.37918E-05	4.68054E-05	positive
PRJNA729271	stage_1D_vs_E10	ordinary_muscle_development	ENSGALG00010023231	ASIC1	-0.693303451	0.201428706	-3.441929726	0.00057758	0.000986453	negative
PRJNA729271	stage_1D_vs_E10	ordinary_muscle_development	ENSGALG00010023233	SEZ6L	0.561224798	0.856815314	0.655012567	0.512459663	0.560195073	positive
PRJNA729271	stage_1D_vs_E10	ordinary_muscle_development	ENSGALG00010023234	ENSGALG00010023234	0.159666859	0.092877174	1.719118395	0.085592816	0.110124966	positive
PRJNA729271	stage_1D_vs_E10	ordinary_muscle_development	ENSGALG00010023235	ENSGALG00010023235	-1.058991402	0.363557757	-2.912856026	0.003581397	0.005597019	negative
PRJNA729271	stage_1D_vs_E10	ordinary_muscle_development	ENSGALG00010023237	GAD1	-4.150451133	0.67596305	-6.140056227	8.24923E-10	2.3464E-09	negative
PRJNA729271	stage_1D_vs_E10	ordinary_muscle_development	ENSGALG00010023238	NUCB2	-0.284807545	0.078117209	-3.645900163	0.000266457	0.000471991	negative
PRJNA729271	stage_1D_vs_E10	ordinary_muscle_development	ENSGALG00010023239	RBPMS2	1.1897991	0.195441919	6.087737489	1.14517E-09	3.21817E-09	positive
PRJNA729271	stage_1D_vs_E10	ordinary_muscle_development	ENSGALG00010023240	PIWIL1	-2.486005091	0.441502099	-5.630788843	1.79387E-08	4.59159E-08	negative
PRJNA729271	stage_1D_vs_E10	ordinary_muscle_development	ENSGALG00010023243	HK1	0.663617882	0.114352078	5.803286596	6.50275E-09	1.72601E-08	positive
PRJNA729271	stage_1D_vs_E10	ordinary_muscle_development	ENSGALG00010023244	PPIB	-1.084786953	0.118658202	-9.142115177	6.12426E-20	3.26364E-19	negative
PRJNA729271	stage_1D_vs_E10	ordinary_muscle_development	ENSGALG00010023245	TANC2	0.077698228	0.143143181	0.542800765	0.587266986	0.63157998	positive
PRJNA729271	stage_1D_vs_E10	ordinary_muscle_development	ENSGALG00010023246	SP5	-5.620116329	0.938208278	-5.990265124	2.09499E-09	5.77757E-09	negative
PRJNA729271	stage_1D_vs_E10	ordinary_muscle_development	ENSGALG00010023248	PRDM10	-0.00714573	0.120469992	-0.059315433	0.952700869	0.960119523	negative
PRJNA729271	stage_1D_vs_E10	ordinary_muscle_development	ENSGALG00010023249	IL12RB2	0.496890485	0.468407129	1.060808971	0.288776725	0.33518727	positive
PRJNA729271	stage_1D_vs_E10	ordinary_muscle_development	ENSGALG00010023250	FZD10	-4.278823296	0.413839377	-10.33933341	4.6778E-25	3.22266E-24	negative
PRJNA729271	stage_1D_vs_E10	ordinary_muscle_development	ENSGALG00010023251	ENSGALG00010023251	4.049258564	1.022942735	3.9584411	7.54405E-05	0.000141649	positive
PRJNA729271	stage_1D_vs_E10	ordinary_muscle_development	ENSGALG00010023252	GLG1	0.204028346	0.079630772	2.562179693	0.010401749	0.015330284	positive
PRJNA729271	stage_1D_vs_E10	ordinary_muscle_development	ENSGALG00010023254	MYO3B	-1.513086545	0.317420234	-4.766824487	1.87152E-06	4.0855E-06	negative
PRJNA729271	stage_1D_vs_E10	ordinary_muscle_development	ENSGALG00010023255	SNX22	1.852318168	0.311656445	5.943461773	2.79065E-09	7.62928E-09	positive
PRJNA729271	stage_1D_vs_E10	ordinary_muscle_development	ENSGALG00010023257	PAK6	1.637950223	0.290154297	5.645100682	1.65085E-08	4.23898E-08	positive
PRJNA729271	stage_1D_vs_E10	ordinary_muscle_development	ENSGALG00010023258	ENSGALG00010023258	-0.081056015	0.344053172	-0.235591535	0.813749643	0.839302391	negative
PRJNA729271	stage_1D_vs_E10	ordinary_muscle_development	ENSGALG00010023259	TSPAN15	1.79955728	0.287818032	6.252413265	4.04158E-10	1.1733E-09	positive
PRJNA729271	stage_1D_vs_E10	ordinary_muscle_development	ENSGALG00010023260	ENSGALG00010023260	-2.307985411	0.231417017	-9.973274413	1.9954E-23	1.26413E-22	negative
PRJNA729271	stage_1D_vs_E10	ordinary_muscle_development	ENSGALG00010023261	SNX1	-0.372840645	0.09599672	-3.883889412	0.000102799	0.000190448	negative
PRJNA729271	stage_1D_vs_E10	ordinary_muscle_development	ENSGALG00010023262	POLD4	0.766947897	0.214756468	3.571244685	0.000355289	0.000621745	positive
PRJNA729271	stage_1D_vs_E10	ordinary_muscle_development	ENSGALG00010023263	SRP14	-1.185771797	0.0729643	-16.25139695	2.18332E-59	5.88239E-58	negative
PRJNA729271	stage_1D_vs_E10	ordinary_muscle_development	ENSGALG00010023264	RACGAP1	-1.709874979	0.228556732	-7.48118406	7.36559E-14	2.74458E-13	negative
PRJNA729271	stage_1D_vs_E10	ordinary_muscle_development	ENSGALG00010023265	RAB5C	-0.525104389	0.071169179	-7.378255575	1.60377E-13	5.84821E-13	negative
PRJNA729271	stage_1D_vs_E10	ordinary_muscle_development	ENSGALG00010023266	RFWD3	-1.00267713	0.138950184	-7.216090687	5.35034E-13	1.88924E-12	negative
PRJNA729271	stage_1D_vs_E10	ordinary_muscle_development	ENSGALG00010023267	DSCAML1	-2.586985467	0.794322329	-3.256845958	0.001126576	0.001868388	negative
PRJNA729271	stage_1D_vs_E10	ordinary_muscle_development	ENSGALG00010023268	KCNJ11	3.432953774	0.179599787	19.11446463	1.91374E-81	9.33994E-80	positive
PRJNA729271	stage_1D_vs_E10	ordinary_muscle_development	ENSGALG00010023269	OPRL1	-1.20065267	0.481647694	-2.492802696	0.012673925	0.018479886	negative
PRJNA729271	stage_1D_vs_E10	ordinary_muscle_development	ENSGALG00010023271	PATL2	-0.595452333	0.420251284	-1.416895929	0.156513357	0.192243124	negative
PRJNA729271	stage_1D_vs_E10	ordinary_muscle_development	ENSGALG00010023273	HDAC7	1.242823736	0.112609601	11.03656988	2.54566E-28	2.02299E-27	positive
PRJNA729271	stage_1D_vs_E10	ordinary_muscle_development	ENSGALG00010023274	ZBTB38	1.55837484	0.114205324	13.6453782	2.15095E-42	3.06568E-41	positive
PRJNA729271	stage_1D_vs_E10	ordinary_muscle_development	ENSGALG00010023275	TWIST3	-8.185252443	0.634465321	-12.90102418	4.44155E-38	5.43293E-37	negative
PRJNA729271	stage_1D_vs_E10	ordinary_muscle_development	ENSGALG00010023276	ABCC8	2.278323563	0.16158221	14.10008912	3.7929E-45	5.9889E-44	positive
PRJNA729271	stage_1D_vs_E10	ordinary_muscle_development	ENSGALG00010023278	ENSGALG00010023278	1.102775719	0.233661326	4.719547466	2.3637E-06	5.12441E-06	positive

PRJNA729271	stage_1D_vs_E10	ordinary_muscle_development	ENSGALG00010023279	FA2H	1.202914634	0.410259382	2.932083183	0.003366966	0.005280693	positive
PRJNA729271	stage_1D_vs_E10	ordinary_muscle_development	ENSGALG00010023281	B2M	2.017782462	0.199578831	10.11020283	4.97811E-24	3.23868E-23	positive
PRJNA729271	stage_1D_vs_E10	ordinary_muscle_development	ENSGALG00010023282	SERBP1	-0.262436663	0.087253433	-3.007751735	0.00263188	0.004176605	negative
PRJNA729271	stage_1D_vs_E10	ordinary_muscle_development	ENSGALG00010023285	SLC48A1	2.262374638	0.110681542	20.44039681	7.31421E-93	4.73301E-91	positive
PRJNA729271	stage_1D_vs_E10	ordinary_muscle_development	ENSGALG00010023286	HCRT	-1.885394735	0.679236668	-2.775755231	0.005507365	0.008420808	negative
PRJNA729271	stage_1D_vs_E10	ordinary_muscle_development	ENSGALG00010023287	RGS19	-0.91471774	0.206233122	-4.43535806	9.19194E-06	1.88534E-05	negative
PRJNA729271	stage_1D_vs_E10	ordinary_muscle_development	ENSGALG00010023289	NFRKB	-0.036302809	0.086372181	-0.420306738	0.674261389	0.712534896	negative
PRJNA729271	stage_1D_vs_E10	ordinary_muscle_development	ENSGALG00010023290	SLC16A14	-3.321580418	0.249466111	-13.31475606	1.89992E-40	2.53352E-39	negative
PRJNA729271	stage_1D_vs_E10	ordinary_muscle_development	ENSGALG00010023293	COL11A1	-2.804957688	0.489135312	-5.734522984	9.77872E-09	2.55208E-08	negative
PRJNA729271	stage_1D_vs_E10	ordinary_muscle_development	ENSGALG00010023295	ENSGALG00010023295	1.457242854	0.494224384	2.948545038	0.003192736	0.005016662	positive
PRJNA729271	stage_1D_vs_E10	ordinary_muscle_development	ENSGALG00010023299	PASK	-1.091569772	0.137950322	-7.912774369	2.51715E-15	1.03039E-14	negative
PRJNA729271	stage_1D_vs_E10	ordinary_muscle_development	ENSGALG00010023304	ENSGALG00010023304	-0.983283351	0.175797626	-5.59326865	2.22834E-08	5.65572E-08	negative
PRJNA729271	stage_1D_vs_E10	ordinary_muscle_development	ENSGALG00010023305	PLCB2	0.712733234	0.160290905	4.446498286	8.72814E-06	1.79428E-05	positive
PRJNA729271	stage_1D_vs_E10	ordinary_muscle_development	ENSGALG00010023309	PIK3R3	-1.612142421	0.201756427	-7.990538114	1.34351E-15	5.60247E-15	negative
PRJNA729271	stage_1D_vs_E10	ordinary_muscle_development	ENSGALG00010023311	ETS1	-0.117109228	0.133532793	-0.877007255	0.380482697	0.429457771	negative
PRJNA729271	stage_1D_vs_E10	ordinary_muscle_development	ENSGALG00010023312	UBR3	1.409067787	0.11421984	12.33645384	5.76391E-35	6.24339E-34	positive
PRJNA729271	stage_1D_vs_E10	ordinary_muscle_development	ENSGALG00010023317	RAPGEF3	1.256945184	0.148487918	8.464966019	2.56227E-17	1.18318E-16	positive
PRJNA729271	stage_1D_vs_E10	ordinary_muscle_development	ENSGALG00010023319	EIF2AK4	-0.432524973	0.062495192	-6.920931943	4.48682E-12	1.48417E-11	negative
PRJNA729271	stage_1D_vs_E10	ordinary_muscle_development	ENSGALG00010023320	ENSGALG00010023320	-0.602641056	0.140288921	-4.295713798	1.74132E-05	3.46852E-05	negative
PRJNA729271	stage_1D_vs_E10	ordinary_muscle_development	ENSGALG00010023322	PRKAB1	-0.333613096	0.091576187	-3.643011422	0.000269467	0.000476972	negative
PRJNA729271	stage_1D_vs_E10	ordinary_muscle_development	ENSGALG00010023324	LAG3	-0.042178415	1.250262793	-0.03373564	0.973087959	0.976997296	negative
PRJNA729271	stage_1D_vs_E10	ordinary_muscle_development	ENSGALG00010023328	TCEA2	-1.214756214	0.109776065	-11.06576565	1.83886E-28	1.47587E-27	negative
PRJNA729271	stage_1D_vs_E10	ordinary_muscle_development	ENSGALG00010023330	TSPAN1	0.011013104	1.460988293	0.007538119	0.993985508	0.995152641	positive
PRJNA729271	stage_1D_vs_E10	ordinary_muscle_development	ENSGALG00010023331	CD4	-2.774898198	0.336763193	-8.239909406	1.72341E-16	7.58835E-16	negative
PRJNA729271	stage_1D_vs_E10	ordinary_muscle_development	ENSGALG00010023332	PPP1R7	-0.423065934	0.07570796	-5.588130158	2.29528E-08	5.82051E-08	negative
PRJNA729271	stage_1D_vs_E10	ordinary_muscle_development	ENSGALG00010023333	ENDOU	-1.066042884	0.533287376	-1.999002664	0.045608066	0.061166628	negative
PRJNA729271	stage_1D_vs_E10	ordinary_muscle_development	ENSGALG00010023334	RPS17	0.239829135	0.151628105	1.581693152	0.113719637	0.143236543	positive
PRJNA729271	stage_1D_vs_E10	ordinary_muscle_development	ENSGALG00010023335	WDR59	0.244931879	0.067649637	3.620594124	0.000293927	0.000518622	positive
PRJNA729271	stage_1D_vs_E10	ordinary_muscle_development	ENSGALG00010023336	GPR162	-1.424306169	0.152555495	-9.336315108	9.97452E-21	5.55652E-20	negative
PRJNA729271	stage_1D_vs_E10	ordinary_muscle_development	ENSGALG00010023337	FLI1	0.104537211	0.116707856	0.895717004	0.370403927	0.419584629	positive
PRJNA729271	stage_1D_vs_E10	ordinary_muscle_development	ENSGALG00010023338	HDLBP	0.004849449	0.124067268	0.039087254	0.968820823	0.973117444	positive
PRJNA729271	stage_1D_vs_E10	ordinary_muscle_development	ENSGALG00010023340	P3H3	-0.364608125	0.158101949	-2.306158322	0.021101787	0.029771307	negative
PRJNA729271	stage_1D_vs_E10	ordinary_muscle_development	ENSGALG00010023342	FITM2	2.064308777	0.133897528	15.41707913	1.25661E-53	2.74315E-52	positive
PRJNA729271	stage_1D_vs_E10	ordinary_muscle_development	ENSGALG00010023343	TMEM233	5.55594668	0.332362388	16.71653256	9.93331E-63	2.94444E-61	positive
PRJNA729271	stage_1D_vs_E10	ordinary_muscle_development	ENSGALG00010023347	STAT5A	2.123108356	0.169202574	12.5477308	4.09065E-36	4.63959E-35	positive
PRJNA729271	stage_1D_vs_E10	ordinary_muscle_development	ENSGALG00010023349	ENSGALG00010023349	3.550212002	0.561416369	6.323670268	2.55422E-10	7.53122E-10	positive
PRJNA729271	stage_1D_vs_E10	ordinary_muscle_development	ENSGALG00010023350	ZNRF1	0.247972551	0.231232542	1.072394695	0.283542789	0.329719433	positive
PRJNA729271	stage_1D_vs_E10	ordinary_muscle_development	ENSGALG00010023351	GNB3	0.514907054	0.367036569	1.402876707	0.160653608	0.19696135	positive
PRJNA729271	stage_1D_vs_E10	ordinary_muscle_development	ENSGALG00010023354	SEPTIN2	-3.349505451	0.172696763	-19.39529961	8.45587E-84	4.40892E-82	negative
PRJNA729271	stage_1D_vs_E10	ordinary_muscle_development	ENSGALG00010023356	ENSGALG00010023356	0.551392348	0.184702987	2.985291982	0.002833078	0.004478728	positive
PRJNA729271	stage_1D_vs_E10	ordinary_muscle_development	ENSGALG00010023359	CDCA3	-1.476018202	0.268864059	-5.489830833	4.02319E-08	9.99934E-08	negative
PRJNA729271	stage_1D_vs_E10	ordinary_muscle_development	ENSGALG00010023362	GADD45A	1.064327113	0.148447455	7.169722847	7.51497E-13	2.62481E-12	positive
PRJNA729271	stage_1D_vs_E10	ordinary_muscle_development	ENSGALG00010023363	CPEB1	-1.859028911	0.240916945	-7.716472211	1.19594E-14	4.68263E-14	negative
PRJNA729271	stage_1D_vs_E10	ordinary_muscle_development	ENSGALG00010023364	CTRB1	7.360378468	2.217349052	3.31944962	0.000901951	0.001511245	positive
PRJNA729271	stage_1D_vs_E10	ordinary_muscle_development	ENSGALG00010023366	GDAP1L1	-3.200211705	0.453928427	-7.050035897	1.78872E-12	6.07769E-12	negative
PRJNA729271	stage_1D_vs_E10	ordinary_muscle_development	ENSGALG00010023368	ENSGALG00010023368	-1.61602841	0.218478821	-7.396727983	1.39581E-13	5.11174E-13	negative
PRJNA729271	stage_1D_vs_E10	ordinary_muscle_development	ENSGALG00010023369	ACE	4.482521128	0.476239012	9.412335019	4.85235E-21	2.7496E-20	positive
PRJNA729271	stage_1D_vs_E10	ordinary_muscle_development	ENSGALG00010023374	ENSGALG00010023374	-1.950757794	0.21859097	-8.924237777	4.48779E-19	2.27529E-18	negative
PRJNA729271	stage_1D_vs_E10	ordinary_muscle_development	ENSGALG00010023375	ST14	-0.818585611	0.150336456	-5.445023981	5.17984E-08	1.27842E-07	negative
PRJNA729271	stage_1D_vs_E10	ordinary_muscle_development	ENSGALG00010023377	OSER1	0.665504025	0.150218288	4.430246382	9.41255E-06	1.92841E-05	positive
PRJNA729271	stage_1D_vs_E10	ordinary_muscle_development	ENSGALG00010023379	CCDC60	1.726469846	0.47369378	3.64469604	0.000267708	0.000474148	positive
PRJNA729271	stage_1D_vs_E10	ordinary_muscle_development	ENSGALG00010023380	USP5	-0.934407692	0.097144557	-9.618734406	6.66461E-22	3.96078E-21	negative
PRJNA729271	stage_1D_vs_E10	ordinary_muscle_development	ENSGALG00010023381	HOXB7	-0.761876958	0.165533397	-4.602557376	4.17335E-06	8.81688E-06	negative
PRJNA729271	stage_1D_vs_E10	ordinary_muscle_development	ENSGALG00010023383	HOXB6	-0.373900207	0.17174629	-2.177049692	0.02947686	0.040676608	negative
PRJNA729271	stage_1D_vs_E10	ordinary_muscle_development	ENSGALG00010023384	AP3B2	-2.802175922	0.243531538	-11.50641903	1.22458E-30	1.09638E-29	negative
PRJNA729271	stage_1D_vs_E10	ordinary_muscle_development	ENSGALG00010023387	ENSGALG00010023387	2.117913714	0.182543772	11.6022239	4.01507E-31	3.65798E-30	positive
PRJNA729271	stage_1D_vs_E10	ordinary_muscle_development	ENSGALG00010023388	FARP2	-0.376013676	0.082651862	-4.549367272	5.38075E-06	1.12643E-05	negative
PRJNA729271	stage_1D_vs_E10	ordinary_muscle_development	ENSGALG00010023389	GNG12	-0.007091174	0.1093784	-0.064831578	0.948308099	0.95622441	negative
PRJNA729271	stage_1D_vs_E10	ordinary_muscle_development	ENSGALG00010023390	HSPB8	2.36587771	0.250775495	9.434246014	3.93816E-21	2.24209E-20	positive
PRJNA729271	stage_1D_vs_E10	ordinary_muscle_development	ENSGALG00010023392	TPI1	1.551810182	0.149567556	10.37531286	3.21159E-25	2.23271E-24	positive
PRJNA729271	stage_1D_vs_E10	ordinary_muscle_development	ENSGALG00010023394	HOXB5	-1.728802291	0.180142469	-9.596861298	8.24159E-22	4.87202E-21	negative
PRJNA729271	stage_1D_vs_E10	ordinary_muscle_development	ENSGALG00010023396	ENSGALG00010023396	0.464719115	0.137859793	3.370954697	0.000749082	0.001266675	positive
PRJNA729271	stage_1D_vs_E10	ordinary_muscle_development	ENSGALG00010023397	ENSGALG00010023397	-0.78993702	0.256012045	-3.085546313	0.002031786	0.003268309	negative
PRJNA729271	stage_1D_vs_E10	ordinary_muscle_development	ENSGALG00010023401	LRRC23	1.30876068	0.367796899	3.55837878	0.000373151	0.000651508	positive
PRJNA729271	stage_1D_vs_E10	ordinary_muscle_development	ENSGALG00010023405	DNAJA2	-0.103509795	0.076588662	-1.351502848	0.176534408	0.214687998	negative
PRJNA729271	stage_1D_vs_E10	ordinary_muscle_development	ENSGALG00010023408	ZBTB44	0.469110541	0.126477151	3.70905368	0.000208035	0.000372971	positive
PRJNA729271	stage_1D_vs_E10	ordinary_muscle_development	ENSGALG00010023411	ENSGALG00010023411	2.957116632	0.18093409	16.34361242	4.83042E-60	1.3362E-58	positive
PRJNA729271	stage_1D_vs_E10	ordinary_muscle_development	ENSGALG00010023412	GSS	-0.790425789	0.107510565	-7.352075518	1.95152E-13	7.08069E-13	negative
PRJNA729271	stage_1D_vs_E10	ordinary_muscle_development	ENSGALG00010023414	SUDS3	-0.656461801	0.0762107	-8.613774755	7.06935E-18	3.36591E-17	negative
PRJNA729271	stage_1D_vs_E10	ordinary_muscle_development	ENSGALG00010023415	HOXB4	0.048060881	0.165614689	0.290196969	0.771665555	0.801009182	positive
PRJNA729271	stage_1D_vs_E10	ordinary_muscle_development	ENSGALG00010023421	OLFM3	-0.795617512	0.253155793	-3.142797974	0.001673413	0.002720516	negative
PRJNA729271	stage_1D_vs_E10	ordinary_muscle_development	ENSGALG00010023422	WLS	-1.997320419	0.15566293	-12.83106019	1.09851E-37	1.3247E-36	negative
PRJNA729271	stage_1D_vs_E10	ordinary_muscle_development	ENSGALG00010023426	KCNJ1	-0.388921725	0.473495082	-0.821384931	0.411427044	0.461117684	negative
PRJNA729271	stage_1D_vs_E10	ordinary_muscle_development	ENSGALG00010023428	HOXB3	-1.037917736	0.179309974	-5.788399352	7.10603E-09	1.88166E-08	negative
PRJNA729271	stage_1D_vs_E10	ordinary_muscle_development	ENSGALG00010023429	BMF	0.843907748	0.146063244	5.777687299	7.57344E-09	2.00104E-08	positive
PRJNA729271	stage_1D_vs_E10	ordinary_muscle_development	ENSGALG00010023431	BCAR1	0.214085032	0.173494232	1.233960517	0.217217617	0.259376337	positive
PRJNA729271	stage_1D_vs_E10	ordinary_muscle_development	ENSGALG00010023432	TMOD3	-0.774532737	0.174981899	-4.426359191	9.58369E-06	1.96062E-05	negative
PRJNA729271	stage_1D_vs_E10	ordinary_muscle_development	ENSGALG00010023434	ATN1	0.79404991	0.182227802	4.357457535	1.31582E-05	2.6586E-05	positive
PRJNA729271	stage_1D_vs_E10	ordinary_muscle_development	ENSGALG00010023437	KCNJ5	1.534404205	0.245068999	6.261111009	3.82244E-10	1.11168E-09	positive
PRJNA729271	stage_1D_vs_E10	ordinary_muscle_development	ENSGALG00010023438	HOXB2	0.140327572	0.228373112	0.614466262	0.538907261	0.585303517	positive
PRJNA729271	stage_1D_vs_E10	ordinary_muscle_development	ENSGALG00010023439	ACSS2	2.138108923	0.131659705	16.23966061	2.64383E-59	7.07053E-58	positive
PRJNA729271	stage_1D_vs_E10	ordinary_muscle_development	ENSGALG00010023441	ENSGALG00010023441	1.082677557	0.149948237	7.220342024	5.1857E-13	1.83442E-12	positive
PRJNA729271	stage_1D_vs_E10	ordinary_muscle_development	ENSGALG00010023442	STK25	-0.227895395	0.112546319	-2.024903145	0.042877311	0.057793065	negative
PRJNA729271	stage_1D_vs_E10	ordinary_muscle_development	ENSGALG00010023443	ENSGALG00010023443	0.77033667	0.179838261	4.283497099	1.83978E-05	3.65511E-05	positive
PRJNA729271	stage_1D_vs_E10	ordinary_muscle_development	ENSGALG00010023447	C12orf57	0.236826136	0.11376152	2.081777173	0.037362828	0.050809099	positive
PRJNA729271	stage_1D_vs_E10	ordinary_muscle_development	ENSGALG00010023448	FAM219B	-0.120861698	0.119667242	-1.009981479	0.312504163	0.359932288	negative
PRJNA729271	stage_1D_vs_E10	ordinary_muscle_development	ENSGALG00010023453	ARHGAP32	-0.374339713	0.173572638	-2.156674684	0.031031021	0.042703375	negative
PRJNA729271	stage_1D_vs_E10	ordinary_muscle_development	ENSGALG00010023454	HOXB1	1.519019946	0.385116585	3.944311941	8.00294E-05	0.000149913	positive
PRJNA729271	stage_1D_vs_E10	ordinary_muscle_development	ENSGALG00010023455	CFDP1	-1.152276522	0.097608843	-11.80504238	3.67606E-32	3.52177E-31	negative
PRJNA729271	stage_1D_vs_E10	ordinary_muscle_development	ENSGALG00010023456	MPI	0.09874059	0.145038165	0.6807904	0.496004125	0.544170436	positive
PRJNA729271	stage_1D_vs_E10	ordinary_muscle_development	ENSGALG00010023469	ENSGALG00010023469	0.599174287	0.213637787	2.80462691	0.005037485	0.00774484	positive
PRJNA729271	stage_1D_vs_E10	ordinary_muscle_development	ENSGALG00010023472	PTPN6	-0.131288693	0.197771391	-0.66384067	0.506792293	0.554788481	negative
PRJNA729271	stage_1D_vs_E10	ordinary_muscle_development	ENSGALG00010023473	BOK	-0.605475638	0.200196725	-3.024403304	0.002491241	0.003961226	negative
PRJNA729271	stage_1D_vs_E10	ordinary_muscle_development	ENSGALG00010023475	ENSGALG00010023475	-2.785865807	0.536345917	-5.194158694	2.05648E-07	4.84536E-07	negative
PRJNA729271	stage_1D_vs_E10	ordinary_muscle_development	ENSGALG00010023476	SCAMP2	-0.157749845	0.151174216	-1.043497029	0.296718156	0.343634522	negative
PRJNA729271	stage_1D_vs_E10	ordinary_muscle_development	ENSGALG00010023478	TAOK3	1.371310031	0.122055082	11.23517361	2.73955E-29	2.29024E-28	positive
PRJNA729271	stage_1D_vs_E10	ordinary_muscle_development	ENSGALG00010023479	MCCC2L	0.740452295	0.156103649	4.743337532	2.10226E-06	4.57747E-06	positive
PRJNA729271	stage_1D_vs_E10	ordinary_muscle_development	ENSGALG00010023482	MTCL2	0.515288138	0.097780486	5.269846368	1.36538E-07	3.26372E-07	positive
PRJNA729271	stage_1D_vs_E10	ordinary_muscle_development	ENSGALG00010023485	BARX2	-3.64616439	0.304129415	-11.98885806	4.0647E-33	4.06891E-32	negative
PRJNA729271	stage_1D_vs_E10	ordinary_muscle_development	ENSGALG00010023487	GRN	-0.463437058	0.147063714	-3.151267207	0.001625637	0.002647002	negative
PRJNA729271	stage_1D_vs_E10	ordinary_muscle_development	ENSGALG00010023489	ETV5	0.24683493	0.216785302	1.138614692	0.254863898	0.299643922	positive
PRJNA729271	stage_1D_vs_E10	ordinary_muscle_development	ENSGALG00010023490	PHB2	0.467851744	0.102783435	4.551820498	5.31837E-06	1.11385E-05	positive
PRJNA729271	stage_1D_vs_E10	ordinary_muscle_development	ENSGALG00010023496	PLEKHA3	-0.404050284	0.105979243	-3.812541702	0.000137545	0.000251732	negative
PRJNA729271	stage_1D_vs_E10	ordinary_muscle_development	ENSGALG00010023499	PEBP1	-0.606577039	0.139913865	-4.335360463	1.45521E-05	2.92354E-05	negative
PRJNA729271	stage_1D_vs_E10	ordinary_muscle_development	ENSGALG00010023501	ENSGALG00010023501	4.841937427	2.984732464	1.622234986	0.104753043	0.132750075	positive
PRJNA729271	stage_1D_vs_E10	ordinary_muscle_development	ENSGALG00010023506	RPE65	-0.824311918	0.547803401	-1.504758673	0.132386132	0.164715621	negative
PRJNA729271	stage_1D_vs_E10	ordinary_muscle_development	ENSGALG00010023510	ASPHD2	-0.551203694	0.25076224	-2.198112818	0.027941067	0.038664035	negative
PRJNA729271	stage_1D_vs_E10	ordinary_muscle_development	ENSGALG00010023513	CDCA9	-1.225212679	0.214521865	-5.711365039	1.12074E-08	2.91182E-08	negative
PRJNA729271	stage_1D_vs_E10	ordinary_muscle_development	ENSGALG00010023514	ULK3	0.160867169	0.106651788	1.508340099	0.131467505	0.163741321	positive
PRJNA729271	stage_1D_vs_E10	ordinary_muscle_development	ENSGALG00010023518	METTL5	-0.787524601	0.121354662	-6.489446591	8.61522E-11	2.62018E-10	negative
PRJNA729271	stage_1D_vs_E10	ordinary_muscle_development	ENSGALG00010023519	DGKG	0.490193038	0.234740617	2.088232726	0.036776847	0.050073304	positive
PRJNA729271	stage_1D_vs_E10	ordinary_muscle_development	ENSGALG00010023522	EMG1	-0.865561635	0.160264889	-5.400818874	6.63374E-08	1.62563E-07	negative
PRJNA729271	stage_1D_vs_E10	ordinary_muscle_development	ENSGALG00010023526	KNSTRN	-2.146067266	0.18821092	-11.4024588	4.06474E-30	3.5344E-29	negative
PRJNA729271	stage_1D_vs_E10	ordinary_muscle_development	ENSGALG00010023527	TMEM98	1.063979481	0.222665199	4.778382452	1.76711E-06	3.86281E-06	positive
PRJNA729271	stage_1D_vs_E10	ordinary_muscle_development	ENSGALG00010023533	FKBP7	-0.780118559	0.162782127	-4.79240919	1.6479E-06	3.61641E-06	negative
PRJNA729271	stage_1D_vs_E10	ordinary_muscle_development	ENSGALG00010023536	ENSGALG00010023536	-0.099088288	0.192424145	-0.514947268	0.606589869	0.649181937	negative
PRJNA729271	stage_1D_vs_E10	ordinary_muscle_development	ENSGALG00010023541	PRSS2	5.664359828	2.494312369	2.270910372	0.023152404	0.032503061	positive
PRJNA729271	stage_1D_vs_E10	ordinary_muscle_development	ENSGALG00010023543	ENSGALG00010023543	-2.00550187	0.757627258	-2.647082517	0.008118954	0.012147423	negative
PRJNA729271	stage_1D_vs_E10	ordinary_muscle_development	ENSGALG00010023544	SSB	-0.918646608	0.102542177	-8.958719545	3.2847E-19	1.6818E-18	negative
PRJNA729271	stage_1D_vs_E10	ordinary_muscle_development	ENSGALG00010023545	PTPRT	0.995919908	0.127386922	7.818070292	5.36392E-15	2.14964E-14	positive
PRJNA729271	stage_1D_vs_E10	ordinary_muscle_development	ENSGALG00010023546	SLC11A2	1.119314887	0.15418414	7.259598101	3.88242E-13	1.38406E-12	positive
PRJNA729271	stage_1D_vs_E10	ordinary_muscle_development	ENSGALG00010023551	ENSGALG00010023551	-0.42490732	0.179523529	-2.366861446	0.017939647	0.025543732	negative
PRJNA729271	stage_1D_vs_E10	ordinary_muscle_development	ENSGALG00010023552	SGPP2	-0.434788966	0.386005317	-1.126380771	0.260004383	0.305070694	negative
PRJNA729271	stage_1D_vs_E10	ordinary_muscle_development	ENSGALG00010023555	HPS4	-0.578801889	0.167264828	-3.460392095	0.000539389	0.000926137	negative
PRJNA729271	stage_1D_vs_E10	ordinary_muscle_development	ENSGALG00010023556	IVD	0.966552824	0.120770436	8.003223793	1.21203E-15	5.06295E-15	positive
PRJNA729271	stage_1D_vs_E10	ordinary_muscle_development	ENSGALG00010023559	PJVK	0.401210517	0.717574257	0.559120556	0.576079447	0.620884198	positive
PRJNA729271	stage_1D_vs_E10	ordinary_muscle_development	ENSGALG00010023569	LPCAT3	0.598990926	0.101768309	5.885829626	3.96062E-09	1.06867E-08	positive
PRJNA729271	stage_1D_vs_E10	ordinary_muscle_development	ENSGALG00010023571	APLP2	1.251511991	0.09855475	12.69864715	6.01607E-37	7.05525E-36	positive
PRJNA729271	stage_1D_vs_E10	ordinary_muscle_development	ENSGALG00010023572	MYBL2	-1.124049154	0.128080301	-8.776128305	1.69199E-18	8.33629E-18	negative
PRJNA729271	stage_1D_vs_E10	ordinary_muscle_development	ENSGALG00010023574	DEPDC1	-1.956505014	0.204419433	-9.571032374	1.05843E-21	6.21889E-21	negative
PRJNA729271	stage_1D_vs_E10	ordinary_muscle_development	ENSGALG00010023578	SRRD	-0.42133048	0.10234956	-4.116583204	3.84531E-05	7.4218E-05	negative

PRJNA729271	stage_1D_vs_E10	ordinary_muscle_development	ENSGALG00010023580	RDM1	-1.284439699	0.163540032	-7.853977296	4.03047E-15	1.63235E-14	negative
PRJNA729271	stage_1D_vs_E10	ordinary_muscle_development	ENSGALG00010023585	TFIP11	-0.252798611	0.096335775	-2.624140518	0.008686794	0.012939582	negative
PRJNA729271	stage_1D_vs_E10	ordinary_muscle_development	ENSGALG00010023588	FARSB	-0.593928414	0.134779202	-4.406677051	1.04969E-05	2.13937E-05	negative
PRJNA729271	stage_1D_vs_E10	ordinary_muscle_development	ENSGALG00010023590	S1PR1	1.045207282	0.11494854	9.092827852	9.6496E-20	5.10105E-19	positive
PRJNA729271	stage_1D_vs_E10	ordinary_muscle_development	ENSGALG00010023592	BAHD1	-0.79030834	0.10518246	-7.51368942	5.74839E-14	2.16086E-13	negative
PRJNA729271	stage_1D_vs_E10	ordinary_muscle_development	ENSGALG00010023593	CSK	-0.678797496	0.082369668	-8.240867205	1.70967E-16	7.53013E-16	negative
PRJNA729271	stage_1D_vs_E10	ordinary_muscle_development	ENSGALG00010023597	MOGAT1	-1.491857745	0.351028307	-4.249964227	2.13805E-05	4.22451E-05	negative
PRJNA729271	stage_1D_vs_E10	ordinary_muscle_development	ENSGALG00010023598	IL10RA	0.518580047	0.451550155	1.148443959	0.250785335	0.295323213	positive
PRJNA729271	stage_1D_vs_E10	ordinary_muscle_development	ENSGALG00010023600	CHST14	-2.307818766	0.084968014	-27.16102972	1.8759E-162	4.1833E-160	negative
PRJNA729271	stage_1D_vs_E10	ordinary_muscle_development	ENSGALG00010023601	TPST2	0.862589301	0.104972654	8.217276265	2.08177E-16	9.11915E-16	positive
PRJNA729271	stage_1D_vs_E10	ordinary_muscle_development	ENSGALG00010023603	C1S	1.449164338	0.393543057	3.682352696	0.000231091	0.000412166	positive
PRJNA729271	stage_1D_vs_E10	ordinary_muscle_development	ENSGALG00010023604	ENSGALG00010023604	0.124021928	0.414080085	0.299511936	0.764549466	0.794646817	positive
PRJNA729271	stage_1D_vs_E10	ordinary_muscle_development	ENSGALG00010023605	INSYN1	1.347080438	0.314799766	4.279165947	1.87595E-05	3.7239E-05	positive
PRJNA729271	stage_1D_vs_E10	ordinary_muscle_development	ENSGALG00010023606	LRRC7	-2.565562509	0.302051843	-8.493782013	2.00019E-17	9.28661E-17	negative
PRJNA729271	stage_1D_vs_E10	ordinary_muscle_development	ENSGALG00010023612	GPT2	2.199159414	0.185132889	11.87881543	1.52513E-32	1.49169E-31	positive
PRJNA729271	stage_1D_vs_E10	ordinary_muscle_development	ENSGALG00010023613	COPB1	-0.827191287	0.051613148	-16.02675528	8.31105E-58	2.11349E-56	negative
PRJNA729271	stage_1D_vs_E10	ordinary_muscle_development	ENSGALG00010023615	PRSS3	1.33880397	5.150489839	0.259937212	0.794912206	0.822371882	positive
PRJNA729271	stage_1D_vs_E10	ordinary_muscle_development	ENSGALG00010023617	ENSGALG00010023617	0.809030962	0.296216655	2.731213618	0.006310156	0.009574553	positive
PRJNA729271	stage_1D_vs_E10	ordinary_muscle_development	ENSGALG00010023620	IRS1	1.78749149	0.140892421	12.68692441	6.98762E-37	8.188E-36	positive
PRJNA729271	stage_1D_vs_E10	ordinary_muscle_development	ENSGALG00010023621	CD79B	2.873783326	0.932505533	3.081786889	0.002057621	0.003305465	positive
PRJNA729271	stage_1D_vs_E10	ordinary_muscle_development	ENSGALG00010023623	OSBPL6	0.285746041	0.217866623	1.311564102	0.189667252	0.229178627	positive
PRJNA729271	stage_1D_vs_E10	ordinary_muscle_development	ENSGALG00010023624	PSMA1	-0.113596995	0.070998246	-1.599997209	0.109599203	0.138323068	negative
PRJNA729271	stage_1D_vs_E10	ordinary_muscle_development	ENSGALG00010023626	LOXL1	0.94321225	0.194909455	4.839232919	1.30341E-06	2.88222E-06	positive
PRJNA729271	stage_1D_vs_E10	ordinary_muscle_development	ENSGALG00010023627	IFT52	-0.453766627	0.101941815	-4.451231597	8.53792E-06	1.75667E-05	negative
PRJNA729271	stage_1D_vs_E10	ordinary_muscle_development	ENSGALG00010023631	ENSGALG00010023631	3.404206158	0.439910943	7.738398439	1.00677E-14	3.96337E-14	positive
PRJNA729271	stage_1D_vs_E10	ordinary_muscle_development	ENSGALG00010023635	SCN4A	10.24942674	0.317259542	32.30612595	5.7379E-229	4.3774E-226	positive
PRJNA729271	stage_1D_vs_E10	ordinary_muscle_development	ENSGALG00010023636	PDE3B	0.027813869	0.081208062	0.342501327	0.731973646	0.765951342	positive
PRJNA729271	stage_1D_vs_E10	ordinary_muscle_development	ENSGALG00010023637	RHBDD1	0.495841813	0.104445705	4.747364303	2.06085E-06	4.49E-06	positive
PRJNA729271	stage_1D_vs_E10	ordinary_muscle_development	ENSGALG00010023638	DCTN2	-0.143539381	0.131338222	-1.092898764	0.274438251	0.320141965	negative
PRJNA729271	stage_1D_vs_E10	ordinary_muscle_development	ENSGALG00010023639	KLHL23	0.947825602	0.216493561	4.37807757	1.19731E-05	2.42659E-05	positive
PRJNA729271	stage_1D_vs_E10	ordinary_muscle_development	ENSGALG00010023641	LRRC40	-1.549420436	0.111975671	-13.83711674	1.5219E-43	2.25561E-42	negative
PRJNA729271	stage_1D_vs_E10	ordinary_muscle_development	ENSGALG00010023642	PAX6	2.080585756	0.540008077	3.852878956	0.000116737	0.000215363	positive
PRJNA729271	stage_1D_vs_E10	ordinary_muscle_development	ENSGALG00010023643	SCN4B	4.239525955	0.146876933	28.86447764	3.3349E-183	1.1509E-180	positive
PRJNA729271	stage_1D_vs_E10	ordinary_muscle_development	ENSGALG00010023646	DPH5	-0.565717418	0.144187228	-3.923491883	8.72747E-05	0.000162896	negative
PRJNA729271	stage_1D_vs_E10	ordinary_muscle_development	ENSGALG00010023648	SCN2B	2.928905368	0.357999642	8.1813081	2.80779E-16	1.22256E-15	positive
PRJNA729271	stage_1D_vs_E10	ordinary_muscle_development	ENSGALG00010023652	SLC30A7	-0.853796082	0.09594572	-8.898740645	5.64839E-19	2.84975E-18	negative
PRJNA729271	stage_1D_vs_E10	ordinary_muscle_development	ENSGALG00010023655	C1R	0.517166589	0.185669536	2.785414342	0.005345936	0.008187801	positive
PRJNA729271	stage_1D_vs_E10	ordinary_muscle_development	ENSGALG00010023658	ENSGALG00010023658	-0.444639401	0.299490867	-1.484650954	0.13763631	0.170705769	negative
PRJNA729271	stage_1D_vs_E10	ordinary_muscle_development	ENSGALG00010023660	CRYBB1	0.452191538	0.48572665	0.93095888	0.351874835	0.400850812	positive
PRJNA729271	stage_1D_vs_E10	ordinary_muscle_development	ENSGALG00010023663	PHOSPHO2	0.323014236	0.091169746	3.5429981	0.000395606	0.000689053	positive
PRJNA729271	stage_1D_vs_E10	ordinary_muscle_development	ENSGALG00010023664	SLC6A9	-0.727450647	0.325686304	-2.233592991	0.025509866	0.035544122	negative
PRJNA729271	stage_1D_vs_E10	ordinary_muscle_development	ENSGALG00010023665	C16orf87	0.172054052	0.136010017	1.265010145	0.205867691	0.246697436	positive
PRJNA729271	stage_1D_vs_E10	ordinary_muscle_development	ENSGALG00010023666	ENSGALG00010023666	1.256231015	0.251529203	4.994374407	5.90268E-07	1.34169E-06	positive
PRJNA729271	stage_1D_vs_E10	ordinary_muscle_development	ENSGALG00010023668	RCN1	-0.283882433	0.161046463	-1.762736216	0.07794499	0.100975299	negative
PRJNA729271	stage_1D_vs_E10	ordinary_muscle_development	ENSGALG00010023670	EXTL2	0.651175585	0.126030853	5.166795036	2.38142E-07	5.58013E-07	positive
PRJNA729271	stage_1D_vs_E10	ordinary_muscle_development	ENSGALG00010023671	ENSGALG00010023671	0.231320371	0.859310621	0.269192962	0.787781196	0.815634888	positive
PRJNA729271	stage_1D_vs_E10	ordinary_muscle_development	ENSGALG00010023676	ENSGALG00010023676	-1.128666921	0.302448662	-3.731763639	0.000190144	0.000342462	negative
PRJNA729271	stage_1D_vs_E10	ordinary_muscle_development	ENSGALG00010023677	MN1	1.176481669	0.16085538	7.313909358	2.5948E-13	9.33987E-13	positive
PRJNA729271	stage_1D_vs_E10	ordinary_muscle_development	ENSGALG00010023680	MPZL3	-8.1438973	0.873424139	-9.324103763	1.11925E-20	6.21594E-20	negative
PRJNA729271	stage_1D_vs_E10	ordinary_muscle_development	ENSGALG00010023686	MYLK3	0.733614579	0.219714412	3.338946098	0.000840969	0.001414135	positive
PRJNA729271	stage_1D_vs_E10	ordinary_muscle_development	ENSGALG00010023689	WT1	-1.579276709	0.231910151	-6.809864527	9.76907E-12	3.16431E-11	negative
PRJNA729271	stage_1D_vs_E10	ordinary_muscle_development	ENSGALG00010023693	SRSF11	-0.41872668	0.068562297	-6.107244065	1.01366E-09	2.86358E-09	negative
PRJNA729271	stage_1D_vs_E10	ordinary_muscle_development	ENSGALG00010023694	PPCDC	1.302923498	0.151849667	8.580351345	9.45838E-18	4.4716E-17	positive
PRJNA729271	stage_1D_vs_E10	ordinary_muscle_development	ENSGALG00010023695	CLSTN3	-1.213729976	0.132025892	-9.193120799	3.81606E-20	2.05551E-19	negative
PRJNA729271	stage_1D_vs_E10	ordinary_muscle_development	ENSGALG00010023696	LRRC4C	-1.283497356	0.531589109	-2.414453821	0.01575882	0.022636419	negative
PRJNA729271	stage_1D_vs_E10	ordinary_muscle_development	ENSGALG00010023698	ENSGALG00010023698	-1.54489565	0.142786895	-10.81958994	2.78018E-27	2.10437E-26	negative
PRJNA729271	stage_1D_vs_E10	ordinary_muscle_development	ENSGALG00010023699	PPIG	-0.087203138	0.202908364	-0.429766108	0.667365789	0.706144033	negative
PRJNA729271	stage_1D_vs_E10	ordinary_muscle_development	ENSGALG00010023702	COL4A4	2.059421559	0.247274551	8.328481638	8.18858E-17	3.67472E-16	positive
PRJNA729271	stage_1D_vs_E10	ordinary_muscle_development	ENSGALG00010023703	PITPNB	-0.786987083	0.080026885	-9.834033721	8.03351E-23	4.98057E-22	negative
PRJNA729271	stage_1D_vs_E10	ordinary_muscle_development	ENSGALG00010023704	ANKRD13C	-0.059648345	0.068301412	-0.873310576	0.382493817	0.431526143	negative
PRJNA729271	stage_1D_vs_E10	ordinary_muscle_development	ENSGALG00010023706	CDK5RAP3	-0.770167987	0.129226779	-5.959817244	2.5252E-09	6.92579E-09	negative
PRJNA729271	stage_1D_vs_E10	ordinary_muscle_development	ENSGALG00010023707	MPZL2	-5.058600821	0.393549064	-12.85379964	8.18871E-38	9.91607E-37	negative
PRJNA729271	stage_1D_vs_E10	ordinary_muscle_development	ENSGALG00010023710	CDC14A	0.683305304	0.119670416	5.709893292	1.13047E-08	2.93606E-08	positive
PRJNA729271	stage_1D_vs_E10	ordinary_muscle_development	ENSGALG00010023711	ENSGALG00010023711	-0.288105177	0.343084566	-0.839749748	0.401048714	0.45050322	negative
PRJNA729271	stage_1D_vs_E10	ordinary_muscle_development	ENSGALG00010023712	ARHGAP40	-1.140483202	0.16490453	-6.916021079	4.64505E-12	1.53511E-11	negative
PRJNA729271	stage_1D_vs_E10	ordinary_muscle_development	ENSGALG00010023714	EIF3M	0.132745493	0.110369781	1.202734042	0.229079256	0.272172444	positive
PRJNA729271	stage_1D_vs_E10	ordinary_muscle_development	ENSGALG00010023715	CD3E	-0.499794755	0.361796435	-1.381425317	0.167148226	0.204267223	negative
PRJNA729271	stage_1D_vs_E10	ordinary_muscle_development	ENSGALG00010023716	FASTKD1	-0.625998128	0.105893302	-5.91159321	3.38814E-09	9.19512E-09	negative
PRJNA729271	stage_1D_vs_E10	ordinary_muscle_development	ENSGALG00010023720	SCAMP5	-0.849690052	0.09607154	-8.844347144	9.2065E-19	4.59849E-18	negative
PRJNA729271	stage_1D_vs_E10	ordinary_muscle_development	ENSGALG00010023724	CYP2R1	0.175133883	0.119103609	1.470433055	0.141444504	0.175174136	positive
PRJNA729271	stage_1D_vs_E10	ordinary_muscle_development	ENSGALG00010023726	PNPO	0.041784994	0.185347588	0.22544126	0.821636016	0.846574784	positive
PRJNA729271	stage_1D_vs_E10	ordinary_muscle_development	ENSGALG00010023730	SP2	1.453061995	0.177199929	8.200127429	2.40132E-16	1.04904E-15	positive
PRJNA729271	stage_1D_vs_E10	ordinary_muscle_development	ENSGALG00010023731	ACTR5	0.273298687	0.09618528	2.841377473	0.004491911	0.006951025	positive
PRJNA729271	stage_1D_vs_E10	ordinary_muscle_development	ENSGALG00010023732	TM2D1	0.184321038	0.077481774	2.378895431	0.017364601	0.024785809	positive
PRJNA729271	stage_1D_vs_E10	ordinary_muscle_development	ENSGALG00010023733	ORC6	-1.507523403	0.189763585	-7.944218612	1.95419E-15	8.06088E-15	negative
PRJNA729271	stage_1D_vs_E10	ordinary_muscle_development	ENSGALG00010023736	KIF5A	-1.714842015	0.537070726	-3.1929538	0.001408255	0.002309908	negative
PRJNA729271	stage_1D_vs_E10	ordinary_muscle_development	ENSGALG00010023738	ENSGALG00010023738	-0.123863422	0.40344534	-0.307014135	0.758832613	0.789808325	negative
PRJNA729271	stage_1D_vs_E10	ordinary_muscle_development	ENSGALG00010023740	PPP1R16B	0.594330665	0.100247173	5.928652614	3.0543E-09	8.31246E-09	positive
PRJNA729271	stage_1D_vs_E10	ordinary_muscle_development	ENSGALG00010023743	CD3D	0.622213544	0.230622261	2.697976944	0.006976227	0.010519132	positive
PRJNA729271	stage_1D_vs_E10	ordinary_muscle_development	ENSGALG00010023744	MT4	0.67929035	0.317131979	2.14197998	0.032195093	0.044225538	positive
PRJNA729271	stage_1D_vs_E10	ordinary_muscle_development	ENSGALG00010023746	TTC28	-1.096289603	0.088890751	-12.33299972	6.01645E-35	6.50809E-34	negative
PRJNA729271	stage_1D_vs_E10	ordinary_muscle_development	ENSGALG00010023748	ENSGALG00010023748	-0.944927818	0.1719293	-5.496025506	3.88446E-08	9.66947E-08	negative
PRJNA729271	stage_1D_vs_E10	ordinary_muscle_development	ENSGALG00010023749	RPP25	1.089807583	0.236798309	4.602260842	4.17929E-06	8.82559E-06	positive
PRJNA729271	stage_1D_vs_E10	ordinary_muscle_development	ENSGALG00010023750	ENSGALG00010023750	-1.553033559	0.163676212	-9.488450034	2.34495E-21	1.35257E-20	negative
PRJNA729271	stage_1D_vs_E10	ordinary_muscle_development	ENSGALG00010023751	PRRG4	-2.079972977	0.314737565	-6.608594618	3.87986E-11	1.20839E-10	negative
PRJNA729271	stage_1D_vs_E10	ordinary_muscle_development	ENSGALG00010023752	C1QL1	-0.952520831	0.272871607	-3.490728998	0.000481705	0.000831326	negative
PRJNA729271	stage_1D_vs_E10	ordinary_muscle_development	ENSGALG00010023759	CALCA	-1.742214557	0.284277757	-6.128564448	8.86755E-10	2.51734E-09	negative
PRJNA729271	stage_1D_vs_E10	ordinary_muscle_development	ENSGALG00010023760	ZW10	-0.556711943	0.091944303	-6.054882421	1.4052E-09	3.92531E-09	negative
PRJNA729271	stage_1D_vs_E10	ordinary_muscle_development	ENSGALG00010023762	BBS5	2.840233528	0.152074894	18.67654451	7.68349E-78	3.44805E-76	positive
PRJNA729271	stage_1D_vs_E10	ordinary_muscle_development	ENSGALG00010023764	COX5A	1.942606015	0.176724103	10.99230939	4.1614E-28	3.26935E-27	positive
PRJNA729271	stage_1D_vs_E10	ordinary_muscle_development	ENSGALG00010023766	ENSGALG00010023766	-0.904432188	0.331521575	-2.728124671	0.006369553	0.009657602	negative
PRJNA729271	stage_1D_vs_E10	ordinary_muscle_development	ENSGALG00010023767	ENSGALG00010023767	1.674601917	0.209853747	7.979852341	1.46508E-15	6.09541E-15	positive
PRJNA729271	stage_1D_vs_E10	ordinary_muscle_development	ENSGALG00010023770	SCRN2	-0.490095181	0.14194237	-3.452775799	0.00055485	0.000950656	negative
PRJNA729271	stage_1D_vs_E10	ordinary_muscle_development	ENSGALG00010023773	OVOA	0.523427444	0.538686183	0.97167416	0.331212676	0.379670046	positive
PRJNA729271	stage_1D_vs_E10	ordinary_muscle_development	ENSGALG00010023775	ENSGALG00010023775	0.738573532	0.140432635	5.259272773	1.44626E-07	3.4525E-07	positive
PRJNA729271	stage_1D_vs_E10	ordinary_muscle_development	ENSGALG00010023777	OAZ2	-0.309499523	0.132670951	-2.332835641	0.019656773	0.027846455	negative
PRJNA729271	stage_1D_vs_E10	ordinary_muscle_development	ENSGALG00010023778	FANCF	-2.678458774	0.218135979	-12.27884911	1.1766E-34	1.26332E-33	negative
PRJNA729271	stage_1D_vs_E10	ordinary_muscle_development	ENSGALG00010023781	COL4A3	2.901956444	0.225679451	12.85875359	7.68051E-38	9.31624E-37	positive
PRJNA729271	stage_1D_vs_E10	ordinary_muscle_development	ENSGALG00010023783	VPS35	-0.163744025	0.062783455	-2.608076057	0.009105272	0.013533728	negative
PRJNA729271	stage_1D_vs_E10	ordinary_muscle_development	ENSGALG00010023786	ENSGALG00010023786	-1.712303701	0.334195088	-5.12366508	2.99653E-07	6.96404E-07	negative
PRJNA729271	stage_1D_vs_E10	ordinary_muscle_development	ENSGALG00010023788	RTCA	-0.378213773	0.104843329	-3.607418595	0.000309259	0.000544281	negative
PRJNA729271	stage_1D_vs_E10	ordinary_muscle_development	ENSGALG00010023791	VTI1B	-0.163703415	0.081928823	-1.998117507	0.045703922	0.061278175	negative
PRJNA729271	stage_1D_vs_E10	ordinary_muscle_development	ENSGALG00010023792	ZNF609	-0.092546338	0.176663474	-0.523856662	0.60037824	0.643341656	negative
PRJNA729271	stage_1D_vs_E10	ordinary_muscle_development	ENSGALG00010023793	RBM45	-0.030688939	0.064379183	-0.476690408	0.633582594	0.67488093	negative
PRJNA729271	stage_1D_vs_E10	ordinary_muscle_development	ENSGALG00010023794	MT3	1.270023617	0.464959894	2.731469172	0.006305264	0.009569134	positive
PRJNA729271	stage_1D_vs_E10	ordinary_muscle_development	ENSGALG00010023798	ARCN1	-0.729533779	0.056863132	-12.82964458	1.11876E-37	1.348E-36	negative
PRJNA729271	stage_1D_vs_E10	ordinary_muscle_development	ENSGALG00010023799	NMT1	-0.206411501	0.127071459	-1.624373429	0.104296134	0.132239515	negative
PRJNA729271	stage_1D_vs_E10	ordinary_muscle_development	ENSGALG00010023800	PEX5	0.309383888	0.072048102	4.29412959	1.7538E-05	3.49242E-05	positive
PRJNA729271	stage_1D_vs_E10	ordinary_muscle_development	ENSGALG00010023802	ENSGALG00010023802	0.976286536	0.287612619	3.394449582	0.000687667	0.001166226	positive
PRJNA729271	stage_1D_vs_E10	ordinary_muscle_development	ENSGALG00010023805	GALNT6	-2.160061808	0.385684646	-5.600590621	2.13623E-08	5.43143E-08	negative
PRJNA729271	stage_1D_vs_E10	ordinary_muscle_development	ENSGALG00010023806	PATJ	-1.147886685	0.172505379	-6.654208062	2.8483E-11	8.94218E-11	negative
PRJNA729271	stage_1D_vs_E10	ordinary_muscle_development	ENSGALG00010023807	MRPL10	0.633500934	0.125108195	5.063624603	4.11359E-07	9.45552E-07	positive
PRJNA729271	stage_1D_vs_E10	ordinary_muscle_development	ENSGALG00010023808	DHX35	-0.125094318	0.114682825	-1.090785114	0.275367448	0.321011031	negative
PRJNA729271	stage_1D_vs_E10	ordinary_muscle_development	ENSGALG00010023809	CDON	-0.511803586	0.096772938	-5.288705685	1.23185E-07	2.9577E-07	negative
PRJNA729271	stage_1D_vs_E10	ordinary_muscle_development	ENSGALG00010023811	MRPL44	0.354962865	0.084511338	4.200180378	2.66702E-05	5.2213E-05	positive
PRJNA729271	stage_1D_vs_E10	ordinary_muscle_development	ENSGALG00010023813	CELA1	0.859209315	5.322023468	0.161444105	0.871743632	0.890794779	positive
PRJNA729271	stage_1D_vs_E10	ordinary_muscle_development	ENSGALG00010023815	PLCD3	0.764430158	0.135363469	5.647241186	1.63043E-08	4.18729E-08	positive
PRJNA729271	stage_1D_vs_E10	ordinary_muscle_development	ENSGALG00010023817	BBS2	0.112009952	0.11610088	0.96476403	0.334663016	0.383231191	positive
PRJNA729271	stage_1D_vs_E10	ordinary_muscle_development	ENSGALG00010023818	ALOX5	0.817665996	0.190065923	4.302012589	1.69254E-05	3.37506E-05	positive
PRJNA729271	stage_1D_vs_E10	ordinary_muscle_development	ENSGALG00010023819	BIN2	-0.086910643	0.246518753	-0.352551853	0.724424438	0.758977537	negative
PRJNA729271	stage_1D_vs_E10	ordinary_muscle_development	ENSGALG00010023820	CYP1C1	-0.959468432	0.913033288	-1.050858106	0.293323764	0.339975049	negative
PRJNA729271	stage_1D_vs_E10	ordinary_muscle_development	ENSGALG00010023821	KANK4	0.086822204	0.123365811	0.703778483	0.481570735	0.530100836	positive
PRJNA729271	stage_1D_vs_E10	ordinary_muscle_development	ENSGALG00010023822	CHEK2	-0.886828578	0.124129391	-7.144388397	9.03975E-13	3.13803E-12	negative
PRJNA729271	stage_1D_vs_E10	ordinary_muscle_development	ENSGALG00010023823	MUC2	2.812519468	0.520127241	5.407368136	6.39576E-08	1.5697E-07	positive
PRJNA729271	stage_1D_vs_E10	ordinary_muscle_development	ENSGALG00010023826	DBT	1.675786675	0.126585204	13.23840876	5.26526E-40	6.88808E-39	positive
PRJNA729271	stage_1D_vs_E10	ordinary_muscle_development	ENSGALG00010023828	CHEK1	-1.559578449	0.16879469	-9.239499443	2.47653E-20	1.34397E-19	negative
PRJNA729271	stage_1D_vs_E10	ordinary_muscle_development	ENSGALG00010023829	USP1	-0.770751921	0.100164965	-7.694825409	1.41688E-14	5.52235E-14	negative
PRJNA729271	stage_1D_vs_E10	ordinary_muscle_development	ENSGALG00010023830	AGFG1	0.855918817	0.112463627	7.610627902	2.72767E-14	1.04597E-13	positive
PRJNA729271	stage_1D_vs_E10	ordinary_muscle_development	ENSGALG00010023831	SATB2	-4.953208734	0.566766152	-8.739422284	2.34306E-18	1.14506E-17	negative
PRJNA729271	stage_1D_vs_E10	ordinary_muscle_development	ENSGALG00010023832	ENSGALG00010023832	-0.436325386	0.491314815	-0.888076998	0.374499335	0.423760176	negative
PRJNA729271	stage_1D_vs_E10	ordinary_muscle_development	ENSGALG00010023833	PIP4K2C	-0.020398844	0.107412279	-0.189911658	0.849378357	0.871378011	negative

PRJNA729271	stage_1D_vs_E10	ordinary_muscle_development	ENSGALG00010023835	INAFM2	0.192207998	0.121463243	1.582437562	0.113549716	0.143047378	positive
PRJNA729271	stage_1D_vs_E10	ordinary_muscle_development	ENSGALG00010023836	NDUFS3	1.48115947	0.14748628	10.04269325	9.89353E-24	6.36232E-23	positive
PRJNA729271	stage_1D_vs_E10	ordinary_muscle_development	ENSGALG00010023837	STT3A	-0.704222443	0.092616386	-7.603648473	2.87897E-14	1.10253E-13	negative
PRJNA729271	stage_1D_vs_E10	ordinary_muscle_development	ENSGALG00010023840	SMAGP	-4.851496483	0.807616111	-6.007181402	1.88776E-09	5.22396E-09	negative
PRJNA729271	stage_1D_vs_E10	ordinary_muscle_development	ENSGALG00010023841	OSBPL7	0.106763087	0.148738718	0.717789483	0.472887099	0.521651331	positive
PRJNA729271	stage_1D_vs_E10	ordinary_muscle_development	ENSGALG00010023847	PIK3AP1	-0.21069099	0.188310717	-1.11884758	0.263205169	0.308350091	negative
PRJNA729271	stage_1D_vs_E10	ordinary_muscle_development	ENSGALG00010023850	DAZAP2	1.627158642	0.197475969	8.239780522	1.72527E-16	7.59422E-16	positive
PRJNA729271	stage_1D_vs_E10	ordinary_muscle_development	ENSGALG00010023851	HSCB	0.178511959	0.149173224	1.196675607	0.231433018	0.274721284	positive
PRJNA729271	stage_1D_vs_E10	ordinary_muscle_development	ENSGALG00010023852	LRRC39	2.84686271	0.190178794	14.96940146	1.16364E-50	2.31054E-49	positive
PRJNA729271	stage_1D_vs_E10	ordinary_muscle_development	ENSGALG00010023854	SHCBP1	-1.427857579	0.217975471	-6.550542468	5.73285E-11	1.76428E-10	negative
PRJNA729271	stage_1D_vs_E10	ordinary_muscle_development	ENSGALG00010023857	ACBD4	0.574164482	0.15186697	3.780706759	0.000156384	0.000284772	positive
PRJNA729271	stage_1D_vs_E10	ordinary_muscle_development	ENSGALG00010023859	ENSGALG00010023859	3.650226163	0.740312019	4.93065906	8.19527E-07	1.84285E-06	positive
PRJNA729271	stage_1D_vs_E10	ordinary_muscle_development	ENSGALG00010023860	TRIP4	-0.110259708	0.11459632	-0.962157492	0.335970498	0.384485423	negative
PRJNA729271	stage_1D_vs_E10	ordinary_muscle_development	ENSGALG00010023861	ENSGALG00010023861	-0.456407306	0.152485023	-2.993128744	0.002761332	0.004371506	negative
PRJNA729271	stage_1D_vs_E10	ordinary_muscle_development	ENSGALG00010023862	TRMT13	0.111113097	0.12701547	0.874799711	0.381682895	0.430710816	positive
PRJNA729271	stage_1D_vs_E10	ordinary_muscle_development	ENSGALG00010023863	EYA2	-3.482999782	0.236038281	-14.75608012	2.81154E-49	5.26528E-48	negative
PRJNA729271	stage_1D_vs_E10	ordinary_muscle_development	ENSGALG00010023864	EPHA1	-7.61434653	0.962869911	-7.90797017	2.61619E-15	1.06912E-14	negative
PRJNA729271	stage_1D_vs_E10	ordinary_muscle_development	ENSGALG00010023866	AMBRA1	-0.039632504	0.07427383	-0.533599839	0.59361842	0.63713432	negative
PRJNA729271	stage_1D_vs_E10	ordinary_muscle_development	ENSGALG00010023867	ENSGALG00010023867	0.750403845	0.491626214	1.526370692	0.126917572	0.158509499	positive
PRJNA729271	stage_1D_vs_E10	ordinary_muscle_development	ENSGALG00010023869	DOCK7	-0.655028954	0.046579537	-14.06259055	6.44819E-45	1.0061E-43	negative
PRJNA729271	stage_1D_vs_E10	ordinary_muscle_development	ENSGALG00010023871	UQCRFS1	1.316664231	0.140256338	9.387556052	6.14086E-21	3.4608E-20	positive
PRJNA729271	stage_1D_vs_E10	ordinary_muscle_development	ENSGALG00010023874	HEXIM1	1.021548213	0.224409006	4.552171187	5.30951E-06	1.11216E-05	positive
PRJNA729271	stage_1D_vs_E10	ordinary_muscle_development	ENSGALG00010023878	ENSGALG00010023878	2.613937074	1.493786691	1.749873051	0.080140222	0.103606182	positive
PRJNA729271	stage_1D_vs_E10	ordinary_muscle_development	ENSGALG00010023879	EI24	0.400745358	0.116886116	3.428511201	0.000606901	0.001034337	positive
PRJNA729271	stage_1D_vs_E10	ordinary_muscle_development	ENSGALG00010023880	ENSGALG00010023880	-1.597628102	0.195035757	-8.191462561	2.58071E-16	1.12571E-15	negative
PRJNA729271	stage_1D_vs_E10	ordinary_muscle_development	ENSGALG00010023881	VSTM2B	-1.282451944	0.223667975	-5.733730732	9.82453E-09	2.56357E-08	negative
PRJNA729271	stage_1D_vs_E10	ordinary_muscle_development	ENSGALG00010023883	TBX21	5.459409287	0.343250168	15.90504474	5.84615E-57	1.45103E-55	positive
PRJNA729271	stage_1D_vs_E10	ordinary_muscle_development	ENSGALG00010023884	C2orf69	-0.90781344	0.125950894	-7.207677619	5.69142E-13	2.00528E-12	negative
PRJNA729271	stage_1D_vs_E10	ordinary_muscle_development	ENSGALG00010023887	RASA2	0.223289278	0.127490057	1.751425035	0.079872724	0.103315646	positive
PRJNA729271	stage_1D_vs_E10	ordinary_muscle_development	ENSGALG00010023888	CTH	-1.811857218	0.259672254	-6.977477149	3.00528E-12	1.0079E-11	negative
PRJNA729271	stage_1D_vs_E10	ordinary_muscle_development	ENSGALG00010023889	SAMD8	1.594171152	0.190506485	8.368067622	5.85708E-17	2.64893E-16	positive
PRJNA729271	stage_1D_vs_E10	ordinary_muscle_development	ENSGALG00010023890	SASS6	-0.738533466	0.119186183	-6.196468806	5.7744E-10	1.65841E-09	negative
PRJNA729271	stage_1D_vs_E10	ordinary_muscle_development	ENSGALG00010023891	ENSGALG00010023891	3.79310995	0.265798279	14.27063398	3.33533E-46	5.48136E-45	positive
PRJNA729271	stage_1D_vs_E10	ordinary_muscle_development	ENSGALG00010023896	POP4	-0.932390801	0.129974344	-7.173652698	7.30227E-13	2.55421E-12	negative
PRJNA729271	stage_1D_vs_E10	ordinary_muscle_development	ENSGALG00010023902	PTGER3	1.742917174	0.232800803	7.486731809	7.06098E-14	2.63446E-13	positive
PRJNA729271	stage_1D_vs_E10	ordinary_muscle_development	ENSGALG00010023903	TBKBP1	-0.492395346	0.396786031	-1.240959378	0.214620756	0.256486755	negative
PRJNA729271	stage_1D_vs_E10	ordinary_muscle_development	ENSGALG00010023909	FOXRED1	0.290929039	0.152786638	1.904152372	0.056890343	0.075143568	positive
PRJNA729271	stage_1D_vs_E10	ordinary_muscle_development	ENSGALG00010023910	RNF7	-0.055126385	0.100958461	-0.546030362	0.585045067	0.629470624	negative
PRJNA729271	stage_1D_vs_E10	ordinary_muscle_development	ENSGALG00010023911	TFCP2	-0.711600892	0.121968379	-5.834306402	5.40148E-09	1.44348E-08	negative
PRJNA729271	stage_1D_vs_E10	ordinary_muscle_development	ENSGALG00010023913	ZMYND8	-0.505504521	0.082948494	-6.094197699	1.09988E-09	3.09627E-09	negative
PRJNA729271	stage_1D_vs_E10	ordinary_muscle_development	ENSGALG00010023914	CCDC117	-0.05698421	0.064352715	-0.885498142	0.375888021	0.425132402	negative
PRJNA729271	stage_1D_vs_E10	ordinary_muscle_development	ENSGALG00010023917	ZRANB2	-0.143119833	0.079412616	-1.802230436	0.071509158	0.093095495	negative
PRJNA729271	stage_1D_vs_E10	ordinary_muscle_development	ENSGALG00010023918	MFSD14A	-0.310768835	0.079613707	-3.903458903	9.48277E-05	0.000176312	negative
PRJNA729271	stage_1D_vs_E10	ordinary_muscle_development	ENSGALG00010023920	ENSGALG00010023920	-0.018798921	0.38786356	-0.048467872	0.961343369	0.967014027	negative
PRJNA729271	stage_1D_vs_E10	ordinary_muscle_development	ENSGALG00010023921	GRK7	0.390784867	0.180885918	2.160394077	0.030742176	0.042350108	positive
PRJNA729271	stage_1D_vs_E10	ordinary_muscle_development	ENSGALG00010023923	CSNK1G1	-0.604037314	0.096435453	-6.263643644	3.76084E-10	1.09465E-09	negative
PRJNA729271	stage_1D_vs_E10	ordinary_muscle_development	ENSGALG00010023925	NCOA3	-0.180588571	0.076692958	-2.354695625	0.018537883	0.026328299	negative
PRJNA729271	stage_1D_vs_E10	ordinary_muscle_development	ENSGALG00010023926	ZYX	0.239341974	0.137353061	1.742531047	0.081415564	0.105114331	positive
PRJNA729271	stage_1D_vs_E10	ordinary_muscle_development	ENSGALG00010023927	KPNB1	-0.470689199	0.130259138	-3.613483137	0.000302111	0.000532092	negative
PRJNA729271	stage_1D_vs_E10	ordinary_muscle_development	ENSGALG00010023930	SRPRA	0.355242808	0.131134392	2.708998021	0.006748674	0.010193388	positive
PRJNA729271	stage_1D_vs_E10	ordinary_muscle_development	ENSGALG00010023931	CSRNP2	-1.38916584	0.148345915	-9.364368684	7.65063E-21	4.28998E-20	negative
PRJNA729271	stage_1D_vs_E10	ordinary_muscle_development	ENSGALG00010023937	XBP1	-0.06654243	0.110002607	-0.604916851	0.545234246	0.591289122	negative
PRJNA729271	stage_1D_vs_E10	ordinary_muscle_development	ENSGALG00010023940	ATP1B3	-0.063691245	0.071580781	-0.889781361	0.373583297	0.422756646	negative
PRJNA729271	stage_1D_vs_E10	ordinary_muscle_development	ENSGALG00010023942	NEGR1	3.330053866	0.219962695	15.1391756	8.93264E-52	1.83658E-50	positive
PRJNA729271	stage_1D_vs_E10	ordinary_muscle_development	ENSGALG00010023945	LETMD1	0.764323009	0.208034797	3.674015218	0.000238769	0.000425178	positive
PRJNA729271	stage_1D_vs_E10	ordinary_muscle_development	ENSGALG00010023946	ZNRF3	-0.13384506	0.124319999	-1.076617285	0.281651279	0.327782841	negative
PRJNA729271	stage_1D_vs_E10	ordinary_muscle_development	ENSGALG00010023947	ENSGALG00010023947	-7.686787804	0.655571842	-11.72531721	9.45462E-32	8.91058E-31	negative
PRJNA729271	stage_1D_vs_E10	ordinary_muscle_development	ENSGALG00010023948	SULF2	-1.208905798	0.220725162	-5.476973195	4.32663E-08	1.07311E-07	negative
PRJNA729271	stage_1D_vs_E10	ordinary_muscle_development	ENSGALG00010023953	SLC35A3	-0.191465224	0.154620811	-1.238288839	0.215608981	0.257540352	negative
PRJNA729271	stage_1D_vs_E10	ordinary_muscle_development	ENSGALG00010023954	PLEKHF1	0.961204009	0.094474904	10.1741729	2.58587E-24	1.70918E-23	positive
PRJNA729271	stage_1D_vs_E10	ordinary_muscle_development	ENSGALG00010023956	KREMEN1	-0.174647371	0.12963615	-1.347211951	0.17791198	0.216151964	negative
PRJNA729271	stage_1D_vs_E10	ordinary_muscle_development	ENSGALG00010023957	YBX3	2.050715408	0.13882434	14.77201631	2.21972E-49	4.18943E-48	positive
PRJNA729271	stage_1D_vs_E10	ordinary_muscle_development	ENSGALG00010023962	FPGT	-1.696901696	0.119144088	-14.24243304	4.9957E-46	8.17299E-45	negative
PRJNA729271	stage_1D_vs_E10	ordinary_muscle_development	ENSGALG00010023966	PEAK1	0.857167998	0.097777727	8.766495423	1.84314E-18	9.0625E-18	positive
PRJNA729271	stage_1D_vs_E10	ordinary_muscle_development	ENSGALG00010023967	PRPH	-3.690952614	0.413448949	-8.927226978	4.36821E-19	2.21699E-18	negative
PRJNA729271	stage_1D_vs_E10	ordinary_muscle_development	ENSGALG00010023968	TYW5	-0.347603854	0.119202851	-2.916069979	0.00354471	0.005545663	negative
PRJNA729271	stage_1D_vs_E10	ordinary_muscle_development	ENSGALG00010023972	ENSGALG00010023972	0.947505852	0.515639836	1.837534236	0.066131067	0.08652914	positive
PRJNA729271	stage_1D_vs_E10	ordinary_muscle_development	ENSGALG00010023974	PREX1	-0.267818028	0.103156522	-2.59622971	0.009425303	0.013983599	negative
PRJNA729271	stage_1D_vs_E10	ordinary_muscle_development	ENSGALG00010023978	TFDP2	-0.178965987	0.139569268	-1.282273596	0.199746687	0.239917818	negative
PRJNA729271	stage_1D_vs_E10	ordinary_muscle_development	ENSGALG00010023979	FAM118B	-0.700911362	0.115540326	-6.066378634	1.30827E-09	3.66016E-09	negative
PRJNA729271	stage_1D_vs_E10	ordinary_muscle_development	ENSGALG00010023981	ENSGALG00010023981	-0.036729539	0.371763828	-0.098798045	0.921298621	0.93301359	negative
PRJNA729271	stage_1D_vs_E10	ordinary_muscle_development	ENSGALG00010023984	FMNL1	1.099042199	0.208515488	5.270794075	1.35835E-07	3.24744E-07	positive
PRJNA729271	stage_1D_vs_E10	ordinary_muscle_development	ENSGALG00010023986	SYCE3	-0.382101132	0.606051426	-0.630476418	0.528382927	0.575295615	negative
PRJNA729271	stage_1D_vs_E10	ordinary_muscle_development	ENSGALG00010023987	TNNI3K	-1.035171453	0.444019406	-2.331365339	0.019734104	0.02795054	negative
PRJNA729271	stage_1D_vs_E10	ordinary_muscle_development	ENSGALG00010023989	ENSGALG00010023989	-0.076219993	0.099771689	-0.763944096	0.444900558	0.49412563	negative
PRJNA729271	stage_1D_vs_E10	ordinary_muscle_development	ENSGALG00010023991	TSPAN3	0.419361611	0.105332747	3.981303264	6.85384E-05	0.000129273	positive
PRJNA729271	stage_1D_vs_E10	ordinary_muscle_development	ENSGALG00010023992	FAM131B	-0.499212327	0.132586154	-3.765192019	0.000166421	0.000302176	negative
PRJNA729271	stage_1D_vs_E10	ordinary_muscle_development	ENSGALG00010023993	C19orf12	-0.598939163	0.175665186	-3.409549589	0.000650702	0.001105607	negative
PRJNA729271	stage_1D_vs_E10	ordinary_muscle_development	ENSGALG00010023996	MYO7B	2.218190353	0.501652287	4.421768645	9.78962E-06	2.00085E-05	positive
PRJNA729271	stage_1D_vs_E10	ordinary_muscle_development	ENSGALG00010023997	ARFGEF2	0.169418611	0.06178711	2.741973388	0.006107129	0.00929081	positive
PRJNA729271	stage_1D_vs_E10	ordinary_muscle_development	ENSGALG00010023999	DGCR14	0.1686632	0.091078326	1.851847832	0.064047677	0.084015482	positive
PRJNA729271	stage_1D_vs_E10	ordinary_muscle_development	ENSGALG00010024001	ENSGALG00010024001	2.754484272	0.456353519	6.035856324	1.58122E-09	4.40003E-09	positive
PRJNA729271	stage_1D_vs_E10	ordinary_muscle_development	ENSGALG00010024002	DGCR2	-0.499566276	0.0602894	-8.28613778	1.16985E-16	5.19516E-16	negative
PRJNA729271	stage_1D_vs_E10	ordinary_muscle_development	ENSGALG00010024003	ERICH3	-1.105695697	0.301230656	-3.670594858	0.000241987	0.000430538	negative
PRJNA729271	stage_1D_vs_E10	ordinary_muscle_development	ENSGALG00010024004	EIF4B	0.47871271	0.125052291	3.828100278	0.000129136	0.00023706	positive
PRJNA729271	stage_1D_vs_E10	ordinary_muscle_development	ENSGALG00010024005	STYK1	3.890737493	0.249457592	15.59678925	7.65483E-55	1.7446E-53	positive
PRJNA729271	stage_1D_vs_E10	ordinary_muscle_development	ENSGALG00010024008	CCNE1	-0.731083001	0.106613963	-6.857291283	7.01784E-12	2.29211E-11	negative
PRJNA729271	stage_1D_vs_E10	ordinary_muscle_development	ENSGALG00010024009	CSE1L	-0.903729376	0.061282833	-14.7468603	3.22314E-49	6.02055E-48	negative
PRJNA729271	stage_1D_vs_E10	ordinary_muscle_development	ENSGALG00010024010	ENSGALG00010024010	1.116905704	0.088502074	12.62010774	1.63597E-36	1.88053E-35	positive
PRJNA729271	stage_1D_vs_E10	ordinary_muscle_development	ENSGALG00010024011	AGPS	-0.053798086	0.070436682	-0.763779386	0.444998723	0.494196789	negative
PRJNA729271	stage_1D_vs_E10	ordinary_muscle_development	ENSGALG00010024013	PTPRJ	-0.09265847	0.120638741	-0.768065621	0.442448201	0.491665644	negative
PRJNA729271	stage_1D_vs_E10	ordinary_muscle_development	ENSGALG00010024016	CRYZ	-0.201700978	0.103701795	-1.945009517	0.051773833	0.068880185	negative
PRJNA729271	stage_1D_vs_E10	ordinary_muscle_development	ENSGALG00010024017	TYW3	-1.353281951	0.128544765	-10.52770956	6.4382E-26	4.58358E-25	negative
PRJNA729271	stage_1D_vs_E10	ordinary_muscle_development	ENSGALG00010024018	KRT18	-8.442204263	1.242153966	-6.796423388	1.07248E-11	3.46382E-11	negative
PRJNA729271	stage_1D_vs_E10	ordinary_muscle_development	ENSGALG00010024019	MAP3K14	0.862492479	0.106116354	8.127799759	4.37153E-16	1.88419E-15	positive
PRJNA729271	stage_1D_vs_E10	ordinary_muscle_development	ENSGALG00010024020	MAIP1	-0.146957377	0.122185492	-1.202739985	0.229076956	0.272172444	negative
PRJNA729271	stage_1D_vs_E10	ordinary_muscle_development	ENSGALG00010024021	IGLL1	2.33610475	1.432497123	1.630791932	0.102934231	0.130662201	positive
PRJNA729271	stage_1D_vs_E10	ordinary_muscle_development	ENSGALG00010024023	LHX8	-2.816723632	0.475710434	-5.921088612	3.19817E-09	8.69095E-09	negative
PRJNA729271	stage_1D_vs_E10	ordinary_muscle_development	ENSGALG00010024024	AGL	3.213215554	0.122639509	26.20049262	2.6233E-151	4.8132E-149	positive
PRJNA729271	stage_1D_vs_E10	ordinary_muscle_development	ENSGALG00010024025	CLCN1	8.417789319	0.33298785	25.27956895	5.3594E-141	8.3531E-139	positive
PRJNA729271	stage_1D_vs_E10	ordinary_muscle_development	ENSGALG00010024026	TIRAP	0.15137957	0.104706737	1.445748142	0.148247856	0.182990094	positive
PRJNA729271	stage_1D_vs_E10	ordinary_muscle_development	ENSGALG00010024029	SCAPER	0.135795659	0.147081983	0.923265078	0.355869082	0.404701267	positive
PRJNA729271	stage_1D_vs_E10	ordinary_muscle_development	ENSGALG00010024030	STAU1	-0.05641886	0.089579387	-0.629819666	0.528812577	0.575676929	negative
PRJNA729271	stage_1D_vs_E10	ordinary_muscle_development	ENSGALG00010024031	LIMS2	0.687860352	0.115115909	5.975371747	2.29566E-09	6.31416E-09	positive
PRJNA729271	stage_1D_vs_E10	ordinary_muscle_development	ENSGALG00010024034	LRP2	-0.548679978	0.245252151	-2.237207607	0.025272774	0.035240872	negative
PRJNA729271	stage_1D_vs_E10	ordinary_muscle_development	ENSGALG00010024035	OSBPL5	-0.601560309	0.136853624	-4.395647643	1.10443E-05	2.24495E-05	negative
PRJNA729271	stage_1D_vs_E10	ordinary_muscle_development	ENSGALG00010024038	NKD1	-1.439663564	0.163034563	-8.830419358	1.04284E-18	5.19271E-18	negative
PRJNA729271	stage_1D_vs_E10	ordinary_muscle_development	ENSGALG00010024039	MRPL45	-0.141219225	0.106444863	-1.326688969	0.184611591	0.223629034	negative
PRJNA729271	stage_1D_vs_E10	ordinary_muscle_development	ENSGALG00010024040	ENSGALG00010024040	-5.078594356	0.918601861	-5.528613179	3.22772E-08	8.07348E-08	negative
PRJNA729271	stage_1D_vs_E10	ordinary_muscle_development	ENSGALG00010024042	DCPS	-0.27879288	0.129996951	-2.144610922	0.031983968	0.04394385	negative
PRJNA729271	stage_1D_vs_E10	ordinary_muscle_development	ENSGALG00010024045	PSTPIP1	0.787538133	0.249972267	3.150502021	0.001629901	0.002653623	positive
PRJNA729271	stage_1D_vs_E10	ordinary_muscle_development	ENSGALG00010024046	ENSGALG00010024046	-2.378492105	0.54564938	-4.359011835	1.30651E-05	2.64016E-05	negative
PRJNA729271	stage_1D_vs_E10	ordinary_muscle_development	ENSGALG00010024049	RCN2	-0.662010835	0.143940482	-4.599198409	4.2412E-06	8.94849E-06	negative
PRJNA729271	stage_1D_vs_E10	ordinary_muscle_development	ENSGALG00010024051	ENSGALG00010024051	-7.730412603	0.863132829	-8.956225908	3.35981E-19	1.71844E-18	negative
PRJNA729271	stage_1D_vs_E10	ordinary_muscle_development	ENSGALG00010024052	ENSGALG00010024052	-0.053587677	0.169283948	-0.316554979	0.751581302	0.783527831	negative
PRJNA729271	stage_1D_vs_E10	ordinary_muscle_development	ENSGALG00010024053	ISL2	-1.367341125	0.570144606	-2.398235659	0.016474263	0.023591626	negative
PRJNA729271	stage_1D_vs_E10	ordinary_muscle_development	ENSGALG00010024054	ENSGALG00010024054	-6.396787374	1.032516126	-6.195338951	5.81597E-10	1.66936E-09	negative
PRJNA729271	stage_1D_vs_E10	ordinary_muscle_development	ENSGALG00010024057	HSD3B1	-2.336214347	0.751410744	-3.109104261	0.001876555	0.003031728	negative
PRJNA729271	stage_1D_vs_E10	ordinary_muscle_development	ENSGALG00010024058	SLC44A5	0.813972742	0.104588279	7.782638259	7.10275E-15	2.82067E-14	positive
PRJNA729271	stage_1D_vs_E10	ordinary_muscle_development	ENSGALG00010024060	FRRS1	0.479656125	0.161315572	2.973402489	0.00294518	0.004646825	positive
PRJNA729271	stage_1D_vs_E10	ordinary_muscle_development	ENSGALG00010024061	ENSGALG00010024061	-2.134862671	1.453496704	-1.468777098	0.141893258	0.175654862	negative
PRJNA729271	stage_1D_vs_E10	ordinary_muscle_development	ENSGALG00010024062	ST3GAL4	0.144962539	0.131916518	1.098896033	0.271813417	0.317378402	positive
PRJNA729271	stage_1D_vs_E10	ordinary_muscle_development	ENSGALG00010024063	BRD7	-0.376436571	0.104200214	-3.612627615	0.00030311	0.000533784	negative
PRJNA729271	stage_1D_vs_E10	ordinary_muscle_development	ENSGALG00010024064	GPR17	0.280291948	0.209172328	1.340004919	0.180243746	0.218795167	positive
PRJNA729271	stage_1D_vs_E10	ordinary_muscle_development	ENSGALG00010024066	ENSGALG00010024066	0.859482265	2.266538334	0.379204822	0.704535771	0.741038096	positive
PRJNA729271	stage_1D_vs_E10	ordinary_muscle_development	ENSGALG00010024068	CASP2	-1.235155017	0.085143619	-14.50672449	1.09842E-47	1.92058E-46	negative
PRJNA729271	stage_1D_vs_E10	ordinary_muscle_development	ENSGALG00010024069	ENSGALG00010024069	0.85947071	2.364835876	0.363437784	0.716277881	0.751588815	positive
PRJNA729271	stage_1D_vs_E10	ordinary_muscle_development	ENSGALG00010024073	RABGGTB	-0.199883537	0.12517891	-1.596782853	0.110314119	0.139201111	negative
PRJNA729271	stage_1D_vs_E10	ordinary_muscle_development	ENSGALG00010024074	ADCY7	-1.557344622	0.231175058	-6.736646388	1.62084E-11	5.17719E-11	negative
PRJNA729271	stage_1D_vs_E10	ordinary_muscle_development	ENSGALG00010024075	SPATS2L	-0.175817288	0.136551306	-1.287554793	0.197900985	0.237867861	negative
PRJNA729271	stage_1D_vs_E10	ordinary_muscle_development	ENSGALG00010024077	SFT2D3	0.527642577	0.114639804	4.602612367	4.17224E-06	8.81584E-06	positive

PRJNA729271	stage_1D_vs_E10	ordinary_muscle_development	ENSGALG00010024078	KIRREL3	-0.251218626	0.229933831	-1.09256922	0.274582983	0.320251073	negative
PRJNA729271	stage_1D_vs_E10	ordinary_muscle_development	ENSGALG00010024080	TENT4B	0.326851201	0.086648054	3.772170124	0.000161834	0.000294216	positive
PRJNA729271	stage_1D_vs_E10	ordinary_muscle_development	ENSGALG00010024081	CARS	-0.404784541	0.098999981	-4.088733503	4.33735E-05	8.32934E-05	negative
PRJNA729271	stage_1D_vs_E10	ordinary_muscle_development	ENSGALG00010024083	PALMD	1.048424739	0.189558809	5.530867946	3.1865E-08	7.97726E-08	positive
PRJNA729271	stage_1D_vs_E10	ordinary_muscle_development	ENSGALG00010024086	GABARAPL1	0.287942216	0.214961526	1.339505822	0.18040606	0.218965657	positive
PRJNA729271	stage_1D_vs_E10	ordinary_muscle_development	ENSGALG00010024089	ENSGALG00010024089	1.070551952	1.934833016	0.553304571	0.580054872	0.624592139	positive
PRJNA729271	stage_1D_vs_E10	ordinary_muscle_development	ENSGALG00010024091	ETFA	1.517423602	0.115807724	13.10295675	3.16676E-39	4.05497E-38	positive
PRJNA729271	stage_1D_vs_E10	ordinary_muscle_development	ENSGALG00010024095	RBM12	-0.63586216	0.052428278	-12.12822901	7.48507E-34	7.73866E-33	negative
PRJNA729271	stage_1D_vs_E10	ordinary_muscle_development	ENSGALG00010024096	MYO1D	0.066058645	0.219833896	0.300493446	0.763800795	0.794153398	positive
PRJNA729271	stage_1D_vs_E10	ordinary_muscle_development	ENSGALG00010024099	ENSGALG00010024099	0.859489398	2.081202727	0.412977259	0.679623275	0.717490122	positive
PRJNA729271	stage_1D_vs_E10	ordinary_muscle_development	ENSGALG00010024101	CNEP1R1	-0.197089763	0.090192287	-2.185217496	0.028872903	0.039884946	negative
PRJNA729271	stage_1D_vs_E10	ordinary_muscle_development	ENSGALG00010024102	SGF29	0.331084222	0.089864938	3.684242465	0.000229384	0.000409321	positive
PRJNA729271	stage_1D_vs_E10	ordinary_muscle_development	ENSGALG00010024104	PLPPR5	-0.152483191	0.161273229	-0.945495984	0.34440572	0.393300316	negative
PRJNA729271	stage_1D_vs_E10	ordinary_muscle_development	ENSGALG00010024106	WDR33	0.170677155	0.06290826	2.713111987	0.006665458	0.010079701	positive
PRJNA729271	stage_1D_vs_E10	ordinary_muscle_development	ENSGALG00010024107	NFE2L2	0.983841903	0.062800345	15.66618622	2.57579E-55	6.01647E-54	positive
PRJNA729271	stage_1D_vs_E10	ordinary_muscle_development	ENSGALG00010024110	CPNE1	-0.673425932	0.126045877	-5.342704979	9.15697E-08	2.21772E-07	negative
PRJNA729271	stage_1D_vs_E10	ordinary_muscle_development	ENSGALG00010024112	NAP1L4	-0.146670344	0.090621688	-1.61849053	0.105556926	0.133675314	negative
PRJNA729271	stage_1D_vs_E10	ordinary_muscle_development	ENSGALG00010024113	ENSGALG00010024113	0.832813983	0.164506822	5.062489036	4.13818E-07	9.50902E-07	positive
PRJNA729271	stage_1D_vs_E10	ordinary_muscle_development	ENSGALG00010024115	POLR2D	-0.616088384	0.073035257	-8.435492771	3.2981E-17	1.51189E-16	negative
PRJNA729271	stage_1D_vs_E10	ordinary_muscle_development	ENSGALG00010024116	MCAM	2.240191223	0.181706088	12.32865257	6.34997E-35	6.86374E-34	positive
PRJNA729271	stage_1D_vs_E10	ordinary_muscle_development	ENSGALG00010024118	TMEM266	-0.928077941	0.130846105	-7.092896956	1.31333E-12	4.50041E-12	negative
PRJNA729271	stage_1D_vs_E10	ordinary_muscle_development	ENSGALG00010024119	AMMECR1L	-0.216740875	0.073329553	-2.95570977	0.003119505	0.004908513	negative
PRJNA729271	stage_1D_vs_E10	ordinary_muscle_development	ENSGALG00010024121	NRG4	-0.690645055	0.172685643	-3.999435292	6.34938E-05	0.000120275	negative
PRJNA729271	stage_1D_vs_E10	ordinary_muscle_development	ENSGALG00010024122	SOCS7	0.423348509	0.105221214	4.023414031	5.73605E-05	0.000109041	positive
PRJNA729271	stage_1D_vs_E10	ordinary_muscle_development	ENSGALG00010024123	PLPPR4	-0.402285283	0.390250537	-1.030838511	0.302616555	0.349655398	negative
PRJNA729271	stage_1D_vs_E10	ordinary_muscle_development	ENSGALG00010024124	DIDO1	0.213438694	0.066480205	3.210560095	0.001324766	0.002180118	positive
PRJNA729271	stage_1D_vs_E10	ordinary_muscle_development	ENSGALG00010024125	ENSGALG00010024125	-3.239277991	1.676827863	-1.931789221	0.053385527	0.0708305	negative
PRJNA729271	stage_1D_vs_E10	ordinary_muscle_development	ENSGALG00010024126	CDK5R1	-1.017353313	0.284329735	-3.578075691	0.000346133	0.000606382	negative
PRJNA729271	stage_1D_vs_E10	ordinary_muscle_development	ENSGALG00010024128	PHLDA2	-2.227113255	0.234346491	-9.503505861	2.0294E-21	1.17243E-20	negative
PRJNA729271	stage_1D_vs_E10	ordinary_muscle_development	ENSGALG00010024129	GID8	0.470039891	0.100655094	4.669807271	3.01482E-06	6.46831E-06	positive
PRJNA729271	stage_1D_vs_E10	ordinary_muscle_development	ENSGALG00010024131	ENSGALG00010024131	-0.555089167	1.302045091	-0.426321002	0.669873957	0.708487632	negative
PRJNA729271	stage_1D_vs_E10	ordinary_muscle_development	ENSGALG00010024132	ENSGALG00010024132	-1.592215165	0.150926909	-10.54957776	5.10259E-26	3.65967E-25	negative
PRJNA729271	stage_1D_vs_E10	ordinary_muscle_development	ENSGALG00010024133	PSMD11	0.086146088	0.141095312	0.610552446	0.541495913	0.5876307	positive
PRJNA729271	stage_1D_vs_E10	ordinary_muscle_development	ENSGALG00010024135	FBXO22	-0.255954885	0.088060541	-2.906578607	0.003654051	0.005706871	negative
PRJNA729271	stage_1D_vs_E10	ordinary_muscle_development	ENSGALG00010024136	SAP130	0.375635276	0.173553407	2.164378579	0.030435306	0.041947301	positive
PRJNA729271	stage_1D_vs_E10	ordinary_muscle_development	ENSGALG00010024137	ENSGALG00010024137	0.085132859	0.747300102	0.113920578			positive
PRJNA729271	stage_1D_vs_E10	ordinary_muscle_development	ENSGALG00010024138	CBL	0.647233079	0.148298918	4.364381679	1.27483E-05	2.57937E-05	positive
PRJNA729271	stage_1D_vs_E10	ordinary_muscle_development	ENSGALG00010024139	SLC22A18	-2.940765207	0.819878111	-3.586832197	0.00033472	0.000587238	negative
PRJNA729271	stage_1D_vs_E10	ordinary_muscle_development	ENSGALG00010024140	KRT6A	-5.200916741	1.054095903	-4.934007167	8.05595E-07	1.81237E-06	negative
PRJNA729271	stage_1D_vs_E10	ordinary_muscle_development	ENSGALG00010024141	YTHDF1	0.00446435	0.094987053	0.046999568	0.962513572	0.967922452	positive
PRJNA729271	stage_1D_vs_E10	ordinary_muscle_development	ENSGALG00010024142	DNAJC6	-1.791989679	0.264123708	-6.784660467	1.1636E-11	3.74891E-11	negative
PRJNA729271	stage_1D_vs_E10	ordinary_muscle_development	ENSGALG00010024143	UBE2Q2	1.617916123	0.116621575	13.87321452	9.2057E-44	1.37563E-42	positive
PRJNA729271	stage_1D_vs_E10	ordinary_muscle_development	ENSGALG00010024144	HNRNPA3	-0.713557125	0.126650925	-5.634045914	1.7603E-08	4.50725E-08	negative
PRJNA729271	stage_1D_vs_E10	ordinary_muscle_development	ENSGALG00010024145	RAP1GAP3	0.556187665	0.178255027	3.120179413	0.001807409	0.002927195	positive
PRJNA729271	stage_1D_vs_E10	ordinary_muscle_development	ENSGALG00010024146	CDKN1C	-0.855551712	0.239518316	-3.571967798	0.000354309	0.000620105	negative
PRJNA729271	stage_1D_vs_E10	ordinary_muscle_development	ENSGALG00010024147	ENSGALG00010024147	-0.637706015	0.275239648	-2.316911895	0.020508532	0.028970974	negative
PRJNA729271	stage_1D_vs_E10	ordinary_muscle_development	ENSGALG00010024148	UGGT1	-0.333903447	0.058352528	-5.722176245	1.05168E-08	2.73732E-08	negative
PRJNA729271	stage_1D_vs_E10	ordinary_muscle_development	ENSGALG00010024149	TCIRG1	0.569227192	0.152935271	3.72201382	0.00019764	0.000355169	positive
PRJNA729271	stage_1D_vs_E10	ordinary_muscle_development	ENSGALG00010024150	NHERF4	1.249877888	0.347405873	3.597745416	0.000320988	0.000564034	positive
PRJNA729271	stage_1D_vs_E10	ordinary_muscle_development	ENSGALG00010024151	LEPROT	-0.519618098	0.148245513	-3.505118551	0.000456404	0.000789072	negative
PRJNA729271	stage_1D_vs_E10	ordinary_muscle_development	ENSGALG00010024152	ZNF423	-0.766508752	0.107497791	-7.1304605	1.00034E-12	3.45977E-12	negative
PRJNA729271	stage_1D_vs_E10	ordinary_muscle_development	ENSGALG00010024153	KCNQ1	1.309587384	0.202754808	6.45897079	1.05417E-10	3.18604E-10	positive
PRJNA729271	stage_1D_vs_E10	ordinary_muscle_development	ENSGALG00010024154	VPS29L	0.12916417	0.502599925	0.25699202	0.797184933	0.824370094	positive
PRJNA729271	stage_1D_vs_E10	ordinary_muscle_development	ENSGALG00010024155	SNX7	-0.923320366	0.165742394	-5.570815905	2.53549E-08	6.40531E-08	negative
PRJNA729271	stage_1D_vs_E10	ordinary_muscle_development	ENSGALG00010024157	CHRNB4	-3.152153676	0.398092901	-7.918135866	2.41098E-15	9.88603E-15	negative
PRJNA729271	stage_1D_vs_E10	ordinary_muscle_development	ENSGALG00010024158	ENSGALG00010024158	-0.153086274	0.348648235	-0.439085183	0.660599815	0.699802259	negative
PRJNA729271	stage_1D_vs_E10	ordinary_muscle_development	ENSGALG00010024159	NLRX1	0.268072734	0.161961063	1.655167781	0.097890469	0.12466371	positive
PRJNA729271	stage_1D_vs_E10	ordinary_muscle_development	ENSGALG00010024162	SRCIN1	-0.9014752	0.310504531	-2.903259404	0.003693006	0.005763984	negative
PRJNA729271	stage_1D_vs_E10	ordinary_muscle_development	ENSGALG00010024163	HS6ST1	-1.404886221	0.097582537	-14.39690195	5.41154E-47	9.16359E-46	negative
PRJNA729271	stage_1D_vs_E10	ordinary_muscle_development	ENSGALG00010024164	SNAP29	0.925814032	0.11228791	8.24500192	1.65158E-16	7.2809E-16	positive
PRJNA729271	stage_1D_vs_E10	ordinary_muscle_development	ENSGALG00010024165	LEPR	-0.081025656	0.262053363	-0.309195252	0.757173005	0.788449908	negative
PRJNA729271	stage_1D_vs_E10	ordinary_muscle_development	ENSGALG00010024166	ENSGALG00010024166	3.356185765	0.935723864	3.586726699	0.000334855	0.000587404	positive
PRJNA729271	stage_1D_vs_E10	ordinary_muscle_development	ENSGALG00010024167	CHRNA3	2.885550007	0.38499216	7.495087705	6.6254E-14	2.47577E-13	positive
PRJNA729271	stage_1D_vs_E10	ordinary_muscle_development	ENSGALG00010024168	BIRC7	-2.002297451	0.380822967	-5.257816951	1.45776E-07	3.47764E-07	negative
PRJNA729271	stage_1D_vs_E10	ordinary_muscle_development	ENSGALG00010024169	FAM168B	-0.534156172	0.072216589	-7.396585444	1.39731E-13	5.11594E-13	negative
PRJNA729271	stage_1D_vs_E10	ordinary_muscle_development	ENSGALG00010024170	NUP160	-0.099279742	0.084137553	-1.17996946	0.238012362	0.281677758	negative
PRJNA729271	stage_1D_vs_E10	ordinary_muscle_development	ENSGALG00010024171	ENSGALG00010024171	-0.665812059	0.436300535	-1.526039977	0.126999909	0.158573307	negative
PRJNA729271	stage_1D_vs_E10	ordinary_muscle_development	ENSGALG00010024172	ABCG4	-3.669837433	0.429443923	-8.545556782	1.27918E-17	5.99086E-17	negative
PRJNA729271	stage_1D_vs_E10	ordinary_muscle_development	ENSGALG00010024173	CBLN1	-0.422547135	0.400307236	-1.055557075	0.291170621	0.337695483	negative
PRJNA729271	stage_1D_vs_E10	ordinary_muscle_development	ENSGALG00010024174	PLEKHB2	0.501792209	0.101434952	4.946935922	7.53908E-07	1.69952E-06	positive
PRJNA729271	stage_1D_vs_E10	ordinary_muscle_development	ENSGALG00010024175	DHRS9	3.192162417	0.413770236	7.714818856	1.21154E-14	4.74117E-14	positive
PRJNA729271	stage_1D_vs_E10	ordinary_muscle_development	ENSGALG00010024177	ENSGALG00010024177	0.55581972	0.418223777	1.329000766	0.183847717	0.222796811	positive
PRJNA729271	stage_1D_vs_E10	ordinary_muscle_development	ENSGALG00010024178	N4BP1	0.756413762	0.120620578	6.271017532	3.58696E-10	1.04593E-09	positive
PRJNA729271	stage_1D_vs_E10	ordinary_muscle_development	ENSGALG00010024179	ATG4C	-0.387474353	0.115765268	-3.347069102	0.000816708	0.001375894	negative
PRJNA729271	stage_1D_vs_E10	ordinary_muscle_development	ENSGALG00010024180	HINFP	0.11789903	0.140394814	0.839767699	0.401038648	0.45050322	positive
PRJNA729271	stage_1D_vs_E10	ordinary_muscle_development	ENSGALG00010024181	FOXD3	2.755371153	0.312440843	8.818857111	1.15634E-18	5.74602E-18	positive
PRJNA729271	stage_1D_vs_E10	ordinary_muscle_development	ENSGALG00010024183	MTX2	0.011670382	0.106529032	0.109551185	0.912765326	0.925919975	positive
PRJNA729271	stage_1D_vs_E10	ordinary_muscle_development	ENSGALG00010024184	ENSGALG00010024184	-3.181092072	0.223544303	-14.2302534	5.94659E-46	9.68492E-45	negative
PRJNA729271	stage_1D_vs_E10	ordinary_muscle_development	ENSGALG00010024185	CHRNA5	0.164563269	0.155237573	1.060073703	0.28911107	0.33554848	positive
PRJNA729271	stage_1D_vs_E10	ordinary_muscle_development	ENSGALG00010024186	HAO2	-1.53689	0.735573403	-2.089376794	0.036673818	0.049937717	negative
PRJNA729271	stage_1D_vs_E10	ordinary_muscle_development	ENSGALG00010024188	TSSC4	-0.850168614	0.083286765	-10.20772767	1.83103E-24	1.22026E-23	negative
PRJNA729271	stage_1D_vs_E10	ordinary_muscle_development	ENSGALG00010024189	KAT7	-0.290777049	0.140409456	-2.070922126	0.038366074	0.05206106	negative
PRJNA729271	stage_1D_vs_E10	ordinary_muscle_development	ENSGALG00010024190	WARS2	-0.538875887	0.104580898	-5.152718115	2.56738E-07	5.99648E-07	negative
PRJNA729271	stage_1D_vs_E10	ordinary_muscle_development	ENSGALG00010024191	DPYD	-0.189402343	0.107713909	-1.758383332	0.078682307	0.10188247	negative
PRJNA729271	stage_1D_vs_E10	ordinary_muscle_development	ENSGALG00010024192	ALG6	0.24882347	0.088560627	2.809639887	0.004959696	0.007629289	positive
PRJNA729271	stage_1D_vs_E10	ordinary_muscle_development	ENSGALG00010024193	GSTK1	0.728884528	0.126040343	5.782946263	7.34035E-09	1.94193E-08	positive
PRJNA729271	stage_1D_vs_E10	ordinary_muscle_development	ENSGALG00010024194	NKAIN4	-0.139939047	0.085417087	-1.638302721	0.10135856	0.128797434	negative
PRJNA729271	stage_1D_vs_E10	ordinary_muscle_development	ENSGALG00010024195	PSMA4	-0.310299517	0.12110482	-2.562239206	0.010399966	0.015329216	negative
PRJNA729271	stage_1D_vs_E10	ordinary_muscle_development	ENSGALG00010024196	MRPL21	-0.015706008	0.109035693	-0.144044651	0.885465211	0.902525718	negative
PRJNA729271	stage_1D_vs_E10	ordinary_muscle_development	ENSGALG00010024197	KRT7	-2.946836315	0.303834758	-9.698812393	3.05028E-22	1.83918E-21	negative
PRJNA729271	stage_1D_vs_E10	ordinary_muscle_development	ENSGALG00010024198	PI4KA	0.104516075	0.064336013	1.624534533	0.104261776	0.13220842	positive
PRJNA729271	stage_1D_vs_E10	ordinary_muscle_development	ENSGALG00010024199	SLC35B1	-0.542139879	0.102226097	-5.303341262	1.13702E-07	2.73818E-07	negative
PRJNA729271	stage_1D_vs_E10	ordinary_muscle_development	ENSGALG00010024200	GCHFR	-3.72660007	0.753225635	-4.947521562	7.51644E-07	1.69468E-06	negative
PRJNA729271	stage_1D_vs_E10	ordinary_muscle_development	ENSGALG00010024201	IREB2	-0.056819617	0.069467485	-0.817931107	0.413396527	0.462931293	negative
PRJNA729271	stage_1D_vs_E10	ordinary_muscle_development	ENSGALG00010024203	SPOP	0.101848574	0.071950722	1.415532332	0.156912475	0.192680017	positive
PRJNA729271	stage_1D_vs_E10	ordinary_muscle_development	ENSGALG00010024204	PTBP2	0.728873725	0.106975101	6.813489457	9.52593E-12	3.08831E-11	positive
PRJNA729271	stage_1D_vs_E10	ordinary_muscle_development	ENSGALG00010024205	ANKRD13D	-0.597317358	0.171064505	-3.491766785	0.000479837	0.0008283	negative
PRJNA729271	stage_1D_vs_E10	ordinary_muscle_development	ENSGALG00010024206	KRT80	-1.942217645	0.587948543	-3.30338032	0.000955267	0.00159707	negative
PRJNA729271	stage_1D_vs_E10	ordinary_muscle_development	ENSGALG00010024207	KCTD18	-0.566597483	0.12278084	-4.614706024	3.93652E-06	8.34331E-06	negative
PRJNA729271	stage_1D_vs_E10	ordinary_muscle_development	ENSGALG00010024208	SERPIND1	3.179026514	0.577358868	5.506153433	3.66759E-08	9.14689E-08	positive
PRJNA729271	stage_1D_vs_E10	ordinary_muscle_development	ENSGALG00010024209	RWDD3	-0.139466479	0.143667916	-0.970755917	0.33166984	0.38016403	negative
PRJNA729271	stage_1D_vs_E10	ordinary_muscle_development	ENSGALG00010024210	NXPH3	-3.243112618	1.189279575	-2.726955618	0.006392164	0.00969087	negative
PRJNA729271	stage_1D_vs_E10	ordinary_muscle_development	ENSGALG00010024211	TRPM5	2.270867621	0.662093586	3.429828758	0.000603962	0.001029449	positive
PRJNA729271	stage_1D_vs_E10	ordinary_muscle_development	ENSGALG00010024212	RIMS4	-0.343214245	0.200069261	-1.715477148	0.086257782	0.110891933	negative
PRJNA729271	stage_1D_vs_E10	ordinary_muscle_development	ENSGALG00010024213	ENSGALG00010024213	-0.180520996	0.187944909	-0.960499523	0.336803871	0.385347865	negative
PRJNA729271	stage_1D_vs_E10	ordinary_muscle_development	ENSGALG00010024214	CRABP1	-9.057198983	0.456260154	-19.85095322	1.0813E-87	6.17066E-86	negative
PRJNA729271	stage_1D_vs_E10	ordinary_muscle_development	ENSGALG00010024215	ENSGALG00010024215	0.977567935	0.154826782	6.313945982	2.72009E-10	8.00562E-10	positive
PRJNA729271	stage_1D_vs_E10	ordinary_muscle_development	ENSGALG00010024218	NGFR	-2.509097375	0.231881364	-10.82060814	2.74947E-27	2.08221E-26	negative
PRJNA729271	stage_1D_vs_E10	ordinary_muscle_development	ENSGALG00010024220	SIAH1	2.039711278	0.200493965	10.17342982	2.60568E-24	1.72149E-23	positive
PRJNA729271	stage_1D_vs_E10	ordinary_muscle_development	ENSGALG00010024222	ENSGALG00010024222	0.771432859	0.283457559	2.721510979	0.006498423	0.0098427	positive
PRJNA729271	stage_1D_vs_E10	ordinary_muscle_development	ENSGALG00010024223	ENSGALG00010024223	-1.299072675	0.285213678	-4.554734842	5.24517E-06	1.09963E-05	negative
PRJNA729271	stage_1D_vs_E10	ordinary_muscle_development	ENSGALG00010024225	BCDIN3D	0.443869224	0.174355935	2.545764928	0.010903859	0.01604746	positive
PRJNA729271	stage_1D_vs_E10	ordinary_muscle_development	ENSGALG00010024226	POU2AF1	-0.960929253	1.487648373	-0.645938429	0.518319231	0.565831438	negative
PRJNA729271	stage_1D_vs_E10	ordinary_muscle_development	ENSGALG00010024227	MUC6	2.358910254	0.411807069	5.728192719	1.01506E-08	2.64438E-08	positive
PRJNA729271	stage_1D_vs_E10	ordinary_muscle_development	ENSGALG00010024230	LONP2	0.317155441	0.150432582	2.10828955	0.035005951	0.047841907	positive
PRJNA729271	stage_1D_vs_E10	ordinary_muscle_development	ENSGALG00010024233	FAIM2	-3.980795391	0.267872321	-14.86079406	5.92191E-50	1.14146E-48	negative
PRJNA729271	stage_1D_vs_E10	ordinary_muscle_development	ENSGALG00010024234	ENSGALG00010024234	1.286864953	0.270125741	4.763947885	1.89842E-06	4.14109E-06	positive
PRJNA729271	stage_1D_vs_E10	ordinary_muscle_development	ENSGALG00010024238	ATF7	1.794946641	0.200528724	8.951069961	3.52053E-19	1.7962E-18	positive
PRJNA729271	stage_1D_vs_E10	ordinary_muscle_development	ENSGALG00010024240	WDR61	-0.617709898	0.126120685	-4.89776834	9.69312E-07	2.16891E-06	negative
PRJNA729271	stage_1D_vs_E10	ordinary_muscle_development	ENSGALG00010024242	KMT2A	0.585089668	0.124540446	4.697989171	2.62735E-06	5.66803E-06	positive
PRJNA729271	stage_1D_vs_E10	ordinary_muscle_development	ENSGALG00010024243	MPP3	1.444865208	0.1984963	7.279053591	3.3617E-13	1.20256E-12	positive
PRJNA729271	stage_1D_vs_E10	ordinary_muscle_development	ENSGALG00010024245	TARBP2	-0.362280979	0.166261685	-2.178980563	0.029333112	0.04049752	negative
PRJNA729271	stage_1D_vs_E10	ordinary_muscle_development	ENSGALG00010024250	AP2A2	-0.154801531	0.05344072	-2.896696181	0.003771148	0.005878983	negative
PRJNA729271	stage_1D_vs_E10	ordinary_muscle_development	ENSGALG00010024251	ENSGALG00010024251	1.318977427	0.207125182	6.368020596	1.91483E-10	5.68527E-10	positive
PRJNA729271	stage_1D_vs_E10	ordinary_muscle_development	ENSGALG00010024254	ENSGALG00010024254	3.499365459	0.297592582	11.75891361	6.35467E-32	6.04402E-31	positive
PRJNA729271	stage_1D_vs_E10	ordinary_muscle_development	ENSGALG00010024255	CFAP97D1	2.008913611	0.664282819	3.024184206	0.002493046	0.003963661	positive
PRJNA729271	stage_1D_vs_E10	ordinary_muscle_development	ENSGALG00010024258	DNAJA4	1.395385245	0.150541856	9.269084875	1.8775E-20	1.02851E-19	positive
PRJNA729271	stage_1D_vs_E10	ordinary_muscle_development	ENSGALG00010024259	ENSGALG00010024259	1.346545754	0.13472046	9.995109568	1.6011E-23	1.02103E-22	positive
PRJNA729271	stage_1D_vs_E10	ordinary_muscle_development	ENSGALG00010024263	PHKB	2.332463915	0.131481582	17.73985289	2.06475E-70	7.71354E-69	positive
PRJNA729271	stage_1D_vs_E10	ordinary_muscle_development	ENSGALG00010024264	AMER3	0.113858415	0.304743138	0.37362093	0.70868636	0.744809222	positive
PRJNA729271	stage_1D_vs_E10	ordinary_muscle_development	ENSGALG00010024266	ENSGALG00010024266	0.699143749	0.239008477	2.925183905	0.003442527	0.005396283	positive

PRJNA729271	stage_1D_vs_E10	ordinary_muscle_development	ENSGALG00010024267	MAP3K12	-2.276574532	0.215976666	-10.54083562	5.59991E-26	4.0025E-25	negative
PRJNA729271	stage_1D_vs_E10	ordinary_muscle_development	ENSGALG00010024270	RAB18L	2.111815917	0.77399371	2.72846651	0.006362955	0.009649616	positive
PRJNA729271	stage_1D_vs_E10	ordinary_muscle_development	ENSGALG00010024274	ACSBG1	0.141928489	0.31422104	0.451683594	0.651496939	0.691371852	positive
PRJNA729271	stage_1D_vs_E10	ordinary_muscle_development	ENSGALG00010024277	ITGB3BP	-1.823737181	0.189851986	-9.606100111	7.53489E-22	4.46701E-21	negative
PRJNA729271	stage_1D_vs_E10	ordinary_muscle_development	ENSGALG00010024280	ENSGALG00010024280	1.968145601	0.12464925	15.78947009	3.67655E-56	8.82312E-55	positive
PRJNA729271	stage_1D_vs_E10	ordinary_muscle_development	ENSGALG00010024282	HIC2	-1.223621916	0.151474477	-8.078073211	6.57981E-16	2.79854E-15	negative
PRJNA729271	stage_1D_vs_E10	ordinary_muscle_development	ENSGALG00010024283	UBE4A	0.321573633	0.110238325	2.917076561	0.00353329	0.005530782	positive
PRJNA729271	stage_1D_vs_E10	ordinary_muscle_development	ENSGALG00010024285	CCND2	-0.475213084	0.09704904	-4.896628361	9.7495E-07	2.18018E-06	negative
PRJNA729271	stage_1D_vs_E10	ordinary_muscle_development	ENSGALG00010024286	ADA	1.850712478	0.17849768	10.36827189	3.4572E-25	2.39771E-24	positive
PRJNA729271	stage_1D_vs_E10	ordinary_muscle_development	ENSGALG00010024288	CHID1	-0.15819766	0.135542005	-1.167148588	0.243150369	0.287172215	negative
PRJNA729271	stage_1D_vs_E10	ordinary_muscle_development	ENSGALG00010024290	SOST	-0.426390891	0.464165043	-0.918619136	0.358294819	0.407204281	negative
PRJNA729271	stage_1D_vs_E10	ordinary_muscle_development	ENSGALG00010024291	ENSGALG00010024291	3.505100549	0.495786573	7.069777078	1.55183E-12	5.29514E-12	positive
PRJNA729271	stage_1D_vs_E10	ordinary_muscle_development	ENSGALG00010024300	MEOX1	-3.826159209	0.264043659	-14.4906309	1.38861E-47	2.41632E-46	negative
PRJNA729271	stage_1D_vs_E10	ordinary_muscle_development	ENSGALG00010024304	PKIG	-0.384577947	0.132183308	-2.909428975	0.003620896	0.005656919	negative
PRJNA729271	stage_1D_vs_E10	ordinary_muscle_development	ENSGALG00010024305	ENSGALG00010024305	5.667334191	0.719092345	7.881232824	3.24167E-15	1.31952E-14	positive
PRJNA729271	stage_1D_vs_E10	ordinary_muscle_development	ENSGALG00010024309	ABCB11	-0.101382114	0.403276985	-0.251395736	0.801508164	0.828249864	negative
PRJNA729271	stage_1D_vs_E10	ordinary_muscle_development	ENSGALG00010024311	KLHL6	1.613385475	0.124127357	12.99782347	1.25875E-38	1.57154E-37	positive
PRJNA729271	stage_1D_vs_E10	ordinary_muscle_development	ENSGALG00010024313	SERINC3	2.224737769	0.106717025	20.84707457	1.62031E-96	1.13461E-94	positive
PRJNA729271	stage_1D_vs_E10	ordinary_muscle_development	ENSGALG00010024314	ITFG1	0.306111639	0.146269333	2.092794393	0.036367513	0.049562532	positive
PRJNA729271	stage_1D_vs_E10	ordinary_muscle_development	ENSGALG00010024321	UBE2L3	-0.586510518	0.082400391	-7.117812312	1.09654E-12	3.77626E-12	negative
PRJNA729271	stage_1D_vs_E10	ordinary_muscle_development	ENSGALG00010024322	IDH3A	1.502099889	0.158828689	9.457358713	3.1582E-21	1.80941E-20	positive
PRJNA729271	stage_1D_vs_E10	ordinary_muscle_development	ENSGALG00010024323	TTPAL	-1.074920804	0.101399321	-10.60086788	2.95228E-26	2.13433E-25	negative
PRJNA729271	stage_1D_vs_E10	ordinary_muscle_development	ENSGALG00010024325	PCBP2	0.787529122	0.168807305	4.665254998	3.08235E-06	6.60731E-06	positive
PRJNA729271	stage_1D_vs_E10	ordinary_muscle_development	ENSGALG00010024326	KLHL24	3.064915799	0.178331656	17.18660539	3.34556E-66	1.08731E-64	positive
PRJNA729271	stage_1D_vs_E10	ordinary_muscle_development	ENSGALG00010024327	PPP1R1B	3.438579759	0.417037045	8.24526215	1.64799E-16	7.26728E-16	positive
PRJNA729271	stage_1D_vs_E10	ordinary_muscle_development	ENSGALG00010024328	YDJC	-2.054989952	0.152618187	-13.46490873	2.51629E-41	3.48363E-40	negative
PRJNA729271	stage_1D_vs_E10	ordinary_muscle_development	ENSGALG00010024330	SDF2L1	-1.372817154	0.102169755	-13.43662965	3.68858E-41	5.06306E-40	negative
PRJNA729271	stage_1D_vs_E10	ordinary_muscle_development	ENSGALG00010024332	EFCAB7	-1.445373471	0.169471513	-8.528710476	1.47988E-17	6.90404E-17	negative
PRJNA729271	stage_1D_vs_E10	ordinary_muscle_development	ENSGALG00010024333	ETV4	-3.710122559	0.235612677	-15.7467018	7.2358E-56	1.71939E-54	negative
PRJNA729271	stage_1D_vs_E10	ordinary_muscle_development	ENSGALG00010024334	USP28	1.122825942	0.123001359	9.128565301	6.9413E-20	3.68685E-19	positive
PRJNA729271	stage_1D_vs_E10	ordinary_muscle_development	ENSGALG00010024336	TSPAN4	-0.093007743	0.116787254	-0.796386077	0.425807668	0.475250801	negative
PRJNA729271	stage_1D_vs_E10	ordinary_muscle_development	ENSGALG00010024338	NDUFA9	0.824612741	0.113870765	7.241654544	4.43244E-13	1.57587E-12	positive
PRJNA729271	stage_1D_vs_E10	ordinary_muscle_development	ENSGALG00010024339	TOP3B	-0.14304485	0.086277396	-1.657964393	0.097324654	0.124019416	negative
PRJNA729271	stage_1D_vs_E10	ordinary_muscle_development	ENSGALG00010024340	NETO2	-1.62853806	0.379738982	-4.288572248	1.79825E-05	3.57554E-05	negative
PRJNA729271	stage_1D_vs_E10	ordinary_muscle_development	ENSGALG00010024342	HNF4A	-0.782437949	1.483042092	-0.527589846	0.597784042	0.64103571	negative
PRJNA729271	stage_1D_vs_E10	ordinary_muscle_development	ENSGALG00010024343	POLR2L	-0.041027546	0.126809675	-0.323536406	0.746289041	0.778794791	negative
PRJNA729271	stage_1D_vs_E10	ordinary_muscle_development	ENSGALG00010024344	YEATS2	0.082433754	0.132853726	0.620485075	0.534938477	0.581690414	positive
PRJNA729271	stage_1D_vs_E10	ordinary_muscle_development	ENSGALG00010024345	STARD3	0.264399783	0.09161831	2.885883639	0.003903164	0.006076293	positive
PRJNA729271	stage_1D_vs_E10	ordinary_muscle_development	ENSGALG00010024346	PPM1F	0.436563647	0.090162662	4.841956058	1.28567E-06	2.84516E-06	positive
PRJNA729271	stage_1D_vs_E10	ordinary_muscle_development	ENSGALG00010024347	OTOG	-0.66710057	0.275816033	-2.418643191	0.015578513	0.022410733	negative
PRJNA729271	stage_1D_vs_E10	ordinary_muscle_development	ENSGALG00010024349	R3HDML	-4.08461455	0.567552999	-7.196886562	6.1603E-13	2.16522E-12	negative
PRJNA729271	stage_1D_vs_E10	ordinary_muscle_development	ENSGALG00010024350	PGM1	3.975763544	0.135947797	29.24478103	5.2317E-188	1.9956E-185	positive
PRJNA729271	stage_1D_vs_E10	ordinary_muscle_development	ENSGALG00010024351	SP1	1.576660884	0.152703378	10.32499024	5.43243E-25	3.7266E-24	positive
PRJNA729271	stage_1D_vs_E10	ordinary_muscle_development	ENSGALG00010024354	C2CD2L	-0.027184389	0.07742183	-0.351120465	0.725497976	0.75970232	negative
PRJNA729271	stage_1D_vs_E10	ordinary_muscle_development	ENSGALG00010024356	PARL	-0.106780275	0.11792819	-0.905468616	0.365217179	0.414259567	negative
PRJNA729271	stage_1D_vs_E10	ordinary_muscle_development	ENSGALG00010024358	MAPK3	0.484187682	0.061685585	7.849284134	4.18419E-15	1.68847E-14	positive
PRJNA729271	stage_1D_vs_E10	ordinary_muscle_development	ENSGALG00010024360	FTL	-1.573120591	0.245269302	-6.413850324	1.4189E-10	4.25463E-10	negative
PRJNA729271	stage_1D_vs_E10	ordinary_muscle_development	ENSGALG00010024361	H2AX	-1.122908796	0.21401596	-5.246846067	1.54725E-07	3.68508E-07	negative
PRJNA729271	stage_1D_vs_E10	ordinary_muscle_development	ENSGALG00010024362	DHX8	-0.004712952	0.057230122	-0.082350905	0.934367675	0.94426964	negative
PRJNA729271	stage_1D_vs_E10	ordinary_muscle_development	ENSGALG00010024363	EDEM2	-0.501837582	0.1131986	-4.433249008	9.28236E-06	1.90335E-05	negative
PRJNA729271	stage_1D_vs_E10	ordinary_muscle_development	ENSGALG00010024365	ENSGALG00010024365	-0.319383241	0.109382431	-2.919876956	0.003501696	0.005483695	negative
PRJNA729271	stage_1D_vs_E10	ordinary_muscle_development	ENSGALG00010024366	PARD6A	0.352268089	0.429585496	0.82001858	0.412205515	0.461811636	positive
PRJNA729271	stage_1D_vs_E10	ordinary_muscle_development	ENSGALG00010024367	ENSGALG00010024367	-1.970991803	0.234346504	-8.410587597	4.07962E-17	1.85839E-16	negative
PRJNA729271	stage_1D_vs_E10	ordinary_muscle_development	ENSGALG00010024368	DPAGT1	0.012571875	0.079061213	0.159014443	0.873657495	0.892121549	positive
PRJNA729271	stage_1D_vs_E10	ordinary_muscle_development	ENSGALG00010024369	MYOD1	1.604937139	0.275393386	5.827798417	5.61634E-09	1.49869E-08	positive
PRJNA729271	stage_1D_vs_E10	ordinary_muscle_development	ENSGALG00010024370	HERPUD1	0.469832142	0.091187697	5.152363283	2.57224E-07	6.00687E-07	positive
PRJNA729271	stage_1D_vs_E10	ordinary_muscle_development	ENSGALG00010024371	YPEL1	1.292341716	0.104255619	12.39589511	2.75057E-35	3.0273E-34	positive
PRJNA729271	stage_1D_vs_E10	ordinary_muscle_development	ENSGALG00010024373	TCAP	9.338298613	0.478660855	19.50921725	9.16751E-85	4.84974E-83	positive
PRJNA729271	stage_1D_vs_E10	ordinary_muscle_development	ENSGALG00010024374	ENSGALG00010024374	0.503169322	0.591069571	0.85128612	0.394610434	0.443848703	positive
PRJNA729271	stage_1D_vs_E10	ordinary_muscle_development	ENSGALG00010024377	CIB2	-0.87514208	0.172421772	-5.075589184	3.86297E-07	8.89355E-07	negative
PRJNA729271	stage_1D_vs_E10	ordinary_muscle_development	ENSGALG00010024378	ZDHHC1	-0.640557314	0.17808868	-3.596844641	0.000322101	0.000565819	negative
PRJNA729271	stage_1D_vs_E10	ordinary_muscle_development	ENSGALG00010024382	ENSGALG00010024382	0.894273782	0.428305826	2.087932798	0.036803897	0.050100722	positive
PRJNA729271	stage_1D_vs_E10	ordinary_muscle_development	ENSGALG00010024383	DUSP28	1.270038299	0.188980288	6.720480295	1.81127E-11	5.76384E-11	positive
PRJNA729271	stage_1D_vs_E10	ordinary_muscle_development	ENSGALG00010024386	TPPP3	2.389131861	0.267409431	8.93435901	4.09546E-19	2.08147E-18	positive
PRJNA729271	stage_1D_vs_E10	ordinary_muscle_development	ENSGALG00010024387	PPIL2	0.103918544	0.064614781	1.608278202	0.107774262	0.136185854	positive
PRJNA729271	stage_1D_vs_E10	ordinary_muscle_development	ENSGALG00010024389	AAAS	-0.237364768	0.107424762	-2.209590814	0.027133573	0.037622073	negative
PRJNA729271	stage_1D_vs_E10	ordinary_muscle_development	ENSGALG00010024391	TBC1D2B	-0.382534309	0.110607937	-3.458470688	0.000543251	0.000932437	negative
PRJNA729271	stage_1D_vs_E10	ordinary_muscle_development	ENSGALG00010024392	CHMP4B	-0.297535578	0.085097012	-3.496428048	0.000471532	0.000814351	negative
PRJNA729271	stage_1D_vs_E10	ordinary_muscle_development	ENSGALG00010024393	OGFOD1	-0.770104501	0.103398755	-7.447908806	9.48313E-14	3.51465E-13	negative
PRJNA729271	stage_1D_vs_E10	ordinary_muscle_development	ENSGALG00010024396	ENSGALG00010024396	0.790466004	0.32185889	2.455939633	0.014051676	0.020337398	positive
PRJNA729271	stage_1D_vs_E10	ordinary_muscle_development	ENSGALG00010024398	PHB	0.107634131	0.087903928	1.224451889	0.220781805	0.263263595	positive
PRJNA729271	stage_1D_vs_E10	ordinary_muscle_development	ENSGALG00010024399	ROR1	-0.506681465	0.167931358	-3.017193883	0.002551266	0.004052224	negative
PRJNA729271	stage_1D_vs_E10	ordinary_muscle_development	ENSGALG00010024400	KCNA1	-2.137099856	0.353830148	-6.03990323	1.54207E-09	4.29603E-09	negative
PRJNA729271	stage_1D_vs_E10	ordinary_muscle_development	ENSGALG00010024401	KCNC1	3.244784768	0.205522806	15.78795477	3.76593E-56	9.02268E-55	positive
PRJNA729271	stage_1D_vs_E10	ordinary_muscle_development	ENSGALG00010024402	GPC1	-0.687206172	0.119457215	-5.752738942	8.7809E-09	2.30245E-08	negative
PRJNA729271	stage_1D_vs_E10	ordinary_muscle_development	ENSGALG00010024403	HMBS	-1.133696814	0.077510252	-14.62641122	1.90578E-48	3.46169E-47	negative
PRJNA729271	stage_1D_vs_E10	ordinary_muscle_development	ENSGALG00010024406	NUDT21	-1.209485763	0.065891287	-18.35577691	2.96811E-75	1.25798E-73	negative
PRJNA729271	stage_1D_vs_E10	ordinary_muscle_development	ENSGALG00010024407	HIRA	-0.223593714	0.080745457	-2.769118205	0.005620824	0.008581614	negative
PRJNA729271	stage_1D_vs_E10	ordinary_muscle_development	ENSGALG00010024408	ENSGALG00010024408	-2.339103325	1.394701643	-1.677135277	0.093516032	0.119544482	negative
PRJNA729271	stage_1D_vs_E10	ordinary_muscle_development	ENSGALG00010024410	MYG1	-0.132880725	0.107455549	-1.236611105	0.216231495	0.25826265	negative
PRJNA729271	stage_1D_vs_E10	ordinary_muscle_development	ENSGALG00010024412	PIGU	-0.472331416	0.100858339	-4.683117164	2.82545E-06	6.07639E-06	negative
PRJNA729271	stage_1D_vs_E10	ordinary_muscle_development	ENSGALG00010024413	AMFR	0.403540403	0.092519676	4.361671148	1.29073E-05	2.60972E-05	positive
PRJNA729271	stage_1D_vs_E10	ordinary_muscle_development	ENSGALG00010024415	SIK2	0.564689491	0.059873005	9.431454044	4.04443E-21	2.30169E-20	positive
PRJNA729271	stage_1D_vs_E10	ordinary_muscle_development	ENSGALG00010024416	VDR	-1.638736757	0.330735818	-4.954820935	7.23969E-07	1.63406E-06	negative
PRJNA729271	stage_1D_vs_E10	ordinary_muscle_development	ENSGALG00010024419	PGAP3	0.499304816	0.124281556	4.017529503	5.88115E-05	0.000111741	positive
PRJNA729271	stage_1D_vs_E10	ordinary_muscle_development	ENSGALG00010024420	TMEM106C	-0.693230694	0.168841882	-4.105798183	4.02921E-05	7.76022E-05	negative
PRJNA729271	stage_1D_vs_E10	ordinary_muscle_development	ENSGALG00010024421	DAPK2	-0.18836392	0.260961757	-0.721806603	0.470413389	0.519120066	negative
PRJNA729271	stage_1D_vs_E10	ordinary_muscle_development	ENSGALG00010024423	SERGEF	0.688069923	0.116563799	5.90294695	3.57065E-09	9.67956E-09	positive
PRJNA729271	stage_1D_vs_E10	ordinary_muscle_development	ENSGALG00010024424	SAMHD1	0.080074011	0.180267741	0.444194898	0.656901681	0.69624085	positive
PRJNA729271	stage_1D_vs_E10	ordinary_muscle_development	ENSGALG00010024425	MRPL40	0.429516183	0.088681233	4.843371806	1.27654E-06	2.8254E-06	positive
PRJNA729271	stage_1D_vs_E10	ordinary_muscle_development	ENSGALG00010024426	PHAF1	1.024735039	0.104614393	9.795354252	1.17883E-22	7.26497E-22	positive
PRJNA729271	stage_1D_vs_E10	ordinary_muscle_development	ENSGALG00010024427	C22orf39	0.30298407	0.13504145	2.243637555	0.024855731	0.034702737	positive
PRJNA729271	stage_1D_vs_E10	ordinary_muscle_development	ENSGALG00010024428	SAAL1	-1.336749451	0.064074263	-20.86250222	1.17368E-96	8.25846E-95	negative
PRJNA729271	stage_1D_vs_E10	ordinary_muscle_development	ENSGALG00010024430	ZNF652	0.053979506	0.063752944	0.846698249	0.397163335	0.446546892	positive
PRJNA729271	stage_1D_vs_E10	ordinary_muscle_development	ENSGALG00010024431	FEZ1	-2.753383648	0.155621433	-17.69283056	4.7622E-70	1.74755E-68	negative
PRJNA729271	stage_1D_vs_E10	ordinary_muscle_development	ENSGALG00010024433	ENSGALG00010024433	-0.932128377	0.169085161	-5.512774576	3.53221E-08	8.81531E-08	negative
PRJNA729271	stage_1D_vs_E10	ordinary_muscle_development	ENSGALG00010024434	B3GNT9	-0.906457964	0.158567037	-5.716559904	1.08702E-08	2.82676E-08	negative
PRJNA729271	stage_1D_vs_E10	ordinary_muscle_development	ENSGALG00010024437	HERC1	0.215570849	0.081478684	2.645733059	0.008151411	0.01219221	positive
PRJNA729271	stage_1D_vs_E10	ordinary_muscle_development	ENSGALG00010024438	RBL1	-1.37487769	0.120747385	-11.3863972	4.88785E-30	4.22981E-29	negative
PRJNA729271	stage_1D_vs_E10	ordinary_muscle_development	ENSGALG00010024439	UFD1	0.319275598	0.112397678	2.840588897	0.004503032	0.006966747	positive
PRJNA729271	stage_1D_vs_E10	ordinary_muscle_development	ENSGALG00010024440	PKNOX2	2.059998839	0.205509165	10.0238782	1.19711E-23	7.68134E-23	positive
PRJNA729271	stage_1D_vs_E10	ordinary_muscle_development	ENSGALG00010024441	SAA	4.05941044	0.790953486	5.132299825	2.86223E-07	6.66046E-07	positive
PRJNA729271	stage_1D_vs_E10	ordinary_muscle_development	ENSGALG00010024446	GRM4	-2.661239248	0.515143147	-5.16601893	2.39133E-07	5.60153E-07	negative
PRJNA729271	stage_1D_vs_E10	ordinary_muscle_development	ENSGALG00010024449	CACHD1	-0.299587695	0.083688456	-3.579797147	0.000343861	0.000602619	negative
PRJNA729271	stage_1D_vs_E10	ordinary_muscle_development	ENSGALG00010024451	PHOSPHO1	1.321354429	0.269039473	4.91137755	9.04388E-07	2.0277E-06	positive
PRJNA729271	stage_1D_vs_E10	ordinary_muscle_development	ENSGALG00010024452	SLC16A1	0.430539	0.251431535	1.71235084	0.086832033	0.111570811	positive
PRJNA729271	stage_1D_vs_E10	ordinary_muscle_development	ENSGALG00010024453	HTR3A	1.199990072	0.657367273	1.825448454	0.067933383	0.088735188	positive
PRJNA729271	stage_1D_vs_E10	ordinary_muscle_development	ENSGALG00010024454	HPS5	-0.186224134	0.12074098	-1.542344067	0.122990006	0.154162931	negative
PRJNA729271	stage_1D_vs_E10	ordinary_muscle_development	ENSGALG00010024457	CHD6	0.258685666	0.1003306	2.578332695	0.009927836	0.014682582	positive
PRJNA729271	stage_1D_vs_E10	ordinary_muscle_development	ENSGALG00010024459	AHCYL1	-0.132519008	0.091089776	-1.454817584	0.145719769	0.180145676	negative
PRJNA729271	stage_1D_vs_E10	ordinary_muscle_development	ENSGALG00010024460	IMP4	-0.251237356	0.15561861	-1.614442872	0.106431399	0.134653323	negative
PRJNA729271	stage_1D_vs_E10	ordinary_muscle_development	ENSGALG00010024462	ZBTB32	7.292814443	0.428447047	17.02150707	5.68874E-65	1.80434E-63	positive
PRJNA729271	stage_1D_vs_E10	ordinary_muscle_development	ENSGALG00010024463	FBXL22	0.354864046	0.228324165	1.554211515	0.120133972	0.150765534	positive
PRJNA729271	stage_1D_vs_E10	ordinary_muscle_development	ENSGALG00010024465	SENP1	-0.121628111	0.156648915	-0.776437618	0.437490641	0.486828408	negative
PRJNA729271	stage_1D_vs_E10	ordinary_muscle_development	ENSGALG00010024466	ADGRG3	3.896060624	0.688146817	5.661670628	1.49906E-08	3.85743E-08	positive
PRJNA729271	stage_1D_vs_E10	ordinary_muscle_development	ENSGALG00010024467	ENSGALG00010024467	0.590578073	0.292235187	2.020899946	0.043290123	0.058306109	positive
PRJNA729271	stage_1D_vs_E10	ordinary_muscle_development	ENSGALG00010024469	GTF2H1	0.12401479	0.12755917	0.972213839	0.330944177	0.379392269	positive
PRJNA729271	stage_1D_vs_E10	ordinary_muscle_development	ENSGALG00010024471	CCDC61	-0.77676693	0.117520637	-6.60962154	3.85304E-11	1.2003E-10	negative
PRJNA729271	stage_1D_vs_E10	ordinary_muscle_development	ENSGALG00010024472	LDHA	3.869707002	0.201277843	19.22569791	2.25584E-82	1.12753E-80	positive
PRJNA729271	stage_1D_vs_E10	ordinary_muscle_development	ENSGALG00010024473	RAVER2	-1.10473481	0.059682112	-18.510317	1.70494E-76	7.33328E-75	negative
PRJNA729271	stage_1D_vs_E10	ordinary_muscle_development	ENSGALG00010024474	STRIP1	-0.268508322	0.104173209	-2.577518008	0.00995127	0.014714236	negative
PRJNA729271	stage_1D_vs_E10	ordinary_muscle_development	ENSGALG00010024475	ADGRG1	0.857930422	0.157262052	5.455419235	4.88574E-08	1.20872E-07	positive
PRJNA729271	stage_1D_vs_E10	ordinary_muscle_development	ENSGALG00010024476	KCNB1	-0.552643513	0.170444584	-3.242364756	0.001185422	0.001961718	negative
PRJNA729271	stage_1D_vs_E10	ordinary_muscle_development	ENSGALG00010024479	ERBB2	0.831730663	0.12741956	6.527495935	6.68783E-11	2.04688E-10	positive
PRJNA729271	stage_1D_vs_E10	ordinary_muscle_development	ENSGALG00010024480	CDC45	-1.761894015	0.215574125	-8.173031032	3.00737E-16	1.30632E-15	negative
PRJNA729271	stage_1D_vs_E10	ordinary_muscle_development	ENSGALG00010024482	USP3	-0.787365055	0.057124079	-13.78341796	3.20721E-43	4.72444E-42	negative
PRJNA729271	stage_1D_vs_E10	ordinary_muscle_development	ENSGALG00010024486	GPR37L1	0.908071097	0.281742619	3.22305195	0.001268325	0.002092225	positive
PRJNA729271	stage_1D_vs_E10	ordinary_muscle_development	ENSGALG00010024488	ADGRG5	-1.150942504	0.604733725	-1.903221957	0.057011589	0.075283136	negative
PRJNA729271	stage_1D_vs_E10	ordinary_muscle_development	ENSGALG00010024490	TGM3	5.589284875	0.805134051	6.942055012	3.86437E-12	1.2862E-11	positive
PRJNA729271	stage_1D_vs_E10	ordinary_muscle_development	ENSGALG00010024492	NTF3	-2.414054203	0.251613114	-9.594309957	8.44805E-22	4.99203E-21	negative
PRJNA729271	stage_1D_vs_E10	ordinary_muscle_development	ENSGALG00010024493	JAK1	0.697658344	0.05992953	11.64131177	2.54077E-31	2.33684E-30	positive

PRJNA729271	stage_1D_vs_E10	ordinary_muscle_development	ENSGALG00010024494	DDAH1	0.335147062	0.160257283	2.091306275	0.036500618	0.049729904	positive
PRJNA729271	stage_1D_vs_E10	ordinary_muscle_development	ENSGALG00010024495	CLDN5	0.474469439	0.168284145	2.819454204	0.004810539	0.007406923	positive
PRJNA729271	stage_1D_vs_E10	ordinary_muscle_development	ENSGALG00010024496	UEVLD	-0.044940009	0.208245416	-0.215803114	0.829141221	0.853337262	negative
PRJNA729271	stage_1D_vs_E10	ordinary_muscle_development	ENSGALG00010024497	ABI3	1.666692251	0.177580473	9.385560355	6.25828E-21	3.5256E-20	positive
PRJNA729271	stage_1D_vs_E10	ordinary_muscle_development	ENSGALG00010024498	CCDC102A	-0.768044026	0.118702584	-6.470322696	9.77938E-11	2.96057E-10	negative
PRJNA729271	stage_1D_vs_E10	ordinary_muscle_development	ENSGALG00010024499	CA12	-1.93104713	0.608870638	-3.17152283	0.001516419	0.002476397	negative
PRJNA729271	stage_1D_vs_E10	ordinary_muscle_development	ENSGALG00010024501	ARL8A	0.404451513	0.089332792	4.527469731	5.96941E-06	1.24499E-05	positive
PRJNA729271	stage_1D_vs_E10	ordinary_muscle_development	ENSGALG00010024502	VPS8	-0.052291305	0.073369157	-0.712715083	0.476022081	0.524750176	negative
PRJNA729271	stage_1D_vs_E10	ordinary_muscle_development	ENSGALG00010024504	CASC3	0.203953092	0.186750495	1.092115405	0.274782379	0.320457847	positive
PRJNA729271	stage_1D_vs_E10	ordinary_muscle_development	ENSGALG00010024505	DNASE2B	0.486864727	0.211397164	2.303080696	0.021274303	0.030005938	positive
PRJNA729271	stage_1D_vs_E10	ordinary_muscle_development	ENSGALG00010024506	RAB8B	-0.174191333	0.081398633	-2.139978604	0.032356496	0.04443041	negative
PRJNA729271	stage_1D_vs_E10	ordinary_muscle_development	ENSGALG00010024508	RPS27L	0.765094206	0.19013851	4.023878203	5.72475E-05	0.000108841	positive
PRJNA729271	stage_1D_vs_E10	ordinary_muscle_development	ENSGALG00010024509	SEPTIN5	-0.490473948	0.107911771	-4.545138517	5.48991E-06	1.14829E-05	negative
PRJNA729271	stage_1D_vs_E10	ordinary_muscle_development	ENSGALG00010024510	CNN3	-0.65753775	0.101058073	-6.506533653	7.69047E-11	2.34631E-10	negative
PRJNA729271	stage_1D_vs_E10	ordinary_muscle_development	ENSGALG00010024511	ENSGALG00010024511	-1.011230643	0.170788552	-5.920950968	3.20085E-09	8.6966E-09	negative
PRJNA729271	stage_1D_vs_E10	ordinary_muscle_development	ENSGALG00010024512	CCNDBP1	0.10189528	0.114962601	0.88633416	0.375437487	0.424689119	positive
PRJNA729271	stage_1D_vs_E10	ordinary_muscle_development	ENSGALG00010024513	SPTY2D1	0.367867009	0.138737449	2.651533619	0.008012714	0.012000856	positive
PRJNA729271	stage_1D_vs_E10	ordinary_muscle_development	ENSGALG00010024514	FAF1	0.227961714	0.068236371	3.340765492	0.000835478	0.001405227	positive
PRJNA729271	stage_1D_vs_E10	ordinary_muscle_development	ENSGALG00010024515	C3orf70	-1.040796898	0.156233242	-6.661814616	2.70467E-11	8.50417E-11	negative
PRJNA729271	stage_1D_vs_E10	ordinary_muscle_development	ENSGALG00010024516	EHHADH	-0.976490469	0.244859535	-3.987961794	6.66434E-05	0.000125912	negative
PRJNA729271	stage_1D_vs_E10	ordinary_muscle_development	ENSGALG00010024517	LACTB	-0.041374106	0.097205592	-0.425635038	0.670373805	0.708861125	negative
PRJNA729271	stage_1D_vs_E10	ordinary_muscle_development	ENSGALG00010024518	ENSGALG00010024518	-0.489003394	1.33961853	-0.365031823	0.715087652	0.750394231	negative
PRJNA729271	stage_1D_vs_E10	ordinary_muscle_development	ENSGALG00010024519	MIEN1	0.185489454	0.14020382	1.322998576	0.185835851	0.224961639	positive
PRJNA729271	stage_1D_vs_E10	ordinary_muscle_development	ENSGALG00010024520	MAP3K13	-0.697156845	0.137590705	-5.066889125	4.0437E-07	9.29928E-07	negative
PRJNA729271	stage_1D_vs_E10	ordinary_muscle_development	ENSGALG00010024521	HEPACAM	1.204360575	0.403289401	2.986343234	0.002823356	0.004464333	positive
PRJNA729271	stage_1D_vs_E10	ordinary_muscle_development	ENSGALG00010024522	DNTTIP2	0.024070812	0.074894796	0.32139499	0.74791108	0.780262782	positive
PRJNA729271	stage_1D_vs_E10	ordinary_muscle_development	ENSGALG00010024523	TOX3	-1.685401995	0.260250059	-6.476086887	9.41319E-11	2.85508E-10	negative
PRJNA729271	stage_1D_vs_E10	ordinary_muscle_development	ENSGALG00010024524	TMEM41A	-0.048280126	0.13465161	-0.358555877	0.719927359	0.755090237	negative
PRJNA729271	stage_1D_vs_E10	ordinary_muscle_development	ENSGALG00010024525	GP1BB	0.31662615	0.18898912	1.675367075	0.093862233	0.119913014	positive
PRJNA729271	stage_1D_vs_E10	ordinary_muscle_development	ENSGALG00010024526	TGM6	1.770749428	1.526165567	1.160260372	0.245942814	0.289997648	positive
PRJNA729271	stage_1D_vs_E10	ordinary_muscle_development	ENSGALG00010024527	ATP6V0B	0.73952916	0.099013048	7.46900711	8.08022E-14	3.00315E-13	positive
PRJNA729271	stage_1D_vs_E10	ordinary_muscle_development	ENSGALG00010024528	TBX1	-0.744579991	0.210081366	-3.544245751	0.000393738	0.000686295	negative
PRJNA729271	stage_1D_vs_E10	ordinary_muscle_development	ENSGALG00010024529	GRB7	-5.71791081	1.212304553	-4.71656301	2.39862E-06	5.19779E-06	negative
PRJNA729271	stage_1D_vs_E10	ordinary_muscle_development	ENSGALG00010024530	ENSGALG00010024530	-0.891883195	0.59102792	-1.509037331	0.131289243	0.163575518	negative
PRJNA729271	stage_1D_vs_E10	ordinary_muscle_development	ENSGALG00010024531	CEP63	-0.957601466	0.128902022	-7.428909597	1.09497E-13	4.0437E-13	negative
PRJNA729271	stage_1D_vs_E10	ordinary_muscle_development	ENSGALG00010024532	TMEM86A	0.542812462	0.214362537	2.532217006	0.011334382	0.016643893	positive
PRJNA729271	stage_1D_vs_E10	ordinary_muscle_development	ENSGALG00010024533	CDKN2C	2.121442289	0.223080685	9.509753353	1.91115E-21	1.10587E-20	positive
PRJNA729271	stage_1D_vs_E10	ordinary_muscle_development	ENSGALG00010024534	VWF	3.547228253	0.125477969	28.26972955	8.1445E-176	2.3148E-173	positive
PRJNA729271	stage_1D_vs_E10	ordinary_muscle_development	ENSGALG00010024535	ENSGALG00010024535	1.737348372	0.151498441	11.46776399	1.91545E-30	1.69917E-29	positive
PRJNA729271	stage_1D_vs_E10	ordinary_muscle_development	ENSGALG00010024536	URI1	-0.455580822	0.062101827	-7.33602926	2.20024E-13	7.95522E-13	negative
PRJNA729271	stage_1D_vs_E10	ordinary_muscle_development	ENSGALG00010024537	EPB42	-0.523099864	0.179043039	-2.921643117	0.003481903	0.005455646	negative
PRJNA729271	stage_1D_vs_E10	ordinary_muscle_development	ENSGALG00010024540	PTPN5	-2.168777994	0.292913484	-7.404158951	1.31984E-13	4.8421E-13	negative
PRJNA729271	stage_1D_vs_E10	ordinary_muscle_development	ENSGALG00010024541	WIPF2	0.783792719	0.201477944	3.890215984	0.000100155	0.000185669	positive
PRJNA729271	stage_1D_vs_E10	ordinary_muscle_development	ENSGALG00010024542	ZNFX1	1.708526342	0.42530908	4.017140531	5.89086E-05	0.000111897	positive
PRJNA729271	stage_1D_vs_E10	ordinary_muscle_development	ENSGALG00010024544	ZNF536	-0.617675755	0.242734652	-2.544654214	0.0109386	0.016095321	negative
PRJNA729271	stage_1D_vs_E10	ordinary_muscle_development	ENSGALG00010024545	PHACTR3	-2.224723084	0.305391174	-7.284830967	3.22074E-13	1.15385E-12	negative
PRJNA729271	stage_1D_vs_E10	ordinary_muscle_development	ENSGALG00010024546	KY	4.209635579	0.402436799	10.46036444	1.31349E-25	9.26027E-25	positive
PRJNA729271	stage_1D_vs_E10	ordinary_muscle_development	ENSGALG00010024547	PFKM	6.734810072	0.197799886	34.04860439	4.2565E-254	4.407E-251	positive
PRJNA729271	stage_1D_vs_E10	ordinary_muscle_development	ENSGALG00010024548	CDC6	-1.448208062	0.283617558	-5.106200316	3.28701E-07	7.61106E-07	negative
PRJNA729271	stage_1D_vs_E10	ordinary_muscle_development	ENSGALG00010024549	GNB1L	-0.320355472	0.132926655	-2.410016802	0.015951786	0.022890915	negative
PRJNA729271	stage_1D_vs_E10	ordinary_muscle_development	ENSGALG00010024551	RNF11	1.010950492	0.126767125	7.974863323	1.5255E-15	6.33583E-15	positive
PRJNA729271	stage_1D_vs_E10	ordinary_muscle_development	ENSGALG00010024552	CD81	1.582427459	0.066121214	23.93222015	1.4154E-126	1.7686E-124	positive
PRJNA729271	stage_1D_vs_E10	ordinary_muscle_development	ENSGALG00010024553	ASB8	0.031086208	0.112390774	0.276590392	0.782094643	0.81044194	positive
PRJNA729271	stage_1D_vs_E10	ordinary_muscle_development	ENSGALG00010024554	ST6GALNAC3	-1.763268862	0.210747637	-8.366731367	5.92385E-17	2.67746E-16	negative
PRJNA729271	stage_1D_vs_E10	ordinary_muscle_development	ENSGALG00010024555	EPHB1	-0.096197438	0.162448588	-0.592171583	0.553735696	0.599477102	negative
PRJNA729271	stage_1D_vs_E10	ordinary_muscle_development	ENSGALG00010024556	ENSGALG00010024556	-0.982019846	0.213007433	-4.610260917	4.02164E-06	8.51002E-06	negative
PRJNA729271	stage_1D_vs_E10	ordinary_muscle_development	ENSGALG00010024557	TSPAN32	0.302665245	0.295910207	1.022827998	0.306389178	0.35359165	positive
PRJNA729271	stage_1D_vs_E10	ordinary_muscle_development	ENSGALG00010024558	PFDN5	0.082472752	0.108147483	0.762595206	0.445704845	0.494943057	positive
PRJNA729271	stage_1D_vs_E10	ordinary_muscle_development	ENSGALG00010024559	ENSGALG00010024559	-1.347900917	0.296015716	-4.553477544	5.27663E-06	1.10575E-05	negative
PRJNA729271	stage_1D_vs_E10	ordinary_muscle_development	ENSGALG00010024560	A4GNT	1.150655591	0.427377491	2.692363579	0.007094755	0.010685628	positive
PRJNA729271	stage_1D_vs_E10	ordinary_muscle_development	ENSGALG00010024561	ROBO3	-1.184729873	0.549308039	-2.156767767	0.031023764	0.042697442	negative
PRJNA729271	stage_1D_vs_E10	ordinary_muscle_development	ENSGALG00010024563	ESPL1	-0.976531926	0.161897139	-6.031804697	1.62139E-09	4.50921E-09	negative
PRJNA729271	stage_1D_vs_E10	ordinary_muscle_development	ENSGALG00010024564	PPP1R3D	1.357784116	0.201673174	6.732596554	1.66662E-11	5.31872E-11	positive
PRJNA729271	stage_1D_vs_E10	ordinary_muscle_development	ENSGALG00010024565	FAM217B	0.902087783	0.137520965	6.559638258	5.39385E-11	1.66455E-10	positive
PRJNA729271	stage_1D_vs_E10	ordinary_muscle_development	ENSGALG00010024566	MTSS2	1.364075552	0.215732485	6.322995607	2.56541E-10	7.56111E-10	positive
PRJNA729271	stage_1D_vs_E10	ordinary_muscle_development	ENSGALG00010024567	IKZF3	0.059827743	0.46385877	0.12897836	0.897374772	0.912472844	positive
PRJNA729271	stage_1D_vs_E10	ordinary_muscle_development	ENSGALG00010024568	DZIP1L	-0.065493494	0.270357474	-0.242247764	0.808588186	0.834463008	negative
PRJNA729271	stage_1D_vs_E10	ordinary_muscle_development	ENSGALG00010024569	ASCL2	-0.687371951	0.267550754	-2.569127322	0.010195499	0.015052328	negative
PRJNA729271	stage_1D_vs_E10	ordinary_muscle_development	ENSGALG00010024570	TPM1	3.630547767	0.164154529	22.11664697	2.1858E-108	1.8528E-106	positive
PRJNA729271	stage_1D_vs_E10	ordinary_muscle_development	ENSGALG00010024571	KCNA3	-4.493968153	0.466689561	-9.629459334	6.00446E-22	3.58315E-21	negative
PRJNA729271	stage_1D_vs_E10	ordinary_muscle_development	ENSGALG00010024573	ENSGALG00010024573	0.534517521	0.281530058	1.898616169	0.057614961	0.076003718	positive
PRJNA729271	stage_1D_vs_E10	ordinary_muscle_development	ENSGALG00010024575	TXNRD2	0.631288001	0.122839111	5.139144985	2.75991E-07	6.43063E-07	positive
PRJNA729271	stage_1D_vs_E10	ordinary_muscle_development	ENSGALG00010024576	CSAD	0.136761569	0.153490084	0.891012407	0.372922514	0.422140714	positive
PRJNA729271	stage_1D_vs_E10	ordinary_muscle_development	ENSGALG00010024578	ENSGALG00010024578	1.477337897	0.597556096	2.472299937	0.013424683	0.019494168	positive
PRJNA729271	stage_1D_vs_E10	ordinary_muscle_development	ENSGALG00010024579	ZNF740	-0.481213806	0.105884065	-4.544723555	5.50074E-06	1.15004E-05	negative
PRJNA729271	stage_1D_vs_E10	ordinary_muscle_development	ENSGALG00010024580	IGF2	0.362998466	0.192808081	1.882693217	0.059741955	0.078666392	positive
PRJNA729271	stage_1D_vs_E10	ordinary_muscle_development	ENSGALG00010024581	RARA	0.569596002	0.171967632	3.312227988	0.000925561	0.00154956	positive
PRJNA729271	stage_1D_vs_E10	ordinary_muscle_development	ENSGALG00010024582	TLN2	0.962703481	0.107383413	8.965104133	3.09988E-19	1.58885E-18	positive
PRJNA729271	stage_1D_vs_E10	ordinary_muscle_development	ENSGALG00010024583	ITGB7	-0.030712544	0.587889948	-0.052241995	0.958335871	0.964859238	negative
PRJNA729271	stage_1D_vs_E10	ordinary_muscle_development	ENSGALG00010024585	TBCCD1	-0.91476233	0.124225545	-7.363721582	1.78853E-13	6.49904E-13	negative
PRJNA729271	stage_1D_vs_E10	ordinary_muscle_development	ENSGALG00010024586	DLGAP4	-0.078192114	0.095861399	-0.815678836	0.414683847	0.464157711	negative
PRJNA729271	stage_1D_vs_E10	ordinary_muscle_development	ENSGALG00010024587	ENSGALG00010024587	2.97862216	0.17878444	16.66041046	2.54273E-62	7.5065E-61	positive
PRJNA729271	stage_1D_vs_E10	ordinary_muscle_development	ENSGALG00010024588	TTC39A	-4.988015331	0.421734327	-11.82738755	2.81776E-32	2.71204E-31	negative
PRJNA729271	stage_1D_vs_E10	ordinary_muscle_development	ENSGALG00010024589	ADORA1	0.911509718	0.470871242	1.935793984	0.052892938	0.070247676	positive
PRJNA729271	stage_1D_vs_E10	ordinary_muscle_development	ENSGALG00010024590	MRPL23	-0.501857475	0.104788736	-4.789231109	1.67422E-06	3.67137E-06	negative
PRJNA729271	stage_1D_vs_E10	ordinary_muscle_development	ENSGALG00010024592	COMT	1.048282901	0.197726393	5.301684241	1.14739E-07	2.76178E-07	positive
PRJNA729271	stage_1D_vs_E10	ordinary_muscle_development	ENSGALG00010024593	SRSF6	-0.194080133	0.074416803	-2.608014907	0.009106899	0.013534758	negative
PRJNA729271	stage_1D_vs_E10	ordinary_muscle_development	ENSGALG00010024594	TNNI2	4.625302624	0.183208427	25.24612384	1.2492E-140	1.9263E-138	positive
PRJNA729271	stage_1D_vs_E10	ordinary_muscle_development	ENSGALG00010024595	MYBPHL	4.268660039	0.316451626	13.48913922	1.81204E-41	2.51585E-40	positive
PRJNA729271	stage_1D_vs_E10	ordinary_muscle_development	ENSGALG00010024596	EPS15	0.976512933	0.066533366	14.67704091	9.04525E-49	1.66596E-47	positive
PRJNA729271	stage_1D_vs_E10	ordinary_muscle_development	ENSGALG00010024597	DNAJB11	-1.162971916	0.100894947	-11.52656256	9.69374E-31	8.70036E-30	negative
PRJNA729271	stage_1D_vs_E10	ordinary_muscle_development	ENSGALG00010024598	LRRC3C	0.78793755	0.23748949	3.317778614	0.000907364	0.001520138	positive
PRJNA729271	stage_1D_vs_E10	ordinary_muscle_development	ENSGALG00010024599	ENSGALG00010024599	0.287738827	0.20767376	1.385532899	0.16588958	0.202850946	positive
PRJNA729271	stage_1D_vs_E10	ordinary_muscle_development	ENSGALG00010024600	CD9	3.299332156	0.332079457	9.93536965	2.92091E-23	1.84001E-22	positive
PRJNA729271	stage_1D_vs_E10	ordinary_muscle_development	ENSGALG00010024601	ENSGALG00010024601	-1.048504749	0.510948434	-2.052075471	0.040162328	0.054350943	negative
PRJNA729271	stage_1D_vs_E10	ordinary_muscle_development	ENSGALG00010024602	PROCR	0.67072956	0.242547807	2.765349925	0.005686176	0.008677735	positive
PRJNA729271	stage_1D_vs_E10	ordinary_muscle_development	ENSGALG00010024603	ENSGALG00010024603	0.171893806	1.088014775	0.157988485	0.874465872	0.892821217	positive
PRJNA729271	stage_1D_vs_E10	ordinary_muscle_development	ENSGALG00010024604	MFSD5	0.357478581	0.169983849	2.103014978	0.03546446	0.048422885	positive
PRJNA729271	stage_1D_vs_E10	ordinary_muscle_development	ENSGALG00010024605	LSP1P1	-1.091563289	0.195975623	-5.569893201	2.54896E-08	6.4379E-08	negative
PRJNA729271	stage_1D_vs_E10	ordinary_muscle_development	ENSGALG00010024607	RARG	-0.735664889	0.141800662	-5.188021557	2.1254E-07	4.99719E-07	negative
PRJNA729271	stage_1D_vs_E10	ordinary_muscle_development	ENSGALG00010024608	DENND2D	0.96905188	0.333773871	2.903318579	0.003692308	0.005763516	positive
PRJNA729271	stage_1D_vs_E10	ordinary_muscle_development	ENSGALG00010024609	PRR33	11.63385666	0.836609477	13.90595849	5.82809E-44	8.7998E-43	positive
PRJNA729271	stage_1D_vs_E10	ordinary_muscle_development	ENSGALG00010024610	PFKL	-1.681497078	0.14185874	-11.85332027	2.0683E-32	2.00535E-31	negative
PRJNA729271	stage_1D_vs_E10	ordinary_muscle_development	ENSGALG00010024611	ENSGALG00010024611	-0.991221485	0.234348092	-4.229697261	2.34006E-05	4.60545E-05	negative
PRJNA729271	stage_1D_vs_E10	ordinary_muscle_development	ENSGALG00010024612	ENSGALG00010024612	-2.852576996	0.73243878	-3.894628565	9.83493E-05	0.000182508	negative
PRJNA729271	stage_1D_vs_E10	ordinary_muscle_development	ENSGALG00010024613	ENSGALG00010024613	0.169285746	0.09508823	1.7803018	0.075026583	0.097342492	positive
PRJNA729271	stage_1D_vs_E10	ordinary_muscle_development	ENSGALG00010024614	ENSGALG00010024614	-6.743536864	0.889407881	-7.582052068	3.40132E-14	1.29708E-13	negative
PRJNA729271	stage_1D_vs_E10	ordinary_muscle_development	ENSGALG00010024615	SYT8	-3.36514032	0.713684112	-4.715167769	2.41512E-06	5.22963E-06	negative
PRJNA729271	stage_1D_vs_E10	ordinary_muscle_development	ENSGALG00010024616	ARVCF	-0.784297536	0.098503082	-7.962162437	1.69058E-15	7.00143E-15	negative
PRJNA729271	stage_1D_vs_E10	ordinary_muscle_development	ENSGALG00010024618	VPS13C	-0.002875772	0.122676929	-0.023441831	0.981297838	0.984013294	negative
PRJNA729271	stage_1D_vs_E10	ordinary_muscle_development	ENSGALG00010024619	ENSGALG00010024619	-1.879835445	0.234693623	-8.009742317	1.14949E-15	4.81001E-15	negative
PRJNA729271	stage_1D_vs_E10	ordinary_muscle_development	ENSGALG00010024620	MSANTD2	-0.284308519	0.125441163	-2.266469086	0.023422678	0.032844318	negative
PRJNA729271	stage_1D_vs_E10	ordinary_muscle_development	ENSGALG00010024621	THRA	1.665684921	0.184430302	9.031514364	1.69312E-19	8.7995E-19	positive
PRJNA729271	stage_1D_vs_E10	ordinary_muscle_development	ENSGALG00010024622	PRMT8	-0.236334141	0.210627209	-1.122049434	0.261841422	0.306995989	negative
PRJNA729271	stage_1D_vs_E10	ordinary_muscle_development	ENSGALG00010024623	NRDC	0.317820425	0.109497741	2.902529517	0.003701623	0.005776811	positive
PRJNA729271	stage_1D_vs_E10	ordinary_muscle_development	ENSGALG00010024624	CTSD	-0.156368385	0.128807216	-1.213972244	0.224758323	0.267520048	negative
PRJNA729271	stage_1D_vs_E10	ordinary_muscle_development	ENSGALG00010024625	SPRYD3	0.062230812	0.079758437	0.78024112	0.435248963	0.484631565	positive
PRJNA729271	stage_1D_vs_E10	ordinary_muscle_development	ENSGALG00010024626	TSHZ3	-1.249569656	0.094533868	-13.218222	6.88728E-40	8.97762E-39	negative
PRJNA729271	stage_1D_vs_E10	ordinary_muscle_development	ENSGALG00010024627	CEPT1	0.573942039	0.077829178	7.374381395	1.6511E-13	6.01491E-13	positive
PRJNA729271	stage_1D_vs_E10	ordinary_muscle_development	ENSGALG00010024628	TANGO2	0.674063442	0.110178166	6.117940318	9.47926E-10	2.68573E-09	positive
PRJNA729271	stage_1D_vs_E10	ordinary_muscle_development	ENSGALG00010024629	ENSGALG00010024629	0.809151232	0.172167627	4.699787337	2.60433E-06	5.62337E-06	positive
PRJNA729271	stage_1D_vs_E10	ordinary_muscle_development	ENSGALG00010024630	CFAP410	-0.39411036	0.096662546	-4.077177537	4.55857E-05	8.74721E-05	negative
PRJNA729271	stage_1D_vs_E10	ordinary_muscle_development	ENSGALG00010024631	EPB41L1	-0.470401534	0.149992562	-3.13616574	0.001711724	0.002779371	negative
PRJNA729271	stage_1D_vs_E10	ordinary_muscle_development	ENSGALG00010024632	IFITM10	0.369047137	0.348567613	1.058753375	0.289712113	0.336165312	positive
PRJNA729271	stage_1D_vs_E10	ordinary_muscle_development	ENSGALG00010024633	NRGN	-1.378648671	0.335854663	-4.104896616	4.04496E-05	7.78848E-05	negative
PRJNA729271	stage_1D_vs_E10	ordinary_muscle_development	ENSGALG00010024634	ENSGALG00010024634	1.87039579	0.163059386	11.47064172	1.8528E-30	1.64561E-29	positive
PRJNA729271	stage_1D_vs_E10	ordinary_muscle_development	ENSGALG00010024635	ENSGALG00010024635	-1.115237024	0.189118618	-5.897023976	3.70116E-09	1.00202E-08	negative
PRJNA729271	stage_1D_vs_E10	ordinary_muscle_development	ENSGALG00010024636	PANX3	-1.372148443	0.296447477	-4.628639298	3.68076E-06	7.82641E-06	negative
PRJNA729271	stage_1D_vs_E10	ordinary_muscle_development	ENSGALG00010024637	DUSP8	2.585021441	0.276490402	9.349407502	8.81401E-21	4.93088E-20	positive
PRJNA729271	stage_1D_vs_E10	ordinary_muscle_development	ENSGALG00010024638	ZNF507	0.286010042	0.129096888	2.215468136	0.026727949	0.037109351	positive
PRJNA729271	stage_1D_vs_E10	ordinary_muscle_development	ENSGALG00010024639	AAR2	-0.231409741	0.075396507	-3.069236874	0.002146064	0.003439159	negative

PRJNA729271	stage_1D_vs_E10	ordinary_muscle_development	ENSGALG00010024640	GSDMA	-1.237868315	0.298180068	-4.151412005	3.3043E-05	6.41434E-05	negative
PRJNA729271	stage_1D_vs_E10	ordinary_muscle_development	ENSGALG00010024641	TRA2B	-1.22043898	0.087413672	-13.96164875	2.67181E-44	4.08953E-43	negative
PRJNA729271	stage_1D_vs_E10	ordinary_muscle_development	ENSGALG00010024642	RAB3B	0.393111627	0.224772054	1.748934622	0.080302321	0.103787975	positive
PRJNA729271	stage_1D_vs_E10	ordinary_muscle_development	ENSGALG00010024643	ETV7	1.771146862	0.384757701	4.603278525	4.15891E-06	8.79024E-06	positive
PRJNA729271	stage_1D_vs_E10	ordinary_muscle_development	ENSGALG00010024644	EIF6	-0.617485991	0.075509071	-8.177639937	2.89457E-16	1.25921E-15	negative
PRJNA729271	stage_1D_vs_E10	ordinary_muscle_development	ENSGALG00010024645	PARP11	0.227182109	0.118211699	1.921824247	0.054627876	0.072405913	positive
PRJNA729271	stage_1D_vs_E10	ordinary_muscle_development	ENSGALG00010024646	RORA	1.835596547	0.192033783	9.558716783	1.19226E-21	6.97971E-21	positive
PRJNA729271	stage_1D_vs_E10	ordinary_muscle_development	ENSGALG00010024647	RPUSD4	-0.295973366	0.132774804	-2.229138042	0.025804721	0.035916981	negative
PRJNA729271	stage_1D_vs_E10	ordinary_muscle_development	ENSGALG00010024648	MMP24	-1.411364307	0.123235901	-11.45254179	2.2835E-30	2.01702E-29	negative
PRJNA729271	stage_1D_vs_E10	ordinary_muscle_development	ENSGALG00010024649	NCOR2	0.656384064	0.129291643	5.076771014	3.83903E-07	8.84124E-07	positive
PRJNA729271	stage_1D_vs_E10	ordinary_muscle_development	ENSGALG00010024650	PRKAG1	1.045221704	0.120312202	8.687578515	3.70247E-18	1.7913E-17	positive
PRJNA729271	stage_1D_vs_E10	ordinary_muscle_development	ENSGALG00010024651	PSMD3	-0.46830089	0.127891246	-3.661711832	0.000250536	0.000445093	negative
PRJNA729271	stage_1D_vs_E10	ordinary_muscle_development	ENSGALG00010024652	PUS3	-0.686908098	0.125956858	-5.453518827	4.93827E-08	1.2213E-07	negative
PRJNA729271	stage_1D_vs_E10	ordinary_muscle_development	ENSGALG00010024653	RFLNA	-2.818717667	0.253737571	-11.10879109	1.13688E-28	9.21135E-28	negative
PRJNA729271	stage_1D_vs_E10	ordinary_muscle_development	ENSGALG00010024654	CCDC92	-2.490835952	0.28491399	-8.742413629	2.28182E-18	1.11589E-17	negative
PRJNA729271	stage_1D_vs_E10	ordinary_muscle_development	ENSGALG00010024655	IGF2BP2	-0.106011033	0.165206915	-0.641686413	0.521076813	0.568279919	negative
PRJNA729271	stage_1D_vs_E10	ordinary_muscle_development	ENSGALG00010024656	HYLS1	-0.887940222	0.18340785	-4.841342523	1.28965E-06	2.85309E-06	negative
PRJNA729271	stage_1D_vs_E10	ordinary_muscle_development	ENSGALG00010024657	DPY19L3	-0.247135039	0.127785646	-1.933981211	0.053115438	0.070517336	negative
PRJNA729271	stage_1D_vs_E10	ordinary_muscle_development	ENSGALG00010024659	BEND5	0.548281416	0.107286822	5.110426498	3.21432E-07	7.44989E-07	positive
PRJNA729271	stage_1D_vs_E10	ordinary_muscle_development	ENSGALG00010024660	KMT2D	1.454238248	0.156634797	9.284260458	1.62834E-20	8.95424E-20	positive
PRJNA729271	stage_1D_vs_E10	ordinary_muscle_development	ENSGALG00010024662	MOB2	-0.296423628	0.071883489	-4.123667799	3.72887E-05	7.20569E-05	negative
PRJNA729271	stage_1D_vs_E10	ordinary_muscle_development	ENSGALG00010024663	ICE2	-0.772711433	0.123521401	-6.255688678	3.95766E-10	1.14985E-09	negative
PRJNA729271	stage_1D_vs_E10	ordinary_muscle_development	ENSGALG00010024664	DNAH10	-3.790920658	0.249380722	-15.20133804	3.46471E-52	7.21566E-51	negative
PRJNA729271	stage_1D_vs_E10	ordinary_muscle_development	ENSGALG00010024665	MAP1LC3A	2.572383677	0.147701539	17.41609263	6.22883E-68	2.12941E-66	positive
PRJNA729271	stage_1D_vs_E10	ordinary_muscle_development	ENSGALG00010024666	MED24	-0.715815092	0.113731908	-6.293880994	3.09625E-10	9.07587E-10	negative
PRJNA729271	stage_1D_vs_E10	ordinary_muscle_development	ENSGALG00010024667	RBM7	0.149219476	0.136001988	1.097185992	0.272560095	0.318198984	positive
PRJNA729271	stage_1D_vs_E10	ordinary_muscle_development	ENSGALG00010024668	ENSGALG00010024668	1.939616617	0.183832354	10.55100787	5.02551E-26	3.60618E-25	positive
PRJNA729271	stage_1D_vs_E10	ordinary_muscle_development	ENSGALG00010024669	STIL	-1.315640792	0.180915481	-7.272129413	3.53864E-13	1.26399E-12	negative
PRJNA729271	stage_1D_vs_E10	ordinary_muscle_development	ENSGALG00010024670	SENP2	0.644908243	0.093645927	6.886666203	5.7115E-12	1.87558E-11	positive
PRJNA729271	stage_1D_vs_E10	ordinary_muscle_development	ENSGALG00010024671	KCTD20	0.969077389	0.280211112	3.458383155	0.000543428	0.000932629	positive
PRJNA729271	stage_1D_vs_E10	ordinary_muscle_development	ENSGALG00010024672	DYNLRB1	-0.870774743	0.105847113	-8.226721701	1.92407E-16	8.44365E-16	negative
PRJNA729271	stage_1D_vs_E10	ordinary_muscle_development	ENSGALG00010024673	REXO2	-0.040464463	0.098784572	-0.409623306	0.682082298	0.719981278	negative
PRJNA729271	stage_1D_vs_E10	ordinary_muscle_development	ENSGALG00010024674	DHH	3.143077319	0.313632946	10.02151516	1.22609E-23	7.85682E-23	positive
PRJNA729271	stage_1D_vs_E10	ordinary_muscle_development	ENSGALG00010024675	NR1D1	3.719774957	0.415507731	8.952360391	3.47961E-19	1.77657E-18	positive
PRJNA729271	stage_1D_vs_E10	ordinary_muscle_development	ENSGALG00010024676	ENSGALG00010024676	1.033629721	0.162454737	6.362570527	1.98405E-10	5.88596E-10	positive
PRJNA729271	stage_1D_vs_E10	ordinary_muscle_development	ENSGALG00010024677	LMBR1L	-2.641374499	0.371041135	-7.118818511	1.08856E-12	3.75058E-12	negative
PRJNA729271	stage_1D_vs_E10	ordinary_muscle_development	ENSGALG00010024678	AMOTL2	1.289348177	0.090052845	14.31768398	1.69684E-46	2.81416E-45	positive
PRJNA729271	stage_1D_vs_E10	ordinary_muscle_development	ENSGALG00010024679	MSL1	0.041283577	0.111115094	0.37153888	0.710236203	0.746005345	positive
PRJNA729271	stage_1D_vs_E10	ordinary_muscle_development	ENSGALG00010024680	BRSK1	-0.327606164	0.254707989	-1.286202936	0.198372247	0.238365723	negative
PRJNA729271	stage_1D_vs_E10	ordinary_muscle_development	ENSGALG00010024681	ATP6V0A2	0.417923991	0.085685487	4.877418633	1.07483E-06	2.39651E-06	positive
PRJNA729271	stage_1D_vs_E10	ordinary_muscle_development	ENSGALG00010024682	ENSGALG00010024682	-1.21771744	0.179166599	-6.796565027	1.07143E-11	3.46119E-11	negative
PRJNA729271	stage_1D_vs_E10	ordinary_muscle_development	ENSGALG00010024683	SHISA4	2.565286558	0.175263201	14.63676654	1.63669E-48	2.98412E-47	positive
PRJNA729271	stage_1D_vs_E10	ordinary_muscle_development	ENSGALG00010024684	ENSGALG00010024684	0.830879832	0.296906802	2.798453345	0.005134799	0.007881913	positive
PRJNA729271	stage_1D_vs_E10	ordinary_muscle_development	ENSGALG00010024685	ENSGALG00010024685	-0.884924841	0.219028075	-4.040234763	5.33977E-05	0.000101695	negative
PRJNA729271	stage_1D_vs_E10	ordinary_muscle_development	ENSGALG00010024686	CMPK1	-1.246707122	0.133677426	-9.326235278	1.09698E-20	6.09925E-20	negative
PRJNA729271	stage_1D_vs_E10	ordinary_muscle_development	ENSGALG00010024687	RYK	-0.11689756	0.106890429	-1.093620465	0.27412147	0.319815736	negative
PRJNA729271	stage_1D_vs_E10	ordinary_muscle_development	ENSGALG00010024688	CADM1	-0.47753335	0.129006159	-3.701632199	0.000214217	0.000383248	negative
PRJNA729271	stage_1D_vs_E10	ordinary_muscle_development	ENSGALG00010024689	FOXD2	0.809960405	0.301851168	2.683310491	0.007289728	0.010972441	positive
PRJNA729271	stage_1D_vs_E10	ordinary_muscle_development	ENSGALG00010024690	TUBA1C	-2.57043734	0.649314854	-3.958691723	7.53614E-05	0.000141553	negative
PRJNA729271	stage_1D_vs_E10	ordinary_muscle_development	ENSGALG00010024691	TCTN2	-0.938105794	0.151697852	-6.184041394	6.2481E-10	1.79126E-09	negative
PRJNA729271	stage_1D_vs_E10	ordinary_muscle_development	ENSGALG00010024692	DRAM2	1.886591872	0.104017123	18.13732027	1.61727E-73	6.52989E-72	positive
PRJNA729271	stage_1D_vs_E10	ordinary_muscle_development	ENSGALG00010024693	ANXA2	0.020318253	0.201602358	0.100783806	0.919722082	0.931677376	positive
PRJNA729271	stage_1D_vs_E10	ordinary_muscle_development	ENSGALG00010024694	GTF2H3	-0.673402993	0.097066763	-6.937523953	3.99032E-12	1.32599E-11	negative
PRJNA729271	stage_1D_vs_E10	ordinary_muscle_development	ENSGALG00010024695	TRABD2B	-0.082071507	0.191802485	-0.427895955	0.668726872	0.707326037	negative
PRJNA729271	stage_1D_vs_E10	ordinary_muscle_development	ENSGALG00010024696	TRADD	-0.058773289	0.114338566	-0.514028564	0.60723202	0.649821198	negative
PRJNA729271	stage_1D_vs_E10	ordinary_muscle_development	ENSGALG00010024697	IGF2BP1	-1.784620776	0.122176954	-14.6068528	2.53982E-48	4.57895E-47	negative
PRJNA729271	stage_1D_vs_E10	ordinary_muscle_development	ENSGALG00010024698	ENSGALG00010024698	-0.306203451	0.313254539	-0.977490867	0.328326197	0.376688952	negative
PRJNA729271	stage_1D_vs_E10	ordinary_muscle_development	ENSGALG00010024699	FBXL8	-0.927285398	0.134960197	-6.870806483	6.38399E-12	2.08979E-11	negative
PRJNA729271	stage_1D_vs_E10	ordinary_muscle_development	ENSGALG00010024700	TOLLIP	0.765998995	0.083887867	9.131225131	6.77284E-20	3.60133E-19	positive
PRJNA729271	stage_1D_vs_E10	ordinary_muscle_development	ENSGALG00010024701	EIF2B1	-0.300281651	0.101035554	-2.972039438	0.002958287	0.004666489	negative
PRJNA729271	stage_1D_vs_E10	ordinary_muscle_development	ENSGALG00010024702	INAVA	-3.571504479	0.949268706	-3.762374612	0.000168308	0.000305372	negative
PRJNA729271	stage_1D_vs_E10	ordinary_muscle_development	ENSGALG00010024703	HSF4	-0.515758787	0.585425244	-0.880998543	0.378318609	0.427414515	negative
PRJNA729271	stage_1D_vs_E10	ordinary_muscle_development	ENSGALG00010024705	SLCO2A1	2.901862156	0.232595792	12.47598733	1.00947E-35	1.12643E-34	positive
PRJNA729271	stage_1D_vs_E10	ordinary_muscle_development	ENSGALG00010024706	ENSGALG00010024706	1.644645593	0.94897005	1.733084824	0.083080593	0.107073282	positive
PRJNA729271	stage_1D_vs_E10	ordinary_muscle_development	ENSGALG00010024707	ENSGALG00010024707	-0.650807873	0.375691754	-1.732292144	0.08322156	0.107235889	negative
PRJNA729271	stage_1D_vs_E10	ordinary_muscle_development	ENSGALG00010024708	BNIP2	0.762325186	0.062233061	12.24952099	1.68991E-34	1.80112E-33	positive
PRJNA729271	stage_1D_vs_E10	ordinary_muscle_development	ENSGALG00010024709	TEAD4	1.350952331	0.096194403	14.04398066	8.38664E-45	1.30574E-43	positive
PRJNA729271	stage_1D_vs_E10	ordinary_muscle_development	ENSGALG00010024710	HSDL1	-0.698731792	0.12757685	-5.47694813	4.32724E-08	1.07311E-07	negative
PRJNA729271	stage_1D_vs_E10	ordinary_muscle_development	ENSGALG00010024711	DDX55	-0.059230455	0.090317717	-0.655801069	0.511952129	0.559802814	negative
PRJNA729271	stage_1D_vs_E10	ordinary_muscle_development	ENSGALG00010024712	LRIF1	0.879066898	0.097387525	9.026483579	1.77277E-19	9.20353E-19	positive
PRJNA729271	stage_1D_vs_E10	ordinary_muscle_development	ENSGALG00010024713	RND2	-1.302029122	0.409361544	-3.1806337	0.001469533	0.002402807	negative
PRJNA729271	stage_1D_vs_E10	ordinary_muscle_development	ENSGALG00010024714	SRPRB	-0.50349017	0.174761834	-2.881007585	0.003964061	0.006163813	negative
PRJNA729271	stage_1D_vs_E10	ordinary_muscle_development	ENSGALG00010024715	DNAAF1	-0.658413529	0.181346439	-3.630694565	0.00028266	0.000499531	negative
PRJNA729271	stage_1D_vs_E10	ordinary_muscle_development	ENSGALG00010024716	GTF2A2	-0.489129365	0.07726062	-6.330901414	2.43733E-10	7.19534E-10	negative
PRJNA729271	stage_1D_vs_E10	ordinary_muscle_development	ENSGALG00010024717	TF	1.107788334	0.652979389	1.696513478	0.08978868	0.115084173	positive
PRJNA729271	stage_1D_vs_E10	ordinary_muscle_development	ENSGALG00010024718	ENSGALG00010024718	1.138659953	0.192264473	5.922362753	3.17349E-09	8.62872E-09	positive
PRJNA729271	stage_1D_vs_E10	ordinary_muscle_development	ENSGALG00010024719	PYM1	-0.852275895	0.134923917	-6.31671475	2.67182E-10	7.86835E-10	negative
PRJNA729271	stage_1D_vs_E10	ordinary_muscle_development	ENSGALG00010024720	BUD13	-0.022358216	0.106227241	-0.210475355	0.833296685	0.857066306	negative
PRJNA729271	stage_1D_vs_E10	ordinary_muscle_development	ENSGALG00010024721	ENSGALG00010024721	1.255380403	0.748719314	1.676703644	0.093600448	0.119621859	positive
PRJNA729271	stage_1D_vs_E10	ordinary_muscle_development	ENSGALG00010024722	BRCA1	-1.653586238	0.260967655	-6.336364714	2.3525E-10	6.95339E-10	negative
PRJNA729271	stage_1D_vs_E10	ordinary_muscle_development	ENSGALG00010024723	ITCH	0.657674501	0.099967408	6.578889229	4.73976E-11	1.46905E-10	positive
PRJNA729271	stage_1D_vs_E10	ordinary_muscle_development	ENSGALG00010024724	HOXD3	-0.949382149	0.238307453	-3.983854206	6.78065E-05	0.000127943	negative
PRJNA729271	stage_1D_vs_E10	ordinary_muscle_development	ENSGALG00010024726	TMED2	-0.118591359	0.058467082	-2.02834406	0.042525145	0.057361063	negative
PRJNA729271	stage_1D_vs_E10	ordinary_muscle_development	ENSGALG00010024728	SLC5A9	-2.901709256	0.906773772	-3.200036597	0.001374101	0.002256952	negative
PRJNA729271	stage_1D_vs_E10	ordinary_muscle_development	ENSGALG00010024729	FOXM1	-1.415972589	0.166767953	-8.490675575	2.05439E-17	9.5291E-17	negative
PRJNA729271	stage_1D_vs_E10	ordinary_muscle_development	ENSGALG00010024731	AOX1	4.031957125	0.197529147	20.4119604	1.30927E-92	8.36027E-91	positive
PRJNA729271	stage_1D_vs_E10	ordinary_muscle_development	ENSGALG00010024732	AHCY	-0.775714894	0.224903432	-3.449102073	0.000562454	0.000962547	negative
PRJNA729271	stage_1D_vs_E10	ordinary_muscle_development	ENSGALG00010024733	MBTPS1	0.26517077	0.087601449	3.027013498	0.002469829	0.003929335	positive
PRJNA729271	stage_1D_vs_E10	ordinary_muscle_development	ENSGALG00010024734	ZPR1	-0.05522298	0.11438259	-0.482791825	0.629243556	0.670998701	negative
PRJNA729271	stage_1D_vs_E10	ordinary_muscle_development	ENSGALG00010024735	OTUD7A	2.899929109	0.177380027	16.34867891	4.44513E-60	1.23197E-58	positive
PRJNA729271	stage_1D_vs_E10	ordinary_muscle_development	ENSGALG00010024736	DGKA	-0.562108711	0.151319321	-3.714718701	0.00020343	0.00036494	negative
PRJNA729271	stage_1D_vs_E10	ordinary_muscle_development	ENSGALG00010024737	STK38	-1.012249719	0.062727756	-16.13719004	1.39761E-58	3.63053E-57	negative
PRJNA729271	stage_1D_vs_E10	ordinary_muscle_development	ENSGALG00010024738	NBR1	0.237785982	0.0781508	3.04265575	0.002345004	0.003740189	positive
PRJNA729271	stage_1D_vs_E10	ordinary_muscle_development	ENSGALG00010024739	ASIP	-8.079263551	0.622126557	-12.98652735	1.45898E-38	1.81372E-37	negative
PRJNA729271	stage_1D_vs_E10	ordinary_muscle_development	ENSGALG00010024741	RILPL2	-0.459410469	0.155490214	-2.954594094	0.003130807	0.004924667	negative
PRJNA729271	stage_1D_vs_E10	ordinary_muscle_development	ENSGALG00010024742	PAK2	-0.323582703	0.070007252	-4.622131201	3.79818E-06	8.06542E-06	negative
PRJNA729271	stage_1D_vs_E10	ordinary_muscle_development	ENSGALG00010024744	KLF13	3.819541384	0.127648844	29.92225592	1.0105E-196	4.5774E-194	positive
PRJNA729271	stage_1D_vs_E10	ordinary_muscle_development	ENSGALG00010024745	PMEL	-3.327116594	0.564969671	-5.889018058	3.88497E-09	1.04904E-08	negative
PRJNA729271	stage_1D_vs_E10	ordinary_muscle_development	ENSGALG00010024746	HOXD4	-0.476324077	0.28434741	-1.675148289	0.09390514	0.119946688	negative
PRJNA729271	stage_1D_vs_E10	ordinary_muscle_development	ENSGALG00010024747	TULP3	0.130647554	0.086182412	1.515942187	0.129533972	0.161569135	positive
PRJNA729271	stage_1D_vs_E10	ordinary_muscle_development	ENSGALG00010024748	TRPM1	1.313258372	0.384887551	3.412057286	0.000644746	0.001096	positive
PRJNA729271	stage_1D_vs_E10	ordinary_muscle_development	ENSGALG00010024749	APOA5	1.263647655	0.68965909	1.832278692	0.06690991	0.087477149	positive
PRJNA729271	stage_1D_vs_E10	ordinary_muscle_development	ENSGALG00010024750	APOA4	2.199466517	1.587458308	1.385527107	0.16589135	0.202850946	positive
PRJNA729271	stage_1D_vs_E10	ordinary_muscle_development	ENSGALG00010024751	MTMR10	0.443974799	0.056926572	7.79907837	6.23609E-15	2.48535E-14	positive
PRJNA729271	stage_1D_vs_E10	ordinary_muscle_development	ENSGALG00010024752	MPP2	-0.51331746	0.221387345	-2.31863958	0.020414587	0.028846699	negative
PRJNA729271	stage_1D_vs_E10	ordinary_muscle_development	ENSGALG00010024753	HOXD8	-0.779998998	0.172085576	-4.532622762	5.82558E-06	1.21586E-05	negative
PRJNA729271	stage_1D_vs_E10	ordinary_muscle_development	ENSGALG00010024754	EIF2S2	-0.364041489	0.144963868	-2.511256737	0.012030217	0.01760327	negative
PRJNA729271	stage_1D_vs_E10	ordinary_muscle_development	ENSGALG00010024755	PIGX	0.018212773	0.158205333	0.115121103	0.908349133	0.921960695	positive
PRJNA729271	stage_1D_vs_E10	ordinary_muscle_development	ENSGALG00010024756	CDK2	-1.515334434	0.164480366	-9.212859103	3.17552E-20	1.71815E-19	negative
PRJNA729271	stage_1D_vs_E10	ordinary_muscle_development	ENSGALG00010024757	PPFIA1	-0.001563516	0.074944005	-0.020862455	0.983355377	0.985599239	negative
PRJNA729271	stage_1D_vs_E10	ordinary_muscle_development	ENSGALG00010024758	SPATA6	-0.549108569	0.107207741	-5.121911564	3.02454E-07	7.02462E-07	negative
PRJNA729271	stage_1D_vs_E10	ordinary_muscle_development	ENSGALG00010024759	APOC3	4.045295056	0.959971868	4.213972504			positive
PRJNA729271	stage_1D_vs_E10	ordinary_muscle_development	ENSGALG00010024760	ENSGALG00010024760	3.175686135	0.182864754	17.3663107	1.4847E-67	4.99322E-66	positive
PRJNA729271	stage_1D_vs_E10	ordinary_muscle_development	ENSGALG00010024761	NECAB2	0.33511187	0.233972035	1.432273177	0.152065656	0.1872243	positive
PRJNA729271	stage_1D_vs_E10	ordinary_muscle_development	ENSGALG00010024762	FAN1	-0.286150911	0.08788352	-3.256024672	0.001129839	0.001873499	negative
PRJNA729271	stage_1D_vs_E10	ordinary_muscle_development	ENSGALG00010024763	HOXD9	-1.018023685	0.302696314	-3.363184943	0.000770487	0.001300898	negative
PRJNA729271	stage_1D_vs_E10	ordinary_muscle_development	ENSGALG00010024764	ENSGALG00010024764	0.567729832	0.25758552	2.204044049	0.027521246	0.038123133	positive
PRJNA729271	stage_1D_vs_E10	ordinary_muscle_development	ENSGALG00010024765	SNRNP35	-0.427650775	0.134093208	-3.189205335	0.001426645	0.002337956	negative
PRJNA729271	stage_1D_vs_E10	ordinary_muscle_development	ENSGALG00010024767	CTTN	-1.074328064	0.069282759	-15.50642727	3.13864E-54	6.99916E-53	negative
PRJNA729271	stage_1D_vs_E10	ordinary_muscle_development	ENSGALG00010024769	RALY	3.381901311	0.203912624	16.58505121	8.93895E-62	2.6123E-60	positive
PRJNA729271	stage_1D_vs_E10	ordinary_muscle_development	ENSGALG00010024770	CDC27	-0.372376375	0.07268872	-5.12289834	3.00887E-07	6.99159E-07	negative
PRJNA729271	stage_1D_vs_E10	ordinary_muscle_development	ENSGALG00010024771	AOX2	4.203567906	0.264350517	15.90149304	6.18724E-57	1.53306E-55	positive
PRJNA729271	stage_1D_vs_E10	ordinary_muscle_development	ENSGALG00010024772	ELAPOR1	-0.713716888	0.234569475	-3.042667368	0.002344914	0.003740189	negative
PRJNA729271	stage_1D_vs_E10	ordinary_muscle_development	ENSGALG00010024773	APOA1	8.278481855	0.264035982	31.35361246	8.6862E-216	5.2461E-213	positive
PRJNA729271	stage_1D_vs_E10	ordinary_muscle_development	ENSGALG00010024775	ITFG2	0.499707342	0.094015967	5.315132736	1.0658E-07	2.56965E-07	positive
PRJNA729271	stage_1D_vs_E10	ordinary_muscle_development	ENSGALG00010024776	MPHOSPH10	-0.444485068	0.10675309	-4.163674038	3.13167E-05	6.09392E-05	negative
PRJNA729271	stage_1D_vs_E10	ordinary_muscle_development	ENSGALG00010024778	RAB5B	0.278558215	0.139734271	1.993485303	0.046208331	0.06191438	positive
PRJNA729271	stage_1D_vs_E10	ordinary_muscle_development	ENSGALG00010024779	ENSGALG00010024779	-2.554714578	0.942326902	-2.711070407	0.006706638	0.010135828	negative
PRJNA729271	stage_1D_vs_E10	ordinary_muscle_development	ENSGALG00010024780	HOXD11	-2.340734768	0.777902135	-3.009035022	0.002620789	0.004159459	negative
PRJNA729271	stage_1D_vs_E10	ordinary_muscle_development	ENSGALG00010024781	CEP19	-1.481873521	0.172183686	-8.606352626	7.5422E-18	3.5844E-17	negative
PRJNA729271	stage_1D_vs_E10	ordinary_muscle_development	ENSGALG00010024782	HOXD12	-5.153711932	2.178365665	-2.365861717	0.017988162	0.025600236	negative

PRJNA729271	stage_1D_vs_E10	ordinary_muscle_development	ENSGALG00010024783	UBE2T	-1.835106763	0.235887667	-7.779579083	7.27662E-15	2.88893E-14	negative
PRJNA729271	stage_1D_vs_E10	ordinary_muscle_development	ENSGALG00010024784	ENSGALG00010024784	0.747138608	0.136938249	5.456025701	4.86909E-08	1.20481E-07	positive
PRJNA729271	stage_1D_vs_E10	ordinary_muscle_development	ENSGALG00010024785	MCEE	0.680370352	0.199743655	3.406217596	0.000658697	0.001118927	positive
PRJNA729271	stage_1D_vs_E10	ordinary_muscle_development	ENSGALG00010024786	PDCD5	-0.038193294	0.097637575	-0.391174132	0.695668528	0.732987956	negative
PRJNA729271	stage_1D_vs_E10	ordinary_muscle_development	ENSGALG00010024787	SARS1	0.100949875	0.099643739	1.013108064	0.311008547	0.358323574	positive
PRJNA729271	stage_1D_vs_E10	ordinary_muscle_development	ENSGALG00010024788	SIK3	0.902694527	0.16543819	5.456385417	4.85925E-08	1.20257E-07	positive
PRJNA729271	stage_1D_vs_E10	ordinary_muscle_development	ENSGALG00010024789	SUOX	0.764707307	0.17272777	4.427240087	9.54465E-06	1.95437E-05	positive
PRJNA729271	stage_1D_vs_E10	ordinary_muscle_development	ENSGALG00010024791	SHANK2	-1.569372552	0.382078345	-4.107462705	4.00029E-05	7.70657E-05	negative
PRJNA729271	stage_1D_vs_E10	ordinary_muscle_development	ENSGALG00010024792	BZW1	-0.53167618	0.068915921	-7.714852691	1.21122E-14	4.74117E-14	negative
PRJNA729271	stage_1D_vs_E10	ordinary_muscle_development	ENSGALG00010024793	FKBP4	-1.22200593	0.117118727	-10.43390722	1.736E-25	1.21856E-24	negative
PRJNA729271	stage_1D_vs_E10	ordinary_muscle_development	ENSGALG00010024794	DDX6	-0.397567232	0.064307456	-6.182288326	6.3179E-10	1.8102E-09	negative
PRJNA729271	stage_1D_vs_E10	ordinary_muscle_development	ENSGALG00010024795	NRROS	0.045303793	0.215749834	0.209982978	0.833680959	0.857400702	positive
PRJNA729271	stage_1D_vs_E10	ordinary_muscle_development	ENSGALG00010024797	IKZF4	1.245779386	0.22927047	5.433666984	5.52076E-08	1.36048E-07	positive
PRJNA729271	stage_1D_vs_E10	ordinary_muscle_development	ENSGALG00010024798	FBXO45	-0.083910369	0.084481523	-0.993239309	0.320593346	0.368663273	negative
PRJNA729271	stage_1D_vs_E10	ordinary_muscle_development	ENSGALG00010024799	ENSGALG00010024799	1.161634927	0.185417723	6.264961682	3.72917E-10	1.0863E-09	positive
PRJNA729271	stage_1D_vs_E10	ordinary_muscle_development	ENSGALG00010024800	ENSGALG00010024800	-0.060772918	0.130671938	-0.465080103	0.641874094	0.682208901	negative
PRJNA729271	stage_1D_vs_E10	ordinary_muscle_development	ENSGALG00010024801	CXCR5	0.780164828	1.212548435	0.643409208	0.51995861	0.567316526	positive
PRJNA729271	stage_1D_vs_E10	ordinary_muscle_development	ENSGALG00010024802	CD59	0.176809797	0.164180743	1.076921657	0.281515267	0.327677169	positive
PRJNA729271	stage_1D_vs_E10	ordinary_muscle_development	ENSGALG00010024803	CLK1	0.219716254	0.137105613	1.602532886	0.109037821	0.13768649	positive
PRJNA729271	stage_1D_vs_E10	ordinary_muscle_development	ENSGALG00010024804	WDR53	0.042176901	0.105105441	0.401281801	0.688212661	0.725803955	positive
PRJNA729271	stage_1D_vs_E10	ordinary_muscle_development	ENSGALG00010024805	ENSGALG00010024805	1.138453944	0.364877919	3.120095474	0.001807924	0.002927702	positive
PRJNA729271	stage_1D_vs_E10	ordinary_muscle_development	ENSGALG00010024806	ANKRD27	0.357019151	0.074017212	4.823461205	1.41088E-06	3.11369E-06	positive
PRJNA729271	stage_1D_vs_E10	ordinary_muscle_development	ENSGALG00010024807	PPIL3	-0.693586172	0.19333076	-3.587562425	0.000333784	0.000585809	negative
PRJNA729271	stage_1D_vs_E10	ordinary_muscle_development	ENSGALG00010024808	CELSR2	-0.792565016	0.24841357	-3.190506126	0.001420238	0.002328246	negative
PRJNA729271	stage_1D_vs_E10	ordinary_muscle_development	ENSGALG00010024809	KANSL1	0.482586518	0.170504306	2.830347983	0.00464974	0.007176106	positive
PRJNA729271	stage_1D_vs_E10	ordinary_muscle_development	ENSGALG00010024810	ADIPOR1	0.318484752	0.11362093	2.80304651	0.005062237	0.007779593	positive
PRJNA729271	stage_1D_vs_E10	ordinary_muscle_development	ENSGALG00010024811	NIF3L1	-1.372442408	0.142898727	-9.604301183	7.66763E-22	4.54384E-21	negative
PRJNA729271	stage_1D_vs_E10	ordinary_muscle_development	ENSGALG00010024812	SBNO1	0.27277958	0.111828571	2.439265534	0.01471715	0.021243287	positive
PRJNA729271	stage_1D_vs_E10	ordinary_muscle_development	ENSGALG00010024813	RNF168	-0.047440042	0.095149952	-0.498581877	0.618073975	0.660315095	negative
PRJNA729271	stage_1D_vs_E10	ordinary_muscle_development	ENSGALG00010024814	APBA2	-2.470061642	0.150528447	-16.4092681	1.64173E-60	4.57631E-59	negative
PRJNA729271	stage_1D_vs_E10	ordinary_muscle_development	ENSGALG00010024816	NFIA	0.113072986	0.12238376	0.923921485	0.355527196	0.404407652	positive
PRJNA729271	stage_1D_vs_E10	ordinary_muscle_development	ENSGALG00010024817	UCKL1	2.693922716	0.220178544	12.23517365	2.01673E-34	2.13376E-33	positive
PRJNA729271	stage_1D_vs_E10	ordinary_muscle_development	ENSGALG00010024818	FBXO3	-0.183934765	0.076565955	-2.402304862	0.016292124	0.023356181	negative
PRJNA729271	stage_1D_vs_E10	ordinary_muscle_development	ENSGALG00010024820	NUDT19	0.342913968	0.140283531	2.444434957	0.014507924	0.020962157	positive
PRJNA729271	stage_1D_vs_E10	ordinary_muscle_development	ENSGALG00010024821	NECAP1	0.067640961	0.105739803	0.639692518	0.52237252	0.569564441	positive
PRJNA729271	stage_1D_vs_E10	ordinary_muscle_development	ENSGALG00010024822	RPS26	0.105289274	0.137153136	0.767676754	0.44267925	0.491884686	positive
PRJNA729271	stage_1D_vs_E10	ordinary_muscle_development	ENSGALG00010024823	ASB10	8.232205547	0.871569037	9.445270764	3.54487E-21	2.02215E-20	positive
PRJNA729271	stage_1D_vs_E10	ordinary_muscle_development	ENSGALG00010024824	DNAJC5	-0.907054978	0.106856806	-8.488509206	2.09305E-17	9.70528E-17	negative
PRJNA729271	stage_1D_vs_E10	ordinary_muscle_development	ENSGALG00010024825	UBXN7	-0.201925691	0.07453925	-2.708984751	0.006748944	0.010193388	negative
PRJNA729271	stage_1D_vs_E10	ordinary_muscle_development	ENSGALG00010024826	RBM33	0.5839082	0.111314736	5.245560641	1.55808E-07	3.70843E-07	positive
PRJNA729271	stage_1D_vs_E10	ordinary_muscle_development	ENSGALG00010024827	ENSGALG00010024827	0.855803676	0.348167946	2.458019717	0.01397055	0.020226042	positive
PRJNA729271	stage_1D_vs_E10	ordinary_muscle_development	ENSGALG00010024828	TIMM17B	0.660992331	0.133091184	4.966462169	6.81853E-07	1.54307E-06	positive
PRJNA729271	stage_1D_vs_E10	ordinary_muscle_development	ENSGALG00010024829	ERBB3	0.220299693	0.200228891	1.100239288	0.271227877	0.316771257	positive
PRJNA729271	stage_1D_vs_E10	ordinary_muscle_development	ENSGALG00010024830	ORC2	-0.669506889	0.106071482	-6.31184628	2.75726E-10	8.10844E-10	negative
PRJNA729271	stage_1D_vs_E10	ordinary_muscle_development	ENSGALG00010024833	MAPT	1.427169741	0.202575759	7.045116094	1.85307E-12	6.2934E-12	positive
PRJNA729271	stage_1D_vs_E10	ordinary_muscle_development	ENSGALG00010024834	INSIG1	-0.771734805	0.26462923	-2.916287076	0.003542244	0.005542404	negative
PRJNA729271	stage_1D_vs_E10	ordinary_muscle_development	ENSGALG00010024835	ENTREP2	0.318945359	0.132090978	2.414588511	0.015752995	0.022630294	positive
PRJNA729271	stage_1D_vs_E10	ordinary_muscle_development	ENSGALG00010024836	TXNDC12	-0.751667054	0.106423292	-7.062993832	1.62953E-12	5.55243E-12	negative
PRJNA729271	stage_1D_vs_E10	ordinary_muscle_development	ENSGALG00010024837	TNFRSF1A	1.269233552	0.155817927	8.145619532	3.77345E-16	1.63321E-15	positive
PRJNA729271	stage_1D_vs_E10	ordinary_muscle_development	ENSGALG00010024838	EN2	-4.405296848	0.847201354	-5.199822722	1.99479E-07	4.70766E-07	negative
PRJNA729271	stage_1D_vs_E10	ordinary_muscle_development	ENSGALG00010024839	PA2G4	-0.870767811	0.099197587	-8.778114824	1.66238E-18	8.19875E-18	negative
PRJNA729271	stage_1D_vs_E10	ordinary_muscle_development	ENSGALG00010024840	BTF3L4	-0.603124949	0.101672627	-5.932028788	2.99214E-09	8.15093E-09	negative
PRJNA729271	stage_1D_vs_E10	ordinary_muscle_development	ENSGALG00010024841	ENSGALG00010024841	-1.784223428	0.207280888	-8.607756595	7.45042E-18	3.54311E-17	negative
PRJNA729271	stage_1D_vs_E10	ordinary_muscle_development	ENSGALG00010024842	ZFYVE9	0.098952482	0.075409197	1.312207087	0.189450271	0.228954618	positive
PRJNA729271	stage_1D_vs_E10	ordinary_muscle_development	ENSGALG00010024843	BCL9L	0.704223991	0.133031828	5.293650412	1.19899E-07	2.88214E-07	positive
PRJNA729271	stage_1D_vs_E10	ordinary_muscle_development	ENSGALG00010024844	TM4SF19	1.359012831	0.387853503	3.503933366	0.00045844	0.000792403	positive
PRJNA729271	stage_1D_vs_E10	ordinary_muscle_development	ENSGALG00010024845	HYCC2	0.969370002	0.108278723	8.952543685	3.47383E-19	1.77425E-18	positive
PRJNA729271	stage_1D_vs_E10	ordinary_muscle_development	ENSGALG00010024846	LMO2	0.272671287	0.143056824	1.906034807	0.05664569	0.074875002	positive
PRJNA729271	stage_1D_vs_E10	ordinary_muscle_development	ENSGALG00010024847	PPIL1	-0.479416983	0.150428105	-3.187017364	0.001437481	0.002354383	negative
PRJNA729271	stage_1D_vs_E10	ordinary_muscle_development	ENSGALG00010024848	ESYT1	-0.078434519	0.085779136	-0.914377584	0.360518476	0.409506724	negative
PRJNA729271	stage_1D_vs_E10	ordinary_muscle_development	ENSGALG00010024849	CENATAC	0.207640675	0.19180156	1.082580742	0.278994559	0.325030231	positive
PRJNA729271	stage_1D_vs_E10	ordinary_muscle_development	ENSGALG00010024850	CDK2AP1	-0.159458264	0.116661885	-1.366841139	0.171675105	0.209182132	negative
PRJNA729271	stage_1D_vs_E10	ordinary_muscle_development	ENSGALG00010024851	CPNE5	-3.647540339	0.897952193	-4.062065183	4.86405E-05	9.30628E-05	negative
PRJNA729271	stage_1D_vs_E10	ordinary_muscle_development	ENSGALG00010024852	CEP89	-0.490987042	0.091932323	-5.340744441	9.25657E-08	2.24071E-07	negative
PRJNA729271	stage_1D_vs_E10	ordinary_muscle_development	ENSGALG00010024853	TJP1	0.640218548	0.067144454	9.534943104	1.49968E-21	8.73707E-21	positive
PRJNA729271	stage_1D_vs_E10	ordinary_muscle_development	ENSGALG00010024854	C12orf65	0.546875958	0.150662564	3.62980653	0.000283634	0.000501188	positive
PRJNA729271	stage_1D_vs_E10	ordinary_muscle_development	ENSGALG00010024855	CAPRIN1	-0.578356599	0.071859553	-8.048430197	8.38627E-16	3.54193E-15	negative
PRJNA729271	stage_1D_vs_E10	ordinary_muscle_development	ENSGALG00010024856	SORT1	1.91869404	0.132732387	14.45535705	2.31924E-47	3.98783E-46	positive
PRJNA729271	stage_1D_vs_E10	ordinary_muscle_development	ENSGALG00010024857	SLC37A4	0.723452101	0.141614014	5.108619396	3.24521E-07	7.51908E-07	positive
PRJNA729271	stage_1D_vs_E10	ordinary_muscle_development	ENSGALG00010024858	SLC25A39	-0.614624387	0.123104793	-4.992692593	5.95433E-07	1.35279E-06	negative
PRJNA729271	stage_1D_vs_E10	ordinary_muscle_development	ENSGALG00010024859	TARSL2	-0.178803737	0.103469535	-1.728081005	0.083973702	0.108147385	negative
PRJNA729271	stage_1D_vs_E10	ordinary_muscle_development	ENSGALG00010024860	NEU4	-0.978423463	0.432976656	-2.259760311	0.023836131	0.033382098	negative
PRJNA729271	stage_1D_vs_E10	ordinary_muscle_development	ENSGALG00010024861	ORC1	0.011623068	0.063063592	0.184307099	0.853772545	0.875083654	positive
PRJNA729271	stage_1D_vs_E10	ordinary_muscle_development	ENSGALG00010024862	METTL16	0.729222028	0.118961297	6.129909878	8.79289E-10	2.49761E-09	positive
PRJNA729271	stage_1D_vs_E10	ordinary_muscle_development	ENSGALG00010024863	LMOD1	1.766905548	0.287356229	6.14883329	7.8055E-10	2.2228E-09	positive
PRJNA729271	stage_1D_vs_E10	ordinary_muscle_development	ENSGALG00010024864	MPHOSPH9	-0.445582617	0.127801719	-3.486515045	0.000489358	0.00084343	negative
PRJNA729271	stage_1D_vs_E10	ordinary_muscle_development	ENSGALG00010024865	SNAP47	0.571950812	0.142819195	4.004719488	6.20911E-05	0.00011771	positive
PRJNA729271	stage_1D_vs_E10	ordinary_muscle_development	ENSGALG00010024866	TRAPPC4	0.114096935	0.085652326	1.33209384	0.182829354	0.221710992	positive
PRJNA729271	stage_1D_vs_E10	ordinary_muscle_development	ENSGALG00010024867	TM2D3	0.351793597	0.105126127	3.346395486	0.000818695	0.00137892	positive
PRJNA729271	stage_1D_vs_E10	ordinary_muscle_development	ENSGALG00010024868	FAAP24	-1.474169802	0.202435059	-7.282186231	3.28453E-13	1.17641E-12	negative
PRJNA729271	stage_1D_vs_E10	ordinary_muscle_development	ENSGALG00010024869	NDUFB3	1.439908176	0.121688302	11.83275753	2.64313E-32	2.54736E-31	positive
PRJNA729271	stage_1D_vs_E10	ordinary_muscle_development	ENSGALG00010024870	TPD52L2	-0.082340783	0.082810678	-0.994325672	0.320064341	0.368113356	negative
PRJNA729271	stage_1D_vs_E10	ordinary_muscle_development	ENSGALG00010024871	POLQ	-0.37079486	0.131133244	-2.827619069	0.004689557	0.007232936	negative
PRJNA729271	stage_1D_vs_E10	ordinary_muscle_development	ENSGALG00010024872	RUNDC3A	-1.619232184	0.211262867	-7.66453758	1.79477E-14	6.95592E-14	negative
PRJNA729271	stage_1D_vs_E10	ordinary_muscle_development	ENSGALG00010024873	HYOU1	-0.798478058	0.108545595	-7.35615347	1.89285E-13	6.86953E-13	negative
PRJNA729271	stage_1D_vs_E10	ordinary_muscle_development	ENSGALG00010024874	JMJD4	-0.87160261	0.083070381	-10.49233917	9.368E-26	6.63033E-25	negative
PRJNA729271	stage_1D_vs_E10	ordinary_muscle_development	ENSGALG00010024876	CFLAR	0.341426076	0.094891139	3.598081742	0.000320573	0.000563442	positive
PRJNA729271	stage_1D_vs_E10	ordinary_muscle_development	ENSGALG00010024877	PRPF38A	-0.756277701	0.079093987	-9.56175973	1.15771E-21	6.78296E-21	negative
PRJNA729271	stage_1D_vs_E10	ordinary_muscle_development	ENSGALG00010024878	RHPN2	-1.830740885	0.276914396	-6.611215994	3.81176E-11	1.18795E-10	negative
PRJNA729271	stage_1D_vs_E10	ordinary_muscle_development	ENSGALG00010024879	D2HGDH	0.308461127	0.149633822	2.061439872	0.039261094	0.05318594	positive
PRJNA729271	stage_1D_vs_E10	ordinary_muscle_development	ENSGALG00010024880	NAT10	-0.816672506	0.137923541	-5.92119737	3.19606E-09	8.68683E-09	negative
PRJNA729271	stage_1D_vs_E10	ordinary_muscle_development	ENSGALG00010024881	CASP10	-0.102165527	0.291982139	-0.349903346	0.726411236	0.760569984	negative
PRJNA729271	stage_1D_vs_E10	ordinary_muscle_development	ENSGALG00010024882	MYL6	-1.419886121	0.164615031	-8.625494973	6.3816E-18	3.04682E-17	negative
PRJNA729271	stage_1D_vs_E10	ordinary_muscle_development	ENSGALG00010024883	ING5	0.801804251	0.123641673	6.484902943	8.87889E-11	2.69697E-10	positive
PRJNA729271	stage_1D_vs_E10	ordinary_muscle_development	ENSGALG00010024884	ZBTB46	0.914750119	0.133287727	6.862973378	6.74418E-12	2.20521E-11	positive
PRJNA729271	stage_1D_vs_E10	ordinary_muscle_development	ENSGALG00010024885	SLC4A1	-0.231057857	0.233896581	-0.987863338	0.323219583	0.371418095	negative
PRJNA729271	stage_1D_vs_E10	ordinary_muscle_development	ENSGALG00010024886	ABTB2	-1.611735814	0.140317541	-11.48634589	1.5451E-30	1.374E-29	negative
PRJNA729271	stage_1D_vs_E10	ordinary_muscle_development	ENSGALG00010024887	CASP18	0.607900839	0.259404113	2.343451041	0.01910627	0.027103678	positive
PRJNA729271	stage_1D_vs_E10	ordinary_muscle_development	ENSGALG00010024888	PITPNM2	0.42814758	0.126782239	3.377031218	0.000732727	0.001240178	positive
PRJNA729271	stage_1D_vs_E10	ordinary_muscle_development	ENSGALG00010024889	SLC2A4RG	-0.010508314	0.47809443	-0.021979578	0.982464246	0.984774169	negative
PRJNA729271	stage_1D_vs_E10	ordinary_muscle_development	ENSGALG00010024890	SMARCC2	0.570520287	0.144982333	3.935102115	8.31613E-05	0.000155539	positive
PRJNA729271	stage_1D_vs_E10	ordinary_muscle_development	ENSGALG00010024891	PKP1	-1.404274654	0.46522718	-3.018470793	0.002540539	0.004035629	negative
PRJNA729271	stage_1D_vs_E10	ordinary_muscle_development	ENSGALG00010024893	ARL6IP4	-0.05328729	0.139077135	-0.383149177	0.701609158	0.738442111	negative
PRJNA729271	stage_1D_vs_E10	ordinary_muscle_development	ENSGALG00010024894	TUT4	0.743353696	0.121906272	6.097747741	1.07573E-09	3.03184E-09	positive
PRJNA729271	stage_1D_vs_E10	ordinary_muscle_development	ENSGALG00010024895	ENSGALG00010024895	1.272776565	0.403251322	3.156286158	0.00159792	0.002603333	positive
PRJNA729271	stage_1D_vs_E10	ordinary_muscle_development	ENSGALG00010024896	RNF41	-0.038251198	0.101371321	-0.377337473	0.705922828	0.742227723	negative
PRJNA729271	stage_1D_vs_E10	ordinary_muscle_development	ENSGALG00010024897	ADAL	-0.486915541	0.192891646	-2.524295639	0.011593039	0.016994448	negative
PRJNA729271	stage_1D_vs_E10	ordinary_muscle_development	ENSGALG00010024898	NABP2	-0.622266818	0.314220527	-1.980350626	0.047664144	0.063753046	negative
PRJNA729271	stage_1D_vs_E10	ordinary_muscle_development	ENSGALG00010024899	OGFOD2	-0.055497797	0.122916334	-0.451508727	0.651622937	0.691430649	negative
PRJNA729271	stage_1D_vs_E10	ordinary_muscle_development	ENSGALG00010024900	DTYMK	-1.11297459	0.102370856	-10.8719867	1.5675E-27	1.19836E-26	negative
PRJNA729271	stage_1D_vs_E10	ordinary_muscle_development	ENSGALG00010024901	ZGPAT	-0.451979245	0.130894144	-3.453013489	0.000554361	0.000949931	negative
PRJNA729271	stage_1D_vs_E10	ordinary_muscle_development	ENSGALG00010024902	LARP6	1.224798604	0.115046012	10.64616308	1.81701E-26	1.32951E-25	positive
PRJNA729271	stage_1D_vs_E10	ordinary_muscle_development	ENSGALG00010024903	CASP8	-1.042879591	0.112879122	-9.238905946	2.4903E-20	1.35093E-19	negative
PRJNA729271	stage_1D_vs_E10	ordinary_muscle_development	ENSGALG00010024904	ALS2CL	1.123416963	0.159920279	7.024856185	2.14287E-12	7.25383E-12	positive
PRJNA729271	stage_1D_vs_E10	ordinary_muscle_development	ENSGALG00010024905	GPX7	-1.200263578	0.208242347	-5.763782431	8.22494E-09	2.16489E-08	negative
PRJNA729271	stage_1D_vs_E10	ordinary_muscle_development	ENSGALG00010024907	PHLDB1	0.866325868	0.132948842	6.516234801	7.20941E-11	2.20233E-10	positive
PRJNA729271	stage_1D_vs_E10	ordinary_muscle_development	ENSGALG00010024908	ENSGALG00010024908	0.517036673	0.12678551	4.078042292	4.54165E-05	8.7159E-05	positive
PRJNA729271	stage_1D_vs_E10	ordinary_muscle_development	ENSGALG00010024910	UBTF	-0.605390586	0.123476274	-4.902889977	9.44369E-07	2.11473E-06	negative
PRJNA729271	stage_1D_vs_E10	ordinary_muscle_development	ENSGALG00010024911	CAT	1.08232526	0.153183778	7.065534445	1.59999E-12	5.45434E-12	positive
PRJNA729271	stage_1D_vs_E10	ordinary_muscle_development	ENSGALG00010024915	STXBP5L	-0.583375954	0.174546883	-3.34223072	0.000831079	0.001398154	negative
PRJNA729271	stage_1D_vs_E10	ordinary_muscle_development	ENSGALG00010024917	TRAK2	1.151392708	0.121893394	9.445899145	3.52366E-21	2.01084E-20	positive
PRJNA729271	stage_1D_vs_E10	ordinary_muscle_development	ENSGALG00010024918	ATG4B	-0.550421375	0.07503925	-7.335112989	2.21535E-13	8.00584E-13	negative
PRJNA729271	stage_1D_vs_E10	ordinary_muscle_development	ENSGALG00010024919	ANKRD52	-0.09594673	0.122624549	-0.782443083	0.433954223	0.483487045	negative
PRJNA729271	stage_1D_vs_E10	ordinary_muscle_development	ENSGALG00010024920	VIPR2	1.151574432	0.298547449	3.85725765	0.000114666	0.000211677	positive
PRJNA729271	stage_1D_vs_E10	ordinary_muscle_development	ENSGALG00010024921	STRADB	-0.800407918	0.109944649	-7.280098917	3.33575E-13	1.19358E-12	negative
PRJNA729271	stage_1D_vs_E10	ordinary_muscle_development	ENSGALG00010024924	MNX1	-2.872378897	0.591909918	-4.8527298	1.21774E-06	2.70059E-06	negative
PRJNA729271	stage_1D_vs_E10	ordinary_muscle_development	ENSGALG00010024925	GTF2E1	-0.577189502	0.083160701	-6.940652192	3.90294E-12	1.29785E-11	negative
PRJNA729271	stage_1D_vs_E10	ordinary_muscle_development	ENSGALG00010024926	ELF5	-4.497101605	0.758746396	-5.92701544	3.0849E-09	8.39257E-09	negative
PRJNA729271	stage_1D_vs_E10	ordinary_muscle_development	ENSGALG00010024927	NOM1	-0.152669331	0.11228717	-1.359632911	0.173946118	0.211717943	negative
PRJNA729271	stage_1D_vs_E10	ordinary_muscle_development	ENSGALG00010024928	THAP4	0.563918213	0.085312208	6.610052943	3.84183E-11	1.19706E-10	positive
PRJNA729271	stage_1D_vs_E10	ordinary_muscle_development	ENSGALG00010024930	TMEM237	0.35932266	0.099138376	3.624455769	0.000289571	0.000511121	positive

PRJNA729271	stage_1D_vs_E10	ordinary_muscle_development	ENSGALG00010024931	UBE3C	0.303435427	0.088530246	3.427477501	0.000609217	0.001038039	positive
PRJNA729271	stage_1D_vs_E10	ordinary_muscle_development	ENSGALG00010024932	ANKK1	-1.56554929	1.757202222	-0.89093291	0.372965163	0.422156024	negative
PRJNA729271	stage_1D_vs_E10	ordinary_muscle_development	ENSGALG00010024933	CS	1.729139086	0.156627937	11.03978712	2.45614E-28	1.954E-27	positive
PRJNA729271	stage_1D_vs_E10	ordinary_muscle_development	ENSGALG00010024934	G6PC3	-0.321008132	0.225303658	-1.424779936	0.154220824	0.189636142	negative
PRJNA729271	stage_1D_vs_E10	ordinary_muscle_development	ENSGALG00010024935	KLHL30	4.279140059	0.387490832	11.04320336	2.3645E-28	1.88523E-27	positive
PRJNA729271	stage_1D_vs_E10	ordinary_muscle_development	ENSGALG00010024936	EHF	0.414453541	0.525500778	0.788683021	0.430297294	0.479818392	positive
PRJNA729271	stage_1D_vs_E10	ordinary_muscle_development	ENSGALG00010024937	ILKAP	-0.868457244	0.126175651	-6.882922634	5.86369E-12	1.92345E-11	negative
PRJNA729271	stage_1D_vs_E10	ordinary_muscle_development	ENSGALG00010024938	ENSGALG00010024938	-0.012256814	0.178891158	-0.068515484	0.945375294	0.953598809	negative
PRJNA729271	stage_1D_vs_E10	ordinary_muscle_development	ENSGALG00010024939	COQ10A	2.060721606	0.202875563	10.15756444	3.06641E-24	2.01943E-23	positive
PRJNA729271	stage_1D_vs_E10	ordinary_muscle_development	ENSGALG00010024940	RABL3	-0.270516927	0.090955787	-2.974158504	0.002937933	0.00463741	negative
PRJNA729271	stage_1D_vs_E10	ordinary_muscle_development	ENSGALG00010024941	PDHX	0.734963557	0.117871992	6.235268799	4.51003E-10	1.30537E-09	positive
PRJNA729271	stage_1D_vs_E10	ordinary_muscle_development	ENSGALG00010024942	DNAJB6	0.365791739	0.065839331	5.555824081	2.76305E-08	6.95148E-08	positive
PRJNA729271	stage_1D_vs_E10	ordinary_muscle_development	ENSGALG00010024943	ABCB9	0.166146156	0.177049723	0.938415228	0.348031063	0.396877528	positive
PRJNA729271	stage_1D_vs_E10	ordinary_muscle_development	ENSGALG00010024944	ENSGALG00010024944	0.027383048	0.112074008	0.24433005	0.806975212	0.833305243	positive
PRJNA729271	stage_1D_vs_E10	ordinary_muscle_development	ENSGALG00010024945	APIP	0.081055122	0.081700012	0.992106601	0.321145527	0.369239661	positive
PRJNA729271	stage_1D_vs_E10	ordinary_muscle_development	ENSGALG00010024946	KIF1A	-2.227847798	0.19220311	-11.59111209	4.57158E-31	4.15195E-30	negative
PRJNA729271	stage_1D_vs_E10	ordinary_muscle_development	ENSGALG00010024947	MPP4	1.287353743	0.311128356	4.137693395	3.50815E-05	6.79457E-05	positive
PRJNA729271	stage_1D_vs_E10	ordinary_muscle_development	ENSGALG00010024949	PAN2	-0.319487147	0.114086626	-2.800390878	0.005104076	0.007839735	negative
PRJNA729271	stage_1D_vs_E10	ordinary_muscle_development	ENSGALG00010024950	NCAM1	0.87989431	0.154548746	5.693312527	1.24598E-08	3.22797E-08	positive
PRJNA729271	stage_1D_vs_E10	ordinary_muscle_development	ENSGALG00010024953	NCAPG2	-1.067796186	0.218691578	-4.882658014	1.04665E-06	2.33583E-06	negative
PRJNA729271	stage_1D_vs_E10	ordinary_muscle_development	ENSGALG00010024956	VPS37B	-0.26819586	0.13843533	-1.937336807	0.052704186	0.070016238	negative
PRJNA729271	stage_1D_vs_E10	ordinary_muscle_development	ENSGALG00010024960	ARFRP1	-0.488034529	0.100173609	-4.871887236	1.10537E-06	2.46006E-06	negative
PRJNA729271	stage_1D_vs_E10	ordinary_muscle_development	ENSGALG00010024961	LSM12	0.327902864	0.203313623	1.61279337	0.106789407	0.135070895	positive
PRJNA729271	stage_1D_vs_E10	ordinary_muscle_development	ENSGALG00010024963	TNFRSF6B	2.827226434	0.572133869	4.941547053	7.75051E-07	1.74609E-06	positive
PRJNA729271	stage_1D_vs_E10	ordinary_muscle_development	ENSGALG00010024964	ITM2C	-0.723445225	0.170050557	-4.25429494	2.09709E-05	4.14923E-05	negative
PRJNA729271	stage_1D_vs_E10	ordinary_muscle_development	ENSGALG00010024965	STAT2	0.160264074	0.220075894	0.728221848	0.466477794	0.515208444	positive
PRJNA729271	stage_1D_vs_E10	ordinary_muscle_development	ENSGALG00010024966	AGAP3	-0.285000037	0.114649525	-2.485837046	0.012924713	0.018813388	negative
PRJNA729271	stage_1D_vs_E10	ordinary_muscle_development	ENSGALG00010024968	TMEM101	0.693988126	0.131963029	5.258958744	1.44873E-07	3.45783E-07	positive
PRJNA729271	stage_1D_vs_E10	ordinary_muscle_development	ENSGALG00010024970	CAB39	0.149194062	0.077784844	1.918035095	0.055106561	0.072986989	positive
PRJNA729271	stage_1D_vs_E10	ordinary_muscle_development	ENSGALG00010024973	RTEL1	-1.187942282	0.083083271	-14.2982127	2.24501E-46	3.7021E-45	negative
PRJNA729271	stage_1D_vs_E10	ordinary_muscle_development	ENSGALG00010024974	CD44	-1.636397762	0.168403082	-9.717148523	2.54817E-22	1.54414E-21	negative
PRJNA729271	stage_1D_vs_E10	ordinary_muscle_development	ENSGALG00010024977	LRP3	1.323179755	0.104135644	12.70630983	5.45484E-37	6.40744E-36	positive
PRJNA729271	stage_1D_vs_E10	ordinary_muscle_development	ENSGALG00010024983	APOF	2.622618093	0.481992114	5.441205402	5.29213E-08	1.30502E-07	positive
PRJNA729271	stage_1D_vs_E10	ordinary_muscle_development	ENSGALG00010024984	SHISAL2A	0.872295587	0.451498	1.932003215	0.053359109	0.070808412	positive
PRJNA729271	stage_1D_vs_E10	ordinary_muscle_development	ENSGALG00010024986	PTS	-1.202794439	0.168986208	-7.117707745	1.09737E-12	3.77733E-12	negative
PRJNA729271	stage_1D_vs_E10	ordinary_muscle_development	ENSGALG00010024989	TIMELESS	-0.910995697	0.157253595	-5.793162928	6.9073E-09	1.83004E-08	negative
PRJNA729271	stage_1D_vs_E10	ordinary_muscle_development	ENSGALG00010024994	NDUFB4	0.935726928	0.104022234	8.995451174	2.35264E-19	1.21271E-18	positive
PRJNA729271	stage_1D_vs_E10	ordinary_muscle_development	ENSGALG00010024996	COA7	-0.207451433	0.124918096	-1.660699616	0.096773791	0.123371688	negative
PRJNA729271	stage_1D_vs_E10	ordinary_muscle_development	ENSGALG00010024997	HES6	0.666899957	0.242526234	2.749805432	0.005963066	0.009079269	positive
PRJNA729271	stage_1D_vs_E10	ordinary_muscle_development	ENSGALG00010025003	ENSGALG00010025003	4.844070516	0.244390157	19.82105403	1.95961E-87	1.10524E-85	positive
PRJNA729271	stage_1D_vs_E10	ordinary_muscle_development	ENSGALG00010025005	HIP1R	-0.430620195	0.122039737	-3.528524439	0.000417883	0.000725589	negative
PRJNA729271	stage_1D_vs_E10	ordinary_muscle_development	ENSGALG00010025006	ENSGALG00010025006	1.174351917	0.265300022	4.426505172	9.57721E-06	1.95965E-05	positive
PRJNA729271	stage_1D_vs_E10	ordinary_muscle_development	ENSGALG00010025007	ZYG11B	0.251612582	0.101379212	2.481895217	0.01306857	0.019013241	positive
PRJNA729271	stage_1D_vs_E10	ordinary_muscle_development	ENSGALG00010025008	ENSGALG00010025008	-1.745337561	0.603325283	-2.892863289	0.003817474	0.005949284	negative
PRJNA729271	stage_1D_vs_E10	ordinary_muscle_development	ENSGALG00010025012	ENSGALG00010025012	-1.180929446	1.101675326	-1.071939635	0.283747147	0.329930602	negative
PRJNA729271	stage_1D_vs_E10	ordinary_muscle_development	ENSGALG00010025026	DENR	-0.556600506	0.116605437	-4.773366647	1.81172E-06	3.95792E-06	negative
PRJNA729271	stage_1D_vs_E10	ordinary_muscle_development	ENSGALG00010025028	HES6	-0.993029424	0.134340763	-7.391869758	1.44778E-13	5.29405E-13	negative
PRJNA729271	stage_1D_vs_E10	ordinary_muscle_development	ENSGALG00010025031	ECHDC2	0.774540494	0.120432935	6.431301331	1.26516E-10	3.80546E-10	positive
PRJNA729271	stage_1D_vs_E10	ordinary_muscle_development	ENSGALG00010025032	FSTL1	-0.725915255	0.181257518	-4.00488357	6.2048E-05	0.000117644	negative
PRJNA729271	stage_1D_vs_E10	ordinary_muscle_development	ENSGALG00010025039	CEBPA	-0.311442067	0.309112711	-1.00753562	0.313677449	0.361197539	negative
PRJNA729271	stage_1D_vs_E10	ordinary_muscle_development	ENSGALG00010025042	BCO2	-3.541599813	0.837301281	-4.229779525	2.3392E-05	4.6044E-05	negative
PRJNA729271	stage_1D_vs_E10	ordinary_muscle_development	ENSGALG00010025044	TMEM9	0.165548012	0.215741909	0.767342852	0.442877697	0.49206747	positive
PRJNA729271	stage_1D_vs_E10	ordinary_muscle_development	ENSGALG00010025047	PER2	0.613061931	0.293046097	2.092032405	0.036435617	0.049650679	positive
PRJNA729271	stage_1D_vs_E10	ordinary_muscle_development	ENSGALG00010025048	ESYT2	1.213100822	0.081924719	14.80750669	1.3101E-49	2.49868E-48	positive
PRJNA729271	stage_1D_vs_E10	ordinary_muscle_development	ENSGALG00010025055	ALS2	0.531526743	0.154262363	3.445602244	0.000569788	0.000973719	positive
PRJNA729271	stage_1D_vs_E10	ordinary_muscle_development	ENSGALG00010025056	RBMS2	0.976464756	0.163531037	5.971128025	2.35619E-09	6.47326E-09	positive
PRJNA729271	stage_1D_vs_E10	ordinary_muscle_development	ENSGALG00010025057	SCP2	-0.134910237	0.066753069	-2.02103421	0.043276224	0.058292804	negative
PRJNA729271	stage_1D_vs_E10	ordinary_muscle_development	ENSGALG00010025060	GPR156	-0.197402782	0.167969729	-1.175228317	0.239903384	0.283660947	negative
PRJNA729271	stage_1D_vs_E10	ordinary_muscle_development	ENSGALG00010025064	GPN3	-0.153638779	0.08295953	-1.851972634	0.064029753	0.083999572	negative
PRJNA729271	stage_1D_vs_E10	ordinary_muscle_development	ENSGALG00010025067	SLC1A2	-1.159290204	0.340496974	-3.404700458	0.000662367	0.001124634	negative
PRJNA729271	stage_1D_vs_E10	ordinary_muscle_development	ENSGALG00010025069	PODN	3.181009402	0.197154479	16.13460375	1.4574E-58	3.77907E-57	positive
PRJNA729271	stage_1D_vs_E10	ordinary_muscle_development	ENSGALG00010025071	CDK15	-0.266812606	0.333080005	-0.801046602	0.423104667	0.472743556	negative
PRJNA729271	stage_1D_vs_E10	ordinary_muscle_development	ENSGALG00010025072	ENSGALG00010025072	-1.777062195	0.799116913	-2.223782486	0.026163082	0.036373861	negative
PRJNA729271	stage_1D_vs_E10	ordinary_muscle_development	ENSGALG00010025073	IL18	1.019921485	0.200342927	5.090878423	3.56409E-07	8.22765E-07	positive
PRJNA729271	stage_1D_vs_E10	ordinary_muscle_development	ENSGALG00010025080	BAZ2A	1.55256082	0.202014617	7.685388532	1.52533E-14	5.93072E-14	positive
PRJNA729271	stage_1D_vs_E10	ordinary_muscle_development	ENSGALG00010025092	COL1A1	3.178256796	0.220962198	14.38371282	6.54851E-47	1.10373E-45	positive
PRJNA729271	stage_1D_vs_E10	ordinary_muscle_development	ENSGALG00010025097	LMBR1	0.11179047	0.12104401	0.92355227	0.355719474	0.40456287	positive
PRJNA729271	stage_1D_vs_E10	ordinary_muscle_development	ENSGALG00010025098	ATP5B	1.490742157	0.138186708	10.78788388	3.92728E-27	2.95873E-26	positive
PRJNA729271	stage_1D_vs_E10	ordinary_muscle_development	ENSGALG00010025102	ARPC3	-0.989232703	0.086278496	-11.46557659	1.96447E-30	1.74052E-29	negative
PRJNA729271	stage_1D_vs_E10	ordinary_muscle_development	ENSGALG00010025103	GSK3B	0.190322434	0.099877436	1.905559876	0.056707332	0.074942813	positive
PRJNA729271	stage_1D_vs_E10	ordinary_muscle_development	ENSGALG00010025113	SDHD	0.882695517	0.120100518	7.349639542	1.98742E-13	7.20552E-13	positive
PRJNA729271	stage_1D_vs_E10	ordinary_muscle_development	ENSGALG00010025114	CEBPG	0.387939782	0.097042858	3.997612896	6.39845E-05	0.000121141	positive
PRJNA729271	stage_1D_vs_E10	ordinary_muscle_development	ENSGALG00010025115	MGAT4F	-0.927535745	0.263306415	-3.522647727	0.000427259	0.000740715	negative
PRJNA729271	stage_1D_vs_E10	ordinary_muscle_development	ENSGALG00010025116	ANAPC7	-0.130530264	0.069320162	-1.883005758	0.059699588	0.078617745	negative
PRJNA729271	stage_1D_vs_E10	ordinary_muscle_development	ENSGALG00010025121	DYNC2I1	-0.638341618	0.154730913	-4.12549506	3.69938E-05	7.15062E-05	negative
PRJNA729271	stage_1D_vs_E10	ordinary_muscle_development	ENSGALG00010025125	PIK3CB	0.733311256	0.131252553	5.58702469	2.30993E-08	5.85664E-08	positive
PRJNA729271	stage_1D_vs_E10	ordinary_muscle_development	ENSGALG00010025126	ENSGALG00010025126	-0.5447287	0.133764066	-4.072309673	4.65492E-05	8.9262E-05	negative
PRJNA729271	stage_1D_vs_E10	ordinary_muscle_development	ENSGALG00010025127	PAMR1	0.554054095	0.155615718	3.560399302	0.000370291	0.000646827	positive
PRJNA729271	stage_1D_vs_E10	ordinary_muscle_development	ENSGALG00010025130	ATP2A2	-0.87653721	0.125302197	-6.99538579	2.6453E-12	8.90263E-12	negative
PRJNA729271	stage_1D_vs_E10	ordinary_muscle_development	ENSGALG00010025131	SUMO1	-0.415755442	0.080868894	-5.141104586	2.73128E-07	6.36903E-07	negative
PRJNA729271	stage_1D_vs_E10	ordinary_muscle_development	ENSGALG00010025138	CFAP91	-1.597264435	0.294281518	-5.427674981	5.70929E-08	1.40527E-07	negative
PRJNA729271	stage_1D_vs_E10	ordinary_muscle_development	ENSGALG00010025139	COPB2	-0.834140187	0.046861742	-17.8000252	7.06371E-71	2.68033E-69	negative
PRJNA729271	stage_1D_vs_E10	ordinary_muscle_development	ENSGALG00010025144	PIH1D2	-0.400820108	0.161535452	-2.481313566	0.013089917	0.019038565	negative
PRJNA729271	stage_1D_vs_E10	ordinary_muscle_development	ENSGALG00010025145	CPT2	0.021989719	0.094294422	0.233202751	0.815603969	0.841077087	positive
PRJNA729271	stage_1D_vs_E10	ordinary_muscle_development	ENSGALG00010025147	COX17	1.415063924	0.113719498	12.44345914	1.51785E-35	1.68206E-34	positive
PRJNA729271	stage_1D_vs_E10	ordinary_muscle_development	ENSGALG00010025149	IFT81	-1.173017331	0.141730046	-8.276419629	1.26934E-16	5.62836E-16	negative
PRJNA729271	stage_1D_vs_E10	ordinary_muscle_development	ENSGALG00010025154	TMEM183A	0.306217361	0.122078709	2.508360081	0.012129299	0.017739299	positive
PRJNA729271	stage_1D_vs_E10	ordinary_muscle_development	ENSGALG00010025155	CZIB	0.454919789	0.090939824	5.002426563	5.66132E-07	1.28885E-06	positive
PRJNA729271	stage_1D_vs_E10	ordinary_muscle_development	ENSGALG00010025156	DLAT	1.496970396	0.114992279	13.01800792	9.66598E-39	1.21306E-37	positive
PRJNA729271	stage_1D_vs_E10	ordinary_muscle_development	ENSGALG00010025157	NOP58	-0.868302466	0.140063579	-6.199345132	5.66986E-10	1.63E-09	negative
PRJNA729271	stage_1D_vs_E10	ordinary_muscle_development	ENSGALG00010025160	MRPS22	0.553186357	0.130120139	4.251350794	2.12485E-05	4.20072E-05	positive
PRJNA729271	stage_1D_vs_E10	ordinary_muscle_development	ENSGALG00010025161	ENSGALG00010025161	-0.907112627	0.104514362	-8.679310742	3.98174E-18	1.92065E-17	negative
PRJNA729271	stage_1D_vs_E10	ordinary_muscle_development	ENSGALG00010025163	LDLRAD3	-0.940826975	0.134621112	-6.988703023	2.77439E-12	9.31976E-12	negative
PRJNA729271	stage_1D_vs_E10	ordinary_muscle_development	ENSGALG00010025164	C11orf52	1.943109218	0.123651074	15.7144548	1.20413E-55	2.84728E-54	positive
PRJNA729271	stage_1D_vs_E10	ordinary_muscle_development	ENSGALG00010025167	PTGES3	-0.56986688	0.188622263	-3.021206889	0.002517693	0.004000653	negative
PRJNA729271	stage_1D_vs_E10	ordinary_muscle_development	ENSGALG00010025168	POPDC2	2.563917814	0.211009989	12.15069402	5.68807E-34	5.89761E-33	positive
PRJNA729271	stage_1D_vs_E10	ordinary_muscle_development	ENSGALG00010025169	RBP2	2.78800412	2.195923538	1.269627139	0.204217477	0.244821134	positive
PRJNA729271	stage_1D_vs_E10	ordinary_muscle_development	ENSGALG00010025170	COMMD9	-0.156517034	0.096082864	-1.628979682	0.10331732	0.131091085	negative
PRJNA729271	stage_1D_vs_E10	ordinary_muscle_development	ENSGALG00010025172	RBP1	3.195322636	0.96549731	3.309509619	0.000934596	0.001563592	positive
PRJNA729271	stage_1D_vs_E10	ordinary_muscle_development	ENSGALG00010025182	PLA1A	2.689417866	0.19430724	13.84105844	1.44069E-43	2.13745E-42	positive
PRJNA729271	stage_1D_vs_E10	ordinary_muscle_development	ENSGALG00010025186	TMEM120B	-0.627108299	0.206268212	-3.040256624	0.002363766	0.00376887	negative
PRJNA729271	stage_1D_vs_E10	ordinary_muscle_development	ENSGALG00010025188	PPFIA4	-4.094191693	0.468564731	-8.737729108	2.37843E-18	1.16118E-17	negative
PRJNA729271	stage_1D_vs_E10	ordinary_muscle_development	ENSGALG00010025190	PRR5L	0.14635138	0.240768068	0.60785212	0.543285558	0.589440431	positive
PRJNA729271	stage_1D_vs_E10	ordinary_muscle_development	ENSGALG00010025192	CRYAB	1.094385321	0.182017866	6.012515946	1.82666E-09	5.06067E-09	positive
PRJNA729271	stage_1D_vs_E10	ordinary_muscle_development	ENSGALG00010025195	P2RX7	3.578449767	0.405381435	8.827364701	1.07171E-18	5.32913E-18	positive
PRJNA729271	stage_1D_vs_E10	ordinary_muscle_development	ENSGALG00010025200	PEPD	-0.765668354	0.080982941	-9.454686914	3.2399E-21	1.85549E-20	negative
PRJNA729271	stage_1D_vs_E10	ordinary_muscle_development	ENSGALG00010025204	TRAF6	0.877219603	0.116877584	7.505456343	6.1215E-14	2.29517E-13	positive
PRJNA729271	stage_1D_vs_E10	ordinary_muscle_development	ENSGALG00010025207	BMPR2	0.199778967	0.097411115	2.050884723	0.040278174	0.054497539	positive
PRJNA729271	stage_1D_vs_E10	ordinary_muscle_development	ENSGALG00010025210	HSPB2	-0.033557393	0.296742875	-0.113085759	0.909962565	0.923210428	negative
PRJNA729271	stage_1D_vs_E10	ordinary_muscle_development	ENSGALG00010025212	P2RX4	0.800295749	0.179220746	4.465419132	7.99122E-06	1.64747E-05	positive
PRJNA729271	stage_1D_vs_E10	ordinary_muscle_development	ENSGALG00010025214	LRP8	-1.716352778	0.204324549	-8.400130028	4.45985E-17	2.02905E-16	negative
PRJNA729271	stage_1D_vs_E10	ordinary_muscle_development	ENSGALG00010025215	MYOG	-1.995051303	0.21531762	-9.265620277	1.93947E-20	1.06085E-19	negative
PRJNA729271	stage_1D_vs_E10	ordinary_muscle_development	ENSGALG00010025216	DPP6	-2.510237255	0.260925065	-9.620529375	6.54932E-22	3.89387E-21	negative
PRJNA729271	stage_1D_vs_E10	ordinary_muscle_development	ENSGALG00010025219	NACA	-0.575235747	0.125490815	-4.583887251	4.5641E-06	9.60742E-06	negative
PRJNA729271	stage_1D_vs_E10	ordinary_muscle_development	ENSGALG00010025222	FAM117B	-1.03807389	0.140432568	-7.391973967	1.44665E-13	5.29124E-13	negative
PRJNA729271	stage_1D_vs_E10	ordinary_muscle_development	ENSGALG00010025225	DMRTB1	-1.223881429	0.903641226	-1.35438866	0.175612414	0.213620505	negative
PRJNA729271	stage_1D_vs_E10	ordinary_muscle_development	ENSGALG00010025228	FDXACB1	0.429365804	0.104955199	4.090943622	4.29622E-05	8.25254E-05	positive
PRJNA729271	stage_1D_vs_E10	ordinary_muscle_development	ENSGALG00010025229	ENSGALG00010025229	-1.6877325	0.166856673	-10.11486369	4.74677E-24	3.09095E-23	negative
PRJNA729271	stage_1D_vs_E10	ordinary_muscle_development	ENSGALG00010025230	CHST8	-1.384278674	0.248117532	-5.579124795	2.41732E-08	6.12249E-08	negative
PRJNA729271	stage_1D_vs_E10	ordinary_muscle_development	ENSGALG00010025232	PRIM1	-0.748693462	0.150551818	-4.972995166	6.59263E-07	1.49336E-06	negative
PRJNA729271	stage_1D_vs_E10	ordinary_muscle_development	ENSGALG00010025233	WDR12	-0.778323096	0.075303205	-10.33585619	4.85061E-25	3.33854E-24	negative
PRJNA729271	stage_1D_vs_E10	ordinary_muscle_development	ENSGALG00010025236	ENSGALG00010025236	1.350818925	0.243940459	5.537494388	3.0683E-08	7.69063E-08	positive
PRJNA729271	stage_1D_vs_E10	ordinary_muscle_development	ENSGALG00010025238	IGSF9B	1.002563451	0.281158391	3.565831517	0.000362704	0.000634262	positive
PRJNA729271	stage_1D_vs_E10	ordinary_muscle_development	ENSGALG00010025239	RAG2	-2.237736509	0.633822991	-3.530538558	0.000414715	0.000720346	negative
PRJNA729271	stage_1D_vs_E10	ordinary_muscle_development	ENSGALG00010025242	CLSTN2	-3.484211729	0.305701067	-11.39744706	4.3056E-30	3.73711E-29	negative
PRJNA729271	stage_1D_vs_E10	ordinary_muscle_development	ENSGALG00010025243	PAXIP1	-0.275352358	0.087835062	-3.134879769	0.001719246	0.002790333	negative
PRJNA729271	stage_1D_vs_E10	ordinary_muscle_development	ENSGALG00010025245	ENSGALG00010025245	0.77159533	0.068316248	11.29446285	1.39756E-29	1.18189E-28	positive
PRJNA729271	stage_1D_vs_E10	ordinary_muscle_development	ENSGALG00010025247	SGCA	3.997393186	0.139543454	28.6462251	1.7864E-180	5.8849E-178	positive
PRJNA729271	stage_1D_vs_E10	ordinary_muscle_development	ENSGALG00010025248	C11orf74	-1.100471742	0.220337562	-4.994480888	5.89942E-07	1.34116E-06	negative
PRJNA729271	stage_1D_vs_E10	ordinary_muscle_development	ENSGALG00010025250	GLIS1	-4.85852797	0.490009697	-9.915166986	3.57658E-23	2.24621E-22	negative
PRJNA729271	stage_1D_vs_E10	ordinary_muscle_development	ENSGALG00010025252	PPP1R9B	-0.361647867	0.145273758	-2.48942322	0.012795055	0.018643378	negative

PRJNA729271	stage_1D_vs_E10	ordinary_muscle_development	ENSGALG00010025256	ANAPC5	-1.172157435	0.122691007	-9.553735545	1.25102E-21	7.31487E-21	negative
PRJNA729271	stage_1D_vs_E10	ordinary_muscle_development	ENSGALG00010025257	LPCAT2	-0.250691076	0.166124859	-1.509052147	0.131285457	0.163575518	negative
PRJNA729271	stage_1D_vs_E10	ordinary_muscle_development	ENSGALG00010025259	SHH	-5.473696172	0.913020573	-5.995150968	2.03296E-09	5.61399E-09	negative
PRJNA729271	stage_1D_vs_E10	ordinary_muscle_development	ENSGALG00010025262	CARF	-0.480273717	0.111392722	-4.31153588	1.62124E-05	3.24048E-05	negative
PRJNA729271	stage_1D_vs_E10	ordinary_muscle_development	ENSGALG00010025263	SAMD14	-2.803113809	0.881237966	-3.180881803	0.001468275	0.002401563	negative
PRJNA729271	stage_1D_vs_E10	ordinary_muscle_development	ENSGALG00010025268	ZBTB39	-0.702046797	0.106450241	-6.595070054	4.25055E-11	1.32101E-10	negative
PRJNA729271	stage_1D_vs_E10	ordinary_muscle_development	ENSGALG00010025270	NDC1	-1.518071876	0.120120981	-12.63785784	1.30563E-36	1.50918E-35	negative
PRJNA729271	stage_1D_vs_E10	ordinary_muscle_development	ENSGALG00010025271	RNF34	0.389256495	0.117819313	3.303842859	0.000953693	0.001594622	positive
PRJNA729271	stage_1D_vs_E10	ordinary_muscle_development	ENSGALG00010025272	NCAPD3	-1.552004596	0.182941949	-8.483590608	2.18349E-17	1.0115E-16	negative
PRJNA729271	stage_1D_vs_E10	ordinary_muscle_development	ENSGALG00010025274	PDK2	3.792617201	0.268998072	14.09904974	3.84918E-45	6.07115E-44	positive
PRJNA729271	stage_1D_vs_E10	ordinary_muscle_development	ENSGALG00010025280	HSPB11	-0.747882358	0.156372215	-4.782706166	1.72951E-06	3.78746E-06	negative
PRJNA729271	stage_1D_vs_E10	ordinary_muscle_development	ENSGALG00010025286	ITGA3	-0.294350816	0.174798712	-1.683941564	0.092192963	0.117957189	negative
PRJNA729271	stage_1D_vs_E10	ordinary_muscle_development	ENSGALG00010025289	KDM2B	-0.923876521	0.073819595	-12.51532905	6.15523E-36	6.92702E-35	negative
PRJNA729271	stage_1D_vs_E10	ordinary_muscle_development	ENSGALG00010025291	DUS2	-1.073896312	0.096313793	-11.14997429	7.16271E-29	5.87237E-28	negative
PRJNA729271	stage_1D_vs_E10	ordinary_muscle_development	ENSGALG00010025292	ATP1A1	0.628421937	0.127498118	4.928872262	8.27056E-07	1.8595E-06	positive
PRJNA729271	stage_1D_vs_E10	ordinary_muscle_development	ENSGALG00010025297	SPSB4	-0.780659775	0.144586816	-5.399245919	6.69216E-08	1.63967E-07	negative
PRJNA729271	stage_1D_vs_E10	ordinary_muscle_development	ENSGALG00010025298	ENSGALG00010025298	-5.294686495	0.367191963	-14.41939646	3.90744E-47	6.63991E-46	negative
PRJNA729271	stage_1D_vs_E10	ordinary_muscle_development	ENSGALG00010025301	MORN3	0.839236305	0.238918976	3.51263981	0.000443679	0.00076762	positive
PRJNA729271	stage_1D_vs_E10	ordinary_muscle_development	ENSGALG00010025303	TMCC2	2.148547204	0.188200219	11.4162843	3.46744E-30	3.03322E-29	positive
PRJNA729271	stage_1D_vs_E10	ordinary_muscle_development	ENSGALG00010025304	DDX28	-0.041651973	0.091198494	-0.456717776	0.647873912	0.688030798	negative
PRJNA729271	stage_1D_vs_E10	ordinary_muscle_development	ENSGALG00010025305	GPR182	3.87827117	0.383034989	10.12510941	4.275E-24	2.79505E-23	positive
PRJNA729271	stage_1D_vs_E10	ordinary_muscle_development	ENSGALG00010025311	ENSGALG00010025311	0.337599725	0.662098529	0.509893484	0.610126087	0.652677316	positive
PRJNA729271	stage_1D_vs_E10	ordinary_muscle_development	ENSGALG00010025312	PLEKHM3	0.651816033	0.112483929	5.794748085	6.84238E-09	1.81383E-08	positive
PRJNA729271	stage_1D_vs_E10	ordinary_muscle_development	ENSGALG00010025313	DIO1	-2.804242971	1.077424753	-2.602727443	0.009248545	0.013736823	negative
PRJNA729271	stage_1D_vs_E10	ordinary_muscle_development	ENSGALG00010025315	LRRC42	-0.719229055	0.122230758	-5.884190408	4.00007E-09	1.07872E-08	negative
PRJNA729271	stage_1D_vs_E10	ordinary_muscle_development	ENSGALG00010025316	SLC25A36	-0.24612058	0.109907223	-2.239348551	0.025133246	0.035059802	negative
PRJNA729271	stage_1D_vs_E10	ordinary_muscle_development	ENSGALG00010025318	NUAK2	-3.062289449	0.484265913	-6.323570103	2.55588E-10	7.53457E-10	negative
PRJNA729271	stage_1D_vs_E10	ordinary_muscle_development	ENSGALG00010025319	LDLRAD1	-0.433811234	0.364153936	-1.191285308	0.233541599	0.276929441	negative
PRJNA729271	stage_1D_vs_E10	ordinary_muscle_development	ENSGALG00010025320	JAM3	-1.404612535	0.144578214	-9.715243382	2.59628E-22	1.57263E-21	negative
PRJNA729271	stage_1D_vs_E10	ordinary_muscle_development	ENSGALG00010025323	KLHDC8A	0.094533061	0.168246375	0.561872792	0.574202692	0.618999556	positive
PRJNA729271	stage_1D_vs_E10	ordinary_muscle_development	ENSGALG00010025325	DPEP2	0.11438326	0.246794449	0.463475821	0.643023342	0.683229977	positive
PRJNA729271	stage_1D_vs_E10	ordinary_muscle_development	ENSGALG00010025327	TMEM59	0.964319524	0.070970763	13.58756034	4.74639E-42	6.70555E-41	positive
PRJNA729271	stage_1D_vs_E10	ordinary_muscle_development	ENSGALG00010025329	MYO1A	-0.585136873	0.205955235	-2.841087649	0.004495995	0.006956603	negative
PRJNA729271	stage_1D_vs_E10	ordinary_muscle_development	ENSGALG00010025330	USH1C	-2.129544155	0.383779957	-5.548867563	2.87526E-08	7.22303E-08	negative
PRJNA729271	stage_1D_vs_E10	ordinary_muscle_development	ENSGALG00010025331	ENSGALG00010025331	-0.412752147	0.367972671	-1.121692394	0.261993251	0.307124317	negative
PRJNA729271	stage_1D_vs_E10	ordinary_muscle_development	ENSGALG00010025333	FZD5	0.21246072	0.199481136	1.065066724	0.286845736	0.333159371	positive
PRJNA729271	stage_1D_vs_E10	ordinary_muscle_development	ENSGALG00010025334	RHOF	0.515283314	0.229797542	2.242336052	0.024939661	0.034816565	positive
PRJNA729271	stage_1D_vs_E10	ordinary_muscle_development	ENSGALG00010025336	ADAM10	-0.163442918	0.063232564	-2.584790314	0.009743824	0.014426631	negative
PRJNA729271	stage_1D_vs_E10	ordinary_muscle_development	ENSGALG00010025337	NEMP1	-0.320526802	0.145572845	-2.201830995	0.027677249	0.038323704	negative
PRJNA729271	stage_1D_vs_E10	ordinary_muscle_development	ENSGALG00010025338	FADD	-0.835507506	0.095814668	-8.720037566	2.7811E-18	1.35411E-17	negative
PRJNA729271	stage_1D_vs_E10	ordinary_muscle_development	ENSGALG00010025340	METTL21A	-0.130689749	0.11936275	-1.094895593	0.27356238	0.31926624	negative
PRJNA729271	stage_1D_vs_E10	ordinary_muscle_development	ENSGALG00010025341	VPS26B	-1.161939628	0.131495565	-8.836340807	9.89048E-19	4.93163E-18	negative
PRJNA729271	stage_1D_vs_E10	ordinary_muscle_development	ENSGALG00010025342	TCEANC2	-0.946468831	0.120077279	-7.882164218	3.21759E-15	1.31045E-14	negative
PRJNA729271	stage_1D_vs_E10	ordinary_muscle_development	ENSGALG00010025343	NAB2	0.535075743	0.294482076	1.817006147	0.069216172	0.090304988	positive
PRJNA729271	stage_1D_vs_E10	ordinary_muscle_development	ENSGALG00010025344	ENSGALG00010025344	2.706301316	0.52764961	5.128974355	2.91325E-07	6.7781E-07	positive
PRJNA729271	stage_1D_vs_E10	ordinary_muscle_development	ENSGALG00010025345	CCM2	-0.346497822	0.089334422	-3.878659705	0.000105034	0.000194415	negative
PRJNA729271	stage_1D_vs_E10	ordinary_muscle_development	ENSGALG00010025346	PXYLP1	-3.263211358	0.132851658	-24.56282	3.1553E-133	4.3147E-131	negative
PRJNA729271	stage_1D_vs_E10	ordinary_muscle_development	ENSGALG00010025348	HIPK1	0.540155679	0.092594636	5.833552559	5.42595E-09	1.44949E-08	positive
PRJNA729271	stage_1D_vs_E10	ordinary_muscle_development	ENSGALG00010025349	SETD1A	0.395223478	0.125952101	3.137887132	0.001701704	0.002764649	positive
PRJNA729271	stage_1D_vs_E10	ordinary_muscle_development	ENSGALG00010025351	CREB1	-0.249996458	0.066626325	-3.752217408	0.000175277	0.000317541	negative
PRJNA729271	stage_1D_vs_E10	ordinary_muscle_development	ENSGALG00010025353	SLC12A4	-0.220364933	0.077533072	-2.842205605	0.004480259	0.006933734	negative
PRJNA729271	stage_1D_vs_E10	ordinary_muscle_development	ENSGALG00010025354	ENSGALG00010025354	-4.053524988	1.705941377	-2.376122089	0.017495673	0.024960608	negative
PRJNA729271	stage_1D_vs_E10	ordinary_muscle_development	ENSGALG00010025355	STAT6	1.245493869	0.139922218	8.901330253	5.51813E-19	2.78694E-18	positive
PRJNA729271	stage_1D_vs_E10	ordinary_muscle_development	ENSGALG00010025357	OLFML3	0.04855798	0.120228031	0.403882353	0.686299234	0.724010727	positive
PRJNA729271	stage_1D_vs_E10	ordinary_muscle_development	ENSGALG00010025358	INCENP	-1.645326272	0.204112849	-8.060865742	7.5756E-16	3.20983E-15	negative
PRJNA729271	stage_1D_vs_E10	ordinary_muscle_development	ENSGALG00010025359	CDCP2	1.046872839	0.383237023	2.73165894	0.006301634	0.009565628	positive
PRJNA729271	stage_1D_vs_E10	ordinary_muscle_development	ENSGALG00010025360	THYN1	0.060179877	0.111405885	0.540185799	0.589068905	0.633142057	positive
PRJNA729271	stage_1D_vs_E10	ordinary_muscle_development	ENSGALG00010025362	G6PC2	-0.862800021	0.244605678	-3.527309862	0.000419805	0.00072849	negative
PRJNA729271	stage_1D_vs_E10	ordinary_muscle_development	ENSGALG00010025364	EPHA4	-2.89284921	0.182995667	-15.80829347	2.72756E-56	6.56743E-55	negative
PRJNA729271	stage_1D_vs_E10	ordinary_muscle_development	ENSGALG00010025365	SIKE1	-0.283835225	0.091512043	-3.101616073	0.001924674	0.003103588	negative
PRJNA729271	stage_1D_vs_E10	ordinary_muscle_development	ENSGALG00010025368	LRP1	1.606647411	0.138682382	11.58508664	4.9047E-31	4.44058E-30	positive
PRJNA729271	stage_1D_vs_E10	ordinary_muscle_development	ENSGALG00010025370	HPD	4.352028972	0.487834285	8.921121594	4.61589E-19	2.3386E-18	positive
PRJNA729271	stage_1D_vs_E10	ordinary_muscle_development	ENSGALG00010025373	CYB5RL	-0.610586596	0.089502881	-6.821976996	8.9796E-12	2.91445E-11	negative
PRJNA729271	stage_1D_vs_E10	ordinary_muscle_development	ENSGALG00010025377	MYO1G	0.51594626	0.302373204	1.706322696	0.087948015	0.112884661	positive
PRJNA729271	stage_1D_vs_E10	ordinary_muscle_development	ENSGALG00010025378	PAX3	-1.514714541	0.260807233	-5.80779346	6.33015E-09	1.68266E-08	negative
PRJNA729271	stage_1D_vs_E10	ordinary_muscle_development	ENSGALG00010025379	ACAD8	1.457072945	0.185238932	7.865910956	3.66421E-15	1.4865E-14	positive
PRJNA729271	stage_1D_vs_E10	ordinary_muscle_development	ENSGALG00010025380	SPC25	-1.86580955	0.224523372	-8.310090526	9.56291E-17	4.27295E-16	negative
PRJNA729271	stage_1D_vs_E10	ordinary_muscle_development	ENSGALG00010025381	MRPL37	0.902153324	0.121651774	7.415866581	1.20832E-13	4.44421E-13	positive
PRJNA729271	stage_1D_vs_E10	ordinary_muscle_development	ENSGALG00010025382	MAB21L3	-1.153112255	0.326018544	-3.536952963	0.000404772	0.000704173	negative
PRJNA729271	stage_1D_vs_E10	ordinary_muscle_development	ENSGALG00010025386	TPCN2	-0.481393655	0.097585191	-4.933060542	8.09511E-07	1.8209E-06	negative
PRJNA729271	stage_1D_vs_E10	ordinary_muscle_development	ENSGALG00010025387	PSMD9	-0.198962076	0.068810977	-2.891429315	0.003834938	0.005973932	negative
PRJNA729271	stage_1D_vs_E10	ordinary_muscle_development	ENSGALG00010025389	SNED1	-0.917152749	0.148722604	-6.166868559	6.96556E-10	1.98908E-09	negative
PRJNA729271	stage_1D_vs_E10	ordinary_muscle_development	ENSGALG00010025390	BCL7A	-0.729152738	0.086912303	-8.389522726	4.8812E-17	2.21657E-16	negative
PRJNA729271	stage_1D_vs_E10	ordinary_muscle_development	ENSGALG00010025392	SLC6A2	-0.485445053	0.24125862	-2.01213558	0.044205647	0.059450812	negative
PRJNA729271	stage_1D_vs_E10	ordinary_muscle_development	ENSGALG00010025398	TSPAN2	-0.034609165	0.144134101	-0.240117813	0.810238925	0.835960796	negative
PRJNA729271	stage_1D_vs_E10	ordinary_muscle_development	ENSGALG00010025400	SLC22A15	-0.769118456	0.156149951	-4.925511979	8.41398E-07	1.89057E-06	negative
PRJNA729271	stage_1D_vs_E10	ordinary_muscle_development	ENSGALG00010025403	ENSGALG00010025403	0.662044659	0.127492712	5.192803953	2.0715E-07	4.87838E-07	positive
PRJNA729271	stage_1D_vs_E10	ordinary_muscle_development	ENSGALG00010025404	CERS6	-2.308330806	0.287697131	-8.023475229	1.02795E-15	4.31387E-15	negative
PRJNA729271	stage_1D_vs_E10	ordinary_muscle_development	ENSGALG00010025405	TAMALIN	1.886096956	0.16340482	11.54248051	8.05678E-31	7.26263E-30	positive
PRJNA729271	stage_1D_vs_E10	ordinary_muscle_development	ENSGALG00010025406	ENSGALG00010025406	0.222200215	0.147683049	1.504574939	0.132433393	0.164760281	positive
PRJNA729271	stage_1D_vs_E10	ordinary_muscle_development	ENSGALG00010025407	MLXIP	2.043812494	0.127692534	16.0057321	1.16534E-57	2.93258E-56	positive
PRJNA729271	stage_1D_vs_E10	ordinary_muscle_development	ENSGALG00010025413	NECAB3	0.817310865	0.120988624	6.755270331	1.42569E-11	4.57402E-11	positive
PRJNA729271	stage_1D_vs_E10	ordinary_muscle_development	ENSGALG00010025414	MMP2	-0.452334631	0.221412242	-2.042952215	0.041057169	0.055468698	negative
PRJNA729271	stage_1D_vs_E10	ordinary_muscle_development	ENSGALG00010025415	ENSGALG00010025415	2.591534635	0.282880087	9.161248005	5.13005E-20	2.75001E-19	positive
PRJNA729271	stage_1D_vs_E10	ordinary_muscle_development	ENSGALG00010025416	ENSGALG00010025416	-0.323717185	0.692887822	-0.467199992	0.640356799	0.680995731	negative
PRJNA729271	stage_1D_vs_E10	ordinary_muscle_development	ENSGALG00010025417	B3GAT1	-1.168898132	0.186584041	-6.264727275	3.73478E-10	1.0875E-09	negative
PRJNA729271	stage_1D_vs_E10	ordinary_muscle_development	ENSGALG00010025418	CASQ2	3.651402732	0.209421413	17.43567038	4.42342E-68	1.53391E-66	positive
PRJNA729271	stage_1D_vs_E10	ordinary_muscle_development	ENSGALG00010025419	CCND1	-0.34592051	0.134370286	-2.57438248	0.010041919	0.014837055	negative
PRJNA729271	stage_1D_vs_E10	ordinary_muscle_development	ENSGALG00010025420	STK39	-1.845591167	0.209832809	-8.795531891	1.42372E-18	7.03848E-18	negative
PRJNA729271	stage_1D_vs_E10	ordinary_muscle_development	ENSGALG00010025421	NR4A1	6.226694573	0.397736494	15.65532624	3.05542E-55	7.10889E-54	positive
PRJNA729271	stage_1D_vs_E10	ordinary_muscle_development	ENSGALG00010025422	SSBP3	-1.278127655	0.157736343	-8.102937037	5.3648E-16	2.29863E-15	negative
PRJNA729271	stage_1D_vs_E10	ordinary_muscle_development	ENSGALG00010025423	NGF	-0.376278483	0.341094072	-1.103151633	0.269961321	0.3154191	negative
PRJNA729271	stage_1D_vs_E10	ordinary_muscle_development	ENSGALG00010025424	IRX6	-2.970308832	0.393037351	-7.557319479	4.1146E-14	1.56047E-13	negative
PRJNA729271	stage_1D_vs_E10	ordinary_muscle_development	ENSGALG00010025425	ENSGALG00010025425	0.301051076	0.138584097	2.17233494	0.029830408	0.041133159	positive
PRJNA729271	stage_1D_vs_E10	ordinary_muscle_development	ENSGALG00010025426	ENSGALG00010025426	1.363228627	0.152850575	8.918701362	4.71788E-19	2.38943E-18	positive
PRJNA729271	stage_1D_vs_E10	ordinary_muscle_development	ENSGALG00010025427	SLC35G2	-1.037157743	0.133585671	-7.763989448	8.22987E-15	3.25579E-14	negative
PRJNA729271	stage_1D_vs_E10	ordinary_muscle_development	ENSGALG00010025428	VANGL1	-0.115074029	0.106630241	-1.079187554	0.280504127	0.326605134	negative
PRJNA729271	stage_1D_vs_E10	ordinary_muscle_development	ENSGALG00010025429	SCN3B	-3.435982321	0.241261029	-14.24176269	5.04386E-46	8.24247E-45	negative
PRJNA729271	stage_1D_vs_E10	ordinary_muscle_development	ENSGALG00010025430	IRX5	-5.8582024	0.993623947	-5.895794296	3.72883E-09	1.00876E-08	negative
PRJNA729271	stage_1D_vs_E10	ordinary_muscle_development	ENSGALG00010025432	IRX3	-3.965793395	0.672574489	-5.89643744	3.71433E-09	1.00521E-08	negative
PRJNA729271	stage_1D_vs_E10	ordinary_muscle_development	ENSGALG00010025433	CHPF2	-0.109303684	0.122100576	-0.895193847	0.370683473	0.419868481	negative
PRJNA729271	stage_1D_vs_E10	ordinary_muscle_development	ENSGALG00010025434	SYT6	0.351281079	0.235590859	1.491064129	0.135944659	0.168723164	positive
PRJNA729271	stage_1D_vs_E10	ordinary_muscle_development	ENSGALG00010025435	SCLY	-0.433267155	0.10061271	-4.306286502	1.66018E-05	3.31464E-05	negative
PRJNA729271	stage_1D_vs_E10	ordinary_muscle_development	ENSGALG00010025438	EXOSC6	0.404206462	0.118504018	3.410909345	0.000647466	0.001100366	positive
PRJNA729271	stage_1D_vs_E10	ordinary_muscle_development	ENSGALG00010025442	ABCF2	0.244391954	0.147270117	1.659480946	0.097018917	0.123662384	positive
PRJNA729271	stage_1D_vs_E10	ordinary_muscle_development	ENSGALG00010025445	B3GNT4	0.928707465	0.298293848	3.113397986	0.001849464	0.002991635	positive
PRJNA729271	stage_1D_vs_E10	ordinary_muscle_development	ENSGALG00010025448	CHD1L	-0.610941015	0.108893964	-5.610421293	2.01835E-08	5.14164E-08	negative
PRJNA729271	stage_1D_vs_E10	ordinary_muscle_development	ENSGALG00010025449	ENSGALG00010025449	0.322152367	0.115123826	2.798311855	0.005137049	0.007884532	positive
PRJNA729271	stage_1D_vs_E10	ordinary_muscle_development	ENSGALG00010025451	LRRC43	2.082856177	0.313848582	6.636500193	3.21219E-11	1.00628E-10	positive
PRJNA729271	stage_1D_vs_E10	ordinary_muscle_development	ENSGALG00010025452	TRIM33	0.303643532	0.125920384	2.411392999	0.015891714	0.02280697	positive
PRJNA729271	stage_1D_vs_E10	ordinary_muscle_development	ENSGALG00010025458	BCAS2	0.14767357	0.115429934	1.27933514	0.200779063	0.241057941	positive
PRJNA729271	stage_1D_vs_E10	ordinary_muscle_development	ENSGALG00010025459	DIABLO	-0.338497905	0.078327795	-4.321555398	1.54933E-05	3.10187E-05	negative
PRJNA729271	stage_1D_vs_E10	ordinary_muscle_development	ENSGALG00010025464	VPS33A	-0.605638036	0.091263244	-6.636165999	3.21947E-11	1.00835E-10	negative
PRJNA729271	stage_1D_vs_E10	ordinary_muscle_development	ENSGALG00010025465	ENSGALG00010025465	-0.203828798	0.082692251	-2.464908089	0.01370484	0.019873115	negative
PRJNA729271	stage_1D_vs_E10	ordinary_muscle_development	ENSGALG00010025470	ORAOV1	0.114976739	0.123838801	0.928438728	0.353180037	0.402021724	positive
PRJNA729271	stage_1D_vs_E10	ordinary_muscle_development	ENSGALG00010025472	ZCCHC8	0.665750951	0.095989082	6.935694561	4.0423E-12	1.34203E-11	positive
PRJNA729271	stage_1D_vs_E10	ordinary_muscle_development	ENSGALG00010025473	PARS2	0.297928128	0.078955817	3.773352496	0.000161068	0.000292898	positive
PRJNA729271	stage_1D_vs_E10	ordinary_muscle_development	ENSGALG00010025477	ENSGALG00010025477	0.141118466	0.070571456	1.999653591	0.045537683	0.061083538	positive
PRJNA729271	stage_1D_vs_E10	ordinary_muscle_development	ENSGALG00010025480	FGF19	-3.682308394	0.640655868	-5.747716638	9.04567E-09	2.36887E-08	negative
PRJNA729271	stage_1D_vs_E10	ordinary_muscle_development	ENSGALG00010025481	TMEM39A	-0.110240428	0.089609561	-1.230230656	0.218610743	0.260932372	negative
PRJNA729271	stage_1D_vs_E10	ordinary_muscle_development	ENSGALG00010025483	FGF4	-1.766369434	0.364685891	-4.843536531	1.27548E-06	2.82391E-06	negative
PRJNA729271	stage_1D_vs_E10	ordinary_muscle_development	ENSGALG00010025485	DENND2C	1.016972205	0.159794808	6.364238119	1.96261E-10	5.82356E-10	positive
PRJNA729271	stage_1D_vs_E10	ordinary_muscle_development	ENSGALG00010025486	B3GALT1	-0.701493664	0.184336065	-3.805515012	0.000141509	0.000258661	negative
PRJNA729271	stage_1D_vs_E10	ordinary_muscle_development	ENSGALG00010025487	FGF3	-6.668104926	0.932916964	-7.147586748	8.83167E-13	3.0677E-12	negative
PRJNA729271	stage_1D_vs_E10	ordinary_muscle_development	ENSGALG00010025488	SLC4A8	-2.576677051	0.196822636	-13.09136544	3.68916E-39	4.70725E-38	negative
PRJNA729271	stage_1D_vs_E10	ordinary_muscle_development	ENSGALG00010025491	ZMYND11	0.069711212	0.063663159	1.095000831	0.273516273	0.319238133	positive
PRJNA729271	stage_1D_vs_E10	ordinary_muscle_development	ENSGALG00010025496	ATG101	0.142247263	0.111705148	1.273417251	0.202870019	0.243326514	positive
PRJNA729271	stage_1D_vs_E10	ordinary_muscle_development	ENSGALG00010025499	STAC3	4.137248058	0.158737489	26.06345913	9.469E-150	1.6945E-147	positive
PRJNA729271	stage_1D_vs_E10	ordinary_muscle_development	ENSGALG00010025504	AMPD1	3.234909643	0.197986652	16.33902898	5.20756E-60	1.43778E-58	positive
PRJNA729271	stage_1D_vs_E10	ordinary_muscle_development	ENSGALG00010025505	ENSGALG00010025505	-0.708151805	0.258819766	-2.736080845	0.006217575	0.009446934	negative
PRJNA729271	stage_1D_vs_E10	ordinary_muscle_development	ENSGALG00010025506	R3HDM2	0.67578635	0.176312644	3.832886483	0.000126648	0.000232582	positive

PRJNA729271	stage_1D_vs_E10	ordinary_muscle_development	ENSGALG00010025508	ENSGALG00010025508	0.528826821	0.1318583	4.010569085	6.05726E-05	0.000114921	positive
PRJNA729271	stage_1D_vs_E10	ordinary_muscle_development	ENSGALG00010025512	NRAS	-0.43396335	0.077842499	-5.574889777	2.47687E-08	6.27003E-08	negative
PRJNA729271	stage_1D_vs_E10	ordinary_muscle_development	ENSGALG00010025513	RSRC2	0.043108735	0.087274372	0.49394495	0.621345058	0.663454631	positive
PRJNA729271	stage_1D_vs_E10	ordinary_muscle_development	ENSGALG00010025520	ACOT11	1.10446697	0.295463023	3.738088642	0.000185425	0.000334669	positive
PRJNA729271	stage_1D_vs_E10	ordinary_muscle_development	ENSGALG00010025522	SUMO3	-0.52373162	0.10550474	-4.964057717	6.90354E-07	1.56086E-06	negative
PRJNA729271	stage_1D_vs_E10	ordinary_muscle_development	ENSGALG00010025523	B4GALT4	-1.378215157	0.133547699	-10.32002173	5.72102E-25	3.91531E-24	negative
PRJNA729271	stage_1D_vs_E10	ordinary_muscle_development	ENSGALG00010025527	KNTC1	-1.505993984	0.238723013	-6.308541287	2.81678E-10	8.27506E-10	negative
PRJNA729271	stage_1D_vs_E10	ordinary_muscle_development	ENSGALG00010025531	ENSGALG00010025531	0.099657224	0.094234955	1.057539895	0.290265257	0.336780189	positive
PRJNA729271	stage_1D_vs_E10	ordinary_muscle_development	ENSGALG00010025533	INHBC	-2.417318915	0.649945516	-3.719263933	0.000199804	0.000358702	negative
PRJNA729271	stage_1D_vs_E10	ordinary_muscle_development	ENSGALG00010025538	ANO1	4.543653636	0.208372283	21.80546072	2.0592E-105	1.6222E-103	positive
PRJNA729271	stage_1D_vs_E10	ordinary_muscle_development	ENSGALG00010025541	TTC21B	-0.164173929	0.127489712	-1.287742569	0.19783559	0.23783917	negative
PRJNA729271	stage_1D_vs_E10	ordinary_muscle_development	ENSGALG00010025544	CSDE1	-0.061136101	0.08601082	-0.710795465	0.477210991	0.525820853	negative
PRJNA729271	stage_1D_vs_E10	ordinary_muscle_development	ENSGALG00010025548	ENSGALG00010025548	0.455694105	0.193774668	2.351670164	0.018689339	0.026535603	positive
PRJNA729271	stage_1D_vs_E10	ordinary_muscle_development	ENSGALG00010025549	DHCR24	-1.722522157	0.24666678	-6.983194711	2.88542E-12	9.68152E-12	negative
PRJNA729271	stage_1D_vs_E10	ordinary_muscle_development	ENSGALG00010025553	UBE2G2	-0.408136429	0.09645599	-4.231322818	2.32321E-05	4.57602E-05	negative
PRJNA729271	stage_1D_vs_E10	ordinary_muscle_development	ENSGALG00010025554	DIP2C	0.127096314	0.07056496	1.801125014	0.071683178	0.093313665	positive
PRJNA729271	stage_1D_vs_E10	ordinary_muscle_development	ENSGALG00010025557	VPS29	-0.668915947	0.077776916	-8.600443147	7.94089E-18	3.7714E-17	negative
PRJNA729271	stage_1D_vs_E10	ordinary_muscle_development	ENSGALG00010025558	GALNT3	-2.436364279	0.223001948	-10.92530491	8.72469E-28	6.76642E-27	negative
PRJNA729271	stage_1D_vs_E10	ordinary_muscle_development	ENSGALG00010025559	SLC6A17	-4.456206288	1.172403152	-3.800916331	0.000144162	0.00026341	negative
PRJNA729271	stage_1D_vs_E10	ordinary_muscle_development	ENSGALG00010025562	GRAMD1B	1.865110809	0.181267362	10.2892809	7.87624E-25	5.35237E-24	positive
PRJNA729271	stage_1D_vs_E10	ordinary_muscle_development	ENSGALG00010025563	TSPEAR	0.07132431	0.232647257	0.306577054	0.759165322	0.790070458	positive
PRJNA729271	stage_1D_vs_E10	ordinary_muscle_development	ENSGALG00010025565	ENSGALG00010025565	1.4086815	0.243716178	5.780008163	7.4697E-09	1.97435E-08	positive
PRJNA729271	stage_1D_vs_E10	ordinary_muscle_development	ENSGALG00010025568	MYL3	-0.731899325	0.960721956	-0.761822212	0.446166122	0.49537939	negative
PRJNA729271	stage_1D_vs_E10	ordinary_muscle_development	ENSGALG00010025569	PPTC7	1.871392832	0.192667811	9.7130539	2.65268E-22	1.60411E-21	positive
PRJNA729271	stage_1D_vs_E10	ordinary_muscle_development	ENSGALG00010025571	SRSF3	-0.856433963	0.228859267	-3.742186088	0.000182426	0.000329709	negative
PRJNA729271	stage_1D_vs_E10	ordinary_muscle_development	ENSGALG00010025572	ENSGALG00010025572	-0.314753474	0.11194335	-2.811721048	0.004927722	0.007581714	negative
PRJNA729271	stage_1D_vs_E10	ordinary_muscle_development	ENSGALG00010025573	CLMP	-1.30242797	0.168266545	-7.740266923	9.92083E-15	3.90768E-14	negative
PRJNA729271	stage_1D_vs_E10	ordinary_muscle_development	ENSGALG00010025574	ARHGAP31	-0.446297685	0.099324534	-4.493327732	7.01187E-06	1.45404E-05	negative
PRJNA729271	stage_1D_vs_E10	ordinary_muscle_development	ENSGALG00010025575	TRPM2	-1.113099502	0.47163652	-2.360079119	0.018271038	0.025972217	negative
PRJNA729271	stage_1D_vs_E10	ordinary_muscle_development	ENSGALG00010025577	PTH2R	-1.723321353	0.187539238	-9.189124192	3.96053E-20	2.13174E-19	negative
PRJNA729271	stage_1D_vs_E10	ordinary_muscle_development	ENSGALG00010025578	ORMDL2	-0.049480176	0.146865792	-0.336907426	0.736186687	0.769859752	negative
PRJNA729271	stage_1D_vs_E10	ordinary_muscle_development	ENSGALG00010025579	CSRNP3	-1.065709284	0.218661951	-4.873775607	1.09485E-06	2.43852E-06	negative
PRJNA729271	stage_1D_vs_E10	ordinary_muscle_development	ENSGALG00010025580	RASSF7	-0.177491532	0.151240631	-1.173570434	0.240567129	0.284352976	negative
PRJNA729271	stage_1D_vs_E10	ordinary_muscle_development	ENSGALG00010025582	DMAP1	-0.46232236	0.114398329	-4.041338415	5.3147E-05	0.000101231	negative
PRJNA729271	stage_1D_vs_E10	ordinary_muscle_development	ENSGALG00010025585	SARNP	-0.893256231	0.096302289	-9.275545137	1.76713E-20	9.70616E-20	negative
PRJNA729271	stage_1D_vs_E10	ordinary_muscle_development	ENSGALG00010025586	TCTN1	0.487135897	0.124569472	3.910555998	9.20839E-05	0.000171518	positive
PRJNA729271	stage_1D_vs_E10	ordinary_muscle_development	ENSGALG00010025587	LRRC56	-1.927317034	0.237758528	-8.106195185	5.22295E-16	2.24183E-15	negative
PRJNA729271	stage_1D_vs_E10	ordinary_muscle_development	ENSGALG00010025588	ENSGALG00010025588	-0.52994216	0.309786273	-1.710670246	0.087142003	0.111909572	negative
PRJNA729271	stage_1D_vs_E10	ordinary_muscle_development	ENSGALG00010025590	HRAS	0.256407238	0.113819826	2.252746702	0.024275124	0.033960808	positive
PRJNA729271	stage_1D_vs_E10	ordinary_muscle_development	ENSGALG00010025591	OTOS	1.71007208	0.34003877	5.029050299	4.92915E-07	1.12819E-06	positive
PRJNA729271	stage_1D_vs_E10	ordinary_muscle_development	ENSGALG00010025592	HSPA8	-0.545623741	0.208198089	-2.620695241	0.008775067	0.013067043	negative
PRJNA729271	stage_1D_vs_E10	ordinary_muscle_development	ENSGALG00010025594	UPK1B	-5.840539127	0.611810853	-9.546315006	1.34392E-21	7.83906E-21	negative
PRJNA729271	stage_1D_vs_E10	ordinary_muscle_development	ENSGALG00010025595	ERI3	0.062078527	0.092295548	0.67260587	0.501198066	0.549286705	positive
PRJNA729271	stage_1D_vs_E10	ordinary_muscle_development	ENSGALG00010025597	SCN2A	-6.709252173	0.941504108	-7.126099736	1.03253E-12	3.56685E-12	negative
PRJNA729271	stage_1D_vs_E10	ordinary_muscle_development	ENSGALG00010025598	ENSGALG00010025598	2.309865785	0.395388021	5.842022678	5.15708E-09	1.37944E-08	positive
PRJNA729271	stage_1D_vs_E10	ordinary_muscle_development	ENSGALG00010025599	COPS9	0.656590664	0.142305129	4.613963438	3.95062E-06	8.37196E-06	positive
PRJNA729271	stage_1D_vs_E10	ordinary_muscle_development	ENSGALG00010025600	ENSGALG00010025600	-1.37624855	0.112228462	-12.26291909	1.43245E-34	1.53348E-33	negative
PRJNA729271	stage_1D_vs_E10	ordinary_muscle_development	ENSGALG00010025604	HVCN1	-0.253378958	0.134600189	-1.882456177	0.059774105	0.078701576	negative
PRJNA729271	stage_1D_vs_E10	ordinary_muscle_development	ENSGALG00010025606	CCDC12	-0.614547228	0.131317861	-4.679844928	2.87092E-06	6.17052E-06	negative
PRJNA729271	stage_1D_vs_E10	ordinary_muscle_development	ENSGALG00010025608	ENSGALG00010025608	-1.427656247	0.495346746	-2.882135107	0.003949903	0.006144435	negative
PRJNA729271	stage_1D_vs_E10	ordinary_muscle_development	ENSGALG00010025610	RNH1	-1.467747091	0.134335851	-10.92595227	8.66269E-28	6.72553E-27	negative
PRJNA729271	stage_1D_vs_E10	ordinary_muscle_development	ENSGALG00010025612	TMEM53	-0.205978255	0.151285899	-1.361516545	0.173350508	0.211046163	negative
PRJNA729271	stage_1D_vs_E10	ordinary_muscle_development	ENSGALG00010025613	CDKN1A	1.200074114	0.276589581	4.338826183	1.43246E-05	2.87942E-05	positive
PRJNA729271	stage_1D_vs_E10	ordinary_muscle_development	ENSGALG00010025618	ADIPOQ	7.351265108	0.221621291	33.17039198	2.8783E-241	2.6076E-238	positive
PRJNA729271	stage_1D_vs_E10	ordinary_muscle_development	ENSGALG00010025621	PPP1CC	-0.42017272	0.117357133	-3.580291272	0.000343211	0.000601699	negative
PRJNA729271	stage_1D_vs_E10	ordinary_muscle_development	ENSGALG00010025623	ENSGALG00010025623	0.86667808	0.40575088	2.135985709	0.032680579	0.044854573	positive
PRJNA729271	stage_1D_vs_E10	ordinary_muscle_development	ENSGALG00010025624	ARMH1	0.53534214	0.358941986	1.491444748	0.135844767	0.168613624	positive
PRJNA729271	stage_1D_vs_E10	ordinary_muscle_development	ENSGALG00010025628	ENSGALG00010025628	-2.137442214	0.273602988	-7.812203472	5.61967E-15	2.24827E-14	negative
PRJNA729271	stage_1D_vs_E10	ordinary_muscle_development	ENSGALG00010025629	ST6GAL1	2.892658177	0.143379713	20.17480791	1.62996E-90	9.80341E-89	positive
PRJNA729271	stage_1D_vs_E10	ordinary_muscle_development	ENSGALG00010025635	CHKA	-1.252954921	0.164055463	-7.637386162	2.21676E-14	8.55938E-14	negative
PRJNA729271	stage_1D_vs_E10	ordinary_muscle_development	ENSGALG00010025637	C6orf89	1.132078373	0.126404948	8.955965688	3.36775E-19	1.72189E-18	positive
PRJNA729271	stage_1D_vs_E10	ordinary_muscle_development	ENSGALG00010025639	MYL2	-2.646455671	0.434765249	-6.087091083	1.14981E-09	3.23055E-09	negative
PRJNA729271	stage_1D_vs_E10	ordinary_muscle_development	ENSGALG00010025640	SLC38A11	-3.679580042	0.647192177	-5.685451977	1.30467E-08	3.37399E-08	negative
PRJNA729271	stage_1D_vs_E10	ordinary_muscle_development	ENSGALG00010025642	RPS8	0.023044513	0.11754862	0.196042392	0.844576986	0.867007324	positive
PRJNA729271	stage_1D_vs_E10	ordinary_muscle_development	ENSGALG00010025644	ENSGALG00010025644	5.027461307	0.281848672	17.83744898	3.61842E-71	1.38024E-69	positive
PRJNA729271	stage_1D_vs_E10	ordinary_muscle_development	ENSGALG00010025645	NBEAL2	0.572163582	0.187591177	3.050055925	0.002287988	0.00365488	positive
PRJNA729271	stage_1D_vs_E10	ordinary_muscle_development	ENSGALG00010025647	JHY	-4.569957777	0.529826642	-8.625383124	6.38784E-18	3.04879E-17	negative
PRJNA729271	stage_1D_vs_E10	ordinary_muscle_development	ENSGALG00010025648	ENSGALG00010025648	-0.827618977	0.638807284	-1.295569098	0.195123981	0.235086203	negative
PRJNA729271	stage_1D_vs_E10	ordinary_muscle_development	ENSGALG00010025650	PPP1R1A	4.947879019	0.456358354	10.84209148	2.17442E-27	1.6519E-26	positive
PRJNA729271	stage_1D_vs_E10	ordinary_muscle_development	ENSGALG00010025652	KMT5B	0.067129166	0.076212167	0.880819533	0.378415506	0.427474558	positive
PRJNA729271	stage_1D_vs_E10	ordinary_muscle_development	ENSGALG00010025653	IGSF11	1.106733798	0.193748748	5.712211356	1.11517E-08	2.89841E-08	positive
PRJNA729271	stage_1D_vs_E10	ordinary_muscle_development	ENSGALG00010025654	CRTAM	0.023768233	0.903699033	0.026301049	0.979017218	0.982202005	positive
PRJNA729271	stage_1D_vs_E10	ordinary_muscle_development	ENSGALG00010025658	PDE1B	-2.371507377	0.406653935	-5.831758088	5.48464E-09	1.46463E-08	negative
PRJNA729271	stage_1D_vs_E10	ordinary_muscle_development	ENSGALG00010025659	COBLL1	1.633453012	0.129816579	12.5827766	2.62634E-36	2.99754E-35	positive
PRJNA729271	stage_1D_vs_E10	ordinary_muscle_development	ENSGALG00010025660	KIF2C	-1.884450563	0.198928178	-9.473019779	2.71868E-21	1.56192E-20	negative
PRJNA729271	stage_1D_vs_E10	ordinary_muscle_development	ENSGALG00010025661	SNRPE	-0.690968168	0.129418319	-5.339029087	9.34457E-08	2.26051E-07	negative
PRJNA729271	stage_1D_vs_E10	ordinary_muscle_development	ENSGALG00010025664	GPR55	1.171206564	0.682060541	1.717159246	0.08595008	0.110555187	positive
PRJNA729271	stage_1D_vs_E10	ordinary_muscle_development	ENSGALG00010025666	CUX2	-2.705188518	0.343238065	-7.881376781	3.23793E-15	1.31837E-14	negative
PRJNA729271	stage_1D_vs_E10	ordinary_muscle_development	ENSGALG00010025674	GRB14	3.463479524	0.42274588	8.19281674	2.55183E-16	1.11345E-15	positive
PRJNA729271	stage_1D_vs_E10	ordinary_muscle_development	ENSGALG00010025679	ENSGALG00010025679	-0.936473675	1.367029135	-0.685042952	0.493316817	0.541632121	negative
PRJNA729271	stage_1D_vs_E10	ordinary_muscle_development	ENSGALG00010025685	NCKAP1L	0.528481662	0.197582773	2.674735525	0.007478822	0.011241888	positive
PRJNA729271	stage_1D_vs_E10	ordinary_muscle_development	ENSGALG00010025688	BEST4	1.515504482	0.507237972	2.98775834	0.002810317	0.0044442	positive
PRJNA729271	stage_1D_vs_E10	ordinary_muscle_development	ENSGALG00010025691	ENSGALG00010025691	-3.03364186	0.172124008	-17.62474564	1.59083E-69	5.76475E-68	negative
PRJNA729271	stage_1D_vs_E10	ordinary_muscle_development	ENSGALG00010025692	ENSGALG00010025692	0.407900314	1.234696571	0.330364823	0.741124318	0.774185428	positive
PRJNA729271	stage_1D_vs_E10	ordinary_muscle_development	ENSGALG00010025696	MTCH1	-0.017095545	0.104380645	-0.163780794	0.869903712	0.889165383	negative
PRJNA729271	stage_1D_vs_E10	ordinary_muscle_development	ENSGALG00010025701	ENSGALG00010025701	0.859418661	3.255211182	0.264013182	0.791769762	0.81947181	positive
PRJNA729271	stage_1D_vs_E10	ordinary_muscle_development	ENSGALG00010025702	GTSF2	0.042804969	0.175616875	0.243740639	0.807431697	0.833598465	positive
PRJNA729271	stage_1D_vs_E10	ordinary_muscle_development	ENSGALG00010025703	UBASH3B	-1.464101772	0.14255309	-10.27057201	9.56359E-25	6.45364E-24	negative
PRJNA729271	stage_1D_vs_E10	ordinary_muscle_development	ENSGALG00010025705	ACSL3	-0.695240563	0.08291584	-8.384894418	5.07713E-17	2.30338E-16	negative
PRJNA729271	stage_1D_vs_E10	ordinary_muscle_development	ENSGALG00010025706	ENSGALG00010025706	0.859448696	3.056422803	0.281194308	0.77856137	0.807300026	positive
PRJNA729271	stage_1D_vs_E10	ordinary_muscle_development	ENSGALG00010025708	HOXC8	-2.706121341	0.245968217	-11.00191471	3.74103E-28	2.94868E-27	negative
PRJNA729271	stage_1D_vs_E10	ordinary_muscle_development	ENSGALG00010025710	ENSGALG00010025710	-0.105555512	2.061446255	-0.051204591	0.959162494	0.965557354	negative
PRJNA729271	stage_1D_vs_E10	ordinary_muscle_development	ENSGALG00010025719	HOXC11	-7.116498271	2.679982774	-2.65542687	0.007920811	0.01187057	negative
PRJNA729271	stage_1D_vs_E10	ordinary_muscle_development	ENSGALG00010025720	PHETA1	-2.075744211	0.26318196	-7.887106747	3.09273E-15	1.26031E-14	negative
PRJNA729271	stage_1D_vs_E10	ordinary_muscle_development	ENSGALG00010025723	SETD2	0.551538299	0.121899379	4.52453739	6.05278E-06	1.26201E-05	positive
PRJNA729271	stage_1D_vs_E10	ordinary_muscle_development	ENSGALG00010025727	GLI1	-2.037017335	0.213725388	-9.531003103	1.55771E-21	9.06422E-21	negative
PRJNA729271	stage_1D_vs_E10	ordinary_muscle_development	ENSGALG00010025734	PLK3	-0.079303429	0.18250447	-0.434528693	0.663904563	0.702892166	negative
PRJNA729271	stage_1D_vs_E10	ordinary_muscle_development	ENSGALG00010025736	KIF9	1.601172791	0.318238448	5.031361858	4.87008E-07	1.11502E-06	positive
PRJNA729271	stage_1D_vs_E10	ordinary_muscle_development	ENSGALG00010025737	ENSGALG00010025737	-0.091290156	0.150390233	-0.607021841	0.543836418	0.589993928	negative
PRJNA729271	stage_1D_vs_E10	ordinary_muscle_development	ENSGALG00010025738	SOX13	0.282699498	0.152698273	1.851360151	0.064117758	0.084099801	positive
PRJNA729271	stage_1D_vs_E10	ordinary_muscle_development	ENSGALG00010025743	SH2B3	0.136847537	0.109172304	1.25350049	0.210023621	0.251345144	positive
PRJNA729271	stage_1D_vs_E10	ordinary_muscle_development	ENSGALG00010025745	COX14	0.650591526	0.228544122	2.846678013	0.004417802	0.006842188	positive
PRJNA729271	stage_1D_vs_E10	ordinary_muscle_development	ENSGALG00010025748	ETNK2	1.320205117	0.195364908	6.757636944	1.4026E-11	4.50393E-11	positive
PRJNA729271	stage_1D_vs_E10	ordinary_muscle_development	ENSGALG00010025753	SORL1	-0.632011085	0.171362508	-3.688152637	0.000225888	0.000403332	negative
PRJNA729271	stage_1D_vs_E10	ordinary_muscle_development	ENSGALG00010025755	ENSGALG00010025755	-0.281163959	0.265544503	-1.058820486	0.289681542	0.336156749	negative
PRJNA729271	stage_1D_vs_E10	ordinary_muscle_development	ENSGALG00010025782	ENSGALG00010025782	0.859344637	4.564438882	0.188269502	0.850665391	0.872148916	positive
PRJNA729271	stage_1D_vs_E10	ordinary_muscle_development	ENSGALG00010025784	ENSGALG00010025784	0.376342095	2.459378008	0.153023282	0.878379915	0.896249251	positive
PRJNA729271	stage_1D_vs_E10	ordinary_muscle_development	ENSGALG00010025787	KCNE4	3.245029176	0.289428801	11.21183921	3.56703E-29	2.96809E-28	positive
PRJNA729271	stage_1D_vs_E10	ordinary_muscle_development	ENSGALG00010025790	LRP5	0.544943684	0.077402001	7.040434064	1.91642E-12	6.50246E-12	positive
PRJNA729271	stage_1D_vs_E10	ordinary_muscle_development	ENSGALG00010025791	FIGN	1.524633601	0.23510303	6.48495938	8.87557E-11	2.69653E-10	positive
PRJNA729271	stage_1D_vs_E10	ordinary_muscle_development	ENSGALG00010025794	ENSGALG00010025794	1.342257849	4.091796531	0.328036313	0.742884206	0.775520819	positive
PRJNA729271	stage_1D_vs_E10	ordinary_muscle_development	ENSGALG00010025799	SC5D	0.384716965	0.269770272	1.426091033	0.153842067	0.189202508	positive
PRJNA729271	stage_1D_vs_E10	ordinary_muscle_development	ENSGALG00010025800	ARL2BP	-0.987235135	0.138445367	-7.130864353	9.97406E-13	3.45159E-12	negative
PRJNA729271	stage_1D_vs_E10	ordinary_muscle_development	ENSGALG00010025801	PTPN7	-0.148066905	0.393632266	-0.376155405	0.706801366	0.743097542	negative
PRJNA729271	stage_1D_vs_E10	ordinary_muscle_development	ENSGALG00010025804	ENSGALG00010025804	0.375732336	2.257954404	0.166403863	0.867839134	0.887430564	positive
PRJNA729271	stage_1D_vs_E10	ordinary_muscle_development	ENSGALG00010025805	ATXN2	0.247856245	0.093651711	2.646574666	0.008131155	0.012164423	positive
PRJNA729271	stage_1D_vs_E10	ordinary_muscle_development	ENSGALG00010025809	ENSGALG00010025809	1.150611305	0.19272155	5.970330284	2.36774E-09	6.50253E-09	positive
PRJNA729271	stage_1D_vs_E10	ordinary_muscle_development	ENSGALG00010025822	KLF7	0.2434373	0.096474369	2.523336528	0.011624709	0.017037428	positive
PRJNA729271	stage_1D_vs_E10	ordinary_muscle_development	ENSGALG00010025829	ENSGALG00010025829	0.41647982	0.258131676	1.613439411	0.106649077	0.134905172	positive
PRJNA729271	stage_1D_vs_E10	ordinary_muscle_development	ENSGALG00010025830	TBCEL	0.356336515	0.190843912	1.867162082	0.061878957	0.081303071	positive
PRJNA729271	stage_1D_vs_E10	ordinary_muscle_development	ENSGALG00010025831	BRAP	-0.107183132	0.069884315	-1.533722293	0.125097987	0.156439938	negative
PRJNA729271	stage_1D_vs_E10	ordinary_muscle_development	ENSGALG00010025832	ENKD1	-0.024501061	0.150672489	-0.162611378	0.870824428	0.889980969	negative
PRJNA729271	stage_1D_vs_E10	ordinary_muscle_development	ENSGALG00010025835	PPP6R3	0.227282977	0.11910336	1.908283504	0.056354584	0.074530994	positive
PRJNA729271	stage_1D_vs_E10	ordinary_muscle_development	ENSGALG00010025836	ENSGALG00010025836	0.663702065	0.113304966	5.857660864	4.69432E-09	1.26031E-08	positive
PRJNA729271	stage_1D_vs_E10	ordinary_muscle_development	ENSGALG00010025837	ENSGALG00010025837	1.819484456	0.179821625	10.11827393	4.58429E-24	2.99186E-23	positive
PRJNA729271	stage_1D_vs_E10	ordinary_muscle_development	ENSGALG00010025846	GRIK4	-1.912901627	0.261620228	-7.311749719	2.63686E-13	9.47948E-13	negative
PRJNA729271	stage_1D_vs_E10	ordinary_muscle_development	ENSGALG00010025847	DEF6	-0.132500234	0.126021736	-1.051407789	0.29307134	0.33973681	negative
PRJNA729271	stage_1D_vs_E10	ordinary_muscle_development	ENSGALG00010025849	OBSCN	4.676589376	0.229071013	20.4154568	1.21887E-92	7.81747E-91	positive
PRJNA729271	stage_1D_vs_E10	ordinary_muscle_development	ENSGALG00010025852	BTBD19	0.208834274	0.236124407	0.88442477	0.376466956	0.425621132	positive
PRJNA729271	stage_1D_vs_E10	ordinary_muscle_development	ENSGALG00010025858	ENSGALG00010025858	0.859438735	2.976788379	0.288713414	0.772800697	0.802072612	positive
PRJNA729271	stage_1D_vs_E10	ordinary_muscle_development	ENSGALG00010025862	PTCH2	-2.888161216	0.280768214	-10.28663886	8.0953E-25	5.48837E-24	negative

PRJNA729271	stage_1D_vs_E10	ordinary_muscle_development	ENSGALG00010025865	LGR6	-3.037391749	0.580034674	-5.236569267	1.63589E-07	3.88725E-07	negative
PRJNA729271	stage_1D_vs_E10	ordinary_muscle_development	ENSGALG00010025869	FASTKD2	-0.195260992	0.09279589	-2.104198721	0.035361116	0.048295428	negative
PRJNA729271	stage_1D_vs_E10	ordinary_muscle_development	ENSGALG00010025870	ENSGALG00010025870	1.949341791	0.162771964	11.97590629	4.75216E-33	4.74398E-32	positive
PRJNA729271	stage_1D_vs_E10	ordinary_muscle_development	ENSGALG00010025871	ENSGALG00010025871	-0.708488818	1.597227315	-0.443574193	0.657350467	0.696614638	negative
PRJNA729271	stage_1D_vs_E10	ordinary_muscle_development	ENSGALG00010025874	MDH1B	-1.561270287	0.29811935	-5.237064569	1.63151E-07	3.8781E-07	negative
PRJNA729271	stage_1D_vs_E10	ordinary_muscle_development	ENSGALG00010025876	ENSGALG00010025876	1.342202367	1.784677425	0.752070009	0.452008959	0.501060711	positive
PRJNA729271	stage_1D_vs_E10	ordinary_muscle_development	ENSGALG00010025877	ENSGALG00010025877	0.859429198	3.503381349	0.245314202	0.806213156	0.832696288	positive
PRJNA729271	stage_1D_vs_E10	ordinary_muscle_development	ENSGALG00010025881	GAL	3.655442153	0.531057483	6.883326701	5.84707E-12	1.9188E-11	positive
PRJNA729271	stage_1D_vs_E10	ordinary_muscle_development	ENSGALG00010025882	LDHD	3.457993158	0.170919874	20.23166218	5.15361E-91	3.13872E-89	positive
PRJNA729271	stage_1D_vs_E10	ordinary_muscle_development	ENSGALG00010025886	ALDH2	-0.125144821	0.10998789	-1.137805456	0.255201726	0.300012086	negative
PRJNA729271	stage_1D_vs_E10	ordinary_muscle_development	ENSGALG00010025887	ZNF76	-0.216536629	0.113291429	-1.911324015	0.055962955	0.074042549	negative
PRJNA729271	stage_1D_vs_E10	ordinary_muscle_development	ENSGALG00010025889	TESMIN	2.337312184	0.534356669	4.374067587	1.21953E-05	2.47024E-05	positive
PRJNA729271	stage_1D_vs_E10	ordinary_muscle_development	ENSGALG00010025890	SCG2	0.287566306	0.512309854	0.561313244	0.574584014	0.619364564	positive
PRJNA729271	stage_1D_vs_E10	ordinary_muscle_development	ENSGALG00010025891	NUP93	-1.595533986	0.10888028	-14.65402165	1.26972E-48	2.32089E-47	negative
PRJNA729271	stage_1D_vs_E10	ordinary_muscle_development	ENSGALG00010025892	ZC3H11A	0.359550435	0.085809685	4.190091542	2.78842E-05	5.45233E-05	positive
PRJNA729271	stage_1D_vs_E10	ordinary_muscle_development	ENSGALG00010025894	DYTN	-0.092407063	0.456703698	-0.202334826	0.839654976	0.862687757	negative
PRJNA729271	stage_1D_vs_E10	ordinary_muscle_development	ENSGALG00010025897	MAPKAPK5	0.104716367	0.120923999	0.865968442	0.386507468	0.43554581	positive
PRJNA729271	stage_1D_vs_E10	ordinary_muscle_development	ENSGALG00010025898	ENSGALG00010025898	0.859496149	2.00257413	0.429195672	0.667780833	0.706531618	positive
PRJNA729271	stage_1D_vs_E10	ordinary_muscle_development	ENSGALG00010025899	ENSGALG00010025899	-0.027059738	0.169860696	-0.159305469	0.873428212	0.8920131	negative
PRJNA729271	stage_1D_vs_E10	ordinary_muscle_development	ENSGALG00010025900	FAM237A	-0.543378575	0.604590257	-0.898755098	0.368783122	0.417944594	negative
PRJNA729271	stage_1D_vs_E10	ordinary_muscle_development	ENSGALG00010025902	ENSGALG00010025902	1.343943702	1.873306446	0.717417967	0.473116237	0.521784969	positive
PRJNA729271	stage_1D_vs_E10	ordinary_muscle_development	ENSGALG00010025904	RPL10A	0.047449587	0.138828494	0.341785649	0.732512211	0.766459575	positive
PRJNA729271	stage_1D_vs_E10	ordinary_muscle_development	ENSGALG00010025905	CPT1A	0.685891523	0.174893912	3.921757562	8.79054E-05	0.000164052	positive
PRJNA729271	stage_1D_vs_E10	ordinary_muscle_development	ENSGALG00010025907	ARHGEF12	0.481299858	0.071305482	6.749829661	1.48019E-11	4.7457E-11	positive
PRJNA729271	stage_1D_vs_E10	ordinary_muscle_development	ENSGALG00010025910	HEATR3	-0.494996338	0.113243311	-4.371086781	1.2363E-05	2.50351E-05	negative
PRJNA729271	stage_1D_vs_E10	ordinary_muscle_development	ENSGALG00010025913	EIF2B3	-0.735325113	0.078425877	-9.3760522	6.84892E-21	3.84638E-20	negative
PRJNA729271	stage_1D_vs_E10	ordinary_muscle_development	ENSGALG00010025914	ENSGALG00010025914	-4.941100023	1.762371208	-2.80366588	0.005052523	0.007765489	negative
PRJNA729271	stage_1D_vs_E10	ordinary_muscle_development	ENSGALG00010025917	SCUBE3	-4.546868068	0.371273181	-12.24669139	1.7499E-34	1.86232E-33	negative
PRJNA729271	stage_1D_vs_E10	ordinary_muscle_development	ENSGALG00010025919	RGS9BP	-1.065617605	0.322467913	-3.304569426	0.000951224	0.001590678	negative
PRJNA729271	stage_1D_vs_E10	ordinary_muscle_development	ENSGALG00010025920	GTSF1	-0.220215579	0.262243081	-0.839738377	0.401055091	0.45050322	negative
PRJNA729271	stage_1D_vs_E10	ordinary_muscle_development	ENSGALG00010025924	TMEM116	2.532516846	0.167615057	15.10912497	1.41E-51	2.86245E-50	positive
PRJNA729271	stage_1D_vs_E10	ordinary_muscle_development	ENSGALG00010025925	KLHL18	0.278243273	0.09142307	3.043468939	0.002338676	0.003730916	positive
PRJNA729271	stage_1D_vs_E10	ordinary_muscle_development	ENSGALG00010025926	ENSGALG00010025926	0.742739433	0.414527956	1.791771634	0.073169554	0.095154557	positive
PRJNA729271	stage_1D_vs_E10	ordinary_muscle_development	ENSGALG00010025927	ENSGALG00010025927	-2.782439276	1.160011456	-2.39863086	0.016456495	0.023568511	negative
PRJNA729271	stage_1D_vs_E10	ordinary_muscle_development	ENSGALG00010025929	NELFE	-0.005925977	0.090212801	-0.065688875	0.94762553	0.95560262	negative
PRJNA729271	stage_1D_vs_E10	ordinary_muscle_development	ENSGALG00010025931	TMEM136	-1.773001249	0.191605179	-9.253409854	2.17443E-20	1.18535E-19	negative
PRJNA729271	stage_1D_vs_E10	ordinary_muscle_development	ENSGALG00010025932	AP1S3	-0.985204536	0.315964172	-3.118089403	0.001820276	0.002946058	negative
PRJNA729271	stage_1D_vs_E10	ordinary_muscle_development	ENSGALG00010025933	SKIC2	0.146736922	0.102160017	1.436343942	0.150904496	0.18596843	positive
PRJNA729271	stage_1D_vs_E10	ordinary_muscle_development	ENSGALG00010025935	CATH3	2.156598586	0.372737079	5.785843979	7.21492E-09	1.90944E-08	positive
PRJNA729271	stage_1D_vs_E10	ordinary_muscle_development	ENSGALG00010025936	GOSR2	-0.455964556	0.067858558	-6.719337508	1.82553E-11	5.80795E-11	negative
PRJNA729271	stage_1D_vs_E10	ordinary_muscle_development	ENSGALG00010025938	ERP29	-0.726240475	0.08977606	-8.089466954	5.99264E-16	2.55857E-15	negative
PRJNA729271	stage_1D_vs_E10	ordinary_muscle_development	ENSGALG00010025939	TMEM136-1	-3.118662459	0.713493053	-4.370978028	1.23691E-05	2.50441E-05	negative
PRJNA729271	stage_1D_vs_E10	ordinary_muscle_development	ENSGALG00010025940	HECTD3	0.329479664	0.088175291	3.73664391	0.000186493	0.000336346	positive
PRJNA729271	stage_1D_vs_E10	ordinary_muscle_development	ENSGALG00010025941	ENSGALG00010025941	-3.991187219	0.829831283	-4.80963697	1.51205E-06	3.32834E-06	negative
PRJNA729271	stage_1D_vs_E10	ordinary_muscle_development	ENSGALG00010025942	ENSGALG00010025942	-1.077844619	1.318310545	-0.817595386	0.413588263	0.463110226	negative
PRJNA729271	stage_1D_vs_E10	ordinary_muscle_development	ENSGALG00010025943	CATH1	3.804680423	0.71322178	5.334498373	9.5809E-08	2.3169E-07	positive
PRJNA729271	stage_1D_vs_E10	ordinary_muscle_development	ENSGALG00010025946	KRT5	-9.199517933	1.256988511	-7.318696913	2.5039E-13	9.02389E-13	negative
PRJNA729271	stage_1D_vs_E10	ordinary_muscle_development	ENSGALG00010025948	WNT9B	-7.557110888	0.891083629	-8.480809931	2.23632E-17	1.03564E-16	negative
PRJNA729271	stage_1D_vs_E10	ordinary_muscle_development	ENSGALG00010025949	GPATCH1	0.120794996	0.121859126	0.991267539	0.321554959	0.369622453	positive
PRJNA729271	stage_1D_vs_E10	ordinary_muscle_development	ENSGALG00010025951	TBRG4	0.469450958	0.113251442	4.145209545	3.39503E-05	6.58605E-05	positive
PRJNA729271	stage_1D_vs_E10	ordinary_muscle_development	ENSGALG00010025953	POU2F3	-2.97321253	1.082342719	-2.747015782	0.006014024	0.009153011	negative
PRJNA729271	stage_1D_vs_E10	ordinary_muscle_development	ENSGALG00010025954	TUBA1A	-2.302068271	0.158695531	-14.50619469	1.10693E-47	1.93313E-46	negative
PRJNA729271	stage_1D_vs_E10	ordinary_muscle_development	ENSGALG00010025956	ENSGALG00010025956	1.713315046	0.488109627	3.510102958	0.000447933	0.000774704	positive
PRJNA729271	stage_1D_vs_E10	ordinary_muscle_development	ENSGALG00010025957	ENSGALG00010025957	-0.301076986	0.114977478	-2.618573578	0.008829825	0.013144532	negative
PRJNA729271	stage_1D_vs_E10	ordinary_muscle_development	ENSGALG00010025958	HOXC9	-4.817483283	0.459281933	-10.48916349	9.68823E-26	6.85029E-25	negative
PRJNA729271	stage_1D_vs_E10	ordinary_muscle_development	ENSGALG00010025959	UROD	-0.478629765	0.104212222	-4.592837174	4.3726E-06	9.21367E-06	negative
PRJNA729271	stage_1D_vs_E10	ordinary_muscle_development	ENSGALG00010025960	CMKLR2	1.98859583	0.158064046	12.5809498	2.68779E-36	3.06526E-35	positive
PRJNA729271	stage_1D_vs_E10	ordinary_muscle_development	ENSGALG00010025962	ENSGALG00010025962	0.922957181	0.183631405	5.02614017	5.0045E-07	1.14435E-06	positive
PRJNA729271	stage_1D_vs_E10	ordinary_muscle_development	ENSGALG00010025963	OAF	0.210977691	0.24543664	0.859601447	0.390008777	0.439218182	positive
PRJNA729271	stage_1D_vs_E10	ordinary_muscle_development	ENSGALG00010025964	HOXC10	-9.529149975	1.094367161	-8.70745241	3.10781E-18	1.50964E-17	negative
PRJNA729271	stage_1D_vs_E10	ordinary_muscle_development	ENSGALG00010025968	FADS2	-0.135542493	0.156369809	-0.866807306	0.386047599	0.435095245	negative
PRJNA729271	stage_1D_vs_E10	ordinary_muscle_development	ENSGALG00010025970	KLHL12	-0.264427928	0.110678407	-2.389155518	0.016887152	0.024156644	negative
PRJNA729271	stage_1D_vs_E10	ordinary_muscle_development	ENSGALG00010025971	TRAFD1	-0.154856908	0.199033298	-0.778045232	0.436542348	0.485885075	negative
PRJNA729271	stage_1D_vs_E10	ordinary_muscle_development	ENSGALG00010025972	WNT3	-7.781135955	1.318245216	-5.902646835	3.57715E-09	9.69537E-09	negative
PRJNA729271	stage_1D_vs_E10	ordinary_muscle_development	ENSGALG00010025974	EEF1B2	0.463355049	0.095535206	4.850097334	1.23401E-06	2.73501E-06	positive
PRJNA729271	stage_1D_vs_E10	ordinary_muscle_development	ENSGALG00010025976	NAA25	-0.367831794	0.098865336	-3.720533484	0.000198802	0.000357081	negative
PRJNA729271	stage_1D_vs_E10	ordinary_muscle_development	ENSGALG00010025978	TRIM29	-5.723400986	0.566362164	-10.10554967	5.2202E-24	3.39313E-23	negative
PRJNA729271	stage_1D_vs_E10	ordinary_muscle_development	ENSGALG00010025980	HECTD4	0.109013506	0.063910193	1.705729584	0.088058439	0.113016387	positive
PRJNA729271	stage_1D_vs_E10	ordinary_muscle_development	ENSGALG00010025982	SERPINE2	-0.509953652	0.198854019	-2.564462393	0.010333578	0.01523911	negative
PRJNA729271	stage_1D_vs_E10	ordinary_muscle_development	ENSGALG00010025983	RNF26	-0.734885866	0.082714288	-8.884630292	6.41337E-19	3.2256E-18	negative
PRJNA729271	stage_1D_vs_E10	ordinary_muscle_development	ENSGALG00010025984	NDUFS1	1.463301472	0.116873132	12.52042657	5.77241E-36	6.51137E-35	positive
PRJNA729271	stage_1D_vs_E10	ordinary_muscle_development	ENSGALG00010025985	CHCHD10	2.365456325	0.318377316	7.429726327	1.08823E-13	4.02086E-13	positive
PRJNA729271	stage_1D_vs_E10	ordinary_muscle_development	ENSGALG00010025986	RAB3IL1	-0.242599691	0.108101865	-2.244176745	0.024821032	0.034660969	negative
PRJNA729271	stage_1D_vs_E10	ordinary_muscle_development	ENSGALG00010025988	NSF	-0.27445774	0.059116463	-4.642661725	3.43949E-06	7.33492E-06	negative
PRJNA729271	stage_1D_vs_E10	ordinary_muscle_development	ENSGALG00010025990	FAM124B	0.99051106	0.238329643	4.156054815	3.2379E-05	6.29218E-05	positive
PRJNA729271	stage_1D_vs_E10	ordinary_muscle_development	ENSGALG00010025992	MMP11	-1.330759705	0.306107472	-4.347361069	1.37785E-05	2.77504E-05	negative
PRJNA729271	stage_1D_vs_E10	ordinary_muscle_development	ENSGALG00010025994	INO80D	0.843913004	0.106862361	7.897195911	2.85248E-15	1.16404E-14	positive
PRJNA729271	stage_1D_vs_E10	ordinary_muscle_development	ENSGALG00010025996	CUL3	0.774114134	0.096710041	8.004485637	1.19967E-15	5.01419E-15	positive
PRJNA729271	stage_1D_vs_E10	ordinary_muscle_development	ENSGALG00010025997	CCDC43	0.167476258	0.148367152	1.128796068	0.258983872	0.304039137	positive
PRJNA729271	stage_1D_vs_E10	ordinary_muscle_development	ENSGALG00010025998	SMARCB1	-0.960689039	0.143707124	-6.685048104	2.30849E-11	7.29009E-11	negative
PRJNA729271	stage_1D_vs_E10	ordinary_muscle_development	ENSGALG00010025999	DBF4B	-1.330731143	0.158684753	-8.386005063	5.02942E-17	2.28245E-16	negative
PRJNA729271	stage_1D_vs_E10	ordinary_muscle_development	ENSGALG00010026001	ANKS1A	0.945823301	0.091684513	10.31606397	5.96173E-25	4.07427E-24	positive
PRJNA729271	stage_1D_vs_E10	ordinary_muscle_development	ENSGALG00010026002	FTH1	0.962968478	0.14185285	6.788502845	1.13303E-11	3.65449E-11	positive
PRJNA729271	stage_1D_vs_E10	ordinary_muscle_development	ENSGALG00010026003	NECTIN1	-0.029219548	0.155130901	-0.18835414	0.850599047	0.872148916	negative
PRJNA729271	stage_1D_vs_E10	ordinary_muscle_development	ENSGALG00010026005	DERL3	-0.056424174	0.115706856	-0.487647629	0.625799453	0.667719033	negative
PRJNA729271	stage_1D_vs_E10	ordinary_muscle_development	ENSGALG00010026006	ITGA2B	0.190732928	0.293879468	0.649017534	0.516327043	0.563915673	positive
PRJNA729271	stage_1D_vs_E10	ordinary_muscle_development	ENSGALG00010026008	NRP2	1.115028448	0.100216147	11.12623551	9.35014E-29	7.60978E-28	positive
PRJNA729271	stage_1D_vs_E10	ordinary_muscle_development	ENSGALG00010026009	ENSGALG00010026009	-1.497352904	0.307356805	-4.871708968	1.10637E-06	2.4619E-06	negative
PRJNA729271	stage_1D_vs_E10	ordinary_muscle_development	ENSGALG00010026010	ZSWIM5	-0.185135267	0.127126496	-1.456307477	0.14530764	0.179697486	negative
PRJNA729271	stage_1D_vs_E10	ordinary_muscle_development	ENSGALG00010026011	GJC2	-0.401928734	0.164049476	-2.450045824	0.014283804	0.020658924	negative
PRJNA729271	stage_1D_vs_E10	ordinary_muscle_development	ENSGALG00010026013	RABIF	-0.904385643	0.11339456	-7.975564624	1.51686E-15	6.30176E-15	negative
PRJNA729271	stage_1D_vs_E10	ordinary_muscle_development	ENSGALG00010026016	SLC2A11	-1.91678313	0.412042052	-4.65191143	3.28872E-06	7.02891E-06	negative
PRJNA729271	stage_1D_vs_E10	ordinary_muscle_development	ENSGALG00010026017	PLEKHM1	0.734654378	0.14453004	5.083056617	3.71409E-07	8.56302E-07	positive
PRJNA729271	stage_1D_vs_E10	ordinary_muscle_development	ENSGALG00010026023	THY1	-0.142069839	0.29986076	-0.47378603	0.635652498	0.676538876	negative
PRJNA729271	stage_1D_vs_E10	ordinary_muscle_development	ENSGALG00010026030	IBA57	0.125036008	0.083795162	1.492162613	0.135656521	0.168437662	positive
PRJNA729271	stage_1D_vs_E10	ordinary_muscle_development	ENSGALG00010026036	TUB5A	-2.056919093	0.207439634	-9.915747812	3.55584E-23	2.23415E-22	negative
PRJNA729271	stage_1D_vs_E10	ordinary_muscle_development	ENSGALG00010026040	GUK1	-0.77259285	0.117475299	-6.576640862	4.81195E-11	1.49005E-10	negative
PRJNA729271	stage_1D_vs_E10	ordinary_muscle_development	ENSGALG00010026051	TAF11	-0.487580417	0.128952412	-3.781088006	0.000156144	0.000284372	negative
PRJNA729271	stage_1D_vs_E10	ordinary_muscle_development	ENSGALG00010026061	MPEG1	1.37963568	0.343769307	4.013260203	5.98858E-05	0.000113678	positive
PRJNA729271	stage_1D_vs_E10	ordinary_muscle_development	ENSGALG00010026062	ITGA4	-2.360930388	0.161465462	-14.62189104	2.03664E-48	3.69475E-47	negative
PRJNA729271	stage_1D_vs_E10	ordinary_muscle_development	ENSGALG00010026064	ENSGALG00010026064	-0.096633129	0.149851738	-0.644858247	0.519019051	0.566445866	negative
PRJNA729271	stage_1D_vs_E10	ordinary_muscle_development	ENSGALG00010026066	SELENOP2	1.940349206	0.566813482	3.423258739	0.000618752	0.001053171	positive
PRJNA729271	stage_1D_vs_E10	ordinary_muscle_development	ENSGALG00010026068	BLTP3A	0.855731385	0.110680261	7.731562752	1.06234E-14	4.17647E-14	positive
PRJNA729271	stage_1D_vs_E10	ordinary_muscle_development	ENSGALG00010026073	HPDL	-0.146383356	0.226912226	-0.645110044	0.518855875	0.566369148	negative
PRJNA729271	stage_1D_vs_E10	ordinary_muscle_development	ENSGALG00010026076	C1orf35	0.088835344	0.133502847	0.665419099	0.505782472	0.553906013	positive
PRJNA729271	stage_1D_vs_E10	ordinary_muscle_development	ENSGALG00010026078	UBE2E3	0.510749471	0.106129557	4.812509224	1.49047E-06	3.28184E-06	positive
PRJNA729271	stage_1D_vs_E10	ordinary_muscle_development	ENSGALG00010026081	KPNA1	-0.060868876	0.108198898	-0.562564658	0.573731363	0.618537457	negative
PRJNA729271	stage_1D_vs_E10	ordinary_muscle_development	ENSGALG00010026083	MUTYH	-1.197925554	0.118142936	-10.13962913	3.68496E-24	2.41471E-23	negative
PRJNA729271	stage_1D_vs_E10	ordinary_muscle_development	ENSGALG00010026088	SNRPC	-0.50767943	0.081925803	-6.196819719	5.76154E-10	1.65504E-09	negative
PRJNA729271	stage_1D_vs_E10	ordinary_muscle_development	ENSGALG00010026090	USP2	4.156803049	0.23391779	17.77035877	1.19921E-70	4.50326E-69	positive
PRJNA729271	stage_1D_vs_E10	ordinary_muscle_development	ENSGALG00010026091	DTX4	0.21201725	0.162690223	1.303196016	0.19250785	0.232281802	positive
PRJNA729271	stage_1D_vs_E10	ordinary_muscle_development	ENSGALG00010026093	TOE1	-0.62698033	0.12651395	-4.955819723	7.2026E-07	1.62594E-06	negative
PRJNA729271	stage_1D_vs_E10	ordinary_muscle_development	ENSGALG00010026094	C6orf106	0.555072529	0.078083999	7.108659116	1.17176E-12	4.02289E-12	positive
PRJNA729271	stage_1D_vs_E10	ordinary_muscle_development	ENSGALG00010026095	MIF	-1.799655438	0.199758461	-9.009157509	2.07645E-19	1.07417E-18	negative
PRJNA729271	stage_1D_vs_E10	ordinary_muscle_development	ENSGALG00010026097	ARF1	-0.793722189	0.056033455	-14.16514806	1.50549E-45	2.40862E-44	negative
PRJNA729271	stage_1D_vs_E10	ordinary_muscle_development	ENSGALG00010026098	STAT3	1.175385375	0.10798039	10.88517442	1.35639E-27	1.03971E-26	positive
PRJNA729271	stage_1D_vs_E10	ordinary_muscle_development	ENSGALG00010026102	IPO9	-0.950778045	0.05862039	-16.2192377	3.68743E-59	9.82524E-58	negative
PRJNA729271	stage_1D_vs_E10	ordinary_muscle_development	ENSGALG00010026104	DDX51	-0.668556517	0.103971072	-6.430216662	1.27422E-10	3.83192E-10	negative
PRJNA729271	stage_1D_vs_E10	ordinary_muscle_development	ENSGALG00010026105	CNTF	3.061197897	0.181056053	16.9074596	3.96433E-64	1.23047E-62	positive
PRJNA729271	stage_1D_vs_E10	ordinary_muscle_development	ENSGALG00010026106	TESK2	0.192748012	0.119203739	1.616962811	0.105886309	0.134033887	positive
PRJNA729271	stage_1D_vs_E10	ordinary_muscle_development	ENSGALG00010026111	ENSGALG00010026111	-2.020687779	0.15975741	-12.64847608	1.14066E-36	1.32377E-35	negative
PRJNA729271	stage_1D_vs_E10	ordinary_muscle_development	ENSGALG00010026112	ZFP91	0.451049592	0.12072762	3.73609282	0.000186902	0.000337	positive
PRJNA729271	stage_1D_vs_E10	ordinary_muscle_development	ENSGALG00010026113	MFRP	3.856872042	0.466745715	8.26332609	1.41669E-16	6.27018E-16	positive
PRJNA729271	stage_1D_vs_E10	ordinary_muscle_development	ENSGALG00010026114	CWC22	-0.254765405	0.082840984	-3.075354622	0.002102524	0.003374982	negative
PRJNA729271	stage_1D_vs_E10	ordinary_muscle_development	ENSGALG00010026115	MMACHC	0.838450547	0.174768948	4.797480091	1.60674E-06	3.52768E-06	positive
PRJNA729271	stage_1D_vs_E10	ordinary_muscle_development	ENSGALG00010026116	DLX3	-4.888418384	0.759606231	-6.435463773	1.23097E-10	3.70569E-10	negative
PRJNA729271	stage_1D_vs_E10	ordinary_muscle_development	ENSGALG00010026117	LZTR1	-0.146779464	0.071312965	-2.058243738	0.039566742	0.053584969	negative
PRJNA729271	stage_1D_vs_E10	ordinary_muscle_development	ENSGALG00010026120	C1QTNF5	0.233095499	0.196018507	1.189150462	0.234380464	0.277787803	positive
PRJNA729271	stage_1D_vs_E10	ordinary_muscle_development	ENSGALG00010026121	ENSGALG00010026121	0.646509393	0.258491873	2.501082092	0.012381447	0.018080705	positive
PRJNA729271	stage_1D_vs_E10	ordinary_muscle_development	ENSGALG00010026124	PACSIN1	-1.273567092	0.313565013	-4.061572683	4.87432E-05	9.32348E-05	negative
PRJNA729271	stage_1D_vs_E10	ordinary_muscle_development	ENSGALG00010026127	ENSGALG00010026127	2.135672988	0.58802299	3.631954911	0.000281282	0.000497339	positive

PRJNA729271	stage_1D_vs_E10	ordinary_muscle_development	ENSGALG00010026128	PRDX1	-1.502794867	0.142118867	-10.57421089	3.92467E-26	2.82323E-25	negative
PRJNA729271	stage_1D_vs_E10	ordinary_muscle_development	ENSGALG00010026132	DDTL	-0.095586414	0.18539142	-0.515592437	0.606139094	0.648939077	negative
PRJNA729271	stage_1D_vs_E10	ordinary_muscle_development	ENSGALG00010026137	LPXN	0.279240888	0.370454816	0.753778532	0.450982217	0.500266873	positive
PRJNA729271	stage_1D_vs_E10	ordinary_muscle_development	ENSGALG00010026141	WNT3A	-7.405101845	0.966025017	-7.665538381	1.78083E-14	6.90374E-14	negative
PRJNA729271	stage_1D_vs_E10	ordinary_muscle_development	ENSGALG00010026143	RNPEP	-0.37769148	0.141371468	-2.671624512	0.007548506	0.011337229	negative
PRJNA729271	stage_1D_vs_E10	ordinary_muscle_development	ENSGALG00010026148	RPS10	0.249633183	0.134085116	1.861751621	0.062638109	0.082248336	positive
PRJNA729271	stage_1D_vs_E10	ordinary_muscle_development	ENSGALG00010026149	LASP1	-0.393647493	0.128866089	-3.054701946	0.002252843	0.003602312	negative
PRJNA729271	stage_1D_vs_E10	ordinary_muscle_development	ENSGALG00010026154	ZNF385B	-1.71334339	0.178694538	-9.588112802	8.97108E-22	5.29462E-21	negative
PRJNA729271	stage_1D_vs_E10	ordinary_muscle_development	ENSGALG00010026162	MED1	0.044997068	0.151328793	0.297346378	0.766202077	0.796193212	positive
PRJNA729271	stage_1D_vs_E10	ordinary_muscle_development	ENSGALG00010026163	CTNND1	-0.200674195	0.081564148	-2.460323571	0.01388118	0.020106696	negative
PRJNA729271	stage_1D_vs_E10	ordinary_muscle_development	ENSGALG00010026166	WNT9A	-1.905263446	0.244388769	-7.796035203	6.38826E-15	2.54459E-14	negative
PRJNA729271	stage_1D_vs_E10	ordinary_muscle_development	ENSGALG00010026167	CABIN1	-0.313126528	0.059001936	-5.307055143	1.1141E-07	2.68478E-07	negative
PRJNA729271	stage_1D_vs_E10	ordinary_muscle_development	ENSGALG00010026173	CDK12	0.106895339	0.162120325	0.659358033	0.509665884	0.557555245	positive
PRJNA729271	stage_1D_vs_E10	ordinary_muscle_development	ENSGALG00010026183	SESTD1	0.176921271	0.168315936	1.05112609	0.293200683	0.339859568	positive
PRJNA729271	stage_1D_vs_E10	ordinary_muscle_development	ENSGALG00010026184	NUDT3	-0.032743837	0.104433406	-0.313537958	0.753871987	0.785294607	negative
PRJNA729271	stage_1D_vs_E10	ordinary_muscle_development	ENSGALG00010026192	SMIM29	0.96835539	0.128150369	7.556399539	4.1438E-14	1.57113E-13	positive
PRJNA729271	stage_1D_vs_E10	ordinary_muscle_development	ENSGALG00010026199	TBX6	-1.634694568	0.442137661	-3.69725249	0.000217946	0.000389582	negative
PRJNA729271	stage_1D_vs_E10	ordinary_muscle_development	ENSGALG00010026203	TMX2	0.492501312	0.102929579	4.784837517	1.71126E-06	3.74863E-06	positive
PRJNA729271	stage_1D_vs_E10	ordinary_muscle_development	ENSGALG00010026205	ENSGALG00010026205	-0.530045823	1.364842328	-0.388356818	0.697751996	0.734809298	negative
PRJNA729271	stage_1D_vs_E10	ordinary_muscle_development	ENSGALG00010026209	MED19	1.111828018	0.170993054	6.50218235	7.91631E-11	2.41318E-10	positive
PRJNA729271	stage_1D_vs_E10	ordinary_muscle_development	ENSGALG00010026213	AKR1A1	-1.436821539	0.124170175	-11.57139015	5.75427E-31	5.20001E-30	negative
PRJNA729271	stage_1D_vs_E10	ordinary_muscle_development	ENSGALG00010026218	FTSJ3	-0.519212786	0.167239394	-3.104608145	0.001905313	0.003074077	negative
PRJNA729271	stage_1D_vs_E10	ordinary_muscle_development	ENSGALG00010026220	ENSGALG00010026220	-0.039833461	0.096365181	-0.413359473	0.679343262	0.717246747	negative
PRJNA729271	stage_1D_vs_E10	ordinary_muscle_development	ENSGALG00010026221	CRKL	0.603089279	0.145651553	4.14063061	3.46352E-05	6.71173E-05	positive
PRJNA729271	stage_1D_vs_E10	ordinary_muscle_development	ENSGALG00010026229	CRHR2	0.818859333	0.212268582	3.857656767	0.000114479	0.000211359	positive
PRJNA729271	stage_1D_vs_E10	ordinary_muscle_development	ENSGALG00010026232	PSMC5	-0.202092929	0.131145504	-1.54098252	0.12332104	0.154484356	negative
PRJNA729271	stage_1D_vs_E10	ordinary_muscle_development	ENSGALG00010026234	ZDHHC5	1.102547713	0.159083751	6.930611743	4.19025E-12	1.38892E-11	positive
PRJNA729271	stage_1D_vs_E10	ordinary_muscle_development	ENSGALG00010026237	KLHL22	0.564557886	0.139209087	4.055467193	5.00342E-05	9.5578E-05	positive
PRJNA729271	stage_1D_vs_E10	ordinary_muscle_development	ENSGALG00010026239	GIP	-2.719342794	0.97964383	-2.775848437	0.005505787	0.008419283	negative
PRJNA729271	stage_1D_vs_E10	ordinary_muscle_development	ENSGALG00010026242	CCDC141	2.068046345	0.253399518	8.161208688	3.31689E-16	1.43861E-15	positive
PRJNA729271	stage_1D_vs_E10	ordinary_muscle_development	ENSGALG00010026244	CLP1	-0.708189411	0.129038207	-5.48821489	4.06016E-08	1.00826E-07	negative
PRJNA729271	stage_1D_vs_E10	ordinary_muscle_development	ENSGALG00010026249	YPEL4	-0.925177938	0.616485674	-1.500729016	0.133425665	0.165838194	negative
PRJNA729271	stage_1D_vs_E10	ordinary_muscle_development	ENSGALG00010026252	ENO2	-0.308031703	0.349782762	-0.880637176	0.37851423	0.427535554	negative
PRJNA729271	stage_1D_vs_E10	ordinary_muscle_development	ENSGALG00010026254	ENSGALG00010026254	-1.024916385	0.575179638	-1.781906587	0.074764466	0.097050115	negative
PRJNA729271	stage_1D_vs_E10	ordinary_muscle_development	ENSGALG00010026259	MED15	0.078674637	0.087556596	0.898557516	0.368888397	0.417998539	positive
PRJNA729271	stage_1D_vs_E10	ordinary_muscle_development	ENSGALG00010026260	ENSGALG00010026260	18.68748269	3.173145464	5.889261273	3.87926E-09	1.04789E-08	positive
PRJNA729271	stage_1D_vs_E10	ordinary_muscle_development	ENSGALG00010026261	IFT70A	-0.986965885	0.108889211	-9.063945609	1.25815E-19	6.59563E-19	negative
PRJNA729271	stage_1D_vs_E10	ordinary_muscle_development	ENSGALG00010026262	NAV1	0.538370552	0.159604842	3.373146744	0.000743143	0.00125722	positive
PRJNA729271	stage_1D_vs_E10	ordinary_muscle_development	ENSGALG00010026264	FZD7	-0.75825188	0.13908149	-5.451853282	4.98476E-08	1.23153E-07	negative
PRJNA729271	stage_1D_vs_E10	ordinary_muscle_development	ENSGALG00010026266	ENSGALG00010026266	-0.937746421	0.310451413	-3.020589956	0.002522828	0.004008373	negative
PRJNA729271	stage_1D_vs_E10	ordinary_muscle_development	ENSGALG00010026268	GARS1	-0.677648942	0.151019212	-4.487170435	7.21753E-06	1.49497E-05	negative
PRJNA729271	stage_1D_vs_E10	ordinary_muscle_development	ENSGALG00010026272	LRRC58	1.289323349	0.094628528	13.62510205	2.84013E-42	4.02421E-41	positive
PRJNA729271	stage_1D_vs_E10	ordinary_muscle_development	ENSGALG00010026277	SMARCD2	0.862393926	0.123403127	6.98842846	2.77982E-12	9.33585E-12	positive
PRJNA729271	stage_1D_vs_E10	ordinary_muscle_development	ENSGALG00010026287	NASP	-1.300990941	0.089313903	-14.56649964	4.58805E-48	8.15997E-47	negative
PRJNA729271	stage_1D_vs_E10	ordinary_muscle_development	ENSGALG00010026291	SERPING1	1.793929314	0.168784533	10.62851724	2.19576E-26	1.60018E-25	positive
PRJNA729271	stage_1D_vs_E10	ordinary_muscle_development	ENSGALG00010026295	SMPD4	-0.105592639	0.12851617	-0.82162921	0.411287959	0.460997446	negative
PRJNA729271	stage_1D_vs_E10	ordinary_muscle_development	ENSGALG00010026298	SNF8	-0.612847041	0.117027385	-5.236783191	1.63399E-07	3.88338E-07	negative
PRJNA729271	stage_1D_vs_E10	ordinary_muscle_development	ENSGALG00010026300	HMGA1	-1.367916032	0.109375181	-12.50664014	6.86677E-36	7.72179E-35	negative
PRJNA729271	stage_1D_vs_E10	ordinary_muscle_development	ENSGALG00010026305	ATP5MC1	1.895604982	0.166940629	11.3549649	7.0068E-30	6.01324E-29	positive
PRJNA729271	stage_1D_vs_E10	ordinary_muscle_development	ENSGALG00010026315	SYT2	-0.889295666	0.272861121	-3.259151263	0.001117461	0.00185412	negative
PRJNA729271	stage_1D_vs_E10	ordinary_muscle_development	ENSGALG00010026316	ENSGALG00010026316	0.303498342	0.351841144	0.862600485	0.388357164	0.437460141	positive
PRJNA729271	stage_1D_vs_E10	ordinary_muscle_development	ENSGALG00010026320	ENSGALG00010026320	0.786028541	0.174460651	4.505477528	6.62238E-06	1.37602E-05	positive
PRJNA729271	stage_1D_vs_E10	ordinary_muscle_development	ENSGALG00010026321	GPBP1L1	0.352584066	0.047277922	7.457689643	8.80528E-14	3.26928E-13	positive
PRJNA729271	stage_1D_vs_E10	ordinary_muscle_development	ENSGALG00010026325	TMEM69	0.055090223	0.137907334	0.399472754	0.689544896	0.727048414	positive
PRJNA729271	stage_1D_vs_E10	ordinary_muscle_development	ENSGALG00010026329	ENSGALG00010026329	3.575164044	0.268486443	13.31599469	1.86867E-40	2.49414E-39	positive
PRJNA729271	stage_1D_vs_E10	ordinary_muscle_development	ENSGALG00010026334	ENSGALG00010026334	0.731175224	0.12700468	5.757073063	8.55849E-09	2.24697E-08	positive
PRJNA729271	stage_1D_vs_E10	ordinary_muscle_development	ENSGALG00010026335	IPP	-0.823262639	0.101941805	-8.075809931	6.70302E-16	2.84844E-15	negative
PRJNA729271	stage_1D_vs_E10	ordinary_muscle_development	ENSGALG00010026344	TIMM10	0.269652938	0.171697992	1.570507233	0.116297153	0.146353598	positive
PRJNA729271	stage_1D_vs_E10	ordinary_muscle_development	ENSGALG00010026346	FZD2	0.876449721	0.162858045	5.381678989	7.37943E-08	1.80166E-07	positive
PRJNA729271	stage_1D_vs_E10	ordinary_muscle_development	ENSGALG00010026354	ENSGALG00010026354	1.538779788	0.245072288	6.278881224	3.41018E-10	9.95981E-10	positive
PRJNA729271	stage_1D_vs_E10	ordinary_muscle_development	ENSGALG00010026355	PPP1R12B	2.135268768	0.137119644	15.57230394	1.12289E-54	2.54714E-53	positive
PRJNA729271	stage_1D_vs_E10	ordinary_muscle_development	ENSGALG00010026360	CTDSPL	1.671384086	0.126451054	13.21763667	6.94107E-40	9.03961E-39	positive
PRJNA729271	stage_1D_vs_E10	ordinary_muscle_development	ENSGALG00010026362	ENSGALG00010026362	-0.259332596	0.381919695	-0.679023886	0.497122727	0.54527385	negative
PRJNA729271	stage_1D_vs_E10	ordinary_muscle_development	ENSGALG00010026367	RBP	3.460417937	0.521160757	6.639828286	3.14049E-11	9.84033E-11	positive
PRJNA729271	stage_1D_vs_E10	ordinary_muscle_development	ENSGALG00010026377	ENSGALG00010026377	-4.116200728	0.221111269	-18.61596988	2.38502E-77	1.05399E-75	negative
PRJNA729271	stage_1D_vs_E10	ordinary_muscle_development	ENSGALG00010026388	SNRPD3	-0.374359182	0.079708353	-4.69661169	2.64513E-06	5.70467E-06	negative
PRJNA729271	stage_1D_vs_E10	ordinary_muscle_development	ENSGALG00010026393	SLC43A3	0.105195082	0.120795064	0.870855801	0.383832893	0.432868418	positive
PRJNA729271	stage_1D_vs_E10	ordinary_muscle_development	ENSGALG00010026402	TFEB	2.309709949	0.164271072	14.06035716	6.65495E-45	1.03724E-43	positive
PRJNA729271	stage_1D_vs_E10	ordinary_muscle_development	ENSGALG00010026403	GUCD1	1.012721427	0.116559641	8.688439849	3.67451E-18	1.77836E-17	positive
PRJNA729271	stage_1D_vs_E10	ordinary_muscle_development	ENSGALG00010026409	VILL	-3.071098164	0.268678912	-11.43036551	2.94865E-30	2.58878E-29	negative
PRJNA729271	stage_1D_vs_E10	ordinary_muscle_development	ENSGALG00010026410	ENSGALG00010026410	2.86603398	0.315296414	9.089966943	9.90692E-20	5.22945E-19	positive
PRJNA729271	stage_1D_vs_E10	ordinary_muscle_development	ENSGALG00010026416	ENSGALG00010026416	-3.820688595	1.639169416	-2.330868644	0.019760287	0.027982157	negative
PRJNA729271	stage_1D_vs_E10	ordinary_muscle_development	ENSGALG00010026422	P2RX3	-3.291813492	0.370928119	-8.87453207	7.0228E-19	3.52478E-18	negative
PRJNA729271	stage_1D_vs_E10	ordinary_muscle_development	ENSGALG00010026427	PGC	1.290013933	1.069324816	1.206381741	0.227670333	0.270609387	positive
PRJNA729271	stage_1D_vs_E10	ordinary_muscle_development	ENSGALG00010026433	VPS11	-0.321673836	0.072036688	-4.465416806	7.9913E-06	1.64747E-05	negative
PRJNA729271	stage_1D_vs_E10	ordinary_muscle_development	ENSGALG00010026434	ENSGALG00010026434	-0.144587753	0.145233499	-0.99555374	0.319467021	0.367513846	negative
PRJNA729271	stage_1D_vs_E10	ordinary_muscle_development	ENSGALG00010026436	PLCD1	-0.419727098	0.075968047	-5.525047916	3.29396E-08	8.23631E-08	negative
PRJNA729271	stage_1D_vs_E10	ordinary_muscle_development	ENSGALG00010026438	TOMM6	-0.120657885	0.121741956	-0.991095338	0.321639029	0.369689773	negative
PRJNA729271	stage_1D_vs_E10	ordinary_muscle_development	ENSGALG00010026443	UPB1	-0.488920968	0.37227971	-1.313316184	0.189076427	0.228540928	negative
PRJNA729271	stage_1D_vs_E10	ordinary_muscle_development	ENSGALG00010026448	ADORA2A	-1.117437235	0.116993846	-9.551247958	1.28143E-21	7.48363E-21	negative
PRJNA729271	stage_1D_vs_E10	ordinary_muscle_development	ENSGALG00010026457	SSRP1	-1.124100213	0.09877133	-11.38083503	5.20985E-30	4.49772E-29	negative
PRJNA729271	stage_1D_vs_E10	ordinary_muscle_development	ENSGALG00010026458	DLEC1	2.466831118	0.175021785	14.09442327	4.10992E-45	6.47535E-44	positive
PRJNA729271	stage_1D_vs_E10	ordinary_muscle_development	ENSGALG00010026480	USP49	0.192481907	0.212603852	0.905354747	0.365277481	0.414295547	positive
PRJNA729271	stage_1D_vs_E10	ordinary_muscle_development	ENSGALG00010026481	DIXDC1	-1.617138942	0.137593096	-11.75305296	6.81127E-32	6.46135E-31	negative
PRJNA729271	stage_1D_vs_E10	ordinary_muscle_development	ENSGALG00010026484	TNKS1BP1	0.280774633	0.111029296	2.528833764	0.011444222	0.016788179	positive
PRJNA729271	stage_1D_vs_E10	ordinary_muscle_development	ENSGALG00010026485	ACAA1	0.756571596	0.104685131	7.227116099	4.93358E-13	1.74761E-12	positive
PRJNA729271	stage_1D_vs_E10	ordinary_muscle_development	ENSGALG00010026492	SPECC1L	-0.466384959	0.075229105	-6.199528175	5.66327E-10	1.62843E-09	negative
PRJNA729271	stage_1D_vs_E10	ordinary_muscle_development	ENSGALG00010026498	ENSGALG00010026498	-0.35438805	0.087690657	-4.041343297	5.31459E-05	0.000101231	negative
PRJNA729271	stage_1D_vs_E10	ordinary_muscle_development	ENSGALG00010026507	APLNR	0.283417651	0.286548726	0.98907315	0.322627353	0.370766946	positive
PRJNA729271	stage_1D_vs_E10	ordinary_muscle_development	ENSGALG00010026508	CSRP1	1.916746731	0.148327798	12.92237026	3.3661E-38	4.12788E-37	positive
PRJNA729271	stage_1D_vs_E10	ordinary_muscle_development	ENSGALG00010026516	ENSGALG00010026516	1.495910028	0.166926672	8.961479988	3.20349E-19	1.6408E-18	positive
PRJNA729271	stage_1D_vs_E10	ordinary_muscle_development	ENSGALG00010026522	MED20	0.323570605	0.128396923	2.52008068	0.011732794	0.01718715	positive
PRJNA729271	stage_1D_vs_E10	ordinary_muscle_development	ENSGALG00010026536	BCR	0.072713949	0.096369048	0.75453635	0.450527227	0.49980041	positive
PRJNA729271	stage_1D_vs_E10	ordinary_muscle_development	ENSGALG00010026537	FJX1	-2.610535339	0.177159316	-14.73552394	3.81229E-49	7.0936E-48	negative
PRJNA729271	stage_1D_vs_E10	ordinary_muscle_development	ENSGALG00010026539	ENSGALG00010026539	-0.31205719	0.375534448	-0.830968214	0.405991586	0.455624655	negative
PRJNA729271	stage_1D_vs_E10	ordinary_muscle_development	ENSGALG00010026550	MYD88	0.457304204	0.105127727	4.349986601	1.36146E-05	2.74431E-05	positive
PRJNA729271	stage_1D_vs_E10	ordinary_muscle_development	ENSGALG00010026553	PLPP3	1.512218279	0.12741104	11.86881674	1.71881E-32	1.67547E-31	positive
PRJNA729271	stage_1D_vs_E10	ordinary_muscle_development	ENSGALG00010026559	RAB36	-0.654318575	0.434110849	-1.507261511	0.131743639	0.164042956	negative
PRJNA729271	stage_1D_vs_E10	ordinary_muscle_development	ENSGALG00010026560	TNNI1	-3.297825362	0.588561215	-5.603198572	2.10432E-08	5.35312E-08	negative
PRJNA729271	stage_1D_vs_E10	ordinary_muscle_development	ENSGALG00010026565	RSPH14	-1.941595714	0.446047874	-4.35288638	1.34357E-05	2.71164E-05	negative
PRJNA729271	stage_1D_vs_E10	ordinary_muscle_development	ENSGALG00010026566	ENSGALG00010026566	-0.325122205	0.123761717	-2.627001417	0.008614098	0.012837893	negative
PRJNA729271	stage_1D_vs_E10	ordinary_muscle_development	ENSGALG00010026568	BYSL	0.17049613	0.110596494	1.541605206	0.12316956	0.154334501	positive
PRJNA729271	stage_1D_vs_E10	ordinary_muscle_development	ENSGALG00010026569	GNAZ	-0.542512114	0.202887495	-2.673955408	0.007496242	0.011264246	negative
PRJNA729271	stage_1D_vs_E10	ordinary_muscle_development	ENSGALG00010026581	DNER	-3.470914289	0.198860863	-17.45398381	3.2104E-68	1.12132E-66	negative
PRJNA729271	stage_1D_vs_E10	ordinary_muscle_development	ENSGALG00010026584	ENSGALG00010026584	-0.727988202	0.111113334	-6.551762734	5.68618E-11	1.75104E-10	negative
PRJNA729271	stage_1D_vs_E10	ordinary_muscle_development	ENSGALG00010026588	CCND3	0.260137571	0.144061203	1.805743425	0.070958426	0.09243663	positive
PRJNA729271	stage_1D_vs_E10	ordinary_muscle_development	ENSGALG00010026595	PRKAA2	3.07317959	0.261026966	11.77341803	5.35116E-32	5.10296E-31	positive
PRJNA729271	stage_1D_vs_E10	ordinary_muscle_development	ENSGALG00010026601	ENSGALG00010026601	0.90502191	0.458453017	1.974077773	0.0483729	0.064659276	positive
PRJNA729271	stage_1D_vs_E10	ordinary_muscle_development	ENSGALG00010026612	MFF	-0.142627431	0.081735212	-1.744993709	0.080985964	0.10459862	negative
PRJNA729271	stage_1D_vs_E10	ordinary_muscle_development	ENSGALG00010026625	ENSGALG00010026625	3.553400388	0.639583156	5.555806708	2.76333E-08	6.95148E-08	positive
PRJNA729271	stage_1D_vs_E10	ordinary_muscle_development	ENSGALG00010026628	FYB2	-4.882023937	1.093081549	-4.466294342	7.95861E-06	1.64143E-05	negative
PRJNA729271	stage_1D_vs_E10	ordinary_muscle_development	ENSGALG00010026630	XIRP1	1.832202257	0.243747496	7.516804416	5.61313E-14	2.11111E-13	positive
PRJNA729271	stage_1D_vs_E10	ordinary_muscle_development	ENSGALG00010026633	SLC5A1	-0.975595543	0.521852796	-1.869484173	0.061555484	0.080922069	negative
PRJNA729271	stage_1D_vs_E10	ordinary_muscle_development	ENSGALG00010026635	C8A	-0.602577279	2.653507162	-0.227087113	0.820356005	0.845376105	negative
PRJNA729271	stage_1D_vs_E10	ordinary_muscle_development	ENSGALG00010026638	LAD1	-4.799364732	0.593869918	-8.081508397	6.39705E-16	2.72321E-15	negative
PRJNA729271	stage_1D_vs_E10	ordinary_muscle_development	ENSGALG00010026643	YWHAH	-1.416797906	0.112391103	-12.60596136	1.95771E-36	2.24501E-35	negative
PRJNA729271	stage_1D_vs_E10	ordinary_muscle_development	ENSGALG00010026659	LIPH	-0.237403494	0.25181265	-0.94278268	0.34579433	0.394637337	negative
PRJNA729271	stage_1D_vs_E10	ordinary_muscle_development	ENSGALG00010026666	ENSGALG00010026666	0.302722021	0.437867928	0.691354634	0.489342706	0.537716816	positive
PRJNA729271	stage_1D_vs_E10	ordinary_muscle_development	ENSGALG00010026671	TAF8	0.726915735	0.15459997	4.701913828	2.57734E-06	5.56843E-06	positive
PRJNA729271	stage_1D_vs_E10	ordinary_muscle_development	ENSGALG00010026673	DEPDC5	0.335127342	0.121626487	2.755381262	0.005862378	0.00893347	positive
PRJNA729271	stage_1D_vs_E10	ordinary_muscle_development	ENSGALG00010026681	PLXDC1	2.279954613	0.257812309	8.843466882	9.27935E-19	4.63328E-18	positive
PRJNA729271	stage_1D_vs_E10	ordinary_muscle_development	ENSGALG00010026686	ENSGALG00010026686	0.61485997	0.398489157	1.542977919	0.122836134	0.154010013	positive
PRJNA729271	stage_1D_vs_E10	ordinary_muscle_development	ENSGALG00010026690	KRT14	-4.839552133	0.440833245	-10.97819229	4.86563E-28	3.81228E-27	negative
PRJNA729271	stage_1D_vs_E10	ordinary_muscle_development	ENSGALG00010026692	NMNAT3	1.931487337	0.53197472	3.630787826	0.000282557	0.000499411	positive
PRJNA729271	stage_1D_vs_E10	ordinary_muscle_development	ENSGALG00010026696	CSRNP1	-0.309127551	0.15074403	-2.050678564	0.04029826	0.054519626	negative
PRJNA729271	stage_1D_vs_E10	ordinary_muscle_development	ENSGALG00010026697	CHN1	-1.159036522	0.116385073	-9.958635506	2.31214E-23	1.46224E-22	negative
PRJNA729271	stage_1D_vs_E10	ordinary_muscle_development	ENSGALG00010026700	C8B	-2.11957743	1.054053809	-2.01088162	0.044337962	0.059601109	negative
PRJNA729271	stage_1D_vs_E10	ordinary_muscle_development	ENSGALG00010026704	WDFY1	-0.189852547	0.069483104	-2.732355576	0.006288323	0.009548423	negative
PRJNA729271	stage_1D_vs_E10	ordinary_muscle_development	ENSGALG00010026706	KRT24	-8.362934307	0.622684628	-13.43044927	4.00971E-41	5.49346E-40	negative
PRJNA729271	stage_1D_vs_E10	ordinary_muscle_development	ENSGALG00010026707	GORASP1	-0.484679404	0.08200422	-5.910420272	3.41236E-09	9.25911E-09	negative
PRJNA729271	stage_1D_vs_E10	ordinary_muscle_development	ENSGALG00010026715	WDR48	-0.076950616	0.066197788	-1.162434852	0.245058873	0.289237714	negative

PRJNA729271	stage_1D_vs_E10	ordinary_muscle_development	ENSGALG00010026719	ENSGALG00010026719	-5.035267279	1.080669774	-4.659394944	3.1714E-06	6.78917E-06	negative
PRJNA729271	stage_1D_vs_E10	ordinary_muscle_development	ENSGALG00010026724	OLA1	0.424007825	0.097922165	4.33004953	1.49076E-05	2.98956E-05	positive
PRJNA729271	stage_1D_vs_E10	ordinary_muscle_development	ENSGALG00010026731	ENSGALG00010026731	-4.587873223	0.700282101	-6.551464359	5.69756E-11	1.75417E-10	negative
PRJNA729271	stage_1D_vs_E10	ordinary_muscle_development	ENSGALG00010026737	SCN11A	-5.522916729	1.025995482	-5.38298348	7.32613E-08	1.78956E-07	negative
PRJNA729271	stage_1D_vs_E10	ordinary_muscle_development	ENSGALG00010026740	DAB1	-3.14073164	0.139161561	-22.56895947	8.748E-113	8.5678E-111	negative
PRJNA729271	stage_1D_vs_E10	ordinary_muscle_development	ENSGALG00010026743	TNNT2	-5.020203192	0.588120675	-8.536008696	1.38938E-17	6.4881E-17	negative
PRJNA729271	stage_1D_vs_E10	ordinary_muscle_development	ENSGALG00010026746	HOXD10	-2.462571432	0.429562982	-5.732736609	9.8823E-09	2.57633E-08	negative
PRJNA729271	stage_1D_vs_E10	ordinary_muscle_development	ENSGALG00010026755	ENSGALG00010026755	-1.294881756	0.208502449	-6.210391118	5.28529E-10	1.52276E-09	negative
PRJNA729271	stage_1D_vs_E10	ordinary_muscle_development	ENSGALG00010026759	OMA1	-0.700615415	0.111125479	-6.304723468	2.88709E-10	8.47647E-10	negative
PRJNA729271	stage_1D_vs_E10	ordinary_muscle_development	ENSGALG00010026767	ENSGALG00010026767	14.91739787	2.614392523	5.705875358	1.15747E-08	3.00403E-08	positive
PRJNA729271	stage_1D_vs_E10	ordinary_muscle_development	ENSGALG00010026770	ENSGALG00010026770	-0.645750711	0.576195254	-1.120715082	0.26240916	0.307586995	negative
PRJNA729271	stage_1D_vs_E10	ordinary_muscle_development	ENSGALG00010026775	BTG2	3.560534134	0.365919919	9.730364341	2.2379E-22	1.35896E-21	positive
PRJNA729271	stage_1D_vs_E10	ordinary_muscle_development	ENSGALG00010026780	MYSM1	0.844541956	0.077395307	10.91205641	1.00947E-27	7.79969E-27	positive
PRJNA729271	stage_1D_vs_E10	ordinary_muscle_development	ENSGALG00010026782	HOXB9	-4.897209462	0.630196637	-7.770922874	7.79163E-15	3.08832E-14	negative
PRJNA729271	stage_1D_vs_E10	ordinary_muscle_development	ENSGALG00010026786	ENSGALG00010026786	2.861658611	0.569563869	5.024298009	5.05277E-07	1.15448E-06	positive
PRJNA729271	stage_1D_vs_E10	ordinary_muscle_development	ENSGALG00010026796	DCAKD	0.20009375	0.213653212	0.936535181	0.348997708	0.397823354	positive
PRJNA729271	stage_1D_vs_E10	ordinary_muscle_development	ENSGALG00010026801	FMOD	3.63762676	0.489895718	7.425308346	1.12517E-13	4.14787E-13	positive
PRJNA729271	stage_1D_vs_E10	ordinary_muscle_development	ENSGALG00010026822	PRELP	0.621629127	0.199753347	3.111983538	0.001858349	0.003005329	positive
PRJNA729271	stage_1D_vs_E10	ordinary_muscle_development	ENSGALG00010026825	ADAM23	-0.602373446	0.135473028	-4.446445556	8.73028E-06	1.79446E-05	negative
PRJNA729271	stage_1D_vs_E10	ordinary_muscle_development	ENSGALG00010026827	FAM117A	0.835298239	0.128934405	6.478474385	9.26547E-11	2.81204E-10	positive
PRJNA729271	stage_1D_vs_E10	ordinary_muscle_development	ENSGALG00010026834	ENSGALG00010026834	-0.116563178	2.099079829	-0.055530607	0.955715747	0.96268935	negative
PRJNA729271	stage_1D_vs_E10	ordinary_muscle_development	ENSGALG00010026836	ENSGALG00010026836	2.271536041	0.604677446	3.756607849	0.000172232	0.000312141	positive
PRJNA729271	stage_1D_vs_E10	ordinary_muscle_development	ENSGALG00010026839	FGGY	-0.016923427	0.117465123	-0.144071927	0.885443672	0.902525718	negative
PRJNA729271	stage_1D_vs_E10	ordinary_muscle_development	ENSGALG00010026846	MICAL1	-0.208714245	0.2267196	-0.920583156	0.3572681	0.406164793	negative
PRJNA729271	stage_1D_vs_E10	ordinary_muscle_development	ENSGALG00010026851	ENSGALG00010026851	0.859516734	1.37856128	0.623488231	0.532963734	0.579760549	positive
PRJNA729271	stage_1D_vs_E10	ordinary_muscle_development	ENSGALG00010026854	SMPD2	0.449212724	0.145698363	3.083169323	0.002048086	0.003290877	positive
PRJNA729271	stage_1D_vs_E10	ordinary_muscle_development	ENSGALG00010026856	UBE2Z	0.131415352	0.076346893	1.721292729	0.085197718	0.109645825	positive
PRJNA729271	stage_1D_vs_E10	ordinary_muscle_development	ENSGALG00010026874	TEAD3	0.701688431	0.160193283	4.380261252	1.18537E-05	2.40408E-05	positive
PRJNA729271	stage_1D_vs_E10	ordinary_muscle_development	ENSGALG00010026885	TULP1	5.308536264	0.620140206	8.560219461	1.12653E-17	5.29648E-17	positive
PRJNA729271	stage_1D_vs_E10	ordinary_muscle_development	ENSGALG00010026886	JUN	2.363186177	0.192587285	12.27072791	1.30079E-34	1.39563E-33	positive
PRJNA729271	stage_1D_vs_E10	ordinary_muscle_development	ENSGALG00010026889	ENSGALG00010026889	0.246480339	0.258199501	0.954611989	0.339773952	0.388531353	positive
PRJNA729271	stage_1D_vs_E10	ordinary_muscle_development	ENSGALG00010026893	FKBP5	4.378409657	0.385997171	11.34311334	8.02382E-30	6.86166E-29	positive
PRJNA729271	stage_1D_vs_E10	ordinary_muscle_development	ENSGALG00010026894	IP6K1	-0.084768579	0.065501495	-1.294147247	0.195614564	0.235540215	negative
PRJNA729271	stage_1D_vs_E10	ordinary_muscle_development	ENSGALG00010026897	ENSGALG00010026897	-0.18483009	0.215825806	-0.856385494	0.391784586	0.440978224	negative
PRJNA729271	stage_1D_vs_E10	ordinary_muscle_development	ENSGALG00010026898	PNPLA2	5.625001813	0.271483021	20.71953451	2.30907E-95	1.54953E-93	positive
PRJNA729271	stage_1D_vs_E10	ordinary_muscle_development	ENSGALG00010026899	HOOK1	-0.087909175	0.166237614	-0.528816391	0.596932826	0.640265008	negative
PRJNA729271	stage_1D_vs_E10	ordinary_muscle_development	ENSGALG00010026901	FAM212A	-0.371345439	0.172440388	-2.153471369	0.031281652	0.043023773	negative
PRJNA729271	stage_1D_vs_E10	ordinary_muscle_development	ENSGALG00010026907	ENSGALG00010026907	-0.429145438	0.132556992	-3.237440992	0.001206069	0.00199361	negative
PRJNA729271	stage_1D_vs_E10	ordinary_muscle_development	ENSGALG00010026909	ENSGALG00010026909	0.173283239	0.155202059	1.116500898	0.264207786	0.309345061	positive
PRJNA729271	stage_1D_vs_E10	ordinary_muscle_development	ENSGALG00010026916	ENSGALG00010026916	0.2820032	0.215645416	1.307717106	0.190969279	0.230602249	positive
PRJNA729271	stage_1D_vs_E10	ordinary_muscle_development	ENSGALG00010026917	AMT	-1.664090981	0.145978262	-11.39958072	4.20138E-30	3.65102E-29	negative
PRJNA729271	stage_1D_vs_E10	ordinary_muscle_development	ENSGALG00010026919	NICN1	-0.643802953	0.170545805	-3.774956252	0.000160036	0.000291202	negative
PRJNA729271	stage_1D_vs_E10	ordinary_muscle_development	ENSGALG00010026920	GATA2	2.248489133	0.197541218	11.38237964	5.11837E-30	4.42402E-29	positive
PRJNA729271	stage_1D_vs_E10	ordinary_muscle_development	ENSGALG00010026924	TPH1	-1.229702677	0.365377247	-3.36556993	0.000763857	0.001290907	negative
PRJNA729271	stage_1D_vs_E10	ordinary_muscle_development	ENSGALG00010026942	CDK18	-1.166893393	0.182007075	-6.411252943	1.44329E-10	4.3224E-10	negative
PRJNA729271	stage_1D_vs_E10	ordinary_muscle_development	ENSGALG00010026943	EEFSEC	-0.156327045	0.069792575	-2.239880762	0.025098665	0.035014932	negative
PRJNA729271	stage_1D_vs_E10	ordinary_muscle_development	ENSGALG00010026950	LHFPL5	1.728412534	0.263523111	6.558865104	5.42188E-11	1.67284E-10	positive
PRJNA729271	stage_1D_vs_E10	ordinary_muscle_development	ENSGALG00010026951	RUVBL1	-1.091339265	0.10640472	-10.25649299	1.10653E-24	7.45658E-24	negative
PRJNA729271	stage_1D_vs_E10	ordinary_muscle_development	ENSGALG00010026954	MFSD4A	1.575105776	0.172745984	9.118045685	7.64912E-20	4.05686E-19	positive
PRJNA729271	stage_1D_vs_E10	ordinary_muscle_development	ENSGALG00010026958	CLTCL1	-0.231564578	0.084242097	-2.748798837	0.005981408	0.00910624	negative
PRJNA729271	stage_1D_vs_E10	ordinary_muscle_development	ENSGALG00010026960	SEC61A1	-1.324304782	0.102354486	-12.93841469	2.73209E-38	3.36177E-37	negative
PRJNA729271	stage_1D_vs_E10	ordinary_muscle_development	ENSGALG00010026964	SRPK1	-0.542476647	0.059912412	-9.05449516	1.37201E-19	7.16659E-19	negative
PRJNA729271	stage_1D_vs_E10	ordinary_muscle_development	ENSGALG00010026972	TNNT3	4.65393026	0.158358233	29.38862198	7.6757E-190	3.0905E-187	positive
PRJNA729271	stage_1D_vs_E10	ordinary_muscle_development	ENSGALG00010026973	MGLL	2.533412437	0.123095126	20.58093209	4.06784E-94	2.66803E-92	positive
PRJNA729271	stage_1D_vs_E10	ordinary_muscle_development	ENSGALG00010026977	ELK4	0.615092362	0.096106165	6.400134307	1.5524E-10	4.64056E-10	positive
PRJNA729271	stage_1D_vs_E10	ordinary_muscle_development	ENSGALG00010026980	LIMK2	-1.443586898	0.119963663	-12.03353464	2.36801E-33	2.40367E-32	negative
PRJNA729271	stage_1D_vs_E10	ordinary_muscle_development	ENSGALG00010026982	ABTB1	1.727845262	0.084519234	20.44321961	6.90315E-93	4.48705E-91	positive
PRJNA729271	stage_1D_vs_E10	ordinary_muscle_development	ENSGALG00010026984	PGR2/3	3.942445724	0.882226091	4.468747596	7.86789E-06	1.62403E-05	positive
PRJNA729271	stage_1D_vs_E10	ordinary_muscle_development	ENSGALG00010026999	MLEC	-0.760612769	0.082670809	-9.200499847	3.56289E-20	1.922E-19	negative
PRJNA729271	stage_1D_vs_E10	ordinary_muscle_development	ENSGALG00010027001	SLC26A8	0.331943116	0.24970045	1.329365311	0.183727476	0.222669713	positive
PRJNA729271	stage_1D_vs_E10	ordinary_muscle_development	ENSGALG00010027003	SEPTIN2L	-0.572834003	0.082227828	-6.966425104	3.25096E-12	1.08778E-11	negative
PRJNA729271	stage_1D_vs_E10	ordinary_muscle_development	ENSGALG00010027012	RILPL1	2.163572963	0.191986394	11.26940781	1.85815E-29	1.56229E-28	positive
PRJNA729271	stage_1D_vs_E10	ordinary_muscle_development	ENSGALG00010027015	ENSGALG00010027015	1.285570107	0.520992856	2.467538838	0.013604546	0.019739529	positive
PRJNA729271	stage_1D_vs_E10	ordinary_muscle_development	ENSGALG00010027025	ORAI1	1.817819171	0.12684472	14.33105906	1.3997E-46	2.32668E-45	positive
PRJNA729271	stage_1D_vs_E10	ordinary_muscle_development	ENSGALG00010027026	SLC45A3	1.771272513	0.195238512	9.072352017	1.16475E-19	6.12371E-19	positive
PRJNA729271	stage_1D_vs_E10	ordinary_muscle_development	ENSGALG00010027027	ARHGEF3	0.122760386	0.102304614	1.199949654	0.230158894	0.27336555	positive
PRJNA729271	stage_1D_vs_E10	ordinary_muscle_development	ENSGALG00010027036	NUCKS1	-1.036861802	0.065703912	-15.78082301	4.21654E-56	1.0069E-54	negative
PRJNA729271	stage_1D_vs_E10	ordinary_muscle_development	ENSGALG00010027040	CLIP1	1.217195569	0.107926788	11.27797454	1.68583E-29	1.41988E-28	positive
PRJNA729271	stage_1D_vs_E10	ordinary_muscle_development	ENSGALG00010027041	MAPK14	0.779717089	0.073569419	10.59838584	3.03168E-26	2.19063E-25	positive
PRJNA729271	stage_1D_vs_E10	ordinary_muscle_development	ENSGALG00010027043	IL17RD	-2.258133391	0.176090007	-12.82374527	1.20725E-37	1.451E-36	negative
PRJNA729271	stage_1D_vs_E10	ordinary_muscle_development	ENSGALG00010027045	MAPK13	1.561764066	0.179626075	8.694528701	3.48272E-18	1.6878E-17	positive
PRJNA729271	stage_1D_vs_E10	ordinary_muscle_development	ENSGALG00010027046	HESX1	0.057333177	0.297175047	0.19292729	0.847015912	0.869203231	positive
PRJNA729271	stage_1D_vs_E10	ordinary_muscle_development	ENSGALG00010027050	GSTT1	3.373429127	0.151217838	22.30840737	3.0621E-110	2.7398E-108	positive
PRJNA729271	stage_1D_vs_E10	ordinary_muscle_development	ENSGALG00010027052	APPL1	0.491371478	0.083954809	5.852809187	4.83339E-09	1.29573E-08	positive
PRJNA729271	stage_1D_vs_E10	ordinary_muscle_development	ENSGALG00010027059	ASB14	5.744796093	0.2119973	27.09843984	1.0272E-161	2.256E-159	positive
PRJNA729271	stage_1D_vs_E10	ordinary_muscle_development	ENSGALG00010027063	RAB7L1	0.209138308	0.08818848	2.371492363	0.017716413	0.025243232	positive
PRJNA729271	stage_1D_vs_E10	ordinary_muscle_development	ENSGALG00010027073	DNAH12	-1.885792462	0.32058163	-5.882409621	4.04336E-09	1.08918E-08	negative
PRJNA729271	stage_1D_vs_E10	ordinary_muscle_development	ENSGALG00010027074	BRPF3	0.379212715	0.079199014	4.788098917	1.68369E-06	3.69102E-06	positive
PRJNA729271	stage_1D_vs_E10	ordinary_muscle_development	ENSGALG00010027075	SLC41A1	1.525803854	0.094218717	16.19427541	5.53458E-59	1.46127E-57	positive
PRJNA729271	stage_1D_vs_E10	ordinary_muscle_development	ENSGALG00010027076	VDHAP	2.184117162	0.171008465	12.77198273	2.35089E-37	2.79084E-36	positive
PRJNA729271	stage_1D_vs_E10	ordinary_muscle_development	ENSGALG00010027077	ARF4	-0.730169939	0.10447145	-6.989181618	2.76494E-12	9.29018E-12	negative
PRJNA729271	stage_1D_vs_E10	ordinary_muscle_development	ENSGALG00010027078	PDE12	-0.511635785	0.120315935	-4.252435756	2.11458E-05	4.18213E-05	negative
PRJNA729271	stage_1D_vs_E10	ordinary_muscle_development	ENSGALG00010027079	PM20D1	0.333732562	0.094769519	3.521517937	0.000429084	0.000743523	positive
PRJNA729271	stage_1D_vs_E10	ordinary_muscle_development	ENSGALG00010027080	TBC1D22B	0.212991255	0.156253819	1.36311071	0.172847622	0.210504645	positive
PRJNA729271	stage_1D_vs_E10	ordinary_muscle_development	ENSGALG00010027081	PDE4B	1.039941534	0.29111737	3.57224144	0.000353939	0.000619607	positive
PRJNA729271	stage_1D_vs_E10	ordinary_muscle_development	ENSGALG00010027082	SLC26A9	-5.638515162	0.747856208	-7.53957125	4.71519E-14	1.78172E-13	negative
PRJNA729271	stage_1D_vs_E10	ordinary_muscle_development	ENSGALG00010027083	DENND6A	-0.462593485	0.086874769	-5.324831268	1.01047E-07	2.44031E-07	negative
PRJNA729271	stage_1D_vs_E10	ordinary_muscle_development	ENSGALG00010027084	OSBPL9	0.271374376	0.093780587	2.893715895	0.003807125	0.005933793	positive
PRJNA729271	stage_1D_vs_E10	ordinary_muscle_development	ENSGALG00010027085	PIM1	-0.125542536	0.152359734	-0.823987626	0.409946589	0.45967168	negative
PRJNA729271	stage_1D_vs_E10	ordinary_muscle_development	ENSGALG00010027086	CACNA1S	5.549545169	0.201094362	27.59672181	1.2182E-167	3.0445E-165	positive
PRJNA729271	stage_1D_vs_E10	ordinary_muscle_development	ENSGALG00010027087	RAB7B	-0.099115835	0.337723434	-0.293482256	0.769153561	0.798687647	negative
PRJNA729271	stage_1D_vs_E10	ordinary_muscle_development	ENSGALG00010027088	AGBL4	0.005948602	0.260093102	0.022871048	0.981753134	0.984176442	positive
PRJNA729271	stage_1D_vs_E10	ordinary_muscle_development	ENSGALG00010027092	ENSGALG00010027092	-1.532593763	0.198805514	-7.709010343	1.26797E-14	4.95531E-14	negative
PRJNA729271	stage_1D_vs_E10	ordinary_muscle_development	ENSGALG00010027093	SLMAP	0.61213165	0.076028164	8.05138018	8.18655E-16	3.46262E-15	positive
PRJNA729271	stage_1D_vs_E10	ordinary_muscle_development	ENSGALG00010027095	SCN5A	-2.324817741	0.404997644	-5.740324112	9.44955E-09	2.47062E-08	negative
PRJNA729271	stage_1D_vs_E10	ordinary_muscle_development	ENSGALG00010027096	YIPF1	0.577252168	0.110422051	5.227689256	1.71642E-07	4.06926E-07	positive
PRJNA729271	stage_1D_vs_E10	ordinary_muscle_development	ENSGALG00010027097	ENSGALG00010027097	-0.358417324	0.148247836	-2.41769009	0.015619373	0.022462825	negative
PRJNA729271	stage_1D_vs_E10	ordinary_muscle_development	ENSGALG00010027098	EXOG	-0.447962905	0.147418846	-3.038708523	0.002375946	0.00378704	negative
PRJNA729271	stage_1D_vs_E10	ordinary_muscle_development	ENSGALG00010027099	GNA11	-0.3771835	0.085250874	-4.424394519	9.67131E-06	1.97779E-05	negative
PRJNA729271	stage_1D_vs_E10	ordinary_muscle_development	ENSGALG00010027100	MIER2	-0.254943649	0.088550979	-2.879060764	0.003988615	0.006199997	negative
PRJNA729271	stage_1D_vs_E10	ordinary_muscle_development	ENSGALG00010027101	RNF220	-0.002494713	0.078503984	-0.031778176	0.974648951	0.978429015	negative
PRJNA729271	stage_1D_vs_E10	ordinary_muscle_development	ENSGALG00010027102	DUS3L	-0.597606556	0.141969829	-4.209391251	2.5606E-05	5.02312E-05	negative
PRJNA729271	stage_1D_vs_E10	ordinary_muscle_development	ENSGALG00010027103	ACVR2B	0.584088785	0.175529478	3.327582308	0.000876031	0.001469344	positive
PRJNA729271	stage_1D_vs_E10	ordinary_muscle_development	ENSGALG00010027105	CHERP	-0.195883409	0.127926656	-1.531216518	0.12571589	0.157144862	negative
PRJNA729271	stage_1D_vs_E10	ordinary_muscle_development	ENSGALG00010027106	CYP2J23	0.924992735	0.140938912	6.563075622	5.27091E-11	1.6273E-10	positive
PRJNA729271	stage_1D_vs_E10	ordinary_muscle_development	ENSGALG00010027107	KIF21B	-1.35280249	0.203227749	-6.656583558	2.80266E-11	8.80081E-11	negative
PRJNA729271	stage_1D_vs_E10	ordinary_muscle_development	ENSGALG00010027108	ENSGALG00010027108	0.119346537	0.140637715	0.848609756	0.396098476	0.445418729	positive
PRJNA729271	stage_1D_vs_E10	ordinary_muscle_development	ENSGALG00010027109	C19orf44	0.137162616	0.12367196	1.109084188	0.267393866	0.312646131	positive
PRJNA729271	stage_1D_vs_E10	ordinary_muscle_development	ENSGALG00010027110	IKBKE	-0.672479257	0.218454332	-3.078351662	0.002081491	0.00334196	negative
PRJNA729271	stage_1D_vs_E10	ordinary_muscle_development	ENSGALG00010027111	CALR3	-2.802072329	0.836927039	-3.348048513	0.000813828	0.0013712	negative
PRJNA729271	stage_1D_vs_E10	ordinary_muscle_development	ENSGALG00010027112	XYLB	-1.429109354	0.186425464	-7.665848462	1.77653E-14	6.89076E-14	negative
PRJNA729271	stage_1D_vs_E10	ordinary_muscle_development	ENSGALG00010027113	PIAS4	-0.446624619	0.133807495	-3.337814665	0.000844401	0.001419576	negative
PRJNA729271	stage_1D_vs_E10	ordinary_muscle_development	ENSGALG00010027114	FLNB	-0.731610481	0.113634504	-6.43827757	1.20837E-10	3.63917E-10	negative
PRJNA729271	stage_1D_vs_E10	ordinary_muscle_development	ENSGALG00010027115	C9ORF152	-1.379145716	0.648505336	-2.126652842	0.033448938	0.045852314	negative
PRJNA729271	stage_1D_vs_E10	ordinary_muscle_development	ENSGALG00010027116	CCDC94	-0.020777049	0.102641332	-0.202423804	0.839585421	0.862677442	negative
PRJNA729271	stage_1D_vs_E10	ordinary_muscle_development	ENSGALG00010027117	RASSF5	0.400954293	0.248862571	1.611147437	0.107147591	0.135464835	positive
PRJNA729271	stage_1D_vs_E10	ordinary_muscle_development	ENSGALG00010027118	ABHD6	1.73483011	0.157127414	11.04091301	2.42556E-28	1.93072E-27	positive
PRJNA729271	stage_1D_vs_E10	ordinary_muscle_development	ENSGALG00010027119	NUB1	0.907354095	0.098092535	9.24998109	2.24532E-20	1.22307E-19	positive
PRJNA729271	stage_1D_vs_E10	ordinary_muscle_development	ENSGALG00010027121	ELF3	-1.506998975	0.939954091	-1.603268702	0.108875343	0.13750528	negative
PRJNA729271	stage_1D_vs_E10	ordinary_muscle_development	ENSGALG00010027122	DNASE1L3	3.81562488	0.248344415	15.36424676	2.84324E-53	6.12374E-52	positive
PRJNA729271	stage_1D_vs_E10	ordinary_muscle_development	ENSGALG00010027123	WDR86	-1.47304072	0.220786593	-6.671785196	2.5271E-11	7.96139E-11	negative
PRJNA729271	stage_1D_vs_E10	ordinary_muscle_development	ENSGALG00010027124	IP6K2	1.211914233	0.173102561	7.00113406	2.53899E-12	8.54881E-12	positive
PRJNA729271	stage_1D_vs_E10	ordinary_muscle_development	ENSGALG00010027125	CRYGN	1.090093912	0.40649572	2.681686079	0.007325216	0.011024713	positive
PRJNA729271	stage_1D_vs_E10	ordinary_muscle_development	ENSGALG00010027126	ENSGALG00010027126	8.117381056	0.534218757	15.19486343	3.8246E-52	7.95374E-51	positive
PRJNA729271	stage_1D_vs_E10	ordinary_muscle_development	ENSGALG00010027127	S1PR4	1.059925109	0.66142649	1.602483608	0.109048709	0.137688244	positive
PRJNA729271	stage_1D_vs_E10	ordinary_muscle_development	ENSGALG00010027128	EIF2D	0.286812587	0.089904222	3.190201539	0.001421736	0.002330438	positive
PRJNA729271	stage_1D_vs_E10	ordinary_muscle_development	ENSGALG00010027129	NCLN	0.919413909	0.128370696	7.162179027	7.94046E-13	2.76809E-12	positive
PRJNA729271	stage_1D_vs_E10	ordinary_muscle_development	ENSGALG00010027130	NCKIPSD	0.358065091	0.143145561	2.501405481	0.012370146	0.018067842	positive
PRJNA729271	stage_1D_vs_E10	ordinary_muscle_development	ENSGALG00010027131	UBA52	0.397842354	0.152216496	2.613661234	0.008957781	0.013324067	positive
PRJNA729271	stage_1D_vs_E10	ordinary_muscle_development	ENSGALG00010027133	CELF5	-3.020128568	0.294821833	-10.24391083	1.26036E-24	8.48142E-24	negative
PRJNA729271	stage_1D_vs_E10	ordinary_muscle_development	ENSGALG00010027134	DYRK3	0.972486364	0.148400809	6.553106908	5.63521E-11	1.73718E-10	positive

PRJNA729271	stage_1D_vs_E10	ordinary_muscle_development	ENSGALG00010027135	PRKAG2	-0.017541277	0.139150792	-0.126059482	0.89968484	0.914575479	negative
PRJNA729271	stage_1D_vs_E10	ordinary_muscle_development	ENSGALG00010027136	MAPKAPK2	1.876682235	0.143774435	13.05296199	6.11239E-39	7.73116E-38	positive
PRJNA729271	stage_1D_vs_E10	ordinary_muscle_development	ENSGALG00010027137	FKBP8	0.210131625	0.090438049	2.323486924	0.020153009	0.028490966	positive
PRJNA729271	stage_1D_vs_E10	ordinary_muscle_development	ENSGALG00010027139	HSD11B1L	0.140668179	0.138223056	1.017689693	0.308825447	0.356091389	positive
PRJNA729271	stage_1D_vs_E10	ordinary_muscle_development	ENSGALG00010027141	PIGR	2.759071875	1.554818836	1.77452949	0.075975609	0.09850326	positive
PRJNA729271	stage_1D_vs_E10	ordinary_muscle_development	ENSGALG00010027143	GALNT11	-0.857075313	0.120091515	-7.136851512	9.54929E-13	3.30745E-12	negative
PRJNA729271	stage_1D_vs_E10	ordinary_muscle_development	ENSGALG00010027144	ENSGALG00010027144	-2.456438869	0.672728401	-3.651457061	0.000260757	0.000462458	negative
PRJNA729271	stage_1D_vs_E10	ordinary_muscle_development	ENSGALG00010027145	CELSR3	-1.138769691	0.168082055	-6.775081905	1.24336E-11	4.00144E-11	negative
PRJNA729271	stage_1D_vs_E10	ordinary_muscle_development	ENSGALG00010027146	KXD1	0.109048245	0.127043532	0.858353377	0.390697364	0.439856954	positive
PRJNA729271	stage_1D_vs_E10	ordinary_muscle_development	ENSGALG00010027147	KMT2C	1.2961418	0.10330893	12.54627069	4.16676E-36	4.72222E-35	positive
PRJNA729271	stage_1D_vs_E10	ordinary_muscle_development	ENSGALG00010027148	ELL	1.185336762	0.165255549	7.17275012	7.35059E-13	2.56987E-12	positive
PRJNA729271	stage_1D_vs_E10	ordinary_muscle_development	ENSGALG00010027149	C1orf116	1.243309621	0.228324979	5.445350869	5.17034E-08	1.27629E-07	positive
PRJNA729271	stage_1D_vs_E10	ordinary_muscle_development	ENSGALG00010027150	YOD1	-0.841548512	0.090734533	-9.274842626	1.77881E-20	9.76663E-20	negative
PRJNA729271	stage_1D_vs_E10	ordinary_muscle_development	ENSGALG00010027151	MICOS13	1.258770266	0.143211327	8.789599884	1.50094E-18	7.41515E-18	positive
PRJNA729271	stage_1D_vs_E10	ordinary_muscle_development	ENSGALG00010027152	C4BPS	1.708260407	0.439931325	3.883016073	0.000103169	0.000191085	positive
PRJNA729271	stage_1D_vs_E10	ordinary_muscle_development	ENSGALG00010027153	ENSGALG00010027153	0.965102435	0.22889421	4.216368929	2.48267E-05	4.8762E-05	positive
PRJNA729271	stage_1D_vs_E10	ordinary_muscle_development	ENSGALG00010027154	PLPP2	0.214804944	0.136812613	1.57006682	0.116399567	0.146408515	positive
PRJNA729271	stage_1D_vs_E10	ordinary_muscle_development	ENSGALG00010027155	ENSGALG00010027155	0.589065814	0.294155707	2.002564626	0.04522404	0.060707766	positive
PRJNA729271	stage_1D_vs_E10	ordinary_muscle_development	ENSGALG00010027156	ISYNA1	-0.691972422	0.146406743	-4.726369889	2.28569E-06	4.96123E-06	negative
PRJNA729271	stage_1D_vs_E10	ordinary_muscle_development	ENSGALG00010027157	ENSGALG00010027157	0.658299091	0.252637958	2.605701442	0.009168634	0.013623716	positive
PRJNA729271	stage_1D_vs_E10	ordinary_muscle_development	ENSGALG00010027158	XRCC2	-0.832727365	0.136426122	-6.103870392	1.0353E-09	2.92187E-09	negative
PRJNA729271	stage_1D_vs_E10	ordinary_muscle_development	ENSGALG00010027159	TMUB1	0.217161771	0.164918147	1.316785173	0.187910644	0.227226561	positive
PRJNA729271	stage_1D_vs_E10	ordinary_muscle_development	ENSGALG00010027160	FASTK	0.628081998	0.16644179	3.77358354	0.000160919	0.000292663	positive
PRJNA729271	stage_1D_vs_E10	ordinary_muscle_development	ENSGALG00010027161	C4BPG	0.572371989	0.203492907	2.812736799	0.004912184	0.007559413	positive
PRJNA729271	stage_1D_vs_E10	ordinary_muscle_development	ENSGALG00010027162	SLC4A2	0.23295567	0.130313157	1.7876604	0.073830814	0.09593101	positive
PRJNA729271	stage_1D_vs_E10	ordinary_muscle_development	ENSGALG00010027163	ENSGALG00010027163	-0.390641732	0.078542788	-4.973616826	6.57151E-07	1.48881E-06	negative
PRJNA729271	stage_1D_vs_E10	ordinary_muscle_development	ENSGALG00010027164	ENSGALG00010027164	1.67222277	0.462329469	3.616950429	0.000298094	0.000525399	positive
PRJNA729271	stage_1D_vs_E10	ordinary_muscle_development	ENSGALG00010027165	ENSGALG00010027165	-2.538738495	0.217590557	-11.6675031	1.86826E-31	1.73482E-30	negative
PRJNA729271	stage_1D_vs_E10	ordinary_muscle_development	ENSGALG00010027166	CDK5	0.020933007	0.099133436	0.211159907	0.832762495	0.856638447	positive
PRJNA729271	stage_1D_vs_E10	ordinary_muscle_development	ENSGALG00010027167	ENSGALG00010027167	-1.99415901	0.443226899	-4.49918318	6.82151E-06	1.41598E-05	negative
PRJNA729271	stage_1D_vs_E10	ordinary_muscle_development	ENSGALG00010027168	SSBP4	-0.588773373	0.179902365	-3.272738371	0.00106511	0.001771517	negative
PRJNA729271	stage_1D_vs_E10	ordinary_muscle_development	ENSGALG00010027169	ABCB8	0.172102054	0.111603226	1.542088517	0.123052085	0.154227408	positive
PRJNA729271	stage_1D_vs_E10	ordinary_muscle_development	ENSGALG00010027170	TLE2	-0.360781298	0.168911715	-2.135916382	0.03268623	0.044857688	negative
PRJNA729271	stage_1D_vs_E10	ordinary_muscle_development	ENSGALG00010027171	ATG9B	4.048557582	0.27364919	14.79469967	1.5849E-49	3.00696E-48	positive
PRJNA729271	stage_1D_vs_E10	ordinary_muscle_development	ENSGALG00010027172	ENSGALG00010027172	-1.654988566	0.516834568	-3.202163069	0.001363998	0.002241372	negative
PRJNA729271	stage_1D_vs_E10	ordinary_muscle_development	ENSGALG00010027173	ENSGALG00010027173	-0.320660561	0.097108005	-3.302102246	0.000959631	0.001603811	negative
PRJNA729271	stage_1D_vs_E10	ordinary_muscle_development	ENSGALG00010027174	SHISA5	-0.403935895	0.132761373	-3.042570933	0.002345665	0.003740832	negative
PRJNA729271	stage_1D_vs_E10	ordinary_muscle_development	ENSGALG00010027175	NOS3	1.145976787	0.162777348	7.040149042	1.92034E-12	6.51425E-12	positive
PRJNA729271	stage_1D_vs_E10	ordinary_muscle_development	ENSGALG00010027176	ENSGALG00010027176	0.532574532	0.17040509	3.125344027	0.001775972	0.002879821	positive
PRJNA729271	stage_1D_vs_E10	ordinary_muscle_development	ENSGALG00010027178	C4BPM	0.859722604	0.080536592	10.67493147	1.33356E-26	9.82712E-26	positive
PRJNA729271	stage_1D_vs_E10	ordinary_muscle_development	ENSGALG00010027183	CD34	0.396520192	0.156826892	2.528394118	0.011458564	0.016807518	positive
PRJNA729271	stage_1D_vs_E10	ordinary_muscle_development	ENSGALG00010027184	MPND	-0.465285254	0.344413894	-1.350947977	0.176712098	0.214850014	negative
PRJNA729271	stage_1D_vs_E10	ordinary_muscle_development	ENSGALG00010027185	ATRIP	-0.962336488	0.081539891	-11.80203303	3.80994E-32	3.64763E-31	negative
PRJNA729271	stage_1D_vs_E10	ordinary_muscle_development	ENSGALG00010027186	PLXNA2	0.875093951	0.122855365	7.122960839	1.05633E-12	3.64472E-12	positive
PRJNA729271	stage_1D_vs_E10	ordinary_muscle_development	ENSGALG00010027187	ENSGALG00010027187	-0.747783201	0.137823746	-5.425648505	5.77445E-08	1.42049E-07	negative
PRJNA729271	stage_1D_vs_E10	ordinary_muscle_development	ENSGALG00010027188	KCNH2	2.160663815	0.252788473	8.547319394	1.2598E-17	5.90583E-17	positive
PRJNA729271	stage_1D_vs_E10	ordinary_muscle_development	ENSGALG00010027189	ENSGALG00010027189	0.433248268	0.260721548	1.661727888	0.096567347	0.123130163	positive
PRJNA729271	stage_1D_vs_E10	ordinary_muscle_development	ENSGALG00010027190	IPPK	0.033785905	0.114286526	0.295624572	0.767516806	0.797394296	positive
PRJNA729271	stage_1D_vs_E10	ordinary_muscle_development	ENSGALG00010027192	SH3GL1	-1.062335417	0.116602353	-9.110754526	8.1811E-20	4.33425E-19	negative
PRJNA729271	stage_1D_vs_E10	ordinary_muscle_development	ENSGALG00010027193	CAMK1G	-4.187906521	0.726584775	-5.763823662	8.22293E-09	2.16476E-08	negative
PRJNA729271	stage_1D_vs_E10	ordinary_muscle_development	ENSGALG00010027194	ENSGALG00010027194	-2.217025142	0.266747123	-8.311336668	9.463E-17	4.23222E-16	negative
PRJNA729271	stage_1D_vs_E10	ordinary_muscle_development	ENSGALG00010027195	CENPP	-1.452426618	0.19527113	-7.437999771	1.02221E-13	3.78177E-13	negative
PRJNA729271	stage_1D_vs_E10	ordinary_muscle_development	ENSGALG00010027196	ENSGALG00010027196	-0.314147637	0.337146807	-0.931782923	0.351448722	0.400428331	negative
PRJNA729271	stage_1D_vs_E10	ordinary_muscle_development	ENSGALG00010027197	ENSGALG00010027197	-2.146096644	0.560912056	-3.826084001	0.000130198	0.000238828	negative
PRJNA729271	stage_1D_vs_E10	ordinary_muscle_development	ENSGALG00010027198	ECM2	1.519041062	0.202919897	7.485914816	7.10505E-14	2.65022E-13	positive
PRJNA729271	stage_1D_vs_E10	ordinary_muscle_development	ENSGALG00010027199	LAMB3	1.206762098	0.286675246	4.20950925	2.55926E-05	5.02118E-05	positive
PRJNA729271	stage_1D_vs_E10	ordinary_muscle_development	ENSGALG00010027200	G0S2	6.701599782	0.316967175	21.14288261	3.20869E-99	2.33718E-97	positive
PRJNA729271	stage_1D_vs_E10	ordinary_muscle_development	ENSGALG00010027201	ASPN	0.173031911	0.316196384	0.547229252	0.584221242	0.628677573	positive
PRJNA729271	stage_1D_vs_E10	ordinary_muscle_development	ENSGALG00010027202	CHAF1A	-1.448814214	0.08474133	-17.09690192	1.56506E-65	5.01892E-64	negative
PRJNA729271	stage_1D_vs_E10	ordinary_muscle_development	ENSGALG00010027203	ENSGALG00010027203	-0.233177128	0.295360071	-0.789467334	0.429838915	0.479417903	negative
PRJNA729271	stage_1D_vs_E10	ordinary_muscle_development	ENSGALG00010027204	OMD	-5.512762756	0.973573322	-5.662401209	1.49269E-08	3.84241E-08	negative
PRJNA729271	stage_1D_vs_E10	ordinary_muscle_development	ENSGALG00010027205	OGN	1.624361246	0.221828763	7.322590749	2.43229E-13	8.77015E-13	positive
PRJNA729271	stage_1D_vs_E10	ordinary_muscle_development	ENSGALG00010027206	ENSGALG00010027206	7.372841177	0.966274157	7.63017527	2.34435E-14	9.02944E-14	positive
PRJNA729271	stage_1D_vs_E10	ordinary_muscle_development	ENSGALG00010027207	NOL8	0.101396336	0.123240749	0.822750081	0.410650129	0.460353722	positive
PRJNA729271	stage_1D_vs_E10	ordinary_muscle_development	ENSGALG00010027208	UBXN6	0.519483503	0.094381038	5.504108805	3.7104E-08	9.25048E-08	positive
PRJNA729271	stage_1D_vs_E10	ordinary_muscle_development	ENSGALG00010027209	TRAF3IP3	0.488993208	0.257402933	1.899718864	0.057470023	0.07584013	positive
PRJNA729271	stage_1D_vs_E10	ordinary_muscle_development	ENSGALG00010027210	ENSGALG00010027210	-1.898194448	0.279919845	-6.781207134	1.19176E-11	3.83793E-11	negative
PRJNA729271	stage_1D_vs_E10	ordinary_muscle_development	ENSGALG00010027211	HDGFRP2	-0.404821611	0.101428179	-3.991214436	6.57358E-05	0.000124294	negative
PRJNA729271	stage_1D_vs_E10	ordinary_muscle_development	ENSGALG00010027212	IRF6	-4.976405066	0.599415473	-8.302096439	1.0229E-16	4.55653E-16	negative
PRJNA729271	stage_1D_vs_E10	ordinary_muscle_development	ENSGALG00010027213	KCNC4	1.560917282	0.330476267	4.723235637	2.32122E-06	5.03532E-06	positive
PRJNA729271	stage_1D_vs_E10	ordinary_muscle_development	ENSGALG00010027214	PLIN4	1.525020279	0.266096965	5.731069805	9.97992E-09	2.60084E-08	positive
PRJNA729271	stage_1D_vs_E10	ordinary_muscle_development	ENSGALG00010027215	ENSGALG00010027215	-2.533797557	0.201534391	-12.57253188	2.98993E-36	3.40181E-35	negative
PRJNA729271	stage_1D_vs_E10	ordinary_muscle_development	ENSGALG00010027216	ENSGALG00010027216	-0.501344907	0.700854518	-0.715333773	0.474402824	0.523084508	negative
PRJNA729271	stage_1D_vs_E10	ordinary_muscle_development	ENSGALG00010027217	SEMA6B	0.306756932	0.220527123	1.391016794	0.164220334	0.200977182	positive
PRJNA729271	stage_1D_vs_E10	ordinary_muscle_development	ENSGALG00010027218	IARS1	-0.311668426	0.164437063	-1.895366045	0.058043924	0.076534765	negative
PRJNA729271	stage_1D_vs_E10	ordinary_muscle_development	ENSGALG00010027219	ENSGALG00010027219	-0.032449466	0.137850777	-0.235395595	0.813901704	0.839381374	negative
PRJNA729271	stage_1D_vs_E10	ordinary_muscle_development	ENSGALG00010027220	ANKRD24	-1.500534254	0.610129804	-2.459368881	0.013918153	0.020154208	negative
PRJNA729271	stage_1D_vs_E10	ordinary_muscle_development	ENSGALG00010027221	LRRC61	-1.206480465	0.277123234	-4.353588288	1.33927E-05	2.7041E-05	negative
PRJNA729271	stage_1D_vs_E10	ordinary_muscle_development	ENSGALG00010027222	GUCA1B	1.769198277	0.899004794	1.967951993	0.049073562	0.065553523	positive
PRJNA729271	stage_1D_vs_E10	ordinary_muscle_development	ENSGALG00010027223	SIRT6	-0.413090731	0.082998588	-4.977081453	6.45502E-07	1.46333E-06	negative
PRJNA729271	stage_1D_vs_E10	ordinary_muscle_development	ENSGALG00010027224	RARRES2	0.901848328	0.120290579	7.497248235	6.51715E-14	2.43658E-13	positive
PRJNA729271	stage_1D_vs_E10	ordinary_muscle_development	ENSGALG00010027225	CREB3L3	-1.18462935	0.747906769	-1.583926499	0.113210446	0.142644769	negative
PRJNA729271	stage_1D_vs_E10	ordinary_muscle_development	ENSGALG00010027227	ENSGALG00010027227	0.397116152	0.184099926	2.1570685	0.031000328	0.04267329	positive
PRJNA729271	stage_1D_vs_E10	ordinary_muscle_development	ENSGALG00010027229	MDM4	0.486256498	0.079730652	6.098739801	1.06908E-09	3.01426E-09	positive
PRJNA729271	stage_1D_vs_E10	ordinary_muscle_development	ENSGALG00010027230	MAP2K2	-0.884171306	0.082500013	-10.71722627	8.44994E-27	6.28435E-26	negative
PRJNA729271	stage_1D_vs_E10	ordinary_muscle_development	ENSGALG00010027231	ENSGALG00010027231	0.516317829	0.153458853	3.364535946	0.000766725	0.00129515	positive
PRJNA729271	stage_1D_vs_E10	ordinary_muscle_development	ENSGALG00010027232	ARRDC2	1.52180633	0.32765461	4.644544231	3.40828E-06	7.27585E-06	positive
PRJNA729271	stage_1D_vs_E10	ordinary_muscle_development	ENSGALG00010027233	ENSGALG00010027233	0.58866713	0.267244188	2.202731274	0.027613695	0.038240232	positive
PRJNA729271	stage_1D_vs_E10	ordinary_muscle_development	ENSGALG00010027234	SLC6A11	-2.44174572	0.38519364	-6.339008408	2.31249E-10	6.83792E-10	negative
PRJNA729271	stage_1D_vs_E10	ordinary_muscle_development	ENSGALG00010027236	PEX11G	-0.159188429	0.281701348	-0.565096441	0.572008169	0.617000924	negative
PRJNA729271	stage_1D_vs_E10	ordinary_muscle_development	ENSGALG00010027237	CTC-575C13.4	0.672064758	0.151252905	4.443318007	8.85821E-06	1.81955E-05	positive
PRJNA729271	stage_1D_vs_E10	ordinary_muscle_development	ENSGALG00010027239	ENSGALG00010027239	-0.020263871	0.082277054	-0.24628824	0.805459113	0.832095349	negative
PRJNA729271	stage_1D_vs_E10	ordinary_muscle_development	ENSGALG00010027240	SLC6A1	1.555380352	0.301118181	5.165348519	2.39991E-07	5.61983E-07	positive
PRJNA729271	stage_1D_vs_E10	ordinary_muscle_development	ENSGALG00010027241	LRRN2	-1.831493839	0.268097075	-6.831457744	8.40561E-12	2.73121E-11	negative
PRJNA729271	stage_1D_vs_E10	ordinary_muscle_development	ENSGALG00010027242	ENSGALG00010027242	0.36331868	0.18805	1.932032328	0.053355516	0.070808412	positive
PRJNA729271	stage_1D_vs_E10	ordinary_muscle_development	ENSGALG00010027243	HRH1	0.461736406	0.198161467	2.330101875	0.019800767	0.028031265	positive
PRJNA729271	stage_1D_vs_E10	ordinary_muscle_development	ENSGALG00010027244	ARHGEF18	0.232229808	0.089967272	2.581269882	0.009843759	0.014564182	positive
PRJNA729271	stage_1D_vs_E10	ordinary_muscle_development	ENSGALG00010027245	LRIG2	0.421848468	0.065141719	6.475857146	9.42752E-11	2.85823E-10	positive
PRJNA729271	stage_1D_vs_E10	ordinary_muscle_development	ENSGALG00010027246	ZNF767	-0.558405845	0.098103144	-5.692028035	1.25539E-08	3.25119E-08	negative
PRJNA729271	stage_1D_vs_E10	ordinary_muscle_development	ENSGALG00010027247	ATG7	-0.162067445	0.079305982	-2.0435715	0.040995897	0.0553955	negative
PRJNA729271	stage_1D_vs_E10	ordinary_muscle_development	ENSGALG00010027248	ENSGALG00010027248	-0.733408639	0.602910444	-1.216447062	0.223814665	0.266507317	negative
PRJNA729271	stage_1D_vs_E10	ordinary_muscle_development	ENSGALG00010027249	C2CD4C	6.063466485	0.958603451	6.325312596	2.5272E-10	7.45395E-10	positive
PRJNA729271	stage_1D_vs_E10	ordinary_muscle_development	ENSGALG00010027250	ENSGALG00010027250	-0.181692993	0.169849618	-1.069728594	0.284741492	0.330976095	negative
PRJNA729271	stage_1D_vs_E10	ordinary_muscle_development	ENSGALG00010027251	ENSGALG00010027251	-0.178010649	0.201718313	-0.882471436	0.37752192	0.42668072	negative
PRJNA729271	stage_1D_vs_E10	ordinary_muscle_development	ENSGALG00010027252	MAGI3	0.045828462	0.071360485	0.642210632	0.52073643	0.567994173	positive
PRJNA729271	stage_1D_vs_E10	ordinary_muscle_development	ENSGALG00010027253	VGLL4	-0.792003607	0.087653294	-9.035639941	1.63046E-19	8.48294E-19	negative
PRJNA729271	stage_1D_vs_E10	ordinary_muscle_development	ENSGALG00010027254	INSR	1.622235714	0.132457101	12.24725367	1.73782E-34	1.85082E-33	positive
PRJNA729271	stage_1D_vs_E10	ordinary_muscle_development	ENSGALG00010027255	ENSGALG00010027255	-0.670267012	0.183422613	-3.65422234	0.000257963	0.000457559	negative
PRJNA729271	stage_1D_vs_E10	ordinary_muscle_development	ENSGALG00010027256	PHTF1	-1.361229921	0.087613206	-15.53681206	1.95468E-54	4.39273E-53	negative
PRJNA729271	stage_1D_vs_E10	ordinary_muscle_development	ENSGALG00010027257	TAFA3	2.081604876	0.262480255	7.930519877	2.1823E-15	8.9763E-15	positive
PRJNA729271	stage_1D_vs_E10	ordinary_muscle_development	ENSGALG00010027258	ENSGALG00010027258	-1.187127582	0.171053144	-6.940109703	3.91796E-12	1.30254E-11	negative
PRJNA729271	stage_1D_vs_E10	ordinary_muscle_development	ENSGALG00010027259	ENSGALG00010027259	-0.085161662	0.081678111	-1.04264975	0.297110539	0.343979015	negative
PRJNA729271	stage_1D_vs_E10	ordinary_muscle_development	ENSGALG00010027260	LEMD2	0.742415548	0.100164904	7.411932895	1.24472E-13	4.57575E-13	positive
PRJNA729271	stage_1D_vs_E10	ordinary_muscle_development	ENSGALG00010027261	ENSGALG00010027261	-0.290224697	0.110937723	-2.616104695	0.008893928	0.013234523	negative
PRJNA729271	stage_1D_vs_E10	ordinary_muscle_development	ENSGALG00010027262	USE1	-0.840583667	0.135935594	-6.183690702	6.262E-10	1.79489E-09	negative
PRJNA729271	stage_1D_vs_E10	ordinary_muscle_development	ENSGALG00010027263	TAMM41	-1.124849818	0.125885469	-8.935501704	4.05335E-19	2.0608E-18	negative
PRJNA729271	stage_1D_vs_E10	ordinary_muscle_development	ENSGALG00010027264	ENSGALG00010027264	0.701028503	0.094015654	7.456508288	8.88455E-14	3.29786E-13	positive
PRJNA729271	stage_1D_vs_E10	ordinary_muscle_development	ENSGALG00010027265	IP6K3	4.857591744	0.207612058	23.3974452	4.5373E-121	5.2615E-119	positive
PRJNA729271	stage_1D_vs_E10	ordinary_muscle_development	ENSGALG00010027267	MYO9B	-0.727335801	0.095787716	-7.593205352	3.12087E-14	1.19296E-13	negative
PRJNA729271	stage_1D_vs_E10	ordinary_muscle_development	ENSGALG00010027268	UQCRC1	1.574313195	0.126529884	12.4422243	1.54151E-35	1.70696E-34	positive
PRJNA729271	stage_1D_vs_E10	ordinary_muscle_development	ENSGALG00010027269	CDC25A	-0.864521838	0.09328111	-9.267919676	1.89812E-20	1.03902E-19	negative
PRJNA729271	stage_1D_vs_E10	ordinary_muscle_development	ENSGALG00010027270	ENSGALG00010027270	-2.528712253	0.407008106	-6.212928473	5.20061E-10	1.49867E-09	negative
PRJNA729271	stage_1D_vs_E10	ordinary_muscle_development	ENSGALG00010027271	INKA2	-0.008055909	0.142591087	-0.05649658	0.95494622	0.962114788	negative
PRJNA729271	stage_1D_vs_E10	ordinary_muscle_development	ENSGALG00010027272	DDX20	-0.175382145	0.102048687	-1.71861246	0.085684961	0.110223954	negative
PRJNA729271	stage_1D_vs_E10	ordinary_muscle_development	ENSGALG00010027273	HAUS8	-1.818665661	0.134042395	-13.56783918	6.21267E-42	8.73449E-41	negative
PRJNA729271	stage_1D_vs_E10	ordinary_muscle_development	ENSGALG00010027274	COPG1	-0.850054505	0.060865682	-13.96607209	2.51098E-44	3.8515E-43	negative
PRJNA729271	stage_1D_vs_E10	ordinary_muscle_development	ENSGALG00010027275	CPAMD8	0.239317332	0.2271761	1.053444141	0.292137482	0.338789728	positive
PRJNA729271	stage_1D_vs_E10	ordinary_muscle_development	ENSGALG00010027276	ENSGALG00010027276	0.246251598	0.138298467	1.78058082	0.074980955	0.097292001	positive
PRJNA729271	stage_1D_vs_E10	ordinary_muscle_development	ENSGALG00010027277	KCND3	1.516921258	0.389308062	3.896454779	9.7611E-05	0.000181208	positive
PRJNA729271	stage_1D_vs_E10	ordinary_muscle_development	ENSGALG00010027278	H1-10	-1.205064537	0.129410644	-9.311942974	1.25515E-20	6.94404E-20	negative
PRJNA729271	stage_1D_vs_E10	ordinary_muscle_development	ENSGALG00010027279	F2RL3	0.97443443	0.344682537	2.827049029	0.004697913	0.007245053	positive

PRJNA729271	stage_1D_vs_E10	ordinary_muscle_development	ENSGALG00010027280	HMCES	-0.818558631	0.125985065	-6.497267206	8.17921E-11	2.49123E-10	negative
PRJNA729271	stage_1D_vs_E10	ordinary_muscle_development	ENSGALG00010027281	ENSGALG00010027281	-0.409101192	0.250279543	-1.634577026	0.102137747	0.12974206	negative
PRJNA729271	stage_1D_vs_E10	ordinary_muscle_development	ENSGALG00010027282	ENSGALG00010027282	0.575838018	0.129501692	4.44656751	8.72533E-06	1.79395E-05	positive
PRJNA729271	stage_1D_vs_E10	ordinary_muscle_development	ENSGALG00010027283	RAB7A	0.237799091	0.062755595	3.789289106	0.000151079	0.000275562	positive
PRJNA729271	stage_1D_vs_E10	ordinary_muscle_development	ENSGALG00010027284	CTTNBP2NL	1.294270884	0.089470978	14.46581797	1.99222E-47	3.43369E-46	positive
PRJNA729271	stage_1D_vs_E10	ordinary_muscle_development	ENSGALG00010027285	PTPRS	0.060603706	0.111772147	0.542207585	0.587675511	0.631878683	positive
PRJNA729271	stage_1D_vs_E10	ordinary_muscle_development	ENSGALG00010027286	RPN1	0.330745206	0.062691321	5.275773476	1.32197E-07	3.16413E-07	positive
PRJNA729271	stage_1D_vs_E10	ordinary_muscle_development	ENSGALG00010027287	DHX30	-0.14745868	0.088931104	-1.658122667	0.09729271	0.12398961	negative
PRJNA729271	stage_1D_vs_E10	ordinary_muscle_development	ENSGALG00010027288	DAG1	1.692145813	0.167682379	10.09137527	6.03184E-24	3.91543E-23	positive
PRJNA729271	stage_1D_vs_E10	ordinary_muscle_development	ENSGALG00010027289	SMARCC1	-0.801748219	0.13923892	-5.758075556	8.50783E-09	2.23529E-08	negative
PRJNA729271	stage_1D_vs_E10	ordinary_muscle_development	ENSGALG00010027290	CSPG5	-3.355110081	0.273236059	-12.2791629	1.17205E-34	1.25936E-33	negative
PRJNA729271	stage_1D_vs_E10	ordinary_muscle_development	ENSGALG00010027291	MST1	1.539049156	0.273814324	5.620776638	1.90101E-08	4.85296E-08	positive
PRJNA729271	stage_1D_vs_E10	ordinary_muscle_development	ENSGALG00010027292	KDM4B	0.283953143	0.060183935	4.718088685	2.38071E-06	5.15974E-06	positive
PRJNA729271	stage_1D_vs_E10	ordinary_muscle_development	ENSGALG00010027293	ELP6	0.148704545	0.147413302	1.008759336	0.313090067	0.360549815	positive
PRJNA729271	stage_1D_vs_E10	ordinary_muscle_development	ENSGALG00010027295	ENSGALG00010027295	1.864195993	0.239763395	7.775148461	7.5359E-15	2.98859E-14	positive
PRJNA729271	stage_1D_vs_E10	ordinary_muscle_development	ENSGALG00010027297	ENSGALG00010027297	0.882655774	2.0965901	0.42099587	0.673758097	0.712128017	positive
PRJNA729271	stage_1D_vs_E10	ordinary_muscle_development	ENSGALG00010027299	PGPEP1	-0.10431781	0.162960201	-0.640142865	0.522079723	0.569288015	negative
PRJNA729271	stage_1D_vs_E10	ordinary_muscle_development	ENSGALG00010027301	SCAP	-0.042266624	0.063520687	-0.665399349	0.505795101	0.553906013	negative
PRJNA729271	stage_1D_vs_E10	ordinary_muscle_development	ENSGALG00010027303	APEH	-0.316314327	0.140455104	-2.252067163	0.024318028	0.034014264	negative
PRJNA729271	stage_1D_vs_E10	ordinary_muscle_development	ENSGALG00010027304	PTPN23	1.051037527	0.136087507	7.723247723	1.13402E-14	4.44863E-14	positive
PRJNA729271	stage_1D_vs_E10	ordinary_muscle_development	ENSGALG00010027305	LSM4	-1.160369574	0.130568587	-8.887050077	6.27527E-19	3.15834E-18	negative
PRJNA729271	stage_1D_vs_E10	ordinary_muscle_development	ENSGALG00010027306	ENSGALG00010027306	2.149087208	0.321144866	6.691955679	2.20208E-11	6.96619E-11	positive
PRJNA729271	stage_1D_vs_E10	ordinary_muscle_development	ENSGALG00010027307	JUND	0.703076155	0.176096656	3.992558242	6.53643E-05	0.00012364	positive
PRJNA729271	stage_1D_vs_E10	ordinary_muscle_development	ENSGALG00010027309	ENSGALG00010027309	1.557515215	0.697188458	2.23399455	0.025483432	0.035514118	positive
PRJNA729271	stage_1D_vs_E10	ordinary_muscle_development	ENSGALG00010027310	ARHGEF2	0.135739565	0.131422437	1.032849245	0.301674454	0.348672845	positive
PRJNA729271	stage_1D_vs_E10	ordinary_muscle_development	ENSGALG00010027311	NFASC	3.436615717	0.238310538	14.42074589	3.83179E-47	6.52665E-46	positive
PRJNA729271	stage_1D_vs_E10	ordinary_muscle_development	ENSGALG00010027312	ENSGALG00010027312	0.609736932	0.728140668	0.837388926	0.402373988	0.451844667	positive
PRJNA729271	stage_1D_vs_E10	ordinary_muscle_development	ENSGALG00010027313	BSN	-0.694745233	0.233929614	-2.969890049	0.002979064	0.00469824	negative
PRJNA729271	stage_1D_vs_E10	ordinary_muscle_development	ENSGALG00010027314	ENSGALG00010027314	3.961850543	0.348273702	11.37568104	5.52697E-30	4.76299E-29	positive
PRJNA729271	stage_1D_vs_E10	ordinary_muscle_development	ENSGALG00010027315	RAB3A	0.317693739	0.225789563	1.40703465	0.159417114	0.195511555	positive
PRJNA729271	stage_1D_vs_E10	ordinary_muscle_development	ENSGALG00010027316	TRAIP	-2.311790083	0.210729835	-10.97039765	5.30386E-28	4.14668E-27	negative
PRJNA729271	stage_1D_vs_E10	ordinary_muscle_development	ENSGALG00010027317	MPV17L2	0.402278124	0.146439759	2.747055352	0.006013299	0.009152868	positive
PRJNA729271	stage_1D_vs_E10	ordinary_muscle_development	ENSGALG00010027319	MST1R	2.011333734	0.320172915	6.282023367	3.34195E-10	9.7684E-10	positive
PRJNA729271	stage_1D_vs_E10	ordinary_muscle_development	ENSGALG00010027320	MON1A	0.282064902	0.16132709	1.748403828	0.080394126	0.103869583	positive
PRJNA729271	stage_1D_vs_E10	ordinary_muscle_development	ENSGALG00010027321	IFI30	0.842366416	0.205170655	4.105686627	4.03116E-05	7.76294E-05	positive
PRJNA729271	stage_1D_vs_E10	ordinary_muscle_development	ENSGALG00010027322	CRABP2	-5.890610473	0.196362454	-29.99865995	1.0216E-197	4.777E-195	negative
PRJNA729271	stage_1D_vs_E10	ordinary_muscle_development	ENSGALG00010027323	ISG20L2	-0.127961588	0.12851047	-0.995728896	0.319381886	0.36744507	negative
PRJNA729271	stage_1D_vs_E10	ordinary_muscle_development	ENSGALG00010027324	PIK3R2	-0.545379209	0.097277662	-5.606417747	2.06557E-08	5.25917E-08	negative
PRJNA729271	stage_1D_vs_E10	ordinary_muscle_development	ENSGALG00010027325	METTL25B	-0.435045893	0.296522311	-1.467160737	0.142332335	0.176138239	negative
PRJNA729271	stage_1D_vs_E10	ordinary_muscle_development	ENSGALG00010027326	MRPL24	0.18887263	0.117878	1.602272091	0.109095454	0.137735268	positive
PRJNA729271	stage_1D_vs_E10	ordinary_muscle_development	ENSGALG00010027327	RBM6	-0.070037735	0.08809415	-0.795032765	0.426594443	0.476018972	negative
PRJNA729271	stage_1D_vs_E10	ordinary_muscle_development	ENSGALG00010027328	RBM15	0.225404662	0.130517984	1.727000794	0.084167521	0.108387368	positive
PRJNA729271	stage_1D_vs_E10	ordinary_muscle_development	ENSGALG00010027329	MINDY4	-1.275396991	0.120127912	-10.61699124	2.4844E-26	1.80327E-25	negative
PRJNA729271	stage_1D_vs_E10	ordinary_muscle_development	ENSGALG00010027330	ENSGALG00010027330	-0.679469739	0.204198985	-3.327488322	0.000876327	0.00146967	negative
PRJNA729271	stage_1D_vs_E10	ordinary_muscle_development	ENSGALG00010027331	SLC16A4	0.34327867	0.165283169	2.076912438	0.037809642	0.051363707	positive
PRJNA729271	stage_1D_vs_E10	ordinary_muscle_development	ENSGALG00010027332	HDGF	0.07332818	0.176938097	0.414428442	0.678560363	0.716576749	positive
PRJNA729271	stage_1D_vs_E10	ordinary_muscle_development	ENSGALG00010027333	PROK1	-0.911898037	0.312742577	-2.915810337	0.003547661	0.005549681	negative
PRJNA729271	stage_1D_vs_E10	ordinary_muscle_development	ENSGALG00010027334	PRCC	-0.166684595	0.11416467	-1.460036582	0.14428002	0.178457112	negative
PRJNA729271	stage_1D_vs_E10	ordinary_muscle_development	ENSGALG00010027335	MAST3	-0.286262945	0.177305651	-1.61451676	0.106415384	0.134644815	negative
PRJNA729271	stage_1D_vs_E10	ordinary_muscle_development	ENSGALG00010027336	RAD54L2	-0.267870414	0.138215596	-1.938062147	0.052615641	0.069905015	negative
PRJNA729271	stage_1D_vs_E10	ordinary_muscle_development	ENSGALG00010027337	AQP1	-0.747331513	0.26566753	-2.813032943	0.004907662	0.007553256	negative
PRJNA729271	stage_1D_vs_E10	ordinary_muscle_development	ENSGALG00010027339	LAMTOR5	0.04642811	0.108957449	0.426112305	0.670026014	0.708596751	positive
PRJNA729271	stage_1D_vs_E10	ordinary_muscle_development	ENSGALG00010027340	ENSGALG00010027340	1.361315623	0.800561583	1.700450849	0.089046154	0.114182944	positive
PRJNA729271	stage_1D_vs_E10	ordinary_muscle_development	ENSGALG00010027341	ENSGALG00010027341	0.998614017	0.650347789	1.535507669	0.124659175	0.155958462	positive
PRJNA729271	stage_1D_vs_E10	ordinary_muscle_development	ENSGALG00010027346	SIN3B	0.195129803	0.088689882	2.200136001	0.027797247	0.038472367	positive
PRJNA729271	stage_1D_vs_E10	ordinary_muscle_development	ENSGALG00010027348	ENSGALG00010027348	0.554336146	0.205869083	2.692663403	0.007088379	0.010678243	positive
PRJNA729271	stage_1D_vs_E10	ordinary_muscle_development	ENSGALG00010027350	INSRR	1.943352985	0.333968672	5.818967917	5.92121E-09	1.57772E-08	positive
PRJNA729271	stage_1D_vs_E10	ordinary_muscle_development	ENSGALG00010027351	TMEM38A	3.330107323	0.147884086	22.51836165	2.7432E-112	2.616E-110	positive
PRJNA729271	stage_1D_vs_E10	ordinary_muscle_development	ENSGALG00010027354	ENSGALG00010027354	2.714146456	0.846785947	3.205233229	0.001349531	0.002218354	positive
PRJNA729271	stage_1D_vs_E10	ordinary_muscle_development	ENSGALG00010027355	ADCYAP1R1	-0.726293808	0.24257146	-2.994143695	0.002752163	0.004357942	negative
PRJNA729271	stage_1D_vs_E10	ordinary_muscle_development	ENSGALG00010027356	ENSGALG00010027356	1.557100254	0.214073686	7.273664888	3.49863E-13	1.25031E-12	positive
PRJNA729271	stage_1D_vs_E10	ordinary_muscle_development	ENSGALG00010027358	RNF126	-0.193857365	0.100577974	-1.927433583	0.053925618	0.071507806	negative
PRJNA729271	stage_1D_vs_E10	ordinary_muscle_development	ENSGALG00010027359	ENSGALG00010027359	0.838631976	0.116587698	7.193142932	6.33166E-13	2.22383E-12	positive
PRJNA729271	stage_1D_vs_E10	ordinary_muscle_development	ENSGALG00010027360	SMIM7	0.246636345	0.113717633	2.168848736	0.03009417	0.041481076	positive
PRJNA729271	stage_1D_vs_E10	ordinary_muscle_development	ENSGALG00010027361	FANCD2	-0.240256626	0.192805697	-1.246107502	0.212724931	0.254367916	negative
PRJNA729271	stage_1D_vs_E10	ordinary_muscle_development	ENSGALG00010027362	ENSGALG00010027362	0.106883648	0.144411671	0.740131644	0.459220119	0.508083019	positive
PRJNA729271	stage_1D_vs_E10	ordinary_muscle_development	ENSGALG00010027363	VIPR1	-0.159069154	0.257948467	-0.616670285	0.537452225	0.583898216	negative
PRJNA729271	stage_1D_vs_E10	ordinary_muscle_development	ENSGALG00010027364	FSTL3	3.424486132	0.229391275	14.92858057	2.1479E-50	4.20159E-49	positive
PRJNA729271	stage_1D_vs_E10	ordinary_muscle_development	ENSGALG00010027365	PLEKHA6	2.12084751	0.165367822	12.8250314	1.18738E-37	1.42831E-36	positive
PRJNA729271	stage_1D_vs_E10	ordinary_muscle_development	ENSGALG00010027366	USP4	0.40282174	0.082290911	4.895093961	9.82588E-07	2.19692E-06	positive
PRJNA729271	stage_1D_vs_E10	ordinary_muscle_development	ENSGALG00010027367	SEC22C	-0.530714167	0.094561798	-5.612352724	1.99594E-08	5.08903E-08	negative
PRJNA729271	stage_1D_vs_E10	ordinary_muscle_development	ENSGALG00010027368	EMC3	0.503360923	0.088782864	5.669572911	1.43154E-08	3.69089E-08	positive
PRJNA729271	stage_1D_vs_E10	ordinary_muscle_development	ENSGALG00010027369	ENSGALG00010027369	0.836405604	0.101271871	8.259012088	1.46883E-16	6.49305E-16	positive
PRJNA729271	stage_1D_vs_E10	ordinary_muscle_development	ENSGALG00010027370	NUP210L	-1.615830886	0.364543642	-4.432475844	9.31571E-06	1.90992E-05	negative
PRJNA729271	stage_1D_vs_E10	ordinary_muscle_development	ENSGALG00010027372	PIK3C2B	0.160286266	0.172323828	0.930145687	0.352295659	0.401172564	positive
PRJNA729271	stage_1D_vs_E10	ordinary_muscle_development	ENSGALG00010027373	MLN	7.946935002	1.044865863	7.60569876	2.83369E-14	1.08576E-13	positive
PRJNA729271	stage_1D_vs_E10	ordinary_muscle_development	ENSGALG00010027374	NKTR	-0.039094257	0.118456956	-0.330029224	0.741377881	0.774333384	negative
PRJNA729271	stage_1D_vs_E10	ordinary_muscle_development	ENSGALG00010027375	FUT7	0.154489516	0.461993157	0.334397844	0.738079366	0.771560682	positive
PRJNA729271	stage_1D_vs_E10	ordinary_muscle_development	ENSGALG00010027376	DSTYK	0.248263724	0.079545742	3.121018399	0.001802268	0.002919521	positive
PRJNA729271	stage_1D_vs_E10	ordinary_muscle_development	ENSGALG00010027378	ZBTB47	0.560057393	0.164187305	3.411088295	0.000647041	0.001099773	positive
PRJNA729271	stage_1D_vs_E10	ordinary_muscle_development	ENSGALG00010027379	OPTC	0.51368553	0.599601121	0.856712092	0.391604019	0.44084345	positive
PRJNA729271	stage_1D_vs_E10	ordinary_muscle_development	ENSGALG00010027380	GPX1	-0.070326126	0.132545666	-0.53058035	0.595709616	0.639047579	negative
PRJNA729271	stage_1D_vs_E10	ordinary_muscle_development	ENSGALG00010027381	KLHL40	1.593270725	0.196877488	8.092701429	5.83558E-16	2.49371E-15	positive
PRJNA729271	stage_1D_vs_E10	ordinary_muscle_development	ENSGALG00010027382	PALM	0.568191737	0.198747128	2.858867666	0.004251561	0.006592467	positive
PRJNA729271	stage_1D_vs_E10	ordinary_muscle_development	ENSGALG00010027383	ASS1	1.522909009	0.284352876	5.35570109	8.52253E-08	2.06959E-07	positive
PRJNA729271	stage_1D_vs_E10	ordinary_muscle_development	ENSGALG00010027384	HHATL	2.284386469	0.218393101	10.45997541	1.3189E-25	9.29384E-25	positive
PRJNA729271	stage_1D_vs_E10	ordinary_muscle_development	ENSGALG00010027385	CNTN2	2.830495378	0.57276069	4.941846444	7.73862E-07	1.74368E-06	positive
PRJNA729271	stage_1D_vs_E10	ordinary_muscle_development	ENSGALG00010027386	RHOA	-0.591072772	0.066069249	-8.94626138	3.67726E-19	1.87287E-18	negative
PRJNA729271	stage_1D_vs_E10	ordinary_muscle_development	ENSGALG00010027387	ENSGALG00010027387	-0.812047518	0.262909058	-3.088701181	0.002010335	0.0032356	negative
PRJNA729271	stage_1D_vs_E10	ordinary_muscle_development	ENSGALG00010027388	URM1	0.595015437	0.143912664	4.134559251	3.55636E-05	6.8852E-05	positive
PRJNA729271	stage_1D_vs_E10	ordinary_muscle_development	ENSGALG00010027391	TPM3	-0.958914139	0.114539119	-8.371935705	5.66794E-17	2.56499E-16	negative
PRJNA729271	stage_1D_vs_E10	ordinary_muscle_development	ENSGALG00010027392	RBBP5	-0.281573331	0.126925689	-2.218410885	0.02652683	0.036844231	negative
PRJNA729271	stage_1D_vs_E10	ordinary_muscle_development	ENSGALG00010027393	TEX264	0.667040126	0.189363175	3.522544058	0.000427426	0.000740916	positive
PRJNA729271	stage_1D_vs_E10	ordinary_muscle_development	ENSGALG00010027394	SLC27A4	-0.457881033	0.086631139	-5.285409374	1.25424E-07	3.00798E-07	negative
PRJNA729271	stage_1D_vs_E10	ordinary_muscle_development	ENSGALG00010027395	BCL2L15	-0.315554424	0.273106345	-1.155426924	0.247915642	0.292133747	negative
PRJNA729271	stage_1D_vs_E10	ordinary_muscle_development	ENSGALG00010027397	RFX5	-0.346997582	0.130435394	-2.660302329	0.007807054	0.011706139	negative
PRJNA729271	stage_1D_vs_E10	ordinary_muscle_development	ENSGALG00010027399	MAPKAP1	-0.087642424	0.111549409	-0.785682547	0.432053491	0.481554429	negative
PRJNA729271	stage_1D_vs_E10	ordinary_muscle_development	ENSGALG00010027401	ENSGALG00010027401	1.380953784	0.310211747	4.451648899	8.52134E-06	1.7535E-05	positive
PRJNA729271	stage_1D_vs_E10	ordinary_muscle_development	ENSGALG00010027402	AP4B1	0.167931221	0.08616918	1.9488548	0.051312767	0.068336876	positive
PRJNA729271	stage_1D_vs_E10	ordinary_muscle_development	ENSGALG00010027403	ACKR2	0.275475531	0.373243696	0.738058094	0.460479148	0.509320507	positive
PRJNA729271	stage_1D_vs_E10	ordinary_muscle_development	ENSGALG00010027405	DCLRE1B	-0.968085769	0.170294037	-5.684789577	1.30974E-08	3.38649E-08	negative
PRJNA729271	stage_1D_vs_E10	ordinary_muscle_development	ENSGALG00010027407	ENSGALG00010027407	-1.683878107	1.157147701	-1.455197211	0.145614673	0.180031106	negative
PRJNA729271	stage_1D_vs_E10	ordinary_muscle_development	ENSGALG00010027410	ALAS1	0.174290202	0.087572524	1.990238428	0.046564677	0.062368785	positive
PRJNA729271	stage_1D_vs_E10	ordinary_muscle_development	ENSGALG00010027412	ENSGALG00010027412	1.261192978	0.272386481	4.630159953	3.65383E-06	7.77257E-06	positive
PRJNA729271	stage_1D_vs_E10	ordinary_muscle_development	ENSGALG00010027414	PBX3	-1.473432518	0.073419061	-20.06880077	1.38301E-89	8.1491E-88	negative
PRJNA729271	stage_1D_vs_E10	ordinary_muscle_development	ENSGALG00010027417	WNT2B	-2.17703401	0.366852968	-5.934350276	2.95011E-09	8.044E-09	negative
PRJNA729271	stage_1D_vs_E10	ordinary_muscle_development	ENSGALG00010027418	ENSGALG00010027418	0.781632546	0.984097043	0.794263687	0.427041939	0.476444959	positive
PRJNA729271	stage_1D_vs_E10	ordinary_muscle_development	ENSGALG00010027419	PTBP1	-1.361735717	0.084531825	-16.10914846	2.20033E-58	5.66497E-57	negative
PRJNA729271	stage_1D_vs_E10	ordinary_muscle_development	ENSGALG00010027421	PLPPR3	-2.728557849	0.256222209	-10.64918554	1.75897E-26	1.28769E-25	negative
PRJNA729271	stage_1D_vs_E10	ordinary_muscle_development	ENSGALG00010027423	PEX11B	1.721125241	0.20048621	8.58475621	9.10293E-18	4.30917E-17	positive
PRJNA729271	stage_1D_vs_E10	ordinary_muscle_development	ENSGALG00010027425	CFD	3.236299014	0.238484017	13.57029733	6.00778E-42	8.47109E-41	positive
PRJNA729271	stage_1D_vs_E10	ordinary_muscle_development	ENSGALG00010027427	UBAC1	1.617458652	0.115739478	13.97499524	2.21528E-44	3.40515E-43	positive
PRJNA729271	stage_1D_vs_E10	ordinary_muscle_development	ENSGALG00010027438	ST7L	0.440137954	0.082376596	5.342997609	9.1422E-08	2.21451E-07	positive
PRJNA729271	stage_1D_vs_E10	ordinary_muscle_development	ENSGALG00010027440	TWF2	0.266358183	0.190409582	1.398869641	0.161852078	0.198296498	positive
PRJNA729271	stage_1D_vs_E10	ordinary_muscle_development	ENSGALG00010027443	MED16	-0.300725533	0.156208335	-1.925156771	0.054209748	0.071864853	negative
PRJNA729271	stage_1D_vs_E10	ordinary_muscle_development	ENSGALG00010027444	SELENBP1	0.7915477	0.121040585	6.539523073	6.17153E-11	1.89446E-10	positive
PRJNA729271	stage_1D_vs_E10	ordinary_muscle_development	ENSGALG00010027445	PPM1M	0.586353777	0.162968801	3.597951113	0.000320734	0.000563656	positive
PRJNA729271	stage_1D_vs_E10	ordinary_muscle_development	ENSGALG00010027448	ADAMTSL5	6.21031933	0.310650152	19.99136101	6.54851E-89	3.81207E-87	positive
PRJNA729271	stage_1D_vs_E10	ordinary_muscle_development	ENSGALG00010027450	CAMSAP1	0.643556676	0.142698908	4.509892082	6.48606E-06	1.34867E-05	positive
PRJNA729271	stage_1D_vs_E10	ordinary_muscle_development	ENSGALG00010027455	R3HDM4	0.314913369	0.148565548	2.119693113	0.034031934	0.046581009	positive
PRJNA729271	stage_1D_vs_E10	ordinary_muscle_development	ENSGALG00010027457	CAPZA1	-0.910645526	0.128308014	-7.097339414	1.27181E-12	4.36021E-12	negative
PRJNA729271	stage_1D_vs_E10	ordinary_muscle_development	ENSGALG00010027460	WDR82	-0.343316104	0.073034216	-4.700756991	2.59199E-06	5.59757E-06	negative
PRJNA729271	stage_1D_vs_E10	ordinary_muscle_development	ENSGALG00010027467	GLYCTK	-0.423072434	0.136443081	-3.100724711	0.001930477	0.003112598	negative
PRJNA729271	stage_1D_vs_E10	ordinary_muscle_development	ENSGALG00010027468	BSG	0.60528308	0.115178543	5.255172208	1.47886E-07	3.52567E-07	positive
PRJNA729271	stage_1D_vs_E10	ordinary_muscle_development	ENSGALG00010027471	ENSGALG00010027471	0.909234984	0.129390744	7.027048094	2.10949E-12	7.14249E-12	positive
PRJNA729271	stage_1D_vs_E10	ordinary_muscle_development	ENSGALG00010027473	DNAH1	-2.639324602	0.393271493	-6.711202442	1.93027E-11	6.13043E-11	negative
PRJNA729271	stage_1D_vs_E10	ordinary_muscle_development	ENSGALG00010027475	ARID3A	-0.871420193	0.169006085	-5.156146853	2.52083E-07	5.89251E-07	negative
PRJNA729271	stage_1D_vs_E10	ordinary_muscle_development	ENSGALG00010027479	HCN2	-0.262817542	0.26818579	-0.9799831	0.327094461	0.375424358	negative
PRJNA729271	stage_1D_vs_E10	ordinary_muscle_development	ENSGALG00010027487	MPZ	8.127201471	0.530449425	15.32135034	5.50616E-53	1.1789E-51	positive
PRJNA729271	stage_1D_vs_E10	ordinary_muscle_development	ENSGALG00010027490	ATP2B4	0.324326295	0.067688081	4.791483078	1.65553E-06	3.63149E-06	positive
PRJNA729271	stage_1D_vs_E10	ordinary_muscle_development	ENSGALG00010027492	PCP4L1	3.683292892	0.486250313	7.574890533	3.59431E-14	1.36996E-13	positive

PRJNA729271	stage_1D_vs_E10	ordinary_muscle_development	ENSGALG00010027497	KCNT1	3.314909696	0.33887596	9.782073936	1.34427E-22	8.24947E-22	positive
PRJNA729271	stage_1D_vs_E10	ordinary_muscle_development	ENSGALG00010027499	MOV10	0.780414196	0.402887256	1.937053565	0.052738796	0.070055796	positive
PRJNA729271	stage_1D_vs_E10	ordinary_muscle_development	ENSGALG00010027501	BAP1	-0.282306425	0.066200194	-4.264435004	2.00409E-05	3.9701E-05	negative
PRJNA729271	stage_1D_vs_E10	ordinary_muscle_development	ENSGALG00010027502	THOP1	0.489452073	0.138754918	3.527457492	0.000419571	0.000728172	positive
PRJNA729271	stage_1D_vs_E10	ordinary_muscle_development	ENSGALG00010027510	RHOC	2.444003616	0.181868103	13.43833018	3.60479E-41	4.95745E-40	positive
PRJNA729271	stage_1D_vs_E10	ordinary_muscle_development	ENSGALG00010027511	POLRMT	0.2525095	0.08117642	3.110626205	0.001866911	0.003018299	positive
PRJNA729271	stage_1D_vs_E10	ordinary_muscle_development	ENSGALG00010027513	SEMA3G	1.569007548	0.181127368	8.662454286	4.61714E-18	2.22049E-17	positive
PRJNA729271	stage_1D_vs_E10	ordinary_muscle_development	ENSGALG00010027516	PPM1J	0.356606629	0.231599069	1.539758473	0.123619238	0.154817768	positive
PRJNA729271	stage_1D_vs_E10	ordinary_muscle_development	ENSGALG00010027520	FANCE	0.019278954	0.157054422	0.122753338	0.902302426	0.916657812	positive
PRJNA729271	stage_1D_vs_E10	ordinary_muscle_development	ENSGALG00010027522	PSMD5	-0.034552854	0.104716643	-0.329965252	0.741426218	0.774333384	negative
PRJNA729271	stage_1D_vs_E10	ordinary_muscle_development	ENSGALG00010027524	ENSGALG00010027524	0.97853117	0.111220945	8.798083594	1.39172E-18	6.88499E-18	positive
PRJNA729271	stage_1D_vs_E10	ordinary_muscle_development	ENSGALG00010027525	ENSGALG00010027525	5.099074642	0.26411217	19.3064736	4.73842E-83	2.41843E-81	positive
PRJNA729271	stage_1D_vs_E10	ordinary_muscle_development	ENSGALG00010027527	ENSGALG00010027527	-2.187299574	0.555544757	-3.93721576	8.24325E-05	0.000154195	negative
PRJNA729271	stage_1D_vs_E10	ordinary_muscle_development	ENSGALG00010027528	ENSGALG00010027528	-1.376525155	2.472318782	-0.556774946			negative
PRJNA729271	stage_1D_vs_E10	ordinary_muscle_development	ENSGALG00010027529	ACY1	-0.126618664	0.14413129	-0.878495321	0.379674976	0.428679606	negative
PRJNA729271	stage_1D_vs_E10	ordinary_muscle_development	ENSGALG00010027530	PHF19	0.969764177	0.134862949	7.190738328	6.44419E-13	2.2628E-12	positive
PRJNA729271	stage_1D_vs_E10	ordinary_muscle_development	ENSGALG00010027531	UFC1	-0.45289272	0.17289509	-2.619465479	0.008806769	0.013112903	negative
PRJNA729271	stage_1D_vs_E10	ordinary_muscle_development	ENSGALG00010027533	WDR18	-0.914188564	0.093365421	-9.791511151	1.22452E-22	7.5337E-22	negative
PRJNA729271	stage_1D_vs_E10	ordinary_muscle_development	ENSGALG00010027534	KCNN3	1.27095936	0.185434457	6.853954654	7.18359E-12	2.34465E-11	positive
PRJNA729271	stage_1D_vs_E10	ordinary_muscle_development	ENSGALG00010027536	USP21	0.10173668	0.311731778	0.326359669	0.744152248	0.776676759	positive
PRJNA729271	stage_1D_vs_E10	ordinary_muscle_development	ENSGALG00010027537	SGTA	-0.530994115	0.067933195	-7.816416025	5.43485E-15	2.1774E-14	negative
PRJNA729271	stage_1D_vs_E10	ordinary_muscle_development	ENSGALG00010027538	ABHD14A	0.730024194	0.13401736	5.44723604	5.11586E-08	1.26306E-07	positive
PRJNA729271	stage_1D_vs_E10	ordinary_muscle_development	ENSGALG00010027539	DDR2L	0.509149505	0.107401943	4.740598645	2.13088E-06	4.6363E-06	positive
PRJNA729271	stage_1D_vs_E10	ordinary_muscle_development	ENSGALG00010027540	PPOX	-1.061195755	0.119509848	-8.879567473	6.71208E-19	3.37233E-18	negative
PRJNA729271	stage_1D_vs_E10	ordinary_muscle_development	ENSGALG00010027543	TUBB4B	-0.730200046	0.152150648	-4.79919116	1.59308E-06	3.49873E-06	negative
PRJNA729271	stage_1D_vs_E10	ordinary_muscle_development	ENSGALG00010027545	PPARD	0.020875559	0.202374525	0.103153095	0.917841466	0.930097319	positive
PRJNA729271	stage_1D_vs_E10	ordinary_muscle_development	ENSGALG00010027547	B4GALT3	0.658715124	0.223183675	2.951448504	0.003162873	0.004972973	positive
PRJNA729271	stage_1D_vs_E10	ordinary_muscle_development	ENSGALG00010027548	PCBP4	0.153983015	0.14618056	1.053375465	0.292168945	0.338799108	positive
PRJNA729271	stage_1D_vs_E10	ordinary_muscle_development	ENSGALG00010027553	FGF22	-0.570259594	0.309837802	-1.840510069	0.065693387	0.086026348	negative
PRJNA729271	stage_1D_vs_E10	ordinary_muscle_development	ENSGALG00010027554	ENSGALG00010027554	0.457151544	0.14472451	3.158770722	0.001584361	0.002582403	positive
PRJNA729271	stage_1D_vs_E10	ordinary_muscle_development	ENSGALG00010027555	ENSGALG00010027555	0.723216233	0.347549369	2.080902162	0.037442862	0.050908385	positive
PRJNA729271	stage_1D_vs_E10	ordinary_muscle_development	ENSGALG00010027557	C6orf132	-4.983600697	1.086066893	-4.588668275	4.46083E-06	9.39684E-06	negative
PRJNA729271	stage_1D_vs_E10	ordinary_muscle_development	ENSGALG00010027559	ENSGALG00010027559	-0.535247294	0.291612298	-1.835475726	0.066435232	0.086895749	negative
PRJNA729271	stage_1D_vs_E10	ordinary_muscle_development	ENSGALG00010027561	ADAMTS4	1.202566384	0.338245606	3.555305262	0.00037754	0.000659014	positive
PRJNA729271	stage_1D_vs_E10	ordinary_muscle_development	ENSGALG00010027562	SLC39A3	0.422792474	0.144439586	2.927123264	0.003421133	0.005363327	positive
PRJNA729271	stage_1D_vs_E10	ordinary_muscle_development	ENSGALG00010027563	WDR77	-0.889477415	0.122627447	-7.253493694	4.06156E-13	1.44685E-12	negative
PRJNA729271	stage_1D_vs_E10	ordinary_muscle_development	ENSGALG00010027567	PARP3	1.328462749	0.128713161	10.32111041	5.65651E-25	3.87482E-24	positive
PRJNA729271	stage_1D_vs_E10	ordinary_muscle_development	ENSGALG00010027568	NELFB	-0.533742199	0.076204881	-7.004042186	2.48681E-12	8.38287E-12	negative
PRJNA729271	stage_1D_vs_E10	ordinary_muscle_development	ENSGALG00010027569	ATP5PB	1.096078498	0.125299884	8.747641765	2.17858E-18	1.06684E-17	positive
PRJNA729271	stage_1D_vs_E10	ordinary_muscle_development	ENSGALG00010027570	NDUFS2	1.405442114	0.137217289	10.24245654	1.27946E-24	8.60591E-24	positive
PRJNA729271	stage_1D_vs_E10	ordinary_muscle_development	ENSGALG00010027571	RBM10	0.372065667	0.10151695	3.665059558	0.000247281	0.000439473	positive
PRJNA729271	stage_1D_vs_E10	ordinary_muscle_development	ENSGALG00010027574	GNG7	-0.297002004	0.095315523	-3.115987752	0.001833299	0.002966142	negative
PRJNA729271	stage_1D_vs_E10	ordinary_muscle_development	ENSGALG00010027575	FCER1G	0.21413246	0.350482337	0.610965055	0.541222715	0.587466172	positive
PRJNA729271	stage_1D_vs_E10	ordinary_muscle_development	ENSGALG00010027576	GADD45B	1.552367254	0.417105683	3.721760015	0.000197839	0.000355438	positive
PRJNA729271	stage_1D_vs_E10	ordinary_muscle_development	ENSGALG00010027578	ENSGALG00010027578	1.14360816	0.241673205	4.73204367	2.22271E-06	4.82813E-06	positive
PRJNA729271	stage_1D_vs_E10	ordinary_muscle_development	ENSGALG00010027579	C26H6ORF125	-0.833848939	0.159399003	-5.231205487	1.68408E-07	3.99849E-07	negative
PRJNA729271	stage_1D_vs_E10	ordinary_muscle_development	ENSGALG00010027583	GLE1	-0.871518154	0.071836453	-12.13197649	7.15025E-34	7.39778E-33	negative
PRJNA729271	stage_1D_vs_E10	ordinary_muscle_development	ENSGALG00010027586	HJV	4.044796713	0.288957798	13.99788047	1.60589E-44	2.47632E-43	positive
PRJNA729271	stage_1D_vs_E10	ordinary_muscle_development	ENSGALG00010027590	SEMA3F	-1.056693931	0.236220274	-4.473341396	7.70067E-06	1.59073E-05	negative
PRJNA729271	stage_1D_vs_E10	ordinary_muscle_development	ENSGALG00010027592	MAP1S	-0.083636796	0.169073226	-0.494677945	0.620827472	0.662999647	negative
PRJNA729271	stage_1D_vs_E10	ordinary_muscle_development	ENSGALG00010027593	ENSGALG00010027593	3.189044181	0.434668231	7.336731675	2.18873E-13	7.91557E-13	positive
PRJNA729271	stage_1D_vs_E10	ordinary_muscle_development	ENSGALG00010027594	ITPR3	0.805412422	0.137836328	5.843252183	5.11914E-09	1.3698E-08	positive
PRJNA729271	stage_1D_vs_E10	ordinary_muscle_development	ENSGALG00010027595	GNAT1	-0.112931652	0.437988683	-0.257841483	0.796529248	0.823985972	negative
PRJNA729271	stage_1D_vs_E10	ordinary_muscle_development	ENSGALG00010027597	EXOSC2	-0.428776957	0.115725303	-3.705127121	0.000211285	0.000378516	negative
PRJNA729271	stage_1D_vs_E10	ordinary_muscle_development	ENSGALG00010027600	KIRREL1	0.367666852	0.140768923	2.611846745	0.009005461	0.013390866	positive
PRJNA729271	stage_1D_vs_E10	ordinary_muscle_development	ENSGALG00010027605	FCHO1	-0.354612654	0.33134292	-1.070228555	0.284516444	0.330765999	negative
PRJNA729271	stage_1D_vs_E10	ordinary_muscle_development	ENSGALG00010027607	ABL1	-0.28628671	0.07888855	-3.629002048	0.000284519	0.000502572	negative
PRJNA729271	stage_1D_vs_E10	ordinary_muscle_development	ENSGALG00010027608	GNAI2	-0.186842511	0.144040025	-1.297156896	0.194577208	0.234505416	negative
PRJNA729271	stage_1D_vs_E10	ordinary_muscle_development	ENSGALG00010027609	RPL36	0.223827557	0.186789686	1.198286486	0.230805506	0.274043726	positive
PRJNA729271	stage_1D_vs_E10	ordinary_muscle_development	ENSGALG00010027612	BAK1	-1.217901089	0.164350485	-7.410389369	1.25929E-13	4.62698E-13	negative
PRJNA729271	stage_1D_vs_E10	ordinary_muscle_development	ENSGALG00010027614	LONP1	-0.19234671	0.079228382	-2.427750062	0.015192809	0.021888275	negative
PRJNA729271	stage_1D_vs_E10	ordinary_muscle_development	ENSGALG00010027616	SLC38A3	4.847900149	0.266303749	18.20440071	4.76237E-74	1.95554E-72	positive
PRJNA729271	stage_1D_vs_E10	ordinary_muscle_development	ENSGALG00010027618	PPP1R26	0.351860537	0.078080851	4.506361485	6.59487E-06	1.3705E-05	positive
PRJNA729271	stage_1D_vs_E10	ordinary_muscle_development	ENSGALG00010027619	JAK3	0.307903342	0.373986264	0.823301204	0.410336729	0.460037968	positive
PRJNA729271	stage_1D_vs_E10	ordinary_muscle_development	ENSGALG00010027621	TSPO2	-2.465631843	0.397545512	-6.202137283	5.57015E-10	1.60292E-09	negative
PRJNA729271	stage_1D_vs_E10	ordinary_muscle_development	ENSGALG00010027622	FIBCD1	-3.864921952	1.222693808	-3.160989224	0.001572343	0.002563968	negative
PRJNA729271	stage_1D_vs_E10	ordinary_muscle_development	ENSGALG00010027623	APOBEC2	2.337516408	0.182666337	12.79664579	1.71187E-37	2.04563E-36	positive
PRJNA729271	stage_1D_vs_E10	ordinary_muscle_development	ENSGALG00010027624	ENSGALG00010027624	0.965276047	0.433060733	2.2289623	0.025816413	0.035929804	positive
PRJNA729271	stage_1D_vs_E10	ordinary_muscle_development	ENSGALG00010027626	SEMA3B	2.567858754	0.208458724	12.31830791	7.21932E-35	7.79181E-34	positive
PRJNA729271	stage_1D_vs_E10	ordinary_muscle_development	ENSGALG00010027627	OARD1	1.125180014	0.093201448	12.07255932	1.47475E-33	1.50858E-32	positive
PRJNA729271	stage_1D_vs_E10	ordinary_muscle_development	ENSGALG00010027628	PIERCE1	-0.870042677	0.251277291	-3.46248033	0.000535221	0.000919198	negative
PRJNA729271	stage_1D_vs_E10	ordinary_muscle_development	ENSGALG00010027632	LAMC3	-0.847437143	0.373450263	-2.269210193	0.023255547	0.032638376	negative
PRJNA729271	stage_1D_vs_E10	ordinary_muscle_development	ENSGALG00010027634	OASL	2.911313035	0.713049121	4.082906705	4.44759E-05	8.53652E-05	positive
PRJNA729271	stage_1D_vs_E10	ordinary_muscle_development	ENSGALG00010027635	CD48	0.130675491	0.235121861	0.555777719	0.578362819	0.62297459	positive
PRJNA729271	stage_1D_vs_E10	ordinary_muscle_development	ENSGALG00010027636	SLC1A6	1.048714715	0.595384074	1.761408744	0.078169246	0.101247719	positive
PRJNA729271	stage_1D_vs_E10	ordinary_muscle_development	ENSGALG00010027638	MRPS2	0.867164417	0.140388338	6.176897806	6.53734E-10	1.87048E-09	positive
PRJNA729271	stage_1D_vs_E10	ordinary_muscle_development	ENSGALG00010027641	HYAL3	0.456397302	0.165618385	2.755716419	0.005856375	0.00892526	positive
PRJNA729271	stage_1D_vs_E10	ordinary_muscle_development	ENSGALG00010027642	DIPK1B	-0.926867455	0.167949515	-5.518726593	3.41465E-08	8.53221E-08	negative
PRJNA729271	stage_1D_vs_E10	ordinary_muscle_development	ENSGALG00010027644	NAA80	2.259338982	0.138754209	16.2830302	1.30256E-59	3.54233E-58	positive
PRJNA729271	stage_1D_vs_E10	ordinary_muscle_development	ENSGALG00010027645	SLAMF1	-1.357726472	1.134516278	-1.196744814	0.231406034	0.274711749	negative
PRJNA729271	stage_1D_vs_E10	ordinary_muscle_development	ENSGALG00010027648	NFYA	-0.027124811	0.071770134	-0.377940091	0.705475099	0.741918418	negative
PRJNA729271	stage_1D_vs_E10	ordinary_muscle_development	ENSGALG00010027649	FBLN2	-0.701096713	0.191609783	-3.65898182	0.000253219	0.00044953	negative
PRJNA729271	stage_1D_vs_E10	ordinary_muscle_development	ENSGALG00010027650	CCDC124	0.343661265	0.191229556	1.797113752	0.072317563	0.094088778	positive
PRJNA729271	stage_1D_vs_E10	ordinary_muscle_development	ENSGALG00010027653	WNT7A	-6.228830604	0.563212252	-11.05947284	1.97253E-28	1.57966E-27	negative
PRJNA729271	stage_1D_vs_E10	ordinary_muscle_development	ENSGALG00010027654	AIF1L	0.178908341	0.091424552	1.956896017	0.050359695	0.067153981	positive
PRJNA729271	stage_1D_vs_E10	ordinary_muscle_development	ENSGALG00010027663	COPA	-0.606011027	0.070562065	-8.588340259	8.82346E-18	4.18098E-17	negative
PRJNA729271	stage_1D_vs_E10	ordinary_muscle_development	ENSGALG00010027664	TREMB1	0.959811344	0.325342668	2.950155137	0.003176144	0.004991674	positive
PRJNA729271	stage_1D_vs_E10	ordinary_muscle_development	ENSGALG00010027665	TRAF1	-0.809289218	0.140318974	-5.767496673	8.04577E-09	2.1212E-08	negative
PRJNA729271	stage_1D_vs_E10	ordinary_muscle_development	ENSGALG00010027667	PTPDC1	0.504036917	0.148879818	3.385528849	0.000710412	0.001203955	positive
PRJNA729271	stage_1D_vs_E10	ordinary_muscle_development	ENSGALG00010027668	RANBP3	-0.293893628	0.053460039	-5.497445061	3.85333E-08	9.59362E-08	negative
PRJNA729271	stage_1D_vs_E10	ordinary_muscle_development	ENSGALG00010027671	BARX1	-3.538675926	0.58140424	-6.086429509	1.15456E-09	3.2433E-09	negative
PRJNA729271	stage_1D_vs_E10	ordinary_muscle_development	ENSGALG00010027672	CNTRL	-1.058731805	0.174653609	-6.061894805	1.34527E-09	3.76079E-09	negative
PRJNA729271	stage_1D_vs_E10	ordinary_muscle_development	ENSGALG00010027674	TREM2	-0.945369578	0.295404489	-3.200254607	0.001373062	0.002255547	negative
PRJNA729271	stage_1D_vs_E10	ordinary_muscle_development	ENSGALG00010027685	ENSGALG00010027685	0.192103243	0.330810928	0.580704042	0.561439937	0.607001707	positive
PRJNA729271	stage_1D_vs_E10	ordinary_muscle_development	ENSGALG00010027692	PHF2	0.916262318	0.111306354	8.23189588	1.84273E-16	8.09652E-16	positive
PRJNA729271	stage_1D_vs_E10	ordinary_muscle_development	ENSGALG00010027693	RAB14	-0.364765093	0.059815374	-6.098182938	1.07281E-09	3.02419E-09	negative
PRJNA729271	stage_1D_vs_E10	ordinary_muscle_development	ENSGALG00010027695	TREMB2	0.052451704	0.313919934	0.167086247	0.867302187	0.886944067	positive
PRJNA729271	stage_1D_vs_E10	ordinary_muscle_development	ENSGALG00010027697	CATH2	4.21451631	0.874266445	4.820631437	1.43105E-06	3.15579E-06	positive
PRJNA729271	stage_1D_vs_E10	ordinary_muscle_development	ENSGALG00010027698	FAM120A	0.090340344	0.066369715	1.361168175	0.173460549	0.211144664	positive
PRJNA729271	stage_1D_vs_E10	ordinary_muscle_development	ENSGALG00010027699	VANGL2	-1.311911102	0.120312353	-10.90420951	1.10046E-27	8.47561E-27	negative
PRJNA729271	stage_1D_vs_E10	ordinary_muscle_development	ENSGALG00010027700	TCF3	-1.054497391	0.096014347	-10.98270645	4.62846E-28	3.63038E-27	negative
PRJNA729271	stage_1D_vs_E10	ordinary_muscle_development	ENSGALG00010027702	FOXP4	-2.395549585	0.247827824	-9.666184965	4.19734E-22	2.52241E-21	negative
PRJNA729271	stage_1D_vs_E10	ordinary_muscle_development	ENSGALG00010027703	NCSTN	0.070678053	0.100017487	0.706656953	0.479779687	0.528370047	positive
PRJNA729271	stage_1D_vs_E10	ordinary_muscle_development	ENSGALG00010027704	MDFI	-2.604395145	0.177553373	-14.66823803	1.02985E-48	1.89197E-47	negative
PRJNA729271	stage_1D_vs_E10	ordinary_muscle_development	ENSGALG00010027705	S100A11	-0.074087727	0.229738304	-0.322487482	0.74708342	0.779455421	negative
PRJNA729271	stage_1D_vs_E10	ordinary_muscle_development	ENSGALG00010027706	GSN	2.4925659	0.171917494	14.49861698	1.23616E-47	2.15621E-46	positive
PRJNA729271	stage_1D_vs_E10	ordinary_muscle_development	ENSGALG00010027707	HIGD1C	0.878782979	0.211559767	4.153828453	3.26958E-05	6.35119E-05	positive
PRJNA729271	stage_1D_vs_E10	ordinary_muscle_development	ENSGALG00010027709	PSMA5	0.524248955	0.107098867	4.895000003	9.83057E-07	2.19763E-06	positive
PRJNA729271	stage_1D_vs_E10	ordinary_muscle_development	ENSGALG00010027710	ENSGALG00010027710	3.639009931	0.283495563	12.83621474	1.02778E-37	1.24147E-36	positive
PRJNA729271	stage_1D_vs_E10	ordinary_muscle_development	ENSGALG00010027711	WNK2	1.540653001	0.185846187	8.289936047	1.13309E-16	5.03655E-16	positive
PRJNA729271	stage_1D_vs_E10	ordinary_muscle_development	ENSGALG00010027712	STOM	1.916112459	0.156682571	12.22926354	2.16896E-34	2.28648E-33	positive
PRJNA729271	stage_1D_vs_E10	ordinary_muscle_development	ENSGALG00010027713	ENSGALG00010027713	0.603077333	0.139580955	4.320627656	1.55586E-05	3.11365E-05	positive
PRJNA729271	stage_1D_vs_E10	ordinary_muscle_development	ENSGALG00010027714	SYPL2	7.476171949	0.162030179	46.14061396	0	0	positive
PRJNA729271	stage_1D_vs_E10	ordinary_muscle_development	ENSGALG00010027715	NINJ1	-0.471436992	0.123649262	-3.812695558	0.000137459	0.000251607	negative
PRJNA729271	stage_1D_vs_E10	ordinary_muscle_development	ENSGALG00010027717	ATXN7L2	1.382602386	0.22638079	6.10741921	1.01255E-09	2.86156E-09	positive
PRJNA729271	stage_1D_vs_E10	ordinary_muscle_development	ENSGALG00010027718	ENSGALG00010027718	1.503149313	0.494634295	3.038910421	0.002374354	0.003784919	positive
PRJNA729271	stage_1D_vs_E10	ordinary_muscle_development	ENSGALG00010027719	GPR61	-4.061003198	1.038034403	-3.912204821	9.14573E-05	0.000170417	negative
PRJNA729271	stage_1D_vs_E10	ordinary_muscle_development	ENSGALG00010027720	DAB2IP	1.042215338	0.086585045	12.03689775	2.27345E-33	2.3093E-32	positive
PRJNA729271	stage_1D_vs_E10	ordinary_muscle_development	ENSGALG00010027721	SUSD3	-2.175388312	0.201496705	-10.79614834	3.58947E-27	2.70704E-26	negative
PRJNA729271	stage_1D_vs_E10	ordinary_muscle_development	ENSGALG00010027722	GNA13	-0.870949891	0.085135083	-10.23021139	1.45202E-24	9.73499E-24	negative
PRJNA729271	stage_1D_vs_E10	ordinary_muscle_development	ENSGALG00010027723	ENSGALG00010027723	-0.366579249	1.245694534	-0.294276999	0.768546247	0.798248775	negative
PRJNA729271	stage_1D_vs_E10	ordinary_muscle_development	ENSGALG00010027724	TTLL11	-0.19824611	0.110199022	-1.798982483	0.072021452	0.093745595	negative
PRJNA729271	stage_1D_vs_E10	ordinary_muscle_development	ENSGALG00010027725	BTN1A1	0.918425059	0.272356317	3.372145239	0.000745851	0.001261654	positive
PRJNA729271	stage_1D_vs_E10	ordinary_muscle_development	ENSGALG00010027727	GSTM2	0.591126896	0.171462297	3.447561972	0.000565671	0.000967594	positive
PRJNA729271	stage_1D_vs_E10	ordinary_muscle_development	ENSGALG00010027728	CRNN	0.375422806	1.679237626	0.22356741	0.823093925	0.847866973	positive
PRJNA729271	stage_1D_vs_E10	ordinary_muscle_development	ENSGALG00010027729	EPS8L3	0.475484853	0.250814672	1.895761716	0.05799156	0.076479635	positive
PRJNA729271	stage_1D_vs_E10	ordinary_muscle_development	ENSGALG00010027730	ENSGALG00010027730	-1.553855361	0.141917373	-10.94901441	6.71745E-28	5.23492E-27	negative
PRJNA729271	stage_1D_vs_E10	ordinary_muscle_development	ENSGALG00010027731	OLFML2A	2.482111198	0.10145431	24.46531061	3.4584E-132	4.685E-130	positive
PRJNA729271	stage_1D_vs_E10	ordinary_muscle_development	ENSGALG00010027732	EEF2	0.763815703	0.117035815	6.526341534	6.73955E-11	2.06184E-10	positive
PRJNA729271	stage_1D_vs_E10	ordinary_muscle_development	ENSGALG00010027733	RPL35	-0.107953321	0.154060675	-0.700719516	0.483478074	0.532038771	negative
PRJNA729271	stage_1D_vs_E10	ordinary_muscle_development	ENSGALG00010027734	CSF1	1.092551366	0.138473405	7.889972582	3.02253E-15	1.23239E-14	positive

PRJNA729271	stage_1D_vs_E10	ordinary_muscle_development	ENSGALG00010027736	ENSGALG00010027736	0.859505887	1.658492684	0.518245209	0.604287196	0.647124188	positive
PRJNA729271	stage_1D_vs_E10	ordinary_muscle_development	ENSGALG00010027738	ENSGALG00010027738	0.857597674	0.247452736	3.465702934	0.000528847	0.000908467	positive
PRJNA729271	stage_1D_vs_E10	ordinary_muscle_development	ENSGALG00010027739	FGD3	-1.994893753	0.07461941	-26.73424704	1.8828E-157	3.8987E-155	negative
PRJNA729271	stage_1D_vs_E10	ordinary_muscle_development	ENSGALG00010027740	MARCHF2	-0.099068761	0.144756739	-0.684380991	0.493734616	0.542008729	negative
PRJNA729271	stage_1D_vs_E10	ordinary_muscle_development	ENSGALG00010027741	RAB11B	-0.844196198	0.06151026	-13.72447791	7.24491E-43	1.05225E-41	negative
PRJNA729271	stage_1D_vs_E10	ordinary_muscle_development	ENSGALG00010027743	C1orf74	0.835431464	0.188863473	4.423467648	9.71292E-06	1.98602E-05	positive
PRJNA729271	stage_1D_vs_E10	ordinary_muscle_development	ENSGALG00010027744	ENSGALG00010027744	0.859518261	1.332846629	0.644874093	0.519008782	0.566445866	positive
PRJNA729271	stage_1D_vs_E10	ordinary_muscle_development	ENSGALG00010027745	BICD2	0.013099364	0.1385425	0.094551235	0.924671285	0.935906032	positive
PRJNA729271	stage_1D_vs_E10	ordinary_muscle_development	ENSGALG00010027746	UQCR11	2.254215664	0.184308409	12.23067181	2.13168E-34	2.24882E-33	positive
PRJNA729271	stage_1D_vs_E10	ordinary_muscle_development	ENSGALG00010027748	ENSGALG00010027748	0.859453518	2.889617951	0.29742808	0.766139709	0.79618548	positive
PRJNA729271	stage_1D_vs_E10	ordinary_muscle_development	ENSGALG00010027751	ANGPTL4	0.283152305	0.208939354	1.355188957	0.175357362	0.213346061	positive
PRJNA729271	stage_1D_vs_E10	ordinary_muscle_development	ENSGALG00010027752	SCAI	-1.135186157	0.084694618	-13.40328564	5.78445E-41	7.88023E-40	negative
PRJNA729271	stage_1D_vs_E10	ordinary_muscle_development	ENSGALG00010027759	ENSGALG00010027759	-1.985200402	0.994884387	-1.995408138	0.045998384	0.06165587	negative
PRJNA729271	stage_1D_vs_E10	ordinary_muscle_development	ENSGALG00010027766	ENSGALG00010027766	-2.134642285	0.753841519	-2.831685748	0.004630333	0.007149199	negative
PRJNA729271	stage_1D_vs_E10	ordinary_muscle_development	ENSGALG00010027767	ENSGALG00010027767	-1.907412124	1.179035241	-1.617773632	0.105711391	0.133835847	negative
PRJNA729271	stage_1D_vs_E10	ordinary_muscle_development	ENSGALG00010027772	ENSGALG00010027772	-2.272816101	1.170293228	-1.942091134	0.052126069	0.069324467	negative
PRJNA729271	stage_1D_vs_E10	ordinary_muscle_development	ENSGALG00010027775	MVB12B	0.943952661	0.087675199	10.76647292	4.95626E-27	3.7204E-26	positive
PRJNA729271	stage_1D_vs_E10	ordinary_muscle_development	ENSGALG00010027776	TKT	-2.02188616	0.129475947	-15.61592099	5.67201E-55	1.30088E-53	negative
PRJNA729271	stage_1D_vs_E10	ordinary_muscle_development	ENSGALG00010027784	PEAR1	1.921870725	0.189346299	10.15003056	3.3126E-24	2.17563E-23	positive
PRJNA729271	stage_1D_vs_E10	ordinary_muscle_development	ENSGALG00010027787	DCP1A	0.232986797	0.136467388	1.707270883	0.087771715	0.112678329	positive
PRJNA729271	stage_1D_vs_E10	ordinary_muscle_development	ENSGALG00010027789	UAP1L1	-1.331930378	0.134480785	-9.904243056	3.9898E-23	2.50031E-22	negative
PRJNA729271	stage_1D_vs_E10	ordinary_muscle_development	ENSGALG00010027801	MBD3	-2.393540212	0.128808186	-18.58220573	4.47719E-77	1.96657E-75	negative
PRJNA729271	stage_1D_vs_E10	ordinary_muscle_development	ENSGALG00010027803	SAPCD2	-2.415187957	0.172172367	-14.02773278	1.0547E-44	1.63332E-43	negative
PRJNA729271	stage_1D_vs_E10	ordinary_muscle_development	ENSGALG00010027811	MEX3D	-1.069930532	0.100566654	-10.6390189	1.96184E-26	1.43331E-25	negative
PRJNA729271	stage_1D_vs_E10	ordinary_muscle_development	ENSGALG00010027813	LRRC71	-2.341702647	0.315241919	-7.428271757	1.10026E-13	4.06117E-13	negative
PRJNA729271	stage_1D_vs_E10	ordinary_muscle_development	ENSGALG00010027814	ENSGALG00010027814	0.320021984	0.130991674	2.443071175	0.014562865	0.021037346	positive
PRJNA729271	stage_1D_vs_E10	ordinary_muscle_development	ENSGALG00010027816	ZMYND19	-0.714537767	0.119388452	-5.984982278	2.16413E-09	5.96371E-09	negative
PRJNA729271	stage_1D_vs_E10	ordinary_muscle_development	ENSGALG00010027821	NMRK2	2.099059543	0.226991218	9.247316111	2.302E-20	1.25113E-19	positive
PRJNA729271	stage_1D_vs_E10	ordinary_muscle_development	ENSGALG00010027822	DPH7	-0.17812135	0.090433744	-1.969633704	0.048880366	0.065313505	negative
PRJNA729271	stage_1D_vs_E10	ordinary_muscle_development	ENSGALG00010027826	ARHGEF11	0.450093053	0.164447938	2.73699421	0.006200338	0.009423708	positive
PRJNA729271	stage_1D_vs_E10	ordinary_muscle_development	ENSGALG00010027830	CACNA1D	-0.724387249	0.323073047	-2.24217791	0.024949876	0.034827471	negative
PRJNA729271	stage_1D_vs_E10	ordinary_muscle_development	ENSGALG00010027831	ATCAY	-1.557540943	0.20251556	-7.690969251	1.46025E-14	5.6868E-14	negative
PRJNA729271	stage_1D_vs_E10	ordinary_muscle_development	ENSGALG00010027838	ENSGALG00010027838	-0.571025708	0.611352794	-0.934036309	0.350285164	0.399196749	negative
PRJNA729271	stage_1D_vs_E10	ordinary_muscle_development	ENSGALG00010027842	SLAMF8	1.279884453	0.186325674	6.86907191	6.46209E-12	2.1144E-11	positive
PRJNA729271	stage_1D_vs_E10	ordinary_muscle_development	ENSGALG00010027847	NRTN	-1.810792123	0.379964219	-4.76569117	1.88207E-06	4.10668E-06	negative
PRJNA729271	stage_1D_vs_E10	ordinary_muscle_development	ENSGALG00010027860	ENSGALG00010027860	0.731232309	0.270962139	2.698651221	0.00696211	0.010502215	positive
PRJNA729271	stage_1D_vs_E10	ordinary_muscle_development	ENSGALG00010027861	PNPLA7	-0.142824954	0.07907442	-1.806209316	0.07088565	0.092360788	negative
PRJNA729271	stage_1D_vs_E10	ordinary_muscle_development	ENSGALG00010027863	CHDH	-1.517070251	0.150828179	-10.05826806	8.44713E-24	5.44667E-23	negative
PRJNA729271	stage_1D_vs_E10	ordinary_muscle_development	ENSGALG00010027864	ACTR8	-0.538934158	0.082394832	-6.540873294	6.11606E-11	1.87876E-10	negative
PRJNA729271	stage_1D_vs_E10	ordinary_muscle_development	ENSGALG00010027865	SELENOK	-0.147721751	0.121845741	-1.212366963	0.225371941	0.268127256	negative
PRJNA729271	stage_1D_vs_E10	ordinary_muscle_development	ENSGALG00010027866	KCNA2	-0.32892877	0.136355178	-2.412293943	0.015852495	0.022752938	negative
PRJNA729271	stage_1D_vs_E10	ordinary_muscle_development	ENSGALG00010027867	UBAP2L	-0.044446202	0.167357774	-0.265575963	0.7905658	0.818342588	negative
PRJNA729271	stage_1D_vs_E10	ordinary_muscle_development	ENSGALG00010027868	CACNA2D3	-3.666748911	0.308427185	-11.88853997	1.35759E-32	1.33051E-31	negative
PRJNA729271	stage_1D_vs_E10	ordinary_muscle_development	ENSGALG00010027869	NSMF	-0.691402391	0.164895781	-4.192965917	2.75331E-05	5.38586E-05	negative
PRJNA729271	stage_1D_vs_E10	ordinary_muscle_development	ENSGALG00010027870	HAX1	1.296123291	0.12106751	10.7057896	9.5612E-27	7.09261E-26	positive
PRJNA729271	stage_1D_vs_E10	ordinary_muscle_development	ENSGALG00010027872	KDM5B	-0.387263767	0.091665462	-4.224751152	2.39205E-05	4.70458E-05	negative
PRJNA729271	stage_1D_vs_E10	ordinary_muscle_development	ENSGALG00010027873	RFX2	0.323121155	0.197762544	1.633884498	0.102283105	0.129903935	positive
PRJNA729271	stage_1D_vs_E10	ordinary_muscle_development	ENSGALG00010027874	FRS3	-0.097794945	0.133283072	-0.733738677	0.463108022	0.511876679	negative
PRJNA729271	stage_1D_vs_E10	ordinary_muscle_development	ENSGALG00010027875	WNT5A	-0.520203309	0.168653662	-3.084447169	0.002039308	0.003278954	negative
PRJNA729271	stage_1D_vs_E10	ordinary_muscle_development	ENSGALG00010027876	ANAPC2	-0.08920244	0.109160985	-0.817164113	0.413834648	0.463350319	negative
PRJNA729271	stage_1D_vs_E10	ordinary_muscle_development	ENSGALG00010027877	OAZ1	0.270724224	0.114386787	2.366743854	0.017945348	0.025549339	positive
PRJNA729271	stage_1D_vs_E10	ordinary_muscle_development	ENSGALG00010027879	ENSGALG00010027879	5.212968187	0.415251112	12.55377296	3.79011E-36	4.30209E-35	positive
PRJNA729271	stage_1D_vs_E10	ordinary_muscle_development	ENSGALG00010027880	AMH	1.889177506	0.335714652	5.627331116	1.83019E-08	4.67795E-08	positive
PRJNA729271	stage_1D_vs_E10	ordinary_muscle_development	ENSGALG00010027881	ENSGALG00010027881	-1.268487714	0.292714223	-4.333536319	1.46733E-05	2.94666E-05	negative
PRJNA729271	stage_1D_vs_E10	ordinary_muscle_development	ENSGALG00010027882	CHIA	3.447999246	1.403131372	2.457360241	0.013996226	0.02026119	positive
PRJNA729271	stage_1D_vs_E10	ordinary_muscle_development	ENSGALG00010027883	ATP8B2	0.147416898	0.117051263	1.259421677	0.207878064	0.248921316	positive
PRJNA729271	stage_1D_vs_E10	ordinary_muscle_development	ENSGALG00010027884	SF3A2	-0.372274594	0.18884108	-1.971364457	0.048682204	0.065054719	negative
PRJNA729271	stage_1D_vs_E10	ordinary_muscle_development	ENSGALG00010027885	PLEKHJ1	-0.739398425	0.072600533	-10.18447657	2.32608E-24	1.54239E-23	negative
PRJNA729271	stage_1D_vs_E10	ordinary_muscle_development	ENSGALG00010027886	IL6R	2.850555679	0.191718363	14.86845409	5.28189E-50	1.02218E-48	positive
PRJNA729271	stage_1D_vs_E10	ordinary_muscle_development	ENSGALG00010027887	ERC2	-1.782196946	0.266870387	-6.67813677	2.41999E-11	7.63556E-11	negative
PRJNA729271	stage_1D_vs_E10	ordinary_muscle_development	ENSGALG00010027888	PFKFB2	0.099213055	0.143657362	0.69062284	0.48980259	0.538181363	positive
PRJNA729271	stage_1D_vs_E10	ordinary_muscle_development	ENSGALG00010027889	RBM8A	-0.75385324	0.163143239	-4.620805888	3.82252E-06	8.11237E-06	negative
PRJNA729271	stage_1D_vs_E10	ordinary_muscle_development	ENSGALG00010027890	ENSGALG00010027890	2.849672118	0.884937091	3.220197397	0.001281024	0.002112341	positive
PRJNA729271	stage_1D_vs_E10	ordinary_muscle_development	ENSGALG00010027891	CCDC66	-0.910709898	0.141637372	-6.429870053	1.27713E-10	3.83987E-10	negative
PRJNA729271	stage_1D_vs_E10	ordinary_muscle_development	ENSGALG00010027892	SHE	0.208898988	0.167455696	1.247488099	0.212218581	0.253804318	positive
PRJNA729271	stage_1D_vs_E10	ordinary_muscle_development	ENSGALG00010027893	DOT1L	1.181576491	0.157299625	7.511629445	5.83959E-14	2.19344E-13	positive
PRJNA729271	stage_1D_vs_E10	ordinary_muscle_development	ENSGALG00010027894	RSBN1	0.252856296	0.066044874	3.82855292	0.000128899	0.000236655	positive
PRJNA729271	stage_1D_vs_E10	ordinary_muscle_development	ENSGALG00010027895	LIX1L	-0.046612478	0.177402061	-0.262750486	0.792742902	0.820303281	negative
PRJNA729271	stage_1D_vs_E10	ordinary_muscle_development	ENSGALG00010027896	TOR4A	1.450753422	0.154867512	9.36770664	7.41256E-21	4.1597E-20	positive
PRJNA729271	stage_1D_vs_E10	ordinary_muscle_development	ENSGALG00010027897	AP3D1	-0.05047461	0.097967426	-0.515218293	0.606400488	0.649075105	negative
PRJNA729271	stage_1D_vs_E10	ordinary_muscle_development	ENSGALG00010027898	TASOR	0.061148001	0.091611553	0.667470415	0.504471701	0.552540223	positive
PRJNA729271	stage_1D_vs_E10	ordinary_muscle_development	ENSGALG00010027899	PMVK	-0.533151736	0.177133447	-3.009887437	0.002613445	0.004148258	negative
PRJNA729271	stage_1D_vs_E10	ordinary_muscle_development	ENSGALG00010027901	MOB3A	-0.392550738	0.096608125	-4.063330484	4.83775E-05	9.25962E-05	negative
PRJNA729271	stage_1D_vs_E10	ordinary_muscle_development	ENSGALG00010027902	UBE2Q1	-0.089827165	0.070308617	-1.277612455	0.201386107	0.241726699	negative
PRJNA729271	stage_1D_vs_E10	ordinary_muscle_development	ENSGALG00010027903	NDUFA8	0.409537133	0.125554293	3.261832976	0.001106943	0.00183709	positive
PRJNA729271	stage_1D_vs_E10	ordinary_muscle_development	ENSGALG00010027904	MORN5	-1.928252757	0.549165712	-3.511240262	0.000446021	0.000771581	negative
PRJNA729271	stage_1D_vs_E10	ordinary_muscle_development	ENSGALG00010027905	SYN2	1.091267142	0.257587643	4.236488705	2.27042E-05	4.4757E-05	positive
PRJNA729271	stage_1D_vs_E10	ordinary_muscle_development	ENSGALG00010027906	LHX6	1.610825399	0.173415975	9.288794759	1.56045E-20	8.59702E-20	positive
PRJNA729271	stage_1D_vs_E10	ordinary_muscle_development	ENSGALG00010027907	MKNK2	2.303669497	0.188323556	12.2325085	2.08402E-34	2.20174E-33	positive
PRJNA729271	stage_1D_vs_E10	ordinary_muscle_development	ENSGALG00010027908	PBXIP1	1.903483697	0.105868324	17.97972833	2.80865E-72	1.10629E-70	positive
PRJNA729271	stage_1D_vs_E10	ordinary_muscle_development	ENSGALG00010027909	TIMP4	0.732919917	0.249441249	2.938246668	0.003300743	0.005180755	positive
PRJNA729271	stage_1D_vs_E10	ordinary_muscle_development	ENSGALG00010027910	CHRNB2	-1.138470123	0.209652362	-5.430275673	5.62671E-08	1.38548E-07	negative
PRJNA729271	stage_1D_vs_E10	ordinary_muscle_development	ENSGALG00010027911	PTPN22	-1.027445656	0.419739879	-2.447815199	0.014372535	0.020778964	negative
PRJNA729271	stage_1D_vs_E10	ordinary_muscle_development	ENSGALG00010027912	RGS3	-0.178770334	0.128510461	-1.391095579	0.164196445	0.200964914	negative
PRJNA729271	stage_1D_vs_E10	ordinary_muscle_development	ENSGALG00010027913	PYGO2	-0.315065753	0.222931201	-1.413286929	0.157571376	0.193411558	negative
PRJNA729271	stage_1D_vs_E10	ordinary_muscle_development	ENSGALG00010027914	BTBD2	1.013576989	0.125338229	8.086734593	6.12856E-16	2.61429E-15	positive
PRJNA729271	stage_1D_vs_E10	ordinary_muscle_development	ENSGALG00010027915	GSTM3	-0.180446865	0.083832479	-2.152469621	0.031360385	0.043123877	negative
PRJNA729271	stage_1D_vs_E10	ordinary_muscle_development	ENSGALG00010027916	ADAR	-0.047958957	0.128654145	-0.372774291	0.709316439	0.745255276	negative
PRJNA729271	stage_1D_vs_E10	ordinary_muscle_development	ENSGALG00010027917	PPARG	0.636207699	0.451974672	1.407618035	0.159244204	0.19533255	positive
PRJNA729271	stage_1D_vs_E10	ordinary_muscle_development	ENSGALG00010027918	CSNK1G2	0.031858301	0.101047661	0.315279946	0.75254911	0.784223248	positive
PRJNA729271	stage_1D_vs_E10	ordinary_muscle_development	ENSGALG00010027919	TSEN2	-0.150747081	0.117727286	-1.280476991	0.200377432	0.240635532	negative
PRJNA729271	stage_1D_vs_E10	ordinary_muscle_development	ENSGALG00010027920	SCAMP4	-0.658282902	0.113841413	-5.782455475	7.36181E-09	1.9469E-08	negative
PRJNA729271	stage_1D_vs_E10	ordinary_muscle_development	ENSGALG00010027921	MKRN2OS	0.549521724	0.232254081	2.366036889	0.017979653	0.025595666	positive
PRJNA729271	stage_1D_vs_E10	ordinary_muscle_development	ENSGALG00010027922	SHC1	-0.201826805	0.11341386	-1.779560318	0.075147945	0.097482501	negative
PRJNA729271	stage_1D_vs_E10	ordinary_muscle_development	ENSGALG00010027923	AMPD2	-0.337683877	0.153462364	-2.200434488	0.027776083	0.038450418	negative
PRJNA729271	stage_1D_vs_E10	ordinary_muscle_development	ENSGALG00010027925	MKRN2	-0.588748192	0.069903898	-8.422251213	3.69318E-17	1.6866E-16	negative
PRJNA729271	stage_1D_vs_E10	ordinary_muscle_development	ENSGALG00010027926	CKS1B	-1.655978606	0.249032924	-6.649637239	2.93816E-11	9.21833E-11	negative
PRJNA729271	stage_1D_vs_E10	ordinary_muscle_development	ENSGALG00010027927	ENSGALG00010027927	0.873951728	0.195721204	4.465288952	7.99608E-06	1.64822E-05	positive
PRJNA729271	stage_1D_vs_E10	ordinary_muscle_development	ENSGALG00010027928	FLAD1	0.377615671	0.160343468	2.355042435	0.018520591	0.026306317	positive
PRJNA729271	stage_1D_vs_E10	ordinary_muscle_development	ENSGALG00010027929	CDK5RAP2	-0.92595778	0.137332685	-6.742442851	1.55746E-11	4.98241E-11	negative
PRJNA729271	stage_1D_vs_E10	ordinary_muscle_development	ENSGALG00010027930	RAF1	0.968521626	0.089216504	10.85585719	1.87044E-27	1.4247E-26	positive
PRJNA729271	stage_1D_vs_E10	ordinary_muscle_development	ENSGALG00010027931	ABHD17A	-0.304408571	0.173086791	-1.758704808	0.07862766	0.101823277	negative
PRJNA729271	stage_1D_vs_E10	ordinary_muscle_development	ENSGALG00010027932	POLE3	-1.469006583	0.075173065	-19.54166133	4.85752E-85	2.58859E-83	negative
PRJNA729271	stage_1D_vs_E10	ordinary_muscle_development	ENSGALG00010027933	ENSGALG00010027933	2.664903841	0.217840308	12.2332908	2.06404E-34	2.18222E-33	positive
PRJNA729271	stage_1D_vs_E10	ordinary_muscle_development	ENSGALG00010027934	CNBP	0.065042724	0.08508633	0.764432127	0.444609769	0.493840506	positive
PRJNA729271	stage_1D_vs_E10	ordinary_muscle_development	ENSGALG00010027935	ISY1	-0.372722737	0.086346832	-4.316576837	1.58468E-05	3.17001E-05	negative
PRJNA729271	stage_1D_vs_E10	ordinary_muscle_development	ENSGALG00010027936	ENSGALG00010027936	0.017004658	0.084586544	0.201032665	0.840673032	0.863488918	positive
PRJNA729271	stage_1D_vs_E10	ordinary_muscle_development	ENSGALG00010027937	HDHD3	0.020768197	0.093250639	0.222713722	0.823758321	0.848399664	positive
PRJNA729271	stage_1D_vs_E10	ordinary_muscle_development	ENSGALG00010027938	ENSGALG00010027938	-0.244778225	0.109635692	-2.232650889	0.025571975	0.035627238	negative
PRJNA729271	stage_1D_vs_E10	ordinary_muscle_development	ENSGALG00010027939	BSPRY	-2.659931729	0.487321829	-5.45826509	4.80809E-08	1.19073E-07	negative
PRJNA729271	stage_1D_vs_E10	ordinary_muscle_development	ENSGALG00010027940	ZBTB7B	1.145274355	0.409440891	2.797166524	0.005155295	0.007909188	positive
PRJNA729271	stage_1D_vs_E10	ordinary_muscle_development	ENSGALG00010027941	KLF2	1.137223873	0.150361815	7.563249153	3.93124E-14	1.49288E-13	positive
PRJNA729271	stage_1D_vs_E10	ordinary_muscle_development	ENSGALG00010027942	KHDC4	0.576388595	0.122945455	4.688165133	2.75666E-06	5.93461E-06	positive
PRJNA729271	stage_1D_vs_E10	ordinary_muscle_development	ENSGALG00010027943	RAB43	-0.291467502	0.0940435	-3.099283867	0.001939891	0.003126733	negative
PRJNA729271	stage_1D_vs_E10	ordinary_muscle_development	ENSGALG00010027944	WDR31	-2.48279349	0.300458094	-8.263360316	1.41628E-16	6.27018E-16	negative
PRJNA729271	stage_1D_vs_E10	ordinary_muscle_development	ENSGALG00010027946	AP1M1	-0.668872604	0.092773615	-7.209728813	5.60634E-13	1.97674E-12	negative
PRJNA729271	stage_1D_vs_E10	ordinary_muscle_development	ENSGALG00010027947	GP9	0.82857238	0.351095056	2.359965956	0.018276612	0.025977593	positive
PRJNA729271	stage_1D_vs_E10	ordinary_muscle_development	ENSGALG00010027948	PRPF4	-0.277109312	0.11353457	-2.440748341	0.014656865	0.021164699	negative
PRJNA729271	stage_1D_vs_E10	ordinary_muscle_development	ENSGALG00010027950	CDC26	-0.363283751	0.128693933	-2.82285064	0.004759874	0.007333586	negative
PRJNA729271	stage_1D_vs_E10	ordinary_muscle_development	ENSGALG00010027951	FAM32A	0.277654238	0.104339067	2.661076479	0.007789126	0.011682883	positive
PRJNA729271	stage_1D_vs_E10	ordinary_muscle_development	ENSGALG00010027952	CIB3	-0.2383867	0.50408305	-0.472911557	0.636276279	0.676954247	negative
PRJNA729271	stage_1D_vs_E10	ordinary_muscle_development	ENSGALG00010027953	SLC31A1	-1.350506666	0.120347167	-11.22175704	3.18871E-29	2.66093E-28	negative
PRJNA729271	stage_1D_vs_E10	ordinary_muscle_development	ENSGALG00010027954	CFAP92	0.067507614	0.223694367	0.301785044	0.762815933	0.793186295	positive
PRJNA729271	stage_1D_vs_E10	ordinary_muscle_development	ENSGALG00010027955	ENSGALG00010027955	0.248749423	0.116212265	2.140474781	0.032316417	0.044379579	positive
PRJNA729271	stage_1D_vs_E10	ordinary_muscle_development	ENSGALG00010027956	RAB8A	-0.759273722	0.064573808	-11.75823063	6.40627E-32	6.08911E-31	negative
PRJNA729271	stage_1D_vs_E10	ordinary_muscle_development	ENSGALG00010027957	ENTREP3	-1.701519004	0.519339702	-3.276312204	0.001051722	0.001750254	negative
PRJNA729271	stage_1D_vs_E10	ordinary_muscle_development	ENSGALG00010027958	ENSGALG00010027958	0.714188745	0.138879402	5.14251022	2.71092E-07	6.32358E-07	positive
PRJNA729271	stage_1D_vs_E10	ordinary_muscle_development	ENSGALG00010027959	TPM4	-2.664212352	0.172874339	-15.41126563	1.37492E-53	2.99242E-52	negative
PRJNA729271	stage_1D_vs_E10	ordinary_muscle_development	ENSGALG00010027960	PPP6C	0.071078083	0.119996403	0.592335113	0.553626208	0.599448112	positive
PRJNA729271	stage_1D_vs_E10	ordinary_muscle_development	ENSGALG00010027961	TINCR	-4.578113584	1.03520552	-4.422419987	9.76015E-06	1.99511E-05	negative
PRJNA729271	stage_1D_vs_E10	ordinary_muscle_development	ENSGALG00010027962	SCAMP3	0.439110542	0.127409807	3.446442258	0.00056802	0.000971072	positive

PRJNA729271	stage_1D_vs_E10	ordinary_muscle_development	ENSGALG00010027964	RABEPK	-0.247865406	0.135992664	-1.822638058	0.068358228	0.08925795	negative
PRJNA729271	stage_1D_vs_E10	ordinary_muscle_development	ENSGALG00010027965	ACAD9	2.165980665	0.109166839	19.84101294	1.31777E-87	7.4906E-86	positive
PRJNA729271	stage_1D_vs_E10	ordinary_muscle_development	ENSGALG00010027966	CLK2	0.459283965	0.09747282	4.711918294	2.45396E-06	5.30906E-06	positive
PRJNA729271	stage_1D_vs_E10	ordinary_muscle_development	ENSGALG00010027968	FKBP15	-0.0793927	0.097047725	-0.818078939	0.413312114	0.462908085	negative
PRJNA729271	stage_1D_vs_E10	ordinary_muscle_development	ENSGALG00010027969	HSPA5	-0.665731408	0.129420249	-5.143950897	2.6902E-07	6.27626E-07	negative
PRJNA729271	stage_1D_vs_E10	ordinary_muscle_development	ENSGALG00010027970	GRIN3B	0.348457166	0.174377448	1.998292606	0.045684947	0.0612584	positive
PRJNA729271	stage_1D_vs_E10	ordinary_muscle_development	ENSGALG00010027971	ENSGALG00010027971	1.451322865	0.377405897	3.845522491	0.000120296	0.000221561	positive
PRJNA729271	stage_1D_vs_E10	ordinary_muscle_development	ENSGALG00010027972	TMEM259	0.468576792	0.105421567	4.444790635	8.79775E-06	1.80807E-05	positive
PRJNA729271	stage_1D_vs_E10	ordinary_muscle_development	ENSGALG00010027973	GAPVD1	0.864570071	0.064238818	13.45868585	2.73741E-41	3.78254E-40	positive
PRJNA729271	stage_1D_vs_E10	ordinary_muscle_development	ENSGALG00010027974	ENSGALG00010027974	-0.292686789	0.316417534	-0.925001802	0.35496497	0.403799815	negative
PRJNA729271	stage_1D_vs_E10	ordinary_muscle_development	ENSGALG00010027975	IQSEC1	-0.01723996	0.117601024	-0.146597025	0.883450102	0.900725134	negative
PRJNA729271	stage_1D_vs_E10	ordinary_muscle_development	ENSGALG00010027976	LRSAM1	-0.105750893	0.098070359	-1.078316569	0.280892505	0.327004808	negative
PRJNA729271	stage_1D_vs_E10	ordinary_muscle_development	ENSGALG00010027977	BOLA1	0.836918433	0.155974724	5.365731138	8.0622E-08	1.96175E-07	positive
PRJNA729271	stage_1D_vs_E10	ordinary_muscle_development	ENSGALG00010027979	RPL12	0.183781355	0.119553572	1.537230148	0.12423696	0.155497344	positive
PRJNA729271	stage_1D_vs_E10	ordinary_muscle_development	ENSGALG00010027980	NUP210	1.04491921	0.14184247	7.366758424	1.74827E-13	6.36233E-13	positive
PRJNA729271	stage_1D_vs_E10	ordinary_muscle_development	ENSGALG00010027981	CNN2	-2.334793368	0.166296467	-14.0399457	8.87806E-45	1.38077E-43	negative
PRJNA729271	stage_1D_vs_E10	ordinary_muscle_development	ENSGALG00010027982	ENTPD2	1.18061469	0.249433593	4.733182388	2.21027E-06	4.80327E-06	positive
PRJNA729271	stage_1D_vs_E10	ordinary_muscle_development	ENSGALG00010027983	POGZ	0.219514771	0.145501615	1.508675837	0.131381643	0.163676572	positive
PRJNA729271	stage_1D_vs_E10	ordinary_muscle_development	ENSGALG00010027984	ARHGAP45	0.643214906	0.171728723	3.74552897	0.000180014	0.000325553	positive
PRJNA729271	stage_1D_vs_E10	ordinary_muscle_development	ENSGALG00010027985	PSMB4	-0.151211754	0.094926409	-1.592936627	0.111174409	0.14022564	negative
PRJNA729271	stage_1D_vs_E10	ordinary_muscle_development	ENSGALG00010027986	CGN	-0.931181391	0.267344041	-3.483082653	0.000495675	0.00085381	negative
PRJNA729271	stage_1D_vs_E10	ordinary_muscle_development	ENSGALG00010027987	TMEM141	0.242207238	0.138530364	1.748405415	0.080393851	0.103869583	positive
PRJNA729271	stage_1D_vs_E10	ordinary_muscle_development	ENSGALG00010027988	HDAC11	0.642855023	0.128389069	5.007085302	5.52604E-07	1.25924E-06	positive
PRJNA729271	stage_1D_vs_E10	ordinary_muscle_development	ENSGALG00010027989	ENSGALG00010027989	0.500089954	0.138616245	3.607729776	0.000308888	0.000543695	positive
PRJNA729271	stage_1D_vs_E10	ordinary_muscle_development	ENSGALG00010027990	ENSGALG00010027990	1.071455778	0.190330391	5.629451895	1.80783E-08	4.62406E-08	positive
PRJNA729271	stage_1D_vs_E10	ordinary_muscle_development	ENSGALG00010027991	ENSGALG00010027991	0.297461865	0.145525056	2.044059449	0.040947675	0.055336243	positive
PRJNA729271	stage_1D_vs_E10	ordinary_muscle_development	ENSGALG00010027993	CLIC3	2.039678266	0.158148469	12.89723688	4.66527E-38	5.70178E-37	positive
PRJNA729271	stage_1D_vs_E10	ordinary_muscle_development	ENSGALG00010027994	TUFT1	-0.37509228	0.158818649	-2.361764703	0.018188182	0.025862046	negative
PRJNA729271	stage_1D_vs_E10	ordinary_muscle_development	ENSGALG00010027995	POLR2E	-0.143982348	0.082291646	-1.749659345	0.080177113	0.103644631	negative
PRJNA729271	stage_1D_vs_E10	ordinary_muscle_development	ENSGALG00010027996	GPX4	0.261282735	0.121979148	2.142027872	0.032191239	0.044224435	positive
PRJNA729271	stage_1D_vs_E10	ordinary_muscle_development	ENSGALG00010027998	SNX27	0.248564594	0.092290002	2.693299247	0.007074874	0.010660114	positive
PRJNA729271	stage_1D_vs_E10	ordinary_muscle_development	ENSGALG00010027999	ENSGALG00010027999	-1.832848378	0.519832776	-3.525842273	0.000422138	0.000732451	negative
PRJNA729271	stage_1D_vs_E10	ordinary_muscle_development	ENSGALG00010028000	MRPL9	0.750011281	0.166092519	4.515623494	6.31307E-06	1.31387E-05	positive
PRJNA729271	stage_1D_vs_E10	ordinary_muscle_development	ENSGALG00010028001	SBNO2	0.741810065	0.115911529	6.399795354	1.55585E-10	4.64992E-10	positive
PRJNA729271	stage_1D_vs_E10	ordinary_muscle_development	ENSGALG00010028002	ABCA2	0.781932932	0.119421094	6.547695277	5.84318E-11	1.79709E-10	positive
PRJNA729271	stage_1D_vs_E10	ordinary_muscle_development	ENSGALG00010028004	ENSGALG00010028004	-1.898182698	0.315346434	-6.019356781	1.75112E-09	4.85788E-09	negative
PRJNA729271	stage_1D_vs_E10	ordinary_muscle_development	ENSGALG00010028005	IER5L	0.047759796	0.143240852	0.333423012	0.738814995	0.772162618	positive
PRJNA729271	stage_1D_vs_E10	ordinary_muscle_development	ENSGALG00010028006	ENSGALG00010028006	2.609536547	0.820406153	3.180786171	0.00146876	0.002402085	positive
PRJNA729271	stage_1D_vs_E10	ordinary_muscle_development	ENSGALG00010028007	STK11	0.629361754	0.119685255	5.258473596	1.45256E-07	3.46582E-07	positive
PRJNA729271	stage_1D_vs_E10	ordinary_muscle_development	ENSGALG00010028008	NTMT1	0.42511583	0.112869432	3.766438986	0.000165593	0.00030071	positive
PRJNA729271	stage_1D_vs_E10	ordinary_muscle_development	ENSGALG00010028009	RRP9	-0.712744847	0.178919923	-3.983596871	6.788E-05	0.000128065	negative
PRJNA729271	stage_1D_vs_E10	ordinary_muscle_development	ENSGALG00010028010	ASB6	-0.429726102	0.07188564	-5.977912989	2.26014E-09	6.22355E-09	negative
PRJNA729271	stage_1D_vs_E10	ordinary_muscle_development	ENSGALG00010028011	ENSGALG00010028011	-0.473349723	0.090607378	-5.224185226	1.74924E-07	4.14571E-07	negative
PRJNA729271	stage_1D_vs_E10	ordinary_muscle_development	ENSGALG00010028012	PRRX2	-3.646160378	0.254370507	-14.33405317	1.34062E-46	2.2336E-45	negative
PRJNA729271	stage_1D_vs_E10	ordinary_muscle_development	ENSGALG00010028013	CBARP	-0.247229469	0.216591738	-1.14145383	0.253681115	0.298418094	negative
PRJNA729271	stage_1D_vs_E10	ordinary_muscle_development	ENSGALG00010028014	ENSGALG00010028014	0.903512399	0.292035724	3.093842037	0.001975827	0.00318324	positive
PRJNA729271	stage_1D_vs_E10	ordinary_muscle_development	ENSGALG00010028015	DUSP7	-0.379121456	0.101248925	-3.744449214	0.00018079	0.000326915	negative
PRJNA729271	stage_1D_vs_E10	ordinary_muscle_development	ENSGALG00010028016	PRF1	1.894022849	1.390868017	1.361755986	0.173274906	0.210971841	positive
PRJNA729271	stage_1D_vs_E10	ordinary_muscle_development	ENSGALG00010028017	ENSGALG00010028017	-1.465463365	1.332193103	-1.100038247	0.271315458	0.316822489	negative
PRJNA729271	stage_1D_vs_E10	ordinary_muscle_development	ENSGALG00010028019	RPL29	0.439145912	0.227120835	1.933534244	0.053170419	0.070583865	positive
PRJNA729271	stage_1D_vs_E10	ordinary_muscle_development	ENSGALG00010028020	ENSGALG00010028020	0.454045241	0.150059927	3.025759446	0.002480095	0.003945235	positive
PRJNA729271	stage_1D_vs_E10	ordinary_muscle_development	ENSGALG00010028021	ATP5F1D	1.461525362	0.145093407	10.07299636	7.2728E-24	4.69992E-23	positive
PRJNA729271	stage_1D_vs_E10	ordinary_muscle_development	ENSGALG00010028022	ENSGALG00010028022	-0.705216264	0.14333492	-4.920058999	8.65181E-07	1.9419E-06	negative
PRJNA729271	stage_1D_vs_E10	ordinary_muscle_development	ENSGALG00010028023	ENSGALG00010028023	0.304591105	0.918970636	0.331448137	0.74030601	0.773497845	positive
PRJNA729271	stage_1D_vs_E10	ordinary_muscle_development	ENSGALG00010028024	RBM3	-1.313399773	0.112863999	-11.63701251	2.67212E-31	2.45297E-30	negative
PRJNA729271	stage_1D_vs_E10	ordinary_muscle_development	ENSGALG00010028025	TNNC1	-0.304697654	0.237358546	-1.283702056	0.199246225	0.23933654	negative
PRJNA729271	stage_1D_vs_E10	ordinary_muscle_development	ENSGALG00010028026	ANKMY1	0.8262861	0.164669593	5.017842616	5.2255E-07	1.19338E-06	positive
PRJNA729271	stage_1D_vs_E10	ordinary_muscle_development	ENSGALG00010028027	ENSGALG00010028027	1.083665971	0.230655123	4.698208987	2.62453E-06	5.66278E-06	positive
PRJNA729271	stage_1D_vs_E10	ordinary_muscle_development	ENSGALG00010028028	FAM174C	-0.189140251	0.16145706	-1.171458535	0.241414515	0.285215046	negative
PRJNA729271	stage_1D_vs_E10	ordinary_muscle_development	ENSGALG00010028029	EFNA2	-4.364286867	0.166994052	-26.13438515	1.4832E-150	2.6874E-148	negative
PRJNA729271	stage_1D_vs_E10	ordinary_muscle_development	ENSGALG00010028030	PFDN2	0.152692909	0.134948274	1.13149212	0.257848018	0.302857856	positive
PRJNA729271	stage_1D_vs_E10	ordinary_muscle_development	ENSGALG00010028031	NISCH	0.775324841	0.080574457	9.622464326	6.42725E-22	3.82757E-21	positive
PRJNA729271	stage_1D_vs_E10	ordinary_muscle_development	ENSGALG00010028032	ENSGALG00010028032	-3.507997597	0.269178155	-13.03225217	8.02048E-39	1.00917E-37	negative
PRJNA729271	stage_1D_vs_E10	ordinary_muscle_development	ENSGALG00010028033	ENSGALG00010028033	1.98037562	0.300109904	6.598834602	4.14402E-11	1.28873E-10	positive
PRJNA729271	stage_1D_vs_E10	ordinary_muscle_development	ENSGALG00010028034	MUM1L1	-0.575276759	0.076333981	-7.536312815	4.83446E-14	1.82583E-13	negative
PRJNA729271	stage_1D_vs_E10	ordinary_muscle_development	ENSGALG00010028035	ENSGALG00010028035	1.235696614	1.043903127	1.183727285	0.236521034	0.280095784	positive
PRJNA729271	stage_1D_vs_E10	ordinary_muscle_development	ENSGALG00010028036	NDUFS7	1.838378841	0.121957652	15.07391143	2.40446E-51	4.85413E-50	positive
PRJNA729271	stage_1D_vs_E10	ordinary_muscle_development	ENSGALG00010028037	ENSGALG00010028037	0.241135773	0.272629489	0.884481624	0.376436278	0.425619645	positive
PRJNA729271	stage_1D_vs_E10	ordinary_muscle_development	ENSGALG00010028039	STAB1	1.706812148	0.162356893	10.51271748	7.54865E-26	5.36361E-25	positive
PRJNA729271	stage_1D_vs_E10	ordinary_muscle_development	ENSGALG00010028042	DPP7	-0.178214035	0.191935461	-0.928510208	0.353142975	0.402011107	negative
PRJNA729271	stage_1D_vs_E10	ordinary_muscle_development	ENSGALG00010028043	NT5DC2	0.755865622	0.115775851	6.528698457	6.63437E-11	2.03094E-10	positive
PRJNA729271	stage_1D_vs_E10	ordinary_muscle_development	ENSGALG00010028044	GAMT	3.410920714	0.153426355	22.23164802	1.6979E-109	1.5098E-107	positive
PRJNA729271	stage_1D_vs_E10	ordinary_muscle_development	ENSGALG00010028045	ENSGALG00010028045	-1.029194945	0.600631059	-1.713522684	0.086616424	0.111313508	negative
PRJNA729271	stage_1D_vs_E10	ordinary_muscle_development	ENSGALG00010028046	SMIM4	1.955841857	0.203251686	9.622758363	6.4089E-22	3.81821E-21	positive
PRJNA729271	stage_1D_vs_E10	ordinary_muscle_development	ENSGALG00010028047	ENSGALG00010028047	1.309164989	0.287850924	4.548065965	5.41412E-06	1.13309E-05	positive
PRJNA729271	stage_1D_vs_E10	ordinary_muscle_development	ENSGALG00010028048	S100A10	0.384101992	0.218745243	1.755933004	0.079099849	0.102361602	positive
PRJNA729271	stage_1D_vs_E10	ordinary_muscle_development	ENSGALG00010028049	MAN1B1	0.142041881	0.059903912	2.371162014	0.017732256	0.025263324	positive
PRJNA729271	stage_1D_vs_E10	ordinary_muscle_development	ENSGALG00010028050	PBRM1	-0.325223734	0.087171122	-3.730865522	0.000190823	0.000343643	negative
PRJNA729271	stage_1D_vs_E10	ordinary_muscle_development	ENSGALG00010028051	GNL3	-0.458672785	0.092519368	-4.957586674	7.13742E-07	1.61223E-06	negative
PRJNA729271	stage_1D_vs_E10	ordinary_muscle_development	ENSGALG00010028052	ENSGALG00010028052	-2.682407489	0.214843954	-12.48537571	8.97188E-36	1.00423E-34	negative
PRJNA729271	stage_1D_vs_E10	ordinary_muscle_development	ENSGALG00010028053	ORM1	-2.954772914	2.282400991	-1.294589744	0.195461792	0.23541493	negative
PRJNA729271	stage_1D_vs_E10	ordinary_muscle_development	ENSGALG00010028054	RPS15	-0.346808601	0.140794943	-2.463217741	0.013769627	0.019957078	negative
PRJNA729271	stage_1D_vs_E10	ordinary_muscle_development	ENSGALG00010028055	ENSGALG00010028055	-0.43151311	0.117930653	-3.659041135	0.000253161	0.000449481	negative
PRJNA729271	stage_1D_vs_E10	ordinary_muscle_development	ENSGALG00010028056	SLC25A25	0.089678488	0.237561876	0.377495284	0.705805568	0.742158267	positive
PRJNA729271	stage_1D_vs_E10	ordinary_muscle_development	ENSGALG00010028057	APC2	-0.958539306	0.369721195	-2.592600371	0.009525338	0.014124785	negative
PRJNA729271	stage_1D_vs_E10	ordinary_muscle_development	ENSGALG00010028058	GLT8D1	-0.706158948	0.11447223	-6.16882319	6.88001E-10	1.96543E-09	negative
PRJNA729271	stage_1D_vs_E10	ordinary_muscle_development	ENSGALG00010028059	GZMM	-0.398078186	0.710381167	-0.560372663	0.575225274	0.620009693	negative
PRJNA729271	stage_1D_vs_E10	ordinary_muscle_development	ENSGALG00010028060	ENSGALG00010028060	0.004685979	0.142970879	0.032775758	0.97385341	0.9776981	positive
PRJNA729271	stage_1D_vs_E10	ordinary_muscle_development	ENSGALG00010028061	NAIF1	-0.816639201	0.095465368	-8.554297908	1.18588E-17	5.57192E-17	negative
PRJNA729271	stage_1D_vs_E10	ordinary_muscle_development	ENSGALG00010028062	ENSGALG00010028062	1.023879104	0.486135338	2.106160619	0.035190403	0.048078502	positive
PRJNA729271	stage_1D_vs_E10	ordinary_muscle_development	ENSGALG00010028064	SPCS1	-0.433251341	0.087751224	-4.93726837	7.92244E-07	1.784E-06	negative
PRJNA729271	stage_1D_vs_E10	ordinary_muscle_development	ENSGALG00010028065	REEP6	0.937669336	0.804033481	1.166206829	0.243530832	0.287574698	positive
PRJNA729271	stage_1D_vs_E10	ordinary_muscle_development	ENSGALG00010028066	ENSGALG00010028066	1.861645332	0.182725432	10.18821142	2.23844E-24	1.48563E-23	positive
PRJNA729271	stage_1D_vs_E10	ordinary_muscle_development	ENSGALG00010028067	FAM102A	-0.155612859	0.111710366	-1.39300285	0.163618929	0.200325735	negative
PRJNA729271	stage_1D_vs_E10	ordinary_muscle_development	ENSGALG00010028068	POLE4	-0.347335951	0.108919778	-3.188915344	0.001428077	0.002340038	negative
PRJNA729271	stage_1D_vs_E10	ordinary_muscle_development	ENSGALG00010028069	NEK4	-1.099574223	0.115280948	-9.538212848	1.45315E-21	8.4728E-21	negative
PRJNA729271	stage_1D_vs_E10	ordinary_muscle_development	ENSGALG00010028070	MEX3A	-2.985755859	0.140353286	-21.27314545	2.01318E-100	1.48883E-98	negative
PRJNA729271	stage_1D_vs_E10	ordinary_muscle_development	ENSGALG00010028071	DPM2	-0.636220381	0.114862967	-5.538951332	3.04288E-08	7.62826E-08	negative
PRJNA729271	stage_1D_vs_E10	ordinary_muscle_development	ENSGALG00010028072	ENSGALG00010028072	-7.746205239	0.991157905	-7.815308944	5.48284E-15	2.1948E-14	negative
PRJNA729271	stage_1D_vs_E10	ordinary_muscle_development	ENSGALG00010028073	ST6GALNAC4	2.019937606	0.122038142	16.55169091	1.55668E-61	4.51283E-60	positive
PRJNA729271	stage_1D_vs_E10	ordinary_muscle_development	ENSGALG00010028074	ENSGALG00010028074	-0.14427917	0.472096485	-0.305613735	0.759898761	0.790663452	negative
PRJNA729271	stage_1D_vs_E10	ordinary_muscle_development	ENSGALG00010028075	RAB25	-1.303330967	0.523885359	-2.487817125	0.012852981	0.018720253	negative
PRJNA729271	stage_1D_vs_E10	ordinary_muscle_development	ENSGALG00010028076	ST6GALNAC6	1.292361428	0.152495513	8.474750543	2.35584E-17	1.09029E-16	positive
PRJNA729271	stage_1D_vs_E10	ordinary_muscle_development	ENSGALG00010028078	LAMTOR2	0.583977792	0.130699663	4.468089507	7.89213E-06	1.62842E-05	positive
PRJNA729271	stage_1D_vs_E10	ordinary_muscle_development	ENSGALG00010028079	ITIH3	2.135335933	1.598994943	1.335423819	0.181737673	0.220479375	positive
PRJNA729271	stage_1D_vs_E10	ordinary_muscle_development	ENSGALG00010028080	MUSTN1	2.597220666	0.405045924	6.412163436	1.43469E-10	4.29843E-10	positive
PRJNA729271	stage_1D_vs_E10	ordinary_muscle_development	ENSGALG00010028081	UBQLN4	-0.253102647	0.162102508	-1.56137403	0.118435526	0.148814402	negative
PRJNA729271	stage_1D_vs_E10	ordinary_muscle_development	ENSGALG00010028082	AK1	4.216359413	0.137219934	30.72701815	2.4801E-207	1.2839E-204	positive
PRJNA729271	stage_1D_vs_E10	ordinary_muscle_development	ENSGALG00010028083	ELAVL1	-0.411163863	0.049186133	-8.359345194	6.30673E-17	2.84785E-16	negative
PRJNA729271	stage_1D_vs_E10	ordinary_muscle_development	ENSGALG00010028084	ENG	1.446958715	0.192171233	7.529528182	5.0924E-14	1.92025E-13	positive
PRJNA729271	stage_1D_vs_E10	ordinary_muscle_development	ENSGALG00010028085	ENSGALG00010028085	-0.341254546	0.284520418	-1.199402661	0.230371413	0.27355072	negative
PRJNA729271	stage_1D_vs_E10	ordinary_muscle_development	ENSGALG00010028086	MATK	-1.256080365	0.322293509	-3.897318224	9.72638E-05	0.00018061	negative
PRJNA729271	stage_1D_vs_E10	ordinary_muscle_development	ENSGALG00010028087	STIMATE	-0.618648476	0.088969542	-6.953486108	3.56368E-12	1.1894E-11	negative
PRJNA729271	stage_1D_vs_E10	ordinary_muscle_development	ENSGALG00010028088	NAXE	0.484381174	0.114955543	4.213639116	2.51289E-05	4.9342E-05	positive
PRJNA729271	stage_1D_vs_E10	ordinary_muscle_development	ENSGALG00010028091	FPGS	-0.487401808	0.097199858	-5.014429189	5.31911E-07	1.21361E-06	negative
PRJNA729271	stage_1D_vs_E10	ordinary_muscle_development	ENSGALG00010028093	SFMBT1	-0.565478828	0.140039409	-4.037997825	5.39094E-05	0.000102656	negative
PRJNA729271	stage_1D_vs_E10	ordinary_muscle_development	ENSGALG00010028095	CDK9	-0.123221503	0.092996207	-1.325016439	0.185165699	0.224244031	negative
PRJNA729271	stage_1D_vs_E10	ordinary_muscle_development	ENSGALG00010028096	TOR2A	-0.325336528	0.148138473	-2.196164991	0.028080136	0.038849057	negative
PRJNA729271	stage_1D_vs_E10	ordinary_muscle_development	ENSGALG00010028097	TTC24	2.82981892	0.215050563	13.15885381	1.51365E-39	1.95198E-38	positive
PRJNA729271	stage_1D_vs_E10	ordinary_muscle_development	ENSGALG00010028098	RFT1	-0.377778332	0.118830799	-3.179128097	0.001477188	0.002415051	negative
PRJNA729271	stage_1D_vs_E10	ordinary_muscle_development	ENSGALG00010028099	PCASP2	1.2318852	0.264154672	4.66349957	3.10877E-06	6.66296E-06	positive
PRJNA729271	stage_1D_vs_E10	ordinary_muscle_development	ENSGALG00010028100	ENSGALG00010028100	1.333468961	0.4574156	2.915224058	0.003554333	0.005559518	positive
PRJNA729271	stage_1D_vs_E10	ordinary_muscle_development	ENSGALG00010028101	MRPL54	0.084770122	0.127659827	0.66403131	0.506670272	0.554738696	positive
PRJNA729271	stage_1D_vs_E10	ordinary_muscle_development	ENSGALG00010028102	CERCAM	0.395274235	0.179250987	2.205143982	0.027443993	0.038023387	positive
PRJNA729271	stage_1D_vs_E10	ordinary_muscle_development	ENSGALG00010028103	APBA3	0.563819468	0.128755005	4.379010116	1.1922E-05	2.41691E-05	positive
PRJNA729271	stage_1D_vs_E10	ordinary_muscle_development	ENSGALG00010028104	GOLGA1	0.732290358	0.11389621	6.429453247	1.28064E-10	3.84961E-10	positive
PRJNA729271	stage_1D_vs_E10	ordinary_muscle_development	ENSGALG00010028105	PRKCD	-1.148601948	0.14342016	-8.008650579	1.15974E-15	4.85149E-15	negative
PRJNA729271	stage_1D_vs_E10	ordinary_muscle_development	ENSGALG00010028106	ARPC5L	0.097312421	0.104265643	0.933312432	0.350658678	0.399591001	positive
PRJNA729271	stage_1D_vs_E10	ordinary_muscle_development	ENSGALG00010028107	NOTCH1	0.00076357	0.124026134	0.006156528	0.995087832	0.996118655	positive
PRJNA729271	stage_1D_vs_E10	ordinary_muscle_development	ENSGALG00010028108	IQGAP3	-1.173709141	0.209167283	-5.611341912	2.00764E-08	5.11705E-08	negative
PRJNA729271	stage_1D_vs_E10	ordinary_muscle_development	ENSGALG00010028109	TJP3	-0.176031663	0.21567115	-0.816204033	0.414383451	0.46389312	negative

PRJNA729271	stage_1D_vs_E10	ordinary_muscle_development	ENSGALG00010028110	COQ4	0.070786097	0.101922877	0.69450647	0.487364645	0.535793123	positive
PRJNA729271	stage_1D_vs_E10	ordinary_muscle_development	ENSGALG00010028111	TRUB2	-0.46812423	0.089231984	-5.24614839	1.55312E-07	3.69784E-07	negative
PRJNA729271	stage_1D_vs_E10	ordinary_muscle_development	ENSGALG00010028112	SWI5	-0.134806086	0.081689766	-1.650220003	0.098897947	0.125857105	negative
PRJNA729271	stage_1D_vs_E10	ordinary_muscle_development	ENSGALG00010028113	PIP5K1C	-0.333579146	0.094721121	-3.521697635	0.000428793	0.000743108	negative
PRJNA729271	stage_1D_vs_E10	ordinary_muscle_development	ENSGALG00010028114	PFKFB4	0.014114831	0.090281158	0.156343048	0.875762626	0.893956286	positive
PRJNA729271	stage_1D_vs_E10	ordinary_muscle_development	ENSGALG00010028115	CACTIN	0.027895163	0.175371714	0.15906307	0.873619183	0.892121549	positive
PRJNA729271	stage_1D_vs_E10	ordinary_muscle_development	ENSGALG00010028116	HYAL1	1.415944252	0.147312996	9.611808103	7.1286E-22	4.23133E-21	positive
PRJNA729271	stage_1D_vs_E10	ordinary_muscle_development	ENSGALG00010028117	ENSGALG00010028117	0.13748902	0.238920169	0.575460084	0.564980178	0.610328491	positive
PRJNA729271	stage_1D_vs_E10	ordinary_muscle_development	ENSGALG00010028118	ENSGALG00010028118	0.041510476	0.077355583	0.536619008	0.591530811	0.635269994	positive
PRJNA729271	stage_1D_vs_E10	ordinary_muscle_development	ENSGALG00010028119	HYAL2	0.336727679	0.127125467	2.648782237	0.008078237	0.012092744	positive
PRJNA729271	stage_1D_vs_E10	ordinary_muscle_development	ENSGALG00010028120	TBXA2R	0.625104964	0.225018321	2.778018083	0.005469157	0.008365918	positive
PRJNA729271	stage_1D_vs_E10	ordinary_muscle_development	ENSGALG00010028121	TUSC2	-0.084522819	0.09662547	-0.874746775	0.381711703	0.430710816	negative
PRJNA729271	stage_1D_vs_E10	ordinary_muscle_development	ENSGALG00010028122	SEC16A	0.279978674	0.088342676	3.169234688	0.001528409	0.002495134	positive
PRJNA729271	stage_1D_vs_E10	ordinary_muscle_development	ENSGALG00010028123	RASSF1	0.218713711	0.105654539	2.070083445	0.038444531	0.052152876	positive
PRJNA729271	stage_1D_vs_E10	ordinary_muscle_development	ENSGALG00010028124	SUGP2	-0.67512608	0.089290853	-7.560976971	4.00053E-14	1.5184E-13	negative
PRJNA729271	stage_1D_vs_E10	ordinary_muscle_development	ENSGALG00010028125	HMG20B	-1.074934267	0.121406395	-8.854016814	8.44247E-19	4.22415E-18	negative
PRJNA729271	stage_1D_vs_E10	ordinary_muscle_development	ENSGALG00010028126	ZMYND10	0.086564834	0.083632788	1.035058568	0.30064156	0.347595072	positive
PRJNA729271	stage_1D_vs_E10	ordinary_muscle_development	ENSGALG00010028127	MFSD12	-0.376044039	0.173331338	-2.169509818	0.030044	0.041415861	negative
PRJNA729271	stage_1D_vs_E10	ordinary_muscle_development	ENSGALG00010028128	DNM1	-2.037990116	0.143462934	-14.20569108	8.44653E-46	1.36339E-44	negative
PRJNA729271	stage_1D_vs_E10	ordinary_muscle_development	ENSGALG00010028129	CYB561D2	0.628539287	0.104875893	5.993172212	2.05787E-09	5.68059E-09	positive
PRJNA729271	stage_1D_vs_E10	ordinary_muscle_development	ENSGALG00010028130	TMEM115	0.345203052	0.126824442	2.721896861	0.00649084	0.009832242	positive
PRJNA729271	stage_1D_vs_E10	ordinary_muscle_development	ENSGALG00010028131	TMEM161A	1.363641559	0.202288972	6.741057325	1.57238E-11	5.02683E-11	positive
PRJNA729271	stage_1D_vs_E10	ordinary_muscle_development	ENSGALG00010028132	CACNA2D2	0.87242025	0.127498683	6.842582421	7.77781E-12	2.53404E-11	positive
PRJNA729271	stage_1D_vs_E10	ordinary_muscle_development	ENSGALG00010028133	ENSGALG00010028133	0.665575317	0.3039416	2.189813163	0.02853779	0.039440814	positive
PRJNA729271	stage_1D_vs_E10	ordinary_muscle_development	ENSGALG00010028134	INPP5E	-0.861672407	0.070887218	-12.15553991	5.36065E-34	5.56791E-33	negative
PRJNA729271	stage_1D_vs_E10	ordinary_muscle_development	ENSGALG00010028136	C3orf18	-1.105711998	0.162957402	-6.785282439	1.1586E-11	3.73363E-11	negative
PRJNA729271	stage_1D_vs_E10	ordinary_muscle_development	ENSGALG00010028137	NUP214	-0.229791696	0.065853753	-3.489424466	0.000484062	0.000834897	negative
PRJNA729271	stage_1D_vs_E10	ordinary_muscle_development	ENSGALG00010028138	ENSGALG00010028138	-0.695540134	0.50870316	-1.367280938	0.171537264	0.209031748	negative
PRJNA729271	stage_1D_vs_E10	ordinary_muscle_development	ENSGALG00010028139	FZR1	-0.419225673	0.086244165	-4.860916363	1.16844E-06	2.59364E-06	negative
PRJNA729271	stage_1D_vs_E10	ordinary_muscle_development	ENSGALG00010028140	HEMK1	-0.371153689	0.106084436	-3.49866298	0.000467597	0.000807845	negative
PRJNA729271	stage_1D_vs_E10	ordinary_muscle_development	ENSGALG00010028141	CISH	2.943791394	0.186140244	15.81491105	2.45556E-56	5.92236E-55	positive
PRJNA729271	stage_1D_vs_E10	ordinary_muscle_development	ENSGALG00010028142	PMPCA	0.610241612	0.121663949	5.01579652	5.28142E-07	1.20558E-06	positive
PRJNA729271	stage_1D_vs_E10	ordinary_muscle_development	ENSGALG00010028144	FAM78A	1.187439139	0.184951213	6.420283047	1.36021E-10	4.08373E-10	positive
PRJNA729271	stage_1D_vs_E10	ordinary_muscle_development	ENSGALG00010028145	ENSGALG00010028145	0.187635405	0.134902933	1.390891959	0.164258192	0.200989573	positive
PRJNA729271	stage_1D_vs_E10	ordinary_muscle_development	ENSGALG00010028146	MAPKAPK3	2.274516158	0.104021363	21.86585613	5.4925E-106	4.3986E-104	positive
PRJNA729271	stage_1D_vs_E10	ordinary_muscle_development	ENSGALG00010028147	SLC25A42	3.305455174	0.197004543	16.77857336	3.50147E-63	1.04863E-61	positive
PRJNA729271	stage_1D_vs_E10	ordinary_muscle_development	ENSGALG00010028148	ENSGALG00010028148	5.746452893	0.255825999	22.46234908	9.6926E-112	9.0641E-110	positive
PRJNA729271	stage_1D_vs_E10	ordinary_muscle_development	ENSGALG00010028149	SNAPC4	0.361247882	0.086940506	4.155115936	3.25123E-05	6.31638E-05	positive
PRJNA729271	stage_1D_vs_E10	ordinary_muscle_development	ENSGALG00010028150	ARMC6	-0.276025209	0.081729938	-3.377283969	0.000732054	0.001239183	negative
PRJNA729271	stage_1D_vs_E10	ordinary_muscle_development	ENSGALG00010028151	DOCK3	-0.217809279	0.231454476	-0.941045872	0.346681352	0.395556219	negative
PRJNA729271	stage_1D_vs_E10	ordinary_muscle_development	ENSGALG00010028152	ENSGALG00010028152	0.277548139	0.333662232	0.831823661	0.405508487	0.455188222	positive
PRJNA729271	stage_1D_vs_E10	ordinary_muscle_development	ENSGALG00010028153	MANF	-0.6503745	0.147774459	-4.4011293	1.07689E-05	2.19265E-05	negative
PRJNA729271	stage_1D_vs_E10	ordinary_muscle_development	ENSGALG00010028154	ENSGALG00010028154	-0.680574645	0.127448529	-5.339996077	9.29486E-08	2.24923E-07	negative
PRJNA729271	stage_1D_vs_E10	ordinary_muscle_development	ENSGALG00010028155	CIZ1	-1.155807931	0.079760387	-14.49100206	1.38113E-47	2.40619E-46	negative
PRJNA729271	stage_1D_vs_E10	ordinary_muscle_development	ENSGALG00010028156	DOHH	-0.424460429	0.098214934	-4.321750391	1.54796E-05	3.09956E-05	negative
PRJNA729271	stage_1D_vs_E10	ordinary_muscle_development	ENSGALG00010028157	PRRC2B	-0.083545315	0.144971185	-0.57628911	0.564419783	0.609814009	negative
PRJNA729271	stage_1D_vs_E10	ordinary_muscle_development	ENSGALG00010028158	DDX49	-0.502907978	0.098558756	-5.102621016	3.34981E-07	7.75029E-07	negative
PRJNA729271	stage_1D_vs_E10	ordinary_muscle_development	ENSGALG00010028159	DCAF1	0.283049358	0.089718919	3.154845828	0.001605829	0.002615631	positive
PRJNA729271	stage_1D_vs_E10	ordinary_muscle_development	ENSGALG00010028160	ENSGALG00010028160	3.592744149	0.553187526	6.494622492	8.32419E-11	2.53432E-10	positive
PRJNA729271	stage_1D_vs_E10	ordinary_muscle_development	ENSGALG00010028161	ENSGALG00010028161	0.236038436	0.595018026	0.396691236	0.691595164	0.728908013	positive
PRJNA729271	stage_1D_vs_E10	ordinary_muscle_development	ENSGALG00010028162	CARD9	-0.157513567	0.178403689	-0.882905329	0.377287424	0.42644894	negative
PRJNA729271	stage_1D_vs_E10	ordinary_muscle_development	ENSGALG00010028163	GBP	0.634180965	0.364009298	1.742210895	0.081471548	0.105177244	positive
PRJNA729271	stage_1D_vs_E10	ordinary_muscle_development	ENSGALG00010028164	BBLN	0.453549764	0.139319747	3.255459286	0.001132091	0.001876892	positive
PRJNA729271	stage_1D_vs_E10	ordinary_muscle_development	ENSGALG00010028165	NFIC	2.251367838	0.153824293	14.63597068	1.65595E-48	3.01546E-47	positive
PRJNA729271	stage_1D_vs_E10	ordinary_muscle_development	ENSGALG00010028168	PTGES2	0.634320955	0.137648539	4.608265091	4.06043E-06	8.58959E-06	positive
PRJNA729271	stage_1D_vs_E10	ordinary_muscle_development	ENSGALG00010028169	DNLZ	0.74817775	0.143665807	5.207764934	1.91129E-07	4.51575E-07	positive
PRJNA729271	stage_1D_vs_E10	ordinary_muscle_development	ENSGALG00010028170	COPE	-1.33738572	0.094804333	-14.10679949	3.44878E-45	5.4634E-44	negative
PRJNA729271	stage_1D_vs_E10	ordinary_muscle_development	ENSGALG00010028171	RNF123	1.552928895	0.158035453	9.826458951	8.66107E-23	5.35361E-22	positive
PRJNA729271	stage_1D_vs_E10	ordinary_muscle_development	ENSGALG00010028172	CERS1	0.25510974	0.210160957	1.213877893	0.224794356	0.267520048	positive
PRJNA729271	stage_1D_vs_E10	ordinary_muscle_development	ENSGALG00010028173	GDF3	0.370238427	0.227053985	1.630618495	0.102970844	0.130697232	positive
PRJNA729271	stage_1D_vs_E10	ordinary_muscle_development	ENSGALG00010028174	GMPPB	-0.986694809	0.155461156	-6.346889715	2.19712E-10	6.50606E-10	negative
PRJNA729271	stage_1D_vs_E10	ordinary_muscle_development	ENSGALG00010028175	POMT1	0.444817583	0.091389004	4.867298736	1.13134E-06	2.51631E-06	positive
PRJNA729271	stage_1D_vs_E10	ordinary_muscle_development	ENSGALG00010028176	UPF1	0.290135936	0.094438155	3.072232164	0.002124644	0.003407093	positive
PRJNA729271	stage_1D_vs_E10	ordinary_muscle_development	ENSGALG00010028177	ENSGALG00010028177	4.499090856	0.388286839	11.58702897	4.79477E-31	4.34648E-30	positive
PRJNA729271	stage_1D_vs_E10	ordinary_muscle_development	ENSGALG00010028178	ENSGALG00010028178	-0.64426971	0.162719328	-3.959392653	7.51406E-05	0.000141156	negative
PRJNA729271	stage_1D_vs_E10	ordinary_muscle_development	ENSGALG00010028179	MCM2	-1.252309014	0.143122705	-8.74989763	2.13546E-18	1.04608E-17	negative
PRJNA729271	stage_1D_vs_E10	ordinary_muscle_development	ENSGALG00010028180	ODF2	-1.224139764	0.105505723	-11.60259106	3.99788E-31	3.6446E-30	negative
PRJNA729271	stage_1D_vs_E10	ordinary_muscle_development	ENSGALG00010028181	BGLAP	-0.858980658	0.279807514	-3.06989847	0.002141316	0.003432309	negative
PRJNA729271	stage_1D_vs_E10	ordinary_muscle_development	ENSGALG00010028182	SMG5	0.117391861	0.146560241	0.800980268	0.423143069	0.472750023	positive
PRJNA729271	stage_1D_vs_E10	ordinary_muscle_development	ENSGALG00010028183	CRTC1	0.55395237	0.095410406	5.805995324	6.39847E-09	1.69989E-08	positive
PRJNA729271	stage_1D_vs_E10	ordinary_muscle_development	ENSGALG00010028184	TMEM79	0.496128645	0.249694693	1.986941092	0.046928925	0.06283924	positive
PRJNA729271	stage_1D_vs_E10	ordinary_muscle_development	ENSGALG00010028185	GLMP	0.106535425	0.087289296	1.220486698	0.222280429	0.264898037	positive
PRJNA729271	stage_1D_vs_E10	ordinary_muscle_development	ENSGALG00010028186	CCT3	-0.244016782	0.132258863	-1.844993798	0.065038441	0.085222582	negative
PRJNA729271	stage_1D_vs_E10	ordinary_muscle_development	ENSGALG00010028187	TPRA1	0.55592773	0.069322005	8.019498704	1.06178E-15	4.45196E-15	positive
PRJNA729271	stage_1D_vs_E10	ordinary_muscle_development	ENSGALG00010028188	ENSGALG00010028188	0.392174349	0.108642635	3.609764696	0.000306475	0.000539513	positive
PRJNA729271	stage_1D_vs_E10	ordinary_muscle_development	ENSGALG00010028189	GPSM1	0.546435905	0.10542095	5.183371119	2.17911E-07	5.11932E-07	positive
PRJNA729271	stage_1D_vs_E10	ordinary_muscle_development	ENSGALG00010028190	ILF2	-0.684563162	0.08049382	-8.504543138	1.82312E-17	8.47263E-17	negative
PRJNA729271	stage_1D_vs_E10	ordinary_muscle_development	ENSGALG00010028191	KLHL26	0.186861704	0.100091403	1.866910633	0.061914069	0.081341831	positive
PRJNA729271	stage_1D_vs_E10	ordinary_muscle_development	ENSGALG00010028192	ENSGALG00010028192	2.399209873	0.307390383	7.805090881	5.94587E-15	2.37425E-14	positive
PRJNA729271	stage_1D_vs_E10	ordinary_muscle_development	ENSGALG00010028193	BRINP1	-5.31603157	0.255262585	-20.82573742	2.53002E-96	1.76311E-94	negative
PRJNA729271	stage_1D_vs_E10	ordinary_muscle_development	ENSGALG00010028194	ENSGALG00010028194	2.871105379	0.180747937	15.88458175	8.10367E-57	1.99766E-55	positive
PRJNA729271	stage_1D_vs_E10	ordinary_muscle_development	ENSGALG00010028195	ENSGALG00010028195	1.571584369	0.298851199	5.258752089	1.45036E-07	3.46115E-07	positive
PRJNA729271	stage_1D_vs_E10	ordinary_muscle_development	ENSGALG00010028196	ENSGALG00010028196	-1.617657717	0.097480258	-16.59472133	7.60962E-62	2.22831E-60	negative
PRJNA729271	stage_1D_vs_E10	ordinary_muscle_development	ENSGALG00010028197	INTS3	0.295978645	0.153244265	1.931417435	0.053431451	0.070878455	positive
PRJNA729271	stage_1D_vs_E10	ordinary_muscle_development	ENSGALG00010028198	UCK1	-0.307752371	0.091968231	-3.346289987	0.000819007	0.001379285	negative
PRJNA729271	stage_1D_vs_E10	ordinary_muscle_development	ENSGALG00010028199	DPP9	0.040696473	0.106367743	0.382601641	0.702015154	0.738815774	positive
PRJNA729271	stage_1D_vs_E10	ordinary_muscle_development	ENSGALG00010028201	ENSGALG00010028201	3.422043774	0.159834622	21.40990313	1.0804E-101	8.11397E-100	positive
PRJNA729271	stage_1D_vs_E10	ordinary_muscle_development	ENSGALG00010028202	QSOX2	-0.503030198	0.066990617	-7.508964973	5.95967E-14	2.2368E-13	negative
PRJNA729271	stage_1D_vs_E10	ordinary_muscle_development	ENSGALG00010028205	PRRT1B	-0.508597823	0.44712352	-1.137488414	0.255334165	0.300143438	negative
PRJNA729271	stage_1D_vs_E10	ordinary_muscle_development	ENSGALG00010028206	RBM15B	-0.360808511	0.094699028	-3.810055074	0.000138936	0.000254085	negative
PRJNA729271	stage_1D_vs_E10	ordinary_muscle_development	ENSGALG00010028207	ENSGALG00010028207	0.134350465	0.273525701	0.491180407	0.623298855	0.665246808	positive
PRJNA729271	stage_1D_vs_E10	ordinary_muscle_development	ENSGALG00010028211	GATAD2B	0.607054606	0.143437628	4.232185195	2.31432E-05	4.55912E-05	positive
PRJNA729271	stage_1D_vs_E10	ordinary_muscle_development	ENSGALG00010028216	NKX2-3	-0.708018984	1.582958047	-0.4472759	0.654675866	0.694186297	negative
PRJNA729271	stage_1D_vs_E10	ordinary_muscle_development	ENSGALG00010028221	TNFAIP8L1	-1.804981509	0.191261283	-9.437255061	3.82673E-21	2.1795E-20	negative
PRJNA729271	stage_1D_vs_E10	ordinary_muscle_development	ENSGALG00010028232	C9orf69	0.55013707	0.108547887	5.068150877	4.01699E-07	9.23933E-07	positive
PRJNA729271	stage_1D_vs_E10	ordinary_muscle_development	ENSGALG00010028239	EDMPN2	0.859508746	1.64549537	0.522340422	0.60143333	0.644376976	positive
PRJNA729271	stage_1D_vs_E10	ordinary_muscle_development	ENSGALG00010028242	ENSGALG00010028242	0.231105599	0.279457091	0.826980622	0.408248045	0.457908799	positive
PRJNA729271	stage_1D_vs_E10	ordinary_muscle_development	ENSGALG00010028245	JAC1	-0.54765442	1.48470997	-0.368862897	0.71222992	0.747774476	negative
PRJNA729271	stage_1D_vs_E10	ordinary_muscle_development	ENSGALG00010028247	MVP	-0.079174392	0.093305907	-0.848546397	0.396133745	0.445423833	negative
PRJNA729271	stage_1D_vs_E10	ordinary_muscle_development	ENSGALG00010028262	C1orf43	1.017893837	0.142428205	7.146715349	8.88789E-13	3.08649E-12	positive
PRJNA729271	stage_1D_vs_E10	ordinary_muscle_development	ENSGALG00010028270	RAPGEF1	0.904006909	0.18098752	4.994857711	5.88792E-07	1.33875E-06	positive
PRJNA729271	stage_1D_vs_E10	ordinary_muscle_development	ENSGALG00010028279	EDBETA	0.080374047	1.629315883	0.049329935	0.960656364	0.966438525	positive
PRJNA729271	stage_1D_vs_E10	ordinary_muscle_development	ENSGALG00010028283	ENSGALG00010028283	1.103069939	0.151098579	7.300332956	2.87056E-13	1.0312E-12	positive
PRJNA729271	stage_1D_vs_E10	ordinary_muscle_development	ENSGALG00010028286	KCTD12A	0.384747266	1.447828087	0.265740988	0.790438694	0.818269452	positive
PRJNA729271	stage_1D_vs_E10	ordinary_muscle_development	ENSGALG00010028292	ENSGALG00010028292	-0.953851208	0.490417741	-1.944976963	0.051777751	0.068880185	negative
PRJNA729271	stage_1D_vs_E10	ordinary_muscle_development	ENSGALG00010028301	FBN3	-2.363373988	0.184817723	-12.78759391	1.92337E-37	2.29648E-36	negative
PRJNA729271	stage_1D_vs_E10	ordinary_muscle_development	ENSGALG00010028311	ENSGALG00010028311	-3.29483922	0.258713965	-12.73545174	3.75665E-37	4.43065E-36	negative
PRJNA729271	stage_1D_vs_E10	ordinary_muscle_development	ENSGALG00010028314	MEF2D	1.818947661	0.213866075	8.505078049	1.81473E-17	8.43907E-17	positive
PRJNA729271	stage_1D_vs_E10	ordinary_muscle_development	ENSGALG00010028316	TIMM44	0.025535007	0.079819758	0.319908347	0.74903781	0.781325781	positive
PRJNA729271	stage_1D_vs_E10	ordinary_muscle_development	ENSGALG00010028318	NACC2	1.08632803	0.190488572	5.702851466	1.1782E-08	3.05618E-08	positive
PRJNA729271	stage_1D_vs_E10	ordinary_muscle_development	ENSGALG00010028321	RHBG	0.174565208	0.192068426	0.908869887	0.363418811	0.41238106	positive
PRJNA729271	stage_1D_vs_E10	ordinary_muscle_development	ENSGALG00010028322	STRBP	-1.80821701	0.114468112	-15.79668763	3.27899E-56	7.88208E-55	negative
PRJNA729271	stage_1D_vs_E10	ordinary_muscle_development	ENSGALG00010028326	CRB2	-1.819957604	0.257310433	-7.073003554	1.51616E-12	5.17951E-12	negative
PRJNA729271	stage_1D_vs_E10	ordinary_muscle_development	ENSGALG00010028328	HNRNPM	-1.302400041	0.165904374	-7.85030562	4.15025E-15	1.67757E-14	negative
PRJNA729271	stage_1D_vs_E10	ordinary_muscle_development	ENSGALG00010028329	HAPLN2	1.957318796	0.55528571	3.524885944	0.000423665	0.000734836	positive
PRJNA729271	stage_1D_vs_E10	ordinary_muscle_development	ENSGALG00010028337	ENSGALG00010028337	3.201830547	0.198490191	16.13092584	1.54684E-58	4.00383E-57	positive
PRJNA729271	stage_1D_vs_E10	ordinary_muscle_development	ENSGALG00010028339	BCAN	-2.158180145	0.419345167	-5.146548279	2.65323E-07	6.19201E-07	negative
PRJNA729271	stage_1D_vs_E10	ordinary_muscle_development	ENSGALG00010028344	LSM7	-1.728541066	0.095225445	-18.15209222	1.23602E-73	5.01852E-72	negative
PRJNA729271	stage_1D_vs_E10	ordinary_muscle_development	ENSGALG00010028346	DENND1A	0.44103666	0.073055656	6.036995389	1.5701E-09	4.37078E-09	positive
PRJNA729271	stage_1D_vs_E10	ordinary_muscle_development	ENSGALG00010028348	NES	-3.777339234	0.277009104	-13.63615558	2.44093E-42	3.47216E-41	negative
PRJNA729271	stage_1D_vs_E10	ordinary_muscle_development	ENSGALG00010028353	ENSGALG00010028353	1.341954067	1.620473986	0.828124413	0.407600045	0.457288131	positive
PRJNA729271	stage_1D_vs_E10	ordinary_muscle_development	ENSGALG00010028355	RBM18	-0.514025073	0.074611612	-6.889344131	5.60502E-12	1.84145E-11	negative
PRJNA729271	stage_1D_vs_E10	ordinary_muscle_development	ENSGALG00010028357	SPPL2C	-0.591319977	0.093280007	-6.339193104	2.30972E-10	6.83112E-10	negative
PRJNA729271	stage_1D_vs_E10	ordinary_muscle_development	ENSGALG00010028360	ENSGALG00010028360	-3.042704931	0.623970376	-4.876361199	1.08061E-06	2.40901E-06	negative
PRJNA729271	stage_1D_vs_E10	ordinary_muscle_development	ENSGALG00010028362	TMPRSS9	-3.467906418	0.461541819	-7.513742583	5.74605E-14	2.16055E-13	negative
PRJNA729271	stage_1D_vs_E10	ordinary_muscle_development	ENSGALG00010028364	MHM2	6.088557555	0.67372101	9.037208977	1.60723E-19	8.36509E-19	positive
PRJNA729271	stage_1D_vs_E10	ordinary_muscle_development	ENSGALG00010028366	MRRF	-0.168437615	0.087481369	-1.925411273	0.054177926	0.071829237	negative
PRJNA729271	stage_1D_vs_E10	ordinary_muscle_development	ENSGALG00010028368	TIMM13	0.311363163	0.120732827	2.578943699	0.009910294	0.014661125	positive
PRJNA729271	stage_1D_vs_E10	ordinary_muscle_development	ENSGALG00010028370	S100A12	4.516818983	0.656640099	6.87868284	6.04085E-12	1.97925E-11	positive
PRJNA729271	stage_1D_vs_E10	ordinary_muscle_development	ENSGALG00010028372	PTGS1	1.011147796	0.159828719	6.326446237	2.50872E-10	7.40306E-10	positive
PRJNA729271	stage_1D_vs_E10	ordinary_muscle_development	ENSGALG00010028374	FDPS	-0.553330614	0.133080033	-4.157878548	3.21217E-05	6.2472E-05	negative

PRJNA729271	stage_1D_vs_E10	ordinary_muscle_development	ENSGALG00010028375	LMNB2	-1.09004715	0.152574186	-7.144374651	9.04065E-13	3.13803E-12	negative
PRJNA729271	stage_1D_vs_E10	ordinary_muscle_development	ENSGALG00010028377	PDCL	0.008727091	0.058373448	0.14950446	0.88115559	0.898575368	positive
PRJNA729271	stage_1D_vs_E10	ordinary_muscle_development	ENSGALG00010028380	RUSC1	1.185810171	0.140307726	8.451495925	2.87594E-17	1.32423E-16	positive
PRJNA729271	stage_1D_vs_E10	ordinary_muscle_development	ENSGALG00010028384	ENSGALG00010028384	-0.758124671	1.16546189	-0.650492888	0.515373894	0.562992282	negative
PRJNA729271	stage_1D_vs_E10	ordinary_muscle_development	ENSGALG00010028386	NCAN	-6.723212253	0.854900416	-7.86432212	3.71101E-15	1.50507E-14	negative
PRJNA729271	stage_1D_vs_E10	ordinary_muscle_development	ENSGALG00010028391	MED26	0.688867875	0.111672084	6.16866679	6.88682E-10	1.96698E-09	positive
PRJNA729271	stage_1D_vs_E10	ordinary_muscle_development	ENSGALG00010028392	ASH1L	1.434929648	0.120592694	11.89897663	1.19807E-32	1.17736E-31	positive
PRJNA729271	stage_1D_vs_E10	ordinary_muscle_development	ENSGALG00010028394	ZBTB26	-0.016679908	0.103495534	-0.161165486	0.871963064	0.890956197	negative
PRJNA729271	stage_1D_vs_E10	ordinary_muscle_development	ENSGALG00010028399	SLC35E1	0.010345578	0.079127291	0.130746015	0.89597623	0.911316782	positive
PRJNA729271	stage_1D_vs_E10	ordinary_muscle_development	ENSGALG00010028405	S100A6	-0.509425726	0.280258752	-1.817697832	0.069110329	0.090183131	negative
PRJNA729271	stage_1D_vs_E10	ordinary_muscle_development	ENSGALG00010028411	LHX2	-6.092151825	0.98243113	-6.201098111	5.60705E-10	1.6129E-09	negative
PRJNA729271	stage_1D_vs_E10	ordinary_muscle_development	ENSGALG00010028412	ENSGALG00010028412	0.201258606	0.319363792	0.630186048	0.528572866	0.575459193	positive
PRJNA729271	stage_1D_vs_E10	ordinary_muscle_development	ENSGALG00010028414	KBTBD12	6.072401156	0.350995472	17.30051136	4.66232E-67	1.55715E-65	positive
PRJNA729271	stage_1D_vs_E10	ordinary_muscle_development	ENSGALG00010028415	FSD1	-0.701522004	0.171355756	-4.09395063	4.24085E-05	8.15158E-05	negative
PRJNA729271	stage_1D_vs_E10	ordinary_muscle_development	ENSGALG00010028418	ENSGALG00010028418	1.773440372	0.124027813	14.29873133	2.22835E-46	3.6788E-45	positive
PRJNA729271	stage_1D_vs_E10	ordinary_muscle_development	ENSGALG00010028419	ENSGALG00010028419	3.553988549	0.36293798	9.792275106	1.2153E-22	7.48016E-22	positive
PRJNA729271	stage_1D_vs_E10	ordinary_muscle_development	ENSGALG00010028420	ENSGALG00010028420	2.923059634	0.830846583	3.518170135	0.000434534	0.000752427	positive
PRJNA729271	stage_1D_vs_E10	ordinary_muscle_development	ENSGALG00010028421	ZBTB7A	1.69572942	0.18745427	9.046096532	1.4817E-19	7.72287E-19	positive
PRJNA729271	stage_1D_vs_E10	ordinary_muscle_development	ENSGALG00010028422	RXRA	1.771508042	0.208159203	8.510351781	1.73406E-17	8.07429E-17	positive
PRJNA729271	stage_1D_vs_E10	ordinary_muscle_development	ENSGALG00010028425	DAP3	0.525126381	0.129452983	4.056502745	4.9813E-05	9.5193E-05	positive
PRJNA729271	stage_1D_vs_E10	ordinary_muscle_development	ENSGALG00010028426	GPR21	0.004145109	0.1465133	0.028291693	0.977429506	0.98088069	positive
PRJNA729271	stage_1D_vs_E10	ordinary_muscle_development	ENSGALG00010028427	ENSGALG00010028427	-0.099228276	0.208873296	-0.475064443	0.634741042	0.675843655	negative
PRJNA729271	stage_1D_vs_E10	ordinary_muscle_development	ENSGALG00010028428	RABGAP1	0.234639133	0.067110688	3.496300539	0.000471757	0.000814644	positive
PRJNA729271	stage_1D_vs_E10	ordinary_muscle_development	ENSGALG00010028429	REXO1	-0.208056937	0.088257683	-2.35738046	0.01840438	0.026154067	negative
PRJNA729271	stage_1D_vs_E10	ordinary_muscle_development	ENSGALG00010028430	NR2C2AP	-0.312212847	0.116615756	-2.677278421	0.007422293	0.011163862	negative
PRJNA729271	stage_1D_vs_E10	ordinary_muscle_development	ENSGALG00010028431	ENSGALG00010028431	-0.810853685	0.273357179	-2.966279092	0.003014268	0.004751176	negative
PRJNA729271	stage_1D_vs_E10	ordinary_muscle_development	ENSGALG00010028432	RFXANK	0.121327071	0.134888006	0.899465224	0.368404911	0.417581263	positive
PRJNA729271	stage_1D_vs_E10	ordinary_muscle_development	ENSGALG00010028433	MSTO1	-0.653655155	0.093485184	-6.992072192	2.70855E-12	9.10916E-12	negative
PRJNA729271	stage_1D_vs_E10	ordinary_muscle_development	ENSGALG00010028434	COL5A1	0.973665067	0.19587271	4.970907218	6.66403E-07	1.50906E-06	positive
PRJNA729271	stage_1D_vs_E10	ordinary_muscle_development	ENSGALG00010028435	ATP8B3	1.808334546	0.325310123	5.558801952	2.71633E-08	6.83918E-08	positive
PRJNA729271	stage_1D_vs_E10	ordinary_muscle_development	ENSGALG00010028436	S100A1	2.859739804	0.185293275	15.43358656	9.73089E-54	2.13388E-52	positive
PRJNA729271	stage_1D_vs_E10	ordinary_muscle_development	ENSGALG00010028437	ENSGALG00010028437	0.265160199	0.066594957	3.981685878	6.84282E-05	0.000129082	positive
PRJNA729271	stage_1D_vs_E10	ordinary_muscle_development	ENSGALG00010028438	MED27	-0.255175869	0.070830681	-3.602617759	0.000315029	0.000554033	negative
PRJNA729271	stage_1D_vs_E10	ordinary_muscle_development	ENSGALG00010028439	BORCS8	0.155018811	0.118663235	1.306376071	0.191424699	0.231090282	positive
PRJNA729271	stage_1D_vs_E10	ordinary_muscle_development	ENSGALG00010028440	PSMB7	-0.205592273	0.078393584	-2.622565026	0.008727061	0.012998228	negative
PRJNA729271	stage_1D_vs_E10	ordinary_muscle_development	ENSGALG00010028442	CHTOP	-0.588043802	0.090527342	-6.495759079	8.26158E-11	2.51579E-10	negative
PRJNA729271	stage_1D_vs_E10	ordinary_muscle_development	ENSGALG00010028443	ENSGALG00010028443	0.588440488	0.324727713	1.812104308	0.069970081	0.091239324	positive
PRJNA729271	stage_1D_vs_E10	ordinary_muscle_development	ENSGALG00010028444	MEF2B	-0.49447925	0.333852971	-1.481128801	0.138572254	0.171822483	negative
PRJNA729271	stage_1D_vs_E10	ordinary_muscle_development	ENSGALG00010028445	SYT11	-2.447663426	0.204060734	-11.9947791	3.78425E-33	3.8013E-32	negative
PRJNA729271	stage_1D_vs_E10	ordinary_muscle_development	ENSGALG00010028446	ENSGALG00010028446	-0.30999372	0.219887326	-1.409784396	0.158603356	0.194628822	negative
PRJNA729271	stage_1D_vs_E10	ordinary_muscle_development	ENSGALG00010028447	OLFM1	-2.027659746	0.158694593	-12.77711929	2.20071E-37	2.62114E-36	negative
PRJNA729271	stage_1D_vs_E10	ordinary_muscle_development	ENSGALG00010028448	RIT1	-0.08409041	0.057019931	-1.474754686	0.140278508	0.17384892	negative
PRJNA729271	stage_1D_vs_E10	ordinary_muscle_development	ENSGALG00010028449	EFCAB12	-0.967536391	0.333985377	-2.896942375	0.00376819	0.005875004	negative
PRJNA729271	stage_1D_vs_E10	ordinary_muscle_development	ENSGALG00010028450	PTGDS	8.100306488	0.432881294	18.71253528	3.91309E-78	1.77807E-76	positive
PRJNA729271	stage_1D_vs_E10	ordinary_muscle_development	ENSGALG00010028451	TMEM59L	-2.087539023	0.344797502	-6.054391381	1.4095E-09	3.93654E-09	negative
PRJNA729271	stage_1D_vs_E10	ordinary_muscle_development	ENSGALG00010028452	GOLPH3L	-3.44084941	0.882530163	-3.898846244	9.66521E-05	0.000179589	negative
PRJNA729271	stage_1D_vs_E10	ordinary_muscle_development	ENSGALG00010028453	ENSGALG00010028453	0.063482165	0.094805852	0.669601756	0.503111694	0.551258902	positive
PRJNA729271	stage_1D_vs_E10	ordinary_muscle_development	ENSGALG00010028454	C8G	2.432344097	0.219519754	11.0802971	1.56353E-28	1.26188E-27	positive
PRJNA729271	stage_1D_vs_E10	ordinary_muscle_development	ENSGALG00010028455	ENSA	1.011786928	0.0692516	14.61030407	2.41438E-48	4.35821E-47	positive
PRJNA729271	stage_1D_vs_E10	ordinary_muscle_development	ENSGALG00010028456	FBXW5	-0.060216096	0.093403311	-0.644689087	0.519128689	0.5664963	negative
PRJNA729271	stage_1D_vs_E10	ordinary_muscle_development	ENSGALG00010028457	KANK3	1.455040493	0.104266238	13.95504927	2.93099E-44	4.47208E-43	positive
PRJNA729271	stage_1D_vs_E10	ordinary_muscle_development	ENSGALG00010028458	TRAF2	-0.205288464	0.109393641	-1.876603271	0.060572489	0.079687622	negative
PRJNA729271	stage_1D_vs_E10	ordinary_muscle_development	ENSGALG00010028459	MCL1	1.902682309	0.209863823	9.066271073	1.2316E-19	6.45876E-19	positive
PRJNA729271	stage_1D_vs_E10	ordinary_muscle_development	ENSGALG00010028460	CRLF1	-1.452460483	0.249557335	-5.820147438	5.87957E-09	1.5672E-08	negative
PRJNA729271	stage_1D_vs_E10	ordinary_muscle_development	ENSGALG00010028461	ENSGALG00010028461	1.94131212	0.317923571	6.106222677	1.02017E-09	2.88139E-09	positive
PRJNA729271	stage_1D_vs_E10	ordinary_muscle_development	ENSGALG00010028462	EDF1	0.071060007	0.10573239	0.672074159	0.501536486	0.549616041	positive
PRJNA729271	stage_1D_vs_E10	ordinary_muscle_development	ENSGALG00010028463	RPS28	1.328712027	0.169237614	7.851162623	4.12198E-15	1.66708E-14	positive
PRJNA729271	stage_1D_vs_E10	ordinary_muscle_development	ENSGALG00010028464	ENSGALG00010028464	0.232355107	0.111371614	2.086304572	0.036951043	0.050291584	positive
PRJNA729271	stage_1D_vs_E10	ordinary_muscle_development	ENSGALG00010028465	ENSGALG00010028465	-0.053471603	0.137393773	-0.389185056	0.697139257	0.734283658	negative
PRJNA729271	stage_1D_vs_E10	ordinary_muscle_development	ENSGALG00010028466	MAMDC4	-0.295541523	0.697457452	-0.423741293	0.671754497	0.710165665	negative
PRJNA729271	stage_1D_vs_E10	ordinary_muscle_development	ENSGALG00010028467	ENSGALG00010028467	0.646467791	0.246518977	2.622385506	0.00873166	0.013003741	positive
PRJNA729271	stage_1D_vs_E10	ordinary_muscle_development	ENSGALG00010028468	PHPT1	-0.924375738	0.104024152	-8.886164606	6.32546E-19	3.18249E-18	negative
PRJNA729271	stage_1D_vs_E10	ordinary_muscle_development	ENSGALG00010028470	AJM1	1.69075225	0.438176675	3.858608517	0.000114034	0.000210564	positive
PRJNA729271	stage_1D_vs_E10	ordinary_muscle_development	ENSGALG00010028471	TPGS1	-0.020024281	0.167165755	-0.119786978	0.904651898	0.918593994	negative
PRJNA729271	stage_1D_vs_E10	ordinary_muscle_development	ENSGALG00010028472	RPRD2	0.957055488	0.13787314	6.941565911	3.87777E-12	1.29007E-11	positive
PRJNA729271	stage_1D_vs_E10	ordinary_muscle_development	ENSGALG00010028473	CDC34	1.438656558	0.138272938	10.40446942	2.3657E-25	1.65019E-24	positive
PRJNA729271	stage_1D_vs_E10	ordinary_muscle_development	ENSGALG00010028474	PEX19	0.27365699	0.120176868	2.27711867	0.02277914	0.03201936	positive
PRJNA729271	stage_1D_vs_E10	ordinary_muscle_development	ENSGALG00010028475	AMBP	2.09549226	1.444700677	1.450468109			positive
PRJNA729271	stage_1D_vs_E10	ordinary_muscle_development	ENSGALG00010028476	ENSGALG00010028476	1.219309253	0.155296781	7.851477975	4.11163E-15	1.66336E-14	positive
PRJNA729271	stage_1D_vs_E10	ordinary_muscle_development	ENSGALG00010028477	NRARP	-0.001114324	0.212372722	-0.005247021	0.995813502	0.996776239	negative
PRJNA729271	stage_1D_vs_E10	ordinary_muscle_development	ENSGALG00010028478	PEA15	0.155227042	0.159918989	0.970660473	0.331717381	0.380188459	positive
PRJNA729271	stage_1D_vs_E10	ordinary_muscle_development	ENSGALG00010028479	CASQ1	7.679483327	0.314888719	24.38792774	2.297E-131	3.0268E-129	positive
PRJNA729271	stage_1D_vs_E10	ordinary_muscle_development	ENSGALG00010028480	F11R	0.015534303	0.216913902	0.071615064	0.942908252	0.951466896	positive
PRJNA729271	stage_1D_vs_E10	ordinary_muscle_development	ENSGALG00010028481	ENSGALG00010028481	0.934578803	0.142137479	6.575175047	4.8596E-11	1.50416E-10	positive
PRJNA729271	stage_1D_vs_E10	ordinary_muscle_development	ENSGALG00010028482	CD320	0.948926307	0.174048582	5.452077209	4.97848E-08	1.23019E-07	positive
PRJNA729271	stage_1D_vs_E10	ordinary_muscle_development	ENSGALG00010028483	EXD3	0.24805908	0.091430672	2.713083861	0.006666023	0.010079701	positive
PRJNA729271	stage_1D_vs_E10	ordinary_muscle_development	ENSGALG00010028484	NOXA1	-2.053056392	0.630238154	-3.257588228	0.001123633	0.001863935	negative
PRJNA729271	stage_1D_vs_E10	ordinary_muscle_development	ENSGALG00010028485	USF1	0.927282505	0.178394418	5.197934526	2.01515E-07	4.75417E-07	positive
PRJNA729271	stage_1D_vs_E10	ordinary_muscle_development	ENSGALG00010028486	ARHGAP30	0.454163998	0.23404236	1.940520502	0.052316465	0.069558536	positive
PRJNA729271	stage_1D_vs_E10	ordinary_muscle_development	ENSGALG00010028488	PTGES	1.878847114	0.128702728	14.59834723	2.87737E-48	5.17462E-47	positive
PRJNA729271	stage_1D_vs_E10	ordinary_muscle_development	ENSGALG00010028489	ENSGALG00010028489	0.340679182	0.857343426	0.397366063	0.691097537	0.728436504	positive
PRJNA729271	stage_1D_vs_E10	ordinary_muscle_development	ENSGALG00010028490	NECTIN4	-1.966927581	0.355487355	-5.533045143	3.14718E-08	7.88155E-08	negative
PRJNA729271	stage_1D_vs_E10	ordinary_muscle_development	ENSGALG00010028491	TOR1B	-0.341933547	0.098310128	-3.478111107	0.000504961	0.000869391	negative
PRJNA729271	stage_1D_vs_E10	ordinary_muscle_development	ENSGALG00010028492	ENSGALG00010028492	2.032862934	0.247117634	8.226296532	1.93091E-16	8.47109E-16	positive
PRJNA729271	stage_1D_vs_E10	ordinary_muscle_development	ENSGALG00010028493	TOR1A	-0.223814565	0.086345763	-2.592073527	0.009539938	0.014144988	negative
PRJNA729271	stage_1D_vs_E10	ordinary_muscle_development	ENSGALG00010028494	ENSGALG00010028494	-0.94706774	0.195478119	-4.844878533	1.26689E-06	2.80574E-06	negative
PRJNA729271	stage_1D_vs_E10	ordinary_muscle_development	ENSGALG00010028495	LINGO3	-3.734247809	0.365315497	-10.2219803	1.58078E-24	1.05787E-23	negative
PRJNA729271	stage_1D_vs_E10	ordinary_muscle_development	ENSGALG00010028496	ENSGALG00010028496	0.609144862	0.14605131	4.170759325	3.03586E-05	5.91542E-05	positive
PRJNA729271	stage_1D_vs_E10	ordinary_muscle_development	ENSGALG00010028498	C9orf78	0.060485004	0.103960165	0.581809425	0.560695055	0.60628682	positive
PRJNA729271	stage_1D_vs_E10	ordinary_muscle_development	ENSGALG00010028500	VPS45	0.50183727	0.069321844	7.23923712	4.51216E-13	1.60342E-12	positive
PRJNA729271	stage_1D_vs_E10	ordinary_muscle_development	ENSGALG00010028501	USP20	0.699341385	0.087083647	8.030685535	9.69295E-16	4.07599E-15	positive
PRJNA729271	stage_1D_vs_E10	ordinary_muscle_development	ENSGALG00010028502	PLEKHO1	-0.720047914	0.150963375	-4.769686102	1.84513E-06	4.02971E-06	negative
PRJNA729271	stage_1D_vs_E10	ordinary_muscle_development	ENSGALG00010028503	NDOR1	-0.11624974	0.126306442	-0.920378545	0.357374976	0.406254434	negative
PRJNA729271	stage_1D_vs_E10	ordinary_muscle_development	ENSGALG00010028506	SHD	-6.583221327	0.565866992	-11.633867	2.77247E-31	2.54348E-30	negative
PRJNA729271	stage_1D_vs_E10	ordinary_muscle_development	ENSGALG00010028507	ANP32E	-0.815129623	0.056756457	-14.36188344	8.97544E-47	1.50752E-45	negative
PRJNA729271	stage_1D_vs_E10	ordinary_muscle_development	ENSGALG00010028510	CILP2	2.073495612	0.582450851	3.559949493	0.000370926	0.000647858	positive
PRJNA729271	stage_1D_vs_E10	ordinary_muscle_development	ENSGALG00010028511	YJEFN3	1.767790249	0.388865938	4.546014643	5.46712E-06	1.14369E-05	positive
PRJNA729271	stage_1D_vs_E10	ordinary_muscle_development	ENSGALG00010028512	NDUFA13	1.492251063	0.12203867	12.22769033	2.21137E-34	2.32949E-33	positive
PRJNA729271	stage_1D_vs_E10	ordinary_muscle_development	ENSGALG00010028513	ENSGALG00010028513	-1.629781405	1.315732802	-1.238687218	0.215461354	0.257385225	negative
PRJNA729271	stage_1D_vs_E10	ordinary_muscle_development	ENSGALG00010028514	FNBP1	1.319620109	0.058415782	22.59013018	5.4188E-113	5.3798E-111	positive
PRJNA729271	stage_1D_vs_E10	ordinary_muscle_development	ENSGALG00010028515	ENSGALG00010028515	0.859436841	3.388812944	0.253609997	0.799796872	0.826717226	positive
PRJNA729271	stage_1D_vs_E10	ordinary_muscle_development	ENSGALG00010028516	GATAD2A	-0.472690602	0.062950674	-7.50890458	5.96242E-14	2.23726E-13	negative
PRJNA729271	stage_1D_vs_E10	ordinary_muscle_development	ENSGALG00010028517	RNF208	-1.07419875	0.33199385	-3.235598342	0.001213881	0.002006065	negative
PRJNA729271	stage_1D_vs_E10	ordinary_muscle_development	ENSGALG00010028518	MAU2	0.744324173	0.114228273	6.516111576	7.21533E-11	2.20367E-10	positive
PRJNA729271	stage_1D_vs_E10	ordinary_muscle_development	ENSGALG00010028519	ENSGALG00010028519	0.859490451	2.100649177	0.409154684	0.682426147	0.72029178	positive
PRJNA729271	stage_1D_vs_E10	ordinary_muscle_development	ENSGALG00010028523	SUGP1	-0.194132542	0.112939938	-1.718900734	0.085632448	0.110166179	negative
PRJNA729271	stage_1D_vs_E10	ordinary_muscle_development	ENSGALG00010028528	UHRF1	-1.699660118	0.183768944	-9.24889744	2.2682E-20	1.23461E-19	negative
PRJNA729271	stage_1D_vs_E10	ordinary_muscle_development	ENSGALG00010028534	ENSGALG00010028534	0.378867347	4.345747042	0.087181178	0.9305275	0.940932079	positive
PRJNA729271	stage_1D_vs_E10	ordinary_muscle_development	ENSGALG00010028535	TICAM1	0.196818578	0.106779471	1.843224878	0.065296184	0.085529386	positive
PRJNA729271	stage_1D_vs_E10	ordinary_muscle_development	ENSGALG00010028536	GPR107	-0.064670281	0.063461245	-1.019051561	0.308178489	0.355515097	negative
PRJNA729271	stage_1D_vs_E10	ordinary_muscle_development	ENSGALG00010028537	ENSGALG00010028537	0.859476486	2.123141423	0.404813583	0.685614544	0.723393712	positive
PRJNA729271	stage_1D_vs_E10	ordinary_muscle_development	ENSGALG00010028538	FEM1A	1.092876575	0.117010975	9.339949292	9.63803E-21	5.3732E-20	positive
PRJNA729271	stage_1D_vs_E10	ordinary_muscle_development	ENSGALG00010028539	ENSGALG00010028539	1.342978639	3.347138525	0.401231867	0.688249421	0.725803955	positive
PRJNA729271	stage_1D_vs_E10	ordinary_muscle_development	ENSGALG00010028541	ENSGALG00010028541	0.08428078	0.297413525	0.283379108	0.776886241	0.805966653	positive
PRJNA729271	stage_1D_vs_E10	ordinary_muscle_development	ENSGALG00010028542	ENSGALG00010028542	0.859487342	1.996452615	0.430507258	0.666826688	0.705625116	positive
PRJNA729271	stage_1D_vs_E10	ordinary_muscle_development	ENSGALG00010028543	ANP32B	-1.28901587	0.093669891	-13.7612616	4.35854E-43	6.36224E-42	negative
PRJNA729271	stage_1D_vs_E10	ordinary_muscle_development	ENSGALG00010028544	ZNF414	1.29069251	0.155477299	8.301485299	1.02818E-16	4.57863E-16	positive
PRJNA729271	stage_1D_vs_E10	ordinary_muscle_development	ENSGALG00010028545	ENSGALG00010028545	3.540151621	1.438881757	2.460349229	0.013880188	0.020106696	positive
PRJNA729271	stage_1D_vs_E10	ordinary_muscle_development	ENSGALG00010028546	ENSGALG00010028546	1.342659086	1.730374514	0.775935542	0.437787046	0.487046069	positive
PRJNA729271	stage_1D_vs_E10	ordinary_muscle_development	ENSGALG00010028547	ZNF618	0.010497037	0.104477908	0.100471357	0.919970122	0.931863516	positive
PRJNA729271	stage_1D_vs_E10	ordinary_muscle_development	ENSGALG00010028548	MYO1F	-0.134719505	0.253505538	-0.531426278	0.595123415	0.638503445	negative
PRJNA729271	stage_1D_vs_E10	ordinary_muscle_development	ENSGALG00010028549	ADAMTS10	-0.764010404	0.177153582	-4.312700852	1.61272E-05	3.22478E-05	negative
PRJNA729271	stage_1D_vs_E10	ordinary_muscle_development	ENSGALG00010028550	ZAP70	-3.725508627	1.002085838	-3.717753993	0.000201002	0.000360808	negative
PRJNA729271	stage_1D_vs_E10	ordinary_muscle_development	ENSGALG00010028551	NCS1	-3.688254617	0.255222941	-14.45110928	2.46681E-47	4.23654E-46	negative
PRJNA729271	stage_1D_vs_E10	ordinary_muscle_development	ENSGALG00010028552	ENSGALG00010028552	-2.714624379	0.697675646	-3.890954765	9.98505E-05	0.000185152	negative
PRJNA729271	stage_1D_vs_E10	ordinary_muscle_development	ENSGALG00010028553	NFILZ	1.375174765	1.15233049	1.193385732	0.23271834	0.276043563	positive
PRJNA729271	stage_1D_vs_E10	ordinary_muscle_development	ENSGALG00010028554	ENSGALG00010028554	-4.861740296	1.078663514	-4.507188971	6.56921E-06	1.36556E-05	negative
PRJNA729271	stage_1D_vs_E10	ordinary_muscle_development	ENSGALG00010028555	ENSGALG00010028555	-0.632720826	1.415274453	-0.447065815	0.654827541	0.694245553	negative
PRJNA729271	stage_1D_vs_E10	ordinary_muscle_development	ENSGALG00010028556	SPTAN1	0.516253668	0.093300472	5.533237502	3.14373E-08	7.87427E-08	positive
PRJNA729271	stage_1D_vs_E10	ordinary_muscle_development	ENSGALG00010028558	ENSGALG00010028558	-0.054711334	1.589021421	-0.034430835	0.972533595	0.976575993	negative
PRJNA729271	stage_1D_vs_E10	ordinary_muscle_development	ENSGALG00010028559	DYNC2I2	-0.81043916	0.123762726	-6.548329891	5.81841E-11	1.78985E-10	negative

PRJNA729271	stage_1D_vs_E10	ordinary_muscle_development	ENSGALG00010028560	ENSGALG00010028560	0.859400432	3.504971005	0.245194734	0.806305654	0.832732487	positive
PRJNA729271	stage_1D_vs_E10	ordinary_muscle_development	ENSGALG00010028561	SET	-1.162056292	0.051526234	-22.5527115	1.263E-112	1.2287E-110	negative
PRJNA729271	stage_1D_vs_E10	ordinary_muscle_development	ENSGALG00010028562	ACSBG2	1.703157085	0.199269692	8.546995104	1.26335E-17	5.91861E-17	positive
PRJNA729271	stage_1D_vs_E10	ordinary_muscle_development	ENSGALG00010028563	ENSGALG00010028563	0.859474661	2.300871649	0.373543071	0.708744295	0.744816107	positive
PRJNA729271	stage_1D_vs_E10	ordinary_muscle_development	ENSGALG00010028565	PKN1	0.392022293	0.118913434	3.296703145	0.000978268	0.001632888	positive
PRJNA729271	stage_1D_vs_E10	ordinary_muscle_development	ENSGALG00010028566	ZDHHC12	-0.052040451	0.12682604	-0.410329386	0.681564338	0.719486933	negative
PRJNA729271	stage_1D_vs_E10	ordinary_muscle_development	ENSGALG00010028567	ENSGALG00010028567	-3.564786782	1.200756272	-2.968784644	0.002989801	0.00471466	negative
PRJNA729271	stage_1D_vs_E10	ordinary_muscle_development	ENSGALG00010028568	MLLT1	-0.862700988	0.064822606	-13.30864392	2.0619E-40	2.74195E-39	negative
PRJNA729271	stage_1D_vs_E10	ordinary_muscle_development	ENSGALG00010028569	ZER1	0.521242787	0.068441137	7.615928204	2.61803E-14	1.00605E-13	positive
PRJNA729271	stage_1D_vs_E10	ordinary_muscle_development	ENSGALG00010028572	TBC1D13	0.171288019	0.067962626	2.520326663	0.011724597	0.017178614	positive
PRJNA729271	stage_1D_vs_E10	ordinary_muscle_development	ENSGALG00010028573	KCNN1	2.267140062	0.261749265	8.661495431	4.65615E-18	2.2385E-17	positive
PRJNA729271	stage_1D_vs_E10	ordinary_muscle_development	ENSGALG00010028574	ENDOG	0.151962154	0.117899616	1.288911349	0.197428908	0.237429024	positive
PRJNA729271	stage_1D_vs_E10	ordinary_muscle_development	ENSGALG00010028575	SETDB1	0.114974687	0.118560249	0.969757468	0.332167397	0.380674132	positive
PRJNA729271	stage_1D_vs_E10	ordinary_muscle_development	ENSGALG00010028577	CERS2	0.291193478	0.132049325	2.205187169	0.027440963	0.038022824	positive
PRJNA729271	stage_1D_vs_E10	ordinary_muscle_development	ENSGALG00010028578	SPOUT1	-0.271695138	0.122273287	-2.222031852	0.026281153	0.036520498	negative
PRJNA729271	stage_1D_vs_E10	ordinary_muscle_development	ENSGALG00010028579	OCLN	0.37821196	0.272103952	1.389953938	0.164542865	0.201320911	positive
PRJNA729271	stage_1D_vs_E10	ordinary_muscle_development	ENSGALG00010028580	PRUNE1	0.605759925	0.15204766	3.984013459	6.7761E-05	0.000127873	positive
PRJNA729271	stage_1D_vs_E10	ordinary_muscle_development	ENSGALG00010028581	NR2F6	-0.062528944	0.124183581	-0.503520223	0.614598566	0.656976859	negative
PRJNA729271	stage_1D_vs_E10	ordinary_muscle_development	ENSGALG00010028582	KYAT1	0.579761261	0.209478122	2.767645875	0.005646277	0.008619567	positive
PRJNA729271	stage_1D_vs_E10	ordinary_muscle_development	ENSGALG00010028583	USHBP1	1.360495079	0.195964952	6.942542878	3.85104E-12	1.28206E-11	positive
PRJNA729271	stage_1D_vs_E10	ordinary_muscle_development	ENSGALG00010028584	LRRC8A	0.485296826	0.075118519	6.460415295	1.04416E-10	3.15643E-10	positive
PRJNA729271	stage_1D_vs_E10	ordinary_muscle_development	ENSGALG00010028585	MINDY1	0.839692684	0.121105867	6.933542595	4.1043E-12	1.36106E-11	positive
PRJNA729271	stage_1D_vs_E10	ordinary_muscle_development	ENSGALG00010028586	BABAM1	-0.43226142	0.12166594	-3.552854807	0.000381075	0.000665023	negative
PRJNA729271	stage_1D_vs_E10	ordinary_muscle_development	ENSGALG00010028587	ANKLE1	-0.463340412	0.182263034	-2.542152411	0.011017212	0.016201125	negative
PRJNA729271	stage_1D_vs_E10	ordinary_muscle_development	ENSGALG00010028588	ABHD8	-3.980224986	0.869963869	-4.575161253	4.75853E-06	1.00065E-05	negative
PRJNA729271	stage_1D_vs_E10	ordinary_muscle_development	ENSGALG00010028589	PHYHD1	-0.142473877	0.190669305	-0.747230277	0.454924571	0.503830352	negative
PRJNA729271	stage_1D_vs_E10	ordinary_muscle_development	ENSGALG00010028590	ENSGALG00010028590	0.903285012	0.228278798	3.956937827	7.59167E-05	0.000142504	positive
PRJNA729271	stage_1D_vs_E10	ordinary_muscle_development	ENSGALG00010028591	GABPB2	-0.649543469	0.121108227	-5.363330672	8.17012E-08	1.98735E-07	negative
PRJNA729271	stage_1D_vs_E10	ordinary_muscle_development	ENSGALG00010028592	SEMA6C	2.770009454	0.159069561	17.41382477	6.4806E-68	2.21027E-66	positive
PRJNA729271	stage_1D_vs_E10	ordinary_muscle_development	ENSGALG00010028593	DOLK	-0.009374675	0.11514154	-0.081418701	0.935108978	0.944755324	negative
PRJNA729271	stage_1D_vs_E10	ordinary_muscle_development	ENSGALG00010028594	ENSGALG00010028594	0.411203108	0.145138901	2.833169513	0.004608894	0.00711913	positive
PRJNA729271	stage_1D_vs_E10	ordinary_muscle_development	ENSGALG00010028595	SCNM1	0.698903803	0.111667391	6.258799458	3.87952E-10	1.12738E-09	positive
PRJNA729271	stage_1D_vs_E10	ordinary_muscle_development	ENSGALG00010028596	DDA1	-0.493580349	0.08888268	-5.553166795	2.8054E-08	7.05487E-08	negative
PRJNA729271	stage_1D_vs_E10	ordinary_muscle_development	ENSGALG00010028597	NUP188	-0.507055146	0.055726529	-9.098990224	9.11756E-20	4.82332E-19	negative
PRJNA729271	stage_1D_vs_E10	ordinary_muscle_development	ENSGALG00010028598	TMOD4	6.392804123	0.250588865	25.51112605	1.4836E-143	2.4437E-141	positive
PRJNA729271	stage_1D_vs_E10	ordinary_muscle_development	ENSGALG00010028599	VPS72	0.074585373	0.118565336	0.629065595	0.529306114	0.576084406	positive
PRJNA729271	stage_1D_vs_E10	ordinary_muscle_development	ENSGALG00010028600	PSMD4	0.01604529	0.13284886	0.120778526	0.903866459	0.917860749	positive
PRJNA729271	stage_1D_vs_E10	ordinary_muscle_development	ENSGALG00010028601	ANO8	3.959420617	0.221742664	17.85592611	2.59933E-71	9.94124E-70	positive
PRJNA729271	stage_1D_vs_E10	ordinary_muscle_development	ENSGALG00010028602	ZNF687	0.641134818	0.168395602	3.807313323	0.000140485	0.000256852	positive
PRJNA729271	stage_1D_vs_E10	ordinary_muscle_development	ENSGALG00010028603	SH3GLB2	0.213932417	0.337471851	0.633926702	0.526128658	0.573122653	positive
PRJNA729271	stage_1D_vs_E10	ordinary_muscle_development	ENSGALG00010028604	PI4KB	-0.214123129	0.140534129	-1.523637932	0.127599182	0.159251778	negative
PRJNA729271	stage_1D_vs_E10	ordinary_muscle_development	ENSGALG00010028605	GTPBP3	0.953158653	0.215617571	4.420598222	9.8428E-06	2.01087E-05	positive
PRJNA729271	stage_1D_vs_E10	ordinary_muscle_development	ENSGALG00010028606	PRPF3	-0.203808731	0.136230786	-1.496054867	0.134639358	0.167246336	negative
PRJNA729271	stage_1D_vs_E10	ordinary_muscle_development	ENSGALG00010028607	ENSGALG00010028607	1.75610992	0.238386394	7.366653314	1.74965E-13	6.36547E-13	positive
PRJNA729271	stage_1D_vs_E10	ordinary_muscle_development	ENSGALG00010028608	MIGA2	1.271724968	0.1566514	8.118184483	4.73209E-16	2.03596E-15	positive
PRJNA729271	stage_1D_vs_E10	ordinary_muscle_development	ENSGALG00010028609	MRPS21	0.357677579	0.145075094	2.46546509	0.013683551	0.019848201	positive
PRJNA729271	stage_1D_vs_E10	ordinary_muscle_development	ENSGALG00010028610	ENSGALG00010028610	2.374895489	0.529758762	4.482975384	7.36094E-06	1.52272E-05	positive
PRJNA729271	stage_1D_vs_E10	ordinary_muscle_development	ENSGALG00010028612	DIRAS1	-1.066156398	0.630815627	-1.690123633	0.091004304	0.116539217	negative
PRJNA729271	stage_1D_vs_E10	ordinary_muscle_development	ENSGALG00010028613	ENSGALG00010028613	1.25729912	0.364723535	3.447266215	0.00056629	0.00096854	positive
PRJNA729271	stage_1D_vs_E10	ordinary_muscle_development	ENSGALG00010028618	DOLPP1	-1.821127108	0.108534703	-16.77921488	3.46385E-63	1.03951E-61	negative
PRJNA729271	stage_1D_vs_E10	ordinary_muscle_development	ENSGALG00010028626	CRAT	2.791467134	0.172822715	16.15220048	1.09582E-58	2.86797E-57	positive
PRJNA729271	stage_1D_vs_E10	ordinary_muscle_development	ENSGALG00010028642	APH1A	0.112757658	0.139214756	0.809954784	0.417966163	0.467290362	positive
PRJNA729271	stage_1D_vs_E10	ordinary_muscle_development	ENSGALG00010028649	ENSGALG00010028649	0.388682754	0.171925936	2.260756938	0.023774312	0.03329874	positive
PRJNA729271	stage_1D_vs_E10	ordinary_muscle_development	ENSGALG00010028658	PTPA	-0.584602752	0.082067349	-7.12345117	1.05258E-12	3.63264E-12	negative
PRJNA729271	stage_1D_vs_E10	ordinary_muscle_development	ENSGALG00010028662	FCRL4	0.324123918	0.224034695	1.446757692	0.1479648	0.182656568	positive
PRJNA729271	stage_1D_vs_E10	ordinary_muscle_development	ENSGALG00010028669	ZBTB34	0.639043889	0.110439779	5.786356102	7.19296E-09	1.90398E-08	positive
PRJNA729271	stage_1D_vs_E10	ordinary_muscle_development	ENSGALG00010028671	CADM3	3.855651766	0.211898039	18.1957879	5.5732E-74	2.28202E-72	positive
PRJNA729271	stage_1D_vs_E10	ordinary_muscle_development	ENSGALG00010028675	ZBTB43	0.269585399	0.069826307	3.860799904	0.000113016	0.000208738	positive
PRJNA729271	stage_1D_vs_E10	ordinary_muscle_development	ENSGALG00010028678	ENSGALG00010028678	0.596659134	0.369280872	1.615732576	0.106152145	0.134335196	positive
PRJNA729271	stage_1D_vs_E10	ordinary_muscle_development	ENSGALG00010028686	TAGLN2	0.119155823	0.119497703	0.997139024	0.318697033	0.36668626	positive
PRJNA729271	stage_1D_vs_E10	ordinary_muscle_development	ENSGALG00010028689	CFAP45	-0.36961665	0.648299839	-0.570132256	0.568587999	0.613847423	negative
PRJNA729271	stage_1D_vs_E10	ordinary_muscle_development	ENSGALG00010028692	RALGPS1	0.729343521	0.10965486	6.651264895	2.90585E-11	9.11891E-11	positive
PRJNA729271	stage_1D_vs_E10	ordinary_muscle_development	ENSGALG00010028695	VSIG8	0.836733765	0.954021626	0.877059537	0.380454301	0.429457771	positive
PRJNA729271	stage_1D_vs_E10	ordinary_muscle_development	ENSGALG00010028700	ANGPTL2	1.918684627	0.197508141	9.714458457	2.61636E-22	1.58281E-21	positive
PRJNA729271	stage_1D_vs_E10	ordinary_muscle_development	ENSGALG00010028707	ENSGALG00010028707	-4.481834392	1.23336907	-3.633814485	0.000279262	0.000493827	negative
PRJNA729271	stage_1D_vs_E10	ordinary_muscle_development	ENSGALG00010028712	FBXW2	-0.024893061	0.084627814	-0.294147508	0.768645189	0.798248775	negative
PRJNA729271	stage_1D_vs_E10	ordinary_muscle_development	ENSGALG00010028726	SLC2A8	0.126756174	0.111561849	1.13619642	0.255874369	0.300705284	positive
PRJNA729271	stage_1D_vs_E10	ordinary_muscle_development	ENSGALG00010028727	ENSGALG00010028727	-0.665246333	0.176784947	-3.763025895	0.00016787	0.000304616	negative
PRJNA729271	stage_1D_vs_E10	ordinary_muscle_development	ENSGALG00010028734	ENSGALG00010028734	4.275844888	0.35368197	12.08951898	1.19987E-33	1.23174E-32	positive
PRJNA729271	stage_1D_vs_E10	ordinary_muscle_development	ENSGALG00010028735	ENSGALG00010028735	-0.558041836	0.335560014	-1.663016488	0.096309135	0.12283334	negative
PRJNA729271	stage_1D_vs_E10	ordinary_muscle_development	ENSGALG00010028738	MIDN	-0.405201224	0.18403159	-2.201802549	0.027679259	0.038323704	negative
PRJNA729271	stage_1D_vs_E10	ordinary_muscle_development	ENSGALG00010028739	GARNL3	-0.283925965	0.07754497	-3.661436249	0.000250805	0.000445517	negative
PRJNA729271	stage_1D_vs_E10	ordinary_muscle_development	ENSGALG00010028740	DAZAP1	-0.487287518	0.106060602	-4.594425358	4.33943E-06	9.14644E-06	negative
PRJNA729271	stage_1D_vs_E10	ordinary_muscle_development	ENSGALG00010028742	ENSGALG00010028742	0.072033479	0.140939958	0.511093373	0.609285676	0.6519131	positive
PRJNA729271	stage_1D_vs_E10	ordinary_muscle_development	ENSGALG00010028743	ARRDC1	1.334881804	0.185871377	7.181750232	6.88246E-13	2.41027E-12	positive
PRJNA729271	stage_1D_vs_E10	ordinary_muscle_development	ENSGALG00010028744	HOMER3	-1.14045559	0.20899651	-5.456816444	4.84747E-08	1.20007E-07	negative
PRJNA729271	stage_1D_vs_E10	ordinary_muscle_development	ENSGALG00010028745	ENSGALG00010028745	0.619595629	0.137221181	4.515306035	6.32254E-06	1.31542E-05	positive
PRJNA729271	stage_1D_vs_E10	ordinary_muscle_development	ENSGALG00010028746	MYDGF	-0.632006992	0.124142327	-5.09098714	3.56204E-07	8.22424E-07	negative
PRJNA729271	stage_1D_vs_E10	ordinary_muscle_development	ENSGALG00010028747	ENSGALG00010028747	-2.353256734	0.186257745	-12.63441012	1.36413E-36	1.57555E-35	negative
PRJNA729271	stage_1D_vs_E10	ordinary_muscle_development	ENSGALG00010028748	PIAS3	1.089633302	0.196236604	5.552650626	2.8137E-08	7.07452E-08	positive
PRJNA729271	stage_1D_vs_E10	ordinary_muscle_development	ENSGALG00010028750	OTUD7B	0.300569264	0.120082328	2.503026621	0.012313628	0.017990731	positive
PRJNA729271	stage_1D_vs_E10	ordinary_muscle_development	ENSGALG00010028751	EHMT1	-0.276741727	0.095113167	-2.909604784	0.003618861	0.005654348	negative
PRJNA729271	stage_1D_vs_E10	ordinary_muscle_development	ENSGALG00010028752	MTMR11	1.834876181	0.132631795	13.83436139	1.58135E-43	2.34133E-42	positive
PRJNA729271	stage_1D_vs_E10	ordinary_muscle_development	ENSGALG00010028753	SV2A	3.512194068	0.60902802	5.766884203	8.07505E-09	2.12776E-08	positive
PRJNA729271	stage_1D_vs_E10	ordinary_muscle_development	ENSGALG00010028756	ENSGALG00010028756	-4.197634241	0.814426945	-5.154095483	2.54858E-07	5.95449E-07	negative
PRJNA729271	stage_1D_vs_E10	ordinary_muscle_development	ENSGALG00010028757	MAT2A	-2.843486573	1.366069393	-2.081059613	0.037387285	0.050837589	negative
PRJNA729271	stage_1D_vs_E10	ordinary_muscle_development	ENSGALG00010028760	SSR2	-0.071477146	0.097303975	-0.734575813	0.462597873	0.511429804	negative
PRJNA729271	stage_1D_vs_E10	ordinary_muscle_development	ENSGALG00010028761	CTSS	0.685791346	0.238386136	2.876808846	0.004017189	0.006241066	positive
PRJNA729271	stage_1D_vs_E10	ordinary_muscle_development	ENSGALG00010028762	CACNA1B	-1.898126188	0.604301587	-3.141024662	0.001683579	0.002736736	negative
PRJNA729271	stage_1D_vs_E10	ordinary_muscle_development	ENSGALG00010028763	CTSK	0.598799946	0.217553404	2.752427379	0.005915526	0.009011619	positive
PRJNA729271	stage_1D_vs_E10	ordinary_muscle_development	ENSGALG00010028764	ARNT	0.313946087	0.125394088	2.503675351	0.012291076	0.017961402	positive
PRJNA729271	stage_1D_vs_E10	ordinary_muscle_development	ENSGALG00010028768	NTNG2	-0.881187954	0.351781703	-2.504928332	0.012247621	0.017901509	negative
PRJNA729271	stage_1D_vs_E10	ordinary_muscle_development	ENSGALG00010028771	SETX	0.14991804	0.071697345	2.090984528	0.036529451	0.049755158	positive
PRJNA729271	stage_1D_vs_E10	ordinary_muscle_development	ENSGALG00010028772	TTF1	-0.009051036	0.071774094	-0.126104495	0.899649209	0.914575479	negative
PRJNA729271	stage_1D_vs_E10	ordinary_muscle_development	ENSGALG00010028782	MLLT11	-0.011433382	0.255812278	-0.044694422	0.96435088	0.969568288	negative
PRJNA729271	stage_1D_vs_E10	ordinary_muscle_development	ENSGALG00010028783	DDX31	-0.129315102	0.073191602	-1.76680246	0.077261317	0.100125416	negative
PRJNA729271	stage_1D_vs_E10	ordinary_muscle_development	ENSGALG00010028786	GTF3C4	-0.038771955	0.064570136	-0.60046265	0.548197946	0.59419197	negative
PRJNA729271	stage_1D_vs_E10	ordinary_muscle_development	ENSGALG00010028800	ENSGALG00010028800	0.581458989	0.403221572	1.442033437	0.149292941	0.184201667	positive
PRJNA729271	stage_1D_vs_E10	ordinary_muscle_development	ENSGALG00010028806	SPACA9	0.031450968	0.435392141	0.072235956	0.942414131	0.951141403	positive
PRJNA729271	stage_1D_vs_E10	ordinary_muscle_development	ENSGALG00010028818	TSC1	1.286827323	0.116690724	11.02767454	2.81038E-28	2.22604E-27	positive
PRJNA729271	stage_1D_vs_E10	ordinary_muscle_development	ENSGALG00010028823	GFI1B	0.715841522	0.310757058	2.303540671	0.021248442	0.029972378	positive
PRJNA729271	stage_1D_vs_E10	ordinary_muscle_development	ENSGALG00010028826	CEL	5.496214534	2.732594338	2.011353993	0.044288079	0.059539576	positive
PRJNA729271	stage_1D_vs_E10	ordinary_muscle_development	ENSGALG00010028829	GTF3C5	-0.52956718	0.114028788	-4.644153351	3.41474E-06	7.28798E-06	negative
PRJNA729271	stage_1D_vs_E10	ordinary_muscle_development	ENSGALG00010028833	GBGT1	1.571952363	0.167950769	9.359602046	8.00376E-21	4.48452E-20	positive
PRJNA729271	stage_1D_vs_E10	ordinary_muscle_development	ENSGALG00010028849	ENSGALG00010028849	2.197661221	0.334280586	6.574301083	4.88822E-11	1.5127E-10	positive
PRJNA729271	stage_1D_vs_E10	ordinary_muscle_development	ENSGALG00010028853	ENSGALG00010028853	1.34022889	1.440009812	0.930708165	0.352004545	0.400935555	positive
PRJNA729271	stage_1D_vs_E10	ordinary_muscle_development	ENSGALG00010028854	RPL7A	0.262068	0.140334999	1.867445765	0.061839364	0.081265782	positive
PRJNA729271	stage_1D_vs_E10	ordinary_muscle_development	ENSGALG00010028855	SURF6	-0.430223535	0.169043694	-2.545043389	0.010926416	0.016079025	negative
PRJNA729271	stage_1D_vs_E10	ordinary_muscle_development	ENSGALG00010028856	ETV3	0.105699929	0.10911447	0.968706798	0.3326915	0.381244627	positive
PRJNA729271	stage_1D_vs_E10	ordinary_muscle_development	ENSGALG00010028857	MED22	0.370499242	0.078148563	4.740960414	2.12707E-06	4.62873E-06	positive
PRJNA729271	stage_1D_vs_E10	ordinary_muscle_development	ENSGALG00010028858	ENSGALG00010028858	0.573215067	0.457068704	1.25411139	0.209801521	0.251100078	positive
PRJNA729271	stage_1D_vs_E10	ordinary_muscle_development	ENSGALG00010028859	SURF1	0.697344502	0.108530265	6.425345995	1.3157E-10	3.95173E-10	positive
PRJNA729271	stage_1D_vs_E10	ordinary_muscle_development	ENSGALG00010028860	SURF2	0.219208319	0.172103889	1.273697648	0.20277059	0.243227384	positive
PRJNA729271	stage_1D_vs_E10	ordinary_muscle_development	ENSGALG00010028861	SURF4	-0.363085203	0.074656954	-4.863380869	1.15397E-06	2.56429E-06	negative
PRJNA729271	stage_1D_vs_E10	ordinary_muscle_development	ENSGALG00010028862	ENSGALG00010028862	-0.591286411	1.406930892	-0.420266848	0.674290525	0.712534896	negative
PRJNA729271	stage_1D_vs_E10	ordinary_muscle_development	ENSGALG00010028865	STKLD1	0.999895994	0.16663852	6.00038929	1.96845E-09	5.44102E-09	positive
PRJNA729271	stage_1D_vs_E10	ordinary_muscle_development	ENSGALG00010028866	HORMAD1	-1.172881313	0.496218875	-2.363637039	0.018096532	0.025741829	negative
PRJNA729271	stage_1D_vs_E10	ordinary_muscle_development	ENSGALG00010028867	REXO4	-0.395327991	0.117413474	-3.366972962	0.000759981	0.001284657	negative
PRJNA729271	stage_1D_vs_E10	ordinary_muscle_development	ENSGALG00010028868	ENSGALG00010028868	0.370522479	4.241825273	0.087349774	0.930393491	0.940932079	positive
PRJNA729271	stage_1D_vs_E10	ordinary_muscle_development	ENSGALG00010028869	ADAMTS13	4.141936441	0.162198282	25.53625349	7.8046E-144	1.3003E-141	positive
PRJNA729271	stage_1D_vs_E10	ordinary_muscle_development	ENSGALG00010028870	CACFD1	-2.430212233	0.228339458	-10.64297978	1.88018E-26	1.37435E-25	negative
PRJNA729271	stage_1D_vs_E10	ordinary_muscle_development	ENSGALG00010028871	PIP5K1A	0.046352404	0.108037013	0.429041892	0.667892738	0.706598441	positive
PRJNA729271	stage_1D_vs_E10	ordinary_muscle_development	ENSGALG00010028872	SLC2A6	0.447950919	0.355002519	1.26182462	0.207011901	0.247966077	positive
PRJNA729271	stage_1D_vs_E10	ordinary_muscle_development	ENSGALG00010028873	TMEM8C	-1.584280365	0.282659847	-5.60490066	2.08374E-08	5.30358E-08	negative
PRJNA729271	stage_1D_vs_E10	ordinary_muscle_development	ENSGALG00010028874	CRPL1	3.076248463	0.487069868	6.315825846	2.68723E-10	7.91211E-10	positive
PRJNA729271	stage_1D_vs_E10	ordinary_muscle_development	ENSGALG00010028875	DUSP23	0.994131894	0.119878297	8.292842967	1.10573E-16	4.92097E-16	positive
PRJNA729271	stage_1D_vs_E10	ordinary_muscle_development	ENSGALG00010028876	ADAMTSL2	3.360071961	0.19459509	17.26699252	8.33757E-67	2.72806E-65	positive
PRJNA729271	stage_1D_vs_E10	ordinary_muscle_development	ENSGALG00010028877	FAM163B	-3.237085871	0.632638103	-5.116805102	3.10754E-07	7.20932E-07	negative
PRJNA729271	stage_1D_vs_E10	ordinary_muscle_development	ENSGALG00010028878	IGSF9	0.835581733	0.155821978	5.362412576	8.21177E-08	1.99647E-07	positive
PRJNA729271	stage_1D_vs_E10	ordinary_muscle_development	ENSGALG00010028881	SARDH	-1.022327567	0.230859694	-4.428350176	9.49566E-06	1.94489E-05	negative
PRJNA729271	stage_1D_vs_E10	ordinary_muscle_development	ENSGALG00010028884	VAV2	-0.826537233	0.129327819	-6.391024306	1.64778E-10	4.91453E-10	negative

PRJNA729271	stage_1D_vs_E10	ordinary_muscle_development	ENSGALG00010028885	BRD3	-0.005812275	0.140292204	-0.041429781	0.966953271	0.971712955	negative
PRJNA729271	stage_1D_vs_E10	ordinary_muscle_development	ENSGALG00010028886	ENSGALG00010028886	-0.098557521	1.859824927	-0.052992903	0.957737563	0.964323838	negative
PRJNA729271	stage_1D_vs_E10	ordinary_muscle_development	ENSGALG00010028887	SF3B4	0.187484406	0.173641149	1.079723366	0.280265386	0.326405814	positive
PRJNA729271	stage_1D_vs_E10	ordinary_muscle_development	ENSGALG00010028888	WDR5B	-1.049169941	0.081707695	-12.84052796	9.72093E-38	1.17519E-36	negative
PRJNA729271	stage_1D_vs_E10	ordinary_muscle_development	ENSGALG00010028889	FUBP3	-0.722662841	0.08246949	-8.762790273	1.90476E-18	9.35597E-18	negative
PRJNA729271	stage_1D_vs_E10	ordinary_muscle_development	ENSGALG00010028890	ENSGALG00010028890	-4.35351371	0.298353143	-14.5918145	3.16662E-48	5.67369E-47	negative
PRJNA729271	stage_1D_vs_E10	ordinary_muscle_development	ENSGALG00010028891	SLC31A2	0.742555158	0.237972808	3.120336163	0.001806448	0.002925965	positive
PRJNA729271	stage_1D_vs_E10	ordinary_muscle_development	ENSGALG00010028892	NIBAN2	-0.023682075	0.1408083	-0.168186643	0.866436448	0.886121239	negative
PRJNA729271	stage_1D_vs_E10	ordinary_muscle_development	ENSGALG00010028893	STXBP1	1.397643417	0.196292879	7.120194203	1.07775E-12	3.7151E-12	positive
PRJNA729271	stage_1D_vs_E10	ordinary_muscle_development	ENSGALG00010028894	AKNA	-1.492402565	0.159781165	-9.340290939	9.60698E-21	5.35795E-20	negative
PRJNA729271	stage_1D_vs_E10	ordinary_muscle_development	ENSGALG00010028895	WHRN	-5.371136485	0.936368514	-5.736135301	9.68613E-09	2.52928E-08	negative
PRJNA729271	stage_1D_vs_E10	ordinary_muscle_development	ENSGALG00010028896	ATP6V1G1	0.26962875	0.098933918	2.725341865	0.006423494	0.009735314	positive
PRJNA729271	stage_1D_vs_E10	ordinary_muscle_development	ENSGALG00010028897	TMEM268	-0.688686427	0.285891446	-2.40890882	0.016000295	0.022955981	negative
PRJNA729271	stage_1D_vs_E10	ordinary_muscle_development	ENSGALG00010028898	TNFSF15	-1.659034376	0.252580445	-6.568340527	5.08791E-11	1.57214E-10	negative
PRJNA729271	stage_1D_vs_E10	ordinary_muscle_development	ENSGALG00010028899	TNFSF8	-3.202373993	0.972617568	-3.292531512	0.000992898	0.001655781	negative
PRJNA729271	stage_1D_vs_E10	ordinary_muscle_development	ENSGALG00010028900	TNC	-3.857618955	0.550173841	-7.011636444	2.35547E-12	7.96048E-12	negative
PRJNA729271	stage_1D_vs_E10	ordinary_muscle_development	ENSGALG00010028901	PAPPA	0.330702126	0.110367532	2.99637149	0.002732134	0.004327645	positive
PRJNA729271	stage_1D_vs_E10	ordinary_muscle_development	ENSGALG00010028902	ENSGALG00010028902	-0.577854119	0.418360895	-1.381233587	0.167207151	0.204309403	negative
PRJNA729271	stage_1D_vs_E10	ordinary_muscle_development	ENSGALG00010028903	ASTN2	-3.720549416	0.237489837	-15.66614158	2.5776E-55	6.01647E-54	negative
PRJNA729271	stage_1D_vs_E10	ordinary_muscle_development	ENSGALG00010028904	TRIM32	-0.31326339	0.081551516	-3.841294523	0.000122387	0.000225184	negative
PRJNA729271	stage_1D_vs_E10	ordinary_muscle_development	ENSGALG00010028905	COLGALT1	0.200245029	0.085786621	2.334222122	0.019584094	0.027746207	positive
PRJNA729271	stage_1D_vs_E10	ordinary_muscle_development	ENSGALG00010028909	ENSGALG00010028909	-0.971015912	0.134235866	-7.233654742	4.70166E-13	1.66872E-12	negative
PRJNA729271	stage_1D_vs_E10	ordinary_muscle_development	ENSGALG00010028911	LMX1B	-4.201214832	0.563858025	-7.450838065	9.27491E-14	3.43924E-13	negative
PRJNA729271	stage_1D_vs_E10	ordinary_muscle_development	ENSGALG00010028912	EXFABP	-0.031190727	0.498877821	-0.062521775	0.950147322	0.957812465	negative
PRJNA729271	stage_1D_vs_E10	ordinary_muscle_development	ENSGALG00010028914	TLR4	0.120208758	0.233259107	0.515344327	0.606312429	0.649028776	positive
PRJNA729271	stage_1D_vs_E10	ordinary_muscle_development	ENSGALG00010028915	ADGRD2	-0.08429714	0.4989706	-0.168942098	0.865842186	0.885638451	negative
PRJNA729271	stage_1D_vs_E10	ordinary_muscle_development	ENSGALG00010028917	NR6A1	0.15672287	0.125956685	1.244260039	0.213403873	0.255095591	positive
PRJNA729271	stage_1D_vs_E10	ordinary_muscle_development	ENSGALG00010028918	AGPAT2	3.322110416	0.18269062	18.18435134	6.8663E-74	2.80358E-72	positive
PRJNA729271	stage_1D_vs_E10	ordinary_muscle_development	ENSGALG00010028919	EGFL7	1.35779101	0.168514403	8.057418149	7.79227E-16	3.29971E-15	positive
PRJNA729271	stage_1D_vs_E10	ordinary_muscle_development	ENSGALG00010028921	MRPL41	-0.371691295	0.121840245	-3.050644675	0.002283506	0.003648124	negative
PRJNA729271	stage_1D_vs_E10	ordinary_muscle_development	ENSGALG00010028923	ENSGALG00010028923	1.599435636	0.181828301	8.796406444	1.41267E-18	6.98625E-18	positive
PRJNA729271	stage_1D_vs_E10	ordinary_muscle_development	ENSGALG00010028924	TMEM203	-0.071847155	0.120626601	-0.595616176	0.551431668	0.597339662	negative
PRJNA729271	stage_1D_vs_E10	ordinary_muscle_development	ENSGALG00010028925	ENSGALG00010028925	0.983747564	0.331850227	2.9644324	0.003032419	0.004778746	positive
PRJNA729271	stage_1D_vs_E10	ordinary_muscle_development	ENSGALG00010028926	ENSGALG00010028926	-1.418382827	0.640728218	-2.213704325	0.026849125	0.037254599	negative
PRJNA729271	stage_1D_vs_E10	ordinary_muscle_development	ENSGALG00010029140	NPDC1	0.810733522	0.189990538	4.267231052	1.97914E-05	3.92229E-05	positive
PRJNA729271	stage_1D_vs_E10	ordinary_muscle_development	ENSGALG00010029141	C5	-1.748879678	0.577064777	-3.030647074	0.002440303	0.003885346	negative
PRJNA729271	stage_1D_vs_E10	ordinary_muscle_development	ENSGALG00010029142	ENSGALG00010029142	0.936176938	0.180372558	5.190240393	2.10023E-07	4.94121E-07	positive
PRJNA729271	stage_1D_vs_E10	ordinary_muscle_development	ENSGALG00010029143	SH2D3C	-0.072195775	0.10916701	-0.661333263	0.508398614	0.556378854	negative
PRJNA729271	stage_1D_vs_E10	ordinary_muscle_development	ENSGALG00010029144	ENSGALG00010029144	4.844907539	0.219037596	22.11906827	2.0716E-108	1.7664E-106	positive
PRJNA729271	stage_1D_vs_E10	ordinary_muscle_development	ENSGALG00010029145	RC3H2	1.098153819	0.133146138	8.247733193	1.61427E-16	7.12078E-16	positive
PRJNA729271	stage_1D_vs_E10	ordinary_muscle_development	ENSGALG00010029146	NEK6	-1.877592434	0.059305803	-31.65950635	5.611E-220	3.6969E-217	negative
PRJNA729271	stage_1D_vs_E10	ordinary_muscle_development	ENSGALG00010029147	RABL6	-0.240875712	0.103023216	-2.338072151	0.019383506	0.02747276	negative
PRJNA729271	stage_1D_vs_E10	ordinary_muscle_development	ENSGALG00010029148	RALGDS	0.136268955	0.118653953	1.148456932	0.250779983	0.295323213	positive
PRJNA729271	stage_1D_vs_E10	ordinary_muscle_development	ENSGALG00010029149	ENSGALG00010029149	-0.722446464	0.209736855	-3.444537502	0.000572037	0.000977331	negative
PRJNA729271	stage_1D_vs_E10	ordinary_muscle_development	ENSGALG00010029150	NARF	-0.71132918	0.123660673	-5.752266767	8.80547E-09	2.30847E-08	negative
PRJNA729271	stage_1D_vs_E10	ordinary_muscle_development	ENSGALG00010029151	CYBC1	-1.118077259	0.114619353	-9.754698775	1.76123E-22	1.074E-21	negative
PRJNA729271	stage_1D_vs_E10	ordinary_muscle_development	ENSGALG00010029152	HEXD	-0.40363087	0.129394596	-3.119379644	0.001812323	0.002933514	negative
PRJNA729271	stage_1D_vs_E10	ordinary_muscle_development	ENSGALG00010029153	OGFOD3	-0.670742553	0.077817318	-8.619450882	6.7276E-18	3.20778E-17	negative
PRJNA729271	stage_1D_vs_E10	ordinary_muscle_development	ENSGALG00010029154	UTS2R	-0.488575627	0.141746818	-3.44681901	0.000567228	0.000969916	negative
PRJNA729271	stage_1D_vs_E10	ordinary_muscle_development	ENSGALG00010029155	ENSGALG00010029155	0.022943573	0.579802697	0.039571346	0.968434872	0.972797191	positive
PRJNA729271	stage_1D_vs_E10	ordinary_muscle_development	ENSGALG00010029156	ENSGALG00010029156	-7.422589715	1.276113028	-5.816561347	6.00705E-09	1.6E-08	negative
PRJNA729271	stage_1D_vs_E10	ordinary_muscle_development	ENSGALG00010029158	CD7	-2.471041767	0.52670769	-4.691486023	2.71228E-06	5.84341E-06	negative
PRJNA729271	stage_1D_vs_E10	ordinary_muscle_development	ENSGALG00010029159	SSC4D	-4.662371749	1.067842225	-4.366161628	1.26449E-05	2.55953E-05	negative
PRJNA729271	stage_1D_vs_E10	ordinary_muscle_development	ENSGALG00010029160	DUS1L	0.217797497	0.092184198	2.362633754	0.018145592	0.025804018	positive
PRJNA729271	stage_1D_vs_E10	ordinary_muscle_development	ENSGALG00010029162	ELN	3.019367292	0.310790939	9.715107217	2.59975E-22	1.57408E-21	positive
PRJNA729271	stage_1D_vs_E10	ordinary_muscle_development	ENSGALG00010029163	ANKFY1	-0.874249571	0.094519149	-9.249443992	2.25663E-20	1.22877E-19	negative
PRJNA729271	stage_1D_vs_E10	ordinary_muscle_development	ENSGALG00010029164	CYB5D2	0.122743753	0.082958345	1.479582952	0.138984579	0.172278945	positive
PRJNA729271	stage_1D_vs_E10	ordinary_muscle_development	ENSGALG00010029165	RAC3	-0.312837798	0.120453082	-2.597175531	0.009399388	0.013948006	negative
PRJNA729271	stage_1D_vs_E10	ordinary_muscle_development	ENSGALG00010029166	ZZEF1	0.608391773	0.123734607	4.916908753	8.79215E-07	1.97218E-06	positive
PRJNA729271	stage_1D_vs_E10	ordinary_muscle_development	ENSGALG00010029167	ENSGALG00010029167	0.802275809	0.126700037	6.332088204	2.41865E-10	7.14602E-10	positive
PRJNA729271	stage_1D_vs_E10	ordinary_muscle_development	ENSGALG00010029168	RFNG	0.706105328	0.079634401	8.866837959	7.52523E-19	3.77303E-18	positive
PRJNA729271	stage_1D_vs_E10	ordinary_muscle_development	ENSGALG00010029169	SBDS	0.88527897	0.077619587	11.40535531	3.93169E-30	3.42076E-29	positive
PRJNA729271	stage_1D_vs_E10	ordinary_muscle_development	ENSGALG00010029170	DCXR	-0.086109719	0.139982863	-0.615144719	0.538459152	0.584904482	negative
PRJNA729271	stage_1D_vs_E10	ordinary_muscle_development	ENSGALG00010029171	ENSGALG00010029171	0.0406529	0.146329607	0.277817327	0.781152589	0.809697281	positive
PRJNA729271	stage_1D_vs_E10	ordinary_muscle_development	ENSGALG00010029172	LRRC45	-0.125630477	0.073393916	-1.711728775	0.086946663	0.111688398	negative
PRJNA729271	stage_1D_vs_E10	ordinary_muscle_development	ENSGALG00010029173	ZNHIT3	-0.254131589	0.136075384	-1.867579431	0.061820716	0.081248642	negative
PRJNA729271	stage_1D_vs_E10	ordinary_muscle_development	ENSGALG00010029174	CALN1	-5.610422542	0.361173614	-15.53386604	2.0466E-54	4.59219E-53	negative
PRJNA729271	stage_1D_vs_E10	ordinary_muscle_development	ENSGALG00010029175	CENPX	-0.146481218	0.129685318	-1.129512733	0.258681601	0.30373348	negative
PRJNA729271	stage_1D_vs_E10	ordinary_muscle_development	ENSGALG00010029176	ENSGALG00010029176	-0.313902499	0.512672388	-0.612286727	0.54034807	0.58669253	negative
PRJNA729271	stage_1D_vs_E10	ordinary_muscle_development	ENSGALG00010029177	ASPSCR1	0.337815416	0.080277714	4.208084623	2.57544E-05	5.05156E-05	positive
PRJNA729271	stage_1D_vs_E10	ordinary_muscle_development	ENSGALG00010029178	NOTUM	-0.05772051	0.206849218	-0.279046306	0.780209288	0.808893043	negative
PRJNA729271	stage_1D_vs_E10	ordinary_muscle_development	ENSGALG00010029179	PIGW	0.257990414	0.148171378	1.741162272	0.081655138	0.105395479	positive
PRJNA729271	stage_1D_vs_E10	ordinary_muscle_development	ENSGALG00010029180	CA4	6.205187326	0.427275116	14.52269766	8.70172E-48	1.53444E-46	positive
PRJNA729271	stage_1D_vs_E10	ordinary_muscle_development	ENSGALG00010029181	MYO19	0.412595801	0.101834745	4.051621089	5.0864E-05	9.71375E-05	positive
PRJNA729271	stage_1D_vs_E10	ordinary_muscle_development	ENSGALG00010029182	HEATR6	-0.479132958	0.067281512	-7.121316799	1.06901E-12	3.68584E-12	negative
PRJNA729271	stage_1D_vs_E10	ordinary_muscle_development	ENSGALG00010029183	ENSGALG00010029183	0.698172856	0.13921448	5.015087932	5.30092E-07	1.20984E-06	positive
PRJNA729271	stage_1D_vs_E10	ordinary_muscle_development	ENSGALG00010029184	DYNLL2	0.017489057	0.095849768	0.182463222	0.855219214	0.87594704	positive
PRJNA729271	stage_1D_vs_E10	ordinary_muscle_development	ENSGALG00010029186	ZNF302	0.045813808	0.14042443	0.326252406	0.744233394	0.776705526	positive
PRJNA729271	stage_1D_vs_E10	ordinary_muscle_development	ENSGALG00010029187	SRSF1	-1.177039425	0.052354107	-22.48227493	6.1885E-112	5.8249E-110	negative
PRJNA729271	stage_1D_vs_E10	ordinary_muscle_development	ENSGALG00010029188	ENSGALG00010029188	0.449641526	0.256648978	1.751970842	0.079778822	0.103203393	positive
PRJNA729271	stage_1D_vs_E10	ordinary_muscle_development	ENSGALG00010029189	ENSGALG00010029189	0.275271444	0.344260767	0.799601555	0.423941687	0.4734598	positive
PRJNA729271	stage_1D_vs_E10	ordinary_muscle_development	ENSGALG00010029190	DHRS7C	6.424521753	0.162789484	39.46521363	0	0	positive
PRJNA729271	stage_1D_vs_E10	ordinary_muscle_development	ENSGALG00010029192	DTX2	1.165581085	0.10007658	11.64689166	2.37982E-31	2.19297E-30	positive
PRJNA729271	stage_1D_vs_E10	ordinary_muscle_development	ENSGALG00010029194	DHRS11	-1.641320696	0.132428601	-12.39400461	2.81621E-35	3.09719E-34	negative
PRJNA729271	stage_1D_vs_E10	ordinary_muscle_development	ENSGALG00010029196	MRM1	-0.324578705	0.098033882	-3.310882907	0.000930021	0.001556478	negative
PRJNA729271	stage_1D_vs_E10	ordinary_muscle_development	ENSGALG00010029197	LHX1	2.475682537	0.587959737	4.210632772	2.54656E-05	4.99762E-05	positive
PRJNA729271	stage_1D_vs_E10	ordinary_muscle_development	ENSGALG00010029198	PPM1D	0.194185766	0.174607144	1.112129559	0.266082449	0.311288547	positive
PRJNA729271	stage_1D_vs_E10	ordinary_muscle_development	ENSGALG00010029199	APPBP2	-0.217221892	0.089568136	-2.425213944	0.015299368	0.022032202	negative
PRJNA729271	stage_1D_vs_E10	ordinary_muscle_development	ENSGALG00010029200	GAS7	1.695598172	0.124185463	13.65375732	1.91732E-42	2.74079E-41	positive
PRJNA729271	stage_1D_vs_E10	ordinary_muscle_development	ENSGALG00010029201	PIGS	-0.527531492	0.109869781	-4.801424797	1.57541E-06	3.46045E-06	negative
PRJNA729271	stage_1D_vs_E10	ordinary_muscle_development	ENSGALG00010029202	PSMD12	-0.266710684	0.14333611	-1.860736176	0.062781443	0.082414147	negative
PRJNA729271	stage_1D_vs_E10	ordinary_muscle_development	ENSGALG00010029203	ALDOC	-1.773936588	0.173278891	-10.23746505	1.3472E-24	9.04477E-24	negative
PRJNA729271	stage_1D_vs_E10	ordinary_muscle_development	ENSGALG00010029204	HELZ	0.746792372	0.097131957	7.688431263	1.4895E-14	5.7945E-14	positive
PRJNA729271	stage_1D_vs_E10	ordinary_muscle_development	ENSGALG00010029205	CACNG1	4.443798097	0.114037228	38.96795956	0	0	positive
PRJNA729271	stage_1D_vs_E10	ordinary_muscle_development	ENSGALG00010029206	CACNG4	-1.616779556	0.325184462	-4.971884409	6.63052E-07	1.50171E-06	negative
PRJNA729271	stage_1D_vs_E10	ordinary_muscle_development	ENSGALG00010029207	SPAG5	-1.253225986	0.220292993	-5.688905339	1.27856E-08	3.30942E-08	negative
PRJNA729271	stage_1D_vs_E10	ordinary_muscle_development	ENSGALG00010029208	CACNG5	0.311687443	0.241770758	1.289185861	0.197333479	0.237333952	positive
PRJNA729271	stage_1D_vs_E10	ordinary_muscle_development	ENSGALG00010029209	PRKCA	0.823918875	0.096182274	8.56622368	1.06934E-17	5.0374E-17	positive
PRJNA729271	stage_1D_vs_E10	ordinary_muscle_development	ENSGALG00010029210	RSKR	0.572011556	0.307408515	1.860753778	0.062778956	0.082414147	positive
PRJNA729271	stage_1D_vs_E10	ordinary_muscle_development	ENSGALG00010029211	SDF2	-1.008970834	0.168403502	-5.991388664	2.08057E-09	5.73997E-09	negative
PRJNA729271	stage_1D_vs_E10	ordinary_muscle_development	ENSGALG00010029212	SUPT6H	0.139662174	0.113531987	1.230157048	0.218638301	0.260943777	positive
PRJNA729271	stage_1D_vs_E10	ordinary_muscle_development	ENSGALG00010029213	APOH	0.4934815	1.557208842	0.316901296	0.751318499	0.78336654	positive
PRJNA729271	stage_1D_vs_E10	ordinary_muscle_development	ENSGALG00010029214	ENSGALG00010029214	4.468424487	0.377527192	11.8360335	2.54193E-32	2.45471E-31	positive
PRJNA729271	stage_1D_vs_E10	ordinary_muscle_development	ENSGALG00010029215	ENSGALG00010029215	7.143961518	0.237026277	30.1399558	1.4524E-199	7.0176E-197	positive
PRJNA729271	stage_1D_vs_E10	ordinary_muscle_development	ENSGALG00010029216	RAB34	-1.828038307	0.13689054	-13.35401486	1.12233E-40	1.51755E-39	negative
PRJNA729271	stage_1D_vs_E10	ordinary_muscle_development	ENSGALG00010029217	CEP112	-0.712594259	0.090781659	-7.849539967	4.17566E-15	1.68652E-14	negative
PRJNA729271	stage_1D_vs_E10	ordinary_muscle_development	ENSGALG00010029218	RPL23A	0.785628442	0.150213906	5.230064671	1.69451E-07	4.02192E-07	positive
PRJNA729271	stage_1D_vs_E10	ordinary_muscle_development	ENSGALG00010029219	TLCD1	0.009021426	0.203263793	0.044382846	0.964599235	0.969750722	positive
PRJNA729271	stage_1D_vs_E10	ordinary_muscle_development	ENSGALG00010029220	NEK8	0.1233793	0.125923312	0.979797129	0.32718627	0.37547027	positive
PRJNA729271	stage_1D_vs_E10	ordinary_muscle_development	ENSGALG00010029221	AXIN2	0.16582447	0.117690489	1.408987866	0.158838756	0.194884694	positive
PRJNA729271	stage_1D_vs_E10	ordinary_muscle_development	ENSGALG00010029222	TRAF4	0.081257136	0.229201401	0.354522859	0.722947076	0.757818764	positive
PRJNA729271	stage_1D_vs_E10	ordinary_muscle_development	ENSGALG00010029223	NME2	-0.187596724	0.14584545	-1.286270666	0.198348616	0.238357088	negative
PRJNA729271	stage_1D_vs_E10	ordinary_muscle_development	ENSGALG00010029224	FAM222B	0.098976367	0.160068439	0.618337801	0.536352689	0.582791022	positive
PRJNA729271	stage_1D_vs_E10	ordinary_muscle_development	ENSGALG00010029226	MBTD1	-0.424537526	0.075271273	-5.640100232	1.69951E-08	4.35776E-08	negative
PRJNA729271	stage_1D_vs_E10	ordinary_muscle_development	ENSGALG00010029227	UTP18	-0.790971019	0.080511428	-9.824332234	8.84583E-23	5.46549E-22	negative
PRJNA729271	stage_1D_vs_E10	ordinary_muscle_development	ENSGALG00010029228	ERAL1	0.107695147	0.11828862	0.91044385	0.362588481	0.411586189	positive
PRJNA729271	stage_1D_vs_E10	ordinary_muscle_development	ENSGALG00010029229	CBX4	0.149976256	0.222056883	0.675395664	0.499424419	0.547591298	positive
PRJNA729271	stage_1D_vs_E10	ordinary_muscle_development	ENSGALG00010029230	PPM1E	-0.35349318	0.166356327	-2.124915747	0.033593644	0.046037616	negative
PRJNA729271	stage_1D_vs_E10	ordinary_muscle_development	ENSGALG00010029231	CBX8	-0.360432869	0.107154252	-3.363682361	0.0007691	0.001298859	negative
PRJNA729271	stage_1D_vs_E10	ordinary_muscle_development	ENSGALG00010029232	CA10	-2.346475538	0.385161833	-6.092180833	1.11383E-09	3.13311E-09	negative
PRJNA729271	stage_1D_vs_E10	ordinary_muscle_development	ENSGALG00010029233	CBX2	-2.533909376	0.162318467	-15.61072764	6.15312E-55	1.409E-53	negative
PRJNA729271	stage_1D_vs_E10	ordinary_muscle_development	ENSGALG00010029234	TOM1L1	-0.697115539	0.228798834	-3.046849174	0.002312537	0.003692468	negative
PRJNA729271	stage_1D_vs_E10	ordinary_muscle_development	ENSGALG00010029235	TRIM37	-0.303358396	0.073842435	-4.108185196	3.9878E-05	7.68455E-05	negative
PRJNA729271	stage_1D_vs_E10	ordinary_muscle_development	ENSGALG00010029237	COX11	-0.060002986	0.104152635	-0.57610627	0.564543353	0.609902058	negative
PRJNA729271	stage_1D_vs_E10	ordinary_muscle_development	ENSGALG00010029238	CAMKK1	0.170376472	0.244346367	0.697274421	0.485631072	0.53423839	positive
PRJNA729271	stage_1D_vs_E10	ordinary_muscle_development	ENSGALG00010029239	RBFOX3	-2.129618316	0.50347732	-4.229819757	2.33879E-05	4.6042E-05	negative
PRJNA729271	stage_1D_vs_E10	ordinary_muscle_development	ENSGALG00010029240	MIS12	-1.894793782	0.249282224	-7.600998401	2.93855E-14	1.12505E-13	negative
PRJNA729271	stage_1D_vs_E10	ordinary_muscle_development	ENSGALG00010029241	STXBP4	-1.660724241	0.095314951	-17.42354399	5.46827E-68	1.88272E-66	negative
PRJNA729271	stage_1D_vs_E10	ordinary_muscle_development	ENSGALG00010029242	DERL2	0.091876357	0.096912239	0.948036677	0.343110783	0.392099558	positive
PRJNA729271	stage_1D_vs_E10	ordinary_muscle_development	ENSGALG00010029243	NME1	-0.265087356	0.339615237	-0.780552009	0.435066027	0.484539536	negative
PRJNA729271	stage_1D_vs_E10	ordinary_muscle_development	ENSGALG00010029244	HLF	2.527411253	0.182033216	13.88434103	7.8822E-44	1.18396E-42	positive

PRJNA729271	stage_1D_vs_E10	ordinary_muscle_development	ENSGALG00010029245	SKA2	-1.378459951	0.115289473	-11.95651181	6.0031E-33	5.96811E-32	negative
PRJNA729271	stage_1D_vs_E10	ordinary_muscle_development	ENSGALG00010029246	DHX33	-1.151244095	0.135309957	-8.508199411	1.76655E-17	8.22028E-17	negative
PRJNA729271	stage_1D_vs_E10	ordinary_muscle_development	ENSGALG00010029247	CCDC92B	6.423583421	0.527538259	12.17652619	4.14566E-34	4.32001E-33	positive
PRJNA729271	stage_1D_vs_E10	ordinary_muscle_development	ENSGALG00010029248	C1QBP	0.002741897	0.081237426	0.033751644	0.973075196	0.976997296	positive
PRJNA729271	stage_1D_vs_E10	ordinary_muscle_development	ENSGALG00010029249	PRR11	-1.133678925	0.271377617	-4.177496053	2.94736E-05	5.75147E-05	negative
PRJNA729271	stage_1D_vs_E10	ordinary_muscle_development	ENSGALG00010029250	SPAG9	0.674516509	0.08714885	7.739821096	9.95568E-15	3.92034E-14	positive
PRJNA729271	stage_1D_vs_E10	ordinary_muscle_development	ENSGALG00010029251	ENGASE	-1.217137949	0.198369087	-6.135723893	8.47723E-10	2.40936E-09	negative
PRJNA729271	stage_1D_vs_E10	ordinary_muscle_development	ENSGALG00010029252	RPAIN	1.027340599	0.137959354	7.446690408	9.57109E-14	3.54617E-13	positive
PRJNA729271	stage_1D_vs_E10	ordinary_muscle_development	ENSGALG00010029253	NUP88	-0.383809413	0.085523223	-4.48778004	7.19692E-06	1.49091E-05	negative
PRJNA729271	stage_1D_vs_E10	ordinary_muscle_development	ENSGALG00010029254	MMD	0.009742292	0.118777282	0.082021509	0.934629609	0.944402662	positive
PRJNA729271	stage_1D_vs_E10	ordinary_muscle_development	ENSGALG00010029255	RAP1GAP2	1.673107073	0.183244669	9.130454296	6.82124E-20	3.62574E-19	positive
PRJNA729271	stage_1D_vs_E10	ordinary_muscle_development	ENSGALG00010029256	RABEP1	0.110162866	0.096594914	1.140462381	0.254093717	0.29878222	positive
PRJNA729271	stage_1D_vs_E10	ordinary_muscle_development	ENSGALG00010029257	YPEL2	0.275166301	0.161633697	1.702406772	0.088679142	0.11375258	positive
PRJNA729271	stage_1D_vs_E10	ordinary_muscle_development	ENSGALG00010029258	TMEM100	0.770250172	0.233845818	3.293837695	0.000988295	0.001648486	positive
PRJNA729271	stage_1D_vs_E10	ordinary_muscle_development	ENSGALG00010029259	PTRH2	-0.791028372	0.109236484	-7.241430203	4.43978E-13	1.57809E-12	negative
PRJNA729271	stage_1D_vs_E10	ordinary_muscle_development	ENSGALG00010029260	DHX40	-0.778915163	0.105487145	-7.383981821	1.53624E-13	5.60903E-13	negative
PRJNA729271	stage_1D_vs_E10	ordinary_muscle_development	ENSGALG00010029261	IFT22	-1.438501018	0.096482413	-14.90946346	2.86042E-50	5.57282E-49	negative
PRJNA729271	stage_1D_vs_E10	ordinary_muscle_development	ENSGALG00010029262	CLTC	-0.38373726	0.065073367	-5.896994043	3.70183E-09	1.00202E-08	negative
PRJNA729271	stage_1D_vs_E10	ordinary_muscle_development	ENSGALG00010029263	TOB1	2.30600635	0.188378506	12.24134533	1.86909E-34	1.98334E-33	positive
PRJNA729271	stage_1D_vs_E10	ordinary_muscle_development	ENSGALG00010029264	COL26A1	-6.002225036	0.181529512	-33.06473409	9.5537E-240	8.146E-237	negative
PRJNA729271	stage_1D_vs_E10	ordinary_muscle_development	ENSGALG00010029266	CANT1	0.148721091	0.111132566	1.338231419	0.18082101	0.219422398	positive
PRJNA729271	stage_1D_vs_E10	ordinary_muscle_development	ENSGALG00010029267	ENSGALG00010029267	3.717987218	0.274708264	13.53431152	9.80972E-42	1.36723E-40	positive
PRJNA729271	stage_1D_vs_E10	ordinary_muscle_development	ENSGALG00010029268	PCTP	0.460929344	0.180502818	2.553585305	0.010662016	0.015699504	positive
PRJNA729271	stage_1D_vs_E10	ordinary_muscle_development	ENSGALG00010029269	WSCD1	1.012759907	0.153093246	6.615314102	3.70764E-11	1.15649E-10	positive
PRJNA729271	stage_1D_vs_E10	ordinary_muscle_development	ENSGALG00010029270	TIMP2	0.698582991	0.149445564	4.674497996	2.94674E-06	6.32597E-06	positive
PRJNA729271	stage_1D_vs_E10	ordinary_muscle_development	ENSGALG00010029271	MYL10	-2.838182804	0.799744101	-3.548863692	0.000386897	0.000674859	negative
PRJNA729271	stage_1D_vs_E10	ordinary_muscle_development	ENSGALG00010029273	SLC13A5	-2.887857577	0.463315916	-6.233020444	4.57526E-10	1.32399E-09	negative
PRJNA729271	stage_1D_vs_E10	ordinary_muscle_development	ENSGALG00010029274	VMP1	-0.312697392	0.147146477	-2.125075627	0.033580303	0.046023685	negative
PRJNA729271	stage_1D_vs_E10	ordinary_muscle_development	ENSGALG00010029275	PCYT2	0.161898526	0.071113528	2.276620639	0.022808889	0.032051852	positive
PRJNA729271	stage_1D_vs_E10	ordinary_muscle_development	ENSGALG00010029276	TUBD1	0.561000452	0.171162838	3.277583269	0.001046998	0.001742793	positive
PRJNA729271	stage_1D_vs_E10	ordinary_muscle_development	ENSGALG00010029277	WFIKKN2	1.795022636	0.744112987	2.41229849	0.015852298	0.022752938	positive
PRJNA729271	stage_1D_vs_E10	ordinary_muscle_development	ENSGALG00010029278	MED31	-0.479154767	0.0700898	-6.836298103	8.12658E-12	2.64291E-11	negative
PRJNA729271	stage_1D_vs_E10	ordinary_muscle_development	ENSGALG00010029279	ENSGALG00010029279	-0.071680492	0.159041182	-0.45070397	0.652202921	0.691867779	negative
PRJNA729271	stage_1D_vs_E10	ordinary_muscle_development	ENSGALG00010029280	USP36	0.847572964	0.182338272	4.648354689	3.34593E-06	7.14802E-06	positive
PRJNA729271	stage_1D_vs_E10	ordinary_muscle_development	ENSGALG00010029281	KIAA0753	-0.339539237	0.131252664	-2.586913116	0.009684001	0.014345385	negative
PRJNA729271	stage_1D_vs_E10	ordinary_muscle_development	ENSGALG00010029282	LUC7L3	0.164746344	0.14033888	1.173918044	0.240427854	0.284234707	positive
PRJNA729271	stage_1D_vs_E10	ordinary_muscle_development	ENSGALG00010029283	RPS6KB1	0.000574856	0.077434169	0.0074238	0.994076719	0.995175222	positive
PRJNA729271	stage_1D_vs_E10	ordinary_muscle_development	ENSGALG00010029284	CYTH1	0.334724354	0.093614695	3.575553539	0.000349488	0.00061211	positive
PRJNA729271	stage_1D_vs_E10	ordinary_muscle_development	ENSGALG00010029285	CUX1	0.300433436	0.069340317	4.332738114	1.47266E-05	2.95695E-05	positive
PRJNA729271	stage_1D_vs_E10	ordinary_muscle_development	ENSGALG00010029286	MAFG	0.987451203	0.182948428	5.397429278	6.76025E-08	1.65551E-07	positive
PRJNA729271	stage_1D_vs_E10	ordinary_muscle_development	ENSGALG00010029287	ANKRD40	0.747843477	0.122740472	6.092884174	1.10894E-09	3.12058E-09	positive
PRJNA729271	stage_1D_vs_E10	ordinary_muscle_development	ENSGALG00010029288	PYCR1	-1.118933418	0.293521313	-3.812102799	0.00013779	0.000252084	negative
PRJNA729271	stage_1D_vs_E10	ordinary_muscle_development	ENSGALG00010029289	PITPNM3	-2.080556464	0.231302231	-8.99496928	2.36298E-19	1.21761E-18	negative
PRJNA729271	stage_1D_vs_E10	ordinary_muscle_development	ENSGALG00010029290	ENSGALG00010029290	-1.972887885	0.19864055	-9.931949365	3.02287E-23	1.90176E-22	negative
PRJNA729271	stage_1D_vs_E10	ordinary_muscle_development	ENSGALG00010029291	RNFT1	-0.971712436	0.116000037	-8.376828654	5.43729E-17	2.46292E-16	negative
PRJNA729271	stage_1D_vs_E10	ordinary_muscle_development	ENSGALG00010029292	ENSGALG00010029292	-0.451402243	0.25204204	-1.790979963	0.07329651	0.09529401	negative
PRJNA729271	stage_1D_vs_E10	ordinary_muscle_development	ENSGALG00010029293	DNAH17	-1.568901314	0.142689926	-10.99517921	4.03112E-28	3.17043E-27	negative
PRJNA729271	stage_1D_vs_E10	ordinary_muscle_development	ENSGALG00010029294	PGS1	-0.863561922	0.076732511	-11.25418562	2.20857E-29	1.85261E-28	negative
PRJNA729271	stage_1D_vs_E10	ordinary_muscle_development	ENSGALG00010029295	SOCS3	2.625684709	0.247275726	10.61844908	2.44591E-26	1.77712E-25	positive
PRJNA729271	stage_1D_vs_E10	ordinary_muscle_development	ENSGALG00010029296	FBXO39	-0.806776131	0.157398319	-5.12569725	2.96439E-07	6.89154E-07	negative
PRJNA729271	stage_1D_vs_E10	ordinary_muscle_development	ENSGALG00010029297	SH2B2	2.804153514	0.251968638	11.12897835	9.06687E-29	7.39169E-28	positive
PRJNA729271	stage_1D_vs_E10	ordinary_muscle_development	ENSGALG00010029298	ENDOV	0.226402574	0.145367806	1.557446456	0.119364524	0.14985179	positive
PRJNA729271	stage_1D_vs_E10	ordinary_muscle_development	ENSGALG00010029299	PRKRIP1	-0.203100332	0.090986712	-2.23219773	0.025601897	0.035665498	negative
PRJNA729271	stage_1D_vs_E10	ordinary_muscle_development	ENSGALG00010029300	TEKT1	1.595756136	0.343697942	4.642902801	3.43548E-06	7.32759E-06	positive
PRJNA729271	stage_1D_vs_E10	ordinary_muscle_development	ENSGALG00010029301	RNF213	2.621465281	0.464541022	5.643129786	1.66986E-08	4.28705E-08	positive
PRJNA729271	stage_1D_vs_E10	ordinary_muscle_development	ENSGALG00010029303	SLC26A11	-0.221751456	0.15284024	-1.450870901	0.146815812	0.181392363	negative
PRJNA729271	stage_1D_vs_E10	ordinary_muscle_development	ENSGALG00010029304	ORAI3	0.008291949	0.116566799	0.071134739	0.94329052	0.951694584	positive
PRJNA729271	stage_1D_vs_E10	ordinary_muscle_development	ENSGALG00010029305	SMTNL2	2.672581337	0.18988243	14.07492698	5.41608E-45	8.48714E-44	positive
PRJNA729271	stage_1D_vs_E10	ordinary_muscle_development	ENSGALG00010029306	CEP95	-1.359307025	0.160483742	-8.470060641	2.45265E-17	1.13437E-16	negative
PRJNA729271	stage_1D_vs_E10	ordinary_muscle_development	ENSGALG00010029307	SGSH	0.335366634	0.164486266	2.038873162	0.041462688	0.055995683	positive
PRJNA729271	stage_1D_vs_E10	ordinary_muscle_development	ENSGALG00010029308	GAA	0.374928349	0.136305989	2.750637386	0.005947944	0.0090591	positive
PRJNA729271	stage_1D_vs_E10	ordinary_muscle_development	ENSGALG00010029309	ALKBH4	1.074976973	0.171723423	6.259932144	3.85145E-10	1.11945E-09	positive
PRJNA729271	stage_1D_vs_E10	ordinary_muscle_development	ENSGALG00010029310	LRWD1	-0.844432038	0.091354169	-9.243497528	2.38569E-20	1.29564E-19	negative
PRJNA729271	stage_1D_vs_E10	ordinary_muscle_development	ENSGALG00010029311	ENSGALG00010029311	0.362272938	0.166283718	2.17864348	0.029358163	0.040528246	positive
PRJNA729271	stage_1D_vs_E10	ordinary_muscle_development	ENSGALG00010029312	SMURF2	0.815861491	0.092608798	8.809762269	1.25412E-18	6.22335E-18	positive
PRJNA729271	stage_1D_vs_E10	ordinary_muscle_development	ENSGALG00010029313	CCDC40	-2.377039424	0.282374273	-8.418045314	3.82818E-17	1.7466E-16	negative
PRJNA729271	stage_1D_vs_E10	ordinary_muscle_development	ENSGALG00010029314	MED13	0.837866455	0.12530236	6.686757175	2.2817E-11	7.21021E-11	positive
PRJNA729271	stage_1D_vs_E10	ordinary_muscle_development	ENSGALG00010029315	TBC1D16	-1.366802614	0.099403586	-13.75003331	5.09061E-43	7.41592E-42	negative
PRJNA729271	stage_1D_vs_E10	ordinary_muscle_development	ENSGALG00010029316	INTS2	-0.442854483	0.094645492	-4.679086923	2.88155E-06	6.19245E-06	negative
PRJNA729271	stage_1D_vs_E10	ordinary_muscle_development	ENSGALG00010029317	KPNA2	-2.548620993	0.204873504	-12.43997367	1.58556E-35	1.75441E-34	negative
PRJNA729271	stage_1D_vs_E10	ordinary_muscle_development	ENSGALG00010029318	C17orf58	2.972896436	0.203323362	14.62151915	2.04779E-48	3.71035E-47	positive
PRJNA729271	stage_1D_vs_E10	ordinary_muscle_development	ENSGALG00010029319	BRIP1	-0.93306474	0.123888858	-7.531466149	5.01737E-14	1.89393E-13	negative
PRJNA729271	stage_1D_vs_E10	ordinary_muscle_development	ENSGALG00010029320	TBX4	-3.939898145	0.693290569	-5.682895915	1.32433E-08	3.42177E-08	negative
PRJNA729271	stage_1D_vs_E10	ordinary_muscle_development	ENSGALG00010029321	TBX2	-0.862367448	0.171622066	-5.024805182	5.03944E-07	1.15179E-06	negative
PRJNA729271	stage_1D_vs_E10	ordinary_muscle_development	ENSGALG00010029322	BPTF	0.16881982	0.096496294	1.74949537	0.080205428	0.103671989	positive
PRJNA729271	stage_1D_vs_E10	ordinary_muscle_development	ENSGALG00010029323	NOL11	0.094606222	0.090973266	1.039934333	0.29837041	0.345272162	positive
PRJNA729271	stage_1D_vs_E10	ordinary_muscle_development	ENSGALG00010029324	BCAS3	0.877409207	0.126128191	6.956487676	3.48861E-12	1.16488E-11	positive
PRJNA729271	stage_1D_vs_E10	ordinary_muscle_development	ENSGALG00010029325	LRRC75A	-0.376393944	0.165715507	-2.271326021	0.023127249	0.032470891	negative
PRJNA729271	stage_1D_vs_E10	ordinary_muscle_development	ENSGALG00010029326	NCBP3	-0.189350764	0.080033808	-2.365884733	0.017987044	0.025600236	negative
PRJNA729271	stage_1D_vs_E10	ordinary_muscle_development	ENSGALG00010029327	CRK	-0.193938594	0.054217119	-3.577073031	0.000347463	0.000608638	negative
PRJNA729271	stage_1D_vs_E10	ordinary_muscle_development	ENSGALG00010029328	ENSGALG00010029328	0.575606642	0.080778713	7.125721891	1.03537E-12	3.5754E-12	positive
PRJNA729271	stage_1D_vs_E10	ordinary_muscle_development	ENSGALG00010029329	ENSGALG00010029329	1.583902449	0.175998982	8.999497804	2.26753E-19	1.1705E-18	positive
PRJNA729271	stage_1D_vs_E10	ordinary_muscle_development	ENSGALG00010029330	AFMID	0.472532638	0.12788713	3.694919407	0.000219957	0.000392983	positive
PRJNA729271	stage_1D_vs_E10	ordinary_muscle_development	ENSGALG00010029331	TK1	-1.49029783	0.277048856	-5.379187808	7.48226E-08	1.82585E-07	negative
PRJNA729271	stage_1D_vs_E10	ordinary_muscle_development	ENSGALG00010029332	YWHAE	-0.690693017	0.148678437	-4.645549365	3.39173E-06	7.24158E-06	negative
PRJNA729271	stage_1D_vs_E10	ordinary_muscle_development	ENSGALG00010029333	ENSGALG00010029333	-0.994875334	0.175165806	-5.679620676	1.34994E-08	3.48546E-08	negative
PRJNA729271	stage_1D_vs_E10	ordinary_muscle_development	ENSGALG00010029335	RNF222	-1.063941513	1.507705825	-0.705669167	0.4803939	0.528925716	negative
PRJNA729271	stage_1D_vs_E10	ordinary_muscle_development	ENSGALG00010029336	SYNGR2	1.655452487	0.110358367	15.00069754	7.26518E-51	1.45053E-49	positive
PRJNA729271	stage_1D_vs_E10	ordinary_muscle_development	ENSGALG00010029337	MYO1C	1.458618412	0.133503418	10.92570091	8.68671E-28	6.74057E-27	positive
PRJNA729271	stage_1D_vs_E10	ordinary_muscle_development	ENSGALG00010029338	INPP5K	0.276639964	0.119740244	2.310334056	0.020869667	0.029458158	positive
PRJNA729271	stage_1D_vs_E10	ordinary_muscle_development	ENSGALG00010029339	PITPNA	0.601467479	0.126854876	4.741382412	2.12265E-06	4.61979E-06	positive
PRJNA729271	stage_1D_vs_E10	ordinary_muscle_development	ENSGALG00010029340	NDEL1	0.863141494	0.103425814	8.345513164	7.09053E-17	3.19382E-16	positive
PRJNA729271	stage_1D_vs_E10	ordinary_muscle_development	ENSGALG00010029341	SLC43A2	3.716571018	0.326037913	11.39919888	4.21984E-30	3.66487E-29	positive
PRJNA729271	stage_1D_vs_E10	ordinary_muscle_development	ENSGALG00010029342	TMC6	-0.164580626	0.127172981	-1.294147742	0.195614393	0.235540215	negative
PRJNA729271	stage_1D_vs_E10	ordinary_muscle_development	ENSGALG00010029343	SCARF1	1.464960108	0.130302319	11.24277848	2.51345E-29	2.1047E-28	positive
PRJNA729271	stage_1D_vs_E10	ordinary_muscle_development	ENSGALG00010029344	ENSGALG00010029344	0.949563913	0.165167205	5.749106877	8.97161E-09	2.35075E-08	positive
PRJNA729271	stage_1D_vs_E10	ordinary_muscle_development	ENSGALG00010029345	RILP	1.757561789	0.243979181	7.203736722	5.85844E-13	2.06262E-12	positive
PRJNA729271	stage_1D_vs_E10	ordinary_muscle_development	ENSGALG00010029346	ARL16	-0.492097843	0.121516535	-4.049636873	5.12972E-05	9.79131E-05	negative
PRJNA729271	stage_1D_vs_E10	ordinary_muscle_development	ENSGALG00010029347	PRPF8	-0.145099284	0.069841474	-2.077551843	0.037750656	0.05129319	negative
PRJNA729271	stage_1D_vs_E10	ordinary_muscle_development	ENSGALG00010029348	HGS	-0.029198883	0.123950505	-0.23556889	0.813767216	0.839302391	negative
PRJNA729271	stage_1D_vs_E10	ordinary_muscle_development	ENSGALG00010029349	MYH10	-1.629965134	0.106989582	-15.23480227	2.07755E-52	4.37704E-51	negative
PRJNA729271	stage_1D_vs_E10	ordinary_muscle_development	ENSGALG00010029350	MRPL12	0.633823155	0.121428244	5.219734181	1.7918E-07	4.24312E-07	positive
PRJNA729271	stage_1D_vs_E10	ordinary_muscle_development	ENSGALG00010029351	ENSGALG00010029351	-0.837319709	0.193352008	-4.330545702	1.4874E-05	2.98366E-05	negative
PRJNA729271	stage_1D_vs_E10	ordinary_muscle_development	ENSGALG00010029352	AATF	-0.509989135	0.10921914	-4.669411738	3.02063E-06	6.47981E-06	negative
PRJNA729271	stage_1D_vs_E10	ordinary_muscle_development	ENSGALG00010029353	GCGR	5.399403328	0.883924618	6.108443206	1.00608E-09	2.84381E-09	positive
PRJNA729271	stage_1D_vs_E10	ordinary_muscle_development	ENSGALG00010029354	TLCD2	0.71843335	0.271688833	2.644324181	0.008185422	0.012239291	positive
PRJNA729271	stage_1D_vs_E10	ordinary_muscle_development	ENSGALG00010029355	MCRIP1	0.301221741	0.172625664	1.744941822	0.080994997	0.104599294	positive
PRJNA729271	stage_1D_vs_E10	ordinary_muscle_development	ENSGALG00010029357	ARHGDIA	-0.859998827	0.097412369	-8.828435617	1.0615E-18	5.2838E-18	negative
PRJNA729271	stage_1D_vs_E10	ordinary_muscle_development	ENSGALG00010029359	ENSGALG00010029359	-1.456133095	0.513313852	-2.836730571	0.004557806	0.00704397	negative
PRJNA729271	stage_1D_vs_E10	ordinary_muscle_development	ENSGALG00010029360	ACACA	-0.294911875	0.085415146	-3.452688308	0.00055503	0.000950852	negative
PRJNA729271	stage_1D_vs_E10	ordinary_muscle_development	ENSGALG00010029361	ALYREF	-1.431026293	0.134681451	-10.62526639	2.27362E-26	1.65609E-25	negative
PRJNA729271	stage_1D_vs_E10	ordinary_muscle_development	ENSGALG00010029362	TADA2A	-0.287371989	0.111501089	-2.577302098	0.009957488	0.014721929	negative
PRJNA729271	stage_1D_vs_E10	ordinary_muscle_development	ENSGALG00010029363	PIK3R6	1.575373374	0.192045716	8.203116439	2.34234E-16	1.02451E-15	positive
PRJNA729271	stage_1D_vs_E10	ordinary_muscle_development	ENSGALG00010029364	ANAPC11	-0.795959441	0.132354255	-6.013856079	1.81161E-09	5.02187E-09	negative
PRJNA729271	stage_1D_vs_E10	ordinary_muscle_development	ENSGALG00010029365	PIK3R5	0.407632615	0.192927991	2.112874407	0.034611519	0.047325155	positive
PRJNA729271	stage_1D_vs_E10	ordinary_muscle_development	ENSGALG00010029366	MGAT5B	0.879772509	0.347009888	2.535295217	0.01123526	0.016506699	positive
PRJNA729271	stage_1D_vs_E10	ordinary_muscle_development	ENSGALG00010029367	DUSP14	-0.27330848	0.116717016	-2.341633546	0.019199556	0.027225353	negative
PRJNA729271	stage_1D_vs_E10	ordinary_muscle_development	ENSGALG00010029368	DDX52	-0.071104216	0.089332088	-0.79595382	0.426058878	0.475494567	negative
PRJNA729271	stage_1D_vs_E10	ordinary_muscle_development	ENSGALG00010029369	MFSD11	0.100524624	0.084781598	1.185689182	0.235745068	0.279268124	positive
PRJNA729271	stage_1D_vs_E10	ordinary_muscle_development	ENSGALG00010029370	SRSF2	-0.980323829	0.125558903	-7.807680765	5.82499E-15	2.32855E-14	negative
PRJNA729271	stage_1D_vs_E10	ordinary_muscle_development	ENSGALG00010029371	AUTS2	-1.361486861	0.170754458	-7.973360581	1.54417E-15	6.40788E-15	negative
PRJNA729271	stage_1D_vs_E10	ordinary_muscle_development	ENSGALG00010029372	METTL23	-0.905526729	0.152052404	-5.955359493	2.59501E-09	7.11321E-09	negative
PRJNA729271	stage_1D_vs_E10	ordinary_muscle_development	ENSGALG00010029373	GATSL2	2.279139793	0.168272403	13.54434687	8.55716E-42	1.19726E-40	positive
PRJNA729271	stage_1D_vs_E10	ordinary_muscle_development	ENSGALG00010029374	RCC1L	0.005748466	0.116375505	0.04939584	0.960603844	0.966438525	positive
PRJNA729271	stage_1D_vs_E10	ordinary_muscle_development	ENSGALG00010029375	JMJD6	0.076435811	0.160748827	0.475498402	0.634431772	0.675686153	positive
PRJNA729271	stage_1D_vs_E10	ordinary_muscle_development	ENSGALG00010029376	ENSGALG00010029376	-1.126857045	0.208513024	-5.404252566	6.50792E-08	1.59669E-07	negative
PRJNA729271	stage_1D_vs_E10	ordinary_muscle_development	ENSGALG00010029377	NCF1C	0.56451697	0.30200944	1.869203063	0.061594568	0.080958766	positive
PRJNA729271	stage_1D_vs_E10	ordinary_muscle_development	ENSGALG00010029378	ST6GALNAC1	-3.120382439	0.374371989	-8.334978387	7.75124E-17	3.48493E-16	negative
PRJNA729271	stage_1D_vs_E10	ordinary_muscle_development	ENSGALG00010029379	GTF2I	0.195452065	0.11227278	1.74086778	0.081706758	0.105443327	positive
PRJNA729271	stage_1D_vs_E10	ordinary_muscle_development	ENSGALG00010029380	RGS9	-0.04498778	0.148394461	-0.303163468	0.761765288	0.792264321	negative
PRJNA729271	stage_1D_vs_E10	ordinary_muscle_development	ENSGALG00010029382	GTF2IRD1	0.235904447	0.20622903	1.143895441	0.252667002	0.297321659	positive

PRJNA729271	stage_1D_vs_E10	ordinary_muscle_development	ENSGALG00010029383	GNA13	-0.035280322	0.07883903	-0.447498172	0.654515407	0.694066927	negative
PRJNA729271	stage_1D_vs_E10	ordinary_muscle_development	ENSGALG00010029384	WDR81	-0.131695263	0.074601435	-1.765318088	0.077510321	0.10043913	negative
PRJNA729271	stage_1D_vs_E10	ordinary_muscle_development	ENSGALG00010029385	CLIP2	-0.741338698	0.122499358	-6.051776199	1.43257E-09	3.99714E-09	negative
PRJNA729271	stage_1D_vs_E10	ordinary_muscle_development	ENSGALG00010029386	SLC16A6	-0.410643413	0.238191176	-1.724007665	0.084706459	0.1090717	negative
PRJNA729271	stage_1D_vs_E10	ordinary_muscle_development	ENSGALG00010029387	SERPINF1	1.934045949	0.175898127	10.99526173	4.02743E-28	3.16925E-27	positive
PRJNA729271	stage_1D_vs_E10	ordinary_muscle_development	ENSGALG00010029388	ST6GALNAC2	1.928683621	0.312091435	6.179867194	6.41555E-10	1.83745E-09	positive
PRJNA729271	stage_1D_vs_E10	ordinary_muscle_development	ENSGALG00010029389	SMYD4	0.015241415	0.095495327	0.159603773	0.873193207	0.891835931	positive
PRJNA729271	stage_1D_vs_E10	ordinary_muscle_development	ENSGALG00010029390	RPA1	-1.056885607	0.079604319	-13.27673703	3.15861E-40	4.16218E-39	negative
PRJNA729271	stage_1D_vs_E10	ordinary_muscle_development	ENSGALG00010029391	ARSG	-0.020659652	0.140623409	-0.146914744	0.883199314	0.900532784	negative
PRJNA729271	stage_1D_vs_E10	ordinary_muscle_development	ENSGALG00010029392	RTN4RL1	2.443780946	0.50856931	4.805207267	1.54591E-06	3.39876E-06	positive
PRJNA729271	stage_1D_vs_E10	ordinary_muscle_development	ENSGALG00010029394	CYGB	-0.71919829	0.143052153	-5.027525098	4.9685E-07	1.13666E-06	negative
PRJNA729271	stage_1D_vs_E10	ordinary_muscle_development	ENSGALG00010029395	DPH1	-0.249326783	0.116742475	-2.135698959	0.032703959	0.044877771	negative
PRJNA729271	stage_1D_vs_E10	ordinary_muscle_development	ENSGALG00010029396	WIPI1	0.653011087	0.15458032	4.224412828	2.39565E-05	4.71101E-05	positive
PRJNA729271	stage_1D_vs_E10	ordinary_muscle_development	ENSGALG00010029397	OVCA2	-0.738557362	0.155220583	-4.758114858	1.95409E-06	4.26126E-06	negative
PRJNA729271	stage_1D_vs_E10	ordinary_muscle_development	ENSGALG00010029398	RHBDF2	-0.022998608	0.125622472	-0.183077182	0.854737459	0.875577348	negative
PRJNA729271	stage_1D_vs_E10	ordinary_muscle_development	ENSGALG00010029399	PRKAR1A	-0.755239373	0.053540908	-14.10583797	3.49612E-45	5.5263E-44	negative
PRJNA729271	stage_1D_vs_E10	ordinary_muscle_development	ENSGALG00010029400	AANAT	-0.33490326	0.702198232	-0.476935493	0.633408057	0.674744601	negative
PRJNA729271	stage_1D_vs_E10	ordinary_muscle_development	ENSGALG00010029401	HIC1	-1.078402006	0.202352464	-5.329324812	9.85786E-08	2.38149E-07	negative
PRJNA729271	stage_1D_vs_E10	ordinary_muscle_development	ENSGALG00010029402	SMG6	0.380492712	0.088290918	4.309533986	1.63599E-05	3.2677E-05	positive
PRJNA729271	stage_1D_vs_E10	ordinary_muscle_development	ENSGALG00010029403	FAM20A	2.270676412	0.156274174	14.53008099	7.8128E-48	1.38106E-46	positive
PRJNA729271	stage_1D_vs_E10	ordinary_muscle_development	ENSGALG00010029404	SRR	0.555157275	0.180013804	3.083970577	0.002042578	0.003283483	positive
PRJNA729271	stage_1D_vs_E10	ordinary_muscle_development	ENSGALG00010029405	POLDIP2	0.754659117	0.078628586	9.597770376	8.16924E-22	4.83122E-21	positive
PRJNA729271	stage_1D_vs_E10	ordinary_muscle_development	ENSGALG00010029406	TMEM199	-0.261724821	0.104022974	-2.516029023	0.011868541	0.017375467	negative
PRJNA729271	stage_1D_vs_E10	ordinary_muscle_development	ENSGALG00010029407	UBE2O	0.370705582	0.188549744	1.96608902	0.049288328	0.06582828	positive
PRJNA729271	stage_1D_vs_E10	ordinary_muscle_development	ENSGALG00010029408	KIF12	2.142147462	0.45967592	4.660125471	3.16017E-06	6.76612E-06	positive
PRJNA729271	stage_1D_vs_E10	ordinary_muscle_development	ENSGALG00010029409	ENSGALG00010029409	4.459273331	0.18855749	23.64940969	1.1969E-123	1.4458E-121	positive
PRJNA729271	stage_1D_vs_E10	ordinary_muscle_development	ENSGALG00010029410	VTN	1.769321684	0.884813668	1.999654559	0.045537578	0.061083538	positive
PRJNA729271	stage_1D_vs_E10	ordinary_muscle_development	ENSGALG00010029411	SARM1	-1.879192619	0.184678288	-10.17549295	2.55105E-24	1.68693E-23	negative
PRJNA729271	stage_1D_vs_E10	ordinary_muscle_development	ENSGALG00010029412	SLC46A1	0.392251104	0.106801876	3.672698621	0.000240002	0.000427166	positive
PRJNA729271	stage_1D_vs_E10	ordinary_muscle_development	ENSGALG00010029413	B3GNTL1	-0.164553887	0.219004405	-0.751372499	0.452428509	0.501487439	negative
PRJNA729271	stage_1D_vs_E10	ordinary_muscle_development	ENSGALG00010029416	UNC119	-2.444357136	0.451548372	-5.413278599	6.18811E-08	1.51951E-07	negative
PRJNA729271	stage_1D_vs_E10	ordinary_muscle_development	ENSGALG00010029417	ENSGALG00010029417	2.326196562	0.210452993	11.05328334	2.11341E-28	1.68968E-27	positive
PRJNA729271	stage_1D_vs_E10	ordinary_muscle_development	ENSGALG00010029418	ABCA5	0.608527856	0.131859581	4.614968846	3.93154E-06	8.33519E-06	positive
PRJNA729271	stage_1D_vs_E10	ordinary_muscle_development	ENSGALG00010029419	RFC2	-0.441761835	0.132552603	-3.332728487	0.000859988	0.001443939	negative
PRJNA729271	stage_1D_vs_E10	ordinary_muscle_development	ENSGALG00010029420	LAT2	-0.244194013	0.330637233	-0.7385557	0.460176833	0.509024969	negative
PRJNA729271	stage_1D_vs_E10	ordinary_muscle_development	ENSGALG00010029421	TBCD	-1.001010849	0.094100402	-10.6376894	1.99003E-26	1.45244E-25	negative
PRJNA729271	stage_1D_vs_E10	ordinary_muscle_development	ENSGALG00010029422	EIF4H	-0.499765729	0.087278782	-5.726085058	1.02775E-08	2.67598E-08	negative
PRJNA729271	stage_1D_vs_E10	ordinary_muscle_development	ENSGALG00010029423	MYBBP1A	-0.717488462	0.120655913	-5.946566915	2.73825E-09	7.48885E-09	negative
PRJNA729271	stage_1D_vs_E10	ordinary_muscle_development	ENSGALG00010029424	MAP2K6	-0.521045213	0.21844187	-2.385280863	0.017066086	0.024398158	negative
PRJNA729271	stage_1D_vs_E10	ordinary_muscle_development	ENSGALG00010029425	SPNS2	1.311813276	0.165331163	7.934458644	2.11415E-15	8.70835E-15	positive
PRJNA729271	stage_1D_vs_E10	ordinary_muscle_development	ENSGALG00010029426	ZNF750	-6.847862631	1.11331863	-6.150856051	7.70658E-10	2.1955E-09	negative
PRJNA729271	stage_1D_vs_E10	ordinary_muscle_development	ENSGALG00010029427	SPNS3	-1.737667703	0.336825574	-5.158954177	2.48333E-07	5.80954E-07	negative
PRJNA729271	stage_1D_vs_E10	ordinary_muscle_development	ENSGALG00010029428	FN3K	1.213459643	0.252464513	4.806456282	1.53629E-06	3.37914E-06	positive
PRJNA729271	stage_1D_vs_E10	ordinary_muscle_development	ENSGALG00010029429	UBE2G1	0.289805361	0.089996647	3.22017954	0.001281103	0.002112341	positive
PRJNA729271	stage_1D_vs_E10	ordinary_muscle_development	ENSGALG00010029430	FN3KRP	-0.199347033	0.09587947	-2.079141995	0.037604301	0.051108706	negative
PRJNA729271	stage_1D_vs_E10	ordinary_muscle_development	ENSGALG00010029432	ENSGALG00010029432	8.160239706	5.430558858	1.502651922			positive
PRJNA729271	stage_1D_vs_E10	ordinary_muscle_development	ENSGALG00010029436	WDR45B	-0.413747141	0.079734734	-5.189045218	2.11375E-07	4.97222E-07	negative
PRJNA729271	stage_1D_vs_E10	ordinary_muscle_development	ENSGALG00010029437	ENSGALG00010029437	-7.306890685	2.926270176	-2.496997969	0.012524967	0.018275559	negative
PRJNA729271	stage_1D_vs_E10	ordinary_muscle_development	ENSGALG00010029438	ENSGALG00010029438	-0.369535875	0.26874099	-1.375063312	0.169111817	0.206336346	negative
PRJNA729271	stage_1D_vs_E10	ordinary_muscle_development	ENSGALG00010029439	ENSGALG00010029439	-1.003611746	0.208996668	-4.802046639	1.57052E-06	3.45129E-06	negative
PRJNA729271	stage_1D_vs_E10	ordinary_muscle_development	ENSGALG00010029440	FOXK2	1.103154084	0.181250191	6.086360936	1.15506E-09	3.24406E-09	positive
PRJNA729271	stage_1D_vs_E10	ordinary_muscle_development	ENSGALG00010029442	ENSGALG00010029442	-0.330299176	0.280482626	-1.177610109	0.238952078	0.282720625	negative
PRJNA729271	stage_1D_vs_E10	ordinary_muscle_development	ENSGALG00010029443	ENSGALG00010029443	-0.210456314	0.31530258	-0.667474127	0.504469331	0.552540223	negative
PRJNA729271	stage_1D_vs_E10	ordinary_muscle_development	ENSGALG00010029445	SPHK1	-0.20312113	0.138163711	-1.470148193	0.141521622	0.17525467	negative
PRJNA729271	stage_1D_vs_E10	ordinary_muscle_development	ENSGALG00010029446	PRPSAP1	-0.1108029	0.080686697	-1.37324868	0.169675051	0.206919053	negative
PRJNA729271	stage_1D_vs_E10	ordinary_muscle_development	ENSGALG00010029449	UBALD2	1.249514831	0.18151566	6.883785285	5.82827E-12	1.91306E-11	positive
PRJNA729271	stage_1D_vs_E10	ordinary_muscle_development	ENSGALG00010029451	ENSGALG00010029451	-1.328459157	0.222263593	-5.976953491	2.27349E-09	6.25674E-09	negative
PRJNA729271	stage_1D_vs_E10	ordinary_muscle_development	ENSGALG00010029452	ENSGALG00010029452	-0.125654009	0.938465763	-0.133893013	0.893487175	0.909167891	negative
PRJNA729271	stage_1D_vs_E10	ordinary_muscle_development	ENSGALG00010029453	ENSGALG00010029453	-0.720623769	1.516757403	-0.475108127	0.634709906	0.675843655	negative
PRJNA729271	stage_1D_vs_E10	ordinary_muscle_development	ENSGALG00010029454	ENSGALG00010029454	-0.411425542	0.289553794	-1.420895013	0.155347283	0.190891732	negative
PRJNA729271	stage_1D_vs_E10	ordinary_muscle_development	ENSGALG00010029455	ENSGALG00010029455	-0.340723981	0.25229111	-1.350519172	0.176849508	0.214999045	negative
PRJNA729271	stage_1D_vs_E10	ordinary_muscle_development	ENSGALG00010029456	RNF157	-3.36831697	0.547048178	-6.157258363	7.4015E-10	2.11036E-09	negative
PRJNA729271	stage_1D_vs_E10	ordinary_muscle_development	ENSGALG00010029457	ENSGALG00010029457	-0.484607053	0.190557321	-2.543103832	0.010987257	0.016163635	negative
PRJNA729271	stage_1D_vs_E10	ordinary_muscle_development	ENSGALG00010029460	ENSGALG00010029460	-0.676547859	0.279970374	-2.416498035	0.015670611	0.022520871	negative
PRJNA729271	stage_1D_vs_E10	ordinary_muscle_development	ENSGALG00010029461	ENSGALG00010029461	-0.357888621	0.201086465	-1.779774787	0.075112825	0.097445664	negative
PRJNA729271	stage_1D_vs_E10	ordinary_muscle_development	ENSGALG00010029462	ENSGALG00010029462	0.694084173	1.806055563	0.384309424	0.700749124	0.737697622	positive
PRJNA729271	stage_1D_vs_E10	ordinary_muscle_development	ENSGALG00010029463	ENSGALG00010029463	0.010526859	0.415796003	0.025317366	0.979801823	0.982785096	positive
PRJNA729271	stage_1D_vs_E10	ordinary_muscle_development	ENSGALG00010029464	ENSGALG00010029464	0.490125564	0.509522015	0.961932064	0.336083731	0.384584643	positive
PRJNA729271	stage_1D_vs_E10	ordinary_muscle_development	ENSGALG00010029465	ENSGALG00010029465	-0.995264534	2.37599386	-0.418883462	0.675301304	0.713498972	negative
PRJNA729271	stage_1D_vs_E10	ordinary_muscle_development	ENSGALG00010029466	SLC39A11	-0.853169919	0.118576142	-7.195122905	6.24045E-13	2.19286E-12	negative
PRJNA729271	stage_1D_vs_E10	ordinary_muscle_development	ENSGALG00010029468	ENSGALG00010029468	-1.364571126	0.2408873	-5.664769894	1.47222E-08	3.79172E-08	negative
PRJNA729271	stage_1D_vs_E10	ordinary_muscle_development	ENSGALG00010029469	ENSGALG00010029469	-1.496786282	0.398931627	-3.751987006	0.000175439	0.000317793	negative
PRJNA729271	stage_1D_vs_E10	ordinary_muscle_development	ENSGALG00010029470	SSTR2	2.270183657	0.919180744	2.469790269	0.013519229	0.019619666	positive
PRJNA729271	stage_1D_vs_E10	ordinary_muscle_development	ENSGALG00010029472	ENSGALG00010029472	-0.463469825	0.795053198	-0.582941903	0.559932411	0.60555251	negative
PRJNA729271	stage_1D_vs_E10	ordinary_muscle_development	ENSGALG00010029473	COG1	0.76834794	0.100191669	7.668780754	1.73639E-14	6.7441E-14	positive
PRJNA729271	stage_1D_vs_E10	ordinary_muscle_development	ENSGALG00010029474	ENSGALG00010029474	-1.200293891	1.079366493	-1.112035531	0.266122874	0.311301464	negative
PRJNA729271	stage_1D_vs_E10	ordinary_muscle_development	ENSGALG00010029477	FAM104A	-0.455394806	0.05882139	-7.741993259	9.78702E-15	3.85602E-14	negative
PRJNA729271	stage_1D_vs_E10	ordinary_muscle_development	ENSGALG00010029479	ENSGALG00010029479	-0.517106226	0.547430665	-0.944605882	0.344860123	0.393716726	negative
PRJNA729271	stage_1D_vs_E10	ordinary_muscle_development	ENSGALG00010029480	ENSGALG00010029480	0.517822968	0.115065253	4.500254885	6.7872E-06	1.40906E-05	positive
PRJNA729271	stage_1D_vs_E10	ordinary_muscle_development	ENSGALG00010029481	ENSGALG00010029481	-0.556875047	0.275527526	-2.021123094	0.043267024	0.058285829	negative
PRJNA729271	stage_1D_vs_E10	ordinary_muscle_development	ENSGALG00010029482	DDX25	-0.116584232	0.183543603	-0.635185481	0.525307451	0.572333998	negative
PRJNA729271	stage_1D_vs_E10	ordinary_muscle_development	ENSGALG00010029484	ENSGALG00010029484	-2.856016754	0.660093021	-4.326688302	1.51368E-05	3.03343E-05	negative
PRJNA729271	stage_1D_vs_E10	ordinary_muscle_development	ENSGALG00010029487	CDC42EP4	-1.509251351	0.143070826	-10.54898047	5.13513E-26	3.68119E-25	negative
PRJNA729271	stage_1D_vs_E10	ordinary_muscle_development	ENSGALG00010029489	ENSGALG00010029489	-3.51099191	1.156938797	-3.034725708	0.002407545	0.003835297	negative
PRJNA729271	stage_1D_vs_E10	ordinary_muscle_development	ENSGALG00010029490	ENSGALG00010029490	-1.132967038	0.600349081	-1.887180431	0.059136061	0.077903954	negative
PRJNA729271	stage_1D_vs_E10	ordinary_muscle_development	ENSGALG00010029491	ENSGALG00010029491	-0.569245367	0.19952124	-2.853056486	0.004330092	0.006710648	negative
PRJNA729271	stage_1D_vs_E10	ordinary_muscle_development	ENSGALG00010029492	SDK2	-0.319811832	0.225567582	-1.417809372	0.156246427	0.191931522	negative
PRJNA729271	stage_1D_vs_E10	ordinary_muscle_development	ENSGALG00010029494	MYH7	-2.300780523	0.505227043	-4.553953621	5.2647E-06	1.10357E-05	negative
PRJNA729271	stage_1D_vs_E10	ordinary_muscle_development	ENSGALG00010029495	RPL38	0.071873846	0.188618545	0.381053975	0.703163198	0.739862845	positive
PRJNA729271	stage_1D_vs_E10	ordinary_muscle_development	ENSGALG00010029496	TTYH2	0.186459809	0.144345657	1.291759051	0.196440601	0.236417013	positive
PRJNA729271	stage_1D_vs_E10	ordinary_muscle_development	ENSGALG00010029497	ENSGALG00010029497	-0.891647679	0.593509019	-1.502332147	0.13301135	0.165394143	negative
PRJNA729271	stage_1D_vs_E10	ordinary_muscle_development	ENSGALG00010029498	ENSGALG00010029498	-0.215303633	0.148972666	-1.445255957	0.148386004	0.183145021	negative
PRJNA729271	stage_1D_vs_E10	ordinary_muscle_development	ENSGALG00010029501	ENSGALG00010029501	-0.656273828	0.390540761	-1.680423387	0.092874969	0.11879833	negative
PRJNA729271	stage_1D_vs_E10	ordinary_muscle_development	ENSGALG00010029502	BTBD17	0.217142094	0.451328145	0.481117998	0.630432637	0.672068918	positive
PRJNA729271	stage_1D_vs_E10	ordinary_muscle_development	ENSGALG00010029504	GPR142	-2.400276776	0.502306908	-4.778506408	1.76602E-06	3.86101E-06	negative
PRJNA729271	stage_1D_vs_E10	ordinary_muscle_development	ENSGALG00010029505	VEZF1	0.646735026	0.109016019	5.932476984	2.98398E-09	8.13176E-09	positive
PRJNA729271	stage_1D_vs_E10	ordinary_muscle_development	ENSGALG00010029506	OXLD1	-0.940677263	0.158611291	-5.930708052	3.01631E-09	8.21214E-09	negative
PRJNA729271	stage_1D_vs_E10	ordinary_muscle_development	ENSGALG00010029507	VPS37D	-0.067949009	0.170312527	-0.398966596	0.689917818	0.727351718	negative
PRJNA729271	stage_1D_vs_E10	ordinary_muscle_development	ENSGALG00010029508	ENSGALG00010029508	-1.011466099	0.142698162	-7.088150833	1.35916E-12	4.65414E-12	negative
PRJNA729271	stage_1D_vs_E10	ordinary_muscle_development	ENSGALG00010029509	DNAJC30	0.904167719	0.168276608	5.373104006	7.73927E-08	1.88634E-07	positive
PRJNA729271	stage_1D_vs_E10	ordinary_muscle_development	ENSGALG00010029510	PDE6G	-1.402910017	0.322302712	-4.352771369	1.34427E-05	2.71269E-05	negative
PRJNA729271	stage_1D_vs_E10	ordinary_muscle_development	ENSGALG00010029511	CUEDC1	0.045072578	0.072781576	0.619285549	0.535728263	0.582374469	positive
PRJNA729271	stage_1D_vs_E10	ordinary_muscle_development	ENSGALG00010029512	TSPAN10	-2.269098162	0.754976632	-3.005521054	0.002651262	0.004204522	negative
PRJNA729271	stage_1D_vs_E10	ordinary_muscle_development	ENSGALG00010029513	WBSCR22	-0.366512944	0.085314675	-4.296012888	1.73897E-05	3.46433E-05	negative
PRJNA729271	stage_1D_vs_E10	ordinary_muscle_development	ENSGALG00010029514	MRPS23	0.235323202	0.097347757	2.417345905	0.015634152	0.022479619	positive
PRJNA729271	stage_1D_vs_E10	ordinary_muscle_development	ENSGALG00010029515	NPLOC4	-0.031429518	0.152758684	-0.205746195	0.836989172	0.860375721	negative
PRJNA729271	stage_1D_vs_E10	ordinary_muscle_development	ENSGALG00010029516	ENSGALG00010029516	3.464424445	0.226510157	15.29478628	8.28323E-53	1.76567E-51	positive
PRJNA729271	stage_1D_vs_E10	ordinary_muscle_development	ENSGALG00010029517	MSI2	0.050896577	0.112589757	0.452053349	0.65123055	0.691139758	positive
PRJNA729271	stage_1D_vs_E10	ordinary_muscle_development	ENSGALG00010029518	FAAP100	-0.084868626	0.186508412	-0.455039134	0.649081085	0.689033836	negative
PRJNA729271	stage_1D_vs_E10	ordinary_muscle_development	ENSGALG00010029519	STX1A	-0.179295609	0.110944256	-1.616087352	0.106075428	0.134249833	negative
PRJNA729271	stage_1D_vs_E10	ordinary_muscle_development	ENSGALG00010029520	MKS1	-0.380144005	0.109336228	-3.476834827	0.00050737	0.000873021	negative
PRJNA729271	stage_1D_vs_E10	ordinary_muscle_development	ENSGALG00010029521	ACTG1	-0.628353026	0.098390326	-6.386329362	1.69915E-10	5.06252E-10	negative
PRJNA729271	stage_1D_vs_E10	ordinary_muscle_development	ENSGALG00010029522	AKAP1	1.591449048	0.111855326	14.22774491	6.16375E-46	1.00273E-44	positive
PRJNA729271	stage_1D_vs_E10	ordinary_muscle_development	ENSGALG00010029523	FSCN2	-0.732082129	0.833112727	-0.878731179	0.379547049	0.42856855	negative
PRJNA729271	stage_1D_vs_E10	ordinary_muscle_development	ENSGALG00010029524	ENSGALG00010029524	1.221951862	0.147737844	8.271082245	1.32748E-16	5.88257E-16	positive
PRJNA729271	stage_1D_vs_E10	ordinary_muscle_development	ENSGALG00010029525	SLC38A10	-0.966563832	0.138400516	-6.983816686	2.87267E-12	9.6432E-12	negative
PRJNA729271	stage_1D_vs_E10	ordinary_muscle_development	ENSGALG00010029526	BCL7B	0.628413042	0.112190919	5.601282617	2.12772E-08	5.41074E-08	positive
PRJNA729271	stage_1D_vs_E10	ordinary_muscle_development	ENSGALG00010029527	NDUFAF8	1.239401074	0.152331304	8.136220447	4.07809E-16	1.76296E-15	positive
PRJNA729271	stage_1D_vs_E10	ordinary_muscle_development	ENSGALG00010029528	TBL2	-0.18110807	0.095915465	-1.888205099	0.058998422	0.077729697	negative
PRJNA729271	stage_1D_vs_E10	ordinary_muscle_development	ENSGALG00010029529	TEPSIN	0.199291157	0.130565866	1.526364912	0.126919011	0.158509499	positive
PRJNA729271	stage_1D_vs_E10	ordinary_muscle_development	ENSGALG00010029530	ENSGALG00010029530	2.812414615	0.358290647	7.849533989	4.17586E-15	1.68652E-14	positive
PRJNA729271	stage_1D_vs_E10	ordinary_muscle_development	ENSGALG00010029531	CEP131	-0.157713401	0.170875278	-0.922973778	0.35602087	0.404810364	negative
PRJNA729271	stage_1D_vs_E10	ordinary_muscle_development	ENSGALG00010029532	EVI2A	0.424893453	0.478879259	0.887266353	0.374935516	0.424187505	positive
PRJNA729271	stage_1D_vs_E10	ordinary_muscle_development	ENSGALG00010029533	MLXIPL	-1.015726884	0.780334595	-1.301655585	0.193034149	0.232858688	negative
PRJNA729271	stage_1D_vs_E10	ordinary_muscle_development	ENSGALG00010029534	NF1	0.082556389	0.090760495	0.909607082	0.363029763	0.412004103	positive
PRJNA729271	stage_1D_vs_E10	ordinary_muscle_development	ENSGALG00010029536	BAZ1B	-0.555093268	0.077334507	-7.177821245	7.08311E-13	2.47875E-12	negative
PRJNA729271	stage_1D_vs_E10	ordinary_muscle_development	ENSGALG00010029537	MYH1B	3.92399249	0.206075835	19.04149747	7.7286E-81	3.7218E-79	positive
PRJNA729271	stage_1D_vs_E10	ordinary_muscle_development	ENSGALG00010029538	FZD9	-0.359871356	0.173609049	-2.072883632	0.038183111	0.051832196	negative
PRJNA729271	stage_1D_vs_E10	ordinary_muscle_development	ENSGALG00010029539	FKBP6	-2.638384431	0.433710372	-6.083286459	1.17744E-09	3.30563E-09	negative
PRJNA729271	stage_1D_vs_E10	ordinary_muscle_development	ENSGALG00010029541	ENSGALG00010029541	0.126504635	0.239817101	0.527504644	0.597843192	0.641051714	positive
PRJNA729271	stage_1D_vs_E10	ordinary_muscle_development	ENSGALG00010029542	AATK	2.620218601	0.138006076	18.9862553	2.21577E-80	1.0635E-78	positive

PRJNA729271	stage_1D_vs_E10	ordinary_muscle_development	ENSGALG00010029543	NSUN5	0.000200167	0.134033445	0.001493414	0.998808428	0.99899921	positive
PRJNA729271	stage_1D_vs_E10	ordinary_muscle_development	ENSGALG00010029544	ENSGALG00010029544	-0.595769395	0.17729175	-3.360389847	0.000778326	0.001313521	negative
PRJNA729271	stage_1D_vs_E10	ordinary_muscle_development	ENSGALG00010029545	OMG	0.594185362	0.324540424	1.830851623	0.067122693	0.08773951	positive
PRJNA729271	stage_1D_vs_E10	ordinary_muscle_development	ENSGALG00010029546	WSB1	0.375841894	0.165252333	2.274351515	0.02294486	0.032227301	positive
PRJNA729271	stage_1D_vs_E10	ordinary_muscle_development	ENSGALG00010029547	HIP1	-0.103771913	0.10024994	-1.035131926	0.300607305	0.347583191	negative
PRJNA729271	stage_1D_vs_E10	ordinary_muscle_development	ENSGALG00010029548	BAIAP2	1.078359032	0.219393151	4.915190048	8.86964E-07	1.98894E-06	positive
PRJNA729271	stage_1D_vs_E10	ordinary_muscle_development	ENSGALG00010029549	KSR1	1.967849485	0.123575449	15.92427537	4.29966E-57	1.06901E-55	positive
PRJNA729271	stage_1D_vs_E10	ordinary_muscle_development	ENSGALG00010029550	CHMP6	0.45215257	0.239459833	1.888218847	0.058996577	0.077729697	positive
PRJNA729271	stage_1D_vs_E10	ordinary_muscle_development	ENSGALG00010029552	NOS2	0.583613965	0.123299792	4.733292366	2.20907E-06	4.80139E-06	positive
PRJNA729271	stage_1D_vs_E10	ordinary_muscle_development	ENSGALG00010029553	MYH1C	4.098016306	0.194793084	21.03779163	2.95838E-98	2.12286E-96	positive
PRJNA729271	stage_1D_vs_E10	ordinary_muscle_development	ENSGALG00010029554	ENSGALG00010029554	4.249681453	0.372089004	11.42114227	3.27896E-30	2.87529E-29	positive
PRJNA729271	stage_1D_vs_E10	ordinary_muscle_development	ENSGALG00010029555	LYRM9	1.095270361	0.153105018	7.153719573	8.44575E-13	2.93928E-12	positive
PRJNA729271	stage_1D_vs_E10	ordinary_muscle_development	ENSGALG00010029556	NPTX1	2.969200429	0.555538973	5.344720307	9.05568E-08	2.19428E-07	positive
PRJNA729271	stage_1D_vs_E10	ordinary_muscle_development	ENSGALG00010029557	ENSGALG00010029557	5.022036031	0.544716942	9.219533376	2.98396E-20	1.6151E-19	positive
PRJNA729271	stage_1D_vs_E10	ordinary_muscle_development	ENSGALG00010029558	ENSGALG00010029558	0.614119632	0.677509725	0.906436631	0.364704791	0.413743128	positive
PRJNA729271	stage_1D_vs_E10	ordinary_muscle_development	ENSGALG00010029560	ENSGALG00010029560	2.970400031	0.170648053	17.4065861	7.35406E-68	2.50228E-66	positive
PRJNA729271	stage_1D_vs_E10	ordinary_muscle_development	ENSGALG00010029561	SCO1	-0.474944662	0.104665374	-4.537743896	5.68593E-06	1.1874E-05	negative
PRJNA729271	stage_1D_vs_E10	ordinary_muscle_development	ENSGALG00010029562	ENSGALG00010029562	0.446219156	0.824070547	0.541481743	0.588175578	0.632322555	positive
PRJNA729271	stage_1D_vs_E10	ordinary_muscle_development	ENSGALG00010029563	ADPRM	1.119918377	0.123822531	9.044544344	1.50291E-19	7.83056E-19	positive
PRJNA729271	stage_1D_vs_E10	ordinary_muscle_development	ENSGALG00010029564	NLK	0.194845962	0.087790308	2.2194473	0.026456309	0.036756848	positive
PRJNA729271	stage_1D_vs_E10	ordinary_muscle_development	ENSGALG00010029566	TMEM97	-0.260630493	0.144404867	-1.804859485	0.071096673	0.092591759	negative
PRJNA729271	stage_1D_vs_E10	ordinary_muscle_development	ENSGALG00010029567	IFT20	-0.561698467	0.119660844	-4.694087438	2.67799E-06	5.77041E-06	negative
PRJNA729271	stage_1D_vs_E10	ordinary_muscle_development	ENSGALG00010029568	ENSGALG00010029568	-2.856740778	1.14667287	-2.491330224	0.012726577	0.01855106	negative
PRJNA729271	stage_1D_vs_E10	ordinary_muscle_development	ENSGALG00010029569	TMEM220	1.316134605	0.199284374	6.6043041	3.99389E-11	1.24284E-10	positive
PRJNA729271	stage_1D_vs_E10	ordinary_muscle_development	ENSGALG00010029570	TNFAIP1	-0.589190626	0.07904303	-7.454049133	9.05183E-14	3.35823E-13	negative
PRJNA729271	stage_1D_vs_E10	ordinary_muscle_development	ENSGALG00010029571	EPX	2.259016859	0.698742729	3.232973688	0.001225088	0.002023665	positive
PRJNA729271	stage_1D_vs_E10	ordinary_muscle_development	ENSGALG00010029572	TSPOAP1	-0.034631258	0.117750384	-0.294107386	0.768675847	0.798248775	negative
PRJNA729271	stage_1D_vs_E10	ordinary_muscle_development	ENSGALG00010029573	MAP2K4	-0.600975295	0.061119745	-9.832751947	8.13644E-23	5.04007E-22	negative
PRJNA729271	stage_1D_vs_E10	ordinary_muscle_development	ENSGALG00010029574	SGSM2	-0.212824566	0.131078388	-1.623643456	0.104451925	0.132391628	negative
PRJNA729271	stage_1D_vs_E10	ordinary_muscle_development	ENSGALG00010029575	ANKFN1	-5.231730041	0.908411202	-5.759209078	8.4509E-09	2.22154E-08	negative
PRJNA729271	stage_1D_vs_E10	ordinary_muscle_development	ENSGALG00010029576	ATP2A3	1.578267677	0.130164633	12.12516515	7.77034E-34	8.01645E-33	positive
PRJNA729271	stage_1D_vs_E10	ordinary_muscle_development	ENSGALG00010029577	NOG	-4.778015891	0.403547151	-11.84004368	2.42327E-32	2.34168E-31	negative
PRJNA729271	stage_1D_vs_E10	ordinary_muscle_development	ENSGALG00010029578	C17orf67	-1.978316482	0.525903321	-3.761749361	0.000168729	0.000306099	negative
PRJNA729271	stage_1D_vs_E10	ordinary_muscle_development	ENSGALG00010029579	GALNT17	0.417824679	0.183696464	2.2745385	0.022933629	0.032214648	positive
PRJNA729271	stage_1D_vs_E10	ordinary_muscle_development	ENSGALG00010029580	P2RX1	2.274385057	0.321095275	7.083209369	1.40854E-12	4.81868E-12	positive
PRJNA729271	stage_1D_vs_E10	ordinary_muscle_development	ENSGALG00010029581	DGKE	-0.012707449	0.104820707	-0.121230329	0.903508602	0.917625924	negative
PRJNA729271	stage_1D_vs_E10	ordinary_muscle_development	ENSGALG00010029582	SCPEP1	-0.693461918	0.150630619	-4.603724802	4.15001E-06	8.77269E-06	negative
PRJNA729271	stage_1D_vs_E10	ordinary_muscle_development	ENSGALG00010029583	LIMK1	-1.184102228	0.203995226	-5.804558526	6.45358E-09	1.71359E-08	negative
PRJNA729271	stage_1D_vs_E10	ordinary_muscle_development	ENSGALG00010029584	TRIM25	0.166325011	0.233390157	0.712647926	0.476063647	0.524756089	positive
PRJNA729271	stage_1D_vs_E10	ordinary_muscle_development	ENSGALG00010029585	ENSGALG00010029585	1.023508367	0.343393191	2.980572693	0.002877099	0.004546338	positive
PRJNA729271	stage_1D_vs_E10	ordinary_muscle_development	ENSGALG00010029586	COIL	-0.559799575	0.188974107	-2.962308348	0.003053418	0.00481027	negative
PRJNA729271	stage_1D_vs_E10	ordinary_muscle_development	ENSGALG00010029588	RAB11FIP4	-2.997355482	0.147823254	-20.27661687	2.0688E-91	1.26529E-89	negative
PRJNA729271	stage_1D_vs_E10	ordinary_muscle_development	ENSGALG00010029590	UTP6	-0.30070355	0.098167421	-3.063170513	0.002190052	0.003503842	negative
PRJNA729271	stage_1D_vs_E10	ordinary_muscle_development	ENSGALG00010029591	SUZ12	-0.67867044	0.079077608	-8.582333924	9.29673E-18	4.39948E-17	negative
PRJNA729271	stage_1D_vs_E10	ordinary_muscle_development	ENSGALG00010029592	MNT	1.028066632	0.164105902	6.264653624	3.73655E-10	1.08779E-09	positive
PRJNA729271	stage_1D_vs_E10	ordinary_muscle_development	ENSGALG00010029593	PAFAH1B1	0.095854016	0.078911183	1.214707628	0.224477622	0.267252824	positive
PRJNA729271	stage_1D_vs_E10	ordinary_muscle_development	ENSGALG00010029594	CRLF3	-0.806563526	0.068101436	-11.8435612	2.32372E-32	2.24849E-31	negative
PRJNA729271	stage_1D_vs_E10	ordinary_muscle_development	ENSGALG00010029595	CLUH	1.867256679	0.134608151	13.87179505	9.38972E-44	1.40169E-42	positive
PRJNA729271	stage_1D_vs_E10	ordinary_muscle_development	ENSGALG00010029596	ATAD5	-1.022575222	0.128662034	-7.947761987	1.89911E-15	7.84262E-15	negative
PRJNA729271	stage_1D_vs_E10	ordinary_muscle_development	ENSGALG00010029597	ADAP2	0.582991292	0.210973702	2.763336318	0.005721378	0.008726863	positive
PRJNA729271	stage_1D_vs_E10	ordinary_muscle_development	ENSGALG00010029598	SUPT4H1	-0.208389978	0.149501042	-1.393903176	0.163346845	0.200009505	negative
PRJNA729271	stage_1D_vs_E10	ordinary_muscle_development	ENSGALG00010029599	RNF43	-2.808158972	0.383905548	-7.314713174	2.57932E-13	9.28741E-13	negative
PRJNA729271	stage_1D_vs_E10	ordinary_muscle_development	ENSGALG00010029600	RHOT1	1.174925575	0.108434329	10.83536543	2.34029E-27	1.77605E-26	positive
PRJNA729271	stage_1D_vs_E10	ordinary_muscle_development	ENSGALG00010029601	HSF5	-2.196758403	0.535503317	-4.102231176	4.09185E-05	7.8725E-05	negative
PRJNA729271	stage_1D_vs_E10	ordinary_muscle_development	ENSGALG00010029602	MTMR4	-0.407368937	0.086395174	-4.715181623	2.41495E-06	5.22963E-06	negative
PRJNA729271	stage_1D_vs_E10	ordinary_muscle_development	ENSGALG00010029603	RHBDL3	-0.265794139	0.425888819	-0.624092785	0.532566652	0.579372082	negative
PRJNA729271	stage_1D_vs_E10	ordinary_muscle_development	ENSGALG00010029604	ENSGALG00010029604	-0.487816331	0.122891746	-3.969480019	7.20296E-05	0.000135558	negative
PRJNA729271	stage_1D_vs_E10	ordinary_muscle_development	ENSGALG00010029605	C17orf75	-0.396094147	0.091859602	-4.311951456	1.6182E-05	3.23484E-05	negative
PRJNA729271	stage_1D_vs_E10	ordinary_muscle_development	ENSGALG00010029606	ABHD11	0.960923579	0.204152543	4.706890067	2.51524E-06	5.43669E-06	positive
PRJNA729271	stage_1D_vs_E10	ordinary_muscle_development	ENSGALG00010029607	ZNF207	-0.434157233	0.064829376	-6.696921352	2.12856E-11	6.73951E-11	negative
PRJNA729271	stage_1D_vs_E10	ordinary_muscle_development	ENSGALG00010029608	ENSGALG00010029608	-2.192017561	0.319605016	-6.858520516	6.95773E-12	2.27299E-11	negative
PRJNA729271	stage_1D_vs_E10	ordinary_muscle_development	ENSGALG00010029609	METTL27	0.048251263	0.139660146	0.345490568	0.7297256	0.763654074	positive
PRJNA729271	stage_1D_vs_E10	ordinary_muscle_development	ENSGALG00010029610	ERN1	0.145457243	0.078925251	1.842974722	0.065332701	0.085569486	positive
PRJNA729271	stage_1D_vs_E10	ordinary_muscle_development	ENSGALG00010029611	YWHAG	0.894208277	0.130473262	6.853574935	7.20269E-12	2.34983E-11	positive
PRJNA729271	stage_1D_vs_E10	ordinary_muscle_development	ENSGALG00010029612	HSPB1	2.607741483	0.195316532	13.35136074	1.16305E-40	1.56968E-39	positive
PRJNA729271	stage_1D_vs_E10	ordinary_muscle_development	ENSGALG00010029614	MDH2	1.403281455	0.128482988	10.92192419	9.05567E-28	7.0156E-27	positive
PRJNA729271	stage_1D_vs_E10	ordinary_muscle_development	ENSGALG00010029615	ENSGALG00010029615	-1.866005745	0.331661101	-5.626242395	1.84177E-08	4.70672E-08	negative
PRJNA729271	stage_1D_vs_E10	ordinary_muscle_development	ENSGALG00010029616	TMEM120A	2.422906444	0.132516002	18.28387824	1.11228E-74	4.63292E-73	positive
PRJNA729271	stage_1D_vs_E10	ordinary_muscle_development	ENSGALG00010029617	POR	0.126825136	0.094366738	1.343960161	0.17896127	0.217292981	positive
PRJNA729271	stage_1D_vs_E10	ordinary_muscle_development	ENSGALG00010029618	TEX2	1.83546248	0.112129943	16.36906643	3.18055E-60	8.84877E-59	positive
PRJNA729271	stage_1D_vs_E10	ordinary_muscle_development	ENSGALG00010029619	ENSGALG00010029619	0.005191423	0.105261201	0.049319436	0.960664731	0.966438525	positive
PRJNA729271	stage_1D_vs_E10	ordinary_muscle_development	ENSGALG00010029620	ENSGALG00010029620	0.386394897	0.392306164	0.984932006	0.324657471	0.37289303	positive
PRJNA729271	stage_1D_vs_E10	ordinary_muscle_development	ENSGALG00010029621	TAF15	-2.136631333	0.122652548	-17.42019529	5.79789E-68	1.98677E-66	negative
PRJNA729271	stage_1D_vs_E10	ordinary_muscle_development	ENSGALG00010029622	MMP28	-0.499501447	0.158832876	-3.144824045	0.001661868	0.002702352	negative
PRJNA729271	stage_1D_vs_E10	ordinary_muscle_development	ENSGALG00010029623	ENSGALG00010029623	0.055723044	0.3941558	0.141373141	0.887575171	0.904104154	positive
PRJNA729271	stage_1D_vs_E10	ordinary_muscle_development	ENSGALG00010029624	RASL10B	-1.165249974	0.292238648	-3.987323306	6.68229E-05	0.000126218	negative
PRJNA729271	stage_1D_vs_E10	ordinary_muscle_development	ENSGALG00010029625	PECAM1	2.311809003	0.174292058	13.26399511	3.74406E-40	4.9247E-39	positive
PRJNA729271	stage_1D_vs_E10	ordinary_muscle_development	ENSGALG00010029626	AP2B1	-0.337519051	0.108731438	-3.104153283	0.001908244	0.003077779	negative
PRJNA729271	stage_1D_vs_E10	ordinary_muscle_development	ENSGALG00010029627	PEX12	-0.473436529	0.147659286	-3.206276705	0.001344646	0.002210827	negative
PRJNA729271	stage_1D_vs_E10	ordinary_muscle_development	ENSGALG00010029628	UNC45B	0.768700588	0.160682543	4.783970757	1.71866E-06	3.76427E-06	positive
PRJNA729271	stage_1D_vs_E10	ordinary_muscle_development	ENSGALG00010029629	ENSGALG00010029629	1.056071742	0.262411372	4.024489243	5.70991E-05	0.000108587	positive
PRJNA729271	stage_1D_vs_E10	ordinary_muscle_development	ENSGALG00010029630	RAD51D	0.082674284	0.11904188	0.694497468	0.487370288	0.535793123	positive
PRJNA729271	stage_1D_vs_E10	ordinary_muscle_development	ENSGALG00010029631	LIG3	-0.273217078	0.086554417	-3.156593145	0.001596239	0.002600886	negative
PRJNA729271	stage_1D_vs_E10	ordinary_muscle_development	ENSGALG00010029632	TMEM132E	1.536624866	0.225473369	6.815105816	9.41944E-12	3.05447E-11	positive
PRJNA729271	stage_1D_vs_E10	ordinary_muscle_development	ENSGALG00010029633	POLG2	0.380891789	0.174613843	2.181337875	0.029158435	0.040271702	positive
PRJNA729271	stage_1D_vs_E10	ordinary_muscle_development	ENSGALG00010029634	DDX5	-0.444957651	0.091159908	-4.88106733	1.05513E-06	2.35439E-06	negative
PRJNA729271	stage_1D_vs_E10	ordinary_muscle_development	ENSGALG00010029635	ENSGALG00010029635	0.061180866	0.582313891	0.105065099	0.916324153	0.92888444	positive
PRJNA729271	stage_1D_vs_E10	ordinary_muscle_development	ENSGALG00010029636	SPATA20	0.483807599	0.075704725	6.39071869	1.65108E-10	4.92335E-10	positive
PRJNA729271	stage_1D_vs_E10	ordinary_muscle_development	ENSGALG00010029638	EPN3	1.037930051	0.196145906	5.291622296	1.21236E-07	2.91236E-07	positive
PRJNA729271	stage_1D_vs_E10	ordinary_muscle_development	ENSGALG00010029639	ENSGALG00010029639	-1.985673235	0.784573121	-2.530896334	0.011377147	0.016701615	negative
PRJNA729271	stage_1D_vs_E10	ordinary_muscle_development	ENSGALG00010029641	ENSGALG00010029641	-3.391243189	0.757045564	-4.479576064	7.47914E-06	1.54607E-05	negative
PRJNA729271	stage_1D_vs_E10	ordinary_muscle_development	ENSGALG00010029642	NAT9	0.674639043	0.144796351	4.659226837	3.17399E-06	6.79371E-06	positive
PRJNA729271	stage_1D_vs_E10	ordinary_muscle_development	ENSGALG00010029644	MYCBPAP	0.624740467	0.162385029	3.847278731	0.000119437	0.000220092	positive
PRJNA729271	stage_1D_vs_E10	ordinary_muscle_development	ENSGALG00010029645	ENSGALG00010029645	-1.451773487	0.384646276	-3.774307926	0.000160452	0.000291851	negative
PRJNA729271	stage_1D_vs_E10	ordinary_muscle_development	ENSGALG00010029646	MRPS17	-0.488499986	0.108993974	-4.481899023	7.39817E-06	1.5302E-05	negative
PRJNA729271	stage_1D_vs_E10	ordinary_muscle_development	ENSGALG00010029647	RSAD1	0.998191685	0.148071712	6.741271998	1.57006E-11	5.02052E-11	positive
PRJNA729271	stage_1D_vs_E10	ordinary_muscle_development	ENSGALG00010029648	NIPSNAP2	1.169521451	0.121839459	9.598872673	8.08236E-22	4.78179E-21	positive
PRJNA729271	stage_1D_vs_E10	ordinary_muscle_development	ENSGALG00010029649	PSPH	1.510050824	0.260236649	5.80260633	6.5292E-09	1.73239E-08	positive
PRJNA729271	stage_1D_vs_E10	ordinary_muscle_development	ENSGALG00010029650	ACSF2	-0.858173237	0.136341383	-6.294297599	3.08795E-10	9.05519E-10	negative
PRJNA729271	stage_1D_vs_E10	ordinary_muscle_development	ENSGALG00010029651	ENSGALG00010029651	3.344100871	0.274987359	12.1609258	5.01867E-34	5.21848E-33	positive
PRJNA729271	stage_1D_vs_E10	ordinary_muscle_development	ENSGALG00010029652	LRRC59	-1.033531467	0.102228958	-10.10996776	4.99007E-24	3.245E-23	negative
PRJNA729271	stage_1D_vs_E10	ordinary_muscle_development	ENSGALG00010029653	CCT6A	-0.735503047	0.090321295	-8.14318539	3.85012E-16	1.6654E-15	negative
PRJNA729271	stage_1D_vs_E10	ordinary_muscle_development	ENSGALG00010029654	EME1	-1.914011348	0.127633513	-14.99615029	7.7803E-51	1.55124E-49	negative
PRJNA729271	stage_1D_vs_E10	ordinary_muscle_development	ENSGALG00010029655	PHKG1	2.877558173	0.128484993	22.39606441	4.2992E-111	3.9693E-109	positive
PRJNA729271	stage_1D_vs_E10	ordinary_muscle_development	ENSGALG00010029656	MRPL27	-0.133223634	0.113933607	-1.169309377	0.242279007	0.286213056	negative
PRJNA729271	stage_1D_vs_E10	ordinary_muscle_development	ENSGALG00010029657	CHCHD2	1.545264809	0.146756865	10.52942094	6.32222E-26	4.50544E-25	positive
PRJNA729271	stage_1D_vs_E10	ordinary_muscle_development	ENSGALG00010029658	XYLT2	0.282473234	0.124890106	2.261774309	0.023711351	0.033220185	positive
PRJNA729271	stage_1D_vs_E10	ordinary_muscle_development	ENSGALG00010029659	VKORC1L1	-0.919426493	0.146392634	-6.280551588	3.37374E-10	9.85736E-10	negative
PRJNA729271	stage_1D_vs_E10	ordinary_muscle_development	ENSGALG00010029660	CD300LG	-0.811338312	0.323670455	-2.506680176	0.012187093	0.017818429	negative
PRJNA729271	stage_1D_vs_E10	ordinary_muscle_development	ENSGALG00010029661	KRABZFP	-1.853549709	1.006517887	-1.841546715	0.065541481	0.08583518	negative
PRJNA729271	stage_1D_vs_E10	ordinary_muscle_development	ENSGALG00010029663	GUSB	-0.051220328	0.153819452	-0.332989926	0.739141888	0.77244857	negative
PRJNA729271	stage_1D_vs_E10	ordinary_muscle_development	ENSGALG00010029664	RAB37	1.081276502	0.398393523	2.71409157	0.006645779	0.010053284	positive
PRJNA729271	stage_1D_vs_E10	ordinary_muscle_development	ENSGALG00010029665	ASL1	0.579454388	0.191305423	3.02894909	0.00245406	0.003905105	positive
PRJNA729271	stage_1D_vs_E10	ordinary_muscle_development	ENSGALG00010029666	SLC9A3R1	-0.659337724	0.200977062	-3.280661575	0.001035639	0.001724479	negative
PRJNA729271	stage_1D_vs_E10	ordinary_muscle_development	ENSGALG00010029667	TMEM104	-0.197685318	0.093281675	-2.119229941	0.034071039	0.046625728	negative
PRJNA729271	stage_1D_vs_E10	ordinary_muscle_development	ENSGALG00010029668	CRCP	-0.046335559	0.181595441	-0.255158162	0.798600944	0.825716576	negative
PRJNA729271	stage_1D_vs_E10	ordinary_muscle_development	ENSGALG00010029669	HID1	0.527772688	0.073517956	7.17882698	7.03121E-13	2.46118E-12	positive
PRJNA729271	stage_1D_vs_E10	ordinary_muscle_development	ENSGALG00010029670	TPST1	0.616992084	0.169293189	3.644518047	0.000267893	0.000474418	positive
PRJNA729271	stage_1D_vs_E10	ordinary_muscle_development	ENSGALG00010029671	GRIN2C	2.978779037	0.199144296	14.95789287	1.38337E-50	2.7356E-49	positive
PRJNA729271	stage_1D_vs_E10	ordinary_muscle_development	ENSGALG00010029672	KCTD7	-1.939444173	0.23964103	-8.093122336	5.81544E-16	2.48584E-15	negative
PRJNA729271	stage_1D_vs_E10	ordinary_muscle_development	ENSGALG00010029673	TMEM248	-0.519806399	0.090329215	-5.754576729	8.68591E-09	2.27919E-08	negative
PRJNA729271	stage_1D_vs_E10	ordinary_muscle_development	ENSGALG00010029674	FDXR	-0.587895877	0.11283017	-5.210449248	1.88384E-07	4.45307E-07	negative
PRJNA729271	stage_1D_vs_E10	ordinary_muscle_development	ENSGALG00010029675	DHRS13	-0.232373123	0.227710758	-1.020474945	0.307503266	0.354792633	negative
PRJNA729271	stage_1D_vs_E10	ordinary_muscle_development	ENSGALG00010029676	FADS6	-2.021298499	1.152665076	-1.753587006	0.079501299	0.102862745	negative
PRJNA729271	stage_1D_vs_E10	ordinary_muscle_development	ENSGALG00010029677	PHF12	0.371812727	0.099909189	3.721506804	0.000198038	0.000355751	positive
PRJNA729271	stage_1D_vs_E10	ordinary_muscle_development	ENSGALG00010029682	CDR2L	-0.979966532	0.12453907	-7.868747814	3.58208E-15	1.454E-14	negative
PRJNA729271	stage_1D_vs_E10	ordinary_muscle_development	ENSGALG00010029683	MRPL58	0.037257292	0.110640236	0.336742699	0.736310872	0.769934071	positive
PRJNA729271	stage_1D_vs_E10	ordinary_muscle_development	ENSGALG00010029684	ENSGALG00010029684	0.000796475	0.548716058	0.001451525	0.998841851	0.99899921	positive
PRJNA729271	stage_1D_vs_E10	ordinary_muscle_development	ENSGALG00010029685	ATP5PD	1.20006209	0.114886048	10.44567296	1.53364E-25	1.07913E-24	positive
PRJNA729271	stage_1D_vs_E10	ordinary_muscle_development	ENSGALG00010029686	KCTD2	-0.01124524	0.059625491	-0.188597857	0.850408013	0.872128495	negative
PRJNA729271	stage_1D_vs_E10	ordinary_muscle_development	ENSGALG00010029687	SLC16A5	-0.766162023	0.442293271	-1.732248879	0.08322926	0.107236277	negative

PRJNA729271	stage_1D_vs_E10	ordinary_muscle_development	ENSGALG00010029689	ENSGALG00010029689	1.710454901	0.175669723	9.736765506	2.10136E-22	1.27819E-21	positive
PRJNA729271	stage_1D_vs_E10	ordinary_muscle_development	ENSGALG00010029690	ENSGALG00010029690	1.635207149	0.160387095	10.19537855	2.07933E-24	1.3813E-23	positive
PRJNA729271	stage_1D_vs_E10	ordinary_muscle_development	ENSGALG00010029691	NUFIP2	1.016654717	0.139928785	7.265515197	3.71619E-13	1.3261E-12	positive
PRJNA729271	stage_1D_vs_E10	ordinary_muscle_development	ENSGALG00010029692	CRYBA1	-1.624919506	0.589862354	-2.754743534	0.005873816	0.008949959	negative
PRJNA729271	stage_1D_vs_E10	ordinary_muscle_development	ENSGALG00010029693	HN1	-1.564231442	0.127086902	-12.30836077	8.1665E-35	8.79446E-34	negative
PRJNA729271	stage_1D_vs_E10	ordinary_muscle_development	ENSGALG00010029694	ARMC7	-0.070157542	0.091652228	-0.765475576	0.443988405	0.493225929	negative
PRJNA729271	stage_1D_vs_E10	ordinary_muscle_development	ENSGALG00010029695	TAOK1	0.444133151	0.086142132	5.155817962	2.52526E-07	5.90191E-07	positive
PRJNA729271	stage_1D_vs_E10	ordinary_muscle_development	ENSGALG00010029696	SUMO2	-0.236083903	0.107679186	-2.19247482	0.028345239	0.039193384	negative
PRJNA729271	stage_1D_vs_E10	ordinary_muscle_development	ENSGALG00010029697	GIT1	0.579944505	0.166748072	3.477968291	0.00050523	0.000869648	positive
PRJNA729271	stage_1D_vs_E10	ordinary_muscle_development	ENSGALG00010029698	NUP85	-0.648555615	0.085732149	-7.564905622	3.88146E-14	1.47475E-13	negative
PRJNA729271	stage_1D_vs_E10	ordinary_muscle_development	ENSGALG00010029699	ANKRD13B	-1.03244916	0.175253155	-5.891187284	3.83431E-09	1.03613E-08	negative
PRJNA729271	stage_1D_vs_E10	ordinary_muscle_development	ENSGALG00010029700	GGA3	0.286483121	0.079168148	3.618666465	0.000296125	0.000521991	positive
PRJNA729271	stage_1D_vs_E10	ordinary_muscle_development	ENSGALG00010029701	MRPS7	0.240693416	0.065526796	3.673205948	0.000239526	0.000426475	positive
PRJNA729271	stage_1D_vs_E10	ordinary_muscle_development	ENSGALG00010029702	CORO6	3.310592797	0.170258674	19.44448828	3.24496E-84	1.69804E-82	positive
PRJNA729271	stage_1D_vs_E10	ordinary_muscle_development	ENSGALG00010029703	MIF4GD	-0.019718252	0.115011887	-0.171445337	0.863873609	0.883874353	negative
PRJNA729271	stage_1D_vs_E10	ordinary_muscle_development	ENSGALG00010029704	SLC25A19	0.974799443	0.098993264	9.847129001	7.05299E-23	4.38392E-22	positive
PRJNA729271	stage_1D_vs_E10	ordinary_muscle_development	ENSGALG00010029705	GRB2	-0.858980176	0.067942927	-12.64267252	1.22808E-36	1.42067E-35	negative
PRJNA729271	stage_1D_vs_E10	ordinary_muscle_development	ENSGALG00010029706	SSH2	2.517937072	0.215639787	11.67658856	1.679E-31	1.56107E-30	positive
PRJNA729271	stage_1D_vs_E10	ordinary_muscle_development	ENSGALG00010029707	NSRP1	0.772980891	0.220805519	3.500731747	0.000463983	0.000801792	positive
PRJNA729271	stage_1D_vs_E10	ordinary_muscle_development	ENSGALG00010029708	TMEM94	0.032294947	0.084647702	0.381521838	0.70281607	0.739604976	positive
PRJNA729271	stage_1D_vs_E10	ordinary_muscle_development	ENSGALG00010029709	SLC6A4	1.363897136	0.194629954	7.007642479	2.42367E-12	8.17954E-12	positive
PRJNA729271	stage_1D_vs_E10	ordinary_muscle_development	ENSGALG00010029710	BLMH	-0.998828208	0.083322081	-11.98755715	4.12903E-33	4.13046E-32	negative
PRJNA729271	stage_1D_vs_E10	ordinary_muscle_development	ENSGALG00010029711	CASKIN2	1.78712436	0.110284733	16.20463975	4.67619E-59	1.23915E-57	positive
PRJNA729271	stage_1D_vs_E10	ordinary_muscle_development	ENSGALG00010029713	CPD	0.319202398	0.116458174	2.740918796	0.006126765	0.009317748	positive
PRJNA729271	stage_1D_vs_E10	ordinary_muscle_development	ENSGALG00010029716	GOSR1	0.199720095	0.062257308	3.207978327	0.001336716	0.002199035	positive
PRJNA729271	stage_1D_vs_E10	ordinary_muscle_development	ENSGALG00010029717	TRARG1	-0.460669915	0.426715277	-1.079572118	0.280332763	0.326458054	negative
PRJNA729271	stage_1D_vs_E10	ordinary_muscle_development	ENSGALG00010029718	ABR	0.742027621	0.109352503	6.785648273	1.15566E-11	3.72501E-11	positive
PRJNA729271	stage_1D_vs_E10	ordinary_muscle_development	ENSGALG00010029720	TIMM22	0.168717387	0.106887625	1.578455753	0.114460946	0.144120171	positive
PRJNA729271	stage_1D_vs_E10	ordinary_muscle_development	ENSGALG00010029721	EXOC7	-0.101922535	0.105296679	-0.967955834	0.333066428	0.381613933	negative
PRJNA729271	stage_1D_vs_E10	ordinary_muscle_development	ENSGALG00010029722	SPECC1	1.914197542	0.141555127	13.52262956	1.14991E-41	1.59961E-40	positive
PRJNA729271	stage_1D_vs_E10	ordinary_muscle_development	ENSGALG00010029723	ADORA2B	0.35449975	0.182005521	1.94774174	0.051445873	0.068495263	positive
PRJNA729271	stage_1D_vs_E10	ordinary_muscle_development	ENSGALG00010029724	CACNA1G	-1.52836102	0.213492455	-7.158852618	8.13551E-13	2.83404E-12	negative
PRJNA729271	stage_1D_vs_E10	ordinary_muscle_development	ENSGALG00010029725	TSEN54	-0.138504001	0.065830945	-2.103934568	0.035384155	0.048322341	negative
PRJNA729271	stage_1D_vs_E10	ordinary_muscle_development	ENSGALG00010029726	SEPTIN9	-1.227777108	0.125481265	-9.784545178	1.31184E-22	8.06065E-22	negative
PRJNA729271	stage_1D_vs_E10	ordinary_muscle_development	ENSGALG00010029727	ZSWIM7	-0.048052813	0.139034024	-0.345619089	0.729628998	0.76360811	negative
PRJNA729271	stage_1D_vs_E10	ordinary_muscle_development	ENSGALG00010029728	TNRC6C	0.891554379	0.10287947	8.666008686	4.47533E-18	2.15443E-17	positive
PRJNA729271	stage_1D_vs_E10	ordinary_muscle_development	ENSGALG00010029729	RASL10A	3.912428167	0.772926914	5.061834563	4.15241E-07	9.53719E-07	positive
PRJNA729271	stage_1D_vs_E10	ordinary_muscle_development	ENSGALG00010029730	TTC19	1.007766226	0.132190201	7.623607613	2.46682E-14	9.49207E-14	positive
PRJNA729271	stage_1D_vs_E10	ordinary_muscle_development	ENSGALG00010029731	NCOR1	0.395208319	0.184610913	2.140763585	0.032293108	0.044353855	positive
PRJNA729271	stage_1D_vs_E10	ordinary_muscle_development	ENSGALG00010029732	PIGL	0.282419701	0.179602782	1.572468409	0.11584196	0.145833699	positive
PRJNA729271	stage_1D_vs_E10	ordinary_muscle_development	ENSGALG00010029733	ENSGALG00010029733	2.377245968	0.164283071	14.47042571	1.86315E-47	3.21888E-46	positive
PRJNA729271	stage_1D_vs_E10	ordinary_muscle_development	ENSGALG00010029734	SEC14L1	-0.194709135	0.083740613	-2.325145792	0.020064166	0.028379204	negative
PRJNA729271	stage_1D_vs_E10	ordinary_muscle_development	ENSGALG00010029735	SRP68	-0.301871007	0.114101146	-2.64564395	0.008153559	0.012194164	negative
PRJNA729271	stage_1D_vs_E10	ordinary_muscle_development	ENSGALG00010029736	ENSGALG00010029736	1.389900939	0.133066998	10.44512136	1.54258E-25	1.0849E-24	positive
PRJNA729271	stage_1D_vs_E10	ordinary_muscle_development	ENSGALG00010029737	ENSGALG00010029737	-5.2207943	0.532875039	-9.797408235	1.15511E-22	7.12181E-22	negative
PRJNA729271	stage_1D_vs_E10	ordinary_muscle_development	ENSGALG00010029738	TRPV2	0.872042613	0.166793884	5.228264914	1.71108E-07	4.05728E-07	positive
PRJNA729271	stage_1D_vs_E10	ordinary_muscle_development	ENSGALG00010029739	P2RX5	2.851268601	0.202644647	14.07028826	5.78332E-45	9.05283E-44	positive
PRJNA729271	stage_1D_vs_E10	ordinary_muscle_development	ENSGALG00010029740	HASPIN	-1.675708954	0.209182749	-8.01074164	1.14019E-15	4.77246E-15	negative
PRJNA729271	stage_1D_vs_E10	ordinary_muscle_development	ENSGALG00010029741	EMC6	0.122815831	0.163939386	0.749153904	0.453764449	0.502737575	positive
PRJNA729271	stage_1D_vs_E10	ordinary_muscle_development	ENSGALG00010029742	TAX1BP3	-1.204576726	0.119035854	-10.11944457	4.52979E-24	2.95896E-23	negative
PRJNA729271	stage_1D_vs_E10	ordinary_muscle_development	ENSGALG00010029743	MYOCD	3.718079468	0.253674111	14.65691337	1.2168E-48	2.22977E-47	positive
PRJNA729271	stage_1D_vs_E10	ordinary_muscle_development	ENSGALG00010029744	CTNS	0.273022911	0.090117793	3.029622712	0.002448594	0.003896835	positive
PRJNA729271	stage_1D_vs_E10	ordinary_muscle_development	ENSGALG00010029745	SHPK	0.942630388	0.167365556	5.63216478	1.77962E-08	4.5559E-08	positive
PRJNA729271	stage_1D_vs_E10	ordinary_muscle_development	ENSGALG00010029746	TRPV1	0.382608517	0.311495345	1.228296097	0.219335836	0.261668555	positive
PRJNA729271	stage_1D_vs_E10	ordinary_muscle_development	ENSGALG00010029747	TRPV3	0.267298867	0.337458895	0.792093115	0.428306384	0.477782133	positive
PRJNA729271	stage_1D_vs_E10	ordinary_muscle_development	ENSGALG00010029749	ASPA	0.580705235	0.298287952	1.946794131	0.051559421	0.068621229	positive
PRJNA729271	stage_1D_vs_E10	ordinary_muscle_development	ENSGALG00010029750	TEN1	-2.230798587	0.239910545	-9.298459937	1.42495E-20	7.86542E-20	negative
PRJNA729271	stage_1D_vs_E10	ordinary_muscle_development	ENSGALG00010029752	ENSGALG00010029752	0.820155511	0.16916514	4.848253662	1.24553E-06	2.7597E-06	positive
PRJNA729271	stage_1D_vs_E10	ordinary_muscle_development	ENSGALG00010029753	AKAP10	-0.04293928	0.074286416	-0.578023316	0.563248378	0.608729887	negative
PRJNA729271	stage_1D_vs_E10	ordinary_muscle_development	ENSGALG00010029754	NLE1	-0.287825946	0.177281892	-1.623549603	0.104471969	0.132405455	negative
PRJNA729271	stage_1D_vs_E10	ordinary_muscle_development	ENSGALG00010029755	ACOX1	0.435970503	0.11907326	3.66136361	0.000250876	0.000445589	positive
PRJNA729271	stage_1D_vs_E10	ordinary_muscle_development	ENSGALG00010029756	ELAC2	-0.191034457	0.082771686	-2.307968664	0.021000879	0.029634711	negative
PRJNA729271	stage_1D_vs_E10	ordinary_muscle_development	ENSGALG00010029757	ULK2	0.772831927	0.209124122	3.695565669	0.000219398	0.000392033	positive
PRJNA729271	stage_1D_vs_E10	ordinary_muscle_development	ENSGALG00010029758	DNAH9	1.011893606	0.302109724	3.349424155	0.000809797	0.001364885	positive
PRJNA729271	stage_1D_vs_E10	ordinary_muscle_development	ENSGALG00010029759	ALDH3A2	0.354802425	0.107627981	3.296563037	0.000978756	0.001633515	positive
PRJNA729271	stage_1D_vs_E10	ordinary_muscle_development	ENSGALG00010029760	SHISA6	-3.707247036	0.448763478	-8.261026611	1.44425E-16	6.39023E-16	negative
PRJNA729271	stage_1D_vs_E10	ordinary_muscle_development	ENSGALG00010029761	MRPL38	0.23799555	0.118121203	2.014841916	0.043921216	0.059084735	positive
PRJNA729271	stage_1D_vs_E10	ordinary_muscle_development	ENSGALG00010029762	SLC47A2	5.563026247	0.461978374	12.04174601	2.1437E-33	2.17903E-32	positive
PRJNA729271	stage_1D_vs_E10	ordinary_muscle_development	ENSGALG00010029763	GEMIN4	-0.561357121	0.097681555	-5.74680781	9.0944E-09	2.38034E-08	negative
PRJNA729271	stage_1D_vs_E10	ordinary_muscle_development	ENSGALG00010029764	NXN	-1.137789615	0.125593418	-9.059309248	1.31279E-19	6.87461E-19	negative
PRJNA729271	stage_1D_vs_E10	ordinary_muscle_development	ENSGALG00010029765	KCNJ16	-1.342524116	0.302440132	-4.438974778	9.03884E-06	1.85525E-05	negative
PRJNA729271	stage_1D_vs_E10	ordinary_muscle_development	ENSGALG00010029766	ENSGALG00010029766	0.222704437	0.146146542	1.523843355	0.127547846	0.159215125	positive
PRJNA729271	stage_1D_vs_E10	ordinary_muscle_development	ENSGALG00010029767	GLOD4	-0.719731965	0.087840458	-8.193627169	2.53469E-16	1.1063E-15	negative
PRJNA729271	stage_1D_vs_E10	ordinary_muscle_development	ENSGALG00010029768	KCNJ2	2.336446353	0.174807555	13.3658202	9.57735E-41	1.29742E-39	positive
PRJNA729271	stage_1D_vs_E10	ordinary_muscle_development	ENSGALG00010029770	TLCD3A	-0.893895295	0.230993785	-3.869780711	0.000108933	0.000201427	negative
PRJNA729271	stage_1D_vs_E10	ordinary_muscle_development	ENSGALG00010029771	SOX9	-3.705629522	0.260286699	-14.23672257	5.42105E-46	8.84888E-45	negative
PRJNA729271	stage_1D_vs_E10	ordinary_muscle_development	ENSGALG00010029772	VPS53	0.073647033	0.068481091	1.075436036	0.282179555	0.328318563	positive
PRJNA729271	stage_1D_vs_E10	ordinary_muscle_development	ENSGALG00010029773	TRIM65	0.776168227	0.13316572	5.828588817	5.5898E-09	1.49216E-08	positive
PRJNA729271	stage_1D_vs_E10	ordinary_muscle_development	ENSGALG00010029774	RFLNB	-0.913194487	0.12559534	-7.27092653	3.5703E-13	1.27498E-12	negative
PRJNA729271	stage_1D_vs_E10	ordinary_muscle_development	ENSGALG00010029775	ENSGALG00010029775	-0.134558143	0.142057483	-0.947209115	0.343532231	0.39248835	negative
PRJNA729271	stage_1D_vs_E10	ordinary_muscle_development	ENSGALG00010029776	WBP2	0.970766334	0.135959741	7.140101356	9.32621E-13	3.23328E-12	positive
PRJNA729271	stage_1D_vs_E10	ordinary_muscle_development	ENSGALG00010029777	ENSGALG00010029777	-0.310501214	0.138274128	-2.245548162	0.024732964	0.034551302	negative
PRJNA729271	stage_1D_vs_E10	ordinary_muscle_development	ENSGALG00010029778	DOC2B	0.316129184	0.345441165	0.915146242	0.360114862	0.409080323	positive
PRJNA729271	stage_1D_vs_E10	ordinary_muscle_development	ENSGALG00010029780	TEX14	-0.186735378	0.257875924	-0.72412878	0.468986677	0.517703289	negative
PRJNA729271	stage_1D_vs_E10	ordinary_muscle_development	ENSGALG00010029781	UNC13D	0.609178637	0.252425278	2.413302827	0.015808679	0.022699039	positive
PRJNA729271	stage_1D_vs_E10	ordinary_muscle_development	ENSGALG00010029782	UNK	0.293111109	0.119953636	2.443536675	0.014544091	0.02101232	positive
PRJNA729271	stage_1D_vs_E10	ordinary_muscle_development	ENSGALG00010029783	RAD51C	-0.353245992	0.121089982	-2.917218967	0.003531677	0.005528855	negative
PRJNA729271	stage_1D_vs_E10	ordinary_muscle_development	ENSGALG00010029784	CLDN3	-6.742973414	0.967407285	-6.97014951	3.16604E-12	1.06059E-11	negative
PRJNA729271	stage_1D_vs_E10	ordinary_muscle_development	ENSGALG00010029785	ENSGALG00010029785	2.575115482	0.242395895	10.62359359	2.31475E-26	1.68266E-25	positive
PRJNA729271	stage_1D_vs_E10	ordinary_muscle_development	ENSGALG00010029787	H3-3B	-0.665089056	0.06398892	-10.39381595	2.64552E-25	1.84094E-24	negative
PRJNA729271	stage_1D_vs_E10	ordinary_muscle_development	ENSGALG00010029791	GALK1	-0.304771021	0.111431537	-2.735051762	0.006237047	0.009474533	negative
PRJNA729271	stage_1D_vs_E10	ordinary_muscle_development	ENSGALG00010029804	ITGB4	1.6118842	0.224854043	7.168580032	7.57796E-13	2.64553E-12	positive
PRJNA729271	stage_1D_vs_E10	ordinary_muscle_development	ENSGALG00010029812	SAP30BP	-0.384795144	0.115353672	-3.335785832	0.000850587	0.001428816	negative
PRJNA729271	stage_1D_vs_E10	ordinary_muscle_development	ENSGALG00010029835	RECQL5	-0.431739811	0.108470432	-3.980253435	6.88418E-05	0.000129795	negative
PRJNA729271	stage_1D_vs_E10	ordinary_muscle_development	ENSGALG00010029841	SMIM5	0.996769764	0.130076891	7.662927331	1.81742E-14	7.03996E-14	positive
PRJNA729271	stage_1D_vs_E10	ordinary_muscle_development	ENSGALG00010029859	CSNK1D	-0.200297203	0.052175218	-3.838933732	0.00012357	0.000227187	negative
PRJNA729271	stage_1D_vs_E10	ordinary_muscle_development	ENSGALG00010029864	SLC16A3	1.565375605	0.338325556	4.626832286	3.71301E-06	7.89035E-06	positive
PRJNA729271	stage_1D_vs_E10	ordinary_muscle_development	ENSGALG00010029879	ENSGALG00010029879	0.415908559	0.302258395	1.376003331	0.168820602	0.206033058	positive
PRJNA729271	stage_1D_vs_E10	ordinary_muscle_development	ENSGALG00010029895	FASN	-1.611985456	0.168385839	-9.573165227	1.03682E-21	6.09683E-21	negative
PRJNA729271	stage_1D_vs_E10	ordinary_muscle_development	ENSGALG00010029907	NTN1	0.243051716	0.264754543	0.918026612	0.358604934	0.407524776	positive
PRJNA729271	stage_1D_vs_E10	ordinary_muscle_development	ENSGALG00010029921	STX8	-0.33017209	0.105233044	-3.13753243	0.001703764	0.002767686	negative
PRJNA729271	stage_1D_vs_E10	ordinary_muscle_development	ENSGALG00010029925	GGNBP2	0.44682624	0.124821613	3.579718515	0.000343965	0.000602728	positive
PRJNA729271	stage_1D_vs_E10	ordinary_muscle_development	ENSGALG00010029926	BLTP2	0.365054992	0.132059116	2.764330119	0.00570398	0.008703073	positive
PRJNA729271	stage_1D_vs_E10	ordinary_muscle_development	ENSGALG00010029927	ENSGALG00010029927	-2.821296212	0.8337996	-3.383662227	0.000715259	0.001211886	negative
PRJNA729271	stage_1D_vs_E10	ordinary_muscle_development	ENSGALG00010029928	USP43	0.748045146	0.131946587	5.669302713	1.4338E-08	3.69605E-08	positive
PRJNA729271	stage_1D_vs_E10	ordinary_muscle_development	ENSGALG00010029929	GDPD1	-0.369549381	0.09479009	-3.898607751	9.67473E-05	0.000179743	negative
PRJNA729271	stage_1D_vs_E10	ordinary_muscle_development	ENSGALG00010029930	ARHGAP44	1.160033299	0.213605806	5.430719895	5.61272E-08	1.38244E-07	positive
PRJNA729271	stage_1D_vs_E10	ordinary_muscle_development	ENSGALG00010029932	RASA4B	2.360428334	0.264111909	8.937227964	3.99055E-19	2.02958E-18	positive
PRJNA729271	stage_1D_vs_E10	ordinary_muscle_development	ENSGALG00010029933	TVP23B	-0.150029405	0.094410359	-1.589120169	0.112033265	0.141259758	negative
PRJNA729271	stage_1D_vs_E10	ordinary_muscle_development	ENSGALG00010029934	TEKT3	3.033894682	0.438433663	6.919848854	4.52126E-12	1.49488E-11	positive
PRJNA729271	stage_1D_vs_E10	ordinary_muscle_development	ENSGALG00010029935	SYNRG	-0.09434777	0.073217369	-1.288598202	0.197537809	0.237520575	negative
PRJNA729271	stage_1D_vs_E10	ordinary_muscle_development	ENSGALG00010029936	SERPINF2	2.614774112	0.500326741	5.226133044			positive
PRJNA729271	stage_1D_vs_E10	ordinary_muscle_development	ENSGALG00010029937	PMP22	3.287956864	0.123571284	26.60777447	5.5181E-156	1.0809E-153	positive
PRJNA729271	stage_1D_vs_E10	ordinary_muscle_development	ENSGALG00010029938	ENSGALG00010029938	-2.141996597	0.319580574	-6.702524398	2.04849E-11	6.49309E-11	negative
PRJNA729271	stage_1D_vs_E10	ordinary_muscle_development	ENSGALG00010029939	ENSGALG00010029939	2.172458808	1.497663439	1.450565429	0.146900908	0.18148203	positive
PRJNA729271	stage_1D_vs_E10	ordinary_muscle_development	ENSGALG00010029941	COX10	1.132209154	0.172179084	6.575764773	4.84037E-11	1.49853E-10	positive
PRJNA729271	stage_1D_vs_E10	ordinary_muscle_development	ENSGALG00010029942	ENSGALG00010029942	-2.382296428	1.545537005	-1.541403681	0.123218569	0.15436933	negative
PRJNA729271	stage_1D_vs_E10	ordinary_muscle_development	ENSGALG00010029943	HS3ST3A1	-1.870036598	0.200429324	-9.330154709	1.05716E-20	5.88012E-20	negative
PRJNA729271	stage_1D_vs_E10	ordinary_muscle_development	ENSGALG00010029945	CCL4	1.426562205	0.732278854	1.948113341	0.051401403	0.068442342	positive
PRJNA729271	stage_1D_vs_E10	ordinary_muscle_development	ENSGALG00010029946	METRNL	-0.763986366	0.137059796	-5.574109893	2.48799E-08	6.29488E-08	negative
PRJNA729271	stage_1D_vs_E10	ordinary_muscle_development	ENSGALG00010029947	CCL5	1.825815515	1.226022257	1.489218899	0.136429736	0.169310704	positive
PRJNA729271	stage_1D_vs_E10	ordinary_muscle_development	ENSGALG00010029948	MYADML2	1.887118298	0.218203853	8.648418769	5.22179E-18	2.50214E-17	positive
PRJNA729271	stage_1D_vs_E10	ordinary_muscle_development	ENSGALG00010029950	RFFL	-0.238488921	0.104777826	-2.276139241	0.022837677	0.032089194	negative
PRJNA729271	stage_1D_vs_E10	ordinary_muscle_development	ENSGALG00010029956	CCLI5	4.957273351	0.181755769	27.27436595	8.5451E-164	1.9661E-161	positive
PRJNA729271	stage_1D_vs_E10	ordinary_muscle_development	ENSGALG00010029962	ENSGALG00010029962	-0.550853416	0.761961689	-0.722941093	0.469716076	0.518429492	negative
PRJNA729271	stage_1D_vs_E10	ordinary_muscle_development	ENSGALG00010029966	RABGEF1	0.25520422	0.080445178	3.172399237	0.00151185	0.002469213	positive
PRJNA729271	stage_1D_vs_E10	ordinary_muscle_development	ENSGALG00010030004	FLOT2	0.594395831	0.070238247	8.46256648	2.61557E-17	1.20702E-16	positive
PRJNA729271	stage_1D_vs_E10	ordinary_muscle_development	ENSGALG00010030014	ENSGALG00010030014	-0.569017523	0.306805839	-1.854650242	0.063646189	0.083526619	negative
PRJNA729271	stage_1D_vs_E10	ordinary_muscle_development	ENSGALG00010030102	GPS1	-0.004669023	0.083207857	-0.056112762	0.955251977	0.96235595	negative
PRJNA729271	stage_1D_vs_E10	ordinary_muscle_development	ENSGALG00010030103	C1QTNF1	2.23298911	0.208452601	10.71221515	8.92015E-27	6.62724E-26	positive
PRJNA729271	stage_1D_vs_E10	ordinary_muscle_development	ENSGALG00010030104	P4HB	-1.279468607	0.11009712	-11.62127226	3.21315E-31	2.94032E-30	negative

PRJNA729271	stage_1D_vs_E10	ordinary_muscle_development	ENSGALG00010030105	RAB40B	0.251387105	0.194943962	1.289535222	0.197212079	0.237207625	positive
PRJNA729271	stage_1D_vs_E10	ordinary_muscle_development	ENSGALG00010030106	GPRC5C	0.186900864	0.165849666	1.126929399	0.259772332	0.304865999	positive
PRJNA729271	stage_1D_vs_E10	ordinary_muscle_development	ENSGALG00010030107	RPTOR	0.818939954	0.137820822	5.9420626	2.81458E-09	7.69035E-09	positive
PRJNA729271	stage_1D_vs_E10	ordinary_muscle_development	ENSGALG00010030108	LLGL2	0.604127015	0.118359726	5.104160319	3.32267E-07	7.69116E-07	positive
PRJNA729271	stage_1D_vs_E10	ordinary_muscle_development	ENSGALG00010030109	FBF1	-0.004838639	0.089470914	-0.054080582	0.956870963	0.963719053	negative
PRJNA729271	stage_1W_vs_E10	ordinary_muscle_development	ENSGALG00010000007	ND1	0.667305156	0.243195414	2.743905184	0.006071307	0.008398091	positive
PRJNA729271	stage_1W_vs_E10	ordinary_muscle_development	ENSGALG00010000011	ND2	0.191457491	0.253242363	0.756024737	0.449634365	0.487796581	positive
PRJNA729271	stage_1W_vs_E10	ordinary_muscle_development	ENSGALG00010000017	COX1	2.869849391	0.272385035	10.53600245	5.89518E-26	2.81829E-25	positive
PRJNA729271	stage_1W_vs_E10	ordinary_muscle_development	ENSGALG00010000020	COX2	3.22753283	0.238926203	13.50849254	1.39346E-41	1.18049E-40	positive
PRJNA729271	stage_1W_vs_E10	ordinary_muscle_development	ENSGALG00010000022	ATP8	0.177813404	0.277311923	0.641203602	0.52139041	0.558371185	positive
PRJNA729271	stage_1W_vs_E10	ordinary_muscle_development	ENSGALG00010000023	ATP6	1.045171881	0.198738247	5.259037431	1.44811E-07	2.86285E-07	positive
PRJNA729271	stage_1W_vs_E10	ordinary_muscle_development	ENSGALG00010000024	COX3	2.855447375	0.221478463	12.89266386	4.95038E-38	3.69304E-37	positive
PRJNA729271	stage_1W_vs_E10	ordinary_muscle_development	ENSGALG00010000026	ND3	2.239514231	0.31774276	7.048199098	1.81248E-12	4.73197E-12	positive
PRJNA729271	stage_1W_vs_E10	ordinary_muscle_development	ENSGALG00010000028	ND4L	1.196870058	0.277680877	4.310235801	1.63081E-05	2.78187E-05	positive
PRJNA729271	stage_1W_vs_E10	ordinary_muscle_development	ENSGALG00010000029	ND4	1.313919069	0.254944707	5.15374132	2.5534E-07	4.96466E-07	positive
PRJNA729271	stage_1W_vs_E10	ordinary_muscle_development	ENSGALG00010000033	ND5	0.272386352	0.232651865	1.170789461	0.241683415	0.274137343	positive
PRJNA729271	stage_1W_vs_E10	ordinary_muscle_development	ENSGALG00010000034	CYTB	1.247483499	0.219107762	5.693470131	1.24483E-08	2.62417E-08	positive
PRJNA729271	stage_1W_vs_E10	ordinary_muscle_development	ENSGALG00010000037	ND6	0.136545154	0.191228048	0.714043549	0.475200251	0.513648594	positive
PRJNA729271	stage_1W_vs_E10	ordinary_muscle_development	ENSGALG00010000039	PDIA3	-1.060355051	0.06604251	-16.05564423	5.21942E-58	7.24669E-57	negative
PRJNA729271	stage_1W_vs_E10	ordinary_muscle_development	ENSGALG00010000042	CKMT1B	3.833817402	0.449117763	8.536329937	1.38552E-17	4.61152E-17	positive
PRJNA729271	stage_1W_vs_E10	ordinary_muscle_development	ENSGALG00010000043	PPIP5K1	0.7434538	0.122209563	6.083433908	1.17635E-09	2.63747E-09	positive
PRJNA729271	stage_1W_vs_E10	ordinary_muscle_development	ENSGALG00010000044	MAP1A	-0.525246967	0.132933553	-3.951199328	7.77605E-05	0.000126219	negative
PRJNA729271	stage_1W_vs_E10	ordinary_muscle_development	ENSGALG00010000045	TP53BP1	-1.143462592	0.073870841	-15.47921457	4.79289E-54	6.03063E-53	negative
PRJNA729271	stage_1W_vs_E10	ordinary_muscle_development	ENSGALG00010000046	TUBGCP4	-0.373565424	0.062027948	-6.022533957	1.71707E-09	3.82229E-09	negative
PRJNA729271	stage_1W_vs_E10	ordinary_muscle_development	ENSGALG00010000047	IQGAP1	-1.208651667	0.097537847	-12.39161723	2.90133E-35	1.98934E-34	negative
PRJNA729271	stage_1W_vs_E10	ordinary_muscle_development	ENSGALG00010000048	MAN2A2	3.200621778	0.146728622	21.81320688	1.7385E-105	7.2414E-104	positive
PRJNA729271	stage_1W_vs_E10	ordinary_muscle_development	ENSGALG00010000049	FES	1.256883407	0.191933684	6.54852958	5.81063E-11	1.39816E-10	positive
PRJNA729271	stage_1W_vs_E10	ordinary_muscle_development	ENSGALG00010000050	CIB1	0.59234437	0.13036482	4.543744005	5.52638E-06	9.76174E-06	positive
PRJNA729271	stage_1W_vs_E10	ordinary_muscle_development	ENSGALG00010000051	GDPGP1	0.210170951	0.189638534	1.108271336	0.267744651	0.301597662	positive
PRJNA729271	stage_1W_vs_E10	ordinary_muscle_development	ENSGALG00010000052	TTLL13	1.086971535	0.131783499	8.248161155	1.6085E-16	5.11861E-16	positive
PRJNA729271	stage_1W_vs_E10	ordinary_muscle_development	ENSGALG00010000053	HDDC3	0.842355394	0.116285864	7.243833147	4.36178E-13	1.17429E-12	positive
PRJNA729271	stage_1W_vs_E10	ordinary_muscle_development	ENSGALG00010000054	VPS33B	0.825708426	0.0991043	8.331711364	7.9682E-17	2.56722E-16	positive
PRJNA729271	stage_1W_vs_E10	ordinary_muscle_development	ENSGALG00010000055	PRC1	-1.580498136	0.213674035	-7.396772081	1.39535E-13	3.84663E-13	negative
PRJNA729271	stage_1W_vs_E10	ordinary_muscle_development	ENSGALG00010000056	UNC45A	0.152050545	0.08213908	1.851135224	0.064150102	0.078995474	positive
PRJNA729271	stage_1W_vs_E10	ordinary_muscle_development	ENSGALG00010000058	FURIN	1.822382907	0.17167877	10.61507433	2.53593E-26	1.23433E-25	positive
PRJNA729271	stage_1W_vs_E10	ordinary_muscle_development	ENSGALG00010000059	MFAP1	-0.180105851	0.110867554	-1.624513623	0.104266235	0.125059088	negative
PRJNA729271	stage_1W_vs_E10	ordinary_muscle_development	ENSGALG00010000060	HYPK	0.078013716	0.246588459	0.31637213	0.751720069	0.776523831	positive
PRJNA729271	stage_1W_vs_E10	ordinary_muscle_development	ENSGALG00010000061	SERINC4	3.521160658	0.330046232	10.66868915	1.42624E-26	7.01506E-26	positive
PRJNA729271	stage_1W_vs_E10	ordinary_muscle_development	ENSGALG00010000063	SERF2	0.635564657	0.127428016	4.987636743	6.11224E-07	1.15889E-06	positive
PRJNA729271	stage_1W_vs_E10	ordinary_muscle_development	ENSGALG00010000069	ENSGALG00010000069	1.757502418	0.234123129	7.506744098	6.06161E-14	1.70973E-13	positive
PRJNA729271	stage_1W_vs_E10	ordinary_muscle_development	ENSGALG00010000076	ENSGALG00010000076	1.259538077	0.192184614	6.553792476	5.60939E-11	1.35243E-10	positive
PRJNA729271	stage_1W_vs_E10	ordinary_muscle_development	ENSGALG00010000078	ENSGALG00010000078	0.862104802	0.469480553	1.836295022	0.066314036	0.081542411	positive
PRJNA729271	stage_1W_vs_E10	ordinary_muscle_development	ENSGALG00010000081	ENSGALG00010000081	9.211939997	0.298752252	30.83471314	8.9803E-209	1.4792E-206	positive
PRJNA729271	stage_1W_vs_E10	ordinary_muscle_development	ENSGALG00010000084	ENSGALG00010000084	3.063385087	0.2549052	12.01774263	2.86697E-33	1.83312E-32	positive
PRJNA729271	stage_1W_vs_E10	ordinary_muscle_development	ENSGALG00010000085	ENSGALG00010000085	-0.895934996	0.155553171	-5.759670424	8.42783E-09	1.7957E-08	negative
PRJNA729271	stage_1W_vs_E10	ordinary_muscle_development	ENSGALG00010000089	ENSGALG00010000089	1.491946462	0.297547859	5.014139463	5.32713E-07	1.01361E-06	positive
PRJNA729271	stage_1W_vs_E10	ordinary_muscle_development	ENSGALG00010000091	ENSGALG00010000091	3.374808621	0.887695761	3.801762687	0.00014367	0.000228485	positive
PRJNA729271	stage_1W_vs_E10	ordinary_muscle_development	ENSGALG00010000092	INPPL1	1.91904819	0.169881309	11.29640575	1.36699E-29	7.58016E-29	positive
PRJNA729271	stage_1W_vs_E10	ordinary_muscle_development	ENSGALG00010000093	ENSGALG00010000093	1.949118726	0.328199352	5.938825643	2.87071E-09	6.29896E-09	positive
PRJNA729271	stage_1W_vs_E10	ordinary_muscle_development	ENSGALG00010000098	ENSGALG00010000098	1.125783112	0.192963914	5.83416397	5.40609E-09	1.16557E-08	positive
PRJNA729271	stage_1W_vs_E10	ordinary_muscle_development	ENSGALG00010000105	CACNA1F	0.6931612	0.286921505	2.415856559	0.015698244	0.020751118	positive
PRJNA729271	stage_1W_vs_E10	ordinary_muscle_development	ENSGALG00010000107	ENSGALG00010000107	-1.506020573	0.577520851	-2.607733679	0.009114384	0.012379403	negative
PRJNA729271	stage_1W_vs_E10	ordinary_muscle_development	ENSGALG00010000121	ENSGALG00010000121	0.045502172	0.138100437	0.329486082	0.741788312	0.767193834	positive
PRJNA729271	stage_1W_vs_E10	ordinary_muscle_development	ENSGALG00010000123	ENSGALG00010000123	0.181119165	0.253777454	0.713692892	0.475417103	0.513840118	positive
PRJNA729271	stage_1W_vs_E10	ordinary_muscle_development	ENSGALG00010000124	ENSGALG00010000124	1.950836195	0.181579774	10.74368665	6.34586E-27	3.1566E-26	positive
PRJNA729271	stage_1W_vs_E10	ordinary_muscle_development	ENSGALG00010000126	ENSGALG00010000126	1.120152892	0.199773585	5.607112122	2.0573E-08	4.28704E-08	positive
PRJNA729271	stage_1W_vs_E10	ordinary_muscle_development	ENSGALG00010000127	ENSGALG00010000127	1.316590603	0.206090402	6.388412997	1.67616E-10	3.93393E-10	positive
PRJNA729271	stage_1W_vs_E10	ordinary_muscle_development	ENSGALG00010000133	USP39	0.450926812	0.139887097	3.223505398	0.001266319	0.001866134	positive
PRJNA729271	stage_1W_vs_E10	ordinary_muscle_development	ENSGALG00010000134	ENSGALG00010000134	0.979458123	0.126612878	7.735849138	1.02715E-14	3.01083E-14	positive
PRJNA729271	stage_1W_vs_E10	ordinary_muscle_development	ENSGALG00010000137	ENSGALG00010000137	-0.198537103	0.546308581	-0.363415677	0.716294393	0.743532457	negative
PRJNA729271	stage_1W_vs_E10	ordinary_muscle_development	ENSGALG00010000140	SEPHS3	-0.008501995	0.127043412	-0.066921968	0.946643825	0.95335248	negative
PRJNA729271	stage_1W_vs_E10	ordinary_muscle_development	ENSGALG00010000141	ENSGALG00010000141	-0.322657919	0.139877561	-2.306716783	0.021070614	0.027473109	negative
PRJNA729271	stage_1W_vs_E10	ordinary_muscle_development	ENSGALG00010000143	SCARF2	-1.988898488	0.161643955	-12.30419344	8.59911E-35	5.80176E-34	negative
PRJNA729271	stage_1W_vs_E10	ordinary_muscle_development	ENSGALG00010000145	ENSGALG00010000145	0.579756497	0.280521898	2.066706741	0.038761796	0.049052927	positive
PRJNA729271	stage_1W_vs_E10	ordinary_muscle_development	ENSGALG00010000146	ACADM	1.710792886	0.16305069	10.49239892	9.36207E-26	4.44346E-25	positive
PRJNA729271	stage_1W_vs_E10	ordinary_muscle_development	ENSGALG00010000147	ENSGALG00010000147	0.006386304	0.111119804	0.057472241	0.954169018	0.960130504	positive
PRJNA729271	stage_1W_vs_E10	ordinary_muscle_development	ENSGALG00010000148	ENSGALG00010000148	0.794026686	0.297698514	2.667217501	0.007648216	0.01046845	positive
PRJNA729271	stage_1W_vs_E10	ordinary_muscle_development	ENSGALG00010000149	ENSGALG00010000149	-0.805707268	0.141649951	-5.688016552	1.28523E-08	2.7058E-08	negative
PRJNA729271	stage_1W_vs_E10	ordinary_muscle_development	ENSGALG00010000150	B3GAT3	0.518380773	0.125906033	4.117203602	3.83497E-05	6.36673E-05	positive
PRJNA729271	stage_1W_vs_E10	ordinary_muscle_development	ENSGALG00010000151	TBX5	-4.3769787	0.255396905	-17.1379473	7.73329E-66	1.3004E-64	negative
PRJNA729271	stage_1W_vs_E10	ordinary_muscle_development	ENSGALG00010000153	NF2	0.841611427	0.093260361	9.024320927	1.80813E-19	6.63516E-19	positive
PRJNA729271	stage_1W_vs_E10	ordinary_muscle_development	ENSGALG00010000154	ENSGALG00010000154	0.095085223	3.977182234	0.023907686	0.980926244	0.983912941	positive
PRJNA729271	stage_1W_vs_E10	ordinary_muscle_development	ENSGALG00010000155	MAZ	-0.047986313	0.16560524	-0.289763253	0.771997362	0.795429469	negative
PRJNA729271	stage_1W_vs_E10	ordinary_muscle_development	ENSGALG00010000156	ENSGALG00010000156	0.722212634	0.51807147	1.394040545	0.163305361	0.190588665	positive
PRJNA729271	stage_1W_vs_E10	ordinary_muscle_development	ENSGALG00010000157	ENSGALG00010000157	1.827739469	5.338751658	0.342353342			positive
PRJNA729271	stage_1W_vs_E10	ordinary_muscle_development	ENSGALG00010000161	CDC42EP2	0.755615101	0.32940086	2.293907495	0.021795813	0.028365084	positive
PRJNA729271	stage_1W_vs_E10	ordinary_muscle_development	ENSGALG00010000166	PFDN6	0.646259357	0.129306256	4.997897075	5.79589E-07	1.10049E-06	positive
PRJNA729271	stage_1W_vs_E10	ordinary_muscle_development	ENSGALG00010000171	DPF2	0.090490881	0.201414381	0.449277158	0.653231733	0.684443688	positive
PRJNA729271	stage_1W_vs_E10	ordinary_muscle_development	ENSGALG00010000172	ENSGALG00010000172	0.093479386	1.123453109	0.083207198	0.933686789	0.941939728	positive
PRJNA729271	stage_1W_vs_E10	ordinary_muscle_development	ENSGALG00010000173	NIPSNAP1	-0.556539068	0.127601078	-4.361554587	1.29142E-05	2.22185E-05	negative
PRJNA729271	stage_1W_vs_E10	ordinary_muscle_development	ENSGALG00010000174	ENSGALG00010000174	0.688840381	0.097674392	7.05241534	1.75838E-12	4.59405E-12	positive
PRJNA729271	stage_1W_vs_E10	ordinary_muscle_development	ENSGALG00010000175	ENSGALG00010000175	-2.692741754	1.130159972	-2.382620001	0.017189928	0.022610527	negative
PRJNA729271	stage_1W_vs_E10	ordinary_muscle_development	ENSGALG00010000176	ENSGALG00010000176	-0.242213493	0.512047137	-0.473029678	0.636192006	0.668759553	negative
PRJNA729271	stage_1W_vs_E10	ordinary_muscle_development	ENSGALG00010000177	ENSGALG00010000177	0.953200033	0.208706554	4.567178243	4.94334E-06	8.76605E-06	positive
PRJNA729271	stage_1W_vs_E10	ordinary_muscle_development	ENSGALG00010000184	ENSGALG00010000184	2.276938561	3.481181596	0.654070607	0.513066317	0.550310512	positive
PRJNA729271	stage_1W_vs_E10	ordinary_muscle_development	ENSGALG00010000186	THOC5	-0.083432296	0.079627655	-1.047780399	0.294739795	0.329827324	negative
PRJNA729271	stage_1W_vs_E10	ordinary_muscle_development	ENSGALG00010000188	ENSGALG00010000188	1.146146322	1.329808231	0.861888425	0.388748919	0.426693592	positive
PRJNA729271	stage_1W_vs_E10	ordinary_muscle_development	ENSGALG00010000189	ENSGALG00010000189	1.758609308	0.570880647	3.080520102	0.002066394	0.002990155	positive
PRJNA729271	stage_1W_vs_E10	ordinary_muscle_development	ENSGALG00010000191	NEFH	2.64405371	0.40472073	6.53303257	6.44511E-11	1.54544E-10	positive
PRJNA729271	stage_1W_vs_E10	ordinary_muscle_development	ENSGALG00010000192	ENSGALG00010000192	0.391782865	0.721707533	0.542855446	0.587229334	0.622487143	positive
PRJNA729271	stage_1W_vs_E10	ordinary_muscle_development	ENSGALG00010000196	KSR2	-3.759013145	1.016376219	-3.698446574	0.000216923	0.000340439	negative
PRJNA729271	stage_1W_vs_E10	ordinary_muscle_development	ENSGALG00010000197	ENSGALG00010000197	-0.577032851	0.528690519	-1.091437865	0.275080259	0.309379915	negative
PRJNA729271	stage_1W_vs_E10	ordinary_muscle_development	ENSGALG00010000200	GAL3ST1	-2.655199088	0.274219136	-9.682763661	3.56934E-22	1.45781E-21	negative
PRJNA729271	stage_1W_vs_E10	ordinary_muscle_development	ENSGALG00010000202	ENSGALG00010000202	-0.410481485	0.301758336	-1.360298744	0.173735406	0.201705623	negative
PRJNA729271	stage_1W_vs_E10	ordinary_muscle_development	ENSGALG00010000205	RNF215	0.473238053	0.113245679	4.178861912	2.92971E-05	4.90882E-05	positive
PRJNA729271	stage_1W_vs_E10	ordinary_muscle_development	ENSGALG00010000210	CCDC157	-1.172454673	0.568390945	-2.062760999	0.039135341	0.049517002	negative
PRJNA729271	stage_1W_vs_E10	ordinary_muscle_development	ENSGALG00010000215	SF3A1	-0.630542651	0.090754191	-6.947807556	3.71007E-12	9.53669E-12	negative
PRJNA729271	stage_1W_vs_E10	ordinary_muscle_development	ENSGALG00010000221	TBC1D10A	0.22668763	0.087729337	2.583943284	0.009767786	0.013219799	positive
PRJNA729271	stage_1W_vs_E10	ordinary_muscle_development	ENSGALG00010000223	CASTOR1	1.190440698	0.153280249	7.766432421	8.07276E-15	2.3769E-14	positive
PRJNA729271	stage_1W_vs_E10	ordinary_muscle_development	ENSGALG00010000224	MYBPC2	11.51081654	0.251740599	45.72491119	0	0	positive
PRJNA729271	stage_1W_vs_E10	ordinary_muscle_development	ENSGALG00010000225	LIF	2.692762377	0.476563979	5.650369091	1.60104E-08	3.35653E-08	positive
PRJNA729271	stage_1W_vs_E10	ordinary_muscle_development	ENSGALG00010000226	ENSGALG00010000226	0.978895906	0.433887618	2.25610473	0.024064073	0.03119935	positive
PRJNA729271	stage_1W_vs_E10	ordinary_muscle_development	ENSGALG00010000227	ENSGALG00010000227	0.888022331	0.27901356	3.18272105	0.001458981	0.002140479	positive
PRJNA729271	stage_1W_vs_E10	ordinary_muscle_development	ENSGALG00010000231	ENSGALG00010000231	3.366307943	0.442714388	7.603791596	2.87578E-14	8.23153E-14	positive
PRJNA729271	stage_1W_vs_E10	ordinary_muscle_development	ENSGALG00010000232	ENSGALG00010000232	1.138592205	0.16120777	7.062886629	1.63079E-12	4.26684E-12	positive
PRJNA729271	stage_1W_vs_E10	ordinary_muscle_development	ENSGALG00010000233	HORMAD2	-1.763277534	0.557374207	-3.163543474	0.001558611	0.002280644	negative
PRJNA729271	stage_1W_vs_E10	ordinary_muscle_development	ENSGALG00010000234	ENSGALG00010000234	1.643538969	4.544451214	0.361658403			positive
PRJNA729271	stage_1W_vs_E10	ordinary_muscle_development	ENSGALG00010000235	ENSGALG00010000235	-1.927617844	0.239148966	-8.060322711	7.60933E-16	2.35577E-15	negative
PRJNA729271	stage_1W_vs_E10	ordinary_muscle_development	ENSGALG00010000239	ENSGALG00010000239	1.744549065	4.619125197	0.377679537	0.705668671	0.733553312	positive
PRJNA729271	stage_1W_vs_E10	ordinary_muscle_development	ENSGALG00010000242	ENSGALG00010000242	2.449672284	0.194247838	12.61106588	1.83494E-36	1.30765E-35	positive
PRJNA729271	stage_1W_vs_E10	ordinary_muscle_development	ENSGALG00010000246	ENSGALG00010000246	2.354836554	4.249331692	0.554166331	0.579465018	0.614976236	positive
PRJNA729271	stage_1W_vs_E10	ordinary_muscle_development	ENSGALG00010000247	ENSGALG00010000247	3.960663452	0.22410725	17.67307153	6.76132E-70	1.27445E-68	positive
PRJNA729271	stage_1W_vs_E10	ordinary_muscle_development	ENSGALG00010000249	ENSGALG00010000249	-1.317803875	4.407161315	-0.299014214			negative
PRJNA729271	stage_1W_vs_E10	ordinary_muscle_development	ENSGALG00010000254	ENSGALG00010000254	-2.254803492	0.655333512	-3.440696148	0.00058022	0.00088075	negative
PRJNA729271	stage_1W_vs_E10	ordinary_muscle_development	ENSGALG00010000255	ENSGALG00010000255	1.900143165	0.143938554	13.20107163	8.64957E-40	6.88877E-39	positive
PRJNA729271	stage_1W_vs_E10	ordinary_muscle_development	ENSGALG00010000256	ENSGALG00010000256	-1.272794276	0.436051572	-2.918907666	0.003512603	0.004973156	negative
PRJNA729271	stage_1W_vs_E10	ordinary_muscle_development	ENSGALG00010000262	HNRNPKL	-0.501030501	3.471594632	-0.14432287	0.88524552	0.898322165	negative
PRJNA729271	stage_1W_vs_E10	ordinary_muscle_development	ENSGALG00010000263	MTMR3	1.010624898	0.091505813	11.04437922	2.33375E-28	1.23279E-27	positive
PRJNA729271	stage_1W_vs_E10	ordinary_muscle_development	ENSGALG00010000267	ENSGALG00010000267	0.866976791	0.120271151	7.208518289	5.6564E-13	1.5144E-12	positive
PRJNA729271	stage_1W_vs_E10	ordinary_muscle_development	ENSGALG00010000269	ENSGALG00010000269	-1.33755647	0.50434638	-2.652059228	0.008000251	0.010927595	negative
PRJNA729271	stage_1W_vs_E10	ordinary_muscle_development	ENSGALG00010000271	RTN4R	-3.905728929	0.492254414	-7.934370565	2.11566E-15	6.40753E-15	negative
PRJNA729271	stage_1W_vs_E10	ordinary_muscle_development	ENSGALG00010000272	ENSGALG00010000272	1.56164919	0.334597858	4.667242038	3.0527E-06	5.5029E-06	positive
PRJNA729271	stage_1W_vs_E10	ordinary_muscle_development	ENSGALG00010000277	ENSGALG00010000277	3.057260112	0.578467077	5.285106501	1.25632E-07	2.49251E-07	positive
PRJNA729271	stage_1W_vs_E10	ordinary_muscle_development	ENSGALG00010000279	ENSGALG00010000279	-0.92567583	0.136628951	-6.775107501	1.24314E-11	3.10902E-11	negative
PRJNA729271	stage_1W_vs_E10	ordinary_muscle_development	ENSGALG00010000281	OSBP2	2.440497809	0.20331068	12.00378558	3.39409E-33	2.16252E-32	positive
PRJNA729271	stage_1W_vs_E10	ordinary_muscle_development	ENSGALG00010000282	ENSGALG00010000282	0.386647771	4.937874625	0.078302468	0.937587454	0.945217009	positive
PRJNA729271	stage_1W_vs_E10	ordinary_muscle_development	ENSGALG00010000284	ENSGALG00010000284	-0.652343191	0.154190483	-4.230761697	2.32901E-05	3.92547E-05	negative
PRJNA729271	stage_1W_vs_E10	ordinary_muscle_development	ENSGALG00010000285	MYLPF	6.729259844	0.145687814	46.18958633	0	0	positive
PRJNA729271	stage_1W_vs_E10	ordinary_muscle_development	ENSGALG00010000286	TCN2	2.459849951	0.136402914	18.0337053	1.05948E-72	2.14186E-71	positive
PRJNA729271	stage_1W_vs_E10	ordinary_muscle_development	ENSGALG00010000287	IQCD	-2.913866794	0.26603144	-10.95309182	6.42175E-28	3.33393E-27	negative
PRJNA729271	stage_1W_vs_E10	ordinary_muscle_development	ENSGALG00010000288	VARS2	1.05672861	0.151660344	6.967731857	3.22091E-12	8.31078E-12	positive

PRJNA729271	stage_1W_vs_E10	ordinary_muscle_development	ENSGALG00010000289	QPRT	-2.852335876	0.472285956	-6.039425566	1.54664E-09	3.44794E-09	negative
PRJNA729271	stage_1W_vs_E10	ordinary_muscle_development	ENSGALG00010000292	ABHD16A	0.639983579	0.135332001	4.728989254	2.2564E-06	4.10165E-06	positive
PRJNA729271	stage_1W_vs_E10	ordinary_muscle_development	ENSGALG00010000293	ENSGALG00010000293	0.998699591	0.370515434	2.695433174	0.007029718	0.009663862	positive
PRJNA729271	stage_1W_vs_E10	ordinary_muscle_development	ENSGALG00010000294	DUSP21	-1.557723095	0.320029979	-4.867428666	1.1306E-06	2.10643E-06	negative
PRJNA729271	stage_1W_vs_E10	ordinary_muscle_development	ENSGALG00010000298	SLC35E4	1.518640872	0.213007086	7.129532185	1.00711E-12	2.66728E-12	positive
PRJNA729271	stage_1W_vs_E10	ordinary_muscle_development	ENSGALG00010000301	UQCR10	2.209862639	0.154630387	14.29125723	2.4809E-46	2.41671E-45	positive
PRJNA729271	stage_1W_vs_E10	ordinary_muscle_development	ENSGALG00010000302	ENSGALG00010000302	0.003002619	0.236328003	0.012705301	0.989862909	0.991641638	positive
PRJNA729271	stage_1W_vs_E10	ordinary_muscle_development	ENSGALG00010000303	ENSGALG00010000303	-0.126765416	0.113086348	-1.120961274	0.262304347	0.296067708	negative
PRJNA729271	stage_1W_vs_E10	ordinary_muscle_development	ENSGALG00010000306	ENSGALG00010000306	2.185878217	0.204731083	10.67682633	1.30662E-26	6.43545E-26	positive
PRJNA729271	stage_1W_vs_E10	ordinary_muscle_development	ENSGALG00010000307	ZMAT5	1.321426584	0.111598424	11.84090722	2.39844E-32	1.48253E-31	positive
PRJNA729271	stage_1W_vs_E10	ordinary_muscle_development	ENSGALG00010000308	ENSGALG00010000308	0.14519539	0.348679259	0.416415335	0.677106109	0.706802035	positive
PRJNA729271	stage_1W_vs_E10	ordinary_muscle_development	ENSGALG00010000309	ENSGALG00010000309	0.84334466	4.281836989	0.19695861	0.843859928	0.860845215	positive
PRJNA729271	stage_1W_vs_E10	ordinary_muscle_development	ENSGALG00010000312	CABP7	-1.651603874	0.513730698	-3.214921518	0.001304802	0.001920892	negative
PRJNA729271	stage_1W_vs_E10	ordinary_muscle_development	ENSGALG00010000313	ENSGALG00010000313	-0.155768905	3.825620853	-0.040717288	0.967521279	0.972081579	negative
PRJNA729271	stage_1W_vs_E10	ordinary_muscle_development	ENSGALG00010000318	ENSGALG00010000318	0.440889033	4.729556772	0.093219947	0.925728828	0.934822304	positive
PRJNA729271	stage_1W_vs_E10	ordinary_muscle_development	ENSGALG00010000323	AP1B1	1.43685341	0.144153776	9.967504502	2.11475E-23	9.09595E-23	positive
PRJNA729271	stage_1W_vs_E10	ordinary_muscle_development	ENSGALG00010000324	ENSGALG00010000324	-0.055361569	0.175354301	-0.31571264	0.752220632	0.776930174	negative
PRJNA729271	stage_1W_vs_E10	ordinary_muscle_development	ENSGALG00010000326	ENSGALG00010000326	1.253773291	1.783044125	0.703164478	0.481953254	0.520172183	positive
PRJNA729271	stage_1W_vs_E10	ordinary_muscle_development	ENSGALG00010000331	ENSGALG00010000331	-0.189285921	0.172632958	-1.096464561	0.272875524	0.307067054	negative
PRJNA729271	stage_1W_vs_E10	ordinary_muscle_development	ENSGALG00010000338	ENSGALG00010000338	-0.784582784	0.194375326	-4.036431983	5.42702E-05	8.90275E-05	negative
PRJNA729271	stage_1W_vs_E10	ordinary_muscle_development	ENSGALG00010000340	NAA10	0.250568448	0.132809031	1.886682297	0.05920307	0.073239609	positive
PRJNA729271	stage_1W_vs_E10	ordinary_muscle_development	ENSGALG00010000341	EWSR1	-1.006570668	0.148243811	-6.789967576	1.12159E-11	2.8127E-11	negative
PRJNA729271	stage_1W_vs_E10	ordinary_muscle_development	ENSGALG00010000346	ENSGALG00010000346	-0.551012027	0.115220568	-4.782236689	1.73355E-06	3.18195E-06	negative
PRJNA729271	stage_1W_vs_E10	ordinary_muscle_development	ENSGALG00010000349	CHIR-IG1-5	1.22779282	0.460028119	2.668951677	0.00760884	0.010415538	positive
PRJNA729271	stage_1W_vs_E10	ordinary_muscle_development	ENSGALG00010000350	ENSGALG00010000350	0.41584526	0.165599437	2.511151402	0.012033807	0.016115118	positive
PRJNA729271	stage_1W_vs_E10	ordinary_muscle_development	ENSGALG00010000353	ENSGALG00010000353	1.270350496	0.110384195	11.50844552	1.19615E-30	6.93805E-30	positive
PRJNA729271	stage_1W_vs_E10	ordinary_muscle_development	ENSGALG00010000356	ENSGALG00010000356	0.62868036	0.123582122	5.087146519	3.63491E-07	6.99894E-07	positive
PRJNA729271	stage_1W_vs_E10	ordinary_muscle_development	ENSGALG00010000366	EMID1	-0.060076619	0.216230095	-0.27783653	0.781137848	0.803818906	negative
PRJNA729271	stage_1W_vs_E10	ordinary_muscle_development	ENSGALG00010000367	HSD17B10	1.229766466	0.138616387	8.871725022	7.20214E-19	2.56373E-18	positive
PRJNA729271	stage_1W_vs_E10	ordinary_muscle_development	ENSGALG00010000373	ENSGALG00010000373	1.217043455	0.514557807	2.365222017	0.018019265	0.023643455	positive
PRJNA729271	stage_1W_vs_E10	ordinary_muscle_development	ENSGALG00010000376	ASCC2	-0.264782056	0.084276195	-3.141836854	0.001678916	0.002451484	negative
PRJNA729271	stage_1W_vs_E10	ordinary_muscle_development	ENSGALG00010000377	ENSGALG00010000377	0.227166985	0.253777095	0.895143765	0.370710241	0.408285461	positive
PRJNA729271	stage_1W_vs_E10	ordinary_muscle_development	ENSGALG00010000381	ENSGALG00010000381	-1.491227534	0.31573101	-4.723094941	2.32282E-06	4.21974E-06	negative
PRJNA729271	stage_1W_vs_E10	ordinary_muscle_development	ENSGALG00010000382	ENSGALG00010000382	-0.541903448	0.537948854	-1.007351245	0.313766012	0.349768388	negative
PRJNA729271	stage_1W_vs_E10	ordinary_muscle_development	ENSGALG00010000384	ENSGALG00010000384	-0.548298647	0.150565737	-3.64158977	0.00027096	0.000422682	negative
PRJNA729271	stage_1W_vs_E10	ordinary_muscle_development	ENSGALG00010000386	ENSGALG00010000386	0.228777844	0.368122469	0.6214721	0.534289046	0.570708896	positive
PRJNA729271	stage_1W_vs_E10	ordinary_muscle_development	ENSGALG00010000387	DTX1	-2.689344527	0.568566597	-4.730043132	2.24472E-06	4.08195E-06	negative
PRJNA729271	stage_1W_vs_E10	ordinary_muscle_development	ENSGALG00010000388	ENSGALG00010000388	-0.033910869	0.184058659	-0.184239467	0.853825599	0.870276497	negative
PRJNA729271	stage_1W_vs_E10	ordinary_muscle_development	ENSGALG00010000391	ENSGALG00010000391	2.174663661	0.663338334	3.278362714	0.001044111	0.00155145	positive
PRJNA729271	stage_1W_vs_E10	ordinary_muscle_development	ENSGALG00010000402	PFKFB1	10.39834301	0.318259893	32.67248955	3.8415E-234	8.3109E-232	positive
PRJNA729271	stage_1W_vs_E10	ordinary_muscle_development	ENSGALG00010000405	ENSGALG00010000405	2.069950105	0.402571991	5.141813513	2.72099E-07	5.27918E-07	positive
PRJNA729271	stage_1W_vs_E10	ordinary_muscle_development	ENSGALG00010000406	NSMCE3	-0.258312046	0.098605564	-2.619649786	0.008802011	0.011973081	negative
PRJNA729271	stage_1W_vs_E10	ordinary_muscle_development	ENSGALG00010000407	WSB2	0.820411052	0.085103936	9.640107079	5.41315E-22	2.19786E-21	positive
PRJNA729271	stage_1W_vs_E10	ordinary_muscle_development	ENSGALG00010000408	ENSGALG00010000408	10.53612493	0.349887323	30.11290848	3.2837E-199	4.7597E-197	positive
PRJNA729271	stage_1W_vs_E10	ordinary_muscle_development	ENSGALG00010000411	ENSGALG00010000411	3.599066505	0.908929496	3.959676214	7.50514E-05	0.000121972	positive
PRJNA729271	stage_1W_vs_E10	ordinary_muscle_development	ENSGALG00010000412	RFC5	-0.748719502	0.106940377	-7.001279818	2.53635E-12	6.57563E-12	negative
PRJNA729271	stage_1W_vs_E10	ordinary_muscle_development	ENSGALG00010000422	ITIH6	-1.839879903	0.560954338	-3.279910288	0.001038401	0.00154344	negative
PRJNA729271	stage_1W_vs_E10	ordinary_muscle_development	ENSGALG00010000423	ENSGALG00010000423	2.033807497	0.203889471	9.975049145	1.96004E-23	8.44719E-23	positive
PRJNA729271	stage_1W_vs_E10	ordinary_muscle_development	ENSGALG00010000428	GNL3L	-0.378609617	0.147547095	-2.56602557	0.010287124	0.013890253	negative
PRJNA729271	stage_1W_vs_E10	ordinary_muscle_development	ENSGALG00010000431	AIFM3	-1.777892444	0.991442564	-1.793237963	0.072934882	0.089176813	negative
PRJNA729271	stage_1W_vs_E10	ordinary_muscle_development	ENSGALG00010000432	ENSGALG00010000432	1.372475132	0.181798519	7.549429649	4.37168E-14	1.2408E-13	positive
PRJNA729271	stage_1W_vs_E10	ordinary_muscle_development	ENSGALG00010000434	TP53	0.15139427	0.220016814	0.688103183	0.491387806	0.529526894	positive
PRJNA729271	stage_1W_vs_E10	ordinary_muscle_development	ENSGALG00010000435	P2RX6	-0.072725257	0.304278672	-0.239008725	0.811098817	0.831109667	negative
PRJNA729271	stage_1W_vs_E10	ordinary_muscle_development	ENSGALG00010000436	ENSGALG00010000436	1.385317697	0.380757296	3.638322129	0.00027442	0.000427942	positive
PRJNA729271	stage_1W_vs_E10	ordinary_muscle_development	ENSGALG00010000440	ENSGALG00010000440	-2.594201129	0.495275951	-5.237890362	1.62422E-07	3.19619E-07	negative
PRJNA729271	stage_1W_vs_E10	ordinary_muscle_development	ENSGALG00010000441	SLC7A4	-1.24445209	0.211502044	-5.883877366	4.00765E-09	8.70927E-09	negative
PRJNA729271	stage_1W_vs_E10	ordinary_muscle_development	ENSGALG00010000442	DGCR6	-0.620299833	0.130631704	-4.74846317	2.04968E-06	3.74042E-06	negative
PRJNA729271	stage_1W_vs_E10	ordinary_muscle_development	ENSGALG00010000443	TBX3	-1.18675843	0.158059903	-7.508282645	5.99081E-14	1.69108E-13	negative
PRJNA729271	stage_1W_vs_E10	ordinary_muscle_development	ENSGALG00010000444	RBM19	-0.349457904	0.118934195	-2.938245838	0.003300752	0.004686951	negative
PRJNA729271	stage_1W_vs_E10	ordinary_muscle_development	ENSGALG00010000449	PLBD2	1.491187781	0.107444942	13.87862238	8.5369E-44	7.71942E-43	positive
PRJNA729271	stage_1W_vs_E10	ordinary_muscle_development	ENSGALG00010000450	ENSGALG00010000450	-0.064443823	0.193235827	-0.333498316	0.738758161	0.764387147	negative
PRJNA729271	stage_1W_vs_E10	ordinary_muscle_development	ENSGALG00010000452	SLC8B1	1.627246303	0.226634664	7.18004154	6.96902E-13	1.85862E-12	positive
PRJNA729271	stage_1W_vs_E10	ordinary_muscle_development	ENSGALG00010000453	ACHE	-0.649845922	0.24798586	-2.620495873	0.008780199	0.011944532	negative
PRJNA729271	stage_1W_vs_E10	ordinary_muscle_development	ENSGALG00010000457	TPCN1	-0.324358773	0.082084351	-3.951529945	7.76532E-05	0.000126059	negative
PRJNA729271	stage_1W_vs_E10	ordinary_muscle_development	ENSGALG00010000458	ENSGALG00010000458	1.06792705	0.199158091	5.362207715	8.22109E-08	1.65369E-07	positive
PRJNA729271	stage_1W_vs_E10	ordinary_muscle_development	ENSGALG00010000462	ENSGALG00010000462	-0.186651185	0.22457308	-0.831137839	0.405895766	0.444034651	negative
PRJNA729271	stage_1W_vs_E10	ordinary_muscle_development	ENSGALG00010000465	ENSGALG00010000465	2.147325822	0.191313161	11.22414065	3.10389E-29	1.69585E-28	positive
PRJNA729271	stage_1W_vs_E10	ordinary_muscle_development	ENSGALG00010000466	FBXO21	0.349900586	0.078966488	4.43100096	9.37967E-06	1.63137E-05	positive
PRJNA729271	stage_1W_vs_E10	ordinary_muscle_development	ENSGALG00010000467	RNASEK	1.391326252	0.198851887	6.996796838	2.61881E-12	6.78334E-12	positive
PRJNA729271	stage_1W_vs_E10	ordinary_muscle_development	ENSGALG00010000468	TESC	-3.854493448	0.361216095	-10.67087958	1.39301E-26	6.85395E-26	negative
PRJNA729271	stage_1W_vs_E10	ordinary_muscle_development	ENSGALG00010000469	ENSGALG00010000469	7.455124995	0.385181116	19.35485589	1.85513E-83	4.81039E-82	positive
PRJNA729271	stage_1W_vs_E10	ordinary_muscle_development	ENSGALG00010000470	FBXW8	0.293409963	0.108469116	2.70500926	0.006830249	0.009402132	positive
PRJNA729271	stage_1W_vs_E10	ordinary_muscle_development	ENSGALG00010000471	ENSGALG00010000471	-0.012389998	0.202739263	-0.061112966	0.951269243	0.957671859	negative
PRJNA729271	stage_1W_vs_E10	ordinary_muscle_development	ENSGALG00010000475	RNFT2	-0.806693946	0.112668053	-7.159917364	8.07257E-13	2.14543E-12	negative
PRJNA729271	stage_1W_vs_E10	ordinary_muscle_development	ENSGALG00010000476	ENSGALG00010000476	8.745614391	0.214021322	40.86328553	0	0	positive
PRJNA729271	stage_1W_vs_E10	ordinary_muscle_development	ENSGALG00010000479	C12orf49	0.452223401	0.114241126	3.958499162	7.54222E-05	0.000122534	positive
PRJNA729271	stage_1W_vs_E10	ordinary_muscle_development	ENSGALG00010000483	ENSGALG00010000483	1.174872467	0.244390513	4.807357096	1.52939E-06	2.81719E-06	positive
PRJNA729271	stage_1W_vs_E10	ordinary_muscle_development	ENSGALG00010000485	MED13L	0.666454856	0.126117155	5.284410816	1.2611E-07	2.50132E-07	positive
PRJNA729271	stage_1W_vs_E10	ordinary_muscle_development	ENSGALG00010000491	ENSGALG00010000491	0.961661184	0.156442005	6.147077838	7.89234E-10	1.78359E-09	positive
PRJNA729271	stage_1W_vs_E10	ordinary_muscle_development	ENSGALG00010000495	ENSGALG00010000495	1.193898937	0.55112334	2.16630081	0.030288208	0.038793636	positive
PRJNA729271	stage_1W_vs_E10	ordinary_muscle_development	ENSGALG00010000501	ENSGALG00010000501	1.159857684	0.161236058	7.19353785	6.31336E-13	1.68624E-12	positive
PRJNA729271	stage_1W_vs_E10	ordinary_muscle_development	ENSGALG00010000507	TRPT1	2.247856902	0.282755952	7.94981285	1.86793E-15	5.67387E-15	positive
PRJNA729271	stage_1W_vs_E10	ordinary_muscle_development	ENSGALG00010000509	ENSGALG00010000509	-4.6348669	1.158226018	-4.001694685	6.28904E-05	0.000102761	negative
PRJNA729271	stage_1W_vs_E10	ordinary_muscle_development	ENSGALG00010000511	FERMT3	0.130382273	0.162478649	0.802457882	0.422288142	0.46026518	positive
PRJNA729271	stage_1W_vs_E10	ordinary_muscle_development	ENSGALG00010000525	ENSGALG00010000525	-2.591213149	0.552523618	-4.689778071	2.73502E-06	4.94685E-06	negative
PRJNA729271	stage_1W_vs_E10	ordinary_muscle_development	ENSGALG00010000526	POLD1	-2.030372349	0.170408803	-11.91471516	9.92046E-33	6.19281E-32	negative
PRJNA729271	stage_1W_vs_E10	ordinary_muscle_development	ENSGALG00010000529	STIP1	0.305805389	0.192657147	1.587303629	0.112443894	0.134267587	positive
PRJNA729271	stage_1W_vs_E10	ordinary_muscle_development	ENSGALG00010000532	ENSGALG00010000532	2.108233415	0.472624334	4.460695867	8.1694E-06	1.42549E-05	positive
PRJNA729271	stage_1W_vs_E10	ordinary_muscle_development	ENSGALG00010000546	FLRT1	-4.88882832	0.898082072	-5.443632015	5.2205E-08	1.06399E-07	negative
PRJNA729271	stage_1W_vs_E10	ordinary_muscle_development	ENSGALG00010000555	OTUB1	-0.499933573	0.102335784	-4.88522735	1.0331E-06	1.92973E-06	negative
PRJNA729271	stage_1W_vs_E10	ordinary_muscle_development	ENSGALG00010000561	ENSGALG00010000561	0.298804839	0.275861158	1.08317112	0.278732476	0.313171633	positive
PRJNA729271	stage_1W_vs_E10	ordinary_muscle_development	ENSGALG00010000564	NAA40	-0.920299795	0.090758467	-10.14009856	3.66729E-24	1.62462E-23	negative
PRJNA729271	stage_1W_vs_E10	ordinary_muscle_development	ENSGALG00010000569	SIPA1	0.630770729	0.233898145	2.696775253	0.007001451	0.009627742	positive
PRJNA729271	stage_1W_vs_E10	ordinary_muscle_development	ENSGALG00010000574	ENSGALG00010000574	4.764966292	0.170788599	27.89979148	2.6836E-171	2.9694E-169	positive
PRJNA729271	stage_1W_vs_E10	ordinary_muscle_development	ENSGALG00010000575	ENSGALG00010000575	1.703925932	0.157829796	10.79597118	3.5964E-27	1.80943E-26	positive
PRJNA729271	stage_1W_vs_E10	ordinary_muscle_development	ENSGALG00010000576	NOS1	3.507694501	0.427024598	8.214268029	2.13462E-16	6.74398E-16	positive
PRJNA729271	stage_1W_vs_E10	ordinary_muscle_development	ENSGALG00010000577	ENSGALG00010000577	0.109018532	0.270275598	0.403360617	0.686682954	0.715870931	positive
PRJNA729271	stage_1W_vs_E10	ordinary_muscle_development	ENSGALG00010000578	ENSGALG00010000578	2.272766487	0.360878277	6.297875584	3.01753E-10	6.9737E-10	positive
PRJNA729271	stage_1W_vs_E10	ordinary_muscle_development	ENSGALG00010000579	ENSGALG00010000579	-3.111430038	0.997142866	-3.120345282	0.001806392	0.00262888	negative
PRJNA729271	stage_1W_vs_E10	ordinary_muscle_development	ENSGALG00010000580	ENSGALG00010000580	-0.358573321	0.551064669	-0.650691908	0.515245388	0.552484236	negative
PRJNA729271	stage_1W_vs_E10	ordinary_muscle_development	ENSGALG00010000581	ENSGALG00010000581	-0.093887677	0.131016	-0.716612298	0.473613354	0.51212425	negative
PRJNA729271	stage_1W_vs_E10	ordinary_muscle_development	ENSGALG00010000583	CCS	0.255752106	0.140168707	1.824602022	0.068061108	0.083563083	positive
PRJNA729271	stage_1W_vs_E10	ordinary_muscle_development	ENSGALG00010000584	RBM14	1.088600939	0.209621115	5.193183615	2.06728E-07	4.04608E-07	positive
PRJNA729271	stage_1W_vs_E10	ordinary_muscle_development	ENSGALG00010000585	SPTBN2	-4.743899754	0.793581556	-5.977835193	2.26122E-09	4.99108E-09	negative
PRJNA729271	stage_1W_vs_E10	ordinary_muscle_development	ENSGALG00010000586	ENSGALG00010000586	4.431992126	0.251597755	17.61538819	1.87697E-69	3.49701E-68	positive
PRJNA729271	stage_1W_vs_E10	ordinary_muscle_development	ENSGALG00010000589	ENSGALG00010000589	2.152017984	0.304086108	7.077001964	1.47306E-12	3.86673E-12	positive
PRJNA729271	stage_1W_vs_E10	ordinary_muscle_development	ENSGALG00010000590	DNAH5	-0.509871844	0.45878206	-1.111359593	0.266413598	0.300238307	negative
PRJNA729271	stage_1W_vs_E10	ordinary_muscle_development	ENSGALG00010000591	SBK2	4.087828735	0.304662626	13.41755892	4.77174E-41	3.95915E-40	positive
PRJNA729271	stage_1W_vs_E10	ordinary_muscle_development	ENSGALG00010000592	CCT5	-0.849377084	0.119200385	-7.125623665	1.0361E-12	2.74308E-12	negative
PRJNA729271	stage_1W_vs_E10	ordinary_muscle_development	ENSGALG00010000593	ATPSCKMT	-1.192801267	0.15328575	-7.781553536	7.16393E-15	2.11532E-14	negative
PRJNA729271	stage_1W_vs_E10	ordinary_muscle_development	ENSGALG00010000595	MYO10	-1.670068181	0.140515739	-11.88527486	1.4117E-32	8.78222E-32	negative
PRJNA729271	stage_1W_vs_E10	ordinary_muscle_development	ENSGALG00010000596	ENSGALG00010000596	-2.725128657	0.803439007	-3.39183016	0.000694275	0.001048173	negative
PRJNA729271	stage_1W_vs_E10	ordinary_muscle_development	ENSGALG00010000597	CDH9	-4.938067469	0.974709565	-5.066193713	4.05849E-07	7.78764E-07	negative
PRJNA729271	stage_1W_vs_E10	ordinary_muscle_development	ENSGALG00010000598	ENSGALG00010000598	-2.682925672	0.570192812	-4.705295496	2.53499E-06	4.59882E-06	negative
PRJNA729271	stage_1W_vs_E10	ordinary_muscle_development	ENSGALG00010000599	RETREG1	6.016432519	0.374576482	16.06196014	4.71415E-58	6.56404E-57	positive
PRJNA729271	stage_1W_vs_E10	ordinary_muscle_development	ENSGALG00010000601	ENSGALG00010000601	-2.760114043	0.378607794	-7.290166987	3.09571E-13	8.39363E-13	negative
PRJNA729271	stage_1W_vs_E10	ordinary_muscle_development	ENSGALG00010000603	ZNF622	0.05443516	0.108818893	0.500236293	0.616908706	0.651044172	positive
PRJNA729271	stage_1W_vs_E10	ordinary_muscle_development	ENSGALG00010000604	ENSGALG00010000604	-0.679694324	0.135751737	-5.006892296	5.53159E-07	1.05113E-06	negative
PRJNA729271	stage_1W_vs_E10	ordinary_muscle_development	ENSGALG00010000605	MARCHF11	-4.08525474	0.995235784	-4.104810944	4.04646E-05	6.70708E-05	negative
PRJNA729271	stage_1W_vs_E10	ordinary_muscle_development	ENSGALG00010000606	UBE2QL1	-0.11174828	0.156739068	-0.712957411	0.475872111	0.514221429	negative
PRJNA729271	stage_1W_vs_E10	ordinary_muscle_development	ENSGALG00010000607	ENSGALG00010000607	-2.034747521	0.298801411	-6.809698507	9.78035E-12	2.46207E-11	negative
PRJNA729271	stage_1W_vs_E10	ordinary_muscle_development	ENSGALG00010000608	ENSGALG00010000608	-4.265159917	1.199374277	-3.556154238	0.000376323	0.000579682	negative
PRJNA729271	stage_1W_vs_E10	ordinary_muscle_development	ENSGALG00010000610	ENSGALG00010000610	-0.400642099	0.080312067	-4.988566645	6.08289E-07	1.15378E-06	negative
PRJNA729271	stage_1W_vs_E10	ordinary_muscle_development	ENSGALG00010000611	C7orf57	1.611180367	0.320184734	5.032033688	4.85304E-07	9.2559E-07	positive
PRJNA729271	stage_1W_vs_E10	ordinary_muscle_development	ENSGALG00010000612	ENSGALG00010000612	-1.349772641	0.465279542	-2.900992888	0.003719823	0.005253199	negative
PRJNA729271	stage_1W_vs_E10	ordinary_muscle_development	ENSGALG00010000613	ENSGALG00010000613	0.072738894	0.176721201	0.411602531	0.680630776	0.710123307	positive
PRJNA729271	stage_1W_vs_E10	ordinary_muscle_development	ENSGALG00010000614	KAT8	1.998778611	0.182044355	10.97962422	4.78912E-28	2.50066E-27	positive
PRJNA729271	stage_1W_vs_E10	ordinary_muscle_development	ENSGALG00010000615	ENSGALG00010000615	-1.40942412	0.621944303	-2.266158101	0.023441705	0.030430549	negative
PRJNA729271	stage_1W_vs_E10	ordinary_muscle_development	ENSGALG00010000616	PSENEN	1.198038193	0.497212005	2.4095118	0.015973879	0.021091399	positive
PRJNA729271	stage_1W_vs_E10	ordinary_muscle_development	ENSGALG00010000619	ENSGALG00010000619	-0.359824156	0.325848858	-1.104267047	0.269477313	0.303455069	negative
PRJNA729271	stage_1W_vs_E10	ordinary_muscle_development	ENSGALG00010000621	UPP1	-0.573129425	0.239898226	-2.38905237	0.016891894	0.022234654	negative

PRJNA729271	stage_1W_vs_E10	ordinary_muscle_development	ENSGALG00010000622	ENSGALG00010000622	-0.674425103	0.468945081	-1.438175022	0.150384401	0.17640381	negative
PRJNA729271	stage_1W_vs_E10	ordinary_muscle_development	ENSGALG00010000623	ENSGALG00010000623	-0.002387336	0.252847357	-0.009441807	0.99246664	0.993700625	negative
PRJNA729271	stage_1W_vs_E10	ordinary_muscle_development	ENSGALG00010000625	ENSGALG00010000625	2.799475455	0.358748063	7.803458043	6.02334E-15	1.78435E-14	positive
PRJNA729271	stage_1W_vs_E10	ordinary_muscle_development	ENSGALG00010000626	ABCA13	-1.124070949	0.700821846	-1.603932518	0.108728928	0.130078063	negative
PRJNA729271	stage_1W_vs_E10	ordinary_muscle_development	ENSGALG00010000627	ENSGALG00010000627	0.666658836	0.232283846	2.870018074	0.004104483	0.005770561	positive
PRJNA729271	stage_1W_vs_E10	ordinary_muscle_development	ENSGALG00010000630	ENSGALG00010000630	0.584532631	0.371354705	1.574054732	0.115474797	0.137693911	positive
PRJNA729271	stage_1W_vs_E10	ordinary_muscle_development	ENSGALG00010000632	ENSGALG00010000632	-0.588272911	0.430492713	-1.366510727	0.171778716	0.199648272	negative
PRJNA729271	stage_1W_vs_E10	ordinary_muscle_development	ENSGALG00010000633	VWC2	-5.704946087	0.901279271	-6.329831683	2.45429E-10	5.69654E-10	negative
PRJNA729271	stage_1W_vs_E10	ordinary_muscle_development	ENSGALG00010000634	ENSGALG00010000634	0.683209433	0.194736531	3.508378366	0.000450847	0.000689713	positive
PRJNA729271	stage_1W_vs_E10	ordinary_muscle_development	ENSGALG00010000635	ENSGALG00010000635	-0.05116657	0.127410667	-0.401587807	0.687987405	0.717111698	negative
PRJNA729271	stage_1W_vs_E10	ordinary_muscle_development	ENSGALG00010000636	ENSGALG00010000636	0.523074685	0.14357446	3.64322933	0.000269239	0.000420088	positive
PRJNA729271	stage_1W_vs_E10	ordinary_muscle_development	ENSGALG00010000639	SCN1B	4.795694071	0.266111751	18.02135399	1.32462E-72	2.66671E-71	positive
PRJNA729271	stage_1W_vs_E10	ordinary_muscle_development	ENSGALG00010000641	ENSGALG00010000641	-1.889277995	0.504587706	-3.744201402	0.000180968	0.000285683	negative
PRJNA729271	stage_1W_vs_E10	ordinary_muscle_development	ENSGALG00010000643	VKORC1	0.264758962	0.212418461	1.246402785	0.212616559	0.24359329	positive
PRJNA729271	stage_1W_vs_E10	ordinary_muscle_development	ENSGALG00010000645	ENSGALG00010000645	-1.471774358	0.547990539	-2.685765999	0.007236375	0.009931943	negative
PRJNA729271	stage_1W_vs_E10	ordinary_muscle_development	ENSGALG00010000646	ENSGALG00010000646	-1.133438193	0.542583582	-2.088965149	0.03671086	0.046557982	negative
PRJNA729271	stage_1W_vs_E10	ordinary_muscle_development	ENSGALG00010000649	PAPD7	-0.147391482	0.084106865	-1.752431056	0.079699715	0.0969981	negative
PRJNA729271	stage_1W_vs_E10	ordinary_muscle_development	ENSGALG00010000650	BCKDK	1.875721069	0.167838291	11.1757636	5.35875E-29	2.89185E-28	positive
PRJNA729271	stage_1W_vs_E10	ordinary_muscle_development	ENSGALG00010000651	ENSGALG00010000651	-4.054616571	0.651058254	-6.227732383	4.73235E-10	1.08263E-09	negative
PRJNA729271	stage_1W_vs_E10	ordinary_muscle_development	ENSGALG00010000652	SRD5A1	-0.21561696	0.231549149	-0.931193056	0.351753709	0.389360062	negative
PRJNA729271	stage_1W_vs_E10	ordinary_muscle_development	ENSGALG00010000653	NSUN2	-0.409905606	0.092045221	-4.453306761	8.45578E-06	1.4744E-05	negative
PRJNA729271	stage_1W_vs_E10	ordinary_muscle_development	ENSGALG00010000654	ENSGALG00010000654	-0.90643318	0.17118923	-5.294919415	1.19069E-07	2.36652E-07	negative
PRJNA729271	stage_1W_vs_E10	ordinary_muscle_development	ENSGALG00010000655	ENSGALG00010000655	-3.815961154	1.072781447	-3.557072286	0.000375011	0.000577722	negative
PRJNA729271	stage_1W_vs_E10	ordinary_muscle_development	ENSGALG00010000656	ENSGALG00010000656	1.30282524	0.409450257	3.181888926	0.001463179	0.002146421	positive
PRJNA729271	stage_1W_vs_E10	ordinary_muscle_development	ENSGALG00010000659	ENSGALG00010000659	2.810243074	0.545415306	5.152482971	2.5706E-07	4.99676E-07	positive
PRJNA729271	stage_1W_vs_E10	ordinary_muscle_development	ENSGALG00010000660	ENSGALG00010000660	1.775090474	0.175695619	10.10321421	5.34607E-24	2.35679E-23	positive
PRJNA729271	stage_1W_vs_E10	ordinary_muscle_development	ENSGALG00010000661	ENSGALG00010000661	-2.73954051	0.832998631	-3.288769522	0.001006264	0.001498284	negative
PRJNA729271	stage_1W_vs_E10	ordinary_muscle_development	ENSGALG00010000670	ENSGALG00010000670	-3.19606556	0.680806785	-4.694526595	2.67225E-06	4.83997E-06	negative
PRJNA729271	stage_1W_vs_E10	ordinary_muscle_development	ENSGALG00010000674	GRB10	1.482044867	0.169121332	8.763204812	1.89776E-18	6.59508E-18	positive
PRJNA729271	stage_1W_vs_E10	ordinary_muscle_development	ENSGALG00010000684	COBL	1.76237969	0.135242788	13.03122863	8.12881E-39	6.25741E-38	positive
PRJNA729271	stage_1W_vs_E10	ordinary_muscle_development	ENSGALG00010000686	ENSGALG00010000686	-0.36030328	0.134659797	-2.675655896	0.007458318	0.010221075	negative
PRJNA729271	stage_1W_vs_E10	ordinary_muscle_development	ENSGALG00010000687	IKZF1	0.697076611	0.288334093	2.417600374	0.015623224	0.020660399	positive
PRJNA729271	stage_1W_vs_E10	ordinary_muscle_development	ENSGALG00010000688	ENSGALG00010000688	1.256984092	0.286719191	4.384024974	1.16506E-05	2.01307E-05	positive
PRJNA729271	stage_1W_vs_E10	ordinary_muscle_development	ENSGALG00010000695	FIGNL1	-2.430742632	0.156894975	-15.49280108	3.8801E-54	4.89914E-53	negative
PRJNA729271	stage_1W_vs_E10	ordinary_muscle_development	ENSGALG00010000697	ENSGALG00010000697	0.788029131	0.247430796	3.18484661	0.001448308	0.002125465	positive
PRJNA729271	stage_1W_vs_E10	ordinary_muscle_development	ENSGALG00010000702	DDC	-2.932208326	0.559199831	-5.243578709	1.57492E-07	3.10422E-07	negative
PRJNA729271	stage_1W_vs_E10	ordinary_muscle_development	ENSGALG00010000704	ENSGALG00010000704	-0.477397696	0.096200767	-4.962514429	6.95864E-07	1.31524E-06	negative
PRJNA729271	stage_1W_vs_E10	ordinary_muscle_development	ENSGALG00010000705	ENSGALG00010000705	4.782288367	1.010943707	4.730518953	2.23947E-06	4.07342E-06	positive
PRJNA729271	stage_1W_vs_E10	ordinary_muscle_development	ENSGALG00010000710	ENSGALG00010000710	1.292277615	0.498500785	2.592328146	0.00953288	0.012913934	positive
PRJNA729271	stage_1W_vs_E10	ordinary_muscle_development	ENSGALG00010000711	SPRY2	-0.204888439	0.111127938	-1.843716746	0.065224431	0.080270685	negative
PRJNA729271	stage_1W_vs_E10	ordinary_muscle_development	ENSGALG00010000714	FBXL7	-1.843117323	0.100099122	-18.41292193	1.03486E-75	2.21569E-74	negative
PRJNA729271	stage_1W_vs_E10	ordinary_muscle_development	ENSGALG00010000715	PCDH20	-2.434982055	0.279863617	-8.706201972	3.14227E-18	1.08136E-17	negative
PRJNA729271	stage_1W_vs_E10	ordinary_muscle_development	ENSGALG00010000717	NDFIP2	0.533774863	0.067315109	7.92949563	2.20038E-15	6.65994E-15	positive
PRJNA729271	stage_1W_vs_E10	ordinary_muscle_development	ENSGALG00010000718	EEF1G	0.400233065	0.150908333	2.652160136	0.00799786	0.010925359	positive
PRJNA729271	stage_1W_vs_E10	ordinary_muscle_development	ENSGALG00010000720	RBM26	-0.253295306	0.051669153	-4.902253896	9.47433E-07	1.77521E-06	negative
PRJNA729271	stage_1W_vs_E10	ordinary_muscle_development	ENSGALG00010000721	ENSGALG00010000721	-1.536926598	0.1572812	-9.771839188	1.48727E-22	6.17531E-22	negative
PRJNA729271	stage_1W_vs_E10	ordinary_muscle_development	ENSGALG00010000722	ANKH	0.478355007	0.170554889	2.804698301	0.00503637	0.00703161	positive
PRJNA729271	stage_1W_vs_E10	ordinary_muscle_development	ENSGALG00010000724	OBI1	-1.784908164	0.148607566	-12.0108835	3.11499E-33	1.98819E-32	negative
PRJNA729271	stage_1W_vs_E10	ordinary_muscle_development	ENSGALG00010000725	ENSGALG00010000725	-4.110560803	0.963916547	-4.264436394	2.00407E-05	3.39358E-05	negative
PRJNA729271	stage_1W_vs_E10	ordinary_muscle_development	ENSGALG00010000727	ENSGALG00010000727	-0.250049556	0.14934811	-1.674273316	0.094076896	0.113589722	negative
PRJNA729271	stage_1W_vs_E10	ordinary_muscle_development	ENSGALG00010000728	OTULIN	-1.195237235	0.316375014	-3.777912867	0.000158148	0.000250696	negative
PRJNA729271	stage_1W_vs_E10	ordinary_muscle_development	ENSGALG00010000729	EDNRB	-0.699594743	0.157644371	-4.437803516	9.08815E-06	1.58219E-05	negative
PRJNA729271	stage_1W_vs_E10	ordinary_muscle_development	ENSGALG00010000730	INTS5	2.192414074	0.221056259	9.917900918	3.47998E-23	1.48534E-22	positive
PRJNA729271	stage_1W_vs_E10	ordinary_muscle_development	ENSGALG00010000731	ENSGALG00010000731	-1.78160617	0.455520291	-3.911145578	9.18594E-05	0.000148175	negative
PRJNA729271	stage_1W_vs_E10	ordinary_muscle_development	ENSGALG00010000732	ENSGALG00010000732	0.160580755	0.14428063	1.112975147	0.265719103	0.2995255	positive
PRJNA729271	stage_1W_vs_E10	ordinary_muscle_development	ENSGALG00010000733	ENSGALG00010000733	-2.432036138	0.528623668	-4.600694761	4.21084E-06	7.50752E-06	negative
PRJNA729271	stage_1W_vs_E10	ordinary_muscle_development	ENSGALG00010000734	SLAIN1	-1.306068372	0.343684023	-3.800201009	0.000144579	0.000229889	negative
PRJNA729271	stage_1W_vs_E10	ordinary_muscle_development	ENSGALG00010000735	ENSGALG00010000735	1.566674774	0.536166708	2.921991895	0.003478006	0.004927543	positive
PRJNA729271	stage_1W_vs_E10	ordinary_muscle_development	ENSGALG00010000737	ENSGALG00010000737	-3.489282671	1.07616883	-3.242319024	0.001185612	0.001752366	negative
PRJNA729271	stage_1W_vs_E10	ordinary_muscle_development	ENSGALG00010000741	FAM105A	-1.334931915	0.199757827	-6.682751498	2.34497E-11	5.78461E-11	negative
PRJNA729271	stage_1W_vs_E10	ordinary_muscle_development	ENSGALG00010000753	TRIO	-0.347625304	0.068532735	-5.07239794	3.92834E-07	7.54489E-07	negative
PRJNA729271	stage_1W_vs_E10	ordinary_muscle_development	ENSGALG00010000757	SCEL	-5.350021925	0.553276242	-9.669712013	4.05518E-22	1.65205E-21	negative
PRJNA729271	stage_1W_vs_E10	ordinary_muscle_development	ENSGALG00010000760	CDH10	-1.811598147	0.326143497	-5.554604531	2.78241E-08	5.74598E-08	negative
PRJNA729271	stage_1W_vs_E10	ordinary_muscle_development	ENSGALG00010000762	ENSGALG00010000762	0.564012773	0.306954059	1.837450125	0.066143473	0.081339581	positive
PRJNA729271	stage_1W_vs_E10	ordinary_muscle_development	ENSGALG00010000768	ENSGALG00010000768	-2.43359816	0.408567485	-5.956416626	2.57829E-09	5.67019E-09	negative
PRJNA729271	stage_1W_vs_E10	ordinary_muscle_development	ENSGALG00010000770	ENSGALG00010000770	-4.60527918	0.964312033	-4.77571473	1.7907E-06	3.2831E-06	negative
PRJNA729271	stage_1W_vs_E10	ordinary_muscle_development	ENSGALG00010000777	MYCBP2	-0.308716698	0.082738264	-3.731244544	0.000190536	0.000300263	negative
PRJNA729271	stage_1W_vs_E10	ordinary_muscle_development	ENSGALG00010000783	DAP	-1.685646383	0.163703558	-10.29694412	7.27352E-25	3.32062E-24	negative
PRJNA729271	stage_1W_vs_E10	ordinary_muscle_development	ENSGALG00010000784	FBXL3	0.433446481	0.095797601	4.524606867	6.05079E-06	1.06556E-05	positive
PRJNA729271	stage_1W_vs_E10	ordinary_muscle_development	ENSGALG00010000785	CLN5	0.065117788	0.094094783	0.692044609	0.488909315	0.527130357	positive
PRJNA729271	stage_1W_vs_E10	ordinary_muscle_development	ENSGALG00010000786	ANKRD33B	1.367709023	0.319228603	4.284418778	1.83218E-05	3.1145E-05	positive
PRJNA729271	stage_1W_vs_E10	ordinary_muscle_development	ENSGALG00010000787	SNRNP48	-0.166808263	0.107832764	-1.546916331	0.121883423	0.144858987	negative
PRJNA729271	stage_1W_vs_E10	ordinary_muscle_development	ENSGALG00010000788	GATD3AL1	1.130790622	0.104408136	10.83048376	2.46848E-27	1.25151E-26	positive
PRJNA729271	stage_1W_vs_E10	ordinary_muscle_development	ENSGALG00010000789	ROPN1L	1.115933192	0.228058791	4.893182087	9.92185E-07	1.8569E-06	positive
PRJNA729271	stage_1W_vs_E10	ordinary_muscle_development	ENSGALG00010000790	MARCHF6	1.256765133	0.098978507	12.69735391	6.11629E-37	4.41292E-36	positive
PRJNA729271	stage_1W_vs_E10	ordinary_muscle_development	ENSGALG00010000791	ACOD1	0.393404587	0.518089312	0.759337392	0.447650755	0.48579005	positive
PRJNA729271	stage_1W_vs_E10	ordinary_muscle_development	ENSGALG00010000792	ENSGALG00010000792	-2.236930469	1.832728758	-1.22054639	0.222257815	0.253631478	negative
PRJNA729271	stage_1W_vs_E10	ordinary_muscle_development	ENSGALG00010000793	ENSGALG00010000793	0.201749747	1.415093565	0.142569899	0.886629871	0.899412134	positive
PRJNA729271	stage_1W_vs_E10	ordinary_muscle_development	ENSGALG00010000795	ENSGALG00010000795	-1.70984174	0.257947942	-6.628631053	3.38814E-11	8.28181E-11	negative
PRJNA729271	stage_1W_vs_E10	ordinary_muscle_development	ENSGALG00010000796	ENSGALG00010000796	-1.823710428	0.21508218	-8.479133095	2.26878E-17	7.48429E-17	negative
PRJNA729271	stage_1W_vs_E10	ordinary_muscle_development	ENSGALG00010000797	ENSGALG00010000797	3.484280929	0.411795446	8.461193446	2.64655E-17	8.69881E-17	positive
PRJNA729271	stage_1W_vs_E10	ordinary_muscle_development	ENSGALG00010000798	ENSGALG00010000798	-5.877413239	0.763151481	-7.701502759	1.34475E-14	3.91803E-14	negative
PRJNA729271	stage_1W_vs_E10	ordinary_muscle_development	ENSGALG00010000799	ENSGALG00010000799	-2.908270933	0.306115582	-9.500564835	2.08755E-21	8.31749E-21	negative
PRJNA729271	stage_1W_vs_E10	ordinary_muscle_development	ENSGALG00010000800	LMO7	0.23780527	0.151716684	1.567429918	0.117014235	0.139380503	positive
PRJNA729271	stage_1W_vs_E10	ordinary_muscle_development	ENSGALG00010000802	SEMA5A	-2.526157679	0.127943429	-19.74433303	8.97419E-87	2.50156E-85	negative
PRJNA729271	stage_1W_vs_E10	ordinary_muscle_development	ENSGALG00010000803	MTRR	-0.030274665	0.098529196	-0.307265925	0.75864097	0.783000719	negative
PRJNA729271	stage_1W_vs_E10	ordinary_muscle_development	ENSGALG00010000804	ENSGALG00010000804	-0.444314504	0.481181432	-0.923382481	0.355807918	0.393397084	negative
PRJNA729271	stage_1W_vs_E10	ordinary_muscle_development	ENSGALG00010000805	FASTKD3	0.557670355	0.129821849	4.295658785	1.74175E-05	2.96602E-05	positive
PRJNA729271	stage_1W_vs_E10	ordinary_muscle_development	ENSGALG00010000806	CFAP90	1.493270805	0.428725368	3.483047463	0.00049574	0.000756236	positive
PRJNA729271	stage_1W_vs_E10	ordinary_muscle_development	ENSGALG00010000807	ENSGALG00010000807	1.322828867	0.785893101	1.683217303	0.092333032	0.111605012	positive
PRJNA729271	stage_1W_vs_E10	ordinary_muscle_development	ENSGALG00010000808	ADCY2	-0.905950551	0.176754807	-5.125464868	2.96805E-07	5.74313E-07	negative
PRJNA729271	stage_1W_vs_E10	ordinary_muscle_development	ENSGALG00010000810	UCHL3	-0.834374545	0.138107422	-6.041489524	1.52698E-09	3.40516E-09	negative
PRJNA729271	stage_1W_vs_E10	ordinary_muscle_development	ENSGALG00010000811	ENSGALG00010000811	10.67438974	1.044016034	10.22435421	1.54253E-24	6.96106E-24	positive
PRJNA729271	stage_1W_vs_E10	ordinary_muscle_development	ENSGALG00010000812	ENSGALG00010000812	-1.160160103	0.097103325	-11.94768664	6.67577E-33	4.20196E-32	negative
PRJNA729271	stage_1W_vs_E10	ordinary_muscle_development	ENSGALG00010000813	ENSGALG00010000813	-2.030162631	0.474715717	-4.276586088	1.89781E-05	3.22079E-05	negative
PRJNA729271	stage_1W_vs_E10	ordinary_muscle_development	ENSGALG00010000814	CDH18	-2.282864559	0.301313138	-7.576385741	3.55314E-14	1.01284E-13	negative
PRJNA729271	stage_1W_vs_E10	ordinary_muscle_development	ENSGALG00010000815	ENSGALG00010000815	-1.434478794	0.173059204	-8.2889483	1.14254E-16	3.65669E-16	negative
PRJNA729271	stage_1W_vs_E10	ordinary_muscle_development	ENSGALG00010000816	TBC1D4	1.564868433	0.103772082	15.0798596	2.19736E-51	2.52383E-50	positive
PRJNA729271	stage_1W_vs_E10	ordinary_muscle_development	ENSGALG00010000817	ENSGALG00010000817	1.415011634	0.115313772	12.27096827	1.29693E-34	8.69117E-34	positive
PRJNA729271	stage_1W_vs_E10	ordinary_muscle_development	ENSGALG00010000818	KLF12	-1.57904107	0.082405114	-19.16193055	7.69649E-82	1.92015E-80	negative
PRJNA729271	stage_1W_vs_E10	ordinary_muscle_development	ENSGALG00010000820	KLF5	0.259300525	0.194200157	1.335223047	0.181803356	0.210499213	positive
PRJNA729271	stage_1W_vs_E10	ordinary_muscle_development	ENSGALG00010000821	ENSGALG00010000821	2.663019614	0.148716781	17.90665177	1.04644E-71	2.06369E-70	positive
PRJNA729271	stage_1W_vs_E10	ordinary_muscle_development	ENSGALG00010000822	BASP1	-4.321491187	0.167839773	-25.74771824	3.4188E-146	2.6643E-144	negative
PRJNA729271	stage_1W_vs_E10	ordinary_muscle_development	ENSGALG00010000823	PIBF1	-0.325439563	0.162260159	-2.005665255	0.044891975	0.056401939	negative
PRJNA729271	stage_1W_vs_E10	ordinary_muscle_development	ENSGALG00010000843	DIS3	-0.720543618	0.084010128	-8.576866049	9.7493E-18	3.27045E-17	negative
PRJNA729271	stage_1W_vs_E10	ordinary_muscle_development	ENSGALG00010000853	ENSGALG00010000853	0.610985847	0.305231179	2.001715057	0.045315386	0.056889531	positive
PRJNA729271	stage_1W_vs_E10	ordinary_muscle_development	ENSGALG00010000857	ENSGALG00010000857	-0.001443162	0.781519752	-0.00184661			negative
PRJNA729271	stage_1W_vs_E10	ordinary_muscle_development	ENSGALG00010000859	BORA	-2.636402107	0.196211174	-13.43655439	3.69233E-41	3.08297E-40	negative
PRJNA729271	stage_1W_vs_E10	ordinary_muscle_development	ENSGALG00010000864	MZT1	-0.30980632	0.129456394	-2.393132628	0.016705201	0.022010898	negative
PRJNA729271	stage_1W_vs_E10	ordinary_muscle_development	ENSGALG00010000870	DACH1	0.16390393	0.144420367	1.134908689	0.256413579	0.289847526	positive
PRJNA729271	stage_1W_vs_E10	ordinary_muscle_development	ENSGALG00010000901	PCDH9	-2.6773938	0.253803983	-10.5490614	5.13071E-26	2.4642E-25	negative
PRJNA729271	stage_1W_vs_E10	ordinary_muscle_development	ENSGALG00010001066	SLC33A1	-0.596124793	0.08861493	-6.727137186	1.73034E-11	4.2962E-11	negative
PRJNA729271	stage_1W_vs_E10	ordinary_muscle_development	ENSGALG00010001070	C3orf33	0.153695987	0.180371259	0.852109077	0.394153555	0.432298409	positive
PRJNA729271	stage_1W_vs_E10	ordinary_muscle_development	ENSGALG00010001082	P2RY1	3.423923639	0.179917661	19.03050323	9.53328E-81	2.33027E-79	positive
PRJNA729271	stage_1W_vs_E10	ordinary_muscle_development	ENSGALG00010001104	MBNL1	2.390548967	0.122555292	19.50588121	9.78561E-85	2.60261E-83	positive
PRJNA729271	stage_1W_vs_E10	ordinary_muscle_development	ENSGALG00010001111	SUCNR1	4.948964139	0.416974192	11.86875407	1.7201E-32	1.06733E-31	positive
PRJNA729271	stage_1W_vs_E10	ordinary_muscle_development	ENSGALG00010001112	TSC22D2	-0.194463681	0.09068856	-2.144302217	0.032008679	0.040852849	negative
PRJNA729271	stage_1W_vs_E10	ordinary_muscle_development	ENSGALG00010001113	IGSF10	1.065579983	0.298384917	3.571158998	0.000355405	0.000548801	positive
PRJNA729271	stage_1W_vs_E10	ordinary_muscle_development	ENSGALG00010001114	MED12L	0.624990313	0.102140086	6.118952299	9.41926E-10	2.12171E-09	positive
PRJNA729271	stage_1W_vs_E10	ordinary_muscle_development	ENSGALG00010001115	PFN2	-1.234716623	0.096004536	-12.86102379	7.45823E-38	5.52694E-37	negative
PRJNA729271	stage_1W_vs_E10	ordinary_muscle_development	ENSGALG00010001116	RNF13	0.298117007	0.070406998	4.234195701	2.29371E-05	3.86867E-05	positive
PRJNA729271	stage_1W_vs_E10	ordinary_muscle_development	ENSGALG00010001117	P2RY13	-0.360902679	0.324886243	-1.110858604	0.266629216	0.300434584	negative
PRJNA729271	stage_1W_vs_E10	ordinary_muscle_development	ENSGALG00010001118	P2RY12	-0.756558145	0.346352269	-2.184360294	0.028935783	0.037127041	negative
PRJNA729271	stage_1W_vs_E10	ordinary_muscle_development	ENSGALG00010001119	COMMD2	-0.694947605	0.099534211	-6.981997405	2.91013E-12	7.5218E-12	negative
PRJNA729271	stage_1W_vs_E10	ordinary_muscle_development	ENSGALG00010001120	GPR87	-2.887579386	1.13697725	-2.539698473	0.011094807	0.014916913	negative
PRJNA729271	stage_1W_vs_E10	ordinary_muscle_development	ENSGALG00010001121	WWTR1	0.276861439	0.09193598	3.01145905	0.002599955	0.003726893	positive
PRJNA729271	stage_1W_vs_E10	ordinary_muscle_development	ENSGALG00010001122	P2RY14	2.945549845	0.424467474	6.939400596	3.93767E-12	1.01056E-11	positive
PRJNA729271	stage_1W_vs_E10	ordinary_muscle_development	ENSGALG00010001123	CP	-0.331316501	0.153564975	-2.157500446	0.030966692	0.039603159	negative
PRJNA729271	stage_1W_vs_E10	ordinary_muscle_development	ENSGALG00010001124	GPR171	2.107428019	0.609074377	3.460050364	0.000540074	0.000821471	positive
PRJNA729271	stage_1W_vs_E10	ordinary_muscle_development	ENSGALG00010001125	CLRN1	2.561007659	0.26162954	9.7886793	1.2593E-22	5.23775E-22	positive
PRJNA729271	stage_1W_vs_E10	ordinary_muscle_development	ENSGALG00010001126	MINDY4B	3.99232318	1.187784915	3.361149928	0.000776187	0.001167583	positive

PRJNA729271	stage_1W_vs_E10	ordinary_muscle_development	ENSGALG00010001127	SIAH2	0.827715127	0.099996	8.27748235	1.25807E-16	4.01667E-16	positive
PRJNA729271	stage_1W_vs_E10	ordinary_muscle_development	ENSGALG00010001129	TM4SF4	0.789645081	0.978775263	0.806768531	0.419799853	0.457862969	positive
PRJNA729271	stage_1W_vs_E10	ordinary_muscle_development	ENSGALG00010001131	HPS3	0.416635875	0.099863292	4.172062291	3.01855E-05	5.053E-05	positive
PRJNA729271	stage_1W_vs_E10	ordinary_muscle_development	ENSGALG00010001132	SELENOT	0.501060174	0.094450562	5.3049994	1.12673E-07	2.24279E-07	positive
PRJNA729271	stage_1W_vs_E10	ordinary_muscle_development	ENSGALG00010001133	EIF2A	0.290621281	0.073060684	3.977806714	6.95539E-05	0.000113292	positive
PRJNA729271	stage_1W_vs_E10	ordinary_muscle_development	ENSGALG00010001134	ENSGALG00010001134	0.151959351	0.646317097	0.235115784	0.814118868	0.833614933	positive
PRJNA729271	stage_1W_vs_E10	ordinary_muscle_development	ENSGALG00010001135	GYG1	2.398095181	0.159187022	15.06464003	2.76668E-51	3.1652E-50	positive
PRJNA729271	stage_1W_vs_E10	ordinary_muscle_development	ENSGALG00010001136	SERP1	-0.936853527	0.126979045	-7.3780168	1.60665E-13	4.41653E-13	negative
PRJNA729271	stage_1W_vs_E10	ordinary_muscle_development	ENSGALG00010001138	SPTSSB	-6.941841949	0.994755276	-6.978441952	2.98471E-12	7.71184E-12	negative
PRJNA729271	stage_1W_vs_E10	ordinary_muscle_development	ENSGALG00010001140	NMD3	1.315164032	0.119863127	10.97221523	5.1983E-28	2.70846E-27	positive
PRJNA729271	stage_1W_vs_E10	ordinary_muscle_development	ENSGALG00010001141	MTAP	-1.306168249	0.114064209	-11.45116648	2.32002E-30	1.32658E-29	negative
PRJNA729271	stage_1W_vs_E10	ordinary_muscle_development	ENSGALG00010001142	PPM1L	2.343087271	0.145953843	16.05361825	5.39265E-58	7.48005E-57	positive
PRJNA729271	stage_1W_vs_E10	ordinary_muscle_development	ENSGALG00010001143	CDKN2A	2.81993022	0.422050915	6.681492973	2.3652E-11	5.83352E-11	positive
PRJNA729271	stage_1W_vs_E10	ordinary_muscle_development	ENSGALG00010001144	CDKN2B	6.456598183	1.365150509	4.72958706	2.24977E-06	4.09011E-06	positive
PRJNA729271	stage_1W_vs_E10	ordinary_muscle_development	ENSGALG00010001145	TRIM36	-4.244200097	0.240585604	-17.64112246	1.19072E-69	2.22991E-68	negative
PRJNA729271	stage_1W_vs_E10	ordinary_muscle_development	ENSGALG00010001146	DIPK1C	1.119735551	0.242915523	4.609567717	4.03507E-06	7.20211E-06	positive
PRJNA729271	stage_1W_vs_E10	ordinary_muscle_development	ENSGALG00010001147	PGGT1B	-0.238219645	0.113622477	-2.096589089	0.036029966	0.045731555	negative
PRJNA729271	stage_1W_vs_E10	ordinary_muscle_development	ENSGALG00010001149	CIDEA	2.683557466	0.620359111	4.325812936	1.5197E-05	2.59704E-05	positive
PRJNA729271	stage_1W_vs_E10	ordinary_muscle_development	ENSGALG00010001150	ENSGALG00010001150	-0.117101482	0.642091586	-0.182375046	0.855288408	0.871461091	negative
PRJNA729271	stage_1W_vs_E10	ordinary_muscle_development	ENSGALG00010001151	CCDC112	2.197915894	0.214531469	10.2451911	1.24379E-24	5.63573E-24	positive
PRJNA729271	stage_1W_vs_E10	ordinary_muscle_development	ENSGALG00010001152	FEM1C	2.692323396	0.197395603	13.63922681	2.34029E-42	2.02281E-41	positive
PRJNA729271	stage_1W_vs_E10	ordinary_muscle_development	ENSGALG00010001153	TSHZ1	-2.100222195	0.117444978	-17.88260541	1.61128E-71	3.169E-70	negative
PRJNA729271	stage_1W_vs_E10	ordinary_muscle_development	ENSGALG00010001154	PTGR3	-0.110212229	0.08488663	-1.298346152	0.194168414	0.223921645	negative
PRJNA729271	stage_1W_vs_E10	ordinary_muscle_development	ENSGALG00010001155	CYB5A	-0.232497106	0.094432407	-2.462047859	0.013814623	0.018404684	negative
PRJNA729271	stage_1W_vs_E10	ordinary_muscle_development	ENSGALG00010001156	ALDH7A1	0.595222449	0.181082842	3.287017383	0.001012546	0.001506864	positive
PRJNA729271	stage_1W_vs_E10	ordinary_muscle_development	ENSGALG00010001157	TIMM21	1.772166331	0.091115005	19.44977481	2.92714E-84	7.70033E-83	positive
PRJNA729271	stage_1W_vs_E10	ordinary_muscle_development	ENSGALG00010001158	FRMPD1	-0.179599891	0.365430891	-0.4914743	0.623091025	0.656851229	negative
PRJNA729271	stage_1W_vs_E10	ordinary_muscle_development	ENSGALG00010001159	RTTN	-1.04507367	0.115281679	-9.065392499	1.24156E-19	4.5886E-19	negative
PRJNA729271	stage_1W_vs_E10	ordinary_muscle_development	ENSGALG00010001160	TRMT10B	-0.175369906	0.128228766	-1.367633104	0.171426949	0.199280907	negative
PRJNA729271	stage_1W_vs_E10	ordinary_muscle_development	ENSGALG00010001161	B3GALNT1	0.358219848	0.098359615	3.641940305	0.000270591	0.000422152	positive
PRJNA729271	stage_1W_vs_E10	ordinary_muscle_development	ENSGALG00010001162	ENSGALG00010001162	0.188654444	0.419854358	0.449333061	0.653191412	0.684443688	positive
PRJNA729271	stage_1W_vs_E10	ordinary_muscle_development	ENSGALG00010001163	ARL14	-1.518169846	0.582779201	-2.605051525	0.009186044	0.012470892	negative
PRJNA729271	stage_1W_vs_E10	ordinary_muscle_development	ENSGALG00010001164	KPNA4	1.127511118	0.074537035	15.12685765	1.07718E-51	1.2531E-50	positive
PRJNA729271	stage_1W_vs_E10	ordinary_muscle_development	ENSGALG00010001165	ENSGALG00010001165	2.382596875	0.158255197	15.05540999	3.18121E-51	3.62798E-50	positive
PRJNA729271	stage_1W_vs_E10	ordinary_muscle_development	ENSGALG00010001166	TRIM59	-0.409101832	0.096833828	-4.224782206	2.39172E-05	4.02788E-05	negative
PRJNA729271	stage_1W_vs_E10	ordinary_muscle_development	ENSGALG00010001167	SMC4	-1.446192957	0.154144987	-9.382030418	6.47143E-21	2.52431E-20	negative
PRJNA729271	stage_1W_vs_E10	ordinary_muscle_development	ENSGALG00010001168	DOK6	-2.013820366	0.172627306	-11.66571162	1.90801E-31	1.14284E-30	negative
PRJNA729271	stage_1W_vs_E10	ordinary_muscle_development	ENSGALG00010001169	ENSGALG00010001169	-0.468355642	0.104267098	-4.491883369	7.05961E-06	1.23705E-05	negative
PRJNA729271	stage_1W_vs_E10	ordinary_muscle_development	ENSGALG00010001170	DCAF10	0.108859572	0.069500469	1.566314212	0.117275075	0.139656771	positive
PRJNA729271	stage_1W_vs_E10	ordinary_muscle_development	ENSGALG00010001171	POLR1E	0.004808093	0.127270753	0.037778461	0.969864318	0.974030575	positive
PRJNA729271	stage_1W_vs_E10	ordinary_muscle_development	ENSGALG00010001172	TMEM200C	-2.537935676	0.290566583	-8.734437547	2.44872E-18	8.46105E-18	negative
PRJNA729271	stage_1W_vs_E10	ordinary_muscle_development	ENSGALG00010001173	IFT80	0.129749506	0.110704749	1.172031766	0.241184303	0.273614031	positive
PRJNA729271	stage_1W_vs_E10	ordinary_muscle_development	ENSGALG00010001174	ENSGALG00010001174	-2.17035722	0.145352281	-14.93170394	2.04961E-50	2.28356E-49	negative
PRJNA729271	stage_1W_vs_E10	ordinary_muscle_development	ENSGALG00010001177	SEH1L	-0.33205359	0.071350214	-4.653855594	3.25785E-06	5.86106E-06	negative
PRJNA729271	stage_1W_vs_E10	ordinary_muscle_development	ENSGALG00010001178	ZBTB5	-0.494115108	0.065539766	-7.53916502	4.7299E-14	1.33985E-13	negative
PRJNA729271	stage_1W_vs_E10	ordinary_muscle_development	ENSGALG00010001179	GRHPR	2.222698834	0.183389115	12.12012413	8.26339E-34	5.38327E-33	positive
PRJNA729271	stage_1W_vs_E10	ordinary_muscle_development	ENSGALG00010001180	SPIRE1	-0.505845846	0.168246371	-3.006578042	0.002642062	0.003784632	negative
PRJNA729271	stage_1W_vs_E10	ordinary_muscle_development	ENSGALG00010001181	MFSD1	0.685500944	0.104650881	6.55035997	5.73986E-11	1.38228E-10	positive
PRJNA729271	stage_1W_vs_E10	ordinary_muscle_development	ENSGALG00010001182	ZCCHC7	-0.447661377	0.085638357	-5.227346629	1.7196E-07	3.37928E-07	negative
PRJNA729271	stage_1W_vs_E10	ordinary_muscle_development	ENSGALG00010001183	RARRES1	1.293268713	0.188492562	6.861112729	6.83262E-12	1.73478E-11	positive
PRJNA729271	stage_1W_vs_E10	ordinary_muscle_development	ENSGALG00010001185	PTPN2	-0.803689791	0.068616563	-11.71276659	1.09643E-31	6.63301E-31	negative
PRJNA729271	stage_1W_vs_E10	ordinary_muscle_development	ENSGALG00010001186	ENSGALG00010001186	-0.549711228	0.390419966	-1.407999784	0.159131134	0.18595661	negative
PRJNA729271	stage_1W_vs_E10	ordinary_muscle_development	ENSGALG00010001187	GFM1	1.534843225	0.181582916	8.452575047	2.84947E-17	9.34882E-17	positive
PRJNA729271	stage_1W_vs_E10	ordinary_muscle_development	ENSGALG00010001188	PSMG2	0.454267951	0.110770691	4.100976049	4.11411E-05	6.81221E-05	positive
PRJNA729271	stage_1W_vs_E10	ordinary_muscle_development	ENSGALG00010001190	LXN	-0.348741635	0.436515998	-0.798920627	0.424336438	0.462323862	negative
PRJNA729271	stage_1W_vs_E10	ordinary_muscle_development	ENSGALG00010001191	TOMM5	0.165466117	0.151240415	1.094060189	0.273928581	0.308132453	positive
PRJNA729271	stage_1W_vs_E10	ordinary_muscle_development	ENSGALG00010001192	ENSGALG00010001192	3.637117644	0.220540661	16.49182343	4.20078E-61	6.34275E-60	positive
PRJNA729271	stage_1W_vs_E10	ordinary_muscle_development	ENSGALG00010001193	PRELID3A	1.412700981	0.227035698	6.222373801	4.89689E-10	1.11939E-09	positive
PRJNA729271	stage_1W_vs_E10	ordinary_muscle_development	ENSGALG00010001194	POT1	-1.287707307	0.113004329	-11.39520338	4.41797E-30	2.50542E-29	negative
PRJNA729271	stage_1W_vs_E10	ordinary_muscle_development	ENSGALG00010001195	ZNF608	-1.3087423	0.143976244	-9.089987805	9.90502E-20	3.68042E-19	negative
PRJNA729271	stage_1W_vs_E10	ordinary_muscle_development	ENSGALG00010001196	LSMEM1	5.054356117	0.205535602	24.59114663	1.5711E-133	1.0543E-131	positive
PRJNA729271	stage_1W_vs_E10	ordinary_muscle_development	ENSGALG00010001197	CEP76	-0.696358932	0.145462791	-4.787196266	1.69127E-06	3.10513E-06	negative
PRJNA729271	stage_1W_vs_E10	ordinary_muscle_development	ENSGALG00010001198	IFRD1	1.583957998	0.176034724	8.997986111	2.29896E-19	8.38114E-19	positive
PRJNA729271	stage_1W_vs_E10	ordinary_muscle_development	ENSGALG00010001199	AFG3L2	0.786687731	0.089945952	8.746227207	2.20605E-18	7.63713E-18	positive
PRJNA729271	stage_1W_vs_E10	ordinary_muscle_development	ENSGALG00010001200	CTTNBP2	1.092452356	0.127705548	8.554462781	1.18419E-17	3.94957E-17	positive
PRJNA729271	stage_1W_vs_E10	ordinary_muscle_development	ENSGALG00010001201	TUBB6	-1.601272926	0.166173509	-9.636150378	5.62583E-22	2.28292E-21	negative
PRJNA729271	stage_1W_vs_E10	ordinary_muscle_development	ENSGALG00010001202	CFTR	3.600518153	0.246403571	14.6122807	2.34533E-48	2.42479E-47	positive
PRJNA729271	stage_1W_vs_E10	ordinary_muscle_development	ENSGALG00010001203	TMX3	-1.039962347	0.100807694	-10.31629938	5.94713E-25	2.72624E-24	negative
PRJNA729271	stage_1W_vs_E10	ordinary_muscle_development	ENSGALG00010001204	SNX2	0.140159411	0.081640409	1.716789673	0.086017608	0.104301927	positive
PRJNA729271	stage_1W_vs_E10	ordinary_muscle_development	ENSGALG00010001205	CEP192	0.153600696	0.10422191	1.473785088	0.140539465	0.165525273	positive
PRJNA729271	stage_1W_vs_E10	ordinary_muscle_development	ENSGALG00010001206	RSRC1	0.203968221	0.076849448	2.654127333	0.007951382	0.010864939	positive
PRJNA729271	stage_1W_vs_E10	ordinary_muscle_development	ENSGALG00010001208	MOV10L1	-0.720749244	0.642202258	-1.122308797	0.261731167	0.295489778	negative
PRJNA729271	stage_1W_vs_E10	ordinary_muscle_development	ENSGALG00010001209	CDH7	-1.777666264	0.248836256	-7.14391984	9.07063E-13	2.40804E-12	negative
PRJNA729271	stage_1W_vs_E10	ordinary_muscle_development	ENSGALG00010001210	PANX2	-4.03172788	0.305799733	-13.18420996	1.08181E-39	8.58756E-39	negative
PRJNA729271	stage_1W_vs_E10	ordinary_muscle_development	ENSGALG00010001213	PP1C	-0.718684792	0.218982638	-3.281925902	0.001031007	0.001532921	negative
PRJNA729271	stage_1W_vs_E10	ordinary_muscle_development	ENSGALG00010001214	TES	-2.955181895	0.174415057	-16.9433875	2.15358E-64	3.51534E-63	negative
PRJNA729271	stage_1W_vs_E10	ordinary_muscle_development	ENSGALG00010001215	PRDM6	-3.087921516	0.738982203	-4.178614183	2.93291E-05	4.9136E-05	negative
PRJNA729271	stage_1W_vs_E10	ordinary_muscle_development	ENSGALG00010001216	SHOX2	-5.375268681	0.613345977	-8.76384436	1.88702E-18	6.56248E-18	negative
PRJNA729271	stage_1W_vs_E10	ordinary_muscle_development	ENSGALG00010001218	ENSGALG00010001218	-0.922675591	0.113748463	-8.111543378	4.99808E-16	1.55767E-15	negative
PRJNA729271	stage_1W_vs_E10	ordinary_muscle_development	ENSGALG00010001219	TFEC	0.323147687	0.412471671	0.783442135	0.433367521	0.471313192	positive
PRJNA729271	stage_1W_vs_E10	ordinary_muscle_development	ENSGALG00010001220	PLXNB2	-0.99082564	0.083073204	-11.92713887	8.54605E-33	5.34866E-32	negative
PRJNA729271	stage_1W_vs_E10	ordinary_muscle_development	ENSGALG00010001221	GRAMD2B	-0.993889532	0.157635751	-6.304975387	2.8824E-10	6.67204E-10	negative
PRJNA729271	stage_1W_vs_E10	ordinary_muscle_development	ENSGALG00010001222	ZNF800	-0.622428409	0.1025073	-6.072039824	1.26296E-09	2.82901E-09	negative
PRJNA729271	stage_1W_vs_E10	ordinary_muscle_development	ENSGALG00010001223	GRM8	-4.104416982	0.697473698	-5.8846907	3.98799E-09	8.67046E-09	negative
PRJNA729271	stage_1W_vs_E10	ordinary_muscle_development	ENSGALG00010001224	DNAJB9	2.265610223	0.188322273	12.03049534	2.45682E-33	1.57156E-32	positive
PRJNA729271	stage_1W_vs_E10	ordinary_muscle_development	ENSGALG00010001225	SOCS6	0.080328012	0.097756573	0.821714686	0.411239299	0.449236087	positive
PRJNA729271	stage_1W_vs_E10	ordinary_muscle_development	ENSGALG00010001226	VEPH1	-2.59031604	0.659413518	-3.928211915	8.55798E-05	0.0001384	negative
PRJNA729271	stage_1W_vs_E10	ordinary_muscle_development	ENSGALG00010001227	ENSGALG00010001227	-4.335555926	0.995401478	-4.355585181	1.32712E-05	2.28111E-05	negative
PRJNA729271	stage_1W_vs_E10	ordinary_muscle_development	ENSGALG00010001228	THAP5	0.28498303	0.109711671	2.597563488	0.009388776	0.012734192	positive
PRJNA729271	stage_1W_vs_E10	ordinary_muscle_development	ENSGALG00010001229	ZNF407	0.304190026	0.103284691	2.945160832	0.003227868	0.004584807	positive
PRJNA729271	stage_1W_vs_E10	ordinary_muscle_development	ENSGALG00010001230	PTX3	-5.6061389	0.601192472	-9.32503177	1.1095E-20	4.30352E-20	negative
PRJNA729271	stage_1W_vs_E10	ordinary_muscle_development	ENSGALG00010001231	CNDP1	0.255238841	0.597649346	0.427071229	0.669327448	0.69958908	positive
PRJNA729271	stage_1W_vs_E10	ordinary_muscle_development	ENSGALG00010001232	TSPAN12	-0.159544528	0.103556496	-1.540652054	0.123401492	0.146591102	negative
PRJNA729271	stage_1W_vs_E10	ordinary_muscle_development	ENSGALG00010001233	CNDP2	-0.476788252	0.096708498	-4.930158807	8.21628E-07	1.54669E-06	negative
PRJNA729271	stage_1W_vs_E10	ordinary_muscle_development	ENSGALG00010001234	AASS	1.839554177	0.098387078	18.69711163	5.22599E-78	1.17992E-76	positive
PRJNA729271	stage_1W_vs_E10	ordinary_muscle_development	ENSGALG00010001235	CCDC102B	-1.916919588	0.321048198	-5.970815598	2.3607E-09	5.20353E-09	negative
PRJNA729271	stage_1W_vs_E10	ordinary_muscle_development	ENSGALG00010001237	MELK	-2.8162921	0.275039938	-10.23957508	1.31814E-24	5.96703E-24	negative
PRJNA729271	stage_1W_vs_E10	ordinary_muscle_development	ENSGALG00010001238	GOLIM4	-0.892320475	0.122606856	-7.277900299	3.39056E-13	9.16905E-13	negative
PRJNA729271	stage_1W_vs_E10	ordinary_muscle_development	ENSGALG00010001239	ENSGALG00010001239	0.453720611	0.24864022	1.824807792	0.06803004	0.083533467	positive
PRJNA729271	stage_1W_vs_E10	ordinary_muscle_development	ENSGALG00010001242	ENSGALG00010001242	-2.121964551	0.661118399	-3.209665895	0.001328926	0.001955306	negative
PRJNA729271	stage_1W_vs_E10	ordinary_muscle_development	ENSGALG00010001244	ENSGALG00010001244	0.490926985	0.155425727	3.158595394	0.001585314	0.002318314	positive
PRJNA729271	stage_1W_vs_E10	ordinary_muscle_development	ENSGALG00010001245	SERPINI1	-1.470654183	0.227531539	-6.463517932	1.02296E-10	2.4296E-10	negative
PRJNA729271	stage_1W_vs_E10	ordinary_muscle_development	ENSGALG00010001248	PTPRZ1	-0.867681888	0.245380407	-3.536068333	0.00040613	0.000623871	negative
PRJNA729271	stage_1W_vs_E10	ordinary_muscle_development	ENSGALG00010001249	PDCD10	0.269709182	0.139182208	1.937813643	0.052645963	0.065575598	positive
PRJNA729271	stage_1W_vs_E10	ordinary_muscle_development	ENSGALG00010001250	ENSGALG00010001250	-2.468832088	0.706308183	-3.495403489	0.000473346	0.000722758	negative
PRJNA729271	stage_1W_vs_E10	ordinary_muscle_development	ENSGALG00010001251	ZNF277	-0.168129979	0.123303865	-1.363541841	0.172711807	0.200613643	negative
PRJNA729271	stage_1W_vs_E10	ordinary_muscle_development	ENSGALG00010001252	SNCAIP	-2.005461618	0.123995849	-16.17361899	7.74142E-59	1.09582E-57	negative
PRJNA729271	stage_1W_vs_E10	ordinary_muscle_development	ENSGALG00010001253	ENSGALG00010001253	-3.118269988	1.057327042	-2.949201018	0.003185967	0.004527953	negative
PRJNA729271	stage_1W_vs_E10	ordinary_muscle_development	ENSGALG00010001254	NETO1	-2.892155617	0.447237959	-6.466704262	1.00163E-10	2.38059E-10	negative
PRJNA729271	stage_1W_vs_E10	ordinary_muscle_development	ENSGALG00010001258	ENSGALG00010001258	-1.206395542	0.131465124	-9.17654437	4.45147E-20	1.67682E-19	negative
PRJNA729271	stage_1W_vs_E10	ordinary_muscle_development	ENSGALG00010001259	ENSGALG00010001259	0.523794024	0.173011825	3.027504187	0.002465823	0.003542329	positive
PRJNA729271	stage_1W_vs_E10	ordinary_muscle_development	ENSGALG00010001260	ENSGALG00010001260	-5.471372211	1.10682976	-4.943282527	7.6818E-07	1.44871E-06	negative
PRJNA729271	stage_1W_vs_E10	ordinary_muscle_development	ENSGALG00010001262	ENSGALG00010001262	0.836631327	0.144356199	5.795603749	6.80758E-09	1.45884E-08	positive
PRJNA729271	stage_1W_vs_E10	ordinary_muscle_development	ENSGALG00010001264	FAM3C	0.569432899	0.11314459	5.032789447	4.83394E-07	9.22189E-07	positive
PRJNA729271	stage_1W_vs_E10	ordinary_muscle_development	ENSGALG00010001265	OPN1LW	3.133762592	0.555639475	5.639920731	1.70128E-08	3.56E-08	positive
PRJNA729271	stage_1W_vs_E10	ordinary_muscle_development	ENSGALG00010001267	BCHE	-0.773507649	0.251405467	-3.076733611	0.002092822	0.003027189	negative
PRJNA729271	stage_1W_vs_E10	ordinary_muscle_development	ENSGALG00010001268	CBLN2	-3.613338266	0.432802144	-8.348706954	6.90143E-17	2.22947E-16	negative
PRJNA729271	stage_1W_vs_E10	ordinary_muscle_development	ENSGALG00010001270	DRAP1	0.357128332	0.110198878	3.240761966	0.001192107	0.001761246	positive
PRJNA729271	stage_1W_vs_E10	ordinary_muscle_development	ENSGALG00010001271	ENSGALG00010001271	0.864991128	0.18479206	4.680889033	2.85634E-06	5.15663E-06	positive
PRJNA729271	stage_1W_vs_E10	ordinary_muscle_development	ENSGALG00010001273	ENSGALG00010001273	0.428998812	0.558924248	0.767543747	0.442758293	0.480840748	positive
PRJNA729271	stage_1W_vs_E10	ordinary_muscle_development	ENSGALG00010001274	DLGAP1	-2.191974212	0.482893142	-4.539253147	5.64538E-06	9.96345E-06	negative
PRJNA729271	stage_1W_vs_E10	ordinary_muscle_development	ENSGALG00010001276	ENSGALG00010001276	-2.476109548	1.195677875	-2.070883471			negative
PRJNA729271	stage_1W_vs_E10	ordinary_muscle_development	ENSGALG00010001277	ENSGALG00010001277	1.151029576	0.161361545	7.133233494	9.8038E-13	2.59887E-12	positive
PRJNA729271	stage_1W_vs_E10	ordinary_muscle_development	ENSGALG00010001279	LPIN2	-1.122206925	0.139406193	-8.049907239	8.28568E-16	2.55697E-15	negative
PRJNA729271	stage_1W_vs_E10	ordinary_muscle_development	ENSGALG00010001281	LRRN3	-0.205151009	0.146945739	-1.396100424	0.162684256	0.189909664	negative
PRJNA729271	stage_1W_vs_E10	ordinary_muscle_development	ENSGALG00010001283	ENSGALG00010001283	1.779548035	0.175252463	10.154197	3.17412E-24	1.41002E-23	positive
PRJNA729271	stage_1W_vs_E10	ordinary_muscle_development	ENSGALG00010001285	ENSGALG00010001285	1.802508332	0.213129421	8.457341676	2.73542E-17	8.98275E-17	positive
PRJNA729271	stage_1W_vs_E10	ordinary_muscle_development	ENSGALG00010001286	SRFBP1	-0.515140542	0.112211547	-4.590797983	4.41555E-06	7.86185E-06	negative
PRJNA729271	stage_1W_vs_E10	ordinary_muscle_development	ENSGALG00010001287	PLPP4	-5.353368501	0.445904061	-12.00565092	3.31843E-33	2.11524E-32	negative
PRJNA729271	stage_1W_vs_E10	ordinary_muscle_development	ENSGALG00010001288	TGIF1	-0.459838309	0.155513977	-2.956893773	0.003107552	0.004421276	negative
PRJNA729271	stage_1W_vs_E10	ordinary_muscle_development	ENSGALG00010001289	ENSGALG00010001289	1.407406325	0.353894294	3.976911611	6.98161E-05	0.000113681	positive

PRJNA729271	stage_1W_vs_E10	ordinary_muscle_development	ENSGALG00010001291	ENSGALG00010001291	1.635715543	0.142505178	11.4782885	1.69605E-30	9.75563E-30	positive
PRJNA729271	stage_1W_vs_E10	ordinary_muscle_development	ENSGALG00010001292	LOX	0.928653767	0.142133755	6.533660963	6.41811E-11	1.53948E-10	positive
PRJNA729271	stage_1W_vs_E10	ordinary_muscle_development	ENSGALG00010001295	ENSGALG00010001295	0.79101453	0.127690603	6.194774811	5.83684E-10	1.3286E-09	positive
PRJNA729271	stage_1W_vs_E10	ordinary_muscle_development	ENSGALG00010001297	MYL12A	-0.583439457	0.222966983	-2.616707861	0.008878229	0.012071094	negative
PRJNA729271	stage_1W_vs_E10	ordinary_muscle_development	ENSGALG00010001298	DOCK4	0.43147341	0.132288956	3.26159812	0.001107861	0.0016418	positive
PRJNA729271	stage_1W_vs_E10	ordinary_muscle_development	ENSGALG00010001300	MRPL49	0.499291248	0.180771566	2.762001008	0.00574483	0.007967022	positive
PRJNA729271	stage_1W_vs_E10	ordinary_muscle_development	ENSGALG00010001301	GMPS	-1.091366321	0.077520888	-14.07835163	5.15993E-45	4.82537E-44	negative
PRJNA729271	stage_1W_vs_E10	ordinary_muscle_development	ENSGALG00010001302	ENSGALG00010001302	-2.127901359	0.465820022	-4.568076212	4.92221E-06	8.72965E-06	negative
PRJNA729271	stage_1W_vs_E10	ordinary_muscle_development	ENSGALG00010001304	SYVN1	0.591818096	0.176583815	3.351485499	0.000803792	0.001208106	positive
PRJNA729271	stage_1W_vs_E10	ordinary_muscle_development	ENSGALG00010001305	WNT16	-2.19474298	0.398234178	-5.511186884	3.56422E-08	7.31672E-08	negative
PRJNA729271	stage_1W_vs_E10	ordinary_muscle_development	ENSGALG00010001306	CEP120	-0.273536425	0.086225624	-3.17233336	0.001512193	0.002215247	negative
PRJNA729271	stage_1W_vs_E10	ordinary_muscle_development	ENSGALG00010001307	CALU	-1.149113393	0.157114619	-7.313854038	2.59587E-13	7.06083E-13	negative
PRJNA729271	stage_1W_vs_E10	ordinary_muscle_development	ENSGALG00010001308	MGMT	-0.340686164	0.140531276	-2.424272899	0.015339074	0.02030338	negative
PRJNA729271	stage_1W_vs_E10	ordinary_muscle_development	ENSGALG00010001313	EMILIN2	1.123143222	0.176445837	6.365371032	1.94818E-10	4.55246E-10	positive
PRJNA729271	stage_1W_vs_E10	ordinary_muscle_development	ENSGALG00010001320	CPED1	-0.558468587	0.199580638	-2.798210247	0.005138665	0.007168221	negative
PRJNA729271	stage_1W_vs_E10	ordinary_muscle_development	ENSGALG00010001330	ENSGALG00010001330	1.525063409	0.14087384	10.82573886	2.59974E-27	1.31667E-26	positive
PRJNA729271	stage_1W_vs_E10	ordinary_muscle_development	ENSGALG00010001333	PLCH1	-1.224199464	0.298385688	-4.102741906	4.08283E-05	6.76427E-05	negative
PRJNA729271	stage_1W_vs_E10	ordinary_muscle_development	ENSGALG00010001334	EBF3	-1.486693282	0.159895912	-9.297881757	1.43271E-20	5.5276E-20	negative
PRJNA729271	stage_1W_vs_E10	ordinary_muscle_development	ENSGALG00010001343	ENSGALG00010001343	-0.203756572	0.299751416	-0.679751825	0.496661615	0.534494774	negative
PRJNA729271	stage_1W_vs_E10	ordinary_muscle_development	ENSGALG00010001347	MYOM1	3.07151648	0.164788793	18.63911023	1.54794E-77	3.45723E-76	positive
PRJNA729271	stage_1W_vs_E10	ordinary_muscle_development	ENSGALG00010001349	ING3	0.777317509	0.104680971	7.425585551	1.12282E-13	3.1131E-13	positive
PRJNA729271	stage_1W_vs_E10	ordinary_muscle_development	ENSGALG00010001351	CAPN1	0.541154461	0.157730644	3.430877144	0.000601633	0.000912533	positive
PRJNA729271	stage_1W_vs_E10	ordinary_muscle_development	ENSGALG00010001353	GLRX3	0.383072664	0.078623286	4.872254547	1.10332E-06	2.05719E-06	positive
PRJNA729271	stage_1W_vs_E10	ordinary_muscle_development	ENSGALG00010001358	TCERG1L	-3.096986817	0.56142098	-5.516336095	3.4614E-08	7.1127E-08	negative
PRJNA729271	stage_1W_vs_E10	ordinary_muscle_development	ENSGALG00010001363	HIBADH	1.670266589	0.210349245	7.940444905	2.01457E-15	6.10649E-15	positive
PRJNA729271	stage_1W_vs_E10	ordinary_muscle_development	ENSGALG00010001364	SMCHD1	-0.054579714	0.184323876	-0.296107674	0.767147854	0.790882514	negative
PRJNA729271	stage_1W_vs_E10	ordinary_muscle_development	ENSGALG00010001365	ENSGALG00010001365	1.354783105	0.27505169	4.925558202	8.41199E-07	1.5823E-06	positive
PRJNA729271	stage_1W_vs_E10	ordinary_muscle_development	ENSGALG00010001367	ENSGALG00010001367	0.428317703	0.434748416	0.985208196	0.324521815	0.361036435	positive
PRJNA729271	stage_1W_vs_E10	ordinary_muscle_development	ENSGALG00010001368	WIPF3	0.758655623	0.141718776	5.353247066	8.63898E-08	1.73582E-07	positive
PRJNA729271	stage_1W_vs_E10	ordinary_muscle_development	ENSGALG00010001369	PPP2R2D	0.339198943	0.123978179	2.735956819	0.006219919	0.008592178	positive
PRJNA729271	stage_1W_vs_E10	ordinary_muscle_development	ENSGALG00010001372	ENSGALG00010001372	1.036835215	0.292823051	3.540825119	0.000398878	0.000613121	positive
PRJNA729271	stage_1W_vs_E10	ordinary_muscle_development	ENSGALG00010001373	IMPA2	1.514186274	0.139650372	10.84269418	2.16014E-27	1.09749E-26	positive
PRJNA729271	stage_1W_vs_E10	ordinary_muscle_development	ENSGALG00010001378	POLA2	-2.094348386	0.137606545	-15.21983123	2.61204E-52	3.11105E-51	negative
PRJNA729271	stage_1W_vs_E10	ordinary_muscle_development	ENSGALG00010001380	SCRN1	-4.412659921	0.193959664	-22.75039994	1.422E-114	6.9167E-113	negative
PRJNA729271	stage_1W_vs_E10	ordinary_muscle_development	ENSGALG00010001381	MDFIC	-0.798813872	0.1309421	-6.100512138	1.05729E-09	2.37604E-09	negative
PRJNA729271	stage_1W_vs_E10	ordinary_muscle_development	ENSGALG00010001382	BNIP3	2.510118038	0.192882937	13.01368633	1.02286E-38	7.83636E-38	positive
PRJNA729271	stage_1W_vs_E10	ordinary_muscle_development	ENSGALG00010001385	MME	3.712398504	0.251988349	14.73242122	3.99145E-49	4.24165E-48	positive
PRJNA729271	stage_1W_vs_E10	ordinary_muscle_development	ENSGALG00010001386	FKBP14	-1.924940226	0.186021225	-10.34796016	4.27497E-25	1.97784E-24	negative
PRJNA729271	stage_1W_vs_E10	ordinary_muscle_development	ENSGALG00010001388	MPPE1	0.740268559	0.165179165	4.481609774	7.40821E-06	1.2961E-05	positive
PRJNA729271	stage_1W_vs_E10	ordinary_muscle_development	ENSGALG00010001395	GNAL	-0.004480195	0.106742028	-0.041972178	0.966520878	0.9712111	negative
PRJNA729271	stage_1W_vs_E10	ordinary_muscle_development	ENSGALG00010001397	KLHL7	-0.038960086	0.063854149	-0.610141805	0.541767876	0.578058547	negative
PRJNA729271	stage_1W_vs_E10	ordinary_muscle_development	ENSGALG00010001398	WDR11	-0.510118377	0.097300819	-5.242693559	1.58249E-07	3.1183E-07	negative
PRJNA729271	stage_1W_vs_E10	ordinary_muscle_development	ENSGALG00010001400	SNX24	1.754561044	0.15888851	11.04271823	2.37731E-28	1.25534E-27	positive
PRJNA729271	stage_1W_vs_E10	ordinary_muscle_development	ENSGALG00010001402	NUP42	0.406894817	0.130320262	3.12226825	0.001794633	0.002613605	positive
PRJNA729271	stage_1W_vs_E10	ordinary_muscle_development	ENSGALG00010001406	EML3	1.057419597	0.159355834	6.635587605	3.23212E-11	7.91289E-11	positive
PRJNA729271	stage_1W_vs_E10	ordinary_muscle_development	ENSGALG00010001407	FOXP2	-1.786816631	0.152718725	-11.70004946	1.27379E-31	7.68997E-31	negative
PRJNA729271	stage_1W_vs_E10	ordinary_muscle_development	ENSGALG00010001409	GPNMB	-3.937692635	1.010329137	-3.89743549	9.72167E-05	0.000156538	negative
PRJNA729271	stage_1W_vs_E10	ordinary_muscle_development	ENSGALG00010001411	FGFR2	-5.044457768	0.370544818	-13.61362383	3.32347E-42	2.85897E-41	negative
PRJNA729271	stage_1W_vs_E10	ordinary_muscle_development	ENSGALG00010001412	ENSGALG00010001412	-2.798686343	0.271601712	-10.30437667	6.73267E-25	3.07952E-24	negative
PRJNA729271	stage_1W_vs_E10	ordinary_muscle_development	ENSGALG00010001414	CCDC126	1.762682689	0.17068551	10.32707868	5.31548E-25	2.44441E-24	positive
PRJNA729271	stage_1W_vs_E10	ordinary_muscle_development	ENSGALG00010001415	MC5R	-0.537270228	0.268017004	-2.004612464	0.045004493	0.056528607	negative
PRJNA729271	stage_1W_vs_E10	ordinary_muscle_development	ENSGALG00010001416	AP4M1	0.526894736	0.092229439	5.712869346	1.11087E-08	2.34998E-08	positive
PRJNA729271	stage_1W_vs_E10	ordinary_muscle_development	ENSGALG00010001418	RNMT	-1.264961556	0.06876942	-18.39424499	1.46081E-75	3.1139E-74	negative
PRJNA729271	stage_1W_vs_E10	ordinary_muscle_development	ENSGALG00010001419	KCND2	-3.670969503	0.317147416	-11.57496266	5.51953E-31	3.23778E-30	negative
PRJNA729271	stage_1W_vs_E10	ordinary_muscle_development	ENSGALG00010001420	FAM221A	-3.336540648	0.530760416	-6.28634041	3.25037E-10	7.50105E-10	negative
PRJNA729271	stage_1W_vs_E10	ordinary_muscle_development	ENSGALG00010001422	AP1S1	-1.215501271	0.141116683	-8.613448446	7.08951E-18	2.39484E-17	negative
PRJNA729271	stage_1W_vs_E10	ordinary_muscle_development	ENSGALG00010001423	FAM210A	1.526603683	0.136473075	11.18611629	4.76866E-29	2.58496E-28	positive
PRJNA729271	stage_1W_vs_E10	ordinary_muscle_development	ENSGALG00010001426	ATE1	0.501684394	0.090952115	5.51591784	3.46964E-08	7.12661E-08	positive
PRJNA729271	stage_1W_vs_E10	ordinary_muscle_development	ENSGALG00010001427	ASB11	9.026136086	0.462703519	19.50738587	9.50184E-85	2.53179E-83	positive
PRJNA729271	stage_1W_vs_E10	ordinary_muscle_development	ENSGALG00010001432	NSMCE4A	0.632563269	0.112740883	5.610770912	2.01427E-08	4.19918E-08	positive
PRJNA729271	stage_1W_vs_E10	ordinary_muscle_development	ENSGALG00010001436	ENSGALG00010001436	-0.331880053	1.265426013	-0.262267449	0.793115255	0.81504471	negative
PRJNA729271	stage_1W_vs_E10	ordinary_muscle_development	ENSGALG00010001440	CDCA7L	-1.759188226	0.151726901	-11.59443849	4.3974E-31	2.59002E-30	negative
PRJNA729271	stage_1W_vs_E10	ordinary_muscle_development	ENSGALG00010001441	HMGCLL1	-1.191431854	0.306334399	-3.889317879	0.000100526	0.000161526	negative
PRJNA729271	stage_1W_vs_E10	ordinary_muscle_development	ENSGALG00010001442	LDLRAD4	0.664354959	0.113992953	5.828035356	5.60837E-09	1.20882E-08	positive
PRJNA729271	stage_1W_vs_E10	ordinary_muscle_development	ENSGALG00010001443	ANKRD26	-0.255652009	0.145945691	-1.751692759	0.079826653	0.097136277	negative
PRJNA729271	stage_1W_vs_E10	ordinary_muscle_development	ENSGALG00010001448	GPR37	-6.249737946	0.398612116	-15.67874555	2.11388E-55	2.76291E-54	negative
PRJNA729271	stage_1W_vs_E10	ordinary_muscle_development	ENSGALG00010001450	GPR149	1.505820128	0.382087556	3.94103421	8.11311E-05	0.000131514	positive
PRJNA729271	stage_1W_vs_E10	ordinary_muscle_development	ENSGALG00010001453	TAX1BP1	1.056591372	0.073476397	14.38001074	6.90838E-47	6.83529E-46	positive
PRJNA729271	stage_1W_vs_E10	ordinary_muscle_development	ENSGALG00010001454	VEGFD	2.786572482	0.164259225	16.96448091	1.50423E-64	2.4665E-63	positive
PRJNA729271	stage_1W_vs_E10	ordinary_muscle_development	ENSGALG00010001456	LSM8	-1.02794192	0.116530046	-8.821260722	1.13178E-18	3.98571E-18	negative
PRJNA729271	stage_1W_vs_E10	ordinary_muscle_development	ENSGALG00010001457	NDC80	-2.95770723	0.228323061	-12.95404511	2.22892E-38	1.688E-37	negative
PRJNA729271	stage_1W_vs_E10	ordinary_muscle_development	ENSGALG00010001458	JAZF1	-1.440489803	0.162880104	-8.843865951	9.24625E-19	3.27048E-18	negative
PRJNA729271	stage_1W_vs_E10	ordinary_muscle_development	ENSGALG00010001460	MALSU1	0.270708944	0.106248224	2.547891465	0.010837618	0.014596848	positive
PRJNA729271	stage_1W_vs_E10	ordinary_muscle_development	ENSGALG00010001461	ASB9	-3.356628184	0.237541495	-14.13070247	2.45681E-45	2.32299E-44	negative
PRJNA729271	stage_1W_vs_E10	ordinary_muscle_development	ENSGALG00010001462	DHX36	-0.359939107	0.109057517	-3.300452061	0.000965292	0.001440534	negative
PRJNA729271	stage_1W_vs_E10	ordinary_muscle_development	ENSGALG00010001463	DSEL	-0.520264577	0.159253091	-3.266904107	0.001087305	0.001614142	negative
PRJNA729271	stage_1W_vs_E10	ordinary_muscle_development	ENSGALG00010001465	TACC2	1.21599926	0.157454364	7.722867936	1.13741E-14	3.32931E-14	positive
PRJNA729271	stage_1W_vs_E10	ordinary_muscle_development	ENSGALG00010001466	PRKX	-0.534666746	0.131640995	-4.061552005	4.87476E-05	8.02312E-05	negative
PRJNA729271	stage_1W_vs_E10	ordinary_muscle_development	ENSGALG00010001468	ANOS1	-0.447072189	0.152781815	-2.926213362	0.003431155	0.004865447	negative
PRJNA729271	stage_1W_vs_E10	ordinary_muscle_development	ENSGALG00010001469	ARHGEF26	-0.471175583	0.130114478	-3.621238711	0.000293196	0.000456142	negative
PRJNA729271	stage_1W_vs_E10	ordinary_muscle_development	ENSGALG00010001470	GPR85	-3.237896152	0.334735673	-9.67299398	3.92718E-22	1.60035E-21	negative
PRJNA729271	stage_1W_vs_E10	ordinary_muscle_development	ENSGALG00010001471	BMP5	-2.461482905	0.371634345	-6.623399951	3.5103E-11	8.57461E-11	negative
PRJNA729271	stage_1W_vs_E10	ordinary_muscle_development	ENSGALG00010001472	ENSGALG00010001472	0.620616723	0.190106308	3.264577214	0.001096276	0.001626793	positive
PRJNA729271	stage_1W_vs_E10	ordinary_muscle_development	ENSGALG00010001475	BMT2	-1.126929462	0.138757573	-8.121570854	4.60188E-16	1.4379E-15	negative
PRJNA729271	stage_1W_vs_E10	ordinary_muscle_development	ENSGALG00010001476	IGF2BP3	-0.95162851	0.193470107	-4.918736656	8.71046E-07	1.6361E-06	negative
PRJNA729271	stage_1W_vs_E10	ordinary_muscle_development	ENSGALG00010001477	NLGN4	-1.344516016	0.226450209	-5.937358249	2.89651E-09	6.35366E-09	negative
PRJNA729271	stage_1W_vs_E10	ordinary_muscle_development	ENSGALG00010001479	TMEM168	-0.183363858	0.155414405	-1.179838238	0.238064558	0.270286345	negative
PRJNA729271	stage_1W_vs_E10	ordinary_muscle_development	ENSGALG00010001480	JAKMIP3	-1.000918488	0.301052723	-3.32472823	0.000885048	0.001325834	negative
PRJNA729271	stage_1W_vs_E10	ordinary_muscle_development	ENSGALG00010001482	TRA2A	-1.086170037	0.066336263	-16.37369959	2.9474E-60	4.355E-59	negative
PRJNA729271	stage_1W_vs_E10	ordinary_muscle_development	ENSGALG00010001483	ENSGALG00010001483	-3.450050928	0.434210607	-7.945570349	1.93299E-15	5.86657E-15	negative
PRJNA729271	stage_1W_vs_E10	ordinary_muscle_development	ENSGALG00010001486	WNT2	1.559500598	0.263344754	5.921897352	3.18248E-09	6.95988E-09	positive
PRJNA729271	stage_1W_vs_E10	ordinary_muscle_development	ENSGALG00010001487	CDH19	0.297178322	0.232102959	1.280372826	0.200414046	0.230705017	positive
PRJNA729271	stage_1W_vs_E10	ordinary_muscle_development	ENSGALG00010001488	GRPR	-3.936157323	0.479575116	-8.207592906	2.25667E-16	7.11715E-16	negative
PRJNA729271	stage_1W_vs_E10	ordinary_muscle_development	ENSGALG00010001491	PHF3	0.253943017	0.088350485	2.874268515	0.004049645	0.005697331	positive
PRJNA729271	stage_1W_vs_E10	ordinary_muscle_development	ENSGALG00010001492	ENSGALG00010001492	2.586484561	0.275484876	9.388844125	6.06623E-21	2.37008E-20	positive
PRJNA729271	stage_1W_vs_E10	ordinary_muscle_development	ENSGALG00010001495	CCNL1	1.212201625	0.107878253	11.23675612	2.69089E-29	1.47577E-28	positive
PRJNA729271	stage_1W_vs_E10	ordinary_muscle_development	ENSGALG00010001496	PTP4A1	1.335088944	0.19015058	7.021219404	2.1994E-12	5.72461E-12	positive
PRJNA729271	stage_1W_vs_E10	ordinary_muscle_development	ENSGALG00010001497	LEKR1	-0.93997848	0.293298978	-3.204847446	0.001351341	0.001987589	negative
PRJNA729271	stage_1W_vs_E10	ordinary_muscle_development	ENSGALG00010001499	TIPARP	1.635193172	0.186431109	8.77103174	1.77037E-18	6.16713E-18	positive
PRJNA729271	stage_1W_vs_E10	ordinary_muscle_development	ENSGALG00010001501	CAPZA2	1.402713084	0.106277653	13.19857034	8.94159E-40	7.11352E-39	positive
PRJNA729271	stage_1W_vs_E10	ordinary_muscle_development	ENSGALG00010001502	LGSN	2.63677833	0.917601157	2.873556022	0.004058791	0.00570909	positive
PRJNA729271	stage_1W_vs_E10	ordinary_muscle_development	ENSGALG00010001504	RBMS3	-0.72862709	0.100055105	-7.282257979	3.28279E-13	8.8859E-13	negative
PRJNA729271	stage_1W_vs_E10	ordinary_muscle_development	ENSGALG00010001505	SSR3	-0.276329015	0.097054189	-2.847162181	0.004411088	0.006186022	negative
PRJNA729271	stage_1W_vs_E10	ordinary_muscle_development	ENSGALG00010001506	DPYSL4	-2.139762475	0.281690816	-7.596138592	3.05098E-14	8.71407E-14	negative
PRJNA729271	stage_1W_vs_E10	ordinary_muscle_development	ENSGALG00010001507	METTL4	-0.398607585	0.100575696	-3.963259531	7.39333E-05	0.00012025	negative
PRJNA729271	stage_1W_vs_E10	ordinary_muscle_development	ENSGALG00010001508	CD109	-1.749431371	0.29168862	-5.997598985	2.00256E-09	4.43569E-09	negative
PRJNA729271	stage_1W_vs_E10	ordinary_muscle_development	ENSGALG00010001510	AP1S2	0.088321782	0.098384822	0.897717557	0.369336147	0.407050445	positive
PRJNA729271	stage_1W_vs_E10	ordinary_muscle_development	ENSGALG00010001512	PLCL2	2.436244562	0.172893033	14.0910511	4.31097E-45	4.0445E-44	positive
PRJNA729271	stage_1W_vs_E10	ordinary_muscle_development	ENSGALG00010001513	ZRSR2	0.051157289	0.093316999	0.548209754	0.583547886	0.618946773	positive
PRJNA729271	stage_1W_vs_E10	ordinary_muscle_development	ENSGALG00010001514	MET	0.578293028	0.153001395	3.779658537	0.000157044	0.000249026	positive
PRJNA729271	stage_1W_vs_E10	ordinary_muscle_development	ENSGALG00010001516	CLTRN	-1.545050675	0.585567374	-2.638553213	0.008326063	0.011351231	negative
PRJNA729271	stage_1W_vs_E10	ordinary_muscle_development	ENSGALG00010001517	CAV2	2.741332273	0.163984139	16.71705746	9.84622E-63	1.53463E-61	positive
PRJNA729271	stage_1W_vs_E10	ordinary_muscle_development	ENSGALG00010001518	SLC17A5	-1.346009582	0.167752072	-8.023803024	1.02521E-15	3.15172E-15	negative
PRJNA729271	stage_1W_vs_E10	ordinary_muscle_development	ENSGALG00010001520	KCNAB1	-2.603654667	0.220426417	-11.81189942	3.38819E-32	2.08101E-31	negative
PRJNA729271	stage_1W_vs_E10	ordinary_muscle_development	ENSGALG00010001521	TBC1D5	-0.297137526	0.074812612	-3.971757158	7.13444E-05	0.000116143	negative
PRJNA729271	stage_1W_vs_E10	ordinary_muscle_development	ENSGALG00010001524	ACE2	4.500280554	1.451630961	3.100154705	0.001934196	0.002806704	positive
PRJNA729271	stage_1W_vs_E10	ordinary_muscle_development	ENSGALG00010001525	EEF1A1	-1.556747527	0.176711566	-8.809539519	1.25661E-18	4.4103E-18	negative
PRJNA729271	stage_1W_vs_E10	ordinary_muscle_development	ENSGALG00010001526	PPP1R3A	6.839062246	0.149171705	45.84691352	0	0	positive
PRJNA729271	stage_1W_vs_E10	ordinary_muscle_development	ENSGALG00010001527	STK32C	-0.560293687	0.231925053	-2.415839421	0.015698983	0.020751118	negative
PRJNA729271	stage_1W_vs_E10	ordinary_muscle_development	ENSGALG00010001528	NR1D2	3.578943883	0.180181813	19.86295851	8.51442E-88	2.42946E-86	positive
PRJNA729271	stage_1W_vs_E10	ordinary_muscle_development	ENSGALG00010001531	ENSGALG00010001531	14.89177363	5.497641601	2.70875672			positive
PRJNA729271	stage_1W_vs_E10	ordinary_muscle_development	ENSGALG00010001532	ENSGALG00010001532	-1.726465199	0.277551948	-6.220331775	4.96105E-10	1.13334E-09	negative
PRJNA729271	stage_1W_vs_E10	ordinary_muscle_development	ENSGALG00010001533	ENSGALG00010001533	0.731904164	0.149408603	4.898674841	9.64852E-07	1.80738E-06	positive
PRJNA729271	stage_1W_vs_E10	ordinary_muscle_development	ENSGALG00010001534	SLITRK3	-3.695373067	0.424655489	-8.702049454	3.25943E-18	1.11983E-17	negative
PRJNA729271	stage_1W_vs_E10	ordinary_muscle_development	ENSGALG00010001535	RAP2B	-0.970105228	0.106609863	-9.099582358	9.06799E-20	3.37459E-19	negative
PRJNA729271	stage_1W_vs_E10	ordinary_muscle_development	ENSGALG00010001540	TRABD	-0.557144006	0.130375356	-4.273384351	1.92528E-05	3.26474E-05	negative
PRJNA729271	stage_1W_vs_E10	ordinary_muscle_development	ENSGALG00010001542	MTO1	0.785859345	0.127831865	6.147601321	7.86635E-10	1.77799E-09	positive
PRJNA729271	stage_1W_vs_E10	ordinary_muscle_development	ENSGALG00010001545	SELENOO	2.225177292	0.128991034	17.25063531	1.10676E-66	1.89627E-65	positive
PRJNA729271	stage_1W_vs_E10	ordinary_muscle_development	ENSGALG00010001546	CGAS	-1.568553685	0.176190912	-8.902579955	5.45633E-19	1.9596E-18	negative
PRJNA729271	stage_1W_vs_E10	ordinary_muscle_development	ENSGALG00010001547	PWWP2B	1.514643374	0.108934765	13.90413221	5.97876E-44	5.42996E-43	positive
PRJNA729271	stage_1W_vs_E10	ordinary_muscle_development	ENSGALG00010001548	AKAP17A	0.178829793	0.09565561	1.869517052	0.061550913	0.075969047	positive

PRJNA729271	stage_1W_vs_E10	ordinary_muscle_development	ENSGALG00010001549	ENSGALG00010001549	-1.907159464	0.542416211	-3.516044365	0.000438028	0.00067081	negative
PRJNA729271	stage_1W_vs_E10	ordinary_muscle_development	ENSGALG00010001551	FHIP1B	2.351149355	0.159679159	14.72420927	4.50711E-49	4.76865E-48	positive
PRJNA729271	stage_1W_vs_E10	ordinary_muscle_development	ENSGALG00010001552	TUBGCP6	0.200188959	0.108390648	1.846920953	0.064758598	0.079704158	positive
PRJNA729271	stage_1W_vs_E10	ordinary_muscle_development	ENSGALG00010001553	CNGA4	0.158874194	0.4127157	0.384948267	0.700275744	0.728626582	positive
PRJNA729271	stage_1W_vs_E10	ordinary_muscle_development	ENSGALG00010001554	ENSGALG00010001554	0.290099369	0.184809585	1.569720364	0.116480182	0.13878999	positive
PRJNA729271	stage_1W_vs_E10	ordinary_muscle_development	ENSGALG00010001556	EPB41L3	-1.377980813	0.117743019	-11.703291	1.22605E-31	7.41098E-31	negative
PRJNA729271	stage_1W_vs_E10	ordinary_muscle_development	ENSGALG00010001557	THRB	-2.069849822	0.332456898	-6.225919311	4.7874E-10	1.09488E-09	negative
PRJNA729271	stage_1W_vs_E10	ordinary_muscle_development	ENSGALG00010001558	CCKBR	-3.299652296	0.871349791	-3.786828585	0.000152582	0.000242217	negative
PRJNA729271	stage_1W_vs_E10	ordinary_muscle_development	ENSGALG00010001559	HDAC10	-2.009794061	0.135506284	-14.83174064	9.13336E-50	9.92414E-49	negative
PRJNA729271	stage_1W_vs_E10	ordinary_muscle_development	ENSGALG00010001567	INPP5A	-0.364804937	0.083160243	-4.386770931	1.15046E-05	1.98901E-05	negative
PRJNA729271	stage_1W_vs_E10	ordinary_muscle_development	ENSGALG00010001570	SMPD1	2.680725063	0.161879797	16.559973	1.35655E-61	2.06329E-60	positive
PRJNA729271	stage_1W_vs_E10	ordinary_muscle_development	ENSGALG00010001585	ENSGALG00010001585	-0.539822679	0.138603151	-3.894735985	9.83057E-05	0.000158204	negative
PRJNA729271	stage_1W_vs_E10	ordinary_muscle_development	ENSGALG00010001586	ZBTB14	0.113439446	0.095615873	1.186408096	0.235461176	0.267519184	positive
PRJNA729271	stage_1W_vs_E10	ordinary_muscle_development	ENSGALG00010001588	ENSGALG00010001588	1.148689844	0.59037988	1.945679185	0.051693289	0.064466508	positive
PRJNA729271	stage_1W_vs_E10	ordinary_muscle_development	ENSGALG00010001595	AKAIN1	1.905649562	0.252864212	7.536256513	4.83655E-14	1.36925E-13	positive
PRJNA729271	stage_1W_vs_E10	ordinary_muscle_development	ENSGALG00010001599	TBLX1	-0.638498185	0.075409873	-8.467037034	2.51714E-17	8.28473E-17	negative
PRJNA729271	stage_1W_vs_E10	ordinary_muscle_development	ENSGALG00010001604	ENSGALG00010001604	6.885470948	0.419025811	16.43209264	1.12702E-60	1.68761E-59	positive
PRJNA729271	stage_1W_vs_E10	ordinary_muscle_development	ENSGALG00010001605	APBB1	-0.104738961	0.113646637	-0.921619534	0.356727066	0.394323101	negative
PRJNA729271	stage_1W_vs_E10	ordinary_muscle_development	ENSGALG00010001606	PIGA	-0.485741405	0.1112231	-4.367270882	1.25809E-05	2.16785E-05	negative
PRJNA729271	stage_1W_vs_E10	ordinary_muscle_development	ENSGALG00010001608	ENSGALG00010001608	-1.028522399	0.522953891	-1.966755417	0.049211415	0.061493056	negative
PRJNA729271	stage_1W_vs_E10	ordinary_muscle_development	ENSGALG00010001609	ENSGALG00010001609	0.851462505	0.467825242	1.820043957	0.068752311	0.084354558	positive
PRJNA729271	stage_1W_vs_E10	ordinary_muscle_development	ENSGALG00010001611	MAPK12	-1.825525998	0.166771963	-10.94624037	6.92631E-28	3.58945E-27	negative
PRJNA729271	stage_1W_vs_E10	ordinary_muscle_development	ENSGALG00010001612	HPX	5.76256011	0.925850046	6.224074981	4.84405E-10	1.10766E-09	positive
PRJNA729271	stage_1W_vs_E10	ordinary_muscle_development	ENSGALG00010001616	OPNVA	-2.583755757	0.772766262	-3.343515218	0.000827242	0.001241676	negative
PRJNA729271	stage_1W_vs_E10	ordinary_muscle_development	ENSGALG00010001617	TRIM3	-0.371224156	0.093728472	-3.96063381	7.47511E-05	0.000121525	negative
PRJNA729271	stage_1W_vs_E10	ordinary_muscle_development	ENSGALG00010001620	MOSPD2	0.322230677	0.143779329	2.241147451	0.025016525	0.032356075	positive
PRJNA729271	stage_1W_vs_E10	ordinary_muscle_development	ENSGALG00010001621	DLGAP2	-1.346611881	0.263192581	-5.116450768	3.11339E-07	6.01874E-07	negative
PRJNA729271	stage_1W_vs_E10	ordinary_muscle_development	ENSGALG00010001627	ARFIP2	0.244064037	0.118910404	2.052503647	0.040120741	0.050675335	positive
PRJNA729271	stage_1W_vs_E10	ordinary_muscle_development	ENSGALG00010001628	MAPK11	-2.526701067	0.216881356	-11.65015338	2.29046E-31	1.36458E-30	negative
PRJNA729271	stage_1W_vs_E10	ordinary_muscle_development	ENSGALG00010001632	FANCB	-0.606586364	0.27483969	-2.207055193	0.027310204	0.035147066	negative
PRJNA729271	stage_1W_vs_E10	ordinary_muscle_development	ENSGALG00010001633	B3GAT2	-2.720726791	0.429240475	-6.338467475	2.32062E-10	5.39493E-10	negative
PRJNA729271	stage_1W_vs_E10	ordinary_muscle_development	ENSGALG00010001635	TIMM10B	0.49484343	0.14836619	3.335284342	0.000852122	0.001278227	positive
PRJNA729271	stage_1W_vs_E10	ordinary_muscle_development	ENSGALG00010001639	ENSGALG00010001639	-0.613330224	0.160341344	-3.825153314	0.000130691	0.000208309	negative
PRJNA729271	stage_1W_vs_E10	ordinary_muscle_development	ENSGALG00010001642	ENSGALG00010001642	2.157279918	0.119306758	18.08179151	4.43438E-73	9.00228E-72	positive
PRJNA729271	stage_1W_vs_E10	ordinary_muscle_development	ENSGALG00010001647	ARL8BL	-0.784895689	0.155711623	-5.040700716	4.6383E-07	8.86618E-07	negative
PRJNA729271	stage_1W_vs_E10	ordinary_muscle_development	ENSGALG00010001649	UBE2E2	-0.720743352	0.232827353	-3.095612877	0.001964066	0.002848338	negative
PRJNA729271	stage_1W_vs_E10	ordinary_muscle_development	ENSGALG00010001654	RPS11	0.888607134	0.16938322	5.246134371	1.55324E-07	3.06315E-07	positive
PRJNA729271	stage_1W_vs_E10	ordinary_muscle_development	ENSGALG00010001657	SMAP1	-0.922697162	0.098332074	-9.383481108	6.38298E-21	2.49115E-20	negative
PRJNA729271	stage_1W_vs_E10	ordinary_muscle_development	ENSGALG00010001661	TCTN3	-0.587982572	0.106015138	-5.546213339	2.91923E-08	6.01909E-08	negative
PRJNA729271	stage_1W_vs_E10	ordinary_muscle_development	ENSGALG00010001664	SPAM1	0.572187594	2.0670095	0.276819044	0.781919057	0.80450857	positive
PRJNA729271	stage_1W_vs_E10	ordinary_muscle_development	ENSGALG00010001666	PIEZO2	-3.870994207	0.18502293	-20.92169984	3.39785E-97	1.20126E-95	negative
PRJNA729271	stage_1W_vs_E10	ordinary_muscle_development	ENSGALG00010001668	UBE2E1	-0.216182519	0.086330886	-2.504115601	0.012275792	0.016425515	negative
PRJNA729271	stage_1W_vs_E10	ordinary_muscle_development	ENSGALG00010001670	SHOX	-2.134926194	0.169818647	-12.57180079	3.01771E-36	2.13478E-35	negative
PRJNA729271	stage_1W_vs_E10	ordinary_muscle_development	ENSGALG00010001672	ENSGALG00010001672	1.924517197	0.563450494	3.415592348	0.000636434	0.000963559	positive
PRJNA729271	stage_1W_vs_E10	ordinary_muscle_development	ENSGALG00010001674	ENSGALG00010001674	0.323011015	0.104791929	3.082403565	0.002053362	0.002972188	positive
PRJNA729271	stage_1W_vs_E10	ordinary_muscle_development	ENSGALG00010001677	SDHAF4	0.473302314	0.106960443	4.425022026	9.64324E-06	1.6754E-05	positive
PRJNA729271	stage_1W_vs_E10	ordinary_muscle_development	ENSGALG00010001678	SYAP1	0.625338065	0.058677893	10.65713231	1.61501E-26	7.922E-26	positive
PRJNA729271	stage_1W_vs_E10	ordinary_muscle_development	ENSGALG00010001681	NAPG	-0.05895035	0.09200796	-0.640709237	0.521711612	0.558664281	negative
PRJNA729271	stage_1W_vs_E10	ordinary_muscle_development	ENSGALG00010001686	WASL	0.131976926	0.086605535	1.523885591	0.127537293	0.151083121	positive
PRJNA729271	stage_1W_vs_E10	ordinary_muscle_development	ENSGALG00010001688	ENSGALG00010001688	-0.565792325	0.166504455	-3.398061189	0.000678652	0.001025442	negative
PRJNA729271	stage_1W_vs_E10	ordinary_muscle_development	ENSGALG00010001689	ILK	0.635415724	0.066910597	9.496488585	2.17087E-21	8.63998E-21	positive
PRJNA729271	stage_1W_vs_E10	ordinary_muscle_development	ENSGALG00010001691	CTPS2	0.980977792	0.134068233	7.31700398	2.53568E-13	6.901E-13	positive
PRJNA729271	stage_1W_vs_E10	ordinary_muscle_development	ENSGALG00010001692	ALDH18A1	-1.084326483	0.255248556	-4.248119952	2.15572E-05	3.64313E-05	negative
PRJNA729271	stage_1W_vs_E10	ordinary_muscle_development	ENSGALG00010001693	NKIRAS1	1.919966783	0.192759547	9.960423832	2.27093E-23	9.76188E-23	positive
PRJNA729271	stage_1W_vs_E10	ordinary_muscle_development	ENSGALG00010001694	ENSGALG00010001694	-0.172380692	0.216399767	-0.796584459	0.425692406	0.463661815	negative
PRJNA729271	stage_1W_vs_E10	ordinary_muscle_development	ENSGALG00010001695	LMOD2	0.614482521	0.471180125	1.304135062	0.192187536	0.221760734	positive
PRJNA729271	stage_1W_vs_E10	ordinary_muscle_development	ENSGALG00010001696	FAM135A	0.261392864	0.090805998	2.878585871	0.003994625	0.005623746	positive
PRJNA729271	stage_1W_vs_E10	ordinary_muscle_development	ENSGALG00010001697	TAF10	-0.132135596	0.118532131	-1.114766052	0.264950687	0.298682549	negative
PRJNA729271	stage_1W_vs_E10	ordinary_muscle_development	ENSGALG00010001698	RPL15	0.756825189	0.176426738	4.289741997	1.78881E-05	3.04293E-05	positive
PRJNA729271	stage_1W_vs_E10	ordinary_muscle_development	ENSGALG00010001699	ASB15	7.651007551	0.249581795	30.65531097	2.2453E-206	3.6161E-204	positive
PRJNA729271	stage_1W_vs_E10	ordinary_muscle_development	ENSGALG00010001700	ENSGALG00010001700	2.22848011	0.198546077	11.22484033	3.07942E-29	1.68375E-28	positive
PRJNA729271	stage_1W_vs_E10	ordinary_muscle_development	ENSGALG00010001701	NDUFA5	2.502987122	0.168822292	14.82616481	9.92433E-50	1.07675E-48	positive
PRJNA729271	stage_1W_vs_E10	ordinary_muscle_development	ENSGALG00010001702	PLEKHA1	0.262279678	0.083701634	3.133507269	0.001727306	0.002518337	positive
PRJNA729271	stage_1W_vs_E10	ordinary_muscle_development	ENSGALG00010001704	ENSGALG00010001704	-1.667020674	0.147284812	-11.31834745	1.0646E-29	5.94657E-29	negative
PRJNA729271	stage_1W_vs_E10	ordinary_muscle_development	ENSGALG00010001705	HTRA1	1.920602392	0.154720567	12.41336187	2.21167E-35	1.52513E-34	positive
PRJNA729271	stage_1W_vs_E10	ordinary_muscle_development	ENSGALG00010001706	IQUB	2.42352174	0.34407971	7.043489259	1.87484E-12	4.89039E-12	positive
PRJNA729271	stage_1W_vs_E10	ordinary_muscle_development	ENSGALG00010001707	ENSGALG00010001707	5.559805023	1.040916382	5.341259989	9.23028E-08	1.85052E-07	positive
PRJNA729271	stage_1W_vs_E10	ordinary_muscle_development	ENSGALG00010001708	AADAC	3.499766441	0.250274167	13.98373028	1.95943E-44	1.79987E-43	positive
PRJNA729271	stage_1W_vs_E10	ordinary_muscle_development	ENSGALG00010001710	LCMT2	-0.753053088	0.081509891	-9.238793952	2.49291E-20	9.50164E-20	negative
PRJNA729271	stage_1W_vs_E10	ordinary_muscle_development	ENSGALG00010001711	CREB5	-0.82747713	0.177527917	-4.661109904	3.14509E-06	5.66311E-06	negative
PRJNA729271	stage_1W_vs_E10	ordinary_muscle_development	ENSGALG00010001712	ATP6AP1	1.884018959	0.15882194	11.86246027	1.85445E-32	1.14873E-31	positive
PRJNA729271	stage_1W_vs_E10	ordinary_muscle_development	ENSGALG00010001713	PNPLA4	0.449604928	0.224521973	2.002498563	0.045231137	0.056796389	positive
PRJNA729271	stage_1W_vs_E10	ordinary_muscle_development	ENSGALG00010001716	TRIL	-1.385759839	0.152052904	-9.113668987	7.9642E-20	2.96993E-19	negative
PRJNA729271	stage_1W_vs_E10	ordinary_muscle_development	ENSGALG00010001717	STARD10	0.468016579	0.504424344	0.927823142	0.353499318	0.391142947	positive
PRJNA729271	stage_1W_vs_E10	ordinary_muscle_development	ENSGALG00010001721	TM4SF1	-0.218043949	0.162270569	-1.343706078	0.179043451	0.207552369	negative
PRJNA729271	stage_1W_vs_E10	ordinary_muscle_development	ENSGALG00010001725	MTA2	0.187325874	0.185750995	1.008478439	0.313224834	0.349191968	positive
PRJNA729271	stage_1W_vs_E10	ordinary_muscle_development	ENSGALG00010001727	COL9A1	-10.68185253	0.802730266	-13.30690143	2.11055E-40	1.71386E-39	negative
PRJNA729271	stage_1W_vs_E10	ordinary_muscle_development	ENSGALG00010001728	STS	0.442527351	0.325023273	1.361525122	0.1733478	0.201303987	positive
PRJNA729271	stage_1W_vs_E10	ordinary_muscle_development	ENSGALG00010001729	ENSGALG00010001729	2.254097707	0.242611434	9.290978858	1.52876E-20	5.89501E-20	positive
PRJNA729271	stage_1W_vs_E10	ordinary_muscle_development	ENSGALG00010001731	ENSGALG00010001731	1.308248752	0.19057368	6.864792425	6.6588E-12	1.69184E-11	positive
PRJNA729271	stage_1W_vs_E10	ordinary_muscle_development	ENSGALG00010001738	PUDP	0.893117478	0.119682455	7.462392701	8.49652E-14	2.37205E-13	positive
PRJNA729271	stage_1W_vs_E10	ordinary_muscle_development	ENSGALG00010001741	SATB1	0.304831381	0.196791055	1.549010351	0.121379232	0.144283415	positive
PRJNA729271	stage_1W_vs_E10	ordinary_muscle_development	ENSGALG00010001745	CADPS2	-1.092707617	0.1124862	-9.714148197	2.62434E-22	1.07518E-21	negative
PRJNA729271	stage_1W_vs_E10	ordinary_muscle_development	ENSGALG00010001749	VAPA	1.550634183	0.105950757	14.63542336	1.66933E-48	1.7358E-47	positive
PRJNA729271	stage_1W_vs_E10	ordinary_muscle_development	ENSGALG00010001751	AVPR2C	-0.351436509	0.432031852	-0.81345046	0.415959839	0.453913865	negative
PRJNA729271	stage_1W_vs_E10	ordinary_muscle_development	ENSGALG00010001752	ARRB1	1.900341495	0.105650611	17.98703741	2.46172E-72	4.93535E-71	positive
PRJNA729271	stage_1W_vs_E10	ordinary_muscle_development	ENSGALG00010001753	PNPLA8	1.44931152	0.204229775	7.096475154	1.27979E-12	3.37283E-12	positive
PRJNA729271	stage_1W_vs_E10	ordinary_muscle_development	ENSGALG00010001754	APCDD1	-4.507365516	0.208544021	-21.61349669	1.341E-103	5.4144E-102	negative
PRJNA729271	stage_1W_vs_E10	ordinary_muscle_development	ENSGALG00010001755	CUZD1	1.033951743	1.447780644	0.714163259	0.475126233	0.513606887	positive
PRJNA729271	stage_1W_vs_E10	ordinary_muscle_development	ENSGALG00010001756	ENSGALG00010001756	0.421380058	0.219279031	1.921661442	0.054648372	0.067900579	positive
PRJNA729271	stage_1W_vs_E10	ordinary_muscle_development	ENSGALG00010001758	COL19A1	0.058592442	0.281211024	0.208357555	0.834949795	0.852886754	positive
PRJNA729271	stage_1W_vs_E10	ordinary_muscle_development	ENSGALG00010001759	C10orf88	0.532415242	0.087885622	6.058047148	1.37784E-09	3.08064E-09	positive
PRJNA729271	stage_1W_vs_E10	ordinary_muscle_development	ENSGALG00010001760	RAB5A	0.465010125	0.095926302	4.847576898	1.24979E-06	2.31836E-06	positive
PRJNA729271	stage_1W_vs_E10	ordinary_muscle_development	ENSGALG00010001761	ELOB	1.079472213	0.148610648	7.263760878	3.76473E-13	1.0162E-12	positive
PRJNA729271	stage_1W_vs_E10	ordinary_muscle_development	ENSGALG00010001763	ENSGALG00010001763	0.247495002	0.268059647	0.923283326	0.355859575	0.393424189	positive
PRJNA729271	stage_1W_vs_E10	ordinary_muscle_development	ENSGALG00010001764	ENSGALG00010001764	0.029839949	0.439596108	0.067880376	0.94588086	0.952716494	positive
PRJNA729271	stage_1W_vs_E10	ordinary_muscle_development	ENSGALG00010001767	ENSGALG00010001767	0.452830721	0.175774181	2.57620726	0.009989075	0.013506683	positive
PRJNA729271	stage_1W_vs_E10	ordinary_muscle_development	ENSGALG00010001771	ENSGALG00010001771	0.615283836	0.1352197	4.550252918	5.35815E-06	9.47497E-06	positive
PRJNA729271	stage_1W_vs_E10	ordinary_muscle_development	ENSGALG00010001773	NRCAM	-1.893015556	0.24889031	-7.605822633	2.83097E-14	8.10807E-14	negative
PRJNA729271	stage_1W_vs_E10	ordinary_muscle_development	ENSGALG00010001776	LMBRD1	1.057784123	0.131274265	8.057817893	7.76684E-16	2.40248E-15	positive
PRJNA729271	stage_1W_vs_E10	ordinary_muscle_development	ENSGALG00010001778	KAT2B	3.173197068	0.147103484	21.57118911	3.3497E-103	1.3339E-101	positive
PRJNA729271	stage_1W_vs_E10	ordinary_muscle_development	ENSGALG00010001779	ENSGALG00010001779	0.348157227	0.621449496	0.560234145	0.57531974	0.610979531	positive
PRJNA729271	stage_1W_vs_E10	ordinary_muscle_development	ENSGALG00010001780	ENSGALG00010001780	0.114220362	0.238857398	0.478194786	0.632511573	0.665282291	positive
PRJNA729271	stage_1W_vs_E10	ordinary_muscle_development	ENSGALG00010001783	SGO1	-2.740480935	0.250899125	-10.92264044	8.98452E-28	4.63289E-27	negative
PRJNA729271	stage_1W_vs_E10	ordinary_muscle_development	ENSGALG00010001785	CNTN1	-0.100215335	0.213410831	-0.469588795	0.638648832	0.671007163	negative
PRJNA729271	stage_1W_vs_E10	ordinary_muscle_development	ENSGALG00010001786	P2RY8	-0.30429408	0.209811492	-1.45032132	0.146968937	0.17260693	negative
PRJNA729271	stage_1W_vs_E10	ordinary_muscle_development	ENSGALG00010001788	HOXA1	-1.286488984	0.40440834	-3.181163334	0.001466849	0.002151369	negative
PRJNA729271	stage_1W_vs_E10	ordinary_muscle_development	ENSGALG00010001790	HOXA2	-1.957729091	0.266313027	-7.351232929	1.96387E-13	5.37505E-13	negative
PRJNA729271	stage_1W_vs_E10	ordinary_muscle_development	ENSGALG00010001793	IKZF5	0.969298557	0.103054654	9.405674768	5.16974E-21	2.02309E-20	positive
PRJNA729271	stage_1W_vs_E10	ordinary_muscle_development	ENSGALG00010001794	ENSGALG00010001794	-2.205576705	0.65085141	-3.388756127	0.000702104	0.001059663	negative
PRJNA729271	stage_1W_vs_E10	ordinary_muscle_development	ENSGALG00010001795	ENSGALG00010001795	1.742754502	0.475393593	3.665919207	0.000246452	0.000385446	positive
PRJNA729271	stage_1W_vs_E10	ordinary_muscle_development	ENSGALG00010001796	SLCO2B1	2.425652013	0.155412991	15.60778152	6.4439E-55	8.3026E-54	positive
PRJNA729271	stage_1W_vs_E10	ordinary_muscle_development	ENSGALG00010001798	PSTK	0.733903064	0.128107071	5.728825581	1.01128E-08	2.14463E-08	positive
PRJNA729271	stage_1W_vs_E10	ordinary_muscle_development	ENSGALG00010001800	ENSGALG00010001800	0.812310859	0.18393575	4.416275017	1.00416E-05	1.74253E-05	positive
PRJNA729271	stage_1W_vs_E10	ordinary_muscle_development	ENSGALG00010001801	HOXA3	-1.8441813	0.158729994	-11.61835423	3.32479E-31	1.97188E-30	negative
PRJNA729271	stage_1W_vs_E10	ordinary_muscle_development	ENSGALG00010001803	PPP4R1	-0.596626502	0.09699848	-6.150885078	7.70517E-10	1.74238E-09	negative
PRJNA729271	stage_1W_vs_E10	ordinary_muscle_development	ENSGALG00010001805	ENSGALG00010001805	2.106834947	0.255723356	8.238727114	1.74052E-16	5.5278E-16	positive
PRJNA729271	stage_1W_vs_E10	ordinary_muscle_development	ENSGALG00010001806	ENSGALG00010001806	4.683364731	0.901274984	5.196377149	2.0321E-07	3.97829E-07	positive
PRJNA729271	stage_1W_vs_E10	ordinary_muscle_development	ENSGALG00010001807	ASMTL	0.563463096	0.102892247	5.476244436	4.34447E-08	8.88949E-08	positive
PRJNA729271	stage_1W_vs_E10	ordinary_muscle_development	ENSGALG00010001808	HOXA4	-1.249047936	0.156003422	-8.006541886	1.17979E-15	3.61774E-15	negative
PRJNA729271	stage_1W_vs_E10	ordinary_muscle_development	ENSGALG00010001811	ACADSB	0.444344745	0.149012238	2.981934585	0.002864332	0.004089686	positive
PRJNA729271	stage_1W_vs_E10	ordinary_muscle_development	ENSGALG00010001813	HOXA5	-0.362744415	0.103666223	-3.49915724	0.000466731	0.000713185	negative
PRJNA729271	stage_1W_vs_E10	ordinary_muscle_development	ENSGALG00010001817	SLC25A6	-1.317799139	0.113035075	-11.65832061	2.08107E-31	1.24289E-30	negative
PRJNA729271	stage_1W_vs_E10	ordinary_muscle_development	ENSGALG00010001818	ADGRB3	-1.922033331	0.264983702	-7.253402058	4.06431E-13	1.09543E-12	negative
PRJNA729271	stage_1W_vs_E10	ordinary_muscle_development	ENSGALG00010001819	HOXA6	1.29585806	0.141419527	9.163218734	5.0372E-20	1.89352E-19	positive
PRJNA729271	stage_1W_vs_E10	ordinary_muscle_development	ENSGALG00010001820	PGM2L1	3.089615321	0.153310405	20.15267866	2.54939E-90	7.54151E-89	positive
PRJNA729271	stage_1W_vs_E10	ordinary_muscle_development	ENSGALG00010001822	KCNE3	3.921819483	0.299387085	13.09949452	3.31458E-39	2.58723E-38	positive
PRJNA729271	stage_1W_vs_E10	ordinary_muscle_development	ENSGALG00010001823	RALBP1	-0.324980558	0.076451147	-4.250826469	2.12983E-05	3.60022E-05	negative
PRJNA729271	stage_1W_vs_E10	ordinary_muscle_development	ENSGALG00010001825	LIPT2	2.940587731	0.141821729	20.73439493	1.69574E-95	5.83842E-94	positive
PRJNA729271	stage_1W_vs_E10	ordinary_muscle_development	ENSGALG00010001826	HOXA7	2.06361124	0.132735902	15.54674509	1.674E-54	2.14163E-53	positive
PRJNA729271	stage_1W_vs_E10	ordinary_muscle_development	ENSGALG00010001827	PDZRN4	-3.226248634	0.208793531	-15.45186105	7.32962E-54	9.15887E-53	negative

PRJNA729271	stage_1W_vs_E10	ordinary_muscle_development	ENSGALG00010001828	ENSGALG00010001828	-3.421242283	1.120296978	-3.053870848	0.002259093	0.003255996	negative
PRJNA729271	stage_1W_vs_E10	ordinary_muscle_development	ENSGALG00010001829	POLD3	-0.381277885	0.121772844	-3.131058402	0.001741775	0.002538155	negative
PRJNA729271	stage_1W_vs_E10	ordinary_muscle_development	ENSGALG00010001831	CSF2RA	1.294180702	0.252331031	5.128900313	2.9144E-07	5.64158E-07	positive
PRJNA729271	stage_1W_vs_E10	ordinary_muscle_development	ENSGALG00010001832	BUB3	-1.112916644	0.087128648	-12.77325739	2.31269E-37	1.69391E-36	negative
PRJNA729271	stage_1W_vs_E10	ordinary_muscle_development	ENSGALG00010001834	ZCRB1	0.279358082	0.084341271	3.312234649	0.000925539	0.001383916	positive
PRJNA729271	stage_1W_vs_E10	ordinary_muscle_development	ENSGALG00010001835	MECP2	0.781083355	0.190223679	4.106131058	4.02341E-05	6.67041E-05	positive
PRJNA729271	stage_1W_vs_E10	ordinary_muscle_development	ENSGALG00010001836	TWSG1	-1.274390227	0.140533003	-9.068262957	1.20929E-19	4.47275E-19	negative
PRJNA729271	stage_1W_vs_E10	ordinary_muscle_development	ENSGALG00010001837	TAB3	0.806234877	0.138745827	5.810876585	6.21466E-09	1.33672E-08	positive
PRJNA729271	stage_1W_vs_E10	ordinary_muscle_development	ENSGALG00010001838	PPHLN1	-1.110804367	0.067875441	-16.36533561	3.38158E-60	4.98131E-59	negative
PRJNA729271	stage_1W_vs_E10	ordinary_muscle_development	ENSGALG00010001839	ENSGALG00010001839	4.300842351	0.610603348	7.043594445	1.87343E-12	4.88757E-12	positive
PRJNA729271	stage_1W_vs_E10	ordinary_muscle_development	ENSGALG00010001840	HOXA9	0.696270387	0.144388058	4.822215885	1.41972E-06	2.62251E-06	positive
PRJNA729271	stage_1W_vs_E10	ordinary_muscle_development	ENSGALG00010001842	GPR26	-0.531849158	0.530880363	-1.001824883	0.316428177	0.352546228	negative
PRJNA729271	stage_1W_vs_E10	ordinary_muscle_development	ENSGALG00010001843	PRICKLE1	-2.513289086	0.123998338	-20.26873208	2.42833E-91	7.53718E-90	negative
PRJNA729271	stage_1W_vs_E10	ordinary_muscle_development	ENSGALG00010001844	HOXA10	-0.750371892	0.165424011	-4.536051858	5.73171E-06	1.01097E-05	negative
PRJNA729271	stage_1W_vs_E10	ordinary_muscle_development	ENSGALG00010001845	ANKRD12	1.00894395	0.13409792	7.523934387	5.3152E-14	1.50212E-13	positive
PRJNA729271	stage_1W_vs_E10	ordinary_muscle_development	ENSGALG00010001848	HOXA11	-0.909481751	0.365140713	-2.490770595	0.012746639	0.017031945	negative
PRJNA729271	stage_1W_vs_E10	ordinary_muscle_development	ENSGALG00010001849	NDUFV2	1.110365402	0.094456077	11.75536227	6.62759E-32	4.02965E-31	positive
PRJNA729271	stage_1W_vs_E10	ordinary_muscle_development	ENSGALG00010001850	MYOM2	4.929712395	0.18778828	26.25143797	6.8825E-152	5.903E-150	positive
PRJNA729271	stage_1W_vs_E10	ordinary_muscle_development	ENSGALG00010001852	CPXM2	-0.858752572	0.382527385	-2.244944034	0.024771726	0.032056617	negative
PRJNA729271	stage_1W_vs_E10	ordinary_muscle_development	ENSGALG00010001853	ENSGALG00010001853	1.353689783	0.18542315	7.300543574	2.86607E-13	7.77827E-13	positive
PRJNA729271	stage_1W_vs_E10	ordinary_muscle_development	ENSGALG00010001854	CHRDL2	0.720741876	0.301728381	2.388710913	0.0169076	0.022251286	positive
PRJNA729271	stage_1W_vs_E10	ordinary_muscle_development	ENSGALG00010001856	CHST15	-0.290320289	0.116838298	-2.484804165	0.012962272	0.017307308	negative
PRJNA729271	stage_1W_vs_E10	ordinary_muscle_development	ENSGALG00010001857	HNRNPA2B1	-0.48063557	0.187718568	-2.56040505	0.010455023	0.014105134	negative
PRJNA729271	stage_1W_vs_E10	ordinary_muscle_development	ENSGALG00010001858	ADAMTS20	1.305143813	0.160602823	8.126530968	4.41751E-16	1.38178E-15	positive
PRJNA729271	stage_1W_vs_E10	ordinary_muscle_development	ENSGALG00010001860	RNF169	0.508077573	0.129059199	3.936779208	8.25825E-05	0.000133702	positive
PRJNA729271	stage_1W_vs_E10	ordinary_muscle_development	ENSGALG00010001861	CBX3	-1.285474533	0.074846374	-17.17484042	4.09778E-66	6.91471E-65	negative
PRJNA729271	stage_1W_vs_E10	ordinary_muscle_development	ENSGALG00010001864	ENSGALG00010001864	0.458759626	0.19576273	2.343447227	0.019106465	0.025008869	positive
PRJNA729271	stage_1W_vs_E10	ordinary_muscle_development	ENSGALG00010001865	CYBB	0.321464339	0.320624343	1.002619876	0.316044302	0.352145603	positive
PRJNA729271	stage_1W_vs_E10	ordinary_muscle_development	ENSGALG00010001866	PUS7L	0.001838956	0.119291706	0.015415624	0.987700599	0.989680643	positive
PRJNA729271	stage_1W_vs_E10	ordinary_muscle_development	ENSGALG00010001868	ENSGALG00010001868	0.478418162	0.121986227	3.921903099	8.78523E-05	0.000141964	positive
PRJNA729271	stage_1W_vs_E10	ordinary_muscle_development	ENSGALG00010001869	CRLF2	6.341929737	0.936063983	6.775102826	1.24318E-11	3.10902E-11	positive
PRJNA729271	stage_1W_vs_E10	ordinary_muscle_development	ENSGALG00010001870	PLP2	1.562549055	0.164478213	9.500036648	2.09816E-21	8.35749E-21	positive
PRJNA729271	stage_1W_vs_E10	ordinary_muscle_development	ENSGALG00010001871	ENSGALG00010001871	-0.959510477	0.124231376	-7.72357605	1.13111E-14	3.31153E-14	negative
PRJNA729271	stage_1W_vs_E10	ordinary_muscle_development	ENSGALG00010001873	ENSGALG00010001873	-0.302166516	0.217441882	-1.389642667	0.164637413	0.192019576	negative
PRJNA729271	stage_1W_vs_E10	ordinary_muscle_development	ENSGALG00010001877	OAT	0.987459822	0.136655191	7.225922536	4.97711E-13	1.33549E-12	positive
PRJNA729271	stage_1W_vs_E10	ordinary_muscle_development	ENSGALG00010001878	XK	-0.41049341	0.201791192	-2.0342484	0.041926557	0.052831908	negative
PRJNA729271	stage_1W_vs_E10	ordinary_muscle_development	ENSGALG00010001879	IRAK4	0.544334546	0.165583379	3.287374317	0.001011263	0.001505109	positive
PRJNA729271	stage_1W_vs_E10	ordinary_muscle_development	ENSGALG00010001880	MTCL1	2.082174678	0.144069073	14.4526138	2.4135E-47	2.416E-46	positive
PRJNA729271	stage_1W_vs_E10	ordinary_muscle_development	ENSGALG00010001883	KBTBD11	-1.582153253	0.090496611	-17.48301115	1.93031E-68	3.51948E-67	negative
PRJNA729271	stage_1W_vs_E10	ordinary_muscle_development	ENSGALG00010001885	SMPX	3.917145155	0.371236033	10.5516297	4.99235E-26	2.40093E-25	positive
PRJNA729271	stage_1W_vs_E10	ordinary_muscle_development	ENSGALG00010001886	NEU3	-0.375029825	0.08548489	-4.387089027	1.14878E-05	1.98635E-05	negative
PRJNA729271	stage_1W_vs_E10	ordinary_muscle_development	ENSGALG00010001888	SNX10	1.561622239	0.200015432	7.807508778	5.83294E-15	1.73007E-14	positive
PRJNA729271	stage_1W_vs_E10	ordinary_muscle_development	ENSGALG00010001890	KLHL34	6.969154343	0.250752487	27.79296204	5.2763E-170	5.7075E-168	positive
PRJNA729271	stage_1W_vs_E10	ordinary_muscle_development	ENSGALG00010001892	RAB12	1.494115977	0.070495716	21.19442237	1.0751E-99	4.02675E-98	positive
PRJNA729271	stage_1W_vs_E10	ordinary_muscle_development	ENSGALG00010001895	P4HA3	-1.610292078	0.240663223	-6.691060049	2.2156E-11	5.47105E-11	negative
PRJNA729271	stage_1W_vs_E10	ordinary_muscle_development	ENSGALG00010001896	ARHGEF10	-0.408055439	0.134579639	-3.032074106	0.002428796	0.003489829	negative
PRJNA729271	stage_1W_vs_E10	ordinary_muscle_development	ENSGALG00010001897	TLR7	1.82813385	0.179036759	10.21094139	1.77138E-24	7.97148E-24	positive
PRJNA729271	stage_1W_vs_E10	ordinary_muscle_development	ENSGALG00010001898	LHPP	0.715790433	0.13146417	5.444756791	5.18762E-08	1.05759E-07	positive
PRJNA729271	stage_1W_vs_E10	ordinary_muscle_development	ENSGALG00010001899	TWF1	-0.603872506	0.11300588	-5.343726432	9.1055E-08	1.82702E-07	negative
PRJNA729271	stage_1W_vs_E10	ordinary_muscle_development	ENSGALG00010001901	PRPS2	1.644658925	0.181637989	9.054597719	1.37072E-19	5.05949E-19	positive
PRJNA729271	stage_1W_vs_E10	ordinary_muscle_development	ENSGALG00010001902	WDR45	0.653101018	0.141620685	4.611621666	3.9954E-06	7.13306E-06	positive
PRJNA729271	stage_1W_vs_E10	ordinary_muscle_development	ENSGALG00010001903	NARS2	-0.405607399	0.119144212	-3.404339934	0.000663242	0.001002888	negative
PRJNA729271	stage_1W_vs_E10	ordinary_muscle_development	ENSGALG00010001906	SKAP2	-0.271861436	0.128399605	-2.117307419	0.034233766	0.043562324	negative
PRJNA729271	stage_1W_vs_E10	ordinary_muscle_development	ENSGALG00010001908	FAM83B	-4.289916733	0.439606964	-9.7585277	1.69599E-22	7.01983E-22	negative
PRJNA729271	stage_1W_vs_E10	ordinary_muscle_development	ENSGALG00010001910	FAM53B	-0.127881814	0.097191778	-1.315767813	0.188251986	0.217531292	negative
PRJNA729271	stage_1W_vs_E10	ordinary_muscle_development	ENSGALG00010001911	USP35	-0.511006981	0.358536435	-1.425258166	0.154082588	0.180391496	negative
PRJNA729271	stage_1W_vs_E10	ordinary_muscle_development	ENSGALG00010001912	TMEM117	5.254341401	0.205011846	25.62945261	7.1661E-145	5.4384E-143	positive
PRJNA729271	stage_1W_vs_E10	ordinary_muscle_development	ENSGALG00010001914	ENSGALG00010001914	-0.552169516	0.154726268	-3.568686333	0.000358776	0.000553652	negative
PRJNA729271	stage_1W_vs_E10	ordinary_muscle_development	ENSGALG00010001915	EEF1AKMT2	-0.177605465	0.167372483	-1.061138972	0.288626749	0.323511037	negative
PRJNA729271	stage_1W_vs_E10	ordinary_muscle_development	ENSGALG00010001918	CNKSR2	-0.668120806	0.146597165	-4.557528826	5.1759E-06	9.16164E-06	negative
PRJNA729271	stage_1W_vs_E10	ordinary_muscle_development	ENSGALG00010001921	ENSGALG00010001921	-0.346236139	0.296913145	-1.166119265	0.243566228	0.276100139	negative
PRJNA729271	stage_1W_vs_E10	ordinary_muscle_development	ENSGALG00010001923	RAPGEF5	2.084223678	0.100921503	20.65192854	9.37997E-95	3.13278E-93	positive
PRJNA729271	stage_1W_vs_E10	ordinary_muscle_development	ENSGALG00010001925	FRMPD4	-3.035205031	0.217385915	-13.9622893	2.6479E-44	2.42766E-43	negative
PRJNA729271	stage_1W_vs_E10	ordinary_muscle_development	ENSGALG00010001928	KCTD21	-0.042000461	0.170338453	-0.246570636	0.805240532	0.825982698	negative
PRJNA729271	stage_1W_vs_E10	ordinary_muscle_development	ENSGALG00010001929	POLR2I	-0.315095641	0.125480396	-2.511114489	0.012035066	0.016115314	negative
PRJNA729271	stage_1W_vs_E10	ordinary_muscle_development	ENSGALG00010001935	ABRAXAS2	0.683012122	0.129022995	5.293723977	1.1985E-07	2.3814E-07	positive
PRJNA729271	stage_1W_vs_E10	ordinary_muscle_development	ENSGALG00010001938	ALG8	-1.040745671	0.107098351	-9.717662868	2.53534E-22	1.03989E-21	negative
PRJNA729271	stage_1W_vs_E10	ordinary_muscle_development	ENSGALG00010001940	PTPRM	1.100026165	0.110570458	9.94864435	2.55642E-23	1.09793E-22	positive
PRJNA729271	stage_1W_vs_E10	ordinary_muscle_development	ENSGALG00010001947	TINAG	5.499283812	0.30310899	18.14292545	1.46046E-73	2.99426E-72	positive
PRJNA729271	stage_1W_vs_E10	ordinary_muscle_development	ENSGALG00010001948	EMX2	-2.738881093	0.27432093	-9.984222102	1.78697E-23	7.71587E-23	negative
PRJNA729271	stage_1W_vs_E10	ordinary_muscle_development	ENSGALG00010001952	ENSGALG00010001952	0.32872624	0.072662375	4.524022763	6.06752E-06	1.06825E-05	positive
PRJNA729271	stage_1W_vs_E10	ordinary_muscle_development	ENSGALG00010001957	NDUFC2	1.696393832	0.111390229	15.22928754	2.26041E-52	2.6989E-51	positive
PRJNA729271	stage_1W_vs_E10	ordinary_muscle_development	ENSGALG00010001958	RAB11FIP2	-0.691623423	0.112641394	-6.140046747	8.24972E-10	1.8629E-09	negative
PRJNA729271	stage_1W_vs_E10	ordinary_muscle_development	ENSGALG00010001966	ARHGAP6	0.585632749	0.142852121	4.09957334	4.13912E-05	6.85206E-05	positive
PRJNA729271	stage_1W_vs_E10	ordinary_muscle_development	ENSGALG00010001967	LAMA1	-5.174588306	0.245443735	-21.08258462	1.14933E-98	4.1858E-97	negative
PRJNA729271	stage_1W_vs_E10	ordinary_muscle_development	ENSGALG00010001969	PLEKHA8	-2.40959315	0.132276809	-18.21629327	3.83254E-74	7.92477E-73	negative
PRJNA729271	stage_1W_vs_E10	ordinary_muscle_development	ENSGALG00010001971	MLIP	4.828653818	0.204306163	23.6344012	1.7078E-123	1.0104E-121	positive
PRJNA729271	stage_1W_vs_E10	ordinary_muscle_development	ENSGALG00010001972	FAM204A	0.073758757	0.158678126	0.464832546	0.642051379	0.67414232	positive
PRJNA729271	stage_1W_vs_E10	ordinary_muscle_development	ENSGALG00010001974	ENSGALG00010001974	0.934784198	0.401713937	2.326989713	0.019965813	0.02610069	positive
PRJNA729271	stage_1W_vs_E10	ordinary_muscle_development	ENSGALG00010001976	THRSP	8.501021392	1.424389304	5.968186766	2.39905E-09	5.28563E-09	positive
PRJNA729271	stage_1W_vs_E10	ordinary_muscle_development	ENSGALG00010001982	DMD	3.405746138	0.121764241	27.97000268	3.7656E-172	4.1987E-170	positive
PRJNA729271	stage_1W_vs_E10	ordinary_muscle_development	ENSGALG00010001987	KCTD14	-3.392907291	0.516470668	-6.569409455	5.05152E-11	1.22199E-10	negative
PRJNA729271	stage_1W_vs_E10	ordinary_muscle_development	ENSGALG00010001991	TOMM7	1.200025214	0.130086997	9.224789863	2.84117E-20	1.07893E-19	positive
PRJNA729271	stage_1W_vs_E10	ordinary_muscle_development	ENSGALG00010001995	LRRC1	-1.557033961	0.144043159	-10.80949608	3.1037E-27	1.56481E-26	negative
PRJNA729271	stage_1W_vs_E10	ordinary_muscle_development	ENSGALG00010001997	INTS4	-0.320570864	0.078118156	-4.103666572	4.06653E-05	6.73959E-05	negative
PRJNA729271	stage_1W_vs_E10	ordinary_muscle_development	ENSGALG00010002005	COLQ	-0.442543717	0.219129468	-2.019553647	0.043429707	0.054650022	negative
PRJNA729271	stage_1W_vs_E10	ordinary_muscle_development	ENSGALG00010002006	ENSGALG00010002006	3.702204032	0.400150799	9.25202209	2.20285E-20	8.40936E-20	positive
PRJNA729271	stage_1W_vs_E10	ordinary_muscle_development	ENSGALG00010002012	MID1	-2.505005282	0.143803884	-17.41959411	5.85913E-68	1.0537E-66	negative
PRJNA729271	stage_1W_vs_E10	ordinary_muscle_development	ENSGALG00010002017	ARHGAP28	-1.62264836	0.118902112	-13.64692628	2.10575E-42	1.82335E-41	negative
PRJNA729271	stage_1W_vs_E10	ordinary_muscle_development	ENSGALG00010002022	ERNI	-2.538144816	0.773598443	-3.280959054	0.001034548	0.001538027	negative
PRJNA729271	stage_1W_vs_E10	ordinary_muscle_development	ENSGALG00010002023	CLCN4	-0.065318907	0.243569638	-0.268173436	0.788565821	0.810829366	negative
PRJNA729271	stage_1W_vs_E10	ordinary_muscle_development	ENSGALG00010002024	KLHL31	4.961917312	0.16576999	29.93254271	7.425E-197	1.0349E-194	positive
PRJNA729271	stage_1W_vs_E10	ordinary_muscle_development	ENSGALG00010002027	ENSGALG00010002027	6.310672114	0.538009243	11.72967229	8.98057E-32	5.44431E-31	positive
PRJNA729271	stage_1W_vs_E10	ordinary_muscle_development	ENSGALG00010002034	WWC3	-0.48613383	0.100087534	-4.857086698	1.19125E-06	2.21545E-06	negative
PRJNA729271	stage_1W_vs_E10	ordinary_muscle_development	ENSGALG00010002039	AAMDC	1.802201045	0.122828127	14.67254362	9.66528E-49	1.01154E-47	positive
PRJNA729271	stage_1W_vs_E10	ordinary_muscle_development	ENSGALG00010002041	GCLC	0.933106258	0.138333244	6.745350776	1.52658E-11	3.7981E-11	positive
PRJNA729271	stage_1W_vs_E10	ordinary_muscle_development	ENSGALG00010002046	FAM126A	-0.939833831	0.088922698	-10.56911063	4.14406E-26	1.99827E-25	negative
PRJNA729271	stage_1W_vs_E10	ordinary_muscle_development	ENSGALG00010002050	ENSGALG00010002050	6.092282231	1.100987697	5.533469855	3.13957E-08	6.46605E-08	positive
PRJNA729271	stage_1W_vs_E10	ordinary_muscle_development	ENSGALG00010002054	RSF1	-0.115053155	0.100030321	-1.150182806	0.250068586	0.283006258	negative
PRJNA729271	stage_1W_vs_E10	ordinary_muscle_development	ENSGALG00010002056	PRLHR	-2.596481756	0.505939606	-5.131999398	2.8668E-07	5.55242E-07	negative
PRJNA729271	stage_1W_vs_E10	ordinary_muscle_development	ENSGALG00010002062	GSTA4	-0.510541947	0.365337598	-1.397452517	0.162277536	0.189495922	negative
PRJNA729271	stage_1W_vs_E10	ordinary_muscle_development	ENSGALG00010002065	CLNS1A	-0.358713603	0.063036253	-5.69059213	1.266E-08	2.66763E-08	negative
PRJNA729271	stage_1W_vs_E10	ordinary_muscle_development	ENSGALG00010002072	NEURL4	0.682224337	0.159971687	4.264656749	2.0021E-05	3.39063E-05	positive
PRJNA729271	stage_1W_vs_E10	ordinary_muscle_development	ENSGALG00010002079	ARX	2.868308819	0.189081817	15.16967027	5.61582E-52	6.60725E-51	positive
PRJNA729271	stage_1W_vs_E10	ordinary_muscle_development	ENSGALG00010002084	HACL1	-0.927012655	0.093351708	-9.930323471	3.07257E-23	1.3161E-22	negative
PRJNA729271	stage_1W_vs_E10	ordinary_muscle_development	ENSGALG00010002087	POLA1	-1.732469335	0.075839008	-22.84404016	1.675E-115	8.3148E-114	negative
PRJNA729271	stage_1W_vs_E10	ordinary_muscle_development	ENSGALG00010002088	OZFL1	-0.127071424	0.137489933	-0.924223482	0.355369972	0.393002804	negative
PRJNA729271	stage_1W_vs_E10	ordinary_muscle_development	ENSGALG00010002090	AQP11	-0.302371233	0.30204149	-1.001091716	0.31678247	0.352913834	negative
PRJNA729271	stage_1W_vs_E10	ordinary_muscle_development	ENSGALG00010002097	PCYT1B	-0.926389261	0.446727485	-2.073723451	0.038105002	0.04827233	negative
PRJNA729271	stage_1W_vs_E10	ordinary_muscle_development	ENSGALG00010002098	ENSGALG00010002098	0.75687604	0.429670439	1.761526909	0.078149263	0.095199056	positive
PRJNA729271	stage_1W_vs_E10	ordinary_muscle_development	ENSGALG00010002100	BTD	0.600485568	0.116937252	5.135109264	2.8198E-07	5.46431E-07	positive
PRJNA729271	stage_1W_vs_E10	ordinary_muscle_development	ENSGALG00010002102	PAK1	-1.71799988	0.276233973	-6.219364907	4.99171E-10	1.13981E-09	negative
PRJNA729271	stage_1W_vs_E10	ordinary_muscle_development	ENSGALG00010002106	ENSGALG00010002106	0.000866332	0.88965187	0.000973787	0.99922303	0.99942988	positive
PRJNA729271	stage_1W_vs_E10	ordinary_muscle_development	ENSGALG00010002113	GDPD4	-7.335087345	0.400620063	-18.30933603	6.97147E-75	1.46664E-73	negative
PRJNA729271	stage_1W_vs_E10	ordinary_muscle_development	ENSGALG00010002118	ENSGALG00010002118	2.348080432	0.263200636	8.921256691	4.61027E-19	1.66151E-18	positive
PRJNA729271	stage_1W_vs_E10	ordinary_muscle_development	ENSGALG00010002122	ENSGALG00010002122	1.925185944	0.513173524	3.751530145	0.000175759	0.000277761	positive
PRJNA729271	stage_1W_vs_E10	ordinary_muscle_development	ENSGALG00010002126	ENSGALG00010002126	3.175687014	0.296345389	10.71616813	8.54714E-27	4.23558E-26	positive
PRJNA729271	stage_1W_vs_E10	ordinary_muscle_development	ENSGALG00010002134	ANKRD28	1.039942796	0.120503168	8.630003747	6.135E-18	2.07821E-17	positive
PRJNA729271	stage_1W_vs_E10	ordinary_muscle_development	ENSGALG00010002135	ELOVL5	-0.181894743	0.168386069	-1.080224412	0.28004226	0.314496983	negative
PRJNA729271	stage_1W_vs_E10	ordinary_muscle_development	ENSGALG00010002136	ACAP1	-1.06395684	0.261806191	-4.063910166	4.82574E-05	7.94425E-05	negative
PRJNA729271	stage_1W_vs_E10	ordinary_muscle_development	ENSGALG00010002137	PDK3	1.927500956	0.131405734	14.6683169	1.02865E-48	1.07578E-47	positive
PRJNA729271	stage_1W_vs_E10	ordinary_muscle_development	ENSGALG00010002138	MYO7A	-1.225407909	0.174791805	-7.010671386	2.37177E-12	6.1644E-12	negative
PRJNA729271	stage_1W_vs_E10	ordinary_muscle_development	ENSGALG00010002140	ENSGALG00010002140	-0.431389318	0.463345115	-0.931032407	0.351836801	0.389422299	negative
PRJNA729271	stage_1W_vs_E10	ordinary_muscle_development	ENSGALG00010002142	CLDN34	0.395324376	0.257302805	1.53641689	0.124436167	0.147699168	positive
PRJNA729271	stage_1W_vs_E10	ordinary_muscle_development	ENSGALG00010002145	ENSGALG00010002145	-0.338207662	0.484442454	-0.698137951	0.485090926	0.523285925	negative
PRJNA729271	stage_1W_vs_E10	ordinary_muscle_development	ENSGALG00010002146	CACUL1	0.280917488	0.08886584	3.161141419	0.001571522	0.002298608	positive
PRJNA729271	stage_1W_vs_E10	ordinary_muscle_development	ENSGALG00010002148	POLR2A	0.843804387	0.177713585	4.748114141	2.05322E-06	3.74593E-06	positive
PRJNA729271	stage_1W_vs_E10	ordinary_muscle_development	ENSGALG00010002149	ENSGALG00010002149	-2.686360482	0.372403871	-7.213567549	5.45047E-13	1.46034E-12	negative
PRJNA729271	stage_1W_vs_E10	ordinary_muscle_development	ENSGALG00010002150	EIF3A	0.118641933	0.132464258	0.895652414	0.370438433	0.40804811	positive
PRJNA729271	stage_1W_vs_E10	ordinary_muscle_development	ENSGALG00010002159	GALNT15	1.240800914	0.189576455	6.545121398	5.94471E-11	1.42923E-10	positive
PRJNA729271	stage_1W_vs_E10	ordinary_muscle_development	ENSGALG00010002160	CAPN5	-0.117298001	0.166672594	-0.703762978	0.481580393	0.519885886	negative

PRJNA729271	stage_1W_vs_E10	ordinary_muscle_development	ENSGALG00010002161	CDKL5	-0.584912871	0.17568463	-3.329334334	0.000870538	0.001304907	negative
PRJNA729271	stage_1W_vs_E10	ordinary_muscle_development	ENSGALG00010002173	GCM1	-0.834407614	0.901560377	-0.925514957	0.354698107	0.392349593	negative
PRJNA729271	stage_1W_vs_E10	ordinary_muscle_development	ENSGALG00010002174	ENSGALG00010002174	-0.903039115	0.472630741	-1.91066521	0.056047618	0.06955567	negative
PRJNA729271	stage_1W_vs_E10	ordinary_muscle_development	ENSGALG00010002177	FBXO9	2.084509897	0.154662925	13.47776073	2.11427E-41	1.78176E-40	positive
PRJNA729271	stage_1W_vs_E10	ordinary_muscle_development	ENSGALG00010002181	FIS1	-0.169207634	0.262546474	-0.64448641	0.519260067	0.556402348	negative
PRJNA729271	stage_1W_vs_E10	ordinary_muscle_development	ENSGALG00010002185	ENSGALG00010002185	-0.096003709	0.340328923	-0.282090949	0.777873773	0.800858039	negative
PRJNA729271	stage_1W_vs_E10	ordinary_muscle_development	ENSGALG00010002186	CILK1	-0.444284793	0.075667998	-5.871501887	4.31865E-09	9.36547E-09	negative
PRJNA729271	stage_1W_vs_E10	ordinary_muscle_development	ENSGALG00010002192	ENSGALG00010002192	0.338689892	0.19578532	1.729904425	0.083647353	0.101580796	positive
PRJNA729271	stage_1W_vs_E10	ordinary_muscle_development	ENSGALG00010002194	SHROOM2	-2.570051136	0.135432281	-18.97665107	2.66021E-80	6.39466E-79	negative
PRJNA729271	stage_1W_vs_E10	ordinary_muscle_development	ENSGALG00010002196	ENSGALG00010002196	-1.247952652	0.102594203	-12.16396853	4.83514E-34	3.16555E-33	negative
PRJNA729271	stage_1W_vs_E10	ordinary_muscle_development	ENSGALG00010002197	GSTA2	-1.255857613	0.190415668	-6.595348093	4.24259E-11	1.03234E-10	negative
PRJNA729271	stage_1W_vs_E10	ordinary_muscle_development	ENSGALG00010002202	SFXN4	0.496537807	0.100753542	4.928241709	8.29729E-07	1.56133E-06	positive
PRJNA729271	stage_1W_vs_E10	ordinary_muscle_development	ENSGALG00010002206	DPH3P1	1.250432747	0.108751075	11.49811851	1.34822E-30	7.80763E-30	positive
PRJNA729271	stage_1W_vs_E10	ordinary_muscle_development	ENSGALG00010002210	GPR143	-5.615204881	1.231349375	-4.560204433	5.11038E-06	9.0512E-06	negative
PRJNA729271	stage_1W_vs_E10	ordinary_muscle_development	ENSGALG00010002211	ACER3	-0.809495298	0.17493683	-4.627357759	3.7036E-06	6.63172E-06	negative
PRJNA729271	stage_1W_vs_E10	ordinary_muscle_development	ENSGALG00010002215	YLEC18	0.322632607	0.355220883	0.908259121	0.363741335	0.401311513	positive
PRJNA729271	stage_1W_vs_E10	ordinary_muscle_development	ENSGALG00010002225	PRDX3	1.311897183	0.105309968	12.45748341	1.27325E-35	8.86447E-35	positive
PRJNA729271	stage_1W_vs_E10	ordinary_muscle_development	ENSGALG00010002226	OXNAD1	0.367164626	0.13271024	2.76666387	0.005663311	0.007860739	positive
PRJNA729271	stage_1W_vs_E10	ordinary_muscle_development	ENSGALG00010002227	ENSGALG00010002227	-0.130138972	1.185308391	-0.109793344	0.91257327	0.923274206	negative
PRJNA729271	stage_1W_vs_E10	ordinary_muscle_development	ENSGALG00010002231	TMEM14A	-1.199756629	0.139606231	-8.593861617	8.40943E-18	2.83081E-17	negative
PRJNA729271	stage_1W_vs_E10	ordinary_muscle_development	ENSGALG00010002235	MXRA5	-1.29663727	0.201354606	-6.439570941	1.19812E-10	2.83399E-10	negative
PRJNA729271	stage_1W_vs_E10	ordinary_muscle_development	ENSGALG00010002238	ENSGALG00010002238	0.100765729	0.409104567	0.246308004	0.805443814	0.826132755	positive
PRJNA729271	stage_1W_vs_E10	ordinary_muscle_development	ENSGALG00010002239	AGPAT5	1.571879408	0.127295263	12.34829461	4.9755E-35	3.38114E-34	positive
PRJNA729271	stage_1W_vs_E10	ordinary_muscle_development	ENSGALG00010002243	ARSH	-0.308893583	0.177913044	-1.73620537	0.082527528	0.100304924	negative
PRJNA729271	stage_1W_vs_E10	ordinary_muscle_development	ENSGALG00010002247	RFTN1	-1.164282467	0.18711766	-6.222194471	4.90249E-10	1.12049E-09	negative
PRJNA729271	stage_1W_vs_E10	ordinary_muscle_development	ENSGALG00010002251	GRK5	-0.162364816	0.15034374	-1.079957279	0.280161205	0.314606187	negative
PRJNA729271	stage_1W_vs_E10	ordinary_muscle_development	ENSGALG00010002253	ENSGALG00010002253	-0.551738695	0.322520001	-1.710711563	0.087134372	0.105594241	negative
PRJNA729271	stage_1W_vs_E10	ordinary_muscle_development	ENSGALG00010002254	ENSGALG00010002254	3.399643653	0.467788739	7.267476462	3.66265E-13	9.8938E-13	positive
PRJNA729271	stage_1W_vs_E10	ordinary_muscle_development	ENSGALG00010002259	TASL	0.625242137	0.538533561	1.161008675	0.245638372	0.278296717	positive
PRJNA729271	stage_1W_vs_E10	ordinary_muscle_development	ENSGALG00010002261	NROB1	-3.49498925	0.454348858	-7.692303363	1.44509E-14	4.20109E-14	negative
PRJNA729271	stage_1W_vs_E10	ordinary_muscle_development	ENSGALG00010002269	MCPH1	-2.358874479	0.155332337	-15.18598458	4.37938E-52	5.16931E-51	negative
PRJNA729271	stage_1W_vs_E10	ordinary_muscle_development	ENSGALG00010002270	IMMP2L	0.59693592	0.241353235	2.473287416	0.013387643	0.017852243	positive
PRJNA729271	stage_1W_vs_E10	ordinary_muscle_development	ENSGALG00010002271	ENSGALG00010002271	3.383819035	0.681348413	4.966356376	6.82225E-07	1.29064E-06	positive
PRJNA729271	stage_1W_vs_E10	ordinary_muscle_development	ENSGALG00010002274	ENSGALG00010002274	4.442758439	0.777718468	5.712553605	1.11293E-08	2.354E-08	positive
PRJNA729271	stage_1W_vs_E10	ordinary_muscle_development	ENSGALG00010002275	IL1RAPL1	-3.731581741	0.35404087	-10.5399745	5.65142E-26	2.70891E-25	negative
PRJNA729271	stage_1W_vs_E10	ordinary_muscle_development	ENSGALG00010002277	RGS10	-2.133023424	0.18044472	-11.82092458	3.04317E-32	1.87307E-31	negative
PRJNA729271	stage_1W_vs_E10	ordinary_muscle_development	ENSGALG00010002279	ENSGALG00010002279	-0.721430172	1.050978346	-0.686436761	0.49243773	0.530393917	negative
PRJNA729271	stage_1W_vs_E10	ordinary_muscle_development	ENSGALG00010002280	ST7	2.444997849	0.139848196	17.48322765	1.92299E-68	3.51055E-67	positive
PRJNA729271	stage_1W_vs_E10	ordinary_muscle_development	ENSGALG00010002281	TGFBR2	1.88939574	0.154898232	12.19765856	3.19891E-34	2.10477E-33	positive
PRJNA729271	stage_1W_vs_E10	ordinary_muscle_development	ENSGALG00010002282	TSKU	-1.823552046	0.166595588	-10.94598043	6.94621E-28	3.59847E-27	negative
PRJNA729271	stage_1W_vs_E10	ordinary_muscle_development	ENSGALG00010002283	ENSGALG00010002283	-1.376782188	0.938805862	-1.466524916	0.142505338	0.167704383	negative
PRJNA729271	stage_1W_vs_E10	ordinary_muscle_development	ENSGALG00010002285	CAV1	2.639511952	0.157434294	16.76580038	4.34129E-63	6.83989E-62	positive
PRJNA729271	stage_1W_vs_E10	ordinary_muscle_development	ENSGALG00010002288	ANGPT2	-0.596639135	0.178185095	-3.348423363	0.000812728	0.001221029	negative
PRJNA729271	stage_1W_vs_E10	ordinary_muscle_development	ENSGALG00010002289	ENSGALG00010002289	4.600089285	0.937391831	4.907328113	9.23255E-07	1.73148E-06	positive
PRJNA729271	stage_1W_vs_E10	ordinary_muscle_development	ENSGALG00010002291	RNASEH1	0.592603869	0.11592374	5.112014729	3.18741E-07	6.15692E-07	positive
PRJNA729271	stage_1W_vs_E10	ordinary_muscle_development	ENSGALG00010002293	GYG2	-3.099595047	0.385049919	-8.049852477	8.28939E-16	2.55757E-15	negative
PRJNA729271	stage_1W_vs_E10	ordinary_muscle_development	ENSGALG00010002294	DAZL	2.663278474	0.80726427	3.29914078	0.000969813	0.001446982	positive
PRJNA729271	stage_1W_vs_E10	ordinary_muscle_development	ENSGALG00010002295	YAF2	0.321784947	0.114018872	2.822207769	0.004769427	0.006670479	positive
PRJNA729271	stage_1W_vs_E10	ordinary_muscle_development	ENSGALG00010002298	ENSGALG00010002298	-2.491212029	0.537724942	-4.632874233	3.60624E-06	6.46936E-06	negative
PRJNA729271	stage_1W_vs_E10	ordinary_muscle_development	ENSGALG00010002303	ENSGALG00010002303	2.971117758	0.781307509	3.802750804	0.000143098	0.00022761	positive
PRJNA729271	stage_1W_vs_E10	ordinary_muscle_development	ENSGALG00010002308	GXYLT1	-1.297704165	0.104827994	-12.37936655	3.38E-35	2.31318E-34	negative
PRJNA729271	stage_1W_vs_E10	ordinary_muscle_development	ENSGALG00010002309	GADL1	7.526786729	0.325458781	23.12669736	2.4951E-118	1.32E-116	positive
PRJNA729271	stage_1W_vs_E10	ordinary_muscle_development	ENSGALG00010002310	PRRG1	-0.48992649	0.10629186	-4.609256895	4.04111E-06	7.21111E-06	negative
PRJNA729271	stage_1W_vs_E10	ordinary_muscle_development	ENSGALG00010002312	ENSGALG00010002312	-0.052659616	0.191354989	-0.275193329	0.783167712	0.805736105	negative
PRJNA729271	stage_1W_vs_E10	ordinary_muscle_development	ENSGALG00010002315	ENSGALG00010002315	-0.82985838	0.181465559	-4.573090256	4.80583E-06	8.5316E-06	negative
PRJNA729271	stage_1W_vs_E10	ordinary_muscle_development	ENSGALG00010002324	SNTG2	-3.246658332	0.404495567	-8.026437355	1.00344E-15	3.08677E-15	negative
PRJNA729271	stage_1W_vs_E10	ordinary_muscle_development	ENSGALG00010002328	ENSGALG00010002328	-0.121267131	0.257940235	-0.470136545	0.638257466	0.670644579	negative
PRJNA729271	stage_1W_vs_E10	ordinary_muscle_development	ENSGALG00010002329	STT3B	-2.68839E-05	0.072552156	-0.000370546	0.999704347	0.99977332	negative
PRJNA729271	stage_1W_vs_E10	ordinary_muscle_development	ENSGALG00010002330	KHDRBS2	-2.297803693	0.294221134	-7.809784645	5.72858E-15	1.70016E-14	negative
PRJNA729271	stage_1W_vs_E10	ordinary_muscle_development	ENSGALG00010002331	ENSGALG00010002331	-3.480031914	0.37061561	-9.389868706	6.00751E-21	2.34904E-20	negative
PRJNA729271	stage_1W_vs_E10	ordinary_muscle_development	ENSGALG00010002332	ENSGALG00010002332	-2.262829409	0.624089325	-3.625810149	0.000288057	0.000448195	negative
PRJNA729271	stage_1W_vs_E10	ordinary_muscle_development	ENSGALG00010002333	ENSGALG00010002333	0.908216605	0.700340603	1.296821291	0.194692683	0.224461023	positive
PRJNA729271	stage_1W_vs_E10	ordinary_muscle_development	ENSGALG00010002334	OSBPL10	-0.95547693	0.169216126	-5.646488627	1.63758E-08	3.43016E-08	negative
PRJNA729271	stage_1W_vs_E10	ordinary_muscle_development	ENSGALG00010002335	TMEM47	0.384955762	0.134324781	2.865858103	0.004158806	0.0058441	positive
PRJNA729271	stage_1W_vs_E10	ordinary_muscle_development	ENSGALG00010002336	PRIM2	-2.21386974	0.125202106	-17.68236819	5.73365E-70	1.08215E-68	negative
PRJNA729271	stage_1W_vs_E10	ordinary_muscle_development	ENSGALG00010002338	CD99	-0.166980396	0.148385361	-1.125315831	0.260455224	0.294140901	negative
PRJNA729271	stage_1W_vs_E10	ordinary_muscle_development	ENSGALG00010002339	ZNFY4	0.000388291	0.312055348	0.001244301	0.999007192	0.999282951	positive
PRJNA729271	stage_1W_vs_E10	ordinary_muscle_development	ENSGALG00010002340	RAB23	-0.981995706	0.139026207	-7.063385582	1.62494E-12	4.2523E-12	negative
PRJNA729271	stage_1W_vs_E10	ordinary_muscle_development	ENSGALG00010002341	FAM49A	-0.678447007	0.111497809	-6.084846114	1.16603E-09	2.61514E-09	negative
PRJNA729271	stage_1W_vs_E10	ordinary_muscle_development	ENSGALG00010002342	BAG2	3.563102004	0.1232666	28.90565651	1.0137E-183	1.2889E-181	positive
PRJNA729271	stage_1W_vs_E10	ordinary_muscle_development	ENSGALG00010002343	GPD1L	-0.209052633	0.082069413	-2.547266101	0.010857061	0.014621676	negative
PRJNA729271	stage_1W_vs_E10	ordinary_muscle_development	ENSGALG00010002345	SCML2	-2.239496149	0.115029779	-19.46883822	2.01796E-84	5.32794E-83	negative
PRJNA729271	stage_1W_vs_E10	ordinary_muscle_development	ENSGALG00010002346	FBXO15	-0.822556015	0.374676102	-2.195378917	0.028136428	0.036152604	negative
PRJNA729271	stage_1W_vs_E10	ordinary_muscle_development	ENSGALG00010002347	ZNF451	-0.653763877	0.063397108	-10.31220347	6.20618E-25	2.84229E-24	negative
PRJNA729271	stage_1W_vs_E10	ordinary_muscle_development	ENSGALG00010002348	THAP12	-0.29513189	0.154247839	-1.913361589	0.055701778	0.069144239	negative
PRJNA729271	stage_1W_vs_E10	ordinary_muscle_development	ENSGALG00010002349	ZBED1	0.566114069	0.130002767	4.354630927	1.33291E-05	2.29052E-05	positive
PRJNA729271	stage_1W_vs_E10	ordinary_muscle_development	ENSGALG00010002350	BEND6	0.941230701	0.246847317	3.813007625	0.000137286	0.000218509	positive
PRJNA729271	stage_1W_vs_E10	ordinary_muscle_development	ENSGALG00010002352	ENSGALG00010002352	0.147107313	0.107024983	1.374513775	0.169282237	0.196977284	positive
PRJNA729271	stage_1W_vs_E10	ordinary_muscle_development	ENSGALG00010002353	CMTM8	0.573332807	0.13820993	4.148275081	3.3499E-05	5.59539E-05	positive
PRJNA729271	stage_1W_vs_E10	ordinary_muscle_development	ENSGALG00010002355	UFM1	-0.993690802	0.101360926	-9.803489741	1.08762E-22	4.53539E-22	negative
PRJNA729271	stage_1W_vs_E10	ordinary_muscle_development	ENSGALG00010002356	WNT11	-5.601709345	0.339904827	-16.48022887	5.08918E-61	7.66816E-60	negative
PRJNA729271	stage_1W_vs_E10	ordinary_muscle_development	ENSGALG00010002357	RAB31	-1.174201643	0.14433698	-8.135140692	4.1146E-16	1.28842E-15	negative
PRJNA729271	stage_1W_vs_E10	ordinary_muscle_development	ENSGALG00010002358	FREM2	-2.640611596	0.234425999	-11.26415845	1.97227E-29	1.087E-28	negative
PRJNA729271	stage_1W_vs_E10	ordinary_muscle_development	ENSGALG00010002359	CMTM6	-0.057639213	0.116344919	-0.495416676	0.620306026	0.654343632	negative
PRJNA729271	stage_1W_vs_E10	ordinary_muscle_development	ENSGALG00010002360	DHRSX	1.316569803	0.16292137	8.081013602	6.42306E-16	1.99448E-15	positive
PRJNA729271	stage_1W_vs_E10	ordinary_muscle_development	ENSGALG00010002361	ENSGALG00010002361	0.460149558	0.099190153	4.639064917	3.49989E-06	6.2848E-06	positive
PRJNA729271	stage_1W_vs_E10	ordinary_muscle_development	ENSGALG00010002362	TRPC4	-1.238442936	0.37327579	-3.317769242	0.000907394	0.001357981	negative
PRJNA729271	stage_1W_vs_E10	ordinary_muscle_development	ENSGALG00010002364	VSNL1	-1.181392306	0.399488053	-2.957265671	0.003103806	0.004416814	negative
PRJNA729271	stage_1W_vs_E10	ordinary_muscle_development	ENSGALG00010002365	RAI2	1.318690765	0.13826747	9.537245195	1.46677E-21	5.87802E-21	positive
PRJNA729271	stage_1W_vs_E10	ordinary_muscle_development	ENSGALG00010002366	ENSGALG00010002366	3.684079869	0.756704177	4.86858667	1.12399E-06	2.09439E-06	positive
PRJNA729271	stage_1W_vs_E10	ordinary_muscle_development	ENSGALG00010002367	STK31	-7.514564191	1.023871724	-7.339360989	2.14616E-13	5.85849E-13	negative
PRJNA729271	stage_1W_vs_E10	ordinary_muscle_development	ENSGALG00010002368	UVRAG	1.018476803	0.095280846	10.68920826	1.14343E-26	5.6451E-26	positive
PRJNA729271	stage_1W_vs_E10	ordinary_muscle_development	ENSGALG00010002369	GEMIN8	-0.61240995	0.121542944	-5.038630234	4.68875E-07	8.95907E-07	negative
PRJNA729271	stage_1W_vs_E10	ordinary_muscle_development	ENSGALG00010002370	ENSGALG00010002370	-0.23692196	0.158891337	-1.491094256	0.13593675	0.160456286	negative
PRJNA729271	stage_1W_vs_E10	ordinary_muscle_development	ENSGALG00010002371	BAG3	3.022904512	0.154603787	19.55259035	3.92097E-85	1.06432E-83	positive
PRJNA729271	stage_1W_vs_E10	ordinary_muscle_development	ENSGALG00010002372	SMC6	-0.457016729	0.127610589	-3.581338604	0.000341838	0.000528753	negative
PRJNA729271	stage_1W_vs_E10	ordinary_muscle_development	ENSGALG00010002373	POSTN	-0.27589248	0.255620421	-1.079305316	0.280451644	0.314883546	negative
PRJNA729271	stage_1W_vs_E10	ordinary_muscle_development	ENSGALG00010002374	DST	1.31155236	0.114211151	11.48357536	1.59544E-30	9.18787E-30	positive
PRJNA729271	stage_1W_vs_E10	ordinary_muscle_development	ENSGALG00010002375	NPY	-4.224874906	0.544976487	-7.752398509	9.01729E-15	2.65015E-14	negative
PRJNA729271	stage_1W_vs_E10	ordinary_muscle_development	ENSGALG00010002376	GEN1	-1.764985889	0.194059832	-9.095060384	9.45339E-20	3.51531E-19	negative
PRJNA729271	stage_1W_vs_E10	ordinary_muscle_development	ENSGALG00010002377	ART1A	5.703075842	0.242295235	23.53771355	1.6773E-122	9.7643E-121	positive
PRJNA729271	stage_1W_vs_E10	ordinary_muscle_development	ENSGALG00010002378	ENSGALG00010002378	0.79681034	0.447527414	1.780472692	0.074998635	0.091561123	positive
PRJNA729271	stage_1W_vs_E10	ordinary_muscle_development	ENSGALG00010002379	NHS	-2.170426238	0.139992262	-15.50390144	3.26452E-54	4.1363E-53	negative
PRJNA729271	stage_1W_vs_E10	ordinary_muscle_development	ENSGALG00010002380	CMPK2	3.500430414	0.74220961	4.7162289	2.40256E-06	4.36241E-06	positive
PRJNA729271	stage_1W_vs_E10	ordinary_muscle_development	ENSGALG00010002381	TDRD3	0.882214867	0.096254784	9.165413185	4.93575E-20	1.85635E-19	positive
PRJNA729271	stage_1W_vs_E10	ordinary_muscle_development	ENSGALG00010002382	ART1D	2.32247586	0.532336297	4.362798239	1.28409E-05	2.21004E-05	positive
PRJNA729271	stage_1W_vs_E10	ordinary_muscle_development	ENSGALG00010002383	INPP5F	0.727380011	0.124051112	5.863550905	4.53072E-09	9.81509E-09	positive
PRJNA729271	stage_1W_vs_E10	ordinary_muscle_development	ENSGALG00010002384	ENSGALG00010002384	-1.234879867	0.310591339	-3.975899232	7.01138E-05	0.000114152	negative
PRJNA729271	stage_1W_vs_E10	ordinary_muscle_development	ENSGALG00010002385	PALS2	-2.21011496	0.127255844	-17.36749278	1.45444E-67	2.58359E-66	negative
PRJNA729271	stage_1W_vs_E10	ordinary_muscle_development	ENSGALG00010002386	MHCY2B2	-1.528209453	0.430995194	-3.545769129	0.000391469	0.000602308	negative
PRJNA729271	stage_1W_vs_E10	ordinary_muscle_development	ENSGALG00010002387	MCMBP	-0.038804055	0.108927544	-0.356237308	0.721662854	0.748676143	negative
PRJNA729271	stage_1W_vs_E10	ordinary_muscle_development	ENSGALG00010002388	ENSGALG00010002388	3.779209488	0.729243526	5.182369611	2.19085E-07	4.28098E-07	positive
PRJNA729271	stage_1W_vs_E10	ordinary_muscle_development	ENSGALG00010002389	COL21A1	-1.812405565	0.323725007	-5.598596106	2.16095E-08	4.49267E-08	negative
PRJNA729271	stage_1W_vs_E10	ordinary_muscle_development	ENSGALG00010002391	REPS2	-0.701897107	0.162165147	-4.328285825	1.50274E-05	2.56957E-05	negative
PRJNA729271	stage_1W_vs_E10	ordinary_muscle_development	ENSGALG00010002392	DFNA5	-2.056247412	0.209455019	-9.817131258	9.50088E-23	3.97561E-22	negative
PRJNA729271	stage_1W_vs_E10	ordinary_muscle_development	ENSGALG00010002393	GPM6B	-1.054123855	0.107402283	-9.814724844	9.73031E-23	4.06692E-22	negative
PRJNA729271	stage_1W_vs_E10	ordinary_muscle_development	ENSGALG00010002395	CLN8	-0.20049933	0.091618149	-2.188423713	0.028638753	0.036771966	negative
PRJNA729271	stage_1W_vs_E10	ordinary_muscle_development	ENSGALG00010002396	SEC23IP	0.007478955	0.088349204	0.084652204	0.932537895	0.94110818	positive
PRJNA729271	stage_1W_vs_E10	ordinary_muscle_development	ENSGALG00010002398	ENSGALG00010002398	-2.570645282	0.441933225	-5.816818329	5.99783E-09	1.29104E-08	negative
PRJNA729271	stage_1W_vs_E10	ordinary_muscle_development	ENSGALG00010002399	CSMD1	-1.210431281	0.393723244	-3.074320098	0.002109829	0.003049572	negative
PRJNA729271	stage_1W_vs_E10	ordinary_muscle_development	ENSGALG00010002400	RBBP7	-2.564182859	0.163883432	-15.64638249	3.51649E-55	4.56734E-54	negative
PRJNA729271	stage_1W_vs_E10	ordinary_muscle_development	ENSGALG00010002401	ZRANB1	0.412760853	0.161505017	2.555715354	0.010596977	0.014287338	positive
PRJNA729271	stage_1W_vs_E10	ordinary_muscle_development	ENSGALG00010002402	TXLNG	0.250468975	0.126381372	1.981850417	0.047495985	0.059462282	positive
PRJNA729271	stage_1W_vs_E10	ordinary_muscle_development	ENSGALG00010002403	ZNFY3	0.330749994	0.262598296	1.259528332	0.207839563	0.238473401	positive
PRJNA729271	stage_1W_vs_E10	ordinary_muscle_development	ENSGALG00010002404	OSBPL3	-1.049821928	0.255469394	-4.109384337	3.96715E-05	6.57864E-05	negative
PRJNA729271	stage_1W_vs_E10	ordinary_muscle_development	ENSGALG00010002405	KCNQ5	1.416568196	0.197102085	7.186977229	6.62415E-13	1.76794E-12	positive
PRJNA729271	stage_1W_vs_E10	ordinary_muscle_development	ENSGALG00010002406	DIAPH3	-1.750289779	0.248590266	-7.040862092	1.91054E-12	4.98081E-12	negative
PRJNA729271	stage_1W_vs_E10	ordinary_muscle_development	ENSGALG00010002408	OFD1	-0.192356661	0.11609967	-1.656823501	0.097555164	0.117505576	negative
PRJNA729271	stage_1W_vs_E10	ordinary_muscle_development	ENSGALG00010002409	CYCS	1.769824073	0.166703409	10.61660398	2.49472E-26	1.21591E-25	positive
PRJNA729271	stage_1W_vs_E10	ordinary_muscle_development	ENSGALG00010002410	RNF121	0.560390266	0.104251007	5.375394254	7.64153E-08	1.54096E-07	positive
PRJNA729271	stage_1W_vs_E10	ordinary_muscle_development	ENSGALG00010002411	ENSGALG00010002411	-1.4667243	0.19273711	-7.609973495	2.74152E-14	7.8612E-14	negative

PRJNA729271	stage_1W_vs_E10	ordinary_muscle_development	ENSGALG00010002413	TSSC1	-0.821964406	0.086278727	-9.526849011	1.6213E-21	6.48116E-21	negative
PRJNA729271	stage_1W_vs_E10	ordinary_muscle_development	ENSGALG00010002414	ENSGALG00010002414	1.34049728	0.360864083	3.71468745	0.000203455	0.000319822	positive
PRJNA729271	stage_1W_vs_E10	ordinary_muscle_development	ENSGALG00010002415	PCDH17	-5.150154475	0.440417373	-11.69380408	1.37105E-31	8.26679E-31	negative
PRJNA729271	stage_1W_vs_E10	ordinary_muscle_development	ENSGALG00010002416	SH3BGRL2	-1.273427693	0.277285534	-4.592477924	4.38014E-06	7.80073E-06	negative
PRJNA729271	stage_1W_vs_E10	ordinary_muscle_development	ENSGALG00010002418	TRAPPC2	-0.237496695	0.083504027	-2.844134622	0.004453223	0.006244507	negative
PRJNA729271	stage_1W_vs_E10	ordinary_muscle_development	ENSGALG00010002419	CTBP2	-1.626669028	0.116139506	-14.00616455	1.42918E-44	1.32033E-43	negative
PRJNA729271	stage_1W_vs_E10	ordinary_muscle_development	ENSGALG00010002420	TMEM272	-4.047197869	1.053600703	-3.841301413	0.000122384	0.000195498	negative
PRJNA729271	stage_1W_vs_E10	ordinary_muscle_development	ENSGALG00010002421	ENSGALG00010002421	-0.389081066	0.191230661	-2.034616547	0.041889471	0.052798946	negative
PRJNA729271	stage_1W_vs_E10	ordinary_muscle_development	ENSGALG00010002422	TRAPPC12	0.347763367	0.103185369	3.370277886	0.000750924	0.001131225	positive
PRJNA729271	stage_1W_vs_E10	ordinary_muscle_development	ENSGALG00010002423	ENSGALG00010002423	2.217090603	0.568601354	3.899200358	9.65109E-05	0.000155419	positive
PRJNA729271	stage_1W_vs_E10	ordinary_muscle_development	ENSGALG00010002424	CASK	-0.377755952	0.057073714	-6.618737913	3.62278E-11	8.84343E-11	negative
PRJNA729271	stage_1W_vs_E10	ordinary_muscle_development	ENSGALG00010002425	FDX2	0.673747111	0.133527593	5.04575193	4.51741E-07	8.63965E-07	positive
PRJNA729271	stage_1W_vs_E10	ordinary_muscle_development	ENSGALG00010002427	CYB5R4	-0.19957937	0.113866675	-1.752746091	0.0796456	0.096940379	negative
PRJNA729271	stage_1W_vs_E10	ordinary_muscle_development	ENSGALG00010002428	IBSP	-4.539032475	0.763030875	-5.948687824	2.70301E-09	5.93907E-09	negative
PRJNA729271	stage_1W_vs_E10	ordinary_muscle_development	ENSGALG00010002429	ENSGALG00010002429	-0.077751329	0.550631744	-0.141203863	0.887708895	0.90031769	negative
PRJNA729271	stage_1W_vs_E10	ordinary_muscle_development	ENSGALG00010002430	NUMA1	1.535295632	0.174215282	8.812634646	1.22238E-18	4.2933E-18	positive
PRJNA729271	stage_1W_vs_E10	ordinary_muscle_development	ENSGALG00010002431	BCKDHB	1.458702843	0.147344777	9.899929089	4.1657E-23	1.76969E-22	positive
PRJNA729271	stage_1W_vs_E10	ordinary_muscle_development	ENSGALG00010002432	RAVER1	-0.398109091	0.179322715	-2.220070619	0.026413974	0.034045043	negative
PRJNA729271	stage_1W_vs_E10	ordinary_muscle_development	ENSGALG00010002433	GRHL1	-1.272093636	0.326546948	-3.895591868	9.79592E-05	0.000157681	negative
PRJNA729271	stage_1W_vs_E10	ordinary_muscle_development	ENSGALG00010002434	GPR34	2.182939484	0.247073495	8.835182764	9.99347E-19	3.53048E-18	positive
PRJNA729271	stage_1W_vs_E10	ordinary_muscle_development	ENSGALG00010002435	ATP7B	-1.753965187	0.680515129	-2.577408074	0.009954436	0.013461101	negative
PRJNA729271	stage_1W_vs_E10	ordinary_muscle_development	ENSGALG00010002436	ENSGALG00010002436	3.767602514	0.874181504	4.309862994	1.63356E-05	2.78602E-05	positive
PRJNA729271	stage_1W_vs_E10	ordinary_muscle_development	ENSGALG00010002437	TTK	-2.852591608	0.198249719	-14.38888094	6.07713E-47	6.01694E-46	negative
PRJNA729271	stage_1W_vs_E10	ordinary_muscle_development	ENSGALG00010002438	RARB	-1.976066804	0.095419076	-20.7093474	2.85295E-95	9.66204E-94	negative
PRJNA729271	stage_1W_vs_E10	ordinary_muscle_development	ENSGALG00010002439	GPR82	1.905536702	0.964910494	1.974832602	0.048287148	0.060384972	positive
PRJNA729271	stage_1W_vs_E10	ordinary_muscle_development	ENSGALG00010002440	LRRC51	0.73641875	0.26672865	2.760928569	0.005763728	0.007991701	positive
PRJNA729271	stage_1W_vs_E10	ordinary_muscle_development	ENSGALG00010002441	USO1	-0.331564605	0.068181139	-4.86299597	1.15622E-06	2.15279E-06	negative
PRJNA729271	stage_1W_vs_E10	ordinary_muscle_development	ENSGALG00010002442	ENSGALG00010002442	-0.252337644	0.469572101	-0.537377845	0.591006644	0.62580417	negative
PRJNA729271	stage_1W_vs_E10	ordinary_muscle_development	ENSGALG00010002443	TYK2	0.524589518	0.137986471	3.801746038	0.00014368	0.000228485	positive
PRJNA729271	stage_1W_vs_E10	ordinary_muscle_development	ENSGALG00010002444	KLF11	1.503932344	0.180927833	8.312332722	9.38389E-17	3.01395E-16	positive
PRJNA729271	stage_1W_vs_E10	ordinary_muscle_development	ENSGALG00010002445	EDRF1	1.034812416	0.11099385	9.323150941	1.12936E-20	4.37818E-20	positive
PRJNA729271	stage_1W_vs_E10	ordinary_muscle_development	ENSGALG00010002446	RIMS1	-5.404691314	0.354641908	-15.23985516	1.92296E-52	2.30358E-51	negative
PRJNA729271	stage_1W_vs_E10	ordinary_muscle_development	ENSGALG00010002447	YLEC12	-0.184288895	0.569785392	-0.323435625	0.746365353	0.771555603	negative
PRJNA729271	stage_1W_vs_E10	ordinary_muscle_development	ENSGALG00010002448	RAB9A	0.487501796	0.135827613	3.589121426	0.000331794	0.000513875	positive
PRJNA729271	stage_1W_vs_E10	ordinary_muscle_development	ENSGALG00010002449	ALG11	-0.85121825	0.14375021	-5.921509623	3.19E-09	6.97526E-09	negative
PRJNA729271	stage_1W_vs_E10	ordinary_muscle_development	ENSGALG00010002453	TOP2B	-0.999609763	0.063405562	-15.76533253	5.3888E-56	7.13991E-55	negative
PRJNA729271	stage_1W_vs_E10	ordinary_muscle_development	ENSGALG00010002454	NEK5	-3.537309635	0.736477852	-4.80300884	1.56299E-06	2.87689E-06	negative
PRJNA729271	stage_1W_vs_E10	ordinary_muscle_development	ENSGALG00010002455	NYX	2.474404765	0.352985497	7.009933222	2.38432E-12	6.19367E-12	positive
PRJNA729271	stage_1W_vs_E10	ordinary_muscle_development	ENSGALG00010002456	ELOVL4	-3.684872971	0.671147701	-5.490405413	4.01012E-08	8.22044E-08	negative
PRJNA729271	stage_1W_vs_E10	ordinary_muscle_development	ENSGALG00010002457	ENSGALG00010002457	1.460235285	0.442870865	3.297203319	0.000976528	0.001456393	positive
PRJNA729271	stage_1W_vs_E10	ordinary_muscle_development	ENSGALG00010002458	ENSGALG00010002458	-0.56666361	0.198507128	-2.854626007	0.004308754	0.00604719	negative
PRJNA729271	stage_1W_vs_E10	ordinary_muscle_development	ENSGALG00010002459	G3BP2	-0.833613237	0.074703692	-11.15892954	6.47667E-29	3.48476E-28	negative
PRJNA729271	stage_1W_vs_E10	ordinary_muscle_development	ENSGALG00010002460	LAMTOR1	1.350661579	0.180228337	7.494168814	6.67198E-14	1.87751E-13	positive
PRJNA729271	stage_1W_vs_E10	ordinary_muscle_development	ENSGALG00010002461	TOMT	0.543059323	0.323736771	1.677471857	0.093450248	0.112861302	positive
PRJNA729271	stage_1W_vs_E10	ordinary_muscle_development	ENSGALG00010002462	RRM2	-3.51394633	0.276997911	-12.68582249	7.0866E-37	5.09778E-36	negative
PRJNA729271	stage_1W_vs_E10	ordinary_muscle_development	ENSGALG00010002463	ASRGL1	0.407579214	0.101687857	4.008140467	6.11987E-05	0.000100121	positive
PRJNA729271	stage_1W_vs_E10	ordinary_muscle_development	ENSGALG00010002464	NGLY1	0.937294657	0.082831508	11.31567779	1.09751E-29	6.12332E-29	positive
PRJNA729271	stage_1W_vs_E10	ordinary_muscle_development	ENSGALG00010002466	UROS	0.374594533	0.178233917	2.101701739	0.035579411	0.045199261	positive
PRJNA729271	stage_1W_vs_E10	ordinary_muscle_development	ENSGALG00010002467	NEK3	-1.527132662	0.225208675	-6.780967298	1.19374E-11	2.9895E-11	negative
PRJNA729271	stage_1W_vs_E10	ordinary_muscle_development	ENSGALG00010002468	ENSGALG00010002468	-0.226802208	0.567266218	-0.399816172	0.689291919	0.718229197	negative
PRJNA729271	stage_1W_vs_E10	ordinary_muscle_development	ENSGALG00010002469	MEPE	-4.57011088	1.384328532	-3.301319574	0.000962312	0.001436383	negative
PRJNA729271	stage_1W_vs_E10	ordinary_muscle_development	ENSGALG00010002470	OXSM	0.695604915	0.159884781	4.350663725	1.35726E-05	2.33043E-05	positive
PRJNA729271	stage_1W_vs_E10	ordinary_muscle_development	ENSGALG00010002471	OGFRL1	-0.468330889	0.154423942	-3.032760867	0.002423275	0.003482242	negative
PRJNA729271	stage_1W_vs_E10	ordinary_muscle_development	ENSGALG00010002472	CDC37	0.385861914	0.214370952	1.799972951	0.071864909	0.087935325	positive
PRJNA729271	stage_1W_vs_E10	ordinary_muscle_development	ENSGALG00010002473	ENSGALG00010002473	1.478685286	0.634127308	2.33184294	0.019708955	0.025776532	positive
PRJNA729271	stage_1W_vs_E10	ordinary_muscle_development	ENSGALG00010002474	HPCAL1	-0.391129699	0.142539833	-2.744002787	0.006069502	0.008396396	negative
PRJNA729271	stage_1W_vs_E10	ordinary_muscle_development	ENSGALG00010002475	ENSGALG00010002475	-0.551422246	0.203050525	-2.715689832	0.006613785	0.009116281	negative
PRJNA729271	stage_1W_vs_E10	ordinary_muscle_development	ENSGALG00010002476	DDX3X	0.726626242	0.144960471	5.012581962	5.37045E-07	1.02145E-06	positive
PRJNA729271	stage_1W_vs_E10	ordinary_muscle_development	ENSGALG00010002478	PPME1	-1.349087183	0.058323439	-23.13113222	2.2514E-118	1.1954E-116	negative
PRJNA729271	stage_1W_vs_E10	ordinary_muscle_development	ENSGALG00010002481	CKAP2	-2.691777245	0.246333025	-10.92739088	8.52648E-28	4.39983E-27	negative
PRJNA729271	stage_1W_vs_E10	ordinary_muscle_development	ENSGALG00010002483	COQ2	1.352512308	0.137831234	9.812814313	9.91636E-23	4.14229E-22	positive
PRJNA729271	stage_1W_vs_E10	ordinary_muscle_development	ENSGALG00010002487	BCCIP	-0.596295977	0.118912676	-5.014570343	5.31521E-07	1.01147E-06	negative
PRJNA729271	stage_1W_vs_E10	ordinary_muscle_development	ENSGALG00010002492	RPL8	0.818498909	0.129560381	6.317509279	2.65812E-10	6.15881E-10	positive
PRJNA729271	stage_1W_vs_E10	ordinary_muscle_development	ENSGALG00010002500	OSR1	-3.481557453	0.207483076	-16.77995876	3.42074E-63	5.40126E-62	negative
PRJNA729271	stage_1W_vs_E10	ordinary_muscle_development	ENSGALG00010002501	TCEANC	0.502148528	0.093589406	5.365441991	8.07512E-08	1.62545E-07	positive
PRJNA729271	stage_1W_vs_E10	ordinary_muscle_development	ENSGALG00010002504	ENSGALG00010002504	0.739301227	0.065622963	11.26589222	1.93383E-29	1.06703E-28	positive
PRJNA729271	stage_1W_vs_E10	ordinary_muscle_development	ENSGALG00010002507	C2CD3	-0.662837267	0.112259371	-5.904516131	3.53683E-09	7.71155E-09	negative
PRJNA729271	stage_1W_vs_E10	ordinary_muscle_development	ENSGALG00010002511	VPS36	-0.243950518	0.077649358	-3.141693954	0.001679735	0.002452434	negative
PRJNA729271	stage_1W_vs_E10	ordinary_muscle_development	ENSGALG00010002512	DAXX	1.288362244	0.228246789	5.644601841	1.65564E-08	3.46699E-08	positive
PRJNA729271	stage_1W_vs_E10	ordinary_muscle_development	ENSGALG00010002518	MAST1	0.879356475	0.352361574	2.495608321	0.012574136	0.016809195	positive
PRJNA729271	stage_1W_vs_E10	ordinary_muscle_development	ENSGALG00010002520	TTC32	0.95522187	0.16369714	5.83529968	5.3694E-09	1.15835E-08	positive
PRJNA729271	stage_1W_vs_E10	ordinary_muscle_development	ENSGALG00010002522	HPSE	0.845728924	0.299115991	2.827427987	0.004692357	0.006567125	positive
PRJNA729271	stage_1W_vs_E10	ordinary_muscle_development	ENSGALG00010002523	DNAJB13	5.356087894	0.414701892	12.91551353	3.67981E-38	2.76224E-37	positive
PRJNA729271	stage_1W_vs_E10	ordinary_muscle_development	ENSGALG00010002526	ZBTB22	-0.174600785	0.108219807	-1.613390283	0.106659743	0.127739649	negative
PRJNA729271	stage_1W_vs_E10	ordinary_muscle_development	ENSGALG00010002527	LRRC3B	-1.129605285	0.174447762	-6.475321166	9.46105E-11	2.25038E-10	negative
PRJNA729271	stage_1W_vs_E10	ordinary_muscle_development	ENSGALG00010002528	MED14	-0.112309792	0.116831594	-0.961296413	0.336403151	0.373390395	negative
PRJNA729271	stage_1W_vs_E10	ordinary_muscle_development	ENSGALG00010002530	STAG3	-0.594336088	0.123261462	-4.821751078	1.42303E-06	2.6283E-06	negative
PRJNA729271	stage_1W_vs_E10	ordinary_muscle_development	ENSGALG00010002533	LPAR2	-1.265423964	0.148614225	-8.51482396	1.66844E-17	5.5417E-17	negative
PRJNA729271	stage_1W_vs_E10	ordinary_muscle_development	ENSGALG00010002536	RGL2	2.226503501	0.139668372	15.94135783	3.27167E-57	4.46543E-56	positive
PRJNA729271	stage_1W_vs_E10	ordinary_muscle_development	ENSGALG00010002539	SNAP91	-1.074745923	0.20961284	-5.127290509	2.93942E-07	5.68926E-07	negative
PRJNA729271	stage_1W_vs_E10	ordinary_muscle_development	ENSGALG00010002543	UCP3	11.45760752	0.502862765	22.78476023	6.4937E-115	3.1907E-113	positive
PRJNA729271	stage_1W_vs_E10	ordinary_muscle_development	ENSGALG00010002544	THSD1	0.298776211	0.126681431	2.358484647	0.018349719	0.024057451	positive
PRJNA729271	stage_1W_vs_E10	ordinary_muscle_development	ENSGALG00010002546	ATP6AP2	0.602034526	0.06313003	9.536420652	1.47847E-21	5.92329E-21	positive
PRJNA729271	stage_1W_vs_E10	ordinary_muscle_development	ENSGALG00010002547	PBX4	-1.097133936	0.107315557	-10.22343789	1.55718E-24	7.02501E-24	negative
PRJNA729271	stage_1W_vs_E10	ordinary_muscle_development	ENSGALG00010002551	WDR35	-1.271543934	0.113187274	-11.23398323	2.77672E-29	1.51939E-28	negative
PRJNA729271	stage_1W_vs_E10	ordinary_muscle_development	ENSGALG00010002553	ENSGALG00010002553	0.034272827	0.230162642	0.148906995	0.881627022	0.894899322	positive
PRJNA729271	stage_1W_vs_E10	ordinary_muscle_development	ENSGALG00010002554	EGFL6	-3.343854307	0.293381695	-11.3976242	4.29685E-30	2.43769E-29	negative
PRJNA729271	stage_1W_vs_E10	ordinary_muscle_development	ENSGALG00010002556	ENSGALG00010002556	3.854180068	0.230960487	16.68761666	1.61282E-62	2.50298E-61	positive
PRJNA729271	stage_1W_vs_E10	ordinary_muscle_development	ENSGALG00010002557	ENSGALG00010002557	0.561075159	0.409313148	1.370772381	0.170445923	0.198207903	positive
PRJNA729271	stage_1W_vs_E10	ordinary_muscle_development	ENSGALG00010002559	NEK10	-1.269801004	0.575781827	-2.205350958	0.027429477	0.035294298	negative
PRJNA729271	stage_1W_vs_E10	ordinary_muscle_development	ENSGALG00010002561	PAAF1	-0.643009839	0.084990227	-7.565691515	3.85807E-14	1.0976E-13	negative
PRJNA729271	stage_1W_vs_E10	ordinary_muscle_development	ENSGALG00010002565	MRPS18C	1.018241881	0.114032162	8.929427143	4.2822E-19	1.54558E-18	positive
PRJNA729271	stage_1W_vs_E10	ordinary_muscle_development	ENSGALG00010002566	TMSB4X	-1.295413514	0.115768154	-11.18972249	4.57864E-29	2.48381E-28	negative
PRJNA729271	stage_1W_vs_E10	ordinary_muscle_development	ENSGALG00010002567	COA4	-0.001171905	0.237101599	-0.004942628	0.996056369	0.996812833	negative
PRJNA729271	stage_1W_vs_E10	ordinary_muscle_development	ENSGALG00010002569	GTPBP6	0.376465502	0.116143737	3.241375828	0.001189542	0.001757637	positive
PRJNA729271	stage_1W_vs_E10	ordinary_muscle_development	ENSGALG00010002570	ADAM12	-3.279260056	0.27206987	-12.05300703	1.87001E-33	1.20043E-32	negative
PRJNA729271	stage_1W_vs_E10	ordinary_muscle_development	ENSGALG00010002571	BCOR	0.783484099	0.140930814	5.559352679	2.70777E-08	5.59663E-08	positive
PRJNA729271	stage_1W_vs_E10	ordinary_muscle_development	ENSGALG00010002573	MVB12A	1.221072049	0.094683144	12.89640374	4.71597E-38	3.52542E-37	positive
PRJNA729271	stage_1W_vs_E10	ordinary_muscle_development	ENSGALG00010002575	BGV	0.729549818	0.399424392	1.826502919	0.067774542	0.08323945	positive
PRJNA729271	stage_1W_vs_E10	ordinary_muscle_development	ENSGALG00010002576	HELQ	-0.407385102	0.108025253	-3.771202474	0.000162463	0.00025731	negative
PRJNA729271	stage_1W_vs_E10	ordinary_muscle_development	ENSGALG00010002577	MRPL48	0.488787119	0.102738221	4.757597638	1.95911E-06	3.58279E-06	positive
PRJNA729271	stage_1W_vs_E10	ordinary_muscle_development	ENSGALG00010002579	MATN3	-0.752199304	0.571511864	-1.316156935	0.188121374	0.217415031	negative
PRJNA729271	stage_1W_vs_E10	ordinary_muscle_development	ENSGALG00010002581	ATP13A1	0.225009931	0.077024148	2.921290726	0.003485844	0.004937683	positive
PRJNA729271	stage_1W_vs_E10	ordinary_muscle_development	ENSGALG00010002582	ENSGALG00010002582	-2.766119122	0.468091188	-5.90935953	3.4344E-09	7.49498E-09	negative
PRJNA729271	stage_1W_vs_E10	ordinary_muscle_development	ENSGALG00010002584	ENSGALG00010002584	1.047413466	0.440708067	2.376660526	0.017470158	0.022964536	positive
PRJNA729271	stage_1W_vs_E10	ordinary_muscle_development	ENSGALG00010002586	GPR63	-0.601181608	0.148320415	-4.053262714	5.05082E-05	8.30535E-05	negative
PRJNA729271	stage_1W_vs_E10	ordinary_muscle_development	ENSGALG00010002587	DOCK1	-0.594677936	0.066990016	-8.87711288	6.86182E-19	2.44597E-18	negative
PRJNA729271	stage_1W_vs_E10	ordinary_muscle_development	ENSGALG00010002588	FAM175A	-1.295379814	0.09272409	-13.97026181	2.36755E-44	2.172E-43	negative
PRJNA729271	stage_1W_vs_E10	ordinary_muscle_development	ENSGALG00010002589	SLC4A7	-0.172709955	0.145079523	-1.190450254	0.233869471	0.265773264	negative
PRJNA729271	stage_1W_vs_E10	ordinary_muscle_development	ENSGALG00010002590	ATP6V1C2	-1.770539344	0.263811901	-6.711370256	1.92805E-11	4.77647E-11	negative
PRJNA729271	stage_1W_vs_E10	ordinary_muscle_development	ENSGALG00010002591	ENSGALG00010002591	0.242169593	0.216240254	1.119909858	0.262752177	0.296527001	positive
PRJNA729271	stage_1W_vs_E10	ordinary_muscle_development	ENSGALG00010002592	RPS6KA3	1.497671757	0.100972062	14.8325361	9.02575E-50	9.81457E-49	positive
PRJNA729271	stage_1W_vs_E10	ordinary_muscle_development	ENSGALG00010002597	ENSGALG00010002597	0.002615637	0.116267028	0.022496811	0.982051656	0.984769198	positive
PRJNA729271	stage_1W_vs_E10	ordinary_muscle_development	ENSGALG00010002598	NMT2	0.373375219	0.102207873	3.653096458	0.000259097	0.000404656	positive
PRJNA729271	stage_1W_vs_E10	ordinary_muscle_development	ENSGALG00010002599	KIFC1	-1.487666973	0.22002946	-6.761217214	1.36837E-11	3.4121E-11	negative
PRJNA729271	stage_1W_vs_E10	ordinary_muscle_development	ENSGALG00010002600	TRPA1	-2.648415069	0.621870448	-4.258789071	2.05537E-05	3.47679E-05	negative
PRJNA729271	stage_1W_vs_E10	ordinary_muscle_development	ENSGALG00010002601	PLCXD1	1.090485053	0.189599503	5.751518519	8.84454E-09	1.88144E-08	positive
PRJNA729271	stage_1W_vs_E10	ordinary_muscle_development	ENSGALG00010002603	INSYN2A	0.327540654	0.256457176	1.277174845	0.201540526	0.231888389	positive
PRJNA729271	stage_1W_vs_E10	ordinary_muscle_development	ENSGALG00010002604	BLEC3	0.067889186	0.390194945	0.173987866	0.861874998	0.877124068	positive
PRJNA729271	stage_1W_vs_E10	ordinary_muscle_development	ENSGALG00010002605	EOMES	1.213497772	0.73790288	1.644522341	0.100068353	0.120332735	positive
PRJNA729271	stage_1W_vs_E10	ordinary_muscle_development	ENSGALG00010002608	RPP38	-0.615110922	0.132526673	-4.641412236	3.46036E-06	6.21613E-06	negative
PRJNA729271	stage_1W_vs_E10	ordinary_muscle_development	ENSGALG00010002609	PDIA6	-1.882555486	0.125888716	-14.9541241	1.46395E-50	1.63861E-49	negative
PRJNA729271	stage_1W_vs_E10	ordinary_muscle_development	ENSGALG00010002611	NPS	0.180952216	0.396129987	0.456800096	0.647814737	0.67940631	positive
PRJNA729271	stage_1W_vs_E10	ordinary_muscle_development	ENSGALG00010002612	JPH1	4.807690353	0.21432595	22.43167636	1.9322E-111	8.9479E-110	positive
PRJNA729271	stage_1W_vs_E10	ordinary_muscle_development	ENSGALG00010002613	ENSGALG00010002613	0.09749286	0.104359899	0.934198487	0.350201516	0.387701136	positive
PRJNA729271	stage_1W_vs_E10	ordinary_muscle_development	ENSGALG00010002614	GPAT3	0.323054872	0.14509519	2.226502983	0.025980509	0.033522119	positive
PRJNA729271	stage_1W_vs_E10	ordinary_muscle_development	ENSGALG00010002616	ZNFB3	-0.206867947	0.167368649	-1.236001772	0.216457905	0.247636727	negative
PRJNA729271	stage_1W_vs_E10	ordinary_muscle_development	ENSGALG00010002619	RAB6A	-0.882812647	0.068976998	-12.7986527	1.6682E-37	1.22495E-36	negative
PRJNA729271	stage_1W_vs_E10	ordinary_muscle_development	ENSGALG00010002621	SLC25A15	-0.863839716	0.163846644	-5.272245397	1.34765E-07	2.6686E-07	negative
PRJNA729271	stage_1W_vs_E10	ordinary_muscle_development	ENSGALG00010002622	AZI2	0.105516561	0.080870808	1.304754628	0.191976413	0.221570038	positive
PRJNA729271	stage_1W_vs_E10	ordinary_muscle_development	ENSGALG00010002623	MID1IP1	2.536278895	0.135388869	18.73328969	2.65022E-78	6.04009E-77	positive

PRJNA729271	stage_1W_vs_E10	ordinary_muscle_development	ENSGALG00010002625	FHL5	0.416173676	0.219615031	1.895014533	0.058090476	0.071955346	positive
PRJNA729271	stage_1W_vs_E10	ordinary_muscle_development	ENSGALG00010002626	CHD7	0.802404827	0.135743229	5.911195967	3.39633E-09	7.41524E-09	positive
PRJNA729271	stage_1W_vs_E10	ordinary_muscle_development	ENSGALG00010002627	MRPS31	1.23228361	0.14616103	8.430999742	3.42725E-17	1.11988E-16	positive
PRJNA729271	stage_1W_vs_E10	ordinary_muscle_development	ENSGALG00010002628	TRIM7.2	0.299552107	0.191806099	1.561744436	0.118348206	0.140842139	positive
PRJNA729271	stage_1W_vs_E10	ordinary_muscle_development	ENSGALG00010002632	HPGD	2.916376588	0.272388612	10.70667588	9.47014E-27	4.67858E-26	positive
PRJNA729271	stage_1W_vs_E10	ordinary_muscle_development	ENSGALG00010002633	ENSGALG00010002633	-1.240560316	0.134345305	-9.234117362	2.60424E-20	9.91034E-20	negative
PRJNA729271	stage_1W_vs_E10	ordinary_muscle_development	ENSGALG00010002634	RGN	1.330897813	0.182907477	7.276344481	3.42987E-13	9.27364E-13	positive
PRJNA729271	stage_1W_vs_E10	ordinary_muscle_development	ENSGALG00010002638	FABP9	5.694532104	0.498388891	11.42588089	3.10491E-30	1.7677E-29	positive
PRJNA729271	stage_1W_vs_E10	ordinary_muscle_development	ENSGALG00010002644	KCNF1	-0.774391287	0.499198793	-1.551268348	0.120837389	0.143674674	negative
PRJNA729271	stage_1W_vs_E10	ordinary_muscle_development	ENSGALG00010002646	FOXO1	1.775831587	0.169129292	10.49984637	8.65208E-26	4.11187E-25	positive
PRJNA729271	stage_1W_vs_E10	ordinary_muscle_development	ENSGALG00010002647	FAM168A	-0.719590435	0.09908487	-7.262364426	3.80382E-13	1.02636E-12	negative
PRJNA729271	stage_1W_vs_E10	ordinary_muscle_development	ENSGALG00010002649	UFL1	0.691886091	0.078897996	8.769374697	1.79661E-18	6.25556E-18	positive
PRJNA729271	stage_1W_vs_E10	ordinary_muscle_development	ENSGALG00010002653	EIF1AX	-0.291116807	0.063698438	-4.57023459	4.87179E-06	8.64339E-06	negative
PRJNA729271	stage_1W_vs_E10	ordinary_muscle_development	ENSGALG00010002655	FABP4	6.460205669	0.946390925	6.826149213	8.72242E-12	2.20072E-11	positive
PRJNA729271	stage_1W_vs_E10	ordinary_muscle_development	ENSGALG00010002656	GLRA3	3.68402117	0.530021963	6.950695304	3.6349E-12	9.34846E-12	positive
PRJNA729271	stage_1W_vs_E10	ordinary_muscle_development	ENSGALG00010002658	FBXL12	-0.25196419	0.146763638	-1.716802565	0.086015252	0.104301927	negative
PRJNA729271	stage_1W_vs_E10	ordinary_muscle_development	ENSGALG00010002661	ACBD7	-1.939123695	0.210304637	-9.220546538	2.9559E-20	1.12191E-19	negative
PRJNA729271	stage_1W_vs_E10	ordinary_muscle_development	ENSGALG00010002664	ZNFB1	0.362717623	0.138106144	2.626368478	0.008630134	0.011750309	positive
PRJNA729271	stage_1W_vs_E10	ordinary_muscle_development	ENSGALG00010002667	ENSGALG00010002667	3.707716698	1.563492642	2.371432138			positive
PRJNA729271	stage_1W_vs_E10	ordinary_muscle_development	ENSGALG00010002669	IL4I1	3.730693194	1.128301922	3.306467109	0.000944804	0.001411558	positive
PRJNA729271	stage_1W_vs_E10	ordinary_muscle_development	ENSGALG00010002673	SLC66A3	-0.67123075	0.153055005	-4.385552425	1.15692E-05	1.9997E-05	negative
PRJNA729271	stage_1W_vs_E10	ordinary_muscle_development	ENSGALG00010002678	CCNI	1.484516078	0.172766878	8.592596557	8.50257E-18	2.86083E-17	positive
PRJNA729271	stage_1W_vs_E10	ordinary_muscle_development	ENSGALG00010002681	PLEKHB1	0.098608924	0.158354746	0.622708989	0.533475775	0.570008208	positive
PRJNA729271	stage_1W_vs_E10	ordinary_muscle_development	ENSGALG00010002682	UBL5	0.579367877	0.165681099	3.496885771	0.000470723	0.000718982	positive
PRJNA729271	stage_1W_vs_E10	ordinary_muscle_development	ENSGALG00010002685	CA8	1.535436979	0.177719124	8.639683464	5.6369E-18	1.91306E-17	positive
PRJNA729271	stage_1W_vs_E10	ordinary_muscle_development	ENSGALG00010002689	COG6	-0.526021417	0.062135376	-8.465731586	2.5455E-17	8.37426E-17	negative
PRJNA729271	stage_1W_vs_E10	ordinary_muscle_development	ENSGALG00010002693	MSC	-1.468509628	0.169417092	-8.668013433	4.39725E-18	1.49924E-17	negative
PRJNA729271	stage_1W_vs_E10	ordinary_muscle_development	ENSGALG00010002700	LHFPL6	-1.458334433	0.171297715	-8.513449441	1.68834E-17	5.60525E-17	negative
PRJNA729271	stage_1W_vs_E10	ordinary_muscle_development	ENSGALG00010002701	TMEM196	-3.495039308	0.766285352	-4.561015422	5.09068E-06	9.02072E-06	negative
PRJNA729271	stage_1W_vs_E10	ordinary_muscle_development	ENSGALG00010002703	PIN1	0.377865103	0.096208866	3.927549704	8.58157E-05	0.000138751	positive
PRJNA729271	stage_1W_vs_E10	ordinary_muscle_development	ENSGALG00010002704	KCNB2	-0.710160605	0.266401459	-2.665753437	0.007681601	0.010513153	negative
PRJNA729271	stage_1W_vs_E10	ordinary_muscle_development	ENSGALG00010002705	JADE3	-1.392021629	0.056355136	-24.70088332	1.0464E-134	7.1543E-133	negative
PRJNA729271	stage_1W_vs_E10	ordinary_muscle_development	ENSGALG00010002706	ROCK2	1.489454906	0.144103687	10.33599439	4.84362E-25	2.23167E-24	positive
PRJNA729271	stage_1W_vs_E10	ordinary_muscle_development	ENSGALG00010002707	RELT	-4.997422428	0.337290281	-14.81638432	1.148E-49	1.24273E-48	negative
PRJNA729271	stage_1W_vs_E10	ordinary_muscle_development	ENSGALG00010002712	PTPRE	0.79557107	0.126847105	6.271889826	3.56692E-10	8.21587E-10	positive
PRJNA729271	stage_1W_vs_E10	ordinary_muscle_development	ENSGALG00010002713	OLFM2	0.490856845	0.176348511	2.783447634	0.005378454	0.0074883	positive
PRJNA729271	stage_1W_vs_E10	ordinary_muscle_development	ENSGALG00010002716	NHLRC3	-1.06316501	0.112950817	-9.412636756	4.83844E-21	1.89652E-20	negative
PRJNA729271	stage_1W_vs_E10	ordinary_muscle_development	ENSGALG00010002722	TRIM7.1	2.831079044	0.148967204	19.00471362	1.55895E-80	3.77875E-79	positive
PRJNA729271	stage_1W_vs_E10	ordinary_muscle_development	ENSGALG00010002725	TSPAN7	0.672320217	0.178579947	3.764813612	0.000166673	0.000263719	positive
PRJNA729271	stage_1W_vs_E10	ordinary_muscle_development	ENSGALG00010002728	RP2	-1.169608088	0.113308203	-10.32236018	5.58335E-25	2.56516E-24	negative
PRJNA729271	stage_1W_vs_E10	ordinary_muscle_development	ENSGALG00010002730	E2F6	0.843480539	0.10035653	8.404839596	4.28447E-17	1.39245E-16	positive
PRJNA729271	stage_1W_vs_E10	ordinary_muscle_development	ENSGALG00010002733	RDH8	1.197598213	0.358258684	3.342830938	0.000829284	0.001244406	positive
PRJNA729271	stage_1W_vs_E10	ordinary_muscle_development	ENSGALG00010002739	HEP21	-1.079552241	0.928099955	-1.163185318	0.244754322	0.277381853	negative
PRJNA729271	stage_1W_vs_E10	ordinary_muscle_development	ENSGALG00010002741	PDK4	8.433931777	0.574095655	14.69081276	7.3823E-49	7.75409E-48	positive
PRJNA729271	stage_1W_vs_E10	ordinary_muscle_development	ENSGALG00010002742	PROSER1	-0.204560564	0.123839484	-1.651820224	0.098571205	0.11869981	negative
PRJNA729271	stage_1W_vs_E10	ordinary_muscle_development	ENSGALG00010002747	FUT9	-0.270983834	0.522890656	-0.518241874	0.604289522	0.638796428	negative
PRJNA729271	stage_1W_vs_E10	ordinary_muscle_development	ENSGALG00010002750	ENSGALG00010002750	-2.133939958	0.235006663	-9.0803381	1.08238E-19	4.01667E-19	negative
PRJNA729271	stage_1W_vs_E10	ordinary_muscle_development	ENSGALG00010002752	NDUFA4	2.102803183	0.154121236	13.64382507	2.19726E-42	1.90145E-41	positive
PRJNA729271	stage_1W_vs_E10	ordinary_muscle_development	ENSGALG00010002753	SLC9A7	-0.421463919	0.155864108	-2.704047296	0.006850054	0.0094285	negative
PRJNA729271	stage_1W_vs_E10	ordinary_muscle_development	ENSGALG00010002758	GREB1	-5.300031013	0.445466566	-11.89770775	1.21643E-32	7.58044E-32	negative
PRJNA729271	stage_1W_vs_E10	ordinary_muscle_development	ENSGALG00010002760	RAB2A	0.854234581	0.092470152	9.237949384	2.51266E-20	9.57441E-20	positive
PRJNA729271	stage_1W_vs_E10	ordinary_muscle_development	ENSGALG00010002762	PPAN	-1.138745074	0.149896914	-7.596854706	3.03415E-14	8.66942E-14	negative
PRJNA729271	stage_1W_vs_E10	ordinary_muscle_development	ENSGALG00010002763	SEPTIN11	-2.311723014	0.116338647	-19.87063693	7.30699E-88	2.08905E-86	negative
PRJNA729271	stage_1W_vs_E10	ordinary_muscle_development	ENSGALG00010002765	MAP7D2	-4.423106757	0.447037368	-9.894266288	4.40831E-23	1.87056E-22	negative
PRJNA729271	stage_1W_vs_E10	ordinary_muscle_development	ENSGALG00010002766	NANOS1	-1.129341539	0.332910607	-3.392326696	0.000693018	0.001046493	negative
PRJNA729271	stage_1W_vs_E10	ordinary_muscle_development	ENSGALG00010002768	TRIM39.2	4.127658549	0.939985283	4.391194867	1.12729E-05	1.94966E-05	positive
PRJNA729271	stage_1W_vs_E10	ordinary_muscle_development	ENSGALG00010002774	TBX18	0.214655187	0.116716864	1.839110294	0.065898965	0.081045775	positive
PRJNA729271	stage_1W_vs_E10	ordinary_muscle_development	ENSGALG00010002779	FBXO8	1.083505347	0.083736611	12.93944593	2.69567E-38	2.03403E-37	positive
PRJNA729271	stage_1W_vs_E10	ordinary_muscle_development	ENSGALG00010002781	EIF3G	0.763612915	0.137673022	5.546569011	2.9133E-08	6.00857E-08	positive
PRJNA729271	stage_1W_vs_E10	ordinary_muscle_development	ENSGALG00010002782	GSTA3	-0.302805738	0.147746683	-2.049492633	0.040413967	0.051027915	negative
PRJNA729271	stage_1W_vs_E10	ordinary_muscle_development	ENSGALG00010002784	TRIM27.2	-1.372720669	0.64470993	-2.129206649	0.033237167	0.042368546	negative
PRJNA729271	stage_1W_vs_E10	ordinary_muscle_development	ENSGALG00010002787	CEP44	-0.452179554	0.13139419	-3.441396882	0.000578719	0.000878564	negative
PRJNA729271	stage_1W_vs_E10	ordinary_muscle_development	ENSGALG00010002788	ENSGALG00010002788	4.710715362	0.630388069	7.472722902	7.85521E-14	2.19852E-13	positive
PRJNA729271	stage_1W_vs_E10	ordinary_muscle_development	ENSGALG00010002790	BCLAF3	0.918463142	0.101438733	9.054363337	1.37367E-19	5.06907E-19	positive
PRJNA729271	stage_1W_vs_E10	ordinary_muscle_development	ENSGALG00010002792	ENSGALG00010002792	-0.942883398	0.232209214	-4.060490897	4.89696E-05	8.05875E-05	negative
PRJNA729271	stage_1W_vs_E10	ordinary_muscle_development	ENSGALG00010002796	PRDM8	1.929299759	0.731284546	2.638234008	0.008333905	0.011360853	positive
PRJNA729271	stage_1W_vs_E10	ordinary_muscle_development	ENSGALG00010002800	TUBGCP5	-0.161398784	0.07594097	-2.125318967	0.033560007	0.042761278	negative
PRJNA729271	stage_1W_vs_E10	ordinary_muscle_development	ENSGALG00010002801	FGF5	-2.011222561	0.430931655	-4.667149742	3.05407E-06	5.504E-06	negative
PRJNA729271	stage_1W_vs_E10	ordinary_muscle_development	ENSGALG00010002805	FAM171A1	-1.15874181	0.100458663	-11.53451355	8.83854E-31	5.15552E-30	negative
PRJNA729271	stage_1W_vs_E10	ordinary_muscle_development	ENSGALG00010002807	P2RY6	1.742288693	0.220571726	7.8989666	2.81225E-15	8.47124E-15	positive
PRJNA729271	stage_1W_vs_E10	ordinary_muscle_development	ENSGALG00010002809	SH3KBP1	-1.189817996	0.1113307	-10.6872408	1.16794E-26	5.76218E-26	negative
PRJNA729271	stage_1W_vs_E10	ordinary_muscle_development	ENSGALG00010002810	TRIM27.1	0.514451811	0.311620095	1.650894212	0.098760178	0.118887865	positive
PRJNA729271	stage_1W_vs_E10	ordinary_muscle_development	ENSGALG00010002812	TRIM13	-0.781273181	0.103878395	-7.52103634	5.43437E-14	1.5355E-13	negative
PRJNA729271	stage_1W_vs_E10	ordinary_muscle_development	ENSGALG00010002814	TERF1	-2.549237982	0.157055224	-16.23147528	3.0211E-59	4.33144E-58	negative
PRJNA729271	stage_1W_vs_E10	ordinary_muscle_development	ENSGALG00010002817	SHFM1	0.899443688	0.091311169	9.850314045	6.83301E-23	2.87836E-22	positive
PRJNA729271	stage_1W_vs_E10	ordinary_muscle_development	ENSGALG00010002819	BMP3	-2.519255334	0.352600624	-7.144784109	9.01374E-13	2.39381E-12	negative
PRJNA729271	stage_1W_vs_E10	ordinary_muscle_development	ENSGALG00010002824	NXPH1	-3.157381815	0.583771251	-5.408594226	6.35214E-08	1.28757E-07	negative
PRJNA729271	stage_1W_vs_E10	ordinary_muscle_development	ENSGALG00010002825	TRIM41	0.677286469	0.121136989	5.591078934	2.25663E-08	4.6869E-08	positive
PRJNA729271	stage_1W_vs_E10	ordinary_muscle_development	ENSGALG00010002827	SBSPON	-0.018657203	0.349980004	-0.053309341	0.957485438	0.962866062	negative
PRJNA729271	stage_1W_vs_E10	ordinary_muscle_development	ENSGALG00010002832	LPIN1	3.292738569	0.200902392	16.389743	2.264E-60	3.34864E-59	positive
PRJNA729271	stage_1W_vs_E10	ordinary_muscle_development	ENSGALG00010002834	MRPL53	0.049092942	0.113249106	0.433495186	0.664655063	0.694956007	positive
PRJNA729271	stage_1W_vs_E10	ordinary_muscle_development	ENSGALG00010002835	CYFIP1	-0.917592153	0.08479047	-10.8218784	2.71162E-27	1.3719E-26	negative
PRJNA729271	stage_1W_vs_E10	ordinary_muscle_development	ENSGALG00010002837	DNMT1	-0.218872686	0.130101158	-1.682326962	0.092505456	0.111804101	negative
PRJNA729271	stage_1W_vs_E10	ordinary_muscle_development	ENSGALG00010002838	ENSGALG00010002838	1.252956719	0.260286667	4.813756821	1.48119E-06	2.73258E-06	positive
PRJNA729271	stage_1W_vs_E10	ordinary_muscle_development	ENSGALG00010002840	SRPX	0.574151692	0.169966105	3.378036417	0.000730054	0.0011007	positive
PRJNA729271	stage_1W_vs_E10	ordinary_muscle_development	ENSGALG00010002848	TRIB2	-1.493656114	0.107752201	-13.86195455	1.07701E-43	9.68441E-43	negative
PRJNA729271	stage_1W_vs_E10	ordinary_muscle_development	ENSGALG00010002853	CEP162	-0.067593023	0.168355997	-0.401488655	0.688060389	0.717111698	negative
PRJNA729271	stage_1W_vs_E10	ordinary_muscle_development	ENSGALG00010002855	RACK1	0.745210952	0.125275861	5.948559797	2.70512E-09	5.94282E-09	positive
PRJNA729271	stage_1W_vs_E10	ordinary_muscle_development	ENSGALG00010002857	DCAF15	-0.519432079	0.147921096	-3.511548345	0.000445504	0.000681899	negative
PRJNA729271	stage_1W_vs_E10	ordinary_muscle_development	ENSGALG00010002860	NIPA2	-0.074804886	0.108894775	-0.686946512	0.492116435	0.530115021	negative
PRJNA729271	stage_1W_vs_E10	ordinary_muscle_development	ENSGALG00010002864	RNASEH2B	-1.012865432	0.094964285	-10.66575115	1.47205E-26	7.23544E-26	negative
PRJNA729271	stage_1W_vs_E10	ordinary_muscle_development	ENSGALG00010002866	ENSGALG00010002866	3.349033138	0.252075985	13.285808	2.79821E-40	2.26088E-39	positive
PRJNA729271	stage_1W_vs_E10	ordinary_muscle_development	ENSGALG00010002868	PPP1R9A	0.773297429	0.067322766	11.48641789	1.54382E-30	8.90474E-30	positive
PRJNA729271	stage_1W_vs_E10	ordinary_muscle_development	ENSGALG00010002869	ENSGALG00010002869	-0.298791207	0.325767416	-0.917191814	0.359042136	0.396670408	negative
PRJNA729271	stage_1W_vs_E10	ordinary_muscle_development	ENSGALG00010002870	FABP5	3.987471409	0.216143823	18.44823212	5.3879E-76	1.16044E-74	positive
PRJNA729271	stage_1W_vs_E10	ordinary_muscle_development	ENSGALG00010002878	P2RY2	6.280030343	0.249917611	25.12840259	2.4343E-139	1.7468E-137	positive
PRJNA729271	stage_1W_vs_E10	ordinary_muscle_development	ENSGALG00010002880	RASGEF1B	-1.002712393	0.209490557	-4.78643241	1.69772E-06	3.11657E-06	negative
PRJNA729271	stage_1W_vs_E10	ordinary_muscle_development	ENSGALG00010002881	ENSGALG00010002881	4.823054713	0.313727097	15.37340816	2.46836E-53	3.03983E-52	positive
PRJNA729271	stage_1W_vs_E10	ordinary_muscle_development	ENSGALG00010002883	MAP3K15	5.316203195	0.412562027	12.88582771	5.4092E-38	4.03118E-37	positive
PRJNA729271	stage_1W_vs_E10	ordinary_muscle_development	ENSGALG00010002884	ADHFE1	4.246854834	0.251407687	16.89230306	5.12623E-64	8.27447E-63	positive
PRJNA729271	stage_1W_vs_E10	ordinary_muscle_development	ENSGALG00010002886	HERC2	0.636864678	0.105777149	6.020815318	1.73541E-09	3.85986E-09	positive
PRJNA729271	stage_1W_vs_E10	ordinary_muscle_development	ENSGALG00010002888	ENSGALG00010002888	-4.305387963	0.428835261	-10.03972469	1.01958E-23	4.44611E-23	negative
PRJNA729271	stage_1W_vs_E10	ordinary_muscle_development	ENSGALG00010002890	VXN	0.389288675	0.16531733	2.354796534	0.01853285	0.024279977	positive
PRJNA729271	stage_1W_vs_E10	ordinary_muscle_development	ENSGALG00010002894	FCHSD2	-0.59084888	0.073121672	-8.080352401	6.45799E-16	2.00447E-15	negative
PRJNA729271	stage_1W_vs_E10	ordinary_muscle_development	ENSGALG00010002896	OCA2	-2.043233895	0.27480485	-7.435217733	1.04396E-13	2.89944E-13	negative
PRJNA729271	stage_1W_vs_E10	ordinary_muscle_development	ENSGALG00010002904	PON1	3.928125212	0.178620666	21.99143748	3.4779E-107	1.4871E-105	positive
PRJNA729271	stage_1W_vs_E10	ordinary_muscle_development	ENSGALG00010002907	GABRG3	-1.437404022	0.361929567	-3.971502062	7.14209E-05	0.000116254	negative
PRJNA729271	stage_1W_vs_E10	ordinary_muscle_development	ENSGALG00010002908	S1PR2	-0.136829059	0.182645051	-0.74915284	0.45376509	0.49194652	negative
PRJNA729271	stage_1W_vs_E10	ordinary_muscle_development	ENSGALG00010002911	MYBL1	-0.742238898	0.233638461	-3.17686949	0.00148874	0.002182151	negative
PRJNA729271	stage_1W_vs_E10	ordinary_muscle_development	ENSGALG00010002912	RPS3	-0.032748147	0.192288637	-0.170307238	0.864768519	0.879698202	negative
PRJNA729271	stage_1W_vs_E10	ordinary_muscle_development	ENSGALG00010002913	NXNL1	4.720314221	0.561303936	8.409551251	4.11582E-17	1.33975E-16	positive
PRJNA729271	stage_1W_vs_E10	ordinary_muscle_development	ENSGALG00010002914	SLC27A1	3.176160032	0.304267566	10.4387072	1.65043E-25	7.75713E-25	positive
PRJNA729271	stage_1W_vs_E10	ordinary_muscle_development	ENSGALG00010002916	SDCBP	-1.222220824	0.123082846	-9.930066328	3.08051E-23	1.31872E-22	negative
PRJNA729271	stage_1W_vs_E10	ordinary_muscle_development	ENSGALG00010002917	PGLS	0.179603883	0.0961905	1.867168627	0.061878043	0.076333807	positive
PRJNA729271	stage_1W_vs_E10	ordinary_muscle_development	ENSGALG00010002918	LRATD1	-4.071331091	0.412470345	-9.870603152	5.58279E-23	2.36132E-22	negative
PRJNA729271	stage_1W_vs_E10	ordinary_muscle_development	ENSGALG00010002919	ENSGALG00010002919	1.34729184	0.203044731	6.635443483	3.23528E-11	7.91885E-11	positive
PRJNA729271	stage_1W_vs_E10	ordinary_muscle_development	ENSGALG00010002920	VCPIP1	1.411960726	0.101426827	13.92097896	4.72389E-44	4.30647E-43	positive
PRJNA729271	stage_1W_vs_E10	ordinary_muscle_development	ENSGALG00010002922	TMEM106B	-0.177313633	0.091821036	-1.931078547	0.05347334	0.066520431	negative
PRJNA729271	stage_1W_vs_E10	ordinary_muscle_development	ENSGALG00010002923	SERPINH1	-0.804461819	0.15858431	-5.072770551	3.92065E-07	7.53112E-07	negative
PRJNA729271	stage_1W_vs_E10	ordinary_muscle_development	ENSGALG00010002925	INTS6	0.312514034	0.121721451	2.567452419	0.010244885	0.013837086	positive
PRJNA729271	stage_1W_vs_E10	ordinary_muscle_development	ENSGALG00010002926	GALNTL6	-1.590798814	0.344786092	-4.613871756	3.95236E-06	7.06058E-06	negative
PRJNA729271	stage_1W_vs_E10	ordinary_muscle_development	ENSGALG00010002927	GABRA5	-6.628600757	0.95827601	-6.917214549	4.6061E-12	1.17814E-11	negative
PRJNA729271	stage_1W_vs_E10	ordinary_muscle_development	ENSGALG00010002928	ASB4	12.25122086	0.5455007	22.45867118	1.0529E-111	4.9073E-110	positive
PRJNA729271	stage_1W_vs_E10	ordinary_muscle_development	ENSGALG00010002929	PDHA2	1.016628188	0.112709502	9.019897769	1.88265E-19	6.89639E-19	positive
PRJNA729271	stage_1W_vs_E10	ordinary_muscle_development	ENSGALG00010002930	SERAF	-3.8045594	0.284959005	-13.35125168	1.16475E-40	9.52232E-40	negative
PRJNA729271	stage_1W_vs_E10	ordinary_muscle_development	ENSGALG00010002934	VWDE	-6.423742458	0.899131689	-7.144384449	9.04001E-13	2.40035E-12	negative
PRJNA729271	stage_1W_vs_E10	ordinary_muscle_development	ENSGALG00010002935	SYTL5	-3.340915894	0.680633353	-4.908539786	9.1757E-07	1.72148E-06	negative
PRJNA729271	stage_1W_vs_E10	ordinary_muscle_development	ENSGALG00010002937	GALNT7	-0.093581377	0.108473077	-0.862715238	0.388294052	0.426323458	negative
PRJNA729271	stage_1W_vs_E10	ordinary_muscle_development	ENSGALG00010002939	HMGB2	-2.470125642	0.172141192	-14.34941636	1.07438E-46	1.05652E-45	negative
PRJNA729271	stage_1W_vs_E10	ordinary_muscle_development	ENSGALG00010002940	BG1	1.833367253	0.410778895	4.463148603	8.0764E-06	1.40994E-05	positive
PRJNA729271	stage_1W_vs_E10	ordinary_muscle_development	ENSGALG00010002942	GABRB3	-4.672621163	0.675787837	-6.914331552	4.70075E-12	1.20129E-11	negative

PRJNA729271	stage_1W_vs_E10	ordinary_muscle_development	ENSGALG00010002943	SAP30	0.065294265	0.147986927	0.441216441	0.659056312	0.689649237	positive
PRJNA729271	stage_1W_vs_E10	ordinary_muscle_development	ENSGALG00010002946	GDPD5	-1.109671611	0.120689246	-9.194453062	3.76907E-20	1.42236E-19	negative
PRJNA729271	stage_1W_vs_E10	ordinary_muscle_development	ENSGALG00010002947	ENSGALG00010002947	-0.944523753	0.692483171	-1.363966363	0.172578152	0.200506598	negative
PRJNA729271	stage_1W_vs_E10	ordinary_muscle_development	ENSGALG00010002948	DLX6	-4.84106167	1.603059143	-3.019889622	0.002528668	0.003629374	negative
PRJNA729271	stage_1W_vs_E10	ordinary_muscle_development	ENSGALG00010002949	ENSGALG00010002949	0.051049995	1.183108765	0.043149029	0.965582736	0.970402951	positive
PRJNA729271	stage_1W_vs_E10	ordinary_muscle_development	ENSGALG00010002951	ENSGALG00010002951	-6.004571021	4.350978426	-1.380050745	0.167571021	0.195221182	negative
PRJNA729271	stage_1W_vs_E10	ordinary_muscle_development	ENSGALG00010002952	NBAS	-0.024879182	0.064710736	-0.384467611	0.700631897	0.728944829	negative
PRJNA729271	stage_1W_vs_E10	ordinary_muscle_development	ENSGALG00010002953	WDFY2	-0.890402122	0.13569464	-6.561807598	5.31594E-11	1.28339E-10	negative
PRJNA729271	stage_1W_vs_E10	ordinary_muscle_development	ENSGALG00010002954	CHST7	0.204560656	0.125333153	1.632135243	0.102650999	0.123256665	positive
PRJNA729271	stage_1W_vs_E10	ordinary_muscle_development	ENSGALG00010002955	BLEC1	0.111284658	0.477669561	0.232974146	0.815781482	0.835199363	positive
PRJNA729271	stage_1W_vs_E10	ordinary_muscle_development	ENSGALG00010002956	SCRG1	0.770129037	0.368006947	2.092702441	0.036375725	0.046154249	positive
PRJNA729271	stage_1W_vs_E10	ordinary_muscle_development	ENSGALG00010002957	ADGRG2	-2.602623274	0.296976747	-8.763727469	1.88898E-18	6.56771E-18	negative
PRJNA729271	stage_1W_vs_E10	ordinary_muscle_development	ENSGALG00010002958	ENSGALG00010002958	0.364929551	0.186560887	1.956088215	0.050454763	0.062986977	positive
PRJNA729271	stage_1W_vs_E10	ordinary_muscle_development	ENSGALG00010002959	DLX5	-6.393604558	0.853485834	-7.491166582	6.82641E-14	1.9191E-13	negative
PRJNA729271	stage_1W_vs_E10	ordinary_muscle_development	ENSGALG00010002960	BLEC2	1.924278094	0.695615789	2.766294447	0.005669732	0.007868897	positive
PRJNA729271	stage_1W_vs_E10	ordinary_muscle_development	ENSGALG00010002965	PRSS35	-0.247611339	0.211641258	-1.169957793	0.242017955	0.274495325	negative
PRJNA729271	stage_1W_vs_E10	ordinary_muscle_development	ENSGALG00010002967	OLFM4	-0.135181948	0.809394832	-0.167016075	0.867357401	0.882084159	negative
PRJNA729271	stage_1W_vs_E10	ordinary_muscle_development	ENSGALG00010002972	ME1	3.466412725	0.150021809	23.10605875	4.0242E-118	2.1134E-116	positive
PRJNA729271	stage_1W_vs_E10	ordinary_muscle_development	ENSGALG00010002978	DYNLT3	1.313284264	0.10146021	12.9438355	2.54594E-38	1.92406E-37	positive
PRJNA729271	stage_1W_vs_E10	ordinary_muscle_development	ENSGALG00010002981	KLHL35	-5.165093041	0.358839793	-14.39386919	5.65421E-47	5.60971E-46	negative
PRJNA729271	stage_1W_vs_E10	ordinary_muscle_development	ENSGALG00010002982	HAND2	-3.204180089	0.311148734	-10.29790496	7.20125E-25	3.29074E-24	negative
PRJNA729271	stage_1W_vs_E10	ordinary_muscle_development	ENSGALG00010002983	NSMAF	1.052153169	0.111344265	9.44954972	3.40291E-21	1.34291E-20	positive
PRJNA729271	stage_1W_vs_E10	ordinary_muscle_development	ENSGALG00010002985	ENSGALG00010002985	0.411460377	0.214187234	1.921031289	0.054727763	0.067987566	positive
PRJNA729271	stage_1W_vs_E10	ordinary_muscle_development	ENSGALG00010002987	PCDH8	-1.424458083	0.359130095	-3.966412458	7.29626E-05	0.000118711	negative
PRJNA729271	stage_1W_vs_E10	ordinary_muscle_development	ENSGALG00010002991	BLB1	4.260783746	0.307352938	13.86283723	1.06385E-43	9.57197E-43	positive
PRJNA729271	stage_1W_vs_E10	ordinary_muscle_development	ENSGALG00010002992	ENSGALG00010002992	0.449196778	0.2402089	1.870025539	0.061480271	0.075894782	positive
PRJNA729271	stage_1W_vs_E10	ordinary_muscle_development	ENSGALG00010002994	DUSP4	-4.619289666	0.223252459	-20.69087921	4.18512E-95	1.41406E-93	negative
PRJNA729271	stage_1W_vs_E10	ordinary_muscle_development	ENSGALG00010002995	RWDD2A	-1.048794473	0.130147577	-8.058501725	7.72352E-16	2.38959E-15	negative
PRJNA729271	stage_1W_vs_E10	ordinary_muscle_development	ENSGALG00010002998	LECT1	-6.527621035	0.290889279	-22.44022557	1.5943E-111	7.407E-110	negative
PRJNA729271	stage_1W_vs_E10	ordinary_muscle_development	ENSGALG00010002999	CMTM7	-0.460331445	0.214275156	-2.148319265	0.031688402	0.04047972	negative
PRJNA729271	stage_1W_vs_E10	ordinary_muscle_development	ENSGALG00010003001	MAP6	-1.086921261	0.302359622	-3.594796334	0.000324645	0.000503287	negative
PRJNA729271	stage_1W_vs_E10	ordinary_muscle_development	ENSGALG00010003002	PHKA2	-1.593632645	0.191438923	-8.324496521	8.46879E-17	2.72607E-16	negative
PRJNA729271	stage_1W_vs_E10	ordinary_muscle_development	ENSGALG00010003006	CYP7B1	-1.192351048	0.20197815	-5.903366505	3.56158E-09	7.76317E-09	negative
PRJNA729271	stage_1W_vs_E10	ordinary_muscle_development	ENSGALG00010003009	TNKS	-0.236983042	0.058242327	-4.068914347	4.72327E-05	7.78175E-05	negative
PRJNA729271	stage_1W_vs_E10	ordinary_muscle_development	ENSGALG00010003017	PGM3	-1.702337997	0.157338998	-10.81955537	2.78123E-27	1.40663E-26	negative
PRJNA729271	stage_1W_vs_E10	ordinary_muscle_development	ENSGALG00010003018	MOGAT2	-0.311923991	0.862880652	-0.361491465	0.717732084	0.744864793	negative
PRJNA729271	stage_1W_vs_E10	ordinary_muscle_development	ENSGALG00010003019	TAPBP	3.034022812	0.251873924	12.04579958	2.04088E-33	1.30838E-32	positive
PRJNA729271	stage_1W_vs_E10	ordinary_muscle_development	ENSGALG00010003022	BLB2	5.09786156	0.265965299	19.16739356	6.92948E-82	1.73476E-80	positive
PRJNA729271	stage_1W_vs_E10	ordinary_muscle_development	ENSGALG00010003024	SUGT1	0.887401885	0.071551466	12.40228794	2.53967E-35	1.74467E-34	positive
PRJNA729271	stage_1W_vs_E10	ordinary_muscle_development	ENSGALG00010003025	MFHAS1	1.495947729	0.122655614	12.19632498	3.25171E-34	2.13855E-33	positive
PRJNA729271	stage_1W_vs_E10	ordinary_muscle_development	ENSGALG00010003027	SCIN	-2.548808964	0.256746862	-9.92732274	3.16643E-23	1.35431E-22	negative
PRJNA729271	stage_1W_vs_E10	ordinary_muscle_development	ENSGALG00010003030	PPEF1	-2.084276264	0.822714162	-2.533414838	0.011295718	0.015170151	negative
PRJNA729271	stage_1W_vs_E10	ordinary_muscle_development	ENSGALG00010003034	ERI1	-0.601017451	0.063135026	-9.519556541	1.7392E-21	6.94481E-21	negative
PRJNA729271	stage_1W_vs_E10	ordinary_muscle_development	ENSGALG00010003035	DGAT2	2.867824719	0.45659427	6.280903877	3.3661E-10	7.76443E-10	positive
PRJNA729271	stage_1W_vs_E10	ordinary_muscle_development	ENSGALG00010003042	TOX	-5.157444346	0.384480974	-13.41404306	5.0035E-41	4.1467E-40	negative
PRJNA729271	stage_1W_vs_E10	ordinary_muscle_development	ENSGALG00010003052	ENSGALG00010003052	0.813101463	0.093158063	8.728192049	2.58776E-18	8.93511E-18	positive
PRJNA729271	stage_1W_vs_E10	ordinary_muscle_development	ENSGALG00010003057	DDX1	-0.148776422	0.089732045	-1.658007708	0.097315911	0.117246623	negative
PRJNA729271	stage_1W_vs_E10	ordinary_muscle_development	ENSGALG00010003061	ENSGALG00010003061	-0.405069084	0.3114373	-1.300644092	0.193380307	0.223083769	negative
PRJNA729271	stage_1W_vs_E10	ordinary_muscle_development	ENSGALG00010003063	GGH	-0.079043001	0.088807062	-0.890053105	0.373437372	0.411070375	negative
PRJNA729271	stage_1W_vs_E10	ordinary_muscle_development	ENSGALG00010003064	VAV1	0.620152247	0.26679891	2.324418218	0.02010309	0.026268303	positive
PRJNA729271	stage_1W_vs_E10	ordinary_muscle_development	ENSGALG00010003068	BRD2	0.548014592	0.126155134	4.343973747	1.39928E-05	2.40031E-05	positive
PRJNA729271	stage_1W_vs_E10	ordinary_muscle_development	ENSGALG00010003072	ELF1	0.676725436	0.074925027	9.032034583	1.68509E-19	6.19148E-19	positive
PRJNA729271	stage_1W_vs_E10	ordinary_muscle_development	ENSGALG00010003074	TTPA	1.864670693	0.351569985	5.303839268	1.13392E-07	2.25679E-07	positive
PRJNA729271	stage_1W_vs_E10	ordinary_muscle_development	ENSGALG00010003077	EIF2S3	0.338526363	0.081768118	4.140077707	3.47188E-05	5.7878E-05	positive
PRJNA729271	stage_1W_vs_E10	ordinary_muscle_development	ENSGALG00010003081	DOP1A	0.275379118	0.104028238	2.647157373	0.008117157	0.011081012	positive
PRJNA729271	stage_1W_vs_E10	ordinary_muscle_development	ENSGALG00010003087	TWISTNB	0.938585898	0.177307756	5.293541118	1.1997E-07	2.38346E-07	positive
PRJNA729271	stage_1W_vs_E10	ordinary_muscle_development	ENSGALG00010003090	DMA	1.897112476	0.187564651	10.11444568	4.76708E-24	2.10346E-23	positive
PRJNA729271	stage_1W_vs_E10	ordinary_muscle_development	ENSGALG00010003092	ART1F	1.426677003	1.552466082	0.918974668	0.358108822	0.39566944	positive
PRJNA729271	stage_1W_vs_E10	ordinary_muscle_development	ENSGALG00010003097	TFPI2	1.466894853	0.135672339	10.81204073	3.01879E-27	1.52464E-26	positive
PRJNA729271	stage_1W_vs_E10	ordinary_muscle_development	ENSGALG00010003101	KLHL15	-0.288800154	0.072841697	-3.964764204	7.34685E-05	0.00011952	negative
PRJNA729271	stage_1W_vs_E10	ordinary_muscle_development	ENSGALG00010003103	YTHDF3	0.429842456	0.086337669	4.978620126	6.40392E-07	1.2126E-06	positive
PRJNA729271	stage_1W_vs_E10	ordinary_muscle_development	ENSGALG00010003107	GNG11	0.222584791	0.181357149	1.227328466	0.219699161	0.25108723	positive
PRJNA729271	stage_1W_vs_E10	ordinary_muscle_development	ENSGALG00010003108	PPP1R3B	-0.629739526	0.183679629	-3.428466896	0.000607001	0.000920342	negative
PRJNA729271	stage_1W_vs_E10	ordinary_muscle_development	ENSGALG00010003109	ART5	4.039781504	0.571847767	7.064435223	1.61271E-12	4.22181E-12	positive
PRJNA729271	stage_1W_vs_E10	ordinary_muscle_development	ENSGALG00010003110	BF1	4.776794475	0.256561735	18.61849929	2.27501E-77	5.03454E-76	positive
PRJNA729271	stage_1W_vs_E10	ordinary_muscle_development	ENSGALG00010003113	WBP4	-0.501840022	0.118514685	-4.234412133	2.2915E-05	3.86585E-05	negative
PRJNA729271	stage_1W_vs_E10	ordinary_muscle_development	ENSGALG00010003115	UBE3D	-0.529012483	0.157300456	-3.363070252	0.000770807	0.001159732	negative
PRJNA729271	stage_1W_vs_E10	ordinary_muscle_development	ENSGALG00010003117	ID2	-2.204405164	0.12735368	-17.30931654	4.00136E-67	7.0048E-66	negative
PRJNA729271	stage_1W_vs_E10	ordinary_muscle_development	ENSGALG00010003121	ARMC1	0.159398795	0.08246387	1.932953116	0.053241974	0.066255358	positive
PRJNA729271	stage_1W_vs_E10	ordinary_muscle_development	ENSGALG00010003125	APOO	1.229329745	0.10579121	11.62033922	3.24844E-31	1.92897E-30	positive
PRJNA729271	stage_1W_vs_E10	ordinary_muscle_development	ENSGALG00010003126	ENSGALG00010003126	-3.208602056	0.54883239	-5.846233042	5.02829E-09	1.08703E-08	negative
PRJNA729271	stage_1W_vs_E10	ordinary_muscle_development	ENSGALG00010003131	TAP1	3.372682593	0.347649472	9.701388523	2.97423E-22	1.21715E-21	positive
PRJNA729271	stage_1W_vs_E10	ordinary_muscle_development	ENSGALG00010003132	TPBG	-4.394551469	0.176620779	-24.8812823	1.1864E-136	8.2284E-135	negative
PRJNA729271	stage_1W_vs_E10	ordinary_muscle_development	ENSGALG00010003133	SAT1	2.531477994	0.167076237	15.151634	7.3906E-52	8.66721E-51	positive
PRJNA729271	stage_1W_vs_E10	ordinary_muscle_development	ENSGALG00010003136	NUP98	-0.015965509	0.104946029	-0.152130664	0.87908388	0.892819565	negative
PRJNA729271	stage_1W_vs_E10	ordinary_muscle_development	ENSGALG00010003152	ENSGALG00010003152	1.530670056	0.126848512	12.06691373	1.57947E-33	1.01753E-32	positive
PRJNA729271	stage_1W_vs_E10	ordinary_muscle_development	ENSGALG00010003154	MTFR1	-0.787954512	0.082114328	-9.595822435	8.32505E-22	3.35665E-21	negative
PRJNA729271	stage_1W_vs_E10	ordinary_muscle_development	ENSGALG00010003159	TAP2	3.908782612	0.264658545	14.76915328	2.31604E-49	2.48123E-48	positive
PRJNA729271	stage_1W_vs_E10	ordinary_muscle_development	ENSGALG00010003160	KIDINS220	0.204898489	0.151450543	1.352906929	0.176085365	0.204302999	positive
PRJNA729271	stage_1W_vs_E10	ordinary_muscle_development	ENSGALG00010003164	C3	3.401041781	0.459199033	7.406465468	1.2971E-13	3.5826E-13	positive
PRJNA729271	stage_1W_vs_E10	ordinary_muscle_development	ENSGALG00010003165	BMP2K	0.071400977	0.110019153	0.648986794	0.516346912	0.553583468	positive
PRJNA729271	stage_1W_vs_E10	ordinary_muscle_development	ENSGALG00010003166	PGAP2	0.727719663	0.110060223	6.612013321	3.79128E-11	9.25006E-11	positive
PRJNA729271	stage_1W_vs_E10	ordinary_muscle_development	ENSGALG00010003169	MTRF1	0.568250403	0.108367126	5.243752619	1.57343E-07	3.10171E-07	positive
PRJNA729271	stage_1W_vs_E10	ordinary_muscle_development	ENSGALG00010003170	C4	0.494849985	0.408001944	1.212861832	0.22518265	0.256686263	positive
PRJNA729271	stage_1W_vs_E10	ordinary_muscle_development	ENSGALG00010003171	PAQR3	-0.735213024	0.107684024	-6.827503255	8.64052E-12	2.18082E-11	negative
PRJNA729271	stage_1W_vs_E10	ordinary_muscle_development	ENSGALG00010003176	PRAG1	-1.185221195	0.259786551	-4.562288498	5.05991E-06	8.96837E-06	negative
PRJNA729271	stage_1W_vs_E10	ordinary_muscle_development	ENSGALG00010003178	CENPA	-1.996764802	0.249606879	-7.999638514	1.24785E-15	3.8224E-15	negative
PRJNA729271	stage_1W_vs_E10	ordinary_muscle_development	ENSGALG00010003180	IBTK	0.236182438	0.126947913	1.860467282	0.062819443	0.077448995	positive
PRJNA729271	stage_1W_vs_E10	ordinary_muscle_development	ENSGALG00010003183	NAA16	1.353810834	0.087447079	15.48148717	4.62653E-54	5.82637E-53	positive
PRJNA729271	stage_1W_vs_E10	ordinary_muscle_development	ENSGALG00010003184	BET1	-0.317023706	0.081843931	-3.873515152	0.000107277	0.000172087	negative
PRJNA729271	stage_1W_vs_E10	ordinary_muscle_development	ENSGALG00010003186	CYP21A1	-1.613126718	0.264763967	-6.09269735	1.11024E-09	2.49232E-09	negative
PRJNA729271	stage_1W_vs_E10	ordinary_muscle_development	ENSGALG00010003187	RHOG	0.428926128	0.254239382	1.687095541	0.091584982	0.110765483	positive
PRJNA729271	stage_1W_vs_E10	ordinary_muscle_development	ENSGALG00010003189	ANTXR2	0.365482796	0.126887776	2.880362528	0.003972181	0.005593236	positive
PRJNA729271	stage_1W_vs_E10	ordinary_muscle_development	ENSGALG00010003191	CC2D1A	1.70592569	0.159414648	10.70118534	1.00484E-26	4.9626E-26	positive
PRJNA729271	stage_1W_vs_E10	ordinary_muscle_development	ENSGALG00010003192	RGCC	1.492328278	0.308380662	4.839240785	1.30336E-06	2.4128E-06	positive
PRJNA729271	stage_1W_vs_E10	ordinary_muscle_development	ENSGALG00010003193	ACOT9	0.242586664	0.136982246	1.770935074	0.076571499	0.093379091	positive
PRJNA729271	stage_1W_vs_E10	ordinary_muscle_development	ENSGALG00010003198	PRKG2	-1.556613689	0.34117523	-4.562504989	5.05469E-06	8.96022E-06	negative
PRJNA729271	stage_1W_vs_E10	ordinary_muscle_development	ENSGALG00010003201	MBOAT2	-0.42119165	0.128123664	-3.287383736	0.001011229	0.001505109	negative
PRJNA729271	stage_1W_vs_E10	ordinary_muscle_development	ENSGALG00010003202	FAM46A	0.591014682	0.103242376	5.724535839	1.03717E-08	2.19856E-08	positive
PRJNA729271	stage_1W_vs_E10	ordinary_muscle_development	ENSGALG00010003204	PKD2	-0.431460699	0.143833257	-2.999728349	0.002702205	0.003866962	negative
PRJNA729271	stage_1W_vs_E10	ordinary_muscle_development	ENSGALG00010003205	ENSGALG00010003205	5.680056355	0.264032824	21.51269024	1.1843E-102	4.6523E-101	positive
PRJNA729271	stage_1W_vs_E10	ordinary_muscle_development	ENSGALG00010003206	ENSGALG00010003206	-2.660881758	0.710448085	-3.745357069	0.000180137	0.000284433	negative
PRJNA729271	stage_1W_vs_E10	ordinary_muscle_development	ENSGALG00010003210	EYA1	-0.135072242	0.201379652	-0.670734309	0.502389802	0.540178042	negative
PRJNA729271	stage_1W_vs_E10	ordinary_muscle_development	ENSGALG00010003212	LTB4R	1.492461368	0.787303352	1.895662407	0.058004699	0.07186752	positive
PRJNA729271	stage_1W_vs_E10	ordinary_muscle_development	ENSGALG00010003214	PRDX4	-0.957133856	0.095331801	-10.04002697	1.01646E-23	4.43384E-23	negative
PRJNA729271	stage_1W_vs_E10	ordinary_muscle_development	ENSGALG00010003218	MAP3K7	0.163821452	0.060686868	2.699454709	0.006945321	0.009554183	positive
PRJNA729271	stage_1W_vs_E10	ordinary_muscle_development	ENSGALG00010003220	SPP1	-2.097901295	0.944748568	-2.220592192	0.026378595	0.034008515	negative
PRJNA729271	stage_1W_vs_E10	ordinary_muscle_development	ENSGALG00010003222	ITGA8	-3.237850454	0.3113345	-10.39990895	2.4817E-25	1.15592E-24	negative
PRJNA729271	stage_1W_vs_E10	ordinary_muscle_development	ENSGALG00010003223	AREG	-1.88217377	0.94472127	-1.992305911	0.046337504	0.058097233	negative
PRJNA729271	stage_1W_vs_E10	ordinary_muscle_development	ENSGALG00010003229	STIM1	1.506236188	0.104827377	14.36872917	8.13096E-47	8.02302E-46	positive
PRJNA729271	stage_1W_vs_E10	ordinary_muscle_development	ENSGALG00010003239	RRM1	-2.192466206	0.119731388	-18.31154085	6.69482E-75	1.41049E-73	negative
PRJNA729271	stage_1W_vs_E10	ordinary_muscle_development	ENSGALG00010003240	PLACL2	4.547757925	0.864581857	5.260066344	1.44003E-07	2.84726E-07	positive
PRJNA729271	stage_1W_vs_E10	ordinary_muscle_development	ENSGALG00010003242	ASAP2	-1.491027772	0.128548978	-11.59890803	4.1737E-31	2.46026E-30	negative
PRJNA729271	stage_1W_vs_E10	ordinary_muscle_development	ENSGALG00010003243	VPS50	-0.376952302	0.093143127	-4.047022194	5.18733E-05	8.52402E-05	negative
PRJNA729271	stage_1W_vs_E10	ordinary_muscle_development	ENSGALG00010003245	ENSGALG00010003245	-0.104555775	0.422756122	-0.247319364	0.804661074	0.82550515	negative
PRJNA729271	stage_1W_vs_E10	ordinary_muscle_development	ENSGALG00010003246	VWA8	1.46626722	0.108747308	13.48325074	1.96265E-41	1.65592E-40	positive
PRJNA729271	stage_1W_vs_E10	ordinary_muscle_development	ENSGALG00010003247	ENSGALG00010003247	0.732583613	0.21082475	3.474846347	0.000511146	0.000778835	positive
PRJNA729271	stage_1W_vs_E10	ordinary_muscle_development	ENSGALG00010003248	MRI1	0.405962868	0.095914335	4.232556788	2.3105E-05	3.89652E-05	positive
PRJNA729271	stage_1W_vs_E10	ordinary_muscle_development	ENSGALG00010003249	YJU2B	1.863733815	0.320970772	5.806553053	6.37721E-09	1.36985E-08	positive
PRJNA729271	stage_1W_vs_E10	ordinary_muscle_development	ENSGALG00010003250	CD1G	0.907972718	0.746881247	1.215685521	0.224104742	0.255638485	positive
PRJNA729271	stage_1W_vs_E10	ordinary_muscle_development	ENSGALG00010003251	ELOF1	0.243223368	0.154286495	1.576439777	0.114924487	0.137049075	positive
PRJNA729271	stage_1W_vs_E10	ordinary_muscle_development	ENSGALG00010003252	PHEX	-3.38985013	0.199708006	-16.97403223	1.27844E-64	2.10341E-63	negative
PRJNA729271	stage_1W_vs_E10	ordinary_muscle_development	ENSGALG00010003253	ITGB1BP1	-0.135113272	0.115169734	-1.173166483	0.24072905	0.273140319	negative
PRJNA729271	stage_1W_vs_E10	ordinary_muscle_development	ENSGALG00010003254	DGKH	0.398601454	0.16923584	2.35530165	0.018507675	0.024251378	positive
PRJNA729271	stage_1W_vs_E10	ordinary_muscle_development	ENSGALG00010003255	CALCR	-0.833424648	0.32865954	-2.53582978	0.011218125	0.015071926	negative
PRJNA729271	stage_1W_vs_E10	ordinary_muscle_development	ENSGALG00010003256	ENSGALG00010003256	3.603387621	1.311720052	2.747070624	0.006013018	0.008324613	positive
PRJNA729271	stage_1W_vs_E10	ordinary_muscle_development	ENSGALG00010003257	ENSGALG00010003257	2.664198858	0.792632959	3.361201206	0.000776043	0.001167487	positive
PRJNA729271	stage_1W_vs_E10	ordinary_muscle_development	ENSGALG00010003259	VEGFC	1.69792925	0.138732916	12.23883484	1.9278E-34	1.27946E-33	positive
PRJNA729271	stage_1W_vs_E10	ordinary_muscle_development	ENSGALG00010003260	ACP5	0.966858482	0.267329514	3.616729283	0.000298349	0.00046391	positive
PRJNA729271	stage_1W_vs_E10	ordinary_muscle_development	ENSGALG00010003261	PEX2	-1.15999578	0.173842221	-6.672693035	2.51151E-11	6.18491E-11	negative
PRJNA729271	stage_1W_vs_E10	ordinary_muscle_development	ENSGALG00010003262	ENSGALG00010003262	-1.712055526	0.596999314	-2.867767996	0.004133785	0.005809504	negative

PRJNA729271	stage_1W_vs_E10	ordinary_muscle_development	ENSGALG00010003263	ENSGALG00010003263	1.586022666	0.762860511	2.079046751	0.037613054	0.047665782	positive
PRJNA729271	stage_1W_vs_E10	ordinary_muscle_development	ENSGALG00010003264	ENSGALG00010003264	-1.065256393	0.396910097	-2.68387325	0.007277469	0.009983619	negative
PRJNA729271	stage_1W_vs_E10	ordinary_muscle_development	ENSGALG00010003268	SMS	-0.361508868	0.063801312	-5.666166721	1.46027E-08	3.06675E-08	negative
PRJNA729271	stage_1W_vs_E10	ordinary_muscle_development	ENSGALG00010003269	ENSGALG00010003269	0.624812856	0.429230751	1.455657252	0.145487393	0.171005494	positive
PRJNA729271	stage_1W_vs_E10	ordinary_muscle_development	ENSGALG00010003271	AKAP11	0.185739241	0.084091712	2.208769891	0.02719065	0.035002529	positive
PRJNA729271	stage_1W_vs_E10	ordinary_muscle_development	ENSGALG00010003272	CPSF3	-0.090398721	0.06655467	-1.358262634	0.174380363	0.202405778	negative
PRJNA729271	stage_1W_vs_E10	ordinary_muscle_development	ENSGALG00010003273	NFIX	0.376305486	0.200346704	1.878271406	0.060344046	0.074562011	positive
PRJNA729271	stage_1W_vs_E10	ordinary_muscle_development	ENSGALG00010003274	BACH2	0.293552102	0.147625763	1.988488299	0.046757712	0.058593675	positive
PRJNA729271	stage_1W_vs_E10	ordinary_muscle_development	ENSGALG00010003275	MBTPS2	-0.010724594	0.1294424	-0.082852249	0.933969023	0.942093319	negative
PRJNA729271	stage_1W_vs_E10	ordinary_muscle_development	ENSGALG00010003276	TNFSF11	-1.053925797	0.407829734	-2.584229912	0.009759671	0.013210051	negative
PRJNA729271	stage_1W_vs_E10	ordinary_muscle_development	ENSGALG00010003278	ENSGALG00010003278	0.852087927	0.50007442	1.703922243	0.088395615	0.107068976	positive
PRJNA729271	stage_1W_vs_E10	ordinary_muscle_development	ENSGALG00010003279	ENSGALG00010003279	0.97940989	0.385482985	2.540734426	0.011061991	0.014876931	positive
PRJNA729271	stage_1W_vs_E10	ordinary_muscle_development	ENSGALG00010003280	ENSGALG00010003280	-0.129544949	0.166544018	-0.777842102	0.436662104	0.474682556	negative
PRJNA729271	stage_1W_vs_E10	ordinary_muscle_development	ENSGALG00010003287	ENSGALG00010003287	-0.762697167	1.927477129	-0.395697129	0.692328473	0.721030408	negative
PRJNA729271	stage_1W_vs_E10	ordinary_muscle_development	ENSGALG00010003292	GADD45GIP1	1.378600176	0.123243106	11.18602266	4.7737E-29	2.58672E-28	positive
PRJNA729271	stage_1W_vs_E10	ordinary_muscle_development	ENSGALG00010003294	ENSGALG00010003294	-4.575942339	4.431167651	-1.032671905	0.301757466	0.337055904	negative
PRJNA729271	stage_1W_vs_E10	ordinary_muscle_development	ENSGALG00010003295	IAH1	0.285888197	0.133354113	2.143827366	0.032046722	0.040897802	positive
PRJNA729271	stage_1W_vs_E10	ordinary_muscle_development	ENSGALG00010003296	ENSGALG00010003296	2.060297613	0.557493909	3.695641474	0.000219332	0.000344109	positive
PRJNA729271	stage_1W_vs_E10	ordinary_muscle_development	ENSGALG00010003298	IL7	7.103325268	0.978630473	7.258434586	3.91596E-13	1.05643E-12	positive
PRJNA729271	stage_1W_vs_E10	ordinary_muscle_development	ENSGALG00010003299	RAD23A	2.515036969	0.120179944	20.92726025	3.02384E-97	1.07165E-95	positive
PRJNA729271	stage_1W_vs_E10	ordinary_muscle_development	ENSGALG00010003300	CASP8AP2	-0.024747775	0.111337812	-0.222276466	0.824098672	0.842814555	negative
PRJNA729271	stage_1W_vs_E10	ordinary_muscle_development	ENSGALG00010003303	MTHFD2L	0.162533072	0.192558122	0.844072795	0.398628729	0.43684308	positive
PRJNA729271	stage_1W_vs_E10	ordinary_muscle_development	ENSGALG00010003304	ENSGALG00010003304	-0.591017594	0.684882595	-0.862947311	0.388166437	0.426247917	negative
PRJNA729271	stage_1W_vs_E10	ordinary_muscle_development	ENSGALG00010003305	CALR	-0.812952362	0.078324629	-10.37926855	3.08127E-25	1.43151E-24	negative
PRJNA729271	stage_1W_vs_E10	ordinary_muscle_development	ENSGALG00010003307	COL1A2	0.374989538	0.220385932	1.701513047	0.08884669	0.107588369	positive
PRJNA729271	stage_1W_vs_E10	ordinary_muscle_development	ENSGALG00010003308	MDN1	-1.220764891	0.099700727	-12.24429278	1.80241E-34	1.19954E-33	negative
PRJNA729271	stage_1W_vs_E10	ordinary_muscle_development	ENSGALG00010003311	ENSGALG00010003311	-0.651625766	1.188306845	-0.548364902	0.583441371	0.618879083	negative
PRJNA729271	stage_1W_vs_E10	ordinary_muscle_development	ENSGALG00010003315	EPGN	-0.81015396	0.364066223	-2.225292842	0.026061586	0.033620744	negative
PRJNA729271	stage_1W_vs_E10	ordinary_muscle_development	ENSGALG00010003317	LYRM2	0.806537622	0.17756534	4.542201884	5.56697E-06	9.82985E-06	positive
PRJNA729271	stage_1W_vs_E10	ordinary_muscle_development	ENSGALG00010003320	OR51E2	0.688229455	0.477687191	1.440753421	0.149654357	0.175604299	positive
PRJNA729271	stage_1W_vs_E10	ordinary_muscle_development	ENSGALG00010003323	STMN2	-2.891319783	0.508310376	-5.688099082	1.28461E-08	2.70489E-08	negative
PRJNA729271	stage_1W_vs_E10	ordinary_muscle_development	ENSGALG00010003324	ENSGALG00010003324	-0.517930634	0.587079424	-0.882215612	0.377660221	0.415434841	negative
PRJNA729271	stage_1W_vs_E10	ordinary_muscle_development	ENSGALG00010003330	HEY1	0.454929309	0.120875678	3.76361331	0.000167476	0.00026496	positive
PRJNA729271	stage_1W_vs_E10	ordinary_muscle_development	ENSGALG00010003331	EPSTI1	3.204230222	0.613575501	5.222226469	1.76785E-07	3.47221E-07	positive
PRJNA729271	stage_1W_vs_E10	ordinary_muscle_development	ENSGALG00010003333	ENSGALG00010003333	19.66904326	2.688905105	7.31488933	2.57594E-13	7.00792E-13	positive
PRJNA729271	stage_1W_vs_E10	ordinary_muscle_development	ENSGALG00010003334	TAC1	-5.019698953	1.086612006	-4.619587236	3.84504E-06	6.87309E-06	negative
PRJNA729271	stage_1W_vs_E10	ordinary_muscle_development	ENSGALG00010003339	ENSGALG00010003339	-0.988371434	0.490377941	-2.015529966	0.043849149	0.055163463	negative
PRJNA729271	stage_1W_vs_E10	ordinary_muscle_development	ENSGALG00010003344	ZFAND1	0.519990402	0.190275628	2.73282715	0.006279328	0.008672592	positive
PRJNA729271	stage_1W_vs_E10	ordinary_muscle_development	ENSGALG00010003346	ENSGALG00010003346	-2.589352277	5.47463579	-0.472972518	0.636232786	0.668759553	negative
PRJNA729271	stage_1W_vs_E10	ordinary_muscle_development	ENSGALG00010003347	GPM6A	0.161946962	0.3537949	0.457742502	0.647137449	0.67879422	positive
PRJNA729271	stage_1W_vs_E10	ordinary_muscle_development	ENSGALG00010003348	ADAM17	-0.155148278	0.083613561	-1.85553965	0.063519203	0.078251814	negative
PRJNA729271	stage_1W_vs_E10	ordinary_muscle_development	ENSGALG00010003351	ENSGALG00010003351	-1.919173666	1.652968065	-1.161047032	0.245622773	0.278296717	negative
PRJNA729271	stage_1W_vs_E10	ordinary_muscle_development	ENSGALG00010003352	FARSA	0.930590907	0.100746176	9.236984887	2.53541E-20	9.65854E-20	positive
PRJNA729271	stage_1W_vs_E10	ordinary_muscle_development	ENSGALG00010003356	ENSGALG00010003356	3.215325184	1.109045892	2.899181366	0.003741384	0.005282619	positive
PRJNA729271	stage_1W_vs_E10	ordinary_muscle_development	ENSGALG00010003358	DNAJC15	0.668767452	0.133619246	5.005023394	5.58552E-07	1.06124E-06	positive
PRJNA729271	stage_1W_vs_E10	ordinary_muscle_development	ENSGALG00010003363	HBE1	-5.241783225	0.50814268	-10.31557363	5.99224E-25	2.74605E-24	negative
PRJNA729271	stage_1W_vs_E10	ordinary_muscle_development	ENSGALG00010003364	ANKRD6	-2.866539565	0.170665654	-16.7962299	2.60057E-63	4.13326E-62	negative
PRJNA729271	stage_1W_vs_E10	ordinary_muscle_development	ENSGALG00010003366	CASD1	0.14247695	0.108727209	1.310407495	0.19005802	0.219513227	positive
PRJNA729271	stage_1W_vs_E10	ordinary_muscle_development	ENSGALG00010003371	ENSGALG00010003371	-1.810118612	0.373274142	-4.84930084	1.23897E-06	2.29889E-06	negative
PRJNA729271	stage_1W_vs_E10	ordinary_muscle_development	ENSGALG00010003372	ENOX1	-1.551055198	0.144157094	-10.75947883	5.34717E-27	2.66898E-26	negative
PRJNA729271	stage_1W_vs_E10	ordinary_muscle_development	ENSGALG00010003374	SYCE2	-0.06710768	0.447885022	-0.149832383	0.88089686	0.894410198	negative
PRJNA729271	stage_1W_vs_E10	ordinary_muscle_development	ENSGALG00010003375	YWHAQ	-1.396379194	0.086323552	-16.17610916	7.43467E-59	1.05446E-57	negative
PRJNA729271	stage_1W_vs_E10	ordinary_muscle_development	ENSGALG00010003379	ENSGALG00010003379	-1.915850156	0.47036748	-4.073092293	4.6393E-05	7.64775E-05	negative
PRJNA729271	stage_1W_vs_E10	ordinary_muscle_development	ENSGALG00010003382	ASNS	2.369955422	0.599251138	3.954861779	7.65789E-05	0.000124343	positive
PRJNA729271	stage_1W_vs_E10	ordinary_muscle_development	ENSGALG00010003383	UBE2J1	0.674179568	0.08631693	7.810153778	5.69553E-15	1.69139E-14	positive
PRJNA729271	stage_1W_vs_E10	ordinary_muscle_development	ENSGALG00010003388	PXDN	-0.712516275	0.186213543	-3.82633971	0.000130063	0.000207354	negative
PRJNA729271	stage_1W_vs_E10	ordinary_muscle_development	ENSGALG00010003389	ENSGALG00010003389	-2.285869489	0.389910861	-5.862543771	4.55829E-09	9.87188E-09	negative
PRJNA729271	stage_1W_vs_E10	ordinary_muscle_development	ENSGALG00010003395	ENSGALG00010003395	-0.058110183	1.020335451	-0.056952037	0.954583402	0.9604808	negative
PRJNA729271	stage_1W_vs_E10	ordinary_muscle_development	ENSGALG00010003397	SPATA4	2.006974448	0.401664169	4.996647951	5.83354E-07	1.10735E-06	positive
PRJNA729271	stage_1W_vs_E10	ordinary_muscle_development	ENSGALG00010003400	PKIA	1.18552743	0.114551391	10.34930626	4.21529E-25	1.95085E-24	positive
PRJNA729271	stage_1W_vs_E10	ordinary_muscle_development	ENSGALG00010003401	ENSGALG00010003401	-1.267382476	1.726270809	-0.734173613	0.462842933	0.501113558	negative
PRJNA729271	stage_1W_vs_E10	ordinary_muscle_development	ENSGALG00010003403	GCDH	0.96609396	0.147468655	6.551181726	5.70835E-11	1.37515E-10	positive
PRJNA729271	stage_1W_vs_E10	ordinary_muscle_development	ENSGALG00010003405	GABRR2	-1.14299074	0.24512571	-4.662875793	3.11821E-06	5.61681E-06	negative
PRJNA729271	stage_1W_vs_E10	ordinary_muscle_development	ENSGALG00010003406	C1GALT1	-1.417415021	0.107366003	-13.20171174	8.57638E-40	6.83799E-39	negative
PRJNA729271	stage_1W_vs_E10	ordinary_muscle_development	ENSGALG00010003407	CHMP4C	0.274828813	0.213476958	1.287393337	0.197957226	0.227982678	positive
PRJNA729271	stage_1W_vs_E10	ordinary_muscle_development	ENSGALG00010003408	ENSGALG00010003408	-4.683443802	3.718201946	-1.259599094	0.207814023	0.238462972	negative
PRJNA729271	stage_1W_vs_E10	ordinary_muscle_development	ENSGALG00010003412	ASB5	1.891634163	0.146580229	12.90511125	4.21207E-38	3.15687E-37	positive
PRJNA729271	stage_1W_vs_E10	ordinary_muscle_development	ENSGALG00010003419	ENSGALG00010003419	-4.972756275	1.198587261	-4.148847928	3.34153E-05	5.58205E-05	negative
PRJNA729271	stage_1W_vs_E10	ordinary_muscle_development	ENSGALG00010003420	ENSGALG00010003420	0.426923227	0.631597006	0.67594245	0.499077185	0.53681536	positive
PRJNA729271	stage_1W_vs_E10	ordinary_muscle_development	ENSGALG00010003428	SNX16	1.035403711	0.153935985	6.726196669	1.74155E-11	4.32331E-11	positive
PRJNA729271	stage_1W_vs_E10	ordinary_muscle_development	ENSGALG00010003431	PM20D2	4.538013341	0.285148966	15.91453551	5.02385E-57	6.81841E-56	positive
PRJNA729271	stage_1W_vs_E10	ordinary_muscle_development	ENSGALG00010003432	LACC1	1.283947155	0.315132215	4.074312605	4.61505E-05	7.60962E-05	positive
PRJNA729271	stage_1W_vs_E10	ordinary_muscle_development	ENSGALG00010003437	PNRC1	1.410628779	0.179005183	7.880379543	3.26388E-15	9.79502E-15	positive
PRJNA729271	stage_1W_vs_E10	ordinary_muscle_development	ENSGALG00010003438	KCNS3	-1.038756055	0.175449586	-5.920538659	3.20889E-09	7.01551E-09	negative
PRJNA729271	stage_1W_vs_E10	ordinary_muscle_development	ENSGALG00010003439	GMIP	-0.427147744	0.236406988	-1.80683214	0.070788456	0.086727974	negative
PRJNA729271	stage_1W_vs_E10	ordinary_muscle_development	ENSGALG00010003442	SPCS3	0.429481263	0.089464594	4.800572418	1.58213E-06	2.90896E-06	positive
PRJNA729271	stage_1W_vs_E10	ordinary_muscle_development	ENSGALG00010003447	ZC2HC1A	-0.994587891	0.126832385	-7.841750287	4.44309E-15	1.32707E-14	negative
PRJNA729271	stage_1W_vs_E10	ordinary_muscle_development	ENSGALG00010003449	BORCS6	1.890191823	0.108855318	17.36425789	1.53877E-67	2.73004E-66	positive
PRJNA729271	stage_1W_vs_E10	ordinary_muscle_development	ENSGALG00010003454	BPNT2	-1.1966778	0.120666148	-9.917261977	3.50232E-23	1.494E-22	negative
PRJNA729271	stage_1W_vs_E10	ordinary_muscle_development	ENSGALG00010003456	CDS1	-3.723237922	0.410247224	-9.075595658	1.13057E-19	4.18801E-19	negative
PRJNA729271	stage_1W_vs_E10	ordinary_muscle_development	ENSGALG00010003466	TSC22D1	0.023667279	0.100477968	0.235546952	0.813784241	0.833398271	positive
PRJNA729271	stage_1W_vs_E10	ordinary_muscle_development	ENSGALG00010003468	ILVBL	1.189482971	0.134732699	8.828465365	1.06122E-18	3.74448E-18	positive
PRJNA729271	stage_1W_vs_E10	ordinary_muscle_development	ENSGALG00010003470	METTL3	1.038197229	0.186506717	5.566540679	2.59846E-08	5.37683E-08	positive
PRJNA729271	stage_1W_vs_E10	ordinary_muscle_development	ENSGALG00010003471	ADI1	0.907379736	0.133372804	6.803334001	1.02225E-11	2.56937E-11	positive
PRJNA729271	stage_1W_vs_E10	ordinary_muscle_development	ENSGALG00010003472	FAM110B	-2.884120857	0.13320707	-21.65140983	5.8949E-104	2.3935E-102	negative
PRJNA729271	stage_1W_vs_E10	ordinary_muscle_development	ENSGALG00010003473	ENSGALG00010003473	-0.930859871	0.265661912	-3.503926715	0.000458452	0.000700902	negative
PRJNA729271	stage_1W_vs_E10	ordinary_muscle_development	ENSGALG00010003474	RPS7	0.260200261	0.166112978	1.56640537	0.117253746	0.139642844	positive
PRJNA729271	stage_1W_vs_E10	ordinary_muscle_development	ENSGALG00010003475	ENSGALG00010003475	1.134981989	2.232576104	0.508373259	0.611191603	0.645339618	positive
PRJNA729271	stage_1W_vs_E10	ordinary_muscle_development	ENSGALG00010003478	COLEC11	0.94644753	0.179203136	5.281422793	1.28184E-07	2.54142E-07	positive
PRJNA729271	stage_1W_vs_E10	ordinary_muscle_development	ENSGALG00010003479	SYDE1	0.641192728	0.200807133	3.193077452	0.001407652	0.002068523	positive
PRJNA729271	stage_1W_vs_E10	ordinary_muscle_development	ENSGALG00010003485	RNGTT	-1.430768449	0.105156115	-13.60613641	3.682E-42	3.16364E-41	negative
PRJNA729271	stage_1W_vs_E10	ordinary_muscle_development	ENSGALG00010003489	SGCE	-0.581688844	0.11998591	-4.847976281	1.24727E-06	2.31399E-06	negative
PRJNA729271	stage_1W_vs_E10	ordinary_muscle_development	ENSGALG00010003503	NUFIP1	-0.019745678	0.085694079	-0.230420565	0.817764983	0.836816338	negative
PRJNA729271	stage_1W_vs_E10	ordinary_muscle_development	ENSGALG00010003506	DYNC1I1	-2.107126994	0.176641707	-11.92881926	8.3753E-33	5.24406E-32	negative
PRJNA729271	stage_1W_vs_E10	ordinary_muscle_development	ENSGALG00010003507	WDFY3	0.113331397	0.097649118	1.160598261	0.245805313	0.278464089	positive
PRJNA729271	stage_1W_vs_E10	ordinary_muscle_development	ENSGALG00010003509	GPALPP1	-0.356438346	0.073202138	-4.869234078	1.12032E-06	2.08781E-06	negative
PRJNA729271	stage_1W_vs_E10	ordinary_muscle_development	ENSGALG00010003511	UBXN2B	-1.101473842	0.145432168	-7.573797846	3.62468E-14	1.03282E-13	negative
PRJNA729271	stage_1W_vs_E10	ordinary_muscle_development	ENSGALG00010003512	ENSGALG00010003512	0.275704111	0.442995536	0.62236318	0.533703086	0.570125007	positive
PRJNA729271	stage_1W_vs_E10	ordinary_muscle_development	ENSGALG00010003517	GTF2F2	-0.772075896	0.088688004	-8.705527897	3.161E-18	1.0873E-17	negative
PRJNA729271	stage_1W_vs_E10	ordinary_muscle_development	ENSGALG00010003519	KCTD4	-3.499608606	0.7956282	-4.398547723	1.08978E-05	1.88703E-05	negative
PRJNA729271	stage_1W_vs_E10	ordinary_muscle_development	ENSGALG00010003520	ENSGALG00010003520	1.135007276	1.808471231	0.627605934	0.53026212	0.56682518	positive
PRJNA729271	stage_1W_vs_E10	ordinary_muscle_development	ENSGALG00010003522	ENSGALG00010003522	2.13362773	0.641556373	3.325705767	0.00088195	0.001321466	positive
PRJNA729271	stage_1W_vs_E10	ordinary_muscle_development	ENSGALG00010003525	NOL10	-0.802670111	0.067995597	-11.80473665	3.68944E-32	2.26508E-31	negative
PRJNA729271	stage_1W_vs_E10	ordinary_muscle_development	ENSGALG00010003528	GDAP1	-1.088555492	0.20268654	-5.370635339	7.84597E-08	1.58064E-07	negative
PRJNA729271	stage_1W_vs_E10	ordinary_muscle_development	ENSGALG00010003531	PPM1K	-1.017916973	0.151687721	-6.710608937	1.93814E-11	4.79982E-11	negative
PRJNA729271	stage_1W_vs_E10	ordinary_muscle_development	ENSGALG00010003539	ENSGALG00010003539	0.542546908	0.216160601	2.509925055	0.012075679	0.016166709	positive
PRJNA729271	stage_1W_vs_E10	ordinary_muscle_development	ENSGALG00010003541	CNR1	-1.471210559	0.220267998	-6.679184317	2.40276E-11	5.92514E-11	negative
PRJNA729271	stage_1W_vs_E10	ordinary_muscle_development	ENSGALG00010003543	SOX11	-3.190258317	0.082551925	-38.64547466	0	0	negative
PRJNA729271	stage_1W_vs_E10	ordinary_muscle_development	ENSGALG00010003552	PI15	-3.538209336	0.389751942	-9.078105731	1.10481E-19	4.09675E-19	negative
PRJNA729271	stage_1W_vs_E10	ordinary_muscle_development	ENSGALG00010003556	ENSGALG00010003556	1.13499108	2.007218613	0.565454641	0.571764568	0.607471041	positive
PRJNA729271	stage_1W_vs_E10	ordinary_muscle_development	ENSGALG00010003558	TPT1	2.970536157	0.184625369	16.08953403	3.02102E-58	4.21461E-57	positive
PRJNA729271	stage_1W_vs_E10	ordinary_muscle_development	ENSGALG00010003560	ADGRL3	-3.00910464	0.142377853	-21.13463977	3.82093E-99	1.40214E-97	negative
PRJNA729271	stage_1W_vs_E10	ordinary_muscle_development	ENSGALG00010003571	RPL7	0.13937779	0.157456547	0.885182563	0.376058175	0.413735351	positive
PRJNA729271	stage_1W_vs_E10	ordinary_muscle_development	ENSGALG00010003577	ENSGALG00010003577	2.103194511	1.755449981	1.19809424	0.230880331	0.262602825	positive
PRJNA729271	stage_1W_vs_E10	ordinary_muscle_development	ENSGALG00010003582	RDH10	-0.883971816	0.141345022	-6.254000338	4.0007E-10	9.19457E-10	negative
PRJNA729271	stage_1W_vs_E10	ordinary_muscle_development	ENSGALG00010003583	RSAD2	3.912923332	0.800560157	4.887731795	1.02004E-06	1.90683E-06	positive
PRJNA729271	stage_1W_vs_E10	ordinary_muscle_development	ENSGALG00010003585	PNPLA6	0.857736714	0.143141949	5.992210664	2.07008E-09	4.57963E-09	positive
PRJNA729271	stage_1W_vs_E10	ordinary_muscle_development	ENSGALG00010003589	SLC25A30	3.76804247	0.387390797	9.726721708	2.31948E-22	9.53782E-22	positive
PRJNA729271	stage_1W_vs_E10	ordinary_muscle_development	ENSGALG00010003595	SNX13	0.289634165	0.094380604	3.068789365	0.002149283	0.003103283	positive
PRJNA729271	stage_1W_vs_E10	ordinary_muscle_development	ENSGALG00010003596	CRISPLD1	-3.063730098	0.155408288	-19.71407146	1.63286E-86	4.53415E-85	negative
PRJNA729271	stage_1W_vs_E10	ordinary_muscle_development	ENSGALG00010003597	RNF144A	-0.610087301	0.14496533	-4.208504893	2.57066E-05	4.3182E-05	negative
PRJNA729271	stage_1W_vs_E10	ordinary_muscle_development	ENSGALG00010003604	ODC	-1.520456468	0.216548962	-7.021305733	2.19804E-12	5.7221E-12	negative
PRJNA729271	stage_1W_vs_E10	ordinary_muscle_development	ENSGALG00010003617	COG3	-0.000280962	0.083029634	-0.003383873	0.997300065	0.997781919	negative
PRJNA729271	stage_1W_vs_E10	ordinary_muscle_development	ENSGALG00010003619	HNF4G	-0.471626165	0.517753015	-0.910909548	0.362343035	0.399951292	negative
PRJNA729271	stage_1W_vs_E10	ordinary_muscle_development	ENSGALG00010003627	MCOLN1	0.580431844	0.119790107	4.84540717	1.26352E-06	2.34264E-06	positive
PRJNA729271	stage_1W_vs_E10	ordinary_muscle_development	ENSGALG00010003632	TECRL	0.103888752	0.350563512	0.296347874	0.766964429	0.790749654	positive
PRJNA729271	stage_1W_vs_E10	ordinary_muscle_development	ENSGALG00010003634	CYP4F11	1.703212774	0.299408963	5.688583131	1.28098E-08	2.69802E-08	positive
PRJNA729271	stage_1W_vs_E10	ordinary_muscle_development	ENSGALG00010003639	ENSGALG00010003639	-0.090325225	0.782469413	-0.11543611	0.908099459	0.919068682	negative
PRJNA729271	stage_1W_vs_E10	ordinary_muscle_development	ENSGALG00010003641	ENSGALG00010003641	0.60687946	2.578076982	0.235400054	0.813898244	0.833447902	positive
PRJNA729271	stage_1W_vs_E10	ordinary_muscle_development	ENSGALG00010003650	SPACA1	-3.006663716	0.952417788	-3.156874802	0.001594698	0.002331801	negative
PRJNA729271	stage_1W_vs_E10	ordinary_muscle_development	ENSGALG00010003652	MYCN	-0.943746762	0.142239235	-6.634925734	3.24666E-11	7.94535E-11	negative

PRJNA729271	stage_1W_vs_E10	ordinary_muscle_development	ENSGALG00010003660	IGFBP7	3.405971572	0.164795498	20.66786783	6.743E-95	2.26774E-93	positive
PRJNA729271	stage_1W_vs_E10	ordinary_muscle_development	ENSGALG00010003665	RDH14	2.012819644	0.091079387	22.09961783	3.1875E-108	1.3959E-106	positive
PRJNA729271	stage_1W_vs_E10	ordinary_muscle_development	ENSGALG00010003666	SLC25A13	-0.624776491	0.07825056	-7.984306964	1.41314E-15	4.31322E-15	negative
PRJNA729271	stage_1W_vs_E10	ordinary_muscle_development	ENSGALG00010003669	ENSGALG00010003669	1.135010799	1.676252672	0.677112	0.498334896	0.536056721	positive
PRJNA729271	stage_1W_vs_E10	ordinary_muscle_development	ENSGALG00010003671	ENSGALG00010003671	0.239979335	0.402487678	0.596240204	0.551014771	0.587146887	positive
PRJNA729271	stage_1W_vs_E10	ordinary_muscle_development	ENSGALG00010003674	AKIRIN2	0.617284237	0.13185433	4.68156211	2.84697E-06	5.14036E-06	positive
PRJNA729271	stage_1W_vs_E10	ordinary_muscle_development	ENSGALG00010003676	POLR2B	0.86790034	0.07751493	11.19655713	4.23889E-29	2.30209E-28	positive
PRJNA729271	stage_1W_vs_E10	ordinary_muscle_development	ENSGALG00010003678	ZNFY1	-0.579056404	0.4465252	-1.296805654	0.194698065	0.224461023	negative
PRJNA729271	stage_1W_vs_E10	ordinary_muscle_development	ENSGALG00010003680	TRAPPC5	0.972376866	0.105731973	9.196620826	3.69383E-20	1.39542E-19	positive
PRJNA729271	stage_1W_vs_E10	ordinary_muscle_development	ENSGALG00010003681	NT5C1B	-0.49447186	0.131163551	-3.76988771	0.000163321	0.000258584	negative
PRJNA729271	stage_1W_vs_E10	ordinary_muscle_development	ENSGALG00010003683	ENSGALG00010003683	0.656394468	1.755426205	0.373923134	0.708461504	0.736036061	positive
PRJNA729271	stage_1W_vs_E10	ordinary_muscle_development	ENSGALG00010003691	ERICH1	0.026908623	0.150952028	0.178259433	0.858519236	0.874199953	positive
PRJNA729271	stage_1W_vs_E10	ordinary_muscle_development	ENSGALG00010003692	ZNFY2	-0.462392598	0.260867137	-1.772521456	0.076308037	0.093081292	negative
PRJNA729271	stage_1W_vs_E10	ordinary_muscle_development	ENSGALG00010003695	NOA1	0.984150996	0.100281554	9.813878635	9.81228E-23	4.1E-22	positive
PRJNA729271	stage_1W_vs_E10	ordinary_muscle_development	ENSGALG00010003698	MACC1	-5.595437993	1.284988043	-4.354466971	1.33391E-05	2.29197E-05	negative
PRJNA729271	stage_1W_vs_E10	ordinary_muscle_development	ENSGALG00010003700	REST	-0.459903382	0.067546251	-6.808718099	9.84722E-12	2.47762E-11	negative
PRJNA729271	stage_1W_vs_E10	ordinary_muscle_development	ENSGALG00010003701	SARAF	1.225241847	0.08185481	14.96847705	1.17992E-50	1.32376E-49	positive
PRJNA729271	stage_1W_vs_E10	ordinary_muscle_development	ENSGALG00010003703	TDRP	-1.120800574	0.232387002	-4.82299166	1.41421E-06	2.613E-06	negative
PRJNA729271	stage_1W_vs_E10	ordinary_muscle_development	ENSGALG00010003713	SPERT	0.613177652	0.259453171	2.363346148	0.018110745	0.023761336	positive
PRJNA729271	stage_1W_vs_E10	ordinary_muscle_development	ENSGALG00010003714	ORC3	-0.290168434	0.10847415	-2.675000765	0.007472908	0.010238166	negative
PRJNA729271	stage_1W_vs_E10	ordinary_muscle_development	ENSGALG00010003716	YLEC8	-1.057836992	0.698312196	-1.514848226	0.129810844	0.153575595	negative
PRJNA729271	stage_1W_vs_E10	ordinary_muscle_development	ENSGALG00010003717	LEPROTL1	-0.014011935	0.097533916	-0.143662176	0.885767241	0.898662851	negative
PRJNA729271	stage_1W_vs_E10	ordinary_muscle_development	ENSGALG00010003719	RPGR	-0.796339494	0.115445538	-6.897966825	5.27521E-12	1.34525E-11	negative
PRJNA729271	stage_1W_vs_E10	ordinary_muscle_development	ENSGALG00010003720	ENSGALG00010003720	1.134961339	2.731725995	0.415474078	0.677794888	0.707368198	positive
PRJNA729271	stage_1W_vs_E10	ordinary_muscle_development	ENSGALG00010003722	MHCY11	2.493930224	0.653667033	3.815291424	0.000136022	0.000216593	positive
PRJNA729271	stage_1W_vs_E10	ordinary_muscle_development	ENSGALG00010003724	ENSGALG00010003724	0.543380628	0.213606911	2.543834489	0.010964302	0.014751027	positive
PRJNA729271	stage_1W_vs_E10	ordinary_muscle_development	ENSGALG00010003726	ENPEP	-1.835405517	0.449789806	-4.080584959	4.49225E-05	7.41969E-05	negative
PRJNA729271	stage_1W_vs_E10	ordinary_muscle_development	ENSGALG00010003727	FBXO25	0.654965963	0.152967372	4.281736403	1.85441E-05	3.15008E-05	positive
PRJNA729271	stage_1W_vs_E10	ordinary_muscle_development	ENSGALG00010003729	RARS2	-0.744807891	0.104994916	-7.093751953	1.30524E-12	3.43803E-12	negative
PRJNA729271	stage_1W_vs_E10	ordinary_muscle_development	ENSGALG00010003731	ENSGALG00010003731	1.719400185	0.580024017	2.964360327	0.003033129	0.004321329	positive
PRJNA729271	stage_1W_vs_E10	ordinary_muscle_development	ENSGALG00010003733	STAU2	0.291158082	0.07065342	4.120933996	3.7734E-05	6.27143E-05	positive
PRJNA729271	stage_1W_vs_E10	ordinary_muscle_development	ENSGALG00010003734	ZC3H13	-0.516444503	0.142052792	-3.635581517	0.000277354	0.000432378	negative
PRJNA729271	stage_1W_vs_E10	ordinary_muscle_development	ENSGALG00010003736	ENSGALG00010003736	1.134939642	3.115575725	0.364279267	0.71564948	0.742969433	positive
PRJNA729271	stage_1W_vs_E10	ordinary_muscle_development	ENSGALG00010003737	SRPT2	-0.227579119	0.056456515	-4.0310515	5.55279E-05	9.10391E-05	negative
PRJNA729271	stage_1W_vs_E10	ordinary_muscle_development	ENSGALG00010003740	PITX2	-2.971500597	0.358802578	-8.281714741	1.21414E-16	3.87985E-16	negative
PRJNA729271	stage_1W_vs_E10	ordinary_muscle_development	ENSGALG00010003742	ZFHX4	-1.872291439	0.167069017	-11.20669453	3.78049E-29	2.05699E-28	negative
PRJNA729271	stage_1W_vs_E10	ordinary_muscle_development	ENSGALG00010003743	ARL9	0.041089016	0.176767656	0.232446461	0.816191266	0.835559884	positive
PRJNA729271	stage_1W_vs_E10	ordinary_muscle_development	ENSGALG00010003746	HOPX	3.212300742	0.406851746	7.89550683	2.89138E-15	8.69874E-15	positive
PRJNA729271	stage_1W_vs_E10	ordinary_muscle_development	ENSGALG00010003749	MHCY15	2.954321306	0.946487323	3.121353274	0.001800219	0.002620687	positive
PRJNA729271	stage_1W_vs_E10	ordinary_muscle_development	ENSGALG00010003753	SLCO5A1	1.039189268	0.159510604	6.51486009	7.27574E-11	1.73915E-10	positive
PRJNA729271	stage_1W_vs_E10	ordinary_muscle_development	ENSGALG00010003754	FAM110C	-0.082856226	0.251086759	-0.329990425	0.741407197	0.766909107	negative
PRJNA729271	stage_1W_vs_E10	ordinary_muscle_development	ENSGALG00010003755	SLC35A1	-0.764391779	0.092304643	-8.281184489	1.21956E-16	3.8963E-16	negative
PRJNA729271	stage_1W_vs_E10	ordinary_muscle_development	ENSGALG00010003756	MHCY7	0.381469514	0.378076674	1.008973946	0.312987129	0.348953807	positive
PRJNA729271	stage_1W_vs_E10	ordinary_muscle_development	ENSGALG00010003761	UGT8	3.203708876	0.326377443	9.81596292	9.61159E-23	4.01962E-22	positive
PRJNA729271	stage_1W_vs_E10	ordinary_muscle_development	ENSGALG00010003762	EVI5L	0.055010848	0.099325172	0.553845991	0.57968425	0.615118829	positive
PRJNA729271	stage_1W_vs_E10	ordinary_muscle_development	ENSGALG00010003764	HDAC9	0.393930738	0.193419705	2.036662901	0.041683835	0.052553465	positive
PRJNA729271	stage_1W_vs_E10	ordinary_muscle_development	ENSGALG00010003765	ENSGALG00010003765	-1.143255859	0.901186869	-1.268611315	0.204579727	0.234919048	negative
PRJNA729271	stage_1W_vs_E10	ordinary_muscle_development	ENSGALG00010003767	ENSGALG00010003767	-0.342689583	0.415179147	-0.825401722	0.409143558	0.447115189	negative
PRJNA729271	stage_1W_vs_E10	ordinary_muscle_development	ENSGALG00010003768	ARSJ	-1.993138289	0.135131562	-14.74961338	3.09432E-49	3.30038E-48	negative
PRJNA729271	stage_1W_vs_E10	ordinary_muscle_development	ENSGALG00010003769	C6orf163	-0.901346646	0.577577995	-1.560562649	0.118626977	0.141150717	negative
PRJNA729271	stage_1W_vs_E10	ordinary_muscle_development	ENSGALG00010003772	ENSGALG00010003772	1.491926328	0.44256223	3.371110831	0.000748657	0.001128044	positive
PRJNA729271	stage_1W_vs_E10	ordinary_muscle_development	ENSGALG00010003775	NCOA2	0.633370021	0.114251397	5.543652323	2.96227E-08	6.10696E-08	positive
PRJNA729271	stage_1W_vs_E10	ordinary_muscle_development	ENSGALG00010003777	ENSGALG00010003777	4.319912289	0.8075455	5.349435157	8.82292E-08	1.77172E-07	positive
PRJNA729271	stage_1W_vs_E10	ordinary_muscle_development	ENSGALG00010003778	LCA5	0.004397761	0.125946282	0.034917749	0.972145327	0.97611849	positive
PRJNA729271	stage_1W_vs_E10	ordinary_muscle_development	ENSGALG00010003785	ENSGALG00010003785	1.67076377	1.235692329	1.352087191	0.176347425	0.204590677	positive
PRJNA729271	stage_1W_vs_E10	ordinary_muscle_development	ENSGALG00010003786	ENSGALG00010003786	-0.7170952	0.613964408	-1.167975196	0.24281677	0.275358245	negative
PRJNA729271	stage_1W_vs_E10	ordinary_muscle_development	ENSGALG00010003787	SMIM8	1.543580823	0.187700445	8.223639651	1.97419E-16	6.25209E-16	positive
PRJNA729271	stage_1W_vs_E10	ordinary_muscle_development	ENSGALG00010003788	NUDT9	0.191842447	0.126775975	1.513239769	0.130218766	0.154020482	positive
PRJNA729271	stage_1W_vs_E10	ordinary_muscle_development	ENSGALG00010003790	ELOVL6	-0.457891136	0.181868823	-2.517700007	0.011812388	0.015836161	negative
PRJNA729271	stage_1W_vs_E10	ordinary_muscle_development	ENSGALG00010003791	ITGB8	-1.968192742	0.176982302	-11.12084495	9.93268E-29	5.31074E-28	negative
PRJNA729271	stage_1W_vs_E10	ordinary_muscle_development	ENSGALG00010003793	TRAM1L1	-0.241315312	0.085036418	-2.837787842	0.004542737	0.006364527	negative
PRJNA729271	stage_1W_vs_E10	ordinary_muscle_development	ENSGALG00010003795	LCP1	0.736546811	0.201293103	3.659076233	0.000253126	0.000395586	positive
PRJNA729271	stage_1W_vs_E10	ordinary_muscle_development	ENSGALG00010003796	ENSGALG00010003796	2.490146779	0.303243664	8.211702585	2.18074E-16	6.88517E-16	positive
PRJNA729271	stage_1W_vs_E10	ordinary_muscle_development	ENSGALG00010003797	ENSGALG00010003797	0.335061673	0.174531947	1.91977273	0.054886613	0.068167377	positive
PRJNA729271	stage_1W_vs_E10	ordinary_muscle_development	ENSGALG00010003803	LACTB2	1.365796954	0.13218879	10.33216929	5.04075E-25	2.32102E-24	positive
PRJNA729271	stage_1W_vs_E10	ordinary_muscle_development	ENSGALG00010003804	PSME1	2.921001708	0.254594468	11.47315467	1.79976E-30	1.03399E-29	positive
PRJNA729271	stage_1W_vs_E10	ordinary_muscle_development	ENSGALG00010003806	EGF	4.390484306	0.315299105	13.92482326	4.47647E-44	4.08605E-43	positive
PRJNA729271	stage_1W_vs_E10	ordinary_muscle_development	ENSGALG00010003808	TWIST1	-3.527644028	0.171598778	-20.55751256	6.59273E-94	2.15715E-92	negative
PRJNA729271	stage_1W_vs_E10	ordinary_muscle_development	ENSGALG00010003810	HMGN2P46	0.359755994	0.09112549	3.947918356	7.88337E-05	0.000127947	positive
PRJNA729271	stage_1W_vs_E10	ordinary_muscle_development	ENSGALG00010003812	BG8	0.358835915	0.403307354	0.889733133	0.373609199	0.411228289	positive
PRJNA729271	stage_1W_vs_E10	ordinary_muscle_development	ENSGALG00010003813	LRIT3	-2.556595603	0.645305699	-3.961836394	7.43755E-05	0.000120942	negative
PRJNA729271	stage_1W_vs_E10	ordinary_muscle_development	ENSGALG00010003814	TPP1	1.586113726	0.132181279	11.99953383	3.57303E-33	2.27353E-32	positive
PRJNA729271	stage_1W_vs_E10	ordinary_muscle_development	ENSGALG00010003815	RASAL3	3.425939581	0.582192491	5.884547862	3.99144E-09	8.67664E-09	positive
PRJNA729271	stage_1W_vs_E10	ordinary_muscle_development	ENSGALG00010003816	KLHL8	-0.809804142	0.181455823	-4.462817056	8.08891E-06	1.41179E-05	negative
PRJNA729271	stage_1W_vs_E10	ordinary_muscle_development	ENSGALG00010003817	BF2	4.55357009	0.282110322	16.14109707	1.31189E-58	1.85159E-57	positive
PRJNA729271	stage_1W_vs_E10	ordinary_muscle_development	ENSGALG00010003818	GAB2	0.172681673	0.163231495	1.057894324	0.290103623	0.324990495	positive
PRJNA729271	stage_1W_vs_E10	ordinary_muscle_development	ENSGALG00010003819	PHIP	-0.002592622	0.094178362	-0.027528847	0.978037932	0.981355381	negative
PRJNA729271	stage_1W_vs_E10	ordinary_muscle_development	ENSGALG00010003820	SH3YL1	-0.590594924	0.300907314	-1.962713757	0.049679444	0.062045798	negative
PRJNA729271	stage_1W_vs_E10	ordinary_muscle_development	ENSGALG00010003821	RUBCNL	0.380878778	0.157281567	2.421636468	0.015450798	0.020443929	positive
PRJNA729271	stage_1W_vs_E10	ordinary_muscle_development	ENSGALG00010003822	ENSGALG00010003822	-0.616244497	0.166124804	-3.709527309	0.000207647	0.000326093	negative
PRJNA729271	stage_1W_vs_E10	ordinary_muscle_development	ENSGALG00010003823	PTPN13	-0.565105751	0.149514535	-3.779604097	0.000157078	0.000249053	negative
PRJNA729271	stage_1W_vs_E10	ordinary_muscle_development	ENSGALG00010003824	SP4	0.437090957	0.158398063	2.759446355	0.005789939	0.008027276	positive
PRJNA729271	stage_1W_vs_E10	ordinary_muscle_development	ENSGALG00010003826	ENSGALG00010003826	-0.013432231	0.106616265	-0.125986694	0.899742457	0.911368874	negative
PRJNA729271	stage_1W_vs_E10	ordinary_muscle_development	ENSGALG00010003827	RRH	-1.243582982	0.786192112	-1.581780029	0.113699796	0.135688995	negative
PRJNA729271	stage_1W_vs_E10	ordinary_muscle_development	ENSGALG00010003829	ENSGALG00010003829	-0.666806802	0.138684944	-4.80806915	1.52395E-06	2.80825E-06	negative
PRJNA729271	stage_1W_vs_E10	ordinary_muscle_development	ENSGALG00010003830	DMB2	0.957780158	0.232341725	4.122290816	3.75123E-05	6.23699E-05	positive
PRJNA729271	stage_1W_vs_E10	ordinary_muscle_development	ENSGALG00010003831	IRAK1BP1	0.269460803	0.16582508	1.624970141	0.104168921	0.124983736	positive
PRJNA729271	stage_1W_vs_E10	ordinary_muscle_development	ENSGALG00010003832	ART1B	5.800780587	0.936170882	6.196283926	5.78118E-10	1.31613E-09	positive
PRJNA729271	stage_1W_vs_E10	ordinary_muscle_development	ENSGALG00010003833	ACP1	0.170149553	0.157665048	1.079183722	0.280505835	0.314919996	positive
PRJNA729271	stage_1W_vs_E10	ordinary_muscle_development	ENSGALG00010003834	MAPK10	-1.078230054	0.439019168	-2.455997673	0.014049407	0.018696856	negative
PRJNA729271	stage_1W_vs_E10	ordinary_muscle_development	ENSGALG00010003835	ENSGALG00010003835	2.757126974	0.16319149	16.89504138	4.8937E-64	7.90794E-63	positive
PRJNA729271	stage_1W_vs_E10	ordinary_muscle_development	ENSGALG00010003836	CD1F	2.281131595	0.357570562	6.379528511	1.77634E-10	4.16164E-10	positive
PRJNA729271	stage_1W_vs_E10	ordinary_muscle_development	ENSGALG00010003837	ALKAL2	1.509857648	0.240507861	6.27778919	3.43421E-10	7.91523E-10	positive
PRJNA729271	stage_1W_vs_E10	ordinary_muscle_development	ENSGALG00010003838	GAR1	-2.384346919	0.190751594	-12.49974831	7.4888E-36	5.26687E-35	negative
PRJNA729271	stage_1W_vs_E10	ordinary_muscle_development	ENSGALG00010003839	ENSGALG00010003839	3.353100518	0.731410527	4.584430212	4.55226E-06	8.09533E-06	positive
PRJNA729271	stage_1W_vs_E10	ordinary_muscle_development	ENSGALG00010003841	UBE2W	0.345660302	0.142896979	2.41894758	0.015565483	0.020587798	positive
PRJNA729271	stage_1W_vs_E10	ordinary_muscle_development	ENSGALG00010003842	ENSGALG00010003842	0.151695299	0.144568768	1.049295093	0.294042324	0.329123049	positive
PRJNA729271	stage_1W_vs_E10	ordinary_muscle_development	ENSGALG00010003843	LRCH1	0.215969797	0.10899082	1.98154116	0.047530618	0.059500502	positive
PRJNA729271	stage_1W_vs_E10	ordinary_muscle_development	ENSGALG00010003844	TMEM18	-1.093183985	0.116044966	-9.42034816	4.49595E-21	1.76418E-20	negative
PRJNA729271	stage_1W_vs_E10	ordinary_muscle_development	ENSGALG00010003845	CFI	1.611622633	0.857888131	1.878592995			positive
PRJNA729271	stage_1W_vs_E10	ordinary_muscle_development	ENSGALG00010003848	ESD	0.185663907	0.124018702	1.497063779	0.134376663	0.158666482	positive
PRJNA729271	stage_1W_vs_E10	ordinary_muscle_development	ENSGALG00010003850	PLA2G12A	-0.084611166	0.099366976	-0.851501873	0.394490623	0.432569906	negative
PRJNA729271	stage_1W_vs_E10	ordinary_muscle_development	ENSGALG00010003851	PHF14	-1.534253435	0.065704573	-23.35078615	1.353E-120	7.6611E-119	negative
PRJNA729271	stage_1W_vs_E10	ordinary_muscle_development	ENSGALG00010003852	TCEB1	-0.331241324	0.107664209	-3.076615029	0.002093655	0.003028091	negative
PRJNA729271	stage_1W_vs_E10	ordinary_muscle_development	ENSGALG00010003853	ENSGALG00010003853	0.333074733	0.232262571	1.434043942	0.151559724	0.177667464	positive
PRJNA729271	stage_1W_vs_E10	ordinary_muscle_development	ENSGALG00010003858	SUCLA2	2.627099827	0.124094658	21.17012831	1.80067E-99	6.67536E-98	positive
PRJNA729271	stage_1W_vs_E10	ordinary_muscle_development	ENSGALG00010003860	TMEM70	0.82343093	0.113582782	7.249610481	4.17972E-13	1.12612E-12	positive
PRJNA729271	stage_1W_vs_E10	ordinary_muscle_development	ENSGALG00010003861	NUDT15	-0.656957972	0.105027526	-6.25510281	3.97254E-10	9.13275E-10	negative
PRJNA729271	stage_1W_vs_E10	ordinary_muscle_development	ENSGALG00010003862	PCK2	-0.390780559	0.145450549	-2.686690152	0.007216386	0.009908261	negative
PRJNA729271	stage_1W_vs_E10	ordinary_muscle_development	ENSGALG00010003863	HTR1B	-3.298274863	0.324897567	-10.15173765	3.25515E-24	1.44513E-23	negative
PRJNA729271	stage_1W_vs_E10	ordinary_muscle_development	ENSGALG00010003865	AKAP8L	-0.91883178	0.190951854	-4.811850537	1.49539E-06	2.75807E-06	negative
PRJNA729271	stage_1W_vs_E10	ordinary_muscle_development	ENSGALG00010003868	HBBA	-1.893478879	0.398896689	-4.746790168	2.0667E-06	3.76816E-06	negative
PRJNA729271	stage_1W_vs_E10	ordinary_muscle_development	ENSGALG00010003869	LY96	2.039863213	0.274112349	7.44170491	9.93941E-14	2.76158E-13	positive
PRJNA729271	stage_1W_vs_E10	ordinary_muscle_development	ENSGALG00010003870	MED4	-0.857973178	0.097238731	-8.823368707	1.11067E-18	3.91516E-18	negative
PRJNA729271	stage_1W_vs_E10	ordinary_muscle_development	ENSGALG00010003871	ENSGALG00010003871	-0.793878855	0.207766601	-3.821012856	0.000132905	0.000211768	negative
PRJNA729271	stage_1W_vs_E10	ordinary_muscle_development	ENSGALG00010003874	YES1	-0.764758082	0.098211983	-7.786810349	6.8722E-15	2.03208E-14	negative
PRJNA729271	stage_1W_vs_E10	ordinary_muscle_development	ENSGALG00010003875	AKAP8	-0.815976681	0.112034307	-7.28327513	3.25812E-13	8.82242E-13	negative
PRJNA729271	stage_1W_vs_E10	ordinary_muscle_development	ENSGALG00010003878	ARHGAP24	0.483780045	0.089234011	5.42147598	5.91089E-08	1.20087E-07	positive
PRJNA729271	stage_1W_vs_E10	ordinary_muscle_development	ENSGALG00010003879	CASP6	-1.541250033	0.121087631	-12.72838543	4.11256E-37	2.98505E-36	negative
PRJNA729271	stage_1W_vs_E10	ordinary_muscle_development	ENSGALG00010003882	ENSGALG00010003882	-0.102412357	0.121807906	-0.840769369	0.40047715	0.438736021	negative
PRJNA729271	stage_1W_vs_E10	ordinary_muscle_development	ENSGALG00010003884	TYMS	-3.431247502	0.196867174	-17.42925153	4.94895E-68	8.93338E-67	negative
PRJNA729271	stage_1W_vs_E10	ordinary_muscle_development	ENSGALG00010003887	GIPC1	-0.559774754	0.1437942	-3.892888282	9.90578E-05	0.000159326	negative
PRJNA729271	stage_1W_vs_E10	ordinary_muscle_development	ENSGALG00010003889	ITM2B	0.131788759	0.087831112	1.500479221	0.133490312	0.157735556	positive
PRJNA729271	stage_1W_vs_E10	ordinary_muscle_development	ENSGALG00010003892	OPRK1	-4.79038295	2.563370122	-1.868783173	0.061652987	0.076075598	negative
PRJNA729271	stage_1W_vs_E10	ordinary_muscle_development	ENSGALG00010003894	COPS4	0.464867958	0.124425837	3.73610472	0.000186893	0.000294714	positive
PRJNA729271	stage_1W_vs_E10	ordinary_muscle_development	ENSGALG00010003895	THSD7A	0.474373416	0.161827225	2.931357286	0.003374844	0.004788407	positive
PRJNA729271	stage_1W_vs_E10	ordinary_muscle_development	ENSGALG00010003896	MCUB	-1.360771889	0.136457125	-9.972157104	2.01798E-23	8.68716E-23	negative
PRJNA729271	stage_1W_vs_E10	ordinary_muscle_development	ENSGALG00010003898	NDST4	-4.879992942	0.637582613	-7.653899031	1.94975E-14	5.62198E-14	negative
PRJNA729271	stage_1W_vs_E10	ordinary_muscle_development	ENSGALG00010003899	LIN54	0.373074303	0.165472747	2.254596663	0.024158657	0.031310777	positive
PRJNA729271	stage_1W_vs_E10	ordinary_muscle_development	ENSGALG00010003900	RB1	-0.564910152	0.088221103	-6.403344903	1.52009E-10	3.57517E-10	negative
PRJNA729271	stage_1W_vs_E10	ordinary_muscle_development	ENSGALG00010003902	ENSGALG00010003902	1.200163915	0.124423435	9.645802743	5.12089E-22	2.07978E-21	positive

PRJNA729271	stage_1W_vs_E10	ordinary_muscle_development	ENSGALG00010003903	COL28A1	7.230655244	0.277210146	26.0836602	5.5875E-150	4.6816E-148	positive
PRJNA729271	stage_1W_vs_E10	ordinary_muscle_development	ENSGALG00010003909	DNAJB1	-0.25161707	0.157399329	-1.598590486	0.109911625	0.131373712	negative
PRJNA729271	stage_1W_vs_E10	ordinary_muscle_development	ENSGALG00010003910	THAP9	1.615809109	0.147120992	10.98285897	4.62065E-28	2.41443E-27	positive
PRJNA729271	stage_1W_vs_E10	ordinary_muscle_development	ENSGALG00010003913	PSMB5	1.022977084	0.116729676	8.763641917	1.89042E-18	6.57112E-18	positive
PRJNA729271	stage_1W_vs_E10	ordinary_muscle_development	ENSGALG00010003914	CAMK2D	1.186383477	0.105766449	11.21701148	3.36449E-29	1.83339E-28	positive
PRJNA729271	stage_1W_vs_E10	ordinary_muscle_development	ENSGALG00010003915	MIOS	-0.131400731	0.093010838	-1.412746452	0.157730288	0.184408818	negative
PRJNA729271	stage_1W_vs_E10	ordinary_muscle_development	ENSGALG00010003918	HTR1E	-3.050571743	0.764532835	-3.990112134	6.60421E-05	0.00010779	negative
PRJNA729271	stage_1W_vs_E10	ordinary_muscle_development	ENSGALG00010003920	LPAR6	1.08665476	0.152913353	7.106343176	1.19158E-12	3.14607E-12	positive
PRJNA729271	stage_1W_vs_E10	ordinary_muscle_development	ENSGALG00010003921	COLEC12	-0.772993697	0.168594378	-4.584931644	4.54135E-06	8.07791E-06	negative
PRJNA729271	stage_1W_vs_E10	ordinary_muscle_development	ENSGALG00010003923	ENSGALG00010003923	-0.87098928	0.121236752	-7.184201722	6.7601E-13	1.8039E-12	negative
PRJNA729271	stage_1W_vs_E10	ordinary_muscle_development	ENSGALG00010003925	SEC31A	-0.686705185	0.085932469	-7.991219054	1.33611E-15	4.08671E-15	negative
PRJNA729271	stage_1W_vs_E10	ordinary_muscle_development	ENSGALG00010003928	ENSGALG00010003928	1.41361204	0.15501663	9.119099301	7.57513E-20	2.82702E-19	positive
PRJNA729271	stage_1W_vs_E10	ordinary_muscle_development	ENSGALG00010003929	RCBTB2	0.333315479	0.097816344	3.407564278	0.000655455	0.000991424	positive
PRJNA729271	stage_1W_vs_E10	ordinary_muscle_development	ENSGALG00010003933	TECR	2.770719079	0.206092629	13.44404744	3.33675E-41	2.7925E-40	positive
PRJNA729271	stage_1W_vs_E10	ordinary_muscle_development	ENSGALG00010003937	ENSGALG00010003937	0.853095398	0.207143008	4.118388576	3.81531E-05	6.33554E-05	positive
PRJNA729271	stage_1W_vs_E10	ordinary_muscle_development	ENSGALG00010003948	SCD5	-3.236219525	0.150123001	-21.5571198	4.54E-103	1.798E-101	negative
PRJNA729271	stage_1W_vs_E10	ordinary_muscle_development	ENSGALG00010003958	CYSLTR2	3.292355898	0.365118042	9.017236948	1.92893E-19	7.05878E-19	positive
PRJNA729271	stage_1W_vs_E10	ordinary_muscle_development	ENSGALG00010003960	ASPH	1.619615525	0.195964976	8.264821402	1.39904E-16	4.4589E-16	positive
PRJNA729271	stage_1W_vs_E10	ordinary_muscle_development	ENSGALG00010003962	UMAD1	1.298281337	0.203498629	6.379803866	1.77315E-10	4.15483E-10	positive
PRJNA729271	stage_1W_vs_E10	ordinary_muscle_development	ENSGALG00010003965	SNTG1	-4.450516716	0.594743105	-7.483090899	7.25946E-14	2.0365E-13	negative
PRJNA729271	stage_1W_vs_E10	ordinary_muscle_development	ENSGALG00010003968	DSN1	0.708684222	0.171951802	4.121412011	3.76557E-05	6.2594E-05	positive
PRJNA729271	stage_1W_vs_E10	ordinary_muscle_development	ENSGALG00010003972	PENK	-2.512671635	0.241196147	-10.41754468	2.06208E-25	9.64499E-25	negative
PRJNA729271	stage_1W_vs_E10	ordinary_muscle_development	ENSGALG00010003977	MYO6	-1.462039703	0.120682323	-12.11477926	8.82013E-34	5.74081E-33	negative
PRJNA729271	stage_1W_vs_E10	ordinary_muscle_development	ENSGALG00010003978	ANK2	-0.140057087	0.120102051	-1.166150663	0.243553536	0.276100139	negative
PRJNA729271	stage_1W_vs_E10	ordinary_muscle_development	ENSGALG00010003980	CHCHD7	0.319107499	0.120099869	2.657017877	0.007883526	0.0107773	positive
PRJNA729271	stage_1W_vs_E10	ordinary_muscle_development	ENSGALG00010003981	TMEM150C	0.178472964	0.16533797	1.079443299	0.280390158	0.314838898	positive
PRJNA729271	stage_1W_vs_E10	ordinary_muscle_development	ENSGALG00010003982	GLCCI1	-0.809589636	0.122618025	-6.602533663	4.04189E-11	9.84494E-11	negative
PRJNA729271	stage_1W_vs_E10	ordinary_muscle_development	ENSGALG00010003985	PRMT5	0.887862781	0.133332226	6.659026166	2.75648E-11	6.77436E-11	positive
PRJNA729271	stage_1W_vs_E10	ordinary_muscle_development	ENSGALG00010003986	FNDC3A	0.7904745	0.063312456	12.48529209	8.98131E-36	6.29213E-35	positive
PRJNA729271	stage_1W_vs_E10	ordinary_muscle_development	ENSGALG00010003988	ENOPH1	-0.641986973	0.089446334	-7.177342516	7.10795E-13	1.89428E-12	negative
PRJNA729271	stage_1W_vs_E10	ordinary_muscle_development	ENSGALG00010003990	ICA1	-0.726291415	0.173363684	-4.189409221	2.79682E-05	4.68886E-05	negative
PRJNA729271	stage_1W_vs_E10	ordinary_muscle_development	ENSGALG00010003993	ENSGALG00010003993	1.159034447	0.133764602	8.664732141	4.52576E-18	1.54137E-17	positive
PRJNA729271	stage_1W_vs_E10	ordinary_muscle_development	ENSGALG00010003994	SDR16C5	1.539832728	0.084421583	18.23979926	2.49372E-74	5.17118E-73	positive
PRJNA729271	stage_1W_vs_E10	ordinary_muscle_development	ENSGALG00010003995	MLNR	-3.289201924	0.288975061	-11.38230375	5.12283E-30	2.89834E-29	negative
PRJNA729271	stage_1W_vs_E10	ordinary_muscle_development	ENSGALG00010004000	ENSGALG00010004000	2.353512186	0.172816556	13.61855738	3.10643E-42	2.67545E-41	positive
PRJNA729271	stage_1W_vs_E10	ordinary_muscle_development	ENSGALG00010004001	LARP7	-0.276371501	0.113065916	-2.444339641	0.014511758	0.019259104	negative
PRJNA729271	stage_1W_vs_E10	ordinary_muscle_development	ENSGALG00010004002	CDADC1	1.366144584	0.12141269	11.25207409	2.26209E-29	1.24626E-28	positive
PRJNA729271	stage_1W_vs_E10	ordinary_muscle_development	ENSGALG00010004003	HNRNPDL	0.061910338	0.068069748	0.909513256	0.363079264	0.400672549	positive
PRJNA729271	stage_1W_vs_E10	ordinary_muscle_development	ENSGALG00010004006	NKAIN3	-1.902456259	0.335270184	-5.674397399	1.39178E-08	2.92628E-08	negative
PRJNA729271	stage_1W_vs_E10	ordinary_muscle_development	ENSGALG00010004007	ENSGALG00010004007	-1.315317086	0.162906364	-8.074068174	6.79939E-16	2.10862E-15	negative
PRJNA729271	stage_1W_vs_E10	ordinary_muscle_development	ENSGALG00010004016	ENSGALG00010004016	-3.399808113	0.739490293	-4.597502017	4.27586E-06	7.62158E-06	negative
PRJNA729271	stage_1W_vs_E10	ordinary_muscle_development	ENSGALG00010004018	HNRNPD	-0.662686322	0.162065982	-4.088990866	4.33254E-05	7.16406E-05	negative
PRJNA729271	stage_1W_vs_E10	ordinary_muscle_development	ENSGALG00010004029	ARL4A	-0.422016312	0.194935113	-2.164906598	0.030394839	0.038919893	negative
PRJNA729271	stage_1W_vs_E10	ordinary_muscle_development	ENSGALG00010004034	NEDD8	0.901481917	0.111300142	8.099557629	5.51594E-16	1.71685E-15	positive
PRJNA729271	stage_1W_vs_E10	ordinary_muscle_development	ENSGALG00010004036	ADCYAP1	-2.581993147	0.836180224	-3.087842875	0.00201615	0.002920366	negative
PRJNA729271	stage_1W_vs_E10	ordinary_muscle_development	ENSGALG00010004038	SENP6	0.408347816	0.087523135	4.66559858	3.0772E-06	5.54362E-06	positive
PRJNA729271	stage_1W_vs_E10	ordinary_muscle_development	ENSGALG00010004039	ALPK1	3.429338154	0.224143574	15.29973886	7.6764E-53	9.32686E-52	positive
PRJNA729271	stage_1W_vs_E10	ordinary_muscle_development	ENSGALG00010004040	LGALS1B	-2.356062608	0.225971617	-10.42636521	1.87941E-25	8.81048E-25	negative
PRJNA729271	stage_1W_vs_E10	ordinary_muscle_development	ENSGALG00010004042	PLAG1	-1.311982713	0.124934914	-10.50132959	8.51719E-26	4.05042E-25	negative
PRJNA729271	stage_1W_vs_E10	ordinary_muscle_development	ENSGALG00010004043	CAB39L	0.093317941	0.201678226	0.46270707	0.64357435	0.675447846	positive
PRJNA729271	stage_1W_vs_E10	ordinary_muscle_development	ENSGALG00010004044	RNF138	-0.244600325	0.133741703	-1.828900928	0.067414451	0.082825264	negative
PRJNA729271	stage_1W_vs_E10	ordinary_muscle_development	ENSGALG00010004046	ENSGALG00010004046	0.526810121	0.121022595	4.352989799	1.34293E-05	2.30665E-05	positive
PRJNA729271	stage_1W_vs_E10	ordinary_muscle_development	ENSGALG00010004047	PDE7A	-1.310644808	0.121477144	-10.78922968	3.8702E-27	1.94382E-26	negative
PRJNA729271	stage_1W_vs_E10	ordinary_muscle_development	ENSGALG00010004049	SETDB2	-0.086421873	0.138609078	-0.623493597	0.532960209	0.569499316	negative
PRJNA729271	stage_1W_vs_E10	ordinary_muscle_development	ENSGALG00010004055	FILIP1	2.312426999	0.17523317	13.19628587	9.21686E-40	7.32447E-39	positive
PRJNA729271	stage_1W_vs_E10	ordinary_muscle_development	ENSGALG00010004056	SHROOM3	-2.835720646	0.188905297	-15.01133477	6.18896E-51	7.00305E-50	negative
PRJNA729271	stage_1W_vs_E10	ordinary_muscle_development	ENSGALG00010004057	ENSGALG00010004057	-0.291151027	0.406471801	-0.716288379	0.473813301	0.512302238	negative
PRJNA729271	stage_1W_vs_E10	ordinary_muscle_development	ENSGALG00010004058	TMEM30A	-0.278526011	0.062218563	-4.47657414	7.58503E-06	1.32608E-05	negative
PRJNA729271	stage_1W_vs_E10	ordinary_muscle_development	ENSGALG00010004060	ETV1	-2.229131471	0.095940209	-23.23459055	2.0365E-119	1.1224E-117	negative
PRJNA729271	stage_1W_vs_E10	ordinary_muscle_development	ENSGALG00010004063	ENSGALG00010004063	1.831624061	0.414767792	4.416022883	1.00533E-05	1.74435E-05	positive
PRJNA729271	stage_1W_vs_E10	ordinary_muscle_development	ENSGALG00010004065	TIFA	0.547854864	0.273588469	2.002477904	0.045233357	0.056796389	positive
PRJNA729271	stage_1W_vs_E10	ordinary_muscle_development	ENSGALG00010004070	ARL11	-0.456742076	0.406161542	-1.124533047	0.260786959	0.294492597	negative
PRJNA729271	stage_1W_vs_E10	ordinary_muscle_development	ENSGALG00010004072	SGK3	-0.488043155	0.136104115	-3.585807504	0.000336037	0.000520057	negative
PRJNA729271	stage_1W_vs_E10	ordinary_muscle_development	ENSGALG00010004076	COX7A2	-1.476852865	0.137842428	-10.71406595	8.74354E-27	4.32995E-26	negative
PRJNA729271	stage_1W_vs_E10	ordinary_muscle_development	ENSGALG00010004079	AP1AR	-0.417319615	0.150284601	-2.776862115	0.005488646	0.007632187	negative
PRJNA729271	stage_1W_vs_E10	ordinary_muscle_development	ENSGALG00010004081	RPS20	0.882942882	0.167931101	5.257768684	1.45814E-07	2.88149E-07	positive
PRJNA729271	stage_1W_vs_E10	ordinary_muscle_development	ENSGALG00010004082	FAM241A	1.048408754	0.122143673	8.583406155	9.21044E-18	3.09255E-17	positive
PRJNA729271	stage_1W_vs_E10	ordinary_muscle_development	ENSGALG00010004083	PHF11	-0.339555836	0.185116985	-1.834277049	0.066612879	0.081875153	negative
PRJNA729271	stage_1W_vs_E10	ordinary_muscle_development	ENSGALG00010004085	RB1CC1	1.823350764	0.140454994	12.98174393	1.55304E-38	1.18231E-37	positive
PRJNA729271	stage_1W_vs_E10	ordinary_muscle_development	ENSGALG00010004086	UTP3	-0.273400583	0.258175262	-1.058972809	0.289612163	0.32446501	negative
PRJNA729271	stage_1W_vs_E10	ordinary_muscle_development	ENSGALG00010004087	NCOA7	-0.817862798	0.111576927	-7.330035145	2.30092E-13	6.27387E-13	negative
PRJNA729271	stage_1W_vs_E10	ordinary_muscle_development	ENSGALG00010004094	DGKB	-1.924711433	0.196976514	-9.771273695	1.4956E-22	6.2081E-22	negative
PRJNA729271	stage_1W_vs_E10	ordinary_muscle_development	ENSGALG00010004095	ANKRD50	-1.104987857	0.11389375	-9.701918332	2.95882E-22	1.21119E-21	negative
PRJNA729271	stage_1W_vs_E10	ordinary_muscle_development	ENSGALG00010004096	COL12A1	-3.321755965	0.414251514	-8.018693597	1.06876E-15	3.28352E-15	negative
PRJNA729271	stage_1W_vs_E10	ordinary_muscle_development	ENSGALG00010004097	ENSGALG00010004097	-0.881665577	0.120065189	-7.343224017	2.08509E-13	5.69715E-13	negative
PRJNA729271	stage_1W_vs_E10	ordinary_muscle_development	ENSGALG00010004101	ARID1B	0.945450847	0.166137607	5.690769639	1.26468E-08	2.66524E-08	positive
PRJNA729271	stage_1W_vs_E10	ordinary_muscle_development	ENSGALG00010004102	PCMTD1	3.547190147	0.187452833	18.92310769	7.3585E-80	1.74284E-78	positive
PRJNA729271	stage_1W_vs_E10	ordinary_muscle_development	ENSGALG00010004104	SCARB2	1.197401375	0.094205567	12.71051604	5.16922E-37	3.73705E-36	positive
PRJNA729271	stage_1W_vs_E10	ordinary_muscle_development	ENSGALG00010004108	PERPB	0.936347492	0.241544996	3.87649303	0.000105973	0.000170052	positive
PRJNA729271	stage_1W_vs_E10	ordinary_muscle_development	ENSGALG00010004109	AGMO	-0.017015761	0.500980321	-0.033964929	0.972905117	0.976752186	negative
PRJNA729271	stage_1W_vs_E10	ordinary_muscle_development	ENSGALG00010004113	ECHDC1	0.438069938	0.188890728	2.319171211	0.020385755	0.026613664	positive
PRJNA729271	stage_1W_vs_E10	ordinary_muscle_development	ENSGALG00010004116	SPRY1	-0.470551205	0.095914465	-4.905946215	9.2978E-07	1.74326E-06	negative
PRJNA729271	stage_1W_vs_E10	ordinary_muscle_development	ENSGALG00010004117	STBD1	5.461648788	0.189301968	28.85151615	4.8498E-183	6.1128E-181	positive
PRJNA729271	stage_1W_vs_E10	ordinary_muscle_development	ENSGALG00010004122	TRIM55	2.389186128	0.194737855	12.26872983	1.33328E-34	8.92242E-34	positive
PRJNA729271	stage_1W_vs_E10	ordinary_muscle_development	ENSGALG00010004123	NDUFAF4	-0.015580094	0.110985881	-0.140379061	0.888360501	0.900789469	negative
PRJNA729271	stage_1W_vs_E10	ordinary_muscle_development	ENSGALG00010004125	MANEA	0.21549327	0.09308062	2.315125001	0.020606092	0.026889206	positive
PRJNA729271	stage_1W_vs_E10	ordinary_muscle_development	ENSGALG00010004127	EPHA7	-4.920774922	0.214093358	-22.98424844	6.7002E-117	3.4077E-115	negative
PRJNA729271	stage_1W_vs_E10	ordinary_muscle_development	ENSGALG00010004131	NUDT6	1.361945959	0.156205732	8.718924327	2.80857E-18	9.67679E-18	positive
PRJNA729271	stage_1W_vs_E10	ordinary_muscle_development	ENSGALG00010004133	CRH	3.216092534	0.973590377	3.303332295	0.000955431	0.001426406	positive
PRJNA729271	stage_1W_vs_E10	ordinary_muscle_development	ENSGALG00010004135	NUP54	-0.697828691	0.066199566	-10.54128803	5.57303E-26	2.6731E-25	negative
PRJNA729271	stage_1W_vs_E10	ordinary_muscle_development	ENSGALG00010004137	CROT	-0.925678991	0.084961297	-10.89530206	1.21363E-27	6.22711E-27	negative
PRJNA729271	stage_1W_vs_E10	ordinary_muscle_development	ENSGALG00010004139	PPEF2	1.230333306	0.620396784	1.983139399	0.047351861	0.059297211	positive
PRJNA729271	stage_1W_vs_E10	ordinary_muscle_development	ENSGALG00010004141	KPNA3	0.776572411	0.140912619	5.511021039	3.56758E-08	7.32258E-08	positive
PRJNA729271	stage_1W_vs_E10	ordinary_muscle_development	ENSGALG00010004142	RBBP8	-1.702468188	0.117881373	-14.44221544	2.80673E-47	2.80576E-46	negative
PRJNA729271	stage_1W_vs_E10	ordinary_muscle_development	ENSGALG00010004143	TCF24	1.29653417	0.408606917	3.173059768	0.001508415	0.002210319	positive
PRJNA729271	stage_1W_vs_E10	ordinary_muscle_development	ENSGALG00010004146	NAAA	2.249304787	0.172772703	13.01886668	9.55789E-39	7.33413E-38	positive
PRJNA729271	stage_1W_vs_E10	ordinary_muscle_development	ENSGALG00010004147	SPRYD7	0.803160901	0.091178843	8.808632332	1.26682E-18	4.44506E-18	positive
PRJNA729271	stage_1W_vs_E10	ordinary_muscle_development	ENSGALG00010004148	ENSGALG00010004148	0.461799649	0.154154604	2.995691577	0.002738232	0.00391504	positive
PRJNA729271	stage_1W_vs_E10	ordinary_muscle_development	ENSGALG00010004149	SDAD1	-0.726351316	0.12236336	-5.936019717	2.92025E-09	6.40378E-09	negative
PRJNA729271	stage_1W_vs_E10	ordinary_muscle_development	ENSGALG00010004154	COPS5	1.313151112	0.129072778	10.17372624	2.59776E-24	1.15825E-23	positive
PRJNA729271	stage_1W_vs_E10	ordinary_muscle_development	ENSGALG00010004158	JCHAIN	4.838666538	0.470368882	10.28696141	8.06824E-25	3.67764E-24	positive
PRJNA729271	stage_1W_vs_E10	ordinary_muscle_development	ENSGALG00010004172	ST8SIA6	-0.371395059	0.126813482	-2.928671726	0.003404137	0.004828552	negative
PRJNA729271	stage_1W_vs_E10	ordinary_muscle_development	ENSGALG00010004177	CSPP1	0.183168416	0.12793181	1.431766002	0.1522108	0.178387415	positive
PRJNA729271	stage_1W_vs_E10	ordinary_muscle_development	ENSGALG00010004181	BBS12	-1.620345376	0.135281171	-11.97761199	4.65541E-33	2.94545E-32	negative
PRJNA729271	stage_1W_vs_E10	ordinary_muscle_development	ENSGALG00010004187	ARFGEF1	0.816148699	0.094142809	8.669262221	4.34929E-18	1.48406E-17	positive
PRJNA729271	stage_1W_vs_E10	ordinary_muscle_development	ENSGALG00010004188	BMI1	0.115564022	0.115488887	1.00065058	0.316995768	0.353097184	positive
PRJNA729271	stage_1W_vs_E10	ordinary_muscle_development	ENSGALG00010004191	CABLES1	0.158532698	0.127908026	1.239427297	0.215187298	0.246280291	positive
PRJNA729271	stage_1W_vs_E10	ordinary_muscle_development	ENSGALG00010004200	ABCB1	3.197286271	0.181573857	17.60873684	2.11104E-69	3.92301E-68	positive
PRJNA729271	stage_1W_vs_E10	ordinary_muscle_development	ENSGALG00010004201	CPA6	-6.016198544	0.518905002	-11.59402688	4.41859E-31	2.60144E-30	negative
PRJNA729271	stage_1W_vs_E10	ordinary_muscle_development	ENSGALG00010004210	SYNCRIP	-0.867530391	0.07080745	-12.2519649	1.63974E-34	1.09329E-33	negative
PRJNA729271	stage_1W_vs_E10	ordinary_muscle_development	ENSGALG00010004211	RNF146	0.399534636	0.075166925	5.315298377	1.06483E-07	2.12306E-07	positive
PRJNA729271	stage_1W_vs_E10	ordinary_muscle_development	ENSGALG00010004212	RSPO3	-2.438018478	0.389357702	-6.261641845	3.80945E-10	8.76754E-10	negative
PRJNA729271	stage_1W_vs_E10	ordinary_muscle_development	ENSGALG00010004214	REPS1	0.223294892	0.125580286	1.778104659	0.075386667	0.091996106	positive
PRJNA729271	stage_1W_vs_E10	ordinary_muscle_development	ENSGALG00010004216	SNX14	0.4511456	0.073552516	6.133652886	8.58838E-10	1.93757E-09	positive
PRJNA729271	stage_1W_vs_E10	ordinary_muscle_development	ENSGALG00010004217	CCDC28A	0.130275163	0.11254021	1.157587707	0.247032328	0.279744812	positive
PRJNA729271	stage_1W_vs_E10	ordinary_muscle_development	ENSGALG00010004219	FGF2	-0.558724542	0.183091162	-3.05161941	0.002276105	0.003278559	negative
PRJNA729271	stage_1W_vs_E10	ordinary_muscle_development	ENSGALG00010004222	SPAG6	-2.519628322	0.259272373	-9.718074838	2.5251E-22	1.03604E-21	negative
PRJNA729271	stage_1W_vs_E10	ordinary_muscle_development	ENSGALG00010004224	NT5E	0.035730847	0.259221405	0.137839109	0.890367575	0.902445843	positive
PRJNA729271	stage_1W_vs_E10	ordinary_muscle_development	ENSGALG00010004225	CETN4	-2.130461542	0.132153353	-16.12113121	1.81261E-58	2.54098E-57	negative
PRJNA729271	stage_1W_vs_E10	ordinary_muscle_development	ENSGALG00010004226	RUNDC3B	-0.021131416	0.196439707	-0.10757202	0.914335193	0.924798592	negative
PRJNA729271	stage_1W_vs_E10	ordinary_muscle_development	ENSGALG00010004230	TAF1B	0.361869326	0.115420524	3.135225126	0.001717223	0.002504391	positive
PRJNA729271	stage_1W_vs_E10	ordinary_muscle_development	ENSGALG00010004231	ARMC3	-0.935068627	0.42206509	-2.215460717	0.026728458	0.034428952	negative
PRJNA729271	stage_1W_vs_E10	ordinary_muscle_development	ENSGALG00010004233	NHSL1	-3.262435491	0.128042239	-25.47936933	3.3379E-143	2.494E-141	negative
PRJNA729271	stage_1W_vs_E10	ordinary_muscle_development	ENSGALG00010004235	ADAD1	-1.680649408	0.451350498	-3.72360154	0.000196401	0.00030927	negative
PRJNA729271	stage_1W_vs_E10	ordinary_muscle_development	ENSGALG00010004236	DBF4	-1.645327956	0.128059124	-12.84819003	8.80462E-38	6.50806E-37	negative
PRJNA729271	stage_1W_vs_E10	ordinary_muscle_development	ENSGALG00010004237	RUFY3	0.508998923	0.091183185	5.582157722	2.37553E-08	4.92537E-08	positive
PRJNA729271	stage_1W_vs_E10	ordinary_muscle_development	ENSGALG00010004238	CCNC	-0.9635131	0.125361776	-7.68586032	1.51972E-14	4.41097E-14	negative
PRJNA729271	stage_1W_vs_E10	ordinary_muscle_development	ENSGALG00010004240	ENSGALG00010004240	1.183917716	0.174280867	6.793159442	1.09704E-11	2.75209E-11	positive
PRJNA729271	stage_1W_vs_E10	ordinary_muscle_development	ENSGALG00010004241	PREX2	0.405239695	0.114752	3.531439067	0.000413305	0.00063449	positive
PRJNA729271	stage_1W_vs_E10	ordinary_muscle_development	ENSGALG00010004242	ADAM22	-2.366358766	0.179675769	-13.17016075	1.3032E-39	1.03111E-38	negative
PRJNA729271	stage_1W_vs_E10	ordinary_muscle_development	ENSGALG00010004243	BLTP1	0.337189337	0.112808731	2.989035821	0.002798593	0.003998924	positive

PRJNA729271	stage_1W_vs_E10	ordinary_muscle_development	ENSGALG00010004244	TMEM241	-0.324941338	0.082728482	-3.927804923	8.57247E-05	0.000138619	negative
PRJNA729271	stage_1W_vs_E10	ordinary_muscle_development	ENSGALG00010004245	ARFGEF3	-1.29739663	0.229221011	-5.660024908	1.51351E-08	3.17487E-08	negative
PRJNA729271	stage_1W_vs_E10	ordinary_muscle_development	ENSGALG00010004246	C8orf34	-3.542054995	0.35531575	-9.968753127	2.08834E-23	8.98501E-23	negative
PRJNA729271	stage_1W_vs_E10	ordinary_muscle_development	ENSGALG00010004247	MEOX2	-1.228430677	0.105258837	-11.67057049	1.80211E-31	1.0803E-30	negative
PRJNA729271	stage_1W_vs_E10	ordinary_muscle_development	ENSGALG00010004248	GRSF1	0.56287293	0.159045062	3.539078304	0.000401527	0.000617128	positive
PRJNA729271	stage_1W_vs_E10	ordinary_muscle_development	ENSGALG00010004249	USP45	1.97037627	0.100807005	19.54602534	4.45941E-85	1.20821E-83	positive
PRJNA729271	stage_1W_vs_E10	ordinary_muscle_development	ENSGALG00010004250	MOB1B	-0.875902195	0.089287074	-9.809955159	1.02014E-22	4.25768E-22	negative
PRJNA729271	stage_1W_vs_E10	ordinary_muscle_development	ENSGALG00010004251	CCN2	-0.287054357	0.266232176	-1.078210612	0.280939777	0.315333907	negative
PRJNA729271	stage_1W_vs_E10	ordinary_muscle_development	ENSGALG00010004255	RPS12	0.356607475	0.151082285	2.36035268	0.018257568	0.023943217	positive
PRJNA729271	stage_1W_vs_E10	ordinary_muscle_development	ENSGALG00010004256	ENSGALG00010004256	-4.132584595	0.55214076	-7.484657702	7.17339E-14	2.01352E-13	negative
PRJNA729271	stage_1W_vs_E10	ordinary_muscle_development	ENSGALG00010004257	ENSGALG00010004257	-2.836244684	0.66256574	-4.280699279	1.86307E-05	3.16331E-05	negative
PRJNA729271	stage_1W_vs_E10	ordinary_muscle_development	ENSGALG00010004265	PIP4K2A	0.621969195	0.092552457	6.720180279	1.815E-11	4.50101E-11	positive
PRJNA729271	stage_1W_vs_E10	ordinary_muscle_development	ENSGALG00010004275	ADARB2	-4.474292218	0.975742235	-4.585526853	4.52843E-06	8.05592E-06	negative
PRJNA729271	stage_1W_vs_E10	ordinary_muscle_development	ENSGALG00010004281	VIM	-0.063362374	0.171613928	-0.369214639	0.711967745	0.739358968	negative
PRJNA729271	stage_1W_vs_E10	ordinary_muscle_development	ENSGALG00010004283	DCK	-2.067148015	0.133076144	-15.53357317	2.05597E-54	2.62105E-53	negative
PRJNA729271	stage_1W_vs_E10	ordinary_muscle_development	ENSGALG00010004285	SLC18B1	0.18674928	0.118001615	1.582599353	0.113512811	0.135488159	positive
PRJNA729271	stage_1W_vs_E10	ordinary_muscle_development	ENSGALG00010004296	ENSGALG00010004296	-8.624599321	1.006555331	-8.568430424	1.04905E-17	3.51098E-17	negative
PRJNA729271	stage_1W_vs_E10	ordinary_muscle_development	ENSGALG00010004297	SULF1	-3.408667521	0.186689498	-18.25848562	1.77136E-74	3.68378E-73	negative
PRJNA729271	stage_1W_vs_E10	ordinary_muscle_development	ENSGALG00010004300	VNN2	2.223478166	0.247054266	8.999958589	2.25803E-19	8.23606E-19	positive
PRJNA729271	stage_1W_vs_E10	ordinary_muscle_development	ENSGALG00010004302	CRPPA	0.398695722	0.127666997	3.122934905	0.001790573	0.002608216	positive
PRJNA729271	stage_1W_vs_E10	ordinary_muscle_development	ENSGALG00010004306	PNISR	0.138586781	0.144242148	0.96079255	0.336656485	0.373618846	positive
PRJNA729271	stage_1W_vs_E10	ordinary_muscle_development	ENSGALG00010004307	NPC2	1.072834933	0.140931236	7.612470926	2.68905E-14	7.71531E-14	positive
PRJNA729271	stage_1W_vs_E10	ordinary_muscle_development	ENSGALG00010004308	DSG2	-4.340312779	0.500359581	-8.674387265	4.15783E-18	1.42141E-17	negative
PRJNA729271	stage_1W_vs_E10	ordinary_muscle_development	ENSGALG00010004309	MRPS28	-0.324581304	0.13715822	-2.366473578	0.017958456	0.023567933	negative
PRJNA729271	stage_1W_vs_E10	ordinary_muscle_development	ENSGALG00010004312	PDLIM5	6.269734541	0.181459626	34.55167787	1.345E-261	3.4814E-259	positive
PRJNA729271	stage_1W_vs_E10	ordinary_muscle_development	ENSGALG00010004319	TAAR1	5.840037383	0.821343187	7.110349817	1.15749E-12	3.05719E-12	positive
PRJNA729271	stage_1W_vs_E10	ordinary_muscle_development	ENSGALG00010004323	TRDMT1	-0.166073876	0.15992261	-1.038464015	0.29905408	0.334397045	negative
PRJNA729271	stage_1W_vs_E10	ordinary_muscle_development	ENSGALG00010004325	LTBP2	0.190573263	0.113485258	1.679277696	0.093097936	0.112454548	positive
PRJNA729271	stage_1W_vs_E10	ordinary_muscle_development	ENSGALG00010004326	ENSGALG00010004326	-0.442783769	0.953364849	-0.464443145	0.642330285	0.674346481	negative
PRJNA729271	stage_1W_vs_E10	ordinary_muscle_development	ENSGALG00010004328	ENSGALG00010004328	-0.852278693	0.69775896	-1.22145145	0.221915137	0.253320201	negative
PRJNA729271	stage_1W_vs_E10	ordinary_muscle_development	ENSGALG00010004329	COQ3	1.510213652	0.119595449	12.62768493	1.48586E-36	1.06044E-35	positive
PRJNA729271	stage_1W_vs_E10	ordinary_muscle_development	ENSGALG00010004330	STX7	0.353240368	0.103191388	3.423157454	0.000618982	0.000938116	positive
PRJNA729271	stage_1W_vs_E10	ordinary_muscle_development	ENSGALG00010004332	ENSGALG00010004332	-0.371376699	0.892619968	-0.416052421	0.677371644	0.707028299	negative
PRJNA729271	stage_1W_vs_E10	ordinary_muscle_development	ENSGALG00010004333	DIO2	-0.315794754	0.313737775	-1.006556361	0.314148014	0.350167292	negative
PRJNA729271	stage_1W_vs_E10	ordinary_muscle_development	ENSGALG00010004334	ENSGALG00010004334	1.172844931	0.229045677	5.120572227	3.0461E-07	5.89259E-07	positive
PRJNA729271	stage_1W_vs_E10	ordinary_muscle_development	ENSGALG00010004336	ENSGALG00010004336	-4.340154135	0.624210692	-6.953027539	3.57529E-12	9.20167E-12	negative
PRJNA729271	stage_1W_vs_E10	ordinary_muscle_development	ENSGALG00010004337	BMPR1B	-1.84472325	0.24088725	-7.658036086	1.88798E-14	5.45515E-14	negative
PRJNA729271	stage_1W_vs_E10	ordinary_muscle_development	ENSGALG00010004338	ENSGALG00010004338	1.483282726	0.165137576	8.982103035	2.6564E-19	9.66479E-19	positive
PRJNA729271	stage_1W_vs_E10	ordinary_muscle_development	ENSGALG00010004339	B4GALT6	-4.058057614	0.146778723	-27.64745135	2.9948E-168	3.1456E-166	negative
PRJNA729271	stage_1W_vs_E10	ordinary_muscle_development	ENSGALG00010004340	SLC4A4	0.850787902	0.170280477	4.996391363	5.84131E-07	1.10868E-06	positive
PRJNA729271	stage_1W_vs_E10	ordinary_muscle_development	ENSGALG00010004341	ENSGALG00010004341	-1.892616792	0.173380889	-10.91594813	9.67148E-28	4.98535E-27	negative
PRJNA729271	stage_1W_vs_E10	ordinary_muscle_development	ENSGALG00010004342	ZNF438	-0.310889943	0.13266661	-2.343392524	0.019109267	0.025010278	negative
PRJNA729271	stage_1W_vs_E10	ordinary_muscle_development	ENSGALG00010004344	SOSTDC1	-6.301142122	0.439152155	-14.34842583	1.08983E-46	1.07099E-45	negative
PRJNA729271	stage_1W_vs_E10	ordinary_muscle_development	ENSGALG00010004345	FAXC	-2.606841069	0.195709433	-13.31995617	1.77212E-40	1.44308E-39	negative
PRJNA729271	stage_1W_vs_E10	ordinary_muscle_development	ENSGALG00010004346	CEP128	-0.475873092	0.107802288	-4.414313484	1.01331E-05	1.75735E-05	negative
PRJNA729271	stage_1W_vs_E10	ordinary_muscle_development	ENSGALG00010004350	MOXD1	0.735814419	0.239550371	3.071648011	0.002128806	0.003075248	positive
PRJNA729271	stage_1W_vs_E10	ordinary_muscle_development	ENSGALG00010004352	CHMP2A	0.565865066	0.056325467	10.04634485	9.53382E-24	4.1662E-23	positive
PRJNA729271	stage_1W_vs_E10	ordinary_muscle_development	ENSGALG00010004355	ANKMY2	0.050012131	0.123558741	0.404764011	0.685650986	0.714897931	positive
PRJNA729271	stage_1W_vs_E10	ordinary_muscle_development	ENSGALG00010004356	TRAPPC8	0.505165088	0.077234562	6.540661002	6.12475E-11	1.47179E-10	positive
PRJNA729271	stage_1W_vs_E10	ordinary_muscle_development	ENSGALG00010004358	GC	7.258190417	2.106293741	3.44595356	0.000569048	0.000864154	positive
PRJNA729271	stage_1W_vs_E10	ordinary_muscle_development	ENSGALG00010004362	FBXL4	2.037308229	0.120694511	16.87987471	6.3279E-64	1.02028E-62	positive
PRJNA729271	stage_1W_vs_E10	ordinary_muscle_development	ENSGALG00010004364	TSHR	0.630141363	0.458654475	1.373891234	0.169475452	0.197170453	positive
PRJNA729271	stage_1W_vs_E10	ordinary_muscle_development	ENSGALG00010004365	TPD52	-1.646219432	0.15714148	-10.47603362	1.11315E-25	5.26602E-25	negative
PRJNA729271	stage_1W_vs_E10	ordinary_muscle_development	ENSGALG00010004366	UNC5C	-4.710125695	0.36796168	-12.80058754	1.62715E-37	1.19602E-36	negative
PRJNA729271	stage_1W_vs_E10	ordinary_muscle_development	ENSGALG00010004367	TSPAN13	0.321040028	0.178390468	1.799647881	0.071916255	0.087990725	positive
PRJNA729271	stage_1W_vs_E10	ordinary_muscle_development	ENSGALG00010004368	GJD4	-7.641286741	0.474117966	-16.11684705	1.94271E-58	2.72074E-57	negative
PRJNA729271	stage_1W_vs_E10	ordinary_muscle_development	ENSGALG00010004369	UBE2M	1.057367105	0.233469831	4.528923928	5.92848E-06	1.04505E-05	positive
PRJNA729271	stage_1W_vs_E10	ordinary_muscle_development	ENSGALG00010004370	ATP6V1H	0.3792914	0.07467813	5.079015789	3.79395E-07	7.29355E-07	positive
PRJNA729271	stage_1W_vs_E10	ordinary_muscle_development	ENSGALG00010004372	SYNJ2	-0.67658115	0.190274387	-3.555818323	0.000376804	0.0005803	negative
PRJNA729271	stage_1W_vs_E10	ordinary_muscle_development	ENSGALG00010004373	MMS22L	-1.825865372	0.131760256	-13.85748198	1.14625E-43	1.02943E-42	negative
PRJNA729271	stage_1W_vs_E10	ordinary_muscle_development	ENSGALG00010004374	ILDR1	-3.950251462	0.71109984	-5.555129165	2.77407E-08	5.72956E-08	negative
PRJNA729271	stage_1W_vs_E10	ordinary_muscle_development	ENSGALG00010004375	ENSGALG00010004375	-0.434400729	0.222241845	-1.954630683	0.050626677	0.063196149	negative
PRJNA729271	stage_1W_vs_E10	ordinary_muscle_development	ENSGALG00010004377	AGR2	-3.41879574	0.755840006	-4.52317384	6.09192E-06	1.07228E-05	negative
PRJNA729271	stage_1W_vs_E10	ordinary_muscle_development	ENSGALG00010004378	TRIM28	0.479447635	0.153939155	3.114526868	0.001842402	0.002678866	positive
PRJNA729271	stage_1W_vs_E10	ordinary_muscle_development	ENSGALG00010004379	ZBTB10	-0.495580263	0.061000333	-8.124222283	4.5024E-16	1.40803E-15	negative
PRJNA729271	stage_1W_vs_E10	ordinary_muscle_development	ENSGALG00010004380	ISCA2	0.422920076	0.08441449	5.010041217	5.44184E-07	1.03489E-06	positive
PRJNA729271	stage_1W_vs_E10	ordinary_muscle_development	ENSGALG00010004381	ENSGALG00010004381	0.317894119	0.443855052	0.716211558	0.473860727	0.512315301	positive
PRJNA729271	stage_1W_vs_E10	ordinary_muscle_development	ENSGALG00010004383	ENSGALG00010004383	-0.132312376	0.471834732	-0.280421019	0.779154513	0.80194878	negative
PRJNA729271	stage_1W_vs_E10	ordinary_muscle_development	ENSGALG00010004384	ENSGALG00010004384	1.568614509	0.548802258	2.858250832	0.004259835	0.005981431	positive
PRJNA729271	stage_1W_vs_E10	ordinary_muscle_development	ENSGALG00010004385	ADAMTS3	-3.622951806	0.208573378	-17.37015453	1.38852E-67	2.46951E-66	negative
PRJNA729271	stage_1W_vs_E10	ordinary_muscle_development	ENSGALG00010004386	CFAP44	-1.172724372	0.42343262	-2.769565492	0.005613112	0.007794047	negative
PRJNA729271	stage_1W_vs_E10	ordinary_muscle_development	ENSGALG00010004389	ENSGALG00010004389	-0.073390262	0.135408266	-0.541992484	0.587823684	0.622934953	negative
PRJNA729271	stage_1W_vs_E10	ordinary_muscle_development	ENSGALG00010004390	PNLDC1	-1.60203721	0.653324266	-2.452131801	0.014201263	0.018872954	negative
PRJNA729271	stage_1W_vs_E10	ordinary_muscle_development	ENSGALG00010004393	ZBTB45	2.274083228	0.206628749	11.00564774	3.58927E-28	1.88365E-27	positive
PRJNA729271	stage_1W_vs_E10	ordinary_muscle_development	ENSGALG00010004394	CCNY	-1.114052051	0.090496604	-12.3104293	7.95987E-35	5.37894E-34	negative
PRJNA729271	stage_1W_vs_E10	ordinary_muscle_development	ENSGALG00010004395	BOC	-2.399513089	0.193908065	-12.37448832	3.59176E-35	2.45462E-34	negative
PRJNA729271	stage_1W_vs_E10	ordinary_muscle_development	ENSGALG00010004397	ENSGALG00010004397	0.338244019	0.170596745	1.982710864	0.047399735	0.059352036	positive
PRJNA729271	stage_1W_vs_E10	ordinary_muscle_development	ENSGALG00010004398	COX18	-0.881105072	0.127468673	-6.912326387	4.76769E-12	1.21795E-11	negative
PRJNA729271	stage_1W_vs_E10	ordinary_muscle_development	ENSGALG00010004399	POU3F2	-0.129845588	0.523736196	-0.247921739	0.80419496	0.825085358	negative
PRJNA729271	stage_1W_vs_E10	ordinary_muscle_development	ENSGALG00010004404	RGS20	-1.467797955	0.447140694	-3.282631114	0.001028431	0.001529405	negative
PRJNA729271	stage_1W_vs_E10	ordinary_muscle_development	ENSGALG00010004406	ENSGALG00010004406	-1.555827116	0.156675087	-9.930277633	3.07398E-23	1.31632E-22	negative
PRJNA729271	stage_1W_vs_E10	ordinary_muscle_development	ENSGALG00010004408	SERAC1	-0.1824725	0.110970824	-1.644328606	0.100108343	0.120370837	negative
PRJNA729271	stage_1W_vs_E10	ordinary_muscle_development	ENSGALG00010004416	STXBP6	-0.555599965	0.214002331	-2.596233235	0.009425206	0.012777625	negative
PRJNA729271	stage_1W_vs_E10	ordinary_muscle_development	ENSGALG00010004420	ENSGALG00010004420	1.997578535	0.281593749	7.093831241	1.30449E-12	3.43668E-12	positive
PRJNA729271	stage_1W_vs_E10	ordinary_muscle_development	ENSGALG00010004421	RAP1GDS1	-0.975394585	0.075500356	-12.91907268	3.5135E-38	2.64014E-37	negative
PRJNA729271	stage_1W_vs_E10	ordinary_muscle_development	ENSGALG00010004424	MRPL18	1.135635695	0.144853507	7.839890931	4.50938E-15	1.34576E-14	positive
PRJNA729271	stage_1W_vs_E10	ordinary_muscle_development	ENSGALG00010004425	CREM	2.977855112	0.240755064	12.36881611	3.85461E-35	2.63053E-34	positive
PRJNA729271	stage_1W_vs_E10	ordinary_muscle_development	ENSGALG00010004429	ENSGALG00010004429	0.369016603	0.352146905	1.047905285	0.294682247	0.329788386	positive
PRJNA729271	stage_1W_vs_E10	ordinary_muscle_development	ENSGALG00010004430	TCEA1	-0.746574508	0.094603499	-7.891616223	2.98298E-15	8.96688E-15	negative
PRJNA729271	stage_1W_vs_E10	ordinary_muscle_development	ENSGALG00010004433	AHR1A	-1.69966649	0.160151559	-10.61286262	2.59669E-26	1.26306E-25	negative
PRJNA729271	stage_1W_vs_E10	ordinary_muscle_development	ENSGALG00010004434	TCP1	-0.811197061	0.086758102	-9.350101502	8.75637E-21	3.40278E-20	negative
PRJNA729271	stage_1W_vs_E10	ordinary_muscle_development	ENSGALG00010004436	NOVA1	-0.131440269	0.152923227	-0.859518022	0.390054781	0.428029681	negative
PRJNA729271	stage_1W_vs_E10	ordinary_muscle_development	ENSGALG00010004440	GTF2H5	-0.455692633	0.104797393	-4.348320319	1.37184E-05	2.35491E-05	negative
PRJNA729271	stage_1W_vs_E10	ordinary_muscle_development	ENSGALG00010004443	ANKRD17	-0.379560617	0.093971854	-4.039088304	5.36594E-05	8.80453E-05	negative
PRJNA729271	stage_1W_vs_E10	ordinary_muscle_development	ENSGALG00010004445	EPHA6	-4.54386241	0.634964758	-7.156085991	8.30131E-13	2.20542E-12	negative
PRJNA729271	stage_1W_vs_E10	ordinary_muscle_development	ENSGALG00010004446	ACAT2	-1.57143029	0.127753628	-12.3004749	9.00432E-35	6.07059E-34	negative
PRJNA729271	stage_1W_vs_E10	ordinary_muscle_development	ENSGALG00010004451	CLDND1	-0.572274842	0.085776538	-6.671694296	2.52867E-11	6.2261E-11	negative
PRJNA729271	stage_1W_vs_E10	ordinary_muscle_development	ENSGALG00010004454	SRI	-0.636483219	0.164452694	-3.870311914	0.000108696	0.000174306	negative
PRJNA729271	stage_1W_vs_E10	ordinary_muscle_development	ENSGALG00010004457	AGA	-0.131843434	0.127303802	-1.035659831	0.300360869	0.33565891	negative
PRJNA729271	stage_1W_vs_E10	ordinary_muscle_development	ENSGALG00010004460	ENSGALG00010004460	-2.763694699	0.839957861	-3.290277795	0.001000885	0.001490888	negative
PRJNA729271	stage_1W_vs_E10	ordinary_muscle_development	ENSGALG00010004468	LYPLA1	0.942684975	0.122177324	7.715711458	1.20309E-14	3.51518E-14	positive
PRJNA729271	stage_1W_vs_E10	ordinary_muscle_development	ENSGALG00010004471	C1H3ORF52	0.340848117	0.261737611	1.302251196	0.192830529	0.222484958	positive
PRJNA729271	stage_1W_vs_E10	ordinary_muscle_development	ENSGALG00010004472	NEIL3	-1.57596852	0.212439155	-7.418446577	1.18502E-13	3.27865E-13	negative
PRJNA729271	stage_1W_vs_E10	ordinary_muscle_development	ENSGALG00010004473	JDP2	0.007681294	0.137991151	0.055665123	0.955608584	0.961312126	positive
PRJNA729271	stage_1W_vs_E10	ordinary_muscle_development	ENSGALG00010004477	TULP4	-0.820677805	0.127078989	-6.458013327	1.06086E-10	2.51838E-10	negative
PRJNA729271	stage_1W_vs_E10	ordinary_muscle_development	ENSGALG00010004481	ZNF704	-0.27385936	0.080894876	-3.385373399	0.000710815	0.001072474	negative
PRJNA729271	stage_1W_vs_E10	ordinary_muscle_development	ENSGALG00010004483	STEAP4	1.041963738	0.611669979	1.703473727	0.088479451	0.107152614	positive
PRJNA729271	stage_1W_vs_E10	ordinary_muscle_development	ENSGALG00010004487	ABHD10	-0.478804635	0.101303408	-4.726441546	2.28489E-06	4.15186E-06	negative
PRJNA729271	stage_1W_vs_E10	ordinary_muscle_development	ENSGALG00010004489	LIG1	-2.574949298	0.264515763	-9.734577888	2.14707E-22	8.84394E-22	negative
PRJNA729271	stage_1W_vs_E10	ordinary_muscle_development	ENSGALG00010004494	WTAP	-0.302304729	0.057618794	-5.246634103	1.54903E-07	3.05527E-07	negative
PRJNA729271	stage_1W_vs_E10	ordinary_muscle_development	ENSGALG00010004495	MRPL15	0.869457376	0.113167335	7.682935844	1.55483E-14	4.51198E-14	positive
PRJNA729271	stage_1W_vs_E10	ordinary_muscle_development	ENSGALG00010004501	TAGLN3	-2.186840181	0.339627725	-6.438933045	1.20316E-10	2.84546E-10	negative
PRJNA729271	stage_1W_vs_E10	ordinary_muscle_development	ENSGALG00010004503	ENSGALG00010004503	-0.116014563	0.185485021	-0.62546594	0.531665296	0.5681994	negative
PRJNA729271	stage_1W_vs_E10	ordinary_muscle_development	ENSGALG00010004504	ZNF804B	-1.972235468	0.668021572	-2.952352964	0.003153622	0.004482423	negative
PRJNA729271	stage_1W_vs_E10	ordinary_muscle_development	ENSGALG00010004505	ENSGALG00010004505	1.388273336	0.131040294	10.59424774	3.16878E-26	1.53463E-25	positive
PRJNA729271	stage_1W_vs_E10	ordinary_muscle_development	ENSGALG00010004507	CUL2	0.813459481	0.089884883	9.050014338	1.4295E-19	5.26972E-19	positive
PRJNA729271	stage_1W_vs_E10	ordinary_muscle_development	ENSGALG00010004515	ENSGALG00010004515	-0.629572047	0.256820432	-2.451409506	0.014229796	0.018907406	negative
PRJNA729271	stage_1W_vs_E10	ordinary_muscle_development	ENSGALG00010004516	ENSGALG00010004516	-0.131208857	0.291794055	-0.449662544	0.652953783	0.68425138	negative
PRJNA729271	stage_1W_vs_E10	ordinary_muscle_development	ENSGALG00010004522	TSPAN5	-1.178537714	0.064411259	-18.29707612	8.73106E-75	1.82885E-73	negative
PRJNA729271	stage_1W_vs_E10	ordinary_muscle_development	ENSGALG00010004533	PAG1	-0.294229643	0.114695321	-2.565315134	0.010308214	0.01391484	negative
PRJNA729271	stage_1W_vs_E10	ordinary_muscle_development	ENSGALG00010004534	CUBN	1.372018794	0.213983532	6.411796184	1.43815E-10	3.38629E-10	positive
PRJNA729271	stage_1W_vs_E10	ordinary_muscle_development	ENSGALG00010004542	ERMARD	-0.107560506	0.130192955	-0.82616226	0.408712053	0.446677314	negative
PRJNA729271	stage_1W_vs_E10	ordinary_muscle_development	ENSGALG00010004545	TMPRSS7	0.275155755	0.590805717	0.465729676	0.641409008	0.673663037	positive
PRJNA729271	stage_1W_vs_E10	ordinary_muscle_development	ENSGALG00010004553	BATF	-0.29801407	0.375327503	-0.794010744	0.427189177	0.465117338	negative
PRJNA729271	stage_1W_vs_E10	ordinary_muscle_development	ENSGALG00010004565	ENSGALG00010004565	2.924870243	0.382630233	7.64411693	2.10384E-14	6.06025E-14	positive
PRJNA729271	stage_1W_vs_E10	ordinary_muscle_development	ENSGALG00010004568	RSU1	0.490838615	0.067010531	7.32479818	2.39259E-13	6.51889E-13	positive
PRJNA729271	stage_1W_vs_E10	ordinary_muscle_development	ENSGALG00010004570	ENSGALG00010004570	-1.296449227	0.660283162	-1.963474615	0.049591051	0.06194074	negative
PRJNA729271	stage_1W_vs_E10	ordinary_muscle_development	ENSGALG00010004576	STEAP2	0.681126769	0.210753664	3.231862047	0.001229864	0.001814256	positive
PRJNA729271	stage_1W_vs_E10	ordinary_muscle_development	ENSGALG00010004577	ZPLD1	-4.942612438	0.468197417	-10.55668455	4.73077E-26	2.27664E-25	negative
PRJNA729271	stage_1W_vs_E10	ordinary_muscle_development	ENSGALG00010004583	XKR4	0.186190672	0.113641177	1.63840851	0.101336505	0.121756684	positive
PRJNA729271	stage_1W_vs_E10	ordinary_muscle_development	ENSGALG00010004584	EIF4E	-0.019655717	0.066651662	-0.29490213	0.768068645	0.791662874	negative

PRJNA729271	stage_1W_vs_E10	ordinary_muscle_development	ENSGALG00010004586	ENSGALG00010004586	-5.398653265	0.482952488	-11.17843555	5.19985E-29	2.81029E-28	negative
PRJNA729271	stage_1W_vs_E10	ordinary_muscle_development	ENSGALG00010004588	MINDY3	-0.957867676	0.154844726	-6.185988377	6.17146E-10	1.403E-09	negative
PRJNA729271	stage_1W_vs_E10	ordinary_muscle_development	ENSGALG00010004591	RRS1	0.673323331	0.161477762	4.169758883	3.04922E-05	5.10247E-05	positive
PRJNA729271	stage_1W_vs_E10	ordinary_muscle_development	ENSGALG00010004602	DYNLT2	-0.324508298	0.337588784	-0.961253197	0.336424875	0.373390395	negative
PRJNA729271	stage_1W_vs_E10	ordinary_muscle_development	ENSGALG00010004609	CFAP69	-2.048119042	0.606689375	-3.375894034	0.000735763	0.001109076	negative
PRJNA729271	stage_1W_vs_E10	ordinary_muscle_development	ENSGALG00010004610	TGS1	-0.765606384	0.124422405	-6.153283933	7.58948E-10	1.71702E-09	negative
PRJNA729271	stage_1W_vs_E10	ordinary_muscle_development	ENSGALG00010004622	ENSGALG00010004622	1.987844452	0.223706706	8.885940365	6.33824E-19	2.26937E-18	positive
PRJNA729271	stage_1W_vs_E10	ordinary_muscle_development	ENSGALG00010004623	MBIP	-0.745749648	0.128386531	-5.808628364	6.29867E-09	1.35379E-08	negative
PRJNA729271	stage_1W_vs_E10	ordinary_muscle_development	ENSGALG00010004624	PHF10	0.403794209	0.075871789	5.322059978	1.02599E-07	2.04929E-07	positive
PRJNA729271	stage_1W_vs_E10	ordinary_muscle_development	ENSGALG00010004630	ENSGALG00010004630	2.04627902	0.309886367	6.603320554	4.02049E-11	9.7961E-11	positive
PRJNA729271	stage_1W_vs_E10	ordinary_muscle_development	ENSGALG00010004639	HBS1L	1.21525905	0.079106939	15.36223074	2.93306E-53	3.60294E-52	positive
PRJNA729271	stage_1W_vs_E10	ordinary_muscle_development	ENSGALG00010004641	METAP1	-0.223890609	0.080776901	-2.771715756	0.00557617	0.00774572	negative
PRJNA729271	stage_1W_vs_E10	ordinary_muscle_development	ENSGALG00010004650	GTPBP10	-0.36977026	0.128466788	-2.878333509	0.003997822	0.005627701	negative
PRJNA729271	stage_1W_vs_E10	ordinary_muscle_development	ENSGALG00010004666	C6orf120	-0.0093194	0.117480959	-0.079326902	0.936772609	0.944461221	negative
PRJNA729271	stage_1W_vs_E10	ordinary_muscle_development	ENSGALG00010004669	ELMOD1	-3.209689665	0.389540096	-8.239690083	1.72657E-16	5.4859E-16	negative
PRJNA729271	stage_1W_vs_E10	ordinary_muscle_development	ENSGALG00010004673	RAB18	0.32548123	0.0849322	3.832247701	0.000126978	0.000202591	positive
PRJNA729271	stage_1W_vs_E10	ordinary_muscle_development	ENSGALG00010004689	BBX	0.264439035	0.084036054	3.146733131	0.001651056	0.002412019	positive
PRJNA729271	stage_1W_vs_E10	ordinary_muscle_development	ENSGALG00010004705	ENSGALG00010004705	3.347468577	0.227157859	14.73630979	3.7682E-49	4.01028E-48	positive
PRJNA729271	stage_1W_vs_E10	ordinary_muscle_development	ENSGALG00010004707	OTUD1	4.294764264	0.296311644	14.49407864	1.32063E-47	1.33119E-46	positive
PRJNA729271	stage_1W_vs_E10	ordinary_muscle_development	ENSGALG00010004715	WDR27	-0.57395448	0.342861172	-1.674014225	0.094127804	0.113641722	negative
PRJNA729271	stage_1W_vs_E10	ordinary_muscle_development	ENSGALG00010004716	ADH6	1.746610307	0.957608682	1.823929065	0.068162797	0.083680845	positive
PRJNA729271	stage_1W_vs_E10	ordinary_muscle_development	ENSGALG00010004717	ALKBH8	0.392903558	0.174076985	2.257067809	0.024003838	0.031129608	positive
PRJNA729271	stage_1W_vs_E10	ordinary_muscle_development	ENSGALG00010004721	LAMA2	2.656562142	0.158862165	16.72243448	8.99678E-63	1.40375E-61	positive
PRJNA729271	stage_1W_vs_E10	ordinary_muscle_development	ENSGALG00010004728	ENSGALG00010004728	-0.212448628	0.216811201	-0.979878471	0.327146111	0.363593228	negative
PRJNA729271	stage_1W_vs_E10	ordinary_muscle_development	ENSGALG00010004729	ENSGALG00010004729	6.824394361	1.099183687	6.208602292	5.34579E-10	1.21893E-09	positive
PRJNA729271	stage_1W_vs_E10	ordinary_muscle_development	ENSGALG00010004730	CLDN12	-0.353779803	0.06431545	-5.500696984	3.78293E-08	7.7602E-08	negative
PRJNA729271	stage_1W_vs_E10	ordinary_muscle_development	ENSGALG00010004735	ENSGALG00010004735	2.488415908	0.165163195	15.06640698	2.69369E-51	3.08657E-50	positive
PRJNA729271	stage_1W_vs_E10	ordinary_muscle_development	ENSGALG00010004736	CD47	-0.22891871	0.101443629	-2.256610026	0.024032453	0.031161142	negative
PRJNA729271	stage_1W_vs_E10	ordinary_muscle_development	ENSGALG00010004739	ENSGALG00010004739	-1.359930571	0.81507465	-1.668473643	0.09522174	0.114847655	negative
PRJNA729271	stage_1W_vs_E10	ordinary_muscle_development	ENSGALG00010004746	LYN	-0.37043581	0.142642563	-2.596951447	0.009405522	0.012753324	negative
PRJNA729271	stage_1W_vs_E10	ordinary_muscle_development	ENSGALG00010004751	PTER	1.300137949	0.124443846	10.4475873	1.503E-25	7.08488E-25	positive
PRJNA729271	stage_1W_vs_E10	ordinary_muscle_development	ENSGALG00010004755	PAX9	-3.713634356	0.774072024	-4.797530776	1.60634E-06	2.95255E-06	negative
PRJNA729271	stage_1W_vs_E10	ordinary_muscle_development	ENSGALG00010004757	TRMT10A	0.712048337	0.074171154	9.600070839	7.98895E-22	3.22203E-21	positive
PRJNA729271	stage_1W_vs_E10	ordinary_muscle_development	ENSGALG00010004758	FLT3	3.3771648	0.804220346	4.199302864	2.67738E-05	4.4933E-05	positive
PRJNA729271	stage_1W_vs_E10	ordinary_muscle_development	ENSGALG00010004760	THBS2	-3.234004136	0.241165226	-13.40991065	5.29025E-41	4.37934E-40	negative
PRJNA729271	stage_1W_vs_E10	ordinary_muscle_development	ENSGALG00010004762	IFT57	-1.649741189	0.11709102	-14.08939117	4.4135E-45	4.13801E-44	negative
PRJNA729271	stage_1W_vs_E10	ordinary_muscle_development	ENSGALG00010004765	ENSGALG00010004765	2.691765094	0.199943287	13.46264299	2.59466E-41	2.17901E-40	positive
PRJNA729271	stage_1W_vs_E10	ordinary_muscle_development	ENSGALG00010004769	HTR2A	-1.529418576	0.384305727	-3.979692384	6.90045E-05	0.000112422	negative
PRJNA729271	stage_1W_vs_E10	ordinary_muscle_development	ENSGALG00010004777	CDK14	-1.408058883	0.125214152	-11.24520563	2.44526E-29	1.34513E-28	negative
PRJNA729271	stage_1W_vs_E10	ordinary_muscle_development	ENSGALG00010004778	ENSGALG00010004778	-3.08233737	0.490506253	-6.283992002	3.29988E-10	7.61409E-10	negative
PRJNA729271	stage_1W_vs_E10	ordinary_muscle_development	ENSGALG00010004780	ENSGALG00010004780	0.158563553	0.260233825	0.609311848	0.542317755	0.578474933	positive
PRJNA729271	stage_1W_vs_E10	ordinary_muscle_development	ENSGALG00010004781	ENSGALG00010004781	-1.065557056	0.08377696	-12.71897494	4.63906E-37	3.36048E-36	negative
PRJNA729271	stage_1W_vs_E10	ordinary_muscle_development	ENSGALG00010004787	RIOK3	0.982254325	0.113839201	8.628436595	6.21963E-18	2.10639E-17	positive
PRJNA729271	stage_1W_vs_E10	ordinary_muscle_development	ENSGALG00010004791	FZD1	-1.227800993	0.118804365	-10.3346455	4.91225E-25	2.26257E-24	negative
PRJNA729271	stage_1W_vs_E10	ordinary_muscle_development	ENSGALG00010004798	PTPRK	-2.743772454	0.116870704	-23.47699087	7.0091E-122	4.0316E-120	negative
PRJNA729271	stage_1W_vs_E10	ordinary_muscle_development	ENSGALG00010004800	ZC3H12C	-1.049997682	0.286684309	-3.662557207	0.00024971	0.000390416	negative
PRJNA729271	stage_1W_vs_E10	ordinary_muscle_development	ENSGALG00010004803	RMC1	1.390884961	0.139778289	9.950650912	2.50539E-23	1.07634E-22	positive
PRJNA729271	stage_1W_vs_E10	ordinary_muscle_development	ENSGALG00010004808	THEMIS	1.957651124	0.731788411	2.675160051	0.007469358	0.01023427	positive
PRJNA729271	stage_1W_vs_E10	ordinary_muscle_development	ENSGALG00010004824	ATM	-0.092836198	0.11153741	-0.832332376	0.405221361	0.443430743	negative
PRJNA729271	stage_1W_vs_E10	ordinary_muscle_development	ENSGALG00010004832	C6orf58	4.815209178	1.854624364	2.596325849	0.009422666	0.012775376	positive
PRJNA729271	stage_1W_vs_E10	ordinary_muscle_development	ENSGALG00010004835	HHLA2	-1.457668693	0.413323916	-3.526698158	0.000420776	0.000645686	negative
PRJNA729271	stage_1W_vs_E10	ordinary_muscle_development	ENSGALG00010004837	CENPC	-2.119211792	0.142222979	-14.90062868	3.26493E-50	3.60435E-49	negative
PRJNA729271	stage_1W_vs_E10	ordinary_muscle_development	ENSGALG00010004841	AKAP9	0.323815226	0.194578741	1.664186047	0.096075256	0.115809632	positive
PRJNA729271	stage_1W_vs_E10	ordinary_muscle_development	ENSGALG00010004846	TTLL2	-0.308027117	0.370601641	-0.831154217	0.405886515	0.444034651	negative
PRJNA729271	stage_1W_vs_E10	ordinary_muscle_development	ENSGALG00010004851	MTCL3	-3.833442491	0.520013313	-7.371816055	1.68319E-13	4.62343E-13	negative
PRJNA729271	stage_1W_vs_E10	ordinary_muscle_development	ENSGALG00010004852	ENSGALG00010004852	4.242381051	0.222040022	19.1063801	2.23441E-81	5.51751E-80	positive
PRJNA729271	stage_1W_vs_E10	ordinary_muscle_development	ENSGALG00010004855	ZEB1	-0.120533735	0.092233961	-1.306825965	0.191271825	0.220862351	negative
PRJNA729271	stage_1W_vs_E10	ordinary_muscle_development	ENSGALG00010004859	NPC1	1.972345798	0.191164086	10.31755411	5.86994E-25	2.69341E-24	positive
PRJNA729271	stage_1W_vs_E10	ordinary_muscle_development	ENSGALG00010004866	SLC25A21	-1.52881413	0.314776718	-4.856820856	1.19285E-06	2.21814E-06	negative
PRJNA729271	stage_1W_vs_E10	ordinary_muscle_development	ENSGALG00010004868	NPAT	0.189859875	0.113243734	1.676559644	0.093628624	0.113057889	positive
PRJNA729271	stage_1W_vs_E10	ordinary_muscle_development	ENSGALG00010004871	C11orf87	-0.733519349	0.316782217	-2.315531967	0.020583837	0.026862584	negative
PRJNA729271	stage_1W_vs_E10	ordinary_muscle_development	ENSGALG00010004872	KIAA0408	-1.479217622	0.25796529	-5.734173087	9.79892E-09	2.07928E-08	negative
PRJNA729271	stage_1W_vs_E10	ordinary_muscle_development	ENSGALG00010004876	ENSGALG00010004876	-0.846250312	0.698881264	-1.210864214	0.22594745	0.25749731	negative
PRJNA729271	stage_1W_vs_E10	ordinary_muscle_development	ENSGALG00010004879	ENSGALG00010004879	-2.047551987	0.658496755	-3.109433678	0.001874464	0.002724118	negative
PRJNA729271	stage_1W_vs_E10	ordinary_muscle_development	ENSGALG00010004880	STAP1	1.572379951	0.628697672	2.50101125	0.012383924	0.016562556	positive
PRJNA729271	stage_1W_vs_E10	ordinary_muscle_development	ENSGALG00010004881	MIPOL1	-1.723126753	0.159400252	-10.81006294	3.08458E-27	1.55571E-26	negative
PRJNA729271	stage_1W_vs_E10	ordinary_muscle_development	ENSGALG00010004883	KRIT1	0.16459499	0.073783774	2.230774879	0.025696045	0.033187576	positive
PRJNA729271	stage_1W_vs_E10	ordinary_muscle_development	ENSGALG00010004887	MYH15	-8.145639671	0.750601531	-10.85214903	1.94791E-27	9.91395E-27	negative
PRJNA729271	stage_1W_vs_E10	ordinary_muscle_development	ENSGALG00010004890	ACAT1	0.241345188	0.098329111	2.454463249	0.014109509	0.018764779	positive
PRJNA729271	stage_1W_vs_E10	ordinary_muscle_development	ENSGALG00010004891	ANKIB1	-0.544101447	0.077905579	-6.984114033	2.86659E-12	7.41325E-12	negative
PRJNA729271	stage_1W_vs_E10	ordinary_muscle_development	ENSGALG00010004892	HEY2	-3.199092511	0.388794112	-8.228243205	1.89979E-16	6.02439E-16	negative
PRJNA729271	stage_1W_vs_E10	ordinary_muscle_development	ENSGALG00010004896	UBA6	0.174252094	0.0820746	2.123094041	0.033745972	0.04297934	positive
PRJNA729271	stage_1W_vs_E10	ordinary_muscle_development	ENSGALG00010004899	GATAD1	0.355465956	0.105169618	3.379930079	0.000725043	0.001093399	positive
PRJNA729271	stage_1W_vs_E10	ordinary_muscle_development	ENSGALG00010004901	CUL5	1.32813189	0.092022512	14.43268456	3.22288E-47	3.21733E-46	positive
PRJNA729271	stage_1W_vs_E10	ordinary_muscle_development	ENSGALG00010004902	ANKRD29	2.502058415	0.301208979	8.306719209	9.83844E-17	3.15714E-16	positive
PRJNA729271	stage_1W_vs_E10	ordinary_muscle_development	ENSGALG00010004903	ENSGALG00010004903	1.332650745	0.529030424	2.519043677	0.011767406	0.015777315	positive
PRJNA729271	stage_1W_vs_E10	ordinary_muscle_development	ENSGALG00010004907	ENSGALG00010004907	-0.968227098	0.118510577	-8.169963571	3.08483E-16	9.67429E-16	negative
PRJNA729271	stage_1W_vs_E10	ordinary_muscle_development	ENSGALG00010004917	ENSGALG00010004917	-0.494454525	0.094168315	-5.250752599	1.51479E-07	2.99018E-07	negative
PRJNA729271	stage_1W_vs_E10	ordinary_muscle_development	ENSGALG00010004921	RRAGD	2.76172168	0.132686791	20.81384035	3.24297E-96	1.12997E-94	positive
PRJNA729271	stage_1W_vs_E10	ordinary_muscle_development	ENSGALG00010004924	ENSGALG00010004924	0.096769475	0.376372091	0.257111187	0.797092942	0.818610046	positive
PRJNA729271	stage_1W_vs_E10	ordinary_muscle_development	ENSGALG00010004927	KIF25	-3.350571154	0.281042604	-11.92193321	9.09728E-33	5.68629E-32	negative
PRJNA729271	stage_1W_vs_E10	ordinary_muscle_development	ENSGALG00010004928	HDDC2	-0.50548727	0.122998617	-4.109698823	3.96175E-05	6.57044E-05	negative
PRJNA729271	stage_1W_vs_E10	ordinary_muscle_development	ENSGALG00010004929	ENSGALG00010004929	2.827591216	0.157667444	17.93389392	6.41271E-72	1.27682E-70	positive
PRJNA729271	stage_1W_vs_E10	ordinary_muscle_development	ENSGALG00010004933	SPTLC2	-0.754022148	0.091973323	-8.19827018	2.43871E-16	7.68458E-16	negative
PRJNA729271	stage_1W_vs_E10	ordinary_muscle_development	ENSGALG00010004934	CIP2A	-2.890494771	0.168361714	-17.16836148	4.58172E-66	7.72233E-65	negative
PRJNA729271	stage_1W_vs_E10	ordinary_muscle_development	ENSGALG00010004935	SVIL	-0.333692215	0.069182781	-4.823342027	1.41173E-06	2.60907E-06	negative
PRJNA729271	stage_1W_vs_E10	ordinary_muscle_development	ENSGALG00010004939	DDX10	-0.008796075	0.109148406	-0.080588217	0.935769437	0.943646722	negative
PRJNA729271	stage_1W_vs_E10	ordinary_muscle_development	ENSGALG00010004942	GABRR1	-1.070799247	0.527750057	-2.028989355	0.042459375	0.053480069	negative
PRJNA729271	stage_1W_vs_E10	ordinary_muscle_development	ENSGALG00010004944	ALKBH1	0.457878749	0.096199346	4.759686701	1.93894E-06	3.54725E-06	positive
PRJNA729271	stage_1W_vs_E10	ordinary_muscle_development	ENSGALG00010004946	SH2D1B	1.177825542	0.474383049	2.482857566	0.013033319	0.017398965	positive
PRJNA729271	stage_1W_vs_E10	ordinary_muscle_development	ENSGALG00010004949	RAB39A	-1.333859695	0.336801643	-3.960371703	7.48332E-05	0.000121645	negative
PRJNA729271	stage_1W_vs_E10	ordinary_muscle_development	ENSGALG00010004950	SLIRP	0.501879069	0.109841126	4.569136235	4.89738E-06	8.68668E-06	positive
PRJNA729271	stage_1W_vs_E10	ordinary_muscle_development	ENSGALG00010004951	AFDN	-1.118996788	0.063817922	-17.53420899	7.85348E-69	1.44462E-67	negative
PRJNA729271	stage_1W_vs_E10	ordinary_muscle_development	ENSGALG00010004952	PEX1	1.784329705	0.119874148	14.88502517	4.12331E-50	4.54505E-49	positive
PRJNA729271	stage_1W_vs_E10	ordinary_muscle_development	ENSGALG00010004957	ENSGALG00010004957	0.103668069	0.143323931	0.723313046	0.469487579	0.507890325	positive
PRJNA729271	stage_1W_vs_E10	ordinary_muscle_development	ENSGALG00010004958	TPD52L1	-4.802461266	0.520918008	-9.219226803	2.9925E-20	1.13551E-19	negative
PRJNA729271	stage_1W_vs_E10	ordinary_muscle_development	ENSGALG00010004962	ENSGALG00010004962	-0.477729419	0.09572278	-4.990759949	6.01422E-07	1.1409E-06	negative
PRJNA729271	stage_1W_vs_E10	ordinary_muscle_development	ENSGALG00010004963	ENSGALG00010004963	0.55783962	0.243228523	2.293479449	0.021820417	0.028392006	positive
PRJNA729271	stage_1W_vs_E10	ordinary_muscle_development	ENSGALG00010004965	JCAD	0.523619217	0.100739048	5.197778078	2.01685E-07	3.95003E-07	positive
PRJNA729271	stage_1W_vs_E10	ordinary_muscle_development	ENSGALG00010004966	LAMA3	0.994335761	0.211503938	4.701263584	2.58557E-06	4.68706E-06	positive
PRJNA729271	stage_1W_vs_E10	ordinary_muscle_development	ENSGALG00010004967	SLC35F2	-3.273290184	0.343854487	-9.519405189	1.74173E-21	6.95301E-21	negative
PRJNA729271	stage_1W_vs_E10	ordinary_muscle_development	ENSGALG00010004968	TRAT1	6.15638481	0.942464834	6.532217002	6.48032E-11	1.55337E-10	positive
PRJNA729271	stage_1W_vs_E10	ordinary_muscle_development	ENSGALG00010004969	YTHDC1	-0.123900851	0.077951518	-1.589460402	0.111956487	0.133718629	negative
PRJNA729271	stage_1W_vs_E10	ordinary_muscle_development	ENSGALG00010004970	RBM48	1.110772311	0.164752582	6.742063136	1.56153E-11	3.8824E-11	positive
PRJNA729271	stage_1W_vs_E10	ordinary_muscle_development	ENSGALG00010004973	SLN	10.00190268	0.194904542	51.31692972	0	0	positive
PRJNA729271	stage_1W_vs_E10	ordinary_muscle_development	ENSGALG00010004974	MTPAP	-0.3185097	0.061846989	-5.149962954	2.60538E-07	5.06233E-07	negative
PRJNA729271	stage_1W_vs_E10	ordinary_muscle_development	ENSGALG00010004975	SNW1	0.114412134	0.069379122	1.649085927	0.099130032	0.119303372	positive
PRJNA729271	stage_1W_vs_E10	ordinary_muscle_development	ENSGALG00010004976	ENSGALG00010004976	2.592138421	0.225933893	11.47299498	1.80309E-30	1.03549E-29	positive
PRJNA729271	stage_1W_vs_E10	ordinary_muscle_development	ENSGALG00010004977	RNF217	0.027464609	0.136381908	0.20138015	0.840401335	0.857860377	positive
PRJNA729271	stage_1W_vs_E10	ordinary_muscle_development	ENSGALG00010004980	FNDC1	-0.065773296	0.377641183	-0.174168758	0.861732838	0.877102555	negative
PRJNA729271	stage_1W_vs_E10	ordinary_muscle_development	ENSGALG00010004981	ENSGALG00010004981	0.423822214	0.201380916	2.104579835	0.035327898	0.044911234	positive
PRJNA729271	stage_1W_vs_E10	ordinary_muscle_development	ENSGALG00010004983	CLXN	-1.279144823	0.279600077	-4.574908686	4.76428E-06	8.46094E-06	negative
PRJNA729271	stage_1W_vs_E10	ordinary_muscle_development	ENSGALG00010004987	MAP3K8	0.007287082	0.285971417	0.025481856	0.979670621	0.982857534	positive
PRJNA729271	stage_1W_vs_E10	ordinary_muscle_development	ENSGALG00010004988	HJURP	-2.143276487	0.220092364	-9.738077441	2.07442E-22	8.55682E-22	negative
PRJNA729271	stage_1W_vs_E10	ordinary_muscle_development	ENSGALG00010004990	ENSGALG00010004990	6.104856312	2.376402188	2.568949121	0.010200743	0.013780035	positive
PRJNA729271	stage_1W_vs_E10	ordinary_muscle_development	ENSGALG00010004991	FAM133B	-0.372051335	0.063504722	-5.858640454	4.66672E-09	1.01022E-08	negative
PRJNA729271	stage_1W_vs_E10	ordinary_muscle_development	ENSGALG00010004993	SULT1B	2.793689162	0.244766393	11.41369582	3.57222E-30	2.03216E-29	positive
PRJNA729271	stage_1W_vs_E10	ordinary_muscle_development	ENSGALG00010004995	PIT54	10.47202441	2.194238401	4.772509865	1.81944E-06	3.33538E-06	positive
PRJNA729271	stage_1W_vs_E10	ordinary_muscle_development	ENSGALG00010004996	GPR158	-0.163700732	0.220503395	-0.742395515	0.457847736	0.496038787	negative
PRJNA729271	stage_1W_vs_E10	ordinary_muscle_development	ENSGALG00010004999	ADCK1	-0.064481985	0.188315895	-0.342413926	0.73203941	0.758409781	negative
PRJNA729271	stage_1W_vs_E10	ordinary_muscle_development	ENSGALG00010005003	EXPH5	-0.98350292	0.456933726	-2.152397305	0.031366076	0.040085635	negative
PRJNA729271	stage_1W_vs_E10	ordinary_muscle_development	ENSGALG00010005004	CDK6	-0.893351857	0.098863411	-9.036223304	1.62178E-19	5.96491E-19	negative
PRJNA729271	stage_1W_vs_E10	ordinary_muscle_development	ENSGALG00010005010	NKAIN2	-4.321300537	0.343273057	-12.58852232	2.44202E-36	1.7343E-35	negative
PRJNA729271	stage_1W_vs_E10	ordinary_muscle_development	ENSGALG00010005012	ENSGALG00010005012	8.677971216	1.060158058	8.185544737	2.71075E-16	8.51957E-16	positive
PRJNA729271	stage_1W_vs_E10	ordinary_muscle_development	ENSGALG00010005014	SULT1B1	1.210407457	0.151580073	7.985267649	1.40218E-15	4.28066E-15	positive
PRJNA729271	stage_1W_vs_E10	ordinary_muscle_development	ENSGALG00010005015	NRXN3	-2.684397653	0.418794894	-6.409814659	1.45697E-10	3.43004E-10	negative
PRJNA729271	stage_1W_vs_E10	ordinary_muscle_development	ENSGALG00010005016	STX11	-0.543912787	0.175443679	-3.100213062	0.001933815	0.002806432	negative
PRJNA729271	stage_1W_vs_E10	ordinary_muscle_development	ENSGALG00010005017	ENSGALG00010005017	0.646405434	0.171529026	3.768490085	0.000164238	0.000259979	positive
PRJNA729271	stage_1W_vs_E10	ordinary_muscle_development	ENSGALG00010005023	POGLUT3	-0.236245888	0.180880945	-1.306084995	0.191523655	0.221135525	negative
PRJNA729271	stage_1W_vs_E10	ordinary_muscle_development	ENSGALG00010005025	THOC1	-0.805243505	0.05880141	-13.69428906	1.0983E-42	9.59028E-42	negative
PRJNA729271	stage_1W_vs_E10	ordinary_muscle_development	ENSGALG00010005029	FAT4	-2.682736572	0.198089963	-13.5430212	8.71303E-42	7.42477E-41	negative

PRJNA729271	stage_1W_vs_E10	ordinary_muscle_development	ENSGALG00010005033	USP14	0.775306808	0.134270292	5.774224502	7.73083E-09	1.64986E-08	positive
PRJNA729271	stage_1W_vs_E10	ordinary_muscle_development	ENSGALG00010005035	NECTIN3	-0.203303358	0.098181509	-2.070688866	0.038387881	0.048600828	negative
PRJNA729271	stage_1W_vs_E10	ordinary_muscle_development	ENSGALG00010005037	THNSL1	0.407893452	0.142069692	2.871079999	0.00409072	0.005751769	positive
PRJNA729271	stage_1W_vs_E10	ordinary_muscle_development	ENSGALG00010005042	SLC46A3	1.876104482	0.201566559	9.307617728	1.30732E-20	5.05186E-20	positive
PRJNA729271	stage_1W_vs_E10	ordinary_muscle_development	ENSGALG00010005045	UTRN	-0.245843659	0.09946284	-2.47171365	0.013446718	0.017924423	negative
PRJNA729271	stage_1W_vs_E10	ordinary_muscle_development	ENSGALG00010005048	ROCK1	-0.426842289	0.114992898	-3.711901317	0.000205708	0.000323259	negative
PRJNA729271	stage_1W_vs_E10	ordinary_muscle_development	ENSGALG00010005057	FLVCR2	-2.185122393	0.301715279	-7.242332578	4.41033E-13	1.18692E-12	negative
PRJNA729271	stage_1W_vs_E10	ordinary_muscle_development	ENSGALG00010005060	POMP	0.637370175	0.138195454	4.612092185	3.98636E-06	7.1178E-06	positive
PRJNA729271	stage_1W_vs_E10	ordinary_muscle_development	ENSGALG00010005065	ENKUR	-1.639792446	0.466956605	-3.511659171	0.000445319	0.000681687	negative
PRJNA729271	stage_1W_vs_E10	ordinary_muscle_development	ENSGALG00010005068	PLCXD2	2.599867458	0.258080498	10.07386253	7.20901E-24	3.1598E-23	positive
PRJNA729271	stage_1W_vs_E10	ordinary_muscle_development	ENSGALG00010005070	ALB	7.236519989	1.403599097	5.15568869	2.527E-07	4.91531E-07	positive
PRJNA729271	stage_1W_vs_E10	ordinary_muscle_development	ENSGALG00010005075	TRDN	7.120171621	0.166998134	42.63623467	0	0	positive
PRJNA729271	stage_1W_vs_E10	ordinary_muscle_development	ENSGALG00010005077	ERG28	-0.268317611	0.103375117	-2.595572491	0.009443348	0.012798628	negative
PRJNA729271	stage_1W_vs_E10	ordinary_muscle_development	ENSGALG00010005081	GREB1L	0.146660522	0.179991984	0.814816964	0.415177089	0.453193901	positive
PRJNA729271	stage_1W_vs_E10	ordinary_muscle_development	ENSGALG00010005082	ENSGALG00010005082	-0.554330264	0.087465346	-6.337713037	2.33201E-10	5.41879E-10	negative
PRJNA729271	stage_1W_vs_E10	ordinary_muscle_development	ENSGALG00010005087	ENPP1	1.688454134	0.21431972	7.878202406	3.32124E-15	9.96304E-15	positive
PRJNA729271	stage_1W_vs_E10	ordinary_muscle_development	ENSGALG00010005092	ESCO1	2.204886875	0.13957293	15.79738183	3.24309E-56	4.32064E-55	positive
PRJNA729271	stage_1W_vs_E10	ordinary_muscle_development	ENSGALG00010005104	ENPP3	-0.434129938	0.20064575	-2.163663761	0.030490164	0.039031611	negative
PRJNA729271	stage_1W_vs_E10	ordinary_muscle_development	ENSGALG00010005107	TTLL5	-0.769426708	0.074715886	-10.29803361	7.19163E-25	3.28738E-24	negative
PRJNA729271	stage_1W_vs_E10	ordinary_muscle_development	ENSGALG00010005109	ABHD3	-0.377614075	0.379467232	-0.995116424	0.319679643	0.355922608	negative
PRJNA729271	stage_1W_vs_E10	ordinary_muscle_development	ENSGALG00010005111	RASSF6	-1.846310795	0.430942598	-4.284354355	1.83271E-05	3.11504E-05	negative
PRJNA729271	stage_1W_vs_E10	ordinary_muscle_development	ENSGALG00010005114	PHLDB2	-1.206159491	0.100677315	-11.98044949	4.49878E-33	2.85008E-32	negative
PRJNA729271	stage_1W_vs_E10	ordinary_muscle_development	ENSGALG00010005118	PRTFDC1	-1.675074511	0.141149262	-11.8673983	1.7482E-32	1.08384E-31	negative
PRJNA729271	stage_1W_vs_E10	ordinary_muscle_development	ENSGALG00010005119	MIB1	-1.769018257	0.058183197	-30.40428067	4.822E-203	7.3573E-201	negative
PRJNA729271	stage_1W_vs_E10	ordinary_muscle_development	ENSGALG00010005122	IL8L1	-0.960389348	1.196626351	-0.802580812	0.422217063	0.46026518	negative
PRJNA729271	stage_1W_vs_E10	ordinary_muscle_development	ENSGALG00010005123	MTIF3	2.048715223	0.103282343	19.83606454	1.45405E-87	4.12456E-86	positive
PRJNA729271	stage_1W_vs_E10	ordinary_muscle_development	ENSGALG00010005126	MED23	-0.893098541	0.058995264	-15.13847867	9.0278E-52	1.05616E-50	negative
PRJNA729271	stage_1W_vs_E10	ordinary_muscle_development	ENSGALG00010005127	GATA6	-1.628080386	0.323449084	-5.033498212	4.81609E-07	9.19027E-07	negative
PRJNA729271	stage_1W_vs_E10	ordinary_muscle_development	ENSGALG00010005131	IL8L2	1.166654551	0.721924458	1.616034113	0.106086938	0.127085138	positive
PRJNA729271	stage_1W_vs_E10	ordinary_muscle_development	ENSGALG00010005132	LNX2	-0.655005575	0.097392998	-6.725386683	1.75127E-11	4.34668E-11	negative
PRJNA729271	stage_1W_vs_E10	ordinary_muscle_development	ENSGALG00010005138	ENSGALG00010005138	0.183429996	0.637618651	0.287679785	0.773591866	0.796732563	positive
PRJNA729271	stage_1W_vs_E10	ordinary_muscle_development	ENSGALG00010005140	DCT	-2.289593495	0.966967463	-2.367808208	0.017893809	0.023485219	negative
PRJNA729271	stage_1W_vs_E10	ordinary_muscle_development	ENSGALG00010005141	CCDC170	-1.658808414	0.325797605	-5.091530414	3.55185E-07	6.84173E-07	negative
PRJNA729271	stage_1W_vs_E10	ordinary_muscle_development	ENSGALG00010005143	GTF3A	-0.028674678	0.135224601	-0.212052228	0.832066289	0.850430183	negative
PRJNA729271	stage_1W_vs_E10	ordinary_muscle_development	ENSGALG00010005145	AKAP7	1.84226775	0.174735204	10.5431974	5.461E-26	2.6211E-25	positive
PRJNA729271	stage_1W_vs_E10	ordinary_muscle_development	ENSGALG00010005146	TGFB3	-0.79219549	0.204907379	-3.866114984	0.000110583	0.000177233	negative
PRJNA729271	stage_1W_vs_E10	ordinary_muscle_development	ENSGALG00010005148	TTC39C	-1.555359396	0.092389466	-16.8348132	1.35616E-63	2.17691E-62	negative
PRJNA729271	stage_1W_vs_E10	ordinary_muscle_development	ENSGALG00010005150	ALCAM	-1.973380614	0.188176773	-10.4868448	9.92888E-26	4.70785E-25	negative
PRJNA729271	stage_1W_vs_E10	ordinary_muscle_development	ENSGALG00010005154	CWF19L2	-0.280002315	0.149464984	-1.873363967	0.061018142	0.075349972	negative
PRJNA729271	stage_1W_vs_E10	ordinary_muscle_development	ENSGALG00010005157	IMPA1	-0.164030636	0.109325137	-1.50039268	0.133512715	0.157749168	negative
PRJNA729271	stage_1W_vs_E10	ordinary_muscle_development	ENSGALG00010005158	IFT43	-1.636744674	0.209000966	-7.831278045	4.82935E-15	1.43829E-14	negative
PRJNA729271	stage_1W_vs_E10	ordinary_muscle_development	ENSGALG00010005159	ARHGAP21	0.187250385	0.098445813	1.902065505	0.057162591	0.070878679	positive
PRJNA729271	stage_1W_vs_E10	ordinary_muscle_development	ENSGALG00010005163	GPC6	-4.016755232	0.409864136	-9.800211537	1.1235E-22	4.68099E-22	negative
PRJNA729271	stage_1W_vs_E10	ordinary_muscle_development	ENSGALG00010005165	METTL21C	3.036868305	0.567246365	5.35370254	8.61725E-08	1.73193E-07	positive
PRJNA729271	stage_1W_vs_E10	ordinary_muscle_development	ENSGALG00010005166	CBLB	-0.32094824	0.125239007	-2.562685917	0.010386596	0.014015426	negative
PRJNA729271	stage_1W_vs_E10	ordinary_muscle_development	ENSGALG00010005167	GUCY1A2	0.328083853	0.178086642	1.842270979	0.065435523	0.080509965	positive
PRJNA729271	stage_1W_vs_E10	ordinary_muscle_development	ENSGALG00010005170	GPATCH2L	0.161488216	0.060941729	2.64987915	0.008052057	0.010996284	positive
PRJNA729271	stage_1W_vs_E10	ordinary_muscle_development	ENSGALG00010005172	OSBPL1A	1.316051041	0.080403632	16.36805472	3.23386E-60	4.76855E-59	positive
PRJNA729271	stage_1W_vs_E10	ordinary_muscle_development	ENSGALG00010005174	TPP2	2.270577519	0.184939731	12.2773917	1.19799E-34	8.03926E-34	positive
PRJNA729271	stage_1W_vs_E10	ordinary_muscle_development	ENSGALG00010005176	BAMBI	-4.191649125	0.32552201	-12.87669958	6.08839E-38	4.5257E-37	negative
PRJNA729271	stage_1W_vs_E10	ordinary_muscle_development	ENSGALG00010005177	AASDHPPT	0.886601674	0.127321822	6.963469893	3.31992E-12	8.55659E-12	positive
PRJNA729271	stage_1W_vs_E10	ordinary_muscle_development	ENSGALG00010005178	EPHA5	-0.573826402	0.20072597	-2.858755157	0.004253069	0.005973088	negative
PRJNA729271	stage_1W_vs_E10	ordinary_muscle_development	ENSGALG00010005180	WAC	1.022819837	0.115379177	8.864856387	7.66027E-19	2.71946E-18	positive
PRJNA729271	stage_1W_vs_E10	ordinary_muscle_development	ENSGALG00010005181	IMPACT	1.357246885	0.130378423	10.41005756	2.23087E-25	1.04177E-24	positive
PRJNA729271	stage_1W_vs_E10	ordinary_muscle_development	ENSGALG00010005182	NEPRO	-0.445303754	0.087303098	-5.10066384	3.38464E-07	6.52833E-07	negative
PRJNA729271	stage_1W_vs_E10	ordinary_muscle_development	ENSGALG00010005184	FGF14	-3.07465033	0.333401858	-9.222055182	2.91459E-20	1.10652E-19	negative
PRJNA729271	stage_1W_vs_E10	ordinary_muscle_development	ENSGALG00010005186	ESR1	0.55265324	0.191845105	2.8807263	0.0039676	0.005587328	positive
PRJNA729271	stage_1W_vs_E10	ordinary_muscle_development	ENSGALG00010005187	KBTBD3	1.820777078	0.248690457	7.321459372	2.45289E-13	6.67943E-13	positive
PRJNA729271	stage_1W_vs_E10	ordinary_muscle_development	ENSGALG00010005190	ENSGALG00010005190	2.006395621	0.282274595	7.107956792	1.17773E-12	3.11008E-12	positive
PRJNA729271	stage_1W_vs_E10	ordinary_muscle_development	ENSGALG00010005191	MSANTD4	-0.45141998	0.105798538	-4.26678844	1.98307E-05	3.35993E-05	negative
PRJNA729271	stage_1W_vs_E10	ordinary_muscle_development	ENSGALG00010005193	EPB41L2	0.361621855	0.098559599	3.669067846	0.000243436	0.000380936	positive
PRJNA729271	stage_1W_vs_E10	ordinary_muscle_development	ENSGALG00010005194	ENSGALG00010005194	3.066337134	1.105676855	2.773266999	0.005549657	0.007711107	positive
PRJNA729271	stage_1W_vs_E10	ordinary_muscle_development	ENSGALG00010005198	TRPC3	0.456002358	0.487739409	0.934930313	0.349824214	0.387372191	positive
PRJNA729271	stage_1W_vs_E10	ordinary_muscle_development	ENSGALG00010005203	ENSGALG00010005203	-0.146749219	0.106484751	-1.378124256	0.168164928	0.195834388	negative
PRJNA729271	stage_1W_vs_E10	ordinary_muscle_development	ENSGALG00010005204	ITGBL1	0.875450462	0.310988793	2.815054693	0.004876893	0.006816834	positive
PRJNA729271	stage_1W_vs_E10	ordinary_muscle_development	ENSGALG00010005208	ENSGALG00010005208	0.27427716	0.110123527	2.49063183	0.012751618	0.017035456	positive
PRJNA729271	stage_1W_vs_E10	ordinary_muscle_development	ENSGALG00010005211	ESRRB	2.229310422	0.342292379	6.512883595	7.37216E-11	1.76161E-10	positive
PRJNA729271	stage_1W_vs_E10	ordinary_muscle_development	ENSGALG00010005212	TAF13	0.326866704	0.101283117	3.227257561	0.001249829	0.001842583	positive
PRJNA729271	stage_1W_vs_E10	ordinary_muscle_development	ENSGALG00010005213	ENSGALG00010005213	-0.520207345	0.165248979	-3.148021535	0.001643796	0.002402139	negative
PRJNA729271	stage_1W_vs_E10	ordinary_muscle_development	ENSGALG00010005214	BBS7	-0.441015835	0.120136694	-3.67095031	0.00024165	0.000378223	negative
PRJNA729271	stage_1W_vs_E10	ordinary_muscle_development	ENSGALG00010005215	VASH1	-1.569275437	0.142512506	-11.01149285	3.36383E-28	1.7679E-27	negative
PRJNA729271	stage_1W_vs_E10	ordinary_muscle_development	ENSGALG00010005216	ENSGALG00010005216	-0.59673373	0.167253312	-3.567844015	0.000359931	0.000555375	negative
PRJNA729271	stage_1W_vs_E10	ordinary_muscle_development	ENSGALG00010005217	CCNA2	-1.677979269	0.144463281	-11.61526482	3.44719E-31	2.04197E-30	negative
PRJNA729271	stage_1W_vs_E10	ordinary_muscle_development	ENSGALG00010005218	GTPBP8	0.142500426	0.119668588	1.190792234	0.233735158	0.265662285	positive
PRJNA729271	stage_1W_vs_E10	ordinary_muscle_development	ENSGALG00010005219	NALCN	-0.768596276	0.18483982	-4.158174761	3.20801E-05	5.36147E-05	negative
PRJNA729271	stage_1W_vs_E10	ordinary_muscle_development	ENSGALG00010005220	ANGEL1	0.205426607	0.085682914	2.397521264	0.016506423	0.021762834	positive
PRJNA729271	stage_1W_vs_E10	ordinary_muscle_development	ENSGALG00010005221	ENSGALG00010005221	0.708470508	0.307653143	2.302822268	0.021288845	0.027740184	positive
PRJNA729271	stage_1W_vs_E10	ordinary_muscle_development	ENSGALG00010005222	ABCB1	4.849832691	0.974045101	4.979063787	6.38926E-07	1.20999E-06	positive
PRJNA729271	stage_1W_vs_E10	ordinary_muscle_development	ENSGALG00010005223	GRIA4	-2.939386535	0.461977095	-6.36262396	1.98336E-10	4.62943E-10	negative
PRJNA729271	stage_1W_vs_E10	ordinary_muscle_development	ENSGALG00010005224	ENSGALG00010005224	1.065824826	0.414808359	2.569439123	0.010186329	0.013761845	positive
PRJNA729271	stage_1W_vs_E10	ordinary_muscle_development	ENSGALG00010005226	MYCT1	0.975227409	0.168387147	5.791578658	6.97279E-09	1.49336E-08	positive
PRJNA729271	stage_1W_vs_E10	ordinary_muscle_development	ENSGALG00010005227	EXOSC9	-0.466563637	0.105210679	-4.434565398	9.22582E-06	1.60519E-05	negative
PRJNA729271	stage_1W_vs_E10	ordinary_muscle_development	ENSGALG00010005228	ZNF521	-1.959252846	0.132653147	-14.76974272	2.29588E-49	2.46144E-48	negative
PRJNA729271	stage_1W_vs_E10	ordinary_muscle_development	ENSGALG00010005229	CIPC	-0.250581142	0.074085369	-3.382329684	0.000718738	0.001084204	negative
PRJNA729271	stage_1W_vs_E10	ordinary_muscle_development	ENSGALG00010005231	ENSGALG00010005231	0.969770687	0.430695319	2.25163972	0.024345049	0.031535432	positive
PRJNA729271	stage_1W_vs_E10	ordinary_muscle_development	ENSGALG00010005232	GATA3	-3.492865471	0.512987925	-6.808864893	9.83718E-12	2.47552E-11	negative
PRJNA729271	stage_1W_vs_E10	ordinary_muscle_development	ENSGALG00010005236	TMTC4	-1.704041134	0.092302854	-18.46141319	4.2215E-76	9.13294E-75	negative
PRJNA729271	stage_1W_vs_E10	ordinary_muscle_development	ENSGALG00010005237	RAP2A	-2.234278955	0.113676696	-19.65467881	5.27216E-86	1.45562E-84	negative
PRJNA729271	stage_1W_vs_E10	ordinary_muscle_development	ENSGALG00010005238	TMEM63C	-1.137435995	0.171269238	-6.641215961	3.11106E-11	7.61994E-11	negative
PRJNA729271	stage_1W_vs_E10	ordinary_muscle_development	ENSGALG00010005239	ENSGALG00010005239	-2.483228754	0.214250308	-11.59031589	4.61428E-31	2.71555E-30	negative
PRJNA729271	stage_1W_vs_E10	ordinary_muscle_development	ENSGALG00010005240	VIP	-4.11726156	0.408294878	-10.08403921	6.49979E-24	2.85672E-23	negative
PRJNA729271	stage_1W_vs_E10	ordinary_muscle_development	ENSGALG00010005241	C4orf54	4.137678823	0.187406811	22.0785936	5.0764E-108	2.2097E-106	positive
PRJNA729271	stage_1W_vs_E10	ordinary_muscle_development	ENSGALG00010005242	CCDC80	-0.286028903	0.233987035	-1.22241347	0.221551308	0.253024441	negative
PRJNA729271	stage_1W_vs_E10	ordinary_muscle_development	ENSGALG00010005243	NGB	-2.797843052	0.564371469	-4.957449495	7.14246E-07	1.34928E-06	negative
PRJNA729271	stage_1W_vs_E10	ordinary_muscle_development	ENSGALG00010005244	FBXO5	-1.417856001	0.098501786	-14.39421621	5.62591E-47	5.58545E-46	negative
PRJNA729271	stage_1W_vs_E10	ordinary_muscle_development	ENSGALG00010005245	KIAA1217	1.925220542	0.134863772	14.275298	3.11951E-46	3.0266E-45	positive
PRJNA729271	stage_1W_vs_E10	ordinary_muscle_development	ENSGALG00010005246	ENSGALG00010005246	-1.278728253	0.19950688	-6.409444391	1.46051E-10	3.43726E-10	negative
PRJNA729271	stage_1W_vs_E10	ordinary_muscle_development	ENSGALG00010005247	THAP7	1.301987124	0.116966338	11.13129769	8.834E-29	4.73378E-28	positive
PRJNA729271	stage_1W_vs_E10	ordinary_muscle_development	ENSGALG00010005248	TMEM200A	-2.448359565	0.242833055	-10.08248058	6.60375E-24	2.89889E-23	negative
PRJNA729271	stage_1W_vs_E10	ordinary_muscle_development	ENSGALG00010005249	PDGFD	-1.024396902	0.229074307	-4.471897853	7.75285E-06	1.35476E-05	negative
PRJNA729271	stage_1W_vs_E10	ordinary_muscle_development	ENSGALG00010005250	SLC35A5	-0.945215892	0.093957593	-10.06002663	8.29759E-24	3.63255E-23	negative
PRJNA729271	stage_1W_vs_E10	ordinary_muscle_development	ENSGALG00010005251	MTRF1L	0.16183773	0.103512511	1.563460582	0.1179443	0.140407575	positive
PRJNA729271	stage_1W_vs_E10	ordinary_muscle_development	ENSGALG00010005252	KLHL14	-2.911845166	0.235227882	-12.37882662	3.40281E-35	2.32769E-34	negative
PRJNA729271	stage_1W_vs_E10	ordinary_muscle_development	ENSGALG00010005253	POMT2	-0.49175115	0.096811742	-5.079457708	3.78514E-07	7.27853E-07	negative
PRJNA729271	stage_1W_vs_E10	ordinary_muscle_development	ENSGALG00010005254	ENSGALG00010005254	-2.158826706	0.783907987	-2.753928703	0.005888459	0.008158555	negative
PRJNA729271	stage_1W_vs_E10	ordinary_muscle_development	ENSGALG00010005255	BZW2	1.053963557	0.092866256	11.34926288	7.47905E-30	4.21005E-29	positive
PRJNA729271	stage_1W_vs_E10	ordinary_muscle_development	ENSGALG00010005256	OPRM1	-7.68294245	0.902936113	-8.508843913	1.75676E-17	5.82573E-17	negative
PRJNA729271	stage_1W_vs_E10	ordinary_muscle_development	ENSGALG00010005258	ITIH5	2.545859524	0.278963251	9.126146585	7.09809E-20	2.6524E-19	positive
PRJNA729271	stage_1W_vs_E10	ordinary_muscle_development	ENSGALG00010005259	SLC27A5	2.073850354	0.669142091	3.099267526	0.001939998	0.002814841	positive
PRJNA729271	stage_1W_vs_E10	ordinary_muscle_development	ENSGALG00010005260	ANXA5	-0.496291489	0.150142585	-3.305467855	0.00094818	0.001416309	negative
PRJNA729271	stage_1W_vs_E10	ordinary_muscle_development	ENSGALG00010005261	ATG3	-0.274069422	0.110573617	-2.478614972	0.01318936	0.017599168	negative
PRJNA729271	stage_1W_vs_E10	ordinary_muscle_development	ENSGALG00010005262	ENSGALG00010005262	-0.541952827	0.125349207	-4.323544107	1.53542E-05	2.62329E-05	negative
PRJNA729271	stage_1W_vs_E10	ordinary_muscle_development	ENSGALG00010005263	ENSGALG00010005263	1.451439825	0.329632528	4.403205701	1.06663E-05	1.84805E-05	positive
PRJNA729271	stage_1W_vs_E10	ordinary_muscle_development	ENSGALG00010005264	ITIH2	3.498430835	1.040674979	3.361694			positive
PRJNA729271	stage_1W_vs_E10	ordinary_muscle_development	ENSGALG00010005265	DYNC2H1	-1.23661696	0.096232339	-12.85032637	8.56481E-38	6.33403E-37	negative
PRJNA729271	stage_1W_vs_E10	ordinary_muscle_development	ENSGALG00010005266	SLC19A2	-0.130348386	0.271089766	-0.480831085	0.630636556	0.6637918	negative
PRJNA729271	stage_1W_vs_E10	ordinary_muscle_development	ENSGALG00010005267	CYP51A1	-0.542617677	0.181179499	-2.994917638	0.00274519	0.0039246	negative
PRJNA729271	stage_1W_vs_E10	ordinary_muscle_development	ENSGALG00010005268	MTTP	4.68729694	0.882538265	5.311154347	1.08933E-07	2.17042E-07	positive
PRJNA729271	stage_1W_vs_E10	ordinary_muscle_development	ENSGALG00010005269	KIN	0.161883112	0.106666666	1.517654184	0.129101599	0.152773914	positive
PRJNA729271	stage_1W_vs_E10	ordinary_muscle_development	ENSGALG00010005270	GSTZ1	-0.095041559	0.215523658	-0.440979706	0.659227689	0.689728984	negative
PRJNA729271	stage_1W_vs_E10	ordinary_muscle_development	ENSGALG00010005272	MBNL2	2.391848113	0.092516938	25.85308337	2.2464E-147	1.809E-145	positive
PRJNA729271	stage_1W_vs_E10	ordinary_muscle_development	ENSGALG00010005273	ENSGALG00010005273	2.724957237	0.641736757	4.246222778	2.17404E-05	3.67196E-05	positive
PRJNA729271	stage_1W_vs_E10	ordinary_muscle_development	ENSGALG00010005275	L3MBTL3	0.462693273	0.171622722	2.695990759	0.007017961	0.009648615	positive
PRJNA729271	stage_1W_vs_E10	ordinary_muscle_development	ENSGALG00010005277	ENSGALG00010005277	1.107121071	0.204736888	5.407531003	6.38995E-08	1.29505E-07	positive
PRJNA729271	stage_1W_vs_E10	ordinary_muscle_development	ENSGALG00010005278	DAPP1	-0.154669274	0.131574174	-1.175529127	0.239783091	0.272173524	negative
PRJNA729271	stage_1W_vs_E10	ordinary_muscle_development	ENSGALG00010005279	CCDC181	0.320344853	0.320684707	0.998940225	0.317823649	0.353992146	positive
PRJNA729271	stage_1W_vs_E10	ordinary_muscle_development	ENSGALG00010005280	TMEM244	-4.023045295	0.9252811	-4.347916861	1.37437E-05	2.35868E-05	negative
PRJNA729271	stage_1W_vs_E10	ordinary_muscle_development	ENSGALG00010005281	TMED8	-1.528021911	0.071149336	-21.47626377	2.5956E-102	1.01139E-100	negative
PRJNA729271	stage_1W_vs_E10	ordinary_muscle_development	ENSGALG00010005282	FLT1	1.775486224	0.125445788	14.15341447	1.77905E-45	1.69431E-44	positive
PRJNA729271	stage_1W_vs_E10	ordinary_muscle_development	ENSGALG00010005283	ATP5C1	1.44404726	0.132037496	10.93664526	7.69965E-28	3.98168E-27	positive
PRJNA729271	stage_1W_vs_E10	ordinary_muscle_development	ENSGALG00010005284	ENSGALG00010005284	0.597610419	0.215703032	2.770523959	0.005596618	0.007771889	positive

PRJNA729271	stage_1W_vs_E10	ordinary_muscle_development	ENSGALG00010005285	MPP7	1.058133492	0.127416795	8.304505628	1.00236E-16	3.21514E-16	positive
PRJNA729271	stage_1W_vs_E10	ordinary_muscle_development	ENSGALG00010005286	ENSGALG00010005286	1.267006335	0.615181293	2.059565774	0.039440071	0.049880798	positive
PRJNA729271	stage_1W_vs_E10	ordinary_muscle_development	ENSGALG00010005287	ENSGALG00010005287	-0.30170557	0.400275882	-0.753744064	0.451002917	0.489061666	negative
PRJNA729271	stage_1W_vs_E10	ordinary_muscle_development	ENSGALG00010005288	ENSGALG00010005288	-0.372014534	0.095095622	-3.912004866	9.15331E-05	0.000147682	negative
PRJNA729271	stage_1W_vs_E10	ordinary_muscle_development	ENSGALG00010005291	IFNGR1	0.071737179	0.096409585	0.744087624	0.456823466	0.495003075	positive
PRJNA729271	stage_1W_vs_E10	ordinary_muscle_development	ENSGALG00010005292	TAF3	-0.154392416	0.099074825	-1.558341546	0.119152305	0.141752517	negative
PRJNA729271	stage_1W_vs_E10	ordinary_muscle_development	ENSGALG00010005293	BLZF1	0.207525118	0.096438961	2.15188049	0.031406768	0.040134101	positive
PRJNA729271	stage_1W_vs_E10	ordinary_muscle_development	ENSGALG00010005295	ENSGALG00010005295	1.564345007	0.550230168	2.843073857	0.004468072	0.006262905	positive
PRJNA729271	stage_1W_vs_E10	ordinary_muscle_development	ENSGALG00010005296	PAN3	0.73000036	0.101070972	7.222651022	5.09837E-13	1.36676E-12	positive
PRJNA729271	stage_1W_vs_E10	ordinary_muscle_development	ENSGALG00010005297	IL22RA2	-0.763984647	0.614630747	-1.242997768	0.213868656	0.244887129	negative
PRJNA729271	stage_1W_vs_E10	ordinary_muscle_development	ENSGALG00010005299	ODAD2	-6.286672903	0.936024836	-6.716352667	1.8633E-11	4.6192E-11	negative
PRJNA729271	stage_1W_vs_E10	ordinary_muscle_development	ENSGALG00010005300	QRFPR	-1.605140679	0.875879516	-1.832604428	0.066861419	0.082173671	negative
PRJNA729271	stage_1W_vs_E10	ordinary_muscle_development	ENSGALG00010005301	ENSGALG00010005301	1.188990204	0.365479707	3.253231798	0.001141004	0.001689017	positive
PRJNA729271	stage_1W_vs_E10	ordinary_muscle_development	ENSGALG00010005302	PSPC1	-1.343944542	0.080824455	-16.6279444	4.37343E-62	6.72247E-61	negative
PRJNA729271	stage_1W_vs_E10	ordinary_muscle_development	ENSGALG00010005303	ENSGALG00010005303	1.775112263	0.288515483	6.152571931	7.62364E-10	1.72448E-09	positive
PRJNA729271	stage_1W_vs_E10	ordinary_muscle_development	ENSGALG00010005304	ENSGALG00010005304	0.377562666	0.21109416	1.78859835	0.073679524	0.090064488	positive
PRJNA729271	stage_1W_vs_E10	ordinary_muscle_development	ENSGALG00010005305	SCAF8	0.040905932	0.086441415	0.473221453	0.636055194	0.668669861	positive
PRJNA729271	stage_1W_vs_E10	ordinary_muscle_development	ENSGALG00010005307	TIAM2	-2.302576822	0.158773688	-14.50225695	1.17231E-47	1.18333E-46	negative
PRJNA729271	stage_1W_vs_E10	ordinary_muscle_development	ENSGALG00010005308	ENSGALG00010005308	2.988420794	0.535035883	5.585458639	2.33084E-08	4.83618E-08	positive
PRJNA729271	stage_1W_vs_E10	ordinary_muscle_development	ENSGALG00010005309	ASXL3	-2.788335758	0.147681812	-18.88069846	1.64398E-79	3.86215E-78	negative
PRJNA729271	stage_1W_vs_E10	ordinary_muscle_development	ENSGALG00010005310	LAMTOR3	1.006561163	0.075728936	13.29163226	2.58869E-40	2.09626E-39	positive
PRJNA729271	stage_1W_vs_E10	ordinary_muscle_development	ENSGALG00010005311	YME1L1	0.47284082	0.078177505	6.048297676	1.46384E-09	3.26739E-09	positive
PRJNA729271	stage_1W_vs_E10	ordinary_muscle_development	ENSGALG00010005313	MPHOSPH8	-0.277752566	0.143410015	-1.936772445	0.052773166	0.065722746	negative
PRJNA729271	stage_1W_vs_E10	ordinary_muscle_development	ENSGALG00010005314	IL20RA	-1.972420992	0.649425369	-3.037178845	0.002388037	0.003433647	negative
PRJNA729271	stage_1W_vs_E10	ordinary_muscle_development	ENSGALG00010005315	ENSGALG00010005315	1.636673151	0.524262826	3.121856193	0.001797147	0.002617003	positive
PRJNA729271	stage_1W_vs_E10	ordinary_muscle_development	ENSGALG00010005316	PARP4	0.336928299	0.108017441	3.119202743	0.001813411	0.002638566	positive
PRJNA729271	stage_1W_vs_E10	ordinary_muscle_development	ENSGALG00010005317	CHCHD3	0.789442093	0.109530503	7.207509036	5.69847E-13	1.52538E-12	positive
PRJNA729271	stage_1W_vs_E10	ordinary_muscle_development	ENSGALG00010005318	SLC35D3	-2.888290726	0.508151084	-5.683921214	1.31641E-08	2.76903E-08	negative
PRJNA729271	stage_1W_vs_E10	ordinary_muscle_development	ENSGALG00010005319	NOL4	-4.436701394	1.015353771	-4.369611381	1.24468E-05	2.14551E-05	negative
PRJNA729271	stage_1W_vs_E10	ordinary_muscle_development	ENSGALG00010005321	HS6ST3	-3.236055115	0.295544406	-10.94947171	6.68363E-28	3.46492E-27	negative
PRJNA729271	stage_1W_vs_E10	ordinary_muscle_development	ENSGALG00010005322	TFB1M	0.431010002	0.122528701	3.51762484	0.000435427	0.000667109	positive
PRJNA729271	stage_1W_vs_E10	ordinary_muscle_development	ENSGALG00010005323	DNAJB14	0.860257574	0.063357568	13.5778188	5.42171E-42	4.64192E-41	positive
PRJNA729271	stage_1W_vs_E10	ordinary_muscle_development	ENSGALG00010005324	CENPJ	-2.568979433	0.201275548	-12.7634949	2.62169E-37	1.91539E-36	negative
PRJNA729271	stage_1W_vs_E10	ordinary_muscle_development	ENSGALG00010005326	MASTL	-1.790949755	0.185509923	-9.654199241	4.71831E-22	1.92004E-21	negative
PRJNA729271	stage_1W_vs_E10	ordinary_muscle_development	ENSGALG00010005329	VIPAS39	0.364434231	0.117283532	3.107292428	0.001888095	0.002743103	positive
PRJNA729271	stage_1W_vs_E10	ordinary_muscle_development	ENSGALG00010005331	ENSGALG00010005331	1.054835719	0.845240249	1.247971475	0.212041504	0.24302535	positive
PRJNA729271	stage_1W_vs_E10	ordinary_muscle_development	ENSGALG00010005332	ENSGALG00010005332	1.025218197	0.739758242	1.385882763	0.165782705	0.193277592	positive
PRJNA729271	stage_1W_vs_E10	ordinary_muscle_development	ENSGALG00010005333	PLXNA4	-1.383966331	0.159762185	-8.662665249	4.6086E-18	1.56885E-17	negative
PRJNA729271	stage_1W_vs_E10	ordinary_muscle_development	ENSGALG00010005334	NME7	-0.284932727	0.133967957	-2.126872222	0.033430701	0.042600265	negative
PRJNA729271	stage_1W_vs_E10	ordinary_muscle_development	ENSGALG00010005335	AHSA1	0.498791801	0.133741819	3.72951262	0.00019185	0.000302203	positive
PRJNA729271	stage_1W_vs_E10	ordinary_muscle_development	ENSGALG00010005336	PEX7	-0.106819695	0.127234118	-0.839552295	0.401159457	0.439350686	negative
PRJNA729271	stage_1W_vs_E10	ordinary_muscle_development	ENSGALG00010005337	DCTN1	0.053907184	0.154304688	0.349355449	0.726822475	0.753651318	positive
PRJNA729271	stage_1W_vs_E10	ordinary_muscle_development	ENSGALG00010005338	PCGF1	1.091749975	0.114564439	9.529571161	1.57934E-21	6.31519E-21	positive
PRJNA729271	stage_1W_vs_E10	ordinary_muscle_development	ENSGALG00010005341	UGGT2	-0.298546744	0.119208905	-2.504399687	0.012265938	0.016413846	negative
PRJNA729271	stage_1W_vs_E10	ordinary_muscle_development	ENSGALG00010005342	MAP3K5	0.184339739	0.14579729	1.264356412	0.20610213	0.236592253	positive
PRJNA729271	stage_1W_vs_E10	ordinary_muscle_development	ENSGALG00010005343	RNF17	-4.481421312	1.784097237	-2.511870553	0.012009313	0.016085289	negative
PRJNA729271	stage_1W_vs_E10	ordinary_muscle_development	ENSGALG00010005344	ENSGALG00010005344	0.747720437	0.730921526	1.022983193	0.306315792	0.341936651	positive
PRJNA729271	stage_1W_vs_E10	ordinary_muscle_development	ENSGALG00010005345	NOX3	1.282645784	0.507602331	2.526871342	0.011508365	0.015442858	positive
PRJNA729271	stage_1W_vs_E10	ordinary_muscle_development	ENSGALG00010005346	FBXO30	2.471241227	0.219971727	11.23435843	2.76495E-29	1.51352E-28	positive
PRJNA729271	stage_1W_vs_E10	ordinary_muscle_development	ENSGALG00010005347	DOK1	-0.141836343	0.15182624	-0.934201777	0.350199819	0.387701136	negative
PRJNA729271	stage_1W_vs_E10	ordinary_muscle_development	ENSGALG00010005349	ACBD5	1.25034637	0.103472027	12.0839072	1.28467E-33	8.29455E-33	positive
PRJNA729271	stage_1W_vs_E10	ordinary_muscle_development	ENSGALG00010005350	ARHGAP20	-1.33167171	0.281534506	-4.730047946	2.24467E-06	4.08195E-06	negative
PRJNA729271	stage_1W_vs_E10	ordinary_muscle_development	ENSGALG00010005351	DTNA	2.250027376	0.163123918	13.79336276	2.79425E-43	2.48026E-42	positive
PRJNA729271	stage_1W_vs_E10	ordinary_muscle_development	ENSGALG00010005352	ENSGALG00010005352	2.736616004	0.604551603	4.526687201	5.99155E-06	1.05577E-05	positive
PRJNA729271	stage_1W_vs_E10	ordinary_muscle_development	ENSGALG00010005353	ENSGALG00010005353	0.025470236	0.104930273	0.242734868	0.808210794	0.828443212	positive
PRJNA729271	stage_1W_vs_E10	ordinary_muscle_development	ENSGALG00010005354	M1AP	-0.428102224	0.352489022	-1.214512218	0.224552187	0.25608843	negative
PRJNA729271	stage_1W_vs_E10	ordinary_muscle_development	ENSGALG00010005355	FDX1	0.448632121	0.103077683	4.352369081	1.34674E-05	2.31292E-05	positive
PRJNA729271	stage_1W_vs_E10	ordinary_muscle_development	ENSGALG00010005356	DNAJC3	0.760720749	0.084096766	9.045778893	1.48602E-19	5.4753E-19	positive
PRJNA729271	stage_1W_vs_E10	ordinary_muscle_development	ENSGALG00010005357	TNIP3	-1.580646423	0.524798348	-3.011911966	0.002596079	0.003721705	negative
PRJNA729271	stage_1W_vs_E10	ordinary_muscle_development	ENSGALG00010005358	SHPRH	0.187888589	0.09001794	2.087234939	0.036866903	0.046748819	positive
PRJNA729271	stage_1W_vs_E10	ordinary_muscle_development	ENSGALG00010005359	MEIS2	-2.935870435	0.138818591	-21.14897162	2.82023E-99	1.04018E-97	negative
PRJNA729271	stage_1W_vs_E10	ordinary_muscle_development	ENSGALG00010005360	CAMK1D	-1.035166281	0.152865071	-6.771764643	1.27221E-11	3.17778E-11	negative
PRJNA729271	stage_1W_vs_E10	ordinary_muscle_development	ENSGALG00010005361	ENSGALG00010005361	1.135329385	0.451977775	2.511914186	0.012007828	0.016084786	positive
PRJNA729271	stage_1W_vs_E10	ordinary_muscle_development	ENSGALG00010005362	ATP1B1	1.270536694	0.17430622	7.289107043	3.12016E-13	8.45834E-13	positive
PRJNA729271	stage_1W_vs_E10	ordinary_muscle_development	ENSGALG00010005363	RDX	0.842450239	0.085396092	9.865208388	5.8912E-23	2.48814E-22	positive
PRJNA729271	stage_1W_vs_E10	ordinary_muscle_development	ENSGALG00010005364	MAP7	-2.612589866	0.35450219	-7.369742537	1.70958E-13	4.69503E-13	negative
PRJNA729271	stage_1W_vs_E10	ordinary_muscle_development	ENSGALG00010005366	AUP1	1.286431563	0.113355375	11.34865961	7.53083E-30	4.23591E-29	positive
PRJNA729271	stage_1W_vs_E10	ordinary_muscle_development	ENSGALG00010005367	H2AZ1	-0.843460101	0.095400057	-8.84129552	9.4615E-19	3.3458E-18	negative
PRJNA729271	stage_1W_vs_E10	ordinary_muscle_development	ENSGALG00010005368	CCDC3	-2.740773336	0.251268155	-10.90776242	1.05829E-27	5.44162E-27	negative
PRJNA729271	stage_1W_vs_E10	ordinary_muscle_development	ENSGALG00010005369	DPT	2.974848648	0.313002142	9.504243739	2.01507E-21	8.03533E-21	positive
PRJNA729271	stage_1W_vs_E10	ordinary_muscle_development	ENSGALG00010005371	GRM1	-3.926027766	1.325544304	-2.96182312	0.003058234	0.004355385	negative
PRJNA729271	stage_1W_vs_E10	ordinary_muscle_development	ENSGALG00010005372	CDIN1	-1.008355481	0.110745334	-9.10517351	8.61287E-20	3.20934E-19	negative
PRJNA729271	stage_1W_vs_E10	ordinary_muscle_development	ENSGALG00010005376	HTRA2	0.704553774	0.094543485	7.452166336	9.18199E-14	2.558E-13	positive
PRJNA729271	stage_1W_vs_E10	ordinary_muscle_development	ENSGALG00010005377	EMCN	2.608338465	0.140472706	18.5682937	5.80175E-77	1.27419E-75	positive
PRJNA729271	stage_1W_vs_E10	ordinary_muscle_development	ENSGALG00010005378	ENSGALG00010005378	-6.700724495	1.034455619	-6.477536949	9.32319E-11	2.21795E-10	negative
PRJNA729271	stage_1W_vs_E10	ordinary_muscle_development	ENSGALG00010005379	DZIP1	-0.78615401	0.168817671	-4.656822977	3.21126E-06	5.77796E-06	negative
PRJNA729271	stage_1W_vs_E10	ordinary_muscle_development	ENSGALG00010005380	XCL1	5.809928568	1.024776361	5.669459982	1.43248E-08	3.00925E-08	positive
PRJNA729271	stage_1W_vs_E10	ordinary_muscle_development	ENSGALG00010005381	FGF9	-4.616070177	0.299173063	-15.42943117	1.03781E-53	1.29236E-52	negative
PRJNA729271	stage_1W_vs_E10	ordinary_muscle_development	ENSGALG00010005382	RAB32	-1.314126183	0.12984866	-10.12044472	4.48374E-24	1.97965E-23	negative
PRJNA729271	stage_1W_vs_E10	ordinary_muscle_development	ENSGALG00010005383	OPTN	3.314928736	0.138245937	23.97848941	4.663E-127	2.9645E-125	positive
PRJNA729271	stage_1W_vs_E10	ordinary_muscle_development	ENSGALG00010005384	MAPRE2	0.485213447	0.172791608	2.808084556	0.004983714	0.006959435	positive
PRJNA729271	stage_1W_vs_E10	ordinary_muscle_development	ENSGALG00010005385	PDSS1	0.617446119	0.098758524	6.252079239	4.05024E-10	9.29957E-10	positive
PRJNA729271	stage_1W_vs_E10	ordinary_muscle_development	ENSGALG00010005386	CPOX	-0.213236609	0.140223948	-1.520686108	0.128338626	0.151939049	negative
PRJNA729271	stage_1W_vs_E10	ordinary_muscle_development	ENSGALG00010005387	CLDN10	-3.562303632	0.620591626	-5.74017354	9.45796E-09	2.00933E-08	negative
PRJNA729271	stage_1W_vs_E10	ordinary_muscle_development	ENSGALG00010005388	ENSGALG00010005388	2.636483035	0.464748356	5.672926006	1.40379E-08	2.95026E-08	positive
PRJNA729271	stage_1W_vs_E10	ordinary_muscle_development	ENSGALG00010005389	MCM10	-2.345071867	0.188986243	-12.40869087	2.34455E-35	1.61292E-34	negative
PRJNA729271	stage_1W_vs_E10	ordinary_muscle_development	ENSGALG00010005391	ADGB	-0.159513748	0.393383209	-0.40549201	0.68511589	0.714391398	negative
PRJNA729271	stage_1W_vs_E10	ordinary_muscle_development	ENSGALG00010005392	ENSGALG00010005392	0.661333236	0.106458592	6.212117046	5.22755E-10	1.19253E-09	positive
PRJNA729271	stage_1W_vs_E10	ordinary_muscle_development	ENSGALG00010005394	PHYH	2.783932815	0.13528029	20.57899804	4.23341E-94	1.3978E-92	positive
PRJNA729271	stage_1W_vs_E10	ordinary_muscle_development	ENSGALG00010005395	ENSGALG00010005395	-2.63799992	0.384730522	-6.856747178	7.04461E-12	1.78673E-11	negative
PRJNA729271	stage_1W_vs_E10	ordinary_muscle_development	ENSGALG00010005396	MICU2	0.740837075	0.12255975	6.044701264	1.49687E-09	3.34059E-09	positive
PRJNA729271	stage_1W_vs_E10	ordinary_muscle_development	ENSGALG00010005398	SEPHS1	-1.046477769	0.086934705	-12.0375145	2.25652E-33	1.44471E-32	negative
PRJNA729271	stage_1W_vs_E10	ordinary_muscle_development	ENSGALG00010005399	NDNF	-5.948957023	0.289157559	-20.57341005	4.75054E-94	1.56143E-92	negative
PRJNA729271	stage_1W_vs_E10	ordinary_muscle_development	ENSGALG00010005401	BCLAF1	-1.221966419	0.087218454	-14.01041142	1.34623E-44	1.24528E-43	negative
PRJNA729271	stage_1W_vs_E10	ordinary_muscle_development	ENSGALG00010005402	ABCC4	1.836874574	0.168547158	10.8982827	1.17453E-27	6.02862E-27	positive
PRJNA729271	stage_1W_vs_E10	ordinary_muscle_development	ENSGALG00010005403	ENSGALG00010005403	-0.831294294	0.146832369	-5.661519312	1.50039E-08	3.14825E-08	negative
PRJNA729271	stage_1W_vs_E10	ordinary_muscle_development	ENSGALG00010005404	BEND7	1.84368706	0.186238319	9.899611775	4.17894E-23	1.77479E-22	positive
PRJNA729271	stage_1W_vs_E10	ordinary_muscle_development	ENSGALG00010005405	CEBPD	7.426978676	0.473851986	15.67362573	2.29127E-55	2.98938E-54	positive
PRJNA729271	stage_1W_vs_E10	ordinary_muscle_development	ENSGALG00010005407	DPH6	-0.324757281	0.120905425	-2.686043916	0.007230358	0.009924626	negative
PRJNA729271	stage_1W_vs_E10	ordinary_muscle_development	ENSGALG00010005408	APBB1IP	-0.529197847	0.21332996	-2.480654126	0.013114155	0.017500431	negative
PRJNA729271	stage_1W_vs_E10	ordinary_muscle_development	ENSGALG00010005409	PRKCQ	1.592252151	0.218056819	7.302005785	2.83509E-13	7.69851E-13	positive
PRJNA729271	stage_1W_vs_E10	ordinary_muscle_development	ENSGALG00010005411	STXBP5	0.714597702	0.096632954	7.39496901	1.41441E-13	3.89696E-13	positive
PRJNA729271	stage_1W_vs_E10	ordinary_muscle_development	ENSGALG00010005412	MTFR2	-1.956699914	0.166824643	-11.729082	9.04341E-32	5.48011E-31	negative
PRJNA729271	stage_1W_vs_E10	ordinary_muscle_development	ENSGALG00010005413	GAD2	-2.59330089	0.349970893	-7.410047361	1.26254E-13	3.49114E-13	negative
PRJNA729271	stage_1W_vs_E10	ordinary_muscle_development	ENSGALG00010005414	ENSGALG00010005414	-1.603778167	0.233858968	-6.8578861	6.98869E-12	1.77317E-11	negative
PRJNA729271	stage_1W_vs_E10	ordinary_muscle_development	ENSGALG00010005415	PRKDC	-0.753654424	0.088123128	-8.552288592	1.20672E-17	4.02363E-17	negative
PRJNA729271	stage_1W_vs_E10	ordinary_muscle_development	ENSGALG00010005416	PPP3CA	1.407841803	0.162101492	8.684940441	3.78941E-18	1.29822E-17	positive
PRJNA729271	stage_1W_vs_E10	ordinary_muscle_development	ENSGALG00010005417	LOXL3	0.859489479	0.164303376	5.231112715	1.68493E-07	3.31339E-07	positive
PRJNA729271	stage_1W_vs_E10	ordinary_muscle_development	ENSGALG00010005420	DHRS4	0.046135092	0.118849935	0.38817936	0.697883306	0.726397998	positive
PRJNA729271	stage_1W_vs_E10	ordinary_muscle_development	ENSGALG00010005421	ENSGALG00010005421	0.777016521	0.335418456	2.316558636	0.020527787	0.026791849	positive
PRJNA729271	stage_1W_vs_E10	ordinary_muscle_development	ENSGALG00010005423	ST3GAL6	1.808344396	0.14962739	12.08565089	1.2577E-33	8.12406E-33	positive
PRJNA729271	stage_1W_vs_E10	ordinary_muscle_development	ENSGALG00010005424	ENSGALG00010005424	1.364087216	0.132801067	10.27165859	9.45647E-25	4.30231E-24	positive
PRJNA729271	stage_1W_vs_E10	ordinary_muscle_development	ENSGALG00010005425	ELAPOR2	-0.673261689	0.251970907	-2.671981846	0.007540473	0.01032878	negative
PRJNA729271	stage_1W_vs_E10	ordinary_muscle_development	ENSGALG00010005426	ENSGALG00010005426	2.33938014	0.930197419	2.514928651	0.011905648	0.01595381	positive
PRJNA729271	stage_1W_vs_E10	ordinary_muscle_development	ENSGALG00010005427	ENSGALG00010005427	1.309236106	0.137297765	9.535742326	1.48817E-21	5.96049E-21	positive
PRJNA729271	stage_1W_vs_E10	ordinary_muscle_development	ENSGALG00010005428	ZNF770	0.038959642	0.102989679	0.378286859	0.705217507	0.733136897	positive
PRJNA729271	stage_1W_vs_E10	ordinary_muscle_development	ENSGALG00010005429	ZDHHC20	-2.239839004	0.12081942	-18.53873323	1.00563E-76	2.20189E-75	negative
PRJNA729271	stage_1W_vs_E10	ordinary_muscle_development	ENSGALG00010005430	MCM4	-1.970063703	0.13614953	-14.46985315	1.87873E-47	1.88751E-46	negative
PRJNA729271	stage_1W_vs_E10	ordinary_muscle_development	ENSGALG00010005431	GRM3	-7.084754831	1.035005469	-6.845137582	7.64024E-12	1.93273E-11	negative
PRJNA729271	stage_1W_vs_E10	ordinary_muscle_development	ENSGALG00010005433	GPC5	-1.506647957	0.163701451	-9.203632279	3.4605E-20	1.30795E-19	negative
PRJNA729271	stage_1W_vs_E10	ordinary_muscle_development	ENSGALG00010005434	PRDM5	-1.828891501	0.224651406	-8.141019618	3.91963E-16	1.2279E-15	negative
PRJNA729271	stage_1W_vs_E10	ordinary_muscle_development	ENSGALG00010005435	MAD2L1	-2.597751218	0.174082607	-14.92252016	2.35219E-50	2.61064E-49	negative
PRJNA729271	stage_1W_vs_E10	ordinary_muscle_development	ENSGALG00010005436	AQR	-0.909745309	0.055459386	-16.40381153	1.79607E-60	2.66742E-59	negative
PRJNA729271	stage_1W_vs_E10	ordinary_muscle_development	ENSGALG00010005437	PDE7B	2.98266826	0.157826949	18.89834582	1.17685E-79	2.77824E-78	positive
PRJNA729271	stage_1W_vs_E10	ordinary_muscle_development	ENSGALG00010005438	GPR180	-0.593023666	0.140389392	-4.224134423	2.39861E-05	4.03854E-05	negative
PRJNA729271	stage_1W_vs_E10	ordinary_muscle_development	ENSGALG00010005439	TBX19	1.397489825	0.498379554	2.804067326	0.005046237	0.007044708	positive
PRJNA729271	stage_1W_vs_E10	ordinary_muscle_development	ENSGALG00010005440	ENSGALG00010005440	2.662562631	0.49662566	5.361307009	8.2622E-08	1.66173E-07	positive
PRJNA729271	stage_1W_vs_E10	ordinary_muscle_development	ENSGALG00010005441	UBE2V2	0.131361194	0.095439997	1.376374668	0.168705667	0.19638521	positive
PRJNA729271	stage_1W_vs_E10	ordinary_muscle_development	ENSGALG00010005442	SASH1	0.089610643	0.097970432	0.914670285	0.360364749	0.397868638	positive
PRJNA729271	stage_1W_vs_E10	ordinary_muscle_development	ENSGALG00010005443	BANK1	-0.580992004	0.446236847	-1.301981243	0.192922798	0.222573699	negative
PRJNA729271	stage_1W_vs_E10	ordinary_muscle_development	ENSGALG00010005444	ACTC1	0.145771125	0.42656217	0.34173477	0.732550503	0.758830811	positive

PRJNA729271	stage_1W_vs_E10	ordinary_muscle_development	ENSGALG00010005445	SFT2D2	-1.596746543	0.107397377	-14.86764934	5.34576E-50	5.87466E-49	negative
PRJNA729271	stage_1W_vs_E10	ordinary_muscle_development	ENSGALG00010005446	ENSGALG00010005446	1.730087021	0.386000748	4.482082042	7.39183E-06	1.2937E-05	positive
PRJNA729271	stage_1W_vs_E10	ordinary_muscle_development	ENSGALG00010005447	MYO3A	-4.077615325	0.507245258	-8.038745091	9.07632E-16	2.79739E-15	negative
PRJNA729271	stage_1W_vs_E10	ordinary_muscle_development	ENSGALG00010005448	ENSGALG00010005448	0.504727155	0.147532822	3.421117738	0.000623643	0.000944786	positive
PRJNA729271	stage_1W_vs_E10	ordinary_muscle_development	ENSGALG00010005449	TIPRL	0.278509005	0.085835802	3.244671783	0.001175861	0.001738307	positive
PRJNA729271	stage_1W_vs_E10	ordinary_muscle_development	ENSGALG00010005450	ENSGALG00010005450	2.153882157	0.437107422	4.927580842	8.3254E-07	1.56642E-06	positive
PRJNA729271	stage_1W_vs_E10	ordinary_muscle_development	ENSGALG00010005453	UST	-0.168758944	0.11480912	-1.469908872	0.141586436	0.166690659	negative
PRJNA729271	stage_1W_vs_E10	ordinary_muscle_development	ENSGALG00010005454	GPR161	-1.813124554	0.100726371	-18.00049512	1.93085E-72	3.87641E-71	negative
PRJNA729271	stage_1W_vs_E10	ordinary_muscle_development	ENSGALG00010005455	ENSGALG00010005455	0.970550327	0.726777112	1.335416747	0.181739987	0.210459464	positive
PRJNA729271	stage_1W_vs_E10	ordinary_muscle_development	ENSGALG00010005456	RPL7L1	-0.594898241	0.070568947	-8.430028601	3.45581E-17	1.12896E-16	negative
PRJNA729271	stage_1W_vs_E10	ordinary_muscle_development	ENSGALG00010005457	SEMA3D	-0.85791791	0.160669079	-5.339657841	9.31222E-08	1.86643E-07	negative
PRJNA729271	stage_1W_vs_E10	ordinary_muscle_development	ENSGALG00010005458	PRKD1	0.268770162	0.174767388	1.537873661	0.124079508	0.147312022	positive
PRJNA729271	stage_1W_vs_E10	ordinary_muscle_development	ENSGALG00010005459	ZC3H12D	4.201124724	0.508859578	8.255960797	1.50685E-16	4.79934E-16	positive
PRJNA729271	stage_1W_vs_E10	ordinary_muscle_development	ENSGALG00010005460	TGDS	-1.096326888	0.103414384	-10.60129984	2.93867E-26	1.42605E-25	negative
PRJNA729271	stage_1W_vs_E10	ordinary_muscle_development	ENSGALG00010005462	AHI1	0.937980332	0.150048068	6.251198998	4.07313E-10	9.35066E-10	positive
PRJNA729271	stage_1W_vs_E10	ordinary_muscle_development	ENSGALG00010005463	SKA3	-3.14911047	0.258597155	-12.17766865	4.08801E-34	2.68125E-33	negative
PRJNA729271	stage_1W_vs_E10	ordinary_muscle_development	ENSGALG00010005464	SLC39A8	-4.599165459	0.394492867	-11.65842489	2.07852E-31	1.24188E-30	negative
PRJNA729271	stage_1W_vs_E10	ordinary_muscle_development	ENSGALG00010005465	ENSGALG00010005465	-0.844552091	0.090814779	-9.299720787	1.40815E-20	5.43572E-20	negative
PRJNA729271	stage_1W_vs_E10	ordinary_muscle_development	ENSGALG00010005466	LATS2	0.395669941	0.089625427	4.414706346	1.01147E-05	1.75458E-05	positive
PRJNA729271	stage_1W_vs_E10	ordinary_muscle_development	ENSGALG00010005467	ENSGALG00010005467	2.01347844	0.259137768	7.769915037	7.85388E-15	2.31386E-14	positive
PRJNA729271	stage_1W_vs_E10	ordinary_muscle_development	ENSGALG00010005468	PPIL4	-0.465359947	0.105242238	-4.421798276	9.78828E-06	1.69958E-05	negative
PRJNA729271	stage_1W_vs_E10	ordinary_muscle_development	ENSGALG00010005470	SEMA3A	-2.084270898	0.142040565	-14.67377224	9.49181E-49	9.94102E-48	negative
PRJNA729271	stage_1W_vs_E10	ordinary_muscle_development	ENSGALG00010005472	PFKP	-1.531890797	0.115725354	-13.23729628	5.34382E-40	4.28422E-39	negative
PRJNA729271	stage_1W_vs_E10	ordinary_muscle_development	ENSGALG00010005473	ENSGALG00010005473	2.029356248	0.686962516	2.954100405	0.00313582	0.004458431	positive
PRJNA729271	stage_1W_vs_E10	ordinary_muscle_development	ENSGALG00010005474	COL8A1	0.534861768	0.344654676	1.551877297	0.120691586	0.143513088	positive
PRJNA729271	stage_1W_vs_E10	ordinary_muscle_development	ENSGALG00010005475	TMEM181	-0.62328284	0.092989838	-6.702698399	2.04605E-11	5.06275E-11	negative
PRJNA729271	stage_1W_vs_E10	ordinary_muscle_development	ENSGALG00010005476	NFKB1	0.650368763	0.116104203	5.601595321	2.12388E-08	4.41842E-08	positive
PRJNA729271	stage_1W_vs_E10	ordinary_muscle_development	ENSGALG00010005477	TMEM161B	0.276293473	0.161975829	1.705769775	0.088050953	0.106678248	positive
PRJNA729271	stage_1W_vs_E10	ordinary_muscle_development	ENSGALG00010005479	ENSGALG00010005479	-0.584353523	0.651858823	-0.89644184	0.370016829	0.407645659	negative
PRJNA729271	stage_1W_vs_E10	ordinary_muscle_development	ENSGALG00010005481	ENSGALG00010005481	-1.498888763	0.557935561	-2.686490823	0.007220693	0.009912297	negative
PRJNA729271	stage_1W_vs_E10	ordinary_muscle_development	ENSGALG00010005482	XPO4	-1.464667577	0.076426885	-19.16429758	7.35428E-82	1.83793E-80	negative
PRJNA729271	stage_1W_vs_E10	ordinary_muscle_development	ENSGALG00010005484	GINM1	1.356058961	0.122489708	11.07079915	1.73843E-28	9.21675E-28	positive
PRJNA729271	stage_1W_vs_E10	ordinary_muscle_development	ENSGALG00010005485	PITRM1	-0.431139606	0.096672513	-4.459795181	8.2038E-06	1.43132E-05	negative
PRJNA729271	stage_1W_vs_E10	ordinary_muscle_development	ENSGALG00010005486	IRF5	1.454521342	0.321923777	4.518216566	6.23627E-06	1.09662E-05	positive
PRJNA729271	stage_1W_vs_E10	ordinary_muscle_development	ENSGALG00010005487	ENSGALG00010005487	-1.741494965	0.212645594	-8.189659301	2.61967E-16	8.24942E-16	negative
PRJNA729271	stage_1W_vs_E10	ordinary_muscle_development	ENSGALG00010005489	G2E3	-1.340788246	0.086623551	-15.47833391	4.85894E-54	6.10844E-53	negative
PRJNA729271	stage_1W_vs_E10	ordinary_muscle_development	ENSGALG00010005490	DCBLD2	-0.89586183	0.115376743	-7.764665652	8.18609E-15	2.40978E-14	negative
PRJNA729271	stage_1W_vs_E10	ordinary_muscle_development	ENSGALG00010005491	GGACT	0.408977317	0.172613438	2.369324905	0.017820591	0.023397596	positive
PRJNA729271	stage_1W_vs_E10	ordinary_muscle_development	ENSGALG00010005492	TNPO3	0.686538805	0.096461849	7.117205499	1.10137E-12	2.91268E-12	positive
PRJNA729271	stage_1W_vs_E10	ordinary_muscle_development	ENSGALG00010005493	KLF6	0.497495833	0.181223862	2.745200483	0.006047394	0.008368208	positive
PRJNA729271	stage_1W_vs_E10	ordinary_muscle_development	ENSGALG00010005494	PPDPFL	2.949679193	0.43456874	6.787600954	1.14013E-11	2.85773E-11	positive
PRJNA729271	stage_1W_vs_E10	ordinary_muscle_development	ENSGALG00010005495	TXN	-1.53585818	0.146724202	-10.46765398	1.2162E-25	5.74415E-25	negative
PRJNA729271	stage_1W_vs_E10	ordinary_muscle_development	ENSGALG00010005497	MKX	-3.187869193	0.207150608	-15.38913752	1.93596E-53	2.39026E-52	negative
PRJNA729271	stage_1W_vs_E10	ordinary_muscle_development	ENSGALG00010005498	SMO	0.52484757	0.136819881	3.836047546	0.00012503	0.000199616	positive
PRJNA729271	stage_1W_vs_E10	ordinary_muscle_development	ENSGALG00010005499	PDE5A	-0.845543089	0.113019866	-7.481366917	7.35535E-14	2.0622E-13	negative
PRJNA729271	stage_1W_vs_E10	ordinary_muscle_development	ENSGALG00010005500	KATNA1	0.370288599	0.129707365	2.854800113	0.004306392	0.006045047	positive
PRJNA729271	stage_1W_vs_E10	ordinary_muscle_development	ENSGALG00010005502	SCFD1	-0.814590954	0.073147753	-11.13624033	8.35734E-29	4.48333E-28	negative
PRJNA729271	stage_1W_vs_E10	ordinary_muscle_development	ENSGALG00010005503	DYNLT1	-2.086442371	0.158880892	-13.13211641	2.15547E-39	1.69158E-38	negative
PRJNA729271	stage_1W_vs_E10	ordinary_muscle_development	ENSGALG00010005504	LATS1	-0.36427559	0.080276575	-4.537757031	5.68557E-06	1.00319E-05	negative
PRJNA729271	stage_1W_vs_E10	ordinary_muscle_development	ENSGALG00010005505	FABP2	1.868898099	0.952308528	1.962492244	0.049705203	0.062072621	positive
PRJNA729271	stage_1W_vs_E10	ordinary_muscle_development	ENSGALG00010005507	USP53	1.67216651	0.119237851	14.0237894	1.11499E-44	1.03402E-43	positive
PRJNA729271	stage_1W_vs_E10	ordinary_muscle_development	ENSGALG00010005508	SS18	-1.314785784	0.065432797	-20.09368149	8.38112E-90	2.45423E-88	negative
PRJNA729271	stage_1W_vs_E10	ordinary_muscle_development	ENSGALG00010005509	COCH	0.875293852	0.449944011	1.945339489	0.051734132	0.064506344	positive
PRJNA729271	stage_1W_vs_E10	ordinary_muscle_development	ENSGALG00010005510	MYOZ2	-0.872424861	0.318592348	-2.738373557	0.00617439	0.008532537	negative
PRJNA729271	stage_1W_vs_E10	ordinary_muscle_development	ENSGALG00010005511	AHCYL2	0.923457131	0.098020897	9.421023026	4.46714E-21	1.75335E-20	positive
PRJNA729271	stage_1W_vs_E10	ordinary_muscle_development	ENSGALG00010005512	SMARCAD1	-2.069083815	0.113214231	-18.27582795	1.28919E-74	2.69261E-73	negative
PRJNA729271	stage_1W_vs_E10	ordinary_muscle_development	ENSGALG00010005513	TMEM30CP	0.008825789	0.263541239	0.033489214	0.973284467	0.976991575	positive
PRJNA729271	stage_1W_vs_E10	ordinary_muscle_development	ENSGALG00010005514	EEF1AKMT1	0.15822391	0.144004582	1.098742193	0.271880533	0.305971144	positive
PRJNA729271	stage_1W_vs_E10	ordinary_muscle_development	ENSGALG00010005515	PCCA	-0.107670814	0.097233171	-1.107346529	0.268144135	0.302000717	negative
PRJNA729271	stage_1W_vs_E10	ordinary_muscle_development	ENSGALG00010005516	LARP4B	0.900883621	0.131744261	6.838124228	8.02368E-12	2.02831E-11	positive
PRJNA729271	stage_1W_vs_E10	ordinary_muscle_development	ENSGALG00010005518	HPGDS	-0.461622312	0.652847247	-0.707090846	0.479510024	0.517843675	negative
PRJNA729271	stage_1W_vs_E10	ordinary_muscle_development	ENSGALG00010005519	PTBP3	-1.005383281	0.255301089	-3.938029734	8.21534E-05	0.000133067	negative
PRJNA729271	stage_1W_vs_E10	ordinary_muscle_development	ENSGALG00010005521	SYTL3	1.62964951	0.397940291	4.095211132	4.21784E-05	6.97838E-05	positive
PRJNA729271	stage_1W_vs_E10	ordinary_muscle_development	ENSGALG00010005522	SYNPO2	2.042731515	0.178113349	11.46871655	1.89448E-30	1.08625E-29	positive
PRJNA729271	stage_1W_vs_E10	ordinary_muscle_development	ENSGALG00010005523	ENSGALG00010005523	2.978369414	0.616283314	4.832792556	1.34631E-06	2.4904E-06	positive
PRJNA729271	stage_1W_vs_E10	ordinary_muscle_development	ENSGALG00010005524	STRIP2	3.156548546	0.273209663	11.55357578	7.08117E-31	4.14211E-30	positive
PRJNA729271	stage_1W_vs_E10	ordinary_muscle_development	ENSGALG00010005526	STRN3	0.685050655	0.069691844	9.829710606	8.38594E-23	3.51821E-22	positive
PRJNA729271	stage_1W_vs_E10	ordinary_muscle_development	ENSGALG00010005527	NUP43	-0.513983614	0.095386983	-5.388404142	7.10861E-08	1.43529E-07	negative
PRJNA729271	stage_1W_vs_E10	ordinary_muscle_development	ENSGALG00010005529	ENSGALG00010005529	1.260031379	0.376427156	3.34734452	0.000815897	0.001225283	positive
PRJNA729271	stage_1W_vs_E10	ordinary_muscle_development	ENSGALG00010005530	IL17D	-0.035200093	0.211301478	-0.166587067	0.86769497	0.882365553	negative
PRJNA729271	stage_1W_vs_E10	ordinary_muscle_development	ENSGALG00010005531	SUSD1	-0.664218601	0.258071577	-2.573776659	0.010059519	0.013600664	negative
PRJNA729271	stage_1W_vs_E10	ordinary_muscle_development	ENSGALG00010005532	GTPBP4	0.2902063	0.097356286	2.980868625	0.002874321	0.004103544	positive
PRJNA729271	stage_1W_vs_E10	ordinary_muscle_development	ENSGALG00010005533	OPN4-1	-1.917947628	0.777127887	-2.467994856	0.013587227	0.018110056	negative
PRJNA729271	stage_1W_vs_E10	ordinary_muscle_development	ENSGALG00010005534	ENSGALG00010005534	-0.226853949	0.162464361	-1.396330539	0.162614982	0.189849492	negative
PRJNA729271	stage_1W_vs_E10	ordinary_muscle_development	ENSGALG00010005535	AP4S1	1.07208161	0.152587402	7.02601652	2.12513E-12	5.53627E-12	positive
PRJNA729271	stage_1W_vs_E10	ordinary_muscle_development	ENSGALG00010005536	TAF4B	-1.714347669	0.208381138	-8.226981014	1.91991E-16	6.08419E-16	negative
PRJNA729271	stage_1W_vs_E10	ordinary_muscle_development	ENSGALG00010005537	GRIN3A	-3.738304539	0.346379215	-10.79251982	3.73408E-27	1.8774E-26	negative
PRJNA729271	stage_1W_vs_E10	ordinary_muscle_development	ENSGALG00010005538	ENSGALG00010005538	1.258795285	0.338003088	3.724212376	0.000195926	0.000308556	positive
PRJNA729271	stage_1W_vs_E10	ordinary_muscle_development	ENSGALG00010005539	IFT88	-0.782442076	0.133189611	-5.874647967	4.23743E-09	9.19484E-09	negative
PRJNA729271	stage_1W_vs_E10	ordinary_muscle_development	ENSGALG00010005540	PCMT1	1.761935647	0.137018791	12.85908042	7.64811E-38	5.66476E-37	positive
PRJNA729271	stage_1W_vs_E10	ordinary_muscle_development	ENSGALG00010005542	KCTD1	-0.419695542	0.136529279	-3.074033239	0.002111859	0.003051984	negative
PRJNA729271	stage_1W_vs_E10	ordinary_muscle_development	ENSGALG00010005543	SEC24D	-1.223501035	0.20273654	-6.034931039	1.59031E-09	3.54311E-09	negative
PRJNA729271	stage_1W_vs_E10	ordinary_muscle_development	ENSGALG00010005544	AQP4	8.133279849	0.308676018	26.34892043	5.2807E-153	4.7543E-151	positive
PRJNA729271	stage_1W_vs_E10	ordinary_muscle_development	ENSGALG00010005545	RPL34	1.085125863	0.166897213	6.501761948	7.93849E-11	1.89475E-10	positive
PRJNA729271	stage_1W_vs_E10	ordinary_muscle_development	ENSGALG00010005546	NRF1	1.853473535	0.15151359	12.23305144	2.07013E-34	1.37142E-33	positive
PRJNA729271	stage_1W_vs_E10	ordinary_muscle_development	ENSGALG00010005547	LRP11	-0.79054839	0.220001459	-3.593377934	0.000326419	0.00050582	negative
PRJNA729271	stage_1W_vs_E10	ordinary_muscle_development	ENSGALG00010005548	EZR	-0.822541625	0.180541785	-4.555962621	5.21462E-06	9.22567E-06	negative
PRJNA729271	stage_1W_vs_E10	ordinary_muscle_development	ENSGALG00010005549	RNF20	-1.094635993	0.114465019	-9.563061303	1.14324E-21	4.5942E-21	negative
PRJNA729271	stage_1W_vs_E10	ordinary_muscle_development	ENSGALG00010005550	ENSGALG00010005550	1.079767316	0.597968038	1.805727476	0.070960919	0.086917231	positive
PRJNA729271	stage_1W_vs_E10	ordinary_muscle_development	ENSGALG00010005551	METTL14	-1.698287578	0.085634628	-19.83178565	1.58318E-87	4.48208E-86	negative
PRJNA729271	stage_1W_vs_E10	ordinary_muscle_development	ENSGALG00010005552	WDR37	-0.050959697	0.087347172	-0.58341553	0.559613606	0.595477846	negative
PRJNA729271	stage_1W_vs_E10	ordinary_muscle_development	ENSGALG00010005553	PPP1R14C	0.64087442	0.164352363	3.899392809	9.64342E-05	0.000155317	positive
PRJNA729271	stage_1W_vs_E10	ordinary_muscle_development	ENSGALG00010005554	CRYL1	2.481828129	0.161663821	15.35178439	3.44576E-53	4.22916E-52	positive
PRJNA729271	stage_1W_vs_E10	ordinary_muscle_development	ENSGALG00010005555	PRSS12	-4.499011102	0.188142454	-23.91279062	2.2547E-126	1.4026E-124	negative
PRJNA729271	stage_1W_vs_E10	ordinary_muscle_development	ENSGALG00010005556	IYD	-0.53688305	0.751700193	-0.714224974	0.475088077	0.513603943	negative
PRJNA729271	stage_1W_vs_E10	ordinary_muscle_development	ENSGALG00010005557	CMSS1	-0.860800612	0.170230997	-5.056661978	4.26658E-07	8.17072E-07	negative
PRJNA729271	stage_1W_vs_E10	ordinary_muscle_development	ENSGALG00010005558	TAGAP	1.331591522	0.212106711	6.277932067	3.43106E-10	7.90922E-10	positive
PRJNA729271	stage_1W_vs_E10	ordinary_muscle_development	ENSGALG00010005559	FILIP1L	0.19032224	0.131182932	1.450815569	0.146831223	0.17248712	positive
PRJNA729271	stage_1W_vs_E10	ordinary_muscle_development	ENSGALG00010005560	RSPH3	-1.248891836	0.386437477	-3.231808278	0.001230096	0.001814413	negative
PRJNA729271	stage_1W_vs_E10	ordinary_muscle_development	ENSGALG00010005561	CLYBL	1.244038499	0.195146981	6.374879558	1.83107E-10	4.2857E-10	positive
PRJNA729271	stage_1W_vs_E10	ordinary_muscle_development	ENSGALG00010005562	HECTD1	0.872996035	0.085366575	10.22643863	1.50969E-24	6.81714E-24	positive
PRJNA729271	stage_1W_vs_E10	ordinary_muscle_development	ENSGALG00010005564	TM9SF2	0.103987773	0.058061696	1.790987705	0.0732953	0.089602376	positive
PRJNA729271	stage_1W_vs_E10	ordinary_muscle_development	ENSGALG00010005565	PGAP4	-1.596843887	0.181119263	-8.816532614	1.18059E-18	4.15153E-18	negative
PRJNA729271	stage_1W_vs_E10	ordinary_muscle_development	ENSGALG00010005567	NDST3	-1.968193032	0.24782031	-7.94201667	1.9892E-15	6.03463E-15	negative
PRJNA729271	stage_1W_vs_E10	ordinary_muscle_development	ENSGALG00010005568	PLEKHG1	0.782976839	0.131943407	5.9341869	2.95305E-09	6.4718E-09	positive
PRJNA729271	stage_1W_vs_E10	ordinary_muscle_development	ENSGALG00010005572	ENSGALG00010005572	1.689973049	0.152187801	11.10452371	1.19251E-28	6.35412E-28	positive
PRJNA729271	stage_1W_vs_E10	ordinary_muscle_development	ENSGALG00010005573	CHST9	0.84232011	0.282200103	2.984832756	0.002837334	0.004052735	positive
PRJNA729271	stage_1W_vs_E10	ordinary_muscle_development	ENSGALG00010005575	ENSGALG00010005575	2.239438222	0.455472325	4.91673829	8.79981E-07	1.65246E-06	positive
PRJNA729271	stage_1W_vs_E10	ordinary_muscle_development	ENSGALG00010005578	HEATR5A	0.11213322	0.120566599	0.930052107	0.352344106	0.389924249	positive
PRJNA729271	stage_1W_vs_E10	ordinary_muscle_development	ENSGALG00010005581	GR42L5	-0.061568958	1.008704586	-0.061037651	0.951329224	0.957671859	negative
PRJNA729271	stage_1W_vs_E10	ordinary_muscle_development	ENSGALG00010005583	ALDOB	-0.114877618	1.011050464	-0.113622042			negative
PRJNA729271	stage_1W_vs_E10	ordinary_muscle_development	ENSGALG00010005587	LEF1	-6.397862127	0.243060843	-26.32206017	1.0724E-152	9.4781E-151	negative
PRJNA729271	stage_1W_vs_E10	ordinary_muscle_development	ENSGALG00010005588	PLAGL1	-0.062184893	0.12738009	-0.488183778	0.625419674	0.659066389	negative
PRJNA729271	stage_1W_vs_E10	ordinary_muscle_development	ENSGALG00010005590	TBC1D23	-0.184313944	0.057243543	-3.21982069	0.001282708	0.00188971	negative
PRJNA729271	stage_1W_vs_E10	ordinary_muscle_development	ENSGALG00010005593	ENSGALG00010005593	1.84212042	1.117714711	1.648113245	0.099329435	0.119523506	positive
PRJNA729271	stage_1W_vs_E10	ordinary_muscle_development	ENSGALG00010005594	MRPL50	0.874976349	0.15012133	5.828461226	5.59408E-09	1.20592E-08	positive
PRJNA729271	stage_1W_vs_E10	ordinary_muscle_development	ENSGALG00010005596	PARD3	-0.441280334	0.054694745	-8.06805718	7.14256E-16	2.2141E-15	negative
PRJNA729271	stage_1W_vs_E10	ordinary_muscle_development	ENSGALG00010005597	ENSGALG00010005597	1.313406595	0.511780214	2.566348913	0.010277539	0.013878603	positive
PRJNA729271	stage_1W_vs_E10	ordinary_muscle_development	ENSGALG00010005601	TMEM215	-4.672010043	0.547911577	-8.526941639	1.50268E-17	4.99456E-17	negative
PRJNA729271	stage_1W_vs_E10	ordinary_muscle_development	ENSGALG00010005609	GJB6	-1.465163603	0.544095171	-2.692844345	0.007084534	0.009733679	negative
PRJNA729271	stage_1W_vs_E10	ordinary_muscle_development	ENSGALG00010005610	MTHFD1L	-0.381666666	0.190627511	-2.002159421	0.045267588	0.056834447	negative
PRJNA729271	stage_1W_vs_E10	ordinary_muscle_development	ENSGALG00010005617	DTD2	1.327504371	0.208419716	6.369380001	1.89794E-10	4.43863E-10	positive
PRJNA729271	stage_1W_vs_E10	ordinary_muscle_development	ENSGALG00010005618	PHACTR2	-0.677582967	0.068756672	-9.854795852	6.53494E-23	2.75681E-22	negative
PRJNA729271	stage_1W_vs_E10	ordinary_muscle_development	ENSGALG00010005619	CELF2	0.594767798	0.076877985	7.736516435	1.02178E-14	2.99569E-14	positive
PRJNA729271	stage_1W_vs_E10	ordinary_muscle_development	ENSGALG00010005621	CDH2	-3.300744929	0.146766504	-22.48977011	5.227E-112	2.4519E-110	negative
PRJNA729271	stage_1W_vs_E10	ordinary_muscle_development	ENSGALG00010005629	NRP1	0.262672214	0.173331633	1.515431483	0.129663169	0.153413406	positive
PRJNA729271	stage_1W_vs_E10	ordinary_muscle_development	ENSGALG00010005630	NIT2	-0.279537465	0.165536292	-1.688677826	0.091281187	0.110444141	negative
PRJNA729271	stage_1W_vs_E10	ordinary_muscle_development	ENSGALG00010005632	ITGB1	0.150644223	0.093469897	1.611687054	0.107030056	0.1281408	positive
PRJNA729271	stage_1W_vs_E10	ordinary_muscle_development	ENSGALG00010005634	HADH	1.056254884	0.086688558	12.18447862	3.76051E-34	2.46868E-33	positive
PRJNA729271	stage_1W_vs_E10	ordinary_muscle_development	ENSGALG00010005636	ENSGALG00010005636	2.654810312	0.594212828	4.46777684	7.90367E-06	1.38045E-05	positive
PRJNA729271	stage_1W_vs_E10	ordinary_muscle_development	ENSGALG00010005637	TOPORS	-0.169508955	0.071910925	-2.357207264	0.018412967	0.024129459	negative

PRJNA729271	stage_1W_vs_E10	ordinary_muscle_development	ENSGALG00010005643	ENSGALG00010005643	-3.81466712	1.286170668	-2.965910523	0.003017883	0.004300453	negative
PRJNA729271	stage_1W_vs_E10	ordinary_muscle_development	ENSGALG00010005645	FUCA2	-0.109825932	0.15794218	-0.695355301	0.486832666	0.52508665	negative
PRJNA729271	stage_1W_vs_E10	ordinary_muscle_development	ENSGALG00010005649	TOMM70	-0.497539404	0.079243978	-6.27857679	3.41686E-10	7.87775E-10	negative
PRJNA729271	stage_1W_vs_E10	ordinary_muscle_development	ENSGALG00010005651	USP6NL	-0.017538777	0.121603069	-0.144229727	0.885319068	0.898333909	negative
PRJNA729271	stage_1W_vs_E10	ordinary_muscle_development	ENSGALG00010005656	CYP2U1	1.249670856	0.131504982	9.502840432	2.04242E-21	8.13991E-21	positive
PRJNA729271	stage_1W_vs_E10	ordinary_muscle_development	ENSGALG00010005660	UBAC2	1.299590881	0.100586068	12.9201877	3.46296E-38	2.60486E-37	positive
PRJNA729271	stage_1W_vs_E10	ordinary_muscle_development	ENSGALG00010005668	LNP1	-3.187272032	0.55347248	-5.75868204	8.47732E-09	1.80545E-08	negative
PRJNA729271	stage_1W_vs_E10	ordinary_muscle_development	ENSGALG00010005670	SGMS2	-0.16550143	0.206771665	-0.800406718	0.42347519	0.461454885	negative
PRJNA729271	stage_1W_vs_E10	ordinary_muscle_development	ENSGALG00010005675	GJA3	-0.342520742	0.287856759	-1.189899945	0.23408572	0.265998159	negative
PRJNA729271	stage_1W_vs_E10	ordinary_muscle_development	ENSGALG00010005679	TMEM45A	-2.422168933	0.208684862	-11.60682625	3.80477E-31	2.25011E-30	negative
PRJNA729271	stage_1W_vs_E10	ordinary_muscle_development	ENSGALG00010005680	ENSGALG00010005680	1.889785439	0.466568452	4.050392673	5.11318E-05	8.40502E-05	positive
PRJNA729271	stage_1W_vs_E10	ordinary_muscle_development	ENSGALG00010005681	EPC1	0.156845473	0.066732899	2.350347047	0.018755914	0.024563334	positive
PRJNA729271	stage_1W_vs_E10	ordinary_muscle_development	ENSGALG00010005688	ENSGALG00010005688	1.395028737	0.538506482	2.590551428	0.00958223	0.012978362	positive
PRJNA729271	stage_1W_vs_E10	ordinary_muscle_development	ENSGALG00010005692	SEC24B	1.084863693	0.143662394	7.551479993	4.3034E-14	1.22166E-13	positive
PRJNA729271	stage_1W_vs_E10	ordinary_muscle_development	ENSGALG00010005694	ENSGALG00010005694	0.236632267	0.072701692	3.254838502	0.001134569	0.001679834	positive
PRJNA729271	stage_1W_vs_E10	ordinary_muscle_development	ENSGALG00010005695	KIF5B	-0.179703282	0.083719269	-2.146498448	0.031833232	0.040653983	negative
PRJNA729271	stage_1W_vs_E10	ordinary_muscle_development	ENSGALG00010005696	NUBPL	-1.240159611	0.143231101	-8.658451994	4.78214E-18	1.62602E-17	negative
PRJNA729271	stage_1W_vs_E10	ordinary_muscle_development	ENSGALG00010005699	PRPF18	0.83383648	0.0923506	9.029031558	1.73198E-19	6.36053E-19	positive
PRJNA729271	stage_1W_vs_E10	ordinary_muscle_development	ENSGALG00010005704	AKAP12	-1.884478505	0.093941004	-20.06023373	1.6431E-89	4.7921E-88	negative
PRJNA729271	stage_1W_vs_E10	ordinary_muscle_development	ENSGALG00010005705	ECHDC3	1.859745639	0.122420007	15.1915172	4.02495E-52	4.75482E-51	positive
PRJNA729271	stage_1W_vs_E10	ordinary_muscle_development	ENSGALG00010005712	ENSGALG00010005712	0.642396663	0.108398268	5.926263174	3.09906E-09	6.78153E-09	positive
PRJNA729271	stage_1W_vs_E10	ordinary_muscle_development	ENSGALG00010005714	ARHGAP5	-1.038433824	0.068606526	-15.13607931	9.36319E-52	1.09363E-50	negative
PRJNA729271	stage_1W_vs_E10	ordinary_muscle_development	ENSGALG00010005716	ZBTB2	-0.242822236	0.11215595	-2.16504106	0.030384541	0.038910144	negative
PRJNA729271	stage_1W_vs_E10	ordinary_muscle_development	ENSGALG00010005720	COL25A1	-4.270369124	0.22646223	-18.85687131	2.58045E-79	6.00379E-78	negative
PRJNA729271	stage_1W_vs_E10	ordinary_muscle_development	ENSGALG00010005724	PROSER2	0.468192753	0.11916352	3.928993991	8.5302E-05	0.000137966	positive
PRJNA729271	stage_1W_vs_E10	ordinary_muscle_development	ENSGALG00010005738	ELAVL2	-4.654131623	0.567531175	-8.200662499	2.39066E-16	7.53481E-16	negative
PRJNA729271	stage_1W_vs_E10	ordinary_muscle_development	ENSGALG00010005740	ENSGALG00010005740	1.252596484	0.337035755	3.716509204	0.000201994	0.000317595	positive
PRJNA729271	stage_1W_vs_E10	ordinary_muscle_development	ENSGALG00010005742	GPR183	-1.439201196	0.212719136	-6.76573448	1.32635E-11	3.31016E-11	negative
PRJNA729271	stage_1W_vs_E10	ordinary_muscle_development	ENSGALG00010005743	ETNPPL	5.913619149	0.22326199	26.48735308	1.3557E-154	1.2516E-152	positive
PRJNA729271	stage_1W_vs_E10	ordinary_muscle_development	ENSGALG00010005748	AIG1	0.531452621	0.179452582	2.961521179	0.003061234	0.004359229	positive
PRJNA729271	stage_1W_vs_E10	ordinary_muscle_development	ENSGALG00010005752	RMND1	0.221120001	0.098877952	2.236292291	0.025332631	0.03274739	positive
PRJNA729271	stage_1W_vs_E10	ordinary_muscle_development	ENSGALG00010005755	ZMYM2	0.639195669	0.097150584	6.579432084	4.72249E-11	1.14507E-10	positive
PRJNA729271	stage_1W_vs_E10	ordinary_muscle_development	ENSGALG00010005756	TET2	0.506881392	0.164013771	3.090480691	0.001998328	0.002894839	positive
PRJNA729271	stage_1W_vs_E10	ordinary_muscle_development	ENSGALG00010005757	ARHGAP12	0.367035907	0.133596679	2.747343057	0.006008026	0.008318496	positive
PRJNA729271	stage_1W_vs_E10	ordinary_muscle_development	ENSGALG00010005758	HIVEP2	1.154753961	0.105373937	10.95862971	6.04071E-28	3.1406E-27	positive
PRJNA729271	stage_1W_vs_E10	ordinary_muscle_development	ENSGALG00010005760	GPR18	-3.616479384	0.401828776	-9.000050774	2.25613E-19	8.23122E-19	negative
PRJNA729271	stage_1W_vs_E10	ordinary_muscle_development	ENSGALG00010005763	AKAP6	2.49449098	0.142460722	17.51002629	1.20139E-68	2.20153E-67	positive
PRJNA729271	stage_1W_vs_E10	ordinary_muscle_development	ENSGALG00010005768	CXXC4	-3.445363949	0.663505996	-5.192664373	2.07306E-07	4.05683E-07	negative
PRJNA729271	stage_1W_vs_E10	ordinary_muscle_development	ENSGALG00010005769	TFG	0.053686226	0.105278886	0.509942952	0.610091429	0.644318781	positive
PRJNA729271	stage_1W_vs_E10	ordinary_muscle_development	ENSGALG00010005772	SVEP1	1.680253329	0.140799128	11.93369134	7.89914E-33	4.95234E-32	positive
PRJNA729271	stage_1W_vs_E10	ordinary_muscle_development	ENSGALG00010005777	ENSGALG00010005777	-0.273350238	1.035771925	-0.26390968	0.791849516	0.813859373	negative
PRJNA729271	stage_1W_vs_E10	ordinary_muscle_development	ENSGALG00010005779	ARMT1	0.970123353	0.110740368	8.760340726	1.94661E-18	6.75997E-18	positive
PRJNA729271	stage_1W_vs_E10	ordinary_muscle_development	ENSGALG00010005784	RASL11A	1.032531071	0.4329303	2.384982224	0.017079947	0.022469942	positive
PRJNA729271	stage_1W_vs_E10	ordinary_muscle_development	ENSGALG00010005785	RPL21	0.225356214	0.139822894	1.611726139	0.107021547	0.1281408	positive
PRJNA729271	stage_1W_vs_E10	ordinary_muscle_development	ENSGALG00010005789	RNASET2	0.090008079	0.155688089	0.578130798	0.563175815	0.598872676	positive
PRJNA729271	stage_1W_vs_E10	ordinary_muscle_development	ENSGALG00010005790	MUSK	0.115571194	0.152098448	0.759844665	0.447347441	0.485497242	positive
PRJNA729271	stage_1W_vs_E10	ordinary_muscle_development	ENSGALG00010005792	ADGRG6	-1.665441689	0.16099045	-10.34497197	4.41045E-25	2.03727E-24	negative
PRJNA729271	stage_1W_vs_E10	ordinary_muscle_development	ENSGALG00010005799	UFSP2	-0.420675617	0.098377604	-4.276131979	1.90169E-05	3.22623E-05	negative
PRJNA729271	stage_1W_vs_E10	ordinary_muscle_development	ENSGALG00010005800	FRMD4A	-1.097004565	0.110007039	-9.972130654	2.01851E-23	8.68716E-23	negative
PRJNA729271	stage_1W_vs_E10	ordinary_muscle_development	ENSGALG00010005814	UPF2	-0.60003377	0.124742948	-4.810161841	1.50808E-06	2.78042E-06	negative
PRJNA729271	stage_1W_vs_E10	ordinary_muscle_development	ENSGALG00010005817	VTA1	0.150328027	0.069807104	2.153477487	0.031281172	0.039987705	positive
PRJNA729271	stage_1W_vs_E10	ordinary_muscle_development	ENSGALG00010005821	DOCK9	0.946328077	0.153996129	6.145142	7.9892E-10	1.80464E-09	positive
PRJNA729271	stage_1W_vs_E10	ordinary_muscle_development	ENSGALG00010005824	NMBR	-5.790974828	1.332630883	-4.345520504	1.38946E-05	2.38402E-05	negative
PRJNA729271	stage_1W_vs_E10	ordinary_muscle_development	ENSGALG00010005826	LRP2BP	-0.320318938	0.20981732	-1.526656323	0.126846494	0.150350799	negative
PRJNA729271	stage_1W_vs_E10	ordinary_muscle_development	ENSGALG00010005828	LPAR1	-2.161881373	0.15684121	-13.78388611	3.18647E-43	2.82322E-42	negative
PRJNA729271	stage_1W_vs_E10	ordinary_muscle_development	ENSGALG00010005829	NPAS3	-0.623187846	0.139428175	-4.46959768	7.83669E-06	1.36908E-05	negative
PRJNA729271	stage_1W_vs_E10	ordinary_muscle_development	ENSGALG00010005830	USP12	-0.248084147	0.186766767	-1.328309911	0.184075747	0.213062201	negative
PRJNA729271	stage_1W_vs_E10	ordinary_muscle_development	ENSGALG00010005831	SEC61A2	0.907419417	0.104749761	8.662734966	4.60578E-18	1.56826E-17	positive
PRJNA729271	stage_1W_vs_E10	ordinary_muscle_development	ENSGALG00010005836	NEBL	0.204934369	0.245950563	0.833233991	0.404712776	0.442974529	positive
PRJNA729271	stage_1W_vs_E10	ordinary_muscle_development	ENSGALG00010005839	RPS6KA2	-0.961816625	0.189946615	-5.063615494	4.11379E-07	7.88853E-07	negative
PRJNA729271	stage_1W_vs_E10	ordinary_muscle_development	ENSGALG00010005841	ENSGALG00010005841	0.656478086	0.320898408	2.045750522	0.040780922	0.051473308	positive
PRJNA729271	stage_1W_vs_E10	ordinary_muscle_development	ENSGALG00010005843	GPR12	-0.297335784	0.438372596	-0.678271831	0.497599355	0.535344961	negative
PRJNA729271	stage_1W_vs_E10	ordinary_muscle_development	ENSGALG00010005848	WASF3	0.537411033	0.191826496	2.801547468	0.005085816	0.007097911	positive
PRJNA729271	stage_1W_vs_E10	ordinary_muscle_development	ENSGALG00010005849	SNX25	0.118557719	0.097900973	1.210996336	0.225896809	0.25745984	positive
PRJNA729271	stage_1W_vs_E10	ordinary_muscle_development	ENSGALG00010005851	SLC15A1	1.682069185	0.877874832	1.916069494	0.055356251	0.068732984	positive
PRJNA729271	stage_1W_vs_E10	ordinary_muscle_development	ENSGALG00010005853	COMMD3	-0.451792029	0.140776403	-3.209288065	0.001330641	0.00195754	negative
PRJNA729271	stage_1W_vs_E10	ordinary_muscle_development	ENSGALG00010005858	CFAP97	-1.155143934	0.114915577	-10.0521092	8.99218E-24	3.93306E-23	negative
PRJNA729271	stage_1W_vs_E10	ordinary_muscle_development	ENSGALG00010005859	ENSGALG00010005859	-0.279919275	0.626424311	-0.446852509	0.654981555	0.686029167	negative
PRJNA729271	stage_1W_vs_E10	ordinary_muscle_development	ENSGALG00010005860	ABI3BP	0.233648317	0.212531248	1.099359832	0.271611144	0.305701656	positive
PRJNA729271	stage_1W_vs_E10	ordinary_muscle_development	ENSGALG00010005861	MPC1L	1.382136156	0.131801808	10.48647341	9.96797E-26	4.72484E-25	positive
PRJNA729271	stage_1W_vs_E10	ordinary_muscle_development	ENSGALG00010005866	DNAJC1	1.132449411	0.147873175	7.658247763	1.88487E-14	5.44789E-14	positive
PRJNA729271	stage_1W_vs_E10	ordinary_muscle_development	ENSGALG00010005871	EGLN3	1.091292098	0.196408283	5.556242753	2.75643E-08	5.69477E-08	positive
PRJNA729271	stage_1W_vs_E10	ordinary_muscle_development	ENSGALG00010005872	STK24	0.661841151	0.134903779	4.906023792	9.29413E-07	1.7428E-06	positive
PRJNA729271	stage_1W_vs_E10	ordinary_muscle_development	ENSGALG00010005875	CDK8	-0.863321062	0.153514883	-5.623696177	1.86914E-08	3.9028E-08	negative
PRJNA729271	stage_1W_vs_E10	ordinary_muscle_development	ENSGALG00010005879	FAM107B	-2.466491986	0.125573756	-19.64177918	6.79745E-86	1.87318E-84	negative
PRJNA729271	stage_1W_vs_E10	ordinary_muscle_development	ENSGALG00010005880	SLC25A4	6.256951224	0.179711053	34.81672999	1.358E-265	3.7854E-263	positive
PRJNA729271	stage_1W_vs_E10	ordinary_muscle_development	ENSGALG00010005881	CORO2A	-0.171791661	0.279024705	-0.615686202	0.538101645	0.574442727	negative
PRJNA729271	stage_1W_vs_E10	ordinary_muscle_development	ENSGALG00010005884	MYB	-2.994842919	0.320948084	-9.331237881	1.04642E-20	4.06099E-20	negative
PRJNA729271	stage_1W_vs_E10	ordinary_muscle_development	ENSGALG00010005885	SLITRK5	0.848400427	0.374592131	2.264864522	0.023520995	0.030530744	positive
PRJNA729271	stage_1W_vs_E10	ordinary_muscle_development	ENSGALG00010005887	CCNH	0.065676062	0.084637625	0.775967684	0.437768067	0.475706434	positive
PRJNA729271	stage_1W_vs_E10	ordinary_muscle_development	ENSGALG00010005888	IMPG2	0.089257259	0.229444765	0.389014143	0.697265684	0.725859386	positive
PRJNA729271	stage_1W_vs_E10	ordinary_muscle_development	ENSGALG00010005890	SNX32	-0.883430053	0.089749185	-9.843321221	7.32519E-23	3.08211E-22	negative
PRJNA729271	stage_1W_vs_E10	ordinary_muscle_development	ENSGALG00010005891	RNF6	0.143503941	0.117572863	1.220553255	0.222255214	0.253631478	positive
PRJNA729271	stage_1W_vs_E10	ordinary_muscle_development	ENSGALG00010005892	ENSGALG00010005892	1.25236036	0.847926714	1.47696769	0.139684299	0.164625084	positive
PRJNA729271	stage_1W_vs_E10	ordinary_muscle_development	ENSGALG00010005893	SFT2D1	0.459226851	0.066494544	6.906233518	4.9769E-12	1.2703E-11	positive
PRJNA729271	stage_1W_vs_E10	ordinary_muscle_development	ENSGALG00010005895	MLLT10	0.746905926	0.099026477	7.542487122	4.61092E-14	1.30742E-13	positive
PRJNA729271	stage_1W_vs_E10	ordinary_muscle_development	ENSGALG00010005899	ENSGALG00010005899	-0.655937049	0.088711264	-7.394067257	1.42404E-13	3.92275E-13	negative
PRJNA729271	stage_1W_vs_E10	ordinary_muscle_development	ENSGALG00010005900	SPTSSA	-0.387648711	0.087812691	-4.414495308	1.01246E-05	1.75608E-05	negative
PRJNA729271	stage_1W_vs_E10	ordinary_muscle_development	ENSGALG00010005901	ENSGALG00010005901	0.451383415	0.250870439	1.79926904	0.071976133	0.088056554	positive
PRJNA729271	stage_1W_vs_E10	ordinary_muscle_development	ENSGALG00010005902	EAPP	-0.650447465	0.102976641	-6.31645644	2.67629E-10	6.1999E-10	negative
PRJNA729271	stage_1W_vs_E10	ordinary_muscle_development	ENSGALG00010005903	PRR18	-1.333353043	0.265381876	-5.024280727	5.05323E-07	9.62504E-07	negative
PRJNA729271	stage_1W_vs_E10	ordinary_muscle_development	ENSGALG00010005905	CITED2	0.02113442	0.202178258	0.104533594	0.91674591	0.926978163	positive
PRJNA729271	stage_1W_vs_E10	ordinary_muscle_development	ENSGALG00010005909	BRMS1L	-0.805631986	0.103016396	-7.820424887	5.26453E-15	1.56468E-14	negative
PRJNA729271	stage_1W_vs_E10	ordinary_muscle_development	ENSGALG00010005910	ENSGALG00010005910	1.021347395	0.468156438	2.181636974	0.029136336	0.037371134	positive
PRJNA729271	stage_1W_vs_E10	ordinary_muscle_development	ENSGALG00010005911	TBXT	-2.897065392	0.549094399	-5.276078938	1.31977E-07	2.61447E-07	negative
PRJNA729271	stage_1W_vs_E10	ordinary_muscle_development	ENSGALG00010005913	NFKBIA	2.287267397	0.142658154	16.03320475	7.49175E-58	1.03422E-56	positive
PRJNA729271	stage_1W_vs_E10	ordinary_muscle_development	ENSGALG00010005915	PAPSS1	-2.630649198	0.102334712	-25.70632342	9.9329E-146	7.6179E-144	negative
PRJNA729271	stage_1W_vs_E10	ordinary_muscle_development	ENSGALG00010005926	NUDT5	0.041695564	0.233086497	0.178884513	0.858028384	0.873761516	positive
PRJNA729271	stage_1W_vs_E10	ordinary_muscle_development	ENSGALG00010005934	SLITRK6	-6.181611127	0.345471886	-17.89323931	1.33139E-71	2.62207E-70	negative
PRJNA729271	stage_1W_vs_E10	ordinary_muscle_development	ENSGALG00010005935	ATP8A2	-3.899231764	0.328478668	-11.87057835	1.683E-32	1.04521E-31	negative
PRJNA729271	stage_1W_vs_E10	ordinary_muscle_development	ENSGALG00010005941	TXLNB	5.130362318	0.181337469	28.29179401	4.3604E-176	5.0563E-174	positive
PRJNA729271	stage_1W_vs_E10	ordinary_muscle_development	ENSGALG00010005943	PROX2	-2.607550785	0.95528837	-2.729595447	0.006341209	0.008754722	negative
PRJNA729271	stage_1W_vs_E10	ordinary_muscle_development	ENSGALG00010005949	SLITRK1	0.723120533	0.354731973	2.038498323	0.041500122	0.052328102	positive
PRJNA729271	stage_1W_vs_E10	ordinary_muscle_development	ENSGALG00010005956	TXNL4B	0.14431255	0.112192318	1.286296183	0.198339714	0.228405034	positive
PRJNA729271	stage_1W_vs_E10	ordinary_muscle_development	ENSGALG00010005963	UGCG	1.070952034	0.103709885	10.32642197	5.35198E-25	2.46042E-24	positive
PRJNA729271	stage_1W_vs_E10	ordinary_muscle_development	ENSGALG00010005964	HSPA14	-0.409750949	0.114446866	-3.580272416	0.000343236	0.000530859	negative
PRJNA729271	stage_1W_vs_E10	ordinary_muscle_development	ENSGALG00010005966	PLXDC2	1.427221983	0.153258977	9.31248537	1.24875E-20	4.82943E-20	positive
PRJNA729271	stage_1W_vs_E10	ordinary_muscle_development	ENSGALG00010005967	GJD2	-2.863625816	0.285009841	-10.04746294	9.4263E-24	4.12045E-23	negative
PRJNA729271	stage_1W_vs_E10	ordinary_muscle_development	ENSGALG00010005969	HECA	0.309317038	0.06621219	4.671602585	2.98859E-06	5.38935E-06	positive
PRJNA729271	stage_1W_vs_E10	ordinary_muscle_development	ENSGALG00010005970	SHISA2	-3.730940389	0.124160477	-30.04934003	2.2271E-198	3.1962E-196	negative
PRJNA729271	stage_1W_vs_E10	ordinary_muscle_development	ENSGALG00010005975	ABRACL	-0.742893392	0.108276358	-6.861085893	6.83391E-12	1.73481E-11	negative
PRJNA729271	stage_1W_vs_E10	ordinary_muscle_development	ENSGALG00010005978	VPS16	0.842342156	0.114035133	7.386689802	1.50529E-13	4.14341E-13	positive
PRJNA729271	stage_1W_vs_E10	ordinary_muscle_development	ENSGALG00010005979	SUV39H2	0.142493619	0.09241627	1.54186725	0.123105856	0.146275874	positive
PRJNA729271	stage_1W_vs_E10	ordinary_muscle_development	ENSGALG00010005981	AREL1	-0.176317125	0.101015909	-1.745439181	0.08090845	0.098403087	negative
PRJNA729271	stage_1W_vs_E10	ordinary_muscle_development	ENSGALG00010005984	IDH3B	2.713872141	0.117290362	23.13806594	1.9172E-118	1.0292E-116	positive
PRJNA729271	stage_1W_vs_E10	ordinary_muscle_development	ENSGALG00010005985	ENSGALG00010005985	-3.591871785	0.323044641	-11.11880939	1.01619E-28	5.42929E-28	negative
PRJNA729271	stage_1W_vs_E10	ordinary_muscle_development	ENSGALG00010005988	NUP58	-0.235218035	0.089585216	-2.625634527	0.008648763	0.011773461	negative
PRJNA729271	stage_1W_vs_E10	ordinary_muscle_development	ENSGALG00010005989	FCF1	-0.039885126	0.10008754	-0.398502411	0.690259881	0.719082721	negative
PRJNA729271	stage_1W_vs_E10	ordinary_muscle_development	ENSGALG00010005990	SLC2A12	2.884622796	0.149910719	19.2422718	1.63867E-82	4.17442E-81	positive
PRJNA729271	stage_1W_vs_E10	ordinary_muscle_development	ENSGALG00010005991	FARP1	-1.283800492	0.091682414	-14.00269083	1.50079E-44	1.3856E-43	negative
PRJNA729271	stage_1W_vs_E10	ordinary_muscle_development	ENSGALG00010005997	ENSGALG00010005997	0.514705088	0.237204987	2.169874648	0.030016343	0.038465821	positive
PRJNA729271	stage_1W_vs_E10	ordinary_muscle_development	ENSGALG00010005998	YLPM1	0.372495244	0.133039364	2.799887429	0.005112043	0.007132454	positive
PRJNA729271	stage_1W_vs_E10	ordinary_muscle_development	ENSGALG00010005999	PDE10A	1.358446588	0.29285039	4.638705065	3.50599E-06	6.29497E-06	positive
PRJNA729271	stage_1W_vs_E10	ordinary_muscle_development	ENSGALG00010006001	NOP56	-0.344165504	0.140057881	-2.457309083	0.013998219	0.018632157	negative
PRJNA729271	stage_1W_vs_E10	ordinary_muscle_development	ENSGALG00010006004	DCLRE1C	-1.334115717	0.157124577	-8.490814996	2.05193E-17	6.77357E-17	negative
PRJNA729271	stage_1W_vs_E10	ordinary_muscle_development	ENSGALG00010006009	MALRD1	1.19928332	0.621692925	1.929060591	0.053723341	0.066825695	positive
PRJNA729271	stage_1W_vs_E10	ordinary_muscle_development	ENSGALG00010006010	IPO5	-0.090318375	0.080795535	-1.117863443	0.26362532	0.297373464	negative
PRJNA729271	stage_1W_vs_E10	ordinary_muscle_development	ENSGALG00010006013	TRMT10C	0.162465712	0.105622321	1.538175938	0.124005603	0.147248399	positive
PRJNA729271	stage_1W_vs_E10	ordinary_muscle_development	ENSGALG00010006015	CDC123	-0.597489316	0.112475773	-5.312160103	1.08333E-07	2.15907E-07	negative
PRJNA729271	stage_1W_vs_E10	ordinary_muscle_development	ENSGALG00010006016	MTMR6	0.110134382	0.077029475	1.429769339	0.152783232	0.179014869	positive
PRJNA729271	stage_1W_vs_E10	ordinary_muscle_development	ENSGALG00010006019	SLC10A2	-4.720506613	1.18694319	-3.977028261	6.97819E-05	0.000113638	negative

PRJNA729271	stage_1W_vs_E10	ordinary_muscle_development	ENSGALG00010006021	ENSGALG00010006021	0.42987314	0.125618855	3.422043125	0.000621525	0.000941674	positive
PRJNA729271	stage_1W_vs_E10	ordinary_muscle_development	ENSGALG00010006022	AFG2A	-0.561286822	0.091312477	-6.146879819	7.9022E-10	1.78554E-09	negative
PRJNA729271	stage_1W_vs_E10	ordinary_muscle_development	ENSGALG00010006024	AMER2	-1.310023829	0.361999378	-3.618856577	0.000295908	0.000460212	negative
PRJNA729271	stage_1W_vs_E10	ordinary_muscle_development	ENSGALG00010006025	TNFAIP3	0.77814259	0.150431589	5.172733957	2.30693E-07	4.50236E-07	positive
PRJNA729271	stage_1W_vs_E10	ordinary_muscle_development	ENSGALG00010006028	ARL5B	-0.195677837	0.128235834	-1.525921666	0.127029374	0.150517708	negative
PRJNA729271	stage_1W_vs_E10	ordinary_muscle_development	ENSGALG00010006029	HSDL2	0.221180787	0.134777585	1.641079915	0.100780827	0.121129195	positive
PRJNA729271	stage_1W_vs_E10	ordinary_muscle_development	ENSGALG00010006031	ERCC5	0.853451333	0.096264348	8.865705274	7.60213E-19	2.69948E-18	positive
PRJNA729271	stage_1W_vs_E10	ordinary_muscle_development	ENSGALG00010006033	NSUN6	-0.521065899	0.177441967	-2.936542623	0.003318932	0.004711843	negative
PRJNA729271	stage_1W_vs_E10	ordinary_muscle_development	ENSGALG00010006035	ADH1C	1.306397114	0.69583474	1.877453134	0.060456015	0.074687628	positive
PRJNA729271	stage_1W_vs_E10	ordinary_muscle_development	ENSGALG00010006037	METTL21EP	7.029632011	0.675898023	10.40043287	2.46809E-25	1.15032E-24	positive
PRJNA729271	stage_1W_vs_E10	ordinary_muscle_development	ENSGALG00010006038	ENSGALG00010006038	1.07824689	0.191976464	5.616557716	1.94799E-08	4.06451E-08	positive
PRJNA729271	stage_1W_vs_E10	ordinary_muscle_development	ENSGALG00010006039	PCNP	-1.381789177	0.127097826	-10.87185535	1.56976E-27	8.01749E-27	negative
PRJNA729271	stage_1W_vs_E10	ordinary_muscle_development	ENSGALG00010006041	QKI	-0.077250929	0.085963574	-0.898647236	0.36884059	0.406627955	negative
PRJNA729271	stage_1W_vs_E10	ordinary_muscle_development	ENSGALG00010006047	ZBTB11	0.242826978	0.13501401	1.798531717	0.072092787	0.088191826	positive
PRJNA729271	stage_1W_vs_E10	ordinary_muscle_development	ENSGALG00010006049	AIMP1	0.190507082	0.124150374	1.53448657	0.124909995	0.148188768	positive
PRJNA729271	stage_1W_vs_E10	ordinary_muscle_development	ENSGALG00010006052	RALGAPA1	-0.596128757	0.08195091	-7.274217695	3.48434E-13	9.41739E-13	negative
PRJNA729271	stage_1W_vs_E10	ordinary_muscle_development	ENSGALG00010006053	TMEM243	-1.664682657	0.261218397	-6.37276194	1.85654E-10	4.34391E-10	negative
PRJNA729271	stage_1W_vs_E10	ordinary_muscle_development	ENSGALG00010006054	ENSGALG00010006054	3.976886312	2.180606385	1.823752485	0.0681895	0.083706538	positive
PRJNA729271	stage_1W_vs_E10	ordinary_muscle_development	ENSGALG00010006055	ENSGALG00010006055	2.145311639	0.576258249	3.722830246	0.000197002	0.000310149	positive
PRJNA729271	stage_1W_vs_E10	ordinary_muscle_development	ENSGALG00010006059	BIVM	-0.31133043	0.056018569	-5.557629123	2.73464E-08	5.65135E-08	negative
PRJNA729271	stage_1W_vs_E10	ordinary_muscle_development	ENSGALG00010006060	ENSGALG00010006060	1.659910662	0.339069494	4.895488066	9.8062E-07	1.83597E-06	positive
PRJNA729271	stage_1W_vs_E10	ordinary_muscle_development	ENSGALG00010006061	RPL24	0.593730045	0.159020257	3.733675539	0.000188706	0.000297443	positive
PRJNA729271	stage_1W_vs_E10	ordinary_muscle_development	ENSGALG00010006062	ENSGALG00010006062	1.650860099	0.6477071	2.548775672	0.010810181	0.014561247	positive
PRJNA729271	stage_1W_vs_E10	ordinary_muscle_development	ENSGALG00010006063	POGLUT2	-1.322653075	0.102586398	-12.89306474	4.92471E-38	3.67768E-37	negative
PRJNA729271	stage_1W_vs_E10	ordinary_muscle_development	ENSGALG00010006064	DMTF1	-0.003323613	0.082474698	-0.040298578	0.967855088	0.972282175	negative
PRJNA729271	stage_1W_vs_E10	ordinary_muscle_development	ENSGALG00010006065	WDR54	0.552284734	0.158703638	3.479975258	0.00050146	0.00076472	positive
PRJNA729271	stage_1W_vs_E10	ordinary_muscle_development	ENSGALG00010006066	ARHGAP18	-0.565458668	0.121417663	-4.657136797	3.20637E-06	5.76988E-06	negative
PRJNA729271	stage_1W_vs_E10	ordinary_muscle_development	ENSGALG00010006068	PACRG	-0.113474343	0.27941595	-0.40611262	0.684659853	0.714018604	negative
PRJNA729271	stage_1W_vs_E10	ordinary_muscle_development	ENSGALG00010006069	TTR	4.640231866	1.391676527	3.334274723	0.000855221	0.001282611	positive
PRJNA729271	stage_1W_vs_E10	ordinary_muscle_development	ENSGALG00010006070	TMEM242	0.851985942	0.113833485	7.484493155	7.18238E-14	2.01565E-13	positive
PRJNA729271	stage_1W_vs_E10	ordinary_muscle_development	ENSGALG00010006071	TEX30	-0.550721544	0.244545197	-2.252023554	0.024320784	0.031511025	negative
PRJNA729271	stage_1W_vs_E10	ordinary_muscle_development	ENSGALG00010006072	ENSGALG00010006072	1.13191137	0.232848017	4.861159584	1.167E-06	2.17202E-06	positive
PRJNA729271	stage_1W_vs_E10	ordinary_muscle_development	ENSGALG00010006073	CEP97	0.174035435	0.104551249	1.664594518	0.09599368	0.115720924	positive
PRJNA729271	stage_1W_vs_E10	ordinary_muscle_development	ENSGALG00010006075	SOX21	-4.15817467	0.409040622	-10.16567657	2.82156E-24	1.25494E-23	negative
PRJNA729271	stage_1W_vs_E10	ordinary_muscle_development	ENSGALG00010006076	PODXL	2.391538634	0.163709234	14.60845291	2.48088E-48	2.5631E-47	positive
PRJNA729271	stage_1W_vs_E10	ordinary_muscle_development	ENSGALG00010006077	TBCK	-1.253600691	0.103215302	-12.14549261	6.06164E-34	3.96317E-33	negative
PRJNA729271	stage_1W_vs_E10	ordinary_muscle_development	ENSGALG00010006078	SPATA13	-2.078424275	0.169330409	-12.27437109	1.24354E-34	8.34112E-34	negative
PRJNA729271	stage_1W_vs_E10	ordinary_muscle_development	ENSGALG00010006079	ENSGALG00010006079	0.856755512	0.782000933	1.095593976	0.273256499	0.307448029	positive
PRJNA729271	stage_1W_vs_E10	ordinary_muscle_development	ENSGALG00010006080	ENSGALG00010006080	-0.620873323	0.207848201	-2.987147924	0.002815934	0.004022565	negative
PRJNA729271	stage_1W_vs_E10	ordinary_muscle_development	ENSGALG00010006081	ZDHHC14	0.207170677	0.079127789	2.618178517	0.008840054	0.012022574	positive
PRJNA729271	stage_1W_vs_E10	ordinary_muscle_development	ENSGALG00010006083	CACNB2	-1.11626298	0.177758436	-6.279662488	3.39309E-10	7.82543E-10	negative
PRJNA729271	stage_1W_vs_E10	ordinary_muscle_development	ENSGALG00010006084	TMEM68	-1.653525081	0.155673807	-10.62172958	2.36145E-26	1.15211E-25	negative
PRJNA729271	stage_1W_vs_E10	ordinary_muscle_development	ENSGALG00010006085	DLST	1.168874538	0.089784328	13.01869227	9.57975E-39	7.34701E-38	positive
PRJNA729271	stage_1W_vs_E10	ordinary_muscle_development	ENSGALG00010006086	ENSGALG00010006086	0.362269799	0.295169017	1.227330032	0.219698572	0.25108723	positive
PRJNA729271	stage_1W_vs_E10	ordinary_muscle_development	ENSGALG00010006087	ENSGALG00010006087	1.467730438	0.565653806	2.594750398	0.009465963	0.01282808	positive
PRJNA729271	stage_1W_vs_E10	ordinary_muscle_development	ENSGALG00010006091	RTKN	-0.71852813	0.102382337	-7.018086823	2.24927E-12	5.8504E-12	negative
PRJNA729271	stage_1W_vs_E10	ordinary_muscle_development	ENSGALG00010006096	EDIL3	-0.541095363	0.236794564	-2.285083549	0.022307929	0.029008113	negative
PRJNA729271	stage_1W_vs_E10	ordinary_muscle_development	ENSGALG00010006100	RPS6KL1	-1.815431489	0.210273124	-8.633682973	5.94075E-18	2.01383E-17	negative
PRJNA729271	stage_1W_vs_E10	ordinary_muscle_development	ENSGALG00010006101	ENSGALG00010006101	1.230077882	0.113578036	10.83024433	2.47494E-27	1.25434E-26	positive
PRJNA729271	stage_1W_vs_E10	ordinary_muscle_development	ENSGALG00010006103	NXPE3	-0.653711603	0.122844017	-5.32147693	1.02928E-07	2.05502E-07	negative
PRJNA729271	stage_1W_vs_E10	ordinary_muscle_development	ENSGALG00010006106	SHOC1	0.903752287	0.381988964	2.365912036	0.017985717	0.023601573	positive
PRJNA729271	stage_1W_vs_E10	ordinary_muscle_development	ENSGALG00010006107	PGF	3.902297177	0.18840995	20.71173617	2.71493E-95	9.21614E-94	positive
PRJNA729271	stage_1W_vs_E10	ordinary_muscle_development	ENSGALG00010006108	MKLN1	0.711162687	0.061018846	11.65480395	2.16879E-31	1.29369E-30	positive
PRJNA729271	stage_1W_vs_E10	ordinary_muscle_development	ENSGALG00010006111	WBP1	0.404515296	0.084233879	4.802287427	1.56863E-06	2.88691E-06	positive
PRJNA729271	stage_1W_vs_E10	ordinary_muscle_development	ENSGALG00010006113	EIF2B2	0.735611939	0.167865139	4.382160254	1.17508E-05	2.02868E-05	positive
PRJNA729271	stage_1W_vs_E10	ordinary_muscle_development	ENSGALG00010006118	NPNT	-4.347115138	0.214715393	-20.24594081	3.85748E-91	1.17962E-89	negative
PRJNA729271	stage_1W_vs_E10	ordinary_muscle_development	ENSGALG00010006120	MOGS	1.115357028	0.133396459	8.361219161	6.20734E-17	2.00659E-16	positive
PRJNA729271	stage_1W_vs_E10	ordinary_muscle_development	ENSGALG00010006121	ENSGALG00010006121	-0.734695035	0.326827469	-2.247959873	0.024578749	0.031821093	negative
PRJNA729271	stage_1W_vs_E10	ordinary_muscle_development	ENSGALG00010006123	MLH3	0.385871516	0.10303526	3.745043375	0.000180363	0.000284757	positive
PRJNA729271	stage_1W_vs_E10	ordinary_muscle_development	ENSGALG00010006126	ENSGALG00010006126	5.239868367	0.281539354	18.61149528	2.5928E-77	5.72033E-76	positive
PRJNA729271	stage_1W_vs_E10	ordinary_muscle_development	ENSGALG00010006128	ACYP1	1.936517483	0.117543213	16.47494084	5.55429E-61	8.36027E-60	positive
PRJNA729271	stage_1W_vs_E10	ordinary_muscle_development	ENSGALG00010006131	ENSGALG00010006131	1.2116845	0.125557564	9.65043014	4.895E-22	1.98971E-21	positive
PRJNA729271	stage_1W_vs_E10	ordinary_muscle_development	ENSGALG00010006132	MIPEP	-0.677305169	0.134425598	-5.038513333	4.69162E-07	8.96336E-07	negative
PRJNA729271	stage_1W_vs_E10	ordinary_muscle_development	ENSGALG00010006134	K123	4.102693026	0.710448001	5.774797054	7.70459E-09	1.64523E-08	positive
PRJNA729271	stage_1W_vs_E10	ordinary_muscle_development	ENSGALG00010006135	ZC2HC1C	-0.495541082	0.151770139	-3.265076283	0.001094346	0.001624096	negative
PRJNA729271	stage_1W_vs_E10	ordinary_muscle_development	ENSGALG00010006139	GAREM1	-0.054292128	0.131704959	-0.412225391	0.680174228	0.709698059	negative
PRJNA729271	stage_1W_vs_E10	ordinary_muscle_development	ENSGALG00010006146	CCDC142	1.266434571	0.17910492	7.07090889	1.53922E-12	4.03527E-12	positive
PRJNA729271	stage_1W_vs_E10	ordinary_muscle_development	ENSGALG00010006147	ENSGALG00010006147	2.837467616	0.215886996	13.14330031	1.85937E-39	1.46237E-38	positive
PRJNA729271	stage_1W_vs_E10	ordinary_muscle_development	ENSGALG00010006151	ENSGALG00010006151	-0.583262189	0.162914042	-3.580183653	0.000343353	0.000530982	negative
PRJNA729271	stage_1W_vs_E10	ordinary_muscle_development	ENSGALG00010006155	TNFRSF19	-2.326533955	0.196480327	-11.84105297	2.39428E-32	1.48059E-31	negative
PRJNA729271	stage_1W_vs_E10	ordinary_muscle_development	ENSGALG00010006156	NFKBIZ	1.372044859	0.1976587	6.94148479	3.88E-12	9.96114E-12	positive
PRJNA729271	stage_1W_vs_E10	ordinary_muscle_development	ENSGALG00010006157	SNX9	0.387613267	0.073823941	5.250509013	1.51679E-07	2.99332E-07	positive
PRJNA729271	stage_1W_vs_E10	ordinary_muscle_development	ENSGALG00010006163	ENSGALG00010006163	1.251598465	0.510662618	2.450930262	0.014248756	0.0189304	positive
PRJNA729271	stage_1W_vs_E10	ordinary_muscle_development	ENSGALG00010006172	ENSGALG00010006172	1.135464368	0.19116048	5.939848908	2.85285E-09	6.26072E-09	positive
PRJNA729271	stage_1W_vs_E10	ordinary_muscle_development	ENSGALG00010006173	TBPL1	0.099586298	0.082051755	1.213701006	0.22486192	0.256360983	positive
PRJNA729271	stage_1W_vs_E10	ordinary_muscle_development	ENSGALG00010006175	IL2RA	5.066756111	0.49876877	10.1585272	3.03628E-24	1.34961E-23	positive
PRJNA729271	stage_1W_vs_E10	ordinary_muscle_development	ENSGALG00010006176	MMR1L3	2.693244899	0.214972895	12.5282999	5.22712E-36	3.68876E-35	positive
PRJNA729271	stage_1W_vs_E10	ordinary_muscle_development	ENSGALG00010006177	NEK9	-0.127416648	0.113815767	-1.119499093	0.262927278	0.296678413	negative
PRJNA729271	stage_1W_vs_E10	ordinary_muscle_development	ENSGALG00010006178	RPS23	0.28137875	0.140878506	1.997314986	0.045790976	0.057451762	positive
PRJNA729271	stage_1W_vs_E10	ordinary_muscle_development	ENSGALG00010006180	CD200L	4.06184503	1.085284594	3.742654281			positive
PRJNA729271	stage_1W_vs_E10	ordinary_muscle_development	ENSGALG00010006183	PRKN	3.065467177	0.134697701	22.7581254	1.1924E-114	5.8389E-113	positive
PRJNA729271	stage_1W_vs_E10	ordinary_muscle_development	ENSGALG00010006184	LBX2	-0.598838853	0.284988249	-2.101275595	0.035616781	0.045234841	negative
PRJNA729271	stage_1W_vs_E10	ordinary_muscle_development	ENSGALG00010006187	TCF21	-1.968803936	0.660719445	-2.979788095	0.002884478	0.004117234	negative
PRJNA729271	stage_1W_vs_E10	ordinary_muscle_development	ENSGALG00010006188	TMED10	0.135672502	0.074843543	1.812748254	0.069870658	0.085639708	positive
PRJNA729271	stage_1W_vs_E10	ordinary_muscle_development	ENSGALG00010006193	RBM17	-0.637939477	0.09995932	-6.381990961	1.748E-10	4.0979E-10	negative
PRJNA729271	stage_1W_vs_E10	ordinary_muscle_development	ENSGALG00010006197	ENSGALG00010006197	-1.084291857	0.47462166	-2.284539344	0.022339853	0.029047019	negative
PRJNA729271	stage_1W_vs_E10	ordinary_muscle_development	ENSGALG00010006198	ENSGALG00010006198	0.992532066	0.703628406	1.41059124	0.158365176	0.1851063	positive
PRJNA729271	stage_1W_vs_E10	ordinary_muscle_development	ENSGALG00010006200	GSTCD	-2.015100678	0.10972489	-18.36502802	2.50321E-75	5.31244E-74	negative
PRJNA729271	stage_1W_vs_E10	ordinary_muscle_development	ENSGALG00010006201	SSTR1	-2.258827137	0.367052726	-6.153958214	7.55726E-10	1.71027E-09	negative
PRJNA729271	stage_1W_vs_E10	ordinary_muscle_development	ENSGALG00010006202	SACS	-0.868591085	0.200348311	-4.335405087	1.45492E-05	2.49191E-05	negative
PRJNA729271	stage_1W_vs_E10	ordinary_muscle_development	ENSGALG00010006203	MMR1L4	2.213221016	0.174763713	12.66407642	9.35145E-37	6.70704E-36	positive
PRJNA729271	stage_1W_vs_E10	ordinary_muscle_development	ENSGALG00010006205	CGGBP1	-0.686955245	0.074620313	-9.206008663	3.38477E-20	1.28133E-19	negative
PRJNA729271	stage_1W_vs_E10	ordinary_muscle_development	ENSGALG00010006207	ENSGALG00010006207	0.939427391	0.200882787	4.676495209	2.91819E-06	5.26634E-06	positive
PRJNA729271	stage_1W_vs_E10	ordinary_muscle_development	ENSGALG00010006212	FOS	4.155219242	0.366133549	11.34891696	7.5087E-30	4.2251E-29	positive
PRJNA729271	stage_1W_vs_E10	ordinary_muscle_development	ENSGALG00010006219	CLEC14A	1.950888093	0.183133721	10.65280647	1.69186E-26	8.29338E-26	positive
PRJNA729271	stage_1W_vs_E10	ordinary_muscle_development	ENSGALG00010006220	SGCG	1.619434029	0.170954018	9.472921726	2.72124E-21	1.07948E-20	positive
PRJNA729271	stage_1W_vs_E10	ordinary_muscle_development	ENSGALG00010006223	ATG10	-0.504413199	0.114611944	-4.401052625	1.07727E-05	1.86582E-05	negative
PRJNA729271	stage_1W_vs_E10	ordinary_muscle_development	ENSGALG00010006227	AGPAT4	-1.521510878	0.148421819	-10.2512615	1.16809E-24	5.30102E-24	negative
PRJNA729271	stage_1W_vs_E10	ordinary_muscle_development	ENSGALG00010006229	INTS12	0.361984057	0.13228266	2.736443734	0.006210722	0.008581108	positive
PRJNA729271	stage_1W_vs_E10	ordinary_muscle_development	ENSGALG00010006232	ZNF654	0.067564674	0.103833102	0.650704572	0.515237211	0.552484236	positive
PRJNA729271	stage_1W_vs_E10	ordinary_muscle_development	ENSGALG00010006236	C3orf38	0.471722901	0.083985476	5.616719973	1.94616E-08	4.06128E-08	positive
PRJNA729271	stage_1W_vs_E10	ordinary_muscle_development	ENSGALG00010006237	ENSGALG00010006237	1.334132488	0.310141784	4.301685732	1.69504E-05	2.88884E-05	positive
PRJNA729271	stage_1W_vs_E10	ordinary_muscle_development	ENSGALG00010006239	EYA4	1.581381609	0.162296121	9.743804074	1.96074E-22	8.09021E-22	positive
PRJNA729271	stage_1W_vs_E10	ordinary_muscle_development	ENSGALG00010006241	MRC1	1.874456212	0.244770171	7.658025502	1.88813E-14	5.45515E-14	positive
PRJNA729271	stage_1W_vs_E10	ordinary_muscle_development	ENSGALG00010006242	ENSGALG00010006242	0.848849609	0.554068749	1.532029392	0.125515182	0.148858007	positive
PRJNA729271	stage_1W_vs_E10	ordinary_muscle_development	ENSGALG00010006244	EPHA3	-2.197875467	0.162588702	-13.51800855	1.22446E-41	1.04158E-40	negative
PRJNA729271	stage_1W_vs_E10	ordinary_muscle_development	ENSGALG00010006245	PFKFB3	1.974941541	0.508775328	3.881755722	0.000103705	0.000166468	positive
PRJNA729271	stage_1W_vs_E10	ordinary_muscle_development	ENSGALG00010006246	SUPT20H	-0.243076716	0.100269421	-2.424235756	0.015340643	0.02030338	negative
PRJNA729271	stage_1W_vs_E10	ordinary_muscle_development	ENSGALG00010006249	C9orf72	0.500729533	0.104824798	4.776823266	1.78086E-06	3.26589E-06	positive
PRJNA729271	stage_1W_vs_E10	ordinary_muscle_development	ENSGALG00010006251	SEMA3E	-3.179978538	0.376175182	-8.453451177	2.82816E-17	9.28101E-17	negative
PRJNA729271	stage_1W_vs_E10	ordinary_muscle_development	ENSGALG00010006252	COX7C	1.779705768	0.136510957	13.03709096	7.52747E-39	5.80994E-38	positive
PRJNA729271	stage_1W_vs_E10	ordinary_muscle_development	ENSGALG00010006254	PROS1	1.201312423	0.137330803	8.747581723	2.17974E-18	7.55145E-18	positive
PRJNA729271	stage_1W_vs_E10	ordinary_muscle_development	ENSGALG00010006259	PPA2	0.374007558	0.087368098	4.28082521	1.86202E-05	3.16189E-05	positive
PRJNA729271	stage_1W_vs_E10	ordinary_muscle_development	ENSGALG00010006260	MAP3K4	0.85068908	0.159323297	5.339389125	9.32603E-08	1.86894E-07	positive
PRJNA729271	stage_1W_vs_E10	ordinary_muscle_development	ENSGALG00010006261	PCLO	-3.165149997	0.57949353	-5.461924651	4.71E-08	9.62247E-08	negative
PRJNA729271	stage_1W_vs_E10	ordinary_muscle_development	ENSGALG00010006263	EXOSC8	-1.039947482	0.150917762	-6.890822314	5.54708E-12	1.41334E-11	negative
PRJNA729271	stage_1W_vs_E10	ordinary_muscle_development	ENSGALG00010006266	CENPW	-1.986566638	0.2218711	-8.953697169	3.43771E-19	1.2445E-18	negative
PRJNA729271	stage_1W_vs_E10	ordinary_muscle_development	ENSGALG00010006269	ENSGALG00010006269	3.693118306	1.223527136	3.018419615	0.002540968	0.003646666	positive
PRJNA729271	stage_1W_vs_E10	ordinary_muscle_development	ENSGALG00010006270	STAM	0.022279982	0.092969484	0.239648336	0.81060289	0.830660225	positive
PRJNA729271	stage_1W_vs_E10	ordinary_muscle_development	ENSGALG00010006273	ARL13B	-0.570205198	0.099778288	-5.71472222	1.09883E-08	2.3252E-08	negative
PRJNA729271	stage_1W_vs_E10	ordinary_muscle_development	ENSGALG00010006277	GNG10	-1.091213161	0.161200953	-6.769272387	1.29432E-11	3.23245E-11	negative
PRJNA729271	stage_1W_vs_E10	ordinary_muscle_development	ENSGALG00010006279	HACD1	4.038859984	0.141302461	28.58308312	1.0904E-179	1.3172E-177	positive
PRJNA729271	stage_1W_vs_E10	ordinary_muscle_development	ENSGALG00010006281	TRMT11	0.17774456	0.081362697	2.184595231	0.028918538	0.037108198	positive
PRJNA729271	stage_1W_vs_E10	ordinary_muscle_development	ENSGALG00010006283	DNAJC25	0.725918083	0.106555966	6.812552211	9.58822E-12	2.41539E-11	positive
PRJNA729271	stage_1W_vs_E10	ordinary_muscle_development	ENSGALG00010006285	FZD8	-0.810524149	0.154723344	-5.238538189	1.61853E-07	3.18629E-07	negative
PRJNA729271	stage_1W_vs_E10	ordinary_muscle_development	ENSGALG00010006287	ECPAS	1.578193354	0.122380267	12.89581563	4.75209E-38	3.55059E-37	positive
PRJNA729271	stage_1W_vs_E10	ordinary_muscle_development	ENSGALG00010006288	ARF5	-3.419081238	0.193112746	-17.70510388	3.82973E-70	7.28504E-69	negative
PRJNA729271	stage_1W_vs_E10	ordinary_muscle_development	ENSGALG00010006289	SKIDA1	-1.785528977	0.151282353	-11.80262559	3.7832E-32	2.32067E-31	negative
PRJNA729271	stage_1W_vs_E10	ordinary_muscle_development	ENSGALG00010006291	ENSGALG00010006291	-0.141808619	0.215534788	-0.657938427	0.510577697	0.54812796	negative
PRJNA729271	stage_1W_vs_E10	ordinary_muscle_development	ENSGALG00010006293	SLC22A3	0.374127201	0.362844971	1.031093803	0.302496833	0.337829694	positive
PRJNA729271	stage_1W_vs_E10	ordinary_muscle_development	ENSGALG00010006300	GCC1	-0.100092352	0.120507454	-0.830590549	0.406204976	0.44433938	negative

PRJNA729271	stage_1W_vs_E10	ordinary_muscle_development	ENSGALG00010006301	ENSGALG00010006301	0.556666432	0.14710901	3.78404037	0.000154303	0.000244814	positive
PRJNA729271	stage_1W_vs_E10	ordinary_muscle_development	ENSGALG00010006310	ALG5	-0.473327128	0.111871885	-4.230974806	2.32681E-05	3.92221E-05	negative
PRJNA729271	stage_1W_vs_E10	ordinary_muscle_development	ENSGALG00010006311	ABCD4	-0.283285962	0.116458207	-2.432511787	0.014994503	0.019868848	negative
PRJNA729271	stage_1W_vs_E10	ordinary_muscle_development	ENSGALG00010006323	ANAPC10	0.304400968	0.105810535	2.87684935	0.004016673	0.005653688	positive
PRJNA729271	stage_1W_vs_E10	ordinary_muscle_development	ENSGALG00010006327	DENND6B	0.313757238	0.110843787	2.830625402	0.00464571	0.006504352	positive
PRJNA729271	stage_1W_vs_E10	ordinary_muscle_development	ENSGALG00010006328	SYNDIG1L	-3.635252058	0.509716755	-7.131906153	9.89884E-13	2.62358E-12	negative
PRJNA729271	stage_1W_vs_E10	ordinary_muscle_development	ENSGALG00010006330	STX19	-0.651445814	0.421172339	-1.546744061	0.121924974	0.144896491	negative
PRJNA729271	stage_1W_vs_E10	ordinary_muscle_development	ENSGALG00010006331	SMAD9	-0.944165223	0.156403405	-6.036730605	1.57268E-09	3.50492E-09	negative
PRJNA729271	stage_1W_vs_E10	ordinary_muscle_development	ENSGALG00010006336	ALDH8A1	1.77584209	0.86222179	2.059611704	0.039435676	0.049879592	positive
PRJNA729271	stage_1W_vs_E10	ordinary_muscle_development	ENSGALG00010006339	ENSGALG00010006339	3.638823356	0.714665758	5.09164363	3.54973E-07	6.83856E-07	positive
PRJNA729271	stage_1W_vs_E10	ordinary_muscle_development	ENSGALG00010006341	HHIP	-0.867773348	0.220390685	-3.937432049	8.23582E-05	0.000133369	negative
PRJNA729271	stage_1W_vs_E10	ordinary_muscle_development	ENSGALG00010006342	RFXAP	-0.341132569	0.085586641	-3.985815608	6.72487E-05	0.000109697	negative
PRJNA729271	stage_1W_vs_E10	ordinary_muscle_development	ENSGALG00010006343	SEC23A	0.105659959	0.094777005	1.114826939	0.26492459	0.298676358	positive
PRJNA729271	stage_1W_vs_E10	ordinary_muscle_development	ENSGALG00010006344	HINT3	0.509602358	0.105366536	4.836472528	1.32164E-06	2.44632E-06	positive
PRJNA729271	stage_1W_vs_E10	ordinary_muscle_development	ENSGALG00010006345	SMC2	-2.839877747	0.250286174	-11.34652268	7.71711E-30	4.33732E-29	negative
PRJNA729271	stage_1W_vs_E10	ordinary_muscle_development	ENSGALG00010006346	NSUN3	-0.349708834	0.135044386	-2.589584393	0.009609187	0.01301244	negative
PRJNA729271	stage_1W_vs_E10	ordinary_muscle_development	ENSGALG00010006347	PPP6R2	2.153275639	0.135371576	15.90640889	5.72018E-57	7.74174E-56	positive
PRJNA729271	stage_1W_vs_E10	ordinary_muscle_development	ENSGALG00010006351	IGF2R	1.643792114	0.173897556	9.452646456	3.3037E-21	1.30554E-20	positive
PRJNA729271	stage_1W_vs_E10	ordinary_muscle_development	ENSGALG00010006352	GEMIN2	1.111149266	0.096667117	11.49459408	1.4044E-30	8.11995E-30	positive
PRJNA729271	stage_1W_vs_E10	ordinary_muscle_development	ENSGALG00010006353	ABCE1	-0.393633796	0.080801928	-4.871589145	1.10704E-06	2.06334E-06	negative
PRJNA729271	stage_1W_vs_E10	ordinary_muscle_development	ENSGALG00010006355	PTGR1	-1.495098203	0.153380978	-9.747611604	1.88859E-22	7.79918E-22	negative
PRJNA729271	stage_1W_vs_E10	ordinary_muscle_development	ENSGALG00010006359	SGK1	1.966697907	0.163721092	12.01248957	3.05507E-33	1.95252E-32	positive
PRJNA729271	stage_1W_vs_E10	ordinary_muscle_development	ENSGALG00010006361	SERTM1	-2.383690676	0.268901685	-8.864543462	7.68182E-19	2.72644E-18	negative
PRJNA729271	stage_1W_vs_E10	ordinary_muscle_development	ENSGALG00010006363	TRAPPC6A	-0.204332976	0.102402374	-1.995392959	0.046000038	0.057689095	negative
PRJNA729271	stage_1W_vs_E10	ordinary_muscle_development	ENSGALG00010006372	SBF1	0.1408409	0.113586677	1.23994207	0.214996822	0.246081722	positive
PRJNA729271	stage_1W_vs_E10	ordinary_muscle_development	ENSGALG00010006378	CAAP1	-1.349977664	0.105639539	-12.77909458	2.14553E-37	1.57227E-36	negative
PRJNA729271	stage_1W_vs_E10	ordinary_muscle_development	ENSGALG00010006381	SPART	1.402628144	0.156644645	8.954204231	3.42195E-19	1.2391E-18	positive
PRJNA729271	stage_1W_vs_E10	ordinary_muscle_development	ENSGALG00010006382	PNN	-0.105852313	0.196508371	-0.538665665	0.590117569	0.625045975	negative
PRJNA729271	stage_1W_vs_E10	ordinary_muscle_development	ENSGALG00010006387	OTUD4	-0.299867484	0.136134765	-2.202725253	0.027614119	0.03552371	negative
PRJNA729271	stage_1W_vs_E10	ordinary_muscle_development	ENSGALG00010006404	SMAD1	0.047510647	0.109410906	0.434240505	0.664113802	0.694440165	positive
PRJNA729271	stage_1W_vs_E10	ordinary_muscle_development	ENSGALG00010006408	ENSGALG00010006408	-1.065479836	0.694141675	-1.534960188	0.124793608	0.148087052	negative
PRJNA729271	stage_1W_vs_E10	ordinary_muscle_development	ENSGALG00010006411	RGS17	-2.676270825	0.476853296	-5.612356773	1.99589E-08	4.16266E-08	negative
PRJNA729271	stage_1W_vs_E10	ordinary_muscle_development	ENSGALG00010006421	LMF2	-1.041130893	0.113642166	-9.161484054	5.11884E-20	1.92222E-19	negative
PRJNA729271	stage_1W_vs_E10	ordinary_muscle_development	ENSGALG00010006434	MMAA	0.090782304	0.117315086	0.773833161	0.43902946	0.476969871	positive
PRJNA729271	stage_1W_vs_E10	ordinary_muscle_development	ENSGALG00010006446	LSAMP	-2.305749773	0.1886047	-12.22530387	2.27729E-34	1.5059E-33	negative
PRJNA729271	stage_1W_vs_E10	ordinary_muscle_development	ENSGALG00010006454	SOD2	1.779871647	0.105272335	16.9073066	3.97463E-64	6.44433E-63	positive
PRJNA729271	stage_1W_vs_E10	ordinary_muscle_development	ENSGALG00010006457	NCAPH2	-2.713485182	0.210698371	-12.87852948	5.94576E-38	4.42649E-37	negative
PRJNA729271	stage_1W_vs_E10	ordinary_muscle_development	ENSGALG00010006463	SCO2	0.344207909	0.135302795	2.543982248	0.010959665	0.014747526	positive
PRJNA729271	stage_1W_vs_E10	ordinary_muscle_development	ENSGALG00010006465	PLAA	0.670044566	0.115629474	5.794755784	6.84207E-09	1.46601E-08	positive
PRJNA729271	stage_1W_vs_E10	ordinary_muscle_development	ENSGALG00010006472	DCLK1	-3.30000353	0.329167528	-10.02530093	1.18E-23	5.13173E-23	negative
PRJNA729271	stage_1W_vs_E10	ordinary_muscle_development	ENSGALG00010006478	ENSGALG00010006478	0.916093534	0.131477833	6.96766532	3.22244E-12	8.31273E-12	positive
PRJNA729271	stage_1W_vs_E10	ordinary_muscle_development	ENSGALG00010006480	IFT74	-0.745598854	0.120191141	-6.203442682	5.52412E-10	1.2588E-09	negative
PRJNA729271	stage_1W_vs_E10	ordinary_muscle_development	ENSGALG00010006486	CEP43	0.101470482	0.112889342	0.898849086	0.368733051	0.406540319	positive
PRJNA729271	stage_1W_vs_E10	ordinary_muscle_development	ENSGALG00010006492	GAP43	-4.716722205	0.509639518	-9.255016618	2.14197E-20	8.18557E-20	negative
PRJNA729271	stage_1W_vs_E10	ordinary_muscle_development	ENSGALG00010006496	UBE2H	1.248529849	0.11367588	10.98324328	4.60103E-28	2.40504E-27	positive
PRJNA729271	stage_1W_vs_E10	ordinary_muscle_development	ENSGALG00010006499	CCR6	0.779728604	0.717262173	1.087090096	0.276996994	0.311318247	positive
PRJNA729271	stage_1W_vs_E10	ordinary_muscle_development	ENSGALG00010006503	LRRC19	6.382508345	0.991555159	6.436866661	1.21965E-10	2.88304E-10	positive
PRJNA729271	stage_1W_vs_E10	ordinary_muscle_development	ENSGALG00010006506	ZNF827	-0.45053524	0.106461294	-4.231915867	2.31709E-05	3.90719E-05	negative
PRJNA729271	stage_1W_vs_E10	ordinary_muscle_development	ENSGALG00010006515	FBXO33	-0.029192485	0.103717747	-0.281460847	0.778356954	0.801298583	negative
PRJNA729271	stage_1W_vs_E10	ordinary_muscle_development	ENSGALG00010006516	NBEA	-0.942079727	0.12736674	-7.39659134	1.39725E-13	3.85113E-13	negative
PRJNA729271	stage_1W_vs_E10	ordinary_muscle_development	ENSGALG00010006517	ZBTB20	3.885086986	0.145108456	26.77367734	6.5466E-158	6.3686E-156	positive
PRJNA729271	stage_1W_vs_E10	ordinary_muscle_development	ENSGALG00010006521	ZNF410	-0.715450803	0.104435469	-6.85064961	7.35154E-12	1.8623E-11	negative
PRJNA729271	stage_1W_vs_E10	ordinary_muscle_development	ENSGALG00010006524	TAB2	0.072030431	0.091681192	0.785662019	0.43206552	0.47000298	positive
PRJNA729271	stage_1W_vs_E10	ordinary_muscle_development	ENSGALG00010006526	LSM6	-1.053916086	0.161776794	-6.514630824	7.28686E-11	1.74152E-10	negative
PRJNA729271	stage_1W_vs_E10	ordinary_muscle_development	ENSGALG00010006528	ZC3HC1	-0.251628101	0.081920643	-3.071607994	0.002129091	0.003075354	negative
PRJNA729271	stage_1W_vs_E10	ordinary_muscle_development	ENSGALG00010006531	FAM161B	-1.197288085	0.148979853	-8.036577164	9.23828E-16	2.84549E-15	negative
PRJNA729271	stage_1W_vs_E10	ordinary_muscle_development	ENSGALG00010006532	KLHDC10	0.478071646	0.103102645	4.636851425	3.53757E-06	6.35009E-06	positive
PRJNA729271	stage_1W_vs_E10	ordinary_muscle_development	ENSGALG00010006537	DRD3	-3.74201937	0.455378511	-8.217382416	2.07993E-16	6.57692E-16	negative
PRJNA729271	stage_1W_vs_E10	ordinary_muscle_development	ENSGALG00010006538	TEK	1.462464553	0.105057388	13.92062548	4.74731E-44	4.3251E-43	positive
PRJNA729271	stage_1W_vs_E10	ordinary_muscle_development	ENSGALG00010006542	RFC3	-2.08781021	0.143044192	-14.59556083	2.99738E-48	3.08572E-47	negative
PRJNA729271	stage_1W_vs_E10	ordinary_muscle_development	ENSGALG00010006544	MOB3B	-2.975067104	0.164015774	-18.13890839	1.57121E-73	3.21223E-72	negative
PRJNA729271	stage_1W_vs_E10	ordinary_muscle_development	ENSGALG00010006546	SMOC2	-0.720173085	0.208532105	-3.453535776	0.000553289	0.000840927	negative
PRJNA729271	stage_1W_vs_E10	ordinary_muscle_development	ENSGALG00010006548	TMEM209	-0.907491435	0.081931517	-11.07621906	1.63638E-28	8.67885E-28	negative
PRJNA729271	stage_1W_vs_E10	ordinary_muscle_development	ENSGALG00010006549	ZDHHC23	-0.391661745	0.184318707	-2.124915866	0.033593634	0.042796601	negative
PRJNA729271	stage_1W_vs_E10	ordinary_muscle_development	ENSGALG00010006552	CPA2	6.327215513	1.650055614	3.834546824	0.000125796	0.000200816	positive
PRJNA729271	stage_1W_vs_E10	ordinary_muscle_development	ENSGALG00010006557	COQ6	0.767118884	0.108114448	7.095433541	1.28947E-12	3.39771E-12	positive
PRJNA729271	stage_1W_vs_E10	ordinary_muscle_development	ENSGALG00010006558	USP10	-0.487318728	0.088826204	-5.486204576	4.10661E-08	8.41467E-08	negative
PRJNA729271	stage_1W_vs_E10	ordinary_muscle_development	ENSGALG00010006564	SLC10A7	-1.655921845	0.145324494	-11.39465066	4.4461E-30	2.52038E-29	negative
PRJNA729271	stage_1W_vs_E10	ordinary_muscle_development	ENSGALG00010006566	CPA5	7.298375732	2.803731728	2.603093463	0.009238676	0.012538822	positive
PRJNA729271	stage_1W_vs_E10	ordinary_muscle_development	ENSGALG00010006568	ENTPD5	0.294921906	0.198915536	1.482648924	0.138167712	0.162903935	positive
PRJNA729271	stage_1W_vs_E10	ordinary_muscle_development	ENSGALG00010006570	ENSGALG00010006570	1.388752231	0.486693326	2.853444165	0.004324813	0.006067966	positive
PRJNA729271	stage_1W_vs_E10	ordinary_muscle_development	ENSGALG00010006574	CPA1	7.796230325	2.554102439	3.05243447	0.002269933	0.003270319	positive
PRJNA729271	stage_1W_vs_E10	ordinary_muscle_development	ENSGALG00010006575	SSBP2	-1.554571977	0.118039634	-13.16991524	1.30744E-39	1.0339E-38	negative
PRJNA729271	stage_1W_vs_E10	ordinary_muscle_development	ENSGALG00010006576	CCDC191	-0.482704348	0.11800883	-4.090408743	4.30614E-05	7.12121E-05	negative
PRJNA729271	stage_1W_vs_E10	ordinary_muscle_development	ENSGALG00010006580	ALDH6A1	1.34717351	0.13844387	9.730828187	2.22772E-22	9.1683E-22	positive
PRJNA729271	stage_1W_vs_E10	ordinary_muscle_development	ENSGALG00010006581	CEP41	-1.22656919	0.108132412	-11.3432149	8.01451E-30	4.49401E-29	negative
PRJNA729271	stage_1W_vs_E10	ordinary_muscle_development	ENSGALG00010006585	TTC29	-0.857725879	0.192341333	-4.459394472	8.21915E-06	1.43383E-05	negative
PRJNA729271	stage_1W_vs_E10	ordinary_muscle_development	ENSGALG00010006588	ACOT12	-1.128470541	0.690443745	-1.634413448	0.102172066	0.122719929	negative
PRJNA729271	stage_1W_vs_E10	ordinary_muscle_development	ENSGALG00010006592	STARD13	-0.531671309	0.114810122	-4.630874869	3.64124E-06	6.53053E-06	negative
PRJNA729271	stage_1W_vs_E10	ordinary_muscle_development	ENSGALG00010006598	ENSGALG00010006598	0.611453902	0.151763241	4.028998715	5.60149E-05	9.18168E-05	positive
PRJNA729271	stage_1W_vs_E10	ordinary_muscle_development	ENSGALG00010006602	EDNRA	-0.989297297	0.118764243	-8.329925501	8.08932E-17	2.60566E-16	negative
PRJNA729271	stage_1W_vs_E10	ordinary_muscle_development	ENSGALG00010006603	MEST	-1.255208331	0.235524809	-5.329410239	9.85322E-08	1.97078E-07	negative
PRJNA729271	stage_1W_vs_E10	ordinary_muscle_development	ENSGALG00010006606	SULT	-1.147715227	1.20392555	-0.953310798			negative
PRJNA729271	stage_1W_vs_E10	ordinary_muscle_development	ENSGALG00010006608	KL	3.396330157	0.408453954	8.315086985	9.1685E-17	2.94738E-16	positive
PRJNA729271	stage_1W_vs_E10	ordinary_muscle_development	ENSGALG00010006614	TMEM184C	-0.286036333	0.055346818	-5.168071904	2.36521E-07	4.61051E-07	negative
PRJNA729271	stage_1W_vs_E10	ordinary_muscle_development	ENSGALG00010006615	GRAMD1C	-0.302136078	0.102849974	-2.937638835	0.003307221	0.004695677	negative
PRJNA729271	stage_1W_vs_E10	ordinary_muscle_development	ENSGALG00010006616	COPG2	-0.794278801	0.069382562	-11.44781595	2.41145E-30	1.37777E-29	negative
PRJNA729271	stage_1W_vs_E10	ordinary_muscle_development	ENSGALG00010006619	LIN52	-0.125951749	0.10194677	-1.23546582	0.21665719	0.247806041	negative
PRJNA729271	stage_1W_vs_E10	ordinary_muscle_development	ENSGALG00010006621	DACT2	-2.34804303	0.211086144	-11.12362462	9.62793E-29	5.1497E-28	negative
PRJNA729271	stage_1W_vs_E10	ordinary_muscle_development	ENSGALG00010006623	ENSGALG00010006623	0.405025667	0.18763208	2.158616303	0.030879947	0.039506163	positive
PRJNA729271	stage_1W_vs_E10	ordinary_muscle_development	ENSGALG00010006627	SF3B5	-0.123324999	0.110107574	-1.12004101	0.262696286	0.29648701	negative
PRJNA729271	stage_1W_vs_E10	ordinary_muscle_development	ENSGALG00010006633	FBXO18	-0.116354584	0.095237742	-1.221727662	0.221810632	0.25323907	negative
PRJNA729271	stage_1W_vs_E10	ordinary_muscle_development	ENSGALG00010006634	PRMT9	0.882035223	0.095138152	9.271098983	1.84238E-20	7.06675E-20	positive
PRJNA729271	stage_1W_vs_E10	ordinary_muscle_development	ENSGALG00010006635	ENSGALG00010006635	-2.276571452	0.479512723	-4.747676844	2.05766E-06	3.75262E-06	negative
PRJNA729271	stage_1W_vs_E10	ordinary_muscle_development	ENSGALG00010006641	ENSGALG00010006641	-3.776130544	0.556555152	-6.784827221	1.16226E-11	2.91217E-11	negative
PRJNA729271	stage_1W_vs_E10	ordinary_muscle_development	ENSGALG00010006651	ENSGALG00010006651	0.053603245	0.115733859	0.463159575	0.64324999	0.675156307	positive
PRJNA729271	stage_1W_vs_E10	ordinary_muscle_development	ENSGALG00010006653	CFL2	1.235556939	0.104560399	11.81668152	3.2008E-32	1.96842E-31	positive
PRJNA729271	stage_1W_vs_E10	ordinary_muscle_development	ENSGALG00010006657	PDS5B	-1.384392254	0.088850485	-15.58114468	9.77878E-55	1.25548E-53	negative
PRJNA729271	stage_1W_vs_E10	ordinary_muscle_development	ENSGALG00010006664	ENSGALG00010006664	3.946636016	1.331662881	2.963690039	0.003039743	0.004329901	positive
PRJNA729271	stage_1W_vs_E10	ordinary_muscle_development	ENSGALG00010006670	ANKRD16	0.118743381	0.18792649	0.631860793	0.527477845	0.564181771	positive
PRJNA729271	stage_1W_vs_E10	ordinary_muscle_development	ENSGALG00010006673	BAZ1A	-1.258804097	0.0832693	-15.1172653	1.24611E-51	1.44731E-50	negative
PRJNA729271	stage_1W_vs_E10	ordinary_muscle_development	ENSGALG00010006678	ENSGALG00010006678	-4.451384062	1.326761373	-3.355075111	0.000793434	0.001192785	negative
PRJNA729271	stage_1W_vs_E10	ordinary_muscle_development	ENSGALG00010006681	CRISPLD2	-1.484252853	0.2124452	-6.98652102	2.81786E-12	7.29113E-12	negative
PRJNA729271	stage_1W_vs_E10	ordinary_muscle_development	ENSGALG00010006682	ARHGAP10	-1.321805411	0.123019781	-10.74465744	6.27946E-27	3.12464E-26	negative
PRJNA729271	stage_1W_vs_E10	ordinary_muscle_development	ENSGALG00010006685	ENSGALG00010006685	-0.054931187	0.116580425	-0.471187056	0.637507161	0.669988537	negative
PRJNA729271	stage_1W_vs_E10	ordinary_muscle_development	ENSGALG00010006694	ENSGALG00010006694	-3.008684174	0.717520783	-4.193166586	2.75087E-05	4.6145E-05	negative
PRJNA729271	stage_1W_vs_E10	ordinary_muscle_development	ENSGALG00010006701	LINGO2	-5.93163662	0.690779106	-8.586879026	8.93636E-18	3.00261E-17	negative
PRJNA729271	stage_1W_vs_E10	ordinary_muscle_development	ENSGALG00010006715	IRF8	0.587330372	0.162368499	3.617267983	0.000297729	0.000462996	positive
PRJNA729271	stage_1W_vs_E10	ordinary_muscle_development	ENSGALG00010006716	ENSGALG00010006716	1.052789858	0.097145772	10.83721744	2.29341E-27	1.16397E-26	positive
PRJNA729271	stage_1W_vs_E10	ordinary_muscle_development	ENSGALG00010006720	SRP54	0.015847234	0.091247032	0.173673968	0.862121697	0.877313536	positive
PRJNA729271	stage_1W_vs_E10	ordinary_muscle_development	ENSGALG00010006728	ENSGALG00010006728	0.056062471	0.137411442	0.407989832	0.68328114	0.712641565	positive
PRJNA729271	stage_1W_vs_E10	ordinary_muscle_development	ENSGALG00010006730	HAPLN1	-7.928440569	0.513508734	-15.43973851	8.84582E-54	1.10249E-52	negative
PRJNA729271	stage_1W_vs_E10	ordinary_muscle_development	ENSGALG00010006735	NR3C2	-0.242056079	0.113191947	-2.13845671	0.032479694	0.04144306	negative
PRJNA729271	stage_1W_vs_E10	ordinary_muscle_development	ENSGALG00010006739	VCAN	-4.296251106	0.254128558	-16.90581785	4.0763E-64	6.60178E-63	negative
PRJNA729271	stage_1W_vs_E10	ordinary_muscle_development	ENSGALG00010006740	BRCA2	-1.385765505	0.153912204	-9.003610311	2.18414E-19	7.97259E-19	negative
PRJNA729271	stage_1W_vs_E10	ordinary_muscle_development	ENSGALG00010006748	FAM177A1	1.005837838	0.077068898	13.05115118	6.25944E-39	4.8545E-38	positive
PRJNA729271	stage_1W_vs_E10	ordinary_muscle_development	ENSGALG00010006752	ZAR1L	-0.296258907	0.434978592	-0.681088478	0.495815507	0.533703088	negative
PRJNA729271	stage_1W_vs_E10	ordinary_muscle_development	ENSGALG00010006760	TAS0R2	0.082118035	0.103069675	0.796723522	0.42561162	0.46360866	positive
PRJNA729271	stage_1W_vs_E10	ordinary_muscle_development	ENSGALG00010006761	PPP2R3C	-0.250651003	0.095384913	-2.627784574	0.008594292	0.011704808	negative
PRJNA729271	stage_1W_vs_E10	ordinary_muscle_development	ENSGALG00010006767	ENSGALG00010006767	8.241389384	0.329793022	24.98958085	7.9353E-138	5.5836E-136	positive
PRJNA729271	stage_1W_vs_E10	ordinary_muscle_development	ENSGALG00010006794	PERPA	-5.853847611	0.604578475	-9.682527334	3.57761E-22	1.46036E-21	negative
PRJNA729271	stage_1W_vs_E10	ordinary_muscle_development	ENSGALG00010006803	COX4I1	1.987524951	0.103799739	19.14768736	1.01181E-81	2.51997E-80	positive
PRJNA729271	stage_1W_vs_E10	ordinary_muscle_development	ENSGALG00010006804	PSMA6	0.80545727	0.101919623	7.902867407	2.72559E-15	8.21874E-15	positive
PRJNA729271	stage_1W_vs_E10	ordinary_muscle_development	ENSGALG00010006814	ENSGALG00010006814	0.56846	0.370365585	1.534861832	0.124817771	0.148103601	positive
PRJNA729271	stage_1W_vs_E10	ordinary_muscle_development	ENSGALG00010006816	ENSGALG00010006816	5.34881892	0.505381752	10.58371992	3.54594E-26	1.71385E-25	positive
PRJNA729271	stage_1W_vs_E10	ordinary_muscle_development	ENSGALG00010006819	EMC8	0.950586228	0.131296728	7.239984156	4.48737E-13	1.20699E-12	positive
PRJNA729271	stage_1W_vs_E10	ordinary_muscle_development	ENSGALG00010006821	XRCC4	-1.187030032	0.131346409	-9.037399985	1.60442E-19	5.90257E-19	negative
PRJNA729271	stage_1W_vs_E10	ordinary_muscle_development	ENSGALG00010006828	GTF2A1	0.062107069	0.106061024	0.585578631	0.558158712	0.594060542	positive
PRJNA729271	stage_1W_vs_E10	ordinary_muscle_development	ENSGALG00010006833	LTV1	-0.371238613	0.087020633	-4.266098728	1.98921E-05	3.36919E-05	negative
PRJNA729271	stage_1W_vs_E10	ordinary_muscle_development	ENSGALG00010006835	FRY	2.209577041	0.152103992	14.52675248	8.20176E-48	8.33692E-47	positive
PRJNA729271	stage_1W_vs_E10	ordinary_muscle_development	ENSGALG00010006838	ASB13	1.626940693	0.120734935	13.47531012	2.18565E-41	1.83978E-40	positive

PRJNA729271	stage_1W_vs_E10	ordinary_muscle_development	ENSGALG00010006840	ENSGALG00010006840	-3.30756941	0.294262925	-11.24018395	2.58842E-29	1.42064E-28	negative
PRJNA729271	stage_1W_vs_E10	ordinary_muscle_development	ENSGALG00010006843	STON2	1.738963974	0.191988163	9.057662462	1.33276E-19	4.92187E-19	positive
PRJNA729271	stage_1W_vs_E10	ordinary_muscle_development	ENSGALG00010006845	CALML3	-9.955627603	0.9746483	-10.21458469	1.70608E-24	7.6824E-24	negative
PRJNA729271	stage_1W_vs_E10	ordinary_muscle_development	ENSGALG00010006847	SEL1L	-0.041582133	0.086948676	-0.478237682	0.632481045	0.665282291	negative
PRJNA729271	stage_1W_vs_E10	ordinary_muscle_development	ENSGALG00010006850	IRF2BPL	-0.955538295	0.160377435	-5.958059449	2.55251E-09	5.6152E-09	negative
PRJNA729271	stage_1W_vs_E10	ordinary_muscle_development	ENSGALG00010006851	TMEM167A	-0.682956709	0.10529603	-6.486063257	8.81082E-11	2.09881E-10	negative
PRJNA729271	stage_1W_vs_E10	ordinary_muscle_development	ENSGALG00010006853	NET1	-0.94672576	0.084267622	-11.23475108	2.75269E-29	1.50794E-28	negative
PRJNA729271	stage_1W_vs_E10	ordinary_muscle_development	ENSGALG00010006855	DCLK2	0.195323096	0.12335996	1.583358952	0.113339673	0.135303786	positive
PRJNA729271	stage_1W_vs_E10	ordinary_muscle_development	ENSGALG00010006865	EFCAB2	-1.469684005	0.382586662	-3.841440776	0.000122314	0.000195409	negative
PRJNA729271	stage_1W_vs_E10	ordinary_muscle_development	ENSGALG00010006877	GSE1	1.343067661	0.240339472	5.583564522	2.35638E-08	4.88708E-08	positive
PRJNA729271	stage_1W_vs_E10	ordinary_muscle_development	ENSGALG00010006883	LRBA	-0.676100535	0.071391547	-9.470316393	2.78997E-21	1.10584E-20	negative
PRJNA729271	stage_1W_vs_E10	ordinary_muscle_development	ENSGALG00010006885	RHOU	0.014843355	0.153086958	0.096960284	0.922757934	0.932081969	positive
PRJNA729271	stage_1W_vs_E10	ordinary_muscle_development	ENSGALG00010006901	TUBAL3	-3.544168513	0.263405842	-13.4551629	2.87106E-41	2.40833E-40	negative
PRJNA729271	stage_1W_vs_E10	ordinary_muscle_development	ENSGALG00010006903	B3GLCT	-1.02674513	0.146467073	-7.010074745	2.38191E-12	6.18852E-12	negative
PRJNA729271	stage_1W_vs_E10	ordinary_muscle_development	ENSGALG00010006912	ENSGALG00010006912	-0.912171332	0.183498336	-4.971006008	6.66064E-07	1.26039E-06	negative
PRJNA729271	stage_1W_vs_E10	ordinary_muscle_development	ENSGALG00010006913	HSPH1	-1.751276117	0.187365803	-9.346828976	9.03148E-21	3.50781E-20	negative
PRJNA729271	stage_1W_vs_E10	ordinary_muscle_development	ENSGALG00010006922	SMYD3	-0.483004727	0.160299798	-3.01313372	0.00258565	0.003708221	negative
PRJNA729271	stage_1W_vs_E10	ordinary_muscle_development	ENSGALG00010006923	ENSGALG00010006923	0.204940885	0.228222327	0.897987885	0.369192008	0.406922528	positive
PRJNA729271	stage_1W_vs_E10	ordinary_muscle_development	ENSGALG00010006926	ID4	-3.501098918	0.235665885	-14.85619744	6.34244E-50	6.94888E-49	negative
PRJNA729271	stage_1W_vs_E10	ordinary_muscle_development	ENSGALG00010006927	UCN3	-4.064609321	1.017867541	-3.99325959	6.51712E-05	0.000106416	negative
PRJNA729271	stage_1W_vs_E10	ordinary_muscle_development	ENSGALG00010006939	FBXO31	1.416960558	0.127082498	11.14992686	7.16653E-29	3.85021E-28	positive
PRJNA729271	stage_1W_vs_E10	ordinary_muscle_development	ENSGALG00010006942	ALOX5AP	-0.430676522	0.189732157	-2.269918443	0.023212532	0.030141149	negative
PRJNA729271	stage_1W_vs_E10	ordinary_muscle_development	ENSGALG00010006944	MAB21L2	-6.091277624	0.583916644	-10.43175887	1.7757E-25	8.33242E-25	negative
PRJNA729271	stage_1W_vs_E10	ordinary_muscle_development	ENSGALG00010006946	PLPPR1	-6.593184741	0.500390024	-13.1760915	1.20472E-39	9.53712E-39	negative
PRJNA729271	stage_1W_vs_E10	ordinary_muscle_development	ENSGALG00010006948	ENSGALG00010006948	-9.211424919	0.476238864	-19.34202689	2.37937E-83	6.15873E-82	negative
PRJNA729271	stage_1W_vs_E10	ordinary_muscle_development	ENSGALG00010006953	MAP1LC3B	1.78095806	0.127744593	13.94155338	3.54147E-44	3.24076E-43	positive
PRJNA729271	stage_1W_vs_E10	ordinary_muscle_development	ENSGALG00010006957	PALM2AKAP2	0.126078356	0.092326863	1.365565252	0.172075459	0.199954608	positive
PRJNA729271	stage_1W_vs_E10	ordinary_muscle_development	ENSGALG00010006958	KIF26B	-5.275464574	0.255259694	-20.66704891	6.85836E-95	2.3012E-93	negative
PRJNA729271	stage_1W_vs_E10	ordinary_muscle_development	ENSGALG00010006961	ZCCHC14	0.48130329	0.117603188	4.09260411	4.26556E-05	7.05652E-05	positive
PRJNA729271	stage_1W_vs_E10	ordinary_muscle_development	ENSGALG00010006963	ENSGALG00010006963	0.900155509	0.426792323	2.109118325	0.03493437	0.044426539	positive
PRJNA729271	stage_1W_vs_E10	ordinary_muscle_development	ENSGALG00010006966	PGBD5	-3.571530964	0.297333378	-12.01187364	3.07791E-33	1.96626E-32	negative
PRJNA729271	stage_1W_vs_E10	ordinary_muscle_development	ENSGALG00010006983	GTPBP2	0.678319586	0.124417338	5.451969922	4.98149E-08	1.01657E-07	positive
PRJNA729271	stage_1W_vs_E10	ordinary_muscle_development	ENSGALG00010006988	USPL1	0.056111824	0.104429815	0.537316132	0.591049264	0.62580417	positive
PRJNA729271	stage_1W_vs_E10	ordinary_muscle_development	ENSGALG00010006992	SLC35B4	-1.98667599	0.106694312	-18.62026157	2.20136E-77	4.88648E-76	negative
PRJNA729271	stage_1W_vs_E10	ordinary_muscle_development	ENSGALG00010006998	POLH	1.066100582	0.120243303	8.866195085	7.56878E-19	2.68896E-18	positive
PRJNA729271	stage_1W_vs_E10	ordinary_muscle_development	ENSGALG00010007000	LRGUK	-3.538086461	0.765027955	-4.624780621	3.74995E-06	6.71139E-06	negative
PRJNA729271	stage_1W_vs_E10	ordinary_muscle_development	ENSGALG00010007001	SNX30	-1.366752839	0.094509424	-14.46155079	2.11968E-47	2.12628E-46	negative
PRJNA729271	stage_1W_vs_E10	ordinary_muscle_development	ENSGALG00010007002	EXOC4	0.050333976	0.119790454	0.420183529	0.674351387	0.704132056	positive
PRJNA729271	stage_1W_vs_E10	ordinary_muscle_development	ENSGALG00010007004	ENSGALG00010007004	-0.372843533	0.633383844	-0.588653369	0.556093825	0.592125436	negative
PRJNA729271	stage_1W_vs_E10	ordinary_muscle_development	ENSGALG00010007007	ACSL1	1.845405111	0.216894419	8.508310717	1.76486E-17	5.85124E-17	positive
PRJNA729271	stage_1W_vs_E10	ordinary_muscle_development	ENSGALG00010007010	ENSGALG00010007010	0.836567161	1.007311151	0.830495285	0.406258812	0.444364736	positive
PRJNA729271	stage_1W_vs_E10	ordinary_muscle_development	ENSGALG00010007011	HMGB1	-1.811423592	0.101986208	-17.76145644	1.4054E-70	2.69818E-69	negative
PRJNA729271	stage_1W_vs_E10	ordinary_muscle_development	ENSGALG00010007014	ENSGALG00010007014	-0.498039275	0.624760211	-0.797168683	0.425353071	0.463361849	negative
PRJNA729271	stage_1W_vs_E10	ordinary_muscle_development	ENSGALG00010007017	SRD5A2	1.098546625	0.341894924	3.213111826	0.001313052	0.001932644	positive
PRJNA729271	stage_1W_vs_E10	ordinary_muscle_development	ENSGALG00010007021	SULT6B1	0.06330862	0.247096816	0.256209778	0.797788859	0.819208664	positive
PRJNA729271	stage_1W_vs_E10	ordinary_muscle_development	ENSGALG00010007025	ENSGALG00010007025	1.170824982	0.771733289	1.517136811	0.129232146	0.152915915	positive
PRJNA729271	stage_1W_vs_E10	ordinary_muscle_development	ENSGALG00010007028	KATNAL1	-0.670561459	0.142407223	-4.70876016	2.49228E-06	4.52418E-06	negative
PRJNA729271	stage_1W_vs_E10	ordinary_muscle_development	ENSGALG00010007033	CEBPZOS	1.435130087	0.185387703	7.741236683	9.84544E-15	2.89003E-14	positive
PRJNA729271	stage_1W_vs_E10	ordinary_muscle_development	ENSGALG00010007038	CENPU	-2.135929603	0.209335858	-10.2033623	1.91525E-24	8.58958E-24	negative
PRJNA729271	stage_1W_vs_E10	ordinary_muscle_development	ENSGALG00010007041	NAE1	-0.187284675	0.079668476	-2.350800255	0.018733087	0.024535655	negative
PRJNA729271	stage_1W_vs_E10	ordinary_muscle_development	ENSGALG00010007049	ENSGALG00010007049	-0.723393221	0.534680997	-1.352943578	0.176073656	0.204302999	negative
PRJNA729271	stage_1W_vs_E10	ordinary_muscle_development	ENSGALG00010007051	E2F3	-1.671972872	0.154731881	-10.80561329	3.23786E-27	1.63074E-26	negative
PRJNA729271	stage_1W_vs_E10	ordinary_muscle_development	ENSGALG00010007066	INIP	-1.059714714	0.156116413	-6.787977587	1.13716E-11	2.85077E-11	negative
PRJNA729271	stage_1W_vs_E10	ordinary_muscle_development	ENSGALG00010007067	CASP3	-1.590884381	0.109033311	-14.59081052	3.21357E-48	3.30594E-47	negative
PRJNA729271	stage_1W_vs_E10	ordinary_muscle_development	ENSGALG00010007068	NCAM2	-4.212607366	0.278834163	-15.10793126	1.43577E-51	1.66093E-50	negative
PRJNA729271	stage_1W_vs_E10	ordinary_muscle_development	ENSGALG00010007070	UBL3	0.970974004	0.052406197	18.52784707	1.23115E-76	2.69164E-75	positive
PRJNA729271	stage_1W_vs_E10	ordinary_muscle_development	ENSGALG00010007085	PRIMPOL	-0.358273575	0.168899522	-2.121223139	0.033903029	0.043156618	negative
PRJNA729271	stage_1W_vs_E10	ordinary_muscle_development	ENSGALG00010007090	CEBPZ	0.153491913	0.092836701	1.653353813	0.098258877	0.118343367	positive
PRJNA729271	stage_1W_vs_E10	ordinary_muscle_development	ENSGALG00010007092	DYNC1LI2	0.142138344	0.061570987	2.308527932	0.020969791	0.02734657	positive
PRJNA729271	stage_1W_vs_E10	ordinary_muscle_development	ENSGALG00010007097	ZCCHC9	-0.176381249	0.168712953	-1.045451733	0.295814238	0.330933972	negative
PRJNA729271	stage_1W_vs_E10	ordinary_muscle_development	ENSGALG00010007098	NDUFAF7	0.94241306	0.126780309	7.433434013	1.05814E-13	2.9377E-13	positive
PRJNA729271	stage_1W_vs_E10	ordinary_muscle_development	ENSGALG00010007102	CMTM3	-1.61285124	0.165947057	-9.719071055	2.50052E-22	1.02706E-21	negative
PRJNA729271	stage_1W_vs_E10	ordinary_muscle_development	ENSGALG00010007106	SLC7A1	-1.495518117	0.241254945	-6.198911774	5.68549E-10	1.29476E-09	negative
PRJNA729271	stage_1W_vs_E10	ordinary_muscle_development	ENSGALG00010007108	MBOAT1	-0.306657438	0.191574148	-1.600724532	0.109437945	0.130839906	negative
PRJNA729271	stage_1W_vs_E10	ordinary_muscle_development	ENSGALG00010007110	ARL6	-0.319548272	0.103028628	-3.101548375	0.001925114	0.002794645	negative
PRJNA729271	stage_1W_vs_E10	ordinary_muscle_development	ENSGALG00010007114	CKLF	-0.257615522	0.151598268	-1.699330248	0.089256976	0.108076174	negative
PRJNA729271	stage_1W_vs_E10	ordinary_muscle_development	ENSGALG00010007115	ENSGALG00010007115	1.131493993	0.277958447	4.070730735	4.68659E-05	7.72219E-05	positive
PRJNA729271	stage_1W_vs_E10	ordinary_muscle_development	ENSGALG00010007118	ENSGALG00010007118	-1.303736077	0.137894212	-9.454610608	3.24226E-21	1.28196E-20	negative
PRJNA729271	stage_1W_vs_E10	ordinary_muscle_development	ENSGALG00010007123	ENSGALG00010007123	2.829047098	0.243016461	11.64138055	2.53873E-31	1.51125E-30	positive
PRJNA729271	stage_1W_vs_E10	ordinary_muscle_development	ENSGALG00010007126	ENSGALG00010007126	2.082217237	1.07823096	1.931142134	0.053465478	0.066516359	positive
PRJNA729271	stage_1W_vs_E10	ordinary_muscle_development	ENSGALG00010007130	IRF2	1.825706271	0.121802564	14.9890627	8.65662E-51	9.7345E-50	positive
PRJNA729271	stage_1W_vs_E10	ordinary_muscle_development	ENSGALG00010007133	BLVRA	1.005609055	0.188291999	5.340689259	9.25939E-08	1.8561E-07	positive
PRJNA729271	stage_1W_vs_E10	ordinary_muscle_development	ENSGALG00010007135	TK2	-1.034524776	0.118883503	-8.702004483	3.26073E-18	1.12001E-17	negative
PRJNA729271	stage_1W_vs_E10	ordinary_muscle_development	ENSGALG00010007137	ENSGALG00010007137	1.915234004	0.359079764	5.333728589	9.62163E-08	1.92579E-07	positive
PRJNA729271	stage_1W_vs_E10	ordinary_muscle_development	ENSGALG00010007141	PRKD3	-0.263740902	0.083268593	-3.16735148	0.001538343	0.002251896	negative
PRJNA729271	stage_1W_vs_E10	ordinary_muscle_development	ENSGALG00010007143	CKMT2	11.7426673	0.316764606	37.070642	8.3522E-301	3.0266E-298	positive
PRJNA729271	stage_1W_vs_E10	ordinary_muscle_development	ENSGALG00010007146	ENSGALG00010007146	1.478437144	0.141713753	10.43255938	1.7608E-25	8.26518E-25	positive
PRJNA729271	stage_1W_vs_E10	ordinary_muscle_development	ENSGALG00010007155	FOXL1	-2.303055288	0.768305538	-2.997577361	0.002721348	0.003892051	negative
PRJNA729271	stage_1W_vs_E10	ordinary_muscle_development	ENSGALG00010007160	ENSGALG00010007160	1.573145124	0.293857625	5.353426251	8.63042E-08	1.73434E-07	positive
PRJNA729271	stage_1W_vs_E10	ordinary_muscle_development	ENSGALG00010007162	ENSGALG00010007162	2.246370274	0.411287305	5.461803086	4.71323E-08	9.62771E-08	positive
PRJNA729271	stage_1W_vs_E10	ordinary_muscle_development	ENSGALG00010007165	ENSGALG00010007165	0.596877224	0.326543541	1.827864125	0.067569946	0.08300927	positive
PRJNA729271	stage_1W_vs_E10	ordinary_muscle_development	ENSGALG00010007166	RASGRF2	1.232970136	0.186443636	6.613098537	3.76358E-11	9.18402E-11	positive
PRJNA729271	stage_1W_vs_E10	ordinary_muscle_development	ENSGALG00010007168	MINA	0.007725151	0.107378491	0.071943191	0.942647118	0.949921439	positive
PRJNA729271	stage_1W_vs_E10	ordinary_muscle_development	ENSGALG00010007169	QPCT	0.316493051	0.218377161	1.449295563	0.14725506	0.172914946	positive
PRJNA729271	stage_1W_vs_E10	ordinary_muscle_development	ENSGALG00010007175	FOXC2	-3.881580714	0.492414047	-7.882757891	3.20233E-15	9.61429E-15	negative
PRJNA729271	stage_1W_vs_E10	ordinary_muscle_development	ENSGALG00010007181	GJB2	2.264344952	0.5772561	3.922600301	8.75984E-05	0.00014157	positive
PRJNA729271	stage_1W_vs_E10	ordinary_muscle_development	ENSGALG00010007182	ENSGALG00010007182	1.345505966	0.293252298	4.588219679	4.47042E-06	7.9576E-06	positive
PRJNA729271	stage_1W_vs_E10	ordinary_muscle_development	ENSGALG00010007183	VOPP1	1.976441566	0.314247094	6.289450569	3.18592E-10	7.35465E-10	positive
PRJNA729271	stage_1W_vs_E10	ordinary_muscle_development	ENSGALG00010007184	VGLL3	0.013355576	0.09426313	0.141683981	0.887329628	0.899996009	positive
PRJNA729271	stage_1W_vs_E10	ordinary_muscle_development	ENSGALG00010007187	ENPP6	-1.292197062	0.281729242	-4.586662897	4.50387E-06	8.01517E-06	negative
PRJNA729271	stage_1W_vs_E10	ordinary_muscle_development	ENSGALG00010007188	MAB21L1	-1.982549863	0.222148937	-8.92441751	4.48051E-19	1.61555E-18	negative
PRJNA729271	stage_1W_vs_E10	ordinary_muscle_development	ENSGALG00010007190	ENSGALG00010007190	-0.668491313	0.150288799	-4.448044806	8.66555E-06	1.51041E-05	negative
PRJNA729271	stage_1W_vs_E10	ordinary_muscle_development	ENSGALG00010007201	ENSGALG00010007201	2.06194341	0.257077569	8.020705271	1.0514E-15	3.23155E-15	positive
PRJNA729271	stage_1W_vs_E10	ordinary_muscle_development	ENSGALG00010007203	SLC30A6	0.489640816	0.104539189	4.683801545	2.81603E-06	5.08956E-06	positive
PRJNA729271	stage_1W_vs_E10	ordinary_muscle_development	ENSGALG00010007204	LANCL2	-0.90407584	0.087535452	-10.32811074	5.25861E-25	2.41903E-24	negative
PRJNA729271	stage_1W_vs_E10	ordinary_muscle_development	ENSGALG00010007207	EPM2A	1.888061893	0.102727019	18.37940891	1.9205E-75	4.08176E-74	positive
PRJNA729271	stage_1W_vs_E10	ordinary_muscle_development	ENSGALG00010007214	MTHFSD	1.070981305	0.139237699	7.691748087	1.45138E-14	4.21768E-14	positive
PRJNA729271	stage_1W_vs_E10	ordinary_muscle_development	ENSGALG00010007217	MSH3	-0.160966449	0.143765767	-1.119643798	0.262865584	0.296631891	negative
PRJNA729271	stage_1W_vs_E10	ordinary_muscle_development	ENSGALG00010007223	FOXF1	-1.01068776	0.507058619	-1.993236525	0.046235553	0.057974424	negative
PRJNA729271	stage_1W_vs_E10	ordinary_muscle_development	ENSGALG00010007224	DHFR	-3.155514077	0.219835055	-14.35400771	1.00554E-46	9.90174E-46	negative
PRJNA729271	stage_1W_vs_E10	ordinary_muscle_development	ENSGALG00010007226	ENSGALG00010007226	3.645581934	1.484383495	2.455956933	0.014051	0.018697259	positive
PRJNA729271	stage_1W_vs_E10	ordinary_muscle_development	ENSGALG00010007230	ENSGALG00010007230	-0.429285823	0.347387311	-1.235755622	0.216549415	0.247702318	negative
PRJNA729271	stage_1W_vs_E10	ordinary_muscle_development	ENSGALG00010007243	BEAN1	-1.131306308	0.190459828	-5.939868366	2.85251E-09	6.26072E-09	negative
PRJNA729271	stage_1W_vs_E10	ordinary_muscle_development	ENSGALG00010007251	SPAST	-0.487980706	0.064119269	-7.610515704	2.73004E-14	7.82984E-14	negative
PRJNA729271	stage_1W_vs_E10	ordinary_muscle_development	ENSGALG00010007252	NFATC1	1.08339012	0.153040202	7.079121091	1.45072E-12	3.81013E-12	positive
PRJNA729271	stage_1W_vs_E10	ordinary_muscle_development	ENSGALG00010007259	ANKRD34B	-1.477733437	0.097808426	-15.1084472	1.42457E-51	1.6493E-50	negative
PRJNA729271	stage_1W_vs_E10	ordinary_muscle_development	ENSGALG00010007265	CD200R1A	-1.633350907	0.316245175	-5.164824755	2.40664E-07	4.68938E-07	negative
PRJNA729271	stage_1W_vs_E10	ordinary_muscle_development	ENSGALG00010007270	CDH5	0.449470269	0.108437604	4.144966811	3.39863E-05	5.67221E-05	positive
PRJNA729271	stage_1W_vs_E10	ordinary_muscle_development	ENSGALG00010007271	CADM2	-2.976815311	0.312607019	-9.522547894	1.68984E-21	6.75145E-21	negative
PRJNA729271	stage_1W_vs_E10	ordinary_muscle_development	ENSGALG00010007275	DPY30	0.484012206	0.098129756	4.932369406	8.12381E-07	1.53008E-06	positive
PRJNA729271	stage_1W_vs_E10	ordinary_muscle_development	ENSGALG00010007277	FAM151B	-0.348523599	0.188265677	-1.851232813	0.064136067	0.078984902	negative
PRJNA729271	stage_1W_vs_E10	ordinary_muscle_development	ENSGALG00010007283	STOX2	-0.949976173	0.123056017	-7.719867708	1.16451E-14	3.40587E-14	negative
PRJNA729271	stage_1W_vs_E10	ordinary_muscle_development	ENSGALG00010007284	ENSGALG00010007284	-0.179834626	0.082730518	-2.17373987	0.029724677	0.038112268	negative
PRJNA729271	stage_1W_vs_E10	ordinary_muscle_development	ENSGALG00010007287	ENSGALG00010007287	-0.045527884	0.620620624	-0.073358638	0.941520731	0.9489183	negative
PRJNA729271	stage_1W_vs_E10	ordinary_muscle_development	ENSGALG00010007294	TRAPPC11	0.738159971	0.064135583	11.50936715	1.18344E-30	6.86981E-30	positive
PRJNA729271	stage_1W_vs_E10	ordinary_muscle_development	ENSGALG00010007300	CCDC125	0.278114089	0.257312265	1.080842723	0.27976708	0.314212291	positive
PRJNA729271	stage_1W_vs_E10	ordinary_muscle_development	ENSGALG00010007302	CDH11	-3.333117877	0.175533279	-18.98852402	2.1221E-80	5.1181E-79	negative
PRJNA729271	stage_1W_vs_E10	ordinary_muscle_development	ENSGALG00010007304	F5	1.205446902	0.355871784	3.387306764	0.000705824	0.001065055	positive
PRJNA729271	stage_1W_vs_E10	ordinary_muscle_development	ENSGALG00010007318	RMDN2	1.380058248	0.12340743	11.1829429	4.94234E-29	2.6741E-28	positive
PRJNA729271	stage_1W_vs_E10	ordinary_muscle_development	ENSGALG00010007319	ATP9B	0.192605155	0.101870475	1.890686722	0.058666176	0.072595422	positive
PRJNA729271	stage_1W_vs_E10	ordinary_muscle_development	ENSGALG00010007320	ENSGALG00010007320	1.790256639	0.30277504	5.91282768	3.36284E-09	7.34323E-09	positive
PRJNA729271	stage_1W_vs_E10	ordinary_muscle_development	ENSGALG00010007321	CD200	1.8049589	0.150585051	11.98630868	4.19172E-33	2.65787E-32	positive
PRJNA729271	stage_1W_vs_E10	ordinary_muscle_development	ENSGALG00010007322	AK6	0.013476664	0.108043436	0.124733763	0.900734327	0.912251542	positive
PRJNA729271	stage_1W_vs_E10	ordinary_muscle_development	ENSGALG00010007324	ENSGALG00010007324	-0.133252749	0.142491576	-0.935162291	0.349704669	0.387298989	negative
PRJNA729271	stage_1W_vs_E10	ordinary_muscle_development	ENSGALG00010007326	RWDD4	1.223972031	0.115562233	10.59145365	3.26482E-26	1.57903E-25	positive
PRJNA729271	stage_1W_vs_E10	ordinary_muscle_development	ENSGALG00010007328	NOVA1	0.188891353	0.137884419	1.369925297	0.170710222	0.198495481	positive
PRJNA729271	stage_1W_vs_E10	ordinary_muscle_development	ENSGALG00010007330	MMP13	-1.733540417	0.713866411	-2.428382103	0.015166355	0.020081878	negative
PRJNA729271	stage_1W_vs_E10	ordinary_muscle_development	ENSGALG00010007333	QTRT2	-1.677488077	0.076944162	-21.80136922	2.2518E-105	9.3256E-104	negative
PRJNA729271	stage_1W_vs_E10	ordinary_muscle_development	ENSGALG00010007337	PRORP	-0.749437087	0.101568337	-7.378648828	1.59904E-13	4.39645E-13	negative
PRJNA729271	stage_1W_vs_E10	ordinary_muscle_development	ENSGALG00010007339	ENSGALG00010007339	1.125410399	0.223558538	5.034074787	4.80162E-07	9.16386E-07	positive
PRJNA729271	stage_1W_vs_E10	ordinary_muscle_development	ENSGALG00010007344	RAD17	0.341917709	0.107597986	3.177733346	0.001484312	0.00217632	positive

PRJNA729271	stage_1W_vs_E10	ordinary_muscle_development	ENSGALG00010007353	ING2	-0.862866472	0.127237465	-6.781544019	1.18898E-11	2.97811E-11	negative
PRJNA729271	stage_1W_vs_E10	ordinary_muscle_development	ENSGALG00010007357	NOX4	0.3579929	0.118720071	3.015437039	0.002566092	0.003681994	positive
PRJNA729271	stage_1W_vs_E10	ordinary_muscle_development	ENSGALG00010007360	ROBO1	-3.791339137	0.18826111	-20.13872723	3.37908E-90	9.93505E-89	negative
PRJNA729271	stage_1W_vs_E10	ordinary_muscle_development	ENSGALG00010007365	MEMO1	2.646655199	0.086217584	30.69739456	6.1661E-207	1.0042E-204	positive
PRJNA729271	stage_1W_vs_E10	ordinary_muscle_development	ENSGALG00010007369	ENSGALG00010007369	0.182195548	0.211779877	0.860306233	0.389620258	0.427594865	positive
PRJNA729271	stage_1W_vs_E10	ordinary_muscle_development	ENSGALG00010007374	CMIP	-0.733035707	0.136133146	-5.384696731	7.25669E-08	1.46498E-07	negative
PRJNA729271	stage_1W_vs_E10	ordinary_muscle_development	ENSGALG00010007375	CHORDC1	-0.40717967	0.075152751	-5.418027475	6.02602E-08	1.22352E-07	negative
PRJNA729271	stage_1W_vs_E10	ordinary_muscle_development	ENSGALG00010007376	CDKN2AIP	-0.693216936	0.06141897	-11.28669095	1.52677E-29	8.46292E-29	negative
PRJNA729271	stage_1W_vs_E10	ordinary_muscle_development	ENSGALG00010007381	EGFR	-0.519524299	0.098402562	-5.279581045	1.2948E-07	2.56605E-07	negative
PRJNA729271	stage_1W_vs_E10	ordinary_muscle_development	ENSGALG00010007382	EIF2AK2	0.477325354	0.257814115	1.851432198	0.064107401	0.078956307	positive
PRJNA729271	stage_1W_vs_E10	ordinary_muscle_development	ENSGALG00010007387	ENSGALG00010007387	-2.813092131	0.560333525	-5.020388757	5.1567E-07	9.81955E-07	negative
PRJNA729271	stage_1W_vs_E10	ordinary_muscle_development	ENSGALG00010007388	CYP1B1	-3.529731097	0.20510542	-17.20935066	2.25951E-66	3.83958E-65	negative
PRJNA729271	stage_1W_vs_E10	ordinary_muscle_development	ENSGALG00010007391	NANP	-0.006957318	0.13951504	-0.049867871	0.960227681	0.965447342	negative
PRJNA729271	stage_1W_vs_E10	ordinary_muscle_development	ENSGALG00010007392	NAALAD2	2.151683072	0.200148004	10.75045978	5.89669E-27	2.9372E-26	positive
PRJNA729271	stage_1W_vs_E10	ordinary_muscle_development	ENSGALG00010007393	PLCG2	0.315703364	0.130617025	2.417015419	0.015648354	0.020691744	positive
PRJNA729271	stage_1W_vs_E10	ordinary_muscle_development	ENSGALG00010007399	WWC2	-0.744389276	0.109823906	-6.778025859	1.21829E-11	3.04993E-11	negative
PRJNA729271	stage_1W_vs_E10	ordinary_muscle_development	ENSGALG00010007401	SEC61G	-0.645141684	0.220544411	-2.925223456	0.00344209	0.004879997	negative
PRJNA729271	stage_1W_vs_E10	ordinary_muscle_development	ENSGALG00010007403	SDR42E1	-0.720835211	0.23237202	-3.102074036	0.001921699	0.002790246	negative
PRJNA729271	stage_1W_vs_E10	ordinary_muscle_development	ENSGALG00010007404	FOLH1	1.206813598	0.171957753	7.018081938	2.24935E-12	5.8504E-12	positive
PRJNA729271	stage_1W_vs_E10	ordinary_muscle_development	ENSGALG00010007408	HCLS1	1.067426797	0.249647491	4.275736127	1.90507E-05	3.23159E-05	positive
PRJNA729271	stage_1W_vs_E10	ordinary_muscle_development	ENSGALG00010007411	ENSGALG00010007411	1.409094995	0.57872812	2.434813421	0.01489947	0.019748338	positive
PRJNA729271	stage_1W_vs_E10	ordinary_muscle_development	ENSGALG00010007413	GPATCH11	-0.211926731	0.09874009	-2.146308868	0.031848345	0.040669699	negative
PRJNA729271	stage_1W_vs_E10	ordinary_muscle_development	ENSGALG00010007414	ROBO2	-4.931905374	0.243085606	-20.28875942	1.61622E-91	5.02728E-90	negative
PRJNA729271	stage_1W_vs_E10	ordinary_muscle_development	ENSGALG00010007416	DCTD	-2.617846106	0.253250817	-10.33697003	4.79458E-25	2.21048E-24	negative
PRJNA729271	stage_1W_vs_E10	ordinary_muscle_development	ENSGALG00010007418	NME6	0.67822141	0.145944905	4.647105786	3.36625E-06	6.05231E-06	positive
PRJNA729271	stage_1W_vs_E10	ordinary_muscle_development	ENSGALG00010007419	RABL2A	-0.595630259	0.155371858	-3.833578792	0.000126292	0.00020152	negative
PRJNA729271	stage_1W_vs_E10	ordinary_muscle_development	ENSGALG00010007423	HEATR5B	-0.558638505	0.066696903	-8.375778759	5.48599E-17	1.77776E-16	negative
PRJNA729271	stage_1W_vs_E10	ordinary_muscle_development	ENSGALG00010007425	ARSA	-0.04974318	0.130319328	-0.381702241	0.702682238	0.730868186	negative
PRJNA729271	stage_1W_vs_E10	ordinary_muscle_development	ENSGALG00010007429	C21orf91	0.63381731	0.208785895	3.035728584	0.002399553	0.003449863	positive
PRJNA729271	stage_1W_vs_E10	ordinary_muscle_development	ENSGALG00010007430	MEF2C	0.370790508	0.147014104	2.522142414	0.011664247	0.015643344	positive
PRJNA729271	stage_1W_vs_E10	ordinary_muscle_development	ENSGALG00010007432	NAT	1.521493968	0.189701381	8.020468601	1.05343E-15	3.2371E-15	positive
PRJNA729271	stage_1W_vs_E10	ordinary_muscle_development	ENSGALG00010007433	RNF144B	2.780801177	0.268920876	10.34059245	4.61674E-25	2.13052E-24	positive
PRJNA729271	stage_1W_vs_E10	ordinary_muscle_development	ENSGALG00010007434	ENSGALG00010007434	0.653332126	0.250717552	2.605849173	0.009164681	0.012445386	positive
PRJNA729271	stage_1W_vs_E10	ordinary_muscle_development	ENSGALG00010007436	PNAT3	1.406531531	0.264294533	5.321833627	1.02727E-07	2.05128E-07	positive
PRJNA729271	stage_1W_vs_E10	ordinary_muscle_development	ENSGALG00010007437	TUSC1	0.231442983	0.138895094	1.666315036	0.095650684	0.115326623	positive
PRJNA729271	stage_1W_vs_E10	ordinary_muscle_development	ENSGALG00010007439	HSD17B2	-5.004153781	0.361162804	-13.85567319	1.1755E-43	1.05438E-42	negative
PRJNA729271	stage_1W_vs_E10	ordinary_muscle_development	ENSGALG00010007441	STRN	1.584636291	0.13522491	11.71852357	1.02442E-31	6.19997E-31	positive
PRJNA729271	stage_1W_vs_E10	ordinary_muscle_development	ENSGALG00010007455	TRPC6	1.053535242	0.20914736	5.03728682	4.72177E-07	9.01859E-07	positive
PRJNA729271	stage_1W_vs_E10	ordinary_muscle_development	ENSGALG00010007457	SHANK3	0.301676364	0.179199926	1.683462552	0.092285583	0.111556961	positive
PRJNA729271	stage_1W_vs_E10	ordinary_muscle_development	ENSGALG00010007465	PNAT10	1.041380364	0.200596895	5.191408186	2.0871E-07	4.08265E-07	positive
PRJNA729271	stage_1W_vs_E10	ordinary_muscle_development	ENSGALG00010007467	CHODL	-2.599279467	0.332839664	-7.809404184	5.7459E-15	1.70495E-14	negative
PRJNA729271	stage_1W_vs_E10	ordinary_muscle_development	ENSGALG00010007479	NFX1	-0.363054479	0.097310841	-3.730873933	0.000190817	0.00030064	negative
PRJNA729271	stage_1W_vs_E10	ordinary_muscle_development	ENSGALG00010007480	TENM3	-0.070149314	0.105742003	-0.663400655	0.507073987	0.544689302	negative
PRJNA729271	stage_1W_vs_E10	ordinary_muscle_development	ENSGALG00010007485	CTSC	1.118324537	0.155449017	7.194156362	6.28481E-13	1.67892E-12	positive
PRJNA729271	stage_1W_vs_E10	ordinary_muscle_development	ENSGALG00010007500	MPHOSPH6	0.532200453	0.116430426	4.570974042	4.85462E-06	8.614E-06	positive
PRJNA729271	stage_1W_vs_E10	ordinary_muscle_development	ENSGALG00010007506	RAB38	-0.208209581	0.555345392	-0.374919076	0.707720652	0.735475398	negative
PRJNA729271	stage_1W_vs_E10	ordinary_muscle_development	ENSGALG00010007513	GALNT12	-0.680896387	0.131196574	-5.189894593	2.10413E-07	4.11487E-07	negative
PRJNA729271	stage_1W_vs_E10	ordinary_muscle_development	ENSGALG00010007514	VIT	-4.13246637	0.373676886	-11.05892959	1.98452E-28	1.05022E-27	negative
PRJNA729271	stage_1W_vs_E10	ordinary_muscle_development	ENSGALG00010007521	SLC12A7	-0.906313808	0.138470687	-6.54516725	5.94288E-11	1.42903E-10	negative
PRJNA729271	stage_1W_vs_E10	ordinary_muscle_development	ENSGALG00010007529	CDH13	2.247348699	0.134271226	16.73738129	7.00007E-63	1.09812E-61	positive
PRJNA729271	stage_1W_vs_E10	ordinary_muscle_development	ENSGALG00010007532	DCUN1D5	1.407540063	0.265808021	5.295325775	1.18804E-07	2.36159E-07	positive
PRJNA729271	stage_1W_vs_E10	ordinary_muscle_development	ENSGALG00010007536	GABBR2	-1.263296657	0.213412296	-5.91951206	3.22898E-09	7.05731E-09	negative
PRJNA729271	stage_1W_vs_E10	ordinary_muscle_development	ENSGALG00010007539	RPS3A	0.128318326	0.175354864	0.731763711	0.464312802	0.502554814	positive
PRJNA729271	stage_1W_vs_E10	ordinary_muscle_development	ENSGALG00010007542	MRPL36	1.511583904	0.109531697	13.80042435	2.53357E-43	2.25578E-42	positive
PRJNA729271	stage_1W_vs_E10	ordinary_muscle_development	ENSGALG00010007544	MLYCD	0.841227166	0.102055565	8.242834813	1.68178E-16	5.34826E-16	positive
PRJNA729271	stage_1W_vs_E10	ordinary_muscle_development	ENSGALG00010007547	ENSGALG00010007547	1.349666716	0.563737433	2.39414067	0.016659358	0.021954486	positive
PRJNA729271	stage_1W_vs_E10	ordinary_muscle_development	ENSGALG00010007548	HSBP1	-0.572527345	0.087701577	-6.52813055	6.65956E-11	1.59475E-10	negative
PRJNA729271	stage_1W_vs_E10	ordinary_muscle_development	ENSGALG00010007549	SH3D19	-0.157910976	0.15848478	-0.996379437	0.31906582	0.355293775	negative
PRJNA729271	stage_1W_vs_E10	ordinary_muscle_development	ENSGALG00010007551	FEZ2	0.138695305	0.111466902	1.244273435	0.213398945	0.244407214	positive
PRJNA729271	stage_1W_vs_E10	ordinary_muscle_development	ENSGALG00010007555	NDUFS6	2.314198772	0.129219083	17.90911004	1.00124E-71	1.97993E-70	positive
PRJNA729271	stage_1W_vs_E10	ordinary_muscle_development	ENSGALG00010007564	IRX4	-9.364572528	0.947294196	-9.885601085	4.80688E-23	2.03492E-22	negative
PRJNA729271	stage_1W_vs_E10	ordinary_muscle_development	ENSGALG00010007566	FHIP1A	0.648386233	0.17306813	3.746421915	0.000179375	0.000283321	positive
PRJNA729271	stage_1W_vs_E10	ordinary_muscle_development	ENSGALG00010007567	TRIP13	-2.825894385	0.146904606	-19.23625443	1.84036E-82	4.68001E-81	negative
PRJNA729271	stage_1W_vs_E10	ordinary_muscle_development	ENSGALG00010007569	GPR89A	-0.079024855	0.08258029	-0.956945715	0.338594655	0.375626016	negative
PRJNA729271	stage_1W_vs_E10	ordinary_muscle_development	ENSGALG00010007574	ENSGALG00010007574	0.333864594	0.189306113	1.763622894	0.077795491	0.094799969	positive
PRJNA729271	stage_1W_vs_E10	ordinary_muscle_development	ENSGALG00010007575	MMP27	-3.744391182	0.357200523	-10.48260275	1.03846E-25	4.9191E-25	negative
PRJNA729271	stage_1W_vs_E10	ordinary_muscle_development	ENSGALG00010007577	WFDC1	-0.039337468	0.119801568	-0.328355201	0.742643111	0.767968317	negative
PRJNA729271	stage_1W_vs_E10	ordinary_muscle_development	ENSGALG00010007579	GATB	1.182336384	0.178251968	6.632949941	3.29043E-11	8.0484E-11	positive
PRJNA729271	stage_1W_vs_E10	ordinary_muscle_development	ENSGALG00010007580	BRD9	-1.05955085	0.104031685	-10.18488596	2.31631E-24	1.03499E-23	negative
PRJNA729271	stage_1W_vs_E10	ordinary_muscle_development	ENSGALG00010007581	LPCAT1	-1.170850334	0.120063629	-9.751915225	1.8102E-22	7.47973E-22	negative
PRJNA729271	stage_1W_vs_E10	ordinary_muscle_development	ENSGALG00010007584	ELP2	-0.456273449	0.116123442	-3.92921052	8.52252E-05	0.000137888	negative
PRJNA729271	stage_1W_vs_E10	ordinary_muscle_development	ENSGALG00010007585	KCNG4	-0.989418476	0.633478239	-1.561882342	0.118315709	0.140815026	negative
PRJNA729271	stage_1W_vs_E10	ordinary_muscle_development	ENSGALG00010007586	MMP7	0.670537877	1.577760236	0.42499352	0.670841398	0.700958015	positive
PRJNA729271	stage_1W_vs_E10	ordinary_muscle_development	ENSGALG00010007588	TTC27	-0.985066432	0.104048759	-9.467353972	2.87022E-21	1.13703E-20	negative
PRJNA729271	stage_1W_vs_E10	ordinary_muscle_development	ENSGALG00010007590	FBXW7	-0.74609829	0.140311355	-5.31744767	1.05233E-07	2.09901E-07	negative
PRJNA729271	stage_1W_vs_E10	ordinary_muscle_development	ENSGALG00010007593	SEC61B	-1.448000549	0.119580621	-12.10899001	9.46524E-34	6.14965E-33	negative
PRJNA729271	stage_1W_vs_E10	ordinary_muscle_development	ENSGALG00010007595	ATP2C2	0.379844097	0.164786909	2.305062327	0.021163083	0.027586231	positive
PRJNA729271	stage_1W_vs_E10	ordinary_muscle_development	ENSGALG00010007596	ENSGALG00010007596	-3.086369731	0.514426915	-5.999627233	1.97771E-09	4.38399E-09	negative
PRJNA729271	stage_1W_vs_E10	ordinary_muscle_development	ENSGALG00010007597	SMKR1	-2.661996415	0.915639162	-2.907254872	0.00364616	0.005155198	negative
PRJNA729271	stage_1W_vs_E10	ordinary_muscle_development	ENSGALG00010007601	TMEM154	-0.981416646	0.345838879	-2.837785759	0.004542766	0.006364527	negative
PRJNA729271	stage_1W_vs_E10	ordinary_muscle_development	ENSGALG00010007603	ALG2	-0.217139294	0.105588571	-2.056465873	0.039737632	0.050239597	negative
PRJNA729271	stage_1W_vs_E10	ordinary_muscle_development	ENSGALG00010007604	ENSGALG00010007604	-3.527107034	0.566058845	-6.230990057	4.63497E-10	1.06069E-09	negative
PRJNA729271	stage_1W_vs_E10	ordinary_muscle_development	ENSGALG00010007610	TLDC1	-0.487941116	0.159674484	-3.055849025	0.002244242	0.003235235	negative
PRJNA729271	stage_1W_vs_E10	ordinary_muscle_development	ENSGALG00010007614	BIRC6	0.2648525	0.088001472	3.00963717	0.002615599	0.003747836	positive
PRJNA729271	stage_1W_vs_E10	ordinary_muscle_development	ENSGALG00010007624	TPPP	1.437145468	0.24744979	5.807826577	6.3289E-09	1.35988E-08	positive
PRJNA729271	stage_1W_vs_E10	ordinary_muscle_development	ENSGALG00010007626	DLK2	-3.573147259	0.391674476	-9.122747266	7.32436E-20	2.73414E-19	negative
PRJNA729271	stage_1W_vs_E10	ordinary_muscle_development	ENSGALG00010007633	ENSGALG00010007633	0.554093615	0.102718934	5.394269509	6.88029E-08	1.39074E-07	positive
PRJNA729271	stage_1W_vs_E10	ordinary_muscle_development	ENSGALG00010007640	TMEM123	-1.854094043	0.219467534	-8.448147261	2.95962E-17	9.70581E-17	negative
PRJNA729271	stage_1W_vs_E10	ordinary_muscle_development	ENSGALG00010007642	ZNF318	0.821912168	0.106592334	7.71079998	1.25031E-14	3.65095E-14	positive
PRJNA729271	stage_1W_vs_E10	ordinary_muscle_development	ENSGALG00010007643	COTL1	-1.069229936	0.16419542	-6.511935211	7.41887E-11	1.77248E-10	negative
PRJNA729271	stage_1W_vs_E10	ordinary_muscle_development	ENSGALG00010007650	KLHL36	-0.454129058	0.089409876	-5.079182264	3.79063E-07	7.28812E-07	negative
PRJNA729271	stage_1W_vs_E10	ordinary_muscle_development	ENSGALG00010007651	CTNNAL1	-0.761875142	0.137743454	-5.531116878	3.18198E-08	6.55061E-08	negative
PRJNA729271	stage_1W_vs_E10	ordinary_muscle_development	ENSGALG00010007658	BIRC2	1.096777759	0.118967106	9.219168191	2.99414E-20	1.13583E-19	positive
PRJNA729271	stage_1W_vs_E10	ordinary_muscle_development	ENSGALG00010007660	FAM98A	0.878150753	0.096745011	9.076961589	1.11648E-19	4.13791E-19	positive
PRJNA729271	stage_1W_vs_E10	ordinary_muscle_development	ENSGALG00010007663	ARFIP1	-0.745049348	0.110996663	-6.712358059	1.91504E-11	4.74505E-11	negative
PRJNA729271	stage_1W_vs_E10	ordinary_muscle_development	ENSGALG00010007671	FAM206A	0.347691794	0.101372331	3.429849047	0.000603917	0.000915763	positive
PRJNA729271	stage_1W_vs_E10	ordinary_muscle_development	ENSGALG00010007673	FHDC1	-2.307609732	0.343851975	-6.711055623	1.93221E-11	4.78596E-11	negative
PRJNA729271	stage_1W_vs_E10	ordinary_muscle_development	ENSGALG00010007678	CDH8	0.62400523	0.284064154	2.19670529	0.028041501	0.03603702	positive
PRJNA729271	stage_1W_vs_E10	ordinary_muscle_development	ENSGALG00010007679	TMEM135	1.239329176	0.141605399	8.751990995	2.09621E-18	7.26556E-18	positive
PRJNA729271	stage_1W_vs_E10	ordinary_muscle_development	ENSGALG00010007680	CHMP2B	-0.312557364	0.126180223	-2.477070936	0.013246557	0.017672236	negative
PRJNA729271	stage_1W_vs_E10	ordinary_muscle_development	ENSGALG00010007695	RASGRP3	1.208803893	0.142649821	8.473925056	2.3726E-17	7.81966E-17	positive
PRJNA729271	stage_1W_vs_E10	ordinary_muscle_development	ENSGALG00010007698	ADAMTS18	-2.891994113	0.407375106	-7.099093866	1.25578E-12	3.31195E-12	negative
PRJNA729271	stage_1W_vs_E10	ordinary_muscle_development	ENSGALG00010007699	SLC39A6	-1.797085588	0.108303191	-16.59309916	7.81799E-62	1.19286E-60	negative
PRJNA729271	stage_1W_vs_E10	ordinary_muscle_development	ENSGALG00010007707	ENSGALG00010007707	2.981697683	0.753384886	3.957734935	7.56639E-05	0.000122899	positive
PRJNA729271	stage_1W_vs_E10	ordinary_muscle_development	ENSGALG00010007713	SCLT1	-1.003819921	0.215483938	-4.658444291	3.18608E-06	5.73479E-06	negative
PRJNA729271	stage_1W_vs_E10	ordinary_muscle_development	ENSGALG00010007719	LTBP1	-2.553823099	0.191273617	-13.35167465	1.15816E-40	9.47375E-40	negative
PRJNA729271	stage_1W_vs_E10	ordinary_muscle_development	ENSGALG00010007724	RBM11	1.234668713	0.342999164	3.599626019	0.000318675	0.000494455	positive
PRJNA729271	stage_1W_vs_E10	ordinary_muscle_development	ENSGALG00010007730	COG2	-0.333709783	0.06486613	-5.144592118	2.68103E-07	5.20443E-07	negative
PRJNA729271	stage_1W_vs_E10	ordinary_muscle_development	ENSGALG00010007731	ENSGALG00010007731	-0.42122097	0.271323919	-1.552465299	0.12055093	0.143357595	negative
PRJNA729271	stage_1W_vs_E10	ordinary_muscle_development	ENSGALG00010007741	ENSGALG00010007741	-0.54510258	0.208198337	-2.618188927	0.008839785	0.012022574	negative
PRJNA729271	stage_1W_vs_E10	ordinary_muscle_development	ENSGALG00010007748	CSGALNACT1	-3.609606753	0.276960974	-13.03290749	7.95188E-39	6.12447E-38	negative
PRJNA729271	stage_1W_vs_E10	ordinary_muscle_development	ENSGALG00010007751	HSPA13	-0.952180125	0.100586442	-9.466287008	2.89968E-21	1.14838E-20	negative
PRJNA729271	stage_1W_vs_E10	ordinary_muscle_development	ENSGALG00010007753	PRSS23	0.933061234	0.227704921	4.097677065	4.17317E-05	6.90605E-05	positive
PRJNA729271	stage_1W_vs_E10	ordinary_muscle_development	ENSGALG00010007758	DEK	-0.205512742	0.076773994	-2.676853602	0.00743171	0.010185575	negative
PRJNA729271	stage_1W_vs_E10	ordinary_muscle_development	ENSGALG00010007759	AGT	-1.18352578	1.28706288	-0.919555523			negative
PRJNA729271	stage_1W_vs_E10	ordinary_muscle_development	ENSGALG00010007761	ENSGALG00010007761	1.80924386	0.114901407	15.74605488	7.31018E-56	9.64159E-55	positive
PRJNA729271	stage_1W_vs_E10	ordinary_muscle_development	ENSGALG00010007762	SH2D4A	-0.817282903	0.337492873	-2.421630112	0.015451069	0.020443929	negative
PRJNA729271	stage_1W_vs_E10	ordinary_muscle_development	ENSGALG00010007763	FZD4	0.527104526	0.099854914	5.278703896	1.30101E-07	2.57801E-07	positive
PRJNA729271	stage_1W_vs_E10	ordinary_muscle_development	ENSGALG00010007765	NUDT7	0.596766881	0.09575855	6.231995806	4.6053E-10	1.05406E-09	positive
PRJNA729271	stage_1W_vs_E10	ordinary_muscle_development	ENSGALG00010007766	ENSGALG00010007766	1.946616597	0.127114787	15.31384859	6.17965E-53	7.53356E-52	positive
PRJNA729271	stage_1W_vs_E10	ordinary_muscle_development	ENSGALG00010007767	VAT1L	-2.85988973	0.331258017	-8.633420414	5.95441E-18	2.01798E-17	negative
PRJNA729271	stage_1W_vs_E10	ordinary_muscle_development	ENSGALG00010007770	ENSGALG00010007770	-0.62219214	0.420228993	-1.480602601	0.138712502	0.163506361	negative
PRJNA729271	stage_1W_vs_E10	ordinary_muscle_development	ENSGALG00010007771	CAPN9	-2.928687792	0.605846974	-4.83403882	1.3379E-06	2.47548E-06	negative
PRJNA729271	stage_1W_vs_E10	ordinary_muscle_development	ENSGALG00010007772	CLEC3A	-3.767764649	0.673784018	-5.591947193	2.24537E-08	4.66485E-08	negative
PRJNA729271	stage_1W_vs_E10	ordinary_muscle_development	ENSGALG00010007774	ENSGALG00010007774	0.446652525	0.284982742	1.567296751	0.117045344	0.139406103	positive
PRJNA729271	stage_1W_vs_E10	ordinary_muscle_development	ENSGALG00010007777	KIF13A	0.522988221	0.124754761	4.192130379	2.76347E-05	4.6351E-05	positive
PRJNA729271	stage_1W_vs_E10	ordinary_muscle_development	ENSGALG00010007780	SIRT5	1.167344015	0.141975627	8.222143727	1.99897E-16	6.32783E-16	positive
PRJNA729271	stage_1W_vs_E10	ordinary_muscle_development	ENSGALG00010007781	MBP	4.743429979	0.234934952	20.19039712	1.18905E-90	3.58322E-89	positive
PRJNA729271	stage_1W_vs_E10	ordinary_muscle_development	ENSGALG00010007783	NOL7	-0.16263685	0.109637232	-1.483408942	0.137965793	0.162705571	negative
PRJNA729271	stage_1W_vs_E10	ordinary_muscle_development	ENSGALG00010007784	INTS10	-0.251955341	0.094316185	-2.671390278	0.007553776	0.010346026	negative
PRJNA729271	stage_1W_vs_E10	ordinary_muscle_development	ENSGALG00010007786	SAMSN1	0.551969567	0.234076669	2.358071696	0.018370145	0.024082052	positive

PRJNA729271	stage_1W_vs_E10	ordinary_muscle_development	ENSGALG00010007787	C1orf198	-2.456685662	0.120663666	-20.35977979	3.80289E-92	1.20356E-90	negative
PRJNA729271	stage_1W_vs_E10	ordinary_muscle_development	ENSGALG00010007788	PALM2AKAP2	-0.968739485	0.17431374	-5.557447643	2.73748E-08	5.65642E-08	negative
PRJNA729271	stage_1W_vs_E10	ordinary_muscle_development	ENSGALG00010007789	WWOX	-0.81476996	0.114385801	-7.122999144	1.05603E-12	2.79431E-12	negative
PRJNA729271	stage_1W_vs_E10	ordinary_muscle_development	ENSGALG00010007792	ENSGALG00010007792	1.507703197	0.222630477	6.772222813	1.26819E-11	3.16828E-11	positive
PRJNA729271	stage_1W_vs_E10	ordinary_muscle_development	ENSGALG00010007795	ZNF236	0.026032023	0.116361836	0.223716164	0.822978168	0.841794407	positive
PRJNA729271	stage_1W_vs_E10	ordinary_muscle_development	ENSGALG00010007796	MCUR1	-0.693138538	0.103565648	-6.692745613	2.19022E-11	5.41022E-11	negative
PRJNA729271	stage_1W_vs_E10	ordinary_muscle_development	ENSGALG00010007798	HGSNAT	0.959354158	0.096974048	9.892895837	4.46909E-23	1.89525E-22	positive
PRJNA729271	stage_1W_vs_E10	ordinary_muscle_development	ENSGALG00010007800	MAF	0.460275537	0.114301928	4.026839672	5.65316E-05	9.26218E-05	positive
PRJNA729271	stage_1W_vs_E10	ordinary_muscle_development	ENSGALG00010007801	TTC13	0.535300882	0.159872767	3.348293104	0.00081311	0.001221476	positive
PRJNA729271	stage_1W_vs_E10	ordinary_muscle_development	ENSGALG00010007802	ENSGALG00010007802	5.924423932	0.716095936	8.273226581	1.30382E-16	4.1609E-16	positive
PRJNA729271	stage_1W_vs_E10	ordinary_muscle_development	ENSGALG00010007803	ME3	0.33700654	0.177957341	1.893749024	0.058258331	0.072132443	positive
PRJNA729271	stage_1W_vs_E10	ordinary_muscle_development	ENSGALG00010007805	ENSGALG00010007805	11.75883499	1.515955735	7.756713946	8.71582E-15	2.56367E-14	positive
PRJNA729271	stage_1W_vs_E10	ordinary_muscle_development	ENSGALG00010007806	DYNLRB2	-0.387799061	0.376959921	-1.028754093	0.303595244	0.339004165	negative
PRJNA729271	stage_1W_vs_E10	ordinary_muscle_development	ENSGALG00010007809	ARV1	-0.623989563	0.122701198	-5.085439863	3.66775E-07	7.05842E-07	negative
PRJNA729271	stage_1W_vs_E10	ordinary_muscle_development	ENSGALG00010007811	PRCP	0.237179957	0.183018968	1.295931012	0.194999254	0.2247725	positive
PRJNA729271	stage_1W_vs_E10	ordinary_muscle_development	ENSGALG00010007812	POMK	0.448116843	0.120664334	3.713747297	0.000204213	0.000320978	positive
PRJNA729271	stage_1W_vs_E10	ordinary_muscle_development	ENSGALG00010007817	CDYL2	-1.747921411	0.142850126	-12.23605088	1.99506E-34	1.32229E-33	negative
PRJNA729271	stage_1W_vs_E10	ordinary_muscle_development	ENSGALG00010007818	ENSGALG00010007818	0.575419258	0.223680818	2.572501581	0.010096649	0.01364832	positive
PRJNA729271	stage_1W_vs_E10	ordinary_muscle_development	ENSGALG00010007819	NRIP1	2.304272152	0.166406885	13.84721646	1.32238E-43	1.18393E-42	positive
PRJNA729271	stage_1W_vs_E10	ordinary_muscle_development	ENSGALG00010007821	FAM89A	-2.37541335	0.127574541	-18.61980708	2.22012E-77	4.9206E-76	negative
PRJNA729271	stage_1W_vs_E10	ordinary_muscle_development	ENSGALG00010007826	ENSGALG00010007826	11.54056804	1.372276447	8.409798237	4.10717E-17	1.33723E-16	positive
PRJNA729271	stage_1W_vs_E10	ordinary_muscle_development	ENSGALG00010007830	SMIM19	1.305474391	0.130087944	10.03532192	1.06611E-23	4.64341E-23	positive
PRJNA729271	stage_1W_vs_E10	ordinary_muscle_development	ENSGALG00010007835	GUCY1A1	1.673588746	0.186083678	8.993742826	2.38951E-19	8.70469E-19	positive
PRJNA729271	stage_1W_vs_E10	ordinary_muscle_development	ENSGALG00010007845	TFAP2B	-7.155111703	1.094265546	-6.538734338	6.20416E-11	1.48964E-10	negative
PRJNA729271	stage_1W_vs_E10	ordinary_muscle_development	ENSGALG00010007846	ENSGALG00010007846	0.322552914	0.149157741	2.162495305	0.030580019	0.039136268	positive
PRJNA729271	stage_1W_vs_E10	ordinary_muscle_development	ENSGALG00010007847	CMC2	0.626120284	0.114561736	5.465352632	4.61987E-08	9.445E-08	positive
PRJNA729271	stage_1W_vs_E10	ordinary_muscle_development	ENSGALG00010007849	CXADR	-4.002671202	0.319274009	-12.53678999	4.69636E-36	3.31582E-35	negative
PRJNA729271	stage_1W_vs_E10	ordinary_muscle_development	ENSGALG00010007850	ENSGALG00010007850	-2.481933233	0.303353393	-8.181656421	2.79968E-16	8.79147E-16	negative
PRJNA729271	stage_1W_vs_E10	ordinary_muscle_development	ENSGALG00010007851	AvBD7	1.384063189	0.928673938	1.49036506	0.136128274	0.160656191	positive
PRJNA729271	stage_1W_vs_E10	ordinary_muscle_development	ENSGALG00010007852	SLC20A2	1.36626823	0.207498005	6.584488508	4.56454E-11	1.10844E-10	positive
PRJNA729271	stage_1W_vs_E10	ordinary_muscle_development	ENSGALG00010007853	C1orf131	0.559665595	0.110507952	5.064482575	4.09511E-07	7.85375E-07	positive
PRJNA729271	stage_1W_vs_E10	ordinary_muscle_development	ENSGALG00010007854	FAM181B	0.917419738	0.1173971	7.814671193	5.51067E-15	1.63716E-14	positive
PRJNA729271	stage_1W_vs_E10	ordinary_muscle_development	ENSGALG00010007855	RNF182	-1.794778684	0.226629796	-7.919429471	2.38603E-15	7.20831E-15	negative
PRJNA729271	stage_1W_vs_E10	ordinary_muscle_development	ENSGALG00010007856	PDCD2	1.512186457	0.127843406	11.82842748	2.78307E-32	1.7137E-31	positive
PRJNA729271	stage_1W_vs_E10	ordinary_muscle_development	ENSGALG00010007857	ENSGALG00010007857	-0.31954508	0.340149981	-0.93942407	0.34751306	0.384960015	negative
PRJNA729271	stage_1W_vs_E10	ordinary_muscle_development	ENSGALG00010007859	CENPN	-2.06691917	0.164060248	-12.59853739	2.15096E-36	1.52834E-35	negative
PRJNA729271	stage_1W_vs_E10	ordinary_muscle_development	ENSGALG00010007860	DEFB4A	1.655746701	0.908022392	1.823464615	0.068233052	0.083752907	positive
PRJNA729271	stage_1W_vs_E10	ordinary_muscle_development	ENSGALG00010007861	INTU	-2.420435506	0.182600982	-13.25532576	4.20288E-40	3.37698E-39	negative
PRJNA729271	stage_1W_vs_E10	ordinary_muscle_development	ENSGALG00010007862	ATMIN	0.745150781	0.085588757	8.70617594	3.14299E-18	1.08136E-17	positive
PRJNA729271	stage_1W_vs_E10	ordinary_muscle_development	ENSGALG00010007863	AvBD1	0.839359823	0.854106021	0.982734932	0.325737913	0.362250349	positive
PRJNA729271	stage_1W_vs_E10	ordinary_muscle_development	ENSGALG00010007864	TYR	-3.873597425	1.118398343	-3.463522143	0.000533153	0.000811344	negative
PRJNA729271	stage_1W_vs_E10	ordinary_muscle_development	ENSGALG00010007865	GABRR3	0.106835623	0.43913313	0.243287549	0.807782649	0.828062907	positive
PRJNA729271	stage_1W_vs_E10	ordinary_muscle_development	ENSGALG00010007866	TBP	-0.149685182	0.085577667	-1.749115007	0.080271142	0.097652555	negative
PRJNA729271	stage_1W_vs_E10	ordinary_muscle_development	ENSGALG00010007867	ZNF516	-1.397210613	0.107356271	-13.01470889	1.00926E-38	7.73625E-38	negative
PRJNA729271	stage_1W_vs_E10	ordinary_muscle_development	ENSGALG00010007868	INHBA	-2.524830618	0.306501237	-8.237587051	1.75718E-16	5.57949E-16	negative
PRJNA729271	stage_1W_vs_E10	ordinary_muscle_development	ENSGALG00010007869	GCSH	-0.998569138	0.124531002	-8.018638923	1.06923E-15	3.28428E-15	negative
PRJNA729271	stage_1W_vs_E10	ordinary_muscle_development	ENSGALG00010007870	AvBD3	-3.601946648	0.944579414	-3.813280911	0.000137134	0.000218291	negative
PRJNA729271	stage_1W_vs_E10	ordinary_muscle_development	ENSGALG00010007871	HSPA4L	-1.062487605	0.08901973	-11.93541704	7.73701E-33	4.85489E-32	negative
PRJNA729271	stage_1W_vs_E10	ordinary_muscle_development	ENSGALG00010007872	KLHL2	-0.77014937	0.078366666	-9.827512204	8.57099E-23	3.59169E-22	negative
PRJNA729271	stage_1W_vs_E10	ordinary_muscle_development	ENSGALG00010007873	ENSGALG00010007873	0.396616982	0.767320482	0.516885696	0.605235953	0.639656955	positive
PRJNA729271	stage_1W_vs_E10	ordinary_muscle_development	ENSGALG00010007874	ENSGALG00010007874	-0.154121217	0.567104163	-0.271768798	0.785799798	0.808271932	negative
PRJNA729271	stage_1W_vs_E10	ordinary_muscle_development	ENSGALG00010007875	AvBD4	2.076601848	1.539424661	1.348946721	0.17735408	0.205692702	positive
PRJNA729271	stage_1W_vs_E10	ordinary_muscle_development	ENSGALG00010007877	MSMO1	-0.556312682	0.187784841	-2.962500482	0.003051513	0.00434624	negative
PRJNA729271	stage_1W_vs_E10	ordinary_muscle_development	ENSGALG00010007878	PSMB1	0.504655542	0.122093783	4.133343487	3.57524E-05	5.95462E-05	positive
PRJNA729271	stage_1W_vs_E10	ordinary_muscle_development	ENSGALG00010007879	PLK4	-2.172147879	0.194632407	-11.16025803	6.38061E-29	3.43435E-28	negative
PRJNA729271	stage_1W_vs_E10	ordinary_muscle_development	ENSGALG00010007880	BCO1	1.206618865	0.284118175	4.2468908	2.16758E-05	3.66189E-05	positive
PRJNA729271	stage_1W_vs_E10	ordinary_muscle_development	ENSGALG00010007881	AvBD14	-1.502001955	0.67986756	-2.209256691	0.027156792	0.034962048	negative
PRJNA729271	stage_1W_vs_E10	ordinary_muscle_development	ENSGALG00010007882	CPE	-1.019493044	0.227632123	-4.478687063	7.51035E-06	1.31334E-05	negative
PRJNA729271	stage_1W_vs_E10	ordinary_muscle_development	ENSGALG00010007883	DHTKD1	0.722391728	0.120380253	6.000915514	1.96208E-09	4.35067E-09	positive
PRJNA729271	stage_1W_vs_E10	ordinary_muscle_development	ENSGALG00010007884	GLI3	-2.184950125	0.117257202	-18.6338245	1.70867E-77	3.80448E-76	negative
PRJNA729271	stage_1W_vs_E10	ordinary_muscle_development	ENSGALG00010007885	USP38	1.467407981	0.103193531	14.21996098	6.88913E-46	6.60874E-45	positive
PRJNA729271	stage_1W_vs_E10	ordinary_muscle_development	ENSGALG00010007886	GRM5	-3.357677778	0.96047968	-3.495834266	0.000472582	0.000721745	negative
PRJNA729271	stage_1W_vs_E10	ordinary_muscle_development	ENSGALG00010007887	CDT1	-1.26949551	0.126753674	-10.01545336	1.30363E-23	5.65921E-23	negative
PRJNA729271	stage_1W_vs_E10	ordinary_muscle_development	ENSGALG00010007889	DCAF6	2.737462924	0.096955742	28.23414963	2.2282E-175	2.5233E-173	positive
PRJNA729271	stage_1W_vs_E10	ordinary_muscle_development	ENSGALG00010007890	ENSGALG00010007890	-1.770916584	0.126588258	-13.98958019	1.80474E-44	1.65988E-43	negative
PRJNA729271	stage_1W_vs_E10	ordinary_muscle_development	ENSGALG00010007891	GDI1	-0.803115071	0.056022057	-14.33569419	1.30931E-46	1.28406E-45	negative
PRJNA729271	stage_1W_vs_E10	ordinary_muscle_development	ENSGALG00010007892	ENSGALG00010007892	-0.135456823	0.462609546	-0.292810263	0.769667183	0.793197712	negative
PRJNA729271	stage_1W_vs_E10	ordinary_muscle_development	ENSGALG00010007893	MFSD8	0.872111272	0.147489446	5.913041881	3.35847E-09	7.33589E-09	positive
PRJNA729271	stage_1W_vs_E10	ordinary_muscle_development	ENSGALG00010007894	CD88	1.054744882	0.263126884	4.008502917	6.11049E-05	9.99904E-05	positive
PRJNA729271	stage_1W_vs_E10	ordinary_muscle_development	ENSGALG00010007895	GOLGB1	-1.186666168	0.112601183	-10.53866519	5.73065E-26	2.74507E-25	negative
PRJNA729271	stage_1W_vs_E10	ordinary_muscle_development	ENSGALG00010007896	TIMMDC1	0.877252973	0.10487294	8.364912566	6.01595E-17	1.94646E-16	positive
PRJNA729271	stage_1W_vs_E10	ordinary_muscle_development	ENSGALG00010007897	EFHC1	-1.966538254	0.466038133	-4.219693871	2.44634E-05	4.117E-05	negative
PRJNA729271	stage_1W_vs_E10	ordinary_muscle_development	ENSGALG00010007898	GAB1	0.281269369	0.089600646	3.139144433	0.001694419	0.002472129	positive
PRJNA729271	stage_1W_vs_E10	ordinary_muscle_development	ENSGALG00010007899	APRT	-1.292918654	0.156695659	-8.251145327	1.56884E-16	4.99347E-16	negative
PRJNA729271	stage_1W_vs_E10	ordinary_muscle_development	ENSGALG00010007900	FHOD3	0.513385184	0.200076496	2.565944495	0.010289529	0.013892206	positive
PRJNA729271	stage_1W_vs_E10	ordinary_muscle_development	ENSGALG00010007901	FSTL5	-0.395988911	0.299597494	-1.321736395	0.186255945	0.215413702	negative
PRJNA729271	stage_1W_vs_E10	ordinary_muscle_development	ENSGALG00010007902	C7orf25	2.453461719	0.141691229	17.31555108	3.59068E-67	6.29346E-66	positive
PRJNA729271	stage_1W_vs_E10	ordinary_muscle_development	ENSGALG00010007903	PSMA2	0.065898987	0.103591871	0.636140527	0.524684832	0.561484433	positive
PRJNA729271	stage_1W_vs_E10	ordinary_muscle_development	ENSGALG00010007904	ENSGALG00010007904	0.130863849	0.123547495	1.059218959	0.289500073	0.324389576	positive
PRJNA729271	stage_1W_vs_E10	ordinary_muscle_development	ENSGALG00010007905	MRPL32	0.372350967	0.155693162	2.391569183	0.016776522	0.022098596	positive
PRJNA729271	stage_1W_vs_E10	ordinary_muscle_development	ENSGALG00010007906	NAF1	-0.770056961	0.112719144	-6.831643091	8.39475E-12	2.12026E-11	negative
PRJNA729271	stage_1W_vs_E10	ordinary_muscle_development	ENSGALG00010007907	IRX2	-6.383332251	0.81841518	-7.799625922	6.2091E-15	1.83825E-14	negative
PRJNA729271	stage_1W_vs_E10	ordinary_muscle_development	ENSGALG00010007908	IRX1	-7.738648867	0.992132472	-7.800015709	6.18995E-15	1.83296E-14	negative
PRJNA729271	stage_1W_vs_E10	ordinary_muscle_development	ENSGALG00010007909	GALNS	-0.560529091	0.109534994	-5.117351744	3.09855E-07	5.99167E-07	negative
PRJNA729271	stage_1W_vs_E10	ordinary_muscle_development	ENSGALG00010007910	ANKS6	-0.079852321	0.148799867	-0.536642421	0.591514636	0.626124374	negative
PRJNA729271	stage_1W_vs_E10	ordinary_muscle_development	ENSGALG00010007911	IFI6	5.368417425	0.800931817	6.702714651	2.04583E-11	5.06275E-11	positive
PRJNA729271	stage_1W_vs_E10	ordinary_muscle_development	ENSGALG00010007912	TRAPPC2L	-0.186154372	0.114901843	-1.620116503	0.105207253	0.126114715	negative
PRJNA729271	stage_1W_vs_E10	ordinary_muscle_development	ENSGALG00010007914	PAQR8	-0.069134549	0.127474495	-0.542340249	0.587584133	0.622772157	negative
PRJNA729271	stage_1W_vs_E10	ordinary_muscle_development	ENSGALG00010007915	HECW1	-0.119201761	0.137978062	-0.863918216	0.387632819	0.425790976	negative
PRJNA729271	stage_1W_vs_E10	ordinary_muscle_development	ENSGALG00010007916	MCM3	-3.13374454	0.205891049	-15.22040201	2.58935E-52	3.08657E-51	negative
PRJNA729271	stage_1W_vs_E10	ordinary_muscle_development	ENSGALG00010007917	POGLUT1	-2.840920386	0.084531346	-33.60789232	1.2864E-247	2.9598E-245	negative
PRJNA729271	stage_1W_vs_E10	ordinary_muscle_development	ENSGALG00010007918	NPY1R	-2.098679949	0.755378372	-2.778316175	0.005464142	0.00760176	negative
PRJNA729271	stage_1W_vs_E10	ordinary_muscle_development	ENSGALG00010007920	FAT3	-3.698097435	0.184722859	-20.01970659	3.70899E-89	1.07095E-87	negative
PRJNA729271	stage_1W_vs_E10	ordinary_muscle_development	ENSGALG00010007921	FAM120B	-0.784962164	0.103097663	-7.61377263	2.66209E-14	7.6425E-14	negative
PRJNA729271	stage_1W_vs_E10	ordinary_muscle_development	ENSGALG00010007922	IL17A	-0.471154363	0.494800819	-0.958273198	0.337925015	0.374911833	negative
PRJNA729271	stage_1W_vs_E10	ordinary_muscle_development	ENSGALG00010007923	YAP1	-2.025128663	0.092143166	-21.97806677	4.6693E-107	1.979E-105	negative
PRJNA729271	stage_1W_vs_E10	ordinary_muscle_development	ENSGALG00010007924	ABHD18	0.134774002	0.115893066	1.162916867	0.244863233	0.277483587	positive
PRJNA729271	stage_1W_vs_E10	ordinary_muscle_development	ENSGALG00010007925	STK17A	-0.525498999	0.239203837	-2.196866932	0.028029951	0.036025372	negative
PRJNA729271	stage_1W_vs_E10	ordinary_muscle_development	ENSGALG00010007926	EIF3E	0.469359444	0.131380731	3.572513556	0.000353571	0.000546202	positive
PRJNA729271	stage_1W_vs_E10	ordinary_muscle_development	ENSGALG00010007927	DLL1	-0.038517159	0.165801586	-0.232308748	0.816298218	0.835610358	negative
PRJNA729271	stage_1W_vs_E10	ordinary_muscle_development	ENSGALG00010007928	C11orf70	-2.872828132	0.738329943	-3.890981479	9.98396E-05	0.000160512	negative
PRJNA729271	stage_1W_vs_E10	ordinary_muscle_development	ENSGALG00010007929	LRRC14B	7.895225411	0.329520721	23.95972363	7.3172E-127	4.6316E-125	positive
PRJNA729271	stage_1W_vs_E10	ordinary_muscle_development	ENSGALG00010007930	CBFA2T3	0.474923252	0.197131508	2.409169678	0.015988862	0.02110926	positive
PRJNA729271	stage_1W_vs_E10	ordinary_muscle_development	ENSGALG00010007931	GBE1	2.455807974	0.188796237	13.00771675	1.10597E-38	8.45522E-38	positive
PRJNA729271	stage_1W_vs_E10	ordinary_muscle_development	ENSGALG00010007932	NPY5R	0.632154832	0.180664952	3.499045196	0.000466927	0.000713409	positive
PRJNA729271	stage_1W_vs_E10	ordinary_muscle_development	ENSGALG00010007933	CEP126	0.540751811	0.448728189	1.205076534	0.228173758	0.259768977	positive
PRJNA729271	stage_1W_vs_E10	ordinary_muscle_development	ENSGALG00010007934	PKHD1	-1.459067225	0.366238498	-3.983926408	6.77859E-05	0.000110536	negative
PRJNA729271	stage_1W_vs_E10	ordinary_muscle_development	ENSGALG00010007935	LARP1B	2.322618066	0.122057143	19.02894014	9.82191E-81	2.39678E-79	positive
PRJNA729271	stage_1W_vs_E10	ordinary_muscle_development	ENSGALG00010007936	ENSGALG00010007936	2.100913695	0.139107419	15.10281555	1.55164E-51	1.79354E-50	positive
PRJNA729271	stage_1W_vs_E10	ordinary_muscle_development	ENSGALG00010007937	ANGPTL5	3.333835328	0.401892804	8.295334722	1.0828E-16	3.46701E-16	positive
PRJNA729271	stage_1W_vs_E10	ordinary_muscle_development	ENSGALG00010007938	BCL9	-0.852267734	0.1059012	-8.047762799	8.43212E-16	2.60105E-15	negative
PRJNA729271	stage_1W_vs_E10	ordinary_muscle_development	ENSGALG00010007940	GLIPR2	-2.666697635	0.272956517	-9.769679287	1.51932E-22	6.30478E-22	negative
PRJNA729271	stage_1W_vs_E10	ordinary_muscle_development	ENSGALG00010007941	MRPL19	-0.510690356	0.074098514	-6.89204585	5.49956E-12	1.40148E-11	negative
PRJNA729271	stage_1W_vs_E10	ordinary_muscle_development	ENSGALG00010007942	PGRMC2	-0.483043475	0.086561971	-5.58031977	2.40077E-08	4.97628E-08	negative
PRJNA729271	stage_1W_vs_E10	ordinary_muscle_development	ENSGALG00010007943	BLOC1S4	-0.135435116	0.101197057	-1.338330582	0.180788697	0.209441509	negative
PRJNA729271	stage_1W_vs_E10	ordinary_muscle_development	ENSGALG00010007944	ACSF3	-0.068556641	0.139068695	-0.492969614	0.622034058	0.655832388	negative
PRJNA729271	stage_1W_vs_E10	ordinary_muscle_development	ENSGALG00010007945	ENSGALG00010007945	-1.648568986	0.125781669	-13.10659176	3.01862E-39	2.36003E-38	negative
PRJNA729271	stage_1W_vs_E10	ordinary_muscle_development	ENSGALG00010007946	ENSGALG00010007946	-0.217340902	0.157066413	-1.383751611	0.166434524	0.193959514	negative
PRJNA729271	stage_1W_vs_E10	ordinary_muscle_development	ENSGALG00010007948	ENSGALG00010007948	-3.893134876	0.545374929	-7.138455894	9.43851E-13	2.50341E-12	negative
PRJNA729271	stage_1W_vs_E10	ordinary_muscle_development	ENSGALG00010007950	ARC	-0.691305037	0.562580164	-1.22881161	0.219142448	0.25049048	negative
PRJNA729271	stage_1W_vs_E10	ordinary_muscle_development	ENSGALG00010007951	TGFBR1	-0.617387311	0.114319938	-5.400521738	6.64474E-08	1.34462E-07	negative
PRJNA729271	stage_1W_vs_E10	ordinary_muscle_development	ENSGALG00010007952	EMC2	-0.288320855	0.109730969	-2.627524921	0.008600854	0.011712644	negative
PRJNA729271	stage_1W_vs_E10	ordinary_muscle_development	ENSGALG00010007953	SCGN	-3.590650026	0.70262787	-5.110315405	3.21621E-07	6.21007E-07	negative
PRJNA729271	stage_1W_vs_E10	ordinary_muscle_development	ENSGALG00010007954	PSCA	0.900744863	0.589738538	1.527363067	0.126670755	0.150179339	positive
PRJNA729271	stage_1W_vs_E10	ordinary_muscle_development	ENSGALG00010007955	TMA16	-0.037080971	0.122047151	-0.303824958	0.761261252	0.785279754	negative
PRJNA729271	stage_1W_vs_E10	ordinary_muscle_development	ENSGALG00010007956	HS1BP3	0.117571188	0.13253975	0.8870636	0.37504466	0.412651612	positive
PRJNA729271	stage_1W_vs_E10	ordinary_muscle_development	ENSGALG00010007957	CDH15	0.908776409	0.162997108	5.575414329	2.46941E-08	5.11637E-08	positive
PRJNA729271	stage_1W_vs_E10	ordinary_muscle_development	ENSGALG00010007958	ENSGALG00010007958	0.9348383	0.518188197	1.804051702	0.071223202	0.087194283	positive
PRJNA729271	stage_1W_vs_E10	ordinary_muscle_development	ENSGALG00010007960	TSNAX	-0.855981442	0.06392069	-13.39130476	6.7976E-41	5.60473E-40	negative
PRJNA729271	stage_1W_vs_E10	ordinary_muscle_development	ENSGALG00010007962	ADCY8	-2.492790163	0.505788913	-4.928518794	8.28554E-07	1.55932E-06	negative
PRJNA729271	stage_1W_vs_E10	ordinary_muscle_development	ENSGALG00010007963	LDAH	-0.143001506	0.085527054	-1.672003168	0.094523697	0.114053192	negative

PRJNA729271	stage_1W_vs_E10	ordinary_muscle_development	ENSGALG00010007964	RECQL4	-2.819567658	0.172904486	-16.30708212	8.78921E-60	1.27655E-58	negative
PRJNA729271	stage_1W_vs_E10	ordinary_muscle_development	ENSGALG00010007965	EGLN1	1.903578303	0.157429344	12.09163587	1.16935E-33	7.56748E-33	positive
PRJNA729271	stage_1W_vs_E10	ordinary_muscle_development	ENSGALG00010007966	TMEM74	-1.121615728	0.218441066	-5.13463767	2.82688E-07	5.47656E-07	negative
PRJNA729271	stage_1W_vs_E10	ordinary_muscle_development	ENSGALG00010007968	SLC22A31	0.090894797	0.210476553	0.431852366	0.665848717	0.696153863	positive
PRJNA729271	stage_1W_vs_E10	ordinary_muscle_development	ENSGALG00010007970	SPRTN	1.077363601	0.105893997	10.17398187	2.59095E-24	1.15556E-23	positive
PRJNA729271	stage_1W_vs_E10	ordinary_muscle_development	ENSGALG00010007972	EXOC8	-0.252649164	0.112736762	-2.241053931	0.025022582	0.032361021	negative
PRJNA729271	stage_1W_vs_E10	ordinary_muscle_development	ENSGALG00010007973	CARMIL1	-0.391570421	0.134810259	-2.904604017	0.00367718	0.005197028	negative
PRJNA729271	stage_1W_vs_E10	ordinary_muscle_development	ENSGALG00010007975	ANKRD11	0.535634722	0.176194292	3.040023133	0.0023656	0.003402769	positive
PRJNA729271	stage_1W_vs_E10	ordinary_muscle_development	ENSGALG00010007976	ENSGALG00010007976	0.054673621	0.376061361	0.14538484	0.884407033	0.897534128	positive
PRJNA729271	stage_1W_vs_E10	ordinary_muscle_development	ENSGALG00010007977	APOB	3.86834902	1.377136205	2.8089807	0.004969863	0.00694143	positive
PRJNA729271	stage_1W_vs_E10	ordinary_muscle_development	ENSGALG00010007978	JADE1	1.732715521	0.115371478	15.01857782	5.54854E-51	6.29312E-50	positive
PRJNA729271	stage_1W_vs_E10	ordinary_muscle_development	ENSGALG00010007979	RAB30	-0.664878217	0.090565327	-7.341421235	2.11338E-13	5.77335E-13	negative
PRJNA729271	stage_1W_vs_E10	ordinary_muscle_development	ENSGALG00010007980	MARCHF1	-0.562693591	0.229753816	-2.449115324	0.014320759	0.019021296	negative
PRJNA729271	stage_1W_vs_E10	ordinary_muscle_development	ENSGALG00010007981	TDRD15	-5.296976752	0.960770913	-5.513256783	3.52254E-08	7.23423E-08	negative
PRJNA729271	stage_1W_vs_E10	ordinary_muscle_development	ENSGALG00010007982	RECK	-0.896434403	0.147028701	-6.097002806	1.08076E-09	2.42802E-09	negative
PRJNA729271	stage_1W_vs_E10	ordinary_muscle_development	ENSGALG00010007983	RCHY1	0.153956916	0.087424039	1.761036422	0.078232239	0.095292126	positive
PRJNA729271	stage_1W_vs_E10	ordinary_muscle_development	ENSGALG00010007984	EIF3H	0.267824927	0.12209296	2.19361483	0.028263111	0.036308943	positive
PRJNA729271	stage_1W_vs_E10	ordinary_muscle_development	ENSGALG00010007985	APELA	-4.782050269	0.411515225	-11.62059135	3.23887E-31	1.92407E-30	negative
PRJNA729271	stage_1W_vs_E10	ordinary_muscle_development	ENSGALG00010007986	MYC	0.550774013	0.187614964	2.93566143	0.003328374	0.004724223	positive
PRJNA729271	stage_1W_vs_E10	ordinary_muscle_development	ENSGALG00010007987	ENSGALG00010007987	0.712260013	0.298917063	2.382801455	0.017181458	0.022601437	positive
PRJNA729271	stage_1W_vs_E10	ordinary_muscle_development	ENSGALG00010007988	TARBP1	-0.080444894	0.12380956	-0.649747028	0.515855641	0.553097678	negative
PRJNA729271	stage_1W_vs_E10	ordinary_muscle_development	ENSGALG00010007989	SPG7	0.68710864	0.097217658	7.067734952	1.57483E-12	4.12564E-12	positive
PRJNA729271	stage_1W_vs_E10	ordinary_muscle_development	ENSGALG00010007990	TRHR	-4.110448541	1.154799176	-3.559448799	0.000371634	0.000572702	negative
PRJNA729271	stage_1W_vs_E10	ordinary_muscle_development	ENSGALG00010007991	PCF11	0.984065675	0.109578692	8.98044735	2.69668E-19	9.80183E-19	positive
PRJNA729271	stage_1W_vs_E10	ordinary_muscle_development	ENSGALG00010007992	LAPTM4A	0.515348732	0.098983867	5.206391195	1.92549E-07	3.77467E-07	positive
PRJNA729271	stage_1W_vs_E10	ordinary_muscle_development	ENSGALG00010007993	LRRC14	0.752711251	0.172608423	4.36080255	1.29586E-05	2.22923E-05	positive
PRJNA729271	stage_1W_vs_E10	ordinary_muscle_development	ENSGALG00010007994	FAM65B	1.28284492	0.166203605	7.718514414	1.17693E-14	3.44083E-14	positive
PRJNA729271	stage_1W_vs_E10	ordinary_muscle_development	ENSGALG00010007995	KIAA0319	-4.117166198	0.805041529	-5.114228334	3.15026E-07	6.08759E-07	negative
PRJNA729271	stage_1W_vs_E10	ordinary_muscle_development	ENSGALG00010007996	NUDCD1	-1.114395801	0.07653266	-14.56104891	4.96895E-48	5.0865E-47	negative
PRJNA729271	stage_1W_vs_E10	ordinary_muscle_development	ENSGALG00010007997	TMEM192	-0.634235214	0.14023746	-4.522580583	6.10902E-06	1.07516E-05	negative
PRJNA729271	stage_1W_vs_E10	ordinary_muscle_development	ENSGALG00010007998	RPL13	0.185986528	0.144822199	1.284240468	0.19905783	0.229195587	positive
PRJNA729271	stage_1W_vs_E10	ordinary_muscle_development	ENSGALG00010007999	SDC1	-1.196088426	0.208081629	-5.748169267	9.02149E-09	1.91824E-08	negative
PRJNA729271	stage_1W_vs_E10	ordinary_muscle_development	ENSGALG00010008000	C8orf82	-0.284033207	0.173012883	-1.641688192	0.10065464	0.120997596	negative
PRJNA729271	stage_1W_vs_E10	ordinary_muscle_development	ENSGALG00010008001	ALDH5A1	0.480662173	0.13633924	3.525486672	0.000422705	0.000648578	positive
PRJNA729271	stage_1W_vs_E10	ordinary_muscle_development	ENSGALG00010008002	GJA8	-1.324454944	0.177273876	-7.471235883	7.94451E-14	2.2218E-13	negative
PRJNA729271	stage_1W_vs_E10	ordinary_muscle_development	ENSGALG00010008004	ENY2	-1.350298653	0.172173552	-7.842660154	4.41101E-15	1.31803E-14	negative
PRJNA729271	stage_1W_vs_E10	ordinary_muscle_development	ENSGALG00010008005	ANKRD42	-0.705206514	0.364963129	-1.932267832	0.053326457	0.066349098	negative
PRJNA729271	stage_1W_vs_E10	ordinary_muscle_development	ENSGALG00010008006	CPNE7	0.038194719	0.539051897	0.070855364	0.943512868	0.950595609	positive
PRJNA729271	stage_1W_vs_E10	ordinary_muscle_development	ENSGALG00010008007	CBR4	0.404191265	0.122438363	3.301181545	0.000962786	0.001436942	positive
PRJNA729271	stage_1W_vs_E10	ordinary_muscle_development	ENSGALG00010008008	BTC	4.256260597	0.197294321	21.57315316	3.2105E-103	1.282E-101	positive
PRJNA729271	stage_1W_vs_E10	ordinary_muscle_development	ENSGALG00010008009	GPLD1	-0.220936881	1.282288468	-0.172298891	0.863202556	0.878351776	negative
PRJNA729271	stage_1W_vs_E10	ordinary_muscle_development	ENSGALG00010008010	CERK	-0.696512526	0.093146636	-7.477591862	7.56969E-14	2.12065E-13	negative
PRJNA729271	stage_1W_vs_E10	ordinary_muscle_development	ENSGALG00010008011	ADNP2	-0.166034694	0.100604552	-1.650369595	0.098867366	0.119007014	negative
PRJNA729271	stage_1W_vs_E10	ordinary_muscle_development	ENSGALG00010008012	CCDC90B	-0.133276657	0.079893281	-1.668183544	0.095279297	0.114907513	negative
PRJNA729271	stage_1W_vs_E10	ordinary_muscle_development	ENSGALG00010008013	ENSGALG00010008013	-0.182608758	0.237733008	-0.768125386	0.442412696	0.480501426	negative
PRJNA729271	stage_1W_vs_E10	ordinary_muscle_development	ENSGALG00010008015	ARHGAP39	-1.096711763	0.162252702	-6.759281952	1.38677E-11	3.45739E-11	negative
PRJNA729271	stage_1W_vs_E10	ordinary_muscle_development	ENSGALG00010008016	GSR	-0.802602872	0.144180024	-5.566671803	2.59651E-08	5.37356E-08	negative
PRJNA729271	stage_1W_vs_E10	ordinary_muscle_development	ENSGALG00010008017	GMNN	-2.062964188	0.138706994	-14.87282026	4.94841E-50	5.44212E-49	negative
PRJNA729271	stage_1W_vs_E10	ordinary_muscle_development	ENSGALG00010008018	ENSGALG00010008018	0.333473966	0.208950702	1.595945657	0.110500927	0.132052063	positive
PRJNA729271	stage_1W_vs_E10	ordinary_muscle_development	ENSGALG00010008019	PPP2CB	-0.442977272	0.100062	-4.427027953	9.55404E-06	1.6603E-05	negative
PRJNA729271	stage_1W_vs_E10	ordinary_muscle_development	ENSGALG00010008020	COA6	1.46270395	0.130150848	11.23852797	2.63743E-29	1.447E-28	positive
PRJNA729271	stage_1W_vs_E10	ordinary_muscle_development	ENSGALG00010008022	SLC35F3	0.204048031	0.380614892	0.536101018	0.591888736	0.626372727	positive
PRJNA729271	stage_1W_vs_E10	ordinary_muscle_development	ENSGALG00010008023	ATAD2	-0.410006585	0.077308064	-5.303542248	1.13577E-07	2.26016E-07	negative
PRJNA729271	stage_1W_vs_E10	ordinary_muscle_development	ENSGALG00010008024	MRS2	0.77566257	0.094749493	8.186456131	2.69031E-16	8.46084E-16	positive
PRJNA729271	stage_1W_vs_E10	ordinary_muscle_development	ENSGALG00010008025	TBC1D22A	-0.493692971	0.097942753	-5.040627866	4.64007E-07	8.86838E-07	negative
PRJNA729271	stage_1W_vs_E10	ordinary_muscle_development	ENSGALG00010008026	PURG	0.40730157	0.12272786	3.318737669	0.000904253	0.00135377	positive
PRJNA729271	stage_1W_vs_E10	ordinary_muscle_development	ENSGALG00010008027	C6orf62	0.822138427	0.107107747	7.675807288	1.6438E-14	4.76347E-14	positive
PRJNA729271	stage_1W_vs_E10	ordinary_muscle_development	ENSGALG00010008028	KCNK1	-3.115451972	0.170616803	-18.25993636	1.72492E-74	3.59235E-73	negative
PRJNA729271	stage_1W_vs_E10	ordinary_muscle_development	ENSGALG00010008029	LY6E	-0.096346478	0.314482084	-0.306365554	0.759326332	0.783487698	negative
PRJNA729271	stage_1W_vs_E10	ordinary_muscle_development	ENSGALG00010008030	GJA5	-2.134774113	0.149833468	-14.24764535	4.63653E-46	4.47149E-45	negative
PRJNA729271	stage_1W_vs_E10	ordinary_muscle_development	ENSGALG00010008031	MAP3K21	-2.727995785	0.192975311	-14.13650154	2.26256E-45	2.14212E-44	negative
PRJNA729271	stage_1W_vs_E10	ordinary_muscle_development	ENSGALG00010008032	PUM2	-0.58313015	0.054602584	-10.67953403	1.26907E-26	6.25474E-26	negative
PRJNA729271	stage_1W_vs_E10	ordinary_muscle_development	ENSGALG00010008033	ACOT13	0.641341426	0.119698815	5.357959691	8.4167E-08	1.69186E-07	positive
PRJNA729271	stage_1W_vs_E10	ordinary_muscle_development	ENSGALG00010008034	DPEP1	-1.411565877	0.396245251	-3.562354056	0.000367544	0.00056658	negative
PRJNA729271	stage_1W_vs_E10	ordinary_muscle_development	ENSGALG00010008035	TDP2	-0.79170343	0.107186824	-7.386201029	1.51083E-13	4.15787E-13	negative
PRJNA729271	stage_1W_vs_E10	ordinary_muscle_development	ENSGALG00010008036	SPOCK3	-4.203118582	0.187538748	-22.41200084	3.0062E-111	1.3877E-109	negative
PRJNA729271	stage_1W_vs_E10	ordinary_muscle_development	ENSGALG00010008037	ENSGALG00010008037	2.069662036	0.909561182	2.275451149	0.02287888	0.029726548	positive
PRJNA729271	stage_1W_vs_E10	ordinary_muscle_development	ENSGALG00010008038	WRN	0.046237232	0.075805274	0.60994743	0.541896632	0.5781167	positive
PRJNA729271	stage_1W_vs_E10	ordinary_muscle_development	ENSGALG00010008039	CHMP1A	-0.423852997	0.092257783	-4.594224833	4.34361E-06	7.73852E-06	negative
PRJNA729271	stage_1W_vs_E10	ordinary_muscle_development	ENSGALG00010008040	ENSGALG00010008040	0.317181883	0.277191482	1.14426995	0.252511701	0.285637358	positive
PRJNA729271	stage_1W_vs_E10	ordinary_muscle_development	ENSGALG00010008041	ENSGALG00010008041	-1.839008933	0.218043234	-8.434148132	3.33624E-17	1.09137E-16	negative
PRJNA729271	stage_1W_vs_E10	ordinary_muscle_development	ENSGALG00010008042	ENSGALG00010008042	0.805727726	0.815879653	0.987557077	0.323369616	0.359809825	positive
PRJNA729271	stage_1W_vs_E10	ordinary_muscle_development	ENSGALG00010008043	IL17REL	-4.058261769	0.405343005	-10.01192007	1.35105E-23	5.86154E-23	negative
PRJNA729271	stage_1W_vs_E10	ordinary_muscle_development	ENSGALG00010008045	DCDC2	-4.476899508	0.918753151	-4.872799081	1.10028E-06	2.05179E-06	negative
PRJNA729271	stage_1W_vs_E10	ordinary_muscle_development	ENSGALG00010008046	RHOB	-1.001444373	0.169355268	-5.913275594	3.3537E-09	7.32659E-09	negative
PRJNA729271	stage_1W_vs_E10	ordinary_muscle_development	ENSGALG00010008047	ASAP1	-1.930990075	0.133044824	-14.51383089	9.90317E-48	1.00312E-46	negative
PRJNA729271	stage_1W_vs_E10	ordinary_muscle_development	ENSGALG00010008048	ENSGALG00010008048	1.323130328	0.81430262	1.624863159	0.10419172	0.125000743	positive
PRJNA729271	stage_1W_vs_E10	ordinary_muscle_development	ENSGALG00010008050	CDK10	-0.461954177	0.156299327	-2.955573676	0.003120881	0.004438934	negative
PRJNA729271	stage_1W_vs_E10	ordinary_muscle_development	ENSGALG00010008051	ENSGALG00010008051	-0.025870284	0.078284961	-0.330463012	0.741050136	0.766594471	negative
PRJNA729271	stage_1W_vs_E10	ordinary_muscle_development	ENSGALG00010008052	RBFA	0.907100736	0.121161714	7.486694506	7.06298E-14	1.98292E-13	positive
PRJNA729271	stage_1W_vs_E10	ordinary_muscle_development	ENSGALG00010008054	CRISP3	-5.538333675	0.978118125	-5.662233973	1.49415E-08	3.13607E-08	negative
PRJNA729271	stage_1W_vs_E10	ordinary_muscle_development	ENSGALG00010008056	GTF2E2	-0.389622323	0.070955272	-5.491097572	3.99444E-08	8.18944E-08	negative
PRJNA729271	stage_1W_vs_E10	ordinary_muscle_development	ENSGALG00010008057	BAG1	-0.89501945	0.134481665	-6.655326952	2.82671E-11	6.94343E-11	negative
PRJNA729271	stage_1W_vs_E10	ordinary_muscle_development	ENSGALG00010008058	USP25	3.203388357	0.138012958	23.2107797	3.5439E-119	1.9239E-117	positive
PRJNA729271	stage_1W_vs_E10	ordinary_muscle_development	ENSGALG00010008059	ENSGALG00010008059	0.664835658	0.092767726	7.166669805	7.68441E-13	2.04415E-12	positive
PRJNA729271	stage_1W_vs_E10	ordinary_muscle_development	ENSGALG00010008062	VPS9D1	0.42542107	0.137209377	3.100524752	0.001931781	0.002803761	positive
PRJNA729271	stage_1W_vs_E10	ordinary_muscle_development	ENSGALG00010008063	KCNV1	-1.382370362	0.316750637	-4.364222826	1.27575E-05	2.19647E-05	negative
PRJNA729271	stage_1W_vs_E10	ordinary_muscle_development	ENSGALG00010008064	DDX60	1.460760423	0.631248514	2.314081364	0.020663259	0.026956525	positive
PRJNA729271	stage_1W_vs_E10	ordinary_muscle_development	ENSGALG00010008065	CHMP5	0.018023961	0.086531327	0.208294054	0.834999374	0.852886754	positive
PRJNA729271	stage_1W_vs_E10	ordinary_muscle_development	ENSGALG00010008066	PCNX2	-4.914227389	0.224753382	-21.8649764	5.5994E-106	2.339E-104	negative
PRJNA729271	stage_1W_vs_E10	ordinary_muscle_development	ENSGALG00010008067	MPC2	2.283255221	0.144787379	15.76971164	5.02788E-56	6.66781E-55	positive
PRJNA729271	stage_1W_vs_E10	ordinary_muscle_development	ENSGALG00010008069	DLG2	-3.410632944	0.230296536	-14.80974485	1.2672E-49	1.36871E-48	negative
PRJNA729271	stage_1W_vs_E10	ordinary_muscle_development	ENSGALG00010008071	SMIM18	-1.916988096	0.397716273	-4.819989092	1.43566E-06	2.65128E-06	negative
PRJNA729271	stage_1W_vs_E10	ordinary_muscle_development	ENSGALG00010008072	NRSN1	1.476828726	0.198013488	7.458222881	8.76972E-14	2.44597E-13	positive
PRJNA729271	stage_1W_vs_E10	ordinary_muscle_development	ENSGALG00010008073	ZNF276	0.203828154	0.083346917	2.445539202	0.014463572	0.019196913	positive
PRJNA729271	stage_1W_vs_E10	ordinary_muscle_development	ENSGALG00010008074	RHAG	-1.799460744	0.435693958	-4.130102589	3.62601E-05	6.03711E-05	negative
PRJNA729271	stage_1W_vs_E10	ordinary_muscle_development	ENSGALG00010008075	SDHA	1.653159562	0.136419859	12.11817378	8.46238E-34	5.51043E-33	positive
PRJNA729271	stage_1W_vs_E10	ordinary_muscle_development	ENSGALG00010008076	ENSGALG00010008076	3.981231364	1.756690618	2.266324714	0.023431509	0.030420038	positive
PRJNA729271	stage_1W_vs_E10	ordinary_muscle_development	ENSGALG00010008077	ENSGALG00010008077	1.703414416	1.484988373	1.147089396	0.251344676	0.28436162	positive
PRJNA729271	stage_1W_vs_E10	ordinary_muscle_development	ENSGALG00010008078	GUCY1B1	0.167395891	0.141550589	1.182587035	0.236972853	0.269152289	positive
PRJNA729271	stage_1W_vs_E10	ordinary_muscle_development	ENSGALG00010008079	CCDC127	0.39674669	0.108517998	3.65604507	0.000256136	0.000400204	positive
PRJNA729271	stage_1W_vs_E10	ordinary_muscle_development	ENSGALG00010008081	TOP1MT	-0.43899808	0.141358712	-3.105560831	0.001899186	0.002758939	negative
PRJNA729271	stage_1W_vs_E10	ordinary_muscle_development	ENSGALG00010008083	FANCA	-2.186410952	0.259316039	-8.431452829	3.414E-17	1.1158E-16	negative
PRJNA729271	stage_1W_vs_E10	ordinary_muscle_development	ENSGALG00010008084	CYP2AC1	4.491468213	1.326749961	3.385316259			positive
PRJNA729271	stage_1W_vs_E10	ordinary_muscle_development	ENSGALG00010008086	RHPN1	0.841340106	0.209072969	4.024145771	5.71825E-05	9.36565E-05	positive
PRJNA729271	stage_1W_vs_E10	ordinary_muscle_development	ENSGALG00010008087	CPNE8	-0.578127555	0.184280616	-3.137213061	0.001705621	0.002488223	negative
PRJNA729271	stage_1W_vs_E10	ordinary_muscle_development	ENSGALG00010008088	SLC35A4	0.9021272	0.069498201	12.98058343	1.57675E-38	1.19974E-37	positive
PRJNA729271	stage_1W_vs_E10	ordinary_muscle_development	ENSGALG00010008091	ENSGALG00010008091	2.128857905	0.271110907	7.852350645	4.08311E-15	1.22181E-14	positive
PRJNA729271	stage_1W_vs_E10	ordinary_muscle_development	ENSGALG00010008092	SPIRE2	1.794462474	0.628638779	2.854520804	0.004310181	0.006048022	positive
PRJNA729271	stage_1W_vs_E10	ordinary_muscle_development	ENSGALG00010008093	EFR3A	-0.159853297	0.092813462	-1.722307225	0.08501388	0.103153875	negative
PRJNA729271	stage_1W_vs_E10	ordinary_muscle_development	ENSGALG00010008094	PIM3	0.767385599	0.141865605	5.409243497	6.32915E-08	1.28309E-07	positive
PRJNA729271	stage_1W_vs_E10	ordinary_muscle_development	ENSGALG00010008096	CPSF1	-1.034574408	0.152133614	-6.800432733	1.04305E-11	2.62029E-11	negative
PRJNA729271	stage_1W_vs_E10	ordinary_muscle_development	ENSGALG00010008098	ENSGALG00010008098	0.385124175	0.167091016	2.304876618	0.021173484	0.027597308	positive
PRJNA729271	stage_1W_vs_E10	ordinary_muscle_development	ENSGALG00010008099	PANK3	-0.300658109	0.077045037	-3.902368307	9.52561E-05	0.000153483	negative
PRJNA729271	stage_1W_vs_E10	ordinary_muscle_development	ENSGALG00010008100	ENSGALG00010008100	1.515660705	0.23557234	6.433950214	1.2433E-10	2.93702E-10	positive
PRJNA729271	stage_1W_vs_E10	ordinary_muscle_development	ENSGALG00010008101	CENPQ	-2.678076781	0.210490057	-12.72305601	4.40297E-37	3.19425E-36	negative
PRJNA729271	stage_1W_vs_E10	ordinary_muscle_development	ENSGALG00010008103	RBPMS	0.29711534	0.108127584	2.747821869	0.005999259	0.008307152	positive
PRJNA729271	stage_1W_vs_E10	ordinary_muscle_development	ENSGALG00010008104	TCF25	0.652174397	0.094169942	6.925504914	4.34423E-12	1.11273E-11	positive
PRJNA729271	stage_1W_vs_E10	ordinary_muscle_development	ENSGALG00010008105	MMUT	0.332507995	0.096149497	3.45823955	0.000543718	0.00082664	positive
PRJNA729271	stage_1W_vs_E10	ordinary_muscle_development	ENSGALG00010008106	ENSGALG00010008106	0.336372603	0.374832723	0.89739391	0.369508761	0.407116866	positive
PRJNA729271	stage_1W_vs_E10	ordinary_muscle_development	ENSGALG00010008107	TUBB3	-1.97792255	0.20929456	-9.450425058	3.37458E-21	1.3328E-20	negative
PRJNA729271	stage_1W_vs_E10	ordinary_muscle_development	ENSGALG00010008108	ENSGALG00010008108	2.364640721	0.308579644	7.662983505	1.81663E-14	5.25799E-14	positive
PRJNA729271	stage_1W_vs_E10	ordinary_muscle_development	ENSGALG00010008110	DEF8	0.505892909	0.152689726	3.31320856	0.000922322	0.001379248	positive
PRJNA729271	stage_1W_vs_E10	ordinary_muscle_development	ENSGALG00010008111	SYTL2	-0.855432901	0.2605494	-3.283188917	0.001026399	0.001526539	negative
PRJNA729271	stage_1W_vs_E10	ordinary_muscle_development	ENSGALG00010008113	DBNDD1	-1.988923002	0.195338702	-10.18191985	2.38803E-24	1.06637E-23	negative
PRJNA729271	stage_1W_vs_E10	ordinary_muscle_development	ENSGALG00010008115	ENSGALG00010008115	0.160887602	0.200492517	0.802461878	0.422285832	0.46026518	positive
PRJNA729271	stage_1W_vs_E10	ordinary_muscle_development	ENSGALG00010008116	URAH	1.079522864	0.397385542	2.716563013	0.006596364	0.009093133	positive
PRJNA729271	stage_1W_vs_E10	ordinary_muscle_development	ENSGALG00010008117	ENSGALG00010008117	2.081980896	0.178846744	11.6411451	2.54574E-31	1.5148E-30	positive
PRJNA729271	stage_1W_vs_E10	ordinary_muscle_development	ENSGALG00010008118	ENSGALG00010008118	1.585468345	0.569969742	2.781671074	0.005407982	0.007526519	positive
PRJNA729271	stage_1W_vs_E10	ordinary_muscle_development	ENSGALG00010008119	CCDC83	-2.193931523	0.595695278	-3.682976184	0.000230527	0.00036132	negative
PRJNA729271	stage_1W_vs_E10	ordinary_muscle_development	ENSGALG00010008120	CDC5L	-0.212580493	0.064695545	-3.28585983	0.001016716	0.001512914	negative

PRJNA729271	stage_1W_vs_E10	ordinary_muscle_development	ENSGALG00010008121	MPZL1	-1.208976196	0.13605521	-8.885923544	6.33919E-19	2.26937E-18	negative
PRJNA729271	stage_1W_vs_E10	ordinary_muscle_development	ENSGALG00010008123	ENSGALG00010008123	-0.995191059	0.165628226	-6.008583707	1.87151E-09	4.15302E-09	negative
PRJNA729271	stage_1W_vs_E10	ordinary_muscle_development	ENSGALG00010008124	ENSGALG00010008124	0.35117631	0.211109611	1.663478552	0.096216681	0.115970463	positive
PRJNA729271	stage_1W_vs_E10	ordinary_muscle_development	ENSGALG00010008126	CDH3	-4.300981392	0.331149191	-12.98804742	1.4303E-38	1.09059E-37	negative
PRJNA729271	stage_1W_vs_E10	ordinary_muscle_development	ENSGALG00010008127	TBC1D2	-0.786905081	0.138367773	-5.687054606	1.29249E-08	2.72069E-08	negative
PRJNA729271	stage_1W_vs_E10	ordinary_muscle_development	ENSGALG00010008128	RARS	-0.387923054	0.070271861	-5.520318482	3.38386E-08	6.95829E-08	negative
PRJNA729271	stage_1W_vs_E10	ordinary_muscle_development	ENSGALG00010008129	TANGO6	0.632545232	0.1169784	5.407367794	6.39577E-08	1.29587E-07	positive
PRJNA729271	stage_1W_vs_E10	ordinary_muscle_development	ENSGALG00010008130	RCSD1	1.354230097	0.119817761	11.30241536	1.27658E-29	7.09237E-29	positive
PRJNA729271	stage_1W_vs_E10	ordinary_muscle_development	ENSGALG00010008131	OC90	-4.03109918	0.964925287	-4.177628292	2.94565E-05	4.9338E-05	negative
PRJNA729271	stage_1W_vs_E10	ordinary_muscle_development	ENSGALG00010008132	TDO2	1.709110402	0.640003233	2.670471514	0.00757448	0.01037294	positive
PRJNA729271	stage_1W_vs_E10	ordinary_muscle_development	ENSGALG00010008133	CREG1	3.601920254	0.167051441	21.56174304	4.1084E-103	1.6315E-101	positive
PRJNA729271	stage_1W_vs_E10	ordinary_muscle_development	ENSGALG00010008134	SLC2A13	-0.632718603	0.079920506	-7.916849287	2.43605E-15	7.35482E-15	negative
PRJNA729271	stage_1W_vs_E10	ordinary_muscle_development	ENSGALG00010008135	WWC1	-0.091499911	0.131553094	-0.69553599	0.486719465	0.52500362	negative
PRJNA729271	stage_1W_vs_E10	ordinary_muscle_development	ENSGALG00010008136	ELP1	0.549227347	0.113068811	4.857461075	1.18901E-06	2.21155E-06	positive
PRJNA729271	stage_1W_vs_E10	ordinary_muscle_development	ENSGALG00010008137	HHLA1	-2.179369507	0.893149884	-2.440093814	0.014683448	0.019469135	negative
PRJNA729271	stage_1W_vs_E10	ordinary_muscle_development	ENSGALG00010008139	ENSGALG00010008139	1.520856275	0.3941154	3.858911061	0.000113893	0.000182358	positive
PRJNA729271	stage_1W_vs_E10	ordinary_muscle_development	ENSGALG00010008141	APTX	0.771048903	0.153033128	5.038444375	4.69331E-07	8.96541E-07	positive
PRJNA729271	stage_1W_vs_E10	ordinary_muscle_development	ENSGALG00010008142	CD247	2.154622782	0.328588241	6.557212078	5.4823E-11	1.32229E-10	positive
PRJNA729271	stage_1W_vs_E10	ordinary_muscle_development	ENSGALG00010008143	DCTN6	1.029811512	0.114751783	8.97425279	2.85282E-19	1.03534E-18	positive
PRJNA729271	stage_1W_vs_E10	ordinary_muscle_development	ENSGALG00010008144	NTPCR	0.21686548	0.133370784	1.626034377	0.103942343	0.124722207	positive
PRJNA729271	stage_1W_vs_E10	ordinary_muscle_development	ENSGALG00010008145	ENSGALG00010008145	1.653413846	0.207734501	7.95926454	1.73065E-15	5.2668E-15	positive
PRJNA729271	stage_1W_vs_E10	ordinary_muscle_development	ENSGALG00010008146	SOX4	-2.08956623	0.119972999	-17.41697092	6.13397E-68	1.10081E-66	negative
PRJNA729271	stage_1W_vs_E10	ordinary_muscle_development	ENSGALG00010008147	CDKAL1	0.41941108	0.112563226	3.726004441	0.000194539	0.000306405	positive
PRJNA729271	stage_1W_vs_E10	ordinary_muscle_development	ENSGALG00010008148	ABCD2	2.210896514	0.13803495	16.01693281	9.73345E-58	1.33985E-56	positive
PRJNA729271	stage_1W_vs_E10	ordinary_muscle_development	ENSGALG00010008149	DNAJA1	1.460653741	0.281483193	5.189133061	2.11275E-07	4.13117E-07	positive
PRJNA729271	stage_1W_vs_E10	ordinary_muscle_development	ENSGALG00010008150	TERT	-2.1446782	0.58668162	-3.655608301	0.000256573	0.000400843	negative
PRJNA729271	stage_1W_vs_E10	ordinary_muscle_development	ENSGALG00010008151	SIPA1L2	-0.952839999	0.122400008	-7.784640011	6.9912E-15	2.06642E-14	negative
PRJNA729271	stage_1W_vs_E10	ordinary_muscle_development	ENSGALG00010008152	ENSGALG00010008152	-2.472280633	0.167511889	-14.75883681	2.69896E-49	2.88719E-48	negative
PRJNA729271	stage_1W_vs_E10	ordinary_muscle_development	ENSGALG00010008153	SMU1	-0.46405966	0.061983298	-7.486850104	7.05462E-14	1.98095E-13	negative
PRJNA729271	stage_1W_vs_E10	ordinary_muscle_development	ENSGALG00010008154	KCNQ3	-0.237678375	0.227347875	-1.045439172	0.29582004	0.330933972	negative
PRJNA729271	stage_1W_vs_E10	ordinary_muscle_development	ENSGALG00010008155	MAP10	0.958076137	0.220891451	4.337316498	1.44233E-05	2.47181E-05	positive
PRJNA729271	stage_1W_vs_E10	ordinary_muscle_development	ENSGALG00010008156	HAS3	-3.993697936	1.154238576	-3.460028126	0.000540119	0.000821471	negative
PRJNA729271	stage_1W_vs_E10	ordinary_muscle_development	ENSGALG00010008157	CTSO	-1.356424176	0.158109764	-8.579003244	9.56988E-18	3.211E-17	negative
PRJNA729271	stage_1W_vs_E10	ordinary_muscle_development	ENSGALG00010008158	CLPTM1L	-0.487452213	0.075760289	-6.43413881	1.24175E-10	2.93386E-10	negative
PRJNA729271	stage_1W_vs_E10	ordinary_muscle_development	ENSGALG00010008159	SUPT3H	-1.88128839	0.137305643	-13.70146452	9.9497E-43	8.70373E-42	negative
PRJNA729271	stage_1W_vs_E10	ordinary_muscle_development	ENSGALG00010008160	TXNL4A	0.133641936	0.091276383	1.464145833	0.143154107	0.168426849	positive
PRJNA729271	stage_1W_vs_E10	ordinary_muscle_development	ENSGALG00010008161	TMEFF1	-3.956571337	0.388964705	-10.17205749	2.64267E-24	1.1779E-23	negative
PRJNA729271	stage_1W_vs_E10	ordinary_muscle_development	ENSGALG00010008162	HSBP1L1	1.218880739	0.199371957	6.113601721	9.74072E-10	2.19276E-09	positive
PRJNA729271	stage_1W_vs_E10	ordinary_muscle_development	ENSGALG00010008163	CHTF8	-3.328340206	0.250138358	-13.30599687	2.13625E-40	1.73376E-39	negative
PRJNA729271	stage_1W_vs_E10	ordinary_muscle_development	ENSGALG00010008164	TG	0.712976468	0.29116718	2.448684185	0.01433791	0.019040587	positive
PRJNA729271	stage_1W_vs_E10	ordinary_muscle_development	ENSGALG00010008165	MRPL1	0.652197202	0.084500052	7.718305375	1.17886E-14	3.44578E-14	positive
PRJNA729271	stage_1W_vs_E10	ordinary_muscle_development	ENSGALG00010008166	RUNX2	-4.549607941	0.433074677	-10.50536588	8.16057E-26	3.88465E-25	negative
PRJNA729271	stage_1W_vs_E10	ordinary_muscle_development	ENSGALG00010008167	UTP4	1.253637078	0.172602528	7.263144381	3.78194E-13	1.02065E-12	positive
PRJNA729271	stage_1W_vs_E10	ordinary_muscle_development	ENSGALG00010008168	CAVIN4	5.547254426	0.124850056	44.43133323	0	0	positive
PRJNA729271	stage_1W_vs_E10	ordinary_muscle_development	ENSGALG00010008169	HTR4	-1.593994	0.414523949	-3.845360455	0.000120375	0.000192481	negative
PRJNA729271	stage_1W_vs_E10	ordinary_muscle_development	ENSGALG00010008170	PICALM	-0.321736876	0.071120345	-4.5238374	6.07284E-06	1.06905E-05	negative
PRJNA729271	stage_1W_vs_E10	ordinary_muscle_development	ENSGALG00010008171	KIF21A	0.244219875	0.179894158	1.357575348	0.17459847	0.202642711	positive
PRJNA729271	stage_1W_vs_E10	ordinary_muscle_development	ENSGALG00010008172	SNTB2	-0.479988274	0.134133358	-3.578440742	0.00034565	0.000534307	negative
PRJNA729271	stage_1W_vs_E10	ordinary_muscle_development	ENSGALG00010008174	SLC26A2	-1.214618013	0.126156215	-9.627888836	6.09692E-22	2.46995E-21	negative
PRJNA729271	stage_1W_vs_E10	ordinary_muscle_development	ENSGALG00010008175	ENSGALG00010008175	-1.779853388	0.462428759	-3.84892452	0.000118638	0.000189765	negative
PRJNA729271	stage_1W_vs_E10	ordinary_muscle_development	ENSGALG00010008176	SLA	1.066051316	0.305747832	3.486701143	0.000489017	0.000746295	positive
PRJNA729271	stage_1W_vs_E10	ordinary_muscle_development	ENSGALG00010008177	NREP	-1.601265411	0.184663086	-8.67128045	4.27288E-18	1.45902E-17	negative
PRJNA729271	stage_1W_vs_E10	ordinary_muscle_development	ENSGALG00010008178	PGR	-0.42657665	0.299801773	-1.422862333	0.154776067	0.181159488	negative
PRJNA729271	stage_1W_vs_E10	ordinary_muscle_development	ENSGALG00010008179	SEMA6A	-1.407458689	0.098153722	-14.33933084	1.24248E-46	1.21934E-45	negative
PRJNA729271	stage_1W_vs_E10	ordinary_muscle_development	ENSGALG00010008180	POU2F1	0.681085178	0.15822188	4.30462068	1.67272E-05	2.85119E-05	positive
PRJNA729271	stage_1W_vs_E10	ordinary_muscle_development	ENSGALG00010008181	DISC1	-0.365011363	0.131681406	-2.771927901	0.005572538	0.007741416	negative
PRJNA729271	stage_1W_vs_E10	ordinary_muscle_development	ENSGALG00010008182	CLIC5	6.365970976	0.28468891	22.36114841	9.4061E-111	4.274E-109	positive
PRJNA729271	stage_1W_vs_E10	ordinary_muscle_development	ENSGALG00010008183	ALG10	-0.785537199	0.088102607	-8.916162979	4.82723E-19	1.73797E-18	negative
PRJNA729271	stage_1W_vs_E10	ordinary_muscle_development	ENSGALG00010008184	SLC66A2	-0.517693122	0.087125129	-5.941949578	2.81652E-09	6.1838E-09	negative
PRJNA729271	stage_1W_vs_E10	ordinary_muscle_development	ENSGALG00010008185	PDGFC	-2.077653717	0.093089028	-22.31899675	2.4165E-110	1.0912E-108	negative
PRJNA729271	stage_1W_vs_E10	ordinary_muscle_development	ENSGALG00010008186	NOP53	0.929840844	0.121139157	7.675807422	1.6438E-14	4.76347E-14	positive
PRJNA729271	stage_1W_vs_E10	ordinary_muscle_development	ENSGALG00010008187	ENSGALG00010008187	3.133870585	0.375631714	8.342933959	7.24696E-17	2.33953E-16	positive
PRJNA729271	stage_1W_vs_E10	ordinary_muscle_development	ENSGALG00010008188	VPS4A	1.249725766	0.112624907	11.09635333	1.30667E-28	6.94798E-28	positive
PRJNA729271	stage_1W_vs_E10	ordinary_muscle_development	ENSGALG00010008189	EXOC3	0.522853409	0.113996101	4.586590271	4.50544E-06	8.01698E-06	positive
PRJNA729271	stage_1W_vs_E10	ordinary_muscle_development	ENSGALG00010008190	TSNARE1	-0.738203423	0.172232016	-4.286098712	1.81838E-05	3.09238E-05	negative
PRJNA729271	stage_1W_vs_E10	ordinary_muscle_development	ENSGALG00010008192	HNRNPU	-0.681928496	0.082990931	-8.216903792	2.08824E-16	6.60177E-16	negative
PRJNA729271	stage_1W_vs_E10	ordinary_muscle_development	ENSGALG00010008193	CNOT6L	1.78748575	0.129581815	13.79426391	2.75955E-43	2.45096E-42	positive
PRJNA729271	stage_1W_vs_E10	ordinary_muscle_development	ENSGALG00010008194	PDF	1.483208749	0.161647999	9.175546606	4.49289E-20	1.69154E-19	positive
PRJNA729271	stage_1W_vs_E10	ordinary_muscle_development	ENSGALG00010008195	ENSGALG00010008195	4.324717207	0.407945048	10.60122491	2.94103E-26	1.42671E-25	positive
PRJNA729271	stage_1W_vs_E10	ordinary_muscle_development	ENSGALG00010008196	PDCD6	-0.002724808	0.147240933	-0.018505778	0.985235368	0.987551805	negative
PRJNA729271	stage_1W_vs_E10	ordinary_muscle_development	ENSGALG00010008197	STARD4	-0.243073436	0.158047897	-1.537973236	0.124055159	0.147295177	negative
PRJNA729271	stage_1W_vs_E10	ordinary_muscle_development	ENSGALG00010008198	GLRB	-3.490485275	0.40566884	-8.60427258	7.68023E-18	2.58955E-17	negative
PRJNA729271	stage_1W_vs_E10	ordinary_muscle_development	ENSGALG00010008199	TPRKB	0.711872141	0.112061391	6.352519242	2.11817E-10	4.93536E-10	positive
PRJNA729271	stage_1W_vs_E10	ordinary_muscle_development	ENSGALG00010008200	COG8	0.920761328	0.125157837	7.35680123	1.8837E-13	5.15952E-13	positive
PRJNA729271	stage_1W_vs_E10	ordinary_muscle_development	ENSGALG00010008201	ENPP4	2.244558939	0.24036713	9.33804442	9.81298E-21	3.8093E-20	positive
PRJNA729271	stage_1W_vs_E10	ordinary_muscle_development	ENSGALG00010008202	KCNG2	0.692813293	0.19664516	3.523164746	0.000426426	0.000653941	positive
PRJNA729271	stage_1W_vs_E10	ordinary_muscle_development	ENSGALG00010008203	CXCL13	3.01159932	1.311778881	2.295813237	0.021686562	0.028235581	positive
PRJNA729271	stage_1W_vs_E10	ordinary_muscle_development	ENSGALG00010008204	ENSGALG00010008204	0.354673013	0.154302479	2.298556807	0.021530119	0.028044489	positive
PRJNA729271	stage_1W_vs_E10	ordinary_muscle_development	ENSGALG00010008205	ENSGALG00010008205	-0.250394673	0.17505371	-1.430387699	0.152605777	0.178821401	negative
PRJNA729271	stage_1W_vs_E10	ordinary_muscle_development	ENSGALG00010008206	CXCL13L2	-1.596093197	0.81883683	-1.949220086	0.051269147	0.063959574	negative
PRJNA729271	stage_1W_vs_E10	ordinary_muscle_development	ENSGALG00010008207	CAMK4	-1.193256509	0.163311392	-7.306633612	2.73918E-13	7.44086E-13	negative
PRJNA729271	stage_1W_vs_E10	ordinary_muscle_development	ENSGALG00010008208	ILDR2	1.277262865	0.153478848	8.32207749	8.64348E-17	2.78169E-16	positive
PRJNA729271	stage_1W_vs_E10	ordinary_muscle_development	ENSGALG00010008209	PPARGC1B	2.947791286	0.233466566	12.62618171	1.51451E-36	1.08035E-35	positive
PRJNA729271	stage_1W_vs_E10	ordinary_muscle_development	ENSGALG00010008210	CTDP1	-0.043696331	0.085822158	-0.509149753	0.61064726	0.64485881	negative
PRJNA729271	stage_1W_vs_E10	ordinary_muscle_development	ENSGALG00010008211	ADGRB1	-1.041569203	0.240314101	-4.334199283	1.46292E-05	2.50472E-05	negative
PRJNA729271	stage_1W_vs_E10	ordinary_muscle_development	ENSGALG00010008212	GTSE1	-2.819722142	0.230700437	-12.22243953	2.35898E-34	1.5585E-33	negative
PRJNA729271	stage_1W_vs_E10	ordinary_muscle_development	ENSGALG00010008213	ISCA1	0.868176767	0.117030744	7.418364917	1.18575E-13	3.28005E-13	positive
PRJNA729271	stage_1W_vs_E10	ordinary_muscle_development	ENSGALG00010008214	CCNG2	-0.725835012	0.136248818	-5.32727567	9.96968E-08	1.9927E-07	negative
PRJNA729271	stage_1W_vs_E10	ordinary_muscle_development	ENSGALG00010008215	SELENOW	3.138581602	0.127739594	24.57015478	2.6342E-133	1.7596E-131	positive
PRJNA729271	stage_1W_vs_E10	ordinary_muscle_development	ENSGALG00010008217	ARHGEF37	4.202290601	0.161268323	26.05775593	1.0989E-149	9.1018E-148	positive
PRJNA729271	stage_1W_vs_E10	ordinary_muscle_development	ENSGALG00010008218	ARHGAP42	-0.679070894	0.103637227	-6.552383889	5.66257E-11	1.36457E-10	negative
PRJNA729271	stage_1W_vs_E10	ordinary_muscle_development	ENSGALG00010008219	NIP7	0.00479645	0.11891339	0.04033566	0.967825524	0.972282175	positive
PRJNA729271	stage_1W_vs_E10	ordinary_muscle_development	ENSGALG00010008221	ENSGALG00010008221	1.597427716	0.519713805	3.07366805	0.002114446	0.003055113	positive
PRJNA729271	stage_1W_vs_E10	ordinary_muscle_development	ENSGALG00010008222	STYXL2	3.030152639	0.274828581	11.02560958	2.87564E-28	1.51462E-27	positive
PRJNA729271	stage_1W_vs_E10	ordinary_muscle_development	ENSGALG00010008223	ENSGALG00010008223	-1.553949281	0.154167649	-10.0796068	6.79977E-24	2.98313E-23	negative
PRJNA729271	stage_1W_vs_E10	ordinary_muscle_development	ENSGALG00010008224	ENSGALG00010008224	0.580125608	0.211669541	2.740713684	0.00613059	0.008475242	positive
PRJNA729271	stage_1W_vs_E10	ordinary_muscle_development	ENSGALG00010008226	COX20	-0.569846512	0.165826094	-3.436410378	0.000589477	0.000894522	negative
PRJNA729271	stage_1W_vs_E10	ordinary_muscle_development	ENSGALG00010008227	DUSP1	3.153197704	0.176958185	17.81888588	5.04309E-71	9.77268E-70	positive
PRJNA729271	stage_1W_vs_E10	ordinary_muscle_development	ENSGALG00010008228	TMED6	3.551131631	0.65587546	5.414338309	6.15158E-08	1.24831E-07	positive
PRJNA729271	stage_1W_vs_E10	ordinary_muscle_development	ENSGALG00010008229	PIGY	0.827712149	0.122531022	6.755123172	1.42714E-11	3.55558E-11	positive
PRJNA729271	stage_1W_vs_E10	ordinary_muscle_development	ENSGALG00010008230	TRMU	-0.006312742	0.115564824	-0.054625119	0.956437126	0.962012084	negative
PRJNA729271	stage_1W_vs_E10	ordinary_muscle_development	ENSGALG00010008231	ZCCHC6	-0.053443398	0.110410228	-0.484043906	0.628354709	0.661678279	negative
PRJNA729271	stage_1W_vs_E10	ordinary_muscle_development	ENSGALG00010008232	PYURF	1.033019056	0.127387569	8.109261085	5.09285E-16	1.58618E-15	positive
PRJNA729271	stage_1W_vs_E10	ordinary_muscle_development	ENSGALG00010008233	CYRIB	-0.617879142	0.072056535	-8.574921638	9.91543E-18	3.32541E-17	negative
PRJNA729271	stage_1W_vs_E10	ordinary_muscle_development	ENSGALG00010008234	COMMD10	-1.456747314	0.121977265	-11.94277732	7.08183E-33	4.45148E-32	negative
PRJNA729271	stage_1W_vs_E10	ordinary_muscle_development	ENSGALG00010008236	ENSGALG00010008236	1.78433254	0.65717169	2.715169516	0.006624185	0.009129749	positive
PRJNA729271	stage_1W_vs_E10	ordinary_muscle_development	ENSGALG00010008237	GRIA2	-4.467249032	0.514528493	-8.682218949	3.88121E-18	1.32872E-17	negative
PRJNA729271	stage_1W_vs_E10	ordinary_muscle_development	ENSGALG00010008238	NTAQ1	-0.486709692	0.166374182	-2.925391939	0.003440226	0.004877832	negative
PRJNA729271	stage_1W_vs_E10	ordinary_muscle_development	ENSGALG00010008240	RCAN2	-0.468161571	0.138974161	-3.368695066	0.000755249	0.001137504	negative
PRJNA729271	stage_1W_vs_E10	ordinary_muscle_development	ENSGALG00010008241	HERC3	0.289709147	0.083504597	3.469379596	0.000521662	0.000794524	positive
PRJNA729271	stage_1W_vs_E10	ordinary_muscle_development	ENSGALG00010008242	CNTN5	-7.244064612	1.419347259	-5.103800051			negative
PRJNA729271	stage_1W_vs_E10	ordinary_muscle_development	ENSGALG00010008243	YTHDC2	0.027645402	0.103653483	0.266709822	0.789692591	0.811930352	positive
PRJNA729271	stage_1W_vs_E10	ordinary_muscle_development	ENSGALG00010008245	FBXO32	5.988865474	0.491798038	12.17748957	4.09699E-34	2.68593E-33	positive
PRJNA729271	stage_1W_vs_E10	ordinary_muscle_development	ENSGALG00010008246	TERF2	-0.753709885	0.059824114	-12.59876386	2.1448E-36	1.52471E-35	negative
PRJNA729271	stage_1W_vs_E10	ordinary_muscle_development	ENSGALG00010008247	GASK1B	0.439188673	0.161221218	2.724136927	0.006446977	0.008893969	positive
PRJNA729271	stage_1W_vs_E10	ordinary_muscle_development	ENSGALG00010008248	TPK1	-0.840933656	0.164736542	-5.104718401	3.31287E-07	6.39075E-07	negative
PRJNA729271	stage_1W_vs_E10	ordinary_muscle_development	ENSGALG00010008250	KLHL38	6.479778697	0.592519023	10.93598423	7.75597E-28	4.00937E-27	positive
PRJNA729271	stage_1W_vs_E10	ordinary_muscle_development	ENSGALG00010008251	AHRR	-3.650627389	0.34203332	-10.67330921	1.35705E-26	6.67929E-26	negative
PRJNA729271	stage_1W_vs_E10	ordinary_muscle_development	ENSGALG00010008252	ZFP92	-2.396785873	0.134580662	-17.80928883	5.98656E-71	1.15855E-69	negative
PRJNA729271	stage_1W_vs_E10	ordinary_muscle_development	ENSGALG00010008253	ENSGALG00010008253	-0.441262655	0.731478617	-0.603247511	0.546344046	0.582512463	negative
PRJNA729271	stage_1W_vs_E10	ordinary_muscle_development	ENSGALG00010008255	MSANTD3	-2.60182691	0.137522759	-18.91924597	7.91791E-80	1.87227E-78	negative
PRJNA729271	stage_1W_vs_E10	ordinary_muscle_development	ENSGALG00010008257	DESI2	-0.752903622	0.06029339	-12.48733268	8.75395E-36	6.13581E-35	negative
PRJNA729271	stage_1W_vs_E10	ordinary_muscle_development	ENSGALG00010008258	CCDC82	0.875424911	0.13155024	6.654681234	2.83915E-11	6.97043E-11	positive
PRJNA729271	stage_1W_vs_E10	ordinary_muscle_development	ENSGALG00010008259	MCC	-1.164129966	0.11030786	-10.55346343	4.89584E-26	2.3553E-25	negative
PRJNA729271	stage_1W_vs_E10	ordinary_muscle_development	ENSGALG00010008261	CRX	-0.390846978	0.39812589	-0.981717059	0.326239259	0.362640955	negative
PRJNA729271	stage_1W_vs_E10	ordinary_muscle_development	ENSGALG00010008262	CYB5B	-1.208045927	0.073144716	-16.5158331	2.82237E-61	4.27931E-60	negative
PRJNA729271	stage_1W_vs_E10	ordinary_muscle_development	ENSGALG00010008263	C1orf101	-1.224749472	0.537399654	-2.279029142	0.022665333	0.029459649	negative
PRJNA729271	stage_1W_vs_E10	ordinary_muscle_development	ENSGALG00010008264	CNTNAP2	-4.011664991	0.345634286	-11.60667549	3.81148E-31	2.25316E-30	negative
PRJNA729271	stage_1W_vs_E10	ordinary_muscle_development	ENSGALG00010008265	ENSGALG00010008265	-2.277689109	0.732884995	-3.107839733	0.001884602	0.002738303	negative
PRJNA729271	stage_1W_vs_E10	ordinary_muscle_development	ENSGALG00010008266	ECSCR	0.72175813	0.187753382	3.844181771	0.000120955	0.000193366	positive
PRJNA729271	stage_1W_vs_E10	ordinary_muscle_development	ENSGALG00010008267	MAML2	-0.763083412	0.120109275	-6.353243018	2.10822E-10	4.91297E-10	negative

PRJNA729271	stage_1W_vs_E10	ordinary_muscle_development	ENSGALG00010008268	ENSGALG00010008268	1.48606396	0.240026248	6.191256052	5.96867E-10	1.35818E-09	positive
PRJNA729271	stage_1W_vs_E10	ordinary_muscle_development	ENSGALG00010008269	ENSGALG00010008269	0.665177989	0.316544739	2.101371175	0.035608396	0.045228155	positive
PRJNA729271	stage_1W_vs_E10	ordinary_muscle_development	ENSGALG00010008270	ENSGALG00010008270	0.949132403	0.16258407	5.837794582	5.28963E-09	1.14182E-08	positive
PRJNA729271	stage_1W_vs_E10	ordinary_muscle_development	ENSGALG00010008272	DCP2	-1.247582778	0.086806639	-14.37197426	7.75875E-47	7.66098E-46	negative
PRJNA729271	stage_1W_vs_E10	ordinary_muscle_development	ENSGALG00010008273	TMEM144	0.018396975	0.12149608	0.151420314	0.879644166	0.893263429	positive
PRJNA729271	stage_1W_vs_E10	ordinary_muscle_development	ENSGALG00010008274	NFAT5	-0.925421155	0.110941318	-8.341537446	7.33308E-17	2.36627E-16	negative
PRJNA729271	stage_1W_vs_E10	ordinary_muscle_development	ENSGALG00010008275	CELSR1	-1.773123764	0.174334252	-10.17082844	2.67623E-24	1.19213E-23	negative
PRJNA729271	stage_1W_vs_E10	ordinary_muscle_development	ENSGALG00010008276	DNAJC18	0.968128299	0.093116551	10.39695182	2.55991E-25	1.19158E-24	positive
PRJNA729271	stage_1W_vs_E10	ordinary_muscle_development	ENSGALG00010008277	MZB1	3.947725723	1.084494222	3.640153763	0.000272475	0.000425	positive
PRJNA729271	stage_1W_vs_E10	ordinary_muscle_development	ENSGALG00010008278	CYP39A1	1.017067397	0.160162496	6.350221932	2.15005E-10	5.00802E-10	positive
PRJNA729271	stage_1W_vs_E10	ordinary_muscle_development	ENSGALG00010008279	MAPK15	-0.777135262	0.177537943	-4.377291128	1.20163E-05	2.07279E-05	negative
PRJNA729271	stage_1W_vs_E10	ordinary_muscle_development	ENSGALG00010008282	FAM83H	-3.057239092	0.304390748	-10.0437977	9.78334E-24	4.27137E-23	negative
PRJNA729271	stage_1W_vs_E10	ordinary_muscle_development	ENSGALG00010008283	NQO1	-0.656229575	0.197123743	-3.329023508	0.00087151	0.001306229	negative
PRJNA729271	stage_1W_vs_E10	ordinary_muscle_development	ENSGALG00010008284	BCKDHA	2.549220559	0.127786256	19.94909804	1.52617E-88	4.38055E-87	positive
PRJNA729271	stage_1W_vs_E10	ordinary_muscle_development	ENSGALG00010008285	ADSS2	-1.76218689	0.090863801	-19.39371759	8.72005E-84	2.27743E-82	negative
PRJNA729271	stage_1W_vs_E10	ordinary_muscle_development	ENSGALG00010008289	MTMR2	-0.430089237	0.07071146	-6.082313086	1.18461E-09	2.65557E-09	negative
PRJNA729271	stage_1W_vs_E10	ordinary_muscle_development	ENSGALG00010008290	FAM13A	0.491645147	0.163721706	3.002931975	0.002673922	0.003828756	positive
PRJNA729271	stage_1W_vs_E10	ordinary_muscle_development	ENSGALG00010008291	PTGFRN	-0.999474767	0.16141125	-6.192101011	5.93675E-10	1.35113E-09	negative
PRJNA729271	stage_1W_vs_E10	ordinary_muscle_development	ENSGALG00010008292	REEP5	0.839622359	0.113422686	7.402596326	1.33547E-13	3.68507E-13	positive
PRJNA729271	stage_1W_vs_E10	ordinary_muscle_development	ENSGALG00010008293	PLA2G7	1.305527422	0.112664645	11.58772944	4.75573E-31	2.79766E-30	positive
PRJNA729271	stage_1W_vs_E10	ordinary_muscle_development	ENSGALG00010008294	NOB1	1.064940099	0.153534663	6.936154186	4.02918E-12	1.03331E-11	positive
PRJNA729271	stage_1W_vs_E10	ordinary_muscle_development	ENSGALG00010008295	DMAC2	0.627656188	0.131361852	4.778070497	1.76985E-06	3.24652E-06	positive
PRJNA729271	stage_1W_vs_E10	ordinary_muscle_development	ENSGALG00010008296	PALLD	-0.404275062	0.153144201	-2.639832651	0.008294697	0.011312725	negative
PRJNA729271	stage_1W_vs_E10	ordinary_muscle_development	ENSGALG00010008297	ENSGALG00010008297	0.254558394	0.285223964	0.892485998	0.372132495	0.409727346	positive
PRJNA729271	stage_1W_vs_E10	ordinary_muscle_development	ENSGALG00010008298	ENSGALG00010008298	-1.624952077	0.132972409	-12.22021988	2.42428E-34	1.60091E-33	negative
PRJNA729271	stage_1W_vs_E10	ordinary_muscle_development	ENSGALG00010008299	ANKRD66	1.24216859	0.264733389	4.692149322	2.7035E-06	4.89228E-06	positive
PRJNA729271	stage_1W_vs_E10	ordinary_muscle_development	ENSGALG00010008300	CUL1	0.12556464	0.085104615	1.475415167	0.14010096	0.165074927	positive
PRJNA729271	stage_1W_vs_E10	ordinary_muscle_development	ENSGALG00010008301	FAM91A1	-1.00362333	0.10639643	-9.432866609	3.99032E-21	1.57045E-20	negative
PRJNA729271	stage_1W_vs_E10	ordinary_muscle_development	ENSGALG00010008302	ENSGALG00010008302	0.506645791	0.144751793	3.500100277	0.000465083	0.000710741	positive
PRJNA729271	stage_1W_vs_E10	ordinary_muscle_development	ENSGALG00010008303	TEX10	-1.180592422	0.07219495	-16.35283934	4.15175E-60	6.09106E-59	negative
PRJNA729271	stage_1W_vs_E10	ordinary_muscle_development	ENSGALG00010008306	ENSGALG00010008306	0.30662818	0.308444867	0.994110172	0.320169234	0.356412952	positive
PRJNA729271	stage_1W_vs_E10	ordinary_muscle_development	ENSGALG00010008307	ENSGALG00010008307	1.002295118	0.950996121	1.053942382	0.291909297	0.326937511	positive
PRJNA729271	stage_1W_vs_E10	ordinary_muscle_development	ENSGALG00010008308	WWP2	-1.946772073	0.126190766	-15.42721496	1.07406E-53	1.33635E-52	negative
PRJNA729271	stage_1W_vs_E10	ordinary_muscle_development	ENSGALG00010008309	ENSGALG00010008309	-0.147469936	0.288935912	-0.51038978	0.609778415	0.644082061	negative
PRJNA729271	stage_1W_vs_E10	ordinary_muscle_development	ENSGALG00010008310	EXOSC5	0.953895514	0.177869311	5.362901053	8.18959E-08	1.64758E-07	positive
PRJNA729271	stage_1W_vs_E10	ordinary_muscle_development	ENSGALG00010008311	SRP19	-0.561666695	0.115455887	-4.864773114	1.14588E-06	2.13381E-06	negative
PRJNA729271	stage_1W_vs_E10	ordinary_muscle_development	ENSGALG00010008312	ZBTB18	1.508860864	0.21712594	6.949242753	3.67252E-12	9.44186E-12	positive
PRJNA729271	stage_1W_vs_E10	ordinary_muscle_development	ENSGALG00010008313	PSMD7	1.595179796	0.177700044	8.976811474	2.78727E-19	1.01181E-18	positive
PRJNA729271	stage_1W_vs_E10	ordinary_muscle_development	ENSGALG00010008314	INVS	-0.373327952	0.107947682	-3.458415635	0.000543362	0.000826187	negative
PRJNA729271	stage_1W_vs_E10	ordinary_muscle_development	ENSGALG00010008316	ADGRF5	3.045043027	0.133951331	22.73245821	2.1401E-114	1.0307E-112	positive
PRJNA729271	stage_1W_vs_E10	ordinary_muscle_development	ENSGALG00010008317	CEP57	-0.160975888	0.111833552	-1.4394239	0.150030457	0.176031364	negative
PRJNA729271	stage_1W_vs_E10	ordinary_muscle_development	ENSGALG00010008318	ADPRH	2.691522631	0.132805718	20.26661713	2.53495E-91	7.85129E-90	positive
PRJNA729271	stage_1W_vs_E10	ordinary_muscle_development	ENSGALG00010008319	ERP44	-0.123456613	0.065158124	-1.894723259	0.058129075	0.071997004	negative
PRJNA729271	stage_1W_vs_E10	ordinary_muscle_development	ENSGALG00010008320	GPRIN3	0.155651268	0.115222901	1.350870931	0.176736781	0.205025982	positive
PRJNA729271	stage_1W_vs_E10	ordinary_muscle_development	ENSGALG00010008321	ZFHX3	-0.386078037	0.103239745	-3.73962601	0.000184294	0.000290774	negative
PRJNA729271	stage_1W_vs_E10	ordinary_muscle_development	ENSGALG00010008323	AKT3	0.281579362	0.104967788	2.682531164	0.007306734	0.010019973	positive
PRJNA729271	stage_1W_vs_E10	ordinary_muscle_development	ENSGALG00010008324	SCRIB	0.054513651	0.104017807	0.524079988	0.600222907	0.634775356	positive
PRJNA729271	stage_1W_vs_E10	ordinary_muscle_development	ENSGALG00010008325	AADAT	0.184964295	0.132780321	1.393009853	0.163616811	0.190906035	positive
PRJNA729271	stage_1W_vs_E10	ordinary_muscle_development	ENSGALG00010008327	TGFB1	0.333379641	0.12882759	2.587796934	0.00965919	0.013078932	positive
PRJNA729271	stage_1W_vs_E10	ordinary_muscle_development	ENSGALG00010008328	ENSGALG00010008328	-0.424121031	0.487229696	-0.870474511	0.384041143	0.421973648	negative
PRJNA729271	stage_1W_vs_E10	ordinary_muscle_development	ENSGALG00010008330	EZH2	-2.655461697	0.084035157	-31.59941373	3.7612E-219	6.5685E-217	negative
PRJNA729271	stage_1W_vs_E10	ordinary_muscle_development	ENSGALG00010008331	CCDC97	1.133756543	0.262352151	4.321506559	1.54967E-05	2.6467E-05	positive
PRJNA729271	stage_1W_vs_E10	ordinary_muscle_development	ENSGALG00010008332	DHX38	-0.261581605	0.093711888	-2.791338533	0.005249054	0.007316573	negative
PRJNA729271	stage_1W_vs_E10	ordinary_muscle_development	ENSGALG00010008333	APC	-0.593253461	0.092384155	-6.421593211	1.34855E-10	3.18049E-10	negative
PRJNA729271	stage_1W_vs_E10	ordinary_muscle_development	ENSGALG00010008334	STX17	0.61803619	0.133655214	4.624108351	3.76213E-06	6.73153E-06	positive
PRJNA729271	stage_1W_vs_E10	ordinary_muscle_development	ENSGALG00010008335	HNRNPUL1	-0.37898159	0.189580832	-1.999050144	0.045602929	0.0572406	negative
PRJNA729271	stage_1W_vs_E10	ordinary_muscle_development	ENSGALG00010008336	PAIP2B	0.749164288	0.109047456	6.870075775	6.41678E-12	1.63149E-11	positive
PRJNA729271	stage_1W_vs_E10	ordinary_muscle_development	ENSGALG00010008339	FAM76B	-1.151104897	0.115243105	-9.988492576	1.71165E-23	7.39944E-23	negative
PRJNA729271	stage_1W_vs_E10	ordinary_muscle_development	ENSGALG00010008340	PDIA4	-1.154094613	0.105992998	-10.88840432	1.30914E-27	6.70766E-27	negative
PRJNA729271	stage_1W_vs_E10	ordinary_muscle_development	ENSGALG00010008341	DHODH	-1.652998616	0.11220193	-14.7323546	3.99539E-49	4.24272E-48	negative
PRJNA729271	stage_1W_vs_E10	ordinary_muscle_development	ENSGALG00010008342	PLD3	0.77225544	0.149431698	5.167949321	2.36676E-07	4.61292E-07	positive
PRJNA729271	stage_1W_vs_E10	ordinary_muscle_development	ENSGALG00010008343	TNFRSF21	2.10656213	0.222995238	9.446668669	3.49786E-21	1.37963E-20	positive
PRJNA729271	stage_1W_vs_E10	ordinary_muscle_development	ENSGALG00010008344	MFAP3L	1.198715295	0.289557924	4.139811746	3.47591E-05	5.79318E-05	positive
PRJNA729271	stage_1W_vs_E10	ordinary_muscle_development	ENSGALG00010008346	NR4A3	4.40646965	0.522509954	8.433274078	3.36126E-17	1.09931E-16	positive
PRJNA729271	stage_1W_vs_E10	ordinary_muscle_development	ENSGALG00010008347	PUF60	-0.483474578	0.055093741	-8.775490069	1.70161E-18	5.93619E-18	negative
PRJNA729271	stage_1W_vs_E10	ordinary_muscle_development	ENSGALG00010008348	ENSGALG00010008348	-1.520680812	0.105621999	-14.39738709	5.3737E-47	5.33871E-46	negative
PRJNA729271	stage_1W_vs_E10	ordinary_muscle_development	ENSGALG00010008350	LRRC6	-0.819383137	0.185374025	-4.42016154	9.86271E-06	1.7123E-05	negative
PRJNA729271	stage_1W_vs_E10	ordinary_muscle_development	ENSGALG00010008351	IST1	-0.524272373	0.068962622	-7.602268556	2.90984E-14	8.32573E-14	negative
PRJNA729271	stage_1W_vs_E10	ordinary_muscle_development	ENSGALG00010008352	ENSGALG00010008352	0.745802184	0.15216748	4.901192982	9.52564E-07	1.7846E-06	positive
PRJNA729271	stage_1W_vs_E10	ordinary_muscle_development	ENSGALG00010008354	TMEM71	2.184446514	0.2225364	9.816131247	9.59556E-23	4.01407E-22	positive
PRJNA729271	stage_1W_vs_E10	ordinary_muscle_development	ENSGALG00010008355	MAFA	5.368567653	0.177192121	30.29800437	1.2179E-201	1.8389E-199	positive
PRJNA729271	stage_1W_vs_E10	ordinary_muscle_development	ENSGALG00010008356	ENSGALG00010008356	-0.929653921	0.135804991	-6.845506292	7.62059E-12	1.92843E-11	negative
PRJNA729271	stage_1W_vs_E10	ordinary_muscle_development	ENSGALG00010008358	HPF1	-2.080736671	0.118716871	-17.52688273	8.93348E-69	1.6412E-67	negative
PRJNA729271	stage_1W_vs_E10	ordinary_muscle_development	ENSGALG00010008360	ZC3H3	-0.113494707	0.062967388	-1.802436314	0.071476786	0.087489952	negative
PRJNA729271	stage_1W_vs_E10	ordinary_muscle_development	ENSGALG00010008361	MATR3	-1.040267841	0.057046	-18.23559647	2.693E-74	5.57643E-73	negative
PRJNA729271	stage_1W_vs_E10	ordinary_muscle_development	ENSGALG00010008362	RAMP3	0.581031408	0.262314159	2.215021145	0.026758613	0.03446167	positive
PRJNA729271	stage_1W_vs_E10	ordinary_muscle_development	ENSGALG00010008364	ENSGALG00010008364	-1.399848499	0.601008011	-2.329167788	0.019850179	0.025956548	negative
PRJNA729271	stage_1W_vs_E10	ordinary_muscle_development	ENSGALG00010008366	RPRD1A	-0.369174237	0.108453541	-3.403985093	0.000664104	0.001003983	negative
PRJNA729271	stage_1W_vs_E10	ordinary_muscle_development	ENSGALG00010008367	ENSGALG00010008367	-1.475141943	0.630802509	-2.338516291	0.019360482	0.025334493	negative
PRJNA729271	stage_1W_vs_E10	ordinary_muscle_development	ENSGALG00010008368	SNCA	1.513703398	0.318141428	4.757957512	1.95562E-06	3.57687E-06	positive
PRJNA729271	stage_1W_vs_E10	ordinary_muscle_development	ENSGALG00010008372	CLCN3	-0.097930483	0.069431855	-1.410454655	0.158405477	0.185138477	negative
PRJNA729271	stage_1W_vs_E10	ordinary_muscle_development	ENSGALG00010008373	MACIR	-0.72830435	0.166540631	-4.373133133	1.22476E-05	2.11168E-05	negative
PRJNA729271	stage_1W_vs_E10	ordinary_muscle_development	ENSGALG00010008374	ZNF821	-0.75445553	0.088050616	-8.56842991	1.04906E-17	3.51098E-17	negative
PRJNA729271	stage_1W_vs_E10	ordinary_muscle_development	ENSGALG00010008375	ENSGALG00010008375	-0.799251159	0.098946365	-8.077620221	6.60429E-16	2.04944E-15	negative
PRJNA729271	stage_1W_vs_E10	ordinary_muscle_development	ENSGALG00010008376	MMRN1	1.065818523	0.258451788	4.123858189	3.72578E-05	6.19681E-05	positive
PRJNA729271	stage_1W_vs_E10	ordinary_muscle_development	ENSGALG00010008377	AKT2	0.493257002	0.116610018	4.22997107	2.33721E-05	3.93883E-05	positive
PRJNA729271	stage_1W_vs_E10	ordinary_muscle_development	ENSGALG00010008380	SIL1	0.860022659	0.171409539	5.017355896	5.23875E-07	9.97316E-07	positive
PRJNA729271	stage_1W_vs_E10	ordinary_muscle_development	ENSGALG00010008381	C18orf21	1.214190236	0.11230925	10.81113295	3.04881E-27	1.53856E-26	positive
PRJNA729271	stage_1W_vs_E10	ordinary_muscle_development	ENSGALG00010008382	ENSGALG00010008382	0.766192415	0.12936925	5.922523427	3.17039E-09	6.93447E-09	positive
PRJNA729271	stage_1W_vs_E10	ordinary_muscle_development	ENSGALG00010008383	PLD5	-4.304028024	0.457493373	-9.407847803	5.06399E-21	1.98332E-20	negative
PRJNA729271	stage_1W_vs_E10	ordinary_muscle_development	ENSGALG00010008385	CTNNA1	-1.049567021	0.091696468	-11.4461008	2.45962E-30	1.40418E-29	negative
PRJNA729271	stage_1W_vs_E10	ordinary_muscle_development	ENSGALG00010008386	SESN3	-1.904835397	0.228610898	-8.332216048	7.9343E-17	2.55743E-16	negative
PRJNA729271	stage_1W_vs_E10	ordinary_muscle_development	ENSGALG00010008387	PHF20L1	0.483872767	0.11223931	4.311081099	1.62458E-05	2.77235E-05	positive
PRJNA729271	stage_1W_vs_E10	ordinary_muscle_development	ENSGALG00010008388	ADCY1	-1.242702037	0.436031516	-2.850028021	0.004371538	0.006132337	negative
PRJNA729271	stage_1W_vs_E10	ordinary_muscle_development	ENSGALG00010008389	GALNT1	-1.193537411	0.089030087	-13.40600078	5.57659E-41	4.60848E-40	negative
PRJNA729271	stage_1W_vs_E10	ordinary_muscle_development	ENSGALG00010008391	ENSGALG00010008391	-0.522025765	0.075650384	-6.900503821	5.18184E-12	1.32191E-11	negative
PRJNA729271	stage_1W_vs_E10	ordinary_muscle_development	ENSGALG00010008392	CD36	3.099361187	0.369339264	8.391637409	4.79418E-17	1.55636E-16	positive
PRJNA729271	stage_1W_vs_E10	ordinary_muscle_development	ENSGALG00010008394	CD2AP	0.911125638	0.110833534	8.220667544	2.02374E-16	6.40481E-16	positive
PRJNA729271	stage_1W_vs_E10	ordinary_muscle_development	ENSGALG00010008395	ENDOD1	0.472691711	0.146564087	3.225153722	0.00125905	0.001855611	positive
PRJNA729271	stage_1W_vs_E10	ordinary_muscle_development	ENSGALG00010008396	LRRTM2	-3.15279259	0.486167769	-6.484988907	8.87383E-11	2.11348E-10	negative
PRJNA729271	stage_1W_vs_E10	ordinary_muscle_development	ENSGALG00010008397	ATXN1L	-0.846304086	0.073923427	-11.44838813	2.39559E-30	1.36924E-29	negative
PRJNA729271	stage_1W_vs_E10	ordinary_muscle_development	ENSGALG00010008399	INO80C	-0.485373205	0.113309522	-4.283604732	1.83889E-05	3.12446E-05	negative
PRJNA729271	stage_1W_vs_E10	ordinary_muscle_development	ENSGALG00010008400	MAP1LC3C	-2.36969484	0.269864644	-8.781049671	1.61956E-18	5.65948E-18	negative
PRJNA729271	stage_1W_vs_E10	ordinary_muscle_development	ENSGALG00010008401	PSMD8	1.175033825	0.155678362	7.547830116	4.4257E-14	1.25539E-13	positive
PRJNA729271	stage_1W_vs_E10	ordinary_muscle_development	ENSGALG00010008403	GNAT3	-1.526742436	0.469120025	-3.254481483	0.001135996	0.001681775	negative
PRJNA729271	stage_1W_vs_E10	ordinary_muscle_development	ENSGALG00010008405	LRATD2	0.232454842	0.144867107	1.604607467	0.108580217	0.129910875	positive
PRJNA729271	stage_1W_vs_E10	ordinary_muscle_development	ENSGALG00010008407	GNAI1	-1.278155686	0.194818954	-6.560735796	5.35429E-11	1.29222E-10	negative
PRJNA729271	stage_1W_vs_E10	ordinary_muscle_development	ENSGALG00010008408	SMARCA4	-0.599859759	0.15400305	-3.895116093	9.81517E-05	0.000157973	negative
PRJNA729271	stage_1W_vs_E10	ordinary_muscle_development	ENSGALG00010008409	CWC15	0.230765374	0.084892672	2.718319132	0.006561452	0.009045867	positive
PRJNA729271	stage_1W_vs_E10	ordinary_muscle_development	ENSGALG00010008410	NAPRT	-0.576713639	0.133175895	-4.330465647	1.48794E-05	2.54517E-05	negative
PRJNA729271	stage_1W_vs_E10	ordinary_muscle_development	ENSGALG00010008411	ENSGALG00010008411	-0.18803837	0.436647712	-0.430640915	0.666729487	0.697024443	negative
PRJNA729271	stage_1W_vs_E10	ordinary_muscle_development	ENSGALG00010008412	NEK1	-1.151377743	0.10581887	-10.8806467	1.42548E-27	7.29087E-27	negative
PRJNA729271	stage_1W_vs_E10	ordinary_muscle_development	ENSGALG00010008415	ENSGALG00010008415	1.408520795	0.346572391	4.064145998	4.82086E-05	7.93715E-05	positive
PRJNA729271	stage_1W_vs_E10	ordinary_muscle_development	ENSGALG00010008416	MSX2	-5.74722376	0.542369956	-10.59650096	3.09338E-26	1.49962E-25	negative
PRJNA729271	stage_1W_vs_E10	ordinary_muscle_development	ENSGALG00010008417	IGFBP1	0.627733102	0.87479869	0.717574351	0.473019777	0.511558731	positive
PRJNA729271	stage_1W_vs_E10	ordinary_muscle_development	ENSGALG00010008418	TMEM245	1.784723009	0.088444456	20.17902639	1.49665E-90	4.4915E-89	positive
PRJNA729271	stage_1W_vs_E10	ordinary_muscle_development	ENSGALG00010008422	EXO1	-3.953823379	0.240906047	-16.41230441	1.56164E-60	2.32164E-59	negative
PRJNA729271	stage_1W_vs_E10	ordinary_muscle_development	ENSGALG00010008424	PPIP5K2	-0.918233253	0.121632155	-7.549264045	4.37725E-14	1.24213E-13	negative
PRJNA729271	stage_1W_vs_E10	ordinary_muscle_development	ENSGALG00010008425	MAGI2	-0.753256121	0.110402233	-6.822834137	8.92617E-12	2.25095E-11	negative
PRJNA729271	stage_1W_vs_E10	ordinary_muscle_development	ENSGALG00010008426	AP1G1	-0.574681494	0.056257459	-10.21520529	1.6952E-24	7.63577E-24	negative
PRJNA729271	stage_1W_vs_E10	ordinary_muscle_development	ENSGALG00010008429	IGFBP3	1.474835937	0.118584732	12.43698	1.64611E-35	1.13783E-34	positive
PRJNA729271	stage_1W_vs_E10	ordinary_muscle_development	ENSGALG00010008430	ENSGALG00010008430	0.284138611	0.39880623	0.71247285	0.476172018	0.514507149	positive
PRJNA729271	stage_1W_vs_E10	ordinary_muscle_development	ENSGALG00010008431	SH3RF1	-0.388754363	0.126623681	-3.07015527	0.002139475	0.003090045	negative
PRJNA729271	stage_1W_vs_E10	ordinary_muscle_development	ENSGALG00010008433	EBAG9	0.803178639	0.095412597	8.417951731	3.83124E-17	1.24907E-16	positive
PRJNA729271	stage_1W_vs_E10	ordinary_muscle_development	ENSGALG00010008434	PTCHD4	-1.549829365	0.487753041	-3.17748786	0.001485569	0.002177943	negative
PRJNA729271	stage_1W_vs_E10	ordinary_muscle_development	ENSGALG00010008437	SHKBP1	0.210484534	0.161852328	1.300472697	0.193439008	0.223133728	positive
PRJNA729271	stage_1W_vs_E10	ordinary_muscle_development	ENSGALG00010008438	SYBU	0.085280516	0.265104699	0.321686173	0.747690454	0.772691653	positive
PRJNA729271	stage_1W_vs_E10	ordinary_muscle_development	ENSGALG00010008440	ENSGALG00010008440	0.276287856	0.320076866	0.863192208	0.388031797	0.426132351	positive
PRJNA729271	stage_1W_vs_E10	ordinary_muscle_development	ENSGALG00010008441	PHLPP2	0.122628063	0.086708428	1.414257716	0.157286247	0.183919341	positive
PRJNA729271	stage_1W_vs_E10	ordinary_muscle_development	ENSGALG00010008443	RSFR	4.612859208	0.439354234	10.49918005	8.71337E-26	4.13964E-25	positive
PRJNA729271	stage_1W_vs_E10	ordinary_muscle_development	ENSGALG00010008444	SLC7A11	-2.91903505	0.460399308	-6.340224673	2.2943E-10	5.3346E-10	negative

PRJNA729271	stage_1W_vs_E10	ordinary_muscle_development	ENSGALG00010008447	FRRS1L	-0.114398075	0.155816785	-0.734183259	0.462837055	0.501113558	negative
PRJNA729271	stage_1W_vs_E10	ordinary_muscle_development	ENSGALG00010008448	TNS3	-1.160708138	0.081487313	-14.24403496	4.88246E-46	4.70241E-45	negative
PRJNA729271	stage_1W_vs_E10	ordinary_muscle_development	ENSGALG00010008449	SRA1	0.322072531	0.108954955	2.956015462	0.003116415	0.004433016	positive
PRJNA729271	stage_1W_vs_E10	ordinary_muscle_development	ENSGALG00010008450	GIN1	-0.682522766	0.180360512	-3.78421396	0.000154195	0.00024467	negative
PRJNA729271	stage_1W_vs_E10	ordinary_muscle_development	ENSGALG00010008451	OPN3	0.562926542	0.311835339	1.805204448	0.071042695	0.087002692	positive
PRJNA729271	stage_1W_vs_E10	ordinary_muscle_development	ENSGALG00010008452	ENSGALG00010008452	0.293764772	0.431461622	0.680859564	0.495960356	0.533779727	positive
PRJNA729271	stage_1W_vs_E10	ordinary_muscle_development	ENSGALG00010008453	AMOTL1	1.049749167	0.120204519	8.733025819	2.47948E-18	8.56329E-18	positive
PRJNA729271	stage_1W_vs_E10	ordinary_muscle_development	ENSGALG00010008455	FKBP1B	2.517702296	0.149955031	16.78971538	2.90233E-63	4.59774E-62	positive
PRJNA729271	stage_1W_vs_E10	ordinary_muscle_development	ENSGALG00010008456	NHLRC1	0.714430872	0.127325602	5.611054364	2.01098E-08	4.19291E-08	positive
PRJNA729271	stage_1W_vs_E10	ordinary_muscle_development	ENSGALG00010008457	MARVELD3	0.822255	0.11859987	6.933017705	4.11957E-12	1.05612E-11	positive
PRJNA729271	stage_1W_vs_E10	ordinary_muscle_development	ENSGALG00010008459	EPB41L4B	-2.17792852	0.228316695	-9.539068159	1.44121E-21	5.77881E-21	negative
PRJNA729271	stage_1W_vs_E10	ordinary_muscle_development	ENSGALG00010008464	SF3B6	-0.119538085	0.110460076	-1.082183617	0.279170947	0.313591357	negative
PRJNA729271	stage_1W_vs_E10	ordinary_muscle_development	ENSGALG00010008466	C11orf97	-1.363169256	0.403927865	-3.374783899	0.000738737	0.001113444	negative
PRJNA729271	stage_1W_vs_E10	ordinary_muscle_development	ENSGALG00010008468	EPPK1	-2.934742746	0.311597851	-9.418366447	4.5816E-21	1.7973E-20	negative
PRJNA729271	stage_1W_vs_E10	ordinary_muscle_development	ENSGALG00010008469	NOCT	2.798165005	0.275670209	10.15040767	3.29982E-24	1.46451E-23	positive
PRJNA729271	stage_1W_vs_E10	ordinary_muscle_development	ENSGALG00010008473	CSMD3	-5.408959191	0.594121177	-9.10413465	8.69569E-20	3.23854E-19	negative
PRJNA729271	stage_1W_vs_E10	ordinary_muscle_development	ENSGALG00010008474	ENSGALG00010008474	1.531158069	0.13918031	11.00125488	3.76851E-28	1.97486E-27	positive
PRJNA729271	stage_1W_vs_E10	ordinary_muscle_development	ENSGALG00010008476	RNF166	0.501416707	0.134188919	3.736647633	0.00018649	0.000294111	positive
PRJNA729271	stage_1W_vs_E10	ordinary_muscle_development	ENSGALG00010008486	CTU2	-0.060950032	0.136954204	-0.445039509	0.656291202	0.687202267	negative
PRJNA729271	stage_1W_vs_E10	ordinary_muscle_development	ENSGALG00010008488	CCSER1	-1.303162508	0.158571516	-8.2181374	2.06688E-16	6.53851E-16	negative
PRJNA729271	stage_1W_vs_E10	ordinary_muscle_development	ENSGALG00010008492	ANKRD49	0.93570891	0.122407879	7.644188577	2.10267E-14	6.05808E-14	positive
PRJNA729271	stage_1W_vs_E10	ordinary_muscle_development	ENSGALG00010008497	PAM	-0.865273323	0.130238549	-6.643757379	3.05786E-11	7.49217E-11	negative
PRJNA729271	stage_1W_vs_E10	ordinary_muscle_development	ENSGALG00010008498	ENSGALG00010008498	2.683814477	0.147636959	18.17847305	7.64325E-74	1.57594E-72	positive
PRJNA729271	stage_1W_vs_E10	ordinary_muscle_development	ENSGALG00010008500	ENSGALG00010008500	1.111387903	0.151422566	7.339645166	2.14161E-13	5.84717E-13	positive
PRJNA729271	stage_1W_vs_E10	ordinary_muscle_development	ENSGALG00010008501	ENSGALG00010008501	-1.705385164	0.443700768	-3.843547919	0.000121268	0.000193823	negative
PRJNA729271	stage_1W_vs_E10	ordinary_muscle_development	ENSGALG00010008504	RNF139	1.191245291	0.08723821	13.65508642	1.88266E-42	1.6331E-41	positive
PRJNA729271	stage_1W_vs_E10	ordinary_muscle_development	ENSGALG00010008505	PTPN3	-0.853824324	0.272566636	-3.132534255	0.001733042	0.002525683	negative
PRJNA729271	stage_1W_vs_E10	ordinary_muscle_development	ENSGALG00010008509	MGARP	2.138320381	0.35300796	6.057428217	1.38315E-09	3.09204E-09	positive
PRJNA729271	stage_1W_vs_E10	ordinary_muscle_development	ENSGALG00010008514	PIEZO1	1.68295304	0.166367996	10.11584607	4.69939E-24	2.07423E-23	positive
PRJNA729271	stage_1W_vs_E10	ordinary_muscle_development	ENSGALG00010008515	TAFA5	-1.410145534	0.219826431	-6.414813397	1.40995E-10	3.32098E-10	negative
PRJNA729271	stage_1W_vs_E10	ordinary_muscle_development	ENSGALG00010008516	MRE11	-1.468068654	0.080925318	-18.14103029	1.51171E-73	3.09494E-72	negative
PRJNA729271	stage_1W_vs_E10	ordinary_muscle_development	ENSGALG00010008522	NUDT12	0.631947573	0.150300613	4.204557528	2.61593E-05	4.39272E-05	positive
PRJNA729271	stage_1W_vs_E10	ordinary_muscle_development	ENSGALG00010008527	CHUK	0.484037344	0.054823663	8.82898578	1.05629E-18	3.72801E-18	positive
PRJNA729271	stage_1W_vs_E10	ordinary_muscle_development	ENSGALG00010008529	GPR83	-0.050853587	0.503112714	-0.101077921	0.919488605	0.92936248	negative
PRJNA729271	stage_1W_vs_E10	ordinary_muscle_development	ENSGALG00010008533	ENSGALG00010008533	0.236277169	0.556022169	0.424941993	0.670878961	0.700958015	positive
PRJNA729271	stage_1W_vs_E10	ordinary_muscle_development	ENSGALG00010008534	RAB33B	0.782670152	0.072087117	10.8572819	1.84149E-27	9.38221E-27	positive
PRJNA729271	stage_1W_vs_E10	ordinary_muscle_development	ENSGALG00010008542	MOCOS	2.668064658	0.15426661	17.29515327	5.11667E-67	8.91421E-66	positive
PRJNA729271	stage_1W_vs_E10	ordinary_muscle_development	ENSGALG00010008561	EEF1D	1.430420769	0.114699709	12.47100614	1.07461E-35	7.50309E-35	positive
PRJNA729271	stage_1W_vs_E10	ordinary_muscle_development	ENSGALG00010008580	PANX1	-2.431540859	0.262821142	-9.251694287	2.20962E-20	8.43297E-20	negative
PRJNA729271	stage_1W_vs_E10	ordinary_muscle_development	ENSGALG00010008588	PYCR3	-1.212809794	0.18119634	-6.693345983	2.18125E-11	5.38985E-11	negative
PRJNA729271	stage_1W_vs_E10	ordinary_muscle_development	ENSGALG00010008592	KIAA0513	1.697345658	0.138676889	12.23957117	1.91039E-34	1.26849E-33	positive
PRJNA729271	stage_1W_vs_E10	ordinary_muscle_development	ENSGALG00010008596	ENSGALG00010008596	0.32268815	0.155016077	2.081643114	0.03737508	0.047376544	positive
PRJNA729271	stage_1W_vs_E10	ordinary_muscle_development	ENSGALG00010008618	EIF4EBP3	0.535211779	0.116618514	4.589423769	4.44471E-06	7.91281E-06	positive
PRJNA729271	stage_1W_vs_E10	ordinary_muscle_development	ENSGALG00010008623	TRPS1	-2.008458823	0.133040202	-15.09663085	1.70422E-51	1.96677E-50	negative
PRJNA729271	stage_1W_vs_E10	ordinary_muscle_development	ENSGALG00010008636	AGTPBP1	5.02201998	0.238428744	21.06298052	1.73883E-98	6.28539E-97	positive
PRJNA729271	stage_1W_vs_E10	ordinary_muscle_development	ENSGALG00010008637	RGS7	-4.397566301	0.827102465	-5.316833748	1.05588E-07	2.10581E-07	negative
PRJNA729271	stage_1W_vs_E10	ordinary_muscle_development	ENSGALG00010008653	ENSGALG00010008653	-1.32579895	0.16215106	-8.176319975	2.92644E-16	9.18155E-16	negative
PRJNA729271	stage_1W_vs_E10	ordinary_muscle_development	ENSGALG00010008656	CCDC6	0.001438165	0.07194892	0.019988696	0.98405239	0.986502482	positive
PRJNA729271	stage_1W_vs_E10	ordinary_muscle_development	ENSGALG00010008658	TSTA3	-1.359985158	0.119873952	-11.34512656	7.84127E-30	4.40369E-29	negative
PRJNA729271	stage_1W_vs_E10	ordinary_muscle_development	ENSGALG00010008666	SLC16A9	1.701513069	0.188366208	9.033005894	1.6702E-19	6.14143E-19	positive
PRJNA729271	stage_1W_vs_E10	ordinary_muscle_development	ENSGALG00010008668	ENSGALG00010008668	-0.25711397	0.331090528	-0.776566975	0.437414292	0.475357637	negative
PRJNA729271	stage_1W_vs_E10	ordinary_muscle_development	ENSGALG00010008674	RXFP1	1.209526731	0.273368163	4.424534001	9.66507E-06	1.67899E-05	positive
PRJNA729271	stage_1W_vs_E10	ordinary_muscle_development	ENSGALG00010008686	ENSGALG00010008686	-1.900610112	0.419988851	-4.525382301	6.02864E-06	1.06192E-05	negative
PRJNA729271	stage_1W_vs_E10	ordinary_muscle_development	ENSGALG00010008688	HBEGF	2.489327045	0.228389323	10.89948955	1.15906E-27	5.9513E-27	positive
PRJNA729271	stage_1W_vs_E10	ordinary_muscle_development	ENSGALG00010008690	YIF1B	-0.495180005	0.124724677	-3.970184717	7.18169E-05	0.000116886	negative
PRJNA729271	stage_1W_vs_E10	ordinary_muscle_development	ENSGALG00010008693	C4orf46	-0.450710089	0.105260403	-4.281857892	1.85339E-05	3.14873E-05	negative
PRJNA729271	stage_1W_vs_E10	ordinary_muscle_development	ENSGALG00010008696	PFN4	-0.327947383	0.216929053	-1.511772532	0.130591739	0.154449026	negative
PRJNA729271	stage_1W_vs_E10	ordinary_muscle_development	ENSGALG00010008699	NAA35	0.866884964	0.099713422	8.693764074	3.50625E-18	1.20291E-17	positive
PRJNA729271	stage_1W_vs_E10	ordinary_muscle_development	ENSGALG00010008700	ENSGALG00010008700	0.684113092	0.254846238	2.684415115	0.007265683	0.009968393	positive
PRJNA729271	stage_1W_vs_E10	ordinary_muscle_development	ENSGALG00010008708	ZC3H4	1.544540497	0.24368161	6.338354776	2.32232E-10	5.39716E-10	positive
PRJNA729271	stage_1W_vs_E10	ordinary_muscle_development	ENSGALG00010008716	TATDN1	0.013794946	0.111282922	0.123962835	0.901344701	0.912805942	positive
PRJNA729271	stage_1W_vs_E10	ordinary_muscle_development	ENSGALG00010008729	TMEM254	-0.880798113	0.120172514	-7.329447345	2.31104E-13	6.29908E-13	negative
PRJNA729271	stage_1W_vs_E10	ordinary_muscle_development	ENSGALG00010008731	NDUFB9	1.069145361	0.087451052	12.2256432	2.2678E-34	1.50031E-33	positive
PRJNA729271	stage_1W_vs_E10	ordinary_muscle_development	ENSGALG00010008739	VSTM5	-1.187375078	0.26468957	-4.485915622	7.26015E-06	1.27112E-05	negative
PRJNA729271	stage_1W_vs_E10	ordinary_muscle_development	ENSGALG00010008745	GRAMD4	1.353105125	0.152247667	8.887526191	6.24845E-19	2.23909E-18	positive
PRJNA729271	stage_1W_vs_E10	ordinary_muscle_development	ENSGALG00010008750	PFDN1	1.024886257	0.165566917	6.190163332	6.01019E-10	1.36741E-09	positive
PRJNA729271	stage_1W_vs_E10	ordinary_muscle_development	ENSGALG00010008752	GOLM1	-1.473901471	0.173660536	-8.487256249	2.11573E-17	6.98258E-17	negative
PRJNA729271	stage_1W_vs_E10	ordinary_muscle_development	ENSGALG00010008756	ITSN2	1.017292168	0.073178675	13.9014839	6.20416E-44	5.62762E-43	positive
PRJNA729271	stage_1W_vs_E10	ordinary_muscle_development	ENSGALG00010008762	SAE1	-0.51830096	0.102386574	-5.062196539	4.14453E-07	7.94433E-07	negative
PRJNA729271	stage_1W_vs_E10	ordinary_muscle_development	ENSGALG00010008764	ETFDH	0.550658138	0.123870056	4.445449979	8.77081E-06	1.52804E-05	positive
PRJNA729271	stage_1W_vs_E10	ordinary_muscle_development	ENSGALG00010008768	PRXL2C	2.757026417	0.145093848	19.00167684	1.65182E-80	3.99718E-79	positive
PRJNA729271	stage_1W_vs_E10	ordinary_muscle_development	ENSGALG00010008775	MED17	0.147812691	0.089830071	1.645470041	0.099872916	0.12011765	positive
PRJNA729271	stage_1W_vs_E10	ordinary_muscle_development	ENSGALG00010008780	KARS1	0.461172447	0.057220799	8.059524821	7.65916E-16	2.37069E-15	positive
PRJNA729271	stage_1W_vs_E10	ordinary_muscle_development	ENSGALG00010008782	CCDC9	0.770249446	0.273350508	2.81780872	0.004835261	0.006760596	positive
PRJNA729271	stage_1W_vs_E10	ordinary_muscle_development	ENSGALG00010008790	GPN1	0.438777435	0.094765796	4.630124533	3.65446E-06	6.551E-06	positive
PRJNA729271	stage_1W_vs_E10	ordinary_muscle_development	ENSGALG00010008793	C11orf54	1.086785905	0.113282757	9.593568683	8.50899E-22	3.42891E-21	positive
PRJNA729271	stage_1W_vs_E10	ordinary_muscle_development	ENSGALG00010008799	ENSGALG00010008799	0.283042867	0.175407843	1.613627202	0.106608313	0.127699157	positive
PRJNA729271	stage_1W_vs_E10	ordinary_muscle_development	ENSGALG00010008807	BRD1	-0.012990737	0.062216867	-0.208797671	0.83460619	0.852605308	negative
PRJNA729271	stage_1W_vs_E10	ordinary_muscle_development	ENSGALG00010008811	TERF2IP	1.085547711	0.094135127	11.53180272	9.12135E-31	5.3162E-30	positive
PRJNA729271	stage_1W_vs_E10	ordinary_muscle_development	ENSGALG00010008814	ENSGALG00010008814	0.722121839	0.234928246	3.073797423	0.002113529	0.003054093	positive
PRJNA729271	stage_1W_vs_E10	ordinary_muscle_development	ENSGALG00010008821	ENSGALG00010008821	-0.087682751	0.138457279	-0.633283797	0.526548332	0.563312279	negative
PRJNA729271	stage_1W_vs_E10	ordinary_muscle_development	ENSGALG00010008824	GREM2	-1.150504186	0.308191884	-3.733077492	0.000189154	0.000298118	negative
PRJNA729271	stage_1W_vs_E10	ordinary_muscle_development	ENSGALG00010008825	PPID	0.416313286	0.108709163	3.829606219	0.000128348	0.000204688	positive
PRJNA729271	stage_1W_vs_E10	ordinary_muscle_development	ENSGALG00010008830	DHX34	0.451866541	0.195978034	2.305699942	0.021127404	0.027544678	positive
PRJNA729271	stage_1W_vs_E10	ordinary_muscle_development	ENSGALG00010008832	MTSS1	-0.295506899	0.125333625	-2.357762324	0.01838546	0.024097771	negative
PRJNA729271	stage_1W_vs_E10	ordinary_muscle_development	ENSGALG00010008837	ENSGALG00010008837	-0.626590435	0.098931362	-6.333587506	2.39525E-10	5.5631E-10	negative
PRJNA729271	stage_1W_vs_E10	ordinary_muscle_development	ENSGALG00010008839	ZNF512	-0.376064734	0.074616308	-5.039980474	4.65579E-07	8.89726E-07	negative
PRJNA729271	stage_1W_vs_E10	ordinary_muscle_development	ENSGALG00010008846	SLC35D2	0.696640406	0.283892395	2.453888931	0.014132062	0.018791326	positive
PRJNA729271	stage_1W_vs_E10	ordinary_muscle_development	ENSGALG00010008850	CYSTM1	2.824674579	0.123121463	22.94217854	1.7637E-116	8.9078E-115	positive
PRJNA729271	stage_1W_vs_E10	ordinary_muscle_development	ENSGALG00010008852	KHDRBS3	0.355171645	0.083628177	4.24703323	2.1662E-05	3.65999E-05	positive
PRJNA729271	stage_1W_vs_E10	ordinary_muscle_development	ENSGALG00010008859	ENSGALG00010008859	-1.419010992	0.22990201	-6.172242657	6.73281E-10	1.52702E-09	negative
PRJNA729271	stage_1W_vs_E10	ordinary_muscle_development	ENSGALG00010008860	SQLE	-2.105148952	0.247536119	-8.504411248	1.82519E-17	6.04713E-17	negative
PRJNA729271	stage_1W_vs_E10	ordinary_muscle_development	ENSGALG00010008862	ZNF367	-0.829970732	0.147306761	-5.634301685	1.75769E-08	3.67644E-08	negative
PRJNA729271	stage_1W_vs_E10	ordinary_muscle_development	ENSGALG00010008864	ADAT1	0.821319333	0.137454878	5.975192351	2.29819E-09	5.07113E-09	positive
PRJNA729271	stage_1W_vs_E10	ordinary_muscle_development	ENSGALG00010008866	WASHC5	-0.368217683	0.07386499	-4.985009548	6.19588E-07	1.17428E-06	negative
PRJNA729271	stage_1W_vs_E10	ordinary_muscle_development	ENSGALG00010008871	PRR7	-1.443399574	0.19648479	-7.346113526	2.04053E-13	5.57855E-13	negative
PRJNA729271	stage_1W_vs_E10	ordinary_muscle_development	ENSGALG00010008877	FNDC4	0.515055715	0.253190028	2.034265402	0.041924844	0.052831908	positive
PRJNA729271	stage_1W_vs_E10	ordinary_muscle_development	ENSGALG00010008879	HABP4	-1.187373963	0.174754089	-6.794541793	1.08657E-11	2.72631E-11	negative
PRJNA729271	stage_1W_vs_E10	ordinary_muscle_development	ENSGALG00010008880	FMN2	-1.184650445	0.29349876	-4.036304775	5.42997E-05	8.90657E-05	negative
PRJNA729271	stage_1W_vs_E10	ordinary_muscle_development	ENSGALG00010008884	FAM135B	0.022112764	0.510999671	0.043273538	0.965483485	0.970370483	positive
PRJNA729271	stage_1W_vs_E10	ordinary_muscle_development	ENSGALG00010008888	FBXO46	1.857652768	0.141466748	13.1313739	2.17671E-39	1.70733E-38	positive
PRJNA729271	stage_1W_vs_E10	ordinary_muscle_development	ENSGALG00010008892	ENSGALG00010008892	-0.319006739	0.126818191	-2.515465136	0.011887543	0.015931023	negative
PRJNA729271	stage_1W_vs_E10	ordinary_muscle_development	ENSGALG00010008893	ENSGALG00010008893	5.490303573	0.137985995	39.78884653	0	0	positive
PRJNA729271	stage_1W_vs_E10	ordinary_muscle_development	ENSGALG00010008897	NSMCE2	-0.092668078	0.164441779	-0.563531231	0.5730732	0.608772153	negative
PRJNA729271	stage_1W_vs_E10	ordinary_muscle_development	ENSGALG00010008903	ENSGALG00010008903	2.015591927	0.436128551	4.621554636	3.80875E-06	6.81326E-06	positive
PRJNA729271	stage_1W_vs_E10	ordinary_muscle_development	ENSGALG00010008905	GABARAPL2	0.832433213	0.093549709	8.898298255	5.67095E-19	2.03517E-18	positive
PRJNA729271	stage_1W_vs_E10	ordinary_muscle_development	ENSGALG00010008906	DBN1	-3.682595253	0.215023791	-17.12645486	9.42242E-66	1.58259E-64	negative
PRJNA729271	stage_1W_vs_E10	ordinary_muscle_development	ENSGALG00010008910	TRIB1	2.697132711	0.260781627	10.34249516	4.52597E-25	2.08996E-24	positive
PRJNA729271	stage_1W_vs_E10	ordinary_muscle_development	ENSGALG00010008918	PLAC9	-0.690695541	0.246494671	-2.802070886	0.005077572	0.007087087	negative
PRJNA729271	stage_1W_vs_E10	ordinary_muscle_development	ENSGALG00010008921	OPA3	1.410920411	0.12486476	11.29958858	1.31834E-29	7.31879E-29	positive
PRJNA729271	stage_1W_vs_E10	ordinary_muscle_development	ENSGALG00010008922	ENSGALG00010008922	3.877492194	1.243586659	3.117991147	0.001820883	0.002648374	positive
PRJNA729271	stage_1W_vs_E10	ordinary_muscle_development	ENSGALG00010008928	CFC1	-6.152065531	0.915705804	-6.718386521	1.83748E-11	4.55598E-11	negative
PRJNA729271	stage_1W_vs_E10	ordinary_muscle_development	ENSGALG00010008931	FNIP2	-0.197138636	0.132636205	-1.486310896	0.137196906	0.161864655	negative
PRJNA729271	stage_1W_vs_E10	ordinary_muscle_development	ENSGALG00010008932	VASP	1.76594377	0.119396132	14.790628	1.68375E-49	1.80919E-48	positive
PRJNA729271	stage_1W_vs_E10	ordinary_muscle_development	ENSGALG00010008936	IFT172	-0.593521609	0.095539191	-6.212336543	5.22025E-10	1.19105E-09	negative
PRJNA729271	stage_1W_vs_E10	ordinary_muscle_development	ENSGALG00010008938	ZBED4	-0.116781014	0.090641598	-1.288382111	0.197612983	0.227604305	negative
PRJNA729271	stage_1W_vs_E10	ordinary_muscle_development	ENSGALG00010008939	RANBP9	1.413236872	0.102506239	13.78683765	3.05877E-43	2.7134E-42	positive
PRJNA729271	stage_1W_vs_E10	ordinary_muscle_development	ENSGALG00010008943	ENSGALG00010008943	3.385133542	0.457373503	7.401245422	1.34913E-13	3.72205E-13	positive
PRJNA729271	stage_1W_vs_E10	ordinary_muscle_development	ENSGALG00010008945	FER1L6	1.314305536	0.547195057	2.401895849	0.016310351	0.021518025	positive
PRJNA729271	stage_1W_vs_E10	ordinary_muscle_development	ENSGALG00010008946	COL22A1	-2.691732556	0.622162301	-4.32641539	1.51555E-05	2.59026E-05	negative
PRJNA729271	stage_1W_vs_E10	ordinary_muscle_development	ENSGALG00010008955	KRTCAP3	-1.430059083	0.373294319	-3.830915743	0.000127667	0.000203647	negative
PRJNA729271	stage_1W_vs_E10	ordinary_muscle_development	ENSGALG00010008956	LVRN	-0.47733979	0.484872636	-0.984464278	0.324887287	0.361359824	negative
PRJNA729271	stage_1W_vs_E10	ordinary_muscle_development	ENSGALG00010008960	LRRC2	9.248596471	0.397538493	23.26465646	1.011E-119	5.6147E-118	positive
PRJNA729271	stage_1W_vs_E10	ordinary_muscle_development	ENSGALG00010008966	NRBP1	0.147596037	0.116322108	1.268856284	0.204492326	0.234855897	positive
PRJNA729271	stage_1W_vs_E10	ordinary_muscle_development	ENSGALG00010008969	PPP1R37	0.409633682	0.187495495	2.184765455	0.028906048	0.037095455	positive
PRJNA729271	stage_1W_vs_E10	ordinary_muscle_development	ENSGALG00010008971	ANXA11	0.560878373	0.103851926	5.40075078	6.63626E-08	1.3431E-07	positive
PRJNA729271	stage_1W_vs_E10	ordinary_muscle_development	ENSGALG00010008973	KCNK9	-3.200812355	0.916218001	-3.493505204	0.000476724	0.00072784	negative
PRJNA729271	stage_1W_vs_E10	ordinary_muscle_development	ENSGALG00010008974	SMCO4	-0.159923142	0.101445787	-1.576439459	0.114924561	0.137049075	negative
PRJNA729271	stage_1W_vs_E10	ordinary_muscle_development	ENSGALG00010008980	ENSGALG00010008980	-0.311299037	0.261848488	-1.188851766	0.234498004	0.26644576	negative
PRJNA729271	stage_1W_vs_E10	ordinary_muscle_development	ENSGALG00010008983	ENSGALG00010008983	-0.443162653	0.350880394	-1.263002041	0.206588446	0.237131733	negative
PRJNA729271	stage_1W_vs_E10	ordinary_muscle_development	ENSGALG00010008988	PPM1G	-0.626665338	0.080524132	-7.782329674	7.1201E-15	2.10324E-14	negative

PRJNA729271	stage_1W_vs_E10	ordinary_muscle_development	ENSGALG00010008991	ENSGALG00010008991	0.113660505	0.131758765	0.862640942	0.388334913	0.426336027	positive
PRJNA729271	stage_1W_vs_E10	ordinary_muscle_development	ENSGALG00010008995	ECD	-0.540775445	0.100060244	-5.404498571	6.49899E-08	1.31642E-07	negative
PRJNA729271	stage_1W_vs_E10	ordinary_muscle_development	ENSGALG00010008996	CHRM3	-0.916126854	0.24057979	-3.807995903	0.000140098	0.000222886	negative
PRJNA729271	stage_1W_vs_E10	ordinary_muscle_development	ENSGALG00010008997	CHST6	-1.774091906	0.134422162	-13.19791233	9.02003E-40	7.17199E-39	negative
PRJNA729271	stage_1W_vs_E10	ordinary_muscle_development	ENSGALG00010008999	TRAPPC9	-0.914683511	0.066613523	-13.73119863	6.6032E-43	5.80433E-42	negative
PRJNA729271	stage_1W_vs_E10	ordinary_muscle_development	ENSGALG00010009000	FAM149B1	-0.526394186	0.103608201	-5.080622804	3.76199E-07	7.23499E-07	negative
PRJNA729271	stage_1W_vs_E10	ordinary_muscle_development	ENSGALG00010009002	ENSGALG00010009002	1.694885325	0.095851611	17.68238744	5.73169E-70	1.08215E-68	positive
PRJNA729271	stage_1W_vs_E10	ordinary_muscle_development	ENSGALG00010009003	ALG12	-2.14218874	0.138200609	-15.50057383	3.43808E-54	4.3524E-53	negative
PRJNA729271	stage_1W_vs_E10	ordinary_muscle_development	ENSGALG00010009007	TMEM65	-0.748711443	0.100551699	-7.446034714	9.61875E-14	2.67454E-13	negative
PRJNA729271	stage_1W_vs_E10	ordinary_muscle_development	ENSGALG00010009011	ACP6	-0.472552677	0.12370008	-3.82014851	0.000133371	0.000212465	negative
PRJNA729271	stage_1W_vs_E10	ordinary_muscle_development	ENSGALG00010009012	DEUP1	-2.67724069	0.932137907	-2.872150858	0.004076883	0.005733426	negative
PRJNA729271	stage_1W_vs_E10	ordinary_muscle_development	ENSGALG00010009013	LIPG	-0.654769183	0.225405717	-2.904847275	0.003674323	0.005193498	negative
PRJNA729271	stage_1W_vs_E10	ordinary_muscle_development	ENSGALG00010009015	TMEM231	-0.197230238	0.092420387	-2.134055527	0.032838236	0.041878429	negative
PRJNA729271	stage_1W_vs_E10	ordinary_muscle_development	ENSGALG00010009016	DNAJC9	-1.106249615	0.110726487	-9.990830923	1.67174E-23	7.23341E-23	negative
PRJNA729271	stage_1W_vs_E10	ordinary_muscle_development	ENSGALG00010009019	ZNF513	1.181579325	0.13305476	8.880398776	6.66211E-19	2.37968E-18	positive
PRJNA729271	stage_1W_vs_E10	ordinary_muscle_development	ENSGALG00010009020	ARHGAP35	0.210106608	0.182811178	1.149309413	0.250428418	0.283391359	positive
PRJNA729271	stage_1W_vs_E10	ordinary_muscle_development	ENSGALG00010009021	HNF4beta	-0.214417966	0.436742843	-0.490947865	0.623463322	0.657195902	negative
PRJNA729271	stage_1W_vs_E10	ordinary_muscle_development	ENSGALG00010009024	PMAIP1	0.214229449	0.258459723	0.828869763	0.407178107	0.445168703	positive
PRJNA729271	stage_1W_vs_E10	ordinary_muscle_development	ENSGALG00010009025	UTP23	0.174042663	0.096455217	1.804388286	0.071170458	0.087144432	positive
PRJNA729271	stage_1W_vs_E10	ordinary_muscle_development	ENSGALG00010009026	RAPGEF2	-0.311450879	0.137225167	-2.269633805	0.023229811	0.030160884	negative
PRJNA729271	stage_1W_vs_E10	ordinary_muscle_development	ENSGALG00010009028	PDLIM7	-1.430285628	0.115621363	-12.37042693	3.77808E-35	2.58074E-34	negative
PRJNA729271	stage_1W_vs_E10	ordinary_muscle_development	ENSGALG00010009029	IGSF3	-0.538278545	0.163220212	-3.297866959	0.000974223	0.001453263	negative
PRJNA729271	stage_1W_vs_E10	ordinary_muscle_development	ENSGALG00010009030	ZDHHC7	-0.629548502	0.092441489	-6.810237599	9.74377E-12	2.45372E-11	negative
PRJNA729271	stage_1W_vs_E10	ordinary_muscle_development	ENSGALG00010009031	SPIN1	-1.049602725	0.079882098	-13.13939854	1.95777E-39	1.53893E-38	negative
PRJNA729271	stage_1W_vs_E10	ordinary_muscle_development	ENSGALG00010009034	SNX17	1.768327941	0.118812618	14.88333451	4.22886E-50	4.65786E-49	positive
PRJNA729271	stage_1W_vs_E10	ordinary_muscle_development	ENSGALG00010009035	ENSGALG00010009035	-1.771700198	0.084337679	-21.00722019	5.63377E-98	2.02634E-96	negative
PRJNA729271	stage_1W_vs_E10	ordinary_muscle_development	ENSGALG00010009037	ENSGALG00010009037	1.40620799	0.18589538	7.564512849	3.89321E-14	1.10695E-13	positive
PRJNA729271	stage_1W_vs_E10	ordinary_muscle_development	ENSGALG00010009038	RAD21	-0.964344847	0.124494001	-7.746114981	9.47467E-15	2.78232E-14	negative
PRJNA729271	stage_1W_vs_E10	ordinary_muscle_development	ENSGALG00010009039	SLC36A4	-0.756089314	0.174571249	-4.331121631	1.48352E-05	2.53879E-05	negative
PRJNA729271	stage_1W_vs_E10	ordinary_muscle_development	ENSGALG00010009040	CRELD2	-2.04523036	0.147121978	-13.90159636	6.19442E-44	5.6223E-43	negative
PRJNA729271	stage_1W_vs_E10	ordinary_muscle_development	ENSGALG00010009043	TFAM	-0.4390198	0.083521724	-5.256354649	1.46939E-07	2.90332E-07	negative
PRJNA729271	stage_1W_vs_E10	ordinary_muscle_development	ENSGALG00010009047	SUPT5H	0.438281246	0.109943723	3.986414456	6.70793E-05	0.000109433	positive
PRJNA729271	stage_1W_vs_E10	ordinary_muscle_development	ENSGALG00010009050	PIK3CG	2.186372423	0.091278095	23.95287091	8.6251E-127	5.4122E-125	positive
PRJNA729271	stage_1W_vs_E10	ordinary_muscle_development	ENSGALG00010009051	TROJANZ	2.642134306	0.241156296	10.9561075	6.21139E-28	3.22703E-27	positive
PRJNA729271	stage_1W_vs_E10	ordinary_muscle_development	ENSGALG00010009054	RYR2	-1.884299349	0.503971741	-3.738898825	0.000184828	0.000291585	negative
PRJNA729271	stage_1W_vs_E10	ordinary_muscle_development	ENSGALG00010009057	NXNL2	1.863595825	0.359905873	5.178008933	2.24266E-07	4.37987E-07	positive
PRJNA729271	stage_1W_vs_E10	ordinary_muscle_development	ENSGALG00010009059	MED29	1.639018374	0.125447497	13.06537323	5.19293E-39	4.03168E-38	positive
PRJNA729271	stage_1W_vs_E10	ordinary_muscle_development	ENSGALG00010009065	EIF2B4	0.84944286	0.094040492	9.032735202	1.67433E-19	6.15508E-19	positive
PRJNA729271	stage_1W_vs_E10	ordinary_muscle_development	ENSGALG00010009067	ENSGALG00010009067	-2.00122901	1.623553621	-1.232622677	0.217716572	0.248958798	negative
PRJNA729271	stage_1W_vs_E10	ordinary_muscle_development	ENSGALG00010009068	AGO2	-0.407125016	0.082783766	-4.917933031	8.74628E-07	1.64262E-06	negative
PRJNA729271	stage_1W_vs_E10	ordinary_muscle_development	ENSGALG00010009069	GRID2	-3.899936252	0.37634566	-10.36264441	3.66682E-25	1.69919E-24	negative
PRJNA729271	stage_1W_vs_E10	ordinary_muscle_development	ENSGALG00010009070	ENSGALG00010009070	0.484837691	1.252620981	0.387058574	0.698712845	0.727157	positive
PRJNA729271	stage_1W_vs_E10	ordinary_muscle_development	ENSGALG00010009073	PRKAR2B	0.197317161	0.407129912	0.484654051	0.627921766	0.661332806	positive
PRJNA729271	stage_1W_vs_E10	ordinary_muscle_development	ENSGALG00010009076	TMEM214	0.106858669	0.116251567	0.919201962	0.357989945	0.395568246	positive
PRJNA729271	stage_1W_vs_E10	ordinary_muscle_development	ENSGALG00010009078	PTTG2	-1.708271099	0.170411591	-10.02438325	1.19101E-23	5.17806E-23	negative
PRJNA729271	stage_1W_vs_E10	ordinary_muscle_development	ENSGALG00010009079	KCTD15	-0.725944699	0.097485661	-7.446681832	9.57171E-14	2.66248E-13	negative
PRJNA729271	stage_1W_vs_E10	ordinary_muscle_development	ENSGALG00010009080	TLL1	-1.386460101	0.173411673	-7.995194753	1.29369E-15	3.96031E-15	negative
PRJNA729271	stage_1W_vs_E10	ordinary_muscle_development	ENSGALG00010009081	HBP1	1.44939169	0.121812354	11.89856076	1.20406E-32	7.50659E-32	positive
PRJNA729271	stage_1W_vs_E10	ordinary_muscle_development	ENSGALG00010009083	MED30	0.495190268	0.127260229	3.891162802	9.9765E-05	0.00016041	positive
PRJNA729271	stage_1W_vs_E10	ordinary_muscle_development	ENSGALG00010009085	PAF1	-0.146372513	0.088965143	-1.645279352	0.099912216	0.120154946	negative
PRJNA729271	stage_1W_vs_E10	ordinary_muscle_development	ENSGALG00010009086	UBE2D1	2.211423568	0.170406701	12.97732754	1.64522E-38	1.25052E-37	positive
PRJNA729271	stage_1W_vs_E10	ordinary_muscle_development	ENSGALG00010009088	ENSGALG00010009088	-0.960025683	0.148186528	-6.478495002	9.2642E-11	2.20464E-10	negative
PRJNA729271	stage_1W_vs_E10	ordinary_muscle_development	ENSGALG00010009089	AGBL5	0.210215367	0.152420437	1.379180977	0.167838962	0.195486199	positive
PRJNA729271	stage_1W_vs_E10	ordinary_muscle_development	ENSGALG00010009091	EXT1	-0.22247788	0.111991826	-1.986554624	0.046971774	0.05883649	negative
PRJNA729271	stage_1W_vs_E10	ordinary_muscle_development	ENSGALG00010009092	EED	-1.218840357	0.119438252	-10.20477387	1.8876E-24	8.47346E-24	negative
PRJNA729271	stage_1W_vs_E10	ordinary_muscle_development	ENSGALG00010009093	SLU7	0.716902907	0.131510306	5.451305894	5.00013E-08	1.02023E-07	positive
PRJNA729271	stage_1W_vs_E10	ordinary_muscle_development	ENSGALG00010009094	DARS	0.472043664	0.095064251	4.965522342	6.85164E-07	1.29569E-06	positive
PRJNA729271	stage_1W_vs_E10	ordinary_muscle_development	ENSGALG00010009096	ENSGALG00010009096	0.517975392	1.376581573	0.37627657	0.706711296	0.734533269	positive
PRJNA729271	stage_1W_vs_E10	ordinary_muscle_development	ENSGALG00010009097	C1QTNF2	-0.018149317	0.181995427	-0.099724031	0.920563422	0.93018939	negative
PRJNA729271	stage_1W_vs_E10	ordinary_muscle_development	ENSGALG00010009098	CAD	-2.090064938	0.19852062	-10.52820072	6.4047E-26	3.05784E-25	negative
PRJNA729271	stage_1W_vs_E10	ordinary_muscle_development	ENSGALG00010009099	CXCR4	0.247608961	0.189657116	1.305561143	0.191701842	0.221306005	positive
PRJNA729271	stage_1W_vs_E10	ordinary_muscle_development	ENSGALG00010009100	ENSGALG00010009100	0.248652226	0.153059752	1.624543499	0.104259865	0.125059088	positive
PRJNA729271	stage_1W_vs_E10	ordinary_muscle_development	ENSGALG00010009101	HIKESHI	0.386146532	0.097972057	3.941394558	8.10092E-05	0.000131331	positive
PRJNA729271	stage_1W_vs_E10	ordinary_muscle_development	ENSGALG00010009102	HTRA3	0.957372258	0.217088971	4.410045594	1.03349E-05	1.79192E-05	positive
PRJNA729271	stage_1W_vs_E10	ordinary_muscle_development	ENSGALG00010009103	AP3S1	-1.525641297	0.124436747	-12.26037597	1.47812E-34	9.86892E-34	negative
PRJNA729271	stage_1W_vs_E10	ordinary_muscle_development	ENSGALG00010009104	MTR	0.498421352	0.122828071	4.057878208	4.95206E-05	8.14572E-05	positive
PRJNA729271	stage_1W_vs_E10	ordinary_muscle_development	ENSGALG00010009106	THSD7B	-0.280246696	0.237437031	-1.180299022	0.237881307	0.270099447	negative
PRJNA729271	stage_1W_vs_E10	ordinary_muscle_development	ENSGALG00010009109	ENSGALG00010009109	-0.337840485	0.219863519	-1.536591819	0.124393297	0.147660375	negative
PRJNA729271	stage_1W_vs_E10	ordinary_muscle_development	ENSGALG00010009111	LRFN1	1.074249372	0.242416498	4.431420217	9.36145E-06	1.62839E-05	positive
PRJNA729271	stage_1W_vs_E10	ordinary_muscle_development	ENSGALG00010009112	ENSGALG00010009112	9.074365257	0.264594255	34.29539796	9.1902E-258	2.2578E-255	positive
PRJNA729271	stage_1W_vs_E10	ordinary_muscle_development	ENSGALG00010009113	RPLP1	0.685064799	0.17635992	3.88446988	0.000102553	0.00016471	positive
PRJNA729271	stage_1W_vs_E10	ordinary_muscle_development	ENSGALG00010009114	SETD7	1.46232538	0.149919509	9.75406995	1.77218E-22	7.32469E-22	positive
PRJNA729271	stage_1W_vs_E10	ordinary_muscle_development	ENSGALG00010009115	COG5	0.041102313	0.114747638	0.358197465	0.720195544	0.747207388	positive
PRJNA729271	stage_1W_vs_E10	ordinary_muscle_development	ENSGALG00010009116	S1PR3	-1.22015148	0.185413868	-6.580691576	4.68265E-11	1.13636E-10	negative
PRJNA729271	stage_1W_vs_E10	ordinary_muscle_development	ENSGALG00010009117	ERGIC1	-0.014284233	0.067092257	-0.212904342	0.831401575	0.849810721	negative
PRJNA729271	stage_1W_vs_E10	ordinary_muscle_development	ENSGALG00010009118	ACTN2	4.581529698	0.143903943	31.83741596	1.9659E-222	3.4751E-220	positive
PRJNA729271	stage_1W_vs_E10	ordinary_muscle_development	ENSGALG00010009119	CISD1	1.051422276	0.153061572	6.869276606	6.45283E-12	1.64037E-11	positive
PRJNA729271	stage_1W_vs_E10	ordinary_muscle_development	ENSGALG00010009120	ATG12	0.200567314	0.089207221	2.248330485	0.024555124	0.031793347	positive
PRJNA729271	stage_1W_vs_E10	ordinary_muscle_development	ENSGALG00010009121	SYCN	2.153618844	0.734173172	2.933393544	0.003352786	0.004758041	positive
PRJNA729271	stage_1W_vs_E10	ordinary_muscle_development	ENSGALG00010009123	ENSGALG00010009123	-2.124780127	0.13738135	-15.46629243	5.85855E-54	7.34599E-53	negative
PRJNA729271	stage_1W_vs_E10	ordinary_muscle_development	ENSGALG00010009125	CD72L1	5.758401892	0.945509244	6.090265039	1.12724E-09	2.52892E-09	positive
PRJNA729271	stage_1W_vs_E10	ordinary_muscle_development	ENSGALG00010009126	JPH3	1.886049195	0.135455996	13.92370403	4.54715E-44	4.14795E-43	positive
PRJNA729271	stage_1W_vs_E10	ordinary_muscle_development	ENSGALG00010009127	SCOC	-0.425353109	0.07550765	-5.633245188	1.7685E-08	3.69798E-08	negative
PRJNA729271	stage_1W_vs_E10	ordinary_muscle_development	ENSGALG00010009129	ENSGALG00010009129	1.128230254	0.379246842	2.974923265	0.002930618	0.004180212	positive
PRJNA729271	stage_1W_vs_E10	ordinary_muscle_development	ENSGALG00010009130	HEATR1	-0.531727129	0.073002784	-7.283655529	3.24894E-13	8.79921E-13	negative
PRJNA729271	stage_1W_vs_E10	ordinary_muscle_development	ENSGALG00010009131	CDKL2	-2.648985379	0.320117331	-8.275045181	1.28407E-16	4.09879E-16	negative
PRJNA729271	stage_1W_vs_E10	ordinary_muscle_development	ENSGALG00010009133	BOD1L1	0.39731963	0.129156188	3.076272502	0.002096062	0.003030665	positive
PRJNA729271	stage_1W_vs_E10	ordinary_muscle_development	ENSGALG00010009135	TENM4	-2.924023574	0.187137127	-15.6250319	4.91676E-55	6.3519E-54	negative
PRJNA729271	stage_1W_vs_E10	ordinary_muscle_development	ENSGALG00010009136	KHK	-0.525905624	0.40152427	-1.309772939	0.19027266	0.219743623	negative
PRJNA729271	stage_1W_vs_E10	ordinary_muscle_development	ENSGALG00010009137	IPMK	-0.057040804	0.092399664	-0.617326966	0.537019084	0.573329279	negative
PRJNA729271	stage_1W_vs_E10	ordinary_muscle_development	ENSGALG00010009138	ENSGALG00010009138	0.241409223	0.107072237	2.254638824	0.024156008	0.031310144	positive
PRJNA729271	stage_1W_vs_E10	ordinary_muscle_development	ENSGALG00010009139	CKS2	-2.919969243	0.301818725	-9.674579483	3.86678E-22	1.57662E-21	negative
PRJNA729271	stage_1W_vs_E10	ordinary_muscle_development	ENSGALG00010009141	MAML3	0.0548295	0.117835973	0.465303582	0.64171407	0.673885782	positive
PRJNA729271	stage_1W_vs_E10	ordinary_muscle_development	ENSGALG00010009142	LGALS8	-0.874934517	0.137593718	-6.358826046	2.03301E-10	4.74152E-10	negative
PRJNA729271	stage_1W_vs_E10	ordinary_muscle_development	ENSGALG00010009143	MCCC2	0.881919183	0.135015723	6.531973913	6.49085E-11	1.55564E-10	positive
PRJNA729271	stage_1W_vs_E10	ordinary_muscle_development	ENSGALG00010009144	CREBZF	0.3710966	0.157094965	2.362243748	0.018164695	0.023827805	positive
PRJNA729271	stage_1W_vs_E10	ordinary_muscle_development	ENSGALG00010009147	HNMT	2.053046136	0.22711064	9.039850084	1.56887E-19	5.77469E-19	positive
PRJNA729271	stage_1W_vs_E10	ordinary_muscle_development	ENSGALG00010009149	FUT4	-1.34425545	0.214705207	-6.260935489	3.82675E-10	8.80596E-10	negative
PRJNA729271	stage_1W_vs_E10	ordinary_muscle_development	ENSGALG00010009150	ENSGALG00010009150	0.515649303	0.395359967	1.304252697	0.192147438	0.221732116	positive
PRJNA729271	stage_1W_vs_E10	ordinary_muscle_development	ENSGALG00010009151	CCNJL	0.25689393	0.371690444	0.691150214	0.489471147	0.52769686	positive
PRJNA729271	stage_1W_vs_E10	ordinary_muscle_development	ENSGALG00010009152	RPP25L	0.814495606	0.122560213	6.645677151	3.01826E-11	7.39765E-11	positive
PRJNA729271	stage_1W_vs_E10	ordinary_muscle_development	ENSGALG00010009153	SECISBP2	-0.069086235	0.124690364	-0.554062344	0.57953618	0.61500673	negative
PRJNA729271	stage_1W_vs_E10	ordinary_muscle_development	ENSGALG00010009155	PAK4	0.384107638	0.218572504	1.757346558	0.078858756	0.09600686	positive
PRJNA729271	stage_1W_vs_E10	ordinary_muscle_development	ENSGALG00010009157	ABHD1	1.126396908	0.137452311	8.194819741	2.50969E-16	7.90481E-16	positive
PRJNA729271	stage_1W_vs_E10	ordinary_muscle_development	ENSGALG00010009159	PTK2	0.286707395	0.098897502	2.89903575	0.003743122	0.005284558	positive
PRJNA729271	stage_1W_vs_E10	ordinary_muscle_development	ENSGALG00010009160	ENSGALG00010009160	-3.113199555	0.394165717	-7.898199723	2.8296E-15	8.52174E-15	negative
PRJNA729271	stage_1W_vs_E10	ordinary_muscle_development	ENSGALG00010009163	SAMD12	-0.621196537	0.179519928	-3.460320774	0.000539532	0.000820793	negative
PRJNA729271	stage_1W_vs_E10	ordinary_muscle_development	ENSGALG00010009164	TMEM128	0.387134951	0.10098209	3.833699138	0.000126231	0.000201444	positive
PRJNA729271	stage_1W_vs_E10	ordinary_muscle_development	ENSGALG00010009166	CLGN	-0.302430061	0.229927135	-1.315330013	0.188399016	0.217683837	negative
PRJNA729271	stage_1W_vs_E10	ordinary_muscle_development	ENSGALG00010009167	DCTN3	-0.241049531	0.11105307	-2.170579631	0.029962963	0.038404205	negative
PRJNA729271	stage_1W_vs_E10	ordinary_muscle_development	ENSGALG00010009168	MRPS12	1.07167673	0.174590685	6.138223995	8.34491E-10	1.88381E-09	positive
PRJNA729271	stage_1W_vs_E10	ordinary_muscle_development	ENSGALG00010009170	RPL26L1	0.444605429	0.191289737	2.324251347	0.020112027	0.026277612	positive
PRJNA729271	stage_1W_vs_E10	ordinary_muscle_development	ENSGALG00010009172	SPOPL	0.708326778	0.131978506	5.366985867	8.00633E-08	1.61205E-07	positive
PRJNA729271	stage_1W_vs_E10	ordinary_muscle_development	ENSGALG00010009173	LYAR	-0.219204338	0.142364264	-1.539742712	0.123623081	0.146842298	negative
PRJNA729271	stage_1W_vs_E10	ordinary_muscle_development	ENSGALG00010009178	ENSGALG00010009178	0.620510954	0.256375596	2.420319888	0.015506859	0.020514002	positive
PRJNA729271	stage_1W_vs_E10	ordinary_muscle_development	ENSGALG00010009180	NFKBIB	0.992612194	0.146403814	6.77996132	1.20208E-11	3.00987E-11	positive
PRJNA729271	stage_1W_vs_E10	ordinary_muscle_development	ENSGALG00010009181	ARID3C	-6.748982984	0.933530869	-7.229523099	4.84693E-13	1.30152E-12	negative
PRJNA729271	stage_1W_vs_E10	ordinary_muscle_development	ENSGALG00010009186	PREB	0.110236574	0.097329404	1.13261327	0.257376694	0.290862533	positive
PRJNA729271	stage_1W_vs_E10	ordinary_muscle_development	ENSGALG00010009188	ENSGALG00010009188	-1.285864953	0.500334489	-2.570010628	0.01016954	0.013740443	negative
PRJNA729271	stage_1W_vs_E10	ordinary_muscle_development	ENSGALG00010009189	ENSGALG00010009189	-1.841567599	0.259243664	-7.10361661	1.21534E-12	3.20763E-12	negative
PRJNA729271	stage_1W_vs_E10	ordinary_muscle_development	ENSGALG00010009190	ZBTB49	-0.115187211	0.14014335	-0.821924203	0.411120037	0.449139655	negative
PRJNA729271	stage_1W_vs_E10	ordinary_muscle_development	ENSGALG00010009192	LSM14A	-0.425222607	0.059795877	-7.111236204	1.15008E-12	3.03817E-12	negative
PRJNA729271	stage_1W_vs_E10	ordinary_muscle_development	ENSGALG00010009193	EDARADD	-1.603969902	0.224906269	-7.131726061	9.9118E-13	2.62606E-12	negative
PRJNA729271	stage_1W_vs_E10	ordinary_muscle_development	ENSGALG00010009195	ENSGALG00010009195	2.558804852	0.649449551	3.93995938	8.14954E-05	0.00013206	positive
PRJNA729271	stage_1W_vs_E10	ordinary_muscle_development	ENSGALG00010009197	FH	1.207171716	0.102348959	11.79466533	4.15851E-32	2.54658E-31	positive
PRJNA729271	stage_1W_vs_E10	ordinary_muscle_development	ENSGALG00010009198	KCNN2	0.410644695	0.173073189	2.372664976	0.017660274	0.023199717	positive
PRJNA729271	stage_1W_vs_E10	ordinary_muscle_development	ENSGALG00010009199	SEMA4D	-3.088473883	0.163756945	-18.86010931	2.42718E-79	5.66536E-78	negative
PRJNA729271	stage_1W_vs_E10	ordinary_muscle_development	ENSGALG00010009200	ADGRA3	-2.247333174	0.095759458	-23.46852425	8.5532E-122	4.9003E-120	negative
PRJNA729271	stage_1W_vs_E10	ordinary_muscle_development	ENSGALG00010009202	FABP6	1.226483316	0.287860518	4.260686125	2.038E-05	3.44862E-05	positive
PRJNA729271	stage_1W_vs_E10	ordinary_muscle_development	ENSGALG00010009205	SIRT2	0.847952611	0.097328039	8.712315773	2.97729E-18	1.02508E-17	positive
PRJNA729271	stage_1W_vs_E10	ordinary_muscle_development	ENSGALG00010009208	SLC5A6	0.403400216	0.190476578	2.117846826	0.034188042	0.043507961	positive

PRJNA729271	stage_1W_vs_E10	ordinary_muscle_development	ENSGALG00010009209	SLC6A19	5.965628562	1.692315263	3.525128381	0.000423278	0.000649387	positive
PRJNA729271	stage_1W_vs_E10	ordinary_muscle_development	ENSGALG00010009210	PCDH15	-4.356773711	0.38098641	-11.43550949	2.77901E-30	1.5834E-29	negative
PRJNA729271	stage_1W_vs_E10	ordinary_muscle_development	ENSGALG00010009212	NXPH2	-1.312417029	0.276191363	-4.751839505	2.01574E-06	3.68127E-06	negative
PRJNA729271	stage_1W_vs_E10	ordinary_muscle_development	ENSGALG00010009213	DUS4L	-1.483254656	0.104132965	-14.24385316	4.89518E-46	4.71153E-45	negative
PRJNA729271	stage_1W_vs_E10	ordinary_muscle_development	ENSGALG00010009214	PCDH10	-5.964196215	0.335998046	-17.7506872	1.70258E-70	3.2644E-69	negative
PRJNA729271	stage_1W_vs_E10	ordinary_muscle_development	ENSGALG00010009216	NSG1	-3.228284025	0.225198384	-14.33528947	1.31696E-46	1.29069E-45	negative
PRJNA729271	stage_1W_vs_E10	ordinary_muscle_development	ENSGALG00010009219	TNFRSF11B	0.928153226	0.180923815	5.130077681	2.89623E-07	5.60791E-07	positive
PRJNA729271	stage_1W_vs_E10	ordinary_muscle_development	ENSGALG00010009220	GADD45G	0.507895051	0.190662995	2.663836522	0.00772551	0.010571251	positive
PRJNA729271	stage_1W_vs_E10	ordinary_muscle_development	ENSGALG00010009223	ENSGALG00010009223	-0.182877833	0.109329742	-1.672718061	0.094382812	0.113910386	negative
PRJNA729271	stage_1W_vs_E10	ordinary_muscle_development	ENSGALG00010009224	ENSGALG00010009224	5.911049575	0.44404712	13.31176198	1.97762E-40	1.60772E-39	positive
PRJNA729271	stage_1W_vs_E10	ordinary_muscle_development	ENSGALG00010009225	HNRNPL	-0.125785925	0.124612558	-1.009416119	0.312775111	0.348744249	negative
PRJNA729271	stage_1W_vs_E10	ordinary_muscle_development	ENSGALG00010009227	ENSGALG00010009227	0.522578074	0.173187913	3.017404994	0.002549489	0.003658534	positive
PRJNA729271	stage_1W_vs_E10	ordinary_muscle_development	ENSGALG00010009228	ATP6V0E1	1.199586391	0.099447953	12.06245428	1.66739E-33	1.07226E-32	positive
PRJNA729271	stage_1W_vs_E10	ordinary_muscle_development	ENSGALG00010009232	ECH1	1.084936009	0.163828734	6.622379248	3.53463E-11	8.63259E-11	positive
PRJNA729271	stage_1W_vs_E10	ordinary_muscle_development	ENSGALG00010009234	PCDH18	-1.279331203	0.12902178	-9.915622061	3.56032E-23	1.51829E-22	negative
PRJNA729271	stage_1W_vs_E10	ordinary_muscle_development	ENSGALG00010009235	KLHDC4	1.018203367	0.130103657	7.826093375	5.03264E-15	1.49791E-14	positive
PRJNA729271	stage_1W_vs_E10	ordinary_muscle_development	ENSGALG00010009236	DTWD2	0.087516672	0.115871147	0.755293046	0.450073171	0.488236088	positive
PRJNA729271	stage_1W_vs_E10	ordinary_muscle_development	ENSGALG00010009237	DENND3	-0.488926272	0.177630052	-2.75249749	0.00591426	0.008191801	negative
PRJNA729271	stage_1W_vs_E10	ordinary_muscle_development	ENSGALG00010009239	STX18	0.388548188	0.081919554	4.743045703	2.10529E-06	3.83513E-06	positive
PRJNA729271	stage_1W_vs_E10	ordinary_muscle_development	ENSGALG00010009243	BCAP29	-0.056834243	0.09987017	-0.569081264	0.569300993	0.604986649	negative
PRJNA729271	stage_1W_vs_E10	ordinary_muscle_development	ENSGALG00010009246	RUSC2	1.947013639	0.142116109	13.70016142	1.01299E-42	8.85601E-42	positive
PRJNA729271	stage_1W_vs_E10	ordinary_muscle_development	ENSGALG00010009250	ERO1B	-0.563569768	0.109419115	-5.150560472	2.59709E-07	5.04758E-07	negative
PRJNA729271	stage_1W_vs_E10	ordinary_muscle_development	ENSGALG00010009252	SIRT1	0.22892038	0.096311587	2.376872697	0.017460113	0.022953413	positive
PRJNA729271	stage_1W_vs_E10	ordinary_muscle_development	ENSGALG00010009253	SLC45A4	-0.614411225	0.144303405	-4.257773567	2.06473E-05	3.49221E-05	negative
PRJNA729271	stage_1W_vs_E10	ordinary_muscle_development	ENSGALG00010009254	SLC26A4	-0.708870134	0.791095316	-0.896061599	0.370219865	0.407838345	negative
PRJNA729271	stage_1W_vs_E10	ordinary_muscle_development	ENSGALG00010009256	CREBRF	1.758502042	0.172911006	10.16998328	2.69955E-24	1.20215E-23	positive
PRJNA729271	stage_1W_vs_E10	ordinary_muscle_development	ENSGALG00010009257	ENSGALG00010009257	2.336755746	0.187103776	12.48908922	8.56283E-36	6.01056E-35	positive
PRJNA729271	stage_1W_vs_E10	ordinary_muscle_development	ENSGALG00010009258	DNAJC12	0.147704473	0.167465736	0.881998174	0.377777795	0.41553264	positive
PRJNA729271	stage_1W_vs_E10	ordinary_muscle_development	ENSGALG00010009259	MGAT4D	2.325888541	0.787203186	2.954622876	0.003130515	0.004451762	positive
PRJNA729271	stage_1W_vs_E10	ordinary_muscle_development	ENSGALG00010009263	COLEC10	-0.600131346	0.223526776	-2.684829789	0.007256675	0.009956977	negative
PRJNA729271	stage_1W_vs_E10	ordinary_muscle_development	ENSGALG00010009266	PCDH7	-2.629772202	0.251503595	-10.45620126	1.37248E-25	6.48018E-25	negative
PRJNA729271	stage_1W_vs_E10	ordinary_muscle_development	ENSGALG00010009268	PWWP2A	0.058229635	0.133502902	0.43616756	0.662715163	0.69317768	positive
PRJNA729271	stage_1W_vs_E10	ordinary_muscle_development	ENSGALG00010009269	ENSGALG00010009269	-4.760941047	0.541703822	-8.788826767	1.5113E-18	5.28371E-18	negative
PRJNA729271	stage_1W_vs_E10	ordinary_muscle_development	ENSGALG00010009273	MAL2	0.175143956	0.481139884	0.364018784	0.715843982	0.743118135	positive
PRJNA729271	stage_1W_vs_E10	ordinary_muscle_development	ENSGALG00010009275	CBLL1	0.072638273	0.143882335	0.504844973	0.613667725	0.647906889	positive
PRJNA729271	stage_1W_vs_E10	ordinary_muscle_development	ENSGALG00010009276	ENSGALG00010009276	-0.255147371	0.325268971	-0.784419647	0.432793914	0.470724678	negative
PRJNA729271	stage_1W_vs_E10	ordinary_muscle_development	ENSGALG00010009277	ACTN4	-0.467571003	0.128637931	-3.6347833	0.000278214	0.000433626	negative
PRJNA729271	stage_1W_vs_E10	ordinary_muscle_development	ENSGALG00010009279	ELMOD2	-2.284137509	0.114551932	-19.93975544	1.83962E-88	5.26982E-87	negative
PRJNA729271	stage_1W_vs_E10	ordinary_muscle_development	ENSGALG00010009280	HSD17B4	0.127116438	0.083298594	1.526033418	0.127001542	0.150499155	positive
PRJNA729271	stage_1W_vs_E10	ordinary_muscle_development	ENSGALG00010009284	CCN3	-3.732191384	0.682390436	-5.469290287	4.51841E-08	9.24279E-08	negative
PRJNA729271	stage_1W_vs_E10	ordinary_muscle_development	ENSGALG00010009286	ENSGALG00010009286	0.892276448	0.181708689	4.910477598	9.08548E-07	1.705E-06	positive
PRJNA729271	stage_1W_vs_E10	ordinary_muscle_development	ENSGALG00010009289	GPR137B	-0.464704733	0.151157959	-3.074298809	0.00210998	0.003049572	negative
PRJNA729271	stage_1W_vs_E10	ordinary_muscle_development	ENSGALG00010009294	ACOX3	0.800199414	0.139702447	5.727884042	1.01691E-08	2.15625E-08	positive
PRJNA729271	stage_1W_vs_E10	ordinary_muscle_development	ENSGALG00010009297	CTNNA3	4.950670315	0.203988259	24.26938856	4.1278E-130	2.7074E-128	positive
PRJNA729271	stage_1W_vs_E10	ordinary_muscle_development	ENSGALG00010009299	MAP4K1	3.060424838	0.294203563	10.40240577	2.41751E-25	1.12747E-24	positive
PRJNA729271	stage_1W_vs_E10	ordinary_muscle_development	ENSGALG00010009303	TTC1	0.320903705	0.096427208	3.327937337	0.000874915	0.001311062	positive
PRJNA729271	stage_1W_vs_E10	ordinary_muscle_development	ENSGALG00010009306	TBC1D9	-1.28877432	0.10862176	-11.86478952	1.80355E-32	1.11768E-31	negative
PRJNA729271	stage_1W_vs_E10	ordinary_muscle_development	ENSGALG00010009307	DIRAS2	3.609439442	0.310707662	11.61683436	3.38446E-31	2.00645E-30	positive
PRJNA729271	stage_1W_vs_E10	ordinary_muscle_development	ENSGALG00010009309	TRIM54	3.015808067	0.148816214	20.2653191	2.60268E-91	8.04389E-90	positive
PRJNA729271	stage_1W_vs_E10	ordinary_muscle_development	ENSGALG00010009311	BNIP1	-0.234473381	0.113330005	-2.068943532	0.038551386	0.048790914	negative
PRJNA729271	stage_1W_vs_E10	ordinary_muscle_development	ENSGALG00010009313	UNC13B	-0.705920516	0.192334333	-3.670278233	0.000242287	0.000379178	negative
PRJNA729271	stage_1W_vs_E10	ordinary_muscle_development	ENSGALG00010009317	LRRTM3	-3.294853167	0.534570122	-6.163556531	7.1129E-10	1.61071E-09	negative
PRJNA729271	stage_1W_vs_E10	ordinary_muscle_development	ENSGALG00010009320	GPR20	-1.824868049	0.231091552	-7.896731964	2.86311E-15	8.61907E-15	negative
PRJNA729271	stage_1W_vs_E10	ordinary_muscle_development	ENSGALG00010009322	ENSGALG00010009322	2.411776728	0.166082503	14.52155816	8.8476E-48	8.98081E-47	positive
PRJNA729271	stage_1W_vs_E10	ordinary_muscle_development	ENSGALG00010009323	RNF150	0.610326356	0.107256289	5.690354953	1.26776E-08	2.67056E-08	positive
PRJNA729271	stage_1W_vs_E10	ordinary_muscle_development	ENSGALG00010009326	DLD	0.75987509	0.131779829	5.76624736	8.10561E-09	1.72806E-08	positive
PRJNA729271	stage_1W_vs_E10	ordinary_muscle_development	ENSGALG00010009327	NID1	1.993142191	0.163070674	12.22256669	2.35529E-34	1.55677E-33	positive
PRJNA729271	stage_1W_vs_E10	ordinary_muscle_development	ENSGALG00010009328	ENSGALG00010009328	-1.648532419	0.141542912	-11.64687372	2.38032E-31	1.41753E-30	negative
PRJNA729271	stage_1W_vs_E10	ordinary_muscle_development	ENSGALG00010009330	EIF3K	0.487046022	0.14150524	3.441893886	0.000577657	0.000877044	positive
PRJNA729271	stage_1W_vs_E10	ordinary_muscle_development	ENSGALG00010009332	ENSGALG00010009332	1.135806234	0.162259663	6.999929685	2.56091E-12	6.63575E-12	positive
PRJNA729271	stage_1W_vs_E10	ordinary_muscle_development	ENSGALG00010009333	ADRA1B	2.126688974	0.315576697	6.739055805	1.59419E-11	3.96089E-11	positive
PRJNA729271	stage_1W_vs_E10	ordinary_muscle_development	ENSGALG00010009334	SS18L2	1.042748383	0.163574637	6.374755918	1.83255E-10	4.28846E-10	positive
PRJNA729271	stage_1W_vs_E10	ordinary_muscle_development	ENSGALG00010009335	PTP4A3	3.586534828	0.195026013	18.39003304	1.57884E-75	3.36054E-74	positive
PRJNA729271	stage_1W_vs_E10	ordinary_muscle_development	ENSGALG00010009336	LAMB1	0.861603054	0.109946141	7.836592071	4.62939E-15	1.381E-14	positive
PRJNA729271	stage_1W_vs_E10	ordinary_muscle_development	ENSGALG00010009337	ZNF330	0.260735416	0.099974422	2.60802123	0.009106731	0.012370168	positive
PRJNA729271	stage_1W_vs_E10	ordinary_muscle_development	ENSGALG00010009338	KYNU	-0.541880684	0.584590477	-0.926940661	0.353957344	0.391589964	negative
PRJNA729271	stage_1W_vs_E10	ordinary_muscle_development	ENSGALG00010009340	SYK	0.862090664	0.141419029	6.096001855	1.08754E-09	2.4425E-09	positive
PRJNA729271	stage_1W_vs_E10	ordinary_muscle_development	ENSGALG00010009344	ENPP2	-1.835670508	0.260297515	-7.052201425	1.76109E-12	4.6001E-12	negative
PRJNA729271	stage_1W_vs_E10	ordinary_muscle_development	ENSGALG00010009345	ZFAT	0.490879447	0.124792815	3.933555351	8.36986E-05	0.000135463	positive
PRJNA729271	stage_1W_vs_E10	ordinary_muscle_development	ENSGALG00010009346	STC2	-0.574827418	0.3072359	-1.870964356	0.061350022	0.075746896	negative
PRJNA729271	stage_1W_vs_E10	ordinary_muscle_development	ENSGALG00010009347	TESK1	2.522955329	0.141789762	17.79363539	7.91726E-71	1.52811E-69	positive
PRJNA729271	stage_1W_vs_E10	ordinary_muscle_development	ENSGALG00010009348	ERGIC2	-0.046062844	0.082450347	-0.55867374	0.576384404	0.611886036	negative
PRJNA729271	stage_1W_vs_E10	ordinary_muscle_development	ENSGALG00010009351	GTF3C2	0.576561665	0.121706255	4.737321536	2.16561E-06	3.94354E-06	positive
PRJNA729271	stage_1W_vs_E10	ordinary_muscle_development	ENSGALG00010009352	HSF1	0.945046893	0.181800784	5.198255336	2.01168E-07	3.94044E-07	positive
PRJNA729271	stage_1W_vs_E10	ordinary_muscle_development	ENSGALG00010009355	GARRE1	0.12765281	0.072465803	1.761559314	0.078143783	0.095199056	positive
PRJNA729271	stage_1W_vs_E10	ordinary_muscle_development	ENSGALG00010009357	LYST	-0.115173619	0.117150516	-0.983125155	0.325545845	0.36206453	negative
PRJNA729271	stage_1W_vs_E10	ordinary_muscle_development	ENSGALG00010009359	REEP3	-1.183979569	0.111506376	-10.6180437	2.45655E-26	1.1977E-25	negative
PRJNA729271	stage_1W_vs_E10	ordinary_muscle_development	ENSGALG00010009360	TNFAIP8	-0.703960435	0.131252366	-5.363411389	8.16647E-08	1.64316E-07	negative
PRJNA729271	stage_1W_vs_E10	ordinary_muscle_development	ENSGALG00010009361	LRRK2	1.037354023	0.087776031	11.81819238	3.14376E-32	1.93416E-31	positive
PRJNA729271	stage_1W_vs_E10	ordinary_muscle_development	ENSGALG00010009362	IL15	4.494646968	0.309469209	14.52372916	8.57173E-48	8.70688E-47	positive
PRJNA729271	stage_1W_vs_E10	ordinary_muscle_development	ENSGALG00010009363	TAF2	-0.197238814	0.077361769	-2.549564416	0.010785758	0.01453105	negative
PRJNA729271	stage_1W_vs_E10	ordinary_muscle_development	ENSGALG00010009365	CA5B	0.079831555	0.161927104	0.493009215	0.622006076	0.655832388	positive
PRJNA729271	stage_1W_vs_E10	ordinary_muscle_development	ENSGALG00010009367	DMXL1	0.578562876	0.1192403	4.852074961	1.22176E-06	2.26957E-06	positive
PRJNA729271	stage_1W_vs_E10	ordinary_muscle_development	ENSGALG00010009368	DSCC1	-2.141050975	0.198212158	-10.80181454	3.37468E-27	1.69847E-26	negative
PRJNA729271	stage_1W_vs_E10	ordinary_muscle_development	ENSGALG00010009370	ENSGALG00010009370	1.139939827	0.454018809	2.510776658	0.012046589	0.016129254	positive
PRJNA729271	stage_1W_vs_E10	ordinary_muscle_development	ENSGALG00010009371	PHTF2	2.32030072	0.154528611	15.01534703	5.82562E-51	6.59706E-50	positive
PRJNA729271	stage_1W_vs_E10	ordinary_muscle_development	ENSGALG00010009372	RAB28	-0.369248263	0.098872115	-3.734604676	0.00018801	0.000296412	negative
PRJNA729271	stage_1W_vs_E10	ordinary_muscle_development	ENSGALG00010009373	ARHGAP15	-0.587433864	0.222530682	-2.639788178	0.008295786	0.011313145	negative
PRJNA729271	stage_1W_vs_E10	ordinary_muscle_development	ENSGALG00010009376	CDO1	-0.045873055	0.250103007	-0.183416645	0.854471116	0.870811983	negative
PRJNA729271	stage_1W_vs_E10	ordinary_muscle_development	ENSGALG00010009378	ENSGALG00010009378	-1.044398541	0.084690305	-12.33197286	6.09363E-35	4.12937E-34	negative
PRJNA729271	stage_1W_vs_E10	ordinary_muscle_development	ENSGALG00010009379	RYR1	5.21031428	0.219464628	23.74102073	1.3604E-124	8.182E-123	positive
PRJNA729271	stage_1W_vs_E10	ordinary_muscle_development	ENSGALG00010009381	INPP4B	-1.11940764	0.116445033	-9.613184966	7.03389E-22	2.84397E-21	negative
PRJNA729271	stage_1W_vs_E10	ordinary_muscle_development	ENSGALG00010009383	ASXL2	0.863358071	0.111303106	7.756819223	8.70859E-15	2.56255E-14	positive
PRJNA729271	stage_1W_vs_E10	ordinary_muscle_development	ENSGALG00010009385	GPI	4.095893304	0.177937654	23.01869899	3.029E-117	1.568E-115	positive
PRJNA729271	stage_1W_vs_E10	ordinary_muscle_development	ENSGALG00010009386	TMEM60	0.655269306	0.110972325	5.904799293	3.53076E-09	7.69948E-09	positive
PRJNA729271	stage_1W_vs_E10	ordinary_muscle_development	ENSGALG00010009387	DEPTOR	3.199843345	0.138179323	23.15717919	1.2307E-118	6.6566E-117	positive
PRJNA729271	stage_1W_vs_E10	ordinary_muscle_development	ENSGALG00010009388	TMED7	-0.220458032	0.073756213	-2.989009664	0.002798833	0.003998924	negative
PRJNA729271	stage_1W_vs_E10	ordinary_muscle_development	ENSGALG00010009389	CPEB4	2.475347279	0.1248486	19.82679246	1.7484E-87	4.94016E-86	positive
PRJNA729271	stage_1W_vs_E10	ordinary_muscle_development	ENSGALG00010009391	RSBN1L	0.175684166	0.107448165	1.635059713	0.10203653	0.122567292	positive
PRJNA729271	stage_1W_vs_E10	ordinary_muscle_development	ENSGALG00010009392	MAPRE3	1.612827281	0.105174368	15.33479418	4.47689E-53	5.47617E-52	positive
PRJNA729271	stage_1W_vs_E10	ordinary_muscle_development	ENSGALG00010009393	MARVELD2	-0.608232567	0.135093792	-4.50229843	6.72225E-06	1.17894E-05	negative
PRJNA729271	stage_1W_vs_E10	ordinary_muscle_development	ENSGALG00010009394	BANP	-0.49569707	0.09860861	-5.026914686	4.98434E-07	9.49757E-07	negative
PRJNA729271	stage_1W_vs_E10	ordinary_muscle_development	ENSGALG00010009396	AUH	0.6768239	0.111562779	6.06675366	1.30522E-09	2.92097E-09	positive
PRJNA729271	stage_1W_vs_E10	ordinary_muscle_development	ENSGALG00010009398	WTIP	-0.242507438	0.139695794	-1.73596807	0.08256948	0.100347499	negative
PRJNA729271	stage_1W_vs_E10	ordinary_muscle_development	ENSGALG00010009400	CNTFR	0.640580125	0.281673929	2.274190331	0.022954545	0.029819514	positive
PRJNA729271	stage_1W_vs_E10	ordinary_muscle_development	ENSGALG00010009404	GNG4	-1.040688721	0.409316868	-2.542501425	0.011006215	0.014804667	negative
PRJNA729271	stage_1W_vs_E10	ordinary_muscle_development	ENSGALG00010009407	PTPN12	-0.142666912	0.087823584	-1.624471538	0.10427521	0.125059504	negative
PRJNA729271	stage_1W_vs_E10	ordinary_muscle_development	ENSGALG00010009408	ENSGALG00010009408	-0.315487085	0.092708267	-3.403009205	0.00066648	0.001007365	negative
PRJNA729271	stage_1W_vs_E10	ordinary_muscle_development	ENSGALG00010009410	JMJD1C	-0.22200551	0.1084886	-2.046348751	0.04072207	0.051407979	negative
PRJNA729271	stage_1W_vs_E10	ordinary_muscle_development	ENSGALG00010009411	SMARCA5	-1.640050328	0.097935246	-16.74627253	6.02874E-63	9.46766E-62	negative
PRJNA729271	stage_1W_vs_E10	ordinary_muscle_development	ENSGALG00010009415	HMP19	-4.169385792	0.453409747	-9.195624539	3.72822E-20	1.40731E-19	negative
PRJNA729271	stage_1W_vs_E10	ordinary_muscle_development	ENSGALG00010009416	COL14A1	-4.740493185	0.474249355	-9.995782039	1.59027E-23	6.88499E-23	negative
PRJNA729271	stage_1W_vs_E10	ordinary_muscle_development	ENSGALG00010009424	ENSGALG00010009424	-0.948362611	1.281971549	-0.739768844	0.459440267	0.497652568	negative
PRJNA729271	stage_1W_vs_E10	ordinary_muscle_development	ENSGALG00010009431	SMN	0.400811015	0.149248587	2.685526372	0.007241566	0.009938127	positive
PRJNA729271	stage_1W_vs_E10	ordinary_muscle_development	ENSGALG00010009432	BOP1	-0.076795384	0.181181757	-0.423858258	0.671669188	0.701631945	negative
PRJNA729271	stage_1W_vs_E10	ordinary_muscle_development	ENSGALG00010009435	GTDC1	-0.500637896	0.090758442	-5.516157911	3.46491E-08	7.1189E-08	negative
PRJNA729271	stage_1W_vs_E10	ordinary_muscle_development	ENSGALG00010009437	DPYSL5	-5.522364248	0.319988494	-17.25800883	9.74125E-67	1.67695E-65	negative
PRJNA729271	stage_1W_vs_E10	ordinary_muscle_development	ENSGALG00010009439	B3GALNT2	0.835240795	0.098334365	8.493885099	1.99842E-17	6.60444E-17	positive
PRJNA729271	stage_1W_vs_E10	ordinary_muscle_development	ENSGALG00010009440	DOK3	-0.593184341	0.230996456	-2.567936981	0.010230575	0.013819047	negative
PRJNA729271	stage_1W_vs_E10	ordinary_muscle_development	ENSGALG00010009441	ZNF518B	-0.878639526	0.098810526	-8.892165215	5.99295E-19	2.1486E-18	negative
PRJNA729271	stage_1W_vs_E10	ordinary_muscle_development	ENSGALG00010009444	NFIL3	2.218040529	0.221284142	10.02349516	1.20176E-23	5.22325E-23	positive
PRJNA729271	stage_1W_vs_E10	ordinary_muscle_development	ENSGALG00010009446	ENSGALG00010009446	0.885755477	0.249695297	3.547345459	0.000389134	0.000598842	positive
PRJNA729271	stage_1W_vs_E10	ordinary_muscle_development	ENSGALG00010009447	DDX41	-0.501418619	0.106816924	-4.694187015	2.67669E-06	4.84726E-06	negative
PRJNA729271	stage_1W_vs_E10	ordinary_muscle_development	ENSGALG00010009451	ROR2	-1.071100063	0.13894173	-7.708987541	1.2682E-14	3.69795E-14	negative
PRJNA729271	stage_1W_vs_E10	ordinary_muscle_development	ENSGALG00010009452	TBCE	-0.027154922	0.086045977	-0.315586191	0.75231662	0.776973952	negative
PRJNA729271	stage_1W_vs_E10	ordinary_muscle_development	ENSGALG00010009453	GSAP	-0.248368731	0.115459299	-2.151136664	0.031465414	0.040205499	negative
PRJNA729271	stage_1W_vs_E10	ordinary_muscle_development	ENSGALG00010009457	BDP1	0.268528182	0.129574795	2.072379752	0.03823004	0.048418037	positive
PRJNA729271	stage_1W_vs_E10	ordinary_muscle_development	ENSGALG00010009458	SCX	-3.644241229	0.460030971	-7.921730178	2.34228E-15	7.08058E-15	negative
PRJNA729271	stage_1W_vs_E10	ordinary_muscle_development	ENSGALG00010009461	ZEB2	-0.311076928	0.125836622	-2.472069919	0.013433324	0.017908216	negative
PRJNA729271	stage_1W_vs_E10	ordinary_muscle_development	ENSGALG00010009462	NRBF2	1.789632175	0.119701614	14.9507773	1.53942E-50	1.72175E-49	positive
PRJNA729271	stage_1W_vs_E10	ordinary_muscle_development	ENSGALG00010009463	MRPL13	0.14984615	0.109146633	1.37288844	0.169787031	0.197517096	positive

PRJNA729271	stage_1W_vs_E10	ordinary_muscle_development	ENSGALG00010009464	SPTLC1	0.100564423	0.148076087	0.6791402	0.497049033	0.534792587	positive
PRJNA729271	stage_1W_vs_E10	ordinary_muscle_development	ENSGALG00010009465	GGPS1	0.817512252	0.09840289	8.307807377	9.74866E-17	3.12972E-16	positive
PRJNA729271	stage_1W_vs_E10	ordinary_muscle_development	ENSGALG00010009466	FAM193B	-0.151243757	0.109914164	-1.376016983	0.168816375	0.196482525	negative
PRJNA729271	stage_1W_vs_E10	ordinary_muscle_development	ENSGALG00010009467	EGR2	1.852747621	0.300218119	6.171338443	6.77143E-10	1.53554E-09	positive
PRJNA729271	stage_1W_vs_E10	ordinary_muscle_development	ENSGALG00010009469	DNMT3A	0.578569046	0.210707357	2.745841686	0.006035588	0.008354264	positive
PRJNA729271	stage_1W_vs_E10	ordinary_muscle_development	ENSGALG00010009470	ZNF469	-1.893428638	0.213550872	-8.866405569	7.5545E-19	2.68492E-18	negative
PRJNA729271	stage_1W_vs_E10	ordinary_muscle_development	ENSGALG00010009472	ADO	-0.031594783	0.085132292	-0.371125714	0.7105439	0.737986087	negative
PRJNA729271	stage_1W_vs_E10	ordinary_muscle_development	ENSGALG00010009474	ACVR2A	-0.75324876	0.096067509	-7.840827439	4.47587E-15	1.33631E-14	negative
PRJNA729271	stage_1W_vs_E10	ordinary_muscle_development	ENSGALG00010009477	ZNF365	1.386172485	0.242980648	5.704867836	1.16433E-08	2.46021E-08	positive
PRJNA729271	stage_1W_vs_E10	ordinary_muscle_development	ENSGALG00010009479	MTBP	-2.995704749	0.148635184	-20.15474848	2.44498E-90	7.26229E-89	negative
PRJNA729271	stage_1W_vs_E10	ordinary_muscle_development	ENSGALG00010009482	ARID4B	0.121753242	0.108128304	1.126007135	0.260162499	0.293833211	positive
PRJNA729271	stage_1W_vs_E10	ordinary_muscle_development	ENSGALG00010009484	RTKN2	-1.498486206	0.134677045	-11.12651536	9.32084E-29	4.99097E-28	negative
PRJNA729271	stage_1W_vs_E10	ordinary_muscle_development	ENSGALG00010009486	B4GALT1	-0.345419727	0.129024505	-2.677163748	0.007424834	0.010178075	negative
PRJNA729271	stage_1W_vs_E10	ordinary_muscle_development	ENSGALG00010009489	MROH1	0.002440488	0.138319805	0.017643805	0.98592301	0.988172731	positive
PRJNA729271	stage_1W_vs_E10	ordinary_muscle_development	ENSGALG00010009490	SNTB1	0.809555706	0.159618962	5.071801599	3.94067E-07	7.56757E-07	positive
PRJNA729271	stage_1W_vs_E10	ordinary_muscle_development	ENSGALG00010009491	RBM34	0.182497816	0.10205135	1.788293988	0.073728589	0.090116865	positive
PRJNA729271	stage_1W_vs_E10	ordinary_muscle_development	ENSGALG00010009492	CDC42SE2	-1.659727745	0.128125027	-12.95396986	2.23111E-38	1.68877E-37	negative
PRJNA729271	stage_1W_vs_E10	ordinary_muscle_development	ENSGALG00010009493	ENSGALG00010009493	0.574276402	0.401983024	1.428608591	0.153116764	0.179347676	positive
PRJNA729271	stage_1W_vs_E10	ordinary_muscle_development	ENSGALG00010009494	UBA2	-0.712486839	0.075074821	-9.49035685	2.30245E-21	9.1561E-21	negative
PRJNA729271	stage_1W_vs_E10	ordinary_muscle_development	ENSGALG00010009497	HS3ST1	2.48056957	0.187004143	13.26478406	3.70486E-40	2.98179E-39	positive
PRJNA729271	stage_1W_vs_E10	ordinary_muscle_development	ENSGALG00010009498	ORC4	-1.945485684	0.118961318	-16.35393512	4.07774E-60	5.98854E-59	negative
PRJNA729271	stage_1W_vs_E10	ordinary_muscle_development	ENSGALG00010009499	MXD3	-0.810521041	0.244278384	-3.318021951	0.000906574	0.001357098	negative
PRJNA729271	stage_1W_vs_E10	ordinary_muscle_development	ENSGALG00010009500	CCDC146	-0.967342652	0.735987277	-1.314346975	0.188729466	0.218048271	negative
PRJNA729271	stage_1W_vs_E10	ordinary_muscle_development	ENSGALG00010009501	HGH1	-0.473097365	0.11365538	-4.162560245	3.14699E-05	5.26193E-05	negative
PRJNA729271	stage_1W_vs_E10	ordinary_muscle_development	ENSGALG00010009502	ARID5B	-1.070255006	0.097377156	-10.99082213	4.23055E-28	2.21458E-27	negative
PRJNA729271	stage_1W_vs_E10	ordinary_muscle_development	ENSGALG00010009503	IL11RA	-2.382170706	0.137814737	-17.28531186	6.06922E-67	1.0498E-65	negative
PRJNA729271	stage_1W_vs_E10	ordinary_muscle_development	ENSGALG00010009506	HAS2	-2.825758095	0.261886831	-10.78999691	3.83802E-27	1.92832E-26	negative
PRJNA729271	stage_1W_vs_E10	ordinary_muscle_development	ENSGALG00010009508	TOMM20	1.379278421	0.089876996	15.34628971	3.75031E-53	4.59905E-52	positive
PRJNA729271	stage_1W_vs_E10	ordinary_muscle_development	ENSGALG00010009511	ZFPM1	-1.376209903	0.267512199	-5.144475319	2.6827E-07	5.20697E-07	negative
PRJNA729271	stage_1W_vs_E10	ordinary_muscle_development	ENSGALG00010009514	PRELID1	-1.284681711	0.09232244	-13.91516202	5.12437E-44	4.66569E-43	negative
PRJNA729271	stage_1W_vs_E10	ordinary_muscle_development	ENSGALG00010009515	MBD5	0.639790601	0.095955225	6.667595249	2.60029E-11	6.39918E-11	positive
PRJNA729271	stage_1W_vs_E10	ordinary_muscle_development	ENSGALG00010009518	FGL2	5.831096586	0.288620503	20.20333455	9.15047E-91	2.76902E-89	positive
PRJNA729271	stage_1W_vs_E10	ordinary_muscle_development	ENSGALG00010009520	SPINK4	-0.208069354	0.618486803	-0.336416805	0.736556579	0.762544647	negative
PRJNA729271	stage_1W_vs_E10	ordinary_muscle_development	ENSGALG00010009522	TAF5L	0.763475149	0.080557569	9.477385632	2.60734E-21	1.03515E-20	positive
PRJNA729271	stage_1W_vs_E10	ordinary_muscle_development	ENSGALG00010009524	FAM185A	1.639291223	0.160207455	10.23230301	1.42099E-24	6.4226E-24	positive
PRJNA729271	stage_1W_vs_E10	ordinary_muscle_development	ENSGALG00010009525	ENSGALG00010009525	0.548428399	0.156594682	3.502215988	0.000461405	0.000705195	positive
PRJNA729271	stage_1W_vs_E10	ordinary_muscle_development	ENSGALG00010009526	ENSGALG00010009526	-1.058906207	0.260851951	-4.059414565	4.91959E-05	8.09323E-05	negative
PRJNA729271	stage_1W_vs_E10	ordinary_muscle_development	ENSGALG00010009527	ENSGALG00010009527	-1.962294744	0.540919041	-3.627705063	0.000285952	0.000445078	negative
PRJNA729271	stage_1W_vs_E10	ordinary_muscle_development	ENSGALG00010009530	KIF3C	-1.8413468	0.163893505	-11.23501997	2.74432E-29	1.5045E-28	negative
PRJNA729271	stage_1W_vs_E10	ordinary_muscle_development	ENSGALG00010009531	FBXL13	-2.305200694	0.56251375	-4.098034395	4.16673E-05	6.89619E-05	negative
PRJNA729271	stage_1W_vs_E10	ordinary_muscle_development	ENSGALG00010009534	XPA	0.196820062	0.131225709	1.499859013	0.133650927	0.157886731	positive
PRJNA729271	stage_1W_vs_E10	ordinary_muscle_development	ENSGALG00010009538	CRIM1	1.185603056	0.074688712	15.87392549	9.60416E-57	1.29259E-55	positive
PRJNA729271	stage_1W_vs_E10	ordinary_muscle_development	ENSGALG00010009540	RAB10	0.838116047	0.089905954	9.322141762	1.14015E-20	4.41767E-20	positive
PRJNA729271	stage_1W_vs_E10	ordinary_muscle_development	ENSGALG00010009541	PMPCB	0.658193138	0.091645967	7.181910573	6.87439E-13	1.83372E-12	positive
PRJNA729271	stage_1W_vs_E10	ordinary_muscle_development	ENSGALG00010009542	WDR1	0.07971379	0.14312654	0.556946254	0.577564151	0.613093546	positive
PRJNA729271	stage_1W_vs_E10	ordinary_muscle_development	ENSGALG00010009543	MAF1	2.725799373	0.129407524	21.06368538	1.71315E-98	6.20803E-97	positive
PRJNA729271	stage_1W_vs_E10	ordinary_muscle_development	ENSGALG00010009546	GAREM2	-2.176419728	0.61804602	-3.521452544	0.000429189	0.000658039	negative
PRJNA729271	stage_1W_vs_E10	ordinary_muscle_development	ENSGALG00010009548	PIK3C3	-0.242775015	0.082343019	-2.948337571	0.00319488	0.004540175	negative
PRJNA729271	stage_1W_vs_E10	ordinary_muscle_development	ENSGALG00010009551	HADHA	1.011115381	0.130122759	7.770472971	7.81936E-15	2.30416E-14	positive
PRJNA729271	stage_1W_vs_E10	ordinary_muscle_development	ENSGALG00010009554	ABCB10	0.872843044	0.101253111	8.620407196	6.67164E-18	2.25631E-17	positive
PRJNA729271	stage_1W_vs_E10	ordinary_muscle_development	ENSGALG00010009555	EPC2	-0.079318884	0.077642837	-1.021586615	0.306976595	0.342595145	negative
PRJNA729271	stage_1W_vs_E10	ordinary_muscle_development	ENSGALG00010009557	CIDEC	2.607615731	1.060903061	2.45792083	0.013974398	0.018604652	positive
PRJNA729271	stage_1W_vs_E10	ordinary_muscle_development	ENSGALG00010009558	TMEM26	-3.943219902	0.204772091	-19.2566276	1.24209E-82	3.18658E-81	negative
PRJNA729271	stage_1W_vs_E10	ordinary_muscle_development	ENSGALG00010009560	RAB24	1.406061747	0.141757722	9.918766536	3.44993E-23	1.47382E-22	positive
PRJNA729271	stage_1W_vs_E10	ordinary_muscle_development	ENSGALG00010009562	HADHB	1.555063477	0.18939145	8.210843092	2.19641E-16	6.93011E-16	positive
PRJNA729271	stage_1W_vs_E10	ordinary_muscle_development	ENSGALG00010009565	TMEM8B	0.350219908	0.230374068	1.520222786	0.128454992	0.152058401	positive
PRJNA729271	stage_1W_vs_E10	ordinary_muscle_development	ENSGALG00010009567	NSD1	-0.010787958	0.106639169	-0.101163181	0.919420924	0.929358877	negative
PRJNA729271	stage_1W_vs_E10	ordinary_muscle_development	ENSGALG00010009569	ZC3H18	-0.026923727	0.143479691	-0.18764835	0.851152321	0.867673739	negative
PRJNA729271	stage_1W_vs_E10	ordinary_muscle_development	ENSGALG00010009570	SHARPIN	2.216567113	0.134909213	16.43006481	1.16534E-60	1.7432E-59	positive
PRJNA729271	stage_1W_vs_E10	ordinary_muscle_development	ENSGALG00010009571	DNAJC2	0.806802534	0.184866682	4.36423982	1.27566E-05	2.19647E-05	positive
PRJNA729271	stage_1W_vs_E10	ordinary_muscle_development	ENSGALG00010009574	TAF1C	0.193990975	0.139311823	1.392494692	0.163772648	0.191072483	positive
PRJNA729271	stage_1W_vs_E10	ordinary_muscle_development	ENSGALG00010009575	RHOBTB1	-3.135399249	0.252635033	-12.41078566	2.28401E-35	1.57276E-34	negative
PRJNA729271	stage_1W_vs_E10	ordinary_muscle_development	ENSGALG00010009578	KIF5C	-2.635484336	0.162513311	-16.21703672	3.82195E-59	5.46342E-58	negative
PRJNA729271	stage_1W_vs_E10	ordinary_muscle_development	ENSGALG00010009582	TIGD5	-0.49641	0.135174659	-3.672359926	0.000240321	0.000376223	negative
PRJNA729271	stage_1W_vs_E10	ordinary_muscle_development	ENSGALG00010009583	LYPD6B	-2.934713632	0.317847202	-9.2330957	2.62921E-20	1.00027E-19	negative
PRJNA729271	stage_1W_vs_E10	ordinary_muscle_development	ENSGALG00010009591	LYPD6	-3.227396958	0.547190459	-5.898123597	3.67659E-09	8.00542E-09	negative
PRJNA729271	stage_1W_vs_E10	ordinary_muscle_development	ENSGALG00010009593	NCBP1	-0.9482697	0.063269118	-14.98787609	8.81265E-51	9.90228E-50	negative
PRJNA729271	stage_1W_vs_E10	ordinary_muscle_development	ENSGALG00010009594	HINT2	2.069842463	0.159397086	12.9854473	1.47971E-38	1.12768E-37	positive
PRJNA729271	stage_1W_vs_E10	ordinary_muscle_development	ENSGALG00010009595	ENSGALG00010009595	1.191004213	0.185842864	6.408662601	1.46802E-10	3.45381E-10	positive
PRJNA729271	stage_1W_vs_E10	ordinary_muscle_development	ENSGALG00010009596	URB2	-0.548640937	0.097551837	-5.62409641	1.86481E-08	3.89432E-08	negative
PRJNA729271	stage_1W_vs_E10	ordinary_muscle_development	ENSGALG00010009597	CDK1	-3.120939843	0.260598407	-11.97605113	4.74387E-33	3.0001E-32	negative
PRJNA729271	stage_1W_vs_E10	ordinary_muscle_development	ENSGALG00010009600	LRRC17	-2.273366897	0.217871007	-10.43446273	1.72587E-25	8.10383E-25	negative
PRJNA729271	stage_1W_vs_E10	ordinary_muscle_development	ENSGALG00010009601	ZHX2	0.349250954	0.136265865	2.563011313	0.010376867	0.014003601	positive
PRJNA729271	stage_1W_vs_E10	ordinary_muscle_development	ENSGALG00010009602	TSTD2	0.191466806	0.080786505	2.37003453	0.017786424	0.023356968	positive
PRJNA729271	stage_1W_vs_E10	ordinary_muscle_development	ENSGALG00010009603	HINT1	1.276831267	0.177094679	7.20987935	5.60015E-13	1.49961E-12	positive
PRJNA729271	stage_1W_vs_E10	ordinary_muscle_development	ENSGALG00010009605	ENSGALG00010009605	-0.728152565	0.184518174	-3.946237656	7.93888E-05	0.000128805	negative
PRJNA729271	stage_1W_vs_E10	ordinary_muscle_development	ENSGALG00010009606	CHSY3	-0.601966473	0.2163823	-2.781958012	0.005403203	0.00752059	negative
PRJNA729271	stage_1W_vs_E10	ordinary_muscle_development	ENSGALG00010009608	AFAP1	-1.875853606	0.107677312	-17.42106644	5.71028E-68	1.02821E-66	negative
PRJNA729271	stage_1W_vs_E10	ordinary_muscle_development	ENSGALG00010009609	MMADHC	-0.295574783	0.072333743	-4.086264151	4.38375E-05	7.24708E-05	negative
PRJNA729271	stage_1W_vs_E10	ordinary_muscle_development	ENSGALG00010009612	DERL1	-0.348395119	0.063841831	-5.457160555	4.83809E-08	9.8772E-08	negative
PRJNA729271	stage_1W_vs_E10	ordinary_muscle_development	ENSGALG00010009615	ENSGALG00010009615	2.332291667	0.395265675	5.900567166	3.62254E-09	7.89249E-09	positive
PRJNA729271	stage_1W_vs_E10	ordinary_muscle_development	ENSGALG00010009616	ARMC10	-0.3696393	0.17978259	-2.056035009	0.039779142	0.050287691	negative
PRJNA729271	stage_1W_vs_E10	ordinary_muscle_development	ENSGALG00010009617	SLC25A46	1.118007635	0.097120883	11.51150609	1.15444E-30	6.70688E-30	positive
PRJNA729271	stage_1W_vs_E10	ordinary_muscle_development	ENSGALG00010009619	ENSGALG00010009619	-0.312473659	0.191779086	-1.629341683	0.103240707	0.123941862	negative
PRJNA729271	stage_1W_vs_E10	ordinary_muscle_development	ENSGALG00010009620	TMOD1	-0.303515827	0.232466368	-1.305633285	0.191677296	0.221295293	negative
PRJNA729271	stage_1W_vs_E10	ordinary_muscle_development	ENSGALG00010009623	XPO5	-0.679717798	0.111425593	-6.100194575	1.05939E-09	2.38039E-09	negative
PRJNA729271	stage_1W_vs_E10	ordinary_muscle_development	ENSGALG00010009629	ENSGALG00010009629	0.119314624	0.587784074	0.20299057	0.839142403	0.856816648	positive
PRJNA729271	stage_1W_vs_E10	ordinary_muscle_development	ENSGALG00010009634	TBC1D31	-0.764283959	0.106110533	-7.202715313	5.90251E-13	1.57883E-12	negative
PRJNA729271	stage_1W_vs_E10	ordinary_muscle_development	ENSGALG00010009635	TDRD7	0.360743732	0.140649539	2.564841188	0.010322304	0.013932563	positive
PRJNA729271	stage_1W_vs_E10	ordinary_muscle_development	ENSGALG00010009637	CYBA	0.337053223	0.136627316	2.466953408	0.013626809	0.018159473	positive
PRJNA729271	stage_1W_vs_E10	ordinary_muscle_development	ENSGALG00010009638	RND3	-1.399049179	0.151009129	-9.26466626	1.95688E-20	7.49406E-20	negative
PRJNA729271	stage_1W_vs_E10	ordinary_muscle_development	ENSGALG00010009640	NPR2	-0.0878362	0.188324986	-0.466407573	0.640923792	0.673250987	negative
PRJNA729271	stage_1W_vs_E10	ordinary_muscle_development	ENSGALG00010009643	PSMC2	0.755018951	0.108550268	6.955477555	3.5137E-12	9.04476E-12	positive
PRJNA729271	stage_1W_vs_E10	ordinary_muscle_development	ENSGALG00010009644	SLC27A6	-0.074551673	0.220110593	-0.338700979	0.734835003	0.761034108	negative
PRJNA729271	stage_1W_vs_E10	ordinary_muscle_development	ENSGALG00010009646	MVD	0.768510213	0.167227242	4.595604182	4.31497E-06	7.68939E-06	positive
PRJNA729271	stage_1W_vs_E10	ordinary_muscle_development	ENSGALG00010009647	GALNT2	-2.03968568	0.079865661	-25.53895706	7.2832E-144	5.4699E-142	negative
PRJNA729271	stage_1W_vs_E10	ordinary_muscle_development	ENSGALG00010009649	TENM2	-1.81697667	0.315829653	-5.75302747	8.76592E-09	1.86527E-08	negative
PRJNA729271	stage_1W_vs_E10	ordinary_muscle_development	ENSGALG00010009650	KLHL29	-0.061159151	0.192392925	-0.317886697	0.750570884	0.775557811	negative
PRJNA729271	stage_1W_vs_E10	ordinary_muscle_development	ENSGALG00010009652	CIAO2B	0.268973487	0.08130701	3.308121748	0.00093924	0.001403823	positive
PRJNA729271	stage_1W_vs_E10	ordinary_muscle_development	ENSGALG00010009653	ABLIM2	4.210204602	0.182830356	23.02792978	2.4481E-117	1.2764E-115	positive
PRJNA729271	stage_1W_vs_E10	ordinary_muscle_development	ENSGALG00010009654	NDUFA12	1.827149465	0.139530633	13.09497012	3.51813E-39	2.74463E-38	positive
PRJNA729271	stage_1W_vs_E10	ordinary_muscle_development	ENSGALG00010009657	MSMP	1.012353708	0.756645832	1.337949229	0.180912988	0.209560409	positive
PRJNA729271	stage_1W_vs_E10	ordinary_muscle_development	ENSGALG00010009658	NAPEPLD	1.362228798	0.122833404	11.09005172	1.40207E-28	7.44703E-28	positive
PRJNA729271	stage_1W_vs_E10	ordinary_muscle_development	ENSGALG00010009659	APOLD1	-0.096486976	0.256426994	-0.376274645	0.706712727	0.734533269	negative
PRJNA729271	stage_1W_vs_E10	ordinary_muscle_development	ENSGALG00010009663	FER	-0.629229184	0.123219548	-5.106569465	3.2806E-07	6.32934E-07	negative
PRJNA729271	stage_1W_vs_E10	ordinary_muscle_development	ENSGALG00010009665	DGKQ	-0.275720306	0.175338092	-1.572506595	0.115833111	0.138064381	negative
PRJNA729271	stage_1W_vs_E10	ordinary_muscle_development	ENSGALG00010009666	RRAD	0.895638491	0.365209595	2.452395836	0.014190846	0.018862569	positive
PRJNA729271	stage_1W_vs_E10	ordinary_muscle_development	ENSGALG00010009668	TFAP2A	-7.826703398	0.965742506	-8.104337699	5.30336E-16	1.65139E-15	negative
PRJNA729271	stage_1W_vs_E10	ordinary_muscle_development	ENSGALG00010009671	MINAR2	1.726380962	0.877911134	1.966464366	0.049244995	0.061529713	positive
PRJNA729271	stage_1W_vs_E10	ordinary_muscle_development	ENSGALG00010009672	DDX47	-0.347334692	0.12030255	-2.887176484	0.003887161	0.005479374	negative
PRJNA729271	stage_1W_vs_E10	ordinary_muscle_development	ENSGALG00010009676	ACO1	-0.561445333	0.096385156	-5.825018682	5.71062E-09	1.23013E-08	negative
PRJNA729271	stage_1W_vs_E10	ordinary_muscle_development	ENSGALG00010009677	SLC26A5	3.001580092	0.160614315	18.68812314	6.18509E-78	1.39429E-76	positive
PRJNA729271	stage_1W_vs_E10	ordinary_muscle_development	ENSGALG00010009678	JARID2	-0.592603479	0.101369377	-5.845981251	5.03591E-09	1.08851E-08	negative
PRJNA729271	stage_1W_vs_E10	ordinary_muscle_development	ENSGALG00010009679	ANK3	2.273726064	0.102779288	22.12241497	1.9235E-108	8.4746E-107	positive
PRJNA729271	stage_1W_vs_E10	ordinary_muscle_development	ENSGALG00010009683	ADAMTS19	2.151641976	0.238512236	9.02109684	1.86215E-19	6.82648E-19	positive
PRJNA729271	stage_1W_vs_E10	ordinary_muscle_development	ENSGALG00010009686	MAT2B	1.225398075	0.086751749	14.1253415	2.65112E-45	2.50345E-44	positive
PRJNA729271	stage_1W_vs_E10	ordinary_muscle_development	ENSGALG00010009689	ENSGALG00010009689	-3.454786592	0.68259931	-5.06122192	4.16578E-07	7.984E-07	negative
PRJNA729271	stage_1W_vs_E10	ordinary_muscle_development	ENSGALG00010009690	RBM43	-0.201979386	0.18734541	-1.078112275	0.280983654	0.315358736	negative
PRJNA729271	stage_1W_vs_E10	ordinary_muscle_development	ENSGALG00010009695	ATAD2B	0.344075514	0.076386934	4.504376524	6.65681E-06	1.16802E-05	positive
PRJNA729271	stage_1W_vs_E10	ordinary_muscle_development	ENSGALG00010009697	ENSGALG00010009697	-0.999114037	0.288046111	-3.468590616	0.000523196	0.000796694	negative
PRJNA729271	stage_1W_vs_E10	ordinary_muscle_development	ENSGALG00010009699	POLR1C	-0.331486762	0.109029492	-3.040340335	0.002363109	0.003399829	negative
PRJNA729271	stage_1W_vs_E10	ordinary_muscle_development	ENSGALG00010009700	SH3TC1	0.676934903	0.207405278	3.263826785	0.001099183	0.00163044	positive
PRJNA729271	stage_1W_vs_E10	ordinary_muscle_development	ENSGALG00010009702	ENSGALG00010009702	-0.940123316	0.125695416	-7.479376325	7.46761E-14	2.09327E-13	negative
PRJNA729271	stage_1W_vs_E10	ordinary_muscle_development	ENSGALG00010009704	RGP1	0.664341621	0.129726521	5.121093336	3.03769E-07	5.87712E-07	positive
PRJNA729271	stage_1W_vs_E10	ordinary_muscle_development	ENSGALG00010009705	DTNBP1	-0.315698526	0.072399642	-4.360498438	1.29767E-05	2.23207E-05	negative
PRJNA729271	stage_1W_vs_E10	ordinary_muscle_development	ENSGALG00010009710	ENSGALG00010009710	-1.051177545	0.113891186	-9.229665445	2.71478E-20	1.03175E-19	negative
PRJNA729271	stage_1W_vs_E10	ordinary_muscle_development	ENSGALG00010009712	UBXN2A	3.340509002	0.17790434	18.77699553	1.16488E-78	2.67166E-77	positive
PRJNA729271	stage_1W_vs_E10	ordinary_muscle_development	ENSGALG00010009715	NUDCD2	-0.295679538	0.093266923	-3.170250805	0.001523074	0.002230443	negative
PRJNA729271	stage_1W_vs_E10	ordinary_muscle_development	ENSGALG00010009717	EDN1	2.182926344	0.255135901	8.55593561	1.16917E-17	3.90215E-17	positive
PRJNA729271	stage_1W_vs_E10	ordinary_muscle_development	ENSGALG00010009718	ENSGALG00010009718	-1.17841826	0.187828267	-6.273913273	3.52085E-10	8.11104E-10	negative
PRJNA729271	stage_1W_vs_E10	ordinary_muscle_development	ENSGALG00010009719	YIPF3	0.079294127	0.082332073	0.963101308	0.335496685	0.37241725	positive

PRJNA729271	stage_1W_vs_E10	ordinary_muscle_development	ENSGALG00010009720	RELN	-1.259232003	0.169678355	-7.421288373	1.15986E-13	3.21151E-13	negative
PRJNA729271	stage_1W_vs_E10	ordinary_muscle_development	ENSGALG00010009721	ZHX1	0.691141503	0.156111136	4.427240227	9.54464E-06	1.65887E-05	positive
PRJNA729271	stage_1W_vs_E10	ordinary_muscle_development	ENSGALG00010009722	GBA2	0.690211214	0.095543828	7.224027207	5.04702E-13	1.35375E-12	positive
PRJNA729271	stage_1W_vs_E10	ordinary_muscle_development	ENSGALG00010009724	ISOC1	0.580785964	0.143047671	4.060086824	4.90545E-05	8.07088E-05	positive
PRJNA729271	stage_1W_vs_E10	ordinary_muscle_development	ENSGALG00010009726	PRR16	-0.3107451	0.149731602	-2.075347463	0.037954344	0.048085675	negative
PRJNA729271	stage_1W_vs_E10	ordinary_muscle_development	ENSGALG00010009727	CDH20	2.947526014	0.213632701	13.79716681	2.65067E-43	2.35608E-42	positive
PRJNA729271	stage_1W_vs_E10	ordinary_muscle_development	ENSGALG00010009728	CCDC149	-1.573211904	0.107136879	-14.68413046	8.14726E-49	8.54519E-48	negative
PRJNA729271	stage_1W_vs_E10	ordinary_muscle_development	ENSGALG00010009730	C8orf76	-0.202737544	0.116853679	-1.73496928	0.082746247	0.100545464	negative
PRJNA729271	stage_1W_vs_E10	ordinary_muscle_development	ENSGALG00010009731	NMI	0.42361839	0.242256544	1.748635486	0.080354048	0.097745211	positive
PRJNA729271	stage_1W_vs_E10	ordinary_muscle_development	ENSGALG00010009732	HMMR	-2.492756698	0.24491954	-10.17785963	2.48977E-24	1.11147E-23	negative
PRJNA729271	stage_1W_vs_E10	ordinary_muscle_development	ENSGALG00010009736	WISP1	-1.700457968	0.332780337	-5.109851097	3.22413E-07	6.2237E-07	negative
PRJNA729271	stage_1W_vs_E10	ordinary_muscle_development	ENSGALG00010009738	ENSGALG00010009738	-1.187173362	0.262037561	-4.530546529	5.88313E-06	1.03729E-05	negative
PRJNA729271	stage_1W_vs_E10	ordinary_muscle_development	ENSGALG00010009740	LRRC73	-2.385278636	0.363712399	-6.558144969	5.44812E-11	1.31442E-10	negative
PRJNA729271	stage_1W_vs_E10	ordinary_muscle_development	ENSGALG00010009741	C2H6orf52	0.166062146	0.0702916	2.362474985	0.018153367	0.023815101	positive
PRJNA729271	stage_1W_vs_E10	ordinary_muscle_development	ENSGALG00010009743	CCNG1	1.701225915	0.172700382	9.850736267	6.80437E-23	2.86796E-22	positive
PRJNA729271	stage_1W_vs_E10	ordinary_muscle_development	ENSGALG00010009744	DAPK1	-0.798692985	0.106202639	-7.520462694	5.45827E-14	1.54195E-13	negative
PRJNA729271	stage_1W_vs_E10	ordinary_muscle_development	ENSGALG00010009749	NDRG1	1.056948454	0.167170424	6.322580452	2.57231E-10	5.96475E-10	positive
PRJNA729271	stage_1W_vs_E10	ordinary_muscle_development	ENSGALG00010009751	ENSGALG00010009751	-0.227283569	0.217636111	-1.044328385	0.296333485	0.33146886	negative
PRJNA729271	stage_1W_vs_E10	ordinary_muscle_development	ENSGALG00010009761	PDP2	0.920072819	0.115869276	7.940610783	2.01188E-15	6.0996E-15	positive
PRJNA729271	stage_1W_vs_E10	ordinary_muscle_development	ENSGALG00010009762	MAN2A1	-0.14457235	0.103030911	-1.403193932	0.160559017	0.187549597	negative
PRJNA729271	stage_1W_vs_E10	ordinary_muscle_development	ENSGALG00010009765	STX10	-0.64992113	0.099115572	-6.557205029	5.48256E-11	1.32229E-10	negative
PRJNA729271	stage_1W_vs_E10	ordinary_muscle_development	ENSGALG00010009769	TMCC3	1.700226414	0.22679432	7.496776891	6.54062E-14	1.84233E-13	positive
PRJNA729271	stage_1W_vs_E10	ordinary_muscle_development	ENSGALG00010009772	ENSGALG00010009772	-0.505804962	0.5858701	-0.863339778	0.38795068	0.42607555	negative
PRJNA729271	stage_1W_vs_E10	ordinary_muscle_development	ENSGALG00010009774	PIAS2	-1.000039925	0.063177422	-15.82907154	1.96093E-56	2.61729E-55	negative
PRJNA729271	stage_1W_vs_E10	ordinary_muscle_development	ENSGALG00010009775	KIAA1614	-0.406980724	0.142039254	-2.865269371	0.004166547	0.00585441	negative
PRJNA729271	stage_1W_vs_E10	ordinary_muscle_development	ENSGALG00010009777	ST3GAL1	-2.831185043	0.192805378	-14.6841601	8.1437E-49	8.54519E-48	negative
PRJNA729271	stage_1W_vs_E10	ordinary_muscle_development	ENSGALG00010009778	ENSGALG00010009778	0.167088258	0.111481051	1.498804118	0.133924456	0.158171188	positive
PRJNA729271	stage_1W_vs_E10	ordinary_muscle_development	ENSGALG00010009779	PPP1R3E	2.91230176	0.343979241	8.466504399	2.52867E-17	8.3208E-17	positive
PRJNA729271	stage_1W_vs_E10	ordinary_muscle_development	ENSGALG00010009780	MFSD2B	-1.658529965	0.265667761	-6.242872518	4.29607E-10	9.84842E-10	negative
PRJNA729271	stage_1W_vs_E10	ordinary_muscle_development	ENSGALG00010009784	ENSGALG00010009784	-0.897601252	0.180172253	-4.981906129	6.2961E-07	1.19312E-06	negative
PRJNA729271	stage_1W_vs_E10	ordinary_muscle_development	ENSGALG00010009786	DIPK2B	0.901242381	0.159173101	5.662026925	1.49595E-08	3.1394E-08	positive
PRJNA729271	stage_1W_vs_E10	ordinary_muscle_development	ENSGALG00010009788	PAK1IP1	0.514133284	0.091829379	5.598788655	2.15855E-08	4.48833E-08	positive
PRJNA729271	stage_1W_vs_E10	ordinary_muscle_development	ENSGALG00010009791	GABRG2	0.66570537	0.28422436	2.342182665	0.019171329	0.02508924	positive
PRJNA729271	stage_1W_vs_E10	ordinary_muscle_development	ENSGALG00010009793	TNFAIP6	-2.168615234	0.296030069	-7.325658636	2.37728E-13	6.47842E-13	negative
PRJNA729271	stage_1W_vs_E10	ordinary_muscle_development	ENSGALG00010009795	NDUFA6	1.44262339	0.114400027	12.61034137	1.85189E-36	1.31907E-35	positive
PRJNA729271	stage_1W_vs_E10	ordinary_muscle_development	ENSGALG00010009796	ENSGALG00010009796	-1.25065793	0.200020807	-6.25263914	4.03574E-10	9.26922E-10	negative
PRJNA729271	stage_1W_vs_E10	ordinary_muscle_development	ENSGALG00010009798	YIPF4	0.804779694	0.077352069	10.4041134	2.37456E-25	1.1078E-24	positive
PRJNA729271	stage_1W_vs_E10	ordinary_muscle_development	ENSGALG00010009801	ENSGALG00010009801	0.404886579	0.589137565	0.687253035	0.491923289	0.529946345	positive
PRJNA729271	stage_1W_vs_E10	ordinary_muscle_development	ENSGALG00010009804	XPR1	1.155381121	0.116919148	9.881881088	4.98875E-23	2.11068E-22	positive
PRJNA729271	stage_1W_vs_E10	ordinary_muscle_development	ENSGALG00010009806	WDCP	-0.300914095	0.173564783	-1.733727828	0.082966387	0.100804507	negative
PRJNA729271	stage_1W_vs_E10	ordinary_muscle_development	ENSGALG00010009809	SMDT1	1.503430882	0.089538821	16.79082728	2.84847E-63	4.51734E-62	positive
PRJNA729271	stage_1W_vs_E10	ordinary_muscle_development	ENSGALG00010009811	ENSGALG00010009811	-0.096155652	0.087828579	-1.09481051	0.273599661	0.307786348	negative
PRJNA729271	stage_1W_vs_E10	ordinary_muscle_development	ENSGALG00010009822	ENSGALG00010009822	-0.523289041	0.203352525	-2.573309773	0.0100731	0.013617757	negative
PRJNA729271	stage_1W_vs_E10	ordinary_muscle_development	ENSGALG00010009830	RNASEL	-1.292685244	0.201435209	-6.417374846	1.38644E-10	3.26751E-10	negative
PRJNA729271	stage_1W_vs_E10	ordinary_muscle_development	ENSGALG00010009831	N6AMT1	-0.638826905	0.121867288	-5.241988373	1.58855E-07	3.12854E-07	negative
PRJNA729271	stage_1W_vs_E10	ordinary_muscle_development	ENSGALG00010009833	SOD3	2.752531658	0.312314785	8.813324861	1.21488E-18	4.26797E-18	positive
PRJNA729271	stage_1W_vs_E10	ordinary_muscle_development	ENSGALG00010009840	ENSGALG00010009840	-0.167564172	0.179264634	-0.934730786	0.349927057	0.387456473	negative
PRJNA729271	stage_1W_vs_E10	ordinary_muscle_development	ENSGALG00010009841	GABRA1	-0.04064592	0.237400314	-0.171212577	0.864056619	0.879035702	negative
PRJNA729271	stage_1W_vs_E10	ordinary_muscle_development	ENSGALG00010009842	PHETA2	-0.129778311	0.090231757	-1.438277557	0.150355317	0.176383969	negative
PRJNA729271	stage_1W_vs_E10	ordinary_muscle_development	ENSGALG00010009847	ENSGALG00010009847	-0.829188155	0.119418995	-6.943519785	3.82449E-12	9.82559E-12	negative
PRJNA729271	stage_1W_vs_E10	ordinary_muscle_development	ENSGALG00010009850	RIF1	-1.90113226	0.106327503	-17.87996713	1.68937E-71	3.31807E-70	negative
PRJNA729271	stage_1W_vs_E10	ordinary_muscle_development	ENSGALG00010009852	ENSGALG00010009852	-0.895819094	0.258599092	-3.464123119	0.000531963	0.000809788	negative
PRJNA729271	stage_1W_vs_E10	ordinary_muscle_development	ENSGALG00010009857	ORC5	-1.59519786	0.142984458	-11.1564423	6.66039E-29	3.58228E-28	negative
PRJNA729271	stage_1W_vs_E10	ordinary_muscle_development	ENSGALG00010009858	ST8SIA5	-1.620222208	0.252777322	-6.409681832	1.45824E-10	3.43247E-10	negative
PRJNA729271	stage_1W_vs_E10	ordinary_muscle_development	ENSGALG00010009866	NAGA	0.407557251	0.1161716	3.508234798	0.000451091	0.000690013	positive
PRJNA729271	stage_1W_vs_E10	ordinary_muscle_development	ENSGALG00010009868	LTN1	1.070423339	0.094582634	11.31733488	1.07696E-29	6.01332E-29	positive
PRJNA729271	stage_1W_vs_E10	ordinary_muscle_development	ENSGALG00010009876	SELENOI	-0.349054132	0.109653393	-3.183249716	0.00145632	0.00213679	negative
PRJNA729271	stage_1W_vs_E10	ordinary_muscle_development	ENSGALG00010009883	ENSGALG00010009883	-0.384944768	0.202987843	-1.896393216	0.057908068	0.071753928	negative
PRJNA729271	stage_1W_vs_E10	ordinary_muscle_development	ENSGALG00010009884	GFOD1	0.996609142	0.114510245	8.703231272	3.22566E-18	1.10901E-17	positive
PRJNA729271	stage_1W_vs_E10	ordinary_muscle_development	ENSGALG00010009910	ENSGALG00010009910	-0.040824538	0.178339012	-0.228915351	0.818934709	0.837937664	negative
PRJNA729271	stage_1W_vs_E10	ordinary_muscle_development	ENSGALG00010009914	RWDD2B	-0.426850289	0.125720405	-3.395234762	0.000685698	0.001035872	negative
PRJNA729271	stage_1W_vs_E10	ordinary_muscle_development	ENSGALG00010009941	CACNA1E	-3.095158064	0.488689588	-6.333587084	2.39526E-10	5.5631E-10	negative
PRJNA729271	stage_1W_vs_E10	ordinary_muscle_development	ENSGALG00010009943	DHX15	-0.706028814	0.069178324	-10.20592533	1.86535E-24	8.37614E-24	negative
PRJNA729271	stage_1W_vs_E10	ordinary_muscle_development	ENSGALG00010009948	WBP2NL	0.635125167	0.065256641	9.732728527	2.18648E-22	9.00114E-22	positive
PRJNA729271	stage_1W_vs_E10	ordinary_muscle_development	ENSGALG00010009972	PTK2B	1.160888193	0.268733644	4.319846883	1.56137E-05	2.66605E-05	positive
PRJNA729271	stage_1W_vs_E10	ordinary_muscle_development	ENSGALG00010009985	USP16	-0.759656666	0.116478459	-6.521864007	6.9439E-11	1.66147E-10	negative
PRJNA729271	stage_1W_vs_E10	ordinary_muscle_development	ENSGALG00010009989	TBC1D7	-1.069958672	0.137461685	-7.78368658	7.04411E-15	2.08164E-14	negative
PRJNA729271	stage_1W_vs_E10	ordinary_muscle_development	ENSGALG00010009991	LHFPL3	-5.245875041	0.674826675	-7.773662826	7.62485E-15	2.24913E-14	negative
PRJNA729271	stage_1W_vs_E10	ordinary_muscle_development	ENSGALG00010009993	ENSGALG00010009993	-0.743233764	0.375421824	-1.979729775	0.047733901	0.0597395	negative
PRJNA729271	stage_1W_vs_E10	ordinary_muscle_development	ENSGALG00010009995	SEPTIN3	1.35688784	0.346163472	3.919789203	8.86265E-05	0.000143151	positive
PRJNA729271	stage_1W_vs_E10	ordinary_muscle_development	ENSGALG00010009999	GABRB2	-1.858437064	0.233914102	-7.944955205	1.94261E-15	5.89452E-15	negative
PRJNA729271	stage_1W_vs_E10	ordinary_muscle_development	ENSGALG00010010006	PPARGC1A	1.635256647	0.267167447	6.120718179	9.31545E-10	2.09963E-09	positive
PRJNA729271	stage_1W_vs_E10	ordinary_muscle_development	ENSGALG00010010007	CTIF	0.228307823	0.095678127	2.386207073	0.017023163	0.022399305	positive
PRJNA729271	stage_1W_vs_E10	ordinary_muscle_development	ENSGALG00010010009	ELF2	0.133872603	0.088106595	1.519439079	0.128652012	0.152279186	positive
PRJNA729271	stage_1W_vs_E10	ordinary_muscle_development	ENSGALG00010010011	EPB41L4A	0.347449244	0.124859789	2.782715303	0.005390609	0.007504502	positive
PRJNA729271	stage_1W_vs_E10	ordinary_muscle_development	ENSGALG00010010014	GLUL	3.812985271	0.392360379	9.718069078	2.52525E-22	1.03604E-21	positive
PRJNA729271	stage_1W_vs_E10	ordinary_muscle_development	ENSGALG00010010017	CCDC59	-0.266791171	0.105680994	-2.524495284	0.011586456	0.015541891	negative
PRJNA729271	stage_1W_vs_E10	ordinary_muscle_development	ENSGALG00010010022	KMT2E	0.072954819	0.101760462	0.716926964	0.473419163	0.51195246	positive
PRJNA729271	stage_1W_vs_E10	ordinary_muscle_development	ENSGALG00010010035	ATP10B	-0.931628277	0.313100169	-2.975495923	0.002925152	0.004173237	negative
PRJNA729271	stage_1W_vs_E10	ordinary_muscle_development	ENSGALG00010010037	NAA15	0.598353246	0.138174115	4.330429375	1.48819E-05	2.54529E-05	positive
PRJNA729271	stage_1W_vs_E10	ordinary_muscle_development	ENSGALG00010010042	METTL25	-0.670691564	0.11631128	-5.766350132	8.10067E-09	1.72726E-08	negative
PRJNA729271	stage_1W_vs_E10	ordinary_muscle_development	ENSGALG00010010049	ZBTB7C	-0.419446485	0.299634761	-1.399859225	0.161555478	0.188683157	negative
PRJNA729271	stage_1W_vs_E10	ordinary_muscle_development	ENSGALG00010010052	ZNF346	-0.106391587	0.113631277	-0.936287879	0.349124987	0.386716085	negative
PRJNA729271	stage_1W_vs_E10	ordinary_muscle_development	ENSGALG00010010053	CHRNA2	-7.041201141	0.942351996	-7.471943788	7.90188E-14	2.21073E-13	negative
PRJNA729271	stage_1W_vs_E10	ordinary_muscle_development	ENSGALG00010010059	ENSGALG00010010059	-1.132551303	0.132305871	-8.560098645	1.12771E-17	3.769E-17	negative
PRJNA729271	stage_1W_vs_E10	ordinary_muscle_development	ENSGALG00010010061	CYP2D6	0.951690184	0.203537816	4.675741367	2.92894E-06	5.28506E-06	positive
PRJNA729271	stage_1W_vs_E10	ordinary_muscle_development	ENSGALG00010010062	RGS7BP	-0.465109696	0.173736499	-2.677098362	0.007426283	0.010179099	negative
PRJNA729271	stage_1W_vs_E10	ordinary_muscle_development	ENSGALG00010010064	ENSGALG00010010064	-0.068964262	0.088697739	-0.777519954	0.436852066	0.474817839	negative
PRJNA729271	stage_1W_vs_E10	ordinary_muscle_development	ENSGALG00010010071	GLT8D2	-1.79669874	0.270299732	-6.647060748	2.99004E-11	7.33095E-11	negative
PRJNA729271	stage_1W_vs_E10	ordinary_muscle_development	ENSGALG00010010077	CCT8	-0.500304155	0.115322324	-4.338311424	1.43582E-05	2.46123E-05	negative
PRJNA729271	stage_1W_vs_E10	ordinary_muscle_development	ENSGALG00010010080	CDC42EP3	2.511008014	0.117934547	21.29153908	1.35988E-100	5.13319E-99	positive
PRJNA729271	stage_1W_vs_E10	ordinary_muscle_development	ENSGALG00010010084	HK3	0.671995036	0.222516321	3.019980879	0.002527906	0.00362864	positive
PRJNA729271	stage_1W_vs_E10	ordinary_muscle_development	ENSGALG00010010085	TDG	-1.147641651	0.104002143	-11.03478853	2.59662E-28	1.36965E-27	negative
PRJNA729271	stage_1W_vs_E10	ordinary_muscle_development	ENSGALG00010010094	SLC2A9	1.217065955	0.250112321	4.866077577	1.13835E-06	2.12005E-06	positive
PRJNA729271	stage_1W_vs_E10	ordinary_muscle_development	ENSGALG00010010095	C12orf73	0.733628632	0.098363429	7.458347499	8.76143E-14	2.44413E-13	positive
PRJNA729271	stage_1W_vs_E10	ordinary_muscle_development	ENSGALG00010010100	IRF2BP2	1.266954357	0.162217505	7.810219727	5.70884E-15	1.69499E-14	positive
PRJNA729271	stage_1W_vs_E10	ordinary_muscle_development	ENSGALG00010010106	C7orf73	-1.177200879	0.207504683	-5.673129207	1.40212E-08	2.94718E-08	negative
PRJNA729271	stage_1W_vs_E10	ordinary_muscle_development	ENSGALG00010010107	MGST2	-0.170131987	0.322782334	-0.527079611	0.598138305	0.632709241	negative
PRJNA729271	stage_1W_vs_E10	ordinary_muscle_development	ENSGALG00010010111	CNOT4	0.656622382	0.135225433	4.855760971	1.19925E-06	2.22918E-06	positive
PRJNA729271	stage_1W_vs_E10	ordinary_muscle_development	ENSGALG00010010115	NEB	5.170327351	0.171690043	30.11431106	3.1477E-199	4.6087E-197	positive
PRJNA729271	stage_1W_vs_E10	ordinary_muscle_development	ENSGALG00010010116	DRD5	-4.146586926	0.659008288	-6.29216203	3.13074E-10	7.22959E-10	negative
PRJNA729271	stage_1W_vs_E10	ordinary_muscle_development	ENSGALG00010010119	SRPK2	-1.08975564	0.125898469	-8.655829157	4.89341E-18	1.66307E-17	negative
PRJNA729271	stage_1W_vs_E10	ordinary_muscle_development	ENSGALG00010010122	THBS4	-3.752064486	0.248169087	-15.11898414	1.21401E-51	1.41115E-50	negative
PRJNA729271	stage_1W_vs_E10	ordinary_muscle_development	ENSGALG00010010124	ENSGALG00010010124	-0.06663533	0.15003552	-0.444130365	0.656948334	0.687797251	negative
PRJNA729271	stage_1W_vs_E10	ordinary_muscle_development	ENSGALG00010010125	AHCTF1	0.148550556	0.06659069	2.230800653	0.025694337	0.033187576	positive
PRJNA729271	stage_1W_vs_E10	ordinary_muscle_development	ENSGALG00010010127	FAM13C	1.535570633	0.340298533	4.512422138	6.40915E-06	1.12552E-05	positive
PRJNA729271	stage_1W_vs_E10	ordinary_muscle_development	ENSGALG00010010136	EPHX2	0.092740495	0.118033098	0.785716006	0.432033884	0.47000298	positive
PRJNA729271	stage_1W_vs_E10	ordinary_muscle_development	ENSGALG00010010142	ENSGALG00010010142	0.651690484	0.301358888	2.162506271	0.030579174	0.039136268	positive
PRJNA729271	stage_1W_vs_E10	ordinary_muscle_development	ENSGALG00010010146	PHACTR1	-4.745838726	0.377490418	-12.57207734	3.00717E-36	2.12837E-35	negative
PRJNA729271	stage_1W_vs_E10	ordinary_muscle_development	ENSGALG00010010150	NTRK2	-0.350510504	0.299008068	-1.172244302	0.241098986	0.273538651	negative
PRJNA729271	stage_1W_vs_E10	ordinary_muscle_development	ENSGALG00010010153	ENSGALG00010010153	1.832231368	0.439987511	4.164280402	3.12336E-05	5.22362E-05	positive
PRJNA729271	stage_1W_vs_E10	ordinary_muscle_development	ENSGALG00010010154	IQSEC3	-5.952937939	0.757289618	-7.860847159	3.81544E-15	1.14243E-14	negative
PRJNA729271	stage_1W_vs_E10	ordinary_muscle_development	ENSGALG00010010155	BRINP3	-3.795893442	0.494357556	-7.678437183	1.61041E-14	4.66952E-14	negative
PRJNA729271	stage_1W_vs_E10	ordinary_muscle_development	ENSGALG00010010156	ENSGALG00010010156	-0.561570594	0.098508091	-5.700756048	1.19277E-08	2.51783E-08	negative
PRJNA729271	stage_1W_vs_E10	ordinary_muscle_development	ENSGALG00010010161	SEPSECS	-0.33883247	0.065201946	-5.19666196	2.02899E-07	3.97274E-07	negative
PRJNA729271	stage_1W_vs_E10	ordinary_muscle_development	ENSGALG00010010164	ENSGALG00010010164	1.562547739	0.392087006	3.985206641	6.74214E-05	0.000109954	positive
PRJNA729271	stage_1W_vs_E10	ordinary_muscle_development	ENSGALG00010010167	PUS7	-0.155476743	0.068014017	-2.285951488	0.022257097	0.02894461	negative
PRJNA729271	stage_1W_vs_E10	ordinary_muscle_development	ENSGALG00010010168	SNCB	-3.54311298	0.340795295	-10.39660182	2.56933E-25	1.19558E-24	negative
PRJNA729271	stage_1W_vs_E10	ordinary_muscle_development	ENSGALG00010010171	ENSGALG00010010171	1.386617279	0.388492437	3.56922593	0.000358038	0.000552572	positive
PRJNA729271	stage_1W_vs_E10	ordinary_muscle_development	ENSGALG00010010178	LGI2	-4.40843147	0.226031977	-19.50357431	1.02372E-84	2.71773E-83	negative
PRJNA729271	stage_1W_vs_E10	ordinary_muscle_development	ENSGALG00010010179	CDHR2	1.821355018	0.827408012	2.201277958	0.027716352	0.035644444	positive
PRJNA729271	stage_1W_vs_E10	ordinary_muscle_development	ENSGALG00010010183	NKX3-2	-4.689371159	0.45453226	-10.31691603	5.90907E-25	2.70965E-24	negative
PRJNA729271	stage_1W_vs_E10	ordinary_muscle_development	ENSGALG00010010185	PHYHIPL	-1.444659442	0.346311936	-4.17155544	3.02527E-05	5.06367E-05	negative
PRJNA729271	stage_1W_vs_E10	ordinary_muscle_development	ENSGALG00010010186	TNPO1	0.875579282	0.07201915	12.15758984	5.22784E-34	3.42111E-33	positive
PRJNA729271	stage_1W_vs_E10	ordinary_muscle_development	ENSGALG00010010188	DSP	-5.495497743	0.35831991	-15.3368473	4.33755E-53	5.31021E-52	negative
PRJNA729271	stage_1W_vs_E10	ordinary_muscle_development	ENSGALG00010010190	RNF44	0.487205039	0.178896083	2.723396904	0.006461438	0.008912222	positive
PRJNA729271	stage_1W_vs_E10	ordinary_muscle_development	ENSGALG00010010191	RINT1	-0.146075565	0.093158395	-1.56803437	0.116873112	0.139223845	negative
PRJNA729271	stage_1W_vs_E10	ordinary_muscle_development	ENSGALG00010010195	ENSGALG00010010195	3.258319375	0.281501049	11.57480367	5.52977E-31	3.24248E-30	positive
PRJNA729271	stage_1W_vs_E10	ordinary_muscle_development	ENSGALG00010010196	EFCAB10	-1.012239065	0.375131151	-2.698360464	0.006968194	0.009583829	negative
PRJNA729271	stage_1W_vs_E10	ordinary_muscle_development	ENSGALG00010010197	SCCPDH	-0.610080293	0.121835526	-5.00740885	5.51677E-07	1.04845E-06	negative
PRJNA729271	stage_1W_vs_E10	ordinary_muscle_development	ENSGALG00010010201	RIOK1	-0.309576878	0.098780687	-3.133981826	0.001724515	0.002514521	negative

PRJNA729271	stage_1W_vs_E10	ordinary_muscle_development	ENSGALG00010010205	ARL5A	0.450994702	0.082976741	5.435194217	5.47368E-08	1.11403E-07	positive
PRJNA729271	stage_1W_vs_E10	ordinary_muscle_development	ENSGALG00010010206	SCARA5	1.072403611	0.158675471	6.758471264	1.39455E-11	3.47619E-11	positive
PRJNA729271	stage_1W_vs_E10	ordinary_muscle_development	ENSGALG00010010214	FAF2	-1.164829822	0.080263093	-14.51264558	1.00758E-47	1.01918E-46	negative
PRJNA729271	stage_1W_vs_E10	ordinary_muscle_development	ENSGALG00010010215	ENSGALG00010010215	0.613908624	0.124323649	4.937987498	7.89329E-07	1.48763E-06	positive
PRJNA729271	stage_1W_vs_E10	ordinary_muscle_development	ENSGALG00010010216	ATXN7L1	0.843732244	0.128007163	6.591289286	4.36023E-11	1.06007E-10	positive
PRJNA729271	stage_1W_vs_E10	ordinary_muscle_development	ENSGALG00010010218	PBK	-2.530357552	0.223221044	-11.33565863	8.73723E-30	4.89359E-29	negative
PRJNA729271	stage_1W_vs_E10	ordinary_muscle_development	ENSGALG00010010220	LHX9	-8.713460484	0.902676048	-9.652920894	4.77752E-22	1.94304E-21	negative
PRJNA729271	stage_1W_vs_E10	ordinary_muscle_development	ENSGALG00010010222	PAH	0.92034224	1.061664292	0.86688631	0.386004305	0.424066424	positive
PRJNA729271	stage_1W_vs_E10	ordinary_muscle_development	ENSGALG00010010223	ENSGALG00010010223	1.543365617	0.201462458	7.660810029	1.84764E-14	5.34348E-14	positive
PRJNA729271	stage_1W_vs_E10	ordinary_muscle_development	ENSGALG00010010226	BICC1	-0.577655361	0.164328165	-3.51525475	0.000439332	0.000672736	negative
PRJNA729271	stage_1W_vs_E10	ordinary_muscle_development	ENSGALG00010010231	C1orf53	-0.409364585	0.241166248	-1.697437302	0.089614015	0.108472247	negative
PRJNA729271	stage_1W_vs_E10	ordinary_muscle_development	ENSGALG00010010234	ENSGALG00010010234	1.057036838	0.268912593	3.930782207	8.46699E-05	0.000137005	positive
PRJNA729271	stage_1W_vs_E10	ordinary_muscle_development	ENSGALG00010010243	ESCO2	-0.93093379	0.114464465	-8.132950132	4.18967E-16	1.31136E-15	negative
PRJNA729271	stage_1W_vs_E10	ordinary_muscle_development	ENSGALG00010010246	CLTB	0.801680137	0.066456182	12.06328904	1.65057E-33	1.06239E-32	positive
PRJNA729271	stage_1W_vs_E10	ordinary_muscle_development	ENSGALG00010010252	WDR7	0.988875852	0.095812363	10.32096295	5.66521E-25	2.60194E-24	positive
PRJNA729271	stage_1W_vs_E10	ordinary_muscle_development	ENSGALG00010010253	IL10RB	0.677916402	0.124402288	5.449388552	5.05433E-08	1.03099E-07	positive
PRJNA729271	stage_1W_vs_E10	ordinary_muscle_development	ENSGALG00010010254	WDR41	1.23532891	0.112084132	11.02144331	3.0119E-28	1.58467E-27	positive
PRJNA729271	stage_1W_vs_E10	ordinary_muscle_development	ENSGALG00010010255	CACNB4	0.46303673	0.175351905	2.640614197	0.00827559	0.011287787	positive
PRJNA729271	stage_1W_vs_E10	ordinary_muscle_development	ENSGALG00010010257	ENSGALG00010010257	1.08118575	0.136041729	7.947456723	1.90379E-15	5.78037E-15	positive
PRJNA729271	stage_1W_vs_E10	ordinary_muscle_development	ENSGALG00010010259	IGF1	-0.062662129	0.39695114	-0.157858545	0.874568265	0.888731562	negative
PRJNA729271	stage_1W_vs_E10	ordinary_muscle_development	ENSGALG00010010262	HSPA9	0.379471491	0.145657425	2.605232735	0.009181187	0.012465465	positive
PRJNA729271	stage_1W_vs_E10	ordinary_muscle_development	ENSGALG00010010263	RAB4A	-0.735728677	0.138087903	-5.327973418	9.93147E-08	1.98561E-07	negative
PRJNA729271	stage_1W_vs_E10	ordinary_muscle_development	ENSGALG00010010266	EFNA5	-2.070620023	0.235578799	-8.789500734	1.50226E-18	5.25338E-18	negative
PRJNA729271	stage_1W_vs_E10	ordinary_muscle_development	ENSGALG00010010267	CCSER2	0.799482468	0.107356031	7.447019607	9.54724E-14	2.65619E-13	positive
PRJNA729271	stage_1W_vs_E10	ordinary_muscle_development	ENSGALG00010010270	SSR1	-0.883896697	0.077904489	-11.34590196	7.77207E-30	4.36652E-29	negative
PRJNA729271	stage_1W_vs_E10	ordinary_muscle_development	ENSGALG00010010271	HUNK	-2.189553476	0.114971148	-19.04437345	7.31561E-81	1.79729E-79	negative
PRJNA729271	stage_1W_vs_E10	ordinary_muscle_development	ENSGALG00010010274	MIS18A	-2.08125079	0.101361236	-20.53300532	1.09207E-93	3.52552E-92	negative
PRJNA729271	stage_1W_vs_E10	ordinary_muscle_development	ENSGALG00010010275	PDE8B	-2.965197974	0.91754353	-3.231670081	0.001230691	0.001815106	negative
PRJNA729271	stage_1W_vs_E10	ordinary_muscle_development	ENSGALG00010010279	CCSAP	1.983632823	0.239438821	8.284507978	1.18598E-16	3.79321E-16	positive
PRJNA729271	stage_1W_vs_E10	ordinary_muscle_development	ENSGALG00010010280	HSP90B1	-1.370334006	0.135641262	-10.10263384	5.37781E-24	2.37006E-23	negative
PRJNA729271	stage_1W_vs_E10	ordinary_muscle_development	ENSGALG00010010284	CCDC25	0.598138895	0.094533154	6.327292245	2.49501E-10	5.7892E-10	positive
PRJNA729271	stage_1W_vs_E10	ordinary_muscle_development	ENSGALG00010010285	ENSGALG00010010285	3.243257694	0.572676103	5.663336878	1.48457E-08	3.11687E-08	positive
PRJNA729271	stage_1W_vs_E10	ordinary_muscle_development	ENSGALG00010010287	STAM2	0.15632057	0.097139936	1.609230724	0.107565901	0.128750432	positive
PRJNA729271	stage_1W_vs_E10	ordinary_muscle_development	ENSGALG00010010288	ENSGALG00010010288	1.232986786	0.163257098	7.55242378	4.27232E-14	1.21331E-13	positive
PRJNA729271	stage_1W_vs_E10	ordinary_muscle_development	ENSGALG00010010290	ENSGALG00010010290	0.929015433	0.1653381	5.61888296	1.92196E-08	4.01135E-08	positive
PRJNA729271	stage_1W_vs_E10	ordinary_muscle_development	ENSGALG00010010291	TCF20	-0.536318555	0.117655801	-4.558369	5.15524E-06	9.12619E-06	negative
PRJNA729271	stage_1W_vs_E10	ordinary_muscle_development	ENSGALG00010010293	ENSGALG00010010293	-0.576109404	0.587399188	-0.980780049	0.326701219	0.363126613	negative
PRJNA729271	stage_1W_vs_E10	ordinary_muscle_development	ENSGALG00010010295	ENSGALG00010010295	8.978695392	0.181411847	49.49343469	0	0	positive
PRJNA729271	stage_1W_vs_E10	ordinary_muscle_development	ENSGALG00010010298	KCNIP4	-1.466306685	0.415095402	-3.532457065	0.000411717	0.000632162	negative
PRJNA729271	stage_1W_vs_E10	ordinary_muscle_development	ENSGALG00010010300	DENND1B	1.676524833	0.091517952	18.31908161	5.82882E-75	1.22982E-73	positive
PRJNA729271	stage_1W_vs_E10	ordinary_muscle_development	ENSGALG00010010301	ENSGALG00010010301	-1.351677326	0.543332722	-2.487752479	0.012855317	0.017169245	negative
PRJNA729271	stage_1W_vs_E10	ordinary_muscle_development	ENSGALG00010010302	WDR36	-0.010069236	0.103967096	-0.096850217	0.922845344	0.932105307	negative
PRJNA729271	stage_1W_vs_E10	ordinary_muscle_development	ENSGALG00010010303	ETF1	1.032657717	0.184506605	5.596860446	2.18268E-08	4.53656E-08	positive
PRJNA729271	stage_1W_vs_E10	ordinary_muscle_development	ENSGALG00010010305	URB1	-0.905074285	0.102850594	-8.799893609	1.36946E-18	4.80172E-18	negative
PRJNA729271	stage_1W_vs_E10	ordinary_muscle_development	ENSGALG00010010308	PACRGL	-0.942989614	0.227523698	-4.144577567	3.40441E-05	5.68054E-05	negative
PRJNA729271	stage_1W_vs_E10	ordinary_muscle_development	ENSGALG00010010310	NT5DC3	1.513866597	0.118300455	12.79679434	1.7086E-37	1.25398E-36	positive
PRJNA729271	stage_1W_vs_E10	ordinary_muscle_development	ENSGALG00010010311	CTSV	1.245635209	0.189395472	6.576900684	4.80355E-11	1.16395E-10	positive
PRJNA729271	stage_1W_vs_E10	ordinary_muscle_development	ENSGALG00010010313	GRID1	-3.369643476	0.228567406	-14.74244965	3.44074E-49	3.66448E-48	negative
PRJNA729271	stage_1W_vs_E10	ordinary_muscle_development	ENSGALG00010010314	CRB1	-0.609592145	0.283982457	-2.146583813	0.03182643	0.040648876	negative
PRJNA729271	stage_1W_vs_E10	ordinary_muscle_development	ENSGALG00010010317	ENSGALG00010010317	-0.255255634	0.108559259	-2.351302282	0.018707829	0.024504788	negative
PRJNA729271	stage_1W_vs_E10	ordinary_muscle_development	ENSGALG00010010320	SYPL1	1.784904856	0.574137005	3.108848308	0.001878181	0.002729247	positive
PRJNA729271	stage_1W_vs_E10	ordinary_muscle_development	ENSGALG00010010321	NUP133	-1.104554381	0.073355476	-15.05755867	3.0795E-51	3.51476E-50	negative
PRJNA729271	stage_1W_vs_E10	ordinary_muscle_development	ENSGALG00010010322	ENSGALG00010010322	0.333166114	0.067147632	4.96169566	6.98804E-07	1.32062E-06	positive
PRJNA729271	stage_1W_vs_E10	ordinary_muscle_development	ENSGALG00010010323	FBP2	10.87699168	0.241131711	45.1080931	0	0	positive
PRJNA729271	stage_1W_vs_E10	ordinary_muscle_development	ENSGALG00010010327	FBP1	0.053704164	0.577790365	0.09294749	0.925945278	0.934910616	positive
PRJNA729271	stage_1W_vs_E10	ordinary_muscle_development	ENSGALG00010010330	ALPK2	0.701262323	0.154686958	4.533428877	5.80338E-06	1.02348E-05	positive
PRJNA729271	stage_1W_vs_E10	ordinary_muscle_development	ENSGALG00010010333	WAPL	0.480986208	0.063088224	7.624025192	2.45885E-14	7.06463E-14	positive
PRJNA729271	stage_1W_vs_E10	ordinary_muscle_development	ENSGALG00010010334	ZBTB41	0.299303318	0.086934549	3.442858098	0.000575601	0.000874014	positive
PRJNA729271	stage_1W_vs_E10	ordinary_muscle_development	ENSGALG00010010336	FMNL2	-1.363247879	0.090598121	-15.04719819	3.60168E-51	4.10105E-50	negative
PRJNA729271	stage_1W_vs_E10	ordinary_muscle_development	ENSGALG00010010337	SCARA3	-1.325865705	0.157480089	-8.419259332	3.78872E-17	1.23549E-16	negative
PRJNA729271	stage_1W_vs_E10	ordinary_muscle_development	ENSGALG00010010338	CNST	1.725122909	0.155141282	11.11968967	1.00621E-28	5.37798E-28	positive
PRJNA729271	stage_1W_vs_E10	ordinary_muscle_development	ENSGALG00010010345	ENSGALG00010010345	-0.417292245	0.140997486	-2.959572232	0.003080665	0.004385606	negative
PRJNA729271	stage_1W_vs_E10	ordinary_muscle_development	ENSGALG00010010346	TFB2M	0.609244601	0.139485428	4.367801065	1.25504E-05	2.16311E-05	positive
PRJNA729271	stage_1W_vs_E10	ordinary_muscle_development	ENSGALG00010010347	FBXW11	0.106634779	0.083968766	1.26993386	0.204108191	0.234451876	positive
PRJNA729271	stage_1W_vs_E10	ordinary_muscle_development	ENSGALG00010010349	RREB1	0.675978274	0.082283016	8.215283142	2.11664E-16	6.69009E-16	positive
PRJNA729271	stage_1W_vs_E10	ordinary_muscle_development	ENSGALG00010010351	NAMPTP1	4.671057749	0.212488477	21.98264028	4.2218E-107	1.7946E-105	positive
PRJNA729271	stage_1W_vs_E10	ordinary_muscle_development	ENSGALG00010010352	MALT1	-0.042913173	0.115540874	-0.371411183	0.710331297	0.737818141	negative
PRJNA729271	stage_1W_vs_E10	ordinary_muscle_development	ENSGALG00010010356	STAB2	0.534906353	0.281920413	1.897366523	0.057779581	0.071619208	positive
PRJNA729271	stage_1W_vs_E10	ordinary_muscle_development	ENSGALG00010010358	ASPM	-2.248160048	0.181922262	-12.35780617	4.42057E-35	3.00968E-34	negative
PRJNA729271	stage_1W_vs_E10	ordinary_muscle_development	ENSGALG00010010361	PIGM	-0.876161253	0.168192715	-5.209269915	1.89585E-07	3.71909E-07	negative
PRJNA729271	stage_1W_vs_E10	ordinary_muscle_development	ENSGALG00010010366	CLU	2.222645743	0.235393121	9.442271445	3.64784E-21	1.43722E-20	positive
PRJNA729271	stage_1W_vs_E10	ordinary_muscle_development	ENSGALG00010010368	ENSGALG00010010368	-0.228497691	0.080231789	-2.84796953	0.004399914	0.006170948	negative
PRJNA729271	stage_1W_vs_E10	ordinary_muscle_development	ENSGALG00010010369	ENSGALG00010010369	-0.204648556	0.250686566	-0.816352304	0.414298668	0.452373574	negative
PRJNA729271	stage_1W_vs_E10	ordinary_muscle_development	ENSGALG00010010371	SOCS1L	-2.669838154	0.21810177	-12.24124937	1.8713E-34	1.24367E-33	negative
PRJNA729271	stage_1W_vs_E10	ordinary_muscle_development	ENSGALG00010010374	CMTM4	-0.351353023	0.112561177	-3.121440558	0.001799686	0.002620436	negative
PRJNA729271	stage_1W_vs_E10	ordinary_muscle_development	ENSGALG00010010379	SLC35F6	1.356131443	0.186889073	7.256344217	3.97692E-13	1.07247E-12	positive
PRJNA729271	stage_1W_vs_E10	ordinary_muscle_development	ENSGALG00010010381	MAST4	1.644486065	0.131458651	12.50953096	6.6214E-36	4.65909E-35	positive
PRJNA729271	stage_1W_vs_E10	ordinary_muscle_development	ENSGALG00010010382	SEC11C	0.742114227	0.15170793	4.89172998	9.99535E-07	1.86969E-06	positive
PRJNA729271	stage_1W_vs_E10	ordinary_muscle_development	ENSGALG00010010383	SLIT2	-1.093682232	0.161384829	-6.776859005	1.22817E-11	3.07254E-11	negative
PRJNA729271	stage_1W_vs_E10	ordinary_muscle_development	ENSGALG00010010386	PRPF40A	0.854130505	0.146204762	5.842015642	5.1573E-09	1.11392E-08	positive
PRJNA729271	stage_1W_vs_E10	ordinary_muscle_development	ENSGALG00010010390	KCNK3	-0.38296274	0.465021059	-0.823538488	0.410201839	0.448204105	negative
PRJNA729271	stage_1W_vs_E10	ordinary_muscle_development	ENSGALG00010010393	FANCC	-2.740834888	0.152596886	-17.96127668	3.91694E-72	7.8204E-71	negative
PRJNA729271	stage_1W_vs_E10	ordinary_muscle_development	ENSGALG00010010394	MRPL42	-0.129712635	0.124805439	-1.039318768	0.298656509	0.333978252	negative
PRJNA729271	stage_1W_vs_E10	ordinary_muscle_development	ENSGALG00010010395	GRP	0.00263981	0.851291465	0.003100947	0.997525806	0.997938889	positive
PRJNA729271	stage_1W_vs_E10	ordinary_muscle_development	ENSGALG00010010397	PICK1	-0.325001228	0.092341663	-3.519551372	0.000432277	0.000662493	negative
PRJNA729271	stage_1W_vs_E10	ordinary_muscle_development	ENSGALG00010010399	FGF18	0.409624352	0.182102756	2.249413241	0.024486217	0.031712425	positive
PRJNA729271	stage_1W_vs_E10	ordinary_muscle_development	ENSGALG00010010403	ENSGALG00010010403	-1.902958805	0.492603142	-3.86306672	0.000111972	0.000179421	negative
PRJNA729271	stage_1W_vs_E10	ordinary_muscle_development	ENSGALG00010010404	COP1	-0.074624295	0.116924538	-0.638226126	0.523326492	0.560154889	negative
PRJNA729271	stage_1W_vs_E10	ordinary_muscle_development	ENSGALG00010010405	EVA1C	2.503750403	0.230525069	10.86107647	1.76654E-27	9.00353E-27	positive
PRJNA729271	stage_1W_vs_E10	ordinary_muscle_development	ENSGALG00010010406	PTCH1	-0.839780263	0.094194263	-8.915407754	4.86024E-19	1.74942E-18	negative
PRJNA729271	stage_1W_vs_E10	ordinary_muscle_development	ENSGALG00010010407	SOX10	-1.14764498	0.297399806	-3.858929821	0.000113885	0.000182358	negative
PRJNA729271	stage_1W_vs_E10	ordinary_muscle_development	ENSGALG00010010408	GAS8	-0.497787423	0.11877218	-4.191111275	2.77591E-05	4.65543E-05	negative
PRJNA729271	stage_1W_vs_E10	ordinary_muscle_development	ENSGALG00010010410	CACNA2D1	2.977111415	0.158039068	18.83781936	3.69894E-79	8.57859E-78	positive
PRJNA729271	stage_1W_vs_E10	ordinary_muscle_development	ENSGALG00010010411	CD180	0.643230418	0.449931784	1.429617644	0.152826789	0.179051431	positive
PRJNA729271	stage_1W_vs_E10	ordinary_muscle_development	ENSGALG00010010416	SLC35B3	-0.875717694	0.129275689	-6.774032286	1.25242E-11	3.13158E-11	negative
PRJNA729271	stage_1W_vs_E10	ordinary_muscle_development	ENSGALG00010010417	OTOF	0.001843384	0.674045131	0.002734807	0.997817942	0.998162255	positive
PRJNA729271	stage_1W_vs_E10	ordinary_muscle_development	ENSGALG00010010418	BRINP2	-8.904285956	0.925908954	-9.616805107	6.79076E-22	2.7472E-21	negative
PRJNA729271	stage_1W_vs_E10	ordinary_muscle_development	ENSGALG00010010420	NPM1	-0.972653463	0.11234895	-8.657432625	4.82509E-18	1.64023E-17	negative
PRJNA729271	stage_1W_vs_E10	ordinary_muscle_development	ENSGALG00010010421	POLR2F	0.639753282	0.090589292	7.062129164	1.6397E-12	4.28939E-12	positive
PRJNA729271	stage_1W_vs_E10	ordinary_muscle_development	ENSGALG00010010422	CDH1	-2.858021856	0.900992896	-3.172080345	0.001513511	0.00221689	negative
PRJNA729271	stage_1W_vs_E10	ordinary_muscle_development	ENSGALG00010010423	C21orf59	0.49747652	0.096827503	5.137760508	2.78032E-07	5.38996E-07	positive
PRJNA729271	stage_1W_vs_E10	ordinary_muscle_development	ENSGALG00010010425	BLOC1S5	0.501623598	0.079110572	6.340790971	2.28589E-10	5.31674E-10	positive
PRJNA729271	stage_1W_vs_E10	ordinary_muscle_development	ENSGALG00010010430	ASTN1	-5.982869115	0.770076558	-7.769187433	7.89912E-15	2.32672E-14	negative
PRJNA729271	stage_1W_vs_E10	ordinary_muscle_development	ENSGALG00010010431	SOCS2	-0.120650272	0.24409072	-0.494284549	0.621105235	0.655091347	negative
PRJNA729271	stage_1W_vs_E10	ordinary_muscle_development	ENSGALG00010010433	ENSGALG00010010433	-0.412938496	1.609611739	-0.256545405	0.797529725	0.819000592	negative
PRJNA729271	stage_1W_vs_E10	ordinary_muscle_development	ENSGALG00010010434	PROM1	-2.653750822	0.154996784	-17.1213283	1.029E-65	1.72233E-64	negative
PRJNA729271	stage_1W_vs_E10	ordinary_muscle_development	ENSGALG00010010435	GAS1	-1.301738275	0.166215118	-7.831647849	4.81517E-15	1.43436E-14	negative
PRJNA729271	stage_1W_vs_E10	ordinary_muscle_development	ENSGALG00010010438	LDB3	3.838564355	0.185206996	20.72580645	2.02703E-95	6.92967E-94	positive
PRJNA729271	stage_1W_vs_E10	ordinary_muscle_development	ENSGALG00010010439	HGF	0.364323585	0.242081389	1.504963217	0.132333534	0.156419683	positive
PRJNA729271	stage_1W_vs_E10	ordinary_muscle_development	ENSGALG00010010444	TXNDC5	-2.110875086	0.140445552	-15.0298465	4.68089E-51	5.3132E-50	negative
PRJNA729271	stage_1W_vs_E10	ordinary_muscle_development	ENSGALG00010010445	LMAN1L	-0.888855494	0.083466312	-10.64927243	1.75733E-26	8.60558E-26	negative
PRJNA729271	stage_1W_vs_E10	ordinary_muscle_development	ENSGALG00010010446	C22orf23	-0.808517751	0.614233732	-1.316303076	0.188072339	0.217375692	negative
PRJNA729271	stage_1W_vs_E10	ordinary_muscle_development	ENSGALG00010010447	BTF3	0.472288886	0.096196921	4.909605009	9.126E-07	1.71238E-06	positive
PRJNA729271	stage_1W_vs_E10	ordinary_muscle_development	ENSGALG00010010448	GALNT13	-1.568643776	0.38513517	-4.07296943	4.64175E-05	7.65092E-05	negative
PRJNA729271	stage_1W_vs_E10	ordinary_muscle_development	ENSGALG00010010449	PAPPA2	-3.449816417	0.769899404	-4.480866462	7.43406E-06	1.30031E-05	negative
PRJNA729271	stage_1W_vs_E10	ordinary_muscle_development	ENSGALG00010010451	RANBP17	-3.085243007	0.573937376	-5.375574299	7.6339E-08	1.53963E-07	negative
PRJNA729271	stage_1W_vs_E10	ordinary_muscle_development	ENSGALG00010010453	DRC1	-1.211675193	0.456632814	-2.653500045	0.007966176	0.010884129	negative
PRJNA729271	stage_1W_vs_E10	ordinary_muscle_development	ENSGALG00010010456	BMP6	-0.355919167	0.176920459	-2.011746804	0.044246634	0.055644192	negative
PRJNA729271	stage_1W_vs_E10	ordinary_muscle_development	ENSGALG00010010457	AGGF1	-0.374299193	0.091233524	-4.102649747	4.08445E-05	6.76619E-05	negative
PRJNA729271	stage_1W_vs_E10	ordinary_muscle_development	ENSGALG00010010460	SEMA3C	0.678684966	0.142099402	4.776128231	1.78702E-06	3.27678E-06	positive
PRJNA729271	stage_1W_vs_E10	ordinary_muscle_development	ENSGALG00010010461	SLC7A5	2.754301889	0.261181727	10.54553052	5.3267E-26	2.55749E-25	positive
PRJNA729271	stage_1W_vs_E10	ordinary_muscle_development	ENSGALG00010010463	ACAA2	0.032856041	0.128673899	0.255343478	0.798457822	0.819815996	positive
PRJNA729271	stage_1W_vs_E10	ordinary_muscle_development	ENSGALG00010010465	BMPR1A	-0.768048598	0.098039485	-7.834074205	4.7231E-15	1.4078E-14	negative
PRJNA729271	stage_1W_vs_E10	ordinary_muscle_development	ENSGALG00010010466	CRADD	1.157667596	0.123758453	9.354250737	8.41943E-21	3.27447E-20	positive
PRJNA729271	stage_1W_vs_E10	ordinary_muscle_development	ENSGALG00010010467	FGFBP2	-10.16624943	0.690163352	-14.73020756	4.12437E-49	4.37328E-48	negative
PRJNA729271	stage_1W_vs_E10	ordinary_muscle_development	ENSGALG00010010468	ENSGALG00010010468	2.7367795	1.362115782	2.009212092	0.044514646	0.055942413	positive
PRJNA729271	stage_1W_vs_E10	ordinary_muscle_development	ENSGALG00010010471	KCNJ3	-1.272025777	0.167405667	-7.59846307	2.99668E-14	8.56812E-14	negative
PRJNA729271	stage_1W_vs_E10	ordinary_muscle_development	ENSGALG00010010472	MMRN2	1.455254256	0.136617997	10.65199527	1.70667E-26	8.36033E-26	positive
PRJNA729271	stage_1W_vs_E10	ordinary_muscle_development	ENSGALG00010010473	TRIM35	0.668563976	0.178714553	3.740959898	0.000183319	0.000289298	positive
PRJNA729271	stage_1W_vs_E10	ordinary_muscle_development	ENSGALG00010010475	CCDC71L	0.937322958	0.107076306	8.753784965	2.06314E-18	7.15265E-18	positive
PRJNA729271	stage_1W_vs_E10	ordinary_muscle_development	ENSGALG00010010478	ENSGALG00010010478	0.498548725	0.219293292	2.273433537	0.023000067	0.029875972	positive

PRJNA729271	stage_1W_vs_E10	ordinary_muscle_development	ENSGALG00010010483	FGFBP1	-5.285811429	1.091126541	-4.844361521	1.27019E-06	2.35411E-06	negative
PRJNA729271	stage_1W_vs_E10	ordinary_muscle_development	ENSGALG00010010486	ENSGALG00010010486	-3.422089317	0.482885225	-7.086755069	1.37293E-12	3.61107E-12	negative
PRJNA729271	stage_1W_vs_E10	ordinary_muscle_development	ENSGALG00010010494	ENSGALG00010010494	0.424186693	0.264900806	1.601303897	0.109309626	0.130720045	positive
PRJNA729271	stage_1W_vs_E10	ordinary_muscle_development	ENSGALG00010010496	MRPL17	0.775660047	0.142232438	5.453467995	4.93968E-08	1.00832E-07	positive
PRJNA729271	stage_1W_vs_E10	ordinary_muscle_development	ENSGALG00010010499	NR4A2	3.067594051	0.455147309	6.739782899	1.58623E-11	3.94179E-11	positive
PRJNA729271	stage_1W_vs_E10	ordinary_muscle_development	ENSGALG00010010505	CD38	2.737087732	0.193885627	14.11702238	2.98333E-45	2.80619E-44	positive
PRJNA729271	stage_1W_vs_E10	ordinary_muscle_development	ENSGALG00010010511	GABRP	-4.55054266	0.791761408	-5.74736608	9.06443E-09	1.92681E-08	negative
PRJNA729271	stage_1W_vs_E10	ordinary_muscle_development	ENSGALG00010010512	PLXNC1	-0.721138736	0.200082898	-3.604199772	0.000313116	0.000486194	negative
PRJNA729271	stage_1W_vs_E10	ordinary_muscle_development	ENSGALG00010010515	RXRG	-1.496404052	0.392450467	-3.81297559	0.000137304	0.000218513	negative
PRJNA729271	stage_1W_vs_E10	ordinary_muscle_development	ENSGALG00010010517	STMN4	-7.437575733	0.579056271	-12.84430564	9.25789E-38	6.8396E-37	negative
PRJNA729271	stage_1W_vs_E10	ordinary_muscle_development	ENSGALG00010010521	SNCG	-0.562045568	0.393214636	-1.429360753	0.152900573	0.179123398	negative
PRJNA729271	stage_1W_vs_E10	ordinary_muscle_development	ENSGALG00010010526	ENSGALG00010010526	0.598943178	0.252878706	2.368499855	0.017860387	0.023443477	positive
PRJNA729271	stage_1W_vs_E10	ordinary_muscle_development	ENSGALG00010010528	GPD2	2.459318609	0.1244889	19.75532448	7.21903E-87	2.02007E-85	positive
PRJNA729271	stage_1W_vs_E10	ordinary_muscle_development	ENSGALG00010010534	SYNJ1	0.676757467	0.12081672	5.601521597	2.12478E-08	4.41939E-08	positive
PRJNA729271	stage_1W_vs_E10	ordinary_muscle_development	ENSGALG00010010535	LMX1A	-1.839913401	0.435845287	-4.221482837	2.427E-05	4.08493E-05	negative
PRJNA729271	stage_1W_vs_E10	ordinary_muscle_development	ENSGALG00010010537	ENSGALG00010010537	0.286506078	0.21580194	1.327634392	0.184298917	0.213286452	positive
PRJNA729271	stage_1W_vs_E10	ordinary_muscle_development	ENSGALG00010010544	ENSGALG00010010544	-0.894361479	0.171146913	-5.225694483	1.73503E-07	3.40914E-07	negative
PRJNA729271	stage_1W_vs_E10	ordinary_muscle_development	ENSGALG00010010546	MICALL1	-0.098606339	0.168204207	-0.586229923	0.557721016	0.593638282	negative
PRJNA729271	stage_1W_vs_E10	ordinary_muscle_development	ENSGALG00010010547	BST1	0.878636858	0.165049353	5.323479557	1.01801E-07	2.03419E-07	positive
PRJNA729271	stage_1W_vs_E10	ordinary_muscle_development	ENSGALG00010010567	ERMN	-1.092424195	0.559405178	-1.952831753	0.050839535	0.063445463	negative
PRJNA729271	stage_1W_vs_E10	ordinary_muscle_development	ENSGALG00010010572	AQP3	3.687713048	0.146103124	25.2404804	1.4408E-140	1.039E-138	positive
PRJNA729271	stage_1W_vs_E10	ordinary_muscle_development	ENSGALG00010010573	CEP83	-1.258280165	0.164873958	-7.631770233	2.31552E-14	6.65677E-14	negative
PRJNA729271	stage_1W_vs_E10	ordinary_muscle_development	ENSGALG00010010575	PAXBP1	0.556139109	0.12280739	4.528547593	5.93905E-06	1.04677E-05	positive
PRJNA729271	stage_1W_vs_E10	ordinary_muscle_development	ENSGALG00010010577	PBX1	-0.712891985	0.145968629	-4.883871214	1.04023E-06	1.94256E-06	negative
PRJNA729271	stage_1W_vs_E10	ordinary_muscle_development	ENSGALG00010010578	GLUD2	0.819346994	0.111346909	7.358506858	1.85979E-13	5.09692E-13	positive
PRJNA729271	stage_1W_vs_E10	ordinary_muscle_development	ENSGALG00010010585	FBXL5	1.405921046	0.163213798	8.613984004	7.05645E-18	2.38478E-17	positive
PRJNA729271	stage_1W_vs_E10	ordinary_muscle_development	ENSGALG00010010586	CYTIP	-0.347556797	0.268700358	-1.293473517	0.195847338	0.225678286	negative
PRJNA729271	stage_1W_vs_E10	ordinary_muscle_development	ENSGALG00010010588	SLC25A3	1.057684119	0.100984439	10.47373367	1.14054E-25	5.39383E-25	positive
PRJNA729271	stage_1W_vs_E10	ordinary_muscle_development	ENSGALG00010010593	EIF3L	0.7941816	0.103707076	7.657930696	1.88953E-14	5.45809E-14	positive
PRJNA729271	stage_1W_vs_E10	ordinary_muscle_development	ENSGALG00010010596	SHLD2	0.29306267	0.13407458	2.185818303	0.028828901	0.036999727	positive
PRJNA729271	stage_1W_vs_E10	ordinary_muscle_development	ENSGALG00010010599	F2RL1	-2.454058543	0.207975254	-11.7997622	3.91416E-32	2.39999E-31	negative
PRJNA729271	stage_1W_vs_E10	ordinary_muscle_development	ENSGALG00010010604	HIVEP1	0.292034879	0.100069235	2.918328275	0.003519137	0.00498192	positive
PRJNA729271	stage_1W_vs_E10	ordinary_muscle_development	ENSGALG00010010605	NUF2	-2.379668677	0.26852686	-8.86193908	7.86346E-19	2.79023E-18	negative
PRJNA729271	stage_1W_vs_E10	ordinary_muscle_development	ENSGALG00010010614	IKBIP	-0.612804248	0.145280941	-4.218063594	2.46409E-05	4.14591E-05	negative
PRJNA729271	stage_1W_vs_E10	ordinary_muscle_development	ENSGALG00010010618	APAF1	-0.380156689	0.136838814	-2.778134927	0.005467191	0.007605272	negative
PRJNA729271	stage_1W_vs_E10	ordinary_muscle_development	ENSGALG00010010619	LRRC52	-1.245527097	0.284458168	-4.378594949	1.19447E-05	2.06117E-05	negative
PRJNA729271	stage_1W_vs_E10	ordinary_muscle_development	ENSGALG00010010620	ANKRD54	0.64946727	0.110499292	5.877569486	4.16334E-09	9.03949E-09	positive
PRJNA729271	stage_1W_vs_E10	ordinary_muscle_development	ENSGALG00010010623	SMAD2	-0.48890993	0.077765721	-6.286959393	3.23744E-10	7.4724E-10	negative
PRJNA729271	stage_1W_vs_E10	ordinary_muscle_development	ENSGALG00010010625	FAM169A	-6.206321985	0.413565392	-15.00686977	6.61988E-51	7.48481E-50	negative
PRJNA729271	stage_1W_vs_E10	ordinary_muscle_development	ENSGALG00010010626	ADTRP	1.307492665	0.613358167	2.131695206	0.033031911	0.042110603	positive
PRJNA729271	stage_1W_vs_E10	ordinary_muscle_development	ENSGALG00010010629	ENSGALG00010010629	-1.288029919	0.363025658	-3.548041002	0.000388108	0.00059739	negative
PRJNA729271	stage_1W_vs_E10	ordinary_muscle_development	ENSGALG00010010633	EPCIP	1.309387296	0.326225215	4.013752574	5.9761E-05	9.78245E-05	positive
PRJNA729271	stage_1W_vs_E10	ordinary_muscle_development	ENSGALG00010010635	ALDH9A1	-0.292450206	0.076606053	-3.81758613	0.000134764	0.000214612	negative
PRJNA729271	stage_1W_vs_E10	ordinary_muscle_development	ENSGALG00010010638	ACVR1C	0.942297415	0.435034728	2.166028028	0.030309045	0.038816894	positive
PRJNA729271	stage_1W_vs_E10	ordinary_muscle_development	ENSGALG00010010642	CC2D2A	-0.882549239	0.120747499	-7.309047746	2.69042E-13	7.31389E-13	negative
PRJNA729271	stage_1W_vs_E10	ordinary_muscle_development	ENSGALG00010010645	FAM219A	0.281413149	0.085700225	3.283692081	0.001024568	0.001523973	positive
PRJNA729271	stage_1W_vs_E10	ordinary_muscle_development	ENSGALG00010010646	MCM3AP	-0.381779953	0.058157637	-6.564571274	5.21828E-11	1.26065E-10	negative
PRJNA729271	stage_1W_vs_E10	ordinary_muscle_development	ENSGALG00010010648	CENPM	-2.446596316	0.267868402	-9.133575662	6.62734E-20	2.47969E-19	negative
PRJNA729271	stage_1W_vs_E10	ordinary_muscle_development	ENSGALG00010010651	NUAK1	0.465265852	0.133867867	3.475560346	0.000509787	0.000776928	positive
PRJNA729271	stage_1W_vs_E10	ordinary_muscle_development	ENSGALG00010010655	C1QTNF7	0.478487381	0.250592339	1.909425414	0.056207235	0.069747784	positive
PRJNA729271	stage_1W_vs_E10	ordinary_muscle_development	ENSGALG00010010657	TMEM170B	-1.556454973	0.181357624	-8.582241778	9.30418E-18	3.12258E-17	negative
PRJNA729271	stage_1W_vs_E10	ordinary_muscle_development	ENSGALG00010010659	ADAMTS1	0.103595728	0.152116338	0.681029596	0.495852764	0.533703558	positive
PRJNA729271	stage_1W_vs_E10	ordinary_muscle_development	ENSGALG00010010661	CPEB2	-0.113999193	0.185467108	-0.614659894	0.538779351	0.575028973	negative
PRJNA729271	stage_1W_vs_E10	ordinary_muscle_development	ENSGALG00010010662	MGST3	-0.806405012	0.152967686	-5.271734402	1.35141E-07	2.67568E-07	negative
PRJNA729271	stage_1W_vs_E10	ordinary_muscle_development	ENSGALG00010010663	EFR3B	-1.759772625	0.178632858	-9.851337793	6.76376E-23	2.85168E-22	negative
PRJNA729271	stage_1W_vs_E10	ordinary_muscle_development	ENSGALG00010010666	C12orf75	-1.199247987	0.38420355	-3.121387056	0.001800013	0.002620649	negative
PRJNA729271	stage_1W_vs_E10	ordinary_muscle_development	ENSGALG00010010671	SIGMAR1	-0.830360263	0.143046273	-5.804836734	6.44288E-09	1.38375E-08	negative
PRJNA729271	stage_1W_vs_E10	ordinary_muscle_development	ENSGALG00010010678	KCNIP1	-3.148157878	0.428142715	-7.353057203	1.93724E-13	5.30317E-13	negative
PRJNA729271	stage_1W_vs_E10	ordinary_muscle_development	ENSGALG00010010680	ENSGALG00010010680	-0.261587337	0.592828043	-0.441253311	0.659029622	0.689649237	negative
PRJNA729271	stage_1W_vs_E10	ordinary_muscle_development	ENSGALG00010010682	SMIM13	-0.883481468	0.106103116	-8.326630721	8.31757E-17	2.67799E-16	negative
PRJNA729271	stage_1W_vs_E10	ordinary_muscle_development	ENSGALG00010010685	STIM2	-0.020944986	0.108689235	-0.192705245	0.847189815	0.863938116	negative
PRJNA729271	stage_1W_vs_E10	ordinary_muscle_development	ENSGALG00010010688	IQGAP2	-1.687625437	0.170385307	-9.904759208	3.96925E-23	1.68871E-22	negative
PRJNA729271	stage_1W_vs_E10	ordinary_muscle_development	ENSGALG00010010689	POFUT2	-0.749669314	0.15028964	-4.988163619	6.09559E-07	1.15603E-06	negative
PRJNA729271	stage_1W_vs_E10	ordinary_muscle_development	ENSGALG00010010690	MTPN	-0.890399676	0.135518663	-6.570310379	5.02105E-11	1.21543E-10	negative
PRJNA729271	stage_1W_vs_E10	ordinary_muscle_development	ENSGALG00010010693	KCNMB1	0.404269489	0.338724069	1.193506829	0.232670939	0.264494178	positive
PRJNA729271	stage_1W_vs_E10	ordinary_muscle_development	ENSGALG00010010697	NEDD9	-0.677417048	0.139618281	-4.851922267	1.22271E-06	2.27074E-06	negative
PRJNA729271	stage_1W_vs_E10	ordinary_muscle_development	ENSGALG00010010698	GPRIN2	-2.714998266	0.219978527	-12.34210584	5.37319E-35	3.64639E-34	negative
PRJNA729271	stage_1W_vs_E10	ordinary_muscle_development	ENSGALG00010010699	DNAJC27	-0.543046268	0.153101978	-3.546957883	0.000389707	0.00059966	negative
PRJNA729271	stage_1W_vs_E10	ordinary_muscle_development	ENSGALG00010010706	SREBF2	1.098281762	0.13828928	7.941915391	1.99083E-15	6.0383E-15	positive
PRJNA729271	stage_1W_vs_E10	ordinary_muscle_development	ENSGALG00010010710	UCK2	-0.044080369	0.099676474	-0.442234428	0.658319575	0.689077285	negative
PRJNA729271	stage_1W_vs_E10	ordinary_muscle_development	ENSGALG00010010711	ENSGALG00010010711	-1.042832848	0.428333506	-2.434628237	0.014907097	0.019756639	negative
PRJNA729271	stage_1W_vs_E10	ordinary_muscle_development	ENSGALG00010010712	ADAMTS5	-0.000839074	0.145690436	-0.005759296	0.995404772	0.996358581	negative
PRJNA729271	stage_1W_vs_E10	ordinary_muscle_development	ENSGALG00010010713	NSA2	0.423429729	0.09070384	4.668266871	3.03751E-06	5.47621E-06	positive
PRJNA729271	stage_1W_vs_E10	ordinary_muscle_development	ENSGALG00010010719	SH3BP4	-0.574738734	0.165929092	-3.463761093	0.000532679	0.000810708	negative
PRJNA729271	stage_1W_vs_E10	ordinary_muscle_development	ENSGALG00010010722	TBC1D19	-1.111512044	0.169257614	-6.566984016	5.13446E-11	1.24102E-10	negative
PRJNA729271	stage_1W_vs_E10	ordinary_muscle_development	ENSGALG00010010724	ADCY3	-0.045759049	0.148507946	-0.308125253	0.757987026	0.78255142	negative
PRJNA729271	stage_1W_vs_E10	ordinary_muscle_development	ENSGALG00010010728	ELOVL2	3.018335491	0.846202647	3.566918044	0.000361204	0.000557163	positive
PRJNA729271	stage_1W_vs_E10	ordinary_muscle_development	ENSGALG00010010732	CCKAR	-6.209681481	1.165005816	-5.330172086	9.81197E-08	1.96307E-07	negative
PRJNA729271	stage_1W_vs_E10	ordinary_muscle_development	ENSGALG00010010733	GFM2	-0.208583446	0.126919135	-1.64343577	0.100292804	0.120582631	negative
PRJNA729271	stage_1W_vs_E10	ordinary_muscle_development	ENSGALG00010010734	AMN1	1.353142057	0.142691347	9.483000064	2.47075E-21	9.81732E-21	positive
PRJNA729271	stage_1W_vs_E10	ordinary_muscle_development	ENSGALG00010010740	CCDC134	1.534442153	0.094699976	16.20319474	4.7874E-59	6.8234E-58	positive
PRJNA729271	stage_1W_vs_E10	ordinary_muscle_development	ENSGALG00010010742	LCP2	1.434176864	0.263447463	5.443881846	5.21318E-08	1.06265E-07	positive
PRJNA729271	stage_1W_vs_E10	ordinary_muscle_development	ENSGALG00010010743	KLHL20	-0.212378096	0.084791747	-2.504702437	0.012255445	0.016401318	negative
PRJNA729271	stage_1W_vs_E10	ordinary_muscle_development	ENSGALG00010010745	ATVR1	-0.337280443	0.063981404	-5.271538673	1.35285E-07	2.67803E-07	negative
PRJNA729271	stage_1W_vs_E10	ordinary_muscle_development	ENSGALG00010010748	SF3B1	0.097575511	0.071777615	1.359414235	0.174015363	0.201998293	positive
PRJNA729271	stage_1W_vs_E10	ordinary_muscle_development	ENSGALG00010010749	NCOA4	-0.226467124	0.098407496	-2.301319857	0.021373558	0.027848065	negative
PRJNA729271	stage_1W_vs_E10	ordinary_muscle_development	ENSGALG00010010751	SLC30A5	-0.17041293	0.078770482	-2.163411029	0.03050958	0.039053017	negative
PRJNA729271	stage_1W_vs_E10	ordinary_muscle_development	ENSGALG00010010752	ENSGALG00010010752	-1.278819857	0.26664122	-4.796032116	1.61839E-06	2.97434E-06	negative
PRJNA729271	stage_1W_vs_E10	ordinary_muscle_development	ENSGALG00010010753	ENSGALG00010010753	-1.906744212	1.16387713	-1.638269335			negative
PRJNA729271	stage_1W_vs_E10	ordinary_muscle_development	ENSGALG00010010754	DNAI1	-2.514387554	0.317624632	-7.916223427	2.44833E-15	7.39038E-15	negative
PRJNA729271	stage_1W_vs_E10	ordinary_muscle_development	ENSGALG00010010756	CENPL	-2.795671916	0.206103874	-13.56438315	6.51252E-42	5.56598E-41	negative
PRJNA729271	stage_1W_vs_E10	ordinary_muscle_development	ENSGALG00010010759	ENSGALG00010010759	-0.671878395	0.29738555	-2.259283932	0.023865729	0.03095881	negative
PRJNA729271	stage_1W_vs_E10	ordinary_muscle_development	ENSGALG00010010760	FOXI1	2.373285102	0.669580442	3.544436118	0.000393454	0.000604976	positive
PRJNA729271	stage_1W_vs_E10	ordinary_muscle_development	ENSGALG00010010761	MEI1	0.40063536	0.271802707	1.473993266	0.140483404	0.165472692	positive
PRJNA729271	stage_1W_vs_E10	ordinary_muscle_development	ENSGALG00010010762	MRPL39	0.098672646	0.111127863	0.887919942	0.374583817	0.412175846	positive
PRJNA729271	stage_1W_vs_E10	ordinary_muscle_development	ENSGALG00010010765	ENSGALG00010010765	4.381103589	0.770777515	5.684005442	1.31576E-08	2.76806E-08	positive
PRJNA729271	stage_1W_vs_E10	ordinary_muscle_development	ENSGALG00010010766	CENPO	-2.783937375	0.252817441	-11.01165081	3.35794E-28	1.76544E-27	negative
PRJNA729271	stage_1W_vs_E10	ordinary_muscle_development	ENSGALG00010010768	ENSGALG00010010768	1.287870116	0.091191402	14.12271431	2.75184E-45	2.59518E-44	positive
PRJNA729271	stage_1W_vs_E10	ordinary_muscle_development	ENSGALG00010010769	HEXB	-0.261410888	0.121847818	-2.14538834	0.031921811	0.040756333	negative
PRJNA729271	stage_1W_vs_E10	ordinary_muscle_development	ENSGALG00010010771	ENSGALG00010010771	-0.425270406	0.072080012	-5.899976884	3.63553E-09	7.9184E-09	negative
PRJNA729271	stage_1W_vs_E10	ordinary_muscle_development	ENSGALG00010010774	KPTN	-0.110992219	0.136056184	-0.815782246	0.414624689	0.452695456	negative
PRJNA729271	stage_1W_vs_E10	ordinary_muscle_development	ENSGALG00010010776	GCAT	1.35383945	0.193678952	6.990121729	2.74648E-12	7.1115E-12	positive
PRJNA729271	stage_1W_vs_E10	ordinary_muscle_development	ENSGALG00010010778	UPP2	3.241657913	0.36486607	8.884514572	6.42005E-19	2.29548E-18	positive
PRJNA729271	stage_1W_vs_E10	ordinary_muscle_development	ENSGALG00010010779	DOCK2	0.584655081	0.187441236	3.119137987	0.00181381	0.002638881	positive
PRJNA729271	stage_1W_vs_E10	ordinary_muscle_development	ENSGALG00010010780	PARG	-0.893125742	0.050045575	-17.84624808	3.09119E-71	6.01434E-70	negative
PRJNA729271	stage_1W_vs_E10	ordinary_muscle_development	ENSGALG00010010781	NAPA	1.247918194	0.096961671	12.87022164	6.62119E-38	4.91167E-37	positive
PRJNA729271	stage_1W_vs_E10	ordinary_muscle_development	ENSGALG00010010783	F2RL2	-0.604931312	0.148831389	-4.064541201	4.8127E-05	7.92549E-05	negative
PRJNA729271	stage_1W_vs_E10	ordinary_muscle_development	ENSGALG00010010784	CCDC148	1.368219354	0.293156183	4.667202791	3.05328E-06	5.50327E-06	positive
PRJNA729271	stage_1W_vs_E10	ordinary_muscle_development	ENSGALG00010010786	NABP1	0.890179038	0.153906423	5.783897919	7.29892E-09	1.56113E-08	positive
PRJNA729271	stage_1W_vs_E10	ordinary_muscle_development	ENSGALG00010010789	ENSGALG00010010789	-0.494451937	0.278701083	-1.774129943	0.076041659	0.092764168	negative
PRJNA729271	stage_1W_vs_E10	ordinary_muscle_development	ENSGALG00010010791	H-10	-0.233716958	0.295047591	-0.792133083	0.428283082	0.466168301	negative
PRJNA729271	stage_1W_vs_E10	ordinary_muscle_development	ENSGALG00010010792	XRCC6	-0.997252454	0.149024593	-6.691864983	2.20344E-11	5.44196E-11	negative
PRJNA729271	stage_1W_vs_E10	ordinary_muscle_development	ENSGALG00010010796	CAVIN2	2.118401355	0.146028206	14.50679574	1.09728E-47	1.10914E-46	positive
PRJNA729271	stage_1W_vs_E10	ordinary_muscle_development	ENSGALG00010010799	DARS2	-0.525450302	0.107824044	-4.873220146	1.09794E-06	2.04768E-06	negative
PRJNA729271	stage_1W_vs_E10	ordinary_muscle_development	ENSGALG00010010800	BICRA	0.909702405	0.188974224	4.813896757	1.48015E-06	2.73101E-06	positive
PRJNA729271	stage_1W_vs_E10	ordinary_muscle_development	ENSGALG00010010805	PTRHD1	-0.312590833	0.164244941	-1.903199158	0.057014563	0.07070118	negative
PRJNA729271	stage_1W_vs_E10	ordinary_muscle_development	ENSGALG00010010806	INSYN2B	0.586	0.784302454	0.747160737	0.454966541	0.493165205	positive
PRJNA729271	stage_1W_vs_E10	ordinary_muscle_development	ENSGALG00010010809	CENPH	-2.374276186	0.159854423	-14.85274002	6.67823E-50	7.30573E-49	negative
PRJNA729271	stage_1W_vs_E10	ordinary_muscle_development	ENSGALG00010010810	GALT	0.509112824	0.112679861	4.518223736	6.23606E-06	1.09662E-05	positive
PRJNA729271	stage_1W_vs_E10	ordinary_muscle_development	ENSGALG00010010813	EHD2	1.785646659	0.149253916	11.96381784	5.4975E-33	3.46763E-32	positive
PRJNA729271	stage_1W_vs_E10	ordinary_muscle_development	ENSGALG00010010816	AP2S1	-1.329631259	0.114571974	-11.60520513	3.87757E-31	2.29129E-30	negative
PRJNA729271	stage_1W_vs_E10	ordinary_muscle_development	ENSGALG00010010821	RGS8	-1.758250138	0.423391805	-4.152773194	3.2847E-05	5.48839E-05	negative
PRJNA729271	stage_1W_vs_E10	ordinary_muscle_development	ENSGALG00010010822	OGDHL	-0.381348264	0.343685632	-1.109584541	0.267178096	0.300982863	negative
PRJNA729271	stage_1W_vs_E10	ordinary_muscle_development	ENSGALG00010010823	TMEFF2	-2.032430977	0.179159316	-11.34426622	7.91877E-30	4.44377E-29	negative
PRJNA729271	stage_1W_vs_E10	ordinary_muscle_development	ENSGALG00010010824	PKP4	-0.298064827	0.110040824	-2.708674978	0.006755248	0.009299774	negative
PRJNA729271	stage_1W_vs_E10	ordinary_muscle_development	ENSGALG00010010825	MAK	4.532053855	0.363552855	12.46601092	1.14412E-35	7.97689E-35	positive
PRJNA729271	stage_1W_vs_E10	ordinary_muscle_development	ENSGALG00010010826	NCOA1	0.042157508	0.162636618	0.259212894	0.795470983	0.817152839	positive
PRJNA729271	stage_1W_vs_E10	ordinary_muscle_development	ENSGALG00010010828	SPDL1	-1.17111074	0.126331035	-9.270174475	1.85842E-20	7.1245E-20	negative
PRJNA729271	stage_1W_vs_E10	ordinary_muscle_development	ENSGALG00010010829	LAMB4	-2.989469204	0.260934362	-11.45678624	2.17434E-30	1.24426E-29	negative
PRJNA729271	stage_1W_vs_E10	ordinary_muscle_development	ENSGALG00010010831	DESI1	-0.086341992	0.092947103	-0.928936882	0.352921797	0.39053374	negative
PRJNA729271	stage_1W_vs_E10	ordinary_muscle_development	ENSGALG00010010834	KGD4	1.461703044	0.124636365	11.72774128	9.18778E-32	5.56527E-31	positive

PRJNA729271	stage_1W_vs_E10	ordinary_muscle_development	ENSGALG00010010836	DIP2A	-0.532471649	0.106441005	-5.00250492	5.65901E-07	1.07492E-06	negative
PRJNA729271	stage_1W_vs_E10	ordinary_muscle_development	ENSGALG00010010837	RBPJ	-1.050942037	0.124070202	-8.470543474	2.44251E-17	8.04457E-17	negative
PRJNA729271	stage_1W_vs_E10	ordinary_muscle_development	ENSGALG00010010838	JAM2	1.23514991	0.174208874	7.090051631	1.34062E-12	3.52929E-12	positive
PRJNA729271	stage_1W_vs_E10	ordinary_muscle_development	ENSGALG00010010840	RGS16	2.955516183	0.542806957	5.444875281	5.18417E-08	1.05703E-07	positive
PRJNA729271	stage_1W_vs_E10	ordinary_muscle_development	ENSGALG00010010841	PMM1	0.799304437	0.13278155	6.019695042	1.74746E-09	3.88607E-09	positive
PRJNA729271	stage_1W_vs_E10	ordinary_muscle_development	ENSGALG00010010843	ZNF395	-0.14732979	0.181102472	-0.813516176	0.415922176	0.453906937	negative
PRJNA729271	stage_1W_vs_E10	ordinary_muscle_development	ENSGALG00010010844	CDC14B	0.111744296	0.175042453	0.63838397	0.523223763	0.560086289	positive
PRJNA729271	stage_1W_vs_E10	ordinary_muscle_development	ENSGALG00010010845	FKRP	1.182849792	0.251477229	4.703605958	2.55606E-06	4.6359E-06	positive
PRJNA729271	stage_1W_vs_E10	ordinary_muscle_development	ENSGALG00010010848	RGR	1.523508423	0.565075445	2.696115068	0.007015343	0.00964593	positive
PRJNA729271	stage_1W_vs_E10	ordinary_muscle_development	ENSGALG00010010849	ACBD6	-0.114210127	0.109016783	-1.04763802	0.294805413	0.329875287	negative
PRJNA729271	stage_1W_vs_E10	ordinary_muscle_development	ENSGALG00010010850	ENSGALG00010010850	3.989507827	0.105684771	37.74912679	7.7742E-312	3.0456E-309	positive
PRJNA729271	stage_1W_vs_E10	ordinary_muscle_development	ENSGALG00010010851	PNOC	5.827814479	0.748914571	7.78168125	7.1567E-15	2.11362E-14	positive
PRJNA729271	stage_1W_vs_E10	ordinary_muscle_development	ENSGALG00010010853	SMIM20	0.72127474	0.145823497	4.946217535	7.56694E-07	1.4276E-06	positive
PRJNA729271	stage_1W_vs_E10	ordinary_muscle_development	ENSGALG00010010854	LRIT1	1.833964267	0.557684043	3.288536386	0.001007098	0.001499371	positive
PRJNA729271	stage_1W_vs_E10	ordinary_muscle_development	ENSGALG00010010855	ATP5PF	1.089940021	0.1484801	7.34064714	2.12564E-13	5.80465E-13	positive
PRJNA729271	stage_1W_vs_E10	ordinary_muscle_development	ENSGALG00010010856	CSDC2	-3.986052177	0.658482909	-6.053387449	1.41831E-09	3.16771E-09	negative
PRJNA729271	stage_1W_vs_E10	ordinary_muscle_development	ENSGALG00010010857	SEL1L3	0.240578297	0.060945784	3.947414942	7.89996E-05	0.000128187	positive
PRJNA729271	stage_1W_vs_E10	ordinary_muscle_development	ENSGALG00010010859	ENSGALG00010010859	-2.175246817	0.28258092	-7.697783751	1.38446E-14	4.02968E-14	negative
PRJNA729271	stage_1W_vs_E10	ordinary_muscle_development	ENSGALG00010010861	ENSGALG00010010861	1.169554415	0.236495919	4.945347122	7.60083E-07	1.43381E-06	positive
PRJNA729271	stage_1W_vs_E10	ordinary_muscle_development	ENSGALG00010010862	STRN4	0.763529796	0.149114097	5.120440052	3.04824E-07	5.89594E-07	positive
PRJNA729271	stage_1W_vs_E10	ordinary_muscle_development	ENSGALG00010010865	ENSGALG00010010865	1.377028145	0.156566537	8.795162573	1.42841E-18	5.00358E-18	positive
PRJNA729271	stage_1W_vs_E10	ordinary_muscle_development	ENSGALG00010010867	QSOX1	-0.704295048	0.164098777	-4.291896994	1.77153E-05	3.01531E-05	negative
PRJNA729271	stage_1W_vs_E10	ordinary_muscle_development	ENSGALG00010010868	SLC39A10	-2.727561992	0.113505608	-24.03019593	1.3449E-127	8.6258E-126	negative
PRJNA729271	stage_1W_vs_E10	ordinary_muscle_development	ENSGALG00010010869	ENSGALG00010010869	6.580318467	0.173391878	37.95055769	3.7777E-315	1.5211E-312	positive
PRJNA729271	stage_1W_vs_E10	ordinary_muscle_development	ENSGALG00010010870	NOL6	-0.930167654	0.123392685	-7.538272268	4.76239E-14	1.34878E-13	negative
PRJNA729271	stage_1W_vs_E10	ordinary_muscle_development	ENSGALG00010010871	GCNT2	-5.308702849	1.196590761	-4.436523346	9.14234E-06	1.59124E-05	negative
PRJNA729271	stage_1W_vs_E10	ordinary_muscle_development	ENSGALG00010010874	ENSGALG00010010874	2.01984597	0.263155321	7.675489751	1.64788E-14	4.77433E-14	positive
PRJNA729271	stage_1W_vs_E10	ordinary_muscle_development	ENSGALG00010010876	ENSGALG00010010876	0.557090257	0.158857696	3.506850907	0.000453443	0.000693392	positive
PRJNA729271	stage_1W_vs_E10	ordinary_muscle_development	ENSGALG00010010880	ELP3	0.328061351	0.105975395	3.095636985	0.001963906	0.002848338	positive
PRJNA729271	stage_1W_vs_E10	ordinary_muscle_development	ENSGALG00010010882	CEP350	0.936891739	0.098266012	9.534239948	1.50987E-21	6.04409E-21	positive
PRJNA729271	stage_1W_vs_E10	ordinary_muscle_development	ENSGALG00010010884	DNAH7	-3.404309638	0.325932167	-10.44484094	1.54715E-25	7.28823E-25	negative
PRJNA729271	stage_1W_vs_E10	ordinary_muscle_development	ENSGALG00010010885	SNRPD2	-0.653889914	0.274556496	-2.381622444	0.017236559	0.022669806	negative
PRJNA729271	stage_1W_vs_E10	ordinary_muscle_development	ENSGALG00010010887	GABPA	0.020928207	0.066271371	0.315795588	0.752157667	0.776920501	positive
PRJNA729271	stage_1W_vs_E10	ordinary_muscle_development	ENSGALG00010010888	GNPAT	1.791710896	0.118229989	15.15445377	7.08016E-52	8.30987E-51	positive
PRJNA729271	stage_1W_vs_E10	ordinary_muscle_development	ENSGALG00010010889	CCL19	0.971874052	0.648655302	1.498290462	0.134057801	0.158302878	positive
PRJNA729271	stage_1W_vs_E10	ordinary_muscle_development	ENSGALG00010010890	NOL12	-0.47840165	0.13552661	-3.529946271	0.000415644	0.000637878	negative
PRJNA729271	stage_1W_vs_E10	ordinary_muscle_development	ENSGALG00010010891	POLR3H	-0.63157367	0.108391823	-5.8267649	5.65121E-09	1.21769E-08	negative
PRJNA729271	stage_1W_vs_E10	ordinary_muscle_development	ENSGALG00010010893	CCL21	-0.95620555	0.494290092	-1.934502766	0.053051342	0.066046483	negative
PRJNA729271	stage_1W_vs_E10	ordinary_muscle_development	ENSGALG00010010894	SPAG1	-0.382893189	0.148479697	-2.578757887	0.009915626	0.013413625	negative
PRJNA729271	stage_1W_vs_E10	ordinary_muscle_development	ENSGALG00010010896	LY86	3.410409131	0.292506499	11.65925935	2.05825E-31	1.23028E-30	positive
PRJNA729271	stage_1W_vs_E10	ordinary_muscle_development	ENSGALG00010010897	CEP170	-0.359952395	0.090902886	-3.959746611	7.50293E-05	0.00012195	negative
PRJNA729271	stage_1W_vs_E10	ordinary_muscle_development	ENSGALG00010010898	PHF24	-4.641380818	3.569493153	-1.300291279	0.193501156	0.223187655	negative
PRJNA729271	stage_1W_vs_E10	ordinary_muscle_development	ENSGALG00010010900	SLC34A2	0.659162663	0.989428275	0.666205605	0.505279691	0.542922841	positive
PRJNA729271	stage_1W_vs_E10	ordinary_muscle_development	ENSGALG00010010904	TOR1AIP1	-0.912794144	0.083184543	-10.97312202	5.14641E-28	2.68239E-27	negative
PRJNA729271	stage_1W_vs_E10	ordinary_muscle_development	ENSGALG00010010905	ENC1	0.188068389	0.147956541	1.271105613	0.203691079	0.234084055	positive
PRJNA729271	stage_1W_vs_E10	ordinary_muscle_development	ENSGALG00010010906	ATP6V1C1	0.872519474	0.138965263	6.27868758	3.41443E-10	7.87339E-10	positive
PRJNA729271	stage_1W_vs_E10	ordinary_muscle_development	ENSGALG00010010908	STK17B	0.282638526	0.124458115	2.270952961	0.023149826	0.030062419	positive
PRJNA729271	stage_1W_vs_E10	ordinary_muscle_development	ENSGALG00010010909	SLIT3	-3.395753785	0.330531373	-10.27362018	9.26608E-25	4.21701E-24	negative
PRJNA729271	stage_1W_vs_E10	ordinary_muscle_development	ENSGALG00010010911	ENSGALG00010010911	-2.536094298	0.407830782	-6.218496511	5.01941E-10	1.14595E-09	negative
PRJNA729271	stage_1W_vs_E10	ordinary_muscle_development	ENSGALG00010010912	BAALC	-0.934193003	0.352877556	-2.647357381	0.008112357	0.011075503	negative
PRJNA729271	stage_1W_vs_E10	ordinary_muscle_development	ENSGALG00010010913	IFT56	-2.340889485	0.153089033	-15.29103323	8.77469E-53	1.06434E-51	negative
PRJNA729271	stage_1W_vs_E10	ordinary_muscle_development	ENSGALG00010010915	APP	-1.660694704	0.110450007	-15.03571388	4.28405E-51	4.86656E-50	negative
PRJNA729271	stage_1W_vs_E10	ordinary_muscle_development	ENSGALG00010010916	ENSGALG00010010916	-3.69647636	0.891906766	-4.144464982	3.40608E-05	5.68268E-05	negative
PRJNA729271	stage_1W_vs_E10	ordinary_muscle_development	ENSGALG00010010917	TANC1	0.362748374	0.068621447	5.286224502	1.24867E-07	2.47801E-07	positive
PRJNA729271	stage_1W_vs_E10	ordinary_muscle_development	ENSGALG00010010918	TOR1AIP2	0.909149852	0.119475704	7.609495652	2.75168E-14	7.88875E-14	positive
PRJNA729271	stage_1W_vs_E10	ordinary_muscle_development	ENSGALG00010010919	CDHR1	-1.463707487	0.527438585	-2.77512402	0.005518066	0.007670154	negative
PRJNA729271	stage_1W_vs_E10	ordinary_muscle_development	ENSGALG00010010920	UBN2	0.732104217	0.113769289	6.434989839	1.23482E-10	2.91794E-10	positive
PRJNA729271	stage_1W_vs_E10	ordinary_muscle_development	ENSGALG00010010921	ENSGALG00010010921	0.12805975	0.128434572	0.99708161	0.318724898	0.354968683	positive
PRJNA729271	stage_1W_vs_E10	ordinary_muscle_development	ENSGALG00010010922	DYM	1.098022167	0.101437026	10.82466837	2.6303E-27	1.33168E-26	positive
PRJNA729271	stage_1W_vs_E10	ordinary_muscle_development	ENSGALG00010010923	F13A1	2.288019279	0.23093027	9.907836148	3.84892E-23	1.63896E-22	positive
PRJNA729271	stage_1W_vs_E10	ordinary_muscle_development	ENSGALG00010010924	ENSGALG00010010924	-1.650489455	0.21892199	-7.539167074	4.72983E-14	1.33985E-13	negative
PRJNA729271	stage_1W_vs_E10	ordinary_muscle_development	ENSGALG00010010928	HECW2	-0.438220011	0.116907138	-3.748445279	0.000177934	0.000281076	negative
PRJNA729271	stage_1W_vs_E10	ordinary_muscle_development	ENSGALG00010010929	FMC1	0.282980615	0.149027064	1.898853862	0.057583693	0.071382505	positive
PRJNA729271	stage_1W_vs_E10	ordinary_muscle_development	ENSGALG00010010930	FAM163A	-4.468615359	0.636233544	-7.023545684	2.16307E-12	5.6341E-12	negative
PRJNA729271	stage_1W_vs_E10	ordinary_muscle_development	ENSGALG00010010931	CYYR1	-2.761656366	0.199118731	-13.86939516	9.7092E-44	8.76853E-43	negative
PRJNA729271	stage_1W_vs_E10	ordinary_muscle_development	ENSGALG00010010932	GHITM	1.219480406	0.088129992	13.83729162	1.5182E-43	1.35674E-42	positive
PRJNA729271	stage_1W_vs_E10	ordinary_muscle_development	ENSGALG00010010933	ANAPC4	-0.837519039	0.111774413	-7.492940588	6.73474E-14	1.8948E-13	negative
PRJNA729271	stage_1W_vs_E10	ordinary_muscle_development	ENSGALG00010010934	ENSGALG00010010934	0.078419675	0.118188908	0.663511291	0.50700315	0.544653573	positive
PRJNA729271	stage_1W_vs_E10	ordinary_muscle_development	ENSGALG00010010935	LGALS1A	-0.153613895	0.228793918	-0.671407249	0.501961128	0.539757163	negative
PRJNA729271	stage_1W_vs_E10	ordinary_muscle_development	ENSGALG00010010937	CDK7	0.483972349	0.114899331	4.212142451	2.5296E-05	4.25267E-05	positive
PRJNA729271	stage_1W_vs_E10	ordinary_muscle_development	ENSGALG00010010938	FZD6	0.020333188	0.12095086	0.168111151	0.866495837	0.881269797	positive
PRJNA729271	stage_1W_vs_E10	ordinary_muscle_development	ENSGALG00010010939	ZCCHC4	-0.376128165	0.141777843	-2.652940383	0.007979396	0.010901164	negative
PRJNA729271	stage_1W_vs_E10	ordinary_muscle_development	ENSGALG00010010940	NRG3	-3.192097273	0.387436925	-8.239011479	1.73639E-16	5.51589E-16	negative
PRJNA729271	stage_1W_vs_E10	ordinary_muscle_development	ENSGALG00010010941	RIPK4	-5.641239326	0.792179592	-7.121162152	1.07021E-12	2.83129E-12	negative
PRJNA729271	stage_1W_vs_E10	ordinary_muscle_development	ENSGALG00010010942	NRN1	2.206406802	0.338449299	6.51916494	7.06999E-11	1.69052E-10	positive
PRJNA729271	stage_1W_vs_E10	ordinary_muscle_development	ENSGALG00010010943	WDSUB1	0.141913592	0.103621365	1.369539881	0.170830577	0.198619493	positive
PRJNA729271	stage_1W_vs_E10	ordinary_muscle_development	ENSGALG00010010945	PI4K2B	-0.739277492	0.100404535	-7.362989054	1.79837E-13	4.93234E-13	negative
PRJNA729271	stage_1W_vs_E10	ordinary_muscle_development	ENSGALG00010010946	SH2D4B	-0.003421836	0.337992612	-0.010123995	0.991922358	0.993347321	negative
PRJNA729271	stage_1W_vs_E10	ordinary_muscle_development	ENSGALG00010010947	LUC7L2	-0.381429	0.070537007	-5.407501927	6.39099E-08	1.29508E-07	negative
PRJNA729271	stage_1W_vs_E10	ordinary_muscle_development	ENSGALG00010010948	PRDM15	-0.32960932	0.08518891	-3.869157609	0.000109212	0.000175114	negative
PRJNA729271	stage_1W_vs_E10	ordinary_muscle_development	ENSGALG00010010949	FZD3	-3.100189822	0.239533151	-12.94263368	2.58609E-38	1.95338E-37	negative
PRJNA729271	stage_1W_vs_E10	ordinary_muscle_development	ENSGALG00010010951	ENSGALG00010010951	-0.886391002	0.134417781	-6.594298704	4.27271E-11	1.03914E-10	negative
PRJNA729271	stage_1W_vs_E10	ordinary_muscle_development	ENSGALG00010010952	ENSGALG00010010952	1.754658071	0.476822516	3.679897678	0.000233328	0.000365591	positive
PRJNA729271	stage_1W_vs_E10	ordinary_muscle_development	ENSGALG00010010953	EXTL3	-0.140775262	0.110692227	-1.271771885	0.203454181	0.23384889	negative
PRJNA729271	stage_1W_vs_E10	ordinary_muscle_development	ENSGALG00010010954	MTERF2	1.074757056	0.225220214	4.772027505	1.82381E-06	3.34253E-06	positive
PRJNA729271	stage_1W_vs_E10	ordinary_muscle_development	ENSGALG00010010955	GTF3C3	-0.100444797	0.083536041	-1.202412708	0.229203668	0.260859545	negative
PRJNA729271	stage_1W_vs_E10	ordinary_muscle_development	ENSGALG00010010956	AXDND1	2.442863542	0.585646871	4.171222732	3.0297E-05	5.07048E-05	positive
PRJNA729271	stage_1W_vs_E10	ordinary_muscle_development	ENSGALG00010010957	ZMAT2	0.20923635	0.075334752	2.777421369	0.00547921	0.007619797	positive
PRJNA729271	stage_1W_vs_E10	ordinary_muscle_development	ENSGALG00010010958	LCORL	0.101592766	0.160610072	0.632542932	0.527032164	0.563788282	positive
PRJNA729271	stage_1W_vs_E10	ordinary_muscle_development	ENSGALG00010010959	TMEM263	-1.469938545	0.090638866	-16.21753015	3.79137E-59	5.42507E-58	negative
PRJNA729271	stage_1W_vs_E10	ordinary_muscle_development	ENSGALG00010010960	POLDIP3	-0.560165491	0.070646137	-7.929173672	2.20609E-15	6.67584E-15	negative
PRJNA729271	stage_1W_vs_E10	ordinary_muscle_development	ENSGALG00010010961	C2orf66	1.086028966	0.363478495	2.987876809	0.002809228	0.00401338	positive
PRJNA729271	stage_1W_vs_E10	ordinary_muscle_development	ENSGALG00010010962	ANKDD1B	0.128920888	0.283447729	0.454831261	0.649230638	0.680645024	positive
PRJNA729271	stage_1W_vs_E10	ordinary_muscle_development	ENSGALG00010010963	TSPAN14	-0.622499313	0.074907413	-8.310249831	9.55008E-17	3.06665E-16	negative
PRJNA729271	stage_1W_vs_E10	ordinary_muscle_development	ENSGALG00010010964	INTS9	-0.960575428	0.094706118	-10.14269666	3.57103E-24	1.58245E-23	negative
PRJNA729271	stage_1W_vs_E10	ordinary_muscle_development	ENSGALG00010010965	BAZ2B	0.12213912	0.132209237	0.923831967	0.355573809	0.393198227	positive
PRJNA729271	stage_1W_vs_E10	ordinary_muscle_development	ENSGALG00010010966	ENSGALG00010010966	3.337383609	0.507984454	6.56985382	5.03647E-11	1.21876E-10	positive
PRJNA729271	stage_1W_vs_E10	ordinary_muscle_development	ENSGALG00010010967	PRXL2A	-0.280994737	0.390845667	-0.718940394	0.472177646	0.510686091	negative
PRJNA729271	stage_1W_vs_E10	ordinary_muscle_development	ENSGALG00010010968	RRP7A	0.585206173	0.24606296	2.378278202	0.017393697	0.022870251	positive
PRJNA729271	stage_1W_vs_E10	ordinary_muscle_development	ENSGALG00010010970	TPM2	-1.281011204	0.352513293	-3.633937296	0.000279129	0.000435004	negative
PRJNA729271	stage_1W_vs_E10	ordinary_muscle_development	ENSGALG00010010971	LCORL	0.118989019	0.125019407	0.95176438	0.341216491	0.378297258	positive
PRJNA729271	stage_1W_vs_E10	ordinary_muscle_development	ENSGALG00010010972	EXOSC3	-0.509561355	0.166921851	-3.052694131	0.00226797	0.003267815	negative
PRJNA729271	stage_1W_vs_E10	ordinary_muscle_development	ENSGALG00010010973	PGAP1	-0.576057041	0.113644481	-5.06893986	4.00038E-07	7.67816E-07	negative
PRJNA729271	stage_1W_vs_E10	ordinary_muscle_development	ENSGALG00010010974	NCAPG	-2.315963404	0.193151318	-11.99040951	3.98928E-33	2.53284E-32	negative
PRJNA729271	stage_1W_vs_E10	ordinary_muscle_development	ENSGALG00010010975	ATP8B1	-3.625010555	0.926174125	-3.913962243	9.07938E-05	0.000146554	negative
PRJNA729271	stage_1W_vs_E10	ordinary_muscle_development	ENSGALG00010010976	DYDC2	-1.804994908	1.013475892	-1.78099442	0.074913362	0.091480134	negative
PRJNA729271	stage_1W_vs_E10	ordinary_muscle_development	ENSGALG00010010977	FARS2	0.064709741	0.081854297	0.790547887	0.429207867	0.46703461	positive
PRJNA729271	stage_1W_vs_E10	ordinary_muscle_development	ENSGALG00010010978	SERHL	-0.137438112	0.135962966	-1.010849614	0.312088413	0.34800535	negative
PRJNA729271	stage_1W_vs_E10	ordinary_muscle_development	ENSGALG00010010979	ENSGALG00010010979	-2.827988188	0.473600178	-5.971256594	2.35433E-09	5.19027E-09	negative
PRJNA729271	stage_1W_vs_E10	ordinary_muscle_development	ENSGALG00010010980	POLK	0.416206601	0.125898985	3.305877325	0.000946795	0.001414387	positive
PRJNA729271	stage_1W_vs_E10	ordinary_muscle_development	ENSGALG00010010981	RIC8B	-0.926663734	0.065821477	-14.07844034	5.15346E-45	4.82243E-44	negative
PRJNA729271	stage_1W_vs_E10	ordinary_muscle_development	ENSGALG00010010982	ZBTB21	0.2977171	0.120254605	2.475723061	0.013296667	0.017734191	positive
PRJNA729271	stage_1W_vs_E10	ordinary_muscle_development	ENSGALG00010010983	MED28	0.217171607	0.088967508	2.441021572	0.01464578	0.01942452	positive
PRJNA729271	stage_1W_vs_E10	ordinary_muscle_development	ENSGALG00010010984	MAT1A	-0.812137171	0.390200794	-2.081331417	0.037403581	0.047404469	negative
PRJNA729271	stage_1W_vs_E10	ordinary_muscle_development	ENSGALG00010010985	CERT1	1.146042225	0.107956992	10.61572953	2.5182E-26	1.22611E-25	positive
PRJNA729271	stage_1W_vs_E10	ordinary_muscle_development	ENSGALG00010010986	NARS	-0.330351575	0.130269523	-2.535908382	0.011215607	0.01507094	negative
PRJNA729271	stage_1W_vs_E10	ordinary_muscle_development	ENSGALG00010010987	MARCHF7	-0.363333763	0.081289824	-4.469609417	7.83626E-06	1.36908E-05	negative
PRJNA729271	stage_1W_vs_E10	ordinary_muscle_development	ENSGALG00010010988	ANKRD44	-1.163804823	0.087415507	-13.3134825	1.93259E-40	1.57288E-39	negative
PRJNA729271	stage_1W_vs_E10	ordinary_muscle_development	ENSGALG00010010989	TRIQK	-1.739663646	0.182068991	-9.554969448	1.2362E-21	4.96228E-21	negative
PRJNA729271	stage_1W_vs_E10	ordinary_muscle_development	ENSGALG00010010990	MED18	0.486602046	0.119418393	4.074766309	4.60606E-05	7.59641E-05	positive
PRJNA729271	stage_1W_vs_E10	ordinary_muscle_development	ENSGALG00010010991	C2CD2	-0.719866029	0.163353887	-4.406788487	1.04915E-05	1.81819E-05	negative
PRJNA729271	stage_1W_vs_E10	ordinary_muscle_development	ENSGALG00010010992	PCDHA6	0.941571975	0.293356998	3.209645527	0.001328988	0.001955306	positive
PRJNA729271	stage_1W_vs_E10	ordinary_muscle_development	ENSGALG00010010993	ENSGALG00010010993	0.245097694	0.366570595	0.668623444	0.503735713	0.541384234	positive
PRJNA729271	stage_1W_vs_E10	ordinary_muscle_development	ENSGALG00010010994	NAA20	0.092702266	0.149807791	0.61880804	0.536042825	0.572455665	positive
PRJNA729271	stage_1W_vs_E10	ordinary_muscle_development	ENSGALG00010010995	HEBP1	-1.181404577	0.184625036	-6.398940268	1.56459E-10	3.67803E-10	negative
PRJNA729271	stage_1W_vs_E10	ordinary_muscle_development	ENSGALG00010010996	RFX4	-0.662031607	0.732958607	-0.903231916	0.366402822	0.40412486	negative
PRJNA729271	stage_1W_vs_E10	ordinary_muscle_development	ENSGALG00010010997	LAP3	-0.836528723	0.149749022	-5.586204923	2.32085E-08	4.81615E-08	negative
PRJNA729271	stage_1W_vs_E10	ordinary_muscle_development	ENSGALG00010010998	SOAT1	-0.499666965	0.166643598	-2.9984168	0.002713862	0.003882112	negative
PRJNA729271	stage_1W_vs_E10	ordinary_muscle_development	ENSGALG00010011001	ANGPT1	-2.410263429	0.256919619	-9.381391105	6.5108E-21	2.5383E-20	negative
PRJNA729271	stage_1W_vs_E10	ordinary_muscle_development	ENSGALG00010011002	RPRM	-2.676327751	0.820531874	-3.261698705	0.001107468	0.001641553	negative
PRJNA729271	stage_1W_vs_E10	ordinary_muscle_development	ENSGALG00010011003	FECH	-0.294993369	0.112194656	-2.629299653	0.008556093	0.011654973	negative

PRJNA729271	stage_1W_vs_E10	ordinary_muscle_development	ENSGALG00010011004	UMODL1	-1.667291606	0.428782089	-3.888435756	0.000100892	0.000162097	negative
PRJNA729271	stage_1W_vs_E10	ordinary_muscle_development	ENSGALG00010011006	TMEM64	-0.691839657	0.101544628	-6.813158648	9.54787E-12	2.40564E-11	negative
PRJNA729271	stage_1W_vs_E10	ordinary_muscle_development	ENSGALG00010011007	ENSGALG00010011007	-0.974274412	0.184194729	-5.28937183	1.22737E-07	2.43676E-07	negative
PRJNA729271	stage_1W_vs_E10	ordinary_muscle_development	ENSGALG00010011009	SFTPA1	-0.55063984	1.347988585	-0.408489987	0.68291398	0.712351622	negative
PRJNA729271	stage_1W_vs_E10	ordinary_muscle_development	ENSGALG00010011013	ENSGALG00010011013	3.876292605	0.335779327	11.54416692	7.90032E-31	4.61383E-30	positive
PRJNA729271	stage_1W_vs_E10	ordinary_muscle_development	ENSGALG00010011015	LYRM4	-0.115094663	0.1838131	-0.626150492	0.531216237	0.567761344	negative
PRJNA729271	stage_1W_vs_E10	ordinary_muscle_development	ENSGALG00010011018	QDPR	-0.84608887	0.105444173	-8.024045783	1.02318E-15	3.14683E-15	negative
PRJNA729271	stage_1W_vs_E10	ordinary_muscle_development	ENSGALG00010011019	NECAB1	-2.931393876	0.238671825	-12.2821111	1.1301E-34	7.59779E-34	negative
PRJNA729271	stage_1W_vs_E10	ordinary_muscle_development	ENSGALG00010011020	SFTPA2	-1.019989303	0.935202282	-1.090661692	0.275421773	0.309691925	negative
PRJNA729271	stage_1W_vs_E10	ordinary_muscle_development	ENSGALG00010011021	PPP1R3G	2.193169711	0.183175585	11.97304605	4.9189E-33	3.10808E-32	positive
PRJNA729271	stage_1W_vs_E10	ordinary_muscle_development	ENSGALG00010011022	C5orf22	0.019779676	0.194558281	0.10166453	0.919022957	0.929021394	positive
PRJNA729271	stage_1W_vs_E10	ordinary_muscle_development	ENSGALG00010011025	POLR3B	-0.014005855	0.090824913	-0.154207195	0.877446369	0.891281368	negative
PRJNA729271	stage_1W_vs_E10	ordinary_muscle_development	ENSGALG00010011027	FAM234B	-0.425870135	0.102857131	-4.140404558	3.46694E-05	5.78089E-05	negative
PRJNA729271	stage_1W_vs_E10	ordinary_muscle_development	ENSGALG00010011028	HMBOX1	-1.339501031	0.088815227	-15.08188497	2.13097E-51	2.44952E-50	negative
PRJNA729271	stage_1W_vs_E10	ordinary_muscle_development	ENSGALG00010011029	HMGCR	-0.700319173	0.125757703	-5.568797417	2.56503E-08	5.3107E-08	negative
PRJNA729271	stage_1W_vs_E10	ordinary_muscle_development	ENSGALG00010011033	DROSHA	-0.975931258	0.102180667	-9.5510363	1.28405E-21	5.15292E-21	negative
PRJNA729271	stage_1W_vs_E10	ordinary_muscle_development	ENSGALG00010011034	FAM180A	1.100015679	0.400690196	2.745302204	0.00604552	0.008366413	positive
PRJNA729271	stage_1W_vs_E10	ordinary_muscle_development	ENSGALG00010011038	GSG1	-2.259413631	0.48230822	-4.684584537	2.80528E-06	5.07205E-06	negative
PRJNA729271	stage_1W_vs_E10	ordinary_muscle_development	ENSGALG00010011040	CDH6	-2.676150442	0.224279451	-11.93221417	8.0406E-33	5.03884E-32	negative
PRJNA729271	stage_1W_vs_E10	ordinary_muscle_development	ENSGALG00010011042	SLC13A4	0.811077204	0.494633945	1.639752411	0.101056657	0.121450646	positive
PRJNA729271	stage_1W_vs_E10	ordinary_muscle_development	ENSGALG00010011044	MBL2	1.665201406	0.6103646	2.728207709	0.00636795	0.008789966	positive
PRJNA729271	stage_1W_vs_E10	ordinary_muscle_development	ENSGALG00010011045	ENSGALG00010011045	-1.948569458	0.41943322	-4.64572038	3.38892E-06	6.09156E-06	negative
PRJNA729271	stage_1W_vs_E10	ordinary_muscle_development	ENSGALG00010011049	ABL2	-0.464835221	0.130075695	-3.573574773	0.000352141	0.00054405	negative
PRJNA729271	stage_1W_vs_E10	ordinary_muscle_development	ENSGALG00010011050	SYT10	2.960082263	0.403233766	7.340859103	2.12227E-13	5.79656E-13	positive
PRJNA729271	stage_1W_vs_E10	ordinary_muscle_development	ENSGALG00010011055	TOR3A	-0.833226163	0.138593566	-6.012011845	1.83235E-09	4.06861E-09	negative
PRJNA729271	stage_1W_vs_E10	ordinary_muscle_development	ENSGALG00010011056	ENSGALG00010011056	-1.204489252	0.538090571	-2.238450766	0.025191675	0.032573892	negative
PRJNA729271	stage_1W_vs_E10	ordinary_muscle_development	ENSGALG00010011059	ENSGALG00010011059	-2.138277179	0.209845389	-10.18977443	2.20275E-24	9.85762E-24	negative
PRJNA729271	stage_1W_vs_E10	ordinary_muscle_development	ENSGALG00010011061	KIF13B	0.553906361	0.154920806	3.575416202	0.000349671	0.00054035	positive
PRJNA729271	stage_1W_vs_E10	ordinary_muscle_development	ENSGALG00010011062	TMEM55A	-0.531923366	0.122824099	-4.330773613	1.48586E-05	2.54221E-05	negative
PRJNA729271	stage_1W_vs_E10	ordinary_muscle_development	ENSGALG00010011064	MYLIP	0.719351744	0.096935118	7.420961166	1.16273E-13	3.21884E-13	positive
PRJNA729271	stage_1W_vs_E10	ordinary_muscle_development	ENSGALG00010011067	TMTC2	-0.348390756	0.102816144	-3.388483007	0.000702804	0.001060608	negative
PRJNA729271	stage_1W_vs_E10	ordinary_muscle_development	ENSGALG00010011069	ENSGALG00010011069	-1.497792005	0.545997582	-2.743220948	0.006083973	0.008414809	negative
PRJNA729271	stage_1W_vs_E10	ordinary_muscle_development	ENSGALG00010011076	PKP2	-2.971519702	0.313064745	-9.491709786	2.27275E-21	9.0405E-21	negative
PRJNA729271	stage_1W_vs_E10	ordinary_muscle_development	ENSGALG00010011077	RASGEF1A	0.292229493	0.146777387	1.990970806	0.046484097	0.058275989	positive
PRJNA729271	stage_1W_vs_E10	ordinary_muscle_development	ENSGALG00010011080	EMP1	-0.802789492	0.25777631	-3.114287309	0.001843898	0.002680773	negative
PRJNA729271	stage_1W_vs_E10	ordinary_muscle_development	ENSGALG00010011085	UGT1A1	4.254041785	1.315769541	3.233120735	0.001224458	0.001807017	positive
PRJNA729271	stage_1W_vs_E10	ordinary_muscle_development	ENSGALG00010011086	OTUD6A	0.734467441	0.113125931	6.492476431	8.44367E-11	2.01301E-10	positive
PRJNA729271	stage_1W_vs_E10	ordinary_muscle_development	ENSGALG00010011090	GRIN2B	-2.718215855	0.433748854	-6.266796624	3.68551E-10	8.48633E-10	negative
PRJNA729271	stage_1W_vs_E10	ordinary_muscle_development	ENSGALG00010011091	YARS2	0.918942889	0.098575759	9.322199499	1.13953E-20	4.41644E-20	positive
PRJNA729271	stage_1W_vs_E10	ordinary_muscle_development	ENSGALG00010011094	CSGALNACT2	-0.760853402	0.092082895	-8.262700726	1.42413E-16	4.53787E-16	negative
PRJNA729271	stage_1W_vs_E10	ordinary_muscle_development	ENSGALG00010011095	TNN	-3.423717452	0.494861273	-6.918539885	4.56322E-12	1.16759E-11	negative
PRJNA729271	stage_1W_vs_E10	ordinary_muscle_development	ENSGALG00010011096	LDB2	-0.57496922	0.11500626	-4.999460186	5.7491E-07	1.09189E-06	negative
PRJNA729271	stage_1W_vs_E10	ordinary_muscle_development	ENSGALG00010011099	CCBE1	-0.583566766	0.200286571	-2.913658975	0.003572199	0.005054078	negative
PRJNA729271	stage_1W_vs_E10	ordinary_muscle_development	ENSGALG00010011101	ENSGALG00010011101	-1.341592369	0.84063846	-1.595920758	0.110506486	0.132052063	negative
PRJNA729271	stage_1W_vs_E10	ordinary_muscle_development	ENSGALG00010011104	ABCG1	-0.960589282	0.262410117	-3.660641193	0.000251585	0.000393262	negative
PRJNA729271	stage_1W_vs_E10	ordinary_muscle_development	ENSGALG00010011106	USP40	0.257290162	0.116317527	2.211963818	0.026969165	0.034726663	positive
PRJNA729271	stage_1W_vs_E10	ordinary_muscle_development	ENSGALG00010011107	ENSGALG00010011107	0.438727615	0.198755845	2.207369622	0.027288247	0.035121927	positive
PRJNA729271	stage_1W_vs_E10	ordinary_muscle_development	ENSGALG00010011109	ENSGALG00010011109	-2.590299052	1.272264802	-2.035974781	0.041752888	0.052635946	negative
PRJNA729271	stage_1W_vs_E10	ordinary_muscle_development	ENSGALG00010011111	MRPS14	1.237577189	0.116362315	10.63554976	2.03624E-26	9.95123E-26	positive
PRJNA729271	stage_1W_vs_E10	ordinary_muscle_development	ENSGALG00010011116	TAPT1	1.0994366	0.127328863	8.634621959	5.89216E-18	1.99829E-17	positive
PRJNA729271	stage_1W_vs_E10	ordinary_muscle_development	ENSGALG00010011117	SLC6A15	-6.598138405	0.569758822	-11.58058139	5.16943E-31	3.0361E-30	negative
PRJNA729271	stage_1W_vs_E10	ordinary_muscle_development	ENSGALG00010011118	GMPR2	3.229911463	0.135529501	23.83179629	1.5641E-125	9.6475E-124	positive
PRJNA729271	stage_1W_vs_E10	ordinary_muscle_development	ENSGALG00010011119	ENSGALG00010011119	-0.720949832	0.293685785	-2.45483394	0.014094968	0.018748882	negative
PRJNA729271	stage_1W_vs_E10	ordinary_muscle_development	ENSGALG00010011120	ENSGALG00010011120	-3.579382417	3.111103183	-1.150518709	0.249930293	0.282893925	negative
PRJNA729271	stage_1W_vs_E10	ordinary_muscle_development	ENSGALG00010011121	NUP35	0.06573441	0.10199662	0.644476355	0.519266586	0.556402348	positive
PRJNA729271	stage_1W_vs_E10	ordinary_muscle_development	ENSGALG00010011124	CACYBP	0.257258169	0.067926599	3.787296449	0.000152295	0.000241788	positive
PRJNA729271	stage_1W_vs_E10	ordinary_muscle_development	ENSGALG00010011126	ATXN1	0.465924106	0.14363935	3.243707979	0.001179846	0.001744021	positive
PRJNA729271	stage_1W_vs_E10	ordinary_muscle_development	ENSGALG00010011128	GCNT4	-0.408050004	0.247879226	-1.64616459	0.099729877	0.119965525	negative
PRJNA729271	stage_1W_vs_E10	ordinary_muscle_development	ENSGALG00010011129	ENSGALG00010011129	0.773967504	0.115714075	6.688620228	2.25285E-11	5.56019E-11	positive
PRJNA729271	stage_1W_vs_E10	ordinary_muscle_development	ENSGALG00010011130	DNM1L	0.149459764	0.115221835	1.297147921	0.194580296	0.224360941	positive
PRJNA729271	stage_1W_vs_E10	ordinary_muscle_development	ENSGALG00010011131	RET	-0.712017456	0.496070841	-1.435314065	0.151197621	0.177285999	negative
PRJNA729271	stage_1W_vs_E10	ordinary_muscle_development	ENSGALG00010011133	CDC37L1	1.027704139	0.118418056	8.678610123	4.00635E-18	1.37124E-17	positive
PRJNA729271	stage_1W_vs_E10	ordinary_muscle_development	ENSGALG00010011136	ENSGALG00010011136	-1.413685338	0.411820217	-3.432773042	0.000597442	0.000906418	negative
PRJNA729271	stage_1W_vs_E10	ordinary_muscle_development	ENSGALG00010011139	UBASH3A	4.460981673	0.579378817	7.699594014	1.36499E-14	3.9746E-14	positive
PRJNA729271	stage_1W_vs_E10	ordinary_muscle_development	ENSGALG00010011141	MSX1	-7.823453567	1.119749926	-6.986786412	2.81254E-12	7.27866E-12	negative
PRJNA729271	stage_1W_vs_E10	ordinary_muscle_development	ENSGALG00010011142	DUSP19	0.10280813	0.150683813	0.682277202	0.495063686	0.532933392	positive
PRJNA729271	stage_1W_vs_E10	ordinary_muscle_development	ENSGALG00010011143	AK3	2.34653946	0.163344379	14.36559659	8.5071E-47	8.38846E-46	positive
PRJNA729271	stage_1W_vs_E10	ordinary_muscle_development	ENSGALG00010011144	ATF7IP	-0.309219839	0.063291042	-4.885680947	1.03072E-06	1.92554E-06	negative
PRJNA729271	stage_1W_vs_E10	ordinary_muscle_development	ENSGALG00010011146	BMS1	-0.73669418	0.129175467	-5.703050244	1.17682E-08	2.48587E-08	negative
PRJNA729271	stage_1W_vs_E10	ordinary_muscle_development	ENSGALG00010011148	ENSGALG00010011148	0.124748059	0.183594701	0.679475273	0.49683677	0.534603888	positive
PRJNA729271	stage_1W_vs_E10	ordinary_muscle_development	ENSGALG00010011150	FGD4	1.660262015	0.164608584	10.08612053	6.36349E-24	2.79766E-23	positive
PRJNA729271	stage_1W_vs_E10	ordinary_muscle_development	ENSGALG00010011152	SLC18A3	0.194526753	0.550465457	0.353385941	0.723799125	0.750677469	positive
PRJNA729271	stage_1W_vs_E10	ordinary_muscle_development	ENSGALG00010011154	SLC26A7	-5.364649275	0.702227005	-7.639480165	2.18101E-14	6.27754E-14	negative
PRJNA729271	stage_1W_vs_E10	ordinary_muscle_development	ENSGALG00010011157	ENSGALG00010011157	-4.475157067	1.574356484	-2.842530972	0.004475688	0.006272368	negative
PRJNA729271	stage_1W_vs_E10	ordinary_muscle_development	ENSGALG00010011161	ENSGALG00010011161	-0.164358781	0.095001904	-1.730057762	0.083619956	0.101556033	negative
PRJNA729271	stage_1W_vs_E10	ordinary_muscle_development	ENSGALG00010011166	SOX7	1.234425201	0.140766102	8.769335666	1.79724E-18	6.25623E-18	positive
PRJNA729271	stage_1W_vs_E10	ordinary_muscle_development	ENSGALG00010011170	RBM24	1.325726214	0.142770816	9.285694757	1.60656E-20	6.18513E-20	positive
PRJNA729271	stage_1W_vs_E10	ordinary_muscle_development	ENSGALG00010011175	GPR52	-0.11931631	0.192252899	-0.620621643	0.534848596	0.57126449	negative
PRJNA729271	stage_1W_vs_E10	ordinary_muscle_development	ENSGALG00010011178	RSPO2	-3.976706698	0.324856176	-12.24143786	1.86696E-34	1.24135E-33	negative
PRJNA729271	stage_1W_vs_E10	ordinary_muscle_development	ENSGALG00010011181	ENSGALG00010011181	-0.657304409	0.294390481	-2.232763797	0.025564525	0.033032429	negative
PRJNA729271	stage_1W_vs_E10	ordinary_muscle_development	ENSGALG00010011184	FAM8A1	1.201000736	0.135237091	8.880705203	6.64378E-19	2.37371E-18	positive
PRJNA729271	stage_1W_vs_E10	ordinary_muscle_development	ENSGALG00010011187	STK32B	-8.64988828	0.885049854	-9.773334505	1.46548E-22	6.08656E-22	negative
PRJNA729271	stage_1W_vs_E10	ordinary_muscle_development	ENSGALG00010011190	ENSGALG00010011190	-0.955617613	0.217492196	-4.393801844	1.11385E-05	1.92711E-05	negative
PRJNA729271	stage_1W_vs_E10	ordinary_muscle_development	ENSGALG00010011192	RPL17	0.528100196	0.12349725	4.276210153	1.90102E-05	3.22548E-05	positive
PRJNA729271	stage_1W_vs_E10	ordinary_muscle_development	ENSGALG00010011193	FAM20B	-0.784627628	0.078556033	-9.988126934	1.71797E-23	7.42422E-23	negative
PRJNA729271	stage_1W_vs_E10	ordinary_muscle_development	ENSGALG00010011198	PINX1	-0.89745132	0.068151186	-13.16853553	1.33155E-39	1.05182E-38	negative
PRJNA729271	stage_1W_vs_E10	ordinary_muscle_development	ENSGALG00010011199	ENSGALG00010011199	-4.78603336	1.718897299	-2.784362604	0.005363304	0.007467924	negative
PRJNA729271	stage_1W_vs_E10	ordinary_muscle_development	ENSGALG00010011200	PCDHB4	-1.064156907	0.606044968	-1.755904204	0.079104767	0.096298278	negative
PRJNA729271	stage_1W_vs_E10	ordinary_muscle_development	ENSGALG00010011203	ENSGALG00010011203	-0.729486625	0.105221213	-6.932885536	4.12342E-12	1.05692E-11	negative
PRJNA729271	stage_1W_vs_E10	ordinary_muscle_development	ENSGALG00010011205	CYTL1	-9.683122119	0.996662835	-9.715544497	2.58862E-22	1.06114E-21	negative
PRJNA729271	stage_1W_vs_E10	ordinary_muscle_development	ENSGALG00010011210	NUP153	-0.461092499	0.098430536	-4.684445704	2.80719E-06	5.07485E-06	negative
PRJNA729271	stage_1W_vs_E10	ordinary_muscle_development	ENSGALG00010011211	SLC37A1	0.302406028	0.151251609	1.999357431	0.045569694	0.057203838	positive
PRJNA729271	stage_1W_vs_E10	ordinary_muscle_development	ENSGALG00010011212	NCKAP1	-0.547395344	0.063299892	-8.647650491	5.25706E-18	1.7854E-17	negative
PRJNA729271	stage_1W_vs_E10	ordinary_muscle_development	ENSGALG00010011213	XKR6	-0.286943375	0.191307958	-1.499902976	0.133639537	0.157886143	negative
PRJNA729271	stage_1W_vs_E10	ordinary_muscle_development	ENSGALG00010011218	ENSGALG00010011218	-1.290688952	0.608526064	-2.121008496	0.033921087	0.043175813	negative
PRJNA729271	stage_1W_vs_E10	ordinary_muscle_development	ENSGALG00010011222	RCL1	0.325537759	0.089068592	3.654910794	0.000257271	0.000401891	positive
PRJNA729271	stage_1W_vs_E10	ordinary_muscle_development	ENSGALG00010011231	PLBD1	0.546786946	0.205954211	2.654895689	0.007933294	0.010841245	positive
PRJNA729271	stage_1W_vs_E10	ordinary_muscle_development	ENSGALG00010011235	ENSGALG00010011235	-0.677022816	0.093676478	-7.227244571	4.92892E-13	1.32305E-12	negative
PRJNA729271	stage_1W_vs_E10	ordinary_muscle_development	ENSGALG00010011237	ENSGALG00010011237	-0.770487265	0.266241553	-2.893940695	0.0038044	0.005366366	negative
PRJNA729271	stage_1W_vs_E10	ordinary_muscle_development	ENSGALG00010011239	RALGPS2	-2.135413	0.078185912	-27.31199211	3.0558E-164	3.1414E-162	negative
PRJNA729271	stage_1W_vs_E10	ordinary_muscle_development	ENSGALG00010011243	ALX1	-6.829583691	0.698276931	-9.780623395	1.36368E-22	5.66864E-22	negative
PRJNA729271	stage_1W_vs_E10	ordinary_muscle_development	ENSGALG00010011244	PDE9A	-1.141325905	0.173496764	-6.578369973	4.75634E-11	1.15309E-10	negative
PRJNA729271	stage_1W_vs_E10	ordinary_muscle_development	ENSGALG00010011246	DNAJB5	2.606525459	0.232687861	11.20181107	3.99482E-29	2.17116E-28	positive
PRJNA729271	stage_1W_vs_E10	ordinary_muscle_development	ENSGALG00010011247	ENSGALG00010011247	-0.481515958	0.097222475	-4.952722711	7.31822E-07	1.38158E-06	negative
PRJNA729271	stage_1W_vs_E10	ordinary_muscle_development	ENSGALG00010011250	ENSGALG00010011250	-0.185834713	0.099654983	-1.864780935	0.062212117	0.076739395	negative
PRJNA729271	stage_1W_vs_E10	ordinary_muscle_development	ENSGALG00010011251	FRZB	0.24249646	0.175416147	1.382406717	0.166846855	0.194393148	positive
PRJNA729271	stage_1W_vs_E10	ordinary_muscle_development	ENSGALG00010011253	KDM4C	0.718141112	0.109065993	6.584464068	4.56529E-11	1.10844E-10	positive
PRJNA729271	stage_1W_vs_E10	ordinary_muscle_development	ENSGALG00010011262	ENSGALG00010011262	-1.249224706	0.207658339	-6.015769509	1.79034E-09	3.97899E-09	negative
PRJNA729271	stage_1W_vs_E10	ordinary_muscle_development	ENSGALG00010011267	EVC2	-1.520429863	0.167488553	-9.077813609	1.10778E-19	4.10671E-19	negative
PRJNA729271	stage_1W_vs_E10	ordinary_muscle_development	ENSGALG00010011271	ANGPTL1	-3.098399278	0.163110802	-18.99567187	1.85202E-80	4.47418E-79	negative
PRJNA729271	stage_1W_vs_E10	ordinary_muscle_development	ENSGALG00010011273	RUNX1T1	-1.189183137	0.099531057	-11.94786009	6.66185E-33	4.19659E-32	negative
PRJNA729271	stage_1W_vs_E10	ordinary_muscle_development	ENSGALG00010011274	ENSGALG00010011274	-1.318059851	0.55466819	-2.376303302	0.017487082	0.022984698	negative
PRJNA729271	stage_1W_vs_E10	ordinary_muscle_development	ENSGALG00010011275	WDR4	-0.605010599	0.103934671	-5.821066181	5.84734E-09	1.25921E-08	negative
PRJNA729271	stage_1W_vs_E10	ordinary_muscle_development	ENSGALG00010011278	ENSGALG00010011278	0.668453759	0.524103965	1.275422061	0.202159894	0.232508741	positive
PRJNA729271	stage_1W_vs_E10	ordinary_muscle_development	ENSGALG00010011280	FAM199X	-0.164641485	0.081607875	-2.017470563	0.04364643	0.054913202	negative
PRJNA729271	stage_1W_vs_E10	ordinary_muscle_development	ENSGALG00010011281	ENSGALG00010011281	-1.182335141	0.63596216	-1.859128131	0.063008979	0.077656249	negative
PRJNA729271	stage_1W_vs_E10	ordinary_muscle_development	ENSGALG00010011282	CRY1	1.178373148	0.1268846	9.286967426	1.58747E-20	6.11977E-20	positive
PRJNA729271	stage_1W_vs_E10	ordinary_muscle_development	ENSGALG00010011283	LONRF3	2.364716486	0.139758469	16.92002283	3.20306E-64	5.20498E-63	positive
PRJNA729271	stage_1W_vs_E10	ordinary_muscle_development	ENSGALG00010011285	ENSGALG00010011285	2.891862251	0.2546991	11.35403403	7.08183E-30	3.9911E-29	positive
PRJNA729271	stage_1W_vs_E10	ordinary_muscle_development	ENSGALG00010011287	ENSGALG00010011287	0.379492101	0.11711268	3.240401471	0.001193615	0.001763295	positive
PRJNA729271	stage_1W_vs_E10	ordinary_muscle_development	ENSGALG00010011288	ENSGALG00010011288	-0.639194864	0.30290559	-2.110211519	0.034840141	0.044314482	negative
PRJNA729271	stage_1W_vs_E10	ordinary_muscle_development	ENSGALG00010011293	NDUFV3	1.825733283	0.167269643	10.9149111	9.7825E-28	5.04079E-27	positive
PRJNA729271	stage_1W_vs_E10	ordinary_muscle_development	ENSGALG00010011295	FAM167A	0.029699506	0.363295122	0.081750358	0.934845233	0.942780327	positive
PRJNA729271	stage_1W_vs_E10	ordinary_muscle_development	ENSGALG00010011297	OSR2	-0.276980497	0.184725975	-1.499412832	0.133766567	0.158010462	negative
PRJNA729271	stage_1W_vs_E10	ordinary_muscle_development	ENSGALG00010011300	EVC	-1.200408549	0.16309257	-7.360289611	1.83512E-13	5.03121E-13	negative
PRJNA729271	stage_1W_vs_E10	ordinary_muscle_development	ENSGALG00010011304	COMMD5	-0.903275281	0.093993781	-9.609947305	7.25861E-22	2.93319E-21	negative
PRJNA729271	stage_1W_vs_E10	ordinary_muscle_development	ENSGALG00010011306	ECI2	0.890676296	0.137953709	6.456341787	1.07264E-10	2.54592E-10	positive
PRJNA729271	stage_1W_vs_E10	ordinary_muscle_development	ENSGALG00010011307	ENSGALG00010011307	-1.759580932	0.470768303	-3.737679278	0.000185727	0.000292971	negative
PRJNA729271	stage_1W_vs_E10	ordinary_muscle_development	ENSGALG00010011308	SPIC	4.982898788	1.398029127	3.564231025	0.000364924	0.000562721	positive
PRJNA729271	stage_1W_vs_E10	ordinary_muscle_development	ENSGALG00010011311	DOCK8	-0.412432554	0.193253954	-2.134148076	0.032830662	0.041872454	negative
PRJNA729271	stage_1W_vs_E10	ordinary_muscle_development	ENSGALG00010011312	VPS13B	-0.548960616	0.053496487	-10.2616199	1.04932E-24	4.76946E-24	negative

PRJNA729271	stage_1W_vs_E10	ordinary_muscle_development	ENSGALG00010011313	RASSF9	-1.039533562	0.253784259	-4.09613096	4.20112E-05	6.95152E-05	negative
PRJNA729271	stage_1W_vs_E10	ordinary_muscle_development	ENSGALG00010011316	PARPBP	-2.774338702	0.222906941	-12.4461746	1.4671E-35	1.01749E-34	negative
PRJNA729271	stage_1W_vs_E10	ordinary_muscle_development	ENSGALG00010011317	BLK	0.732199686	0.370186991	1.97791847	0.047937905	0.059979278	positive
PRJNA729271	stage_1W_vs_E10	ordinary_muscle_development	ENSGALG00010011318	PRP4K	-0.087588596	0.09780378	-0.895554295	0.370490856	0.408074844	negative
PRJNA729271	stage_1W_vs_E10	ordinary_muscle_development	ENSGALG00010011321	MAP2	-3.452390865	0.161095529	-21.4307056	6.9125E-102	2.67904E-100	negative
PRJNA729271	stage_1W_vs_E10	ordinary_muscle_development	ENSGALG00010011322	GATA4	-2.258883003	1.243613607	-1.816386529	0.0693111	0.084996988	negative
PRJNA729271	stage_1W_vs_E10	ordinary_muscle_development	ENSGALG00010011323	GUCY2C	0.200566083	0.800131624	0.250666361	0.80207207	0.8230821	positive
PRJNA729271	stage_1W_vs_E10	ordinary_muscle_development	ENSGALG00010011326	COX6C	1.269216199	0.135492887	9.367400947	7.43405E-21	2.8959E-20	positive
PRJNA729271	stage_1W_vs_E10	ordinary_muscle_development	ENSGALG00010011329	PCDHGC5	-1.042111638	0.11730716	-8.883614945	6.4722E-19	2.31298E-18	negative
PRJNA729271	stage_1W_vs_E10	ordinary_muscle_development	ENSGALG00010011333	ENSGALG00010011333	-1.839828507	0.133459984	-13.78561908	3.11086E-43	2.75792E-42	negative
PRJNA729271	stage_1W_vs_E10	ordinary_muscle_development	ENSGALG00010011335	VCP	0.806952847	0.173643463	4.647182416	3.365E-06	6.05081E-06	positive
PRJNA729271	stage_1W_vs_E10	ordinary_muscle_development	ENSGALG00010011339	ENSGALG00010011339	-1.156746981	0.31869656	-3.629618663	0.00028384	0.000441975	negative
PRJNA729271	stage_1W_vs_E10	ordinary_muscle_development	ENSGALG00010011340	PKNOX1	-0.275532856	0.109034893	-2.52701542	0.011503645	0.015437954	negative
PRJNA729271	stage_1W_vs_E10	ordinary_muscle_development	ENSGALG00010011342	RASAL2	-0.050119502	0.067989731	-0.73716282	0.461023341	0.499292687	negative
PRJNA729271	stage_1W_vs_E10	ordinary_muscle_development	ENSGALG00010011343	PXDC1	-0.96558723	0.142083086	-6.795933665	1.07613E-11	2.70058E-11	negative
PRJNA729271	stage_1W_vs_E10	ordinary_muscle_development	ENSGALG00010011345	CRMP1	-2.843432746	0.234852931	-12.10729086	9.66333E-34	6.27554E-33	negative
PRJNA729271	stage_1W_vs_E10	ordinary_muscle_development	ENSGALG00010011347	RGS22	-2.013574037	0.430316361	-4.679287659	2.87873E-06	5.19577E-06	negative
PRJNA729271	stage_1W_vs_E10	ordinary_muscle_development	ENSGALG00010011348	SLC22A23	-0.034558412	0.163689988	-0.211121113	0.832792766	0.85111268	negative
PRJNA729271	stage_1W_vs_E10	ordinary_muscle_development	ENSGALG00010011349	ENSGALG00010011349	-0.621449274	0.091756572	-6.772803958	1.2631E-11	3.15721E-11	negative
PRJNA729271	stage_1W_vs_E10	ordinary_muscle_development	ENSGALG00010011351	KANK1	-2.180349721	0.111761892	-19.50888342	9.22756E-85	2.46323E-83	negative
PRJNA729271	stage_1W_vs_E10	ordinary_muscle_development	ENSGALG00010011352	NTS	-1.403499887	0.558679853	-2.512172003	0.011999059	0.016074525	negative
PRJNA729271	stage_1W_vs_E10	ordinary_muscle_development	ENSGALG00010011353	ENSGALG00010011353	-3.619037198	0.218497399	-16.56329648	1.28364E-61	1.95446E-60	negative
PRJNA729271	stage_1W_vs_E10	ordinary_muscle_development	ENSGALG00010011357	UNC80	-0.729383465	0.424550451	-1.718013638	0.085794128	0.104065764	negative
PRJNA729271	stage_1W_vs_E10	ordinary_muscle_development	ENSGALG00010011359	RNF19A	-0.208717853	0.087042133	-2.3978945	0.016489614	0.021746607	negative
PRJNA729271	stage_1W_vs_E10	ordinary_muscle_development	ENSGALG00010011360	TCF4	0.361438162	0.17301044	2.089111863	0.036697655	0.046546422	positive
PRJNA729271	stage_1W_vs_E10	ordinary_muscle_development	ENSGALG00010011361	NUP37	-1.500917052	0.092704933	-16.19026098	5.90775E-59	8.41187E-58	negative
PRJNA729271	stage_1W_vs_E10	ordinary_muscle_development	ENSGALG00010011363	FDFT1	1.449755661	0.145886905	9.937531101	2.85823E-23	1.22538E-22	positive
PRJNA729271	stage_1W_vs_E10	ordinary_muscle_development	ENSGALG00010011365	PSMG4	-0.217759701	0.179862706	-1.21069957	0.226010567	0.257548992	negative
PRJNA729271	stage_1W_vs_E10	ordinary_muscle_development	ENSGALG00010011367	IER3IP1	0.557325015	0.112821688	4.939874821	7.81727E-07	1.47368E-06	positive
PRJNA729271	stage_1W_vs_E10	ordinary_muscle_development	ENSGALG00010011368	ENSGALG00010011368	-3.38104777	0.334801667	-10.0986587	5.60029E-24	2.46661E-23	negative
PRJNA729271	stage_1W_vs_E10	ordinary_muscle_development	ENSGALG00010011370	MGAT4C	-4.934519345	1.574826278	-3.133373766	0.001728092	0.002519229	negative
PRJNA729271	stage_1W_vs_E10	ordinary_muscle_development	ENSGALG00010011371	ENSGALG00010011371	0.977984658	0.195914446	4.99189661	5.97893E-07	1.1345E-06	positive
PRJNA729271	stage_1W_vs_E10	ordinary_muscle_development	ENSGALG00010011372	VMA21	-0.508645085	0.093028251	-5.46764103	4.56064E-08	9.32654E-08	negative
PRJNA729271	stage_1W_vs_E10	ordinary_muscle_development	ENSGALG00010011374	ENSGALG00010011374	-0.65128516	0.0934895	-6.966399043	3.25156E-12	8.38487E-12	negative
PRJNA729271	stage_1W_vs_E10	ordinary_muscle_development	ENSGALG00010011375	C12orf50	0.565113122	0.137277483	4.116575498	3.84543E-05	6.38264E-05	positive
PRJNA729271	stage_1W_vs_E10	ordinary_muscle_development	ENSGALG00010011377	SEC16B	1.494383893	0.302473622	4.940542859	7.79054E-07	1.46883E-06	positive
PRJNA729271	stage_1W_vs_E10	ordinary_muscle_development	ENSGALG00010011378	TUBB2B	-3.46239872	0.195514254	-17.70918821	3.56172E-70	6.78412E-69	negative
PRJNA729271	stage_1W_vs_E10	ordinary_muscle_development	ENSGALG00010011379	CELF4	-4.518017883	0.917081813	-4.926515628	8.37089E-07	1.57477E-06	negative
PRJNA729271	stage_1W_vs_E10	ordinary_muscle_development	ENSGALG00010011381	RLIG1	-0.195669959	0.120170849	-1.628264765	0.103468757	0.124184784	negative
PRJNA729271	stage_1W_vs_E10	ordinary_muscle_development	ENSGALG00010011382	ENSGALG00010011382	0.009846934	0.197581945	0.049837215	0.96025211	0.965447342	positive
PRJNA729271	stage_1W_vs_E10	ordinary_muscle_development	ENSGALG00010011384	PUM3	-0.732917555	0.081267484	-9.018583055	1.90538E-19	6.97788E-19	negative
PRJNA729271	stage_1W_vs_E10	ordinary_muscle_development	ENSGALG00010011386	CTSB	1.645686966	0.126100491	13.05059917	6.30496E-39	4.88719E-38	positive
PRJNA729271	stage_1W_vs_E10	ordinary_muscle_development	ENSGALG00010011388	GPR50	-0.312990221	0.167462339	-1.869018569	0.061620231	0.076048127	negative
PRJNA729271	stage_1W_vs_E10	ordinary_muscle_development	ENSGALG00010011389	RC3H1	0.643182326	0.085841784	7.492648661	6.74974E-14	1.89865E-13	positive
PRJNA729271	stage_1W_vs_E10	ordinary_muscle_development	ENSGALG00010011394	SERPINC1	4.147997392	0.903409816	4.591490281	4.40092E-06	7.83678E-06	positive
PRJNA729271	stage_1W_vs_E10	ordinary_muscle_development	ENSGALG00010011398	ENSGALG00010011398	0.358549155	0.188105216	1.90610959	0.056635989	0.070249757	positive
PRJNA729271	stage_1W_vs_E10	ordinary_muscle_development	ENSGALG00010011399	JAKMIP1	-3.156190953	0.278029584	-11.35199682	7.24881E-30	4.08203E-29	negative
PRJNA729271	stage_1W_vs_E10	ordinary_muscle_development	ENSGALG00010011400	BPHL	1.710533881	0.197188779	8.674600479	4.15005E-18	1.41908E-17	positive
PRJNA729271	stage_1W_vs_E10	ordinary_muscle_development	ENSGALG00010011401	ANKRD46	-0.762632023	0.069491606	-10.97444816	5.07146E-28	2.64427E-27	negative
PRJNA729271	stage_1W_vs_E10	ordinary_muscle_development	ENSGALG00010011403	SV2C	-5.073427155	0.616259922	-8.23260929	1.83179E-16	5.81383E-16	negative
PRJNA729271	stage_1W_vs_E10	ordinary_muscle_development	ENSGALG00010011406	UBE2R2	-0.331995887	0.116298105	-2.85469731	0.004307786	0.006046418	negative
PRJNA729271	stage_1W_vs_E10	ordinary_muscle_development	ENSGALG00010011407	CEP290	-0.970747884	0.181679469	-5.343189793	9.13251E-08	1.83219E-07	negative
PRJNA729271	stage_1W_vs_E10	ordinary_muscle_development	ENSGALG00010011411	ENSGALG00010011411	-0.581475584	0.227051772	-2.560982368	0.010437665	0.014083027	negative
PRJNA729271	stage_1W_vs_E10	ordinary_muscle_development	ENSGALG00010011412	KANSL1L	0.520126109	0.120467184	4.317575052	1.57753E-05	2.693E-05	positive
PRJNA729271	stage_1W_vs_E10	ordinary_muscle_development	ENSGALG00010011413	ZBTB37	0.620530196	0.104069606	5.962645795	2.48186E-09	5.4656E-09	positive
PRJNA729271	stage_1W_vs_E10	ordinary_muscle_development	ENSGALG00010011414	PABPC1	-0.093472594	0.114249129	-0.818147107	0.413273193	0.451321852	negative
PRJNA729271	stage_1W_vs_E10	ordinary_muscle_development	ENSGALG00010011416	ENSGALG00010011416	0.127414597	0.441889335	0.288340512	0.773086099	0.796268244	positive
PRJNA729271	stage_1W_vs_E10	ordinary_muscle_development	ENSGALG00010011417	WFS1	-0.25681902	0.145576842	-1.764147482	0.077707152	0.094700283	negative
PRJNA729271	stage_1W_vs_E10	ordinary_muscle_development	ENSGALG00010011418	RIPK1	-0.969202989	0.088890122	-10.90338236	1.11051E-27	5.70607E-27	negative
PRJNA729271	stage_1W_vs_E10	ordinary_muscle_development	ENSGALG00010011419	RNF180	-0.028542559	0.150508561	-0.189640765	0.849590641	0.866203583	negative
PRJNA729271	stage_1W_vs_E10	ordinary_muscle_development	ENSGALG00010011420	HMGB3	-1.893776928	0.100020896	-18.9338128	6.00542E-80	1.42469E-78	negative
PRJNA729271	stage_1W_vs_E10	ordinary_muscle_development	ENSGALG00010011421	ENSGALG00010011421	1.185776692	0.116836259	10.14904707	3.34615E-24	1.48462E-23	positive
PRJNA729271	stage_1W_vs_E10	ordinary_muscle_development	ENSGALG00010011422	KATNAL2	-2.259608943	0.260386418	-8.677906308	4.03121E-18	1.37942E-17	negative
PRJNA729271	stage_1W_vs_E10	ordinary_muscle_development	ENSGALG00010011423	GDF6	-0.942869347	0.370358051	-2.545831916	0.010901767	0.014675206	negative
PRJNA729271	stage_1W_vs_E10	ordinary_muscle_development	ENSGALG00010011424	ACADL	1.41678267	0.09711418	14.58883415	3.30802E-48	3.4007E-47	positive
PRJNA729271	stage_1W_vs_E10	ordinary_muscle_development	ENSGALG00010011425	WASHC3	-0.420155849	0.080744595	-5.203516693	1.95552E-07	3.83252E-07	negative
PRJNA729271	stage_1W_vs_E10	ordinary_muscle_development	ENSGALG00010011426	NQO2	0.896471961	0.126680728	7.076624648	1.47708E-12	3.87656E-12	positive
PRJNA729271	stage_1W_vs_E10	ordinary_muscle_development	ENSGALG00010011427	TMTC3	-0.734327634	0.088241276	-8.321815661	8.6626E-17	2.7866E-16	negative
PRJNA729271	stage_1W_vs_E10	ordinary_muscle_development	ENSGALG00010011428	UQCRB	1.820955309	0.162880324	11.1797132	5.12554E-29	2.77115E-28	positive
PRJNA729271	stage_1W_vs_E10	ordinary_muscle_development	ENSGALG00010011429	UBAP2	-0.027130857	0.107478126	-0.252431431	0.800707608	0.821856449	negative
PRJNA729271	stage_1W_vs_E10	ordinary_muscle_development	ENSGALG00010011430	ENSGALG00010011430	-0.469327741	0.342193722	-1.371526452	0.170210903	0.197978418	negative
PRJNA729271	stage_1W_vs_E10	ordinary_muscle_development	ENSGALG00010011431	DRAM1	2.921343084	0.403483124	7.240310467	4.47659E-13	1.20431E-12	positive
PRJNA729271	stage_1W_vs_E10	ordinary_muscle_development	ENSGALG00010011433	RNASE6	4.869273587	0.331345295	14.69546622	6.89225E-49	7.24461E-48	positive
PRJNA729271	stage_1W_vs_E10	ordinary_muscle_development	ENSGALG00010011434	NPL	0.642563912	0.180222332	3.565395609	0.000363308	0.000560288	positive
PRJNA729271	stage_1W_vs_E10	ordinary_muscle_development	ENSGALG00010011436	UBAP2L2	-0.274832939	2.249523696	-0.122173836			negative
PRJNA729271	stage_1W_vs_E10	ordinary_muscle_development	ENSGALG00010011437	DMRT1	-0.680044093	0.514334537	-1.322182439	0.186107407	0.215276264	negative
PRJNA729271	stage_1W_vs_E10	ordinary_muscle_development	ENSGALG00010011438	ENSGALG00010011438	4.846271678	0.440255391	11.00786448	3.50206E-28	1.83855E-27	positive
PRJNA729271	stage_1W_vs_E10	ordinary_muscle_development	ENSGALG00010011439	ENSGALG00010011439	-0.683929183	0.337372356	-2.02722354	0.042639557	0.053702353	negative
PRJNA729271	stage_1W_vs_E10	ordinary_muscle_development	ENSGALG00010011440	FUNDC1	0.14606794	0.111414947	1.31102642	0.189848838	0.219289099	positive
PRJNA729271	stage_1W_vs_E10	ordinary_muscle_development	ENSGALG00010011441	CD99L2	1.200314215	0.079651095	15.06965156	2.56464E-51	2.94101E-50	positive
PRJNA729271	stage_1W_vs_E10	ordinary_muscle_development	ENSGALG00010011442	ENSGALG00010011442	-0.375361365	2.055089157	-0.182649674	0.855072906	0.87130276	negative
PRJNA729271	stage_1W_vs_E10	ordinary_muscle_development	ENSGALG00010011443	ENSGALG00010011443	-1.162443611	0.170958361	-6.799571581	1.04931E-11	2.63463E-11	negative
PRJNA729271	stage_1W_vs_E10	ordinary_muscle_development	ENSGALG00010011445	MTERF3	0.549789398	0.079537799	6.912303404	4.76847E-12	1.21795E-11	positive
PRJNA729271	stage_1W_vs_E10	ordinary_muscle_development	ENSGALG00010011446	ENSGALG00010011446	-0.311367888	0.228776654	-1.361012513	0.173509735	0.201459757	negative
PRJNA729271	stage_1W_vs_E10	ordinary_muscle_development	ENSGALG00010011447	LAMC1	1.622349453	0.130336955	12.44734812	1.44569E-35	1.00313E-34	positive
PRJNA729271	stage_1W_vs_E10	ordinary_muscle_development	ENSGALG00010011448	AvBD10	3.183118612	2.641784171	1.20491244	0.228237106	0.25982069	positive
PRJNA729271	stage_1W_vs_E10	ordinary_muscle_development	ENSGALG00010011449	DUSP22	-1.107080329	0.145414974	-7.613248484	2.67291E-14	7.67053E-14	negative
PRJNA729271	stage_1W_vs_E10	ordinary_muscle_development	ENSGALG00010011450	PTDSS1	-0.993924956	0.070148421	-14.16888574	1.42748E-45	1.36127E-44	negative
PRJNA729271	stage_1W_vs_E10	ordinary_muscle_development	ENSGALG00010011451	CRHBP	-9.165596078	0.808865017	-11.33142847	9.16969E-30	5.13184E-29	negative
PRJNA729271	stage_1W_vs_E10	ordinary_muscle_development	ENSGALG00010011452	KITLG	1.059287401	0.188701402	5.613564019	1.98201E-08	4.1349E-08	positive
PRJNA729271	stage_1W_vs_E10	ordinary_muscle_development	ENSGALG00010011453	GNPTAB	-0.193585001	0.124546201	-1.554322809	0.120107436	0.142841916	negative
PRJNA729271	stage_1W_vs_E10	ordinary_muscle_development	ENSGALG00010011456	KIAA1328	-1.102381494	0.10077758	-10.93875734	7.52239E-28	3.89418E-27	negative
PRJNA729271	stage_1W_vs_E10	ordinary_muscle_development	ENSGALG00010011457	ENSGALG00010011457	0.831495766	0.268761398	3.093806527	0.001976063	0.00286459	positive
PRJNA729271	stage_1W_vs_E10	ordinary_muscle_development	ENSGALG00010011458	TPGS2	-0.919082897	0.131099212	-7.010590526	2.37314E-12	6.16685E-12	negative
PRJNA729271	stage_1W_vs_E10	ordinary_muscle_development	ENSGALG00010011459	ENSGALG00010011459	-0.269882765	0.285898193	-0.943982058	0.345178817	0.382461929	negative
PRJNA729271	stage_1W_vs_E10	ordinary_muscle_development	ENSGALG00010011460	LAMC2	-1.446695606	0.63147768	-2.290968709	0.021965223	0.028567601	negative
PRJNA729271	stage_1W_vs_E10	ordinary_muscle_development	ENSGALG00010011461	IRF4	-1.095581708	0.411878648	-2.659962378	0.007814938	0.010688576	negative
PRJNA729271	stage_1W_vs_E10	ordinary_muscle_development	ENSGALG00010011463	IMP3	-0.185508539	0.12892444	-1.438893503	0.150180699	0.176193381	negative
PRJNA729271	stage_1W_vs_E10	ordinary_muscle_development	ENSGALG00010011464	FANCG	-2.213040696	0.169911044	-13.02470186	8.85463E-39	6.80529E-38	negative
PRJNA729271	stage_1W_vs_E10	ordinary_muscle_development	ENSGALG00010011465	PPP2R2C	-3.096764634	0.364214851	-8.502576511	1.85428E-17	6.1393E-17	negative
PRJNA729271	stage_1W_vs_E10	ordinary_muscle_development	ENSGALG00010011466	MTMR1	-0.095929029	0.066719937	-1.437786573	0.15049462	0.176518815	negative
PRJNA729271	stage_1W_vs_E10	ordinary_muscle_development	ENSGALG00010011467	ENSGALG00010011467	0.039537961	3.975830487	0.009944579			positive
PRJNA729271	stage_1W_vs_E10	ordinary_muscle_development	ENSGALG00010011468	EFHC2	-1.464420256	0.301215989	-4.861694963	1.16385E-06	2.16671E-06	negative
PRJNA729271	stage_1W_vs_E10	ordinary_muscle_development	ENSGALG00010011469	CPS1	1.537817834	0.331263744	4.642276324	3.44592E-06	6.19171E-06	positive
PRJNA729271	stage_1W_vs_E10	ordinary_muscle_development	ENSGALG00010011471	NMNAT2	-2.687182341	0.430254293	-6.245567756	4.22263E-10	9.6816E-10	negative
PRJNA729271	stage_1W_vs_E10	ordinary_muscle_development	ENSGALG00010011475	NDP	-3.005398875	0.333420649	-9.013835473	1.98973E-19	7.27761E-19	negative
PRJNA729271	stage_1W_vs_E10	ordinary_muscle_development	ENSGALG00010011477	SDC2	-0.042130048	0.167337506	-0.25176692	0.801221228	0.822267184	negative
PRJNA729271	stage_1W_vs_E10	ordinary_muscle_development	ENSGALG00010011478	HDAC3	-0.658285315	0.077472345	-8.497036149	1.94493E-17	6.42913E-17	negative
PRJNA729271	stage_1W_vs_E10	ordinary_muscle_development	ENSGALG00010011480	BLTP3B	-0.023917614	0.064679995	-0.36978379	0.711543594	0.738971441	negative
PRJNA729271	stage_1W_vs_E10	ordinary_muscle_development	ENSGALG00010011482	DMRT2	-0.644579449	0.159445855	-4.042622788	5.28566E-05	8.67871E-05	negative
PRJNA729271	stage_1W_vs_E10	ordinary_muscle_development	ENSGALG00010011484	CHPT1	1.056073172	0.078243226	13.49731118	1.62188E-41	1.3716E-40	positive
PRJNA729271	stage_1W_vs_E10	ordinary_muscle_development	ENSGALG00010011485	MTM1	1.126512147	0.076119793	14.79920145	1.48232E-49	1.59512E-48	positive
PRJNA729271	stage_1W_vs_E10	ordinary_muscle_development	ENSGALG00010011487	MAN2B2	-1.027033252	0.159417931	-6.442394799	1.17603E-10	2.78265E-10	negative
PRJNA729271	stage_1W_vs_E10	ordinary_muscle_development	ENSGALG00010011493	ENSGALG00010011493	1.316582781	0.261650365	5.031840043	4.85795E-07	9.26403E-07	positive
PRJNA729271	stage_1W_vs_E10	ordinary_muscle_development	ENSGALG00010011496	ACTR6	-0.809052538	0.087252431	-9.272550086	1.81748E-20	6.97309E-20	negative
PRJNA729271	stage_1W_vs_E10	ordinary_muscle_development	ENSGALG00010011497	CPQ	0.303870297	0.249857402	1.216174885	0.223918309	0.255445922	positive
PRJNA729271	stage_1W_vs_E10	ordinary_muscle_development	ENSGALG00010011499	SMG7	-0.157482141	0.147459599	-1.067968053	0.285534923	0.320169313	negative
PRJNA729271	stage_1W_vs_E10	ordinary_muscle_development	ENSGALG00010011503	NCF2	-0.223968305	0.416946798	-0.53716279	0.591155172	0.625870587	negative
PRJNA729271	stage_1W_vs_E10	ordinary_muscle_development	ENSGALG00010011505	MYL1	6.772346764	0.152228353	44.4880775	0	0	positive
PRJNA729271	stage_1W_vs_E10	ordinary_muscle_development	ENSGALG00010011509	EXOC2	0.264842809	0.067406486	3.929040442	8.52855E-05	0.000137955	positive
PRJNA729271	stage_1W_vs_E10	ordinary_muscle_development	ENSGALG00010011510	ENSGALG00010011510	0.665552832	0.266829802	2.49429721	0.012620682	0.016869862	positive
PRJNA729271	stage_1W_vs_E10	ordinary_muscle_development	ENSGALG00010011515	SERINC5	-0.436136918	0.086369615	-5.049656849	4.42604E-07	8.46937E-07	negative
PRJNA729271	stage_1W_vs_E10	ordinary_muscle_development	ENSGALG00010011523	PIGO	0.089335135	0.120535264	0.741153517	0.458600364	0.496791201	positive
PRJNA729271	stage_1W_vs_E10	ordinary_muscle_development	ENSGALG00010011525	ENSGALG00010011525	-2.378060429	0.25607835	-9.286456383	1.59511E-20	6.14381E-20	negative
PRJNA729271	stage_1W_vs_E10	ordinary_muscle_development	ENSGALG00010011529	SCYL2	0.428649317	0.082040817	5.22482945	1.74315E-07	3.42418E-07	positive
PRJNA729271	stage_1W_vs_E10	ordinary_muscle_development	ENSGALG00010011530	ARPC5	-1.50116285	0.120316472	-12.47678584	9.99401E-36	6.99484E-35	negative
PRJNA729271	stage_1W_vs_E10	ordinary_muscle_development	ENSGALG00010011531	MTDH	0.534575889	0.071825578	7.442695231	9.86515E-14	2.74253E-13	positive
PRJNA729271	stage_1W_vs_E10	ordinary_muscle_development	ENSGALG00010011533	ENSGALG00010011533	-0.6754488	0.274807017	-2.457902305	0.013975119	0.018604652	negative
PRJNA729271	stage_1W_vs_E10	ordinary_muscle_development	ENSGALG00010011536	FCHSD1	-1.087470129	0.185069949	-5.875995183	4.20311E-09	9.12309E-09	negative
PRJNA729271	stage_1W_vs_E10	ordinary_muscle_development	ENSGALG00010011538	ENSGALG00010011538	2.015138752	0.255565694	7.885012722	3.14503E-15	9.44618E-15	positive
PRJNA729271	stage_1W_vs_E10	ordinary_muscle_development	ENSGALG00010011539	LANCL1	0.11604725	0.106353099	1.091150618	0.275206613	0.309473999	positive

PRJNA729271	stage_1W_vs_E10	ordinary_muscle_development	ENSGALG00010011544	STOML2	0.061409254	0.091790866	0.66901269	0.503487383	0.541157468	positive
PRJNA729271	stage_1W_vs_E10	ordinary_muscle_development	ENSGALG00010011545	USP12-like	-0.442106652	0.076818874	-5.755182632	8.65482E-09	1.84244E-08	negative
PRJNA729271	stage_1W_vs_E10	ordinary_muscle_development	ENSGALG00010011546	MAOA	1.724613081	0.126453294	13.63834047	2.3689E-42	2.04632E-41	positive
PRJNA729271	stage_1W_vs_E10	ordinary_muscle_development	ENSGALG00010011549	KIAA0232	0.926844816	0.143799232	6.44540866	1.15289E-10	2.72969E-10	positive
PRJNA729271	stage_1W_vs_E10	ordinary_muscle_development	ENSGALG00010011551	GMDS	-1.559363577	0.156798598	-9.945009721	2.65148E-23	1.13775E-22	negative
PRJNA729271	stage_1W_vs_E10	ordinary_muscle_development	ENSGALG00010011555	ENSGALG00010011555	0.538596148	4.215378863	0.127769334	0.898331519	0.910199593	positive
PRJNA729271	stage_1W_vs_E10	ordinary_muscle_development	ENSGALG00010011556	LAPTM4B	0.018134399	0.143861142	0.126054882	0.899688481	0.911368874	positive
PRJNA729271	stage_1W_vs_E10	ordinary_muscle_development	ENSGALG00010011558	MYBPC1	-0.032722631	0.559965069	-0.058436916			negative
PRJNA729271	stage_1W_vs_E10	ordinary_muscle_development	ENSGALG00010011559	MTX3	0.07803399	0.127161179	0.613662048	0.539438672	0.575658069	positive
PRJNA729271	stage_1W_vs_E10	ordinary_muscle_development	ENSGALG00010011562	ENSGALG00010011562	-7.928881209	0.892338086	-8.885512493	6.36268E-19	2.27671E-18	negative
PRJNA729271	stage_1W_vs_E10	ordinary_muscle_development	ENSGALG00010011564	ARAP3	1.351337902	0.121583108	11.11452009	1.06622E-28	5.69242E-28	positive
PRJNA729271	stage_1W_vs_E10	ordinary_muscle_development	ENSGALG00010011570	RGL1	-1.161936977	0.137180484	-8.470133213	2.45113E-17	8.07112E-17	negative
PRJNA729271	stage_1W_vs_E10	ordinary_muscle_development	ENSGALG00010011571	ATOSB	0.770461201	0.116435982	6.617036998	3.6647E-11	8.94423E-11	positive
PRJNA729271	stage_1W_vs_E10	ordinary_muscle_development	ENSGALG00010011572	PTCD2	1.178407109	0.101144308	11.65075062	2.27447E-31	1.35561E-30	positive
PRJNA729271	stage_1W_vs_E10	ordinary_muscle_development	ENSGALG00010011573	CMAS	0.079494107	0.178201619	0.446090822	0.655531642	0.686506116	positive
PRJNA729271	stage_1W_vs_E10	ordinary_muscle_development	ENSGALG00010011574	GAS2L3	-0.567759842	0.145929073	-3.890656135	9.99735E-05	0.000160674	negative
PRJNA729271	stage_1W_vs_E10	ordinary_muscle_development	ENSGALG00010011575	ENSGALG00010011575	-2.322118819	0.482716499	-4.810523	1.50536E-06	2.77575E-06	negative
PRJNA729271	stage_1W_vs_E10	ordinary_muscle_development	ENSGALG00010011577	ENSGALG00010011577	0.297517052	0.160474596	1.85398225	0.063741699	0.078512571	positive
PRJNA729271	stage_1W_vs_E10	ordinary_muscle_development	ENSGALG00010011578	MYLK4	3.55129306	0.328471636	10.81156686	3.03442E-27	1.53201E-26	positive
PRJNA729271	stage_1W_vs_E10	ordinary_muscle_development	ENSGALG00010011584	ENSGALG00010011584	-2.84638436	1.020444825	-2.789356456	0.00528129	0.007360798	negative
PRJNA729271	stage_1W_vs_E10	ordinary_muscle_development	ENSGALG00010011586	ENSGALG00010011586	0.319966873	0.253028394	1.264549281	0.206032944	0.236531564	positive
PRJNA729271	stage_1W_vs_E10	ordinary_muscle_development	ENSGALG00010011588	COLGALT2	-0.066427401	0.198476811	-0.334685953	0.737861998	0.763568906	negative
PRJNA729271	stage_1W_vs_E10	ordinary_muscle_development	ENSGALG00010011589	RERG	-2.008397326	0.225735876	-8.897111784	5.73188E-19	2.05652E-18	negative
PRJNA729271	stage_1W_vs_E10	ordinary_muscle_development	ENSGALG00010011593	WRNIP1	-0.815143296	0.103268741	-7.893417593	2.94022E-15	8.842E-15	negative
PRJNA729271	stage_1W_vs_E10	ordinary_muscle_development	ENSGALG00010011595	ENSGALG00010011595	0.525462352	0.163358908	3.216612777	0.001297135	0.001909994	positive
PRJNA729271	stage_1W_vs_E10	ordinary_muscle_development	ENSGALG00010011597	TBC1D14	-0.758940767	0.130988198	-5.793962958	6.87446E-09	1.47252E-08	negative
PRJNA729271	stage_1W_vs_E10	ordinary_muscle_development	ENSGALG00010011601	ANO4	-5.109092408	0.940470217	-5.432487194	5.55739E-08	1.13059E-07	negative
PRJNA729271	stage_1W_vs_E10	ordinary_muscle_development	ENSGALG00010011603	ENSGALG00010011603	0.492404747	0.281229913	1.750897486	0.07996357	0.097294716	positive
PRJNA729271	stage_1W_vs_E10	ordinary_muscle_development	ENSGALG00010011605	APPL2	-1.130056753	0.143156596	-7.893850393	2.93003E-15	8.81321E-15	negative
PRJNA729271	stage_1W_vs_E10	ordinary_muscle_development	ENSGALG00010011607	TSEN15	0.560636388	0.156250412	3.588063421	0.000333143	0.000515854	positive
PRJNA729271	stage_1W_vs_E10	ordinary_muscle_development	ENSGALG00010011609	ENSGALG00010011609	-1.656195442	1.002698451	-1.651738307	0.09858791	0.118710064	negative
PRJNA729271	stage_1W_vs_E10	ordinary_muscle_development	ENSGALG00010011612	ENSGALG00010011612	-1.441427944	0.510464327	-2.823758424	0.004746414	0.006638934	negative
PRJNA729271	stage_1W_vs_E10	ordinary_muscle_development	ENSGALG00010011617	MAMLD1	0.640037293	0.098614875	6.490271309	8.56819E-11	2.04203E-10	positive
PRJNA729271	stage_1W_vs_E10	ordinary_muscle_development	ENSGALG00010011619	PCDH1	0.274326422	0.159699401	1.717767383	0.085839053	0.104111545	positive
PRJNA729271	stage_1W_vs_E10	ordinary_muscle_development	ENSGALG00010011621	WASHC4	0.527078704	0.087947671	5.993094515	2.05885E-09	4.55689E-09	positive
PRJNA729271	stage_1W_vs_E10	ordinary_muscle_development	ENSGALG00010011622	BACH1	0.515274195	0.16004741	3.219509731	0.0012841	0.001891376	positive
PRJNA729271	stage_1W_vs_E10	ordinary_muscle_development	ENSGALG00010011625	ERBB4	2.478270311	0.215992132	11.47389162	1.7845E-30	1.02563E-29	positive
PRJNA729271	stage_1W_vs_E10	ordinary_muscle_development	ENSGALG00010011626	SERPINB1	1.249159566	0.579317749	2.156259789	0.031063385	0.03971981	positive
PRJNA729271	stage_1W_vs_E10	ordinary_muscle_development	ENSGALG00010011628	MATN2	2.053181756	0.191762874	10.70687833	9.44946E-27	4.66996E-26	positive
PRJNA729271	stage_1W_vs_E10	ordinary_muscle_development	ENSGALG00010011630	DELE1	0.699658004	0.100793576	6.941494029	3.87975E-12	9.96114E-12	positive
PRJNA729271	stage_1W_vs_E10	ordinary_muscle_development	ENSGALG00010011633	TADA2B	-0.417068203	0.087978787	-4.740554116	2.13135E-06	3.88162E-06	negative
PRJNA729271	stage_1W_vs_E10	ordinary_muscle_development	ENSGALG00010011634	AVD	7.422530484	0.882971461	8.406308487	4.23117E-17	1.37575E-16	positive
PRJNA729271	stage_1W_vs_E10	ordinary_muscle_development	ENSGALG00010011635	PCDH12	-0.323580096	0.19699271	-1.642599346	0.100465856	0.120780674	negative
PRJNA729271	stage_1W_vs_E10	ordinary_muscle_development	ENSGALG00010011636	GRIK1	-0.731416349	0.401938982	-1.819719861	0.068801677	0.084400839	negative
PRJNA729271	stage_1W_vs_E10	ordinary_muscle_development	ENSGALG00010011637	CECR2	-0.040266267	0.155366885	-0.259168917	0.795504913	0.817152839	negative
PRJNA729271	stage_1W_vs_E10	ordinary_muscle_development	ENSGALG00010011638	GRPEL1	0.56864901	0.081302311	6.994253919	2.66674E-12	6.90628E-12	positive
PRJNA729271	stage_1W_vs_E10	ordinary_muscle_development	ENSGALG00010011639	ENSGALG00010011639	-0.502982997	0.147068827	-3.420051736	0.000626092	0.000948298	negative
PRJNA729271	stage_1W_vs_E10	ordinary_muscle_development	ENSGALG00010011641	ENSGALG00010011641	-2.309607738	0.645192756	-3.579717405	0.000343966	0.000531874	negative
PRJNA729271	stage_1W_vs_E10	ordinary_muscle_development	ENSGALG00010011643	C1orf21	0.875703922	0.080240972	10.91342619	9.94366E-28	5.11837E-27	positive
PRJNA729271	stage_1W_vs_E10	ordinary_muscle_development	ENSGALG00010011647	FBXO38	0.53316919	0.082625573	6.452835028	1.09777E-10	2.60513E-10	positive
PRJNA729271	stage_1W_vs_E10	ordinary_muscle_development	ENSGALG00010011648	ENSGALG00010011648	-0.236215397	0.224246389	-1.053374362	0.29216945	0.327153046	negative
PRJNA729271	stage_1W_vs_E10	ordinary_muscle_development	ENSGALG00010011649	MRPS27	0.498980455	0.136760306	3.648576627	0.000263697	0.000411752	positive
PRJNA729271	stage_1W_vs_E10	ordinary_muscle_development	ENSGALG00010011650	RPL30	0.364659527	0.155343655	2.347437535	0.018903042	0.024749309	positive
PRJNA729271	stage_1W_vs_E10	ordinary_muscle_development	ENSGALG00010011651	SERPINB6	-0.314859781	0.129101371	-2.438856993	0.014733798	0.019532321	negative
PRJNA729271	stage_1W_vs_E10	ordinary_muscle_development	ENSGALG00010011653	NR2C1	-0.123201816	0.089432188	-1.377600374	0.168326705	0.195991292	negative
PRJNA729271	stage_1W_vs_E10	ordinary_muscle_development	ENSGALG00010011654	CLDN8	-2.473455284	1.363757507	-1.813706081	0.069722988	0.085465938	negative
PRJNA729271	stage_1W_vs_E10	ordinary_muscle_development	ENSGALG00010011655	SORCS2	-1.227105719	0.18566476	-6.609254859	3.86259E-11	9.41771E-11	negative
PRJNA729271	stage_1W_vs_E10	ordinary_muscle_development	ENSGALG00010011657	ALDH1L2	0.996568586	0.142236124	7.006438033	2.44462E-12	6.34576E-12	positive
PRJNA729271	stage_1W_vs_E10	ordinary_muscle_development	ENSGALG00010011658	TENM1	-2.106339331	0.320992519	-6.561957691	5.31059E-11	1.28231E-10	negative
PRJNA729271	stage_1W_vs_E10	ordinary_muscle_development	ENSGALG00010011659	CD14	0.921207366	0.155217432	5.934947855	2.93939E-09	6.44283E-09	positive
PRJNA729271	stage_1W_vs_E10	ordinary_muscle_development	ENSGALG00010011660	ENSGALG00010011660	-0.149005942	0.085537607	-1.741993348	0.081509608	0.099109284	negative
PRJNA729271	stage_1W_vs_E10	ordinary_muscle_development	ENSGALG00010011662	RIDA	-1.35045498	0.173373735	-7.789270842	6.7397E-15	1.99371E-14	negative
PRJNA729271	stage_1W_vs_E10	ordinary_muscle_development	ENSGALG00010011663	UTP20	-0.336381469	0.137136722	-2.452891281	0.014171316	0.018838337	negative
PRJNA729271	stage_1W_vs_E10	ordinary_muscle_development	ENSGALG00010011664	SERPINB10B	2.009414418	0.5580135	3.601013987	0.000316979	0.00049198	positive
PRJNA729271	stage_1W_vs_E10	ordinary_muscle_development	ENSGALG00010011667	ENSGALG00010011667	-0.513893287	0.411925308	-1.247539973	0.212199573	0.243187287	negative
PRJNA729271	stage_1W_vs_E10	ordinary_muscle_development	ENSGALG00010011669	ENSGALG00010011669	-6.738318945	0.799051257	-8.432899496	3.37204E-17	1.10259E-16	negative
PRJNA729271	stage_1W_vs_E10	ordinary_muscle_development	ENSGALG00010011670	TMCO6	0.44037841	0.170449201	2.583634352	0.009776538	0.013230271	positive
PRJNA729271	stage_1W_vs_E10	ordinary_muscle_development	ENSGALG00010011671	SLC41A2	-2.147034134	0.197451669	-10.87371987	1.538E-27	7.85803E-27	negative
PRJNA729271	stage_1W_vs_E10	ordinary_muscle_development	ENSGALG00010011673	POP1	-1.487872037	0.127392071	-11.67947126	1.62302E-31	9.76169E-31	negative
PRJNA729271	stage_1W_vs_E10	ordinary_muscle_development	ENSGALG00010011674	ENSGALG00010011674	-2.097666505	0.521119401	-4.025308789	5.69006E-05	9.32159E-05	negative
PRJNA729271	stage_1W_vs_E10	ordinary_muscle_development	ENSGALG00010011677	EDEM3	-0.312839662	0.080343449	-3.893779327	9.86944E-05	0.000158776	negative
PRJNA729271	stage_1W_vs_E10	ordinary_muscle_development	ENSGALG00010011679	OVAL	-7.894812411	1.214507677	-6.500421993	8.0095E-11	1.91139E-10	negative
PRJNA729271	stage_1W_vs_E10	ordinary_muscle_development	ENSGALG00010011681	MAP1B	-3.194115978	0.359480479	-8.885366984	6.37101E-19	2.27907E-18	negative
PRJNA729271	stage_1W_vs_E10	ordinary_muscle_development	ENSGALG00010011682	USP15	1.640648084	0.122378413	13.40635197	5.55025E-41	4.59195E-40	positive
PRJNA729271	stage_1W_vs_E10	ordinary_muscle_development	ENSGALG00010011683	NIPAL2	-1.753448539	0.29237075	-5.997345971	2.00568E-09	4.44125E-09	negative
PRJNA729271	stage_1W_vs_E10	ordinary_muscle_development	ENSGALG00010011685	NDUFA2	1.627575686	0.11111213	14.64804692	1.38644E-48	1.44475E-47	positive
PRJNA729271	stage_1W_vs_E10	ordinary_muscle_development	ENSGALG00010011688	PDE3A	0.080453086	0.147885555	0.544022616	0.58642591	0.621680945	positive
PRJNA729271	stage_1W_vs_E10	ordinary_muscle_development	ENSGALG00010011692	ARL1	-0.701048541	0.089902793	-7.797850493	6.29706E-15	1.86391E-14	negative
PRJNA729271	stage_1W_vs_E10	ordinary_muscle_development	ENSGALG00010011696	ZFC3H1	0.327593486	0.109217333	2.999464241	0.002704549	0.003869643	positive
PRJNA729271	stage_1W_vs_E10	ordinary_muscle_development	ENSGALG00010011699	TIAM1	-2.357223619	0.148446777	-15.87925088	8.82258E-57	1.18851E-55	negative
PRJNA729271	stage_1W_vs_E10	ordinary_muscle_development	ENSGALG00010011702	NUP205	-0.317330319	0.083753835	-3.788845225	0.000151349	0.000240364	negative
PRJNA729271	stage_1W_vs_E10	ordinary_muscle_development	ENSGALG00010011703	YWHAZ	-1.268535637	0.094520938	-13.42068396	4.57471E-41	3.80003E-40	negative
PRJNA729271	stage_1W_vs_E10	ordinary_muscle_development	ENSGALG00010011708	IK	0.366538572	0.091540747	4.004102923	6.22533E-05	0.000101789	positive
PRJNA729271	stage_1W_vs_E10	ordinary_muscle_development	ENSGALG00010011712	DCAF12L2	0.067891962	0.076201138	0.890957329	0.372952063	0.410567339	positive
PRJNA729271	stage_1W_vs_E10	ordinary_muscle_development	ENSGALG00010011713	AVPR1A	-3.808179866	0.896054787	-4.249940878	2.13827E-05	3.61406E-05	negative
PRJNA729271	stage_1W_vs_E10	ordinary_muscle_development	ENSGALG00010011715	SLCO1B3	2.493592348	1.746279799	1.427945481	0.153307551	0.179542131	positive
PRJNA729271	stage_1W_vs_E10	ordinary_muscle_development	ENSGALG00010011720	IKZF2	0.50997971	0.174254228	2.926641824	0.003426432	0.004859225	positive
PRJNA729271	stage_1W_vs_E10	ordinary_muscle_development	ENSGALG00010011723	SPRY3	2.010096611	0.155905876	12.89301377	4.92797E-38	3.67821E-37	positive
PRJNA729271	stage_1W_vs_E10	ordinary_muscle_development	ENSGALG00010011724	ARHGEF28	-0.4775209	0.152228586	-3.136867476	0.001707633	0.002490656	negative
PRJNA729271	stage_1W_vs_E10	ordinary_muscle_development	ENSGALG00010011726	WDR55	-0.062322521	0.085092052	-0.732412952	0.463916556	0.502163429	negative
PRJNA729271	stage_1W_vs_E10	ordinary_muscle_development	ENSGALG00010011729	FAM129A	-1.527463509	0.232798885	-6.561300785	5.33404E-11	1.28754E-10	negative
PRJNA729271	stage_1W_vs_E10	ordinary_muscle_development	ENSGALG00010011734	ENSGALG00010011734	-2.371027305	0.30943831	-7.662358637	1.82549E-14	5.28259E-14	negative
PRJNA729271	stage_1W_vs_E10	ordinary_muscle_development	ENSGALG00010011738	FGD6	0.521309203	0.117545734	4.434947892	9.20946E-06	1.60253E-05	positive
PRJNA729271	stage_1W_vs_E10	ordinary_muscle_development	ENSGALG00010011745	AFAP1L1	-0.257444196	0.19210736	-1.340105844	0.180210936	0.20882225	negative
PRJNA729271	stage_1W_vs_E10	ordinary_muscle_development	ENSGALG00010011746	SOD1	0.706122395	0.14234011	4.960811071	7.01994E-07	1.32648E-06	positive
PRJNA729271	stage_1W_vs_E10	ordinary_muscle_development	ENSGALG00010011747	UTP15	-0.302962247	0.116426201	-2.602182702	0.009263249	0.012570995	negative
PRJNA729271	stage_1W_vs_E10	ordinary_muscle_development	ENSGALG00010011751	ZNF706	1.747406001	0.10931474	15.98509043	1.62331E-57	2.23032E-56	positive
PRJNA729271	stage_1W_vs_E10	ordinary_muscle_development	ENSGALG00010011752	RNF2	-2.238503534	0.128489226	-17.42172167	5.64526E-68	1.01776E-66	negative
PRJNA729271	stage_1W_vs_E10	ordinary_muscle_development	ENSGALG00010011758	SERPINB11	0.019414062	0.523923346	0.037055157	0.970441027	0.974542239	positive
PRJNA729271	stage_1W_vs_E10	ordinary_muscle_development	ENSGALG00010011759	CREB3	0.588673566	0.130218723	4.520652265	6.16494E-06	1.08461E-05	positive
PRJNA729271	stage_1W_vs_E10	ordinary_muscle_development	ENSGALG00010011764	PCYOX1L	-0.733064512	0.134147203	-5.46462761	4.6388E-08	9.48235E-08	negative
PRJNA729271	stage_1W_vs_E10	ordinary_muscle_development	ENSGALG00010011770	CHST11	-4.182201656	0.315033525	-13.27541776	3.21473E-40	2.59163E-39	negative
PRJNA729271	stage_1W_vs_E10	ordinary_muscle_development	ENSGALG00010011772	GRHL2	-3.10086884	0.471093211	-6.582283015	4.63279E-11	1.12445E-10	negative
PRJNA729271	stage_1W_vs_E10	ordinary_muscle_development	ENSGALG00010011774	ANKRA2	-0.402433557	0.093760065	-4.292163811	1.7694E-05	3.01204E-05	negative
PRJNA729271	stage_1W_vs_E10	ordinary_muscle_development	ENSGALG00010011775	VEZT	0.553378575	0.079671849	6.945722758	3.76528E-12	9.67518E-12	positive
PRJNA729271	stage_1W_vs_E10	ordinary_muscle_development	ENSGALG00010011776	IL17RA	-1.274748347	0.139513096	-9.137123223	6.41357E-20	2.40157E-19	negative
PRJNA729271	stage_1W_vs_E10	ordinary_muscle_development	ENSGALG00010011780	SERPINB5	-7.052003776	1.038713458	-6.789171471	1.12779E-11	2.82777E-11	negative
PRJNA729271	stage_1W_vs_E10	ordinary_muscle_development	ENSGALG00010011784	GRPEL2	0.566829312	0.133819661	4.235770053	2.2777E-05	3.84434E-05	positive
PRJNA729271	stage_1W_vs_E10	ordinary_muscle_development	ENSGALG00010011789	TXNRD1	-1.043816907	0.136565373	-7.64334973	2.11642E-14	6.09528E-14	negative
PRJNA729271	stage_1W_vs_E10	ordinary_muscle_development	ENSGALG00010011790	AMY2A	3.614588236	1.107658271	3.263270207	0.001101345	0.001633277	positive
PRJNA729271	stage_1W_vs_E10	ordinary_muscle_development	ENSGALG00010011792	HDHD5	1.74713066	0.140750284	12.41298145	2.22221E-35	1.53094E-34	positive
PRJNA729271	stage_1W_vs_E10	ordinary_muscle_development	ENSGALG00010011793	TLN1	-0.015415394	0.114185399	-0.135003199	0.892609345	0.904591516	negative
PRJNA729271	stage_1W_vs_E10	ordinary_muscle_development	ENSGALG00010011796	METAP2	0.479039372	0.089264344	5.366525408	8.02679E-08	1.61595E-07	positive
PRJNA729271	stage_1W_vs_E10	ordinary_muscle_development	ENSGALG00010011797	SCAF4	0.339108083	0.155962088	2.174298169	0.02968275	0.038061877	positive
PRJNA729271	stage_1W_vs_E10	ordinary_muscle_development	ENSGALG00010011802	VPS4B	-0.07223111	0.09297415	-0.776894546	0.437220989	0.475183192	negative
PRJNA729271	stage_1W_vs_E10	ordinary_muscle_development	ENSGALG00010011808	SMARCA2	0.414406219	0.122669741	3.378226897	0.000729549	0.001100053	positive
PRJNA729271	stage_1W_vs_E10	ordinary_muscle_development	ENSGALG00010011810	FGFR4	-0.304163229	0.126072523	-2.412605229	0.015838965	0.020920885	negative
PRJNA729271	stage_1W_vs_E10	ordinary_muscle_development	ENSGALG00010011814	ENSGALG00010011814	4.491834613	0.523890621	8.573993189	9.99573E-18	3.35157E-17	positive
PRJNA729271	stage_1W_vs_E10	ordinary_muscle_development	ENSGALG00010011817	KDSR	0.578077523	0.089850438	6.433775247	1.24473E-10	2.93993E-10	positive
PRJNA729271	stage_1W_vs_E10	ordinary_muscle_development	ENSGALG00010011819	USP44	0.626012482	0.13953349	4.486467603	7.24137E-06	1.26798E-05	positive
PRJNA729271	stage_1W_vs_E10	ordinary_muscle_development	ENSGALG00010011823	ENSGALG00010011823	-3.008285365	0.642409949	-4.682812538	2.82965E-06	5.11164E-06	negative
PRJNA729271	stage_1W_vs_E10	ordinary_muscle_development	ENSGALG00010011825	TAFA2	-6.101665888	0.490333751	-12.44390351	1.50943E-35	1.04585E-34	negative
PRJNA729271	stage_1W_vs_E10	ordinary_muscle_development	ENSGALG00010011837	VLDLR	2.054472306	0.134406011	15.28556865	9.54264E-53	1.15556E-51	positive
PRJNA729271	stage_1W_vs_E10	ordinary_muscle_development	ENSGALG00010011839	CBS	-0.914248613	0.460193487	-1.986661348	0.046959938	0.058826748	negative
PRJNA729271	stage_1W_vs_E10	ordinary_muscle_development	ENSGALG00010011841	KCNMB4	-3.681651615	0.276541669	-13.31318941	1.94019E-40	1.57817E-39	negative
PRJNA729271	stage_1W_vs_E10	ordinary_muscle_development	ENSGALG00010011843	BCL2	0.457769153	0.112292859	4.076565114	4.57059E-05	7.5422E-05	positive
PRJNA729271	stage_1W_vs_E10	ordinary_muscle_development	ENSGALG00010011846	UBAP1	0.5348024	0.105188537	5.084227005	3.69126E-07	7.10179E-07	positive
PRJNA729271	stage_1W_vs_E10	ordinary_muscle_development	ENSGALG00010011850	SPAG16	-2.206078485	0.368869347	-5.980650068	2.22249E-09	4.90932E-09	negative
PRJNA729271	stage_1W_vs_E10	ordinary_muscle_development	ENSGALG00010011856	NFYB	0.048713263	0.128771849	0.378291244	0.705214249	0.733136897	positive
PRJNA729271	stage_1W_vs_E10	ordinary_muscle_development	ENSGALG00010011857	ENSGALG00010011857	0.727811377	0.244293499	2.979249879	0.00288955	0.004124068	positive
PRJNA729271	stage_1W_vs_E10	ordinary_muscle_development	ENSGALG00010011859	NTN4	2.263547348	0.200015925	11.31683561	1.08311E-29	6.04533E-29	positive

PRJNA729271	stage_1W_vs_E10	ordinary_muscle_development	ENSGALG00010011860	CECR1	0.311484068	0.157689701	1.975297471	0.0482344	0.060329418	positive
PRJNA729271	stage_1W_vs_E10	ordinary_muscle_development	ENSGALG00010011861	ENSGALG00010011861	-2.746490367	0.55926166	-4.910921961	9.06492E-07	1.70136E-06	negative
PRJNA729271	stage_1W_vs_E10	ordinary_muscle_development	ENSGALG00010011866	KLF8	1.449277349	0.185160299	7.827149532	4.99055E-15	1.48569E-14	positive
PRJNA729271	stage_1W_vs_E10	ordinary_muscle_development	ENSGALG00010011867	ENSGALG00010011867	0.031906149	0.27756913	0.114948479	0.908485959	0.919395656	positive
PRJNA729271	stage_1W_vs_E10	ordinary_muscle_development	ENSGALG00010011868	SNRPF	-1.826824458	0.126361796	-14.45709479	2.26143E-47	2.26691E-46	negative
PRJNA729271	stage_1W_vs_E10	ordinary_muscle_development	ENSGALG00010011870	AvBD6	-0.283301918	1.564309255	-0.181103523	0.856286319	0.872293921	negative
PRJNA729271	stage_1W_vs_E10	ordinary_muscle_development	ENSGALG00010011871	CCT2	-0.80134516	0.097887461	-8.186392305	2.69173E-16	8.46349E-16	negative
PRJNA729271	stage_1W_vs_E10	ordinary_muscle_development	ENSGALG00010011873	RFX3	-0.200911173	0.19379261	-1.036732892	0.299860358	0.335221032	negative
PRJNA729271	stage_1W_vs_E10	ordinary_muscle_development	ENSGALG00010011874	KCNS2	-7.876218279	0.903969005	-8.712929579	2.96121E-18	1.01978E-17	negative
PRJNA729271	stage_1W_vs_E10	ordinary_muscle_development	ENSGALG00010011875	PSD2	-2.196468047	0.298202599	-7.365690492	1.76232E-13	4.83438E-13	negative
PRJNA729271	stage_1W_vs_E10	ordinary_muscle_development	ENSGALG00010011876	AMDHD1	-0.061581663	0.452824441	-0.135994565	0.891825577	0.903860421	negative
PRJNA729271	stage_1W_vs_E10	ordinary_muscle_development	ENSGALG00010011880	U2AF1	-0.167498051	0.156319175	-1.07151315	0.283938761	0.318478089	negative
PRJNA729271	stage_1W_vs_E10	ordinary_muscle_development	ENSGALG00010011881	RIOK2	-0.49892328	0.096462973	-5.172173988	2.31386E-07	4.51527E-07	negative
PRJNA729271	stage_1W_vs_E10	ordinary_muscle_development	ENSGALG00010011882	ENSGALG00010011882	-0.249727515	0.297530251	-0.839334871	0.40128142	0.439451057	negative
PRJNA729271	stage_1W_vs_E10	ordinary_muscle_development	ENSGALG00010011883	HCFC2	0.991751047	0.106809989	9.285190006	1.61419E-20	6.21122E-20	positive
PRJNA729271	stage_1W_vs_E10	ordinary_muscle_development	ENSGALG00010011885	NCALD	-1.563791763	0.215276562	-7.264105992	3.75513E-13	1.0138E-12	negative
PRJNA729271	stage_1W_vs_E10	ordinary_muscle_development	ENSGALG00010011886	NTNG1	-4.512075429	0.221321904	-20.38693573	2.18404E-92	6.94247E-91	negative
PRJNA729271	stage_1W_vs_E10	ordinary_muscle_development	ENSGALG00010011887	PGM5	2.521767953	0.132206106	19.07451957	4.11184E-81	1.0119E-79	positive
PRJNA729271	stage_1W_vs_E10	ordinary_muscle_development	ENSGALG00010011888	CAND1	-1.437632441	0.070989547	-20.25132579	3.45807E-91	1.06196E-89	negative
PRJNA729271	stage_1W_vs_E10	ordinary_muscle_development	ENSGALG00010011891	HAL	0.079261083	0.563133818	0.140749996	0.888067447	0.900555313	positive
PRJNA729271	stage_1W_vs_E10	ordinary_muscle_development	ENSGALG00010011893	LIX1	-2.876041904	0.479988391	-5.99189888	2.07405E-09	4.58772E-09	negative
PRJNA729271	stage_1W_vs_E10	ordinary_muscle_development	ENSGALG00010011894	VWC2L	-1.125676783	0.337602371	-3.334327249	0.00085506	0.001282501	negative
PRJNA729271	stage_1W_vs_E10	ordinary_muscle_development	ENSGALG00010011895	PHF21B	-3.613676205	0.93545283	-3.863023434	0.000111992	0.000179433	negative
PRJNA729271	stage_1W_vs_E10	ordinary_muscle_development	ENSGALG00010011896	SIK1	4.843349849	0.29811304	16.24668899	2.35758E-59	3.39019E-58	positive
PRJNA729271	stage_1W_vs_E10	ordinary_muscle_development	ENSGALG00010011898	ENSGALG00010011898	1.431399268	0.109719682	13.04596624	6.70021E-39	5.18801E-38	positive
PRJNA729271	stage_1W_vs_E10	ordinary_muscle_development	ENSGALG00010011899	PHLPP1	0.016844936	0.085186961	0.197740777	0.843247884	0.860281396	positive
PRJNA729271	stage_1W_vs_E10	ordinary_muscle_development	ENSGALG00010011902	TP53I3	-1.867780494	0.267303171	-6.987498452	2.79831E-12	7.24312E-12	negative
PRJNA729271	stage_1W_vs_E10	ordinary_muscle_development	ENSGALG00010011903	LTA4H	-0.628148522	0.116279369	-5.402063373	6.58787E-08	1.33367E-07	negative
PRJNA729271	stage_1W_vs_E10	ordinary_muscle_development	ENSGALG00010011904	KIF24	-3.961187722	0.341484688	-11.5998985	4.12567E-31	2.43294E-30	negative
PRJNA729271	stage_1W_vs_E10	ordinary_muscle_development	ENSGALG00010011906	ENSGALG00010011906	-4.278684727	0.977114307	-4.378898862	1.1928E-05	2.05854E-05	negative
PRJNA729271	stage_1W_vs_E10	ordinary_muscle_development	ENSGALG00010011907	BIK	-1.179707937	0.25529043	-4.621042534	3.81816E-06	6.82841E-06	negative
PRJNA729271	stage_1W_vs_E10	ordinary_muscle_development	ENSGALG00010011908	ENSGALG00010011908	-1.436103631	0.222769225	-6.446597953	1.14389E-10	2.71014E-10	negative
PRJNA729271	stage_1W_vs_E10	ordinary_muscle_development	ENSGALG00010011909	C8orf48	-1.678491837	0.218844975	-7.669775536	1.72298E-14	4.99091E-14	negative
PRJNA729271	stage_1W_vs_E10	ordinary_muscle_development	ENSGALG00010011911	NUDT2	-0.290927608	0.139760988	-2.081608122	0.037378279	0.047376544	negative
PRJNA729271	stage_1W_vs_E10	ordinary_muscle_development	ENSGALG00010011912	TCP11X2	1.842622112	0.125327657	14.70243799	6.21801E-49	6.55015E-48	positive
PRJNA729271	stage_1W_vs_E10	ordinary_muscle_development	ENSGALG00010011913	CXXC5	0.691088002	0.111356641	6.206077988	5.43232E-10	1.23846E-09	positive
PRJNA729271	stage_1W_vs_E10	ordinary_muscle_development	ENSGALG00010011915	RRM2B	0.966866234	0.120879047	7.998625531	1.25816E-15	3.85234E-15	positive
PRJNA729271	stage_1W_vs_E10	ordinary_muscle_development	ENSGALG00010011916	MCAT	0.369393309	0.097293264	3.79669971	0.000146635	0.000233057	positive
PRJNA729271	stage_1W_vs_E10	ordinary_muscle_development	ENSGALG00010011917	HSF2BP	-0.544249257	0.179992679	-3.023729956	0.002496792	0.003585397	negative
PRJNA729271	stage_1W_vs_E10	ordinary_muscle_development	ENSGALG00010011921	MEGF10	0.176416997	0.167423206	1.053718904	0.292011631	0.327017857	positive
PRJNA729271	stage_1W_vs_E10	ordinary_muscle_development	ENSGALG00010011922	PIK3R1	-0.244844495	0.17325242	-1.413224103	0.157589842	0.184259479	negative
PRJNA729271	stage_1W_vs_E10	ordinary_muscle_development	ENSGALG00010011923	BARD1	-2.658272085	0.167903354	-15.83215594	1.86712E-56	2.49898E-55	negative
PRJNA729271	stage_1W_vs_E10	ordinary_muscle_development	ENSGALG00010011925	RRP1B	-0.187728548	0.446781304	-0.420179953	0.674353998	0.704132056	negative
PRJNA729271	stage_1W_vs_E10	ordinary_muscle_development	ENSGALG00010011926	DYRK2	0.892548384	0.194896032	4.579612897	4.65837E-06	8.27895E-06	positive
PRJNA729271	stage_1W_vs_E10	ordinary_muscle_development	ENSGALG00010011927	ENSGALG00010011927	-1.222292417	0.497927246	-2.454761066	0.014097826	0.018750962	negative
PRJNA729271	stage_1W_vs_E10	ordinary_muscle_development	ENSGALG00010011929	ELK3	-1.205637747	0.1362291	-8.850074981	8.74605E-19	3.09582E-18	negative
PRJNA729271	stage_1W_vs_E10	ordinary_muscle_development	ENSGALG00010011930	TSPO	-0.172380573	0.152065571	-1.133593698	0.256965018	0.29042557	negative
PRJNA729271	stage_1W_vs_E10	ordinary_muscle_development	ENSGALG00010011931	ENSGALG00010011931	0.204711289	1.298192405	0.157689483			positive
PRJNA729271	stage_1W_vs_E10	ordinary_muscle_development	ENSGALG00010011932	VAV3	-0.077718169	0.167130196	-0.465015724	0.641920196	0.674053408	negative
PRJNA729271	stage_1W_vs_E10	ordinary_muscle_development	ENSGALG00010011935	ST8SIA4	-0.577612045	0.127192708	-4.541235543	5.59255E-06	9.87382E-06	negative
PRJNA729271	stage_1W_vs_E10	ordinary_muscle_development	ENSGALG00010011936	CKAP4	-1.949407128	0.175751915	-11.09181158	1.37475E-28	7.30463E-28	negative
PRJNA729271	stage_1W_vs_E10	ordinary_muscle_development	ENSGALG00010011937	SLC1A1	-1.240239056	0.321431206	-3.858489884	0.00011409	0.000182652	negative
PRJNA729271	stage_1W_vs_E10	ordinary_muscle_development	ENSGALG00010011941	ENSGALG00010011941	-3.266012608	0.246919713	-13.22702254	6.12667E-40	4.901E-39	negative
PRJNA729271	stage_1W_vs_E10	ordinary_muscle_development	ENSGALG00010011943	TTLL12	-0.302036187	0.054730304	-5.518627999	3.41657E-08	7.02356E-08	negative
PRJNA729271	stage_1W_vs_E10	ordinary_muscle_development	ENSGALG00010011945	ENSGALG00010011945	0.554953517	0.470010383	1.18072608	0.237711558	0.269948999	positive
PRJNA729271	stage_1W_vs_E10	ordinary_muscle_development	ENSGALG00010011947	THAP1	0.994095237	0.112738183	8.817733334	1.168E-18	4.10926E-18	positive
PRJNA729271	stage_1W_vs_E10	ordinary_muscle_development	ENSGALG00010011949	ABCA12	-2.808845037	0.456381345	-6.154600901	7.52668E-10	1.70361E-09	negative
PRJNA729271	stage_1W_vs_E10	ordinary_muscle_development	ENSGALG00010011950	PDXK	0.290609986	0.165168668	1.75947406	0.078497022	0.095598583	positive
PRJNA729271	stage_1W_vs_E10	ordinary_muscle_development	ENSGALG00010011952	UBE2D2	1.029364556	0.173617995	5.928904764	3.04962E-09	6.67536E-09	positive
PRJNA729271	stage_1W_vs_E10	ordinary_muscle_development	ENSGALG00010011953	CDK17	0.283393764	0.05853848	4.841153464	1.29088E-06	2.39152E-06	positive
PRJNA729271	stage_1W_vs_E10	ordinary_muscle_development	ENSGALG00010011954	ENSGALG00010011954	1.800534735	0.445351743	4.042949786	5.27829E-05	8.66858E-05	positive
PRJNA729271	stage_1W_vs_E10	ordinary_muscle_development	ENSGALG00010011957	ENSGALG00010011957	3.948288239	0.209849578	18.81484959	5.70702E-79	1.31307E-77	positive
PRJNA729271	stage_1W_vs_E10	ordinary_muscle_development	ENSGALG00010011960	CFAP54	-0.89620038	0.297656118	-3.010858253	0.002605104	0.003733906	negative
PRJNA729271	stage_1W_vs_E10	ordinary_muscle_development	ENSGALG00010011961	AGPAT3	0.247197572	0.103453484	2.389456243	0.016873334	0.022214257	positive
PRJNA729271	stage_1W_vs_E10	ordinary_muscle_development	ENSGALG00010011962	CPSF6	-0.892080285	0.060125006	-14.83709257	8.43326E-50	9.1979E-49	negative
PRJNA729271	stage_1W_vs_E10	ordinary_muscle_development	ENSGALG00010011963	ZCCHC2	0.12787386	0.100884061	1.267532835	0.204964831	0.235323978	positive
PRJNA729271	stage_1W_vs_E10	ordinary_muscle_development	ENSGALG00010011964	ENSGALG00010011964	1.050953285	4.312289994	0.24371118	0.807454514	0.827785075	positive
PRJNA729271	stage_1W_vs_E10	ordinary_muscle_development	ENSGALG00010011965	ATIC	0.107192528	0.146903792	0.729678429	0.465586769	0.503896082	positive
PRJNA729271	stage_1W_vs_E10	ordinary_muscle_development	ENSGALG00010011966	SLC25A24	-0.327966115	0.138320133	-2.371065636	0.017736881	0.02329613	negative
PRJNA729271	stage_1W_vs_E10	ordinary_muscle_development	ENSGALG00010011967	MTMR9	0.437535644	0.137260656	3.187626053	0.001434459	0.002105781	positive
PRJNA729271	stage_1W_vs_E10	ordinary_muscle_development	ENSGALG00010011968	ENSGALG00010011968	5.938937538	0.223828593	26.53341769	3.9906E-155	3.7319E-153	positive
PRJNA729271	stage_1W_vs_E10	ordinary_muscle_development	ENSGALG00010011969	TMEM173	2.683296553	0.289168334	9.279358223	1.70502E-20	6.55205E-20	positive
PRJNA729271	stage_1W_vs_E10	ordinary_muscle_development	ENSGALG00010011971	RNF170	-0.454189987	0.09105865	-4.987884042	6.10442E-07	1.15756E-06	negative
PRJNA729271	stage_1W_vs_E10	ordinary_muscle_development	ENSGALG00010011972	IPO11	-0.088217635	0.081475058	-1.082756335	0.278916592	0.313354208	negative
PRJNA729271	stage_1W_vs_E10	ordinary_muscle_development	ENSGALG00010011973	TRAPPC10	0.004253457	0.050469733	0.084277372	0.932835903	0.941277856	positive
PRJNA729271	stage_1W_vs_E10	ordinary_muscle_development	ENSGALG00010011976	TNFRSF11A	0.091685636	0.090261029	1.015783191	0.309732642	0.345564551	positive
PRJNA729271	stage_1W_vs_E10	ordinary_muscle_development	ENSGALG00010011977	GATD3A	1.327002724	0.118252203	11.22180131	3.18712E-29	1.73935E-28	positive
PRJNA729271	stage_1W_vs_E10	ordinary_muscle_development	ENSGALG00010011978	ENSGALG00010011978	-0.127388613	5.456524398	-0.023346109	0.981374192	0.984226037	negative
PRJNA729271	stage_1W_vs_E10	ordinary_muscle_development	ENSGALG00010011979	NEDD1	0.188613245	0.077060686	2.447593644	0.014381375	0.019096558	positive
PRJNA729271	stage_1W_vs_E10	ordinary_muscle_development	ENSGALG00010011980	AvBD9	20.86473358	2.27876776	9.156147437	5.37832E-20	2.01861E-19	positive
PRJNA729271	stage_1W_vs_E10	ordinary_muscle_development	ENSGALG00010011981	SCUBE1	-2.890682319	0.342410632	-8.442151172	3.1155E-17	1.02055E-16	negative
PRJNA729271	stage_1W_vs_E10	ordinary_muscle_development	ENSGALG00010011982	STK3	-1.084445015	0.106103882	-10.22059693	1.6035E-24	7.22948E-24	negative
PRJNA729271	stage_1W_vs_E10	ordinary_muscle_development	ENSGALG00010011983	LYZ	0.347098208	0.569070264	0.609939106	0.541902146	0.5781167	positive
PRJNA729271	stage_1W_vs_E10	ordinary_muscle_development	ENSGALG00010011984	FN1	-1.068523175	0.2403135	-4.44637182	8.73327E-06	1.52168E-05	negative
PRJNA729271	stage_1W_vs_E10	ordinary_muscle_development	ENSGALG00010011987	H2BC32	-2.094944977	0.375460564	-5.579667151	2.40979E-08	4.99427E-08	negative
PRJNA729271	stage_1W_vs_E10	ordinary_muscle_development	ENSGALG00010011988	HINTW	-2.007430663	3.138194232	-0.639676997	0.522382613	0.559268482	negative
PRJNA729271	stage_1W_vs_E10	ordinary_muscle_development	ENSGALG00010011989	H1-010	-0.582618939	0.136763528	-4.260046141	2.04385E-05	3.4577E-05	negative
PRJNA729271	stage_1W_vs_E10	ordinary_muscle_development	ENSGALG00010011991	CSNK1A1	-0.129361924	0.056452574	-2.291515074	0.021933641	0.028531646	negative
PRJNA729271	stage_1W_vs_E10	ordinary_muscle_development	ENSGALG00010011992	YEATS4	-1.323815711	0.087200532	-15.18128021	4.70508E-52	5.54924E-51	negative
PRJNA729271	stage_1W_vs_E10	ordinary_muscle_development	ENSGALG00010011993	RELCH	-0.346572692	0.052992476	-6.540035849	6.15041E-11	1.47722E-10	negative
PRJNA729271	stage_1W_vs_E10	ordinary_muscle_development	ENSGALG00010011996	LRRC70	1.396276988	0.218554324	6.388695329	1.67307E-10	3.92795E-10	positive
PRJNA729271	stage_1W_vs_E10	ordinary_muscle_development	ENSGALG00010011997	DENND5B	-1.082825619	0.137516555	-7.874147377	3.43074E-15	1.0283E-14	negative
PRJNA729271	stage_1W_vs_E10	ordinary_muscle_development	ENSGALG00010011998	MPPED1	-0.80752653	0.076229	-10.59342948	3.19661E-26	1.54759E-25	negative
PRJNA729271	stage_1W_vs_E10	ordinary_muscle_development	ENSGALG00010012001	EEIG2	-0.453578695	0.254598795	-1.781542976	0.074823791	0.091378452	negative
PRJNA729271	stage_1W_vs_E10	ordinary_muscle_development	ENSGALG00010012004	LGR5	-3.936464858	0.212235719	-18.54760775	8.52632E-77	1.86973E-75	negative
PRJNA729271	stage_1W_vs_E10	ordinary_muscle_development	ENSGALG00010012007	MREG	-1.252939968	0.230975969	-5.424546861	5.81017E-08	1.18135E-07	negative
PRJNA729271	stage_1W_vs_E10	ordinary_muscle_development	ENSGALG00010012008	ZNF366	4.145386199	0.176300827	23.51314096	2.9932E-122	1.7285E-120	positive
PRJNA729271	stage_1W_vs_E10	ordinary_muscle_development	ENSGALG00010012010	HENMT1	-1.938866351	0.19685936	-9.848992477	6.92345E-23	2.91561E-22	negative
PRJNA729271	stage_1W_vs_E10	ordinary_muscle_development	ENSGALG00010012011	HOOK3	0.693865787	0.068349165	10.15178147	3.25369E-24	1.44492E-23	positive
PRJNA729271	stage_1W_vs_E10	ordinary_muscle_development	ENSGALG00010012013	FOXD1	-5.734820633	1.040523295	-5.511477409	3.55834E-08	7.30569E-08	negative
PRJNA729271	stage_1W_vs_E10	ordinary_muscle_development	ENSGALG00010012015	ENSGALG00010012015	2.120227384	0.214996508	9.861682911	6.1018E-23	2.57558E-22	positive
PRJNA729271	stage_1W_vs_E10	ordinary_muscle_development	ENSGALG00010012016	WDR91	-0.552050948	0.064922586	-8.503218716	1.84405E-17	6.10821E-17	negative
PRJNA729271	stage_1W_vs_E10	ordinary_muscle_development	ENSGALG00010012017	N4BP3	-0.323032995	0.130007814	-2.484719852	0.012965342	0.017309813	negative
PRJNA729271	stage_1W_vs_E10	ordinary_muscle_development	ENSGALG00010012019	ENSGALG00010012019	7.887222137	0.300530239	26.24435452	8.291E-152	7.0693E-150	positive
PRJNA729271	stage_1W_vs_E10	ordinary_muscle_development	ENSGALG00010012021	ENSGALG00010012021	0.314353637	0.1301468	2.415377391	0.01571891 4	0.020773674	positive
PRJNA729271	stage_1W_vs_E10	ordinary_muscle_development	ENSGALG00010012024	UBR5	0.31841314	0.07576959	4.202386989	2.64115E-05	4.43404E-05	positive
PRJNA729271	stage_1W_vs_E10	ordinary_muscle_development	ENSGALG00010012026	SYT1	-3.574276409	0.282827835	-12.63764017	1.30925E-36	9.35314E-36	negative
PRJNA729271	stage_1W_vs_E10	ordinary_muscle_development	ENSGALG00010012028	TMEM140	4.909128343	0.170243096	28.83599075	7.5934E-183	9.4884E-181	positive
PRJNA729271	stage_1W_vs_E10	ordinary_muscle_development	ENSGALG00010012031	GRK6	-0.306275699	0.109454147	-2.798210103	0.005138667	0.007168221	negative
PRJNA729271	stage_1W_vs_E10	ordinary_muscle_development	ENSGALG00010012034	ARHGEF9	0.971472661	0.150852121	6.439900588	1.19552E-10	2.82831E-10	positive
PRJNA729271	stage_1W_vs_E10	ordinary_muscle_development	ENSGALG00010012035	ENSGALG00010012035	-0.595872158	0.534017176	-1.115829575	0.264495092	0.298238534	negative
PRJNA729271	stage_1W_vs_E10	ordinary_muscle_development	ENSGALG00010012039	ODF1	-0.745093991	0.653131055	-1.14080319	0.253951833	0.287243997	negative
PRJNA729271	stage_1W_vs_E10	ordinary_muscle_development	ENSGALG00010012040	B4GALT7	0.614825039	0.149525609	4.111837706	3.92522E-05	6.51283E-05	positive
PRJNA729271	stage_1W_vs_E10	ordinary_muscle_development	ENSGALG00010012041	ICOSLG	-0.402330981	0.15327995	-2.624811534	0.008669694	0.011799739	negative
PRJNA729271	stage_1W_vs_E10	ordinary_muscle_development	ENSGALG00010012042	FNDC7	-4.265191402	0.47079477	-9.059555609	1.30983E-19	4.83966E-19	negative
PRJNA729271	stage_1W_vs_E10	ordinary_muscle_development	ENSGALG00010012043	NUP50	-0.02945733	0.09571301	-0.307767255	0.75825944	0.782721164	negative
PRJNA729271	stage_1W_vs_E10	ordinary_muscle_development	ENSGALG00010012044	ENSGALG00010012044	1.908678057	0.641694146	2.974435824	0.002935278	0.004186447	positive
PRJNA729271	stage_1W_vs_E10	ordinary_muscle_development	ENSGALG00010012045	ENSGALG00010012045	1.910489145	0.300160096	6.364900505	1.95416E-10	4.5657E-10	positive
PRJNA729271	stage_1W_vs_E10	ordinary_muscle_development	ENSGALG00010012046	LOXHD1	-0.68538316	0.279128835	-2.455436607	0.014071357	0.018719192	negative
PRJNA729271	stage_1W_vs_E10	ordinary_muscle_development	ENSGALG00010012047	LMAN2	0.05502612	0.090873669	0.605523257	0.544831377	0.580985125	positive
PRJNA729271	stage_1W_vs_E10	ordinary_muscle_development	ENSGALG00010012048	PDE6H	-2.77879375	0.659902105	-4.210918149	2.54335E-05	4.27331E-05	negative
PRJNA729271	stage_1W_vs_E10	ordinary_muscle_development	ENSGALG00010012049	IFNAR1	0.415092502	0.145371507	2.855391055	0.004298387	0.006034394	positive
PRJNA729271	stage_1W_vs_E10	ordinary_muscle_development	ENSGALG00010012051	ARK2C	-2.415994573	0.213541695	-11.31392431	1.11967E-29	6.23977E-29	negative
PRJNA729271	stage_1W_vs_E10	ordinary_muscle_development	ENSGALG00010012052	KLF10	2.009803679	0.123020671	16.33712176	5.37301E-60	7.85891E-59	positive
PRJNA729271	stage_1W_vs_E10	ordinary_muscle_development	ENSGALG00010012054	FAM60A	-1.83053979	0.091270516	-20.05620065	1.7819E-89	5.18648E-88	negative
PRJNA729271	stage_1W_vs_E10	ordinary_muscle_development	ENSGALG00010012055	ENSGALG00010012055	1.406219251	0.156348799	8.994116098	2.38141E-19	8.67735E-19	positive
PRJNA729271	stage_1W_vs_E10	ordinary_muscle_development	ENSGALG00010012058	ENSGALG00010012058	-2.54937316	0.333260905	-7.64978166	2.01321E-14	5.80148E-14	negative
PRJNA729271	stage_1W_vs_E10	ordinary_muscle_development	ENSGALG00010012059	ARHGDIB	-1.002987089	0.146766705	-6.833887089	8.26441E-12	2.08807E-11	negative
PRJNA729271	stage_1W_vs_E10	ordinary_muscle_development	ENSGALG00010012060	ENSGALG00010012060	1.71672956	0.123953671	13.84976776	1.27624E-43	1.14333E-42	positive
PRJNA729271	stage_1W_vs_E10	ordinary_muscle_development	ENSGALG00010012061	AZIN1	1.704365927	0.120048025	14.19736746	9.5119E-46	9.08866E-45	positive
PRJNA729271	stage_1W_vs_E10	ordinary_muscle_development	ENSGALG00010012062	HMGA2	-5.498427361	0.168860493	-32.5619526	1.4183E-232	3.0234E-230	negative

PRJNA729271	stage_1W_vs_E10	ordinary_muscle_development	ENSGALG00010012063	PTPRD	-1.629034379	0.198035385	-8.225976271	1.93607E-16	6.13407E-16	negative
PRJNA729271	stage_1W_vs_E10	ordinary_muscle_development	ENSGALG00010012064	RGS14	-1.111922253	0.177909053	-6.249947567	4.10591E-10	9.42142E-10	negative
PRJNA729271	stage_1W_vs_E10	ordinary_muscle_development	ENSGALG00010012066	STXBP3	-0.849582376	0.12418887	-6.841050857	7.86143E-12	1.98799E-11	negative
PRJNA729271	stage_1W_vs_E10	ordinary_muscle_development	ENSGALG00010012067	ENSGALG00010012067	0.116633151	0.083897623	1.390184225	0.164472943	0.191843188	positive
PRJNA729271	stage_1W_vs_E10	ordinary_muscle_development	ENSGALG00010012069	ERP27	0.015606606	0.512709461	0.030439473	0.975716564	0.979297299	positive
PRJNA729271	stage_1W_vs_E10	ordinary_muscle_development	ENSGALG00010012070	DNAJC28	0.928900086	0.160070617	5.80306431	6.51138E-09	1.39784E-08	positive
PRJNA729271	stage_1W_vs_E10	ordinary_muscle_development	ENSGALG00010012071	ENSGALG00010012071	-0.936259519	0.152266247	-6.148831653	7.80558E-10	1.76481E-09	negative
PRJNA729271	stage_1W_vs_E10	ordinary_muscle_development	ENSGALG00010012072	CAPRIN2	-0.20675722	0.151294145	-1.36659102	0.171753534	0.199644544	negative
PRJNA729271	stage_1W_vs_E10	ordinary_muscle_development	ENSGALG00010012074	KIAA0930	-0.715834316	0.089486117	-7.999389631	1.25037E-15	3.82932E-15	negative
PRJNA729271	stage_1W_vs_E10	ordinary_muscle_development	ENSGALG00010012075	PIGN	-0.316376048	0.097000012	-3.261608351	0.001107821	0.0016418	negative
PRJNA729271	stage_1W_vs_E10	ordinary_muscle_development	ENSGALG00010012077	IFNGR2	0.507458315	0.06897255	7.357395263	1.87533E-13	5.13759E-13	positive
PRJNA729271	stage_1W_vs_E10	ordinary_muscle_development	ENSGALG00010012078	TMBIM4	-0.241944428	0.083311452	-2.904095691	0.003683156	0.005204967	negative
PRJNA729271	stage_1W_vs_E10	ordinary_muscle_development	ENSGALG00010012079	ENSGALG00010012079	-0.859212981	0.373880835	-2.298093137	0.021556489	0.028076315	negative
PRJNA729271	stage_1W_vs_E10	ordinary_muscle_development	ENSGALG00010012081	CHIC1	-0.076026019	0.125598782	-0.605308568	0.544973989	0.581094451	negative
PRJNA729271	stage_1W_vs_E10	ordinary_muscle_development	ENSGALG00010012082	HAUS1	-2.8968426	0.220188025	-13.15622226	1.56728E-39	1.23668E-38	negative
PRJNA729271	stage_1W_vs_E10	ordinary_muscle_development	ENSGALG00010012083	MGP	4.242200394	0.27766272	15.2782498	1.0677E-52	1.29077E-51	positive
PRJNA729271	stage_1W_vs_E10	ordinary_muscle_development	ENSGALG00010012084	TMEM50B	0.359614619	0.106071223	3.390312749	0.000698129	0.001053773	positive
PRJNA729271	stage_1W_vs_E10	ordinary_muscle_development	ENSGALG00010012085	IPO8	-0.271046777	0.096805069	-2.799923394	0.005111474	0.007132346	negative
PRJNA729271	stage_1W_vs_E10	ordinary_muscle_development	ENSGALG00010012086	CIBAR1	-1.709927409	0.134784436	-12.68638616	7.0358E-37	5.06375E-36	negative
PRJNA729271	stage_1W_vs_E10	ordinary_muscle_development	ENSGALG00010012087	TMEM171	0.851489208	0.168498634	5.053389374	4.34038E-07	8.30874E-07	positive
PRJNA729271	stage_1W_vs_E10	ordinary_muscle_development	ENSGALG00010012088	ATP5F1AZ	1.176359274	0.169855975	6.925627856	4.34046E-12	1.11197E-11	positive
PRJNA729271	stage_1W_vs_E10	ordinary_muscle_development	ENSGALG00010012089	ENSGALG00010012089	-1.849636574	0.286792447	-6.44939082	1.12301E-10	2.66285E-10	negative
PRJNA729271	stage_1W_vs_E10	ordinary_muscle_development	ENSGALG00010012090	HELB	0.39744565	0.223508711	1.778211009	0.075369205	0.09198254	positive
PRJNA729271	stage_1W_vs_E10	ordinary_muscle_development	ENSGALG00010012091	ENSGALG00010012091	0.513348891	0.442630398	1.159768721	0.246142982	0.27882483	positive
PRJNA729271	stage_1W_vs_E10	ordinary_muscle_development	ENSGALG00010012092	OC3	8.421278101	0.698144531	12.06237065	1.66908E-33	1.07288E-32	positive
PRJNA729271	stage_1W_vs_E10	ordinary_muscle_development	ENSGALG00010012093	ENSGALG00010012093	-1.73269238	0.069650899	-24.87681276	1.3262E-136	9.154E-135	negative
PRJNA729271	stage_1W_vs_E10	ordinary_muscle_development	ENSGALG00010012094	ENSGALG00010012094	-0.036617965	0.343863192	-0.106489923	0.91519365	0.925602286	negative
PRJNA729271	stage_1W_vs_E10	ordinary_muscle_development	ENSGALG00010012095	RNF38	-0.515551479	0.090465309	-5.698885931	1.20593E-08	2.54476E-08	negative
PRJNA729271	stage_1W_vs_E10	ordinary_muscle_development	ENSGALG00010012096	ENSGALG00010012096	-2.047414994	0.166934289	-12.26479595	1.39964E-34	9.35786E-34	negative
PRJNA729271	stage_1W_vs_E10	ordinary_muscle_development	ENSGALG00010012097	GART	-0.011997238	0.192197069	-0.062421547	0.950227136	0.956761763	negative
PRJNA729271	stage_1W_vs_E10	ordinary_muscle_development	ENSGALG00010012098	ART4	1.426092366	0.54904727	2.597394511	0.009393397	0.01273862	positive
PRJNA729271	stage_1W_vs_E10	ordinary_muscle_development	ENSGALG00010012099	UPK3A	-5.026599544	0.555365281	-9.050979095	1.41692E-19	5.22602E-19	negative
PRJNA729271	stage_1W_vs_E10	ordinary_muscle_development	ENSGALG00010012100	UBE2F	1.205683239	0.111116921	10.85058181	1.9816E-27	1.00783E-26	positive
PRJNA729271	stage_1W_vs_E10	ordinary_muscle_development	ENSGALG00010012101	ENSGALG00010012101	-2.119750313	0.138912721	-15.2595838	1.42152E-52	1.71565E-51	negative
PRJNA729271	stage_1W_vs_E10	ordinary_muscle_development	ENSGALG00010012102	SON	0.716499194	0.104817529	6.835681056	8.16163E-12	2.06246E-11	positive
PRJNA729271	stage_1W_vs_E10	ordinary_muscle_development	ENSGALG00010012103	ENSGALG00010012103	1.639997254	0.207185118	7.915613206	2.46037E-15	7.42518E-15	positive
PRJNA729271	stage_1W_vs_E10	ordinary_muscle_development	ENSGALG00010012105	DONSON	-1.993289568	0.210738087	-9.458610917	3.12061E-21	1.23487E-20	negative
PRJNA729271	stage_1W_vs_E10	ordinary_muscle_development	ENSGALG00010012106	TMTC1	-0.817687066	0.139952344	-5.842610715	5.1389E-09	1.11011E-08	negative
PRJNA729271	stage_1W_vs_E10	ordinary_muscle_development	ENSGALG00010012107	CLCC1	0.065500801	0.085262496	0.768225241	0.44235338	0.480483176	positive
PRJNA729271	stage_1W_vs_E10	ordinary_muscle_development	ENSGALG00010012108	PSTPIP2	-0.715816912	0.350886533	-2.040023896	0.041347946	0.052157208	negative
PRJNA729271	stage_1W_vs_E10	ordinary_muscle_development	ENSGALG00010012110	ENSGALG00010012110	0.374157941	0.113490984	3.296807618	0.000977905	0.001458155	positive
PRJNA729271	stage_1W_vs_E10	ordinary_muscle_development	ENSGALG00010012111	DRD1	-0.320235058	0.332219422	-0.963926359	0.335082848	0.371986358	negative
PRJNA729271	stage_1W_vs_E10	ordinary_muscle_development	ENSGALG00010012112	MBD2	2.235234202	0.189433209	11.79959003	3.92218E-32	2.40389E-31	positive
PRJNA729271	stage_1W_vs_E10	ordinary_muscle_development	ENSGALG00010012113	WDR47	-0.815776456	0.096662212	-8.43945572	3.18819E-17	1.04341E-16	negative
PRJNA729271	stage_1W_vs_E10	ordinary_muscle_development	ENSGALG00010012114	TAL2	0.410215461	0.310875859	1.319547494	0.186986143	0.216223705	positive
PRJNA729271	stage_1W_vs_E10	ordinary_muscle_development	ENSGALG00010012116	RBM12B	-0.544046867	0.078207992	-6.956410089	3.49053E-12	8.98672E-12	negative
PRJNA729271	stage_1W_vs_E10	ordinary_muscle_development	ENSGALG00010012118	ENSGALG00010012118	-1.470939787	0.803674392	-1.830268329	0.067209825	0.08258086	negative
PRJNA729271	stage_1W_vs_E10	ordinary_muscle_development	ENSGALG00010012119	EPG5	0.559227787	0.122622912	4.560548917	5.10201E-06	9.03831E-06	positive
PRJNA729271	stage_1W_vs_E10	ordinary_muscle_development	ENSGALG00010012120	ENSGALG00010012120	0.276871656	0.187552435	1.476236	0.139880548	0.164829571	positive
PRJNA729271	stage_1W_vs_E10	ordinary_muscle_development	ENSGALG00010012121	SREK1	-0.408285495	0.100559993	-4.060118578	4.90478E-05	8.0707E-05	negative
PRJNA729271	stage_1W_vs_E10	ordinary_muscle_development	ENSGALG00010012122	GRIP1	-3.906152981	0.278763246	-14.01243901	1.30833E-44	1.211E-43	negative
PRJNA729271	stage_1W_vs_E10	ordinary_muscle_development	ENSGALG00010012123	ENSGALG00010012123	-1.740650039	0.211576708	-8.227039987	1.91896E-16	6.08252E-16	negative
PRJNA729271	stage_1W_vs_E10	ordinary_muscle_development	ENSGALG00010012124	ERAP1	2.514072911	0.146612985	17.14768241	6.54096E-66	1.10117E-64	positive
PRJNA729271	stage_1W_vs_E10	ordinary_muscle_development	ENSGALG00010012125	RNF152	0.409049503	0.147880075	2.766089365	0.005673299	0.007873094	positive
PRJNA729271	stage_1W_vs_E10	ordinary_muscle_development	ENSGALG00010012127	ENSGALG00010012127	-0.405066606	0.116715739	-3.470539701	0.000519414	0.000791183	negative
PRJNA729271	stage_1W_vs_E10	ordinary_muscle_development	ENSGALG00010012128	ENSGALG00010012128	2.416955225	0.771395274	3.133225346	0.001728966	0.00252025	positive
PRJNA729271	stage_1W_vs_E10	ordinary_muscle_development	ENSGALG00010012129	CRYZL1	-0.887236476	0.099626616	-8.905616876	5.309E-19	1.90763E-18	negative
PRJNA729271	stage_1W_vs_E10	ordinary_muscle_development	ENSGALG00010012130	TMEM67	-2.037897719	0.174291046	-11.69249809	1.3923E-31	8.39144E-31	negative
PRJNA729271	stage_1W_vs_E10	ordinary_muscle_development	ENSGALG00010012131	SLC14A1	0.54198279	0.244659613	2.215252379	0.026742746	0.034444296	positive
PRJNA729271	stage_1W_vs_E10	ordinary_muscle_development	ENSGALG00010012132	MC4R	-5.499988269	1.155122634	-4.761389055	1.92265E-06	3.51879E-06	negative
PRJNA729271	stage_1W_vs_E10	ordinary_muscle_development	ENSGALG00010012133	RAB3IP	-2.805523427	0.243631782	-11.51542462	1.10315E-30	6.41658E-30	negative
PRJNA729271	stage_1W_vs_E10	ordinary_muscle_development	ENSGALG00010012135	CAMSAP2	-0.30211155	0.092358105	-3.271088671	0.001071343	0.001590935	negative
PRJNA729271	stage_1W_vs_E10	ordinary_muscle_development	ENSGALG00010012136	ENSGALG00010012136	0.333651087	0.183716639	1.816117954	0.06935228	0.085033099	positive
PRJNA729271	stage_1W_vs_E10	ordinary_muscle_development	ENSGALG00010012137	ENSGALG00010012137	-0.810184699	1.01289771	-0.799868231	0.423787146	0.461760105	negative
PRJNA729271	stage_1W_vs_E10	ordinary_muscle_development	ENSGALG00010012138	DDX11	-2.583436456	0.184125427	-14.0308511	1.00933E-44	9.37235E-44	negative
PRJNA729271	stage_1W_vs_E10	ordinary_muscle_development	ENSGALG00010012140	GLOD5	-1.011664652	0.796611807	-1.269959399	0.204099093	0.234451876	negative
PRJNA729271	stage_1W_vs_E10	ordinary_muscle_development	ENSGALG00010012141	ENSGALG00010012141	0.032826847	0.094794172	0.346296052	0.729120236	0.755690057	positive
PRJNA729271	stage_1W_vs_E10	ordinary_muscle_development	ENSGALG00010012144	H1-01	0.125350525	0.157534236	0.79570339	0.426204456	0.464184656	positive
PRJNA729271	stage_1W_vs_E10	ordinary_muscle_development	ENSGALG00010012145	ENSGALG00010012145	5.431568711	0.889082022	6.109187427	1.0014E-09	2.25287E-09	positive
PRJNA729271	stage_1W_vs_E10	ordinary_muscle_development	ENSGALG00010012147	ARFGAP3	-0.602041923	0.123176911	-4.887619925	1.02062E-06	1.90766E-06	negative
PRJNA729271	stage_1W_vs_E10	ordinary_muscle_development	ENSGALG00010012148	SETBP1	-0.502910123	0.109706771	-4.584130211	4.5588E-06	8.10596E-06	negative
PRJNA729271	stage_1W_vs_E10	ordinary_muscle_development	ENSGALG00010012149	SFXN1	0.384441281	0.172651094	2.226694728	0.025967683	0.033511535	positive
PRJNA729271	stage_1W_vs_E10	ordinary_muscle_development	ENSGALG00010012151	MYRFL	-0.260111354	0.502170484	-0.517974198	0.604476272	0.638947244	negative
PRJNA729271	stage_1W_vs_E10	ordinary_muscle_development	ENSGALG00010012154	CWC27	-0.058176223	0.113824563	-0.511104292	0.609278031	0.643647333	negative
PRJNA729271	stage_1W_vs_E10	ordinary_muscle_development	ENSGALG00010012156	ENSGALG00010012156	-4.402286481	0.819399055	-5.372579397	7.76182E-08	1.56456E-07	negative
PRJNA729271	stage_1W_vs_E10	ordinary_muscle_development	ENSGALG00010012157	H2BC32	-1.688897341	0.198651301	-8.501818677	1.86643E-17	6.17811E-17	negative
PRJNA729271	stage_1W_vs_E10	ordinary_muscle_development	ENSGALG00010012159	WASHC1	0.211879938	0.101429441	2.088939234	0.036713193	0.046557982	positive
PRJNA729271	stage_1W_vs_E10	ordinary_muscle_development	ENSGALG00010012160	SYT4	-5.180325729	0.507469627	-10.20814932	1.82309E-24	8.18893E-24	negative
PRJNA729271	stage_1W_vs_E10	ordinary_muscle_development	ENSGALG00010012163	PDP1	0.833010341	0.221031702	3.768736936	0.000164076	0.000259751	positive
PRJNA729271	stage_1W_vs_E10	ordinary_muscle_development	ENSGALG00010012168	ENSGALG00010012168	-2.189140328	0.578922836	-3.781402617	0.000155947	0.000247369	negative
PRJNA729271	stage_1W_vs_E10	ordinary_muscle_development	ENSGALG00010012169	H3C33	-2.328877933	0.22844039	-10.19468551	2.09421E-24	9.3777E-24	negative
PRJNA729271	stage_1W_vs_E10	ordinary_muscle_development	ENSGALG00010012171	CDH17	-0.485070933	0.397855554	-1.219213677	0.222763102	0.254188079	negative
PRJNA729271	stage_1W_vs_E10	ordinary_muscle_development	ENSGALG00010012172	CNOT2	-0.489775891	0.089636373	-5.464030675	4.65443E-08	9.51163E-08	negative
PRJNA729271	stage_1W_vs_E10	ordinary_muscle_development	ENSGALG00010012175	PACSIN2	0.047440865	0.154714377	0.306635144	0.759121101	0.783331697	positive
PRJNA729271	stage_1W_vs_E10	ordinary_muscle_development	ENSGALG00010012179	ENSGALG00010012179	-0.86968218	0.180282515	-4.82399627	1.4071E-06	2.60086E-06	negative
PRJNA729271	stage_1W_vs_E10	ordinary_muscle_development	ENSGALG00010012180	DDX59	-0.198050772	0.107019599	-1.85060282	0.064226714	0.079069663	negative
PRJNA729271	stage_1W_vs_E10	ordinary_muscle_development	ENSGALG00010012186	KIF20A	-2.758109509	0.248610828	-11.09408442	1.34025E-28	7.12393E-28	negative
PRJNA729271	stage_1W_vs_E10	ordinary_muscle_development	ENSGALG00010012187	ENSGALG00010012187	-1.249228863	0.84235198	-1.483024785	0.138067826	0.162799409	negative
PRJNA729271	stage_1W_vs_E10	ordinary_muscle_development	ENSGALG00010012188	ADAMTS6	-1.085738291	0.18557139	-5.850784911	4.89259E-09	1.05848E-08	negative
PRJNA729271	stage_1W_vs_E10	ordinary_muscle_development	ENSGALG00010012190	CAST	0.182104378	0.133012129	1.369080993	0.170973959	0.198770255	positive
PRJNA729271	stage_1W_vs_E10	ordinary_muscle_development	ENSGALG00010012191	BEST3	5.196471583	0.373711703	13.9050277	5.9044E-44	5.36579E-43	positive
PRJNA729271	stage_1W_vs_E10	ordinary_muscle_development	ENSGALG00010012193	TTLL1	-1.12589114	0.22380706	-5.030632813	4.88864E-07	9.32011E-07	negative
PRJNA729271	stage_1W_vs_E10	ordinary_muscle_development	ENSGALG00010012194	LRRFIP1	2.031250198	0.159758185	12.71452975	4.91055E-37	3.55182E-36	positive
PRJNA729271	stage_1W_vs_E10	ordinary_muscle_development	ENSGALG00010012195	ITSN1	-0.066401306	0.072145843	-0.920376051	0.357376279	0.394980493	negative
PRJNA729271	stage_1W_vs_E10	ordinary_muscle_development	ENSGALG00010012196	CENPK	-2.971541985	0.264861333	-11.21923671	3.2809E-29	1.78919E-28	negative
PRJNA729271	stage_1W_vs_E10	ordinary_muscle_development	ENSGALG00010012198	TMEM5	1.104297681	0.098541847	11.20638299	3.79382E-29	2.06347E-28	positive
PRJNA729271	stage_1W_vs_E10	ordinary_muscle_development	ENSGALG00010012199	ENSGALG00010012199	1.216835492	0.284843969	4.271937013	1.93782E-05	3.28562E-05	positive
PRJNA729271	stage_1W_vs_E10	ordinary_muscle_development	ENSGALG00010012200	GEM	0.815903129	0.239385621	3.408321371	0.000653639	0.000988883	positive
PRJNA729271	stage_1W_vs_E10	ordinary_muscle_development	ENSGALG00010012205	SLC6A13	-5.364151807	0.511245953	-10.49231154	9.37074E-26	4.44612E-25	negative
PRJNA729271	stage_1W_vs_E10	ordinary_muscle_development	ENSGALG00010012206	RIBC2	-4.37715406	0.898427384	-4.872017638	1.10464E-06	2.0594E-06	negative
PRJNA729271	stage_1W_vs_E10	ordinary_muscle_development	ENSGALG00010012207	GRIA3	-2.97553775	0.277372507	-10.72758719	7.55429E-27	3.7487E-26	negative
PRJNA729271	stage_1W_vs_E10	ordinary_muscle_development	ENSGALG00010012209	KIF14	-1.423540998	0.229256267	-6.209387509	5.31915E-10	1.21304E-09	negative
PRJNA729271	stage_1W_vs_E10	ordinary_muscle_development	ENSGALG00010012210	PPWD1	-1.259577093	0.077374268	-16.27901793	1.39083E-59	2.00598E-58	negative
PRJNA729271	stage_1W_vs_E10	ordinary_muscle_development	ENSGALG00010012211	ENSGALG00010012211	-0.474372977	3.997262673	-0.118674457	0.905533274	0.916855602	negative
PRJNA729271	stage_1W_vs_E10	ordinary_muscle_development	ENSGALG00010012212	RAD54B	-3.268914701	0.203640725	-16.05236233	5.5029E-58	7.62568E-57	negative
PRJNA729271	stage_1W_vs_E10	ordinary_muscle_development	ENSGALG00010012215	RAB17	4.125507554	0.775089698	5.322619517	1.02284E-07	2.04327E-07	positive
PRJNA729271	stage_1W_vs_E10	ordinary_muscle_development	ENSGALG00010012216	BRD8	-0.025221394	0.057788375	-0.436444079	0.662514563	0.693017868	negative
PRJNA729271	stage_1W_vs_E10	ordinary_muscle_development	ENSGALG00010012219	KDM5A	-0.779760951	0.078062288	-9.988958492	1.70362E-23	7.36915E-23	negative
PRJNA729271	stage_1W_vs_E10	ordinary_muscle_development	ENSGALG00010012226	SLCO4C1	-0.67009639	0.554871207	-1.207661132	0.227177634	0.258736529	negative
PRJNA729271	stage_1W_vs_E10	ordinary_muscle_development	ENSGALG00010012228	NHP2	-0.99521583	0.115851193	-8.590466817	8.66167E-18	2.91369E-17	negative
PRJNA729271	stage_1W_vs_E10	ordinary_muscle_development	ENSGALG00010012229	FSBP	-0.220602344	0.203798119	-1.082455253	0.279050288	0.313480115	negative
PRJNA729271	stage_1W_vs_E10	ordinary_muscle_development	ENSGALG00010012230	PTPRQ	-1.363484637	0.420810633	-3.240138269	0.001194718	0.001764744	negative
PRJNA729271	stage_1W_vs_E10	ordinary_muscle_development	ENSGALG00010012232	PCSK1	-0.601197684	0.45690507	-1.315804363	0.188239715	0.217531292	negative
PRJNA729271	stage_1W_vs_E10	ordinary_muscle_development	ENSGALG00010012234	C1GALT1C1	-0.334266222	0.065309763	-5.118166171	3.08521E-07	5.96665E-07	negative
PRJNA729271	stage_1W_vs_E10	ordinary_muscle_development	ENSGALG00010012236	PRLH	-1.733492285	0.609799439	-2.842725286	0.004472961	0.006269151	negative
PRJNA729271	stage_1W_vs_E10	ordinary_muscle_development	ENSGALG00010012240	TRIM14	-0.761437584	0.250275345	-3.042399496	0.002347002	0.00337766	negative
PRJNA729271	stage_1W_vs_E10	ordinary_muscle_development	ENSGALG00010012242	CCDC77	-2.825100436	0.158268376	-17.85006269	2.88712E-71	5.63241E-70	negative
PRJNA729271	stage_1W_vs_E10	ordinary_muscle_development	ENSGALG00010012243	ENSGALG00010012243	0.083306732	1.494721793	0.055733938	0.955553763	0.961312126	positive
PRJNA729271	stage_1W_vs_E10	ordinary_muscle_development	ENSGALG00010012244	ENSGALG00010012244	-1.263823694	0.085336418	-14.80989856	1.26431E-49	1.3666E-48	negative
PRJNA729271	stage_1W_vs_E10	ordinary_muscle_development	ENSGALG00010012245	ENSGALG00010012245	-0.652316875	0.079659586	-8.188805752	2.63831E-16	8.30632E-16	negative
PRJNA729271	stage_1W_vs_E10	ordinary_muscle_development	ENSGALG00010012247	TRIM23	1.38626047	0.125377464	11.05669572	2.03455E-28	1.07592E-27	positive
PRJNA729271	stage_1W_vs_E10	ordinary_muscle_development	ENSGALG00010012248	H2AC39	-2.09573546	0.209951003	-9.982021659	1.82706E-23	7.8819E-23	negative
PRJNA729271	stage_1W_vs_E10	ordinary_muscle_development	ENSGALG00010012250	NANS	-1.079433532	0.112015049	-9.636504578	5.60646E-22	2.27571E-21	negative
PRJNA729271	stage_1W_vs_E10	ordinary_muscle_development	ENSGALG00010012253	MYF6	2.603511818	0.289012699	9.008295577	2.09284E-19	7.64315E-19	positive
PRJNA729271	stage_1W_vs_E10	ordinary_muscle_development	ENSGALG00010012259	IL1RAPL2	-2.206198819	0.23891422	-9.234271696	2.60049E-20	9.90043E-20	negative
PRJNA729271	stage_1W_vs_E10	ordinary_muscle_development	ENSGALG00010012260	ENSGALG00010012260	2.170392138	0.302342194	7.178594922	7.04315E-13	1.87735E-12	positive
PRJNA729271	stage_1W_vs_E10	ordinary_muscle_development	ENSGALG00010012263	SHLD3	0.334855912	0.128990787	2.595967656	0.009432494	0.012786309	positive
PRJNA729271	stage_1W_vs_E10	ordinary_muscle_development	ENSGALG00010012264	MYF5	1.696707236	0.155018997	10.94515685	7.00963E-28	3.63003E-27	positive
PRJNA729271	stage_1W_vs_E10	ordinary_muscle_development	ENSGALG00010012266	H3C33	-1.816138762	0.203031588	-8.94510447	3.71598E-19	1.34356E-18	negative
PRJNA729271	stage_1W_vs_E10	ordinary_muscle_development	ENSGALG00010012268	ENSGALG00010012268	-0.915177183	0.169737393	-5.391724029	6.97849E-08	1.4096E-07	negative
PRJNA729271	stage_1W_vs_E10	ordinary_muscle_development	ENSGALG00010012271	ENSGALG00010012271	0.340003281	0.285555863	1.190671689	0.233782496	0.265695255	positive

PRJNA729271	stage_1W_vs_E10	ordinary_muscle_development	ENSGALG00010012274	B4GALNT3	0.616474185	0.277068695	2.224986785	0.026082125	0.033644247	positive
PRJNA729271	stage_1W_vs_E10	ordinary_muscle_development	ENSGALG00010012275	VIRMA	-0.061234524	0.073756122	-0.83022971	0.406408921	0.444495383	negative
PRJNA729271	stage_1W_vs_E10	ordinary_muscle_development	ENSGALG00010012277	H3C33	-1.77766922	0.210116372	-8.460403185	2.66455E-17	8.75598E-17	negative
PRJNA729271	stage_1W_vs_E10	ordinary_muscle_development	ENSGALG00010012282	ENSGALG00010012282	0.411831068	1.479533249	0.27835202			positive
PRJNA729271	stage_1W_vs_E10	ordinary_muscle_development	ENSGALG00010012283	CUPIN1A	-1.083426973	0.835950978	-1.296041277	0.194961265	0.224746583	negative
PRJNA729271	stage_1W_vs_E10	ordinary_muscle_development	ENSGALG00010012285	FBLN1	-3.21609146	0.200127329	-16.0702263	4.12577E-58	5.7503E-57	negative
PRJNA729271	stage_1W_vs_E10	ordinary_muscle_development	ENSGALG00010012286	ENSGALG00010012286	0.587388924	2.669405391	0.220044856	0.825836221	0.844479437	positive
PRJNA729271	stage_1W_vs_E10	ordinary_muscle_development	ENSGALG00010012288	MCTS1	1.140705586	0.112990423	10.09559535	5.77794E-24	2.54331E-23	positive
PRJNA729271	stage_1W_vs_E10	ordinary_muscle_development	ENSGALG00010012290	ENSGALG00010012290	0.343249668	0.095650108	3.588596766	0.000332463	0.000514855	positive
PRJNA729271	stage_1W_vs_E10	ordinary_muscle_development	ENSGALG00010012291	NR5A2	-0.954326438	0.197492461	-4.832217049	1.35021E-06	2.49729E-06	negative
PRJNA729271	stage_1W_vs_E10	ordinary_muscle_development	ENSGALG00010012293	SRGAP1	-1.526627041	0.107011328	-14.26603208	3.56282E-46	3.44977E-45	negative
PRJNA729271	stage_1W_vs_E10	ordinary_muscle_development	ENSGALG00010012294	ENSGALG00010012294	-0.364832456	0.53828059	-0.677773754	0.497915155	0.535644958	negative
PRJNA729271	stage_1W_vs_E10	ordinary_muscle_development	ENSGALG00010012295	CUL4B	-0.311859331	0.06574327	-4.743593242	2.0996E-06	3.82622E-06	negative
PRJNA729271	stage_1W_vs_E10	ordinary_muscle_development	ENSGALG00010012297	ABLIM3	-0.690441791	0.19902139	-3.469183845	0.000522042	0.00079502	negative
PRJNA729271	stage_1W_vs_E10	ordinary_muscle_development	ENSGALG00010012300	NINJ2	-2.562327683	0.227127712	-11.28144012	1.62071E-29	8.97675E-29	negative
PRJNA729271	stage_1W_vs_E10	ordinary_muscle_development	ENSGALG00010012303	CA9	2.8117453	0.210418286	13.36264707	9.99457E-41	8.19408E-40	positive
PRJNA729271	stage_1W_vs_E10	ordinary_muscle_development	ENSGALG00010012304	ESRP1	-5.408017736	0.742473016	-7.283790282	3.24569E-13	8.79207E-13	negative
PRJNA729271	stage_1W_vs_E10	ordinary_muscle_development	ENSGALG00010012307	ENSGALG00010012307	-1.467356067	0.173006702	-8.481498408	2.22312E-17	7.33534E-17	negative
PRJNA729271	stage_1W_vs_E10	ordinary_muscle_development	ENSGALG00010012308	TRABD2A	2.39636848	0.338881788	7.071399418	1.53379E-12	4.02176E-12	positive
PRJNA729271	stage_1W_vs_E10	ordinary_muscle_development	ENSGALG00010012309	TRAPPC13	-0.111810938	0.125260552	-0.892626899	0.372057009	0.409675353	negative
PRJNA729271	stage_1W_vs_E10	ordinary_muscle_development	ENSGALG00010012311	ENSGALG00010012311	-3.547830886	0.533475568	-6.650409314	2.92279E-11	7.16972E-11	negative
PRJNA729271	stage_1W_vs_E10	ordinary_muscle_development	ENSGALG00010012312	SH3TC2	0.902358207	0.180783318	4.991379843	5.99495E-07	1.13739E-06	positive
PRJNA729271	stage_1W_vs_E10	ordinary_muscle_development	ENSGALG00010012314	ADRB2	5.289405733	0.26153611	20.22438023	5.97363E-91	1.81907E-89	positive
PRJNA729271	stage_1W_vs_E10	ordinary_muscle_development	ENSGALG00010012315	ATP6V1E1	0.654104546	0.100581424	6.503234111	7.86113E-11	1.8766E-10	positive
PRJNA729271	stage_1W_vs_E10	ordinary_muscle_development	ENSGALG00010012316	ENSGALG00010012316	-3.514486283	1.102039623	-3.189074341	0.001427292	0.002096109	negative
PRJNA729271	stage_1W_vs_E10	ordinary_muscle_development	ENSGALG00010012317	ENSGALG00010012317	-5.496096188	1.156723314	-4.751435474	2.01978E-06	3.68817E-06	negative
PRJNA729271	stage_1W_vs_E10	ordinary_muscle_development	ENSGALG00010012318	POLR1D	-0.761050678	0.144642878	-5.261584181	1.42819E-07	2.82424E-07	negative
PRJNA729271	stage_1W_vs_E10	ordinary_muscle_development	ENSGALG00010012320	DPY19L4	-0.542080967	0.061735053	-8.780764655	1.62367E-18	5.67247E-18	negative
PRJNA729271	stage_1W_vs_E10	ordinary_muscle_development	ENSGALG00010012322	ENSGALG00010012322	-2.670525642	0.28338827	-9.423557458	4.36058E-21	1.71385E-20	negative
PRJNA729271	stage_1W_vs_E10	ordinary_muscle_development	ENSGALG00010012323	SMAD7B	0.290787823	3.546929426	0.081982974	0.934660253	0.942659363	positive
PRJNA729271	stage_1W_vs_E10	ordinary_muscle_development	ENSGALG00010012324	ENSGALG00010012324	-1.485050728	0.163774119	-9.067676492	1.21582E-19	4.49459E-19	negative
PRJNA729271	stage_1W_vs_E10	ordinary_muscle_development	ENSGALG00010012325	MLPH	-3.534547562	0.646645164	-5.465976949	4.60364E-08	9.41315E-08	negative
PRJNA729271	stage_1W_vs_E10	ordinary_muscle_development	ENSGALG00010012326	SGTB	-0.089303585	0.116885156	-0.764028451	0.444850288	0.483040297	negative
PRJNA729271	stage_1W_vs_E10	ordinary_muscle_development	ENSGALG00010012327	UBTD2	-1.062700925	0.089950389	-11.81430052	3.29278E-32	2.02412E-31	negative
PRJNA729271	stage_1W_vs_E10	ordinary_muscle_development	ENSGALG00010012329	ATXN10	-0.325512093	0.107968781	-3.014872364	0.002570874	0.003687941	negative
PRJNA729271	stage_1W_vs_E10	ordinary_muscle_development	ENSGALG00010012331	ENSGALG00010012331	-0.732936147	0.264126048	-2.774948373	0.005521047	0.007673562	negative
PRJNA729271	stage_1W_vs_E10	ordinary_muscle_development	ENSGALG00010012334	ARHGEF39	-2.155830041	0.238674724	-9.032502515	1.6779E-19	6.16662E-19	negative
PRJNA729271	stage_1W_vs_E10	ordinary_muscle_development	ENSGALG00010012336	ENSGALG00010012336	0.041103903	4.113358658	0.009992784	0.992027045	0.993347321	positive
PRJNA729271	stage_1W_vs_E10	ordinary_muscle_development	ENSGALG00010012337	ACSS3	0.644586193	0.154379273	4.175341549	2.97539E-05	4.98248E-05	positive
PRJNA729271	stage_1W_vs_E10	ordinary_muscle_development	ENSGALG00010012338	H3C33	-2.230209042	0.225666031	-9.882785763	4.9439E-23	2.09232E-22	negative
PRJNA729271	stage_1W_vs_E10	ordinary_muscle_development	ENSGALG00010012339	WNK1	0.552204405	0.154947003	3.563827605	0.000365486	0.000563467	positive
PRJNA729271	stage_1W_vs_E10	ordinary_muscle_development	ENSGALG00010012340	ENHO	-1.530367492	0.167170404	-9.15453605	5.45919E-20	2.04843E-19	negative
PRJNA729271	stage_1W_vs_E10	ordinary_muscle_development	ENSGALG00010012341	GOPC	-0.658673719	0.081390476	-8.092761558	5.8327E-16	1.81349E-15	negative
PRJNA729271	stage_1W_vs_E10	ordinary_muscle_development	ENSGALG00010012342	ENSGALG00010012342	-0.296969508	1.630763836	-0.182104546	0.855500681	0.871554848	negative
PRJNA729271	stage_1W_vs_E10	ordinary_muscle_development	ENSGALG00010012343	BCL2L13	1.831031461	0.170167976	10.76014126	5.30887E-27	2.65078E-26	positive
PRJNA729271	stage_1W_vs_E10	ordinary_muscle_development	ENSGALG00010012345	H2BC32	-1.654598559	0.165263747	-10.01186643	1.35178E-23	5.86297E-23	negative
PRJNA729271	stage_1W_vs_E10	ordinary_muscle_development	ENSGALG00010012346	NLN	-0.838669627	0.141999798	-5.906132553	3.50232E-09	7.6386E-09	negative
PRJNA729271	stage_1W_vs_E10	ordinary_muscle_development	ENSGALG00010012347	ENSGALG00010012347	-1.058101731	0.47542117	-2.225609201	0.026040369	0.033596364	negative
PRJNA729271	stage_1W_vs_E10	ordinary_muscle_development	ENSGALG00010012350	H2AC32	-1.865062832	0.192302467	-9.698590272	3.05693E-22	1.25064E-21	negative
PRJNA729271	stage_1W_vs_E10	ordinary_muscle_development	ENSGALG00010012352	PTPRC	1.722855942	0.194053187	8.87826668	6.79103E-19	2.42393E-18	positive
PRJNA729271	stage_1W_vs_E10	ordinary_muscle_development	ENSGALG00010012353	PIGG	0.06478459	0.152761786	0.424088978	0.671500923	0.70150673	positive
PRJNA729271	stage_1W_vs_E10	ordinary_muscle_development	ENSGALG00010012354	DCBLD1	0.567021421	0.149830879	3.784409618	0.000154074	0.000244504	positive
PRJNA729271	stage_1W_vs_E10	ordinary_muscle_development	ENSGALG00010012358	STK10	0.276400319	0.149341107	1.850798649	0.064198526	0.079042727	positive
PRJNA729271	stage_1W_vs_E10	ordinary_muscle_development	ENSGALG00010012360	ROS1	0.999679385	0.398486154	2.508692898	0.012117878	0.016221707	positive
PRJNA729271	stage_1W_vs_E10	ordinary_muscle_development	ENSGALG00010012361	WIF1	-7.418707961	0.496449255	-14.94353731	1.71618E-50	1.91797E-49	negative
PRJNA729271	stage_1W_vs_E10	ordinary_muscle_development	ENSGALG00010012362	COL6A3	1.928916828	0.292486941	6.594881885	4.25595E-11	1.03533E-10	positive
PRJNA729271	stage_1W_vs_E10	ordinary_muscle_development	ENSGALG00010012366	INTS8	-0.573755918	0.068469414	-8.379740401	5.30446E-17	1.71932E-16	negative
PRJNA729271	stage_1W_vs_E10	ordinary_muscle_development	ENSGALG00010012367	RAD52	-2.350335272	0.154954709	-15.16788541	5.77062E-52	6.78387E-51	negative
PRJNA729271	stage_1W_vs_E10	ordinary_muscle_development	ENSGALG00010012371	ATP6V1G3	0.085947202	0.447956535	0.191865048	0.847847916	0.864548403	positive
PRJNA729271	stage_1W_vs_E10	ordinary_muscle_development	ENSGALG00010012372	LEMD3	-0.047659346	0.073932947	-0.644629326	0.519167426	0.556401615	negative
PRJNA729271	stage_1W_vs_E10	ordinary_muscle_development	ENSGALG00010012373	WNT7B	-8.611411137	0.964819024	-8.925415987	4.44027E-19	1.60144E-18	negative
PRJNA729271	stage_1W_vs_E10	ordinary_muscle_development	ENSGALG00010012376	VGLL2	1.539992522	0.186157848	8.272509279	1.31169E-16	4.1851E-16	positive
PRJNA729271	stage_1W_vs_E10	ordinary_muscle_development	ENSGALG00010012378	RPS19BP1	-0.319306935	0.092623752	-3.447354805	0.000566105	0.000859864	negative
PRJNA729271	stage_1W_vs_E10	ordinary_muscle_development	ENSGALG00010012382	SH3PXD2B	-1.917605244	0.135429237	-14.15946277	1.63237E-45	1.55564E-44	negative
PRJNA729271	stage_1W_vs_E10	ordinary_muscle_development	ENSGALG00010012384	NRK	-0.18244921	0.122133559	-1.493849943	0.135214844	0.159630165	negative
PRJNA729271	stage_1W_vs_E10	ordinary_muscle_development	ENSGALG00010012387	ERBIN	-0.419066437	0.089336052	-4.690899468	2.72007E-06	4.92104E-06	negative
PRJNA729271	stage_1W_vs_E10	ordinary_muscle_development	ENSGALG00010012390	MMP16	-1.727487116	0.175097931	-9.865833948	5.85459E-23	2.4734E-22	negative
PRJNA729271	stage_1W_vs_E10	ordinary_muscle_development	ENSGALG00010012391	NEURL1B	-1.206260037	0.159218269	-7.576140884	3.55985E-14	1.01455E-13	negative
PRJNA729271	stage_1W_vs_E10	ordinary_muscle_development	ENSGALG00010012393	ENSGALG00010012393	0.263057587	1.605188604	0.16387955	0.869825966	0.884284428	positive
PRJNA729271	stage_1W_vs_E10	ordinary_muscle_development	ENSGALG00010012398	ENSGALG00010012398	-4.043582496	0.835263575	-4.841085638	1.29132E-06	2.39203E-06	negative
PRJNA729271	stage_1W_vs_E10	ordinary_muscle_development	ENSGALG00010012399	ERC1	-0.882383735	0.086366096	-10.21678389	1.66783E-24	7.51481E-24	negative
PRJNA729271	stage_1W_vs_E10	ordinary_muscle_development	ENSGALG00010012401	RIPK2	0.067961107	0.133609719	0.508653921	0.610994826	0.645178845	positive
PRJNA729271	stage_1W_vs_E10	ordinary_muscle_development	ENSGALG00010012404	VAMP7	0.017923932	0.053021417	0.33805079	0.735324914	0.761432678	positive
PRJNA729271	stage_1W_vs_E10	ordinary_muscle_development	ENSGALG00010012405	MSRB3	2.949814098	0.125407042	23.52191754	2.434E-122	1.4113E-120	positive
PRJNA729271	stage_1W_vs_E10	ordinary_muscle_development	ENSGALG00010012406	ENSGALG00010012406	4.952437487	0.238027692	20.80613999	3.80799E-96	1.32367E-94	positive
PRJNA729271	stage_1W_vs_E10	ordinary_muscle_development	ENSGALG00010012407	ATF4	1.437404133	0.12873766	11.16537408	6.02371E-29	3.24345E-28	positive
PRJNA729271	stage_1W_vs_E10	ordinary_muscle_development	ENSGALG00010012410	CACNA1I	-3.337714946	0.449115624	-7.431749794	1.0717E-13	2.97479E-13	negative
PRJNA729271	stage_1W_vs_E10	ordinary_muscle_development	ENSGALG00010012412	PURA	1.422686501	0.131115308	10.85065141	1.98009E-27	1.00742E-26	positive
PRJNA729271	stage_1W_vs_E10	ordinary_muscle_development	ENSGALG00010012413	NEK7	0.177986326	0.073188518	2.431888643	0.015020324	0.019899424	positive
PRJNA729271	stage_1W_vs_E10	ordinary_muscle_development	ENSGALG00010012414	ENSGALG00010012414	7.802188474	0.203317337	38.37443771	3.5573E-322	1.5165E-319	positive
PRJNA729271	stage_1W_vs_E10	ordinary_muscle_development	ENSGALG00010012415	BID	-2.514209048	0.117545487	-21.38924355	1.6827E-101	6.46951E-100	negative
PRJNA729271	stage_1W_vs_E10	ordinary_muscle_development	ENSGALG00010012418	ENSGALG00010012418	-0.618456323	0.844099604	-0.732681688	0.463752595	0.50202344	negative
PRJNA729271	stage_1W_vs_E10	ordinary_muscle_development	ENSGALG00010012419	HACE1	0.672180573	0.096169656	6.989528713	2.75811E-12	7.14035E-12	positive
PRJNA729271	stage_1W_vs_E10	ordinary_muscle_development	ENSGALG00010012426	MIEF1	-1.024576159	0.083618741	-12.25294891	1.61996E-34	1.0806E-33	negative
PRJNA729271	stage_1W_vs_E10	ordinary_muscle_development	ENSGALG00010012427	C7	3.849339404	0.447650292	8.598987814	8.04222E-18	2.70972E-17	positive
PRJNA729271	stage_1W_vs_E10	ordinary_muscle_development	ENSGALG00010012428	CCNE2	-3.274222045	0.236238144	-13.8598365	1.10927E-43	9.96827E-43	negative
PRJNA729271	stage_1W_vs_E10	ordinary_muscle_development	ENSGALG00010012429	CXCR7	0.90532633	0.101340042	8.933549935	4.12553E-19	1.48977E-18	positive
PRJNA729271	stage_1W_vs_E10	ordinary_muscle_development	ENSGALG00010012431	LAMP2	0.57175355	0.079489904	7.192781974	6.34843E-13	1.69529E-12	positive
PRJNA729271	stage_1W_vs_E10	ordinary_muscle_development	ENSGALG00010012433	ENSGALG00010012433	-0.601329347	0.787274814	-0.763811233	0.444979742	0.483144671	negative
PRJNA729271	stage_1W_vs_E10	ordinary_muscle_development	ENSGALG00010012436	ENSGALG00010012436	4.640671652	0.199680932	23.24043465	1.7775E-119	9.8338E-118	positive
PRJNA729271	stage_1W_vs_E10	ordinary_muscle_development	ENSGALG00010012437	ENSGALG00010012437	-0.681639769	3.804132222	-0.179184037	0.857793197	0.873644772	negative
PRJNA729271	stage_1W_vs_E10	ordinary_muscle_development	ENSGALG00010012439	TENT2	0.195671156	0.10260459	1.907040968	0.056515283	0.070118037	positive
PRJNA729271	stage_1W_vs_E10	ordinary_muscle_development	ENSGALG00010012441	FST	0.158956527	0.239258087	0.664372639	0.50645184	0.544101647	positive
PRJNA729271	stage_1W_vs_E10	ordinary_muscle_development	ENSGALG00010012442	CPLX1	-5.742488634	0.429830063	-13.35990459	1.03697E-40	8.49682E-40	negative
PRJNA729271	stage_1W_vs_E10	ordinary_muscle_development	ENSGALG00010012445	SOBP	-1.011025692	0.168612138	-5.996161977	2.02036E-09	4.47305E-09	negative
PRJNA729271	stage_1W_vs_E10	ordinary_muscle_development	ENSGALG00010012447	COPS8	0.265393781	0.116771038	2.272770598	0.023040008	0.02992249	positive
PRJNA729271	stage_1W_vs_E10	ordinary_muscle_development	ENSGALG00010012449	SUB1	-1.496121595	0.109825225	-13.62275007	2.93311E-42	2.52767E-41	negative
PRJNA729271	stage_1W_vs_E10	ordinary_muscle_development	ENSGALG00010012450	PPFIA2	-2.521319533	0.278784771	-9.043964373	1.51091E-19	5.56417E-19	negative
PRJNA729271	stage_1W_vs_E10	ordinary_muscle_development	ENSGALG00010012454	IQCA1	1.961653228	0.421062862	4.658813218	3.18038E-06	5.72523E-06	positive
PRJNA729271	stage_1W_vs_E10	ordinary_muscle_development	ENSGALG00010012456	ENSGALG00010012456	-1.598781348	0.093374692	-17.1222129	1.01348E-65	1.69832E-64	negative
PRJNA729271	stage_1W_vs_E10	ordinary_muscle_development	ENSGALG00010012458	MRPS30	0.658852909	0.107659659	6.11977516	9.37075E-10	2.11111E-09	positive
PRJNA729271	stage_1W_vs_E10	ordinary_muscle_development	ENSGALG00010012464	ENSGALG00010012464	0.795489996	0.238767311	3.331653699	0.000863316	0.001294349	positive
PRJNA729271	stage_1W_vs_E10	ordinary_muscle_development	ENSGALG00010012466	PDSS2	0.56184478	0.121051203	4.641381208	3.46088E-06	6.21629E-06	positive
PRJNA729271	stage_1W_vs_E10	ordinary_muscle_development	ENSGALG00010012469	CETN2	-1.129892758	0.132491304	-8.528052242	1.48832E-17	4.94799E-17	negative
PRJNA729271	stage_1W_vs_E10	ordinary_muscle_development	ENSGALG00010012470	CHD1	0.255541833	0.105555379	2.420926666	0.015481	0.020481663	positive
PRJNA729271	stage_1W_vs_E10	ordinary_muscle_development	ENSGALG00010012474	ASB18	0.173095485	0.967266683	0.178953217	0.857974436	0.873761516	positive
PRJNA729271	stage_1W_vs_E10	ordinary_muscle_development	ENSGALG00010012477	ENSGALG00010012477	-1.057268453	0.264801521	-3.992682703	6.533E-05	0.000106663	negative
PRJNA729271	stage_1W_vs_E10	ordinary_muscle_development	ENSGALG00010012483	ENSGALG00010012483	1.049515644	0.253029128	4.147805645	3.35677E-05	5.60505E-05	positive
PRJNA729271	stage_1W_vs_E10	ordinary_muscle_development	ENSGALG00010012485	C6	5.251714913	1.223648158	4.291850462	1.7719E-05	3.01558E-05	positive
PRJNA729271	stage_1W_vs_E10	ordinary_muscle_development	ENSGALG00010012486	RADX	0.941267316	0.107418107	8.76265036	1.90712E-18	6.62602E-18	positive
PRJNA729271	stage_1W_vs_E10	ordinary_muscle_development	ENSGALG00010012489	GBX2	-2.964006494	0.98909589	-2.996682651	0.002729347	0.003903106	negative
PRJNA729271	stage_1W_vs_E10	ordinary_muscle_development	ENSGALG00010012497	PLCXD3	-0.624853715	0.431496602	-1.448108077	0.147586827	0.173248385	negative
PRJNA729271	stage_1W_vs_E10	ordinary_muscle_development	ENSGALG00010012498	KCNT2	-0.050875341	0.219568845	-0.231705646	0.816766646	0.835971793	negative
PRJNA729271	stage_1W_vs_E10	ordinary_muscle_development	ENSGALG00010012506	TP53INP1	-0.965414805	0.127646687	-7.563179464	3.93334E-14	1.11814E-13	negative
PRJNA729271	stage_1W_vs_E10	ordinary_muscle_development	ENSGALG00010012508	MDM1	-0.971834603	0.070777023	-13.73093367	6.62739E-43	5.82206E-42	negative
PRJNA729271	stage_1W_vs_E10	ordinary_muscle_development	ENSGALG00010012509	MICAL3	0.846520162	0.093351425	9.068101075	1.21109E-19	4.47826E-19	positive
PRJNA729271	stage_1W_vs_E10	ordinary_muscle_development	ENSGALG00010012511	OXCT1	-0.798537946	0.122263307	-6.53129682	6.52026E-11	1.56217E-10	negative
PRJNA729271	stage_1W_vs_E10	ordinary_muscle_development	ENSGALG00010012517	MGAT3	-0.02475415	0.175854058	-0.140765302	0.888055355	0.900555313	negative
PRJNA729271	stage_1W_vs_E10	ordinary_muscle_development	ENSGALG00010012519	ENSGALG00010012519	0.147890377	1.676651527	0.088205792	0.929713114	0.938388105	positive
PRJNA729271	stage_1W_vs_E10	ordinary_muscle_development	ENSGALG00010012521	HOMER1	0.773050527	0.133325932	5.798200823	6.70302E-09	1.43728E-08	positive
PRJNA729271	stage_1W_vs_E10	ordinary_muscle_development	ENSGALG00010012529	BEND3	-0.669207888	0.125442013	-5.334798693	9.56506E-08	1.91499E-07	negative
PRJNA729271	stage_1W_vs_E10	ordinary_muscle_development	ENSGALG00010012532	FAM174A	1.205003431	0.150370208	8.013578296	1.11418E-15	3.41945E-15	positive
PRJNA729271	stage_1W_vs_E10	ordinary_muscle_development	ENSGALG00010012535	JMY	-0.163546665	0.103695714	-1.577178638	0.114754427	0.136877438	negative
PRJNA729271	stage_1W_vs_E10	ordinary_muscle_development	ENSGALG00010012536	FBXO4	0.054366788	0.13586309	0.400158629	0.689039684	0.718017988	positive
PRJNA729271	stage_1W_vs_E10	ordinary_muscle_development	ENSGALG00010012538	RAP1B	-0.793235736	0.088334983	-8.97985953	2.71113E-19	9.85154E-19	negative
PRJNA729271	stage_1W_vs_E10	ordinary_muscle_development	ENSGALG00010012547	ENSGALG00010012547	-2.181624245	0.479535972	-4.54944858	5.37867E-06	9.50778E-06	negative
PRJNA729271	stage_1W_vs_E10	ordinary_muscle_development	ENSGALG00010012550	TAB1	-0.246779756	0.097477818	-2.531650378	0.011352713	0.015245282	negative
PRJNA729271	stage_1W_vs_E10	ordinary_muscle_development	ENSGALG00010012551	PCGF3	-0.813325351	0.156745887	-5.188814636	2.11637E-07	4.13768E-07	negative
PRJNA729271	stage_1W_vs_E10	ordinary_muscle_development	ENSGALG00010012553	ENSGALG00010012553	0.613671888	0.223814452	2.741877852	0.006108905	0.008446874	positive
PRJNA729271	stage_1W_vs_E10	ordinary_muscle_development	ENSGALG00010012555	ENSGALG00010012555	-0.296761628	0.212985769	-1.393340175	0.163516947	0.190804875	negative
PRJNA729271	stage_1W_vs_E10	ordinary_muscle_development	ENSGALG00010012561	OSGIN2	-1.279249028	0.132528501	-9.652633332	4.79094E-22	1.94796E-21	negative

PRJNA729271	stage_1W_vs_E10	ordinary_muscle_development	ENSGALG00010012565	SYNGR1	1.257506316	0.121976832	10.30938658	6.39079E-25	2.92592E-24	positive
PRJNA729271	stage_1W_vs_E10	ordinary_muscle_development	ENSGALG00010012569	C6orf203	1.691607496	0.120515221	14.03646351	9.32513E-45	8.67016E-44	positive
PRJNA729271	stage_1W_vs_E10	ordinary_muscle_development	ENSGALG00010012571	AGAP1	-0.196407804	0.082910888	-2.368902436	0.017840959	0.023420096	negative
PRJNA729271	stage_1W_vs_E10	ordinary_muscle_development	ENSGALG00010012574	PHAX	-0.495190373	0.105510613	-4.693275462	2.68865E-06	4.86785E-06	negative
PRJNA729271	stage_1W_vs_E10	ordinary_muscle_development	ENSGALG00010012578	NDUFS4	0.93468644	0.152821513	6.116196743	9.5835E-10	2.15804E-09	positive
PRJNA729271	stage_1W_vs_E10	ordinary_muscle_development	ENSGALG00010012582	CD24	-2.691145743	0.225585821	-11.92958727	8.29839E-33	5.19815E-32	negative
PRJNA729271	stage_1W_vs_E10	ordinary_muscle_development	ENSGALG00010012583	NDUFAF6	0.562246199	0.101263793	5.552292525	2.81947E-08	5.82086E-08	positive
PRJNA729271	stage_1W_vs_E10	ordinary_muscle_development	ENSGALG00010012586	CDC73	0.995252536	0.133550446	7.45225915	9.17553E-14	2.55719E-13	positive
PRJNA729271	stage_1W_vs_E10	ordinary_muscle_development	ENSGALG00010012587	MBLAC2	0.852675506	0.146593552	5.816596277	6.0058E-09	1.29256E-08	positive
PRJNA729271	stage_1W_vs_E10	ordinary_muscle_development	ENSGALG00010012588	PEX26	0.814187428	0.104272389	7.808274448	5.79762E-15	1.71995E-14	positive
PRJNA729271	stage_1W_vs_E10	ordinary_muscle_development	ENSGALG00010012593	TUBA8B	3.426974718	0.959803511	3.570496128	0.000356306	0.000550016	positive
PRJNA729271	stage_1W_vs_E10	ordinary_muscle_development	ENSGALG00010012599	DMGDH	-2.37791106	0.198501708	-11.97929771	4.56172E-33	2.88743E-32	negative
PRJNA729271	stage_1W_vs_E10	ordinary_muscle_development	ENSGALG00010012600	NUP107	-0.951515366	0.10029746	-9.486933797	2.3793E-21	9.45652E-21	negative
PRJNA729271	stage_1W_vs_E10	ordinary_muscle_development	ENSGALG00010012601	TUBA8A	4.14164634	0.237271643	17.45529425	3.13757E-68	5.67778E-67	positive
PRJNA729271	stage_1W_vs_E10	ordinary_muscle_development	ENSGALG00010012604	ENSGALG00010012604	-0.118182739	2.127947384	-0.055538375			negative
PRJNA729271	stage_1W_vs_E10	ordinary_muscle_development	ENSGALG00010012606	ATP5PO	0.909774924	0.11411881	7.972173211	1.55908E-15	4.75166E-15	positive
PRJNA729271	stage_1W_vs_E10	ordinary_muscle_development	ENSGALG00010012607	LMNB1	-3.703995368	0.166292547	-22.27397103	6.6078E-110	2.9745E-108	negative
PRJNA729271	stage_1W_vs_E10	ordinary_muscle_development	ENSGALG00010012610	RPL3	-1.233551118	0.155063913	-7.955114069	1.78967E-15	5.44069E-15	negative
PRJNA729271	stage_1W_vs_E10	ordinary_muscle_development	ENSGALG00010012614	LHFPL2	-1.30273844	0.145717991	-8.940134514	3.88697E-19	1.40433E-18	negative
PRJNA729271	stage_1W_vs_E10	ordinary_muscle_development	ENSGALG00010012618	B3GALT2	-0.925302376	0.333243945	-2.776651727	0.005492199	0.007636396	negative
PRJNA729271	stage_1W_vs_E10	ordinary_muscle_development	ENSGALG00010012624	PDGFB	0.795574337	0.183548534	4.334408557	1.46153E-05	2.50264E-05	positive
PRJNA729271	stage_1W_vs_E10	ordinary_muscle_development	ENSGALG00010012629	NUS1	0.00256559	0.110683762	0.023179458	0.981507124	0.984291253	positive
PRJNA729271	stage_1W_vs_E10	ordinary_muscle_development	ENSGALG00010012631	GLRX2	0.510056833	0.155056454	3.28949114	0.001003687	0.001494754	positive
PRJNA729271	stage_1W_vs_E10	ordinary_muscle_development	ENSGALG00010012633	USP18	0.985623341	0.542179633	1.817890754	0.069080832	0.084736114	positive
PRJNA729271	stage_1W_vs_E10	ordinary_muscle_development	ENSGALG00010012640	PRRC1	-0.790650739	0.074841204	-10.56437759	4.3585E-26	2.10028E-25	negative
PRJNA729271	stage_1W_vs_E10	ordinary_muscle_development	ENSGALG00010012648	MRPS6	0.36333449	0.130334738	2.787702609	0.005308325	0.007396344	positive
PRJNA729271	stage_1W_vs_E10	ordinary_muscle_development	ENSGALG00010012650	DSE	-1.248577925	0.106693073	-11.70252095	1.23722E-31	7.47544E-31	negative
PRJNA729271	stage_1W_vs_E10	ordinary_muscle_development	ENSGALG00010012653	CBX7	2.167867787	0.250001804	8.67140856	4.26807E-18	1.45807E-17	positive
PRJNA729271	stage_1W_vs_E10	ordinary_muscle_development	ENSGALG00010012654	TENT5B	3.844008007	0.308353136	12.46625235	1.14066E-35	7.9566E-35	positive
PRJNA729271	stage_1W_vs_E10	ordinary_muscle_development	ENSGALG00010012662	SLC35E3	-1.9331645	0.109743574	-17.61528658	1.88034E-69	3.49879E-68	negative
PRJNA729271	stage_1W_vs_E10	ordinary_muscle_development	ENSGALG00010012667	SCAMP1	-0.848514663	0.090032999	-9.424485191	4.32221E-21	1.69968E-20	negative
PRJNA729271	stage_1W_vs_E10	ordinary_muscle_development	ENSGALG00010012669	ERCC8	-0.429666326	0.150308735	-2.858558585	0.004255705	0.005976211	negative
PRJNA729271	stage_1W_vs_E10	ordinary_muscle_development	ENSGALG00010012670	ENSGALG00010012670	-0.348932074	0.220547281	-1.582119138	0.113622375	0.135607767	negative
PRJNA729271	stage_1W_vs_E10	ordinary_muscle_development	ENSGALG00010012671	NBN	0.138817418	0.184027217	0.75433091	0.450650546	0.488752687	positive
PRJNA729271	stage_1W_vs_E10	ordinary_muscle_development	ENSGALG00010012672	RO60	-1.142204192	0.093247049	-12.24922621	1.69606E-34	1.13032E-33	negative
PRJNA729271	stage_1W_vs_E10	ordinary_muscle_development	ENSGALG00010012673	ENSGALG00010012673	-1.286240705	0.329112682	-3.908207663	9.29834E-05	0.000149921	negative
PRJNA729271	stage_1W_vs_E10	ordinary_muscle_development	ENSGALG00010012674	ENSGALG00010012674	-0.099771808	0.140600728	-0.709610894	0.477945462	0.516269429	negative
PRJNA729271	stage_1W_vs_E10	ordinary_muscle_development	ENSGALG00010012676	ENSGALG00010012676	0.27310035	0.210813782	1.295457763	0.195162363	0.224942624	positive
PRJNA729271	stage_1W_vs_E10	ordinary_muscle_development	ENSGALG00010012680	TRPM8	-0.982119366	0.327400688	-2.99974741	0.002702036	0.003866962	negative
PRJNA729271	stage_1W_vs_E10	ordinary_muscle_development	ENSGALG00010012682	ENSGALG00010012682	-1.337975604	0.112708863	-11.87107713	1.673E-32	1.03944E-31	negative
PRJNA729271	stage_1W_vs_E10	ordinary_muscle_development	ENSGALG00010012683	MDM2	1.051833317	0.085896648	12.24533595	1.77939E-34	1.18476E-33	positive
PRJNA729271	stage_1W_vs_E10	ordinary_muscle_development	ENSGALG00010012685	NPTXR	0.433224521	0.256562631	1.688572179	0.091301446	0.110459432	positive
PRJNA729271	stage_1W_vs_E10	ordinary_muscle_development	ENSGALG00010012688	NDUFAF2	0.946180306	0.112096042	8.440800301	3.15172E-17	1.03195E-16	positive
PRJNA729271	stage_1W_vs_E10	ordinary_muscle_development	ENSGALG00010012690	NT5DC1	0.970440658	0.335492914	2.892581688	0.003820898	0.005388589	positive
PRJNA729271	stage_1W_vs_E10	ordinary_muscle_development	ENSGALG00010012693	UCHL5	-0.304171129	0.077819485	-3.908675694	9.28034E-05	0.000149648	negative
PRJNA729271	stage_1W_vs_E10	ordinary_muscle_development	ENSGALG00010012696	DNAL4	-1.373749315	0.097666605	-14.06570157	6.17078E-45	5.75583E-44	negative
PRJNA729271	stage_1W_vs_E10	ordinary_muscle_development	ENSGALG00010012699	RNF128	0.037850765	0.144456847	0.262021258	0.793305053	0.815181962	positive
PRJNA729271	stage_1W_vs_E10	ordinary_muscle_development	ENSGALG00010012702	PTPRO	-3.508022377	0.384911726	-9.113836093	7.95194E-20	2.96612E-19	negative
PRJNA729271	stage_1W_vs_E10	ordinary_muscle_development	ENSGALG00010012704	SMIM15	-0.348456398	0.068633663	-5.07704792	3.83344E-07	7.36751E-07	negative
PRJNA729271	stage_1W_vs_E10	ordinary_muscle_development	ENSGALG00010012705	CPM	1.933360388	0.184634709	10.47127269	1.17058E-25	5.53232E-25	positive
PRJNA729271	stage_1W_vs_E10	ordinary_muscle_development	ENSGALG00010012706	TBC1D8B	1.162687634	0.103686917	11.21344592	3.50285E-29	1.90736E-28	positive
PRJNA729271	stage_1W_vs_E10	ordinary_muscle_development	ENSGALG00010012708	SPP2	2.742343437	1.096012782	2.502108992	0.012345591	0.016514336	positive
PRJNA729271	stage_1W_vs_E10	ordinary_muscle_development	ENSGALG00010012709	PPP1R8	-1.573706746	0.059929486	-26.25930646	5.5962E-152	4.8284E-150	negative
PRJNA729271	stage_1W_vs_E10	ordinary_muscle_development	ENSGALG00010012711	ELL2	-0.161831761	0.126746204	-1.276817419	0.201666714	0.232015162	negative
PRJNA729271	stage_1W_vs_E10	ordinary_muscle_development	ENSGALG00010012712	EEF1E1	0.624705249	0.133027971	4.69604433	2.65248E-06	4.80476E-06	positive
PRJNA729271	stage_1W_vs_E10	ordinary_muscle_development	ENSGALG00010012714	RGS2	0.468062526	0.173244848	2.701739941	0.006897769	0.009491472	positive
PRJNA729271	stage_1W_vs_E10	ordinary_muscle_development	ENSGALG00010012719	COL10A1	-1.783798251	0.770959479	-2.313738012	0.020682097	0.026978672	negative
PRJNA729271	stage_1W_vs_E10	ordinary_muscle_development	ENSGALG00010012720	ACO2	1.453201445	0.119864336	12.12371827	7.90879E-34	5.1569E-33	positive
PRJNA729271	stage_1W_vs_E10	ordinary_muscle_development	ENSGALG00010012722	ENSGALG00010012722	-0.295093355	0.10212252	-2.88960119	0.003857308	0.005438351	negative
PRJNA729271	stage_1W_vs_E10	ordinary_muscle_development	ENSGALG00010012725	ENSGALG00010012725	1.261263402	0.26041203	4.843337698	1.27676E-06	2.36597E-06	positive
PRJNA729271	stage_1W_vs_E10	ordinary_muscle_development	ENSGALG00010012726	IL7R	4.857431667	0.470560832	10.32264341	5.5669E-25	2.55841E-24	positive
PRJNA729271	stage_1W_vs_E10	ordinary_muscle_development	ENSGALG00010012730	CAP2	2.454017681	0.167694002	14.63390253	1.70708E-48	1.7725E-47	positive
PRJNA729271	stage_1W_vs_E10	ordinary_muscle_development	ENSGALG00010012732	MGST1	-1.251289794	0.176259805	-7.099121619	1.25552E-12	3.31188E-12	negative
PRJNA729271	stage_1W_vs_E10	ordinary_muscle_development	ENSGALG00010012733	ENSGALG00010012733	-2.192919661	1.106425653	-1.981985554	0.047480858	0.059448478	negative
PRJNA729271	stage_1W_vs_E10	ordinary_muscle_development	ENSGALG00010012734	TMEM19	-0.792123013	0.146611312	-5.402877856	6.55801E-08	1.32782E-07	negative
PRJNA729271	stage_1W_vs_E10	ordinary_muscle_development	ENSGALG00010012738	RAB21	0.654404799	0.068666882	9.530137085	1.57076E-21	6.28259E-21	positive
PRJNA729271	stage_1W_vs_E10	ordinary_muscle_development	ENSGALG00010012741	ENSGALG00010012741	0.356289386	0.151100665	2.357960415	0.018375652	0.024087094	positive
PRJNA729271	stage_1W_vs_E10	ordinary_muscle_development	ENSGALG00010012742	ENSGALG00010012742	-2.942966288	0.175084904	-16.80879514	2.10404E-63	3.36624E-62	negative
PRJNA729271	stage_1W_vs_E10	ordinary_muscle_development	ENSGALG00010012743	ENSGALG00010012743	0.865910381	0.508225486	1.703791733	0.088420003	0.107089567	positive
PRJNA729271	stage_1W_vs_E10	ordinary_muscle_development	ENSGALG00010012744	ARL4C	-3.205284662	0.237122951	-13.51739528	1.23471E-41	1.04968E-40	negative
PRJNA729271	stage_1W_vs_E10	ordinary_muscle_development	ENSGALG00010012745	MARCHF3	-0.557508049	0.323081914	-1.725593496	0.08442057	0.102476858	negative
PRJNA729271	stage_1W_vs_E10	ordinary_muscle_development	ENSGALG00010012747	TOB2	1.70631862	0.145185876	11.75264882	6.84393E-32	4.15945E-31	positive
PRJNA729271	stage_1W_vs_E10	ordinary_muscle_development	ENSGALG00010012748	ENSGALG00010012748	0.135608447	0.219528853	0.617724939	0.53675667	0.573091332	positive
PRJNA729271	stage_1W_vs_E10	ordinary_muscle_development	ENSGALG00010012749	RGS4	2.531152244	0.176733606	14.3218503	1.59811E-46	1.56518E-45	positive
PRJNA729271	stage_1W_vs_E10	ordinary_muscle_development	ENSGALG00010012751	AP3B1	-0.395450111	0.075298755	-5.251748366	1.50662E-07	2.97487E-07	negative
PRJNA729271	stage_1W_vs_E10	ordinary_muscle_development	ENSGALG00010012753	GTPBP1	-0.494561379	0.053261352	-9.285558166	1.60862E-20	6.19143E-20	negative
PRJNA729271	stage_1W_vs_E10	ordinary_muscle_development	ENSGALG00010012755	CLVS2	-2.913392043	0.404962848	-7.19422055	6.28186E-13	1.67844E-12	negative
PRJNA729271	stage_1W_vs_E10	ordinary_muscle_development	ENSGALG00010012756	RPA2	-2.062624452	0.107830218	-19.1284456	1.46374E-81	3.63303E-80	negative
PRJNA729271	stage_1W_vs_E10	ordinary_muscle_development	ENSGALG00010012758	NAB1	-0.59401816	0.129228305	-4.596656734	4.29324E-06	7.65161E-06	negative
PRJNA729271	stage_1W_vs_E10	ordinary_muscle_development	ENSGALG00010012759	ATP1B4	7.043523169	0.19762127	35.64152366	3.1882E-278	1.027E-275	positive
PRJNA729271	stage_1W_vs_E10	ordinary_muscle_development	ENSGALG00010012760	PLEKHF2	0.613827877	0.105783792	5.802664709	6.52692E-09	1.40097E-08	positive
PRJNA729271	stage_1W_vs_E10	ordinary_muscle_development	ENSGALG00010012761	SMPDL3A	2.190244021	0.22476101	9.744768542	1.94221E-22	8.01604E-22	positive
PRJNA729271	stage_1W_vs_E10	ordinary_muscle_development	ENSGALG00010012763	SMPDL3B	-0.238576706	0.134835245	-1.769394241	0.076828106	0.093676261	negative
PRJNA729271	stage_1W_vs_E10	ordinary_muscle_development	ENSGALG00010012765	PHF5A	0.062914794	0.107073732	0.587583838	0.556811662	0.592757421	positive
PRJNA729271	stage_1W_vs_E10	ordinary_muscle_development	ENSGALG00010012767	JOSD1	-0.909549982	0.072258815	-12.58739139	2.47725E-36	1.7576E-35	negative
PRJNA729271	stage_1W_vs_E10	ordinary_muscle_development	ENSGALG00010012768	EPS8	-1.266928939	0.117538084	-10.77888026	4.33126E-27	2.17012E-26	negative
PRJNA729271	stage_1W_vs_E10	ordinary_muscle_development	ENSGALG00010012769	NEMP2	-1.212750242	0.086537497	-14.01415904	1.27702E-44	1.18277E-43	negative
PRJNA729271	stage_1W_vs_E10	ordinary_muscle_development	ENSGALG00010012771	ITGA2	-1.533078766	0.190369633	-8.053168662	8.06776E-16	2.49184E-15	negative
PRJNA729271	stage_1W_vs_E10	ordinary_muscle_development	ENSGALG00010012772	TOMM22	-0.556024919	0.07471365	-7.442079453	9.91126E-14	2.75482E-13	negative
PRJNA729271	stage_1W_vs_E10	ordinary_muscle_development	ENSGALG00010012773	XKR8	-0.25707863	0.125406315	-2.049965581	0.04036779	0.05097849	negative
PRJNA729271	stage_1W_vs_E10	ordinary_muscle_development	ENSGALG00010012775	KCNE2	2.955950411	0.145477994	20.31888351	8.75448E-92	2.74667E-90	positive
PRJNA729271	stage_1W_vs_E10	ordinary_muscle_development	ENSGALG00010012778	TBC1D15	0.784975181	0.151360869	5.186117022	2.14724E-07	4.1969E-07	positive
PRJNA729271	stage_1W_vs_E10	ordinary_muscle_development	ENSGALG00010012780	FABP7	-2.83957925	0.25430521	-11.16602859	5.9795E-29	3.22204E-28	negative
PRJNA729271	stage_1W_vs_E10	ordinary_muscle_development	ENSGALG00010012781	SPIN1L	-0.51030239	3.185351174	-0.160202867	0.872721271	0.886979023	negative
PRJNA729271	stage_1W_vs_E10	ordinary_muscle_development	ENSGALG00010012786	TEF	1.062088066	0.087807662	12.09561946	1.11399E-33	7.21504E-33	positive
PRJNA729271	stage_1W_vs_E10	ordinary_muscle_development	ENSGALG00010012788	RGS1	2.050269322	0.648432403	3.161885978	0.001567509	0.002293202	positive
PRJNA729271	stage_1W_vs_E10	ordinary_muscle_development	ENSGALG00010012790	PITX1	-4.545220101	0.487175591	-9.329736931	1.06134E-20	4.11781E-20	negative
PRJNA729271	stage_1W_vs_E10	ordinary_muscle_development	ENSGALG00010012791	CFAP418	0.166217087	0.190839951	0.870976368	0.383767057	0.421704457	positive
PRJNA729271	stage_1W_vs_E10	ordinary_muscle_development	ENSGALG00010012792	CBY1	-0.269390512	0.144735374	-1.86126241	0.062707129	0.077323678	negative
PRJNA729271	stage_1W_vs_E10	ordinary_muscle_development	ENSGALG00010012795	TBCA	-0.088355834	0.136330834	-0.648098682	0.516921126	0.554132916	negative
PRJNA729271	stage_1W_vs_E10	ordinary_muscle_development	ENSGALG00010012796	MOCS2	0.871676749	0.121052538	7.200813484	5.98544E-13	1.60042E-12	positive
PRJNA729271	stage_1W_vs_E10	ordinary_muscle_development	ENSGALG00010012797	ZC3H7B	0.062203112	0.070467841	0.882716293	0.377389577	0.415168634	positive
PRJNA729271	stage_1W_vs_E10	ordinary_muscle_development	ENSGALG00010012798	MFSD6	0.64429055	0.095484554	6.747589269	1.50322E-11	3.74127E-11	positive
PRJNA729271	stage_1W_vs_E10	ordinary_muscle_development	ENSGALG00010012801	DMC1	2.987520397	0.424490422	7.037898256	1.95161E-12	5.08513E-12	positive
PRJNA729271	stage_1W_vs_E10	ordinary_muscle_development	ENSGALG00010012803	NUP155	-0.837224249	0.090386213	-9.262742866	1.99247E-20	7.62631E-20	negative
PRJNA729271	stage_1W_vs_E10	ordinary_muscle_development	ENSGALG00010012804	ENSGALG00010012804	0.527435566	0.160573503	3.284698638	0.001020915	0.001518851	positive
PRJNA729271	stage_1W_vs_E10	ordinary_muscle_development	ENSGALG00010012808	EYA3	-0.404131271	0.093101027	-4.340782082	1.41976E-05	2.43487E-05	negative
PRJNA729271	stage_1W_vs_E10	ordinary_muscle_development	ENSGALG00010012809	RHOBTB3	4.458428691	0.238929566	18.66001252	1.04707E-77	2.34579E-76	positive
PRJNA729271	stage_1W_vs_E10	ordinary_muscle_development	ENSGALG00010012812	DECR1	1.532335469	0.23961122	6.395090647	1.60452E-10	3.76946E-10	positive
PRJNA729271	stage_1W_vs_E10	ordinary_muscle_development	ENSGALG00010012818	RGS18	0.550470356	0.196908341	2.795566471	0.005180885	0.007224331	positive
PRJNA729271	stage_1W_vs_E10	ordinary_muscle_development	ENSGALG00010012819	RANGAP1	0.118480683	0.06719971	1.763113009	0.077881432	0.094896717	positive
PRJNA729271	stage_1W_vs_E10	ordinary_muscle_development	ENSGALG00010012821	ZBTB33	0.036352714	0.051966919	0.699535671	0.484217329	0.522421301	positive
PRJNA729271	stage_1W_vs_E10	ordinary_muscle_development	ENSGALG00010012824	HIBCH	1.275734217	0.117182476	10.88673201	1.33339E-27	6.82711E-27	positive
PRJNA729271	stage_1W_vs_E10	ordinary_muscle_development	ENSGALG00010012827	ENSGALG00010012827	0.060921164	0.18855941	0.323087369	0.746629075	0.771704824	positive
PRJNA729271	stage_1W_vs_E10	ordinary_muscle_development	ENSGALG00010012829	RGS5	5.095991552	0.18045787	28.23923147	1.93E-175	2.2028E-173	positive
PRJNA729271	stage_1W_vs_E10	ordinary_muscle_development	ENSGALG00010012830	ARRDC3	0.492675422	0.149423444	3.297176197	0.000976622	0.001456393	positive
PRJNA729271	stage_1W_vs_E10	ordinary_muscle_development	ENSGALG00010012831	ENSGALG00010012831	-1.437136397	0.384537734	-3.737309163	0.000186	0.00029337	negative
PRJNA729271	stage_1W_vs_E10	ordinary_muscle_development	ENSGALG00010012832	CHADL	-3.041562846	0.361867902	-8.405174457	4.27226E-17	1.3888E-16	negative
PRJNA729271	stage_1W_vs_E10	ordinary_muscle_development	ENSGALG00010012833	JAK2	0.381977468	0.105692972	3.614029012	0.000301475	0.000468671	positive
PRJNA729271	stage_1W_vs_E10	ordinary_muscle_development	ENSGALG00010012834	TPH2	-5.597936991	0.968330476	-5.781019112	7.42495E-09	1.58645E-08	negative
PRJNA729271	stage_1W_vs_E10	ordinary_muscle_development	ENSGALG00010012836	ENSGALG00010012836	-1.812928482	1.1533367	-1.57189872			negative
PRJNA729271	stage_1W_vs_E10	ordinary_muscle_development	ENSGALG00010012837	PKIB	0.645479089	0.273467372	2.360351379	0.018257632	0.023943217	positive
PRJNA729271	stage_1W_vs_E10	ordinary_muscle_development	ENSGALG00010012838	C2orf88	-0.136899533	0.236461841	-0.578949791	0.562623052	0.598372672	negative
PRJNA729271	stage_1W_vs_E10	ordinary_muscle_development	ENSGALG00010012839	TGFBI	-1.255083053	0.180121241	-6.967990257	3.215E-12	8.29799E-12	negative
PRJNA729271	stage_1W_vs_E10	ordinary_muscle_development	ENSGALG00010012841	L3MBTL2	-0.890444842	0.095217613	-9.351682087	8.62647E-21	3.3532E-20	negative
PRJNA729271	stage_1W_vs_E10	ordinary_muscle_development	ENSGALG00010012842	DDX17	-0.210777073	0.049198003	-4.284260746	1.83348E-05	3.11599E-05	negative
PRJNA729271	stage_1W_vs_E10	ordinary_muscle_development	ENSGALG00010012843	ENSGALG00010012843	0.067832438	0.159435146	0.425454736	0.670505212	0.700719037	positive
PRJNA729271	stage_1W_vs_E10	ordinary_muscle_development	ENSGALG00010012845	HDHD2	0.24817943	0.102071353	2.43143078	0.015039322	0.019921333	positive
PRJNA729271	stage_1W_vs_E10	ordinary_muscle_development	ENSGALG00010012847	RLN3	-1.237644218	0.830646205	-1.489977576	0.136230132	0.160763312	negative
PRJNA729271	stage_1W_vs_E10	ordinary_muscle_development	ENSGALG00010012848	SERINC1	1.20533151	0.092418856	13.04205187	7.05329E-39	5.44976E-38	positive
PRJNA729271	stage_1W_vs_E10	ordinary_muscle_development	ENSGALG00010012849	TRHDE	-3.686566426	0.361947848	-10.18535251	2.30523E-24	1.03035E-23	negative

PRJNA729271	stage_1W_vs_E10	ordinary_muscle_development	ENSGALG00010012850	CALB1	4.178927484	1.086880799	3.84488114	0.000120611	0.000192836	positive
PRJNA729271	stage_1W_vs_E10	ordinary_muscle_development	ENSGALG00010012851	ENSGALG00010012851	0.580073365	0.239453922	2.42248429	0.015414792	0.020399654	positive
PRJNA729271	stage_1W_vs_E10	ordinary_muscle_development	ENSGALG00010012852	MSTN	0.778788772	0.419098771	1.858246374	0.063134035	0.077797147	positive
PRJNA729271	stage_1W_vs_E10	ordinary_muscle_development	ENSGALG00010012854	LECT2	1.681176325	0.751939087	2.235787916	0.025365667	0.032787171	positive
PRJNA729271	stage_1W_vs_E10	ordinary_muscle_development	ENSGALG00010012856	AVR2	-0.367839987	1.168535765	-0.314787102	0.752923305	0.77749318	negative
PRJNA729271	stage_1W_vs_E10	ordinary_muscle_development	ENSGALG00010012857	NSDHL	-2.1060587	0.226290108	-9.306896894	1.31622E-20	5.0849E-20	negative
PRJNA729271	stage_1W_vs_E10	ordinary_muscle_development	ENSGALG00010012859	FBXL21	0.95383879	0.111259912	8.573067999	1.00764E-17	3.37705E-17	positive
PRJNA729271	stage_1W_vs_E10	ordinary_muscle_development	ENSGALG00010012860	ENSGALG00010012860	1.097288477	0.260318951	4.21516941	2.49591E-05	4.19798E-05	positive
PRJNA729271	stage_1W_vs_E10	ordinary_muscle_development	ENSGALG00010012861	WDR70	-0.314122296	0.082987416	-3.785179862	0.000153597	0.000243801	negative
PRJNA729271	stage_1W_vs_E10	ordinary_muscle_development	ENSGALG00010012863	KCNE1	-0.554116927	0.535045286	-1.03564491	0.300367832	0.33565891	negative
PRJNA729271	stage_1W_vs_E10	ordinary_muscle_development	ENSGALG00010012865	EP300	0.074079725	0.123561545	0.599537062	0.548814803	0.584888653	positive
PRJNA729271	stage_1W_vs_E10	ordinary_muscle_development	ENSGALG00010012867	A4GALT	0.491371102	0.102195047	4.808169417	1.52319E-06	2.8072E-06	positive
PRJNA729271	stage_1W_vs_E10	ordinary_muscle_development	ENSGALG00010012868	CTHRC1	-5.637616823	0.605783599	-9.306321317	1.32337E-20	5.11116E-20	negative
PRJNA729271	stage_1W_vs_E10	ordinary_muscle_development	ENSGALG00010012869	ENSGALG00010012869	2.294114113	0.212614073	10.79003886	3.83627E-27	1.92811E-26	positive
PRJNA729271	stage_1W_vs_E10	ordinary_muscle_development	ENSGALG00010012870	KCNC2	-2.438943615	0.213795879	-11.40781399	3.82214E-30	2.17263E-29	negative
PRJNA729271	stage_1W_vs_E10	ordinary_muscle_development	ENSGALG00010012871	ENSGALG00010012871	0.593216018	0.35458138	1.67300386	0.094326536	0.113853205	positive
PRJNA729271	stage_1W_vs_E10	ordinary_muscle_development	ENSGALG00010012873	PTAFR	1.694181585	0.223042283	7.595786607	3.05928E-14	8.73607E-14	positive
PRJNA729271	stage_1W_vs_E10	ordinary_muscle_development	ENSGALG00010012875	HSF2	-0.532386402	0.133704494	-3.98181381	6.83913E-05	0.000111473	negative
PRJNA729271	stage_1W_vs_E10	ordinary_muscle_development	ENSGALG00010012876	KDELR3	-1.303081669	0.164097491	-7.940899397	2.0072E-15	6.0867E-15	negative
PRJNA729271	stage_1W_vs_E10	ordinary_muscle_development	ENSGALG00010012877	SLC25A32	-0.552772061	0.095974309	-5.759583656	8.43217E-09	1.79636E-08	negative
PRJNA729271	stage_1W_vs_E10	ordinary_muscle_development	ENSGALG00010012878	GDNF	0.661776489	0.314726042	2.102706482	0.035491435	0.045092954	positive
PRJNA729271	stage_1W_vs_E10	ordinary_muscle_development	ENSGALG00010012879	RCAN1	-0.396452072	0.173856722	-2.280337892	0.022587656	0.02936132	negative
PRJNA729271	stage_1W_vs_E10	ordinary_muscle_development	ENSGALG00010012880	CLK4	1.775792359	0.138730665	12.80028718	1.63346E-37	1.20005E-36	positive
PRJNA729271	stage_1W_vs_E10	ordinary_muscle_development	ENSGALG00010012882	ENSGALG00010012882	0.63470745	0.612855719	1.035655589	0.300362848	0.33565891	positive
PRJNA729271	stage_1W_vs_E10	ordinary_muscle_development	ENSGALG00010012883	DCAF13	-0.550347104	0.067037672	-8.209519921	2.22075E-16	7.00538E-16	negative
PRJNA729271	stage_1W_vs_E10	ordinary_muscle_development	ENSGALG00010012884	ENSGALG00010012884	0.412646876	0.20861359	1.978044082	0.047923734	0.059966723	positive
PRJNA729271	stage_1W_vs_E10	ordinary_muscle_development	ENSGALG00010012885	PMS1	0.419656649	0.129295825	3.24570921	0.001171584	0.001732339	positive
PRJNA729271	stage_1W_vs_E10	ordinary_muscle_development	ENSGALG00010012887	KCNJ4	0.493064237	0.411067863	1.199471626	0.230344611	0.262055187	positive
PRJNA729271	stage_1W_vs_E10	ordinary_muscle_development	ENSGALG00010012888	ENSGALG00010012888	1.747625177	3.606065809	0.484634854	0.627935385	0.661332806	positive
PRJNA729271	stage_1W_vs_E10	ordinary_muscle_development	ENSGALG00010012889	RIC1	0.648870958	0.104893346	6.186006875	6.17073E-10	1.403E-09	positive
PRJNA729271	stage_1W_vs_E10	ordinary_muscle_development	ENSGALG00010012890	MFAP3	-0.461615665	0.070310299	-6.565406073	5.18913E-11	1.25382E-10	negative
PRJNA729271	stage_1W_vs_E10	ordinary_muscle_development	ENSGALG00010012891	CAPS2	-3.778880476	0.376497539	-10.03693275	1.04885E-23	4.57098E-23	negative
PRJNA729271	stage_1W_vs_E10	ordinary_muscle_development	ENSGALG00010012892	ORMDL1	-0.188444276	0.166037307	-1.13495141	0.256395678	0.289847526	negative
PRJNA729271	stage_1W_vs_E10	ordinary_muscle_development	ENSGALG00010012893	ENSGALG00010012893	1.724003456	0.187472888	9.196014806	3.71471E-20	1.40257E-19	positive
PRJNA729271	stage_1W_vs_E10	ordinary_muscle_development	ENSGALG00010012894	CLIC6	-0.224932521	0.669160494	-0.336141364	0.736764267	0.762705189	negative
PRJNA729271	stage_1W_vs_E10	ordinary_muscle_development	ENSGALG00010012896	STK38L	0.240243645	0.099508531	2.414302	0.015765389	0.020829397	positive
PRJNA729271	stage_1W_vs_E10	ordinary_muscle_development	ENSGALG00010012897	ENSGALG00010012897	0.736168451	0.301394111	2.442544243	0.014584142	0.019349852	positive
PRJNA729271	stage_1W_vs_E10	ordinary_muscle_development	ENSGALG00010012898	FAM114A2	1.173588928	0.114403637	10.2583183	1.08581E-24	4.93226E-24	positive
PRJNA729271	stage_1W_vs_E10	ordinary_muscle_development	ENSGALG00010012899	ENSGALG00010012899	1.006166547	0.130489652	7.710699909	1.2513E-14	3.65308E-14	positive
PRJNA729271	stage_1W_vs_E10	ordinary_muscle_development	ENSGALG00010012900	RBX1	0.453106843	0.172217059	2.631021837	0.008512857	0.011597167	positive
PRJNA729271	stage_1W_vs_E10	ordinary_muscle_development	ENSGALG00010012903	RGMB	-0.059176687	0.092780783	-0.637811896	0.523596135	0.560402125	negative
PRJNA729271	stage_1W_vs_E10	ordinary_muscle_development	ENSGALG00010012904	ERMP1	-0.726157773	0.138284675	-5.25118038	1.51128E-07	2.98365E-07	negative
PRJNA729271	stage_1W_vs_E10	ordinary_muscle_development	ENSGALG00010012905	NKAIN1	-4.63922099	0.410331234	-11.30603914	1.22495E-29	6.81863E-29	negative
PRJNA729271	stage_1W_vs_E10	ordinary_muscle_development	ENSGALG00010012908	STRAP	-0.195563324	0.082948441	-2.35764919	0.018391064	0.024102936	negative
PRJNA729271	stage_1W_vs_E10	ordinary_muscle_development	ENSGALG00010012910	XPNPEP3	0.35636488	0.099126222	3.595061666	0.000324315	0.000502828	positive
PRJNA729271	stage_1W_vs_E10	ordinary_muscle_development	ENSGALG00010012911	CSNK1E	-0.968845443	0.08591284	-11.27707389	1.70317E-29	9.41551E-29	negative
PRJNA729271	stage_1W_vs_E10	ordinary_muscle_development	ENSGALG00010012912	NKAPL	0.093018678	0.09996092	0.930550445	0.352086159	0.38966854	positive
PRJNA729271	stage_1W_vs_E10	ordinary_muscle_development	ENSGALG00010012913	EGFLAM	-1.238648556	0.155042264	-7.989102619	1.35925E-15	4.15484E-15	negative
PRJNA729271	stage_1W_vs_E10	ordinary_muscle_development	ENSGALG00010012915	ENSGALG00010012915	6.360278301	0.27784579	22.89139706	5.6598E-116	2.8387E-114	positive
PRJNA729271	stage_1W_vs_E10	ordinary_muscle_development	ENSGALG00010012917	RUNX1	-0.627184932	0.256978255	-2.440614804	0.014662285	0.019444631	negative
PRJNA729271	stage_1W_vs_E10	ordinary_muscle_development	ENSGALG00010012918	GLRX	0.360044777	0.282336391	1.275233336	0.202226665	0.232567083	positive
PRJNA729271	stage_1W_vs_E10	ordinary_muscle_development	ENSGALG00010012921	EBF1	-0.635845869	0.141189573	-4.503490276	6.68464E-06	1.17262E-05	negative
PRJNA729271	stage_1W_vs_E10	ordinary_muscle_development	ENSGALG00010012922	SNRNP40	-0.822781003	0.129495397	-6.353747089	2.10132E-10	4.89768E-10	negative
PRJNA729271	stage_1W_vs_E10	ordinary_muscle_development	ENSGALG00010012923	MLANA	-5.322206599	1.007860832	-5.280695938	1.28694E-07	2.55083E-07	negative
PRJNA729271	stage_1W_vs_E10	ordinary_muscle_development	ENSGALG00010012924	GLIPR1	0.070758802	0.202739659	0.349013126	0.727079455	0.753863856	positive
PRJNA729271	stage_1W_vs_E10	ordinary_muscle_development	ENSGALG00010012925	SETD4	-1.52447853	0.198185265	-7.692189061	1.44639E-14	4.20401E-14	negative
PRJNA729271	stage_1W_vs_E10	ordinary_muscle_development	ENSGALG00010012926	OSGEPL1	0.918069056	0.163872872	5.602324805	2.11496E-08	4.40211E-08	positive
PRJNA729271	stage_1W_vs_E10	ordinary_muscle_development	ENSGALG00010012927	ENSGALG00010012927	-0.137385356	3.689867106	-0.037233145			negative
PRJNA729271	stage_1W_vs_E10	ordinary_muscle_development	ENSGALG00010012930	IL6ST	0.439405409	0.090977854	4.82980627	1.36666E-06	2.5274E-06	positive
PRJNA729271	stage_1W_vs_E10	ordinary_muscle_development	ENSGALG00010012932	ATF6	0.666724057	0.118033403	5.6486049	1.61755E-08	3.38919E-08	positive
PRJNA729271	stage_1W_vs_E10	ordinary_muscle_development	ENSGALG00010012933	ZCCHC17	-0.673877221	0.14907059	-4.520524282	6.16867E-06	1.08513E-05	negative
PRJNA729271	stage_1W_vs_E10	ordinary_muscle_development	ENSGALG00010012934	NDUFA1	1.62901935	0.151465566	10.75504744	5.61051E-27	2.79561E-26	positive
PRJNA729271	stage_1W_vs_E10	ordinary_muscle_development	ENSGALG00010012938	UPF3B	0.307520124	0.150435032	2.044205526	0.040933247	0.051661073	positive
PRJNA729271	stage_1W_vs_E10	ordinary_muscle_development	ENSGALG00010012939	GJA1	-3.512226729	0.207633336	-16.91552422	3.45727E-64	5.61177E-63	negative
PRJNA729271	stage_1W_vs_E10	ordinary_muscle_development	ENSGALG00010012940	FBN2	-3.37519535	0.224468363	-15.03639671	4.2401E-51	4.82041E-50	negative
PRJNA729271	stage_1W_vs_E10	ordinary_muscle_development	ENSGALG00010012943	ASNSD1	-0.371450284	0.12338665	-3.010457648	0.002608543	0.003738465	negative
PRJNA729271	stage_1W_vs_E10	ordinary_muscle_development	ENSGALG00010012944	NCK1	-0.466908183	0.053265546	-8.765669743	1.8567E-18	6.46011E-18	negative
PRJNA729271	stage_1W_vs_E10	ordinary_muscle_development	ENSGALG00010012945	CLINT1	-0.057761931	0.063025978	-0.916478139	0.359416168	0.397023118	negative
PRJNA729271	stage_1W_vs_E10	ordinary_muscle_development	ENSGALG00010012948	ST13P5	-0.896364215	0.095461363	-9.389811627	6.01077E-21	2.34968E-20	negative
PRJNA729271	stage_1W_vs_E10	ordinary_muscle_development	ENSGALG00010012949	ENSGALG00010012949	-0.609822167	0.176323458	-3.458542465	0.000543107	0.000825884	negative
PRJNA729271	stage_1W_vs_E10	ordinary_muscle_development	ENSGALG00010012952	BRD10	1.320994126	0.186353178	7.088658953	1.35418E-12	3.56369E-12	positive
PRJNA729271	stage_1W_vs_E10	ordinary_muscle_development	ENSGALG00010012954	RIMS2	-1.502668704	0.3672146	-4.092072334	4.27535E-05	7.07192E-05	negative
PRJNA729271	stage_1W_vs_E10	ordinary_muscle_development	ENSGALG00010012955	RPL39L	0.345610889	0.155187679	2.227051087	0.02594386	0.033486754	positive
PRJNA729271	stage_1W_vs_E10	ordinary_muscle_development	ENSGALG00010012956	FABP3	3.582276018	0.16821866	21.29535468	1.25354E-100	4.74414E-99	positive
PRJNA729271	stage_1W_vs_E10	ordinary_muscle_development	ENSGALG00010012957	DERA	-0.024206659	0.228086642	-0.106129227	0.915479823	0.925771622	negative
PRJNA729271	stage_1W_vs_E10	ordinary_muscle_development	ENSGALG00010012958	OLFML2B	0.745645905	0.205049234	3.636423763	0.000276449	0.000431014	positive
PRJNA729271	stage_1W_vs_E10	ordinary_muscle_development	ENSGALG00010012959	TBC1D32	-0.093450626	0.147414971	-0.633929004	0.526127156	0.562903242	negative
PRJNA729271	stage_1W_vs_E10	ordinary_muscle_development	ENSGALG00010012962	SLC25A17	-0.800108078	0.209885042	-3.812125298	0.000137777	0.000219242	negative
PRJNA729271	stage_1W_vs_E10	ordinary_muscle_development	ENSGALG00010012963	UIMC1	0.075724103	0.090028305	0.841114387	0.400283856	0.438557407	positive
PRJNA729271	stage_1W_vs_E10	ordinary_muscle_development	ENSGALG00010012964	CBR3	-0.401922155	0.072816965	-5.519622451	3.39729E-08	6.98492E-08	negative
PRJNA729271	stage_1W_vs_E10	ordinary_muscle_development	ENSGALG00010012965	ENSGALG00010012965	-0.186775328	0.08463212	-2.206908298	0.027320467	0.035157152	negative
PRJNA729271	stage_1W_vs_E10	ordinary_muscle_development	ENSGALG00010012966	GLIPR1L	0.390773522	0.269677725	1.449038928	0.147326712	0.172971058	positive
PRJNA729271	stage_1W_vs_E10	ordinary_muscle_development	ENSGALG00010012967	ECRG4	-1.718857902	0.209954672	-8.186804716	2.68253E-16	8.43821E-16	negative
PRJNA729271	stage_1W_vs_E10	ordinary_muscle_development	ENSGALG00010012968	LSM11	-1.087702204	0.078787952	-13.80543827	2.36331E-43	2.10548E-42	negative
PRJNA729271	stage_1W_vs_E10	ordinary_muscle_development	ENSGALG00010012969	SLC12A2	0.121412415	0.142357826	0.852867864	0.393732585	0.431869377	positive
PRJNA729271	stage_1W_vs_E10	ordinary_muscle_development	ENSGALG00010012970	UNC5A	-1.638245271	0.197721664	-8.285613407	1.17502E-16	3.75897E-16	negative
PRJNA729271	stage_1W_vs_E10	ordinary_muscle_development	ENSGALG00010012971	ENSGALG00010012971	-1.243219312	0.659051205	-1.886377422	0.059244113	0.073284128	negative
PRJNA729271	stage_1W_vs_E10	ordinary_muscle_development	ENSGALG00010012973	GLDC	-4.68545117	0.342840029	-13.66658142	1.60773E-42	1.3988E-41	negative
PRJNA729271	stage_1W_vs_E10	ordinary_muscle_development	ENSGALG00010012974	SLC49A3	-1.4725541	0.224003301	-6.573805359	4.90453E-11	1.18802E-10	negative
PRJNA729271	stage_1W_vs_E10	ordinary_muscle_development	ENSGALG00010012975	KRR1	-0.286059301	0.093186458	-3.069751825	0.002142367	0.003093914	negative
PRJNA729271	stage_1W_vs_E10	ordinary_muscle_development	ENSGALG00010012976	SLC9A2	2.384841931	0.232800278	10.24415415	1.2572E-24	5.69293E-24	positive
PRJNA729271	stage_1W_vs_E10	ordinary_muscle_development	ENSGALG00010012977	NKRF	-0.198817326	0.099225305	-2.003695777	0.045102659	0.056647001	negative
PRJNA729271	stage_1W_vs_E10	ordinary_muscle_development	ENSGALG00010012978	HSD17B7	-0.193029294	0.210799236	-0.915702059	0.359823185	0.39741215	negative
PRJNA729271	stage_1W_vs_E10	ordinary_muscle_development	ENSGALG00010012979	HTR1A	1.441227212	0.53181007	2.710041224	0.006727485	0.009265073	positive
PRJNA729271	stage_1W_vs_E10	ordinary_muscle_development	ENSGALG00010012980	KICS2	-0.407220601	0.078348513	-5.197553691	2.01928E-07	3.95427E-07	negative
PRJNA729271	stage_1W_vs_E10	ordinary_muscle_development	ENSGALG00010012982	DDR2	-0.752142222	0.243897887	-3.08384067	0.00204347	0.002958164	negative
PRJNA729271	stage_1W_vs_E10	ordinary_muscle_development	ENSGALG00010012985	ARNTL2	-0.217079293	0.170863094	-1.270486724	0.203911309	0.234281422	negative
PRJNA729271	stage_1W_vs_E10	ordinary_muscle_development	ENSGALG00010012986	TMEM184B	0.654330655	0.140234249	4.665983238	3.07145E-06	5.53395E-06	positive
PRJNA729271	stage_1W_vs_E10	ordinary_muscle_development	ENSGALG00010012988	RPL18A	0.666026967	0.134875723	4.938078944	7.88959E-07	1.48712E-06	positive
PRJNA729271	stage_1W_vs_E10	ordinary_muscle_development	ENSGALG00010012991	MFSD9	0.02649295	0.102182418	0.259271119	0.795426062	0.817152839	positive
PRJNA729271	stage_1W_vs_E10	ordinary_muscle_development	ENSGALG00010012992	HARS1	0.279780128	0.126626313	2.209494389	0.027140272	0.034943884	positive
PRJNA729271	stage_1W_vs_E10	ordinary_muscle_development	ENSGALG00010012993	DOP1B	-0.325430148	0.099741666	-3.262730211	0.001103445	0.001635925	negative
PRJNA729271	stage_1W_vs_E10	ordinary_muscle_development	ENSGALG00010012995	SERINC2	1.060102457	0.14907	7.111440662	1.14838E-12	3.03422E-12	positive
PRJNA729271	stage_1W_vs_E10	ordinary_muscle_development	ENSGALG00010012997	UBE2A	0.8656537	0.057378805	15.08664569	1.98269E-51	2.28269E-50	positive
PRJNA729271	stage_1W_vs_E10	ordinary_muscle_development	ENSGALG00010012998	MAFF	0.390077886	0.199812975	1.952214997	0.050912684	0.063520344	positive
PRJNA729271	stage_1W_vs_E10	ordinary_muscle_development	ENSGALG00010013001	ADGRV1	-2.240472315	0.358081051	-6.256885998	3.9274E-10	9.03328E-10	negative
PRJNA729271	stage_1W_vs_E10	ordinary_muscle_development	ENSGALG00010013004	UAP1	-0.508644283	0.154656894	-3.288856198	0.001005954	0.001497977	negative
PRJNA729271	stage_1W_vs_E10	ordinary_muscle_development	ENSGALG00010013005	SLC40A1	0.623736913	0.090868604	6.86416304	6.68822E-12	1.69901E-11	positive
PRJNA729271	stage_1W_vs_E10	ordinary_muscle_development	ENSGALG00010013006	GRIK2	-6.040812367	1.174255433	-5.144376765	2.6841E-07	5.20901E-07	negative
PRJNA729271	stage_1W_vs_E10	ordinary_muscle_development	ENSGALG00010013008	STEEP1	-0.071924401	0.109360328	-0.657682749	0.51074201	0.548263751	negative
PRJNA729271	stage_1W_vs_E10	ordinary_muscle_development	ENSGALG00010013012	TMEM182	2.15086828	0.151481116	14.19892024	9.30348E-46	8.89538E-45	positive
PRJNA729271	stage_1W_vs_E10	ordinary_muscle_development	ENSGALG00010013013	DCSTAMP	3.401492557	1.114700139	3.051486619	0.002277112	0.003279684	positive
PRJNA729271	stage_1W_vs_E10	ordinary_muscle_development	ENSGALG00010013015	PLA2G6	0.968496849	0.148796976	6.508847677	7.57295E-11	1.8087E-10	positive
PRJNA729271	stage_1W_vs_E10	ordinary_muscle_development	ENSGALG00010013016	QRSL1	1.046123725	0.121796545	8.589108394	8.76468E-18	2.94697E-17	positive
PRJNA729271	stage_1W_vs_E10	ordinary_muscle_development	ENSGALG00010013017	SCMH1	1.160053609	0.15761681	7.359961227	1.83964E-13	5.04265E-13	positive
PRJNA729271	stage_1W_vs_E10	ordinary_muscle_development	ENSGALG00010013018	ID1	0.40763497	0.192049833	2.122547902	0.033791755	0.043030087	positive
PRJNA729271	stage_1W_vs_E10	ordinary_muscle_development	ENSGALG00010013019	DPYS	0.463912482	0.940022479	0.493512115	0.621650781	0.655619039	positive
PRJNA729271	stage_1W_vs_E10	ordinary_muscle_development	ENSGALG00010013021	SLC25A5	-1.290481104	0.164127951	-7.862652862	3.76082E-15	1.12654E-14	negative
PRJNA729271	stage_1W_vs_E10	ordinary_muscle_development	ENSGALG00010013027	UHMK1	1.030123714	0.125420344	8.21330202	2.15065E-16	6.79314E-16	positive
PRJNA729271	stage_1W_vs_E10	ordinary_muscle_development	ENSGALG00010013029	MYLK2	9.572033102	0.253702863	37.72930661	1.6434E-311	6.2686E-309	positive
PRJNA729271	stage_1W_vs_E10	ordinary_muscle_development	ENSGALG00010013032	BAIAP2L2	2.302179158	0.378917265	6.075677652	1.23465E-09	2.7669E-09	positive
PRJNA729271	stage_1W_vs_E10	ordinary_muscle_development	ENSGALG00010013033	MAP3K1	-0.784905593	0.12624606	-6.217268026	5.05885E-10	1.15459E-09	negative
PRJNA729271	stage_1W_vs_E10	ordinary_muscle_development	ENSGALG00010013036	ENSGALG00010013036	0.969839029	0.429725577	2.256879925	0.024015579	0.031142048	positive
PRJNA729271	stage_1W_vs_E10	ordinary_muscle_development	ENSGALG00010013037	MORC4	0.770167416	0.089616946	8.593993134	8.3998E-18	2.82823E-17	positive
PRJNA729271	stage_1W_vs_E10	ordinary_muscle_development	ENSGALG00010013040	SLC25A43	2.504003502	0.203508521	12.3041703	8.60158E-35	5.80176E-34	positive
PRJNA729271	stage_1W_vs_E10	ordinary_muscle_development	ENSGALG00010013041	LRP12	0.281666649	0.075013666	3.754871132	0.000173431	0.000274171	positive
PRJNA729271	stage_1W_vs_E10	ordinary_muscle_development	ENSGALG00010013042	SLC25A48	-1.74156771	0.323930249	-5.3763666	7.6004E-08	1.53309E-07	negative
PRJNA729271	stage_1W_vs_E10	ordinary_muscle_development	ENSGALG00010013044	ENSGALG00010013044	0.717119649	0.125793935	5.700748996	1.19282E-08	2.51783E-08	positive
PRJNA729271	stage_1W_vs_E10	ordinary_muscle_development	ENSGALG00010013045	FSD1L	1.08315394	0.144847524	7.477890633	7.5525E-14	2.11625E-13	positive
PRJNA729271	stage_1W_vs_E10	ordinary_muscle_development	ENSGALG00010013046	SLC12A5	-0.821931525	0.383207983	-2.144870569	0.031963197	0.040805579	negative
PRJNA729271	stage_1W_vs_E10	ordinary_muscle_development	ENSGALG00010013047	CTPS1	0.184011769	0.100287923	1.834834775	0.066530174	0.081780434	positive

PRJNA729271	stage_1W_vs_E10	ordinary_muscle_development	ENSGALG00010013048	WDR75	-1.516056878	0.089437487	-16.95102271	1.89135E-64	3.09077E-63	negative
PRJNA729271	stage_1W_vs_E10	ordinary_muscle_development	ENSGALG00010013051	XPOT	0.452101217	0.078580998	5.753314804	8.75103E-09	1.86237E-08	positive
PRJNA729271	stage_1W_vs_E10	ordinary_muscle_development	ENSGALG00010013053	MRTFA	0.828069111	0.174408787	4.747863485	2.05577E-06	3.75011E-06	positive
PRJNA729271	stage_1W_vs_E10	ordinary_muscle_development	ENSGALG00010013056	ENSGALG00010013056	2.55590859	0.561327225	4.553330885	5.28031E-06	9.33961E-06	positive
PRJNA729271	stage_1W_vs_E10	ordinary_muscle_development	ENSGALG00010013058	ENSGALG00010013058	0.471728981	0.276736665	1.704613234	0.088266582	0.106930556	positive
PRJNA729271	stage_1W_vs_E10	ordinary_muscle_development	ENSGALG00010013062	ENSGALG00010013062	0.165519717	0.106089205	1.560193781	0.118714095	0.141242781	positive
PRJNA729271	stage_1W_vs_E10	ordinary_muscle_development	ENSGALG00010013063	PARP12	1.056771037	0.217602928	4.856419189	1.19528E-06	2.22236E-06	positive
PRJNA729271	stage_1W_vs_E10	ordinary_muscle_development	ENSGALG00010013065	FKTN	-0.245549864	0.104352072	-2.353090448	0.018618105	0.024389465	negative
PRJNA729271	stage_1W_vs_E10	ordinary_muscle_development	ENSGALG00010013071	ENSGALG00010013071	-1.268574229	0.226035093	-5.612288838	1.99668E-08	4.1637E-08	negative
PRJNA729271	stage_1W_vs_E10	ordinary_muscle_development	ENSGALG00010013076	RTN4IP1	0.193228206	0.080863728	2.389553532	0.016868865	0.022210392	positive
PRJNA729271	stage_1W_vs_E10	ordinary_muscle_development	ENSGALG00010013078	COL5A2	-1.358003976	0.242052751	-5.610363741	2.01902E-08	4.20846E-08	negative
PRJNA729271	stage_1W_vs_E10	ordinary_muscle_development	ENSGALG00010013079	ENSGALG00010013079	-0.952173446	0.206549776	-4.609898217	4.02866E-06	7.19156E-06	negative
PRJNA729271	stage_1W_vs_E10	ordinary_muscle_development	ENSGALG00010013080	ARHGEF7	-0.241589071	0.099974196	-2.416514273	0.015669912	0.020718359	negative
PRJNA729271	stage_1W_vs_E10	ordinary_muscle_development	ENSGALG00010013081	NCOA5	-0.501143652	0.100944984	-4.964522579	6.88703E-07	1.30221E-06	negative
PRJNA729271	stage_1W_vs_E10	ordinary_muscle_development	ENSGALG00010013082	CHAF1B	-2.27975211	0.117324204	-19.43121736	4.20276E-84	1.1036E-82	negative
PRJNA729271	stage_1W_vs_E10	ordinary_muscle_development	ENSGALG00010013084	SGSM3	0.88735508	0.115318861	7.694795763	1.4172E-14	4.12166E-14	positive
PRJNA729271	stage_1W_vs_E10	ordinary_muscle_development	ENSGALG00010013085	LYSMD3	-0.304067651	0.09091246	-3.344620226	0.000823953	0.001236868	negative
PRJNA729271	stage_1W_vs_E10	ordinary_muscle_development	ENSGALG00010013086	CXCL14	-2.815422864	0.517527447	-5.440142125	5.32381E-08	1.08444E-07	negative
PRJNA729271	stage_1W_vs_E10	ordinary_muscle_development	ENSGALG00010013087	GPN2	-0.092012008	0.092331772	-0.996536786	0.318989403	0.355235971	negative
PRJNA729271	stage_1W_vs_E10	ordinary_muscle_development	ENSGALG00010013089	TBK1	0.458692809	0.076428994	6.00155496	1.95437E-09	4.33423E-09	positive
PRJNA729271	stage_1W_vs_E10	ordinary_muscle_development	ENSGALG00010013093	GPATCH3	0.706985836	0.156270054	4.524128713	6.06448E-06	1.06784E-05	positive
PRJNA729271	stage_1W_vs_E10	ordinary_muscle_development	ENSGALG00010013095	ADSL	3.145460619	0.127553398	24.6599516	2.8783E-134	1.9496E-132	positive
PRJNA729271	stage_1W_vs_E10	ordinary_muscle_development	ENSGALG00010013096	CLDN14	-1.006733203	0.426670795	-2.359508113	0.018299181	0.023993363	negative
PRJNA729271	stage_1W_vs_E10	ordinary_muscle_development	ENSGALG00010013097	ENSGALG00010013097	-0.053684509	0.421261374	-0.12743753	0.898594114	0.91033839	negative
PRJNA729271	stage_1W_vs_E10	ordinary_muscle_development	ENSGALG00010013099	SIM2	0.657599455	0.202034183	3.254892047	0.001134355	0.001679689	positive
PRJNA729271	stage_1W_vs_E10	ordinary_muscle_development	ENSGALG00010013100	ZFPM2	-1.99755814	0.190658557	-10.47714915	1.1001E-25	5.20641E-25	negative
PRJNA729271	stage_1W_vs_E10	ordinary_muscle_development	ENSGALG00010013103	AIRIM	0.463216628	0.143022969	3.238756902	0.001200518	0.001772951	positive
PRJNA729271	stage_1W_vs_E10	ordinary_muscle_development	ENSGALG00010013104	HLCS	-0.521927445	0.110055138	-4.742417807	2.11183E-06	3.84656E-06	negative
PRJNA729271	stage_1W_vs_E10	ordinary_muscle_development	ENSGALG00010013106	SETD9	0.406751763	0.179630864	2.264375696	0.023551017	0.03056424	positive
PRJNA729271	stage_1W_vs_E10	ordinary_muscle_development	ENSGALG00010013109	ENSGALG00010013109	-1.103420499	1.596695519	-0.691065069	0.489524651	0.527715292	negative
PRJNA729271	stage_1W_vs_E10	ordinary_muscle_development	ENSGALG00010013110	ANKRD10	1.392699157	0.154544602	9.011632512	2.03012E-19	7.42157E-19	positive
PRJNA729271	stage_1W_vs_E10	ordinary_muscle_development	ENSGALG00010013111	RFESD	1.668540212	0.280992608	5.938021725	2.88482E-09	6.32896E-09	positive
PRJNA729271	stage_1W_vs_E10	ordinary_muscle_development	ENSGALG00010013112	CD40	0.573045809	0.162077006	3.535639166	0.00040679	0.000624819	positive
PRJNA729271	stage_1W_vs_E10	ordinary_muscle_development	ENSGALG00010013114	PIGP	0.080222438	0.122626285	0.654202625	0.512981271	0.550286723	positive
PRJNA729271	stage_1W_vs_E10	ordinary_muscle_development	ENSGALG00010013115	ENSGALG00010013115	-2.804906966	0.391529153	-7.163979867	7.83679E-13	2.08392E-12	negative
PRJNA729271	stage_1W_vs_E10	ordinary_muscle_development	ENSGALG00010013116	TARS	-0.195349182	0.168054719	-1.162414141	0.245067282	0.277693109	negative
PRJNA729271	stage_1W_vs_E10	ordinary_muscle_development	ENSGALG00010013117	ARSK	-0.431099241	0.133309542	-3.233821341	0.001221458	0.001802957	negative
PRJNA729271	stage_1W_vs_E10	ordinary_muscle_development	ENSGALG00010013119	RNF114	0.565880588	0.078906385	7.171543707	7.41568E-13	1.9752E-12	positive
PRJNA729271	stage_1W_vs_E10	ordinary_muscle_development	ENSGALG00010013121	PCBD2	0.474380616	0.163517395	2.90110184	0.00371853	0.005251885	positive
PRJNA729271	stage_1W_vs_E10	ordinary_muscle_development	ENSGALG00010013123	PPFIBP1	-0.263682613	0.108403833	-2.432410409	0.014998702	0.019872594	negative
PRJNA729271	stage_1W_vs_E10	ordinary_muscle_development	ENSGALG00010013125	POU3F3	-7.859049167	0.878220055	-8.948838185	3.59243E-19	1.29953E-18	negative
PRJNA729271	stage_1W_vs_E10	ordinary_muscle_development	ENSGALG00010013129	RASSF3	-0.353060582	0.111388492	-3.169632475	0.001526319	0.002234746	negative
PRJNA729271	stage_1W_vs_E10	ordinary_muscle_development	ENSGALG00010013131	SPATA2	0.107504272	0.117589502	0.914233582	0.360594122	0.398081629	positive
PRJNA729271	stage_1W_vs_E10	ordinary_muscle_development	ENSGALG00010013132	SKIC3	-0.799732912	0.09008948	-8.87709544	6.86289E-19	2.44597E-18	negative
PRJNA729271	stage_1W_vs_E10	ordinary_muscle_development	ENSGALG00010013133	TXNDC15	0.030557714	0.119694587	0.255297375	0.798493427	0.819815996	positive
PRJNA729271	stage_1W_vs_E10	ordinary_muscle_development	ENSGALG00010013134	ENSGALG00010013134	-0.169334661	5.383977549	-0.031451591	0.974909398	0.978554928	negative
PRJNA729271	stage_1W_vs_E10	ordinary_muscle_development	ENSGALG00010013135	TTC3	-0.583386448	0.085701498	-6.807190777	9.95229E-12	2.50318E-11	negative
PRJNA729271	stage_1W_vs_E10	ordinary_muscle_development	ENSGALG00010013137	TMEM121	-5.512960381	0.299228735	-18.42390031	8.44903E-76	1.81435E-74	negative
PRJNA729271	stage_1W_vs_E10	ordinary_muscle_development	ENSGALG00010013139	TMEM185A	0.931258115	0.08209431	11.34375961	7.96476E-30	4.46785E-29	positive
PRJNA729271	stage_1W_vs_E10	ordinary_muscle_development	ENSGALG00010013143	ENSGALG00010013143	0.091776033	0.435030177	0.210964751	0.832914777	0.851177362	positive
PRJNA729271	stage_1W_vs_E10	ordinary_muscle_development	ENSGALG00010013144	TEDC1	-1.874639119	0.176639178	-10.61281614	2.59798E-26	1.26326E-25	negative
PRJNA729271	stage_1W_vs_E10	ordinary_muscle_development	ENSGALG00010013145	ENSGALG00010013145	-0.5721878	0.296804207	-1.927829144	0.053876381	0.066998812	negative
PRJNA729271	stage_1W_vs_E10	ordinary_muscle_development	ENSGALG00010013146	TNRC6B	0.692998702	0.119447049	5.801723108	6.56369E-09	1.40866E-08	positive
PRJNA729271	stage_1W_vs_E10	ordinary_muscle_development	ENSGALG00010013149	MCTP1	-0.149257297	0.202601634	-0.736703323	0.461302786	0.499558004	negative
PRJNA729271	stage_1W_vs_E10	ordinary_muscle_development	ENSGALG00010013150	GNS	0.58837512	0.103178386	5.702503622	1.1806E-08	2.49349E-08	positive
PRJNA729271	stage_1W_vs_E10	ordinary_muscle_development	ENSGALG00010013152	TBXAS1	-0.207312546	0.226657004	-0.914653165	0.360373739	0.397868638	negative
PRJNA729271	stage_1W_vs_E10	ordinary_muscle_development	ENSGALG00010013154	ENSGALG00010013154	-2.113627276	0.494147715	-4.277318724	1.89158E-05	3.21059E-05	negative
PRJNA729271	stage_1W_vs_E10	ordinary_muscle_development	ENSGALG00010013156	ABCC9	2.156664479	0.129638157	16.63603165	3.82117E-62	5.88607E-61	positive
PRJNA729271	stage_1W_vs_E10	ordinary_muscle_development	ENSGALG00010013158	CRIP1	3.209878544	0.184712978	17.37765576	1.21833E-67	2.16949E-66	positive
PRJNA729271	stage_1W_vs_E10	ordinary_muscle_development	ENSGALG00010013159	ENSGALG00010013159	0.746112852	1.392404376	0.535844949	0.592065714	0.626514274	positive
PRJNA729271	stage_1W_vs_E10	ordinary_muscle_development	ENSGALG00010013160	CRYBG1	-4.766783928	0.606538263	-7.858999533	3.87214E-15	1.15916E-14	negative
PRJNA729271	stage_1W_vs_E10	ordinary_muscle_development	ENSGALG00010013162	IDS	0.001876181	0.112127146	0.016732616	0.986649927	0.988764566	positive
PRJNA729271	stage_1W_vs_E10	ordinary_muscle_development	ENSGALG00010013163	ENSGALG00010013163	-0.779768845	0.496251523	-1.571317788	0.116108852	0.138358912	negative
PRJNA729271	stage_1W_vs_E10	ordinary_muscle_development	ENSGALG00010013164	FAM83F	-2.082379172	0.461914371	-4.508149781	6.53954E-06	1.14786E-05	negative
PRJNA729271	stage_1W_vs_E10	ordinary_muscle_development	ENSGALG00010013165	NUDC	-0.251221263	0.081193297	-3.094113314	0.001974021	0.002861915	negative
PRJNA729271	stage_1W_vs_E10	ordinary_muscle_development	ENSGALG00010013168	VPS26C	0.112516836	0.138097121	0.814765978	0.415206279	0.453193901	positive
PRJNA729271	stage_1W_vs_E10	ordinary_muscle_development	ENSGALG00010013170	COL3A1	2.251954375	0.234907098	9.586574406	9.10581E-22	3.66432E-21	positive
PRJNA729271	stage_1W_vs_E10	ordinary_muscle_development	ENSGALG00010013171	SLC9A8	0.412112828	0.13636164	3.022204974	0.002509406	0.003603154	positive
PRJNA729271	stage_1W_vs_E10	ordinary_muscle_development	ENSGALG00010013172	TNFSF13B	2.229150524	0.424298061	5.253737244	1.49043E-07	2.94371E-07	positive
PRJNA729271	stage_1W_vs_E10	ordinary_muscle_development	ENSGALG00010013173	KCNJ8	0.505286941	0.15547514	3.249953284	0.00115424	0.001707918	positive
PRJNA729271	stage_1W_vs_E10	ordinary_muscle_development	ENSGALG00010013174	ADAMTS12	0.433509818	0.165359224	2.621624657	0.008751175	0.011907283	positive
PRJNA729271	stage_1W_vs_E10	ordinary_muscle_development	ENSGALG00010013175	HIPK2	2.886661151	0.162781133	17.73338899	2.31641E-70	4.4296E-69	positive
PRJNA729271	stage_1W_vs_E10	ordinary_muscle_development	ENSGALG00010013177	OXR1	0.907691964	0.069286142	13.1006279	3.26545E-39	2.55163E-38	positive
PRJNA729271	stage_1W_vs_E10	ordinary_muscle_development	ENSGALG00010013179	TBC1D30	0.092880376	0.186478956	0.498074302	0.618431673	0.652556388	positive
PRJNA729271	stage_1W_vs_E10	ordinary_muscle_development	ENSGALG00010013180	C5orf24	-0.066074373	0.059523973	-1.110046419	0.266979024	0.30080536	negative
PRJNA729271	stage_1W_vs_E10	ordinary_muscle_development	ENSGALG00010013181	DYRK1A	0.759583744	0.079075708	9.605778565	7.55845E-22	3.05265E-21	positive
PRJNA729271	stage_1W_vs_E10	ordinary_muscle_development	ENSGALG00010013183	CLTA	-1.614940619	0.138553788	-11.6556945	2.14623E-31	1.28076E-30	negative
PRJNA729271	stage_1W_vs_E10	ordinary_muscle_development	ENSGALG00010013184	LDHB	-1.725116497	0.114990394	-15.00226613	7.09548E-51	8.01004E-50	negative
PRJNA729271	stage_1W_vs_E10	ordinary_muscle_development	ENSGALG00010013185	ENSGALG00010013185	-0.855366498	0.325468984	-2.628104492	0.008586214	0.011694904	negative
PRJNA729271	stage_1W_vs_E10	ordinary_muscle_development	ENSGALG00010013186	DDX46	-0.951264957	0.080898316	-11.75877324	6.36524E-32	3.87665E-31	negative
PRJNA729271	stage_1W_vs_E10	ordinary_muscle_development	ENSGALG00010013187	KDF1	-3.550641259	0.279704557	-12.69425603	6.36317E-37	4.58648E-36	negative
PRJNA729271	stage_1W_vs_E10	ordinary_muscle_development	ENSGALG00010013188	ENSGALG00010013188	0.808886618	0.363050208	2.228029623	0.02587854	0.033405417	positive
PRJNA729271	stage_1W_vs_E10	ordinary_muscle_development	ENSGALG00010013190	SLC45A2	-3.727380339	0.63253233	-5.892790238	3.79728E-09	8.26327E-09	negative
PRJNA729271	stage_1W_vs_E10	ordinary_muscle_development	ENSGALG00010013191	ABRA	6.406463758	0.418791063	15.29751784	7.94286E-53	9.64253E-52	positive
PRJNA729271	stage_1W_vs_E10	ordinary_muscle_development	ENSGALG00010013192	B4GALT5	0.450802636	0.082265704	5.479836874	4.25718E-08	8.71457E-08	positive
PRJNA729271	stage_1W_vs_E10	ordinary_muscle_development	ENSGALG00010013194	GRAP2	-2.111066753	0.381026675	-5.540469708	3.01661E-08	6.21635E-08	negative
PRJNA729271	stage_1W_vs_E10	ordinary_muscle_development	ENSGALG00010013196	MRPS9	0.398219645	0.113679419	3.503005639	0.00046004	0.000703182	positive
PRJNA729271	stage_1W_vs_E10	ordinary_muscle_development	ENSGALG00010013198	CAMLG	-0.498684021	0.086893648	-5.739015851	9.52283E-09	2.02247E-08	negative
PRJNA729271	stage_1W_vs_E10	ordinary_muscle_development	ENSGALG00010013199	RALYL	-2.880225363	0.567225627	-5.077741955	3.81947E-07	7.34163E-07	negative
PRJNA729271	stage_1W_vs_E10	ordinary_muscle_development	ENSGALG00010013202	ENSGALG00010013202	-0.803593315	0.689247284	-1.165899864	0.243654934	0.276179095	negative
PRJNA729271	stage_1W_vs_E10	ordinary_muscle_development	ENSGALG00010013203	AMACR	1.126892721	0.118867985	9.480203768	2.53787E-21	1.00812E-20	positive
PRJNA729271	stage_1W_vs_E10	ordinary_muscle_development	ENSGALG00010013204	ENSGALG00010013204	-0.007691816	0.378737844	-0.020309076	0.983796815	0.986314486	negative
PRJNA729271	stage_1W_vs_E10	ordinary_muscle_development	ENSGALG00010013205	KCNJ6	-0.693817383	0.364959489	-1.901080542	0.057291464	0.071026323	negative
PRJNA729271	stage_1W_vs_E10	ordinary_muscle_development	ENSGALG00010013206	AFF2	-0.918414372	0.11385227	-8.066719924	7.22119E-16	2.23752E-15	negative
PRJNA729271	stage_1W_vs_E10	ordinary_muscle_development	ENSGALG00010013207	C1QTNF3	-3.464058013	0.200395486	-17.28610796	5.986E-67	1.03664E-65	negative
PRJNA729271	stage_1W_vs_E10	ordinary_muscle_development	ENSGALG00010013208	ENSGALG00010013208	0.524525209	0.305079874	1.719304527	0.085558936	0.103797856	positive
PRJNA729271	stage_1W_vs_E10	ordinary_muscle_development	ENSGALG00010013209	KIAA1549	-1.219805699	0.104975237	-11.61993756	3.26375E-31	1.93727E-30	negative
PRJNA729271	stage_1W_vs_E10	ordinary_muscle_development	ENSGALG00010013212	ENSGALG00010013212	0.829492873	0.152611145	5.435336139	5.46932E-08	1.1133E-07	positive
PRJNA729271	stage_1W_vs_E10	ordinary_muscle_development	ENSGALG00010013213	SH2D1A	2.129079595	0.363618825	5.855251289	4.76289E-09	1.03073E-08	positive
PRJNA729271	stage_1W_vs_E10	ordinary_muscle_development	ENSGALG00010013214	RAI14	0.24837185	0.16922278	1.467721132	0.142179988	0.167362265	positive
PRJNA729271	stage_1W_vs_E10	ordinary_muscle_development	ENSGALG00010013215	SF3A3	-0.899132361	0.082743548	-10.86649515	1.66474E-27	8.48764E-27	negative
PRJNA729271	stage_1W_vs_E10	ordinary_muscle_development	ENSGALG00010013217	TGFBRAP1	0.140314252	0.083979334	1.670818823	0.094757469	0.114306718	positive
PRJNA729271	stage_1W_vs_E10	ordinary_muscle_development	ENSGALG00010013220	CSTF1	0.181209026	0.08663084	2.091738069	0.036461953	0.046253041	positive
PRJNA729271	stage_1W_vs_E10	ordinary_muscle_development	ENSGALG00010013221	C2orf49	0.151055045	0.118677433	1.272820299	0.203081816	0.233476438	positive
PRJNA729271	stage_1W_vs_E10	ordinary_muscle_development	ENSGALG00010013224	ATP6V0A4	0.111998413	0.749047983	0.149521013	0.88114253	0.894596972	positive
PRJNA729271	stage_1W_vs_E10	ordinary_muscle_development	ENSGALG00010013225	GNE	-1.577217661	0.099107671	-15.91418347	5.05218E-57	6.85046E-56	negative
PRJNA729271	stage_1W_vs_E10	ordinary_muscle_development	ENSGALG00010013226	DUSP6	-1.03067243	0.11470628	-8.985318206	2.57987E-19	9.39105E-19	negative
PRJNA729271	stage_1W_vs_E10	ordinary_muscle_development	ENSGALG00010013227	CITED4	1.277826972	0.162937362	7.842443011	4.41864E-15	1.32004E-14	positive
PRJNA729271	stage_1W_vs_E10	ordinary_muscle_development	ENSGALG00010013228	RTFDC1	0.436681101	0.08482335	5.148123717	2.63105E-07	5.11152E-07	positive
PRJNA729271	stage_1W_vs_E10	ordinary_muscle_development	ENSGALG00010013230	ENSGALG00010013230	4.602741085	0.34057344	13.51468008	1.28111E-41	1.0885E-40	positive
PRJNA729271	stage_1W_vs_E10	ordinary_muscle_development	ENSGALG00010013232	FHL2	0.734349151	0.42129139	1.74309081	0.081317754	0.098884299	positive
PRJNA729271	stage_1W_vs_E10	ordinary_muscle_development	ENSGALG00010013233	LRRCC1	-1.23807808	0.134825206	-9.182838438	4.19875E-20	1.58286E-19	negative
PRJNA729271	stage_1W_vs_E10	ordinary_muscle_development	ENSGALG00010013235	GCNT7	0.212578075	0.256826169	0.827711896	0.40783368	0.445750958	positive
PRJNA729271	stage_1W_vs_E10	ordinary_muscle_development	ENSGALG00010013236	MON2	0.048485858	0.082841939	0.585281548	0.55835842	0.594229464	positive
PRJNA729271	stage_1W_vs_E10	ordinary_muscle_development	ENSGALG00010013238	RAD1	0.249497073	0.131076829	1.903441481	0.056982963	0.070674086	positive
PRJNA729271	stage_1W_vs_E10	ordinary_muscle_development	ENSGALG00010013241	SEC24A	0.720258254	0.135480789	5.316312819	1.05891E-07	2.11156E-07	positive
PRJNA729271	stage_1W_vs_E10	ordinary_muscle_development	ENSGALG00010013242	SULT4A1	-0.802295994	0.163087091	-4.919432849	8.67953E-07	1.63093E-06	negative
PRJNA729271	stage_1W_vs_E10	ordinary_muscle_development	ENSGALG00010013244	TRIM24	-0.02206509	0.148334364	-0.148752384	0.881749025	0.894899322	negative
PRJNA729271	stage_1W_vs_E10	ordinary_muscle_development	ENSGALG00010013246	LNPEP	2.483714715	0.174763378	14.21187178	7.73306E-46	7.40361E-45	positive
PRJNA729271	stage_1W_vs_E10	ordinary_muscle_development	ENSGALG00010013247	ATG5	0.034677797	0.092686756	0.374139727	0.708300364	0.735972313	positive
PRJNA729271	stage_1W_vs_E10	ordinary_muscle_development	ENSGALG00010013248	ENSGALG00010013248	-0.256015603	0.685383736	-0.373536152	0.708749444	0.736280598	negative
PRJNA729271	stage_1W_vs_E10	ordinary_muscle_development	ENSGALG00010013249	AURKA	-3.485089823	0.232498678	-14.98971887	8.57153E-51	9.65379E-50	negative
PRJNA729271	stage_1W_vs_E10	ordinary_muscle_development	ENSGALG00010013250	E2F5	-0.060335619	0.09573748	-0.630219419	0.528551036	0.565086651	negative
PRJNA729271	stage_1W_vs_E10	ordinary_muscle_development	ENSGALG00010013251	MTA1	-0.78308951	0.083063282	-9.427625461	4.19477E-21	1.65002E-20	negative
PRJNA729271	stage_1W_vs_E10	ordinary_muscle_development	ENSGALG00010013252	PPM1H	-3.162155508	0.212784411	-14.86084197	5.91768E-50	6.4884E-49	negative
PRJNA729271	stage_1W_vs_E10	ordinary_muscle_development	ENSGALG00010013253	SHB	0.81683970 4	0.098811045	8.266684177	1.37736E-16	4.39174E-16	positive
PRJNA729271	stage_1W_vs_E10	ordinary_muscle_development	ENSGALG00010013254	SAR1B	2.541167268	0.141434539	17.96709123	3.52731E-72	7.05219E-71	positive
PRJNA729271	stage_1W_vs_E10	ordinary_muscle_development	ENSGALG00010013256	BRIX1	-0.365014053	0.117714916	-3.100830923	0.001929784	0.002801144	negative
PRJNA729271	stage_1W_vs_E10	ordinary_muscle_development	ENSGALG00010013257	STX12	-0.731097751	0.090741256	-8.056959889	7.82217E-16	2.41805E-15	negative
PRJNA729271	stage_1W_vs_E10	ordinary_muscle_development	ENSGALG00010013258	RBIS	0.572970622	0.145423056	3.940026002	8.14728E-05	0.000132038	positive
PRJNA729271	stage_1W_vs_E10	ordinary_muscle_development	ENSGALG00010013259	PTPRB	2.638672815	0.139148419	18.96300972	3.44835E-80	8.24815E-79	positive

PRJNA729271	stage_1W_vs_E10	ordinary_muscle_development	ENSGALG00010013260	FAM210B	-0.401961652	0.083576759	-4.809490792	1.51315E-06	2.78941E-06	negative
PRJNA729271	stage_1W_vs_E10	ordinary_muscle_development	ENSGALG00010013261	JADE2	2.812755462	0.196861871	14.28796471	2.601E-46	2.53201E-45	positive
PRJNA729271	stage_1W_vs_E10	ordinary_muscle_development	ENSGALG00010013262	CA13	-2.068038897	0.264977088	-7.804595157	5.96929E-15	1.7687E-14	negative
PRJNA729271	stage_1W_vs_E10	ordinary_muscle_development	ENSGALG00010013263	ATP11A	-0.759330104	0.113706695	-6.677971836	2.42272E-11	5.97131E-11	negative
PRJNA729271	stage_1W_vs_E10	ordinary_muscle_development	ENSGALG00010013264	DNAJC21	0.525641946	0.121583658	4.323294374	1.53716E-05	2.62595E-05	positive
PRJNA729271	stage_1W_vs_E10	ordinary_muscle_development	ENSGALG00010013265	PACS2	1.281319122	0.121936543	10.50808146	7.92901E-26	3.77938E-25	positive
PRJNA729271	stage_1W_vs_E10	ordinary_muscle_development	ENSGALG00010013266	KIAA1755	1.48624458	0.189657191	7.836478938	4.63356E-15	1.38196E-14	positive
PRJNA729271	stage_1W_vs_E10	ordinary_muscle_development	ENSGALG00010013267	PNPLA3	0.096305185	0.179417548	0.536765693	0.591429472	0.626115264	positive
PRJNA729271	stage_1W_vs_E10	ordinary_muscle_development	ENSGALG00010013268	PTPRR	1.956504892	0.258584768	7.56620318	3.84291E-14	1.0935E-13	positive
PRJNA729271	stage_1W_vs_E10	ordinary_muscle_development	ENSGALG00010013269	INPP5B	1.203206753	0.100534135	11.96814153	5.21844E-33	3.29592E-32	positive
PRJNA729271	stage_1W_vs_E10	ordinary_muscle_development	ENSGALG00010013271	GULP1	0.022982418	0.139738171	0.16446772	0.869362957	0.883875714	positive
PRJNA729271	stage_1W_vs_E10	ordinary_muscle_development	ENSGALG00010013273	STAG2	1.11976018	0.116206203	9.635975984	5.63539E-22	2.28553E-21	positive
PRJNA729271	stage_1W_vs_E10	ordinary_muscle_development	ENSGALG00010013274	MTF1	1.148462916	0.119518946	9.609044869	7.32251E-22	2.95819E-21	positive
PRJNA729271	stage_1W_vs_E10	ordinary_muscle_development	ENSGALG00010013275	MAPRE1	-1.547905506	0.076752403	-20.16751856	1.88881E-90	5.6334E-89	negative
PRJNA729271	stage_1W_vs_E10	ordinary_muscle_development	ENSGALG00010013276	POC1B	-1.73700339	0.111743455	-15.54456488	1.73195E-54	2.21186E-53	negative
PRJNA729271	stage_1W_vs_E10	ordinary_muscle_development	ENSGALG00010013277	YRDC	-0.706400339	0.092976064	-7.59765806	3.01538E-14	8.61919E-14	negative
PRJNA729271	stage_1W_vs_E10	ordinary_muscle_development	ENSGALG00010013278	SAMM50	1.130053284	0.102694173	11.00406428	3.65288E-28	1.91634E-27	positive
PRJNA729271	stage_1W_vs_E10	ordinary_muscle_development	ENSGALG00010013280	FRS2	-0.523331913	0.113298137	-4.619068992	3.85466E-06	6.88943E-06	negative
PRJNA729271	stage_1W_vs_E10	ordinary_muscle_development	ENSGALG00010013281	BIRC8	2.231759962	0.138497224	16.11411328	2.03056E-58	2.84102E-57	positive
PRJNA729271	stage_1W_vs_E10	ordinary_muscle_development	ENSGALG00010013282	SEC22B	-1.389507509	0.091120045	-15.24919692	1.6667E-52	2.00655E-51	negative
PRJNA729271	stage_1W_vs_E10	ordinary_muscle_development	ENSGALG00010013283	PAWR	-1.273742202	0.282527814	-4.508378071	6.53251E-06	1.14677E-05	negative
PRJNA729271	stage_1W_vs_E10	ordinary_muscle_development	ENSGALG00010013284	MANEAL	-1.065176108	0.318645615	-3.342823681	0.000829306	0.001244406	negative
PRJNA729271	stage_1W_vs_E10	ordinary_muscle_development	ENSGALG00010013285	ATP2B1	-0.315677654	0.10792468	-2.92498114	0.003444771	0.004883016	negative
PRJNA729271	stage_1W_vs_E10	ordinary_muscle_development	ENSGALG00010013286	RNF14	1.461212806	0.118860976	12.29346126	9.82091E-35	6.61497E-34	positive
PRJNA729271	stage_1W_vs_E10	ordinary_muscle_development	ENSGALG00010013288	CA3A	1.877193568	0.782944037	2.397608869	0.016502476	0.02175961	positive
PRJNA729271	stage_1W_vs_E10	ordinary_muscle_development	ENSGALG00010013289	TM9SF4	-1.319350479	0.076134402	-17.32922897	2.83098E-67	4.97395E-66	negative
PRJNA729271	stage_1W_vs_E10	ordinary_muscle_development	ENSGALG00010013290	AKR1D1	-3.454441024	0.339074851	-10.18784203	2.24696E-24	1.00462E-23	negative
PRJNA729271	stage_1W_vs_E10	ordinary_muscle_development	ENSGALG00010013292	PRDM1	-2.616483261	0.301576883	-8.676007378	4.09906E-18	1.40231E-17	negative
PRJNA729271	stage_1W_vs_E10	ordinary_muscle_development	ENSGALG00010013293	TUBGCP3	-0.857369741	0.058593234	-14.63257242	1.74079E-48	1.80621E-47	negative
PRJNA729271	stage_1W_vs_E10	ordinary_muscle_development	ENSGALG00010013295	CA3B	0.067675625	0.400039897	0.16917219	0.865661204	0.880482713	positive
PRJNA729271	stage_1W_vs_E10	ordinary_muscle_development	ENSGALG00010013296	AGXT2	-0.836251026	0.430288012	-1.943468101	0.051959626	0.064770793	negative
PRJNA729271	stage_1W_vs_E10	ordinary_muscle_development	ENSGALG00010013297	FCHO2	0.412296112	0.126300722	3.264400287	0.001096961	0.001627476	positive
PRJNA729271	stage_1W_vs_E10	ordinary_muscle_development	ENSGALG00010013298	EPYC	-6.908413002	0.405684753	-17.02901811	5.00368E-65	8.27948E-64	negative
PRJNA729271	stage_1W_vs_E10	ordinary_muscle_development	ENSGALG00010013299	PLAGL2	-1.385600862	0.114408252	-12.11102207	9.23364E-34	6.00254E-33	negative
PRJNA729271	stage_1W_vs_E10	ordinary_muscle_development	ENSGALG00010013300	GNPDA1	-1.286908154	0.099655909	-12.91351575	3.77656E-38	2.8334E-37	negative
PRJNA729271	stage_1W_vs_E10	ordinary_muscle_development	ENSGALG00010013301	PARVB	-3.192612243	0.142523532	-22.40059727	3.8834E-111	1.787E-109	negative
PRJNA729271	stage_1W_vs_E10	ordinary_muscle_development	ENSGALG00010013302	ENSGALG00010013302	3.672056225	0.557640839	6.584984395	4.54933E-11	1.10494E-10	positive
PRJNA729271	stage_1W_vs_E10	ordinary_muscle_development	ENSGALG00010013303	TFPI	0.322188424	0.232801423	1.383962434	0.166369958	0.193899858	positive
PRJNA729271	stage_1W_vs_E10	ordinary_muscle_development	ENSGALG00010013304	KCNJ15	0.098222573	0.237153692	0.41417265	0.67874767	0.708260563	positive
PRJNA729271	stage_1W_vs_E10	ordinary_muscle_development	ENSGALG00010013305	THOC2	-0.436849011	0.091207815	-4.78960066	1.67114E-06	3.06932E-06	negative
PRJNA729271	stage_1W_vs_E10	ordinary_muscle_development	ENSGALG00010013306	ARSB	-0.673585016	0.15158434	-4.443631942	8.84529E-06	1.54083E-05	negative
PRJNA729271	stage_1W_vs_E10	ordinary_muscle_development	ENSGALG00010013307	POFUT1	-1.144951362	0.090480314	-12.65414881	1.06119E-36	7.60357E-36	negative
PRJNA729271	stage_1W_vs_E10	ordinary_muscle_development	ENSGALG00010013309	ENSGALG00010013309	0.722497289	0.287068559	2.516810942	0.011842235	0.015873239	positive
PRJNA729271	stage_1W_vs_E10	ordinary_muscle_development	ENSGALG00010013310	MYO16	-2.865578998	0.304360045	-9.415095854	4.72651E-21	1.85314E-20	negative
PRJNA729271	stage_1W_vs_E10	ordinary_muscle_development	ENSGALG00010013311	RSPO1	-3.765984498	0.266188797	-14.14779488	1.92706E-45	1.83165E-44	negative
PRJNA729271	stage_1W_vs_E10	ordinary_muscle_development	ENSGALG00010013312	ENSGALG00010013312	-0.022828455	0.59089357	-0.038633785	0.969182366	0.973413137	negative
PRJNA729271	stage_1W_vs_E10	ordinary_muscle_development	ENSGALG00010013313	GAK	-0.37883913	0.095101114	-3.983540419	6.78961E-05	0.000110704	negative
PRJNA729271	stage_1W_vs_E10	ordinary_muscle_development	ENSGALG00010013314	KIT	0.18100995	0.205927783	0.878997228	0.379402779	0.417161745	positive
PRJNA729271	stage_1W_vs_E10	ordinary_muscle_development	ENSGALG00010013316	KDM7A	1.990053417	0.129503581	15.36678292	2.73412E-53	3.36142E-52	positive
PRJNA729271	stage_1W_vs_E10	ordinary_muscle_development	ENSGALG00010013317	PREP	-0.624318042	0.109784868	-5.68674038	1.29487E-08	2.7253E-08	negative
PRJNA729271	stage_1W_vs_E10	ordinary_muscle_development	ENSGALG00010013318	KERA	-5.37762187	0.4891844	-10.99303632	4.12801E-28	2.16169E-27	negative
PRJNA729271	stage_1W_vs_E10	ordinary_muscle_development	ENSGALG00010013319	ENSGALG00010013319	0.426876715	1.560236104	0.273597511			positive
PRJNA729271	stage_1W_vs_E10	ordinary_muscle_development	ENSGALG00010013320	CALCRL	0.139970983	0.123940333	1.129341672	0.258753728	0.292333226	positive
PRJNA729271	stage_1W_vs_E10	ordinary_muscle_development	ENSGALG00010013322	IRS2	2.712170396	0.510768256	5.309982296	1.09636E-07	2.18353E-07	positive
PRJNA729271	stage_1W_vs_E10	ordinary_muscle_development	ENSGALG00010013323	NOTCH2	-1.737777431	0.109152549	-15.92063071	4.55759E-57	6.19722E-56	negative
PRJNA729271	stage_1W_vs_E10	ordinary_muscle_development	ENSGALG00010013324	PARVG	0.717279372	0.14936544	4.802177609	1.56949E-06	2.88813E-06	positive
PRJNA729271	stage_1W_vs_E10	ordinary_muscle_development	ENSGALG00010013325	CA2	2.485274672	0.316995475	7.840095102	4.50205E-15	1.34385E-14	positive
PRJNA729271	stage_1W_vs_E10	ordinary_muscle_development	ENSGALG00010013326	FGF20	-6.058164771	1.021618393	-5.929968384	3.02993E-09	6.63427E-09	negative
PRJNA729271	stage_1W_vs_E10	ordinary_muscle_development	ENSGALG00010013327	SLF1	-1.468913823	0.100721997	-14.58384328	3.55903E-48	3.65614E-47	negative
PRJNA729271	stage_1W_vs_E10	ordinary_muscle_development	ENSGALG00010013328	SLC37A3	-0.612486722	0.079748218	-7.680255893	1.58771E-14	4.60554E-14	negative
PRJNA729271	stage_1W_vs_E10	ordinary_muscle_development	ENSGALG00010013329	LIFR	-0.57416913	0.122870998	-4.672942682	2.96915E-06	5.35562E-06	negative
PRJNA729271	stage_1W_vs_E10	ordinary_muscle_development	ENSGALG00010013330	ERG	-0.348698174	0.129395657	-2.69482131	0.007042639	0.009678871	negative
PRJNA729271	stage_1W_vs_E10	ordinary_muscle_development	ENSGALG00010013331	KIF3B	-0.687791293	0.150923966	-4.55720393	5.18391E-06	9.1747E-06	negative
PRJNA729271	stage_1W_vs_E10	ordinary_muscle_development	ENSGALG00010013332	LUM	-1.117525804	0.320755552	-3.48404197	0.000493902	0.00075367	negative
PRJNA729271	stage_1W_vs_E10	ordinary_muscle_development	ENSGALG00010013333	PPP1R12A	0.162191248	0.098108125	1.653188754	0.098292455	0.118373972	positive
PRJNA729271	stage_1W_vs_E10	ordinary_muscle_development	ENSGALG00010013334	SH3BGRL3	0.21755998	0.121396887	1.79213805	0.073110855	0.089384434	positive
PRJNA729271	stage_1W_vs_E10	ordinary_muscle_development	ENSGALG00010013335	POPDC3	3.313355723	0.175845681	18.8424061	3.3919E-79	7.8791E-78	positive
PRJNA729271	stage_1W_vs_E10	ordinary_muscle_development	ENSGALG00010013336	MTNR1A	0.80710975	0.697554801	1.157055688	0.247249609	0.279968993	positive
PRJNA729271	stage_1W_vs_E10	ordinary_muscle_development	ENSGALG00010013337	RAB19	0.158601586	0.256341234	0.618712735	0.536105619	0.572480547	positive
PRJNA729271	stage_1W_vs_E10	ordinary_muscle_development	ENSGALG00010013339	ATP6V0D2	2.251583825	0.542803483	4.148064441	3.35298E-05	5.59989E-05	positive
PRJNA729271	stage_1W_vs_E10	ordinary_muscle_development	ENSGALG00010013340	LIN28B	-6.779989907	0.670511598	-10.11166686	4.90427E-24	2.16334E-23	negative
PRJNA729271	stage_1W_vs_E10	ordinary_muscle_development	ENSGALG00010013343	SHISAL1	-5.639048439	0.447215273	-12.60924835	1.87775E-36	1.33684E-35	negative
PRJNA729271	stage_1W_vs_E10	ordinary_muscle_development	ENSGALG00010013344	OSMR	1.056102441	0.12339912	8.558427642	1.14417E-17	3.82138E-17	positive
PRJNA729271	stage_1W_vs_E10	ordinary_muscle_development	ENSGALG00010013345	DCN	-0.017139961	0.21379116	-0.080171512	0.936100847	0.943915254	negative
PRJNA729271	stage_1W_vs_E10	ordinary_muscle_development	ENSGALG00010013346	HMGCS2	1.507132668	0.689394779	2.1861678	0.028803331	0.036970184	positive
PRJNA729271	stage_1W_vs_E10	ordinary_muscle_development	ENSGALG00010013347	ENSGALG00010013347	-0.15552201	0.317360596	-0.490048267	0.624099744	0.657723266	negative
PRJNA729271	stage_1W_vs_E10	ordinary_muscle_development	ENSGALG00010013348	MKRN4P	0.40411674	0.070106992	5.764285842	8.20043E-09	1.74776E-08	positive
PRJNA729271	stage_1W_vs_E10	ordinary_muscle_development	ENSGALG00010013349	ETS2	-0.092869863	0.101374329	-0.916108286	0.359610102	0.397207074	negative
PRJNA729271	stage_1W_vs_E10	ordinary_muscle_development	ENSGALG00010013351	EDAR	-4.735111249	0.446046085	-10.61574444	2.51779E-26	1.22611E-25	negative
PRJNA729271	stage_1W_vs_E10	ordinary_muscle_development	ENSGALG00010013352	ASXL1	-0.389575231	0.088621712	-4.395934366	1.10297E-05	1.90919E-05	negative
PRJNA729271	stage_1W_vs_E10	ordinary_muscle_development	ENSGALG00010013353	NDFIP1	-0.461024876	0.085074295	-5.41908548	5.99047E-08	1.21664E-07	negative
PRJNA729271	stage_1W_vs_E10	ordinary_muscle_development	ENSGALG00010013354	WWP1	4.297196926	0.145351218	29.56423058	4.3102E-192	5.7848E-190	positive
PRJNA729271	stage_1W_vs_E10	ordinary_muscle_development	ENSGALG00010013355	FAM171B	-2.340136732	0.152770096	-15.31802881	5.79484E-53	7.07634E-52	negative
PRJNA729271	stage_1W_vs_E10	ordinary_muscle_development	ENSGALG00010013356	AMER1	-0.713057752	0.065847208	-10.82897466	2.5095E-27	1.27141E-26	negative
PRJNA729271	stage_1W_vs_E10	ordinary_muscle_development	ENSGALG00010013357	RICTOR	-0.111565897	0.110220341	-1.012207872	0.311438673	0.347361001	negative
PRJNA729271	stage_1W_vs_E10	ordinary_muscle_development	ENSGALG00010013358	RPS16	0.336780143	0.174159869	1.933741363	0.053144935	0.066151639	positive
PRJNA729271	stage_1W_vs_E10	ordinary_muscle_development	ENSGALG00010013359	BTG1	0.705734606	0.132509328	5.325923986	1.00441E-07	2.0073E-07	positive
PRJNA729271	stage_1W_vs_E10	ordinary_muscle_development	ENSGALG00010013360	PRR5	-1.076537695	0.116325942	-9.254493668	2.15248E-20	8.22357E-20	negative
PRJNA729271	stage_1W_vs_E10	ordinary_muscle_development	ENSGALG00010013361	ENSGALG00010013361	0.067337871	0.231632502	0.290709943	0.771273167	0.794796285	positive
PRJNA729271	stage_1W_vs_E10	ordinary_muscle_development	ENSGALG00010013362	ENSGALG00010013362	3.843132614	0.358859666	10.70929106	9.20641E-27	4.55295E-26	positive
PRJNA729271	stage_1W_vs_E10	ordinary_muscle_development	ENSGALG00010013363	HS3ST5	1.954977544	0.231760086	8.435350448	3.30212E-17	1.08046E-16	positive
PRJNA729271	stage_1W_vs_E10	ordinary_muscle_development	ENSGALG00010013364	OTOGL	3.01771321	0.357536107	8.440303377	3.16515E-17	1.03611E-16	positive
PRJNA729271	stage_1W_vs_E10	ordinary_muscle_development	ENSGALG00010013365	CEP85	2.492941955	0.227073995	10.97854447	4.8467E-28	2.5289E-27	positive
PRJNA729271	stage_1W_vs_E10	ordinary_muscle_development	ENSGALG00010013367	NOL4L	-0.256524934	0.18084985	-1.418441504	0.156061905	0.182546588	negative
PRJNA729271	stage_1W_vs_E10	ordinary_muscle_development	ENSGALG00010013369	MTMR8	-0.77062838	0.063869837	-12.06560741	1.60474E-33	1.03335E-32	negative
PRJNA729271	stage_1W_vs_E10	ordinary_muscle_development	ENSGALG00010013370	CNKSR1	3.556593937	0.233729799	15.21669021	2.74049E-52	3.25869E-51	positive
PRJNA729271	stage_1W_vs_E10	ordinary_muscle_development	ENSGALG00010013371	FAT1	-1.297807216	0.156692471	-8.282511653	1.20604E-16	3.85481E-16	negative
PRJNA729271	stage_1W_vs_E10	ordinary_muscle_development	ENSGALG00010013372	SPRY4	-2.210894468	0.303638419	-7.281339674	3.30521E-13	8.94462E-13	negative
PRJNA729271	stage_1W_vs_E10	ordinary_muscle_development	ENSGALG00010013373	ENSGALG00010013373	0.6111162	0.564789718	1.082024302	0.27924173	0.313646561	positive
PRJNA729271	stage_1W_vs_E10	ordinary_muscle_development	ENSGALG00010013374	RMDN1	0.632129479	0.166018028	3.807595391	0.000140325	0.000223223	positive
PRJNA729271	stage_1W_vs_E10	ordinary_muscle_development	ENSGALG00010013375	PSMG1	-0.090252292	0.088542684	-1.019308285	0.308056632	0.343774031	negative
PRJNA729271	stage_1W_vs_E10	ordinary_muscle_development	ENSGALG00010013376	ARHGAP8	2.746560449	0.377072358	7.283908224	3.24286E-13	8.78621E-13	positive
PRJNA729271	stage_1W_vs_E10	ordinary_muscle_development	ENSGALG00010013378	SH3RF3	-0.4894855	0.14499318	-3.375920846	0.000735691	0.001109076	negative
PRJNA729271	stage_1W_vs_E10	ordinary_muscle_development	ENSGALG00010013379	ZNF593	0.71403614	0.13178769	5.418079206	6.02427E-08	1.22334E-07	positive
PRJNA729271	stage_1W_vs_E10	ordinary_muscle_development	ENSGALG00010013380	PHGDH	1.023986469	0.609519678	1.679989186	0.09295942	0.112315311	positive
PRJNA729271	stage_1W_vs_E10	ordinary_muscle_development	ENSGALG00010013381	DENND2A	-1.502531071	0.089928739	-16.70801896	1.14579E-62	1.782E-61	negative
PRJNA729271	stage_1W_vs_E10	ordinary_muscle_development	ENSGALG00010013382	ASB12	1.708582811	0.220936763	7.733356773	1.04747E-14	3.06915E-14	positive
PRJNA729271	stage_1W_vs_E10	ordinary_muscle_development	ENSGALG00010013383	SEPTIN10	0.683337211	0.139294176	4.905712734	9.30887E-07	1.74489E-06	positive
PRJNA729271	stage_1W_vs_E10	ordinary_muscle_development	ENSGALG00010013385	PGRMC1	0.066554049	0.155553764	0.427852383	0.668758597	0.699044917	positive
PRJNA729271	stage_1W_vs_E10	ordinary_muscle_development	ENSGALG00010013386	ITGAV	-0.356844507	0.121999368	-2.924970121	0.003444893	0.004883016	negative
PRJNA729271	stage_1W_vs_E10	ordinary_muscle_development	ENSGALG00010013387	NDST1	-0.372788105	0.093125316	-4.003080154	6.25231E-05	0.000102184	negative
PRJNA729271	stage_1W_vs_E10	ordinary_muscle_development	ENSGALG00010013388	SOWAHC	-0.15266209	0.226929563	-0.672728963	0.501119738	0.538932379	negative
PRJNA729271	stage_1W_vs_E10	ordinary_muscle_development	ENSGALG00010013389	NAP1L1	-1.201669342	0.062303139	-19.28746073	6.84539E-83	1.76555E-81	negative
PRJNA729271	stage_1W_vs_E10	ordinary_muscle_development	ENSGALG00010013390	SLC16A2	-3.445374593	0.137775631	-25.00714074	5.1123E-138	3.6148E-136	negative
PRJNA729271	stage_1W_vs_E10	ordinary_muscle_development	ENSGALG00010013391	HMGN1	-0.014257355	0.087750553	-0.162475959	0.870931059	0.88528371	negative
PRJNA729271	stage_1W_vs_E10	ordinary_muscle_development	ENSGALG00010013392	HDAC2	-0.77359075	0.069162769	-11.18507492	4.82498E-29	2.61255E-28	negative
PRJNA729271	stage_1W_vs_E10	ordinary_muscle_development	ENSGALG00010013393	ADCK2	-1.007094669	0.165933801	-6.069255702	1.28504E-09	2.87716E-09	negative
PRJNA729271	stage_1W_vs_E10	ordinary_muscle_development	ENSGALG00010013394	DNMT3B	-7.313583027	0.341510897	-21.41537237	9.6073E-102	3.71354E-100	negative
PRJNA729271	stage_1W_vs_E10	ordinary_muscle_development	ENSGALG00010013396	FYB1	0.108886928	0.189665191	0.574100749	0.565899626	0.601592599	positive
PRJNA729271	stage_1W_vs_E10	ordinary_muscle_development	ENSGALG00010013397	MATN1	-10.51704016	0.575009802	-18.29019285	9.9064E-75	2.07205E-73	negative
PRJNA729271	stage_1W_vs_E10	ordinary_muscle_development	ENSGALG00010013398	CHAMP1	0.971864656	0.155477454	6.250839804	4.08251E-10	9.37071E-10	positive
PRJNA729271	stage_1W_vs_E10	ordinary_muscle_development	ENSGALG00010013399	FGF1	3.734762672	0.322835675	11.56861822	5.94321E-31	3.48209E-30	positive
PRJNA729271	stage_1W_vs_E10	ordinary_muscle_development	ENSGALG00010013400	BBS10	0.339720626	0.165282799	2.055390081	0.039841343	0.050353149	positive
PRJNA729271	stage_1W_vs_E10	ordinary_muscle_development	ENSGALG00010013402	MARCKS	-2.311550326	0.155257884	-14.88845698	3.91705E-50	4.32098E-49	negative
PRJNA729271	stage_1W_vs_E10	ordinary_muscle_development	ENSGALG00010013403	CPNE3	0.716301185	0.103258069	6.936999585	4.00515E-12	1.02752E-11	positive
PRJNA729271	stage_1W_vs_E10	ordinary_muscle_development	ENSGALG00010013404	ENSGALG00010013404	-0.093225782	0.231653662	-0.402436038	0.687363152	0.716528507	negative
PRJNA729271	stage_1W_vs_E10	ordinary_muscle_development	ENSGALG00010013405	RPS14	0.195586608	0.17536238	1.115328202	0.264709804	0.298457422	positive
PRJNA729271	stage_1W_vs_E10	ordinary_muscle_development	ENSGALG00010013406	ENSGALG00010013406	0.70926096	0.281248512	2.521830088	0.011674608	0.015655791	positive
PRJNA729271	stage_1W_vs_E10	ordinary_muscle_development	ENSGALG00010013407	ZC3H15	1.43525163	0.11248553	12.75943337	2.76203E-37	2.01691E-36	positive
PRJNA729271	stage_1W_vs_E10	ordinary_muscle_development	ENSGALG00010013408	RLIM	-0.064643744	0.065315316	-0.989718	0.322311975	0.358743152	negative
PRJNA729271	stage_1W_vs_E10	ordinary_muscle_development	ENSGALG00010013409	DAB2	-0.324390055	0.148024112	-2.191467656	0.028417968	0.036501413	negative

PRJNA729271	stage_1W_vs_E10	ordinary_muscle_development	ENSGALG00010013410	KIAA0825	-0.586635101	0.103830847	-5.649911553	1.6053E-08	3.36499E-08	negative
PRJNA729271	stage_1W_vs_E10	ordinary_muscle_development	ENSGALG00010013411	ENSGALG00010013411	2.008443772	0.440397067	4.560529397	5.10248E-06	9.03831E-06	positive
PRJNA729271	stage_1W_vs_E10	ordinary_muscle_development	ENSGALG00010013412	COMMD7	-0.785642587	0.102002863	-7.70216212	1.33783E-14	3.89864E-14	negative
PRJNA729271	stage_1W_vs_E10	ordinary_muscle_development	ENSGALG00010013413	WRB	-0.007774016	0.083581779	-0.093010897	0.925894904	0.934910616	negative
PRJNA729271	stage_1W_vs_E10	ordinary_muscle_development	ENSGALG00010013414	LAPTM5	1.287190042	0.173788537	7.406645245	1.29534E-13	3.57843E-13	positive
PRJNA729271	stage_1W_vs_E10	ordinary_muscle_development	ENSGALG00010013416	NDUFB2	1.294651145	0.110260724	11.74172542	7.78811E-32	4.72535E-31	positive
PRJNA729271	stage_1W_vs_E10	ordinary_muscle_development	ENSGALG00010013417	AKR1B1	-0.415071721	0.088517162	-4.689166619	2.7432E-06	4.96103E-06	negative
PRJNA729271	stage_1W_vs_E10	ordinary_muscle_development	ENSGALG00010013418	HCN1	-1.290318217	0.767405343	-1.681403744	0.092684519	0.112001842	negative
PRJNA729271	stage_1W_vs_E10	ordinary_muscle_development	ENSGALG00010013419	ENSGALG00010013419	3.283316399	0.332669739	9.869597418	5.63905E-23	2.38442E-22	positive
PRJNA729271	stage_1W_vs_E10	ordinary_muscle_development	ENSGALG00010013420	ENSGALG00010013420	-0.852360052	4.205149148	-0.202694369			negative
PRJNA729271	stage_1W_vs_E10	ordinary_muscle_development	ENSGALG00010013423	ZNF804A	-1.705441206	0.435126327	-3.919416274	8.87637E-05	0.000143355	negative
PRJNA729271	stage_1W_vs_E10	ordinary_muscle_development	ENSGALG00010013425	ENSGALG00010013425	0.576587995	0.222369496	2.592927554	0.009516281	0.012892653	positive
PRJNA729271	stage_1W_vs_E10	ordinary_muscle_development	ENSGALG00010013427	NEXMIF	-2.835055581	0.198868089	-14.25596028	4.11601E-46	3.98009E-45	negative
PRJNA729271	stage_1W_vs_E10	ordinary_muscle_development	ENSGALG00010013428	BPGM	4.373624632	0.152776955	28.62751544	3.0545E-180	3.7206E-178	positive
PRJNA729271	stage_1W_vs_E10	ordinary_muscle_development	ENSGALG00010013429	PDE4DIP	2.453512705	0.187514301	13.08440311	4.04324E-39	3.14752E-38	positive
PRJNA729271	stage_1W_vs_E10	ordinary_muscle_development	ENSGALG00010013431	TMEM175	-0.328782435	0.087571323	-3.754453199	0.00017372	0.000274569	negative
PRJNA729271	stage_1W_vs_E10	ordinary_muscle_development	ENSGALG00010013434	CORIN	-4.304316385	0.428272357	-10.05041843	9.14782E-24	3.99993E-23	negative
PRJNA729271	stage_1W_vs_E10	ordinary_muscle_development	ENSGALG00010013435	SDC3	-2.120605179	0.18071143	-11.73475955	8.45663E-32	5.12882E-31	negative
PRJNA729271	stage_1W_vs_E10	ordinary_muscle_development	ENSGALG00010013436	NPAS2	0.605165779	0.207255091	2.9199079	0.003501348	0.004958191	positive
PRJNA729271	stage_1W_vs_E10	ordinary_muscle_development	ENSGALG00010013437	ENSGALG00010013437	0.494081576	0.124678612	3.96284148	7.4063E-05	0.000120447	positive
PRJNA729271	stage_1W_vs_E10	ordinary_muscle_development	ENSGALG00010013438	RPL37	0.67195977	0.187493825	3.583903468	0.000338497	0.000523697	positive
PRJNA729271	stage_1W_vs_E10	ordinary_muscle_development	ENSGALG00010013440	EEA1	0.006543473	0.098564234	0.066387903	0.947069001	0.953648153	positive
PRJNA729271	stage_1W_vs_E10	ordinary_muscle_development	ENSGALG00010013442	ENSGALG00010013442	1.679278188	0.103594828	16.2100581	4.28164E-59	6.11451E-58	positive
PRJNA729271	stage_1W_vs_E10	ordinary_muscle_development	ENSGALG00010013444	OSBPL8	-1.466417406	0.103830811	-14.12314318	2.73514E-45	2.58111E-44	negative
PRJNA729271	stage_1W_vs_E10	ordinary_muscle_development	ENSGALG00010013446	ATP5I	2.119175846	0.145177057	14.59718146	2.92698E-48	3.0154E-47	positive
PRJNA729271	stage_1W_vs_E10	ordinary_muscle_development	ENSGALG00010013449	LCA5L	-0.83418264	0.237151123	-3.517515025	0.000435608	0.000667315	negative
PRJNA729271	stage_1W_vs_E10	ordinary_muscle_development	ENSGALG00010013450	CD74	4.339497714	0.190119219	22.82513963	2.5811E-115	1.2726E-113	positive
PRJNA729271	stage_1W_vs_E10	ordinary_muscle_development	ENSGALG00010013451	PRKAB2	4.468790219	0.166849774	26.78331591	5.0555E-158	4.9513E-156	positive
PRJNA729271	stage_1W_vs_E10	ordinary_muscle_development	ENSGALG00010013452	LSS	-0.567331688	0.225014897	-2.521307239	0.011691972	0.015677625	negative
PRJNA729271	stage_1W_vs_E10	ordinary_muscle_development	ENSGALG00010013453	PUM1	0.039435294	0.089273854	0.441733974	0.658681722	0.689356792	positive
PRJNA729271	stage_1W_vs_E10	ordinary_muscle_development	ENSGALG00010013457	SH3BGR	3.109159829	0.147572203	21.06873633	1.53986E-98	5.59406E-97	positive
PRJNA729271	stage_1W_vs_E10	ordinary_muscle_development	ENSGALG00010013458	LAMA4	0.779112326	0.143696722	5.421921372	5.89618E-08	1.19817E-07	positive
PRJNA729271	stage_1W_vs_E10	ordinary_muscle_development	ENSGALG00010013459	ATP10D	-1.530222546	0.147854155	-10.34954037	4.205E-25	1.94671E-24	negative
PRJNA729271	stage_1W_vs_E10	ordinary_muscle_development	ENSGALG00010013461	S100B	3.310806018	0.573497036	5.773013306	7.78663E-09	1.66152E-08	positive
PRJNA729271	stage_1W_vs_E10	ordinary_muscle_development	ENSGALG00010013463	ENSGALG00010013463	2.683085949	0.20814934	12.89019678	5.11132E-38	3.81114E-37	positive
PRJNA729271	stage_1W_vs_E10	ordinary_muscle_development	ENSGALG00010013464	ABCB7	-0.048918167	0.074628269	-0.655491111	0.512151608	0.549532723	negative
PRJNA729271	stage_1W_vs_E10	ordinary_muscle_development	ENSGALG00010013465	CALD1	-0.968499023	0.157420482	-6.152306312	7.63642E-10	1.7271E-09	negative
PRJNA729271	stage_1W_vs_E10	ordinary_muscle_development	ENSGALG00010013466	BRAF	-0.129982594	0.062745021	-2.071600152	0.038302746	0.048501642	negative
PRJNA729271	stage_1W_vs_E10	ordinary_muscle_development	ENSGALG00010013468	DEPDC1B	-2.7672372	0.278252302	-9.945064894	2.65001E-23	1.13746E-22	negative
PRJNA729271	stage_1W_vs_E10	ordinary_muscle_development	ENSGALG00010013470	RPL31	0.130425064	0.14635773	0.891138881	0.372854668	0.410491297	positive
PRJNA729271	stage_1W_vs_E10	ordinary_muscle_development	ENSGALG00010013472	ENSGALG00010013472	1.157541496	0.230207345	5.028256135	4.9496E-07	9.43386E-07	positive
PRJNA729271	stage_1W_vs_E10	ordinary_muscle_development	ENSGALG00010013476	ZDHHC17	-1.00413102	0.131164946	-7.655483066	1.92587E-14	5.55753E-14	negative
PRJNA729271	stage_1W_vs_E10	ordinary_muscle_development	ENSGALG00010013477	RFC1	-0.530228075	0.09034032	-5.869229536	4.37825E-09	9.4933E-09	negative
PRJNA729271	stage_1W_vs_E10	ordinary_muscle_development	ENSGALG00010013480	TBC1D8	1.87267574	0.151604505	12.35237526	4.72941E-35	3.21543E-34	positive
PRJNA729271	stage_1W_vs_E10	ordinary_muscle_development	ENSGALG00010013482	ENSGALG00010013482	-1.097780337	0.237664027	-4.619042909	3.85514E-06	6.88944E-06	negative
PRJNA729271	stage_1W_vs_E10	ordinary_muscle_development	ENSGALG00010013483	ENSGALG00010013483	-1.062769802	0.209919865	-5.062740511	4.13272E-07	7.92274E-07	negative
PRJNA729271	stage_1W_vs_E10	ordinary_muscle_development	ENSGALG00010013484	TCOF1	-0.284535135	0.102090666	-2.787082762	0.005318489	0.007409795	negative
PRJNA729271	stage_1W_vs_E10	ordinary_muscle_development	ENSGALG00010013486	PLPP6	2.157712711	0.15969717	13.51127707	1.34174E-41	1.138E-40	positive
PRJNA729271	stage_1W_vs_E10	ordinary_muscle_development	ENSGALG00010013487	ENSGALG00010013487	-0.37576367	0.093746909	-4.008277955	6.11631E-05	0.000100074	negative
PRJNA729271	stage_1W_vs_E10	ordinary_muscle_development	ENSGALG00010013488	UPRT	-0.681213771	0.121087378	-5.625803307	1.84647E-08	3.85767E-08	negative
PRJNA729271	stage_1W_vs_E10	ordinary_muscle_development	ENSGALG00010013491	CNOT11	0.955114483	0.110526136	8.641526034	5.54671E-18	1.88289E-17	positive
PRJNA729271	stage_1W_vs_E10	ordinary_muscle_development	ENSGALG00010013492	B3GALT5	-2.26079181	0.634883842	-3.560953454	0.000369511	0.00056949	negative
PRJNA729271	stage_1W_vs_E10	ordinary_muscle_development	ENSGALG00010013493	ENSGALG00010013493	0.570067725	0.127206983	4.481418473	7.41486E-06	1.29711E-05	positive
PRJNA729271	stage_1W_vs_E10	ordinary_muscle_development	ENSGALG00010013494	ASB1	-0.685411776	0.145286371	-4.71766052	2.38572E-06	4.33292E-06	negative
PRJNA729271	stage_1W_vs_E10	ordinary_muscle_development	ENSGALG00010013497	FAM172A	-0.922390989	0.108982464	-8.463664268	2.59105E-17	8.52219E-17	negative
PRJNA729271	stage_1W_vs_E10	ordinary_muscle_development	ENSGALG00010013498	KDR	0.957349596	0.125073815	7.654276788	1.94403E-14	5.60771E-14	positive
PRJNA729271	stage_1W_vs_E10	ordinary_muscle_development	ENSGALG00010013501	AKR1B10	-1.722957389	0.138520279	-12.43830436	1.61904E-35	1.12019E-34	negative
PRJNA729271	stage_1W_vs_E10	ordinary_muscle_development	ENSGALG00010013502	PAFAH2	1.47245068	0.204346653	7.205651061	5.77672E-13	1.54575E-12	positive
PRJNA729271	stage_1W_vs_E10	ordinary_muscle_development	ENSGALG00010013504	IGSF5	-0.906357743	0.785345545	-1.154087839	0.248464158	0.281300318	negative
PRJNA729271	stage_1W_vs_E10	ordinary_muscle_development	ENSGALG00010013506	RNF149	-0.298859404	0.1047915	-2.851943194	0.004345287	0.006096102	negative
PRJNA729271	stage_1W_vs_E10	ordinary_muscle_development	ENSGALG00010013508	TUBE1	-0.212649543	0.194414669	-1.093793713	0.274045462	0.308240007	negative
PRJNA729271	stage_1W_vs_E10	ordinary_muscle_development	ENSGALG00010013509	ENSGALG00010013509	-0.170698616	0.194783761	-0.87634932	0.38084016	0.418646908	negative
PRJNA729271	stage_1W_vs_E10	ordinary_muscle_development	ENSGALG00010013510	MRPS33	-0.164273538	0.088353925	-1.859267	0.062989302	0.0776386	negative
PRJNA729271	stage_1W_vs_E10	ordinary_muscle_development	ENSGALG00010013511	FAM78B	1.831485184	0.225646079	8.116627568	4.79317E-16	1.49606E-15	positive
PRJNA729271	stage_1W_vs_E10	ordinary_muscle_development	ENSGALG00010013512	ENSGALG00010013512	0.519865352	3.43311421	0.151426757			positive
PRJNA729271	stage_1W_vs_E10	ordinary_muscle_development	ENSGALG00010013514	LMBRD2	0.644965459	0.136148021	4.737237123	2.16652E-06	3.94469E-06	positive
PRJNA729271	stage_1W_vs_E10	ordinary_muscle_development	ENSGALG00010013516	CSRP2	-1.648507285	0.135323397	-12.18198276	3.87739E-34	2.54427E-33	negative
PRJNA729271	stage_1W_vs_E10	ordinary_muscle_development	ENSGALG00010013517	STMN1	-3.268162684	0.218031442	-14.98941005	8.61147E-51	9.69125E-50	negative
PRJNA729271	stage_1W_vs_E10	ordinary_muscle_development	ENSGALG00010013520	CREG2	-0.625348605	0.192458655	-3.249262059	0.001157048	0.001711893	negative
PRJNA729271	stage_1W_vs_E10	ordinary_muscle_development	ENSGALG00010013523	TMEM178B	-2.106796647	0.200524878	-10.50641031	8.07073E-26	3.8444E-25	negative
PRJNA729271	stage_1W_vs_E10	ordinary_muscle_development	ENSGALG00010013524	ZDHHC15	0.313338769	0.122890975	2.549729701	0.010780647	0.014525513	positive
PRJNA729271	stage_1W_vs_E10	ordinary_muscle_development	ENSGALG00010013526	SIRPA	-0.134184612	0.248675967	-0.53959622	0.589475524	0.624457189	negative
PRJNA729271	stage_1W_vs_E10	ordinary_muscle_development	ENSGALG00010013527	ADARB1	0.032727255	0.101258773	0.323204145	0.746540642	0.771668445	positive
PRJNA729271	stage_1W_vs_E10	ordinary_muscle_development	ENSGALG00010013528	PRDX6	1.253491733	0.130931746	9.573627275	1.03219E-21	4.15025E-21	positive
PRJNA729271	stage_1W_vs_E10	ordinary_muscle_development	ENSGALG00010013531	ARSI	-4.653644723	0.272201247	-17.09633874	1.58025E-65	2.63284E-64	negative
PRJNA729271	stage_1W_vs_E10	ordinary_muscle_development	ENSGALG00010013532	WDR19	-0.668635815	0.076939494	-8.690410887	3.61131E-18	1.23837E-17	negative
PRJNA729271	stage_1W_vs_E10	ordinary_muscle_development	ENSGALG00010013533	SKP2	-1.062504952	0.082301041	-12.90998193	3.95394E-38	2.96494E-37	negative
PRJNA729271	stage_1W_vs_E10	ordinary_muscle_development	ENSGALG00010013537	LMO3	-3.075663374	0.474790175	-6.477942334	9.29819E-11	2.21236E-10	negative
PRJNA729271	stage_1W_vs_E10	ordinary_muscle_development	ENSGALG00010013538	E2F7	-2.453974442	0.27350979	-8.972163081	2.90748E-19	1.05491E-18	negative
PRJNA729271	stage_1W_vs_E10	ordinary_muscle_development	ENSGALG00010013539	NSFL1C	0.269421579	0.091930367	2.930713612	0.003381844	0.004797869	positive
PRJNA729271	stage_1W_vs_E10	ordinary_muscle_development	ENSGALG00010013544	ENSGALG00010013544	-1.040580077	0.291787932	-3.566220403	0.000362167	0.000558588	negative
PRJNA729271	stage_1W_vs_E10	ordinary_muscle_development	ENSGALG00010013546	IDUA	-0.330247804	0.132764132	-2.487477591	0.012865256	0.017180936	negative
PRJNA729271	stage_1W_vs_E10	ordinary_muscle_development	ENSGALG00010013549	PCP4	-4.449681017	0.402356701	-11.05904538	1.98196E-28	1.04925E-27	negative
PRJNA729271	stage_1W_vs_E10	ordinary_muscle_development	ENSGALG00010013550	KLHL5	-0.795843764	0.125669619	-6.332825482	2.40712E-10	5.58974E-10	negative
PRJNA729271	stage_1W_vs_E10	ordinary_muscle_development	ENSGALG00010013551	UBE2N	-0.226393878	0.111708097	-2.026655937	0.042697612	0.053766126	negative
PRJNA729271	stage_1W_vs_E10	ordinary_muscle_development	ENSGALG00010013552	FGF16	-0.53544549	0.40645679	-1.317349108	0.187721631	0.217022256	negative
PRJNA729271	stage_1W_vs_E10	ordinary_muscle_development	ENSGALG00010013553	TADA1	0.805304382	0.109950936	7.324215784	2.403E-13	6.54603E-13	positive
PRJNA729271	stage_1W_vs_E10	ordinary_muscle_development	ENSGALG00010013554	ENSGALG00010013554	0.374444367	5.410653885	0.069205012	0.944826434	0.951852874	positive
PRJNA729271	stage_1W_vs_E10	ordinary_muscle_development	ENSGALG00010013556	RASL11B	-2.691208624	0.171797951	-15.66496343	2.62581E-55	3.41661E-54	negative
PRJNA729271	stage_1W_vs_E10	ordinary_muscle_development	ENSGALG00010013558	SNAPC3	-0.42810323	0.087229256	-4.907794126	9.21065E-07	1.72782E-06	negative
PRJNA729271	stage_1W_vs_E10	ordinary_muscle_development	ENSGALG00010013561	AGK	0.294866361	0.108222279	2.724636399	0.006437233	0.008883909	positive
PRJNA729271	stage_1W_vs_E10	ordinary_muscle_development	ENSGALG00010013562	RERGL	0.67611475	0.158418889	4.267892271	1.97329E-05	3.34379E-05	positive
PRJNA729271	stage_1W_vs_E10	ordinary_muscle_development	ENSGALG00010013563	FKBP1A	1.052062594	0.09868422	10.66089991	1.55089E-26	7.61525E-26	positive
PRJNA729271	stage_1W_vs_E10	ordinary_muscle_development	ENSGALG00010013565	ENSGALG00010013565	1.9124019	5.2964272	0.361073952	0.718044165	0.745135322	positive
PRJNA729271	stage_1W_vs_E10	ordinary_muscle_development	ENSGALG00010013569	ENSGALG00010013569	-2.857465969	0.550304778	-5.19251528	2.07472E-07	4.05954E-07	negative
PRJNA729271	stage_1W_vs_E10	ordinary_muscle_development	ENSGALG00010013572	DENND11	0.116201779	0.105184927	1.104737935	0.269273161	0.303248735	positive
PRJNA729271	stage_1W_vs_E10	ordinary_muscle_development	ENSGALG00010013573	ENSGALG00010013573	-1.201998373	0.482600229	-2.490670954	0.012750214	0.017035151	negative
PRJNA729271	stage_1W_vs_E10	ordinary_muscle_development	ENSGALG00010013574	NR2F1	-1.85385395	0.160474608	-11.55231952	7.18546E-31	4.20142E-30	negative
PRJNA729271	stage_1W_vs_E10	ordinary_muscle_development	ENSGALG00010013576	CAMK2A	0.370206849	0.186321879	1.986920973	0.046931155	0.058795773	positive
PRJNA729271	stage_1W_vs_E10	ordinary_muscle_development	ENSGALG00010013579	H2AC32	-1.849945276	0.177129774	-10.44401083	1.56074E-25	7.34749E-25	negative
PRJNA729271	stage_1W_vs_E10	ordinary_muscle_development	ENSGALG00010013580	RANBP3L	-0.827978237	0.315732837	-2.622401414	0.008731252	0.01188129	negative
PRJNA729271	stage_1W_vs_E10	ordinary_muscle_development	ENSGALG00010013582	MAP4K4	-1.52116115	0.107884499	-14.0999046	3.80283E-45	3.5724E-44	negative
PRJNA729271	stage_1W_vs_E10	ordinary_muscle_development	ENSGALG00010013583	FASLG	0.583914084	0.348230202	1.676804827	0.093580654	0.113009379	positive
PRJNA729271	stage_1W_vs_E10	ordinary_muscle_development	ENSGALG00010013587	SLC26A1	0.098415467	0.476295478	0.206626919	0.836301236	0.854096133	positive
PRJNA729271	stage_1W_vs_E10	ordinary_muscle_development	ENSGALG00010013589	DSCAM	-4.885868743	0.831729908	-5.874345383	4.24517E-09	9.21027E-09	negative
PRJNA729271	stage_1W_vs_E10	ordinary_muscle_development	ENSGALG00010013592	H3C33	-1.953760531	0.184133278	-10.6105781	2.66098E-26	1.29346E-25	negative
PRJNA729271	stage_1W_vs_E10	ordinary_muscle_development	ENSGALG00010013596	SDCBP2	0.20607271	0.12148235	1.696318101	0.089825654	0.108719344	positive
PRJNA729271	stage_1W_vs_E10	ordinary_muscle_development	ENSGALG00010013598	PAQR7	2.383882382	0.224561221	10.61573487	2.51805E-26	1.22611E-25	positive
PRJNA729271	stage_1W_vs_E10	ordinary_muscle_development	ENSGALG00010013604	FYN	-1.078054559	0.11244716	-9.587210187	9.04989E-22	3.64384E-21	negative
PRJNA729271	stage_1W_vs_E10	ordinary_muscle_development	ENSGALG00010013609	PIGC	2.403719126	0.160607751	14.96639551	1.21743E-50	1.36478E-49	positive
PRJNA729271	stage_1W_vs_E10	ordinary_muscle_development	ENSGALG00010013612	TWIST2	-5.225414462	0.29648698	-17.62443145	1.59969E-69	2.98808E-68	negative
PRJNA729271	stage_1W_vs_E10	ordinary_muscle_development	ENSGALG00010013614	ATRX	-0.895029842	0.086894414	-10.30019999	7.0315E-25	3.21519E-24	negative
PRJNA729271	stage_1W_vs_E10	ordinary_muscle_development	ENSGALG00010013617	PIK3C2G	0.53804969	0.161184993	3.338087986	0.00084357	0.001265536	positive
PRJNA729271	stage_1W_vs_E10	ordinary_muscle_development	ENSGALG00010013619	IL1R2	0.548957087	0.19918674	2.75599213	0.00585144	0.008110991	positive
PRJNA729271	stage_1W_vs_E10	ordinary_muscle_development	ENSGALG00010013621	PDGFRA	-0.551400504	0.164761674	-3.346655138	0.000817929	0.00122808	negative
PRJNA729271	stage_1W_vs_E10	ordinary_muscle_development	ENSGALG00010013624	SST1	-7.437611563	1.740043611	-4.274382271	1.91668E-05	3.25053E-05	negative
PRJNA729271	stage_1W_vs_E10	ordinary_muscle_development	ENSGALG00010013625	SSBP1	-0.348801503	0.112758749	-3.093343151	0.001979151	0.002868493	negative
PRJNA729271	stage_1W_vs_E10	ordinary_muscle_development	ENSGALG00010013627	MYCBP	0.462438942	0.147279073	3.139882227	0.001690158	0.002466657	positive
PRJNA729271	stage_1W_vs_E10	ordinary_muscle_development	ENSGALG00010013632	SLC1A3	-7.150542154	0.664754505	-10.75666596	5.51287E-27	2.74885E-26	negative
PRJNA729271	stage_1W_vs_E10	ordinary_muscle_development	ENSGALG00010013635	BCL6	0.305409551	0.162090632	1.884190026	0.059539276	0.073624109	positive
PRJNA729271	stage_1W_vs_E10	ordinary_muscle_development	ENSGALG00010013638	COMMD8	-0.923069435	0.120096133	-7.686087901	1.51702E-14	4.40402E-14	negative
PRJNA729271	stage_1W_vs_E10	ordinary_muscle_development	ENSGALG00010013639	ENSGALG00010013639	0.446471716	0.476891125	0.936213096	0.349163482	0.386729172	positive
PRJNA729271	stage_1W_vs_E10	ordinary_muscle_development	ENSGALG00010013640	SNPH	-2.382029973	0.655890766	-3.631747995	0.000281508	0.000438665	negative
PRJNA729271	stage_1W_vs_E10	ordinary_muscle_development	ENSGALG00010013645	RRAGC	-0.177497558	0.121592887	-1.459769254	0.144353501	0.169769087	negative
PRJNA729271	stage_1W_vs_E10	ordinary_muscle_development	ENSGALG00010013646	CLDN1	-0.059261199	0.336396721	-0.176164616	0.860164618	0.875629338	negative
PRJNA729271	stage_1W_vs_E10	ordinary_muscle_development	ENSGALG00010013648	NAV3	-1.244600355	0.119503268	-10.41478089	2.12286E-25	9.92287E-25	negative
PRJNA729271	stage_1W_vs_E10	ordinary_muscle_development	ENSGALG00010013650	BACE2	-1.179550864	0.162618383	-7.25349028	4.06167E-13	1.09492E-12	negative
PRJNA729271	stage_1W_vs_E10	ordinary_muscle_development	ENSGALG00010013651	ENSGALG00010013651	0.32509575	0.158170583	2.055349006	0.039845307	0.050353769	positive
PRJNA729271	stage_1W_vs_E10	ordinary_muscle_development	ENSGALG00010013654	SLC6A7	-3.337657897	0.534336621	-6.246358126	4.20133E-10	9.63429E-10	negative
PRJNA729271	stage_1W_vs_E10	ordinary_muscle_development	ENSGALG00010013657	MAGT1	-0.7391414	0.104962509	-7.041956261	1.89559E-12	4.94273E-12	negative
PRJNA729271	stage_1W_vs_E10	ordinary_muscle_development	ENSGALG00010013661	GABRA4	-1.635595791	0.384252186	-4.256568601	2.07588E-05	3.50985E-05	negative

PRJNA729271	stage_1W_vs_E10	ordinary_muscle_development	ENSGALG00010013663	OPA1	0.471978042	0.106274486	4.441122811	8.94907E-06	1.55835E-05	positive
PRJNA729271	stage_1W_vs_E10	ordinary_muscle_development	ENSGALG00010013664	REV3L	0.475556541	0.09613107	4.946959843	7.53815E-07	1.42236E-06	positive
PRJNA729271	stage_1W_vs_E10	ordinary_muscle_development	ENSGALG00010013666	COX7B	1.234942198	0.143389737	8.612486663	7.14926E-18	2.41446E-17	positive
PRJNA729271	stage_1W_vs_E10	ordinary_muscle_development	ENSGALG00010013667	SOWAHA	-2.139892871	0.545877088	-3.920100178	8.85122E-05	0.000142983	negative
PRJNA729271	stage_1W_vs_E10	ordinary_muscle_development	ENSGALG00010013669	NPR3	-0.671009615	0.257726931	-2.603568095	0.009225894	0.012523818	negative
PRJNA729271	stage_1W_vs_E10	ordinary_muscle_development	ENSGALG00010013672	ENSGALG00010013672	-4.50626146	0.506217129	-8.901835203	5.49308E-19	1.97231E-18	negative
PRJNA729271	stage_1W_vs_E10	ordinary_muscle_development	ENSGALG00010013675	CHRNA6	0.649692517	0.398065173	1.632125997	0.102652946	0.123256665	positive
PRJNA729271	stage_1W_vs_E10	ordinary_muscle_development	ENSGALG00010013677	GABRG1	-4.50324915	0.539467297	-8.347585058	6.96729E-17	2.25024E-16	negative
PRJNA729271	stage_1W_vs_E10	ordinary_muscle_development	ENSGALG00010013678	ATP7A	0.421221629	0.101739384	4.140202293	3.47E-05	5.78532E-05	positive
PRJNA729271	stage_1W_vs_E10	ordinary_muscle_development	ENSGALG00010013679	MFSD4B	1.960767805	0.21333801	9.190897609	3.89577E-20	1.4694E-19	positive
PRJNA729271	stage_1W_vs_E10	ordinary_muscle_development	ENSGALG00010013686	UQCRQ	2.932842235	0.137857462	21.27445406	1.95779E-100	7.37095E-99	positive
PRJNA729271	stage_1W_vs_E10	ordinary_muscle_development	ENSGALG00010013690	CREB3L2	-1.377432086	0.102552663	-13.43146088	3.95531E-41	3.29495E-40	negative
PRJNA729271	stage_1W_vs_E10	ordinary_muscle_development	ENSGALG00010013696	CETN3	-0.617954945	0.11257754	-5.489149466	4.03874E-08	8.27793E-08	negative
PRJNA729271	stage_1W_vs_E10	ordinary_muscle_development	ENSGALG00010013697	HDAC4	-0.244247889	0.06789285	-3.597549479	0.000321229	0.000498258	negative
PRJNA729271	stage_1W_vs_E10	ordinary_muscle_development	ENSGALG00010013698	TFAP2E	-4.832151122	0.803563553	-6.013402553	1.81669E-09	4.0357E-09	negative
PRJNA729271	stage_1W_vs_E10	ordinary_muscle_development	ENSGALG00010013709	PSMF1	0.883907281	0.100179227	8.823259136	1.11176E-18	3.91804E-18	positive
PRJNA729271	stage_1W_vs_E10	ordinary_muscle_development	ENSGALG00010013715	IL1R1	-0.176968643	0.234473921	-0.754747658	0.450400405	0.488554507	negative
PRJNA729271	stage_1W_vs_E10	ordinary_muscle_development	ENSGALG00010013719	NCDN	0.349192929	0.138955953	2.512975665	0.011971759	0.016039435	positive
PRJNA729271	stage_1W_vs_E10	ordinary_muscle_development	ENSGALG00010013720	SLC16A10	0.744415949	0.189820311	3.921687548	8.7931E-05	0.000142076	positive
PRJNA729271	stage_1W_vs_E10	ordinary_muscle_development	ENSGALG00010013732	FNTA	0.271702638	0.075902471	3.579628381	0.000344083	0.000531998	positive
PRJNA729271	stage_1W_vs_E10	ordinary_muscle_development	ENSGALG00010013737	AFF4	0.728100432	0.08456124	8.610332962	7.28487E-18	2.45968E-17	positive
PRJNA729271	stage_1W_vs_E10	ordinary_muscle_development	ENSGALG00010013740	GNPDA2	-1.357852466	0.122329861	-11.09992652	1.25547E-28	6.67818E-28	negative
PRJNA729271	stage_1W_vs_E10	ordinary_muscle_development	ENSGALG00010013742	RSPO4	-5.043599538	1.375038368	-3.667970039	0.000244484	0.000382534	negative
PRJNA729271	stage_1W_vs_E10	ordinary_muscle_development	ENSGALG00010013744	ENSGALG00010013744	-0.419801231	0.315015296	-1.332637613	0.182650756	0.2114297	negative
PRJNA729271	stage_1W_vs_E10	ordinary_muscle_development	ENSGALG00010013746	ENSGALG00010013746	-0.03433539	0.08687169	-0.395242565	0.692663882	0.721276076	negative
PRJNA729271	stage_1W_vs_E10	ordinary_muscle_development	ENSGALG00010013749	NELL2	-3.693289109	0.466248398	-7.921290724	2.35058E-15	7.10269E-15	negative
PRJNA729271	stage_1W_vs_E10	ordinary_muscle_development	ENSGALG00010013751	FUT10	0.108402826	0.17051523	0.635736912	0.524947913	0.56168303	positive
PRJNA729271	stage_1W_vs_E10	ordinary_muscle_development	ENSGALG00010013753	PLEKHA5	0.402875065	0.124063619	3.247326408	0.001164947	0.001722876	positive
PRJNA729271	stage_1W_vs_E10	ordinary_muscle_development	ENSGALG00010013755	ANGPT4	1.955207557	0.153410958	12.74490155	3.32811E-37	2.42538E-36	positive
PRJNA729271	stage_1W_vs_E10	ordinary_muscle_development	ENSGALG00010013757	DGKI	-2.523048855	0.329441616	-7.658561428	1.88027E-14	5.43569E-14	negative
PRJNA729271	stage_1W_vs_E10	ordinary_muscle_development	ENSGALG00010013758	FAM3B	-4.475379597	0.920748614	-4.860587925	1.17038E-06	2.17802E-06	negative
PRJNA729271	stage_1W_vs_E10	ordinary_muscle_development	ENSGALG00010013760	RPF2	-1.096571124	0.116913018	-9.379375693	6.63645E-21	2.5859E-20	negative
PRJNA729271	stage_1W_vs_E10	ordinary_muscle_development	ENSGALG00010013766	DNM3	-0.146270422	0.108917612	-1.342945556	0.179289604	0.207804478	negative
PRJNA729271	stage_1W_vs_E10	ordinary_muscle_development	ENSGALG00010013767	PGK2	5.706937377	0.171874072	33.20417862	9.3697E-242	2.0894E-239	positive
PRJNA729271	stage_1W_vs_E10	ordinary_muscle_development	ENSGALG00010013768	FAM110A	-1.210357097	0.239432735	-5.055102836	4.30159E-07	8.23557E-07	negative
PRJNA729271	stage_1W_vs_E10	ordinary_muscle_development	ENSGALG00010013771	PELO	0.382203875	0.10283046	3.716835199	0.000201734	0.000317254	positive
PRJNA729271	stage_1W_vs_E10	ordinary_muscle_development	ENSGALG00010013772	KIAA0319L	1.784461114	0.142941851	12.48382545	9.14834E-36	6.40605E-35	positive
PRJNA729271	stage_1W_vs_E10	ordinary_muscle_development	ENSGALG00010013775	DBX2	-2.670821222	0.41130724	-6.493494313	8.38679E-11	1.99978E-10	negative
PRJNA729271	stage_1W_vs_E10	ordinary_muscle_development	ENSGALG00010013777	CPLANE1	-1.333877073	0.096561866	-13.81370445	2.10711E-43	1.8807E-42	negative
PRJNA729271	stage_1W_vs_E10	ordinary_muscle_development	ENSGALG00010013778	ENSGALG00010013778	-3.052999487	0.681623334	-4.479012578	7.49891E-06	1.3115E-05	negative
PRJNA729271	stage_1W_vs_E10	ordinary_muscle_development	ENSGALG00010013779	SLC52A3	-0.096867179	0.238817602	-0.405611554	0.685028038	0.714351181	negative
PRJNA729271	stage_1W_vs_E10	ordinary_muscle_development	ENSGALG00010013783	NDUFA10	1.096312478	0.095612462	11.46620904	1.95017E-30	1.1173E-29	positive
PRJNA729271	stage_1W_vs_E10	ordinary_muscle_development	ENSGALG00010013784	ANO6	1.637497616	0.094453828	17.33648766	2.49526E-67	4.39476E-66	positive
PRJNA729271	stage_1W_vs_E10	ordinary_muscle_development	ENSGALG00010013787	ZCCHC10	-0.385798736	0.104643532	-3.686790085	0.000227101	0.000356065	negative
PRJNA729271	stage_1W_vs_E10	ordinary_muscle_development	ENSGALG00010013791	AEBP2	-0.026563237	0.06510958	-0.407977396	0.68329027	0.712641565	negative
PRJNA729271	stage_1W_vs_E10	ordinary_muscle_development	ENSGALG00010013792	FGF10	-0.617160243	0.237858859	-2.594648969	0.009468757	0.012830666	negative
PRJNA729271	stage_1W_vs_E10	ordinary_muscle_development	ENSGALG00010013793	METTL13	-0.908033137	0.131469121	-6.906816802	4.95649E-12	1.26553E-11	negative
PRJNA729271	stage_1W_vs_E10	ordinary_muscle_development	ENSGALG00010013794	GTF3C6	-1.27009373	0.181386721	-7.002131829	2.52097E-12	6.53693E-12	negative
PRJNA729271	stage_1W_vs_E10	ordinary_muscle_development	ENSGALG00010013797	IL1RL1	1.217584649	0.316181931	3.85089889	0.000117685	0.000188283	positive
PRJNA729271	stage_1W_vs_E10	ordinary_muscle_development	ENSGALG00010013802	PYROXD1	0.830297557	0.139921301	5.934032559	2.95583E-09	6.47691E-09	positive
PRJNA729271	stage_1W_vs_E10	ordinary_muscle_development	ENSGALG00010013805	HSPA4	0.858673567	0.145149295	5.915795648	3.30275E-09	7.21745E-09	positive
PRJNA729271	stage_1W_vs_E10	ordinary_muscle_development	ENSGALG00010013808	PLAAT1	1.592033946	0.136244571	11.6851184	1.51868E-31	9.14171E-31	positive
PRJNA729271	stage_1W_vs_E10	ordinary_muscle_development	ENSGALG00010013809	MX1	5.9248621	1.212401773	4.8868801	1.02446E-06	1.91435E-06	positive
PRJNA729271	stage_1W_vs_E10	ordinary_muscle_development	ENSGALG00010013814	AMD1	-0.653788461	0.11122165	-5.878248179	4.14631E-09	9.00521E-09	negative
PRJNA729271	stage_1W_vs_E10	ordinary_muscle_development	ENSGALG00010013815	SRXN1	1.117111449	0.249250235	4.481887236	7.39858E-06	1.29457E-05	positive
PRJNA729271	stage_1W_vs_E10	ordinary_muscle_development	ENSGALG00010013820	MASP1	-3.387195085	0.498000195	-6.801593891	1.03468E-11	2.5997E-11	negative
PRJNA729271	stage_1W_vs_E10	ordinary_muscle_development	ENSGALG00010013823	GUF1	0.145987508	0.112983716	1.292111052	0.196318689	0.22618547	positive
PRJNA729271	stage_1W_vs_E10	ordinary_muscle_development	ENSGALG00010013828	PTN	-7.10953755	0.27574683	-25.78284421	1.3811E-146	1.088E-144	negative
PRJNA729271	stage_1W_vs_E10	ordinary_muscle_development	ENSGALG00010013831	TCERG1	0.066858633	0.113001605	0.591660909	0.554077678	0.590193705	positive
PRJNA729271	stage_1W_vs_E10	ordinary_muscle_development	ENSGALG00010013833	TMPRSS2	-0.961343525	1.212961509	-0.792558971	0.428034821	0.465968061	negative
PRJNA729271	stage_1W_vs_E10	ordinary_muscle_development	ENSGALG00010013836	IL18R1	0.104133559	0.260520862	0.399712936	0.689367963	0.718256801	positive
PRJNA729271	stage_1W_vs_E10	ordinary_muscle_development	ENSGALG00010013837	IFI27L2	2.388003918	0.334871829	7.131098259	9.95712E-13	2.63758E-12	positive
PRJNA729271	stage_1W_vs_E10	ordinary_muscle_development	ENSGALG00010013842	CDK19	0.763240651	0.110890484	6.882832693	5.86739E-12	1.49364E-11	positive
PRJNA729271	stage_1W_vs_E10	ordinary_muscle_development	ENSGALG00010013846	YIPF7	8.45931899	0.31899365	26.51876927	5.8889E-155	5.4718E-153	positive
PRJNA729271	stage_1W_vs_E10	ordinary_muscle_development	ENSGALG00010013848	ITPA	-0.802268876	0.100844168	-7.95553073	1.78365E-15	5.42355E-15	negative
PRJNA729271	stage_1W_vs_E10	ordinary_muscle_development	ENSGALG00010013857	IL18RAP	-0.737476331	0.759988263	-0.970378579	0.331857822	0.368462591	negative
PRJNA729271	stage_1W_vs_E10	ordinary_muscle_development	ENSGALG00010013859	MYOC	0.528632086	0.251972861	2.097972314	0.035907591	0.045588205	positive
PRJNA729271	stage_1W_vs_E10	ordinary_muscle_development	ENSGALG00010013861	TCF15	2.530381045	0.155334686	16.28986486	1.16486E-59	1.68342E-58	positive
PRJNA729271	stage_1W_vs_E10	ordinary_muscle_development	ENSGALG00010013866	KCTD8	-2.972908687	0.421952084	-7.045607301	1.84654E-12	4.8183E-12	negative
PRJNA729271	stage_1W_vs_E10	ordinary_muscle_development	ENSGALG00010013867	RECQL	-1.561686413	0.133564829	-11.69234766	1.39477E-31	8.40282E-31	negative
PRJNA729271	stage_1W_vs_E10	ordinary_muscle_development	ENSGALG00010013871	ENSGALG00010013871	0.356883429	0.238843359	1.494215418	0.135119324	0.15953039	positive
PRJNA729271	stage_1W_vs_E10	ordinary_muscle_development	ENSGALG00010013872	GLS2	2.503104725	0.196833652	12.71685355	4.76671E-37	3.45122E-36	positive
PRJNA729271	stage_1W_vs_E10	ordinary_muscle_development	ENSGALG00010013878	ARID2	-0.578835967	0.082506955	-7.015602137	2.28961E-12	5.95372E-12	negative
PRJNA729271	stage_1W_vs_E10	ordinary_muscle_development	ENSGALG00010013880	ZMYM4	-0.762717695	0.090727704	-8.406668088	4.21823E-17	1.37185E-16	negative
PRJNA729271	stage_1W_vs_E10	ordinary_muscle_development	ENSGALG00010013889	ENSGALG00010013889	0.61755046	0.420084836	1.470061302	0.141545152	0.166655591	positive
PRJNA729271	stage_1W_vs_E10	ordinary_muscle_development	ENSGALG00010013890	SFPQ	-1.13634891	0.07237064	-15.7017945	1.47024E-55	1.92686E-54	negative
PRJNA729271	stage_1W_vs_E10	ordinary_muscle_development	ENSGALG00010013893	CSNK2A1	0.174386332	0.07863603	2.217639074	0.026579451	0.034246147	positive
PRJNA729271	stage_1W_vs_E10	ordinary_muscle_development	ENSGALG00010013897	AMOT	-1.521690856	0.077290061	-19.68805358	2.72979E-86	7.56564E-85	negative
PRJNA729271	stage_1W_vs_E10	ordinary_muscle_development	ENSGALG00010013898	EMB	-2.71697396	0.300292059	-9.047771583	1.45916E-19	5.37769E-19	negative
PRJNA729271	stage_1W_vs_E10	ordinary_muscle_development	ENSGALG00010013899	WNT5B	0.296040518	0.141433679	2.09314019	0.036336642	0.046112732	positive
PRJNA729271	stage_1W_vs_E10	ordinary_muscle_development	ENSGALG00010013901	NFXL1	0.068950148	0.121061607	0.569545947	0.568985699	0.604695924	positive
PRJNA729271	stage_1W_vs_E10	ordinary_muscle_development	ENSGALG00010013908	TBC1D20	1.548448382	0.114996922	13.4651289	2.5088E-41	2.10812E-40	positive
PRJNA729271	stage_1W_vs_E10	ordinary_muscle_development	ENSGALG00010013910	ADIPOR2	1.815010764	0.207824151	8.733396755	2.47136E-18	8.53728E-18	positive
PRJNA729271	stage_1W_vs_E10	ordinary_muscle_development	ENSGALG00010013911	TNIK	0.320724935	0.148002003	2.167031048	0.030232487	0.038725689	positive
PRJNA729271	stage_1W_vs_E10	ordinary_muscle_development	ENSGALG00010013914	NRG1	0.862122579	0.228084553	3.779837647	0.000156931	0.000248874	positive
PRJNA729271	stage_1W_vs_E10	ordinary_muscle_development	ENSGALG00010013915	ENSGALG00010013915	1.926322524	0.189458269	10.16752941	2.76841E-24	1.23244E-23	positive
PRJNA729271	stage_1W_vs_E10	ordinary_muscle_development	ENSGALG00010013917	TRPC5	-4.620926711	0.936549925	-4.933988662	8.05671E-07	1.51783E-06	negative
PRJNA729271	stage_1W_vs_E10	ordinary_muscle_development	ENSGALG00010013921	PPP2R2B	0.947930944	0.105743093	8.964471509	3.11772E-19	1.13063E-18	positive
PRJNA729271	stage_1W_vs_E10	ordinary_muscle_development	ENSGALG00010013922	ENSGALG00010013922	-2.776282986	0.917385585	-3.026298901	0.002475674	0.003555425	negative
PRJNA729271	stage_1W_vs_E10	ordinary_muscle_development	ENSGALG00010013923	FMR1NB	1.489108828	0.516778919	2.881520072	0.00395762	0.005574899	positive
PRJNA729271	stage_1W_vs_E10	ordinary_muscle_development	ENSGALG00010013928	CCNO	3.77300619	0.457074475	8.254685833	1.52302E-16	4.84979E-16	positive
PRJNA729271	stage_1W_vs_E10	ordinary_muscle_development	ENSGALG00010013930	SLC22A16	3.163984444	0.303407031	10.42818434	1.84378E-25	8.64625E-25	positive
PRJNA729271	stage_1W_vs_E10	ordinary_muscle_development	ENSGALG00010013934	STAT1	0.887704122	0.282781497	3.139187434	0.00169417	0.002472015	positive
PRJNA729271	stage_1W_vs_E10	ordinary_muscle_development	ENSGALG00010013936	DLGAP3	-3.10345429	0.45888665	-6.763008453	1.35155E-11	3.37132E-11	negative
PRJNA729271	stage_1W_vs_E10	ordinary_muscle_development	ENSGALG00010013938	ENSGALG00010013938	0.872468749	0.411181396	2.121858524	0.03384962	0.043096201	positive
PRJNA729271	stage_1W_vs_E10	ordinary_muscle_development	ENSGALG00010013940	UPF3A	-0.51175767	0.242128389	-2.113579789	0.034551174	0.043950791	negative
PRJNA729271	stage_1W_vs_E10	ordinary_muscle_development	ENSGALG00010013942	CACNA2D4	1.774168999	0.484642542	3.660778504	0.00025145	0.000393094	positive
PRJNA729271	stage_1W_vs_E10	ordinary_muscle_development	ENSGALG00010013944	METTL24	-2.521033287	0.2317916	-10.87629269	1.49521E-27	7.64213E-27	negative
PRJNA729271	stage_1W_vs_E10	ordinary_muscle_development	ENSGALG00010013945	SCAF11	-0.11050477	0.07691685	-1.436678307	0.150809422	0.176873741	negative
PRJNA729271	stage_1W_vs_E10	ordinary_muscle_development	ENSGALG00010013947	ENSGALG00010013947	0.245311486	0.177878502	1.379095751	0.167865234	0.19550109	positive
PRJNA729271	stage_1W_vs_E10	ordinary_muscle_development	ENSGALG00010013949	PRRC2C	-0.514470413	0.126293581	-4.07360698	4.62906E-05	7.63173E-05	negative
PRJNA729271	stage_1W_vs_E10	ordinary_muscle_development	ENSGALG00010013953	DHX29	-0.929258434	0.082963214	-11.20084904	4.03844E-29	2.19405E-28	negative
PRJNA729271	stage_1W_vs_E10	ordinary_muscle_development	ENSGALG00010013954	LPL	0.56969148	0.334230444	1.704487099	0.088290124	0.106950138	positive
PRJNA729271	stage_1W_vs_E10	ordinary_muscle_development	ENSGALG00010013958	TUSC3	1.768166422	0.170747625	10.35543788	3.95373E-25	1.83155E-24	positive
PRJNA729271	stage_1W_vs_E10	ordinary_muscle_development	ENSGALG00010013959	STK32A	-2.241914322	0.510018885	-4.395747666	1.10392E-05	1.91061E-05	negative
PRJNA729271	stage_1W_vs_E10	ordinary_muscle_development	ENSGALG00010013964	SLC38A1	-1.649917472	0.161931881	-10.18896006	2.22128E-24	9.9344E-24	negative
PRJNA729271	stage_1W_vs_E10	ordinary_muscle_development	ENSGALG00010013965	SLC2A2	2.127139966	1.010155416	2.105755146			positive
PRJNA729271	stage_1W_vs_E10	ordinary_muscle_development	ENSGALG00010013967	MLLT3	0.139777387	0.153587521	0.910082972	0.362778754	0.400371405	positive
PRJNA729271	stage_1W_vs_E10	ordinary_muscle_development	ENSGALG00010013971	ENSGALG00010013971	-0.778646575	0.379259522	-2.053070601	0.040065731	0.050614674	negative
PRJNA729271	stage_1W_vs_E10	ordinary_muscle_development	ENSGALG00010013973	ENSGALG00010013973	0.733052237	0.243334768	3.012525681	0.002590835	0.003714923	positive
PRJNA729271	stage_1W_vs_E10	ordinary_muscle_development	ENSGALG00010013976	CDC40	-0.128113649	0.075449981	-1.697994464	0.089508807	0.108353946	negative
PRJNA729271	stage_1W_vs_E10	ordinary_muscle_development	ENSGALG00010013977	SLC38A2	0.313080645	0.19271286	1.624596538	0.104248555	0.125058579	positive
PRJNA729271	stage_1W_vs_E10	ordinary_muscle_development	ENSGALG00010013980	FMO4	1.3209441	0.371960771	3.551299501	0.000383334	0.000590167	positive
PRJNA729271	stage_1W_vs_E10	ordinary_muscle_development	ENSGALG00010013981	RLF	0.683550451	0.106873937	6.395857318	1.59649E-10	3.7512E-10	positive
PRJNA729271	stage_1W_vs_E10	ordinary_muscle_development	ENSGALG00010013989	ENSGALG00010013989	1.70502744	0.202564164	8.417221504	3.85518E-17	1.2566E-16	positive
PRJNA729271	stage_1W_vs_E10	ordinary_muscle_development	ENSGALG00010013994	CHRM2	-4.890328788	0.563879799	-8.672644064	4.222E-18	1.44267E-17	negative
PRJNA729271	stage_1W_vs_E10	ordinary_muscle_development	ENSGALG00010013995	REM1	0.371686663	0.211395748	1.758250425	0.078704909	0.095827606	positive
PRJNA729271	stage_1W_vs_E10	ordinary_muscle_development	ENSGALG00010014000	STAT4	6.676455285	0.933674949	7.150727662	8.63191E-13	2.29283E-12	positive
PRJNA729271	stage_1W_vs_E10	ordinary_muscle_development	ENSGALG00010014001	CDC16	-0.895679965	0.086890806	-10.3081098	6.47625E-25	2.96411E-24	negative
PRJNA729271	stage_1W_vs_E10	ordinary_muscle_development	ENSGALG00010014002	ZDHHC18	0.230336585	0.271357856	0.848829616	0.395976108	0.434165937	positive
PRJNA729271	stage_1W_vs_E10	ordinary_muscle_development	ENSGALG00010014010	EIF5A2	0.414752313	0.102347458	4.052394859	5.0696E-05	8.33528E-05	positive
PRJNA729271	stage_1W_vs_E10	ordinary_muscle_development	ENSGALG00010014011	SFN	-6.546983396	0.774122978	-8.457291129	2.7366E-17	8.98461E-17	negative
PRJNA729271	stage_1W_vs_E10	ordinary_muscle_development	ENSGALG00010014014	PARP8	0.126236287	0.202324536	0.623929701	0.532673753	0.569235185	positive
PRJNA729271	stage_1W_vs_E10	ordinary_muscle_development	ENSGALG00010014018	DPYSL3	-2.055416042	0.239375113	-8.586590396	8.95883E-18	3.00876E-17	negative
PRJNA729271	stage_1W_vs_E10	ordinary_muscle_development	ENSGALG00010014019	IRAG2	0.437949087	0.235539672	1.859343198	0.062978508	0.077631896	positive
PRJNA729271	stage_1W_vs_E10	ordinary_muscle_development	ENSGALG00010014020	PDXP	-0.943936589	0.107923871	-8.746318898	2.20426E-18	7.63275E-18	negative
PRJNA729271	stage_1W_vs_E10	ordinary_muscle_development	ENSGALG00010014026	SH3BP1	1.934416813	0.338946772	5.707140386	1.1489E-08	2.42901E-08	positive
PRJNA729271	stage_1W_vs_E10	ordinary_muscle_development	ENSGALG00010014030	WASF1	-2.728793367	0.375455481	-7.26795454	3.64972E-13	9.86069E-13	negative
PRJNA729271	stage_1W_vs_E10	ordinary_muscle_development	ENSGALG00010014031	RPL22L1	0.267426598	0.188392964	1.419514785	0.15574899	0.182224683	positive
PRJNA729271	stage_1W_vs_E10	ordinary_muscle_development	ENSGALG00010014032	PSD3	-1.291554902	0.07939691	-16.2670675	1.69063E-59	2.43595E-58	negative
PRJNA729271	stage_1W_vs_E10	ordinary_muscle_development	ENSGALG00010014033	SLC38A4	2.769869421	0.474197797	5.841168897	5.18358E-09	1.11943E-08	positive

PRJNA729271	stage_1W_vs_E10	ordinary_muscle_development	ENSGALG00010014035	PSIP1	-1.021254272	0.11848417	-8.619330923	6.73465E-18	2.27656E-17	negative
PRJNA729271	stage_1W_vs_E10	ordinary_muscle_development	ENSGALG00010014041	SKIV2L2	0.186558286	0.098499462	1.894003085	0.0582246	0.072102997	positive
PRJNA729271	stage_1W_vs_E10	ordinary_muscle_development	ENSGALG00010014042	SGCZ	-3.074211748	0.41103759	-7.479149903	7.48049E-14	2.09648E-13	negative
PRJNA729271	stage_1W_vs_E10	ordinary_muscle_development	ENSGALG00010014045	GOLT1B	-1.756127881	0.112487024	-15.6118263	6.04806E-55	7.79953E-54	negative
PRJNA729271	stage_1W_vs_E10	ordinary_muscle_development	ENSGALG00010014047	HM13	0.343174848	0.167135579	2.053272259	0.04004618	0.050594385	positive
PRJNA729271	stage_1W_vs_E10	ordinary_muscle_development	ENSGALG00010014056	FMO3	4.858935097	0.253809805	19.14400072	1.08602E-81	2.70015E-80	positive
PRJNA729271	stage_1W_vs_E10	ordinary_muscle_development	ENSGALG00010014062	GGA1	0.791750814	0.103747577	7.631511389	2.32017E-14	6.66883E-14	positive
PRJNA729271	stage_1W_vs_E10	ordinary_muscle_development	ENSGALG00010014065	CHUNK-1	-4.188549853	0.990700796	-4.227865638	2.35919E-05	3.9741E-05	negative
PRJNA729271	stage_1W_vs_E10	ordinary_muscle_development	ENSGALG00010014066	BCL2L1	0.068723433	0.116909591	0.587834007	0.556643715	0.592622156	positive
PRJNA729271	stage_1W_vs_E10	ordinary_muscle_development	ENSGALG00010014076	PRRX1	-3.175144321	0.249858608	-12.70776441	5.35433E-37	3.86895E-36	negative
PRJNA729271	stage_1W_vs_E10	ordinary_muscle_development	ENSGALG00010014077	SLITRK2	-0.102588638	0.101062625	-1.015099676	0.310058316	0.345901277	negative
PRJNA729271	stage_1W_vs_E10	ordinary_muscle_development	ENSGALG00010014080	MYO1B	-0.328114667	0.160870122	-2.039624654	0.041387725	0.052198301	negative
PRJNA729271	stage_1W_vs_E10	ordinary_muscle_development	ENSGALG00010014082	HNRNPR	-1.123760643	0.043481511	-25.84456273	2.8008E-147	2.243E-145	negative
PRJNA729271	stage_1W_vs_E10	ordinary_muscle_development	ENSGALG00010014083	ENSGALG00010014083	-0.743679417	0.146216727	-5.086144615	3.65415E-07	7.03413E-07	negative
PRJNA729271	stage_1W_vs_E10	ordinary_muscle_development	ENSGALG00010014089	FIG4	-0.670771194	0.120437497	-5.569454781	2.55538E-08	5.29297E-08	negative
PRJNA729271	stage_1W_vs_E10	ordinary_muscle_development	ENSGALG00010014091	CHIC2	0.586282646	0.098507353	5.951663802	2.6543E-09	5.83471E-09	positive
PRJNA729271	stage_1W_vs_E10	ordinary_muscle_development	ENSGALG00010014092	TYRP1	-2.493482407	1.052476305	-2.369157761	0.017828647	0.023406053	negative
PRJNA729271	stage_1W_vs_E10	ordinary_muscle_development	ENSGALG00010014093	PLD1	0.156575547	0.094176167	1.662581441	0.096396249	0.116177237	positive
PRJNA729271	stage_1W_vs_E10	ordinary_muscle_development	ENSGALG00010014096	JAKMIP2	-3.372730589	0.16603312	-20.31360121	9.74876E-92	3.05202E-90	negative
PRJNA729271	stage_1W_vs_E10	ordinary_muscle_development	ENSGALG00010014099	TPX2	-1.603668061	0.143575033	-11.16954685	5.74733E-29	3.09924E-28	negative
PRJNA729271	stage_1W_vs_E10	ordinary_muscle_development	ENSGALG00010014102	TRIM2	1.074608737	0.137199756	7.83243915	4.78495E-15	1.42565E-14	positive
PRJNA729271	stage_1W_vs_E10	ordinary_muscle_development	ENSGALG00010014104	ALDH1A1	1.830828319	0.524595771	3.489979182	0.000483058	0.000737433	positive
PRJNA729271	stage_1W_vs_E10	ordinary_muscle_development	ENSGALG00010014105	AHR1B	-2.653087787	0.63303313	-4.19107257	2.77639E-05	4.65569E-05	negative
PRJNA729271	stage_1W_vs_E10	ordinary_muscle_development	ENSGALG00010014109	AMIGO2	-1.009534845	0.165121571	-6.113888317	9.72324E-10	2.18916E-09	negative
PRJNA729271	stage_1W_vs_E10	ordinary_muscle_development	ENSGALG00010014111	GORAB	-0.027485721	0.08167432	-0.336528312	0.736472505	0.762512069	negative
PRJNA729271	stage_1W_vs_E10	ordinary_muscle_development	ENSGALG00010014114	ANXA1	1.955836652	0.332252221	5.886602185	3.94216E-09	8.57339E-09	positive
PRJNA729271	stage_1W_vs_E10	ordinary_muscle_development	ENSGALG00010014120	SOX5	-2.848905829	0.141365831	-20.15271871	2.54733E-90	7.54151E-89	negative
PRJNA729271	stage_1W_vs_E10	ordinary_muscle_development	ENSGALG00010014122	ABCA1	-0.076409238	0.140930844	-0.542175409	0.587697674	0.622846954	negative
PRJNA729271	stage_1W_vs_E10	ordinary_muscle_development	ENSGALG00010014125	PLPP1	3.125502527	0.138741109	22.52758792	2.2276E-112	1.0552E-110	positive
PRJNA729271	stage_1W_vs_E10	ordinary_muscle_development	ENSGALG00010014128	COL4A1	3.327411821	0.144538942	23.02086736	2.8812E-117	1.4969E-115	positive
PRJNA729271	stage_1W_vs_E10	ordinary_muscle_development	ENSGALG00010014131	CDC23	-1.021263736	0.119349767	-8.556897579	1.15945E-17	3.87063E-17	negative
PRJNA729271	stage_1W_vs_E10	ordinary_muscle_development	ENSGALG00010014134	RPAP3	-0.101243806	0.113908032	-0.888820599	0.374099504	0.411705436	negative
PRJNA729271	stage_1W_vs_E10	ordinary_muscle_development	ENSGALG00010014135	ZBTB24	0.694510443	0.152981638	4.539828784	5.62999E-06	9.9375E-06	positive
PRJNA729271	stage_1W_vs_E10	ordinary_muscle_development	ENSGALG00010014142	PPIL6	0.38965062	0.523514422	0.744297776	0.456696347	0.494902328	positive
PRJNA729271	stage_1W_vs_E10	ordinary_muscle_development	ENSGALG00010014143	NFATC2	-0.854465516	0.158300797	-5.397733515	6.7488E-08	1.36511E-07	negative
PRJNA729271	stage_1W_vs_E10	ordinary_muscle_development	ENSGALG00010014145	NTMT2	6.889788623	0.251336361	27.41262191	1.9396E-165	2.0227E-163	positive
PRJNA729271	stage_1W_vs_E10	ordinary_muscle_development	ENSGALG00010014147	ENSGALG00010014147	-3.111774164	0.418774073	-7.430675311	1.08045E-13	2.99848E-13	negative
PRJNA729271	stage_1W_vs_E10	ordinary_muscle_development	ENSGALG00010014148	KCNG1	-1.303902596	0.378107839	-3.448493947	0.000563722	0.000856335	negative
PRJNA729271	stage_1W_vs_E10	ordinary_muscle_development	ENSGALG00010014149	LGALS2	3.665342217	0.519269669	7.058648776	1.68129E-12	4.39502E-12	positive
PRJNA729271	stage_1W_vs_E10	ordinary_muscle_development	ENSGALG00010014151	ATP23	0.210071099	0.09033328	2.325511702	0.020044615	0.02619898	positive
PRJNA729271	stage_1W_vs_E10	ordinary_muscle_development	ENSGALG00010014153	FOCAD	0.417292729	0.132533635	3.148579831	0.001640659	0.002397797	positive
PRJNA729271	stage_1W_vs_E10	ordinary_muscle_development	ENSGALG00010014154	DNAI7	-1.820503034	0.49496693	-3.67802963	0.000235043	0.000368199	negative
PRJNA729271	stage_1W_vs_E10	ordinary_muscle_development	ENSGALG00010014155	ENSGALG00010014155	-5.123736572	1.43208803	-3.577808393	0.000346487	0.000535544	negative
PRJNA729271	stage_1W_vs_E10	ordinary_muscle_development	ENSGALG00010014156	CD164	0.407358653	0.092228997	4.416817554	1.00165E-05	1.73837E-05	positive
PRJNA729271	stage_1W_vs_E10	ordinary_muscle_development	ENSGALG00010014159	CDC42EP1	-0.536033421	0.150124026	-3.570603829	0.000356159	0.000549849	negative
PRJNA729271	stage_1W_vs_E10	ordinary_muscle_development	ENSGALG00010014161	GFRA3	0.698512129	0.2500381	2.793622772	0.005212124	0.007265795	positive
PRJNA729271	stage_1W_vs_E10	ordinary_muscle_development	ENSGALG00010014162	COL18A1	1.642464938	0.188240253	8.725365106	2.65323E-18	9.15681E-18	positive
PRJNA729271	stage_1W_vs_E10	ordinary_muscle_development	ENSGALG00010014165	LUZP1	-1.677148192	0.121145506	-13.84408097	1.38136E-43	1.23522E-42	negative
PRJNA729271	stage_1W_vs_E10	ordinary_muscle_development	ENSGALG00010014166	FGF12	-1.993062813	0.215655481	-9.241883405	2.42196E-20	9.23607E-20	negative
PRJNA729271	stage_1W_vs_E10	ordinary_muscle_development	ENSGALG00010014167	SLC38A9	-0.011024525	0.109527625	-0.100655202	0.919824174	0.929572015	negative
PRJNA729271	stage_1W_vs_E10	ordinary_muscle_development	ENSGALG00010014170	CEMIP2	-1.050215497	0.10756714	-9.763348745	1.61725E-22	6.69772E-22	negative
PRJNA729271	stage_1W_vs_E10	ordinary_muscle_development	ENSGALG00010014171	DLC1	-0.265676495	0.132993058	-1.997671905	0.045752242	0.057408133	negative
PRJNA729271	stage_1W_vs_E10	ordinary_muscle_development	ENSGALG00010014173	MND1	-2.291080036	0.20365794	-11.2496475	2.32519E-29	1.28005E-28	negative
PRJNA729271	stage_1W_vs_E10	ordinary_muscle_development	ENSGALG00010014176	ABHD17B	-0.028030251	0.089472213	-0.313284423	0.754064583	0.778612775	negative
PRJNA729271	stage_1W_vs_E10	ordinary_muscle_development	ENSGALG00010014178	LRTM2	-2.560526933	0.541717638	-4.726681861	2.28218E-06	4.14747E-06	negative
PRJNA729271	stage_1W_vs_E10	ordinary_muscle_development	ENSGALG00010014179	CTBPL	-0.83380357	0.042937871	-19.41883824	5.34862E-84	1.40196E-82	negative
PRJNA729271	stage_1W_vs_E10	ordinary_muscle_development	ENSGALG00010014180	SLC19A1	0.117368026	0.143330853	0.818860867	0.412865799	0.450944899	positive
PRJNA729271	stage_1W_vs_E10	ordinary_muscle_development	ENSGALG00010014184	SLC44A1	0.86631592	0.13230046	6.548094567	5.82758E-11	1.402E-10	positive
PRJNA729271	stage_1W_vs_E10	ordinary_muscle_development	ENSGALG00010014186	MOCS3	-0.371009309	0.109358108	-3.392609063	0.000692304	0.001045633	negative
PRJNA729271	stage_1W_vs_E10	ordinary_muscle_development	ENSGALG00010014189	DDX4	-4.244809868	0.856817164	-4.954160638	7.26432E-07	1.37158E-06	negative
PRJNA729271	stage_1W_vs_E10	ordinary_muscle_development	ENSGALG00010014190	HACD4	1.343506792	0.223831776	6.002305914	1.94535E-09	4.31489E-09	positive
PRJNA729271	stage_1W_vs_E10	ordinary_muscle_development	ENSGALG00010014192	KDM1A	-1.830378871	0.062658096	-29.21216848	1.3586E-187	1.8067E-185	negative
PRJNA729271	stage_1W_vs_E10	ordinary_muscle_development	ENSGALG00010014193	CARD10	-2.563932493	0.404510769	-6.338354099	2.32233E-10	5.39716E-10	negative
PRJNA729271	stage_1W_vs_E10	ordinary_muscle_development	ENSGALG00010014194	LRIG3	-1.797088049	0.094034284	-19.11098778	2.0456E-81	5.05989E-80	negative
PRJNA729271	stage_1W_vs_E10	ordinary_muscle_development	ENSGALG00010014197	SH3GL2	-3.107338616	0.374650578	-8.293964552	1.09535E-16	3.50643E-16	negative
PRJNA729271	stage_1W_vs_E10	ordinary_muscle_development	ENSGALG00010014198	KDM3B	-0.738076843	0.046399816	-15.90689154	5.67627E-57	7.68948E-56	negative
PRJNA729271	stage_1W_vs_E10	ordinary_muscle_development	ENSGALG00010014199	LNX1	-0.500383697	0.237888936	-2.103434083	0.035427842	0.045022494	negative
PRJNA729271	stage_1W_vs_E10	ordinary_muscle_development	ENSGALG00010014200	DCP1B	0.217930598	0.089275449	2.441103364	0.014642463	0.019421898	positive
PRJNA729271	stage_1W_vs_E10	ordinary_muscle_development	ENSGALG00010014203	TMEM38B	1.00533549	0.169324736	5.937322054	2.89715E-09	6.3541E-09	positive
PRJNA729271	stage_1W_vs_E10	ordinary_muscle_development	ENSGALG00010014204	KIFAP3	-0.323975319	0.083946573	-3.859303705	0.000113711	0.000182145	negative
PRJNA729271	stage_1W_vs_E10	ordinary_muscle_development	ENSGALG00010014205	DPM1	-0.106089935	0.085769925	-1.236912994	0.216119385	0.247288482	negative
PRJNA729271	stage_1W_vs_E10	ordinary_muscle_development	ENSGALG00010014209	CCDC50	-0.774347595	0.107704868	-7.189532027	6.50138E-13	1.73582E-12	negative
PRJNA729271	stage_1W_vs_E10	ordinary_muscle_development	ENSGALG00010014210	ENSGALG00010014210	0.061324164	0.2550769	0.240414416	0.810009004	0.830126732	positive
PRJNA729271	stage_1W_vs_E10	ordinary_muscle_development	ENSGALG00010014213	ETFRF1	0.808081221	0.151942534	5.318334517	1.04721E-07	2.08938E-07	positive
PRJNA729271	stage_1W_vs_E10	ordinary_muscle_development	ENSGALG00010014214	REEP2	0.062698719	0.134447835	0.466342347	0.640970471	0.673251231	positive
PRJNA729271	stage_1W_vs_E10	ordinary_muscle_development	ENSGALG00010014217	RASA3	0.024457582	0.109825629	0.222694672	0.823773149	0.842548108	positive
PRJNA729271	stage_1W_vs_E10	ordinary_muscle_development	ENSGALG00010014218	IL31RA	1.272423244	0.167229664	7.608836914	2.76573E-14	7.92749E-14	positive
PRJNA729271	stage_1W_vs_E10	ordinary_muscle_development	ENSGALG00010014220	C9orf85	-0.397550995	0.132343705	-3.003928254	0.002665182	0.003816618	negative
PRJNA729271	stage_1W_vs_E10	ordinary_muscle_development	ENSGALG00010014221	SCYL3	1.939140175	0.130675523	14.8393527	8.15389E-50	8.90661E-49	positive
PRJNA729271	stage_1W_vs_E10	ordinary_muscle_development	ENSGALG00010014222	TBX22	-2.200213235	0.432732344	-5.084466792	3.6866E-07	7.09376E-07	negative
PRJNA729271	stage_1W_vs_E10	ordinary_muscle_development	ENSGALG00010014224	NMUR2	-6.394842297	0.967166025	-6.611938524	3.7932E-11	9.25318E-11	negative
PRJNA729271	stage_1W_vs_E10	ordinary_muscle_development	ENSGALG00010014225	ZMPSTE24	-0.287114206	0.076294608	-3.76323064	0.000167732	0.000265337	negative
PRJNA729271	stage_1W_vs_E10	ordinary_muscle_development	ENSGALG00010014226	SLC16A7	0.61719759	0.259942295	2.374363854	0.017579218	0.023099516	positive
PRJNA729271	stage_1W_vs_E10	ordinary_muscle_development	ENSGALG00010014234	GLRA1	-2.556752006	1.003791004	-2.547095955	0.010862356	0.014627448	negative
PRJNA729271	stage_1W_vs_E10	ordinary_muscle_development	ENSGALG00010014236	WDR44	-0.252648074	0.082675551	-3.055898293	0.002243874	0.003235026	negative
PRJNA729271	stage_1W_vs_E10	ordinary_muscle_development	ENSGALG00010014238	PHLDA1	-2.595370014	0.122745678	-21.14428832	3.11452E-99	1.14581E-97	negative
PRJNA729271	stage_1W_vs_E10	ordinary_muscle_development	ENSGALG00010014240	PIGV	0.624530875	0.128763173	4.850229002	1.23319E-06	2.28874E-06	positive
PRJNA729271	stage_1W_vs_E10	ordinary_muscle_development	ENSGALG00010014241	ENSGALG00010014241	-2.904353396	0.881147405	-3.296103897	0.000980358	0.001461512	negative
PRJNA729271	stage_1W_vs_E10	ordinary_muscle_development	ENSGALG00010014243	ENSGALG00010014243	1.202938178	0.143622357	8.37570279	5.48953E-17	1.77851E-16	positive
PRJNA729271	stage_1W_vs_E10	ordinary_muscle_development	ENSGALG00010014244	SESN1	4.51380015	0.369804345	12.20591432	2.89043E-34	1.90527E-33	positive
PRJNA729271	stage_1W_vs_E10	ordinary_muscle_development	ENSGALG00010014246	KRAS	-1.546165671	0.115530557	-13.38317512	7.58372E-41	6.24225E-40	negative
PRJNA729271	stage_1W_vs_E10	ordinary_muscle_development	ENSGALG00010014249	ENSGALG00010014249	0.886631288	0.100543078	8.818421993	1.16084E-18	4.08506E-18	positive
PRJNA729271	stage_1W_vs_E10	ordinary_muscle_development	ENSGALG00010014250	ENSGALG00010014250	5.516249211	0.253003209	21.80307999	2.1692E-105	9.0093E-104	positive
PRJNA729271	stage_1W_vs_E10	ordinary_muscle_development	ENSGALG00010014257	NOP16	0.025821424	0.12406401	0.208129854	0.835127577	0.852957598	positive
PRJNA729271	stage_1W_vs_E10	ordinary_muscle_development	ENSGALG00010014258	ENSGALG00010014258	-1.809859682	0.566614335	-3.194165009	0.001402359	0.002060955	negative
PRJNA729271	stage_1W_vs_E10	ordinary_muscle_development	ENSGALG00010014260	ADNP	-0.579265793	0.075709645	-7.651149275	1.99191E-14	5.74125E-14	negative
PRJNA729271	stage_1W_vs_E10	ordinary_muscle_development	ENSGALG00010014263	ZNF131	-0.289798721	0.120005389	-2.414880892	0.015740356	0.020800115	negative
PRJNA729271	stage_1W_vs_E10	ordinary_muscle_development	ENSGALG00010014265	ZNF462	-1.283159245	0.140136076	-9.15652332	5.35962E-20	2.01211E-19	negative
PRJNA729271	stage_1W_vs_E10	ordinary_muscle_development	ENSGALG00010014267	SCFD2	-0.911565721	0.076610727	-11.89866951	1.20249E-32	7.50004E-32	negative
PRJNA729271	stage_1W_vs_E10	ordinary_muscle_development	ENSGALG00010014272	PARD6B	-0.987639604	0.254537499	-3.880133998	0.000104399	0.000167563	negative
PRJNA729271	stage_1W_vs_E10	ordinary_muscle_development	ENSGALG00010014274	FIRRM	-0.805989281	0.15089513	-5.34138696	9.22381E-08	1.84948E-07	negative
PRJNA729271	stage_1W_vs_E10	ordinary_muscle_development	ENSGALG00010014278	G3BP1	-0.394363023	0.139485842	-2.827262005	0.00469479	0.006569896	negative
PRJNA729271	stage_1W_vs_E10	ordinary_muscle_development	ENSGALG00010014284	TMEM131L	-1.324517864	0.086862201	-15.24849534	1.68471E-52	2.02486E-51	negative
PRJNA729271	stage_1W_vs_E10	ordinary_muscle_development	ENSGALG00010014288	ARMC2	-2.273945275	0.256862381	-8.852776606	8.53684E-19	3.02399E-18	negative
PRJNA729271	stage_1W_vs_E10	ordinary_muscle_development	ENSGALG00010014291	OSTN	-4.302005744	0.53429119	-8.051799882	8.15852E-16	2.51934E-15	negative
PRJNA729271	stage_1W_vs_E10	ordinary_muscle_development	ENSGALG00010014292	NIM1K	2.538563459	0.448445114	5.66081195	1.50658E-08	3.1608E-08	positive
PRJNA729271	stage_1W_vs_E10	ordinary_muscle_development	ENSGALG00010014294	TMPO	-0.428137402	0.102198869	-4.189257717	2.79868E-05	4.69145E-05	negative
PRJNA729271	stage_1W_vs_E10	ordinary_muscle_development	ENSGALG00010014295	CACNA1C	-1.969849307	0.182205995	-10.81111136	3.04953E-27	1.53856E-26	negative
PRJNA729271	stage_1W_vs_E10	ordinary_muscle_development	ENSGALG00010014298	METTL18	-0.363934242	0.224652887	-1.619984713	0.105235561	0.126138217	negative
PRJNA729271	stage_1W_vs_E10	ordinary_muscle_development	ENSGALG00010014300	ELFN2	-0.817138922	0.475714523	-1.717708589	0.085849782	0.104115846	negative
PRJNA729271	stage_1W_vs_E10	ordinary_muscle_development	ENSGALG00010014301	ARID1A	0.127602608	0.119323171	1.069386662	0.284895475	0.319501734	positive
PRJNA729271	stage_1W_vs_E10	ordinary_muscle_development	ENSGALG00010014302	FOXO3	1.770764247	0.233362546	7.588039641	3.24782E-14	9.2635E-14	positive
PRJNA729271	stage_1W_vs_E10	ordinary_muscle_development	ENSGALG00010014303	GABRA2	0.78001748	0.358243311	2.177339969	0.029455211	0.03777679	positive
PRJNA729271	stage_1W_vs_E10	ordinary_muscle_development	ENSGALG00010014305	ARL10	-1.987465484	0.333000062	-5.968363713	2.39645E-09	5.28071E-09	negative
PRJNA729271	stage_1W_vs_E10	ordinary_muscle_development	ENSGALG00010014306	ENSGALG00010014306	-0.81614809	0.332160764	-2.457087587	0.014006853	0.018641937	negative
PRJNA729271	stage_1W_vs_E10	ordinary_muscle_development	ENSGALG00010014308	ENSGALG00010014308	2.834079787	0.198257761	14.29492482	2.3536E-46	2.29424E-45	positive
PRJNA729271	stage_1W_vs_E10	ordinary_muscle_development	ENSGALG00010014310	COL6A1	1.337701038	0.237748169	5.626546121	1.83854E-08	3.84166E-08	positive
PRJNA729271	stage_1W_vs_E10	ordinary_muscle_development	ENSGALG00010014312	KIAA1191	-0.523118433	0.085416022	-6.124359572	9.10491E-10	2.0525E-09	negative
PRJNA729271	stage_1W_vs_E10	ordinary_muscle_development	ENSGALG00010014314	ENSGALG00010014314	0.043362378	0.168569082	0.257238027	0.79699503	0.818567488	positive
PRJNA729271	stage_1W_vs_E10	ordinary_muscle_development	ENSGALG00010014317	RAD23B	1.009176939	0.146236116	6.901010275	5.1634E-12	1.31743E-11	positive
PRJNA729271	stage_1W_vs_E10	ordinary_muscle_development	ENSGALG00010014318	ENSGALG00010014318	0.571272711	0.470049825	1.215345015	0.22423453	0.255746283	positive
PRJNA729271	stage_1W_vs_E10	ordinary_muscle_development	ENSGALG00010014319	ENSGALG00010014319	0.269821274	0.115599739	2.334099331	0.019590521	0.025626894	positive
PRJNA729271	stage_1W_vs_E10	ordinary_muscle_development	ENSGALG00010014320	UTS2B	-2.772319533	1.025524715	-2.703318108	0.006865101	0.009448314	negative
PRJNA729271	stage_1W_vs_E10	ordinary_muscle_development	ENSGALG00010014323	KLF4	0.068966504	0.177821406	0.387841402	0.698133405	0.726606139	positive
PRJNA729271	stage_1W_vs_E10	ordinary_muscle_development	ENSGALG00010014324	COL4A2	3.354510012	0.142206304	23.5890387	4.994E-123	2.9307E-121	positive
PRJNA729271	stage_1W_vs_E10	ordinary_muscle_development	ENSGALG00010014328	RASSF8	-1.039470858	0.092215467	-11.27219649	1.80022E-29	9.94823E-29	negative
PRJNA729271	stage_1W_vs_E10	ordinary_muscle_development	ENSGALG00010014329	IER5	1.159151733	0.154398749	7.50752023	6.02579E-14	1.70062E-13	positive
PRJNA729271	stage_1W_vs_E10	ordinary_muscle_development	ENSGALG00010014334	HMGCS1	0.377556267	0.1580516	2.388816481	0.016902743	0.022246914	positive
PRJNA729271	stage_1W_vs_E10	ordinary_muscle_development	ENSGALG00010014338	BHLHE41	2.018172238	0.219047898	9.213383255	3.16004E-20	1.19783E-19	positive
PRJNA729271	stage_1W_vs_E10	ordinary_muscle_development	ENSGALG00010014340	EFNB1	-2.16142597	0.138269193	-15.63201405	4.40652E-55	5.7029E-54	negative
PRJNA729271	stage_1W_vs_E10	ordinary_muscle_development	ENSGALG00010014344	LSG1	-0.223410248	0.086905905	-2.570714245	0.010148903	0.013713839	negative

PRJNA729271	stage_1W_vs_E10	ordinary_muscle_development	ENSGALG00010014346	AFG1L	0.740069366	0.159618774	4.636480695	3.54391E-06	6.3607E-06	positive
PRJNA729271	stage_1W_vs_E10	ordinary_muscle_development	ENSGALG00010014347	KIAA0040	1.992769252	0.344249144	5.788741347	7.09158E-09	1.51746E-08	positive
PRJNA729271	stage_1W_vs_E10	ordinary_muscle_development	ENSGALG00010014350	PTGER4	-0.897137759	0.178964726	-5.012930657	5.36072E-07	1.01985E-06	negative
PRJNA729271	stage_1W_vs_E10	ordinary_muscle_development	ENSGALG00010014355	RAB20	1.599713961	0.16448981	9.725307377	2.35194E-22	9.66857E-22	positive
PRJNA729271	stage_1W_vs_E10	ordinary_muscle_development	ENSGALG00010014359	TMEM271	-0.992451429	0.486857409	-2.0384848	0.041501473	0.052328102	negative
PRJNA729271	stage_1W_vs_E10	ordinary_muscle_development	ENSGALG00010014361	SSPN	2.47859068	0.149063794	16.6277177	4.39001E-62	6.7408E-61	positive
PRJNA729271	stage_1W_vs_E10	ordinary_muscle_development	ENSGALG00010014362	NAXD	2.661178444	0.106044368	25.09495313	5.646E-139	4.0117E-137	positive
PRJNA729271	stage_1W_vs_E10	ordinary_muscle_development	ENSGALG00010014367	OPHN1	-0.484887066	0.11023473	-4.398677866	1.08912E-05	1.88612E-05	negative
PRJNA729271	stage_1W_vs_E10	ordinary_muscle_development	ENSGALG00010014371	TTC33	-0.420345855	0.102746346	-4.091102715	4.29327E-05	7.10075E-05	negative
PRJNA729271	stage_1W_vs_E10	ordinary_muscle_development	ENSGALG00010014372	CCDC171	-1.093825477	0.128539203	-8.509664358	1.74438E-17	5.78598E-17	negative
PRJNA729271	stage_1W_vs_E10	ordinary_muscle_development	ENSGALG00010014375	H2BC35	-1.738371199	0.246972828	-7.038714394	1.94022E-12	5.05635E-12	negative
PRJNA729271	stage_1W_vs_E10	ordinary_muscle_development	ENSGALG00010014379	CARS2	-0.234527229	0.08974923	-2.613139169	0.008971476	0.012194444	negative
PRJNA729271	stage_1W_vs_E10	ordinary_muscle_development	ENSGALG00010014380	SNX3	0.732334721	0.117415184	6.237138105	4.45649E-10	1.02049E-09	positive
PRJNA729271	stage_1W_vs_E10	ordinary_muscle_development	ENSGALG00010014388	MFNG	1.942548424	0.228727367	8.492855288	2.01621E-17	6.66021E-17	positive
PRJNA729271	stage_1W_vs_E10	ordinary_muscle_development	ENSGALG00010014389	COL6A2	1.36492207	0.264487185	5.16063594	2.46112E-07	4.79231E-07	positive
PRJNA729271	stage_1W_vs_E10	ordinary_muscle_development	ENSGALG00010014390	SPARC	1.396117748	0.159044567	8.778154284	1.66179E-18	5.80286E-18	positive
PRJNA729271	stage_1W_vs_E10	ordinary_muscle_development	ENSGALG00010014393	ATP8A1	1.524341373	0.144064586	10.5809583	3.65204E-26	1.76395E-25	positive
PRJNA729271	stage_1W_vs_E10	ordinary_muscle_development	ENSGALG00010014396	ING1	0.831672234	0.116729649	7.124772839	1.04252E-12	2.75957E-12	positive
PRJNA729271	stage_1W_vs_E10	ordinary_muscle_development	ENSGALG00010014397	PRKAA1	-0.471579411	0.077054645	-6.120064663	9.35374E-10	2.10761E-09	negative
PRJNA729271	stage_1W_vs_E10	ordinary_muscle_development	ENSGALG00010014399	GAS6	2.300458476	0.190252157	12.09162889	1.16945E-33	7.56748E-33	positive
PRJNA729271	stage_1W_vs_E10	ordinary_muscle_development	ENSGALG00010014400	RIPOR3	-1.247824345	0.104199617	-11.97532568	4.78555E-33	3.02514E-32	negative
PRJNA729271	stage_1W_vs_E10	ordinary_muscle_development	ENSGALG00010014403	H2BC37	-0.314629717	0.188150917	-1.672219955	0.094480957	0.114011111	negative
PRJNA729271	stage_1W_vs_E10	ordinary_muscle_development	ENSGALG00010014407	YIPF6	-0.598545163	0.091681801	-6.528505716	6.64291E-11	1.59103E-10	negative
PRJNA729271	stage_1W_vs_E10	ordinary_muscle_development	ENSGALG00010014408	ENSGALG00010014408	-1.561289415	0.387551754	-4.028595914	5.6111E-05	9.19639E-05	negative
PRJNA729271	stage_1W_vs_E10	ordinary_muscle_development	ENSGALG00010014411	ATP13A3	-0.576101383	0.081300787	-7.086049273	1.37995E-12	3.62887E-12	negative
PRJNA729271	stage_1W_vs_E10	ordinary_muscle_development	ENSGALG00010014414	TMEM255B	0.948650106	0.506357008	1.873480748	0.061002029	0.075336492	positive
PRJNA729271	stage_1W_vs_E10	ordinary_muscle_development	ENSGALG00010014416	H2BC32	-2.263029209	0.269964002	-8.382707289	5.1724E-17	1.67727E-16	negative
PRJNA729271	stage_1W_vs_E10	ordinary_muscle_development	ENSGALG00010014418	RAC2	1.752640245	0.298855746	5.864502418	4.50482E-09	9.76189E-09	positive
PRJNA729271	stage_1W_vs_E10	ordinary_muscle_development	ENSGALG00010014427	TMEM267	-0.954786755	0.137924694	-6.922522186	4.43672E-12	1.13582E-11	negative
PRJNA729271	stage_1W_vs_E10	ordinary_muscle_development	ENSGALG00010014431	ENSGALG00010014431	-0.723834807	0.157613044	-4.592480342	4.38009E-06	7.80073E-06	negative
PRJNA729271	stage_1W_vs_E10	ordinary_muscle_development	ENSGALG00010014439	ENSGALG00010014439	-1.170878367	0.841753895	-1.390998454			negative
PRJNA729271	stage_1W_vs_E10	ordinary_muscle_development	ENSGALG00010014442	SHISA3	-5.136568244	0.997838986	-5.147692477	2.6371E-07	5.1226E-07	negative
PRJNA729271	stage_1W_vs_E10	ordinary_muscle_development	ENSGALG00010014449	ENSGALG00010014449	-0.835088499	0.15477474	-5.395508971	6.83296E-08	1.38136E-07	negative
PRJNA729271	stage_1W_vs_E10	ordinary_muscle_development	ENSGALG00010014453	NR2E1	1.043319161	0.343508158	3.03724711	0.002387496	0.00343321	positive
PRJNA729271	stage_1W_vs_E10	ordinary_muscle_development	ENSGALG00010014455	C4orf19	-2.072003132	0.259857657	-7.973608158	1.54108E-15	4.69876E-15	negative
PRJNA729271	stage_1W_vs_E10	ordinary_muscle_development	ENSGALG00010014465	SIMC1	1.845763347	0.148419317	12.4361396	1.66351E-35	1.14931E-34	positive
PRJNA729271	stage_1W_vs_E10	ordinary_muscle_development	ENSGALG00010014469	TMCO3	-0.760546828	0.128844688	-5.90281865	3.57343E-09	7.78666E-09	negative
PRJNA729271	stage_1W_vs_E10	ordinary_muscle_development	ENSGALG00010014470	RPS6KA1	-1.510468002	0.13138776	-11.49626115	1.37754E-30	7.96787E-30	negative
PRJNA729271	stage_1W_vs_E10	ordinary_muscle_development	ENSGALG00010014474	PTPN1	-0.296032909	0.077989507	-3.795804335	0.000147166	0.000233848	negative
PRJNA729271	stage_1W_vs_E10	ordinary_muscle_development	ENSGALG00010014476	C5orf34	0.603009703	0.173913913	3.467288464	0.000525737	0.000800479	positive
PRJNA729271	stage_1W_vs_E10	ordinary_muscle_development	ENSGALG00010014477	ITPR2	-0.21035192	0.098857155	-2.127837071	0.033350595	0.04250566	negative
PRJNA729271	stage_1W_vs_E10	ordinary_muscle_development	ENSGALG00010014482	THOC3	-1.37155707	0.087000271	-15.76497475	5.4194E-56	7.1739E-55	negative
PRJNA729271	stage_1W_vs_E10	ordinary_muscle_development	ENSGALG00010014487	OSTM1	0.109233634	0.084430615	1.293768077	0.195745543	0.225578919	positive
PRJNA729271	stage_1W_vs_E10	ordinary_muscle_development	ENSGALG00010014488	ENSGALG00010014488	-1.9744019	0.410732631	-4.807024696	1.53193E-06	2.82152E-06	negative
PRJNA729271	stage_1W_vs_E10	ordinary_muscle_development	ENSGALG00010014492	CPLX2	-1.464886108	0.324509768	-4.514151051	6.35709E-06	1.11652E-05	negative
PRJNA729271	stage_1W_vs_E10	ordinary_muscle_development	ENSGALG00010014494	KLHL9	-1.265547424	0.069007585	-18.33925105	4.02301E-75	8.50052E-74	negative
PRJNA729271	stage_1W_vs_E10	ordinary_muscle_development	ENSGALG00010014504	SEC63	-0.685255115	0.046309493	-14.7972926	1.52498E-49	1.63981E-48	negative
PRJNA729271	stage_1W_vs_E10	ordinary_muscle_development	ENSGALG00010014505	CYTH4	1.897651454	0.213981076	8.868314397	7.42614E-19	2.64087E-18	positive
PRJNA729271	stage_1W_vs_E10	ordinary_muscle_development	ENSGALG00010014512	HMGN4	-1.175563794	0.111131916	-10.57809343	3.76542E-26	1.81811E-25	negative
PRJNA729271	stage_1W_vs_E10	ordinary_muscle_development	ENSGALG00010014515	ETNK1	-2.232790493	0.114267823	-19.53997575	5.02061E-85	1.35772E-83	negative
PRJNA729271	stage_1W_vs_E10	ordinary_muscle_development	ENSGALG00010014517	DCUN1D2	1.985103435	0.126672481	15.67114982	2.3823E-55	3.10535E-54	positive
PRJNA729271	stage_1W_vs_E10	ordinary_muscle_development	ENSGALG00010014522	FAT2	-6.264973595	0.553690468	-11.3149385	1.1068E-29	6.17278E-29	negative
PRJNA729271	stage_1W_vs_E10	ordinary_muscle_development	ENSGALG00010014537	GKAP1	2.747226921	0.119774024	22.93675066	1.9981E-116	1.0056E-114	positive
PRJNA729271	stage_1W_vs_E10	ordinary_muscle_development	ENSGALG00010014539	PAIP1	-0.404842496	0.085596718	-4.729649739	2.24908E-06	4.08936E-06	negative
PRJNA729271	stage_1W_vs_E10	ordinary_muscle_development	ENSGALG00010014547	DHDDS	-0.115221386	0.107420664	-1.072618448	0.283442344	0.317970496	negative
PRJNA729271	stage_1W_vs_E10	ordinary_muscle_development	ENSGALG00010014550	SCML4	-6.662181118	0.984979851	-6.763774015	1.34443E-11	3.35413E-11	negative
PRJNA729271	stage_1W_vs_E10	ordinary_muscle_development	ENSGALG00010014555	VGLL1	-6.268188137	0.848762538	-7.38509048	1.5235E-13	4.19193E-13	negative
PRJNA729271	stage_1W_vs_E10	ordinary_muscle_development	ENSGALG00010014567	UTP11	0.374973449	0.115378143	3.249952204	0.001154244	0.001707918	positive
PRJNA729271	stage_1W_vs_E10	ordinary_muscle_development	ENSGALG00010014570	KIF27	-1.140847294	0.166364649	-6.857510284	7.0071E-12	1.77753E-11	negative
PRJNA729271	stage_1W_vs_E10	ordinary_muscle_development	ENSGALG00010014572	IL1RAP	-0.426867229	0.128148902	-3.331025244	0.000865268	0.001297141	negative
PRJNA729271	stage_1W_vs_E10	ordinary_muscle_development	ENSGALG00010014573	CEBPB	5.26407962	0.347122253	15.16491546	6.03766E-52	7.09205E-51	positive
PRJNA729271	stage_1W_vs_E10	ordinary_muscle_development	ENSGALG00010014576	HRH2	0.535418943	0.267922653	1.998408636	0.045672376	0.057317844	positive
PRJNA729271	stage_1W_vs_E10	ordinary_muscle_development	ENSGALG00010014578	ENSGALG00010014578	6.582170346	0.300742855	21.88637318	3.503E-106	1.4675E-104	positive
PRJNA729271	stage_1W_vs_E10	ordinary_muscle_development	ENSGALG00010014579	NNT	0.687453436	0.144621964	4.753451132	1.99973E-06	3.65341E-06	positive
PRJNA729271	stage_1W_vs_E10	ordinary_muscle_development	ENSGALG00010014592	QNG1	0.071322604	0.09284265	0.768209476	0.442362745	0.480483176	positive
PRJNA729271	stage_1W_vs_E10	ordinary_muscle_development	ENSGALG00010014599	SSTR3	-1.75912764	0.424204076	-4.146889999	3.37022E-05	5.62674E-05	negative
PRJNA729271	stage_1W_vs_E10	ordinary_muscle_development	ENSGALG00010014602	FRK	-1.561462794	1.175803634	-1.32799623	0.184179352	0.2131651	negative
PRJNA729271	stage_1W_vs_E10	ordinary_muscle_development	ENSGALG00010014603	ENSGALG00010014603	4.986868832	0.221472274	22.51689903	2.8353E-112	1.3387E-110	positive
PRJNA729271	stage_1W_vs_E10	ordinary_muscle_development	ENSGALG00010014611	TMEM189	0.731460897	0.103448216	7.070792753	1.54051E-12	4.03719E-12	positive
PRJNA729271	stage_1W_vs_E10	ordinary_muscle_development	ENSGALG00010014614	GHR	0.797414316	0.090404862	8.820480465	1.1397E-18	4.01163E-18	positive
PRJNA729271	stage_1W_vs_E10	ordinary_muscle_development	ENSGALG00010014617	GRTP1	1.764007823	0.202304263	8.719578112	2.7924E-18	9.62794E-18	positive
PRJNA729271	stage_1W_vs_E10	ordinary_muscle_development	ENSGALG00010014624	GPRC6A	0.753280083	0.412235545	1.827305025	0.06765392	0.083105387	positive
PRJNA729271	stage_1W_vs_E10	ordinary_muscle_development	ENSGALG00010014626	C2CD5	-0.49708696	0.092094448	-5.397577948	6.75465E-08	1.36591E-07	negative
PRJNA729271	stage_1W_vs_E10	ordinary_muscle_development	ENSGALG00010014632	NWD2	-0.974591559	0.16922663	-5.759091003	8.45681E-09	1.80134E-08	negative
PRJNA729271	stage_1W_vs_E10	ordinary_muscle_development	ENSGALG00010014634	CCDC152	2.324453904	0.454996954	5.108724095	3.24342E-07	6.26009E-07	positive
PRJNA729271	stage_1W_vs_E10	ordinary_muscle_development	ENSGALG00010014641	DOCK11	-0.396211158	0.097193703	-4.076510554	4.57166E-05	7.54311E-05	negative
PRJNA729271	stage_1W_vs_E10	ordinary_muscle_development	ENSGALG00010014645	SLC36A1	-0.126168832	0.121651492	-1.037133446	0.29967367	0.335038167	negative
PRJNA729271	stage_1W_vs_E10	ordinary_muscle_development	ENSGALG00010014648	C1QTNF6	-0.247166428	0.275120618	-0.898392966	0.368976085	0.406746395	negative
PRJNA729271	stage_1W_vs_E10	ordinary_muscle_development	ENSGALG00010014653	FAM162B	3.030075044	0.47916728	6.323626782	2.55494E-10	5.92543E-10	positive
PRJNA729271	stage_1W_vs_E10	ordinary_muscle_development	ENSGALG00010014654	LAMP1	1.261060322	0.104435609	12.07500328	1.43159E-33	9.2349E-33	positive
PRJNA729271	stage_1W_vs_E10	ordinary_muscle_development	ENSGALG00010014655	FHL3	-1.355400927	0.134787431	-10.05584061	8.65793E-24	3.78801E-23	negative
PRJNA729271	stage_1W_vs_E10	ordinary_muscle_development	ENSGALG00010014658	SELENOP1	2.248587237	0.173510519	12.95937125	2.07945E-38	1.57619E-37	positive
PRJNA729271	stage_1W_vs_E10	ordinary_muscle_development	ENSGALG00010014668	GP5	-0.373794578	0.156458105	-2.389103314	0.016889552	0.02223359	negative
PRJNA729271	stage_1W_vs_E10	ordinary_muscle_development	ENSGALG00010014672	ISL1	-2.628125967	1.090300886	-2.410459353	0.015932447	0.021038609	negative
PRJNA729271	stage_1W_vs_E10	ordinary_muscle_development	ENSGALG00010014676	UBE2V1	-0.471988897	0.058408033	-8.08089016	6.42957E-16	1.99607E-15	negative
PRJNA729271	stage_1W_vs_E10	ordinary_muscle_development	ENSGALG00010014677	ENSGALG00010014677	-1.147988176	0.090108953	-12.74000126	3.54389E-37	2.58005E-36	negative
PRJNA729271	stage_1W_vs_E10	ordinary_muscle_development	ENSGALG00010014682	IL13RA1	-0.099468762	0.127905598	-0.777673254	0.436761664	0.474755179	negative
PRJNA729271	stage_1W_vs_E10	ordinary_muscle_development	ENSGALG00010014687	CUL4A	2.395072179	0.114189286	20.97457875	1.11954E-97	4.00683E-96	positive
PRJNA729271	stage_1W_vs_E10	ordinary_muscle_development	ENSGALG00010014690	CSF3R	1.991437547	0.373713178	5.328785985	9.88714E-08	1.97729E-07	positive
PRJNA729271	stage_1W_vs_E10	ordinary_muscle_development	ENSGALG00010014691	PABIR2	0.150046241	0.054127592	2.772084159	0.005569863	0.007738442	positive
PRJNA729271	stage_1W_vs_E10	ordinary_muscle_development	ENSGALG00010014696	SPINK5	1.238604032	0.631681731	1.960803949	0.049901897	0.06230752	positive
PRJNA729271	stage_1W_vs_E10	ordinary_muscle_development	ENSGALG00010014697	ENSGALG00010014697	0.346084547	0.709698659	0.487649994	0.625797777	0.659321034	positive
PRJNA729271	stage_1W_vs_E10	ordinary_muscle_development	ENSGALG00010014702	ENSGALG00010014702	0.598111392	0.268426206	2.228215351	0.025866158	0.033392408	positive
PRJNA729271	stage_1W_vs_E10	ordinary_muscle_development	ENSGALG00010014703	PCID2	-1.006216244	0.081136359	-12.40154543	2.56332E-35	1.76008E-34	negative
PRJNA729271	stage_1W_vs_E10	ordinary_muscle_development	ENSGALG00010014705	ARAP2	-0.848153683	0.220895165	-3.839620868	0.000123224	0.000196798	negative
PRJNA729271	stage_1W_vs_E10	ordinary_muscle_development	ENSGALG00010014707	KPNA5	1.685246371	0.129270657	13.03657307	7.57876E-39	5.84331E-38	positive
PRJNA729271	stage_1W_vs_E10	ordinary_muscle_development	ENSGALG00010014710	NPEPL1	1.603569345	0.099350342	16.14055191	1.32353E-58	1.8662E-57	positive
PRJNA729271	stage_1W_vs_E10	ordinary_muscle_development	ENSGALG00010014713	ST8SIA1	-3.597216636	0.146418142	-24.56810745	2.7704E-133	1.842E-131	negative
PRJNA729271	stage_1W_vs_E10	ordinary_muscle_development	ENSGALG00010014716	TMEM35A	-2.437746928	0.1418061	-17.19070566	3.11715E-66	5.28457E-65	negative
PRJNA729271	stage_1W_vs_E10	ordinary_muscle_development	ENSGALG00010014717	ELOVL7	1.952386855	0.18222524	10.71414066	8.73648E-27	4.32793E-26	positive
PRJNA729271	stage_1W_vs_E10	ordinary_muscle_development	ENSGALG00010014719	ENSGALG00010014719	3.023926176	0.90342987	3.347162049	0.000816435	0.001225963	positive
PRJNA729271	stage_1W_vs_E10	ordinary_muscle_development	ENSGALG00010014722	KCTD17	-3.728201664	0.470847965	-7.918058351	2.41248E-15	7.28671E-15	negative
PRJNA729271	stage_1W_vs_E10	ordinary_muscle_development	ENSGALG00010014726	PWP2	-1.802897438	0.064816367	-27.81546595	2.82E-170	3.0966E-168	negative
PRJNA729271	stage_1W_vs_E10	ordinary_muscle_development	ENSGALG00010014728	INTS13	0.931182462	0.111810564	8.32821537	8.20701E-17	2.64298E-16	positive
PRJNA729271	stage_1W_vs_E10	ordinary_muscle_development	ENSGALG00010014729	MRPS15	0.885032978	0.09502163	9.31401597	1.23088E-20	4.76158E-20	positive
PRJNA729271	stage_1W_vs_E10	ordinary_muscle_development	ENSGALG00010014734	ZUP1	-0.706322371	0.100571654	-7.023075993	2.17036E-12	5.65105E-12	negative
PRJNA729271	stage_1W_vs_E10	ordinary_muscle_development	ENSGALG00010014735	STX16	0.795191584	0.10462625	7.600306638	2.9543E-14	8.4496E-14	positive
PRJNA729271	stage_1W_vs_E10	ordinary_muscle_development	ENSGALG00010014736	KIF2A	-1.568147162	0.090963643	-17.23927388	1.34718E-66	2.30276E-65	negative
PRJNA729271	stage_1W_vs_E10	ordinary_muscle_development	ENSGALG00010014737	RMI1	0.189564547	0.113430052	1.671202161	0.094681754	0.114224887	positive
PRJNA729271	stage_1W_vs_E10	ordinary_muscle_development	ENSGALG00010014738	BRWD1	0.499004869	0.086565903	5.764450574	8.19243E-09	1.74631E-08	positive
PRJNA729271	stage_1W_vs_E10	ordinary_muscle_development	ENSGALG00010014739	TRMT2B	1.15836656	0.17476652	6.628080498	3.4008E-11	8.31135E-11	positive
PRJNA729271	stage_1W_vs_E10	ordinary_muscle_development	ENSGALG00010014740	TRMT9B	-2.444756549	0.480879485	-5.083927732	3.69709E-07	7.11205E-07	negative
PRJNA729271	stage_1W_vs_E10	ordinary_muscle_development	ENSGALG00010014742	PROZ	19.47495175	2.862150911	6.804306396	1.01537E-11	2.55296E-11	positive
PRJNA729271	stage_1W_vs_E10	ordinary_muscle_development	ENSGALG00010014744	IL2RB	2.92436925	0.443749982	6.590128154	4.39447E-11	1.06804E-10	positive
PRJNA729271	stage_1W_vs_E10	ordinary_muscle_development	ENSGALG00010014745	LSM10	1.780906329	0.181012068	9.838605504	7.67675E-23	3.22722E-22	positive
PRJNA729271	stage_1W_vs_E10	ordinary_muscle_development	ENSGALG00010014748	FGFR1OP2	0.052083247	0.108527869	0.479906657	0.631293768	0.664338838	positive
PRJNA729271	stage_1W_vs_E10	ordinary_muscle_development	ENSGALG00010014750	LONRF1	1.443335852	0.180341392	8.003353164	1.21076E-15	3.71035E-15	positive
PRJNA729271	stage_1W_vs_E10	ordinary_muscle_development	ENSGALG00010014753	ENSGALG00010014753	-4.350340445	0.652535253	-6.666828227	2.61391E-11	6.4316E-11	negative
PRJNA729271	stage_1W_vs_E10	ordinary_muscle_development	ENSGALG00010014754	DIMT1	0.070849063	0.142199558	0.498236873	0.618317096	0.652482987	positive
PRJNA729271	stage_1W_vs_E10	ordinary_muscle_development	ENSGALG00010014758	FXN	-0.659517554	0.101986746	-6.466698687	1.00167E-10	2.38059E-10	negative
PRJNA729271	stage_1W_vs_E10	ordinary_muscle_development	ENSGALG00010014762	STK40	0.12595129	0.104487572	1.205418853	0.228041647	0.259669181	positive
PRJNA729271	stage_1W_vs_E10	ordinary_muscle_development	ENSGALG00010014764	ENSGALG00010014764	8.225593049	2.265632679	3.630594282	0.000282769	0.000440441	positive
PRJNA729271	stage_1W_vs_E10	ordinary_muscle_development	ENSGALG00010014766	F10	-0.010166546	0.218954227	-0.04643229	0.962965701	0.967974191	negative
PRJNA729271	stage_1W_vs_E10	ordinary_muscle_development	ENSGALG00010014767	LRRC15	0.452969829	0.25163727	1.800090381	0.071846368	0.08792006	positive
PRJNA729271	stage_1W_vs_E10	ordinary_muscle_development	ENSGALG00010014769	CCDC69	1.990378937	0.321771643	6.185687838	6.18323E-10	1.40545E-09	positive
PRJNA729271	stage_1W_vs_E10	ordinary_muscle_development	ENSGALG00010014777	APCDD1L	-1.655084151	0.224278607	-7.37958993	1.58778E-13	4.36631E-13	negative
PRJNA729271	stage_1W_vs_E10	ordinary_muscle_development	ENSGALG00010014780	EVA1B	0.529507004	0.125231453	4.228226935	2.3554E-05	3.96856E-05	positive
PRJNA729271	stage_1W_vs_E10	ordinary_muscle_development	ENSGALG00010014783	ENSGALG00010014783	0.462268031	0.118768076	3.892190955	9.9343E-05	0.000159749	positive
PRJNA729271	stage_1W_vs_E10	ordinary_muscle_development	ENSGALG00010014789	F7	-0.361633456	0.935464098	-0.386581865	0.699065785	0.727472077	negative
PRJNA729271	stage_1W_vs_E10	ordinary_muscle_development	ENSGALG00010014792	ENSGALG00010014792	1.260476947	0.270954537	4.651986865	3.28752E-06	5.91297E-06	positive
PRJNA729271	stage_1W_vs_E10	ordinary_muscle_development	ENSGALG00010014793	TLR2A	0.374736838	0.176176845	2.127049321	0.033415985	0.042585257	positive

PRJNA729271	stage_1W_vs_E10	ordinary_muscle_development	ENSGALG00010014795	THRAP3	-0.481719215	0.095490911	-5.044660364	4.54328E-07	8.68682E-07	negative
PRJNA729271	stage_1W_vs_E10	ordinary_muscle_development	ENSGALG00010014796	PRLR	-1.608674458	0.253432392	-6.347548743	2.18773E-10	5.09333E-10	negative
PRJNA729271	stage_1W_vs_E10	ordinary_muscle_development	ENSGALG00010014797	ZDHHC9	0.189856354	0.092207465	2.059012823	0.03949301	0.049939037	positive
PRJNA729271	stage_1W_vs_E10	ordinary_muscle_development	ENSGALG00010014801	MPST	-0.379156842	0.087407239	-4.337819691	1.43903E-05	2.46645E-05	negative
PRJNA729271	stage_1W_vs_E10	ordinary_muscle_development	ENSGALG00010014802	ENSGALG00010014802	-2.210676054	0.465919957	-4.744755015	2.08759E-06	3.8048E-06	negative
PRJNA729271	stage_1W_vs_E10	ordinary_muscle_development	ENSGALG00010014805	SPEF2	-2.166824626	0.400396631	-5.411695446	6.24308E-08	1.266E-07	negative
PRJNA729271	stage_1W_vs_E10	ordinary_muscle_development	ENSGALG00010014806	MAP7D1	2.26423957	0.225920817	10.02227065	1.21675E-23	5.2868E-23	positive
PRJNA729271	stage_1W_vs_E10	ordinary_muscle_development	ENSGALG00010014807	HES1	-0.000160817	0.225139709	-0.000714297	0.999430073	0.999567992	negative
PRJNA729271	stage_1W_vs_E10	ordinary_muscle_development	ENSGALG00010014810	ENTREP1	3.385215093	0.216274829	15.65237669	3.2004E-55	4.16053E-54	positive
PRJNA729271	stage_1W_vs_E10	ordinary_muscle_development	ENSGALG00010014812	RWDD1	-0.400729406	0.08385035	-4.779102354	1.7608E-06	3.23032E-06	negative
PRJNA729271	stage_1W_vs_E10	ordinary_muscle_development	ENSGALG00010014813	SASH3	2.074588315	0.256306343	8.094174687	5.76539E-16	1.79295E-15	positive
PRJNA729271	stage_1W_vs_E10	ordinary_muscle_development	ENSGALG00010014816	TST	0.815710657	0.108219885	7.53753024	4.78956E-14	1.35621E-13	positive
PRJNA729271	stage_1W_vs_E10	ordinary_muscle_development	ENSGALG00010014818	ENSGALG00010014818	0.584091497	0.436802291	1.337198794	0.181157755	0.209801987	positive
PRJNA729271	stage_1W_vs_E10	ordinary_muscle_development	ENSGALG00010014821	C3AR1L	1.508132114	0.230874477	6.532259999	6.47846E-11	1.55318E-10	positive
PRJNA729271	stage_1W_vs_E10	ordinary_muscle_development	ENSGALG00010014824	ANXA6	0.127420398	0.090758774	1.403945792	0.160334992	0.187303007	positive
PRJNA729271	stage_1W_vs_E10	ordinary_muscle_development	ENSGALG00010014829	ENSGALG00010014829	2.793878511	0.394873204	7.075381377	1.49038E-12	3.91077E-12	positive
PRJNA729271	stage_1W_vs_E10	ordinary_muscle_development	ENSGALG00010014830	TRAPPC3L	-2.42080994	0.536252423	-4.514310491	6.35231E-06	1.11595E-05	negative
PRJNA729271	stage_1W_vs_E10	ordinary_muscle_development	ENSGALG00010014831	VAPB	0.508723106	0.060500644	8.40855684	4.15086E-17	1.35054E-16	positive
PRJNA729271	stage_1W_vs_E10	ordinary_muscle_development	ENSGALG00010014837	APBA1	-1.323018678	0.117654396	-11.24495746	2.45215E-29	1.3484E-28	negative
PRJNA729271	stage_1W_vs_E10	ordinary_muscle_development	ENSGALG00010014839	TM7SF3	0.446836746	0.108722941	4.109866244	3.95888E-05	6.56643E-05	positive
PRJNA729271	stage_1W_vs_E10	ordinary_muscle_development	ENSGALG00010014840	NCF4	0.972330445	0.215368949	4.51471973	6.34006E-06	1.11393E-05	positive
PRJNA729271	stage_1W_vs_E10	ordinary_muscle_development	ENSGALG00010014847	PAICS	-1.538479819	0.092459862	-16.63943454	3.61011E-62	5.56687E-61	negative
PRJNA729271	stage_1W_vs_E10	ordinary_muscle_development	ENSGALG00010014851	CALHM4	-0.296101758	0.485799952	-0.609513766	0.542183951	0.57837477	negative
PRJNA729271	stage_1W_vs_E10	ordinary_muscle_development	ENSGALG00010014856	MMGT1	0.924897433	0.071016626	13.02367463	8.97459E-39	6.89383E-38	positive
PRJNA729271	stage_1W_vs_E10	ordinary_muscle_development	ENSGALG00010014859	TRAPPC3	0.379608209	0.128352234	2.957550455	0.00310094	0.004413169	positive
PRJNA729271	stage_1W_vs_E10	ordinary_muscle_development	ENSGALG00010014861	MED21	-0.384828463	0.098269705	-3.916043738	9.00139E-05	0.000145328	negative
PRJNA729271	stage_1W_vs_E10	ordinary_muscle_development	ENSGALG00010014862	ENSGALG00010014862	-0.891564133	0.348800232	-2.55608813	0.01058563	0.014273369	negative
PRJNA729271	stage_1W_vs_E10	ordinary_muscle_development	ENSGALG00010014865	TLR2	2.057047699	0.375218901	5.48226034	4.19926E-08	8.59842E-08	positive
PRJNA729271	stage_1W_vs_E10	ordinary_muscle_development	ENSGALG00010014866	FAM26E	-0.013583445	0.149948909	-0.090587157	0.927820637	0.936608409	negative
PRJNA729271	stage_1W_vs_E10	ordinary_muscle_development	ENSGALG00010014867	TP63	-1.167258937	0.275627596	-4.234913174	2.2864E-05	3.8585E-05	negative
PRJNA729271	stage_1W_vs_E10	ordinary_muscle_development	ENSGALG00010014870	ENSGALG00010014870	5.114301581	1.903627195	2.686608804	0.007218144	0.009909736	positive
PRJNA729271	stage_1W_vs_E10	ordinary_muscle_development	ENSGALG00010014873	CALHM6	0.576387757	0.707014366	0.81524193	0.414933841	0.452998872	positive
PRJNA729271	stage_1W_vs_E10	ordinary_muscle_development	ENSGALG00010014876	PCNT	-0.264727811	0.103528511	-2.557052249	0.010556336	0.014236518	negative
PRJNA729271	stage_1W_vs_E10	ordinary_muscle_development	ENSGALG00010014877	ENSGALG00010014877	-0.263442563	0.091204061	-2.888495979	0.00387089	0.005456968	negative
PRJNA729271	stage_1W_vs_E10	ordinary_muscle_development	ENSGALG00010014878	TMCO1	1.014874886	0.129315158	7.84807365	4.22476E-15	1.26368E-14	positive
PRJNA729271	stage_1W_vs_E10	ordinary_muscle_development	ENSGALG00010014880	SFRP2	-3.292317926	0.294204202	-11.19058771	4.53418E-29	2.46061E-28	negative
PRJNA729271	stage_1W_vs_E10	ordinary_muscle_development	ENSGALG00010014881	ENSGALG00010014881	0.920305072	0.414794138	2.218703176	0.026506925	0.034158773	positive
PRJNA729271	stage_1W_vs_E10	ordinary_muscle_development	ENSGALG00010014882	XPNPEP2	0.3863247	0.612458379	0.630777066	0.528186301	0.564772826	positive
PRJNA729271	stage_1W_vs_E10	ordinary_muscle_development	ENSGALG00010014883	PPAT	-0.835992699	0.122058553	-6.849111965	7.43098E-12	1.88209E-11	negative
PRJNA729271	stage_1W_vs_E10	ordinary_muscle_development	ENSGALG00010014886	RABGAP1L	0.239814966	0.070450699	3.404011178	0.00066404	0.001003983	positive
PRJNA729271	stage_1W_vs_E10	ordinary_muscle_development	ENSGALG00010014887	MTMR12	-0.411283155	0.132568585	-3.102417934	0.001919468	0.002787565	negative
PRJNA729271	stage_1W_vs_E10	ordinary_muscle_development	ENSGALG00010014889	PVALB	2.616288633	0.411551121	6.357141307	2.05543E-10	4.79225E-10	positive
PRJNA729271	stage_1W_vs_E10	ordinary_muscle_development	ENSGALG00010014893	SLC9A6	-0.315227203	0.090125947	-3.497629839	0.000469412	0.000717054	negative
PRJNA729271	stage_1W_vs_E10	ordinary_muscle_development	ENSGALG00010014894	RAB22A	-0.466752501	0.081872309	-5.700981274	1.1912E-08	2.51514E-08	negative
PRJNA729271	stage_1W_vs_E10	ordinary_muscle_development	ENSGALG00010014897	COL8A2	-4.260902808	0.255833078	-16.65501129	2.78292E-62	4.30506E-61	negative
PRJNA729271	stage_1W_vs_E10	ordinary_muscle_development	ENSGALG00010014898	ENSGALG00010014898	3.120414738	0.639281813	4.88112547	1.05482E-06	1.96955E-06	positive
PRJNA729271	stage_1W_vs_E10	ordinary_muscle_development	ENSGALG00010014900	UBLCP1	-0.30209035	0.08390485	-3.600392009	0.000317738	0.000493053	negative
PRJNA729271	stage_1W_vs_E10	ordinary_muscle_development	ENSGALG00010014902	AASDH	-0.078421897	0.127497666	-0.61508496	0.538498614	0.57482417	negative
PRJNA729271	stage_1W_vs_E10	ordinary_muscle_development	ENSGALG00010014903	MCF2L	1.686011343	0.153771357	10.9644044	5.66725E-28	2.94855E-27	positive
PRJNA729271	stage_1W_vs_E10	ordinary_muscle_development	ENSGALG00010014906	IFT27	-0.743541432	0.111511689	-6.667834038	2.59606E-11	6.38986E-11	negative
PRJNA729271	stage_1W_vs_E10	ordinary_muscle_development	ENSGALG00010014907	ENSGALG00010014907	-0.710453811	0.071432962	-9.945742053	2.63205E-23	1.13008E-22	negative
PRJNA729271	stage_1W_vs_E10	ordinary_muscle_development	ENSGALG00010014908	ENSGALG00010014908	-0.77112056	0.102694502	-7.508878705	5.96359E-14	1.68405E-13	negative
PRJNA729271	stage_1W_vs_E10	ordinary_muscle_development	ENSGALG00010014909	FBXL14	-0.276809513	0.098412968	-2.812734138	0.004912225	0.006864234	negative
PRJNA729271	stage_1W_vs_E10	ordinary_muscle_development	ENSGALG00010014912	ENSGALG00010014912	1.049837759	0.343062688	3.060192193	0.00221195	0.003190588	positive
PRJNA729271	stage_1W_vs_E10	ordinary_muscle_development	ENSGALG00010014914	PTAR1	0.348763743	0.180230438	1.935099014	0.052978147	0.06596669	positive
PRJNA729271	stage_1W_vs_E10	ordinary_muscle_development	ENSGALG00010014916	DYNLT2B	-0.315497732	0.157213795	-2.006806925	0.044770225	0.056258727	negative
PRJNA729271	stage_1W_vs_E10	ordinary_muscle_development	ENSGALG00010014917	EIF3D	0.491760494	0.105016545	4.682695442	2.83127E-06	5.11393E-06	positive
PRJNA729271	stage_1W_vs_E10	ordinary_muscle_development	ENSGALG00010014918	PLRG1	-0.337204963	0.084674516	-3.982366586	6.82324E-05	0.000111227	negative
PRJNA729271	stage_1W_vs_E10	ordinary_muscle_development	ENSGALG00010014922	MAN1A1	-0.199679899	0.165336931	-1.207715047	0.227156888	0.258733231	negative
PRJNA729271	stage_1W_vs_E10	ordinary_muscle_development	ENSGALG00010014923	CIMIP1	-4.287798258	0.60406974	-7.09818416	1.26407E-12	3.33321E-12	negative
PRJNA729271	stage_1W_vs_E10	ordinary_muscle_development	ENSGALG00010014928	SUCO	-1.105012308	0.081991787	-13.47710977	2.133E-41	1.7965E-40	negative
PRJNA729271	stage_1W_vs_E10	ordinary_muscle_development	ENSGALG00010014929	FHL1	0.72755704	0.285019813	2.552654256	0.010690556	0.014410563	positive
PRJNA729271	stage_1W_vs_E10	ordinary_muscle_development	ENSGALG00010014931	RNF145	-0.4748836	0.109123653	-4.351793456	1.35028E-05	2.31873E-05	negative
PRJNA729271	stage_1W_vs_E10	ordinary_muscle_development	ENSGALG00010014934	MAMDC2	0.557400797	0.204806344	2.721599278	0.006496687	0.008959988	positive
PRJNA729271	stage_1W_vs_E10	ordinary_muscle_development	ENSGALG00010014935	PCYT1A	0.129673591	0.132031617	0.982140443	0.326030664	0.362464678	positive
PRJNA729271	stage_1W_vs_E10	ordinary_muscle_development	ENSGALG00010014936	FOXRED2	-0.636358004	0.17245646	-3.689963277	0.000224286	0.000351767	negative
PRJNA729271	stage_1W_vs_E10	ordinary_muscle_development	ENSGALG00010014940	VAMP4	-0.105621925	0.085541656	-1.234742589	0.21692632	0.248074715	negative
PRJNA729271	stage_1W_vs_E10	ordinary_muscle_development	ENSGALG00010014942	PMEPA1	-2.267516456	0.149016511	-15.21654502	2.74658E-52	3.26325E-51	negative
PRJNA729271	stage_1W_vs_E10	ordinary_muscle_development	ENSGALG00010014943	APLN	0.590159507	0.302282664	1.952343212	0.05089747	0.063509595	positive
PRJNA729271	stage_1W_vs_E10	ordinary_muscle_development	ENSGALG00010014944	FGB	2.089887609	0.915551466	2.282654428			positive
PRJNA729271	stage_1W_vs_E10	ordinary_muscle_development	ENSGALG00010014945	TXN2	0.887611716	0.104354186	8.505760507	1.80409E-17	5.97994E-17	positive
PRJNA729271	stage_1W_vs_E10	ordinary_muscle_development	ENSGALG00010014946	PCK1	4.399196474	1.039639806	4.231462135	2.32177E-05	3.91463E-05	positive
PRJNA729271	stage_1W_vs_E10	ordinary_muscle_development	ENSGALG00010014948	RBM38	4.049806547	0.258951856	15.63922579	3.93483E-55	5.10155E-54	positive
PRJNA729271	stage_1W_vs_E10	ordinary_muscle_development	ENSGALG00010014949	AGO3	-0.03080983	0.109651853	-0.280978654	0.778726772	0.801622368	negative
PRJNA729271	stage_1W_vs_E10	ordinary_muscle_development	ENSGALG00010014952	C21orf58	-0.718929023	0.59652454	-1.205196055	0.228127625	0.259736857	negative
PRJNA729271	stage_1W_vs_E10	ordinary_muscle_development	ENSGALG00010014953	ENSGALG00010014953	0.430967793	0.373303023	1.154471749	0.248306814	0.28114414	positive
PRJNA729271	stage_1W_vs_E10	ordinary_muscle_development	ENSGALG00010014956	FGG	4.907152203	1.524164874	3.219567834	0.00128384	0.001891185	positive
PRJNA729271	stage_1W_vs_E10	ordinary_muscle_development	ENSGALG00010014965	ABHD13	-0.195448864	0.099479026	-1.964724337	0.049446152	0.061765078	negative
PRJNA729271	stage_1W_vs_E10	ordinary_muscle_development	ENSGALG00010014967	HNRNPH1	-0.992754664	0.107797334	-9.209454719	3.27786E-20	1.24118E-19	negative
PRJNA729271	stage_1W_vs_E10	ordinary_muscle_development	ENSGALG00010014970	SLC51A	1.04474305	0.50710588	2.060206935	0.039378762	0.049811952	positive
PRJNA729271	stage_1W_vs_E10	ordinary_muscle_development	ENSGALG00010014971	SMC5	-0.59983206	0.125698907	-4.77197513	1.82428E-06	3.34298E-06	negative
PRJNA729271	stage_1W_vs_E10	ordinary_muscle_development	ENSGALG00010014972	FGA	4.02927637	1.657836099	2.430443137	0.015080372	0.019973501	positive
PRJNA729271	stage_1W_vs_E10	ordinary_muscle_development	ENSGALG00010014974	LIG4	0.509754201	0.111749983	4.561559561	5.07751E-06	8.99847E-06	positive
PRJNA729271	stage_1W_vs_E10	ordinary_muscle_development	ENSGALG00010014976	ITGB2	1.521817154	0.212346195	7.166679647	7.68385E-13	2.04415E-12	positive
PRJNA729271	stage_1W_vs_E10	ordinary_muscle_development	ENSGALG00010014980	ENSGALG00010014980	0.206257194	0.727530325	0.283503226	0.776791109	0.799800193	positive
PRJNA729271	stage_1W_vs_E10	ordinary_muscle_development	ENSGALG00010014982	ENSGALG00010014982	1.275128872	0.325003223	3.923434538	8.72955E-05	0.000141096	positive
PRJNA729271	stage_1W_vs_E10	ordinary_muscle_development	ENSGALG00010014984	LPP	0.694117899	0.092431398	7.509546666	5.93325E-14	1.67581E-13	positive
PRJNA729271	stage_1W_vs_E10	ordinary_muscle_development	ENSGALG00010014985	ENSGALG00010014985	0.242026308	0.107780018	2.245558238	0.024732318	0.032008478	positive
PRJNA729271	stage_1W_vs_E10	ordinary_muscle_development	ENSGALG00010014987	KLF9	7.720526335	0.289963511	26.62585481	3.408E-156	3.2287E-154	positive
PRJNA729271	stage_1W_vs_E10	ordinary_muscle_development	ENSGALG00010014990	AGO1	-1.33016613	0.049129889	-27.07447844	1.9675E-161	1.9534E-159	negative
PRJNA729271	stage_1W_vs_E10	ordinary_muscle_development	ENSGALG00010014992	ARL15	-1.249157474	0.098721556	-12.65334061	1.07217E-36	7.67842E-36	negative
PRJNA729271	stage_1W_vs_E10	ordinary_muscle_development	ENSGALG00010014994	ENSGALG00010014994	0.521266589	0.118947383	4.382329195	1.17417E-05	2.02735E-05	positive
PRJNA729271	stage_1W_vs_E10	ordinary_muscle_development	ENSGALG00010014995	LRAT	-1.179577736	0.259913038	-4.538355392	5.66947E-06	1.00047E-05	negative
PRJNA729271	stage_1W_vs_E10	ordinary_muscle_development	ENSGALG00010014997	RAE1	-0.215231673	0.089375251	-2.40817978	0.016032284	0.021162732	negative
PRJNA729271	stage_1W_vs_E10	ordinary_muscle_development	ENSGALG00010014998	NALF1	0.255642072	0.476593027	0.536394905	0.591685652	0.626249253	positive
PRJNA729271	stage_1W_vs_E10	ordinary_muscle_development	ENSGALG00010015002	ADAM19	-1.167614563	0.201324788	-5.799656227	6.6451E-09	1.4255E-08	negative
PRJNA729271	stage_1W_vs_E10	ordinary_muscle_development	ENSGALG00010015006	HSPB3	3.903155809	0.329968895	11.82885982	2.76877E-32	1.70562E-31	positive
PRJNA729271	stage_1W_vs_E10	ordinary_muscle_development	ENSGALG00010015009	AGO4	0.415321001	0.104412915	3.977678452	6.95914E-05	0.00011334	positive
PRJNA729271	stage_1W_vs_E10	ordinary_muscle_development	ENSGALG00010015013	SNX18	-1.514796865	0.114146248	-13.27066712	3.42518E-40	2.75822E-39	negative
PRJNA729271	stage_1W_vs_E10	ordinary_muscle_development	ENSGALG00010015016	ARGLU1	0.233429662	0.079941588	2.920002822	0.003500282	0.00495765	positive
PRJNA729271	stage_1W_vs_E10	ordinary_muscle_development	ENSGALG00010015017	FAM184A	0.455749054	0.188915854	2.412444724	0.01584594	0.020928191	positive
PRJNA729271	stage_1W_vs_E10	ordinary_muscle_development	ENSGALG00010015018	MAP7D3	0.117218985	0.068593788	1.708886301	0.087472013	0.105994551	positive
PRJNA729271	stage_1W_vs_E10	ordinary_muscle_development	ENSGALG00010015020	NIPAL4	-1.222614145	0.821346657	-1.48854827	0.136606362	0.161194173	negative
PRJNA729271	stage_1W_vs_E10	ordinary_muscle_development	ENSGALG00010015021	RBM46	-1.977421681	0.917773905	-2.154584774	0.031194342	0.039880224	negative
PRJNA729271	stage_1W_vs_E10	ordinary_muscle_development	ENSGALG00010015024	CRACD	-1.900706648	0.389449871	-4.880491146	1.05822E-06	1.97539E-06	negative
PRJNA729271	stage_1W_vs_E10	ordinary_muscle_development	ENSGALG00010015025	TRAF3IP1	-1.321153605	0.197328164	-6.695210534	2.15361E-11	5.32344E-11	negative
PRJNA729271	stage_1W_vs_E10	ordinary_muscle_development	ENSGALG00010015027	CLSPN	-2.659768869	0.186316934	-14.27550791	3.11014E-46	3.01952E-45	negative
PRJNA729271	stage_1W_vs_E10	ordinary_muscle_development	ENSGALG00010015028	MCM9	-2.427144401	0.274012264	-8.857794788	8.16128E-19	2.89449E-18	negative
PRJNA729271	stage_1W_vs_E10	ordinary_muscle_development	ENSGALG00010015029	TFRC	-1.785960742	0.183034308	-9.75751904	1.71294E-22	7.08796E-22	negative
PRJNA729271	stage_1W_vs_E10	ordinary_muscle_development	ENSGALG00010015031	MYH9	0.045919439	0.109607866	0.418942922	0.675257847	0.70502503	positive
PRJNA729271	stage_1W_vs_E10	ordinary_muscle_development	ENSGALG00010015032	C1orf216	-1.531520974	0.256873706	-5.962155485	2.48932E-09	5.48036E-09	negative
PRJNA729271	stage_1W_vs_E10	ordinary_muscle_development	ENSGALG00010015033	BMP7	-0.976653982	0.324142916	-3.013035094	0.00258649	0.003709059	negative
PRJNA729271	stage_1W_vs_E10	ordinary_muscle_development	ENSGALG00010015035	SLC35F1	-3.51252744	0.133009229	-26.40814822	1.1046E-153	1.007E-151	negative
PRJNA729271	stage_1W_vs_E10	ordinary_muscle_development	ENSGALG00010015037	CYFIP2	-1.186747559	0.196965054	-6.025168096	1.68934E-09	3.76202E-09	negative
PRJNA729271	stage_1W_vs_E10	ordinary_muscle_development	ENSGALG00010015038	PCSK5	-0.458906941	0.207081409	-2.216070206	0.026686694	0.034378211	negative
PRJNA729271	stage_1W_vs_E10	ordinary_muscle_development	ENSGALG00010015039	FAM114A1	-1.093171217	0.128590828	-8.501160138	1.87705E-17	6.21184E-17	negative
PRJNA729271	stage_1W_vs_E10	ordinary_muscle_development	ENSGALG00010015040	ZC4H2	-1.389845823	0.090412838	-15.37221757	2.51414E-53	3.09359E-52	negative
PRJNA729271	stage_1W_vs_E10	ordinary_muscle_development	ENSGALG00010015041	TFAP2C	-6.847450121	0.712037041	-9.616704922	6.79738E-22	2.74911E-21	negative
PRJNA729271	stage_1W_vs_E10	ordinary_muscle_development	ENSGALG00010015042	EFNB2	-0.400717692	0.133619815	-2.998939126	0.002709214	0.003875845	negative
PRJNA729271	stage_1W_vs_E10	ordinary_muscle_development	ENSGALG00010015043	COQ10B	3.832649931	0.221651582	17.29132678	5.46791E-67	9.51469E-66	positive
PRJNA729271	stage_1W_vs_E10	ordinary_muscle_development	ENSGALG00010015044	ZC3H12B	-0.586408858	0.131579254	-4.456696945	8.32321E-06	1.45163E-05	negative
PRJNA729271	stage_1W_vs_E10	ordinary_muscle_development	ENSGALG00010015046	PSMB2	0.466189476	0.093589897	4.981194478	6.3193E-07	1.19736E-06	positive
PRJNA729271	stage_1W_vs_E10	ordinary_muscle_development	ENSGALG00010015047	NPY2R	-2.651682915	0.212981101	-12.45032023	1.39285E-35	9.67852E-35	negative
PRJNA729271	stage_1W_vs_E10	ordinary_muscle_development	ENSGALG00010015049	DCK2	-2.256645064	0.250506823	-9.008317755	2.09241E-19	7.64315E-19	negative
PRJNA729271	stage_1W_vs_E10	ordinary_muscle_development	ENSGALG00010015050	HIVEP3	-0.817794833	0.180895442	-4.520815036	6.1602E-06	1.0839E-05	negative
PRJNA729271	stage_1W_vs_E10	ordinary_muscle_development	ENSGALG00010015052	ENSGALG00010015052	-0.098068562	0.107883561	-0.909022298	0.363338356	0.400927944	negative
PRJNA729271	stage_1W_vs_E10	ordinary_muscle_development	ENSGALG00010015054	ENSGALG00010015054	-2.373786903	0.178334113	-13.31089641	2.00067E-40	1.62554E-39	negative
PRJNA729271	stage_1W_vs_E10	ordinary_muscle_development	ENSGALG00010015055	ZNF335	1.323704624	0.181808545	7.280761345	3.31941E-13	8.98001E-13	positive
PRJNA729271	stage_1W_vs_E10	ordinary_muscle_development	ENSGALG00010015056	ENSGALG00010015056	-1.259953719	0.511670148	-2.462433509	0.013799776	0.018386593	negative
PRJNA729271	stage_1W_vs_E10	ordinary_muscle_development	ENSGALG00010015057	LAS1L	-0.285615336	0.113130152	-2.524661468	0.011580979	0.015535982	negative
PRJNA729271	stage_1W_vs_E10	ordinary_muscle_development	ENSGALG00010015059	ENSGALG00010015059	1.014584339	0.161155276	6.295694215	3.06027E-10	7.07023E-10	positive

PRJNA729271	stage_1W_vs_E10	ordinary_muscle_development	ENSGALG00010015061	MMP9	-2.729665398	0.550846519	-4.955401015	7.21813E-07	1.36304E-06	negative
PRJNA729271	stage_1W_vs_E10	ordinary_muscle_development	ENSGALG00010015062	ENSGALG00010015062	0.339228327	0.236427453	1.434809381	0.151341424	0.17744026	positive
PRJNA729271	stage_1W_vs_E10	ordinary_muscle_development	ENSGALG00010015063	EEF1AKMT4	-1.632324666	0.23328496	-6.997127733	2.61263E-12	6.76856E-12	negative
PRJNA729271	stage_1W_vs_E10	ordinary_muscle_development	ENSGALG00010015064	RFK	0.175997965	0.146440587	1.201838707	0.229426026	0.261071616	positive
PRJNA729271	stage_1W_vs_E10	ordinary_muscle_development	ENSGALG00010015065	F11	5.523002118	1.207032357	4.575686878	4.7466E-06	8.43161E-06	positive
PRJNA729271	stage_1W_vs_E10	ordinary_muscle_development	ENSGALG00010015066	RUFY1	-0.398727212	0.071400188	-5.584400022	2.34508E-08	4.86434E-08	negative
PRJNA729271	stage_1W_vs_E10	ordinary_muscle_development	ENSGALG00010015068	ESM1	-1.615664406	0.329833741	-4.898420642	9.661E-07	1.80949E-06	negative
PRJNA729271	stage_1W_vs_E10	ordinary_muscle_development	ENSGALG00010015069	HSPD1	-1.753218436	0.103037044	-17.01541864	6.31204E-65	1.04088E-63	negative
PRJNA729271	stage_1W_vs_E10	ordinary_muscle_development	ENSGALG00010015070	EDN2	0.141878906	0.448317971	0.316469372	0.751646269	0.776502935	positive
PRJNA729271	stage_1W_vs_E10	ordinary_muscle_development	ENSGALG00010015071	SNAI1	-2.090294353	0.12991247	-16.09002092	2.99736E-58	4.18562E-57	negative
PRJNA729271	stage_1W_vs_E10	ordinary_muscle_development	ENSGALG00010015072	MAP9	-0.874682689	0.143233157	-6.106705371	1.01709E-09	2.28746E-09	negative
PRJNA729271	stage_1W_vs_E10	ordinary_muscle_development	ENSGALG00010015075	ENSGALG00010015075	3.509020473	0.16691586	21.02269051	4.06713E-98	1.46649E-96	positive
PRJNA729271	stage_1W_vs_E10	ordinary_muscle_development	ENSGALG00010015076	ITK	1.410647903	0.624179951	2.260001943	0.02382113	0.030903722	positive
PRJNA729271	stage_1W_vs_E10	ordinary_muscle_development	ENSGALG00010015080	ASF1A	-1.306805357	0.08008948	-16.31681654	7.49432E-60	1.09176E-58	negative
PRJNA729271	stage_1W_vs_E10	ordinary_muscle_development	ENSGALG00010015083	CEP135	-2.475416837	0.197831486	-12.51275457	6.35805E-36	4.47813E-35	negative
PRJNA729271	stage_1W_vs_E10	ordinary_muscle_development	ENSGALG00010015084	FOXO6	0.621756727	0.203139457	3.060738353	0.002207919	0.003185408	positive
PRJNA729271	stage_1W_vs_E10	ordinary_muscle_development	ENSGALG00010015085	PSMD2	0.675539221	0.163386867	4.134599258	3.55575E-05	5.92284E-05	positive
PRJNA729271	stage_1W_vs_E10	ordinary_muscle_development	ENSGALG00010015086	FOXN3	0.182201215	0.096368181	1.890678172	0.058667318	0.072595422	positive
PRJNA729271	stage_1W_vs_E10	ordinary_muscle_development	ENSGALG00010015087	GABRA3	-6.679052927	0.911238715	-7.329641308	2.3077E-13	6.29115E-13	negative
PRJNA729271	stage_1W_vs_E10	ordinary_muscle_development	ENSGALG00010015088	ENSGALG00010015088	-1.411717199	1.085415891	-1.300623301			negative
PRJNA729271	stage_1W_vs_E10	ordinary_muscle_development	ENSGALG00010015090	GZMA	1.740173831	0.511908436	3.399384944	0.000675376	0.001020598	positive
PRJNA729271	stage_1W_vs_E10	ordinary_muscle_development	ENSGALG00010015091	MSN	-1.095082274	0.121426913	-9.018447795	1.90773E-19	6.9831E-19	negative
PRJNA729271	stage_1W_vs_E10	ordinary_muscle_development	ENSGALG00010015092	HSPE1	-0.729168443	0.12292479	-5.931825794	2.99584E-09	6.56261E-09	negative
PRJNA729271	stage_1W_vs_E10	ordinary_muscle_development	ENSGALG00010015094	CDK5RAP1	1.429202284	0.213255742	6.701823227	2.05835E-11	5.09056E-11	positive
PRJNA729271	stage_1W_vs_E10	ordinary_muscle_development	ENSGALG00010015095	RBFOX2	-0.208503583	0.133156425	-1.565854466	0.117382692	0.139750482	negative
PRJNA729271	stage_1W_vs_E10	ordinary_muscle_development	ENSGALG00010015096	GABRE	-5.162221864	1.209021987	-4.269750194	1.95692E-05	3.31683E-05	negative
PRJNA729271	stage_1W_vs_E10	ordinary_muscle_development	ENSGALG00010015097	MFSD2A	-1.160553159	0.167185018	-6.941729425	3.87329E-12	9.94743E-12	negative
PRJNA729271	stage_1W_vs_E10	ordinary_muscle_development	ENSGALG00010015098	HAVCR1	1.312717157	0.216118504	6.074061846	1.24715E-09	2.79403E-09	positive
PRJNA729271	stage_1W_vs_E10	ordinary_muscle_development	ENSGALG00010015100	MOB4	0.94477921	0.088680243	10.6537733	1.67437E-26	8.21044E-26	positive
PRJNA729271	stage_1W_vs_E10	ordinary_muscle_development	ENSGALG00010015101	CAP1	-1.717254585	0.100383824	-17.10688549	1.31864E-65	2.19951E-64	negative
PRJNA729271	stage_1W_vs_E10	ordinary_muscle_development	ENSGALG00010015103	ITPK1	-1.123262724	0.093761408	-11.98001125	4.52262E-33	2.86393E-32	negative
PRJNA729271	stage_1W_vs_E10	ordinary_muscle_development	ENSGALG00010015104	CNGA2	1.915427121	0.271485199	7.055364807	1.72148E-12	4.49926E-12	positive
PRJNA729271	stage_1W_vs_E10	ordinary_muscle_development	ENSGALG00010015105	ENSGALG00010015105	6.109279997	0.952398928	6.414622922	1.41172E-10	3.32459E-10	positive
PRJNA729271	stage_1W_vs_E10	ordinary_muscle_development	ENSGALG00010015107	MB	-0.375437356	0.567540986	-0.661515847	0.508281555	0.545824652	negative
PRJNA729271	stage_1W_vs_E10	ordinary_muscle_development	ENSGALG00010015108	ENSGALG00010015108	0.938617336	0.087088808	10.77770329	4.38703E-27	2.1973E-26	positive
PRJNA729271	stage_1W_vs_E10	ordinary_muscle_development	ENSGALG00010015109	PPT1	0.990945567	0.107676245	9.203010104	3.4806E-20	1.31521E-19	positive
PRJNA729271	stage_1W_vs_E10	ordinary_muscle_development	ENSGALG00010015110	ENSGALG00010015110	3.127347446	0.709017696	4.410817195	1.02981E-05	1.78576E-05	positive
PRJNA729271	stage_1W_vs_E10	ordinary_muscle_development	ENSGALG00010015111	ADAMTS2	1.11408215	0.142450306	7.820847704	5.24688E-15	1.55975E-14	positive
PRJNA729271	stage_1W_vs_E10	ordinary_muscle_development	ENSGALG00010015112	CBLN4	-2.602324839	0.396107326	-6.569746805	5.04009E-11	1.21943E-10	negative
PRJNA729271	stage_1W_vs_E10	ordinary_muscle_development	ENSGALG00010015115	CLDN2	0.292106975	0.412733241	0.707737942	0.479108011	0.517448075	positive
PRJNA729271	stage_1W_vs_E10	ordinary_muscle_development	ENSGALG00010015117	RASD2	-0.364492994	0.207118286	-1.759830103	0.078436617	0.095533045	negative
PRJNA729271	stage_1W_vs_E10	ordinary_muscle_development	ENSGALG00010015118	GPX8	-1.120369958	0.187338022	-5.980472872	2.22491E-09	4.91392E-09	negative
PRJNA729271	stage_1W_vs_E10	ordinary_muscle_development	ENSGALG00010015119	TLR1A	0.415162914	0.223604346	1.856685349	0.063355933	0.07805731	positive
PRJNA729271	stage_1W_vs_E10	ordinary_muscle_development	ENSGALG00010015121	UNC79	-4.818474565	0.314929206	-15.30018323	7.62417E-53	9.27117E-52	negative
PRJNA729271	stage_1W_vs_E10	ordinary_muscle_development	ENSGALG00010015122	ENSGALG00010015122	-3.500747525	0.214603508	-16.31262951	8.02619E-60	1.16807E-58	negative
PRJNA729271	stage_1W_vs_E10	ordinary_muscle_development	ENSGALG00010015123	CEP85L	2.165263292	0.250533463	8.642611114	5.49427E-18	1.86553E-17	positive
PRJNA729271	stage_1W_vs_E10	ordinary_muscle_development	ENSGALG00010015124	UXS1	0.008484137	0.096318465	0.088084223	0.929809736	0.938420284	positive
PRJNA729271	stage_1W_vs_E10	ordinary_muscle_development	ENSGALG00010015126	MCM5	-3.136673466	0.183283767	-17.11375495	1.17195E-65	1.95707E-64	negative
PRJNA729271	stage_1W_vs_E10	ordinary_muscle_development	ENSGALG00010015127	RPL11	0.871588841	0.15155682	5.750904781	8.87671E-09	1.88801E-08	positive
PRJNA729271	stage_1W_vs_E10	ordinary_muscle_development	ENSGALG00010015128	OCRL	0.639869937	0.106434192	6.011883249	1.8338E-09	4.07122E-09	positive
PRJNA729271	stage_1W_vs_E10	ordinary_muscle_development	ENSGALG00010015129	PRRG3	-1.566459649	0.143371321	-10.92589256	8.66839E-28	4.47147E-27	negative
PRJNA729271	stage_1W_vs_E10	ordinary_muscle_development	ENSGALG00010015131	DOK5	2.505922084	0.240730446	10.40965996	2.2402E-25	1.04579E-24	positive
PRJNA729271	stage_1W_vs_E10	ordinary_muscle_development	ENSGALG00010015132	GCNT1	-0.936880631	0.291017672	-3.219325561	0.001284925	0.001892207	negative
PRJNA729271	stage_1W_vs_E10	ordinary_muscle_development	ENSGALG00010015133	RFTN2	-1.576342371	0.128744579	-12.24395145	1.81001E-34	1.20404E-33	negative
PRJNA729271	stage_1W_vs_E10	ordinary_muscle_development	ENSGALG00010015134	PLN	2.903559089	0.52317125	5.54992097	2.85799E-08	5.89785E-08	positive
PRJNA729271	stage_1W_vs_E10	ordinary_muscle_development	ENSGALG00010015136	TCEB3	1.098859683	0.089970302	12.21358226	2.63047E-34	1.73628E-33	positive
PRJNA729271	stage_1W_vs_E10	ordinary_muscle_development	ENSGALG00010015137	AMN	1.801927875	0.422317077	4.266765356	1.98328E-05	3.35993E-05	positive
PRJNA729271	stage_1W_vs_E10	ordinary_muscle_development	ENSGALG00010015138	ENSGALG00010015138	-0.277903766	0.462337688	-0.601083954	0.54778407	0.583876028	negative
PRJNA729271	stage_1W_vs_E10	ordinary_muscle_development	ENSGALG00010015140	ASCC3	0.50584386	0.072760557	6.952171369	3.59706E-12	9.25442E-12	positive
PRJNA729271	stage_1W_vs_E10	ordinary_muscle_development	ENSGALG00010015141	PFDN4	0.757786739	0.135693554	5.584544849	2.34313E-08	4.86098E-08	positive
PRJNA729271	stage_1W_vs_E10	ordinary_muscle_development	ENSGALG00010015142	HNRNPAB	-0.764789163	0.128693615	-5.942712569	2.80344E-09	6.15601E-09	negative
PRJNA729271	stage_1W_vs_E10	ordinary_muscle_development	ENSGALG00010015143	EXOC1	0.206487139	0.079630059	2.593080324	0.009512055	0.012888132	positive
PRJNA729271	stage_1W_vs_E10	ordinary_muscle_development	ENSGALG00010015145	CLIC2	1.398804199	0.125277859	11.16561382	6.00748E-29	3.23591E-28	positive
PRJNA729271	stage_1W_vs_E10	ordinary_muscle_development	ENSGALG00010015146	ENSGALG00010015146	1.374323332	0.127163777	10.80750635	3.17175E-27	1.59856E-26	positive
PRJNA729271	stage_1W_vs_E10	ordinary_muscle_development	ENSGALG00010015148	ENSGALG00010015148	-0.411608273	0.076943019	-5.349520733	8.81875E-08	1.77128E-07	negative
PRJNA729271	stage_1W_vs_E10	ordinary_muscle_development	ENSGALG00010015149	ENSGALG00010015149	1.213314746	0.138558891	8.7566719	2.011E-18	6.97689E-18	positive
PRJNA729271	stage_1W_vs_E10	ordinary_muscle_development	ENSGALG00010015151	ENSGALG00010015151	-4.57428143	0.515689338	-8.870226879	7.2997E-19	2.59782E-18	negative
PRJNA729271	stage_1W_vs_E10	ordinary_muscle_development	ENSGALG00010015152	BOLL	3.994360071	0.274313695	14.56128563	4.95177E-48	5.0725E-47	positive
PRJNA729271	stage_1W_vs_E10	ordinary_muscle_development	ENSGALG00010015153	ERCC6L	-2.929821568	0.248358729	-11.79673283	4.05761E-32	2.48585E-31	negative
PRJNA729271	stage_1W_vs_E10	ordinary_muscle_development	ENSGALG00010015154	CYP24A1	-2.277297826	0.729620603	-3.121208222	0.001801106	0.002621715	negative
PRJNA729271	stage_1W_vs_E10	ordinary_muscle_development	ENSGALG00010015155	HMOX1	-0.643135365	0.179309649	-3.586730377	0.00033485	0.000518331	negative
PRJNA729271	stage_1W_vs_E10	ordinary_muscle_development	ENSGALG00010015156	CNOT6	-0.725419933	0.074440258	-9.744994828	1.93789E-22	8.00048E-22	negative
PRJNA729271	stage_1W_vs_E10	ordinary_muscle_development	ENSGALG00010015157	CDC42BPB	-0.541902552	0.070191371	-7.720358513	1.16003E-14	3.39347E-14	negative
PRJNA729271	stage_1W_vs_E10	ordinary_muscle_development	ENSGALG00010015158	PIN4	0.781578344	0.163930594	4.767739339	1.86305E-06	3.41272E-06	positive
PRJNA729271	stage_1W_vs_E10	ordinary_muscle_development	ENSGALG00010015160	SIM1	-5.698042962	0.547446827	-10.40839527	2.27016E-25	1.05943E-24	negative
PRJNA729271	stage_1W_vs_E10	ordinary_muscle_development	ENSGALG00010015161	GFPT2	-1.398398347	0.201671297	-6.934047457	4.08967E-12	1.04864E-11	negative
PRJNA729271	stage_1W_vs_E10	ordinary_muscle_development	ENSGALG00010015163	PLK2	-0.738478692	0.2096241	-3.522871141	0.000426899	0.000654596	negative
PRJNA729271	stage_1W_vs_E10	ordinary_muscle_development	ENSGALG00010015165	KLF3	0.396824903	0.118496823	3.348823143	0.000811556	0.001219395	positive
PRJNA729271	stage_1W_vs_E10	ordinary_muscle_development	ENSGALG00010015166	YTHDF2	0.725033822	0.180609303	4.014376934	5.9603E-05	9.75769E-05	positive
PRJNA729271	stage_1W_vs_E10	ordinary_muscle_development	ENSGALG00010015167	RAB3C	-3.592898489	0.478630005	-7.506630282	6.06688E-14	1.71088E-13	negative
PRJNA729271	stage_1W_vs_E10	ordinary_muscle_development	ENSGALG00010015169	ENSGALG00010015169	-1.153702197	0.679425373	-1.698055803	0.08949723	0.108348981	negative
PRJNA729271	stage_1W_vs_E10	ordinary_muscle_development	ENSGALG00010015170	EXOC1L	-2.765650619	0.989581613	-2.794767589	0.005193704	0.00724151	negative
PRJNA729271	stage_1W_vs_E10	ordinary_muscle_development	ENSGALG00010015171	NHSL2	1.634117721	0.201255741	8.11960799	4.67692E-16	1.4604E-15	positive
PRJNA729271	stage_1W_vs_E10	ordinary_muscle_development	ENSGALG00010015172	PPP4R3A	0.311911681	0.076542249	4.075026321	4.60091E-05	7.58879E-05	positive
PRJNA729271	stage_1W_vs_E10	ordinary_muscle_development	ENSGALG00010015173	ENS-1	1.011218112	0.659734832	1.532764474	0.125333897	0.14865517	positive
PRJNA729271	stage_1W_vs_E10	ordinary_muscle_development	ENSGALG00010015174	ST6GAL2	-5.424062955	0.227734005	-23.8175364	2.1982E-125	1.3388E-123	negative
PRJNA729271	stage_1W_vs_E10	ordinary_muscle_development	ENSGALG00010015175	MAPK9	0.00708127	0.069276157	0.102218002	0.918583639	0.928642059	positive
PRJNA729271	stage_1W_vs_E10	ordinary_muscle_development	ENSGALG00010015177	TC2N	0.133692428	0.159656975	0.837372925	0.40238298	0.440524267	positive
PRJNA729271	stage_1W_vs_E10	ordinary_muscle_development	ENSGALG00010015180	PITHD1	1.623654438	0.123626444	13.13355284	2.11496E-39	1.66159E-38	positive
PRJNA729271	stage_1W_vs_E10	ordinary_muscle_development	ENSGALG00010015181	VPS13A	1.496141227	0.119978873	12.470039	1.08773E-35	7.59105E-35	positive
PRJNA729271	stage_1W_vs_E10	ordinary_muscle_development	ENSGALG00010015182	PHYKPL	-1.860856277	0.33819113	-5.502380499	3.74697E-08	7.68752E-08	negative
PRJNA729271	stage_1W_vs_E10	ordinary_muscle_development	ENSGALG00010015183	SLC5A7	-4.651211003	0.74253884	-6.26392958	3.75395E-10	8.64255E-10	negative
PRJNA729271	stage_1W_vs_E10	ordinary_muscle_development	ENSGALG00010015186	ENSGALG00010015186	0.784589577	0.361655279	2.169440411	0.030049264	0.038497797	positive
PRJNA729271	stage_1W_vs_E10	ordinary_muscle_development	ENSGALG00010015188	SULT1C3	-0.416889415	1.176538904	-0.354335427	0.723087521	0.749993103	negative
PRJNA729271	stage_1W_vs_E10	ordinary_muscle_development	ENSGALG00010015190	FBLN5	0.624522225	0.19220627	3.249229194	0.001157182	0.001711916	positive
PRJNA729271	stage_1W_vs_E10	ordinary_muscle_development	ENSGALG00010015191	TOM1	4.725300621	0.1867664	25.30059272	3.1466E-141	2.3035E-139	positive
PRJNA729271	stage_1W_vs_E10	ordinary_muscle_development	ENSGALG00010015192	ENSGALG00010015192	1.193939964	0.232246574	5.140829176	2.73529E-07	5.30621E-07	positive
PRJNA729271	stage_1W_vs_E10	ordinary_muscle_development	ENSGALG00010015193	LYPLA2	-0.66220294	0.107066153	-6.184988622	6.21069E-10	1.41148E-09	negative
PRJNA729271	stage_1W_vs_E10	ordinary_muscle_development	ENSGALG00010015194	TMLHE	-0.157909867	0.11385082	-1.386989277	0.165445033	0.19291495	negative
PRJNA729271	stage_1W_vs_E10	ordinary_muscle_development	ENSGALG00010015196	PDE4D	1.144113306	0.217980438	5.248697149	1.53179E-07	3.02208E-07	positive
PRJNA729271	stage_1W_vs_E10	ordinary_muscle_development	ENSGALG00010015198	NME5	0.525616813	0.220718403	2.381390971	0.017247396	0.022682	positive
PRJNA729271	stage_1W_vs_E10	ordinary_muscle_development	ENSGALG00010015199	ENSGALG00010015199	-0.308536849	0.169427957	-1.821050399	0.068599196	0.084180947	negative
PRJNA729271	stage_1W_vs_E10	ordinary_muscle_development	ENSGALG00010015200	TBC1D1	-0.443229528	0.086130303	-5.146034687	2.6605E-07	5.16597E-07	negative
PRJNA729271	stage_1W_vs_E10	ordinary_muscle_development	ENSGALG00010015201	GCC2	0.242677296	0.108826137	2.229954141	0.025750488	0.033251966	positive
PRJNA729271	stage_1W_vs_E10	ordinary_muscle_development	ENSGALG00010015203	HMGXB4	-0.748677361	0.067212895	-11.13889473	8.11198E-29	4.35332E-28	negative
PRJNA729271	stage_1W_vs_E10	ordinary_muscle_development	ENSGALG00010015204	ENSGALG00010015204	0.277002827	0.139961044	1.979142326	0.047799984	0.05981704	positive
PRJNA729271	stage_1W_vs_E10	ordinary_muscle_development	ENSGALG00010015207	AKR1E2	-0.513798609	0.179149608	-2.867986231	0.004130935	0.005806061	negative
PRJNA729271	stage_1W_vs_E10	ordinary_muscle_development	ENSGALG00010015208	GNA14	2.576796094	0.57376872	4.491001349	7.08891E-06	1.24204E-05	positive
PRJNA729271	stage_1W_vs_E10	ordinary_muscle_development	ENSGALG00010015212	GNAQ	0.325520432	0.097635146	3.334049746	0.000855913	0.001283516	positive
PRJNA729271	stage_1W_vs_E10	ordinary_muscle_development	ENSGALG00010015213	RUBCN	0.477263201	0.119543998	3.992364387	6.54177E-05	0.000106795	positive
PRJNA729271	stage_1W_vs_E10	ordinary_muscle_development	ENSGALG00010015218	PLCL1	-2.015739312	0.163072802	-12.36097793	4.24955E-35	2.89733E-34	negative
PRJNA729271	stage_1W_vs_E10	ordinary_muscle_development	ENSGALG00010015227	FYTTD1	-1.056360069	0.076270851	-13.85011514	1.27008E-43	1.13852E-42	negative
PRJNA729271	stage_1W_vs_E10	ordinary_muscle_development	ENSGALG00010015233	ENSGALG00010015233	0.280684781	0.734087319	0.382358847	0.702195211	0.730414035	positive
PRJNA729271	stage_1W_vs_E10	ordinary_muscle_development	ENSGALG00010015234	TRIP11	0.412398444	0.143606428	2.871726908	0.004082356	0.005740565	positive
PRJNA729271	stage_1W_vs_E10	ordinary_muscle_development	ENSGALG00010015241	ENSGALG00010015241	0.135949715	0.168066103	0.808906215	0.418569094	0.456658062	positive
PRJNA729271	stage_1W_vs_E10	ordinary_muscle_development	ENSGALG00010015248	ENSGALG00010015248	1.264693163	0.131687349	9.603755988	7.70831E-22	3.11231E-21	positive
PRJNA729271	stage_1W_vs_E10	ordinary_muscle_development	ENSGALG00010015250	RORB	-1.544407028	0.558983782	-2.762883431	0.005729322	0.007947797	negative
PRJNA729271	stage_1W_vs_E10	ordinary_muscle_development	ENSGALG00010015253	ENSGALG00010015253	-1.046213992	0.075466299	-13.86332725	1.05661E-43	9.51274E-43	negative
PRJNA729271	stage_1W_vs_E10	ordinary_muscle_development	ENSGALG00010015260	RASGEF1C	0.658866681	0.142301638	4.630070957	3.6554E-06	6.55188E-06	positive
PRJNA729271	stage_1W_vs_E10	ordinary_muscle_development	ENSGALG00010015261	IQCG	-0.88487374	0.470687188	-1.879961391	0.06011334	0.074302282	negative
PRJNA729271	stage_1W_vs_E10	ordinary_muscle_development	ENSGALG00010015283	ENSGALG00010015283	-2.132708663	0.843095819	-2.529615987	0.011418743	0.015331111	negative
PRJNA729271	stage_1W_vs_E10	ordinary_muscle_development	ENSGALG00010015285	ALG13	-0.331551227	0.423528846	-0.78283033	0.433726754	0.47166849	negative
PRJNA729271	stage_1W_vs_E10	ordinary_muscle_development	ENSGALG00010015291	ZFP64	0.310017223	0.092140365	3.364618991	0.000766494	0.001153601	positive
PRJNA729271	stage_1W_vs_E10	ordinary_muscle_development	ENSGALG00010015296	RPL35A	0.204907097	0.195395998	1.048676017	0.294327256	0.329416538	positive
PRJNA729271	stage_1W_vs_E10	ordinary_muscle_development	ENSGALG00010015298	ENSGALG00010015298	3.545852703	0.230422129	15.38850769	1.95489E-53	2.41159E-52	positive
PRJNA729271	stage_1W_vs_E10	ordinary_muscle_development	ENSGALG00010015299	GALE	-0.629799731	0.095843305	-6.571139553	4.99316E-11	1.20888E-10	negative
PRJNA729271	stage_1W_vs_E10	ordinary_muscle_development	ENSGALG00010015300	ATXN3	1.965562771	0.1266942	15.51422854	2.77955E-54	3.52799E-53	positive
PRJNA729271	stage_1W_vs_E10	ordinary_muscle_development	ENSGALG00010015306	BCAT1	1.738378607	0.090549391	19.19812596	3.83688E-82	9.67226E-81	positive
PRJNA729271	stage_1W_vs_E10	ordinary_muscle_development	ENSGALG00010015310	PGM2	-1.748077708	0.096265354	-18.15894949	1.09092E-73	2.24297E-72	negative
PRJNA729271	stage_1W_vs_E10	ordinary_muscle_development	ENSGALG00010015314	GMEB1	-0.014451658	0.068604322	-0.210652288	0.833158608	0.8513065	negative
PRJNA729271	stage_1W_vs_E10	ordinary_muscle_development	ENSGALG00010015318	SALL4	-0.771723158	0.188907214	-4.085196852	4.40395E-05	7.27964E-05	negative

PRJNA729271	stage_1W_vs_E10	ordinary_muscle_development	ENSGALG00010015320	TRPM6	-3.869455402	0.473079903	-8.179285092	2.85533E-16	8.96232E-16	negative
PRJNA729271	stage_1W_vs_E10	ordinary_muscle_development	ENSGALG00010015327	LARGE1	-0.238010299	0.126940913	-1.874969166	0.060796966	0.075096039	negative
PRJNA729271	stage_1W_vs_E10	ordinary_muscle_development	ENSGALG00010015328	ENSGALG00010015328	1.641755796	0.381941325	4.298450279	1.71996E-05	2.92994E-05	positive
PRJNA729271	stage_1W_vs_E10	ordinary_muscle_development	ENSGALG00010015333	MED7	-0.151920446	0.069433278	-2.188006241	0.028669148	0.036804472	negative
PRJNA729271	stage_1W_vs_E10	ordinary_muscle_development	ENSGALG00010015339	ENSGALG00010015339	0.86818401	0.777916881	1.116036984	0.264406304	0.298184811	positive
PRJNA729271	stage_1W_vs_E10	ordinary_muscle_development	ENSGALG00010015348	CPSF2	-0.727033491	0.061807742	-11.76282246	6.06716E-32	3.69821E-31	negative
PRJNA729271	stage_1W_vs_E10	ordinary_muscle_development	ENSGALG00010015353	RELL1	0.651514781	0.09125813	7.139251931	9.38402E-13	2.48941E-12	positive
PRJNA729271	stage_1W_vs_E10	ordinary_muscle_development	ENSGALG00010015356	ENSGALG00010015356	-0.737731849	0.087556744	-8.425757025	3.58425E-17	1.17013E-16	negative
PRJNA729271	stage_1W_vs_E10	ordinary_muscle_development	ENSGALG00010015362	ATP9A	-1.038440995	0.136489706	-7.608200084	2.77939E-14	7.96507E-14	negative
PRJNA729271	stage_1W_vs_E10	ordinary_muscle_development	ENSGALG00010015364	CLOCK	2.810505	0.202217141	13.89845088	6.47271E-44	5.86387E-43	positive
PRJNA729271	stage_1W_vs_E10	ordinary_muscle_development	ENSGALG00010015370	ENSGALG00010015370	1.937403221	0.142553978	13.59066398	4.54934E-42	3.89963E-41	positive
PRJNA729271	stage_1W_vs_E10	ordinary_muscle_development	ENSGALG00010015374	LIMS1	-1.72073304	0.151900784	-11.32800634	9.53504E-30	5.33219E-29	negative
PRJNA729271	stage_1W_vs_E10	ordinary_muscle_development	ENSGALG00010015384	TAF12	0.31190676	0.116348969	2.680786623	0.007344933	0.010070451	positive
PRJNA729271	stage_1W_vs_E10	ordinary_muscle_development	ENSGALG00010015386	SLC24A4	-0.300659723	0.26546506	-1.132577384	0.25739177	0.290862533	negative
PRJNA729271	stage_1W_vs_E10	ordinary_muscle_development	ENSGALG00010015388	PSMD1	0.640267863	0.122228494	5.238286435	1.62074E-07	3.18977E-07	positive
PRJNA729271	stage_1W_vs_E10	ordinary_muscle_development	ENSGALG00010015390	CCNB3	-2.747500051	0.208898742	-13.15230537	1.65064E-39	1.30103E-38	negative
PRJNA729271	stage_1W_vs_E10	ordinary_muscle_development	ENSGALG00010015391	SYN3	-2.875367319	0.59242114	-4.853586622	1.21248E-06	2.2532E-06	negative
PRJNA729271	stage_1W_vs_E10	ordinary_muscle_development	ENSGALG00010015392	TMEM165	-0.628058638	0.08231439	-7.629998116	2.34757E-14	6.74624E-14	negative
PRJNA729271	stage_1W_vs_E10	ordinary_muscle_development	ENSGALG00010015393	DCX	-3.255428551	0.378997818	-8.589570704	8.72949E-18	2.93582E-17	negative
PRJNA729271	stage_1W_vs_E10	ordinary_muscle_development	ENSGALG00010015397	SRD5A3	-0.119596776	0.083727449	-1.428405822	0.153175084	0.179401491	negative
PRJNA729271	stage_1W_vs_E10	ordinary_muscle_development	ENSGALG00010015404	BEND4	-3.545535473	0.362982302	-9.767791576	1.54789E-22	6.41782E-22	negative
PRJNA729271	stage_1W_vs_E10	ordinary_muscle_development	ENSGALG00010015407	TIMP3	-0.512980291	0.175682253	-2.91993234	0.003501074	0.004958191	negative
PRJNA729271	stage_1W_vs_E10	ordinary_muscle_development	ENSGALG00010015409	FAM13B	-0.16583923	0.132592884	-1.250740051	0.211029333	0.241999223	negative
PRJNA729271	stage_1W_vs_E10	ordinary_muscle_development	ENSGALG00010015410	HTR2B	-2.713213278	0.248177333	-10.93255876	8.05447E-28	4.16071E-27	negative
PRJNA729271	stage_1W_vs_E10	ordinary_muscle_development	ENSGALG00010015413	CARNMT1	0.635492353	0.106126824	5.988046459	2.12376E-09	4.69482E-09	positive
PRJNA729271	stage_1W_vs_E10	ordinary_muscle_development	ENSGALG00010015416	CAPN6	1.865538606	0.162459358	11.48310953	1.60406E-30	9.23385E-30	positive
PRJNA729271	stage_1W_vs_E10	ordinary_muscle_development	ENSGALG00010015422	ENSGALG00010015422	-1.667860984	0.548489784	-3.040824158	0.002359316	0.003394707	negative
PRJNA729271	stage_1W_vs_E10	ordinary_muscle_development	ENSGALG00010015430	HMGCL	1.132523892	0.12989612	8.718689138	2.81441E-18	9.6946E-18	positive
PRJNA729271	stage_1W_vs_E10	ordinary_muscle_development	ENSGALG00010015431	RIN3	-1.093207327	0.161650146	-6.762798289	1.35352E-11	3.37564E-11	negative
PRJNA729271	stage_1W_vs_E10	ordinary_muscle_development	ENSGALG00010015436	SLC30A9	0.010255033	0.098687895	0.103913792	0.917237762	0.927410809	positive
PRJNA729271	stage_1W_vs_E10	ordinary_muscle_development	ENSGALG00010015440	ENSGALG00010015440	1.17679667	0.363664219	3.235942964	0.001212416	0.001790157	positive
PRJNA729271	stage_1W_vs_E10	ordinary_muscle_development	ENSGALG00010015443	PAK3	-0.155084116	0.10189014	-1.522071876	0.127991072	0.151558744	negative
PRJNA729271	stage_1W_vs_E10	ordinary_muscle_development	ENSGALG00010015449	HDX	-0.923257131	0.0944551	-9.774560903	1.44784E-22	6.01502E-22	negative
PRJNA729271	stage_1W_vs_E10	ordinary_muscle_development	ENSGALG00010015466	NMRK1	0.553810683	0.125002235	4.430406236	9.40557E-06	1.63568E-05	positive
PRJNA729271	stage_1W_vs_E10	ordinary_muscle_development	ENSGALG00010015469	TMEM33	-0.029552022	0.073162785	-0.403921498	0.686270447	0.715492349	negative
PRJNA729271	stage_1W_vs_E10	ordinary_muscle_development	ENSGALG00010015472	LGMN	0.293780588	0.134285363	2.187733504	0.02868902	0.036826722	positive
PRJNA729271	stage_1W_vs_E10	ordinary_muscle_development	ENSGALG00010015477	ARMC9	-1.479388869	0.108049731	-13.69174039	1.13752E-42	9.92673E-42	negative
PRJNA729271	stage_1W_vs_E10	ordinary_muscle_development	ENSGALG00010015478	FBXO7	0.121684173	0.062693256	1.940945166	0.052264929	0.065128967	positive
PRJNA729271	stage_1W_vs_E10	ordinary_muscle_development	ENSGALG00010015481	GOLGA5	0.080606763	0.071692454	1.124340975	0.260868402	0.294561618	positive
PRJNA729271	stage_1W_vs_E10	ordinary_muscle_development	ENSGALG00010015483	SGCD	1.469053693	0.162192917	9.057446632	1.3354E-19	4.93036E-19	positive
PRJNA729271	stage_1W_vs_E10	ordinary_muscle_development	ENSGALG00010015485	BCAS1	1.515392631	0.186460305	8.127159473	4.39467E-16	1.37494E-15	positive
PRJNA729271	stage_1W_vs_E10	ordinary_muscle_development	ENSGALG00010015486	MTFR1L	2.386479118	0.118452162	20.14719768	2.84786E-90	8.39017E-89	positive
PRJNA729271	stage_1W_vs_E10	ordinary_muscle_development	ENSGALG00010015487	RPS6KA6	0.008818548	0.117943881	0.074769016	0.940398495	0.947853152	positive
PRJNA729271	stage_1W_vs_E10	ordinary_muscle_development	ENSGALG00010015490	RANBP2	0.407917516	0.082866621	4.922579325	8.54109E-07	1.60616E-06	positive
PRJNA729271	stage_1W_vs_E10	ordinary_muscle_development	ENSGALG00010015493	OSTF1	-0.383088917	0.142203381	-2.693950837	0.007061058	0.009703265	negative
PRJNA729271	stage_1W_vs_E10	ordinary_muscle_development	ENSGALG00010015494	CHRDL1	-1.499017986	0.242516642	-6.181093266	6.36592E-10	1.4463E-09	negative
PRJNA729271	stage_1W_vs_E10	ordinary_muscle_development	ENSGALG00010015498	MID2	1.034466403	0.134161686	7.710594844	1.25233E-14	3.65535E-14	positive
PRJNA729271	stage_1W_vs_E10	ordinary_muscle_development	ENSGALG00010015502	BPIFB6	0.884503704	1.420924854	0.622484504	0.53362333	0.570081822	positive
PRJNA729271	stage_1W_vs_E10	ordinary_muscle_development	ENSGALG00010015505	CHGA	-1.27903014	0.498254307	-2.567022747	0.010257588	0.013852953	negative
PRJNA729271	stage_1W_vs_E10	ordinary_muscle_development	ENSGALG00010015507	AMMECR1	-1.434038696	0.073485801	-19.51450036	8.26734E-85	2.21098E-83	negative
PRJNA729271	stage_1W_vs_E10	ordinary_muscle_development	ENSGALG00010015508	FUCA1	1.017847004	0.103581142	9.826567004	8.65179E-23	3.62345E-22	positive
PRJNA729271	stage_1W_vs_E10	ordinary_muscle_development	ENSGALG00010015511	BPIFB4	-2.72732987	0.51246358	-5.321997462	1.02634E-07	2.04971E-07	negative
PRJNA729271	stage_1W_vs_E10	ordinary_muscle_development	ENSGALG00010015516	SELENON	-1.001785001	0.113436542	-8.831237149	1.03524E-18	3.65461E-18	negative
PRJNA729271	stage_1W_vs_E10	ordinary_muscle_development	ENSGALG00010015520	UBE3A	1.003470458	0.078210393	12.83039786	1.10794E-37	8.18113E-37	positive
PRJNA729271	stage_1W_vs_E10	ordinary_muscle_development	ENSGALG00010015521	DKC1	-0.792696655	0.082094618	-9.65589069	4.64108E-22	1.88915E-21	negative
PRJNA729271	stage_1W_vs_E10	ordinary_muscle_development	ENSGALG00010015523	MRPL22	-0.206105547	0.145357614	-1.417920548	0.156213962	0.182709706	negative
PRJNA729271	stage_1W_vs_E10	ordinary_muscle_development	ENSGALG00010015532	NXT2	-0.527248915	0.111351317	-4.735003848	2.19051E-06	3.98687E-06	negative
PRJNA729271	stage_1W_vs_E10	ordinary_muscle_development	ENSGALG00010015533	PSAT1	-0.446859325	0.277125746	-1.612478562	0.106857841	0.127955755	negative
PRJNA729271	stage_1W_vs_E10	ordinary_muscle_development	ENSGALG00010015540	ACSL4	1.197584038	0.151561137	7.901656454	2.75221E-15	8.29555E-15	positive
PRJNA729271	stage_1W_vs_E10	ordinary_muscle_development	ENSGALG00010015541	RASEF	-1.604367408	0.350510464	-4.57723113	4.71171E-06	8.37168E-06	negative
PRJNA729271	stage_1W_vs_E10	ordinary_muscle_development	ENSGALG00010015542	CNGA3	-0.925537414	0.289597443	-3.195944709	0.001393738	0.0020487	negative
PRJNA729271	stage_1W_vs_E10	ordinary_muscle_development	ENSGALG00010015544	RTCB	0.13844047	0.073198548	1.891300769	0.058584206	0.072517341	positive
PRJNA729271	stage_1W_vs_E10	ordinary_muscle_development	ENSGALG00010015551	VWA3B	-2.131617201	0.527568918	-4.040452592	5.33481E-05	8.75644E-05	negative
PRJNA729271	stage_1W_vs_E10	ordinary_muscle_development	ENSGALG00010015553	ASCL4	-7.278012023	1.134180545	-6.416978369	1.39006E-10	3.27517E-10	negative
PRJNA729271	stage_1W_vs_E10	ordinary_muscle_development	ENSGALG00010015555	PNRC2	-0.709570408	0.102698767	-6.909239791	4.87258E-12	1.24433E-11	negative
PRJNA729271	stage_1W_vs_E10	ordinary_muscle_development	ENSGALG00010015557	IRS4	1.640984138	0.19187711	8.552266263	1.20695E-17	4.02363E-17	positive
PRJNA729271	stage_1W_vs_E10	ordinary_muscle_development	ENSGALG00010015558	MPP1	1.035939678	0.259416021	3.993352743	6.51455E-05	0.000106386	positive
PRJNA729271	stage_1W_vs_E10	ordinary_muscle_development	ENSGALG00010015562	IRF9	2.437486468	0.267983807	9.09564832	9.40238E-20	3.49724E-19	positive
PRJNA729271	stage_1W_vs_E10	ordinary_muscle_development	ENSGALG00010015563	B3GNT7	-2.882475683	0.325089982	-8.866701048	7.53448E-19	2.67874E-18	negative
PRJNA729271	stage_1W_vs_E10	ordinary_muscle_development	ENSGALG00010015564	PKD2L2	-0.769540166	0.307972871	-2.498727124	0.012464024	0.016666608	negative
PRJNA729271	stage_1W_vs_E10	ordinary_muscle_development	ENSGALG00010015565	ENSGALG00010015565	-0.900907915	0.456453956	-1.97371039	0.048414683	0.060539237	negative
PRJNA729271	stage_1W_vs_E10	ordinary_muscle_development	ENSGALG00010015567	ENSGALG00010015567	5.044155546	0.426069464	11.83881026	2.45917E-32	1.51748E-31	positive
PRJNA729271	stage_1W_vs_E10	ordinary_muscle_development	ENSGALG00010015570	LIMCH1	1.600532356	0.127801767	12.52355423	5.54933E-36	3.91234E-35	positive
PRJNA729271	stage_1W_vs_E10	ordinary_muscle_development	ENSGALG00010015571	DUSP15	0.890074584	0.300012286	2.966793784	0.003009227	0.004289384	positive
PRJNA729271	stage_1W_vs_E10	ordinary_muscle_development	ENSGALG00010015573	MAN1C1	0.774686599	0.12386447	6.254308445	3.99281E-10	9.1779E-10	positive
PRJNA729271	stage_1W_vs_E10	ordinary_muscle_development	ENSGALG00010015574	C14orf132	0.140389355	0.257837105	0.544488563	0.586105316	0.621386524	positive
PRJNA729271	stage_1W_vs_E10	ordinary_muscle_development	ENSGALG00010015580	GEMIN5	0.077354124	0.071139436	1.087359255	0.276878071	0.311208719	positive
PRJNA729271	stage_1W_vs_E10	ordinary_muscle_development	ENSGALG00010015583	PRDM4	0.787534517	0.134969242	5.834918444	5.38169E-09	1.16083E-08	positive
PRJNA729271	stage_1W_vs_E10	ordinary_muscle_development	ENSGALG00010015584	TTLL9	1.89808044	0.234490579	8.094484859	5.75072E-16	1.78877E-15	positive
PRJNA729271	stage_1W_vs_E10	ordinary_muscle_development	ENSGALG00010015585	BDKRB1	1.971884187	0.297168348	6.635579458	3.2323E-11	7.91289E-11	positive
PRJNA729271	stage_1W_vs_E10	ordinary_muscle_development	ENSGALG00010015588	BDKRB2	-0.926706369	0.463687962	-1.99855602	0.045656413	0.057302771	negative
PRJNA729271	stage_1W_vs_E10	ordinary_muscle_development	ENSGALG00010015589	FRMD3	0.65906077	0.17970009	3.667559496	0.000244877	0.000383066	positive
PRJNA729271	stage_1W_vs_E10	ordinary_muscle_development	ENSGALG00010015596	UCHL1	-2.459120269	0.504182797	-4.877437868	1.07473E-06	2.00543E-06	negative
PRJNA729271	stage_1W_vs_E10	ordinary_muscle_development	ENSGALG00010015605	LDLRAP1	-0.354970238	0.059187481	-5.997387132	2.00518E-09	4.4408E-09	negative
PRJNA729271	stage_1W_vs_E10	ordinary_muscle_development	ENSGALG00010015606	TMEM131	0.171724807	0.108633822	1.580767431	0.113931224	0.135931607	positive
PRJNA729271	stage_1W_vs_E10	ordinary_muscle_development	ENSGALG00010015610	ENSGALG00010015610	1.156147143	0.286580259	4.034287451	5.47682E-05	8.9814E-05	positive
PRJNA729271	stage_1W_vs_E10	ordinary_muscle_development	ENSGALG00010015613	PWP1	-0.282821084	0.080469859	-3.514621351	0.000440381	0.000674272	negative
PRJNA729271	stage_1W_vs_E10	ordinary_muscle_development	ENSGALG00010015614	ATG2B	1.049335503	0.106907239	9.815383052	9.66701E-23	4.04163E-22	positive
PRJNA729271	stage_1W_vs_E10	ordinary_muscle_development	ENSGALG00010015616	NADK2	-0.817972987	0.119364635	-6.852724751	7.24564E-12	1.83643E-11	negative
PRJNA729271	stage_1W_vs_E10	ordinary_muscle_development	ENSGALG00010015617	SRSF10	-0.01688875	0.055892036	-0.302167379	0.76252447	0.786507664	negative
PRJNA729271	stage_1W_vs_E10	ordinary_muscle_development	ENSGALG00010015618	NCL	-1.274309208	0.101132463	-12.60039721	2.10084E-36	1.49419E-35	negative
PRJNA729271	stage_1W_vs_E10	ordinary_muscle_development	ENSGALG00010015620	PDRG1	1.690479768	0.143088813	11.8141994	3.29674E-32	2.0257E-31	positive
PRJNA729271	stage_1W_vs_E10	ordinary_muscle_development	ENSGALG00010015621	IDNK	0.843746737	0.151600521	5.565592588	2.61263E-08	5.40538E-08	positive
PRJNA729271	stage_1W_vs_E10	ordinary_muscle_development	ENSGALG00010015624	ITGA1	1.293218259	0.129268968	10.004089	1.46231E-23	6.33669E-23	positive
PRJNA729271	stage_1W_vs_E10	ordinary_muscle_development	ENSGALG00010015626	XKR7	0.052048871	0.527131489	0.098739825	0.921344848	0.930719463	positive
PRJNA729271	stage_1W_vs_E10	ordinary_muscle_development	ENSGALG00010015628	UBQLN1	0.628554468	0.118418247	5.307919026	1.10884E-07	2.20778E-07	positive
PRJNA729271	stage_1W_vs_E10	ordinary_muscle_development	ENSGALG00010015631	GSKIP	-0.626116111	0.108293946	-5.781635385	7.39779E-09	1.58111E-08	negative
PRJNA729271	stage_1W_vs_E10	ordinary_muscle_development	ENSGALG00010015632	COL4A5	1.261218756	0.161562011	7.806406661	5.88415E-15	1.74454E-14	positive
PRJNA729271	stage_1W_vs_E10	ordinary_muscle_development	ENSGALG00010015633	CCM2L	1.089309673	0.18469996	5.897725545	3.68546E-09	8.02355E-09	positive
PRJNA729271	stage_1W_vs_E10	ordinary_muscle_development	ENSGALG00010015634	ENSGALG00010015634	-1.081958734	1.149097597	-0.941572532	0.346411538	0.38379846	negative
PRJNA729271	stage_1W_vs_E10	ordinary_muscle_development	ENSGALG00010015635	ABTB3	-3.12313252	0.137556255	-22.70440213	4.0531E-114	1.9454E-112	negative
PRJNA729271	stage_1W_vs_E10	ordinary_muscle_development	ENSGALG00010015636	APBB2	0.251426468	0.124308214	2.022605418	0.043113845	0.054280829	positive
PRJNA729271	stage_1W_vs_E10	ordinary_muscle_development	ENSGALG00010015642	ENSGALG00010015642	-1.984958168	0.934376272	-2.124367054	0.033639463	0.042847453	negative
PRJNA729271	stage_1W_vs_E10	ordinary_muscle_development	ENSGALG00010015643	ENSGALG00010015643	-2.31149353	0.121794615	-18.97861844	2.56246E-80	6.1699E-79	negative
PRJNA729271	stage_1W_vs_E10	ordinary_muscle_development	ENSGALG00010015645	MACO1	0.799764822	0.093839039	8.522730331	1.55835E-17	5.17725E-17	positive
PRJNA729271	stage_1W_vs_E10	ordinary_muscle_development	ENSGALG00010015647	PDZD2	-0.21648687	0.137605583	-1.573241905	0.115662816	0.13789542	negative
PRJNA729271	stage_1W_vs_E10	ordinary_muscle_development	ENSGALG00010015650	ENSGALG00010015650	-0.23489774	1.289796307	-0.182120028	0.855488531	0.871554848	negative
PRJNA729271	stage_1W_vs_E10	ordinary_muscle_development	ENSGALG00010015653	HCK	1.863404643	0.522861907	3.563856186	0.000365446	0.000563466	positive
PRJNA729271	stage_1W_vs_E10	ordinary_muscle_development	ENSGALG00010015655	ENSGALG00010015655	5.703244022	0.361990178	15.75524522	6.32137E-56	8.34501E-55	positive
PRJNA729271	stage_1W_vs_E10	ordinary_muscle_development	ENSGALG00010015657	NMUR1	-4.197038096	1.196859156	-3.506710105	0.000453683	0.000693685	negative
PRJNA729271	stage_1W_vs_E10	ordinary_muscle_development	ENSGALG00010015660	KLHL3	-0.172079814	0.126765619	-1.357464392	0.1746337	0.202667373	negative
PRJNA729271	stage_1W_vs_E10	ordinary_muscle_development	ENSGALG00010015668	ZNF217	0.168348379	0.135309112	1.244176212	0.213434717	0.244428871	positive
PRJNA729271	stage_1W_vs_E10	ordinary_muscle_development	ENSGALG00010015669	INPP4A	0.232566598	0.130470527	1.782522101	0.07466413	0.091198835	positive
PRJNA729271	stage_1W_vs_E10	ordinary_muscle_development	ENSGALG00010015670	ENSGALG00010015670	-1.569846424	0.131696626	-11.92017196	9.29166E-33	5.80528E-32	negative
PRJNA729271	stage_1W_vs_E10	ordinary_muscle_development	ENSGALG00010015674	RFKL	1.230156675	0.193374712	6.361517808	1.9977E-10	4.6614E-10	positive
PRJNA729271	stage_1W_vs_E10	ordinary_muscle_development	ENSGALG00010015676	TGM2	2.245086742	0.192362612	11.67111801	1.79055E-31	1.07381E-30	positive
PRJNA729271	stage_1W_vs_E10	ordinary_muscle_development	ENSGALG00010015677	GRHL3	-3.524196318	0.287376132	-12.26335776	1.42472E-34	9.52109E-34	negative
PRJNA729271	stage_1W_vs_E10	ordinary_muscle_development	ENSGALG00010015680	ENSGALG00010015680	-0.383289524	0.104405229	-3.671171713	0.000241441	0.000377936	negative
PRJNA729271	stage_1W_vs_E10	ordinary_muscle_development	ENSGALG00010015684	PDE6D	-1.045316601	0.102395463	-10.20862228	1.81422E-24	8.1567E-24	negative
PRJNA729271	stage_1W_vs_E10	ordinary_muscle_development	ENSGALG00010015686	CNOT8	-1.086062724	0.055372752	-19.61366692	1.18195E-85	3.24263E-84	negative
PRJNA729271	stage_1W_vs_E10	ordinary_muscle_development	ENSGALG00010015691	EXOC3L4	-0.730728842	0.8073798	-0.905062081	0.365432499	0.403085311	negative
PRJNA729271	stage_1W_vs_E10	ordinary_muscle_development	ENSGALG00010015693	COA5	-0.060053504	0.108243709	-0.554799023	0.579032135	0.614561822	negative
PRJNA729271	stage_1W_vs_E10	ordinary_muscle_development	ENSGALG00010015695	ASAH1	0.698767173	0.094104325	7.425452231	1.12395E-13	3.11564E-13	positive
PRJNA729271	stage_1W_vs_E10	ordinary_muscle_development	ENSGALG00010015697	FAXDC2	0.483734531	0.350700557	1.379337788	0.167790631	0.195445612	positive
PRJNA729271	stage_1W_vs_E10	ordinary_muscle_development	ENSGALG00010015698	TNFAIP2	0.255487912	0.200525583	1.274091351	0.202631043	0.233013643	positive
PRJNA729271	stage_1W_vs_E10	ordinary_muscle_development	ENSGALG00010015701	UNC50	-0.202895231	0.087748221	-2.312243256	0.020764282	0.02708344	negative
PRJNA729271	stage_1W_vs_E10	ordinary_muscle_development	ENSGALG00010015703	GK5	0.097105129	0.188473362	0.515219379	0.606399728	0.640793473	positive
PRJNA729271	stage_1W_vs_E10	ordinary_muscle_development	ENSGALG00010015705	NPY6R	-5.175082375	1.176881841	-4.397282885	1.09614E-05	1.89783E-05	negative
PRJNA729271	stage_1W_vs_E10	ordinary_muscle_development	ENSGALG00010015706	ENSGALG00010015706	1.179329106	0.163149825	7.228503649	4.88345E-13	1.31109E-12	positive
PRJNA729271	stage_1W_vs_E10	ordinary_muscle_development	ENSGALG00010015709	HNRNPA0	-1.692824437	0.114125844	-14.83296312	8.96851E-50	9.76698E-49	negative
PRJNA729271	stage_1W_vs_E10	ordinary_muscle_development	ENSGALG00010015711	LBFABP	1.378440727	0.675532967	2.040523251	0.041298238	0.052103573	positive

PRJNA729271	stage_1W_vs_E10	ordinary_muscle_development	ENSGALG00010015716	MGAT4A	-0.146107792	0.132357905	-1.103884133	0.269643402	0.303618513	negative
PRJNA729271	stage_1W_vs_E10	ordinary_muscle_development	ENSGALG00010015719	XRN1	0.503940797	0.099331155	5.073340771	3.90892E-07	7.50957E-07	positive
PRJNA729271	stage_1W_vs_E10	ordinary_muscle_development	ENSGALG00010015729	RHCE	0.445118929	0.19709115	2.25844199	0.023918117	0.031021216	positive
PRJNA729271	stage_1W_vs_E10	ordinary_muscle_development	ENSGALG00010015731	PHF6	-1.790619171	0.071709214	-24.97055919	1.2772E-137	8.9434E-136	negative
PRJNA729271	stage_1W_vs_E10	ordinary_muscle_development	ENSGALG00010015733	COL4A6	0.734861106	0.152650799	4.814001059	1.47938E-06	2.72993E-06	positive
PRJNA729271	stage_1W_vs_E10	ordinary_muscle_development	ENSGALG00010015734	TSHZ2	-1.962428299	0.169539972	-11.57501841	5.51594E-31	3.23699E-30	negative
PRJNA729271	stage_1W_vs_E10	ordinary_muscle_development	ENSGALG00010015738	CRACDL	-3.620475241	0.49293154	-7.344783091	2.06093E-13	5.6322E-13	negative
PRJNA729271	stage_1W_vs_E10	ordinary_muscle_development	ENSGALG00010015742	HPRT1	1.398581537	0.085579119	16.342556	4.91486E-60	7.20333E-59	positive
PRJNA729271	stage_1W_vs_E10	ordinary_muscle_development	ENSGALG00010015746	PAPOLB	-0.149540374	0.072211883	-2.070855473	0.038372304	0.048585478	negative
PRJNA729271	stage_1W_vs_E10	ordinary_muscle_development	ENSGALG00010015749	ATR	-1.427394402	0.135530095	-10.53193687	6.15547E-26	2.94176E-25	negative
PRJNA729271	stage_1W_vs_E10	ordinary_muscle_development	ENSGALG00010015751	MYOM3	-4.367035355	0.545326083	-8.008117511	1.16478E-15	3.57321E-15	negative
PRJNA729271	stage_1W_vs_E10	ordinary_muscle_development	ENSGALG00010015753	RPRD1B	-0.550349309	0.078909109	-6.974471225	3.07023E-12	7.92715E-12	negative
PRJNA729271	stage_1W_vs_E10	ordinary_muscle_development	ENSGALG00010015756	PCM1	-0.058125924	0.103207587	-0.563194294	0.573302588	0.608971201	negative
PRJNA729271	stage_1W_vs_E10	ordinary_muscle_development	ENSGALG00010015758	PCDH19	0.308868471	0.170199769	1.814740841	0.069563745	0.085277951	positive
PRJNA729271	stage_1W_vs_E10	ordinary_muscle_development	ENSGALG00010015759	EIF5	0.94425667	0.091519957	10.31749472	5.87357E-25	2.69422E-24	positive
PRJNA729271	stage_1W_vs_E10	ordinary_muscle_development	ENSGALG00010015764	TTI1	0.404223247	0.096303746	4.197378242	2.70023E-05	4.53059E-05	positive
PRJNA729271	stage_1W_vs_E10	ordinary_muscle_development	ENSGALG00010015765	ENSGALG00010015765	0.187692352	0.390118072	0.481116785	0.630433499	0.663626258	positive
PRJNA729271	stage_1W_vs_E10	ordinary_muscle_development	ENSGALG00010015766	TLE1	-1.123890394	0.1045914	-10.7455335	6.22013E-27	3.09618E-26	negative
PRJNA729271	stage_1W_vs_E10	ordinary_muscle_development	ENSGALG00010015769	TMEM50A	0.16448814	0.09991287	1.646315829	0.099698752	0.119943912	positive
PRJNA729271	stage_1W_vs_E10	ordinary_muscle_development	ENSGALG00010015774	PLS1	-2.150646724	0.384982616	-5.586347629	2.31895E-08	4.81288E-08	negative
PRJNA729271	stage_1W_vs_E10	ordinary_muscle_development	ENSGALG00010015776	SPOCK1	-4.843048718	0.220082558	-22.00559987	2.5453E-107	1.0948E-105	negative
PRJNA729271	stage_1W_vs_E10	ordinary_muscle_development	ENSGALG00010015778	PABIR3	-1.825908347	0.088664623	-20.59342604	3.14336E-94	1.04025E-92	negative
PRJNA729271	stage_1W_vs_E10	ordinary_muscle_development	ENSGALG00010015782	ATG4A	0.528506946	0.081733284	6.466238943	1.00472E-10	2.38745E-10	positive
PRJNA729271	stage_1W_vs_E10	ordinary_muscle_development	ENSGALG00010015785	FGL1	1.735656453	0.190950752	9.089550221	9.94496E-20	3.69432E-19	positive
PRJNA729271	stage_1W_vs_E10	ordinary_muscle_development	ENSGALG00010015788	TRPC1	-0.358376344	0.128836653	-2.781633451	0.005408609	0.007526669	negative
PRJNA729271	stage_1W_vs_E10	ordinary_muscle_development	ENSGALG00010015792	MARK3	0.482809133	0.081379659	5.932798671	2.97814E-09	6.52481E-09	positive
PRJNA729271	stage_1W_vs_E10	ordinary_muscle_development	ENSGALG00010015793	RSRP1	1.905530253	0.23286225	8.183079278	2.76681E-16	8.69201E-16	positive
PRJNA729271	stage_1W_vs_E10	ordinary_muscle_development	ENSGALG00010015795	PSMD10	-0.305281512	0.134804645	-2.264621607	0.02353591	0.030547369	negative
PRJNA729271	stage_1W_vs_E10	ordinary_muscle_development	ENSGALG00010015796	LARP1	0.317577387	0.141019745	2.252006529	0.024321859	0.031511025	positive
PRJNA729271	stage_1W_vs_E10	ordinary_muscle_development	ENSGALG00010015797	PIP5K1B	-0.558581502	0.164658326	-3.392367188	0.000692915	0.001046448	negative
PRJNA729271	stage_1W_vs_E10	ordinary_muscle_development	ENSGALG00010015798	IL22RA1	-3.074380808	0.332731926	-9.239813101	2.46928E-20	9.41406E-20	negative
PRJNA729271	stage_1W_vs_E10	ordinary_muscle_development	ENSGALG00010015800	BVES	2.925761637	0.181433941	16.12576795	1.68156E-58	2.35955E-57	positive
PRJNA729271	stage_1W_vs_E10	ordinary_muscle_development	ENSGALG00010015801	VSTM2L	-2.312617829	0.276504628	-8.36375811	6.07514E-17	1.96517E-16	negative
PRJNA729271	stage_1W_vs_E10	ordinary_muscle_development	ENSGALG00010015802	TRPC7	-3.509517413	0.99968914	-3.51060872	0.000447082	0.000684169	negative
PRJNA729271	stage_1W_vs_E10	ordinary_muscle_development	ENSGALG00010015803	SYF2	0.424522649	0.15986608	2.655489194	0.007919347	0.010823207	positive
PRJNA729271	stage_1W_vs_E10	ordinary_muscle_development	ENSGALG00010015804	CKB	-2.725775825	0.161017626	-16.92843131	2.77682E-64	4.5174E-63	negative
PRJNA729271	stage_1W_vs_E10	ordinary_muscle_development	ENSGALG00010015807	ENSGALG00010015807	-0.978965435	0.946336955	-1.034478713	0.300912423	0.336215646	negative
PRJNA729271	stage_1W_vs_E10	ordinary_muscle_development	ENSGALG00010015809	PCOLCE2	3.391232453	0.232224698	14.60323765	2.67819E-48	2.76301E-47	positive
PRJNA729271	stage_1W_vs_E10	ordinary_muscle_development	ENSGALG00010015810	LIPT1	0.102541886	0.104156904	0.984494377	0.324872495	0.361359824	positive
PRJNA729271	stage_1W_vs_E10	ordinary_muscle_development	ENSGALG00010015811	CTNNBL1	-0.599314223	0.0563679	-10.63218995	2.11096E-26	1.03094E-25	negative
PRJNA729271	stage_1W_vs_E10	ordinary_muscle_development	ENSGALG00010015813	ENSGALG00010015813	1.116811942	0.13873245	8.050113308	8.27174E-16	2.55321E-15	positive
PRJNA729271	stage_1W_vs_E10	ordinary_muscle_development	ENSGALG00010015814	PAQR9	2.339067769	0.407775946	5.736159266	9.68476E-09	2.05595E-08	positive
PRJNA729271	stage_1W_vs_E10	ordinary_muscle_development	ENSGALG00010015816	MITD1	-0.657883936	0.217174889	-3.029281784	0.002451359	0.0035219	negative
PRJNA729271	stage_1W_vs_E10	ordinary_muscle_development	ENSGALG00010015820	ENSGALG00010015820	1.484343622	0.516665163	2.872931499	0.004066823	0.005719833	positive
PRJNA729271	stage_1W_vs_E10	ordinary_muscle_development	ENSGALG00010015822	WFDC2	-0.648883875	0.526876446	-1.231567439	0.21811071	0.24938982	negative
PRJNA729271	stage_1W_vs_E10	ordinary_muscle_development	ENSGALG00010015825	RUNX3	-4.183500521	0.636750428	-6.570078857	5.02886E-11	1.21712E-10	negative
PRJNA729271	stage_1W_vs_E10	ordinary_muscle_development	ENSGALG00010015827	ENSGALG00010015827	1.588238141	0.14982249	10.60079922	2.95444E-26	1.43274E-25	positive
PRJNA729271	stage_1W_vs_E10	ordinary_muscle_development	ENSGALG00010015829	SMAD5	-0.503027435	0.048172935	-10.44211732	1.59219E-25	7.49069E-25	negative
PRJNA729271	stage_1W_vs_E10	ordinary_muscle_development	ENSGALG00010015831	LYG2	3.367860514	0.626610053	5.374731062	7.66971E-08	1.54642E-07	positive
PRJNA729271	stage_1W_vs_E10	ordinary_muscle_development	ENSGALG00010015832	RAB39B	-1.653014028	0.087519123	-18.88746118	1.44637E-79	3.40342E-78	negative
PRJNA729271	stage_1W_vs_E10	ordinary_muscle_development	ENSGALG00010015834	STARD8	-0.122533187	0.133916658	-0.914995856	0.360193805	0.397760872	negative
PRJNA729271	stage_1W_vs_E10	ordinary_muscle_development	ENSGALG00010015837	GDA	2.538999064	0.160377648	15.83137731	1.89038E-56	2.52544E-55	positive
PRJNA729271	stage_1W_vs_E10	ordinary_muscle_development	ENSGALG00010015838	IFNLR1	-0.037538327	1.267648046	-0.029612578	0.976376034	0.979891339	negative
PRJNA729271	stage_1W_vs_E10	ordinary_muscle_development	ENSGALG00010015839	U2SURP	-0.408329322	0.071363547	-5.721819315	1.05389E-08	2.23303E-08	negative
PRJNA729271	stage_1W_vs_E10	ordinary_muscle_development	ENSGALG00010015840	MTUS1	1.649736602	0.145935613	11.30455111	1.2459E-29	6.92722E-29	positive
PRJNA729271	stage_1W_vs_E10	ordinary_muscle_development	ENSGALG00010015843	ENSGALG00010015843	-2.468900145	0.513953621	-4.803741125	1.55728E-06	2.86675E-06	negative
PRJNA729271	stage_1W_vs_E10	ordinary_muscle_development	ENSGALG00010015845	SAP30L	1.250534095	0.12527845	9.982036802	1.82678E-23	7.8819E-23	positive
PRJNA729271	stage_1W_vs_E10	ordinary_muscle_development	ENSGALG00010015848	UBE2B	2.672894263	0.161044304	16.59726045	7.29451E-62	1.11534E-60	positive
PRJNA729271	stage_1W_vs_E10	ordinary_muscle_development	ENSGALG00010015852	ZFAND5	2.187872113	0.285728503	7.657171366	1.90073E-14	5.48935E-14	positive
PRJNA729271	stage_1W_vs_E10	ordinary_muscle_development	ENSGALG00010015855	NIPAL3	-0.945988597	0.081763126	-11.56986833	5.85725E-31	3.43311E-30	negative
PRJNA729271	stage_1W_vs_E10	ordinary_muscle_development	ENSGALG00010015856	TXNDC9	0.804356268	0.133072165	6.044511766	1.49863E-09	3.34401E-09	positive
PRJNA729271	stage_1W_vs_E10	ordinary_muscle_development	ENSGALG00010015858	CHST2	-1.136649826	0.093252545	-12.18894162	3.56015E-34	2.33927E-33	negative
PRJNA729271	stage_1W_vs_E10	ordinary_muscle_development	ENSGALG00010015861	PPP2CA	-1.132714375	0.068010897	-16.65489529	2.78832E-62	4.30881E-61	negative
PRJNA729271	stage_1W_vs_E10	ordinary_muscle_development	ENSGALG00010015862	PDGFRL	0.626563994	0.209150551	2.995755877	0.002737655	0.003914601	positive
PRJNA729271	stage_1W_vs_E10	ordinary_muscle_development	ENSGALG00010015863	ENSGALG00010015863	-3.010916259	0.585988732	-5.138181153	2.7741E-07	5.37935E-07	negative
PRJNA729271	stage_1W_vs_E10	ordinary_muscle_development	ENSGALG00010015865	VRK1	-2.304269908	0.146710832	-15.7062016	1.37154E-55	1.80404E-54	negative
PRJNA729271	stage_1W_vs_E10	ordinary_muscle_development	ENSGALG00010015866	ARHGEF6	-0.286533684	0.07292615	-3.929093781	8.52666E-05	0.00013794	negative
PRJNA729271	stage_1W_vs_E10	ordinary_muscle_development	ENSGALG00010015867	PICALML	0.314331659	0.056804654	5.533554648	3.13805E-08	6.46384E-08	positive
PRJNA729271	stage_1W_vs_E10	ordinary_muscle_development	ENSGALG00010015869	SLC7A2	3.494165625	0.193435541	18.06372088	6.15317E-73	1.24567E-71	positive
PRJNA729271	stage_1W_vs_E10	ordinary_muscle_development	ENSGALG00010015870	EIF5B	0.39642113	0.156075696	2.53992864	0.011087509	0.014908482	positive
PRJNA729271	stage_1W_vs_E10	ordinary_muscle_development	ENSGALG00010015872	TMC1	-1.881336683	0.396261711	-4.747712512	2.0573E-06	3.75243E-06	negative
PRJNA729271	stage_1W_vs_E10	ordinary_muscle_development	ENSGALG00010015874	CLIC4	-1.019421079	0.114846971	-8.876342751	6.90947E-19	2.46197E-18	negative
PRJNA729271	stage_1W_vs_E10	ordinary_muscle_development	ENSGALG00010015875	DNTTIP1	0.794980706	0.139579902	5.695524166	1.22993E-08	2.5939E-08	positive
PRJNA729271	stage_1W_vs_E10	ordinary_muscle_development	ENSGALG00010015876	SKP1	-0.635502526	0.077629469	-8.18635677	2.69253E-16	8.46415E-16	negative
PRJNA729271	stage_1W_vs_E10	ordinary_muscle_development	ENSGALG00010015877	REV1	-0.06760031	0.105607937	-0.640106342	0.522103466	0.559010913	negative
PRJNA729271	stage_1W_vs_E10	ordinary_muscle_development	ENSGALG00010015879	TRMT61A	-1.51643391	0.124901381	-12.14104994	6.39995E-34	4.18059E-33	negative
PRJNA729271	stage_1W_vs_E10	ordinary_muscle_development	ENSGALG00010015881	GAB3	1.552953578	0.177962884	8.726277856	2.63192E-18	9.08541E-18	positive
PRJNA729271	stage_1W_vs_E10	ordinary_muscle_development	ENSGALG00010015886	AFF3	-1.075412891	0.210404915	-5.111158599	3.20189E-07	6.18324E-07	negative
PRJNA729271	stage_1W_vs_E10	ordinary_muscle_development	ENSGALG00010015887	SLC9A9	-0.277222231	0.182093406	-1.522417733	0.127904444	0.151480915	negative
PRJNA729271	stage_1W_vs_E10	ordinary_muscle_development	ENSGALG00010015889	SH3BP5	1.710558052	0.164978255	10.36838495	3.45311E-25	1.60221E-24	positive
PRJNA729271	stage_1W_vs_E10	ordinary_muscle_development	ENSGALG00010015892	UBE2C	-2.039673783	0.224136571	-9.100138258	9.02169E-20	3.35823E-19	negative
PRJNA729271	stage_1W_vs_E10	ordinary_muscle_development	ENSGALG00010015893	TCF7	-2.19929393	0.454360906	-4.840411888	1.2957E-06	2.39925E-06	negative
PRJNA729271	stage_1W_vs_E10	ordinary_muscle_development	ENSGALG00010015894	CEP78	-2.691048981	0.129793437	-20.73332085	1.73402E-95	5.95606E-94	negative
PRJNA729271	stage_1W_vs_E10	ordinary_muscle_development	ENSGALG00010015895	RBMXL1	-1.146793812	0.0854582	-13.41935364	4.65758E-41	3.86664E-40	negative
PRJNA729271	stage_1W_vs_E10	ordinary_muscle_development	ENSGALG00010015896	LONRF2	-0.094706086	0.07856841	-1.205396503	0.228050271	0.259669181	negative
PRJNA729271	stage_1W_vs_E10	ordinary_muscle_development	ENSGALG00010015898	ENSGALG00010015898	2.679528493	0.216627741	12.36927681	3.83257E-35	2.61672E-34	positive
PRJNA729271	stage_1W_vs_E10	ordinary_muscle_development	ENSGALG00010015899	SMARCA1	-0.648158491	0.116105328	-5.582504273	2.3708E-08	4.91627E-08	negative
PRJNA729271	stage_1W_vs_E10	ordinary_muscle_development	ENSGALG00010015900	ENSGALG00010015900	2.416444189	0.156897877	15.40138228	1.60208E-53	1.9831E-52	positive
PRJNA729271	stage_1W_vs_E10	ordinary_muscle_development	ENSGALG00010015902	ENSGALG00010015902	2.547270702	0.445597884	5.716523329	1.08726E-08	2.3017E-08	positive
PRJNA729271	stage_1W_vs_E10	ordinary_muscle_development	ENSGALG00010015904	MTMR7	2.077753037	0.259228619	8.015137553	1.10014E-15	3.3785E-15	positive
PRJNA729271	stage_1W_vs_E10	ordinary_muscle_development	ENSGALG00010015905	ANPEP	0.795583332	0.217503867	3.657789373	0.0002544	0.000397534	positive
PRJNA729271	stage_1W_vs_E10	ordinary_muscle_development	ENSGALG00010015906	BAG5	1.106109521	0.10065663	10.98893853	4.31976E-28	2.26047E-27	positive
PRJNA729271	stage_1W_vs_E10	ordinary_muscle_development	ENSGALG00010015908	CHRD	0.757836335	0.137998352	5.491633234	3.98234E-08	8.16579E-08	positive
PRJNA729271	stage_1W_vs_E10	ordinary_muscle_development	ENSGALG00010015909	TNNC2	4.618831416	0.106576701	43.33809708	0	0	positive
PRJNA729271	stage_1W_vs_E10	ordinary_muscle_development	ENSGALG00010015910	C5orf15	0.435443999	0.092971686	4.683619451	2.81853E-06	5.09346E-06	positive
PRJNA729271	stage_1W_vs_E10	ordinary_muscle_development	ENSGALG00010015911	TM9SF2L	0.375599707	0.07827359	4.798549634	1.59819E-06	2.93795E-06	positive
PRJNA729271	stage_1W_vs_E10	ordinary_muscle_development	ENSGALG00010015912	ENSGALG00010015912	0.054508579	0.271275474	0.200934417	0.840749856	0.858155704	positive
PRJNA729271	stage_1W_vs_E10	ordinary_muscle_development	ENSGALG00010015913	CHST10	-0.973000375	0.10587127	-9.190409996	3.91347E-20	1.4757E-19	negative
PRJNA729271	stage_1W_vs_E10	ordinary_muscle_development	ENSGALG00010015914	SRRM1	-0.663127376	0.118216958	-5.609409904	2.03018E-08	4.23112E-08	negative
PRJNA729271	stage_1W_vs_E10	ordinary_muscle_development	ENSGALG00010015916	COA8	1.618963211	0.144323256	11.21761837	3.34148E-29	1.82154E-28	positive
PRJNA729271	stage_1W_vs_E10	ordinary_muscle_development	ENSGALG00010015917	TMEM63A	1.097215429	0.106788624	10.27464717	9.16793E-25	4.17496E-24	positive
PRJNA729271	stage_1W_vs_E10	ordinary_muscle_development	ENSGALG00010015918	BICD1	-1.295354382	0.195165652	-6.637204684	3.19688E-11	7.82881E-11	negative
PRJNA729271	stage_1W_vs_E10	ordinary_muscle_development	ENSGALG00010015919	ENSGALG00010015919	0.942876683	0.228714678	4.122501849	3.7478E-05	6.232E-05	positive
PRJNA729271	stage_1W_vs_E10	ordinary_muscle_development	ENSGALG00010015921	SNX21	2.516150043	0.10885382	23.1149448	3.2758E-118	1.7267E-116	positive
PRJNA729271	stage_1W_vs_E10	ordinary_muscle_development	ENSGALG00010015923	RCAN3	-0.912660965	0.122297135	-7.462652062	8.47981E-14	2.36784E-13	negative
PRJNA729271	stage_1W_vs_E10	ordinary_muscle_development	ENSGALG00010015927	GLA	2.038474192	0.167480394	12.17141988	4.41338E-34	2.89204E-33	positive
PRJNA729271	stage_1W_vs_E10	ordinary_muscle_development	ENSGALG00010015928	CAPN7	0.529723008	0.12193224	4.344404786	1.39654E-05	2.39588E-05	positive
PRJNA729271	stage_1W_vs_E10	ordinary_muscle_development	ENSGALG00010015929	SLC6A12	-2.170516914	0.834695811	-2.600368765	0.009312363	0.012632915	negative
PRJNA729271	stage_1W_vs_E10	ordinary_muscle_development	ENSGALG00010015931	RBM47	0.975175547	0.277849962	3.509719921	0.000448579	0.000686388	positive
PRJNA729271	stage_1W_vs_E10	ordinary_muscle_development	ENSGALG00010015932	ACOT8	2.186444949	0.141481402	15.45393892	7.09707E-54	8.87593E-53	positive
PRJNA729271	stage_1W_vs_E10	ordinary_muscle_development	ENSGALG00010015933	ENSGALG00010015933	2.247668281	0.751010125	2.992860157	0.002763764	0.003949985	positive
PRJNA729271	stage_1W_vs_E10	ordinary_muscle_development	ENSGALG00010015934	TMC2	-1.87233507	1.288779615	-1.452796931	0.146280144	0.171853679	negative
PRJNA729271	stage_1W_vs_E10	ordinary_muscle_development	ENSGALG00010015935	EPHX1	0.29562767	0.111746125	2.645529509	0.008156317	0.011131326	positive
PRJNA729271	stage_1W_vs_E10	ordinary_muscle_development	ENSGALG00010015936	RPL36AL	0.149807907	0.139554713	1.073470777	0.283059944	0.31756609	positive
PRJNA729271	stage_1W_vs_E10	ordinary_muscle_development	ENSGALG00010015937	ENSGALG00010015937	1.980774217	0.189673684	10.44306292	1.57641E-25	7.41884E-25	positive
PRJNA729271	stage_1W_vs_E10	ordinary_muscle_development	ENSGALG00010015939	SUN2	0.823704165	0.079689585	10.33640931	4.8227E-25	2.22274E-24	positive
PRJNA729271	stage_1W_vs_E10	ordinary_muscle_development	ENSGALG00010015942	AP3S2	0.011765709	0.131226132	0.089659806	0.928557558	0.937287033	positive
PRJNA729271	stage_1W_vs_E10	ordinary_muscle_development	ENSGALG00010015946	TLE4	0.666207283	0.098352347	6.773679564	1.25548E-11	3.13869E-11	positive
PRJNA729271	stage_1W_vs_E10	ordinary_muscle_development	ENSGALG00010015954	CTSA	1.01941616	0.096538267	10.5597106	4.58072E-26	2.20517E-25	positive
PRJNA729271	stage_1W_vs_E10	ordinary_muscle_development	ENSGALG00010015957	ENSGALG00010015957	2.21413527	0.216106495	10.24557487	1.23886E-24	5.61517E-24	positive
PRJNA729271	stage_1W_vs_E10	ordinary_muscle_development	ENSGALG00010015959	TRNAU1AP	1.344333383	0.129280209	10.39860154	2.51598E-25	1.17151E-24	positive
PRJNA729271	stage_1W_vs_E10	ordinary_muscle_development	ENSGALG00010015960	CHRNA9	3.560912324	0.845369223	4.212256879	2.52832E-05	4.25101E-05	positive
PRJNA729271	stage_1W_vs_E10	ordinary_muscle_development	ENSGALG00010015963	DIPK2A	-0.313831217	0.109106606	-2.876372269	0.00402275	0.005660592	negative
PRJNA729271	stage_1W_vs_E10	ordinary_muscle_development	ENSGALG00010015964	KLC1	-0.169501971	0.088146319	-1.922961432	0.054484894	0.067726484	negative
PRJNA729271	stage_1W_vs_E10	ordinary_muscle_development	ENSGALG00010015967	ZSWIM1	-0.228088206	0.211668142	-1.07757456	0.28122366	0.315603666	negative
PRJNA729271	stage_1W_vs_E10	ordinary_muscle_development	ENSGALG00010015970	THPO	0.005686958	0.401246009	0.014173245	0.988691765	0.990536849	positive
PRJNA729271	stage_1W_vs_E10	ordinary_muscle_development	ENSGALG00010015971	ENSGALG00010015971	-1.580028285	0.162291117	-9.735765695	2.12213E-22	8.7437E-22	negative
PRJNA729271	stage_1W_vs_E10	ordinary_muscle_development	ENSGALG00010015972	FSTL4	-2.554948011	0.307712716	-8.303030328	1.01489E-16	3.25389E-16	negative
PRJNA729271	stage_1W_vs_E10	ordinary_muscle_development	ENSGALG00010015974	VPS37A	2.352120239	0.163222251	14.41053669	4.44245E-47	4.41958E-46	positive
PRJNA729271	stage_1W_vs_E10	ordinary_muscle_development	ENSGALG00010015975	BTK	1.160412418	0.177428589	6.540165976	6.14506E-11	1.47642E-10	positive
PRJNA729271	stage_1W_vs_E10	ordinary_muscle_development	ENSGALG00010015979	ENSGALG00010015979	4.057610218	0.202258072	20.06154901	1.60021E-89	4.67641E-88	positive

PRJNA729271	stage_1W_vs_E10	ordinary_muscle_development	ENSGALG00010015980	EXOSC7	-1.323963333	0.094919649	-13.94825351	3.22404E-44	2.95214E-43	negative
PRJNA729271	stage_1W_vs_E10	ordinary_muscle_development	ENSGALG00010015983	CNOT7	-0.471983678	0.065157875	-7.243693556	4.36627E-13	1.17529E-12	negative
PRJNA729271	stage_1W_vs_E10	ordinary_muscle_development	ENSGALG00010015984	PDCL3	0.839696163	0.107571941	7.805903243	5.90769E-15	1.75116E-14	positive
PRJNA729271	stage_1W_vs_E10	ordinary_muscle_development	ENSGALG00010015985	TIMM8A	-0.373830439	0.111577573	-3.350408405	0.000806925	0.001212562	negative
PRJNA729271	stage_1W_vs_E10	ordinary_muscle_development	ENSGALG00010015986	NEURL2	4.050224916	0.22775665	17.78312472	9.55057E-71	1.8409E-69	positive
PRJNA729271	stage_1W_vs_E10	ordinary_muscle_development	ENSGALG00010015987	POLR2H	-0.083171776	0.096083266	-0.865621867	0.38669756	0.424795842	negative
PRJNA729271	stage_1W_vs_E10	ordinary_muscle_development	ENSGALG00010015989	ENAH	-1.609246262	0.163550469	-9.839447536	7.61277E-23	3.20125E-22	negative
PRJNA729271	stage_1W_vs_E10	ordinary_muscle_development	ENSGALG00010015992	ATP10A	-1.165387718	0.158610962	-7.347460111	2.02009E-13	5.5237E-13	negative
PRJNA729271	stage_1W_vs_E10	ordinary_muscle_development	ENSGALG00010015994	ENSGALG00010015994	-0.302486941	0.111037294	-2.724192299	0.006445896	0.008893767	negative
PRJNA729271	stage_1W_vs_E10	ordinary_muscle_development	ENSGALG00010016004	XRCC3	-0.644878231	0.171013521	-3.77091957	0.000162647	0.000257573	negative
PRJNA729271	stage_1W_vs_E10	ordinary_muscle_development	ENSGALG00010016006	PLTP	-1.418765115	0.322935296	-4.393341737	1.11621E-05	1.93073E-05	negative
PRJNA729271	stage_1W_vs_E10	ordinary_muscle_development	ENSGALG00010016008	TAF7	-0.609096511	0.09571489	-6.363654702	1.97009E-10	4.60068E-10	negative
PRJNA729271	stage_1W_vs_E10	ordinary_muscle_development	ENSGALG00010016011	ADD1	-1.277360974	0.0813307	-15.70576631	1.38099E-55	1.81482E-54	negative
PRJNA729271	stage_1W_vs_E10	ordinary_muscle_development	ENSGALG00010016015	ZDHHC3	0.588562601	0.082422723	7.140780803	9.28022E-13	2.46233E-12	positive
PRJNA729271	stage_1W_vs_E10	ordinary_muscle_development	ENSGALG00010016016	ZDHHC2	0.975935491	0.275309717	3.544863948	0.000392816	0.000604059	positive
PRJNA729271	stage_1W_vs_E10	ordinary_muscle_development	ENSGALG00010016017	RAPGEF6	0.287973979	0.07205517	3.99657621	6.42652E-05	0.000104984	positive
PRJNA729271	stage_1W_vs_E10	ordinary_muscle_development	ENSGALG00010016022	EPHB3	-1.929618845	0.169230662	-11.40230039	4.07215E-30	2.31292E-29	negative
PRJNA729271	stage_1W_vs_E10	ordinary_muscle_development	ENSGALG00010016025	PCIF1	0.610729398	0.107715821	5.669820769	1.42947E-08	3.00379E-08	positive
PRJNA729271	stage_1W_vs_E10	ordinary_muscle_development	ENSGALG00010016027	COPS2	1.095668959	0.149825203	7.312981636	2.61279E-13	7.10551E-13	positive
PRJNA729271	stage_1W_vs_E10	ordinary_muscle_development	ENSGALG00010016030	TGM4	0.659599408	0.719200271	0.917128976	0.359075059	0.396676547	positive
PRJNA729271	stage_1W_vs_E10	ordinary_muscle_development	ENSGALG00010016040	RPSA2	0.886868886	0.105886519	8.375654367	5.49179E-17	1.77885E-16	positive
PRJNA729271	stage_1W_vs_E10	ordinary_muscle_development	ENSGALG00010016041	ENSGALG00010016041	0.760300829	0.090421984	8.40836258	4.15774E-17	1.35248E-16	positive
PRJNA729271	stage_1W_vs_E10	ordinary_muscle_development	ENSGALG00010016043	ADAMTSL1	-0.431859549	0.193029014	-2.237277905	0.025268182	0.032666991	negative
PRJNA729271	stage_1W_vs_E10	ordinary_muscle_development	ENSGALG00010016047	SRP9	0.288045462	0.088841401	3.242243579	0.001185926	0.001752651	positive
PRJNA729271	stage_1W_vs_E10	ordinary_muscle_development	ENSGALG00010016049	ZFYVE21	0.513039764	0.122434784	4.19031054	2.78573E-05	4.67081E-05	positive
PRJNA729271	stage_1W_vs_E10	ordinary_muscle_development	ENSGALG00010016053	DRP2	3.305885708	0.45881931	7.205201777	5.7958E-13	1.55057E-12	positive
PRJNA729271	stage_1W_vs_E10	ordinary_muscle_development	ENSGALG00010016057	SOHO1	-3.025585113	0.646588592	-4.679304818	2.87849E-06	5.19577E-06	negative
PRJNA729271	stage_1W_vs_E10	ordinary_muscle_development	ENSGALG00010016059	EPDR1	0.394177548	0.201014444	1.960941412	0.049885858	0.062292859	positive
PRJNA729271	stage_1W_vs_E10	ordinary_muscle_development	ENSGALG00010016060	ENSGALG00010016060	0.44494771	1.37510924	0.323572627	0.746261614	0.771545085	positive
PRJNA729271	stage_1W_vs_E10	ordinary_muscle_development	ENSGALG00010016066	C14orf2	1.348312593	0.149467	9.020804537	1.86713E-19	6.84126E-19	positive
PRJNA729271	stage_1W_vs_E10	ordinary_muscle_development	ENSGALG00010016067	AHSG	5.490448198	0.846841002	6.483446344	8.96508E-11	2.13416E-10	positive
PRJNA729271	stage_1W_vs_E10	ordinary_muscle_development	ENSGALG00010016069	GALK2	0.09410335	0.145376179	0.647309284	0.517431794	0.55462352	positive
PRJNA729271	stage_1W_vs_E10	ordinary_muscle_development	ENSGALG00010016073	SAXO1	4.833220087	0.979611041	4.93381545	8.06386E-07	1.51898E-06	positive
PRJNA729271	stage_1W_vs_E10	ordinary_muscle_development	ENSGALG00010016076	RAB3GAP2	0.245470547	0.090602917	2.709300697	0.00674252	0.009283133	positive
PRJNA729271	stage_1W_vs_E10	ordinary_muscle_development	ENSGALG00010016083	CDH22	-3.039629616	0.61878056	-4.912290096	9.00187E-07	1.68975E-06	negative
PRJNA729271	stage_1W_vs_E10	ordinary_muscle_development	ENSGALG00010016086	TDRD9	-2.28528732	0.370675366	-6.16519879	7.03946E-10	1.59458E-09	negative
PRJNA729271	stage_1W_vs_E10	ordinary_muscle_development	ENSGALG00010016087	IARS2	0.624453115	0.089901827	6.945944676	3.75936E-12	9.66169E-12	positive
PRJNA729271	stage_1W_vs_E10	ordinary_muscle_development	ENSGALG00010016091	HMX1	-4.356786264	0.99559196	-4.376076181	1.20835E-05	2.08413E-05	negative
PRJNA729271	stage_1W_vs_E10	ordinary_muscle_development	ENSGALG00010016094	FGF7	-0.107831401	0.185875965	-0.580125577	0.561829937	0.597616859	negative
PRJNA729271	stage_1W_vs_E10	ordinary_muscle_development	ENSGALG00010016097	FNIP1	1.278317471	0.110153644	11.60485863	3.89331E-31	2.29965E-30	positive
PRJNA729271	stage_1W_vs_E10	ordinary_muscle_development	ENSGALG00010016100	SFRP4	-2.005021494	0.420829253	-4.764453705	1.89366E-06	3.46748E-06	negative
PRJNA729271	stage_1W_vs_E10	ordinary_muscle_development	ENSGALG00010016103	BPNT1	-1.252688167	0.093255216	-13.43290192	3.87907E-41	3.2333E-40	negative
PRJNA729271	stage_1W_vs_E10	ordinary_muscle_development	ENSGALG00010016107	UVSSA	0.217013012	0.091193708	2.379692824	0.017327075	0.022784719	positive
PRJNA729271	stage_1W_vs_E10	ordinary_muscle_development	ENSGALG00010016109	MLH1	0.090441995	0.100343341	0.901325327	0.367415372	0.405149168	positive
PRJNA729271	stage_1W_vs_E10	ordinary_muscle_development	ENSGALG00010016111	EPB41	-0.243644512	0.102777497	-2.370601717	0.017759157	0.023323274	negative
PRJNA729271	stage_1W_vs_E10	ordinary_muscle_development	ENSGALG00010016113	DTWD1	0.333761866	0.148379411	2.249381263	0.02448825	0.031712425	positive
PRJNA729271	stage_1W_vs_E10	ordinary_muscle_development	ENSGALG00010016114	HAUS6	-1.381360871	0.151980124	-9.089088965	9.98724E-20	3.70872E-19	negative
PRJNA729271	stage_1W_vs_E10	ordinary_muscle_development	ENSGALG00010016115	RD3L	-0.720629021	0.472086653	-1.526476159	0.126891324	0.150391638	negative
PRJNA729271	stage_1W_vs_E10	ordinary_muscle_development	ENSGALG00010016117	ENSGALG00010016117	0.599861028	0.236051883	2.541225348	0.01104647	0.014857435	positive
PRJNA729271	stage_1W_vs_E10	ordinary_muscle_development	ENSGALG00010016118	EPRS	0.453375066	0.087496957	5.181609512	2.19979E-07	4.29731E-07	positive
PRJNA729271	stage_1W_vs_E10	ordinary_muscle_development	ENSGALG00010016119	FETUB	3.117492241	1.398385226	2.229351529			positive
PRJNA729271	stage_1W_vs_E10	ordinary_muscle_development	ENSGALG00010016121	SLC35C2	-0.639142434	0.102215838	-6.252870871	4.02975E-10	9.25694E-10	negative
PRJNA729271	stage_1W_vs_E10	ordinary_muscle_development	ENSGALG00010016122	AFG2B	0.587256466	0.130495729	4.500196831	6.78906E-06	1.19051E-05	positive
PRJNA729271	stage_1W_vs_E10	ordinary_muscle_development	ENSGALG00010016127	ENSGALG00010016127	-0.384076325	0.179108901	-2.144373191	0.032002997	0.040849193	negative
PRJNA729271	stage_1W_vs_E10	ordinary_muscle_development	ENSGALG00010016129	CENPI	-2.272153803	0.237061409	-9.584663355	9.27596E-22	3.73175E-21	negative
PRJNA729271	stage_1W_vs_E10	ordinary_muscle_development	ENSGALG00010016131	SMYD1	0.109831465	0.217652183	0.504619175	0.613826339	0.648027151	positive
PRJNA729271	stage_1W_vs_E10	ordinary_muscle_development	ENSGALG00010016137	STARD3NL	-0.091672172	0.141452833	-0.648075901	0.51693586	0.554132916	negative
PRJNA729271	stage_1W_vs_E10	ordinary_muscle_development	ENSGALG00010016139	LURAP1L	-0.06227868	0.192563694	-0.323418599	0.746378246	0.771555603	negative
PRJNA729271	stage_1W_vs_E10	ordinary_muscle_development	ENSGALG00010016141	RRP15	-1.06477727	0.17863422	-5.960656746	2.51226E-09	5.52835E-09	negative
PRJNA729271	stage_1W_vs_E10	ordinary_muscle_development	ENSGALG00010016147	FABP1	4.087658197	0.790829434	5.168824052	2.35572E-07	4.59323E-07	positive
PRJNA729271	stage_1W_vs_E10	ordinary_muscle_development	ENSGALG00010016150	POLG	0.397850405	0.094665646	4.202690449	2.63761E-05	4.42861E-05	positive
PRJNA729271	stage_1W_vs_E10	ordinary_muscle_development	ENSGALG00010016159	ASPG	-2.350346453	0.19670101	-11.94882756	6.58476E-33	4.14983E-32	negative
PRJNA729271	stage_1W_vs_E10	ordinary_muscle_development	ENSGALG00010016165	KK34	3.07321804	0.941993508	3.262462018	0.00110449	0.001637306	positive
PRJNA729271	stage_1W_vs_E10	ordinary_muscle_development	ENSGALG00010016168	PLIN2	1.965217301	0.229281248	8.57120814	1.02405E-17	3.43125E-17	positive
PRJNA729271	stage_1W_vs_E10	ordinary_muscle_development	ENSGALG00010016169	RHOH	-0.327806394	0.12371705	-2.649646052	0.008057614	0.011002837	negative
PRJNA729271	stage_1W_vs_E10	ordinary_muscle_development	ENSGALG00010016173	ENSGALG00010016173	-1.521455096	0.343817043	-4.425188124	9.63583E-06	1.67431E-05	negative
PRJNA729271	stage_1W_vs_E10	ordinary_muscle_development	ENSGALG00010016177	ENSGALG00010016177	3.6466032	1.538894524	2.36962517	0.017806127	0.023380724	positive
PRJNA729271	stage_1W_vs_E10	ordinary_muscle_development	ENSGALG00010016181	FANCI	-2.6866104	0.181151488	-14.83073881	9.2707E-50	1.00658E-48	negative
PRJNA729271	stage_1W_vs_E10	ordinary_muscle_development	ENSGALG00010016182	ELMO2	0.023318447	0.062078827	0.375626409	0.707194654	0.734981467	positive
PRJNA729271	stage_1W_vs_E10	ordinary_muscle_development	ENSGALG00010016190	THNSL2	0.826274772	0.152330113	5.42423791	5.82023E-08	1.18323E-07	positive
PRJNA729271	stage_1W_vs_E10	ordinary_muscle_development	ENSGALG00010016193	TCAIM	-0.107694138	0.135461236	-0.79501813	0.426602956	0.464548858	negative
PRJNA729271	stage_1W_vs_E10	ordinary_muscle_development	ENSGALG00010016197	SNRPN	-1.558814487	0.116863754	-13.33873368	1.3778E-40	1.12387E-39	negative
PRJNA729271	stage_1W_vs_E10	ordinary_muscle_development	ENSGALG00010016199	RLBP1	-6.203333417	0.672004813	-9.231084809	2.67905E-20	1.01843E-19	negative
PRJNA729271	stage_1W_vs_E10	ordinary_muscle_development	ENSGALG00010016200	MICU3	0.910593429	0.117401028	7.756264524	8.74675E-15	2.57221E-14	positive
PRJNA729271	stage_1W_vs_E10	ordinary_muscle_development	ENSGALG00010016205	CD40LG	6.715670242	1.016126671	6.609087658	3.86696E-11	9.42676E-11	positive
PRJNA729271	stage_1W_vs_E10	ordinary_muscle_development	ENSGALG00010016206	KNG1	2.92387594	1.153920091	2.533863447	0.011281268	0.015153553	positive
PRJNA729271	stage_1W_vs_E10	ordinary_muscle_development	ENSGALG00010016209	ADAMTS7	-1.642684712	0.167751748	-9.792355283	1.21434E-22	5.05363E-22	negative
PRJNA729271	stage_1W_vs_E10	ordinary_muscle_development	ENSGALG00010016211	TACC3	-1.963710889	0.162142581	-12.11101286	9.23467E-34	6.00254E-33	negative
PRJNA729271	stage_1W_vs_E10	ordinary_muscle_development	ENSGALG00010016215	SPATA17	-3.186586404	0.426295509	-7.475064444	7.7166E-14	2.16139E-13	negative
PRJNA729271	stage_1W_vs_E10	ordinary_muscle_development	ENSGALG00010016218	N4BP2	-0.110702583	0.075966163	-1.457261751	0.145044141	0.170498323	negative
PRJNA729271	stage_1W_vs_E10	ordinary_muscle_development	ENSGALG00010016221	TMEM200B	-0.601329139	0.203201291	-2.959278135	0.003083607	0.004389362	negative
PRJNA729271	stage_1W_vs_E10	ordinary_muscle_development	ENSGALG00010016224	MOSPD1	2.436395186	0.11506152	21.17471758	1.63358E-99	6.07148E-98	positive
PRJNA729271	stage_1W_vs_E10	ordinary_muscle_development	ENSGALG00010016225	P4HA2	0.849790636	0.156872536	5.417077195	6.05812E-08	1.22987E-07	positive
PRJNA729271	stage_1W_vs_E10	ordinary_muscle_development	ENSGALG00010016229	ENSGALG00010016229	0.685417421	0.223917325	3.061028981	0.002205777	0.003182634	positive
PRJNA729271	stage_1W_vs_E10	ordinary_muscle_development	ENSGALG00010016235	DDX27	-0.061388816	0.123386324	-0.497533389	0.618812963	0.652911188	negative
PRJNA729271	stage_1W_vs_E10	ordinary_muscle_development	ENSGALG00010016236	GPATCH2	-0.944957534	0.067717591	-13.95438802	2.9583E-44	2.71053E-43	negative
PRJNA729271	stage_1W_vs_E10	ordinary_muscle_development	ENSGALG00010016240	EIF4A2	1.291747235	0.100311789	12.87732225	6.03948E-38	4.49165E-37	positive
PRJNA729271	stage_1W_vs_E10	ordinary_muscle_development	ENSGALG00010016241	TOPAZ1	-0.405198022	0.825876337	-0.49062796	0.623689607	0.657338825	negative
PRJNA729271	stage_1W_vs_E10	ordinary_muscle_development	ENSGALG00010016242	PDS5A	0.459120391	0.092200969	4.979561429	6.37285E-07	1.20704E-06	positive
PRJNA729271	stage_1W_vs_E10	ordinary_muscle_development	ENSGALG00010016245	FAM96A	-0.387446714	0.106251398	-3.646509328	0.000265827	0.000414988	negative
PRJNA729271	stage_1W_vs_E10	ordinary_muscle_development	ENSGALG00010016247	KIF26A	-2.928049148	0.168240829	-17.4039154	7.70515E-68	1.37545E-66	negative
PRJNA729271	stage_1W_vs_E10	ordinary_muscle_development	ENSGALG00010016251	MPDZ	-0.475154387	0.073334294	-6.479293127	9.21533E-11	2.19337E-10	negative
PRJNA729271	stage_1W_vs_E10	ordinary_muscle_development	ENSGALG00010016253	EDN3	-0.861619765	0.209964984	-4.103635508	4.06708E-05	6.73972E-05	negative
PRJNA729271	stage_1W_vs_E10	ordinary_muscle_development	ENSGALG00010016257	ENSGALG00010016257	-0.993345885	0.114326629	-8.688665925	3.6672E-18	1.25694E-17	negative
PRJNA729271	stage_1W_vs_E10	ordinary_muscle_development	ENSGALG00010016258	CTNNB1	-2.144530292	0.077383073	-27.71317058	4.8451E-169	5.1987E-167	negative
PRJNA729271	stage_1W_vs_E10	ordinary_muscle_development	ENSGALG00010016260	INTS6L	1.408616082	0.143937394	9.786310839	1.28914E-22	5.36032E-22	positive
PRJNA729271	stage_1W_vs_E10	ordinary_muscle_development	ENSGALG00010016262	SPG11	0.370099602	0.061054724	6.061768498	1.34633E-09	3.01158E-09	positive
PRJNA729271	stage_1W_vs_E10	ordinary_muscle_development	ENSGALG00010016263	HGFAC	1.044376836	1.351803237	0.772580511			positive
PRJNA729271	stage_1W_vs_E10	ordinary_muscle_development	ENSGALG00010016266	PDLIM4	-1.20653072	0.121789819	-9.906663194	3.89436E-23	1.65782E-22	negative
PRJNA729271	stage_1W_vs_E10	ordinary_muscle_development	ENSGALG00010016270	RFC4	-1.591567445	0.112462527	-14.15198008	1.81572E-45	1.72809E-44	negative
PRJNA729271	stage_1W_vs_E10	ordinary_muscle_development	ENSGALG00010016274	ZNF831	0.612839026	0.521982973	1.174059419	0.240371227	0.27275567	positive
PRJNA729271	stage_1W_vs_E10	ordinary_muscle_development	ENSGALG00010016276	ACTR2	-1.503810338	0.108437196	-13.86803042	9.89569E-44	8.92582E-43	negative
PRJNA729271	stage_1W_vs_E10	ordinary_muscle_development	ENSGALG00010016279	PRELID3B	-0.500067476	0.106425158	-4.698771275	2.61731E-06	4.74225E-06	negative
PRJNA729271	stage_1W_vs_E10	ordinary_muscle_development	ENSGALG00010016281	SLC22A4	-2.120606295	0.236546376	-8.964864859	3.10662E-19	1.12689E-18	negative
PRJNA729271	stage_1W_vs_E10	ordinary_muscle_development	ENSGALG00010016286	ULK4	-1.824977557	0.147669352	-12.35853977	4.38041E-35	2.98515E-34	negative
PRJNA729271	stage_1W_vs_E10	ordinary_muscle_development	ENSGALG00010016287	BCL11B	-5.695742731	0.363123543	-15.68541296	1.90321E-55	2.49206E-54	negative
PRJNA729271	stage_1W_vs_E10	ordinary_muscle_development	ENSGALG00010016289	IRF1	4.241845521	0.256381873	16.54502897	1.73881E-61	2.64194E-60	positive
PRJNA729271	stage_1W_vs_E10	ordinary_muscle_development	ENSGALG00010016292	PHKA1	6.66612829	0.147856222	45.08520643	0	0	positive
PRJNA729271	stage_1W_vs_E10	ordinary_muscle_development	ENSGALG00010016295	UBE2K	0.704315785	0.093048433	7.569345992	3.75108E-14	1.068E-13	positive
PRJNA729271	stage_1W_vs_E10	ordinary_muscle_development	ENSGALG00010016299	SRSF4	-0.978003266	0.110219134	-8.873262084	7.10339E-19	2.52982E-18	negative
PRJNA729271	stage_1W_vs_E10	ordinary_muscle_development	ENSGALG00010016303	ENSGALG00010016303	0.667205571	0.203423721	3.27988087	0.001038509	0.001543442	positive
PRJNA729271	stage_1W_vs_E10	ordinary_muscle_development	ENSGALG00010016304	EIF3J	1.476797484	0.099564554	14.83256268	9.02218E-50	9.81457E-49	positive
PRJNA729271	stage_1W_vs_E10	ordinary_muscle_development	ENSGALG00010016305	DENND4C	0.600041036	0.096581267	6.212809754	5.20454E-10	1.18766E-09	positive
PRJNA729271	stage_1W_vs_E10	ordinary_muscle_development	ENSGALG00010016307	RAD50	-0.618772429	0.129187388	-4.789727834	1.67008E-06	3.06777E-06	negative
PRJNA729271	stage_1W_vs_E10	ordinary_muscle_development	ENSGALG00010016308	RPS6	-0.068168167	0.140719591	-0.484425563	0.62808388	0.661441139	negative
PRJNA729271	stage_1W_vs_E10	ordinary_muscle_development	ENSGALG00010016310	HDAC8	-0.611635524	0.093399296	-6.548609602	5.80752E-11	1.39764E-10	negative
PRJNA729271	stage_1W_vs_E10	ordinary_muscle_development	ENSGALG00010016312	MECR	-0.310514391	0.149130055	-2.082171777	0.037326782	0.047323687	negative
PRJNA729271	stage_1W_vs_E10	ordinary_muscle_development	ENSGALG00010016314	ACER2	0.316391293	0.157363082	2.010581437	0.044369686	0.055774747	positive
PRJNA729271	stage_1W_vs_E10	ordinary_muscle_development	ENSGALG00010016315	RPS4Y1	0.390137827	0.136430153	2.859615856	0.004241545	0.005958057	positive
PRJNA729271	stage_1W_vs_E10	ordinary_muscle_development	ENSGALG00010016317	CTDSPL2	-0.825964032	0.057756094	-14.30089834	2.16003E-46	2.10839E-45	negative
PRJNA729271	stage_1W_vs_E10	ordinary_muscle_development	ENSGALG00010016319	RBM41	1.058488806	0.184146708	5.748073458	9.0266E-09	1.91905E-08	positive
PRJNA729271	stage_1W_vs_E10	ordinary_muscle_development	ENSGALG00010016322	EBF4	-0.824266219	0.134412608	-6.132357898	8.6586E-10	1.9531E-09	negative
PRJNA729271	stage_1W_vs_E10	ordinary_muscle_development	ENSGALG00010016324	TUBB1	-1.647906321	0.214916519	-7.667657774	1.75165E-14	5.07297E-14	negative
PRJNA729271	stage_1W_vs_E10	ordinary_muscle_development	ENSGALG00010016328	USH2A	5.009970757	0.60915807	8.22441826	1.96141E-16	6.21297E-16	positive
PRJNA729271	stage_1W_vs_E10	ordinary_muscle_development	ENSGALG00010016332	ENSGALG00010016332	-0.293168501	0.234861739	-1.248259944	0.211935878	0.242942709	negative
PRJNA729271	stage_1W_vs_E10	ordinary_muscle_development	ENSGALG00010016335	ABHD4	2.889705169	0.183072614	15.7844754	3.97944E-56	5.29678E-55	positive
PRJNA729271	stage_1W_vs_E10	ordinary_muscle_development	ENSGALG00010016336	SLC24A2	-0.452533168	0.370761047	-1.220552084	0.222255658	0.253631478	negative
PRJNA729271	stage_1W_vs_E10	ordinary_muscle_development	ENSGALG00010016338	PTPRU	-1.462481172	0.152331367	-9.600656809	7.94366E-22	3.20555E-21	negative
PRJNA729271	stage_1W_vs_E10	ordinary_muscle_development	ENSGALG00010016339	SETD3	0.768293019	0.121871422	6.304127772	2.89821E-10	6.70651E-10	positive
PRJNA729271	stage_1W_vs_E10	ordinary_muscle_development	ENSGALG00010016340	RGS12	-1.884142763	0.101924917	-18.48559522	2.69706E-76	5.86996E-75	negative
PRJNA729271	stage_1W_vs_E10	ordinary_muscle_development	ENSGALG00010016341	SLX4	-0.816676888	0.06511037	-12.54296183	4.34449E-36	3.06888E-35	negative

PRJNA729271	stage_1W_vs_E10	ordinary_muscle_development	ENSGALG00010016344	SMIM14	-0.125695265	0.112290487	-1.119375903	0.262979807	0.296714587	negative
PRJNA729271	stage_1W_vs_E10	ordinary_muscle_development	ENSGALG00010016345	CTSZ	1.040204863	0.110389712	9.423023592	4.38282E-21	1.72212E-20	positive
PRJNA729271	stage_1W_vs_E10	ordinary_muscle_development	ENSGALG00010016346	TRAP1	0.466559734	0.103708785	4.49874843	6.83547E-06	1.1985E-05	positive
PRJNA729271	stage_1W_vs_E10	ordinary_muscle_development	ENSGALG00010016347	ANO10	0.198189273	0.090326997	2.194131097	0.028225986	0.036264463	positive
PRJNA729271	stage_1W_vs_E10	ordinary_muscle_development	ENSGALG00010016350	CASC4	0.101197078	0.062965942	1.607171668	0.108016715	0.129258011	positive
PRJNA729271	stage_1W_vs_E10	ordinary_muscle_development	ENSGALG00010016353	C1orf232	0.418454903	0.14314777	2.923237323	0.003464124	0.004908835	positive
PRJNA729271	stage_1W_vs_E10	ordinary_muscle_development	ENSGALG00010016355	MCF2L2	0.339920266	0.22991162	1.478482322	0.139278725	0.164160443	positive
PRJNA729271	stage_1W_vs_E10	ordinary_muscle_development	ENSGALG00010016361	NELFCD	-1.485231399	0.084695524	-17.53612623	7.59302E-69	1.40027E-67	negative
PRJNA729271	stage_1W_vs_E10	ordinary_muscle_development	ENSGALG00010016362	NUP62	0.085092823	0.168930795	0.503714096	0.614462301	0.648651304	positive
PRJNA729271	stage_1W_vs_E10	ordinary_muscle_development	ENSGALG00010016366	KIF3A	-0.50221673	0.098808375	-5.082734441	3.7204E-07	7.15594E-07	negative
PRJNA729271	stage_1W_vs_E10	ordinary_muscle_development	ENSGALG00010016368	CCNK	-0.745466827	0.15421187	-4.834043098	1.33788E-06	2.47548E-06	negative
PRJNA729271	stage_1W_vs_E10	ordinary_muscle_development	ENSGALG00010016369	MSANTD1	-4.676574774	0.388514236	-12.03707444	2.26859E-33	1.4518E-32	negative
PRJNA729271	stage_1W_vs_E10	ordinary_muscle_development	ENSGALG00010016370	TMEM255A	4.91686447	0.185155048	26.55538983	2.2253E-155	2.0945E-153	positive
PRJNA729271	stage_1W_vs_E10	ordinary_muscle_development	ENSGALG00010016372	DNAAF6	-0.209700283	0.19910948	-1.053190854	0.29225353	0.327221915	negative
PRJNA729271	stage_1W_vs_E10	ordinary_muscle_development	ENSGALG00010016373	SRL	4.15746093	0.131615453	31.58793923	5.4066E-219	9.2198E-217	positive
PRJNA729271	stage_1W_vs_E10	ordinary_muscle_development	ENSGALG00010016374	FRMD5	8.617149551	0.335125108	25.71323172	8.3143E-146	6.4104E-144	positive
PRJNA729271	stage_1W_vs_E10	ordinary_muscle_development	ENSGALG00010016375	CCNI2	-2.219297203	0.146688833	-15.12928526	1.03817E-51	1.21064E-50	negative
PRJNA729271	stage_1W_vs_E10	ordinary_muscle_development	ENSGALG00010016377	FRMPD3	3.530713719	0.277854448	12.70706208	5.40263E-37	3.9019E-36	positive
PRJNA729271	stage_1W_vs_E10	ordinary_muscle_development	ENSGALG00010016378	WDR76	-0.46291244	0.173017863	-2.675518188	0.007461382	0.010224309	negative
PRJNA729271	stage_1W_vs_E10	ordinary_muscle_development	ENSGALG00010016379	TFAP4	-0.78652185	0.15428777	-5.097758888	3.43698E-07	6.62664E-07	negative
PRJNA729271	stage_1W_vs_E10	ordinary_muscle_development	ENSGALG00010016382	UGDH	-1.574466124	0.186393737	-8.446990475	2.98908E-17	9.80021E-17	negative
PRJNA729271	stage_1W_vs_E10	ordinary_muscle_development	ENSGALG00010016383	FAM110D	1.364977094	0.174447069	7.824591718	5.09307E-15	1.51558E-14	positive
PRJNA729271	stage_1W_vs_E10	ordinary_muscle_development	ENSGALG00010016384	GNAS	-0.045411127	0.074627373	-0.608504968	0.542852612	0.579002841	negative
PRJNA729271	stage_1W_vs_E10	ordinary_muscle_development	ENSGALG00010016385	HTT	0.234356091	0.106700944	2.196382539	0.028064574	0.036063476	positive
PRJNA729271	stage_1W_vs_E10	ordinary_muscle_development	ENSGALG00010016387	CCDC85C	-1.133845758	0.16395011	-6.915797494	4.65239E-12	1.18935E-11	negative
PRJNA729271	stage_1W_vs_E10	ordinary_muscle_development	ENSGALG00010016390	ENSGALG00010016390	2.850569798	0.314066678	9.076320394	1.12307E-19	4.16129E-19	positive
PRJNA729271	stage_1W_vs_E10	ordinary_muscle_development	ENSGALG00010016392	STMN3	0.48829236	0.317545886	1.53770646	0.124120403	0.147348505	positive
PRJNA729271	stage_1W_vs_E10	ordinary_muscle_development	ENSGALG00010016393	BLM	-2.158813717	0.127895422	-16.87952303	6.36571E-64	1.02523E-62	negative
PRJNA729271	stage_1W_vs_E10	ordinary_muscle_development	ENSGALG00010016394	PDIK1L	0.57497995	0.095662325	6.010516128	1.84934E-09	4.10444E-09	positive
PRJNA729271	stage_1W_vs_E10	ordinary_muscle_development	ENSGALG00010016395	ENSGALG00010016395	-1.024613751	0.163922085	-6.250614437	4.08841E-10	9.38276E-10	negative
PRJNA729271	stage_1W_vs_E10	ordinary_muscle_development	ENSGALG00010016397	LIAS	0.492729011	0.103592426	4.756419283	1.97057E-06	3.6024E-06	positive
PRJNA729271	stage_1W_vs_E10	ordinary_muscle_development	ENSGALG00010016398	SEPTIN6	-0.505156001	0.068878183	-7.334049504	2.23301E-13	6.09441E-13	negative
PRJNA729271	stage_1W_vs_E10	ordinary_muscle_development	ENSGALG00010016399	ACSM5	7.104950187	0.32722739	21.71257784	1.5605E-104	6.3581E-103	positive
PRJNA729271	stage_1W_vs_E10	ordinary_muscle_development	ENSGALG00010016400	ENSGALG00010016400	0.485546949	0.086679838	5.601613453	2.12366E-08	4.41842E-08	positive
PRJNA729271	stage_1W_vs_E10	ordinary_muscle_development	ENSGALG00010016402	SEPTIN8	-1.140565493	0.078822408	-14.4700666	1.87291E-47	1.88395E-46	negative
PRJNA729271	stage_1W_vs_E10	ordinary_muscle_development	ENSGALG00010016403	GRK4	-1.388170521	0.136694742	-10.15525909	3.13975E-24	1.39518E-23	negative
PRJNA729271	stage_1W_vs_E10	ordinary_muscle_development	ENSGALG00010016404	ESRRG	0.933615282	0.149802157	6.232322034	4.59572E-10	1.05204E-09	positive
PRJNA729271	stage_1W_vs_E10	ordinary_muscle_development	ENSGALG00010016407	RPL9	0.278192621	0.180726706	1.539300013	0.123731071	0.146934448	positive
PRJNA729271	stage_1W_vs_E10	ordinary_muscle_development	ENSGALG00010016409	GMEB2	1.855726555	0.205711885	9.020998248	1.86383E-19	6.8309E-19	positive
PRJNA729271	stage_1W_vs_E10	ordinary_muscle_development	ENSGALG00010016410	TRAK1	1.31766838	0.096672094	13.6302869	2.64538E-42	2.28379E-41	positive
PRJNA729271	stage_1W_vs_E10	ordinary_muscle_development	ENSGALG00010016411	CTSH	0.803799983	0.12346312	6.51044605	7.4928E-11	1.78985E-10	positive
PRJNA729271	stage_1W_vs_E10	ordinary_muscle_development	ENSGALG00010016412	TMSB15B	-3.44003209	0.216087718	-15.9196095	4.63259E-57	6.29329E-56	negative
PRJNA729271	stage_1W_vs_E10	ordinary_muscle_development	ENSGALG00010016415	RASGRF1	3.223312141	0.516479075	6.24093462	4.34964E-10	9.96807E-10	positive
PRJNA729271	stage_1W_vs_E10	ordinary_muscle_development	ENSGALG00010016416	CYP46A1	1.556875946	0.110739857	14.05885818	6.79741E-45	6.33623E-44	positive
PRJNA729271	stage_1W_vs_E10	ordinary_muscle_development	ENSGALG00010016417	NOP14	-0.48896244	0.106789479	-4.578751051	4.67761E-06	8.31211E-06	negative
PRJNA729271	stage_1W_vs_E10	ordinary_muscle_development	ENSGALG00010016418	NFIB	-0.389866837	0.098469028	-3.959283882	7.51748E-05	0.000122145	negative
PRJNA729271	stage_1W_vs_E10	ordinary_muscle_development	ENSGALG00010016421	ENSGALG00010016421	-3.693750248	0.719364553	-5.134740425	2.82534E-07	5.4743E-07	negative
PRJNA729271	stage_1W_vs_E10	ordinary_muscle_development	ENSGALG00010016422	KCTD3	-0.265546949	0.090819716	-2.923890983	0.003456858	0.004899497	negative
PRJNA729271	stage_1W_vs_E10	ordinary_muscle_development	ENSGALG00010016423	TRMT44	-0.444881987	0.109449861	-4.064710394	4.80921E-05	7.92064E-05	negative
PRJNA729271	stage_1W_vs_E10	ordinary_muscle_development	ENSGALG00010016425	TRIM63	6.252793522	0.413852203	15.1087598	1.41783E-51	1.6428E-50	positive
PRJNA729271	stage_1W_vs_E10	ordinary_muscle_development	ENSGALG00010016426	HELZ2	1.843249706	0.368068505	5.007898473	5.50275E-07	1.04593E-06	positive
PRJNA729271	stage_1W_vs_E10	ordinary_muscle_development	ENSGALG00010016428	ADRA1D	1.323780842	0.198362603	6.67354037	2.49705E-11	6.15033E-11	positive
PRJNA729271	stage_1W_vs_E10	ordinary_muscle_development	ENSGALG00010016429	ENSGALG00010016429	-1.339870192	0.444411188	-3.014933532	0.002570356	0.003687747	negative
PRJNA729271	stage_1W_vs_E10	ordinary_muscle_development	ENSGALG00010016431	B3GNT5	-4.314799285	0.163815568	-26.33937264	6.7934E-153	6.0784E-151	negative
PRJNA729271	stage_1W_vs_E10	ordinary_muscle_development	ENSGALG00010016433	TMED3	-0.854868975	0.147820556	-5.783153564	7.33131E-09	1.56736E-08	negative
PRJNA729271	stage_1W_vs_E10	ordinary_muscle_development	ENSGALG00010016434	GPR139	-2.817708556	0.65107212	-4.327797904	1.50608E-05	2.57497E-05	negative
PRJNA729271	stage_1W_vs_E10	ordinary_muscle_development	ENSGALG00010016436	SLC30A2	3.558073783	0.519686369	6.846579006	7.56369E-12	1.9147E-11	positive
PRJNA729271	stage_1W_vs_E10	ordinary_muscle_development	ENSGALG00010016441	ENSGALG00010016441	-1.993280377	0.699566541	-2.849307766	0.004381447	0.006145644	negative
PRJNA729271	stage_1W_vs_E10	ordinary_muscle_development	ENSGALG00010016445	ZDHHC21	-0.539945936	0.143718752	-3.756962312	0.000171988	0.000271921	negative
PRJNA729271	stage_1W_vs_E10	ordinary_muscle_development	ENSGALG00010016447	ID3	-1.118150916	0.113106016	-9.885865956	4.79419E-23	2.03014E-22	negative
PRJNA729271	stage_1W_vs_E10	ordinary_muscle_development	ENSGALG00010016449	FMR1	0.173988224	0.106842753	1.628451345	0.103429218	0.124147608	positive
PRJNA729271	stage_1W_vs_E10	ordinary_muscle_development	ENSGALG00010016450	ENSGALG00010016450	-0.053788137	0.2641798	-0.203604274	0.838662753	0.856387221	negative
PRJNA729271	stage_1W_vs_E10	ordinary_muscle_development	ENSGALG00010016451	USP46	0.184909188	0.147740468	1.251581172	0.210722519	0.241685624	positive
PRJNA729271	stage_1W_vs_E10	ordinary_muscle_development	ENSGALG00010016452	GLCE	-0.815642756	0.11553853	-7.059487026	1.67118E-12	4.36937E-12	negative
PRJNA729271	stage_1W_vs_E10	ordinary_muscle_development	ENSGALG00010016453	MYCL	-1.835867716	0.184014255	-9.976769011	1.92638E-23	8.30542E-23	negative
PRJNA729271	stage_1W_vs_E10	ordinary_muscle_development	ENSGALG00010016454	ANKS3	-0.247606508	0.096061256	-2.577589732	0.009949205	0.013455283	negative
PRJNA729271	stage_1W_vs_E10	ordinary_muscle_development	ENSGALG00010016455	RBM27	-0.652285978	0.092129721	-7.080081956	1.44069E-12	3.78449E-12	negative
PRJNA729271	stage_1W_vs_E10	ordinary_muscle_development	ENSGALG00010016456	LAMP3	-0.799786299	0.36508983	-2.190656198	0.028476681	0.036573586	negative
PRJNA729271	stage_1W_vs_E10	ordinary_muscle_development	ENSGALG00010016457	SMOX	0.809801383	0.178075173	4.547525453	5.42804E-06	9.59271E-06	positive
PRJNA729271	stage_1W_vs_E10	ordinary_muscle_development	ENSGALG00010016458	ZC3H12A	1.423273423	0.088627408	16.05906639	4.9393E-58	6.86434E-57	positive
PRJNA729271	stage_1W_vs_E10	ordinary_muscle_development	ENSGALG00010016459	PAQR5	2.994427911	0.272774174	10.97768117	4.89323E-28	2.55226E-27	positive
PRJNA729271	stage_1W_vs_E10	ordinary_muscle_development	ENSGALG00010016460	LBR	-1.283198856	0.069609358	-18.43428667	6.97324E-76	1.49966E-74	negative
PRJNA729271	stage_1W_vs_E10	ordinary_muscle_development	ENSGALG00010016461	HPCAL4	-3.033870344	0.34525227	-8.787401582	1.53059E-18	5.34986E-18	negative
PRJNA729271	stage_1W_vs_E10	ordinary_muscle_development	ENSGALG00010016462	RNF24	-1.323432703	0.160799472	-8.230329892	1.86699E-16	5.92295E-16	negative
PRJNA729271	stage_1W_vs_E10	ordinary_muscle_development	ENSGALG00010016463	FREM1	-1.659760404	0.143909529	-11.53336001	8.9578E-31	5.22298E-30	negative
PRJNA729271	stage_1W_vs_E10	ordinary_muscle_development	ENSGALG00010016464	PANK2	-0.379215799	0.089694485	-4.22786082	2.35924E-05	3.9741E-05	negative
PRJNA729271	stage_1W_vs_E10	ordinary_muscle_development	ENSGALG00010016465	STK35	-1.891820535	0.229598071	-8.23970572	1.72635E-16	5.4859E-16	negative
PRJNA729271	stage_1W_vs_E10	ordinary_muscle_development	ENSGALG00010016466	TMEM164	0.153308652	0.2338521	0.655579539	0.512094694	0.549512333	positive
PRJNA729271	stage_1W_vs_E10	ordinary_muscle_development	ENSGALG00010016468	TTC39B	-0.325250885	0.167564386	-1.941050197	0.052252189	0.06511869	negative
PRJNA729271	stage_1W_vs_E10	ordinary_muscle_development	ENSGALG00010016469	MAVS	0.317994663	0.103361152	3.076539444	0.002094186	0.003028557	positive
PRJNA729271	stage_1W_vs_E10	ordinary_muscle_development	ENSGALG00010016470	TNIP1	1.466935254	0.158176107	9.274063438	1.79186E-20	6.87846E-20	positive
PRJNA729271	stage_1W_vs_E10	ordinary_muscle_development	ENSGALG00010016471	POMGNT2	0.880373516	0.135757807	6.484883154	8.88006E-11	2.11461E-10	positive
PRJNA729271	stage_1W_vs_E10	ordinary_muscle_development	ENSGALG00010016472	TFDP1	-1.563232759	0.122775857	-12.73241166	3.90586E-37	2.8379E-36	negative
PRJNA729271	stage_1W_vs_E10	ordinary_muscle_development	ENSGALG00010016473	RAB9B	-0.736854551	0.161077454	-4.574535607	4.77277E-06	8.47499E-06	negative
PRJNA729271	stage_1W_vs_E10	ordinary_muscle_development	ENSGALG00010016474	MAPK8IP3	-0.735969503	0.111844634	-6.580284438	4.69549E-11	1.13929E-10	negative
PRJNA729271	stage_1W_vs_E10	ordinary_muscle_development	ENSGALG00010016475	MCCC1	0.761106991	0.083799114	9.082518402	1.06091E-19	3.93801E-19	positive
PRJNA729271	stage_1W_vs_E10	ordinary_muscle_development	ENSGALG00010016477	CNIH3	0.600990079	0.19092959	3.147705279	0.001645575	0.002404497	positive
PRJNA729271	stage_1W_vs_E10	ordinary_muscle_development	ENSGALG00010016479	ENSGALG00010016479	-2.217306759	0.414554111	-5.348654619	8.86105E-08	1.77896E-07	negative
PRJNA729271	stage_1W_vs_E10	ordinary_muscle_development	ENSGALG00010016480	GPX3	4.303652149	0.13324181	32.29956234	7.0945E-229	1.4283E-226	positive
PRJNA729271	stage_1W_vs_E10	ordinary_muscle_development	ENSGALG00010016482	EML1	1.169896451	0.140366065	8.334610323	7.77538E-17	2.50677E-16	positive
PRJNA729271	stage_1W_vs_E10	ordinary_muscle_development	ENSGALG00010016483	WDR26	0.481447026	0.10273063	4.686499322	2.77918E-06	5.02547E-06	positive
PRJNA729271	stage_1W_vs_E10	ordinary_muscle_development	ENSGALG00010016489	CCK	-7.915498773	0.951718599	-8.317057981	9.01736E-17	2.89944E-16	negative
PRJNA729271	stage_1W_vs_E10	ordinary_muscle_development	ENSGALG00010016491	DNAJC8	-0.238401052	0.05407003	-4.409116326	1.03793E-05	1.79941E-05	negative
PRJNA729271	stage_1W_vs_E10	ordinary_muscle_development	ENSGALG00010016496	PLP1	3.216388005	0.329017541	9.775734132	1.43116E-22	5.94744E-22	positive
PRJNA729271	stage_1W_vs_E10	ordinary_muscle_development	ENSGALG00010016498	KIF23	-2.464894827	0.208172171	-11.84065487	2.40567E-32	1.48637E-31	negative
PRJNA729271	stage_1W_vs_E10	ordinary_muscle_development	ENSGALG00010016500	ENSGALG00010016500	0.976164155	0.106735142	9.145667828	5.92626E-20	2.22081E-19	positive
PRJNA729271	stage_1W_vs_E10	ordinary_muscle_development	ENSGALG00010016502	YIPF5	-1.770174404	0.13417884	-13.19264953	9.6725E-40	7.68235E-39	negative
PRJNA729271	stage_1W_vs_E10	ordinary_muscle_development	ENSGALG00010016504	SNRK	0.787455158	0.084851084	9.280437262	1.68784E-20	6.48775E-20	positive
PRJNA729271	stage_1W_vs_E10	ordinary_muscle_development	ENSGALG00010016505	KCTD16	-3.140289246	0.399671132	-7.857183056	3.92868E-15	1.17585E-14	negative
PRJNA729271	stage_1W_vs_E10	ordinary_muscle_development	ENSGALG00010016507	SPATA18	0.072936564	0.141661756	0.51486418	0.606647933	0.641009024	positive
PRJNA729271	stage_1W_vs_E10	ordinary_muscle_development	ENSGALG00010016508	CREBBP	0.188393961	0.11979164	1.572680381	0.115792845	0.138027737	positive
PRJNA729271	stage_1W_vs_E10	ordinary_muscle_development	ENSGALG00010016510	THG1L	-0.554733204	0.112759076	-4.919632407	8.67069E-07	1.62948E-06	negative
PRJNA729271	stage_1W_vs_E10	ordinary_muscle_development	ENSGALG00010016514	GASK1A	-2.529932429	0.257253647	-9.834388961	8.00521E-23	3.3614E-22	negative
PRJNA729271	stage_1W_vs_E10	ordinary_muscle_development	ENSGALG00010016518	TMEM42	0.354547256	0.141625955	2.503405931	0.012300437	0.016455454	positive
PRJNA729271	stage_1W_vs_E10	ordinary_muscle_development	ENSGALG00010016519	MEX3B	-3.19234833	0.181199634	-17.61785199	1.797E-69	3.35231E-68	negative
PRJNA729271	stage_1W_vs_E10	ordinary_muscle_development	ENSGALG00010016520	CHMP1B	0.138220284	0.083147323	1.662353985	0.096441819	0.116222495	positive
PRJNA729271	stage_1W_vs_E10	ordinary_muscle_development	ENSGALG00010016526	EIF1B	1.759411014	0.113181429	15.54505039	1.71887E-54	2.1971E-53	positive
PRJNA729271	stage_1W_vs_E10	ordinary_muscle_development	ENSGALG00010016527	MRPS34	0.166300924	0.097169872	1.711445325	0.086998936	0.105447745	positive
PRJNA729271	stage_1W_vs_E10	ordinary_muscle_development	ENSGALG00010016529	DCUN1D1	0.013885144	0.097661318	0.142176498	0.886940595	0.899664376	positive
PRJNA729271	stage_1W_vs_E10	ordinary_muscle_development	ENSGALG00010016533	CNIH4	0.359317523	0.110119893	3.262966526	0.001102525	0.001634729	positive
PRJNA729271	stage_1W_vs_E10	ordinary_muscle_development	ENSGALG00010016534	TRIT1	-0.143591365	0.145155992	-0.989221069	0.322554992	0.358958512	negative
PRJNA729271	stage_1W_vs_E10	ordinary_muscle_development	ENSGALG00010016535	BNC2	-2.131211929	0.135187742	-15.76483122	5.43172E-56	7.18365E-55	negative
PRJNA729271	stage_1W_vs_E10	ordinary_muscle_development	ENSGALG00010016541	UBE2J2	-0.110747409	0.079367642	-1.395372299	0.1629036	0.190150402	negative
PRJNA729271	stage_1W_vs_E10	ordinary_muscle_development	ENSGALG00010016542	ENOX2	0.026660602	0.076861981	0.346863321	0.728694004	0.755321766	positive
PRJNA729271	stage_1W_vs_E10	ordinary_muscle_development	ENSGALG00010016543	TCEA3	6.035779471	0.173758627	34.73657446	2.2106E-264	6.0457E-262	positive
PRJNA729271	stage_1W_vs_E10	ordinary_muscle_development	ENSGALG00010016544	ADCY9	1.506508066	0.135510301	11.11729553	1.03358E-28	5.52016E-28	positive
PRJNA729271	stage_1W_vs_E10	ordinary_muscle_development	ENSGALG00010016546	C1QTNF12	-1.22356591	0.420123743	-2.9123941	0.003586698	0.005073601	negative
PRJNA729271	stage_1W_vs_E10	ordinary_muscle_development	ENSGALG00010016547	SGCB	2.334880096	0.143209014	16.30400229	9.2436E-60	1.3412E-58	positive
PRJNA729271	stage_1W_vs_E10	ordinary_muscle_development	ENSGALG00010016548	EVL	-0.243004765	0.109361325	-2.222035667	0.026280895	0.033891599	negative
PRJNA729271	stage_1W_vs_E10	ordinary_muscle_development	ENSGALG00010016549	PTPRA	-0.961547148	0.103922787	-9.252515034	2.19271E-20	8.37286E-20	negative
PRJNA729271	stage_1W_vs_E10	ordinary_muscle_development	ENSGALG00010016550	FBLIM1	-1.343173783	0.136539988	-9.837219118	7.78324E-23	3.27104E-22	negative
PRJNA729271	stage_1W_vs_E10	ordinary_muscle_development	ENSGALG00010016552	ENSGALG00010016552	3.52272864	0.442219106	7.966025426	1.63859E-15	4.99189E-15	positive
PRJNA729271	stage_1W_vs_E10	ordinary_muscle_development	ENSGALG00010016553	SLC25A14	0.03662965	0.095305436	0.384339566	0.700726786	0.728991227	positive
PRJNA729271	stage_1W_vs_E10	ordinary_muscle_development	ENSGALG00010016556	ASAP3	-1.091497992	0.197339436	-5.531068783	3.18285E-08	6.55147E-08	negative
PRJNA729271	stage_1W_vs_E10	ordinary_muscle_development	ENSGALG00010016558	NR2F2	-1.241223791	0.110349031	-11.24816208	2.36468E-29	1.30129E-28	negative
PRJNA729271	stage_1W_vs_E10	ordinary_muscle_development	ENSGALG00010016559	ROGDI	-0.366621337	0.11534867	-3.178375082	0.00148103	0.002171728	negative
PRJNA729271	stage_1W_vs_E10	ordinary_muscle_development	ENSGALG00010016560	DCUN1D4	1.46576609	0.169150415	8.665459629	4.49695E-18	1.53192E-17	positive
PRJNA729271	stage_1W_vs_E10	ordinary_muscle_development	ENSGALG00010016561	DEGS2	1.88304991	0.575050648	3.274580974	0.001058188	0.001571722	positive
PRJNA729271	stage_1W_vs_E10	ordinary_muscle_development	ENSGALG00010016562	GLIS2	0.821919545	0.220822349	3.722084954	0.000197585	0.000311033	positive
PRJNA729271	stage_1W_vs_E10	ordinary_muscle_development	ENSGALG00010016564	SPEN	0.879403213	0.165941991	5.299461634	1.16145E-07	2.30998E-07	positive
PRJNA729271	stage_1W_vs_E10	ordinary_muscle_development	ENSGALG00010016565	NVL	-0.368596029	0.097646121	-3.774814852	0.000160127	0.000253693	negative

PRJNA729271	stage_1W_vs_E10	ordinary_muscle_development	ENSGALG00010016566	CNTLN	-2.523041429	0.218844994	-11.52889717	9.43445E-31	5.49206E-30	negative
PRJNA729271	stage_1W_vs_E10	ordinary_muscle_development	ENSGALG00010016567	E2F2	-1.541436476	0.274959753	-5.606044002	2.07003E-08	4.31276E-08	negative
PRJNA729271	stage_1W_vs_E10	ordinary_muscle_development	ENSGALG00010016568	PAM16	2.03395302	0.133446884	15.24166738	1.87036E-52	2.24242E-51	positive
PRJNA729271	stage_1W_vs_E10	ordinary_muscle_development	ENSGALG00010016569	YY1	0.593099394	0.086650899	6.844699839	7.66364E-12	1.93831E-11	positive
PRJNA729271	stage_1W_vs_E10	ordinary_muscle_development	ENSGALG00010016572	RAB33A	0.481788054	0.113911285	4.229502391	2.34209E-05	3.94659E-05	positive
PRJNA729271	stage_1W_vs_E10	ordinary_muscle_development	ENSGALG00010016573	DEGS1	-0.532118479	0.128930749	-4.127165037	3.67263E-05	6.11051E-05	negative
PRJNA729271	stage_1W_vs_E10	ordinary_muscle_development	ENSGALG00010016575	CWH43	-0.226243417	0.395588559	-0.571915977	0.5673789	0.603076714	negative
PRJNA729271	stage_1W_vs_E10	ordinary_muscle_development	ENSGALG00010016576	MRPS26	0.58911326	0.106280478	5.543005359	2.97324E-08	6.12871E-08	positive
PRJNA729271	stage_1W_vs_E10	ordinary_muscle_development	ENSGALG00010016577	ZBTB17	1.687131083	0.184797708	9.129610421	6.87462E-20	2.57089E-19	positive
PRJNA729271	stage_1W_vs_E10	ordinary_muscle_development	ENSGALG00010016580	MARS2	-0.395476968	0.110781568	-3.569880582	0.000357144	0.000551251	negative
PRJNA729271	stage_1W_vs_E10	ordinary_muscle_development	ENSGALG00010016581	H2AFYMACROH2A1	0.429491754	0.076180559	5.637813084	1.72223E-08	3.6028E-08	positive
PRJNA729271	stage_1W_vs_E10	ordinary_muscle_development	ENSGALG00010016583	VPS41	0.047896372	0.08318366	0.575790629	0.564756708	0.600421629	positive
PRJNA729271	stage_1W_vs_E10	ordinary_muscle_development	ENSGALG00010016584	EHBP1	0.018683682	0.160754273	0.116225104	0.907474141	0.918564084	positive
PRJNA729271	stage_1W_vs_E10	ordinary_muscle_development	ENSGALG00010016588	ATP11B	-0.302134904	0.100444623	-3.007974879	0.002629949	0.003767652	negative
PRJNA729271	stage_1W_vs_E10	ordinary_muscle_development	ENSGALG00010016589	FBXO28	0.26082816	0.077334224	3.372739104	0.000744244	0.001121628	positive
PRJNA729271	stage_1W_vs_E10	ordinary_muscle_development	ENSGALG00010016591	HOMER2	-0.277155985	0.203589181	-1.361349281	0.173403336	0.201352348	negative
PRJNA729271	stage_1W_vs_E10	ordinary_muscle_development	ENSGALG00010016592	TMEM129	0.537909727	0.103063559	5.219203877	1.79694E-07	3.52792E-07	positive
PRJNA729271	stage_1W_vs_E10	ordinary_muscle_development	ENSGALG00010016597	FRYL	0.345671401	0.125519649	2.75392262	0.005888569	0.008158555	positive
PRJNA729271	stage_1W_vs_E10	ordinary_muscle_development	ENSGALG00010016598	AIFM1	0.316155822	0.088421832	3.575540282	0.000349505	0.000540151	positive
PRJNA729271	stage_1W_vs_E10	ordinary_muscle_development	ENSGALG00010016599	ENSGALG00010016599	0.454662312	0.275994016	1.647362937	0.099483469	0.119698919	positive
PRJNA729271	stage_1W_vs_E10	ordinary_muscle_development	ENSGALG00010016600	GNL2	-0.073048945	0.109487103	-0.667192239	0.504649348	0.542285736	negative
PRJNA729271	stage_1W_vs_E10	ordinary_muscle_development	ENSGALG00010016602	HSPB7	3.32697269	0.514768745	6.463043296	1.02618E-10	2.43684E-10	positive
PRJNA729271	stage_1W_vs_E10	ordinary_muscle_development	ENSGALG00010016603	ENSGALG00010016603	0.163185656	0.126658783	1.288388004	0.197610932	0.227604305	positive
PRJNA729271	stage_1W_vs_E10	ordinary_muscle_development	ENSGALG00010016604	TP53BP2	-1.339623093	0.063981529	-20.93765365	2.43142E-97	8.63809E-96	negative
PRJNA729271	stage_1W_vs_E10	ordinary_muscle_development	ENSGALG00010016605	WDPCP	-0.514156196	0.168387678	-3.053407478	0.002262585	0.003260705	negative
PRJNA729271	stage_1W_vs_E10	ordinary_muscle_development	ENSGALG00010016606	VASN	-1.065139095	0.181049422	-5.883139986	4.02555E-09	8.74687E-09	negative
PRJNA729271	stage_1W_vs_E10	ordinary_muscle_development	ENSGALG00010016607	FAM103A1	-0.280111481	0.114693306	-2.442265304	0.014595416	0.019363038	negative
PRJNA729271	stage_1W_vs_E10	ordinary_muscle_development	ENSGALG00010016609	SOX2	-1.530090672	0.55303815	-2.766700038	0.005662683	0.00786062	negative
PRJNA729271	stage_1W_vs_E10	ordinary_muscle_development	ENSGALG00010016611	DNALI1	-0.453431917	0.215537121	-2.103730044	0.035402002	0.044993601	negative
PRJNA729271	stage_1W_vs_E10	ordinary_muscle_development	ENSGALG00010016612	GRIA1	-1.315384467	0.339152021	-3.878450915	0.000105124	0.000168708	negative
PRJNA729271	stage_1W_vs_E10	ordinary_muscle_development	ENSGALG00010016613	SNIP1	-0.255108209	0.076836384	-3.320148559	0.000899696	0.00134722	negative
PRJNA729271	stage_1W_vs_E10	ordinary_muscle_development	ENSGALG00010016614	DNAJC19	1.046675788	0.139008779	7.529566106	5.09092E-14	1.43986E-13	positive
PRJNA729271	stage_1W_vs_E10	ordinary_muscle_development	ENSGALG00010016615	BCORL1	1.112233726	0.125044955	8.894670911	5.85927E-19	2.10171E-18	positive
PRJNA729271	stage_1W_vs_E10	ordinary_muscle_development	ENSGALG00010016616	SLBP	-3.329088901	0.248327733	-13.40602944	5.57444E-41	4.60848E-40	negative
PRJNA729271	stage_1W_vs_E10	ordinary_muscle_development	ENSGALG00010016618	TRPM3	-2.828380354	0.550505235	-5.137790111	2.77988E-07	5.38983E-07	negative
PRJNA729271	stage_1W_vs_E10	ordinary_muscle_development	ENSGALG00010016619	SLC25A47	-0.104286094	0.34128134	-0.30557221	0.759930382	0.783999351	negative
PRJNA729271	stage_1W_vs_E10	ordinary_muscle_development	ENSGALG00010016620	ENSGALG00010016620	0.041084025	0.122301616	0.335923816	0.736928317	0.762820535	positive
PRJNA729271	stage_1W_vs_E10	ordinary_muscle_development	ENSGALG00010016622	KIAA1143	-0.724083958	0.1348458	-5.369718271	7.88597E-08	1.58848E-07	negative
PRJNA729271	stage_1W_vs_E10	ordinary_muscle_development	ENSGALG00010016623	DNAJA3	1.733252367	0.141012247	12.29150236	1.00618E-34	6.77096E-34	positive
PRJNA729271	stage_1W_vs_E10	ordinary_muscle_development	ENSGALG00010016624	MDH1	0.99353772	0.154697863	6.422439866	1.34107E-10	3.16336E-10	positive
PRJNA729271	stage_1W_vs_E10	ordinary_muscle_development	ENSGALG00010016626	CAPN2	-0.70539405	0.231527665	-3.046694442	0.002313728	0.003330766	negative
PRJNA729271	stage_1W_vs_E10	ordinary_muscle_development	ENSGALG00010016629	LARS1	-0.349337836	0.071412025	-4.891862916	9.9886E-07	1.86867E-06	negative
PRJNA729271	stage_1W_vs_E10	ordinary_muscle_development	ENSGALG00010016630	ENSGALG00010016630	1.915689821	0.336460487	5.693654656	1.24348E-08	2.62172E-08	positive
PRJNA729271	stage_1W_vs_E10	ordinary_muscle_development	ENSGALG00010016631	FAM53A	1.56818726	0.091303766	17.17549372	4.05191E-66	6.84527E-65	positive
PRJNA729271	stage_1W_vs_E10	ordinary_muscle_development	ENSGALG00010016637	POU6F2	-2.425745902	0.733190123	-3.308481423	0.000938034	0.001402311	negative
PRJNA729271	stage_1W_vs_E10	ordinary_muscle_development	ENSGALG00010016640	OCIAD1	1.281436388	0.103548142	12.37527169	3.55689E-35	2.43194E-34	positive
PRJNA729271	stage_1W_vs_E10	ordinary_muscle_development	ENSGALG00010016643	BTBD1	1.502151646	0.173196086	8.673126967	4.20413E-18	1.4369E-17	positive
PRJNA729271	stage_1W_vs_E10	ordinary_muscle_development	ENSGALG00010016644	NMRAL1	-2.794656089	0.214800502	-13.01047283	1.0668E-38	8.16003E-38	negative
PRJNA729271	stage_1W_vs_E10	ordinary_muscle_development	ENSGALG00010016647	EGR1	1.823201428	0.204578453	8.91199148	5.0124E-19	1.80195E-18	positive
PRJNA729271	stage_1W_vs_E10	ordinary_muscle_development	ENSGALG00010016648	ADRA2C	-2.016572291	0.306138062	-6.587133525	4.48399E-11	1.08943E-10	negative
PRJNA729271	stage_1W_vs_E10	ordinary_muscle_development	ENSGALG00010016651	ENSGALG00010016651	3.169724033	0.179044195	17.70358453	3.93448E-70	7.47449E-69	positive
PRJNA729271	stage_1W_vs_E10	ordinary_muscle_development	ENSGALG00010016652	FXR1	2.576099923	0.098831363	26.06561169	8.9516E-150	7.4571E-148	positive
PRJNA729271	stage_1W_vs_E10	ordinary_muscle_development	ENSGALG00010016653	EPHA2	0.072026934	0.207459514	0.347185494	0.728451968	0.755124886	positive
PRJNA729271	stage_1W_vs_E10	ordinary_muscle_development	ENSGALG00010016654	SMIM3	1.266195942	0.182548149	6.936229971	4.02702E-12	1.03294E-11	positive
PRJNA729271	stage_1W_vs_E10	ordinary_muscle_development	ENSGALG00010016655	TRANK1	0.146341163	0.323859151	0.45186669	0.651365022	0.682734543	positive
PRJNA729271	stage_1W_vs_E10	ordinary_muscle_development	ENSGALG00010016656	CAPN8	-4.000061051	1.085514682	-3.684944219	0.000228753	0.000358617	negative
PRJNA729271	stage_1W_vs_E10	ordinary_muscle_development	ENSGALG00010016657	SLC25A29	1.504630592	0.279444327	5.384366218	7.27004E-08	1.46747E-07	positive
PRJNA729271	stage_1W_vs_E10	ordinary_muscle_development	ENSGALG00010016658	ZAR1	-3.085794671	0.677298011	-4.556036814	5.21278E-06	9.22354E-06	negative
PRJNA729271	stage_1W_vs_E10	ordinary_muscle_development	ENSGALG00010016661	UBE4B	-0.424807298	0.128597314	-3.303391687	0.000955229	0.00142625	negative
PRJNA729271	stage_1W_vs_E10	ordinary_muscle_development	ENSGALG00010016664	TM6SF1	3.545912061	0.248264383	14.28280618	2.80092E-46	2.72478E-45	positive
PRJNA729271	stage_1W_vs_E10	ordinary_muscle_development	ENSGALG00010016665	ENSGALG00010016665	5.692567129	1.004018363	5.669783879	1.42978E-08	3.004E-08	positive
PRJNA729271	stage_1W_vs_E10	ordinary_muscle_development	ENSGALG00010016667	WARS1	-0.083022932	0.14332914	-0.579246703	0.562422722	0.598203504	negative
PRJNA729271	stage_1W_vs_E10	ordinary_muscle_development	ENSGALG00010016671	CASS4	-0.908530753	0.249815821	-3.636802298	0.000276044	0.000430427	negative
PRJNA729271	stage_1W_vs_E10	ordinary_muscle_development	ENSGALG00010016672	DCTN4	0.474349241	0.080352772	5.903333906	3.56228E-09	7.76354E-09	positive
PRJNA729271	stage_1W_vs_E10	ordinary_muscle_development	ENSGALG00010016673	LRPAP1	0.305099109	0.079417729	3.841700228	0.000122185	0.000195224	positive
PRJNA729271	stage_1W_vs_E10	ordinary_muscle_development	ENSGALG00010016674	SLC10A4	0.081978621	0.340113042	0.241033453	0.809529189	0.829735935	positive
PRJNA729271	stage_1W_vs_E10	ordinary_muscle_development	ENSGALG00010016675	SKIL	-0.272239886	0.139943732	-1.945352477	0.05173257	0.064506344	negative
PRJNA729271	stage_1W_vs_E10	ordinary_muscle_development	ENSGALG00010016676	NLRC3	2.046292963	0.430285899	4.755658889	1.978E-06	3.61507E-06	positive
PRJNA729271	stage_1W_vs_E10	ordinary_muscle_development	ENSGALG00010016677	MEAF6	0.792122929	0.087833642	9.018445725	1.90777E-19	6.9831E-19	positive
PRJNA729271	stage_1W_vs_E10	ordinary_muscle_development	ENSGALG00010016678	NOX1	3.048733419	0.314068075	9.707237567	2.80845E-22	1.15028E-21	positive
PRJNA729271	stage_1W_vs_E10	ordinary_muscle_development	ENSGALG00010016680	BPIFB3	-0.799032125	1.010522564	-0.79071181	0.429112183	0.466965547	negative
PRJNA729271	stage_1W_vs_E10	ordinary_muscle_development	ENSGALG00010016681	SUSD4	1.495248776	0.291135279	5.13592438	2.80761E-07	5.4414E-07	positive
PRJNA729271	stage_1W_vs_E10	ordinary_muscle_development	ENSGALG00010016682	GPR141	-1.00319979	0.484484489	-2.070654092	0.038391133	0.048600828	negative
PRJNA729271	stage_1W_vs_E10	ordinary_muscle_development	ENSGALG00010016683	RBM22	-0.467378399	0.074144791	-6.303590459	2.90828E-10	6.72659E-10	negative
PRJNA729271	stage_1W_vs_E10	ordinary_muscle_development	ENSGALG00010016691	ENSGALG00010016691	1.71553499	0.283985875	6.040916621	1.53241E-09	3.41675E-09	positive
PRJNA729271	stage_1W_vs_E10	ordinary_muscle_development	ENSGALG00010016692	PRKCI	-1.7620683	0.0756177	-23.30232615	4.1989E-120	2.3409E-118	negative
PRJNA729271	stage_1W_vs_E10	ordinary_muscle_development	ENSGALG00010016696	DOK7	0.824403795	0.129486513	6.36671556	1.93119E-10	4.51348E-10	positive
PRJNA729271	stage_1W_vs_E10	ordinary_muscle_development	ENSGALG00010016699	SYNPO	2.144954281	0.14480606	14.81260029	1.2145E-49	1.31374E-48	positive
PRJNA729271	stage_1W_vs_E10	ordinary_muscle_development	ENSGALG00010016700	CLUAP1	-0.93295475	0.128129906	-7.281319244	3.30571E-13	8.94462E-13	negative
PRJNA729271	stage_1W_vs_E10	ordinary_muscle_development	ENSGALG00010016702	WDR25	0.230125623	0.098031311	2.347470623	0.018901364	0.024749309	positive
PRJNA729271	stage_1W_vs_E10	ordinary_muscle_development	ENSGALG00010016704	CSTF2	-0.75110489	0.074046022	-10.14375748	3.53245E-24	1.56584E-23	negative
PRJNA729271	stage_1W_vs_E10	ordinary_muscle_development	ENSGALG00010016706	RTN4	2.561930327	0.12542669	20.42571909	9.87933E-93	3.1542E-91	positive
PRJNA729271	stage_1W_vs_E10	ordinary_muscle_development	ENSGALG00010016707	KIF1B	1.574283266	0.130431622	12.06979755	1.52509E-33	9.83371E-33	positive
PRJNA729271	stage_1W_vs_E10	ordinary_muscle_development	ENSGALG00010016712	ENSGALG00010016712	0.345361859	0.158727393	2.175817627	0.0295689	0.037919243	positive
PRJNA729271	stage_1W_vs_E10	ordinary_muscle_development	ENSGALG00010016720	INTS15	0.273922853	0.096992466	2.824166302	0.004740378	0.006631131	positive
PRJNA729271	stage_1W_vs_E10	ordinary_muscle_development	ENSGALG00010016724	PGD	-1.774180785	0.111568726	-15.90213364	6.12429E-57	8.28093E-56	negative
PRJNA729271	stage_1W_vs_E10	ordinary_muscle_development	ENSGALG00010016729	ELMO1	0.700910905	0.094767604	7.396102389	1.4024E-13	3.8646E-13	positive
PRJNA729271	stage_1W_vs_E10	ordinary_muscle_development	ENSGALG00010016731	C4orf48	-2.018485521	0.279800382	-7.214019879	5.43238E-13	1.45577E-12	negative
PRJNA729271	stage_1W_vs_E10	ordinary_muscle_development	ENSGALG00010016732	ARHGAP26	-0.339219524	0.078179516	-4.33898213	1.43144E-05	2.45402E-05	negative
PRJNA729271	stage_1W_vs_E10	ordinary_muscle_development	ENSGALG00010016733	PHC3	0.437850111	0.157871849	2.773452736	0.00554649	0.007707446	positive
PRJNA729271	stage_1W_vs_E10	ordinary_muscle_development	ENSGALG00010016735	ENSGALG00010016735	-1.541513485	0.357165356	-4.315965871	1.58907E-05	2.71238E-05	negative
PRJNA729271	stage_1W_vs_E10	ordinary_muscle_development	ENSGALG00010016736	CENPS	-3.27657203	0.274385787	-11.94147872	7.19329E-33	4.51958E-32	negative
PRJNA729271	stage_1W_vs_E10	ordinary_muscle_development	ENSGALG00010016738	NAA60	1.683662711	0.148858238	11.3105108	1.1641E-29	6.48237E-29	positive
PRJNA729271	stage_1W_vs_E10	ordinary_muscle_development	ENSGALG00010016741	NELFA	-0.319119868	0.089945606	-3.547920586	0.000388285	0.0005976	negative
PRJNA729271	stage_1W_vs_E10	ordinary_muscle_development	ENSGALG00010016742	TRMT12	-0.185541332	0.140363871	-1.321859617	0.186214902	0.215383419	negative
PRJNA729271	stage_1W_vs_E10	ordinary_muscle_development	ENSGALG00010016744	CRYBG2	-7.207893745	1.035850956	-6.95842747	3.44092E-12	8.86214E-12	negative
PRJNA729271	stage_1W_vs_E10	ordinary_muscle_development	ENSGALG00010016745	BEGAIN	0.596467992	0.367367553	1.623627313	0.104455373	0.12525485	positive
PRJNA729271	stage_1W_vs_E10	ordinary_muscle_development	ENSGALG00010016746	HDGFL3	-2.14111201	0.125613983	-17.04517248	3.79623E-65	6.30313E-64	negative
PRJNA729271	stage_1W_vs_E10	ordinary_muscle_development	ENSGALG00010016750	SYTL4	0.644099396	0.126406757	5.095450665	3.47913E-07	6.70522E-07	positive
PRJNA729271	stage_1W_vs_E10	ordinary_muscle_development	ENSGALG00010016751	DFFA	-1.459151675	0.094610215	-15.4227709	1.1506E-53	1.42913E-52	negative
PRJNA729271	stage_1W_vs_E10	ordinary_muscle_development	ENSGALG00010016752	ENSGALG00010016752	1.20465085	0.173151397	6.957211251	3.47074E-12	8.93736E-12	positive
PRJNA729271	stage_1W_vs_E10	ordinary_muscle_development	ENSGALG00010016754	ENSGALG00010016754	-0.080724453	0.185282891	-0.435682173	0.663067342	0.693496004	negative
PRJNA729271	stage_1W_vs_E10	ordinary_muscle_development	ENSGALG00010016756	NUDT16L1	0.199796818	0.106854724	1.869798649	0.061511784	0.075927217	positive
PRJNA729271	stage_1W_vs_E10	ordinary_muscle_development	ENSGALG00010016757	SRPX2	-1.155828825	0.281821018	-4.10128681	4.10859E-05	6.80384E-05	negative
PRJNA729271	stage_1W_vs_E10	ordinary_muscle_development	ENSGALG00010016758	NR3C1	0.18267751	0.068694968	2.659256057	0.007831342	0.010708991	positive
PRJNA729271	stage_1W_vs_E10	ordinary_muscle_development	ENSGALG00010016760	DLK1	1.045308359	0.260703642	4.009565622	6.08305E-05	9.95528E-05	positive
PRJNA729271	stage_1W_vs_E10	ordinary_muscle_development	ENSGALG00010016761	GPR160	3.819388397	0.341591938	11.18114326	5.0436E-29	2.72787E-28	positive
PRJNA729271	stage_1W_vs_E10	ordinary_muscle_development	ENSGALG00010016762	EML6	-0.382258862	0.128880642	-2.965991287	0.00301709	0.004299747	negative
PRJNA729271	stage_1W_vs_E10	ordinary_muscle_development	ENSGALG00010016763	UBALD1	2.161306796	0.171878897	12.57459081	2.91305E-36	2.06276E-35	positive
PRJNA729271	stage_1W_vs_E10	ordinary_muscle_development	ENSGALG00010016766	AOAH	0.622467037	0.340724	1.826895192	0.067715529	0.083174019	positive
PRJNA729271	stage_1W_vs_E10	ordinary_muscle_development	ENSGALG00010016767	SLAIN2	0.923491625	0.092300234	10.00530107	1.44452E-23	6.26144E-23	positive
PRJNA729271	stage_1W_vs_E10	ordinary_muscle_development	ENSGALG00010016768	GALNT10	0.396083048	0.099490074	3.981131307	6.8588E-05	0.000111769	positive
PRJNA729271	stage_1W_vs_E10	ordinary_muscle_development	ENSGALG00010016769	BNC1	-9.517189986	0.632047266	-15.05771879	3.07206E-51	3.50902E-50	negative
PRJNA729271	stage_1W_vs_E10	ordinary_muscle_development	ENSGALG00010016772	TSPAN6	-1.188339256	0.127024183	-9.355220588	8.34255E-21	3.24544E-20	negative
PRJNA729271	stage_1W_vs_E10	ordinary_muscle_development	ENSGALG00010016773	RPS27A	0.675377744	0.161262588	4.188062177	2.81346E-05	4.71568E-05	positive
PRJNA729271	stage_1W_vs_E10	ordinary_muscle_development	ENSGALG00010016774	PEX14	0.289128492	0.117388713	2.46300079	0.013777961	0.018359216	positive
PRJNA729271	stage_1W_vs_E10	ordinary_muscle_development	ENSGALG00010016777	TNMD	-1.169559867	0.487928023	-2.396992612	0.016530257	0.021792276	negative
PRJNA729271	stage_1W_vs_E10	ordinary_muscle_development	ENSGALG00010016778	MTIF2	0.358343961	0.107917994	3.320520961	0.000898496	0.001345702	positive
PRJNA729271	stage_1W_vs_E10	ordinary_muscle_development	ENSGALG00010016779	CTNNA2	-2.691022347	0.155241751	-17.33439827	2.58761E-67	4.55187E-66	negative
PRJNA729271	stage_1W_vs_E10	ordinary_muscle_development	ENSGALG00010016780	PRORSD1P	-1.703017928	0.142675747	-11.93628184	7.65702E-33	4.80886E-32	negative
PRJNA729271	stage_1W_vs_E10	ordinary_muscle_development	ENSGALG00010016781	CDIP1	1.917871974	0.143241967	13.38903688	7.00836E-41	5.77523E-40	positive
PRJNA729271	stage_1W_vs_E10	ordinary_muscle_development	ENSGALG00010016783	CLBA1	-0.308688321	0.307841022	-1.002752392	0.315980344	0.352101406	negative
PRJNA729271	stage_1W_vs_E10	ordinary_muscle_development	ENSGALG00010016785	PDGFRB	-0.002368245	0.194668081	-0.012165553	0.990293532	0.992004475	negative
PRJNA729271	stage_1W_vs_E10	ordinary_muscle_development	ENSGALG00010016786	DISP1	-0.864341249	0.112513942	-7.682081306	1.56524E-14	4.54128E-14	negative
PRJNA729271	stage_1W_vs_E10	ordinary_muscle_development	ENSGALG00010016787	PPP2R5C	-0.363208364	0.075857673	-4.78802408	1.68431E-06	3.09275E-06	negative
PRJNA729271	stage_1W_vs_E10	ordinary_muscle_development	ENSGALG00010016788	ANLN	-2.320667049	0.288969187	-8.030846042	9.68028E-16	2.98037E-15	negative
PRJNA729271	stage_1W_vs_E10	ordinary_muscle_development	ENSGALG00010016789	DISP3	1.675083004	0.111106176	15.07641674	2.31496E-51	2.6568E-50	positive
PRJNA729271	stage_1W_vs_E10	ordinary_muscle_development	ENSGALG00010016791	LRRTM1	-4.453715289	0.59328826	-7.506831984	6.05754E-14	1.70925E-13	negative
PRJNA729271	stage_1W_vs_E10	ordinary_muscle_development	ENSGALG00010016792	HMOX2	-1.130444879	0.124159459	-9.104782596	8.64394E-20	3.22009E-19	negative
PRJNA729271	stage_1W_vs_E10	ordinary_muscle_development	ENSGALG00010016793	SLC9A1	0.639319624	0.155427666	4.113293598	3.90054E-05	6.47336E-05	positive
PRJNA729271	stage_1W_vs_E10	ordinary_muscle_development	ENSGALG00010016795	GUCA2B	3.778716409	1.221991301	3.092261301	0.001986379	0.002878368	positive

PRJNA729271	stage_1W_vs_E10	ordinary_muscle_development	ENSGALG00010016796	BROX	0.236851962	0.086258242	2.745847325	0.006035484	0.008354264	positive
PRJNA729271	stage_1W_vs_E10	ordinary_muscle_development	ENSGALG00010016797	SEC62	-0.501009536	0.109710866	-4.566635517	4.95615E-06	8.78769E-06	negative
PRJNA729271	stage_1W_vs_E10	ordinary_muscle_development	ENSGALG00010016799	WDTC1	1.568417721	0.131957253	11.88580155	1.40283E-32	8.73078E-32	positive
PRJNA729271	stage_1W_vs_E10	ordinary_muscle_development	ENSGALG00010016800	RPL14	-0.265844302	0.169087515	-1.572229046	0.115897442	0.1381297	negative
PRJNA729271	stage_1W_vs_E10	ordinary_muscle_development	ENSGALG00010016802	TMEM222	-0.3627796	0.085664426	-4.234892069	2.28662E-05	3.8585E-05	negative
PRJNA729271	stage_1W_vs_E10	ordinary_muscle_development	ENSGALG00010016804	CSF1R	1.979494721	0.136021558	14.55280145	5.60594E-48	5.72644E-47	positive
PRJNA729271	stage_1W_vs_E10	ordinary_muscle_development	ENSGALG00010016805	ENSGALG00010016805	-6.176078715	1.067002275	-5.788252622	7.11224E-09	1.52165E-08	negative
PRJNA729271	stage_1W_vs_E10	ordinary_muscle_development	ENSGALG00010016807	VSIG4	4.15054413	0.232349466	17.86336853	2.27487E-71	4.462E-70	positive
PRJNA729271	stage_1W_vs_E10	ordinary_muscle_development	ENSGALG00010016808	ENTPD3	-4.183719517	0.809475543	-5.168432267	2.36066E-07	4.60225E-07	negative
PRJNA729271	stage_1W_vs_E10	ordinary_muscle_development	ENSGALG00010016809	SYTL1	-0.684886385	0.508685629	-1.346384378	0.178178586	0.206582835	negative
PRJNA729271	stage_1W_vs_E10	ordinary_muscle_development	ENSGALG00010016810	SH3GL3	-2.123814229	0.20429808	-10.39566415	2.59473E-25	1.20702E-24	negative
PRJNA729271	stage_1W_vs_E10	ordinary_muscle_development	ENSGALG00010016812	LRRC31	-0.852056302	0.13193021	-6.458386591	1.05825E-10	2.51259E-10	negative
PRJNA729271	stage_1W_vs_E10	ordinary_muscle_development	ENSGALG00010016813	ENSGALG00010016813	-2.498216032	0.574437003	-4.34898173	1.36771E-05	2.3481E-05	negative
PRJNA729271	stage_1W_vs_E10	ordinary_muscle_development	ENSGALG00010016814	HMGXB3	0.656737325	0.099583084	6.5948683	4.25634E-11	1.03533E-10	positive
PRJNA729271	stage_1W_vs_E10	ordinary_muscle_development	ENSGALG00010016816	MAP3K6	3.296475939	0.260839766	12.63793475	1.30435E-36	9.32277E-36	positive
PRJNA729271	stage_1W_vs_E10	ordinary_muscle_development	ENSGALG00010016817	GGCT	-0.885806258	0.104052121	-8.513101446	1.69342E-17	5.62081E-17	negative
PRJNA729271	stage_1W_vs_E10	ordinary_muscle_development	ENSGALG00010016820	EIF2AK3	-0.851495843	0.110024792	-7.73912706	1.00102E-14	2.93719E-14	negative
PRJNA729271	stage_1W_vs_E10	ordinary_muscle_development	ENSGALG00010016823	HSF3	1.310124728	0.078710231	16.64491012	3.2946E-62	5.08576E-61	positive
PRJNA729271	stage_1W_vs_E10	ordinary_muscle_development	ENSGALG00010016825	ENSGALG00010016825	4.615472225	0.290302849	15.89881824	6.45713E-57	8.72285E-56	positive
PRJNA729271	stage_1W_vs_E10	ordinary_muscle_development	ENSGALG00010016828	LACTBL1	4.568302205	0.378101842	12.08220033	1.31162E-33	8.46481E-33	positive
PRJNA729271	stage_1W_vs_E10	ordinary_muscle_development	ENSGALG00010016831	AIDA	-0.738763129	0.067921861	-10.87666207	1.48917E-27	7.61392E-27	negative
PRJNA729271	stage_1W_vs_E10	ordinary_muscle_development	ENSGALG00010016832	DYNC1H1	-0.022032882	0.094186082	-0.233929275	0.815039886	0.834499056	negative
PRJNA729271	stage_1W_vs_E10	ordinary_muscle_development	ENSGALG00010016834	ENSGALG00010016834	1.727314913	0.074019234	23.33602787	1.9107E-120	1.0694E-118	positive
PRJNA729271	stage_1W_vs_E10	ordinary_muscle_development	ENSGALG00010016835	CCDC88A	-2.252417813	0.152995878	-14.72208165	4.65119E-49	4.91035E-48	negative
PRJNA729271	stage_1W_vs_E10	ordinary_muscle_development	ENSGALG00010016836	HEPH	2.024338497	0.148812301	13.60330086	3.82761E-42	3.2868E-41	positive
PRJNA729271	stage_1W_vs_E10	ordinary_muscle_development	ENSGALG00010016840	LRRC34	-0.961273265	0.221849232	-4.333002449	1.47089E-05	2.51808E-05	negative
PRJNA729271	stage_1W_vs_E10	ordinary_muscle_development	ENSGALG00010016842	NPY7R	0.526124411	0.433415587	1.213902838	0.224784829	0.256313412	positive
PRJNA729271	stage_1W_vs_E10	ordinary_muscle_development	ENSGALG00010016845	MIA3	1.539865089	0.137393643	11.20768804	3.73831E-29	2.0348E-28	positive
PRJNA729271	stage_1W_vs_E10	ordinary_muscle_development	ENSGALG00010016847	EPHB2	-1.923489204	0.107730284	-17.85467494	2.65824E-71	5.20691E-70	negative
PRJNA729271	stage_1W_vs_E10	ordinary_muscle_development	ENSGALG00010016848	ENSGALG00010016848	0.253784812	0.171095191	1.483295999	0.137995785	0.162727701	positive
PRJNA729271	stage_1W_vs_E10	ordinary_muscle_development	ENSGALG00010016849	WASF2	1.106392456	0.152645652	7.24810985	4.22628E-13	1.13845E-12	positive
PRJNA729271	stage_1W_vs_E10	ordinary_muscle_development	ENSGALG00010016851	TEC	1.634256712	0.138935353	11.76271325	6.07501E-32	3.70144E-31	positive
PRJNA729271	stage_1W_vs_E10	ordinary_muscle_development	ENSGALG00010016852	AHDC1	0.407142617	0.169226248	2.405907011	0.01613237	0.021287092	positive
PRJNA729271	stage_1W_vs_E10	ordinary_muscle_development	ENSGALG00010016854	TAF1A	0.050564904	0.104846322	0.482276372	0.62960963	0.662949086	positive
PRJNA729271	stage_1W_vs_E10	ordinary_muscle_development	ENSGALG00010016856	NOD1	-1.06437367	0.143463284	-7.419136361	1.17886E-13	3.26225E-13	negative
PRJNA729271	stage_1W_vs_E10	ordinary_muscle_development	ENSGALG00010016858	ENSGALG00010016858	-2.794127966	0.198527889	-14.07423399	5.46943E-45	5.10821E-44	negative
PRJNA729271	stage_1W_vs_E10	ordinary_muscle_development	ENSGALG00010016859	RPIA	0.376660296	0.130576048	2.884604821	0.003919053	0.005522717	positive
PRJNA729271	stage_1W_vs_E10	ordinary_muscle_development	ENSGALG00010016860	VSX1	-1.315977275	0.49809485	-2.642021446	0.008241284	0.01124305	negative
PRJNA729271	stage_1W_vs_E10	ordinary_muscle_development	ENSGALG00010016861	FGR	1.125816482	0.121567234	9.260854622	2.02802E-20	7.7583E-20	positive
PRJNA729271	stage_1W_vs_E10	ordinary_muscle_development	ENSGALG00010016862	TXK	4.659087642	0.516275854	9.024415151	1.80658E-19	6.63113E-19	positive
PRJNA729271	stage_1W_vs_E10	ordinary_muscle_development	ENSGALG00010016863	MYNN	-0.41157112	0.083478336	-4.930274606	8.21141E-07	1.54597E-06	negative
PRJNA729271	stage_1W_vs_E10	ordinary_muscle_development	ENSGALG00010016864	ENSGALG00010016864	0.177576596	0.269405788	0.659141727	0.509804763	0.547379262	positive
PRJNA729271	stage_1W_vs_E10	ordinary_muscle_development	ENSGALG00010016865	CFAP36	-1.444873329	0.139889625	-10.3286668	5.22822E-25	2.40581E-24	negative
PRJNA729271	stage_1W_vs_E10	ordinary_muscle_development	ENSGALG00010016866	ZNRF2	-0.212189923	0.108943068	-1.947713852	0.051449212	0.064178685	negative
PRJNA729271	stage_1W_vs_E10	ordinary_muscle_development	ENSGALG00010016867	FLT4	-0.261247101	0.12036215	-2.170508763	0.029968326	0.038407682	negative
PRJNA729271	stage_1W_vs_E10	ordinary_muscle_development	ENSGALG00010016868	NIPAL1	0.902559167	0.547446807	1.648670073	0.099215244	0.119396012	positive
PRJNA729271	stage_1W_vs_E10	ordinary_muscle_development	ENSGALG00010016869	HSP90AA1	-0.866116081	0.156422753	-5.537021074	3.0766E-08	6.33905E-08	negative
PRJNA729271	stage_1W_vs_E10	ordinary_muscle_development	ENSGALG00010016872	EDA2R	2.747582517	0.280072667	9.810248701	1.01718E-22	4.24653E-22	positive
PRJNA729271	stage_1W_vs_E10	ordinary_muscle_development	ENSGALG00010016873	ENTPD6	0.563540806	0.139356784	4.043870626	5.25759E-05	8.63752E-05	positive
PRJNA729271	stage_1W_vs_E10	ordinary_muscle_development	ENSGALG00010016874	ADAMTSL3	-0.881849671	0.145684214	-6.053158734	1.42033E-09	3.17172E-09	negative
PRJNA729271	stage_1W_vs_E10	ordinary_muscle_development	ENSGALG00010016875	SESN2	3.10864565	0.217667996	14.28159266	2.85012E-46	2.7708E-45	positive
PRJNA729271	stage_1W_vs_E10	ordinary_muscle_development	ENSGALG00010016876	MTURN	-1.452585193	0.196783735	-7.381632406	1.5636E-13	4.30147E-13	negative
PRJNA729271	stage_1W_vs_E10	ordinary_muscle_development	ENSGALG00010016877	UBE2D3	1.168432768	0.135444642	8.626644427	6.31782E-18	2.13915E-17	positive
PRJNA729271	stage_1W_vs_E10	ordinary_muscle_development	ENSGALG00010016878	SUCLG1	1.97208353	0.125294945	15.73952991	8.10435E-56	1.06793E-54	positive
PRJNA729271	stage_1W_vs_E10	ordinary_muscle_development	ENSGALG00010016879	RNF130	-0.214840153	0.101111072	-2.124793537	0.033603844	0.042805846	negative
PRJNA729271	stage_1W_vs_E10	ordinary_muscle_development	ENSGALG00010016880	AR	1.574685501	0.201448453	7.816816066	5.41762E-15	1.60985E-14	positive
PRJNA729271	stage_1W_vs_E10	ordinary_muscle_development	ENSGALG00010016881	C1QB	2.061996805	0.291191626	7.081236627	1.42874E-12	3.75377E-12	positive
PRJNA729271	stage_1W_vs_E10	ordinary_muscle_development	ENSGALG00010016882	ENSGALG00010016882	-0.837436514	0.050237692	-16.66948607	2.18465E-62	3.38679E-61	negative
PRJNA729271	stage_1W_vs_E10	ordinary_muscle_development	ENSGALG00010016883	TMEM251	-0.268069435	0.07867049	-3.407496683	0.000655617	0.000991566	negative
PRJNA729271	stage_1W_vs_E10	ordinary_muscle_development	ENSGALG00010016884	SBK1	-0.615940926	0.268335374	-2.295414566	0.021709377	0.028262747	negative
PRJNA729271	stage_1W_vs_E10	ordinary_muscle_development	ENSGALG00010016885	CDCA4	-0.86834691	0.153880101	-5.643009761	1.67103E-08	3.4977E-08	negative
PRJNA729271	stage_1W_vs_E10	ordinary_muscle_development	ENSGALG00010016887	TJP2	-0.023959102	0.139523388	-0.171721044	0.863656841	0.878690665	negative
PRJNA729271	stage_1W_vs_E10	ordinary_muscle_development	ENSGALG00010016888	ENSGALG00010016888	-0.426835465	0.087499486	-4.878148237	1.07086E-06	1.99874E-06	negative
PRJNA729271	stage_1W_vs_E10	ordinary_muscle_development	ENSGALG00010016889	FUNDC2	1.672921036	0.086908841	19.24914669	1.43507E-82	3.67515E-81	positive
PRJNA729271	stage_1W_vs_E10	ordinary_muscle_development	ENSGALG00010016890	UBR7	-0.617884293	0.075667346	-8.165798443	3.19316E-16	1.00119E-15	negative
PRJNA729271	stage_1W_vs_E10	ordinary_muscle_development	ENSGALG00010016891	ENSGALG00010016891	-1.800387319	1.166478217	-1.54343844			negative
PRJNA729271	stage_1W_vs_E10	ordinary_muscle_development	ENSGALG00010016892	MAL	0.348040031	0.456177393	0.762948879	0.445493884	0.48366668	positive
PRJNA729271	stage_1W_vs_E10	ordinary_muscle_development	ENSGALG00010016893	TBC1D9B	-0.189118684	0.05123138	-3.691461863	0.000222969	0.000349738	negative
PRJNA729271	stage_1W_vs_E10	ordinary_muscle_development	ENSGALG00010016894	BTBD7	0.509746617	0.091653621	5.561663724	2.67215E-08	5.52457E-08	positive
PRJNA729271	stage_1W_vs_E10	ordinary_muscle_development	ENSGALG00010016895	ENSGALG00010016895	1.0777957	0.155886755	6.913965843	4.71289E-12	1.20418E-11	positive
PRJNA729271	stage_1W_vs_E10	ordinary_muscle_development	ENSGALG00010016896	SPTBN1	-0.718787555	0.098859301	-7.270813665	3.57329E-13	9.656E-13	negative
PRJNA729271	stage_1W_vs_E10	ordinary_muscle_development	ENSGALG00010016898	ENSGALG00010016898	-2.273145182	0.822733361	-2.76291845	0.005728708	0.007947705	negative
PRJNA729271	stage_1W_vs_E10	ordinary_muscle_development	ENSGALG00010016899	SAXO2	-2.001256143	0.636286639	-3.145211639	0.001659667	0.002424355	negative
PRJNA729271	stage_1W_vs_E10	ordinary_muscle_development	ENSGALG00010016900	CISD2	-0.125942237	0.09019614	-1.396315151	0.162619613	0.189849492	negative
PRJNA729271	stage_1W_vs_E10	ordinary_muscle_development	ENSGALG00010016901	MECOM	-0.629631769	0.192814498	-3.265479384	0.00109279	0.001621952	negative
PRJNA729271	stage_1W_vs_E10	ordinary_muscle_development	ENSGALG00010016902	MRNIP	-1.262963678	0.145309233	-8.691558339	3.57501E-18	1.22621E-17	negative
PRJNA729271	stage_1W_vs_E10	ordinary_muscle_development	ENSGALG00010016903	PPM1A	1.483653548	0.147138083	10.08340951	6.5416E-24	2.87335E-23	positive
PRJNA729271	stage_1W_vs_E10	ordinary_muscle_development	ENSGALG00010016904	EAF1	0.845686286	0.069884569	12.10118765	1.04094E-33	6.74797E-33	positive
PRJNA729271	stage_1W_vs_E10	ordinary_muscle_development	ENSGALG00010016905	C1QC	3.382741221	0.371413721	9.107744348	8.41125E-20	3.13502E-19	positive
PRJNA729271	stage_1W_vs_E10	ordinary_muscle_development	ENSGALG00010016907	SQSTM1	4.04726701	0.159291997	25.40784904	2.0653E-142	1.5274E-140	positive
PRJNA729271	stage_1W_vs_E10	ordinary_muscle_development	ENSGALG00010016908	CMC4	1.945233315	0.158167873	12.29853622	9.22304E-35	6.21515E-34	positive
PRJNA729271	stage_1W_vs_E10	ordinary_muscle_development	ENSGALG00010016909	MRPS5	0.010681861	0.134003859	0.079713084	0.936465453	0.944217219	positive
PRJNA729271	stage_1W_vs_E10	ordinary_muscle_development	ENSGALG00010016910	NSD2	-1.09823663	0.111315042	-9.866021784	5.84364E-23	2.46949E-22	negative
PRJNA729271	stage_1W_vs_E10	ordinary_muscle_development	ENSGALG00010016911	AXIN1	-0.030439837	0.099088507	-0.307198465	0.758692314	0.783000719	negative
PRJNA729271	stage_1W_vs_E10	ordinary_muscle_development	ENSGALG00010016912	CCDC88C	0.795353445	0.15986184	4.97525517	6.51617E-07	1.2337E-06	positive
PRJNA729271	stage_1W_vs_E10	ordinary_muscle_development	ENSGALG00010016913	BRCC3	-0.049593185	0.111158954	-0.446146555	0.655491386	0.686506116	negative
PRJNA729271	stage_1W_vs_E10	ordinary_muscle_development	ENSGALG00010016915	PLOD2	-2.209537792	0.168516818	-13.11167525	2.82293E-39	2.21061E-38	negative
PRJNA729271	stage_1W_vs_E10	ordinary_muscle_development	ENSGALG00010016916	ACYP2	4.030051929	0.157288938	25.62196665	8.6841E-145	6.556E-143	positive
PRJNA729271	stage_1W_vs_E10	ordinary_muscle_development	ENSGALG00010016917	METTL6	1.796463134	0.169560086	10.59484681	3.14856E-26	1.52586E-25	positive
PRJNA729271	stage_1W_vs_E10	ordinary_muscle_development	ENSGALG00010016918	RCOR1	-0.619779649	0.075297235	-8.231107697	1.8549E-16	5.8859E-16	negative
PRJNA729271	stage_1W_vs_E10	ordinary_muscle_development	ENSGALG00010016919	ARHGDIG	0.48912176	0.198226022	2.467495216	0.013606204	0.018133682	positive
PRJNA729271	stage_1W_vs_E10	ordinary_muscle_development	ENSGALG00010016920	C1QA	3.124064796	0.318488401	9.809037914	1.02945E-22	4.29532E-22	positive
PRJNA729271	stage_1W_vs_E10	ordinary_muscle_development	ENSGALG00010016921	EFL1	0.134983392	0.074380007	1.814780594	0.069557634	0.085277671	positive
PRJNA729271	stage_1W_vs_E10	ordinary_muscle_development	ENSGALG00010016922	ENSGALG00010016922	-0.307247453	0.196186543	-1.566098508	0.117325557	0.139693934	negative
PRJNA729271	stage_1W_vs_E10	ordinary_muscle_development	ENSGALG00010016923	ENSGALG00010016923	-2.826410749	0.175263893	-16.12660028	1.65905E-58	2.33023E-57	negative
PRJNA729271	stage_1W_vs_E10	ordinary_muscle_development	ENSGALG00010016924	LETM1	0.322757184	0.117159217	2.754859516	0.005871734	0.008136786	positive
PRJNA729271	stage_1W_vs_E10	ordinary_muscle_development	ENSGALG00010016925	PHACTR4	0.015660334	0.134480538	0.116450559	0.907295468	0.918447364	positive
PRJNA729271	stage_1W_vs_E10	ordinary_muscle_development	ENSGALG00010016926	ENSGALG00010016926	0.048105646	0.184650709	0.260522402	0.794460838	0.816253887	positive
PRJNA729271	stage_1W_vs_E10	ordinary_muscle_development	ENSGALG00010016927	MGAT4B	1.532267487	0.114957825	13.32895331	1.57086E-40	1.28063E-39	positive
PRJNA729271	stage_1W_vs_E10	ordinary_muscle_development	ENSGALG00010016928	RGS11	3.904782134	0.37565388	10.39466762	2.6231E-25	1.21982E-24	positive
PRJNA729271	stage_1W_vs_E10	ordinary_muscle_development	ENSGALG00010016930	C1OH15ORF48	3.869831999	0.39233383	9.86362047	5.98515E-23	2.52708E-22	positive
PRJNA729271	stage_1W_vs_E10	ordinary_muscle_development	ENSGALG00010016931	SLC30A4	0.093673469	0.082594706	1.134134059	0.256738319	0.290191979	positive
PRJNA729271	stage_1W_vs_E10	ordinary_muscle_development	ENSGALG00010016932	OGT	0.765108711	0.092233403	8.295353776	1.08263E-16	3.46701E-16	positive
PRJNA729271	stage_1W_vs_E10	ordinary_muscle_development	ENSGALG00010016934	SLC25A38	-0.695182022	0.142425731	-4.881014234	1.05542E-06	1.97041E-06	negative
PRJNA729271	stage_1W_vs_E10	ordinary_muscle_development	ENSGALG00010016935	PRIMA1	-2.787425922	0.26736853	-10.42540767	1.89843E-25	8.89393E-25	negative
PRJNA729271	stage_1W_vs_E10	ordinary_muscle_development	ENSGALG00010016936	RCC1	-0.746558157	0.164176817	-4.547281227	5.43434E-06	9.6015E-06	negative
PRJNA729271	stage_1W_vs_E10	ordinary_muscle_development	ENSGALG00010016937	DHRS7	-0.408826846	0.172419792	-2.371113209	0.017734598	0.023295242	negative
PRJNA729271	stage_1W_vs_E10	ordinary_muscle_development	ENSGALG00010016938	PSME4	1.955121276	0.166156915	11.76671627	5.7936E-32	3.53444E-31	positive
PRJNA729271	stage_1W_vs_E10	ordinary_muscle_development	ENSGALG00010016939	FAM234A	0.22516896	0.079947037	2.816476619	0.004855358	0.006788041	positive
PRJNA729271	stage_1W_vs_E10	ordinary_muscle_development	ENSGALG00010016940	FGFR3	-5.542314298	0.229402072	-24.15982671	5.8866E-129	3.8092E-127	negative
PRJNA729271	stage_1W_vs_E10	ordinary_muscle_development	ENSGALG00010016941	SLC8A1	1.047299175	0.172537772	6.069970435	1.27934E-09	2.86482E-09	positive
PRJNA729271	stage_1W_vs_E10	ordinary_muscle_development	ENSGALG00010016942	ZBTB40	0.943459551	0.15141807	6.230825365	4.63984E-10	1.06163E-09	positive
PRJNA729271	stage_1W_vs_E10	ordinary_muscle_development	ENSGALG00010016943	PCNX4	-0.699696656	0.111044674	-6.30103753	2.9566E-10	6.83507E-10	negative
PRJNA729271	stage_1W_vs_E10	ordinary_muscle_development	ENSGALG00010016944	OPRD1	-6.412705824	0.903571754	-7.097063178	1.27436E-12	3.35973E-12	negative
PRJNA729271	stage_1W_vs_E10	ordinary_muscle_development	ENSGALG00010016945	BDH2	-0.117283741	0.146233279	-0.802031808	0.422534559	0.460464509	negative
PRJNA729271	stage_1W_vs_E10	ordinary_muscle_development	ENSGALG00010016946	TAF1	0.7004234	0.106337192	6.586814924	4.49362E-11	1.09159E-10	positive
PRJNA729271	stage_1W_vs_E10	ordinary_muscle_development	ENSGALG00010016947	CANX	-1.045399646	0.071949799	-14.52957016	7.87128E-48	8.00661E-47	negative
PRJNA729271	stage_1W_vs_E10	ordinary_muscle_development	ENSGALG00010016948	CCR8	0.280991807	0.595951202	0.471501368	0.637282743	0.669814615	positive
PRJNA729271	stage_1W_vs_E10	ordinary_muscle_development	ENSGALG00010016949	LUC7L	0.421504628	0.094043924	4.481997457	7.39476E-06	1.29406E-05	positive
PRJNA729271	stage_1W_vs_E10	ordinary_muscle_development	ENSGALG00010016950	ENSGALG00010016950	0.963559647	1.136615267	0.847744769	0.396580128	0.434729559	positive
PRJNA729271	stage_1W_vs_E10	ordinary_muscle_development	ENSGALG00010016952	GINS1	-3.015969603	0.230070202	-13.10891016	2.92775E-39	2.29022E-38	negative
PRJNA729271	stage_1W_vs_E10	ordinary_muscle_development	ENSGALG00010016953	GJA4	1.294408188	0.161724044	8.003807957	1.20629E-15	3.69745E-15	positive
PRJNA729271	stage_1W_vs_E10	ordinary_muscle_development	ENSGALG00010016955	ENSGALG00010016955	-7.277632149	0.325209275	-22.37830439	6.4034E-111	2.9372E-109	negative
PRJNA729271	stage_1W_vs_E10	ordinary_muscle_development	ENSGALG00010016956	CENPE	-2.347106408	0.256588978	-9.147339171	5.83531E-20	2.1873E-19	negative
PRJNA729271	stage_1W_vs_E10	ordinary_muscle_development	ENSGALG00010016957	WNT4	-1.719411113	0.2348973	-7.319841965	2.48263E-13	6.75916E-13	negative
PRJNA729271	stage_1W_vs_E10	ordinary_muscle_development	ENSGALG00010016958	TLR15	0.214682029	0.556754716	0.385595348	0.699796379	0.72818008	positive
PRJNA729271	stage_1W_vs_E10	ordinary_muscle_development	ENSGALG00010016959	PERCC1	-0.443696867	1.33182179	-0.333150328	0.739020811	0.764549757	negative
PRJNA729271	stage_1W_vs_E10	ordinary_muscle_development	ENSGALG00010016960	GJB3	-5.453146903	1.067158386	-5.109969594	3.22211E-07	6.22062E-07	negative
PRJNA729271	stage_1W_vs_E10	ordinary_muscle_development	ENSGALG00010016961	EIF4A3	-0.416738281	0.074672046	-5.580914214	2.39258E-08	4.96001E-08	negative
PRJNA729271	stage_1W_vs_E10	ordinary_muscle_development	ENSGALG00010016962	SQOR	2.318063374	0.136197067	17.01992142	5.8449E-65	9.64941E-64	positive
PRJNA729271	stage_1W_vs_E10	ordinary_muscle_development	ENSGALG00010016963	CD8A	3.447288212	0.676369517	5.096752773	3.45529E-07	6.66105E-07	positive

PRJNA729271	stage_1W_vs_E10	ordinary_muscle_development	ENSGALG00010016964	MAML1	0.170423722	0.085704716	1.988498766	0.046756555	0.058593675	positive
PRJNA729271	stage_1W_vs_E10	ordinary_muscle_development	ENSGALG00010016965	SLC25A33	0.640938062	0.338146946	1.89544241	0.058033815	0.071891303	positive
PRJNA729271	stage_1W_vs_E10	ordinary_muscle_development	ENSGALG00010016966	KDM3A	0.556209108	0.15818039	3.516296213	0.000437612	0.000670244	positive
PRJNA729271	stage_1W_vs_E10	ordinary_muscle_development	ENSGALG00010016967	ERLEC1	0.21759848	0.07499352	2.901563774	0.003713052	0.005244659	positive
PRJNA729271	stage_1W_vs_E10	ordinary_muscle_development	ENSGALG00010016968	C15orf61	1.121928833	0.146524773	7.656922498	1.90441E-14	5.49781E-14	positive
PRJNA729271	stage_1W_vs_E10	ordinary_muscle_development	ENSGALG00010016969	CHMP3	1.482591326	0.082682288	17.93118408	6.73304E-72	1.33876E-70	positive
PRJNA729271	stage_1W_vs_E10	ordinary_muscle_development	ENSGALG00010016970	SYNE3	0.920760871	0.099809969	9.225139325	2.83192E-20	1.0757E-19	positive
PRJNA729271	stage_1W_vs_E10	ordinary_muscle_development	ENSGALG00010016972	ASB3	-1.819999577	0.120631818	-15.0872266	1.96531E-51	2.26448E-50	negative
PRJNA729271	stage_1W_vs_E10	ordinary_muscle_development	ENSGALG00010016973	PLSCR1	2.748059635	0.120788837	22.75094045	1.4046E-114	6.855E-113	positive
PRJNA729271	stage_1W_vs_E10	ordinary_muscle_development	ENSGALG00010016974	CFAP96	-4.302873401	0.981532206	-4.383833127	1.16609E-05	2.01438E-05	negative
PRJNA729271	stage_1W_vs_E10	ordinary_muscle_development	ENSGALG00010016975	MALL	1.101693621	0.222289245	4.956126514	7.19124E-07	1.35814E-06	positive
PRJNA729271	stage_1W_vs_E10	ordinary_muscle_development	ENSGALG00010016976	ENSGALG00010016976	6.011148049	0.907710056	6.622321753	3.536E-11	8.6345E-11	positive
PRJNA729271	stage_1W_vs_E10	ordinary_muscle_development	ENSGALG00010016977	CLCN7	1.033558768	0.117377605	8.805417054	1.30367E-18	4.57215E-18	positive
PRJNA729271	stage_1W_vs_E10	ordinary_muscle_development	ENSGALG00010016978	FAM181A	4.927375656	0.349198498	14.11052935	3.27113E-45	3.07491E-44	positive
PRJNA729271	stage_1W_vs_E10	ordinary_muscle_development	ENSGALG00010016979	PLSCR5	-0.199371229	0.478742648	-0.416447603	0.677082501	0.706802035	negative
PRJNA729271	stage_1W_vs_E10	ordinary_muscle_development	ENSGALG00010016980	NPHP1	-0.439654061	0.102135471	-4.304616777	1.67275E-05	2.85119E-05	negative
PRJNA729271	stage_1W_vs_E10	ordinary_muscle_development	ENSGALG00010016981	ENSGALG00010016981	2.110075505	0.441492894	4.779409893	1.7581E-06	3.22579E-06	positive
PRJNA729271	stage_1W_vs_E10	ordinary_muscle_development	ENSGALG00010016982	GLRX5	-0.221686828	0.141252992	-1.56943103	0.116547538	0.138858834	negative
PRJNA729271	stage_1W_vs_E10	ordinary_muscle_development	ENSGALG00010016983	ZFAND2A	1.343601271	0.282886099	4.74961928	2.038E-06	3.72004E-06	positive
PRJNA729271	stage_1W_vs_E10	ordinary_muscle_development	ENSGALG00010016984	RNF103	1.505203593	0.114336723	13.16465573	1.40175E-39	1.10667E-38	positive
PRJNA729271	stage_1W_vs_E10	ordinary_muscle_development	ENSGALG00010016985	ENSGALG00010016985	1.883696319	0.1849768	10.1834193	2.3515E-24	1.05039E-23	positive
PRJNA729271	stage_1W_vs_E10	ordinary_muscle_development	ENSGALG00010016986	GFER	0.057124512	0.087652729	0.651714015	0.51458568	0.551858495	positive
PRJNA729271	stage_1W_vs_E10	ordinary_muscle_development	ENSGALG00010016987	ASB2	6.069754665	0.317833909	19.09725329	2.66124E-81	6.56032E-80	positive
PRJNA729271	stage_1W_vs_E10	ordinary_muscle_development	ENSGALG00010016988	ECI1	1.578248003	0.109240812	14.44742105	2.60249E-47	2.60339E-46	positive
PRJNA729271	stage_1W_vs_E10	ordinary_muscle_development	ENSGALG00010016989	RHOG2	-0.044562781	0.09326017	-0.477832935	0.632769118	0.665504888	negative
PRJNA729271	stage_1W_vs_E10	ordinary_muscle_development	ENSGALG00010016991	PDLIM3	4.626025015	0.199152531	23.2285525	2.3438E-119	1.282E-117	positive
PRJNA729271	stage_1W_vs_E10	ordinary_muscle_development	ENSGALG00010016993	ENSGALG00010016993	0.960493783	0.179922754	5.338367506	9.37872E-08	1.87924E-07	positive
PRJNA729271	stage_1W_vs_E10	ordinary_muscle_development	ENSGALG00010016994	ATL2	1.275517582	0.115515625	11.04194851	2.39776E-28	1.26568E-27	positive
PRJNA729271	stage_1W_vs_E10	ordinary_muscle_development	ENSGALG00010016998	DNASE1L2	0.974501093	0.344648225	2.827523895	0.004690951	0.006565792	positive
PRJNA729271	stage_1W_vs_E10	ordinary_muscle_development	ENSGALG00010016999	ITGB1BP2	3.507532815	0.172300619	20.35705289	4.02051E-92	1.26966E-90	positive
PRJNA729271	stage_1W_vs_E10	ordinary_muscle_development	ENSGALG00010017001	TPCN3	-1.228322229	0.122482961	-10.02851516	1.14221E-23	4.97189E-23	negative
PRJNA729271	stage_1W_vs_E10	ordinary_muscle_development	ENSGALG00010017003	CPZ	0.444924085	0.397958206	1.118017116	0.263559683	0.297332238	positive
PRJNA729271	stage_1W_vs_E10	ordinary_muscle_development	ENSGALG00010017004	CX3CR1	-2.01662	0.447894027	-4.502448972	6.71749E-06	1.17824E-05	negative
PRJNA729271	stage_1W_vs_E10	ordinary_muscle_development	ENSGALG00010017006	DNAH3	0.254497223	0.245661714	1.035966162	0.300217929	0.335569007	positive
PRJNA729271	stage_1W_vs_E10	ordinary_muscle_development	ENSGALG00010017008	RTN1	-0.722171234	0.168512252	-4.28557107	1.8227E-05	3.09913E-05	negative
PRJNA729271	stage_1W_vs_E10	ordinary_muscle_development	ENSGALG00010017010	NONO	-1.670049982	0.055649204	-30.01031225	7.1993E-198	1.0231E-195	negative
PRJNA729271	stage_1W_vs_E10	ordinary_muscle_development	ENSGALG00010017012	SYNGR3	0.846462276	0.182195135	4.645910413	3.3858E-06	6.08671E-06	positive
PRJNA729271	stage_1W_vs_E10	ordinary_muscle_development	ENSGALG00010017016	ZIC1	-6.404165506	0.950698726	-6.736272316	1.62502E-11	4.03678E-11	negative
PRJNA729271	stage_1W_vs_E10	ordinary_muscle_development	ENSGALG00010017017	ENSGALG00010017017	1.379547032	0.26366979	5.232101217	1.67594E-07	3.29617E-07	positive
PRJNA729271	stage_1W_vs_E10	ordinary_muscle_development	ENSGALG00010017018	HP1BP3	-1.139165559	0.083960529	-13.56787017	6.21004E-42	5.31373E-41	negative
PRJNA729271	stage_1W_vs_E10	ordinary_muscle_development	ENSGALG00010017020	GJB5	-3.212591788	0.510470209	-6.293397209	3.10592E-10	7.17456E-10	negative
PRJNA729271	stage_1W_vs_E10	ordinary_muscle_development	ENSGALG00010017022	CCR4	0.633935899	0.597107322	1.061678321	0.288381744	0.323261417	positive
PRJNA729271	stage_1W_vs_E10	ordinary_muscle_development	ENSGALG00010017024	BUB1	-3.101513554	0.219639134	-14.12095145	2.82156E-45	2.65747E-44	negative
PRJNA729271	stage_1W_vs_E10	ordinary_muscle_development	ENSGALG00010017033	LDAF1	0.528438878	0.111199931	4.752151121	2.01264E-06	3.67606E-06	positive
PRJNA729271	stage_1W_vs_E10	ordinary_muscle_development	ENSGALG00010017034	ZMYM3	0.602036941	0.088952345	6.768084015	1.30499E-11	3.25742E-11	positive
PRJNA729271	stage_1W_vs_E10	ordinary_muscle_development	ENSGALG00010017035	PIK3CD	0.451744339	0.205163645	2.201873241	0.027674264	0.035593474	positive
PRJNA729271	stage_1W_vs_E10	ordinary_muscle_development	ENSGALG00010017038	ZNF598	0.095801572	0.13139689	0.729100762	0.465940028	0.504203114	positive
PRJNA729271	stage_1W_vs_E10	ordinary_muscle_development	ENSGALG00010017039	MAP2K5	-0.93425274	0.079510957	-11.74998743	7.06294E-32	4.29075E-31	negative
PRJNA729271	stage_1W_vs_E10	ordinary_muscle_development	ENSGALG00010017041	JKAMP	0.38885325	0.089236927	4.357537455	1.31534E-05	2.26113E-05	positive
PRJNA729271	stage_1W_vs_E10	ordinary_muscle_development	ENSGALG00010017042	HNRNPLL	-1.056483552	0.070891171	-14.90289316	3.15612E-50	3.48688E-49	negative
PRJNA729271	stage_1W_vs_E10	ordinary_muscle_development	ENSGALG00010017045	GLB1	-0.045199732	0.099353223	-0.45493977	0.64915257	0.680612405	negative
PRJNA729271	stage_1W_vs_E10	ordinary_muscle_development	ENSGALG00010017049	TRAF3	-0.127450962	0.092657042	-1.375512957	0.168972472	0.196632625	negative
PRJNA729271	stage_1W_vs_E10	ordinary_muscle_development	ENSGALG00010017050	L3HYPDH	-0.875392382	0.156248845	-5.602552648	2.11218E-08	4.39696E-08	negative
PRJNA729271	stage_1W_vs_E10	ordinary_muscle_development	ENSGALG00010017054	GALM	-0.574848129	0.151765504	-3.787739081	0.000152024	0.000241384	negative
PRJNA729271	stage_1W_vs_E10	ordinary_muscle_development	ENSGALG00010017055	GPR135	-1.002556154	0.315754172	-3.175116093	0.001497766	0.002194936	negative
PRJNA729271	stage_1W_vs_E10	ordinary_muscle_development	ENSGALG00010017056	AGTR1	3.68581804	0.202367075	18.21352625	4.03126E-74	8.32381E-73	positive
PRJNA729271	stage_1W_vs_E10	ordinary_muscle_development	ENSGALG00010017057	NDUFAB1	1.634134761	0.120948018	13.51105029	1.34588E-41	1.14085E-40	positive
PRJNA729271	stage_1W_vs_E10	ordinary_muscle_development	ENSGALG00010017060	CLSTN1	-1.526329418	0.102600918	-14.87637195	4.69266E-50	5.16477E-49	negative
PRJNA729271	stage_1W_vs_E10	ordinary_muscle_development	ENSGALG00010017063	TMPPE	0.049629767	0.112044297	0.442947731	0.657803542	0.688586866	positive
PRJNA729271	stage_1W_vs_E10	ordinary_muscle_development	ENSGALG00010017065	VRK2	-0.635016369	0.136843354	-4.640461904	3.47631E-06	6.24324E-06	negative
PRJNA729271	stage_1W_vs_E10	ordinary_muscle_development	ENSGALG00010017068	ZP2	-4.309620893	1.197543657	-3.598717147	0.000319791	0.00049608	negative
PRJNA729271	stage_1W_vs_E10	ordinary_muscle_development	ENSGALG00010017070	BLOC1S6	0.141182512	0.105848829	1.333812694	0.18226525	0.211017156	positive
PRJNA729271	stage_1W_vs_E10	ordinary_muscle_development	ENSGALG00010017077	CTNNBIP1	-0.720762979	0.10560143	-6.825314594	8.77328E-12	2.21317E-11	negative
PRJNA729271	stage_1W_vs_E10	ordinary_muscle_development	ENSGALG00010017080	PALB2	-1.012182797	0.104411635	-9.694157185	3.19263E-22	1.30579E-21	negative
PRJNA729271	stage_1W_vs_E10	ordinary_muscle_development	ENSGALG00010017083	ENSGALG00010017083	-0.002964621	0.411630385	-0.007202142	0.994253572	0.99535227	negative
PRJNA729271	stage_1W_vs_E10	ordinary_muscle_development	ENSGALG00010017085	ENSGALG00010017085	-0.675534247	0.12059699	-5.601584655	2.12401E-08	4.41842E-08	negative
PRJNA729271	stage_1W_vs_E10	ordinary_muscle_development	ENSGALG00010017089	CRTAP	-0.373880414	0.157500968	-2.373829302	0.017604687	0.023130886	negative
PRJNA729271	stage_1W_vs_E10	ordinary_muscle_development	ENSGALG00010017094	SORBS2	0.217162337	0.148361642	1.463736404	0.143265985	0.168544797	positive
PRJNA729271	stage_1W_vs_E10	ordinary_muscle_development	ENSGALG00010017096	DCTN5	-0.836354644	0.092474544	-9.044160813	1.50819E-19	5.55559E-19	negative
PRJNA729271	stage_1W_vs_E10	ordinary_muscle_development	ENSGALG00010017098	DAAM1	-1.564359144	0.126155782	-12.40021754	2.60615E-35	1.78864E-34	negative
PRJNA729271	stage_1W_vs_E10	ordinary_muscle_development	ENSGALG00010017100	SUSD5	-7.955954287	0.626803851	-12.69289313	6.4749E-37	4.6647E-36	negative
PRJNA729271	stage_1W_vs_E10	ordinary_muscle_development	ENSGALG00010017101	CSMD2	-1.705880296	0.249362195	-6.840974011	7.86565E-12	1.98871E-11	negative
PRJNA729271	stage_1W_vs_E10	ordinary_muscle_development	ENSGALG00010017104	FANCL	-2.645928423	0.181890989	-14.54678124	6.12164E-48	6.24003E-47	negative
PRJNA729271	stage_1W_vs_E10	ordinary_muscle_development	ENSGALG00010017105	PLK1	-3.188158167	0.236136781	-13.50131969	1.536E-41	1.29973E-40	negative
PRJNA729271	stage_1W_vs_E10	ordinary_muscle_development	ENSGALG00010017106	CRYM	-0.69513262	0.117346537	-5.923759135	3.14664E-09	6.88358E-09	negative
PRJNA729271	stage_1W_vs_E10	ordinary_muscle_development	ENSGALG00010017109	ITGB1BP3	1.848803304	0.127364245	14.51587382	9.61249E-48	9.74357E-47	positive
PRJNA729271	stage_1W_vs_E10	ordinary_muscle_development	ENSGALG00010017110	BRF1	-0.382263737	0.073115556	-5.228213472	1.71156E-07	3.36394E-07	negative
PRJNA729271	stage_1W_vs_E10	ordinary_muscle_development	ENSGALG00010017111	GJB1	-1.328063401	0.472951841	-2.808030938	0.004984544	0.006959923	negative
PRJNA729271	stage_1W_vs_E10	ordinary_muscle_development	ENSGALG00010017112	LZIC	-0.581432316	0.063700862	-9.127542391	7.00719E-20	2.61911E-19	negative
PRJNA729271	stage_1W_vs_E10	ordinary_muscle_development	ENSGALG00010017115	LGALSL	3.135589882	0.191545909	16.36991308	3.13662E-60	4.62986E-59	positive
PRJNA729271	stage_1W_vs_E10	ordinary_muscle_development	ENSGALG00010017116	ENSGALG00010017116	-2.362972714	0.303493996	-7.785896087	6.92208E-15	2.04641E-14	negative
PRJNA729271	stage_1W_vs_E10	ordinary_muscle_development	ENSGALG00010017119	BTBD6	-1.748523126	0.152165223	-11.49095101	1.4649E-30	8.46304E-30	negative
PRJNA729271	stage_1W_vs_E10	ordinary_muscle_development	ENSGALG00010017120	ERN2	1.220475334	0.142430448	8.56892152	1.04459E-17	3.49764E-17	positive
PRJNA729271	stage_1W_vs_E10	ordinary_muscle_development	ENSGALG00010017122	NCBP2	-0.755123347	0.192795751	-3.916701177	8.97689E-05	0.000144948	negative
PRJNA729271	stage_1W_vs_E10	ordinary_muscle_development	ENSGALG00010017127	SOX8	-1.3881695	0.136160527	-10.19509497	2.08541E-24	9.34115E-24	negative
PRJNA729271	stage_1W_vs_E10	ordinary_muscle_development	ENSGALG00010017131	TLR3	3.201010178	0.376544139	8.501022452	1.87928E-17	6.2178E-17	positive
PRJNA729271	stage_1W_vs_E10	ordinary_muscle_development	ENSGALG00010017132	TMEM108	-2.652706164	0.307690816	-8.621336829	6.61769E-18	2.23911E-17	negative
PRJNA729271	stage_1W_vs_E10	ordinary_muscle_development	ENSGALG00010017133	WDR20	0.597994227	0.11238539	5.320924972	1.03241E-07	2.0607E-07	positive
PRJNA729271	stage_1W_vs_E10	ordinary_muscle_development	ENSGALG00010017135	ENSGALG00010017135	1.166359357	0.131694802	8.856532961	8.25415E-19	2.92599E-18	positive
PRJNA729271	stage_1W_vs_E10	ordinary_muscle_development	ENSGALG00010017136	ENSGALG00010017136	-0.868298614	0.460965979	-1.883650103	0.05961232	0.073708144	negative
PRJNA729271	stage_1W_vs_E10	ordinary_muscle_development	ENSGALG00010017139	COL9A2	-8.928372472	0.597926929	-14.93221336	2.03401E-50	2.26792E-49	negative
PRJNA729271	stage_1W_vs_E10	ordinary_muscle_development	ENSGALG00010017140	UQCRC2	1.366226542	0.11496873	11.88346207	1.44266E-32	8.97098E-32	positive
PRJNA729271	stage_1W_vs_E10	ordinary_muscle_development	ENSGALG00010017141	AFTPH	0.632084156	0.075744895	8.344907681	7.12695E-17	2.3013E-16	positive
PRJNA729271	stage_1W_vs_E10	ordinary_muscle_development	ENSGALG00010017142	RBP7	1.673176809	0.694556088	2.408987319	0.015996854	0.021117887	positive
PRJNA729271	stage_1W_vs_E10	ordinary_muscle_development	ENSGALG00010017143	FAM149A	-0.133099516	0.274095907	-0.485594686	0.627254565	0.660711788	negative
PRJNA729271	stage_1W_vs_E10	ordinary_muscle_development	ENSGALG00010017144	CPB1	2.890673972	0.724385863	3.990516822	6.59295E-05	0.000107618	positive
PRJNA729271	stage_1W_vs_E10	ordinary_muscle_development	ENSGALG00010017147	SRSF7L	-0.981215745	0.071271201	-13.76735238	4.00628E-43	3.54308E-42	negative
PRJNA729271	stage_1W_vs_E10	ordinary_muscle_development	ENSGALG00010017148	MOK	-0.63217347	0.494193041	-1.279203506	0.200825402	0.231138971	negative
PRJNA729271	stage_1W_vs_E10	ordinary_muscle_development	ENSGALG00010017152	CYP4V2	3.389080448	0.196459761	17.25076136	1.10434E-66	1.89438E-65	positive
PRJNA729271	stage_1W_vs_E10	ordinary_muscle_development	ENSGALG00010017153	SLC35F4	-1.267036658	0.620201263	-2.042944337	0.041057949	0.051813944	negative
PRJNA729271	stage_1W_vs_E10	ordinary_muscle_development	ENSGALG00010017154	PIGZ	0.580747724	0.187909228	3.090575863	0.001997688	0.002894201	positive
PRJNA729271	stage_1W_vs_E10	ordinary_muscle_development	ENSGALG00010017162	BCL11A	-3.743531891	0.247888842	-15.10165546	1.57918E-51	1.82392E-50	negative
PRJNA729271	stage_1W_vs_E10	ordinary_muscle_development	ENSGALG00010017163	CDV3	-0.398607502	0.124772432	-3.194676071	0.001399879	0.002057518	negative
PRJNA729271	stage_1W_vs_E10	ordinary_muscle_development	ENSGALG00010017167	SMAP2	-0.49525985	0.097869032	-5.060434738	4.18302E-07	8.0157E-07	negative
PRJNA729271	stage_1W_vs_E10	ordinary_muscle_development	ENSGALG00010017169	CCDC198	0.468113526	0.243225037	1.924610772	0.054278071	0.067480971	positive
PRJNA729271	stage_1W_vs_E10	ordinary_muscle_development	ENSGALG00010017174	DHX57	-0.933108709	0.099482276	-9.379647744	6.61935E-21	2.57993E-20	negative
PRJNA729271	stage_1W_vs_E10	ordinary_muscle_development	ENSGALG00010017180	ENSGALG00010017180	-4.159825816	0.808785562	-5.143298804	2.69956E-07	5.2383E-07	negative
PRJNA729271	stage_1W_vs_E10	ordinary_muscle_development	ENSGALG00010017183	NFYC	0.255412376	0.093552912	2.730138153	0.006330779	0.008741987	positive
PRJNA729271	stage_1W_vs_E10	ordinary_muscle_development	ENSGALG00010017184	CARD11	1.166437509	0.212883993	5.479216614	4.27213E-08	8.74393E-08	positive
PRJNA729271	stage_1W_vs_E10	ordinary_muscle_development	ENSGALG00010017185	NAA30	0.545550251	0.063945392	8.531502116	1.4446E-17	4.80372E-17	positive
PRJNA729271	stage_1W_vs_E10	ordinary_muscle_development	ENSGALG00010017187	ZNF839	1.54610314	0.137200002	11.26897313	1.86735E-29	1.03113E-28	positive
PRJNA729271	stage_1W_vs_E10	ordinary_muscle_development	ENSGALG00010017188	ENSGALG00010017188	1.360369275	0.156929244	8.668679181	4.37162E-18	1.49133E-17	positive
PRJNA729271	stage_1W_vs_E10	ordinary_muscle_development	ENSGALG00010017190	STK26	-0.358798156	0.068081448	-5.270131066	1.36326E-07	2.69731E-07	negative
PRJNA729271	stage_1W_vs_E10	ordinary_muscle_development	ENSGALG00010017198	TOPBP1	-1.202378499	0.070848899	-16.97102591	1.34561E-64	2.2089E-63	negative
PRJNA729271	stage_1W_vs_E10	ordinary_muscle_development	ENSGALG00010017200	KCNQ4	1.259705265	0.306613283	4.108449746	3.98324E-05	6.60456E-05	positive
PRJNA729271	stage_1W_vs_E10	ordinary_muscle_development	ENSGALG00010017201	AP5M1	-2.078032542	0.160485041	-12.94845008	2.39746E-38	1.81279E-37	negative
PRJNA729271	stage_1W_vs_E10	ordinary_muscle_development	ENSGALG00010017207	ENSGALG00010017207	9.955289318	0.280369379	35.50776243	3.7305E-276	1.1755E-273	positive
PRJNA729271	stage_1W_vs_E10	ordinary_muscle_development	ENSGALG00010017208	CINP	-0.677624836	0.173565043	-3.904155042	9.45552E-05	0.000152405	negative
PRJNA729271	stage_1W_vs_E10	ordinary_muscle_development	ENSGALG00010017211	UBFD1	1.79194183	0.127286499	14.07801958	5.18423E-45	4.84497E-44	positive
PRJNA729271	stage_1W_vs_E10	ordinary_muscle_development	ENSGALG00010017212	TINAGL1	3.470987269	0.134763415	25.75615395	2.7503E-146	2.1549E-144	positive
PRJNA729271	stage_1W_vs_E10	ordinary_muscle_development	ENSGALG00010017215	FRMD7	3.380271033	0.590727177	5.722220284	1.05141E-08	2.22809E-08	positive
PRJNA729271	stage_1W_vs_E10	ordinary_muscle_development	ENSGALG00010017219	EARS2	-1.205791906	0.146964879	-8.204626249	2.31309E-16	7.29351E-16	negative
PRJNA729271	stage_1W_vs_E10	ordinary_muscle_development	ENSGALG00010017221	SPPL2A	0.258898445	0.0756531	3.422178942	0.000621214	0.000941302	positive
PRJNA729271	stage_1W_vs_E10	ordinary_muscle_development	ENSGALG00010017223	NOC2L	-1.146512268	0.077291457	-14.8336222	8.88086E-50	9.67881E-49	negative
PRJNA729271	stage_1W_vs_E10	ordinary_muscle_development	ENSGALG00010017226	PEF1	0.017715115	0.07512229	0.235817022	0.813574659	0.833352037	positive
PRJNA729271	stage_1W_vs_E10	ordinary_muscle_development	ENSGALG00010017228	RAP2C	-0.900186339	0.072535842	-12.41022799	2.29997E-35	1.583E-34	negative
PRJNA729271	stage_1W_vs_E10	ordinary_muscle_development	ENSGALG00010017240	MELTF	0.27887431	0.237487228	1.174270771	0.240286587	0.272680974	positive
PRJNA729271	stage_1W_vs_E10	ordinary_muscle_development	ENSGALG00010017246	GGA2	0.701119231	0.130283798	5.381476749	7.38772E-08	1.49102E-07	positive
PRJNA729271	stage_1W_vs_E10	ordinary_muscle_development	ENSGALG00010017248	TECPR2	0.892749086	0.083689454	10.66740246	1.44613E-26	7.11045E-26	positive
PRJNA729271	stage_1W_vs_E10	ordinary_muscle_development	ENSGALG00010017250	COL16A1	-0.327804255	0.170030679	-1.927912411	0.053866022	0.066991676	negative
PRJNA729271	stage_1W_vs_E10	ordinary_muscle_development	ENSGALG00010017252	EXOC5	-0.748008351	0.088532872	-8.448933515	2.93976E-17	9.64286E-17	negative

PRJNA729271	stage_1W_vs_E10	ordinary_muscle_development	ENSGALG00010017256	CARHSP1	0.300717957	0.128787259	2.334997723	0.01954354	0.025571728	positive
PRJNA729271	stage_1W_vs_E10	ordinary_muscle_development	ENSGALG00010017263	COG7	-0.067918072	0.071408621	-0.951118661	0.341544143	0.378579365	negative
PRJNA729271	stage_1W_vs_E10	ordinary_muscle_development	ENSGALG00010017264	PIAS1	0.505715327	0.086164903	5.869156816	4.38017E-09	9.49605E-09	positive
PRJNA729271	stage_1W_vs_E10	ordinary_muscle_development	ENSGALG00010017266	PMM2	-1.436312707	0.111014993	-12.93800654	2.74664E-38	2.07141E-37	negative
PRJNA729271	stage_1W_vs_E10	ordinary_muscle_development	ENSGALG00010017272	NRXN1	1.254657466	0.13423116	9.346991133	9.01765E-21	3.50337E-20	positive
PRJNA729271	stage_1W_vs_E10	ordinary_muscle_development	ENSGALG00010017278	CCR5	0.450413252	0.232655497	1.935966513	0.052871802	0.065839929	positive
PRJNA729271	stage_1W_vs_E10	ordinary_muscle_development	ENSGALG00010017280	SOS1	0.861613243	0.155344931	5.54645226	2.91524E-08	6.01173E-08	positive
PRJNA729271	stage_1W_vs_E10	ordinary_muscle_development	ENSGALG00010017283	INF2	0.40119606	0.138029869	2.906588721	0.003653933	0.005165684	positive
PRJNA729271	stage_1W_vs_E10	ordinary_muscle_development	ENSGALG00010017284	ENSGALG00010017284	0.906220815	0.196882424	4.602852783	4.16743E-06	7.43195E-06	positive
PRJNA729271	stage_1W_vs_E10	ordinary_muscle_development	ENSGALG00010017285	IL21R	3.458844151	0.853254663	4.053706709	5.04124E-05	8.29054E-05	positive
PRJNA729271	stage_1W_vs_E10	ordinary_muscle_development	ENSGALG00010017289	ADGRB2	-1.444020693	0.318235844	-4.537580288	5.69034E-06	1.00391E-05	negative
PRJNA729271	stage_1W_vs_E10	ordinary_muscle_development	ENSGALG00010017290	DACT1	-1.946105181	0.157649916	-12.34447334	5.21745E-35	3.54391E-34	negative
PRJNA729271	stage_1W_vs_E10	ordinary_muscle_development	ENSGALG00010017296	SCNN1G	2.18663084	0.224143319	9.755503104	1.74732E-22	7.22815E-22	positive
PRJNA729271	stage_1W_vs_E10	ordinary_muscle_development	ENSGALG00010017299	USP31	1.288472808	0.110388153	11.67220192	1.76787E-31	1.06065E-30	positive
PRJNA729271	stage_1W_vs_E10	ordinary_muscle_development	ENSGALG00010017300	TRPM7	-0.623873963	0.104995267	-5.94192464	2.81695E-09	6.18381E-09	negative
PRJNA729271	stage_1W_vs_E10	ordinary_muscle_development	ENSGALG00010017301	LHCGR	-2.942790362	0.6329776	-4.649122434	3.3335E-06	5.99493E-06	negative
PRJNA729271	stage_1W_vs_E10	ordinary_muscle_development	ENSGALG00010017304	ANKRD9	6.319917995	0.297363987	21.25313849	3.0835E-100	1.15791E-98	positive
PRJNA729271	stage_1W_vs_E10	ordinary_muscle_development	ENSGALG00010017305	HS3ST2	4.123929803	0.464483001	8.878537633	6.77451E-19	2.41863E-18	positive
PRJNA729271	stage_1W_vs_E10	ordinary_muscle_development	ENSGALG00010017307	TMEM186	0.891380051	0.156697495	5.688540534	1.2813E-08	2.6983E-08	positive
PRJNA729271	stage_1W_vs_E10	ordinary_muscle_development	ENSGALG00010017310	SAMD11	-2.00680885	0.183147629	-10.9573291	6.12813E-28	3.18491E-27	negative
PRJNA729271	stage_1W_vs_E10	ordinary_muscle_development	ENSGALG00010017312	PTP4A2	2.023152591	0.154554427	13.09022739	3.74485E-39	2.9168E-38	positive
PRJNA729271	stage_1W_vs_E10	ordinary_muscle_development	ENSGALG00010017313	MAP4K3	0.847180315	0.082315429	10.29187757	7.66666E-25	3.499E-24	positive
PRJNA729271	stage_1W_vs_E10	ordinary_muscle_development	ENSGALG00010017314	CALML4	0.859652279	0.220036256	3.906866502	9.35008E-05	0.000150722	positive
PRJNA729271	stage_1W_vs_E10	ordinary_muscle_development	ENSGALG00010017315	HS3ST4	-2.524237042	0.971839786	-2.597379813	0.009393799	0.01273862	negative
PRJNA729271	stage_1W_vs_E10	ordinary_muscle_development	ENSGALG00010017318	ADSS1	4.559257504	0.123637072	36.87613609	1.1151E-297	3.9424E-295	positive
PRJNA729271	stage_1W_vs_E10	ordinary_muscle_development	ENSGALG00010017322	KHDRBS1	-1.124596999	0.07057937	-15.93379197	3.69268E-57	5.02586E-56	negative
PRJNA729271	stage_1W_vs_E10	ordinary_muscle_development	ENSGALG00010017323	ENSGALG00010017323	0.795708954	0.105507851	7.541703744	4.63871E-14	1.31478E-13	positive
PRJNA729271	stage_1W_vs_E10	ordinary_muscle_development	ENSGALG00010017326	STON1	-0.213587828	0.123872184	-1.724259806	0.084660952	0.102751444	negative
PRJNA729271	stage_1W_vs_E10	ordinary_muscle_development	ENSGALG00010017327	CLN6	1.189247112	0.131284475	9.058551025	1.32195E-19	4.88319E-19	positive
PRJNA729271	stage_1W_vs_E10	ordinary_muscle_development	ENSGALG00010017332	FGFR1	0.065954344	0.158792047	0.415350422	0.677885395	0.70741172	positive
PRJNA729271	stage_1W_vs_E10	ordinary_muscle_development	ENSGALG00010017334	KDM8	-0.357617801	0.157937643	-2.264297441	0.023555827	0.030567745	negative
PRJNA729271	stage_1W_vs_E10	ordinary_muscle_development	ENSGALG00010017335	THUMPD2	0.655684871	0.135952234	4.822906176	1.41482E-06	2.61378E-06	positive
PRJNA729271	stage_1W_vs_E10	ordinary_muscle_development	ENSGALG00010017337	ABAT	1.427086839	0.201454498	7.083916484	1.40136E-12	3.68385E-12	positive
PRJNA729271	stage_1W_vs_E10	ordinary_muscle_development	ENSGALG00010017338	MBNL3	-0.019064371	0.075056091	-0.254001649	0.799494284	0.820669191	negative
PRJNA729271	stage_1W_vs_E10	ordinary_muscle_development	ENSGALG00010017340	SIVA1	-0.355497601	0.104770834	-3.393096974	0.000691072	0.001043881	negative
PRJNA729271	stage_1W_vs_E10	ordinary_muscle_development	ENSGALG00010017341	TA3	-0.302238327	0.080619743	-3.748936857	0.000177586	0.000280557	negative
PRJNA729271	stage_1W_vs_E10	ordinary_muscle_development	ENSGALG00010017343	DLG1	0.285652598	0.099306336	2.876479087	0.004021388	0.005659226	positive
PRJNA729271	stage_1W_vs_E10	ordinary_muscle_development	ENSGALG00010017344	RERE	1.484087555	0.153199121	9.687311155	3.41398E-22	1.39514E-21	positive
PRJNA729271	stage_1W_vs_E10	ordinary_muscle_development	ENSGALG00010017346	GM2A	-1.71389922	0.175690079	-9.755241872	1.75183E-22	7.24471E-22	negative
PRJNA729271	stage_1W_vs_E10	ordinary_muscle_development	ENSGALG00010017347	TMEM39B	-0.468227578	0.1187117	-3.944241189	8.00531E-05	0.000129868	negative
PRJNA729271	stage_1W_vs_E10	ordinary_muscle_development	ENSGALG00010017349	ENSGALG00010017349	0.704141217	0.115410121	6.101208557	1.05269E-09	2.36607E-09	positive
PRJNA729271	stage_1W_vs_E10	ordinary_muscle_development	ENSGALG00010017351	CCR2	1.819801501	0.207304308	8.778406587	1.65807E-18	5.79126E-18	positive
PRJNA729271	stage_1W_vs_E10	ordinary_muscle_development	ENSGALG00010017352	GPCPD1	2.662913253	0.238250162	11.17696301	5.28684E-29	2.85517E-28	positive
PRJNA729271	stage_1W_vs_E10	ordinary_muscle_development	ENSGALG00010017354	FEM1B	0.071903544	0.105116611	0.684035979	0.493952446	0.531816141	positive
PRJNA729271	stage_1W_vs_E10	ordinary_muscle_development	ENSGALG00010017356	NSMCE1	-0.242883728	0.118313078	-2.052889941	0.040083253	0.050632396	negative
PRJNA729271	stage_1W_vs_E10	ordinary_muscle_development	ENSGALG00010017359	EHD3	1.074285278	0.095261689	11.27720167	1.7007E-29	9.40544E-29	positive
PRJNA729271	stage_1W_vs_E10	ordinary_muscle_development	ENSGALG00010017361	METTL22	-0.237523121	0.152151408	-1.561097089	0.118500845	0.141012211	negative
PRJNA729271	stage_1W_vs_E10	ordinary_muscle_development	ENSGALG00010017362	ENO1	0.262895411	0.130162902	2.019741468	0.043410211	0.0546309	positive
PRJNA729271	stage_1W_vs_E10	ordinary_muscle_development	ENSGALG00010017363	TIMM9	0.498022937	0.140925912	3.53393446	0.000409423	0.00062873	positive
PRJNA729271	stage_1W_vs_E10	ordinary_muscle_development	ENSGALG00010017364	AKT1	-0.675912407	0.074440601	-9.079889166	1.08685E-19	4.03224E-19	negative
PRJNA729271	stage_1W_vs_E10	ordinary_muscle_development	ENSGALG00010017367	TIMD4	-0.628255414	0.324062293	-1.938687183	0.05253944	0.065448538	negative
PRJNA729271	stage_1W_vs_E10	ordinary_muscle_development	ENSGALG00010017368	ERLIN2	-0.139164669	0.050546344	-2.753209401	0.005901414	0.00817557	negative
PRJNA729271	stage_1W_vs_E10	ordinary_muscle_development	ENSGALG00010017369	CAPN14	-1.053616317	0.5933881	-1.775593943	0.075799869	0.092484773	negative
PRJNA729271	stage_1W_vs_E10	ordinary_muscle_development	ENSGALG00010017371	TMEM114	-3.396922972	1.345936528	-2.523835933	0.011608209	0.01556963	negative
PRJNA729271	stage_1W_vs_E10	ordinary_muscle_development	ENSGALG00010017372	ENSGALG00010017372	0.586422013	0.110917265	5.287021938	1.24324E-07	2.46758E-07	positive
PRJNA729271	stage_1W_vs_E10	ordinary_muscle_development	ENSGALG00010017373	RP1-2705.3	0.132869697	0.106368671	1.249143147	0.211612722	0.242610646	positive
PRJNA729271	stage_1W_vs_E10	ordinary_muscle_development	ENSGALG00010017375	BDH1A	-0.279104569	0.16138126	-1.729473228	0.083724434	0.101665885	negative
PRJNA729271	stage_1W_vs_E10	ordinary_muscle_development	ENSGALG00010017376	GALNT14	-0.968030341	0.293508442	-3.298134574	0.000973295	0.001452028	negative
PRJNA729271	stage_1W_vs_E10	ordinary_muscle_development	ENSGALG00010017377	USP8	0.596659214	0.075256913	7.928297745	2.2217E-15	6.72168E-15	positive
PRJNA729271	stage_1W_vs_E10	ordinary_muscle_development	ENSGALG00010017378	ENSGALG00010017378	-3.420582121	0.688821148	-4.965849454	6.8401E-07	1.29384E-06	negative
PRJNA729271	stage_1W_vs_E10	ordinary_muscle_development	ENSGALG00010017380	MARCKSL1	-1.550034341	0.13532545	-11.45412296	2.24221E-30	1.28259E-29	negative
PRJNA729271	stage_1W_vs_E10	ordinary_muscle_development	ENSGALG00010017381	CA6	-0.844059708	0.72892481	-1.157951679	0.246883756	0.279620257	negative
PRJNA729271	stage_1W_vs_E10	ordinary_muscle_development	ENSGALG00010017383	ENSGALG00010017383	0.203308157	0.151209932	1.344542342	0.178773074	0.207255515	positive
PRJNA729271	stage_1W_vs_E10	ordinary_muscle_development	ENSGALG00010017384	ARID4A	-0.201645841	0.093404943	-2.158834803	0.030862985	0.039487949	negative
PRJNA729271	stage_1W_vs_E10	ordinary_muscle_development	ENSGALG00010017386	ENSGALG00010017386	-4.1089786	0.958679404	-4.286082066	1.81852E-05	3.09238E-05	negative
PRJNA729271	stage_1W_vs_E10	ordinary_muscle_development	ENSGALG00010017387	HS6ST2	-0.590513853	0.114406968	-5.161519979	2.44953E-07	4.77037E-07	negative
PRJNA729271	stage_1W_vs_E10	ordinary_muscle_development	ENSGALG00010017388	PLPBP	0.339691531	0.082750747	4.104996533	4.04321E-05	6.70246E-05	positive
PRJNA729271	stage_1W_vs_E10	ordinary_muscle_development	ENSGALG00010017390	DDX24	-0.799496347	0.102923846	-7.767843661	7.98335E-15	2.35105E-14	negative
PRJNA729271	stage_1W_vs_E10	ordinary_muscle_development	ENSGALG00010017391	CRAMP1	0.240492435	0.11699331	2.055608435	0.039820274	0.050335299	positive
PRJNA729271	stage_1W_vs_E10	ordinary_muscle_development	ENSGALG00010017393	VDAC1	3.960268978	0.163541772	24.21564184	1.5225E-129	9.8961E-128	positive
PRJNA729271	stage_1W_vs_E10	ordinary_muscle_development	ENSGALG00010017395	BRF2	-0.259616558	0.126340782	-2.054891167	0.039889518	0.050400851	negative
PRJNA729271	stage_1W_vs_E10	ordinary_muscle_development	ENSGALG00010017396	CCR8L	3.20382055	0.272475469	11.75819813	6.40874E-32	3.9015E-31	positive
PRJNA729271	stage_1W_vs_E10	ordinary_muscle_development	ENSGALG00010017398	SLC22A5	0.193930837	0.183024048	1.059592106	0.289330207	0.324224302	positive
PRJNA729271	stage_1W_vs_E10	ordinary_muscle_development	ENSGALG00010017399	CPXM1	-0.416971044	0.291150839	-1.432147837	0.152101516	0.178273751	negative
PRJNA729271	stage_1W_vs_E10	ordinary_muscle_development	ENSGALG00010017400	GPC4	-1.390671504	0.119193912	-11.66730314	1.87266E-31	1.12212E-30	negative
PRJNA729271	stage_1W_vs_E10	ordinary_muscle_development	ENSGALG00010017402	SLC2A5	1.770241475	0.228900254	7.733680705	1.04481E-14	3.06197E-14	positive
PRJNA729271	stage_1W_vs_E10	ordinary_muscle_development	ENSGALG00010017403	ITGA11	-1.227188247	0.294323282	-4.16952488	3.05235E-05	5.10663E-05	negative
PRJNA729271	stage_1W_vs_E10	ordinary_muscle_development	ENSGALG00010017405	XCR1	4.200635224	0.938600236	4.47542528	7.62594E-06	1.33307E-05	positive
PRJNA729271	stage_1W_vs_E10	ordinary_muscle_development	ENSGALG00010017406	IFT140	-1.425350679	0.127215218	-11.20424663	3.88646E-29	2.11306E-28	negative
PRJNA729271	stage_1W_vs_E10	ordinary_muscle_development	ENSGALG00010017407	EPN2	0.33066948	0.085108789	3.885256544	0.000102222	0.000164196	positive
PRJNA729271	stage_1W_vs_E10	ordinary_muscle_development	ENSGALG00010017409	GPC3	-1.536957307	0.337667903	-4.551683151	5.32184E-06	9.41192E-06	negative
PRJNA729271	stage_1W_vs_E10	ordinary_muscle_development	ENSGALG00010017411	RAB11FIP1	0.179861668	0.137834512	1.304910257	0.191923408	0.221526501	positive
PRJNA729271	stage_1W_vs_E10	ordinary_muscle_development	ENSGALG00010017413	ZIC3	-6.019216601	0.995874432	-6.044152161	1.50198E-09	3.35096E-09	negative
PRJNA729271	stage_1W_vs_E10	ordinary_muscle_development	ENSGALG00010017414	ENSGALG00010017414	0.201387125	0.197835522	1.017952303	0.308700624	0.34446617	positive
PRJNA729271	stage_1W_vs_E10	ordinary_muscle_development	ENSGALG00010017415	HDAC1	-1.914661168	0.066008745	-29.00617418	5.4997E-185	7.1818E-183	negative
PRJNA729271	stage_1W_vs_E10	ordinary_muscle_development	ENSGALG00010017418	CHGB	-1.551045904	0.289412609	-5.359289326	8.355E-08	1.68016E-07	negative
PRJNA729271	stage_1W_vs_E10	ordinary_muscle_development	ENSGALG00010017419	B9D1	-1.156981049	0.103587193	-11.16915152	5.77297E-29	3.11191E-28	negative
PRJNA729271	stage_1W_vs_E10	ordinary_muscle_development	ENSGALG00010017421	GPR157	3.231053421	0.261451554	12.35813431	4.40256E-35	2.99883E-34	positive
PRJNA729271	stage_1W_vs_E10	ordinary_muscle_development	ENSGALG00010017422	ENSGALG00010017422	-0.492724801	0.239280238	-2.05919555	0.039475509	0.049921262	negative
PRJNA729271	stage_1W_vs_E10	ordinary_muscle_development	ENSGALG00010017423	TMEM204	0.016211168	0.133325545	0.12159086	0.903223052	0.914644274	positive
PRJNA729271	stage_1W_vs_E10	ordinary_muscle_development	ENSGALG00010017424	APOD	3.278754559	0.482848688	6.790438998	1.11793E-11	2.80401E-11	positive
PRJNA729271	stage_1W_vs_E10	ordinary_muscle_development	ENSGALG00010017425	H6PD	0.834886945	0.12547399	6.653864619	2.85495E-11	7.00568E-11	positive
PRJNA729271	stage_1W_vs_E10	ordinary_muscle_development	ENSGALG00010017428	PSMA3	0.198584386	0.107977141	1.839133586	0.06589554	0.081045775	positive
PRJNA729271	stage_1W_vs_E10	ordinary_muscle_development	ENSGALG00010017429	TELO2	-0.448573957	0.105367311	-4.257240241	2.06966E-05	3.49973E-05	negative
PRJNA729271	stage_1W_vs_E10	ordinary_muscle_development	ENSGALG00010017430	CACNA1H	-3.16885811	0.223952665	-14.14967803	1.87614E-45	1.78443E-44	negative
PRJNA729271	stage_1W_vs_E10	ordinary_muscle_development	ENSGALG00010017431	FYCO1	3.774737245	0.117198364	32.20810525	1.3591E-227	2.6622E-225	positive
PRJNA729271	stage_1W_vs_E10	ordinary_muscle_development	ENSGALG00010017433	RNPS1	-0.470404227	0.079153697	-5.94292177	2.79986E-09	6.14909E-09	negative
PRJNA729271	stage_1W_vs_E10	ordinary_muscle_development	ENSGALG00010017434	FGF13	2.628264015	0.148363325	17.715052	3.20931E-70	6.12897E-69	positive
PRJNA729271	stage_1W_vs_E10	ordinary_muscle_development	ENSGALG00010017435	ACTR10	-0.949648864	0.091221835	-10.41032402	2.22463E-25	1.03919E-24	negative
PRJNA729271	stage_1W_vs_E10	ordinary_muscle_development	ENSGALG00010017437	DRG2	0.725444766	0.13921086	5.211121923	1.87702E-07	3.68265E-07	positive
PRJNA729271	stage_1W_vs_E10	ordinary_muscle_development	ENSGALG00010017438	ADRB3	3.991469036	0.778570272	5.126665092	2.94919E-07	5.70742E-07	positive
PRJNA729271	stage_1W_vs_E10	ordinary_muscle_development	ENSGALG00010017439	PPP4R4	-1.959511124	0.277499497	-7.061314157	1.64935E-12	4.31307E-12	negative
PRJNA729271	stage_1W_vs_E10	ordinary_muscle_development	ENSGALG00010017441	SPSB1	1.119930445	0.256505888	4.366100344	1.26484E-05	2.17872E-05	positive
PRJNA729271	stage_1W_vs_E10	ordinary_muscle_development	ENSGALG00010017442	LCK	0.951244441	0.26232631	3.626187705	0.000287636	0.000447589	positive
PRJNA729271	stage_1W_vs_E10	ordinary_muscle_development	ENSGALG00010017445	F9	4.139610227	1.176192323	3.519501144	0.000432359	0.000662548	positive
PRJNA729271	stage_1W_vs_E10	ordinary_muscle_development	ENSGALG00010017447	CAPN13	-0.458729347	0.546990408	-0.838642398	0.401670011	0.439843379	negative
PRJNA729271	stage_1W_vs_E10	ordinary_muscle_development	ENSGALG00010017449	TRMT6	-0.893438485	0.087102172	-10.25736171	1.09662E-24	4.97979E-24	negative
PRJNA729271	stage_1W_vs_E10	ordinary_muscle_development	ENSGALG00010017450	ENSGALG00010017450	1.829179869	0.210162355	8.70365135	3.21373E-18	1.10517E-17	positive
PRJNA729271	stage_1W_vs_E10	ordinary_muscle_development	ENSGALG00010017451	GABPB1	-0.540011801	0.077673281	-6.952349608	3.59252E-12	9.24437E-12	negative
PRJNA729271	stage_1W_vs_E10	ordinary_muscle_development	ENSGALG00010017452	ARMH4	-3.990320871	0.456438568	-8.742295573	2.28421E-18	7.89829E-18	negative
PRJNA729271	stage_1W_vs_E10	ordinary_muscle_development	ENSGALG00010017453	PPP1R2	1.083810709	0.138133121	7.846132047	4.29065E-15	1.28312E-14	positive
PRJNA729271	stage_1W_vs_E10	ordinary_muscle_development	ENSGALG00010017454	ENSGALG00010017454	0.982515936	0.159336966	6.166277421	6.99164E-10	1.58399E-09	positive
PRJNA729271	stage_1W_vs_E10	ordinary_muscle_development	ENSGALG00010017455	CCR9	3.34950929	0.689798162	4.855781698	1.19913E-06	2.22918E-06	positive
PRJNA729271	stage_1W_vs_E10	ordinary_muscle_development	ENSGALG00010017457	ZMAT4	-2.759878691	0.284438068	-9.702916036	2.93003E-22	1.19974E-21	negative
PRJNA729271	stage_1W_vs_E10	ordinary_muscle_development	ENSGALG00010017458	ENSGALG00010017458	1.06033767	0.171780696	6.172624121	6.71658E-10	1.52382E-09	positive
PRJNA729271	stage_1W_vs_E10	ordinary_muscle_development	ENSGALG00010017459	FAM167B	1.244246391	0.301141944	4.131760502	3.59995E-05	5.99441E-05	positive
PRJNA729271	stage_1W_vs_E10	ordinary_muscle_development	ENSGALG00010017462	MCM8	-0.093229709	0.118231916	-0.788532507	0.430385292	0.468280649	negative
PRJNA729271	stage_1W_vs_E10	ordinary_muscle_development	ENSGALG00010017463	PPP1R36	0.457053238	0.34624677	1.320021667	0.186827784	0.216057821	positive
PRJNA729271	stage_1W_vs_E10	ordinary_muscle_development	ENSGALG00010017464	SH3BP2	0.142455318	0.130865757	1.088560677	0.276347665	0.310684822	positive
PRJNA729271	stage_1W_vs_E10	ordinary_muscle_development	ENSGALG00010017465	TMEM234	-0.473870762	0.24915541	-1.901908377	0.057183134	0.070898086	negative
PRJNA729271	stage_1W_vs_E10	ordinary_muscle_development	ENSGALG00010017466	SPIA4	7.767199168	2.719668143	2.855936371			positive
PRJNA729271	stage_1W_vs_E10	ordinary_muscle_development	ENSGALG00010017467	SFRP1	-4.784077347	0.119104474	-40.16706681	0	0	negative
PRJNA729271	stage_1W_vs_E10	ordinary_muscle_development	ENSGALG00010017468	AMDHD2	0.162802095	0.127960285	1.27228613	0.203271475	0.233675948	positive
PRJNA729271	stage_1W_vs_E10	ordinary_muscle_development	ENSGALG00010017470	HSPA2	1.577576743	0.170113163	9.273690024	1.79815E-20	6.90076E-20	positive
PRJNA729271	stage_1W_vs_E10	ordinary_muscle_development	ENSGALG00010017471	CRLS1	0.958717903	0.088910149	10.78299738	4.14166E-27	2.07728E-26	positive
PRJNA729271	stage_1W_vs_E10	ordinary_muscle_development	ENSGALG00010017472	LZTFL1	-1.614142784	0.173555902	-9.30042	1.39892E-20	5.40153E-20	negative
PRJNA729271	stage_1W_vs_E10	ordinary_muscle_development	ENSGALG00010017473	EIF3I	0.56027214	0.104451744	5.36393285	8.14292E-08	1.63887E-07	positive
PRJNA729271	stage_1W_vs_E10	ordinary_muscle_development	ENSGALG00010017475	STUM	3.076978289	0.246710693	12.47201026	1.06115E-35	7.41269E-35	positive
PRJNA729271	stage_1W_vs_E10	ordinary_muscle_development	ENSGALG00010017476	ATP6V0C	1.836651569	0.111812398	16.42618883	1.24225E-60	1.85251E-59	positive
PRJNA729271	stage_1W_vs_E10	ordinary_muscle_development	ENSGALG00010017477	MCF2	1.524201678	0.18212746	8.368873547	5.81716E-17	1.8834E-16	positive
PRJNA729271	stage_1W_vs_E10	ordinary_muscle_development	ENSGALG00010017479	SSTR5	-0.67330543	0.562806589	-1.196335372	0.23156571	0.263320387	negative

PRJNA729271	stage_1W_vs_E10	ordinary_muscle_development	ENSGALG00010017480	DCDC2B	0.593117532	0.20074437	2.954591108	0.003130837	0.004451784	positive
PRJNA729271	stage_1W_vs_E10	ordinary_muscle_development	ENSGALG00010017481	SPIA1	7.217126574	2.485241627	2.90399392			positive
PRJNA729271	stage_1W_vs_E10	ordinary_muscle_development	ENSGALG00010017482	CORO2B	-1.017379777	0.145980258	-6.969296998	3.18529E-12	8.22275E-12	negative
PRJNA729271	stage_1W_vs_E10	ordinary_muscle_development	ENSGALG00010017484	IQCC	-0.171606317	0.137667136	-1.24653074	0.212569611	0.243572847	negative
PRJNA729271	stage_1W_vs_E10	ordinary_muscle_development	ENSGALG00010017485	LBH	-0.877862679	0.108121475	-8.119225915	4.69166E-16	1.46469E-15	negative
PRJNA729271	stage_1W_vs_E10	ordinary_muscle_development	ENSGALG00010017487	C1QTNF8	-0.927198463	0.512681139	-1.808528523	0.070524282	0.086418925	negative
PRJNA729271	stage_1W_vs_E10	ordinary_muscle_development	ENSGALG00010017489	TNFRSF1B	0.613967421	0.179097574	3.428116911	0.000607784	0.000921433	positive
PRJNA729271	stage_1W_vs_E10	ordinary_muscle_development	ENSGALG00010017491	CCDC28B	1.747195617	0.190542031	9.169607397	4.74745E-20	1.78646E-19	positive
PRJNA729271	stage_1W_vs_E10	ordinary_muscle_development	ENSGALG00010017493	YPEL5	0.194143951	0.134503071	1.443416492	0.148903181	0.174751163	positive
PRJNA729271	stage_1W_vs_E10	ordinary_muscle_development	ENSGALG00010017494	TBC1D24	-1.610676126	0.097854189	-16.45996087	7.11464E-61	1.06978E-59	negative
PRJNA729271	stage_1W_vs_E10	ordinary_muscle_development	ENSGALG00010017495	NT5M	0.024366033	0.134883131	0.180645516	0.856645827	0.872598824	positive
PRJNA729271	stage_1W_vs_E10	ordinary_muscle_development	ENSGALG00010017496	PARP1	-1.633472195	0.104015788	-15.70407945	1.41821E-55	1.86205E-54	negative
PRJNA729271	stage_1W_vs_E10	ordinary_muscle_development	ENSGALG00010017499	SNRPD1	-1.186198255	0.09886018	-11.99874671	3.60717E-33	2.29425E-32	negative
PRJNA729271	stage_1W_vs_E10	ordinary_muscle_development	ENSGALG00010017500	ACAP2	0.12776999	0.100765914	1.267988205	0.204802163	0.235155842	positive
PRJNA729271	stage_1W_vs_E10	ordinary_muscle_development	ENSGALG00010017502	NAT8B	1.314175446	0.116884935	11.24332615	2.4979E-29	1.37252E-28	positive
PRJNA729271	stage_1W_vs_E10	ordinary_muscle_development	ENSGALG00010017503	MED9	0.855723536	0.125513002	6.817807915	9.24401E-12	2.33029E-11	positive
PRJNA729271	stage_1W_vs_E10	ordinary_muscle_development	ENSGALG00010017505	TNFRSF8	1.43294621	0.297873018	4.810594195	1.50482E-06	2.77512E-06	positive
PRJNA729271	stage_1W_vs_E10	ordinary_muscle_development	ENSGALG00010017509	RASD1	0.17189566	0.307125456	0.559691997	0.575689542	0.611327466	positive
PRJNA729271	stage_1W_vs_E10	ordinary_muscle_development	ENSGALG00010017510	HDC	3.471791991	0.386581248	8.980756341	2.68912E-19	9.77892E-19	positive
PRJNA729271	stage_1W_vs_E10	ordinary_muscle_development	ENSGALG00010017513	TNIP2	0.30722786	0.146458268	2.097716044	0.035930237	0.04561296	positive
PRJNA729271	stage_1W_vs_E10	ordinary_muscle_development	ENSGALG00010017514	TXLNA	-0.300170834	0.097008664	-3.094268313	0.00197299	0.002860993	negative
PRJNA729271	stage_1W_vs_E10	ordinary_muscle_development	ENSGALG00010017516	AAK1	-0.632409296	0.081345313	-7.774379007	7.58184E-15	2.2369E-14	negative
PRJNA729271	stage_1W_vs_E10	ordinary_muscle_development	ENSGALG00010017517	LIN9	-0.646714653	0.103347646	-6.257662135	3.90791E-10	8.98987E-10	negative
PRJNA729271	stage_1W_vs_E10	ordinary_muscle_development	ENSGALG00010017518	PEMT	1.233903736	0.147110191	8.387615598	4.96101E-17	1.60944E-16	positive
PRJNA729271	stage_1W_vs_E10	ordinary_muscle_development	ENSGALG00010017519	NTN3	-3.395426382	0.233542677	-14.53878337	6.88046E-48	7.0086E-47	negative
PRJNA729271	stage_1W_vs_E10	ordinary_muscle_development	ENSGALG00010017520	ZBTB1	-1.173187777	0.081944794	-14.31680671	1.7184E-46	1.68185E-45	negative
PRJNA729271	stage_1W_vs_E10	ordinary_muscle_development	ENSGALG00010017521	MIIP	0.577533952	0.115853076	4.985054988	6.19442E-07	1.17416E-06	positive
PRJNA729271	stage_1W_vs_E10	ordinary_muscle_development	ENSGALG00010017522	XXYLT1	-0.701666928	0.108100984	-6.490846816	8.53552E-11	2.03457E-10	negative
PRJNA729271	stage_1W_vs_E10	ordinary_muscle_development	ENSGALG00010017524	ACBD3	-0.389567343	0.060403224	-6.449446152	1.1226E-10	2.66231E-10	negative
PRJNA729271	stage_1W_vs_E10	ordinary_muscle_development	ENSGALG00010017525	ENSGALG00010017525	-6.189992561	0.97856559	-6.325577582	2.52287E-10	5.85198E-10	negative
PRJNA729271	stage_1W_vs_E10	ordinary_muscle_development	ENSGALG00010017526	PLEKHA2	0.98140113	0.173293367	5.663235408	1.48545E-08	3.11826E-08	positive
PRJNA729271	stage_1W_vs_E10	ordinary_muscle_development	ENSGALG00010017528	RAI1	0.02107707	0.178581276	0.118025085	0.906047782	0.9172484	positive
PRJNA729271	stage_1W_vs_E10	ordinary_muscle_development	ENSGALG00010017529	MFN2	0.410708689	0.118655867	3.461343306	0.000537487	0.000817854	positive
PRJNA729271	stage_1W_vs_E10	ordinary_muscle_development	ENSGALG00010017530	TLR1B	0.03977355	0.216372918	0.183819449	0.854155098	0.870551129	positive
PRJNA729271	stage_1W_vs_E10	ordinary_muscle_development	ENSGALG00010017531	SACM1L	-0.573804745	0.112666891	-5.092931405	3.52569E-07	6.79316E-07	negative
PRJNA729271	stage_1W_vs_E10	ordinary_muscle_development	ENSGALG00010017532	MIXL1	0.763769009	0.318676998	2.396686972	0.01654405	0.021806494	positive
PRJNA729271	stage_1W_vs_E10	ordinary_muscle_development	ENSGALG00010017533	FAM193A	-0.03728554	0.078578418	-0.474501027	0.635142666	0.667758972	negative
PRJNA729271	stage_1W_vs_E10	ordinary_muscle_development	ENSGALG00010017534	ATP11C	-1.403069338	0.085355033	-16.43803881	1.02172E-60	1.53153E-59	negative
PRJNA729271	stage_1W_vs_E10	ordinary_muscle_development	ENSGALG00010017535	LIMD1	-0.440267603	0.09095666	-4.840410858	1.29571E-06	2.39925E-06	negative
PRJNA729271	stage_1W_vs_E10	ordinary_muscle_development	ENSGALG00010017536	GSC	-1.814710195	0.460348349	-3.942036933	8.07925E-05	0.000131009	negative
PRJNA729271	stage_1W_vs_E10	ordinary_muscle_development	ENSGALG00010017537	MANBA	0.661331475	0.18792796	3.519069084	0.000433064	0.000663558	positive
PRJNA729271	stage_1W_vs_E10	ordinary_muscle_development	ENSGALG00010017538	H3-3A	-0.169847843	0.075991116	-2.235101313	0.025410698	0.032842449	negative
PRJNA729271	stage_1W_vs_E10	ordinary_muscle_development	ENSGALG00010017539	KPNA6	1.463654806	0.13818093	10.59230681	3.23519E-26	1.56525E-25	positive
PRJNA729271	stage_1W_vs_E10	ordinary_muscle_development	ENSGALG00010017540	EIF4G1	1.126297396	0.164465755	6.848218339	7.47754E-12	1.89355E-11	positive
PRJNA729271	stage_1W_vs_E10	ordinary_muscle_development	ENSGALG00010017541	SREBF1	3.158949221	0.226593092	13.94106586	3.56575E-44	3.26092E-43	positive
PRJNA729271	stage_1W_vs_E10	ordinary_muscle_development	ENSGALG00010017542	PLOD1	-0.808761524	0.125469435	-6.44588478	1.14928E-10	2.72158E-10	negative
PRJNA729271	stage_1W_vs_E10	ordinary_muscle_development	ENSGALG00010017543	ZBTB25	-0.135946995	0.179067901	-0.759192428	0.447737455	0.485847763	negative
PRJNA729271	stage_1W_vs_E10	ordinary_muscle_development	ENSGALG00010017544	SDE2	0.582924212	0.068287107	8.536372906	1.38501E-17	4.61086E-17	positive
PRJNA729271	stage_1W_vs_E10	ordinary_muscle_development	ENSGALG00010017545	DICER1	-0.406875456	0.121188082	-3.357388355	0.000786825	0.001183463	negative
PRJNA729271	stage_1W_vs_E10	ordinary_muscle_development	ENSGALG00010017546	NFU1	1.515992135	0.107832905	14.05871554	6.81112E-45	6.34494E-44	positive
PRJNA729271	stage_1W_vs_E10	ordinary_muscle_development	ENSGALG00010017547	RNF4	0.055712789	0.065716969	0.847768693	0.396566802	0.434729559	positive
PRJNA729271	stage_1W_vs_E10	ordinary_muscle_development	ENSGALG00010017550	TM2D2	0.804430395	0.075517698	10.65221025	1.70273E-26	8.34386E-26	positive
PRJNA729271	stage_1W_vs_E10	ordinary_muscle_development	ENSGALG00010017551	NALF2	-0.500667101	0.282487218	-1.772353117	0.076335959	0.093107517	negative
PRJNA729271	stage_1W_vs_E10	ordinary_muscle_development	ENSGALG00010017552	LARS2	0.114234402	0.102924691	1.109883364	0.26704929	0.300861142	positive
PRJNA729271	stage_1W_vs_E10	ordinary_muscle_development	ENSGALG00010017553	EDA	0.641544249	0.108998005	5.885834779	3.9605E-09	8.61197E-09	positive
PRJNA729271	stage_1W_vs_E10	ordinary_muscle_development	ENSGALG00010017554	FAM131A	-2.107813424	0.331992138	-6.348985955	2.16739E-10	5.04679E-10	negative
PRJNA729271	stage_1W_vs_E10	ordinary_muscle_development	ENSGALG00010017555	CELA2A	3.13439673	1.20546667	2.60015213	0.009318244	0.012639711	positive
PRJNA729271	stage_1W_vs_E10	ordinary_muscle_development	ENSGALG00010017556	TOM1L2	-0.254025729	0.115829423	-2.193101913	0.028300037	0.036353158	negative
PRJNA729271	stage_1W_vs_E10	ordinary_muscle_development	ENSGALG00010017558	ENSGALG00010017558	-2.262712917	0.07643871	-29.60166295	1.4224E-192	1.9451E-190	negative
PRJNA729271	stage_1W_vs_E10	ordinary_muscle_development	ENSGALG00010017559	DUSP10	2.766552929	0.149525696	18.50219061	1.9825E-76	4.32776E-75	positive
PRJNA729271	stage_1W_vs_E10	ordinary_muscle_development	ENSGALG00010017561	CFAP99	-1.017588032	0.635948142	-1.600111652	0.109573817	0.130991544	negative
PRJNA729271	stage_1W_vs_E10	ordinary_muscle_development	ENSGALG00010017562	SLC30A10	-2.63991922	0.590051261	-4.474050639	7.67516E-06	1.34151E-05	negative
PRJNA729271	stage_1W_vs_E10	ordinary_muscle_development	ENSGALG00010017563	ALK	1.401027005	0.153659932	9.117711969	7.6727E-20	2.8627E-19	positive
PRJNA729271	stage_1W_vs_E10	ordinary_muscle_development	ENSGALG00010017565	ENSGALG00010017565	-1.222839485	0.192146325	-6.364105494	1.96431E-10	4.58867E-10	negative
PRJNA729271	stage_1W_vs_E10	ordinary_muscle_development	ENSGALG00010017568	CDCP1	-3.118286196	0.260082421	-11.98960769	4.02808E-33	2.55523E-32	negative
PRJNA729271	stage_1W_vs_E10	ordinary_muscle_development	ENSGALG00010017569	CLMN	-1.444039927	0.247055485	-5.845002508	5.06561E-09	1.0946E-08	negative
PRJNA729271	stage_1W_vs_E10	ordinary_muscle_development	ENSGALG00010017570	DRC3	-0.966893328	0.403531693	-2.396077793	0.016571573	0.021840784	negative
PRJNA729271	stage_1W_vs_E10	ordinary_muscle_development	ENSGALG00010017571	ADAM9	0.860547787	0.150106017	5.732933311	9.87085E-09	2.09423E-08	positive
PRJNA729271	stage_1W_vs_E10	ordinary_muscle_development	ENSGALG00010017573	ENSGALG00010017573	0.452740006	0.229197416	1.975327708	0.048230971	0.060329418	positive
PRJNA729271	stage_1W_vs_E10	ordinary_muscle_development	ENSGALG00010017574	DYNC1LI1	-0.314076984	0.056450076	-5.563800918	2.63961E-08	5.45886E-08	negative
PRJNA729271	stage_1W_vs_E10	ordinary_muscle_development	ENSGALG00010017575	CLCN2	2.916680988	0.707940583	4.119951671	3.78952E-05	6.29487E-05	positive
PRJNA729271	stage_1W_vs_E10	ordinary_muscle_development	ENSGALG00010017576	ENSGALG00010017576	-3.660449698	0.311671492	-11.74457656	7.52987E-32	4.57058E-31	negative
PRJNA729271	stage_1W_vs_E10	ordinary_muscle_development	ENSGALG00010017577	CLPX	1.617876794	0.137517109	11.76491282	5.91875E-32	3.60926E-31	positive
PRJNA729271	stage_1W_vs_E10	ordinary_muscle_development	ENSGALG00010017578	BSDC1	0.538267652	0.10006394	5.379237053	7.48022E-08	1.50948E-07	positive
PRJNA729271	stage_1W_vs_E10	ordinary_muscle_development	ENSGALG00010017579	P3H2	0.178869004	0.143317044	1.248065117	0.212007212	0.243005261	positive
PRJNA729271	stage_1W_vs_E10	ordinary_muscle_development	ENSGALG00010017580	LYPLAL1	0.300476712	0.154982415	1.938779392	0.052528207	0.065440168	positive
PRJNA729271	stage_1W_vs_E10	ordinary_muscle_development	ENSGALG00010017581	EFCAB11	-2.439488478	0.48183217	-5.062942306	4.12835E-07	7.9154E-07	negative
PRJNA729271	stage_1W_vs_E10	ordinary_muscle_development	ENSGALG00010017582	CLCN6	-0.183729753	0.103556035	-1.774206139	0.076029059	0.092756604	negative
PRJNA729271	stage_1W_vs_E10	ordinary_muscle_development	ENSGALG00010017583	MTHFD1	-1.19292818	0.106648562	-11.18560027	4.79649E-29	2.5981E-28	negative
PRJNA729271	stage_1W_vs_E10	ordinary_muscle_development	ENSGALG00010017584	WNT11B	-2.654835257	0.869478715	-3.053364286	0.002262911	0.00326085	negative
PRJNA729271	stage_1W_vs_E10	ordinary_muscle_development	ENSGALG00010017585	ENSGALG00010017585	-0.224700514	0.119086342	-1.886870567	0.059177737	0.073214518	negative
PRJNA729271	stage_1W_vs_E10	ordinary_muscle_development	ENSGALG00010017586	ZFYVE28	0.279362949	0.161273022	1.732236089	0.083231536	0.101118189	positive
PRJNA729271	stage_1W_vs_E10	ordinary_muscle_development	ENSGALG00010017587	MB21D2	-0.855970305	0.148962088	-5.746229233	9.12556E-09	1.93952E-08	negative
PRJNA729271	stage_1W_vs_E10	ordinary_muscle_development	ENSGALG00010017588	NOX5	-1.545739157	0.51835011	-2.982036902	0.002863375	0.004088722	negative
PRJNA729271	stage_1W_vs_E10	ordinary_muscle_development	ENSGALG00010017589	ATPAF2	0.922343586	0.144734142	6.372674556	1.8576E-10	4.34569E-10	positive
PRJNA729271	stage_1W_vs_E10	ordinary_muscle_development	ENSGALG00010017590	ENSGALG00010017590	-0.955425732	0.195352464	-4.890779026	1.00438E-06	1.87851E-06	negative
PRJNA729271	stage_1W_vs_E10	ordinary_muscle_development	ENSGALG00010017591	ESR2	-2.474589507	0.283142313	-8.739737546	2.33653E-18	8.07726E-18	negative
PRJNA729271	stage_1W_vs_E10	ordinary_muscle_development	ENSGALG00010017592	GID4	0.867839764	0.111419786	7.788919689	6.75845E-15	1.99885E-14	positive
PRJNA729271	stage_1W_vs_E10	ordinary_muscle_development	ENSGALG00010017593	MTHFR	0.851186134	0.132224334	6.437439375	1.21506E-10	2.87266E-10	positive
PRJNA729271	stage_1W_vs_E10	ordinary_muscle_development	ENSGALG00010017594	CEP68	0.642300014	0.100842343	6.369348368	1.89833E-10	4.43883E-10	positive
PRJNA729271	stage_1W_vs_E10	ordinary_muscle_development	ENSGALG00010017595	CCNF	-2.424210859	0.129220044	-18.76033151	1.59402E-78	3.65014E-77	negative
PRJNA729271	stage_1W_vs_E10	ordinary_muscle_development	ENSGALG00010017596	TDP1	-2.023655254	0.101102256	-20.0159258	4.00134E-89	1.15307E-87	negative
PRJNA729271	stage_1W_vs_E10	ordinary_muscle_development	ENSGALG00010017597	CNOT10	0.152810726	0.13508287	1.131236892	0.257955398	0.29147673	positive
PRJNA729271	stage_1W_vs_E10	ordinary_muscle_development	ENSGALG00010017598	ENSGALG00010017598	1.070119816	0.117095274	9.138881346	6.31017E-20	2.36346E-19	positive
PRJNA729271	stage_1W_vs_E10	ordinary_muscle_development	ENSGALG00010017599	KCNK13	-0.333592554	0.236337149	-1.411511291	0.158093909	0.184819034	negative
PRJNA729271	stage_1W_vs_E10	ordinary_muscle_development	ENSGALG00010017600	MXD4	0.609023326	0.103343351	5.893202787	3.78781E-09	8.24389E-09	positive
PRJNA729271	stage_1W_vs_E10	ordinary_muscle_development	ENSGALG00010017601	ENSGALG00010017601	1.021719874	0.115786089	8.824202345	1.10243E-18	3.888E-18	positive
PRJNA729271	stage_1W_vs_E10	ordinary_muscle_development	ENSGALG00010017603	CYP3A5	2.009594527	1.350877624	1.487621448	0.136850754	0.161469408	positive
PRJNA729271	stage_1W_vs_E10	ordinary_muscle_development	ENSGALG00010017604	GATM	3.735926524	0.241180113	15.4901931	4.04075E-54	5.09753E-53	positive
PRJNA729271	stage_1W_vs_E10	ordinary_muscle_development	ENSGALG00010017605	GFPT1	-1.40396764	0.072878677	-19.26445008	1.06794E-82	2.74951E-81	negative
PRJNA729271	stage_1W_vs_E10	ordinary_muscle_development	ENSGALG00010017606	ENSGALG00010017606	0.468476772	0.260242961	1.800151556	0.071836711	0.087915664	positive
PRJNA729271	stage_1W_vs_E10	ordinary_muscle_development	ENSGALG00010017608	RBBP4	-1.174647336	0.081179005	-14.46984154	1.87905E-47	1.88751E-46	negative
PRJNA729271	stage_1W_vs_E10	ordinary_muscle_development	ENSGALG00010017609	TRIM71	-1.5137351	0.209523412	-7.224658501	5.02363E-13	1.34772E-12	negative
PRJNA729271	stage_1W_vs_E10	ordinary_muscle_development	ENSGALG00010017610	PSMC1	0.642524516	0.118962436	5.401070595	6.62443E-08	1.34089E-07	positive
PRJNA729271	stage_1W_vs_E10	ordinary_muscle_development	ENSGALG00010017612	CLDN11	-0.967861241	0.436106843	-2.21932138	0.026464868	0.034107608	negative
PRJNA729271	stage_1W_vs_E10	ordinary_muscle_development	ENSGALG00010017613	HAUS3	-1.869130293	0.123012284	-15.1946638	3.83627E-52	4.53562E-51	negative
PRJNA729271	stage_1W_vs_E10	ordinary_muscle_development	ENSGALG00010017615	AGTRAP	0.021689193	0.139240598	0.155767742	0.876216099	0.890218851	positive
PRJNA729271	stage_1W_vs_E10	ordinary_muscle_development	ENSGALG00010017616	PDCD7	1.462238143	0.154821143	9.444692865	3.56448E-21	1.40553E-20	positive
PRJNA729271	stage_1W_vs_E10	ordinary_muscle_development	ENSGALG00010017619	POLN	-0.369588475	0.351202078	-1.052352757	0.292637735	0.327601481	negative
PRJNA729271	stage_1W_vs_E10	ordinary_muscle_development	ENSGALG00010017620	UBAP1L	0.600896871	0.326034861	1.84304485	0.065322462	0.080377682	positive
PRJNA729271	stage_1W_vs_E10	ordinary_muscle_development	ENSGALG00010017621	SYNE2	1.517669252	0.155915119	9.733945364	2.16047E-22	8.8966E-22	positive
PRJNA729271	stage_1W_vs_E10	ordinary_muscle_development	ENSGALG00010017622	RAB1B	0.269103938	0.112523287	2.39153996	0.016777857	0.022098596	positive
PRJNA729271	stage_1W_vs_E10	ordinary_muscle_development	ENSGALG00010017625	YBX1	-0.030853309	0.0912645	-0.33806474	0.735314401	0.761432678	negative
PRJNA729271	stage_1W_vs_E10	ordinary_muscle_development	ENSGALG00010017626	COPS7B	-0.890715003	0.141251664	-6.305872631	2.86575E-10	6.63456E-10	negative
PRJNA729271	stage_1W_vs_E10	ordinary_muscle_development	ENSGALG00010017628	NRDE2	-0.099778872	0.104616758	-0.953756114	0.340207109	0.377299315	negative
PRJNA729271	stage_1W_vs_E10	ordinary_muscle_development	ENSGALG00010017629	ENSGALG00010017629	0.238406623	0.140682149	1.694647286	0.090142351	0.109084437	positive
PRJNA729271	stage_1W_vs_E10	ordinary_muscle_development	ENSGALG00010017631	NAT8L	-4.049354771	0.277949853	-14.56865232	4.44575E-48	4.55737E-47	negative
PRJNA729271	stage_1W_vs_E10	ordinary_muscle_development	ENSGALG00010017632	AWAT1	1.212798918	0.591774553	2.04942729	0.040420351	0.051031529	positive
PRJNA729271	stage_1W_vs_E10	ordinary_muscle_development	ENSGALG00010017633	HLX	0.678387024	0.15152266	4.477132496	7.56523E-06	1.32277E-05	positive
PRJNA729271	stage_1W_vs_E10	ordinary_muscle_development	ENSGALG00010017634	PPIH	-0.398861974	0.097600695	-4.086671475	4.37606E-05	7.2352E-05	negative
PRJNA729271	stage_1W_vs_E10	ordinary_muscle_development	ENSGALG00010017636	ZBTB8OS	-0.425247373	0.159592889	-2.664575947	0.007708546	0.010549034	negative
PRJNA729271	stage_1W_vs_E10	ordinary_muscle_development	ENSGALG00010017638	GFRA2	0.003038702	0.259050116	0.011730171	0.990640892	0.99228386	positive
PRJNA729271	stage_1W_vs_E10	ordinary_muscle_development	ENSGALG00010017639	ENSGALG00010017639	-1.081542565	0.141248321	-7.657029517	1.90283E-14	5.49432E-14	negative
PRJNA729271	stage_1W_vs_E10	ordinary_muscle_development	ENSGALG00010017641	GPRC5B	1.574373838	0.159169623	9.89117026	4.54681E-23	1.92708E-22	positive
PRJNA729271	stage_1W_vs_E10	ordinary_muscle_development	ENSGALG00010017642	MTARC1	3.162588888	0.121603968	26.00728355	4.0965E-149	3.3359E-147	positive
PRJNA729271	stage_1W_vs_E10	ordinary_muscle_development	ENSGALG00010017643	CALM1	0.785886231	0.060927478	12.89871589	4.5766E-38	3.423E-37	positive
PRJNA729271	stage_1W_vs_E10	ordinary_muscle_development	ENSGALG00010017644	RBBP6	0.610356887	0.096915496	6.297825552	3.0185E-10	6.97484E-10	positive
PRJNA729271	stage_1W_vs_E10	ordinary_muscle_development	ENSGALG00010017645	SYNC	1.316636073	0.270076834	4.8750426	1.08785E-06	2.02939E-06	positive
PRJNA729271	stage_1W_vs_E10	ordinary_muscle_development	ENSGALG00010017646	MAEA	-0.466576925	0.087071798	-5.358530967	8.39014E-08	1.68699E-07	negative
PRJNA729271	stage_1W_vs_E10	ordinary_muscle_development	ENSGALG00010017647	ITGA9	-0.203249033	0.167200436	-1.215601101	0.224136915	0.255655066	negative
PRJNA729271	stage_1W_vs_E10	ordinary_muscle_development	ENSGALG00010017648	ENSGALG00010017648	-0.822543141	0.156600894	-5.252480478	1.50064E-07	2.96346E-07	negative

PRJNA729271	stage_1W_vs_E10	ordinary_muscle_development	ENSGALG00010017649	ENSGALG00010017649	-0.549413486	0.114049943	-4.817306098	1.45509E-06	2.6858E-06	negative
PRJNA729271	stage_1W_vs_E10	ordinary_muscle_development	ENSGALG00010017651	PPCS	0.241085398	0.122649238	1.965649366	0.049339127	0.061642014	positive
PRJNA729271	stage_1W_vs_E10	ordinary_muscle_development	ENSGALG00010017652	C1orf115	1.980663486	0.170954171	11.5859325	4.85653E-31	2.85579E-30	positive
PRJNA729271	stage_1W_vs_E10	ordinary_muscle_development	ENSGALG00010017653	ENSGALG00010017653	1.543632316	0.172501021	8.948540173	3.60214E-19	1.30272E-18	positive
PRJNA729271	stage_1W_vs_E10	ordinary_muscle_development	ENSGALG00010017654	KBTBD13	6.4656404	0.311940018	20.7271912	1.96955E-95	6.74908E-94	positive
PRJNA729271	stage_1W_vs_E10	ordinary_muscle_development	ENSGALG00010017655	ENSGALG00010017655	-1.183431663	0.112597973	-10.51023948	7.74965E-26	3.6951E-25	negative
PRJNA729271	stage_1W_vs_E10	ordinary_muscle_development	ENSGALG00010017656	FSD2	7.606257569	0.207932798	36.5803647	5.8699E-293	1.9787E-290	positive
PRJNA729271	stage_1W_vs_E10	ordinary_muscle_development	ENSGALG00010017657	DIS3L2	0.03753802	0.095115267	0.394658197	0.693095158	0.721673322	positive
PRJNA729271	stage_1W_vs_E10	ordinary_muscle_development	ENSGALG00010017658	WHAMM	3.654649544	0.161193345	22.6724592	8.3781E-114	4.0079E-112	positive
PRJNA729271	stage_1W_vs_E10	ordinary_muscle_development	ENSGALG00010017659	TNRC6A	-0.695836585	0.079319392	-8.772591002	1.74601E-18	6.08669E-18	negative
PRJNA729271	stage_1W_vs_E10	ordinary_muscle_development	ENSGALG00010017660	CLIP4	4.24017752	0.246758033	17.183544	3.52691E-66	5.97226E-65	positive
PRJNA729271	stage_1W_vs_E10	ordinary_muscle_development	ENSGALG00010017661	NHSL3	-0.126737089	0.174895881	-0.724643075	0.468671026	0.507044822	negative
PRJNA729271	stage_1W_vs_E10	ordinary_muscle_development	ENSGALG00010017662	GOLGA4	0.857741723	0.165389734	5.186184804	2.14646E-07	4.19594E-07	positive
PRJNA729271	stage_1W_vs_E10	ordinary_muscle_development	ENSGALG00010017663	ZMYND12	-0.198067879	0.21976959	-0.901252435	0.367454117	0.40516107	negative
PRJNA729271	stage_1W_vs_E10	ordinary_muscle_development	ENSGALG00010017664	ENSGALG00010017664	0.384489606	0.074600408	5.1539798	2.55004E-07	4.9588E-07	positive
PRJNA729271	stage_1W_vs_E10	ordinary_muscle_development	ENSGALG00010017665	CTBP1	0.042148572	0.062931861	0.669749343	0.503017591	0.540772824	positive
PRJNA729271	stage_1W_vs_E10	ordinary_muscle_development	ENSGALG00010017667	SLC2A1	-0.495416746	0.111388396	-4.447651334	8.68143E-06	1.51283E-05	negative
PRJNA729271	stage_1W_vs_E10	ordinary_muscle_development	ENSGALG00010017668	ENSGALG00010017668	3.298455455	0.616927727	5.346583251	8.96301E-08	1.79918E-07	positive
PRJNA729271	stage_1W_vs_E10	ordinary_muscle_development	ENSGALG00010017669	ENSGALG00010017669	0.893270948	0.175635024	5.085949983	3.6579E-07	7.04041E-07	positive
PRJNA729271	stage_1W_vs_E10	ordinary_muscle_development	ENSGALG00010017670	FAM179A	3.243914499	0.327694768	9.899195263	4.19638E-23	1.78116E-22	positive
PRJNA729271	stage_1W_vs_E10	ordinary_muscle_development	ENSGALG00010017671	ANTXR1	-1.342429992	0.186110901	-7.213064802	5.47064E-13	1.46548E-12	negative
PRJNA729271	stage_1W_vs_E10	ordinary_muscle_development	ENSGALG00010017672	SPRED2	-0.834302458	0.07914581	-10.54133449	5.57028E-26	2.67266E-25	negative
PRJNA729271	stage_1W_vs_E10	ordinary_muscle_development	ENSGALG00010017673	WDR89	-0.524455207	0.091869326	-5.708708549	1.13837E-08	2.40744E-08	negative
PRJNA729271	stage_1W_vs_E10	ordinary_muscle_development	ENSGALG00010017675	ENSGALG00010017675	0.087188886	0.523975919	0.16639865	0.867843236	0.882454417	positive
PRJNA729271	stage_1W_vs_E10	ordinary_muscle_development	ENSGALG00010017676	SPON2	2.245782406	0.262771846	8.546510763	1.26866E-17	4.22642E-17	positive
PRJNA729271	stage_1W_vs_E10	ordinary_muscle_development	ENSGALG00010017677	WDR43	0.152600866	0.109905932	1.388467972	0.164994591	0.192420677	positive
PRJNA729271	stage_1W_vs_E10	ordinary_muscle_development	ENSGALG00010017678	YARS	0.274658837	0.137120886	2.003041589	0.045172825	0.056730211	positive
PRJNA729271	stage_1W_vs_E10	ordinary_muscle_development	ENSGALG00010017679	RASL12	3.527024002	0.177983745	19.81655127	2.14301E-87	6.04337E-86	positive
PRJNA729271	stage_1W_vs_E10	ordinary_muscle_development	ENSGALG00010017681	ENSGALG00010017681	6.377495113	1.232097642	5.176128008	2.26538E-07	4.42305E-07	positive
PRJNA729271	stage_1W_vs_E10	ordinary_muscle_development	ENSGALG00010017684	TRMT61B	0.636913574	0.094598888	6.732780795	1.66451E-11	4.13346E-11	positive
PRJNA729271	stage_1W_vs_E10	ordinary_muscle_development	ENSGALG00010017685	PHC2	1.382760643	0.18309099	7.552313997	4.27592E-14	1.21409E-13	positive
PRJNA729271	stage_1W_vs_E10	ordinary_muscle_development	ENSGALG00010017687	TTC7B	1.135429099	0.095852877	11.84554007	2.26952E-32	1.40404E-31	positive
PRJNA729271	stage_1W_vs_E10	ordinary_muscle_development	ENSGALG00010017688	SPDYA	-0.947373522	0.579960751	-1.633513165	0.102361114	0.122926624	negative
PRJNA729271	stage_1W_vs_E10	ordinary_muscle_development	ENSGALG00010017691	LZTS1	-2.96920682	0.291827234	-10.17453641	2.57624E-24	1.14936E-23	negative
PRJNA729271	stage_1W_vs_E10	ordinary_muscle_development	ENSGALG00010017692	EPCAM	-4.375367139	0.995439167	-4.395413886	1.10562E-05	1.91332E-05	negative
PRJNA729271	stage_1W_vs_E10	ordinary_muscle_development	ENSGALG00010017693	MTFMT	0.079119644	0.117655892	0.672466484	0.50128677	0.539072018	positive
PRJNA729271	stage_1W_vs_E10	ordinary_muscle_development	ENSGALG00010017694	ATP6V1B2	-0.004168544	0.070884898	-0.058807222	0.953105656	0.959200829	negative
PRJNA729271	stage_1W_vs_E10	ordinary_muscle_development	ENSGALG00010017695	FNDC5	-1.642466272	0.208484825	-7.878109457	3.32372E-15	9.96839E-15	negative
PRJNA729271	stage_1W_vs_E10	ordinary_muscle_development	ENSGALG00010017698	ENSGALG00010017698	0.171398967	0.840972689	0.203810385	0.838501677	0.856283064	positive
PRJNA729271	stage_1W_vs_E10	ordinary_muscle_development	ENSGALG00010017699	RNF19B	-0.204017045	0.251995397	-0.809606235	0.418166524	0.4562532	negative
PRJNA729271	stage_1W_vs_E10	ordinary_muscle_development	ENSGALG00010017700	VPS35L	-0.281381931	0.079224101	-3.551721364	0.00038272	0.000589285	negative
PRJNA729271	stage_1W_vs_E10	ordinary_muscle_development	ENSGALG00010017701	PPP2R5E	0.048575499	0.099633319	0.487542722	0.625873775	0.659353178	positive
PRJNA729271	stage_1W_vs_E10	ordinary_muscle_development	ENSGALG00010017703	RPS6KA5	0.571209734	0.14031391	4.070941593	4.68235E-05	7.71608E-05	positive
PRJNA729271	stage_1W_vs_E10	ordinary_muscle_development	ENSGALG00010017704	ZNF362	0.092808457	0.197387475	0.470184124	0.638223476	0.670644579	positive
PRJNA729271	stage_1W_vs_E10	ordinary_muscle_development	ENSGALG00010017705	MAK16	0.977432292	0.10314483	9.476309077	2.63437E-21	1.04531E-20	positive
PRJNA729271	stage_1W_vs_E10	ordinary_muscle_development	ENSGALG00010017706	FGFRL1	-1.202508405	0.144756078	-8.307135874	9.80397E-17	3.14678E-16	negative
PRJNA729271	stage_1W_vs_E10	ordinary_muscle_development	ENSGALG00010017707	AK2	-0.830505863	0.09619099	-8.63392572	5.92815E-18	2.01002E-17	negative
PRJNA729271	stage_1W_vs_E10	ordinary_muscle_development	ENSGALG00010017708	MEIS1	-1.742720983	0.104997697	-16.59770668	7.24049E-62	1.10825E-60	negative
PRJNA729271	stage_1W_vs_E10	ordinary_muscle_development	ENSGALG00010017710	RAB41	-0.464283476	0.117911933	-3.937544426	8.23197E-05	0.000133321	negative
PRJNA729271	stage_1W_vs_E10	ordinary_muscle_development	ENSGALG00010017711	LRRFIP2	1.330167009	0.134059766	9.922194002	3.33347E-23	1.42449E-22	positive
PRJNA729271	stage_1W_vs_E10	ordinary_muscle_development	ENSGALG00010017712	MSH2	-1.92540006	0.075012829	-25.66761012	2.6891E-145	2.0515E-143	negative
PRJNA729271	stage_1W_vs_E10	ordinary_muscle_development	ENSGALG00010017713	ALKBH5	3.220389454	0.149033928	21.60843169	1.4965E-103	6.0086E-102	positive
PRJNA729271	stage_1W_vs_E10	ordinary_muscle_development	ENSGALG00010017714	ATAD3A	-0.996801402	0.155376988	-6.415373459	1.40478E-10	3.30933E-10	negative
PRJNA729271	stage_1W_vs_E10	ordinary_muscle_development	ENSGALG00010017715	CILP	2.268286353	0.168708451	13.44500726	3.29373E-41	2.7581E-40	positive
PRJNA729271	stage_1W_vs_E10	ordinary_muscle_development	ENSGALG00010017717	LLGL1	0.184916549	0.149624902	1.235867466	0.216507832	0.247674297	positive
PRJNA729271	stage_1W_vs_E10	ordinary_muscle_development	ENSGALG00010017718	ATOH8	0.947439616	0.298689248	3.171991026	0.001513977	0.002217348	positive
PRJNA729271	stage_1W_vs_E10	ordinary_muscle_development	ENSGALG00010017719	ENSGALG00010017719	3.216018866	1.781883211	1.804842678	0.071099304	0.087064663	positive
PRJNA729271	stage_1W_vs_E10	ordinary_muscle_development	ENSGALG00010017720	ENSGALG00010017720	-2.177895845	0.394718489	-5.517592682	3.43675E-08	7.06405E-08	negative
PRJNA729271	stage_1W_vs_E10	ordinary_muscle_development	ENSGALG00010017721	VWA1	0.869577143	0.120587773	7.211155148	5.54792E-13	1.4859E-12	positive
PRJNA729271	stage_1W_vs_E10	ordinary_muscle_development	ENSGALG00010017723	KCNK12	-3.046951273	0.457875186	-6.654545533	2.84177E-11	6.97569E-11	negative
PRJNA729271	stage_1W_vs_E10	ordinary_muscle_development	ENSGALG00010017724	FLII	1.941400892	0.127669236	15.20648947	3.20263E-52	3.79265E-51	positive
PRJNA729271	stage_1W_vs_E10	ordinary_muscle_development	ENSGALG00010017725	ENSGALG00010017725	1.160659849	0.399747463	2.903482711	0.003690373	0.005213642	positive
PRJNA729271	stage_1W_vs_E10	ordinary_muscle_development	ENSGALG00010017726	TTI2	0.602838923	0.120327606	5.009980209	5.44356E-07	1.03508E-06	positive
PRJNA729271	stage_1W_vs_E10	ordinary_muscle_development	ENSGALG00010017727	AZIN2	0.492670145	0.153836508	3.202556732	0.001362135	0.002002855	positive
PRJNA729271	stage_1W_vs_E10	ordinary_muscle_development	ENSGALG00010017729	MIEF2	1.9055738	0.13986595	13.62428667	2.87203E-42	2.4765E-41	positive
PRJNA729271	stage_1W_vs_E10	ordinary_muscle_development	ENSGALG00010017730	TARP	4.837886348	0.544347944	8.887488967	6.25054E-19	2.23929E-18	positive
PRJNA729271	stage_1W_vs_E10	ordinary_muscle_development	ENSGALG00010017732	RHOJ	-0.686196098	0.080940724	-8.477760846	2.29569E-17	7.57135E-17	negative
PRJNA729271	stage_1W_vs_E10	ordinary_muscle_development	ENSGALG00010017733	TRIM62	0.002284452	0.131306597	0.017397846	0.986119228	0.988301058	positive
PRJNA729271	stage_1W_vs_E10	ordinary_muscle_development	ENSGALG00010017734	TOP3A	-0.4858653	0.119795473	-4.055790144	4.99651E-05	8.21791E-05	negative
PRJNA729271	stage_1W_vs_E10	ordinary_muscle_development	ENSGALG00010017736	ENSGALG00010017736	7.886246683	4.31841232	1.826191224	0.067821463	0.08329002	positive
PRJNA729271	stage_1W_vs_E10	ordinary_muscle_development	ENSGALG00010017737	ENSGALG00010017737	-0.170583095	0.194460933	-0.877210103	0.38037253	0.418164567	negative
PRJNA729271	stage_1W_vs_E10	ordinary_muscle_development	ENSGALG00010017738	NDUFS5	1.104663571	0.111665038	9.892653896	4.47991E-23	1.89928E-22	positive
PRJNA729271	stage_1W_vs_E10	ordinary_muscle_development	ENSGALG00010017739	MRPL20	-0.266013957	0.099652474	-2.669416486	0.007598317	0.010403098	negative
PRJNA729271	stage_1W_vs_E10	ordinary_muscle_development	ENSGALG00010017741	SMCR8	1.741158831	0.138380798	12.58237314	2.63979E-36	1.87017E-35	positive
PRJNA729271	stage_1W_vs_E10	ordinary_muscle_development	ENSGALG00010017742	DGLUCY	-0.676471905	0.246373153	-2.745720855	0.006037811	0.008356543	negative
PRJNA729271	stage_1W_vs_E10	ordinary_muscle_development	ENSGALG00010017743	KCNH5	-3.581515253	0.444413812	-8.058964765	7.69433E-16	2.38107E-15	negative
PRJNA729271	stage_1W_vs_E10	ordinary_muscle_development	ENSGALG00010017744	ETAA1	-0.9261653	0.099826136	-9.277783771	1.7304E-20	6.64781E-20	negative
PRJNA729271	stage_1W_vs_E10	ordinary_muscle_development	ENSGALG00010017745	SHMT1	-0.183084172	0.185907377	-0.98481392	0.324715482	0.361224168	negative
PRJNA729271	stage_1W_vs_E10	ordinary_muscle_development	ENSGALG00010017746	MSH6	-0.711470463	0.08934383	-7.963285951	1.6753E-15	5.10156E-15	negative
PRJNA729271	stage_1W_vs_E10	ordinary_muscle_development	ENSGALG00010017747	DUSP26	0.393024812	0.128571336	3.056861861	0.002236673	0.003225286	positive
PRJNA729271	stage_1W_vs_E10	ordinary_muscle_development	ENSGALG00010017748	ENSGALG00010017748	-4.671263673	0.913070579	-5.115994073	3.12093E-07	6.03171E-07	negative
PRJNA729271	stage_1W_vs_E10	ordinary_muscle_development	ENSGALG00010017751	ST3GAL5	-0.326541233	0.127799073	-2.555114252	0.010615295	0.014310705	negative
PRJNA729271	stage_1W_vs_E10	ordinary_muscle_development	ENSGALG00010017753	GNRHR	0.260178632	0.366597458	0.709712047	0.47788272	0.516240128	positive
PRJNA729271	stage_1W_vs_E10	ordinary_muscle_development	ENSGALG00010017754	PDZD11	-1.29556926	0.162209237	-7.987025182	1.38234E-15	4.22098E-15	negative
PRJNA729271	stage_1W_vs_E10	ordinary_muscle_development	ENSGALG00010017755	C1D	-0.611759684	0.140236148	-4.362353747	1.28671E-05	2.21427E-05	negative
PRJNA729271	stage_1W_vs_E10	ordinary_muscle_development	ENSGALG00010017756	EIF2B5	-0.074949496	0.118789686	-0.630942795	0.528077929	0.564698604	negative
PRJNA729271	stage_1W_vs_E10	ordinary_muscle_development	ENSGALG00010017757	ENSGALG00010017757	1.291094989	0.138539474	9.319329373	1.17078E-20	4.53392E-20	positive
PRJNA729271	stage_1W_vs_E10	ordinary_muscle_development	ENSGALG00010017759	ENSGALG00010017759	6.206595031	0.840111284	7.387824861	1.4925E-13	4.10977E-13	positive
PRJNA729271	stage_1W_vs_E10	ordinary_muscle_development	ENSGALG00010017761	PARP16	0.681782219	0.107207884	6.359441049	2.02489E-10	4.72334E-10	positive
PRJNA729271	stage_1W_vs_E10	ordinary_muscle_development	ENSGALG00010017762	FBXO11	-0.408397815	0.056095269	-7.280432404	3.32752E-13	9.00026E-13	negative
PRJNA729271	stage_1W_vs_E10	ordinary_muscle_development	ENSGALG00010017763	POLR1A	0.18181578	0.086017362	2.113710257	0.034540022	0.043940461	positive
PRJNA729271	stage_1W_vs_E10	ordinary_muscle_development	ENSGALG00010017764	IGDCC3	-1.828979581	0.246173793	-7.429627488	1.08904E-13	3.02117E-13	negative
PRJNA729271	stage_1W_vs_E10	ordinary_muscle_development	ENSGALG00010017765	SYT16	-4.339966847	0.325358907	-13.33901349	1.37264E-40	1.12029E-39	negative
PRJNA729271	stage_1W_vs_E10	ordinary_muscle_development	ENSGALG00010017766	DNAAF10	1.529414874	0.153151666	9.986276449	1.75034E-23	7.55993E-23	positive
PRJNA729271	stage_1W_vs_E10	ordinary_muscle_development	ENSGALG00010017767	KIF4B	-3.05210586	0.222613884	-13.71031223	8.8078E-43	7.70948E-42	negative
PRJNA729271	stage_1W_vs_E10	ordinary_muscle_development	ENSGALG00010017768	FOXN2	0.299684092	0.082071853	3.651484431	0.000260729	0.000407161	positive
PRJNA729271	stage_1W_vs_E10	ordinary_muscle_development	ENSGALG00010017770	PTCD3	0.16961084	0.069841501	2.428510793	0.015160974	0.020076587	positive
PRJNA729271	stage_1W_vs_E10	ordinary_muscle_development	ENSGALG00010017771	PNO1	1.420761471	0.1566392	9.070280451	1.18711E-19	4.39182E-19	positive
PRJNA729271	stage_1W_vs_E10	ordinary_muscle_development	ENSGALG00010017772	ENSGALG00010017772	2.568233902	0.342860496	7.490608951	6.85548E-14	1.92615E-13	positive
PRJNA729271	stage_1W_vs_E10	ordinary_muscle_development	ENSGALG00010017773	DVL3	-1.69113214	0.08560694	-19.75461507	7.32117E-87	2.04471E-85	negative
PRJNA729271	stage_1W_vs_E10	ordinary_muscle_development	ENSGALG00010017774	ASH2L	1.188387106	0.162633819	7.307133969	2.729E-13	7.41461E-13	positive
PRJNA729271	stage_1W_vs_E10	ordinary_muscle_development	ENSGALG00010017775	PRPSAP2	-1.263969695	0.087088195	-14.51367421	9.92583E-48	1.00471E-46	negative
PRJNA729271	stage_1W_vs_E10	ordinary_muscle_development	ENSGALG00010017776	GPR68	-0.920833712	0.376851284	-2.443493632	0.014545826	0.01930255	negative
PRJNA729271	stage_1W_vs_E10	ordinary_muscle_development	ENSGALG00010017778	PPP3R1	1.37201307	0.078537266	17.4695803	2.44285E-68	4.43168E-67	positive
PRJNA729271	stage_1W_vs_E10	ordinary_muscle_development	ENSGALG00010017779	SNAPC1	0.203778904	0.124294446	1.639485192	0.101112252	0.121507386	positive
PRJNA729271	stage_1W_vs_E10	ordinary_muscle_development	ENSGALG00010017780	TAF9	0.60385524	0.136492778	4.424081991	9.68532E-06	1.68231E-05	positive
PRJNA729271	stage_1W_vs_E10	ordinary_muscle_development	ENSGALG00010017781	CYP26C1	1.482240749	0.300209891	4.937348151	7.9192E-07	1.49231E-06	positive
PRJNA729271	stage_1W_vs_E10	ordinary_muscle_development	ENSGALG00010017782	IGDCC4	-0.856055958	0.117456848	-7.288259249	3.13985E-13	8.51013E-13	negative
PRJNA729271	stage_1W_vs_E10	ordinary_muscle_development	ENSGALG00010017783	STAR	0.781829142	0.383525401	2.038532884	0.04149667	0.052328102	positive
PRJNA729271	stage_1W_vs_E10	ordinary_muscle_development	ENSGALG00010017786	KAZALD1	-3.123697604	0.172359108	-18.12319427	2.09095E-73	4.26879E-72	negative
PRJNA729271	stage_1W_vs_E10	ordinary_muscle_development	ENSGALG00010017787	CNRIP1	1.202907098	0.238948071	5.034177899	4.79904E-07	9.16013E-07	positive
PRJNA729271	stage_1W_vs_E10	ordinary_muscle_development	ENSGALG00010017789	IMMT	2.014748108	0.13001661	15.49608244	3.68698E-54	4.66342E-53	positive
PRJNA729271	stage_1W_vs_E10	ordinary_muscle_development	ENSGALG00010017790	R3HCC1L	0.539572768	0.174891709	3.085182085	0.002034276	0.002945737	positive
PRJNA729271	stage_1W_vs_E10	ordinary_muscle_development	ENSGALG00010017791	CCNL2	0.28119477	0.111714039	2.517094289	0.011832716	0.015861945	positive
PRJNA729271	stage_1W_vs_E10	ordinary_muscle_development	ENSGALG00010017792	LSM1	0.182091571	0.122832481	1.482438268	0.138223718	0.162956713	positive
PRJNA729271	stage_1W_vs_E10	ordinary_muscle_development	ENSGALG00010017793	FAM83G	-3.797429055	0.433015254	-8.769735069	1.79087E-18	6.23707E-18	negative
PRJNA729271	stage_1W_vs_E10	ordinary_muscle_development	ENSGALG00010017795	MACF1	-0.827314636	0.114390563	-7.232367874	4.74644E-13	1.27525E-12	negative
PRJNA729271	stage_1W_vs_E10	ordinary_muscle_development	ENSGALG00010017796	MXI1	1.979193127	0.131924176	15.00250508	7.06998E-51	7.98748E-50	positive
PRJNA729271	stage_1W_vs_E10	ordinary_muscle_development	ENSGALG00010017797	PPP1R21	-0.471683775	0.093210473	-5.060416088	4.18343E-07	8.0157E-07	negative
PRJNA729271	stage_1W_vs_E10	ordinary_muscle_development	ENSGALG00010017798	BAG4	0.949111366	0.156668759	6.058076758	1.37759E-09	3.08055E-09	positive
PRJNA729271	stage_1W_vs_E10	ordinary_muscle_development	ENSGALG00010017799	GRAPL	0.92697356	0.331433704	2.796859672	0.005160194	0.00719754	positive
PRJNA729271	stage_1W_vs_E10	ordinary_muscle_development	ENSGALG00010017801	SMNDC1	-0.059470029	0.071522961	-0.83148165	0.40570159	0.443909695	negative
PRJNA729271	stage_1W_vs_E10	ordinary_muscle_development	ENSGALG00010017803	ENSGALG00010017803	0.761144518	0.118324313	6.432697555	1.25359E-10	2.96037E-10	positive
PRJNA729271	stage_1W_vs_E10	ordinary_muscle_development	ENSGALG00010017804	DUSP5	-0.963580841	0.256242849	-3.760420411	0.000169628	0.000268306	negative
PRJNA729271	stage_1W_vs_E10	ordinary_muscle_development	ENSGALG00010017805	DPP8	1.166873021	0.106250563	10.98227611	4.65056E-28	2.42919E-27	positive
PRJNA729271	stage_1W_vs_E10	ordinary_muscle_development	ENSGALG00010017806	ENSGALG00010017806	3.665498782	0.749114438	4.893109248	9.92553E-07	1.85735E-06	positive
PRJNA729271	stage_1W_vs_E10	ordinary_muscle_development	ENSGALG00010017807	MRPL35	0.742718708	0.146629774	5.065265311	4.07832E-07	7.82362E-07	positive
PRJNA729271	stage_1W_vs_E10	ordinary_muscle_development	ENSGALG00010017808	SFRP5	1.264396529	0.354402697	3.567683142	0.000360152	0.000555617	positive
PRJNA729271	stage_1W_vs_E10	ordinary_muscle_development	ENSGALG00010017809	MOSMO	-0.185529559	0.10493226	-1.768088857	0.07704605	0.093934098	negative
PRJNA729271	stage_1W_vs_E10	ordinary_muscle_development	ENSGALG00010017810	HIF1A	-1.014831079	0.079154634	-12.82086762	1.25291E-37	9.24688E-37	negative
PRJNA729271	stage_1W_vs_E10	ordinary_muscle_development	ENSGALG00010017811	HACD3	-0.948619146	0.06691814	-14.17581461	1.29334E-45	1.23416E-44	negative

PRJNA729271	stage_1W_vs_E10	ordinary_muscle_development	ENSGALG00010017812	ENSGALG00010017812	4.832659221	1.728534537	2.795812937	0.005176936	0.007219519	positive
PRJNA729271	stage_1W_vs_E10	ordinary_muscle_development	ENSGALG00010017813	DDHD2	0.42386771	0.160410378	2.642395814	0.008232179	0.011231686	positive
PRJNA729271	stage_1W_vs_E10	ordinary_muscle_development	ENSGALG00010017814	ENSGALG00010017814	-2.88674344	0.264960417	-10.89499887	1.21768E-27	6.24568E-27	negative
PRJNA729271	stage_1W_vs_E10	ordinary_muscle_development	ENSGALG00010017815	VWA9	-0.658372434	0.086204025	-7.637374626	2.21696E-14	6.37975E-14	negative
PRJNA729271	stage_1W_vs_E10	ordinary_muscle_development	ENSGALG00010017816	PRKCH	0.204592264	0.287391928	0.711892868	0.476531119	0.51485678	positive
PRJNA729271	stage_1W_vs_E10	ordinary_muscle_development	ENSGALG00010017817	REEP1	3.044134261	0.116445125	26.14222169	1.2081E-150	1.0241E-148	positive
PRJNA729271	stage_1W_vs_E10	ordinary_muscle_development	ENSGALG00010017818	SLC38A6	-0.261307879	0.138184042	-1.891013424	0.058622552	0.072552416	negative
PRJNA729271	stage_1W_vs_E10	ordinary_muscle_development	ENSGALG00010017820	FLRT2	-0.353344323	0.134970462	-2.617938163	0.008846283	0.012029916	negative
PRJNA729271	stage_1W_vs_E10	ordinary_muscle_development	ENSGALG00010017821	ENSGALG00010017821	-4.499798574	0.44571626	-10.09565721	5.7743E-24	2.54248E-23	negative
PRJNA729271	stage_1W_vs_E10	ordinary_muscle_development	ENSGALG00010017822	VWA3A	-1.801652197	0.270522405	-6.659900112	2.74014E-11	6.73648E-11	negative
PRJNA729271	stage_1W_vs_E10	ordinary_muscle_development	ENSGALG00010017823	PPIE	-0.108397487	0.090180999	-1.201999177	0.229363847	0.261021352	negative
PRJNA729271	stage_1W_vs_E10	ordinary_muscle_development	ENSGALG00010017824	YAE1	-0.202513911	0.125900325	-1.60852572	0.107720088	0.128913695	negative
PRJNA729271	stage_1W_vs_E10	ordinary_muscle_development	ENSGALG00010017825	PLPP5	-1.455451387	0.152637706	-9.535333185	1.49405E-21	5.98239E-21	negative
PRJNA729271	stage_1W_vs_E10	ordinary_muscle_development	ENSGALG00010017826	PABPC4	4.274655073	0.139807583	30.57527337	2.6097E-205	4.1568E-203	positive
PRJNA729271	stage_1W_vs_E10	ordinary_muscle_development	ENSGALG00010017828	ENSGALG00010017828	1.021732069	0.236155109	4.326529594	1.51477E-05	2.58922E-05	positive
PRJNA729271	stage_1W_vs_E10	ordinary_muscle_development	ENSGALG00010017830	DENND4A	1.036607204	0.127772787	8.112895015	4.94278E-16	1.54143E-15	positive
PRJNA729271	stage_1W_vs_E10	ordinary_muscle_development	ENSGALG00010017831	PLEK	1.02274079	0.184172739	5.553160566	2.8055E-08	5.79284E-08	positive
PRJNA729271	stage_1W_vs_E10	ordinary_muscle_development	ENSGALG00010017832	POU3F1	-3.000640319	0.525829338	-5.706490875	1.15329E-08	2.43758E-08	negative
PRJNA729271	stage_1W_vs_E10	ordinary_muscle_development	ENSGALG00010017834	ENSGALG00010017834	-1.194743765	0.380127596	-3.143007182	0.001672217	0.00244195	negative
PRJNA729271	stage_1W_vs_E10	ordinary_muscle_development	ENSGALG00010017838	B3GNT2	-1.132918244	0.127674367	-8.873498065	7.08835E-19	2.52508E-18	negative
PRJNA729271	stage_1W_vs_E10	ordinary_muscle_development	ENSGALG00010017841	GALC	-1.751480581	0.272758683	-6.421355905	1.35066E-10	3.18493E-10	negative
PRJNA729271	stage_1W_vs_E10	ordinary_muscle_development	ENSGALG00010017843	NSD3	-0.3090562	0.071759727	-4.306819649	1.65619E-05	2.82362E-05	negative
PRJNA729271	stage_1W_vs_E10	ordinary_muscle_development	ENSGALG00010017848	CYP3A4	0.83189358	0.198126325	4.198803857	2.68329E-05	4.50269E-05	positive
PRJNA729271	stage_1W_vs_E10	ordinary_muscle_development	ENSGALG00010017849	FBXO48	3.007503429	0.173965362	17.287944	5.79838E-67	1.00656E-65	positive
PRJNA729271	stage_1W_vs_E10	ordinary_muscle_development	ENSGALG00010017850	TRMT5	-0.650353986	0.09397697	-6.920354886	4.50514E-12	1.15293E-11	negative
PRJNA729271	stage_1W_vs_E10	ordinary_muscle_development	ENSGALG00010017852	LETM2	0.447449987	0.114911853	3.893854085	9.8664E-05	0.000158745	positive
PRJNA729271	stage_1W_vs_E10	ordinary_muscle_development	ENSGALG00010017855	SDR42E2	-0.484729624	0.297827775	-1.627550095	0.103620318	0.124356393	negative
PRJNA729271	stage_1W_vs_E10	ordinary_muscle_development	ENSGALG00010017856	FASTKD5	0.528213376	0.115599093	4.569355702	4.89226E-06	8.67865E-06	positive
PRJNA729271	stage_1W_vs_E10	ordinary_muscle_development	ENSGALG00010017858	GPR65	2.202131038	0.284567517	7.738518657	1.00582E-14	2.95068E-14	positive
PRJNA729271	stage_1W_vs_E10	ordinary_muscle_development	ENSGALG00010017859	EXOC6	2.17030042	0.133689305	16.23391202	2.9035E-59	4.16696E-58	positive
PRJNA729271	stage_1W_vs_E10	ordinary_muscle_development	ENSGALG00010017860	CYSLTR1	-0.121319418	0.208261092	-0.582535207	0.560206234	0.596035558	negative
PRJNA729271	stage_1W_vs_E10	ordinary_muscle_development	ENSGALG00010017861	PRSS56	0.7060936	0.507834444	1.390401159	0.164407095	0.191781815	positive
PRJNA729271	stage_1W_vs_E10	ordinary_muscle_development	ENSGALG00010017865	UBOX5	0.732572585	0.132697865	5.520605656	3.37833E-08	6.94989E-08	positive
PRJNA729271	stage_1W_vs_E10	ordinary_muscle_development	ENSGALG00010017866	RALA	-0.685818553	0.096041967	-7.140821633	9.27747E-13	2.46204E-12	negative
PRJNA729271	stage_1W_vs_E10	ordinary_muscle_development	ENSGALG00010017867	APLF	1.22263641	0.108539374	11.26445049	1.96574E-29	1.08381E-28	positive
PRJNA729271	stage_1W_vs_E10	ordinary_muscle_development	ENSGALG00010017868	CHRND	1.058581681	0.241530921	4.382799839	1.17164E-05	2.02346E-05	positive
PRJNA729271	stage_1W_vs_E10	ordinary_muscle_development	ENSGALG00010017870	RAB11A	0.29895673	0.073850219	4.048149567	5.16242E-05	8.48404E-05	positive
PRJNA729271	stage_1W_vs_E10	ordinary_muscle_development	ENSGALG00010017873	HSPG2	1.858963701	0.109623169	16.95776286	1.68644E-64	2.76214E-63	positive
PRJNA729271	stage_1W_vs_E10	ordinary_muscle_development	ENSGALG00010017876	COMMD1	-0.362332461	0.138046772	-2.624707964	0.008672331	0.01180222	negative
PRJNA729271	stage_1W_vs_E10	ordinary_muscle_development	ENSGALG00010017878	CHRNG	0.252931209	0.250028101	1.011611125	0.311724025	0.347652515	positive
PRJNA729271	stage_1W_vs_E10	ordinary_muscle_development	ENSGALG00010017880	CDK13	0.278310007	0.084435574	3.296122659	0.000980292	0.001461512	positive
PRJNA729271	stage_1W_vs_E10	ordinary_muscle_development	ENSGALG00010017881	KCNK10	-3.965890063	0.728034385	-5.447393895	5.11132E-08	1.04247E-07	negative
PRJNA729271	stage_1W_vs_E10	ordinary_muscle_development	ENSGALG00010017882	LZTS3	2.230415057	0.199622317	11.17317487	5.51729E-29	2.9763E-28	positive
PRJNA729271	stage_1W_vs_E10	ordinary_muscle_development	ENSGALG00010017886	DDRGK1	0.097465562	0.102432052	0.9515143	0.341343363	0.378385748	positive
PRJNA729271	stage_1W_vs_E10	ordinary_muscle_development	ENSGALG00010017887	PROKR1	6.55069305	0.38758011	16.90152016	4.38456E-64	7.0931E-63	positive
PRJNA729271	stage_1W_vs_E10	ordinary_muscle_development	ENSGALG00010017888	MPLKIP	-0.539742276	0.113722249	-4.74614493	2.0733E-06	3.77971E-06	negative
PRJNA729271	stage_1W_vs_E10	ordinary_muscle_development	ENSGALG00010017890	ANAPC1	-0.394224329	0.06545834	-6.022522544	1.71719E-09	3.82229E-09	negative
PRJNA729271	stage_1W_vs_E10	ordinary_muscle_development	ENSGALG00010017891	GOLGA7B	3.956393209	0.326194147	12.12895218	7.41927E-34	4.83989E-33	positive
PRJNA729271	stage_1W_vs_E10	ordinary_muscle_development	ENSGALG00010017894	KAT6A	-0.102685556	0.081340771	-1.262411892	0.206800612	0.237356471	negative
PRJNA729271	stage_1W_vs_E10	ordinary_muscle_development	ENSGALG00010017895	MNAT1	0.546650409	0.080553675	6.786163475	1.15155E-11	2.88583E-11	positive
PRJNA729271	stage_1W_vs_E10	ordinary_muscle_development	ENSGALG00010017898	NHERF2	0.796119677	0.114695544	6.941156139	3.88904E-12	9.98258E-12	positive
PRJNA729271	stage_1W_vs_E10	ordinary_muscle_development	ENSGALG00010017901	HTR7L	-2.382129715	0.72080559	-3.304815817	0.000950388	0.001419462	negative
PRJNA729271	stage_1W_vs_E10	ordinary_muscle_development	ENSGALG00010017903	ENSGALG00010017903	-5.103893819	0.568299433	-8.980994044	2.68331E-19	9.76026E-19	negative
PRJNA729271	stage_1W_vs_E10	ordinary_muscle_development	ENSGALG00010017904	MEGF11	-4.302861928	0.40365757	-10.65968346	1.57131E-26	7.7129E-26	negative
PRJNA729271	stage_1W_vs_E10	ordinary_muscle_development	ENSGALG00010017905	EIF4E2	-0.280399905	0.087918482	-3.189316945	0.001426094	0.002094562	negative
PRJNA729271	stage_1W_vs_E10	ordinary_muscle_development	ENSGALG00010017909	NTHL1	-0.545313292	0.140014715	-3.894685582	9.83262E-05	0.000158219	negative
PRJNA729271	stage_1W_vs_E10	ordinary_muscle_development	ENSGALG00010017915	BFSP1	2.758868612	0.441260689	6.252241998	4.04602E-10	9.29135E-10	positive
PRJNA729271	stage_1W_vs_E10	ordinary_muscle_development	ENSGALG00010017918	SIX4	1.170365159	0.20049863	5.8372726	5.30622E-09	1.14514E-08	positive
PRJNA729271	stage_1W_vs_E10	ordinary_muscle_development	ENSGALG00010017920	AP3M2	-0.890377155	0.082152867	-10.83805337	2.27255E-27	1.15379E-26	negative
PRJNA729271	stage_1W_vs_E10	ordinary_muscle_development	ENSGALG00010017921	SPATA7	0.535000374	0.204885278	2.611219209	0.009022004	0.012259675	positive
PRJNA729271	stage_1W_vs_E10	ordinary_muscle_development	ENSGALG00010017922	MERTK	0.409406876	0.131548548	3.112211277	0.001856916	0.002698886	positive
PRJNA729271	stage_1W_vs_E10	ordinary_muscle_development	ENSGALG00010017927	PCSK2	0.457670103	0.125553801	3.645211047	0.000267172	0.000416973	positive
PRJNA729271	stage_1W_vs_E10	ordinary_muscle_development	ENSGALG00010017930	TSC2	-0.493032382	0.078808015	-6.256119774	3.94674E-10	9.0763E-10	negative
PRJNA729271	stage_1W_vs_E10	ordinary_muscle_development	ENSGALG00010017933	SIX1	1.483595081	0.195595175	7.585029033	3.32413E-14	9.47742E-14	positive
PRJNA729271	stage_1W_vs_E10	ordinary_muscle_development	ENSGALG00010017937	PTPN21	1.630019222	0.119274419	13.66612584	1.61782E-42	1.40589E-41	positive
PRJNA729271	stage_1W_vs_E10	ordinary_muscle_development	ENSGALG00010017941	DIS3L	1.252880163	0.135504609	9.24603354	2.32978E-20	8.88689E-20	positive
PRJNA729271	stage_1W_vs_E10	ordinary_muscle_development	ENSGALG00010017946	PTGDR	0.484513326	0.256633684	1.887956863	0.059031742	0.073040128	positive
PRJNA729271	stage_1W_vs_E10	ordinary_muscle_development	ENSGALG00010017948	PLAT	1.397390688	0.15741421	8.877157192	6.85908E-19	2.44582E-18	positive
PRJNA729271	stage_1W_vs_E10	ordinary_muscle_development	ENSGALG00010017949	SFXN3	1.538907491	0.137692913	11.17637398	5.32203E-29	2.8731E-28	positive
PRJNA729271	stage_1W_vs_E10	ordinary_muscle_development	ENSGALG00010017950	FBLN7	-0.303549137	0.31128523	-0.975147896	0.329486903	0.365942278	negative
PRJNA729271	stage_1W_vs_E10	ordinary_muscle_development	ENSGALG00010017954	PTGER2	0.990978311	0.262308482	3.777911805	0.000158149	0.000250696	positive
PRJNA729271	stage_1W_vs_E10	ordinary_muscle_development	ENSGALG00010017955	IKBKB	0.749291722	0.062990345	11.89534235	1.25139E-32	7.79496E-32	positive
PRJNA729271	stage_1W_vs_E10	ordinary_muscle_development	ENSGALG00010017956	TIPIN	-0.748003515	0.08146745	-9.1816243	4.24637E-20	1.60039E-19	negative
PRJNA729271	stage_1W_vs_E10	ordinary_muscle_development	ENSGALG00010017957	THUMPD1	0.278678448	0.098001192	2.843623028	0.004460379	0.006253936	positive
PRJNA729271	stage_1W_vs_E10	ordinary_muscle_development	ENSGALG00010017958	CAPN10	-1.460632482	0.096025961	-15.21080831	2.99818E-52	3.55345E-51	negative
PRJNA729271	stage_1W_vs_E10	ordinary_muscle_development	ENSGALG00010017959	PDZD7	3.640686284	0.270855632	13.44142729	3.45704E-41	2.88984E-40	positive
PRJNA729271	stage_1W_vs_E10	ordinary_muscle_development	ENSGALG00010017962	TXNDC16	-1.021670959	0.151985952	-6.722140729	1.79074E-11	4.44237E-11	negative
PRJNA729271	stage_1W_vs_E10	ordinary_muscle_development	ENSGALG00010017963	LPAR4	-1.905228308	0.170369043	-11.18294895	4.942E-29	2.6741E-28	negative
PRJNA729271	stage_1W_vs_E10	ordinary_muscle_development	ENSGALG00010017970	AMPH	2.759365614	0.670071321	4.118017779	3.82145E-05	6.34501E-05	positive
PRJNA729271	stage_1W_vs_E10	ordinary_muscle_development	ENSGALG00010017972	RNPEPL1	1.141308472	0.172248642	6.625935955	3.45055E-11	8.43008E-11	positive
PRJNA729271	stage_1W_vs_E10	ordinary_muscle_development	ENSGALG00010017973	PKD1	0.955813153	0.129219155	7.396837983	1.39466E-13	3.84545E-13	positive
PRJNA729271	stage_1W_vs_E10	ordinary_muscle_development	ENSGALG00010017974	MXRA8	-0.131502671	0.184269703	-0.71364239	0.475448338	0.513840118	negative
PRJNA729271	stage_1W_vs_E10	ordinary_muscle_development	ENSGALG00010017976	GPR137C	-1.102465354	0.109791879	-10.04141079	1.0023E-23	4.37338E-23	negative
PRJNA729271	stage_1W_vs_E10	ordinary_muscle_development	ENSGALG00010017979	ENSGALG00010017979	-0.548102204	0.170426364	-3.216064643	0.001299616	0.001913451	negative
PRJNA729271	stage_1W_vs_E10	ordinary_muscle_development	ENSGALG00010017983	SLC4A11	2.12506937	0.202466783	10.49589144	9.0222E-26	4.28355E-25	positive
PRJNA729271	stage_1W_vs_E10	ordinary_muscle_development	ENSGALG00010017985	ABCC5	1.379596892	0.267531764	5.156759226	2.51261E-07	4.88928E-07	positive
PRJNA729271	stage_1W_vs_E10	ordinary_muscle_development	ENSGALG00010017987	ERI2	-2.120156482	0.238042706	-8.906622318	5.26109E-19	1.89089E-18	negative
PRJNA729271	stage_1W_vs_E10	ordinary_muscle_development	ENSGALG00010017991	RAB26	-1.018757825	0.246697856	-4.129577135	3.63431E-05	6.04953E-05	negative
PRJNA729271	stage_1W_vs_E10	ordinary_muscle_development	ENSGALG00010017992	ENSGALG00010017992	2.845145099	0.601167997	4.73269554	2.21558E-06	4.03199E-06	positive
PRJNA729271	stage_1W_vs_E10	ordinary_muscle_development	ENSGALG00010017993	REXO5	-1.103340426	0.097067535	-11.36672968	6.12393E-30	3.45663E-29	negative
PRJNA729271	stage_1W_vs_E10	ordinary_muscle_development	ENSGALG00010017996	ZC3H14	0.34528376	0.091457358	3.77535246	0.000159782	0.000253174	positive
PRJNA729271	stage_1W_vs_E10	ordinary_muscle_development	ENSGALG00010017997	ENSGALG00010017997	-0.658891091	0.373409585	-1.764526453	0.077643386	0.094638486	negative
PRJNA729271	stage_1W_vs_E10	ordinary_muscle_development	ENSGALG00010017999	GPR174	2.366344627	0.582155595	4.064797532	4.80742E-05	7.91858E-05	positive
PRJNA729271	stage_1W_vs_E10	ordinary_muscle_development	ENSGALG00010018000	MAP2K1	0.517843678	0.073039313	7.089930797	1.34179E-12	3.53173E-12	positive
PRJNA729271	stage_1W_vs_E10	ordinary_muscle_development	ENSGALG00010018001	TRAF7	-0.314547192	0.086707148	-3.627696191	0.000285961	0.000445078	negative
PRJNA729271	stage_1W_vs_E10	ordinary_muscle_development	ENSGALG00010018002	ZC3H8	0.234416937	0.079770005	2.938660174	0.003296343	0.004681608	positive
PRJNA729271	stage_1W_vs_E10	ordinary_muscle_development	ENSGALG00010018004	CDC42BPA	-0.227095108	0.062406964	-3.638938574	0.000273764	0.000426965	negative
PRJNA729271	stage_1W_vs_E10	ordinary_muscle_development	ENSGALG00010018006	DCUN1D3	-0.135642937	0.105057455	-1.291131006	0.196658254	0.226558686	negative
PRJNA729271	stage_1W_vs_E10	ordinary_muscle_development	ENSGALG00010018007	DNAAF9	-0.545405067	0.179693187	-3.035201697	0.002403749	0.00345521	negative
PRJNA729271	stage_1W_vs_E10	ordinary_muscle_development	ENSGALG00010018008	ABCF3	2.805349622	0.131596008	21.31789304	7.7468E-101	2.94726E-99	positive
PRJNA729271	stage_1W_vs_E10	ordinary_muscle_development	ENSGALG00010018010	ENSGALG00010018010	-0.439582978	0.119647923	-3.673970823	0.00023881	0.000373898	negative
PRJNA729271	stage_1W_vs_E10	ordinary_muscle_development	ENSGALG00010018011	LIPA	0.257287457	0.185735403	1.385236484	0.165980168	0.193492242	positive
PRJNA729271	stage_1W_vs_E10	ordinary_muscle_development	ENSGALG00010018012	DVL1	1.380156166	0.138036728	9.998470579	1.54769E-23	6.70264E-23	positive
PRJNA729271	stage_1W_vs_E10	ordinary_muscle_development	ENSGALG00010018014	LYRM1	0.195004275	0.097005918	2.0102307	0.044406778	0.055816531	positive
PRJNA729271	stage_1W_vs_E10	ordinary_muscle_development	ENSGALG00010018016	IFIT5	6.453979331	0.857145988	7.529615046	5.08901E-14	1.4396E-13	positive
PRJNA729271	stage_1W_vs_E10	ordinary_muscle_development	ENSGALG00010018019	NAPB	0.686340125	0.353692474	1.940499657	0.052318996	0.065190737	positive
PRJNA729271	stage_1W_vs_E10	ordinary_muscle_development	ENSGALG00010018020	SNAPC5	-1.021464525	0.105144128	-9.714898474	2.60509E-22	1.06759E-21	negative
PRJNA729271	stage_1W_vs_E10	ordinary_muscle_development	ENSGALG00010018021	ATRN	1.189109904	0.070835563	16.78690546	3.04303E-63	4.81537E-62	positive
PRJNA729271	stage_1W_vs_E10	ordinary_muscle_development	ENSGALG00010018022	XPO6	-0.528625885	0.117316616	-4.505976255	6.60685E-06	1.15954E-05	negative
PRJNA729271	stage_1W_vs_E10	ordinary_muscle_development	ENSGALG00010018025	CPTP	-0.181082978	0.134072296	-1.350636802	0.176811806	0.205096601	negative
PRJNA729271	stage_1W_vs_E10	ordinary_muscle_development	ENSGALG00010018026	CCDC39	-2.617732637	0.82956013	-3.155567078	0.001601864	0.002341808	negative
PRJNA729271	stage_1W_vs_E10	ordinary_muscle_development	ENSGALG00010018027	EML5	-1.516538377	0.345877526	-4.384610924	1.16193E-05	2.00813E-05	negative
PRJNA729271	stage_1W_vs_E10	ordinary_muscle_development	ENSGALG00010018030	POLB	0.762306701	0.100690768	7.570770548	3.71017E-14	1.05677E-13	positive
PRJNA729271	stage_1W_vs_E10	ordinary_muscle_development	ENSGALG00010018032	ERO1A	0.149565131	0.123766401	1.208446955	0.226875388	0.258453218	positive
PRJNA729271	stage_1W_vs_E10	ordinary_muscle_development	ENSGALG00010018033	COQ8A	2.408540615	0.213181257	11.29808806	1.34106E-29	7.44206E-29	positive
PRJNA729271	stage_1W_vs_E10	ordinary_muscle_development	ENSGALG00010018035	ITM2A	1.156785739	0.086368692	13.39357712	6.59274E-41	5.44202E-40	positive
PRJNA729271	stage_1W_vs_E10	ordinary_muscle_development	ENSGALG00010018036	RPL4	0.582592432	0.107034311	5.443043717	5.23778E-08	1.06721E-07	positive
PRJNA729271	stage_1W_vs_E10	ordinary_muscle_development	ENSGALG00010018037	GZF1	-0.206406556	0.084341039	-2.447284965	0.014393699	0.019111172	negative
PRJNA729271	stage_1W_vs_E10	ordinary_muscle_development	ENSGALG00010018039	TTC14	0.502161675	0.131332281	3.823596702	0.000131519	0.000209606	positive
PRJNA729271	stage_1W_vs_E10	ordinary_muscle_development	ENSGALG00010018042	ZW1LCH	-1.61498656	0.140630054	-11.48393618	1.58879E-30	9.15323E-30	negative
PRJNA729271	stage_1W_vs_E10	ordinary_muscle_development	ENSGALG00010018043	PSMC6	0.05091963	0.091126194	0.558781482	0.576310862	0.611852775	positive
PRJNA729271	stage_1W_vs_E10	ordinary_muscle_development	ENSGALG00010018044	INTS11	-0.644668526	0.060128599	-10.72149583	8.06877E-27	4.00126E-26	negative
PRJNA729271	stage_1W_vs_E10	ordinary_muscle_development	ENSGALG00010018045	PSEN2	1.201636534	0.073170979	16.42231042	1.32427E-60	1.97279E-59	positive
PRJNA729271	stage_1W_vs_E10	ordinary_muscle_development	ENSGALG00010018046	CAPN15	-0.478200621	0.104006281	-4.597805235	4.26965E-06	7.61143E-06	negative
PRJNA729271	stage_1W_vs_E10	ordinary_muscle_development	ENSGALG00010018048	GFRA4	-4.014221413	0.169527635	-23.67886159	5.9546E-124	3.5519E-122	negative
PRJNA729271	stage_1W_vs_E10	ordinary_muscle_development	ENSGALG00010018049	SUGCT	-1.454995455	0.18073644	-8.05037133	8.25432E-16	2.54838E-15	negative
PRJNA729271	stage_1W_vs_E10	ordinary_muscle_development	ENSGALG00010018051	GNPNAT1	-0.241741618	0.133830898	-1.80632142	0.070868148	0.086810936	negative
PRJNA729271	stage_1W_vs_E10	ordinary_muscle_development	ENSGALG00010018052	PPP1RL	-0.543815505	0.131890289	-4.123241436	3.73578E-05	6.21272E-05	negative
PRJNA729271	stage_1W_vs_E10	ordinary_muscle_development	ENSGALG00010018053	DIAPH2	1.725585503	0.091448684	18.86944055	2.03441E-79	4.76393E-78	positive
PRJNA729271	stage_1W_vs_E10	ordinary_muscle_development	ENSGALG00010018054	SMAD6	-0.775910203	0.159867605	-4.853454859	1.21329E-06	2.25441E-06	negative
PRJNA729271	stage_1W_vs_E10	ordinary_muscle_development	ENSGALG00010018055	PEX5L	-0.138401625	0.495075176	-0.279556785	0.779817566	0.802574241	negative

PRJNA729271	stage_1W_vs_E10	ordinary_muscle_development	ENSGALG00010018057	SSTR4	-0.580012337	0.180694386	-3.209907897	0.001327775	0.001953919	negative
PRJNA729271	stage_1W_vs_E10	ordinary_muscle_development	ENSGALG00010018058	ITPKB	-0.269268927	0.092995757	-2.895496911	0.003785588	0.005341909	negative
PRJNA729271	stage_1W_vs_E10	ordinary_muscle_development	ENSGALG00010018060	CASKIN1	-2.648159074	0.362331768	-7.308658271	2.69823E-13	7.33374E-13	negative
PRJNA729271	stage_1W_vs_E10	ordinary_muscle_development	ENSGALG00010018061	NDUFAF5	0.830564616	0.135280442	6.139576428	8.27418E-10	1.86813E-09	positive
PRJNA729271	stage_1W_vs_E10	ordinary_muscle_development	ENSGALG00010018062	GOLGA7	-0.941785102	0.094870529	-9.927056524	3.17489E-23	1.35752E-22	negative
PRJNA729271	stage_1W_vs_E10	ordinary_muscle_development	ENSGALG00010018063	DECR2	0.324857652	0.098358436	3.302793993	0.000957267	0.001428999	positive
PRJNA729271	stage_1W_vs_E10	ordinary_muscle_development	ENSGALG00010018064	TTC8	-1.92018094	0.164176733	-11.69581651	1.33893E-31	8.07986E-31	negative
PRJNA729271	stage_1W_vs_E10	ordinary_muscle_development	ENSGALG00010018067	ENSGALG00010018067	-0.227753557	0.178806589	-1.273742526	0.20275468	0.233137322	negative
PRJNA729271	stage_1W_vs_E10	ordinary_muscle_development	ENSGALG00010018068	ADAM33	-0.75394754	0.139862672	-5.390627331	7.02121E-08	1.41804E-07	negative
PRJNA729271	stage_1W_vs_E10	ordinary_muscle_development	ENSGALG00010018069	ENSGALG00010018069	-0.481369086	0.370659564	-1.298682489	0.194052916	0.223806255	negative
PRJNA729271	stage_1W_vs_E10	ordinary_muscle_development	ENSGALG00010018071	GPAT4	-0.11473973	0.079926768	-1.43556074	0.151127373	0.177217965	negative
PRJNA729271	stage_1W_vs_E10	ordinary_muscle_development	ENSGALG00010018075	MLST8	1.781798007	0.102211746	17.43241924	4.68225E-68	8.4625E-67	positive
PRJNA729271	stage_1W_vs_E10	ordinary_muscle_development	ENSGALG00010018076	FOXA2	-5.44737115	1.546946622	-3.52136982	0.000429323	0.000658175	negative
PRJNA729271	stage_1W_vs_E10	ordinary_muscle_development	ENSGALG00010018078	NKX6-3	6.072676349	0.692114453	8.774092674	1.72287E-18	6.00891E-18	positive
PRJNA729271	stage_1W_vs_E10	ordinary_muscle_development	ENSGALG00010018080	BRICD5	1.819067744	0.322128819	5.647019571	1.63253E-08	3.42008E-08	positive
PRJNA729271	stage_1W_vs_E10	ordinary_muscle_development	ENSGALG00010018081	DIO3	-5.515941961	0.289770834	-19.03553191	8.66087E-81	2.12418E-79	negative
PRJNA729271	stage_1W_vs_E10	ordinary_muscle_development	ENSGALG00010018082	RAB11FIP3	-0.350267315	0.177322162	-1.975316066	0.048232291	0.060329418	negative
PRJNA729271	stage_1W_vs_E10	ordinary_muscle_development	ENSGALG00010018083	PAX1	-5.915112545	1.051206139	-5.626976788	1.83395E-08	3.83264E-08	negative
PRJNA729271	stage_1W_vs_E10	ordinary_muscle_development	ENSGALG00010018085	PGP	2.913444921	0.159170566	18.30391758	7.70072E-75	1.61537E-73	positive
PRJNA729271	stage_1W_vs_E10	ordinary_muscle_development	ENSGALG00010018087	ENSGALG00010018087	-1.081610987	0.0573405	-18.8629499	2.30021E-79	5.37767E-78	negative
PRJNA729271	stage_1W_vs_E10	ordinary_muscle_development	ENSGALG00010018089	TASP1	0.325695292	0.093627721	3.478620333	0.000504002	0.000768435	positive
PRJNA729271	stage_1W_vs_E10	ordinary_muscle_development	ENSGALG00010018092	ENSGALG00010018092	-4.468138315	0.221005633	-20.21730515	6.8948E-91	2.09518E-89	negative
PRJNA729271	stage_1W_vs_E10	ordinary_muscle_development	ENSGALG00010018095	E4F1	-0.227168275	0.070404618	-3.226610421	0.001252659	0.001846567	negative
PRJNA729271	stage_1W_vs_E10	ordinary_muscle_development	ENSGALG00010018099	SMAD3	-1.825003525	0.131429017	-13.88584932	7.71801E-44	6.9833E-43	negative
PRJNA729271	stage_1W_vs_E10	ordinary_muscle_development	ENSGALG00010018102	ZDHHC4	0.522847932	0.169672021	3.081521207	0.002059458	0.002980713	positive
PRJNA729271	stage_1W_vs_E10	ordinary_muscle_development	ENSGALG00010018103	BTRC	-0.356958957	0.108025079	-3.304408208	0.000951771	0.001421381	negative
PRJNA729271	stage_1W_vs_E10	ordinary_muscle_development	ENSGALG00010018111	NKX2-2	-2.62111562	0.991385701	-2.643890886	0.008195907	0.011184304	negative
PRJNA729271	stage_1W_vs_E10	ordinary_muscle_development	ENSGALG00010018115	KLHL4	-1.917231415	0.202413428	-9.471858838	2.74907E-21	1.09004E-20	negative
PRJNA729271	stage_1W_vs_E10	ordinary_muscle_development	ENSGALG00010018119	GRID2IP	-3.993590592	0.941105977	-4.24350784	2.20053E-05	3.71582E-05	negative
PRJNA729271	stage_1W_vs_E10	ordinary_muscle_development	ENSGALG00010018121	FERMT2	0.086013046	0.159871545	0.53801348	0.590567742	0.625477088	positive
PRJNA729271	stage_1W_vs_E10	ordinary_muscle_development	ENSGALG00010018123	USP13	4.06979625	0.140698897	28.92557333	5.6948E-184	7.305E-182	positive
PRJNA729271	stage_1W_vs_E10	ordinary_muscle_development	ENSGALG00010018128	SEPTIN12	-0.904272977	0.199666287	-4.528921684	5.92855E-06	1.04505E-05	negative
PRJNA729271	stage_1W_vs_E10	ordinary_muscle_development	ENSGALG00010018137	NDUFB5	1.8162859	0.127421441	14.25416234	4.2234E-46	4.07577E-45	positive
PRJNA729271	stage_1W_vs_E10	ordinary_muscle_development	ENSGALG00010018140	CHPF	-1.363891839	0.107822013	-12.64947479	1.12625E-36	8.05776E-36	negative
PRJNA729271	stage_1W_vs_E10	ordinary_muscle_development	ENSGALG00010018142	DDHD1	-1.34877014	0.145529878	-9.267994721	1.89678E-20	7.26774E-20	negative
PRJNA729271	stage_1W_vs_E10	ordinary_muscle_development	ENSGALG00010018143	MRPL47	0.62068465	0.123253598	5.03583393	4.75773E-07	9.08487E-07	positive
PRJNA729271	stage_1W_vs_E10	ordinary_muscle_development	ENSGALG00010018145	ENSGALG00010018145	-1.27694126	0.362652165	-3.521118537	0.00042973	0.00065866	negative
PRJNA729271	stage_1W_vs_E10	ordinary_muscle_development	ENSGALG00010018147	ISM1	0.282589601	0.285561129	0.989594071	0.322372569	0.358783046	positive
PRJNA729271	stage_1W_vs_E10	ordinary_muscle_development	ENSGALG00010018148	XRN2	-0.45493964	0.055359125	-8.217970185	2.06976E-16	6.5462E-16	negative
PRJNA729271	stage_1W_vs_E10	ordinary_muscle_development	ENSGALG00010018149	ENSGALG00010018149	1.797625951	0.29169355	6.162720958	7.15055E-10	1.61898E-09	positive
PRJNA729271	stage_1W_vs_E10	ordinary_muscle_development	ENSGALG00010018150	AAGAB	-1.812278728	0.082146283	-22.06160361	7.3916E-108	3.2078E-106	negative
PRJNA729271	stage_1W_vs_E10	ordinary_muscle_development	ENSGALG00010018154	SLC16A12	-1.978467651	0.264724885	-7.473674601	7.79858E-14	2.18351E-13	negative
PRJNA729271	stage_1W_vs_E10	ordinary_muscle_development	ENSGALG00010018157	FBXL2	-1.21162712	0.15550661	-7.791483065	6.62272E-15	1.95951E-14	negative
PRJNA729271	stage_1W_vs_E10	ordinary_muscle_development	ENSGALG00010018160	ENSGALG00010018160	0.400013481	0.166624754	2.4006846	0.016364434	0.021587411	positive
PRJNA729271	stage_1W_vs_E10	ordinary_muscle_development	ENSGALG00010018162	ENSGALG00010018162	-0.298641562	0.358524829	-0.832973166	0.404859863	0.443068608	negative
PRJNA729271	stage_1W_vs_E10	ordinary_muscle_development	ENSGALG00010018163	CCT7	-0.353487778	0.080940022	-4.367280459	1.25803E-05	2.16785E-05	negative
PRJNA729271	stage_1W_vs_E10	ordinary_muscle_development	ENSGALG00010018165	DACH2	1.649203678	0.178596337	9.23425254	2.60096E-20	9.90043E-20	positive
PRJNA729271	stage_1W_vs_E10	ordinary_muscle_development	ENSGALG00010018166	MSS51	6.467960672	0.236010333	27.40541309	2.364E-165	2.4476E-163	positive
PRJNA729271	stage_1W_vs_E10	ordinary_muscle_development	ENSGALG00010018168	ENSGALG00010018168	-5.795673997	0.496421715	-11.67490024	1.71267E-31	1.02881E-30	negative
PRJNA729271	stage_1W_vs_E10	ordinary_muscle_development	ENSGALG00010018172	ACTL6A	-1.858824906	0.094881148	-19.59108782	1.84212E-85	5.02854E-84	negative
PRJNA729271	stage_1W_vs_E10	ordinary_muscle_development	ENSGALG00010018174	BMP4	1.177007159	0.203320871	5.788914614	7.08427E-09	1.51612E-08	positive
PRJNA729271	stage_1W_vs_E10	ordinary_muscle_development	ENSGALG00010018177	KIF20B	-2.498412016	0.188778672	-13.23460955	5.53838E-40	4.43529E-39	negative
PRJNA729271	stage_1W_vs_E10	ordinary_muscle_development	ENSGALG00010018179	PRADC1	0.580732773	0.117538562	4.940785087	7.78086E-07	1.4672E-06	positive
PRJNA729271	stage_1W_vs_E10	ordinary_muscle_development	ENSGALG00010018183	CYP17A1	0.166189649	0.338081192	0.491567272	0.623025285	0.656829697	positive
PRJNA729271	stage_1W_vs_E10	ordinary_muscle_development	ENSGALG00010018185	GNB4	-0.798925615	0.069818344	-11.44291848	2.55155E-30	1.45495E-29	negative
PRJNA729271	stage_1W_vs_E10	ordinary_muscle_development	ENSGALG00010018186	SPTLC3	-4.547717709	0.705050742	-6.450199166	1.11703E-10	2.64955E-10	negative
PRJNA729271	stage_1W_vs_E10	ordinary_muscle_development	ENSGALG00010018188	UBP1	-0.136365988	0.052268247	-2.608964221	0.009081674	0.012338444	negative
PRJNA729271	stage_1W_vs_E10	ordinary_muscle_development	ENSGALG00010018189	MIS18BP1	-1.197882796	0.120826864	-9.914043619	3.61704E-23	1.54158E-22	negative
PRJNA729271	stage_1W_vs_E10	ordinary_muscle_development	ENSGALG00010018192	IQCH	-1.990595957	0.232865125	-8.548278554	1.24938E-17	4.16316E-17	negative
PRJNA729271	stage_1W_vs_E10	ordinary_muscle_development	ENSGALG00010018193	ANK1	4.539623655	0.246543433	18.4130788	1.03186E-75	2.21255E-74	positive
PRJNA729271	stage_1W_vs_E10	ordinary_muscle_development	ENSGALG00010018198	BTBD3	-0.265153688	0.102285599	-2.592287585	0.009534004	0.012914249	negative
PRJNA729271	stage_1W_vs_E10	ordinary_muscle_development	ENSGALG00010018199	BORCS7	-0.91084697	0.121652067	-7.48731193	7.02985E-14	1.97438E-13	negative
PRJNA729271	stage_1W_vs_E10	ordinary_muscle_development	ENSGALG00010018206	KIZ	0.181734159	0.124985638	1.454040336	0.145935123	0.171476136	positive
PRJNA729271	stage_1W_vs_E10	ordinary_muscle_development	ENSGALG00010018209	GMPPA	0.57086122	0.130866404	4.362167859	1.2878E-05	2.21589E-05	positive
PRJNA729271	stage_1W_vs_E10	ordinary_muscle_development	ENSGALG00010018215	CFAP70	3.386225526	0.264480694	12.80329947	1.5713E-37	1.15673E-36	positive
PRJNA729271	stage_1W_vs_E10	ordinary_muscle_development	ENSGALG00010018216	FANCM	-0.436740856	0.086826718	-5.030028383	4.90407E-07	9.34831E-07	negative
PRJNA729271	stage_1W_vs_E10	ordinary_muscle_development	ENSGALG00010018217	ENSGALG00010018217	-3.309979623	0.689307289	-4.801892679	1.57173E-06	2.89188E-06	negative
PRJNA729271	stage_1W_vs_E10	ordinary_muscle_development	ENSGALG00010018219	SLC24A5	-4.480577751	0.796483466	-5.625449796	1.85025E-08	3.86447E-08	negative
PRJNA729271	stage_1W_vs_E10	ordinary_muscle_development	ENSGALG00010018226	JAG1	-0.321753231	0.102778909	-3.130537507	0.001744867	0.002542405	negative
PRJNA729271	stage_1W_vs_E10	ordinary_muscle_development	ENSGALG00010018229	AS3MT	0.951875142	0.188613111	5.046707187	4.49489E-07	8.59885E-07	positive
PRJNA729271	stage_1W_vs_E10	ordinary_muscle_development	ENSGALG00010018231	SMYD5	-0.900704963	0.081283865	-11.08098102	1.55163E-28	8.2354E-28	negative
PRJNA729271	stage_1W_vs_E10	ordinary_muscle_development	ENSGALG00010018233	RASSF2	-0.12675071	0.186501771	-0.679622018	0.496743824	0.534543558	negative
PRJNA729271	stage_1W_vs_E10	ordinary_muscle_development	ENSGALG00010018240	SOUL	-1.114847348	0.726954126	-1.533586932	0.125131305	0.148439174	negative
PRJNA729271	stage_1W_vs_E10	ordinary_muscle_development	ENSGALG00010018241	RALGAPA2	0.487830415	0.09142779	5.335690785	9.51815E-08	1.90613E-07	positive
PRJNA729271	stage_1W_vs_E10	ordinary_muscle_development	ENSGALG00010018244	CHM	0.317478843	0.081786059	3.881821011	0.000103677	0.000166442	positive
PRJNA729271	stage_1W_vs_E10	ordinary_muscle_development	ENSGALG00010018251	USP48	0.049945716	0.166773226	0.299482822	0.764571677	0.788338772	positive
PRJNA729271	stage_1W_vs_E10	ordinary_muscle_development	ENSGALG00010018255	MFN1	1.162421853	0.112700722	10.31423606	6.07626E-25	2.78367E-24	positive
PRJNA729271	stage_1W_vs_E10	ordinary_muscle_development	ENSGALG00010018258	OSCP1	-0.717008088	0.283593743	-2.528293051	0.011461864	0.015384731	negative
PRJNA729271	stage_1W_vs_E10	ordinary_muscle_development	ENSGALG00010018259	WDR90	-0.544818936	0.130866505	-4.163165647	3.13865E-05	5.24859E-05	negative
PRJNA729271	stage_1W_vs_E10	ordinary_muscle_development	ENSGALG00010018261	PRNP	1.351142938	0.194100033	6.961064973	3.3771E-12	8.70086E-12	positive
PRJNA729271	stage_1W_vs_E10	ordinary_muscle_development	ENSGALG00010018263	SLX4IP	-1.495556409	0.117610607	-12.71616941	4.80861E-37	3.47982E-36	negative
PRJNA729271	stage_1W_vs_E10	ordinary_muscle_development	ENSGALG00010018264	CFAP61	-1.529108764	0.372718845	-4.102579696	4.08569E-05	6.76746E-05	negative
PRJNA729271	stage_1W_vs_E10	ordinary_muscle_development	ENSGALG00010018265	PDCD4	-0.263498004	0.065798441	-4.004623848	6.21162E-05	0.000101577	negative
PRJNA729271	stage_1W_vs_E10	ordinary_muscle_development	ENSGALG00010018267	ADPRHL2	-0.523839954	0.077387586	-6.769043748	1.29636E-11	3.237E-11	negative
PRJNA729271	stage_1W_vs_E10	ordinary_muscle_development	ENSGALG00010018273	PROM2	-5.442189585	1.077725316	-5.049700054	4.42504E-07	8.46858E-07	negative
PRJNA729271	stage_1W_vs_E10	ordinary_muscle_development	ENSGALG00010018274	ZNF711	-0.148760461	0.097597359	-1.524226305	0.127452189	0.150994645	negative
PRJNA729271	stage_1W_vs_E10	ordinary_muscle_development	ENSGALG00010018275	ZNF639	-0.031801367	0.083712887	-0.379886155	0.704029925	0.732059811	negative
PRJNA729271	stage_1W_vs_E10	ordinary_muscle_development	ENSGALG00010018276	ARHGAP17	-0.441969149	0.073143108	-6.042526248	1.5172E-09	3.38387E-09	negative
PRJNA729271	stage_1W_vs_E10	ordinary_muscle_development	ENSGALG00010018278	PUSL1	0.654035149	0.151201304	4.32559198	1.52123E-05	2.59934E-05	positive
PRJNA729271	stage_1W_vs_E10	ordinary_muscle_development	ENSGALG00010018283	POF1B	0.39174826	0.178890493	2.189877466	0.028533125	0.036642832	positive
PRJNA729271	stage_1W_vs_E10	ordinary_muscle_development	ENSGALG00010018284	ENSGALG00010018284	2.140188518	0.166994464	12.81592498	1.33536E-37	9.84042E-37	positive
PRJNA729271	stage_1W_vs_E10	ordinary_muscle_development	ENSGALG00010018285	BBIP1	0.010552139	0.191603982	0.05507265	0.956080585	0.9617202	positive
PRJNA729271	stage_1W_vs_E10	ordinary_muscle_development	ENSGALG00010018287	MKKS	0.112774699	0.102981819	1.095093296	0.273475766	0.307670849	positive
PRJNA729271	stage_1W_vs_E10	ordinary_muscle_development	ENSGALG00010018288	CRNKL1	-0.223712517	0.061953564	-3.610970926	0.000305053	0.000473978	negative
PRJNA729271	stage_1W_vs_E10	ordinary_muscle_development	ENSGALG00010018289	HEYL	-1.486821862	0.21556313	-6.897384821	5.29686E-12	1.35054E-11	negative
PRJNA729271	stage_1W_vs_E10	ordinary_muscle_development	ENSGALG00010018290	RHOT2	-0.023430334	0.134305617	-0.174455357	0.861507613	0.876934891	negative
PRJNA729271	stage_1W_vs_E10	ordinary_muscle_development	ENSGALG00010018294	FKBP3	0.790736477	0.177772608	4.448022023	8.66646E-06	1.51041E-05	positive
PRJNA729271	stage_1W_vs_E10	ordinary_muscle_development	ENSGALG00010018296	PIK3CA	0.692356243	0.096653705	7.163266483	7.8777E-13	2.09441E-12	positive
PRJNA729271	stage_1W_vs_E10	ordinary_muscle_development	ENSGALG00010018298	ENSGALG00010018298	0.302469926	0.197706987	1.529889917	0.12604398	0.149460691	positive
PRJNA729271	stage_1W_vs_E10	ordinary_muscle_development	ENSGALG00010018299	SHOC2	0.368132288	0.083040201	4.433181538	9.28526E-06	1.61534E-05	positive
PRJNA729271	stage_1W_vs_E10	ordinary_muscle_development	ENSGALG00010018301	ENSGALG00010018301	-0.851737894	0.331198171	-2.571686589	0.010120446	0.013677937	negative
PRJNA729271	stage_1W_vs_E10	ordinary_muscle_development	ENSGALG00010018302	RAB11FIP5	0.885295567	0.100932431	8.771170575	1.76818E-18	6.16101E-18	positive
PRJNA729271	stage_1W_vs_E10	ordinary_muscle_development	ENSGALG00010018306	DES	4.034923767	0.150916655	26.73610655	1.7913E-157	1.7196E-155	positive
PRJNA729271	stage_1W_vs_E10	ordinary_muscle_development	ENSGALG00010018308	ACAP3	-1.383576562	0.17995104	-7.688627769	1.48721E-14	4.31834E-14	negative
PRJNA729271	stage_1W_vs_E10	ordinary_muscle_development	ENSGALG00010018309	DPYSL2	-1.57340892	0.124511957	-12.63660906	1.32652E-36	9.47191E-36	negative
PRJNA729271	stage_1W_vs_E10	ordinary_muscle_development	ENSGALG00010018310	APOOL	-0.213180931	0.112551943	-1.894067086	0.058216106	0.072098638	negative
PRJNA729271	stage_1W_vs_E10	ordinary_muscle_development	ENSGALG00010018311	NKX2-3	2.731656027	0.726842857	3.758248431	0.000171107	0.000270586	positive
PRJNA729271	stage_1W_vs_E10	ordinary_muscle_development	ENSGALG00010018312	PRPF39	-0.512434032	0.079466209	-6.448452009	1.12998E-10	2.67895E-10	negative
PRJNA729271	stage_1W_vs_E10	ordinary_muscle_development	ENSGALG00010018313	MYEF2	-1.680916831	0.082698782	-20.32577486	7.60784E-92	2.3921E-90	negative
PRJNA729271	stage_1W_vs_E10	ordinary_muscle_development	ENSGALG00010018314	SLC24A3	-1.639339965	0.133397235	-12.28916003	1.03577E-34	6.96679E-34	negative
PRJNA729271	stage_1W_vs_E10	ordinary_muscle_development	ENSGALG00010018315	CLASP2	0.758193489	0.117274568	6.465114297	1.01222E-10	2.40488E-10	positive
PRJNA729271	stage_1W_vs_E10	ordinary_muscle_development	ENSGALG00010018317	ZMAT3	-0.937599034	0.144971117	-6.467488536	9.96451E-11	2.36896E-10	negative
PRJNA729271	stage_1W_vs_E10	ordinary_muscle_development	ENSGALG00010018319	STUB1	1.021894752	0.201530572	5.070668638	3.9642E-07	7.61075E-07	positive
PRJNA729271	stage_1W_vs_E10	ordinary_muscle_development	ENSGALG00010018322	NT5C1A	5.305952439	0.239996153	22.10848953	2.6189E-108	1.1503E-106	positive
PRJNA729271	stage_1W_vs_E10	ordinary_muscle_development	ENSGALG00010018323	GOT1	2.698237908	0.142274003	18.9650804	3.3152E-80	7.95593E-79	positive
PRJNA729271	stage_1W_vs_E10	ordinary_muscle_development	ENSGALG00010018325	ENSGALG00010018325	-0.114822292	0.20829587	-0.551246129	0.581464962	0.616918066	negative
PRJNA729271	stage_1W_vs_E10	ordinary_muscle_development	ENSGALG00010018326	LCMT1	-1.104357043	0.113413736	-9.737418759	2.0879E-22	8.60756E-22	negative
PRJNA729271	stage_1W_vs_E10	ordinary_muscle_development	ENSGALG00010018327	SNAP25	-1.191950596	0.556236572	-2.142884264	0.032122393	0.040990764	negative
PRJNA729271	stage_1W_vs_E10	ordinary_muscle_development	ENSGALG00010018328	ENSGALG00010018328	3.723365214	0.539597978	6.900257904	5.19082E-12	1.32397E-11	positive
PRJNA729271	stage_1W_vs_E10	ordinary_muscle_development	ENSGALG00010018329	ENSGALG00010018329	1.66900347	0.226501241	7.368628352	1.72393E-13	4.73263E-13	positive
PRJNA729271	stage_1W_vs_E10	ordinary_muscle_development	ENSGALG00010018333	ENSGALG00010018333	1.386124525	0.210423114	6.587320653	4.47834E-11	1.08824E-10	positive
PRJNA729271	stage_1W_vs_E10	ordinary_muscle_development	ENSGALG00010018336	JMJD8	-0.182393555	0.14870003	-1.22658721	0.219977779	0.251385833	negative
PRJNA729271	stage_1W_vs_E10	ordinary_muscle_development	ENSGALG00010018337	TMEM151B	-0.534319445	0.318125366	-1.679587678	0.093037567	0.112390993	negative
PRJNA729271	stage_1W_vs_E10	ordinary_muscle_development	ENSGALG00010018338	PDCD6IP	0.319897168	0.084730984	3.775444998	0.000159722	0.000253107	positive
PRJNA729271	stage_1W_vs_E10	ordinary_muscle_development	ENSGALG00010018339	FAM76A	0.29929974	0.115741019	2.585943535	0.009711284	0.01314824	positive
PRJNA729271	stage_1W_vs_E10	ordinary_muscle_development	ENSGALG00010018340	DNPEP	1.236553142	0.09706266	12.73974093	3.55574E-37	2.58737E-36	positive
PRJNA729271	stage_1W_vs_E10	ordinary_muscle_development	ENSGALG00010018342	BNIP3L	-0.799020952	0.106461671	-7.505245246	6.13137E-14	1.7284E-13	negative
PRJNA729271	stage_1W_vs_E10	ordinary_muscle_development	ENSGALG00010018344	SFXN5	-0.543171735	0.192328651	-2.824185234	0.004740098	0.006631131	negative
PRJNA729271	stage_1W_vs_E10	ordinary_muscle_development	ENSGALG00010018345	WDR24	1.042761376	0.170370679	6.120544803	9.3256E-10	2.10159E-09	positive
PRJNA729271	stage_1W_vs_E10	ordinary_muscle_development	ENSGALG00010018347	CTXN2	-4.849589759	0.960936957	-5.046730407	4.49435E-07	8.59885E-07	negative
PRJNA729271	stage_1W_vs_E10	ordinary_muscle_development	ENSGALG00010018348	TOGARAM1	0.82955006	0.10703254	7.75044729	9.15694E-15	2.69011E-14	positive

PRJNA729271	stage_1W_vs_E10	ordinary_muscle_development	ENSGALG00010018349	KCNMB2	0.640367024	0.194382761	3.294361194	0.000986457	0.001470302	positive
PRJNA729271	stage_1W_vs_E10	ordinary_muscle_development	ENSGALG00010018350	CNNM1	-0.50628115	0.250710227	-2.019387707	0.043446938	0.054666959	negative
PRJNA729271	stage_1W_vs_E10	ordinary_muscle_development	ENSGALG00010018351	RIN2	0.497598075	0.086952119	5.722667592	1.04864E-08	2.22256E-08	positive
PRJNA729271	stage_1W_vs_E10	ordinary_muscle_development	ENSGALG00010018354	AARS2	0.365179634	0.117211867	3.115551722	0.001836011	0.002669842	positive
PRJNA729271	stage_1W_vs_E10	ordinary_muscle_development	ENSGALG00010018355	PRKCB	-0.767983732	0.156636635	-4.902963681	9.44014E-07	1.76904E-06	negative
PRJNA729271	stage_1W_vs_E10	ordinary_muscle_development	ENSGALG00010018356	ADRA1A	-2.117135106	0.44548334	-4.752445073	2.00971E-06	3.67118E-06	negative
PRJNA729271	stage_1W_vs_E10	ordinary_muscle_development	ENSGALG00010018359	CACNG3	-4.680711572	0.86688871	-5.39943769	6.68501E-08	1.3524E-07	negative
PRJNA729271	stage_1W_vs_E10	ordinary_muscle_development	ENSGALG00010018361	ASIC4	-2.340072461	0.3043287	-7.689292741	1.47951E-14	4.29682E-14	negative
PRJNA729271	stage_1W_vs_E10	ordinary_muscle_development	ENSGALG00010018363	TCTE1	0.539638231	0.385176399	1.401015827	0.16120934	0.188294068	positive
PRJNA729271	stage_1W_vs_E10	ordinary_muscle_development	ENSGALG00010018365	HPSE2	-2.334015713	0.268276136	-8.70004969	3.31739E-18	1.1392E-17	negative
PRJNA729271	stage_1W_vs_E10	ordinary_muscle_development	ENSGALG00010018367	PAK5	-1.450525069	0.229429845	-6.322303306	2.57693E-10	5.9745E-10	negative
PRJNA729271	stage_1W_vs_E10	ordinary_muscle_development	ENSGALG00010018370	ZBTB8A	0.319060815	0.110077423	2.898512759	0.003749371	0.005291833	positive
PRJNA729271	stage_1W_vs_E10	ordinary_muscle_development	ENSGALG00010018371	ENSGALG00010018371	0.719997873	0.13633745	5.280998537	1.28482E-07	2.54697E-07	positive
PRJNA729271	stage_1W_vs_E10	ordinary_muscle_development	ENSGALG00010018372	NLGN3	-1.738621108	0.481520222	-3.610691783	0.000305381	0.000474438	negative
PRJNA729271	stage_1W_vs_E10	ordinary_muscle_development	ENSGALG00010018374	HPS1	-0.71080933	0.138569032	-5.129640601	2.90296E-07	5.62019E-07	negative
PRJNA729271	stage_1W_vs_E10	ordinary_muscle_development	ENSGALG00010018376	PYGB	-0.212261014	0.142371683	-1.490893484	0.135989462	0.160505436	negative
PRJNA729271	stage_1W_vs_E10	ordinary_muscle_development	ENSGALG00010018377	RAP1GAP	2.231850426	0.265410905	8.409038154	4.13387E-17	1.34532E-16	positive
PRJNA729271	stage_1W_vs_E10	ordinary_muscle_development	ENSGALG00010018378	ARPP21	0.364804726	0.145268861	2.51123829	0.012030846	0.01611264	positive
PRJNA729271	stage_1W_vs_E10	ordinary_muscle_development	ENSGALG00010018379	SRBD1	0.994598766	0.125948727	7.896854477	2.8603E-15	8.61239E-15	positive
PRJNA729271	stage_1W_vs_E10	ordinary_muscle_development	ENSGALG00010018380	SLC20A1	-1.361356377	0.14333149	-9.497957312	2.14048E-21	8.52369E-21	negative
PRJNA729271	stage_1W_vs_E10	ordinary_muscle_development	ENSGALG00010018381	HAPSTR1	0.467088491	0.067447522	6.925213489	4.35319E-12	1.11483E-11	positive
PRJNA729271	stage_1W_vs_E10	ordinary_muscle_development	ENSGALG00010018383	METRN	-1.626021903	0.165000622	-9.854641046	6.54501E-23	2.76026E-22	negative
PRJNA729271	stage_1W_vs_E10	ordinary_muscle_development	ENSGALG00010018384	LAMP5	-0.620897877	0.243993959	-2.54472643	0.010936338	0.014717503	negative
PRJNA729271	stage_1W_vs_E10	ordinary_muscle_development	ENSGALG00010018385	SPR	-1.162574909	0.14325918	-8.115186115	4.85041E-16	1.5136E-15	negative
PRJNA729271	stage_1W_vs_E10	ordinary_muscle_development	ENSGALG00010018387	NT5DC4	-2.377792575	0.35224331	-6.750426507	1.47411E-11	3.67009E-11	negative
PRJNA729271	stage_1W_vs_E10	ordinary_muscle_development	ENSGALG00010018388	SMC3	-1.163256865	0.066486997	-17.49600539	1.53676E-68	2.809E-67	negative
PRJNA729271	stage_1W_vs_E10	ordinary_muscle_development	ENSGALG00010018391	STAC	-1.956152612	0.228651123	-8.555184784	1.1768E-17	3.92673E-17	negative
PRJNA729271	stage_1W_vs_E10	ordinary_muscle_development	ENSGALG00010018393	PRKCE	-0.13955202	0.077425758	-1.802397852	0.071482833	0.087489966	negative
PRJNA729271	stage_1W_vs_E10	ordinary_muscle_development	ENSGALG00010018394	KLHL28	-0.024544541	0.098303985	-0.249680026	0.802834806	0.823806492	negative
PRJNA729271	stage_1W_vs_E10	ordinary_muscle_development	ENSGALG00010018395	MED12	0.124812734	0.078926846	1.581372388	0.113792917	0.135777769	positive
PRJNA729271	stage_1W_vs_E10	ordinary_muscle_development	ENSGALG00010018396	TBL1XR1	-0.115027807	0.074959365	-1.534535498	0.124897967	0.148186628	negative
PRJNA729271	stage_1W_vs_E10	ordinary_muscle_development	ENSGALG00010018397	FAM173A	2.507279371	0.159056163	15.76348456	5.54874E-56	7.33172E-55	positive
PRJNA729271	stage_1W_vs_E10	ordinary_muscle_development	ENSGALG00010018398	LRFN5	-0.258223208	0.189925976	-1.359599219	0.173956786	0.201946469	negative
PRJNA729271	stage_1W_vs_E10	ordinary_muscle_development	ENSGALG00010018399	ABHD12	-0.279766696	0.163233381	-1.713906153	0.086545963	0.104907487	negative
PRJNA729271	stage_1W_vs_E10	ordinary_muscle_development	ENSGALG00010018401	ENSGALG00010018401	-0.693272656	0.103241146	-6.715080975	1.87962E-11	4.65807E-11	negative
PRJNA729271	stage_1W_vs_E10	ordinary_muscle_development	ENSGALG00010018404	IL2RG	1.816738811	0.257140984	7.065146837	1.60446E-12	4.20099E-12	positive
PRJNA729271	stage_1W_vs_E10	ordinary_muscle_development	ENSGALG00010018405	DCLK3	-2.282264108	0.196672829	-11.60436915	3.91565E-31	2.31191E-30	negative
PRJNA729271	stage_1W_vs_E10	ordinary_muscle_development	ENSGALG00010018406	TMEM260	-0.598678481	0.129802242	-4.612235291	3.98362E-06	7.11378E-06	negative
PRJNA729271	stage_1W_vs_E10	ordinary_muscle_development	ENSGALG00010018410	EXOC6B	0.253525151	0.099977561	2.535820531	0.011218421	0.015071926	positive
PRJNA729271	stage_1W_vs_E10	ordinary_muscle_development	ENSGALG00010018413	SLC15A2	-1.354795771	0.359789844	-3.765519772	0.000166203	0.000263003	negative
PRJNA729271	stage_1W_vs_E10	ordinary_muscle_development	ENSGALG00010018414	EPAS1	1.827773469	0.137686003	13.27494031	3.23529E-40	2.60675E-39	positive
PRJNA729271	stage_1W_vs_E10	ordinary_muscle_development	ENSGALG00010018415	PELI2	-1.797735269	0.131500418	-13.67094718	1.51411E-42	1.31894E-41	negative
PRJNA729271	stage_1W_vs_E10	ordinary_muscle_development	ENSGALG00010018416	DUT	-2.269318915	0.135751023	-16.7167721	9.89347E-63	1.54034E-61	negative
PRJNA729271	stage_1W_vs_E10	ordinary_muscle_development	ENSGALG00010018418	PLCB4	-0.357043093	0.119289877	-2.993071169	0.002761853	0.003947645	negative
PRJNA729271	stage_1W_vs_E10	ordinary_muscle_development	ENSGALG00010018419	USP7	0.607560912	0.133636992	4.546352792	5.45835E-06	9.64276E-06	positive
PRJNA729271	stage_1W_vs_E10	ordinary_muscle_development	ENSGALG00010018420	RHOQ	2.7132421	0.131508365	20.63170742	1.42528E-94	4.7384E-93	positive
PRJNA729271	stage_1W_vs_E10	ordinary_muscle_development	ENSGALG00010018422	ENSGALG00010018422	1.135898096	0.212794575	5.338003087	9.39759E-08	1.88276E-07	positive
PRJNA729271	stage_1W_vs_E10	ordinary_muscle_development	ENSGALG00010018425	CYP26B1	-3.411969206	0.241610491	-14.12177588	2.78874E-45	2.62827E-44	negative
PRJNA729271	stage_1W_vs_E10	ordinary_muscle_development	ENSGALG00010018426	ENSGALG00010018426	3.852339053	0.346069547	11.1316904	8.79516E-29	4.71471E-28	positive
PRJNA729271	stage_1W_vs_E10	ordinary_muscle_development	ENSGALG00010018427	PIGF	0.170146672	0.142148715	1.19696243	0.231321199	0.263083623	positive
PRJNA729271	stage_1W_vs_E10	ordinary_muscle_development	ENSGALG00010018428	PPP2R5D	-0.265966538	0.064273554	-4.138040034	3.50285E-05	5.83675E-05	negative
PRJNA729271	stage_1W_vs_E10	ordinary_muscle_development	ENSGALG00010018430	LOXL4	-2.119281578	0.285563783	-7.421394816	1.15893E-13	3.20954E-13	negative
PRJNA729271	stage_1W_vs_E10	ordinary_muscle_development	ENSGALG00010018434	HSPBAP1	2.060691699	0.158765487	12.97946888	1.59986E-38	1.21668E-37	positive
PRJNA729271	stage_1W_vs_E10	ordinary_muscle_development	ENSGALG00010018436	FOXO4	0.92918173	0.145466674	6.387591766	1.68518E-10	3.95447E-10	positive
PRJNA729271	stage_1W_vs_E10	ordinary_muscle_development	ENSGALG00010018438	PPP2R2A	0.655787231	0.098306377	6.670851408	2.54324E-11	6.26091E-11	positive
PRJNA729271	stage_1W_vs_E10	ordinary_muscle_development	ENSGALG00010018440	MEA1	0.793268258	0.101111417	7.845486498	4.31278E-15	1.28921E-14	positive
PRJNA729271	stage_1W_vs_E10	ordinary_muscle_development	ENSGALG00010018441	CRIPT	0.535648757	0.093645649	5.719953497	1.06553E-08	2.25703E-08	positive
PRJNA729271	stage_1W_vs_E10	ordinary_muscle_development	ENSGALG00010018443	PLCB1	2.084540765	0.146450204	14.23378535	5.65367E-46	5.43435E-45	positive
PRJNA729271	stage_1W_vs_E10	ordinary_muscle_development	ENSGALG00010018444	NUBP2	-0.277163701	0.098421879	-2.816078122	0.004861384	0.006795811	negative
PRJNA729271	stage_1W_vs_E10	ordinary_muscle_development	ENSGALG00010018446	FBN1	1.452336176	0.232083784	6.257809788	3.90422E-10	8.9828E-10	positive
PRJNA729271	stage_1W_vs_E10	ordinary_muscle_development	ENSGALG00010018448	KLHDC3	3.160688775	0.164121245	19.25825493	1.20367E-82	3.09348E-81	positive
PRJNA729271	stage_1W_vs_E10	ordinary_muscle_development	ENSGALG00010018451	PIK3R4	-0.050549167	0.111774853	-0.452240957	0.651095406	0.682501295	negative
PRJNA729271	stage_1W_vs_E10	ordinary_muscle_development	ENSGALG00010018452	SPSB3	0.877348759	0.118554023	7.40041379	1.35761E-13	3.74403E-13	positive
PRJNA729271	stage_1W_vs_E10	ordinary_muscle_development	ENSGALG00010018453	TMX4	-0.215382422	0.092800232	-2.320925461	0.020290867	0.026492174	negative
PRJNA729271	stage_1W_vs_E10	ordinary_muscle_development	ENSGALG00010018455	RBM20	4.612883125	0.246312144	18.72779412	2.93841E-78	6.68639E-77	positive
PRJNA729271	stage_1W_vs_E10	ordinary_muscle_development	ENSGALG00010018458	SEMA5B	-2.073872548	0.10935553	-18.96449628	3.35223E-80	8.0315E-79	negative
PRJNA729271	stage_1W_vs_E10	ordinary_muscle_development	ENSGALG00010018459	SNX12	0.393005456	0.102497994	3.834274607	0.000125935	0.000200995	positive
PRJNA729271	stage_1W_vs_E10	ordinary_muscle_development	ENSGALG00010018462	KTN1	0.826681447	0.116339045	7.105795378	1.19632E-12	3.158E-12	positive
PRJNA729271	stage_1W_vs_E10	ordinary_muscle_development	ENSGALG00010018464	ENSGALG00010018464	-3.820695414	1.179339516	-3.23969083	0.001196594	0.001767335	negative
PRJNA729271	stage_1W_vs_E10	ordinary_muscle_development	ENSGALG00010018465	NAALADL2	-3.074128956	0.210247417	-14.62148263	2.04889E-48	2.12285E-47	negative
PRJNA729271	stage_1W_vs_E10	ordinary_muscle_development	ENSGALG00010018466	DYSF	2.687267153	0.182411389	14.73190442	4.0221E-49	4.26796E-48	positive
PRJNA729271	stage_1W_vs_E10	ordinary_muscle_development	ENSGALG00010018467	TLX1	-6.262277457	1.166590458	-5.368017039	7.9607E-08	1.60309E-07	negative
PRJNA729271	stage_1W_vs_E10	ordinary_muscle_development	ENSGALG00010018469	KLHDC7A	0.611681853	0.198757196	3.077533115	0.002087216	0.003019984	positive
PRJNA729271	stage_1W_vs_E10	ordinary_muscle_development	ENSGALG00010018470	IQCB1	1.33208129	0.157479892	8.458738896	2.70284E-17	8.87981E-17	positive
PRJNA729271	stage_1W_vs_E10	ordinary_muscle_development	ENSGALG00010018471	HHEX	1.523893896	0.18007635	8.462487697	2.61733E-17	8.60668E-17	positive
PRJNA729271	stage_1W_vs_E10	ordinary_muscle_development	ENSGALG00010018472	LMF1	-0.233367078	0.142788227	-1.634357974	0.102183707	0.122723741	negative
PRJNA729271	stage_1W_vs_E10	ordinary_muscle_development	ENSGALG00010018476	ENSGALG00010018476	0.168345874	0.376046162	0.447673427	0.654388902	0.68550749	positive
PRJNA729271	stage_1W_vs_E10	ordinary_muscle_development	ENSGALG00010018480	EME2	-0.147565545	0.24595854	-0.599961055	0.548532191	0.584630449	negative
PRJNA729271	stage_1W_vs_E10	ordinary_muscle_development	ENSGALG00010018482	TWNK	0.45797879	0.127352473	3.596151535	0.00032296	0.000500835	positive
PRJNA729271	stage_1W_vs_E10	ordinary_muscle_development	ENSGALG00010018484	NLGN1	-3.058222891	0.270364879	-11.31146508	1.15151E-29	6.41471E-29	negative
PRJNA729271	stage_1W_vs_E10	ordinary_muscle_development	ENSGALG00010018485	ATP2C1	-0.126254813	0.066974378	-1.885121088	0.059413491	0.073481106	negative
PRJNA729271	stage_1W_vs_E10	ordinary_muscle_development	ENSGALG00010018486	ENSGALG00010018486	0.829567084	0.100203099	8.278856577	1.24364E-16	3.97147E-16	positive
PRJNA729271	stage_1W_vs_E10	ordinary_muscle_development	ENSGALG00010018487	ENSGALG00010018487	-2.224488537	0.137051578	-16.23103189	3.04301E-59	4.35853E-58	negative
PRJNA729271	stage_1W_vs_E10	ordinary_muscle_development	ENSGALG00010018488	ENSGALG00010018488	1.146627575	0.153923556	7.449331378	9.38145E-14	2.61156E-13	positive
PRJNA729271	stage_1W_vs_E10	ordinary_muscle_development	ENSGALG00010018489	SLC7A3	-1.45668082	0.208617409	-6.982546804	2.89876E-12	7.49511E-12	negative
PRJNA729271	stage_1W_vs_E10	ordinary_muscle_development	ENSGALG00010018490	NME3	-0.435065819	0.121374502	-3.584491071	0.000337736	0.000522575	negative
PRJNA729271	stage_1W_vs_E10	ordinary_muscle_development	ENSGALG00010018491	TIA1	0.616261626	0.119536944	5.155407245	2.5308E-07	4.92204E-07	positive
PRJNA729271	stage_1W_vs_E10	ordinary_muscle_development	ENSGALG00010018492	ENSGALG00010018492	0.650450422	0.17246259	3.771545019	0.00016224	0.000257013	positive
PRJNA729271	stage_1W_vs_E10	ordinary_muscle_development	ENSGALG00010018494	HAO1	1.169498086	0.282402139	4.141250807	3.45417E-05	5.76092E-05	positive
PRJNA729271	stage_1W_vs_E10	ordinary_muscle_development	ENSGALG00010018495	ENSGALG00010018495	0.244321427	0.668603598	0.36542045	0.714797578	0.742191325	positive
PRJNA729271	stage_1W_vs_E10	ordinary_muscle_development	ENSGALG00010018496	GNG13	-1.265325685	0.4970192	-2.545828581	0.010901871	0.014675206	negative
PRJNA729271	stage_1W_vs_E10	ordinary_muscle_development	ENSGALG00010018497	ASTE1	-1.193352905	0.117453232	-10.16023895	2.98344E-24	1.32653E-23	negative
PRJNA729271	stage_1W_vs_E10	ordinary_muscle_development	ENSGALG00010018498	ENSGALG00010018498	2.631169867	0.383052158	6.868959778	6.46717E-12	1.64373E-11	positive
PRJNA729271	stage_1W_vs_E10	ordinary_muscle_development	ENSGALG00010018501	ATG14	0.815380261	0.13439329	6.067120309	1.30224E-09	2.91476E-09	positive
PRJNA729271	stage_1W_vs_E10	ordinary_muscle_development	ENSGALG00010018502	ARHGEF10L	1.263502528	0.121823371	10.37159389	3.33908E-25	1.54979E-24	positive
PRJNA729271	stage_1W_vs_E10	ordinary_muscle_development	ENSGALG00010018503	BMP2	-3.635025749	0.206168877	-17.63130207	1.41667E-69	2.64962E-68	negative
PRJNA729271	stage_1W_vs_E10	ordinary_muscle_development	ENSGALG00010018504	SLCO3A1	0.334971297	0.180546502	1.855318674	0.063550733	0.078284004	positive
PRJNA729271	stage_1W_vs_E10	ordinary_muscle_development	ENSGALG00010018505	CHTF18	-1.668642636	0.146592566	-11.38285987	5.09026E-30	2.88104E-29	negative
PRJNA729271	stage_1W_vs_E10	ordinary_muscle_development	ENSGALG00010018506	ENSGALG00010018506	0.944467751	0.142092559	6.646848767	2.99434E-11	7.34027E-11	positive
PRJNA729271	stage_1W_vs_E10	ordinary_muscle_development	ENSGALG00010018508	ENSGALG00010018508	-0.276859485	0.101786615	-2.719998947	0.006528212	0.009001754	negative
PRJNA729271	stage_1W_vs_E10	ordinary_muscle_development	ENSGALG00010018509	ENSGALG00010018509	-1.148141402	0.112360239	-10.21839588	1.64033E-24	7.39321E-24	negative
PRJNA729271	stage_1W_vs_E10	ordinary_muscle_development	ENSGALG00010018510	ENSGALG00010018510	0.876451247	0.125615499	6.977254041	3.01005E-12	7.77432E-12	positive
PRJNA729271	stage_1W_vs_E10	ordinary_muscle_development	ENSGALG00010018511	SOCS5	-0.333048326	0.101199384	-3.291011408	0.000998279	0.001487311	negative
PRJNA729271	stage_1W_vs_E10	ordinary_muscle_development	ENSGALG00010018512	FERMT1	0.078068659	0.171520754	0.455155757	0.648997187	0.680498715	positive
PRJNA729271	stage_1W_vs_E10	ordinary_muscle_development	ENSGALG00010018513	RPUSD1	-0.393710899	0.160572537	-2.451919276	0.014209654	0.018882373	negative
PRJNA729271	stage_1W_vs_E10	ordinary_muscle_development	ENSGALG00010018514	EAF2	-0.724400328	0.244703117	-2.960323256	0.003073164	0.004375357	negative
PRJNA729271	stage_1W_vs_E10	ordinary_muscle_development	ENSGALG00010018515	SEMA4G	-0.038486249	0.177367734	-0.21698563	0.828219544	0.84673736	negative
PRJNA729271	stage_1W_vs_E10	ordinary_muscle_development	ENSGALG00010018516	LRRN4	0.225833214	0.158210621	1.427421326	0.153458487	0.179704377	positive
PRJNA729271	stage_1W_vs_E10	ordinary_muscle_development	ENSGALG00010018517	ECT2	-2.591809645	0.201303889	-12.87510962	6.21508E-38	4.61514E-37	negative
PRJNA729271	stage_1W_vs_E10	ordinary_muscle_development	ENSGALG00010018518	ENSGALG00010018518	-2.271126844	1.055902224	-2.150887452	0.031485084	0.040227086	negative
PRJNA729271	stage_1W_vs_E10	ordinary_muscle_development	ENSGALG00010018519	OGDH	2.985491	0.136061031	21.94229298	1.026E-106	4.3357E-105	positive
PRJNA729271	stage_1W_vs_E10	ordinary_muscle_development	ENSGALG00010018520	ENSGALG00010018520	0.455547291	0.124476486	3.659705592	0.000252505	0.000394658	positive
PRJNA729271	stage_1W_vs_E10	ordinary_muscle_development	ENSGALG00010018521	ENSGALG00010018521	0.907165964	0.158719752	5.71552031	1.09369E-08	2.31465E-08	positive
PRJNA729271	stage_1W_vs_E10	ordinary_muscle_development	ENSGALG00010018522	NEK11	-1.213768046	0.286642994	-4.234424252	2.29138E-05	3.86585E-05	negative
PRJNA729271	stage_1W_vs_E10	ordinary_muscle_development	ENSGALG00010018523	TEX11	-0.094510512	0.173297212	-0.545366605	0.585501404	0.620802159	negative
PRJNA729271	stage_1W_vs_E10	ordinary_muscle_development	ENSGALG00010018524	SEC22A	-0.305528059	0.117139345	-2.608244563	0.009100791	0.012363258	negative
PRJNA729271	stage_1W_vs_E10	ordinary_muscle_development	ENSGALG00010018525	ST8SIA2	-2.176989482	0.29164717	-7.464462913	8.36401E-14	2.33596E-13	negative
PRJNA729271	stage_1W_vs_E10	ordinary_muscle_development	ENSGALG00010018526	ENSGALG00010018526	0.487414665	0.100949227	4.828314958	1.37693E-06	2.54607E-06	positive
PRJNA729271	stage_1W_vs_E10	ordinary_muscle_development	ENSGALG00010018527	CCT4	-0.916064164	0.081625656	-11.22274791	3.15318E-29	1.72148E-28	negative
PRJNA729271	stage_1W_vs_E10	ordinary_muscle_development	ENSGALG00010018528	ENSGALG00010018528	0.014540219	0.703558873	0.020666671	0.983511556	0.986096701	positive
PRJNA729271	stage_1W_vs_E10	ordinary_muscle_development	ENSGALG00010018529	UBR2	0.916491707	0.092579967	9.89946031	4.18527E-23	1.77696E-22	positive
PRJNA729271	stage_1W_vs_E10	ordinary_muscle_development	ENSGALG00010018530	FAM161A	-0.871905933	0.233100045	-3.740479469	0.00018367	0.00028982	negative
PRJNA729271	stage_1W_vs_E10	ordinary_muscle_development	ENSGALG00010018531	ADCY5	0.146096272	0.135661952	1.076914121	0.281518634	0.315910242	positive
PRJNA729271	stage_1W_vs_E10	ordinary_muscle_development	ENSGALG00010018532	MFSD13A	-0.91815118	0.134118824	-6.845803972	7.60476E-12	1.92476E-11	negative
PRJNA729271	stage_1W_vs_E10	ordinary_muscle_development	ENSGALG00010018533	PCYOX1	2.278316989	0.172387817	13.21622967	7.0721E-40	5.64483E-39	positive
PRJNA729271	stage_1W_vs_E10	ordinary_muscle_development	ENSGALG00010018534	NCEH1	2.202977947	0.194858596	11.305521	1.23221E-29	6.85636E-29	positive
PRJNA729271	stage_1W_vs_E10	ordinary_muscle_development	ENSGALG00010018535	FAM174B	-0.061652167	0.103328519	-0.596661668	0.55073329	0.586890092	negative
PRJNA729271	stage_1W_vs_E10	ordinary_muscle_development	ENSGALG00010018536	ENSGALG00010018536	1.735715494	0.237044672	7.322313881	2.43731E-13	6.63826E-13	positive
PRJNA729271	stage_1W_vs_E10	ordinary_muscle_development	ENSGALG00010018537	ENSGALG00010018537	0.315757964	0.663907638	0.475605259	0.634355628	0.667028279	positive
PRJNA729271	stage_1W_vs_E10	ordinary_muscle_development	ENSGALG00010018539	CHD2	-0.473130165	0.088960143	-5.318451018	1.04654E-07	2.08833E-07	negative
PRJNA729271	stage_1W_vs_E10	ordinary_muscle_development	ENSGALG00010018540	TNFSF10	1.076862447	0.108894872	9.889009718	4.64601E-23	1.96797E-22	positive

PRJNA729271	stage_1W_vs_E10	ordinary_muscle_development	ENSGALG00010018542	ZMIZ2	2.826845349	0.262095425	10.78555777	4.02792E-27	2.02163E-26	positive
PRJNA729271	stage_1W_vs_E10	ordinary_muscle_development	ENSGALG00010018543	MRPL3	-0.643523213	0.077855823	-8.265575894	1.39022E-16	4.43176E-16	negative
PRJNA729271	stage_1W_vs_E10	ordinary_muscle_development	ENSGALG00010018544	HACD2	-1.059017071	0.103725764	-10.20977842	1.79274E-24	8.06259E-24	negative
PRJNA729271	stage_1W_vs_E10	ordinary_muscle_development	ENSGALG00010018546	SNRPGP15	-0.306233229	0.135850966	-2.25418514	0.024184523	0.031338697	negative
PRJNA729271	stage_1W_vs_E10	ordinary_muscle_development	ENSGALG00010018548	RGMA	-1.842480517	0.125766654	-14.64999239	1.34731E-48	1.40599E-47	negative
PRJNA729271	stage_1W_vs_E10	ordinary_muscle_development	ENSGALG00010018549	GHSR	-2.585768909	0.558806334	-4.627307802	3.7045E-06	6.6325E-06	negative
PRJNA729271	stage_1W_vs_E10	ordinary_muscle_development	ENSGALG00010018551	FBXO34	1.714491006	0.111163029	15.42321242	1.14276E-53	1.42061E-52	positive
PRJNA729271	stage_1W_vs_E10	ordinary_muscle_development	ENSGALG00010018552	DTD1	0.758149914	0.102892874	7.368342291	1.72763E-13	4.7401E-13	positive
PRJNA729271	stage_1W_vs_E10	ordinary_muscle_development	ENSGALG00010018553	ENSGALG00010018553	-2.526614095	0.684998103	-3.688497943	0.000225582	0.000353722	negative
PRJNA729271	stage_1W_vs_E10	ordinary_muscle_development	ENSGALG00010018554	PPIA	-1.347237337	0.123768835	-10.88510961	1.35735E-27	6.94487E-27	negative
PRJNA729271	stage_1W_vs_E10	ordinary_muscle_development	ENSGALG00010018555	UBN1	-0.805701245	0.077929209	-10.33888642	4.69967E-25	2.16741E-24	negative
PRJNA729271	stage_1W_vs_E10	ordinary_muscle_development	ENSGALG00010018556	MRPL2	2.365231259	0.125654375	18.82331005	4.86483E-79	1.12286E-77	positive
PRJNA729271	stage_1W_vs_E10	ordinary_muscle_development	ENSGALG00010018557	TBCC	0.174419561	0.102651259	1.699146823	0.089291523	0.108108973	positive
PRJNA729271	stage_1W_vs_E10	ordinary_muscle_development	ENSGALG00010018558	ENSGALG00010018558	0.475804692	0.238426815	1.995600591	0.045977415	0.057665712	positive
PRJNA729271	stage_1W_vs_E10	ordinary_muscle_development	ENSGALG00010018559	DLG3	-2.361508377	0.140532828	-16.80396252	2.28272E-63	3.64405E-62	negative
PRJNA729271	stage_1W_vs_E10	ordinary_muscle_development	ENSGALG00010018560	ENSGALG00010018560	1.18349907	0.080074527	14.7799695	1.97256E-49	2.11794E-48	positive
PRJNA729271	stage_1W_vs_E10	ordinary_muscle_development	ENSGALG00010018561	ACTR1A	0.301308447	0.059122559	5.096336384	3.4629E-07	6.67482E-07	positive
PRJNA729271	stage_1W_vs_E10	ordinary_muscle_development	ENSGALG00010018562	ENSGALG00010018562	-6.509187364	0.919628357	-7.078062912	1.46183E-12	3.83864E-12	negative
PRJNA729271	stage_1W_vs_E10	ordinary_muscle_development	ENSGALG00010018564	DLGAP5	-2.627046919	0.155160312	-16.93117836	2.65021E-64	4.32112E-63	negative
PRJNA729271	stage_1W_vs_E10	ordinary_muscle_development	ENSGALG00010018566	ENSGALG00010018566	1.7476772	0.142170683	12.29281005	9.90036E-35	6.66539E-34	positive
PRJNA729271	stage_1W_vs_E10	ordinary_muscle_development	ENSGALG00010018568	FAM136A	-0.345961791	0.193978257	-1.783508088	0.074503633	0.091018133	negative
PRJNA729271	stage_1W_vs_E10	ordinary_muscle_development	ENSGALG00010018573	SEC23B	-0.845444464	0.08021896	-10.53921001	5.69755E-26	2.73011E-25	negative
PRJNA729271	stage_1W_vs_E10	ordinary_muscle_development	ENSGALG00010018574	ENSGALG00010018574	-1.24328011	0.242977095	-5.116861358	3.10662E-07	6.00646E-07	negative
PRJNA729271	stage_1W_vs_E10	ordinary_muscle_development	ENSGALG00010018576	PTK7	-2.478912177	0.11632027	-21.31109367	8.9578E-101	3.39905E-99	negative
PRJNA729271	stage_1W_vs_E10	ordinary_muscle_development	ENSGALG00010018577	NARFL	0.729089386	0.081352644	8.962085974	3.18593E-19	1.15479E-18	positive
PRJNA729271	stage_1W_vs_E10	ordinary_muscle_development	ENSGALG00010018579	FNDC3B	0.070310826	0.101894683	0.690034292	0.490172622	0.528374519	positive
PRJNA729271	stage_1W_vs_E10	ordinary_muscle_development	ENSGALG00010018581	SUFU	-1.438699951	0.09339406	-15.40461948	1.52383E-53	1.88948E-52	negative
PRJNA729271	stage_1W_vs_E10	ordinary_muscle_development	ENSGALG00010018582	RCC2	-2.526590466	0.113151707	-22.32922983	1.9221E-110	8.7066E-109	negative
PRJNA729271	stage_1W_vs_E10	ordinary_muscle_development	ENSGALG00010018583	PPL	1.849005123	0.213239564	8.671022788	4.28256E-18	1.46198E-17	positive
PRJNA729271	stage_1W_vs_E10	ordinary_muscle_development	ENSGALG00010018585	GDPD2	-4.732589524	0.915176422	-5.171231917	2.32556E-07	4.53565E-07	negative
PRJNA729271	stage_1W_vs_E10	ordinary_muscle_development	ENSGALG00010018587	SRF	1.070201896	0.173318914	6.17475537	6.6266E-10	1.50388E-09	positive
PRJNA729271	stage_1W_vs_E10	ordinary_muscle_development	ENSGALG00010018588	POLR3F	0.869661484	0.101915805	8.533136618	1.42432E-17	4.73848E-17	positive
PRJNA729271	stage_1W_vs_E10	ordinary_muscle_development	ENSGALG00010018590	TGFA	-0.595332889	0.530630863	-1.121934157	0.261890436	0.295646564	negative
PRJNA729271	stage_1W_vs_E10	ordinary_muscle_development	ENSGALG00010018593	LGALS3	-0.398232121	0.336072423	-1.184959234	0.236033566	0.26810646	negative
PRJNA729271	stage_1W_vs_E10	ordinary_muscle_development	ENSGALG00010018594	BICRAL	-0.326489006	0.105420268	-3.097023121	0.001954746	0.002835389	negative
PRJNA729271	stage_1W_vs_E10	ordinary_muscle_development	ENSGALG00010018598	ENSGALG00010018598	0.342705018	0.078479591	4.366804315	1.26078E-05	2.17197E-05	positive
PRJNA729271	stage_1W_vs_E10	ordinary_muscle_development	ENSGALG00010018601	CPNE4	-6.003278162	0.639760555	-9.383632858	6.37379E-21	2.48823E-20	negative
PRJNA729271	stage_1W_vs_E10	ordinary_muscle_development	ENSGALG00010018602	MCTP2	-1.537997557	0.227959897	-6.746790024	1.51152E-11	3.76128E-11	negative
PRJNA729271	stage_1W_vs_E10	ordinary_muscle_development	ENSGALG00010018604	FAM43A	0.919671937	0.123995338	7.416988017	1.19814E-13	3.31368E-13	positive
PRJNA729271	stage_1W_vs_E10	ordinary_muscle_development	ENSGALG00010018606	DZANK1	-0.328048683	0.242384658	-1.353421807	0.175920915	0.204144877	negative
PRJNA729271	stage_1W_vs_E10	ordinary_muscle_development	ENSGALG00010018607	HAGHL	0.292420685	0.117043566	2.498391801	0.012475822	0.016680845	positive
PRJNA729271	stage_1W_vs_E10	ordinary_muscle_development	ENSGALG00010018608	TRIM8	-0.228365259	0.127774834	-1.787247547	0.073897488	0.09031569	negative
PRJNA729271	stage_1W_vs_E10	ordinary_muscle_development	ENSGALG00010018609	ENSGALG00010018609	0.66306457	0.124302714	5.334272673	9.59283E-08	1.92029E-07	positive
PRJNA729271	stage_1W_vs_E10	ordinary_muscle_development	ENSGALG00010018611	NUDCD3	1.158688643	0.112055087	10.34034842	4.62852E-25	2.13528E-24	positive
PRJNA729271	stage_1W_vs_E10	ordinary_muscle_development	ENSGALG00010018613	PADI3	1.83058949	0.375749417	4.871835872	1.10566E-06	2.06103E-06	positive
PRJNA729271	stage_1W_vs_E10	ordinary_muscle_development	ENSGALG00010018615	HTR2C	-0.994920072	0.297307578	-3.346433616	0.000818583	0.001228934	negative
PRJNA729271	stage_1W_vs_E10	ordinary_muscle_development	ENSGALG00010018616	ENSGALG00010018616	0.01686514	0.849638671	0.019849779	0.984163208	0.986545346	positive
PRJNA729271	stage_1W_vs_E10	ordinary_muscle_development	ENSGALG00010018618	FAHD1	1.012472392	0.169693502	5.9664771	2.42431E-09	5.34048E-09	positive
PRJNA729271	stage_1W_vs_E10	ordinary_muscle_development	ENSGALG00010018619	MAPK1IP1L	-0.657199328	0.077991368	-8.426564918	3.5596E-17	1.16234E-16	negative
PRJNA729271	stage_1W_vs_E10	ordinary_muscle_development	ENSGALG00010018623	ARRDC4	1.357655836	0.131552911	10.3202265	5.70883E-25	2.62114E-24	positive
PRJNA729271	stage_1W_vs_E10	ordinary_muscle_development	ENSGALG00010018624	ENSGALG00010018624	1.442493614	0.387581222	3.721784065	0.00019782	0.00031137	positive
PRJNA729271	stage_1W_vs_E10	ordinary_muscle_development	ENSGALG00010018627	MYLK	-1.112366249	0.180358743	-6.16752051	6.93691E-10	1.57208E-09	negative
PRJNA729271	stage_1W_vs_E10	ordinary_muscle_development	ENSGALG00010018628	XDH	3.050430691	0.56822628	5.368337926	7.94656E-08	1.60046E-07	positive
PRJNA729271	stage_1W_vs_E10	ordinary_muscle_development	ENSGALG00010018631	KAT14	-0.252913233	0.05262525	-4.8059293	1.54034E-06	2.83629E-06	negative
PRJNA729271	stage_1W_vs_E10	ordinary_muscle_development	ENSGALG00010018633	ENSGALG00010018633	2.308796095	0.177637632	12.99722404	1.26865E-38	9.68358E-38	positive
PRJNA729271	stage_1W_vs_E10	ordinary_muscle_development	ENSGALG00010018638	SEC14L5	2.012066255	0.15771287	12.75778098	2.82124E-37	2.05807E-36	positive
PRJNA729271	stage_1W_vs_E10	ordinary_muscle_development	ENSGALG00010018641	ENSGALG00010018641	-5.681716	1.008660718	-5.632930774	1.77173E-08	3.70366E-08	negative
PRJNA729271	stage_1W_vs_E10	ordinary_muscle_development	ENSGALG00010018644	ESF1	-0.077594261	0.096497613	-0.804105496	0.421336051	0.459434744	negative
PRJNA729271	stage_1W_vs_E10	ordinary_muscle_development	ENSGALG00010018649	HAGH	3.43077863	0.10472801	32.75894042	2.2649E-235	4.9741E-233	positive
PRJNA729271	stage_1W_vs_E10	ordinary_muscle_development	ENSGALG00010018654	ENSGALG00010018654	1.951254759	0.216657408	9.006176033	2.13367E-19	7.7903E-19	positive
PRJNA729271	stage_1W_vs_E10	ordinary_muscle_development	ENSGALG00010018655	BIRC5	-2.513308664	0.212378865	-11.83408085	2.60178E-32	1.6048E-31	negative
PRJNA729271	stage_1W_vs_E10	ordinary_muscle_development	ENSGALG00010018662	PADI1	2.175419384	0.808816388	2.689633168	0.00715306	0.009824105	positive
PRJNA729271	stage_1W_vs_E10	ordinary_muscle_development	ENSGALG00010018665	IL13RA2	-1.094698111	0.333897596	-3.278544448	0.001043439	0.00155061	negative
PRJNA729271	stage_1W_vs_E10	ordinary_muscle_development	ENSGALG00010018668	OVOL2	-4.818479844	0.955615883	-5.042276847	4.60025E-07	8.7946E-07	negative
PRJNA729271	stage_1W_vs_E10	ordinary_muscle_development	ENSGALG00010018670	MRAS	1.374722484	0.213073166	6.451879936	1.10471E-10	2.62118E-10	positive
PRJNA729271	stage_1W_vs_E10	ordinary_muscle_development	ENSGALG00010018672	PKDCC	-1.667972456	0.099391014	-16.78192419	3.30937E-63	5.23111E-62	negative
PRJNA729271	stage_1W_vs_E10	ordinary_muscle_development	ENSGALG00010018673	NAGPA	-0.230581981	0.092777834	-2.48531325	0.012943748	0.017284167	negative
PRJNA729271	stage_1W_vs_E10	ordinary_muscle_development	ENSGALG00010018684	PADI2	5.538315095	0.980407987	5.648990187	1.61393E-08	3.38209E-08	positive
PRJNA729271	stage_1W_vs_E10	ordinary_muscle_development	ENSGALG00010018685	ARL3	-2.289265571	0.106642924	-21.46664293	3.1926E-102	1.24068E-100	negative
PRJNA729271	stage_1W_vs_E10	ordinary_muscle_development	ENSGALG00010018693	ENSGALG00010018693	1.08656961	0.163323291	6.652876045	2.8742E-11	7.05172E-11	positive
PRJNA729271	stage_1W_vs_E10	ordinary_muscle_development	ENSGALG00010018694	MGME1	-1.744313934	0.107125257	-16.28293811	1.30452E-59	1.88337E-58	negative
PRJNA729271	stage_1W_vs_E10	ordinary_muscle_development	ENSGALG00010018698	MRPS16	1.65588462	0.112951636	14.6601207	1.16066E-48	1.21297E-47	positive
PRJNA729271	stage_1W_vs_E10	ordinary_muscle_development	ENSGALG00010018701	SFXN2	-1.840911923	0.129472776	-14.21852514	7.03194E-46	6.73681E-45	negative
PRJNA729271	stage_1W_vs_E10	ordinary_muscle_development	ENSGALG00010018704	PGPEP1L	5.125756839	0.417416513	12.27971745	1.16404E-34	7.81509E-34	positive
PRJNA729271	stage_1W_vs_E10	ordinary_muscle_development	ENSGALG00010018707	SLC45A1	0.260801344	0.099940918	2.609555207	0.009066002	0.012318306	positive
PRJNA729271	stage_1W_vs_E10	ordinary_muscle_development	ENSGALG00010018709	SNX5	-0.778409741	0.116410432	-6.686769626	2.28151E-11	5.62997E-11	negative
PRJNA729271	stage_1W_vs_E10	ordinary_muscle_development	ENSGALG00010018713	ENSGALG00010018713	-0.451006888	0.13843447	-3.257908882	0.001122364	0.001662443	negative
PRJNA729271	stage_1W_vs_E10	ordinary_muscle_development	ENSGALG00010018715	DNAJC13	-0.2761457	0.103407744	-2.670454728	0.007574859	0.01037294	negative
PRJNA729271	stage_1W_vs_E10	ordinary_muscle_development	ENSGALG00010018717	MEIOB	-4.54117782	0.986228426	-4.604590275	4.13279E-06	7.37198E-06	negative
PRJNA729271	stage_1W_vs_E10	ordinary_muscle_development	ENSGALG00010018719	ERRFI1	-0.404700758	0.087717778	-4.613668616	3.95623E-06	7.06622E-06	negative
PRJNA729271	stage_1W_vs_E10	ordinary_muscle_development	ENSGALG00010018721	LRCH2	-0.132762837	0.189351055	-0.701146541	0.483211568	0.521452627	negative
PRJNA729271	stage_1W_vs_E10	ordinary_muscle_development	ENSGALG00010018724	RRBP1	-1.343019974	0.142396241	-9.431568993	4.04E-21	1.58957E-20	negative
PRJNA729271	stage_1W_vs_E10	ordinary_muscle_development	ENSGALG00010018726	EBF2	-1.429495096	0.156923545	-9.109500389	8.27622E-20	3.08549E-19	negative
PRJNA729271	stage_1W_vs_E10	ordinary_muscle_development	ENSGALG00010018727	PARK7	2.548508923	0.131660892	19.35661288	1.79294E-83	4.66582E-82	positive
PRJNA729271	stage_1W_vs_E10	ordinary_muscle_development	ENSGALG00010018729	HS3ST6	-7.304247036	0.792517676	-9.216509936	3.06928E-20	1.16372E-19	negative
PRJNA729271	stage_1W_vs_E10	ordinary_muscle_development	ENSGALG00010018730	IGF1R	-0.621742474	0.088224921	-7.047243208	1.82497E-12	4.76287E-12	negative
PRJNA729271	stage_1W_vs_E10	ordinary_muscle_development	ENSGALG00010018732	ENSGALG00010018732	4.665039026	0.218248506	21.37489557	2.2883E-101	8.77501E-100	positive
PRJNA729271	stage_1W_vs_E10	ordinary_muscle_development	ENSGALG00010018736	EEF2KMT	0.297668164	0.131564032	2.26253451	0.023664399	0.030705887	positive
PRJNA729271	stage_1W_vs_E10	ordinary_muscle_development	ENSGALG00010018747	FLRT3	-3.119430186	0.12805541	-24.36000317	4.542E-131	2.9925E-129	negative
PRJNA729271	stage_1W_vs_E10	ordinary_muscle_development	ENSGALG00010018750	SOCS4	0.285258124	0.126545038	2.254202356	0.024183441	0.031338697	positive
PRJNA729271	stage_1W_vs_E10	ordinary_muscle_development	ENSGALG00010018752	PLS3	-1.143634328	0.141140452	-8.102810442	5.37039E-16	1.6719E-15	negative
PRJNA729271	stage_1W_vs_E10	ordinary_muscle_development	ENSGALG00010018753	SYNM	1.786341695	0.153788442	11.61557835	3.43456E-31	2.03532E-30	positive
PRJNA729271	stage_1W_vs_E10	ordinary_muscle_development	ENSGALG00010018756	EML4	-2.721189106	0.064286783	-42.32890473	0	0	negative
PRJNA729271	stage_1W_vs_E10	ordinary_muscle_development	ENSGALG00010018758	ENSGALG00010018758	-1.219997371	0.137172452	-8.893894866	5.90035E-19	2.11592E-18	negative
PRJNA729271	stage_1W_vs_E10	ordinary_muscle_development	ENSGALG00010018764	TMEM177	0.133115603	0.091767433	1.450575642	0.146898062	0.172540608	positive
PRJNA729271	stage_1W_vs_E10	ordinary_muscle_development	ENSGALG00010018767	NDUFB6	1.847844576	0.168932574	10.93835566	7.55578E-28	3.91007E-27	positive
PRJNA729271	stage_1W_vs_E10	ordinary_muscle_development	ENSGALG00010018774	MSRB1	1.969052973	0.122010844	16.138344	1.37173E-58	1.93228E-57	positive
PRJNA729271	stage_1W_vs_E10	ordinary_muscle_development	ENSGALG00010018780	WDHD1	-3.059748247	0.210775767	-14.51660352	9.51073E-48	9.64717E-47	negative
PRJNA729271	stage_1W_vs_E10	ordinary_muscle_development	ENSGALG00010018783	ENSGALG00010018783	-0.009228838	0.178662195	-0.051655238	0.958803404	0.964124547	negative
PRJNA729271	stage_1W_vs_E10	ordinary_muscle_development	ENSGALG00010018786	DSTN	-1.560918853	0.115047692	-13.56758071	6.23461E-42	5.3316E-41	negative
PRJNA729271	stage_1W_vs_E10	ordinary_muscle_development	ENSGALG00010018796	PER3	0.924283262	0.309832733	2.983168536	0.002852809	0.004074437	positive
PRJNA729271	stage_1W_vs_E10	ordinary_muscle_development	ENSGALG00010018799	ENSGALG00010018799	-0.263404161	0.301347244	-0.8740885	0.38207006	0.419935207	negative
PRJNA729271	stage_1W_vs_E10	ordinary_muscle_development	ENSGALG00010018806	GCH1	0.62697073	0.311758489	2.011078293	0.044317188	0.055713585	positive
PRJNA729271	stage_1W_vs_E10	ordinary_muscle_development	ENSGALG00010018807	RD3	-3.445026047	0.280534584	-12.28021873	1.15685E-34	7.77041E-34	negative
PRJNA729271	stage_1W_vs_E10	ordinary_muscle_development	ENSGALG00010018808	MATCAP2	-1.367247024	0.092518707	-14.77806025	2.02927E-49	2.17722E-48	negative
PRJNA729271	stage_1W_vs_E10	ordinary_muscle_development	ENSGALG00010018811	VAMP3	1.386982056	0.122963721	11.27960386	1.6549E-29	9.16261E-29	positive
PRJNA729271	stage_1W_vs_E10	ordinary_muscle_development	ENSGALG00010018815	ENSGALG00010018815	1.397014245	0.170296857	8.203405933	2.33671E-16	7.36636E-16	positive
PRJNA729271	stage_1W_vs_E10	ordinary_muscle_development	ENSGALG00010018819	LRRC28	0.327446897	0.078529386	4.169737147	3.04951E-05	5.10247E-05	positive
PRJNA729271	stage_1W_vs_E10	ordinary_muscle_development	ENSGALG00010018822	TRAF5	0.216711476	0.20518953	1.056152698	0.290898457	0.325855729	positive
PRJNA729271	stage_1W_vs_E10	ordinary_muscle_development	ENSGALG00010018823	RAB40C	-0.57392491	0.077453744	-7.409905352	1.2639E-13	3.49422E-13	negative
PRJNA729271	stage_1W_vs_E10	ordinary_muscle_development	ENSGALG00010018829	PTPN4	2.293567418	0.138022796	16.61730879	5.22249E-62	8.01058E-61	positive
PRJNA729271	stage_1W_vs_E10	ordinary_muscle_development	ENSGALG00010018831	WBP1L	1.032091156	0.240335576	4.294375286	1.75186E-05	2.98252E-05	positive
PRJNA729271	stage_1W_vs_E10	ordinary_muscle_development	ENSGALG00010018832	EEPD1	1.79181549	0.184009282	9.737636446	2.08344E-22	8.59158E-22	positive
PRJNA729271	stage_1W_vs_E10	ordinary_muscle_development	ENSGALG00010018834	SAMD4A	0.762884528	0.171994886	4.435507047	9.18558E-06	1.59857E-05	positive
PRJNA729271	stage_1W_vs_E10	ordinary_muscle_development	ENSGALG00010018835	CAMK2B	0.948239387	0.178488821	5.312598176	1.08073E-07	2.15418E-07	positive
PRJNA729271	stage_1W_vs_E10	ordinary_muscle_development	ENSGALG00010018837	RCOR3	-1.160436468	0.095544277	-12.14553616	6.05841E-34	3.96285E-33	negative
PRJNA729271	stage_1W_vs_E10	ordinary_muscle_development	ENSGALG00010018838	WFIKKN1	-6.738473295	0.465097576	-14.48830019	1.43654E-47	1.44702E-46	negative
PRJNA729271	stage_1W_vs_E10	ordinary_muscle_development	ENSGALG00010018839	RPL3L	11.17766247	0.249400295	44.81816055	0	0	positive
PRJNA729271	stage_1W_vs_E10	ordinary_muscle_development	ENSGALG00010018843	MCRIP2	3.001187649	0.235670187	12.7346937	3.79332E-37	2.75886E-36	positive
PRJNA729271	stage_1W_vs_E10	ordinary_muscle_development	ENSGALG00010018847	CGRRF1	-0.13358388	0.158186458	-0.844471018	0.398406252	0.436632287	negative
PRJNA729271	stage_1W_vs_E10	ordinary_muscle_development	ENSGALG00010018848	MEF2A	1.706915639	0.11452867	14.90382836	3.11224E-50	3.44103E-49	positive
PRJNA729271	stage_1W_vs_E10	ordinary_muscle_development	ENSGALG00010018849	KCNH1	-2.637179418	0.42411973	-6.218006931	5.03509E-10	1.14935E-09	negative
PRJNA729271	stage_1W_vs_E10	ordinary_muscle_development	ENSGALG00010018852	NDUFB10	2.271583153	0.115705666	19.63242788	8.17151E-86	2.24755E-84	positive
PRJNA729271	stage_1W_vs_E10	ordinary_muscle_development	ENSGALG00010018854	LYSMD4	0.521148491	0.171550677	3.037869038	0.002382575	0.003426473	positive
PRJNA729271	stage_1W_vs_E10	ordinary_muscle_development	ENSGALG00010018855	SYNDIG1	-1.298658307	0.61213552	-2.121520911	0.03387799	0.043128532	negative
PRJNA729271	stage_1W_vs_E10	ordinary_muscle_development	ENSGALG00010018857	ENSGALG00010018857	0.551783816	0.082156602	6.716244383	1.86468E-11	4.62184E-11	positive
PRJNA729271	stage_1W_vs_E10	ordinary_muscle_development	ENSGALG00010018858	EPB41L5	-1.206265792	0.118388477	-10.18904732	2.21928E-24	9.92855E-24	negative
PRJNA729271	stage_1W_vs_E10	ordinary_muscle_development	ENSGALG00010018860	AGTR2	0.9318746	0.215226294	4.329743275	1.49283E-05	2.55293E-05	positive
PRJNA729271	stage_1W_vs_E10	ordinary_muscle_development	ENSGALG00010018864	ABCA3	1.199195127	0.118928472	10.08333079	6.54684E-24	2.87478E-23	positive
PRJNA729271	stage_1W_vs_E10	ordinary_muscle_development	ENSGALG00010018865	ENSGALG00010018865	-2.559194729	0.234603392	-10.90860073	1.04858E-27	5.3936E-27	negative
PRJNA729271	stage_1W_vs_E10	ordinary_muscle_development	ENSGALG00010018867	RPS2	0.591270637	0.155923046	3.792067004	0.000149399	0.000237371	positive

PRJNA729271	stage_1W_vs_E10	ordinary_muscle_development	ENSGALG00010018872	KCTD9	-0.025501486	0.101214183	-0.251955658	0.801075338	0.822175672	negative
PRJNA729271	stage_1W_vs_E10	ordinary_muscle_development	ENSGALG00010018874	ENSGALG00010018874	-0.517611698	0.11712089	-4.419465214	9.89454E-06	1.71762E-05	negative
PRJNA729271	stage_1W_vs_E10	ordinary_muscle_development	ENSGALG00010018876	GMFB	-1.533772851	0.098881768	-15.51117954	2.91475E-54	3.69635E-53	negative
PRJNA729271	stage_1W_vs_E10	ordinary_muscle_development	ENSGALG00010018877	CST7	3.757983305	0.820265365	4.58142385	4.61821E-06	8.21059E-06	positive
PRJNA729271	stage_1W_vs_E10	ordinary_muscle_development	ENSGALG00010018880	TMEM17	-0.148529674	0.256580275	-0.578881888	0.562668872	0.598377499	negative
PRJNA729271	stage_1W_vs_E10	ordinary_muscle_development	ENSGALG00010018881	ENSGALG00010018881	1.687155311	0.628842873	2.682952105	0.007297544	0.010009264	positive
PRJNA729271	stage_1W_vs_E10	ordinary_muscle_development	ENSGALG00010018882	HHAT	-1.150372628	0.166373771	-6.914386924	4.69891E-12	1.20104E-11	negative
PRJNA729271	stage_1W_vs_E10	ordinary_muscle_development	ENSGALG00010018883	TBL3	-1.435995734	0.077043642	-18.63873126	1.55895E-77	3.47645E-76	negative
PRJNA729271	stage_1W_vs_E10	ordinary_muscle_development	ENSGALG00010018885	EFEMP1	0.428649241	0.207594716	2.064836954	0.03893843	0.049272156	positive
PRJNA729271	stage_1W_vs_E10	ordinary_muscle_development	ENSGALG00010018887	ADAMTS17	-3.771067628	0.20924723	-18.0220671	1.30765E-72	2.63622E-71	negative
PRJNA729271	stage_1W_vs_E10	ordinary_muscle_development	ENSGALG00010018891	GNRH1	-2.167168081	0.358576002	-6.043817955	1.50509E-09	3.35739E-09	negative
PRJNA729271	stage_1W_vs_E10	ordinary_muscle_development	ENSGALG00010018892	APMAP	-0.822154922	0.124627008	-6.596924193	4.19775E-11	1.0216E-10	negative
PRJNA729271	stage_1W_vs_E10	ordinary_muscle_development	ENSGALG00010018894	SERTAD4	-1.292491111	0.094071824	-13.73940733	5.89564E-43	5.19182E-42	negative
PRJNA729271	stage_1W_vs_E10	ordinary_muscle_development	ENSGALG00010018895	SEPTIN7	-1.409917681	0.090123577	-15.64427115	3.63507E-55	4.71713E-54	negative
PRJNA729271	stage_1W_vs_E10	ordinary_muscle_development	ENSGALG00010018897	CCDC85A	-0.375243671	0.16724566	-2.243667615	0.024853795	0.032154209	negative
PRJNA729271	stage_1W_vs_E10	ordinary_muscle_development	ENSGALG00010018899	ACSS1	-0.032270784	0.099993811	-0.322727809	0.74690139	0.77193124	negative
PRJNA729271	stage_1W_vs_E10	ordinary_muscle_development	ENSGALG00010018901	TCF7L2	-1.256570705	0.149709886	-8.393371624	4.72395E-17	1.53391E-16	negative
PRJNA729271	stage_1W_vs_E10	ordinary_muscle_development	ENSGALG00010018902	NOXO1	-0.772347363	0.567038345	-1.362072546	0.173174992	0.201119422	negative
PRJNA729271	stage_1W_vs_E10	ordinary_muscle_development	ENSGALG00010018903	CAMTA1	0.055525607	0.096194507	0.577222223	0.563789346	0.599437143	positive
PRJNA729271	stage_1W_vs_E10	ordinary_muscle_development	ENSGALG00010018904	UGP2	3.507160103	0.271155354	12.93413553	2.88854E-38	2.17729E-37	positive
PRJNA729271	stage_1W_vs_E10	ordinary_muscle_development	ENSGALG00010018908	ENSGALG00010018908	-0.568730319	0.247348338	-2.299309243	0.021487385	0.027993857	negative
PRJNA729271	stage_1W_vs_E10	ordinary_muscle_development	ENSGALG00010018909	TTBK1	-0.104605486	0.133713648	-0.782309714	0.43403258	0.47196566	negative
PRJNA729271	stage_1W_vs_E10	ordinary_muscle_development	ENSGALG00010018911	CNIH1	0.469995483	0.093640855	5.01912849	5.19064E-07	9.88288E-07	positive
PRJNA729271	stage_1W_vs_E10	ordinary_muscle_development	ENSGALG00010018913	ENSGALG00010018913	1.188270621	0.219651693	5.409794961	6.30969E-08	1.27933E-07	positive
PRJNA729271	stage_1W_vs_E10	ordinary_muscle_development	ENSGALG00010018915	DOCK5	-1.474556633	0.194276621	-7.589984973	3.19943E-14	9.12727E-14	negative
PRJNA729271	stage_1W_vs_E10	ordinary_muscle_development	ENSGALG00010018916	CERS3	-1.724525713	0.348557223	-4.947611471	7.51297E-07	1.41779E-06	negative
PRJNA729271	stage_1W_vs_E10	ordinary_muscle_development	ENSGALG00010018917	DNAJC11	0.149581484	0.105374778	1.419518851	0.155747805	0.182224683	positive
PRJNA729271	stage_1W_vs_E10	ordinary_muscle_development	ENSGALG00010018919	RALB	0.063603871	0.107698396	0.590573986	0.5548059	0.590882551	positive
PRJNA729271	stage_1W_vs_E10	ordinary_muscle_development	ENSGALG00010018920	ACAD11	-0.475685973	0.117044535	-4.064145112	4.82088E-05	7.93715E-05	negative
PRJNA729271	stage_1W_vs_E10	ordinary_muscle_development	ENSGALG00010018922	EEF2K	1.990952234	0.114013957	17.46235543	2.77255E-68	5.02351E-67	positive
PRJNA729271	stage_1W_vs_E10	ordinary_muscle_development	ENSGALG00010018923	VPS54	-0.384984535	0.093142225	-4.133297582	3.57595E-05	5.95513E-05	negative
PRJNA729271	stage_1W_vs_E10	ordinary_muscle_development	ENSGALG00010018925	LINS1	-0.471597036	0.133930204	-3.521214952	0.000429574	0.00065849	negative
PRJNA729271	stage_1W_vs_E10	ordinary_muscle_development	ENSGALG00010018926	SLC22A7	-2.13991027	1.184605532	-1.806432784	0.070850765	0.086796977	negative
PRJNA729271	stage_1W_vs_E10	ordinary_muscle_development	ENSGALG00010018932	CDKN3	-2.268711707	0.238180167	-9.525191532	1.64738E-21	6.58361E-21	negative
PRJNA729271	stage_1W_vs_E10	ordinary_muscle_development	ENSGALG00010018933	PHF13	0.314425368	0.087493706	3.593691299	0.000326026	0.000505265	positive
PRJNA729271	stage_1W_vs_E10	ordinary_muscle_development	ENSGALG00010018936	SYT14	1.692193863	0.217747925	7.771343226	7.76582E-15	2.28885E-14	positive
PRJNA729271	stage_1W_vs_E10	ordinary_muscle_development	ENSGALG00010018937	INHBB	-2.610267633	0.342761978	-7.615394367	2.62888E-14	7.55014E-14	negative
PRJNA729271	stage_1W_vs_E10	ordinary_muscle_development	ENSGALG00010018941	KLHL21	2.187645977	0.143590812	15.23527831	2.06247E-52	2.46866E-51	positive
PRJNA729271	stage_1W_vs_E10	ordinary_muscle_development	ENSGALG00010018943	ACKR4	2.861197081	0.346648701	8.25388087	1.53332E-16	4.88151E-16	positive
PRJNA729271	stage_1W_vs_E10	ordinary_muscle_development	ENSGALG00010018944	NEFL	-4.112244175	0.208973288	-19.67832451	3.30757E-86	9.14948E-85	negative
PRJNA729271	stage_1W_vs_E10	ordinary_muscle_development	ENSGALG00010018945	HABP2	-0.173975463	0.682226179	-0.255011415	0.798714283	0.819984668	negative
PRJNA729271	stage_1W_vs_E10	ordinary_muscle_development	ENSGALG00010018947	ENSGALG00010018947	0.124392681	0.228056859	0.545445912	0.585446871	0.620802159	positive
PRJNA729271	stage_1W_vs_E10	ordinary_muscle_development	ENSGALG00010018948	POLR3E	1.420601815	0.107306826	13.23869005	5.24558E-40	4.20778E-39	positive
PRJNA729271	stage_1W_vs_E10	ordinary_muscle_development	ENSGALG00010018950	TTL	0.315417014	0.097001349	3.251676587	0.001147265	0.001698111	positive
PRJNA729271	stage_1W_vs_E10	ordinary_muscle_development	ENSGALG00010018951	YKT6	-0.599228256	0.090318261	-6.634630125	3.25317E-11	7.95995E-11	negative
PRJNA729271	stage_1W_vs_E10	ordinary_muscle_development	ENSGALG00010018955	ZBTB48	1.550970265	0.140123596	11.06858734	1.78187E-28	9.44015E-28	positive
PRJNA729271	stage_1W_vs_E10	ordinary_muscle_development	ENSGALG00010018958	PELI3	-0.656513749	0.11867931	-5.531829857	3.16907E-08	6.52496E-08	negative
PRJNA729271	stage_1W_vs_E10	ordinary_muscle_development	ENSGALG00010018959	UBA5	-0.845845933	0.119353851	-7.086875939	1.37173E-12	3.60858E-12	negative
PRJNA729271	stage_1W_vs_E10	ordinary_muscle_development	ENSGALG00010018960	RBFOX1	-0.105008466	0.219082478	-0.479310196	0.63171797	0.664688732	negative
PRJNA729271	stage_1W_vs_E10	ordinary_muscle_development	ENSGALG00010018961	ASB7	-0.2281477	0.099447928	-2.294142314	0.021782325	0.028352623	negative
PRJNA729271	stage_1W_vs_E10	ordinary_muscle_development	ENSGALG00010018968	GLI2	-2.338184272	0.201563753	-11.60022194	4.11011E-31	2.42475E-30	negative
PRJNA729271	stage_1W_vs_E10	ordinary_muscle_development	ENSGALG00010018969	DIEXF	-0.469275824	0.090092881	-5.208800288	1.90066E-07	3.72751E-07	negative
PRJNA729271	stage_1W_vs_E10	ordinary_muscle_development	ENSGALG00010018971	POLR1B	0.174200379	0.099070136	1.758354092	0.078687279	0.095814189	positive
PRJNA729271	stage_1W_vs_E10	ordinary_muscle_development	ENSGALG00010018973	NEFM	-7.462160314	0.891026963	-8.374786199	5.53242E-17	1.79161E-16	negative
PRJNA729271	stage_1W_vs_E10	ordinary_muscle_development	ENSGALG00010018974	TAS1R1	0.735891339	0.295427946	2.490933402	0.0127408	0.017025712	positive
PRJNA729271	stage_1W_vs_E10	ordinary_muscle_development	ENSGALG00010018977	NPHP3	-0.270895133	0.112110016	-2.416333003	0.015677716	0.020726787	negative
PRJNA729271	stage_1W_vs_E10	ordinary_muscle_development	ENSGALG00010018983	MDGA2	-3.522120835	0.228802035	-15.39374787	1.80281E-53	2.22776E-52	negative
PRJNA729271	stage_1W_vs_E10	ordinary_muscle_development	ENSGALG00010018985	NOL9	-0.227539071	0.089336397	-2.546991821	0.010865598	0.014630454	negative
PRJNA729271	stage_1W_vs_E10	ordinary_muscle_development	ENSGALG00010018986	CTDSP1	2.373211286	0.190994252	12.42556395	1.89883E-35	1.31002E-34	positive
PRJNA729271	stage_1W_vs_E10	ordinary_muscle_development	ENSGALG00010018987	MRPS10	0.607230167	0.10660724	5.69595617	1.22682E-08	2.58772E-08	positive
PRJNA729271	stage_1W_vs_E10	ordinary_muscle_development	ENSGALG00010018990	GCK	-1.749732588	0.429091326	-4.077762661	4.54711E-05	7.50688E-05	negative
PRJNA729271	stage_1W_vs_E10	ordinary_muscle_development	ENSGALG00010018991	RYBP	-2.493659535	0.135016451	-18.46930143	3.64774E-76	7.91527E-75	negative
PRJNA729271	stage_1W_vs_E10	ordinary_muscle_development	ENSGALG00010018993	ENSGALG00010018993	-0.313803657	0.526463247	-0.596059951	0.551135178	0.58723202	negative
PRJNA729271	stage_1W_vs_E10	ordinary_muscle_development	ENSGALG00010018998	RPS29	0.845168724	0.439936487	1.921115317	0.054717171	0.067980234	positive
PRJNA729271	stage_1W_vs_E10	ordinary_muscle_development	ENSGALG00010018999	THUMPD3	-0.646102628	0.072346137	-8.93071362	4.23269E-19	1.52809E-18	negative
PRJNA729271	stage_1W_vs_E10	ordinary_muscle_development	ENSGALG00010019000	TRERF1	-0.300157804	0.122040234	-2.45949877	0.013913117	0.01852739	negative
PRJNA729271	stage_1W_vs_E10	ordinary_muscle_development	ENSGALG00010019001	ALDH1A3	0.062661219	0.160028536	0.391562785	0.695381289	0.723949708	positive
PRJNA729271	stage_1W_vs_E10	ordinary_muscle_development	ENSGALG00010019004	ECE2	-0.050792606	0.205704651	-0.246920067	0.804970086	0.825763722	negative
PRJNA729271	stage_1W_vs_E10	ordinary_muscle_development	ENSGALG00010019005	ENSGALG00010019005	-1.788549689	0.221438973	-8.076941744	6.64113E-16	2.05999E-15	negative
PRJNA729271	stage_1W_vs_E10	ordinary_muscle_development	ENSGALG00010019006	ENSGALG00010019006	-1.199951217	0.081447295	-14.73285536	3.96589E-49	4.21757E-48	negative
PRJNA729271	stage_1W_vs_E10	ordinary_muscle_development	ENSGALG00010019008	HERPUD2	0.123292035	0.045263284	2.723886218	0.006451873	0.008899876	positive
PRJNA729271	stage_1W_vs_E10	ordinary_muscle_development	ENSGALG00010019009	NRAP	4.449643387	0.218348372	20.37864236	2.58732E-92	8.2064E-91	positive
PRJNA729271	stage_1W_vs_E10	ordinary_muscle_development	ENSGALG00010019011	VIL1	2.3515896	0.391388135	6.008331347	1.87442E-09	4.15885E-09	positive
PRJNA729271	stage_1W_vs_E10	ordinary_muscle_development	ENSGALG00010019012	ADAM28	-1.247430398	0.378327841	-3.29722073	0.000976467	0.001456393	negative
PRJNA729271	stage_1W_vs_E10	ordinary_muscle_development	ENSGALG00010019013	LRRK1	-0.937677684	0.106828265	-8.777430631	1.67252E-18	5.8389E-18	negative
PRJNA729271	stage_1W_vs_E10	ordinary_muscle_development	ENSGALG00010019015	GSG1L	-4.963647604	1.073981452	-4.621725632	3.80561E-06	6.80848E-06	negative
PRJNA729271	stage_1W_vs_E10	ordinary_muscle_development	ENSGALG00010019016	PLEKHG5	1.064785776	0.142948759	7.448723451	9.42477E-14	2.62312E-13	positive
PRJNA729271	stage_1W_vs_E10	ordinary_muscle_development	ENSGALG00010019017	IRAK2	0.871357867	0.082054117	10.6193071	2.42353E-26	1.182E-25	positive
PRJNA729271	stage_1W_vs_E10	ordinary_muscle_development	ENSGALG00010019018	SH3BGRL	-1.696113843	0.110954928	-15.28651202	9.40544E-53	1.1399E-51	negative
PRJNA729271	stage_1W_vs_E10	ordinary_muscle_development	ENSGALG00010019019	TBX20	-0.936640294	0.32509649	-2.881114753	0.003962713	0.005580988	negative
PRJNA729271	stage_1W_vs_E10	ordinary_muscle_development	ENSGALG00010019020	KCNK2	0.516212283	0.445709882	1.158180028	0.246790577	0.279536564	positive
PRJNA729271	stage_1W_vs_E10	ordinary_muscle_development	ENSGALG00010019021	LRR1	-1.30718099	0.154540929	-8.458477603	2.70891E-17	8.89771E-17	negative
PRJNA729271	stage_1W_vs_E10	ordinary_muscle_development	ENSGALG00010019023	AP2M1	-0.322361901	0.071690991	-4.496546858	6.9066E-06	1.21083E-05	negative
PRJNA729271	stage_1W_vs_E10	ordinary_muscle_development	ENSGALG00010019024	CNPY3	-0.740550859	0.104712001	-7.072263467	1.52427E-12	3.99823E-12	negative
PRJNA729271	stage_1W_vs_E10	ordinary_muscle_development	ENSGALG00010019025	DNAAF2	-0.691832324	0.248271998	-2.786590228	0.005326579	0.007419639	negative
PRJNA729271	stage_1W_vs_E10	ordinary_muscle_development	ENSGALG00010019026	CHSY1	-2.222164464	0.127112037	-17.48193571	1.96707E-68	3.58199E-67	negative
PRJNA729271	stage_1W_vs_E10	ordinary_muscle_development	ENSGALG00010019027	GNLY	1.943965943	0.31465533	6.178080445	6.48857E-10	1.4737E-09	positive
PRJNA729271	stage_1W_vs_E10	ordinary_muscle_development	ENSGALG00010019028	TNFRSF25	6.534838812	0.704128242	9.280750896	1.68288E-20	6.4704E-20	positive
PRJNA729271	stage_1W_vs_E10	ordinary_muscle_development	ENSGALG00010019029	CENPF	-2.16149494	0.260314651	-8.303393329	1.01179E-16	3.24467E-16	negative
PRJNA729271	stage_1W_vs_E10	ordinary_muscle_development	ENSGALG00010019030	CDR2	-2.733938283	0.157012806	-17.41219938	6.66726E-68	1.19311E-66	negative
PRJNA729271	stage_1W_vs_E10	ordinary_muscle_development	ENSGALG00010019032	GNMT	-1.259444726	0.190607129	-6.607542619	3.90752E-11	9.52404E-11	negative
PRJNA729271	stage_1W_vs_E10	ordinary_muscle_development	ENSGALG00010019033	CYP2AB4	-3.638936576	1.168804554	-3.113383297	0.001849556	0.002688729	negative
PRJNA729271	stage_1W_vs_E10	ordinary_muscle_development	ENSGALG00010019034	CASP7	0.673498318	0.16888047	3.988017794	6.66277E-05	0.000108709	positive
PRJNA729271	stage_1W_vs_E10	ordinary_muscle_development	ENSGALG00010019035	ENSGALG00010019035	-2.190429186	0.577764212	-3.791216449	0.000149911	0.000238159	negative
PRJNA729271	stage_1W_vs_E10	ordinary_muscle_development	ENSGALG00010019036	REL	-0.062919766	0.112586994	-0.558854656	0.576260918	0.611844565	negative
PRJNA729271	stage_1W_vs_E10	ordinary_muscle_development	ENSGALG00010019038	PLEKHS1	-1.038839337	0.464896116	-2.234562304	0.025446098	0.03288527	negative
PRJNA729271	stage_1W_vs_E10	ordinary_muscle_development	ENSGALG00010019040	HMGN5	-2.897100205	0.083994078	-34.49171975	1.0676E-260	2.7149E-258	negative
PRJNA729271	stage_1W_vs_E10	ordinary_muscle_development	ENSGALG00010019041	KATNIP	-0.737501831	0.092709515	-7.954974546	1.79168E-15	5.44568E-15	negative
PRJNA729271	stage_1W_vs_E10	ordinary_muscle_development	ENSGALG00010019042	PEX6	0.703198717	0.10563536	6.656849733	2.79759E-11	6.87306E-11	positive
PRJNA729271	stage_1W_vs_E10	ordinary_muscle_development	ENSGALG00010019043	METTL9	-1.101916235	0.104439807	-10.55073028	5.04038E-26	2.42242E-25	negative
PRJNA729271	stage_1W_vs_E10	ordinary_muscle_development	ENSGALG00010019045	LBX1	1.309868066	0.264872236	4.945282617	7.60335E-07	1.4341E-06	positive
PRJNA729271	stage_1W_vs_E10	ordinary_muscle_development	ENSGALG00010019046	SELENOS	0.867356838	0.080805142	10.7339313	7.05303E-27	3.50355E-26	positive
PRJNA729271	stage_1W_vs_E10	ordinary_muscle_development	ENSGALG00010019047	GHRL	-0.630967465	0.456340869	-1.382667008	0.166766992	0.19431572	negative
PRJNA729271	stage_1W_vs_E10	ordinary_muscle_development	ENSGALG00010019049	POLD2	-0.102040534	0.161711956	-0.631001795	0.528039351	0.564698604	negative
PRJNA729271	stage_1W_vs_E10	ordinary_muscle_development	ENSGALG00010019050	TNS1	0.684252392	0.133938787	5.108694856	3.24392E-07	6.26023E-07	positive
PRJNA729271	stage_1W_vs_E10	ordinary_muscle_development	ENSGALG00010019051	IGSF6	1.611240174	0.258096013	6.242793742	4.29824E-10	9.85182E-10	positive
PRJNA729271	stage_1W_vs_E10	ordinary_muscle_development	ENSGALG00010019059	BRWD3	0.687611061	0.07744977	8.878154973	6.79785E-19	2.42517E-18	positive
PRJNA729271	stage_1W_vs_E10	ordinary_muscle_development	ENSGALG00010019062	PUS10	-1.287436728	0.103076022	-12.49016694	8.44762E-36	5.93545E-35	negative
PRJNA729271	stage_1W_vs_E10	ordinary_muscle_development	ENSGALG00010019064	ENSGALG00010019064	-1.63108118	0.172701785	-9.444495205	3.57121E-21	1.4078E-20	negative
PRJNA729271	stage_1W_vs_E10	ordinary_muscle_development	ENSGALG00010019065	CAND2	4.385499666	0.120765407	36.31420428	9.6564E-289	3.1811E-286	positive
PRJNA729271	stage_1W_vs_E10	ordinary_muscle_development	ENSGALG00010019067	GTF3C1	-0.458095266	0.058247267	-7.864665409	3.70085E-15	1.1088E-14	negative
PRJNA729271	stage_1W_vs_E10	ordinary_muscle_development	ENSGALG00010019068	SNRPA1	-0.112243776	0.163673668	-0.685777851	0.492853205	0.530790342	negative
PRJNA729271	stage_1W_vs_E10	ordinary_muscle_development	ENSGALG00010019072	AEBP1	0.92342113	0.211580886	4.364388238	1.27479E-05	2.19533E-05	positive
PRJNA729271	stage_1W_vs_E10	ordinary_muscle_development	ENSGALG00010019076	ESPN	0.235461767	0.382932621	0.614890856	0.538626802	0.574918667	positive
PRJNA729271	stage_1W_vs_E10	ordinary_muscle_development	ENSGALG00010019079	CAPG	0.534562853	0.250657629	2.132641468	0.032954149	0.042018859	positive
PRJNA729271	stage_1W_vs_E10	ordinary_muscle_development	ENSGALG00010019081	PCSK6	0.896364354	0.137129821	6.536611429	6.29283E-11	1.51042E-10	positive
PRJNA729271	stage_1W_vs_E10	ordinary_muscle_development	ENSGALG00010019082	POLR3K	1.948249658	0.174850119	11.14239827	7.79904E-29	4.18693E-28	positive
PRJNA729271	stage_1W_vs_E10	ordinary_muscle_development	ENSGALG00010019083	OTOA	-0.744999522	0.234999172	-3.170221908	0.001523226	0.002230443	negative
PRJNA729271	stage_1W_vs_E10	ordinary_muscle_development	ENSGALG00010019087	PEX13	1.099298992	0.086115347	12.76542487	2.55751E-37	1.86945E-36	positive
PRJNA729271	stage_1W_vs_E10	ordinary_muscle_development	ENSGALG00010019093	RPL32	0.885748176	0.151740365	5.837261407	5.30658E-09	1.14514E-08	positive
PRJNA729271	stage_1W_vs_E10	ordinary_muscle_development	ENSGALG00010019094	SNRNP25	0.257757501	0.168376634	1.530838899	0.125809213	0.149194514	positive
PRJNA729271	stage_1W_vs_E10	ordinary_muscle_development	ENSGALG00010019097	STC1	-2.680039073	0.273604856	-9.795290595	1.17958E-22	4.9118E-22	negative
PRJNA729271	stage_1W_vs_E10	ordinary_muscle_development	ENSGALG00010019101	POLE2	-2.133879526	0.12398361	-17.21098071	2.1968E-66	3.73739E-65	negative
PRJNA729271	stage_1W_vs_E10	ordinary_muscle_development	ENSGALG00010019104	POLL	0.236489389	0.120152912	1.968236843	0.049040793	0.061295705	positive
PRJNA729271	stage_1W_vs_E10	ordinary_muscle_development	ENSGALG00010019108	KDELR2	-0.939933721	0.099935662	-9.405388418	5.18384E-21	2.02806E-20	negative
PRJNA729271	stage_1W_vs_E10	ordinary_muscle_development	ENSGALG00010019110	MBD4	1.16508057	0.107004982	10.88809645	1.31357E-27	6.72799E-27	positive
PRJNA729271	stage_1W_vs_E10	ordinary_muscle_development	ENSGALG00010019113	KLHDC1	1.846053984	0.132701628	13.91131372	5.4077E-44	4.92056E-43	positive
PRJNA729271	stage_1W_vs_E10	ordinary_muscle_development	ENSGALG00010019114	TENT5D	-1.688401468	0.380129638	-4.441646476	8.92731E-06	1.55474E-05	negative
PRJNA729271	stage_1W_vs_E10	ordinary_muscle_development	ENSGALG00010019115	DBNL	-1.118338819	0.103809647	-10.77297585	4.61828E-27	2.30994E-26	negative
PRJNA729271	stage_1W_vs_E10	ordinary_muscle_development	ENSGALG00010019120	DAGLB	2.012179109	0.077241511	26.0504888	1.3283E-149	1.094E-147	positive
PRJNA729271	stage_1W_vs_E10	ordinary_muscle_development	ENSGALG00010019123	PLEKHO2	1.049613456	0.118208804	8.879317095	6.72721E-19	2.40234E-18	positive

PRJNA729271	stage_1W_vs_E10	ordinary_muscle_development	ENSGALG00010019124	BMPER	-1.976474177	0.252293362	-7.83403162	4.7247E-15	1.40799E-14	negative
PRJNA729271	stage_1W_vs_E10	ordinary_muscle_development	ENSGALG00010019125	ENSGALG00010019125	2.402346165	0.196944749	12.1980717	3.18272E-34	2.09507E-33	positive
PRJNA729271	stage_1W_vs_E10	ordinary_muscle_development	ENSGALG00010019127	TMBIM1	1.864636314	0.121677375	15.32442911	5.25139E-53	6.41812E-52	positive
PRJNA729271	stage_1W_vs_E10	ordinary_muscle_development	ENSGALG00010019130	RPS6KC1	-0.825080567	0.084229383	-9.795638288	1.17553E-22	4.89634E-22	negative
PRJNA729271	stage_1W_vs_E10	ordinary_muscle_development	ENSGALG00010019132	ENSGALG00010019132	-0.23601397	0.153317273	-1.539382779	0.123710876	0.146922503	negative
PRJNA729271	stage_1W_vs_E10	ordinary_muscle_development	ENSGALG00010019135	KIAA1841	-1.258405778	0.198191862	-6.349432146	2.16111E-10	5.03299E-10	negative
PRJNA729271	stage_1W_vs_E10	ordinary_muscle_development	ENSGALG00010019139	DPCD	-1.715819682	0.139832878	-12.27050251	1.30441E-34	8.73727E-34	negative
PRJNA729271	stage_1W_vs_E10	ordinary_muscle_development	ENSGALG00010019141	ACOT7	0.188198183	0.15105992	1.245851199	0.212819029	0.243800824	positive
PRJNA729271	stage_1W_vs_E10	ordinary_muscle_development	ENSGALG00010019144	IFT122	-1.210310181	0.074785606	-16.18373162	6.56896E-59	9.32587E-58	negative
PRJNA729271	stage_1W_vs_E10	ordinary_muscle_development	ENSGALG00010019145	RAC1	-0.968458141	0.078181827	-12.38725384	3.06355E-35	2.0976E-34	negative
PRJNA729271	stage_1W_vs_E10	ordinary_muscle_development	ENSGALG00010019146	KLHDC2	1.221102185	0.085301968	14.31505298	1.7623E-46	1.72365E-45	positive
PRJNA729271	stage_1W_vs_E10	ordinary_muscle_development	ENSGALG00010019148	ENSGALG00010019148	0.7034112	0.215695025	3.261137796	0.001109661	0.001644131	positive
PRJNA729271	stage_1W_vs_E10	ordinary_muscle_development	ENSGALG00010019149	COX5B	2.738620474	0.179581765	15.24999194	1.64653E-52	1.98557E-51	positive
PRJNA729271	stage_1W_vs_E10	ordinary_muscle_development	ENSGALG00010019158	ENSGALG00010019158	2.705051828	0.252040324	10.73261526	7.15423E-27	3.5526E-26	positive
PRJNA729271	stage_1W_vs_E10	ordinary_muscle_development	ENSGALG00010019166	PNPT1	-0.423953034	0.109858292	-3.859089971	0.00011381	0.000182265	negative
PRJNA729271	stage_1W_vs_E10	ordinary_muscle_development	ENSGALG00010019169	GPR153	-2.941701583	0.255790268	-11.50044374	1.31239E-30	7.60315E-30	negative
PRJNA729271	stage_1W_vs_E10	ordinary_muscle_development	ENSGALG00010019172	NEMF	0.562997416	0.086395741	6.516494995	7.19692E-11	1.72059E-10	positive
PRJNA729271	stage_1W_vs_E10	ordinary_muscle_development	ENSGALG00010019173	RHO	-1.872882418	0.806409359	-2.322495885	0.02020625	0.026388827	negative
PRJNA729271	stage_1W_vs_E10	ordinary_muscle_development	ENSGALG00010019174	ENSGALG00010019174	0.48550438	0.429816064	1.129563132	0.258660354	0.292250512	positive
PRJNA729271	stage_1W_vs_E10	ordinary_muscle_development	ENSGALG00010019183	ANGEL2	0.850835776	0.07763032	10.96009616	5.94362E-28	3.09124E-27	positive
PRJNA729271	stage_1W_vs_E10	ordinary_muscle_development	ENSGALG00010019184	PNKD	-0.319530219	0.185333578	-1.72408164	0.084693106	0.102781863	negative
PRJNA729271	stage_1W_vs_E10	ordinary_muscle_development	ENSGALG00010019185	ANKDD1A	-0.405637256	0.223342543	-1.816211323	0.069337962	0.085022736	negative
PRJNA729271	stage_1W_vs_E10	ordinary_muscle_development	ENSGALG00010019188	ENSGALG00010019188	0.62912296	0.373036534	1.686491542	0.091701163	0.110887491	positive
PRJNA729271	stage_1W_vs_E10	ordinary_muscle_development	ENSGALG00010019189	ICMT	0.190634329	0.093457265	2.039802133	0.041370037	0.052180534	positive
PRJNA729271	stage_1W_vs_E10	ordinary_muscle_development	ENSGALG00010019193	ENSGALG00010019193	1.683465441	1.194563582	1.409272362	0.158754648	0.185531615	positive
PRJNA729271	stage_1W_vs_E10	ordinary_muscle_development	ENSGALG00010019194	ENSGALG00010019194	0.545456755	0.187744659	2.905311707	0.003668875	0.005186303	positive
PRJNA729271	stage_1W_vs_E10	ordinary_muscle_development	ENSGALG00010019204	ARF6	-0.383616512	0.230294263	-1.665766691	0.095759893	0.115448694	negative
PRJNA729271	stage_1W_vs_E10	ordinary_muscle_development	ENSGALG00010019205	ENSGALG00010019205	-0.276351718	0.267338825	-1.033713373	0.30127018	0.336563488	negative
PRJNA729271	stage_1W_vs_E10	ordinary_muscle_development	ENSGALG00010019206	RNF207	2.330062279	0.281426287	8.279476316	1.23718E-16	3.95173E-16	positive
PRJNA729271	stage_1W_vs_E10	ordinary_muscle_development	ENSGALG00010019208	PLXND1	0.598793934	0.158063233	3.788318908	0.00015167	0.000240848	positive
PRJNA729271	stage_1W_vs_E10	ordinary_muscle_development	ENSGALG00010019209	VCPKMT	0.208638297	0.085595039	2.437504565	0.014789027	0.019603745	positive
PRJNA729271	stage_1W_vs_E10	ordinary_muscle_development	ENSGALG00010019212	RPL22	0.080937398	0.113913765	0.710514646	0.477385052	0.51574095	positive
PRJNA729271	stage_1W_vs_E10	ordinary_muscle_development	ENSGALG00010019213	RHBDF1	-0.387434251	0.111386184	-3.478297205	0.00050461	0.000769281	negative
PRJNA729271	stage_1W_vs_E10	ordinary_muscle_development	ENSGALG00010019215	ENSGALG00010019215	-0.006797575	0.101908759	-0.066702555	0.9468185	0.953462148	negative
PRJNA729271	stage_1W_vs_E10	ordinary_muscle_development	ENSGALG00010019218	RMND5A	1.503914863	0.137279446	10.955135	6.27847E-28	3.26071E-27	positive
PRJNA729271	stage_1W_vs_E10	ordinary_muscle_development	ENSGALG00010019221	VASH2	-2.625886834	0.232844738	-11.27741542	1.69657E-29	9.3862E-29	negative
PRJNA729271	stage_1W_vs_E10	ordinary_muscle_development	ENSGALG00010019223	AAMP	-0.020802466	0.125936006	-0.165182832	0.868800078	0.883365399	negative
PRJNA729271	stage_1W_vs_E10	ordinary_muscle_development	ENSGALG00010019225	PROK2	-4.871911609	0.741923986	-6.566591327	5.14801E-11	1.24409E-10	negative
PRJNA729271	stage_1W_vs_E10	ordinary_muscle_development	ENSGALG00010019227	CLCN5	0.161028727	0.073236716	2.198743159	0.02789619	0.035859815	positive
PRJNA729271	stage_1W_vs_E10	ordinary_muscle_development	ENSGALG00010019230	SPG21	-0.580353287	0.098632814	-5.883977801	4.00522E-09	8.70529E-09	negative
PRJNA729271	stage_1W_vs_E10	ordinary_muscle_development	ENSGALG00010019231	ENSGALG00010019231	3.942331078	0.311111936	12.67174488	8.48067E-37	6.09456E-36	positive
PRJNA729271	stage_1W_vs_E10	ordinary_muscle_development	ENSGALG00010019232	SOS2	0.801084008	0.093565669	8.561730151	1.11186E-17	3.71775E-17	positive
PRJNA729271	stage_1W_vs_E10	ordinary_muscle_development	ENSGALG00010019233	ARPC2	-0.69448639	0.053966714	-12.86879158	6.74491E-38	5.0009E-37	negative
PRJNA729271	stage_1W_vs_E10	ordinary_muscle_development	ENSGALG00010019236	MPG	0.08247598	0.208580891	0.395414844	0.692536756	0.721195508	positive
PRJNA729271	stage_1W_vs_E10	ordinary_muscle_development	ENSGALG00010019237	SORCS3	-3.935296574	0.38436273	-10.23849677	1.33291E-24	6.03202E-24	negative
PRJNA729271	stage_1W_vs_E10	ordinary_muscle_development	ENSGALG00010019239	CYTH3	0.245440757	0.081254146	3.020655188	0.002522284	0.003621287	positive
PRJNA729271	stage_1W_vs_E10	ordinary_muscle_development	ENSGALG00010019240	ENSGALG00010019240	0.502958435	0.205602352	2.446267898	0.01443437	0.019159908	positive
PRJNA729271	stage_1W_vs_E10	ordinary_muscle_development	ENSGALG00010019243	SERTAD2	0.445586024	0.155283776	2.869495036	0.004111278	0.005779553	positive
PRJNA729271	stage_1W_vs_E10	ordinary_muscle_development	ENSGALG00010019246	IL17RC	1.481302815	0.134616896	11.0038402	3.66197E-28	1.92042E-27	positive
PRJNA729271	stage_1W_vs_E10	ordinary_muscle_development	ENSGALG00010019247	HBZ	-7.019615849	0.660087647	-10.63436935	2.06219E-26	1.00746E-25	negative
PRJNA729271	stage_1W_vs_E10	ordinary_muscle_development	ENSGALG00010019248	RMI2	-0.470371188	0.169992389	-2.767013221	0.005657246	0.007853825	negative
PRJNA729271	stage_1W_vs_E10	ordinary_muscle_development	ENSGALG00010019250	CXCR1	-0.493552855	0.274459863	-1.798269697	0.072134279	0.088227692	negative
PRJNA729271	stage_1W_vs_E10	ordinary_muscle_development	ENSGALG00010019252	TATDN3	0.187702915	0.117041345	1.6037317	0.108773205	0.130120295	positive
PRJNA729271	stage_1W_vs_E10	ordinary_muscle_development	ENSGALG00010019253	HBAD	-1.882699138	0.426446433	-4.414854935	1.01078E-05	1.75359E-05	negative
PRJNA729271	stage_1W_vs_E10	ordinary_muscle_development	ENSGALG00010019255	ENSGALG00010019255	2.551669887	0.198451759	12.85788499	7.76729E-38	5.74716E-37	positive
PRJNA729271	stage_1W_vs_E10	ordinary_muscle_development	ENSGALG00010019256	BBS9	-1.40094782	0.108951041	-12.85850793	7.70496E-38	5.70395E-37	negative
PRJNA729271	stage_1W_vs_E10	ordinary_muscle_development	ENSGALG00010019257	SLC1A4	-0.832499886	0.15858931	-5.249407315	1.52589E-07	3.01087E-07	negative
PRJNA729271	stage_1W_vs_E10	ordinary_muscle_development	ENSGALG00010019259	L2HGDH	0.333653893	0.108602811	3.072239933	0.002124589	0.003069462	positive
PRJNA729271	stage_1W_vs_E10	ordinary_muscle_development	ENSGALG00010019260	TRAIL-LIKE	-0.691510004	0.177141219	-3.903721599	9.47247E-05	0.000152661	negative
PRJNA729271	stage_1W_vs_E10	ordinary_muscle_development	ENSGALG00010019262	TMC3	-4.138165792	0.534775556	-7.738135637	1.00885E-14	2.95899E-14	negative
PRJNA729271	stage_1W_vs_E10	ordinary_muscle_development	ENSGALG00010019263	SNRPB2	-0.775217609	0.102729688	-7.546188712	4.48181E-14	1.27106E-13	negative
PRJNA729271	stage_1W_vs_E10	ordinary_muscle_development	ENSGALG00010019264	HK2	1.529433623	0.214308954	7.136582917	9.56796E-13	2.53681E-12	positive
PRJNA729271	stage_1W_vs_E10	ordinary_muscle_development	ENSGALG00010019265	RUFY4	2.644411424	0.714942096	3.69877706	0.000216641	0.000340033	positive
PRJNA729271	stage_1W_vs_E10	ordinary_muscle_development	ENSGALG00010019266	ATP5S	-0.067263344	0.158529926	-0.424294297	0.671351198	0.701400866	negative
PRJNA729271	stage_1W_vs_E10	ordinary_muscle_development	ENSGALG00010019267	STARD5	-0.35703631	0.187586512	-1.903315465	0.056999394	0.070688417	negative
PRJNA729271	stage_1W_vs_E10	ordinary_muscle_development	ENSGALG00010019268	NSL1	-1.334183379	0.095726264	-13.9374851	3.7492E-44	3.42436E-43	negative
PRJNA729271	stage_1W_vs_E10	ordinary_muscle_development	ENSGALG00010019269	SOCS1	0.015228123	0.258111693	0.058998189	0.95295355	0.959173787	positive
PRJNA729271	stage_1W_vs_E10	ordinary_muscle_development	ENSGALG00010019270	ENSGALG00010019270	-1.189632957	0.208103108	-5.71655545	1.08705E-08	2.30161E-08	negative
PRJNA729271	stage_1W_vs_E10	ordinary_muscle_development	ENSGALG00010019271	CDKL1	-0.539679452	0.196991802	-2.739603616	0.006151332	0.008502294	negative
PRJNA729271	stage_1W_vs_E10	ordinary_muscle_development	ENSGALG00010019274	ENSGALG00010019274	-3.54525757	0.985613764	-3.59700493	0.000321902	0.000499248	negative
PRJNA729271	stage_1W_vs_E10	ordinary_muscle_development	ENSGALG00010019275	CTAGE1	2.749166793	0.578192419	4.754761048	1.98681E-06	3.63072E-06	positive
PRJNA729271	stage_1W_vs_E10	ordinary_muscle_development	ENSGALG00010019277	IGFBP5	-0.527642507	0.133122957	-3.963572624	7.38364E-05	0.000120105	negative
PRJNA729271	stage_1W_vs_E10	ordinary_muscle_development	ENSGALG00010019278	JPT2	-2.223378453	0.074237902	-29.94937083	4.4837E-197	6.3098E-195	negative
PRJNA729271	stage_1W_vs_E10	ordinary_muscle_development	ENSGALG00010019280	TLNRD1	0.060810223	0.116597134	0.521541314	0.601989733	0.636551005	positive
PRJNA729271	stage_1W_vs_E10	ordinary_muscle_development	ENSGALG00010019281	ENSGALG00010019281	1.170130361	1.120486268	1.044305847	0.296343908	0.33146886	positive
PRJNA729271	stage_1W_vs_E10	ordinary_muscle_development	ENSGALG00010019282	IGFBP2	-2.691208911	0.157693461	-17.06607801	2.65444E-65	4.41746E-64	negative
PRJNA729271	stage_1W_vs_E10	ordinary_muscle_development	ENSGALG00010019283	NT5C3A	3.54180657	0.255346846	13.87057103	9.55134E-44	8.63134E-43	positive
PRJNA729271	stage_1W_vs_E10	ordinary_muscle_development	ENSGALG00010019284	CHD5	1.270544166	0.131665348	9.649799183	4.92521E-22	2.00143E-21	positive
PRJNA729271	stage_1W_vs_E10	ordinary_muscle_development	ENSGALG00010019285	BATF3	-1.660303865	0.295128315	-5.625701711	1.84755E-08	3.85939E-08	negative
PRJNA729271	stage_1W_vs_E10	ordinary_muscle_development	ENSGALG00010019287	AHSA2	-0.767641935	0.099279351	-7.732140975	1.05753E-14	3.09799E-14	negative
PRJNA729271	stage_1W_vs_E10	ordinary_muscle_development	ENSGALG00010019288	RPL37A	0.620675569	0.156709431	3.960677834	7.47373E-05	0.000121516	positive
PRJNA729271	stage_1W_vs_E10	ordinary_muscle_development	ENSGALG00010019289	FKBP9	-2.181376056	0.166836877	-13.07490345	4.58145E-39	3.55885E-38	negative
PRJNA729271	stage_1W_vs_E10	ordinary_muscle_development	ENSGALG00010019291	ADD2	-0.964404691	0.278270814	-3.465705508	0.000528842	0.000805122	negative
PRJNA729271	stage_1W_vs_E10	ordinary_muscle_development	ENSGALG00010019292	ENSGALG00010019292	-0.665084349	0.097774337	-6.80223836	1.03006E-11	2.58854E-11	negative
PRJNA729271	stage_1W_vs_E10	ordinary_muscle_development	ENSGALG00010019294	SMARCAL1	-0.43738545	0.09816463	-4.45563182	8.36465E-06	1.45868E-05	negative
PRJNA729271	stage_1W_vs_E10	ordinary_muscle_development	ENSGALG00010019295	MAP4K5	0.620806554	0.129549204	4.792052248	1.65084E-06	3.0332E-06	positive
PRJNA729271	stage_1W_vs_E10	ordinary_muscle_development	ENSGALG00010019296	ENSGALG00010019296	0.22244447	0.102450339	2.171241921	0.02991289	0.038346807	positive
PRJNA729271	stage_1W_vs_E10	ordinary_muscle_development	ENSGALG00010019297	SLC25A26	1.498500129	0.146056479	10.25973062	1.07005E-24	4.86218E-24	positive
PRJNA729271	stage_1W_vs_E10	ordinary_muscle_development	ENSGALG00010019298	PGAP6	0.227124375	0.0940269	2.41552551	0.015712522	0.02076712	positive
PRJNA729271	stage_1W_vs_E10	ordinary_muscle_development	ENSGALG00010019300	MESDC2	-0.35967781	0.066332321	-5.422361309	5.88168E-08	1.19539E-07	negative
PRJNA729271	stage_1W_vs_E10	ordinary_muscle_development	ENSGALG00010019302	USP34	-0.226670587	0.094837874	-2.390085074	0.01684447	0.022180287	negative
PRJNA729271	stage_1W_vs_E10	ordinary_muscle_development	ENSGALG00010019305	SNRNP27	0.568238531	0.118341077	4.801701528	1.57323E-06	2.89427E-06	positive
PRJNA729271	stage_1W_vs_E10	ordinary_muscle_development	ENSGALG00010019307	ATF3	4.74030849	0.485949277	9.754739264	1.76052E-22	7.27861E-22	positive
PRJNA729271	stage_1W_vs_E10	ordinary_muscle_development	ENSGALG00010019310	CRELD1	1.642227325	0.096359147	17.04277574	3.95506E-65	6.55934E-64	positive
PRJNA729271	stage_1W_vs_E10	ordinary_muscle_development	ENSGALG00010019312	KBTBD2	0.567449218	0.071813852	7.901667975	2.75196E-15	8.29555E-15	positive
PRJNA729271	stage_1W_vs_E10	ordinary_muscle_development	ENSGALG00010019313	MRPL28	0.286358242	0.099197326	2.886753654	0.003892389	0.005486209	positive
PRJNA729271	stage_1W_vs_E10	ordinary_muscle_development	ENSGALG00010019314	GKN2	-2.585186036	1.23532801	-2.092712232	0.036374851	0.046154249	negative
PRJNA729271	stage_1W_vs_E10	ordinary_muscle_development	ENSGALG00010019316	CLEC16A	0.23623077	0.088055529	2.682747736	0.007302005	0.010014435	positive
PRJNA729271	stage_1W_vs_E10	ordinary_muscle_development	ENSGALG00010019318	NOG2	0.406191255	0.278412046	1.458957187	0.144576892	0.170005572	positive
PRJNA729271	stage_1W_vs_E10	ordinary_muscle_development	ENSGALG00010019319	ACTR3	-1.295568613	0.067543803	-19.18116167	5.31789E-82	1.33592E-80	negative
PRJNA729271	stage_1W_vs_E10	ordinary_muscle_development	ENSGALG00010019322	EGR3	1.148262389	0.31784927	3.612600367	0.000303142	0.000471059	positive
PRJNA729271	stage_1W_vs_E10	ordinary_muscle_development	ENSGALG00010019323	LRIG1	-0.288564464	0.119679847	-2.41113663	0.01590289	0.021001493	negative
PRJNA729271	stage_1W_vs_E10	ordinary_muscle_development	ENSGALG00010019325	SLITRK4	-2.742496654	0.214240239	-12.8010343	1.61782E-37	1.19037E-36	negative
PRJNA729271	stage_1W_vs_E10	ordinary_muscle_development	ENSGALG00010019331	CEMIP	-0.889393739	0.321018719	-2.770535444	0.005596421	0.007771889	negative
PRJNA729271	stage_1W_vs_E10	ordinary_muscle_development	ENSGALG00010019334	ATL1	-1.501301654	0.13216774	-11.35906275	6.68581E-30	3.77085E-29	negative
PRJNA729271	stage_1W_vs_E10	ordinary_muscle_development	ENSGALG00010019335	HIPK3	1.611896572	0.115127224	14.00100272	1.53687E-44	1.41801E-43	positive
PRJNA729271	stage_1W_vs_E10	ordinary_muscle_development	ENSGALG00010019336	DEXI	1.404837532	0.235836239	5.956834877	2.5717E-09	5.65657E-09	positive
PRJNA729271	stage_1W_vs_E10	ordinary_muscle_development	ENSGALG00010019337	XPO1	-0.727871101	0.084255224	-8.638883979	5.67648E-18	1.92604E-17	negative
PRJNA729271	stage_1W_vs_E10	ordinary_muscle_development	ENSGALG00010019344	DNAJC24	-0.577281486	0.08813168	-6.550215366	5.74542E-11	1.38339E-10	negative
PRJNA729271	stage_1W_vs_E10	ordinary_muscle_development	ENSGALG00010019345	ENSGALG00010019345	1.212823975	0.183677873	6.602994453	4.02935E-11	9.81603E-11	positive
PRJNA729271	stage_1W_vs_E10	ordinary_muscle_development	ENSGALG00010019346	POU3F4	-0.818201764	0.540181132	-1.514680383	0.129853365	0.153613362	negative
PRJNA729271	stage_1W_vs_E10	ordinary_muscle_development	ENSGALG00010019350	SAV1	0.249625565	0.088280166	2.827651728	0.004689079	0.006563805	positive
PRJNA729271	stage_1W_vs_E10	ordinary_muscle_development	ENSGALG00010019355	ENTPD4	0.854338888	0.096834924	8.822631836	1.118E-18	3.9391E-18	positive
PRJNA729271	stage_1W_vs_E10	ordinary_muscle_development	ENSGALG00010019356	AVL9	-0.248282167	0.096312589	-2.57787866	0.00994089	0.013445292	negative
PRJNA729271	stage_1W_vs_E10	ordinary_muscle_development	ENSGALG00010019365	ENSGALG00010019365	0.265223668	0.177849364	1.491282635	0.135887306	0.160410986	positive
PRJNA729271	stage_1W_vs_E10	ordinary_muscle_development	ENSGALG00010019371	TMEM87A	1.12575649	0.076561021	14.70404227	6.0724E-49	6.40141E-48	positive
PRJNA729271	stage_1W_vs_E10	ordinary_muscle_development	ENSGALG00010019373	DENND5A	0.05802772	0.06176735	0.939456199	0.347496571	0.384960015	positive
PRJNA729271	stage_1W_vs_E10	ordinary_muscle_development	ENSGALG00010019376	ENSGALG00010019376	3.310372986	0.319941466	10.3468082	4.3267E-25	1.99986E-24	positive
PRJNA729271	stage_1W_vs_E10	ordinary_muscle_development	ENSGALG00010019388	GPR75	-0.546115017	0.138640406	-3.939075427	8.17962E-05	0.000132518	negative
PRJNA729271	stage_1W_vs_E10	ordinary_muscle_development	ENSGALG00010019390	TVP23A	-1.236330925	0.240303375	-5.144875414	2.67698E-07	5.19728E-07	negative
PRJNA729271	stage_1W_vs_E10	ordinary_muscle_development	ENSGALG00010019391	GANC	3.81130479	0.130716585	29.15701018	6.8082E-187	8.9714E-185	positive
PRJNA729271	stage_1W_vs_E10	ordinary_muscle_development	ENSGALG00010019392	NIN	-2.071875635	0.112867366	-18.35672894	2.91655E-75	6.1806E-74	negative
PRJNA729271	stage_1W_vs_E10	ordinary_muscle_development	ENSGALG00010019398	SLC35F5	1.076632885	0.120155469	8.960331947	3.23702E-19	1.17302E-18	positive
PRJNA729271	stage_1W_vs_E10	ordinary_muscle_development	ENSGALG00010019399	CD93	1.011519628	0.151347836	6.683409905	2.33445E-11	5.75964E-11	positive
PRJNA729271	stage_1W_vs_E10	ordinary_muscle_development	ENSGALG00010019403	PACC1	-0.20011326	0.106107293	-1.885951976	0.059301426	0.073348765	negative
PRJNA729271	stage_1W_vs_E10	ordinary_muscle_development	ENSGALG00010019411	LSM5	-0.676058225	0.157920065	-4.281015373	1.86043E-05	3.15956E-05	negative
PRJNA729271	stage_1W_vs_E10	ordinary_muscle_development	ENSGALG00010019416	GPR39	-0.053143881	0.514348235	-0.103322764	0.917706809	0.927820339	negative
PRJNA729271	stage_1W_vs_E10	ordinary_muscle_development	ENSGALG00010019418	ZNF703	0.536160912	0.234024692	2.2910442	0.021960857	0.028564486	positive
PRJNA729271	stage_1W_vs_E10	ordinary_muscle_development	ENSGALG00010019422	IMMP1L	0.984397525	0.139530708	7.05506005	1.72526E-12	4.50832E-12	positive
PRJNA729271	stage_1W_vs_E10	ordinary_muscle_development	ENSGALG00010019423	LYPD1	-1.135149842	0.181935271	-6.23930607	4.39516E-10	1.0066E-09	negative
PRJNA729271	stage_1W_vs_E10	ordinary_muscle_development	ENSGALG00010019426	GRM7	-2.958001923	0.271589399	-10.89144839	1.2661E-27	6.48946E-27	negative

PRJNA729271	stage_1W_vs_E10	ordinary_muscle_development	ENSGALG00010019430	ENSGALG00010019430	0.988371401	0.401458775	2.461949922	0.013818396	0.018408018	positive
PRJNA729271	stage_1W_vs_E10	ordinary_muscle_development	ENSGALG00010019441	LMCD1	2.202344221	0.137953768	15.96436443	2.26335E-57	3.10088E-56	positive
PRJNA729271	stage_1W_vs_E10	ordinary_muscle_development	ENSGALG00010019443	NCKAP5	-0.835929171	0.135331832	-6.176885054	6.53786E-10	1.48467E-09	negative
PRJNA729271	stage_1W_vs_E10	ordinary_muscle_development	ENSGALG00010019451	CAV3	4.058680234	0.142873376	28.40753372	1.6321E-177	1.9079E-175	positive
PRJNA729271	stage_1W_vs_E10	ordinary_muscle_development	ENSGALG00010019458	ELP4	0.091728116	0.100988289	0.908304487	0.363717372	0.401311513	positive
PRJNA729271	stage_1W_vs_E10	ordinary_muscle_development	ENSGALG00010019461	MGAT5	-0.0475289	0.070974407	-0.669662518	0.50307295	0.540792229	negative
PRJNA729271	stage_1W_vs_E10	ordinary_muscle_development	ENSGALG00010019470	TGFB2	-2.019144826	0.201116908	-10.03965728	1.02028E-23	4.44781E-23	negative
PRJNA729271	stage_1W_vs_E10	ordinary_muscle_development	ENSGALG00010019471	NUBP1	0.547692738	0.092350319	5.930599326	3.01831E-09	6.61082E-09	positive
PRJNA729271	stage_1W_vs_E10	ordinary_muscle_development	ENSGALG00010019472	CAPN3	8.187296999	0.255595566	32.03223408	3.8811E-225	7.2125E-223	positive
PRJNA729271	stage_1W_vs_E10	ordinary_muscle_development	ENSGALG00010019473	OXTR	-1.216325448	0.509576084	-2.386935897	0.016989453	0.022356979	negative
PRJNA729271	stage_1W_vs_E10	ordinary_muscle_development	ENSGALG00010019474	TMEM163	-0.775673391	0.547954629	-1.415579593	0.15689863	0.18348089	negative
PRJNA729271	stage_1W_vs_E10	ordinary_muscle_development	ENSGALG00010019477	NCAPH	-2.424918199	0.179683041	-13.4955318	1.66152E-41	1.40348E-40	negative
PRJNA729271	stage_1W_vs_E10	ordinary_muscle_development	ENSGALG00010019481	PYGL	0.067991528	0.098896313	0.687503166	0.491765707	0.529815959	positive
PRJNA729271	stage_1W_vs_E10	ordinary_muscle_development	ENSGALG00010019483	SCUBE2	-3.078829589	0.265783296	-11.58398453	4.96819E-31	2.91909E-30	negative
PRJNA729271	stage_1W_vs_E10	ordinary_muscle_development	ENSGALG00010019488	TEKT5	-2.93486038	1.279534745	-2.293693385	0.021808117	0.028378549	negative
PRJNA729271	stage_1W_vs_E10	ordinary_muscle_development	ENSGALG00010019495	EMP2	0.088458175	0.197021145	0.448978078	0.653447471	0.684620245	positive
PRJNA729271	stage_1W_vs_E10	ordinary_muscle_development	ENSGALG00010019498	ENSGALG00010019498	-1.112901309	0.302722419	-3.676309515	0.000236632	0.000370609	negative
PRJNA729271	stage_1W_vs_E10	ordinary_muscle_development	ENSGALG00010019509	ZNF106	1.178233599	0.222182531	5.302998371	1.13916E-07	2.26659E-07	positive
PRJNA729271	stage_1W_vs_E10	ordinary_muscle_development	ENSGALG00010019515	ENSGALG00010019515	-0.492875564	0.109691694	-4.493280638	7.01343E-06	1.22911E-05	negative
PRJNA729271	stage_1W_vs_E10	ordinary_muscle_development	ENSGALG00010019520	CD6	3.946628916	1.001249323	3.941704455	8.09046E-05	0.000131176	positive
PRJNA729271	stage_1W_vs_E10	ordinary_muscle_development	ENSGALG00010019524	HHIPL2	1.549723138	0.357432355	4.335710283	1.4529E-05	2.48875E-05	positive
PRJNA729271	stage_1W_vs_E10	ordinary_muscle_development	ENSGALG00010019529	SNAP23	-0.198150314	0.067800105	-2.922566473	0.003471595	0.004918941	negative
PRJNA729271	stage_1W_vs_E10	ordinary_muscle_development	ENSGALG00010019534	PPP2R5A	1.500047717	0.104227503	14.39205264	5.80472E-47	5.75116E-46	positive
PRJNA729271	stage_1W_vs_E10	ordinary_muscle_development	ENSGALG00010019540	UNC5D	-1.8040944	0.400540825	-4.504146118	6.66403E-06	1.16915E-05	negative
PRJNA729271	stage_1W_vs_E10	ordinary_muscle_development	ENSGALG00010019546	HSD17B12	-1.199300403	0.078831601	-15.21344717	2.87972E-52	3.41583E-51	negative
PRJNA729271	stage_1W_vs_E10	ordinary_muscle_development	ENSGALG00010019557	TRIM9	-1.928725679	0.369684987	-5.217213969	1.81634E-07	3.56553E-07	negative
PRJNA729271	stage_1W_vs_E10	ordinary_muscle_development	ENSGALG00010019572	NFKBIE	-0.098056756	0.188414753	-0.520430348	0.602763662	0.637276388	negative
PRJNA729271	stage_1W_vs_E10	ordinary_muscle_development	ENSGALG00010019577	MXD1	-0.2370932	0.190825988	-1.242457605	0.214067773	0.245095764	negative
PRJNA729271	stage_1W_vs_E10	ordinary_muscle_development	ENSGALG00010019580	CD5	2.075150657	0.468561435	4.428769639	9.47722E-06	1.64735E-05	positive
PRJNA729271	stage_1W_vs_E10	ordinary_muscle_development	ENSGALG00010019581	SLC35B2	-0.216536102	0.133898936	-1.61716073	0.105843591	0.126846039	negative
PRJNA729271	stage_1W_vs_E10	ordinary_muscle_development	ENSGALG00010019584	RAD18	-1.4447228	0.119273717	-12.11266685	9.0503E-34	5.88797E-33	negative
PRJNA729271	stage_1W_vs_E10	ordinary_muscle_development	ENSGALG00010019585	PDE1C	-0.991151126	0.214257577	-4.625979348	3.72832E-06	6.67433E-06	negative
PRJNA729271	stage_1W_vs_E10	ordinary_muscle_development	ENSGALG00010019586	GLYR1	-0.544957706	0.090620843	-6.013602243	1.81445E-09	4.03135E-09	negative
PRJNA729271	stage_1W_vs_E10	ordinary_muscle_development	ENSGALG00010019587	CCNT2	-0.230584774	0.128449558	-1.795138703	0.072631604	0.088828476	negative
PRJNA729271	stage_1W_vs_E10	ordinary_muscle_development	ENSGALG00010019588	CHMP7	1.077102767	0.092583021	11.63391246	2.77099E-31	1.6468E-30	positive
PRJNA729271	stage_1W_vs_E10	ordinary_muscle_development	ENSGALG00010019591	ENSGALG00010019591	-1.254109664	0.227175675	-5.520439917	3.38152E-08	6.95447E-08	negative
PRJNA729271	stage_1W_vs_E10	ordinary_muscle_development	ENSGALG00010019592	ALKBH3	0.869006388	0.129804144	6.694750732	2.1604E-11	5.33929E-11	positive
PRJNA729271	stage_1W_vs_E10	ordinary_muscle_development	ENSGALG00010019594	TMX1	-0.396769405	0.335789466	-1.181601702	0.237363778	0.269575175	negative
PRJNA729271	stage_1W_vs_E10	ordinary_muscle_development	ENSGALG00010019595	GRIN2A	-2.734767307	0.238165557	-11.48263141	1.61296E-30	9.28138E-30	negative
PRJNA729271	stage_1W_vs_E10	ordinary_muscle_development	ENSGALG00010019598	KCNAB2	-1.21106037	0.138003575	-8.77557242	1.70037E-18	5.93327E-18	negative
PRJNA729271	stage_1W_vs_E10	ordinary_muscle_development	ENSGALG00010019600	MAP3K19	-1.920060692	0.269666767	-7.120123527	1.0783E-12	2.85219E-12	negative
PRJNA729271	stage_1W_vs_E10	ordinary_muscle_development	ENSGALG00010019602	SLC29A1	2.152940221	0.193499759	11.12631988	9.3413E-29	5.00008E-28	positive
PRJNA729271	stage_1W_vs_E10	ordinary_muscle_development	ENSGALG00010019610	CORO7	0.163220725	0.101565168	1.60705416	0.108042488	0.129278179	positive
PRJNA729271	stage_1W_vs_E10	ordinary_muscle_development	ENSGALG00010019613	FRMD6	-0.728777932	0.125362855	-5.81334824	6.12355E-09	1.31732E-08	negative
PRJNA729271	stage_1W_vs_E10	ordinary_muscle_development	ENSGALG00010019614	MICALL2	0.418924175	0.157350025	2.662371209	0.007759226	0.010616384	positive
PRJNA729271	stage_1W_vs_E10	ordinary_muscle_development	ENSGALG00010019617	ANO3	-1.265226473	0.145375186	-8.703180415	3.2271E-18	1.10924E-17	negative
PRJNA729271	stage_1W_vs_E10	ordinary_muscle_development	ENSGALG00010019618	NPHP4	-0.212125192	0.104985453	-2.020519847	0.043329494	0.054542858	negative
PRJNA729271	stage_1W_vs_E10	ordinary_muscle_development	ENSGALG00010019621	ENSGALG00010019621	0.616211757	0.151165989	4.076391532	4.574E-05	7.54611E-05	positive
PRJNA729271	stage_1W_vs_E10	ordinary_muscle_development	ENSGALG00010019622	C2orf42	1.481378278	0.140143527	10.5704367	4.08588E-26	1.97087E-25	positive
PRJNA729271	stage_1W_vs_E10	ordinary_muscle_development	ENSGALG00010019624	SRGAP3	-0.202595488	0.130881857	-1.547926451	0.121640005	0.14458154	negative
PRJNA729271	stage_1W_vs_E10	ordinary_muscle_development	ENSGALG00010019625	INTS1	-0.17005215	0.076510596	-2.222596058	0.02624305	0.033845806	negative
PRJNA729271	stage_1W_vs_E10	ordinary_muscle_development	ENSGALG00010019630	AJAP1	-1.448847758	0.39021268	-3.712969448	0.000204842	0.000321932	negative
PRJNA729271	stage_1W_vs_E10	ordinary_muscle_development	ENSGALG00010019634	GNG2	-1.936880125	0.28577937	-6.777536543	1.22242E-11	3.05922E-11	negative
PRJNA729271	stage_1W_vs_E10	ordinary_muscle_development	ENSGALG00010019635	LRRC57	1.080177033	0.076378069	14.14250249	2.07763E-45	1.9696E-44	positive
PRJNA729271	stage_1W_vs_E10	ordinary_muscle_development	ENSGALG00010019640	MGRN1	0.227985361	0.116345642	1.959552222	0.05004815	0.062484749	positive
PRJNA729271	stage_1W_vs_E10	ordinary_muscle_development	ENSGALG00010019641	CAPN11	0.927374868	0.081760367	11.34259673	8.07133E-30	4.52413E-29	positive
PRJNA729271	stage_1W_vs_E10	ordinary_muscle_development	ENSGALG00010019643	RAB3GAP1	-0.34717367	0.071035249	-4.887343571	1.02206E-06	1.9101E-06	negative
PRJNA729271	stage_1W_vs_E10	ordinary_muscle_development	ENSGALG00010019645	ENSGALG00010019645	4.09252773	0.160796619	25.45157819	6.7811E-143	5.0406E-141	positive
PRJNA729271	stage_1W_vs_E10	ordinary_muscle_development	ENSGALG00010019646	VPS37C	1.399140694	0.12542408	11.15527966	6.74803E-29	3.62807E-28	positive
PRJNA729271	stage_1W_vs_E10	ordinary_muscle_development	ENSGALG00010019648	PRDM16	-1.656833692	0.196650148	-8.42528575	3.59871E-17	1.17458E-16	negative
PRJNA729271	stage_1W_vs_E10	ordinary_muscle_development	ENSGALG00010019649	TMEM63B	-0.020283308	0.076566978	-0.264909351	0.791079295	0.813179098	negative
PRJNA729271	stage_1W_vs_E10	ordinary_muscle_development	ENSGALG00010019650	KBTBD8	0.705218196	0.086960247	8.109661794	5.07608E-16	1.5813E-15	positive
PRJNA729271	stage_1W_vs_E10	ordinary_muscle_development	ENSGALG00010019652	TNFRSF10B	0.390693921	0.323262949	1.208594803	0.226818554	0.258429094	positive
PRJNA729271	stage_1W_vs_E10	ordinary_muscle_development	ENSGALG00010019655	RTRAF	0.651134141	0.098376264	6.618813439	3.62093E-11	8.8404E-11	positive
PRJNA729271	stage_1W_vs_E10	ordinary_muscle_development	ENSGALG00010019657	PPP1R17	-1.882250286	0.272979248	-6.895213844	5.37838E-12	1.37084E-11	negative
PRJNA729271	stage_1W_vs_E10	ordinary_muscle_development	ENSGALG00010019659	MRPL14	1.619216411	0.124590508	12.99630637	1.28396E-38	9.79529E-38	positive
PRJNA729271	stage_1W_vs_E10	ordinary_muscle_development	ENSGALG00010019660	ENSGALG00010019660	1.251191881	0.48301601	2.590373515	0.009587185	0.012983859	positive
PRJNA729271	stage_1W_vs_E10	ordinary_muscle_development	ENSGALG00010019667	ITPRID1	1.298403804	0.600930542	2.160655371	0.030721971	0.039314467	positive
PRJNA729271	stage_1W_vs_E10	ordinary_muscle_development	ENSGALG00010019670	ENSGALG00010019670	-0.92807401	0.123688756	-7.503301379	6.22302E-14	1.75389E-13	negative
PRJNA729271	stage_1W_vs_E10	ordinary_muscle_development	ENSGALG00010019673	NID2	0.148249802	0.132747141	1.116783386	0.264086954	0.297847837	positive
PRJNA729271	stage_1W_vs_E10	ordinary_muscle_development	ENSGALG00010019675	MAFK	1.951190274	0.13710424	14.23143643	5.84683E-46	5.61629E-45	positive
PRJNA729271	stage_1W_vs_E10	ordinary_muscle_development	ENSGALG00010019679	RHOBTB2	-2.074421247	0.177387016	-11.69432406	1.36268E-31	8.21974E-31	negative
PRJNA729271	stage_1W_vs_E10	ordinary_muscle_development	ENSGALG00010019680	CCDC34	-0.406502284	0.203457256	-1.997973883	0.045719492	0.057372005	negative
PRJNA729271	stage_1W_vs_E10	ordinary_muscle_development	ENSGALG00010019682	TMEM158	-1.425729228	0.369721556	-3.856224245	0.000115152	0.000184332	negative
PRJNA729271	stage_1W_vs_E10	ordinary_muscle_development	ENSGALG00010019683	TMEM184A	0.522602978	0.293844632	1.77850102	0.075321605	0.091932187	positive
PRJNA729271	stage_1W_vs_E10	ordinary_muscle_development	ENSGALG00010019684	PIGQ	-0.305108124	0.130817269	-2.332322984	0.019683706	0.025745833	negative
PRJNA729271	stage_1W_vs_E10	ordinary_muscle_development	ENSGALG00010019690	ENSGALG00010019690	-0.198928678	0.091091071	-2.183843894	0.028973721	0.037172427	negative
PRJNA729271	stage_1W_vs_E10	ordinary_muscle_development	ENSGALG00010019692	MMEL1	-0.921554033	0.305670837	-3.01485756	0.002571	0.003687941	negative
PRJNA729271	stage_1W_vs_E10	ordinary_muscle_development	ENSGALG00010019693	HAUS2	-0.638220523	0.120503756	-5.29627079	1.18192E-07	2.35005E-07	negative
PRJNA729271	stage_1W_vs_E10	ordinary_muscle_development	ENSGALG00010019695	PGA5	12.90643582	2.384507498	5.412621195	6.21088E-08	1.25964E-07	positive
PRJNA729271	stage_1W_vs_E10	ordinary_muscle_development	ENSGALG00010019697	EIF4EBP1	3.207466883	0.145153926	22.09700401	3.3775E-108	1.4746E-106	positive
PRJNA729271	stage_1W_vs_E10	ordinary_muscle_development	ENSGALG00010019700	HES5B	-2.148589803	0.259007428	-8.295475627	1.08152E-16	3.4652E-16	negative
PRJNA729271	stage_1W_vs_E10	ordinary_muscle_development	ENSGALG00010019706	NPRL3	0.767248494	0.097842694	7.84165341	4.44652E-15	1.32782E-14	positive
PRJNA729271	stage_1W_vs_E10	ordinary_muscle_development	ENSGALG00010019713	NUDT14	0.488200795	0.114055979	4.280361285	1.8659E-05	3.16775E-05	positive
PRJNA729271	stage_1W_vs_E10	ordinary_muscle_development	ENSGALG00010019716	VEGFA	0.858738701	0.1300934	6.600939806	4.08559E-11	9.94637E-11	positive
PRJNA729271	stage_1W_vs_E10	ordinary_muscle_development	ENSGALG00010019717	ZRANB3	-0.868188078	0.143143228	-6.065170445	1.31814E-09	2.94943E-09	negative
PRJNA729271	stage_1W_vs_E10	ordinary_muscle_development	ENSGALG00010019724	HES5A	-4.509502749	0.326494381	-13.81188471	2.16103E-43	1.92764E-42	negative
PRJNA729271	stage_1W_vs_E10	ordinary_muscle_development	ENSGALG00010019726	PEBP4	6.159078182	0.222245661	27.71292877	4.8777E-169	5.1987E-167	positive
PRJNA729271	stage_1W_vs_E10	ordinary_muscle_development	ENSGALG00010019731	PANK4	1.814035758	0.132983719	13.64103641	2.28293E-42	1.97441E-41	positive
PRJNA729271	stage_1W_vs_E10	ordinary_muscle_development	ENSGALG00010019732	HBA1	-1.233284566	0.403534763	-3.056204019	0.002241587	0.00323205	negative
PRJNA729271	stage_1W_vs_E10	ordinary_muscle_development	ENSGALG00010019735	ENSGALG00010019735	-0.391632269	0.17157739	-2.282540073	0.022457474	0.029194717	negative
PRJNA729271	stage_1W_vs_E10	ordinary_muscle_development	ENSGALG00010019738	ADRA2A	-2.714461684	0.364335104	-7.450453325	9.302E-14	2.58995E-13	negative
PRJNA729271	stage_1W_vs_E10	ordinary_muscle_development	ENSGALG00010019743	KCNU1	2.744877857	0.450239145	6.09648869	1.08424E-09	2.43546E-09	positive
PRJNA729271	stage_1W_vs_E10	ordinary_muscle_development	ENSGALG00010019744	KBP	-0.246868053	0.459163962	-0.537646839	0.590820887	0.625653767	negative
PRJNA729271	stage_1W_vs_E10	ordinary_muscle_development	ENSGALG00010019752	GPAM	-0.41344117	0.168347399	-2.455880945	0.014053971	0.01869852	negative
PRJNA729271	stage_1W_vs_E10	ordinary_muscle_development	ENSGALG00010019761	MRPS18A	0.871118393	0.086944787	10.01921359	1.25498E-23	5.44963E-23	positive
PRJNA729271	stage_1W_vs_E10	ordinary_muscle_development	ENSGALG00010019767	SUCLG2	-1.038284264	0.078938577	-13.1530654	1.63413E-39	1.28872E-38	negative
PRJNA729271	stage_1W_vs_E10	ordinary_muscle_development	ENSGALG00010019771	VWCE	-2.577710643	0.390704131	-6.59760273	4.17859E-11	1.01711E-10	negative
PRJNA729271	stage_1W_vs_E10	ordinary_muscle_development	ENSGALG00010019772	ENSGALG00010019772	0.367925443	0.16489864	2.231221824	0.025666439	0.033158203	positive
PRJNA729271	stage_1W_vs_E10	ordinary_muscle_development	ENSGALG00010019778	ABHD17C	0.336282778	0.131499896	2.557285514	0.010549259	0.014228297	positive
PRJNA729271	stage_1W_vs_E10	ordinary_muscle_development	ENSGALG00010019785	ACSL5	1.417356256	0.216288443	6.553083635	5.63609E-11	1.35864E-10	positive
PRJNA729271	stage_1W_vs_E10	ordinary_muscle_development	ENSGALG00010019786	R3HDM1	0.709376399	0.087236631	8.131634513	4.2354E-16	1.32539E-15	positive
PRJNA729271	stage_1W_vs_E10	ordinary_muscle_development	ENSGALG00010019789	ARNT2	-2.679472676	0.178658005	-14.99777563	7.59213E-51	8.56404E-50	negative
PRJNA729271	stage_1W_vs_E10	ordinary_muscle_development	ENSGALG00010019794	RSPH9	-1.237221683	0.50290936	-2.460128567	0.013888725	0.018498307	negative
PRJNA729271	stage_1W_vs_E10	ordinary_muscle_development	ENSGALG00010019799	DOK2	0.730226315	0.271713475	2.687486571	0.0071992	0.009885601	positive
PRJNA729271	stage_1W_vs_E10	ordinary_muscle_development	ENSGALG00010019800	EXT2	-0.685648029	0.061031163	-11.23439231	2.76389E-29	1.51351E-28	negative
PRJNA729271	stage_1W_vs_E10	ordinary_muscle_development	ENSGALG00010019801	FIBIN	-4.987186545	0.189507571	-26.31655571	1.2398E-152	1.0891E-150	negative
PRJNA729271	stage_1W_vs_E10	ordinary_muscle_development	ENSGALG00010019803	FAH	-1.47224	0.497847969	-2.957208003	0.003104386	0.004417206	negative
PRJNA729271	stage_1W_vs_E10	ordinary_muscle_development	ENSGALG00010019804	ELFN1	-2.910794778	0.315297633	-9.231895443	2.65885E-20	1.01128E-19	negative
PRJNA729271	stage_1W_vs_E10	ordinary_muscle_development	ENSGALG00010019807	ZDHHC6	0.900492814	0.078208349	11.51402407	1.12122E-30	6.51907E-30	positive
PRJNA729271	stage_1W_vs_E10	ordinary_muscle_development	ENSGALG00010019810	ZFAND6	0.531585138	0.12486189	4.25738502	2.06832E-05	3.49788E-05	positive
PRJNA729271	stage_1W_vs_E10	ordinary_muscle_development	ENSGALG00010019811	UBXN4	1.083196805	0.110433085	9.808625826	1.03367E-22	4.31165E-22	positive
PRJNA729271	stage_1W_vs_E10	ordinary_muscle_development	ENSGALG00010019813	ENSGALG00010019813	4.195116992	0.216739309	19.355589	1.82892E-83	4.75093E-82	positive
PRJNA729271	stage_1W_vs_E10	ordinary_muscle_development	ENSGALG00010019816	ENSGALG00010019816	3.038435927	0.714007572	4.255467375	2.08613E-05	3.52676E-05	positive
PRJNA729271	stage_1W_vs_E10	ordinary_muscle_development	ENSGALG00010019820	BBOX1	0.235416187	0.154194147	1.526751771	0.126822748	0.150347243	positive
PRJNA729271	stage_1W_vs_E10	ordinary_muscle_development	ENSGALG00010019823	LCT	-0.985439669	0.211789919	-4.652911109	3.27281E-06	5.88725E-06	negative
PRJNA729271	stage_1W_vs_E10	ordinary_muscle_development	ENSGALG00010019825	BCL2A1	1.919948719	0.282359168	6.79966842	1.0486E-11	2.63377E-11	positive
PRJNA729271	stage_1W_vs_E10	ordinary_muscle_development	ENSGALG00010019830	JAG2	0.028965189	0.098307753	0.294637895	0.768270511	0.791814637	positive
PRJNA729271	stage_1W_vs_E10	ordinary_muscle_development	ENSGALG00010019832	MAD2L1BP	-2.527644215	0.145582122	-17.36232571	1.59144E-67	2.82004E-66	negative
PRJNA729271	stage_1W_vs_E10	ordinary_muscle_development	ENSGALG00010019833	MTHFS	-0.871656147	0.133235032	-6.542244415	6.06024E-11	1.45653E-10	negative
PRJNA729271	stage_1W_vs_E10	ordinary_muscle_development	ENSGALG00010019834	PLCH2	-1.095769114	0.236281722	-4.637553445	3.52557E-06	6.32935E-06	negative
PRJNA729271	stage_1W_vs_E10	ordinary_muscle_development	ENSGALG00010019836	MINAR1	1.012302475	0.491327244	2.060342646	0.039365796	0.049799896	positive
PRJNA729271	stage_1W_vs_E10	ordinary_muscle_development	ENSGALG00010019838	ALX4	-3.803149163	0.218547526	-17.40193188	7.97668E-68	1.42216E-66	negative
PRJNA729271	stage_1W_vs_E10	ordinary_muscle_development	ENSGALG00010019843	MCM6	-2.373380876	0.112153271	-21.16194083	2.14221E-99	7.92126E-98	negative
PRJNA729271	stage_1W_vs_E10	ordinary_muscle_development	ENSGALG00010019846	ENSGALG00010019846	0.328379011	0.072020145	4.559543882	5.12648E-06	9.07639E-06	positive
PRJNA729271	stage_1W_vs_E10	ordinary_muscle_development	ENSGALG00010019848	MAD1L1	-1.127899789	0.12896115	-8.74604319	2.20965E-18	7.64776E-18	negative
PRJNA729271	stage_1W_vs_E10	ordinary_muscle_development	ENSGALG00010019854	CCDC14	-0.527608161	0.148441529	-3.554316406	0.000378963	0.000583562	negative
PRJNA729271	stage_1W_vs_E10	ordinary_muscle_development	ENSGALG00010019855	TTC7A	0.236565741	0.126300126	1.873044383	0.061062256	0.075398024	positive
PRJNA729271	stage_1W_vs_E10	ordinary_muscle_development	ENSGALG00010019859	BIN3	-0.263274619	0.136639307	-1.926785374	0.054006383	0.067154716	negative
PRJNA729271	stage_1W_vs_E10	ordinary_muscle_development	ENSGALG00010019865	ENSGALG00010019865	0.610167384	0.134821286	4.525749602	6.01818E-06	1.06033E-05	positive
PRJNA729271	stage_1W_vs_E10	ordinary_muscle_development	ENSGALG00010019867	PEX11A	-1.256870236	0.383425905	-3.278000308	0.001045453	0.001553125	negative

PRJNA729271	stage_1W_vs_E10	ordinary_muscle_development	ENSGALG00010019871	TAFA1	-4.891462939	0.800849395	-6.107843707	1.00986E-09	2.27156E-09	negative
PRJNA729271	stage_1W_vs_E10	ordinary_muscle_development	ENSGALG00010019872	VTI1A	0.166321498	0.109210206	1.522948309	0.127771638	0.151335994	positive
PRJNA729271	stage_1W_vs_E10	ordinary_muscle_development	ENSGALG00010019873	LGR4	-2.093018766	0.099811771	-20.9696587	1.24151E-97	4.43245E-96	negative
PRJNA729271	stage_1W_vs_E10	ordinary_muscle_development	ENSGALG00010019875	PLEKHH1	-1.621883	0.267640655	-6.059927632	1.36183E-09	3.04578E-09	negative
PRJNA729271	stage_1W_vs_E10	ordinary_muscle_development	ENSGALG00010019876	MRM2	1.500700295	0.222510955	6.74438836	1.53673E-11	3.8227E-11	positive
PRJNA729271	stage_1W_vs_E10	ordinary_muscle_development	ENSGALG00010019878	MCFD2	0.068664878	0.150105388	0.457444455	0.647351618	0.678969732	positive
PRJNA729271	stage_1W_vs_E10	ordinary_muscle_development	ENSGALG00010019891	PIGH	-1.408323931	0.108230477	-13.01226766	1.04203E-38	7.9748E-38	negative
PRJNA729271	stage_1W_vs_E10	ordinary_muscle_development	ENSGALG00010019896	DDB1	0.064100211	0.090940217	0.704860988	0.48089675	0.519225206	positive
PRJNA729271	stage_1W_vs_E10	ordinary_muscle_development	ENSGALG00010019898	NUDT1	-1.183729948	0.187776793	-6.303920352	2.9021E-10	6.71336E-10	negative
PRJNA729271	stage_1W_vs_E10	ordinary_muscle_development	ENSGALG00010019899	ENSGALG00010019899	-0.527324598	0.459480803	-1.147653165	0.251111773	0.284120299	negative
PRJNA729271	stage_1W_vs_E10	ordinary_muscle_development	ENSGALG00010019905	ENSGALG00010019905	-0.577912677	0.124970072	-4.624408609	3.75669E-06	6.72261E-06	negative
PRJNA729271	stage_1W_vs_E10	ordinary_muscle_development	ENSGALG00010019906	ENSGALG00010019906	1.854661095	0.232357163	7.981940685	1.4405E-15	4.39561E-15	positive
PRJNA729271	stage_1W_vs_E10	ordinary_muscle_development	ENSGALG00010019910	ENSGALG00010019910	-0.246466508	0.252330708	-0.976759864	0.32868805	0.365138971	negative
PRJNA729271	stage_1W_vs_E10	ordinary_muscle_development	ENSGALG00010019912	ARG2	-5.458738561	0.237415348	-22.99235753	5.5587E-117	2.8572E-115	negative
PRJNA729271	stage_1W_vs_E10	ordinary_muscle_development	ENSGALG00010019916	NOC3L	0.37024016	0.113119212	3.273008665	0.001064092	0.001580329	positive
PRJNA729271	stage_1W_vs_E10	ordinary_muscle_development	ENSGALG00010019919	LIN7C	-0.038213458	0.107395016	-0.355821519	0.721974232	0.748945573	negative
PRJNA729271	stage_1W_vs_E10	ordinary_muscle_development	ENSGALG00010019921	CALM2	0.23398103	0.112670537	2.076683371	0.037830792	0.047933334	positive
PRJNA729271	stage_1W_vs_E10	ordinary_muscle_development	ENSGALG00010019925	RDH11	-2.263562835	0.151715874	-14.91974954	2.45192E-50	2.71717E-49	negative
PRJNA729271	stage_1W_vs_E10	ordinary_muscle_development	ENSGALG00010019926	KIF7	-1.264013876	0.10308087	-12.26235165	1.44252E-34	9.63563E-34	negative
PRJNA729271	stage_1W_vs_E10	ordinary_muscle_development	ENSGALG00010019931	ZFYVE26	0.836648967	0.134456892	6.22243273	4.89505E-10	1.11914E-09	positive
PRJNA729271	stage_1W_vs_E10	ordinary_muscle_development	ENSGALG00010019932	KALRN	-0.261599503	0.117471805	-2.226913116	0.025953081	0.033495674	negative
PRJNA729271	stage_1W_vs_E10	ordinary_muscle_development	ENSGALG00010019934	TKFC	0.495843128	0.15057158	3.293072487	0.000990989	0.001476602	positive
PRJNA729271	stage_1W_vs_E10	ordinary_muscle_development	ENSGALG00010019935	ENSGALG00010019935	3.907307351	1.24420123	3.140414314	0.001687091	0.002462428	positive
PRJNA729271	stage_1W_vs_E10	ordinary_muscle_development	ENSGALG00010019938	TAFA4	-4.710065994	0.571615864	-8.239914759	1.72333E-16	5.47801E-16	negative
PRJNA729271	stage_1W_vs_E10	ordinary_muscle_development	ENSGALG00010019947	ENSGALG00010019947	-0.725130294	0.290849816	-2.493143381	0.01266177	0.016923224	negative
PRJNA729271	stage_1W_vs_E10	ordinary_muscle_development	ENSGALG00010019948	TICRR	-1.64496312	0.130715323	-12.58431748	2.57559E-36	1.82648E-35	negative
PRJNA729271	stage_1W_vs_E10	ordinary_muscle_development	ENSGALG00010019954	CYB561A3	1.462007237	0.125363029	11.66218821	1.98865E-31	1.19015E-30	positive
PRJNA729271	stage_1W_vs_E10	ordinary_muscle_development	ENSGALG00010019956	SLC29A3	-1.534852092	0.187698499	-8.177220922	2.90465E-16	9.11516E-16	negative
PRJNA729271	stage_1W_vs_E10	ordinary_muscle_development	ENSGALG00010019961	HTATSF1	-0.742340444	0.070652985	-10.50685179	8.03305E-26	3.82771E-25	negative
PRJNA729271	stage_1W_vs_E10	ordinary_muscle_development	ENSGALG00010019962	SNX8	0.683118664	0.142271566	4.801512237	1.57472E-06	2.89628E-06	positive
PRJNA729271	stage_1W_vs_E10	ordinary_muscle_development	ENSGALG00010019967	CEP170B	-0.409365218	0.107836566	-3.796163328	0.000146953	0.000233536	negative
PRJNA729271	stage_1W_vs_E10	ordinary_muscle_development	ENSGALG00010019968	RHCG	1.937424145	0.863305819	2.244192153	0.024820041	0.032113406	positive
PRJNA729271	stage_1W_vs_E10	ordinary_muscle_development	ENSGALG00010019972	PPP1CB	0.037596625	0.09150038	0.410890372	0.681152923	0.710616929	positive
PRJNA729271	stage_1W_vs_E10	ordinary_muscle_development	ENSGALG00010019975	ENSGALG00010019975	0.269295519	0.196456832	1.370761793	0.170449225	0.198207903	positive
PRJNA729271	stage_1W_vs_E10	ordinary_muscle_development	ENSGALG00010019977	ZMIZ1	-0.359697284	0.088941243	-4.044212483	5.24993E-05	8.6259E-05	negative
PRJNA729271	stage_1W_vs_E10	ordinary_muscle_development	ENSGALG00010019979	ENSGALG00010019979	-3.349711393	0.538552657	-6.219840063	4.97662E-10	1.13654E-09	negative
PRJNA729271	stage_1W_vs_E10	ordinary_muscle_development	ENSGALG00010019982	PLCE1	-0.584676272	0.113265834	-5.161982667	2.44348E-07	4.75924E-07	negative
PRJNA729271	stage_1W_vs_E10	ordinary_muscle_development	ENSGALG00010019985	UMPS	-1.875892719	0.089366188	-20.99107912	7.91288E-98	2.83904E-96	negative
PRJNA729271	stage_1W_vs_E10	ordinary_muscle_development	ENSGALG00010019989	ENSGALG00010019989	1.290559507	0.276793931	4.662528191	3.12348E-06	5.62561E-06	positive
PRJNA729271	stage_1W_vs_E10	ordinary_muscle_development	ENSGALG00010019990	MARCHF8	0.441653876	0.10739365	4.112476616	3.91437E-05	6.49557E-05	positive
PRJNA729271	stage_1W_vs_E10	ordinary_muscle_development	ENSGALG00010019991	EIF3B	0.479747636	0.07515554	6.383396854	1.73202E-10	4.06175E-10	positive
PRJNA729271	stage_1W_vs_E10	ordinary_muscle_development	ENSGALG00010019992	CHAC2	0.055042388	0.13788547	0.399189182	0.689753814	0.718607168	positive
PRJNA729271	stage_1W_vs_E10	ordinary_muscle_development	ENSGALG00010019994	PLD4	0.546340307	0.700964692	0.779412021	0.435737045	0.473712478	positive
PRJNA729271	stage_1W_vs_E10	ordinary_muscle_development	ENSGALG00010019996	ITGB5	0.343129054	0.124490613	2.75626447	0.00584657	0.008105015	positive
PRJNA729271	stage_1W_vs_E10	ordinary_muscle_development	ENSGALG00010019997	FSHR	-0.049573172	0.432073135	-0.114733288	0.908656528	0.919504075	negative
PRJNA729271	stage_1W_vs_E10	ordinary_muscle_development	ENSGALG00010019998	PLEK2	-6.119510788	0.86531721	-7.071985526	1.52732E-12	4.00553E-12	negative
PRJNA729271	stage_1W_vs_E10	ordinary_muscle_development	ENSGALG00010019999	ERLIN1	-0.795358564	0.064442709	-12.34210318	5.37337E-35	3.64639E-34	negative
PRJNA729271	stage_1W_vs_E10	ordinary_muscle_development	ENSGALG00010020003	ENSGALG00010020003	0.361946487	0.187088115	1.934631103	0.05303558	0.066032532	positive
PRJNA729271	stage_1W_vs_E10	ordinary_muscle_development	ENSGALG00010020005	EOGT	0.265015871	0.163883162	1.617102498	0.105856158	0.126850612	positive
PRJNA729271	stage_1W_vs_E10	ordinary_muscle_development	ENSGALG00010020006	PLB1	-1.565520676	0.391900845	-3.994685637	6.47801E-05	0.000105801	negative
PRJNA729271	stage_1W_vs_E10	ordinary_muscle_development	ENSGALG00010020008	EIF2S1	-0.254732506	0.079030657	-3.223211298	0.00126762	0.001867861	negative
PRJNA729271	stage_1W_vs_E10	ordinary_muscle_development	ENSGALG00010020009	MACROD2	-0.338655208	0.117013167	-2.894163258	0.003801704	0.005363086	negative
PRJNA729271	stage_1W_vs_E10	ordinary_muscle_development	ENSGALG00010020010	SORBS3	2.365246407	0.207020167	11.42519803	3.12941E-30	1.78095E-29	positive
PRJNA729271	stage_1W_vs_E10	ordinary_muscle_development	ENSGALG00010020011	TLE3	-0.946798569	0.204563041	-4.628395067	3.68511E-06	6.60185E-06	negative
PRJNA729271	stage_1W_vs_E10	ordinary_muscle_development	ENSGALG00010020013	ADAM8	-1.503674421	0.600322476	-2.504777819	0.012252834	0.016399337	negative
PRJNA729271	stage_1W_vs_E10	ordinary_muscle_development	ENSGALG00010020015	ATP6V1D	1.130451885	0.07805459	14.48283674	1.55543E-47	1.56569E-46	positive
PRJNA729271	stage_1W_vs_E10	ordinary_muscle_development	ENSGALG00010020017	ENSGALG00010020017	4.502942663	0.283838155	15.86447268	1.11651E-56	1.50129E-55	positive
PRJNA729271	stage_1W_vs_E10	ordinary_muscle_development	ENSGALG00010020018	ENSGALG00010020018	0.092333206	0.209376986	0.440990235	0.659220067	0.689728984	positive
PRJNA729271	stage_1W_vs_E10	ordinary_muscle_development	ENSGALG00010020019	PEX10	1.498012759	0.097229966	15.40690405	1.47092E-53	1.82543E-52	positive
PRJNA729271	stage_1W_vs_E10	ordinary_muscle_development	ENSGALG00010020023	FOSL2	0.068827084	0.20938852	0.328705147	0.742378563	0.7674952	positive
PRJNA729271	stage_1W_vs_E10	ordinary_muscle_development	ENSGALG00010020025	LOXL2	-0.836619954	0.140487737	-5.95511018	2.59897E-09	5.71394E-09	negative
PRJNA729271	stage_1W_vs_E10	ordinary_muscle_development	ENSGALG00010020027	RER1	-0.081335244	0.070543618	-1.152978049	0.248919394	0.2817717	negative
PRJNA729271	stage_1W_vs_E10	ordinary_muscle_development	ENSGALG00010020030	MRPL33	0.990036527	0.134358966	7.368592908	1.72438E-13	4.73299E-13	positive
PRJNA729271	stage_1W_vs_E10	ordinary_muscle_development	ENSGALG00010020032	CHST12	-0.163847654	0.124766523	-1.31323411	0.189104073	0.21844625	negative
PRJNA729271	stage_1W_vs_E10	ordinary_muscle_development	ENSGALG00010020036	TMF1	0.453495993	0.083619854	5.423305272	5.85069E-08	1.18926E-07	positive
PRJNA729271	stage_1W_vs_E10	ordinary_muscle_development	ENSGALG00010020037	MPP5	-0.361261673	0.090416108	-3.995545474	6.45455E-05	0.00010543	negative
PRJNA729271	stage_1W_vs_E10	ordinary_muscle_development	ENSGALG00010020038	SKI	-0.937379035	0.091804031	-10.2106522	1.77667E-24	7.99279E-24	negative
PRJNA729271	stage_1W_vs_E10	ordinary_muscle_development	ENSGALG00010020041	CDH23	-0.047107239	0.153256356	-0.307375436	0.758557623	0.782973207	negative
PRJNA729271	stage_1W_vs_E10	ordinary_muscle_development	ENSGALG00010020046	BDNF	-1.871881123	0.24986455	-7.491853434	6.80476E-14	1.91339E-13	negative
PRJNA729271	stage_1W_vs_E10	ordinary_muscle_development	ENSGALG00010020048	ENSGALG00010020048	-0.70477135	0.267151121	-2.638099914	0.008337201	0.011364278	negative
PRJNA729271	stage_1W_vs_E10	ordinary_muscle_development	ENSGALG00010020053	LFNG	-0.311759895	0.135935413	-2.29344133	0.021822609	0.02839231	negative
PRJNA729271	stage_1W_vs_E10	ordinary_muscle_development	ENSGALG00010020055	R3HCC1	-1.027233175	0.085202015	-12.05644231	1.79365E-33	1.15192E-32	negative
PRJNA729271	stage_1W_vs_E10	ordinary_muscle_development	ENSGALG00010020056	UACA	-2.442841124	0.166854129	-14.64057936	1.54744E-48	1.61136E-47	negative
PRJNA729271	stage_1W_vs_E10	ordinary_muscle_development	ENSGALG00010020057	RBKS	0.913338814	0.089249039	10.2335983	1.40211E-24	6.33922E-24	positive
PRJNA729271	stage_1W_vs_E10	ordinary_muscle_development	ENSGALG00010020059	PRKCZ	-0.065577957	0.140339544	-0.467280675	0.640299081	0.672692264	negative
PRJNA729271	stage_1W_vs_E10	ordinary_muscle_development	ENSGALG00010020061	UBA3	-0.014124245	0.072734754	-0.194188391	0.846028373	0.862935843	negative
PRJNA729271	stage_1W_vs_E10	ordinary_muscle_development	ENSGALG00010020063	ENSGALG00010020063	1.19130728	0.129252669	9.21688725	3.0585E-20	1.15994E-19	positive
PRJNA729271	stage_1W_vs_E10	ordinary_muscle_development	ENSGALG00010020064	TMEM138	-0.273944099	0.135011009	-2.029050073	0.04245319	0.053476927	negative
PRJNA729271	stage_1W_vs_E10	ordinary_muscle_development	ENSGALG00010020065	TTYH3	-2.313843959	0.113433472	-20.39824686	1.73319E-92	5.52144E-91	negative
PRJNA729271	stage_1W_vs_E10	ordinary_muscle_development	ENSGALG00010020066	ENSGALG00010020066	2.585589472	0.379044614	6.821332836	9.01997E-12	2.27421E-11	positive
PRJNA729271	stage_1W_vs_E10	ordinary_muscle_development	ENSGALG00010020069	TMEM216	-0.820740018	0.208363807	-3.938975913	8.18301E-05	0.000132558	negative
PRJNA729271	stage_1W_vs_E10	ordinary_muscle_development	ENSGALG00010020070	ARL6IP5	0.67438282	0.114473905	5.89114889	3.8352E-09	8.34452E-09	positive
PRJNA729271	stage_1W_vs_E10	ordinary_muscle_development	ENSGALG00010020071	ENSGALG00010020071	0.140612089	0.144063672	0.976041267	0.329044015	0.36547839	positive
PRJNA729271	stage_1W_vs_E10	ordinary_muscle_development	ENSGALG00010020074	ENSGALG00010020074	-0.444763836	0.208113906	-2.137117339	0.032588449	0.041574509	negative
PRJNA729271	stage_1W_vs_E10	ordinary_muscle_development	ENSGALG00010020075	CPSF7	-0.65987222	0.147121527	-4.485218665	7.28392E-06	1.27513E-05	negative
PRJNA729271	stage_1W_vs_E10	ordinary_muscle_development	ENSGALG00010020080	SLC4A1AP	0.212438006	0.102467271	2.07322792	0.038151073	0.048326471	positive
PRJNA729271	stage_1W_vs_E10	ordinary_muscle_development	ENSGALG00010020083	CFAP74	-1.795902608	0.458739007	-3.914867887	9.04537E-05	0.000146021	negative
PRJNA729271	stage_1W_vs_E10	ordinary_muscle_development	ENSGALG00010020084	ENSGALG00010020084	-0.838744146	0.088833585	-9.441745984	3.66619E-21	1.44406E-20	negative
PRJNA729271	stage_1W_vs_E10	ordinary_muscle_development	ENSGALG00010020087	ENSGALG00010020087	3.498779049	0.473614746	7.387394674	1.49734E-13	4.1223E-13	positive
PRJNA729271	stage_1W_vs_E10	ordinary_muscle_development	ENSGALG00010020088	LMOD3	3.913660338	0.194044423	20.16888852	1.83721E-90	5.49081E-89	positive
PRJNA729271	stage_1W_vs_E10	ordinary_muscle_development	ENSGALG00010020090	MORF4L2	-0.604118951	0.053795768	-11.22986019	2.90939E-29	1.59138E-28	negative
PRJNA729271	stage_1W_vs_E10	ordinary_muscle_development	ENSGALG00010020091	SUPT7L	-0.474065715	0.085216351	-5.563083982	2.65048E-08	5.48056E-08	negative
PRJNA729271	stage_1W_vs_E10	ordinary_muscle_development	ENSGALG00010020092	SDHAF2	1.705283536	0.136543545	12.48893556	8.57938E-36	6.01927E-35	positive
PRJNA729271	stage_1W_vs_E10	ordinary_muscle_development	ENSGALG00010020094	CLEC3B	3.673438595	0.227682048	16.13407219	1.47E-58	2.0667E-57	positive
PRJNA729271	stage_1W_vs_E10	ordinary_muscle_development	ENSGALG00010020095	LRFN2	-1.006011069	0.329804233	-3.050327953	0.002285916	0.003292037	negative
PRJNA729271	stage_1W_vs_E10	ordinary_muscle_development	ENSGALG00010020096	GPHN	2.323475963	0.115306873	20.15036836	2.67119E-90	7.88572E-89	positive
PRJNA729271	stage_1W_vs_E10	ordinary_muscle_development	ENSGALG00010020097	RETSAT	1.198406523	0.228538221	5.243790376	1.57311E-07	3.1015E-07	positive
PRJNA729271	stage_1W_vs_E10	ordinary_muscle_development	ENSGALG00010020098	GABRD	1.692080946	0.331027148	5.111607785	3.19428E-07	6.16937E-07	positive
PRJNA729271	stage_1W_vs_E10	ordinary_muscle_development	ENSGALG00010020100	MOCS1	2.55102194	0.157049893	16.24338542	2.48806E-59	3.57428E-58	positive
PRJNA729271	stage_1W_vs_E10	ordinary_muscle_development	ENSGALG00010020101	KIF15	-3.02003373	0.208197912	-14.50559083	1.11672E-47	1.128E-46	negative
PRJNA729271	stage_1W_vs_E10	ordinary_muscle_development	ENSGALG00010020102	TMEM127	0.621143822	0.202357156	3.069542164	0.002143871	0.003095778	positive
PRJNA729271	stage_1W_vs_E10	ordinary_muscle_development	ENSGALG00010020103	PSAP	0.644438102	0.105047816	6.134712071	8.53136E-10	1.9253E-09	positive
PRJNA729271	stage_1W_vs_E10	ordinary_muscle_development	ENSGALG00010020104	BRAT1	0.561533608	0.112017529	5.012908382	5.36134E-07	1.01985E-06	positive
PRJNA729271	stage_1W_vs_E10	ordinary_muscle_development	ENSGALG00010020106	ENSGALG00010020106	1.896589628	0.162447284	11.67510826	1.70848E-31	1.02672E-30	positive
PRJNA729271	stage_1W_vs_E10	ordinary_muscle_development	ENSGALG00010020108	RPAP1	-0.850624831	0.118166396	-7.198534128	6.08633E-13	1.6268E-12	negative
PRJNA729271	stage_1W_vs_E10	ordinary_muscle_development	ENSGALG00010020109	WDFY4	1.920314334	0.359259457	5.34520191	9.03163E-08	1.8127E-07	positive
PRJNA729271	stage_1W_vs_E10	ordinary_muscle_development	ENSGALG00010020111	ADRA2B	1.695865479	0.120528664	14.0702255	5.78845E-45	5.40268E-44	positive
PRJNA729271	stage_1W_vs_E10	ordinary_muscle_development	ENSGALG00010020113	CIAO1	0.318090543	0.103774568	3.065207095	0.002175193	0.003139443	positive
PRJNA729271	stage_1W_vs_E10	ordinary_muscle_development	ENSGALG00010020116	GNA12	-0.171095275	0.07248547	-2.360407895	0.018254851	0.023943217	negative
PRJNA729271	stage_1W_vs_E10	ordinary_muscle_development	ENSGALG00010020117	AKAP13	-1.082525787	0.103052715	-10.5045829	8.22857E-26	3.91445E-25	negative
PRJNA729271	stage_1W_vs_E10	ordinary_muscle_development	ENSGALG00010020118	GNB1	-0.823472793	0.093501171	-8.80708535	1.28442E-18	4.50572E-18	negative
PRJNA729271	stage_1W_vs_E10	ordinary_muscle_development	ENSGALG00010020120	DAAM2	-1.151838139	0.104054788	-11.06953524	1.76313E-28	9.34425E-28	negative
PRJNA729271	stage_1W_vs_E10	ordinary_muscle_development	ENSGALG00010020121	ENSGALG00010020121	-2.367219687	0.277670464	-8.525284432	1.52435E-17	5.06543E-17	negative
PRJNA729271	stage_1W_vs_E10	ordinary_muscle_development	ENSGALG00010020123	ENSGALG00010020123	-0.425148836	0.156065121	-2.724175863	0.006446217	0.008893767	negative
PRJNA729271	stage_1W_vs_E10	ordinary_muscle_development	ENSGALG00010020125	NAV2	0.390542581	0.130073751	3.002470353	0.002677981	0.003834189	positive
PRJNA729271	stage_1W_vs_E10	ordinary_muscle_development	ENSGALG00010020126	NADK	-0.367522245	0.105309352	-3.489929778	0.000483147	0.000737492	negative
PRJNA729271	stage_1W_vs_E10	ordinary_muscle_development	ENSGALG00010020127	FRMD4B	-1.073942143	0.141243449	-7.603482884	2.88265E-14	8.24957E-14	negative
PRJNA729271	stage_1W_vs_E10	ordinary_muscle_development	ENSGALG00010020128	KIF6	0.690649338	0.498766691	1.384714236	0.166139864	0.193662833	positive
PRJNA729271	stage_1W_vs_E10	ordinary_muscle_development	ENSGALG00010020129	MGAT2	-0.426254905	0.117438094	-3.62961362	0.000283846	0.000441975	negative
PRJNA729271	stage_1W_vs_E10	ordinary_muscle_development	ENSGALG00010020130	PDXDC1	-0.246329348	0.074245818	-3.31775385	0.000907444	0.001357981	negative
PRJNA729271	stage_1W_vs_E10	ordinary_muscle_development	ENSGALG00010020131	SNRNP200	-0.470572256	0.12477565	-3.771346856	0.000162369	0.000257189	negative
PRJNA729271	stage_1W_vs_E10	ordinary_muscle_development	ENSGALG00010020132	SHC4	-0.82537698	0.232834665	-3.544905908	0.000392753	0.000604059	negative
PRJNA729271	stage_1W_vs_E10	ordinary_muscle_development	ENSGALG00010020133	ENSGALG00010020133	2.354253365	0.368377203	6.390876924	1.64937E-10	3.87356E-10	positive
PRJNA729271	stage_1W_vs_E10	ordinary_muscle_development	ENSGALG00010020136	KCNK17	0.368343801	0.294429236	1.251043565	0.210918584	0.241891358	positive
PRJNA729271	stage_1W_vs_E10	ordinary_muscle_development	ENSGALG00010020137	NTAN1	-0.257583812	0.083700175	-3.077458456	0.002087739	0.003020439	negative
PRJNA729271	stage_1W_vs_E10	ordinary_muscle_development	ENSGALG00010020138	ENSGALG00010020138	0.271261333	0.085507063	3.172385111	0.001511924	0.002215236	positive
PRJNA729271	stage_1W_vs_E10	ordinary_muscle_development	ENSGALG00010020140	SECISBP2L	0.735269937	0.094565248	7.775265786	7.52892E-15	2.22174E-14	positive
PRJNA729271	stage_1W_vs_E10	ordinary_muscle_development	ENSGALG00010020141	THOC7	0.077635819	0.127660349	0.608143564	0.543092261	0.579215829	positive
PRJNA729271	stage_1W_vs_E10	ordinary_muscle_development	ENSGALG00010020142	SLC35E2B	-1.627173606	0.071561227	-22.73820154	1.8777E-114	9.1026E-113	negative
PRJNA729271	stage_1W_vs_E10	ordinary_muscle_development	ENSGALG00010020143	KCNK5	-1.848762527	0.189630102	-9.749309332	1.85728E-22	7.67204E-22	negative
PRJNA729271	stage_1W_vs_E10	ordinary_muscle_development	ENSGALG00010020147	ABHD2	0.853015926	0.146967168	5.804125768	6.47027E-09	1.38943E-08	positive

PRJNA729271	stage_1W_vs_E10	ordinary_muscle_development	ENSGALG00010020149	FAHD2A	1.817386136	0.171852428	10.57527182	3.88051E-26	1.87306E-25	positive
PRJNA729271	stage_1W_vs_E10	ordinary_muscle_development	ENSGALG00010020152	ATXN7	0.741496572	0.10783338	6.87631762	6.14195E-12	1.56216E-11	positive
PRJNA729271	stage_1W_vs_E10	ordinary_muscle_development	ENSGALG00010020153	AXL	-0.945083201	0.122380773	-7.722481055	1.14087E-14	3.33876E-14	negative
PRJNA729271	stage_1W_vs_E10	ordinary_muscle_development	ENSGALG00010020155	MFGE8	2.531314149	0.134072769	18.88015121	1.6611E-79	3.89606E-78	positive
PRJNA729271	stage_1W_vs_E10	ordinary_muscle_development	ENSGALG00010020160	SYT7	-3.854610306	0.353450823	-10.90564813	1.08319E-27	5.56765E-27	negative
PRJNA729271	stage_1W_vs_E10	ordinary_muscle_development	ENSGALG00010020163	CDC2L1	-0.446082266	0.09655784	-4.619845111	3.84027E-06	6.8654E-06	negative
PRJNA729271	stage_1W_vs_E10	ordinary_muscle_development	ENSGALG00010020164	C1Oorf71	4.752940323	0.147802575	32.15735796	6.9698E-227	1.3293E-224	positive
PRJNA729271	stage_1W_vs_E10	ordinary_muscle_development	ENSGALG00010020166	HAPLN3	-0.244055773	0.176009453	-1.386606056	0.165561921	0.193035718	negative
PRJNA729271	stage_1W_vs_E10	ordinary_muscle_development	ENSGALG00010020168	COX7A2L	1.619887862	0.114518376	14.14522208	1.99885E-45	1.89616E-44	positive
PRJNA729271	stage_1W_vs_E10	ordinary_muscle_development	ENSGALG00010020170	SORCS1	-2.247134122	0.312861523	-7.182519917	6.84381E-13	1.8259E-12	negative
PRJNA729271	stage_1W_vs_E10	ordinary_muscle_development	ENSGALG00010020171	RRN3	-0.254187528	0.076591653	-3.318736683	0.000904257	0.00135377	negative
PRJNA729271	stage_1W_vs_E10	ordinary_muscle_development	ENSGALG00010020175	ACAN	-9.974891246	0.554637265	-17.98453129	2.57557E-72	5.15648E-71	negative
PRJNA729271	stage_1W_vs_E10	ordinary_muscle_development	ENSGALG00010020176	ENSGALG00010020176	0.231944511	0.250701347	0.925182547	0.35487096	0.392510841	positive
PRJNA729271	stage_1W_vs_E10	ordinary_muscle_development	ENSGALG00010020177	KCNG3	-3.895259682	0.305289102	-12.75924902	2.76857E-37	2.02067E-36	negative
PRJNA729271	stage_1W_vs_E10	ordinary_muscle_development	ENSGALG00010020179	PSMD6	1.301000543	0.134363577	9.682687581	3.572E-22	1.45848E-21	positive
PRJNA729271	stage_1W_vs_E10	ordinary_muscle_development	ENSGALG00010020180	TCF7L1	-0.400616477	0.208424189	-1.922120836	0.054590555	0.067840371	negative
PRJNA729271	stage_1W_vs_E10	ordinary_muscle_development	ENSGALG00010020181	LEUTX	2.676056657	0.214556001	12.47253231	1.05422E-35	7.37139E-35	positive
PRJNA729271	stage_1W_vs_E10	ordinary_muscle_development	ENSGALG00010020183	XPNPEP1	-0.354374837	0.068508064	-5.172746332	2.30678E-07	4.50236E-07	negative
PRJNA729271	stage_1W_vs_E10	ordinary_muscle_development	ENSGALG00010020184	RSL1D1	-0.576903603	0.069738593	-8.272372295	1.31319E-16	4.18899E-16	negative
PRJNA729271	stage_1W_vs_E10	ordinary_muscle_development	ENSGALG00010020186	MGA	0.172851096	0.094885884	1.821673444	0.06850455	0.08407192	positive
PRJNA729271	stage_1W_vs_E10	ordinary_muscle_development	ENSGALG00010020187	MMP23B	-1.128188203	0.209153029	-5.394080149	6.88755E-08	1.39182E-07	negative
PRJNA729271	stage_1W_vs_E10	ordinary_muscle_development	ENSGALG00010020193	VSTM4	-2.087661096	0.182019832	-11.46941556	1.87924E-30	1.07794E-29	negative
PRJNA729271	stage_1W_vs_E10	ordinary_muscle_development	ENSGALG00010020194	PRICKLE2	-1.720113824	0.15854856	-10.8491293	2.01334E-27	1.02362E-26	negative
PRJNA729271	stage_1W_vs_E10	ordinary_muscle_development	ENSGALG00010020199	DET1	-0.341887254	0.085413862	-4.002713935	6.262E-05	0.000102331	negative
PRJNA729271	stage_1W_vs_E10	ordinary_muscle_development	ENSGALG00010020202	MAPKBP1	0.110826486	0.091277446	1.214171639	0.224682188	0.256216531	positive
PRJNA729271	stage_1W_vs_E10	ordinary_muscle_development	ENSGALG00010020204	GSPT2	0.228079304	0.086492176	2.636993485	0.008364444	0.01140034	positive
PRJNA729271	stage_1W_vs_E10	ordinary_muscle_development	ENSGALG00010020206	MRPS11	0.648033589	0.081129285	7.987665499	1.37518E-15	4.20089E-15	positive
PRJNA729271	stage_1W_vs_E10	ordinary_muscle_development	ENSGALG00010020211	ADD3	-0.57575961	0.083480679	-6.896920545	5.31419E-12	1.35472E-11	negative
PRJNA729271	stage_1W_vs_E10	ordinary_muscle_development	ENSGALG00010020212	JMJD7	0.202680928	0.139281455	1.455189619	0.145616774	0.171129815	positive
PRJNA729271	stage_1W_vs_E10	ordinary_muscle_development	ENSGALG00010020215	PRMT3	-0.285702004	0.080767877	-3.537322212	0.000404206	0.000621048	negative
PRJNA729271	stage_1W_vs_E10	ordinary_muscle_development	ENSGALG00010020224	MRPL46	0.301295292	0.086712476	3.474647541	0.000511525	0.000779331	positive
PRJNA729271	stage_1W_vs_E10	ordinary_muscle_development	ENSGALG00010020226	TCIM	2.35088782	0.197523329	11.90182363	1.15789E-32	7.22497E-32	positive
PRJNA729271	stage_1W_vs_E10	ordinary_muscle_development	ENSGALG00010020228	TM9SF3	-0.834811277	0.083317627	-10.01962381	1.24978E-23	5.42868E-23	negative
PRJNA729271	stage_1W_vs_E10	ordinary_muscle_development	ENSGALG00010020230	ENSGALG00010020230	0.776259948	0.216366744	3.587704529	0.000333602	0.000516454	positive
PRJNA729271	stage_1W_vs_E10	ordinary_muscle_development	ENSGALG00010020236	SLC35G1	3.118404955	0.2843405	10.96715012	5.4978E-28	2.86245E-27	positive
PRJNA729271	stage_1W_vs_E10	ordinary_muscle_development	ENSGALG00010020237	MIB2	-0.00115142	0.076146436	-0.015121123	0.987935549	0.989847639	negative
PRJNA729271	stage_1W_vs_E10	ordinary_muscle_development	ENSGALG00010020240	SV2B	-1.77380068	0.397990075	-4.456896774	8.31546E-06	1.45045E-05	negative
PRJNA729271	stage_1W_vs_E10	ordinary_muscle_development	ENSGALG00010020244	SLC25A37	-1.720624219	0.216794418	-7.93666292	2.07693E-15	6.29157E-15	negative
PRJNA729271	stage_1W_vs_E10	ordinary_muscle_development	ENSGALG00010020246	ADAMTS9	-0.518834762	0.129534606	-4.005375704	6.1919E-05	0.000101277	negative
PRJNA729271	stage_1W_vs_E10	ordinary_muscle_development	ENSGALG00010020248	NOLC1	-0.067591119	0.080467211	-0.839983371	0.40091771	0.439152287	negative
PRJNA729271	stage_1W_vs_E10	ordinary_muscle_development	ENSGALG00010020250	ENSGALG00010020250	0.661881972	0.243803036	2.714822524	0.00663113	0.009138451	positive
PRJNA729271	stage_1W_vs_E10	ordinary_muscle_development	ENSGALG00010020252	DMTN	0.903888664	0.189088597	4.780238888	1.75087E-06	3.21292E-06	positive
PRJNA729271	stage_1W_vs_E10	ordinary_muscle_development	ENSGALG00010020254	ENSGALG00010020254	-1.404539378	0.135411484	-10.37238008	3.31172E-25	1.53808E-24	negative
PRJNA729271	stage_1W_vs_E10	ordinary_muscle_development	ENSGALG00010020257	ZNF710	1.239960255	0.160579576	7.721780605	1.14716E-14	3.35649E-14	positive
PRJNA729271	stage_1W_vs_E10	ordinary_muscle_development	ENSGALG00010020259	ENSGALG00010020259	-1.021317694	0.199753387	-5.112892991	3.17262E-07	6.12917E-07	negative
PRJNA729271	stage_1W_vs_E10	ordinary_muscle_development	ENSGALG00010020261	SSUT2	0.88258005	0.090714467	9.72920944	2.26345E-22	9.31008E-22	positive
PRJNA729271	stage_1W_vs_E10	ordinary_muscle_development	ENSGALG00010020265	ENSGALG00010020265	-1.312132115	0.229448712	-5.718629254	1.07387E-08	2.27436E-08	negative
PRJNA729271	stage_1W_vs_E10	ordinary_muscle_development	ENSGALG00010020268	CPLANE2	0.3743624	0.098046672	3.818206086	0.000134426	0.000214121	positive
PRJNA729271	stage_1W_vs_E10	ordinary_muscle_development	ENSGALG00010020269	ENSGALG00010020269	-2.649320349	0.826190844	-3.206668734	0.001342815	0.001975249	negative
PRJNA729271	stage_1W_vs_E10	ordinary_muscle_development	ENSGALG00010020272	ENSGALG00010020272	-1.257631905	0.094010562	-13.37755976	8.17887E-41	6.72449E-40	negative
PRJNA729271	stage_1W_vs_E10	ordinary_muscle_development	ENSGALG00010020275	ELOVL3	-4.510688262	0.907820277	-4.96870182	6.74026E-07	1.27529E-06	negative
PRJNA729271	stage_1W_vs_E10	ordinary_muscle_development	ENSGALG00010020277	IDH2	1.696963968	0.124621229	13.61697345	3.17453E-42	2.73247E-41	positive
PRJNA729271	stage_1W_vs_E10	ordinary_muscle_development	ENSGALG00010020282	MAGI1	-0.536467747	0.137435081	-3.903426571	9.48403E-05	0.000152831	negative
PRJNA729271	stage_1W_vs_E10	ordinary_muscle_development	ENSGALG00010020283	FBXO42	0.181342575	0.222506085	0.815000523	0.415072011	0.45311559	positive
PRJNA729271	stage_1W_vs_E10	ordinary_muscle_development	ENSGALG00010020284	HEG1	1.238015516	0.117474993	10.53854516	5.73796E-26	2.74766E-25	positive
PRJNA729271	stage_1W_vs_E10	ordinary_muscle_development	ENSGALG00010020285	ADAMTS14	-3.972434961	0.276970825	-14.34243106	1.18819E-46	1.16686E-45	negative
PRJNA729271	stage_1W_vs_E10	ordinary_muscle_development	ENSGALG00010020287	GALNT16	-0.434947196	0.162654546	-2.674054957	0.007494017	0.010266116	negative
PRJNA729271	stage_1W_vs_E10	ordinary_muscle_development	ENSGALG00010020290	NELL1	-6.25638635	0.69332784	-9.023705652	1.81832E-19	6.67086E-19	negative
PRJNA729271	stage_1W_vs_E10	ordinary_muscle_development	ENSGALG00010020291	SNX29	-0.103488202	0.14637756	-0.70699499	0.479569591	0.51786942	negative
PRJNA729271	stage_1W_vs_E10	ordinary_muscle_development	ENSGALG00010020292	NPM2	-0.899102969	0.249558365	-3.60277632	0.000314836	0.00048876	negative
PRJNA729271	stage_1W_vs_E10	ordinary_muscle_development	ENSGALG00010020293	SEMA4B	0.235003368	0.111093367	2.115368129	0.034398584	0.043764371	positive
PRJNA729271	stage_1W_vs_E10	ordinary_muscle_development	ENSGALG00010020295	LGI1	-0.07549946	0.147947025	-0.510314152	0.60983139	0.644091081	negative
PRJNA729271	stage_1W_vs_E10	ordinary_muscle_development	ENSGALG00010020298	ENSGALG00010020298	-0.008221766	0.174483041	-0.047120717	0.962417016	0.967489746	negative
PRJNA729271	stage_1W_vs_E10	ordinary_muscle_development	ENSGALG00010020300	EHD4	0.83036862	0.128863431	6.443787924	1.16528E-10	2.75812E-10	positive
PRJNA729271	stage_1W_vs_E10	ordinary_muscle_development	ENSGALG00010020302	HAAO	1.412726872	1.490678674	0.947707173	0.343278548	0.380443655	positive
PRJNA729271	stage_1W_vs_E10	ordinary_muscle_development	ENSGALG00010020303	EXD2	-0.471410509	0.074778642	-6.304079597	2.89911E-10	6.70753E-10	negative
PRJNA729271	stage_1W_vs_E10	ordinary_muscle_development	ENSGALG00010020304	ZFP36L2	0.9062192	0.155324264	5.83436982	5.39942E-09	1.16431E-08	positive
PRJNA729271	stage_1W_vs_E10	ordinary_muscle_development	ENSGALG00010020305	XPO7	0.230810413	0.138052229	1.671906449	0.094542771	0.114066711	positive
PRJNA729271	stage_1W_vs_E10	ordinary_muscle_development	ENSGALG00010020307	SHQ1	-1.025638557	0.228473633	-4.489089371	7.15283E-06	1.25308E-05	negative
PRJNA729271	stage_1W_vs_E10	ordinary_muscle_development	ENSGALG00010020309	GXYLT2	-1.460263056	0.160040654	-9.124325734	7.21842E-20	2.69529E-19	negative
PRJNA729271	stage_1W_vs_E10	ordinary_muscle_development	ENSGALG00010020311	SGPL1	-0.664326361	0.134997004	-4.921045231	8.60832E-07	1.61818E-06	negative
PRJNA729271	stage_1W_vs_E10	ordinary_muscle_development	ENSGALG00010020313	FRA10AC1	0.198918739	0.114895733	1.731297885	0.083398648	0.101295744	positive
PRJNA729271	stage_1W_vs_E10	ordinary_muscle_development	ENSGALG00010020314	THADA	-0.601715942	0.080565461	-7.468658816	8.10163E-14	2.26486E-13	negative
PRJNA729271	stage_1W_vs_E10	ordinary_muscle_development	ENSGALG00010020315	ANO5	0.289581152	0.108451606	2.670141656	0.007581925	0.010381637	positive
PRJNA729271	stage_1W_vs_E10	ordinary_muscle_development	ENSGALG00010020316	PDPK1	0.708569794	0.112185604	6.316049211	2.68335E-10	6.21526E-10	positive
PRJNA729271	stage_1W_vs_E10	ordinary_muscle_development	ENSGALG00010020319	NTRK3	-0.553203463	0.162307895	-3.408358318	0.00065355	0.000988853	negative
PRJNA729271	stage_1W_vs_E10	ordinary_muscle_development	ENSGALG00010020321	SZRD1	1.156366492	0.169440177	6.824629854	8.81523E-12	2.22336E-11	positive
PRJNA729271	stage_1W_vs_E10	ordinary_muscle_development	ENSGALG00010020323	ARHGAP25	0.791276149	0.155558167	5.086689848	3.64367E-07	7.01487E-07	positive
PRJNA729271	stage_1W_vs_E10	ordinary_muscle_development	ENSGALG00010020324	PLEKHH2	0.210897489	0.185738721	1.13545247	0.256185786	0.289657798	positive
PRJNA729271	stage_1W_vs_E10	ordinary_muscle_development	ENSGALG00010020329	PPP4R2	0.007120038	0.105870537	0.067252304	0.946380847	0.953153862	positive
PRJNA729271	stage_1W_vs_E10	ordinary_muscle_development	ENSGALG00010020334	CABP4	0.477284751	0.486007327	0.982052585	0.326073943	0.362484991	positive
PRJNA729271	stage_1W_vs_E10	ordinary_muscle_development	ENSGALG00010020336	OSBP	0.805290348	0.134256639	5.998141729	1.99588E-09	4.42224E-09	positive
PRJNA729271	stage_1W_vs_E10	ordinary_muscle_development	ENSGALG00010020338	NECAP2	-0.802595471	0.169200593	-4.743455423	2.10103E-06	3.82834E-06	negative
PRJNA729271	stage_1W_vs_E10	ordinary_muscle_development	ENSGALG00010020340	DCAF5	0.990155976	0.096060458	10.30763332	6.50843E-25	2.9779E-24	positive
PRJNA729271	stage_1W_vs_E10	ordinary_muscle_development	ENSGALG00010020341	DYNC2LI1	-1.407607575	0.13261559	-10.61419378	2.55995E-26	1.2456E-25	negative
PRJNA729271	stage_1W_vs_E10	ordinary_muscle_development	ENSGALG00010020342	CPPED1	-1.191627618	0.108475704	-10.98520288	4.50226E-28	2.35427E-27	negative
PRJNA729271	stage_1W_vs_E10	ordinary_muscle_development	ENSGALG00010020343	PATL1	1.151930466	0.174430032	6.603968679	4.00294E-11	9.75498E-11	positive
PRJNA729271	stage_1W_vs_E10	ordinary_muscle_development	ENSGALG00010020345	AGBL1	-1.31809379	0.457228064	-2.882792844	0.003941666	0.005553503	negative
PRJNA729271	stage_1W_vs_E10	ordinary_muscle_development	ENSGALG00010020346	PCBD1	-1.500059214	0.273492331	-5.484830997	4.13865E-08	8.47672E-08	negative
PRJNA729271	stage_1W_vs_E10	ordinary_muscle_development	ENSGALG00010020350	PDZRN3	0.340942648	0.108063552	3.155019816	0.001604872	0.002345968	positive
PRJNA729271	stage_1W_vs_E10	ordinary_muscle_development	ENSGALG00010020351	CROCC	-0.8123891	0.155014439	-5.240731797	1.59941E-07	3.1495E-07	negative
PRJNA729271	stage_1W_vs_E10	ordinary_muscle_development	ENSGALG00010020352	ABCG5	-0.571672441	0.639881236	-0.893403977	0.371640874	0.409248231	negative
PRJNA729271	stage_1W_vs_E10	ordinary_muscle_development	ENSGALG00010020356	STX3	-0.442225725	0.401070407	-1.102613701	0.270194958	0.304192304	negative
PRJNA729271	stage_1W_vs_E10	ordinary_muscle_development	ENSGALG00010020357	RBP4	1.845097336	0.727328723	2.536813519	0.011186653	0.015033426	positive
PRJNA729271	stage_1W_vs_E10	ordinary_muscle_development	ENSGALG00010020362	CDS2	-0.577027089	0.127602696	-4.522060329	6.12406E-06	1.07768E-05	negative
PRJNA729271	stage_1W_vs_E10	ordinary_muscle_development	ENSGALG00010020364	MRPL16	1.800502144	0.109799094	16.39815116	1.97147E-60	2.92492E-59	positive
PRJNA729271	stage_1W_vs_E10	ordinary_muscle_development	ENSGALG00010020365	UNC5B	-0.656385608	0.141298814	-4.645372394	3.39464E-06	6.10108E-06	negative
PRJNA729271	stage_1W_vs_E10	ordinary_muscle_development	ENSGALG00010020369	CNTN3	-4.878692252	0.38755479	-12.58839365	2.446E-36	1.73628E-35	negative
PRJNA729271	stage_1W_vs_E10	ordinary_muscle_development	ENSGALG00010020370	CEP55	-1.527372171	0.225151535	-6.783751983	1.17094E-11	2.93343E-11	negative
PRJNA729271	stage_1W_vs_E10	ordinary_muscle_development	ENSGALG00010020373	MS4A4A	-1.443184016	0.219342837	-6.579581252	4.71775E-11	1.14412E-10	negative
PRJNA729271	stage_1W_vs_E10	ordinary_muscle_development	ENSGALG00010020374	PCNA	-2.000817922	0.096745359	-20.68128068	5.1067E-95	1.72143E-93	negative
PRJNA729271	stage_1W_vs_E10	ordinary_muscle_development	ENSGALG00010020375	LRPPRC	0.550930835	0.101641442	5.420336644	5.94869E-08	1.20833E-07	positive
PRJNA729271	stage_1W_vs_E10	ordinary_muscle_development	ENSGALG00010020377	TMEM230	-0.306219574	0.100459383	-3.04819287	0.002302221	0.003314531	negative
PRJNA729271	stage_1W_vs_E10	ordinary_muscle_development	ENSGALG00010020379	COL13A1	-2.410852897	0.202016483	-11.93394155	7.87543E-33	4.93961E-32	negative
PRJNA729271	stage_1W_vs_E10	ordinary_muscle_development	ENSGALG00010020382	MFAP2	-3.12195104	0.151795038	-20.56688462	5.43469E-94	1.78226E-92	negative
PRJNA729271	stage_1W_vs_E10	ordinary_muscle_development	ENSGALG00010020383	BAAT	-0.282671466	0.10542202	-2.681332282	0.007332966	0.010054994	negative
PRJNA729271	stage_1W_vs_E10	ordinary_muscle_development	ENSGALG00010020385	SLC12A8	-3.204261328	0.485333688	-6.602181984	4.0515E-11	9.86502E-11	negative
PRJNA729271	stage_1W_vs_E10	ordinary_muscle_development	ENSGALG00010020386	PPM1B	1.052781758	0.071868603	14.64870211	1.37314E-48	1.43191E-47	positive
PRJNA729271	stage_1W_vs_E10	ordinary_muscle_development	ENSGALG00010020388	MYOF	1.390053862	0.166245303	8.361462495	6.19455E-17	2.0029E-16	positive
PRJNA729271	stage_1W_vs_E10	ordinary_muscle_development	ENSGALG00010020389	ACTN1	-1.487609184	0.157900602	-9.421174904	4.46068E-21	1.75129E-20	negative
PRJNA729271	stage_1W_vs_E10	ordinary_muscle_development	ENSGALG00010020390	KLHL25	1.109533776	0.222021843	4.99740818	5.8106E-07	1.10314E-06	positive
PRJNA729271	stage_1W_vs_E10	ordinary_muscle_development	ENSGALG00010020392	SLC23A2	-0.46684198	0.083708379	-5.577004152	2.44696E-08	5.07058E-08	negative
PRJNA729271	stage_1W_vs_E10	ordinary_muscle_development	ENSGALG00010020393	SHISA9	-2.756812587	0.328128282	-8.401630521	4.40321E-17	1.4304E-16	negative
PRJNA729271	stage_1W_vs_E10	ordinary_muscle_development	ENSGALG00010020396	CHL1	-0.245753803	0.193359721	-1.270966886	0.203740429	0.234122207	negative
PRJNA729271	stage_1W_vs_E10	ordinary_muscle_development	ENSGALG00010020399	ATP13A2	0.118183073	0.132111534	0.894570433	0.371016763	0.408592006	positive
PRJNA729271	stage_1W_vs_E10	ordinary_muscle_development	ENSGALG00010020402	SLC3A1	3.32569569	0.309575486	10.74276173	6.40978E-27	3.1862E-26	positive
PRJNA729271	stage_1W_vs_E10	ordinary_muscle_development	ENSGALG00010020405	UBE2I	-0.908353564	0.082336333	-11.0322325	2.6715E-28	1.40812E-27	negative
PRJNA729271	stage_1W_vs_E10	ordinary_muscle_development	ENSGALG00010020413	CYP26A1	0.764595251	0.31163198	2.453519858	0.014146573	0.01880717	positive
PRJNA729271	stage_1W_vs_E10	ordinary_muscle_development	ENSGALG00010020415	TSR3	0.364668089	0.091556312	3.982992331	6.8053E-05	0.000110947	positive
PRJNA729271	stage_1W_vs_E10	ordinary_muscle_development	ENSGALG00010020416	ENSGALG00010020416	-5.635861333	1.103478365	-5.107360064	3.26691E-07	6.30376E-07	negative
PRJNA729271	stage_1W_vs_E10	ordinary_muscle_development	ENSGALG00010020417	ENSGALG00010020417	2.50325088	0.766828354	3.264421388	0.001096879	0.001627476	positive
PRJNA729271	stage_1W_vs_E10	ordinary_muscle_development	ENSGALG00010020422	ERCC4	0.121077698	0.088323221	1.370847859	0.170422388	0.198207903	positive
PRJNA729271	stage_1W_vs_E10	ordinary_muscle_development	ENSGALG00010020428	SEMA4C	0.16039179	0.149959344	1.069568497	0.284813582	0.319434607	positive
PRJNA729271	stage_1W_vs_E10	ordinary_muscle_development	ENSGALG00010020429	ZNF148	0.064247659	0.10222478	0.628493975	0.529680393	0.566245099	positive
PRJNA729271	stage_1W_vs_E10	ordinary_muscle_development	ENSGALG00010020433	GNPTG	-0.176179429	0.071941713	-2.448919008	0.014328566	0.019029922	negative
PRJNA729271	stage_1W_vs_E10	ordinary_muscle_development	ENSGALG00010020436	CNTN4	-3.381106112	0.271499259	-12.45346351	1.33906E-35	9.30916E-35	negative
PRJNA729271	stage_1W_vs_E10	ordinary_muscle_development	ENSGALG00010020437	SNX4	-1.389143175	0.085732674	-16.20319422	4.78744E-59	6.8234E-58	negative
PRJNA729271	stage_1W_vs_E10	ordinary_muscle_development	ENSGALG00010020438	HTR7	-0.340494827	0.288479043	-1.180310443	0.237876766	0.270099447	negative
PRJNA729271	stage_1W_vs_E10	ordinary_muscle_development	ENSGALG00010020440	PAX7	-0.574550354	0.187674406	-3.061420934	0.002202892	0.003178787	negative
PRJNA729271	stage_1W_vs_E10	ordinary_muscle_development	ENSGALG00010020443	PREPL	0.307518235	0.136308577	2.256044645	0.024067835	0.031201437	positive
PRJNA729271	stage_1W_vs_E10	ordinary_muscle_development	ENSGALG00010020444	ENSGALG00010020444	-2.966924005	1.199661159	-2.473135004	0.013393354	0.017858216	negative

PRJNA729271	stage_1W_vs_E10	ordinary_muscle_development	ENSGALG00010020449	PRPF19	-0.377404908	0.097610405	-3.86644135	0.000110435	0.000177036	negative
PRJNA729271	stage_1W_vs_E10	ordinary_muscle_development	ENSGALG00010020450	ANKRD39	1.363233056	0.179458659	7.59636264	3.0457E-14	8.70072E-14	positive
PRJNA729271	stage_1W_vs_E10	ordinary_muscle_development	ENSGALG00010020453	ENSGALG00010020453	-1.316100136	0.148125265	-8.885048341	6.3893E-19	2.28505E-18	negative
PRJNA729271	stage_1W_vs_E10	ordinary_muscle_development	ENSGALG00010020455	ENSGALG00010020455	-0.499933517	0.710344092	-0.703790632	0.481563169	0.519885886	negative
PRJNA729271	stage_1W_vs_E10	ordinary_muscle_development	ENSGALG00010020457	IL5RA	-2.796693351	1.23645313	-2.261867662	0.023705581	0.03075657	negative
PRJNA729271	stage_1W_vs_E10	ordinary_muscle_development	ENSGALG00010020460	MAPK8	0.638738767	0.140684749	4.540213297	5.61973E-06	9.9206E-06	positive
PRJNA729271	stage_1W_vs_E10	ordinary_muscle_development	ENSGALG00010020462	CNNM4	1.030052406	0.180175014	5.716954778	1.0845E-08	2.29654E-08	positive
PRJNA729271	stage_1W_vs_E10	ordinary_muscle_development	ENSGALG00010020467	ALDH4A1	1.401794875	0.130323046	10.75630838	5.5343E-27	2.75859E-26	positive
PRJNA729271	stage_1W_vs_E10	ordinary_muscle_development	ENSGALG00010020468	ENSGALG00010020468	-1.199859361	0.571174398	-2.100688275	0.035668339	0.045296353	negative
PRJNA729271	stage_1W_vs_E10	ordinary_muscle_development	ENSGALG00010020473	TMEM109	3.644826552	0.158411704	23.00856853	3.8259E-117	1.9736E-115	positive
PRJNA729271	stage_1W_vs_E10	ordinary_muscle_development	ENSGALG00010020476	TMEM132A	-0.858758441	0.112463236	-7.63590374	2.24242E-14	6.45046E-14	negative
PRJNA729271	stage_1W_vs_E10	ordinary_muscle_development	ENSGALG00010020478	ENSGALG00010020478	1.311776149	0.725604342	1.807839442	0.070631493	0.086542983	positive
PRJNA729271	stage_1W_vs_E10	ordinary_muscle_development	ENSGALG00010020481	RPP30	-0.266748034	0.123526746	-2.15943545	0.0308164	0.039431826	negative
PRJNA729271	stage_1W_vs_E10	ordinary_muscle_development	ENSGALG00010020484	TRNT1	0.843859302	0.123541536	6.830571555	8.4577E-12	2.13579E-11	positive
PRJNA729271	stage_1W_vs_E10	ordinary_muscle_development	ENSGALG00010020485	LMAN2L	-0.04025571	0.131090515	-0.307083315	0.758779957	0.783035418	negative
PRJNA729271	stage_1W_vs_E10	ordinary_muscle_development	ENSGALG00010020486	MRTFB	1.685011343	0.111637901	15.09354197	1.78592E-51	2.05942E-50	positive
PRJNA729271	stage_1W_vs_E10	ordinary_muscle_development	ENSGALG00010020489	IFFO2	-1.431526283	0.18954462	-7.552450089	4.27145E-14	1.2133E-13	negative
PRJNA729271	stage_1W_vs_E10	ordinary_muscle_development	ENSGALG00010020493	DEPDC7	-0.671319885	0.153476153	-4.374098993	1.21935E-05	2.10285E-05	negative
PRJNA729271	stage_1W_vs_E10	ordinary_muscle_development	ENSGALG00010020497	OSBPL11	-0.672520793	0.070713542	-9.510495014	1.89757E-21	7.57305E-21	negative
PRJNA729271	stage_1W_vs_E10	ordinary_muscle_development	ENSGALG00010020498	FRMPD2	-1.042958978	0.572960512	-1.820298181	0.068713608	0.084314209	negative
PRJNA729271	stage_1W_vs_E10	ordinary_muscle_development	ENSGALG00010020507	ANKRD1	6.409364308	0.585305084	10.95046751	6.61055E-28	3.42826E-27	positive
PRJNA729271	stage_1W_vs_E10	ordinary_muscle_development	ENSGALG00010020509	CRBN	0.915944988	0.102693952	8.919171704	4.69789E-19	1.69224E-18	positive
PRJNA729271	stage_1W_vs_E10	ordinary_muscle_development	ENSGALG00010020514	CAMKMT	-1.493864374	0.143144405	-10.43606539	1.69699E-25	7.97081E-25	negative
PRJNA729271	stage_1W_vs_E10	ordinary_muscle_development	ENSGALG00010020517	UBR4	0.72261489	0.094098009	7.679385507	1.59854E-14	4.63601E-14	positive
PRJNA729271	stage_1W_vs_E10	ordinary_muscle_development	ENSGALG00010020520	ARHGAP22	-0.41714073	0.201529865	-2.069870541	0.038464469	0.048685163	negative
PRJNA729271	stage_1W_vs_E10	ordinary_muscle_development	ENSGALG00010020521	ENSGALG00010020521	0.975613391	0.697372225	1.398985157	0.161817434	0.188973873	positive
PRJNA729271	stage_1W_vs_E10	ordinary_muscle_development	ENSGALG00010020524	ENSGALG00010020524	-0.367027578	0.412988359	-0.888711679	0.374158053	0.41173861	negative
PRJNA729271	stage_1W_vs_E10	ordinary_muscle_development	ENSGALG00010020525	ENSGALG00010020525	0.190785162	0.723770077	0.263599129	0.79208883	0.814047617	positive
PRJNA729271	stage_1W_vs_E10	ordinary_muscle_development	ENSGALG00010020530	SRSF5A	-0.525713965	0.069474389	-7.56701822	3.81888E-14	1.08709E-13	negative
PRJNA729271	stage_1W_vs_E10	ordinary_muscle_development	ENSGALG00010020533	ENSGALG00010020533	-0.45538818	0.100230189	-4.54342336	5.53479E-06	9.77541E-06	negative
PRJNA729271	stage_1W_vs_E10	ordinary_muscle_development	ENSGALG00010020535	LMLN	-0.553714157	0.152497815	-3.630964525	0.000282364	0.000439904	negative
PRJNA729271	stage_1W_vs_E10	ordinary_muscle_development	ENSGALG00010020537	CCDC167	-1.126196516	0.101418005	-11.10450274	1.19279E-28	6.35412E-28	negative
PRJNA729271	stage_1W_vs_E10	ordinary_muscle_development	ENSGALG00010020540	PCGF5	1.615503996	0.161742841	9.988101989	1.7184E-23	7.42422E-23	positive
PRJNA729271	stage_1W_vs_E10	ordinary_muscle_development	ENSGALG00010020544	BMERB1	0.299126632	0.163258676	1.832225031	0.066917901	0.082236115	positive
PRJNA729271	stage_1W_vs_E10	ordinary_muscle_development	ENSGALG00010020545	STYX	1.970806877	0.140988063	13.9785372	2.10776E-44	1.93489E-43	positive
PRJNA729271	stage_1W_vs_E10	ordinary_muscle_development	ENSGALG00010020546	LRRC20	3.866573131	0.235847971	16.39434555	2.09889E-60	3.1076E-59	positive
PRJNA729271	stage_1W_vs_E10	ordinary_muscle_development	ENSGALG00010020548	CMTR1	-0.3569962	0.185418996	-1.925348572	0.054185765	0.06737199	negative
PRJNA729271	stage_1W_vs_E10	ordinary_muscle_development	ENSGALG00010020550	HECTD2	1.643296347	0.212319084	7.739748653	9.96135E-15	2.92346E-14	positive
PRJNA729271	stage_1W_vs_E10	ordinary_muscle_development	ENSGALG00010020554	ZFP36L1	0.621400411	0.210873583	2.946791165	0.0032109	0.004562046	positive
PRJNA729271	stage_1W_vs_E10	ordinary_muscle_development	ENSGALG00010020556	EMC1	0.097834954	0.085782474	1.140500497	0.254077846	0.287335256	positive
PRJNA729271	stage_1W_vs_E10	ordinary_muscle_development	ENSGALG00010020557	ENSGALG00010020557	2.556004327	0.257493079	9.926497195	3.19275E-23	1.36476E-22	positive
PRJNA729271	stage_1W_vs_E10	ordinary_muscle_development	ENSGALG00010020558	EIF4EBP2	0.885231706	0.099808165	8.869331531	7.35863E-19	2.61815E-18	positive
PRJNA729271	stage_1W_vs_E10	ordinary_muscle_development	ENSGALG00010020562	LRRN1	-6.25388992	0.259161029	-24.13128987	1.1739E-128	7.5623E-127	negative
PRJNA729271	stage_1W_vs_E10	ordinary_muscle_development	ENSGALG00010020568	PLA2G4F	0.186081449	0.398622931	0.466810698	0.640635321	0.672996738	positive
PRJNA729271	stage_1W_vs_E10	ordinary_muscle_development	ENSGALG00010020569	DTX3L	2.954378984	0.251077034	11.76682285	5.78629E-32	3.53146E-31	positive
PRJNA729271	stage_1W_vs_E10	ordinary_muscle_development	ENSGALG00010020571	MRTO4	-0.547614174	0.082374944	-6.647824531	2.97457E-11	7.29425E-11	negative
PRJNA729271	stage_1W_vs_E10	ordinary_muscle_development	ENSGALG00010020572	PPP1R3C	2.904615893	0.271900652	10.68263672	1.22735E-26	6.05118E-26	positive
PRJNA729271	stage_1W_vs_E10	ordinary_muscle_development	ENSGALG00010020574	DTL	-2.999241231	0.189479819	-15.82881626	1.96891E-56	2.62551E-55	negative
PRJNA729271	stage_1W_vs_E10	ordinary_muscle_development	ENSGALG00010020576	AKR7L	0.763578895	0.104143815	7.331965873	2.26801E-13	6.18645E-13	positive
PRJNA729271	stage_1W_vs_E10	ordinary_muscle_development	ENSGALG00010020578	TNKS2	0.242309736	0.063139057	3.837715489	0.000124184	0.000198287	positive
PRJNA729271	stage_1W_vs_E10	ordinary_muscle_development	ENSGALG00010020580	ENSGALG00010020580	-0.38461669	0.116297078	-3.307191359	0.000942365	0.001408349	negative
PRJNA729271	stage_1W_vs_E10	ordinary_muscle_development	ENSGALG00010020582	KANSL3	1.165990591	0.147792393	7.889381622	3.03688E-15	9.127E-15	positive
PRJNA729271	stage_1W_vs_E10	ordinary_muscle_development	ENSGALG00010020584	PARP9	2.655636311	0.288469507	9.205951567	3.38657E-20	1.28168E-19	positive
PRJNA729271	stage_1W_vs_E10	ordinary_muscle_development	ENSGALG00010020586	C11orf16	0.705387192	0.097528613	7.232617902	4.73771E-13	1.27314E-12	positive
PRJNA729271	stage_1W_vs_E10	ordinary_muscle_development	ENSGALG00010020588	PALD1	-0.721580112	0.109851043	-6.56871425	5.07516E-11	1.22751E-10	negative
PRJNA729271	stage_1W_vs_E10	ordinary_muscle_development	ENSGALG00010020590	INTS7	-0.883789781	0.079589405	-11.10436471	1.19464E-28	6.3616E-28	negative
PRJNA729271	stage_1W_vs_E10	ordinary_muscle_development	ENSGALG00010020594	PQLC2	0.354664689	0.148339178	2.390903697	0.016806961	0.022134917	positive
PRJNA729271	stage_1W_vs_E10	ordinary_muscle_development	ENSGALG00010020595	ZFYVE27	0.93611346	0.130665089	7.164220123	7.82306E-13	2.08065E-12	positive
PRJNA729271	stage_1W_vs_E10	ordinary_muscle_development	ENSGALG00010020596	SUMF1	0.716388453	0.120084536	5.965701121	2.43586E-09	5.36511E-09	positive
PRJNA729271	stage_1W_vs_E10	ordinary_muscle_development	ENSGALG00010020597	ARID5A	2.878767995	0.236638779	12.16524192	4.76033E-34	3.11798E-33	positive
PRJNA729271	stage_1W_vs_E10	ordinary_muscle_development	ENSGALG00010020599	PARN	-1.518096598	0.069844303	-21.73543927	9.4871E-105	3.8846E-103	negative
PRJNA729271	stage_1W_vs_E10	ordinary_muscle_development	ENSGALG00010020601	AKIP1	-0.770609146	0.170674488	-4.515081034	6.32926E-06	1.1123E-05	negative
PRJNA729271	stage_1W_vs_E10	ordinary_muscle_development	ENSGALG00010020602	DENND2B	-0.977465457	0.097991231	-9.975029868	1.96042E-23	8.44719E-23	negative
PRJNA729271	stage_1W_vs_E10	ordinary_muscle_development	ENSGALG00010020603	VPS39	0.399713258	0.093142269	4.291427088	1.77528E-05	3.02099E-05	positive
PRJNA729271	stage_1W_vs_E10	ordinary_muscle_development	ENSGALG00010020605	STAMBP	-0.129686215	0.088991209	-1.457292434	0.145035675	0.170498323	negative
PRJNA729271	stage_1W_vs_E10	ordinary_muscle_development	ENSGALG00010020607	ENSGALG00010020607	3.841210316	0.238503945	16.10543721	2.33641E-58	3.26579E-57	positive
PRJNA729271	stage_1W_vs_E10	ordinary_muscle_development	ENSGALG00010020608	CAPZB	1.716666634	0.099251925	17.2960538	5.03734E-67	8.79713E-66	positive
PRJNA729271	stage_1W_vs_E10	ordinary_muscle_development	ENSGALG00010020609	AVPI1	-0.066241497	0.233281815	-0.283954826	0.776445	0.799500623	negative
PRJNA729271	stage_1W_vs_E10	ordinary_muscle_development	ENSGALG00010020611	LPGAT1	-0.455581967	0.100187755	-4.547281936	5.43432E-06	9.6015E-06	negative
PRJNA729271	stage_1W_vs_E10	ordinary_muscle_development	ENSGALG00010020612	RPL27A	0.386544615	0.192790517	2.004998076	0.044963253	0.056486596	positive
PRJNA729271	stage_1W_vs_E10	ordinary_muscle_development	ENSGALG00010020613	ACTG2	0.11704789	0.254074496	0.460683351	0.645025801	0.676922169	positive
PRJNA729271	stage_1W_vs_E10	ordinary_muscle_development	ENSGALG00010020614	FAIM	-0.573447558	0.112084628	-5.116201661	3.1175E-07	6.02589E-07	negative
PRJNA729271	stage_1W_vs_E10	ordinary_muscle_development	ENSGALG00010020616	PI4K2A	1.312385376	0.130015355	10.09407983	5.86788E-24	2.58212E-23	positive
PRJNA729271	stage_1W_vs_E10	ordinary_muscle_development	ENSGALG00010020617	DGUOK	0.121995254	0.112360922	1.0857445	0.277592047	0.311962841	positive
PRJNA729271	stage_1W_vs_E10	ordinary_muscle_development	ENSGALG00010020618	ENSGALG00010020618	-0.386183962	0.330216813	-1.169486067	0.242207851	0.274689211	negative
PRJNA729271	stage_1W_vs_E10	ordinary_muscle_development	ENSGALG00010020619	QARS	0.581601767	0.099023306	5.873382628	4.26991E-09	9.26256E-09	positive
PRJNA729271	stage_1W_vs_E10	ordinary_muscle_development	ENSGALG00010020620	MARF1	0.201145904	0.103491504	1.943598241	0.051943918	0.064756781	positive
PRJNA729271	stage_1W_vs_E10	ordinary_muscle_development	ENSGALG00010020622	ABCG2	2.10966651	0.11616646	18.16071963	1.05631E-73	2.17489E-72	positive
PRJNA729271	stage_1W_vs_E10	ordinary_muscle_development	ENSGALG00010020623	NEK2	-1.815612221	0.223810904	-8.112259902	4.96869E-16	1.54917E-15	negative
PRJNA729271	stage_1W_vs_E10	ordinary_muscle_development	ENSGALG00010020624	MINOS1	0.988802979	0.135777495	7.282524807	3.2763E-13	8.86999E-13	positive
PRJNA729271	stage_1W_vs_E10	ordinary_muscle_development	ENSGALG00010020625	SLC30A1	-0.408446405	0.076909033	-5.31077286	1.09161E-07	2.17467E-07	negative
PRJNA729271	stage_1W_vs_E10	ordinary_muscle_development	ENSGALG00010020626	MORN4	-0.960609938	0.121134188	-7.930130689	2.18915E-15	6.62736E-15	negative
PRJNA729271	stage_1W_vs_E10	ordinary_muscle_development	ENSGALG00010020629	TRIM66	-3.894271158	0.267522626	-14.55679175	5.2882E-48	5.40567E-47	negative
PRJNA729271	stage_1W_vs_E10	ordinary_muscle_development	ENSGALG00010020630	QRICH1	0.410065855	0.125798114	3.259713845	0.001115247	0.00165207	positive
PRJNA729271	stage_1W_vs_E10	ordinary_muscle_development	ENSGALG00010020632	BLOC1S2	-0.217777299	0.113592429	-1.917181455	0.055214883	0.068563328	negative
PRJNA729271	stage_1W_vs_E10	ordinary_muscle_development	ENSGALG00010020633	HOGA1	-0.539577552	0.369838881	-1.458953016	0.14457804	0.170005572	negative
PRJNA729271	stage_1W_vs_E10	ordinary_muscle_development	ENSGALG00010020635	NDE1	-0.589342692	0.134424336	-4.384196416	1.16415E-05	2.01172E-05	negative
PRJNA729271	stage_1W_vs_E10	ordinary_muscle_development	ENSGALG00010020636	NBL1	-1.051445019	0.178935032	-5.876127261	4.19976E-09	9.11719E-09	negative
PRJNA729271	stage_1W_vs_E10	ordinary_muscle_development	ENSGALG00010020638	IMPDH2	1.66071644	0.111283422	14.92330497	2.32468E-50	2.58407E-49	positive
PRJNA729271	stage_1W_vs_E10	ordinary_muscle_development	ENSGALG00010020640	PTPN14	-0.364450509	0.09270762	-3.931181792	8.45293E-05	0.000136793	negative
PRJNA729271	stage_1W_vs_E10	ordinary_muscle_development	ENSGALG00010020642	ANKRD2	2.310523271	0.641181181	3.603541929	0.00031391	0.000487374	positive
PRJNA729271	stage_1W_vs_E10	ordinary_muscle_development	ENSGALG00010020646	CEP70	-0.470659465	0.116573765	-4.037439002	5.40379E-05	8.86564E-05	negative
PRJNA729271	stage_1W_vs_E10	ordinary_muscle_development	ENSGALG00010020648	TET3	-0.533727927	0.150046097	-3.557093049	0.000374981	0.000577722	negative
PRJNA729271	stage_1W_vs_E10	ordinary_muscle_development	ENSGALG00010020649	MYH11	3.564124835	0.212056747	16.80741069	2.15376E-63	3.44197E-62	positive
PRJNA729271	stage_1W_vs_E10	ordinary_muscle_development	ENSGALG00010020651	LMO1	-1.061968189	0.196135222	-5.414469555	6.14707E-08	1.24757E-07	negative
PRJNA729271	stage_1W_vs_E10	ordinary_muscle_development	ENSGALG00010020653	NDUFAF3	1.729881779	0.116375056	14.8647127	5.5854E-50	6.13336E-49	positive
PRJNA729271	stage_1W_vs_E10	ordinary_muscle_development	ENSGALG00010020654	SAYSD1	0.08053726	0.149426535	0.538975623	0.589903675	0.624865081	positive
PRJNA729271	stage_1W_vs_E10	ordinary_muscle_development	ENSGALG00010020655	RIC3	-1.778575329	0.141972869	-12.52757196	5.27531E-36	3.72095E-35	negative
PRJNA729271	stage_1W_vs_E10	ordinary_muscle_development	ENSGALG00010020657	DALRD3	0.882585164	0.091934994	9.600100279	7.98667E-22	3.22201E-21	positive
PRJNA729271	stage_1W_vs_E10	ordinary_muscle_development	ENSGALG00010020659	RAD51B	-1.294703222	0.141296394	-9.163030882	5.04598E-20	1.89584E-19	negative
PRJNA729271	stage_1W_vs_E10	ordinary_muscle_development	ENSGALG00010020662	BFAR	-0.392754385	0.088662533	-4.429767261	9.43348E-06	1.64014E-05	negative
PRJNA729271	stage_1W_vs_E10	ordinary_muscle_development	ENSGALG00010020664	ESYT3	1.315284484	0.438701864	2.998128325	0.002716433	0.003885405	positive
PRJNA729271	stage_1W_vs_E10	ordinary_muscle_development	ENSGALG00010020666	TMCO4	0.982001265	0.2064887	4.755714298	1.97746E-06	3.61454E-06	positive
PRJNA729271	stage_1W_vs_E10	ordinary_muscle_development	ENSGALG00010020667	UBTD1	0.110311012	0.15629754	0.70577574	0.480327611	0.518649339	positive
PRJNA729271	stage_1W_vs_E10	ordinary_muscle_development	ENSGALG00010020670	ENSGALG00010020670	0.003369119	0.132674321	0.025393907	0.979740771	0.982859884	positive
PRJNA729271	stage_1W_vs_E10	ordinary_muscle_development	ENSGALG00010020673	MMS19	0.209872465	0.127481912	1.6462921	0.099703635	0.119943912	positive
PRJNA729271	stage_1W_vs_E10	ordinary_muscle_development	ENSGALG00010020674	TRPV4	-8.662038779	0.94523584	-9.163891605	5.00587E-20	1.88223E-19	negative
PRJNA729271	stage_1W_vs_E10	ordinary_muscle_development	ENSGALG00010020675	SMYD2	-1.848602739	0.183804538	-10.05743799	8.51863E-24	3.72819E-23	negative
PRJNA729271	stage_1W_vs_E10	ordinary_muscle_development	ENSGALG00010020676	CYP2C23a	7.089890616	1.374902537	5.156649601	2.51408E-07	4.89148E-07	positive
PRJNA729271	stage_1W_vs_E10	ordinary_muscle_development	ENSGALG00010020677	CYR61	1.125038511	0.233017881	4.828120937	1.37827E-06	2.54822E-06	positive
PRJNA729271	stage_1W_vs_E10	ordinary_muscle_development	ENSGALG00010020679	OTUD3	0.222663159	0.108996642	2.042844208	0.041067863	0.051821944	positive
PRJNA729271	stage_1W_vs_E10	ordinary_muscle_development	ENSGALG00010020680	CFAP221	-0.227495636	0.55432323	-0.410402494	0.681510717	0.710939032	negative
PRJNA729271	stage_1W_vs_E10	ordinary_muscle_development	ENSGALG00010020681	BCL10	-0.336436755	0.088774442	-3.789792955	0.000150773	0.000239475	negative
PRJNA729271	stage_1W_vs_E10	ordinary_muscle_development	ENSGALG00010020682	ZDHHC16	1.219154277	0.166321082	7.330124739	2.29939E-13	6.27086E-13	positive
PRJNA729271	stage_1W_vs_E10	ordinary_muscle_development	ENSGALG00010020684	P4HTM	1.533763919	0.119653457	12.81838364	1.29369E-37	9.54305E-37	positive
PRJNA729271	stage_1W_vs_E10	ordinary_muscle_development	ENSGALG00010020685	RPL6	0.561508936	0.116024069	4.839590094	1.30107E-06	2.40887E-06	positive
PRJNA729271	stage_1W_vs_E10	ordinary_muscle_development	ENSGALG00010020686	C1orf52	0.490288147	0.121712445	4.028249927	5.61936E-05	9.20889E-05	positive
PRJNA729271	stage_1W_vs_E10	ordinary_muscle_development	ENSGALG00010020691	ENSGALG00010020691	1.721844563	0.162381514	10.60369815	2.86425E-26	1.3904E-25	positive
PRJNA729271	stage_1W_vs_E10	ordinary_muscle_development	ENSGALG00010020692	TUB	-0.929839598	0.155194941	-5.991429814	2.08004E-09	4.60027E-09	negative
PRJNA729271	stage_1W_vs_E10	ordinary_muscle_development	ENSGALG00010020693	PTGFR	-0.755902504	0.191214492	-3.953165349	7.71241E-05	0.000125214	negative
PRJNA729271	stage_1W_vs_E10	ordinary_muscle_development	ENSGALG00010020696	MOB1A	-0.866886775	0.102072057	-8.49289026	2.01561E-17	6.65972E-17	negative
PRJNA729271	stage_1W_vs_E10	ordinary_muscle_development	ENSGALG00010020699	PLA2G10	0.3744065	0.411048408	0.910857438	0.362370494	0.399951292	positive
PRJNA729271	stage_1W_vs_E10	ordinary_muscle_development	ENSGALG00010020700	MTHFD2	2.413470514	0.130783117	18.45399142	4.84322E-76	1.04546E-74	positive
PRJNA729271	stage_1W_vs_E10	ordinary_muscle_development	ENSGALG00010020701	PTPN11	-0.89469527	0.085722004	-10.4371717	1.67734E-25	7.88104E-25	negative
PRJNA729271	stage_1W_vs_E10	ordinary_muscle_development	ENSGALG00010020704	PROX1	0.08103049	0.24141754	0.335644583	0.7371389	0.76298403	positive
PRJNA729271	stage_1W_vs_E10	ordinary_muscle_development	ENSGALG00010020705	EXOSC1	2.194928201	0.172559506	12.71983362	4.58836E-37	3.32541E-36	positive
PRJNA729271	stage_1W_vs_E10	ordinary_muscle_development	ENSGALG00010020707	SLC4A5	-1.071726504	0.421112842	-2.544986518	0.010928196	0.014707911	negative
PRJNA729271	stage_1W_vs_E10	ordinary_muscle_development	ENSGALG00010020708	FAM214A	0.36547767	0.133923162	2.729010157	0.006352475	0.00876944	positive
PRJNA729271	stage_1W_vs_E10	ordinary_muscle_development	ENSGALG00010020712	ZC3H7A	-0.926110254	0.071686391	-12.91891308	3.5208E-38	2.64425E-37	negative
PRJNA729271	stage_1W_vs_E10	ordinary_muscle_development	ENSGALG00010020713	SCTR	-2.725145022	1.218013352	-2.23736876	0.025262249	0.032662233	negative
PRJNA729271	stage_1W_vs_E10	ordinary_muscle_development	ENSGALG00010020714	PGAM1	5.684299859	0.144436455	39.35502205	0	0	positive

PRJNA729271	stage_1W_vs_E10	ordinary_muscle_development	ENSGALG00010020715	ENSGALG00010020715	-2.02479595	1.0592362	-1.911562265	0.055932363	0.069418581	negative
PRJNA729271	stage_1W_vs_E10	ordinary_muscle_development	ENSGALG00010020716	RRAS	0.563478399	0.179410545	3.140720618	0.001685327	0.002460102	positive
PRJNA729271	stage_1W_vs_E10	ordinary_muscle_development	ENSGALG00010020719	RPH3A	-1.986064	0.67004188	-2.964089347	0.003035801	0.004324712	negative
PRJNA729271	stage_1W_vs_E10	ordinary_muscle_development	ENSGALG00010020720	ENSGALG00010020720	-5.001869801	0.458286914	-10.91427585	9.85112E-28	5.07254E-27	negative
PRJNA729271	stage_1W_vs_E10	ordinary_muscle_development	ENSGALG00010020721	PTDSS2	0.625133985	0.169875125	3.67996189	0.000233269	0.000365539	positive
PRJNA729271	stage_1W_vs_E10	ordinary_muscle_development	ENSGALG00010020723	RPS24	0.036095416	0.19012101	0.189854958	0.849422788	0.866093368	positive
PRJNA729271	stage_1W_vs_E10	ordinary_muscle_development	ENSGALG00010020724	TACR1	-3.4649102	0.288808496	-11.99725857	3.6726E-33	2.33382E-32	negative
PRJNA729271	stage_1W_vs_E10	ordinary_muscle_development	ENSGALG00010020725	FOPNL	-0.690705011	0.112047555	-6.164391654	7.07546E-10	1.60248E-09	negative
PRJNA729271	stage_1W_vs_E10	ordinary_muscle_development	ENSGALG00010020728	TMEM37	1.456003999	0.20899541	6.966679304	3.24509E-12	8.36968E-12	positive
PRJNA729271	stage_1W_vs_E10	ordinary_muscle_development	ENSGALG00010020730	RRP12	0.680189301	0.461751393	1.473063884	0.140733811	0.165740704	positive
PRJNA729271	stage_1W_vs_E10	ordinary_muscle_development	ENSGALG00010020732	ARIH2	1.782395236	0.142725106	12.48830907	8.6472E-36	6.06391E-35	positive
PRJNA729271	stage_1W_vs_E10	ordinary_muscle_development	ENSGALG00010020733	TMEM229B	-1.726929401	0.111781378	-15.44916902	7.64223E-54	9.54127E-53	negative
PRJNA729271	stage_1W_vs_E10	ordinary_muscle_development	ENSGALG00010020736	LRRTM4	-0.609518872	0.231149762	-2.63690028	0.008366742	0.0114024	negative
PRJNA729271	stage_1W_vs_E10	ordinary_muscle_development	ENSGALG00010020737	POLR3A	0.014770879	0.11589423	0.127451372	0.898583159	0.91033839	positive
PRJNA729271	stage_1W_vs_E10	ordinary_muscle_development	ENSGALG00010020739	PKP3	-4.680077419	0.775365229	-6.035965042	1.58015E-09	3.52104E-09	negative
PRJNA729271	stage_1W_vs_E10	ordinary_muscle_development	ENSGALG00010020742	THAP10	-1.962358241	0.222093814	-8.83571768	9.94577E-19	3.51448E-18	negative
PRJNA729271	stage_1W_vs_E10	ordinary_muscle_development	ENSGALG00010020743	PURB	0.88505888	0.167973553	5.269037072	1.37141E-07	2.71306E-07	positive
PRJNA729271	stage_1W_vs_E10	ordinary_muscle_development	ENSGALG00010020744	CHST3	-1.745857806	0.120011017	-14.54747951	6.05948E-48	6.18537E-47	negative
PRJNA729271	stage_1W_vs_E10	ordinary_muscle_development	ENSGALG00010020745	ABCC1	0.962013978	0.166378515	5.78208055	7.37824E-09	1.57716E-08	positive
PRJNA729271	stage_1W_vs_E10	ordinary_muscle_development	ENSGALG00010020747	DBI	-0.240502303	0.12116628	-1.984894659	0.047156193	0.059057285	negative
PRJNA729271	stage_1W_vs_E10	ordinary_muscle_development	ENSGALG00010020750	AIFM2	1.214367751	0.28502411	4.260579047	2.03898E-05	3.44987E-05	positive
PRJNA729271	stage_1W_vs_E10	ordinary_muscle_development	ENSGALG00010020753	SYDE2	1.829696135	0.095132812	19.233071	1.9569E-82	4.95895E-81	positive
PRJNA729271	stage_1W_vs_E10	ordinary_muscle_development	ENSGALG00010020754	SLC25A20	-0.062941694	0.114833171	-0.548114222	0.583613476	0.618971049	negative
PRJNA729271	stage_1W_vs_E10	ordinary_muscle_development	ENSGALG00010020756	MACROH2A2	-4.58468962	0.171391616	-26.74978934	1.2418E-157	1.2E-155	negative
PRJNA729271	stage_1W_vs_E10	ordinary_muscle_development	ENSGALG00010020759	ARHGAP19	-2.555793417	0.17436947	-14.65734468	1.2091E-48	1.26267E-47	negative
PRJNA729271	stage_1W_vs_E10	ordinary_muscle_development	ENSGALG00010020760	TXNDC11	-0.634721732	0.097180059	-6.531398896	6.51582E-11	1.56136E-10	negative
PRJNA729271	stage_1W_vs_E10	ordinary_muscle_development	ENSGALG00010020762	TTBK2	-0.300827467	0.142933599	-2.104665863	0.035320404	0.044905645	negative
PRJNA729271	stage_1W_vs_E10	ordinary_muscle_development	ENSGALG00010020764	PRKAR2A	0.034126323	0.09898857	0.34475014	0.73028222	0.756751557	positive
PRJNA729271	stage_1W_vs_E10	ordinary_muscle_development	ENSGALG00010020765	GLT1D1	-3.400098658	0.244783391	-13.89023429	7.25975E-44	6.57277E-43	negative
PRJNA729271	stage_1W_vs_E10	ordinary_muscle_development	ENSGALG00010020766	C2orf76	-0.185930017	0.123051916	-1.51098839	0.130791409	0.154659934	negative
PRJNA729271	stage_1W_vs_E10	ordinary_muscle_development	ENSGALG00010020767	UBXN10	-0.328787964	0.176362087	-1.864278032	0.06228267	0.076819886	negative
PRJNA729271	stage_1W_vs_E10	ordinary_muscle_development	ENSGALG00010020768	DNAH8	-0.796619871	0.701909421	-1.13493258	0.256403568	0.289847526	negative
PRJNA729271	stage_1W_vs_E10	ordinary_muscle_development	ENSGALG00010020770	B4GALNT4	-4.951397114	0.207857876	-23.82107053	2.0204E-125	1.2409E-123	negative
PRJNA729271	stage_1W_vs_E10	ordinary_muscle_development	ENSGALG00010020771	LINGO1	3.451624284	0.199915533	17.26541321	8.56883E-67	1.47687E-65	positive
PRJNA729271	stage_1W_vs_E10	ordinary_muscle_development	ENSGALG00010020773	ZCCHC24	0.892324	0.089474566	9.972934641	2.00224E-23	8.62223E-23	positive
PRJNA729271	stage_1W_vs_E10	ordinary_muscle_development	ENSGALG00010020775	UBR1	1.082198409	0.080924648	13.37291458	8.70614E-41	7.14584E-40	positive
PRJNA729271	stage_1W_vs_E10	ordinary_muscle_development	ENSGALG00010020776	MCOLN3	-0.373382302	0.449944794	-0.829840254	0.406629108	0.4446691	negative
PRJNA729271	stage_1W_vs_E10	ordinary_muscle_development	ENSGALG00010020778	SLIT1	-0.524391152	0.213193835	-2.459691908	0.013905633	0.018519125	negative
PRJNA729271	stage_1W_vs_E10	ordinary_muscle_development	ENSGALG00010020779	SLC15A4	0.119923729	0.065718836	1.824799952	0.068031223	0.083533467	positive
PRJNA729271	stage_1W_vs_E10	ordinary_muscle_development	ENSGALG00010020781	SNN	0.008256566	0.13703706	0.060250605	0.951956042	0.958236308	positive
PRJNA729271	stage_1W_vs_E10	ordinary_muscle_development	ENSGALG00010020782	PPIF	-0.085787384	0.158175676	-0.542355095	0.587573908	0.622772157	negative
PRJNA729271	stage_1W_vs_E10	ordinary_muscle_development	ENSGALG00010020786	DDX19	-0.246772537	0.131380401	-1.878305553	0.060339377	0.074562011	negative
PRJNA729271	stage_1W_vs_E10	ordinary_muscle_development	ENSGALG00010020787	TMEM132C	-3.991691594	0.306348267	-13.02991407	8.27007E-39	6.36278E-38	negative
PRJNA729271	stage_1W_vs_E10	ordinary_muscle_development	ENSGALG00010020790	PRPH2L	2.371289374	0.307583185	7.709424609	1.26386E-14	3.68754E-14	positive
PRJNA729271	stage_1W_vs_E10	ordinary_muscle_development	ENSGALG00010020794	STEAP3	0.369267623	0.140588175	2.626590912	0.008624495	0.011743735	positive
PRJNA729271	stage_1W_vs_E10	ordinary_muscle_development	ENSGALG00010020797	DGCR8	-0.751332754	0.066569668	-11.28641276	1.53161E-29	8.48649E-29	negative
PRJNA729271	stage_1W_vs_E10	ordinary_muscle_development	ENSGALG00010020798	C1QL2	-1.902705543	0.593779703	-3.204396399	0.00135346	0.001990503	negative
PRJNA729271	stage_1W_vs_E10	ordinary_muscle_development	ENSGALG00010020803	ABCC6	2.067617338	0.15743687	13.13299316	2.13065E-39	1.67301E-38	positive
PRJNA729271	stage_1W_vs_E10	ordinary_muscle_development	ENSGALG00010020806	HTD2	0.759840286	0.172640151	4.401295316	1.07607E-05	1.86395E-05	positive
PRJNA729271	stage_1W_vs_E10	ordinary_muscle_development	ENSGALG00010020809	ENSGALG00010020809	0.515197236	0.221472982	2.32623064	0.02000625	0.026151194	positive
PRJNA729271	stage_1W_vs_E10	ordinary_muscle_development	ENSGALG00010020810	IFITM5	1.007263009	0.242350448	4.156225068	3.23549E-05	5.40678E-05	positive
PRJNA729271	stage_1W_vs_E10	ordinary_muscle_development	ENSGALG00010020811	MCOLN2	0.414998915	0.661335913	0.627516072	0.530321004	0.566846321	positive
PRJNA729271	stage_1W_vs_E10	ordinary_muscle_development	ENSGALG00010020813	NPY4R	-0.505236599	0.552317903	-0.914756874	0.36031928	0.397868638	negative
PRJNA729271	stage_1W_vs_E10	ordinary_muscle_development	ENSGALG00010020816	TRMT2A	0.548244468	0.089238815	6.143565058	8.06896E-10	1.82237E-09	positive
PRJNA729271	stage_1W_vs_E10	ordinary_muscle_development	ENSGALG00010020818	TMEM62	0.732140444	0.195426335	3.746375546	0.000179408	0.000283343	positive
PRJNA729271	stage_1W_vs_E10	ordinary_muscle_development	ENSGALG00010020820	VWA5B1	5.24272E-06	0.444174449	1.18033E-05	0.999990582	0.999990582	positive
PRJNA729271	stage_1W_vs_E10	ordinary_muscle_development	ENSGALG00010020827	ENSGALG00010020827	-0.385089414	0.092017802	-4.184944698	2.85236E-05	4.77976E-05	negative
PRJNA729271	stage_1W_vs_E10	ordinary_muscle_development	ENSGALG00010020828	CAMK2N1	-0.599133806	0.231823694	-2.584437323	0.009753803	0.013204574	negative
PRJNA729271	stage_1W_vs_E10	ordinary_muscle_development	ENSGALG00010020831	MUL1	1.512165206	0.142519718	10.61021747	2.67127E-26	1.29802E-25	positive
PRJNA729271	stage_1W_vs_E10	ordinary_muscle_development	ENSGALG00010020833	RANBP1	-2.401982946	0.084323328	-28.48539064	1.7771E-178	2.1289E-176	negative
PRJNA729271	stage_1W_vs_E10	ordinary_muscle_development	ENSGALG00010020835	PTGR2	-0.409490784	0.115717726	-3.538704037	0.000402096	0.000617938	negative
PRJNA729271	stage_1W_vs_E10	ordinary_muscle_development	ENSGALG00010020839	PGGHG	-1.439722965	0.150949933	-9.537751621	1.45962E-21	5.85101E-21	negative
PRJNA729271	stage_1W_vs_E10	ordinary_muscle_development	ENSGALG00010020840	LPAR3	-4.00261689	0.371274497	-10.78074825	4.24419E-27	2.12723E-26	negative
PRJNA729271	stage_1W_vs_E10	ordinary_muscle_development	ENSGALG00010020843	HMG20A	-1.595154027	0.085224754	-18.71702704	3.59674E-78	8.14605E-77	negative
PRJNA729271	stage_1W_vs_E10	ordinary_muscle_development	ENSGALG00010020845	RAB35	0.062115788	0.092802873	0.669330439	0.503284714	0.54097975	positive
PRJNA729271	stage_1W_vs_E10	ordinary_muscle_development	ENSGALG00010020847	MIDEAS	-0.147967339	0.090830823	-1.629043259	0.103303862	0.12400741	negative
PRJNA729271	stage_1W_vs_E10	ordinary_muscle_development	ENSGALG00010020854	ENSGALG00010020854	12.3025691	0.380981943	32.29173806	9.1363E-229	1.8141E-226	positive
PRJNA729271	stage_1W_vs_E10	ordinary_muscle_development	ENSGALG00010020855	DNAL1	0.081310684	0.100268779	0.810927238	0.417407457	0.455459282	positive
PRJNA729271	stage_1W_vs_E10	ordinary_muscle_development	ENSGALG00010020856	VTG2	-5.186489558	0.986898223	-5.255343904	1.47748E-07	2.91892E-07	negative
PRJNA729271	stage_1W_vs_E10	ordinary_muscle_development	ENSGALG00010020861	CDA	1.219751345	0.177967742	6.85377772	7.19248E-12	1.82392E-11	positive
PRJNA729271	stage_1W_vs_E10	ordinary_muscle_development	ENSGALG00010020865	BICDL1	-3.64338526	1.048584069	-3.474576208	0.000511661	0.000779456	negative
PRJNA729271	stage_1W_vs_E10	ordinary_muscle_development	ENSGALG00010020866	LCOR	-0.411281896	0.098516127	-4.174767187	2.98291E-05	4.99449E-05	negative
PRJNA729271	stage_1W_vs_E10	ordinary_muscle_development	ENSGALG00010020870	RIOX1	0.868049148	0.146260061	5.934970523	2.93898E-09	6.44283E-09	positive
PRJNA729271	stage_1W_vs_E10	ordinary_muscle_development	ENSGALG00010020873	PINK1	3.078279666	0.136158806	22.6080102	3.6147E-113	1.7235E-111	positive
PRJNA729271	stage_1W_vs_E10	ordinary_muscle_development	ENSGALG00010020875	ENSGALG00010020875	0.940344594	0.172462326	5.452463825	4.96767E-08	1.01389E-07	positive
PRJNA729271	stage_1W_vs_E10	ordinary_muscle_development	ENSGALG00010020878	XYLT1	-1.106570877	0.126510676	-8.746857698	2.19376E-18	7.59822E-18	negative
PRJNA729271	stage_1W_vs_E10	ordinary_muscle_development	ENSGALG00010020880	PXK	1.972306827	0.164483628	11.99090053	3.9657E-33	2.51897E-32	positive
PRJNA729271	stage_1W_vs_E10	ordinary_muscle_development	ENSGALG00010020886	GBF1	-0.29084864	0.086407197	-3.366023317	0.000762603	0.001148102	negative
PRJNA729271	stage_1W_vs_E10	ordinary_muscle_development	ENSGALG00010020887	ARPP19	-1.000559655	0.071341351	-14.02496087	1.09673E-44	1.01774E-43	negative
PRJNA729271	stage_1W_vs_E10	ordinary_muscle_development	ENSGALG00010020889	PPRC1	0.888525795	0.280662095	3.165820433	0.001546462	0.002263554	positive
PRJNA729271	stage_1W_vs_E10	ordinary_muscle_development	ENSGALG00010020890	RPS15A	-0.123318021	0.188383019	-0.654613254	0.512716788	0.550098434	negative
PRJNA729271	stage_1W_vs_E10	ordinary_muscle_development	ENSGALG00010020894	PSMD13	0.751204329	0.120759613	6.22065865	4.95072E-10	1.13116E-09	positive
PRJNA729271	stage_1W_vs_E10	ordinary_muscle_development	ENSGALG00010020896	MARCO	3.597559615	0.391279548	9.194346176	3.77282E-20	1.4234E-19	positive
PRJNA729271	stage_1W_vs_E10	ordinary_muscle_development	ENSGALG00010020898	ENSGALG00010020898	0.345239758	0.127848185	2.700388407	0.006925857	0.009529216	positive
PRJNA729271	stage_1W_vs_E10	ordinary_muscle_development	ENSGALG00010020900	PDHB	0.644387859	0.116063344	5.552035969	2.82361E-08	5.82858E-08	positive
PRJNA729271	stage_1W_vs_E10	ordinary_muscle_development	ENSGALG00010020903	AGMAT	2.280512496	0.376579918	6.055852656	1.39676E-09	3.12101E-09	positive
PRJNA729271	stage_1W_vs_E10	ordinary_muscle_development	ENSGALG00010020905	KCTD6	-0.346878233	0.088036677	-3.940155905	8.14287E-05	0.000131981	negative
PRJNA729271	stage_1W_vs_E10	ordinary_muscle_development	ENSGALG00010020906	SIRT3	1.766476973	0.108597892	16.26621785	1.71424E-59	2.46753E-58	positive
PRJNA729271	stage_1W_vs_E10	ordinary_muscle_development	ENSGALG00010020912	PDE8A	1.286491472	0.14372306	8.951183407	3.51691E-19	1.27285E-18	positive
PRJNA729271	stage_1W_vs_E10	ordinary_muscle_development	ENSGALG00010020913	TMEM132B	-3.205322222	0.274471433	-11.67816334	1.64818E-31	9.90892E-31	negative
PRJNA729271	stage_1W_vs_E10	ordinary_muscle_development	ENSGALG00010020915	EN1	-5.129426646	0.439445189	-11.67250608	1.76156E-31	1.0573E-30	negative
PRJNA729271	stage_1W_vs_E10	ordinary_muscle_development	ENSGALG00010020916	ARL6IP1	-0.494462039	0.098242572	-5.033073034	4.82679E-07	9.20947E-07	negative
PRJNA729271	stage_1W_vs_E10	ordinary_muscle_development	ENSGALG00010020918	ENSGALG00010020918	-3.939365689	0.374998716	-10.50501114	8.19131E-26	3.898E-25	negative
PRJNA729271	stage_1W_vs_E10	ordinary_muscle_development	ENSGALG00010020920	ACOX2	2.288688519	0.205248243	11.15083126	7.09405E-29	3.81269E-28	positive
PRJNA729271	stage_1W_vs_E10	ordinary_muscle_development	ENSGALG00010020921	LDB1	-0.085152891	0.150753189	-0.564849683	0.57217601	0.607863623	negative
PRJNA729271	stage_1W_vs_E10	ordinary_muscle_development	ENSGALG00010020922	INSIG2	0.013724258	0.080648847	0.170173021	0.864874068	0.879743833	positive
PRJNA729271	stage_1W_vs_E10	ordinary_muscle_development	ENSGALG00010020924	DNAJC16	1.085202969	0.119396462	9.089071384	9.98885E-20	3.70872E-19	positive
PRJNA729271	stage_1W_vs_E10	ordinary_muscle_development	ENSGALG00010020925	RIC8A	0.000591401	0.104049861	0.005683828	0.995464986	0.996358581	positive
PRJNA729271	stage_1W_vs_E10	ordinary_muscle_development	ENSGALG00010020927	ENSGALG00010020927	-0.639714917	0.1190391	-5.373989859	7.70132E-08	1.55258E-07	negative
PRJNA729271	stage_1W_vs_E10	ordinary_muscle_development	ENSGALG00010020928	AACS	-1.214507592	0.15269183	-7.953978866	1.80615E-15	5.4885E-15	negative
PRJNA729271	stage_1W_vs_E10	ordinary_muscle_development	ENSGALG00010020932	CASP9	-0.144227435	0.12396383	-1.163463852	0.244641356	0.277275507	negative
PRJNA729271	stage_1W_vs_E10	ordinary_muscle_development	ENSGALG00010020934	ABCD3	0.236021416	0.153679129	1.535806572	0.124585827	0.147864699	positive
PRJNA729271	stage_1W_vs_E10	ordinary_muscle_development	ENSGALG00010020935	BRI3BP	-0.167958466	0.065511064	-2.56381831	0.010352772	0.013972387	negative
PRJNA729271	stage_1W_vs_E10	ordinary_muscle_development	ENSGALG00010020936	BET1L	-0.387256184	0.126566283	-3.059710497	0.00221551	0.003195405	negative
PRJNA729271	stage_1W_vs_E10	ordinary_muscle_development	ENSGALG00010020938	SMG1	0.103721795	0.08011887	1.294598823	0.195458659	0.225266221	positive
PRJNA729271	stage_1W_vs_E10	ordinary_muscle_development	ENSGALG00010020939	CTRC	8.500848305	2.112388054	4.02428346	5.7149E-05	9.36123E-05	positive
PRJNA729271	stage_1W_vs_E10	ordinary_muscle_development	ENSGALG00010020940	ENSGALG00010020940	-1.239491442	0.135427143	-9.15245948	5.56518E-20	2.08658E-19	negative
PRJNA729271	stage_1W_vs_E10	ordinary_muscle_development	ENSGALG00010020941	ENSGALG00010020941	2.741717475	0.183902494	14.90853884	2.9003E-50	3.20915E-49	positive
PRJNA729271	stage_1W_vs_E10	ordinary_muscle_development	ENSGALG00010020943	FAM107A	0.708808309	0.37795693	1.875367937	0.060742123	0.075034692	positive
PRJNA729271	stage_1W_vs_E10	ordinary_muscle_development	ENSGALG00010020944	MYO5A	-1.619225697	0.157551141	-10.27746095	8.90424E-25	4.05616E-24	negative
PRJNA729271	stage_1W_vs_E10	ordinary_muscle_development	ENSGALG00010020945	NUMB	0.353804156	0.08668652	4.081420686	4.47613E-05	7.39474E-05	positive
PRJNA729271	stage_1W_vs_E10	ordinary_muscle_development	ENSGALG00010020946	CTBS	0.139476672	0.216789012	0.643375192	0.519980677	0.557067251	positive
PRJNA729271	stage_1W_vs_E10	ordinary_muscle_development	ENSGALG00010020949	HPS6	0.638318844	0.109873927	5.809557017	6.26384E-09	1.3467E-08	positive
PRJNA729271	stage_1W_vs_E10	ordinary_muscle_development	ENSGALG00010020950	EFHD2	-2.604163038	0.302579022	-8.606555134	7.52889E-18	2.5403E-17	negative
PRJNA729271	stage_1W_vs_E10	ordinary_muscle_development	ENSGALG00010020951	ENSGALG00010020951	-1.379059147	0.269668118	-5.113912453	3.15554E-07	6.09697E-07	negative
PRJNA729271	stage_1W_vs_E10	ordinary_muscle_development	ENSGALG00010020952	GNG5	-1.333551306	0.100348186	-13.28924183	2.67273E-40	2.1619E-39	negative
PRJNA729271	stage_1W_vs_E10	ordinary_muscle_development	ENSGALG00010020954	PLIN1	8.717713366	1.406525949	6.198046595	5.71682E-10	1.30169E-09	positive
PRJNA729271	stage_1W_vs_E10	ordinary_muscle_development	ENSGALG00010020955	FHAD1	-0.91034123	0.962719381	-0.945593543	0.344355939	0.381608512	negative
PRJNA729271	stage_1W_vs_E10	ordinary_muscle_development	ENSGALG00010020957	RPF1	0.064932509	0.101351934	0.640663739	0.521741178	0.558664281	positive
PRJNA729271	stage_1W_vs_E10	ordinary_muscle_development	ENSGALG00010020959	ENSGALG00010020959	-2.02023466	0.388266365	-5.203218318	1.95867E-07	3.838E-07	negative
PRJNA729271	stage_1W_vs_E10	ordinary_muscle_development	ENSGALG00010020960	CLEC19A	2.878488975	0.370091035	7.777786281	7.38046E-15	2.17837E-14	positive
PRJNA729271	stage_1W_vs_E10	ordinary_muscle_development	ENSGALG00010020962	MYO5C	-4.113701704	0.401536792	-10.24489357	1.24762E-24	5.65133E-24	negative
PRJNA729271	stage_1W_vs_E10	ordinary_muscle_development	ENSGALG00010020963	CCDC93	1.225118069	0.099169926	12.35372575	4.65067E-35	3.16337E-34	positive
PRJNA729271	stage_1W_vs_E10	ordinary_muscle_development	ENSGALG00010020965	DHX37	-0.523396744	0.093691215	-5.586401519	2.31823E-08	4.81208E-08	negative
PRJNA729271	stage_1W_vs_E10	ordinary_muscle_development	ENSGALG00010020966	PAPLN	1.381709244	0.246985181	5.594300186	2.21513E-08	4.60269E-08	positive
PRJNA729271	stage_1W_vs_E10	ordinary_muscle_development	ENSGALG00010020967	VPS51	0.438392739	0.074584052	5.877834819	4.15667E-09	9.02637E-09	positive
PRJNA729271	stage_1W_vs_E10	ordinary_muscle_development	ENSGALG00010020969	RBP3	-1.716320157	0.358445692	-4.788229282	1.68259E-06	3.08998E-06	negative
PRJNA729271	stage_1W_vs_E10	ordinary_muscle_development	ENSGALG00010020970	ARMH3	0.651174777	0.078447991	8.300719655	1.03483E-16	3.31634E-16	positive
PRJNA729271	stage_1W_vs_E10	ordinary_muscle_development	ENSGALG00010020971	TMEM51	-1.426640823	0.301265774	-4.735489207	2.18527E-06	3.97784E-06	negative
PRJNA729271	stage_1W_vs_E10	ordinary_muscle_development	ENSGALG00010020973	GNB5	-1.310766994	0.165994532	-7.8964468	2.86967E-15	8.63701E-15	negative
PRJNA729271	stage_1W_vs_E10	ordinary_muscle_development	ENSGALG00010020975	HCCS	0.975311319	0.130195327	7.491139205	6.82784E-14	1.91913E-13	positive

PRJNA729271	stage_1W_vs_E10	ordinary_muscle_development	ENSGALG00010020976	GDF10	-1.711666772	0.314699497	-5.439051502	5.3565E-08	1.09079E-07	negative
PRJNA729271	stage_1W_vs_E10	ordinary_muscle_development	ENSGALG00010020978	BCL2L10	1.724139947	0.115400628	14.94047288	1.79696E-50	2.00669E-49	positive
PRJNA729271	stage_1W_vs_E10	ordinary_muscle_development	ENSGALG00010020979	ENSGALG00010020979	0.897394227	0.243919208	3.67906339	0.000234092	0.000366749	positive
PRJNA729271	stage_1W_vs_E10	ordinary_muscle_development	ENSGALG00010020981	CIT	-2.365420805	0.237993542	-9.939012572	2.81605E-23	1.20801E-22	negative
PRJNA729271	stage_1W_vs_E10	ordinary_muscle_development	ENSGALG00010020982	TLCD4	0.4154313	0.151551163	2.741195066	0.006121615	0.008463641	positive
PRJNA729271	stage_1W_vs_E10	ordinary_muscle_development	ENSGALG00010020984	PSEN1	-1.223922545	0.061072395	-20.04052	2.44203E-89	7.07945E-88	negative
PRJNA729271	stage_1W_vs_E10	ordinary_muscle_development	ENSGALG00010020986	CFAP20DC	-2.325373234	0.337826592	-6.883333905	5.84678E-12	1.48865E-11	negative
PRJNA729271	stage_1W_vs_E10	ordinary_muscle_development	ENSGALG00010020987	NADSYN1	0.039242478	0.092979691	0.422054295	0.672985388	0.702905548	positive
PRJNA729271	stage_1W_vs_E10	ordinary_muscle_development	ENSGALG00010020989	MAPK4	0.862007667	0.152831088	5.640263888	1.6979E-08	3.55343E-08	positive
PRJNA729271	stage_1W_vs_E10	ordinary_muscle_development	ENSGALG00010020990	UBB	-0.950974272	0.106684701	-8.913876673	4.92786E-19	1.77244E-18	negative
PRJNA729271	stage_1W_vs_E10	ordinary_muscle_development	ENSGALG00010020991	ALG14	1.08234089	0.136194293	7.947035579	1.91028E-15	5.79884E-15	positive
PRJNA729271	stage_1W_vs_E10	ordinary_muscle_development	ENSGALG00010020992	KCNH7	4.332688571	0.427390907	10.13753101	3.76495E-24	1.66635E-23	positive
PRJNA729271	stage_1W_vs_E10	ordinary_muscle_development	ENSGALG00010020994	DHCR7	-1.555242913	0.146377223	-10.62489698	2.28264E-26	1.11441E-25	negative
PRJNA729271	stage_1W_vs_E10	ordinary_muscle_development	ENSGALG00010020998	SYT17	-0.438330917	0.297173085	-1.475002073	0.140211986	0.165179839	negative
PRJNA729271	stage_1W_vs_E10	ordinary_muscle_development	ENSGALG00010020999	IFIH1	2.350836726	0.335398506	7.009085259	2.39881E-12	6.23021E-12	positive
PRJNA729271	stage_1W_vs_E10	ordinary_muscle_development	ENSGALG00010021000	NFKB2	1.420129158	0.11401319	12.45583211	1.29989E-35	9.04553E-35	positive
PRJNA729271	stage_1W_vs_E10	ordinary_muscle_development	ENSGALG00010021002	SLC44A3	-0.489538888	0.16174459	-3.026616768	0.002473073	0.00355204	negative
PRJNA729271	stage_1W_vs_E10	ordinary_muscle_development	ENSGALG00010021003	KAZN	-1.09998557	0.124431857	-8.840063889	9.56639E-19	3.38207E-18	negative
PRJNA729271	stage_1W_vs_E10	ordinary_muscle_development	ENSGALG00010021004	PSD	0.388277978	0.170518081	2.277048723	0.022783316	0.029610344	positive
PRJNA729271	stage_1W_vs_E10	ordinary_muscle_development	ENSGALG00010021010	LEO1	-0.587086885	0.113075714	-5.191980366	2.08069E-07	4.07067E-07	negative
PRJNA729271	stage_1W_vs_E10	ordinary_muscle_development	ENSGALG00010021011	SCARB1	0.503803544	0.107567943	4.683584434	2.81901E-06	5.09369E-06	positive
PRJNA729271	stage_1W_vs_E10	ordinary_muscle_development	ENSGALG00010021012	KCNIP2	-1.572366715	0.751708292	-2.091724585	0.03646316	0.046253041	negative
PRJNA729271	stage_1W_vs_E10	ordinary_muscle_development	ENSGALG00010021013	FAP	-0.477919223	0.296797305	-1.610254593	0.107342288	0.128504002	negative
PRJNA729271	stage_1W_vs_E10	ordinary_muscle_development	ENSGALG00010021014	FBXL15	0.827255032	0.08987682	9.204320267	3.4384E-20	1.29994E-19	positive
PRJNA729271	stage_1W_vs_E10	ordinary_muscle_development	ENSGALG00010021015	RBM25	0.009701421	0.135212304	0.071749544	0.942801227	0.950010691	positive
PRJNA729271	stage_1W_vs_E10	ordinary_muscle_development	ENSGALG00010021016	FHIT	-0.559668157	0.220291613	-2.540578599	0.011066922	0.014882181	negative
PRJNA729271	stage_1W_vs_E10	ordinary_muscle_development	ENSGALG00010021017	COQ7	0.510104408	0.156640489	3.256529726	0.001127831	0.00167037	positive
PRJNA729271	stage_1W_vs_E10	ordinary_muscle_development	ENSGALG00010021018	F3	-0.418447933	0.223894679	-1.868949874	0.061629789	0.076053447	negative
PRJNA729271	stage_1W_vs_E10	ordinary_muscle_development	ENSGALG00010021019	VSIG10	-1.116010093	0.097980161	-11.39016391	4.68108E-30	2.65152E-29	negative
PRJNA729271	stage_1W_vs_E10	ordinary_muscle_development	ENSGALG00010021020	ZDHHC13	-0.191520318	0.120547817	-1.588749778	0.112116896	0.133888153	negative
PRJNA729271	stage_1W_vs_E10	ordinary_muscle_development	ENSGALG00010021021	GCG	0.953718904	0.353864695	2.695151334	0.007035667	0.009670206	positive
PRJNA729271	stage_1W_vs_E10	ordinary_muscle_development	ENSGALG00010021022	ZFYVE1	1.79228051	0.077431285	23.14672319	1.5685E-118	8.452E-117	positive
PRJNA729271	stage_1W_vs_E10	ordinary_muscle_development	ENSGALG00010021023	MGEA5	0.835982055	0.097109914	8.608616959	7.39473E-18	2.49619E-17	positive
PRJNA729271	stage_1W_vs_E10	ordinary_muscle_development	ENSGALG00010021024	ENSGALG00010021024	1.063631022	0.185151871	5.744640972	9.21162E-09	1.95752E-08	positive
PRJNA729271	stage_1W_vs_E10	ordinary_muscle_development	ENSGALG00010021025	CUEDC2	0.623202213	0.115411275	5.399838197	6.6701E-08	1.34957E-07	positive
PRJNA729271	stage_1W_vs_E10	ordinary_muscle_development	ENSGALG00010021026	ADGRA2	-0.733233578	0.135246326	-5.421467613	5.91117E-08	1.20087E-07	negative
PRJNA729271	stage_1W_vs_E10	ordinary_muscle_development	ENSGALG00010021027	PLA2G4A	-0.151928597	0.167503334	-0.907018345	0.364397096	0.402004407	negative
PRJNA729271	stage_1W_vs_E10	ordinary_muscle_development	ENSGALG00010021028	ENSGALG00010021028	0.835030134	0.320514421	2.605281008	0.009179894	0.012464877	positive
PRJNA729271	stage_1W_vs_E10	ordinary_muscle_development	ENSGALG00010021029	PRDM2	-0.898694895	0.076495353	-11.74835931	7.20033E-32	4.37238E-31	negative
PRJNA729271	stage_1W_vs_E10	ordinary_muscle_development	ENSGALG00010021030	CSRP3	0.841647401	0.551580887	1.525882098	0.12703923	0.150517708	positive
PRJNA729271	stage_1W_vs_E10	ordinary_muscle_development	ENSGALG00010021031	DDX54	0.16432732	0.109662681	1.498479872	0.134008618	0.158257693	positive
PRJNA729271	stage_1W_vs_E10	ordinary_muscle_development	ENSGALG00010021032	TMC7	-0.062820026	0.119992399	-0.523533378	0.600603129	0.635131127	negative
PRJNA729271	stage_1W_vs_E10	ordinary_muscle_development	ENSGALG00010021033	RNF122	-2.735965538	0.236723932	-11.55762119	6.75543E-31	3.95476E-30	negative
PRJNA729271	stage_1W_vs_E10	ordinary_muscle_development	ENSGALG00010021034	NPM3	0.466247832	0.277476801	1.680312842	0.092896464	0.112248603	positive
PRJNA729271	stage_1W_vs_E10	ordinary_muscle_development	ENSGALG00010021036	E2F8	-2.33504866	0.203995268	-11.44658245	2.446E-30	1.39696E-29	negative
PRJNA729271	stage_1W_vs_E10	ordinary_muscle_development	ENSGALG00010021038	PTGS2	-0.916747042	0.30435824	-3.012065789	0.002594764	0.003720188	negative
PRJNA729271	stage_1W_vs_E10	ordinary_muscle_development	ENSGALG00010021040	FGF8	-4.787090606	0.683397979	-7.004835763	2.47276E-12	6.41421E-12	negative
PRJNA729271	stage_1W_vs_E10	ordinary_muscle_development	ENSGALG00010021041	GLTP	-0.015332314	0.183978375	-0.083337588	0.933583113	0.941900691	negative
PRJNA729271	stage_1W_vs_E10	ordinary_muscle_development	ENSGALG00010021042	LRRC38	0.366987141	0.098089406	3.741353493	0.000183032	0.000288909	positive
PRJNA729271	stage_1W_vs_E10	ordinary_muscle_development	ENSGALG00010021043	PTPRG	-0.799253559	0.091151109	-8.768445824	1.81149E-18	6.30434E-18	negative
PRJNA729271	stage_1W_vs_E10	ordinary_muscle_development	ENSGALG00010021044	EIF4G2	0.113837173	0.068033699	1.673246862	0.094278708	0.113804953	positive
PRJNA729271	stage_1W_vs_E10	ordinary_muscle_development	ENSGALG00010021045	DPP4	1.943258928	0.260203708	7.468221503	8.12859E-14	2.27196E-13	positive
PRJNA729271	stage_1W_vs_E10	ordinary_muscle_development	ENSGALG00010021046	ENSGALG00010021046	-2.358198981	0.254623018	-9.261531015	2.01522E-20	7.71134E-20	negative
PRJNA729271	stage_1W_vs_E10	ordinary_muscle_development	ENSGALG00010021047	TMC5	-1.118887343	0.520367823	-2.150185491	0.031540545	0.040294395	negative
PRJNA729271	stage_1W_vs_E10	ordinary_muscle_development	ENSGALG00010021048	API5	-0.40299387	0.075874261	-5.311338325	1.08823E-07	2.16853E-07	negative
PRJNA729271	stage_1W_vs_E10	ordinary_muscle_development	ENSGALG00010021050	CTR9	-0.202330731	0.094879597	-2.132499897	0.032965773	0.042029983	negative
PRJNA729271	stage_1W_vs_E10	ordinary_muscle_development	ENSGALG00010021051	GDE1	0.783917617	0.134929753	5.809820309	6.25399E-09	1.34478E-08	positive
PRJNA729271	stage_1W_vs_E10	ordinary_muscle_development	ENSGALG00010021052	CEP15	-2.122007406	0.15665016	-13.54615533	8.34899E-42	7.12293E-41	negative
PRJNA729271	stage_1W_vs_E10	ordinary_muscle_development	ENSGALG00010021053	ENSGALG00010021053	-4.156249124	0.546986238	-7.598452816	2.99692E-14	8.56812E-14	negative
PRJNA729271	stage_1W_vs_E10	ordinary_muscle_development	ENSGALG00010021055	IRAG1	-0.176124973	0.222538739	-0.791435115	0.428690126	0.466576316	negative
PRJNA729271	stage_1W_vs_E10	ordinary_muscle_development	ENSGALG00010021057	ZDHHC8	-0.829334722	0.082711099	-10.02688548	1.16122E-23	5.05158E-23	negative
PRJNA729271	stage_1W_vs_E10	ordinary_muscle_development	ENSGALG00010021058	CCP110	-0.190849466	0.094897937	-2.011102358	0.044314646	0.055713585	negative
PRJNA729271	stage_1W_vs_E10	ordinary_muscle_development	ENSGALG00010021059	ENSGALG00010021059	-2.508632101	1.385081825	-1.811179712	0.070113038	0.085929524	negative
PRJNA729271	stage_1W_vs_E10	ordinary_muscle_development	ENSGALG00010021062	MYO9A	-1.556642425	0.102302343	-15.21609754	2.76542E-52	3.28295E-51	negative
PRJNA729271	stage_1W_vs_E10	ordinary_muscle_development	ENSGALG00010021064	LYVE1	2.458534538	0.285939143	8.598104178	8.10437E-18	2.72939E-17	positive
PRJNA729271	stage_1W_vs_E10	ordinary_muscle_development	ENSGALG00010021065	CNNM2	-0.737519007	0.115073393	-6.409118487	1.46363E-10	3.44405E-10	negative
PRJNA729271	stage_1W_vs_E10	ordinary_muscle_development	ENSGALG00010021067	SLC4A10	-1.082580532	0.230170424	-4.703386786	2.55881E-06	4.6403E-06	negative
PRJNA729271	stage_1W_vs_E10	ordinary_muscle_development	ENSGALG00010021069	PGAM5	0.373768747	0.07107933	5.258473154	1.45256E-07	2.87126E-07	positive
PRJNA729271	stage_1W_vs_E10	ordinary_muscle_development	ENSGALG00010021070	RNF141	0.042473679	0.086139195	0.493081912	0.621954711	0.655832388	positive
PRJNA729271	stage_1W_vs_E10	ordinary_muscle_development	ENSGALG00010021071	HIF1AN	0.757399263	0.095661583	7.917486213	2.4236E-15	7.31878E-15	positive
PRJNA729271	stage_1W_vs_E10	ordinary_muscle_development	ENSGALG00010021072	ENSGALG00010021072	0.66995116	0.089899456	7.452227114	9.17776E-14	2.55732E-13	positive
PRJNA729271	stage_1W_vs_E10	ordinary_muscle_development	ENSGALG00010021073	NDUFB8	1.014173832	0.107355358	9.446886052	3.4906E-21	1.37715E-20	positive
PRJNA729271	stage_1W_vs_E10	ordinary_muscle_development	ENSGALG00010021074	TTC17	0.393549154	0.069540774	5.659257622	1.52029E-08	3.18863E-08	positive
PRJNA729271	stage_1W_vs_E10	ordinary_muscle_development	ENSGALG00010021075	DHRS3	-2.022979889	0.135423471	-14.93817782	1.85992E-50	2.07541E-49	negative
PRJNA729271	stage_1W_vs_E10	ordinary_muscle_development	ENSGALG00010021077	SEC31B	0.579130485	0.078802197	7.349166777	1.99446E-13	5.45672E-13	positive
PRJNA729271	stage_1W_vs_E10	ordinary_muscle_development	ENSGALG00010021078	ENSGALG00010021078	-3.190155441	0.296008664	-10.7772367	4.40933E-27	2.20771E-26	negative
PRJNA729271	stage_1W_vs_E10	ordinary_muscle_development	ENSGALG00010021080	TTLL7	2.979351709	0.173091853	17.21254731	2.13816E-66	3.64191E-65	positive
PRJNA729271	stage_1W_vs_E10	ordinary_muscle_development	ENSGALG00010021081	KCNH6	-0.42257733	0.211725605	-1.995872579	0.045947794	0.057633548	negative
PRJNA729271	stage_1W_vs_E10	ordinary_muscle_development	ENSGALG00010021082	SENP8	0.800968281	0.163015999	4.913433553	8.94951E-07	1.68013E-06	positive
PRJNA729271	stage_1W_vs_E10	ordinary_muscle_development	ENSGALG00010021083	SCD	-1.334629921	0.563547169	-2.36826657			negative
PRJNA729271	stage_1W_vs_E10	ordinary_muscle_development	ENSGALG00010021084	ENSGALG00010021084	-2.371654763	0.721956973	-3.285036162	0.001019693	0.001517188	negative
PRJNA729271	stage_1W_vs_E10	ordinary_muscle_development	ENSGALG00010021085	AMPD3	1.80754937	0.184090905	9.818786923	9.34614E-23	3.91312E-22	positive
PRJNA729271	stage_1W_vs_E10	ordinary_muscle_development	ENSGALG00010021086	PSMD14	0.075757333	0.109327587	0.692938857	0.488347923	0.526642597	positive
PRJNA729271	stage_1W_vs_E10	ordinary_muscle_development	ENSGALG00010021087	THBS1	-2.863890197	0.356711169	-8.028596942	9.85937E-16	3.03422E-15	negative
PRJNA729271	stage_1W_vs_E10	ordinary_muscle_development	ENSGALG00010021088	ANKLE2	-0.304292808	0.087568637	-3.474906298	0.000511032	0.000778743	negative
PRJNA729271	stage_1W_vs_E10	ordinary_muscle_development	ENSGALG00010021090	ADM	3.40841617	0.255754103	13.32692665	1.61411E-40	1.31515E-39	positive
PRJNA729271	stage_1W_vs_E10	ordinary_muscle_development	ENSGALG00010021091	TANK	0.389738067	0.081807329	4.764097197	1.89701E-06	3.47274E-06	positive
PRJNA729271	stage_1W_vs_E10	ordinary_muscle_development	ENSGALG00010021092	VPS13D	1.04320975	0.094704349	11.01543662	3.21972E-28	1.69339E-27	positive
PRJNA729271	stage_1W_vs_E10	ordinary_muscle_development	ENSGALG00010021093	GRAMD2	3.011608396	0.219255996	13.73558053	6.21559E-43	5.47024E-42	positive
PRJNA729271	stage_1W_vs_E10	ordinary_muscle_development	ENSGALG00010021094	ENSGALG00010021094	3.290977321	0.314352645	10.46906199	1.19824E-25	5.6612E-25	positive
PRJNA729271	stage_1W_vs_E10	ordinary_muscle_development	ENSGALG00010021095	NT5C2	-0.082813594	0.106080984	-0.780663895	0.435000201	0.472982366	negative
PRJNA729271	stage_1W_vs_E10	ordinary_muscle_development	ENSGALG00010021096	ZFAND4	-0.920413321	0.105710588	-8.706917051	3.12251E-18	1.07482E-17	negative
PRJNA729271	stage_1W_vs_E10	ordinary_muscle_development	ENSGALG00010021097	KATNBL1	-0.478411817	0.130130232	-3.676407928	0.000236541	0.000370506	negative
PRJNA729271	stage_1W_vs_E10	ordinary_muscle_development	ENSGALG00010021098	SDK1	-0.913595478	0.142730761	-6.400830997	1.54534E-10	3.63336E-10	negative
PRJNA729271	stage_1W_vs_E10	ordinary_muscle_development	ENSGALG00010021099	HMCN1	-2.445493542	0.265037382	-9.226975931	2.7838E-20	1.0577E-19	negative
PRJNA729271	stage_1W_vs_E10	ordinary_muscle_development	ENSGALG00010021100	EMC7	1.089259577	0.085289415	12.77133373	2.37057E-37	1.73455E-36	positive
PRJNA729271	stage_1W_vs_E10	ordinary_muscle_development	ENSGALG00010021101	TMEM240	-4.210267871	0.632665834	-6.654805186	2.83676E-11	6.96574E-11	negative
PRJNA729271	stage_1W_vs_E10	ordinary_muscle_development	ENSGALG00010021102	GOLGA3	1.224554003	0.112061899	10.92747857	8.51825E-28	4.39715E-27	positive
PRJNA729271	stage_1W_vs_E10	ordinary_muscle_development	ENSGALG00010021103	RBMS1	-0.445055148	0.074661674	-5.960958571	2.50763E-09	5.51898E-09	negative
PRJNA729271	stage_1W_vs_E10	ordinary_muscle_development	ENSGALG00010021104	INA	-1.770564962	0.377350098	-4.692101509	2.70413E-06	4.89282E-06	negative
PRJNA729271	stage_1W_vs_E10	ordinary_muscle_development	ENSGALG00010021105	ENSGALG00010021105	2.873457679	0.408023031	7.042390888	1.88969E-12	4.92822E-12	positive
PRJNA729271	stage_1W_vs_E10	ordinary_muscle_development	ENSGALG00010021106	ITGB6	-2.046151606	0.20752144	-9.859952834	6.20785E-23	2.61959E-22	negative
PRJNA729271	stage_1W_vs_E10	ordinary_muscle_development	ENSGALG00010021108	PCGF6	0.227231047	0.10595946	2.144509305	0.031992101	0.040838881	positive
PRJNA729271	stage_1W_vs_E10	ordinary_muscle_development	ENSGALG00010021110	PKLR	5.704630496	0.168773337	33.8005434	1.9363E-250	4.601E-248	positive
PRJNA729271	stage_1W_vs_E10	ordinary_muscle_development	ENSGALG00010021111	AVEN	0.53072504	0.078935962	6.723488578	1.77424E-11	4.40296E-11	positive
PRJNA729271	stage_1W_vs_E10	ordinary_muscle_development	ENSGALG00010021112	TAF5	-1.064477808	0.072875643	-14.60677081	2.54288E-48	2.62529E-47	negative
PRJNA729271	stage_1W_vs_E10	ordinary_muscle_development	ENSGALG00010021113	SYP	-0.498449112	0.30281676	-1.64604202	0.099755108	0.119985918	negative
PRJNA729271	stage_1W_vs_E10	ordinary_muscle_development	ENSGALG00010021114	SBF2	-0.574378557	0.09900969	-5.80123581	6.58279E-09	1.41255E-08	negative
PRJNA729271	stage_1W_vs_E10	ordinary_muscle_development	ENSGALG00010021115	ALPL	-0.376314535	0.244249792	-1.540695414	0.123390933	0.146590573	negative
PRJNA729271	stage_1W_vs_E10	ordinary_muscle_development	ENSGALG00010021116	FAM21C	0.505540763	0.064218608	7.872184989	3.485E-15	1.04435E-14	positive
PRJNA729271	stage_1W_vs_E10	ordinary_muscle_development	ENSGALG00010021118	USMG5	1.179160996	0.179776876	6.559024857	5.41608E-11	1.30691E-10	positive
PRJNA729271	stage_1W_vs_E10	ordinary_muscle_development	ENSGALG00010021120	PDCD11	-1.538761717	0.088657216	-17.35630539	1.76737E-67	3.12415E-66	negative
PRJNA729271	stage_1W_vs_E10	ordinary_muscle_development	ENSGALG00010021121	PLA2R1	1.94645893	0.091272441	21.32581211	6.5408E-101	2.49499E-99	positive
PRJNA729271	stage_1W_vs_E10	ordinary_muscle_development	ENSGALG00010021122	CYP1A1	3.780128956	0.917353793	4.120688206	3.77742E-05	6.27622E-05	positive
PRJNA729271	stage_1W_vs_E10	ordinary_muscle_development	ENSGALG00010021123	RYR3	7.623197242	0.216118261	35.27326749	1.5094E-272	4.4651E-270	positive
PRJNA729271	stage_1W_vs_E10	ordinary_muscle_development	ENSGALG00010021124	CALHM2	-0.06938346	0.134792097	-0.51474427	0.606731733	0.64105084	negative
PRJNA729271	stage_1W_vs_E10	ordinary_muscle_development	ENSGALG00010021125	LY75	2.045021212	0.124086774	16.48057357	5.06025E-61	7.6325E-60	positive
PRJNA729271	stage_1W_vs_E10	ordinary_muscle_development	ENSGALG00010021126	ECE1	0.594828421	0.104277545	5.704281026	1.16835E-08	2.46834E-08	positive
PRJNA729271	stage_1W_vs_E10	ordinary_muscle_development	ENSGALG00010021127	SWAP70	-0.361416655	0.078049773	-4.630591989	3.64622E-06	6.53865E-06	negative
PRJNA729271	stage_1W_vs_E10	ordinary_muscle_development	ENSGALG00010021129	TXNRD3	0.020173009	0.085509716	0.235914819	0.813498769	0.833333191	positive
PRJNA729271	stage_1W_vs_E10	ordinary_muscle_development	ENSGALG00010021130	ENSGALG00010021130	4.455674374	1.102095114	4.042912737	5.27913E-05	8.66896E-05	positive
PRJNA729271	stage_1W_vs_E10	ordinary_muscle_development	ENSGALG00010021131	EDC3	-0.504838213	0.116829677	-4.32114705	1.5522E-05	2.6507E-05	negative
PRJNA729271	stage_1W_vs_E10	ordinary_muscle_development	ENSGALG00010021133	ENSGALG00010021133	-0.617401441	0.103335171	-5.974746385	2.30448E-09	5.08347E-09	negative
PRJNA729271	stage_1W_vs_E10	ordinary_muscle_development	ENSGALG00010021134	CHFR	-0.913221711	0.094261568	-9.688165888	3.38554E-22	1.38391E-21	negative
PRJNA729271	stage_1W_vs_E10	ordinary_muscle_development	ENSGALG00010021135	CLK3	1.271012516	0.1046962	12.14000615	6.48212E-34	4.23235E-33	positive
PRJNA729271	stage_1W_vs_E10	ordinary_muscle_development	ENSGALG00010021136	WEE1	-2.058256449	0.150441325	-13.68145652	1.31041E-42	1.14218E-41	negative
PRJNA729271	stage_1W_vs_E10	ordinary_muscle_development	ENSGALG00010021137	PLAU	0.092734759	0.193405192	0.479484331	0.631594113	0.664606654	positive
PRJNA729271	stage_1W_vs_E10	ordinary_muscle_development	ENSGALG00010021138	DDX18	-0.341820536	0.099631025	-3.430864393	0.000601661	0.000912533	negative
PRJNA729271	stage_1W_vs_E10	ordinary_muscle_development	ENSGALG00010021139	ARHGAP29	1.036949521	0.095585854	10.84835753	2.03041E-27	1.03194E-26	positive
PRJNA729271	stage_1W_vs_E10	ordinary_muscle_development	ENSGALG00010021140	SLC11A1	-0.201644393	0.449964505	-0.448133998	0.654056493	0.685208794	negative
PRJNA729271	stage_1W_vs_E10	ordinary_muscle_development	ENSGALG00010021141	SGSM1	-0.719613568	0.15939174	-4.514748189	6.3392E-06	1.11391E-05	negative

PRJNA729271	stage_1W_vs_E10	ordinary_muscle_development	ENSGALG00010021142	WNT6	-6.603760942	0.744280237	-8.872680765	7.14058E-19	2.54244E-18	negative
PRJNA729271	stage_1W_vs_E10	ordinary_muscle_development	ENSGALG00010021143	ABCA4	-1.053976776	0.361022805	-2.919418832	0.003506847	0.004965493	negative
PRJNA729271	stage_1W_vs_E10	ordinary_muscle_development	ENSGALG00010021144	FOXK1	0.484491468	0.108530544	4.464102454	8.04051E-06	1.40418E-05	positive
PRJNA729271	stage_1W_vs_E10	ordinary_muscle_development	ENSGALG00010021145	EIF4G3	0.436673121	0.157318123	2.775733099	0.00550774	0.007656535	positive
PRJNA729271	stage_1W_vs_E10	ordinary_muscle_development	ENSGALG00010021146	AP5Z1	0.288795388	0.085151366	3.391553208	0.000694977	0.001049124	positive
PRJNA729271	stage_1W_vs_E10	ordinary_muscle_development	ENSGALG00010021147	ARID3B	-0.372598749	0.085854007	-4.339910977	1.4254E-05	2.44425E-05	negative
PRJNA729271	stage_1W_vs_E10	ordinary_muscle_development	ENSGALG00010021148	GPATCH8	0.273967884	0.105072786	2.607410481	0.009122992	0.012389935	positive
PRJNA729271	stage_1W_vs_E10	ordinary_muscle_development	ENSGALG00010021149	ENSGALG00010021149	-3.667721728	1.345538149	-2.725840014	0.006413808	0.008852423	negative
PRJNA729271	stage_1W_vs_E10	ordinary_muscle_development	ENSGALG00010021150	ENSGALG00010021150	0.627727005	0.255829625	2.453691606	0.014139819	0.018799915	positive
PRJNA729271	stage_1W_vs_E10	ordinary_muscle_development	ENSGALG00010021151	WNT10A	-6.148600587	0.963663626	-6.38044274	1.76577E-10	4.13887E-10	negative
PRJNA729271	stage_1W_vs_E10	ordinary_muscle_development	ENSGALG00010021152	STK11IP	1.10400047	0.126359081	8.737009335	2.39363E-18	8.27268E-18	positive
PRJNA729271	stage_1W_vs_E10	ordinary_muscle_development	ENSGALG00010021153	PTPRF	-2.840400613	0.160988831	-17.64346379	1.14239E-69	2.14494E-68	negative
PRJNA729271	stage_1W_vs_E10	ordinary_muscle_development	ENSGALG00010021154	CHST13	-2.961282134	0.216624194	-13.67013575	1.53109E-42	1.33292E-41	negative
PRJNA729271	stage_1W_vs_E10	ordinary_muscle_development	ENSGALG00010021155	RADIL	-2.519736132	0.232996611	-10.81447548	2.93969E-27	1.48522E-26	negative
PRJNA729271	stage_1W_vs_E10	ordinary_muscle_development	ENSGALG00010021156	DDOST	-1.215872147	0.081267681	-14.96132458	1.31385E-50	1.47173E-49	negative
PRJNA729271	stage_1W_vs_E10	ordinary_muscle_development	ENSGALG00010021157	ENSGALG00010021157	6.632094389	0.809297442	8.194878727	2.50846E-16	7.90266E-16	positive
PRJNA729271	stage_1W_vs_E10	ordinary_muscle_development	ENSGALG00010021158	ACTG1L	-2.445865299	0.129432358	-18.89686118	1.21043E-79	2.85287E-78	negative
PRJNA729271	stage_1W_vs_E10	ordinary_muscle_development	ENSGALG00010021159	MMD2	2.097148508	0.237972647	8.812561162	1.22319E-18	4.29508E-18	positive
PRJNA729271	stage_1W_vs_E10	ordinary_muscle_development	ENSGALG00010021160	ZNF143	0.566338747	0.164147874	3.450174119	0.000560225	0.000851112	positive
PRJNA729271	stage_1W_vs_E10	ordinary_muscle_development	ENSGALG00010021161	ENSGALG00010021161	-2.076507275	0.113644583	-18.27194239	1.38434E-74	2.8872E-73	negative
PRJNA729271	stage_1W_vs_E10	ordinary_muscle_development	ENSGALG00010021162	WIPI2	-0.030382923	0.094899681	-0.320158331	0.748848309	0.773833053	negative
PRJNA729271	stage_1W_vs_E10	ordinary_muscle_development	ENSGALG00010021163	INHA	-3.565132498	0.558910103	-6.378722582	1.78571E-10	4.18292E-10	negative
PRJNA729271	stage_1W_vs_E10	ordinary_muscle_development	ENSGALG00010021164	SLC41A3	1.223003378	0.212071848	5.766929407	8.07288E-09	1.72184E-08	positive
PRJNA729271	stage_1W_vs_E10	ordinary_muscle_development	ENSGALG00010021165	PTRF	3.420109666	0.105585232	32.39193198	3.5655E-230	7.383E-228	positive
PRJNA729271	stage_1W_vs_E10	ordinary_muscle_development	ENSGALG00010021166	NEURL1	-0.003032098	0.304122958	-0.009969973	0.992045244	0.993347321	negative
PRJNA729271	stage_1W_vs_E10	ordinary_muscle_development	ENSGALG00010021167	KIAA1671	-0.150192199	0.126692203	-1.185488896	0.235824202	0.267889641	negative
PRJNA729271	stage_1W_vs_E10	ordinary_muscle_development	ENSGALG00010021168	IVNS1ABP	0.816046677	0.105609051	7.727052416	1.10065E-14	3.22368E-14	positive
PRJNA729271	stage_1W_vs_E10	ordinary_muscle_development	ENSGALG00010021169	CAMK2G	2.96376242	0.139902898	21.1844248	1.32939E-99	4.96639E-98	positive
PRJNA729271	stage_1W_vs_E10	ordinary_muscle_development	ENSGALG00010021170	UBL7	0.145457798	0.137101532	1.060949473	0.288712864	0.323581043	positive
PRJNA729271	stage_1W_vs_E10	ordinary_muscle_development	ENSGALG00010021171	ENSGALG00010021171	-0.756636066	0.214330816	-3.530225289	0.000415206	0.000637273	negative
PRJNA729271	stage_1W_vs_E10	ordinary_muscle_development	ENSGALG00010021172	SLC29A4	-1.344111774	0.285784713	-4.703231882	2.56075E-06	4.64325E-06	negative
PRJNA729271	stage_1W_vs_E10	ordinary_muscle_development	ENSGALG00010021173	KLF15	2.761992189	0.287193799	9.617172092	6.76659E-22	2.73818E-21	positive
PRJNA729271	stage_1W_vs_E10	ordinary_muscle_development	ENSGALG00010021175	IPO7	0.619978642	0.097575809	6.353815053	2.10039E-10	4.8963E-10	positive
PRJNA729271	stage_1W_vs_E10	ordinary_muscle_development	ENSGALG00010021176	ENSGALG00010021176	0.385075694	0.117843871	3.267676901	0.001084341	0.001609907	positive
PRJNA729271	stage_1W_vs_E10	ordinary_muscle_development	ENSGALG00010021177	OBSL1	0.731058113	0.18789416	3.890797413	9.99153E-05	0.000160598	positive
PRJNA729271	stage_1W_vs_E10	ordinary_muscle_development	ENSGALG00010021178	NDST2	-0.750164818	0.098552668	-7.611816405	2.7027E-14	7.75295E-14	negative
PRJNA729271	stage_1W_vs_E10	ordinary_muscle_development	ENSGALG00010021180	TMEM198	0.222878469	0.140124589	1.590573574	0.111705576	0.13342994	positive
PRJNA729271	stage_1W_vs_E10	ordinary_muscle_development	ENSGALG00010021181	DAGLA	0.204951223	0.128467555	1.595353964	0.110633104	0.13219247	positive
PRJNA729271	stage_1W_vs_E10	ordinary_muscle_development	ENSGALG00010021182	ZSWIM8	0.076276849	0.069903335	1.091176103	0.275195401	0.309473999	positive
PRJNA729271	stage_1W_vs_E10	ordinary_muscle_development	ENSGALG00010021183	CFAP100	3.1169848	0.454965126	6.851041144	7.33144E-12	1.85753E-11	positive
PRJNA729271	stage_1W_vs_E10	ordinary_muscle_development	ENSGALG00010021184	DRAXIN	-1.880238139	0.485630198	-3.871748803	0.000108057	0.00017332	negative
PRJNA729271	stage_1W_vs_E10	ordinary_muscle_development	ENSGALG00010021186	SWT1	0.337574074	0.090743357	3.720096835	0.000199146	0.000313423	positive
PRJNA729271	stage_1W_vs_E10	ordinary_muscle_development	ENSGALG00010021187	SEMA7A	2.268224485	0.277984516	8.159535354	3.36316E-16	1.05403E-15	positive
PRJNA729271	stage_1W_vs_E10	ordinary_muscle_development	ENSGALG00010021188	TNRC18	-0.55658227	0.133630779	-4.165075388	3.1125E-05	5.20605E-05	negative
PRJNA729271	stage_1W_vs_E10	ordinary_muscle_development	ENSGALG00010021189	ENSGALG00010021189	4.209458003	0.359708778	11.70240556	1.23891E-31	7.48249E-31	positive
PRJNA729271	stage_1W_vs_E10	ordinary_muscle_development	ENSGALG00010021190	ENSGALG00010021190	0.759600257	0.101943772	7.451168805	9.25169E-14	2.57693E-13	positive
PRJNA729271	stage_1W_vs_E10	ordinary_muscle_development	ENSGALG00010021191	MYRF	-1.510037744	0.218163978	-6.921572277	4.46658E-12	1.14327E-11	negative
PRJNA729271	stage_1W_vs_E10	ordinary_muscle_development	ENSGALG00010021192	ENSGALG00010021192	-2.435406528	0.17841409	-13.65030375	2.01039E-42	1.74286E-41	negative
PRJNA729271	stage_1W_vs_E10	ordinary_muscle_development	ENSGALG00010021193	USP37	0.166864369	0.156711253	1.064788691	0.286971564	0.321730437	positive
PRJNA729271	stage_1W_vs_E10	ordinary_muscle_development	ENSGALG00010021194	CYP11A1	0.250321392	0.364001927	0.687692491	0.49164645	0.529726848	positive
PRJNA729271	stage_1W_vs_E10	ordinary_muscle_development	ENSGALG00010021195	GCLM	-0.543561769	0.090862785	-5.982226604	2.20108E-09	4.86277E-09	negative
PRJNA729271	stage_1W_vs_E10	ordinary_muscle_development	ENSGALG00010021196	ATP6V0A1	2.781476793	0.119804329	23.21683054	3.0787E-119	1.6776E-117	positive
PRJNA729271	stage_1W_vs_E10	ordinary_muscle_development	ENSGALG00010021197	CHCHD1	0.765157323	0.105182751	7.274551341	3.47574E-13	9.3959E-13	positive
PRJNA729271	stage_1W_vs_E10	ordinary_muscle_development	ENSGALG00010021199	TMEM258	-0.676491348	0.137455028	-4.921546753	8.58629E-07	1.61445E-06	negative
PRJNA729271	stage_1W_vs_E10	ordinary_muscle_development	ENSGALG00010021200	FTO	-0.654890922	0.167493632	-3.9099452	9.23171E-05	0.00014888	negative
PRJNA729271	stage_1W_vs_E10	ordinary_muscle_development	ENSGALG00010021201	TRMT1L	-0.133843807	0.089134421	-1.50159507	0.133201717	0.157420211	negative
PRJNA729271	stage_1W_vs_E10	ordinary_muscle_development	ENSGALG00010021202	UROC1	-2.220580919	0.617842898	-3.59408666	0.000325531	0.000504553	negative
PRJNA729271	stage_1W_vs_E10	ordinary_muscle_development	ENSGALG00010021203	FUT11	0.188324823	0.08160974	2.307626789	0.021019903	0.027409455	positive
PRJNA729271	stage_1W_vs_E10	ordinary_muscle_development	ENSGALG00010021204	NAGLU	0.358307261	0.095520651	3.751097343	0.000176062	0.00027818	positive
PRJNA729271	stage_1W_vs_E10	ordinary_muscle_development	ENSGALG00010021206	HSD17B1	0.90616931	0.407515979	2.223641176	0.026172596	0.033757944	positive
PRJNA729271	stage_1W_vs_E10	ordinary_muscle_development	ENSGALG00010021207	MAD2L2	0.168203676	0.121169042	1.388173693	0.165084161	0.192509647	positive
PRJNA729271	stage_1W_vs_E10	ordinary_muscle_development	ENSGALG00010021210	ENSGALG00010021210	-0.617219964	0.064172608	-9.618121817	6.70441E-22	2.71378E-21	negative
PRJNA729271	stage_1W_vs_E10	ordinary_muscle_development	ENSGALG00010021211	FEN1	-2.659617132	0.158548257	-16.77481154	3.73041E-63	5.88381E-62	negative
PRJNA729271	stage_1W_vs_E10	ordinary_muscle_development	ENSGALG00010021212	MYO18B	4.231830153	0.161041366	26.27790773	3.4307E-152	2.9777E-150	positive
PRJNA729271	stage_1W_vs_E10	ordinary_muscle_development	ENSGALG00010021213	FBXO44	0.357066929	0.20305097	1.758508858	0.078660966	0.095790196	positive
PRJNA729271	stage_1W_vs_E10	ordinary_muscle_development	ENSGALG00010021214	FBXL18	-0.267592297	0.116078395	-2.30527221	0.021151333	0.027573394	negative
PRJNA729271	stage_1W_vs_E10	ordinary_muscle_development	ENSGALG00010021215	SH3PXD2A	-0.718305228	0.111865128	-6.421171981	1.35229E-10	3.18827E-10	negative
PRJNA729271	stage_1W_vs_E10	ordinary_muscle_development	ENSGALG00010021217	FBXO2	-1.805357945	0.223821497	-8.066061424	7.26023E-16	2.24865E-15	negative
PRJNA729271	stage_1W_vs_E10	ordinary_muscle_development	ENSGALG00010021218	DPF3	0.338162056	0.174596848	1.936816504	0.052767778	0.065721682	positive
PRJNA729271	stage_1W_vs_E10	ordinary_muscle_development	ENSGALG00010021219	STRA6	-8.394320711	0.847166213	-9.908705732	3.81557E-23	1.62523E-22	negative
PRJNA729271	stage_1W_vs_E10	ordinary_muscle_development	ENSGALG00010021220	SYNPO2L	3.267927179	0.205779896	15.88069215	8.62217E-57	1.16259E-55	positive
PRJNA729271	stage_1W_vs_E10	ordinary_muscle_development	ENSGALG00010021222	ENSGALG00010021222	4.020413513	0.305114975	13.17671645	1.19479E-39	9.46364E-39	positive
PRJNA729271	stage_1W_vs_E10	ordinary_muscle_development	ENSGALG00010021223	DFFB	0.323555576	0.111222131	2.909093484	0.003624785	0.005125976	positive
PRJNA729271	stage_1W_vs_E10	ordinary_muscle_development	ENSGALG00010021224	MYOZ1	8.115042024	0.140954809	57.57194161	0	0	positive
PRJNA729271	stage_1W_vs_E10	ordinary_muscle_development	ENSGALG00010021227	CHCHD4	-1.020697572	0.098645723	-10.34710416	4.31335E-25	1.99432E-24	negative
PRJNA729271	stage_1W_vs_E10	ordinary_muscle_development	ENSGALG00010021228	ESRP2	-5.500997596	0.932320115	-5.900331344	3.62772E-09	7.90259E-09	negative
PRJNA729271	stage_1W_vs_E10	ordinary_muscle_development	ENSGALG00010021229	GLO1	2.195291158	0.178227902	12.31732592	7.30775E-35	4.94287E-34	positive
PRJNA729271	stage_1W_vs_E10	ordinary_muscle_development	ENSGALG00010021230	C1orf174	-0.058744253	0.059558477	-0.986329003	0.323971692	0.360452078	negative
PRJNA729271	stage_1W_vs_E10	ordinary_muscle_development	ENSGALG00010021231	STN1	-1.621926712	0.122653463	-13.22365202	6.40757E-40	5.11723E-39	negative
PRJNA729271	stage_1W_vs_E10	ordinary_muscle_development	ENSGALG00010021232	ACTB	-1.470182345	0.109425847	-13.43542125	3.74929E-41	3.12872E-40	negative
PRJNA729271	stage_1W_vs_E10	ordinary_muscle_development	ENSGALG00010021233	COASY	0.59387425	0.061699837	9.625215837	6.25754E-22	2.53431E-21	positive
PRJNA729271	stage_1W_vs_E10	ordinary_muscle_development	ENSGALG00010021234	ISLR2	-1.6762548	0.342488685	-4.894336287	9.8638E-07	1.84628E-06	negative
PRJNA729271	stage_1W_vs_E10	ordinary_muscle_development	ENSGALG00010021235	XRCC5	-1.88944727	0.140739807	-13.42510917	4.30949E-41	3.58382E-40	negative
PRJNA729271	stage_1W_vs_E10	ordinary_muscle_development	ENSGALG00010021236	BTBD9	0.235836163	0.073962275	3.188600714	0.001429632	0.00209912	positive
PRJNA729271	stage_1W_vs_E10	ordinary_muscle_development	ENSGALG00010021237	FSCN1	-3.946376199	0.22416532	-17.60475798	2.26474E-69	4.20325E-68	negative
PRJNA729271	stage_1W_vs_E10	ordinary_muscle_development	ENSGALG00010021238	NDUFS8	2.282916102	0.146691726	15.56267803	1.30521E-54	1.67277E-53	positive
PRJNA729271	stage_1W_vs_E10	ordinary_muscle_development	ENSGALG00010021239	USP54	0.107502827	0.070446153	1.526028345	0.127002805	0.150499155	positive
PRJNA729271	stage_1W_vs_E10	ordinary_muscle_development	ENSGALG00010021240	SLK	-0.173541547	0.078863921	-2.200518878	0.027770102	0.03570608	negative
PRJNA729271	stage_1W_vs_E10	ordinary_muscle_development	ENSGALG00010021241	TMEM43	-0.517059836	0.172000297	-3.006156645	0.002645727	0.003789507	negative
PRJNA729271	stage_1W_vs_E10	ordinary_muscle_development	ENSGALG00010021242	BCAR3	3.574155939	0.224261386	15.93745587	3.48246E-57	4.74419E-56	positive
PRJNA729271	stage_1W_vs_E10	ordinary_muscle_development	ENSGALG00010021243	DYL1	-0.880052412	0.094957347	-9.267870652	1.89899E-20	7.27428E-20	negative
PRJNA729271	stage_1W_vs_E10	ordinary_muscle_development	ENSGALG00010021244	PPP3CB	0.697479095	0.087292219	7.990163386	1.3476E-15	4.12098E-15	positive
PRJNA729271	stage_1W_vs_E10	ordinary_muscle_development	ENSGALG00010021245	ZFAND3	1.153629433	0.070228414	16.42681871	1.22942E-60	1.83526E-59	positive
PRJNA729271	stage_1W_vs_E10	ordinary_muscle_development	ENSGALG00010021246	PECR	0.473602129	0.115453124	4.102116171	4.09389E-05	6.78027E-05	positive
PRJNA729271	stage_1W_vs_E10	ordinary_muscle_development	ENSGALG00010021247	GNAO1	-1.075637786	0.221934398	-4.846647465	1.25565E-06	2.32868E-06	negative
PRJNA729271	stage_1W_vs_E10	ordinary_muscle_development	ENSGALG00010021248	ANXA7	0.281708864	0.168812012	1.66877262	0.095162451	0.114785697	positive
PRJNA729271	stage_1W_vs_E10	ordinary_muscle_development	ENSGALG00010021249	ENSGALG00010021249	-0.37320876	0.382289405	-0.976246672	0.32894224	0.365393346	negative
PRJNA729271	stage_1W_vs_E10	ordinary_muscle_development	ENSGALG00010021250	ISLR	-0.321169792	0.14713727	-2.182790205	0.029051263	0.037268613	negative
PRJNA729271	stage_1W_vs_E10	ordinary_muscle_development	ENSGALG00010021251	RNF216	-0.01694882	0.084898302	-0.199636733	0.841764697	0.859131058	negative
PRJNA729271	stage_1W_vs_E10	ordinary_muscle_development	ENSGALG00010021252	XPC	0.83302107	0.131239633	6.347328573	2.19086E-10	5.09981E-10	positive
PRJNA729271	stage_1W_vs_E10	ordinary_muscle_development	ENSGALG00010021253	TMEM169	-2.356341011	0.120308187	-19.58587419	2.04073E-85	5.56023E-84	negative
PRJNA729271	stage_1W_vs_E10	ordinary_muscle_development	ENSGALG00010021254	FNBP1L	-0.604709428	0.108589989	-5.568740096	2.56588E-08	5.31168E-08	negative
PRJNA729271	stage_1W_vs_E10	ordinary_muscle_development	ENSGALG00010021255	EIF3F	1.200508949	0.091684615	13.09389743	3.56818E-39	2.78218E-38	positive
PRJNA729271	stage_1W_vs_E10	ordinary_muscle_development	ENSGALG00010021256	ENSGALG00010021256	0.41312344	0.291438175	1.417533721	0.156326942	0.182827096	positive
PRJNA729271	stage_1W_vs_E10	ordinary_muscle_development	ENSGALG00010021257	RGS6	-1.45922658	0.344627495	-4.23421405	2.29352E-05	3.86867E-05	negative
PRJNA729271	stage_1W_vs_E10	ordinary_muscle_development	ENSGALG00010021258	LSM3	-1.311603893	0.115611477	-11.34492805	7.85909E-30	4.41199E-29	negative
PRJNA729271	stage_1W_vs_E10	ordinary_muscle_development	ENSGALG00010021259	CEP104	-1.128345294	0.136232039	-8.282525155	1.2059E-16	3.85481E-16	negative
PRJNA729271	stage_1W_vs_E10	ordinary_muscle_development	ENSGALG00010021260	KDM4A	0.334475789	0.067534099	4.952694907	7.31927E-07	1.3816E-06	positive
PRJNA729271	stage_1W_vs_E10	ordinary_muscle_development	ENSGALG00010021261	JUP	0.385343688	0.128369048	3.001842688	0.002683508	0.003841723	positive
PRJNA729271	stage_1W_vs_E10	ordinary_muscle_development	ENSGALG00010021262	MDGA1	-2.540551476	0.381458857	-6.660093033	2.73654E-11	6.72879E-11	negative
PRJNA729271	stage_1W_vs_E10	ordinary_muscle_development	ENSGALG00010021263	STOML1	-0.251479792	0.245947063	-1.022495608	0.30654639	0.342167714	negative
PRJNA729271	stage_1W_vs_E10	ordinary_muscle_development	ENSGALG00010021264	CBFB	-1.985617425	0.170886607	-11.6195029	3.28039E-31	1.94635E-30	negative
PRJNA729271	stage_1W_vs_E10	ordinary_muscle_development	ENSGALG00010021265	SIPA1L1	-0.927935301	0.090538751	-10.24904028	1.19524E-24	5.42255E-24	negative
PRJNA729271	stage_1W_vs_E10	ordinary_muscle_development	ENSGALG00010021266	COQ5	0.082605375	0.099638855	0.829047814	0.407077352	0.445092118	positive
PRJNA729271	stage_1W_vs_E10	ordinary_muscle_development	ENSGALG00010021267	LRRC47	0.471331467	0.078327408	6.017452608	1.77183E-09	3.93906E-09	positive
PRJNA729271	stage_1W_vs_E10	ordinary_muscle_development	ENSGALG00010021269	NUDT13	0.228542768	0.1723946	1.325695633	0.184940535	0.213977734	positive
PRJNA729271	stage_1W_vs_E10	ordinary_muscle_development	ENSGALG00010021271	ENSGALG00010021271	-0.74312659	0.473711339	-1.568732956	0.116710176	0.139041177	negative
PRJNA729271	stage_1W_vs_E10	ordinary_muscle_development	ENSGALG00010021272	ENSGALG00010021272	0.404386984	0.3599956	1.123310907	0.261305473	0.295032157	positive
PRJNA729271	stage_1W_vs_E10	ordinary_muscle_development	ENSGALG00010021275	MAP3K2	0.088057143	0.092377423	0.953232295	0.340472387	0.377535744	positive
PRJNA729271	stage_1W_vs_E10	ordinary_muscle_development	ENSGALG00010021277	ENSGALG00010021277	-0.513387374	0.252357927	-2.034361989	0.041915111	0.052826671	negative
PRJNA729271	stage_1W_vs_E10	ordinary_muscle_development	ENSGALG00010021287	COL17A1	-6.777803787	0.479724495	-14.1285339	2.53364E-45	2.39408E-44	negative
PRJNA729271	stage_1W_vs_E10	ordinary_muscle_development	ENSGALG00010021290	ERCC3	0.209608617	0.067915068	3.086334481	0.002026407	0.002934929	positive
PRJNA729271	stage_1W_vs_E10	ordinary_muscle_development	ENSGALG00010021293	PCNX1	0.188516506	0.068947471	2.734204782	0.006253114	0.008637211	positive
PRJNA729271	stage_1W_vs_E10	ordinary_muscle_development	ENSGALG00010021296	GATC	0.29244554	0.121963056	2.397820695	0.016492936	0.02174901	positive
PRJNA729271	stage_1W_vs_E10	ordinary_muscle_development	ENSGALG00010021298	RPGRIP1L	-1.469228941	0.149427247	-9.832403164	8.16468E-23	3.42638E-22	negative
PRJNA729271	stage_1W_vs_E10	ordinary_muscle_development	ENSGALG00010021299	CYP27C1	-4.320289699	0.180376772	-23.9514748	8.9189E-127	5.5724E-125	negative
PRJNA729271	stage_1W_vs_E10	ordinary_muscle_development	ENSGALG00010021301	SMURF1	-0.237465037	0.081608632	-2.909802946	0.003616567	0.005115353	negative
PRJNA729271	stage_1W_vs_E10	ordinary_muscle_development	ENSGALG00010021302	P4HA1	-0.838935605	0.060489251	-13.86916833	9.73996E-44	8.79083E-43	negative
PRJNA729271	stage_1W_vs_E10	ordinary_muscle_development	ENSGALG00010021307	MAP3K9	-0.642257016	0.140086751	-4.5847092	4.54619E-06	8.08552E-06	negative
PRJNA729271	stage_1W_vs_E10	ordinary_muscle_development	ENSGALG00010021309	ST3GAL3	0.043979206	0.127015141	0.346251679	0.72915358	0.755690057	positive
PRJNA729271	stage_1W_vs_E10	ordinary_muscle_development	ENSGALG00010021311	TRIAP1	0.432442227	0.120373511	3.592503217	0.000327517	0.000507413	positive

PRJNA729271	stage_1W_vs_E10	ordinary_muscle_development	ENSGALG00010021313	KPNA7	2.283506312	1.315807128	1.735441513	0.082662632	0.100452285	positive
PRJNA729271	stage_1W_vs_E10	ordinary_muscle_development	ENSGALG00010021315	COX6A1	0.437336031	0.135257249	3.233364826	0.001223412	0.001805657	positive
PRJNA729271	stage_1W_vs_E10	ordinary_muscle_development	ENSGALG00010021318	TP73	-2.13079042	0.27214882	-7.82950454	4.89796E-15	1.45842E-14	negative
PRJNA729271	stage_1W_vs_E10	ordinary_muscle_development	ENSGALG00010021320	SLC6A6	0.374569619	0.195863913	1.912397302	0.055825254	0.069291578	positive
PRJNA729271	stage_1W_vs_E10	ordinary_muscle_development	ENSGALG00010021325	ARPC1A	0.338937744	0.063695233	5.321241929	1.03061E-07	2.05739E-07	positive
PRJNA729271	stage_1W_vs_E10	ordinary_muscle_development	ENSGALG00010021327	IGSF1	1.656398141	0.454405366	3.645199345	0.000267185	0.000416973	positive
PRJNA729271	stage_1W_vs_E10	ordinary_muscle_development	ENSGALG00010021329	PSMC3IP	-2.322084335	0.172621998	-13.45184485	3.00286E-41	2.51743E-40	negative
PRJNA729271	stage_1W_vs_E10	ordinary_muscle_development	ENSGALG00010021330	COMMD4	-0.062333479	0.130208068	-0.478722096	0.632136344	0.665080664	negative
PRJNA729271	stage_1W_vs_E10	ordinary_muscle_development	ENSGALG00010021332	OIT3	0.682351895	0.129152719	5.283294854	1.26881E-07	2.51592E-07	positive
PRJNA729271	stage_1W_vs_E10	ordinary_muscle_development	ENSGALG00010021335	NFATC3	-0.034059184	0.102079492	-0.33365354	0.738641012	0.764320493	negative
PRJNA729271	stage_1W_vs_E10	ordinary_muscle_development	ENSGALG00010021338	PDIA5	-1.037439209	0.158764231	-6.534464341	6.38376E-11	1.53174E-10	negative
PRJNA729271	stage_1W_vs_E10	ordinary_muscle_development	ENSGALG00010021341	L3MBTL1	-0.948932876	0.160719848	-5.904266891	3.54218E-09	7.72206E-09	negative
PRJNA729271	stage_1W_vs_E10	ordinary_muscle_development	ENSGALG00010021342	SFR1	-3.252426163	0.195562914	-16.63109886	4.14914E-62	6.38448E-61	negative
PRJNA729271	stage_1W_vs_E10	ordinary_muscle_development	ENSGALG00010021345	NEIL1	0.647958784	0.153596163	4.218587044	2.45838E-05	4.13678E-05	positive
PRJNA729271	stage_1W_vs_E10	ordinary_muscle_development	ENSGALG00010021346	RPS21	0.532957698	0.278476862	1.913831166	0.055641732	0.069075617	positive
PRJNA729271	stage_1W_vs_E10	ordinary_muscle_development	ENSGALG00010021348	SLC4A3	0.187714915	0.116347495	1.61339885	0.106657883	0.127739649	positive
PRJNA729271	stage_1W_vs_E10	ordinary_muscle_development	ENSGALG00010021350	CABLES2	-0.518133095	0.136997242	-3.782069538	0.00015553	0.000246734	negative
PRJNA729271	stage_1W_vs_E10	ordinary_muscle_development	ENSGALG00010021352	CFAP43	-1.187020107	0.236655663	-5.015811133	5.28102E-07	1.00523E-06	negative
PRJNA729271	stage_1W_vs_E10	ordinary_muscle_development	ENSGALG00010021353	IPO13	1.612117472	0.178091624	9.052180207	1.40142E-19	5.17016E-19	positive
PRJNA729271	stage_1W_vs_E10	ordinary_muscle_development	ENSGALG00010021354	ENSGALG00010021354	0.315494752	0.112213919	2.811547406	0.004930382	0.006888943	positive
PRJNA729271	stage_1W_vs_E10	ordinary_muscle_development	ENSGALG00010021358	MMP15	1.029560976	0.129126892	7.973249892	1.54555E-15	4.71142E-15	positive
PRJNA729271	stage_1W_vs_E10	ordinary_muscle_development	ENSGALG00010021360	PTPRN	-0.6448899	0.277761914	-2.321736231	0.020247143	0.026437468	negative
PRJNA729271	stage_1W_vs_E10	ordinary_muscle_development	ENSGALG00010021361	WRAP73	-0.963024636	0.094720704	-10.16699193	2.78373E-24	1.23887E-23	negative
PRJNA729271	stage_1W_vs_E10	ordinary_muscle_development	ENSGALG00010021362	ARPC1B	0.329156656	0.121280096	2.7140204	0.006647207	0.009158866	positive
PRJNA729271	stage_1W_vs_E10	ordinary_muscle_development	ENSGALG00010021363	ENSGALG00010021363	0.122774263	0.073995262	1.659217894	0.097071893	0.116962352	positive
PRJNA729271	stage_1W_vs_E10	ordinary_muscle_development	ENSGALG00010021367	GRIP2	-0.895936723	0.181557944	-4.934715075	8.02679E-07	1.51239E-06	negative
PRJNA729271	stage_1W_vs_E10	ordinary_muscle_development	ENSGALG00010021372	CD86	3.319162005	0.573311132	5.789460239	7.0613E-09	1.51187E-08	positive
PRJNA729271	stage_1W_vs_E10	ordinary_muscle_development	ENSGALG00010021373	CEP250	-0.601594637	0.154279325	-3.899385984	9.64369E-05	0.000155317	negative
PRJNA729271	stage_1W_vs_E10	ordinary_muscle_development	ENSGALG00010021375	TUBG1	-0.651482137	0.117449432	-5.546916041	2.90752E-08	5.99752E-08	negative
PRJNA729271	stage_1W_vs_E10	ordinary_muscle_development	ENSGALG00010021378	KIF11	-2.664957899	0.187107904	-14.2428932	4.96291E-46	4.77354E-45	negative
PRJNA729271	stage_1W_vs_E10	ordinary_muscle_development	ENSGALG00010021381	DNAJB2	2.263723385	0.146971769	15.40243685	1.57616E-53	1.95269E-52	positive
PRJNA729271	stage_1W_vs_E10	ordinary_muscle_development	ENSGALG00010021382	TPRG1L	0.498149523	0.086810791	5.738336417	9.56111E-09	2.03E-08	positive
PRJNA729271	stage_1W_vs_E10	ordinary_muscle_development	ENSGALG00010021383	MSI1	-0.966282978	0.119822966	-8.064255225	7.36837E-16	2.28166E-15	negative
PRJNA729271	stage_1W_vs_E10	ordinary_muscle_development	ENSGALG00010021384	ARTN	1.189434948	0.383401352	3.102323301	0.001920081	0.002788177	positive
PRJNA729271	stage_1W_vs_E10	ordinary_muscle_development	ENSGALG00010021385	GDF5	-7.688905036	0.646438408	-11.89425774	1.26775E-32	7.89349E-32	negative
PRJNA729271	stage_1W_vs_E10	ordinary_muscle_development	ENSGALG00010021387	SLC2A14	0.884729194	0.094041943	9.407814909	5.06558E-21	1.9834E-20	positive
PRJNA729271	stage_1W_vs_E10	ordinary_muscle_development	ENSGALG00010021388	DPH2	-0.868425916	0.152721333	-5.686343205	1.29788E-08	2.73125E-08	negative
PRJNA729271	stage_1W_vs_E10	ordinary_muscle_development	ENSGALG00010021390	GSTO2	-0.933178945	0.215197065	-4.336392528	1.4484E-05	2.48192E-05	negative
PRJNA729271	stage_1W_vs_E10	ordinary_muscle_development	ENSGALG00010021391	ENSGALG00010021391	2.183726683	0.362892166	6.017563582	1.77062E-09	3.93697E-09	positive
PRJNA729271	stage_1W_vs_E10	ordinary_muscle_development	ENSGALG00010021393	CCDC174	0.237667852	0.113280701	2.098043617	0.035901293	0.045584201	positive
PRJNA729271	stage_1W_vs_E10	ordinary_muscle_development	ENSGALG00010021394	MAN2C1	-0.20495972	0.097590916	-2.100192597	0.035711902	0.045347702	negative
PRJNA729271	stage_1W_vs_E10	ordinary_muscle_development	ENSGALG00010021396	MED6	0.284640643	0.078634646	3.61978668	0.000294846	0.000458611	positive
PRJNA729271	stage_1W_vs_E10	ordinary_muscle_development	ENSGALG00010021397	ENSGALG00010021397	4.241386915	1.725633284	2.457872685	0.013976271	0.018604652	positive
PRJNA729271	stage_1W_vs_E10	ordinary_muscle_development	ENSGALG00010021400	B4GALT2	0.233766076	0.09208038	2.538717546	0.011125961	0.014957411	positive
PRJNA729271	stage_1W_vs_E10	ordinary_muscle_development	ENSGALG00010021401	PDAP1	-0.587076283	0.094981211	-6.180972837	6.37078E-10	1.44718E-09	negative
PRJNA729271	stage_1W_vs_E10	ordinary_muscle_development	ENSGALG00010021402	ENSGALG00010021402	0.207448323	0.088404766	2.346573977	0.018946905	0.024804496	positive
PRJNA729271	stage_1W_vs_E10	ordinary_muscle_development	ENSGALG00010021404	UQCC1	0.264156854	0.094041903	2.80892715	0.004970689	0.006941916	positive
PRJNA729271	stage_1W_vs_E10	ordinary_muscle_development	ENSGALG00010021405	IDE	1.500685336	0.113853534	13.18084107	1.13123E-39	8.96998E-39	positive
PRJNA729271	stage_1W_vs_E10	ordinary_muscle_development	ENSGALG00010021407	SIN3A	-0.215175098	0.111808376	-1.924498916	0.054292077	0.067492594	negative
PRJNA729271	stage_1W_vs_E10	ordinary_muscle_development	ENSGALG00010021408	TTC9	0.103749724	0.217531153	0.476941913	0.633403485	0.66612374	positive
PRJNA729271	stage_1W_vs_E10	ordinary_muscle_development	ENSGALG00010021409	MEGF6	-2.350992267	0.273765735	-8.587605979	8.88002E-18	2.98506E-17	negative
PRJNA729271	stage_1W_vs_E10	ordinary_muscle_development	ENSGALG00010021411	MCU	1.294914563	0.128920496	10.04428775	9.73484E-24	4.25148E-23	positive
PRJNA729271	stage_1W_vs_E10	ordinary_muscle_development	ENSGALG00010021412	ZNF319	0.63447989	0.123450819	5.139535682	2.75418E-07	5.34215E-07	positive
PRJNA729271	stage_1W_vs_E10	ordinary_muscle_development	ENSGALG00010021413	SYNJ2BP	1.138180405	0.151873504	7.494265767	6.66705E-14	1.87648E-13	positive
PRJNA729271	stage_1W_vs_E10	ordinary_muscle_development	ENSGALG00010021414	BUD31	0.548331771	0.111250974	4.928781766	8.27439E-07	1.55743E-06	positive
PRJNA729271	stage_1W_vs_E10	ordinary_muscle_development	ENSGALG00010021415	SIRT4	0.863106929	0.149289766	5.781420586	7.40724E-09	1.5829E-08	positive
PRJNA729271	stage_1W_vs_E10	ordinary_muscle_development	ENSGALG00010021416	PLEKHH3	1.331008577	0.1216917	10.93754612	7.62354E-28	3.94373E-27	positive
PRJNA729271	stage_1W_vs_E10	ordinary_muscle_development	ENSGALG00010021417	STK16	-1.373348335	0.16603131	-8.271622588	1.32148E-16	4.21449E-16	negative
PRJNA729271	stage_1W_vs_E10	ordinary_muscle_development	ENSGALG00010021419	MANSC1	-1.065143216	0.200669976	-5.307935139	1.10874E-07	2.20778E-07	negative
PRJNA729271	stage_1W_vs_E10	ordinary_muscle_development	ENSGALG00010021420	PLXNB3	-1.280525788	0.190548131	-6.720222246	1.81448E-11	4.50048E-11	negative
PRJNA729271	stage_1W_vs_E10	ordinary_muscle_development	ENSGALG00010021421	GLB1L	-0.227083711	0.169125716	-1.342691794	0.179371793	0.207883116	negative
PRJNA729271	stage_1W_vs_E10	ordinary_muscle_development	ENSGALG00010021424	PTCD1	0.522686533	0.106913074	4.888892566	1.01405E-06	1.89611E-06	positive
PRJNA729271	stage_1W_vs_E10	ordinary_muscle_development	ENSGALG00010021425	PTPN9	-1.840856936	0.129467499	-14.21868003	7.01639E-46	6.72636E-45	negative
PRJNA729271	stage_1W_vs_E10	ordinary_muscle_development	ENSGALG00010021426	SLC38A7	-0.435983794	0.146624359	-2.973474518	0.002944489	0.004198757	negative
PRJNA729271	stage_1W_vs_E10	ordinary_muscle_development	ENSGALG00010021427	RPLPO	1.158586399	0.135373703	8.558430282	1.14415E-17	3.82138E-17	positive
PRJNA729271	stage_1W_vs_E10	ordinary_muscle_development	ENSGALG00010021429	MED8	1.485404213	0.112674518	13.18314238	1.09724E-39	8.70521E-39	positive
PRJNA729271	stage_1W_vs_E10	ordinary_muscle_development	ENSGALG00010021431	CPSF4	-0.401364724	0.106993891	-3.751286346	0.00017593	0.000278001	negative
PRJNA729271	stage_1W_vs_E10	ordinary_muscle_development	ENSGALG00010021432	SYT9	-0.646099146	0.299486664	-2.157355311	0.03097799	0.039614113	negative
PRJNA729271	stage_1W_vs_E10	ordinary_muscle_development	ENSGALG00010021433	ENSGALG00010021433	6.186527784	0.2693629	22.96726012	9.9065E-117	5.0208E-115	positive
PRJNA729271	stage_1W_vs_E10	ordinary_muscle_development	ENSGALG00010021434	ANKZF1	0.947668534	0.176411834	5.371910221	7.79069E-08	1.57016E-07	positive
PRJNA729271	stage_1W_vs_E10	ordinary_muscle_development	ENSGALG00010021435	GCN1	0.479954092	0.089014691	5.391852565	6.9735E-08	1.40879E-07	positive
PRJNA729271	stage_1W_vs_E10	ordinary_muscle_development	ENSGALG00010021438	GOT2	1.941600343	0.109948623	17.65916021	8.65173E-70	1.62866E-68	positive
PRJNA729271	stage_1W_vs_E10	ordinary_muscle_development	ENSGALG00010021439	ITPRIP	-0.158320046	0.186646521	-0.848234644	0.396307307	0.434496212	negative
PRJNA729271	stage_1W_vs_E10	ordinary_muscle_development	ENSGALG00010021441	ENSGALG00010021441	-0.382810362	0.326903414	-1.171019771	0.24159083	0.274053771	negative
PRJNA729271	stage_1W_vs_E10	ordinary_muscle_development	ENSGALG00010021443	SNUPN	0.101618262	0.134676322	0.754536953	0.450526865	0.488655111	positive
PRJNA729271	stage_1W_vs_E10	ordinary_muscle_development	ENSGALG00010021444	ATG9A	2.552676208	0.245708082	10.38906083	2.78078E-25	1.29232E-24	positive
PRJNA729271	stage_1W_vs_E10	ordinary_muscle_development	ENSGALG00010021446	HOXB8	-1.822137535	0.647548439	-2.813901518	0.004894422	0.006840676	negative
PRJNA729271	stage_1W_vs_E10	ordinary_muscle_development	ENSGALG00010021447	OCSTAMP	0.835695812	0.664443483	1.257737991	0.208486528	0.239177857	positive
PRJNA729271	stage_1W_vs_E10	ordinary_muscle_development	ENSGALG00010021448	SZT2	0.507967295	0.100271439	5.065922048	4.06428E-07	7.79772E-07	positive
PRJNA729271	stage_1W_vs_E10	ordinary_muscle_development	ENSGALG00010021450	OLFML1	2.662006128	0.232806299	11.43442482	2.81395E-30	1.60268E-29	positive
PRJNA729271	stage_1W_vs_E10	ordinary_muscle_development	ENSGALG00010021452	SLC25A1	-0.955694864	0.120107685	-7.956983462	1.76284E-15	5.36252E-15	negative
PRJNA729271	stage_1W_vs_E10	ordinary_muscle_development	ENSGALG00010021454	ATP5J2	1.222604273	0.174201935	7.018316276	2.24558E-12	5.84269E-12	positive
PRJNA729271	stage_1W_vs_E10	ordinary_muscle_development	ENSGALG00010021455	CFAP58	-0.125791463	0.515086152	-0.244214415	0.807064763	0.827444034	negative
PRJNA729271	stage_1W_vs_E10	ordinary_muscle_development	ENSGALG00010021457	USB1	1.018750566	0.112567275	9.050148567	1.42774E-19	5.26459E-19	positive
PRJNA729271	stage_1W_vs_E10	ordinary_muscle_development	ENSGALG00010021458	USP30	0.32077485	0.080162868	4.001539106	6.29318E-05	0.000102817	positive
PRJNA729271	stage_1W_vs_E10	ordinary_muscle_development	ENSGALG00010021459	ARHGEF16	0.200148457	0.125667139	1.592687302	0.111230358	0.132873251	positive
PRJNA729271	stage_1W_vs_E10	ordinary_muscle_development	ENSGALG00010021461	SNX33	0.723358486	0.109559471	6.602427708	4.04479E-11	9.85033E-11	positive
PRJNA729271	stage_1W_vs_E10	ordinary_muscle_development	ENSGALG00010021462	SETD5	-0.569664601	0.095371166	-5.973132423	2.32741E-09	5.13326E-09	negative
PRJNA729271	stage_1W_vs_E10	ordinary_muscle_development	ENSGALG00010021464	ENSGALG00010021464	2.349853601	0.403253604	5.827235215	5.63532E-09	1.21445E-08	positive
PRJNA729271	stage_1W_vs_E10	ordinary_muscle_development	ENSGALG00010021465	ABCB6	2.045374799	0.211425408	9.674214756	3.88059E-22	1.58181E-21	positive
PRJNA729271	stage_1W_vs_E10	ordinary_muscle_development	ENSGALG00010021466	HES4	-0.826988806	0.18201998	-4.543395751	5.53552E-06	9.7755E-06	negative
PRJNA729271	stage_1W_vs_E10	ordinary_muscle_development	ENSGALG00010021467	LHFPL4	0.744186748	0.248948336	2.989322035	0.002795973	0.003995625	positive
PRJNA729271	stage_1W_vs_E10	ordinary_muscle_development	ENSGALG00010021469	CSPG4	-0.621960719	0.162311243	-3.831901651	0.000127157	0.000202854	negative
PRJNA729271	stage_1W_vs_E10	ordinary_muscle_development	ENSGALG00010021470	ENSGALG00010021470	-0.974352621	0.600145808	-1.623526497	0.104476904	0.125270305	negative
PRJNA729271	stage_1W_vs_E10	ordinary_muscle_development	ENSGALG00010021473	SLC8A3	1.671788432	0.131419856	12.72097293	4.52194E-37	3.27892E-36	positive
PRJNA729271	stage_1W_vs_E10	ordinary_muscle_development	ENSGALG00010021474	PPFIBP2	0.474401054	0.181011163	2.620838662	0.008771376	0.011933649	positive
PRJNA729271	stage_1W_vs_E10	ordinary_muscle_development	ENSGALG00010021476	ALKBH2	0.234366078	0.120378181	1.946914927	0.051544935	0.064292559	positive
PRJNA729271	stage_1W_vs_E10	ordinary_muscle_development	ENSGALG00010021477	ZFAND2B	0.961724339	0.116673727	8.242852623	1.68153E-16	5.34826E-16	positive
PRJNA729271	stage_1W_vs_E10	ordinary_muscle_development	ENSGALG00010021478	SLC13A3	0.157338313	0.194583574	0.808589903	0.418751075	0.456822219	positive
PRJNA729271	stage_1W_vs_E10	ordinary_muscle_development	ENSGALG00010021479	SKAP1	0.320534699	0.458788194	0.698655072	0.484767618	0.522976081	positive
PRJNA729271	stage_1W_vs_E10	ordinary_muscle_development	ENSGALG00010021480	ENSGALG00010021480	0.036274723	0.07528141	0.48185499	0.629908962	0.663170425	positive
PRJNA729271	stage_1W_vs_E10	ordinary_muscle_development	ENSGALG00010021481	MICU1	0.269778841	0.175250887	1.539386446	0.123709981	0.146922503	positive
PRJNA729271	stage_1W_vs_E10	ordinary_muscle_development	ENSGALG00010021483	UNG	-2.20320217	0.159685248	-13.79715529	2.65109E-43	2.35608E-42	negative
PRJNA729271	stage_1W_vs_E10	ordinary_muscle_development	ENSGALG00010021484	MARCHF5	0.870483118	0.070882477	12.28065329	1.15065E-34	7.73237E-34	positive
PRJNA729271	stage_1W_vs_E10	ordinary_muscle_development	ENSGALG00010021485	SMOC1	-3.649185508	0.235505405	-15.49512425	3.74237E-54	4.72935E-53	negative
PRJNA729271	stage_1W_vs_E10	ordinary_muscle_development	ENSGALG00010021487	KLHL17	-2.136464706	0.164726639	-12.96975836	1.81602E-38	1.37818E-37	negative
PRJNA729271	stage_1W_vs_E10	ordinary_muscle_development	ENSGALG00010021488	HYI	-0.1697067	0.132762687	-1.27827105	0.201153872	0.231480261	negative
PRJNA729271	stage_1W_vs_E10	ordinary_muscle_development	ENSGALG00010021489	CNPPD1	1.319413317	0.08488949	15.5427169	1.78263E-54	2.27458E-53	positive
PRJNA729271	stage_1W_vs_E10	ordinary_muscle_development	ENSGALG00010021490	ACACB	6.828287203	0.328683285	20.7746713	7.33645E-96	2.54406E-94	positive
PRJNA729271	stage_1W_vs_E10	ordinary_muscle_development	ENSGALG00010021492	PARP6	0.878353739	0.155069065	5.664274422	1.47648E-08	3.10033E-08	positive
PRJNA729271	stage_1W_vs_E10	ordinary_muscle_development	ENSGALG00010021493	UNC93B1	0.37895513	0.133769158	2.8329036	0.004612729	0.006460674	positive
PRJNA729271	stage_1W_vs_E10	ordinary_muscle_development	ENSGALG00010021494	SLC2A10	-1.57542088	0.249372168	-6.317548962	2.65744E-10	6.15821E-10	negative
PRJNA729271	stage_1W_vs_E10	ordinary_muscle_development	ENSGALG00010021496	MTMR14	1.129544404	0.114919835	9.828976864	8.44726E-23	3.54291E-22	positive
PRJNA729271	stage_1W_vs_E10	ordinary_muscle_development	ENSGALG00010021498	B3GALT6	-1.618612682	0.115747724	-13.98396989	1.95284E-44	1.79495E-43	negative
PRJNA729271	stage_1W_vs_E10	ordinary_muscle_development	ENSGALG00010021501	SLC23A3	4.375063753	0.553039349	7.910944789	2.55443E-15	7.70581E-15	positive
PRJNA729271	stage_1W_vs_E10	ordinary_muscle_development	ENSGALG00010021502	DNAJB12	1.433957911	0.145626773	9.846801407	7.07601E-23	2.97899E-22	positive
PRJNA729271	stage_1W_vs_E10	ordinary_muscle_development	ENSGALG00010021503	KCNS1	-2.676239188	0.66728369	-4.01064679	6.05526E-05	9.91091E-05	negative
PRJNA729271	stage_1W_vs_E10	ordinary_muscle_development	ENSGALG00010021504	ODR4	-0.862648528	0.079991904	-10.7841979	4.08794E-27	2.05104E-26	negative
PRJNA729271	stage_1W_vs_E10	ordinary_muscle_development	ENSGALG00010021505	IHH	-4.555641231	0.567433036	-8.028508985	9.86644E-16	3.03575E-15	negative
PRJNA729271	stage_1W_vs_E10	ordinary_muscle_development	ENSGALG00010021506	MAX	0.341360327	0.254855209	1.339428486	0.180431221	0.209060795	positive
PRJNA729271	stage_1W_vs_E10	ordinary_muscle_development	ENSGALG00010021509	DDIT4	2.23792934	0.27781069	8.055591162	7.90956E-16	2.44454E-15	positive
PRJNA729271	stage_1W_vs_E10	ordinary_muscle_development	ENSGALG00010021510	NTSR1	1.448907374	0.390487817	3.710505964	0.000206845	0.00032494	positive
PRJNA729271	stage_1W_vs_E10	ordinary_muscle_development	ENSGALG00010021512	CPNE9	2.856492639	0.397385135	7.188222177	6.56404E-13	1.75222E-12	positive
PRJNA729271	stage_1W_vs_E10	ordinary_muscle_development	ENSGALG00010021513	SDF4	-0.302556939	0.097645672	-3.098518674	0.001944907	0.0028214	negative
PRJNA729271	stage_1W_vs_E10	ordinary_muscle_development	ENSGALG00010021514	TPR	-0.500183867	0.079358141	-6.302867737	2.92188E-10	6.75697E-10	negative
PRJNA729271	stage_1W_vs_E10	ordinary_muscle_development	ENSGALG00010021516	SRSF5	-0.497997993	0.152292689	-3.270005918	0.001075452	0.001596874	negative
PRJNA729271	stage_1W_vs_E10	ordinary_muscle_development	ENSGALG00010021518	CPEB3	2.311233327	0.145959097	15.83480145	1.79023E-56	2.39828E-55	positive
PRJNA729271	stage_1W_vs_E10	ordinary_muscle_development	ENSGALG00010021519	FNTB	1.299992137	0.09885473	13.15053051	1.68985E-39	1.33121E-38	positive
PRJNA729271	stage_1W_vs_E10	ordinary_muscle_development	ENSGALG00010021521	ENSGALG00010021521	1.438522232	0.260380194	5.524699141	3.30051E-08	6.7912E-08	positive
PRJNA729271	stage_1W_vs_E10	ordinary_muscle_development	ENSGALG00010021523	CELF6	-0.804651736	0.155386884	-5.178376155	2.23826E-07	4.37185E-07	negative
PRJNA729271	stage_1W_vs_E10	ordinary_muscle_development	ENSGALG00010021525	ENSGALG00010021525	2.250720037	0.737925781	3.050062886	0.002287935	0.003294617	positive

PRJNA729271	stage_1W_vs_E10	ordinary_muscle_development	ENSGALG00010021526	KCNJ12	3.430592888	0.213814584	16.04470954	6.22495E-58	8.61626E-57	positive
PRJNA729271	stage_1W_vs_E10	ordinary_muscle_development	ENSGALG00010021528	BRPF1	0.835734138	0.166070472	5.032406581	4.84361E-07	9.23912E-07	positive
PRJNA729271	stage_1W_vs_E10	ordinary_muscle_development	ENSGALG00010021529	BTAF1	0.605532024	0.07538303	8.032736654	9.53221E-16	2.9354E-15	positive
PRJNA729271	stage_1W_vs_E10	ordinary_muscle_development	ENSGALG00010021530	MLF2	-0.292670503	0.060725998	-4.819525645	1.439E-06	2.65711E-06	negative
PRJNA729271	stage_1W_vs_E10	ordinary_muscle_development	ENSGALG00010021531	RAB15	-1.440771705	0.292229007	-4.930283001	8.21106E-07	1.54597E-06	negative
PRJNA729271	stage_1W_vs_E10	ordinary_muscle_development	ENSGALG00010021532	DPP10	-2.052350144	0.18611381	-11.02739312	2.81919E-28	1.48543E-27	negative
PRJNA729271	stage_1W_vs_E10	ordinary_muscle_development	ENSGALG00010021533	SSX2IP	-4.686584027	0.350890413	-13.35626123	1.08898E-40	8.91794E-40	negative
PRJNA729271	stage_1W_vs_E10	ordinary_muscle_development	ENSGALG00010021534	KIFC3	1.762524378	0.169093107	10.42339581	1.93904E-25	9.08121E-25	positive
PRJNA729271	stage_1W_vs_E10	ordinary_muscle_development	ENSGALG00010021535	SUSD6	0.250888402	0.086019217	2.916655261	0.003538066	0.00500774	positive
PRJNA729271	stage_1W_vs_E10	ordinary_muscle_development	ENSGALG00010021538	TNFRSF18	1.743604648	0.417467526	4.176623425	2.95868E-05	4.95507E-05	positive
PRJNA729271	stage_1W_vs_E10	ordinary_muscle_development	ENSGALG00010021539	HEXA	-0.793969957	0.121456912	-6.537050422	6.27439E-11	1.50625E-10	negative
PRJNA729271	stage_1W_vs_E10	ordinary_muscle_development	ENSGALG00010021540	STK4	-1.252984879	0.079144632	-15.83158394	1.88418E-56	2.51948E-55	negative
PRJNA729271	stage_1W_vs_E10	ordinary_muscle_development	ENSGALG00010021541	CYP27A1	3.778194155	0.275147176	13.73153892	6.57225E-43	5.78063E-42	positive
PRJNA729271	stage_1W_vs_E10	ordinary_muscle_development	ENSGALG00010021543	PLEKHD1	0.050126605	0.270482253	0.185323084	0.852975633	0.869471294	positive
PRJNA729271	stage_1W_vs_E10	ordinary_muscle_development	ENSGALG00010021545	CYB5R2	-1.4559147	0.143749203	-10.12815842	4.1438E-24	1.83123E-23	negative
PRJNA729271	stage_1W_vs_E10	ordinary_muscle_development	ENSGALG00010021546	OGG1	-1.017895878	0.181101195	-5.620591715	1.90305E-08	3.97244E-08	negative
PRJNA729271	stage_1W_vs_E10	ordinary_muscle_development	ENSGALG00010021549	ANAPC16	0.901787915	0.090061919	10.01297689	1.33669E-23	5.80098E-23	positive
PRJNA729271	stage_1W_vs_E10	ordinary_muscle_development	ENSGALG00010021550	MAP2K3	1.984885511	0.17098811	11.60832473	3.73869E-31	2.21211E-30	positive
PRJNA729271	stage_1W_vs_E10	ordinary_muscle_development	ENSGALG00010021552	ENSGALG00010021552	0.979887084	0.172707466	5.673681095	1.39761E-08	2.93813E-08	positive
PRJNA729271	stage_1W_vs_E10	ordinary_muscle_development	ENSGALG00010021553	SLC39A9	-1.112015296	0.087806131	-12.66443796	9.30847E-37	6.67951E-36	negative
PRJNA729271	stage_1W_vs_E10	ordinary_muscle_development	ENSGALG00010021554	SNX11	0.767324032	0.147309275	5.208932244	1.8993E-07	3.72536E-07	positive
PRJNA729271	stage_1W_vs_E10	ordinary_muscle_development	ENSGALG00010021556	CAMK1	0.065033681	0.14254949	0.456218261	0.648233035	0.679795821	positive
PRJNA729271	stage_1W_vs_E10	ordinary_muscle_development	ENSGALG00010021558	CHURC1	2.153084396	0.179414528	12.00061344	3.52673E-33	2.24506E-32	positive
PRJNA729271	stage_1W_vs_E10	ordinary_muscle_development	ENSGALG00010021559	MYO1H	-0.158160461	0.597189033	-0.264841536	0.791131538	0.813179098	negative
PRJNA729271	stage_1W_vs_E10	ordinary_muscle_development	ENSGALG00010021561	TGIF2	-1.655090306	0.099738865	-16.59423634	7.67133E-62	1.17172E-60	negative
PRJNA729271	stage_1W_vs_E10	ordinary_muscle_development	ENSGALG00010021562	LRP6	-0.407699543	0.082973216	-4.913628326	8.94062E-07	1.67868E-06	negative
PRJNA729271	stage_1W_vs_E10	ordinary_muscle_development	ENSGALG00010021564	PRKAG3	11.92707295	0.629607258	18.94367131	4.98004E-80	1.18531E-78	positive
PRJNA729271	stage_1W_vs_E10	ordinary_muscle_development	ENSGALG00010021565	CBX1	-2.155679906	0.106832252	-20.17817534	1.52264E-90	4.56005E-89	negative
PRJNA729271	stage_1W_vs_E10	ordinary_muscle_development	ENSGALG00010021566	ENSGALG00010021566	-0.266658773	0.098374499	-2.710649357	0.00671516	0.009249857	negative
PRJNA729271	stage_1W_vs_E10	ordinary_muscle_development	ENSGALG00010021568	TADA3	1.223893125	0.140626471	8.703148945	3.228E-18	1.10929E-17	positive
PRJNA729271	stage_1W_vs_E10	ordinary_muscle_development	ENSGALG00010021570	KATNB1	-0.673056364	0.104831821	-6.420344129	1.35967E-10	3.20513E-10	negative
PRJNA729271	stage_1W_vs_E10	ordinary_muscle_development	ENSGALG00010021572	MYL9	0.678955417	0.11221914	6.050263968	1.44609E-09	3.22825E-09	positive
PRJNA729271	stage_1W_vs_E10	ordinary_muscle_development	ENSGALG00010021573	NATD1	1.66062126	0.19459476	8.533740888	1.4169E-17	4.71487E-17	positive
PRJNA729271	stage_1W_vs_E10	ordinary_muscle_development	ENSGALG00010021574	TTLL4	2.791789565	0.224280174	12.44777689	1.43795E-35	9.98231E-35	positive
PRJNA729271	stage_1W_vs_E10	ordinary_muscle_development	ENSGALG00010021575	ADGRL2	-0.303480174	0.083037993	-3.654714699	0.000257468	0.000402155	negative
PRJNA729271	stage_1W_vs_E10	ordinary_muscle_development	ENSGALG00010021576	ERH	-1.51399229	0.122856076	-12.32330006	6.78594E-35	4.59207E-34	negative
PRJNA729271	stage_1W_vs_E10	ordinary_muscle_development	ENSGALG00010021577	ARIH1	0.549477817	0.076561986	7.176901305	7.13091E-13	1.90005E-12	positive
PRJNA729271	stage_1W_vs_E10	ordinary_muscle_development	ENSGALG00010021578	ENSGALG00010021578	3.99123011	0.977481232	4.083178253	4.44239E-05	7.34152E-05	positive
PRJNA729271	stage_1W_vs_E10	ordinary_muscle_development	ENSGALG00010021579	C20H20ORF24	0.647293804	0.111813733	5.789036684	7.07912E-09	1.51524E-08	positive
PRJNA729271	stage_1W_vs_E10	ordinary_muscle_development	ENSGALG00010021580	KCTD10	-1.444939252	0.111286459	-12.9839629	1.50868E-38	1.14915E-37	negative
PRJNA729271	stage_1W_vs_E10	ordinary_muscle_development	ENSGALG00010021582	STK36	-0.198640544	0.148468899	-1.337926968	0.180920245	0.209560409	negative
PRJNA729271	stage_1W_vs_E10	ordinary_muscle_development	ENSGALG00010021583	NFE2L1	2.616220769	0.220729347	11.8526186	2.0857E-32	1.29087E-31	positive
PRJNA729271	stage_1W_vs_E10	ordinary_muscle_development	ENSGALG00010021584	OVCH2	0.596281277	0.372446541	1.600984867	0.109380271	0.130781739	positive
PRJNA729271	stage_1W_vs_E10	ordinary_muscle_development	ENSGALG00010021586	BBS4	-0.355570991	0.124098961	-2.865221346	0.004167178	0.00585473	negative
PRJNA729271	stage_1W_vs_E10	ordinary_muscle_development	ENSGALG00010021588	RNF25	0.908015705	0.138047725	6.577549209	4.78266E-11	1.15927E-10	positive
PRJNA729271	stage_1W_vs_E10	ordinary_muscle_development	ENSGALG00010021593	DCAF7	-1.111063136	0.05776521	-19.23412265	1.91761E-82	4.8679E-81	negative
PRJNA729271	stage_1W_vs_E10	ordinary_muscle_development	ENSGALG00010021594	ADPGK	-1.807922365	0.092543446	-19.53593088	5.4346E-85	1.46694E-83	negative
PRJNA729271	stage_1W_vs_E10	ordinary_muscle_development	ENSGALG00010021595	UBE3B	0.246745616	0.084446837	2.921904786	0.003478979	0.00492844	positive
PRJNA729271	stage_1W_vs_E10	ordinary_muscle_development	ENSGALG00010021597	GGT7	-1.043914052	0.189617032	-5.505381254	3.6837E-08	7.55879E-08	negative
PRJNA729271	stage_1W_vs_E10	ordinary_muscle_development	ENSGALG00010021599	ARPC4	-0.327831074	0.103700519	-3.16132529	0.00157053	0.00229739	negative
PRJNA729271	stage_1W_vs_E10	ordinary_muscle_development	ENSGALG00010021600	FMN1	-0.229959508	0.132209026	-1.739363156	0.081970905	0.099645108	negative
PRJNA729271	stage_1W_vs_E10	ordinary_muscle_development	ENSGALG00010021601	LPAR5	0.346877749	0.386458115	0.897581746	0.369408574	0.407099314	positive
PRJNA729271	stage_1W_vs_E10	ordinary_muscle_development	ENSGALG00010021602	PLEKHG4	0.000736522	0.098301049	0.007492509	0.994021898	0.995189074	positive
PRJNA729271	stage_1W_vs_E10	ordinary_muscle_development	ENSGALG00010021604	SPTB	2.598551866	0.189284968	13.72825265	6.87722E-43	6.03787E-42	positive
PRJNA729271	stage_1W_vs_E10	ordinary_muscle_development	ENSGALG00010021605	MAP3K3	0.032366625	0.112542481	0.287594736	0.773656975	0.796743009	positive
PRJNA729271	stage_1W_vs_E10	ordinary_muscle_development	ENSGALG00010021606	RNF8	0.176735995	0.093955785	1.881054962	0.059964443	0.074130882	positive
PRJNA729271	stage_1W_vs_E10	ordinary_muscle_development	ENSGALG00010021607	SLC9A5	0.818694738	0.19110264	4.284057717	1.83515E-05	3.11847E-05	positive
PRJNA729271	stage_1W_vs_E10	ordinary_muscle_development	ENSGALG00010021609	ENSGALG00010021609	1.249538339	0.204289539	6.116506716	9.56489E-10	2.15418E-09	positive
PRJNA729271	stage_1W_vs_E10	ordinary_muscle_development	ENSGALG00010021610	TMEM11	0.255295194	0.090059675	2.834733688	0.00458639	0.006425024	positive
PRJNA729271	stage_1W_vs_E10	ordinary_muscle_development	ENSGALG00010021611	GREM1	-3.029917032	0.610827608	-4.960347228	7.03673E-07	1.32948E-06	negative
PRJNA729271	stage_1W_vs_E10	ordinary_muscle_development	ENSGALG00010021612	BCS1L	0.174522478	0.132550513	1.316648832	0.187956362	0.217258969	positive
PRJNA729271	stage_1W_vs_E10	ordinary_muscle_development	ENSGALG00010021613	ADGRL4	2.50191401	0.149560243	16.72846976	8.13003E-63	1.27262E-61	positive
PRJNA729271	stage_1W_vs_E10	ordinary_muscle_development	ENSGALG00010021615	FLVCR1	-1.322335371	0.178057331	-7.426458456	1.11544E-13	3.09382E-13	negative
PRJNA729271	stage_1W_vs_E10	ordinary_muscle_development	ENSGALG00010021617	SORBS1	0.81398558	0.111514036	7.299400202	2.89053E-13	7.84318E-13	positive
PRJNA729271	stage_1W_vs_E10	ordinary_muscle_development	ENSGALG00010021618	NEO1	-2.261491808	0.10821966	-20.89723634	5.67361E-97	1.99609E-95	negative
PRJNA729271	stage_1W_vs_E10	ordinary_muscle_development	ENSGALG00010021619	FHOD1	4.643681046	0.178552958	26.00730394	4.0943E-149	3.3359E-147	positive
PRJNA729271	stage_1W_vs_E10	ordinary_muscle_development	ENSGALG00010021621	ZNF518A	0.021434153	0.137496706	0.155888486	0.876120921	0.890184547	positive
PRJNA729271	stage_1W_vs_E10	ordinary_muscle_development	ENSGALG00010021622	PTHLH	-2.166480826	0.264617326	-8.187222133	2.67325E-16	8.41265E-16	negative
PRJNA729271	stage_1W_vs_E10	ordinary_muscle_development	ENSGALG00010021623	FAR1	1.137002969	0.129551746	8.77643878	1.68733E-18	5.88918E-18	positive
PRJNA729271	stage_1W_vs_E10	ordinary_muscle_development	ENSGALG00010021624	HSD11B2	1.727508989	0.35985564	4.800561108	1.58222E-06	2.90896E-06	positive
PRJNA729271	stage_1W_vs_E10	ordinary_muscle_development	ENSGALG00010021625	HCN4	-2.487895292	0.956506337	-2.601023325	0.009294613	0.012610017	negative
PRJNA729271	stage_1W_vs_E10	ordinary_muscle_development	ENSGALG00010021626	ZNF142	1.601924471	0.211987906	7.556678579	4.13492E-14	1.17475E-13	positive
PRJNA729271	stage_1W_vs_E10	ordinary_muscle_development	ENSGALG00010021627	NCOA6	0.332813086	0.103970474	3.201034615	0.001369351	0.002013261	positive
PRJNA729271	stage_1W_vs_E10	ordinary_muscle_development	ENSGALG00010021628	REC114	-0.718810544	0.584327266	-1.230150614	0.21864071	0.249936679	negative
PRJNA729271	stage_1W_vs_E10	ordinary_muscle_development	ENSGALG00010021629	DHRS7B	2.072365533	0.100598671	20.6003271	2.72596E-94	9.04182E-93	positive
PRJNA729271	stage_1W_vs_E10	ordinary_muscle_development	ENSGALG00010021630	VTG1	-1.402287598	0.291252676	-4.814677126	1.47438E-06	2.72105E-06	negative
PRJNA729271	stage_1W_vs_E10	ordinary_muscle_development	ENSGALG00010021632	LIMD2	-4.009516431	0.171662771	-23.35693644	1.1717E-120	6.6603E-119	negative
PRJNA729271	stage_1W_vs_E10	ordinary_muscle_development	ENSGALG00010021633	CCNJ	-1.255267114	0.110345449	-11.37579413	5.51981E-30	3.1205E-29	negative
PRJNA729271	stage_1W_vs_E10	ordinary_muscle_development	ENSGALG00010021634	PLCD4	5.632314838	0.241301138	23.34143505	1.6838E-120	9.4601E-119	positive
PRJNA729271	stage_1W_vs_E10	ordinary_muscle_development	ENSGALG00010021635	FOXJ2	0.532226292	0.113908964	4.672382885	2.97725E-06	5.36958E-06	positive
PRJNA729271	stage_1W_vs_E10	ordinary_muscle_development	ENSGALG00010021636	C1orf159	-0.053920073	0.198155923	-0.272109317	0.785537966	0.808059954	negative
PRJNA729271	stage_1W_vs_E10	ordinary_muscle_development	ENSGALG00010021637	ATP6V0D1	0.333024644	0.103067469	3.231132451	0.001233008	0.001818339	positive
PRJNA729271	stage_1W_vs_E10	ordinary_muscle_development	ENSGALG00010021638	SRC	-1.436346924	0.179804076	-7.988400222	1.36701E-15	4.1777E-15	negative
PRJNA729271	stage_1W_vs_E10	ordinary_muscle_development	ENSGALG00010021639	SCG5	0.146757691	0.347048288	0.42287398	0.672387208	0.702331381	positive
PRJNA729271	stage_1W_vs_E10	ordinary_muscle_development	ENSGALG00010021640	PLEKHG3	-0.055443623	0.108514412	-0.510933263	0.609397788	0.643726931	negative
PRJNA729271	stage_1W_vs_E10	ordinary_muscle_development	ENSGALG00010021642	NPTN	-0.392584101	0.090888035	-4.319425579	1.56436E-05	2.67083E-05	negative
PRJNA729271	stage_1W_vs_E10	ordinary_muscle_development	ENSGALG00010021643	ENSGALG00010021643	0.794329506	0.12810748	6.200492803	5.62866E-10	1.28202E-09	positive
PRJNA729271	stage_1W_vs_E10	ordinary_muscle_development	ENSGALG00010021644	TARDBP	-0.590634821	0.154240801	-3.829303381	0.000128507	0.000204918	negative
PRJNA729271	stage_1W_vs_E10	ordinary_muscle_development	ENSGALG00010021645	ASCC1	0.170076004	0.138160648	1.231001785	0.218322197	0.249611945	positive
PRJNA729271	stage_1W_vs_E10	ordinary_muscle_development	ENSGALG00010021646	RPUSD3	0.971937347	0.164451396	5.910179963	3.41734E-09	7.45887E-09	positive
PRJNA729271	stage_1W_vs_E10	ordinary_muscle_development	ENSGALG00010021647	ARHGAP11B	-2.061934345	0.139649109	-14.76510919	2.45924E-49	2.63269E-48	negative
PRJNA729271	stage_1W_vs_E10	ordinary_muscle_development	ENSGALG00010021648	BRE	-0.046852875	0.067395484	-0.695193088	0.486934304	0.525157197	negative
PRJNA729271	stage_1W_vs_E10	ordinary_muscle_development	ENSGALG00010021649	PXN	0.642572655	0.096229611	6.677494058	2.43062E-11	5.98978E-11	positive
PRJNA729271	stage_1W_vs_E10	ordinary_muscle_development	ENSGALG00010021650	CD276	-1.63152792	0.112154175	-14.54718843	6.08532E-48	6.20737E-47	negative
PRJNA729271	stage_1W_vs_E10	ordinary_muscle_development	ENSGALG00010021651	RNF113B	0.568929201	0.152793863	3.723508202	0.000196473	0.000309351	positive
PRJNA729271	stage_1W_vs_E10	ordinary_muscle_development	ENSGALG00010021652	BORCS5	-0.052187158	0.096431514	-0.541183647	0.588381008	0.623479984	negative
PRJNA729271	stage_1W_vs_E10	ordinary_muscle_development	ENSGALG00010021655	USP22	-0.563328517	0.093694371	-6.012405146	1.82791E-09	4.05999E-09	negative
PRJNA729271	stage_1W_vs_E10	ordinary_muscle_development	ENSGALG00010021656	SETD6	0.052159828	0.078695132	0.662808829	0.507452999	0.54501565	positive
PRJNA729271	stage_1W_vs_E10	ordinary_muscle_development	ENSGALG00010021657	JAGN1	0.993637822	0.141721431	7.011203683	2.36276E-12	6.14209E-12	positive
PRJNA729271	stage_1W_vs_E10	ordinary_muscle_development	ENSGALG00010021658	STRADA	-0.320761309	0.090517655	-3.543632539	0.000394655	0.000606759	negative
PRJNA729271	stage_1W_vs_E10	ordinary_muscle_development	ENSGALG00010021659	SPOCK2	-1.382154547	0.150686916	-9.172359385	4.62777E-20	1.74187E-19	negative
PRJNA729271	stage_1W_vs_E10	ordinary_muscle_development	ENSGALG00010021660	RNF223	2.335176539	0.457898748	5.09976616	3.40073E-07	6.55849E-07	positive
PRJNA729271	stage_1W_vs_E10	ordinary_muscle_development	ENSGALG00010021661	DUSP16	0.108990147	0.12144473	0.897446495	0.369480712	0.407116866	positive
PRJNA729271	stage_1W_vs_E10	ordinary_muscle_development	ENSGALG00010021662	RASGRP1	1.196738536	0.16122912	7.422595448	1.14847E-13	3.18284E-13	positive
PRJNA729271	stage_1W_vs_E10	ordinary_muscle_development	ENSGALG00010021663	CNOT9	0.68275	0.139607875	4.890483416	1.00589E-06	1.88109E-06	positive
PRJNA729271	stage_1W_vs_E10	ordinary_muscle_development	ENSGALG00010021664	TYSND1	0.149846435	0.162891182	0.919917415	0.357615918	0.395215212	positive
PRJNA729271	stage_1W_vs_E10	ordinary_muscle_development	ENSGALG00010021665	CNOT1	0.294305974	0.091416027	3.219413294	0.001284532	0.00189182	positive
PRJNA729271	stage_1W_vs_E10	ordinary_muscle_development	ENSGALG00010021666	CCDC47	1.002217266	0.092062936	10.88621882	1.34093E-27	6.86326E-27	positive
PRJNA729271	stage_1W_vs_E10	ordinary_muscle_development	ENSGALG00010021667	TBX15	0.385834361	0.191601129	2.013737405	0.044037111	0.055390309	positive
PRJNA729271	stage_1W_vs_E10	ordinary_muscle_development	ENSGALG00010021668	PYGO1	-0.504461027	0.129901193	-3.883421049	0.000102997	0.000165386	negative
PRJNA729271	stage_1W_vs_E10	ordinary_muscle_development	ENSGALG00010021669	ENSGALG00010021669	-0.565101553	0.074221726	-7.613694548	2.6637E-14	7.64561E-14	negative
PRJNA729271	stage_1W_vs_E10	ordinary_muscle_development	ENSGALG00010021670	BLCAP	1.876940805	0.108556771	17.28994682	5.60039E-67	9.73353E-66	positive
PRJNA729271	stage_1W_vs_E10	ordinary_muscle_development	ENSGALG00010021671	TNFRSF13B	1.600160123	0.783762972	2.041637816	0.041187471	0.051968349	positive
PRJNA729271	stage_1W_vs_E10	ordinary_muscle_development	ENSGALG00010021674	ING4	-1.352378749	0.127469701	-10.60941334	2.69435E-26	1.3088E-25	negative
PRJNA729271	stage_1W_vs_E10	ordinary_muscle_development	ENSGALG00010021675	DDX42	-0.555148179	0.059637372	-9.308729723	1.2937E-20	5.00193E-20	negative
PRJNA729271	stage_1W_vs_E10	ordinary_muscle_development	ENSGALG00010021676	TP53INP2	2.734519379	0.27570328	9.918341844	3.46464E-23	1.47923E-22	positive
PRJNA729271	stage_1W_vs_E10	ordinary_muscle_development	ENSGALG00010021677	ENSGALG00010021677	-0.637491932	0.154675248	-4.121486415	3.76436E-05	6.2581E-05	negative
PRJNA729271	stage_1W_vs_E10	ordinary_muscle_development	ENSGALG00010021678	DNAAF4	0.210148379	0.165518461	1.269637105	0.204213926	0.234554743	positive
PRJNA729271	stage_1W_vs_E10	ordinary_muscle_development	ENSGALG00010021679	MASP2	-1.933673575	1.09703121	-1.762642264	0.077960844	0.094985495	negative
PRJNA729271	stage_1W_vs_E10	ordinary_muscle_development	ENSGALG00010021680	PIGT	1.198207774	0.116862142	10.25317312	1.14521E-24	5.19882E-24	positive
PRJNA729271	stage_1W_vs_E10	ordinary_muscle_development	ENSGALG00010021681	BTBD10	-0.364405014	0.081070335	-4.49492423	6.95947E-06	1.2198E-05	negative
PRJNA729271	stage_1W_vs_E10	ordinary_muscle_development	ENSGALG00010021682	MYL4	-6.225692928	0.276796137	-22.49197911	4.9731E-112	2.3404E-110	negative
PRJNA729271	stage_1W_vs_E10	ordinary_muscle_development	ENSGALG00010021683	HFM1	-1.30240344	0.443202905	-2.938616657	0.003296806	0.004681806	negative
PRJNA729271	stage_1W_vs_E10	ordinary_muscle_development	ENSGALG00010021684	BIN1	2.606590866	0.121186815	21.50886514	1.2861E-102	5.0385E-101	positive
PRJNA729271	stage_1W_vs_E10	ordinary_muscle_development	ENSGALG00010021685	DBNDD2	1.952775855	0.138657079	14.08349199	4.79792E-45	4.49262E-44	positive
PRJNA729271	stage_1W_vs_E10	ordinary_muscle_development	ENSGALG00010021686	CCPG1	2.769278141	0.164864599	16.79728792	2.55461E-63	4.06466E-62	positive
PRJNA729271	stage_1W_vs_E10	ordinary_muscle_development	ENSGALG00010021687	ZNF384	-0.153448669	0.10685721	-1.436016048	0.150997775	0.177080319	negative
PRJNA729271	stage_1W_vs_E10	ordinary_muscle_development	ENSGALG00010021688	ITPR1	-0.331266069	0.117532864	-2.818497382	0.0048249	0.006746762	negative
PRJNA729271	stage_1W_vs_E10	ordinary_muscle_development	ENSGALG00010021689	MPRIP	0.419336594	0.109242411	3.838587891	0.000123744	0.000197606	positive
PRJNA729271	stage_1W_vs_E10	ordinary_muscle_development	ENSGALG00010021691	PIGB	-1.146101586	0.15403401	-7.440574877	1.00248E-13	2.78478E-13	negative
PRJNA729271	stage_1W_vs_E10	ordinary_muscle_development	ENSGALG00010021693	PPA1	0.333468842	0.090915357	3.667904433	0.000244547	0.000382591	positive

PRJNA729271	stage_1W_vs_E10	ordinary_muscle_development	ENSGALG00010021694	ENSGALG00010021694	3.057413507	1.117328301	2.736360928	0.006212285	0.00858245	positive
PRJNA729271	stage_1W_vs_E10	ordinary_muscle_development	ENSGALG00010021695	FNBP4	0.30492226	0.070742251	4.310327356	1.63013E-05	2.78116E-05	positive
PRJNA729271	stage_1W_vs_E10	ordinary_muscle_development	ENSGALG00010021697	PIANP	-1.887243567	0.254885621	-7.404276318	1.31868E-13	3.6408E-13	negative
PRJNA729271	stage_1W_vs_E10	ordinary_muscle_development	ENSGALG00010021698	GYPC	-0.441679343	0.135349643	-3.263247187	0.001101434	0.001633277	negative
PRJNA729271	stage_1W_vs_E10	ordinary_muscle_development	ENSGALG00010021699	SYS1	-0.311191224	0.085738937	-3.629520446	0.000283948	0.000442087	negative
PRJNA729271	stage_1W_vs_E10	ordinary_muscle_development	ENSGALG00010021700	FLCN	0.975970471	0.077565284	12.5825682	2.63328E-36	1.86647E-35	positive
PRJNA729271	stage_1W_vs_E10	ordinary_muscle_development	ENSGALG00010021701	DR1	-0.2575589	0.076727199	-3.35681353	0.000788463	0.00118568	negative
PRJNA729271	stage_1W_vs_E10	ordinary_muscle_development	ENSGALG00010021702	PLD6	-0.235452365	0.322883228	-0.729218318	0.465868128	0.504162947	negative
PRJNA729271	stage_1W_vs_E10	ordinary_muscle_development	ENSGALG00010021703	CD82	-1.054227214	0.174803659	-6.030921884	1.63027E-09	3.63103E-09	negative
PRJNA729271	stage_1W_vs_E10	ordinary_muscle_development	ENSGALG00010021704	COPS7A	1.761532241	0.101897787	17.28724733	5.86887E-67	1.01757E-65	positive
PRJNA729271	stage_1W_vs_E10	ordinary_muscle_development	ENSGALG00010021705	CCDC18	-0.632154278	0.19269459	-3.280602113	0.001035858	0.001539817	negative
PRJNA729271	stage_1W_vs_E10	ordinary_muscle_development	ENSGALG00010021706	ENSGALG00010021706	2.143660848	0.159002896	13.48189813	1.99897E-41	1.68558E-40	positive
PRJNA729271	stage_1W_vs_E10	ordinary_muscle_development	ENSGALG00010021707	DLX2	-2.501386752	0.949041385	-2.635698286	0.008396435	0.011441791	negative
PRJNA729271	stage_1W_vs_E10	ordinary_muscle_development	ENSGALG00010021708	CNTNAP5	-5.795830966	0.413688473	-14.01013406	1.3515E-44	1.24936E-43	negative
PRJNA729271	stage_1W_vs_E10	ordinary_muscle_development	ENSGALG00010021710	ENSGALG00010021710	-1.590036086	0.330630157	-4.809107853	1.51605E-06	2.79441E-06	negative
PRJNA729271	stage_1W_vs_E10	ordinary_muscle_development	ENSGALG00010021711	ENSGALG00010021711	0.281924211	0.226022345	1.247328934	0.212276912	0.243256688	positive
PRJNA729271	stage_1W_vs_E10	ordinary_muscle_development	ENSGALG00010021713	RAB27A	0.214517365	0.080776447	2.655692015	0.007914586	0.01081772	positive
PRJNA729271	stage_1W_vs_E10	ordinary_muscle_development	ENSGALG00010021714	TSPAN18	0.198204716	0.118495053	1.67268346	0.094389627	0.113910386	positive
PRJNA729271	stage_1W_vs_E10	ordinary_muscle_development	ENSGALG00010021715	MMAB	0.067786443	0.130501772	0.519429294	0.603461408	0.637967552	positive
PRJNA729271	stage_1W_vs_E10	ordinary_muscle_development	ENSGALG00010021716	TFCP2L1	-0.573608218	0.390867493	-1.467526025	0.142233015	0.167411088	negative
PRJNA729271	stage_1W_vs_E10	ordinary_muscle_development	ENSGALG00010021717	SDC4	0.861351765	0.198180585	4.346297423	1.38455E-05	2.37588E-05	positive
PRJNA729271	stage_1W_vs_E10	ordinary_muscle_development	ENSGALG00010021718	SST7	-4.086675559	0.522501995	-7.821358765	5.22562E-15	1.55375E-14	negative
PRJNA729271	stage_1W_vs_E10	ordinary_muscle_development	ENSGALG00010021719	BHLHE40	4.145573539	0.213768835	19.39278724	8.87923E-84	2.31483E-82	positive
PRJNA729271	stage_1W_vs_E10	ordinary_muscle_development	ENSGALG00010021720	CACNB1	2.448479956	0.260271567	9.407404677	5.08538E-21	1.99062E-20	positive
PRJNA729271	stage_1W_vs_E10	ordinary_muscle_development	ENSGALG00010021721	TP53I11	-0.611010215	0.1433654	-4.261908478	2.02688E-05	3.43181E-05	negative
PRJNA729271	stage_1W_vs_E10	ordinary_muscle_development	ENSGALG00010021722	AARS1	1.224389741	0.158722398	7.714032542	1.21903E-14	3.56033E-14	positive
PRJNA729271	stage_1W_vs_E10	ordinary_muscle_development	ENSGALG00010021723	COPS3	1.356906977	0.106002682	12.80068531	1.62511E-37	1.19513E-36	positive
PRJNA729271	stage_1W_vs_E10	ordinary_muscle_development	ENSGALG00010021725	SH2D6	0.69898319	0.216089744	3.234689335	0.001217752	0.001797669	positive
PRJNA729271	stage_1W_vs_E10	ordinary_muscle_development	ENSGALG00010021726	ARNTL	-0.33628432	0.147786165	-2.275479036	0.022877209	0.029726548	negative
PRJNA729271	stage_1W_vs_E10	ordinary_muscle_development	ENSGALG00010021727	MVK	-0.758142373	0.103686209	-7.311892143	2.63407E-13	7.16203E-13	negative
PRJNA729271	stage_1W_vs_E10	ordinary_muscle_development	ENSGALG00010021728	TMED5	-0.011477792	0.06489948	-0.176854911	0.859622356	0.875200257	negative
PRJNA729271	stage_1W_vs_E10	ordinary_muscle_development	ENSGALG00010021729	RBPJL	-2.895057655	0.626466124	-4.621251722	3.81432E-06	6.82237E-06	negative
PRJNA729271	stage_1W_vs_E10	ordinary_muscle_development	ENSGALG00010021730	ARL8B	0.13838223	0.072175309	1.917307069	0.055198932	0.068549394	positive
PRJNA729271	stage_1W_vs_E10	ordinary_muscle_development	ENSGALG00010021731	SRM	-0.406143885	0.119182433	-3.407749576	0.00065501	0.000990854	negative
PRJNA729271	stage_1W_vs_E10	ordinary_muscle_development	ENSGALG00010021732	CASR	-2.164488562	0.319962673	-6.764815857	1.33479E-11	3.33065E-11	negative
PRJNA729271	stage_1W_vs_E10	ordinary_muscle_development	ENSGALG00010021733	ENSGALG00010021733	2.174323777	0.586519877	3.70716128	0.000209595	0.000329118	positive
PRJNA729271	stage_1W_vs_E10	ordinary_muscle_development	ENSGALG00010021734	PARD3B	0.049161319	0.064444885	0.762842843	0.445557127	0.483699113	positive
PRJNA729271	stage_1W_vs_E10	ordinary_muscle_development	ENSGALG00010021735	PIGBOS1	1.661528406	0.144615386	11.48929204	1.49331E-30	8.62026E-30	positive
PRJNA729271	stage_1W_vs_E10	ordinary_muscle_development	ENSGALG00010021736	EDEM1	0.258810277	0.083130848	3.11328806	0.001850153	0.002689327	positive
PRJNA729271	stage_1W_vs_E10	ordinary_muscle_development	ENSGALG00010021738	PRDM11	-0.480130763	0.073922448	-6.495060425	8.30001E-11	1.97941E-10	negative
PRJNA729271	stage_1W_vs_E10	ordinary_muscle_development	ENSGALG00010021739	EXOSC10	0.9543578	0.109739939	8.696540265	3.42156E-18	1.17442E-17	positive
PRJNA729271	stage_1W_vs_E10	ordinary_muscle_development	ENSGALG00010021740	PDLIM1	0.467266215	0.159376108	2.931846057	0.003369537	0.004781345	positive
PRJNA729271	stage_1W_vs_E10	ordinary_muscle_development	ENSGALG00010021741	RPL19	0.381868199	0.146026372	2.615063247	0.008921093	0.012127098	positive
PRJNA729271	stage_1W_vs_E10	ordinary_muscle_development	ENSGALG00010021742	MATN4	-7.862531501	0.508470225	-15.46311095	6.15526E-54	7.7047E-53	negative
PRJNA729271	stage_1W_vs_E10	ordinary_muscle_development	ENSGALG00010021743	SYT13	-6.265208733	0.630839806	-9.931536776	3.03541E-23	1.30057E-22	negative
PRJNA729271	stage_1W_vs_E10	ordinary_muscle_development	ENSGALG00010021744	LITAF	2.087879473	0.35059188	5.955298995	2.59597E-09	5.70822E-09	positive
PRJNA729271	stage_1W_vs_E10	ordinary_muscle_development	ENSGALG00010021745	RSL24D1	0.155441383	0.125734114	1.236270561	0.21635801	0.247541981	positive
PRJNA729271	stage_1W_vs_E10	ordinary_muscle_development	ENSGALG00010021746	MTOR	0.476846387	0.110631597	4.31021879	1.63093E-05	2.78187E-05	positive
PRJNA729271	stage_1W_vs_E10	ordinary_muscle_development	ENSGALG00010021747	AKTIP	1.167800379	0.115118156	10.14436314	3.51061E-24	1.55663E-23	positive
PRJNA729271	stage_1W_vs_E10	ordinary_muscle_development	ENSGALG00010021748	CDCA7	-2.676435185	0.179444223	-14.9151371	2.62737E-50	2.90938E-49	negative
PRJNA729271	stage_1W_vs_E10	ordinary_muscle_development	ENSGALG00010021749	CLASP1	0.335073895	0.090964134	3.683582518	0.000229979	0.0003605	positive
PRJNA729271	stage_1W_vs_E10	ordinary_muscle_development	ENSGALG00010021750	HELLS	-3.096586904	0.15337951	-20.18905201	1.22186E-90	3.67446E-89	negative
PRJNA729271	stage_1W_vs_E10	ordinary_muscle_development	ENSGALG00010021751	MTCH2	-0.429562465	0.09300039	-4.618931854	3.8572E-06	6.89228E-06	negative
PRJNA729271	stage_1W_vs_E10	ordinary_muscle_development	ENSGALG00010021752	CSTB	-0.21979496	0.110411203	-1.990694366	0.046514498	0.058309059	negative
PRJNA729271	stage_1W_vs_E10	ordinary_muscle_development	ENSGALG00010021753	MTF2	-1.662761212	0.098762154	-16.83601614	1.32887E-63	2.13548E-62	negative
PRJNA729271	stage_1W_vs_E10	ordinary_muscle_development	ENSGALG00010021754	SLC17A9	-2.336133401	0.513470702	-4.549691721	5.37246E-06	9.49796E-06	negative
PRJNA729271	stage_1W_vs_E10	ordinary_muscle_development	ENSGALG00010021755	ENSGALG00010021755	2.569218124	0.153633159	16.72307036	8.90128E-63	1.39034E-61	positive
PRJNA729271	stage_1W_vs_E10	ordinary_muscle_development	ENSGALG00010021756	USP42	-1.777316281	0.13151949	-13.5137102	1.29811E-41	1.10229E-40	negative
PRJNA729271	stage_1W_vs_E10	ordinary_muscle_development	ENSGALG00010021757	ZP4	-0.496243686	0.400047149	-1.240462999	0.214804192	0.245880657	negative
PRJNA729271	stage_1W_vs_E10	ordinary_muscle_development	ENSGALG00010021758	RASSF10	-5.581645029	0.550595576	-10.13746799	3.76738E-24	1.66692E-23	negative
PRJNA729271	stage_1W_vs_E10	ordinary_muscle_development	ENSGALG00010021759	NIFK	-0.06906189	0.093183783	-0.741136358	0.458610766	0.496791201	negative
PRJNA729271	stage_1W_vs_E10	ordinary_muscle_development	ENSGALG00010021760	CSTA	1.754990629	0.393408646	4.460986427	8.15833E-06	1.42373E-05	positive
PRJNA729271	stage_1W_vs_E10	ordinary_muscle_development	ENSGALG00010021761	MAFB	-0.492565932	0.170411646	-2.890447591	0.003846937	0.005424256	negative
PRJNA729271	stage_1W_vs_E10	ordinary_muscle_development	ENSGALG00010021762	LAMB2	3.527355748	0.19847147	17.77260856	1.15207E-70	2.21769E-69	positive
PRJNA729271	stage_1W_vs_E10	ordinary_muscle_development	ENSGALG00010021764	FAM222A	-0.975046232	0.775688995	-1.257006659	0.208751224	0.239462567	negative
PRJNA729271	stage_1W_vs_E10	ordinary_muscle_development	ENSGALG00010021765	EIF2AK1	0.504937407	0.10418288	4.846644723	1.25567E-06	2.32868E-06	positive
PRJNA729271	stage_1W_vs_E10	ordinary_muscle_development	ENSGALG00010021766	AGRN	-0.865955404	0.149695518	-5.784778413	7.2608E-09	1.55321E-08	negative
PRJNA729271	stage_1W_vs_E10	ordinary_muscle_development	ENSGALG00010021767	CCDC58	0.351324871	0.212783421	1.651091377	0.098719918	0.11884927	positive
PRJNA729271	stage_1W_vs_E10	ordinary_muscle_development	ENSGALG00010021768	DIPK1A	1.316542345	0.148801628	8.847634029	8.93942E-19	3.1635E-18	positive
PRJNA729271	stage_1W_vs_E10	ordinary_muscle_development	ENSGALG00010021769	ENSGALG00010021769	-1.079748883	0.185333325	-5.825983446	5.67772E-09	1.22323E-08	negative
PRJNA729271	stage_1W_vs_E10	ordinary_muscle_development	ENSGALG00010021770	RALGAPB	0.268419186	0.097815982	2.744124017	0.006067261	0.008394097	positive
PRJNA729271	stage_1W_vs_E10	ordinary_muscle_development	ENSGALG00010021771	ANKRD61	1.408676737	0.143242274	9.834224887	8.01827E-23	3.36591E-22	positive
PRJNA729271	stage_1W_vs_E10	ordinary_muscle_development	ENSGALG00010021772	TSN	-0.770847664	0.083670195	-9.212930203	3.17341E-20	1.20258E-19	negative
PRJNA729271	stage_1W_vs_E10	ordinary_muscle_development	ENSGALG00010021773	ENSGALG00010021773	-3.028165337	0.996159871	-3.039838711	0.002367049	0.003404482	negative
PRJNA729271	stage_1W_vs_E10	ordinary_muscle_development	ENSGALG00010021774	FBXL20	1.140331569	0.071543824	15.93892389	3.40161E-57	4.63841E-56	positive
PRJNA729271	stage_1W_vs_E10	ordinary_muscle_development	ENSGALG00010021775	CCDC36	2.47462415	0.314255872	7.874551818	3.41966E-15	1.0254E-14	positive
PRJNA729271	stage_1W_vs_E10	ordinary_muscle_development	ENSGALG00010021776	PMS2	-0.298489606	0.093897111	-3.17890085	0.001478347	0.002168012	negative
PRJNA729271	stage_1W_vs_E10	ordinary_muscle_development	ENSGALG00010021778	RBL2	0.684204267	0.103776243	6.593072214	4.30817E-11	1.04759E-10	positive
PRJNA729271	stage_1W_vs_E10	ordinary_muscle_development	ENSGALG00010021779	UNC13C	0.0489889	0.247160045	0.198207199	0.842882955	0.860030159	positive
PRJNA729271	stage_1W_vs_E10	ordinary_muscle_development	ENSGALG00010021781	RAPGEF4	0.497874215	0.264582047	1.881738468	0.059871534	0.074022338	positive
PRJNA729271	stage_1W_vs_E10	ordinary_muscle_development	ENSGALG00010021782	ADAM11	-2.015592834	0.357983041	-5.630414305	1.79777E-08	3.75756E-08	negative
PRJNA729271	stage_1W_vs_E10	ordinary_muscle_development	ENSGALG00010021783	GIT2	-0.910332407	0.048888784	-18.62047538	2.19259E-77	4.87448E-76	negative
PRJNA729271	stage_1W_vs_E10	ordinary_muscle_development	ENSGALG00010021784	ANGPTL7	5.028863221	0.843487446	5.96198941	2.49185E-09	5.4851E-09	positive
PRJNA729271	stage_1W_vs_E10	ordinary_muscle_development	ENSGALG00010021785	CCDC71	-0.041493457	0.090143855	-0.460302668	0.645298987	0.677110816	negative
PRJNA729271	stage_1W_vs_E10	ordinary_muscle_development	ENSGALG00010021786	FAM162A	0.641253726	0.172804	3.71087317	0.000206546	0.000324504	positive
PRJNA729271	stage_1W_vs_E10	ordinary_muscle_development	ENSGALG00010021788	RPL5	0.357308671	0.123912277	2.883561498	0.003932059	0.005540506	positive
PRJNA729271	stage_1W_vs_E10	ordinary_muscle_development	ENSGALG00010021789	CHD9	0.116919659	0.090940215	1.285676069	0.198556136	0.228636098	positive
PRJNA729271	stage_1W_vs_E10	ordinary_muscle_development	ENSGALG00010021790	PHF21A	0.426010906	0.128112344	3.325291641	0.000883261	0.001323294	positive
PRJNA729271	stage_1W_vs_E10	ordinary_muscle_development	ENSGALG00010021791	PLEKHM2	2.367792265	0.183964087	12.87094835	6.55918E-38	4.86817E-37	positive
PRJNA729271	stage_1W_vs_E10	ordinary_muscle_development	ENSGALG00010021792	KLHDC8B	3.916615425	0.258057753	15.17728253	5.00074E-52	5.88836E-51	positive
PRJNA729271	stage_1W_vs_E10	ordinary_muscle_development	ENSGALG00010021793	ENSGALG00010021793	-0.479506697	0.162357742	-2.953395956	0.003142985	0.004467743	negative
PRJNA729271	stage_1W_vs_E10	ordinary_muscle_development	ENSGALG00010021794	ENSGALG00010021794	-1.179491946	0.20106718	-5.866158489	4.46007E-09	9.66639E-09	negative
PRJNA729271	stage_1W_vs_E10	ordinary_muscle_development	ENSGALG00010021795	NDRG3	-0.032565599	0.106399294	-0.306069689	0.759551586	0.783664335	negative
PRJNA729271	stage_1W_vs_E10	ordinary_muscle_development	ENSGALG00010021797	SLC25A34	1.414199197	0.165156926	8.562760464	1.10197E-17	3.68551E-17	positive
PRJNA729271	stage_1W_vs_E10	ordinary_muscle_development	ENSGALG00010021798	RANBP10	1.216820385	0.180237688	6.751198385	1.46629E-11	3.65124E-11	positive
PRJNA729271	stage_1W_vs_E10	ordinary_muscle_development	ENSGALG00010021799	ENSGALG00010021799	0.494317552	0.207163166	2.386126652	0.017026886	0.022402171	positive
PRJNA729271	stage_1W_vs_E10	ordinary_muscle_development	ENSGALG00010021800	TMEM82	1.521829083	0.332856555	4.572026784	4.83029E-06	8.57293E-06	positive
PRJNA729271	stage_1W_vs_E10	ordinary_muscle_development	ENSGALG00010021801	VAMP1	3.118910853	0.241698363	12.90414556	4.2652E-38	3.19504E-37	positive
PRJNA729271	stage_1W_vs_E10	ordinary_muscle_development	ENSGALG00010021803	TLL2	-3.394971319	0.173970766	-19.51460812	8.24993E-85	2.2104E-83	negative
PRJNA729271	stage_1W_vs_E10	ordinary_muscle_development	ENSGALG00010021805	FUT8	-0.702134725	0.109771887	-6.396307339	1.59179E-10	3.74077E-10	negative
PRJNA729271	stage_1W_vs_E10	ordinary_muscle_development	ENSGALG00010021807	CDC42	-0.146954419	0.083002056	-1.770491321	0.076645329	0.093461264	negative
PRJNA729271	stage_1W_vs_E10	ordinary_muscle_development	ENSGALG00010021810	ANKRD13A	-1.667297794	0.098160849	-16.98536444	1.05397E-64	1.73606E-63	negative
PRJNA729271	stage_1W_vs_E10	ordinary_muscle_development	ENSGALG00010021812	TEAD1	0.00509697	0.08335867	0.061145047	0.951243694	0.957671859	positive
PRJNA729271	stage_1W_vs_E10	ordinary_muscle_development	ENSGALG00010021817	EVI5	-0.005476139	0.100916607	-0.054264001	0.95672483	0.962234695	negative
PRJNA729271	stage_1W_vs_E10	ordinary_muscle_development	ENSGALG00010021821	PPP1R19L	-1.461510632	0.120723327	-12.10628194	9.7829E-34	6.35034E-33	negative
PRJNA729271	stage_1W_vs_E10	ordinary_muscle_development	ENSGALG00010021823	ENSGALG00010021823	0.658034879	0.248414397	2.648940184	0.008074462	0.011024805	positive
PRJNA729271	stage_1W_vs_E10	ordinary_muscle_development	ENSGALG00010021826	FBXO40	7.242218384	0.207438592	34.91258936	4.789E-267	1.3611E-264	positive
PRJNA729271	stage_1W_vs_E10	ordinary_muscle_development	ENSGALG00010021827	CCZ1	-0.648124144	0.096831452	-6.693322564	2.1816E-11	5.38985E-11	negative
PRJNA729271	stage_1W_vs_E10	ordinary_muscle_development	ENSGALG00010021829	ERCC6	0.177999263	0.096564369	1.843322383	0.065281955	0.080334658	positive
PRJNA729271	stage_1W_vs_E10	ordinary_muscle_development	ENSGALG00010021831	ENSGALG00010021831	1.264343498	0.363527435	3.477986461	0.000505195	0.000770092	positive
PRJNA729271	stage_1W_vs_E10	ordinary_muscle_development	ENSGALG00010021835	NUDT8	0.692151836	0.170973886	4.048289775	5.15932E-05	8.47992E-05	positive
PRJNA729271	stage_1W_vs_E10	ordinary_muscle_development	ENSGALG00010021837	ENSGALG00010021837	0.741565314	0.323132089	2.29492935	0.021737173	0.028296392	positive
PRJNA729271	stage_1W_vs_E10	ordinary_muscle_development	ENSGALG00010021839	C1orf50	0.293713308	0.120281574	2.441881156	0.014610956	0.01938188	positive
PRJNA729271	stage_1W_vs_E10	ordinary_muscle_development	ENSGALG00010021840	ENSGALG00010021840	0.556866166	0.2189393	2.543472854	0.010975658	0.014764935	positive
PRJNA729271	stage_1W_vs_E10	ordinary_muscle_development	ENSGALG00010021841	CENPT	-1.849950007	0.095708217	-19.32906144	3.05933E-83	7.90462E-82	negative
PRJNA729271	stage_1W_vs_E10	ordinary_muscle_development	ENSGALG00010021842	ENSGALG00010021842	-1.954243784	0.185461872	-10.53717273	5.8223E-26	2.78621E-25	negative
PRJNA729271	stage_1W_vs_E10	ordinary_muscle_development	ENSGALG00010021843	ENSGALG00010021843	0.868233439	0.389173529	2.230967358	0.025683291	0.033177017	positive
PRJNA729271	stage_1W_vs_E10	ordinary_muscle_development	ENSGALG00010021847	P3H1	-1.242795985	0.151883554	-8.18255795	2.77881E-16	8.72781E-16	negative
PRJNA729271	stage_1W_vs_E10	ordinary_muscle_development	ENSGALG00010021848	LARGE2	1.860866913	0.136163509	13.66641423	1.61142E-42	1.40117E-41	positive
PRJNA729271	stage_1W_vs_E10	ordinary_muscle_development	ENSGALG00010021851	ENSGALG00010021851	1.628510707	0.212732708	7.655196615	1.93017E-14	5.56882E-14	positive
PRJNA729271	stage_1W_vs_E10	ordinary_muscle_development	ENSGALG00010021853	ENSGALG00010021853	0.355642099	0.590068632	0.602713108	0.54669956	0.582848641	positive
PRJNA729271	stage_1W_vs_E10	ordinary_muscle_development	ENSGALG00010021854	TAPBPL	1.444038287	0.153917129	9.381920651	6.47817E-21	2.52626E-20	positive
PRJNA729271	stage_1W_vs_E10	ordinary_muscle_development	ENSGALG00010021860	ENSGALG00010021860	1.57475088	0.258001908	6.103640447	1.03679E-09	2.33106E-09	positive
PRJNA729271	stage_1W_vs_E10	ordinary_muscle_development	ENSGALG00010021862	PDK1	-0.208227016	0.106578467	-1.953743768	0.050731527	0.063321578	negative
PRJNA729271	stage_1W_vs_E10	ordinary_muscle_development	ENSGALG00010021863	CLDN19	2.181128545	0.290739035	7.502014806	6.28443E-14	1.77085E-13	positive
PRJNA729271	stage_1W_vs_E10	ordinary_muscle_development	ENSGALG00010021864	GFI1	1.435364633	0.510578331	2.811252547	0.004934903	0.006894595	positive
PRJNA729271	stage_1W_vs_E10	ordinary_muscle_development	ENSGALG00010021866	SCNN1A	1.678392554	0.364181797	4.608666786	4.05259E-06	7.23071E-06	positive
PRJNA729271	stage_1W_vs_E10	ordinary_muscle_development	ENSGALG00010021867	CXCL12	-0.143731665	0.182370404	-0.788130433	0.430620416	0.468501308	negative
PRJNA729271	stage_1W_vs_E10	ordinary_muscle_development	ENSGALG00010021869	GJC3	0.161055379	0.121733578	1.323015235	0.185830311	0.214972894	positive
PRJNA729271	stage_1W_vs_E10	ordinary_muscle_development	ENSGALG00010021870	ENSGALG00010021870	-0.706730561	0.170386821	-4.147800625	3.35685E-05	5.60505E-05	negative
PRJNA729271	stage_1W_vs_E10	ordinary_muscle_development	ENSGALG00010021871	ENSGALG00010021871	4.677503639	0.250757236	18.65351412	1.18244E-77	2.64497E-76	positive

PRJNA729271	stage_1W_vs_E10	ordinary_muscle_development	ENSGALG00010021874	TMCC1	0.532482651	0.078971025	6.742759738	1.55406E-11	3.86449E-11	positive
PRJNA729271	stage_1W_vs_E10	ordinary_muscle_development	ENSGALG00010021877	PLEKHN1	0.623588571	0.149264551	4.177740575	2.94419E-05	4.93194E-05	positive
PRJNA729271	stage_1W_vs_E10	ordinary_muscle_development	ENSGALG00010021879	CREBL2	1.378660163	0.15404349	8.949811248	3.56091E-19	1.28845E-18	positive
PRJNA729271	stage_1W_vs_E10	ordinary_muscle_development	ENSGALG00010021881	PHF20	-0.802254178	0.0500745	-16.02121182	9.08621E-58	1.25314E-56	negative
PRJNA729271	stage_1W_vs_E10	ordinary_muscle_development	ENSGALG00010021887	NUTF2	-0.661441399	0.110646024	-5.977995198	2.259E-09	4.98694E-09	negative
PRJNA729271	stage_1W_vs_E10	ordinary_muscle_development	ENSGALG00010021892	NDUFV1	1.626555456	0.121690539	13.36632631	9.51242E-41	7.8032E-40	positive
PRJNA729271	stage_1W_vs_E10	ordinary_muscle_development	ENSGALG00010021897	ENSGALG00010021897	-0.281273575	0.221405015	-1.270402911	0.203941147	0.234297133	negative
PRJNA729271	stage_1W_vs_E10	ordinary_muscle_development	ENSGALG00010021902	LMTK2	1.20560762	0.118910961	10.13874259	3.71855E-24	1.64682E-23	positive
PRJNA729271	stage_1W_vs_E10	ordinary_muscle_development	ENSGALG00010021903	MORC2	-0.717135764	0.080199261	-8.941924786	3.8245E-19	1.38245E-18	negative
PRJNA729271	stage_1W_vs_E10	ordinary_muscle_development	ENSGALG00010021905	RBSN	-0.226584168	0.073539438	-3.081124552	0.002062204	0.002984389	negative
PRJNA729271	stage_1W_vs_E10	ordinary_muscle_development	ENSGALG00010021908	EDC4	0.34356409	0.108300407	3.172325013	0.001512237	0.002215247	positive
PRJNA729271	stage_1W_vs_E10	ordinary_muscle_development	ENSGALG00010021913	ENSGALG00010021913	1.302848837	0.175963729	7.404076082	1.32067E-13	3.6456E-13	positive
PRJNA729271	stage_1W_vs_E10	ordinary_muscle_development	ENSGALG00010021916	MRPS25	0.75213766	0.148242175	5.073709022	3.90135E-07	7.49604E-07	positive
PRJNA729271	stage_1W_vs_E10	ordinary_muscle_development	ENSGALG00010021919	PARVA	0.078988613	0.124902641	0.632401458	0.527124582	0.563845533	positive
PRJNA729271	stage_1W_vs_E10	ordinary_muscle_development	ENSGALG00010021922	MRPL51	-0.215625583	0.138608209	-1.555648	0.119791818	0.14248994	negative
PRJNA729271	stage_1W_vs_E10	ordinary_muscle_development	ENSGALG00010021924	ENSGALG00010021924	0.901624578	0.46611124	1.934354937	0.053069503	0.066063418	positive
PRJNA729271	stage_1W_vs_E10	ordinary_muscle_development	ENSGALG00010021928	ENSGALG00010021928	5.100798794	0.877345793	5.813897817	6.10347E-09	1.31319E-08	positive
PRJNA729271	stage_1W_vs_E10	ordinary_muscle_development	ENSGALG00010021931	ITGA6	0.733060112	0.121733239	6.021856632	1.72428E-09	3.83687E-09	positive
PRJNA729271	stage_1W_vs_E10	ordinary_muscle_development	ENSGALG00010021932	RPAP2	1.448884527	0.161362541	8.97906364	2.73081E-19	9.92057E-19	positive
PRJNA729271	stage_1W_vs_E10	ordinary_muscle_development	ENSGALG00010021933	PRR14L	-0.565342376	0.090101174	-6.274528431	3.50696E-10	8.08161E-10	negative
PRJNA729271	stage_1W_vs_E10	ordinary_muscle_development	ENSGALG00010021934	EFTUD2	-1.053772068	0.067464173	-15.6197285	5.34327E-55	6.89676E-54	negative
PRJNA729271	stage_1W_vs_E10	ordinary_muscle_development	ENSGALG00010021935	CETP	4.997672072	0.297558218	16.79561099	2.62784E-63	4.17203E-62	positive
PRJNA729271	stage_1W_vs_E10	ordinary_muscle_development	ENSGALG00010021938	CABP2	0.217817299	0.163128392	1.335250696	0.181794309	0.210499213	positive
PRJNA729271	stage_1W_vs_E10	ordinary_muscle_development	ENSGALG00010021943	OCM3	-4.134276055	0.569360774	-7.261259024			negative
PRJNA729271	stage_1W_vs_E10	ordinary_muscle_development	ENSGALG00010021945	C1QTNF4	-2.711710409	0.225862074	-12.00604583	3.30262E-33	2.10609E-32	negative
PRJNA729271	stage_1W_vs_E10	ordinary_muscle_development	ENSGALG00010021947	CDK2AP2	2.081044236	0.244699004	8.504506366	1.8237E-17	6.04356E-17	positive
PRJNA729271	stage_1W_vs_E10	ordinary_muscle_development	ENSGALG00010021949	ENSGALG00010021949	-1.448636984	0.249875619	-5.797432301	6.73379E-09	1.44345E-08	negative
PRJNA729271	stage_1W_vs_E10	ordinary_muscle_development	ENSGALG00010021951	PSKH1	-0.092834916	0.1440528	-0.644450621	0.519283268	0.556402348	negative
PRJNA729271	stage_1W_vs_E10	ordinary_muscle_development	ENSGALG00010021953	PITPNM1	0.056392316	0.179146809	0.3147827	0.752926648	0.77749318	positive
PRJNA729271	stage_1W_vs_E10	ordinary_muscle_development	ENSGALG00010021958	BHLHA15	-1.03222654	0.430005796	-2.400494478	0.016372938	0.021596663	negative
PRJNA729271	stage_1W_vs_E10	ordinary_muscle_development	ENSGALG00010021960	CDKN1B	0.932917221	0.144681614	6.448070326	1.13283E-10	2.68527E-10	positive
PRJNA729271	stage_1W_vs_E10	ordinary_muscle_development	ENSGALG00010021965	PEX16	0.888253774	0.100544178	8.834462542	1.00581E-18	3.55243E-18	positive
PRJNA729271	stage_1W_vs_E10	ordinary_muscle_development	ENSGALG00010021966	ADGRA1	-0.233459147	0.159092157	-1.467445988	0.142254772	0.167423102	negative
PRJNA729271	stage_1W_vs_E10	ordinary_muscle_development	ENSGALG00010021969	CCDC103	-0.60283256	0.283682119	-2.125028399	0.033584243	0.042788398	negative
PRJNA729271	stage_1W_vs_E10	ordinary_muscle_development	ENSGALG00010021970	ERGIC3	-0.166622131	0.090596682	-1.839163715	0.06589111	0.081045775	negative
PRJNA729271	stage_1W_vs_E10	ordinary_muscle_development	ENSGALG00010021971	ICOS	0.191912993	0.373351793	0.514027245	0.607232942	0.641533637	positive
PRJNA729271	stage_1W_vs_E10	ordinary_muscle_development	ENSGALG00010021972	PISD	0.073102083	0.183869308	0.397576318	0.69094252	0.719690416	positive
PRJNA729271	stage_1W_vs_E10	ordinary_muscle_development	ENSGALG00010021974	GFAP	4.777258498	0.791262183	6.037516515	1.56504E-09	3.48843E-09	positive
PRJNA729271	stage_1W_vs_E10	ordinary_muscle_development	ENSGALG00010021978	NR2C2	0.455187478	0.073779715	6.169547819	6.84856E-10	1.55279E-09	positive
PRJNA729271	stage_1W_vs_E10	ordinary_muscle_development	ENSGALG00010021980	GLMN	0.213016743	0.132318395	1.609880046	0.107424046	0.128591258	positive
PRJNA729271	stage_1W_vs_E10	ordinary_muscle_development	ENSGALG00010021983	CTLA4	-2.542645857	0.581675021	-4.371248145	1.23538E-05	2.12974E-05	negative
PRJNA729271	stage_1W_vs_E10	ordinary_muscle_development	ENSGALG00010021988	FAM180B	3.834211227	0.263080688	14.57427852	4.09425E-48	4.20001E-47	positive
PRJNA729271	stage_1W_vs_E10	ordinary_muscle_development	ENSGALG00010021989	NCAPD2	-1.904300889	0.152464851	-12.49009771	8.45497E-36	5.93773E-35	negative
PRJNA729271	stage_1W_vs_E10	ordinary_muscle_development	ENSGALG00010021994	ENSGALG00010021994	-4.923758384	0.41783633	-11.78394034	4.72323E-32	2.88996E-31	negative
PRJNA729271	stage_1W_vs_E10	ordinary_muscle_development	ENSGALG00010021995	CD28	3.556397684	0.47529153	7.482560621	7.28883E-14	2.04395E-13	positive
PRJNA729271	stage_1W_vs_E10	ordinary_muscle_development	ENSGALG00010021999	ANKRD22	-4.538060622	0.655986262	-6.917920217	4.58322E-12	1.1725E-11	negative
PRJNA729271	stage_1W_vs_E10	ordinary_muscle_development	ENSGALG00010022002	KIF18B	-1.728961213	0.217096348	-7.96402716	1.66529E-15	5.07214E-15	negative
PRJNA729271	stage_1W_vs_E10	ordinary_muscle_development	ENSGALG00010022005	NLRC5	3.977993903	0.181686635	21.89480752	2.9113E-106	1.2232E-104	positive
PRJNA729271	stage_1W_vs_E10	ordinary_muscle_development	ENSGALG00010022006	SFI1	-0.021627312	0.108631034	-0.199089627	0.842192636	0.859490274	negative
PRJNA729271	stage_1W_vs_E10	ordinary_muscle_development	ENSGALG00010022012	ENSGALG00010022012	-0.692858113	2.998349023	-0.231079873	0.817252751	0.836351216	negative
PRJNA729271	stage_1W_vs_E10	ordinary_muscle_development	ENSGALG00010022017	CNP1	2.827579845	0.583977753	4.841930755	1.28584E-06	2.38249E-06	positive
PRJNA729271	stage_1W_vs_E10	ordinary_muscle_development	ENSGALG00010022019	BTBD8	0.082446133	0.154596036	0.533300433	0.593825628	0.628330716	positive
PRJNA729271	stage_1W_vs_E10	ordinary_muscle_development	ENSGALG00010022021	TECPR1	1.511273704	0.077613035	19.47190574	1.90067E-84	5.02741E-83	positive
PRJNA729271	stage_1W_vs_E10	ordinary_muscle_development	ENSGALG00010022022	STAMBPL1	-1.267965636	0.255493264	-4.962814341	6.9479E-07	1.31338E-06	negative
PRJNA729271	stage_1W_vs_E10	ordinary_muscle_development	ENSGALG00010022024	MICAL2	3.29850636	0.182916411	18.03286183	1.07577E-72	2.17176E-71	positive
PRJNA729271	stage_1W_vs_E10	ordinary_muscle_development	ENSGALG00010022027	EIF4ENIF1	-0.596671203	0.068243361	-8.743285681	2.26427E-18	7.83121E-18	negative
PRJNA729271	stage_1W_vs_E10	ordinary_muscle_development	ENSGALG00010022028	FGD5	0.391183058	0.115323622	3.392046248	0.000693727	0.001047456	positive
PRJNA729271	stage_1W_vs_E10	ordinary_muscle_development	ENSGALG00010022029	ENSGALG00010022029	0.757170755	0.638655009	1.185570839	0.235791824	0.267873853	positive
PRJNA729271	stage_1W_vs_E10	ordinary_muscle_development	ENSGALG00010022030	ZNF341	0.120985296	0.157933304	0.766053095	0.44364471	0.481767312	positive
PRJNA729271	stage_1W_vs_E10	ordinary_muscle_development	ENSGALG00010022032	KBTBD4	0.373278866	0.081859075	4.56001811	5.11492E-06	9.05813E-06	positive
PRJNA729271	stage_1W_vs_E10	ordinary_muscle_development	ENSGALG00010022033	RAPH1	-1.066671073	0.119538097	-8.923272967	4.52707E-19	1.63193E-18	negative
PRJNA729271	stage_1W_vs_E10	ordinary_muscle_development	ENSGALG00010022034	ENSGALG00010022034	1.965783772	0.105010513	18.71987591	3.40944E-78	7.73394E-77	positive
PRJNA729271	stage_1W_vs_E10	ordinary_muscle_development	ENSGALG00010022037	DRG1	1.015619314	0.09307108	10.91229749	1.00679E-27	5.18049E-27	positive
PRJNA729271	stage_1W_vs_E10	ordinary_muscle_development	ENSGALG00010022038	GAPDH	5.458328455	0.137180696	39.78933346	0	0	positive
PRJNA729271	stage_1W_vs_E10	ordinary_muscle_development	ENSGALG00010022041	MAPK8IP1	-0.517811118	0.153564712	-3.371940807	0.000746405	0.001124768	negative
PRJNA729271	stage_1W_vs_E10	ordinary_muscle_development	ENSGALG00010022042	AIP	0.413664001	0.084872429	4.873950298	1.09389E-06	2.04039E-06	positive
PRJNA729271	stage_1W_vs_E10	ordinary_muscle_development	ENSGALG00010022044	PXMP4	0.811547673	0.058608176	13.84700445	1.32629E-43	1.1867E-42	positive
PRJNA729271	stage_1W_vs_E10	ordinary_muscle_development	ENSGALG00010022046	CRY2	1.376545813	0.103191703	13.33969472	1.36015E-40	1.11073E-39	positive
PRJNA729271	stage_1W_vs_E10	ordinary_muscle_development	ENSGALG00010022049	EPHX4	-2.814588574	0.679063749	-4.144807582	3.40099E-05	5.6755E-05	negative
PRJNA729271	stage_1W_vs_E10	ordinary_muscle_development	ENSGALG00010022050	ENSGALG00010022050	0.335407331	0.201103438	1.667834893	0.095348507	0.114981415	positive
PRJNA729271	stage_1W_vs_E10	ordinary_muscle_development	ENSGALG00010022051	BAIAP2L1	-0.215134624	0.114567956	-1.877790545	0.060409824	0.074636925	negative
PRJNA729271	stage_1W_vs_E10	ordinary_muscle_development	ENSGALG00010022052	IFFO1	0.614642437	0.104341609	5.890674333	3.84623E-09	8.36726E-09	positive
PRJNA729271	stage_1W_vs_E10	ordinary_muscle_development	ENSGALG00010022054	ENSGALG00010022054	2.051448099	0.43088398	4.76102198	1.92615E-06	3.52475E-06	positive
PRJNA729271	stage_1W_vs_E10	ordinary_muscle_development	ENSGALG00010022055	E2F1	-1.467280183	0.164812689	-8.902713705	5.44976E-19	1.95773E-18	negative
PRJNA729271	stage_1W_vs_E10	ordinary_muscle_development	ENSGALG00010022058	NLRP3	-0.625905551	0.129458794	-4.834785892	1.33289E-06	2.46683E-06	negative
PRJNA729271	stage_1W_vs_E10	ordinary_muscle_development	ENSGALG00010022059	NPTX2	-1.37271634	0.250689522	-5.475762724	4.35631E-08	8.91245E-08	negative
PRJNA729271	stage_1W_vs_E10	ordinary_muscle_development	ENSGALG00010022062	MITF	1.967004731	0.174937054	11.24407145	2.47689E-29	1.36149E-28	positive
PRJNA729271	stage_1W_vs_E10	ordinary_muscle_development	ENSGALG00010022065	PABPC1L	0.087942331	0.252397834	0.348427441	0.727519197	0.754211898	positive
PRJNA729271	stage_1W_vs_E10	ordinary_muscle_development	ENSGALG00010022066	DAD1	-0.619310647	0.140141943	-4.419166978	9.90821E-06	1.71979E-05	negative
PRJNA729271	stage_1W_vs_E10	ordinary_muscle_development	ENSGALG00010022067	TMEM130	-0.489106492	0.233296206	-2.096504272	0.036037481	0.045737089	negative
PRJNA729271	stage_1W_vs_E10	ordinary_muscle_development	ENSGALG00010022069	NOP2	0.164651583	0.134403986	1.225049851	0.220556438	0.252000114	positive
PRJNA729271	stage_1W_vs_E10	ordinary_muscle_development	ENSGALG00010022071	ACTA2	-0.910089112	0.198438408	-4.586254861	4.51268E-06	8.02888E-06	negative
PRJNA729271	stage_1W_vs_E10	ordinary_muscle_development	ENSGALG00010022072	SMTN	0.970326746	0.31259498	3.104102145	0.001908574	0.0027723	positive
PRJNA729271	stage_1W_vs_E10	ordinary_muscle_development	ENSGALG00010022073	PTPMT1	0.704908506	0.133720093	5.271522714	1.35297E-07	2.67803E-07	positive
PRJNA729271	stage_1W_vs_E10	ordinary_muscle_development	ENSGALG00010022076	YWHAB	-1.443925431	0.070312772	-20.53574886	1.03211E-93	3.35436E-92	negative
PRJNA729271	stage_1W_vs_E10	ordinary_muscle_development	ENSGALG00010022079	ABI2	-0.784315343	0.077302617	-10.14603863	3.45088E-24	1.53061E-23	negative
PRJNA729271	stage_1W_vs_E10	ordinary_muscle_development	ENSGALG00010022081	FAS	0.899187875	0.179297661	5.015056365	5.30179E-07	1.00905E-06	positive
PRJNA729271	stage_1W_vs_E10	ordinary_muscle_development	ENSGALG00010022082	TGFBR3	1.171720638	0.144425991	8.112948576	4.9406E-16	1.54108E-15	positive
PRJNA729271	stage_1W_vs_E10	ordinary_muscle_development	ENSGALG00010022084	SELENOM	-1.333441907	0.203046847	-6.567163823	5.12826E-11	1.23973E-10	negative
PRJNA729271	stage_1W_vs_E10	ordinary_muscle_development	ENSGALG00010022085	MLLT6	0.681765933	0.177378534	3.843565042	0.00012126	0.000193823	positive
PRJNA729271	stage_1W_vs_E10	ordinary_muscle_development	ENSGALG00010022087	SLC35C1	-0.133437718	0.074773388	-1.784561631	0.07433245	0.090824316	negative
PRJNA729271	stage_1W_vs_E10	ordinary_muscle_development	ENSGALG00010022088	DKK3	-1.511548302	0.270550133	-5.586943485	2.31101E-08	4.79778E-08	negative
PRJNA729271	stage_1W_vs_E10	ordinary_muscle_development	ENSGALG00010022090	TRRAP	-1.099231862	0.091262982	-12.04466305	2.0692E-33	1.32595E-32	negative
PRJNA729271	stage_1W_vs_E10	ordinary_muscle_development	ENSGALG00010022092	CTRL	4.927856628	1.798528257	2.739938397	0.00614507	0.008494449	positive
PRJNA729271	stage_1W_vs_E10	ordinary_muscle_development	ENSGALG00010022093	CHD4	-0.730330548	0.136475204	-5.351379064	8.72865E-08	1.75359E-07	negative
PRJNA729271	stage_1W_vs_E10	ordinary_muscle_development	ENSGALG00010022097	INPP5J	-0.852094184	0.293856242	-2.899697411	0.003735231	0.005274444	negative
PRJNA729271	stage_1W_vs_E10	ordinary_muscle_development	ENSGALG00010022098	CDC7	-2.961794392	0.175507143	-16.87563449	6.79905E-64	1.09381E-62	negative
PRJNA729271	stage_1W_vs_E10	ordinary_muscle_development	ENSGALG00010022100	ENTPD1	-0.397971937	0.166277479	-2.39342056	0.016692096	0.02199563	negative
PRJNA729271	stage_1W_vs_E10	ordinary_muscle_development	ENSGALG00010022101	ENSGALG00010022101	2.163781575	0.178793026	12.10215872	1.0287E-33	6.67159E-33	positive
PRJNA729271	stage_1W_vs_E10	ordinary_muscle_development	ENSGALG00010022104	EXOC3L1	0.52815799	0.141377128	3.735809309	0.000187112	0.000295028	positive
PRJNA729271	stage_1W_vs_E10	ordinary_muscle_development	ENSGALG00010022105	DLG5	-1.332227617	0.085379139	-15.60366648	6.87309E-55	8.83987E-54	negative
PRJNA729271	stage_1W_vs_E10	ordinary_muscle_development	ENSGALG00010022108	USP47	0.95560909	0.087934315	10.86730582	1.65001E-27	8.41554E-27	positive
PRJNA729271	stage_1W_vs_E10	ordinary_muscle_development	ENSGALG00010022111	PCGF2	-1.618567524	0.187643459	-8.625760439	6.36682E-18	2.15473E-17	negative
PRJNA729271	stage_1W_vs_E10	ordinary_muscle_development	ENSGALG00010022115	E2F4	-0.086122786	0.185437138	-0.464431166	0.642338866	0.674346481	negative
PRJNA729271	stage_1W_vs_E10	ordinary_muscle_development	ENSGALG00010022116	CYP20A1	-0.842028036	0.104323568	-8.071311678	6.95469E-16	2.15633E-15	negative
PRJNA729271	stage_1W_vs_E10	ordinary_muscle_development	ENSGALG00010022117	FAM20C	-0.365147704	0.122691596	-2.976142746	0.00291899	0.004165264	negative
PRJNA729271	stage_1W_vs_E10	ordinary_muscle_development	ENSGALG00010022120	FOXL3	-4.817156612	0.841381008	-5.725297534	1.03253E-08	2.18904E-08	negative
PRJNA729271	stage_1W_vs_E10	ordinary_muscle_development	ENSGALG00010022122	PSMB3	-0.0244811	0.157991708	-0.154951805	0.876859312	0.890809906	negative
PRJNA729271	stage_1W_vs_E10	ordinary_muscle_development	ENSGALG00010022124	ENSGALG00010022124	0.458510468	0.155660054	2.945588527	0.003223409	0.004578921	positive
PRJNA729271	stage_1W_vs_E10	ordinary_muscle_development	ENSGALG00010022125	CBFA2T2	0.553571886	0.186584694	2.966866548	0.003008515	0.004288791	positive
PRJNA729271	stage_1W_vs_E10	ordinary_muscle_development	ENSGALG00010022129	ELMO3	-0.341087642	0.254692623	-1.339212883	0.18050138	0.209125369	negative
PRJNA729271	stage_1W_vs_E10	ordinary_muscle_development	ENSGALG00010022134	CH25H	-0.851110051	0.248815179	-3.420651645	0.000624713	0.000946307	negative
PRJNA729271	stage_1W_vs_E10	ordinary_muscle_development	ENSGALG00010022135	CHST1	-4.086039576	0.268269107	-15.23112229	2.19786E-52	2.62855E-51	negative
PRJNA729271	stage_1W_vs_E10	ordinary_muscle_development	ENSGALG00010022137	PDDC1	-0.864197487	0.129866454	-6.654509013	2.84247E-11	6.97624E-11	negative
PRJNA729271	stage_1W_vs_E10	ordinary_muscle_development	ENSGALG00010022138	SPAG17	0.906946938	0.209370901	4.331771677	1.47914E-05	2.53161E-05	positive
PRJNA729271	stage_1W_vs_E10	ordinary_muscle_development	ENSGALG00010022142	TMEM208	1.584342731	0.104117945	15.21680755	2.73558E-52	3.25552E-51	positive
PRJNA729271	stage_1W_vs_E10	ordinary_muscle_development	ENSGALG00010022143	MATCAP1	-0.47192355	0.198700905	-2.375044793	0.017546821	0.023059036	negative
PRJNA729271	stage_1W_vs_E10	ordinary_muscle_development	ENSGALG00010022144	SNTA1	4.399333648	0.186501438	23.58873849	5.0295E-123	2.9396E-121	positive
PRJNA729271	stage_1W_vs_E10	ordinary_muscle_development	ENSGALG00010022148	TALDO1	-0.04604611	0.085863616	-0.536270328	0.591771735	0.626294633	negative
PRJNA729271	stage_1W_vs_E10	ordinary_muscle_development	ENSGALG00010022149	NBEAL1	-0.120092718	0.106598785	-1.126586182	0.259917484	0.29357936	negative
PRJNA729271	stage_1W_vs_E10	ordinary_muscle_development	ENSGALG00010022150	CPNE2	-0.938547013	0.132688386	-7.073316988	1.51273E-12	3.9687E-12	negative
PRJNA729271	stage_1W_vs_E10	ordinary_muscle_development	ENSGALG00010022151	PIP4K2B	-0.216349396	0.154867779	-1.396994242	0.162415303	0.189641519	negative
PRJNA729271	stage_1W_vs_E10	ordinary_muscle_development	ENSGALG00010022152	ZNF644	-0.396864776	0.100750551	-3.939082954	8.17936E-05	0.000132518	negative
PRJNA729271	stage_1W_vs_E10	ordinary_muscle_development	ENSGALG00010022154	CPN1	1.358901512	0.19452493	6.985744775	2.83349E-12	7.32894E-12	positive
PRJNA729271	stage_1W_vs_E10	ordinary_muscle_development	ENSGALG00010022157	EPS8L2	0.289848684	0.137846415	2.102692949	0.035492619	0.045092954	positive
PRJNA729271	stage_1W_vs_E10	ordinary_muscle_development	ENSGALG00010022158	CWC25	-0.339041857	0.080483863	-4.212544514	2.5251E-05	4.24609E-05	negative
PRJNA729271	stage_1W_vs_E10	ordinary_muscle_development	ENSGALG00010022159	KIAA2013	0.248515302	0.089666538	2.771550099	0.005579009	0.00774892	positive
PRJNA729271	stage_1W_vs_E10	ordinary_muscle_development	ENSGALG00010022161	CELF1	0.16136787	0.094315155	1.710943168	0.087091605	0.105551238	positive
PRJNA729271	stage_1W_vs_E10	ordinary_muscle_development	ENSGALG00010022162	POLE	-2.502557151	0.162058036	-15.44235145	8.4946E-54	1.05963E-52	negative
PRJNA729271	stage_1W_vs_E10	ordinary_muscle_development	ENSGALG00010022164	PDGFA	-0.49142099	0.108797282	-4.51684986	6.27664E-06	1.10359E-05	negative
PRJNA729271	stage_1W_vs_E10	ordinary_muscle_development	ENSGALG00010022165	FAM192A	0.023283549	0.089169383	0.261115955	0.794003088	0.81584141	positive

PRJNA729271	stage_1W_vs_E10	ordinary_muscle_development	ENSGALG00010022166	SDHB	1.608366668	0.10773831	14.92845648	2.1519E-50	2.39568E-49	positive
PRJNA729271	stage_1W_vs_E10	ordinary_muscle_development	ENSGALG00010022167	WDR3	-0.64052213	0.072324546	-8.856220529	8.2773E-19	2.93348E-18	negative
PRJNA729271	stage_1W_vs_E10	ordinary_muscle_development	ENSGALG00010022169	TOX2	-2.373367715	0.323672982	-7.332609916	2.25713E-13	6.15794E-13	negative
PRJNA729271	stage_1W_vs_E10	ordinary_muscle_development	ENSGALG00010022170	RSPRY1	-1.24502189	0.146473574	-8.499976206	1.8963E-17	6.27123E-17	negative
PRJNA729271	stage_1W_vs_E10	ordinary_muscle_development	ENSGALG00010022171	FOXP1	-1.015335482	0.095834318	-10.59469614	3.15364E-26	1.52781E-25	negative
PRJNA729271	stage_1W_vs_E10	ordinary_muscle_development	ENSGALG00010022172	GIPC2	0.569045392	0.18549605	3.067695468	0.002157163	0.003114041	positive
PRJNA729271	stage_1W_vs_E10	ordinary_muscle_development	ENSGALG00010022173	RPL23	0.405502663	0.10404852	3.89724585	9.72928E-05	0.000156643	positive
PRJNA729271	stage_1W_vs_E10	ordinary_muscle_development	ENSGALG00010022174	KCNMA1	1.402420213	0.12482658	11.23494863	2.74654E-29	1.50514E-28	positive
PRJNA729271	stage_1W_vs_E10	ordinary_muscle_development	ENSGALG00010022176	ARL2BPL	-1.638185049	0.15290898	-10.71346532	8.80047E-27	4.35665E-26	negative
PRJNA729271	stage_1W_vs_E10	ordinary_muscle_development	ENSGALG00010022178	ENSGALG00010022178	0.147315048	0.338222135	0.435557088	0.663158111	0.693540896	positive
PRJNA729271	stage_1W_vs_E10	ordinary_muscle_development	ENSGALG00010022180	PLLP	1.63525171	0.382253523	4.2779245	1.88644E-05	3.20224E-05	positive
PRJNA729271	stage_1W_vs_E10	ordinary_muscle_development	ENSGALG00010022181	ENSGALG00010022181	-1.710915541	0.23630073	-7.240415797	4.47311E-13	1.2036E-12	negative
PRJNA729271	stage_1W_vs_E10	ordinary_muscle_development	ENSGALG00010022182	CNTNAP1	-1.12579455	0.30493795	-3.691880764	0.000222602	0.000349201	negative
PRJNA729271	stage_1W_vs_E10	ordinary_muscle_development	ENSGALG00010022184	PRKAR1B	-1.224120952	0.094743101	-12.92042316	3.45238E-38	2.59824E-37	negative
PRJNA729271	stage_1W_vs_E10	ordinary_muscle_development	ENSGALG00010022186	DNAJB4	0.884318035	0.112711904	7.845826451	4.30111E-15	1.28599E-14	positive
PRJNA729271	stage_1W_vs_E10	ordinary_muscle_development	ENSGALG00010022190	ACVR1B	1.342488905	0.163248816	8.223575159	1.97525E-16	6.25409E-16	positive
PRJNA729271	stage_1W_vs_E10	ordinary_muscle_development	ENSGALG00010022193	GDAP2	-0.197541435	0.081631462	-2.419917894	0.015524012	0.020534819	negative
PRJNA729271	stage_1W_vs_E10	ordinary_muscle_development	ENSGALG00010022194	ENSGALG00010022194	-1.563804646	0.09301577	-16.81225292	1.98482E-63	3.17901E-62	negative
PRJNA729271	stage_1W_vs_E10	ordinary_muscle_development	ENSGALG00010022196	PLXNA1	-0.615604925	0.109070579	-5.64409697	1.6605E-08	3.47667E-08	negative
PRJNA729271	stage_1W_vs_E10	ordinary_muscle_development	ENSGALG00010022199	RBM39	-0.299360976	0.061779175	-4.845661584	1.2619E-06	2.33994E-06	negative
PRJNA729271	stage_1W_vs_E10	ordinary_muscle_development	ENSGALG00010022200	DNMBP	-0.013884609	0.110270491	-0.125914091	0.899799928	0.911368874	negative
PRJNA729271	stage_1W_vs_E10	ordinary_muscle_development	ENSGALG00010022202	CX3CL1	2.151714468	0.160061687	13.44303251	3.38284E-41	2.82944E-40	positive
PRJNA729271	stage_1W_vs_E10	ordinary_muscle_development	ENSGALG00010022203	DNAAF5	0.134644037	0.131795448	1.021613718	0.306963762	0.342595145	positive
PRJNA729271	stage_1W_vs_E10	ordinary_muscle_development	ENSGALG00010022204	ENSGALG00010022204	0.746222761	0.332227223	2.246121662	0.024696216	0.031964609	positive
PRJNA729271	stage_1W_vs_E10	ordinary_muscle_development	ENSGALG00010022205	GALNT18	-0.695023182	0.108940801	-6.379824402	1.77291E-10	4.15483E-10	negative
PRJNA729271	stage_1W_vs_E10	ordinary_muscle_development	ENSGALG00010022207	ENSGALG00010022207	3.525494417	0.305619843	11.53555472	8.73225E-31	5.09762E-30	positive
PRJNA729271	stage_1W_vs_E10	ordinary_muscle_development	ENSGALG00010022208	CCL17	2.000622273	0.707869151	2.826260008	0.004709502	0.006589848	positive
PRJNA729271	stage_1W_vs_E10	ordinary_muscle_development	ENSGALG00010022209	ZFYVE19	-0.4744271	0.121304562	-3.911040862	9.18992E-05	0.000148223	negative
PRJNA729271	stage_1W_vs_E10	ordinary_muscle_development	ENSGALG00010022210	EZH1	1.106962208	0.082759379	13.37567077	8.38934E-41	6.88972E-40	positive
PRJNA729271	stage_1W_vs_E10	ordinary_muscle_development	ENSGALG00010022211	ENSGALG00010022211	0.625228551	0.489958308	1.276085212	0.201925399	0.232275903	positive
PRJNA729271	stage_1W_vs_E10	ordinary_muscle_development	ENSGALG00010022213	IDH1	-0.529355768	0.080447543	-6.580135961	4.70018E-11	1.14024E-10	negative
PRJNA729271	stage_1W_vs_E10	ordinary_muscle_development	ENSGALG00010022214	ENSGALG00010022214	-2.546709294	0.287388229	-8.86156437	7.88994E-19	2.79894E-18	negative
PRJNA729271	stage_1W_vs_E10	ordinary_muscle_development	ENSGALG00010022215	ENSGALG00010022215	1.682214629	0.128601854	13.08079611	4.23976E-39	3.29519E-38	positive
PRJNA729271	stage_1W_vs_E10	ordinary_muscle_development	ENSGALG00010022216	ROMO1	1.004862221	0.144018625	6.9773074	3.00891E-12	7.77297E-12	positive
PRJNA729271	stage_1W_vs_E10	ordinary_muscle_development	ENSGALG00010022218	ENSGALG00010022218	5.842481986	0.246473561	23.70429493	3.2561E-124	1.9503E-122	positive
PRJNA729271	stage_1W_vs_E10	ordinary_muscle_development	ENSGALG00010022219	EIF4E3	0.802430746	0.136843003	5.863878518	4.52178E-09	9.7972E-09	positive
PRJNA729271	stage_1W_vs_E10	ordinary_muscle_development	ENSGALG00010022221	CUTC	0.734109466	0.081374364	9.021384986	1.85726E-19	6.81027E-19	positive
PRJNA729271	stage_1W_vs_E10	ordinary_muscle_development	ENSGALG00010022222	ENSGALG00010022222	-0.117820181	0.109737239	-1.073657248	0.282976329	0.317496857	negative
PRJNA729271	stage_1W_vs_E10	ordinary_muscle_development	ENSGALG00010022223	DOK4	-1.459144132	0.177701193	-8.211223039	2.18947E-16	6.91122E-16	negative
PRJNA729271	stage_1W_vs_E10	ordinary_muscle_development	ENSGALG00010022224	NHEJ1	1.497151977	0.140085512	10.68741482	1.16575E-26	5.75334E-26	positive
PRJNA729271	stage_1W_vs_E10	ordinary_muscle_development	ENSGALG00010022225	ENSGALG00010022225	-0.484600912	0.579753605	-0.83587391	0.403225841	0.441380348	negative
PRJNA729271	stage_1W_vs_E10	ordinary_muscle_development	ENSGALG00010022226	FBRSL1	-0.04230068	0.133543211	-0.316756499	0.751428374	0.776333164	negative
PRJNA729271	stage_1W_vs_E10	ordinary_muscle_development	ENSGALG00010022227	TMEM80	0.442052937	0.158670512	2.785980411	0.00533661	0.007432897	positive
PRJNA729271	stage_1W_vs_E10	ordinary_muscle_development	ENSGALG00010022228	FUBP1	-1.763324945	0.055814323	-31.59269596	4.6515E-219	8.0267E-217	negative
PRJNA729271	stage_1W_vs_E10	ordinary_muscle_development	ENSGALG00010022229	RAPSN	0.347526817	0.178966266	1.941856555	0.052154468	0.065002495	positive
PRJNA729271	stage_1W_vs_E10	ordinary_muscle_development	ENSGALG00010022230	NFS1	-0.228029615	0.087309136	-2.611749749	0.009008017	0.012241815	negative
PRJNA729271	stage_1W_vs_E10	ordinary_muscle_development	ENSGALG00010022231	ZNF503	-2.839674737	0.214152916	-13.26003305	3.94722E-40	3.17508E-39	negative
PRJNA729271	stage_1W_vs_E10	ordinary_muscle_development	ENSGALG00010022232	GPR27	-3.949235566	0.211439447	-18.67785608	7.49703E-78	1.6848E-76	negative
PRJNA729271	stage_1W_vs_E10	ordinary_muscle_development	ENSGALG00010022233	FAM46C	3.030729448	0.164114737	18.46713772	3.7969E-76	8.22661E-75	positive
PRJNA729271	stage_1W_vs_E10	ordinary_muscle_development	ENSGALG00010022234	COX15	0.775954537	0.0930223	8.341596973	7.32939E-17	2.36561E-16	positive
PRJNA729271	stage_1W_vs_E10	ordinary_muscle_development	ENSGALG00010022235	RAMP2	1.348418761	0.112860948	11.94761152	6.6818E-33	4.20368E-32	positive
PRJNA729271	stage_1W_vs_E10	ordinary_muscle_development	ENSGALG00010022236	SHF	1.601013405	0.181572723	8.817477545	1.17067E-18	4.11766E-18	positive
PRJNA729271	stage_1W_vs_E10	ordinary_muscle_development	ENSGALG00010022237	SUN1	0.712525178	0.091087411	7.822433133	5.1812E-15	1.54086E-14	positive
PRJNA729271	stage_1W_vs_E10	ordinary_muscle_development	ENSGALG00010022238	COMTD1	0.518331802	0.164290123	3.15497848	0.001605099	0.002346064	positive
PRJNA729271	stage_1W_vs_E10	ordinary_muscle_development	ENSGALG00010022239	DEAF1	0.706299666	0.113187721	6.240073193	4.37366E-10	1.00215E-09	positive
PRJNA729271	stage_1W_vs_E10	ordinary_muscle_development	ENSGALG00010022240	ENSGALG00010022240	-1.179499499	0.135759701	-8.688141549	3.68417E-18	1.26246E-17	negative
PRJNA729271	stage_1W_vs_E10	ordinary_muscle_development	ENSGALG00010022241	POLR2C	0.819562885	0.09398595	8.720057465	2.78061E-18	9.58955E-18	positive
PRJNA729271	stage_1W_vs_E10	ordinary_muscle_development	ENSGALG00010022242	USP19	1.270736752	0.12554387	10.12185421	4.41962E-24	1.95193E-23	positive
PRJNA729271	stage_1W_vs_E10	ordinary_muscle_development	ENSGALG00010022243	ENSGALG00010022243	2.600344425	0.556876899	4.669513907	3.01913E-06	5.44375E-06	positive
PRJNA729271	stage_1W_vs_E10	ordinary_muscle_development	ENSGALG00010022244	ENTPD7	-1.372228612	0.179112926	-7.661248381	1.84134E-14	5.32634E-14	negative
PRJNA729271	stage_1W_vs_E10	ordinary_muscle_development	ENSGALG00010022245	PIKFYVE	0.703690321	0.100261893	7.018522192	2.24227E-12	5.83514E-12	positive
PRJNA729271	stage_1W_vs_E10	ordinary_muscle_development	ENSGALG00010022246	RHOV	-4.134900297	0.472106482	-8.758406104	1.98031E-18	6.87369E-18	negative
PRJNA729271	stage_1W_vs_E10	ordinary_muscle_development	ENSGALG00010022247	GALNT9	-2.704095633	0.241680308	-11.18872965	4.6302E-29	2.51084E-28	negative
PRJNA729271	stage_1W_vs_E10	ordinary_muscle_development	ENSGALG00010022248	FIGNL2	-2.926041436	0.236804542	-12.3563569	4.50097E-35	3.06298E-34	negative
PRJNA729271	stage_1W_vs_E10	ordinary_muscle_development	ENSGALG00010022250	SLC25A28	2.38641862	0.126731943	18.83044299	4.25189E-79	9.84523E-78	positive
PRJNA729271	stage_1W_vs_E10	ordinary_muscle_development	ENSGALG00010022251	WNK4	-0.75231144	0.155095349	-4.850638297	1.23065E-06	2.2849E-06	negative
PRJNA729271	stage_1W_vs_E10	ordinary_muscle_development	ENSGALG00010022252	COQ9	4.189568238	0.131451249	31.87165033	6.5991E-223	1.1809E-220	positive
PRJNA729271	stage_1W_vs_E10	ordinary_muscle_development	ENSGALG00010022253	ALPK3	3.874171091	0.217256323	17.83225929	3.97047E-71	7.71474E-70	positive
PRJNA729271	stage_1W_vs_E10	ordinary_muscle_development	ENSGALG00010022255	NOC4L	-2.434525161	0.094639674	-25.72414979	6.2761E-146	4.8648E-144	negative
PRJNA729271	stage_1W_vs_E10	ordinary_muscle_development	ENSGALG00010022256	PSMC3	0.34240505	0.092044308	3.720002418	0.000199221	0.000313506	positive
PRJNA729271	stage_1W_vs_E10	ordinary_muscle_development	ENSGALG00010022257	KCNK18	-1.008848877	1.156296039	-0.872483207	0.382944825	0.420864754	negative
PRJNA729271	stage_1W_vs_E10	ordinary_muscle_development	ENSGALG00010022258	ENSGALG00010022258	4.665739267	0.680124451	6.860125756	6.88E-12	1.7462E-11	positive
PRJNA729271	stage_1W_vs_E10	ordinary_muscle_development	ENSGALG00010022259	GET4	0.670620047	0.084962986	7.893084751	2.94807E-15	8.86379E-15	positive
PRJNA729271	stage_1W_vs_E10	ordinary_muscle_development	ENSGALG00010022264	SCN8A	-1.358779096	0.262432301	-5.177636637	2.24714E-07	4.38803E-07	negative
PRJNA729271	stage_1W_vs_E10	ordinary_muscle_development	ENSGALG00010022266	COA3	0.36145409	0.145912787	2.477192704	0.013242038	0.017667834	positive
PRJNA729271	stage_1W_vs_E10	ordinary_muscle_development	ENSGALG00010022269	CNTD1	-0.490626581	0.40037217	-1.225426284	0.220414649	0.251865221	negative
PRJNA729271	stage_1W_vs_E10	ordinary_muscle_development	ENSGALG00010022271	SLC18A2	-1.727590313	0.251090325	-6.880353971	5.9704E-12	1.51933E-11	negative
PRJNA729271	stage_1W_vs_E10	ordinary_muscle_development	ENSGALG00010022274	CCDC91	0.353598238	0.174738415	2.023586166	0.04301275	0.054158253	positive
PRJNA729271	stage_1W_vs_E10	ordinary_muscle_development	ENSGALG00010022275	VDAC2	2.002539107	0.124027865	16.14588064	1.21403E-58	1.71682E-57	positive
PRJNA729271	stage_1W_vs_E10	ordinary_muscle_development	ENSGALG00010022276	BECN1	0.763878018	0.088187225	8.662003112	4.63546E-18	1.57762E-17	positive
PRJNA729271	stage_1W_vs_E10	ordinary_muscle_development	ENSGALG00010022279	EP400	0.422170129	0.149026639	2.832850095	0.004613502	0.006461131	positive
PRJNA729271	stage_1W_vs_E10	ordinary_muscle_development	ENSGALG00010022280	PDZD8	-0.069972237	0.06832555	-1.024100612	0.305787756	0.341373499	negative
PRJNA729271	stage_1W_vs_E10	ordinary_muscle_development	ENSGALG00010022282	TMT1A	2.157459944	0.223911695	9.635316008	5.67172E-22	2.29963E-21	positive
PRJNA729271	stage_1W_vs_E10	ordinary_muscle_development	ENSGALG00010022290	NEXN	1.864679644	0.220067286	8.473225073	2.38691E-17	7.86502E-17	positive
PRJNA729271	stage_1W_vs_E10	ordinary_muscle_development	ENSGALG00010022293	DNAJC10	-0.721600048	0.064181768	-11.24306901	2.50519E-29	1.37548E-28	negative
PRJNA729271	stage_1W_vs_E10	ordinary_muscle_development	ENSGALG00010022294	ENSGALG00010022294	-0.215246062	0.77183812	-0.27887462	0.780341046	0.803055979	negative
PRJNA729271	stage_1W_vs_E10	ordinary_muscle_development	ENSGALG00010022296	CIAPIN1	-0.015940276	0.097491358	-0.163504501	0.87012123	0.884522563	negative
PRJNA729271	stage_1W_vs_E10	ordinary_muscle_development	ENSGALG00010022297	VPS18	-0.473877856	0.058534294	-8.095730337	5.69218E-16	1.77094E-15	negative
PRJNA729271	stage_1W_vs_E10	ordinary_muscle_development	ENSGALG00010022299	MRGBP	-0.096945524	0.080242366	-1.208158833	0.226986173	0.258559103	negative
PRJNA729271	stage_1W_vs_E10	ordinary_muscle_development	ENSGALG00010022302	ENSGALG00010022302	-0.990600417	0.156073837	-6.34699853	2.19556E-10	5.10994E-10	negative
PRJNA729271	stage_1W_vs_E10	ordinary_muscle_development	ENSGALG00010022303	DUSP13A	5.431723547	0.203569414	26.68241483	7.531E-157	7.1817E-155	positive
PRJNA729271	stage_1W_vs_E10	ordinary_muscle_development	ENSGALG00010022304	ADAP1	-1.990909853	0.195027051	-10.20837796	1.8188E-24	8.17472E-24	negative
PRJNA729271	stage_1W_vs_E10	ordinary_muscle_development	ENSGALG00010022307	ENSGALG00010022307	8.217336371	0.63491396	12.94244085	2.5926E-38	1.95727E-37	positive
PRJNA729271	stage_1W_vs_E10	ordinary_muscle_development	ENSGALG00010022308	OGFR	0.219454132	0.181594542	1.208484186	0.226861075	0.258453218	positive
PRJNA729271	stage_1W_vs_E10	ordinary_muscle_development	ENSGALG00010022309	WSCD2	-1.082827632	0.356912963	-3.033870283	0.002414382	0.003469807	negative
PRJNA729271	stage_1W_vs_E10	ordinary_muscle_development	ENSGALG00010022310	DCLRE1A	-1.976018587	0.103814461	-19.03413611	8.89471E-81	2.17785E-79	negative
PRJNA729271	stage_1W_vs_E10	ordinary_muscle_development	ENSGALG00010022311	DLL4	1.245338356	0.141161777	8.82206488	1.12368E-18	3.95814E-18	positive
PRJNA729271	stage_1W_vs_E10	ordinary_muscle_development	ENSGALG00010022312	ZNF592	0.05724336	0.055321268	1.034744162	0.300788406	0.336102987	positive
PRJNA729271	stage_1W_vs_E10	ordinary_muscle_development	ENSGALG00010022313	ENSGALG00010022313	-1.037721596	0.4065439	-2.552544995	0.01069391	0.014412666	negative
PRJNA729271	stage_1W_vs_E10	ordinary_muscle_development	ENSGALG00010022314	DUSP13B	6.754056698	0.551895861	12.23791874	1.94968E-34	1.2928E-33	positive
PRJNA729271	stage_1W_vs_E10	ordinary_muscle_development	ENSGALG00010022316	INO80	0.170516587	0.069583524	2.450531071	0.014264565	0.018948394	positive
PRJNA729271	stage_1W_vs_E10	ordinary_muscle_development	ENSGALG00010022317	PSME3	-1.280201476	0.074104382	-17.27565145	7.17586E-67	1.23826E-65	negative
PRJNA729271	stage_1W_vs_E10	ordinary_muscle_development	ENSGALG00010022320	COL9A3	-9.47507023	0.576762607	-16.42802449	1.20522E-60	1.80099E-59	negative
PRJNA729271	stage_1W_vs_E10	ordinary_muscle_development	ENSGALG00010022322	ENSGALG00010022322	2.884710837	0.371107547	7.773247575	7.6499E-15	2.2556E-14	positive
PRJNA729271	stage_1W_vs_E10	ordinary_muscle_development	ENSGALG00010022323	DUSP29	7.094209043	0.381264315	18.60706276	2.8164E-77	6.20421E-76	positive
PRJNA729271	stage_1W_vs_E10	ordinary_muscle_development	ENSGALG00010022325	MIGA1	0.112331662	0.107699497	1.043010086	0.296943621	0.332114027	positive
PRJNA729271	stage_1W_vs_E10	ordinary_muscle_development	ENSGALG00010022326	COX19	1.287840319	0.093922068	13.71179693	8.62939E-43	7.55788E-42	positive
PRJNA729271	stage_1W_vs_E10	ordinary_muscle_development	ENSGALG00010022328	ENSGALG00010022328	-0.982047727	0.252229459	-3.893469586	9.88206E-05	0.000158962	negative
PRJNA729271	stage_1W_vs_E10	ordinary_muscle_development	ENSGALG00010022332	KAT6B	-1.487981032	0.063050421	-23.59985889	3.867E-123	2.2785E-121	negative
PRJNA729271	stage_1W_vs_E10	ordinary_muscle_development	ENSGALG00010022333	ENSGALG00010022333	1.307517605	0.132159827	9.893457276	4.44409E-23	1.8852E-22	positive
PRJNA729271	stage_1W_vs_E10	ordinary_muscle_development	ENSGALG00010022334	PDE1A	-1.932935351	0.124503363	-15.52516576	2.34398E-54	2.98296E-53	negative
PRJNA729271	stage_1W_vs_E10	ordinary_muscle_development	ENSGALG00010022340	GFOD2	-0.810713119	0.095644799	-8.476290718	2.32487E-17	7.66584E-17	negative
PRJNA729271	stage_1W_vs_E10	ordinary_muscle_development	ENSGALG00010022343	GATA5	-2.542773461	0.60381225	-4.211198865	2.54019E-05	4.26949E-05	negative
PRJNA729271	stage_1W_vs_E10	ordinary_muscle_development	ENSGALG00010022345	USP33	-0.219117293	0.123090391	-1.780133211	0.075054163	0.091621196	negative
PRJNA729271	stage_1W_vs_E10	ordinary_muscle_development	ENSGALG00010022347	SEC11A	-1.625035594	0.106478098	-15.26168872	1.3764E-52	1.66257E-51	negative
PRJNA729271	stage_1W_vs_E10	ordinary_muscle_development	ENSGALG00010022348	G6PC1	3.849586114	0.611771765	6.292520074	3.12353E-10	7.21408E-10	positive
PRJNA729271	stage_1W_vs_E10	ordinary_muscle_development	ENSGALG00010022353	SLC39A13	0.116523777	0.095341632	1.222170997	0.221642969	0.253109181	positive
PRJNA729271	stage_1W_vs_E10	ordinary_muscle_development	ENSGALG00010022354	FAR2	-0.54577728	0.137940134	-3.956624267	7.60164E-05	0.000123457	negative
PRJNA729271	stage_1W_vs_E10	ordinary_muscle_development	ENSGALG00010022356	TCFL5	-1.38985264	0.249671186	-5.566732246	2.59561E-08	5.37246E-08	negative
PRJNA729271	stage_1W_vs_E10	ordinary_muscle_development	ENSGALG00010022357	ENSGALG00010022357	-5.628792667	0.904837173	-6.220779641	4.94691E-10	1.13046E-09	negative
PRJNA729271	stage_1W_vs_E10	ordinary_muscle_development	ENSGALG00010022359	ZZZ3	-0.508723247	0.07979849	-6.375098629	1.82845E-10	4.28027E-10	negative
PRJNA729271	stage_1W_vs_E10	ordinary_muscle_development	ENSGALG00010022363	KIF18A	-2.021119498	0.203926605	-9.911014296	3.72842E-23	1.58858E-22	negative
PRJNA729271	stage_1W_vs_E10	ordinary_muscle_development	ENSGALG00010022365	PLCG1	-1.696542302	0.111398468	-15.229494	2.25329E-52	2.69261E-51	negative
PRJNA729271	stage_1W_vs_E10	ordinary_muscle_development	ENSGALG00010022366	AARSD1	1.205413239	0.112131498	10.74999678	5.92637E-27	2.95097E-26	positive
PRJNA729271	stage_1W_vs_E10	ordinary_muscle_development	ENSGALG00010022373	AK5	-3.96870762	0.234100695	-16.95299376	1.82899E-64	2.99223E-63	negative
PRJNA729271	stage_1W_vs_E10	ordinary_muscle_development	ENSGALG00010022375	NMB	-2.024153251	0.443259163	-4.566523203	4.9588E-06	8.79132E-06	negative
PRJNA729271	stage_1W_vs_E10	ordinary_muscle_development	ENSGALG00010022377	ZHX3	1.02242738	0.089941894	11.36764338	6.06017E-30	3.42198E-29	positive
PRJNA729271	stage_1W_vs_E10	ordinary_muscle_development	ENSGALG00010022379	METTL15	-1.045067195	0.125981315	-8.295414214	1.08207E-16	3.46623E-16	negative
PRJNA729271	stage_1W_vs_E10	ordinary_muscle_development	ENSGALG00010022382	CALB2	-6.372348789	1.098760477	-5.799579547	6.64814E-09	1.42594E-08	negative
PRJNA729271	stage_1W_vs_E10	ordinary_muscle_development	ENSGALG00010022385	ENSGALG00010022385	1.888714582	0.656896974	2.8752067	0.004037631	0.00568098	positive
PRJNA729271	stage_1W_vs_E10	ordinary_muscle_development	ENSGALG00010022386	SPI1	1.453731636	0.244916539	5.935620532	2.92736E-09	6.41841E-09	positive

PRJNA729271	stage_1W_vs_E10	ordinary_muscle_development	ENSGALG00010022388	CYP2W1	-0.117757576	0.437655755	-0.269064384	0.787880138	0.810239276	negative
PRJNA729271	stage_1W_vs_E10	ordinary_muscle_development	ENSGALG00010022391	ENSGALG00010022391	0.733105067	0.179890804	4.07527817	4.59594E-05	7.58144E-05	positive
PRJNA729271	stage_1W_vs_E10	ordinary_muscle_development	ENSGALG00010022392	EMILIN3	-3.957431426	0.192696411	-20.53713096	1.00316E-93	3.2676E-92	negative
PRJNA729271	stage_1W_vs_E10	ordinary_muscle_development	ENSGALG00010022395	PTGES3L	1.836885456	0.133281705	13.78197749	3.27185E-43	2.8971E-42	positive
PRJNA729271	stage_1W_vs_E10	ordinary_muscle_development	ENSGALG00010022396	PPP1R1C	-1.201193542	0.335200876	-3.583503586	0.000339016	0.000524444	negative
PRJNA729271	stage_1W_vs_E10	ordinary_muscle_development	ENSGALG00010022397	ATF1	-0.326365287	0.047780464	-6.830517359	8.46089E-12	2.13622E-11	negative
PRJNA729271	stage_1W_vs_E10	ordinary_muscle_development	ENSGALG00010022400	NHLRC2	-0.100423423	0.072529177	-1.384593437	0.16617682	0.193690334	negative
PRJNA729271	stage_1W_vs_E10	ordinary_muscle_development	ENSGALG00010022401	LCAT	1.569019993	0.438345409	3.579414679	0.000344365	0.000532377	positive
PRJNA729271	stage_1W_vs_E10	ordinary_muscle_development	ENSGALG00010022406	ITPRID2	0.504785597	0.105043072	4.805510616	1.54357E-06	2.84187E-06	positive
PRJNA729271	stage_1W_vs_E10	ordinary_muscle_development	ENSGALG00010022407	RUNDC1	0.813482868	0.095123723	8.551840123	1.21142E-17	4.03759E-17	positive
PRJNA729271	stage_1W_vs_E10	ordinary_muscle_development	ENSGALG00010022408	RAD51AP1	-0.438560724	0.138076446	-3.176216788	0.001492094	0.002186846	negative
PRJNA729271	stage_1W_vs_E10	ordinary_muscle_development	ENSGALG00010022409	CARNS1	10.83492856	0.338177518	32.03917466	3.1067E-225	5.8483E-223	positive
PRJNA729271	stage_1W_vs_E10	ordinary_muscle_development	ENSGALG00010022411	ADRB1	0.387857093	0.295344719	1.313235239	0.189103693	0.21844625	positive
PRJNA729271	stage_1W_vs_E10	ordinary_muscle_development	ENSGALG00010022412	CHAC1	6.926078029	0.650390682	10.64910402	1.76051E-26	8.61825E-26	positive
PRJNA729271	stage_1W_vs_E10	ordinary_muscle_development	ENSGALG00010022415	DIP2B	-0.642983334	0.109921266	-5.849489872	4.93083E-09	1.06627E-08	negative
PRJNA729271	stage_1W_vs_E10	ordinary_muscle_development	ENSGALG00010022416	CCDC186	-0.347541941	0.087561199	-3.969131797	7.2135E-05	0.00011739	negative
PRJNA729271	stage_1W_vs_E10	ordinary_muscle_development	ENSGALG00010022419	DUOX2	1.361868682	0.759027617	1.794228105	0.072776768	0.088998503	positive
PRJNA729271	stage_1W_vs_E10	ordinary_muscle_development	ENSGALG00010022421	CMKLR1	1.250652529	0.17711065	7.061419106	1.64811E-12	4.31059E-12	positive
PRJNA729271	stage_1W_vs_E10	ordinary_muscle_development	ENSGALG00010022422	RPS6KB2	2.557498409	0.152604499	16.75899744	4.86763E-63	7.66084E-62	positive
PRJNA729271	stage_1W_vs_E10	ordinary_muscle_development	ENSGALG00010022425	VWA2	-4.205868615	0.576014743	-7.301668342	2.84221E-13	7.7164E-13	negative
PRJNA729271	stage_1W_vs_E10	ordinary_muscle_development	ENSGALG00010022426	EXD1	-1.465724274	0.245405941	-5.97265195	2.33428E-09	5.14762E-09	negative
PRJNA729271	stage_1W_vs_E10	ordinary_muscle_development	ENSGALG00010022427	RPL27	0.452517852	0.13866579	3.263370528	0.001100955	0.001632901	positive
PRJNA729271	stage_1W_vs_E10	ordinary_muscle_development	ENSGALG00010022429	ADK	-0.649440268	0.130846562	-4.96337281	6.92794E-07	1.30978E-06	negative
PRJNA729271	stage_1W_vs_E10	ordinary_muscle_development	ENSGALG00010022431	LARP4	-0.069872122	0.132848543	-0.525953244	0.598920693	0.633490619	negative
PRJNA729271	stage_1W_vs_E10	ordinary_muscle_development	ENSGALG00010022433	LNPK	1.760481505	0.106732163	16.49438607	4.02631E-61	6.08565E-60	positive
PRJNA729271	stage_1W_vs_E10	ordinary_muscle_development	ENSGALG00010022435	PIGK	0.59676401	0.163709571	3.645260364	0.000267121	0.000416963	positive
PRJNA729271	stage_1W_vs_E10	ordinary_muscle_development	ENSGALG00010022436	AFAP1L2	-0.226294908	0.156004486	-1.450566671	0.146900562	0.172540608	negative
PRJNA729271	stage_1W_vs_E10	ordinary_muscle_development	ENSGALG00010022438	RPN2	-0.731595968	0.101364654	-7.217466243	5.29651E-13	1.41962E-12	negative
PRJNA729271	stage_1W_vs_E10	ordinary_muscle_development	ENSGALG00010022439	IFI35	1.700765976	0.203967786	8.338404837	7.52994E-17	2.42926E-16	positive
PRJNA729271	stage_1W_vs_E10	ordinary_muscle_development	ENSGALG00010022440	SORD	1.281684514	0.123235737	10.40026656	2.47241E-25	1.15196E-24	positive
PRJNA729271	stage_1W_vs_E10	ordinary_muscle_development	ENSGALG00010022441	FICD	-0.148475446	0.12139165	-1.223110866	0.221287825	0.252763359	negative
PRJNA729271	stage_1W_vs_E10	ordinary_muscle_development	ENSGALG00010022448	FERRY3	-0.819258316	0.122470615	-6.689427617	2.24045E-11	5.53148E-11	negative
PRJNA729271	stage_1W_vs_E10	ordinary_muscle_development	ENSGALG00010022449	ENSGALG00010022449	-0.392850198	0.102452418	-3.834464867	0.000125838	0.000200861	negative
PRJNA729271	stage_1W_vs_E10	ordinary_muscle_development	ENSGALG00010022451	CHP1	-0.194390387	0.121382197	-1.601473625	0.109272057	0.130695476	negative
PRJNA729271	stage_1W_vs_E10	ordinary_muscle_development	ENSGALG00010022452	AP3M1	-0.351623755	0.08164007	-4.30699969	1.65484E-05	2.82199E-05	negative
PRJNA729271	stage_1W_vs_E10	ordinary_muscle_development	ENSGALG00010022453	ENSGALG00010022453	2.727848712	0.2336735	11.6737615	1.73575E-31	1.04224E-30	positive
PRJNA729271	stage_1W_vs_E10	ordinary_muscle_development	ENSGALG00010022454	ABLIM1	1.671458676	0.140715574	11.87827775	1.53497E-32	9.54093E-32	positive
PRJNA729271	stage_1W_vs_E10	ordinary_muscle_development	ENSGALG00010022455	C7orf50	-1.46820573	0.156753559	-9.366331064	7.50977E-21	2.92383E-20	negative
PRJNA729271	stage_1W_vs_E10	ordinary_muscle_development	ENSGALG00010022456	ATP5G3	0.719672978	0.158172095	4.549936442	5.36621E-06	9.48808E-06	positive
PRJNA729271	stage_1W_vs_E10	ordinary_muscle_development	ENSGALG00010022459	FGF6	2.609980493	0.749305321	3.483200266	0.000495458	0.000755884	positive
PRJNA729271	stage_1W_vs_E10	ordinary_muscle_development	ENSGALG00010022460	VAT1	-1.1061216	0.119492762	-9.256808397	2.10634E-20	8.05366E-20	negative
PRJNA729271	stage_1W_vs_E10	ordinary_muscle_development	ENSGALG00010022462	ITGA5	-0.0140944	0.13038322	-0.108099796	0.91391653	0.924447907	negative
PRJNA729271	stage_1W_vs_E10	ordinary_muscle_development	ENSGALG00010022463	DUSP12	0.495481624	0.114948368	4.310471146	1.62907E-05	2.77968E-05	positive
PRJNA729271	stage_1W_vs_E10	ordinary_muscle_development	ENSGALG00010022466	NEDD4	-0.00404354	0.137767451	-0.029350473	0.976585073	0.979965429	negative
PRJNA729271	stage_1W_vs_E10	ordinary_muscle_development	ENSGALG00010022467	SART3	-0.923616875	0.117182912	-7.881839278	3.22597E-15	9.68325E-15	negative
PRJNA729271	stage_1W_vs_E10	ordinary_muscle_development	ENSGALG00010022468	MYBPC3	0.627257434	0.429188272	1.461497145	0.143879059	0.169238575	positive
PRJNA729271	stage_1W_vs_E10	ordinary_muscle_development	ENSGALG00010022470	ENSGALG00010022470	-0.810613645	0.181605971	-4.463584775	8.05997E-06	1.40724E-05	negative
PRJNA729271	stage_1W_vs_E10	ordinary_muscle_development	ENSGALG00010022471	ST6GALNAC5	-2.357777169	0.164221038	-14.35733937	9.58363E-47	9.44355E-46	negative
PRJNA729271	stage_1W_vs_E10	ordinary_muscle_development	ENSGALG00010022472	QSER1	-0.039712938	0.088083492	-0.450855631	0.652093603	0.683448791	negative
PRJNA729271	stage_1W_vs_E10	ordinary_muscle_development	ENSGALG00010022473	ZNF385A	-0.211790798	0.531956833	-0.39813531	0.690530448	0.719312888	negative
PRJNA729271	stage_1W_vs_E10	ordinary_muscle_development	ENSGALG00010022474	VCL	-0.349377268	0.119813136	-2.916018059	0.0035453	0.005017489	negative
PRJNA729271	stage_1W_vs_E10	ordinary_muscle_development	ENSGALG00010022476	OIP5	-1.199007867	0.115083791	-10.41856422	2.0401E-25	9.54774E-25	negative
PRJNA729271	stage_1W_vs_E10	ordinary_muscle_development	ENSGALG00010022478	ATP6V1A	-0.348571523	0.097093026	-3.590077852	0.000330579	0.000512103	negative
PRJNA729271	stage_1W_vs_E10	ordinary_muscle_development	ENSGALG00010022479	ENSGALG00010022479	1.033145258	0.644781683	1.60231794	0.109085321	0.130482895	positive
PRJNA729271	stage_1W_vs_E10	ordinary_muscle_development	ENSGALG00010022480	PIF1	-3.102808834	0.268057401	-11.57516569	5.50648E-31	3.23274E-30	negative
PRJNA729271	stage_1W_vs_E10	ordinary_muscle_development	ENSGALG00010022481	CORO1B	-0.318746461	0.148525509	-2.146072164	0.031867222	0.04069022	negative
PRJNA729271	stage_1W_vs_E10	ordinary_muscle_development	ENSGALG00010022482	PRTG	-0.114928597	0.301443668	-0.38126061	0.703009879	0.731104046	negative
PRJNA729271	stage_1W_vs_E10	ordinary_muscle_development	ENSGALG00010022483	TOP2A	-2.815767507	0.239065761	-11.77821321	5.05533E-32	3.08795E-31	negative
PRJNA729271	stage_1W_vs_E10	ordinary_muscle_development	ENSGALG00010022485	ATF2	-0.38214613	0.058229513	-6.562756805	5.2822E-11	1.27567E-10	negative
PRJNA729271	stage_1W_vs_E10	ordinary_muscle_development	ENSGALG00010022486	FAM183A	-1.80477976	0.693822822	-2.601211295	0.009289522	0.012604289	negative
PRJNA729271	stage_1W_vs_E10	ordinary_muscle_development	ENSGALG00010022489	NUSAP1	-1.987976768	0.239876315	-8.28750754	1.15646E-16	3.70043E-16	negative
PRJNA729271	stage_1W_vs_E10	ordinary_muscle_development	ENSGALG00010022493	ENSGALG00010022493	-0.197759458	0.164057106	-1.205430617	0.228037108	0.259669181	negative
PRJNA729271	stage_1W_vs_E10	ordinary_muscle_development	ENSGALG00010022494	FHIP2A	0.237876639	0.065499755	3.631717982	0.000281541	0.000438668	positive
PRJNA729271	stage_1W_vs_E10	ordinary_muscle_development	ENSGALG00010022496	TUBGCP2	0.183113205	0.072143702	2.538173132	0.011143284	0.014979311	positive
PRJNA729271	stage_1W_vs_E10	ordinary_muscle_development	ENSGALG00010022497	CORO1C	-0.459390343	0.14975057	-3.067703468	0.002157106	0.003114041	negative
PRJNA729271	stage_1W_vs_E10	ordinary_muscle_development	ENSGALG00010022498	NAA50	0.469927514	0.113841524	4.127909534	3.66076E-05	6.09146E-05	positive
PRJNA729271	stage_1W_vs_E10	ordinary_muscle_development	ENSGALG00010022500	ENSGALG00010022500	-0.206974826	0.439306288	-0.471140141	0.637540661	0.669988537	negative
PRJNA729271	stage_1W_vs_E10	ordinary_muscle_development	ENSGALG00010022502	ENSGALG00010022502	-1.591590818	0.512838372	-3.103494017	0.001912501	0.002777726	negative
PRJNA729271	stage_1W_vs_E10	ordinary_muscle_development	ENSGALG00010022504	RAD9A	1.766266581	0.142111036	12.42877842	1.824E-35	1.259E-34	positive
PRJNA729271	stage_1W_vs_E10	ordinary_muscle_development	ENSGALG00010022505	ZNF511	1.583307372	0.118036654	13.41369247	5.02721E-41	4.16397E-40	positive
PRJNA729271	stage_1W_vs_E10	ordinary_muscle_development	ENSGALG00010022506	EBNA1BP2	-1.152275092	0.087118527	-13.22652179	6.16762E-40	4.93103E-39	negative
PRJNA729271	stage_1W_vs_E10	ordinary_muscle_development	ENSGALG00010022507	ENSGALG00010022507	-0.214732253	0.218587963	-0.982360829	0.325922117	0.362399592	negative
PRJNA729271	stage_1W_vs_E10	ordinary_muscle_development	ENSGALG00010022508	USF3	0.126355191	0.124722976	1.01308673	0.311018736	0.346919321	positive
PRJNA729271	stage_1W_vs_E10	ordinary_muscle_development	ENSGALG00010022511	IGFBP4	-1.12563318	0.27200077	-4.138345572	3.49819E-05	5.82965E-05	negative
PRJNA729271	stage_1W_vs_E10	ordinary_muscle_development	ENSGALG00010022512	PRAP1	2.800899052	0.831066997	3.370244594	0.000751015	0.001131244	positive
PRJNA729271	stage_1W_vs_E10	ordinary_muscle_development	ENSGALG00010022513	NDUFAF1	0.436385363	0.08479735	5.14621461	2.65795E-07	5.16171E-07	positive
PRJNA729271	stage_1W_vs_E10	ordinary_muscle_development	ENSGALG00010022515	CHRNA1	-2.353258423	0.173593518	-13.55614228	7.28689E-42	6.22413E-41	negative
PRJNA729271	stage_1W_vs_E10	ordinary_muscle_development	ENSGALG00010022516	TRUB1	-0.512654866	0.152990049	-3.350903343	0.000805484	0.001210523	negative
PRJNA729271	stage_1W_vs_E10	ordinary_muscle_development	ENSGALG00010022517	HNRNPA1	-0.334191584	0.181409383	-1.842195696	0.06544653	0.080516674	negative
PRJNA729271	stage_1W_vs_E10	ordinary_muscle_development	ENSGALG00010022518	ECHS1	0.802790755	0.112001424	7.167683461	7.62774E-13	2.03019E-12	positive
PRJNA729271	stage_1W_vs_E10	ordinary_muscle_development	ENSGALG00010022520	TMOD2	-3.144702604	0.215656159	-14.58202081	3.65534E-48	3.75242E-47	negative
PRJNA729271	stage_1W_vs_E10	ordinary_muscle_development	ENSGALG00010022521	RTF1	-0.199686992	0.091259945	-2.188112124	0.028661436	0.036797831	negative
PRJNA729271	stage_1W_vs_E10	ordinary_muscle_development	ENSGALG00010022522	TNS4	-2.419508491	0.398928576	-6.065016734	1.3194E-09	2.9518E-09	negative
PRJNA729271	stage_1W_vs_E10	ordinary_muscle_development	ENSGALG00010022524	SOX18	1.29998116	0.227850083	5.705423245	1.16054E-08	2.45256E-08	positive
PRJNA729271	stage_1W_vs_E10	ordinary_muscle_development	ENSGALG00010022525	ISCU	1.038256434	0.19408773	5.349418182	8.82374E-08	1.77172E-07	positive
PRJNA729271	stage_1W_vs_E10	ordinary_muscle_development	ENSGALG00010022526	PPP1CA	2.338803621	0.137311327	17.03285277	4.68629E-65	7.76318E-64	positive
PRJNA729271	stage_1W_vs_E10	ordinary_muscle_development	ENSGALG00010022528	SIDT1	-3.553232318	0.513771406	-6.915979118	4.64643E-12	1.18804E-11	negative
PRJNA729271	stage_1W_vs_E10	ordinary_muscle_development	ENSGALG00010022529	CCR7	-1.682807949	0.388375459	-4.332940999	1.47131E-05	2.51849E-05	negative
PRJNA729271	stage_1W_vs_E10	ordinary_muscle_development	ENSGALG00010022531	ENSGALG00010022531	-1.560303061	0.448447679	-3.479342486	0.000502646	0.000766448	negative
PRJNA729271	stage_1W_vs_E10	ordinary_muscle_development	ENSGALG00010022532	CBX5	-2.961881688	0.180037153	-16.4515026	8.18131E-61	1.22889E-59	negative
PRJNA729271	stage_1W_vs_E10	ordinary_muscle_development	ENSGALG00010022533	TMEM119	-1.159122052	0.134455154	-8.620882272	6.64402E-18	2.24749E-17	negative
PRJNA729271	stage_1W_vs_E10	ordinary_muscle_development	ENSGALG00010022534	CFAP57	-1.32309299	0.564022548	-2.345815774	0.018985489	0.024852765	negative
PRJNA729271	stage_1W_vs_E10	ordinary_muscle_development	ENSGALG00010022536	SMUG1	0.471256208	0.099352532	4.743273262	2.10292E-06	3.83131E-06	positive
PRJNA729271	stage_1W_vs_E10	ordinary_muscle_development	ENSGALG00010022538	WIPF1	-0.291354931	0.158760505	-1.835185208	0.066478252	0.08172354	negative
PRJNA729271	stage_1W_vs_E10	ordinary_muscle_development	ENSGALG00010022539	LYSMD2	0.627776987	0.102020477	6.153440996	7.58196E-10	1.71559E-09	positive
PRJNA729271	stage_1W_vs_E10	ordinary_muscle_development	ENSGALG00010022540	GPR146	1.112099183	0.238685275	4.659270176	3.17332E-06	5.71325E-06	positive
PRJNA729271	stage_1W_vs_E10	ordinary_muscle_development	ENSGALG00010022541	ITPKA	-2.182506383	0.129917025	-16.79923311	2.4722E-63	3.94219E-62	negative
PRJNA729271	stage_1W_vs_E10	ordinary_muscle_development	ENSGALG00010022542	ENSGALG00010022542	0.160211159	0.750216421	0.213553256	0.830895455	0.849353288	positive
PRJNA729271	stage_1W_vs_E10	ordinary_muscle_development	ENSGALG00010022544	SPICE1	-0.260974548	0.070534475	-3.699957312	0.000215636	0.000338493	negative
PRJNA729271	stage_1W_vs_E10	ordinary_muscle_development	ENSGALG00010022545	CSTF3	-0.528165633	0.066998475	-7.883248629	3.18977E-15	9.57858E-15	negative
PRJNA729271	stage_1W_vs_E10	ordinary_muscle_development	ENSGALG00010022546	MTG1	1.374781212	0.156952644	8.759210291	1.96623E-18	6.82647E-18	positive
PRJNA729271	stage_1W_vs_E10	ordinary_muscle_development	ENSGALG00010022547	SMARCE1	-0.994765678	0.062113409	-16.01531291	9.99029E-58	1.3739E-56	negative
PRJNA729271	stage_1W_vs_E10	ordinary_muscle_development	ENSGALG00010022548	ENSGALG00010022548	-0.826370716	0.119035146	-6.9422414	3.85927E-12	9.91319E-12	negative
PRJNA729271	stage_1W_vs_E10	ordinary_muscle_development	ENSGALG00010022550	TRIM45	-1.581367484	0.173187989	-9.130930438	6.79131E-20	2.54039E-19	negative
PRJNA729271	stage_1W_vs_E10	ordinary_muscle_development	ENSGALG00010022551	GPER1	-1.569532216	0.640388561	-2.450906078	0.014249713	0.0189304	negative
PRJNA729271	stage_1W_vs_E10	ordinary_muscle_development	ENSGALG00010022552	GPR155	1.149487726	0.105134209	10.93352715	7.96895E-28	4.118E-27	positive
PRJNA729271	stage_1W_vs_E10	ordinary_muscle_development	ENSGALG00010022553	PRPF6	0.298868228	0.169395291	1.764324303	0.077677394	0.094671978	positive
PRJNA729271	stage_1W_vs_E10	ordinary_muscle_development	ENSGALG00010022554	SCG3	-3.515435343	0.358670572	-9.801292937	1.11154E-22	4.63247E-22	negative
PRJNA729271	stage_1W_vs_E10	ordinary_muscle_development	ENSGALG00010022557	WNT1	0.257704096	0.385899883	0.6678004	0.504261011	0.541908612	positive
PRJNA729271	stage_1W_vs_E10	ordinary_muscle_development	ENSGALG00010022558	NYAP2	-2.035866585	0.639879473	-3.181640718	0.001464434	0.002148043	negative
PRJNA729271	stage_1W_vs_E10	ordinary_muscle_development	ENSGALG00010022559	SSH1	0.194168238	0.087052393	2.230475587	0.025715886	0.033210244	positive
PRJNA729271	stage_1W_vs_E10	ordinary_muscle_development	ENSGALG00010022560	SAMD10	-3.465848458	1.448819092	-2.392188561	0.016748235	0.022063589	negative
PRJNA729271	stage_1W_vs_E10	ordinary_muscle_development	ENSGALG00010022562	GHRH	-5.29548675	1.216494837	-4.353069645	1.34244E-05	2.30608E-05	negative
PRJNA729271	stage_1W_vs_E10	ordinary_muscle_development	ENSGALG00010022565	ARF3	-1.517970728	0.175252293	-8.661631177	4.65061E-18	1.58241E-17	negative
PRJNA729271	stage_1W_vs_E10	ordinary_muscle_development	ENSGALG00010022567	PAOX	0.836620189	0.124063053	6.743508011	1.54607E-11	3.84529E-11	positive
PRJNA729271	stage_1W_vs_E10	ordinary_muscle_development	ENSGALG00010022568	SCRN3	2.988938505	0.166767088	17.92283207	7.82416E-72	1.55358E-70	positive
PRJNA729271	stage_1W_vs_E10	ordinary_muscle_development	ENSGALG00010022569	KIAA1549L	-3.587047538	0.271236146	-13.22481383	6.30933E-40	5.04155E-39	negative
PRJNA729271	stage_1W_vs_E10	ordinary_muscle_development	ENSGALG00010022571	RIPOR1	0.984375148	0.107279928	9.175762573	4.48389E-20	1.6886E-19	positive
PRJNA729271	stage_1W_vs_E10	ordinary_muscle_development	ENSGALG00010022572	ENSGALG00010022572	-6.324228743	0.529857173	-11.93572355	7.70857E-33	4.83914E-32	negative
PRJNA729271	stage_1W_vs_E10	ordinary_muscle_development	ENSGALG00010022573	AP4E1	-0.190142399	0.082334351	-2.309393301	0.020921766	0.027286396	negative
PRJNA729271	stage_1W_vs_E10	ordinary_muscle_development	ENSGALG00010022574	ITPRIPL2	-0.367264118	0.113099563	-3.247263812	0.001165203	0.001723079	negative
PRJNA729271	stage_1W_vs_E10	ordinary_muscle_development	ENSGALG00010022575	TMEM41B	0.437420129	0.109673661	3.988379029	6.65263E-05	0.000108556	positive
PRJNA729271	stage_1W_vs_E10	ordinary_muscle_development	ENSGALG00010022578	ZNF512B	-0.355434314	0.135388242	-2.625296765	0.008657347	0.011784041	negative
PRJNA729271	stage_1W_vs_E10	ordinary_muscle_development	ENSGALG00010022579	TNFAIP8L3	-0.406242463	0.14743047	-2.755485094	0.005860517	0.008122019	negative
PRJNA729271	stage_1W_vs_E10	ordinary_muscle_development	ENSGALG00010022581	TMEM125	-2.830706354	0.751248408	-3.768003133	0.000164559	0.000260458	negative
PRJNA729271	stage_1W_vs_E10	ordinary_muscle_development	ENSGALG00010022582	ENSGALG00010022582	0.955727259	0.214829443	4.448772233	8.63625E-06	1.50568E-05	positive
PRJNA729271	stage_1W_vs_E10	ordinary_muscle_development	ENSGALG00010022583	FKBP11	-0.264325167	0.122131162	-2.164272926	0.030443409	0.038978643	negative
PRJNA729271	stage_1W_vs_E10	ordinary_muscle_development	ENSGALG00010022585	CIR1	0.256111073	0.093792312	2.730619045	0.00632155	0.008730075	positive
PRJNA729271	stage_1W_vs_E10	ordinary_muscle_development	ENSGALG00010022587	ENSGALG00010022587	-1.608909242	0.876346045	-1.835929142	0.066368137	0.081595093	negative
PRJNA729271	stage_1W_vs_E10	ordinary_muscle_development	ENSGALG00010022588	TCP11L1	-0.364437386	0.078950138	-4.616044976	3.91122E-06	6.98794E-06	negative
PRJNA729271	stage_1W_vs_E10	ordinary_muscle_development	ENSGALG00010022590	ENSGALG00010022590	-0.953879393	0.162359317	-5.875113351	4.22554E-09	9.17042E-09	negative

PRJNA729271	stage_1W_vs_E10	ordinary_muscle_development	ENSGALG00010022592	CCDC65	-0.249615877	0.428514216	-0.58251481	0.560219969	0.596035558	negative
PRJNA729271	stage_1W_vs_E10	ordinary_muscle_development	ENSGALG00010022593	MANBAL	-2.3319657	0.149914256	-15.55532982	1.46399E-54	1.87461E-53	negative
PRJNA729271	stage_1W_vs_E10	ordinary_muscle_development	ENSGALG00010022597	SLCO4A1	1.676697842	0.226291776	7.409451072	1.26823E-13	3.50487E-13	positive
PRJNA729271	stage_1W_vs_E10	ordinary_muscle_development	ENSGALG00010022599	RNLS	-1.635456245	0.287402556	-5.690472161	1.26689E-08	2.66911E-08	negative
PRJNA729271	stage_1W_vs_E10	ordinary_muscle_development	ENSGALG00010022603	ATRNL1	-0.530024829	0.169969678	-3.118349305	0.001818671	0.002645423	negative
PRJNA729271	stage_1W_vs_E10	ordinary_muscle_development	ENSGALG00010022608	P3H4	-2.107090125	0.208963507	-10.08353158	6.53347E-24	2.87065E-23	negative
PRJNA729271	stage_1W_vs_E10	ordinary_muscle_development	ENSGALG00010022609	RBBP8NL	-7.927894204	1.061065745	-7.471633346	7.92054E-14	2.21552E-13	negative
PRJNA729271	stage_1W_vs_E10	ordinary_muscle_development	ENSGALG00010022614	DAO	-0.95373483	0.206997402	-4.607472461	4.07593E-06	7.27146E-06	negative
PRJNA729271	stage_1W_vs_E10	ordinary_muscle_development	ENSGALG00010022616	GLDN	3.285102513	0.331721096	9.903206487	4.03138E-23	1.71391E-22	positive
PRJNA729271	stage_1W_vs_E10	ordinary_muscle_development	ENSGALG00010022619	MADD	0.157788762	0.131920135	1.196093093	0.231660233	0.263407207	positive
PRJNA729271	stage_1W_vs_E10	ordinary_muscle_development	ENSGALG00010022622	MPPED2	-1.283775546	0.137206223	-9.35654022	8.23904E-21	3.20604E-20	negative
PRJNA729271	stage_1W_vs_E10	ordinary_muscle_development	ENSGALG00010022623	TOP1	-1.151907846	0.126722355	-9.09001292	9.90273E-20	3.68042E-19	negative
PRJNA729271	stage_1W_vs_E10	ordinary_muscle_development	ENSGALG00010022625	ENSGALG00010022625	0.327287071	0.123663832	2.646586852	0.008130862	0.011098677	positive
PRJNA729271	stage_1W_vs_E10	ordinary_muscle_development	ENSGALG00010022627	CTCF	-0.773055645	0.098814674	-7.823287879	5.14612E-15	1.53106E-14	negative
PRJNA729271	stage_1W_vs_E10	ordinary_muscle_development	ENSGALG00010022628	SVOP	1.212416909	0.553869482	2.188993885	0.028597284	0.036721974	positive
PRJNA729271	stage_1W_vs_E10	ordinary_muscle_development	ENSGALG00010022630	ENSGALG00010022630	-3.126285418	0.331087654	-9.442470535	3.64092E-21	1.43489E-20	negative
PRJNA729271	stage_1W_vs_E10	ordinary_muscle_development	ENSGALG00010022637	DMXL2	-0.868092907	0.110965776	-7.823068882	5.15509E-15	1.53341E-14	negative
PRJNA729271	stage_1W_vs_E10	ordinary_muscle_development	ENSGALG00010022642	SEC13	-0.515750696	0.06881155	-7.495118079	6.62387E-14	1.86505E-13	negative
PRJNA729271	stage_1W_vs_E10	ordinary_muscle_development	ENSGALG00010022643	SP3	-0.064663174	0.077013044	-0.839639245	0.401110688	0.439330469	negative
PRJNA729271	stage_1W_vs_E10	ordinary_muscle_development	ENSGALG00010022644	ARMC8	0.857907933	0.085047251	10.08742702	6.27939E-24	2.76236E-23	positive
PRJNA729271	stage_1W_vs_E10	ordinary_muscle_development	ENSGALG00010022645	ENSGALG00010022645	-0.456800263	0.659493472	-0.692653199	0.488527216	0.526796756	negative
PRJNA729271	stage_1W_vs_E10	ordinary_muscle_development	ENSGALG00010022647	SVIP	-1.115684088	0.135598592	-8.227844193	1.90613E-16	6.04316E-16	negative
PRJNA729271	stage_1W_vs_E10	ordinary_muscle_development	ENSGALG00010022649	PATZ1	-3.019801642	0.100198549	-30.13817737	1.5325E-199	2.2667E-197	negative
PRJNA729271	stage_1W_vs_E10	ordinary_muscle_development	ENSGALG00010022650	NR1H3	1.402424583	0.098550195	14.2305612	5.92047E-46	5.68326E-45	positive
PRJNA729271	stage_1W_vs_E10	ordinary_muscle_development	ENSGALG00010022652	CARMIL2	-0.77710819	0.274259356	-2.833479235	0.00460443	0.006449673	negative
PRJNA729271	stage_1W_vs_E10	ordinary_muscle_development	ENSGALG00010022655	NRIP3	-8.894913439	0.65666228	-13.54564396	8.40734E-42	7.16849E-41	negative
PRJNA729271	stage_1W_vs_E10	ordinary_muscle_development	ENSGALG00010022656	FKBP10	-1.96525602	0.211988447	-9.270580778	1.85135E-20	7.09929E-20	negative
PRJNA729271	stage_1W_vs_E10	ordinary_muscle_development	ENSGALG00010022657	MRPS35	0.797825992	0.11113411	7.178947935	7.02499E-13	1.87286E-12	positive
PRJNA729271	stage_1W_vs_E10	ordinary_muscle_development	ENSGALG00010022659	TIE1	1.311765137	0.150946361	8.690273356	3.61568E-18	1.23958E-17	positive
PRJNA729271	stage_1W_vs_E10	ordinary_muscle_development	ENSGALG00010022660	METAP1D	0.704564127	0.183973307	3.829708441	0.000128295	0.000204626	positive
PRJNA729271	stage_1W_vs_E10	ordinary_muscle_development	ENSGALG00010022661	ENSGALG00010022661	-1.184712282	0.406379605	-2.915284787	0.003553641	0.005028803	negative
PRJNA729271	stage_1W_vs_E10	ordinary_muscle_development	ENSGALG00010022662	GFRA1	-4.506610092	0.170493647	-26.43271563	5.7664E-154	5.2902E-152	negative
PRJNA729271	stage_1W_vs_E10	ordinary_muscle_development	ENSGALG00010022664	TRPC4AP	0.998883395	0.097311944	10.26475633	1.01577E-24	4.61844E-24	positive
PRJNA729271	stage_1W_vs_E10	ordinary_muscle_development	ENSGALG00010022666	ENSGALG00010022666	-0.424858995	0.103103619	-4.120699142	3.77724E-05	6.27622E-05	negative
PRJNA729271	stage_1W_vs_E10	ordinary_muscle_development	ENSGALG00010022667	PTEN	0.466167749	0.042082304	11.07752441	1.6127E-28	8.5564E-28	positive
PRJNA729271	stage_1W_vs_E10	ordinary_muscle_development	ENSGALG00010022669	MANSC4	-6.485809101	1.139271136	-5.692946038	1.24866E-08	2.63186E-08	negative
PRJNA729271	stage_1W_vs_E10	ordinary_muscle_development	ENSGALG00010022671	DBR1	-0.321802255	0.056441448	-5.701523744	1.18741E-08	2.50751E-08	negative
PRJNA729271	stage_1W_vs_E10	ordinary_muscle_development	ENSGALG00010022673	KLHL42	0.112828861	0.150652778	0.748933161	0.453897492	0.492053261	positive
PRJNA729271	stage_1W_vs_E10	ordinary_muscle_development	ENSGALG00010022674	ENSGALG00010022674	3.067008413	0.324341951	9.456095351	3.19657E-21	1.26424E-20	positive
PRJNA729271	stage_1W_vs_E10	ordinary_muscle_development	ENSGALG00010022681	PPP2R3A	0.747383017	0.093275802	8.01261425	1.12295E-15	3.44564E-15	positive
PRJNA729271	stage_1W_vs_E10	ordinary_muscle_development	ENSGALG00010022682	EIF1	1.338429631	0.155530109	8.605598234	7.59197E-18	2.56099E-17	positive
PRJNA729271	stage_1W_vs_E10	ordinary_muscle_development	ENSGALG00010022684	ENSGALG00010022684	-2.452043591	0.538166327	-4.556293226	5.20642E-06	9.21342E-06	negative
PRJNA729271	stage_1W_vs_E10	ordinary_muscle_development	ENSGALG00010022685	ATAD1	0.655343616	0.107889577	6.074206917	1.24602E-09	2.79194E-09	positive
PRJNA729271	stage_1W_vs_E10	ordinary_muscle_development	ENSGALG00010022689	MPL	-0.107993652	0.440698388	-0.245051161	0.806416818	0.826955202	negative
PRJNA729271	stage_1W_vs_E10	ordinary_muscle_development	ENSGALG00010022691	ASIC2	-3.861796805	0.596393075	-6.475254264	9.46524E-11	2.251E-10	negative
PRJNA729271	stage_1W_vs_E10	ordinary_muscle_development	ENSGALG00010022695	PIK3IP1	3.086982254	0.202441217	15.24878334	1.67729E-52	2.01762E-51	positive
PRJNA729271	stage_1W_vs_E10	ordinary_muscle_development	ENSGALG00010022698	SEMA6D	0.93057195	0.116939126	7.957746753	1.752E-15	5.33067E-15	positive
PRJNA729271	stage_1W_vs_E10	ordinary_muscle_development	ENSGALG00010022702	CDC20	-2.57819097	0.234667183	-10.98658509	4.43386E-28	2.31934E-27	negative
PRJNA729271	stage_1W_vs_E10	ordinary_muscle_development	ENSGALG00010022707	SALL1	-4.140434421	0.542356141	-7.634161591	2.27295E-14	6.53698E-14	negative
PRJNA729271	stage_1W_vs_E10	ordinary_muscle_development	ENSGALG00010022708	SPRED1	-0.747757216	0.131246559	-5.697347195	1.21686E-08	2.56745E-08	negative
PRJNA729271	stage_1W_vs_E10	ordinary_muscle_development	ENSGALG00010022709	RNF5	-0.3640717	0.08515618	-4.275340893	1.90845E-05	3.23695E-05	negative
PRJNA729271	stage_1W_vs_E10	ordinary_muscle_development	ENSGALG00010022711	LRRC49	-0.739393595	0.195766814	-3.776909784	0.000158786	0.000251652	negative
PRJNA729271	stage_1W_vs_E10	ordinary_muscle_development	ENSGALG00010022712	DDX23	0.325033365	0.201512831	1.612966104	0.106751872	0.127839424	positive
PRJNA729271	stage_1W_vs_E10	ordinary_muscle_development	ENSGALG00010022713	PNLIPRP1	7.863665087	2.789490735	2.819032517	0.004816864	0.006736174	positive
PRJNA729271	stage_1W_vs_E10	ordinary_muscle_development	ENSGALG00010022717	ELOVL1	1.235218696	0.170791071	7.232337681	4.7475E-13	1.2753E-12	positive
PRJNA729271	stage_1W_vs_E10	ordinary_muscle_development	ENSGALG00010022718	C12orf43	-1.09304479	0.146378602	-7.467244371	8.18916E-14	2.28845E-13	negative
PRJNA729271	stage_1W_vs_E10	ordinary_muscle_development	ENSGALG00010022719	MSL2	0.133291296	0.09754793	1.366418499	0.171807646	0.19965941	positive
PRJNA729271	stage_1W_vs_E10	ordinary_muscle_development	ENSGALG00010022724	LUZP2	-4.235061693	0.451029667	-9.389763028	6.01354E-21	2.35013E-20	negative
PRJNA729271	stage_1W_vs_E10	ordinary_muscle_development	ENSGALG00010022725	KRT222	-6.993384513	0.91234631	-7.665274071	1.7845E-14	5.16603E-14	negative
PRJNA729271	stage_1W_vs_E10	ordinary_muscle_development	ENSGALG00010022727	ACP2	0.35205271	0.083348702	4.22385355	2.4016E-05	4.04311E-05	positive
PRJNA729271	stage_1W_vs_E10	ordinary_muscle_development	ENSGALG00010022729	ENSGALG00010022729	-0.417362842	0.133231796	-3.132606901	0.001732613	0.002525312	negative
PRJNA729271	stage_1W_vs_E10	ordinary_muscle_development	ENSGALG00010022731	LRRC8B	-0.485268676	0.17692895	-2.742731914	0.006093041	0.008426546	negative
PRJNA729271	stage_1W_vs_E10	ordinary_muscle_development	ENSGALG00010022733	PCCB	1.128574162	0.114678861	9.841169957	7.48354E-23	3.14782E-22	positive
PRJNA729271	stage_1W_vs_E10	ordinary_muscle_development	ENSGALG00010022734	ENSGALG00010022734	1.432109695	0.118778688	12.0569584	1.78245E-33	1.14524E-32	positive
PRJNA729271	stage_1W_vs_E10	ordinary_muscle_development	ENSGALG00010022737	CYLD	0.41751789	0.079455459	5.254741406	1.48233E-07	2.92809E-07	positive
PRJNA729271	stage_1W_vs_E10	ordinary_muscle_development	ENSGALG00010022742	IFT46	-0.809235652	0.127087451	-6.36754962	1.92072E-10	4.48973E-10	negative
PRJNA729271	stage_1W_vs_E10	ordinary_muscle_development	ENSGALG00010022743	ZNF326	-1.747878195	0.153726744	-11.37003329	5.89651E-30	3.33216E-29	negative
PRJNA729271	stage_1W_vs_E10	ordinary_muscle_development	ENSGALG00010022745	PAPSS2	-0.840430916	0.238450007	-3.52455815	0.00042419	0.000650718	negative
PRJNA729271	stage_1W_vs_E10	ordinary_muscle_development	ENSGALG00010022747	NT5C3B	-0.135066736	0.137477439	-0.982464737	0.325870947	0.362370493	negative
PRJNA729271	stage_1W_vs_E10	ordinary_muscle_development	ENSGALG00010022748	ENSGALG00010022748	-0.711501814	0.334232413	-2.128763659	0.033273819	0.042411538	negative
PRJNA729271	stage_1W_vs_E10	ordinary_muscle_development	ENSGALG00010022749	HSPA12A	-1.270179468	0.131227309	-9.679231234	3.69484E-22	1.50736E-21	negative
PRJNA729271	stage_1W_vs_E10	ordinary_muscle_development	ENSGALG00010022751	SLC25A12	4.125390671	0.190884735	21.61194646	1.3868E-103	5.5837E-102	positive
PRJNA729271	stage_1W_vs_E10	ordinary_muscle_development	ENSGALG00010022754	SNX20	-0.158232031	0.186810365	-0.847019548	0.396984226	0.435139621	negative
PRJNA729271	stage_1W_vs_E10	ordinary_muscle_development	ENSGALG00010022755	MINPP1	-0.898229908	0.116795465	-7.690623147	1.4642E-14	4.25408E-14	negative
PRJNA729271	stage_1W_vs_E10	ordinary_muscle_development	ENSGALG00010022759	THSD4	-0.963537434	0.121634188	-7.921600446	2.34473E-15	7.08649E-15	negative
PRJNA729271	stage_1W_vs_E10	ordinary_muscle_development	ENSGALG00010022760	ELAVL4	-3.84062478	0.168186782	-22.83547333	2.0378E-115	1.0081E-113	negative
PRJNA729271	stage_1W_vs_E10	ordinary_muscle_development	ENSGALG00010022764	MFAP5	2.318807356	0.222922509	10.40185383	2.43156E-25	1.13366E-24	positive
PRJNA729271	stage_1W_vs_E10	ordinary_muscle_development	ENSGALG00010022767	CACNB3	-1.249328375	0.494433624	-2.526786841	0.011511134	0.015445144	negative
PRJNA729271	stage_1W_vs_E10	ordinary_muscle_development	ENSGALG00010022768	ENSGALG00010022768	1.075028046	0.54432262	1.974983228	0.048270051	0.0603688	positive
PRJNA729271	stage_1W_vs_E10	ordinary_muscle_development	ENSGALG00010022769	FXYD6	1.971756303	0.097168125	20.29221311	1.50658E-91	4.70645E-90	positive
PRJNA729271	stage_1W_vs_E10	ordinary_muscle_development	ENSGALG00010022770	HAT1	-1.182572908	0.092739681	-12.75153085	3.05683E-37	2.22881E-36	negative
PRJNA729271	stage_1W_vs_E10	ordinary_muscle_development	ENSGALG00010022771	SGMS1	1.262245899	0.219519461	5.750040994	8.92218E-09	1.8974E-08	positive
PRJNA729271	stage_1W_vs_E10	ordinary_muscle_development	ENSGALG00010022776	CDH4	-0.08493232	0.17752784	-0.478416906	0.632353503	0.665212572	negative
PRJNA729271	stage_1W_vs_E10	ordinary_muscle_development	ENSGALG00010022780	DYNC1I2	-0.893918994	0.058370289	-15.31462335	6.10646E-53	7.4506E-52	negative
PRJNA729271	stage_1W_vs_E10	ordinary_muscle_development	ENSGALG00010022782	TMPRSS13	-0.305151342	0.249870923	-1.221235901	0.221996715	0.253393368	negative
PRJNA729271	stage_1W_vs_E10	ordinary_muscle_development	ENSGALG00010022783	STAG1	-0.041327866	0.055368236	-0.746418334	0.455414748	0.493587316	negative
PRJNA729271	stage_1W_vs_E10	ordinary_muscle_development	ENSGALG00010022784	DMRTA2	-2.547363816	0.845236616	-3.013787818	0.002580082	0.003700602	negative
PRJNA729271	stage_1W_vs_E10	ordinary_muscle_development	ENSGALG00010022786	KRT40	0.75568877	0.550626521	1.372416224	0.169933902	0.19767209	positive
PRJNA729271	stage_1W_vs_E10	ordinary_muscle_development	ENSGALG00010022787	ENO4	-2.205025613	0.377598317	-5.839606563	5.23242E-09	1.12981E-08	negative
PRJNA729271	stage_1W_vs_E10	ordinary_muscle_development	ENSGALG00010022788	CYBRD1	1.701356846	0.173583841	9.801354995	1.11085E-22	4.63095E-22	positive
PRJNA729271	stage_1W_vs_E10	ordinary_muscle_development	ENSGALG00010022789	ENSGALG00010022789	-0.53059073	0.337131018	-1.573841328	0.115524137	0.137741413	negative
PRJNA729271	stage_1W_vs_E10	ordinary_muscle_development	ENSGALG00010022792	DDB2	-0.740419127	0.126659384	-5.845750242	5.0429E-09	1.08986E-08	negative
PRJNA729271	stage_1W_vs_E10	ordinary_muscle_development	ENSGALG00010022795	TAF4	0.414580288	0.110690514	3.745400349	0.000180106	0.000284415	positive
PRJNA729271	stage_1W_vs_E10	ordinary_muscle_development	ENSGALG00010022796	RIMKLB	-2.385817906	0.140150956	-17.02320106	5.52649E-65	9.13416E-64	negative
PRJNA729271	stage_1W_vs_E10	ordinary_muscle_development	ENSGALG00010022797	EFHD1	0.571945721	0.132667278	4.311128776	1.62423E-05	2.77208E-05	positive
PRJNA729271	stage_1W_vs_E10	ordinary_muscle_development	ENSGALG00010022799	DCAF17	-0.348252551	0.155376326	-2.241348866	0.025003486	0.032342096	negative
PRJNA729271	stage_1W_vs_E10	ordinary_muscle_development	ENSGALG00010022800	KLHL10	-0.430970939	0.44901765	-0.959808461	0.337151623	0.374082423	negative
PRJNA729271	stage_1W_vs_E10	ordinary_muscle_development	ENSGALG00010022801	LSM14B	-0.438672505	0.103303059	-4.246461923	2.17173E-05	3.66847E-05	negative
PRJNA729271	stage_1W_vs_E10	ordinary_muscle_development	ENSGALG00010022803	ENSGALG00010022803	0.75949077	0.156050409	4.866957888	1.13329E-06	2.11118E-06	positive
PRJNA729271	stage_1W_vs_E10	ordinary_muscle_development	ENSGALG00010022804	SNX19	-0.408353172	0.117031085	-3.489271004	0.00048434	0.000739234	negative
PRJNA729271	stage_1W_vs_E10	ordinary_muscle_development	ENSGALG00010022805	ENSGALG00010022805	-1.312803395	0.284802202	-4.609526848	4.03586E-06	7.20264E-06	negative
PRJNA729271	stage_1W_vs_E10	ordinary_muscle_development	ENSGALG00010022806	SPPL3	-0.41466359	0.06383392	-6.495975668	8.2497E-11	1.96806E-10	negative
PRJNA729271	stage_1W_vs_E10	ordinary_muscle_development	ENSGALG00010022807	ADCY6	-0.498580943	0.149149814	-3.342819737	0.000829318	0.001244406	negative
PRJNA729271	stage_1W_vs_E10	ordinary_muscle_development	ENSGALG00010022808	PSMA7	0.576028529	0.13664163	4.215615167	2.49098E-05	4.19018E-05	positive
PRJNA729271	stage_1W_vs_E10	ordinary_muscle_development	ENSGALG00010022809	MYO1E	-0.679766566	0.15680151	-4.335204196	1.45625E-05	2.49389E-05	negative
PRJNA729271	stage_1W_vs_E10	ordinary_muscle_development	ENSGALG00010022811	GIGYF2	-0.04127245	0.098156846	-0.420474488	0.674138863	0.704008849	negative
PRJNA729271	stage_1W_vs_E10	ordinary_muscle_development	ENSGALG00010022813	USP24	1.954241921	0.1218467	16.03852976	6.8763E-58	9.50162E-57	positive
PRJNA729271	stage_1W_vs_E10	ordinary_muscle_development	ENSGALG00010022815	LRRC8D	0.788643221	0.236256063	3.338086695	0.000843574	0.001265536	positive
PRJNA729271	stage_1W_vs_E10	ordinary_muscle_development	ENSGALG00010022816	METTL8	0.236571381	0.140891507	1.679103199	0.093131933	0.11248624	positive
PRJNA729271	stage_1W_vs_E10	ordinary_muscle_development	ENSGALG00010022817	KRT23	-5.026069894	1.0555672	-4.761487373	1.92171E-06	3.51752E-06	negative
PRJNA729271	stage_1W_vs_E10	ordinary_muscle_development	ENSGALG00010022819	PACSIN3	2.030020136	0.108408928	18.72558083	3.06312E-78	6.95924E-77	positive
PRJNA729271	stage_1W_vs_E10	ordinary_muscle_development	ENSGALG00010022820	SHTN1	-1.993533639	0.185155609	-10.76680124	4.93862E-27	2.46761E-26	negative
PRJNA729271	stage_1W_vs_E10	ordinary_muscle_development	ENSGALG00010022821	ENSGALG00010022821	4.333355999	1.372031238	3.15835083			positive
PRJNA729271	stage_1W_vs_E10	ordinary_muscle_development	ENSGALG00010022823	IGHMBP2	0.500381661	0.11400156	4.389252745	1.13741E-05	1.96692E-05	positive
PRJNA729271	stage_1W_vs_E10	ordinary_muscle_development	ENSGALG00010022824	PUS1	-1.252137385	0.085755354	-14.6012736	2.75647E-48	2.84176E-47	negative
PRJNA729271	stage_1W_vs_E10	ordinary_muscle_development	ENSGALG00010022825	SS18L1	1.955448061	0.258672836	7.5595416	4.04492E-14	1.14963E-13	positive
PRJNA729271	stage_1W_vs_E10	ordinary_muscle_development	ENSGALG00010022826	SNORC	-5.520228858	0.583446606	-9.461412237	3.03812E-21	1.20255E-20	negative
PRJNA729271	stage_1W_vs_E10	ordinary_muscle_development	ENSGALG00010022827	CMTR2	-0.31094246	0.0948551	-3.278078443	0.001045163	0.001552854	negative
PRJNA729271	stage_1W_vs_E10	ordinary_muscle_development	ENSGALG00010022828	PRKG1	-1.636320533	0.171848504	-9.52187826	1.70077E-21	6.79323E-21	negative
PRJNA729271	stage_1W_vs_E10	ordinary_muscle_development	ENSGALG00010022830	MTG2	-0.46112071	0.130580178	-3.531322424	0.000413487	0.000634703	negative
PRJNA729271	stage_1W_vs_E10	ordinary_muscle_development	ENSGALG00010022831	LRRC8C	0.598705999	0.089875701	6.661489093	2.71067E-11	6.66743E-11	positive
PRJNA729271	stage_1W_vs_E10	ordinary_muscle_development	ENSGALG00010022833	HRH3	-0.883923561	0.554801218	-1.593225704	0.111109567	0.132750838	negative
PRJNA729271	stage_1W_vs_E10	ordinary_muscle_development	ENSGALG00010022834	CHRNA7	-2.717108143	0.251185269	-10.81714767	2.85525E-27	1.44356E-26	negative
PRJNA729271	stage_1W_vs_E10	ordinary_muscle_development	ENSGALG00010022837	ITGB3	-1.359075437	0.152926394	-8.887121461	6.27124E-19	2.24615E-18	negative
PRJNA729271	stage_1W_vs_E10	ordinary_muscle_development	ENSGALG00010022838	ARFGAP2	0.616943429	0.079177575	7.791896029	6.6011E-15	1.95351E-14	positive
PRJNA729271	stage_1W_vs_E10	ordinary_muscle_development	ENSGALG00010022839	SYT12	-1.87879325	0.31787061	-5.910559808	3.40947E-09	7.44281E-09	negative
PRJNA729271	stage_1W_vs_E10	ordinary_muscle_development	ENSGALG00010022840	MARVELD1	-0.10510872	0.197652101	-0.531786502	0.594873873	0.629347988	negative
PRJNA729271	stage_1W_vs_E10	ordinary_muscle_development	ENSGALG00010022841	ENSGALG00010022841	-6.882316484	0.5008442	-13.74143193	5.73306E-43	5.05171E-42	negative
PRJNA729271	stage_1W_vs_E10	ordinary_muscle_development	ENSGALG00010022842	PHC1	-1.180317281	0.14414148	-8.188602462	2.64277E-16	8.31855E-16	negative
PRJNA729271	stage_1W_vs_E10	ordinary_muscle_development	ENSGALG00010022845	HYDIN	2.363379546	0.321536435	7.350269793	1.97807E-13	5.4129E-13	positive
PRJNA729271	stage_1W_vs_E10	ordinary_muscle_development	ENSGALG00010022848	SGIP1	-2.833296097	0.955612621	-2.964900249	0.003027811	0.004314177	negative

PRJNA729271	stage_1W_vs_E10	ordinary_muscle_development	ENSGALG00010022850	ULK1	3.254403475	0.2494832	13.04457968	6.82323E-39	5.27762E-38	positive
PRJNA729271	stage_1W_vs_E10	ordinary_muscle_development	ENSGALG00010022858	HERC4	0.678429314	0.127142729	5.335966261	9.50371E-08	1.9035E-07	positive
PRJNA729271	stage_1W_vs_E10	ordinary_muscle_development	ENSGALG00010022860	MOB3C	-1.367549841	0.133590137	-10.23690727	1.35499E-24	6.13E-24	negative
PRJNA729271	stage_1W_vs_E10	ordinary_muscle_development	ENSGALG00010022862	ACADS	0.758524769	0.122823531	6.175728387	6.58591E-10	1.49488E-09	positive
PRJNA729271	stage_1W_vs_E10	ordinary_muscle_development	ENSGALG00010022863	FAM81A	-1.750751496	0.389463142	-4.495294434	6.94737E-06	1.21783E-05	negative
PRJNA729271	stage_1W_vs_E10	ordinary_muscle_development	ENSGALG00010022864	CSTPP1	-1.798700702	0.22033126	-8.163620111	3.2513E-16	1.0192E-15	negative
PRJNA729271	stage_1W_vs_E10	ordinary_muscle_development	ENSGALG00010022866	KLHL11	-0.624622388	0.099888815	-6.253176441	4.02187E-10	9.2403E-10	negative
PRJNA729271	stage_1W_vs_E10	ordinary_muscle_development	ENSGALG00010022867	ATPAF1	-0.39351257	0.134287682	-2.930369818	0.003385588	0.004802711	negative
PRJNA729271	stage_1W_vs_E10	ordinary_muscle_development	ENSGALG00010022868	VAC14	-0.583219751	0.102697156	-5.67902532	1.35464E-08	2.84904E-08	negative
PRJNA729271	stage_1W_vs_E10	ordinary_muscle_development	ENSGALG00010022872	KRT13	-4.030075415	0.510405337	-7.895833219	2.88382E-15	8.6778E-15	negative
PRJNA729271	stage_1W_vs_E10	ordinary_muscle_development	ENSGALG00010022873	NGEF	-0.002461867	0.37345003	-0.006592226	0.994740203	0.995770666	negative
PRJNA729271	stage_1W_vs_E10	ordinary_muscle_development	ENSGALG00010022874	EFCAB14	0.69107289	0.076873327	8.989761694	2.47767E-19	9.02358E-19	positive
PRJNA729271	stage_1W_vs_E10	ordinary_muscle_development	ENSGALG00010022875	SPON1	-0.741834172	0.244506925	-3.03400066	0.002413339	0.003468651	negative
PRJNA729271	stage_1W_vs_E10	ordinary_muscle_development	ENSGALG00010022877	KYAT3	0.207453556	0.101397824	2.045936963	0.040762573	0.051454628	positive
PRJNA729271	stage_1W_vs_E10	ordinary_muscle_development	ENSGALG00010022879	ENSGALG00010022879	0.583453167	0.281817582	2.070322096	0.038422192	0.048635898	positive
PRJNA729271	stage_1W_vs_E10	ordinary_muscle_development	ENSGALG00010022881	CCNB1	-2.570614721	0.212452234	-12.09973024	1.05959E-33	6.86577E-33	negative
PRJNA729271	stage_1W_vs_E10	ordinary_muscle_development	ENSGALG00010022885	M6PR	-0.020081074	0.099493922	-0.201832168	0.840047932	0.857620424	negative
PRJNA729271	stage_1W_vs_E10	ordinary_muscle_development	ENSGALG00010022886	PLA2G15	2.936214728	0.281120325	10.4446903	1.5496E-25	7.29743E-25	positive
PRJNA729271	stage_1W_vs_E10	ordinary_muscle_development	ENSGALG00010022888	OSBPL2	1.066538005	0.090617561	11.76966135	5.59487E-32	3.41607E-31	positive
PRJNA729271	stage_1W_vs_E10	ordinary_muscle_development	ENSGALG00010022890	MYPN	5.864557469	0.166024038	35.32354438	2.5554E-273	7.7167E-271	positive
PRJNA729271	stage_1W_vs_E10	ordinary_muscle_development	ENSGALG00010022891	NTM	-1.381146982	0.23038381	-5.994982808	2.03507E-09	4.50494E-09	negative
PRJNA729271	stage_1W_vs_E10	ordinary_muscle_development	ENSGALG00010022900	MMP17	0.01685686	0.199927753	0.084314759	0.932806179	0.941277856	positive
PRJNA729271	stage_1W_vs_E10	ordinary_muscle_development	ENSGALG00010022911	SMPD3	-5.444710342	0.381271489	-14.28040254	2.89922E-46	2.81663E-45	negative
PRJNA729271	stage_1W_vs_E10	ordinary_muscle_development	ENSGALG00010022917	CYP4B7	-0.190667127	0.474917501	-0.401474207	0.688071024	0.717111698	negative
PRJNA729271	stage_1W_vs_E10	ordinary_muscle_development	ENSGALG00010022919	ACLY	-2.203965989	0.126115842	-17.47572678	2.19333E-68	3.984E-67	negative
PRJNA729271	stage_1W_vs_E10	ordinary_muscle_development	ENSGALG00010022921	ENSGALG00010022921	0.71685736	0.198769751	3.606471078	0.000310389	0.000482063	positive
PRJNA729271	stage_1W_vs_E10	ordinary_muscle_development	ENSGALG00010022922	ATOH7	0.498453107	0.427372963	1.166318765	0.243485589	0.276051906	positive
PRJNA729271	stage_1W_vs_E10	ordinary_muscle_development	ENSGALG00010022923	CD63	1.698031814	0.126434398	13.43014122	4.02643E-41	3.35227E-40	positive
PRJNA729271	stage_1W_vs_E10	ordinary_muscle_development	ENSGALG00010022928	RNF111	0.336931163	0.083167324	4.051244473	5.09459E-05	8.37543E-05	positive
PRJNA729271	stage_1W_vs_E10	ordinary_muscle_development	ENSGALG00010022929	LRP4	-0.383524745	0.116634155	-3.288271291	0.001008047	0.00150063	negative
PRJNA729271	stage_1W_vs_E10	ordinary_muscle_development	ENSGALG00010022930	GTF2B	-0.124255384	0.117830048	-1.054530538	0.291640087	0.326661237	negative
PRJNA729271	stage_1W_vs_E10	ordinary_muscle_development	ENSGALG00010022931	RDH5	1.456252731	0.120655079	12.06955187	1.52965E-33	9.85873E-33	positive
PRJNA729271	stage_1W_vs_E10	ordinary_muscle_development	ENSGALG00010022932	OPCML	-1.71230399	0.223766139	-7.652203327	1.97565E-14	5.6955E-14	negative
PRJNA729271	stage_1W_vs_E10	ordinary_muscle_development	ENSGALG00010022933	UNC119B	-0.980871175	0.077438048	-12.66652767	9.06385E-37	6.50721E-36	negative
PRJNA729271	stage_1W_vs_E10	ordinary_muscle_development	ENSGALG00010022937	BLOC1S1	1.485755663	0.160480489	9.258170101	2.07966E-20	7.95373E-20	positive
PRJNA729271	stage_1W_vs_E10	ordinary_muscle_development	ENSGALG00010022942	NEU2	-2.249295545	0.52433806	-4.289781187	1.78849E-05	3.04275E-05	negative
PRJNA729271	stage_1W_vs_E10	ordinary_muscle_development	ENSGALG00010022945	KCNA4	-6.63942654	0.651386117	-10.19276642	2.13598E-24	9.56177E-24	negative
PRJNA729271	stage_1W_vs_E10	ordinary_muscle_development	ENSGALG00010022946	KRT17	-6.323333236	0.554226348	-11.40929741	3.75752E-30	2.13673E-29	negative
PRJNA729271	stage_1W_vs_E10	ordinary_muscle_development	ENSGALG00010022949	INSC	-3.149402507	0.274539407	-11.47158634	1.83268E-30	1.05207E-29	negative
PRJNA729271	stage_1W_vs_E10	ordinary_muscle_development	ENSGALG00010022950	ITGA7	4.201970744	0.154092006	27.26923254	9.831E-164	1.0035E-161	positive
PRJNA729271	stage_1W_vs_E10	ordinary_muscle_development	ENSGALG00010022953	ENSGALG00010022953	-1.696570019	1.166613399	-1.454269273	0.145871666	0.171415468	negative
PRJNA729271	stage_1W_vs_E10	ordinary_muscle_development	ENSGALG00010022954	ADRM1	0.923216877	0.143264707	6.444133373	1.16263E-10	2.75229E-10	positive
PRJNA729271	stage_1W_vs_E10	ordinary_muscle_development	ENSGALG00010022956	PBLD	1.022665296	0.121094808	8.445162241	3.03623E-17	9.95031E-17	positive
PRJNA729271	stage_1W_vs_E10	ordinary_muscle_development	ENSGALG00010022957	SLTM	0.250673452	0.142828575	1.755065126	0.079248169	0.096464747	positive
PRJNA729271	stage_1W_vs_E10	ordinary_muscle_development	ENSGALG00010022958	SFSWAP	0.011209856	0.097815146	0.114602456	0.908760233	0.919544822	positive
PRJNA729271	stage_1W_vs_E10	ordinary_muscle_development	ENSGALG00010022959	ADAMTS8	-0.43478653	0.265258715	-1.639103657	0.101191673	0.121592747	negative
PRJNA729271	stage_1W_vs_E10	ordinary_muscle_development	ENSGALG00010022967	ADAMTS15	-1.550885293	0.164037019	-9.454483501	3.24621E-21	1.28317E-20	negative
PRJNA729271	stage_1W_vs_E10	ordinary_muscle_development	ENSGALG00010022969	TTN	4.954214752	0.575497415	8.608578642	7.3972E-18	2.49645E-17	positive
PRJNA729271	stage_1W_vs_E10	ordinary_muscle_development	ENSGALG00010022971	CKAP5	-1.238432208	0.05862488	-21.12468647	4.71747E-99	1.72676E-97	negative
PRJNA729271	stage_1W_vs_E10	ordinary_muscle_development	ENSGALG00010022972	ENSGALG00010022972	0.787831388	1.09063687	0.722359027			positive
PRJNA729271	stage_1W_vs_E10	ordinary_muscle_development	ENSGALG00010022974	INPP5D	1.02883554	0.15610043	6.590856531	4.37296E-11	1.06299E-10	positive
PRJNA729271	stage_1W_vs_E10	ordinary_muscle_development	ENSGALG00010022976	MINDY2	-0.368278448	0.066590314	-5.530510745	3.193E-08	6.57142E-08	negative
PRJNA729271	stage_1W_vs_E10	ordinary_muscle_development	ENSGALG00010022978	ARL14EP	-0.162092567	0.086963258	-1.863920143	0.06233292	0.076875323	negative
PRJNA729271	stage_1W_vs_E10	ordinary_muscle_development	ENSGALG00010022979	LAMA5	-0.171524032	0.149281453	-1.148997604	0.250556969	0.283514696	negative
PRJNA729271	stage_1W_vs_E10	ordinary_muscle_development	ENSGALG00010022981	HNRNPH3	-1.753789879	0.129985913	-13.4921534	1.73945E-41	1.46845E-40	negative
PRJNA729271	stage_1W_vs_E10	ordinary_muscle_development	ENSGALG00010022983	PKN2	0.061318819	0.061617283	0.995156169	0.319660316	0.355922608	positive
PRJNA729271	stage_1W_vs_E10	ordinary_muscle_development	ENSGALG00010022986	ALG9	-0.604994461	0.108017746	-5.600880278	2.13266E-08	4.43514E-08	negative
PRJNA729271	stage_1W_vs_E10	ordinary_muscle_development	ENSGALG00010022987	F2	4.095078395	1.379754139	2.967976888			positive
PRJNA729271	stage_1W_vs_E10	ordinary_muscle_development	ENSGALG00010022990	SLC25A22	-1.482322422	0.102804479	-14.41885063	3.93847E-47	3.92628E-46	negative
PRJNA729271	stage_1W_vs_E10	ordinary_muscle_development	ENSGALG00010022991	TAL1	0.978715573	0.176351804	5.549790531	2.86012E-08	5.90142E-08	positive
PRJNA729271	stage_1W_vs_E10	ordinary_muscle_development	ENSGALG00010022992	SLC7A6	-0.13427193	0.107434155	-1.24980673	0.211370157	0.24237089	negative
PRJNA729271	stage_1W_vs_E10	ordinary_muscle_development	ENSGALG00010022994	LIPC	-2.364939711	0.879076041	-2.690256133	0.00713972	0.009806712	negative
PRJNA729271	stage_1W_vs_E10	ordinary_muscle_development	ENSGALG00010022997	ARHGAP1	-1.730924872	0.097433881	-17.76512283	1.31652E-70	2.53089E-69	negative
PRJNA729271	stage_1W_vs_E10	ordinary_muscle_development	ENSGALG00010022999	ENSGALG00010022999	1.742200868	0.492653055	3.536364689	0.000405674	0.000623238	positive
PRJNA729271	stage_1W_vs_E10	ordinary_muscle_development	ENSGALG00010023002	ATG16L1	1.179986979	0.133723092	8.824107834	1.10336E-18	3.89034E-18	positive
PRJNA729271	stage_1W_vs_E10	ordinary_muscle_development	ENSGALG00010023003	ENSGALG00010023003	-6.770275593	0.812588998	-8.331734259	7.96666E-17	2.56722E-16	negative
PRJNA729271	stage_1W_vs_E10	ordinary_muscle_development	ENSGALG00010023005	IRF7	2.535171209	0.34758153	7.293745474	3.01455E-13	8.17662E-13	positive
PRJNA729271	stage_1W_vs_E10	ordinary_muscle_development	ENSGALG00010023007	LMO4	-0.048675357	0.142379553	-0.341870419	0.732448412	0.758779283	negative
PRJNA729271	stage_1W_vs_E10	ordinary_muscle_development	ENSGALG00010023008	CDHR5	1.139303381	0.835258918	1.364012231	0.172563716	0.200505897	positive
PRJNA729271	stage_1W_vs_E10	ordinary_muscle_development	ENSGALG00010023012	ENSGALG00010023012	0.89864541	0.51746622	1.736626229	0.082453167	0.100222948	positive
PRJNA729271	stage_1W_vs_E10	ordinary_muscle_development	ENSGALG00010023013	ENSGALG00010023013	0.212666777	0.441511618	0.48167878	0.630034153	0.663254052	positive
PRJNA729271	stage_1W_vs_E10	ordinary_muscle_development	ENSGALG00010023015	METTL2B	-0.073619967	0.102031793	-0.721539475	0.470577662	0.508993599	negative
PRJNA729271	stage_1W_vs_E10	ordinary_muscle_development	ENSGALG00010023017	BUB1B	-2.578191749	0.201955699	-12.76612525	2.53461E-37	1.85364E-36	negative
PRJNA729271	stage_1W_vs_E10	ordinary_muscle_development	ENSGALG00010023019	CABP1	0.019435542	0.331013728	0.058715215	0.953178941	0.959200829	positive
PRJNA729271	stage_1W_vs_E10	ordinary_muscle_development	ENSGALG00010023024	PRMT7	0.210773497	0.112181132	1.878867628	0.06026257	0.074480385	positive
PRJNA729271	stage_1W_vs_E10	ordinary_muscle_development	ENSGALG00010023026	PHRF1	0.171867435	0.091370785	1.880988938	0.059973424	0.074135662	positive
PRJNA729271	stage_1W_vs_E10	ordinary_muscle_development	ENSGALG00010023028	TMEM61	-1.867430698	0.312146798	-5.982539975	2.19685E-09	4.85416E-09	negative
PRJNA729271	stage_1W_vs_E10	ordinary_muscle_development	ENSGALG00010023030	HS2ST1	-0.795065431	0.085615385	-9.286478486	1.59477E-20	6.14381E-20	negative
PRJNA729271	stage_1W_vs_E10	ordinary_muscle_development	ENSGALG00010023041	CD151	1.192185147	0.119419738	9.983149928	1.80639E-23	7.7974E-23	positive
PRJNA729271	stage_1W_vs_E10	ordinary_muscle_development	ENSGALG00010023042	ENSGALG00010023042	-4.844248788	1.029218704	-4.706724402	2.51729E-06	4.56729E-06	negative
PRJNA729271	stage_1W_vs_E10	ordinary_muscle_development	ENSGALG00010023043	CNP	0.925558196	0.145970315	6.340728915	2.28681E-10	5.31803E-10	positive
PRJNA729271	stage_1W_vs_E10	ordinary_muscle_development	ENSGALG00010023046	SLC7A6OS	-0.70045385	0.075845634	-9.235256	2.57669E-20	9.81323E-20	negative
PRJNA729271	stage_1W_vs_E10	ordinary_muscle_development	ENSGALG00010023048	ARFGAP1	-0.500833307	0.084001041	-5.962227384	2.48823E-09	5.47878E-09	negative
PRJNA729271	stage_1W_vs_E10	ordinary_muscle_development	ENSGALG00010023050	RUFY2	-0.352410678	0.094454456	-3.731011658	0.000190712	0.000300508	negative
PRJNA729271	stage_1W_vs_E10	ordinary_muscle_development	ENSGALG00010023052	RAD54L	-1.881918268	0.136279323	-13.80927224	2.24084E-43	1.9976E-42	negative
PRJNA729271	stage_1W_vs_E10	ordinary_muscle_development	ENSGALG00010023053	MAN1A2	-1.083435791	0.048916347	-22.14874684	1.0726E-108	4.7546E-107	negative
PRJNA729271	stage_1W_vs_E10	ordinary_muscle_development	ENSGALG00010023054	AQP9	2.012171072	0.23077695	8.719116319	2.80381E-18	9.66269E-18	positive
PRJNA729271	stage_1W_vs_E10	ordinary_muscle_development	ENSGALG00010023056	SAG	1.906926369	0.432702292	4.407017031	1.04804E-05	1.81649E-05	positive
PRJNA729271	stage_1W_vs_E10	ordinary_muscle_development	ENSGALG00010023057	PAFAH1B2	-1.735257015	0.07590841	-22.85987827	1.1655E-115	5.8057E-114	negative
PRJNA729271	stage_1W_vs_E10	ordinary_muscle_development	ENSGALG00010023058	PPARA	1.110588165	0.204118752	5.44089239	5.30143E-08	1.08003E-07	positive
PRJNA729271	stage_1W_vs_E10	ordinary_muscle_development	ENSGALG00010023059	SELENOF	-0.656364481	0.09370278	-7.004749273	2.47429E-12	6.41703E-12	negative
PRJNA729271	stage_1W_vs_E10	ordinary_muscle_development	ENSGALG00010023060	ALDH1A2	-2.037549718	0.168340943	-12.10370862	1.00945E-33	6.54971E-33	negative
PRJNA729271	stage_1W_vs_E10	ordinary_muscle_development	ENSGALG00010023062	STX2	0.05052493	0.103584719	0.487764323	0.625716785	0.659283622	positive
PRJNA729271	stage_1W_vs_E10	ordinary_muscle_development	ENSGALG00010023063	ENSGALG00010023063	-0.652937286	0.104642532	-6.239693095	4.3843E-10	1.00443E-09	negative
PRJNA729271	stage_1W_vs_E10	ordinary_muscle_development	ENSGALG00010023064	ATG13	0.777997588	0.068555285	11.34846987	7.54719E-30	4.24346E-29	positive
PRJNA729271	stage_1W_vs_E10	ordinary_muscle_development	ENSGALG00010023065	DNA2	-2.595185228	0.229719848	-11.2971746	1.35508E-29	7.51698E-29	negative
PRJNA729271	stage_1W_vs_E10	ordinary_muscle_development	ENSGALG00010023066	TLR21	1.638275383	0.185357629	8.838456747	9.70498E-19	3.43023E-18	positive
PRJNA729271	stage_1W_vs_E10	ordinary_muscle_development	ENSGALG00010023068	CDPF1	-0.690124084	0.127938388	-5.394190868	6.8833E-08	1.39115E-07	negative
PRJNA729271	stage_1W_vs_E10	ordinary_muscle_development	ENSGALG00010023069	ENSGALG00010023069	0.039195331	0.283620708	0.138196296	0.890085283	0.902222809	positive
PRJNA729271	stage_1W_vs_E10	ordinary_muscle_development	ENSGALG00010023070	SIDT2	2.495651893	0.199685009	12.49794315	7.66078E-36	5.38521E-35	positive
PRJNA729271	stage_1W_vs_E10	ordinary_muscle_development	ENSGALG00010023071	POLR2M	0.802100093	0.081462379	9.846263977	7.11393E-23	2.99409E-22	positive
PRJNA729271	stage_1W_vs_E10	ordinary_muscle_development	ENSGALG00010023073	POMGNT1	-0.111780463	0.08648445	-1.292492038	0.196186801	0.226051485	negative
PRJNA729271	stage_1W_vs_E10	ordinary_muscle_development	ENSGALG00010023074	CCNT1	1.032239229	0.164099032	6.290343178	3.16765E-10	7.31365E-10	positive
PRJNA729271	stage_1W_vs_E10	ordinary_muscle_development	ENSGALG00010023075	PKDREJ	-2.253692833	0.314820575	-7.158658011	8.14706E-13	2.16483E-12	negative
PRJNA729271	stage_1W_vs_E10	ordinary_muscle_development	ENSGALG00010023076	DNAJC7	0.982101952	0.080041723	12.26987527	1.31456E-34	8.80116E-34	positive
PRJNA729271	stage_1W_vs_E10	ordinary_muscle_development	ENSGALG00010023077	KANSL2	0.693705933	0.150977429	4.594765841	4.33235E-06	7.71942E-06	positive
PRJNA729271	stage_1W_vs_E10	ordinary_muscle_development	ENSGALG00010023078	TAGLN	2.008935274	0.196696877	10.21335623	1.72783E-24	7.77791E-24	positive
PRJNA729271	stage_1W_vs_E10	ordinary_muscle_development	ENSGALG00010023079	SLC25A16	0.84858359	0.095185037	8.915094399	4.87401E-19	1.75351E-18	positive
PRJNA729271	stage_1W_vs_E10	ordinary_muscle_development	ENSGALG00010023080	MCRS1	0.542936459	0.111728953	4.859407033	1.17738E-06	2.19077E-06	positive
PRJNA729271	stage_1W_vs_E10	ordinary_muscle_development	ENSGALG00010023081	PRLHR2	-6.722877118	0.712565027	-9.434755942	3.91906E-21	1.54282E-20	negative
PRJNA729271	stage_1W_vs_E10	ordinary_muscle_development	ENSGALG00010023083	HARBI1	0.666796649	0.154295916	4.321544386	1.54941E-05	2.64656E-05	positive
PRJNA729271	stage_1W_vs_E10	ordinary_muscle_development	ENSGALG00010023084	ENSGALG00010023084	0.900062625	0.24133162	3.729567733	0.000191809	0.00030217	positive
PRJNA729271	stage_1W_vs_E10	ordinary_muscle_development	ENSGALG00010023086	PCSK7	0.486187469	0.091393859	5.31969516	1.03941E-07	2.07439E-07	positive
PRJNA729271	stage_1W_vs_E10	ordinary_muscle_development	ENSGALG00010023087	SH3GLB1	1.23085312	0.117777381	10.45067494	1.45486E-25	6.86019E-25	positive
PRJNA729271	stage_1W_vs_E10	ordinary_muscle_development	ENSGALG00010023089	ENSGALG00010023089	1.647110358	0.732877653	2.247456109	0.024610893	0.031859863	positive
PRJNA729271	stage_1W_vs_E10	ordinary_muscle_development	ENSGALG00010023090	LRRC41	-0.33336115	0.094598667	-3.52395189	0.000425161	0.000652139	negative
PRJNA729271	stage_1W_vs_E10	ordinary_muscle_development	ENSGALG00010023091	ENSGALG00010023091	0.991004905	0.131137639	7.556982955	4.12526E-14	1.17223E-13	positive
PRJNA729271	stage_1W_vs_E10	ordinary_muscle_development	ENSGALG00010023093	POP5	-0.011938164	0.129091665	-0.092478197	0.926318113	0.935221916	negative
PRJNA729271	stage_1W_vs_E10	ordinary_muscle_development	ENSGALG00010023094	CCDC32	-0.398585943	0.102644793	-3.883157937	0.000103109	0.000165547	negative
PRJNA729271	stage_1W_vs_E10	ordinary_muscle_development	ENSGALG00010023096	SPATS2	0.331709962	0.170760408	1.942546081	0.052071027	0.064904079	positive
PRJNA729271	stage_1W_vs_E10	ordinary_muscle_development	ENSGALG00010023097	LURAP1	1.127231664	0.211978869	5.317660528	1.0511E-07	2.09685E-07	positive
PRJNA729271	stage_1W_vs_E10	ordinary_muscle_development	ENSGALG00010023101	CLCA2	2.222213307	1.185920485	1.873829937	0.060953869	0.075283429	positive
PRJNA729271	stage_1W_vs_E10	ordinary_muscle_development	ENSGALG00010023102	RNF10	0.987689892	0.101443396	9.736364607	2.10967E-22	8.69481E-22	positive
PRJNA729271	stage_1W_vs_E10	ordinary_muscle_development	ENSGALG00010023103	DGKD	0.52022335	0.125503611	4.145086716	3.39685E-05	5.66989E-05	positive
PRJNA729271	stage_1W_vs_E10	ordinary_muscle_development	ENSGALG00010023104	ENSGALG00010023104	0.989297683	0.157939242	6.263786429	3.7574E-10	8.64912E-10	positive
PRJNA729271	stage_1W_vs_E10	ordinary_muscle_development	ENSGALG00010023107	ZC3HAV1	0.402160362	0.292210995	1.376267044	0.168738972	0.196408207	positive
PRJNA729271	stage_1W_vs_E10	ordinary_muscle_development	ENSGALG00010023108	TET1	-0.384653835	0.061925503	-6.21155772	5.24619E-10	1.19659E-09	negative
PRJNA729271	stage_1W_vs_E10	ordinary_muscle_development	ENSGALG00010023110	LIMA1	-2.009683142	0.155490247	-12.924818	3.26067E-38	2.45524E-37	negative
PRJNA729271	stage_1W_vs_E10	ordinary_muscle_development	ENSGALG00010023112	PCMT1L	2.537549098	0.130285331	19.47685956	1.72544E-84	4.57225E-83	positive
PRJNA729271	stage_1W_vs_E10	ordinary_muscle_development	ENSGALG00010023113	MYZAP	-0.190746793	0.133247009	-1.431527761	0.152279017	0.178452934	negative
PRJNA729271	stage_1W_vs_E10	ordinary_muscle_development	ENSGALG00010023114	UQCRHL	1.772694792	0.167372565	10.5913104	3.26982E-26	1.58092E-25	positive

PRJNA729271	stage_1W_vs_E10	ordinary_muscle_development	ENSGALG00010023115	RPLP2	0.500974282	0.136353157	3.674093762	0.000238695	0.000373759	positive
PRJNA729271	stage_1W_vs_E10	ordinary_muscle_development	ENSGALG00010023116	PROC	4.148997983	1.012110639	4.09935221	4.14308E-05	6.85782E-05	positive
PRJNA729271	stage_1W_vs_E10	ordinary_muscle_development	ENSGALG00010023117	RPUSD2	-0.247247905	0.104051372	-2.376209941	0.017491508	0.022988431	negative
PRJNA729271	stage_1W_vs_E10	ordinary_muscle_development	ENSGALG00010023118	PIDD1	0.908888737	0.135729016	6.696348105	2.13692E-11	5.28308E-11	positive
PRJNA729271	stage_1W_vs_E10	ordinary_muscle_development	ENSGALG00010023119	NDRG4	-0.916100714	0.174475918	-5.250585445	1.51617E-07	2.99249E-07	negative
PRJNA729271	stage_1W_vs_E10	ordinary_muscle_development	ENSGALG00010023120	CHRM4	-0.885569534	0.249804264	-3.545053718	0.000392533	0.000603817	negative
PRJNA729271	stage_1W_vs_E10	ordinary_muscle_development	ENSGALG00010023122	ENSGALG00010023122	2.204186918	0.667912298	3.30011429	0.000966455	0.001442121	positive
PRJNA729271	stage_1W_vs_E10	ordinary_muscle_development	ENSGALG00010023123	RNF214	0.6926758	0.142677816	4.85482479	1.20493E-06	2.23945E-06	positive
PRJNA729271	stage_1W_vs_E10	ordinary_muscle_development	ENSGALG00010023124	NSUN4	1.730796274	0.132942866	13.0190986	9.52891E-39	7.31576E-38	positive
PRJNA729271	stage_1W_vs_E10	ordinary_muscle_development	ENSGALG00010023125	COL20A1	0.777236383	0.282614954	2.750160143	0.005956615	0.008249678	positive
PRJNA729271	stage_1W_vs_E10	ordinary_muscle_development	ENSGALG00010023126	CGNL1	-0.131411637	0.138077597	-0.951723088	0.341237437	0.378297258	negative
PRJNA729271	stage_1W_vs_E10	ordinary_muscle_development	ENSGALG00010023127	CERS5	-1.283407744	0.098900381	-12.97677244	1.65718E-38	1.25895E-37	negative
PRJNA729271	stage_1W_vs_E10	ordinary_muscle_development	ENSGALG00010023128	IWS1	0.199028251	0.13727888	1.449809697	0.147111595	0.172760477	positive
PRJNA729271	stage_1W_vs_E10	ordinary_muscle_development	ENSGALG00010023130	DYNLT5	-0.229168567	0.514162182	-0.445712608	0.655804856	0.686742623	negative
PRJNA729271	stage_1W_vs_E10	ordinary_muscle_development	ENSGALG00010023131	SOX6	1.094861103	0.136950771	7.994559623	1.30038E-15	3.97994E-15	positive
PRJNA729271	stage_1W_vs_E10	ordinary_muscle_development	ENSGALG00010023134	KNL1	-2.943012838	0.217814808	-13.511537	1.33701E-41	1.13465E-40	negative
PRJNA729271	stage_1W_vs_E10	ordinary_muscle_development	ENSGALG00010023135	GINS3	-1.546389498	0.071514238	-21.62351925	1.0793E-103	4.3698E-102	negative
PRJNA729271	stage_1W_vs_E10	ordinary_muscle_development	ENSGALG00010023136	MDK	-5.723391556	0.415904901	-13.76129866	4.35631E-43	3.84794E-42	negative
PRJNA729271	stage_1W_vs_E10	ordinary_muscle_development	ENSGALG00010023137	ETV6	-0.223082612	0.114612993	-1.946398968	0.051606833	0.064364227	negative
PRJNA729271	stage_1W_vs_E10	ordinary_muscle_development	ENSGALG00010023140	CCAR1	-0.188314748	0.083630212	-2.251755012	0.024337758	0.031528805	negative
PRJNA729271	stage_1W_vs_E10	ordinary_muscle_development	ENSGALG00010023142	TLK2	1.149094696	0.168064977	6.837204984	8.07532E-12	2.04101E-11	positive
PRJNA729271	stage_1W_vs_E10	ordinary_muscle_development	ENSGALG00010023144	C11orf58	0.193814953	0.106373794	1.822017863	0.068452275	0.08401488	positive
PRJNA729271	stage_1W_vs_E10	ordinary_muscle_development	ENSGALG00010023147	RAD51	-3.223392172	0.196590218	-16.39650334	2.02567E-60	3.00226E-59	negative
PRJNA729271	stage_1W_vs_E10	ordinary_muscle_development	ENSGALG00010023149	ADGRD1	-0.970234915	0.185562774	-5.228607514	1.70792E-07	3.35769E-07	negative
PRJNA729271	stage_1W_vs_E10	ordinary_muscle_development	ENSGALG00010023151	ENSGALG00010023151	-0.957326319	0.506202675	-1.891191742	0.058598753	0.072529155	negative
PRJNA729271	stage_1W_vs_E10	ordinary_muscle_development	ENSGALG00010023155	CFAP263	-0.840303695	0.351538773	-2.390358498	0.016831934	0.022165793	negative
PRJNA729271	stage_1W_vs_E10	ordinary_muscle_development	ENSGALG00010023157	STOX1	-3.197995782	0.397657177	-8.042092461	8.83172E-16	2.72258E-15	negative
PRJNA729271	stage_1W_vs_E10	ordinary_muscle_development	ENSGALG00010023158	ENSGALG00010023158	1.411102937	0.502064084	2.810603231	0.004944872	0.006907191	positive
PRJNA729271	stage_1W_vs_E10	ordinary_muscle_development	ENSGALG00010023159	DAW1	-0.072401976	0.504824661	-0.143420046	0.885958452	0.898793937	negative
PRJNA729271	stage_1W_vs_E10	ordinary_muscle_development	ENSGALG00010023160	ENSGALG00010023160	-0.974607499	0.11059425	-8.812460855	1.22428E-18	4.29788E-18	negative
PRJNA729271	stage_1W_vs_E10	ordinary_muscle_development	ENSGALG00010023161	ODF2L	-1.623986361	0.457585484	-3.549033825	0.000386647	0.000595205	negative
PRJNA729271	stage_1W_vs_E10	ordinary_muscle_development	ENSGALG00010023162	CHRNA4	-2.979503984	0.298633042	-9.977141043	1.91917E-23	8.27681E-23	negative
PRJNA729271	stage_1W_vs_E10	ordinary_muscle_development	ENSGALG00010023163	CRTAC1	-1.539106036	0.287709548	-5.349513237	8.81911E-08	1.77128E-07	negative
PRJNA729271	stage_1W_vs_E10	ordinary_muscle_development	ENSGALG00010023164	NKIRAS2	1.03246314	0.132651028	7.78330296	7.06552E-15	2.08754E-14	positive
PRJNA729271	stage_1W_vs_E10	ordinary_muscle_development	ENSGALG00010023165	CSNK2A2	0.475044597	0.129547167	3.666962449	0.000245449	0.000383919	positive
PRJNA729271	stage_1W_vs_E10	ordinary_muscle_development	ENSGALG00010023166	KDM2A	-0.121040028	0.114091482	-1.06090329	0.288733854	0.323581043	negative
PRJNA729271	stage_1W_vs_E10	ordinary_muscle_development	ENSGALG00010023167	GPD1	12.30674764	0.2098684	58.64030802	0	0	positive
PRJNA729271	stage_1W_vs_E10	ordinary_muscle_development	ENSGALG00010023168	SPHKAP	-3.945589199	0.445805313	-8.850475944	8.71468E-19	3.08548E-18	negative
PRJNA729271	stage_1W_vs_E10	ordinary_muscle_development	ENSGALG00010023169	RAN	-0.999297293	0.097047363	-10.29700615	7.26884E-25	3.31953E-24	negative
PRJNA729271	stage_1W_vs_E10	ordinary_muscle_development	ENSGALG00010023170	TCF12	-1.25008634	0.064951609	-19.24642615	1.51243E-82	3.85963E-81	negative
PRJNA729271	stage_1W_vs_E10	ordinary_muscle_development	ENSGALG00010023171	ENSGALG00010023171	-0.7675711	0.114986092	-6.675338635	2.46662E-11	6.07642E-11	negative
PRJNA729271	stage_1W_vs_E10	ordinary_muscle_development	ENSGALG00010023172	DHX58	3.062227477	0.323960802	9.452462933	3.3095E-21	1.30747E-20	positive
PRJNA729271	stage_1W_vs_E10	ordinary_muscle_development	ENSGALG00010023174	KCNQ2	-2.410332624	0.366987452	-6.567888392	5.10338E-11	1.23412E-10	negative
PRJNA729271	stage_1W_vs_E10	ordinary_muscle_development	ENSGALG00010023175	GRK2	0.419541301	0.137711433	3.046524835	0.002315034	0.003332315	positive
PRJNA729271	stage_1W_vs_E10	ordinary_muscle_development	ENSGALG00010023177	LZTS2	0.651816549	0.170888954	3.814269646	0.000136586	0.000217467	positive
PRJNA729271	stage_1W_vs_E10	ordinary_muscle_development	ENSGALG00010023178	CFAP20	-0.350151195	0.098838256	-3.542668694	0.0003961	0.000608916	negative
PRJNA729271	stage_1W_vs_E10	ordinary_muscle_development	ENSGALG00010023179	KAT2A	-0.272466742	0.128719748	-2.116743896	0.034281589	0.04361935	negative
PRJNA729271	stage_1W_vs_E10	ordinary_muscle_development	ENSGALG00010023180	EEF1A2	7.192914712	0.121643725	59.1309967	0	0	positive
PRJNA729271	stage_1W_vs_E10	ordinary_muscle_development	ENSGALG00010023181	PLEKHA7	0.366278939	0.164867388	2.22165792	0.026306432	0.033921514	positive
PRJNA729271	stage_1W_vs_E10	ordinary_muscle_development	ENSGALG00010023182	EPHB6	-2.552961919	0.147946743	-17.25595212	1.00944E-66	1.73569E-65	negative
PRJNA729271	stage_1W_vs_E10	ordinary_muscle_development	ENSGALG00010023183	ENSGALG00010023183	-1.170277662	0.538852301	-2.171796723	0.029870999	0.038296491	negative
PRJNA729271	stage_1W_vs_E10	ordinary_muscle_development	ENSGALG00010023184	SMARCD1	-0.850105345	0.054780989	-15.51825483	2.61055E-54	3.31929E-53	negative
PRJNA729271	stage_1W_vs_E10	ordinary_muscle_development	ENSGALG00010023185	FBXW4	2.669898874	0.116132466	22.99011599	5.8533E-117	2.998E-115	positive
PRJNA729271	stage_1W_vs_E10	ordinary_muscle_development	ENSGALG00010023186	MIER1	-0.022905854	0.099072676	-0.231202533	0.817157463	0.836312746	negative
PRJNA729271	stage_1W_vs_E10	ordinary_muscle_development	ENSGALG00010023187	ENSGALG00010023187	3.203864963	0.230495085	13.89992745	6.34056E-44	5.74774E-43	positive
PRJNA729271	stage_1W_vs_E10	ordinary_muscle_development	ENSGALG00010023188	KIF1BP	0.692929979	0.057497589	12.05146153	1.90541E-33	1.22262E-32	positive
PRJNA729271	stage_1W_vs_E10	ordinary_muscle_development	ENSGALG00010023189	RMDN3	-0.787710316	0.086802672	-9.074724334	1.13966E-19	4.22057E-19	negative
PRJNA729271	stage_1W_vs_E10	ordinary_muscle_development	ENSGALG00010023190	SF3B3	-1.122676974	0.056540533	-19.85614414	9.75161E-88	2.77156E-86	negative
PRJNA729271	stage_1W_vs_E10	ordinary_muscle_development	ENSGALG00010023193	PPDPF	6.267408655	0.312529994	20.05378293	1.87065E-89	5.43389E-88	positive
PRJNA729271	stage_1W_vs_E10	ordinary_muscle_development	ENSGALG00010023194	MRC2	-0.066863051	0.19328684	-0.345926559	0.729397908	0.755889231	negative
PRJNA729271	stage_1W_vs_E10	ordinary_muscle_development	ENSGALG00010023195	ENSGALG00010023195	-4.093316243	0.195205726	-20.96924274	1.25242E-97	4.46038E-96	negative
PRJNA729271	stage_1W_vs_E10	ordinary_muscle_development	ENSGALG00010023196	DGKZ	1.141676808	0.127154297	8.978672625	2.74053E-19	9.95339E-19	positive
PRJNA729271	stage_1W_vs_E10	ordinary_muscle_development	ENSGALG00010023198	PTK6	-2.159168294	0.599891545	-3.599264424	0.000319119	0.00049509	negative
PRJNA729271	stage_1W_vs_E10	ordinary_muscle_development	ENSGALG00010023199	RPS13	0.627991521	0.196721418	3.192288504	0.001411503	0.002073762	positive
PRJNA729271	stage_1W_vs_E10	ordinary_muscle_development	ENSGALG00010023200	VPS26A	0.53129967	0.082417261	6.446461122	1.14492E-10	2.71214E-10	positive
PRJNA729271	stage_1W_vs_E10	ordinary_muscle_development	ENSGALG00010023201	ZNF280D	-0.095390303	0.118169041	-0.807235992	0.419530533	0.457638101	negative
PRJNA729271	stage_1W_vs_E10	ordinary_muscle_development	ENSGALG00010023202	PID1	-0.685659443	0.158139254	-4.335795342	1.45234E-05	2.48808E-05	negative
PRJNA729271	stage_1W_vs_E10	ordinary_muscle_development	ENSGALG00010023203	COG4	0.209153068	0.063302175	3.304042378	0.000953014	0.001423091	positive
PRJNA729271	stage_1W_vs_E10	ordinary_muscle_development	ENSGALG00010023204	SRGN	3.897191282	0.312787998	12.45952948	1.24101E-35	8.64826E-35	positive
PRJNA729271	stage_1W_vs_E10	ordinary_muscle_development	ENSGALG00010023205	DNAJC17	-0.810512516	0.116165324	-6.977232852	3.01051E-12	7.77432E-12	negative
PRJNA729271	stage_1W_vs_E10	ordinary_muscle_development	ENSGALG00010023206	HSPB9	2.719719689	0.314082843	8.659243102	4.74907E-18	1.61515E-17	positive
PRJNA729271	stage_1W_vs_E10	ordinary_muscle_development	ENSGALG00010023207	MNS1	-1.02040065	0.166577112	-6.125695419	9.02884E-10	2.03567E-09	negative
PRJNA729271	stage_1W_vs_E10	ordinary_muscle_development	ENSGALG00010023208	BACE1	-0.667402541	0.0793316	-8.41282086	4.00266E-17	1.30379E-16	negative
PRJNA729271	stage_1W_vs_E10	ordinary_muscle_development	ENSGALG00010023209	PIK3C2A	-0.820045642	0.108072737	-7.58790481	3.2512E-14	9.27131E-14	negative
PRJNA729271	stage_1W_vs_E10	ordinary_muscle_development	ENSGALG00010023210	ZNHIT6	0.163636677	0.136283577	1.200707237	0.229864786	0.261529833	positive
PRJNA729271	stage_1W_vs_E10	ordinary_muscle_development	ENSGALG00010023211	TLK1	-0.041881322	0.076467398	-0.547701669	0.583896766	0.619226194	negative
PRJNA729271	stage_1W_vs_E10	ordinary_muscle_development	ENSGALG00010023212	RIMBP2	1.652860412	0.21437382	7.71017847	1.25642E-14	3.66656E-14	positive
PRJNA729271	stage_1W_vs_E10	ordinary_muscle_development	ENSGALG00010023213	FCSK	1.004637918	0.113039091	8.887526529	6.24843E-19	2.23909E-18	positive
PRJNA729271	stage_1W_vs_E10	ordinary_muscle_development	ENSGALG00010023214	SUPV3L1	0.296342578	0.08852695	3.347484317	0.000815486	0.001224792	positive
PRJNA729271	stage_1W_vs_E10	ordinary_muscle_development	ENSGALG00010023215	TRIP12	0.48149046	0.056188911	8.56913679	1.04264E-17	3.49192E-17	positive
PRJNA729271	stage_1W_vs_E10	ordinary_muscle_development	ENSGALG00010023216	SLC35D1	-0.368040077	0.123768355	-2.973620163	0.002943091	0.004197177	negative
PRJNA729271	stage_1W_vs_E10	ordinary_muscle_development	ENSGALG00010023217	CREB3L1	-0.961049287	0.20758383	-4.629692428	3.66209E-06	6.56306E-06	negative
PRJNA729271	stage_1W_vs_E10	ordinary_muscle_development	ENSGALG00010023218	SSH3	0.35172674	0.130669004	2.691738129	0.007108073	0.00976417	positive
PRJNA729271	stage_1W_vs_E10	ordinary_muscle_development	ENSGALG00010023219	TEX9	0.213299841	0.149645534	1.425367236	0.154051074	0.180369169	positive
PRJNA729271	stage_1W_vs_E10	ordinary_muscle_development	ENSGALG00010023220	ST3GAL2	-1.032262181	0.088313002	-11.68867732	1.45637E-31	8.77029E-31	negative
PRJNA729271	stage_1W_vs_E10	ordinary_muscle_development	ENSGALG00010023221	AMY1A	2.921909112	0.135797415	21.51667695	1.0868E-102	4.2807E-101	positive
PRJNA729271	stage_1W_vs_E10	ordinary_muscle_development	ENSGALG00010023222	ENSGALG00010023222	0.154318526	0.523283851	0.294904048	0.768067179	0.791662874	positive
PRJNA729271	stage_1W_vs_E10	ordinary_muscle_development	ENSGALG00010023223	CEP164	-1.66464377	0.104202704	-15.97505355	1.90691E-57	2.61748E-56	negative
PRJNA729271	stage_1W_vs_E10	ordinary_muscle_development	ENSGALG00010023225	PDPR	-0.107481293	0.121985357	-0.881099963	0.378263717	0.416035555	negative
PRJNA729271	stage_1W_vs_E10	ordinary_muscle_development	ENSGALG00010023226	ENSGALG00010023226	-0.057484651	0.215936664	-0.2662107	0.790076943	0.812267911	negative
PRJNA729271	stage_1W_vs_E10	ordinary_muscle_development	ENSGALG00010023227	RNPC3	0.190152155	0.124010226	1.533358671	0.125187506	0.148493691	positive
PRJNA729271	stage_1W_vs_E10	ordinary_muscle_development	ENSGALG00010023228	GORASP2	0.992510373	0.101637066	9.765240257	1.58735E-22	6.57767E-22	positive
PRJNA729271	stage_1W_vs_E10	ordinary_muscle_development	ENSGALG00010023229	HKDC1	0.435301893	0.389358719	1.117997034	0.26356826	0.297332238	positive
PRJNA729271	stage_1W_vs_E10	ordinary_muscle_development	ENSGALG00010023230	ENSGALG00010023230	0.502906248	0.083511589	6.021993522	1.72282E-09	3.83422E-09	positive
PRJNA729271	stage_1W_vs_E10	ordinary_muscle_development	ENSGALG00010023231	ASIC1	-2.556111015	0.270877343	-9.436414976	3.85752E-21	1.51901E-20	negative
PRJNA729271	stage_1W_vs_E10	ordinary_muscle_development	ENSGALG00010023233	SEZ6L	-1.347975367	1.102476591	-1.222679355	0.221450828	0.252929615	negative
PRJNA729271	stage_1W_vs_E10	ordinary_muscle_development	ENSGALG00010023234	ENSGALG00010023234	-0.64109482	0.096273471	-6.659101544	2.75507E-11	6.77203E-11	negative
PRJNA729271	stage_1W_vs_E10	ordinary_muscle_development	ENSGALG00010023235	ENSGALG00010023235	0.394694804	0.353319965	1.117103031	0.263950275	0.297716849	positive
PRJNA729271	stage_1W_vs_E10	ordinary_muscle_development	ENSGALG00010023237	GAD1	-4.311587768	0.680946404	-6.331757888	2.42384E-10	5.62676E-10	negative
PRJNA729271	stage_1W_vs_E10	ordinary_muscle_development	ENSGALG00010023238	NUCB2	-0.472268209	0.078409225	-6.023120442	1.71086E-09	3.80936E-09	negative
PRJNA729271	stage_1W_vs_E10	ordinary_muscle_development	ENSGALG00010023239	RBPMS2	0.617864094	0.20457579	3.020220982	0.002525903	0.003626123	positive
PRJNA729271	stage_1W_vs_E10	ordinary_muscle_development	ENSGALG00010023240	PIWIL1	-3.675251196	0.524958195	-7.001035955	2.54077E-12	6.58591E-12	negative
PRJNA729271	stage_1W_vs_E10	ordinary_muscle_development	ENSGALG00010023243	HK1	2.013531918	0.114107478	17.64592423	1.0937E-69	2.05619E-68	positive
PRJNA729271	stage_1W_vs_E10	ordinary_muscle_development	ENSGALG00010023244	PPIB	-1.58020901	0.118958412	-13.28370969	2.87776E-40	2.32256E-39	negative
PRJNA729271	stage_1W_vs_E10	ordinary_muscle_development	ENSGALG00010023245	TANC2	-1.084197548	0.147552657	-7.347868709	2.01392E-13	5.50893E-13	negative
PRJNA729271	stage_1W_vs_E10	ordinary_muscle_development	ENSGALG00010023246	SP5	-4.177243234	0.750640023	-5.564908753	2.6229E-08	5.42584E-08	negative
PRJNA729271	stage_1W_vs_E10	ordinary_muscle_development	ENSGALG00010023248	PRDM10	-0.008601513	0.121056151	-0.071053913	0.943354848	0.950502469	negative
PRJNA729271	stage_1W_vs_E10	ordinary_muscle_development	ENSGALG00010023249	IL12RB2	1.839228185	0.431555051	4.261862257	2.0273E-05	3.43212E-05	positive
PRJNA729271	stage_1W_vs_E10	ordinary_muscle_development	ENSGALG00010023250	FZD10	-4.714714194	0.437245628	-10.78275893	4.15241E-27	2.08195E-26	negative
PRJNA729271	stage_1W_vs_E10	ordinary_muscle_development	ENSGALG00010023251	ENSGALG00010023251	8.082448383	0.923663352	8.75042662	2.12548E-18	7.36524E-18	positive
PRJNA729271	stage_1W_vs_E10	ordinary_muscle_development	ENSGALG00010023252	GLG1	-0.246823725	0.080012262	-3.084823741	0.002036728	0.002948699	negative
PRJNA729271	stage_1W_vs_E10	ordinary_muscle_development	ENSGALG00010023254	MYO3B	-1.26611717	0.318069663	-3.98062851	6.87333E-05	0.000111993	negative
PRJNA729271	stage_1W_vs_E10	ordinary_muscle_development	ENSGALG00010023255	SNX22	1.543126666	0.321074229	4.806136792	1.53875E-06	2.83371E-06	positive
PRJNA729271	stage_1W_vs_E10	ordinary_muscle_development	ENSGALG00010023257	PAK6	2.6573954	0.288456558	9.212463102	3.18726E-20	1.20751E-19	positive
PRJNA729271	stage_1W_vs_E10	ordinary_muscle_development	ENSGALG00010023258	ENSGALG00010023258	-0.2594541	0.357118172	-0.726521696	0.467519012	0.505873998	negative
PRJNA729271	stage_1W_vs_E10	ordinary_muscle_development	ENSGALG00010023259	TSPAN15	2.098931028	0.287770505	7.293767059	3.01406E-13	8.17662E-13	positive
PRJNA729271	stage_1W_vs_E10	ordinary_muscle_development	ENSGALG00010023260	ENSGALG00010023260	-3.394479379	0.267394631	-12.69464301	6.3318E-37	4.56614E-36	negative
PRJNA729271	stage_1W_vs_E10	ordinary_muscle_development	ENSGALG00010023261	SNX1	-0.688559312	0.096458738	-7.138381945	9.44359E-13	2.5043E-12	negative
PRJNA729271	stage_1W_vs_E10	ordinary_muscle_development	ENSGALG00010023262	POLD4	2.187188188	0.207508922	10.54021274	5.63712E-26	2.70295E-25	positive
PRJNA729271	stage_1W_vs_E10	ordinary_muscle_development	ENSGALG00010023263	SRP14	-0.852951531	0.073131699	-11.663226	1.96455E-31	1.17622E-30	negative
PRJNA729271	stage_1W_vs_E10	ordinary_muscle_development	ENSGALG00010023264	RACGAP1	-2.264448031	0.230630229	-9.818522232	9.37071E-23	3.92228E-22	negative
PRJNA729271	stage_1W_vs_E10	ordinary_muscle_development	ENSGALG00010023265	RAB5C	-0.605931681	0.071759536	-8.443918636	3.06873E-17	1.00545E-16	negative
PRJNA729271	stage_1W_vs_E10	ordinary_muscle_development	ENSGALG00010023266	RFWD3	-1.221827944	0.142981178	-8.545376117	1.28118E-17	4.26718E-17	negative
PRJNA729271	stage_1W_vs_E10	ordinary_muscle_development	ENSGALG00010023267	DSCAML1	-3.528463166	0.864213881	-4.082858705	4.44851E-05	7.34998E-05	negative
PRJNA729271	stage_1W_vs_E10	ordinary_muscle_development	ENSGALG00010023268	KCNJ11	3.311939277	0.179985463	18.40114871	1.28609E-75	2.7455E-74	positive
PRJNA729271	stage_1W_vs_E10	ordinary_muscle_development	ENSGALG00010023269	OPRL1	-2.492821573	0.568640055	-4.383830421	1.16611E-05	2.01438E-05	negative
PRJNA729271	stage_1W_vs_E10	ordinary_muscle_development	ENSGALG00010023271	PATL2	-0.532769562	0.422757805	-1.260224069	0.207588542	0.238241957	negative
PRJNA729271	stage_1W_vs_E10	ordinary_muscle_development	ENSGALG00010023273	HDAC7	0.390042311	0.114168154	3.416384497	0.000634586	0.000960861	positive
PRJNA729271	stage_1W_vs_E10	ordinary_muscle_development	ENSGALG00010023274	ZBTB38	0.762344348	0.114976462	6.630438404	3.34691E-11	8.18379E-11	positive
PRJNA729271	stage_1W_vs_E10	ordinary_muscle_development	ENSGALG00010023275	TWIST3	-9.448209819	0.886118457	-10.66246814	1.52496E-26	7.49044E-26	negative
PRJNA729271	stage_1W_vs_E10	ordinary_muscle_development	ENSGALG00010023276	ABCC8	1.824302685	0.16188853	11.26888166	1.86929E-29	1.03181E-28	positive

PRJNA729271	stage_1W_vs_E10	ordinary_muscle_development	ENSGALG00010023278	ENSGALG00010023278	-0.603308372	0.303504576	-1.987806511	0.046833093	0.058683064	negative
PRJNA729271	stage_1W_vs_E10	ordinary_muscle_development	ENSGALG00010023279	FA2H	0.677940485	0.41842304	1.620227427	0.105183432	0.126096588	positive
PRJNA729271	stage_1W_vs_E10	ordinary_muscle_development	ENSGALG00010023281	B2M	4.201104751	0.199103346	21.1001213	7.93324E-99	2.89653E-97	positive
PRJNA729271	stage_1W_vs_E10	ordinary_muscle_development	ENSGALG00010023282	SERBP1	-0.193175963	0.08728366	-2.213197334	0.026884044	0.034620133	negative
PRJNA729271	stage_1W_vs_E10	ordinary_muscle_development	ENSGALG00010023285	SLC48A1	2.298643843	0.110913629	20.72462927	2.07721E-95	7.08451E-94	positive
PRJNA729271	stage_1W_vs_E10	ordinary_muscle_development	ENSGALG00010023286	HCRT	0.388592551	0.490481492	0.79226751	0.428204712	0.466118001	positive
PRJNA729271	stage_1W_vs_E10	ordinary_muscle_development	ENSGALG00010023287	RGS19	-0.799315529	0.20816817	-3.83975863	0.000123155	0.000196709	negative
PRJNA729271	stage_1W_vs_E10	ordinary_muscle_development	ENSGALG00010023289	NFRKB	-0.165275537	0.087114553	-1.897220745	0.05779881	0.071636918	negative
PRJNA729271	stage_1W_vs_E10	ordinary_muscle_development	ENSGALG00010023290	SLC16A14	-4.604988309	0.294672507	-15.62747863	4.73158E-55	6.11814E-54	negative
PRJNA729271	stage_1W_vs_E10	ordinary_muscle_development	ENSGALG00010023293	COL11A1	-4.215074542	0.48933838	-8.613823721	7.06633E-18	2.38756E-17	negative
PRJNA729271	stage_1W_vs_E10	ordinary_muscle_development	ENSGALG00010023295	ENSGALG00010023295	0.831221439	0.514307686	1.616194863	0.106052189	0.127054011	positive
PRJNA729271	stage_1W_vs_E10	ordinary_muscle_development	ENSGALG00010023299	PASK	-1.192935868	0.14035665	-8.499318479	1.90707E-17	6.30542E-17	negative
PRJNA729271	stage_1W_vs_E10	ordinary_muscle_development	ENSGALG00010023304	ENSGALG00010023304	0.330383513	0.174294344	1.895549245	0.058019674	0.07187993	positive
PRJNA729271	stage_1W_vs_E10	ordinary_muscle_development	ENSGALG00010023305	PLCB2	0.979603054	0.160314462	6.110509559	9.93135E-10	2.23463E-09	positive
PRJNA729271	stage_1W_vs_E10	ordinary_muscle_development	ENSGALG00010023309	PIK3R3	-1.736223623	0.202751705	-8.563299729	1.09682E-17	3.66916E-17	negative
PRJNA729271	stage_1W_vs_E10	ordinary_muscle_development	ENSGALG00010023311	ETS1	-0.567374571	0.134095246	-4.23113114	2.32519E-05	3.91994E-05	negative
PRJNA729271	stage_1W_vs_E10	ordinary_muscle_development	ENSGALG00010023312	UBR3	2.266537058	0.114116339	19.86163493	8.74181E-88	2.48944E-86	positive
PRJNA729271	stage_1W_vs_E10	ordinary_muscle_development	ENSGALG00010023317	RAPGEF3	1.111576933	0.148902261	7.465144781	8.32081E-14	2.32434E-13	positive
PRJNA729271	stage_1W_vs_E10	ordinary_muscle_development	ENSGALG00010023319	EIF2AK4	-0.143438524	0.062446075	-2.296998232	0.021618871	0.028149976	negative
PRJNA729271	stage_1W_vs_E10	ordinary_muscle_development	ENSGALG00010023320	ENSGALG00010023320	-0.595753578	0.140406873	-4.243051381	2.20501E-05	3.72296E-05	negative
PRJNA729271	stage_1W_vs_E10	ordinary_muscle_development	ENSGALG00010023322	PRKAB1	-0.347062303	0.092297963	-3.760237936	0.000169752	0.000268473	negative
PRJNA729271	stage_1W_vs_E10	ordinary_muscle_development	ENSGALG00010023324	LAG3	3.804520676	0.97734789	3.892698512	9.91353E-05	0.000159433	positive
PRJNA729271	stage_1W_vs_E10	ordinary_muscle_development	ENSGALG00010023328	TCEA2	-1.523447972	0.11203688	-13.5977365	4.13019E-42	3.54453E-41	negative
PRJNA729271	stage_1W_vs_E10	ordinary_muscle_development	ENSGALG00010023330	TSPAN1	3.276780283	1.334266334	2.455866719	0.014054527	0.01869852	positive
PRJNA729271	stage_1W_vs_E10	ordinary_muscle_development	ENSGALG00010023331	CD4	-2.790259717	0.342375109	-8.149715444	3.64782E-16	1.143E-15	negative
PRJNA729271	stage_1W_vs_E10	ordinary_muscle_development	ENSGALG00010023332	PPP1R7	1.059445821	0.074318535	14.25547228	4.14489E-46	4.00534E-45	positive
PRJNA729271	stage_1W_vs_E10	ordinary_muscle_development	ENSGALG00010023333	ENDOU	-1.226452689	0.566836066	-2.163681465	0.030488804	0.039031611	negative
PRJNA729271	stage_1W_vs_E10	ordinary_muscle_development	ENSGALG00010023334	RPS17	0.265677185	0.151640783	1.752016702	0.079770936	0.097076628	positive
PRJNA729271	stage_1W_vs_E10	ordinary_muscle_development	ENSGALG00010023335	WDR59	0.177566019	0.069367878	2.559772985	0.010474056	0.014129496	positive
PRJNA729271	stage_1W_vs_E10	ordinary_muscle_development	ENSGALG00010023336	GPR162	-2.062473355	0.15752043	-13.09337054	3.59303E-39	2.80005E-38	negative
PRJNA729271	stage_1W_vs_E10	ordinary_muscle_development	ENSGALG00010023337	FLI1	0.080237707	0.117031735	0.685606403	0.492961343	0.53086736	positive
PRJNA729271	stage_1W_vs_E10	ordinary_muscle_development	ENSGALG00010023338	HDLBP	1.069838162	0.124023817	8.626070306	6.3496E-18	2.1494E-17	positive
PRJNA729271	stage_1W_vs_E10	ordinary_muscle_development	ENSGALG00010023340	P3H3	-1.033807801	0.159922397	-6.464434121	1.01679E-10	2.41532E-10	negative
PRJNA729271	stage_1W_vs_E10	ordinary_muscle_development	ENSGALG00010023342	FITM2	-0.437167719	0.137130174	-3.187976123	0.001432724	0.002103447	negative
PRJNA729271	stage_1W_vs_E10	ordinary_muscle_development	ENSGALG00010023343	TMEM233	5.757415984	0.332774529	17.30125198	4.60277E-67	8.0479E-66	positive
PRJNA729271	stage_1W_vs_E10	ordinary_muscle_development	ENSGALG00010023347	STAT5A	3.121150515	0.169131831	18.45395096	4.84685E-76	1.04546E-74	positive
PRJNA729271	stage_1W_vs_E10	ordinary_muscle_development	ENSGALG00010023349	ENSGALG00010023349	3.16087252	0.567547831	5.569350013	2.55691E-08	5.29539E-08	positive
PRJNA729271	stage_1W_vs_E10	ordinary_muscle_development	ENSGALG00010023350	ZNRF1	1.776527731	0.230576847	7.70470998	1.31141E-14	3.82318E-14	positive
PRJNA729271	stage_1W_vs_E10	ordinary_muscle_development	ENSGALG00010023351	GNB3	0.300810343	0.378708906	0.794304909	0.427017947	0.464965831	positive
PRJNA729271	stage_1W_vs_E10	ordinary_muscle_development	ENSGALG00010023354	SEPTIN2	-3.557993037	0.173273842	-20.53393049	1.07147E-93	3.46674E-92	negative
PRJNA729271	stage_1W_vs_E10	ordinary_muscle_development	ENSGALG00010023356	ENSGALG00010023356	-0.073539824	0.192934846	-0.381164034	0.703081535	0.73112611	negative
PRJNA729271	stage_1W_vs_E10	ordinary_muscle_development	ENSGALG00010023359	CDCA3	-2.148382384	0.272658397	-7.879391972	3.28978E-15	9.8707E-15	negative
PRJNA729271	stage_1W_vs_E10	ordinary_muscle_development	ENSGALG00010023362	GADD45A	1.035888264	0.148718367	6.965435958	3.27388E-12	8.43943E-12	positive
PRJNA729271	stage_1W_vs_E10	ordinary_muscle_development	ENSGALG00010023363	CPEB1	-1.654068574	0.24337014	-6.796514043	1.07181E-11	2.69066E-11	negative
PRJNA729271	stage_1W_vs_E10	ordinary_muscle_development	ENSGALG00010023364	CTRB1	8.758961222	2.213079962	3.957815068	7.56385E-05	0.000122871	positive
PRJNA729271	stage_1W_vs_E10	ordinary_muscle_development	ENSGALG00010023366	GDAP1L1	-4.743171739	0.538147734	-8.813884076	1.20883E-18	4.24776E-18	negative
PRJNA729271	stage_1W_vs_E10	ordinary_muscle_development	ENSGALG00010023368	ENSGALG00010023368	-1.648976283	0.22994278	-7.171246168	7.43181E-13	1.97913E-12	negative
PRJNA729271	stage_1W_vs_E10	ordinary_muscle_development	ENSGALG00010023369	ACE	4.173106269	0.476267095	8.762113349	1.91623E-18	6.65608E-18	positive
PRJNA729271	stage_1W_vs_E10	ordinary_muscle_development	ENSGALG00010023374	ENSGALG00010023374	-1.109911316	0.209839531	-5.28933377	1.22763E-07	2.43693E-07	negative
PRJNA729271	stage_1W_vs_E10	ordinary_muscle_development	ENSGALG00010023375	ST14	-0.803949415	0.151967428	-5.290274552	1.22133E-07	2.42542E-07	negative
PRJNA729271	stage_1W_vs_E10	ordinary_muscle_development	ENSGALG00010023377	OSER1	1.569503885	0.14922376	10.51778808	7.15332E-26	3.41301E-25	positive
PRJNA729271	stage_1W_vs_E10	ordinary_muscle_development	ENSGALG00010023379	CCDC60	-0.111281669	0.562678396	-0.197771355	0.843223959	0.860281396	negative
PRJNA729271	stage_1W_vs_E10	ordinary_muscle_development	ENSGALG00010023380	USP5	-0.400289594	0.096927168	-4.129797693	3.63083E-05	6.04443E-05	negative
PRJNA729271	stage_1W_vs_E10	ordinary_muscle_development	ENSGALG00010023381	HOXB7	-0.426788194	0.165194787	-2.583545176	0.009779066	0.01323136	negative
PRJNA729271	stage_1W_vs_E10	ordinary_muscle_development	ENSGALG00010023383	HOXB6	-0.120299525	0.171850399	-0.700024704	0.483911877	0.522130613	negative
PRJNA729271	stage_1W_vs_E10	ordinary_muscle_development	ENSGALG00010023384	AP3B2	-1.928239545	0.232246372	-8.302560477	1.01891E-16	3.26606E-16	negative
PRJNA729271	stage_1W_vs_E10	ordinary_muscle_development	ENSGALG00010023387	ENSGALG00010023387	1.91732881	0.18259996	10.50016007	8.62338E-26	4.09957E-25	positive
PRJNA729271	stage_1W_vs_E10	ordinary_muscle_development	ENSGALG00010023388	FARP2	-0.985293794	0.085562551	-11.51547937	1.10245E-30	6.41508E-30	negative
PRJNA729271	stage_1W_vs_E10	ordinary_muscle_development	ENSGALG00010023389	GNG12	-0.239205158	0.110253755	-2.169587397	0.030038117	0.038490321	negative
PRJNA729271	stage_1W_vs_E10	ordinary_muscle_development	ENSGALG00010023390	HSPB8	2.953346553	0.250732102	11.7788928	5.01474E-32	3.06574E-31	positive
PRJNA729271	stage_1W_vs_E10	ordinary_muscle_development	ENSGALG00010023392	TPI1	5.131872254	0.149505631	34.32561189	3.2564E-258	8.1381E-256	positive
PRJNA729271	stage_1W_vs_E10	ordinary_muscle_development	ENSGALG00010023394	HOXB5	-1.131688721	0.177884845	-6.361917582	1.9925E-10	4.65003E-10	negative
PRJNA729271	stage_1W_vs_E10	ordinary_muscle_development	ENSGALG00010023396	ENSGALG00010023396	-0.220527732	0.148798021	-1.482060922	0.138324086	0.163061778	negative
PRJNA729271	stage_1W_vs_E10	ordinary_muscle_development	ENSGALG00010023397	ENSGALG00010023397	0.051995832	0.247261467	0.210286837	0.833443808	0.851537887	positive
PRJNA729271	stage_1W_vs_E10	ordinary_muscle_development	ENSGALG00010023401	LRRC23	1.578916179	0.368327616	4.286716801	1.81333E-05	3.08428E-05	positive
PRJNA729271	stage_1W_vs_E10	ordinary_muscle_development	ENSGALG00010023405	DNAJA2	0.242141493	0.076488004	3.165744714	0.001546865	0.002263914	positive
PRJNA729271	stage_1W_vs_E10	ordinary_muscle_development	ENSGALG00010023408	ZBTB44	0.264392303	0.127211323	2.078370841	0.037675216	0.047740385	positive
PRJNA729271	stage_1W_vs_E10	ordinary_muscle_development	ENSGALG00010023411	ENSGALG00010023411	4.643492862	0.180055353	25.78925196	1.1705E-146	9.2711E-145	positive
PRJNA729271	stage_1W_vs_E10	ordinary_muscle_development	ENSGALG00010023412	GSS	-0.801270159	0.108835272	-7.362228632	1.80865E-13	4.95958E-13	negative
PRJNA729271	stage_1W_vs_E10	ordinary_muscle_development	ENSGALG00010023414	SUDS3	-0.737560784	0.076933913	-9.586939707	9.07364E-22	3.65239E-21	negative
PRJNA729271	stage_1W_vs_E10	ordinary_muscle_development	ENSGALG00010023415	HOXB4	0.264998508	0.165846295	1.59785606	0.110075015	0.131558158	positive
PRJNA729271	stage_1W_vs_E10	ordinary_muscle_development	ENSGALG00010023421	OLFM3	-1.396632709	0.254688875	-5.483681641	4.16564E-08	8.5308E-08	negative
PRJNA729271	stage_1W_vs_E10	ordinary_muscle_development	ENSGALG00010023422	WLS	-3.015198322	0.157079339	-19.19538457	4.04478E-82	1.01787E-80	negative
PRJNA729271	stage_1W_vs_E10	ordinary_muscle_development	ENSGALG00010023426	KCNJ1	-0.573552581	0.502842411	-1.140620934	0.254027702	0.287307392	negative
PRJNA729271	stage_1W_vs_E10	ordinary_muscle_development	ENSGALG00010023428	HOXB3	-1.822477086	0.195563647	-9.319099521	1.17332E-20	4.54253E-20	negative
PRJNA729271	stage_1W_vs_E10	ordinary_muscle_development	ENSGALG00010023429	BMF	0.924228693	0.146516687	6.308009788	2.82646E-10	6.54571E-10	positive
PRJNA729271	stage_1W_vs_E10	ordinary_muscle_development	ENSGALG00010023431	BCAR1	0.14162151	0.173870199	0.814524341	0.415344633	0.453310779	positive
PRJNA729271	stage_1W_vs_E10	ordinary_muscle_development	ENSGALG00010023432	TMOD3	-1.300493948	0.175221707	-7.421991104	1.15373E-13	3.19573E-13	negative
PRJNA729271	stage_1W_vs_E10	ordinary_muscle_development	ENSGALG00010023434	ATN1	-1.046542085	0.193888762	-5.397641796	6.75225E-08	1.36562E-07	negative
PRJNA729271	stage_1W_vs_E10	ordinary_muscle_development	ENSGALG00010023437	KCNJ5	-1.247722172	0.257517744	-4.84518913	1.26491E-06	2.34491E-06	negative
PRJNA729271	stage_1W_vs_E10	ordinary_muscle_development	ENSGALG00010023438	HOXB2	-0.15331945	0.238289904	-0.643415634	0.519954442	0.557067251	negative
PRJNA729271	stage_1W_vs_E10	ordinary_muscle_development	ENSGALG00010023439	ACSS2	0.887307788	0.132864061	6.67831304	2.41708E-11	5.95844E-11	positive
PRJNA729271	stage_1W_vs_E10	ordinary_muscle_development	ENSGALG00010023441	ENSGALG00010023441	1.829540044	0.147385775	12.4132742	2.2141E-35	1.52607E-34	positive
PRJNA729271	stage_1W_vs_E10	ordinary_muscle_development	ENSGALG00010023442	STK25	-0.026778611	0.112587039	-0.237848081	0.811998925	0.831914364	negative
PRJNA729271	stage_1W_vs_E10	ordinary_muscle_development	ENSGALG00010023443	ENSGALG00010023443	1.723441303	0.177863755	9.689671166	3.33601E-22	1.36405E-21	positive
PRJNA729271	stage_1W_vs_E10	ordinary_muscle_development	ENSGALG00010023447	C12orf57	1.034591922	0.113190313	9.140286798	6.22869E-20	2.33355E-19	positive
PRJNA729271	stage_1W_vs_E10	ordinary_muscle_development	ENSGALG00010023448	FAM219B	0.229678961	0.119086264	1.928677199	0.053770949	0.066879175	positive
PRJNA729271	stage_1W_vs_E10	ordinary_muscle_development	ENSGALG00010023453	ARHGAP32	-1.85712186	0.187479375	-9.905739577	3.93051E-23	1.67272E-22	negative
PRJNA729271	stage_1W_vs_E10	ordinary_muscle_development	ENSGALG00010023454	HOXB1	1.405120429	0.393478091	3.571025836	0.000355586	0.000549022	positive
PRJNA729271	stage_1W_vs_E10	ordinary_muscle_development	ENSGALG00010023455	CFDP1	-0.852309936	0.097469359	-8.744388425	2.24227E-18	7.75882E-18	negative
PRJNA729271	stage_1W_vs_E10	ordinary_muscle_development	ENSGALG00010023456	MPI	1.710427233	0.143524421	11.91732547	9.61456E-33	6.00444E-32	positive
PRJNA729271	stage_1W_vs_E10	ordinary_muscle_development	ENSGALG00010023469	ENSGALG00010023469	1.435986626	0.210732575	6.814260342	9.475E-12	2.38769E-11	positive
PRJNA729271	stage_1W_vs_E10	ordinary_muscle_development	ENSGALG00010023472	PTPN6	0.569746852	0.196553531	2.898685401	0.003747307	0.005289435	positive
PRJNA729271	stage_1W_vs_E10	ordinary_muscle_development	ENSGALG00010023473	BOK	-0.602674072	0.201314863	-2.9936889	0.002756268	0.00394005	negative
PRJNA729271	stage_1W_vs_E10	ordinary_muscle_development	ENSGALG00010023475	ENSGALG00010023475	-3.55447821	0.619228851	-5.740168923	9.45822E-09	2.00933E-08	negative
PRJNA729271	stage_1W_vs_E10	ordinary_muscle_development	ENSGALG00010023476	SCAMP2	0.421242672	0.15074764	2.794356658	0.005200309	0.007250022	positive
PRJNA729271	stage_1W_vs_E10	ordinary_muscle_development	ENSGALG00010023478	TAOK3	0.823049059	0.123228714	6.679036338	2.40519E-11	5.93012E-11	positive
PRJNA729271	stage_1W_vs_E10	ordinary_muscle_development	ENSGALG00010023479	MCCC2L	0.93590201	0.156259874	5.989394374	2.10624E-09	4.6575E-09	positive
PRJNA729271	stage_1W_vs_E10	ordinary_muscle_development	ENSGALG00010023482	MTCL2	-0.662297319	0.099925232	-6.627928753	3.4043E-11	8.31849E-11	negative
PRJNA729271	stage_1W_vs_E10	ordinary_muscle_development	ENSGALG00010023485	BARX2	-5.746217836	0.332460843	-17.2838936	6.22035E-67	1.07466E-65	negative
PRJNA729271	stage_1W_vs_E10	ordinary_muscle_development	ENSGALG00010023487	GRN	0.078852665	0.146655905	0.53767126	0.590804024	0.625653767	positive
PRJNA729271	stage_1W_vs_E10	ordinary_muscle_development	ENSGALG00010023489	ETV5	-0.418227279	0.217769909	-1.920500773	0.054794676	0.068064857	negative
PRJNA729271	stage_1W_vs_E10	ordinary_muscle_development	ENSGALG00010023490	PHB2	0.630907027	0.102813561	6.13641842	8.44027E-10	1.90504E-09	positive
PRJNA729271	stage_1W_vs_E10	ordinary_muscle_development	ENSGALG00010023496	PLEKHA3	0.108836504	0.104934368	1.037186442	0.299648976	0.335036401	positive
PRJNA729271	stage_1W_vs_E10	ordinary_muscle_development	ENSGALG00010023499	PEBP1	0.518427488	0.139736695	3.710031138	0.000207234	0.000325515	positive
PRJNA729271	stage_1W_vs_E10	ordinary_muscle_development	ENSGALG00010023501	ENSGALG00010023501	7.145216584	2.967267208	2.408012519	0.016039631	0.021170502	positive
PRJNA729271	stage_1W_vs_E10	ordinary_muscle_development	ENSGALG00010023506	RPE65	-2.116813079	0.727897927	-2.908118021	0.003636111	0.005141491	negative
PRJNA729271	stage_1W_vs_E10	ordinary_muscle_development	ENSGALG00010023510	ASPHD2	-1.018334594	0.258645643	-3.937180549	8.24446E-05	0.000133494	negative
PRJNA729271	stage_1W_vs_E10	ordinary_muscle_development	ENSGALG00010023513	CDCA9	-2.027206863	0.222072534	-9.128579858	6.94037E-20	2.59481E-19	negative
PRJNA729271	stage_1W_vs_E10	ordinary_muscle_development	ENSGALG00010023514	ULK3	1.104483678	0.10344968	10.67653059	1.31079E-26	6.45378E-26	positive
PRJNA729271	stage_1W_vs_E10	ordinary_muscle_development	ENSGALG00010023518	METTL5	0.187983522	0.119609642	1.571641879	0.116033628	0.138280642	positive
PRJNA729271	stage_1W_vs_E10	ordinary_muscle_development	ENSGALG00010023519	DGKG	0.22483827	0.243443894	0.923573256	0.355708543	0.393317212	positive
PRJNA729271	stage_1W_vs_E10	ordinary_muscle_development	ENSGALG00010023522	EMG1	-0.943413729	0.161562696	-5.839304187	5.24193E-09	1.13169E-08	negative
PRJNA729271	stage_1W_vs_E10	ordinary_muscle_development	ENSGALG00010023526	KNSTRN	-2.719144152	0.192370698	-14.1349186	2.31401E-45	2.1894E-44	negative
PRJNA729271	stage_1W_vs_E10	ordinary_muscle_development	ENSGALG00010023527	TMEM98	0.214926671	0.224830867	0.955948237	0.339098387	0.376127267	positive
PRJNA729271	stage_1W_vs_E10	ordinary_muscle_development	ENSGALG00010023533	FKBP7	-1.167766655	0.163517773	-7.141527407	9.22995E-13	2.44988E-12	negative
PRJNA729271	stage_1W_vs_E10	ordinary_muscle_development	ENSGALG00010023536	ENSGALG00010023536	-0.710639469	0.227952114	-3.117494527	0.001823953	0.002652574	negative
PRJNA729271	stage_1W_vs_E10	ordinary_muscle_development	ENSGALG00010023541	PRSS2	8.320334728	2.481105617	3.353478655	0.000798026	0.001199563	positive
PRJNA729271	stage_1W_vs_E10	ordinary_muscle_development	ENSGALG00010023543	ENSGALG00010023543	-2.152734193	0.810483125	-2.656112294	0.007904729	0.010805266	negative
PRJNA729271	stage_1W_vs_E10	ordinary_muscle_development	ENSGALG00010023544	SSB	-0.702758877	0.102632554	-6.847329135	7.52415E-12	1.90502E-11	negative
PRJNA729271	stage_1W_vs_E10	ordinary_muscle_development	ENSGALG00010023545	PTPRT	-1.929295011	0.139232888	-13.8566041	1.16035E-43	1.04144E-42	negative
PRJNA729271	stage_1W_vs_E10	ordinary_muscle_development	ENSGALG00010023546	SLC11A2	1.247028171	0.154157047	8.089336119	5.99908E-16	1.86442E-15	positive
PRJNA729271	stage_1W_vs_E10	ordinary_muscle_development	ENSGALG00010023551	ENSGALG00010023551	0.067428061	0.177967494	0.37887852	0.704778076	0.732785181	positive
PRJNA729271	stage_1W_vs_E10	ordinary_muscle_development	ENSGALG00010023552	SGPP2	0.394160703	0.357804239	1.101609933	0.270631288	0.304636214	positive
PRJNA729271	stage_1W_vs_E10	ordinary_muscle_development	ENSGALG00010023555	HPS4	-1.153056386	0.171904972	-6.707522039	1.97957E-11	4.90075E-11	negative
PRJNA729271	stage_1W_vs_E10	ordinary_muscle_development	ENSGALG00010023556	IVD	0.325645639	0.121528922	2.679573159	0.007371609	0.01010607	positive
PRJNA729271	stage_1W_vs_E10	ordinary_muscle_development	ENSGALG00010023559	PJVK	2.502703755	0.682857987	3.665042807	0.000247297	0.000386727	positive
PRJNA729271	stage_1W_vs_E10	ordinary_muscle_development	ENSGALG00010023569	LPCAT3	0.873109479	0.101733406	8.582328193	9.29719E-18	3.12095E-17	positive
PRJNA729271	stage_1W_vs_E10	ordinary_muscle_development	ENSGALG00010023571	APLP2	0.551325458	0.098673146	5.5873911	2.30506E-08	4.78612E-08	positive
PRJNA729271	stage_1W_vs_E10	ordinary_muscle_development	ENSGALG00010023572	MYBL2	-1.739679079	0.129977129	-13.38450155	7.44954E-41	6.13529E-40	negative
PRJNA729271	stage_1W_vs_E10	ordinary_muscle_development	ENSGALG00010023574	DEPDC1	-2.497782468	0.210962804	-11.83991881	2.42688E-32	1.49819E-31	negative

PRJNA729271	stage_1W_vs_E10	ordinary_muscle_development	ENSGALG00010023578	SRRD	-0.029527684	0.101878953	-0.289831051	0.771945492	0.795429469	negative
PRJNA729271	stage_1W_vs_E10	ordinary_muscle_development	ENSGALG00010023580	RDM1	-1.746745831	0.16975549	-10.28977518	7.83591E-25	3.57399E-24	negative
PRJNA729271	stage_1W_vs_E10	ordinary_muscle_development	ENSGALG00010023585	TFIP11	0.242763408	0.095934937	2.530500514	0.011389992	0.015293926	positive
PRJNA729271	stage_1W_vs_E10	ordinary_muscle_development	ENSGALG00010023588	FARSB	0.559504424	0.134052498	4.173770961	2.99599E-05	5.01581E-05	positive
PRJNA729271	stage_1W_vs_E10	ordinary_muscle_development	ENSGALG00010023590	S1PR1	1.255422796	0.115023419	10.91449729	9.82715E-28	5.06199E-27	positive
PRJNA729271	stage_1W_vs_E10	ordinary_muscle_development	ENSGALG00010023592	BAHD1	-0.956360961	0.106249014	-9.00112785	2.23411E-19	8.15291E-19	negative
PRJNA729271	stage_1W_vs_E10	ordinary_muscle_development	ENSGALG00010023593	CSK	-0.616636588	0.082939162	-7.434806071	1.04721E-13	2.90793E-13	negative
PRJNA729271	stage_1W_vs_E10	ordinary_muscle_development	ENSGALG00010023597	MOGAT1	0.290115176	0.33984642	0.853665535	0.393290335	0.431416938	positive
PRJNA729271	stage_1W_vs_E10	ordinary_muscle_development	ENSGALG00010023598	IL10RA	2.491232822	0.379308535	6.567826963	5.10548E-11	1.23443E-10	positive
PRJNA729271	stage_1W_vs_E10	ordinary_muscle_development	ENSGALG00010023600	CHST14	-2.336367566	0.085856175	-27.21257457	4.611E-163	4.6414E-161	negative
PRJNA729271	stage_1W_vs_E10	ordinary_muscle_development	ENSGALG00010023601	TPST2	0.736106189	0.106966594	6.881645585	5.91651E-12	1.50588E-11	positive
PRJNA729271	stage_1W_vs_E10	ordinary_muscle_development	ENSGALG00010023603	C1S	2.456258916	0.393191432	6.246979763	4.18465E-10	9.59755E-10	positive
PRJNA729271	stage_1W_vs_E10	ordinary_muscle_development	ENSGALG00010023604	ENSGALG00010023604	1.058819216	0.391723409	2.702976622	0.006872158	0.009457128	positive
PRJNA729271	stage_1W_vs_E10	ordinary_muscle_development	ENSGALG00010023605	INSYN1	4.169338693	0.303079975	13.75656273	4.65121E-43	4.10093E-42	positive
PRJNA729271	stage_1W_vs_E10	ordinary_muscle_development	ENSGALG00010023606	LRRC7	-3.214373188	0.349525063	-9.196402571	3.70134E-20	1.39789E-19	negative
PRJNA729271	stage_1W_vs_E10	ordinary_muscle_development	ENSGALG00010023612	GPT2	0.406754921	0.187356009	2.171026822	0.029929145	0.038364251	positive
PRJNA729271	stage_1W_vs_E10	ordinary_muscle_development	ENSGALG00010023613	COPB1	-0.587389871	0.05166146	-11.36998194	5.89998E-30	3.33282E-29	negative
PRJNA729271	stage_1W_vs_E10	ordinary_muscle_development	ENSGALG00010023615	PRSS3	3.873444499	5.115073949	0.757260704	0.448893688	0.487029492	positive
PRJNA729271	stage_1W_vs_E10	ordinary_muscle_development	ENSGALG00010023617	ENSGALG00010023617	1.212790165	0.293706687	4.129256216	3.63939E-05	6.05729E-05	positive
PRJNA729271	stage_1W_vs_E10	ordinary_muscle_development	ENSGALG00010023620	IRS1	1.73885277	0.140997589	12.33250004	6.05388E-35	4.10435E-34	positive
PRJNA729271	stage_1W_vs_E10	ordinary_muscle_development	ENSGALG00010023621	CD79B	2.372343778	0.982721463	2.41405512	0.015776076	0.020841616	positive
PRJNA729271	stage_1W_vs_E10	ordinary_muscle_development	ENSGALG00010023623	OSBPL6	0.523022423	0.217981562	2.39938836	0.016422487	0.02166005	positive
PRJNA729271	stage_1W_vs_E10	ordinary_muscle_development	ENSGALG00010023624	PSMA1	0.802557562	0.070447661	11.39225284	4.57018E-30	2.58971E-29	positive
PRJNA729271	stage_1W_vs_E10	ordinary_muscle_development	ENSGALG00010023626	LOXL1	1.073767073	0.195015059	5.506072601	3.66927E-08	7.53024E-08	positive
PRJNA729271	stage_1W_vs_E10	ordinary_muscle_development	ENSGALG00010023627	IFT52	-0.172269632	0.102044438	-1.688182478	0.091376206	0.110540653	negative
PRJNA729271	stage_1W_vs_E10	ordinary_muscle_development	ENSGALG00010023631	ENSGALG00010023631	3.23235513	0.447456878	7.223836062	5.05412E-13	1.3554E-12	positive
PRJNA729271	stage_1W_vs_E10	ordinary_muscle_development	ENSGALG00010023635	SCN4A	10.13987671	0.317291909	31.95756474	4.2415E-224	7.685E-222	positive
PRJNA729271	stage_1W_vs_E10	ordinary_muscle_development	ENSGALG00010023636	PDE3B	0.068321675	0.081558678	0.837699633	0.40219942	0.440356567	positive
PRJNA729271	stage_1W_vs_E10	ordinary_muscle_development	ENSGALG00010023637	RHBDD1	0.356361453	0.106159718	3.356842507	0.00078838	0.001185678	positive
PRJNA729271	stage_1W_vs_E10	ordinary_muscle_development	ENSGALG00010023638	DCTN2	0.897500246	0.130681777	6.86783014	6.51858E-12	1.6565E-11	positive
PRJNA729271	stage_1W_vs_E10	ordinary_muscle_development	ENSGALG00010023639	KLHL23	1.651264617	0.215560238	7.660339551	1.85442E-14	5.36203E-14	positive
PRJNA729271	stage_1W_vs_E10	ordinary_muscle_development	ENSGALG00010023641	LRRC40	-1.995463173	0.115376669	-17.29520521	5.11206E-67	8.91421E-66	negative
PRJNA729271	stage_1W_vs_E10	ordinary_muscle_development	ENSGALG00010023642	PAX6	0.411291769	0.703619747	0.584536989	0.558859087	0.59471863	positive
PRJNA729271	stage_1W_vs_E10	ordinary_muscle_development	ENSGALG00010023643	SCN4B	3.834421951	0.148631896	25.79810973	9.3109E-147	7.4155E-145	positive
PRJNA729271	stage_1W_vs_E10	ordinary_muscle_development	ENSGALG00010023646	DPH5	1.092198224	0.141887667	7.69762619	1.38617E-14	4.03384E-14	positive
PRJNA729271	stage_1W_vs_E10	ordinary_muscle_development	ENSGALG00010023648	SCN2B	2.68267267	0.358998614	7.472654666	7.85929E-14	2.19924E-13	positive
PRJNA729271	stage_1W_vs_E10	ordinary_muscle_development	ENSGALG00010023652	SLC30A7	-1.328440507	0.097697995	-13.59741826	4.1482E-42	3.55788E-41	negative
PRJNA729271	stage_1W_vs_E10	ordinary_muscle_development	ENSGALG00010023655	C1R	0.714849757	0.185715016	3.849175864	0.000118516	0.000189591	positive
PRJNA729271	stage_1W_vs_E10	ordinary_muscle_development	ENSGALG00010023658	ENSGALG00010023658	-0.630491735	0.320571429	-1.96677457	0.049209206	0.061493056	negative
PRJNA729271	stage_1W_vs_E10	ordinary_muscle_development	ENSGALG00010023660	CRYBB1	0.583138719	0.499890197	1.166533614	0.243398766	0.275975056	positive
PRJNA729271	stage_1W_vs_E10	ordinary_muscle_development	ENSGALG00010023663	PHOSPHO2	1.011113502	0.089291382	11.32375241	1.00094E-29	5.59529E-29	positive
PRJNA729271	stage_1W_vs_E10	ordinary_muscle_development	ENSGALG00010023664	SLC6A9	-0.243049718	0.325315596	-0.747119785	0.454991258	0.493165205	negative
PRJNA729271	stage_1W_vs_E10	ordinary_muscle_development	ENSGALG00010023665	C16orf87	-0.450111209	0.140220148	-3.210032324	0.0013272	0.001953271	negative
PRJNA729271	stage_1W_vs_E10	ordinary_muscle_development	ENSGALG00010023666	ENSGALG00010023666	1.645541368	0.251117991	6.552861325	5.64449E-11	1.36044E-10	positive
PRJNA729271	stage_1W_vs_E10	ordinary_muscle_development	ENSGALG00010023668	RCN1	-1.132636761	0.16144581	-7.015584755	2.28989E-12	5.95372E-12	negative
PRJNA729271	stage_1W_vs_E10	ordinary_muscle_development	ENSGALG00010023670	EXTL2	0.670100673	0.126670933	5.290090285	1.22256E-07	2.42754E-07	positive
PRJNA729271	stage_1W_vs_E10	ordinary_muscle_development	ENSGALG00010023671	ENSGALG00010023671	4.134347794	0.649072903	6.369620071	1.89497E-10	4.4324E-10	positive
PRJNA729271	stage_1W_vs_E10	ordinary_muscle_development	ENSGALG00010023676	ENSGALG00010023676	0.788484968	0.25625106	3.077001784	0.00209094	0.003024769	positive
PRJNA729271	stage_1W_vs_E10	ordinary_muscle_development	ENSGALG00010023677	MN1	0.968418866	0.160965813	6.016301536	1.78447E-09	3.96655E-09	positive
PRJNA729271	stage_1W_vs_E10	ordinary_muscle_development	ENSGALG00010023680	MPZL3	-5.453469975	0.494653965	-11.02481808	2.90104E-28	1.52745E-27	negative
PRJNA729271	stage_1W_vs_E10	ordinary_muscle_development	ENSGALG00010023686	MYLK3	-0.50612004	0.226537697	-2.234153725	0.02547296	0.032917051	negative
PRJNA729271	stage_1W_vs_E10	ordinary_muscle_development	ENSGALG00010023689	WT1	-2.139521472	0.248986824	-8.592910408	8.47937E-18	2.85369E-17	negative
PRJNA729271	stage_1W_vs_E10	ordinary_muscle_development	ENSGALG00010023693	SRSF11	-0.499862799	0.068995779	-7.244831599	4.32977E-13	1.16589E-12	negative
PRJNA729271	stage_1W_vs_E10	ordinary_muscle_development	ENSGALG00010023694	PPCDC	1.633918028	0.151661591	10.77344643	4.59473E-27	2.29895E-26	positive
PRJNA729271	stage_1W_vs_E10	ordinary_muscle_development	ENSGALG00010023695	CLSTN3	-1.029262973	0.132410484	-7.773274004	7.6483E-15	2.25559E-14	negative
PRJNA729271	stage_1W_vs_E10	ordinary_muscle_development	ENSGALG00010023696	LRRC4C	0.233082316	0.529492632	0.440199358	0.659792725	0.690270339	positive
PRJNA729271	stage_1W_vs_E10	ordinary_muscle_development	ENSGALG00010023698	ENSGALG00010023698	-1.837709785	0.153272707	-11.98980448	4.01853E-33	2.55029E-32	negative
PRJNA729271	stage_1W_vs_E10	ordinary_muscle_development	ENSGALG00010023699	PPIG	-0.150931179	0.203253654	-0.742575479	0.457738739	0.495957768	negative
PRJNA729271	stage_1W_vs_E10	ordinary_muscle_development	ENSGALG00010023702	COL4A4	0.662943122	0.259062455	2.559008876	0.010497106	0.014159274	positive
PRJNA729271	stage_1W_vs_E10	ordinary_muscle_development	ENSGALG00010023703	PITPNB	-1.17835467	0.081814976	-14.40267692	4.97775E-47	4.94873E-46	negative
PRJNA729271	stage_1W_vs_E10	ordinary_muscle_development	ENSGALG00010023704	ANKRD13C	-0.360837411	0.069008809	-5.228860132	1.70558E-07	3.35356E-07	negative
PRJNA729271	stage_1W_vs_E10	ordinary_muscle_development	ENSGALG00010023706	CDK5RAP3	-0.439934772	0.128983847	-3.41077416	0.000647787	0.000980338	negative
PRJNA729271	stage_1W_vs_E10	ordinary_muscle_development	ENSGALG00010023707	MPZL2	-1.694233512	0.282361685	-6.000224543	1.97045E-09	4.36856E-09	negative
PRJNA729271	stage_1W_vs_E10	ordinary_muscle_development	ENSGALG00010023710	CDC14A	0.574530352	0.120430796	4.770626542	1.83654E-06	3.36459E-06	positive
PRJNA729271	stage_1W_vs_E10	ordinary_muscle_development	ENSGALG00010023711	ENSGALG00010023711	-0.685831702	0.359694622	-1.906705468	0.056558739	0.070165946	negative
PRJNA729271	stage_1W_vs_E10	ordinary_muscle_development	ENSGALG00010023712	ARHGAP40	-2.233223295	0.166295337	-13.42925988	4.07463E-41	3.39046E-40	negative
PRJNA729271	stage_1W_vs_E10	ordinary_muscle_development	ENSGALG00010023714	EIF3M	-0.205202679	0.110518039	-1.856734719	0.063348905	0.078055286	negative
PRJNA729271	stage_1W_vs_E10	ordinary_muscle_development	ENSGALG00010023715	CD3E	1.243674165	0.3467887	3.586259195	0.000335456	0.000519213	positive
PRJNA729271	stage_1W_vs_E10	ordinary_muscle_development	ENSGALG00010023716	FASTKD1	-0.622908052	0.10801588	-5.766819221	8.07816E-09	1.72272E-08	negative
PRJNA729271	stage_1W_vs_E10	ordinary_muscle_development	ENSGALG00010023720	SCAMP5	-1.29597156	0.100258904	-12.92624906	3.20056E-38	2.41124E-37	negative
PRJNA729271	stage_1W_vs_E10	ordinary_muscle_development	ENSGALG00010023724	CYP2R1	-0.284005502	0.124860807	-2.27457686	0.022931326	0.02979202	negative
PRJNA729271	stage_1W_vs_E10	ordinary_muscle_development	ENSGALG00010023726	PNPO	0.844266735	0.180570316	4.6755566	2.93157E-06	5.28917E-06	positive
PRJNA729271	stage_1W_vs_E10	ordinary_muscle_development	ENSGALG00010023730	SP2	0.856220666	0.186638481	4.587589133	4.48394E-06	7.98069E-06	positive
PRJNA729271	stage_1W_vs_E10	ordinary_muscle_development	ENSGALG00010023731	ACTR5	0.167981186	0.098002999	1.71404129	0.086521143	0.104886173	positive
PRJNA729271	stage_1W_vs_E10	ordinary_muscle_development	ENSGALG00010023732	TM2D1	0.401209618	0.077755608	5.159880116	2.47108E-07	4.81106E-07	positive
PRJNA729271	stage_1W_vs_E10	ordinary_muscle_development	ENSGALG00010023733	ORC6	-1.982907941	0.193908695	-10.22598777	1.51674E-24	6.84681E-24	negative
PRJNA729271	stage_1W_vs_E10	ordinary_muscle_development	ENSGALG00010023736	KIF5A	-1.778609194	0.565097441	-3.147438063	0.00164708	0.002406453	negative
PRJNA729271	stage_1W_vs_E10	ordinary_muscle_development	ENSGALG00010023738	ENSGALG00010023738	0.315892678	0.397934369	0.793831101	0.427293764	0.465196268	positive
PRJNA729271	stage_1W_vs_E10	ordinary_muscle_development	ENSGALG00010023740	PPP1R16B	0.144829838	0.105391727	1.374205003	0.169378048	0.19707295	positive
PRJNA729271	stage_1W_vs_E10	ordinary_muscle_development	ENSGALG00010023743	CD3D	1.226457599	0.226563876	5.413297205	6.18747E-08	1.25541E-07	positive
PRJNA729271	stage_1W_vs_E10	ordinary_muscle_development	ENSGALG00010023744	MT4	1.685738077	0.316297304	5.3295999	9.84294E-08	1.96899E-07	positive
PRJNA729271	stage_1W_vs_E10	ordinary_muscle_development	ENSGALG00010023746	TTC28	-1.790595194	0.090426018	-19.80176982	2.87416E-87	8.07384E-86	negative
PRJNA729271	stage_1W_vs_E10	ordinary_muscle_development	ENSGALG00010023748	ENSGALG00010023748	-1.076697356	0.175128273	-6.148049864	7.84414E-10	1.77325E-09	negative
PRJNA729271	stage_1W_vs_E10	ordinary_muscle_development	ENSGALG00010023749	RPP25	0.762321315	0.24782046	3.076103224	0.002097252	0.003032084	positive
PRJNA729271	stage_1W_vs_E10	ordinary_muscle_development	ENSGALG00010023750	ENSGALG00010023750	-1.911057335	0.16625995	-11.49439376	1.40766E-30	8.13557E-30	negative
PRJNA729271	stage_1W_vs_E10	ordinary_muscle_development	ENSGALG00010023751	PRRG4	-1.136803864	0.290499081	-3.913278692	9.10513E-05	0.000146937	negative
PRJNA729271	stage_1W_vs_E10	ordinary_muscle_development	ENSGALG00010023752	C1QL1	-1.846551982	0.322024585	-5.734195674	9.79762E-09	2.07928E-08	negative
PRJNA729271	stage_1W_vs_E10	ordinary_muscle_development	ENSGALG00010023759	CALCA	-2.393977472	0.323084583	-7.40975458	1.26533E-13	3.49752E-13	negative
PRJNA729271	stage_1W_vs_E10	ordinary_muscle_development	ENSGALG00010023760	ZW10	-0.798480239	0.093808614	-8.511800786	1.71253E-17	5.68294E-17	negative
PRJNA729271	stage_1W_vs_E10	ordinary_muscle_development	ENSGALG00010023762	BBS5	2.738409112	0.152093079	18.00482393	1.78567E-72	3.58991E-71	positive
PRJNA729271	stage_1W_vs_E10	ordinary_muscle_development	ENSGALG00010023764	COX5A	2.515995602	0.176600733	14.24680158	4.69287E-46	4.52282E-45	positive
PRJNA729271	stage_1W_vs_E10	ordinary_muscle_development	ENSGALG00010023766	ENSGALG00010023766	-2.534601533	0.333458006	-7.600961706	2.93938E-14	8.40859E-14	negative
PRJNA729271	stage_1W_vs_E10	ordinary_muscle_development	ENSGALG00010023767	ENSGALG00010023767	2.580100544	0.209284593	12.32819151	6.3864E-35	4.32574E-34	positive
PRJNA729271	stage_1W_vs_E10	ordinary_muscle_development	ENSGALG00010023770	SCRN2	0.069274245	0.139776709	0.4956065	0.620172066	0.654297554	positive
PRJNA729271	stage_1W_vs_E10	ordinary_muscle_development	ENSGALG00010023773	OVOA	1.424123716	0.518940545	2.744290711	0.006064181	0.008390636	positive
PRJNA729271	stage_1W_vs_E10	ordinary_muscle_development	ENSGALG00010023775	ENSGALG00010023775	1.777070501	0.134476584	13.21472073	7.21536E-40	5.75601E-39	positive
PRJNA729271	stage_1W_vs_E10	ordinary_muscle_development	ENSGALG00010023777	OAZ2	0.70511772	0.132233319	5.332375569	9.69362E-08	1.93993E-07	positive
PRJNA729271	stage_1W_vs_E10	ordinary_muscle_development	ENSGALG00010023778	FANCF	-2.797197226	0.224771916	-12.44460283	1.49627E-35	1.03723E-34	negative
PRJNA729271	stage_1W_vs_E10	ordinary_muscle_development	ENSGALG00010023781	COL4A3	1.644486952	0.239954509	6.853327997	7.21514E-12	1.82902E-11	positive
PRJNA729271	stage_1W_vs_E10	ordinary_muscle_development	ENSGALG00010023783	VPS35	-0.156988078	0.063055196	-2.489692991	0.012785348	0.01707737	negative
PRJNA729271	stage_1W_vs_E10	ordinary_muscle_development	ENSGALG00010023786	ENSGALG00010023786	-3.532609502	0.5415276	-6.523415435	6.87242E-11	1.64491E-10	negative
PRJNA729271	stage_1W_vs_E10	ordinary_muscle_development	ENSGALG00010023788	RTCA	-0.252483248	0.10533871	-2.396870507	0.016535766	0.021797557	negative
PRJNA729271	stage_1W_vs_E10	ordinary_muscle_development	ENSGALG00010023791	VTI1B	0.750420404	0.079966294	9.384208838	6.33905E-21	2.47534E-20	positive
PRJNA729271	stage_1W_vs_E10	ordinary_muscle_development	ENSGALG00010023792	ZNF609	-0.082016644	0.176860786	-0.463735608	0.642837182	0.674792254	negative
PRJNA729271	stage_1W_vs_E10	ordinary_muscle_development	ENSGALG00010023793	RBM45	-0.033915272	0.065121442	-0.520800389	0.602505832	0.637050262	negative
PRJNA729271	stage_1W_vs_E10	ordinary_muscle_development	ENSGALG00010023794	MT3	0.843347778	0.473168411	1.782341674	0.074693531	0.091227058	positive
PRJNA729271	stage_1W_vs_E10	ordinary_muscle_development	ENSGALG00010023798	ARCN1	-0.927283307	0.057325706	-16.17569808	7.48446E-59	1.06048E-57	negative
PRJNA729271	stage_1W_vs_E10	ordinary_muscle_development	ENSGALG00010023799	NMT1	0.482579185	0.126599022	3.81187135	0.000137919	0.000219444	positive
PRJNA729271	stage_1W_vs_E10	ordinary_muscle_development	ENSGALG00010023800	PEX5	0.867521659	0.071429287	12.14518157	6.08474E-34	3.97648E-33	positive
PRJNA729271	stage_1W_vs_E10	ordinary_muscle_development	ENSGALG00010023802	ENSGALG00010023802	2.211040829	0.275863206	8.01498998	1.10146E-15	3.38184E-15	positive
PRJNA729271	stage_1W_vs_E10	ordinary_muscle_development	ENSGALG00010023805	GALNT6	-0.814765022	0.336015226	-2.42478602	0.015317412	0.020278189	negative
PRJNA729271	stage_1W_vs_E10	ordinary_muscle_development	ENSGALG00010023806	PATJ	-1.761163216	0.176575687	-9.973984805	1.98117E-23	8.53405E-23	negative
PRJNA729271	stage_1W_vs_E10	ordinary_muscle_development	ENSGALG00010023807	MRPL10	1.70166323	0.124074433	13.71485801	8.27282E-43	7.24997E-42	positive
PRJNA729271	stage_1W_vs_E10	ordinary_muscle_development	ENSGALG00010023808	DHX35	-0.414894496	0.117452696	-3.532439095	0.000411745	0.000632162	negative
PRJNA729271	stage_1W_vs_E10	ordinary_muscle_development	ENSGALG00010023809	CDON	-1.493455117	0.100069724	-14.92414542	2.29558E-50	2.55368E-49	negative
PRJNA729271	stage_1W_vs_E10	ordinary_muscle_development	ENSGALG00010023811	MRPL44	1.12862673	0.083920381	13.44877984	3.12994E-41	2.62246E-40	positive
PRJNA729271	stage_1W_vs_E10	ordinary_muscle_development	ENSGALG00010023813	CELA1	4.549570731	5.27480758	0.862509326	0.388407303	0.426383207	positive
PRJNA729271	stage_1W_vs_E10	ordinary_muscle_development	ENSGALG00010023815	PLCD3	0.282734849	0.140393255	2.013877722	0.044022373	0.055376577	positive
PRJNA729271	stage_1W_vs_E10	ordinary_muscle_development	ENSGALG00010023817	BBS2	0.304082099	0.116396844	2.612460007	0.008989321	0.012217553	positive
PRJNA729271	stage_1W_vs_E10	ordinary_muscle_development	ENSGALG00010023818	ALOX5	1.181793092	0.189403319	6.239558506	4.38808E-10	1.00514E-09	positive
PRJNA729271	stage_1W_vs_E10	ordinary_muscle_development	ENSGALG00010023819	BIN2	1.115876917	0.234578831	4.756937828	1.96552E-06	3.59407E-06	positive
PRJNA729271	stage_1W_vs_E10	ordinary_muscle_development	ENSGALG00010023820	CYP1C1	-0.657902369	0.911596037	-0.721703849	0.470476575	0.508922235	negative
PRJNA729271	stage_1W_vs_E10	ordinary_muscle_development	ENSGALG00010023821	KANK4	-0.490513405	0.124430374	-3.942071298	8.0781E-05	0.000131005	negative
PRJNA729271	stage_1W_vs_E10	ordinary_muscle_development	ENSGALG00010023822	CHEK2	-1.211839698	0.13053961	-9.283310213	1.64293E-20	6.31847E-20	negative
PRJNA729271	stage_1W_vs_E10	ordinary_muscle_development	ENSGALG00010023823	MUC2	3.799775407	0.515656424	7.368812314	1.72155E-13	4.727E-13	positive
PRJNA729271	stage_1W_vs_E10	ordinary_muscle_development	ENSGALG00010023826	DBT	1.342615856	0.126983271	10.57317116	3.96844E-26	1.91487E-25	positive
PRJNA729271	stage_1W_vs_E10	ordinary_muscle_development	ENSGALG00010023828	CHEK1	-2.355546322	0.184324587	-12.77933864	2.1388E-37	1.56813E-36	negative
PRJNA729271	stage_1W_vs_E10	ordinary_muscle_development	ENSGALG00010023829	USP1	-1.143691297	0.101209278	-11.30026137	1.30828E-29	7.26572E-29	negative
PRJNA729271	stage_1W_vs_E10	ordinary_muscle_development	ENSGALG00010023830	AGFG1	1.498543519	0.1120337	13.37582817	8.3716E-41	6.87905E-40	positive
PRJNA729271	stage_1W_vs_E10	ordinary_muscle_development	ENSGALG00010023831	SATB2	-3.793925411	0.535702868	-7.082145037	1.4194E-12	3.72991E-12	negative
PRJNA729271	stage_1W_vs_E10	ordinary_muscle_development	ENSGALG00010023832	ENSGALG00010023832	-0.866447035	0.547827684	-1.581605057	0.113739759	0.135725513	negative

PRJNA729271	stage_1W_vs_E10	ordinary_muscle_development	ENSGALG00010023833	PIP4K2C	-0.000521139	0.108482631	-0.004803897	0.996167059	0.996854783	negative
PRJNA729271	stage_1W_vs_E10	ordinary_muscle_development	ENSGALG00010023835	INAFM2	0.928587843	0.120452948	7.709133391	1.26675E-14	3.69522E-14	positive
PRJNA729271	stage_1W_vs_E10	ordinary_muscle_development	ENSGALG00010023836	NDUFS3	2.377494498	0.147283312	16.14232098	1.28613E-58	1.817E-57	positive
PRJNA729271	stage_1W_vs_E10	ordinary_muscle_development	ENSGALG00010023837	STT3A	-0.842782561	0.093246328	-9.038238562	1.59217E-19	5.85896E-19	negative
PRJNA729271	stage_1W_vs_E10	ordinary_muscle_development	ENSGALG00010023840	SMAGP	-1.967578917	0.608729619	-3.23227071	0.001228107	0.001811974	negative
PRJNA729271	stage_1W_vs_E10	ordinary_muscle_development	ENSGALG00010023841	OSBPL7	0.358131242	0.149093382	2.402059952	0.016303036	0.021510332	positive
PRJNA729271	stage_1W_vs_E10	ordinary_muscle_development	ENSGALG00010023847	PIK3AP1	0.258179421	0.187097386	1.379919985	0.167611283	0.195252394	positive
PRJNA729271	stage_1W_vs_E10	ordinary_muscle_development	ENSGALG00010023850	DAZAP2	1.69560815	0.201170236	8.428722779	3.49458E-17	1.14137E-16	positive
PRJNA729271	stage_1W_vs_E10	ordinary_muscle_development	ENSGALG00010023851	HSCB	0.830698375	0.148460989	5.595398381	2.20116E-08	4.5743E-08	positive
PRJNA729271	stage_1W_vs_E10	ordinary_muscle_development	ENSGALG00010023852	LRRC39	2.980690454	0.190271522	15.66545761	2.60548E-55	3.39321E-54	positive
PRJNA729271	stage_1W_vs_E10	ordinary_muscle_development	ENSGALG00010023854	SHCBP1	-2.307776493	0.221611157	-10.41362957	2.1487E-25	1.00404E-24	negative
PRJNA729271	stage_1W_vs_E10	ordinary_muscle_development	ENSGALG00010023857	ACBD4	1.149338041	0.15059079	7.632193447	2.30793E-14	6.63627E-14	positive
PRJNA729271	stage_1W_vs_E10	ordinary_muscle_development	ENSGALG00010023859	ENSGALG00010023859	3.240105926	0.766973348	4.224535225	2.39434E-05	4.03183E-05	positive
PRJNA729271	stage_1W_vs_E10	ordinary_muscle_development	ENSGALG00010023860	TRIP4	0.488231331	0.113933339	4.285236737	1.82545E-05	3.10343E-05	positive
PRJNA729271	stage_1W_vs_E10	ordinary_muscle_development	ENSGALG00010023861	ENSGALG00010023861	-0.240243562	0.153715223	-1.562913275	0.118072995	0.140549237	negative
PRJNA729271	stage_1W_vs_E10	ordinary_muscle_development	ENSGALG00010023862	TRMT13	-0.680459845	0.134692757	-5.05194088	4.37343E-07	8.37091E-07	negative
PRJNA729271	stage_1W_vs_E10	ordinary_muscle_development	ENSGALG00010023863	EYA2	-4.340197559	0.245035492	-17.71252616	3.35664E-70	6.4019E-69	negative
PRJNA729271	stage_1W_vs_E10	ordinary_muscle_development	ENSGALG00010023864	EPHA1	-7.345646145	0.981573634	-7.483540601	7.23465E-14	2.02993E-13	negative
PRJNA729271	stage_1W_vs_E10	ordinary_muscle_development	ENSGALG00010023866	AMBRA1	-0.073288775	0.074834363	-0.979346542	0.327408781	0.363857263	negative
PRJNA729271	stage_1W_vs_E10	ordinary_muscle_development	ENSGALG00010023867	ENSGALG00010023867	0.833722271	0.502411951	1.659439567	0.097027249	0.116918278	positive
PRJNA729271	stage_1W_vs_E10	ordinary_muscle_development	ENSGALG00010023869	DOCK7	-1.140268845	0.047879152	-23.81556052	2.3043E-125	1.3975E-123	negative
PRJNA729271	stage_1W_vs_E10	ordinary_muscle_development	ENSGALG00010023871	UQCRFS1	1.809109253	0.140227057	12.90128516	4.42653E-38	3.31418E-37	positive
PRJNA729271	stage_1W_vs_E10	ordinary_muscle_development	ENSGALG00010023874	HEXIM1	2.156791407	0.223893317	9.633120969	5.79423E-22	2.34864E-21	positive
PRJNA729271	stage_1W_vs_E10	ordinary_muscle_development	ENSGALG00010023878	ENSGALG00010023878	4.231267833	1.435626413	2.947332115	0.003205287	0.004554518	positive
PRJNA729271	stage_1W_vs_E10	ordinary_muscle_development	ENSGALG00010023879	EI24	0.988064454	0.116689892	8.467438234	2.50849E-17	8.25813E-17	positive
PRJNA729271	stage_1W_vs_E10	ordinary_muscle_development	ENSGALG00010023880	ENSGALG00010023880	-2.268607596	0.201146492	-11.27838506	1.67798E-29	9.28687E-29	negative
PRJNA729271	stage_1W_vs_E10	ordinary_muscle_development	ENSGALG00010023881	VSTM2B	-1.669930396	0.234414992	-7.123820802	1.04976E-12	2.7782E-12	negative
PRJNA729271	stage_1W_vs_E10	ordinary_muscle_development	ENSGALG00010023883	TBX21	7.220192089	0.340913423	21.17896101	1.49289E-99	5.56283E-98	positive
PRJNA729271	stage_1W_vs_E10	ordinary_muscle_development	ENSGALG00010023884	C2orf69	-0.591326253	0.125970422	-4.694167451	2.67695E-06	4.84726E-06	negative
PRJNA729271	stage_1W_vs_E10	ordinary_muscle_development	ENSGALG00010023887	RASA2	-0.416505099	0.128906065	-3.231074496	0.001233258	0.001818522	negative
PRJNA729271	stage_1W_vs_E10	ordinary_muscle_development	ENSGALG00010023888	CTH	-0.290038301	0.258615141	-1.12150549	0.262072756	0.295829343	negative
PRJNA729271	stage_1W_vs_E10	ordinary_muscle_development	ENSGALG00010023889	SAMD8	2.872274676	0.189705329	15.14071691	8.72573E-52	1.02164E-50	positive
PRJNA729271	stage_1W_vs_E10	ordinary_muscle_development	ENSGALG00010023890	SASS6	-0.804975297	0.120105601	-6.702229472	2.05263E-11	5.07816E-11	negative
PRJNA729271	stage_1W_vs_E10	ordinary_muscle_development	ENSGALG00010023891	ENSGALG00010023891	4.142321597	0.265489126	15.60260363	6.98849E-55	8.98033E-54	positive
PRJNA729271	stage_1W_vs_E10	ordinary_muscle_development	ENSGALG00010023896	POP4	-0.784640537	0.131293636	-5.97622674	2.28365E-09	5.03982E-09	negative
PRJNA729271	stage_1W_vs_E10	ordinary_muscle_development	ENSGALG00010023902	PTGER3	0.053512379	0.235273607	0.227447436	0.820075838	0.838944122	positive
PRJNA729271	stage_1W_vs_E10	ordinary_muscle_development	ENSGALG00010023903	TBKBP1	-0.390983738	0.407302447	-0.959934664	0.337088099	0.374040572	negative
PRJNA729271	stage_1W_vs_E10	ordinary_muscle_development	ENSGALG00010023909	FOXRED1	0.688366096	0.152444927	4.515506745	6.31655E-06	1.11034E-05	positive
PRJNA729271	stage_1W_vs_E10	ordinary_muscle_development	ENSGALG00010023910	RNF7	0.784065226	0.100485798	7.802746691	6.05741E-15	1.79408E-14	positive
PRJNA729271	stage_1W_vs_E10	ordinary_muscle_development	ENSGALG00010023911	TFCP2	-1.267912861	0.124033225	-10.22236467	1.57452E-24	7.10103E-24	negative
PRJNA729271	stage_1W_vs_E10	ordinary_muscle_development	ENSGALG00010023913	ZMYND8	-1.235950291	0.084534666	-14.62063261	2.07464E-48	2.14799E-47	negative
PRJNA729271	stage_1W_vs_E10	ordinary_muscle_development	ENSGALG00010023914	CCDC117	-0.30726436	0.065901219	-4.662498877	3.12393E-06	5.62571E-06	negative
PRJNA729271	stage_1W_vs_E10	ordinary_muscle_development	ENSGALG00010023917	ZRANB2	0.124084474	0.07942442	1.562296259	0.118218213	0.140710543	positive
PRJNA729271	stage_1W_vs_E10	ordinary_muscle_development	ENSGALG00010023918	MFSD14A	-0.796095104	0.081004508	-9.827787643	8.54758E-23	3.58292E-22	negative
PRJNA729271	stage_1W_vs_E10	ordinary_muscle_development	ENSGALG00010023920	ENSGALG00010023920	0.300424439	0.382462485	0.785500409	0.43216023	0.470070729	positive
PRJNA729271	stage_1W_vs_E10	ordinary_muscle_development	ENSGALG00010023921	GRK7	1.111422244	0.179099343	6.205618753	5.44821E-10	1.24189E-09	positive
PRJNA729271	stage_1W_vs_E10	ordinary_muscle_development	ENSGALG00010023923	CSNK1G1	-0.882849096	0.0993583	-8.885509259	6.36286E-19	2.27671E-18	negative
PRJNA729271	stage_1W_vs_E10	ordinary_muscle_development	ENSGALG00010023925	NCOA3	-0.786609165	0.078161597	-10.06388294	7.97879E-24	3.49404E-23	negative
PRJNA729271	stage_1W_vs_E10	ordinary_muscle_development	ENSGALG00010023926	ZYX	-0.921945825	0.14140849	-6.519734613	7.04319E-11	1.68439E-10	negative
PRJNA729271	stage_1W_vs_E10	ordinary_muscle_development	ENSGALG00010023927	KPNB1	-0.076476062	0.130189231	-0.587422333	0.556920098	0.592829318	negative
PRJNA729271	stage_1W_vs_E10	ordinary_muscle_development	ENSGALG00010023930	SRPRA	0.966589772	0.131018707	7.377494382	1.61296E-13	4.4322E-13	positive
PRJNA729271	stage_1W_vs_E10	ordinary_muscle_development	ENSGALG00010023931	CSRNP2	-1.599857557	0.154309951	-10.36781845	3.47364E-25	1.61121E-24	negative
PRJNA729271	stage_1W_vs_E10	ordinary_muscle_development	ENSGALG00010023937	XBP1	-0.485043876	0.110571732	-4.386689667	1.15089E-05	1.98952E-05	negative
PRJNA729271	stage_1W_vs_E10	ordinary_muscle_development	ENSGALG00010023940	ATP1B3	0.176289528	0.071618146	2.461520411	0.013834953	0.018428381	positive
PRJNA729271	stage_1W_vs_E10	ordinary_muscle_development	ENSGALG00010023942	NEGR1	1.564352031	0.220843072	7.083545889	1.40512E-12	3.69305E-12	positive
PRJNA729271	stage_1W_vs_E10	ordinary_muscle_development	ENSGALG00010023945	LETMD1	0.82186498	0.210741347	3.899875331	9.62422E-05	0.000155038	positive
PRJNA729271	stage_1W_vs_E10	ordinary_muscle_development	ENSGALG00010023946	ZNRF3	-0.542106338	0.127067716	-4.266279096	1.9876E-05	3.36687E-05	negative
PRJNA729271	stage_1W_vs_E10	ordinary_muscle_development	ENSGALG00010023947	ENSGALG00010023947	-9.46659817	0.943980893	-10.028379	1.14379E-23	4.97725E-23	negative
PRJNA729271	stage_1W_vs_E10	ordinary_muscle_development	ENSGALG00010023948	SULF2	-1.738409417	0.221122882	-7.861734608	3.7885E-15	1.13459E-14	negative
PRJNA729271	stage_1W_vs_E10	ordinary_muscle_development	ENSGALG00010023953	SLC35A3	0.46107113	0.154691853	2.980577983	0.00287705	0.004107035	positive
PRJNA729271	stage_1W_vs_E10	ordinary_muscle_development	ENSGALG00010023954	PLEKHF1	0.433603474	0.096143032	4.509983351	6.48327E-06	1.1384E-05	positive
PRJNA729271	stage_1W_vs_E10	ordinary_muscle_development	ENSGALG00010023956	KREMEN1	-1.811800937	0.132079733	-13.71747878	7.97921E-43	6.99689E-42	negative
PRJNA729271	stage_1W_vs_E10	ordinary_muscle_development	ENSGALG00010023957	YBX3	2.961521809	0.138797628	21.33697702	5.152E-101	1.9704E-99	positive
PRJNA729271	stage_1W_vs_E10	ordinary_muscle_development	ENSGALG00010023962	FPGT	-1.341457236	0.118678314	-11.3033055	1.2637E-29	7.02352E-29	negative
PRJNA729271	stage_1W_vs_E10	ordinary_muscle_development	ENSGALG00010023966	PEAK1	0.668481969	0.098169149	6.809491347	9.79444E-12	2.46519E-11	positive
PRJNA729271	stage_1W_vs_E10	ordinary_muscle_development	ENSGALG00010023967	PRPH	-2.85851603	0.368175386	-7.764006328	8.22878E-15	2.42185E-14	negative
PRJNA729271	stage_1W_vs_E10	ordinary_muscle_development	ENSGALG00010023968	TYW5	0.691958915	0.113399374	6.101964167	1.04773E-09	2.35528E-09	positive
PRJNA729271	stage_1W_vs_E10	ordinary_muscle_development	ENSGALG00010023972	ENSGALG00010023972	2.143727461	0.509111457	4.210723274	2.54554E-05	4.2765E-05	positive
PRJNA729271	stage_1W_vs_E10	ordinary_muscle_development	ENSGALG00010023974	PREX1	-0.675281484	0.103968464	-6.49506071	8.29999E-11	1.97941E-10	negative
PRJNA729271	stage_1W_vs_E10	ordinary_muscle_development	ENSGALG00010023978	TFDP2	0.170834232	0.139834873	1.221685469	0.221826593	0.25323907	positive
PRJNA729271	stage_1W_vs_E10	ordinary_muscle_development	ENSGALG00010023979	FAM118B	-0.651370452	0.116958213	-5.56925791	2.55827E-08	5.29744E-08	negative
PRJNA729271	stage_1W_vs_E10	ordinary_muscle_development	ENSGALG00010023981	ENSGALG00010023981	1.156787976	0.365573766	3.164307958	0.001554522	0.002274891	positive
PRJNA729271	stage_1W_vs_E10	ordinary_muscle_development	ENSGALG00010023984	FMNL1	2.093285209	0.202846924	10.31953145	5.75031E-25	2.63935E-24	positive
PRJNA729271	stage_1W_vs_E10	ordinary_muscle_development	ENSGALG00010023986	SYCE3	-0.311753401	0.619327754	-0.503373858	0.614701448	0.648856502	negative
PRJNA729271	stage_1W_vs_E10	ordinary_muscle_development	ENSGALG00010023987	TNNI3K	-1.962073182	0.478526061	-4.100243104	4.12716E-05	6.83304E-05	negative
PRJNA729271	stage_1W_vs_E10	ordinary_muscle_development	ENSGALG00010023989	ENSGALG00010023989	-0.036480777	0.100645511	-0.362467999	0.717002333	0.744210262	negative
PRJNA729271	stage_1W_vs_E10	ordinary_muscle_development	ENSGALG00010023991	TSPAN3	-0.204320792	0.106425858	-1.919841635	0.054877906	0.068162403	negative
PRJNA729271	stage_1W_vs_E10	ordinary_muscle_development	ENSGALG00010023992	FAM131B	-2.113477373	0.149396507	-14.14676561	1.95546E-45	1.85743E-44	negative
PRJNA729271	stage_1W_vs_E10	ordinary_muscle_development	ENSGALG00010023993	C19orf12	-0.549002724	0.176013995	-3.119085634	0.001814132	0.002639085	negative
PRJNA729271	stage_1W_vs_E10	ordinary_muscle_development	ENSGALG00010023996	MYO7B	3.841561662	0.473580605	8.111737724	4.99009E-16	1.55551E-15	positive
PRJNA729271	stage_1W_vs_E10	ordinary_muscle_development	ENSGALG00010023997	ARFGEF2	-0.240112293	0.063355543	-3.789917692	0.000150697	0.000239381	negative
PRJNA729271	stage_1W_vs_E10	ordinary_muscle_development	ENSGALG00010023999	DGCR14	0.090574456	0.092206425	0.982300922	0.325951621	0.362404598	positive
PRJNA729271	stage_1W_vs_E10	ordinary_muscle_development	ENSGALG00010024001	ENSGALG00010024001	5.989012008	0.448600759	13.35042771	1.17771E-40	9.62282E-40	positive
PRJNA729271	stage_1W_vs_E10	ordinary_muscle_development	ENSGALG00010024002	DGCR2	-0.931994887	0.061922103	-15.05108578	3.39614E-51	3.87005E-50	negative
PRJNA729271	stage_1W_vs_E10	ordinary_muscle_development	ENSGALG00010024003	ERICH3	-1.484914542	0.308864406	-4.807658352	1.52708E-06	2.81331E-06	negative
PRJNA729271	stage_1W_vs_E10	ordinary_muscle_development	ENSGALG00010024004	EIF4B	0.564998003	0.125095302	4.516540532	6.28581E-06	1.10507E-05	positive
PRJNA729271	stage_1W_vs_E10	ordinary_muscle_development	ENSGALG00010024005	STYK1	4.86466541	0.249090234	19.5297316	6.13611E-85	1.64709E-83	positive
PRJNA729271	stage_1W_vs_E10	ordinary_muscle_development	ENSGALG00010024008	CCNE1	0.239472628	0.102983143	2.325357547	0.02005285	0.02620738	positive
PRJNA729271	stage_1W_vs_E10	ordinary_muscle_development	ENSGALG00010024009	CSE1L	-0.953364353	0.061602413	-15.4760877	5.03156E-54	6.31997E-53	negative
PRJNA729271	stage_1W_vs_E10	ordinary_muscle_development	ENSGALG00010024010	ENSGALG00010024010	1.749939849	0.08835332	19.80615827	2.63434E-87	7.41452E-86	positive
PRJNA729271	stage_1W_vs_E10	ordinary_muscle_development	ENSGALG00010024011	AGPS	0.845234964	0.06972068	12.12316011	7.96285E-34	5.18981E-33	positive
PRJNA729271	stage_1W_vs_E10	ordinary_muscle_development	ENSGALG00010024013	PTPRJ	-0.649380272	0.123105852	-5.274974853	1.32774E-07	2.62954E-07	negative
PRJNA729271	stage_1W_vs_E10	ordinary_muscle_development	ENSGALG00010024016	CRYZ	0.155925632	0.103453109	1.507210685	0.131756662	0.155750515	positive
PRJNA729271	stage_1W_vs_E10	ordinary_muscle_development	ENSGALG00010024017	TYW3	-1.756862138	0.138796215	-12.65785333	1.0123E-36	7.2568E-36	negative
PRJNA729271	stage_1W_vs_E10	ordinary_muscle_development	ENSGALG00010024018	KRT18	-4.298036468	0.970477258	-4.428786385	9.47648E-06	1.64735E-05	negative
PRJNA729271	stage_1W_vs_E10	ordinary_muscle_development	ENSGALG00010024019	MAP3K14	1.166777572	0.106054438	11.00168544	3.75055E-28	1.96616E-27	positive
PRJNA729271	stage_1W_vs_E10	ordinary_muscle_development	ENSGALG00010024020	MAIP1	0.289742453	0.122067593	2.373623056	0.017614522	0.023141712	positive
PRJNA729271	stage_1W_vs_E10	ordinary_muscle_development	ENSGALG00010024021	IGLL1	7.39641592	1.21720179	6.07657332	1.22778E-09	2.75192E-09	positive
PRJNA729271	stage_1W_vs_E10	ordinary_muscle_development	ENSGALG00010024023	LHX8	-5.928169466	0.6827486	-8.682799881	3.86143E-18	1.32227E-17	negative
PRJNA729271	stage_1W_vs_E10	ordinary_muscle_development	ENSGALG00010024024	AGL	5.430019196	0.122580195	44.29768777	0	0	positive
PRJNA729271	stage_1W_vs_E10	ordinary_muscle_development	ENSGALG00010024025	CLCN1	8.407712933	0.333071018	25.24300364	1.3517E-140	9.7967E-139	positive
PRJNA729271	stage_1W_vs_E10	ordinary_muscle_development	ENSGALG00010024026	TIRAP	0.646312139	0.103802153	6.226384713	4.77321E-10	1.09181E-09	positive
PRJNA729271	stage_1W_vs_E10	ordinary_muscle_development	ENSGALG00010024029	SCAPER	0.084159516	0.147305863	0.571324954	0.567779389	0.60345815	positive
PRJNA729271	stage_1W_vs_E10	ordinary_muscle_development	ENSGALG00010024030	STAU1	0.360059365	0.089546777	4.020908151	5.79742E-05	9.49425E-05	positive
PRJNA729271	stage_1W_vs_E10	ordinary_muscle_development	ENSGALG00010024031	LIMS2	0.668188963	0.115209369	5.799779739	6.64021E-09	1.42466E-08	positive
PRJNA729271	stage_1W_vs_E10	ordinary_muscle_development	ENSGALG00010024034	LRP2	-1.068152608	0.248729603	-4.294432976	1.7514E-05	2.9821E-05	negative
PRJNA729271	stage_1W_vs_E10	ordinary_muscle_development	ENSGALG00010024035	OSBPL5	-1.222026268	0.138049772	-8.852070142	8.59107E-19	3.04245E-18	negative
PRJNA729271	stage_1W_vs_E10	ordinary_muscle_development	ENSGALG00010024038	NKD1	-3.189993055	0.171371904	-18.61444604	2.45385E-77	5.42203E-76	negative
PRJNA729271	stage_1W_vs_E10	ordinary_muscle_development	ENSGALG00010024039	MRPL45	0.01261796	0.106687063	0.118270762	0.905853123	0.917115389	positive
PRJNA729271	stage_1W_vs_E10	ordinary_muscle_development	ENSGALG00010024040	ENSGALG00010024040	-4.442157568	0.853735519	-5.203201075	1.95885E-07	3.838E-07	negative
PRJNA729271	stage_1W_vs_E10	ordinary_muscle_development	ENSGALG00010024042	DCPS	-0.091428409	0.130249188	-0.701949934	0.482710387	0.520950566	negative
PRJNA729271	stage_1W_vs_E10	ordinary_muscle_development	ENSGALG00010024045	PSTPIP1	1.16636998	0.249012736	4.683977211	2.81361E-06	5.08584E-06	positive
PRJNA729271	stage_1W_vs_E10	ordinary_muscle_development	ENSGALG00010024046	ENSGALG00010024046	-8.460867458	0.986823518	-8.573840511	1.0009E-17	3.35524E-17	negative
PRJNA729271	stage_1W_vs_E10	ordinary_muscle_development	ENSGALG00010024049	RCN2	-1.062233048	0.144361714	-7.358135469	1.86497E-13	5.11015E-13	negative
PRJNA729271	stage_1W_vs_E10	ordinary_muscle_development	ENSGALG00010024051	ENSGALG00010024051	-6.289416122	0.73496717	-8.557410963	1.1543E-17	3.85433E-17	negative
PRJNA729271	stage_1W_vs_E10	ordinary_muscle_development	ENSGALG00010024052	ENSGALG00010024052	0.336484256	0.168526471	1.996625545	0.045865876	0.057535774	positive
PRJNA729271	stage_1W_vs_E10	ordinary_muscle_development	ENSGALG00010024053	ISL2	-0.570533741	0.535563915	-1.065295336	0.286742303	0.321498273	negative
PRJNA729271	stage_1W_vs_E10	ordinary_muscle_development	ENSGALG00010024054	ENSGALG00010024054	-5.636036675	1.032516126	-5.458545908	4.8005E-08	9.80183E-08	negative
PRJNA729271	stage_1W_vs_E10	ordinary_muscle_development	ENSGALG00010024057	HSD3B1	-2.323502423	0.773017233	-3.0057576	0.002649201	0.003794108	negative
PRJNA729271	stage_1W_vs_E10	ordinary_muscle_development	ENSGALG00010024058	SLC44A5	0.577887289	0.105106034	5.498136209	3.83826E-08	7.8726E-08	positive
PRJNA729271	stage_1W_vs_E10	ordinary_muscle_development	ENSGALG00010024060	FRRS1	0.793498246	0.161286474	4.919806506	8.66298E-07	1.62824E-06	positive
PRJNA729271	stage_1W_vs_E10	ordinary_muscle_development	ENSGALG00010024061	ENSGALG00010024061	-1.859363316	1.453496704	-1.279234628	0.200814445	0.231138971	negative
PRJNA729271	stage_1W_vs_E10	ordinary_muscle_development	ENSGALG00010024062	ST3GAL4	-0.457882544	0.134773548	-3.397421456	0.000680241	0.001027736	negative
PRJNA729271	stage_1W_vs_E10	ordinary_muscle_development	ENSGALG00010024063	BRD7	0.280478332	0.103891238	2.699730373	0.006939569	0.009547177	positive
PRJNA729271	stage_1W_vs_E10	ordinary_muscle_development	ENSGALG00010024064	GPR17	-1.606588751	0.226770043	-7.084660427	1.39386E-12	3.66478E-12	negative
PRJNA729271	stage_1W_vs_E10	ordinary_muscle_development	ENSGALG00010024066	ENSGALG00010024066	1.134981266	2.266538334	0.50075538	0.616543291	0.650705913	positive
PRJNA729271	stage_1W_vs_E10	ordinary_muscle_development	ENSGALG00010024068	CASP2	-1.673510619	0.088372905	-18.93691984	5.66137E-80	1.34527E-78	negative
PRJNA729271	stage_1W_vs_E10	ordinary_muscle_development	ENSGALG00010024069	ENSGALG00010024069	1.134969647	2.364835876	0.479935905	0.631272969	0.664338838	positive
PRJNA729271	stage_1W_vs_E10	ordinary_muscle_development	ENSGALG00010024073	RABGGTB	0.450766124	0.124943757	3.607752273	0.000308861	0.000479741	positive
PRJNA729271	stage_1W_vs_E10	ordinary_muscle_development	ENSGALG00010024074	ADCY7	-1.014401468	0.22899401	-4.429816602	9.43133E-06	1.63996E-05	negative
PRJNA729271	stage_1W_vs_E10	ordinary_muscle_development	ENSGALG00010024075	SPATS2L	-0.560055282	0.137380574	-4.076670118	4.56852E-05	7.54051E-05	negative

PRJNA729271	stage_1W_vs_E10	ordinary_muscle_development	ENSGALG00010024077	SFT2D3	0.807537097	0.114552679	7.049482444	1.79584E-12	4.68938E-12	positive
PRJNA729271	stage_1W_vs_E10	ordinary_muscle_development	ENSGALG00010024078	KIRREL3	-2.171240649	0.253641778	-8.560264279	1.12609E-17	3.76446E-17	negative
PRJNA729271	stage_1W_vs_E10	ordinary_muscle_development	ENSGALG00010024080	TENT4B	0.203017865	0.087395465	2.322979408	0.020180259	0.026357258	positive
PRJNA729271	stage_1W_vs_E10	ordinary_muscle_development	ENSGALG00010024081	CARS	-0.021527454	0.098831576	-0.217819593	0.827569678	0.846132643	negative
PRJNA729271	stage_1W_vs_E10	ordinary_muscle_development	ENSGALG00010024083	PALMD	-0.328923441	0.19053307	-1.72633255	0.084287603	0.10232402	negative
PRJNA729271	stage_1W_vs_E10	ordinary_muscle_development	ENSGALG00010024086	GABARAPL1	0.277428143	0.215031254	1.290175909	0.196989589	0.226922363	positive
PRJNA729271	stage_1W_vs_E10	ordinary_muscle_development	ENSGALG00010024089	ENSGALG00010024089	1.782854403	1.936858367	0.920487752	0.357317931	0.394946119	positive
PRJNA729271	stage_1W_vs_E10	ordinary_muscle_development	ENSGALG00010024091	ETFA	-0.324152474	0.116847453	-2.774150949	0.005534599	0.00769166	negative
PRJNA729271	stage_1W_vs_E10	ordinary_muscle_development	ENSGALG00010024095	RBM12	-1.558254417	0.054163612	-28.76939652	5.1815E-182	6.4193E-180	negative
PRJNA729271	stage_1W_vs_E10	ordinary_muscle_development	ENSGALG00010024096	MYO1D	-0.480332962	0.221611828	-2.167451827	0.030200419	0.038688031	negative
PRJNA729271	stage_1W_vs_E10	ordinary_muscle_development	ENSGALG00010024099	ENSGALG00010024099	1.134988509	2.081202727	0.545352211	0.585511301	0.620802159	positive
PRJNA729271	stage_1W_vs_E10	ordinary_muscle_development	ENSGALG00010024101	CNEP1R1	0.010163893	0.090387099	0.112448497	0.910467804	0.92120835	positive
PRJNA729271	stage_1W_vs_E10	ordinary_muscle_development	ENSGALG00010024102	SGF29	0.677370454	0.089894761	7.535149382	4.87776E-14	1.38038E-13	positive
PRJNA729271	stage_1W_vs_E10	ordinary_muscle_development	ENSGALG00010024104	PLPPR5	-0.677708775	0.166075242	-4.080733322	4.48938E-05	7.4158E-05	negative
PRJNA729271	stage_1W_vs_E10	ordinary_muscle_development	ENSGALG00010024106	WDR33	-0.184177449	0.064092883	-2.873602204	0.004058197	0.005708809	negative
PRJNA729271	stage_1W_vs_E10	ordinary_muscle_development	ENSGALG00010024107	NFE2L2	1.632948068	0.062473023	26.13845108	1.3335E-150	1.1238E-148	positive
PRJNA729271	stage_1W_vs_E10	ordinary_muscle_development	ENSGALG00010024110	CPNE1	-1.137767931	0.127026335	-8.956945259	3.33797E-19	1.2093E-18	negative
PRJNA729271	stage_1W_vs_E10	ordinary_muscle_development	ENSGALG00010024112	NAP1L4	0.44205086	0.090509262	4.884040033	1.03934E-06	1.94115E-06	positive
PRJNA729271	stage_1W_vs_E10	ordinary_muscle_development	ENSGALG00010024113	ENSGALG00010024113	1.474039905	0.162451841	9.073703908	1.15038E-19	4.25921E-19	positive
PRJNA729271	stage_1W_vs_E10	ordinary_muscle_development	ENSGALG00010024115	POLR2D	-0.552952445	0.074147063	-7.457509782	8.8173E-14	2.4583E-13	negative
PRJNA729271	stage_1W_vs_E10	ordinary_muscle_development	ENSGALG00010024116	MCAM	0.950588107	0.182626813	5.205085115	1.93908E-07	3.8008E-07	positive
PRJNA729271	stage_1W_vs_E10	ordinary_muscle_development	ENSGALG00010024118	TMEM266	-1.631487867	0.146410489	-11.14324446	7.72527E-29	4.14886E-28	negative
PRJNA729271	stage_1W_vs_E10	ordinary_muscle_development	ENSGALG00010024119	AMMECR1L	-0.839012551	0.076043465	-11.03332872	2.63912E-28	1.39156E-27	negative
PRJNA729271	stage_1W_vs_E10	ordinary_muscle_development	ENSGALG00010024121	NRG4	0.321829065	0.169662639	1.896876452	0.057844246	0.071687102	positive
PRJNA729271	stage_1W_vs_E10	ordinary_muscle_development	ENSGALG00010024122	SOCS7	0.831156723	0.104272537	7.971003171	1.57391E-15	4.79586E-15	positive
PRJNA729271	stage_1W_vs_E10	ordinary_muscle_development	ENSGALG00010024123	PLPPR4	0.455971325	0.381920339	1.19389118	0.232520538	0.264343937	positive
PRJNA729271	stage_1W_vs_E10	ordinary_muscle_development	ENSGALG00010024124	DIDO1	-0.016382172	0.067039423	-0.244366242	0.806947185	0.827382008	negative
PRJNA729271	stage_1W_vs_E10	ordinary_muscle_development	ENSGALG00010024125	ENSGALG00010024125	-2.96377875	1.676827863	-1.767491354	0.077145976	0.094048017	negative
PRJNA729271	stage_1W_vs_E10	ordinary_muscle_development	ENSGALG00010024126	CDK5R1	-1.221935957	0.296007847	-4.128052585	3.65848E-05	6.08837E-05	negative
PRJNA729271	stage_1W_vs_E10	ordinary_muscle_development	ENSGALG00010024128	PHLDA2	-2.07666029	0.235550508	-8.816199594	1.1841E-18	4.16289E-18	negative
PRJNA729271	stage_1W_vs_E10	ordinary_muscle_development	ENSGALG00010024129	GID8	-0.121450833	0.101472106	-1.196888865	0.231349875	0.263095594	negative
PRJNA729271	stage_1W_vs_E10	ordinary_muscle_development	ENSGALG00010024131	ENSGALG00010024131	-0.279589704	1.302045091	-0.214731199	0.829976897	0.84847416	negative
PRJNA729271	stage_1W_vs_E10	ordinary_muscle_development	ENSGALG00010024132	ENSGALG00010024132	-1.560952706	0.152910567	-10.20827231	1.82078E-24	8.18109E-24	negative
PRJNA729271	stage_1W_vs_E10	ordinary_muscle_development	ENSGALG00010024133	PSMD11	1.1563915	0.140515037	8.229663692	1.8774E-16	5.95468E-16	positive
PRJNA729271	stage_1W_vs_E10	ordinary_muscle_development	ENSGALG00010024135	FBXO22	0.918626617	0.087224196	10.53178657	6.16531E-26	2.94549E-25	positive
PRJNA729271	stage_1W_vs_E10	ordinary_muscle_development	ENSGALG00010024136	SAP130	0.080409643	0.174880964	0.459796428	0.645662347	0.67734499	positive
PRJNA729271	stage_1W_vs_E10	ordinary_muscle_development	ENSGALG00010024137	ENSGALG00010024137	2.139028382	0.717516075	2.981157435			positive
PRJNA729271	stage_1W_vs_E10	ordinary_muscle_development	ENSGALG00010024138	CBL	0.278152232	0.15040913	1.849304172	0.064413904	0.079293379	positive
PRJNA729271	stage_1W_vs_E10	ordinary_muscle_development	ENSGALG00010024139	SLC22A18	-3.571815583	0.960788098	-3.717589331	0.000201133	0.000316378	negative
PRJNA729271	stage_1W_vs_E10	ordinary_muscle_development	ENSGALG00010024140	KRT6A	-5.893883264	1.054095903	-5.591410844	2.25232E-08	4.67861E-08	negative
PRJNA729271	stage_1W_vs_E10	ordinary_muscle_development	ENSGALG00010024141	YTHDF1	-0.211030027	0.095761083	-2.203713876	0.027544473	0.035439121	negative
PRJNA729271	stage_1W_vs_E10	ordinary_muscle_development	ENSGALG00010024142	DNAJC6	-2.267419587	0.281105676	-8.066075422	7.2594E-16	2.24865E-15	negative
PRJNA729271	stage_1W_vs_E10	ordinary_muscle_development	ENSGALG00010024143	UBE2Q2	2.585219326	0.116373091	22.21492359	2.464E-109	1.0989E-107	positive
PRJNA729271	stage_1W_vs_E10	ordinary_muscle_development	ENSGALG00010024144	HNRNPA3	-1.830965246	0.127000892	-14.41694788	4.04854E-47	4.03323E-46	negative
PRJNA729271	stage_1W_vs_E10	ordinary_muscle_development	ENSGALG00010024145	RAP1GAP3	-0.682684891	0.188176693	-3.62789291	0.000285744	0.000444835	negative
PRJNA729271	stage_1W_vs_E10	ordinary_muscle_development	ENSGALG00010024146	CDKN1C	-1.427108049	0.241584802	-5.907275772	3.47811E-09	7.58809E-09	negative
PRJNA729271	stage_1W_vs_E10	ordinary_muscle_development	ENSGALG00010024147	ENSGALG00010024147	-0.693068358	0.285046295	-2.431423843	0.01503961	0.019921333	negative
PRJNA729271	stage_1W_vs_E10	ordinary_muscle_development	ENSGALG00010024148	UGGT1	-0.569053225	0.058946288	-9.653758459	4.73864E-22	1.92777E-21	negative
PRJNA729271	stage_1W_vs_E10	ordinary_muscle_development	ENSGALG00010024149	TCIRG1	0.64843746	0.153959336	4.211744965	2.53406E-05	4.25967E-05	positive
PRJNA729271	stage_1W_vs_E10	ordinary_muscle_development	ENSGALG00010024150	NHERF4	1.368624119	0.354000092	3.866168821	0.000110558	0.000177214	positive
PRJNA729271	stage_1W_vs_E10	ordinary_muscle_development	ENSGALG00010024151	LEPROT	-0.845636718	0.148449181	-5.696472762	1.22311E-08	2.58027E-08	negative
PRJNA729271	stage_1W_vs_E10	ordinary_muscle_development	ENSGALG00010024152	ZNF423	-1.274001745	0.108129574	-11.7821767	4.82312E-32	2.94984E-31	negative
PRJNA729271	stage_1W_vs_E10	ordinary_muscle_development	ENSGALG00010024153	KCNQ1	1.960514928	0.201731996	9.718413399	2.51672E-22	1.03313E-21	positive
PRJNA729271	stage_1W_vs_E10	ordinary_muscle_development	ENSGALG00010024154	VPS29L	0.301917347	0.501496376	0.602032958	0.547152199	0.583288308	positive
PRJNA729271	stage_1W_vs_E10	ordinary_muscle_development	ENSGALG00010024155	SNX7	-1.245235467	0.167012504	-7.455491545	8.92283E-14	2.48724E-13	negative
PRJNA729271	stage_1W_vs_E10	ordinary_muscle_development	ENSGALG00010024157	CHRNB4	-3.150637872	0.406239759	-7.755611809	8.79186E-15	2.58495E-14	negative
PRJNA729271	stage_1W_vs_E10	ordinary_muscle_development	ENSGALG00010024158	ENSGALG00010024158	0.382287262	0.342600906	1.115838447	0.264491293	0.298238534	positive
PRJNA729271	stage_1W_vs_E10	ordinary_muscle_development	ENSGALG00010024159	NLRX1	0.344512821	0.163369597	2.108793966	0.03496237	0.044458246	positive
PRJNA729271	stage_1W_vs_E10	ordinary_muscle_development	ENSGALG00010024162	SRCIN1	-2.990414245	0.506215821	-5.907389937	3.4757E-09	7.58397E-09	negative
PRJNA729271	stage_1W_vs_E10	ordinary_muscle_development	ENSGALG00010024163	HS6ST1	-1.35880345	0.098236898	-13.83190503	1.63629E-43	1.46137E-42	negative
PRJNA729271	stage_1W_vs_E10	ordinary_muscle_development	ENSGALG00010024164	SNAP29	1.381686453	0.112186043	12.3160281	7.42627E-35	5.0207E-34	positive
PRJNA729271	stage_1W_vs_E10	ordinary_muscle_development	ENSGALG00010024165	LEPR	-1.367741793	0.282972274	-4.833483419	1.34164E-06	2.48209E-06	negative
PRJNA729271	stage_1W_vs_E10	ordinary_muscle_development	ENSGALG00010024166	ENSGALG00010024166	5.039264418	0.899190892	5.604220934	2.09194E-08	4.3567E-08	positive
PRJNA729271	stage_1W_vs_E10	ordinary_muscle_development	ENSGALG00010024167	CHRNA3	0.881891235	0.443494629	1.98850488	0.04675588	0.058593675	positive
PRJNA729271	stage_1W_vs_E10	ordinary_muscle_development	ENSGALG00010024168	BIRC7	-2.567095624	0.471411877	-5.445547193	5.16464E-08	1.0532E-07	negative
PRJNA729271	stage_1W_vs_E10	ordinary_muscle_development	ENSGALG00010024169	FAM168B	-1.152928876	0.073056954	-15.78123385	4.18918E-56	5.56574E-55	negative
PRJNA729271	stage_1W_vs_E10	ordinary_muscle_development	ENSGALG00010024170	NUP160	-0.905000421	0.086888163	-10.41569288	2.10261E-25	9.83139E-25	negative
PRJNA729271	stage_1W_vs_E10	ordinary_muscle_development	ENSGALG00010024171	ENSGALG00010024171	-2.328240174	0.641986163	-3.626620491	0.000287155	0.000446887	negative
PRJNA729271	stage_1W_vs_E10	ordinary_muscle_development	ENSGALG00010024172	ABCG4	-4.955319295	0.558145554	-8.878184652	6.79604E-19	2.42512E-18	negative
PRJNA729271	stage_1W_vs_E10	ordinary_muscle_development	ENSGALG00010024173	CBLN1	-1.035788544	0.412165664	-2.513039377	0.011969597	0.016038021	negative
PRJNA729271	stage_1W_vs_E10	ordinary_muscle_development	ENSGALG00010024174	PLEKHB2	0.350353733	0.101851141	3.439860646	0.000582014	0.000883289	positive
PRJNA729271	stage_1W_vs_E10	ordinary_muscle_development	ENSGALG00010024175	DHRS9	4.509485248	0.401905753	11.22025553	3.24332E-29	1.76936E-28	positive
PRJNA729271	stage_1W_vs_E10	ordinary_muscle_development	ENSGALG00010024177	ENSGALG00010024177	0.216469574	0.445469226	0.48593609	0.627012479	0.660504788	positive
PRJNA729271	stage_1W_vs_E10	ordinary_muscle_development	ENSGALG00010024178	N4BP1	1.441017191	0.120275988	11.98092169	4.47323E-33	2.83513E-32	positive
PRJNA729271	stage_1W_vs_E10	ordinary_muscle_development	ENSGALG00010024179	ATG4C	-0.57713148	0.117961021	-4.8925609	9.95323E-07	1.8623E-06	negative
PRJNA729271	stage_1W_vs_E10	ordinary_muscle_development	ENSGALG00010024180	HINFP	0.935707176	0.13816274	6.77250013	1.26576E-11	3.16276E-11	positive
PRJNA729271	stage_1W_vs_E10	ordinary_muscle_development	ENSGALG00010024181	FOXD3	2.503585938	0.312774533	8.004443055	1.20009E-15	3.6792E-15	positive
PRJNA729271	stage_1W_vs_E10	ordinary_muscle_development	ENSGALG00010024183	MTX2	0.699163654	0.106255889	6.579999098	4.70451E-11	1.1411E-10	positive
PRJNA729271	stage_1W_vs_E10	ordinary_muscle_development	ENSGALG00010024184	ENSGALG00010024184	-3.35992902	0.226908348	-14.80742798	1.31164E-49	1.41565E-48	negative
PRJNA729271	stage_1W_vs_E10	ordinary_muscle_development	ENSGALG00010024185	CHRNA5	0.432695752	0.155313902	2.785943475	0.005337218	0.00743303	positive
PRJNA729271	stage_1W_vs_E10	ordinary_muscle_development	ENSGALG00010024186	HAO2	-0.540086755	0.70907835	-0.7616743	0.446254418	0.48434727	negative
PRJNA729271	stage_1W_vs_E10	ordinary_muscle_development	ENSGALG00010024188	TSSC4	-1.532626461	0.085841963	-17.85404721	2.68829E-71	5.25868E-70	negative
PRJNA729271	stage_1W_vs_E10	ordinary_muscle_development	ENSGALG00010024189	KAT7	0.171789371	0.140233866	1.225020577	0.220567468	0.252000114	positive
PRJNA729271	stage_1W_vs_E10	ordinary_muscle_development	ENSGALG00010024190	WARS2	-1.266766557	0.114088811	-11.10333739	1.20845E-28	6.43281E-28	negative
PRJNA729271	stage_1W_vs_E10	ordinary_muscle_development	ENSGALG00010024191	DPYD	-0.675949072	0.111692238	-6.051889403	1.43157E-09	3.19633E-09	negative
PRJNA729271	stage_1W_vs_E10	ordinary_muscle_development	ENSGALG00010024192	ALG6	-1.00124262	0.097323021	-10.28782919	7.99587E-25	3.6458E-24	negative
PRJNA729271	stage_1W_vs_E10	ordinary_muscle_development	ENSGALG00010024193	GSTK1	0.437640715	0.126459941	3.460706308	0.00053876	0.000819705	positive
PRJNA729271	stage_1W_vs_E10	ordinary_muscle_development	ENSGALG00010024194	NKAIN4	-0.730728611	0.086884751	-8.410320562	4.08892E-17	1.33158E-16	negative
PRJNA729271	stage_1W_vs_E10	ordinary_muscle_development	ENSGALG00010024195	PSMA4	0.881674579	0.120515271	7.315874344	2.55711E-13	6.958E-13	positive
PRJNA729271	stage_1W_vs_E10	ordinary_muscle_development	ENSGALG00010024196	MRPL21	0.309199081	0.109195317	2.831614852	0.00463136	0.006485514	positive
PRJNA729271	stage_1W_vs_E10	ordinary_muscle_development	ENSGALG00010024197	KRT7	-4.342808528	0.310296741	-13.9956627	1.65678E-44	1.5267E-43	negative
PRJNA729271	stage_1W_vs_E10	ordinary_muscle_development	ENSGALG00010024198	PI4KA	0.339667478	0.064363591	5.277323321	1.31084E-07	2.59714E-07	positive
PRJNA729271	stage_1W_vs_E10	ordinary_muscle_development	ENSGALG00010024199	SLC35B1	-0.065360733	0.101750513	-0.642362685	0.520637722	0.557688722	negative
PRJNA729271	stage_1W_vs_E10	ordinary_muscle_development	ENSGALG00010024200	GCHFR	-3.799417607	0.805962874	-4.714134766	2.4274E-06	4.40696E-06	negative
PRJNA729271	stage_1W_vs_E10	ordinary_muscle_development	ENSGALG00010024201	IREB2	-0.002781907	0.069622826	-0.03995682	0.968127551	0.972488486	negative
PRJNA729271	stage_1W_vs_E10	ordinary_muscle_development	ENSGALG00010024203	SPOP	-0.010876799	0.07297183	-0.149054768	0.881510417	0.894899322	negative
PRJNA729271	stage_1W_vs_E10	ordinary_muscle_development	ENSGALG00010024204	PTBP2	0.10340021	0.108429846	0.953613914	0.34027911	0.377350294	positive
PRJNA729271	stage_1W_vs_E10	ordinary_muscle_development	ENSGALG00010024205	ANKRD13D	0.773180459	0.16802651	4.601538513	4.19382E-06	7.47809E-06	positive
PRJNA729271	stage_1W_vs_E10	ordinary_muscle_development	ENSGALG00010024206	KRT80	-5.015624644	0.923161185	-5.433097413	5.53842E-08	1.12704E-07	negative
PRJNA729271	stage_1W_vs_E10	ordinary_muscle_development	ENSGALG00010024207	KCTD18	-0.935264146	0.126819193	-7.374783951	1.64612E-13	4.52246E-13	negative
PRJNA729271	stage_1W_vs_E10	ordinary_muscle_development	ENSGALG00010024208	SERPIND1	2.759288139	0.579664111	4.760150037	1.93449E-06	3.53957E-06	positive
PRJNA729271	stage_1W_vs_E10	ordinary_muscle_development	ENSGALG00010024209	RWDD3	-0.292540722	0.144143238	-2.029514015	0.042405963	0.053422078	negative
PRJNA729271	stage_1W_vs_E10	ordinary_muscle_development	ENSGALG00010024210	NXPH3	-3.834884004	1.247172595	-3.074862308	0.002105998	0.003044424	negative
PRJNA729271	stage_1W_vs_E10	ordinary_muscle_development	ENSGALG00010024211	TRPM5	0.470529261	0.733582995	0.641412443	0.521254751	0.558267352	positive
PRJNA729271	stage_1W_vs_E10	ordinary_muscle_development	ENSGALG00010024212	RIMS4	-0.160843024	0.20046749	-0.802339688	0.42235649	0.460305062	negative
PRJNA729271	stage_1W_vs_E10	ordinary_muscle_development	ENSGALG00010024213	ENSGALG00010024213	-0.294615161	0.190008964	-1.55053296	0.121013649	0.143872444	negative
PRJNA729271	stage_1W_vs_E10	ordinary_muscle_development	ENSGALG00010024214	CRABP1	-9.964122626	0.52412973	-19.01079459	1.38834E-80	3.3765E-79	negative
PRJNA729271	stage_1W_vs_E10	ordinary_muscle_development	ENSGALG00010024215	ENSGALG00010024215	0.727917925	0.159069486	4.576100324	4.73723E-06	8.41601E-06	positive
PRJNA729271	stage_1W_vs_E10	ordinary_muscle_development	ENSGALG00010024218	NGFR	-3.224653662	0.236952582	-13.60885642	3.54751E-42	3.04989E-41	negative
PRJNA729271	stage_1W_vs_E10	ordinary_muscle_development	ENSGALG00010024220	SIAH1	2.712105359	0.200435162	13.53108574	1.02498E-41	8.72411E-41	positive
PRJNA729271	stage_1W_vs_E10	ordinary_muscle_development	ENSGALG00010024222	ENSGALG00010024222	-0.309771315	0.307801773	-1.006398734	0.314223802	0.35019791	negative
PRJNA729271	stage_1W_vs_E10	ordinary_muscle_development	ENSGALG00010024223	ENSGALG00010024223	-3.450891857	0.409640908	-8.424187603	3.63262E-17	1.18512E-16	negative
PRJNA729271	stage_1W_vs_E10	ordinary_muscle_development	ENSGALG00010024225	BCDIN3D	0.17721288	0.17889812	0.990579888	0.321890763	0.358301843	positive
PRJNA729271	stage_1W_vs_E10	ordinary_muscle_development	ENSGALG00010024226	POU2AF1	-1.614248934	1.489806763	-1.083529068	0.278573656	0.313017453	negative
PRJNA729271	stage_1W_vs_E10	ordinary_muscle_development	ENSGALG00010024227	MUC6	1.297190507	0.450939782	2.876637988	0.004019364	0.005656926	positive
PRJNA729271	stage_1W_vs_E10	ordinary_muscle_development	ENSGALG00010024230	LONP2	0.88862211	0.150244235	5.914517177	3.32851E-09	7.27264E-09	positive
PRJNA729271	stage_1W_vs_E10	ordinary_muscle_development	ENSGALG00010024233	FAIM2	-4.463582843	0.301561497	-14.80156744	1.43108E-49	1.54113E-48	negative
PRJNA729271	stage_1W_vs_E10	ordinary_muscle_development	ENSGALG00010024234	ENSGALG00010024234	0.60421782	0.270638346	2.232565447	0.025577615	0.033046397	positive
PRJNA729271	stage_1W_vs_E10	ordinary_muscle_development	ENSGALG00010024238	ATF7	1.618382269	0.202756007	7.981920219	1.44074E-15	4.39561E-15	positive
PRJNA729271	stage_1W_vs_E10	ordinary_muscle_development	ENSGALG00010024240	WDR61	-0.703013697	0.126840822	-5.542487689	2.98204E-08	6.14599E-08	negative
PRJNA729271	stage_1W_vs_E10	ordinary_muscle_development	ENSGALG00010024242	KMT2A	0.039782112	0.125270131	0.317570612	0.750810669	0.775750278	positive
PRJNA729271	stage_1W_vs_E10	ordinary_muscle_development	ENSGALG00010024243	MPP3	-0.135938263	0.208556296	-0.651806084	0.514526276	0.551835618	negative
PRJNA729271	stage_1W_vs_E10	ordinary_muscle_development	ENSGALG00010024245	TARBP2	0.826942932	0.163918048	5.044855891	4.53863E-07	8.67909E-07	positive
PRJNA729271	stage_1W_vs_E10	ordinary_muscle_development	ENSGALG00010024250	AP2A2	-0.414675295	0.0538766	-7.696760633	1.39559E-14	4.06043E-14	negative
PRJNA729271	stage_1W_vs_E10	ordinary_muscle_development	ENSGALG00010024251	ENSGALG00010024251	3.089311553	0.206073442	14.9913134	8.36819E-51	9.43211E-50	positive
PRJNA729271	stage_1W_vs_E10	ordinary_muscle_development	ENSGALG00010024254	ENSGALG00010024254	1.789889869	0.321651565	5.564685719	2.62625E-08	5.43201E-08	positive
PRJNA729271	stage_1W_vs_E10	ordinary_muscle_development	ENSGALG00010024255	CFAP97D1	0.017292814	0.694621775	0.024895294	0.980138481	0.983190815	positive
PRJNA729271	stage_1W_vs_E10	ordinary_muscle_development	ENSGALG00010024258	DNAJA4	0.311617755	0.151999214	2.050127402	0.040352	0.05096299	positive
PRJNA729271	stage_1W_vs_E10	ordinary_muscle_development	ENSGALG00010024259	ENSGALG00010024259	1.751002192	0.134703131	12.99897175	1.23999E-38	9.46981E-38	positive
PRJNA729271	stage_1W_vs_E10	ordinary_muscle_development	ENSGALG00010024263	PHKB	5.073041052	0.131300058	38.63700551	0	0	positive
PRJNA729271	stage_1W_vs_E10	ordinary_muscle_development	ENSGALG00010024264	AMER3	-0.470222077	0.305073285	-1.541341374	0.123233724	0.146415806	negative

PRJNA729271	stage_1W_vs_E10	ordinary_muscle_development	ENSGALG00010024266	ENSGALG00010024266	0.200977657	0.245529634	0.818547454	0.413044657	0.451106262	positive
PRJNA729271	stage_1W_vs_E10	ordinary_muscle_development	ENSGALG00010024267	MAP3K12	-1.751065614	0.208912965	-8.381794866	5.21266E-17	1.68995E-16	negative
PRJNA729271	stage_1W_vs_E10	ordinary_muscle_development	ENSGALG00010024270	RAB18L	-0.310952369	0.77487516	-0.401293505	0.688204045	0.717198765	negative
PRJNA729271	stage_1W_vs_E10	ordinary_muscle_development	ENSGALG00010024274	ACSBG1	-0.001658117	0.317046879	-0.005229879	0.99582718	0.99665228	negative
PRJNA729271	stage_1W_vs_E10	ordinary_muscle_development	ENSGALG00010024277	ITGB3BP	-2.166101962	0.195042904	-11.10577167	1.17597E-28	6.27372E-28	negative
PRJNA729271	stage_1W_vs_E10	ordinary_muscle_development	ENSGALG00010024280	ENSGALG00010024280	3.264423213	0.123998909	26.3262253	9.6086E-153	8.5446E-151	positive
PRJNA729271	stage_1W_vs_E10	ordinary_muscle_development	ENSGALG00010024282	HIC2	-2.488897407	0.152704429	-16.29878988	1.00665E-59	1.45769E-58	negative
PRJNA729271	stage_1W_vs_E10	ordinary_muscle_development	ENSGALG00010024283	UBE4A	0.477667234	0.110303271	4.330490201	1.48778E-05	2.54517E-05	positive
PRJNA729271	stage_1W_vs_E10	ordinary_muscle_development	ENSGALG00010024285	CCND2	-1.064325409	0.097925687	-10.86870502	1.62491E-27	8.2904E-27	negative
PRJNA729271	stage_1W_vs_E10	ordinary_muscle_development	ENSGALG00010024286	ADA	1.038303249	0.179816472	5.774238796	7.73017E-09	1.64986E-08	positive
PRJNA729271	stage_1W_vs_E10	ordinary_muscle_development	ENSGALG00010024288	CHID1	-0.081354696	0.137189841	-0.593008167	0.553175687	0.589319534	negative
PRJNA729271	stage_1W_vs_E10	ordinary_muscle_development	ENSGALG00010024290	SOST	0.013480235	0.459116953	0.029361222	0.9765765	0.979965429	positive
PRJNA729271	stage_1W_vs_E10	ordinary_muscle_development	ENSGALG00010024291	ENSGALG00010024291	2.772974191	0.519565488	5.337102356	9.44437E-08	1.89188E-07	positive
PRJNA729271	stage_1W_vs_E10	ordinary_muscle_development	ENSGALG00010024300	MEOX1	-3.780010861	0.276952381	-13.64859494	2.05809E-42	1.78314E-41	negative
PRJNA729271	stage_1W_vs_E10	ordinary_muscle_development	ENSGALG00010024304	PKIG	-0.690846989	0.133309028	-5.18229711	2.1917E-07	4.28207E-07	negative
PRJNA729271	stage_1W_vs_E10	ordinary_muscle_development	ENSGALG00010024305	ENSGALG00010024305	5.266869724	0.723083227	7.283905267	3.24293E-13	8.78621E-13	positive
PRJNA729271	stage_1W_vs_E10	ordinary_muscle_development	ENSGALG00010024309	ABCB11	1.066851399	0.395809602	2.695365131	0.007031154	0.009664919	positive
PRJNA729271	stage_1W_vs_E10	ordinary_muscle_development	ENSGALG00010024311	KLHL6	1.421558367	0.128453619	11.06670543	1.81968E-28	9.63693E-28	positive
PRJNA729271	stage_1W_vs_E10	ordinary_muscle_development	ENSGALG00010024313	SERINC3	2.470117631	0.106763915	23.1362595	1.9992E-118	1.0693E-116	positive
PRJNA729271	stage_1W_vs_E10	ordinary_muscle_development	ENSGALG00010024314	ITFG1	2.141541818	0.145422228	14.72637195	4.3652E-49	4.62526E-48	positive
PRJNA729271	stage_1W_vs_E10	ordinary_muscle_development	ENSGALG00010024321	UBE2L3	0.054483573	0.082213846	0.662705561	0.507519148	0.54504631	positive
PRJNA729271	stage_1W_vs_E10	ordinary_muscle_development	ENSGALG00010024322	IDH3A	2.236430036	0.158779593	14.08512262	4.68844E-45	4.39295E-44	positive
PRJNA729271	stage_1W_vs_E10	ordinary_muscle_development	ENSGALG00010024323	TTPAL	-1.175366093	0.103471733	-11.35929651	6.66794E-30	3.76223E-29	negative
PRJNA729271	stage_1W_vs_E10	ordinary_muscle_development	ENSGALG00010024325	PCBP2	0.491226144	0.169609304	2.896221686	0.003776855	0.005330105	positive
PRJNA729271	stage_1W_vs_E10	ordinary_muscle_development	ENSGALG00010024326	KLHL24	2.652033402	0.178422268	14.8638028	5.6618E-50	6.21255E-49	positive
PRJNA729271	stage_1W_vs_E10	ordinary_muscle_development	ENSGALG00010024327	PPP1R1B	4.146881468	0.415159887	9.988637139	1.70915E-23	7.39087E-23	positive
PRJNA729271	stage_1W_vs_E10	ordinary_muscle_development	ENSGALG00010024328	YDJC	-2.848923425	0.159047825	-17.91236956	9.44286E-72	1.86987E-70	negative
PRJNA729271	stage_1W_vs_E10	ordinary_muscle_development	ENSGALG00010024330	SDF2L1	-1.822462119	0.104637275	-17.41694933	6.13628E-68	1.10081E-66	negative
PRJNA729271	stage_1W_vs_E10	ordinary_muscle_development	ENSGALG00010024332	EFCAB7	-0.902405931	0.168016696	-5.370930107	7.83316E-08	1.57828E-07	negative
PRJNA729271	stage_1W_vs_E10	ordinary_muscle_development	ENSGALG00010024333	ETV4	-4.765732915	0.250664636	-19.01238641	1.34684E-80	3.28108E-79	negative
PRJNA729271	stage_1W_vs_E10	ordinary_muscle_development	ENSGALG00010024334	USP28	1.77811295	0.122623444	14.50059545	1.20103E-47	1.21148E-46	positive
PRJNA729271	stage_1W_vs_E10	ordinary_muscle_development	ENSGALG00010024336	TSPAN4	-0.657920366	0.117414688	-5.60339067	2.10199E-08	4.377E-08	negative
PRJNA729271	stage_1W_vs_E10	ordinary_muscle_development	ENSGALG00010024338	NDUFA9	0.854339483	0.114033348	7.492014382	6.78245E-14	1.90748E-13	positive
PRJNA729271	stage_1W_vs_E10	ordinary_muscle_development	ENSGALG00010024339	TOP3B	0.103886378	0.086370646	1.202797281	0.229054777	0.260710561	positive
PRJNA729271	stage_1W_vs_E10	ordinary_muscle_development	ENSGALG00010024340	NETO2	-1.263056227	0.378931534	-3.333204321	0.000858519	0.00128729	negative
PRJNA729271	stage_1W_vs_E10	ordinary_muscle_development	ENSGALG00010024342	HNF4A	1.992521256	1.351572145	1.47422486	0.140421059	0.165412698	positive
PRJNA729271	stage_1W_vs_E10	ordinary_muscle_development	ENSGALG00010024343	POLR2L	0.984267996	0.126108406	7.8049357	5.95319E-15	1.76429E-14	positive
PRJNA729271	stage_1W_vs_E10	ordinary_muscle_development	ENSGALG00010024344	YEATS2	0.410933536	0.13281119	3.094118322	0.001973987	0.002861915	positive
PRJNA729271	stage_1W_vs_E10	ordinary_muscle_development	ENSGALG00010024345	STARD3	0.62183845	0.091312033	6.810038377	9.75727E-12	2.45669E-11	positive
PRJNA729271	stage_1W_vs_E10	ordinary_muscle_development	ENSGALG00010024346	PPM1F	0.43387477	0.091067913	4.764299036	1.89511E-06	3.46971E-06	positive
PRJNA729271	stage_1W_vs_E10	ordinary_muscle_development	ENSGALG00010024347	OTOG	-1.514357519	0.290339971	-5.215807915	1.83018E-07	3.5922E-07	negative
PRJNA729271	stage_1W_vs_E10	ordinary_muscle_development	ENSGALG00010024349	R3HDML	-5.26881028	0.809702068	-6.507097472	7.66167E-11	1.82959E-10	negative
PRJNA729271	stage_1W_vs_E10	ordinary_muscle_development	ENSGALG00010024350	PGM1	7.97523702	0.135807861	58.72441354	0	0	positive
PRJNA729271	stage_1W_vs_E10	ordinary_muscle_development	ENSGALG00010024351	SP1	1.183702696	0.153396771	7.716607665	1.19467E-14	3.49127E-14	positive
PRJNA729271	stage_1W_vs_E10	ordinary_muscle_development	ENSGALG00010024354	C2CD2L	-0.152712021	0.079010038	-1.932817975	0.053258625	0.06627039	negative
PRJNA729271	stage_1W_vs_E10	ordinary_muscle_development	ENSGALG00010024356	PARL	0.412817178	0.117657949	3.508621241	0.000450436	0.000689156	positive
PRJNA729271	stage_1W_vs_E10	ordinary_muscle_development	ENSGALG00010024358	MAPK3	0.428271703	0.061847171	6.924677306	4.3697E-12	1.11886E-11	positive
PRJNA729271	stage_1W_vs_E10	ordinary_muscle_development	ENSGALG00010024360	FTL	-1.28210711	0.24522126	-5.22836849	1.71012E-07	3.36157E-07	negative
PRJNA729271	stage_1W_vs_E10	ordinary_muscle_development	ENSGALG00010024361	H2AX	-2.215077068	0.217643654	-10.17754036	2.49795E-24	1.11477E-23	negative
PRJNA729271	stage_1W_vs_E10	ordinary_muscle_development	ENSGALG00010024362	DHX8	-0.209954843	0.058385855	-3.595988173	0.000323162	0.000501095	negative
PRJNA729271	stage_1W_vs_E10	ordinary_muscle_development	ENSGALG00010024363	EDEM2	-0.45329791	0.114943529	-3.943657479	8.02483E-05	0.000130155	negative
PRJNA729271	stage_1W_vs_E10	ordinary_muscle_development	ENSGALG00010024365	ENSGALG00010024365	-0.202488748	0.111474533	-1.816457468	0.069300227	0.084990844	negative
PRJNA729271	stage_1W_vs_E10	ordinary_muscle_development	ENSGALG00010024366	PARD6A	-0.034501334	0.473746587	-0.072826559	0.941944136	0.949279027	negative
PRJNA729271	stage_1W_vs_E10	ordinary_muscle_development	ENSGALG00010024367	ENSGALG00010024367	-2.468197128	0.238089396	-10.3666823	3.51518E-25	1.62996E-24	negative
PRJNA729271	stage_1W_vs_E10	ordinary_muscle_development	ENSGALG00010024368	DPAGT1	-0.074190588	0.079973639	-0.927688032	0.353569418	0.391190651	negative
PRJNA729271	stage_1W_vs_E10	ordinary_muscle_development	ENSGALG00010024369	MYOD1	1.752449918	0.275410545	6.363045819	1.97792E-10	4.61747E-10	positive
PRJNA729271	stage_1W_vs_E10	ordinary_muscle_development	ENSGALG00010024370	HERPUD1	0.177403583	0.09306096	1.906315853	0.056609239	0.070222586	positive
PRJNA729271	stage_1W_vs_E10	ordinary_muscle_development	ENSGALG00010024371	YPEL1	0.629992138	0.105485381	5.972317034	2.33907E-09	5.15742E-09	positive
PRJNA729271	stage_1W_vs_E10	ordinary_muscle_development	ENSGALG00010024373	TCAP	13.29873047	0.478223073	27.80863411	3.4109E-170	3.7174E-168	positive
PRJNA729271	stage_1W_vs_E10	ordinary_muscle_development	ENSGALG00010024374	ENSGALG00010024374	1.777103498	0.581002095	3.058686902	0.002223094	0.003206023	positive
PRJNA729271	stage_1W_vs_E10	ordinary_muscle_development	ENSGALG00010024377	CIB2	-0.176980449	0.171117736	-1.034261281	0.301014032	0.336303253	negative
PRJNA729271	stage_1W_vs_E10	ordinary_muscle_development	ENSGALG00010024378	ZDHHC1	-0.417582493	0.178893431	-2.334252809	0.019582488	0.025620378	negative
PRJNA729271	stage_1W_vs_E10	ordinary_muscle_development	ENSGALG00010024382	ENSGALG00010024382	1.510797495	0.420299414	3.594574355	0.000324922	0.000503663	positive
PRJNA729271	stage_1W_vs_E10	ordinary_muscle_development	ENSGALG00010024383	DUSP28	1.740981661	0.188082273	9.256489925	2.11263E-20	8.07558E-20	positive
PRJNA729271	stage_1W_vs_E10	ordinary_muscle_development	ENSGALG00010024386	TPPP3	2.010432112	0.267845095	7.50595083	6.09843E-14	1.71945E-13	positive
PRJNA729271	stage_1W_vs_E10	ordinary_muscle_development	ENSGALG00010024387	PPIL2	-0.229912012	0.066376151	-3.463774383	0.000532653	0.000810708	negative
PRJNA729271	stage_1W_vs_E10	ordinary_muscle_development	ENSGALG00010024389	AAAS	0.616982777	0.106574212	5.789231422	7.07092E-09	1.51371E-08	positive
PRJNA729271	stage_1W_vs_E10	ordinary_muscle_development	ENSGALG00010024391	TBC1D2B	-0.66950394	0.111321435	-6.014151199	1.80832E-09	4.01833E-09	negative
PRJNA729271	stage_1W_vs_E10	ordinary_muscle_development	ENSGALG00010024392	CHMP4B	0.286861466	0.084832457	3.381506035	0.000720896	0.001087346	positive
PRJNA729271	stage_1W_vs_E10	ordinary_muscle_development	ENSGALG00010024393	OGFOD1	-0.732584259	0.104550945	-7.006959717	2.43552E-12	6.32329E-12	negative
PRJNA729271	stage_1W_vs_E10	ordinary_muscle_development	ENSGALG00010024396	ENSGALG00010024396	0.488574865	0.329280361	1.483765576	0.137871123	0.162620386	positive
PRJNA729271	stage_1W_vs_E10	ordinary_muscle_development	ENSGALG00010024398	PHB	0.148035637	0.088130701	1.679728353	0.093010181	0.112367275	positive
PRJNA729271	stage_1W_vs_E10	ordinary_muscle_development	ENSGALG00010024399	ROR1	0.039977641	0.167618211	0.23850416	0.81149009	0.831451817	positive
PRJNA729271	stage_1W_vs_E10	ordinary_muscle_development	ENSGALG00010024400	KCNA1	-3.041674491	0.399470985	-7.614256365	2.65214E-14	7.61544E-14	negative
PRJNA729271	stage_1W_vs_E10	ordinary_muscle_development	ENSGALG00010024401	KCNC1	2.380978402	0.213986005	11.12679498	9.29165E-29	4.97718E-28	positive
PRJNA729271	stage_1W_vs_E10	ordinary_muscle_development	ENSGALG00010024402	GPC1	-1.779518172	0.120213813	-14.80294254	1.40212E-49	1.51106E-48	negative
PRJNA729271	stage_1W_vs_E10	ordinary_muscle_development	ENSGALG00010024403	HMBS	-0.844210252	0.077770595	-10.8551343	1.88529E-27	9.59865E-27	negative
PRJNA729271	stage_1W_vs_E10	ordinary_muscle_development	ENSGALG00010024406	NUDT21	-1.284923802	0.06683252	-19.22602662	2.24159E-82	5.67047E-81	negative
PRJNA729271	stage_1W_vs_E10	ordinary_muscle_development	ENSGALG00010024407	HIRA	-0.861682934	0.082620092	-10.42945983	1.8192E-25	8.53373E-25	negative
PRJNA729271	stage_1W_vs_E10	ordinary_muscle_development	ENSGALG00010024408	ENSGALG00010024408	-3.025743739	1.394701643	-2.169455923	0.030048087	0.038497797	negative
PRJNA729271	stage_1W_vs_E10	ordinary_muscle_development	ENSGALG00010024410	MYG1	0.711121648	0.105509838	6.739861078	1.58538E-11	3.94035E-11	positive
PRJNA729271	stage_1W_vs_E10	ordinary_muscle_development	ENSGALG00010024412	PIGU	-0.608227729	0.102589898	-5.928729272	3.05288E-09	6.68149E-09	negative
PRJNA729271	stage_1W_vs_E10	ordinary_muscle_development	ENSGALG00010024413	AMFR	1.532256768	0.091978945	16.65877742	2.61312E-62	4.0467E-61	positive
PRJNA729271	stage_1W_vs_E10	ordinary_muscle_development	ENSGALG00010024415	SIK2	0.985870532	0.059613402	16.53773309	1.9627E-61	2.97899E-60	positive
PRJNA729271	stage_1W_vs_E10	ordinary_muscle_development	ENSGALG00010024416	VDR	-0.524629084	0.307197258	-1.707792211	0.087674905	0.106231526	negative
PRJNA729271	stage_1W_vs_E10	ordinary_muscle_development	ENSGALG00010024419	PGAP3	0.854817458	0.123804916	6.904551802	5.03622E-12	1.28521E-11	positive
PRJNA729271	stage_1W_vs_E10	ordinary_muscle_development	ENSGALG00010024420	TMEM106C	0.164823591	0.162115188	1.016706661	0.309292996	0.345100607	positive
PRJNA729271	stage_1W_vs_E10	ordinary_muscle_development	ENSGALG00010024421	DAPK2	-0.739203044	0.261693078	-2.82469468	0.004732569	0.006621485	negative
PRJNA729271	stage_1W_vs_E10	ordinary_muscle_development	ENSGALG00010024423	SERGEF	-0.044823544	0.119695184	-0.374480768	0.708046662	0.735761443	negative
PRJNA729271	stage_1W_vs_E10	ordinary_muscle_development	ENSGALG00010024424	SAMHD1	0.855883919	0.179632694	4.764633329	1.89197E-06	3.46483E-06	positive
PRJNA729271	stage_1W_vs_E10	ordinary_muscle_development	ENSGALG00010024425	MRPL40	1.106108088	0.088155686	12.54721207	4.11753E-36	2.90998E-35	positive
PRJNA729271	stage_1W_vs_E10	ordinary_muscle_development	ENSGALG00010024426	PHAF1	1.652061768	0.104298842	15.83969427	1.65624E-56	2.22083E-55	positive
PRJNA729271	stage_1W_vs_E10	ordinary_muscle_development	ENSGALG00010024427	C22orf39	0.37747879	0.135658866	2.782558934	0.005393207	0.007507398	positive
PRJNA729271	stage_1W_vs_E10	ordinary_muscle_development	ENSGALG00010024428	SAAL1	-1.742822514	0.066042727	-26.38931791	1.8172E-153	1.6463E-151	negative
PRJNA729271	stage_1W_vs_E10	ordinary_muscle_development	ENSGALG00010024430	ZNF652	-0.342668462	0.065415312	-5.238352506	1.62016E-07	3.18906E-07	negative
PRJNA729271	stage_1W_vs_E10	ordinary_muscle_development	ENSGALG00010024431	FEZ1	-3.379824799	0.16195927	-20.86836278	1.03829E-96	3.64408E-95	negative
PRJNA729271	stage_1W_vs_E10	ordinary_muscle_development	ENSGALG00010024433	ENSGALG00010024433	-1.0375197	0.169214648	-6.131382323	8.71188E-10	1.96481E-09	negative
PRJNA729271	stage_1W_vs_E10	ordinary_muscle_development	ENSGALG00010024434	B3GNT9	-1.182933654	0.159843232	-7.400586415	1.35584E-13	3.73986E-13	negative
PRJNA729271	stage_1W_vs_E10	ordinary_muscle_development	ENSGALG00010024437	HERC1	0.152603577	0.081571878	1.870786602	0.061374665	0.075770869	positive
PRJNA729271	stage_1W_vs_E10	ordinary_muscle_development	ENSGALG00010024438	RBL1	-2.377257383	0.123979147	-19.17465513	6.02668E-82	1.51136E-80	negative
PRJNA729271	stage_1W_vs_E10	ordinary_muscle_development	ENSGALG00010024439	UFD1	1.059299916	0.11199879	9.458137132	3.13478E-21	1.24014E-20	positive
PRJNA729271	stage_1W_vs_E10	ordinary_muscle_development	ENSGALG00010024440	PKNOX2	2.419701048	0.20543784	11.77826369	5.05231E-32	3.0874E-31	positive
PRJNA729271	stage_1W_vs_E10	ordinary_muscle_development	ENSGALG00010024441	SAA	4.742330227	0.789927752	6.003498694	1.9311E-09	4.28395E-09	positive
PRJNA729271	stage_1W_vs_E10	ordinary_muscle_development	ENSGALG00010024446	GRM4	-3.344851724	0.530319174	-6.307242671	2.8405E-10	6.57717E-10	negative
PRJNA729271	stage_1W_vs_E10	ordinary_muscle_development	ENSGALG00010024449	CACHD1	-0.709449081	0.084925621	-8.353769748	6.61182E-17	2.13639E-16	negative
PRJNA729271	stage_1W_vs_E10	ordinary_muscle_development	ENSGALG00010024451	PHOSPHO1	2.487281013	0.267832094	9.286717579	1.5912E-20	6.13252E-20	positive
PRJNA729271	stage_1W_vs_E10	ordinary_muscle_development	ENSGALG00010024452	SLC16A1	0.386662651	0.25180381	1.535571088	0.12464361	0.147921166	positive
PRJNA729271	stage_1W_vs_E10	ordinary_muscle_development	ENSGALG00010024453	HTR3A	-0.433130918	0.898210596	-0.482215329	0.629652988	0.662949086	negative
PRJNA729271	stage_1W_vs_E10	ordinary_muscle_development	ENSGALG00010024454	HPS5	0.108979092	0.120758514	0.90245473	0.366815359	0.404518307	positive
PRJNA729271	stage_1W_vs_E10	ordinary_muscle_development	ENSGALG00010024457	CHD6	-0.487466326	0.101170263	-4.818276768	1.44803E-06	2.67345E-06	negative
PRJNA729271	stage_1W_vs_E10	ordinary_muscle_development	ENSGALG00010024459	AHCYL1	-0.57272238	0.094327089	-6.071663857	1.26592E-09	2.83521E-09	negative
PRJNA729271	stage_1W_vs_E10	ordinary_muscle_development	ENSGALG00010024460	IMP4	0.209345918	0.155301632	1.347995612	0.177659794	0.205997817	positive
PRJNA729271	stage_1W_vs_E10	ordinary_muscle_development	ENSGALG00010024462	ZBTB32	6.720221249	0.428633875	15.67823179	2.13104E-55	2.78284E-54	positive
PRJNA729271	stage_1W_vs_E10	ordinary_muscle_development	ENSGALG00010024463	FBXL22	-0.926955043	0.229233621	-4.043713296	5.26112E-05	8.64234E-05	negative
PRJNA729271	stage_1W_vs_E10	ordinary_muscle_development	ENSGALG00010024465	SENP1	-0.465794487	0.160088678	-2.909602928	0.003618882	0.005118128	negative
PRJNA729271	stage_1W_vs_E10	ordinary_muscle_development	ENSGALG00010024466	ADGRG3	2.505761754	0.743954654	3.36816463	0.000756704	0.001139577	positive
PRJNA729271	stage_1W_vs_E10	ordinary_muscle_development	ENSGALG00010024467	ENSGALG00010024467	0.807677126	0.293768819	2.749363015	0.005971122	0.008268979	positive
PRJNA729271	stage_1W_vs_E10	ordinary_muscle_development	ENSGALG00010024469	GTF2H1	0.690687101	0.127591376	5.413274198	6.18826E-08	1.25541E-07	positive
PRJNA729271	stage_1W_vs_E10	ordinary_muscle_development	ENSGALG00010024471	CCDC61	-0.366387293	0.116869395	-3.135014896	0.001718454	0.002505934	negative
PRJNA729271	stage_1W_vs_E10	ordinary_muscle_development	ENSGALG00010024472	LDHA	8.151325055	0.201250137	40.50345095	0	0	positive
PRJNA729271	stage_1W_vs_E10	ordinary_muscle_development	ENSGALG00010024473	RAVER2	-1.278647556	0.061350268	-20.84175998	1.81059E-96	6.32396E-95	negative
PRJNA729271	stage_1W_vs_E10	ordinary_muscle_development	ENSGALG00010024474	STRIP1	0.502156168	0.103359827	4.858330208	1.1838E-06	2.20215E-06	positive
PRJNA729271	stage_1W_vs_E10	ordinary_muscle_development	ENSGALG00010024475	ADGRG1	1.269447919	0.156983303	8.086515555	6.13959E-16	1.90768E-15	positive
PRJNA729271	stage_1W_vs_E10	ordinary_muscle_development	ENSGALG00010024476	KCNB1	-0.753069393	0.177553065	-4.241376473	2.22153E-05	3.75042E-05	negative
PRJNA729271	stage_1W_vs_E10	ordinary_muscle_development	ENSGALG00010024479	ERBB2	1.02380454	0.127512516	8.02905138	9.82292E-16	3.02364E-15	positive
PRJNA729271	stage_1W_vs_E10	ordinary_muscle_development	ENSGALG00010024480	CDC45	-2.345219973	0.217811547	-10.76719761	4.91741E-27	2.45786E-26	negative
PRJNA729271	stage_1W_vs_E10	ordinary_muscle_development	ENSGALG00010024482	USP3	-1.300400289	0.059699925	-21.78227668	3.4167E-105	1.411E-103	negative
PRJNA729271	stage_1W_vs_E10	ordinary_muscle_development	ENSGALG00010024486	GPR37L1	-0.670556855	0.311516217	-2.15255818	0.0313534	0.040072968	negative
PRJNA729271	stage_1W_vs_E10	ordinary_muscle_development	ENSGALG00010024488	ADGRG5	0.035312591	0.519112764	0.068024895	0.945765817	0.952666819	positive
PRJNA729271	stage_1W_vs_E10	ordinary_muscle_development	ENSGALG00010024490	TGM3	1.076562534	0.893616976	1.204724802	0.228309558	0.259882758	positive
PRJNA729271	stage_1W_vs_E10	ordinary_muscle_development	ENSGALG00010024492	NTF3	-2.619457385	0.257538239	-10.17113962	2.66769E-24	1.18869E-23	negative

PRJNA729271	stage_1W_vs_E10	ordinary_muscle_development	ENSGALG00010024493	JAK1	0.168086041	0.06031425	2.786837947	0.005322509	0.007414682	positive
PRJNA729271	stage_1W_vs_E10	ordinary_muscle_development	ENSGALG00010024494	DDAH1	-0.372210611	0.174250337	-2.136068237	0.032673852	0.041676125	negative
PRJNA729271	stage_1W_vs_E10	ordinary_muscle_development	ENSGALG00010024495	CLDN5	0.338191177	0.170697281	1.98123353	0.047565091	0.059538514	positive
PRJNA729271	stage_1W_vs_E10	ordinary_muscle_development	ENSGALG00010024496	UEVLD	1.593903604	0.20790547	7.666482284	1.76778E-14	5.11864E-14	positive
PRJNA729271	stage_1W_vs_E10	ordinary_muscle_development	ENSGALG00010024497	ABI3	1.357310188	0.181938595	7.460265322	8.63484E-14	2.40974E-13	positive
PRJNA729271	stage_1W_vs_E10	ordinary_muscle_development	ENSGALG00010024498	CCDC102A	-1.180066257	0.121823967	-9.686651035	3.43611E-22	1.40379E-21	negative
PRJNA729271	stage_1W_vs_E10	ordinary_muscle_development	ENSGALG00010024499	CA12	-1.865396088	0.621911238	-2.999457115	0.002704612	0.003869643	negative
PRJNA729271	stage_1W_vs_E10	ordinary_muscle_development	ENSGALG00010024501	ARL8A	0.552311063	0.08954322	6.168094709	6.91177E-10	1.56663E-09	positive
PRJNA729271	stage_1W_vs_E10	ordinary_muscle_development	ENSGALG00010024502	VPS8	0.346864655	0.07323014	4.73663787	2.17293E-06	3.95587E-06	positive
PRJNA729271	stage_1W_vs_E10	ordinary_muscle_development	ENSGALG00010024504	CASC3	1.026982609	0.18630468	5.512382228	3.54009E-08	7.26925E-08	positive
PRJNA729271	stage_1W_vs_E10	ordinary_muscle_development	ENSGALG00010024505	DNASE2B	1.427516885	0.210637027	6.777141254	1.22577E-11	3.06707E-11	positive
PRJNA729271	stage_1W_vs_E10	ordinary_muscle_development	ENSGALG00010024506	RAB8B	-0.061152757	0.081541826	-0.749955706	0.453281382	0.491495634	negative
PRJNA729271	stage_1W_vs_E10	ordinary_muscle_development	ENSGALG00010024508	RPS27L	3.083375669	0.188874315	16.32501314	6.55266E-60	9.57468E-59	positive
PRJNA729271	stage_1W_vs_E10	ordinary_muscle_development	ENSGALG00010024509	SEPTIN5	-0.355914076	0.108157455	-3.290703129	0.000999373	0.001488789	negative
PRJNA729271	stage_1W_vs_E10	ordinary_muscle_development	ENSGALG00010024510	CNN3	-1.065667145	0.101266472	-10.52339557	6.73999E-26	3.21686E-25	negative
PRJNA729271	stage_1W_vs_E10	ordinary_muscle_development	ENSGALG00010024511	ENSGALG00010024511	-0.812137986	0.171066399	-4.747501493	2.05945E-06	3.7554E-06	negative
PRJNA729271	stage_1W_vs_E10	ordinary_muscle_development	ENSGALG00010024512	CCNDBP1	0.197610982	0.115236047	1.714836522	0.086375203	0.104726773	positive
PRJNA729271	stage_1W_vs_E10	ordinary_muscle_development	ENSGALG00010024513	SPTY2D1	0.892682077	0.138660207	6.437911044	1.21129E-10	2.86422E-10	positive
PRJNA729271	stage_1W_vs_E10	ordinary_muscle_development	ENSGALG00010024514	FAF1	0.735894291	0.068031072	10.81703205	2.85886E-27	1.44488E-26	positive
PRJNA729271	stage_1W_vs_E10	ordinary_muscle_development	ENSGALG00010024515	C3orf70	-1.493992091	0.159235271	-9.382293755	6.45528E-21	2.51869E-20	negative
PRJNA729271	stage_1W_vs_E10	ordinary_muscle_development	ENSGALG00010024516	EHHADH	0.26118177	0.240782275	1.084721747	0.278044913	0.312447555	positive
PRJNA729271	stage_1W_vs_E10	ordinary_muscle_development	ENSGALG00010024517	LACTB	0.146480391	0.097614106	1.500606803	0.133457291	0.157709394	positive
PRJNA729271	stage_1W_vs_E10	ordinary_muscle_development	ENSGALG00010024518	ENSGALG00010024518	-0.213503949	1.33961853	-0.159376676	0.873372114	0.887578264	negative
PRJNA729271	stage_1W_vs_E10	ordinary_muscle_development	ENSGALG00010024519	MIEN1	1.343033608	0.138031334	9.729918329	2.24773E-22	9.24805E-22	positive
PRJNA729271	stage_1W_vs_E10	ordinary_muscle_development	ENSGALG00010024520	MAP3K13	-0.724436567	0.142370086	-5.08840436	3.61089E-07	6.95453E-07	negative
PRJNA729271	stage_1W_vs_E10	ordinary_muscle_development	ENSGALG00010024521	HEPACAM	-0.544432573	0.453279319	-1.201097317	0.229713454	0.26137817	negative
PRJNA729271	stage_1W_vs_E10	ordinary_muscle_development	ENSGALG00010024522	DNTTIP2	0.40634879	0.074864652	5.427779064	5.70596E-08	1.16049E-07	positive
PRJNA729271	stage_1W_vs_E10	ordinary_muscle_development	ENSGALG00010024523	TOX3	-2.916965999	0.346679745	-8.414007567	3.96235E-17	1.29124E-16	negative
PRJNA729271	stage_1W_vs_E10	ordinary_muscle_development	ENSGALG00010024524	TMEM41A	0.961461424	0.13253492	7.254400766	4.03444E-13	1.08778E-12	positive
PRJNA729271	stage_1W_vs_E10	ordinary_muscle_development	ENSGALG00010024525	GP1BB	0.008587427	0.195538198	0.043916877	0.964970662	0.969922318	positive
PRJNA729271	stage_1W_vs_E10	ordinary_muscle_development	ENSGALG00010024526	TGM6	0.172673136	1.583567651	0.109040581	0.913170303	0.92381376	positive
PRJNA729271	stage_1W_vs_E10	ordinary_muscle_development	ENSGALG00010024527	ATP6V0B	1.205238338	0.098761407	12.20353554	2.97615E-34	1.95999E-33	positive
PRJNA729271	stage_1W_vs_E10	ordinary_muscle_development	ENSGALG00010024528	TBX1	-1.29644624	0.219879864	-5.896157191	3.72065E-09	8.09893E-09	negative
PRJNA729271	stage_1W_vs_E10	ordinary_muscle_development	ENSGALG00010024529	GRB7	-5.440601723	1.212304553	-4.487817612	7.19565E-06	1.26043E-05	negative
PRJNA729271	stage_1W_vs_E10	ordinary_muscle_development	ENSGALG00010024530	ENSGALG00010024530	0.771450973	0.534306757	1.443835331	0.148785301	0.174626959	positive
PRJNA729271	stage_1W_vs_E10	ordinary_muscle_development	ENSGALG00010024531	CEP63	-1.161969315	0.133261841	-8.719445133	2.79568E-18	9.63697E-18	negative
PRJNA729271	stage_1W_vs_E10	ordinary_muscle_development	ENSGALG00010024532	TMEM86A	0.747137546	0.214461812	3.483778949	0.000494388	0.000754331	positive
PRJNA729271	stage_1W_vs_E10	ordinary_muscle_development	ENSGALG00010024533	CDKN2C	2.258608494	0.223645577	10.09905283	5.57783E-24	2.45747E-23	positive
PRJNA729271	stage_1W_vs_E10	ordinary_muscle_development	ENSGALG00010024534	VWF	4.037341144	0.125453584	32.18195148	3.1571E-227	6.1016E-225	positive
PRJNA729271	stage_1W_vs_E10	ordinary_muscle_development	ENSGALG00010024535	ENSGALG00010024535	2.113589285	0.151067327	13.99104178	1.76803E-44	1.62715E-43	positive
PRJNA729271	stage_1W_vs_E10	ordinary_muscle_development	ENSGALG00010024536	URI1	0.083165812	0.061659761	1.348785842	0.177405763	0.205736182	positive
PRJNA729271	stage_1W_vs_E10	ordinary_muscle_development	ENSGALG00010024537	EPB42	-0.746696518	0.181368143	-4.117021366	3.83801E-05	6.37104E-05	negative
PRJNA729271	stage_1W_vs_E10	ordinary_muscle_development	ENSGALG00010024540	PTPN5	-2.323019894	0.306826819	-7.57111096	3.70046E-14	1.05421E-13	negative
PRJNA729271	stage_1W_vs_E10	ordinary_muscle_development	ENSGALG00010024541	WIPF2	1.135592703	0.201214831	5.64368291	1.66451E-08	3.48455E-08	positive
PRJNA729271	stage_1W_vs_E10	ordinary_muscle_development	ENSGALG00010024542	ZNFX1	2.687090874	0.425042075	6.321940888	2.58298E-10	5.98758E-10	positive
PRJNA729271	stage_1W_vs_E10	ordinary_muscle_development	ENSGALG00010024544	ZNF536	-1.302497418	0.246501115	-5.283941291	1.26434E-07	2.5074E-07	negative
PRJNA729271	stage_1W_vs_E10	ordinary_muscle_development	ENSGALG00010024545	PHACTR3	-1.821476917	0.302514751	-6.021117688	1.73217E-09	3.85325E-09	negative
PRJNA729271	stage_1W_vs_E10	ordinary_muscle_development	ENSGALG00010024546	KY	7.016262315	0.401447356	17.47741568	2.12933E-68	3.87261E-67	positive
PRJNA729271	stage_1W_vs_E10	ordinary_muscle_development	ENSGALG00010024547	PFKM	10.29363735	0.197736324	52.05739207	0	0	positive
PRJNA729271	stage_1W_vs_E10	ordinary_muscle_development	ENSGALG00010024548	CDC6	-2.518030577	0.304799102	-8.261279503	1.44119E-16	4.59123E-16	negative
PRJNA729271	stage_1W_vs_E10	ordinary_muscle_development	ENSGALG00010024549	GNB1L	-0.093848811	0.133424704	-0.703384069	0.481816432	0.520063235	negative
PRJNA729271	stage_1W_vs_E10	ordinary_muscle_development	ENSGALG00010024551	RNF11	1.681390764	0.12657336	13.28392302	2.86957E-40	2.31724E-39	positive
PRJNA729271	stage_1W_vs_E10	ordinary_muscle_development	ENSGALG00010024552	CD81	0.784475161	0.066252917	11.84061307	2.40687E-32	1.48648E-31	positive
PRJNA729271	stage_1W_vs_E10	ordinary_muscle_development	ENSGALG00010024553	ASB8	0.089642991	0.113363174	0.790759363	0.429084428	0.466965547	positive
PRJNA729271	stage_1W_vs_E10	ordinary_muscle_development	ENSGALG00010024554	ST6GALNAC3	-3.047181578	0.214527501	-14.20415359	8.63395E-46	8.26067E-45	negative
PRJNA729271	stage_1W_vs_E10	ordinary_muscle_development	ENSGALG00010024555	EPHB1	-0.942120989	0.163149978	-5.774570115	7.71498E-09	1.64696E-08	negative
PRJNA729271	stage_1W_vs_E10	ordinary_muscle_development	ENSGALG00010024556	ENSGALG00010024556	0.073137362	0.203322937	0.359710336	0.719063762	0.746086559	positive
PRJNA729271	stage_1W_vs_E10	ordinary_muscle_development	ENSGALG00010024557	TSPAN32	0.040693179	0.309812463	0.13134778	0.895500196	0.907457728	positive
PRJNA729271	stage_1W_vs_E10	ordinary_muscle_development	ENSGALG00010024558	PFDN5	0.370501014	0.108151082	3.42577259	0.000613053	0.000929325	positive
PRJNA729271	stage_1W_vs_E10	ordinary_muscle_development	ENSGALG00010024559	ENSGALG00010024559	-2.525622719	0.360509638	-7.005700969	2.45752E-12	6.37698E-12	negative
PRJNA729271	stage_1W_vs_E10	ordinary_muscle_development	ENSGALG00010024560	A4GNT	-0.339778384	0.510202848	-0.665967243	0.505432039	0.543046283	negative
PRJNA729271	stage_1W_vs_E10	ordinary_muscle_development	ENSGALG00010024561	ROBO3	-1.195231578	0.563210748	-2.122174662	0.033823074	0.043066185	negative
PRJNA729271	stage_1W_vs_E10	ordinary_muscle_development	ENSGALG00010024563	ESPL1	-1.340873296	0.165052645	-8.123912816	4.5139E-16	1.41132E-15	negative
PRJNA729271	stage_1W_vs_E10	ordinary_muscle_development	ENSGALG00010024564	PPP1R3D	2.429767678	0.198535889	12.23843047	1.93743E-34	1.28526E-33	positive
PRJNA729271	stage_1W_vs_E10	ordinary_muscle_development	ENSGALG00010024565	FAM217B	0.536771973	0.138820222	3.866669903	0.000110332	0.000176889	positive
PRJNA729271	stage_1W_vs_E10	ordinary_muscle_development	ENSGALG00010024566	MTSS2	-0.299318228	0.22551458	-1.327267742	0.184420131	0.213392655	negative
PRJNA729271	stage_1W_vs_E10	ordinary_muscle_development	ENSGALG00010024567	IKZF3	1.780982204	0.409059158	4.353849975	1.33767E-05	2.29816E-05	positive
PRJNA729271	stage_1W_vs_E10	ordinary_muscle_development	ENSGALG00010024568	DZIP1L	0.120842187	0.272092276	0.444122078	0.656954325	0.687797251	positive
PRJNA729271	stage_1W_vs_E10	ordinary_muscle_development	ENSGALG00010024569	ASCL2	-1.524057776	0.291680517	-5.225092821	1.74068E-07	3.41978E-07	negative
PRJNA729271	stage_1W_vs_E10	ordinary_muscle_development	ENSGALG00010024570	TPM1	6.525205777	0.164135227	39.75505997	0	0	positive
PRJNA729271	stage_1W_vs_E10	ordinary_muscle_development	ENSGALG00010024571	KCNA3	-3.430667258	0.417776169	-8.211735164	2.18015E-16	6.8848E-16	negative
PRJNA729271	stage_1W_vs_E10	ordinary_muscle_development	ENSGALG00010024573	ENSGALG00010024573	1.271024884	0.273814347	4.641922147	3.45183E-06	6.20157E-06	positive
PRJNA729271	stage_1W_vs_E10	ordinary_muscle_development	ENSGALG00010024575	TXNRD2	0.530194269	0.125426618	4.227127198	2.36694E-05	3.98661E-05	positive
PRJNA729271	stage_1W_vs_E10	ordinary_muscle_development	ENSGALG00010024576	CSAD	0.092577215	0.156522821	0.591461454	0.554211275	0.590292632	positive
PRJNA729271	stage_1W_vs_E10	ordinary_muscle_development	ENSGALG00010024578	ENSGALG00010024578	2.002657663	0.588397987	3.403576672	0.000665097	0.00100538	positive
PRJNA729271	stage_1W_vs_E10	ordinary_muscle_development	ENSGALG00010024579	ZNF740	-0.12917381	0.105722192	-1.221823049	0.22177455	0.253236601	negative
PRJNA729271	stage_1W_vs_E10	ordinary_muscle_development	ENSGALG00010024580	IGF2	-0.167663981	0.194103602	-0.863786034	0.387705441	0.425838476	negative
PRJNA729271	stage_1W_vs_E10	ordinary_muscle_development	ENSGALG00010024581	RARA	0.933462463	0.17187904	5.430926677	5.60622E-08	1.14036E-07	positive
PRJNA729271	stage_1W_vs_E10	ordinary_muscle_development	ENSGALG00010024582	TLN2	1.397040319	0.10735431	13.01336031	1.02724E-38	7.86571E-38	positive
PRJNA729271	stage_1W_vs_E10	ordinary_muscle_development	ENSGALG00010024583	ITGB7	1.875940424	0.504980109	3.714879834	0.0002033	0.000319614	positive
PRJNA729271	stage_1W_vs_E10	ordinary_muscle_development	ENSGALG00010024585	TBCCD1	-1.380707484	0.132094955	-10.45238622	1.42884E-25	6.74188E-25	negative
PRJNA729271	stage_1W_vs_E10	ordinary_muscle_development	ENSGALG00010024586	DLGAP4	0.329676783	0.095646642	3.446820273	0.000567226	0.000861477	positive
PRJNA729271	stage_1W_vs_E10	ordinary_muscle_development	ENSGALG00010024587	ENSGALG00010024587	3.43576867	0.178506666	19.24728492	1.48757E-82	3.80289E-81	positive
PRJNA729271	stage_1W_vs_E10	ordinary_muscle_development	ENSGALG00010024588	TTC39A	-4.549813739	0.414668293	-10.97217659	5.20052E-28	2.70864E-27	negative
PRJNA729271	stage_1W_vs_E10	ordinary_muscle_development	ENSGALG00010024589	ADORA1	0.410609222	0.490733471	0.836725527	0.402746867	0.440889346	positive
PRJNA729271	stage_1W_vs_E10	ordinary_muscle_development	ENSGALG00010024590	MRPL23	0.535965638	0.103874066	5.159763749	2.47262E-07	4.8134E-07	positive
PRJNA729271	stage_1W_vs_E10	ordinary_muscle_development	ENSGALG00010024592	COMT	-0.122771014	0.198294139	-0.619135869	0.535826856	0.572267188	negative
PRJNA729271	stage_1W_vs_E10	ordinary_muscle_development	ENSGALG00010024593	SRSF6	-0.823115209	0.075158427	-10.95173552	6.51865E-28	3.38181E-27	negative
PRJNA729271	stage_1W_vs_E10	ordinary_muscle_development	ENSGALG00010024594	TNNI2	6.363240296	0.183198354	34.73415656	2.4044E-264	6.4541E-262	positive
PRJNA729271	stage_1W_vs_E10	ordinary_muscle_development	ENSGALG00010024595	MYBPHL	5.947753237	0.316335416	18.80204659	7.2658E-79	1.66906E-77	positive
PRJNA729271	stage_1W_vs_E10	ordinary_muscle_development	ENSGALG00010024596	EPS15	0.95918139	0.066723399	14.37548762	7.37488E-47	7.29187E-46	positive
PRJNA729271	stage_1W_vs_E10	ordinary_muscle_development	ENSGALG00010024597	DNAJB11	-1.623606032	0.102862755	-15.78419739	3.99701E-56	5.31529E-55	negative
PRJNA729271	stage_1W_vs_E10	ordinary_muscle_development	ENSGALG00010024598	LRRC3C	1.108805407	0.236304937	4.692265086	2.70197E-06	4.89013E-06	positive
PRJNA729271	stage_1W_vs_E10	ordinary_muscle_development	ENSGALG00010024599	ENSGALG00010024599	0.241863349	0.217526577	1.111879533	0.26618995	0.300009589	positive
PRJNA729271	stage_1W_vs_E10	ordinary_muscle_development	ENSGALG00010024600	CD9	1.239433496	0.333440338	3.717107246	0.000201517	0.000316947	positive
PRJNA729271	stage_1W_vs_E10	ordinary_muscle_development	ENSGALG00010024601	ENSGALG00010024601	-1.028356526	0.515414078	-1.995204574	0.046020572	0.057709854	negative
PRJNA729271	stage_1W_vs_E10	ordinary_muscle_development	ENSGALG00010024602	PROCR	1.102972351	0.241272279	4.571483944	4.84282E-06	8.59412E-06	positive
PRJNA729271	stage_1W_vs_E10	ordinary_muscle_development	ENSGALG00010024603	ENSGALG00010024603	0.577381981	1.07594419	0.536628188	0.591524469	0.626124374	positive
PRJNA729271	stage_1W_vs_E10	ordinary_muscle_development	ENSGALG00010024604	MFSD5	1.446658936	0.168252487	8.598142974	8.10163E-18	2.7291E-17	positive
PRJNA729271	stage_1W_vs_E10	ordinary_muscle_development	ENSGALG00010024605	LSP1P1	-1.510260131	0.196273196	-7.694683544	1.41845E-14	4.12445E-14	negative
PRJNA729271	stage_1W_vs_E10	ordinary_muscle_development	ENSGALG00010024607	RARG	-0.819379581	0.142790395	-5.738338217	9.561E-09	2.03E-08	negative
PRJNA729271	stage_1W_vs_E10	ordinary_muscle_development	ENSGALG00010024608	DENND2D	1.312137063	0.333421908	3.935365473	8.30702E-05	0.000134476	positive
PRJNA729271	stage_1W_vs_E10	ordinary_muscle_development	ENSGALG00010024609	PRR33	14.7926692	0.836400761	17.68610202	5.36616E-70	1.01544E-68	positive
PRJNA729271	stage_1W_vs_E10	ordinary_muscle_development	ENSGALG00010024610	PFKL	-0.885111438	0.138326622	-6.398706387	1.56699E-10	3.68307E-10	negative
PRJNA729271	stage_1W_vs_E10	ordinary_muscle_development	ENSGALG00010024611	ENSGALG00010024611	-1.947231857	0.281544562	-6.916247455	4.63764E-12	1.186E-11	negative
PRJNA729271	stage_1W_vs_E10	ordinary_muscle_development	ENSGALG00010024612	ENSGALG00010024612	-1.538871364	0.60321082	-2.551133557	0.010737317	0.014468477	negative
PRJNA729271	stage_1W_vs_E10	ordinary_muscle_development	ENSGALG00010024613	ENSGALG00010024613	-0.235043158	0.096076979	-2.446404543	0.0144289	0.019154401	negative
PRJNA729271	stage_1W_vs_E10	ordinary_muscle_development	ENSGALG00010024614	ENSGALG00010024614	-5.036623913	0.76929514	-6.547063217	5.86795E-11	1.41148E-10	negative
PRJNA729271	stage_1W_vs_E10	ordinary_muscle_development	ENSGALG00010024615	SYT8	-2.714712465	0.680471067	-3.989460534	6.62237E-05	0.000108074	negative
PRJNA729271	stage_1W_vs_E10	ordinary_muscle_development	ENSGALG00010024616	ARVCF	-1.193493526	0.099477616	-11.99760883	3.6571E-33	2.32498E-32	negative
PRJNA729271	stage_1W_vs_E10	ordinary_muscle_development	ENSGALG00010024618	VPS13C	-0.053368703	0.122869143	-0.434353995	0.6640314	0.694404094	negative
PRJNA729271	stage_1W_vs_E10	ordinary_muscle_development	ENSGALG00010024619	ENSGALG00010024619	-0.257122138	0.214537538	-1.198494864	0.230724421	0.262446087	negative
PRJNA729271	stage_1W_vs_E10	ordinary_muscle_development	ENSGALG00010024620	MSANTD2	-0.726193027	0.132096886	-5.497427289	3.85372E-08	7.90318E-08	negative
PRJNA729271	stage_1W_vs_E10	ordinary_muscle_development	ENSGALG00010024621	THRA	1.488227816	0.184749002	8.055403815	7.92168E-16	2.44777E-15	positive
PRJNA729271	stage_1W_vs_E10	ordinary_muscle_development	ENSGALG00010024622	PRMT8	-1.096647798	0.213689318	-5.131972939	2.86721E-07	5.55246E-07	negative
PRJNA729271	stage_1W_vs_E10	ordinary_muscle_development	ENSGALG00010024623	NRDC	1.176390931	0.109361334	10.75691827	5.4978E-27	2.74228E-26	positive
PRJNA729271	stage_1W_vs_E10	ordinary_muscle_development	ENSGALG00010024624	CTSD	0.867524151	0.128694965	6.740933132	1.57373E-11	3.91205E-11	positive
PRJNA729271	stage_1W_vs_E10	ordinary_muscle_development	ENSGALG00010024625	SPRYD3	0.527213795	0.079298799	6.648446148	2.96203E-11	7.26475E-11	positive
PRJNA729271	stage_1W_vs_E10	ordinary_muscle_development	ENSGALG00010024626	TSHZ3	-1.861164712	0.100716201	-18.47929814	3.03098E-76	6.58682E-75	negative
PRJNA729271	stage_1W_vs_E10	ordinary_muscle_development	ENSGALG00010024627	CEPT1	0.372321805	0.078929134	4.717165724	2.39153E-06	4.34292E-06	positive
PRJNA729271	stage_1W_vs_E10	ordinary_muscle_development	ENSGALG00010024628	TANGO2	0.192513569	0.111603864	1.724972265	0.084532471	0.1026041	positive
PRJNA729271	stage_1W_vs_E10	ordinary_muscle_development	ENSGALG00010024629	ENSGALG00010024629	1.220113766	0.172007587	7.093371772	1.30883E-12	3.44686E-12	positive
PRJNA729271	stage_1W_vs_E10	ordinary_muscle_development	ENSGALG00010024630	CFAP410	-0.899273016	0.09910984	-9.073498812	1.15255E-19	4.26615E-19	negative
PRJNA729271	stage_1W_vs_E10	ordinary_muscle_development	ENSGALG00010024631	EPB41L1	-1.027653269	0.150853919	-6.812241098	9.60899E-12	2.4202E-11	negative
PRJNA729271	stage_1W_vs_E10	ordinary_muscle_development	ENSGALG00010024632	IFITM10	-1.092079364	0.441529611	-2.473400056	0.013383423	0.017848258	negative
PRJNA729271	stage_1W_vs_E10	ordinary_muscle_development	ENSGALG00010024633	NRGN	4.154173669	0.278415267	14.92078261	2.41425E-50	2.67747E-49	positive
PRJNA729271	stage_1W_vs_E10	ordinary_muscle_development	ENSGALG00010024634	ENSGALG00010024634	1.68970696	0.164460208	10.27426014	9.2048E-25	4.19044E-24	positive
PRJNA729271	stage_1W_vs_E10	ordinary_muscle_development	ENSGALG00010024635	ENSGALG00010024635	-0.227820044	0.178927988	-1.273249908	0.202929376	0.233319688	negative
PRJNA729271	stage_1W_vs_E10	ordinary_muscle_development	ENSGALG00010024636	PANX3	-1.830838512	0.312270646	-5.862986271	4.54616E-09	9.84707E-09	negative
PRJNA729271	stage_1W_vs_E10	ordinary_muscle_development	ENSGALG00010024637	DUSP8	2.339514365	0.276718553	8.454490461	2.80309E-17	9.2008E-17	positive
PRJNA729271	stage_1W_vs_E10	ordinary_muscle_development	ENSGALG00010024638	ZNF507	0.447521119	0.129340848	3.460013814	0.000540148	0.000821471	positive

PRJNA729271	stage_1W_vs_E10	ordinary_muscle_development	ENSGALG00010024639	AAR2	-0.387973538	0.076737088	-5.05588038	4.2841E-07	8.20317E-07	negative
PRJNA729271	stage_1W_vs_E10	ordinary_muscle_development	ENSGALG00010024640	GSDMA	-0.797594281	0.296749014	-2.687773993	0.007193006	0.009878032	negative
PRJNA729271	stage_1W_vs_E10	ordinary_muscle_development	ENSGALG00010024641	TRA2B	-1.490667829	0.088740485	-16.79805822	2.52165E-63	4.01663E-62	negative
PRJNA729271	stage_1W_vs_E10	ordinary_muscle_development	ENSGALG00010024642	RAB3B	0.66892453	0.224785265	2.975837982	0.002921892	0.004168995	positive
PRJNA729271	stage_1W_vs_E10	ordinary_muscle_development	ENSGALG00010024643	ETV7	1.507931471	0.394693423	3.820513297	0.000133174	0.000212174	positive
PRJNA729271	stage_1W_vs_E10	ordinary_muscle_development	ENSGALG00010024644	EIF6	-0.530534822	0.075707534	-7.007688562	2.42287E-12	6.29157E-12	negative
PRJNA729271	stage_1W_vs_E10	ordinary_muscle_development	ENSGALG00010024645	PARP11	0.467709692	0.118212729	3.956508702	7.60531E-05	0.000123503	positive
PRJNA729271	stage_1W_vs_E10	ordinary_muscle_development	ENSGALG00010024646	RORA	1.559914283	0.192679672	8.095894437	5.68451E-16	1.76893E-15	positive
PRJNA729271	stage_1W_vs_E10	ordinary_muscle_development	ENSGALG00010024647	RPUSD4	-0.181244746	0.134253413	-1.350019646	0.177009681	0.2053097	negative
PRJNA729271	stage_1W_vs_E10	ordinary_muscle_development	ENSGALG00010024648	MMP24	-1.749717909	0.127073163	-13.76937403	3.89574E-43	3.44742E-42	negative
PRJNA729271	stage_1W_vs_E10	ordinary_muscle_development	ENSGALG00010024649	NCOR2	-0.107526451	0.129904355	-0.82773554	0.407820287	0.445750958	negative
PRJNA729271	stage_1W_vs_E10	ordinary_muscle_development	ENSGALG00010024650	PRKAG1	1.229243965	0.120460159	10.20456865	1.8916E-24	8.48877E-24	positive
PRJNA729271	stage_1W_vs_E10	ordinary_muscle_development	ENSGALG00010024651	PSMD3	0.761287426	0.127551352	5.968477918	2.39477E-09	5.27782E-09	positive
PRJNA729271	stage_1W_vs_E10	ordinary_muscle_development	ENSGALG00010024652	PUS3	0.249379569	0.122242464	2.04004042	0.0413463	0.052157208	positive
PRJNA729271	stage_1W_vs_E10	ordinary_muscle_development	ENSGALG00010024653	RFLNA	-3.484073961	0.259669697	-13.41732978	4.78651E-41	3.96913E-40	negative
PRJNA729271	stage_1W_vs_E10	ordinary_muscle_development	ENSGALG00010024654	CCDC92	-0.878110914	0.275041083	-3.192653638	0.001409719	0.002071351	negative
PRJNA729271	stage_1W_vs_E10	ordinary_muscle_development	ENSGALG00010024655	IGF2BP2	-1.685114562	0.167248486	-10.07551459	7.08887E-24	3.10902E-23	negative
PRJNA729271	stage_1W_vs_E10	ordinary_muscle_development	ENSGALG00010024656	HYLS1	-0.88034599	0.187381781	-4.698140807	2.6254E-06	4.75631E-06	negative
PRJNA729271	stage_1W_vs_E10	ordinary_muscle_development	ENSGALG00010024657	DPY19L3	-1.021372606	0.129267731	-7.901218632	2.7619E-15	8.32301E-15	negative
PRJNA729271	stage_1W_vs_E10	ordinary_muscle_development	ENSGALG00010024659	BEND5	1.013062302	0.107058898	9.462663248	3.00198E-21	1.18857E-20	positive
PRJNA729271	stage_1W_vs_E10	ordinary_muscle_development	ENSGALG00010024660	KMT2D	1.181991396	0.157073527	7.525083424	5.26867E-14	1.48926E-13	positive
PRJNA729271	stage_1W_vs_E10	ordinary_muscle_development	ENSGALG00010024662	MOB2	0.440020789	0.07127538	6.173531325	6.67813E-10	1.51533E-09	positive
PRJNA729271	stage_1W_vs_E10	ordinary_muscle_development	ENSGALG00010024663	ICE2	-1.158488143	0.125196187	-9.253382034	2.17499E-20	8.30739E-20	negative
PRJNA729271	stage_1W_vs_E10	ordinary_muscle_development	ENSGALG00010024664	DNAH10	-4.349337119	0.277333918	-15.68267288	1.98713E-55	2.59959E-54	negative
PRJNA729271	stage_1W_vs_E10	ordinary_muscle_development	ENSGALG00010024665	MAP1LC3A	3.637804312	0.147491571	24.66448963	2.573E-134	1.751E-132	positive
PRJNA729271	stage_1W_vs_E10	ordinary_muscle_development	ENSGALG00010024666	MED24	-1.204604611	0.116424702	-10.3466411	4.33426E-25	2.00271E-24	negative
PRJNA729271	stage_1W_vs_E10	ordinary_muscle_development	ENSGALG00010024667	RBM7	1.44486793	0.134883253	10.71198906	8.94196E-27	4.42519E-26	positive
PRJNA729271	stage_1W_vs_E10	ordinary_muscle_development	ENSGALG00010024668	ENSGALG00010024668	1.008927001	0.184742237	5.461268738	4.72744E-08	9.65402E-08	positive
PRJNA729271	stage_1W_vs_E10	ordinary_muscle_development	ENSGALG00010024669	STIL	-1.907166789	0.189191855	-10.08059669	6.73161E-24	2.95412E-23	negative
PRJNA729271	stage_1W_vs_E10	ordinary_muscle_development	ENSGALG00010024670	SENP2	0.718324644	0.094462815	7.604311232	2.86425E-14	8.20176E-14	positive
PRJNA729271	stage_1W_vs_E10	ordinary_muscle_development	ENSGALG00010024671	KCTD20	1.835352238	0.280145271	6.551430376	5.69886E-11	1.37309E-10	positive
PRJNA729271	stage_1W_vs_E10	ordinary_muscle_development	ENSGALG00010024672	DYNLRB1	-0.514061873	0.105743887	-4.861386194	1.16567E-06	2.16981E-06	negative
PRJNA729271	stage_1W_vs_E10	ordinary_muscle_development	ENSGALG00010024673	REXO2	1.094158414	0.097751859	11.19322361	4.40135E-29	2.38942E-28	positive
PRJNA729271	stage_1W_vs_E10	ordinary_muscle_development	ENSGALG00010024674	DHH	2.459944249	0.32509219	7.566912774	3.82198E-14	1.08776E-13	positive
PRJNA729271	stage_1W_vs_E10	ordinary_muscle_development	ENSGALG00010024675	NR1D1	5.027870495	0.408535407	12.30706178	8.29898E-35	5.60287E-34	positive
PRJNA729271	stage_1W_vs_E10	ordinary_muscle_development	ENSGALG00010024676	ENSGALG00010024676	0.653395347	0.167010096	3.912310464	9.14173E-05	0.000147511	positive
PRJNA729271	stage_1W_vs_E10	ordinary_muscle_development	ENSGALG00010024677	LMBR1L	-2.565431017	0.381583784	-6.723113315	1.77882E-11	4.41356E-11	negative
PRJNA729271	stage_1W_vs_E10	ordinary_muscle_development	ENSGALG00010024678	AMOTL2	0.40471296	0.091797387	4.408763395	1.03963E-05	1.80212E-05	positive
PRJNA729271	stage_1W_vs_E10	ordinary_muscle_development	ENSGALG00010024679	MSL1	0.380687748	0.110992481	3.429851693	0.000603911	0.000915763	positive
PRJNA729271	stage_1W_vs_E10	ordinary_muscle_development	ENSGALG00010024680	BRSK1	-1.859902693	0.284812035	-6.530281247	6.56463E-11	1.57254E-10	negative
PRJNA729271	stage_1W_vs_E10	ordinary_muscle_development	ENSGALG00010024681	ATP6V0A2	1.050445731	0.085218261	12.32653322	6.51916E-35	4.4136E-34	positive
PRJNA729271	stage_1W_vs_E10	ordinary_muscle_development	ENSGALG00010024682	ENSGALG00010024682	-2.029246268	0.179357506	-11.31397463	1.11903E-29	6.23859E-29	negative
PRJNA729271	stage_1W_vs_E10	ordinary_muscle_development	ENSGALG00010024683	SHISA4	3.809438482	0.173237353	21.98970613	3.6132E-107	1.5404E-105	positive
PRJNA729271	stage_1W_vs_E10	ordinary_muscle_development	ENSGALG00010024684	ENSGALG00010024684	-0.177374524	0.31705318	-0.559447232	0.575856534	0.61146	negative
PRJNA729271	stage_1W_vs_E10	ordinary_muscle_development	ENSGALG00010024685	ENSGALG00010024685	-1.208035919	0.222122486	-5.438602556	5.37001E-08	1.09339E-07	negative
PRJNA729271	stage_1W_vs_E10	ordinary_muscle_development	ENSGALG00010024686	CMPK1	-1.483535662	0.134373324	-11.04040313	2.43936E-28	1.28717E-27	negative
PRJNA729271	stage_1W_vs_E10	ordinary_muscle_development	ENSGALG00010024687	RYK	-0.213538574	0.107295773	-1.990186275	0.04657042	0.058374112	negative
PRJNA729271	stage_1W_vs_E10	ordinary_muscle_development	ENSGALG00010024688	CADM1	-2.922005364	0.135244951	-21.60528247	1.6021E-103	6.4149E-102	negative
PRJNA729271	stage_1W_vs_E10	ordinary_muscle_development	ENSGALG00010024689	FOXD2	1.162305445	0.301510395	3.854943193	0.000115757	0.000185279	positive
PRJNA729271	stage_1W_vs_E10	ordinary_muscle_development	ENSGALG00010024690	TUBA1C	-2.935733612	0.649677668	-4.518754077	6.22046E-06	1.09411E-05	negative
PRJNA729271	stage_1W_vs_E10	ordinary_muscle_development	ENSGALG00010024691	TCTN2	-1.120041301	0.153258311	-7.308192921	2.70759E-13	7.3578E-13	negative
PRJNA729271	stage_1W_vs_E10	ordinary_muscle_development	ENSGALG00010024692	DRAM2	1.45186947	0.106008861	13.69573697	1.07662E-42	9.40667E-42	positive
PRJNA729271	stage_1W_vs_E10	ordinary_muscle_development	ENSGALG00010024693	ANXA2	0.219249924	0.201628673	1.08739457	0.27686247	0.311208719	positive
PRJNA729271	stage_1W_vs_E10	ordinary_muscle_development	ENSGALG00010024694	GTF2H3	-0.615306575	0.097961361	-6.281114993	3.36153E-10	7.75512E-10	negative
PRJNA729271	stage_1W_vs_E10	ordinary_muscle_development	ENSGALG00010024695	TRABD2B	-0.320690719	0.1953495	-1.641625495	0.100667641	0.121003189	negative
PRJNA729271	stage_1W_vs_E10	ordinary_muscle_development	ENSGALG00010024696	TRADD	-0.750353296	0.118725585	-6.320064018	2.61455E-10	6.05979E-10	negative
PRJNA729271	stage_1W_vs_E10	ordinary_muscle_development	ENSGALG00010024697	IGF2BP1	-1.628857525	0.122637744	-13.28186143	2.94969E-40	2.37929E-39	negative
PRJNA729271	stage_1W_vs_E10	ordinary_muscle_development	ENSGALG00010024698	ENSGALG00010024698	-1.932328303	0.379823703	-5.087434742	3.62939E-07	6.98924E-07	negative
PRJNA729271	stage_1W_vs_E10	ordinary_muscle_development	ENSGALG00010024699	FBXL8	-0.769016966	0.135660154	-5.668701855	1.43883E-08	3.02216E-08	negative
PRJNA729271	stage_1W_vs_E10	ordinary_muscle_development	ENSGALG00010024700	TOLLIP	0.747265549	0.084170414	8.878007327	6.80688E-19	2.4278E-18	positive
PRJNA729271	stage_1W_vs_E10	ordinary_muscle_development	ENSGALG00010024701	EIF2B1	0.268899411	0.100141342	2.6851988	0.007248668	0.009946931	positive
PRJNA729271	stage_1W_vs_E10	ordinary_muscle_development	ENSGALG00010024702	INAVA	-2.317499889	0.804281656	-2.881453123	0.003958461	0.005575541	negative
PRJNA729271	stage_1W_vs_E10	ordinary_muscle_development	ENSGALG00010024703	HSF4	0.039031332	0.568393798	0.068669525	0.945252677	0.952216106	positive
PRJNA729271	stage_1W_vs_E10	ordinary_muscle_development	ENSGALG00010024705	SLCO2A1	2.468399315	0.235438295	10.4842728	1.02028E-25	4.83455E-25	positive
PRJNA729271	stage_1W_vs_E10	ordinary_muscle_development	ENSGALG00010024706	ENSGALG00010024706	1.65823041	0.960495994	1.726431366	0.084269837	0.102311022	positive
PRJNA729271	stage_1W_vs_E10	ordinary_muscle_development	ENSGALG00010024707	ENSGALG00010024707	-0.810213691	0.375887682	-2.155467525	0.031125267	0.039795426	negative
PRJNA729271	stage_1W_vs_E10	ordinary_muscle_development	ENSGALG00010024708	BNIP2	0.555547404	0.063015509	8.816042517	1.18576E-18	4.16772E-18	positive
PRJNA729271	stage_1W_vs_E10	ordinary_muscle_development	ENSGALG00010024709	TEAD4	1.118621559	0.096714264	11.56625209	6.10935E-31	3.57798E-30	positive
PRJNA729271	stage_1W_vs_E10	ordinary_muscle_development	ENSGALG00010024710	HSDL1	-0.625130603	0.128881477	-4.850430151	1.23194E-06	2.28674E-06	negative
PRJNA729271	stage_1W_vs_E10	ordinary_muscle_development	ENSGALG00010024711	DDX55	-0.652229635	0.093718859	-6.959427833	3.41657E-12	8.801E-12	negative
PRJNA729271	stage_1W_vs_E10	ordinary_muscle_development	ENSGALG00010024712	LRIF1	0.598002922	0.09827917	6.084737196	1.16683E-09	2.61651E-09	positive
PRJNA729271	stage_1W_vs_E10	ordinary_muscle_development	ENSGALG00010024713	RND2	-1.04253487	0.410913808	-2.53711326	0.011177079	0.015021952	negative
PRJNA729271	stage_1W_vs_E10	ordinary_muscle_development	ENSGALG00010024714	SRPRB	-0.486326275	0.1749647	-2.779567961	0.005443126	0.00757325	negative
PRJNA729271	stage_1W_vs_E10	ordinary_muscle_development	ENSGALG00010024715	DNAAF1	-0.371556647	0.181276597	-2.049666931	0.040396944	0.051010864	negative
PRJNA729271	stage_1W_vs_E10	ordinary_muscle_development	ENSGALG00010024716	GTF2A2	-0.232022944	0.077313869	-3.001052025	0.002690486	0.003851333	negative
PRJNA729271	stage_1W_vs_E10	ordinary_muscle_development	ENSGALG00010024717	TF	2.205737522	0.652599878	3.379923284	0.000725061	0.001093399	positive
PRJNA729271	stage_1W_vs_E10	ordinary_muscle_development	ENSGALG00010024718	ENSGALG00010024718	1.239988887	0.192352613	6.446436397	1.14511E-10	2.71214E-10	positive
PRJNA729271	stage_1W_vs_E10	ordinary_muscle_development	ENSGALG00010024719	PYM1	-0.737721741	0.136720234	-5.395849008	6.82003E-08	1.37894E-07	negative
PRJNA729271	stage_1W_vs_E10	ordinary_muscle_development	ENSGALG00010024720	BUD13	-0.232055364	0.108057695	-2.147513555	0.031752419	0.040557924	negative
PRJNA729271	stage_1W_vs_E10	ordinary_muscle_development	ENSGALG00010024721	ENSGALG00010024721	0.978388196	0.765019675	1.278905927	0.200930185	0.23124121	positive
PRJNA729271	stage_1W_vs_E10	ordinary_muscle_development	ENSGALG00010024722	BRCA1	-3.037514919	0.26333013	-11.53500708	8.78799E-31	5.1281E-30	negative
PRJNA729271	stage_1W_vs_E10	ordinary_muscle_development	ENSGALG00010024723	ITCH	0.38693097	0.100560199	3.847754605	0.000119205	0.000190652	positive
PRJNA729271	stage_1W_vs_E10	ordinary_muscle_development	ENSGALG00010024724	HOXD3	-1.741796904	0.258699111	-6.732906419	1.66307E-11	4.1306E-11	negative
PRJNA729271	stage_1W_vs_E10	ordinary_muscle_development	ENSGALG00010024726	TMED2	-0.326346475	0.058744313	-5.555371418	2.77022E-08	5.72244E-08	negative
PRJNA729271	stage_1W_vs_E10	ordinary_muscle_development	ENSGALG00010024728	SLC5A9	-3.365418602	1.033836003	-3.255273171	0.001132833	0.001677607	negative
PRJNA729271	stage_1W_vs_E10	ordinary_muscle_development	ENSGALG00010024729	FOXM1	-2.047244718	0.169329485	-12.09030265	1.18848E-33	7.68721E-33	negative
PRJNA729271	stage_1W_vs_E10	ordinary_muscle_development	ENSGALG00010024731	AOX1	4.98544945	0.197385486	25.25742678	9.386E-141	6.8367E-139	positive
PRJNA729271	stage_1W_vs_E10	ordinary_muscle_development	ENSGALG00010024732	AHCY	-1.18479972	0.224962049	-5.266664873	1.38924E-07	2.74797E-07	negative
PRJNA729271	stage_1W_vs_E10	ordinary_muscle_development	ENSGALG00010024733	MBTPS1	-0.483415754	0.088469179	-5.464227895	4.64926E-08	9.5024E-08	negative
PRJNA729271	stage_1W_vs_E10	ordinary_muscle_development	ENSGALG00010024734	ZPR1	0.396613618	0.113957889	3.480352434	0.000500755	0.000763725	positive
PRJNA729271	stage_1W_vs_E10	ordinary_muscle_development	ENSGALG00010024735	OTUD7A	2.470157582	0.178150902	13.86553512	1.02459E-43	9.23598E-43	positive
PRJNA729271	stage_1W_vs_E10	ordinary_muscle_development	ENSGALG00010024736	DGKA	-0.254188372	0.151118392	-1.682047888	0.092559554	0.111860158	negative
PRJNA729271	stage_1W_vs_E10	ordinary_muscle_development	ENSGALG00010024737	STK38	-1.035729849	0.063532197	-16.30244021	9.48293E-60	1.37455E-58	negative
PRJNA729271	stage_1W_vs_E10	ordinary_muscle_development	ENSGALG00010024738	NBR1	0.203988839	0.078415909	2.601370591	0.009285209	0.012599617	positive
PRJNA729271	stage_1W_vs_E10	ordinary_muscle_development	ENSGALG00010024739	ASIP	-8.566065	0.6441695	-13.2978432	2.38242E-40	1.93139E-39	negative
PRJNA729271	stage_1W_vs_E10	ordinary_muscle_development	ENSGALG00010024741	RILPL2	-1.034127913	0.158583757	-6.521020372	6.98307E-11	1.67029E-10	negative
PRJNA729271	stage_1W_vs_E10	ordinary_muscle_development	ENSGALG00010024742	PAK2	-0.664911204	0.070822586	-9.38840616	6.0915E-21	2.37931E-20	negative
PRJNA729271	stage_1W_vs_E10	ordinary_muscle_development	ENSGALG00010024744	KLF13	2.976588245	0.130056992	22.88679901	6.2892E-116	3.1435E-114	positive
PRJNA729271	stage_1W_vs_E10	ordinary_muscle_development	ENSGALG00010024745	PMEL	-5.982225112	0.639217588	-9.358667881	8.07483E-21	3.14298E-20	negative
PRJNA729271	stage_1W_vs_E10	ordinary_muscle_development	ENSGALG00010024746	HOXD4	-1.126147	0.321026941	-3.507951687	0.000451571	0.000690675	negative
PRJNA729271	stage_1W_vs_E10	ordinary_muscle_development	ENSGALG00010024747	TULP3	0.130598868	0.086566303	1.508657093	0.131386436	0.155325535	positive
PRJNA729271	stage_1W_vs_E10	ordinary_muscle_development	ENSGALG00010024748	TRPM1	1.248464585	0.386202023	3.232672306	0.001226382	0.001809671	positive
PRJNA729271	stage_1W_vs_E10	ordinary_muscle_development	ENSGALG00010024749	APOA5	2.525218701	0.668421879	3.777881576	0.000158168	0.000250699	positive
PRJNA729271	stage_1W_vs_E10	ordinary_muscle_development	ENSGALG00010024750	APOA4	5.355263227	1.550834186	3.453150101	0.000554081	0.000842042	positive
PRJNA729271	stage_1W_vs_E10	ordinary_muscle_development	ENSGALG00010024751	MTMR10	0.945420184	0.056523246	16.72621891	8.4431E-63	1.3202E-61	positive
PRJNA729271	stage_1W_vs_E10	ordinary_muscle_development	ENSGALG00010024752	MPP2	-0.273461206	0.220151038	-1.2421527	0.214180228	0.24520515	negative
PRJNA729271	stage_1W_vs_E10	ordinary_muscle_development	ENSGALG00010024753	HOXD8	-1.124561709	0.177181122	-6.346961201	2.19609E-10	5.11035E-10	negative
PRJNA729271	stage_1W_vs_E10	ordinary_muscle_development	ENSGALG00010024754	EIF2S2	1.950144203	0.144487904	13.49693743	1.63013E-41	1.37777E-40	positive
PRJNA729271	stage_1W_vs_E10	ordinary_muscle_development	ENSGALG00010024755	PIGX	0.482815186	0.157443804	3.066587409	0.002165175	0.003125295	positive
PRJNA729271	stage_1W_vs_E10	ordinary_muscle_development	ENSGALG00010024756	CDK2	-2.287807509	0.168787219	-13.55438828	7.46317E-42	6.37095E-41	negative
PRJNA729271	stage_1W_vs_E10	ordinary_muscle_development	ENSGALG00010024757	PPFIA1	-0.557831955	0.0760726	-7.332889338	2.25243E-13	6.14627E-13	negative
PRJNA729271	stage_1W_vs_E10	ordinary_muscle_development	ENSGALG00010024758	SPATA6	-0.153336949	0.106911313	-1.43424437	0.151502539	0.177614794	negative
PRJNA729271	stage_1W_vs_E10	ordinary_muscle_development	ENSGALG00010024759	APOC3	3.660165833	0.962205101	3.803935177			positive
PRJNA729271	stage_1W_vs_E10	ordinary_muscle_development	ENSGALG00010024760	ENSGALG00010024760	2.606128738	0.185460091	14.05223478	7.46409E-45	6.94875E-44	positive
PRJNA729271	stage_1W_vs_E10	ordinary_muscle_development	ENSGALG00010024761	NECAB2	0.429377623	0.234389537	1.831897568	0.066966683	0.082289087	positive
PRJNA729271	stage_1W_vs_E10	ordinary_muscle_development	ENSGALG00010024762	FAN1	-0.363272706	0.090043745	-4.034402441	5.47414E-05	8.97802E-05	negative
PRJNA729271	stage_1W_vs_E10	ordinary_muscle_development	ENSGALG00010024763	HOXD9	-1.125953144	0.311566701	-3.613843011	0.000301692	0.000468907	negative
PRJNA729271	stage_1W_vs_E10	ordinary_muscle_development	ENSGALG00010024764	ENSGALG00010024764	-0.043088121	0.265996932	-0.161987285	0.871315867	0.885612754	negative
PRJNA729271	stage_1W_vs_E10	ordinary_muscle_development	ENSGALG00010024765	SNRNP35	-0.203211407	0.134247013	-1.513712691	0.130098725	0.153891057	negative
PRJNA729271	stage_1W_vs_E10	ordinary_muscle_development	ENSGALG00010024767	CTTN	-1.336977713	0.069902255	-19.12638899	1.52263E-81	3.77274E-80	negative
PRJNA729271	stage_1W_vs_E10	ordinary_muscle_development	ENSGALG00010024769	RALY	4.176392538	0.203829193	20.48966818	2.66201E-93	8.55563E-92	positive
PRJNA729271	stage_1W_vs_E10	ordinary_muscle_development	ENSGALG00010024770	CDC27	0.557733803	0.071995246	7.746814303	9.42266E-15	2.76761E-14	positive
PRJNA729271	stage_1W_vs_E10	ordinary_muscle_development	ENSGALG00010024771	AOX2	4.996575843	0.263671263	18.95002051	4.41411E-80	1.05408E-78	positive
PRJNA729271	stage_1W_vs_E10	ordinary_muscle_development	ENSGALG00010024772	ELAPOR1	-0.068286869	0.227950104	-0.29956937	0.764505651	0.78832677	negative
PRJNA729271	stage_1W_vs_E10	ordinary_muscle_development	ENSGALG00010024773	APOA1	6.25241208	0.264061212	23.67788907	6.0935E-124	3.6199E-122	positive
PRJNA729271	stage_1W_vs_E10	ordinary_muscle_development	ENSGALG00010024775	ITFG2	0.808610668	0.093985916	8.60353023	7.73009E-18	2.60576E-17	positive
PRJNA729271	stage_1W_vs_E10	ordinary_muscle_development	ENSGALG00010024776	MPHOSPH10	-0.189237809	0.106803102	-1.771838141	0.076421429	0.093203922	negative
PRJNA729271	stage_1W_vs_E10	ordinary_muscle_development	ENSGALG00010024778	RAB5B	0.338455298	0.140206642	2.413974784	0.015779554	0.020844312	positive
PRJNA729271	stage_1W_vs_E10	ordinary_muscle_development	ENSGALG00010024779	ENSGALG00010024779	-5.664399847	1.204783999	-4.70158954	2.58144E-06	4.68017E-06	negative
PRJNA729271	stage_1W_vs_E10	ordinary_muscle_development	ENSGALG00010024780	HOXD11	-4.598086521	0.922289865	-4.985511276	6.17982E-07	1.17155E-06	negative
PRJNA729271	stage_1W_vs_E10	ordinary_muscle_development	ENSGALG00010024781	CEP19	-0.959562883	0.166004117	-5.780355937	7.45427E-09	1.59248E-08	negative

PRJNA729271	stage_1W_vs_E10	ordinary_muscle_development	ENSGALG00010024782	HOXD12	-3.439085292	2.167748945	-1.586477668	0.112630994	0.134479922	negative
PRJNA729271	stage_1W_vs_E10	ordinary_muscle_development	ENSGALG00010024783	UBE2T	-2.715140557	0.245000117	-11.08220108	1.53063E-28	8.1269E-28	negative
PRJNA729271	stage_1W_vs_E10	ordinary_muscle_development	ENSGALG00010024784	ENSGALG00010024784	0.428474103	0.139789892	3.065129375	0.002175759	0.003139946	positive
PRJNA729271	stage_1W_vs_E10	ordinary_muscle_development	ENSGALG00010024785	MCEE	1.117104494	0.199910794	5.588014901	2.2968E-08	4.76964E-08	positive
PRJNA729271	stage_1W_vs_E10	ordinary_muscle_development	ENSGALG00010024786	PDCD5	1.453712066	0.096798204	15.01796527	5.60005E-51	6.34657E-50	positive
PRJNA729271	stage_1W_vs_E10	ordinary_muscle_development	ENSGALG00010024787	SARS1	1.325868648	0.098977976	13.39559265	6.41618E-41	5.29929E-40	positive
PRJNA729271	stage_1W_vs_E10	ordinary_muscle_development	ENSGALG00010024788	SIK3	0.919447732	0.165736315	5.547654008	2.89528E-08	5.97311E-08	positive
PRJNA729271	stage_1W_vs_E10	ordinary_muscle_development	ENSGALG00010024789	SUOX	0.904185835	0.173606829	5.208238869	1.90641E-07	3.73829E-07	positive
PRJNA729271	stage_1W_vs_E10	ordinary_muscle_development	ENSGALG00010024791	SHANK2	-2.390021869	0.419319442	-5.699764028	1.19973E-08	2.53205E-08	negative
PRJNA729271	stage_1W_vs_E10	ordinary_muscle_development	ENSGALG00010024792	BZW1	-0.70572111	0.06916658	-10.20320953	1.91826E-24	8.60045E-24	negative
PRJNA729271	stage_1W_vs_E10	ordinary_muscle_development	ENSGALG00010024793	FKBP4	-1.278495334	0.117351522	-10.8945782	1.22332E-27	6.27239E-27	negative
PRJNA729271	stage_1W_vs_E10	ordinary_muscle_development	ENSGALG00010024794	DDX6	-1.326068768	0.065492157	-20.24774917	3.71846E-91	1.13952E-89	negative
PRJNA729271	stage_1W_vs_E10	ordinary_muscle_development	ENSGALG00010024795	NRROS	0.692974618	0.212302628	3.264088745	0.001098168	0.0016291	positive
PRJNA729271	stage_1W_vs_E10	ordinary_muscle_development	ENSGALG00010024797	IKZF4	0.967995758	0.234560317	4.126852197	3.67763E-05	6.11742E-05	positive
PRJNA729271	stage_1W_vs_E10	ordinary_muscle_development	ENSGALG00010024798	FBXO45	-0.654492216	0.085651696	-7.641322315	2.15002E-14	6.18958E-14	negative
PRJNA729271	stage_1W_vs_E10	ordinary_muscle_development	ENSGALG00010024799	ENSGALG00010024799	1.467016794	0.185410602	7.912259503	2.52759E-15	7.62643E-15	positive
PRJNA729271	stage_1W_vs_E10	ordinary_muscle_development	ENSGALG00010024800	ENSGALG00010024800	0.756640211	0.130276676	5.80794841	6.3243E-09	1.35909E-08	positive
PRJNA729271	stage_1W_vs_E10	ordinary_muscle_development	ENSGALG00010024801	CXCR5	1.999487653	1.118819961	1.787139775	0.0739149	0.090329355	positive
PRJNA729271	stage_1W_vs_E10	ordinary_muscle_development	ENSGALG00010024802	CD59	0.206211429	0.164402298	1.254309895	0.209729389	0.2405656	positive
PRJNA729271	stage_1W_vs_E10	ordinary_muscle_development	ENSGALG00010024803	CLK1	0.369914461	0.137339151	2.693437796	0.007071934	0.009717289	positive
PRJNA729271	stage_1W_vs_E10	ordinary_muscle_development	ENSGALG00010024804	WDR53	0.263017415	0.105257604	2.498797274	0.012461558	0.016664847	positive
PRJNA729271	stage_1W_vs_E10	ordinary_muscle_development	ENSGALG00010024805	ENSGALG00010024805	0.923271363	0.372075953	2.481405628	0.013086536	0.01746679	positive
PRJNA729271	stage_1W_vs_E10	ordinary_muscle_development	ENSGALG00010024806	ANKRD27	0.652667131	0.073611373	8.866389855	7.55556E-19	2.68492E-18	positive
PRJNA729271	stage_1W_vs_E10	ordinary_muscle_development	ENSGALG00010024807	PPIL3	-0.608936809	0.195123323	-3.1207792	0.001803732	0.002625273	negative
PRJNA729271	stage_1W_vs_E10	ordinary_muscle_development	ENSGALG00010024808	CELSR2	-0.071900609	0.243086084	-0.295782496	0.767396191	0.791082269	negative
PRJNA729271	stage_1W_vs_E10	ordinary_muscle_development	ENSGALG00010024809	KANSL1	0.835778274	0.170387694	4.905156306	9.3353E-07	1.74961E-06	positive
PRJNA729271	stage_1W_vs_E10	ordinary_muscle_development	ENSGALG00010024810	ADIPOR1	1.401082408	0.112455134	12.45903459	1.24873E-35	8.69791E-35	positive
PRJNA729271	stage_1W_vs_E10	ordinary_muscle_development	ENSGALG00010024811	NIF3L1	-0.843464751	0.140755389	-5.992415322	2.06747E-09	4.57457E-09	negative
PRJNA729271	stage_1W_vs_E10	ordinary_muscle_development	ENSGALG00010024812	SBNO1	0.521952223	0.111839013	4.666995983	3.05635E-06	5.50744E-06	positive
PRJNA729271	stage_1W_vs_E10	ordinary_muscle_development	ENSGALG00010024813	RNF168	-0.213180371	0.096003882	-2.220539081	0.026382196	0.034010132	negative
PRJNA729271	stage_1W_vs_E10	ordinary_muscle_development	ENSGALG00010024814	APBA2	-3.604577334	0.173984761	-20.71777611	2.39495E-95	8.149E-94	negative
PRJNA729271	stage_1W_vs_E10	ordinary_muscle_development	ENSGALG00010024816	NFIA	-0.994992986	0.123118418	-8.081593318	6.3926E-16	1.98544E-15	negative
PRJNA729271	stage_1W_vs_E10	ordinary_muscle_development	ENSGALG00010024817	UCKL1	3.912089298	0.219422042	17.82906249	4.20412E-71	8.15779E-70	positive
PRJNA729271	stage_1W_vs_E10	ordinary_muscle_development	ENSGALG00010024818	FBXO3	0.087789869	0.076576229	1.146437621	0.251614124	0.284644246	positive
PRJNA729271	stage_1W_vs_E10	ordinary_muscle_development	ENSGALG00010024820	NUDT19	0.787237157	0.139600319	5.639221771	1.7082E-08	3.57396E-08	positive
PRJNA729271	stage_1W_vs_E10	ordinary_muscle_development	ENSGALG00010024821	NECAP1	-0.801277023	0.106740947	-7.506744534	6.06159E-14	1.70973E-13	negative
PRJNA729271	stage_1W_vs_E10	ordinary_muscle_development	ENSGALG00010024822	RPS26	1.070335306	0.137102572	7.806821518	5.86482E-15	1.73917E-14	positive
PRJNA729271	stage_1W_vs_E10	ordinary_muscle_development	ENSGALG00010024823	ASB10	12.8123413	0.865288723	14.80701293	1.31976E-49	1.42336E-48	positive
PRJNA729271	stage_1W_vs_E10	ordinary_muscle_development	ENSGALG00010024824	DNAJC5	-1.472898289	0.115218666	-12.78350412	2.02726E-37	1.4871E-36	negative
PRJNA729271	stage_1W_vs_E10	ordinary_muscle_development	ENSGALG00010024825	UBXN7	-0.596564664	0.076373839	-7.811112733	5.66853E-15	1.68372E-14	negative
PRJNA729271	stage_1W_vs_E10	ordinary_muscle_development	ENSGALG00010024826	RBM33	0.591673457	0.111586561	5.302372014	1.14308E-07	2.27407E-07	positive
PRJNA729271	stage_1W_vs_E10	ordinary_muscle_development	ENSGALG00010024827	ENSGALG00010024827	0.015784809	0.366898463	0.043022281	0.965683773	0.970437208	positive
PRJNA729271	stage_1W_vs_E10	ordinary_muscle_development	ENSGALG00010024828	TIMM17B	1.272846159	0.132999615	9.570299585	1.06596E-21	4.28484E-21	positive
PRJNA729271	stage_1W_vs_E10	ordinary_muscle_development	ENSGALG00010024829	ERBB3	-2.107553324	0.212504794	-9.917674241	3.48789E-23	1.48828E-22	negative
PRJNA729271	stage_1W_vs_E10	ordinary_muscle_development	ENSGALG00010024830	ORC2	-1.562923363	0.116109003	-13.46082833	2.65918E-41	2.2319E-40	negative
PRJNA729271	stage_1W_vs_E10	ordinary_muscle_development	ENSGALG00010024833	MAPT	2.972533805	0.200618633	14.81683811	1.14027E-49	1.23529E-48	positive
PRJNA729271	stage_1W_vs_E10	ordinary_muscle_development	ENSGALG00010024834	INSIG1	1.996849627	0.258447297	7.726332029	1.1069E-14	3.24131E-14	positive
PRJNA729271	stage_1W_vs_E10	ordinary_muscle_development	ENSGALG00010024835	ENTREP2	-1.185447829	0.144212679	-8.220135941	2.03273E-16	6.43186E-16	negative
PRJNA729271	stage_1W_vs_E10	ordinary_muscle_development	ENSGALG00010024836	TXNDC12	-0.992200176	0.107100375	-9.26420821	1.9653E-20	7.52431E-20	negative
PRJNA729271	stage_1W_vs_E10	ordinary_muscle_development	ENSGALG00010024837	TNFRSF1A	0.558868647	0.156471845	3.57168822	0.000354688	0.00054781	positive
PRJNA729271	stage_1W_vs_E10	ordinary_muscle_development	ENSGALG00010024838	EN2	-5.482919877	0.975319979	-5.62166263	1.89128E-08	3.94846E-08	negative
PRJNA729271	stage_1W_vs_E10	ordinary_muscle_development	ENSGALG00010024839	PA2G4	-0.600613484	0.099199966	-6.054573494	1.4079E-09	3.14543E-09	negative
PRJNA729271	stage_1W_vs_E10	ordinary_muscle_development	ENSGALG00010024840	BTF3L4	-0.919881352	0.101868494	-9.030086899	1.71536E-19	6.30108E-19	negative
PRJNA729271	stage_1W_vs_E10	ordinary_muscle_development	ENSGALG00010024841	ENSGALG00010024841	-1.878745065	0.213720363	-8.790669454	1.48671E-18	5.20278E-18	negative
PRJNA729271	stage_1W_vs_E10	ordinary_muscle_development	ENSGALG00010024842	ZFYVE9	-0.063351075	0.076193622	-0.831448531	0.405720293	0.443909695	negative
PRJNA729271	stage_1W_vs_E10	ordinary_muscle_development	ENSGALG00010024843	BCL9L	-0.154186549	0.13519603	-1.140466543	0.254091984	0.287335256	negative
PRJNA729271	stage_1W_vs_E10	ordinary_muscle_development	ENSGALG00010024844	TM4SF19	2.055852468	0.378957369	5.42502307	5.79471E-08	1.17837E-07	positive
PRJNA729271	stage_1W_vs_E10	ordinary_muscle_development	ENSGALG00010024845	HYCC2	0.369492447	0.111391463	3.317062516	0.000909693	0.001361205	positive
PRJNA729271	stage_1W_vs_E10	ordinary_muscle_development	ENSGALG00010024846	LMO2	0.509846633	0.143155462	3.561489215	0.000368757	0.00056839	positive
PRJNA729271	stage_1W_vs_E10	ordinary_muscle_development	ENSGALG00010024847	PPIL1	0.062684849	0.149945645	0.41805048	0.67591021	0.705655322	positive
PRJNA729271	stage_1W_vs_E10	ordinary_muscle_development	ENSGALG00010024848	ESYT1	0.081686234	0.085981914	0.950039731	0.342092065	0.379157706	positive
PRJNA729271	stage_1W_vs_E10	ordinary_muscle_development	ENSGALG00010024849	CENATAC	0.695708671	0.189830219	3.664899486	0.000247436	0.000386902	positive
PRJNA729271	stage_1W_vs_E10	ordinary_muscle_development	ENSGALG00010024850	CDK2AP1	-0.479347853	0.117404981	-4.082857892	4.44852E-05	7.34998E-05	negative
PRJNA729271	stage_1W_vs_E10	ordinary_muscle_development	ENSGALG00010024851	CPNE5	-5.184117619	0.988739155	-5.24316003	1.5785E-07	3.11085E-07	negative
PRJNA729271	stage_1W_vs_E10	ordinary_muscle_development	ENSGALG00010024852	CEP89	-0.96390548	0.097076023	-9.929387772	3.10154E-23	1.32733E-22	negative
PRJNA729271	stage_1W_vs_E10	ordinary_muscle_development	ENSGALG00010024853	TJP1	0.062698333	0.067663336	0.926621949	0.354122855	0.391743172	positive
PRJNA729271	stage_1W_vs_E10	ordinary_muscle_development	ENSGALG00010024854	C12orf65	1.409880647	0.149606354	9.423935621	4.3449E-21	1.70814E-20	positive
PRJNA729271	stage_1W_vs_E10	ordinary_muscle_development	ENSGALG00010024855	CAPRIN1	0.176108438	0.071762277	2.454053091	0.014125612	0.018784473	positive
PRJNA729271	stage_1W_vs_E10	ordinary_muscle_development	ENSGALG00010024856	SORT1	4.01483085	0.131474814	30.53688179	8.445E-205	1.3305E-202	positive
PRJNA729271	stage_1W_vs_E10	ordinary_muscle_development	ENSGALG00010024857	SLC37A4	1.269249182	0.141600426	8.963597211	3.14255E-19	1.13935E-18	positive
PRJNA729271	stage_1W_vs_E10	ordinary_muscle_development	ENSGALG00010024858	SLC25A39	0.580787055	0.121704927	4.772091539	1.82323E-06	3.34189E-06	positive
PRJNA729271	stage_1W_vs_E10	ordinary_muscle_development	ENSGALG00010024859	TARSL2	0.610022518	0.102870854	5.92998401	3.02964E-09	6.63427E-09	positive
PRJNA729271	stage_1W_vs_E10	ordinary_muscle_development	ENSGALG00010024860	NEU4	-3.74581109	0.740326041	-5.059677604	4.19966E-07	8.04574E-07	negative
PRJNA729271	stage_1W_vs_E10	ordinary_muscle_development	ENSGALG00010024861	ORC1	0.296552832	0.063094438	4.700142246	2.5998E-06	4.7117E-06	positive
PRJNA729271	stage_1W_vs_E10	ordinary_muscle_development	ENSGALG00010024862	METTL16	0.562616932	0.119595851	4.704318149	2.54716E-06	4.62033E-06	positive
PRJNA729271	stage_1W_vs_E10	ordinary_muscle_development	ENSGALG00010024863	LMOD1	0.755715722	0.295555316	2.556934963	0.010559896	0.014239994	positive
PRJNA729271	stage_1W_vs_E10	ordinary_muscle_development	ENSGALG00010024864	MPHOSPH9	-0.936213275	0.131819981	-7.102210684	1.22777E-12	3.23985E-12	negative
PRJNA729271	stage_1W_vs_E10	ordinary_muscle_development	ENSGALG00010024865	SNAP47	0.911528425	0.14264105	6.390365364	1.6549E-10	3.88592E-10	positive
PRJNA729271	stage_1W_vs_E10	ordinary_muscle_development	ENSGALG00010024866	TRAPPC4	0.774736146	0.084637838	9.153543646	5.50959E-20	2.06681E-19	positive
PRJNA729271	stage_1W_vs_E10	ordinary_muscle_development	ENSGALG00010024867	TM2D3	0.561913715	0.105379903	5.332266417	9.69945E-08	1.94083E-07	positive
PRJNA729271	stage_1W_vs_E10	ordinary_muscle_development	ENSGALG00010024868	FAAP24	-2.380921751	0.225979113	-10.53602575	5.89372E-26	2.81829E-25	negative
PRJNA729271	stage_1W_vs_E10	ordinary_muscle_development	ENSGALG00010024869	NDUFB3	2.072898208	0.12154635	17.05438471	3.24269E-65	5.39023E-64	positive
PRJNA729271	stage_1W_vs_E10	ordinary_muscle_development	ENSGALG00010024870	TPD52L2	0.135150932	0.082863886	1.630999209	0.102890486	0.123531649	positive
PRJNA729271	stage_1W_vs_E10	ordinary_muscle_development	ENSGALG00010024871	POLQ	-1.201125605	0.137046781	-8.764347442	1.87862E-18	6.53481E-18	negative
PRJNA729271	stage_1W_vs_E10	ordinary_muscle_development	ENSGALG00010024872	RUNDC3A	-1.134935446	0.206810011	-5.487816775	4.06932E-08	8.33943E-08	negative
PRJNA729271	stage_1W_vs_E10	ordinary_muscle_development	ENSGALG00010024873	HYOU1	-1.256367327	0.109167005	-11.50867271	1.193E-30	6.92257E-30	negative
PRJNA729271	stage_1W_vs_E10	ordinary_muscle_development	ENSGALG00010024874	JMJD4	0.225433384	0.081630201	2.761641897	0.005751152	0.007975026	positive
PRJNA729271	stage_1W_vs_E10	ordinary_muscle_development	ENSGALG00010024876	CFLAR	0.023602097	0.096001809	0.245850543	0.80579793	0.826437486	positive
PRJNA729271	stage_1W_vs_E10	ordinary_muscle_development	ENSGALG00010024877	PRPF38A	-1.036812327	0.08174457	-12.68356211	7.29401E-37	5.24438E-36	negative
PRJNA729271	stage_1W_vs_E10	ordinary_muscle_development	ENSGALG00010024878	RHPN2	-1.469722058	0.275863533	-5.327714189	9.94565E-08	1.98817E-07	negative
PRJNA729271	stage_1W_vs_E10	ordinary_muscle_development	ENSGALG00010024879	D2HGDH	0.710318608	0.149336917	4.756483674	1.96994E-06	3.6017E-06	positive
PRJNA729271	stage_1W_vs_E10	ordinary_muscle_development	ENSGALG00010024880	NAT10	-1.278126598	0.13949188	-9.162731175	5.06001E-20	1.90062E-19	negative
PRJNA729271	stage_1W_vs_E10	ordinary_muscle_development	ENSGALG00010024881	CASP10	1.417839893	0.285170627	4.971900184	6.62998E-07	1.25475E-06	positive
PRJNA729271	stage_1W_vs_E10	ordinary_muscle_development	ENSGALG00010024882	MYL6	-1.485772633	0.164857517	-9.012465187	2.01476E-19	7.36728E-19	negative
PRJNA729271	stage_1W_vs_E10	ordinary_muscle_development	ENSGALG00010024883	ING5	1.48727176	0.123033181	12.08837926	1.21663E-33	7.86577E-33	positive
PRJNA729271	stage_1W_vs_E10	ordinary_muscle_development	ENSGALG00010024884	ZBTB46	1.514856242	0.133015373	11.38858019	4.76694E-30	2.69909E-29	positive
PRJNA729271	stage_1W_vs_E10	ordinary_muscle_development	ENSGALG00010024885	SLC4A1	-0.922558425	0.238010114	-3.876131182	0.00010613	0.000170286	negative
PRJNA729271	stage_1W_vs_E10	ordinary_muscle_development	ENSGALG00010024886	ABTB2	-2.019679607	0.145672284	-13.86454274	1.03886E-43	9.35878E-43	negative
PRJNA729271	stage_1W_vs_E10	ordinary_muscle_development	ENSGALG00010024887	CASP18	1.394463648	0.255026136	5.467924456	4.55336E-08	9.31296E-08	positive
PRJNA729271	stage_1W_vs_E10	ordinary_muscle_development	ENSGALG00010024888	PITPNM2	0.036440963	0.129897229	0.280536877	0.779065637	0.801914246	positive
PRJNA729271	stage_1W_vs_E10	ordinary_muscle_development	ENSGALG00010024889	SLC2A4RG	0.472064103	0.46952314	1.005411795	0.314698602	0.350700103	positive
PRJNA729271	stage_1W_vs_E10	ordinary_muscle_development	ENSGALG00010024890	SMARCC2	0.320875722	0.145819675	2.200496761	0.027771669	0.03570608	positive
PRJNA729271	stage_1W_vs_E10	ordinary_muscle_development	ENSGALG00010024891	PKP1	-1.155929655	0.465787575	-2.481667003	0.013076942	0.017455592	negative
PRJNA729271	stage_1W_vs_E10	ordinary_muscle_development	ENSGALG00010024893	ARL6IP4	0.225395037	0.139104226	1.620332067	0.105160964	0.12608008	positive
PRJNA729271	stage_1W_vs_E10	ordinary_muscle_development	ENSGALG00010024894	TUT4	0.863593704	0.122013546	7.07785102	1.46407E-12	3.84381E-12	positive
PRJNA729271	stage_1W_vs_E10	ordinary_muscle_development	ENSGALG00010024895	ENSGALG00010024895	1.450998462	0.407891828	3.55731192	0.000374669	0.000577318	positive
PRJNA729271	stage_1W_vs_E10	ordinary_muscle_development	ENSGALG00010024896	RNF41	0.96213326	0.099973493	9.623883628	6.33915E-22	2.56665E-21	positive
PRJNA729271	stage_1W_vs_E10	ordinary_muscle_development	ENSGALG00010024897	ADAL	0.57071123	0.191994277	2.972542926	0.002953439	0.004211106	positive
PRJNA729271	stage_1W_vs_E10	ordinary_muscle_development	ENSGALG00010024898	NABP2	0.642651907	0.308461488	2.083410516	0.037213821	0.047184598	positive
PRJNA729271	stage_1W_vs_E10	ordinary_muscle_development	ENSGALG00010024899	OGFOD2	-0.498062614	0.129612872	-3.842694069	0.000121691	0.000194478	negative
PRJNA729271	stage_1W_vs_E10	ordinary_muscle_development	ENSGALG00010024900	DTYMK	-1.699748844	0.106598283	-15.9453679	3.06828E-57	4.19177E-56	negative
PRJNA729271	stage_1W_vs_E10	ordinary_muscle_development	ENSGALG00010024901	ZGPAT	-0.650654	0.132263297	-4.9193844	8.68168E-07	1.63112E-06	negative
PRJNA729271	stage_1W_vs_E10	ordinary_muscle_development	ENSGALG00010024902	LARP6	0.696502797	0.117793367	5.912920323	3.36095E-09	7.3402E-09	positive
PRJNA729271	stage_1W_vs_E10	ordinary_muscle_development	ENSGALG00010024903	CASP8	-1.183398337	0.115973321	-10.20405662	1.9016E-24	8.53101E-24	negative
PRJNA729271	stage_1W_vs_E10	ordinary_muscle_development	ENSGALG00010024904	ALS2CL	1.234886385	0.16036986	7.700239842	1.35811E-14	3.95536E-14	positive
PRJNA729271	stage_1W_vs_E10	ordinary_muscle_development	ENSGALG00010024905	GPX7	-2.283329856	0.21078134	-10.83269447	2.40959E-27	1.2225E-26	negative
PRJNA729271	stage_1W_vs_E10	ordinary_muscle_development	ENSGALG00010024907	PHLDB1	0.780667548	0.133436439	5.850482471	4.90149E-09	1.06025E-08	positive
PRJNA729271	stage_1W_vs_E10	ordinary_muscle_development	ENSGALG00010024908	ENSGALG00010024908	1.324570222	0.125811264	10.5282324	6.40254E-26	3.05782E-25	positive
PRJNA729271	stage_1W_vs_E10	ordinary_muscle_development	ENSGALG00010024910	UBTF	-0.581811022	0.123611971	-4.70675307	2.51693E-06	4.56722E-06	negative
PRJNA729271	stage_1W_vs_E10	ordinary_muscle_development	ENSGALG00010024911	CAT	0.882280641	0.153271403	5.756329122	8.59627E-09	1.83051E-08	positive
PRJNA729271	stage_1W_vs_E10	ordinary_muscle_development	ENSGALG00010024915	STXBP5L	0.133292554	0.172476272	0.77281676	0.439630833	0.477587419	positive
PRJNA729271	stage_1W_vs_E10	ordinary_muscle_development	ENSGALG00010024917	TRAK2	0.720187638	0.123014095	5.854513141	4.78409E-09	1.03516E-08	positive
PRJNA729271	stage_1W_vs_E10	ordinary_muscle_development	ENSGALG00010024918	ATG4B	-0.807996512	0.076588535	-10.54983633	5.08857E-26	2.44477E-25	negative
PRJNA729271	stage_1W_vs_E10	ordinary_muscle_development	ENSGALG00010024919	ANKRD52	-0.540609101	0.124133775	-4.355052462	1.33035E-05	2.28639E-05	negative
PRJNA729271	stage_1W_vs_E10	ordinary_muscle_development	ENSGALG00010024920	VIPR2	0.836895738	0.299230176	2.796829345	0.005160678	0.00719754	positive
PRJNA729271	stage_1W_vs_E10	ordinary_muscle_development	ENSGALG00010024921	STRADB	-0.825499291	0.111168612	-7.425650821	1.12227E-13	3.11216E-13	negative
PRJNA729271	stage_1W_vs_E10	ordinary_muscle_development	ENSGALG00010024924	MNX1	-3.764140541	0.721782189	-5.215064324	1.83753E-07	3.60616E-07	negative
PRJNA729271	stage_1W_vs_E10	ordinary_muscle_development	ENSGALG00010024925	GTF2E1	-0.552793047	0.084107378	-6.572467993	4.9488E-11	1.19834E-10	negative
PRJNA729271	stage_1W_vs_E10	ordinary_muscle_development	ENSGALG00010024926	ELF5	-5.996997085	0.999617173	-5.999293774	1.98178E-09	4.39233E-09	negative
PRJNA729271	stage_1W_vs_E10	ordinary_muscle_development	ENSGALG00010024927	NOM1	-0.294485995	0.113953443	-2.584265885	0.009758653	0.013209906	negative
PRJNA729271	stage_1W_vs_E10	ordinary_muscle_development	ENSGALG00010024928	THAP4	1.355793559	0.084416073	16.06084608	4.79959E-58	6.6766E-57	positive

PRJNA729271	stage_1W_vs_E10	ordinary_muscle_development	ENSGALG00010024930	TMEM237	-0.073540928	0.100027822	-0.73520473	0.462214819	0.500508277	negative
PRJNA729271	stage_1W_vs_E10	ordinary_muscle_development	ENSGALG00010024931	UBE3C	0.564651808	0.088537645	6.377533589	1.79963E-10	4.21415E-10	positive
PRJNA729271	stage_1W_vs_E10	ordinary_muscle_development	ENSGALG00010024932	ANKK1	-0.808121773	1.757202222	-0.459891163	0.645594344	0.67732267	negative
PRJNA729271	stage_1W_vs_E10	ordinary_muscle_development	ENSGALG00010024933	CS	1.621699528	0.156727957	10.34722557	4.30789E-25	1.99243E-24	positive
PRJNA729271	stage_1W_vs_E10	ordinary_muscle_development	ENSGALG00010024934	G6PC3	0.983746266	0.221470896	4.441876047	8.91779E-06	1.55327E-05	positive
PRJNA729271	stage_1W_vs_E10	ordinary_muscle_development	ENSGALG00010024935	KLHL30	4.16049429	0.387564342	10.73497697	6.97364E-27	3.4653E-26	positive
PRJNA729271	stage_1W_vs_E10	ordinary_muscle_development	ENSGALG00010024936	EHF	0.195372893	0.536182406	0.364377665	0.715576011	0.742946371	positive
PRJNA729271	stage_1W_vs_E10	ordinary_muscle_development	ENSGALG00010024937	ILKAP	-0.832024553	0.127023109	-6.550182549	5.74668E-11	1.38346E-10	negative
PRJNA729271	stage_1W_vs_E10	ordinary_muscle_development	ENSGALG00010024938	ENSGALG00010024938	0.025503652	0.182071483	0.14007494	0.888600781	0.900907066	positive
PRJNA729271	stage_1W_vs_E10	ordinary_muscle_development	ENSGALG00010024939	COQ10A	2.892771332	0.202233005	14.30415043	2.0614E-46	2.01347E-45	positive
PRJNA729271	stage_1W_vs_E10	ordinary_muscle_development	ENSGALG00010024940	RABL3	-0.383954474	0.092480207	-4.151747549	3.29946E-05	5.51241E-05	negative
PRJNA729271	stage_1W_vs_E10	ordinary_muscle_development	ENSGALG00010024941	PDHX	0.756044256	0.118110335	6.401169346	1.54191E-10	3.6259E-10	positive
PRJNA729271	stage_1W_vs_E10	ordinary_muscle_development	ENSGALG00010024942	DNAJB6	0.149311234	0.066359419	2.250038303	0.024446514	0.031664035	positive
PRJNA729271	stage_1W_vs_E10	ordinary_muscle_development	ENSGALG00010024943	ABCB9	0.255947525	0.180118763	1.420993134	0.155318755	0.181750655	positive
PRJNA729271	stage_1W_vs_E10	ordinary_muscle_development	ENSGALG00010024944	ENSGALG00010024944	0.504680515	0.111918828	4.509344197	6.50283E-06	1.1417E-05	positive
PRJNA729271	stage_1W_vs_E10	ordinary_muscle_development	ENSGALG00010024945	APIP	0.726594125	0.080653167	9.008872794	2.08185E-19	7.60687E-19	positive
PRJNA729271	stage_1W_vs_E10	ordinary_muscle_development	ENSGALG00010024946	KIF1A	-4.104220004	0.276545794	-14.84101403	7.95444E-50	8.6953E-49	negative
PRJNA729271	stage_1W_vs_E10	ordinary_muscle_development	ENSGALG00010024947	MPP4	1.386643256	0.312398457	4.438700717	9.05036E-06	1.57579E-05	positive
PRJNA729271	stage_1W_vs_E10	ordinary_muscle_development	ENSGALG00010024949	PAN2	-0.365274238	0.115730378	-3.156252023	0.001598107	0.002336551	negative
PRJNA729271	stage_1W_vs_E10	ordinary_muscle_development	ENSGALG00010024950	NCAM1	-1.333673654	0.155316716	-8.586800477	8.94247E-18	3.00397E-17	negative
PRJNA729271	stage_1W_vs_E10	ordinary_muscle_development	ENSGALG00010024953	NCAPG2	-2.159630833	0.223222797	-9.67477722	3.85931E-22	1.57402E-21	negative
PRJNA729271	stage_1W_vs_E10	ordinary_muscle_development	ENSGALG00010024956	VPS37B	-0.885538028	0.139152664	-6.363787841	1.96838E-10	4.59743E-10	negative
PRJNA729271	stage_1W_vs_E10	ordinary_muscle_development	ENSGALG00010024960	ARFRP1	-0.874746954	0.10408448	-8.404201574	4.30782E-17	1.39973E-16	negative
PRJNA729271	stage_1W_vs_E10	ordinary_muscle_development	ENSGALG00010024961	LSM12	0.930902125	0.202685521	4.592839793	4.37255E-06	7.78912E-06	positive
PRJNA729271	stage_1W_vs_E10	ordinary_muscle_development	ENSGALG00010024963	TNFRSF6B	1.015681709	0.580181717	1.750626878	0.080010202	0.097343284	positive
PRJNA729271	stage_1W_vs_E10	ordinary_muscle_development	ENSGALG00010024964	ITM2C	-1.565488938	0.173949154	-8.999692719	2.2635E-19	8.25396E-19	negative
PRJNA729271	stage_1W_vs_E10	ordinary_muscle_development	ENSGALG00010024965	STAT2	1.132748515	0.218112073	5.193424182	2.06461E-07	4.04139E-07	positive
PRJNA729271	stage_1W_vs_E10	ordinary_muscle_development	ENSGALG00010024966	AGAP3	0.027049004	0.114775852	0.235668074	0.813690245	0.833398271	positive
PRJNA729271	stage_1W_vs_E10	ordinary_muscle_development	ENSGALG00010024968	TMEM101	1.597008309	0.130277976	12.25846732	1.51335E-34	1.00995E-33	positive
PRJNA729271	stage_1W_vs_E10	ordinary_muscle_development	ENSGALG00010024970	CAB39	0.422638372	0.077790133	5.433058891	5.53961E-08	1.12713E-07	positive
PRJNA729271	stage_1W_vs_E10	ordinary_muscle_development	ENSGALG00010024973	RTEL1	-1.895471122	0.091411281	-20.73563685	1.65253E-95	5.7032E-94	negative
